# On the Use of Bone Remodelling Models to Estimate the Density Distribution of Bones. Uniqueness of the Solution

**DOI:** 10.1371/journal.pone.0148603

**Published:** 2016-02-09

**Authors:** Javier Martínez-Reina, Joaquín Ojeda, Juana Mayo

**Affiliations:** Department of Mechanical Engineering, Universidad de Sevilla, Sevilla, Spain; University of Zaragoza, SPAIN

## Abstract

Bone remodelling models are widely used in a phenomenological manner to estimate numerically the distribution of apparent density in bones from the loads they are daily subjected to. These simulations start from an arbitrary initial distribution, usually homogeneous, and the density changes locally until a bone remodelling equilibrium is achieved. The bone response to mechanical stimulus is traditionally formulated with a mathematical relation that considers the existence of a range of stimulus, called *dead* or *lazy zone*, for which no net bone mass change occurs. Implementing a relation like that leads to different solutions depending on the starting density. The non-uniqueness of the solution has been shown in this paper using two different bone remodelling models: one isotropic and another anisotropic. It has also been shown that the problem of non-uniqueness is only mitigated by removing the dead zone, but it is not completely solved unless the bone formation and bone resorption rates are limited to certain maximum values.

## Introduction

Finite element (FE) models of bones are extensively used in many clinical applications. The elastic properties of bone are needed for those FE models, but they are not directly available. These properties have been traditionally correlated with the apparent density or the porosity (see e.g. [1–3]), which can be easily estimated from CT scans. This could solve the problem of assigning elastic properties to a FE model of a bone. However, the anisotropy of bone has a strong influence on its elastic behaviour as well [4, 5], but it is not so easily assessed through imaging techniques. These techniques have only recently been applied to that purpose, but only to quantify the spatial orientation of trabecular bone [6]. An approximate and simple way of getting a map of the elastic properties in bones has been the use of bone remodelling models (BRM). These models predict the changes induced in bone geometry, apparent density and anisotropy under changes in the mechanical environment of the bone. Therefore, they can be indirectly used to predict the distribution of density and anisotropy to subsequently relate them with the elastic properties.

The distribution of apparent density and anisotropy is related to the loads the bone is usually subjected to [7]. A mechanical stimulus is usually defined to measure the intensity of those loads, by means of the strains and/or stresses. Lanyon and Rubin [8] showed that the strain rate has also an effect on bone remodelling and recent BRMs have included this effect [9]. As established in the mechanostat theory [10], bone is resorbed from sites where it is in disuse (low mechanical stimulus) and is deposited where the loads are frequent and intense (high mechanical stimulus). The combination of these effects gives shape to bones and establish their distribution of density and anisotropy. Many authors have applied BRM *in silico* following a phenomenological approach whose goal is to predict the distribution of elastic properties from the loads as follows: starting from an unrealistic situation (isotropic with an homogeneous distribution of elastic properties or apparent density) and applying the normal loads of daily activity, anisotropy and density change until an equilibrium state is reached, with a distribution of density [11, 12] and anisotropy [12–15] similar to the physiological ones.

**Fig 1:**
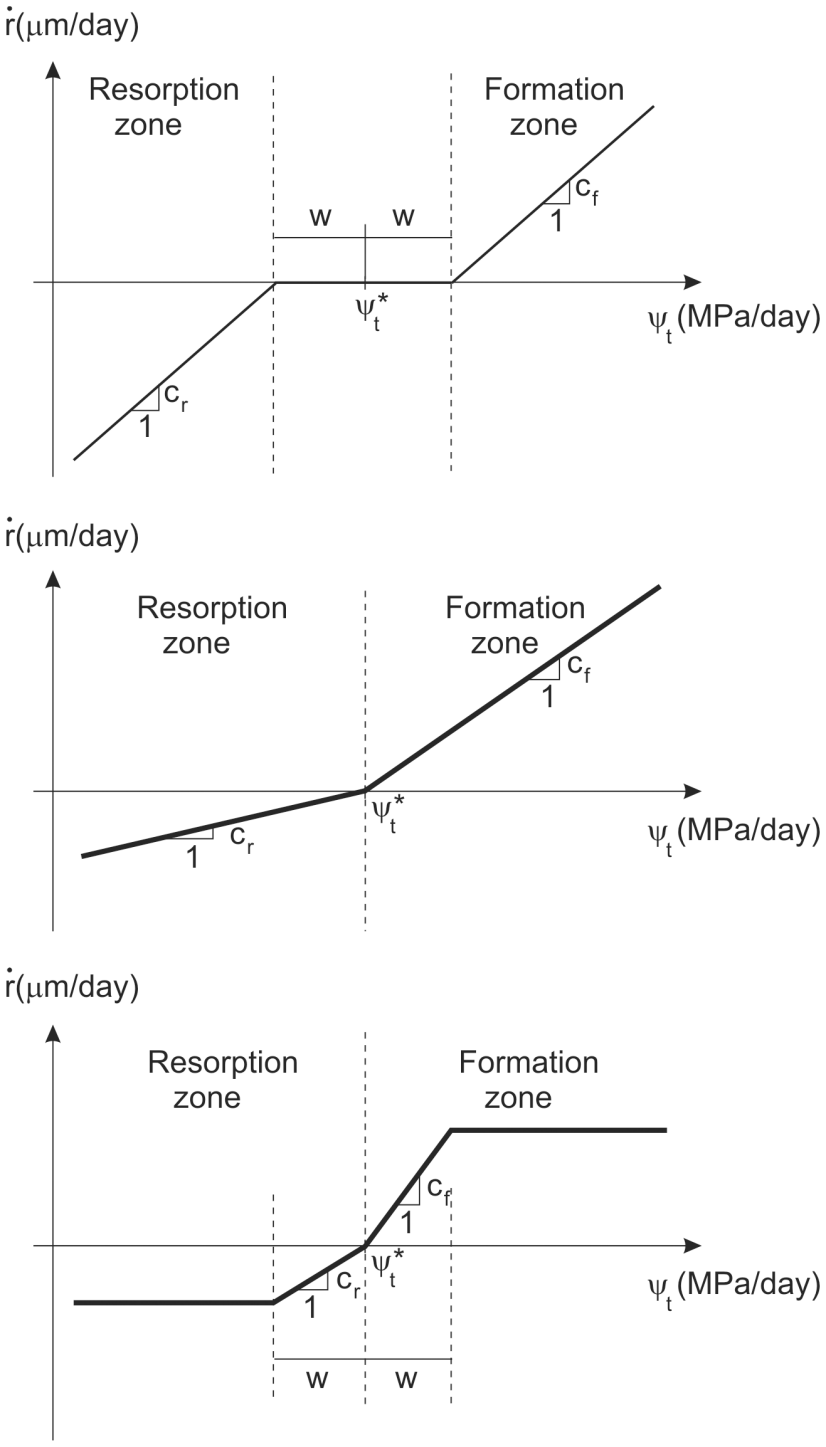
Different RRR analyzed: (a) with dead zone (*DZ*), (b) bilinear (*BL*), (c) with saturation (*S*).

**Fig 2:**
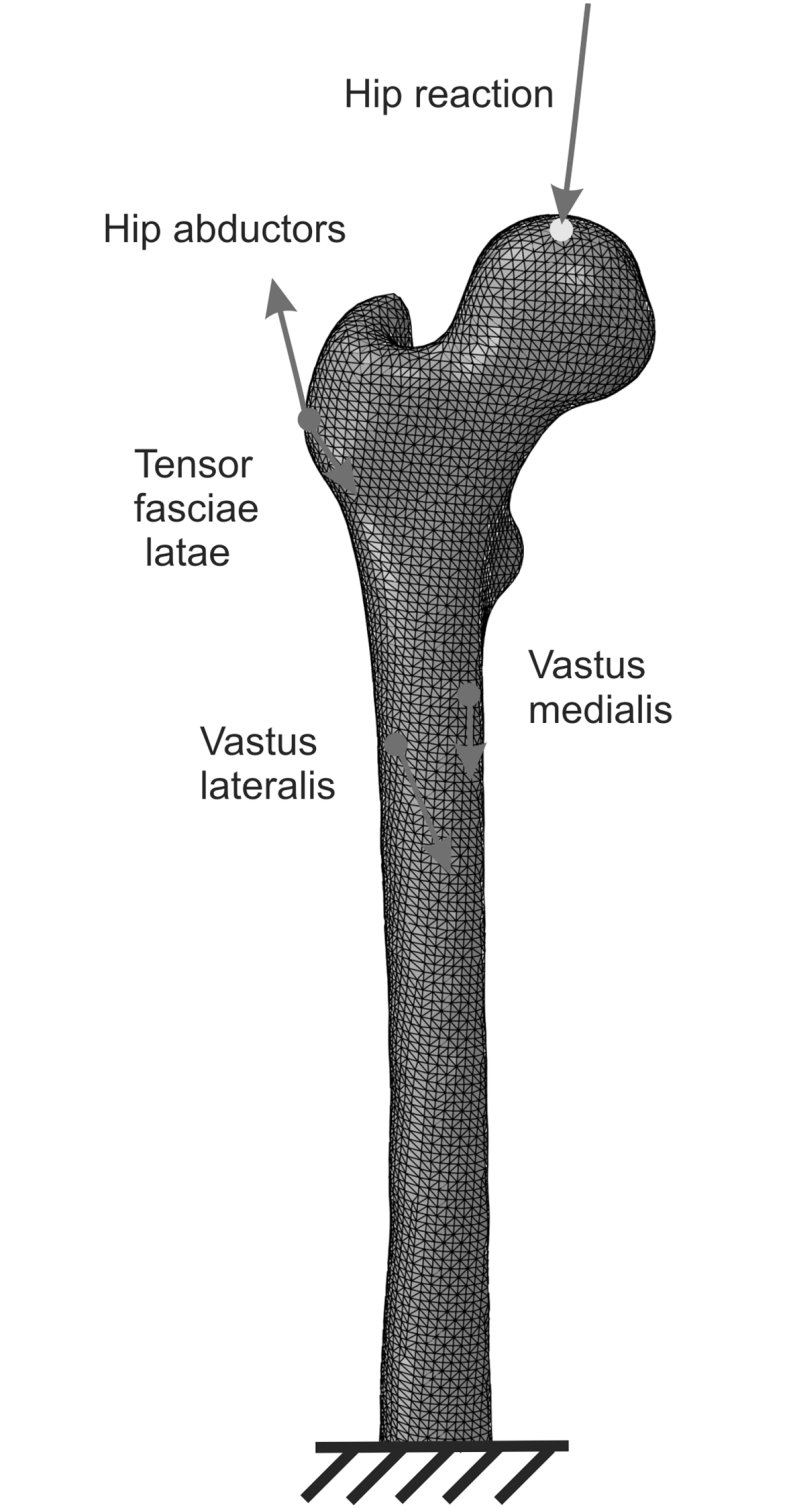
FE model of the femur and location of the insertion points of the muscles (red); point of application of the resultant of hip reaction (yellow).

One feature of most BRM (e.g. [16, 17]) is the presence of the well-known *lazy zone* (originally termed *dead zone* [11]), a range of mechanical stimulus (around a reference value that bone senses as normal) within which no net change of bone density is seen. In those models, if the mechanical stimulus is different to that reference value, bone is assumed to respond, but in a “lazily” way, such that only if the stimulus is well over (or below) that reference value, net bone formation (or resorption) is produced (see Fig 1a). Evidences of the existence of the dead zone are still lacking and it has been recently debated by Christen et al. [18]. These authors have suggested that bone remodelling response correlates with tissue loads following a linear relationship without a lazy zone. Actually, the lazy or dead zone seems more like a mathematical approximation than a mechanobiological fact. For example, the behaviour of actual bones shown by Beaupré et al. [19] (see Fig 2 of that work) exhibits a range of stimulus where the remodelling response is certainly low though not exactly zero. In the subsequent work Beaupré et al. [11] approximated that response by introducing a dead zone with no net mass change.

The inclusion of the dead zone in the BRM has a serious drawback: the final bone density distribution depends on the starting density, making the solution of the problem to be non-unique. An example may help to illustrate the problem. Let us assume that a point must reach a density of 1 *g*/*cm*^3^ at the remodelling equilibrium, because with that density (and the stiffness derived from it) the external loads would produce a mechanical stimulus equal to the reference value. If the starting density was 0.5 *g*/*cm*^3^, that point would increase its density, since its stiffness is too low, the strain level is high and, consequently, its mechanical stimulus is higher than the reference value. The density will rise until the stiffness is such that the mechanical stimulus has entered the dead zone, though not necessarily equal to the reference value. That may happen when the density is 0.8 *g*/*cm*^3^, for example. On the contrary, if the initial density was 1.5 *g*/*cm*^3^, the local stiffness is too high and the mechanical stimulus lower than the reference value. The density would decrease and the mechanical stimulus would increase, again not necessarily until the reference value, but until the limit of the dead zone. This may happen when the density is 1.2 *g*/*cm*^3^. Thus, a unique solution of the density distribution is not guaranteed.

Cowin and Hegedus [7] formulated this phenomenological approach of bone remodelling (that they termed adaptive elasticity) in a general manner using the continuum mechanics theory. Monnier and Trabucho [20] provided the conditions needed for the existence of solution of the problem of adaptive elasticity. Cowin and Nachlinger [21] had previously established the sufficient conditions needed for uniqueness of the solution, provided that such solution existed. One of these conditions is that the remodelling response must be differentiable, which is not met if the dead zone is implemented as done by Beaupré et al. [19].

In the subsequent work Beaupré et al. [11] proposed another remodelling rate relation (RRR) with no dead zone. In this case, bone would not stop changing its density unless it had reached exactly the reference mechanical stimulus. This relation suggests a biunivocal correspondence between density and mechanical stimulus and might seem the remedy for the problem of non-uniqueness. However, the solution is still non-unique as will be shown later. For this reason, a different RRR has been tried here to solve the problem. The objective of this paper is to analyse the effect of different RRR on the estimation of bone density with BRM. It must be clear from the beginning that it is not an objective of this paper to obtain a realistic distribution of density in the femur, for which a more complex analysis should be performed. This work just uses a simple FE model, which is enough to highlight the problems arising from the inclusion of the dead zone in BRMs based on the mechanostat theory.

## Materials and Methods

Two BRM were chosen from the literature to illustrate the effect of the RRR on the predicted density: an isotropic model, IBRM [19] and an anisotropic one, ABRM [13]. Both are briefly described next. For a more detailed description, the reader is advised to consult the original papers.

The reason to choose an isotropic and an anisotropic model is that the density distribution is influenced by the relationship between density and stiffness, which, in turn, depends on whether anisotropy is considered or not. Thus, the effect of the RRR could be different in each model and must be analysed separately.

### Description of IBRM

The bone remodelling response is expressed as a function of the mechanical stimulus, called daily stress stimulus, which is computed from the strain energy density (SED) produced by the loads. The daily stress stimulus measured at the continuum level is defined as:

$$\begin{array}{c} \psi ={\left(\sum_{day}n_{i}{\bar{\sigma }}_{i}^{m}\right)}^{1/m} \end{array}$$ (1)

where *n*_*i*_ is the number of cycles of load *i*, *m* is a constant and ${\bar{\sigma }}_{i}$ is the local effective stress, calculated at the continuum level as ${\bar{\sigma }}_{i}=\sqrt{2\;E\;U_{i}}$, where *E* represents the Young’s modulus and *U*_*i*_ is the SED produced by the load *i* in a certain point.

The daily stress stimulus at the continuum level, *ψ*, is computed through FE analysis at each integration point of the mesh, but the variable that controls the bone remodelling response is the daily stress stimulus measured at the tissue level, *ψ*_*t*_, related to *ψ* by:

$$\begin{array}{c} \psi_{t}={\left(\frac{\hat{\rho }}{\rho }\right)}^{2}\psi \end{array}$$ (2)

being *ρ* the apparent density and $\hat{\rho }=2.1\;g/cm^{3}$ the apparent density of the cortical bone of maximum density, assumed equal to the density of the fully mineralized tissue [19]. The remodelling response is measured in terms of the bone resorption/formation rate, $\dot{r}\left(\mu m/day\right)$, which gives the net tissue volume formed or resorbed per unit time and unit surface available for remodelling and is defined as a function of *ψ*_*t*_ in the RRR, addressed later on. The density change rate is given by:

$$\begin{array}{c} \dot{\rho }=\dot{r}\;S_{v}\;\hat{\rho } \end{array}$$ (3)

where the specific surface, *S*_*v*_, is the free bone surface per unit volume and was correlated with porosity by Martin [22]. Finally, the Young’s modulus and the Poisson’s ratio were related to the apparent density by Jacobs [23]:

$$\begin{array}{c} \begin{array}{c} E\;\left(\text{MPa}\right)=B\;\rho^{\beta }\\ \text{with}\left\{\begin{array}{cc} B=2014\;,\;\beta =2.5 & \;\text{if}\;\;\rho \leq 1.2g/cm^{3}\\ B=1763\;,\;\beta =3.2 & \;\text{if}\;\;\rho \;>1.2g/cm^{3} \end{array}\right. \end{array} \end{array}$$ (4a)

$$\begin{array}{c} \nu =\left\{\begin{array}{cc} 0.2 & \;\text{if}\;\;\rho \leq 1.2g/cm^{3}\\ 0.32 & \;\text{if}\;\;\rho \;>1.2g/cm^{3} \end{array}\right. \end{array}$$ (4b)

This algorithm was applied by Beaupré et al. [11] starting from an ideal situation: homogeneous apparent density equal to 0.57 *g*/*cm*^3^. By applying normal loads on the proximal femur the apparent density changed following Eq (3). This implied a change in the local stiffness and, consequently, in the distribution of stresses and mechanical stimulus. For this reason, the remodelling algorithm was applied iteratively until a remodelling equilibrium was achieved with no further changes in the apparent density.

The RRR used by Beaupré et al. [11] was given by the following piecewise linear function (see Fig 1a):

$$\begin{array}{c} \dot{r}=\left\{\begin{array}{cc} c_{f}\left(\psi_{t}-\psi_{t}^{*}-w\right) & \;\text{if}\;\;\psi_{t}>\psi_{t}^{*}+w\\ 0 & \;\text{if}\;\;\psi_{t}^{*}-w<\psi_{t}<\psi_{t}^{*}+w\\ -c_{r}\left(\psi_{t}^{*}-w-\psi_{t}\right) & \;\text{if}\;\;\psi_{t}<\psi_{t}^{*}-w \end{array}\right. \end{array}$$ (5)

where *c*_*r*_ and *c*_*f*_ are the slopes of the resorption and formation ramps, respectively. This relation exhibited the so called dead zone, a range of stimulus of width 2w around the reference stimulus, $\psi_{t}^{*}$, within which no net bone mass change was produced. This relation with dead zone has been named here *DZ* (see Fig 1).

### Description of ABRM

This ABRM, proposed by Doblaré and García [24], is an extension of the IBRM to the anisotropic case. The anisotropy is measured with the fabric tensor $\hat{H}$, normalized such that $det(\hat{H})=1$. Then, a tensor **H** was defined to consider jointly the porosity and the orientation of that porosity (defined by $\hat{H}$):

$$\begin{array}{c} H\left(\rho ,\hat{H}\right)={\left(\frac{\rho^{\beta (\rho )}\;B\left(\rho \right)}{{\hat{\rho }}^{\beta (\hat{\rho })}\;B\left(\hat{\rho }\right)}\right)}^{1/4}\;{\hat{H}}^{1/2} \end{array}$$ (6)

where *β*(*ρ*) and *B*(*ρ*) represent the constants in the relationship Eq (4a), which depend on *ρ* (e.g. $\beta (\hat{\rho })=3.2$, $B(\hat{\rho })=1763$). The mechanical stimulus, **Y**, is represented in this model as a tensorial function of porosity and anisotropy (through the tensor **H**) and of the strain tensor, *ε*, which is the mechanical variable driving the remodelling process:

$$\begin{array}{c} Y=2\left[2\hat{G}\;sym\left[\left(H\varepsilon H\right)\left(H\varepsilon \right)\right]+\hat{\lambda }\;tr\left(H^{2}\varepsilon \right)\;sym\left(H\varepsilon \right)\right] \end{array}$$ (7)

where $\hat{G}$ and $\hat{\lambda }$ are the Lamé constants of the cortical bone of density $\hat{\rho }$, obtained from the relationships Eq (4). To weight the relative influence of the spherical and deviatoric parts of the stimulus, a new stimulus tensor, **J**, is defined as:

$$\begin{array}{c} J=\frac{\left(1-\omega \right)}{3}\;tr\left(Y\right)1+\omega \;dev\left(Y\right) \end{array}$$ (8)

where *ω* ∈ [0, 1] is a parameter to be chosen *a priori*, *tr*(•) and *dev*(•) represent the trace and deviatoric part of a tensor, respectively, and **1** is the second order identity tensor. If *ω* = 0, the model is purely isotropic and if *ω* = 1, **J** = *dev*(**Y**) and the spherical part of the stimulus has no influence on the remodelling response.

Doblaré and García [24] defined two functions, *g*^*r*^ and *g*^*f*^, to propose a RRR analogous to Eq (5). These functions depend on the stimulus tensor, **J**, the reference stimulus, $\psi_{t}^{*}$, and the width of the dead zone, *w*. They are used to establish the remodelling criteria, that, like the inequalities in Eq (5), defines the domains of the stimulus **J** for which formation, resorption or no net remodelling response (dead zone) take place:

$$\begin{array}{c} \begin{array}{cc} g^{r}\left(J,\psi_{t}^{*},w\right)\leq 0\;g^{f}\left(J,\psi_{t}^{*},w\right)>0 & \;\;\text{formation}\\ g^{r}\left(J,\psi_{t}^{*},w\right)\leq 0\;g^{f}\left(J,\psi_{t}^{*},w\right)\leq 0 & \;\;\text{dead zone}\\ g^{f}\left(J,\psi_{t}^{*},w\right)\leq 0\;g^{r}\left(J,\psi_{t}^{*},w\right)>0 & \;\;\text{resorption} \end{array} \end{array}$$ (9)

In fact, *g*^*r*^ and *g*^*f*^ measure how far **J** is from the lower and upper limit of the dead zone, respectively. Thus, they also provide the remodelling response, $\dot{r}$, in a linear way:

$$\begin{array}{c} \dot{r}=\left\{\begin{array}{cc} c_{f}\frac{g^{f}}{\rho^{2-\beta /2}} & \text{in formation}\\ 0 & \text{in the dead zone}\\ -c_{r}\frac{g^{r}}{\rho^{2-\beta /2}} & \text{in resorption} \end{array}\right. \end{array}$$ (10)

Note that this relation is analogous to Eq (5). This value of $\dot{r}$ is used to calculate the evolution of apparent density and anisotropy, through $\dot{H}$:

$$\begin{array}{c} \dot{H}=\left\{\begin{array}{cc} \frac{3\beta k\dot{r}S_{v}}{4\;tr(H^{-2}J^{-3}H\hat{\omega })}\frac{\hat{\rho }}{\rho }\;J^{-3}\hat{\omega } & \;\text{in formation}\\ 0 & \;\text{in the dead zone}\\ \frac{3\beta k\dot{r}S_{v}}{4\;tr(H^{-2}JH\hat{\omega })}\frac{\hat{\rho }}{\rho }\;J\hat{\omega } & \;\text{in resorption} \end{array}\right. \end{array}$$ (11)

with:

$$\begin{array}{c} \hat{\omega }=\frac{1}{3}\left(1-2\omega \right)1⊗1+\omega I \end{array}$$ (12)

where **I** is the fourth rank identity tensor.

The mechanical properties in this model are still given as a function of the apparent density, following the relationships Eq (4), but corrected with the anisotropy through tensor **H** (see [24]).

### Description of the RRRs

The RRR named *DZ* exhibited the referred problem of non-uniqueness of the solution. Apart from it, Beaupré et al. [11] tried a different RRR by making zero the width of the dead zone. This resulted in a bilinear relation (named here *BL*, see Fig 1b) or linear if the slopes of the resorption and formation zones are assumed equal, as those authors did. In principle, this *BL* relation might seem adequate to solve the problem of non-uniqueness, since the density of an element will not stop changing until its mechanical stimulus equals the reference mechanical stimulus. This suggests a biunivocal correspondence between density and mechanical stimulus, and, therefore, the existence of a unique bone density distribution in equilibrium with the external loads. However, that is not what occured in the simulations, as shown later on. For this reason a third RRR was tried in this paper.

Adams et al. [25] reported evidences of saturation in the bone remodelling response for high mechanical stimuli. This saturation and that corresponding to the resorption response were considered in the RRR named *S* and depicted in Fig 1c, which is analogous to that used in other BRM found in the literature [26, 27].

The effect of the three studied RRR (*DZ*, *BL* and *S*) was tested on both models: IBRM and ABRM. The values adopted for the parameters of the models are given in Table 1.

**Table 1:** Constants taken from: a) Doblaré and García [24], b) Beaupré et al. [11].

Parameter	Value
$\psi_{t}^{*}$	50 *MPa*/*day*^a^
*c*_*r*_	0.02 *μm*/*day*^a^
*c*_*f*_	0.02 *μm*/*day*^a^
w	12.5 *MPa*/*day*^a^ (in *DZ*, *S*)
	0 (in *BL*)
*ω*	0.1^a^
*n*	6000 *cycles*/*day*^a^
*k*	1^b^
*m*	4^b^

### FE model of a human femur

A male 28 years old person with no bone and gait pathologies was selected for this study. The subject signed an informed consent for its participation in the study and the study protocol was approved by the medical ethics committee of the Universitary Hospitals *Virgen del Rocío* and *Virgen Macarena* (approval number 20151012181252).

The proximal end of the right femur of the subject was CT scanned and meshed (see Fig 2) with 339168 type C3D4 elements (four-noded tetrahedra) from the library of Abaqus FEA. This mesh was selected after a convergence analysis performed on the SED (basis of the mechanical stimulus). The rigid body motion was prevented by constraining six degrees of freedom of nodes placed in the mid-length of the diaphysis. The loads simulate the reactions at the hip joint and the muscle forces applied in the femur during walking. Only those muscles that are predominant during the gait cycle have been considered, i.e. hip abductors, tensor fasciae latae (TFL), vastus lateralis and vastus medialis [28]. The applied loads (see Table 2) were taken from [29] and correspond to the instant at 25% of the gait cycle. This is the instant of maximum loading of the femur during walking [28] and provides the amplitude of SED (in IBRM) or strains (in ABRM) to evaluate the mechanical stimulus. Therefore, this is the most representative instant of the gait cycle from the perspective of both BRM.

**Table 2:** Components of the hip reaction and muscle forces corresponding to the instant at 25% of the gait cycle. The axes are: x, postero-anterior; y, latero-medial; z, vertical upward (see Fig 2). Data taken from Heller et al. [29].

Loads (N)			
Component	x	y	z
Hip reaction	-451.4	225.7	-1806
Hip abductor	468	0	694
TFL	-117	158.8	-75.2
Vastus medialis	-8.4	-33.4	-167
Vastus lateralis	-8.4	–108	-543

Bone was assumed initially isotropic ($\hat{H}=1$) and with a homogeneous distribution of density, *ρ*_0_, in all cases. The application of loads produced changes of density and, in the case of ABRM, also of anisotropy. The density was forced to remain in the range between *ρ*_*min*_ = 0.05 *g*/*cm*^3^, value assumed to model a void, and *ρ*_*max*_ = 2.1 *g*/*cm*^3^, corresponding to the maximum density of cortical bone, fully mineralized and with the minimum porosity. The minimum value was defined to avoid the numerical problems derived from elements with null stiffness. Loads were applied in steps of 1 day of activity. The density and elastic properties were updated at the end of that day and these steps were repeated until a remodelling equilibrium was achieved, with no further changes of density. That remodelling equilibrium was assumed when the following criterion was met:

$$\begin{array}{c} h=\frac{\int_{femur}|\rho_{i}-\rho_{i-1}|\;dV}{\int_{femur}\rho_{i}\;dV}\cdot 100<h^{*} \end{array}$$ (13)

where *ρ*_*i*_ is the apparent density obtained in a given point in the step *i* of the simulation. Two tolerances were compared: h* = 0.05% and h* = 0.02%, for the reasons stated later on. In any of those cases, the directionality of the stiffness tensor converged long before the density.

## Results

**Fig 3:**
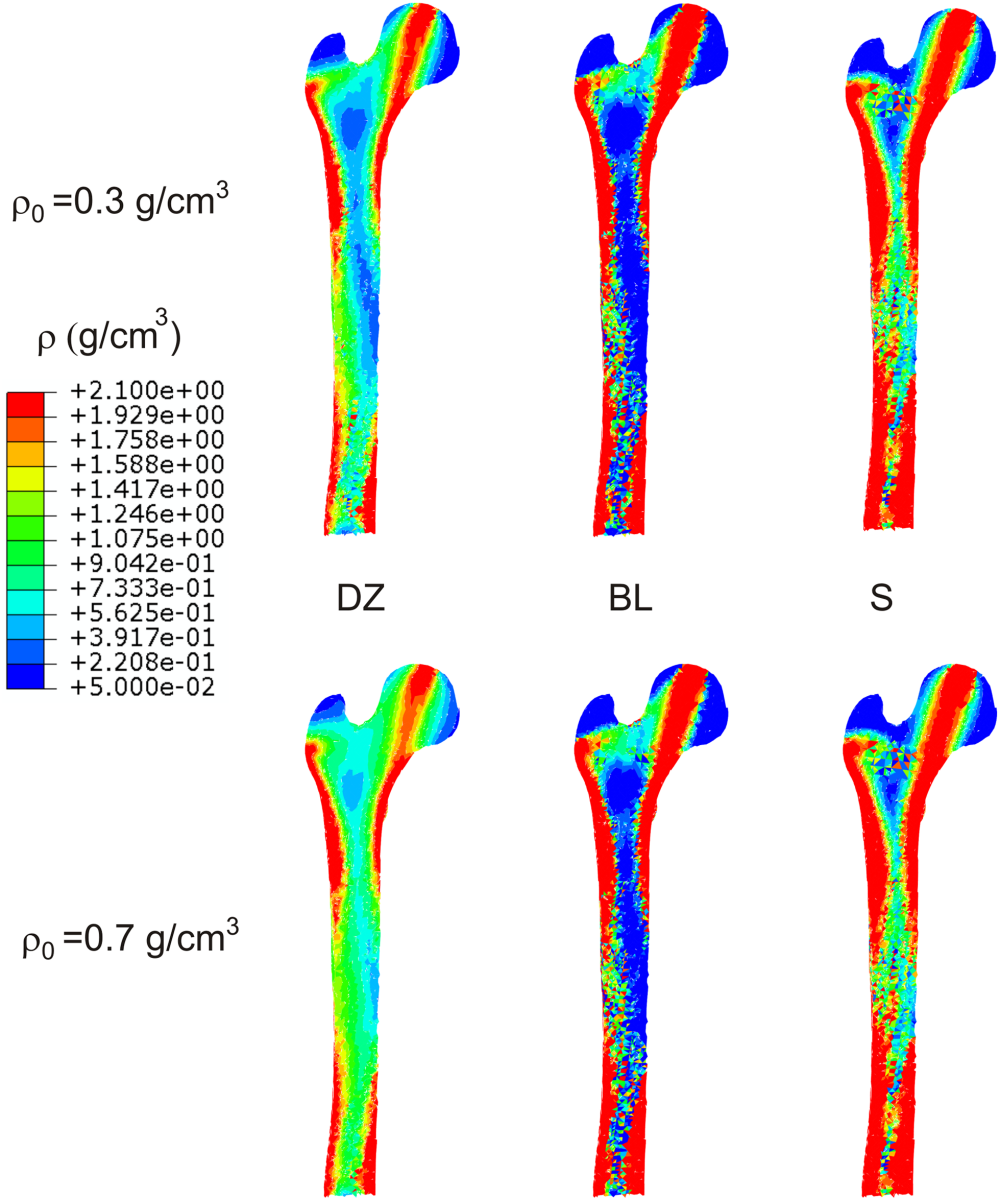
Distribution of density obtained in a frontal section of the femur in the cases that implemented IBRM.

**Fig 4:**
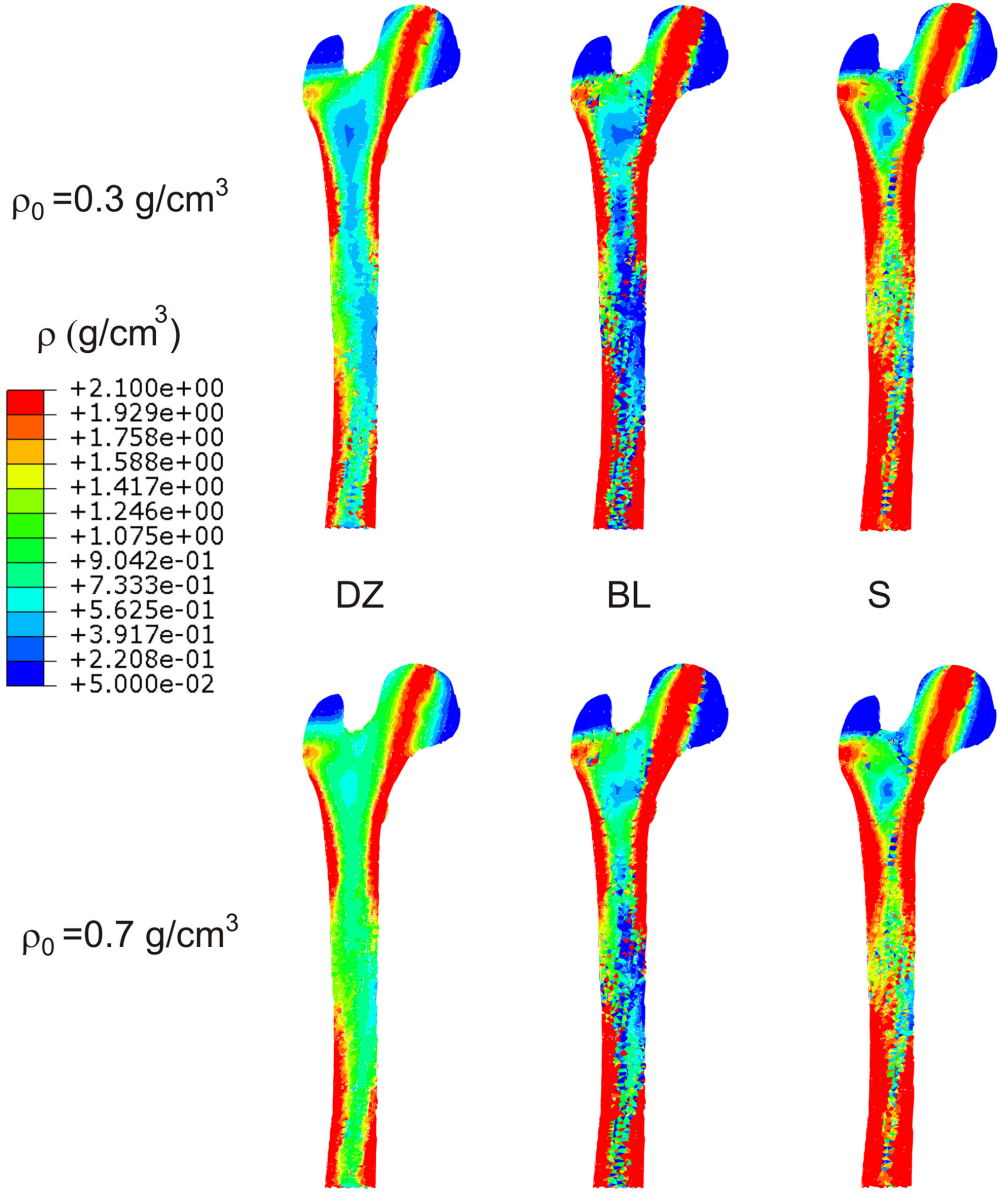
Distribution of density obtained in a frontal section of the femur in the cases that implemented ABRM.

To show the problem of non-uniqueness, two different values of the initial density were tested: *ρ*_0_ = 0.3 *g*/*cm*^3^ and *ρ*_0_ = 0.7 *g*/*cm*^3^. Fig 3 compares the distribution of density in a frontal section of the femur, obtained at the remodelling equilibrium with each *ρ*_0_ and using the IBRM with the three RRR. Fig 4 compares the distributions obtained with the ABRM.

The simulations with *BL* and *S* clearly exhibited the well-known “checkerboard” phenomenon, typical of element-based finite element simulations of bone remodelling. This phenomenon is unstable if the exponent *β* in Eq (4a) is greater than 1 [30]. It appears more clearly in long-term simulations and can be avoided with the use of a node-based approach [31]. This approach has not been used here, precisely to illustrate the appearance of this problem. The longer the simulation needs to be to reach the convergence for a certain tolerance, h*, the more likely is the appearance of checkerboard. Thus, it is hardly noticeable in *DZ* cases where the convergence was much faster and it is very clear in *BL* and *S* cases (see in Table 3 the number of steps needed for convergence). The IBRM simulations were longer than the ABRM ones for a given h* and, consequently, the checkerboard was more evident. To reduce as much as possible the presence of checkerboard, the results obtained for the laxer criterion of convergence (h* = 0.05%) were chosen for Figs 3 and 4.

**Table 3:** Differences in density and days of activity needed for convergence in each case. The results are given for h* = 0.05% (and for h* = 0.02% in parentheses).

Bone remodelling model	RRR	*diff*(%)	Days for convergence with *ρ*_0_ = 0.3 *g*/*cm*^3^	Days for convergence with *ρ*_0_ = 0.7 *g*/*cm*^3^
IBRM	DZ	12.48 (10.55)	175 (355)	173 (373)
	BL	5.36 (4.16)	370 (625)	410 (710)
	S	0.18 (1.04)	1038 (1500)	739 (1250)
ABRM	DZ	14.19 (12.80)	150 (280)	140 (299)
	BL	10.02 (8.53)	230 (509)	240 (530)
	S	1.26 (0.98)	990 (1209)	714 (880)

**Fig 5:**
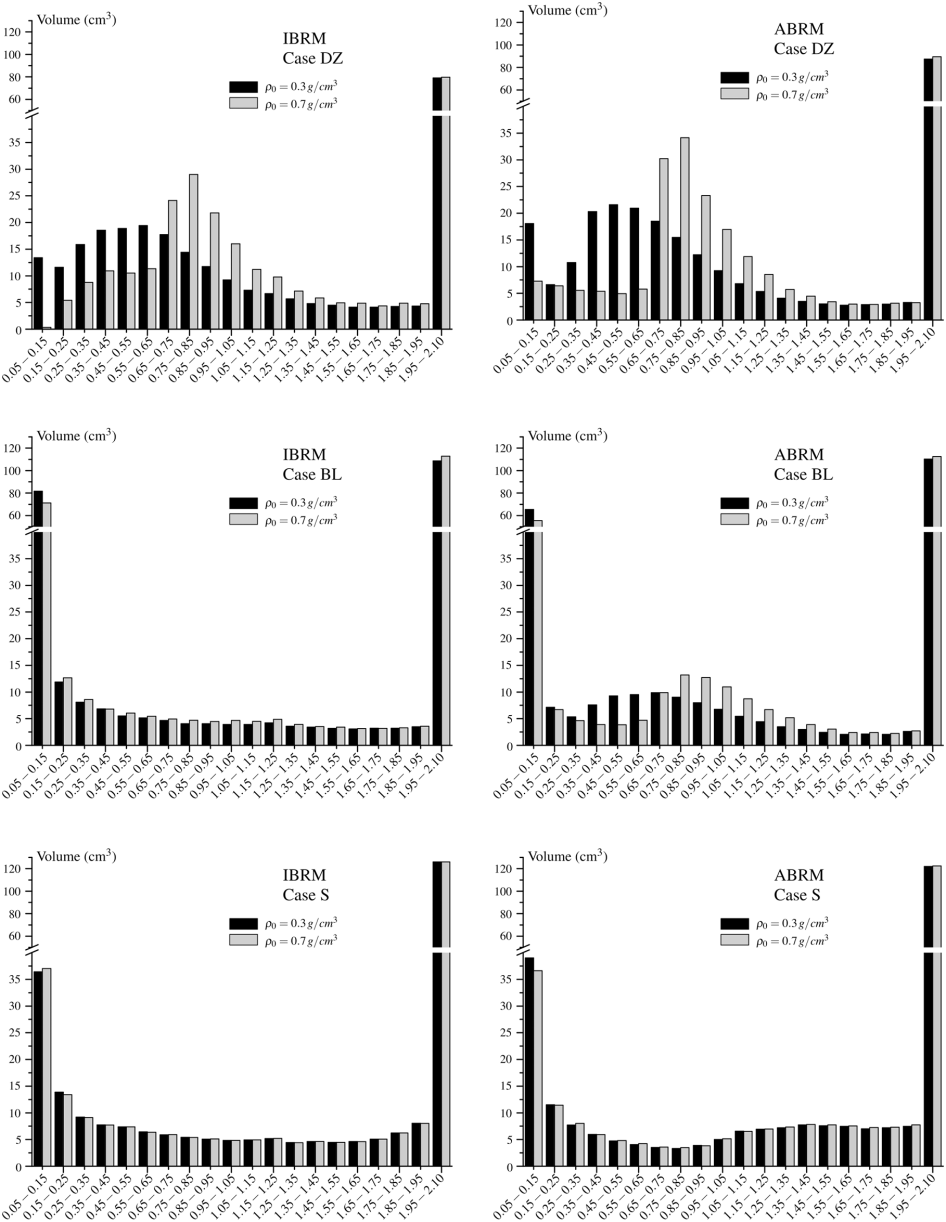
Histograms with the occurrence of density in the elements of the femur. The groups are named after their range of apparent density (*g*/*cm*^3^).

The volume of the elements having a density within given ranges was summed and plotted in the histograms of Fig 5. These histograms show clearly the different distributions of density obtained starting from *ρ*_0_ = 0.3 *g*/*cm*^3^ and *ρ*_0_ = 0.7 *g*/*cm*^3^ in *DZ* and, to a lesser extent, in *BL*. This difference can be summarized in the following parameter:

$$\begin{array}{c} diff=\frac{\int_{femur}|\rho_{0.7}-\rho_{0.3}|\;dV}{\int_{femur}\;\rho_{0.3}\;dV}\cdot 100 \end{array}$$ (14)

where *ρ*_0.3_ and *ρ*_0.7_ are the final distributions of density, obtained by starting from *ρ*_0_ = 0.3 *g*/*cm*^3^ and *ρ*_0_ = 0.7 *g*/*cm*^3^, respectively. This parameter is given in Table 3 along with the days needed for convergence in each case.

## Discussion

The final distributions of density shown in Figs 3 and 4 illustrate the problem of non-uniqueness of the estimated density in *DZ* cases. They are different starting from *ρ*_0_ = 0.3 *g*/*cm*^3^ and *ρ*_0_ = 0.7 *g*/*cm*^3^, with the first case leading to a lighter bone. That difference is reduced using the relation *BL* and almost eliminated using *S*. Furthermore, this evidence is independent of the BRM and, consequently, it is not an effect of the anisotropy, but of the RRR.

It must be said that the ABRM has been recently enhanced by Mengoni and Ponthot [15] to correct some inconsistencies observed in the original model, particularly in the resorption criterion and response. This enhanced model has been tested as well, providing similar results to ABRM. This fact reinforces the idea that non-uniqueness of the solution is caused by the dead zone, regardless of the BRM.

The uniqueness of the solution can be more precisely analysed in the histograms of Fig 5. These are very different with *DZ*. It can be seen that the ranges close to the corresponding *ρ*_0_ are predominant (particularly in the simulations with *ρ*_0_ = 0.7 *g*/*cm*^3^), because the dead zone “traps” many of the elements that are initially within it, not letting them to change their density. The differences between *ρ*_0_ = 0.3 *g*/*cm*^3^ and *ρ*_0_ = 0.7 *g*/*cm*^3^ fall using *BL* (more with IBRM) and are almost negligible using *S*. These conclusions are confirmed by the parameter *diff*, shown in Table 3.

The use of *BL* might suggest the existence of a unique value of density in equilibrium with the external loads. Indeed, *BL* removed the dead zone trying to eliminate that “trapping” effect, so that a point would not stop changing its density until it reached its reference mechanical stimulus. However, that RRR was not entirely satisfactory. Cowin and Nachlinger [21] proved the uniqueness of the solution for the adaptive elasticity problem under certain conditions. One of them, that the remodelling response must be a differentiable function, is not met with *DZ*. It would be met with *BL*, provided that the resorption and formation slopes, *c*_*r*_ and *c*_*f*_, were equal, as assumed here, but the solution remained non-unique. In that case, non-uniqueness may be due to the aforementioned instability of the numerical solution. This problem was first investigated by Weinans et al. [30], who noticed that instability in FE simulations of bone remodelling for *β* > 1 (see Eq (4a)). Such instability leads to the well-known problem of checkerboard in which the density and stiffness rise in some elements to unload the neighboring ones and finally get a patched distribution of density [30].

This undesired unloading effect is occurring at a global scale with *BL*, in which the formation rate is unlimited. Those points initially bearing a very high mechanical stimulus increase their density too fast; faster than with *S* for the same value of stimulus. This way, the stiffness in these points (let us name it zone A) rise faster than in other areas (zone B), which are then unloaded by the stiffer elements of zone A. The mechanical stimulus in zone B may then drop, leading to a local standstill of density increase or even resorption. This yields a distribution of density which is excessively polarized between areas of maximum (zone A) and minimum density (zone B). This polarization enhances the instability problems and so the final distribution of density exhibits a marked checkerboard and is still non-unique.

The relation *S* limited the resorption and formation rates to solve this problem. The changes of density were more gradual and this prevented high stressed areas from increasing their density too quickly, what might unload less stressed areas and lead to the referred polarization of density. But, more importantly, this RRR solved the problem of non-uniqueness. The histograms for *ρ*_0_ = 0.3 *g*/*cm*^3^ and *ρ*_0_ = 0.7 *g*/*cm*^3^ were almost identical and the parameter *diff* was very stable around 1% in any case.

The problem of non-uniqueness was more evident in the cases of ABRM. It must be noted that the remodelling equilibrium is achieved when the distribution of stiffness is such that the mechanical stimulus equals the reference value (or is within the dead zone) in the whole bone. In IBRM, stiffness is exclusively dependent on the density, but in ABRM it depends on the density and the anisotropy. Therefore, that distribution of stiffness can be obtained with different densities in ABRM, thus, worsening the problem of non-uniqueness.

In the simulation *S*-IBRM the parameter *diff* reached a minimum around 1000 days of simulation, but rose subsequently due to the appearance of checkerboard. This trend in long-term simulations has been recently analyzed by Garijo et al. [32] and is seen in all the simulations if they are continued beyond the days represented in this paper. Eventually, *diff* oscillates around the values given in Table 3. A node-based approach, or another method intended to eliminate the discontinuous element-based solution, should be used to avoid this problem [31]. In any case, the conclusion of this study was the same regardless of the presence of checkerboard: the parameter *diff* was progressively reduced from *DZ* to *BL* and *S* for all the values of h* checked (not all shown).

The main limitation of this study is that the obtained apparent density does not resemble completely the actual distribution of bone density, since the applied load is very simplistic and does not represent the whole set of loads the femur is subjected to during the daily activity. Other authors have applied the walking loads applied here and other loads to obtain a distribution of density closer to the actual one, with good results (see e.g. [33], among many others). Nonetheless, obtaining the actual density distribution was not the objective of the present study. For this reason, we have selected this only load, since walking is the most representative activity and the conclusions of the study are the same with a more complicated and realistic set of loads.

## Conclusions

This paper studies a phenomenological and very typical application of BRM: the obtention of a map of bone apparent density from the loads the bone is daily subjected to. These simulations usually start from a homogeneous distribution of density, *ρ*_0_, but the solution may depend on that initial value. The uniqueness of this solution has been addressed in this paper.

The RRR defined in most BRM includes a dead zone, which allegedly characterizes a normal situation within which bone responds with no net variation of mass. The existence of a lazy or dead zone has not been empirically shown to date. On the contrary, recent works have argued just the opposite: that bone remodelling response correlates with tissue loading following a linear relationship without a ‘lazy zone’ [18]. The lazy or dead zone seems more like a mathematical approximation of the remodelling response of certain bones, which exhibit a range of stimulus for which the remodelling response is certainly low, though not exactly zero.

In the phenomenological estimation of bone density this dead zone is one of the causes of non-uniqueness of the final density distribution. Removing the dead zone mitigated but did not solve the problem in FE simulations. Additionally, it was necessary to saturate or limit the bone response under disuse and overload. That way, the elements of the mesh reached their bone remodelling equilibrium state gradually and the uniqueness of density distribution was practically achieved.

It must be clear that the intention of this paper is not to propose a RRR for mechanobiological use. It is only intended for its use in this phenomenological estimation of bone density that starts from an unreal density distribution and implements BRMs based on the mechanostat theory. The true remodelling response has a mechanobiological basis and governs the behaviour of bone in every situation. This mechanobiological response of bone is more complex than that depicted by the BRMs used here and involves other variables like load frequency, hormonal response, cellular interactions, pathologies, etc., covered by more complicated models [9, 34]. However, mechanobiological models should be applied only once the distribution of density (and anisotropy) is correctly established, for example, to predict variations of bone density due to changes in the normal activity or due to the onset of diseases, for example.

## Supporting Information

S1 File List with the elements of the mesh, their volume and the density reached at the end of the simulation: IBRM, *DZ* and *ρ*_0_ = 0.3 *g*/*cm*^3^. (PDF)

S2 File idem for simulation: IBRM, *DZ* and *ρ*_0_ = 0.7 *g*/*cm*^3^. (PDF)

S3 File idem for simulation: IBRM, *BL* and *ρ*_0_ = 0.3 *g*/*cm*^3^. (PDF)

S4 File idem for simulation: IBRM, *BL* and *ρ*_0_ = 0.7 *g*/*cm*^3^. (PDF)

S5 File idem for simulation: IBRM, *S* and *ρ*_0_ = 0.3 *g*/*cm*^3^. (PDF)

S6 File idem for simulation: IBRM, *S* and *ρ*_0_ = 0.7 *g*/*cm*^3^. (PDF)

S7 File idem for simulation: ABRM, *DZ* and *ρ*_0_ = 0.3 *g*/*cm*^3^. (PDF)

S8 File idem for simulation: ABRM, *DZ* and *ρ*_0_ = 0.7 *g*/*cm*^3^. (PDF)

S9 File idem for simulation: ABRM, *BL* and *ρ*_0_ = 0.3 *g*/*cm*^3^. (PDF)

S10 File idem for simulation: ABRM, *BL* and *ρ*_0_ = 0.7 *g*/*cm*^3^. (PDF)

S11 File idem for simulation: ABRM, *S* and *ρ*_0_ = 0.3 *g*/*cm*^3^. (PDF)

S12 File idem for simulation: ABRM, *S* and *ρ*_0_ = 0.7 *g*/*cm*^3^. (PDF)
