# Supplementary material for: On the Use of Bone Remodelling Models to Estimate the Density Distribution of Bones. Uniqueness of the Solution
Source: PLoS One. 2016 Feb 9;11(2):e0148603. doi: 10.1371/journal.pone.0148603 (PMC4747586; doi:10.1371/journal.pone.0148603)
Supplement: S1 File — (PDF) [file pone.0148603.s001.pdf]

N° Elem	Volume (mm3)	Density (g/cm3)
1	1.32415	2.1
2	1.75823	2.1
3	0.984099	2.1
4	1.46442	2.1
5	1.53082	2.1
6	1.32068	2.1
7	1.91404	0.163355
8	1.17036	0.153875
9	0.929209	0.125823
10	1.27254	0.112647
11	2.0926	0.119619
12	2.20407	0.107397
13	1.21488	0.132889
14	1.26024	0.107975
15	0.794454	0.123955
16	1.95087	0.0824035
17	0.953925	2.1
18	0.98949	2.1
19	1.27875	2.1
20	1.04598	2.1
21	0.594005	2.1
22	0.578861	2.1
23	1.09506	2.1
24	1.45327	2.02278
25	2.58396	1.59491
26	1.58581	2.1
27	1.5858	2.1
28	0.636946	1.86816
29	0.94202	2.1
30	0.946119	1.48644
31	0.636953	2.1
32	0.946107	2.07515
33	1.09505	2.1
34	1.27389	1.43146
35	1.00232	2.1
36	1.00231	2.1
37	1.83583	2.1
38	1.00231	2.1
39	1.28828	0.116261
40	1.81696	0.106797
41	1.28828	0.103513
42	1.28828	0.0979267
43	1.0044	0.134571
44	0.85148	0.140309
45	0.851539	0.147728
46	1.57767	0.110323
47	1.52227	0.106666
48	0.730657	0.108116
49	0.94613	0.137206
50	1.28228	2.1
51	1.64268	1.80849
52	1.10101	2.1
53	1.3939	2.1
54	0.959471	2.1
55	1.10101	2.1

56	1.2873	2.1
57	1.59659	2.1
58	1.80841	0.348345
59	1.80841	0.569113
60	0.946129	0.268206
61	1.36991	0.264164
62	1.32262	0.643531
63	1.32261	0.376822
64	1.38464	0.485363
65	0.962877	0.352737
66	0.962878	0.352031
67	1.82129	0.362899
68	1.82129	0.400393
69	0.94613	0.396203
70	1.39751	0.393463
71	0.946131	0.422709
72	1.34493	0.312417
73	1.39751	0.344854
74	0.837577	0.806589
75	0.823478	0.551808
76	1.10889	1.57057
77	1.01686	0.828169
78	1.1942	1.58121
79	0.836129	0.766588
80	0.937165	0.55114
81	0.969438	0.940305
82	0.895386	0.683849
83	1.30554	1.67886
84	1.15798	0.733185
85	1.22578	1.90177
86	1.15255	0.895057
87	0.9765	0.550515
88	2.02178	1.70549
89	1.63755	1.59204
90	0.727713	0.962174
91	0.810945	0.952701
92	1.00559	1.63722
93	0.879098	1.01238
94	1.07852	1.73481
95	0.657764	0.827508
96	0.829406	0.739375
97	0.720201	0.221414
98	0.946131	0.282078
99	1.11827	0.225731
100	1.50754	0.188773
101	1.64835	0.157565
102	1.71903	0.284853
103	0.851554	0.234143
104	1.75626	0.157849
105	2.1646	0.226665
106	1.5563	0.24379
107	1.5563	0.185976
108	2.10876	0.292431
109	1.05438	0.384068
110	0.946133	0.340721
111	1.05438	0.563708
112	1.20256	0.241376
113	1.47879	0.410377

114	1.47879	0.762455
115	0.77815	0.508883
116	0.947114	2.1
117	1.31257	2.1
118	1.09235	2.1
119	0.946132	2.1
120	1.54246	2.1
121	1.54246	2.1
122	1.09235	2.1
123	0.946131	2.1
124	1.29507	1.47059
125	1.0353	2.1
126	1.03529	2.1
127	1.28667	2.1
128	1.44777	2.1
129	1.61121	2.1
130	1.22176	0.440895
131	0.946129	0.406747
132	1.08848	0.313952
133	0.946131	0.426373
134	1.61201	0.347218
135	1.61201	0.293984
136	1.19993	0.366961
137	1.35822	0.377901
138	1.1539	0.142321
139	1.00765	0.115561
140	1.28228	2.1
141	2.65339	2.1
142	1.28228	2.1
143	1.55998	2.1
144	1.00509	2.1
145	0.93845	2.1
146	1.27674	2.1
147	0.810958	2.1
148	1.91381	2.1
149	1.1861	2.1
150	1.24886	2.1
151	2.02842	2.1
152	0.991436	0.0934488
153	1.34588	0.093391
154	2.02729	0.0890397
155	0.72904	0.111481
156	1.06139	0.110573
157	0.835368	0.11352
158	1.63819	0.0871515
159	1.10745	0.0941227
160	1.79925	0.0909748
161	1.00085	0.108614
162	0.946133	1.80215
163	1.41123	1.69443
164	1.8327	1.84667
165	1.8327	2.1
166	0.946132	0.883468
167	1.41123	1.98858
168	1.3676	2.04864
169	1.11048	2.1
170	1.11048	2.1
171	1.65815	2.1

172	1.9004	2.1
173	1.88691	2.1
174	1.82232	2.1
175	1.46591	2.1
176	1.09115	2.1
177	1.02051	0.876627
178	1.03373	0.947684
179	1.10307	2.1
180	1.05556	2.1
181	1.82533	0.90389
182	1.19496	0.749661
183	1.28597	2.1
184	1.20421	2.1
185	2.23644	2.1
186	1.08378	2.1
187	0.983724	2.1
188	0.602075	2.01121
189	1.45876	2.1
190	1.45875	2.1
191	0.602103	1.65112
192	1.11822	2.1
193	0.946133	2.1
194	0.602103	2.1
195	1.20421	2.08712
196	1.26371	0.702888
197	1.55179	0.483797
198	1.14042	0.284896
199	1.56876	0.328794
200	2.27496	0.557382
201	1.13748	0.4461
202	1.02884	0.671847
203	1.25124	0.541122
204	0.82703	0.489148
205	0.607775	0.486343
206	1.49242	0.523766
207	1.35674	0.583682
208	1.6697	2.1
209	1.81655	2.1
210	0.900108	1.01935
211	0.851537	0.762472
212	0.703076	0.959452
213	0.639143	0.817139
214	1.42358	1.04332
215	1.4976	1.17535
216	0.86284	0.835732
217	1.2965	0.946835
218	1.27835	1.78245
219	1.00012	0.877405
220	1.74332	1.17431
221	1.08576	2.1
222	1.19 2.1	
223	1.08576	2.1
224	1.20854	2.1
225	0.946131	2.1
226	0.946131	2.1
227	1.75985	2.1
228	1.75985	2.1
229	1.41232	2.1

230	1.29365	2.1
231	0.973559	2.1
232	1.23698	0.0868175
233	1.45055	0.0926799
234	1.26947	0.0996955
235	0.856218	0.109303
236	1.75303	0.0792731
237	1.14938	0.0928
238	0.984711	0.0831087
239	2.05771	0.0900684
240	0.625171	0.0847844
241	0.965675	0.0817471
242	1.17631	0.0850475
243	1.4677	0.105758
244	1.35403	0.14237
245	0.975149	0.152636
246	0.946131	0.159715
247	1.80893	0.114586
248	1.80893	0.109554
249	0.975149	0.115935
250	0.946132	0.151282
251	1.9503	0.146489
252	2.80207	0.127151
253	1.76411	0.144026
254	2.77469	0.12393
255	1.9503	0.119846
256	1.26784	0.141111
257	2.31093	0.184418
258	0.851541	0.225668
259	1.39521	0.153088
260	1.45613	0.198042
261	0.851541	0.236978
262	0.996013	0.157522
263	1.07989	0.221421
264	0.946131	0.229635
265	1.08213	0.176747
266	1.74289	0.161931
267	1.75838	0.167317
268	2.11163	0.213243
269	1.23773	2.1
270	1.25398	2.1
271	1.0304	2.1
272	1.32368	2.1
273	1.11712	0.903792
274	1.07999	0.80939
275	1.1766	2.1
276	1.35735	2.1
277	1.089	0.127224
278	1.81193	0.147083
279	1.11081	0.0745282
280	1.78069	0.0773452
281	0.717512	0.0801285
282	0.810946	0.0743717
283	0.703901	0.0756601
284	1.09028	0.0719958
285	0.958031	0.0747072
286	0.892585	0.0746092
287	0.873431	0.0764738

288	0.997755	0.0726768
289	1.85479	0.0733468
290	0.983029	0.308719
291	1.27922	0.280729
292	1.64346	0.591104
293	1.27922	0.424119
294	1.09795	1.60443
295	1.07508	1.02564
296	1.02975	1.19151
297	1.01121	1.3352
298	1.72369	0.08239
299	1.12035	0.0769414
300	2.13511	0.0839763
301	0.9705	0.0844079
302	0.946131	0.0954825
303	0.86182	0.0843878
304	1.51379	0.0799466
305	1.51379	0.0769843
306	0.946132	0.085192
307	0.861849	0.0844897
308	1.34591	0.535016
309	1.14721	0.318302
310	1.15539	2.1
311	1.90938	1.90404
312	2.19436	0.990856
313	1.41563	2.09454
314	0.785641	2.1
315	0.736384	1.83656
316	1.46489	2.1
317	1.46488	2.1
318	0.736372	2.1
319	0.946132	2.1
320	1.90941	1.00018
321	1.18907	1.95829
322	1.20409	2.07832
323	0.973559	2.1
324	1.29461	2.1
325	0.946129	2.1
326	0.94613	2.1
327	1.78751	2.1
328	1.78751	2.1
329	1.38885	2.1
330	1.34479	2.1
331	1.07283	0.969698
332	1.6869	2.1
333	1.52544	2.1
334	1.07797	2.1
335	1.13819	0.795573
336	0.776179	2.1
337	1.61092	0.914779
338	1.36155	2.1
339	0.77618	2.1
340	1.2933	0.856385
341	1.15946	2.09387
342	0.69857	1.85224
343	1.87607	2.1
344	1.07612	2.1
345	1.31976	1.08152

346	1.01243	1.8536
347	1.41279	1.13581
348	1.35146	0.294364
349	1.90664	0.260969
350	1.35145	0.277368
351	1.22811	0.30123
352	1.93147	0.26143
353	1.42486	2.1
354	1.56558	2.1
355	1.0476	0.0733666
356	2.31605	0.0676832
357	1.89367	0.072359
358	0.804706	0.0691269
359	0.783919	0.0634406
360	1.3116	0.0594514
361	1.03891	0.059337
362	0.960473	0.063984
363	1.08462	0.0641988
364	0.785994	0.0640931
365	1.1318	0.05931
366	1.29576	0.0581944
367	1.3946	0.0789323
368	1.11924	0.0755116
369	0.834322	2.1
370	1.0256	2.1
371	1.74321	0.862229
372	1.41798	0.827917
373	0.871608	0.834508
374	1.47253	0.832546
375	1.47252	0.8363
376	0.817064	0.79496
377	0.817064	0.819268
378	0.946129	0.783356
379	1.63413	0.783542
380	1.9194	0.17045
381	1.93562	0.153541
382	2.06307	0.175171
383	1.46021	0.138236
384	0.955002	1.28527
385	0.833409	1.51234
386	0.838039	1.20315
387	1.48702	1.32671
388	1.1697	1.23938
389	1.23549	1.37524
390	1.18373	1.25038
391	2.02254	1.2827
392	1.68867	1.38793
393	0.875481	0.944499
394	0.821635	1.37441
395	0.858878	0.815656
396	1.30245	1.03979
397	1.07476	0.976158
398	1.11907	1.05247
399	1.10453	0.936927
400	1.70233	1.06174
401	1.70233	0.931919
402	2.6558	1.26664
403	1.7818	2.1

404	1.87059	2.1
405	1.34412	0.120487
406	1.98068	0.144702
407	2.56369	0.13643
408	1.49512	0.105909
409	1.35977	0.269662
410	1.34254	0.356772
411	1.75618	0.303385
412	1.75618	0.246426
413	0.946129	0.488851
414	0.946129	0.347799
415	1.17665	0.332176
416	1.01976	0.306601
417	1.27376	0.208035
418	1.35978	0.26033
419	1.68741	0.224964
420	1.68741	0.191344
421	0.946132	0.348187
422	0.946133	0.265678
423	1.14241	0.253319
424	0.996831	0.225877
425	0.996828	0.25895
426	1.12774	0.336505
427	1.13664	2.1
428	1.09627	2.1
429	2.8814	2.1
430	3.49751	2.1
431	1.79109	0.441844
432	1.54197	0.342826
433	2.75153	0.397092
434	1.93019	0.357986
435	1.51569	0.883174
436	1.55145	1.09182
437	1.78595	2.1
438	3.74452	2.1
439	1.66537	2.1
440	3.55161	2.1
441	1.35002	0.718392
442	1.19901	0.676071
443	1.61028	1.84067
444	1.35002	0.792789
445	1.65712	0.736076
446	1.10585	0.845271
447	0.961347	0.94075
448	0.810944	1.08101
449	1.16148	1.64243
450	1.08066	1.3598
451	0.934577	0.733688
452	1.05164	1.27792
453	0.79216	0.77654
454	2.99194	2.1
455	1.2832	2.1
456	2.97842	2.1
457	1.39517	2.1
458	1.03391	2.1
459	1.74416	2.1
460	1.05071	2.1
461	0.923782	2.05426

462	1.55369	2.1
463	1.86444	2.1
464	1.01617	1.89269
465	1.27174	2.1
466	1.20573	2.1
467	1.01616	2.1
468	1.13621	1.88352
469	1.75366	1.9492
470	2.27009	1.27954
471	1.6813	1.50508
472	1.47904	0.124991
473	2.62714	0.144319
474	1.80085	0.147107
475	1.80085	0.117501
476	2.74104	0.163621
477	1.37052	0.162961
478	0.946131	0.191228
479	1.37052	0.160171
480	1.31357	0.153563
481	1.7837	0.148188
482	1.7837	0.200915
483	0.900397	0.151887
484	1.74487	0.181446
485	2.88672	2.1
486	2.88249	2.1
487	1.0961	0.351393
488	1.20143	0.340476
489	1.66739	0.326331
490	1.85795	0.147096
491	1.45789	0.101287
492	2.11407	0.125054
493	0.713524	0.121524
494	1.58391	0.120639
495	1.58391	0.0953658
496	1.04765	0.111955
497	1.24979	0.124711
498	0.713551	0.096538
499	0.946127	0.133289
500	1.36768	0.114998
501	1.14433	0.0775904
502	1.66923	0.0795425
503	0.699479	0.0883828
504	0.70351	0.0822431
505	0.758416	0.0799602
506	1.07678	0.0767778
507	0.909874	0.0785289
508	0.940218	0.0813977
509	0.876604	0.0866073
510	1.27148	0.083626
511	2.42348	0.0829484
512	1.19837	0.0764555
513	0.764764	0.0863922
514	0.829259	0.0832333
515	0.740584	0.0790864
516	1.2772	0.0747385
517	0.964465	0.0772536
518	1.06231	0.077295
519	1.06924	0.0818212

520	1.17566	0.166352
521	1.3063	0.168442
522	2.51253	0.217854
523	2.42267	0.192827
524	1.89226	0.198538
525	1.46492	0.207805
526	0.946132	0.217607
527	0.653151	0.206089
528	0.653124	0.24277
529	1.53995	0.146079
530	1.53995	0.140189
531	0.936806	0.191968
532	1.25626	0.174855
533	1.1893	0.270257
534	1.62206	0.253879
535	1.04921	2.1
536	1.22927	2.1
537	0.801765	2.1
538	0.774917	2.1
539	0.844623	2.1
540	0.737481	2.1
541	3.24379	2.1
542	2.48573	2.1
543	1.40967	1.00531
544	1.47203	1.35388
545	2.07659	0.946303
546	1.40967	1.32431
547	2.06638	0.95295
548	1.17418	1.52852
549	1.04921	2.1
550	1.74593	1.83864
551	1.74593	1.14293
552	0.946132	0.81505
553	1.38651	1.68537
554	1.20761	0.498869
555	1.20761	0.713013
556	1.30555	0.690546
557	1.46812	0.807687
558	2.41602	1.07
559	1.46812	1.15319
560	1.46812	0.593383
561	0.734059	0.648085
562	0.946132	1.47245
563	1.21261	1.06191
564	1.34225	1.11196
565	0.734084	0.977769
566	1.82077	0.998228
567	1.82077	0.985289
568	1.13751	0.512662
569	0.946131	0.899406
570	1.13751	0.711495
571	1.13748	0.684654
572	0.784392	0.68543
573	1.4906	0.617401
574	1.4906	0.446025
575	1.17879	1.10117
576	1.28291	1.05062
577	0.626495	2.1

578	1.45334	2.1
579	1.45334	2.1
580	0.626494	2.1
581	1.3852	2.1
582	0.946116	2.1
583	0.626499	2.1
584	1.47203	0.877407
585	1.42639	2.1
586	1.42639	2.1
587	0.795469	2.1
588	0.795469	2.1
589	0.946132	2.1
590	0.850877	2.1
591	1.37098	2.1
592	1.87194	2.1
593	1.76988	2.1
594	3.16295	0.0824528
595	3.26725	0.0730697
596	1.13322	2.1
597	1.20761	2.1
598	0.686933	1.05527
599	0.493248	0.801232
600	1.2053	1.99527
601	0.947149	0.925343
602	1.27591	1.02241
603	1.75732	1.97299
604	0.493261	1.10486
605	1.34337	2.08888
606	2.73207	0.776953
607	1.19857	0.995009
608	0.671688	0.666123
609	0.946134	1.9963
610	1.36603	0.751986
611	2.07817	0.979094
612	1.82573	1.83875
613	0.671699	1.11089
614	1.47772	0.756333
615	0.975349	1.90856
616	1.22615	2.1
617	1.47868	2.1
618	2.29351	1.22025
619	1.47642	2.1
620	2.94443	1.09265
621	1.80454	2.1
622	0.949135	1.57401
623	0.949134	1.61779
624	1.10476	0.151363
625	0.982857	0.220827
626	1.10938	0.250289
627	1.19052	0.417242
628	1.01012	0.06273
629	1.35562	0.0627124
630	0.691742	0.0609328
631	2.39596	0.0620968
632	2.35604	0.0639263
633	0.974081	0.0581853
634	3.22444	0.0579326
635	1.3294	0.598789

636	1.37265	0.930973
637	1.3294	0.80912
638	1.51113	2.1
639	1.43643	2.1
640	1.72726	2.1
641	1.634	2.1
642	0.755566	2.1
643	1.29319	2.1
644	0.755567	2.1
645	0.946131	2.1
646	1.29319	2.1
647	0.973563	2.1
648	0.904398	2.1
649	0.904406	2.1
650	1.1538	2.1
651	1.10437	2.1
652	0.935418	2.1
653	1.16649	2.1
654	0.975667	2.1
655	1.17952	2.1
656	1.95053	2.1
657	0.948676	2.1
658	1.44287	2.1
659	0.746095	1.8966
660	1.51224	2.1
661	1.27354	2.1
662	0.746066	1.73903
663	1.20573	2.1
664	1.12442	2.1
665	0.671487	2.1
666	1.04355	2.1
667	0.948675	2.1
668	0.967673	0.899332
669	1.12931	0.0896513
670	1.38212	0.0917116
671	2.40113	0.0871282
672	1.08054	0.0922943
673	1.04072	0.113971
674	0.691062	0.0953028
675	1.58052	0.0921961
676	1.33502	0.0883585
677	0.946131	0.101631
678	0.691062	0.104925
679	1.02663	0.0910065
680	1.80569	0.0883348
681	1.26062	0.0810525
682	1.18203	0.0836735
683	0.946131	0.104819
684	0.795065	0.090482
685	1.58753	0.0836935
686	1.58753	0.0797984
687	0.946131	0.0917845
688	0.795093	0.0856973
689	0.805057	2.1
690	0.812415	2.1
691	1.23868	0.314576
692	1.34448	0.33363
693	1.67201	2.1

694	1.61202	2.1
695	1.41103	2.1
696	1.41103	2.1
697	0.841088	2.1
698	1.38302	2.1
699	0.841089	2.1
700	0.869097	2.1
701	1.38302	2.1
702	0.949941	2.1
703	0.94994	2.1
704	1.88608	2.1
705	0.854942	2.1
706	1.68893	2.1
707	1.70002	2.1
708	2.02107	0.188472
709	2.04982	0.178945
710	3.11063	0.237508
711	2.03805	0.190217
712	1.67638	2.1
713	0.967492	2.1
714	2.00209	2.1
715	0.967491	2.1
716	0.870742	2.1
717	1.30266	0.181318
718	0.651334	0.197712
719	1.69413	0.148268
720	1.69413	0.197937
721	0.946132	0.203896
722	1.03079	0.192085
723	1.31468	0.169951
724	0.946132	0.212788
725	0.919055	0.143696
726	1.16815	0.160809
727	2.79004	0.152647
728	1.2136	0.12854
729	2.62935	0.142531
730	0.947692	0.377302
731	1.51835	0.361285
732	0.947693	0.204955
733	0.947692	0.242303
734	0.980841	2.1
735	1.43577	2.1
736	0.980841	2.1
737	1.67858	2.1
738	1.34124	0.546097
739	1.39818	0.427449
740	1.2065	2.1
741	0.946133	2.1
742	1.2079	2.1
743	1.65229	2.1
744	1.65229	2.1
745	0.946132	1.99963
746	1.2079	2.1
747	1.39053	2.1
748	1.20651	2.1
749	1.36919	2.1
750	1.12717	2.1
751	1.09483	2.1

752	0.994294	2.1
753	0.97356	2.1
754	1.06827	2.1
755	1.40571	2.1
756	1.29259	2.1
757	0.946131	2.1
758	1.7787	2.1
759	1.7787	2.1
760	1.29259	2.1
761	0.946132	2.1
762	1.09408	0.71659
763	1.03824	0.466706
764	0.851541	0.3161
765	1.23608	0.318846
766	0.851539	0.314527
767	1.5021	0.394754
768	1.54839	0.372459
769	1.12373	0.287568
770	1.22617	0.263097
771	1.42762	0.428192
772	2.24746	0.443008
773	1.05475	0.285736
774	1.46845	0.329069
775	1.05475	0.195805
776	0.929726	0.231688
777	1.52072	0.263834
778	1.82488	0.239951
779	1.02271	0.213546
780	1.19225	0.250757
781	1.43071	0.219301
782	0.94613	0.244702
783	1.05475	0.213212
784	1.39223	2.1
785	1.71255	2.1
786	0.946132	0.731314
787	1.10531	0.737138
788	1.46355	0.710866
789	1.46355	0.656849
790	0.78086	0.913813
791	1.10531	0.744989
792	1.1391	0.718433
793	1.56166	0.808285
794	2.21062	0.724086
795	1.0361	1.06484
796	1.73577	2.1
797	1.73577	1.50929
798	0.946116	2.1
799	1.28174	2.1
800	0.946118	1.19043
801	1.22883	2.1
802	1.28172	2.1
803	1.03609	1.22041
804	0.553567	0.0728193
805	1.60581	0.0717107
806	1.60581	0.0682427
807	0.553594	0.0705961
808	0.966748	0.0758731
809	0.966748	0.07204

810	0.946133	0.0786739
811	1.05602	0.0713783
812	2.38525	0.0755429
813	1.14503	0.0654591
814	1.24678	0.0660094
815	0.56262	0.0649973
816	1.24678	0.0676929
817	0.978467	0.0678582
818	1.66262	0.0645993
819	1.66262	0.0629787
820	0.562633	0.0686712
821	1.09522	0.0674894
822	2.49356	0.066775
823	0.592547	0.0676404
824	1.04321	0.9482
825	1.25133	1.14431
826	1.70508	1.15625
827	1.81043	0.841544
828	0.494934	0.954905
829	0.601047	0.856325
830	0.993428	0.913925
831	0.946573	1.00987
832	1.35039	0.81335
833	0.816895	1.31395
834	1.5063	0.812264
835	0.816895	0.700574
836	0.946132	0.775998
837	1.9067	1.11113
838	2.14504	1.30016
839	0.816895	0.677067
840	2.29624	0.702516
841	2.76933	0.768491
842	1.254	1.23051
843	0.946128	2.1
844	1.38813	2.1
845	1.79998	2.1
846	1.79998	2.1
847	0.94613	2.1
848	1.38813	2.1
849	1.35798	2.1
850	1.27809	2.1
851	1.35798	2.1
852	1.68994	2.1
853	1.68994	2.1
854	0.946132	2.1
855	0.946132	2.1
856	1.18067	2.1
857	0.958256	2.1
858	0.958255	2.1
859	0.958257	2.1
860	0.958255	2.1
861	1.06457	2.1
862	1.14195	1.1858
863	1.70423	0.532736
864	1.14195	1.21642
865	1.64285	1.97349
866	2.02057	2.1
867	0.946132	2.1

868	1.44186	2.1
869	0.946133	1.79864
870	1.16366	2.1
871	1.17233	2.1
872	1.31259	0.544849
873	1.2016	2.1
874	1.17129	2.1
875	1.05724	0.319393
876	1.32697	0.704076
877	1.05724	0.528948
878	1.04292	0.805694
879	3.39037	2.1
880	2.57275	2.1
881	0.629212	2.1
882	2.32525	2.1
883	1.76949	0.816973
884	1.65809	1.1253
885	1.27665	2.1
886	1.19861	2.1
887	1.20045	2.1
888	1.1635	2.1
889	0.953633	0.206982
890	0.953634	0.219893
891	1.21408	2.1
892	1.25716	2.1
893	2.01791	2.1
894	1.89318	2.1
895	0.947921	0.975522
896	0.939049	2.1
897	0.947921	1.31094
898	0.740753	2.1
899	0.916104	1.49704
900	1.16941	2.1
901	1.72038	2.1
902	2.04441	2.1
903	0.973182	2.1
904	1.11751	1.62158
905	0.611089	2.1
906	1.53584	0.354108
907	0.767923	0.400485
908	1.38925	0.427994
909	1.38926	0.374613
910	0.946131	0.459305
911	0.961347	0.420484
912	1.19583	0.500971
913	0.946131	0.486234
914	1.3663	0.579243
915	1.21067	0.368727
916	1.00166	0.111779
917	1.40707	0.125366
918	1.50435	0.124524
919	1.47954	0.59549
920	1.1233	2.1
921	2.36036	1.21148
922	1.04024	0.622979
923	0.739729	0.651742
924	1.45533	0.565824
925	1.45532	0.551272

926	0.946118	0.619518
927	1.15163	0.405786
928	1.15165	0.672118
929	0.739748	0.568473
930	1.04025	0.84071
931	1.47608	0.303879
932	0.946131	0.397443
933	1.02028	0.330047
934	0.946129	0.349135
935	1.78223	0.268539
936	1.78223	0.247535
937	1.34153	0.299896
938	1.38683	0.25049
939	1.3407	0.29089
940	1.3407	0.301087
941	2.04729	0.285394
942	0.851529	0.366983
943	1.26671	0.320303
944	0.946116	0.362253
945	1.4869	0.261111
946	1.29553	0.251391
947	1.03645	0.317124
948	1.20731	0.261984
949	0.73665	2.1
950	0.693596	2.1
951	2.11152	1.34021
952	1.73627	1.0618
953	1.06191	2.1
954	1.52226	2.1
955	1.76869	2.1
956	1.1881	2.1
957	1.32349	2.1
958	1.51041	2.1
959	1.61591	2.1
960	0.946132	2.1
961	0.946132	2.1
962	1.13537	2.1
963	3.0048	2.1
964	0.985109	2.1
965	1.68899	2.1
966	3.23261	2.1
967	1.08831	2.1
968	2.20623	2.1
969	0.91749	2.1
970	1.15515	2.1
971	0.550438	2.1
972	1.66294	2.1
973	1.08257	2.1
974	0.87851	2.1
975	2.09046	2.1
976	0.738711	2.1
977	1.31242	0.111125
978	1.26311	0.112831
979	1.67652	0.0736593
980	0.968594	2.1
981	0.968594	2.1
982	0.981099	0.073996
983	1.32418	0.0816367

984	2.67175	0.0806048
985	2.09678	0.0765936
986	1.2511	0.0769466
987	0.912698	0.0894779
988	0.810946	0.0961022
989	0.810959	0.0915525
990	1.67479	0.0811077
991	1.65677	0.0837089
992	1.28469	0.0840751
993	1.48817	0.0879482
994	1.08131	0.0918081
995	1.04349	0.49744
996	1.42492	0.450975
997	1.05724	0.413916
998	1.65723	0.269382
999	1.95313	0.27843
1000	1.9293	0.27305
1001	2.02729	0.28428
1002	0.963393	0.824991
1003	0.963392	0.69963
1004	2.90171	2.1
1005	2.65447	2.1
1006	1.64158	2.07293
1007	1.6488	2.1
1008	1.15991	0.437894
1009	0.946132	0.518055
1010	1.58199	0.484222
1011	1.58199	0.508675
1012	1.03333	0.515872
1013	1.25436	0.493745
1014	1.25436	0.526265
1015	0.946132	0.540138
1016	1.3771	0.279374
1017	0.687745	0.272855
1018	1.37709	0.241191
1019	0.812386	0.288021
1020	1.50172	0.289326
1021	1.50172	0.214115
1022	0.687758	0.283746
1023	1.17506	0.218653
1024	2.00539	0.265469
1025	2.2441	0.318141
1026	1.09667	0.226997
1027	1.43577	2.1
1028	2.24741	2.1
1029	1.26103	2.1
1030	0.851544	2.06507
1031	1.5543	2.1
1032	1.59756	2.1
1033	0.851543	2.1
1034	1.44673	2.1
1035	1.31947	2.1
1036	1.1237	2.1
1037	1.26103	2.1
1038	1.089	0.127644
1039	0.946128	0.18711
1040	0.965824	0.18028
1041	0.94613	0.199724

1042	1.51344	0.137489
1043	1.51344	0.132567
1044	1.13508	0.15867
1045	1.32449	0.155038
1046	1.089	0.153852
1047	3.33706	0.194884
1048	2.7542	0.248803
1049	1.27256	0.318222
1050	0.954172	0.289349
1051	1.55527	0.262749
1052	1.05765	0.396641
1053	1.57326	0.334685
1054	1.38251	0.203166
1055	0.94613	0.322831
1056	2.38829	0.213704
1057	2.00539	0.249089
1058	1.53798	0.237259
1059	1.20969	2.1
1060	1.15864	2.1
1061	1.7986	2.1
1062	1.74533	2.1
1063	0.944827	1.55038
1064	0.946131	1.00811
1065	0.851543	0.933926
1066	1.52816	0.969965
1067	1.5314	1.24403
1068	1.39266	0.688901
1069	0.851528	0.68838
1070	1.39589	0.651547
1071	1.7348	0.9528
1072	1.85439	1.29352
1073	1.86367	1.0835
1074	1.90905	0.887442
1075	1.22176	0.430141
1076	1.59492	0.421397
1077	2.97929	2.1
1078	2.1905	2.1
1079	1.24128	2.1
1080	0.994293	2.1
1081	2.49767	0.0600403
1082	2.37105	0.0618694
1083	1.10834	0.88766
1084	1.53214	1.00277
1085	1.417	1.66372
1086	2.13599	0.710907
1087	1.61727	2.1
1088	1.75004	2.1
1089	1.96301	2.1
1090	0.966631	2.1
1091	1.37791	2.1
1092	1.47302	2.1
1093	1.47302	2.1
1094	0.871519	2.1
1095	0.94613	2.1
1096	0.87155	2.1
1097	2.38731	2.1
1098	2.12399	1.28722
1099	1.74865	1.23859

1100	1.46724	0.763475
1101	2.38056	0.685205
1102	1.46724	0.7766
1103	0.818887	0.740624
1104	0.810944	1.39883
1105	0.805227	0.721821
1106	1.06558	0.978677
1107	0.841487	0.777643
1108	1.08456	1.04332
1109	0.903083	0.972663
1110	1.08929	0.0869512
1111	1.68454	0.0904164
1112	1.08929	0.0871556
1113	1.07658	0.101541
1114	0.904439	0.113502
1115	1.58501	0.0817165
1116	1.19729	0.090924
1117	1.05427	0.0883618
1118	1.47764	0.0955908
1119	0.810959	0.093596
1120	1.60946	0.82429
1121	2.28887	0.817053
1122	0.946133	0.896619
1123	1.14444	0.767635
1124	1.67964	0.663728
1125	1.67964	0.69962
1126	0.755334	0.9498
1127	1.14444	0.85072
1128	1.29054	0.779902
1129	1.37762	0.743554
1130	0.946132	0.853088
1131	0.533645	0.67145
1132	1.48361	0.608763
1133	1.48361	0.705311
1134	0.533645	0.827527
1135	0.872823	0.870083
1136	1.14444	0.695142
1137	1.37762	0.719033
1138	2.10101	0.721425
1139	1.02311	0.753465
1140	1.04083	2.1
1141	1.04084	2.1
1142	1.04084	2.1
1143	1.30116	2.1
1144	1.23477	0.0958597
1145	1.81893	0.120356
1146	1.23477	0.10358
1147	1.23477	0.108438
1148	1.11967	0.096408
1149	0.946131	0.115391
1150	1.11966	0.114166
1151	1.12628	0.0952136
1152	0.629641	0.11603
1153	1.6163	0.110712
1154	1.6163	0.0869337
1155	1.11117	2.1
1156	1.13006	2.1
1157	1.15149	2.1

1158	1.13179	2.1
1159	0.585368	0.507485
1160	0.986036	1.13693
1161	1.94788	0.516368
1162	1.34857	0.532266
1163	2.31806	2.1
1164	2.06363	2.1
1165	1.00423	2.1
1166	0.829065	2.1
1167	0.551816	2.1
1168	0.99406	2.1
1169	1.98339	2.1
1170	1.68073	2.1
1171	2.558	2.1
1172	0.711814	0.874361
1173	2.97316	1.38926
1174	2.41579	2.1
1175	1.15248	0.225574
1176	1.14305	0.219476
1177	1.15483	0.0827467
1178	1.90981	0.0843824
1179	0.998957	0.0822361
1180	1.83742	0.0857066
1181	1.27581	2.1
1182	0.94613	2.1
1183	1.6315	2.1
1184	1.6315	2.1
1185	0.946132	2.1
1186	1.28441	2.1
1187	1.28441	2.1
1188	1.00365	2.1
1189	0.968536	2.1
1190	1.57575	2.1
1191	1.27581	2.1
1192	1.99297	2.1
1193	1.32784	2.1
1194	1.4259	2.1
1195	2.27952	2.1
1196	1.5685	2.1
1197	1.54231	2.1
1198	3.54638	2.1
1199	1.3294	2.1
1200	2.98237	2.1
1201	2.01633	0.935591
1202	2.0985	0.988276
1203	2.26047	0.682651
1204	1.7913	0.597014
1205	1.09504	2.1
1206	1.74126	2.1
1207	1.05983	2.1
1208	2.37664	2.1
1209	2.28084	2.1
1210	2.19948	2.1
1211	1.33593	0.0847644
1212	1.31508	0.0841173
1213	1.30647	2.1
1214	1.28846	2.1
1215	1.2955	1.81679

1216	1.09044	1.33968
1217	1.4099	1.29081
1218	1.14984	1.49303
1219	0.946132	0.805631
1220	1.70727	1.22584
1221	1.70727	2.1
1222	1.14984	1.00435
1223	0.946132	1.24259
1224	0.911818	2.1
1225	2.12209	2.1
1226	2.4158	2.1
1227	1.20867	2.1
1228	2.84085	2.1
1229	1.42284	2.1
1230	2.45689	2.1
1231	1.41562	2.1
1232	0.918358	2.1
1233	1.11222	2.1
1234	0.999083	2.1
1235	0.696781	2.1
1236	1.30869	2.1
1237	1.66132	2.1
1238	0.696807	2.1
1239	0.745315	2.1
1240	1.23296	2.1
1241	0.780548	2.1
1242	1.93242	0.878553
1243	1.27362	0.871626
1244	2.77828	1.31612
1245	1.02653	0.629579
1246	0.808576	0.640451
1247	0.744012	0.763913
1248	0.605174	0.542706
1249	1.16501	0.43406
1250	0.803432	0.457134
1251	0.961016	0.479786
1252	1.10425	0.583849
1253	1.3756	0.679117
1254	2.17765	0.880703
1255	1.84578	0.781913
1256	1.32801	0.64195
1257	1.76377	0.63836
1258	0.946131	0.561471
1259	1.3022	0.719187
1260	0.694814	0.752946
1261	1.53028	0.583722
1262	1.53028	0.590873
1263	1.3022	0.670406
1264	0.922889	0.686796
1265	1.57766	1.84498
1266	2.06911	1.00292
1267	1.57766	1.22256
1268	1.37721	0.13268
1269	1.72174	0.180095
1270	1.4468	0.174641
1271	1.37721	0.112121
1272	1.73972	0.397913
1273	1.62324	0.414235

1274	0.946125	0.359163
1275	1.34259	0.254455
1276	1.04187	0.227276
1277	1.05019	0.292476
1278	1.2361	0.365804
1279	1.69055	0.274239
1280	2.25056	0.350975
1281	1.10574	0.568893
1282	1.21632	0.732331
1283	1.10574	0.758042
1284	1.46181	1.09015
1285	0.946131	0.723678
1286	1.74111	1.15434
1287	1.74111	0.854321
1288	0.94613	0.849569
1289	1.26891	0.770189
1290	1.26891	0.611786
1291	1.23452	1.01388
1292	1.00255	0.811076
1293	1.72286	0.333608
1294	1.12233	0.206268
1295	1.30682	0.374596
1296	1.97397	0.43904
1297	1.67425	2.1
1298	1.77775	2.1
1299	1.73571	1.69839
1300	0.426265	1.99908
1301	1.31817	1.35734
1302	1.81042	2.1
1303	1.85456	2.1
1304	0.466532	1.42964
1305	1.28025	1.25215
1306	0.964467	1.29476
1307	1.27921	2.1
1308	2.11851	1.42026
1309	2.37121	1.79272
1310	1.49357	0.516139
1311	1.42486	2.1
1312	1.00278	2.1
1313	1.00278	2.1
1314	1.12503	2.1
1315	0.568697	2.1
1316	0.555211	2.1
1317	0.982272	1.12628
1318	1.57558	0.97296
1319	0.851539	1.56789
1320	1.07307	2.09689
1321	1.58292	1.99732
1322	1.50731	2.1
1323	0.851541	0.861188
1324	1.00304	1.99411
1325	1.36003	2.07472
1326	1.13127	2.1
1327	0.672234	2.1
1328	0.763044	2.1
1329	0.966979	2.1
1330	1.31502	2.1
1331	1.67969	2.1

1332	1.62408	2.1
1333	0.661955	2.1
1334	1.35048	2.1
1335	1.40447	2.1
1336	1.40447	2.1
1337	0.607961	2.1
1338	0.946131	2.1
1339	0.607963	2.1
1340	1.01531	2.1
1341	1.01531	2.1
1342	1.91802	2.1
1343	0.777749	2.1
1344	1.22585	0.140142
1345	1.20672	0.131676
1346	1.03166	0.292954
1347	0.553805	2.1
1348	2.00551	2.1
1349	2.07203	1.12702
1350	0.589479	2.1
1351	1.22352	2.1
1352	0.984227	2.1
1353	0.802455	2.1
1354	1.40942	2.1
1355	1.6662	2.1
1356	0.802485	2.1
1357	0.936669	2.1
1358	1.31127	0.727861
1359	0.946132	0.662034
1360	1.09729	0.518332
1361	0.946133	0.678212
1362	1.68703	0.572653
1363	1.68703	0.528923
1364	1.3689	0.620323
1365	1.26426	0.643044
1366	1.26923	2.1
1367	1.23614	2.1
1368	0.828371	2.1
1369	2.04556	2.1
1370	0.772648	2.1
1371	0.791059	2.1
1372	0.809958	2.1
1373	1.70012	2.1
1374	0.267032	2.1
1375	1.67604	2.1
1376	0.803823	2.1
1377	0.267305	2.1
1378	0.80246	2.1
1379	0.874582	2.1
1380	1.00188	2.1
1381	0.709588	2.1
1382	1.50679	1.95993
1383	1.22456	1.93322
1384	0.870622	2.1
1385	1.05754	1.93545
1386	1.1689	2.1
1387	1.54556	2.05003
1388	1.89226	2.08608
1389	0.995311	2.1

1390	1.26195	2.1
1391	1.9	2.1
1392	0.845055	2.1
1393	0.737836	2.1
1394	0.823707	2.1
1395	0.626587	2.1
1396	1.16109	2.1
1397	0.85517	2.1
1398	0.932036	2.1
1399	1.08321	2.1
1400	1.43863	2.1
1401	2.72297	2.1
1402	2.90798	2.1
1403	1.47818	1.17668
1404	1.47819	1.08257
1405	0.694357	0.992304
1406	0.694382	1.02708
1407	0.946132	0.767029
1408	0.694382	0.898486
1409	1.22641	0.823516
1410	1.31155	1.32334
1411	2.45282	1.11595
1412	0.787843	0.8988
1413	0.808984	0.70722
1414	1.14232	1.139
1415	0.795037	1.09419
1416	0.887382	1.02551
1417	1.14493	0.979023
1418	0.78393	0.8051
1419	1.09129	1.0362
1420	0.791482	0.948726
1421	1.1137	1.25466
1422	1.11819	1.04995
1423	0.960173	0.971169
1424	1.09447	1.26821
1425	0.874365	0.908133
1426	1.2474	0.317692
1427	1.75038	0.431577
1428	1.75038	0.546545
1429	0.94613	0.736764
1430	1.30076	0.450371
1431	1.12817	0.920554
1432	0.94613	0.644687
1433	1.30076	0.609472
1434	1.51841	0.524482
1435	1.51841	0.614779
1436	0.946132	0.918737
1437	1.39575	0.563143
1438	0.970358	1.15559
1439	0.946133	0.782737
1440	1.39575	0.724957
1441	1.06486	0.405641
1442	1.06486	0.402885
1443	1.38622	0.440144
1444	1.04521	0.0999498
1445	1.00809	0.118967
1446	1.28952	0.128302
1447	0.849316	0.110235

1448	0.946132	0.120616
1449	0.849344	0.138605
1450	1.53631	0.129937
1451	1.53631	0.135896
1452	1.77843	0.0927736
1453	1.69863	0.101605
1454	1.91646	2.1
1455	1.8582	2.1
1456	2.8349	0.0697967
1457	1.36307	0.0678558
1458	1.23886	0.0935082
1459	1.12971	0.0975495
1460	1.59662	0.0965206
1461	1.59662	0.0914052
1462	0.640304	0.128889
1463	0.640333	0.0878978
1464	0.886276	0.13559
1465	0.886276	0.125931
1466	1.35065	0.115421
1467	2.09002	0.101713
1468	1.35543	0.107829
1469	1.94688	2.1
1470	1.97221	2.1
1471	0.719563	2.1
1472	1.20358	2.1
1473	0.874264	2.1
1474	1.27676	2.1
1475	1.46557	2.1
1476	1.46557	2.1
1477	0.68546	2.1
1478	0.94613	2.1
1479	0.685489	2.1
1480	1.39615	0.241514
1481	1.05404	0.168513
1482	1.06474	0.240539
1483	1.00713	0.250729
1484	0.714535	0.265964
1485	0.714563	0.291283
1486	1.41646	0.181201
1487	1.52582	0.189624
1488	0.721694	0.274479
1489	0.93665	0.25343
1490	1.21067	0.387019
1491	1.21067	0.377628
1492	1.73577	0.47636
1493	1.17799	0.416617
1494	3.21432	0.364847
1495	2.41452	0.360186
1496	1.36208	0.273559
1497	2.53256	0.323273
1498	0.834593	0.237816
1499	2.5853	0.29014
1500	1.77259	0.242105
1501	0.615724	0.394929
1502	1.11374	0.673118
1503	0.615726	0.350275
1504	0.946129	0.902969
1505	0.615714	0.841669

1506	1.75489	0.684491
1507	1.75489	0.58189
1508	1.23146	0.412392
1509	2.2772	0.436782
1510	0.74463	0.415413
1511	1.3546	0.341163
1512	1.80252	0.268808
1513	2.38304	0.382882
1514	1.19151	0.47783
1515	1.1231	0.481971
1516	1.82345	0.419152
1517	1.79805	0.357897
1518	0.761337	0.694046
1519	0.946127	0.479798
1520	0.557726	0.517338
1521	1.05353	0.243199
1522	1.05353	0.204758
1523	1.68599	0.280349
1524	1.05353	0.219918
1525	1.40588	2.1
1526	1.09373	2.1
1527	1.18382	0.947145
1528	1.46903	0.893084
1529	1.25103	2.1
1530	1.10378	2.1
1531	1.58941	2.1
1532	1.29639	2.1
1533	1.14874	0.085774
1534	0.899817	0.0917573
1535	0.666898	0.0879436
1536	1.76519	0.0805382
1537	1.17289	0.0830777
1538	1.03526	0.0825104
1539	1.97708	0.0847387
1540	1.31711	0.0756555
1541	0.992517	0.0801382
1542	1.70193	0.0811732
1543	1.13852	0.0890843
1544	1.23943	0.227788
1545	1.40014	0.704466
1546	1.04598	0.944032
1547	1.1483	0.782244
1548	1.11433	0.944258
1549	1.32037	0.552612
1550	1.00381	0.675863
1551	1.11435	0.461396
1552	0.973502	0.807544
1553	0.946128	1.52791
1554	1.83697	1.03437
1555	1.83697	2.1
1556	1.31721	1.51287
1557	1.38614	1.19532
1558	1.39696	1.02565
1559	1.31721	1.37477
1560	1.0756	1.6454
1561	1.08542	0.872897
1562	0.946127	1.86464
1563	1.81052	0.760997

1564	1.81052	2.09068
1565	1.24315	1.67993
1566	1.39696	0.707953
1567	1.35969	1.22301
1568	1.24315	1.12199
1569	1.0756	0.494529
1570	1.20669	0.220247
1571	1.18508	0.253549
1572	1.71059	2.1
1573	1.66762	2.1
1574	2.50853	2.1
1575	1.8236	2.1
1576	1.78872	2.1
1577	1.41721	2.1
1578	1.01335	1.37681
1579	1.42439	0.930566
1580	0.946129	1.00705
1581	1.33266	1.22262
1582	1.79776	1.33706
1583	1.79776	1.75584
1584	0.946131	0.74698
1585	1.33266	1.27838
1586	1.41123	1.12181
1587	1.25346	2.1
1588	0.946132	0.803451
1589	0.827287	1.77979
1590	1.57878	1.15348
1591	1.57878	1.26982
1592	0.827287	1.01788
1593	1.07341	1.05883
1594	1.33266	1.0539
1595	1.25346	1.06114
1596	1.61149	2.05686
1597	1.15644	1.70878
1598	1.27558	0.77344
1599	1.3764	0.562456
1600	1.89526	0.949553
1601	1.37641	0.55016
1602	1.2064	0.541783
1603	1.38426	1.60508
1604	1.01595	2.1
1605	1.15067	2.1
1606	1.82276	1.33017
1607	1.82276	0.463909
1608	0.946131	0.556763
1609	1.40609	0.497106
1610	0.946132	0.965438
1611	1.29143	0.701785
1612	1.40609	0.698841
1613	1.30272	0.903973
1614	1.19548	0.662015
1615	2.95451	2.1
1616	1.46124	2.1
1617	2.93215	2.1
1618	1.42105	2.1
1619	1.75477	0.980993
1620	2.31301	1.02959
1621	2.10275	0.672411

1622	1.80099	0.730807
1623	0.97541	2.1
1624	1.49733	2.1
1625	0.97541	2.1
1626	1.30751	2.1
1627	1.28614	0.164616
1628	1.74759	0.123562
1629	1.19756	0.31411
1630	1.71053	0.290637
1631	1.19757	0.351953
1632	1.28954	0.290631
1633	2.02703	0.271875
1634	2.29453	0.133243
1635	1.28265	0.102928
1636	0.793267	0.144501
1637	1.19974	0.198167
1638	0.509551	0.120309
1639	1.04072	0.188216
1640	1.35302	0.119203
1641	1.53133	0.207467
1642	0.566153	0.159319
1643	1.09634	0.141658
1644	1.12792	0.180657
1645	1.68908	2.1
1646	1.68908	2.1
1647	0.768212	1.8195
1648	0.768213	2.1
1649	0.946132	2.1
1650	0.768212	1.81125
1651	1.32461	1.80249
1652	1.87291	1.86424
1653	2.64921	1.91011
1654	1.53642	2.1
1655	2.26537	2.1
1656	1.53642	2.1
1657	1.79874	2.07018
1658	1.79874	2.06833
1659	0.726635	1.31354
1660	0.726636	2.03668
1661	0.946128	1.33533
1662	0.726634	1.36713
1663	1.23339	1.06573
1664	0.992674	2.1
1665	1.8023	2.1
1666	1.12324	0.0669295
1667	1.29163	0.0637506
1668	0.996831	0.235446
1669	1.0019	0.221792
1670	0.993831	0.30311
1671	1.07283	1.01253
1672	1.04788	2.1
1673	1.44489	0.952402
1674	1.04788	1.61189
1675	1.07652	2.1
1676	0.991406	2.1
1677	1.37	1.87489
1678	1.14195	1.87909
1679	1.47904	0.131652

1680	1.26593	1.39891
1681	1.75366	1.25053
1682	1.57621	0.328782
1683	1.24538	0.210154
1684	0.969118	2.1
1685	0.946133	0.838911
1686	1.69387	2.04744
1687	1.69387	2.1
1688	0.94613	1.47458
1689	1.28713	2.1
1690	1.28713	1.89446
1691	1.23877	1.90421
1692	0.969118	2.08691
1693	1.22615	2.1
1694	0.98084	2.1
1695	1.01843	2.1
1696	1.50876	2.1
1697	1.85391	2.1
1698	1.84322	2.1
1699	1.70377	2.1
1700	1.78919	2.1
1701	2.66787	2.1
1702	1.41549	2.1
1703	1.41549	2.1
1704	0.86707	2.1
1705	0.896172	2.1
1706	0.946126	2.1
1707	0.867074	2.1
1708	0.946118	2.1
1709	1.54468	0.217753
1710	1.54468	0.220063
1711	2.27259	0.243098
1712	0.946131	0.363983
1713	1.25837	0.326034
1714	0.663171	0.400447
1715	1.68931	0.236006
1716	1.68931	0.235323
1717	1.25837	0.312378
1718	1.09411	0.304145
1719	1.73571	0.19535
1720	2.34894	0.23018
1721	0.946128	0.265401
1722	1.30497	0.23952
1723	0.772312	0.298073
1724	1.66895	0.189573
1725	1.66895	0.167523
1726	1.30497	0.221923
1727	1.13629	0.20112
1728	1.22852	0.763471
1729	1.578	0.717598
1730	2.07781	1.90054
1731	1.5478	1.86428
1732	1.70048	1.24148
1733	1.42329	1.16102
1734	1.05481	1.5085
1735	1.1032	0.951413
1736	1.19267	2.1
1737	1.77324	2.1

1738	1.01121	0.30729
1739	0.991867	0.304138
1740	2.0744	0.969944
1741	1.75142	2.1
1742	1.35418	0.955257
1743	1.40041	2.1
1744	1.50045	2.1
1745	1.37704	2.1
1746	1.50045	2.1
1747	1.87875	2.1
1748	1.35308	0.618032
1749	1.29983	0.620322
1750	1.62784	2.1
1751	1.44807	2.1
1752	1.00965	2.1
1753	2.69264	2.1
1754	1.04075	2.1
1755	0.946131	2.1
1756	0.946132	2.1
1757	1.52704	2.1
1758	1.83246	2.1
1759	1.56757	2.1
1760	1.16687	2.1
1761	2.04857	2.1
1762	1.32707	0.987703
1763	1.25519	0.196863
1764	1.32101	0.301848
1765	1.25055	0.698006
1766	0.827853	0.452569
1767	1.11835	0.278385
1768	1.07281	0.969715
1769	1.54302	0.517002
1770	0.724064	0.683909
1771	1.32709	1.84377
1772	3.44123	0.792761
1773	2.46889	0.28139
1774	0.730076	0.144389
1775	1.25809	0.259079
1776	0.730077	0.168641
1777	0.946129	0.23691
1778	1.72777	0.130806
1779	1.72777	0.243993
1780	0.73005	0.199637
1781	1.7522	0.121751
1782	2.51618	0.146551
1783	0.628177	0.147584
1784	1.1068	0.25402
1785	0.747435	0.158753
1786	0.946133	0.236658
1787	1.84668	0.148063
1788	1.8563	0.232252
1789	0.628177	0.210732
1790	1.45501	0.173968
1791	2.19809	0.251365
1792	1.45501	0.18626
1793	1.48974	0.156612
1794	1.7709	1.86801
1795	2.63288	1.72804

1796	1.5362	2.1
1797	1.56327	2.1
1798	3.03945	0.16937
1799	0.899979	0.219721
1800	0.869386	0.227683
1801	1.85864	0.197062
1802	1.78028	0.211371
1803	1.28719	0.229962
1804	1.09532	0.185188
1805	0.881898	0.230446
1806	1.28911	0.216574
1807	1.39644	0.224912
1808	0.688829	0.212792
1809	2.14611	0.180529
1810	1.80313	0.179327
1811	0.68883	0.215463
1812	0.946131	0.239602
1813	1.25334	0.14363
1814	1.88987	0.186695
1815	1.94432	0.202671
1816	1.37592	0.164671
1817	1.11691	1.1005
1818	1.02923	1.21857
1819	0.45824	0.0938343
1820	0.646512	0.128935
1821	2.46292	0.109195
1822	0.493451	0.117161
1823	2.00826	2.1
1824	2.49784	2.1
1825	1.17054	2.1
1826	1.01485	2.1
1827	1.26045	2.1
1828	1.17054	2.1
1829	1.44247	2.1
1830	2.02687	0.0841239
1831	1.66365	0.0915669
1832	1.64656	0.0847067
1833	1.56597	0.0945001
1834	1.21419	2.1
1835	1.5008	2.1
1836	1.10711	2.1
1837	1.44287	2.1
1838	1.44287	2.1
1839	0.750368	2.1
1840	1.25876	2.1
1841	0.82542	2.1
1842	1.04074	2.1
1843	1.25874	2.1
1844	1.04159	0.0932091
1845	1.2489	0.0925565
1846	0.980547	0.334849
1847	1.62235	0.304635
1848	1.15991	0.343237
1849	0.980547	0.254643
1850	0.94613	0.354601
1851	1.54662	0.316982
1852	1.54661	0.331702
1853	1.07912	0.372748

1854	1.1032	0.314601
1855	1.1032	0.323388
1856	0.94613	0.338279
1857	1.12468	2.1
1858	1.2724	2.1
1859	1.06521	1.33854
1860	1.0752	1.50466
1861	1.94936	0.273562
1862	1.62772	0.281629
1863	1.94733	0.253135
1864	1.42901	0.281119
1865	1.33022	2.1
1866	1.70839	2.1
1867	1.75048	2.1
1868	1.41201	2.1
1869	1.52314	2.1
1870	1.44039	2.1
1871	1.51113	2.1
1872	2.58638	2.1
1873	0.987247	0.265998
1874	1.38622	0.444142
1875	0.858359	0.295633
1876	1.19967	0.341271
1877	1.06414	0.25292
1878	0.946132	0.441496
1879	0.94613	0.267349
1880	0.687079	0.542365
1881	0.946132	0.427714
1882	0.822611	0.416719
1883	1.70242	0.29149
1884	1.70242	0.410512
1885	0.946132	0.560128
1886	1.11155	0.357429
1887	0.946132	0.638498
1888	1.11155	0.491549
1889	1.37981	0.510158
1890	1.18348	0.309475
1891	2.44179	0.398562
1892	2.00403	0.52889
1893	1.10961	2.1
1894	1.85415	2.1
1895	1.44825	2.1
1896	0.932272	2.1
1897	0.810942	2.1
1898	1.00189	2.1
1899	1.11779	2.1
1900	1.3359	2.1
1901	0.965279	2.1
1902	0.833779	2.1
1903	1.05337	2.1
1904	0.526656	2.1
1905	1.44599	2.1
1906	1.44599	2.1
1907	1.24853	2.1
1908	0.946132	2.1
1909	1.24853	2.1
1910	0.724122	2.1
1911	1.91547	2.1

1912	2.49705	2.1
1913	1.05931	2.1
1914	1.1729	2.1
1915	1.34526	2.1
1916	1.5379	2.1
1917	1.90855	2.1
1918	2.08597	2.1
1919	1.68165	2.1
1920	1.45887	2.1
1921	1.50022	2.1
1922	1.4748	2.04574
1923	2.63645	1.90299
1924	1.74431	2.03795
1925	1.25288	1.93942
1926	1.25289	1.72743
1927	0.970025	1.60655
1928	0.747542	2.1
1929	0.810959	1.49005
1930	1.15369	1.73107
1931	0.977472	1.60384
1932	1.12695	1.7493
1933	0.996762	1.59883
1934	1.28249	0.668452
1935	0.946132	0.681397
1936	0.528621	0.74055
1937	1.59186	0.584466
1938	1.44072	0.607555
1939	0.633488	0.627555
1940	0.598506	0.708827
1941	1.51618	0.722001
1942	1.19999	0.569891
1943	1.46511	0.572173
1944	1.44056	0.539046
1945	0.912754	0.441719
1946	1.4725	0.386011
1947	1.4725	0.345752
1948	0.912753	0.42376
1949	1.23351	0.458934
1950	1.23351	0.481401
1951	0.946131	0.529364
1952	1.17129	0.30552
1953	1.82551	0.293345
1954	1.27575	1.77802
1955	0.946129	1.98174
1956	0.946129	2.0985
1957	0.946128	1.91527
1958	0.792754	2.04812
1959	0.94613	1.54984
1960	0.792752	1.49124
1961	2.08054	2.1
1962	1.20091	2.1
1963	2.1669	1.97377
1964	1.24383	1.61545
1965	0.904669	1.89095
1966	0.83804	1.36242
1967	1.90486	1.70753
1968	1.29162	1.51016
1969	1.37383	1.82282

1970	1.86533	1.51311
1971	1.76472	1.86773
1972	1.60428	1.49391
1973	2.43993	1.86391
1974	1.37833	2.1
1975	1.18632	2.1
1976	0.946132	2.1
1977	1.27544	2.1
1978	1.05085	2.1
1979	0.961178	2.1
1980	0.859844	2.1
1981	1.0499	2.1
1982	1.75592	2.1
1983	2.08668	2.1
1984	1.75592	2.1
1985	1.4402	2.1
1986	1.72285	2.1
1987	0.83312	0.132141
1988	1.07295	0.119695
1989	1.37607	0.143591
1990	1.32097	0.116667
1991	1.11332	0.155763
1992	1.18568	0.118007
1993	0.877482	0.129302
1994	1.4458	0.100309
1995	2.34112	0.087316
1996	2.68021	0.115515
1997	0.681677	0.0783023
1998	0.946131	0.0981764
1999	0.681649	0.0852973
2000	1.83115	0.0859552
2001	1.68029	0.0917127
2002	1.05482	0.0778222
2003	1.31485	0.0909797
2004	2.08247	1.11798
2005	1.33153	1.04166
2006	0.935062	1.17192
2007	0.806958	1.63799
2008	0.827683	1.04604
2009	1.01041	1.28363
2010	0.741832	0.998828
2011	1.15668	1.36618
2012	0.908649	1.16056
2013	0.59561	0.349784
2014	1.19028	0.779116
2015	0.59561	0.707237
2016	0.946132	0.889513
2017	0.595623	1.07869
2018	1.58164	0.818106
2019	1.58164	0.889433
2020	0.946131	1.3287
2021	1.55201	2.1
2022	1.55201	2.1
2023	0.946127	2.1
2024	1.13312	2.1
2025	1.13312	2.1
2026	1.08108	2.1
2027	1.04973	2.1

2028	1.20149	2.1
2029	1.86902	0.924418
2030	0.613447	2.1
2031	1.22618	1.03524
2032	1.38663	1.20676
2033	1.38662	2.1
2034	0.613447	1.08065
2035	1.22617	1.80724
2036	0.773899	1.56513
2037	1.23515	1.30533
2038	1.87823	1.1654
2039	1.70303	0.637457
2040	1.4714	2.1
2041	1.74067	2.1
2042	0.735699	2.1
2043	1.73461	2.1
2044	1.47224	2.1
2045	1.22827	2.1
2046	1.1634	2.1
2047	1.36471	2.1
2048	0.735701	2.1
2049	2.8386	0.622426
2050	2.39056	1.98252
2051	2.44128	0.78871
2052	1.54102	2.1
2053	1.54102	1.72193
2054	0.936141	1.00843
2055	1.40594	2.02752
2056	1.07121	0.723893
2057	0.936142	1.29539
2058	1.07121	1.0073
2059	1.22691	0.214647
2060	1.42907	0.203932
2061	2.18048	0.187815
2062	0.947662	0.190415
2063	2.18394	2.1
2064	1.21292	2.1
2065	2.18394	2.1
2066	2.19064	2.1
2067	1.27229	2.1
2068	1.20859	0.734011
2069	1.21073	0.689677
2070	1.73899	2.1
2071	1.3164	2.1
2072	1.61245	2.1
2073	1.41444	2.1
2074	2.55116	2.1
2075	1.26193	2.1
2076	1.89553	2.1
2077	0.916438	2.1
2078	1.26113	2.1
2079	0.810934	2.1
2080	2.00419	2.1
2081	1.27872	2.1
2082	1.35306	2.1
2083	1.96749	2.1
2084	1.07955	2.1
2085	1.31854	2.1

2086	1.29505	0.672555
2087	1.29504	0.664981
2088	0.612469	0.746658
2089	0.990053	0.730443
2090	1.04072	0.697223
2091	0.985266	0.668579
2092	0.990039	0.640767
2093	1.06892	0.796074
2094	1.57158	0.566131
2095	1.895	0.669605
2096	1.05698	0.593925
2097	0.925053	2.1
2098	0.624712	2.1
2099	1.42465	2.1
2100	1.49014	2.1
2101	0.687198	2.1
2102	1.22058	2.1
2103	0.851488	2.1
2104	0.588128	2.1
2105	1.12452	2.1
2106	1.02783	2.1
2107	1.7083	2.1
2108	1.06977	1.09657
2109	0.818569	0.805624
2110	1.14386	1.35548
2111	0.983791	0.919622
2112	1.12214	1.49978
2113	1.0915	0.956951
2114	0.810934	0.898594
2115	0.764114	1.125
2116	1.87956	0.917595
2117	1.87956	1.3797
2118	0.764141	0.871506
2119	1.37675	0.814121
2120	0.764141	0.851879
2121	0.946132	1.01346
2122	1.9637	1.63825
2123	2.10107	0.725373
2124	1.67814	1.15211
2125	3.02886	1.10815
2126	0.908982	0.186721
2127	0.621727	0.212398
2128	0.525963	0.196115
2129	0.946128	0.229493
2130	1.60979	0.189083
2131	1.45596	0.208201
2132	0.556172	0.213519
2133	2.06779	0.152519
2134	2.17911	0.182827
2135	1.00205	0.124282
2136	1.30685	0.14704
2137	1.16534	0.159086
2138	1.65532	2.1
2139	2.20346	2.1
2140	0.993889	2.1
2141	1.09328	2.1
2142	2.09372	2.1
2143	2.10036	2.1

2144	1.33727	1.46382
2145	2.53383	1.49287
2146	0.893379	1.53896
2147	1.52828	1.30558
2148	1.09599	0.0827532
2149	1.09599	0.0811636
2150	1.50524	2.1
2151	1.54711	2.1
2152	1.41469	2.1
2153	1.99459	1.8026
2154	1.79805	1.84196
2155	1.79805	2.1
2156	0.707344	2.1
2157	1.30318	1.69772
2158	0.707344	2.1
2159	0.946129	2.1
2160	1.30318	2.1
2161	2.40442	2.1
2162	1.89226	2.1
2163	1.40794	2.06383
2164	0.898721	1.99484
2165	0.791921	1.10459
2166	0.810933	1.84479
2167	1.10904	1.43177
2168	0.991102	2.01238
2169	0.808502	1.02722
2170	1.1398	1.67174
2171	1.31346	2.1
2172	1.11833	2.1
2173	1.669	2.1
2174	1.25715	2.1
2175	1.3199	2.1
2176	1.63092	2.1
2177	1.63092	2.1
2178	0.946131	2.1
2179	0.946131	2.1
2180	1.13534	2.1
2181	0.979853	2.1
2182	2.19994	2.1
2183	0.725844	2.1
2184	2.19994	2.1
2185	2.35055	2.1
2186	1.52204	2.1
2187	1.40118	2.1
2188	3.42285	1.84975
2189	2.05217	2.1
2190	1.50319	2.1
2191	0.947516	1.0234
2192	0.903572	1.00153
2193	0.76996	1.09829
2194	1.90959	2.01031
2195	1.18897	1.66626
2196	1.24166	1.59896
2197	2.26785	1.28714
2198	2.05217	2.1
2199	2.73306	2.1
2200	1.42729	0.169397
2201	1.387	0.169194

2202	1.59458	0.486161
2203	1.91051	0.512873
2204	1.68108	0.492294
2205	2.03357	0.569658
2206	0.928982	0.229325
2207	1.55687	0.239057
2208	1.55687	0.150237
2209	0.92898	0.197089
2210	1.3495	0.257832
2211	1.3495	0.196497
2212	0.946133	0.264019
2213	1.85795	0.176342
2214	2.2727	0.243137
2215	1.20324	0.896231
2216	1.07784	0.259705
2217	0.957792	0.170076
2218	1.2745	0.170334
2219	1.4659	0.193624
2220	0.946129	0.279745
2221	0.95778	0.267485
2222	0.851553	0.335941
2223	1.74103	0.202013
2224	2.28994	0.220661
2225	2.08213	0.172833
2226	2.1556	0.188125
2227	2.20372	0.155455
2228	0.838027	0.120971
2229	1.11373	0.108312
2230	1.2909	0.0932844
2231	0.998424	0.110372
2232	1.40621	0.0936321
2233	1.19478	0.100231
2234	1.11783	0.108986
2235	1.6695	0.0950466
2236	1.7498	0.10728
2237	0.477652	0.111738
2238	1.77291	0.118893
2239	0.590448	0.105508
2240	1.07668	0.109412
2241	0.810946	0.144372
2242	1.1946	0.125938
2243	2.03914	0.11511
2244	1.25585	0.134759
2245	2.02622	0.116886
2246	1.39091	0.117127
2247	1.31871	0.13177
2248	1.15789	2.1
2249	1.15755	2.1
2250	1.82268	2.1
2251	1.87119	2.1
2252	3.10636	2.1
2253	2.96285	2.1
2254	1.73443	0.640904
2255	2.33812	0.669672
2256	1.59457	0.530807
2257	1.73443	0.983561
2258	2.00665	2.1
2259	1.99841	2.1

2260	1.19901	2.07614
2261	1.16661	2.0778
2262	2.15883	0.172269
2263	1.20951	0.170327
2264	2.49192	0.223452
2265	0.65112	0.159784
2266	2.45317	0.0837002
2267	1.73091	0.0796445
2268	2.0795	0.0831646
2269	1.45891	0.0738659
2270	1.6531	0.0752028
2271	1.78304	0.0717718
2272	0.775132	0.0953881
2273	1.35051	0.0825735
2274	1.0461	0.0826564
2275	1.37837	0.081964
2276	1.00259	0.0849166
2277	1.25796	0.525524
2278	1.10719	0.65
2279	1.51067	2.1
2280	2.65632	1.35626
2281	1.30521	2.1
2282	2.456 2.05301	
2283	3.19218	1.99867
2284	2.67387	1.57583
2285	0.988862	2.1
2286	1.917 2.1	
2287	0.946132	2.1
2288	1.16202	2.1
2289	1.53871	2.1
2290	1.53871	2.1
2291	0.946132	2.1
2292	1.16202	2.1
2293	1.32282	2.1
2294	2.46356	2.1
2295	2.32404	2.1
2296	1.37562	0.233655
2297	0.985546	0.249306
2298	1.28983	0.193339
2299	1.05566	0.217422
2300	0.895312	0.232342
2301	1.53325	0.194721
2302	1.04342	0.231254
2303	1.80881	0.211433
2304	0.876865	0.19781
2305	0.5689	0.206814
2306	1.09506	0.221499
2307	1.51789	2.1
2308	1.49872	2.1
2309	0.949364	0.379997
2310	2.01233	0.388544
2311	1.55063	0.368451
2312	1.57783	0.581294
2313	1.57783	0.618121
2314	0.674605	0.525345
2315	0.98537	0.363618
2316	0.674633	0.320854
2317	0.98537	0.327015

2318	1.26706	0.591785
2319	0.980664	0.679475
2320	1.03298	0.386072
2321	1.84936	0.625795
2322	1.51696	0.388592
2323	1.97074	0.346313
2324	1.03298	0.365137
2325	1.51324	0.286288
2326	1.51324	0.326605
2327	0.758454	0.279731
2328	1.28632	0.252507
2329	0.758482	0.199814
2330	0.758482	0.230652
2331	0.985372	0.29723
2332	1.14105	0.139456
2333	1.59787	0.326592
2334	2.46244	0.491837
2335	1.70367	0.19683
2336	2.13757	1.15298
2337	1.98142	1.1725
2338	1.35878	0.115931
2339	2.39947	0.144002
2340	1.24554	0.12119
2341	1.7522	0.124234
2342	1.49693	1.77505
2343	1.49652	1.92603
2344	1.6211	1.7425
2345	1.45938	1.8795
2346	0.737431	1.97089
2347	1.18643	1.89516
2348	1.04072	2.09555
2349	0.9459	1.80889
2350	0.66364	1.97752
2351	0.985109	1.5317
2352	0.638368	1.91871
2353	0.83804	1.42334
2354	0.998024	1.68112
2355	0.914471	1.3521
2356	1.14281	1.75657
2357	0.833972	1.52078
2358	0.991865	0.452971
2359	1.42208	0.457394
2360	1.70718	2.1
2361	1.79888	2.1
2362	1.16851	0.484
2363	1.16851	0.433411
2364	1.51021	0.452505
2365	0.946132	0.326258
2366	1.14897	0.27302
2367	0.946132	0.342535
2368	1.77177	0.370121
2369	1.77177	0.283915
2370	1.33038	0.311113
2371	1.38752	0.316903
2372	1.1726	0.360302
2373	1.51688	0.356406
2374	1.06455	2.1
2375	0.98511	2.1

2376	1.31144	2.1
2377	1.79825	2.1
2378	1.79825	2.1
2379	1.73044	2.1
2380	1.59589	0.0956873
2381	1.59589	0.0939936
2382	2.78009	2.1
2383	1.68761	2.1
2384	3.05511	2.1
2385	2.25318	2.1
2386	0.578998	2.1
2387	1.03512	2.1
2388	2.26156	2.1
2389	1.44859	1.02782
2390	1.35897	1.27366
2391	1.18301	1.7249
2392	1.25173	1.69323
2393	1.98851	2.1
2394	2.70685	2.1
2395	0.682342	2.1
2396	1.52491	2.1
2397	1.37119	2.1
2398	0.682313	2.1
2399	1.35342	2.1
2400	0.947555	2.1
2401	0.614107	2.1
2402	1.23007	2.1
2403	1.5735	2.1
2404	1.58713	2.1
2405	1.57979	0.0766245
2406	1.30723	0.0759969
2407	0.935658	0.801099
2408	0.8952	0.765356
2409	1.27352	1.08568
2410	1.0985	0.901147
2411	1.07163	0.753608
2412	1.15823	0.93199
2413	0.836451	0.712638
2414	2.08051	0.596458
2415	1.92601	1.10547
2416	2.0644	0.709231
2417	1.37259	2.1
2418	1.37259	2.1
2419	0.752217	2.1
2420	1.28569	2.1
2421	0.752186	2.1
2422	0.839124	2.1
2423	1.28568	2.1
2424	1.04199	2.1
2425	1.04199	2.1
2426	2.59268	2.1
2427	1.04199	2.1
2428	1.5098	2.1
2429	1.5098	2.1
2430	0.520997	2.1
2431	1.29634	2.1
2432	0.520996	2.1
2433	0.73446	2.1

2434	1.29634	2.1
2435	0.983724	2.1
2436	1.23914	2.1
2437	2.65095	1.99544
2438	1.52851	2.1
2439	2.399	2.1
2440	1.68137	2.1
2441	1.11285	2.1
2442	1.04921	2.1
2443	1.30711	2.1
2444	1.04921	2.1
2445	1.07496	0.989651
2446	1.55831	0.654189
2447	1.07497	0.45784
2448	1.07497	0.76881
2449	1.27391	2.1
2450	2.5615	1.88386
2451	1.2739	2.1
2452	1.75253	1.7273
2453	1.12907	0.296329
2454	1.9194	0.292866
2455	1.96617	0.214438
2456	1.01225	1.80438
2457	0.947979	1.37771
2458	1.01225	0.849818
2459	1.06561	1.85827
2460	1.01225	1.13541
2461	3.56876	2.1
2462	3.68767	2.1
2463	1.52301	2.1
2464	1.61013	2.1
2465	1.43726	2.1
2466	2.04275	2.1
2467	0.995734	2.1
2468	1.16199	2.1
2469	1.73704	0.147356
2470	1.04019	0.183883
2471	0.956294	2.1
2472	1.81488	2.1
2473	1.63719	2.1
2474	1.50103	0.0848071
2475	1.75289	0.0954301
2476	1.31491	0.0922511
2477	1.03567	0.0905552
2478	0.672257	0.0915776
2479	1.04072	0.115944
2480	0.746903	0.104821
2481	1.43317	0.0975373
2482	1.39986	0.0897804
2483	1.06385	0.103614
2484	1.1624	0.107512
2485	1.61667	0.0808185
2486	1.27258	0.07863
2487	2.559	0.0816949
2488	1.66093	0.0780435
2489	1.10719	0.608422
2490	2.03235	0.741397
2491	1.17105	1.02185

2492	1.10719	0.750474
2493	1.3028	2.1
2494	0.946131	1.9827
2495	0.946127	1.93249
2496	1.52912	2.1
2497	1.52912	2.1
2498	0.946128	1.38881
2499	0.949997	1.77991
2500	1.31565	2.1
2501	1.46643	2.1
2502	1.5482	2.1
2503	1.47102	2.1
2504	1.47102	2.1
2505	0.774101	2.1
2506	1.30583	2.1
2507	0.774103	2.1
2508	0.939287	2.1
2509	1.30584	2.1
2510	0.958661	2.1
2511	2.28835	2.1
2512	1.43213	0.145114
2513	2.13255	0.182454
2514	1.43213	0.141685
2515	1.10476	0.137381
2516	1.93165	0.174432
2517	1.03235	0.886718
2518	1.31	0.901473
2519	1.22082	2.1
2520	1.35827	2.1
2521	0.790936	2.1
2522	0.716644	2.1
2523	1.43257	2.1
2524	1.43257	2.1
2525	0.716615	2.1
2526	0.946131	2.1
2527	0.730157	2.1
2528	1.47212	2.1
2529	1.47212	2.1
2530	0.946131	2.1
2531	0.790792	2.1
2532	1.41149	2.1
2533	0.790791	2.1
2534	1.01006	2.1
2535	1.53045	0.509747
2536	1.53045	0.625606
2537	0.946132	0.909552
2538	1.40779	0.563319
2539	1.06879	1.09122
2540	0.990713	0.739513
2541	1.06879	0.793464
2542	1.06486	0.403685
2543	1.18347	0.398288
2544	1.38622	0.433806
2545	1.18347	0.320275
2546	1.7304	0.381351
2547	1.7304	0.477754
2548	0.946131	0.675348
2549	1.26874	0.475988

2550	1.13337	0.936755
2551	1.13337	0.614981
2552	1.40779	0.62395
2553	1.695 2.1	
2554	1.77071	2.1
2555	0.649313	2.1
2556	0.692829	2.1
2557	1.40395	2.1
2558	1.34243	2.1
2559	0.813292	0.287573
2560	0.632691	0.341
2561	1.75766	0.437651
2562	2.25727	0.70159
2563	1.74206	0.284073
2564	0.826073	0.365928
2565	1.30022	0.380726
2566	1.45641	0.331565
2567	0.946131	0.351643
2568	0.752245	0.342023
2569	0.960952	0.381005
2570	0.743477	0.316163
2571	1.04355	0.315598
2572	1.6253	0.289973
2573	0.960577	0.298091
2574	1.34852	0.915031
2575	1.03235	0.950668
2576	1.78168	0.660025
2577	1.2966	0.114815
2578	2.21116	0.105142
2579	1.6395	0.112396
2580	1.6395	0.107752
2581	0.94613	0.143448
2582	1.22187	0.127241
2583	0.962737	0.146649
2584	0.962735	0.130574
2585	1.36376	0.134502
2586	1.2966	0.0967656
2587	1.2966	0.0978215
2588	1.19669	1.02506
2589	2.37636	1.0647
2590	1.08894	1.0351
2591	1.62551	1.35727
2592	2.51472	1.14695
2593	1.89226	0.962377
2594	0.980038	1.06307
2595	1.10187	0.638767
2596	1.01479	0.644162
2597	1.81663	2.1
2598	1.83247	2.1
2599	0.921851	0.543745
2600	0.983148	2.1
2601	1.82816	2.1
2602	1.26103	2.1
2603	2.34818	2.1
2604	1.23152	2.1
2605	2.15738	2.1
2606	1.23152	2.1
2607	1.51042	2.1

2608	0.842995	2.1
2609	1.22257	2.1
2610	0.810959	2.1
2611	1.68242	2.1
2612	0.95701	2.1
2613	1.12389	2.1
2614	1.95255	2.1
2615	1.57974	2.1
2616	1.94632	2.1
2617	0.81534	2.1
2618	1.13996	2.1
2619	1.18958	0.0984652
2620	1.25929	0.0880497
2621	3.02933	0.570139
2622	2.37434	0.251847
2623	1.10609	0.551211
2624	1.64132	0.481868
2625	1.64132	0.484302
2626	0.946132	0.416722
2627	1.40029	0.390624
2628	1.08946	0.341596
2629	1.08946	0.391453
2630	1.18717	0.223714
2631	2.71421	0.226518
2632	1.08189	0.336634
2633	0.94613	0.320459
2634	1.37109	0.340798
2635	1.17925	0.250701
2636	0.851541	0.33619
2637	0.942512	0.328047
2638	1.18541	0.303362
2639	0.851526	0.342575
2640	1.0819	0.306006
2641	1.86009	0.360704
2642	0.946132	0.276861
2643	1.67409	0.183564
2644	1.67409	0.303306
2645	0.946131	0.305597
2646	1.31716	0.271396
2647	1.30307	0.25829
2648	0.946131	0.313145
2649	1.18601	0.261015
2650	3.15646	2.1
2651	0.985109	2.1
2652	3.37048	2.1
2653	1.10667	2.1
2654	1.77065	1.04811
2655	1.45837	0.898092
2656	1.77063	0.921307
2657	2.58104	0.86213
2658	1.37652	1.56994
2659	1.54168	1.44495
2660	1.00224	0.0853292
2661	1.05851	0.0851994
2662	1.39093	0.0795392
2663	1.43358	0.085231
2664	0.575741	0.0813842
2665	0.518161	0.0782175

2666	1.04071	0.0866245
2667	1.13092	1.15952
2668	1.17042	1.6331
2669	1.53347	1.41622
2670	1.52938	1.2213
2671	1.00613	0.386712
2672	1.81618	0.549503
2673	1.81618	0.386228
2674	0.946127	0.558878
2675	1.36479	0.533735
2676	0.94613	0.380183
2677	1.31724	0.452928
2678	1.3648	0.450481
2679	1.00613	0.385531
2680	0.781375	0.294705
2681	1.13431	0.352023
2682	1.03547	0.365646
2683	1.40668	0.392843
2684	0.94613	0.386506
2685	0.713285	0.42285
2686	1.72885	0.440853
2687	1.72885	0.420205
2688	0.713298	0.3742
2689	3.30075	0.307525
2690	2.81337	0.3755
2691	1.39296	0.488993
2692	0.830164	1.08491
2693	1.50493	2.1
2694	1.50494	2.1
2695	0.830136	0.980974
2696	1.28298	2.1
2697	1.0521	2.09955
2698	0.830147	2.1
2699	2.0799	1.31006
2700	1.52115	1.01038
2701	2.5142	1.00086
2702	1.31253	0.816131
2703	1.94446	1.1599
2704	1.18817	1.12362
2705	0.598348	1.61063
2706	1.69315	1.23177
2707	1.69315	1.50056
2708	0.598377	0.824946
2709	0.598347	0.954327
2710	0.94613	0.825345
2711	1.28418	0.886405
2712	2.05205	0.987831
2713	1.28418	1.4933
2714	1.0737	2.1
2715	1.81024	2.1
2716	2.73834	2.1
2717	1.71369	2.1
2718	1.71368	2.1
2719	0.946118	2.1
2720	1.29063	2.1
2721	1.36917	2.1
2722	0.94613	2.1
2723	1.29062	2.1

2724	0.947921	1.93826
2725	1.58661	0.724168
2726	1.37658	1.32518
2727	1.2364	1.27713
2728	1.66685	0.851813
2729	1.66685	1.51279
2730	0.946133	0.801248
2731	0.946133	1.08413
2732	1.11978	1.2253
2733	1.09194	0.992495
2734	1.21858	1.53582
2735	1.37658	1.1947
2736	1.64903	0.719621
2737	1.64903	1.8003
2738	0.946132	0.801563
2739	0.946132	1.11343
2740	1.05496	0.96094
2741	1.22851	0.72311
2742	1.22852	1.65368
2743	1.58482	0.677194
2744	1.60578	0.143058
2745	1.06336	0.130979
2746	1.68062	0.139847
2747	1.14721	0.115352
2748	1.11924	0.0783804
2749	1.18421	0.0856439
2750	2.01276	0.0831861
2751	1.23502	1.77045
2752	1.34926	1.62912
2753	0.546751	1.28834
2754	0.790446	1.70237
2755	1.04072	1.27725
2756	0.555868	1.22499
2757	0.930305	1.20985
2758	1.09344	1.3819
2759	1.18351	1.60624
2760	1.97215	1.28798
2761	1.07416	1.68762
2762	1.33204	1.21747
2763	1.22038	1.15729
2764	0.597449	0.931558
2765	0.821422	1.33928
2766	1.04071	1.11772
2767	0.608026	0.818939
2768	0.929427	1.03838
2769	1.64929	0.0778591
2770	1.68541	0.074013
2771	0.496093	0.102451
2772	1.3385	0.0859968
2773	0.790798	0.0859255
2774	1.22971	0.0857369
2775	0.898853	0.0872442
2776	2.14361	0.0843889
2777	2.10813	0.0832203
2778	1.05417	0.0972894
2779	1.80768	0.143658
2780	1.09725	2.1
2781	1.41131	2.1

2782	1.1656	2.1
2783	0.946129	2.1
2784	1.68365	2.1
2785	1.68365	2.1
2786	1.1656	2.1
2787	0.94613	2.1
2788	1.21269	0.0714577
2789	1.48867	0.0753254
2790	1.27806	0.0720157
2791	1.68264	0.075775
2792	1.37813	1.54329
2793	1.50743	1.15787
2794	1.04121	0.988722
2795	1.12434	0.66173
2796	1.39553	0.608315
2797	1.44721	1.39828
2798	0.946132	0.676889
2799	1.23943	0.702132
2800	1.05292	0.80721
2801	1.28607	0.687164
2802	1.16915	0.72986
2803	1.76306	0.892293
2804	1.42266	0.978652
2805	1.36844	2.1
2806	1.36844	2.1
2807	1.67352	2.1
2808	1.11256	0.992592
2809	1.03217	0.92031
2810	1.18422	0.0906726
2811	1.04159	0.0974245
2812	1.60139	0.0890891
2813	1.04159	0.0927955
2814	0.988805	2.1
2815	1.05481	2.1
2816	1.52938	1.52842
2817	1.94724	1.36356
2818	0.896336	0.13821
2819	0.911484	0.149699
2820	1.18315	0.0984848
2821	1.10515	0.115375
2822	1.04067	0.0998766
2823	1.12758	0.130835
2824	0.904106	0.131301
2825	1.6901	0.0983532
2826	1.68671	0.100797
2827	1.99908	0.112004
2828	0.772426	0.810564
2829	0.810945	1.55108
2830	0.667562	1.10858
2831	0.496617	0.528086
2832	0.847689	0.957491
2833	0.914378	1.31106
2834	0.934003	0.733518
2835	1.07459	0.983126
2836	1.40225	2.1
2837	1.07043	2.1
2838	0.94613	2.1
2839	1.63072	2.1

2840	1.63072	2.1
2841	1.07043	2.1
2842	0.946131	2.1
2843	1.53082	2.1
2844	1.69916	2.1
2845	1.75211	0.546574
2846	1.96831	1.51544
2847	1.75211	0.697938
2848	0.946127	0.718961
2849	0.817065	0.743313
2850	1.41798	0.740835
2851	1.41798	0.630811
2852	0.791656	0.729348
2853	0.791656	0.760426
2854	1.41798	0.708335
2855	1.58331	0.704264
2856	1.41776	0.601074
2857	0.791647	0.596411
2858	1.4512	0.562419
2859	1.45119	0.573845
2860	0.75821	0.642181
2861	0.758248	0.630355
2862	0.946104	0.590601
2863	1.36482	0.603451
2864	1.29389	1.9244
2865	1.04164	1.45103
2866	1.40574	1.50244
2867	1.64833	1.29864
2868	1.71183	0.286816
2869	1.71183	0.35265
2870	0.946131	0.600768
2871	1.35721	0.414634
2872	0.946131	0.792911
2873	0.946131	0.623892
2874	1.30076	0.59606
2875	1.2474	0.327211
2876	2.85225	0.490842
2877	2.71441	0.350877
2878	1.2474	0.260842
2879	1.38622	0.449741
2880	0.858359	0.283632
2881	1.3682	0.315885
2882	1.57364	0.267275
2883	0.729695	0.456632
2884	0.729723	0.255847
2885	0.822611	0.602765
2886	0.946133	0.462048
2887	0.729723	0.323392
2888	0.974704	0.627736
2889	0.974702	0.639926
2890	1.81603	2.1
2891	1.96348	2.1
2892	1.43612	0.872632
2893	1.43612	0.982226
2894	0.634295	0.890576
2895	0.93796	1.18547
2896	0.946133	1.014
2897	0.634326	0.739603

2898	0.946133	0.866969
2899	1.2654	0.647957
2900	1.26865	0.917199
2901	2.2649	0.814079
2902	1.00864	0.850021
2903	1.09689	0.64388
2904	0.832021	0.65597
2905	1.53229	0.613902
2906	1.1145	0.652257
2907	0.929583	0.6547
2908	1.613	0.587519
2909	0.644729	0.642563
2910	1.03471	0.971688
2911	2.7739	2.1
2912	1.36133	0.980713
2913	2.50215	2.1
2914	2.52195	1.8635
2915	1.0465	2.1
2916	1.42673	2.1
2917	1.42673	2.1
2918	0.946132	2.1
2919	1.31904	2.1
2920	0.986499	2.1
2921	0.946132	2.1
2922	1.31904	2.1
2923	0.966978	2.1
2924	1.42237	0.148868
2925	2.60925	0.180321
2926	0.753659	0.209336
2927	1.77241	0.159153
2928	1.77241	0.187682
2929	0.946129	0.223072
2930	0.75366	0.15815
2931	1.22144	0.210384
2932	0.94613	0.222131
2933	1.50732	0.157515
2934	1.18514	0.175602
2935	0.902187	0.196245
2936	1.89858	0.137555
2937	1.24294	0.160127
2938	1.67211	0.172375
2939	1.41786	0.145711
2940	0.810959	0.195984
2941	1.50732	0.160362
2942	2.44288	0.192745
2943	1.40969	0.101932
2944	2.06654	0.115388
2945	1.40969	0.103588
2946	1.40882	0.128982
2947	1.94063	0.137124
2948	0.867921	0.10405
2949	1.29289	0.109272
2950	0.867918	0.104483
2951	0.946128	0.113786
2952	0.867944	0.113555
2953	1.66589	0.109395
2954	1.66589	0.100399
2955	0.968595	2.1

2956	1.5534	0.859118
2957	1.27793	2.1
2958	1.13433	2.1
2959	1.3439	0.0749681
2960	2.53709	0.082749
2961	0.571998	0.0715859
2962	0.64837	0.0749537
2963	1.99755	0.0730606
2964	1.99854	0.0765109
2965	0.764229	1.99123
2966	1.61842	2.1
2967	1.61842	2.1
2968	1.18315	2.1
2969	1.1995	2.1
2970	1.18315	2.1
2971	0.764256	2.1
2972	1.52364	2.1
2973	2.36631	2.1
2974	0.641912	1.42758
2975	1.7181	2.1
2976	1.459 2.1	
2977	0.634845	1.33068
2978	1.18315	2.1
2979	1.17615	2.00134
2980	0.571367	2.03226
2981	1.3324	0.25365
2982	0.798214	0.262695
2983	0.946128	0.282181
2984	0.627252	0.233942
2985	1.50337	0.250766
2986	1.50337	0.208065
2987	0.62725	0.273113
2988	1.07763	0.208984
2989	1.07763	0.239484
2990	2.16109	0.293403
2991	1.07763	0.225143
2992	1.12486	2.1
2993	1.11827	2.1
2994	1.0491	2.1
2995	1.09034	2.1
2996	1.09431	2.1
2997	0.946131	2.1
2998	0.748607	2.1
2999	1.44002	2.1
3000	1.44002	2.1
3001	0.748635	2.1
3002	0.782505	2.1
3003	1.40615	2.1
3004	1.19539	2.1
3005	1.29389	1.87919
3006	1.4867	1.12205
3007	1.50103	0.0836167
3008	1.63025	0.0844079
3009	2.72109	0.100085
3010	0.915755	0.0851808
3011	0.946132	0.109511
3012	0.915755	0.105049
3013	1.70236	0.0913804

3014	1.70236	0.0817757
3015	1.26155	0.099258
3016	1.35657	0.103938
3017	1.83151	0.0854648
3018	1.83151	0.0861628
3019	2.52309	0.0993591
3020	0.983607	2.1
3021	1.06838	0.587413
3022	0.983609	0.863632
3023	0.983596	0.83224
3024	2.74384	0.289122
3025	1.6261	0.24316
3026	1.15477	2.1
3027	1.02136	2.1
3028	1.83561	2.1
3029	1.83561	2.1
3030	0.946131	1.82098
3031	1.39523	2.1
3032	1.29313	0.704532
3033	1.29313	1.74242
3034	1.38651	1.53058
3035	1.06313	2.1
3036	0.974888	2.1
3037	1.0894	2.1
3038	0.974888	2.1
3039	1.82282	2.1
3040	1.82282	2.1
3041	0.946131	2.1
3042	1.37372	2.1
3043	1.32374	1.8754
3044	1.32374	2.1
3045	1.39523	2.1
3046	1.40882	0.131811
3047	0.861998	0.128833
3048	1.24448	0.13876
3049	0.861999	0.128895
3050	0.946129	0.153568
3051	0.862011	0.15163
3052	1.67537	0.140977
3053	1.67537	0.124905
3054	1.72399	0.0954346
3055	2.58579	0.10154
3056	1.72399	0.0948772
3057	1.50951	0.109397
3058	2.48897	0.124388
3059	1.26755	2.1
3060	1.03824	2.1
3061	1.27875	2.1
3062	1.48901	2.1
3063	1.18902	2.1
3064	1.11764	2.1
3065	1.87436	2.1
3066	0.95345	1.89592
3067	0.724191	1.37993
3068	0.86668	1.35714
3069	0.811523	1.53818
3070	1.04074	0.994743
3071	0.843097	1.23287

3072	0.833434	1.44646
3073	0.965727	1.24973
3074	0.85346	1.44024
3075	1.00185	0.805144
3076	1.00184	1.24594
3077	0.980651	1.4251
3078	0.980664	1.22776
3079	0.475737	0.947275
3080	0.719696	1.0095
3081	0.946109	0.962723
3082	0.946116	1.08385
3083	0.719693	0.95003
3084	0.946117	1.07838
3085	1.14879	1.38552
3086	0.719683	1.29538
3087	0.900977	0.998522
3088	0.972364	1.159
3089	1.22961	1.11423
3090	0.405384	0.993997
3091	1.34406	2.1
3092	1.2187	2.1
3093	0.963571	0.403059
3094	0.946131	0.403597
3095	1.57246	0.694118
3096	1.57246	1.96911
3097	0.946131	0.730625
3098	1.1235	0.932024
3099	1.1235	1.00455
3100	1.07439	1.06953
3101	1.19612	1.01665
3102	1.24844	0.974774
3103	1.00007	1.27524
3104	1.02429	2.1
3105	1.10459	2.1
3106	1.06724	0.39736
3107	0.67519	0.5985
3108	0.49762	0.410443
3109	1.43435	0.324869
3110	0.833315	0.46161
3111	0.58135	0.445948
3112	1.14749	0.748165
3113	1.85091	0.992147
3114	1.2802	0.65107
3115	1.27737	0.676173
3116	2.3655	0.691036
3117	1.30584	2.1
3118	2.06424	2.1
3119	0.946131	0.276104
3120	1.04257	0.278246
3121	0.94613	0.26348
3122	1.67201	0.309512
3123	1.67201	0.234229
3124	1.27696	0.26696
3125	1.34118	0.198216
3126	1.3413	0.132122
3127	2.36238	0.115927
3128	0.6036	0.160835
3129	1.11745	0.164682

3130	1.46126	0.134309
3131	1.39612	0.11699
3132	0.670678	0.16316
3133	1.03402	0.171785
3134	1.11271	0.157898
3135	0.838028	0.124078
3136	0.807019	0.162583
3137	1.27787	0.10791
3138	0.994705	0.124373
3139	1.20014	0.109976
3140	0.964793	0.148213
3141	0.863868	0.138077
3142	1.3413	0.113633
3143	1.79549	0.1195
3144	1.35877	2.1
3145	1.63648	1.56404
3146	1.08171	2.1
3147	1.27839	2.1
3148	1.52601	2.1
3149	1.27841	2.1
3150	1.71255	2.1
3151	2.16307	2.1
3152	1.91592	2.1
3153	1.78508	2.1
3154	0.946128	2.1
3155	1.41199	2.1
3156	0.946128	2.1
3157	0.901088	2.1
3158	1.55244	2.1
3159	1.59475	2.1
3160	1.40921	2.1
3161	0.960106	2.1
3162	1.40921	2.1
3163	2.0073	2.1
3164	2.2973	0.435263
3165	1.90705	0.429953
3166	1.53671	0.291194
3167	1.86035	0.288683
3168	1.33747	2.05037
3169	1.14007	2.1
3170	1.3212	2.1
3171	1.66867	0.265341
3172	1.30203	0.221968
3173	1.27696	2.1
3174	1.05025	2.1
3175	1.05025	2.1
3176	1.3704	2.1
3177	1.15677	2.1
3178	0.946129	2.1
3179	1.65879	2.1
3180	1.65879	2.1
3181	1.15676	2.1
3182	0.94613	2.1
3183	0.867217	1.13496
3184	1.28684	0.95279
3185	1.58866	1.04916
3186	1.58866	0.766463
3187	0.867246	1.46582

3188	1.28684	0.847355
3189	1.16906	1.10775
3190	1.13438	1.05284
3191	1.37224	1.13357
3192	1.61519	1.19351
3193	0.883939	1.54364
3194	1.40092	1.06931
3195	1.80383	1.66595
3196	1.80383	1.40453
3197	0.883935	1.47539
3198	1.40092	0.977609
3199	1.28684	1.49138
3200	1.76788	0.84358
3201	2.57368	1.02892
3202	1.37224	1.72112
3203	1.65411	0.800801
3204	1.77042	2.1
3205	1.77042	2.1
3206	1.90446	2.1
3207	1.28667	2.1
3208	1.55635	2.1
3209	1.30646	0.997057
3210	2.31836	1.27254
3211	1.30645	0.953269
3212	1.86429	1.19416
3213	1.48676	0.285244
3214	0.743379	0.359778
3215	1.41848	0.363084
3216	1.41848	0.331054
3217	0.946131	0.368859
3218	0.812649	0.352497
3219	1.34921	0.347162
3220	0.812649	0.325194
3221	0.949366	0.296759
3222	1.32974	0.291748
3223	0.794204	0.0522355
3224	0.634352	2.1
3225	0.838025	2.1
3226	1.08802	1.93352
3227	0.838038	1.87109
3228	1.18314	2.1
3229	1.12495	2.1
3230	1.22774	2.06618
3231	1.04343	2.04421
3232	0.921111	0.072766
3233	2.1609	0.0700808
3234	1.09987	0.0681948
3235	2.5043	0.0657812
3236	1.05492	0.0646332
3237	0.590344	0.0674743
3238	1.05492	0.0654634
3239	1.25215	0.0651527
3240	1.71674	0.0619163
3241	1.71674	0.062016
3242	0.590331	0.0662814
3243	1.17078	0.0639978
3244	0.625542	0.0658007
3245	0.724673	2.1

3246	1.08011	2.1
3247	1.87598	2.1
3248	1.50738	2.1
3249	1.44951	0.245191
3250	1.50102	0.242583
3251	1.8042	0.467967
3252	0.687769	0.600983
3253	1.66483	0.490496
3254	1.66483	0.539723
3255	0.946132	0.602134
3256	0.687798	0.504787
3257	1.20885	0.61886
3258	0.687799	0.570338
3259	0.886063	0.432446
3260	0.798751	0.438814
3261	0.584813	0.406403
3262	1.49483	0.277393
3263	0.888005	0.319702
3264	1.03589	0.334931
3265	1.70435	0.350623
3266	0.710414	2.03301
3267	0.917609	0.951349
3268	0.810947	0.974464
3269	0.81096	0.994892
3270	1.48658	1.12309
3271	1.53653	1.08603
3272	1.19329	1.05463
3273	0.946153	1.0726
3274	1.50798	1.06352
3275	1.85017	2.1
3276	1.19768	2.1
3277	0.864304	1.52292
3278	0.792906	1.06348
3279	0.989025	1.07614
3280	1.19768	1.31339
3281	1.54548	1.16331
3282	1.18798	1.40521
3283	1.31875	0.996094
3284	1.05725	1.1312
3285	2.17365	0.0865547
3286	2.48106	0.0790261
3287	1.87451	0.0923205
3288	1.27189	1.06769
3289	2.79399	1.62581
3290	0.859565	1.16262
3291	0.926174	1.0201
3292	0.604744	0.992569
3293	1.41832	0.662476
3294	1.05494	0.845918
3295	0.874749	0.891936
3296	1.50067	0.769637
3297	0.917817	1.22264
3298	0.63591	1.20373
3299	1.72849	1.1142
3300	1.72849	0.836962
3301	0.635943	0.9393
3302	1.39699	1.16801
3303	0.967411	1.60103

3304	0.635943	1.02751
3305	1.39908	0.974501
3306	1.26971	1.11015
3307	1.02969	1.03123
3308	1.15326	1.82652
3309	2.30741	1.40494
3310	0.93545	1.12914
3311	0.802747	0.847092
3312	1.15466	1.20903
3313	0.859164	1.38152
3314	1.02616	1.30438
3315	1.23149	1.11549
3316	0.83804	0.923361
3317	1.13266	1.60618
3318	1.64142	1.64503
3319	1.21223	2.1
3320	1.43051	2.1
3321	1.32418	0.893851
3322	0.621319	0.791737
3323	1.47134	1.47901
3324	1.79103	0.637934
3325	3.25951	0.138602
3326	3.16764	0.110503
3327	1.65583	0.148375
3328	1.4732	0.155573
3329	1.76476	2.1
3330	1.80114	0.957726
3331	0.754121	1.60962
3332	0.94613	0.740788
3333	0.754119	1.6529
3334	1.45636	1.79548
3335	1.45636	0.644662
3336	1.30029	0.943346
3337	0.910183	0.979192
3338	1.29099	1.23514
3339	1.08039	2.05173
3340	1.05498	1.91992
3341	1.24445	1.73223
3342	1.2873	2.1
3343	0.987703	1.6091
3344	0.964782	1.3756
3345	1.73645	0.136137
3346	0.871279	0.168873
3347	0.884744	0.189875
3348	1.85193	0.131815
3349	1.68681	0.152742
3350	1.33689	0.166142
3351	1.23493	0.13796
3352	0.892482	0.183991
3353	2.95185	0.147504
3354	2.10496	0.148536
3355	1.87334	2.1
3356	0.94411	2.1
3357	0.754196	2.1
3358	1.43122	2.1
3359	1.58684	2.1
3360	0.838027	2.1
3361	1.1536	2.1

3362	1.24878	2.1
3363	1.23125	2.1
3364	2.86862	2.1
3365	1.57636	2.1
3366	0.998451	0.642899
3367	1.5176	0.600214
3368	1.13271	0.109667
3369	1.60248	0.107879
3370	1.22083	2.1
3371	1.79996	2.1
3372	1.19087	2.1
3373	2.00342	2.1
3374	1.3715	2.1
3375	2.79227	2.1
3376	2.7873	2.1
3377	0.987265	0.0668964
3378	1.62715	2.09916
3379	1.70117	1.5325
3380	1.27806	0.0686658
3381	1.45246	2.1
3382	1.45244	2.1
3383	1.87214	2.1
3384	0.995796	2.1
3385	1.68189	0.575671
3386	1.49155	1.19661
3387	1.58054	2.1
3388	1.59169	2.1
3389	0.736364	0.0779762
3390	0.70236	0.0766616
3391	2.24367	0.0835995
3392	2.57085	0.0866485
3393	1.70952	0.0800273
3394	2.60516	0.0803657
3395	1.45158	2.1
3396	1.00371	2.1
3397	1.65748	2.1
3398	1.23706	0.413165
3399	1.55834	0.68308
3400	1.72671	0.625034
3401	0.597656	0.548318
3402	1.39137	0.610243
3403	0.855578	0.37342
3404	1.48398	0.520764
3405	0.745117	0.461991
3406	1.23706	0.711717
3407	1.57916	0.490733
3408	0.893025	0.411767
3409	3.22433	0.494229
3410	2.47324	1.01272
3411	1.63852	2.1
3412	1.63852	2.1
3413	0.946132	2.1
3414	1.19095	2.1
3415	0.946133	2.1
3416	1.16684	2.1
3417	1.19095	2.1
3418	1.06698	0.325089
3419	1.48692	0.310535

3420	1.08334	0.819793
3421	1.07283	2.1
3422	1.28517	0.831635
3423	1.2746	0.0740416
3424	1.30723	0.0760306
3425	2.02069	0.0764125
3426	1.05579	0.0745249
3427	1.59475	2.1
3428	2.40113	2.1
3429	1.58875	2.1
3430	1.6881	2.1
3431	1.6881	2.1
3432	0.819953	2.1
3433	0.819954	2.1
3434	0.946131	2.1
3435	0.946131	2.1
3436	1.34782	2.1
3437	1.65619	2.1
3438	1.65619	2.05614
3439	0.79437	2.1
3440	1.25 2.1	
3441	0.94613	2.1
3442	0.946131	2.04628
3443	0.794371	2.1
3444	1.58875	2.1
3445	2.5 2.1	
3446	1.04147	2.1
3447	0.946131	0.734743
3448	1.79686	2.02914
3449	1.79686	2.1
3450	1.25144	0.754297
3451	1.35714	1.32508
3452	1.38585	1.33571
3453	1.25144	1.9056
3454	1.15564	1.86426
3455	1.34389	1.42868
3456	0.946133	1.03534
3457	1.74353	1.76031
3458	1.74353	2.1
3459	1.14553	0.974075
3460	1.38585	0.947536
3461	1.30382	1.11459
3462	1.14553	1.72871
3463	1.15564	1.54508
3464	1.90618	2.1
3465	2.11056	2.1
3466	1.032 2.1	
3467	0.953636	2.1
3468	1.23131	1.19216
3469	1.39763	1.27498
3470	2.01024	0.951345
3471	1.9186	0.956723
3472	1.83658	0.19047
3473	1.81656	0.183405
3474	2.2049	0.220802
3475	2.16081	0.194648
3476	1.12219	0.216618
3477	0.599771	0.253842

3478	1.61401	0.180225
3479	1.05912	0.217156
3480	0.951648	0.215788
3481	1.89328	0.181745
3482	0.913682	0.243889
3483	1.00763	0.194385
3484	1.29129	2.1
3485	1.37138	2.1
3486	1.41986	0.133913
3487	1.41986	0.13781
3488	0.944166	0.199408
3489	1.41789	0.159201
3490	0.944167	0.201956
3491	0.944166	0.154184
3492	0.946132	0.152953
3493	1.98713	0.117025
3494	1.23626	0.117565
3495	0.932791	0.148167
3496	0.810251	0.11146
3497	0.986748	0.119238
3498	0.898155	0.144478
3499	0.92624	0.120162
3500	1.1349	0.114297
3501	0.934572	0.131129
3502	1.23104	1.14964
3503	1.23104	1.17729
3504	0.799994	0.511478
3505	0.94613	0.946485
3506	0.802822	0.374478
3507	1.40944	1.09831
3508	0.800035	0.775339
3509	1.41223	0.837794
3510	1.41223	0.860374
3511	1.6	0.636201
3512	1.60565	0.576607
3513	1.23042	2.1
3514	1.0645	1.99737
3515	1.00319	2.1
3516	1.29435	2.1
3517	1.2243	2.1
3518	1.2243	2.1
3519	1.05655	1.32592
3520	1.29764	1.47763
3521	2.55252	2.1
3522	1.15676	2.1
3523	0.855295	2.1
3524	0.810959	2.1
3525	1.65857	2.1
3526	1.00849	2.0629
3527	1.16021	2.1
3528	1.75869	2.1
3529	1.13775	2.1
3530	1.20091	2.1
3531	1.61582	2.1
3532	1.88031	2.1
3533	0.968536	2.1
3534	0.946131	2.1
3535	1.61481	2.1

3536	1.61481	2.1
3537	1.09547	2.1
3538	1.18486	2.1
3539	1.37609	2.1
3540	1.09546	2.1
3541	1.02461	2.1
3542	1.83223	2.1
3543	1.74002	2.1
3544	1.70939	1.59755
3545	1.7094	2.1
3546	0.726287	2.06965
3547	1.01712	1.51326
3548	1.30318	2.1
3549	0.946131	2.1
3550	0.87155	2.1
3551	1.77093	1.71416
3552	2.02356	1.72552
3553	1.32449	2.1
3554	1.10003	2.1
3555	1.5161	2.1
3556	1.5161	2.1
3557	0.946132	2.1
3558	1.09206	2.1
3559	0.946132	2.1
3560	1.04404	2.1
3561	1.09206	2.1
3562	0.635628	1.07513
3563	1.14623	0.660764
3564	1.73197	0.715096
3565	1.73197	1.01958
3566	0.529655	0.711345
3567	1.14623	0.867093
3568	0.992085	0.807407
3569	1.16915	1.38529
3570	2.29245	0.593897
3571	0.584573	0.920841
3572	0.956756	0.677092
3573	1.52471	0.669855
3574	1.51409	1.28424
3575	0.584575	0.754374
3576	0.94613	0.844545
3577	1.14622	0.612778
3578	1.28096	0.985768
3579	1.34695	1.22641
3580	1.04193	2.1
3581	1.05378	2.1
3582	0.798209	2.1
3583	1.32007	1.76918
3584	1.64327	0.0956395
3585	2.64674	1.9914
3586	2.38936	0.185873
3587	2.88475	0.174902
3588	1.07745	1.62677
3589	1.08911	1.74458
3590	1.49127	2.1
3591	1.49127	2.1
3592	0.709714	2.1
3593	1.37199	2.1

3594	0.82899	2.1
3595	0.709714	2.1
3596	0.946132	2.1
3597	1.00133	2.1
3598	1.40182	2.1
3599	1.53278	2.1
3600	1.40182	2.1
3601	1.31764	0.0863835
3602	1.00691	0.0878278
3603	1.24094	0.092121
3604	0.64868	0.0868754
3605	0.946133	0.095847
3606	0.64868	0.095381
3607	1.71762	0.0914822
3608	1.71762	0.0856557
3609	1.41662	0.0795722
3610	0.975477	0.0767154
3611	1.73584	0.0840183
3612	2.48188	0.0862157
3613	2.2045	0.547501
3614	2.08134	0.545426
3615	0.993366	0.326502
3616	0.993368	0.67406
3617	1.14187	0.571214
3618	0.946132	0.532846
3619	0.946133	0.971409
3620	1.68951	0.68088
3621	1.68951	1.20142
3622	1.34612	0.577948
3623	1.28953	0.53574
3624	1.34611	0.906908
3625	1.31892	0.50284
3626	0.946131	0.638114
3627	1.80988	0.474707
3628	1.80988	1.58912
3629	1.31892	0.718206
3630	0.946131	0.646361
3631	2.13214	2.1
3632	1.94691	2.1
3633	1.52661	0.0732332
3634	1.53175	0.0757646
3635	0.956005	0.0722135
3636	0.955991	0.0734568
3637	1.40572	0.0796711
3638	0.955992	0.0740038
3639	1.51084	0.833516
3640	1.67115	0.93201
3641	1.73143	2.1
3642	1.55234	2.1
3643	1.69377	2.1
3644	2.51483	2.1
3645	1.25987	2.1
3646	1.4472	2.1
3647	1.83151	0.624112
3648	2.01001	0.966561
3649	0.993998	0.618344
3650	1.83151	0.55809
3651	1.40915	0.290434

3652	1.24653	0.400257
3653	1.74472	2.1
3654	1.74472	2.1
3655	1.74244	2.1
3656	1.04788	1.53041
3657	1.28522	1.28095
3658	1.03785	2.1
3659	0.896519	2.1
3660	1.60428	2.1
3661	1.73305	2.1
3662	1.39072	0.326567
3663	2.27501	0.559202
3664	1.39073	0.491641
3665	1.39073	0.407522
3666	1.06838	1.84116
3667	0.968462	0.774063
3668	1.06837	1.27997
3669	1.13455	1.1822
3670	1.23527	0.590175
3671	2.71686	0.501474
3672	1.86881	0.502994
3673	1.10586	0.856742
3674	1.03852	0.982222
3675	1.03899	2.1
3676	0.963973	2.1
3677	1.00319	2.1
3678	1.00319	2.1
3679	0.987132	2.1
3680	0.974889	2.1
3681	0.987131	2.1
3682	1.7677	2.1
3683	1.7677	2.1
3684	0.946131	2.1
3685	1.31185	2.1
3686	0.946132	2.1
3687	1.30203	2.1
3688	1.31185	2.1
3689	1.82958	2.1
3690	1.82958	2.1
3691	0.946132	2.1
3692	1.40199	2.1
3693	0.946133	2.1
3694	1.36994	2.1
3695	1.40199	2.1
3696	0.97489	2.1
3697	1.05939	1.79214
3698	1.01922	1.77721
3699	0.810941	1.43548
3700	1.05372	2.03513
3701	0.754065	2.08325
3702	0.926131	1.82977
3703	1.35905	1.76487
3704	0.810931	1.66378
3705	1.75647	0.990441
3706	1.68265	2.1
3707	1.41995	2.1
3708	1.36457	1.25874
3709	0.933392	1.11406

3710	1.42255	2.1
3711	1.3868	2.1
3712	1.08074	2.1
3713	1.07196	2.1
3714	1.13537	0.981559
3715	1.01179	0.751868
3716	1.60196	0.659009
3717	1.72798	0.7566
3718	1.3715	0.806928
3719	1.56409	0.996129
3720	1.56409	0.687137
3721	1.97819	1.17879
3722	1.47954	0.610329
3723	0.798655	0.360381
3724	1.47952	0.551036
3725	2.30327	0.393306
3726	1.49111	2.1
3727	1.96013	1.75031
3728	1.54599	2.1
3729	2.06632	2.1
3730	1.54601	2.1
3731	1.10557	0.899055
3732	1.17624	0.722933
3733	1.60497	2.1
3734	1.61587	2.1
3735	1.65977	0.075969
3736	1.69044	0.0766624
3737	1.00659	2.1
3738	1.06226	2.1
3739	1.15171	2.1
3740	1.50853	2.1
3741	1.80252	2.1
3742	1.41432	2.1
3743	1.04687	2.1
3744	0.940878	2.09581
3745	1.52031	2.1
3746	1.52031	2.1
3747	0.940877	2.1
3748	0.946132	2.1
3749	1.50853	2.01245
3750	0.963973	2.1
3751	1.70562	0.139715
3752	2.43203	0.134737
3753	1.36004	0.124104
3754	1.06648	0.126555
3755	1.66009	0.100437
3756	1.66009	0.117585
3757	0.766427	0.114476
3758	0.766452	0.108354
3759	0.946132	0.12852
3760	1.98012	0.1
3761	1.20357	2.1
3762	1.20356	2.1
3763	1.67324	2.1
3764	1.52008	2.1
3765	1.19964	2.1
3766	1.36861	2.1
3767	1.19964	2.1

3768	2.85415	2.1
3769	1.43687	2.1
3770	0.935852	2.1
3771	1.09972	2.1
3772	0.810933	1.94233
3773	2.25974	2.1
3774	1.74149	2.1
3775	1.36984	2.1
3776	1.87602	2.1
3777	0.890748	2.1
3778	1.20587	1.80651
3779	0.810956	1.59681
3780	2.03174	1.94134
3781	1.34403	2.01572
3782	1.58825	1.89149
3783	1.63096	1.83411
3784	2.53735	2.06769
3785	1.98241	1.9565
3786	2.53014	2.1
3787	1.1775	1.06276
3788	1.06538	0.932496
3789	0.946132	1.1168
3790	1.4101	1.73272
3791	1.75162	2.1
3792	1.75162	1.03387
3793	0.946132	1.08268
3794	1.4101	0.980379
3795	1.28765	1.03798
3796	1.1427	0.951472
3797	1.1427	0.746821
3798	1.3109	2.07464
3799	1.02721	1.20377
3800	0.846447	1.44147
3801	0.862076	2.1
3802	0.644098	1.50046
3803	0.581308	1.46698
3804	1.02306	1.28313
3805	0.322641	0.823222
3806	0.589346	0.834174
3807	2.25572	1.29586
3808	1.31372	2.1
3809	1.18873	2.1
3810	1.78225	2.1
3811	1.764	2.1
3812	1.17503	2.1
3813	1.06821	2.1
3814	1.06821	1.70899
3815	1.7777	1.91498
3816	1.9096	1.05177
3817	1.70239	1.22816
3818	1.16182	0.0950918
3819	1.20132	0.103806
3820	1.23475	2.1
3821	1.56859	1.16076
3822	2.46263	2.1
3823	0.528744	2.1
3824	2.93337	2.1
3825	2.42504	2.1

3826	1.38646	0.217111
3827	1.61963	0.344381
3828	1.88016	0.684274
3829	0.622283	0.867526
3830	1.03243	0.706096
3831	0.710307	1.17834
3832	0.798124	0.793464
3833	0.931073	0.975557
3834	0.892862	1.42142
3835	1.06791	0.681861
3836	0.552787	1.17014
3837	0.946132	0.886151
3838	1.36665	0.965055
3839	1.36665	1.49838
3840	0.506966	0.943272
3841	0.506964	1.59446
3842	1.36665	0.869145
3843	0.653469	0.898366
3844	1.36665	0.695936
3845	1.21339	0.760018
3846	1.21339	1.32928
3847	0.552757	0.803617
3848	0.552786	1.41065
3849	1.21336	0.603119
3850	1.70744	1.46254
3851	1.0775	0.302847
3852	1.59527	0.548074
3853	1.48312	0.561971
3854	2.01868	0.448415
3855	1.18947	0.194092
3856	1.48312	0.564923
3857	0.785626	0.20781
3858	1.19601	0.246401
3859	0.785626	0.261799
3860	0.946132	0.245292
3861	0.7856	0.31669
3862	1.59087	0.263909
3863	1.51158	0.267177
3864	1.74098	0.280373
3865	2.39202	0.234951
3866	0.870489	0.331199
3867	1.34534	0.459754
3868	0.870488	0.247823
3869	0.946133	0.528711
3870	0.870489	0.648945
3871	1.67086	0.485439
3872	1.67086	0.484329
3873	2.11311	1.91866
3874	1.98765	1.18003
3875	0.946133	0.849683
3876	1.30881	1.6837
3877	1.72211	2.1
3878	1.72211	1.18473
3879	0.94613	1.47847
3880	1.30881	1.13903
3881	1.35943	0.883111
3882	1.01294	0.356311
3883	1.01294	0.122254

3884	1.38963	0.56827
3885	1.01294	0.343678
3886	1.60056	0.319145
3887	1.60056	0.369413
3888	0.946132	0.503967
3889	1.204	0.380176
3890	0.946132	0.308114
3891	1.11288	0.27324
3892	1.204	0.345189
3893	1.14599	0.546115
3894	1.00065	0.511642
3895	1.74685	0.59801
3896	1.74685	1.29119
3897	0.946128	0.560895
3898	1.38463	0.398885
3899	1.2275	1.57072
3900	1.2275	0.742687
3901	1.30835	0.774859
3902	2.04407	2.1
3903	2.50425	2.1
3904	1.19647	0.571165
3905	1.19647	0.647278
3906	1.19645	0.714053
3907	1.75446	0.607663
3908	1.1315	0.425438
3909	1.1315	0.524247
3910	1.45569	0.444206
3911	1.1315	0.38945
3912	1.14357	2.1
3913	1.25793	2.1
3914	1.14357	2.1
3915	2.08181	2.08148
3916	1.4714	2.1
3917	0.71295	2.05751
3918	1.68285	2.1
3919	1.68285	2.1
3920	1.25603	2.1
3921	1.13976	2.1
3922	1.25603	2.1
3923	0.71295	2.1
3924	1.61095	2.1
3925	2.51207	2.1
3926	1.63762	2.1
3927	2.74451	2.1
3928	0.671255	1.45102
3929	1.87601	1.97117
3930	1.87601	2.05014
3931	0.671226	1.48903
3932	1.25603	1.92643
3933	1.29123	1.98505
3934	0.671226	1.95446
3935	1.63723	2.1
3936	0.872928	0.11724
3937	1.47904	0.136589
3938	1.18793	0.126739
3939	2.57464	0.140713
3940	1.14986	1.63973
3941	1.19611	1.69983

3942	0.96029	0.654531
3943	1.7961	2.1
3944	0.928721	2.1
3945	1.34352	2.1
3946	1.37935	2.1
3947	1.37935	2.1
3948	0.892888	2.1
3949	0.946131	2.1
3950	0.892888	2.1
3951	1.75422	2.1
3952	1.05556	2.1
3953	1.27362	0.494581
3954	1.27421	0.228422
3955	1.3003	0.90388
3956	1.31415	0.743152
3957	1.01431	2.1
3958	0.673951	2.1
3959	1.63384	1.88927
3960	1.63661	2.1
3961	1.52245	2.1
3962	1.50714	2.1
3963	0.996778	0.0983265
3964	1.56091	0.10863
3965	1.06543	2.1
3966	2.66689	2.1
3967	1.01474	2.1
3968	2.76422	2.1
3969	1.45321	0.427126
3970	0.974716	0.290193
3971	0.810943	0.291472
3972	0.909982	0.444293
3973	0.937793	0.334898
3974	1.05138	0.306304
3975	1.25044	0.314523
3976	0.92095	0.295327
3977	0.830414	0.364443
3978	2.1402	0.35762
3979	1.75938	2.1
3980	1.05556	2.1
3981	1.78558	2.1
3982	1.379 2.1	
3983	0.970214	0.64094
3984	0.9702	0.605053
3985	1.08894	0.969663
3986	2.17251	0.912709
3987	1.51569	0.882155
3988	1.06012	0.807294
3989	0.98966	0.799866
3990	1.55926	0.48448
3991	1.92645	0.699434
3992	1.37427	0.160419
3993	2.16338	0.199082
3994	1.5456	0.136967
3995	1.5456	0.117716
3996	1.94002	0.128347
3997	1.33135	0.142663
3998	1.31948	0.20695
3999	2.23255	0.162256

4000	2.34061	0.144148
4001	1.52903	2.1
4002	3.38327	2.1
4003	1.35977	2.1
4004	2.22414	2.1
4005	1.6812	0.141
4006	2.33397	0.159317
4007	1.16699	0.195493
4008	0.946131	0.182049
4009	1.58115	0.195521
4010	1.58115	0.173084
4011	0.519726	0.287433
4012	0.946131	0.221634
4013	0.519697	0.212938
4014	1.26868	0.150812
4015	1.16699	0.154353
4016	1.5951	0.136486
4017	1.5951	0.125306
4018	0.840568	0.189282
4019	0.946131	0.156973
4020	0.840598	0.146737
4021	1.30047	2.1
4022	1.5586	2.1
4023	1.31825	0.766491
4024	1.31924	0.713706
4025	1.15818	1.76094
4026	1.52782	0.957392
4027	1.15818	1.15257
4028	1.15818	1.0871
4029	0.99518	0.434425
4030	0.94613	0.435144
4031	0.922045	0.468521
4032	1.29322	0.577428
4033	1.29321	0.391843
4034	1.29321	0.564876
4035	0.922056	0.458211
4036	1.06848	0.504457
4037	1.84409	0.54636
4038	1.9186	1.15538
4039	2.72244	0.890194
4040	0.946132	0.851198
4041	1.36122	0.928395
4042	1.79658	0.985514
4043	1.79658	0.787291
4044	0.946133	1.16493
4045	1.36122	0.905685
4046	1.38149	0.829644
4047	1.59106	0.766735
4048	0.946116	0.847682
4049	1.29052	0.832742
4050	1.76641	0.786887
4051	1.76641	0.748233
4052	0.885309	1.11797
4053	1.29054	0.87429
4054	1.36118	0.810891
4055	1.59103	0.895808
4056	2.27816	0.736236
4057	1.56164	0.758097

4058	1.41114	0.0657998
4059	2.04128	0.0599274
4060	0.986723	2.1
4061	1.03645	0.334246
4062	1.79224	0.291627
4063	1.2627	2.1
4064	1.23036	2.1
4065	1.45257	2.1
4066	1.41469	2.1
4067	1.41469	2.1
4068	2.60636	2.1
4069	1.45789	0.0959303
4070	0.851541	0.0961162
4071	1.57877	0.0961114
4072	1.53134	0.0804671
4073	0.851479	0.0906482
4074	1.08982	0.101975
4075	0.716284	0.0844714
4076	0.946131	0.103709
4077	1.73193	0.0872804
4078	2.57309	0.0966207
4079	1.36307	0.078705
4080	2.42175	0.0823399
4081	1.72948	0.0832539
4082	0.978698	2.1
4083	0.978699	2.1
4084	2.08398	2.1
4085	0.978698	2.1
4086	1.44096	2.1
4087	1.44096	2.1
4088	0.638164	2.1
4089	0.681588	2.1
4090	0.946133	2.1
4091	0.638165	2.1
4092	0.94613	2.1
4093	1.55432	2.1
4094	0.901147	2.1
4095	0.85777	2.1
4096	1.36073	2.1
4097	1.44004	2.1
4098	0.946133	2.1
4099	1.09982	2.1
4100	1.27562	2.1
4101	1.55432	2.1
4102	1.62229	2.1
4103	1.0101	2.1
4104	2.28459	1.89429
4105	2.50554	1.55231
4106	0.946131	0.876875
4107	1.25276	2.00129
4108	1.73721	2.1
4109	1.73721	1.22536
4110	0.93406	1.66046
4111	1.25277	1.39105
4112	1.4185	1.04228
4113	1.53064	1.044
4114	0.946133	1.02959
4115	0.609462	1.99506

4116	1.65123	1.99265
4117	1.65123	1.41677
4118	0.609466	1.81732
4119	1.00792	1.52905
4120	1.25277	1.21725
4121	1.53064	1.71935
4122	2.15223	1.18456
4123	1.30664	1.41914
4124	1.10674	2.1
4125	1.22862	2.1
4126	1.49358	0.424571
4127	2.0207	0.283657
4128	2.09374	2.1
4129	1.88175	2.1
4130	1.10938	0.273538
4131	1.02478	0.449511
4132	1.13272	0.113517
4133	1.13272	0.10741
4134	1.03935	1.29923
4135	0.810945	1.5073
4136	0.527573	0.84432
4137	0.925011	1.21909
4138	1.03193	0.97492
4139	1.38587	1.14168
4140	0.703469	1.0475
4141	0.912282	1.27452
4142	1.75505	2.1
4143	1.78529	2.1
4144	1.60017	0.750314
4145	1.35608	0.463021
4146	1.01479	0.68252
4147	1.47561	0.452035
4148	1.01479	0.651299
4149	1.28418	0.443358
4150	2.62605	2.1
4151	1.71233	2.1
4152	3.22222	2.1
4153	1.97526	2.1
4154	0.949712	0.435991
4155	1.78986	0.378007
4156	2.72879	0.332822
4157	2.66428	0.35598
4158	1.68108	0.457192
4159	1.67265	0.412791
4160	2.24877	0.479463
4161	1.15558	0.531558
4162	1.39708	2.1
4163	1.39327	2.1
4164	1.69788	0.133884
4165	1.1273	0.123866
4166	1.17302	0.418653
4167	1.04349	0.367202
4168	0.979853	2.1
4169	1.19094	2.1
4170	1.39408	2.1
4171	1.61857	2.1
4172	1.66523	2.1
4173	0.946132	2.1

4174	0.838554	2.1
4175	1.05151	2.1
4176	1.18901	2.1
4177	1.18901	2.1
4178	1.61149	2.1
4179	1.22857	0.70495
4180	1.25848	0.685966
4181	1.09154	2.1
4182	2.20358	2.1
4183	1.23013	2.1
4184	1.55698	2.1
4185	1.55828	1.69871
4186	1.52391	2.00973
4187	0.60346	1.36022
4188	1.06336	2.04466
4189	0.870714	1.0359
4190	0.543109	1.35707
4191	1.10367	1.05576
4192	1.20686	1.41119
4193	1.20684	1.97593
4194	2.5118	1.20186
4195	1.20684	2.0787
4196	1.52468	1.60066
4197	1.52469	1.80627
4198	0.532308	1.36906
4199	0.903301	1.89139
4200	0.946119	1.23048
4201	0.532315	1.24887
4202	0.946107	1.28973
4203	1.46129	2.1
4204	0.804766	2.1
4205	1.4809	2.1
4206	1.4809	2.1
4207	1.11822	2.1
4208	1.16744	2.1
4209	1.11822	2.1
4210	0.804737	2.1
4211	1.04193	2.1
4212	0.983723	2.1
4213	1.8014	2.1
4214	1.1729	2.1
4215	1.03951	2.1
4216	1.03951	2.1
4217	1.17583	2.1
4218	1.42866	2.1
4219	1.78503	2.1
4220	1.17822	1.17157
4221	1.89121	2.1
4222	1.44472	2.1
4223	1.62143	2.1
4224	1.43837	2.1
4225	1.01121	0.288393
4226	0.962703	0.693615
4227	0.946129	0.507254
4228	1.10941	0.436853
4229	0.946131	0.522828
4230	1.60326	0.58681
4231	1.60326	0.470231

4232	1.14215	0.501962
4233	1.40724	0.515085
4234	0.962703	0.544167
4235	1.53336	0.144519
4236	1.40272	0.135229
4237	1.64984	0.12652
4238	1.67345	0.135385
4239	1.08212	2.1
4240	1.40905	2.1
4241	0.946132	0.0788208
4242	0.946132	0.0758289
4243	0.81268	0.0910849
4244	0.946131	0.0780948
4245	0.812681	0.0841454
4246	0.946131	0.0836447
4247	0.946132	0.0860945
4248	0.997755	0.0772798
4249	1.94585	0.0722996
4250	1.2925	0.0726551
4251	2.52454	0.0774314
4252	1.69182	0.0734687
4253	1.69182	0.0713738
4254	0.92361	0.0849262
4255	1.35316	0.0767723
4256	1.26227	0.0795621
4257	0.946131	0.0805957
4258	1.35316	0.0766241
4259	1.48676	0.253103
4260	1.33917	0.247485
4261	0.757292	2.1
4262	1.02415	2.1
4263	1.48495	2.1
4264	0.97587	2.1
4265	0.946132	2.1
4266	0.747365	2.1
4267	1.55432	2.1
4268	1.55432	2.1
4269	0.946132	2.1
4270	0.747394	2.1
4271	1.17464	0.145682
4272	1.0372	0.149966
4273	2.16553	0.0993958
4274	2.00616	0.132894
4275	1.12184	0.108102
4276	1.33177	0.11101
4277	0.936674	0.101875
4278	0.946129	0.112614
4279	0.851552	0.122953
4280	1.60205	0.110074
4281	1.77391	0.108559
4282	1.03327	0.147927
4283	1.41512	0.163329
4284	1.03327	0.130646
4285	0.946133	0.164073
4286	0.946131	0.179338
4287	1.80077	0.170731
4288	1.80077	0.161591
4289	1.54848	2.1

4290	1.29931	2.1
4291	1.04482	0.48038
4292	2.08069	0.487532
4293	0.946127	0.414674
4294	0.515539	0.528183
4295	0.515566	0.575561
4296	1.38423	0.424992
4297	1.38423	0.444803
4298	0.972058	0.493037
4299	0.927737	0.516945
4300	0.94971	0.365803
4301	2.19682	0.416537
4302	0.946132	0.497018
4303	1.09841	0.419398
4304	0.522382	0.544275
4305	1.5962	0.339374
4306	1.72791	0.335344
4307	1.07428	0.422192
4308	1.05215	0.419198
4309	1.54632	1.26706
4310	1.15693	1.32264
4311	1.41238	2.1
4312	1.41238	2.1
4313	1.77966	2.1
4314	1.77966	2.1
4315	0.946129	2.1
4316	1.40525	2.1
4317	0.946131	2.1
4318	0.94613	2.1
4319	1.32054	2.1
4320	1.41238	2.1
4321	1.94026	2.1
4322	1.0171	0.52692
4323	1.1211	0.44847
4324	0.993022	2.1
4325	0.773813	2.1
4326	1.4073	2.1
4327	1.4073	2.1
4328	0.94613	2.1
4329	0.787615	2.1
4330	0.94613	2.1
4331	0.787615	2.1
4332	1.764	2.1
4333	1.90964	2.1
4334	1.05787	2.1
4335	1.403	0.509008
4336	0.744189	0.626271
4337	2.32834	2.1
4338	2.34986	2.1
4339	1.17106	1.41118
4340	1.94826	0.154159
4341	2.40606	0.137031
4342	1.26387	0.163357
4343	0.954013	0.16877
4344	0.946131	0.187909
4345	1.71	0.127984
4346	1.51926	0.123224
4347	0.953997	0.126052

4348	0.851554	0.171111
4349	1.96502	0.135243
4350	1.72954	0.159751
4351	2.89117	0.14087
4352	0.971958	0.163183
4353	0.985575	0.185819
4354	1.41943	0.129319
4355	1.20021	0.148413
4356	1.29599	0.160572
4357	1.15687	0.135605
4358	0.810958	0.183563
4359	1.12919	0.0723476
4360	1.98859	2.1
4361	1.82044	2.1
4362	1.90488	2.1
4363	0.851542	2.1
4364	1.02973	2.1
4365	1.39286	2.1
4366	1.44873	2.1
4367	0.946132	2.1
4368	1.11826	2.1
4369	1.26842	2.1
4370	2.09461	2.1
4371	1.76952	0.0811505
4372	1.6473	0.0755279
4373	0.946131	0.111183
4374	1.36486	0.0923686
4375	1.04243	0.0969921
4376	1.05017	0.0967129
4377	1.24059	0.0952992
4378	2.11646	0.0792427
4379	1.50368	0.0946881
4380	2.38796	0.0857826
4381	1.50368	0.090991
4382	1.71844	0.0908567
4383	1.71844	0.0865246
4384	0.914836	0.124804
4385	1.24416	0.10506
4386	0.914805	0.111032
4387	1.24416	0.104797
4388	1.38911	0.103013
4389	1.46859	0.112549
4390	1.35348	0.114575
4391	0.946131	0.137061
4392	1.1226	0.118656
4393	0.857078	0.146517
4394	0.9746	0.127241
4395	1.37624	0.127729
4396	1.15131	0.0945139
4397	1.139	0.095526
4398	1.90456	0.101677
4399	2.09692	0.113654
4400	1.16655	2.1
4401	1.23017	2.1
4402	2.14592	2.1
4403	1.23017	2.1
4404	1.0752	0.796529
4405	1.52482	0.424308

4406	1.09252	2.1
4407	1.10725	2.1
4408	1.14305	0.184038
4409	0.851541	0.210531
4410	1.48073	0.186101
4411	1.45607	0.144529
4412	0.946131	0.226088
4413	0.932823	0.145154
4414	1.45828	0.204888
4415	0.851528	0.206583
4416	1.14305	0.181206
4417	1.92737	0.239873
4418	1.08211	0.22152
4419	1.69831	0.0522355
4420	1.69831	0.0522355
4421	0.946131	0.0522355
4422	1.26868	0.0522355
4423	1.23377	0.0522355
4424	1.23377	0.0522355
4425	0.94723	0.0522355
4426	1.64322	0.0602813
4427	1.64322	0.0574151
4428	0.946131	0.196662
4429	1.32068	0.0620379
4430	0.946129	0.0620378
4431	1.18789	0.214267
4432	1.32068	0.0644292
4433	1.32813	2.1
4434	1.32461	2.1
4435	1.42729	0.141256
4436	2.47846	0.173599
4437	1.00024	0.230566
4438	1.23923	0.175471
4439	0.946131	0.237962
4440	0.712304	0.163993
4441	1.77501	0.249312
4442	1.77501	0.146123
4443	0.593593	0.31727
4444	1.23923	0.245495
4445	1.23175	0.198564
4446	0.94613	0.2755
4447	0.713646	0.180522
4448	1.75735	0.271547
4449	1.75735	0.152117
4450	0.713633	0.358085
4451	1.37157	0.175905
4452	1.47169	0.15934
4453	2.21808	0.224567
4454	1.47169	0.207392
4455	1.04759	2.1
4456	0.987132	2.1
4457	1.11181	2.1
4458	2.61682	1.60338
4459	1.11181	2.1
4460	1.06461	1.18341
4461	1.69733	2.1
4462	2.79705	2.1
4463	1.08245	2.1

4464	1.40583	1.98696
4465	1.39852	1.79952
4466	2.0239	1.71881
4467	2.0239	1.90015
4468	0.780455	2.1
4469	0.946132	2.07129
4470	0.780455	2.0389
4471	1.01885	2.1
4472	0.564217	2.1
4473	0.733461	2.1
4474	0.537126	2.1
4475	0.683838	2.1
4476	0.779662	2.1
4477	0.634141	2.1
4478	0.685337	2.1
4479	1.58848	1.61863
4480	1.17129	0.175856
4481	0.663168	0.288563
4482	1.55587	0.246385
4483	1.31342	0.21631
4484	0.596868	0.278215
4485	1.1666	0.321312
4486	0.867261	0.333288
4487	0.946132	0.375132
4488	1.28843	0.217674
4489	1.31248	0.276011
4490	2.59921	0.251306
4491	1.32634	0.16097
4492	1.35564	0.0769764
4493	1.52661	0.0822237
4494	1.12323	0.0688892
4495	1.17701	0.944742
4496	1.36405	0.930788
4497	1.19837	0.941149
4498	2.25636	0.870914
4499	1.0823	0.268702
4500	1.31774	0.303538
4501	1.0823	0.303808
4502	1.01121	0.260577
4503	1.27408	0.293975
4504	0.946131	0.282577
4505	0.566074	0.236547
4506	0.79576	0.267672
4507	1.24596	0.26786
4508	1.47567	0.275664
4509	1.47567	0.173294
4510	0.56605	0.266706
4511	1.6567	0.199659
4512	0.967792	0.176892
4513	1.00366	0.257547
4514	1.00366	0.219563
4515	1.56409	0.102356
4516	1.56409	0.0968928
4517	0.774911	0.133599
4518	1.09483	0.119509
4519	0.774912	0.118386
4520	1.09483	0.118537
4521	1.24417	0.115496

4522	1.13387	0.0987987
4523	1.13387	0.100481
4524	1.30572	0.1238
4525	2.18966	0.112815
4526	0.98575	0.0952496
4527	1.54982	0.112642
4528	2.48833	0.101733
4529	1.54982	0.104593
4530	1.28954	0.293721
4531	1.64002	0.0903214
4532	1.64002	0.0835127
4533	1.85594	0.0828138
4534	1.12968	2.1
4535	1.23536	1.51399
4536	1.4882	1.79582
4537	1.4882	2.1
4538	0.876836	1.557
4539	0.946131	2.09141
4540	0.876835	2.1
4541	1.43166	2.1
4542	2.25936	2.1
4543	1.0305	1.69168
4544	0.992095	2.1
4545	1.12968	1.91896
4546	1.53813	2.02949
4547	1.53812	2.1
4548	0.583653	2.1
4549	0.94613	2.1
4550	0.583651	2.1
4551	0.964033	2.04614
4552	0.964034	2.1
4553	1.54457	1.62715
4554	3.54109	0.160362
4555	0.99974	0.193397
4556	0.78814	0.225537
4557	1.65729	0.155582
4558	1.1283	0.182351
4559	1.09407	0.193298
4560	1.55091	0.158836
4561	0.870773	0.210672
4562	1.19967	0.171654
4563	1.56521	0.170936
4564	1.30896	1.12206
4565	1.17989	2.1
4566	1.72526	1.95664
4567	0.851537	1.48641
4568	1.58944	1.94119
4569	1.56915	2.06441
4570	0.891039	1.39039
4571	1.4172	1.42916
4572	0.963818	1.22808
4573	0.851474	1.72779
4574	1.17989	2.08176
4575	1.56261	2.1
4576	1.56261	2.1
4577	0.761574	2.1
4578	1.31323	2.1
4579	0.761573	2.1

4580	0.946133	2.1
4581	1.31323	2.1
4582	1.16759	2.1
4583	1.16759	2.1
4584	2.11829	2.1
4585	1.16759	2.1
4586	1.10748	0.267421
4587	1.47688	0.26316
4588	0.946133	0.264457
4589	0.953319	0.262485
4590	0.946132	0.261235
4591	1.51168	0.2597
4592	1.51168	0.26273
4593	1.25797	0.252824
4594	1.19984	0.227805
4595	1.95729	2.1
4596	2.34853	2.1
4597	1.94343	1.29634
4598	2.00551	1.36169
4599	1.20067	0.96132
4600	1.87244	0.9689
4601	1.4178	1.57334
4602	1.33538	2.1
4603	0.946131	1.68028
4604	1.22895	1.04879
4605	1.12433	2.1
4606	0.956757	2.1
4607	0.91826	2.1
4608	1.44221	2.1
4609	1.14206	2.1
4610	1.80514	0.888858
4611	0.890304	1.16432
4612	1.1362	0.730643
4613	1.32819	0.774952
4614	1.46311	1.76434
4615	0.8654	0.780699
4616	1.1699	1.11146
4617	1.00267	1.08892
4618	1.02882	0.924061
4619	1.00832	2.1
4620	0.974137	2.1
4621	1.02368	2.1
4622	0.974138	2.1
4623	0.995734	0.47507
4624	1.44605	0.376087
4625	1.18549	0.362597
4626	1.33621	0.586403
4627	1.5176	0.602326
4628	1.91762	0.610027
4629	1.33621	0.567409
4630	1.6638	0.578286
4631	1.66774	2.1
4632	1.11833	2.1
4633	0.926499	2.1
4634	1.51229	2.1
4635	1.51229	2.1
4636	0.926528	2.1
4637	1.40335	2.1

4638	1.03544	2.1
4639	0.926527	2.1
4640	1.85305	2.1
4641	1.5812	2.1
4642	1.15529	2.1
4643	1.31086	2.1
4644	0.999206	2.1
4645	1.46714	2.1
4646	1.73087	2.1
4647	0.772299	2.1
4648	1.24341	2.1
4649	0.997867	2.1
4650	0.772311	2.1
4651	0.936657	2.1
4652	1.35986	2.1
4653	0.959414	2.1
4654	1.36541	2.1
4655	1.49268	2.1
4656	1.49268	2.1
4657	0.832137	2.1
4658	0.946132	2.1
4659	0.832138	2.1
4660	1.64523	2.1
4661	1.11764	2.1
4662	1.05776	2.1
4663	0.972465	2.1
4664	0.972464	2.1
4665	1.98425	2.1
4666	0.969822	2.1
4667	1.30566	2.1
4668	1.52724	2.1
4669	2.32237	0.379295
4670	2.30274	0.433782
4671	2.27761	0.260374
4672	3.33012	0.243754
4673	1.24001	0.840019
4674	1.41821	1.13758
4675	0.978584	1.99697
4676	1.18076	2.1
4677	1.07645	2.1
4678	0.877351	0.392299
4679	0.946132	0.757993
4680	0.877353	0.521068
4681	1.58444	1.98253
4682	1.49614	1.24796
4683	1.28355	0.863067
4684	1.13491	0.79224
4685	1.56875	0.425589
4686	0.499592	0.227247
4687	0.946131	0.166619
4688	1.20538	0.158834
4689	1.09913	0.184102
4690	1.8049	0.182878
4691	1.8049	0.149841
4692	0.499606	0.222722
4693	2.41076	0.197082
4694	0.860202	0.148707
4695	0.906798	0.164099

4696	1.8626	0.222896
4697	1.33986	0.158439
4698	1.2992	0.235837
4699	1.58702	0.170281
4700	1.05939	0.189152
4701	1.02074	1.81761
4702	1.07866	1.67227
4703	1.5082	0.214668
4704	1.5522	0.165294
4705	1.65903	0.272328
4706	1.86766	2.1
4707	2.11684	2.1
4708	0.946132	2.1
4709	1.6585	2.1
4710	1.6585	2.1
4711	0.94613	2.1
4712	1.1872	2.1
4713	1.1872	2.1
4714	1.05842	2.1
4715	1.20455	2.1
4716	1.34141	2.1
4717	1.34141	2.1
4718	1.42416	2.1
4719	1.75818	1.58814
4720	2.63715	0.95195
4721	1.80454	2.00769
4722	2.26198	2.1
4723	0.696255	2.1
4724	0.624075	1.78711
4725	1.50905	0.797237
4726	1.50905	1.97704
4727	0.624075	1.89406
4728	1.31857	0.732454
4729	0.946131	1.59712
4730	0.624075	0.983242
4731	0.914628	1.65234
4732	1.14721	2.1
4733	1.16153	2.1
4734	1.47664	2.1
4735	1.54791	2.1
4736	1.19098	2.1
4737	1.13341	2.1
4738	1.00503	0.539175
4739	1.00503	1.03042
4740	1.22655	1.65774
4741	0.999489	2.1
4742	1.3268	1.36225
4743	0.946132	2.1
4744	1.60061	2.1
4745	1.60061	2.1
4746	1.08034	2.1
4747	1.40372	2.1
4748	1.14302	2.1
4749	1.08034	2.1
4750	0.999491	2.1
4751	1.17152	0.291335
4752	1.17152	0.174118
4753	1.17152	0.198375

4754	2.05423	0.224787
4755	1.40984	1.71128
4756	1.2489	2.1
4757	0.885089	2.1
4758	0.810931	2.1
4759	1.84128	2.1
4760	1.11206	2.1
4761	1.21926	2.1
4762	1.99084	2.1
4763	0.976334	0.79929
4764	0.599363	0.655939
4765	1.02621	0.514584
4766	0.462959	0.647425
4767	1.42612	0.476112
4768	0.299682	0.508581
4769	1.51379	2.1
4770	0.719531	1.78563
4771	1.3702	2.1
4772	1.33293	2.1
4773	0.851539	2.1
4774	0.62998	2.1
4775	0.851543	2.1
4776	0.629966	2.1
4777	1.43132	2.1
4778	1.13328	1.1685
4779	1.93212	0.875057
4780	1.62345	1.34363
4781	1.38511	1.48562
4782	1.40013	0.973805
4783	1.04132	0.87602
4784	0.83473	1.29202
4785	0.868732	1.25593
4786	0.810595	1.58141
4787	0.729919	1.20023
4788	0.992428	1.33659
4789	0.577107	0.946138
4790	1.18464	1.36879
4791	0.791199	1.28471
4792	0.810943	0.147899
4793	0.860723	0.150147
4794	1.33348	0.112769
4795	1.57467	0.122226
4796	0.981032	0.144933
4797	0.731569	0.117434
4798	0.770312	0.150727
4799	1.46055	2.1
4800	1.42249	2.1
4801	1.75999	0.176192
4802	1.6868	0.152319
4803	1.17042	0.762549
4804	1.01485	2.1
4805	1.19952	0.287924
4806	1.40592	0.367621
4807	1.19952	0.312646
4808	2.20145	0.291731
4809	1.33649	0.281032
4810	1.11612	0.471059
4811	0.957588	0.34323

4812	0.946126	0.465626
4813	1.50648	0.267038
4814	1.50648	0.38315
4815	0.946128	0.33247
4816	2.10671	0.152447
4817	1.91518	0.221794
4818	2.23223	0.267898
4819	1.53556	2.1
4820	1.56253	2.1
4821	1.27806	0.0763197
4822	1.44593	0.079151
4823	1.03818	0.564028
4824	1.03818	0.534026
4825	1.38969	0.76733
4826	1.85548	0.618762
4827	2.6044	0.704253
4828	1.85548	0.592556
4829	1.49346	1.98731
4830	1.83192	1.76196
4831	0.834043	1.86549
4832	0.810943	1.21452
4833	0.887113	1.25799
4834	0.893827	1.37378
4835	0.991061	1.3841
4836	0.91939	1.4517
4837	0.793307	1.41123
4838	1.75291	1.66001
4839	1.35435	1.44797
4840	1.16989	0.498477
4841	1.36717	0.422277
4842	2.12276	0.390491
4843	2.30493	0.390278
4844	2.47857	0.450351
4845	1.92269	0.404993
4846	1.66426	0.443048
4847	0.956698	0.717324
4848	0.956698	1.39617
4849	0.857957	2.03584
4850	1.4706	1.20899
4851	1.65706	2.1
4852	1.47059	1.74231
4853	1.04713	0.303092
4854	0.946131	0.339965
4855	1.47374	0.424619
4856	1.47374	0.310204
4857	0.951675	0.27625
4858	1.2582	0.423652
4859	1.2582	0.322909
4860	0.94613	0.394364
4861	1.4468	0.187173
4862	1.90335	0.280815
4863	1.14886	2.1
4864	2.59689	2.1
4865	1.02747	2.1
4866	0.946131	2.1
4867	0.934061	2.1
4868	1.63979	2.1
4869	1.65206	2.1

4870	1.48	2.1
4871	1.27467	2.1
4872	2.20449	2.1
4873	1.1333	0.079811
4874	1.95334	2.1
4875	2.20984	2.1
4876	1.46389	0.205194
4877	1.4639	0.225162
4878	1.4639	0.290033
4879	0.946132	0.294422
4880	1.5144	0.220313
4881	1.5144	0.280688
4882	0.946132	0.311987
4883	1.35732	0.222023
4884	1.10321	0.289698
4885	0.946132	0.298903
4886	0.980549	0.24571
4887	1.32819	2.08906
4888	1.30171	1.14282
4889	1.68377	0.929647
4890	1.68377	2.1
4891	0.946132	0.811398
4892	0.946132	1.58626
4893	1.1952	1.1448
4894	0.990019	1.24864
4895	1.08195	1.63602
4896	1.2269	1.88359
4897	1.19219	1.73082
4898	1.01485	2.1
4899	1.57125	0.147451
4900	2.10696	0.134728
4901	1.29666	0.0941083
4902	1.57125	0.150724
4903	1.47712	2.1
4904	1.45182	2.1
4905	1.17816	0.0840051
4906	1.4516	0.0937935
4907	1.17816	0.0757622
4908	1.17816	0.0739523
4909	0.596954	0.911568
4910	1.65133	0.651063
4911	0.583859	0.595955
4912	1.04072	0.945813
4913	1.28795	0.666142
4914	1.59423	0.646736
4915	0.483559	0.695439
4916	1.74088	0.605836
4917	1.09545	0.831761
4918	1.02519	1.21269
4919	2.47579	0.793723
4920	1.54732	0.931238
4921	2.17663	0.739856
4922	1.75006	0.763629
4923	1.32354	0.888764
4924	1.09962	2.1
4925	1.85057	2.03727
4926	1.91046	2.1
4927	2.17037	2.1

4928	1.99528	0.217314
4929	1.97969	0.196077
4930	3.82765	2.1
4931	1.85767	2.1
4932	2.77042	2.1
4933	1.37236	0.151154
4934	2.23254	0.132325
4935	1.10089	0.123753
4936	0.994294	2.1
4937	1.81921	2.1
4938	1.00073	2.1
4939	0.681043	2.1
4940	0.946131	2.1
4941	0.681073	2.1
4942	1.51804	2.1
4943	1.60653	2.1
4944	1.30188	2.1
4945	0.919622	2.1
4946	1.00965	2.1
4947	1.93821	2.1
4948	2.60377	2.1
4949	1.36416	0.0982189
4950	1.36416	0.108807
4951	1.36416	0.114251
4952	2.44132	0.105455
4953	2.15871	0.642398
4954	2.22171	0.514534
4955	1.70747	0.439013
4956	1.49515	0.409149
4957	0.773007	0.452423
4958	0.946125	0.489566
4959	0.946131	0.50351
4960	0.773034	0.37076
4961	1.15844	0.450484
4962	1.98104	0.377844
4963	2.44283	0.444411
4964	1.05417	0.0976688
4965	1.1596	0.107419
4966	0.829921	0.0973307
4967	0.946131	0.113712
4968	0.951911	0.111908
4969	1.39027	0.094672
4970	0.829946	0.111426
4971	1.51223	0.105202
4972	1.51223	0.085848
4973	1.65984	0.086302
4974	1.90382	0.0859236
4975	1.93228	0.996523
4976	2.47924	0.951071
4977	1.73904	1.07979
4978	1.40547	0.683721
4979	2.37589	0.779378
4980	0.662605	1.04462
4981	2.33293	0.85045
4982	1.72249	2.1
4983	1.68478	2.1
4984	1.00514	0.475271
4985	1.4537	2.1

4986	1.92698	2.1
4987	1.46041	0.0575007
4988	2.04817	0.0559809
4989	1.65954	2.1
4990	1.42342	2.1
4991	1.04405	0.0916346
4992	1.17056	0.0713405
4993	1.11339	0.0671033
4994	0.946133	0.0781504
4995	0.929724	0.0753038
4996	1.35424	0.0686941
4997	1.35424	0.0686714
4998	0.929713	0.0832048
4999	1.85945	0.0768393
5000	1.38905	2.1
5001	1.39056	2.1
5002	1.52852	2.1
5003	1.06472	2.1
5004	0.749096	2.05334
5005	1.46076	2.1
5006	1.29263	2.1
5007	0.94613	2.1
5008	0.69097	2.1
5009	0.982284	2.1
5010	0.67419	2.1
5011	1.52852	2.1
5012	1.91121	2.1
5013	1.00775	1.84209
5014	1.61201	2.02472
5015	0.529987	2.1
5016	0.506125	2.1
5017	1.44696	2.1
5018	2.32981	2.1
5019	1.32784	2.1
5020	1.94158	2.1
5021	1.31595	2.1
5022	0.663922	2.1
5023	1.44663	2.1
5024	1.44663	2.1
5025	1.13976	2.1
5026	0.970788	2.1
5027	1.13976	2.1
5028	0.663921	2.1
5029	0.966114	2.1
5030	1.05498	0.909575
5031	1.38086	2.1
5032	0.946127	2.1
5033	0.922513	2.1
5034	1.49903	2.1
5035	1.49903	2.1
5036	0.92251	2.1
5037	1.06105	2.1
5038	1.36049	2.1
5039	1.38086	2.1
5040	1.52534	2.1
5041	1.04101	2.1
5042	1.30861	0.126472
5043	0.949112	0.140514

5044	0.894275	0.172408
5045	1.10232	0.120043
5046	0.830889	0.133225
5047	1.26554	0.136727
5048	0.948347	0.129859
5049	0.81096	0.143683
5050	1.18338	0.149201
5051	0.883764	0.178607
5052	1.54793	0.121824
5053	1.02867	0.149261
5054	1.8573	0.143506
5055	1.06065	0.133367
5056	0.64842	0.144476
5057	1.99319	0.158041
5058	1.9476	0.167405
5059	2.51436	0.0556237
5060	1.68337	0.145589
5061	1.92321	0.160691
5062	0.810942	0.100971
5063	0.921001	0.119756
5064	0.99758	0.149991
5065	0.960785	0.119534
5066	1.01565	0.155231
5067	0.908624	0.120233
5068	0.912835	0.124516
5069	1.18351	0.119932
5070	1.40239	2.1
5071	1.26091	2.1
5072	0.947979	2.00503
5073	0.946131	1.70239
5074	1.81959	1.52057
5075	1.81959	0.738061
5076	0.94613	1.63651
5077	1.38415	0.834379
5078	1.38414	1.24916
5079	1.33266	1.633
5080	0.946132	2.1
5081	1.76225	1.82263
5082	1.76225	2.1
5083	1.27599	2.1
5084	1.38158	1.23969
5085	1.3268	2.1
5086	1.27599	2.1
5087	0.947979	1.76048
5088	1.0142	0.544621
5089	1.01422	0.556263
5090	1.11435	0.78355
5091	1.42186	0.79401
5092	1.24001	1.12581
5093	1.77342	1.14547
5094	1.42186	1.28143
5095	1.99817	0.921216
5096	1.27361	2.1
5097	1.68914	2.1
5098	1.30116	2.1
5099	1.32819	1.21731
5100	1.68322	1.17536
5101	1.68322	2.1

5102	0.94613	0.783281
5103	0.94613	1.74435
5104	1.14666	1.17343
5105	1.07612	1.1003
5106	1.31151	2.1
5107	1.22095	1.29094
5108	1.57256	1.47742
5109	1.6679	2.1
5110	0.946132	0.909137
5111	0.946133	1.83214
5112	1.13537	1.69649
5113	1.07855	1.32189
5114	1.07855	2.1
5115	1.43895	1.08185
5116	1.38481	0.112641
5117	1.72385	0.110501
5118	0.550441	0.110753
5119	0.79807	0.1191
5120	1.11627	0.145435
5121	1.30349	0.137485
5122	0.94613	0.146688
5123	0.686176	0.153518
5124	1.11627	0.1158
5125	1.73363	0.114764
5126	1.73363	0.103937
5127	0.686139	0.160283
5128	1.24932	0.111254
5129	0.899582	0.102732
5130	2.00472	0.0931599
5131	1.17121	0.11371
5132	1.2874	0.0990442
5133	2.0194	0.10465
5134	0.88601	0.131366
5135	1.77409	0.124578
5136	1.73293	0.108623
5137	2.74045	0.117052
5138	1.83787	0.105379
5139	1.45448	0.127482
5140	1.27778	0.113953
5141	1.1192	2.1
5142	1.25953	2.1
5143	1.93611	2.1
5144	1.92104	2.00764
5145	1.15506	0.0944372
5146	1.15506	0.0986502
5147	1.23943	0.5279
5148	0.851543	2.1
5149	1.6061	2.1
5150	1.80058	2.1
5151	0.869471	2.1
5152	1.3931	2.06704
5153	1.06454	2.1
5154	0.93667	2.1
5155	2.16916	2.1
5156	3.10246	2.1
5157	1.91539	1.96323
5158	1.91539	1.65265
5159	0.620263	1.22021

5160	0.620265	1.92971
5161	0.946132	1.33846
5162	0.620265	1.1816
5163	1.30226	1.08098
5164	1.87292	1.91116
5165	1.18769	2.1
5166	2.60452	1.8315
5167	1.26574	2.1
5168	2.46679	1.45322
5169	1.36459	2.1
5170	1.26865	1.09557
5171	1.1744	0.795862
5172	1.15841	0.466423
5173	1.02247	0.356662
5174	1.93649	2.1
5175	1.64718	2.1
5176	1.59654	2.1
5177	1.57823	2.1
5178	1.00665	0.24868
5179	1.28239	0.249636
5180	0.887895	2.1
5181	1.22083	2.1
5182	1.42127	2.1
5183	1.07374	2.1
5184	1.00503	1.34933
5185	1.23082	0.873942
5186	2.78663	2.1
5187	2.20883	2.1
5188	2.54588	2.1
5189	0.946128	2.1
5190	1.13716	2.1
5191	1.58724	2.1
5192	1.58724	2.1
5193	0.946131	2.1
5194	1.13716	2.1
5195	1.39621	2.1
5196	1.02438	2.1
5197	1.49543	0.109488
5198	2.78336	0.0984398
5199	1.27148	0.0868905
5200	1.0906	0.0885312
5201	1.04072	0.103976
5202	0.63574	0.0880357
5203	1.45506	0.0836354
5204	1.5863	0.0871107
5205	0.928352	0.0971155
5206	0.647687	0.0986313
5207	2.2375	1.86315
5208	1.50998	1.52748
5209	2.75021	1.51097
5210	1.91912	1.41242
5211	1.99872	1.39099
5212	0.918127	1.76567
5213	1.03031	1.24567
5214	0.951169	1.09727
5215	1.27975	1.25148
5216	1.12735	1.36687
5217	1.15741	1.2612

5218	1.24998	1.23815
5219	1.65953	0.413829
5220	0.967555	0.702098
5221	0.967542	0.405099
5222	1.03824	0.297297
5223	1.48693	0.345609
5224	1.48693	0.301017
5225	2.02901	0.298632
5226	0.946127	0.380794
5227	1.07139	0.333449
5228	0.946126	0.33443
5229	1.84644	0.291003
5230	1.84644	0.251444
5231	1.38683	0.289593
5232	1.40574	0.228656
5233	1.36866	1.08201
5234	0.649325	0.959929
5235	1.93631	2.1
5236	1.50145	2.1
5237	1.6287	2.1
5238	1.31302	2.1
5239	1.18041	2.04281
5240	1.18041	2.05843
5241	0.974196	2.1
5242	0.946131	2.1
5243	1.74169	2.1
5244	1.74169	2.1
5245	1.23163	2.1
5246	1.27451	2.1
5247	1.41331	2.1
5248	1.23163	2.1
5249	1.28037	2.1
5250	1.28158	2.1
5251	1.28037	2.1
5252	2.28015	0.0972853
5253	2.06561	0.0903348
5254	2.61343	0.10891
5255	1.11712	0.107456
5256	1.63689	1.21805
5257	1.01393	0.892951
5258	0.996775	2.1
5259	1.54277	2.1
5260	1.26397	2.1
5261	1.46129	2.1
5262	1.62391	2.1
5263	1.08848	0.105575
5264	1.11833	2.1
5265	1.24041	2.1
5266	2.31884	2.1
5267	1.335	2.1
5268	1.48589	0.144284
5269	1.77131	0.125087
5270	1.8422	1.20773
5271	2.47453	1.6154
5272	1.23726	1.34167
5273	1.21996	1.33203
5274	1.65512	1.47143
5275	1.65512	1.44352

5276	0.802107	1.54974
5277	0.946129	1.15202
5278	0.802137	1.1858
5279	1.3279	1.1274
5280	1.23726	1.15301
5281	1.79759	1.19742
5282	1.79759	1.12071
5283	0.767577	1.38605
5284	0.946131	1.0195
5285	0.767577	1.00972
5286	1.03534	0.120005
5287	1.09962	0.477649
5288	0.929217	1.02995
5289	1.70126	1.05868
5290	0.991407	2.1
5291	1.41077	1.42366
5292	0.991406	2.1
5293	1.62966	2.1
5294	1.62966	2.1
5295	0.946128	2.1
5296	1.18364	2.1
5297	0.946131	2.1
5298	1.14186	2.1
5299	1.18364	2.1
5300	1.16858	1.23282
5301	1.66137	0.367192
5302	1.90165	0.165578
5303	1.66139	0.161241
5304	1.26893	0.508339
5305	1.15918	0.585717
5306	0.653228	0.718484
5307	0.946133	0.633759
5308	1.77488	1.77846
5309	1.77489	1.06492
5310	0.653228	0.932829
5311	1.56043	0.690522
5312	1.60894	1.04265
5313	1.56043	0.580125
5314	1.06848	1.33952
5315	1.04307	2.1
5316	1.14188	2.1
5317	1.09195	2.1
5318	0.615121	2.1
5319	1.50867	2.1
5320	1.27152	1.78818
5321	0.615094	1.58813
5322	1.07297	2.1
5323	1.1372	2.1
5324	0.553608	2.1
5325	1.23019	2.00464
5326	0.992675	2.1
5327	1.40984	1.10596
5328	2.55682	2.1
5329	3.66069	2.1
5330	3.58726	2.1
5331	1.4375	0.2478
5332	1.88424	0.362491
5333	1.56714	2.1

5334	1.6721	2.1
5335	1.18474	2.1
5336	1.2295	2.1
5337	1.28754	2.1
5338	1.36838	2.1
5339	2.78661	2.1
5340	2.64055	2.1
5341	1.35065	1.04362
5342	2.26675	0.60164
5343	1.35065	0.770283
5344	1.21558	0.630917
5345	1.49709	0.0828189
5346	1.4971	0.0766219
5347	0.85472	0.0987422
5348	0.854718	0.0766218
5349	0.946129	0.10812
5350	0.854717	0.0981615
5351	1.1604	0.100377
5352	1.25265	0.0878877
5353	2.32081	0.090387
5354	1.15371	0.0630265
5355	1.14945	0.544589
5356	0.995725	0.522014
5357	1.00307	0.318819
5358	1.10907	0.349458
5359	2.30315	0.331455
5360	1.10908	0.351196
5361	1.27558	0.790689
5362	1.71613	1.22043
5363	1.32977	2.1
5364	0.815276	2.1
5365	1.41697	2.1
5366	1.41697	2.1
5367	0.815276	2.1
5368	1.38897	2.1
5369	0.946131	2.1
5370	0.815275	2.1
5371	1.44684	2.1
5372	1.21489	1.54299
5373	1.22476	1.4668
5374	0.870366	0.725921
5375	0.862572	0.663858
5376	0.835435	0.627864
5377	1.6993	0.591865
5378	1.23618	0.551517
5379	1.18624	0.625623
5380	1.53058	0.624788
5381	2.16108	0.866415
5382	2.21589	0.780113
5383	1.04037	1.37965
5384	1.03933	1.36037
5385	1.57753	0.164971
5386	1.87644	0.192487
5387	1.24728	0.221299
5388	1.30394	0.167368
5389	1.3538	2.1
5390	1.69858	2.1
5391	1.1726	0.410351

5392	0.94612	0.298282
5393	1.09805	0.261164
5394	0.946118	0.318636
5395	1.72576	0.317196
5396	1.72575	0.255674
5397	1.34152	0.280316
5398	1.33034	0.267393
5399	0.784151	0.985922
5400	0.946131	1.77075
5401	0.784151	1.16065
5402	1.47963	2.1
5403	1.47963	2.1
5404	1.3335	2.1
5405	0.930281	2.07055
5406	1.29435	0.937503
5407	0.883308	0.841908
5408	1.31589	2.1
5409	1.67846	2.1
5410	0.993598	0.128977
5411	2.23193	0.117971
5412	0.9936	0.116508
5413	0.615629	0.112322
5414	1.65925	2.1
5415	2.05992	2.1
5416	1.65925	2.1
5417	4.08735	1.35871
5418	2.72856	1.49436
5419	0.869602	2.1
5420	3.42833	0.912814
5421	1.14397	0.365726
5422	1.34563	0.373379
5423	1.08189	0.353209
5424	0.782128	1.62181
5425	1.0022	1.85342
5426	0.900427	1.55409
5427	1.51499	1.65549
5428	1.05588	1.61465
5429	1.13739	1.89602
5430	1.37004	1.61924
5431	1.14603	1.84151
5432	0.851541	1.14295
5433	0.946799	1.63221
5434	1.14854	1.57295
5435	1.33793	1.24078
5436	0.94613	1.26357
5437	1.24336	1.40036
5438	0.851473	1.40877
5439	2.08792	1.47837
5440	1.01869	1.61558
5441	0.986953	0.242769
5442	0.896238	0.29706
5443	0.906574	0.312195
5444	0.852145	0.269669
5445	2.14191	0.233666
5446	1.15979	0.245623
5447	1.18327	0.22963
5448	2.32742	0.252004
5449	2.35961	0.257016

5450	2.38126	0.293087
5451	1.13632	0.249592
5452	1.17897	0.107211
5453	1.76199	0.0971516
5454	1.00573	1.26702
5455	0.946127	0.583066
5456	1.7077	1.24119
5457	1.7077	2.1
5458	0.946127	1.20373
5459	1.24887	2.1
5460	1.24887	1.43324
5461	1.22441	1.57198
5462	1.08542	1.54174
5463	1.21743	1.54418
5464	1.12572	2.1
5465	1.05499	0.87296
5466	1.00521	0.909454
5467	2.86191	0.0700409
5468	2.27972	0.0674981
5469	1.01169	2.1
5470	1.03373	2.1
5471	1.39319	2.1
5472	0.636837	1.97405
5473	1.63908	1.73755
5474	1.45713	1.70356
5475	1.04072	1.7452
5476	1.25025	1.76568
5477	1.38914	1.59524
5478	0.636807	1.44425
5479	1.50211	2.1
5480	1.36555	1.85289
5481	2.77545	1.28296
5482	0.682805	2.1
5483	1.50267	1.82883
5484	1.50267	2.0369
5485	0.797749	2.1
5486	1.38773	1.74263
5487	1.38773	2.06262
5488	0.682776	2.05796
5489	2.01245	0.0847345
5490	0.925918	0.114287
5491	0.75689	0.0904942
5492	1.25823	0.0824498
5493	1.16505	0.0982498
5494	0.838779	0.0916483
5495	1.14722	0.0881943
5496	0.756849	0.0942404
5497	1.52298	0.106318
5498	2.11034	0.100149
5499	1.92174	0.0966384
5500	1.92174	0.094251
5501	0.576083	0.131286
5502	1.23146	0.112955
5503	1.26637	0.104962
5504	1.23146	0.112298
5505	1.26637	0.109489
5506	0.946133	0.734383
5507	1.37034	0.606145

5508	1.84145	0.655282
5509	1.84145	0.494546
5510	0.946132	0.794559
5511	1.37034	0.677792
5512	1.41724	0.674519
5513	1.0685	0.495506
5514	1.14547	0.519537
5515	1.24976	2.1
5516	1.30682	2.1
5517	2.12095	2.1
5518	1.46392	2.1
5519	1.46392	2.1
5520	0.935738	2.1
5521	1.32949	2.1
5522	1.07017	2.1
5523	0.935766	2.1
5524	1.07017	2.1
5525	1.78095	2.1
5526	1.05619	0.739723
5527	1.05793	0.751339
5528	1.77677	2.1
5529	2.04766	2.1
5530	1.02524	2.1
5531	1.18803	2.1
5532	1.70047	2.1
5533	1.556 2.1	
5534	1.24201	1.13776
5535	1.3592	2.1
5536	2.11824	0.186924
5537	2.20248	0.308774
5538	3.43878	0.20398
5539	3.12466	0.168482
5540	2.09351	2.1
5541	0.946128	1.54033
5542	1.51269	2.1
5543	1.51269	2.1
5544	0.946131	2.1
5545	1.01306	2.1
5546	1.04676	2.1
5547	1.01306	2.1
5548	1.07444	2.1
5549	2.17465	2.1
5550	1.14808	2.1
5551	1.39067	1.04952
5552	1.20958	1.08184
5553	0.662832	0.673955
5554	1.93512	0.956814
5555	1.15471	2.1
5556	1.75119	2.1
5557	1.75118	2.1
5558	0.946131	2.1
5559	1.28606	2.1
5560	1.27087	2.1
5561	0.946131	2.1
5562	1.28606	2.1
5563	1.58916	0.112604
5564	0.927822	0.175834
5565	2.24367	0.0902365

5566	1.8734	0.0896275
5567	3.51668	0.082327
5568	2.68986	0.0817075
5569	2.32395	2.1
5570	1.39564	2.1
5571	2.10862	2.1
5572	1.36763	2.1
5573	0.970963	0.0872396
5574	0.97096	0.0939871
5575	1.11897	0.191385
5576	1.26885	0.275378
5577	1.11605	0.199036
5578	2.06612	0.161299
5579	1.717	0.173717
5580	0.59502	0.269669
5581	0.825755	0.137431
5582	0.709624	0.308801
5583	1.65741	0.157067
5584	0.307486	0.159046
5585	1.4785	0.186419
5586	0.983143	2.1
5587	1.08247	2.1
5588	1.42353	2.1
5589	1.42353	2.1
5590	0.781667	2.1
5591	0.797634	2.1
5592	0.946131	2.1
5593	0.781638	2.1
5594	0.946131	2.1
5595	0.98315	2.1
5596	1.083	2.1
5597	1.61975	0.0522355
5598	1.11946	0.0522355
5599	1.18977	0.123766
5600	0.996484	0.136932
5601	2.24343	0.0869271
5602	1.4787	0.0989405
5603	1.66127	0.0948064
5604	1.86028	0.113635
5605	1.09128	0.114689
5606	1.03115	0.143484
5607	0.838025	0.144926
5608	1.3736	0.0942173
5609	1.44009	0.102769
5610	0.92917	0.101469
5611	1.03889	0.140147
5612	0.956974	0.1215
5613	3.23468	0.10493
5614	0.953922	0.148378
5615	0.957104	0.155322
5616	1.66231	0.0997726
5617	1.26778	0.111587
5618	1.47615	0.107147
5619	1.28621	0.139023
5620	1.08043	0.133373
5621	2.35026	2.1
5622	2.35025	2.1
5623	2.60659	2.1

5624	1.57627	2.1
5625	1.5493	2.1
5626	1.24682	0.412927
5627	2.14277	0.473485
5628	1.24682	0.630344
5629	1.00307	0.38949
5630	2.04056	0.444625
5631	1.45408	2.1
5632	1.54249	2.1
5633	1.24474	1.31606
5634	1.37813	1.48362
5635	0.924158	0.213145
5636	0.916623	0.23045
5637	1.35344	0.196582
5638	1.00859	0.223029
5639	1.34786	0.209351
5640	0.952457	0.194615
5641	0.85404	0.204026
5642	1.06341	2.1
5643	1.09194	2.1
5644	1.93707	2.1
5645	2.14087	2.1
5646	1.93707	2.1
5647	1.07519	0.654295
5648	1.07519	0.61485
5649	1.62518	2.1
5650	1.3592	2.1
5651	1.13183	2.1
5652	1.20357	2.1
5653	1.4858	2.1
5654	1.23694	2.1
5655	1.44011	2.1
5656	1.23989	2.1
5657	2.21604	1.09451
5658	1.85574	0.341355
5659	1.55649	1.01704
5660	1.48861	0.278406
5661	1.15968	0.0845701
5662	0.946132	0.099464
5663	0.663056	0.0831691
5664	1.70837	0.0794291
5665	1.70837	0.0820103
5666	0.946131	0.0879782
5667	0.663026	0.0817655
5668	2.09604	0.0837685
5669	0.81094	0.0899648
5670	1.06559	0.0822288
5671	0.810956	0.0816804
5672	1.15474	0.0742334
5673	0.990051	0.0785258
5674	0.894971	0.0766246
5675	1.32536	0.0794821
5676	0.946133	2.1
5677	1.33125	2.1
5678	1.75823	2.1
5679	1.75823	2.1
5680	0.946133	2.1
5681	1.33125	2.1

5682	1.37312	2.1
5683	1.27985	2.1
5684	1.37312	2.1
5685	1.70684	2.1
5686	1.70684	2.1
5687	0.946132	2.1
5688	0.946132	2.1
5689	1.25081	2.1
5690	0.956293	2.1
5691	0.956294	2.1
5692	1.36035	2.1
5693	1.41944	2.1
5694	0.830638	2.1
5695	0.747568	2.1
5696	0.888498	2.1
5697	1.04071	2.1
5698	1.17128	2.1
5699	1.92339	2.1
5700	2.91208	2.1
5701	1.82208	1.00827
5702	1.89653	1.86686
5703	1.82588	1.24806
5704	1.26703	0.589001
5705	1.26703	0.933714
5706	0.6157	0.527323
5707	1.26706	1.19958
5708	0.61567	0.331609
5709	0.615671	0.377266
5710	1.26706	0.615155
5711	1.89158	0.926805
5712	1.23134	0.484632
5713	2.49497	0.106961
5714	1.68103	0.0871997
5715	2.0554	2.1
5716	1.72647	2.1
5717	1.67375	2.1
5718	1.65037	2.1
5719	1.04349	0.370913
5720	1.69055	0.185774
5721	1.53463	0.123279
5722	1.19022	1.9974
5723	1.89226	1.3649
5724	1.15009	0.764782
5725	1.7054	0.546907
5726	1.31612	0.279786
5727	1.31611	0.437858
5728	1.67345	0.641264
5729	1.06577	0.280782
5730	1.40193	0.190019
5731	1.72411	0.173533
5732	1.46123	0.192898
5733	0.808808	0.262103
5734	0.897714	0.191953
5735	0.851554	0.293594
5736	1.3499	0.235552
5737	1.29001	0.234805
5738	1.22083	2.1
5739	1.06191	2.1

5740	1.05758	1.24244
5741	1.348	1.71893
5742	1.43485	1.21523
5743	1.34799	1.65577
5744	1.36307	0.0724567
5745	2.28188	0.167303
5746	1.7926	0.154274
5747	1.38957	2.1
5748	1.47446	2.1
5749	0.953638	2.1
5750	1.11868	1.20107
5751	0.953636	2.1
5752	1.03408	1.71948
5753	1.52443	0.0989219
5754	1.38539	0.0962311
5755	0.946132	0.124636
5756	1.12747	0.104558
5757	1.04393	0.131113
5758	1.05017	0.114614
5759	1.20529	0.114327
5760	1.3663	0.591727
5761	1.89226	0.551771
5762	1.17885	0.722433
5763	0.867881	0.465764
5764	1.32917	0.429595
5765	1.32917	0.492753
5766	0.946127	0.522404
5767	1.19583	0.514542
5768	1.00122	0.548323
5769	0.946128	0.540215
5770	1.71405	0.83068
5771	0.871815	1.35507
5772	1.16435	2.1
5773	0.686619	1.93528
5774	1.63102	2.1
5775	1.06589	1.82106
5776	1.05375	2.1
5777	1.9023	2.03489
5778	1.27394	0.731306
5779	1.88345	0.692765
5780	2.05636	0.823292
5781	0.672977	0.660303
5782	1.41274	0.672019
5783	1.65606	0.652909
5784	0.869329	0.659094
5785	1.2207	0.692316
5786	1.35631	0.683788
5787	0.908776	0.651945
5788	0.955081	0.605774
5789	1.2243	2.1
5790	1.23169	2.1
5791	1.52228	2.1
5792	1.23169	2.1
5793	0.990892	1.17259
5794	1.08376	0.759049
5795	1.79348	1.83169
5796	1.50043	0.932938
5797	1.32599	1.99888

5798	1.37924	1.0363
5799	0.838014	0.769855
5800	0.87737	1.44902
5801	0.947086	0.871678
5802	1.27199	1.61614
5803	1.04918	0.94914
5804	1.04851	1.84991
5805	1.1699	1.28807
5806	0.810934	1.1676
5807	1.83602	2.04111
5808	2.21909	1.9219
5809	0.619441	1.24958
5810	2.03755	0.915606
5811	1.83472	1.71862
5812	1.20416	1.49002
5813	1.26691	0.78855
5814	0.655795	0.929826
5815	0.94613	1.10646
5816	2.35169	1.45865
5817	3.54705	2.1
5818	2.60741	2.1
5819	1.59019	0.0802364
5820	1.24526	0.076801
5821	1.59019	0.0830346
5822	2.36406	0.0794009
5823	1.16776	0.645276
5824	1.67311	0.755248
5825	1.67311	0.673644
5826	0.94613	0.584017
5827	1.38645	0.672797
5828	1.13563	0.312142
5829	1.13563	0.553534
5830	1.23279	0.482481
5831	1.05042	1.12965
5832	1.17418	0.699898
5833	1.28909	2.08475
5834	0.973448	1.93157
5835	1.02958	1.7819
5836	3.3005	2.08459
5837	2.86325	2.1
5838	1.35879	1.61164
5839	1.69332	1.83465
5840	1.69332	2.1
5841	0.946128	1.50075
5842	1.39659	1.08841
5843	1.12035	2.1
5844	1.12035	2.1
5845	1.24286	2.1
5846	1.10407	1.33889
5847	1.14599	0.90487
5848	1.32259	0.558797
5849	0.788592	0.0921144
5850	2.4588	2.1
5851	0.909581	1.68835
5852	1.35656	1.21527
5853	0.986895	1.45333
5854	2.30602	1.19897
5855	1.46873	1.44909

5856	1.55584	1.16701
5857	2.1547	1.24137
5858	1.87794	1.67208
5859	0.808286	1.74184
5860	0.928464	1.45975
5861	0.810959	1.43888
5862	1.50962	1.06335
5863	1.1053	1.31373
5864	1.0712	1.26736
5865	1.40504	1.24708
5866	1.87794	1.16958
5867	2.80496	1.25134
5868	1.41479	1.55989
5869	1.38634	2.1
5870	1.38634	2.1
5871	1.67762	2.1
5872	0.946131	2.1
5873	1.188 2.1	
5874	1.62585	2.1
5875	1.62585	2.1
5876	0.946129	2.1
5877	1.18801	2.1
5878	1.38397	2.1
5879	1.04546	2.1
5880	1.16817	2.1
5881	1.06007	2.1
5882	1.19785	2.1
5883	1.43652	1.87843
5884	1.06994	2.1
5885	1.89111	1.20717
5886	1.07144	2.1
5887	1.51754	2.1
5888	2.0873	2.1
5889	0.758799	2.04096
5890	1.59518	2.1
5891	1.59518	2.1
5892	0.946132	2.09067
5893	0.758771	2.1
5894	1.06957	2.1
5895	0.758771	2.1
5896	1.33916	2.1
5897	2.03045	1.12193
5898	2.80184	1.38687
5899	1.76788	1.70677
5900	1.27442	1.75674
5901	1.06756	2.1
5902	0.966979	2.1
5903	1.7054	2.1
5904	1.07156	2.1
5905	0.986096	2.1
5906	1.32813	2.1
5907	1.3331	2.1
5908	2.08369	0.877839
5909	2.23818	1.81368
5910	1.10335	0.991927
5911	0.622314	1.5802
5912	1.72451	1.09528
5913	1.72451	1.75203

5914	0.622283	0.772437
5915	0.622314	0.824556
5916	0.946131	0.795592
5917	1.31254	2.01117
5918	1.15704	2.08024
5919	1.24463	0.922623
5920	2.2067	1.39968
5921	1.30001	0.747043
5922	1.29175	0.809499
5923	1.1233	0.0901763
5924	1.8689	0.097977
5925	1.29494	0.102137
5926	1.11895	0.0967849
5927	0.851539	0.108557
5928	0.851474	0.10727
5929	1.58352	0.0893395
5930	1.58228	0.0835094
5931	0.851527	0.116833
5932	2.6389	0.087679
5933	1.77548	0.114732
5934	1.24555	2.1
5935	2.0512	2.1
5936	1.46101	2.1
5937	2.60635	2.1
5938	1.10476	0.170594
5939	1.44103	0.159841
5940	0.652391	2.1
5941	0.618322	2.1
5942	2.22915	2.1
5943	1.39067	2.1
5944	1.99737	2.1
5945	1.42174	2.1
5946	0.946132	0.76543
5947	1.29735	1.281
5948	1.61765	2.1
5949	1.61765	0.980102
5950	0.946132	1.55932
5951	1.29735	0.914626
5952	1.26643	0.84211
5953	1.01335	1.19964
5954	1.01335	0.552034
5955	0.947669	2.1
5956	2.17167	2.04718
5957	2.59733	1.60738
5958	1.50954	1.72213
5959	1.02638	1.21175
5960	0.555785	1.92159
5961	1.91101	1.87996
5962	2.09809	1.58212
5963	0.641974	1.73588
5964	1.2022	1.59261
5965	1.36359	1.41359
5966	1.8128	1.77351
5967	2.34348	0.368301
5968	1.66343	0.380691
5969	0.974717	0.707502
5970	1.53908	0.625316
5971	1.27737	0.918016

5972	1.09408	0.645328
5973	2.07382	0.600029
5974	1.10466	1.071
5975	1.05654	1.53838
5976	1.23498	1.11265
5977	1.48104	1.30881
5978	1.23498	1.22374
5979	1.17261	2.1
5980	1.18059	2.0038
5981	2.43005	2.1
5982	1.04546	2.1
5983	2.81222	2.1
5984	1.16817	2.1
5985	1.75766	0.47656
5986	1.47021	0.565355
5987	1.07236	0.4011
5988	1.81759	0.658368
5989	1.42913	0.521943
5990	1.00636	0.616476
5991	1.00636	0.356122
5992	1.79397	0.452608
5993	0.946131	0.336855
5994	1.03691	0.359342
5995	0.851539	0.338031
5996	1.46001	0.534749
5997	1.37878	0.501519
5998	0.933258	0.32727
5999	1.32423	0.35796
6000	1.32614	0.0582771
6001	1.11356	0.0651975
6002	1.32614	0.0595953
6003	1.28895	0.0599966
6004	1.66895	0.0564981
6005	1.66895	0.0561833
6006	0.946132	0.0631858
6007	1.29576	0.0558943
6008	1.1318	0.0565719
6009	1.37621	1.30834
6010	1.94071	1.21132
6011	1.10517	2.1
6012	1.97098	2.1
6013	0.811 2.1	
6014	0.956127	2.1
6015	1.18647	1.93669
6016	0.988455	1.37608
6017	1.12457	0.138216
6018	1.7453	0.160378
6019	0.92492	2.1
6020	1.24353	2.1
6021	1.84536	0.714654
6022	1.67662	0.632191
6023	1.38247	0.105217
6024	1.02663	0.0964217
6025	0.946132	0.655723
6026	1.41723	0.532521
6027	1.72976	0.601508
6028	1.72976	0.428117
6029	0.946131	0.793245

6030	1.41723	0.638252
6031	1.25866	0.605896
6032	1.34254	0.366601
6033	1.25866	0.499769
6034	1.65507	0.419647
6035	1.65507	0.333027
6036	0.946132	0.679709
6037	0.946132	0.483339
6038	1.11929	0.458323
6039	0.961779	0.425753
6040	0.961779	0.426426
6041	0.946132	2.1
6042	1.69361	2.1
6043	1.69361	2.1
6044	0.94613	2.1
6045	1.36523	2.1
6046	1.36523	2.1
6047	1.19802	2.1
6048	0.974196	2.1
6049	1.09518	2.1
6050	0.946131	2.1
6051	1.7161	2.1
6052	1.7161	2.1
6053	0.94613	2.1
6054	1.297	2.1
6055	1.297	2.1
6056	1.14599	2.1
6057	0.974197	2.1
6058	0.974197	2.1
6059	1.80778	2.1
6060	2.20473	2.1
6061	1.80777	2.1
6062	1.3515	2.1
6063	0.583237	2.1
6064	1.39359	2.1
6065	1.39359	2.1
6066	1.07296	2.1
6067	0.946114	2.1
6068	1.07297	2.1
6069	0.903862	2.1
6070	0.956572	2.1
6071	1.03437	2.1
6072	0.975409	2.1
6073	1.31167	0.160264
6074	2.70246	0.141997
6075	1.12018	0.153658
6076	0.655836	0.163017
6077	0.655837	0.160279
6078	1.81557	0.119232
6079	1.81557	0.115316
6080	0.655836	0.157195
6081	0.946131	0.172529
6082	1.01847	0.134857
6083	1.61334	0.12754
6084	0.810946	0.173343
6085	0.8311	0.1803
6086	1.2466	0.129206
6087	1.20286	0.142736

6088	0.97508	0.174346
6089	0.870805	0.136247
6090	0.760087	0.181092
6091	0.853389	2.1
6092	0.820894	2.1
6093	0.928291	2.1
6094	1.06922	2.06963
6095	1.32829	2.1
6096	1.12173	2.1
6097	1.16615	2.1
6098	0.957748	2.1
6099	0.612491	1.71672
6100	0.548141	2.1
6101	0.979614	2.1
6102	0.658184	2.07378
6103	0.678456	1.43295
6104	0.529544	1.59045
6105	0.739013	2.08202
6106	0.694037	1.94235
6107	0.475716	1.59547
6108	1.58794	1.43924
6109	1.58794	1.15497
6110	0.475743	1.25751
6111	1.08301	1.90316
6112	0.980664	2.04255
6113	0.475737	1.67845
6114	1.32709	1.93835
6115	1.59396	2.1
6116	1.19719	0.232244
6117	1.34921	0.240949
6118	1.69061	0.222964
6119	1.69061	0.166548
6120	0.946132	0.279442
6121	1.34921	0.253706
6122	1.28753	0.247039
6123	0.95895	0.178418
6124	1.01051	0.206907
6125	1.12468	0.220048
6126	1.22588	0.278912
6127	1.34254	0.255936
6128	1.74561	0.239633
6129	1.74561	0.195151
6130	0.946128	0.301387
6131	1.34253	0.270264
6132	1.3492	0.271022
6133	1.01051	0.180958
6134	1.922	2.1
6135	1.922	2.1
6136	2.12556	2.1
6137	0.946131	2.1
6138	1.06278	2.1
6139	1.47631	2.1
6140	1.47631	2.1
6141	0.946129	2.1
6142	1.06278	2.1
6143	1.35966	2.1
6144	0.991985	2.1
6145	0.738507	1.36533

6146	0.536935	1.76886
6147	0.799004	1.48707
6148	1.04281	1.27065
6149	0.879197	1.1498
6150	1.06538	1.24223
6151	0.715942	1.38405
6152	1.00636	0.403108
6153	1.03824	0.304315
6154	1.70147	0.358101
6155	1.21223	2.1
6156	1.01243	1.19689
6157	1.01242	2.1
6158	1.06935	2.1
6159	1.54241	2.1
6160	0.998971	0.0763365
6161	1.60312	0.0888881
6162	1.44836	0.0788028
6163	2.05632	0.0940849
6164	1.44836	0.0775079
6165	0.973765	0.0796507
6166	0.946131	0.101164
6167	0.851541	0.0924627
6168	1.73854	0.085306
6169	1.91752	0.0781641
6170	1.49622	0.0910091
6171	1.23351	0.0949458
6172	3.33289	2.1
6173	1.86606	2.1
6174	2.71233	2.1
6175	0.788816	2.1
6176	0.963972	2.1
6177	1.25866	2.1
6178	1.10817	2.1
6179	1.41908	2.1
6180	1.48734	2.1
6181	1.40822	0.561813
6182	1.40822	0.536874
6183	2.09871	0.628979
6184	0.946133	0.520707
6185	1.14926	0.380213
6186	0.946133	0.544651
6187	1.78098	0.427333
6188	1.78098	0.375564
6189	1.35822	0.461736
6190	1.3689	0.478517
6191	1.22176	0.392259
6192	2.03426	0.489156
6193	0.946131	0.569413
6194	1.01713	0.464603
6195	0.704112	0.583625
6196	1.36237	0.466683
6197	1.36237	0.45372
6198	1.01713	0.536853
6199	1.04936	0.551658
6200	1.87377	2.1
6201	1.60501	2.1
6202	0.981584	0.104837
6203	1.69435	0.110434

6204	0.85154	2.1
6205	1.47841	2.1
6206	1.4952	2.1
6207	1.31941	2.1
6208	1.04023	2.1
6209	1.1995	2.1
6210	0.914791	2.1
6211	1.1427	1.34749
6212	1.64787	1.16504
6213	1.07526	0.62301
6214	1.07527	0.681739
6215	1.07527	0.69509
6216	1.58551	0.725279
6217	2.77362	1.06051
6218	2.17974	0.752921
6219	2.71723	0.908248
6220	1.29868	0.221148
6221	1.3594	0.204668
6222	1.04786	0.183147
6223	0.527876	0.198706
6224	1.4085	0.207218
6225	1.48538	0.14332
6226	0.76829	0.171498
6227	1.18514	0.204282
6228	0.617954	0.142114
6229	0.864161	0.175962
6230	1.18835	0.155155
6231	1.16817	2.1
6232	1.04263	2.1
6233	1.50524	2.1
6234	1.85355	2.1
6235	1.23963	2.1
6236	1.06279	2.1
6237	0.94613	2.1
6238	1.35229	2.1
6239	1.15947	2.1
6240	0.965593	2.1
6241	0.851553	2.1
6242	2.21296	2.1
6243	1.11764	2.1
6244	2.46399	2.1
6245	1.39292	2.1
6246	1.34701	1.74424
6247	1.34701	2.1
6248	1.47001	2.1
6249	0.963166	0.0862918
6250	0.893445	0.0815199
6251	1.21056	0.0804898
6252	1.67375	2.1
6253	2.0977	2.1
6254	1.09154	2.1
6255	1.09154	2.1
6256	1.57122	0.937813
6257	1.60976	0.484446
6258	1.69533	0.368087
6259	1.6951	0.303035
6260	1.5831	0.274534
6261	1.20969	2.1

6262	1.12076	2.1
6263	1.49808	2.1
6264	1.50201	2.1
6265	1.00278	2.1
6266	1.22811	0.270206
6267	1.11545	2.1
6268	1.09646	2.1
6269	1.03107	0.0522355
6270	1.44293	0.167435
6271	1.66752	0.910427
6272	1.11023	0.934564
6273	2.57831	1.63805
6274	2.20191	1.05701
6275	2.18051	1.91404
6276	1.48035	0.482109
6277	1.0961	0.396709
6278	1.63241	0.382855
6279	1.0961	0.502883
6280	1.20959	2.1
6281	2.86136	2.1
6282	1.64933	2.1
6283	1.47217	2.1
6284	0.915178	2.1
6285	1.19074	2.1
6286	0.897276	2.1
6287	0.915177	2.1
6288	0.987037	2.1
6289	1.04288	2.1
6290	2.39323	2.1
6291	0.976218	0.07539
6292	2.23933	0.088517
6293	0.976215	0.0774404
6294	1.07384	0.0813083
6295	1.07652	2.1
6296	1.55678	2.1
6297	1.54168	2.1
6298	1.69136	1.49979
6299	1.64391	1.33659
6300	1.76477	1.21244
6301	0.611486	1.37127
6302	0.683421	0.973748
6303	0.946131	1.16483
6304	1.1019	1.14597
6305	1.12946	0.996292
6306	1.54226	1.61084
6307	1.19496	1.21026
6308	1.96199	1.07145
6309	2.0775	1.42443
6310	1.22297	1.12037
6311	1.3469	0.875566
6312	1.25366	1.0716
6313	1.65443	1.13079
6314	1.65443	1.19161
6315	0.946132	1.26662
6316	0.946132	1.42885
6317	1.12873	1.241
6318	1.07468	0.971789
6319	1.2854	0.634906

6320	1.3469	0.904285
6321	1.68616	0.86625
6322	1.68616	0.735159
6323	0.946132	1.228
6324	0.946132	1.16689
6325	1.16109	0.961251
6326	1.17879	0.803315
6327	1.17879	0.838977
6328	1.45304	1.09606
6329	2.21058	2.1
6330	2.81552	2.01243
6331	1.45535	0.332254
6332	1.49906	0.240745
6333	1.04413	2.1
6334	1.74586	1.81691
6335	1.34526	2.1
6336	1.17402	0.898952
6337	1.74564	0.775183
6338	1.52724	2.1
6339	1.68142	2.1
6340	1.09651	2.1
6341	1.49149	2.1
6342	1.36814	2.1
6343	1.46783	1.06984
6344	2.18116	1.36234
6345	1.23602	1.06633
6346	1.74538	0.889869
6347	0.851539	2.1
6348	1.45775	1.08483
6349	1.28919	2.1
6350	0.851483	2.1
6351	1.26102	1.02475
6352	0.869889	2.08643
6353	0.85151	1.83025
6354	1.23451	0.780402
6355	1.23602	0.979285
6356	1.11955	2.1
6357	1.08022	2.1
6358	0.977776	2.1
6359	1.27896	2.1
6360	1.64268	1.90918
6361	1.97548	2.1
6362	1.72527	1.56746
6363	0.946132	2.1
6364	1.69702	1.24825
6365	1.69702	2.1
6366	1.13635	2.1
6367	1.40274	1.03243
6368	1.24041	2.1
6369	1.13635	1.96916
6370	1.78427	0.10755
6371	1.68338	0.135717
6372	1.00681	0.0919929
6373	1.12988	0.0860798
6374	0.946132	0.0953279
6375	0.957543	0.0889372
6376	1.40515	0.0911028
6377	1.40515	0.0854288

6378	0.797932	0.10441
6379	1.30187	0.104516
6380	1.14846	0.100518
6381	0.665137	0.250581
6382	1.09575	0.371393
6383	0.596894	0.316451
6384	1.04072	0.448001
6385	0.663171	0.485151
6386	1.47218	0.392422
6387	1.32889	0.360925
6388	1.32639	0.187403
6389	1.94887	0.205888
6390	1.32639	0.19264
6391	1.19323	0.259921
6392	2.19794	0.326383
6393	0.754756	0.164054
6394	1.11015	0.19122
6395	0.754755	0.168133
6396	0.946133	0.21953
6397	0.754768	0.221395
6398	1.59986	0.19511
6399	1.59986	0.165053
6400	1.84082	2.1
6401	1.79044	2.1
6402	1.75922	2.1
6403	1.86766	2.1
6404	1.58995	2.1
6405	1.58995	2.1
6406	1.74351	2.1
6407	1.04442	2.1
6408	1.42278	2.1
6409	1.08576	2.1
6410	0.994294	2.1
6411	1.44884	1.30397
6412	1.71036	0.972166
6413	0.714101	0.657427
6414	1.17541	1.05754
6415	1.00872	0.469325
6416	0.714072	0.680174
6417	0.936677	0.648387
6418	0.966979	2.1
6419	1.4908	2.1
6420	1.03108	0.544592
6421	0.949712	0.368325
6422	0.810946	0.468671
6423	0.88209	0.480159
6424	0.646702	0.419474
6425	0.972663	0.366017
6426	0.862301	0.38221
6427	0.868143	0.33235
6428	1.11094	0.399693
6429	1.35809	0.40017
6430	1.32455	2.1
6431	1.32328	2.1
6432	1.69586	2.1
6433	1.78301	2.1
6434	1.67906	2.1
6435	1.60635	2.1

6436	1.57234	2.1
6437	1.47012	2.1
6438	1.22811	0.240854
6439	1.08732	0.302676
6440	2.08849	0.26328
6441	1.08732	0.277044
6442	1.30635	0.0746974
6443	0.77315	0.0734382
6444	1.25732	0.0677829
6445	2.43845	2.06905
6446	2.38577	1.02926
6447	0.946132	0.884998
6448	1.19288	1.96039
6449	1.54508	2.1
6450	1.54508	1.38128
6451	0.94515	1.87909
6452	1.19288	1.46875
6453	1.29735	1.08001
6454	1.12087	0.760238
6455	1.54699	1.08414
6456	1.43635	2.1
6457	1.33385	2.1
6458	1.43635	2.1
6459	1.02368	2.1
6460	1.035 2.1	
6461	1.00128	2.1
6462	1.04009	2.1
6463	1.14052	2.1
6464	0.443552	2.1
6465	0.984351	2.1
6466	0.591443	2.1
6467	0.929561	2.1
6468	1.52079	2.1
6469	1.035 2.1	
6470	3.5282	1.69916
6471	1.88288	1.65138
6472	2.73877	1.04329
6473	0.906059	2.1
6474	1.23107	0.13592
6475	0.906712	0.151193
6476	0.841322	0.172667
6477	1.53005	0.1314
6478	1.10007	0.15324
6479	1.38033	0.148545
6480	1.03442	0.13655
6481	0.787223	0.144918
6482	1.42589	0.145684
6483	2.35665	0.14467
6484	1.17793	0.266464
6485	1.81356	0.281363
6486	1.42964	0.266184
6487	1.95641	0.279163
6488	1.39139	0.437381
6489	1.39139	0.589946
6490	0.564533	0.400166
6491	1.20016	0.631344
6492	0.755765	0.26996
6493	0.755766	0.344836

6494	1.20016	0.45884
6495	1.91011	0.729649
6496	1.4695	0.368138
6497	1.70048	2.1
6498	1.93765	1.2519
6499	0.741307	2.1
6500	0.780371	2.1
6501	0.946133	0.792893
6502	1.28918	1.11932
6503	1.5111	1.56495
6504	1.5111	0.781762
6505	0.946131	1.24769
6506	1.28918	0.824732
6507	1.16805	0.719138
6508	1.01179	0.633317
6509	0.946132	0.805592
6510	1.35328	1.13179
6511	1.69633	1.46009
6512	1.69633	0.805361
6513	0.946133	1.29164
6514	1.35328	0.847116
6515	1.28918	0.71567
6516	1.01179	1.03022
6517	1.58147	1.03484
6518	1.27737	0.691657
6519	2.17223	0.173116
6520	0.697168	0.151473
6521	1.62495	0.360671
6522	1.62495	0.362957
6523	1.93948	0.594939
6524	0.601592	2.1
6525	1.93528	2.1
6526	2.13757	2.1
6527	1.67561	2.1
6528	2.70875	2.1
6529	1.93528	2.1
6530	3.98475	2.1
6531	1.12376	2.1
6532	1.19016	2.1
6533	1.67323	0.18331
6534	1.74019	0.163552
6535	1.84719	0.197372
6536	1.00764	0.166175
6537	1.23475	2.1
6538	1.06757	2.1
6539	1.57916	1.45168
6540	1.06758	2.1
6541	1.28014	2.1
6542	1.33339	2.1
6543	1.2679	2.1
6544	1.23504	2.1
6545	1.50108	2.1
6546	1.3715	2.1
6547	0.58831	0.905003
6548	0.865971	1.04994
6549	0.813704	1.25098
6550	0.658856	1.2381
6551	0.495869	1.21721

6552	1.13845	1.00818
6553	0.780804	1.23896
6554	0.642023	1.04682
6555	0.707748	0.882953
6556	1.64476	0.846186
6557	1.64476	1.09129
6558	0.99611	0.857638
6559	1.35639	0.869
6560	1.3564	1.16957
6561	0.946129	1.20909
6562	1.99222	0.813508
6563	1.4777	2.1
6564	1.03968	2.1
6565	1.82516	2.1
6566	0.851539	2.1
6567	1.63693	2.1
6568	1.59416	2.1
6569	0.878566	2.02821
6570	1.40796	2.1
6571	1.13172	1.6898
6572	0.851527	0.928975
6573	1.14366	2.1
6574	0.823822	0.2878
6575	0.882001	0.302633
6576	1.09756	0.13258
6577	1.11857	0.18734
6578	1.0934	0.177655
6579	0.960104	0.279797
6580	0.982001	0.364047
6581	1.74319	0.222656
6582	1.03043	0.208489
6583	0.967194	0.198416
6584	1.16938	2.1
6585	2.48377	2.1
6586	1.1181	2.1
6587	2.57349	2.1
6588	1.0465	2.1
6589	1.44709	2.1
6590	0.946132	2.1
6591	1.29302	2.1
6592	1.73502	2.1
6593	1.73502	2.1
6594	0.946132	2.1
6595	1.29302	2.1
6596	1.38813	2.1
6597	1.03933	2.1
6598	1.01976	0.347708
6599	1.11764	0.432801
6600	0.961779	0.540842
6601	1.0685	0.451156
6602	1.08426	1.07164
6603	0.99695	1.37253
6604	2.25593	1.45496
6605	1.47992	1.63253
6606	1.88989	1.06571
6607	1.65813	0.98804
6608	1.20278	1.26806
6609	0.981842	1.07307

6610	0.802655	1.84892
6611	0.953489	1.85546
6612	0.961719	1.74075
6613	0.975614	1.62643
6614	0.991617	1.09341
6615	0.940748	1.28739
6616	2.25019	1.89315
6617	2.30712	1.27709
6618	1.98823	1.77042
6619	0.522844	1.21233
6620	0.946122	1.76549
6621	0.946132	1.42287
6622	0.593846	1.13973
6623	1.26501	1.21193
6624	1.21171	0.142537
6625	0.851542	0.197772
6626	0.940327	0.211272
6627	0.940356	0.214609
6628	1.52117	0.158071
6629	1.58072	0.139524
6630	1.35169	0.170006
6631	1.13889	0.164408
6632	1.35007	0.124391
6633	1.35007	0.125971
6634	1.78381	0.144806
6635	0.946132	0.196504
6636	1.06627	0.186572
6637	0.946131	0.21121
6638	1.73005	0.142999
6639	1.73005	0.140529
6640	1.32449	0.166649
6641	1.35169	0.16546
6642	1.42446	0.584184
6643	1.86697	0.563569
6644	1.13413	0.535647
6645	1.42446	0.444453
6646	1.33575	0.216786
6647	1.20473	0.239799
6648	1.87517	0.107534
6649	1.87517	0.106168
6650	0.488712	0.164015
6651	1.13242	0.137167
6652	0.488713	0.148634
6653	0.488713	0.140165
6654	1.23146	0.136019
6655	1.57932	0.109318
6656	1.01698	0.118655
6657	1.42085	0.118555
6658	0.246725	0.106725
6659	0.996283	0.109883
6660	1.36965	0.116869
6661	0.86361	0.148299
6662	0.810945	0.103463
6663	1.72938	0.116787
6664	1.21649	0.147445
6665	1.1865	0.114719
6666	1.62387	0.119034
6667	1.05302	0.132029

6668	1.10049	0.594848
6669	0.946129	2.03718
6670	1.70184	0.872058
6671	1.70184	2.1
6672	1.12538	1.98323
6673	1.35969	0.803322
6674	1.28828	1.60488
6675	1.12538	1.17907
6676	0.99747	1.33746
6677	1.34851	0.831909
6678	0.923724	2.1
6679	1.47154	0.873249
6680	1.47154	2.08726
6681	1.10698	2.1
6682	1.28828	0.798521
6683	1.10698	1.84596
6684	0.923695	1.37033
6685	0.99747	0.650782
6686	1.65677	2.1
6687	1.77677	2.1
6688	1.05689	0.278301
6689	2.11031	0.266531
6690	1.07367	0.19115
6691	0.656239	0.262811
6692	1.4563	0.242797
6693	1.4563	0.218695
6694	0.656238	0.247646
6695	0.946133	0.263615
6696	0.812937	0.305337
6697	0.946131	0.317618
6698	1.05689	0.203059
6699	1.34009	2.1
6700	1.398 2.1	
6701	3.29047	2.1
6702	2.53897	2.1
6703	1.24509	2.1
6704	3.06162	2.1
6705	1.01595	2.1
6706	1.52661	2.1
6707	1.5266	2.1
6708	0.946131	2.1
6709	1.31049	2.1
6710	1.16225	2.1
6711	1.05978	2.1
6712	1.16225	2.1
6713	1.06561	0.891451
6714	0.953927	2.1
6715	1.15114	1.09042
6716	1.1807	0.469753
6717	0.507259	0.39721
6718	1.47709	0.391838
6719	1.36192	0.356152
6720	0.487293	0.375222
6721	1.65569	0.466446
6722	0.679725	0.270093
6723	0.721928	0.370516
6724	1.03931	0.371423
6725	1.0775	0.424331

6726	1.0775	0.427157
6727	2.30207	0.737275
6728	1.01814	0.699839
6729	2.33963	0.63476
6730	2.42589	0.730204
6731	1.33962	2.1
6732	1.27956	2.1
6733	2.14624	0.089174
6734	1.6812	0.100452
6735	1.25698	0.0925525
6736	1.35725	0.100809
6737	1.86387	0.0897556
6738	1.86387	0.0867626
6739	0.750365	0.109714
6740	0.946128	0.0996016
6741	0.750365	0.0883887
6742	1.72271	0.0873126
6743	1.72272	0.11139
6744	2.53736	0.12733
6745	1.07327	2.1
6746	1.05376	2.1
6747	1.00387	0.878567
6748	2.0554	0.815242
6749	1.00387	0.85538
6750	1.00387	0.822403
6751	1.27119	2.1
6752	1.51748	2.1
6753	3.15271	2.1
6754	3.45801	2.1
6755	1.99871	2.1
6756	1.41186	2.1
6757	1.07624	2.1
6758	1.5954	2.1
6759	1.19323	0.862741
6760	1.21286	0.789924
6761	0.946114	2.1
6762	1.38395	2.1
6763	1.73389	2.1
6764	1.7339	2.1
6765	0.946115	2.1
6766	1.38397	2.1
6767	1.29604	2.1
6768	1.04544	2.1
6769	1.60578	0.134305
6770	1.93407	0.132817
6771	1.0525	0.821395
6772	1.0525	1.16322
6773	1.0525	1.4233
6774	1.38506	1.51795
6775	2.34737	0.0822623
6776	1.3531	0.0735691
6777	1.2925	0.071521
6778	0.871466	0.0843995
6779	1.26623	0.0771685
6780	0.700631	0.0785604
6781	1.88774	0.0730394
6782	1.14167	0.075911
6783	1.18606	0.0744833

6784	2.1238	0.0763673
6785	1.42762	0.718653
6786	1.67791	0.178405
6787	1.47118	0.169944
6788	0.946128	2.1
6789	1.31641	2.1
6790	1.75933	2.1
6791	1.75933	2.1
6792	0.946131	2.1
6793	1.31641	2.1
6794	1.38905	2.1
6795	1.38437	2.1
6796	1.51829	2.1
6797	0.947114	2.1
6798	1.13335	2.1
6799	1.47521	2.1
6800	1.14911	2.1
6801	1.53822	2.1
6802	1.39702	2.1
6803	0.739166	2.1
6804	0.706056	2.1
6805	0.964033	2.08429
6806	1.58993	1.99341
6807	2.51892	1.61847
6808	0.595905	0.146473
6809	1.18155	0.119278
6810	1.73364	0.113631
6811	0.952735	0.122382
6812	2.10381	0.113384
6813	1.12115	0.112136
6814	1.27826	0.117114
6815	2.74021	0.108806
6816	1.78416	2.1
6817	1.78416	2.1
6818	0.946132	2.1
6819	1.41126	2.1
6820	1.21275	2.1
6821	0.946133	2.1
6822	1.41126	2.1
6823	1.33125	2.1
6824	1.33125	2.1
6825	1.78918	2.1
6826	1.0465	2.1
6827	1.99715	2.1
6828	1.17683	2.1
6829	1.25889	2.1
6830	0.860834	0.278477
6831	0.946131	0.26081
6832	1.05008	0.182686
6833	0.970444	0.303245
6834	1.19401	0.251698
6835	1.25989	0.176931
6836	0.702942	0.330886
6837	1.40591	0.399963
6838	1.72168	0.367932
6839	0.446352	0.3622
6840	1.04072	0.294655
6841	0.711038	0.37949

6842	0.980737	0.371148
6843	1.05099	0.291111
6844	1.26003	0.20216
6845	0.495934	0.390976
6846	1.03114	0.198516
6847	1.09911	0.225617
6848	1.24825	0.211695
6849	2.19827	0.193266
6850	2.0953	0.120839
6851	2.30437	0.141969
6852	0.775923	0.128619
6853	2.2283	0.106319
6854	1.19583	2.1
6855	1.14496	2.1
6856	1.62183	2.1
6857	1.62316	2.1
6858	1.02305	0.493274
6859	1.21136	0.36152
6860	1.44426	0.438698
6861	1.21137	0.287165
6862	1.20143	0.402498
6863	1.15009	0.772283
6864	1.24965	2.1
6865	1.41077	2.1
6866	1.00052	0.145445
6867	1.79553	0.148031
6868	1.06336	0.112478
6869	1.00053	0.13249
6870	1.18041	2.1
6871	1.80818	1.04855
6872	1.73571	1.20563
6873	1.94429	1.3673
6874	1.4375	0.18031
6875	1.44421	0.152046
6876	1.66953	0.224648
6877	1.09529	0.190381
6878	0.952395	2.1
6879	0.717337	2.1
6880	1.03609	1.12962
6881	1.33949	0.508741
6882	1.2774	0.464282
6883	2.24983	0.413439
6884	1.28727	0.433927
6885	0.583592	0.400141
6886	0.583591	0.504657
6887	1.82859	0.463915
6888	1.82859	0.448586
6889	0.583593	0.501001
6890	0.946128	0.402449
6891	0.692233	0.560942
6892	1.12226	0.52002
6893	2.57454	0.545546
6894	0.810944	0.498523
6895	0.810945	0.477856
6896	1.12226	0.505363
6897	1.45867	0.503488
6898	1.22403	0.453255
6899	1.15262	0.543874

6900	1.08132	0.49678
6901	0.846671	0.472181
6902	0.480954	0.523316
6903	0.917975	0.499016
6904	0.917975	0.668821
6905	0.720957	0.516452
6906	0.795998	0.470063
6907	0.69316	0.616988
6908	0.641306	0.549972
6909	0.690817	0.442202
6910	0.961899	2.1
6911	2.49579	2.1
6912	2.31106	2.1
6913	1.70482	0.349592
6914	1.93156	0.376246
6915	1.23868	0.357915
6916	1.73878	0.415142
6917	1.26267	2.1
6918	1.26441	2.1
6919	1.58095	2.1
6920	1.58095	2.1
6921	0.946132	2.1
6922	0.946132	2.1
6923	1.11077	2.1
6924	1.62905	2.1
6925	1.49692	2.1
6926	3.49966	2.1
6927	2.39213	2.1
6928	2.12603	2.1
6929	0.946129	2.1
6930	0.461661	2.1
6931	1.52841	2.1
6932	1.39091	2.1
6933	0.550679	2.1
6934	0.509894	2.1
6935	1.49508	2.1
6936	2.05862	2.1
6937	2.47851	2.1
6938	1.07213	2.1
6939	1.09148	0.615571
6940	1.99776	0.6556
6941	1.38333	0.644962
6942	1.04412	2.1
6943	1.04412	2.1
6944	1.1893	0.254964
6945	0.946132	0.270642
6946	0.965737	0.271761
6947	0.946132	0.266336
6948	1.54061	0.263486
6949	1.54061	0.266422
6950	1.22878	0.261828
6951	1.25797	0.236838
6952	1.00665	0.251524
6953	1.00665	0.272282
6954	1.80369	0.253888
6955	0.946128	0.269076
6956	1.04424	0.277972
6957	0.85154	0.278609

6958	1.39322	0.262857
6959	1.35464	0.27168
6960	0.939857	0.259003
6961	1.28272	0.247226
6962	1.19323	0.273723
6963	1.03498	0.145491
6964	2.22029	0.165564
6965	0.540005	0.14403
6966	0.970382	0.15613
6967	2.14514	0.190717
6968	1.31542	0.191099
6969	1.05575	0.246933
6970	0.810943	0.28353
6971	1.31542	0.173283
6972	1.70493	0.195314
6973	1.22582	0.228163
6974	1.31681	0.261035
6975	1.40775	0.188716
6976	0.928633	0.322913
6977	1.2545	0.190898
6978	2.66481	0.220396
6979	1.32051	0.209795
6980	1.32051	0.241729
6981	2.66305	2.1
6982	1.3551	2.1
6983	2.45325	2.1
6984	1.3685	2.1
6985	1.51748	2.1
6986	1.78941	2.1
6987	1.02963	0.114761
6988	1.28643	0.139701
6989	0.86131	0.149171
6990	0.851539	0.163633
6991	1.46585	0.118253
6992	1.38822	0.111344
6993	0.996402	0.12327
6994	0.851553	0.1469
6995	1.73156	0.138162
6996	1.91695	0.122386
6997	1.92535	0.143274
6998	2.57287	0.126104
6999	1.77743	0.122184
7000	1.02836	0.362646
7001	1.36362	0.379671
7002	1.36936	0.287011
7003	1.78684	0.255221
7004	1.78685	0.295367
7005	0.946132	0.427522
7006	1.27581	0.402088
7007	0.946132	0.479595
7008	1.02836	0.231307
7009	1.55398	2.1
7010	1.07167	2.1
7011	1.87557	2.1
7012	0.751639	2.1
7013	1.50884	2.1
7014	1.33793	2.1
7015	0.751664	2.1

7016	1.37277	2.1
7017	0.979515	2.1
7018	0.676508	2.1
7019	1.09578	2.1
7020	0.953925	2.1
7021	0.94613	2.1
7022	1.64707	2.1
7023	1.64707	2.1
7024	1.16658	2.1
7025	1.40638	2.1
7026	1.18682	2.1
7027	1.16658	2.1
7028	0.543765	0.219654
7029	1.57462	0.219418
7030	1.57462	0.175903
7031	1.00083	0.219535
7032	1.16834	0.276226
7033	1.16834	0.246471
7034	0.889626	0.251215
7035	1.25944	0.249351
7036	0.824061	0.271608
7037	1.9397	0.164924
7038	1.21893	0.196666
7039	1.91364	0.18291
7040	1.33672	0.244301
7041	0.928069	0.329424
7042	1.41484	0.243531
7043	0.538379	0.226219
7044	1.41484	0.22283
7045	1.35594	0.253527
7046	2.11414	0.23483
7047	2.11414	0.160343
7048	0.538378	0.23896
7049	1.38532	0.15869
7050	0.962877	0.503797
7051	1.07497	0.375906
7052	1.00613	0.397211
7053	0.962878	0.36517
7054	0.711447	2.1
7055	1.40008	0.26692
7056	2.51674	0.319479
7057	1.40008	0.263017
7058	1.7263	0.190657
7059	1.40008	0.314257
7060	1.9216	0.0976568
7061	2.7013	0.102301
7062	2.58584	0.0908806
7063	0.979296	0.102936
7064	1.46459	0.383572
7065	0.899254	0.324901
7066	1.15038	1.80096
7067	1.24687	1.38308
7068	1.16738	1.7908
7069	0.942323	2.1
7070	1.65573	1.33897
7071	0.844791	2.09467
7072	0.54674	1.47762
7073	0.795811	1.80372

7074	1.08131	1.92335
7075	1.03553	1.8244
7076	0.758334	2.00891
7077	0.708665	1.56872
7078	1.22626	1.58331
7079	0.835439	1.59056
7080	0.861131	1.399
7081	0.68296	1.71693
7082	1.65224	1.69343
7083	2.82624	1.76721
7084	1.0151	1.18604
7085	1.15905	1.43596
7086	1.71076	1.30068
7087	1.4529	1.04008
7088	0.494895	1.96725
7089	0.946132	1.43495
7090	0.613132	1.27369
7091	0.863631	1.3366
7092	1.78391	2.1
7093	1.1706	0.0848538
7094	2.02734	0.0960355
7095	1.1706	0.0705394
7096	0.681532	0.0793305
7097	1.50316	0.0762957
7098	1.50316	0.0712445
7099	0.681534	0.0761947
7100	0.973819	0.0826719
7101	0.97382	0.0792489
7102	0.94613	0.0841158
7103	1.1706	0.0748411
7104	1.67727	0.124149
7105	1.60329	0.111388
7106	1.3227	2.1
7107	1.40846	2.1
7108	1.07889	2.1
7109	1.07721	2.1
7110	0.953523	0.734729
7111	1.79675	0.739191
7112	2.09859	0.818008
7113	1.21309	2.05263
7114	1.54567	0.689031
7115	1.19722	0.714846
7116	1.82793	0.757075
7117	1.1599	0.557137
7118	1.73621	0.522063
7119	0.933474	0.603617
7120	1.51477	0.5846
7121	1.51477	0.621489
7122	0.93347	0.586992
7123	1.27374	0.592034
7124	1.27376	0.637609
7125	0.946105	0.675075
7126	1.1972	0.560189
7127	0.851541	0.0906149
7128	1.36971	0.0868238
7129	1.27832	0.0848857
7130	0.853489	0.0853901
7131	0.973847	0.092731

7132	0.976308	0.0926303
7133	0.851528	0.0992372
7134	2.72654	0.0795834
7135	1.77867	0.0737757
7136	3.23032	2.1
7137	2.78208	2.1
7138	1.0227	0.0945204
7139	1.02269	0.084362
7140	1.02269	0.0849989
7141	1.24293	0.097843
7142	0.831695	2.1
7143	0.67513	2.1
7144	0.956294	2.1
7145	1.08623	2.1
7146	1.05556	2.1
7147	1.38859	2.1
7148	1.13179	2.1
7149	1.02438	2.1
7150	1.06659	2.1
7151	1.36595	1.78823
7152	1.06659	2.02379
7153	1.06659	0.686005
7154	0.946131	1.00441
7155	0.875912	2.1
7156	1.65971	2.1
7157	1.65971	1.43097
7158	0.875912	2.09417
7159	1.1229	1.85114
7160	1.41273	1.7179
7161	1.54699	1.32051
7162	1.93655	1.39452
7163	1.42307	0.943847
7164	1.97907	1.2474
7165	0.996774	2.1
7166	1.15505	0.107514
7167	1.28463	0.0982963
7168	1.86353	0.127158
7169	2.71398	0.13316
7170	1.56787	0.164182
7171	1.66295	0.177279
7172	1.73432	1.58635
7173	1.05019	0.0807195
7174	1.42117	0.081438
7175	0.888299	0.078912
7176	1.38127	0.0841994
7177	1.72993	0.0796349
7178	0.664129	0.0801313
7179	0.99302	0.619837
7180	0.993023	0.61413
7181	1.13491	2.1
7182	0.876316	1.43832
7183	1.2588	2.1
7184	0.895613	2.00105
7185	2.07526	2.1
7186	1.23369	1.83727
7187	1.25146	2.1
7188	2.15282	2.05334
7189	1.22239	1.46916

7190	1.10962	2.1
7191	1.77402	1.48889
7192	1.0305	2.1
7193	1.50108	1.21015
7194	1.20761	2.1
7195	1.3626	2.1
7196	1.14825	2.1
7197	1.78711	2.1
7198	1.17933	2.1
7199	1.14824	2.1
7200	1.09651	0.192002
7201	1.23851	0.168641
7202	1.83284	0.21097
7203	1.23851	0.134236
7204	1.53521	2.1
7205	1.46279	2.1
7206	1.9233	0.313586
7207	1.2966	0.23631
7208	2.11443	0.219387
7209	1.30265	0.246462
7210	1.13528	0.289169
7211	0.946131	0.466057
7212	1.41169	0.334549
7213	1.33879	0.283233
7214	0.946128	0.281661
7215	1.80435	0.297926
7216	1.80435	0.191978
7217	1.234	2.1
7218	1.45633	2.1
7219	1.234	2.1
7220	1.98448	2.1
7221	2.02612	2.1
7222	1.23527	0.717471
7223	1.44472	0.622563
7224	0.993366	0.784095
7225	1.22025	0.705354
7226	0.993368	0.654499
7227	2.43669	2.1
7228	2.56707	2.1
7229	1.80348	1.16758
7230	1.74406	0.813573
7231	1.90937	1.00374
7232	1.49357	0.329007
7233	2.5832	0.580456
7234	1.82274	0.56102
7235	1.6957	0.501449
7236	2.22495	0.4788
7237	2.49959	0.514136
7238	2.01183	0.306277
7239	1.37427	0.224998
7240	1.15069	0.965248
7241	1.39379	0.833848
7242	1.69661	0.389977
7243	1.39379	0.486449
7244	1.39654	2.1
7245	1.93505	2.1
7246	1.5525	2.1
7247	1.5525	2.1

7248	0.615065	2.1
7249	1.38908	2.1
7250	0.615065	2.1
7251	0.77849	2.1
7252	1.38908	2.1
7253	1.56839	0.984775
7254	1.09142	1.20177
7255	2.07313	0.95736
7256	0.998288	0.125261
7257	1.31481	0.131384
7258	1.88175	0.131191
7259	2.23267	0.1553
7260	1.06935	2.1
7261	1.06936	2.1
7262	1.17989	1.4585
7263	1.00757	0.947728
7264	1.65318	0.792181
7265	1.4185	0.365576
7266	2.22495	0.389793
7267	1.02011	0.226937
7268	0.59676	0.311277
7269	1.43606	0.28921
7270	1.43606	0.24696
7271	0.89771	0.284105
7272	1.13511	0.348416
7273	0.59676	0.184723
7274	0.897709	0.299167
7275	1.79542	0.271787
7276	2.38566	2.1
7277	1.63838	0.883356
7278	1.63837	1.28752
7279	0.916275	1.98824
7280	1.36183	0.977782
7281	1.19282	2.0855
7282	0.916275	1.59986
7283	1.19283	1.81854
7284	2.01582	2.1
7285	1.00215	0.124181
7286	2.60643	2.1
7287	2.15971	2.1
7288	1.28267	2.1
7289	1.29954	2.1
7290	3.40327	2.1
7291	3.49128	2.1
7292	1.3864	2.1
7293	1.3201	2.1
7294	1.45666	0.0730818
7295	1.55798	0.0698699
7296	0.857202	0.0735531
7297	1.24374	0.0744976
7298	1.04157	0.0758385
7299	0.85723	0.0735942
7300	1.02722	0.0741805
7301	1.01361	0.0778017
7302	0.974077	0.0653825
7303	1.01814	0.0697927
7304	0.974077	0.0646081
7305	1.43987	0.0624679

7306	1.43986	0.0598431
7307	0.927881	0.0610445
7308	0.946132	0.0635359
7309	0.927849	0.0624348
7310	0.927852	0.0603171
7311	1.2493	0.0624549
7312	1.22482	0.679574
7313	1.41319	0.802421
7314	1.22482	0.521169
7315	0.999492	2.1
7316	1.14657	2.1
7317	1.3617	0.0787637
7318	0.983269	0.0841485
7319	1.33174	0.0891236
7320	1.62816	0.0904133
7321	1.62817	0.0827567
7322	0.81938	0.0807684
7323	1.33174	0.0823736
7324	1.01332	0.090293
7325	0.874788	0.0688129
7326	1.16725	2.1
7327	1.63991	2.1
7328	1.08848	0.118236
7329	1.15354	0.102542
7330	1.12665	2.1
7331	1.58742	2.1
7332	1.57523	2.1
7333	1.32201	0.824122
7334	1.32097	0.726175
7335	0.851542	0.447721
7336	0.877188	0.360707
7337	0.797489	0.46554
7338	1.44721	0.350578
7339	1.61761	0.336913
7340	0.943799	0.395931
7341	1.36079	0.392665
7342	1.16361	1.72121
7343	1.15852	1.19054
7344	1.07624	2.1
7345	1.07624	2.1
7346	0.946132	0.801947
7347	1.37938	1.08384
7348	1.78653	1.37051
7349	1.78653	0.818078
7350	0.946131	1.29426
7351	1.37938	0.886666
7352	1.35328	0.748293
7353	1.44847	0.721545
7354	0.946133	0.831107
7355	1.38149	1.02089
7356	1.81474	1.1851
7357	1.81474	0.821062
7358	0.946133	1.29377
7359	1.38149	0.911341
7360	1.37938	0.788525
7361	1.44847	0.972218
7362	1.93665	0.794267
7363	1.5515	0.742359

7364	1.05025	2.1
7365	1.05025	2.1
7366	1.71468	1.10116
7367	1.04759	2.1
7368	1.04646	0.097304
7369	1.00739	0.0994637
7370	1.37086	0.0878948
7371	1.12547	0.0981917
7372	1.03853	0.0953214
7373	1.34824	0.0864851
7374	0.799786	0.0924421
7375	2.10105	0.0969456
7376	2.72007	0.0904468
7377	0.589918	0.0798158
7378	0.589918	0.0843814
7379	1.1509	0.0823202
7380	0.619832	0.077299
7381	1.1509	0.085427
7382	0.705903	0.0842866
7383	1.53703	0.0793753
7384	1.53703	0.0824317
7385	0.705904	0.0820161
7386	0.946133	0.0849479
7387	0.946132	0.0819827
7388	0.946132	0.0894954
7389	0.588467	0.0740654
7390	1.1078	0.0800029
7391	0.408061	0.0764454
7392	1.38703	0.086183
7393	1.68328	0.149434
7394	1.87031	0.169793
7395	1.86268	0.235608
7396	1.27472	0.24859
7397	0.947862	0.99602
7398	1.19854	1.15155
7399	0.946129	0.828982
7400	0.963111	0.69042
7401	0.946131	0.854768
7402	1.39055	0.735058
7403	1.39055	0.714405
7404	1.26426	0.777838
7405	1.07242	0.797294
7406	1.31126	0.710862
7407	1.76597	0.862282
7408	0.94613	1.15704
7409	1.27214	0.939453
7410	1.90751	0.907388
7411	1.90751	0.851048
7412	0.767114	1.1155
7413	1.27214	1.06053
7414	1.40248	0.986439
7415	1.41479	1.10394
7416	2.22625	1.11418
7417	0.913373	1.19771
7418	1.0158	0.873625
7419	1.61259	0.801674
7420	1.69051	0.719018
7421	0.560949	1.11096

7422	0.898559	1.11101
7423	1.28011	0.980503
7424	1.51026	0.178389
7425	1.98252	0.218335
7426	1.05129	0.189789
7427	1.2951	0.196588
7428	1.46041	0.0587839
7429	1.7155	2.1
7430	1.72196	2.1
7431	0.933885	2.1
7432	0.986723	2.1
7433	1.28072	0.383527
7434	1.40765	0.768326
7435	1.28072	0.662001
7436	1.61687	2.1
7437	1.60965	2.1
7438	1.21424	2.1
7439	2.07297	2.1
7440	1.19943	2.1
7441	2.38784	2.1
7442	1.10278	0.189681
7443	1.43678	0.202597
7444	1.1142	0.292955
7445	1.18034	0.287217
7446	0.94613	0.331854
7447	0.752356	0.305508
7448	0.752329	0.311939
7449	1.46634	0.273858
7450	1.31789	0.262338
7451	0.958159	0.314802
7452	1.22752	0.265466
7453	1.50472	0.274638
7454	1.98974	2.1
7455	2.54186	2.1
7456	0.76512	2.1
7457	2.08777	2.1
7458	1.7753	2.1
7459	3.07372	2.1
7460	1.9756	2.1
7461	1.48162	2.1
7462	1.23204	2.1
7463	0.946131	0.93025
7464	1.13505	2.02673
7465	1.47432	2.1
7466	1.47432	1.24191
7467	0.876831	1.65405
7468	1.13505	1.41782
7469	1.2161	1.11031
7470	1.05267	1.3648
7471	1.05267	0.969221
7472	1.5795	1.16225
7473	0.98186	2.1
7474	1.56735	2.1
7475	0.859099	2.1
7476	1.69858	2.1
7477	1.93586	2.1
7478	1.09233	2.1
7479	1.08732	0.260835

7480	1.3357	0.268122
7481	1.84826	2.1
7482	0.924132	1.28825
7483	1.39188	2.1
7484	1.43685	2.1
7485	0.851541	2.00783
7486	1.16251	2.1
7487	0.946131	2.1
7488	1.05685	2.1
7489	2.13781	2.1
7490	1.98859	2.1
7491	1.99731	2.1
7492	1.51379	2.1
7493	1.48796	2.1
7494	0.851542	2.1
7495	0.802558	2.1
7496	1.09244	2.1
7497	1.33891	2.1
7498	0.844958	2.1
7499	0.710864	2.1
7500	1.26087	2.1
7501	1.03679	2.1
7502	1.51026	0.175672
7503	1.05248	0.470849
7504	0.827258	0.428849
7505	1.05248	0.449124
7506	1.28435	0.466665
7507	1.50953	0.49998
7508	1.50953	0.256057
7509	0.827298	0.471694
7510	1.00366	0.247038
7511	2.10495	0.323144
7512	1.17885	2.1
7513	1.25185	2.1
7514	1.02784	2.1
7515	1.76764	2.1
7516	1.23746	0.195059
7517	1.24082	0.210185
7518	1.46506	0.203011
7519	1.33061	0.149849
7520	0.768407	1.16946
7521	0.972058	0.814413
7522	1.16536	1.78276
7523	1.13325	1.06475
7524	0.978906	2.03198
7525	1.02291	0.990897
7526	0.838015	0.812226
7527	1.01052	0.216304
7528	1.08166	0.252129
7529	1.42607	0.230981
7530	1.47354	0.297824
7531	1.21703	2.1
7532	1.29897	2.1
7533	1.83293	2.1
7534	1.29897	2.1
7535	1.24688	0.15999
7536	0.782563	2.1
7537	0.946129	2.1

7538	0.782591	2.1
7539	1.49487	2.1
7540	1.49487	2.1
7541	1.37713	2.1
7542	0.900337	2.1
7543	1.31284	2.1
7544	0.924468	2.1
7545	1.40602	1.27628
7546	1.25669	1.32735
7547	0.986901	2.1
7548	0.958565	2.1
7549	1.37266	2.1
7550	1.64146	2.1
7551	0.49345	2.1
7552	1.08276	2.1
7553	1.08276	2.1
7554	1.26929	1.92928
7555	1.09847	2.1
7556	0.827081	0.389083
7557	1.07236	0.492669
7558	1.25063	0.387154
7559	1.43548	0.221465
7560	1.25063	0.177031
7561	1.3659	0.125996
7562	1.72441	0.119185
7563	1.3659	0.109904
7564	1.4677	0.124933
7565	1.86114	0.116693
7566	1.40104	0.144197
7567	0.962675	0.155286
7568	0.946131	0.168831
7569	1.74134	0.118501
7570	1.74134	0.112761
7571	0.962676	0.121386
7572	0.946131	0.150704
7573	1.05548	0.184135
7574	0.902187	0.215189
7575	2.14178	0.14635
7576	1.74998	0.171796
7577	1.38541	0.18473
7578	1.64468	0.14804
7579	0.949317	0.205399
7580	2.06654	0.162972
7581	0.839626	0.189417
7582	0.810945	0.20791
7583	1.29203	0.137304
7584	1.18091	0.164983
7585	1.06872	0.190513
7586	1.11956	0.149251
7587	0.926911	0.21414
7588	0.901716	0.198525
7589	0.909146	0.22524
7590	1.33776	0.154824
7591	1.02048	0.18338
7592	1.08365	0.200518
7593	1.21042	0.159989
7594	0.865634	0.215892
7595	0.590354	2.1

7596	0.61173	2.1
7597	3.46825	2.1
7598	3.28404	2.1
7599	1.56004	0.15136
7600	1.69344	0.227038
7601	1.7151	0.413513
7602	0.946131	0.16131
7603	1.40265	0.186555
7604	1.13537	0.399605
7605	0.965708	0.201346
7606	1.13537	0.31779
7607	1.29681	0.200813
7608	1.40915	0.249396
7609	1.61652	0.477761
7610	1.33171	0.502333
7611	1.37681	2.1
7612	0.81094	0.0855327
7613	0.598606	0.0787372
7614	0.723111	0.0842976
7615	1.04562	0.076157
7616	0.979052	0.0794308
7617	0.798185	0.0777696
7618	0.880117	0.0851857
7619	0.979341	0.512903
7620	0.800229	0.336136
7621	1.39126	0.508039
7622	1.13005	0.525692
7623	0.913877	0.541426
7624	1.26959	0.486442
7625	0.699741	0.53594
7626	1.19496	1.25986
7627	1.13392	0.124189
7628	1.11913	0.113001
7629	1.06866	0.106567
7630	0.615546	0.129873
7631	0.831006	0.150605
7632	1.53286	0.116616
7633	0.690977	0.127561
7634	1.01956	0.135969
7635	2.33818	0.0968676
7636	1.6585	2.1
7637	1.67061	2.1
7638	1.41414	2.1
7639	1.16756	2.1
7640	1.00898	0.0990998
7641	0.966964	2.1
7642	2.85443	2.1
7643	0.966977	2.1
7644	2.08427	2.1
7645	2.94901	2.1
7646	1.10735	0.245071
7647	1.65815	2.1
7648	1.4502	2.1
7649	1.45026	2.1
7650	1.53446	2.1
7651	0.946129	0.824015
7652	1.81075	2.1
7653	1.81075	0.818956

7654	0.946131	1.41001
7655	1.37075	2.1
7656	1.37075	0.855147
7657	1.34245	1.54158
7658	0.973502	0.881263
7659	1.08542	1.93514
7660	1.5814	0.0909479
7661	1.43836	0.0849901
7662	0.946132	0.117611
7663	1.13747	0.0991292
7664	1.04429	0.115149
7665	1.05017	0.106257
7666	1.24732	0.105058
7667	1.13387	0.0972458
7668	2.19489	0.0975553
7669	1.10748	1.85322
7670	1.25006	1.94629
7671	1.58834	1.15209
7672	1.58834	0.436171
7673	0.94613	1.39582
7674	1.16548	0.973761
7675	0.946132	0.434787
7676	1.08998	0.629531
7677	1.16548	0.602386
7678	0.995732	0.442526
7679	0.657014	2.1
7680	0.957173	1.95231
7681	1.01485	2.1
7682	1.12468	2.1
7683	1.43871	2.1
7684	1.23716	2.1
7685	1.89277	0.72921
7686	2.06653	0.576787
7687	0.967544	0.416087
7688	1.89016	0.492996
7689	2.22165	1.62231
7690	0.83514	1.34716
7691	1.76565	2.08523
7692	1.76565	2.1
7693	0.94613	1.27208
7694	0.835112	2.1
7695	1.23426	1.36153
7696	1.36653	1.729
7697	0.895892	0.907239
7698	0.859278	0.86661
7699	0.791461	0.934295
7700	1.86249	1.1113
7701	1.14607	0.949419
7702	1.19904	1.07505
7703	1.84898	0.988824
7704	1.03452	0.787437
7705	0.946131	0.462483
7706	0.940472	0.42731
7707	1.53478	0.754451
7708	1.84175	0.623302
7709	1.55687	0.583569
7710	1.17787	0.425573
7711	1.60589	0.481337

7712	2.35574	0.307542
7713	1.3305	0.418829
7714	1.15008	0.795717
7715	1.12457	0.110852
7716	1.12457	0.123891
7717	1.01778	2.1
7718	1.35733	2.1
7719	1.50683	2.1
7720	1.12064	2.1
7721	1.42716	0.71951
7722	1.42717	0.791553
7723	1.45927	1.10287
7724	1.04321	1.56425
7725	1.32634	0.268285
7726	2.27022	0.236548
7727	1.19352	0.179975
7728	1.32634	0.172037
7729	1.40924	1.62278
7730	1.22927	1.20102
7731	1.69237	1.43002
7732	1.69237	2.1
7733	0.946132	0.902385
7734	0.946133	1.42347
7735	1.16912	1.29815
7736	1.0417	1.23758
7737	1.14605	1.62011
7738	1.1233	2.1
7739	1.96882	1.7827
7740	1.03804	0.297644
7741	1.15338	0.305854
7742	1.15338	0.304452
7743	2.33051	0.340934
7744	1.81507	1.02024
7745	2.42949	1.78772
7746	0.725879	0.696006
7747	1.88394	0.76288
7748	2.2699	0.212808
7749	2.78952	1.7863
7750	1.88394	0.334327
7751	1.79606	2.1
7752	1.20091	2.1
7753	1.9614	2.05022
7754	1.19901	1.48512
7755	3.07915	2.1
7756	1.32592	2.1
7757	2.86461	2.1
7758	1.34817	2.1
7759	1.44859	2.1
7760	2.03969	2.1
7761	0.946132	2.1
7762	1.50709	2.1
7763	1.50709	2.1
7764	0.946132	2.1
7765	1.29322	2.1
7766	1.29322	2.1
7767	1.01985	2.1
7768	0.968537	2.1
7769	1.401	2.1

7770	1.28936	2.1
7771	1.56002	0.0608906
7772	1.92548	0.0601649
7773	1.46041	0.0609819
7774	2.22712	0.0591885
7775	1.56001	0.0604418
7776	1.50524	2.1
7777	1.21304	2.1
7778	1.60665	1.47257
7779	1.85178	0.996459
7780	2.17534	2.09897
7781	2.31012	2.1
7782	2.17533	1.32134
7783	0.986724	2.1
7784	2.01895	2.1
7785	2.00433	2.1
7786	1.45752	0.202368
7787	1.12695	0.180826
7788	0.652605	1.89359
7789	1.5133	2.1
7790	1.5133	2.1
7791	0.652603	1.62462
7792	1.228	2.1
7793	0.946132	2.1
7794	0.652604	2.1
7795	0.946132	1.02037
7796	0.716815	2.1
7797	1.62203	1.52159
7798	1.62203	2.1
7799	0.716814	2.08867
7800	1.00191	1.7858
7801	1.33694	1.39381
7802	1.38216	1.98753
7803	2.53338	1.82415
7804	1.46932	0.0982206
7805	1.50848	0.0971366
7806	1.50108	1.173
7807	1.18382	0.808883
7808	2.02115	0.649823
7809	0.921823	0.476777
7810	2.08356	0.0839302
7811	1.543	0.0702955
7812	1.00763	0.648195
7813	1.4185	0.663423
7814	1.08028	0.561623
7815	0.946131	0.765693
7816	1.56599	0.645323
7817	1.56599	0.803828
7818	1.08028	0.65927
7819	0.946131	0.506946
7820	1.37518	2.1
7821	1.63453	2.1
7822	1.04627	1.08633
7823	1.04626	1.02785
7824	1.35111	1.13484
7825	1.22297	1.06905
7826	1.64499	1.10473
7827	1.87598	0.901268

7828	1.06994	2.1
7829	1.37063	2.08899
7830	1.99943	0.0988412
7831	2.38807	0.0975828
7832	0.99972	0.128903
7833	1.41388	0.136075
7834	1.66179	0.122862
7835	1.66179	0.101155
7836	0.946129	0.151607
7837	1.41388	0.135445
7838	1.19404	0.13351
7839	1.31023	0.839619
7840	1.20871	0.878067
7841	1.69731	2.1
7842	1.67971	2.1
7843	0.998971	0.0762206
7844	1.32988	0.0867355
7845	0.701079	0.0878754
7846	0.946131	0.0932124
7847	0.582785	0.0872859
7848	1.48592	0.0805644
7849	1.48592	0.074485
7850	1.23351	0.083785
7851	0.835199	0.0866255
7852	0.92427	0.0762912
7853	1.68262	0.0773785
7854	2.46702	0.0880569
7855	1.08715	1.74948
7856	1.01606	1.52884
7857	1.29579	1.4529
7858	1.01606	1.86541
7859	1.63823	2.1
7860	1.67687	2.1
7861	1.11048	1.82921
7862	1.39477	2.1
7863	0.991114	2.1
7864	2.90978	0.797251
7865	0.906811	0.809956
7866	0.872849	0.560393
7867	1.17091	0.745811
7868	0.685763	0.789215
7869	1.6386	1.59904
7870	1.06907	0.900266
7871	1.05516	0.922226
7872	1.9189	0.994001
7873	1.04615	0.677696
7874	1.2966	2.1
7875	1.37392	2.1
7876	1.35504	2.1
7877	1.30347	2.1
7878	1.50877	2.1
7879	1.11193	2.1
7880	1.40984	1.99533
7881	1.67682	2.02881
7882	1.00371	2.1
7883	1.15412	2.1
7884	1.41638	2.1
7885	1.03796	2.1

7886	0.946117	2.1
7887	1.50823	2.1
7888	1.50822	2.1
7889	0.966923	2.1
7890	0.946131	2.1
7891	2.07593	2.1
7892	1.9339	2.1
7893	1.1817	0.729218
7894	0.729178	0.726622
7895	1.5079	0.774941
7896	1.26991	0.927006
7897	0.994049	0.715799
7898	1.17452	0.763666
7899	1.05711	0.854744
7900	1.04071	0.853289
7901	1.80299	0.702046
7902	1.60417	0.633444
7903	0.943405	0.214475
7904	1.01129	0.162296
7905	1.42972	0.177506
7906	1.15904	0.182596
7907	0.680409	0.155876
7908	1.2816	0.126016
7909	0.806176	0.156352
7910	1.18954	0.159972
7911	1.26055	0.199612
7912	1.06313	2.08446
7913	1.19352	2.1
7914	1.55766	2.1
7915	1.53729	2.1
7916	0.993021	0.250297
7917	1.09056	0.551127
7918	1.03408	1.8543
7919	0.993023	0.542042
7920	0.659901	2.1
7921	0.632905	2.1
7922	1.34072	0.233324
7923	1.17793	0.319713
7924	2.24885	0.28312
7925	0.946132	0.289279
7926	1.12443	0.287325
7927	0.904207	0.28786
7928	1.51237	0.258081
7929	1.51237	0.27641
7930	1.12443	0.270788
7931	1.29215	0.254174
7932	1.6943	1.81917
7933	2.43858	1.95562
7934	2.0589	1.35026
7935	1.67204	2.1
7936	1.67204	1.73098
7937	0.555875	1.65553
7938	0.91951	2.1
7939	0.946131	1.24013
7940	0.555904	1.56204
7941	0.946131	1.5418
7942	1.6943	2.1
7943	1.04744	1.41853

7944	1.50938	2.1
7945	1.60861	2.1
7946	1.92406	0.147906
7947	1.18716	0.147236
7948	0.962705	0.584075
7949	1.35382	0.730865
7950	1.43632	0.108671
7951	1.09386	0.1206
7952	1.92547	0.112896
7953	0.994164	0.112651
7954	0.903974	2.04473
7955	0.928829	1.69165
7956	0.810958	1.39807
7957	1.92853	1.67868
7958	1.598	1.62815
7959	1.28967	1.77978
7960	1.44993	1.59325
7961	0.946132	0.871581
7962	0.882984	0.810187
7963	0.882955	0.923333
7964	1.56071	0.639867
7965	1.56071	0.696772
7966	1.35946	0.796779
7967	1.08421	0.823524
7968	1.41943	0.0996821
7969	1.26265	0.0821423
7970	1.03235	0.550736
7971	1.50778	2.1
7972	1.70387	2.1
7973	1.65608	2.1
7974	1.39076	2.1
7975	1.6104	2.1
7976	1.37456	2.1
7977	1.51898	0.460206
7978	1.9726	0.372501
7979	2.13757	1.36021
7980	1.74856	1.43544
7981	0.635949	0.691695
7982	2.46448	0.90944
7983	1.23224	0.87522
7984	1.12786	1.00347
7985	1.82721	0.82584
7986	1.82721	0.671838
7987	0.532892	1.54213
7988	0.946131	1.07523
7989	0.53292	1.01108
7990	1.41394	2.1
7991	1.45506	2.1
7992	1.39523	2.1
7993	1.09006	2.1
7994	1.80264	0.134425
7995	1.80264	0.547531
7996	0.946131	0.28448
7997	1.34268	0.231969
7998	0.946132	0.493221
7999	1.23732	0.339449
8000	1.34268	0.349074
8001	1.2064	0.62749

8002	1.58164	2.1
8003	1.73853	2.1
8004	1.01243	0.958578
8005	0.910672	1.41848
8006	1.47793	1.49998
8007	1.47793	2.1
8008	0.910702	1.37906
8009	1.30382	0.99728
8010	1.08478	0.955017
8011	0.910702	1.65783
8012	2.60709	1.17031
8013	1.01243	0.992068
8014	1.85516	1.43374
8015	2.52823	0.6994
8016	1.24001	0.700604
8017	1.89793	1.0892
8018	1.73584	0.0833744
8019	1.24613	1.99145
8020	1.37074	1.69544
8021	1.23763	1.42238
8022	1.04072	1.92692
8023	0.75499	1.25662
8024	1.72329	1.56555
8025	1.46195	1.4256
8026	0.946132	1.30137
8027	0.747438	1.86481
8028	1.24613	1.3998
8029	2.59886	1.219
8030	1.87279	2.1
8031	1.07977	2.1
8032	1.29606	2.1
8033	1.45178	2.1
8034	1.45177	2.1
8035	0.92406	2.1
8036	0.946131	2.1
8037	0.924047	2.1
8038	2.04529	2.1
8039	1.04544	2.1
8040	1.08276	2.1
8041	1.67491	2.1
8042	0.961826	0.927663
8043	0.810942	0.912822
8044	0.826622	1.62923
8045	0.914378	1.5887
8046	0.89022	0.805381
8047	0.922383	1.04859
8048	0.988165	0.713298
8049	1.31357	0.145948
8050	0.946131	0.206133
8051	1.31357	0.16973
8052	1.10564	0.158885
8053	1.6797	0.147495
8054	1.6797	0.209325
8055	0.739506	0.196536
8056	1.33957	0.157132
8057	1.33957	0.175182
8058	1.33957	0.136201
8059	2.34223	0.184817

8060	1.5515	0.887175
8061	2.22137	0.767966
8062	0.94612	0.723734
8063	1.13908	0.779847
8064	1.45423	0.673065
8065	1.45422	0.829563
8066	0.795519	1.00519
8067	1.1391	0.757571
8068	1.11065	0.762531
8069	1.12266	1.801
8070	1.24925	1.8959
8071	1.20242	1.13103
8072	2.18297	1.23038
8073	3.00107	1.22448
8074	2.58297	0.623727
8075	0.884783	1.5468
8076	0.851538	0.794403
8077	0.713587	1.64271
8078	1.47949	0.840273
8079	1.66673	1.82208
8080	0.696199	1.4501
8081	0.9383	0.969352
8082	1.29149	1.00236
8083	1.32266	0.989147
8084	2.11428	0.0815562
8085	2.17439	0.0750962
8086	1.40489	0.0776155
8087	1.21071	0.0717734
8088	0.946118	0.0994046
8089	1.0746	0.0866768
8090	0.973119	0.0904644
8091	1.31333	0.0873685
8092	1.08723	0.0869532
8093	0.994292	2.1
8094	1.4964	2.1
8095	1.15096	2.1
8096	1.10101	2.1
8097	1.50438	2.1
8098	1.67825	2.1
8099	2.02103	2.1
8100	2.01978	2.1
8101	1.50931	0.131262
8102	1.50931	0.116808
8103	0.65023	0.106296
8104	1.34831	0.115683
8105	0.811233	0.122472
8106	1.34831	0.116641
8107	0.94613	0.129208
8108	1.00754	0.981648
8109	0.714132	1.81138
8110	1.28877	1.27558
8111	1.39905	1.20554
8112	1.03282	1.2136
8113	0.997769	0.975845
8114	1.11743	1.0826
8115	1.86863	0.962318
8116	3.30723	1.31409
8117	1.66635	1.83164

8118	1.35609	0.0859491
8119	1.38212	0.0931764
8120	1.95597	2.1
8121	1.64257	2.1
8122	2.93316	2.1
8123	3.13417	2.1
8124	0.733947	2.1
8125	1.15259	2.1
8126	1.07976	2.1
8127	1.30237	2.1
8128	1.99943	0.108545
8129	0.907431	0.115259
8130	0.878161	0.129635
8131	1.07544	0.0871271
8132	1.03401	0.104922
8133	0.991975	0.0883832
8134	1.03635	0.104824
8135	0.788549	0.10196
8136	1.44984	0.0974653
8137	1.84007	0.113858
8138	2.31127	0.106089
8139	1.27362	2.1
8140	1.27737	1.05564
8141	1.87783	0.829431
8142	2.35499	0.0924678
8143	2.69662	0.116029
8144	2.24065	0.0749541
8145	0.996082	2.1
8146	0.996083	2.1
8147	1.44859	2.1
8148	1.03968	1.86291
8149	1.60018	0.924692
8150	1.73082	1.45292
8151	0.946132	1.12062
8152	1.49742	1.42064
8153	1.49742	0.460826
8154	1.00501	0.870388
8155	1.41585	1.76461
8156	1.41585	0.953486
8157	0.946131	1.32585
8158	1.03697	1.07792
8159	2.95786	2.1
8160	2.32185	1.61861
8161	1.65783	0.492561
8162	1.772	1.33016
8163	0.946131	0.487854
8164	1.40404	0.309451
8165	1.13537	1.29867
8166	1.04559	0.713983
8167	1.16091	0.755469
8168	2.63751	1.60253
8169	1.68455	0.0901961
8170	1.9682	0.100249
8171	2.16834	0.476661
8172	2.45569	0.560777
8173	0.946128	0.407408
8174	1.36428	0.565688
8175	0.663978	0.609845

8176	1.58218	0.432385
8177	1.58219	0.431244
8178	1.36428	0.516997
8179	0.881886	0.535153
8180	1.73849	2.1
8181	1.35668	2.1
8182	1.1233	2.1
8183	1.15009	2.1
8184	1.16482	0.568771
8185	1.29313	0.597037
8186	1.47608	0.306569
8187	2.05949	0.289348
8188	0.946119	0.287024
8189	1.02974	0.276718
8190	0.946119	0.262635
8191	1.66758	0.261422
8192	1.66757	0.253904
8193	1.32082	0.255699
8194	1.29287	0.217188
8195	1.36005	1.84547
8196	1.47109	2.1
8197	2.45958	2.1
8198	2.2975	2.1
8199	0.953521	0.854599
8200	0.953521	0.718474
8201	1.67054	2.1
8202	1.67054	2.1
8203	0.946131	2.1
8204	1.38154	2.1
8205	1.13326	2.1
8206	1.13326	2.1
8207	1.23513	2.1
8208	1.50998	2.1
8209	2.47982	0.127627
8210	1.45091	0.122118
8211	0.958953	0.129341
8212	0.79239	0.13758
8213	2.11929	0.144431
8214	1.26511	0.159271
8215	1.27733	0.126751
8216	2.18127	0.134582
8217	1.30169	0.135052
8218	1.19259	0.141575
8219	0.963395	0.984177
8220	0.999947	1.70311
8221	1.62241	2.1
8222	1.62027	2.1
8223	0.981586	0.0825377
8224	0.981585	0.0774248
8225	0.721961	2.1
8226	0.891321	2.1
8227	1.04442	2.1
8228	1.66554	2.1
8229	2.43434	2.1
8230	1.73944	0.0777389
8231	1.88032	0.0761997
8232	0.711391	0.0842111
8233	1.40837	0.0787709

8234	0.946133	0.0853883
8235	0.92252	0.0827425
8236	1.13964	0.0823561
8237	1.83394	0.0805463
8238	2.81674	0.0782828
8239	1.10049	1.43143
8240	1.25092	0.706664
8241	0.760299	0.887453
8242	1.48017	0.984843
8243	1.48017	1.06886
8244	0.760271	0.79154
8245	1.35861	0.927498
8246	0.946129	0.845115
8247	0.760269	0.794351
8248	2.26155	2.1
8249	2.15634	2.1
8250	1.39824	2.1
8251	1.07817	2.1
8252	0.946132	2.1
8253	1.53028	2.1
8254	1.53028	2.1
8255	1.00807	2.1
8256	0.946133	2.1
8257	2.06126	0.757507
8258	1.97385	1.59562
8259	1.75754	0.72309
8260	2.01613	2.1
8261	1.97995	2.1
8262	1.47696	2.1
8263	1.50172	2.1
8264	1.5177	0.0981011
8265	1.44146	0.167259
8266	1.8807	0.155602
8267	2.07068	0.177977
8268	1.84267	0.172806
8269	1.08975	2.1
8270	1.21327	2.1
8271	1.10106	0.0753012
8272	1.34496	0.0724598
8273	2.08452	0.436766
8274	1.70537	0.313651
8275	2.62464	0.959305
8276	1.5726	0.684505
8277	1.5726	2.1
8278	0.94613	2.1
8279	1.15038	2.1
8280	0.946132	0.67421
8281	1.03974	0.938556
8282	1.15038	1.00218
8283	0.99747	0.80528
8284	1.03472	1.32248
8285	1.58407	0.830136
8286	1.21148	0.45581
8287	1.35025	0.816709
8288	1.3428	0.465732
8289	1.00775	2.1
8290	1.00775	2.1
8291	1.3879	2.1

8292	1.39958	2.1
8293	0.853042	2.1
8294	1.06997	2.1
8295	0.853042	2.1
8296	0.853042	2.1
8297	1.12491	2.1
8298	1.17879	2.1
8299	1.07162	2.05216
8300	1.61179	1.80309
8301	1.76016	2.1
8302	0.946132	0.676328
8303	1.23449	0.551496
8304	1.70392	0.568122
8305	1.70392	0.486519
8306	0.94613	0.692085
8307	1.23449	0.580987
8308	1.41556	0.581004
8309	1.14547	0.548121
8310	1.1721	0.396405
8311	0.96478	0.490389
8312	1.41843	0.505799
8313	1.2358	0.4374
8314	1.70678	0.429044
8315	1.70678	0.43609
8316	0.946118	0.61067
8317	1.23582	0.563821
8318	0.946105	0.637335
8319	0.964769	0.345425
8320	1.1357	0.36485
8321	0.964767	0.350278
8322	0.619856	0.0837066
8323	1.72366	0.0833019
8324	1.72367	0.0772861
8325	0.619831	0.0805083
8326	1.19262	0.0863272
8327	0.619829	0.0722551
8328	0.946129	0.0895414
8329	1.57459	2.1
8330	1.86512	2.1
8331	0.93841	2.1
8332	1.57459	2.1
8333	1.10476	0.136782
8334	1.00013	0.725913
8335	2.13446	0.760522
8336	0.759781	2.1
8337	1.96011	1.15245
8338	0.946129	0.848357
8339	1.32056	1.50609
8340	1.75702	1.03876
8341	1.75702	1.86556
8342	0.946124	1.45457
8343	1.32056	1.16072
8344	1.38258	0.885456
8345	1.96011	1.26858
8346	2.31099	1.18954
8347	0.97356	2.1
8348	1.38885	2.1
8349	1.3771	2.1

8350	0.946131	2.1
8351	1.84843	2.1
8352	1.84843	2.1
8353	1.3771	2.1
8354	0.946131	2.1
8355	1.00278	2.1
8356	1.05447	0.528769
8357	1.05446	0.542992
8358	1.24913	0.372678
8359	1.05447	0.500191
8360	1.80882	0.512492
8361	1.80882	1.64361
8362	0.946129	1.18873
8363	1.33997	1.62248
8364	0.946131	0.392354
8365	1.28831	0.656971
8366	1.33997	0.614969
8367	1.44391	0.0777831
8368	1.44391	0.0758603
8369	0.850964	0.0871316
8370	0.850963	0.0805645
8371	0.946132	0.0914364
8372	0.921299	0.0882094
8373	1.37358	0.0820159
8374	1.46297	0.0759102
8375	1.46298	0.0720509
8376	0.823988	0.0903246
8377	0.921287	0.0833995
8378	0.94612	0.100091
8379	0.823995	0.0879893
8380	0.946107	0.0843249
8381	1.44437	2.1
8382	1.06203	2.1
8383	2.21397	2.04966
8384	0.637677	2.1
8385	1.8892	2.1
8386	2.075	2.1
8387	1.32051	0.183477
8388	2.23637	0.258575
8389	1.10904	0.313327
8390	1.11819	0.277311
8391	0.94613	0.330845
8392	0.735845	0.238695
8393	1.4914	0.369995
8394	1.4914	0.195826
8395	0.735831	0.397806
8396	1.61683	2.1
8397	1.61683	2.1
8398	0.946131	2.1
8399	1.3281	2.1
8400	1.10441	2.1
8401	1.10442	2.1
8402	1.23487	2.1
8403	1.09914	2.1
8404	1.20691	0.0923056
8405	2.27379	0.104845
8406	1.1369	0.117794
8407	1.26868	0.122612

8408	1.54422	0.112942
8409	1.54422	0.104797
8410	0.861357	0.14294
8411	0.94613	0.123677
8412	0.861359	0.116653
8413	1.2858	0.584338
8414	1.31011	0.615143
8415	1.294 0.529043	
8416	1.03079	0.655173
8417	1.81979	1.91825
8418	1.57829	2.1
8419	1.19023	1.72267
8420	1.19022	1.95255
8421	1.46458	1.71002
8422	1.46458	2.04811
8423	0.789173	2.08634
8424	1.34386	2.0453
8425	0.909896	2.08781
8426	0.946131	1.83512
8427	0.909893	1.85837
8428	1.27725	2.1
8429	1.68451	2.1
8430	2.26155	2.1
8431	2.34217	2.1
8432	1.55485	0.689968
8433	0.90854	0.654105
8434	0.858256	0.618663
8435	1.47042	0.565927
8436	1.62771	0.562048
8437	0.777398	0.69688
8438	0.982525	0.625467
8439	1.23865	0.604179
8440	2.09928	0.606634
8441	0.946128	0.428302
8442	0.474856	0.424839
8443	0.474855	0.462468
8444	1.58747	0.259388
8445	1.58747	0.271901
8446	0.963917	0.36909
8447	1.09841	0.366578
8448	2.23904	1.41378
8449	2.60055	0.799115
8450	1.02599	2.1
8451	2.17837	1.7734
8452	2.07122	2.1
8453	2.7309	0.78082
8454	2.08482	1.96889
8455	1.18322	0.343916
8456	1.35381	0.826861
8457	1.01139	0.529831
8458	0.946133	0.795744
8459	1.59089	0.811941
8460	1.59089	0.215788
8461	0.946133	0.634108
8462	1.09634	0.155753
8463	2.02279	0.225257
8464	1.03108	0.56336
8465	0.808978	1.22233

8466	1.07317	1.30246
8467	0.831695	1.26103
8468	1.22583	1.29314
8469	1.10215	1.15181
8470	1.07508	1.37101
8471	1.22204	1.21948
8472	1.04679	1.94413
8473	1.92535	0.121882
8474	1.11081	0.086924
8475	0.946131	0.289499
8476	0.714824	0.296143
8477	0.714795	0.284984
8478	1.4231	0.275052
8479	1.4231	0.276424
8480	0.828617	0.317222
8481	1.30928	0.261244
8482	1.49894	2.1
8483	1.55702	2.1
8484	0.946131	2.1
8485	1.10689	2.1
8486	0.946227	2.1
8487	0.946131	2.1
8488	1.21754	2.1
8489	1.504 2.1	
8490	1.504 2.1	
8491	0.723573	2.1
8492	1.24512	2.1
8493	0.723544	2.1
8494	0.946131	2.1
8495	1.24512	2.1
8496	1.29383	0.329231
8497	1.29383	0.303026
8498	1.61969	0.293469
8499	0.946132	0.271097
8500	1.12818	0.253997
8501	0.946132	0.291692
8502	1.80922	0.276051
8503	1.80922	0.233624
8504	1.41383	0.254112
8505	1.34153	0.219233
8506	1.47689	0.284882
8507	1.79375	0.269097
8508	1.24333	0.410129
8509	0.768989	0.381821
8510	1.24333	0.318536
8511	1.0027	0.502547
8512	1.47705	0.472185
8513	1.47705	0.259107
8514	0.768977	0.396573
8515	1.28436	0.473429
8516	0.928583	0.516609
8517	1.28436	0.331496
8518	1.24333	0.534202
8519	1.5991	0.537955
8520	1.5991	0.211448
8521	0.928583	0.524299
8522	1.85717	0.267406
8523	2.48665	0.335379

8524	1.18423	0.0870835
8525	1.9493	1.17313
8526	1.8519	0.989471
8527	1.68802	2.1
8528	1.0302	0.155033
8529	0.900473	0.142266
8530	1.99663	0.166865
8531	2.61301	0.144252
8532	0.745772	0.220878
8533	0.946131	0.167784
8534	1.48881	0.170438
8535	1.48881	0.151069
8536	0.745745	0.200394
8537	0.809786	0.19671
8538	1.3065	0.190571
8539	0.946132	0.26885
8540	0.768357	0.293846
8541	0.768387	0.301848
8542	1.76897	0.192631
8543	1.76896	0.190466
8544	1.40271	0.234194
8545	1.00777	0.235841
8546	1.37427	0.191937
8547	1.1967	1.94581
8548	0.946132	1.20614
8549	1.59397	2.1
8550	1.59397	2.1
8551	0.946131	2.04088
8552	1.14989	2.1
8553	1.14989	2.1
8554	1.10046	2.1
8555	1.09506	0.210217
8556	2.53251	0.246659
8557	1.9212	0.877865
8558	1.99586	0.827801
8559	0.913967	0.624345
8560	1.55028	0.657336
8561	1.55028	0.768325
8562	0.913966	0.604701
8563	1.16318	0.587261
8564	1.16318	0.595845
8565	0.946132	0.582338
8566	1.20478	2.1
8567	1.09662	2.1
8568	1.03472	2.1
8569	1.84774	2.1
8570	1.7984	2.1
8571	2.1221	2.1
8572	1.68223	1.8637
8573	1.68224	1.67599
8574	0.514872	1.52699
8575	0.888991	1.7632
8576	0.946127	0.901064
8577	0.514845	1.49293
8578	0.946129	1.1138
8579	1.10127	1.45022
8580	2.61623	0.99957
8581	0.983581	1.03426

8582	0.869663	0.738081
8583	1.1818	1.68075
8584	1.10406	0.872296
8585	1.07133	1.84374
8586	1.09405	0.926515
8587	0.838014	0.809593
8588	0.842998	1.32839
8589	1.03818	0.99835
8590	1.46338	0.523383
8591	1.903 2.1	
8592	1.23942	2.1
8593	0.965933	2.1
8594	0.810075	1.82567
8595	1.40571	2.1
8596	1.66219	2.1
8597	0.810047	2.1
8598	0.936678	2.1
8599	1.45808	2.1
8600	1.6384	2.1
8601	1.54699	2.1
8602	0.719877	2.1
8603	1.33256	2.1
8604	0.753547	2.1
8605	1.04073	2.1
8606	1.25822	2.1
8607	0.851542	2.1
8608	0.714102	2.1
8609	1.45886	2.1
8610	1.80171	2.1
8611	0.597337	2.1
8612	0.957713	2.1
8613	1.3033	2.1
8614	0.596613	0.270374
8615	1.1386	0.545479
8616	0.596612	0.397502
8617	0.946133	0.710829
8618	0.596652	0.74891
8619	1.64092	0.564602
8620	1.64092	0.529704
8621	1.991 2.1	
8622	1.23013	2.1
8623	2.77816	2.1
8624	2.74107	0.741788
8625	2.07681	0.654167
8626	1.47764	0.199246
8627	1.72677	0.205952
8628	1.52161	2.1
8629	1.28526	2.1
8630	1.90502	2.1
8631	1.87359	0.175247
8632	1.93489	0.223321
8633	2.70895	2.1
8634	2.83141	2.1
8635	1.41766	0.587937
8636	1.11337	0.56553
8637	1.41765	1.30647
8638	1.29172	0.581411
8639	1.76324	0.653392

8640	1.76324	1.0605
8641	0.946131	0.587437
8642	1.52428	2.1
8643	1.71459	2.1
8644	1.93852	0.613149
8645	1.07119	1.15957
8646	1.29698	0.96946
8647	1.42203	0.623788
8648	1.42203	1.10624
8649	0.946132	0.716676
8650	0.946132	0.834529
8651	0.96926	0.909717
8652	0.769913	1.50978
8653	0.946119	1.33126
8654	1.37867	0.936283
8655	1.94028	0.874614
8656	0.94612	0.6863
8657	0.946132	1.20717
8658	1.37865	0.820528
8659	2.14234	0.990652
8660	3.06459	1.10532
8661	1.44421	0.120728
8662	1.33924	0.0965721
8663	1.33671	0.153434
8664	1.10733	0.150939
8665	0.68396	0.179328
8666	1.76008	0.129211
8667	1.76008	0.124714
8668	0.68393	0.130843
8669	0.946131	0.160609
8670	3.79242	2.1
8671	2.46293	2.1
8672	3.56947	2.1
8673	1.35487	1.41934
8674	1.38616	1.71832
8675	0.954048	0.174355
8676	1.36663	0.303424
8677	0.909487	0.38408
8678	0.887288	1.60949
8679	0.810947	0.924213
8680	0.810934	1.40019
8681	1.07046	1.58427
8682	1.13979	1.82419
8683	0.927838	0.971487
8684	0.919995	1.38457
8685	1.61447	2.1
8686	2.56305	2.1
8687	0.943414	1.2046
8688	1.17016	1.97491
8689	1.7238	2.06042
8690	1.89334	1.66396
8691	0.946128	0.814593
8692	1.25184	1.40678
8693	1.55639	1.5702
8694	3.12098	1.05388
8695	3.57307	0.806701
8696	2.81281	2.05824
8697	0.851542	1.95866

8698	1.27689	1.65551
8699	1.52568	2.01596
8700	0.839689	2.06905
8701	1.36471	1.8795
8702	0.827865	2.1
8703	0.93667	2.1
8704	0.900861	2.02452
8705	0.943152	2.0473
8706	1.00667	2.0649
8707	1.18141	2.07956
8708	1.15298	2.09387
8709	1.20335	2.03084
8710	1.00713	2.08805
8711	0.597537	2.1
8712	1.0577	2.1
8713	0.967342	0.838529
8714	0.838027	1.26864
8715	0.893587	0.669765
8716	1.06436	0.948497
8717	0.850048	0.619331
8718	1.22026	1.09339
8719	0.988077	0.817603
8720	1.18769	0.378329
8721	1.3991	1.94792
8722	1.52984	2.1
8723	1.35469	1.06907
8724	1.25083	0.935385
8725	1.6594	1.26617
8726	1.6594	1.04381
8727	0.946129	0.849944
8728	1.02602	0.847996
8729	0.94613	0.785874
8730	1.29724	0.341238
8731	1.14501	0.284836
8732	1.13078	0.211763
8733	1.48381	0.211273
8734	1.45921	0.188998
8735	0.851539	0.298464
8736	0.890836	0.308599
8737	0.946131	0.36909
8738	1.60428	2.1
8739	1.50097	2.1
8740	0.714507	0.311978
8741	1.51396	0.323259
8742	1.51396	0.295494
8743	0.946132	0.319858
8744	0.813862	0.31446
8745	1.41461	0.30655
8746	0.813862	0.300749
8747	0.921311	2.1
8748	1.25084	0.943098
8749	1.30165	0.825443
8750	1.60636	1.33421
8751	1.60636	0.938818
8752	0.94613	0.754783
8753	0.972232	0.752456
8754	0.946131	0.707801
8755	2.35003	2.1

8756	0.92546	1.66815
8757	1.00713	2.1
8758	0.810947	2.1
8759	0.810934	2.1
8760	1.056	2.1
8761	0.926811	2.1
8762	0.766408	2.1
8763	1.34292	2.1
8764	1.57932	0.117287
8765	2.67595	0.109233
8766	1.77737	0.106215
8767	1.77738	0.103964
8768	0.789633	0.143831
8769	1.22903	0.125364
8770	0.789663	0.136516
8771	0.789662	0.128712
8772	1.33797	0.129494
8773	0.946128	2.1
8774	1.47351	2.1
8775	1.47351	2.1
8776	0.94613	2.1
8777	1.30082	2.1
8778	1.30082	2.1
8779	0.996486	2.1
8780	0.946127	2.04761
8781	1.72122	2.1
8782	1.72122	2.1
8783	0.94613	2.1
8784	1.36653	2.1
8785	1.36653	2.1
8786	1.02608	2.1
8787	0.709123	0.604676
8788	0.946127	0.893006
8789	1.41342	1.03403
8790	1.818	1.17185
8791	1.818	0.984744
8792	0.946127	0.612334
8793	1.41342	0.984238
8794	1.35071	0.819002
8795	1.8993	1.20606
8796	1.4748	2.1
8797	1.18711	0.118318
8798	0.946131	0.163218
8799	1.18711	0.136525
8800	1.20416	0.124159
8801	1.91358	0.156842
8802	1.91358	0.112029
8803	0.477691	0.143331
8804	2.16798	0.0776774
8805	2.22307	0.076658
8806	0.758971	0.152144
8807	1.80694	0.148128
8808	1.80694	0.113608
8809	0.946132	0.162686
8810	0.75897	0.14361
8811	1.25124	0.134259
8812	0.946131	0.172244
8813	1.76837	2.1

8814	1.76837	2.1
8815	0.946131	2.1
8816	1.36422	2.1
8817	0.946131	2.1
8818	1.14184	2.1
8819	1.36422	2.1
8820	0.793303	0.0824703
8821	1.79584	0.0834505
8822	1.35333	1.92653
8823	1.19967	1.33421
8824	1.55088	1.48426
8825	1.64246	2.1
8826	0.946132	0.872573
8827	0.886293	1.74224
8828	1.25018	1.76767
8829	1.5795	1.43893
8830	2.15414	1.28018
8831	0.94613	0.903152
8832	1.37758	1.42669
8833	1.71567	0.940627
8834	1.71567	1.82341
8835	0.946125	1.14887
8836	1.37759	0.908184
8837	1.28421	0.809844
8838	0.933959	0.939174
8839	0.946133	0.159099
8840	1.7813	0.169744
8841	1.7813	0.130587
8842	1.05704	0.142835
8843	1.37794	0.18104
8844	1.37794	0.157689
8845	0.946132	0.181392
8846	0.946132	0.117869
8847	1.68197	0.121591
8848	1.68197	0.103341
8849	1.01367	0.108249
8850	1.25017	0.127922
8851	1.25017	0.115092
8852	0.946132	0.13226
8853	0.838953	2.1
8854	1.4045	2.1
8855	1.56388	2.1
8856	1.3182	2.1
8857	0.928518	2.1
8858	1.04073	2.1
8859	0.835676	2.1
8860	1.47507	2.1
8861	1.47506	2.1
8862	0.797664	2.1
8863	1.34285	2.1
8864	0.929875	2.1
8865	0.797663	2.1
8866	0.946133	2.1
8867	1.83628	0.458935
8868	1.83627	0.457039
8869	0.946131	0.563744
8870	1.36748	0.415964
8871	0.946133	0.461289

8872	1.21139	0.459703
8873	1.36748	0.397854
8874	1.06795	2.1
8875	1.08192	2.1
8876	0.827866	2.1
8877	1.02548	2.1
8878	0.95006	2.1
8879	1.53933	2.1
8880	0.586511	2.1
8881	0.868145	2.1
8882	1.28401	2.1
8883	1.37719	2.1
8884	1.37719	2.1
8885	0.77497	2.1
8886	0.946132	2.1
8887	0.774968	2.1
8888	1.7053	0.409272
8889	1.7053	0.623553
8890	0.94613	0.760281
8891	1.25715	0.606795
8892	0.946132	0.455605
8893	1.19655	0.533313
8894	1.25716	0.515063
8895	0.851542	2.1
8896	0.80043	2.1
8897	1.27239	2.1
8898	1.02719	2.1
8899	0.946131	2.1
8900	0.981318	2.1
8901	1.06628	2.1
8902	1.26022	2.1
8903	0.946132	2.1
8904	0.946132	2.1
8905	1.76447	2.1
8906	1.76447	2.1
8907	1.35357	2.1
8908	1.35703	2.1
8909	0.946131	2.1
8910	1.50538	2.01932
8911	1.50538	2.1
8912	1.10311	2.1
8913	1.3484	1.63094
8914	1.10312	2.1
8915	0.966428	2.1
8916	0.946133	2.1
8917	1.4008	2.1
8918	1.71908	2.1
8919	1.71908	2.1
8920	0.946133	2.1
8921	1.40081	2.1
8922	1.26441	2.1
8923	0.851543	2.1
8924	0.961732	2.1
8925	1.54576	2.1
8926	1.35364	2.1
8927	0.946133	2.1
8928	1.02913	2.1
8929	1.2735	2.1

8930	1.32608	2.1
8931	0.967643	2.1
8932	0.94613	2.1
8933	1.44873	2.1
8934	1.44873	2.1
8935	0.967641	2.1
8936	0.946131	2.1
8937	1.40488	2.1
8938	1.24272	2.1
8939	0.946131	2.1
8940	1.78483	2.1
8941	1.78483	2.1
8942	1.24272	2.1
8943	0.946131	2.1
8944	0.94613	1.68534
8945	1.5627	0.705833
8946	1.5627	1.90187
8947	1.03656	1.64041
8948	1.31649	0.588933
8949	1.19233	1.17577
8950	1.03656	0.931956
8951	0.912059	1.81665
8952	1.47954	0.759932
8953	1.47954	2.01495
8954	0.912061	1.76509
8955	1.34647	0.694146
8956	1.04514	1.40156
8957	0.912061	0.959045
8958	0.761972	0.316478
8959	0.946132	0.3041
8960	0.761974	0.421652
8961	1.70225	0.55275
8962	1.70225	0.482909
8963	1.33728	0.382929
8964	1.01188	0.386357
8965	0.94613	0.947269
8966	0.746361	1.35122
8967	1.6369	1.50378
8968	1.62454	1.32301
8969	0.736738	0.874385
8970	0.958487	0.967455
8971	1.40641	1.09602
8972	1.2284	2.1
8973	0.92609	2.1
8974	0.595102	2.1
8975	1.80871	2.1
8976	1.1516	2.1
8977	0.962474	2.1
8978	2.24024	2.1
8979	0.946131	2.1
8980	1.73689	2.1
8981	1.73689	2.1
8982	0.946132	2.1
8983	1.30492	2.1
8984	1.30492	2.1
8985	1.17949	2.1
8986	0.946131	1.79489
8987	1.8566	2.1

8988	1.8566	2.1
8989	1.22392	1.89397
8990	1.39826	2.1
8991	1.40447	2.1
8992	1.22392	2.1
8993	0.946131	1.29137
8994	1.74134	2.1
8995	1.74134	2.1
8996	1.09148	1.25098
8997	1.40447	2.1
8998	1.283 2.1	
8999	1.09148	2.1
9000	1.33607	2.1
9001	1.41022	1.72618
9002	1.80016	1.97003
9003	1.80015	2.1
9004	0.946127	1.29802
9005	0.946129	2.1
9006	1.20541	2.1
9007	1.13078	2.1
9008	1.33607	2.1
9009	1.54624	2.1
9010	1.85553	2.1
9011	0.920606	2.1
9012	0.946131	2.1
9013	1.01271	2.1
9014	1.32664	1.28301
9015	0.954692	0.957998
9016	0.946116	0.900521
9017	1.47771	0.806479
9018	1.4777	2.1
9019	0.95468	1.03868
9020	0.94613	0.82447
9021	1.31381	1.08891
9022	1.14005	0.683994
9023	0.946128	0.963254
9024	1.69433	0.57384
9025	1.69433	1.2507
9026	1.14005	0.877873
9027	0.94613	0.687756
9028	1.521 2.1	
9029	1.411 2.1	
9030	0.94612	1.97634
9031	1.35115	2.1
9032	1.04127	1.41465
9033	1.05017	2.05298
9034	1.03564	1.95556
9035	0.607156	1.40417
9036	1.54549	2.1
9037	1.36715	2.1
9038	0.607127	1.32303
9039	1.2571	2.1
9040	0.998701	1.7378
9041	0.546436	1.91054
9042	0.946133	2.1
9043	1.75286	2.1
9044	1.75286	2.1
9045	1.12206	1.98314

9046	1.34237	2.1
9047	1.35663	2.1
9048	1.12206	2.1
9049	0.919455	2.01446
9050	1.66517	2.1
9051	1.99822	2.1
9052	1.228	2.08871
9053	1.35662	2.1
9054	1.228	2.1
9055	1.01143	2.1
9056	0.851543	0.914915
9057	0.986856	2.09353
9058	1.42762	1.40926
9059	1.23095	2.1
9060	0.946132	1.86932
9061	1.00659	1.44147
9062	1.1779	1.06894
9063	1.40025	2.1
9064	1.40025	2.1
9065	0.889308	2.1
9066	1.34652	2.1
9067	0.943042	2.1
9068	0.943041	2.1
9069	1.34652	2.1
9070	1.39514	1.25505
9071	1.30809	1.1135
9072	1.75711	1.04576
9073	1.7571	1.93733
9074	0.946125	0.65069
9075	0.946127	1.14679
9076	1.18396	1.14189
9077	1.4844	0.830762
9078	1.4844	1.16778
9079	0.803322	1.48969
9080	0.946133	0.747364
9081	0.803324	2.05119
9082	0.803324	1.31273
9083	1.36183	1.34458
9084	0.946131	0.301557
9085	1.01462	0.307348
9086	0.946132	0.299608
9087	1.59749	0.396694
9088	1.59749	0.304928
9089	1.16009	0.324448
9090	1.38354	0.295745
9091	0.851539	0.90636
9092	1.36005	1.48373
9093	1.5532	1.11014
9094	1.80402	1.75512
9095	0.851537	1.53009
9096	0.902632	1.32151
9097	1.44437	1.16604
9098	0.633098	2.1
9099	1.00307	0.48913
9100	0.94613	0.68115
9101	1.16623	0.494536
9102	0.946128	0.692002
9103	1.64588	0.703329

9104	1.64588	0.614411
9105	1.35059	0.664442
9106	1.24142	0.691253
9107	0.946131	0.290842
9108	1.13343	0.262542
9109	0.946127	0.314201
9110	1.63367	0.284667
9111	1.63367	0.247429
9112	1.33035	0.269496
9113	1.24945	0.267057
9114	1.78733	1.26823
9115	1.70856	1.54923
9116	0.637995	1.05192
9117	0.712454	1.05832
9118	0.946132	1.10074
9119	0.637964	0.959496
9120	1.13021	1.08814
9121	0.94613	0.286332
9122	1.20509	0.2843
9123	0.946127	0.308385
9124	1.70545	0.293293
9125	1.70545	0.248863
9126	1.28508	0.277963
9127	1.3665	0.261334
9128	1.58612	2.1
9129	1.51845	2.1
9130	0.797374	2.1
9131	1.20057	2.1
9132	0.946132	2.1
9133	0.946132	2.1
9134	0.899819	2.1
9135	1.50471	0.884385
9136	1.50472	0.999134
9137	0.59387	1.00797
9138	0.593844	0.780782
9139	0.946126	0.910737
9140	0.966137	0.858985
9141	1.13245	0.810929
9142	1.20801	0.880387
9143	0.946131	1.1773
9144	1.47644	0.941924
9145	0.733223	0.915646
9146	0.563609	0.841961
9147	1.69218	0.918328
9148	1.6173	1.11455
9149	1.8126	1.84699
9150	1.8126	2.01071
9151	0.678938	1.88643
9152	0.678935	1.99859
9153	0.946133	2.09813
9154	0.946132	1.86382
9155	0.678936	2.1
9156	1.21997	1.54579
9157	1.28439	1.50797
9158	1.65282	1.80381
9159	1.86735	1.79345
9160	0.851539	1.62059
9161	0.94613	1.19969

9162	0.87688	1.21538
9163	0.804877	0.587354
9164	1.40747	0.688173
9165	1.41312	0.391908
9166	0.885349	0.513509
9167	1.25405	0.675154
9168	1.34463	0.514698
9169	0.804878	0.504591
9170	0.810299	0.211012
9171	1.04072	0.344984
9172	1.25601	0.274408
9173	1.04943	0.219349
9174	0.900294	0.305825
9175	1.38777	0.264704
9176	1.33131	0.193117
9177	1.39168	0.104173
9178	0.946132	0.128206
9179	0.747712	0.112399
9180	1.61944	0.100391
9181	1.61944	0.0941721
9182	0.946132	0.127405
9183	0.747712	0.123596
9184	1.41201	0.35429
9185	1.12082	0.340029
9186	0.946128	0.371461
9187	1.79415	0.35239
9188	1.79415	0.341207
9189	1.12082	0.31825
9190	0.94613	0.331924
9191	0.711721	0.188515
9192	1.12316	0.224781
9193	0.707038	0.204517
9194	1.04072	0.241997
9195	0.704409	0.27245
9196	1.49036	0.237099
9197	1.27689	0.212526
9198	0.793044	0.65955
9199	0.810886	0.968342
9200	0.885957	0.707812
9201	1.38254	0.694082
9202	1.35991	0.624342
9203	1.01375	0.805929
9204	1.03169	0.781337
9205	0.946131	0.718237
9206	1.3279	0.936571
9207	1.42284	0.813444
9208	1.42284	0.716353
9209	0.851192	1.14562
9210	0.946131	1.03187
9211	0.851165	0.851052
9212	0.748062	0.0979089
9213	1.27511	0.104179
9214	0.74806	0.0926838
9215	0.94613	0.107616
9216	0.748059	0.10379
9217	1.86293	0.103719
9218	1.86293	0.0946306
9219	0.851542	0.255753

9220	1.38186	0.225879
9221	0.851542	0.28538
9222	1.67869	0.165252
9223	1.66579	0.15476
9224	1.25626	0.20491
9225	1.23046	0.187789
9226	1.46023	0.075515
9227	1.27083	0.0717825
9228	0.946131	0.0869358
9229	0.999304	0.0810113
9230	0.946803	0.0840361
9231	0.851554	0.0822604
9232	0.999227	0.0808896
9233	0.750097	0.210745
9234	1.91815	0.193901
9235	0.946132	0.216929
9236	0.768117	0.175014
9237	1.78003	0.178079
9238	1.99572	0.159501
9239	0.650453	0.218764
9240	0.946132	0.14557
9241	1.23845	0.147535
9242	0.946131	0.150771
9243	1.75477	0.111974
9244	1.75477	0.114486
9245	1.33393	0.127496
9246	1.36697	0.12842
9247	0.749018	1.14075
9248	1.02366	1.45693
9249	1.43924	1.25629
9250	1.00018	1.25305
9251	1.06438	0.905707
9252	0.946127	1.12198
9253	0.74906	0.979271
9254	0.946131	1.13783
9255	1.26488	0.0906606
9256	0.946131	0.115534
9257	0.94613	0.10237
9258	1.49051	0.0897589
9259	1.59837	0.0874285
9260	0.907797	0.0996688
9261	1.04455	0.102924
9262	1.25214	0.0666849
9263	0.68452	0.0691729
9264	1.25215	0.0684094
9265	1.24677	0.0671634
9266	1.8144	0.063981
9267	1.81441	0.0632726
9268	0.684518	0.0692289
9269	0.851542	0.582561
9270	1.52275	0.570962
9271	1.45223	0.565009
9272	1.0269	0.559892
9273	1.29689	0.607282
9274	1.32822	0.63807
9275	0.851553	0.68345
9276	1.21756	0.111865
9277	0.946131	0.119254

9278	0.946127	0.111385
9279	1.78014	0.09862
9280	1.78014	0.0867064
9281	1.32076	0.104323
9282	1.40551	0.107896
9283	0.711214	0.1879
9284	1.82057	0.165797
9285	1.82057	0.141082
9286	0.946129	0.202019
9287	1.22716	0.143522
9288	1.30463	0.19227
9289	0.94613	0.192775
9290	0.568927	0.105856
9291	0.946132	0.110666
9292	0.568927	0.106951
9293	1.99266	0.0895162
9294	1.99266	0.0865557
9295	1.26201	0.106008
9296	1.29957	0.106686
9297	1.21614	0.167923
9298	0.851484	0.168768
9299	0.851543	0.186508
9300	1.85815	0.126178
9301	1.81937	0.120902
9302	0.697705	0.117631
9303	0.946132	0.174707
9304	0.593295	0.232446
9305	1.53642	0.206598
9306	1.53642	0.178852
9307	0.908276	0.253988
9308	0.593296	0.177673
9309	0.593295	0.195549
9310	0.94613	0.23536
9311	0.867593	0.380728
9312	1.42107	0.295884
9313	1.42107	0.2911
9314	0.867621	0.352273
9315	1.05516	0.363625
9316	0.867624	0.278021
9317	0.946129	0.406033
9318	0.766681	0.138822
9319	1.18119	0.15037
9320	1.57018	0.125705
9321	1.57018	0.109288
9322	0.766652	0.161193
9323	1.18119	0.145977
9324	1.15564	0.142815
9325	0.946131	0.252772
9326	1.239	0.203688
9327	0.851541	0.270463
9328	0.851487	0.192119
9329	1.40021	0.253162
9330	1.38373	0.1789
9331	0.851554	0.282464
9332	1.12266	0.239849
9333	0.946131	0.282365
9334	1.08234	0.293969
9335	1.2882	0.24153

9336	1.49963	0.52735
9337	1.46188	0.302724
9338	0.851555	0.281035
9339	1.33007	0.403431
9340	1.08054	0.406438
9341	0.946129	0.483171
9342	0.91151	0.315464
9343	1.49914	0.452486
9344	1.49914	0.300073
9345	0.911471	0.496033
9346	0.946132	0.937836
9347	1.35943	1.64929
9348	1.68117	1.30689
9349	1.68117	2.07496
9350	0.946132	1.392
9351	1.35943	1.06457
9352	1.26787	0.843197
9353	1.81612	0.693608
9354	1.81612	0.301178
9355	0.946131	0.305822
9356	1.41492	0.247006
9357	0.946132	0.697367
9358	1.25037	0.504247
9359	1.41492	0.437497
9360	1.30541	0.112994
9361	1.23646	0.137641
9362	0.94613	0.128193
9363	1.77304	0.103212
9364	1.77304	0.0999317
9365	1.23646	0.120984
9366	0.946132	0.125054
9367	0.946131	2.1
9368	1.26689	2.1
9369	1.66806	2.1
9370	1.66806	2.1
9371	0.94613	1.60816
9372	1.26689	2.1
9373	1.3473	2.1
9374	1.39384	2.1
9375	1.4181	2.1
9376	1.86581	2.1
9377	1.86581	2.1
9378	0.946128	2.1
9379	0.946129	2.1
9380	1.32068	2.1
9381	1.20328	2.1
9382	1.39384	2.1
9383	1.651	2.1
9384	1.651	2.1
9385	0.946129	2.1
9386	0.946131	2.1
9387	1.12913	2.1
9388	1.21474	1.76805
9389	0.946132	2.1
9390	0.94613	2.02656
9391	1.79724	2.1
9392	1.79724	2.1
9393	1.40987	2.1

9394	1.3335	2.1
9395	1.65642	0.693482
9396	1.65642	2.1
9397	0.946132	2.1
9398	1.19063	2.1
9399	0.946133	0.650402
9400	1.15203	1.82381
9401	1.19063	1.83324
9402	0.946131	0.701896
9403	1.77815	1.46243
9404	1.77815	2.1
9405	0.946131	1.23422
9406	1.31745	2.1
9407	1.31745	1.23242
9408	1.2597	1.56787
9409	0.946131	0.641942
9410	1.85184	0.962925
9411	1.85184	2.1
9412	0.946128	1.22226
9413	1.40684	2.1
9414	1.40684	0.908712
9415	1.34049	1.20412
9416	0.946132	1.43748
9417	1.71333	1.16207
9418	1.71333	1.9605
9419	1.1848	1.46606
9420	1.34297	1.03211
9421	1.31649	1.04767
9422	1.1848	1.4328
9423	1.36604	0.678789
9424	0.954559	0.707343
9425	1.36604	0.729815
9426	1.41766	0.643739
9427	1.83756	0.80751
9428	1.83756	1.74431
9429	0.946131	0.763703
9430	0.946127	0.384273
9431	1.14874	0.304008
9432	0.946127	0.409506
9433	1.66393	0.386937
9434	1.66393	0.313232
9435	1.35559	0.366611
9436	1.25447	0.386603
9437	0.94613	0.283576
9438	1.20732	0.271545
9439	0.946129	0.304612
9440	1.75073	0.28802
9441	1.75073	0.245308
9442	1.3665	0.267558
9443	1.33035	0.248182
9444	1.73256	2.1
9445	1.73256	2.1
9446	0.946131	1.57489
9447	1.34921	1.57517
9448	1.19462	2.1
9449	1.19462	2.1
9450	1.32949	2.1
9451	1.75754	0.422566

9452	1.75754	0.46304
9453	0.946132	0.550939
9454	1.3201	0.49008
9455	0.946132	0.501644
9456	1.25479	0.369383
9457	1.3201	0.413781
9458	1.8087	0.61727
9459	1.8087	0.270688
9460	0.946131	0.352509
9461	1.38357	0.37575
9462	0.946133	0.608932
9463	1.31034	0.430868
9464	1.38357	0.492823
9465	1.12702	0.263602
9466	0.946131	0.44207
9467	1.23923	0.347867
9468	1.4117	0.309202
9469	0.946131	0.322481
9470	1.70479	0.309823
9471	1.70479	0.18253
9472	0.946119	2.1
9473	1.55309	2.1
9474	1.55309	2.1
9475	0.946119	2.1
9476	1.08006	2.1
9477	1.08005	2.1
9478	1.02681	2.1
9479	1.36581	2.1
9480	1.93075	2.1
9481	0.946133	2.1
9482	0.772538	2.1
9483	0.946131	2.1
9484	0.946131	2.1
9485	1.33748	2.1
9486	1.44991	2.1
9487	1.31183	2.1
9488	0.946132	2.1
9489	0.944104	2.1
9490	0.915236	2.1
9491	1.05017	2.1
9492	0.944148	2.1
9493	0.946132	0.326395
9494	1.60171	0.313066
9495	1.60171	0.249855
9496	0.946131	0.366227
9497	1.16234	0.248843
9498	1.3855	0.374285
9499	0.946132	0.373386
9500	0.946133	0.373392
9501	1.73285	0.38807
9502	1.73285	0.310659
9503	0.946133	0.413935
9504	1.3855	0.315316
9505	1.29348	0.443537
9506	0.946132	0.445754
9507	1.34828	2.09814
9508	1.20943	1.85472
9509	0.946129	0.905818

9510	1.71013	2.1
9511	1.71013	2.1
9512	1.20943	1.86968
9513	0.94613	1.83927
9514	1.19613	0.102925
9515	0.946118	0.110178
9516	0.946117	0.103021
9517	1.70868	0.0908295
9518	1.70869	0.082777
9519	1.40551	0.0970369
9520	1.2493	0.100284
9521	0.946131	2.1
9522	1.73123	2.1
9523	1.73123	2.1
9524	0.946132	2.1
9525	1.37811	2.1
9526	1.37811	2.1
9527	1.25144	2.1
9528	0.946132	2.1
9529	1.75139	2.1
9530	1.75139	2.1
9531	1.23374	2.1
9532	1.29926	2.1
9533	1.39827	2.1
9534	1.23374	2.1
9535	1.41375	0.120778
9536	1.25697	0.150362
9537	0.946118	0.147969
9538	1.80377	0.106759
9539	1.80377	0.102018
9540	1.25699	0.133836
9541	0.946106	0.139036
9542	0.946133	1.50184
9543	1.81806	0.990814
9544	1.81806	1.86942
9545	0.946133	1.75664
9546	1.37089	2.00717
9547	1.37089	1.02213
9548	1.34999	1.71541
9549	0.946131	1.98471
9550	1.80855	0.932117
9551	1.80855	2.1
9552	1.31184	1.73841
9553	1.3933	1.11523
9554	1.36139	1.2235
9555	1.31184	1.34111
9556	1.01175	1.07943
9557	0.94613	0.772105
9558	0.919796	1.37753
9559	1.50484	0.65551
9560	1.80584	1.62289
9561	1.53512	0.76591
9562	1.14539	0.849718
9563	0.946133	2.1
9564	1.72797	1.09227
9565	1.72797	2.1
9566	1.2111	2.1
9567	1.39731	1.06587

9568	1.27679	1.7995
9569	1.21111	1.6194
9570	0.946119	0.488536
9571	1.67673	0.9729
9572	1.67673	2.1
9573	0.946118	1.0208
9574	1.21519	1.64266
9575	1.2152	1.43464
9576	1.19808	1.71259
9577	0.946127	2.1
9578	1.54442	2.1
9579	1.54442	2.1
9580	1.04806	2.1
9581	1.13485	2.1
9582	1.3557	2.1
9583	1.04805	2.1
9584	0.946129	2.1
9585	1.32282	2.1
9586	1.77749	2.1
9587	1.77749	2.1
9588	0.946131	2.1
9589	1.32282	2.1
9590	1.4008	2.1
9591	0.946128	0.295364
9592	1.02366	0.282465
9593	0.94613	0.270947
9594	1.66684	0.259248
9595	1.66684	0.261959
9596	1.29215	0.265749
9597	1.32082	0.230776
9598	0.946133	0.274305
9599	1.01451	0.274449
9600	0.946132	0.257507
9601	1.62374	0.279747
9602	1.62374	0.249126
9603	1.2929	0.256634
9604	1.27697	0.208042
9605	1.37869	2.1
9606	1.17215	2.1
9607	0.946132	2.1
9608	1.70508	2.1
9609	1.70508	2.1
9610	1.17215	2.1
9611	0.946132	2.1
9612	1.03784	2.1
9613	0.946132	2.1
9614	0.946132	2.1
9615	1.56718	2.1
9616	1.56717	2.1
9617	1.35703	2.1
9618	1.15628	2.1
9619	1.35356	2.1
9620	1.28976	2.1
9621	0.946131	2.1
9622	1.78612	2.1
9623	1.78612	2.1
9624	1.28976	2.1
9625	0.94613	2.1

9626	0.946132	2.1
9627	1.36373	2.1
9628	1.92382	2.1
9629	0.946131	2.1
9630	1.92382	2.1
9631	1.33471	2.1
9632	0.946132	2.1
9633	0.94613	0.689384
9634	1.29149	1.03218
9635	1.75878	0.849599
9636	1.75878	1.68939
9637	0.946128	0.968057
9638	1.29149	0.710768
9639	1.41342	0.637274
9640	1.41022	2.1
9641	1.37034	1.04358
9642	1.83443	1.22355
9643	1.83443	2.1
9644	0.946128	0.870174
9645	0.946131	1.419
9646	1.26928	1.09731
9647	0.946128	0.852905
9648	1.38259	1.52004
9649	1.81405	0.963452
9650	1.81405	1.87016
9651	0.946126	1.25449
9652	1.38259	0.978676
9653	1.37759	0.797508
9654	1.68131	0.234241
9655	1.68131	0.400157
9656	0.946131	0.254734
9657	1.38896	0.212445
9658	1.17438	0.617547
9659	1.17438	0.326202
9660	1.23848	0.465675
9661	1.80088	0.414634
9662	1.80088	0.197074
9663	0.946132	0.289626
9664	1.40332	0.392098
9665	1.28185	0.394375
9666	1.28185	0.256122
9667	1.34369	0.234169
9668	1.71939	2.1
9669	1.71939	2.1
9670	0.946131	2.1
9671	1.28308	2.1
9672	0.946133	2.1
9673	1.24024	2.1
9674	1.28309	2.1
9675	1.8525	1.43136
9676	1.8525	2.1
9677	0.946132	2.1
9678	1.38244	2.1
9679	0.946132	0.683502
9680	1.33469	1.67031
9681	1.38244	1.73107
9682	1.26654	1.17951
9683	1.39514	1.06585

9684	1.71556	1.00624
9685	1.71556	1.94535
9686	0.946126	0.682924
9687	0.946129	1.26177
9688	1.1908	1.1202
9689	1.49995	2.1
9690	1.63875	2.1
9691	0.93608	2.1
9692	0.937208	2.1
9693	0.943708	2.1
9694	0.936134	2.1
9695	1.14341	2.1
9696	1.17802	0.0715838
9697	1.3542	0.0760808
9698	1.73981	0.0767621
9699	1.73981	0.0709351
9700	0.94613	0.0712501
9701	1.3542	0.0711843
9702	1.33174	0.0767122
9703	1.59452	2.1
9704	1.59452	2.1
9705	0.946132	2.1
9706	1.25	2.1
9707	0.975266	2.1
9708	0.946132	2.1
9709	1.25	2.1
9710	0.946131	2.1
9711	1.48685	2.1
9712	1.48685	2.1
9713	0.946132	2.1
9714	1.17273	2.1
9715	1.17273	2.1
9716	0.955602	2.1
9717	1.32827	0.319257
9718	1.11166	0.319088
9719	0.946127	0.340424
9720	1.6136	0.302669
9721	1.6136	0.293372
9722	1.11166	0.308174
9723	0.946129	0.316
9724	0.851541	0.691486
9725	1.22107	0.684228
9726	1.64594	0.61297
9727	1.5271	0.618654
9728	0.94613	0.766599
9729	1.22112	0.703497
9730	1.25002	0.642397
9731	1.65795	0.429674
9732	1.65795	1.04719
9733	0.946132	1.00071
9734	1.2366	0.612456
9735	0.946132	0.410417
9736	1.10023	0.566619
9737	1.2366	0.474709
9738	1.35856	0.290391
9739	0.946132	0.288251
9740	1.35856	0.258226
9741	1.08019	0.286596

9742	1.49261	0.25183
9743	1.49261	0.277837
9744	0.94613	0.326863
9745	1.29322	0.399323
9746	0.946132	0.379423
9747	1.29322	0.343129
9748	1.35856	0.400647
9749	1.70565	0.329092
9750	1.70565	0.385209
9751	0.946132	0.388552
9752	1.66635	2.1
9753	1.66635	2.1
9754	0.946133	2.1
9755	1.39558	2.1
9756	1.14793	2.1
9757	1.14793	2.1
9758	1.21691	2.1
9759	1.35381	0.302595
9760	1.3365	0.685978
9761	1.09905	0.44037
9762	0.946132	0.629022
9763	1.74418	0.598809
9764	1.74418	0.246698
9765	0.946132	0.417087
9766	1.84759	0.545404
9767	1.84759	0.291221
9768	0.94613	0.435788
9769	1.3904	0.534155
9770	1.34522	0.22196
9771	1.34522	0.338639
9772	1.40331	0.170869
9773	1.86047	0.567177
9774	1.86047	1.7214
9775	0.946132	0.749468
9776	1.41619	1.64874
9777	0.946132	0.318787
9778	1.3659	0.61607
9779	1.41619	0.640525
9780	1.28464	1.93175
9781	1.14496	1.30134
9782	1.57806	1.46113
9783	1.7753	1.84821
9784	0.851541	1.30043
9785	0.946131	1.59103
9786	0.872786	1.52382
9787	0.85062	0.703434
9788	1.52635	0.807785
9789	1.63785	0.645307
9790	1.69972	0.985921
9791	0.946132	1.11857
9792	1.37493	0.705878
9793	1.23854	0.698766
9794	1.41134	1.87121
9795	1.27079	1.55068
9796	1.736	1.20048
9797	1.736	2.1
9798	0.946132	0.788342
9799	0.946133	1.42968

9800	1.21177	1.62792
9801	0.946131	0.154754
9802	0.946129	0.14032
9803	0.94613	0.154861
9804	0.946131	0.12118
9805	0.946131	0.126464
9806	1.35403	0.128666
9807	0.946131	0.155051
9808	1.37052	0.251741
9809	0.946132	0.599057
9810	0.94613	0.354567
9811	0.946133	0.251591
9812	0.946131	0.338152
9813	0.946131	0.326076
9814	0.946131	0.335309
9815	1.24249	0.224711
9816	0.946132	0.227091
9817	0.946133	0.175455
9818	0.946132	0.24333
9819	0.946132	0.229309
9820	0.946132	0.205454
9821	0.946132	0.221104
9822	0.946132	2.1
9823	0.946132	1.68838
9824	0.946131	2.1
9825	0.946132	2.1
9826	0.946132	2.1
9827	0.946132	2.01293
9828	1.20608	2.1
9829	1.41232	2.1
9830	0.946128	2.1
9831	0.946129	2.1
9832	0.94613	2.1
9833	0.94613	2.1
9834	0.94613	2.1
9835	0.94613	2.1
9836	0.946132	0.20087
9837	0.946131	0.200832
9838	0.946131	0.213429
9839	0.946132	0.131995
9840	0.946132	0.153482
9841	0.946131	0.193705
9842	0.946132	0.173175
9843	0.946116	0.525237
9844	0.94612	1.17019
9845	0.946118	0.867131
9846	0.946124	2.1
9847	0.946118	0.797458
9848	1.41561	1.37648
9849	0.946131	0.772492
9850	0.945846	1.9104
9851	1.03508	2.1
9852	0.77579	1.20431
9853	0.658908	2.1
9854	1.17474	2.1
9855	0.94303	2.1
9856	0.849189	2.1
9857	0.94298	2.1

9858	1.03963	2.1
9859	0.946131	1.31807
9860	0.946131	1.26296
9861	0.946131	2.1
9862	0.946131	0.98935
9863	0.946131	0.550141
9864	0.946131	1.06328
9865	1.32819	1.2814
9866	0.946133	0.952515
9867	0.946129	2.1
9868	0.946128	1.65009
9869	0.94613	1.61427
9870	0.946131	2.1
9871	0.946131	0.929443
9872	0.946131	1.52094
9873	0.954072	0.0589299
9874	1.28895	0.0586356
9875	0.946133	0.0640066
9876	1.15803	0.0648793
9877	1.29689	0.0585536
9878	1.29689	0.0607924
9879	0.946132	0.0657998
9880	0.946132	1.04336
9881	0.946132	1.06767
9882	0.946132	0.863593
9883	0.946132	1.14444
9884	0.946132	1.39486
9885	0.946131	1.39757
9886	1.0264	1.39441
9887	0.946126	0.365496
9888	0.946132	0.340327
9889	0.946131	0.258037
9890	0.94613	0.344438
9891	0.946129	0.460688
9892	0.94613	0.39382
9893	1.35977	0.364654
9894	0.946132	0.660109
9895	0.946133	0.627026
9896	0.876632	0.957846
9897	0.946132	0.882716
9898	1.01566	1.19044
9899	1.01566	0.841061
9900	0.876606	0.939709
9901	0.946131	0.121637
9902	0.946128	0.114468
9903	0.946128	0.122976
9904	0.94613	0.116533
9905	0.94613	0.128722
9906	1.24979	0.102681
9907	0.94613	0.130562
9908	0.946131	0.0872215
9909	0.946131	0.103905
9910	0.921762	0.0927603
9911	0.9705	0.0820187
9912	0.9705	0.0866532
9913	0.946131	0.0990551
9914	0.921791	0.0966303
9915	0.810944	0.311447

9916	0.810945	0.337098
9917	0.810959	0.292215
9918	2.42267	0.189973
9919	1.35168	0.256227
9920	1.08132	0.290592
9921	1.08132	0.221593
9922	1.08131	0.272986
9923	0.946133	0.264729
9924	0.946132	0.26775
9925	0.946132	0.268196
9926	0.946133	0.254833
9927	0.946133	0.264964
9928	0.946132	0.294417
9929	0.946133	0.277936
9930	0.946131	0.746353
9931	0.946131	0.986424
9932	0.94613	0.614436
9933	0.851514	0.663168
9934	0.946131	0.813361
9935	0.965268	0.860998
9936	0.851513	0.63875
9937	0.946122	2.1
9938	0.946123	2.1
9939	0.946126	2.1
9940	0.946124	2.1
9941	0.946124	2.1
9942	0.946118	2.1
9943	0.94613	2.1
9944	0.946131	0.581016
9945	1.30448	0.685732
9946	0.946132	0.75747
9947	0.946129	0.632671
9948	0.946131	1.26944
9949	0.946131	0.594256
9950	0.94613	1.0111
9951	0.946133	0.755484
9952	0.946128	2.1
9953	0.946128	1.51205
9954	0.94613	0.920155
9955	1.36503	2.1
9956	0.946131	1.34075
9957	0.946131	2.1
9958	0.810947	0.066658
9959	0.810947	0.0663901
9960	2.48233	0.0634244
9961	1.3517	0.0637383
9962	1.08132	0.0665868
9963	1.08132	0.0649956
9964	1.08133	0.0645901
9965	0.810934	0.0669627
9966	1.48874	0.0576097
9967	2.48233	0.0585075
9968	2.2369	0.0608734
9969	0.946133	0.635578
9970	0.946132	0.515959
9971	0.946132	0.716831
9972	0.946133	0.755198
9973	0.946133	0.792461

9974	1.41851	0.649568
9975	0.946132	0.616126
9976	1.03925	2.1
9977	0.946131	2.1
9978	0.94613	2.1
9979	0.946128	2.1
9980	0.946133	2.1
9981	0.944743	2.1
9982	1.13703	2.1
9983	0.946132	1.93288
9984	0.946128	2.1
9985	0.946128	2.1
9986	0.946131	2.1
9987	0.94613	2.1
9988	0.94613	2.1
9989	0.94613	2.1
9990	0.946131	0.103287
9991	0.946132	0.119693
9992	0.946131	0.104448
9993	0.946132	0.0917289
9994	0.946132	0.0960819
9995	0.946131	0.105548
9996	1.20057	0.0979832
9997	0.946129	2.1
9998	0.94613	2.1
9999	0.94613	2.1
10000	0.946128	2.1
10001	0.946132	2.1
10002	0.94613	2.1
10003	1.38302	2.1
10004	0.856567	0.212291
10005	1.14319	0.209273
10006	0.716526	0.201543
10007	1.20631	0.193058
10008	0.626936	0.175603
10009	0.946131	0.206629
10010	0.94613	0.235037
10011	1.39502	0.170787
10012	0.881445	0.143982
10013	0.946128	0.187923
10014	0.94613	0.162069
10015	0.881447	0.177523
10016	0.946117	0.15508
10017	0.946116	0.148919
10018	0.88146	0.22403
10019	0.946132	2.1
10020	1.39053	2.1
10021	0.946132	2.1
10022	0.946132	2.1
10023	0.946132	2.1
10024	0.946132	2.1
10025	0.946131	2.1
10026	0.946131	2.1
10027	0.946131	2.1
10028	0.946131	2.1
10029	0.946132	2.1
10030	0.946132	2.1
10031	1.40571	2.1

10032	0.946132	2.1
10033	0.94613	0.372039
10034	0.946127	0.49506
10035	0.946128	0.419415
10036	0.946131	0.377625
10037	0.946127	0.407594
10038	1.25819	0.378509
10039	0.946129	0.345947
10040	0.946129	0.767933
10041	1.29054	0.782317
10042	0.94613	0.726833
10043	0.94613	0.7543
10044	0.946131	1.24155
10045	0.946131	0.869983
10046	0.94613	0.799518
10047	0.810944	0.0743437
10048	0.810941	0.0763079
10049	1.61558	0.0702678
10050	1.3517	0.0709231
10051	1.08131	0.0750552
10052	1.08132	0.074956
10053	1.08133	0.0716328
10054	0.810931	0.0746227
10055	1.02036	1.31111
10056	0.946132	1.02
10057	1.02036	0.914404
10058	0.946132	0.733919
10059	1.1576	0.854174
10060	0.964656	1.03827
10061	0.927608	0.688784
10062	0.946128	2.1
10063	1.38813	2.1
10064	0.946129	2.1
10065	0.946131	2.1
10066	0.946131	2.1
10067	0.94613	2.1
10068	0.946131	2.1
10069	0.946131	1.62277
10070	0.94613	0.547909
10071	0.94613	1.87328
10072	1.41665	1.25956
10073	0.946132	0.563213
10074	0.946131	0.952548
10075	0.94613	0.639803
10076	0.946118	0.344326
10077	0.946118	0.321019
10078	0.946116	0.335756
10079	0.946126	0.235503
10080	0.94612	0.271241
10081	0.946131	0.330965
10082	0.946105	0.275135
10083	0.946132	2.1
10084	0.946132	2.1
10085	0.946132	2.1
10086	0.946131	2.1
10087	0.946132	2.1
10088	0.946132	2.1
10089	0.946132	2.1

10090	0.864997	0.0812595
10091	0.786573	0.0831759
10092	0.978561	0.0816092
10093	1.47385	0.0748496
10094	1.68652	0.0763497
10095	1.27803	0.0749621
10096	1.19771	0.0807486
10097	1.27595	0.0796851
10098	0.946127	0.444102
10099	1.3771	0.384241
10100	0.946127	0.366453
10101	0.946131	0.541475
10102	0.946129	0.338105
10103	0.946129	0.46532
10104	0.946129	0.412219
10105	0.95697	0.0651367
10106	1.48133	0.0692474
10107	1.24499	0.0650609
10108	1.5295	0.0690751
10109	0.938161	0.0655726
10110	0.808717	0.0650165
10111	1.3542	0.0690841
10112	0.946133	0.770589
10113	1.14444	0.708744
10114	0.946132	0.677483
10115	0.946132	0.744161
10116	0.946133	1.15411
10117	0.946132	0.866341
10118	0.946132	0.766137
10119	0.946131	0.121268
10120	0.946132	0.169822
10121	0.946131	0.115545
10122	0.946132	0.116704
10123	0.946131	0.145445
10124	0.946132	0.111598
10125	0.946132	0.139566
10126	0.946132	2.1
10127	0.946133	2.1
10128	0.946133	2.1
10129	0.946132	2.1
10130	1.29322	2.1
10131	0.946132	2.1
10132	0.946132	2.1
10133	0.946131	1.98521
10134	0.946132	2.1
10135	0.946131	2.1
10136	0.946132	1.96715
10137	1.28441	2.1
10138	0.946131	2.07593
10139	0.946131	2.1
10140	0.946113	2.1
10141	0.946118	2.1
10142	0.946118	1.254
10143	1.28073	2.1
10144	0.946117	1.65277
10145	0.946131	2.1
10146	0.946105	2.06224
10147	0.810945	1.96369

10148	0.810942	2.1
10149	2.75904	2.1
10150	1.3517	2.1
10151	1.08131	2.1
10152	1.08133	2.1
10153	1.08132	2.1
10154	0.81093	2.1
10155	0.946127	2.03248
10156	1.37952	2.1
10157	1.86965	2.1
10158	0.946127	2.1
10159	1.86965	2.1
10160	1.28228	2.1
10161	1.37952	2.1
10162	0.946128	2.1
10163	0.946132	2.1
10164	1.14042	2.1
10165	0.946132	2.1
10166	1.14042	2.1
10167	1.09974	2.1
10168	1.09974	2.1
10169	0.946132	2.1
10170	0.946132	0.313651
10171	0.946132	0.255406
10172	0.946133	0.308845
10173	0.946132	0.242719
10174	0.946132	0.314862
10175	0.946132	0.244307
10176	0.946133	0.330637
10177	1.31817	1.33591
10178	0.946131	1.0391
10179	0.946131	1.26312
10180	0.946131	1.21953
10181	0.946131	0.862781
10182	0.946131	1.0785
10183	0.946131	1.09374
10184	0.946132	2.1
10185	0.946132	2.1
10186	0.946132	2.1
10187	0.946132	2.1
10188	0.946132	2.1
10189	0.946132	2.1
10190	1.35048	2.1
10191	0.946131	2.02157
10192	0.946131	2.1
10193	0.94613	2.1
10194	0.946133	2.1
10195	0.946133	2.1
10196	1.35943	2.1
10197	0.946131	2.1
10198	0.946133	2.1
10199	0.946132	2.1
10200	0.946132	2.1
10201	0.946133	2.1
10202	0.946133	2.1
10203	0.946133	2.1
10204	0.990712	2.1
10205	0.965725	1.71369

10206	0.946129	0.857276
10207	0.946133	1.25911
10208	1.21721	1.01039
10209	0.877931	0.760048
10210	0.946131	1.22008
10211	0.946131	0.825558
10212	0.946132	0.532303
10213	0.946131	0.465836
10214	0.946131	0.736446
10215	0.946133	0.520135
10216	0.946128	0.95076
10217	0.946131	0.668139
10218	1.39575	0.646969
10219	0.810943	0.46378
10220	0.810942	0.488427
10221	2.58794	0.352049
10222	1.21652	0.324442
10223	0.946154	0.43043
10224	0.979952	0.416462
10225	1.0813	0.408468
10226	0.810959	0.428005
10227	0.924071	0.351856
10228	1.16453	0.253886
10229	1.28586	0.241268
10230	0.946131	0.386463
10231	1.1227	0.227704
10232	1.16462	0.299652
10233	0.851553	0.431625
10234	0.709578	0.34645
10235	0.810943	0.576326
10236	0.810959	0.464425
10237	1.82615	0.289626
10238	1.21651	0.34729
10239	1.08132	0.360105
10240	0.946155	0.378635
10241	0.979937	0.475211
10242	0.946128	1.23892
10243	0.946132	2.1
10244	0.946132	0.894689
10245	0.94613	1.83717
10246	0.94613	1.68053
10247	0.94613	0.818688
10248	0.94613	1.44001
10249	0.946131	1.50859
10250	1.29735	1.87145
10251	0.946131	2.1
10252	0.946131	0.933235
10253	0.946131	1.35221
10254	0.946131	0.859482
10255	0.946131	1.74843
10256	0.946128	2.0053
10257	1.33266	1.14302
10258	0.94613	1.17991
10259	0.946131	1.73053
10260	0.94613	0.68369
10261	0.946131	1.1035
10262	0.946131	0.987087
10263	0.946131	0.408072

10264	0.946131	1.3879
10265	0.946131	0.333899
10266	1.40608	0.434373
10267	0.946131	1.16041
10268	0.946131	0.627033
10269	0.946131	0.711986
10270	0.946131	0.905167
10271	0.946131	1.5284
10272	0.946131	1.05902
10273	1.2334	1.07009
10274	0.946129	1.17131
10275	0.946131	1.07718
10276	0.946131	1.47011
10277	0.946133	1.63018
10278	0.946133	2.09721
10279	0.946132	1.09392
10280	1.29198	1.74979
10281	0.946132	1.46846
10282	0.946132	1.46369
10283	0.946132	1.7775
10284	0.946131	2.1
10285	0.946128	2.1
10286	0.946128	2.1
10287	0.946131	2.1
10288	0.946127	2.1
10289	0.94613	2.1
10290	0.946129	2.1
10291	0.946123	2.1
10292	0.946126	2.1
10293	0.946125	2.1
10294	1.38639	2.1
10295	0.946126	2.1
10296	0.946132	2.1
10297	0.946119	2.1
10298	0.946128	0.389576
10299	0.946132	0.378378
10300	0.946131	0.419665
10301	0.94613	0.232271
10302	0.94613	0.27608
10303	0.946131	0.361657
10304	0.94613	0.315653
10305	0.946131	0.360413
10306	0.946131	0.337714
10307	0.946131	0.396601
10308	0.946131	0.188228
10309	0.946131	0.240437
10310	0.946131	0.338541
10311	0.946131	0.281857
10312	0.810945	2.1
10313	0.810943	2.1
10314	0.810933	2.1
10315	2.24688	2.1
10316	1.3517	2.1
10317	1.08132	2.1
10318	1.08132	2.1
10319	1.08133	2.1
10320	1.25809	0.246367
10321	0.946131	0.522358

10322	1.25809	0.245763
10323	0.946129	0.487083
10324	0.94613	0.258459
10325	0.946131	0.430426
10326	0.946131	0.336988
10327	0.946131	0.267843
10328	0.946131	0.263527
10329	0.946129	0.296085
10330	1.28911	0.251007
10331	0.946131	0.256833
10332	0.946131	0.279635
10333	0.946131	0.291875
10334	0.94613	0.431761
10335	0.946131	0.469507
10336	0.94613	0.374209
10337	0.946128	0.441513
10338	0.946132	0.364079
10339	1.38954	0.421022
10340	0.946131	0.419301
10341	0.946131	0.295183
10342	0.946131	0.481763
10343	0.946131	0.565902
10344	1.37981	0.410708
10345	0.946131	0.746911
10346	0.946131	0.514589
10347	0.946131	0.536633
10348	0.946131	1.48615
10349	0.946127	2.1
10350	0.946127	2.1
10351	0.946131	1.7757
10352	0.946127	2.1
10353	0.946129	2.08023
10354	0.946128	2.07286
10355	0.94613	2.1
10356	0.946126	2.1
10357	0.946127	2.1
10358	0.946131	2.1
10359	0.946126	1.95934
10360	0.946128	2.1
10361	0.946128	2.1
10362	0.946133	0.428514
10363	0.946132	0.369879
10364	0.946132	0.393561
10365	0.946132	0.37543
10366	0.946132	0.448556
10367	1.13511	0.27535
10368	0.946132	0.331261
10369	0.946132	1.61817
10370	0.946129	1.63486
10371	0.94613	1.29456
10372	0.946131	1.74071
10373	0.946131	1.86335
10374	0.94613	2.1
10375	1.27575	2.03346
10376	0.946129	2.1
10377	0.946131	2.1
10378	0.946131	2.1
10379	0.946128	2.1

10380	0.946133	2.1
10381	0.946131	2.1
10382	0.94613	2.1
10383	1.15581	0.096778
10384	0.946132	0.0920535
10385	0.946131	0.107747
10386	0.94613	0.101196
10387	1.15581	0.0953136
10388	0.94613	0.0966281
10389	0.946131	0.118587
10390	0.611407	0.977996
10391	0.946131	0.745259
10392	1.37335	0.859124
10393	1.15086	0.984056
10394	0.617549	0.825441
10395	0.851553	0.916003
10396	1.14592	0.985433
10397	1.22618	1.20966
10398	0.946132	0.734876
10399	0.946132	1.28054
10400	0.946132	0.768617
10401	0.946131	0.788112
10402	0.946132	0.988216
10403	0.946132	0.891438
10404	0.946131	2.05117
10405	0.94613	2.1
10406	0.946131	2.1
10407	0.946133	2.1
10408	0.946129	2.1
10409	0.946131	2.1
10410	0.946131	2.1
10411	0.810945	0.715527
10412	0.810943	1.0302
10413	2.50715	0.996154
10414	1.3517	0.777242
10415	1.08133	0.725769
10416	1.08132	0.952229
10417	1.08132	0.507587
10418	0.810932	0.697564
10419	1.07811	1.04749
10420	0.94613	0.721438
10421	0.946128	1.16725
10422	1.25358	0.551435
10423	0.946131	0.74982
10424	1.25358	0.742716
10425	0.94613	0.767332
10426	0.946129	2.1
10427	1.09197	2.1
10428	0.946131	2.1
10429	1.09197	2.1
10430	1.09532	2.1
10431	1.09532	2.1
10432	0.946131	2.1
10433	1.58788	2.1
10434	1.34255	2.1
10435	0.594912	2.1
10436	0.58894	2.1
10437	0.946129	2.1

10438	1.16679	2.1
10439	1.04071	2.1
10440	0.535418	2.1
10441	0.946131	2.01873
10442	0.946128	2.1
10443	0.946131	1.91588
10444	1.20221	2.1
10445	0.946128	2.1
10446	0.94613	2.1
10447	1.20221	2.1
10448	0.946132	2.1
10449	0.946133	2.1
10450	0.946132	2.1
10451	0.946132	2.1
10452	0.946133	2.1
10453	0.946133	2.1
10454	0.946132	2.1
10455	0.946132	0.844029
10456	0.946132	1.07403
10457	0.946133	1.28781
10458	0.946132	0.910801
10459	1.411	1.15667
10460	0.946132	1.01601
10461	0.946133	1.11471
10462	1.05575	0.183305
10463	0.563734	0.190371
10464	1.05575	0.188706
10465	0.773833	0.151087
10466	0.751686	0.163108
10467	0.75884	0.189029
10468	0.815612	0.157235
10469	0.793465	0.173058
10470	0.768563	0.20775
10471	0.946129	0.225386
10472	0.946125	0.182633
10473	0.946126	0.232073
10474	0.94613	0.201662
10475	0.946124	0.251785
10476	1.31678	0.166729
10477	0.946128	0.20463
10478	0.946132	0.368252
10479	1.29914	0.286048
10480	0.946132	0.296793
10481	0.946132	0.299655
10482	0.946132	0.382151
10483	1.29914	0.197317
10484	0.946132	0.311513
10485	0.810946	0.331678
10486	0.810943	0.323094
10487	2.21082	0.214392
10488	1.35168	0.227398
10489	1.08132	0.267934
10490	1.08132	0.276342
10491	1.0813	0.313105
10492	0.81096	0.323527
10493	0.625813	0.109832
10494	0.946132	0.122182
10495	0.946131	0.14015

10496	0.946132	0.127967
10497	0.946131	0.121264
10498	1.39301	0.131429
10499	0.625812	0.127401
10500	0.810946	1.28982
10501	0.810945	2.1
10502	0.810931	1.89741
10503	2.70043	2.0972
10504	1.3517	1.92065
10505	1.08131	1.34783
10506	1.08131	2.1
10507	1.08133	1.4127
10508	0.810943	2.1
10509	0.810944	1.96471
10510	0.810958	2.1
10511	2.44802	2.1
10512	1.35168	2.1
10513	1.08132	2.1
10514	1.08132	2.04938
10515	1.0813	2.1
10516	0.946131	0.40268
10517	0.946129	0.430098
10518	0.946128	0.383156
10519	0.946131	0.391526
10520	1.3492	0.386605
10521	1.3492	0.40497
10522	0.94613	0.386851
10523	0.946132	0.51415
10524	0.946132	0.421767
10525	0.946133	0.499183
10526	0.946133	0.393247
10527	0.946132	0.58194
10528	0.946132	0.392628
10529	0.946132	0.554948
10530	0.946129	0.328323
10531	0.946133	0.285228
10532	0.946131	0.346298
10533	0.94613	0.332236
10534	0.946131	0.390733
10535	0.946131	0.294104
10536	0.946131	0.319101
10537	0.946119	0.282444
10538	0.946119	0.258303
10539	0.946119	0.307516
10540	0.946125	0.271759
10541	0.94612	0.330935
10542	0.946132	0.26533
10543	0.946107	0.279123
10544	0.946133	2.1
10545	0.946132	2.1
10546	0.946132	2.1
10547	0.946132	2.1
10548	0.946132	2.1
10549	0.946133	2.1
10550	1.3868	2.1
10551	0.810947	2.1
10552	0.810946	2.1
10553	0.81096	2.1

10554	2.66094	2.1
10555	1.35168	2.1
10556	1.08132	2.1
10557	1.08132	2.1
10558	1.0813	2.1
10559	0.810946	2.1
10560	0.810946	2.1
10561	0.810933	2.1
10562	2.75737	2.1
10563	1.3517	2.1
10564	1.08132	2.1
10565	1.08132	2.1
10566	1.08133	2.1
10567	0.946133	2.1
10568	0.946132	2.1
10569	0.946132	1.14383
10570	0.946132	2.1
10571	0.946133	1.19469
10572	0.946132	2.1
10573	0.946133	2.1
10574	0.946133	2.1
10575	0.946133	2.1
10576	0.946132	2.1
10577	0.946132	2.1
10578	0.946133	2.1
10579	0.946132	2.1
10580	1.28569	2.1
10581	0.946132	1.21882
10582	0.946133	2.08867
10583	0.946133	2.1
10584	0.946132	1.6188
10585	0.946132	2.1
10586	0.946132	1.82703
10587	0.946133	1.76536
10588	0.946132	0.241206
10589	0.946131	0.214631
10590	0.94613	0.209487
10591	0.946133	0.172537
10592	0.946128	0.24895
10593	0.946131	0.170248
10594	0.946131	0.240551
10595	0.946131	1.59366
10596	0.94613	1.4523
10597	0.946131	1.94803
10598	0.94613	1.44063
10599	1.41833	1.07909
10600	0.946131	0.938833
10601	0.946131	1.71437
10602	1.26155	0.100856
10603	0.946131	0.129953
10604	0.946131	0.115024
10605	0.946132	0.0981847
10606	0.946132	0.106683
10607	0.946131	0.106885
10608	0.946132	0.105355
10609	0.810946	2.05304
10610	0.810946	2.1
10611	0.810959	2.1

10612	2.47938	2.1
10613	1.35168	2.1
10614	1.08132	2.1
10615	1.08132	2.1
10616	1.08131	2.1
10617	0.946131	0.513388
10618	0.94613	0.459776
10619	0.94613	0.693255
10620	0.946132	0.441131
10621	0.946128	0.880889
10622	0.94613	0.621823
10623	0.94613	0.652411
10624	0.94613	0.123219
10625	0.946131	0.126183
10626	0.94613	0.179114
10627	0.946129	0.13703
10628	0.946132	0.152608
10629	0.94613	0.141493
10630	0.946131	0.147969
10631	0.946132	2.1
10632	0.946131	2.1
10633	0.946128	2.1
10634	1.40756	2.1
10635	0.946131	2.1
10636	0.946129	2.1
10637	0.94613	2.1
10638	0.946131	1.05312
10639	0.946131	1.081
10640	0.946131	1.48195
10641	0.946132	1.17291
10642	1.31947	1.16363
10643	0.946131	1.15504
10644	0.946131	1.21859
10645	0.946131	2.1
10646	1.25768	2.1
10647	0.946132	2.1
10648	0.946132	2.1
10649	0.946129	2.1
10650	0.946131	2.1
10651	0.94613	2.1
10652	0.946132	0.346868
10653	0.946129	0.320354
10654	0.946129	0.392059
10655	0.94613	0.356062
10656	1.31716	0.346312
10657	1.31716	0.341049
10658	0.946131	0.389297
10659	0.810943	1.04732
10660	0.810942	2.00596
10661	0.81093	1.58596
10662	2.37052	2.1
10663	1.3517	1.53546
10664	1.08132	0.901711
10665	1.08132	1.86167
10666	1.08133	1.09072
10667	0.946128	0.96841
10668	1.10335	0.821987
10669	0.94613	0.744917

10670	0.946131	1.17779
10671	0.946131	0.678121
10672	1.10335	0.970784
10673	0.94613	1.09662
10674	0.946132	0.988493
10675	1.18818	0.866573
10676	0.946132	0.825184
10677	0.946132	1.02075
10678	0.946132	0.773237
10679	1.18818	0.935682
10680	0.946132	0.98791
10681	0.946125	2.1
10682	0.94612	2.1
10683	0.946119	2.1
10684	0.946119	2.1
10685	0.946119	2.1
10686	0.946132	2.1
10687	1.31522	2.1
10688	1.28803	1.56499
10689	0.946132	0.72114
10690	0.946132	1.88547
10691	0.946133	1.89987
10692	0.946133	0.675596
10693	0.946133	1.40084
10694	0.946132	1.06949
10695	0.946133	0.974587
10696	0.946132	1.20272
10697	0.946132	0.935405
10698	0.946132	0.77129
10699	0.946133	0.765499
10700	0.946132	0.736704
10701	1.37658	0.842749
10702	1.02239	1.5159
10703	0.946128	0.913737
10704	0.946132	1.31367
10705	1.02239	1.02568
10706	0.94613	0.856422
10707	0.96283	1.31845
10708	0.92943	0.878489
10709	0.946132	0.0824745
10710	0.946131	0.0927512
10711	0.946126	0.113867
10712	0.946129	0.096248
10713	0.946128	0.100099
10714	0.946128	0.0940782
10715	0.946129	0.0977634
10716	0.810946	0.117032
10717	0.810944	0.098121
10718	2.22493	0.0827575
10719	1.21651	0.0857861
10720	1.0813	0.0956676
10721	0.946154	0.0918039
10722	0.979951	0.0912507
10723	0.70959	0.0935781
10724	0.946132	0.132019
10725	0.946131	0.171827
10726	0.946131	0.122649
10727	0.946133	0.127702

10728	0.946131	0.163553
10729	0.946132	0.134519
10730	0.946132	0.149701
10731	0.946132	1.7484
10732	0.946131	1.45771
10733	0.946132	2.02395
10734	0.946132	1.09686
10735	0.946132	0.783166
10736	0.946132	1.13016
10737	1.40924	1.52022
10738	0.946131	2.1
10739	0.946128	2.1
10740	0.946127	2.1
10741	0.94613	2.1
10742	1.41743	2.1
10743	0.946128	2.1
10744	0.946129	2.1
10745	1.37675	0.638178
10746	0.946128	0.713202
10747	0.94613	0.632147
10748	0.94613	0.933047
10749	0.94613	0.64112
10750	0.946129	0.670243
10751	0.94613	0.841553
10752	0.946132	0.356657
10753	0.946132	0.476904
10754	0.946132	0.576865
10755	1.35721	0.355623
10756	0.946132	0.742854
10757	0.946132	0.563194
10758	1.35721	0.504948
10759	0.946133	0.792213
10760	0.946133	0.660753
10761	0.946132	0.778372
10762	1.10003	0.590676
10763	0.946133	0.594489
10764	0.946132	0.709758
10765	0.946132	0.667827
10766	0.946132	0.210391
10767	0.946131	0.161248
10768	0.946131	0.184218
10769	0.946131	0.226406
10770	1.0616	0.164196
10771	1.0616	0.172332
10772	0.946132	0.222562
10773	0.946132	0.235425
10774	0.946131	0.178887
10775	0.946131	0.205461
10776	0.946131	0.25726
10777	1.09402	0.183475
10778	1.09402	0.192765
10779	0.946131	0.254833
10780	0.946132	0.117038
10781	0.946126	0.112419
10782	1.24093	0.111057
10783	0.946129	0.135844
10784	0.946129	0.122866
10785	0.946128	0.125143

10786	0.946129	0.121475
10787	0.946131	0.149369
10788	0.946131	0.149461
10789	1.2929	0.143288
10790	0.946131	0.190618
10791	0.946131	0.170304
10792	0.946131	0.172858
10793	0.946131	0.160396
10794	0.810946	0.0973686
10795	0.810945	0.0816086
10796	0.810959	0.0895417
10797	2.41233	0.0817028
10798	1.35168	0.0828185
10799	1.08132	0.0869099
10800	1.08132	0.0832421
10801	1.0813	0.090164
10802	0.810946	1.19561
10803	0.810943	1.79386
10804	0.750103	1.55484
10805	2.22778	1.87774
10806	1.27059	1.67095
10807	1.00021	1.37599
10808	1.02049	1.8244
10809	1.08133	1.35413
10810	0.946128	1.51865
10811	0.946132	2.1
10812	0.946132	2.1
10813	0.94613	1.86003
10814	0.94613	2.09679
10815	0.94613	1.95143
10816	0.946131	1.94549
10817	0.946126	0.411147
10818	0.946131	0.377367
10819	0.946127	0.476811
10820	1.3324	0.338498
10821	0.946127	0.367653
10822	0.946128	0.420795
10823	0.946128	0.536339
10824	0.94613	2.1
10825	0.946131	2.1
10826	0.94613	2.1
10827	0.946128	2.1
10828	0.946133	1.80596
10829	0.946131	2.1
10830	0.946131	2.1
10831	0.946133	2.1
10832	0.946132	2.1
10833	0.946133	2.1
10834	0.946133	2.1
10835	0.946132	2.1
10836	0.946132	2.1
10837	1.36541	2.1
10838	0.946131	0.637161
10839	0.94613	2.06177
10840	0.946131	0.889378
10841	0.94613	0.820679
10842	1.39509	1.33651
10843	0.946131	0.576549

10844	0.946131	1.43924
10845	0.946133	2.1
10846	0.946133	2.1
10847	0.946132	2.1
10848	0.946132	2.1
10849	0.946133	2.1
10850	0.946132	2.1
10851	1.29319	2.1
10852	1.01196	0.132884
10853	0.946131	0.185391
10854	0.946132	0.125093
10855	0.946132	0.151208
10856	0.946132	0.189064
10857	0.946132	0.168942
10858	0.946132	0.162979
10859	0.946112	2.1
10860	0.946118	1.77704
10861	0.946116	1.3464
10862	0.946117	1.41986
10863	0.946118	2.1
10864	0.94613	2.1
10865	0.946105	2.1
10866	1.28684	1.0204
10867	0.946131	1.0695
10868	0.946132	1.07088
10869	0.946132	1.28557
10870	0.946132	1.30907
10871	0.946132	1.13031
10872	0.946132	1.34876
10873	0.94613	1.93737
10874	0.94613	2.02965
10875	0.946128	1.65583
10876	0.946131	1.48168
10877	0.946131	1.77791
10878	0.94613	1.67548
10879	1.10185	1.96278
10880	0.810947	0.0737032
10881	0.810945	0.0748148
10882	1.57348	0.0706129
10883	1.3517	0.0709714
10884	1.08131	0.0743561
10885	1.08132	0.0749703
10886	1.08133	0.070093
10887	0.810932	0.0742593
10888	0.946119	0.438711
10889	0.946116	0.390979
10890	0.946117	0.435908
10891	0.946121	0.309614
10892	0.946118	0.349007
10893	0.94613	0.406243
10894	0.946105	0.355873
10895	0.94612	0.535107
10896	0.946122	0.371599
10897	0.946119	0.458006
10898	0.946119	0.541923
10899	1.20884	0.367949
10900	0.946132	0.492399
10901	0.946107	0.446537

10902	2.67849	0.950597
10903	1.17709	0.929748
10904	0.605418	1.15818
10905	0.286984	0.973913
10906	0.172654	1.0105
10907	0.810946	0.702102
10908	0.940878	0.66776
10909	0.941235	0.66217
10910	1.26696	0.69294
10911	1.56837	0.721188
10912	1.13737	0.689106
10913	1.34115	0.714738
10914	1.24889	0.657232
10915	0.946132	1.19006
10916	0.946129	0.93805
10917	0.946128	1.09508
10918	0.94613	1.00375
10919	0.94613	1.2511
10920	0.946131	1.14356
10921	0.94613	1.10265
10922	0.946116	2.1
10923	0.94612	2.06565
10924	0.946118	2.1
10925	0.946123	2.1
10926	0.946118	2.1
10927	0.946131	2.1
10928	1.23836	2.1
10929	0.810946	0.100479
10930	0.810946	0.0994989
10931	0.810959	0.100396
10932	2.80677	0.0898053
10933	1.35168	0.0942359
10934	1.08132	0.0883301
10935	1.08132	0.0988702
10936	1.08131	0.098692
10937	0.810947	0.426009
10938	0.810947	0.686293
10939	2.41574	0.647332
10940	1.21653	0.634108
10941	1.08133	0.540522
10942	0.946153	0.681725
10943	0.979949	0.54489
10944	0.709567	0.519398
10945	0.94613	2.08532
10946	0.946131	2.1
10947	0.946128	2.1
10948	1.34782	2.1
10949	0.946128	2.1
10950	0.946129	2.1
10951	1.34782	2.1
10952	0.946132	1.78805
10953	0.946132	2.01066
10954	0.94613	2.0922
10955	1.16023	2.00637
10956	0.946131	2.09969
10957	0.946131	2.1
10958	1.16023	2.1
10959	0.946131	0.888006

10960	0.946127	2.1
10961	0.946127	1.65758
10962	0.946131	1.4616
10963	0.946128	2.1
10964	0.94613	1.04295
10965	0.946129	1.47802
10966	0.946133	0.301464
10967	0.946132	0.249638
10968	0.946132	0.27157
10969	0.946133	0.327361
10970	1.20045	0.251812
10971	1.20045	0.255744
10972	0.946132	0.333862
10973	0.918871	0.179266
10974	0.971961	0.147279
10975	1.37324	0.112972
10976	1.78255	0.113979
10977	1.25155	0.14919
10978	1.42172	0.114044
10979	1.40554	0.138667
10980	1.08862	0.147985
10981	1.03803	0.132142
10982	0.946132	0.134623
10983	0.946126	0.155265
10984	0.943647	0.140425
10985	0.948611	0.157748
10986	0.946129	0.140732
10987	0.946129	0.146092
10988	1.1699	0.736683
10989	0.946132	0.62876
10990	0.946133	0.914625
10991	0.946133	0.648906
10992	0.946132	0.65176
10993	0.946133	0.715872
10994	0.946132	0.826282
10995	0.946132	2.1
10996	0.946131	2.1
10997	0.946131	2.1
10998	0.946132	2.1
10999	0.946131	2.1
11000	0.946131	2.1
11001	0.946132	2.1
11002	0.946132	0.781257
11003	1.38623	0.700728
11004	0.946132	0.642315
11005	0.946132	0.650235
11006	0.946133	0.731254
11007	0.946132	0.617312
11008	0.946133	0.855284
11009	0.946132	2.1
11010	1.40098	2.1
11011	0.946131	2.1
11012	0.946131	2.1
11013	0.946132	2.1
11014	0.946132	2.1
11015	0.946132	2.1
11016	1.01777	0.648197
11017	0.946132	0.465313

11018	0.946133	0.934859
11019	1.68068	0.573792
11020	1.68068	1.20657
11021	1.41894	0.500212
11022	1.20787	0.430394
11023	0.946132	0.501963
11024	0.946133	2.1
11025	0.946133	2.1
11026	0.946132	2.1
11027	1.40199	2.1
11028	0.946133	2.1
11029	0.946132	2.1
11030	0.946132	2.1
11031	0.810946	1.08328
11032	0.810947	0.780762
11033	2.37827	1.31571
11034	1.3517	1.08385
11035	1.08132	1.10378
11036	1.08132	1.1358
11037	1.08133	1.03039
11038	0.810934	1.00018
11039	0.946124	1.74809
11040	0.94613	1.38385
11041	1.40248	1.07465
11042	0.946131	1.07332
11043	0.946125	0.743412
11044	0.946127	1.32867
11045	0.946128	0.811669
11046	1.25703	1.72467
11047	0.87821	1.08269
11048	1.18912	1.67409
11049	0.946119	0.977732
11050	0.87825	1.12702
11051	1.1891	1.25938
11052	0.946107	1.52314
11053	0.946132	1.95109
11054	1.28543	1.73169
11055	0.946132	1.65533
11056	0.946132	1.40701
11057	0.946131	0.949848
11058	0.946132	0.830328
11059	0.946132	0.907562
11060	0.946115	2.1
11061	0.946119	2.1
11062	0.946116	2.1
11063	0.946123	2.1
11064	0.946118	2.1
11065	1.41636	2.1
11066	0.946129	2.1
11067	0.946132	0.674268
11068	0.946132	1.57817
11069	0.946132	0.883901
11070	0.946131	2.1
11071	0.946131	1.13927
11072	1.41432	1.84815
11073	0.946132	0.850341
11074	0.810947	0.161596
11075	0.810947	0.164077

11076	2.44433	0.120976
11077	1.21654	0.120133
11078	0.946154	0.15746
11079	0.97995	0.147066
11080	1.08133	0.126249
11081	0.810934	0.147681
11082	0.851542	0.122114
11083	0.835698	0.130318
11084	1.22216	0.112986
11085	0.946133	0.117779
11086	1.01217	0.115663
11087	1.09996	0.123054
11088	0.851529	0.128875
11089	0.946132	1.47532
11090	0.946132	1.91823
11091	0.946129	2.1
11092	0.94613	1.53328
11093	0.946131	2.055
11094	0.946131	1.90268
11095	1.2254	2.08267
11096	0.800897	1.14578
11097	0.838029	1.92268
11098	0.838013	1.41529
11099	0.93679	0.988518
11100	1.20217	1.12443
11101	1.16923	1.24325
11102	0.94948	1.20221
11103	0.958118	1.36122
11104	0.36751	1.24876
11105	0.800535	1.61963
11106	0.272087	0.829679
11107	0.428084	1.045
11108	0.986335	1.58854
11109	0.946132	0.398821
11110	0.946133	0.570172
11111	1.19601	0.393178
11112	0.946132	0.471155
11113	0.946132	0.499779
11114	0.946133	0.542934
11115	0.946133	0.384305
11116	0.946132	1.39417
11117	1.36956	2.07705
11118	0.946132	2.1
11119	0.946132	1.27227
11120	0.946132	2.07291
11121	0.946133	1.45673
11122	0.946132	1.7714
11123	0.946132	0.121684
11124	0.946131	0.553179
11125	0.946131	0.22236
11126	1.34268	0.164339
11127	0.946133	0.38618
11128	0.946132	0.247554
11129	0.946131	0.324036
11130	0.946132	1.74973
11131	0.946131	1.81169
11132	0.946131	1.91596
11133	0.946132	1.93372

11134	0.946132	1.71289
11135	0.946132	1.95899
11136	0.946132	1.78265
11137	0.810946	0.964227
11138	0.810943	0.862541
11139	2.51472	0.960363
11140	1.3517	0.874149
11141	1.08132	0.837114
11142	1.08132	0.942391
11143	1.08133	0.906229
11144	0.810933	0.894388
11145	0.902316	0.687763
11146	1.04517	0.753257
11147	0.974924	0.456777
11148	0.530063	0.898691
11149	1.1272	0.809736
11150	0.68207	0.751568
11151	0.477061	0.879623
11152	0.810946	0.151153
11153	0.810943	0.230837
11154	0.810959	0.186906
11155	1.99211	0.149152
11156	1.35168	0.151082
11157	1.08132	0.155619
11158	1.08132	0.161587
11159	1.08131	0.199259
11160	0.94613	0.214585
11161	0.946131	0.208635
11162	0.946132	0.172815
11163	0.946132	0.213987
11164	0.946129	0.307418
11165	0.946131	0.23485
11166	1.16699	0.219127
11167	0.810943	1.09799
11168	0.810942	1.01602
11169	1.6943	1.51029
11170	1.27059	1.53407
11171	1.00021	1.48229
11172	1.02049	1.26989
11173	1.08133	1.09381
11174	0.750103	1.08793
11175	0.957813	1.14308
11176	0.946128	1.20929
11177	0.946132	0.935363
11178	1.04832	1.29066
11179	0.870714	0.946362
11180	0.94613	0.992095
11181	0.94613	1.07472
11182	1.86374	1.93458
11183	2.0815	1.10215
11184	1.15918	0.743025
11185	0.946129	0.708484
11186	1.15918	0.490815
11187	0.946131	0.86256
11188	0.946131	0.486933
11189	0.946131	0.709666
11190	0.94613	0.72426
11191	0.946119	0.778136

11192	1.3612	0.852163
11193	0.946125	0.811199
11194	0.946119	0.770469
11195	0.94612	1.20763
11196	0.946132	0.873011
11197	0.946107	0.830914
11198	0.810947	0.348837
11199	0.810947	0.3994
11200	2.50398	0.33571
11201	1.3517	0.326586
11202	1.08132	0.331049
11203	1.08133	0.371763
11204	1.08132	0.319203
11205	0.810934	0.350701
11206	0.946132	2.1
11207	0.946132	2.1
11208	0.946132	2.1
11209	1.39754	2.1
11210	0.946132	2.1
11211	0.946132	2.1
11212	0.946131	2.1
11213	0.946129	2.1
11214	1.41732	2.1
11215	0.946129	2.1
11216	0.946131	2.1
11217	0.946131	2.1
11218	0.946131	2.1
11219	0.946131	2.1
11220	0.946132	1.08048
11221	1.25276	1.62475
11222	0.946131	1.93877
11223	0.94613	1.0643
11224	0.94613	1.4506
11225	0.94613	1.12415
11226	0.946131	1.31893
11227	1.21261	0.912069
11228	0.94613	1.07899
11229	0.94613	1.57943
11230	0.946128	0.837687
11231	0.946131	1.27975
11232	0.94613	1.20764
11233	0.946129	1.33514
11234	0.946128	2.1
11235	0.946133	2.1
11236	0.946132	2.1
11237	0.946131	2.1
11238	0.94613	2.1
11239	0.946131	2.1
11240	1.39407	2.1
11241	0.946116	1.26589
11242	0.946119	1.18324
11243	0.946119	0.962309
11244	1.15369	1.22814
11245	0.946119	0.841025
11246	0.946131	1.06887
11247	0.946106	1.00102
11248	0.946131	1.8092
11249	0.946131	2.1

11250	0.946131	2.1
11251	0.946133	2.1
11252	0.946129	2.1
11253	0.946131	2.1
11254	0.946131	2.1
11255	1.19564	0.0710475
11256	0.498881	0.0821339
11257	1.33964	0.0724552
11258	0.498878	0.078437
11259	0.923583	0.0729745
11260	0.831259	0.0767678
11261	0.553735	0.0742623
11262	0.946132	0.0756857
11263	0.946132	0.0809049
11264	0.946131	0.0951666
11265	0.946131	0.0813762
11266	0.946132	0.0848578
11267	0.946131	0.0866855
11268	1.28673	0.081086
11269	0.946132	0.162097
11270	0.946132	0.17075
11271	1.33178	0.155555
11272	0.946132	0.17868
11273	0.946132	0.166648
11274	0.946131	0.180036
11275	0.946131	0.161228
11276	0.946128	0.542913
11277	0.946132	0.468492
11278	0.946129	0.579552
11279	0.946131	0.316583
11280	0.946131	0.36982
11281	0.946129	0.480992
11282	0.94613	0.426822
11283	0.946131	2.1
11284	0.946131	2.1
11285	0.946129	2.1
11286	1.40525	2.1
11287	0.94613	2.1
11288	0.94613	2.1
11289	1.40525	2.1
11290	0.946131	2.1
11291	0.946126	2.1
11292	0.946127	2.1
11293	0.946127	2.1
11294	1.4073	2.1
11295	0.946129	2.1
11296	0.946129	2.1
11297	0.946131	0.197183
11298	0.946131	0.167271
11299	0.946132	0.210432
11300	0.946131	0.146906
11301	0.946131	0.159054
11302	1.05016	0.159391
11303	0.946132	0.20621
11304	0.946128	0.197526
11305	0.946132	0.171448
11306	0.946131	0.208716
11307	0.946129	0.149714

11308	0.94613	0.160184
11309	1.13398	0.16377
11310	0.94613	0.213715
11311	0.946132	0.0865306
11312	0.946132	0.101838
11313	0.946131	0.131289
11314	0.946131	0.107631
11315	0.946132	0.10819
11316	0.946131	0.102574
11317	0.946132	0.109201
11318	0.946131	0.280179
11319	0.94613	0.208055
11320	0.946131	0.239517
11321	0.94613	0.304834
11322	1.38645	0.212113
11323	1.38645	0.221046
11324	0.946131	0.266586
11325	0.946131	0.358069
11326	0.946131	0.269356
11327	0.94613	0.394746
11328	1.23923	0.248654
11329	0.94613	0.25607
11330	0.946131	0.35397
11331	0.946131	0.451552
11332	0.946132	0.0962792
11333	0.946131	0.110445
11334	0.946131	0.0966093
11335	0.946132	0.084904
11336	0.946132	0.0911287
11337	0.946132	0.0972015
11338	1.40511	0.0917223
11339	0.946132	2.1
11340	0.946132	1.69372
11341	0.946132	2.1
11342	0.946132	1.67945
11343	0.946132	1.24827
11344	0.946131	2.02376
11345	1.12968	2.01853
11346	0.946132	1.26173
11347	0.946128	2.1
11348	0.946128	1.89253
11349	0.94613	1.52221
11350	0.94613	2.1
11351	0.94613	1.32837
11352	0.94613	1.75831
11353	0.946133	2.1
11354	0.946133	2.1
11355	0.946132	2.1
11356	0.946132	2.1
11357	0.946132	2.1
11358	0.946133	2.1
11359	1.31323	2.1
11360	0.94613	1.29341
11361	0.946131	0.589941
11362	0.946131	0.567335
11363	0.94613	0.467479
11364	0.946131	1.33709
11365	0.946131	0.802644

11366	0.946131	0.767959
11367	0.946132	0.458681
11368	0.946132	0.334194
11369	0.946132	0.541723
11370	1.36899	0.435535
11371	0.946132	0.344933
11372	0.946132	0.333098
11373	0.946133	0.394684
11374	1.03543	0.246708
11375	0.946131	0.243785
11376	0.946131	0.213041
11377	0.946132	0.174045
11378	0.94613	0.279119
11379	0.950987	0.164432
11380	0.941275	0.274842
11381	0.946131	0.187612
11382	0.94613	0.209497
11383	1.28952	0.2112
11384	0.946132	0.185235
11385	0.94613	0.25544
11386	0.946131	0.247792
11387	0.946131	0.195946
11388	0.94613	2.1
11389	1.35966	2.1
11390	0.946131	2.1
11391	0.946131	2.1
11392	0.946129	2.1
11393	0.94613	2.1
11394	0.94613	2.1
11395	0.810946	2.1
11396	0.810942	0.806816
11397	0.810932	1.85502
11398	2.6655	1.64868
11399	1.3517	1.26206
11400	1.08132	2.1
11401	1.08131	0.767102
11402	1.08133	1.48944
11403	0.94613	2.1
11404	0.946131	2.1
11405	0.94613	1.34371
11406	0.946128	2.1
11407	0.946131	1.59158
11408	0.94613	2.1
11409	0.94613	2.1
11410	0.946128	2.1
11411	0.946128	2.1
11412	0.946133	2.1
11413	1.25375	2.1
11414	0.94613	2.1
11415	0.946131	2.1
11416	1.25375	2.1
11417	0.94613	2.1
11418	0.94613	2.1
11419	0.946132	2.1
11420	0.946132	2.1
11421	0.946128	2.1
11422	0.94613	2.1
11423	1.31453	2.1

11424	0.946131	0.180754
11425	0.946129	0.140983
11426	0.94613	0.180646
11427	0.94613	0.129651
11428	0.94613	0.137485
11429	1.12018	0.13274
11430	0.94613	0.1767
11431	0.946131	0.239818
11432	0.946129	0.179148
11433	0.946131	0.239279
11434	1.16373	0.165481
11435	0.94613	0.165603
11436	0.94613	0.216609
11437	0.946131	0.252269
11438	1.36811	0.400176
11439	0.946132	0.429805
11440	1.36811	0.338872
11441	0.946129	0.424008
11442	0.94613	0.475184
11443	0.946129	0.426316
11444	0.946129	0.415084
11445	0.946132	0.351428
11446	0.946133	0.276826
11447	0.946132	0.328524
11448	0.946133	0.370008
11449	1.35732	0.286745
11450	1.35732	0.298762
11451	0.946133	0.342833
11452	1.36399	0.589705
11453	0.946129	0.795973
11454	0.94613	0.629025
11455	0.946131	0.844143
11456	0.94613	1.27443
11457	0.946131	0.749032
11458	0.946131	0.912479
11459	0.946133	2.1
11460	0.946132	2.08705
11461	0.946132	2.1
11462	1.38302	2.1
11463	0.946133	2.1
11464	0.946132	2.1
11465	0.946133	2.1
11466	0.810943	2.1
11467	0.810944	2.1
11468	0.810945	2.1
11469	2.80542	2.1
11470	1.35169	2.1
11471	1.08132	2.1
11472	1.08132	2.1
11473	1.08132	2.1
11474	1.30189	2.1
11475	0.946131	2.1
11476	0.946132	2.1
11477	0.946131	2.1
11478	0.946131	2.1
11479	0.946131	2.1
11480	0.946132	2.1
11481	0.810946	0.833623

11482	0.810947	0.677689
11483	2.33293	0.733425
11484	1.24357	0.714549
11485	1.08132	0.7388
11486	0.973183	0.795289
11487	1.00023	0.758998
11488	0.810934	0.787627
11489	1.06584	0.670267
11490	0.946129	0.730813
11491	0.84731	0.649309
11492	1.16646	0.713877
11493	0.946126	0.789403
11494	1.2831	0.671329
11495	0.851527	0.716757
11496	0.946131	1.96852
11497	0.946128	2.1
11498	0.946128	2.1
11499	0.94613	2.08634
11500	1.31961	2.1
11501	0.94613	2.1
11502	0.94613	2.1
11503	0.946131	2.1
11504	1.3605	2.1
11505	0.946133	2.1
11506	0.946132	2.1
11507	0.94613	2.1
11508	0.946131	2.1
11509	0.946131	2.1
11510	0.946128	1.71934
11511	0.946133	1.34307
11512	0.946132	2.1
11513	0.946131	1.12109
11514	0.946131	0.672613
11515	0.946131	1.27019
11516	1.30116	1.60892
11517	0.946132	0.12346
11518	0.946129	0.126544
11519	0.94613	0.120582
11520	1.3035	0.117934
11521	0.946131	0.105311
11522	0.94613	0.121657
11523	0.946131	0.155923
11524	0.946132	2.1
11525	1.2051	2.1
11526	1.2051	2.1
11527	0.946132	2.1
11528	1.37869	2.1
11529	0.946132	2.1
11530	0.946131	2.1
11531	0.810946	2.1
11532	0.810945	2.1
11533	2.69247	2.1
11534	1.35169	2.1
11535	1.08132	2.1
11536	1.08132	2.1
11537	1.08132	2.1
11538	0.810946	2.1
11539	1.22993	2.1

11540	0.946131	2.1
11541	0.946131	2.1
11542	1.34623	2.1
11543	0.946131	2.1
11544	1.34623	2.1
11545	0.946131	2.1
11546	0.946131	0.115906
11547	0.946132	0.146474
11548	0.946132	0.121481
11549	0.946132	0.105795
11550	0.946131	0.110108
11551	0.946132	0.134237
11552	1.32299	0.104861
11553	0.94613	0.267725
11554	0.946131	0.302122
11555	0.946128	0.270732
11556	0.946132	0.231591
11557	0.946133	0.256187
11558	0.946131	0.303373
11559	0.946131	0.25938
11560	0.946132	2.1
11561	0.946129	2.1
11562	0.946128	2.1
11563	0.94613	2.1
11564	0.94613	2.1
11565	0.946131	2.1
11566	0.94613	2.1
11567	0.94613	2.1
11568	0.94613	2.1
11569	0.946131	2.1
11570	0.946132	2.1
11571	0.946129	2.1
11572	0.946131	2.1
11573	0.946131	2.1
11574	0.946132	1.3276
11575	0.946131	1.35897
11576	1.13731	1.15621
11577	0.94775	1.46859
11578	0.944514	1.72215
11579	0.946132	1.88911
11580	1.039	1.86519
11581	0.946131	1.25956
11582	0.946128	1.16495
11583	0.94613	0.985066
11584	0.946131	1.23535
11585	0.946128	1.40225
11586	0.946129	1.64765
11587	1.23726	1.59434
11588	0.946132	2.1
11589	0.946132	1.5703
11590	0.946129	2.1
11591	1.39214	2.1
11592	0.946131	1.61659
11593	0.946131	2.1
11594	0.94613	2.1
11595	0.946125	0.436078
11596	0.946119	0.200363
11597	0.946118	0.19846

11598	0.946118	0.190751
11599	0.94612	0.438509
11600	0.946106	0.230549
11601	0.946131	0.255818
11602	1.26893	0.62233
11603	0.946131	0.59777
11604	1.26893	0.705324
11605	0.94613	0.649512
11606	0.946131	0.733245
11607	0.946131	0.582417
11608	0.946131	0.84673
11609	0.946133	0.0889254
11610	0.946133	0.0944102
11611	0.946132	0.113768
11612	1.28924	0.0886509
11613	0.946133	0.118762
11614	0.946132	0.103512
11615	0.946132	0.105441
11616	0.946133	0.527489
11617	0.946132	0.516701
11618	0.946132	0.517481
11619	0.946133	0.399404
11620	0.946133	0.484267
11621	0.946132	0.534628
11622	0.946133	0.468718
11623	0.946132	0.692241
11624	0.946133	0.476326
11625	0.946133	0.677107
11626	0.946132	0.70603
11627	1.15388	0.646244
11628	0.946132	0.701992
11629	0.946133	0.67641
11630	0.946132	0.524725
11631	0.946133	0.45596
11632	0.946132	0.519524
11633	0.946133	0.363584
11634	0.946133	0.465975
11635	0.946132	0.511283
11636	0.946132	0.422036
11637	1.0644	1.01526
11638	1.12486	1.58107
11639	1.08674	1.69029
11640	0.946132	1.14312
11641	0.946132	1.59556
11642	0.946133	1.31307
11643	0.946132	1.24713
11644	0.808978	1.02975
11645	0.911537	1.42219
11646	0.932832	1.24781
11647	1.14604	1.602
11648	1.56953	1.36272
11649	1.25866	1.16737
11650	1.18488	1.50277
11651	1.341	1.06626
11652	0.946131	0.361267
11653	0.946131	0.245539
11654	0.946131	0.330268
11655	0.94613	0.393307

11656	1.33922	0.247764
11657	0.946131	0.383539
11658	0.946131	0.342314
11659	0.946132	0.145436
11660	0.946133	0.131168
11661	0.946132	0.155658
11662	0.946132	0.131118
11663	0.946132	0.172075
11664	0.946133	0.143648
11665	0.946132	0.15061
11666	0.946129	0.727926
11667	0.946133	2.1
11668	0.946133	0.884915
11669	0.946131	1.12782
11670	1.40496	1.82707
11671	0.946131	0.778744
11672	0.946131	2.01953
11673	0.94613	0.852079
11674	0.946127	0.567517
11675	0.946127	0.7752
11676	0.94613	0.875844
11677	1.38913	0.691464
11678	0.946128	0.807944
11679	0.946128	0.730818
11680	1.98863	0.0857329
11681	2.59915	0.106308
11682	1.40338	0.0897646
11683	0.946131	0.0999556
11684	1.22266	0.0942699
11685	1.40338	0.0923525
11686	0.946132	0.0981122
11687	0.946132	0.0996205
11688	1.22266	0.0903357
11689	0.94613	0.103404
11690	0.94613	0.112989
11691	0.946124	0.162022
11692	1.21428	0.103767
11693	0.946126	0.174083
11694	0.946127	0.143766
11695	0.946127	0.133581
11696	1.03411	0.133195
11697	0.946131	0.218121
11698	1.03411	0.132092
11699	0.946129	0.236775
11700	1.14258	0.139136
11701	0.952143	0.195397
11702	0.940118	0.172879
11703	0.810943	2.1
11704	0.810945	2.1
11705	0.810958	2.1
11706	2.82603	2.1
11707	1.35168	2.1
11708	1.08132	2.1
11709	1.08132	2.1
11710	1.0813	2.1
11711	0.946133	0.257964
11712	0.946132	0.24403
11713	0.946132	0.286491

11714	0.946132	0.225393
11715	0.946132	0.222088
11716	1.11539	0.226714
11717	0.946133	0.275983
11718	0.946133	2.07401
11719	0.946132	2.1
11720	0.946133	2.1
11721	0.946133	2.1
11722	0.946132	2.1
11723	1.41195	2.1
11724	0.946133	2.1
11725	0.946131	0.0909746
11726	0.946131	0.103769
11727	0.946131	0.0909907
11728	0.946131	0.0834343
11729	0.946131	0.0892939
11730	0.946131	0.093931
11731	1.15968	0.0897659
11732	0.946131	1.19453
11733	0.94613	1.30654
11734	0.94613	2.08643
11735	1.34386	1.39982
11736	0.946132	1.8997
11737	0.94613	2.03372
11738	1.34386	1.73603
11739	0.946131	1.14462
11740	0.935202	1.08982
11741	0.946131	1.85626
11742	1.25054	1.24144
11743	0.850155	1.65703
11744	0.946131	1.64523
11745	0.935205	1.46505
11746	0.946131	0.341118
11747	1.40193	0.27052
11748	0.946132	0.288354
11749	0.946132	0.288857
11750	0.946129	0.362173
11751	1.40193	0.224794
11752	0.946131	0.31323
11753	0.808779	0.250162
11754	0.561937	0.181123
11755	1.40193	0.195747
11756	1.40193	0.166533
11757	0.561936	0.216782
11758	0.561937	0.153928
11759	1.40193	0.182151
11760	0.946132	0.0968244
11761	0.946132	0.101139
11762	0.946132	0.12631
11763	0.946132	0.10353
11764	0.946133	0.126522
11765	0.946132	0.110086
11766	0.946133	0.114648
11767	0.946127	0.462544
11768	0.946131	0.455797
11769	0.946131	0.361061
11770	0.946127	0.463988
11771	1.19583	0.369137

11772	1.19583	0.362167
11773	0.946129	0.427924
11774	0.589428	0.550811
11775	1.08737	0.422242
11776	1.10961	0.555373
11777	0.872901	0.57616
11778	0.541327	0.476195
11779	0.541312	0.440921
11780	0.85153	0.538615
11781	0.946132	1.17213
11782	0.946131	2.1
11783	0.946131	2.1
11784	0.946132	1.43937
11785	0.946132	2.1
11786	0.946132	1.77312
11787	0.946132	1.79764
11788	0.946132	1.06952
11789	1.2778	1.93606
11790	0.946131	2.1
11791	0.946131	1.10258
11792	0.94613	1.80394
11793	0.94613	1.20043
11794	0.94613	1.46329
11795	0.946118	0.74364
11796	0.946112	0.801096
11797	0.946118	0.691604
11798	0.946119	0.694487
11799	0.946117	0.746786
11800	1.35631	0.613329
11801	0.946105	0.613712
11802	0.946133	0.735576
11803	0.946133	1.58498
11804	0.946132	0.813239
11805	0.946132	1.5471
11806	0.946133	0.436313
11807	0.946132	0.711666
11808	0.946132	0.689948
11809	0.94613	1.37545
11810	0.946131	0.594032
11811	0.946128	0.621459
11812	0.946128	0.493891
11813	0.94613	1.29986
11814	0.946129	0.78094
11815	0.946129	0.740215
11816	0.946132	1.0875
11817	1.15139	1.1345
11818	0.946132	1.0164
11819	0.946132	1.2075
11820	0.946132	1.31169
11821	0.946132	1.31747
11822	0.946132	1.16429
11823	0.946133	2.1
11824	1.36151	2.1
11825	0.946132	2.1
11826	0.946132	2.1
11827	0.946132	1.61677
11828	0.946132	2.1
11829	0.946133	2.1

11830	0.946127	2.1
11831	1.38397	2.1
11832	0.94613	2.1
11833	0.946131	2.1
11834	0.946128	2.1
11835	0.94613	2.1
11836	0.946129	2.1
11837	0.946131	0.27172
11838	0.946127	0.289395
11839	0.946127	0.290511
11840	0.946132	0.3007
11841	0.946131	0.32794
11842	0.946128	0.270319
11843	0.946129	0.282815
11844	1.0268	0.0566381
11845	1.23823	0.0577578
11846	1.2171	0.0607981
11847	0.883446	0.0610129
11848	1.23827	0.0575406
11849	1.32614	0.0571445
11850	0.94613	0.0630021
11851	0.946132	2.1
11852	0.946129	2.1
11853	0.946129	2.1
11854	0.94613	2.1
11855	1.36523	2.1
11856	0.946131	2.1
11857	0.94613	2.1
11858	0.946132	0.212032
11859	0.946131	0.173298
11860	0.946132	0.224051
11861	0.946131	0.150263
11862	0.946131	0.158286
11863	1.01653	0.159526
11864	0.946131	0.239461
11865	0.94612	2.06882
11866	0.946121	1.68706
11867	0.946119	1.93033
11868	0.946119	1.86992
11869	0.946119	2.1
11870	0.946132	2.02135
11871	0.946107	2.02347
11872	0.810942	0.781232
11873	0.810944	0.618895
11874	2.60324	0.844917
11875	1.21651	0.79137
11876	1.0813	0.756297
11877	0.946153	0.617076
11878	0.979948	1.89141
11879	0.709588	0.907119
11880	1.3492	0.262829
11881	0.946132	0.284832
11882	0.946131	0.2358
11883	0.94613	0.284305
11884	0.946129	0.360539
11885	0.946129	0.286459
11886	0.94613	0.314591
11887	0.946131	0.23705

11888	0.94613	0.282422
11889	0.946128	0.248087
11890	0.946133	0.240904
11891	0.946133	0.285608
11892	0.946131	0.276502
11893	0.946131	0.250051
11894	1.23351	0.0856128
11895	0.946131	0.107869
11896	0.946131	0.0964849
11897	0.946132	0.0818278
11898	0.946132	0.0909775
11899	0.946131	0.0912885
11900	0.946132	0.0931225
11901	1.35657	0.09311
11902	0.946132	0.12418
11903	0.946131	0.106057
11904	0.946132	0.0893894
11905	0.946132	0.0981367
11906	0.946131	0.0993185
11907	0.946131	0.101466
11908	0.946132	0.602758
11909	0.946132	0.520286
11910	0.946132	0.608715
11911	0.946131	0.414104
11912	0.946131	0.544456
11913	0.946132	0.530818
11914	0.946132	0.509071
11915	0.946133	0.478674
11916	0.946132	0.382428
11917	0.946133	0.501761
11918	0.946132	0.303968
11919	0.946132	0.428872
11920	0.946132	0.421074
11921	0.946133	0.398037
11922	0.946131	0.9989
11923	1.4101	1.54032
11924	0.946131	2.1
11925	0.946131	0.73057
11926	0.946131	1.13232
11927	0.946131	0.715752
11928	0.946131	1.59441
11929	0.810944	1.16252
11930	0.810939	1.10828
11931	0.709586	0.875139
11932	2.71261	0.899187
11933	1.21651	0.993912
11934	0.946151	0.823273
11935	0.979943	1.03106
11936	1.0813	1.24099
11937	0.946132	2.1
11938	0.946131	2.1
11939	0.946132	2.1
11940	0.946131	2.1
11941	0.946131	2.1
11942	1.37733	2.1
11943	0.946131	2.1
11944	0.946133	2.06901
11945	0.946132	1.01767

11946	0.946132	2.07966
11947	0.946133	2.1
11948	0.946133	1.94224
11949	0.946132	1.53602
11950	1.32582	2.07292
11951	0.946132	2.1
11952	0.946132	2.1
11953	0.946132	1.90861
11954	0.946132	2.1
11955	0.946132	1.59313
11956	0.946132	2.1
11957	0.946131	2.1
11958	0.946133	1.50146
11959	0.946132	1.1699
11960	0.946132	1.34587
11961	0.946132	1.06599
11962	0.946132	1.05188
11963	0.946133	1.09336
11964	1.3469	1.11141
11965	0.946126	0.736055
11966	0.94613	2.1
11967	0.946131	1.26976
11968	0.946127	0.952797
11969	1.41206	2.1
11970	0.946128	1.51336
11971	0.946128	2.1
11972	0.946127	2.1
11973	0.94613	1.88441
11974	0.946131	0.877475
11975	0.94613	2.1
11976	0.946128	0.950543
11977	0.946129	1.65767
11978	0.946129	1.56088
11979	0.946131	0.213614
11980	0.94613	0.206165
11981	1.24448	0.191819
11982	0.946129	0.315829
11983	0.946131	0.257018
11984	0.94613	0.260329
11985	0.946131	0.228785
11986	0.946132	0.289734
11987	0.946133	0.2938
11988	1.11015	0.253488
11989	0.946133	0.489033
11990	0.946132	0.405062
11991	0.946132	0.403144
11992	0.946132	0.32407
11993	1.38775	2.1
11994	0.946131	2.1
11995	0.946131	2.1
11996	0.946132	2.1
11997	0.946132	2.1
11998	0.946131	2.1
11999	0.946131	2.1
12000	0.946129	1.21938
12001	0.946129	1.79185
12002	0.946133	0.794845
12003	0.946131	1.78026

12004	0.946131	0.555938
12005	0.946131	0.87685
12006	0.946131	0.818962
12007	0.946132	2.1
12008	0.946133	2.1
12009	0.946132	2.1
12010	1.40774	2.1
12011	0.946132	2.1
12012	0.946133	2.1
12013	0.946132	2.1
12014	0.610968	0.0731077
12015	0.688233	0.0741101
12016	0.715226	0.0753128
12017	0.792409	0.0746444
12018	1.13418	0.0708358
12019	0.956667	0.0740751
12020	0.757321	0.0743282
12021	0.968845	0.0703747
12022	0.595679	0.0718577
12023	0.946133	1.08807
12024	1.28917	1.21168
12025	0.946131	1.50644
12026	0.94613	0.892351
12027	0.946131	1.17311
12028	0.946131	1.03066
12029	0.946131	1.28642
12030	0.946132	0.40217
12031	0.946128	0.427778
12032	0.94613	0.541593
12033	1.40668	0.441448
12034	0.946131	0.501828
12035	0.946131	0.438573
12036	0.94613	0.446534
12037	0.810946	2.1
12038	0.810945	2.1
12039	2.78163	2.1
12040	1.3517	2.1
12041	1.08132	2.1
12042	1.08133	2.1
12043	1.08132	2.1
12044	0.810933	2.1
12045	0.946131	2.1
12046	1.39081	2.1
12047	0.94613	2.1
12048	1.30339	2.1
12049	1.30339	2.1
12050	1.39081	2.1
12051	0.946131	2.1
12052	0.946127	1.08427
12053	0.821631	1.38765
12054	0.946131	0.840934
12055	0.946131	0.958604
12056	0.946128	0.899269
12057	0.996109	0.788864
12058	0.827876	0.777474
12059	0.946131	2.1
12060	0.946129	2.1
12061	0.946129	2.1

12062	0.94613	2.1
12063	0.94613	2.1
12064	0.94613	2.1
12065	0.94613	2.1
12066	0.946132	0.484431
12067	1.41723	0.544148
12068	0.946132	0.501072
12069	0.946133	0.426785
12070	0.946132	0.747326
12071	0.946132	0.602151
12072	0.946133	0.587779
12073	0.946133	0.499343
12074	0.946132	0.427003
12075	0.946133	0.336583
12076	0.946133	0.442966
12077	0.946132	0.634685
12078	0.946132	0.536651
12079	1.34254	0.496662
12080	1.26501	0.936379
12081	0.946132	1.06949
12082	0.946133	1.06804
12083	0.946133	1.36411
12084	0.946132	1.26739
12085	0.946133	1.06672
12086	0.946132	1.46009
12087	0.946131	0.20676
12088	0.946131	0.207936
12089	0.946131	0.219212
12090	0.946131	0.139379
12091	0.946131	0.15738
12092	0.946131	0.195847
12093	0.946131	0.175237
12094	0.878843	0.124474
12095	0.82561	0.142049
12096	0.586974	0.124469
12097	1.05343	0.142469
12098	0.80631	0.107601
12099	0.782663	0.126447
12100	0.789405	0.113132
12101	0.902491	0.107686
12102	1.05344	0.126536
12103	0.94613	0.119465
12104	0.946131	0.142608
12105	0.946131	0.197141
12106	0.94613	0.163281
12107	0.946131	0.146372
12108	0.946131	0.147263
12109	0.946131	0.146163
12110	0.946128	1.93419
12111	0.946133	2.1
12112	0.946133	0.802695
12113	0.94613	2.00757
12114	0.946131	1.01645
12115	0.946131	1.15952
12116	0.946131	1.34436
12117	0.946131	0.424906
12118	0.946131	0.408198
12119	0.94613	0.382818

12120	0.946128	0.387795
12121	0.946133	0.421843
12122	1.2996	0.325499
12123	0.946131	0.369216
12124	0.946132	2.1
12125	0.946132	2.1
12126	0.946132	1.25348
12127	0.946132	2.1
12128	0.946131	1.75104
12129	0.946132	2.05519
12130	0.946132	2.1
12131	1.99211	0.121368
12132	2.55024	0.108299
12133	0.946131	0.127711
12134	0.946131	0.127608
12135	0.946131	0.119587
12136	0.946131	0.129607
12137	0.946131	0.158446
12138	0.946131	0.137597
12139	1.35726	0.131055
12140	0.946128	2.1
12141	0.946132	1.53465
12142	0.946131	1.73475
12143	0.94613	1.56113
12144	0.94613	1.43624
12145	0.94613	2.02001
12146	1.39852	1.98738
12147	1.31666	0.122753
12148	0.946131	0.179833
12149	0.946128	0.139864
12150	0.94613	0.115615
12151	0.946128	0.131008
12152	0.946128	0.132745
12153	0.946128	0.134049
12154	1.32275	0.159805
12155	0.946131	0.255569
12156	0.946129	0.179462
12157	0.946128	0.168872
12158	0.946129	0.18484
12159	0.946128	0.171735
12160	0.946129	0.175747
12161	0.946132	2.1
12162	0.946131	2.1
12163	0.946131	2.1
12164	0.946132	2.1
12165	0.94613	2.1
12166	0.946131	2.1
12167	1.41125	2.1
12168	0.946132	2.1
12169	0.946132	2.1
12170	0.946131	2.1
12171	0.946132	2.1
12172	0.946132	2.1
12173	0.946132	2.1
12174	1.31904	2.1
12175	1.02425	0.259096
12176	0.946132	0.249905
12177	0.94613	0.177376

12178	0.946131	0.318916
12179	1.1534	0.203508
12180	0.961161	0.174943
12181	0.9311	0.303177
12182	1.40092	1.04975
12183	0.946133	1.06887
12184	0.946132	1.11101
12185	0.946131	1.32625
12186	0.946131	0.986065
12187	0.94613	1.10968
12188	0.946131	1.36102
12189	0.946113	1.25702
12190	0.946119	0.518028
12191	0.946117	1.25959
12192	1.40015	1.00396
12193	0.946118	0.524129
12194	0.946131	0.687291
12195	0.946105	0.593359
12196	0.946132	0.431839
12197	0.946133	0.425623
12198	0.946132	0.593354
12199	1.03547	0.450158
12200	0.946133	0.418271
12201	0.946133	0.488709
12202	0.946132	0.423355
12203	0.946133	2.1
12204	1.36694	2.1
12205	0.946133	2.1
12206	0.946132	2.1
12207	0.946133	2.1
12208	0.946133	2.1
12209	0.946132	2.1
12210	0.946132	0.27441
12211	0.946133	0.275713
12212	0.946132	0.279758
12213	0.946133	0.264625
12214	0.946133	0.271736
12215	0.946132	0.301603
12216	0.946132	0.283613
12217	0.491552	0.177708
12218	0.913078	0.264827
12219	0.946132	0.375711
12220	0.491552	0.161826
12221	0.946132	0.322856
12222	0.49154	0.323459
12223	1.53168	0.271833
12224	1.53168	0.198512
12225	0.946127	2.1
12226	1.38752	2.1
12227	0.946129	2.1
12228	0.94613	2.1
12229	0.946128	2.1
12230	0.946129	2.1
12231	0.946129	2.1
12232	0.946132	2.1
12233	0.946132	2.1
12234	0.946132	2.1
12235	0.946132	2.1

12236	0.946132	2.1
12237	0.946131	2.1
12238	0.946132	2.1
12239	0.94613	0.381825
12240	0.94613	0.320558
12241	0.94613	0.322442
12242	0.946131	0.319741
12243	0.946129	0.388216
12244	1.16834	0.21622
12245	0.94613	0.321892
12246	0.946127	0.38657
12247	1.35594	0.293217
12248	0.946131	0.314683
12249	0.946128	0.410208
12250	0.946127	0.20498
12251	0.946129	0.35182
12252	0.94613	0.330563
12253	0.94613	0.601902
12254	0.946131	0.364496
12255	0.946128	0.312872
12256	0.946128	0.290861
12257	0.946131	0.598776
12258	0.94613	0.411747
12259	0.94613	0.475836
12260	0.946132	0.40978
12261	0.946132	0.38511
12262	0.946129	0.402299
12263	1.39751	0.37359
12264	0.94613	0.457152
12265	0.946131	0.350713
12266	0.946131	0.372355
12267	0.946132	0.322623
12268	0.700041	0.3044
12269	0.700013	0.360219
12270	1.7697	0.204556
12271	1.7697	0.205504
12272	1.21134	0.255503
12273	0.946131	0.262365
12274	1.25837	0.247613
12275	0.946133	2.08152
12276	0.946132	1.52505
12277	0.946132	1.29746
12278	0.946132	1.61081
12279	0.946132	1.95899
12280	0.946133	1.96278
12281	1.40583	1.83364
12282	0.946131	1.08342
12283	0.946131	1.58767
12284	0.946131	1.1052
12285	0.946131	2.02804
12286	0.946131	1.65265
12287	0.827865	1.47644
12288	0.946131	1.46836
12289	0.946132	0.0972733
12290	0.946129	0.0900279
12291	0.946129	0.0992648
12292	0.946131	0.0945892
12293	0.94613	0.103698

12294	1.21087	0.0877043
12295	0.946131	0.0979835
12296	0.94613	0.689236
12297	0.946131	0.930668
12298	0.94613	0.631956
12299	0.946128	0.673105
12300	0.946132	0.644628
12301	1.17452	0.616635
12302	0.94613	0.607315
12303	0.946119	0.571228
12304	0.946114	0.631211
12305	0.946119	0.538545
12306	0.946119	0.564629
12307	0.946119	0.544643
12308	1.27376	0.53528
12309	0.946106	0.531805
12310	0.946133	2.1
12311	1.39621	2.1
12312	0.946132	2.1
12313	0.946132	2.1
12314	0.946133	2.1
12315	0.946132	2.1
12316	0.946132	2.1
12317	0.946132	2.1
12318	0.946132	2.1
12319	0.946131	2.1
12320	0.946131	2.1
12321	0.946132	2.1
12322	0.946132	2.1
12323	1.34352	2.1
12324	0.946132	1.75849
12325	1.41272	2.00845
12326	0.946131	2.1
12327	0.94613	1.466
12328	0.94613	1.8156
12329	0.94613	1.47274
12330	0.94613	1.90844
12331	0.946132	2.1
12332	0.946131	1.46594
12333	0.946132	2.1
12334	0.946132	1.30644
12335	0.946132	0.949186
12336	0.946132	1.77961
12337	1.23536	1.84899
12338	0.946132	2.1
12339	0.946131	2.1
12340	0.946131	2.1
12341	0.946132	2.1
12342	0.946132	2.1
12343	1.41131	2.1
12344	0.946131	2.1
12345	1.41894	1.02378
12346	0.946132	0.52946
12347	0.946133	0.588786
12348	0.946132	0.636107
12349	0.946132	0.625159
12350	0.946133	0.651852
12351	0.946132	0.574331

12352	0.946128	0.291937
12353	0.946133	0.665867
12354	0.946131	0.622494
12355	0.946129	1.86681
12356	0.94613	0.465475
12357	1.4099	0.733289
12358	0.946131	0.560043
12359	0.810947	2.1
12360	0.810945	0.922214
12361	0.810933	1.78307
12362	2.62883	1.8596
12363	1.3517	1.58056
12364	1.08132	2.1
12365	1.08132	0.854468
12366	1.08133	1.38672
12367	0.946131	0.970146
12368	0.946126	1.84418
12369	0.946127	0.879972
12370	0.94613	1.10742
12371	0.946128	1.29103
12372	0.946129	0.70074
12373	0.946129	0.988341
12374	1.21347	2.08428
12375	0.946129	2.1
12376	0.94613	2.08518
12377	1.21347	2.1
12378	1.33064	2.1
12379	0.94613	2.1
12380	1.33064	2.1
12381	0.94613	0.0618153
12382	0.946129	0.0724774
12383	0.94613	0.0701309
12384	0.946132	0.0718365
12385	0.946126	0.0703861
12386	0.946129	0.0698527
12387	0.94613	0.0721716
12388	0.946132	0.873157
12389	1.37938	1.02809
12390	0.946131	1.19082
12391	0.94613	0.793143
12392	0.946131	1.34204
12393	0.94613	0.950241
12394	0.94613	1.02245
12395	0.946133	0.10028
12396	0.946133	0.0915129
12397	1.2968	0.0903908
12398	0.946132	0.0993563
12399	0.946133	0.101478
12400	1.2968	0.0875614
12401	0.946133	0.0996825
12402	1.43643	0.0964823
12403	0.946129	0.755816
12404	0.946133	0.673073
12405	0.946131	0.767695
12406	0.94613	0.57131
12407	0.946131	0.684994
12408	0.946131	0.684947
12409	0.946131	0.658747

12410	0.872447	1.0955
12411	0.946132	1.00723
12412	0.872447	0.929615
12413	0.827867	1.06598
12414	0.946132	1.22985
12415	0.827867	1.29041
12416	0.872447	1.27936
12417	0.946117	0.876083
12418	1.27213	0.854878
12419	0.946124	0.757459
12420	0.946119	0.877939
12421	0.946113	1.25444
12422	0.946129	1.01799
12423	0.946104	0.921808
12424	0.94613	0.482046
12425	0.946131	0.633361
12426	0.94613	0.534431
12427	0.946129	0.628032
12428	0.946132	0.205444
12429	0.946131	0.445801
12430	0.94613	0.39662
12431	0.810942	2.1
12432	0.810943	2.1
12433	2.80212	2.1
12434	1.3517	2.1
12435	1.08133	2.1
12436	1.08132	2.1
12437	1.08132	2.1
12438	0.810932	2.1
12439	1.29521	2.1
12440	0.946129	2.1
12441	0.946131	2.1
12442	1.40106	2.1
12443	0.94613	2.1
12444	1.40106	2.1
12445	0.946131	2.1
12446	0.946132	0.437108
12447	1.24596	0.330239
12448	0.94613	0.372423
12449	0.946131	0.410439
12450	0.946131	0.282374
12451	0.946131	0.402233
12452	0.946131	0.405461
12453	0.946131	2.1
12454	0.946131	2.1
12455	0.946131	2.1
12456	0.946128	2.1
12457	0.946133	2.1
12458	0.94613	2.1
12459	0.946131	2.1
12460	0.879548	0.74861
12461	1.21527	0.730164
12462	0.967506	0.77384
12463	0.946132	1.00382
12464	0.946133	0.632365
12465	0.967511	1.19517
12466	0.946132	1.00632
12467	0.946132	1.03056

12468	0.946132	0.77028
12469	0.974253	0.856055
12470	0.974253	1.27541
12471	0.91801	0.749083
12472	0.918009	0.81471
12473	0.946132	1.03655
12474	0.946132	0.167185
12475	0.946131	0.151261
12476	0.946131	0.171983
12477	0.946133	0.125189
12478	0.946132	0.135775
12479	1.28644	0.139692
12480	0.946132	0.16214
12481	0.946131	0.173604
12482	0.946131	0.153523
12483	0.946131	0.178363
12484	0.946131	0.127727
12485	0.946131	0.134892
12486	1.40103	0.138106
12487	0.946131	0.164732
12488	0.946128	0.270174
12489	0.946132	0.214477
12490	0.946129	0.313158
12491	1.03327	0.191247
12492	0.946129	0.198637
12493	0.946129	0.259153
12494	0.94613	0.29964
12495	0.946134	0.33978
12496	0.946133	0.386716
12497	0.946132	0.23556
12498	0.946133	0.358477
12499	0.946132	0.250564
12500	0.946132	0.24713
12501	0.946133	0.172224
12502	0.856654	0.0908531
12503	0.946131	0.114001
12504	0.946131	0.0915579
12505	0.946132	0.0950916
12506	0.856616	0.103092
12507	1.03565	0.0858911
12508	1.03565	0.100998
12509	1.15844	0.497857
12510	0.946132	0.521818
12511	0.946132	0.423432
12512	0.946132	0.483039
12513	0.946132	0.435509
12514	0.946132	0.483845
12515	0.946131	0.481839
12516	1.4582	2.1
12517	1.35067	2.1
12518	0.946131	2.1
12519	1.04106	2.1
12520	0.938748	2.1
12521	0.943015	2.1
12522	1.05017	2.1
12523	0.938793	2.1
12524	0.946131	0.0944504
12525	0.946132	0.107988

12526	0.946131	0.150835
12527	0.94613	0.118244
12528	0.946133	0.118824
12529	0.946131	0.113771
12530	0.946131	0.119254
12531	0.946132	0.420895
12532	0.946133	0.557825
12533	0.946132	0.415785
12534	0.946133	0.649823
12535	0.946133	0.568524
12536	0.946132	0.521325
12537	0.946133	0.587937
12538	0.946128	0.808157
12539	0.946132	0.997871
12540	0.946132	0.81906
12541	0.94613	0.851783
12542	1.23426	0.9204
12543	0.94613	0.899372
12544	0.946131	0.917931
12545	0.810943	0.46697
12546	0.810946	0.5478
12547	2.60388	0.55508
12548	1.3517	0.507361
12549	1.08133	0.467416
12550	1.08132	0.550162
12551	1.08132	0.467045
12552	0.810933	0.389577
12553	0.810945	0.650291
12554	0.810945	0.388585
12555	2.72377	0.943948
12556	1.3517	0.768242
12557	1.08133	0.679576
12558	1.08132	0.487589
12559	1.08132	1.64198
12560	0.810932	0.782233
12561	1.25104	0.347014
12562	0.946131	0.656783
12563	0.946131	0.414265
12564	1.36188	1.79766
12565	0.946131	0.714082
12566	1.36188	0.846942
12567	0.946132	0.852275
12568	0.946132	1.92678
12569	0.946132	1.93347
12570	0.946132	1.9272
12571	0.946132	1.95959
12572	0.946132	1.85279
12573	0.946132	2.1
12574	1.25488	2.1
12575	1.32132	0.101853
12576	0.946126	0.128942
12577	0.946127	0.0915083
12578	0.946128	0.122577
12579	0.946128	0.132863
12580	0.946128	0.1168
12581	0.946128	0.123756
12582	0.946133	2.1
12583	1.41123	1.22714

12584	0.946134	1.46853
12585	0.946133	2.09331
12586	0.946132	0.838382
12587	0.946133	1.46934
12588	0.946132	1.3027
12589	1.32163	0.186759
12590	0.946133	0.210576
12591	0.946133	0.172415
12592	0.946132	0.201986
12593	0.946132	0.158805
12594	0.946132	0.177218
12595	0.946133	0.208724
12596	0.946133	1.86654
12597	0.946133	2.1
12598	0.946132	1.61689
12599	0.946132	2.1
12600	0.946132	0.721765
12601	0.946133	1.67016
12602	0.946132	1.65887
12603	0.94613	0.325347
12604	0.946131	0.31451
12605	0.946128	0.317511
12606	0.946133	0.279535
12607	0.946133	0.276811
12608	0.946131	0.325272
12609	0.946131	0.290946
12610	0.946133	0.601687
12611	0.946132	0.43137
12612	0.946132	0.610477
12613	0.946134	0.493505
12614	0.946133	0.729829
12615	0.946133	0.448312
12616	0.946132	0.555163
12617	0.94613	1.67892
12618	0.946128	1.98702
12619	0.946127	1.3269
12620	0.94613	1.21242
12621	0.946131	1.59167
12622	0.946128	1.52863
12623	1.21099	1.8448
12624	0.946132	0.769416
12625	0.946131	0.723833
12626	0.946131	0.816765
12627	0.946133	0.493678
12628	0.946133	0.603686
12629	0.946131	0.729544
12630	0.946132	0.647706
12631	1.34794	0.780729
12632	0.517994	0.784769
12633	0.517965	0.67247
12634	0.946132	0.874659
12635	0.9198	1.45543
12636	0.946132	1.03522
12637	0.9198	1.01206
12638	0.946132	0.959586
12639	0.94613	0.984905
12640	0.946131	0.62134
12641	0.946131	0.972166

12642	0.946131	1.28968
12643	0.946131	0.994381
12644	1.23224	0.826742
12645	0.946132	1.58032
12646	0.946132	1.71873
12647	0.946133	1.37313
12648	0.946132	0.925392
12649	0.946132	1.17289
12650	0.946133	1.34679
12651	1.37511	1.44718
12652	0.946133	0.718267
12653	0.946129	0.751369
12654	0.851104	1.01455
12655	0.946131	0.872874
12656	1.0412	0.958675
12657	1.0412	0.971786
12658	0.851066	0.887406
12659	1.31357	0.201939
12660	0.946131	0.327438
12661	0.94613	0.221719
12662	0.946131	0.278305
12663	0.94613	0.26177
12664	0.946131	0.234841
12665	0.946131	0.227294
12666	0.946133	0.802335
12667	1.38149	0.933884
12668	0.946132	0.988661
12669	0.946132	0.767542
12670	0.946133	1.34585
12671	0.946132	0.910618
12672	0.946133	0.913579
12673	0.810944	1.12955
12674	0.810945	2.1
12675	0.810958	1.83339
12676	2.65782	1.98834
12677	1.35168	1.69284
12678	1.08132	1.12476
12679	1.08132	1.83337
12680	1.0813	1.07326
12681	0.946113	0.0796311
12682	0.946119	0.0894385
12683	0.946118	0.11023
12684	0.946118	0.0926938
12685	0.946119	0.0949553
12686	0.946131	0.0902067
12687	0.946106	0.0943419
12688	0.946132	0.0836828
12689	0.946132	0.114817
12690	0.946128	0.103497
12691	0.946131	0.108648
12692	0.94613	0.109445
12693	0.94613	0.102584
12694	0.94613	0.117828
12695	1.00099	0.114191
12696	0.946132	0.159272
12697	1.17876	0.109841
12698	0.982282	0.131747
12699	0.909981	0.174855

12700	0.946132	0.147405
12701	0.946131	0.146246
12702	0.946132	0.944557
12703	1.35328	1.11555
12704	0.946132	1.4054
12705	0.946132	0.787254
12706	0.946133	1.29302
12707	0.946132	0.940075
12708	0.946133	1.20001
12709	0.946133	0.0792613
12710	0.946133	0.103858
12711	0.946132	0.0882196
12712	0.946132	0.0987318
12713	0.946132	0.0945615
12714	0.946132	0.0921669
12715	0.946133	0.103107
12716	0.900195	0.0723845
12717	0.946132	0.0723538
12718	0.900167	0.0770085
12719	1.41963	0.08178
12720	0.900195	0.0719919
12721	0.946133	0.0746479
12722	1.35385	0.0843332
12723	0.946132	1.00255
12724	0.946129	1.48939
12725	0.946129	1.25068
12726	0.946131	0.959322
12727	0.946131	1.30298
12728	1.41585	1.01893
12729	0.94613	1.80364
12730	0.810946	2.1
12731	0.810946	1.50724
12732	2.68895	2.1
12733	1.3517	2.1
12734	1.08133	2.1
12735	1.08132	1.73307
12736	1.08132	2.1
12737	0.810933	2.1
12738	1.19886	1.67502
12739	0.946131	2.08507
12740	0.946131	1.83284
12741	1.34447	2.1
12742	0.946131	2.1
12743	1.34447	2.1
12744	0.946131	2.1
12745	0.946133	0.0973686
12746	0.946133	0.107721
12747	0.946132	0.138624
12748	1.35065	0.0989034
12749	0.946132	0.15222
12750	0.946133	0.12645
12751	0.946132	0.12201
12752	0.946131	0.696562
12753	0.946131	0.681053
12754	0.94613	0.740436
12755	0.946131	0.416561
12756	0.946131	0.507625
12757	0.946131	0.649709

12758	0.946131	0.576252
12759	0.946127	0.30675
12760	0.946131	0.316005
12761	0.946127	0.303846
12762	0.94613	0.230831
12763	0.946131	0.264048
12764	0.946128	0.31383
12765	0.946129	0.262875
12766	0.810946	0.0957334
12767	0.810947	0.103504
12768	0.810959	0.0970142
12769	2.4391	0.0894145
12770	1.35168	0.0905138
12771	1.08132	0.0900257
12772	1.08132	0.0951781
12773	1.08131	0.0994527
12774	0.946132	0.0812333
12775	0.946132	0.0834479
12776	0.946132	0.093132
12777	1.40837	0.0851508
12778	0.946132	0.0938933
12779	0.946132	0.0910519
12780	1.40837	0.0864367
12781	0.946127	1.62376
12782	0.946132	2.1
12783	0.946131	0.724996
12784	0.946127	1.9072
12785	0.946131	1.3023
12786	0.946129	0.977875
12787	0.94613	1.42428
12788	0.946133	1.63117
12789	0.946132	1.31412
12790	0.946132	1.28986
12791	0.946132	1.06377
12792	0.946132	1.44467
12793	0.946132	0.870503
12794	0.946133	1.99494
12795	0.946132	0.818575
12796	1.24344	0.722538
12797	0.946132	0.637884
12798	0.946132	1.02225
12799	0.946132	0.639968
12800	1.24344	0.85687
12801	0.946132	0.993941
12802	0.946131	0.412761
12803	0.946128	0.377508
12804	0.946128	0.370567
12805	0.946131	0.403581
12806	1.41232	0.357219
12807	0.946129	0.411543
12808	0.94613	0.36335
12809	0.946128	2.01996
12810	0.946133	1.78078
12811	0.946133	0.83822
12812	0.946131	2.06113
12813	0.946131	0.962968
12814	0.946131	1.43675
12815	0.94613	1.30906

12816	0.946133	0.526265
12817	1.41556	0.51017
12818	0.946133	0.459478
12819	0.946133	0.448832
12820	0.946132	0.69839
12821	0.946132	0.555311
12822	0.946132	0.611886
12823	0.94612	0.480656
12824	1.41843	0.471547
12825	0.946114	0.472385
12826	0.94612	0.423771
12827	0.946119	0.629529
12828	1.41845	0.462439
12829	0.946107	0.571681
12830	0.946129	0.0982663
12831	0.946129	0.0887424
12832	0.94613	0.100095
12833	0.946132	0.0965426
12834	0.946126	0.104099
12835	1.1509	0.0864514
12836	0.94613	0.0966423
12837	0.946131	0.0840402
12838	0.946132	0.0787635
12839	0.946132	0.0845279
12840	0.946132	0.0827432
12841	0.946132	0.0874067
12842	1.19263	0.0756153
12843	0.946132	0.08541
12844	0.94613	0.493831
12845	0.946131	0.58431
12846	0.946128	0.495574
12847	0.946133	0.684408
12848	0.946133	0.633807
12849	0.946131	0.509395
12850	0.946131	0.616304
12851	0.946133	1.02136
12852	1.22848	1.33288
12853	1.00336	1.33258
12854	0.827866	1.07312
12855	0.946132	1.50493
12856	0.827865	1.26792
12857	0.946133	1.16614
12858	0.946131	0.558529
12859	0.94613	0.378412
12860	0.946127	0.56226
12861	1.41498	0.516851
12862	0.94613	0.403315
12863	0.946128	0.475666
12864	0.946128	0.433681
12865	0.94613	0.0764819
12866	0.94613	0.0844552
12867	0.94613	0.0961374
12868	1.37358	0.0844274
12869	0.946129	0.102517
12870	0.94613	0.0969033
12871	1.37357	0.0885849
12872	0.946111	0.0790679
12873	0.946118	0.0852534

12874	0.946118	0.101167
12875	1.36568	0.0798419
12876	0.946116	0.108246
12877	0.94613	0.090589
12878	0.946105	0.0904757
12879	0.946132	0.383379
12880	0.946131	0.296571
12881	0.94613	0.487695
12882	1.23175	0.26785
12883	0.946131	0.265715
12884	0.946131	0.392562
12885	0.946131	0.430099
12886	0.946132	0.102624
12887	0.946126	0.10675
12888	0.946128	0.0983658
12889	0.946129	0.106768
12890	0.946129	0.122221
12891	0.946128	0.108286
12892	1.25698	0.103553
12893	0.810943	2.1
12894	0.810944	2.1
12895	2.81171	2.1
12896	1.3517	2.1
12897	1.08132	2.1
12898	1.08133	2.1
12899	1.08132	2.1
12900	0.810932	2.1
12901	0.946132	0.455037
12902	0.946131	0.438592
12903	0.946131	0.48569
12904	0.946132	0.248456
12905	0.946132	0.316323
12906	0.946132	0.426474
12907	0.946131	0.370916
12908	0.810936	0.757888
12909	0.810935	1.7103
12910	2.80914	1.21359
12911	1.35167	1.0013
12912	1.0813	0.927592
12913	1.0813	1.64584
12914	1.0813	0.719904
12915	0.810934	0.938047
12916	1.23851	2.01202
12917	0.946132	0.827584
12918	0.946131	2.03432
12919	1.40459	0.766453
12920	0.946132	1.21844
12921	1.40459	1.09216
12922	0.946132	1.15902
12923	1.19974	0.182624
12924	0.946131	0.326684
12925	1.19974	0.178367
12926	0.946132	0.308688
12927	0.946132	0.215212
12928	0.946132	0.300548
12929	0.946132	0.226311
12930	0.939141	0.919964
12931	1.14375	1.2503

12932	1.03306	1.2625
12933	0.946132	1.01908
12934	0.946126	1.35054
12935	0.946129	1.1778
12936	0.94613	1.06013
12937	0.946132	0.291437
12938	0.946131	0.293617
12939	0.946132	0.302513
12940	0.946132	0.276903
12941	0.946132	0.275759
12942	0.946132	0.32827
12943	0.946132	0.290768
12944	0.946131	2.1
12945	0.94613	2.1
12946	0.94613	2.1
12947	0.946131	2.1
12948	0.946129	2.1
12949	0.94613	2.1
12950	1.24512	2.1
12951	0.946133	0.255937
12952	0.946129	0.241473
12953	0.946131	0.29935
12954	0.946132	0.239281
12955	0.946131	0.280562
12956	0.946131	0.273972
12957	0.946131	0.260627
12958	0.946132	0.257001
12959	0.946133	0.253766
12960	0.946132	0.268066
12961	0.946132	0.246318
12962	0.946133	0.275846
12963	0.946132	0.267262
12964	0.946132	0.266563
12965	0.946132	0.520681
12966	1.41392	0.407889
12967	0.946132	0.411505
12968	0.946132	0.606316
12969	0.946132	0.331825
12970	0.946132	0.525455
12971	0.946132	0.489425
12972	0.946132	0.709985
12973	1.24333	0.608901
12974	0.946132	0.539666
12975	0.946133	0.815626
12976	0.946132	0.396282
12977	0.946132	0.783287
12978	0.946133	0.615088
12979	1.30649	0.219996
12980	0.94613	0.233708
12981	0.94613	0.210521
12982	0.946128	0.219336
12983	0.946126	0.253407
12984	0.946127	0.236734
12985	0.946127	0.246816
12986	0.709578	0.302285
12987	0.810943	0.302752
12988	2.53303	0.196238
12989	1.21651	0.202338

12990	0.979951	0.220883
12991	1.08132	0.220029
12992	0.946141	0.308114
12993	0.81096	0.271645
12994	0.946131	0.275998
12995	0.946131	0.310377
12996	0.946131	0.289916
12997	0.946131	0.186483
12998	0.946131	0.213998
12999	0.946131	0.29437
13000	0.946131	0.250701
13001	0.946132	0.272696
13002	0.946129	0.239292
13003	0.946129	0.265821
13004	0.94613	0.279046
13005	1.20634	0.248574
13006	1.20634	0.242587
13007	0.946131	0.289636
13008	0.946133	0.62263
13009	0.946132	0.610754
13010	0.946132	0.563739
13011	0.946132	0.656772
13012	0.946132	0.53944
13013	1.30107	0.628613
13014	0.946133	0.649457
13015	0.810946	2.1
13016	0.810944	2.1
13017	2.73936	2.1
13018	1.35168	2.1
13019	1.0813	2.1
13020	1.08132	2.1
13021	1.08132	2.1
13022	0.810958	2.1
13023	0.946129	1.64072
13024	0.946129	1.96727
13025	0.946133	0.872281
13026	1.30812	1.64791
13027	0.946131	1.01347
13028	0.946131	1.28454
13029	0.946131	1.15715
13030	1.00901	1.07587
13031	0.94613	1.33604
13032	0.94613	0.760711
13033	1.17001	1.18228
13034	0.917259	0.733253
13035	0.94613	0.946157
13036	0.94613	0.908625
13037	0.946126	2.1
13038	0.946127	2.1
13039	0.946131	2.1
13040	0.946127	2.1
13041	0.946131	2.1
13042	0.946129	2.1
13043	1.398	2.1
13044	0.946133	2.1
13045	1.3033	2.1
13046	0.946132	2.1
13047	0.946132	2.1

13048	0.946132	2.1
13049	0.946132	2.1
13050	0.946133	2.1
13051	0.946131	0.573667
13052	0.946131	0.683838
13053	1.09897	0.459938
13054	0.946129	0.862618
13055	0.946131	0.750574
13056	0.94613	0.787987
13057	0.94613	0.594391
13058	0.946132	2.1
13059	0.946131	2.1
13060	0.946131	2.1
13061	0.946132	2.1
13062	0.94613	2.1
13063	0.946131	2.1
13064	1.39858	2.1
13065	1.07117	0.701418
13066	1.08878	0.877118
13067	0.946119	0.995989
13068	1.08878	1.06897
13069	0.946119	0.598171
13070	0.946132	0.909008
13071	1.07116	0.717583
13072	0.946132	0.184896
13073	0.946132	0.168608
13074	0.946132	0.19475
13075	0.946132	0.13609
13076	0.946132	0.148619
13077	1.10733	0.158039
13078	0.946132	0.177776
13079	0.946128	0.194504
13080	0.946132	0.165681
13081	0.94613	0.206138
13082	0.946129	0.138614
13083	0.94613	0.153178
13084	1.3367	0.155567
13085	0.94613	0.189538
13086	0.810943	2.1
13087	0.810944	2.1
13088	2.70234	2.1
13089	1.21653	2.1
13090	1.08132	2.1
13091	0.946155	2.1
13092	0.979951	2.1
13093	0.709578	2.1
13094	1.27174	2.1
13095	0.946129	2.1
13096	0.94613	2.1
13097	1.35117	2.1
13098	0.94613	2.1
13099	1.35117	2.1
13100	0.94613	2.1
13101	0.946133	1.0026
13102	0.946133	0.962514
13103	0.946132	1.53305
13104	0.946133	0.902853
13105	0.946132	2.01577

13106	0.946133	1.43935
13107	0.997296	1.39472
13108	0.810943	0.783917
13109	0.810944	1.39119
13110	0.810945	2.1
13111	2.78833	2.1
13112	1.35169	2.1
13113	1.08132	1.14079
13114	1.08132	2.1
13115	1.08132	2.01055
13116	0.810946	2.06203
13117	0.810943	0.936803
13118	0.810933	1.45522
13119	2.70049	1.87266
13120	1.3517	1.19483
13121	1.08132	1.79174
13122	1.08132	0.687711
13123	1.08133	0.990704
13124	0.946127	1.08011
13125	1.35469	1.05352
13126	0.94613	1.03402
13127	0.946131	1.10026
13128	0.946128	0.943983
13129	1.35469	1.16177
13130	0.946129	1.19637
13131	0.946131	0.319346
13132	1.2722	0.281244
13133	0.946132	0.288135
13134	0.946132	0.281957
13135	0.946129	0.345447
13136	1.2722	0.229943
13137	0.94613	0.288694
13138	0.946133	0.350813
13139	0.946132	0.343043
13140	0.946132	0.36717
13141	0.946132	0.359467
13142	1.41461	0.348645
13143	1.41461	0.344397
13144	0.946133	0.374747
13145	0.946131	0.860351
13146	1.30165	0.690353
13147	0.946132	0.610285
13148	0.946132	0.982225
13149	0.946129	0.684565
13150	1.30165	0.908477
13151	0.946131	0.996141
13152	0.946132	0.79763
13153	0.946129	1.12028
13154	0.94613	0.786457
13155	1.39356	1.40874
13156	0.946131	0.905587
13157	0.946131	0.884248
13158	0.94613	1.20903
13159	0.810946	1.5545
13160	0.810947	1.85251
13161	0.810934	1.98106
13162	2.35003	2.1
13163	1.3517	2.1

13164	1.08132	2.1
13165	1.08132	2.02801
13166	1.08133	2.1
13167	0.946131	0.104627
13168	0.946131	0.115698
13169	0.946125	0.152165
13170	0.946127	0.125028
13171	0.946129	0.135274
13172	0.946128	0.122098
13173	0.946128	0.126901
13174	0.946131	0.103403
13175	0.946131	0.123454
13176	0.946131	0.157128
13177	0.946131	0.134873
13178	0.946132	0.122821
13179	0.946132	0.123442
13180	0.946131	0.124052
13181	0.946129	2.01942
13182	0.946132	2.1
13183	0.946132	2.1
13184	0.94613	2.1
13185	1.30082	2.1
13186	0.94613	2.1
13187	0.946131	2.1
13188	1.20416	0.146932
13189	0.946132	0.25001
13190	0.946131	0.185734
13191	0.946132	0.174157
13192	0.946131	0.164204
13193	0.946131	0.177381
13194	0.946132	0.160073
13195	1.18711	0.140144
13196	0.946131	0.257127
13197	0.946131	0.1801
13198	0.946132	0.166261
13199	0.946131	0.158346
13200	0.946131	0.176471
13201	0.946132	0.17615
13202	0.946133	0.0850368
13203	0.946133	0.0913813
13204	0.946132	0.0953497
13205	1.30212	0.0892752
13206	0.946133	0.104273
13207	0.946132	0.0933965
13208	1.30212	0.0900894
13209	0.946131	0.215946
13210	0.946131	0.181016
13211	0.946131	0.224891
13212	0.946132	0.206966
13213	1.31467	0.197912
13214	1.31467	0.189743
13215	0.946131	0.253165
13216	0.946133	0.156982
13217	0.946132	0.13763
13218	0.946132	0.165229
13219	0.946132	0.143537
13220	1.25124	0.147921
13221	1.25124	0.140704

13222	0.946132	0.184103
13223	0.946132	2.1
13224	0.946132	2.1
13225	0.946132	2.1
13226	1.36422	2.1
13227	0.946132	2.1
13228	0.946131	2.1
13229	0.946132	2.1
13230	0.946131	1.21065
13231	1.41851	1.55063
13232	0.946132	1.90264
13233	0.946132	1.04157
13234	0.946132	1.38594
13235	0.946132	1.06204
13236	0.946132	1.39867
13237	0.946133	0.177424
13238	0.946132	0.189364
13239	0.946132	0.175389
13240	0.946132	0.165558
13241	0.946132	0.185693
13242	1.37794	0.150726
13243	0.946132	0.173234
13244	0.946132	2.1
13245	0.946132	2.1
13246	0.946131	2.1
13247	0.946131	2.1
13248	0.946132	2.1
13249	0.946132	2.1
13250	1.4045	2.1
13251	0.946133	2.1
13252	0.946132	2.1
13253	0.946131	2.1
13254	0.946132	2.1
13255	0.946131	2.1
13256	0.946131	2.1
13257	0.946132	2.1
13258	0.946132	2.1
13259	0.946132	2.1
13260	0.946131	2.1
13261	0.946132	2.1
13262	0.946131	2.1
13263	0.946131	2.1
13264	0.946131	2.1
13265	0.946128	2.1
13266	1.31641	2.1
13267	0.946129	2.1
13268	0.94613	2.1
13269	0.94613	2.1
13270	0.94613	2.1
13271	0.94613	2.1
13272	0.946132	2.1
13273	0.946131	2.1
13274	0.946131	1.00002
13275	0.946132	2.1
13276	0.946132	1.30054
13277	0.946132	1.72363
13278	0.946132	2.04049
13279	0.946132	2.1

13280	1.41507	2.1
13281	0.946132	2.1
13282	0.946132	2.1
13283	0.946133	2.1
13284	0.946133	2.1
13285	0.946132	2.1
13286	0.946132	2.1
13287	0.946131	2.1
13288	0.946131	2.1
13289	0.946133	2.1
13290	0.946132	2.1
13291	1.32608	2.1
13292	0.946132	2.1
13293	0.94613	1.59519
13294	0.94613	2.1
13295	0.94613	0.785994
13296	0.946131	1.80559
13297	0.946129	1.2927
13298	0.94613	0.87924
13299	0.94613	1.2538
13300	1.33728	0.732704
13301	0.946131	0.634758
13302	0.94613	0.480008
13303	0.946132	0.685913
13304	0.946132	0.906513
13305	0.946131	0.581301
13306	0.946131	0.611779
13307	0.946127	2.1
13308	1.40641	1.57513
13309	0.946129	1.84867
13310	0.94613	2.03405
13311	0.946128	1.08017
13312	0.946129	1.62164
13313	0.946129	1.46433
13314	0.946129	2.1
13315	0.946128	2.1
13316	0.946133	2.1
13317	1.37467	2.1
13318	0.946131	2.1
13319	0.946131	2.1
13320	1.37467	2.1
13321	0.946131	2.1
13322	0.946131	2.1
13323	0.946132	2.1
13324	0.946132	2.1
13325	0.946129	2.1
13326	0.946131	2.1
13327	1.34912	2.1
13328	0.946132	1.95956
13329	0.946133	2.1
13330	0.946133	2.1
13331	0.946132	2.1
13332	0.946132	2.1
13333	0.946133	2.1
13334	0.946132	2.1
13335	0.946127	2.1
13336	0.946131	1.34345
13337	0.946132	2.1

13338	0.946131	1.30433
13339	0.946127	1.03186
13340	0.94613	1.96885
13341	1.33607	2.1
13342	0.946117	0.46468
13343	0.946117	0.712058
13344	0.946115	1.14099
13345	0.946124	1.87015
13346	0.946119	0.508253
13347	1.32662	0.791974
13348	0.94613	0.588466
13349	0.946119	2.1
13350	0.946114	2.1
13351	0.946118	2.1
13352	0.946114	2.1
13353	0.946118	2.0197
13354	0.946104	2.1
13355	0.946129	2.1
13356	0.946132	1.36218
13357	0.946133	2.1
13358	0.946133	2.1
13359	0.946133	1.7345
13360	0.946132	2.1
13361	0.946132	2.1
13362	0.946133	2.1
13363	0.946133	1.90165
13364	0.946132	2.1
13365	0.946132	2.1
13366	0.946133	2.1
13367	0.946132	2.1
13368	0.946132	2.1
13369	0.946132	2.1
13370	0.946133	1.48161
13371	1.30881	1.88003
13372	0.946132	2.1
13373	0.946131	1.20667
13374	0.946131	1.29335
13375	0.946131	1.02533
13376	0.946131	1.65535
13377	0.946128	2.1
13378	0.946128	2.1
13379	0.946132	2.1
13380	0.94613	2.1
13381	0.94613	2.1
13382	0.94613	2.1
13383	1.34652	2.1
13384	0.946133	0.903094
13385	0.946133	0.830385
13386	0.946132	1.54485
13387	0.946133	0.831369
13388	0.946132	1.88068
13389	0.946132	1.05946
13390	0.946132	1.2702
13391	0.946132	0.271538
13392	0.946128	0.270045
13393	0.94613	0.283922
13394	0.946131	0.276335
13395	0.94613	0.342126

13396	0.94613	0.227863
13397	0.94613	0.271326
13398	0.946128	0.45717
13399	0.946132	0.511032
13400	0.94613	0.441936
13401	0.94613	0.413879
13402	0.946131	0.44167
13403	0.94613	0.480832
13404	0.94613	0.447398
13405	0.946129	0.913579
13406	1.32056	1.28008
13407	0.946131	1.61565
13408	0.94613	0.864061
13409	0.946124	1.33294
13410	0.946128	0.944626
13411	0.946127	1.13892
13412	0.946129	0.909375
13413	1.20351	1.26366
13414	0.946132	1.4209
13415	0.946132	0.941967
13416	0.946126	1.40028
13417	0.946129	1.09548
13418	0.946129	1.07696
13419	0.946127	1.16753
13420	0.946127	1.56251
13421	0.946131	0.865733
13422	1.25592	1.23823
13423	0.946127	0.908853
13424	0.946129	1.02369
13425	0.94613	1.09683
13426	1.02332	1.68223
13427	0.946133	1.07013
13428	0.946132	1.18489
13429	1.02332	1.23584
13430	0.946132	0.850588
13431	0.96196	1.29308
13432	0.930306	0.950695
13433	0.946133	1.75878
13434	0.946133	1.87488
13435	0.946132	2.1
13436	1.20057	1.97377
13437	0.946132	2.1
13438	0.946133	2.1
13439	1.20057	2.1
13440	0.946128	1.40484
13441	0.946129	0.808713
13442	0.946129	1.15213
13443	1.13245	0.809876
13444	0.946126	0.676494
13445	0.946128	1.00584
13446	0.946129	0.813114
13447	0.946131	1.34533
13448	0.946131	1.72465
13449	0.94613	2.09313
13450	1.25	1.63282
13451	0.946129	2.08075
13452	0.946131	2.1
13453	1.25	2.1

13454	0.540425	1.75295
13455	0.748493	1.811
13456	1.46266	2.06656
13457	1.20046	2.04617
13458	0.600454	1.76962
13459	1.41283	2.04385
13460	0.582567	1.81047
13461	0.946131	1.48555
13462	0.946131	1.28516
13463	0.946132	1.30127
13464	0.946132	1.33719
13465	0.94613	1.22326
13466	0.946131	1.95094
13467	1.28439	1.86438
13468	0.946132	0.656192
13469	0.946131	0.62653
13470	0.946131	0.723561
13471	0.946132	0.598454
13472	0.946132	0.766906
13473	1.39335	0.532511
13474	0.946132	0.629771
13475	0.946131	0.421509
13476	0.946131	0.668471
13477	0.94613	0.345122
13478	0.946132	0.358095
13479	0.946131	0.532251
13480	0.946131	0.47302
13481	0.946131	0.38766
13482	0.946131	0.420366
13483	0.94613	0.433923
13484	0.946126	0.476764
13485	0.946131	0.375866
13486	0.946125	0.555694
13487	0.946127	0.378112
13488	0.946128	0.493726
13489	0.946131	0.125036
13490	0.946131	0.16345
13491	0.946131	0.129599
13492	0.946131	0.111703
13493	0.946131	0.119698
13494	0.946131	0.144126
13495	1.39168	0.114665
13496	0.946131	0.36022
13497	0.946126	0.325761
13498	0.946127	0.394837
13499	0.946131	0.333962
13500	0.94613	0.374864
13501	1.412	0.343393
13502	0.946128	0.363218
13503	0.948901	0.352674
13504	0.943364	0.412355
13505	1.03768	0.343077
13506	0.946132	0.598894
13507	0.946133	0.564072
13508	1.03767	0.54531
13509	0.946132	0.416027
13510	1.00902	0.668804
13511	0.88324	0.604388

13512	0.971584	0.463086
13513	0.946132	0.67253
13514	0.946129	0.917898
13515	0.946131	0.838558
13516	0.971572	0.857649
13517	0.946131	0.111818
13518	0.946125	0.109466
13519	1.33587	0.104424
13520	0.946126	0.133242
13521	0.946127	0.115015
13522	0.946126	0.116578
13523	0.946128	0.115298
13524	0.946132	0.130729
13525	0.946132	0.130207
13526	1.27512	0.11284
13527	0.946133	0.174741
13528	0.946132	0.142719
13529	0.946133	0.138216
13530	0.946133	0.147671
13531	0.946128	0.275468
13532	0.946133	0.274422
13533	0.946131	0.29226
13534	0.94613	0.155831
13535	0.946131	0.199723
13536	0.94613	0.258139
13537	0.946131	0.223685
13538	0.946131	0.242161
13539	0.946131	0.232439
13540	0.946131	0.258542
13541	0.946131	0.13984
13542	0.946131	0.180718
13543	0.946131	0.228683
13544	0.946131	0.201431
13545	0.94613	0.0758359
13546	0.946131	0.0791402
13547	0.946131	0.0908552
13548	0.94613	0.0795787
13549	0.946132	0.0848422
13550	0.946131	0.0851561
13551	1.35316	0.0805492
13552	0.94613	0.400589
13553	0.94613	0.354157
13554	0.946128	0.416889
13555	0.946132	0.361284
13556	0.946132	0.410607
13557	1.28727	0.364043
13558	0.94613	0.381722
13559	0.946131	0.210257
13560	0.946132	0.210745
13561	0.946132	0.207712
13562	1.39502	0.200207
13563	0.946132	0.194163
13564	0.946132	0.207433
13565	0.946132	0.25581
13566	0.946125	0.487613
13567	0.946131	0.455686
13568	0.94613	0.323102
13569	0.946129	0.491869

13570	0.946126	0.710551
13571	0.946127	0.618826
13572	1.36173	0.572167
13573	0.946129	1.11463
13574	1.02363	1.11153
13575	0.94613	0.931641
13576	0.946131	1.13782
13577	0.946131	0.743479
13578	1.02363	0.883821
13579	0.946131	0.934478
13580	0.946128	0.0659774
13581	1.24678	0.0651669
13582	0.94613	0.0677002
13583	0.94613	0.0685317
13584	0.94613	0.06303
13585	0.946131	0.0654252
13586	0.94613	0.0720492
13587	0.946129	0.0652518
13588	1.25215	0.0651825
13589	0.946132	0.0668882
13590	0.946126	0.0676499
13591	0.946129	0.0631293
13592	0.94613	0.0644005
13593	0.946129	0.0705198
13594	0.946129	0.626025
13595	0.946133	0.688731
13596	0.946133	0.577892
13597	0.946131	0.583663
13598	0.946131	0.624317
13599	1.29689	0.520111
13600	0.946131	0.536372
13601	0.946128	0.218412
13602	0.946132	0.155666
13603	0.946132	0.19005
13604	0.94613	0.235378
13605	1.22716	0.162564
13606	1.22716	0.177857
13607	0.946131	0.222294
13608	0.946127	0.247016
13609	0.946131	0.186673
13610	0.946131	0.223542
13611	0.946128	0.266318
13612	1.30463	0.19323
13613	1.30463	0.203628
13614	0.946129	0.256947
13615	1.29958	0.107495
13616	0.946132	0.141806
13617	0.946132	0.111236
13618	0.946132	0.0951218
13619	0.946132	0.10524
13620	0.946132	0.107442
13621	0.946133	0.107856
13622	1.26201	0.100466
13623	0.946133	0.144412
13624	0.946132	0.116809
13625	0.946132	0.0983815
13626	0.946132	0.107664
13627	0.946133	0.111986

13628	0.946132	0.110721
13629	0.946133	0.202575
13630	0.946132	0.166832
13631	0.946132	0.212232
13632	0.946133	0.142868
13633	0.946132	0.154385
13634	1.40427	0.15392
13635	0.946133	0.199358
13636	0.946132	0.206169
13637	0.946132	0.160499
13638	0.946132	0.21261
13639	0.946132	0.141456
13640	0.946132	0.149762
13641	1.35123	0.145679
13642	0.946131	0.192579
13643	0.946128	0.277351
13644	0.946132	0.212961
13645	0.946132	0.242971
13646	0.946129	0.306211
13647	1.22144	0.215109
13648	1.22144	0.22081
13649	0.946131	0.282197
13650	0.946127	0.487218
13651	0.946132	0.484469
13652	0.946131	0.445647
13653	0.946127	0.452761
13654	0.946131	0.480225
13655	1.23351	0.378423
13656	0.94613	0.418051
13657	1.18119	0.153354
13658	0.946131	0.193534
13659	0.946132	0.142151
13660	0.946132	0.170887
13661	0.94613	0.217481
13662	0.946131	0.181541
13663	0.946131	0.187849
13664	0.946132	0.329574
13665	0.946132	0.287954
13666	0.946133	0.378714
13667	1.30563	0.252463
13668	0.946132	0.275286
13669	0.946132	0.326261
13670	0.946133	0.412814
13671	1.20256	0.473999
13672	0.946132	1.0727
13673	0.946133	0.73058
13674	0.946132	0.371159
13675	0.946132	0.689605
13676	0.946132	0.819843
13677	0.946133	0.576491
13678	0.94613	0.716143
13679	0.94613	0.616611
13680	0.946128	0.860142
13681	1.33007	0.563882
13682	0.946129	0.630228
13683	0.94613	0.761305
13684	0.946131	0.841735
13685	0.946127	2.1

13686	0.946127	2.1
13687	0.946131	2.1
13688	1.30136	2.1
13689	0.946128	2.1
13690	0.946129	2.1
13691	1.30136	2.1
13692	0.810942	2.1
13693	0.810942	2.1
13694	0.81093	2.1
13695	2.3208	2.1
13696	1.3517	2.1
13697	1.08132	2.1
13698	1.08132	2.1
13699	1.08133	2.1
13700	0.946132	2.1
13701	0.946132	2.1
13702	0.946132	2.1
13703	0.946132	2.1
13704	0.946132	2.1
13705	1.39823	2.1
13706	0.946132	2.1
13707	1.37713	2.1
13708	0.946131	2.1
13709	0.946131	2.1
13710	0.946131	2.1
13711	0.946131	2.1
13712	0.946131	2.1
13713	0.946131	2.1
13714	0.946128	2.08208
13715	0.946128	2.1
13716	0.946133	1.69624
13717	0.946131	2.1
13718	0.946131	0.921697
13719	0.946131	2.01946
13720	0.94613	1.99019
13721	0.946129	2.1
13722	0.946129	2.1
13723	0.946133	2.1
13724	0.946131	2.1
13725	0.946131	2.1
13726	0.946131	2.1
13727	0.946131	2.1
13728	0.946132	1.06839
13729	1.33694	1.86535
13730	0.946133	2.1
13731	0.946133	0.939869
13732	0.946132	1.59057
13733	0.946132	0.965136
13734	0.946133	1.49143
13735	0.946131	1.56271
13736	0.94613	2.1
13737	0.946131	2.1
13738	0.946133	1.90887
13739	0.946129	2.1
13740	0.946131	2.1
13741	0.946131	2.1
13742	0.946132	1.54897
13743	1.35943	1.82323

13744	0.946132	2.1
13745	0.946132	1.11799
13746	0.946131	1.26303
13747	0.946132	0.9872
13748	0.946132	1.7663
13749	0.946132	0.134214
13750	0.946132	0.161788
13751	0.946133	0.120448
13752	0.946133	0.138959
13753	0.946132	0.134306
13754	0.946132	0.127188
13755	0.946132	0.157646
13756	1.29322	0.373753
13757	0.946131	0.396744
13758	0.946132	0.34123
13759	0.946129	0.419617
13760	0.946131	0.361646
13761	0.946131	0.343692
13762	0.946131	0.409871
13763	0.946132	0.312198
13764	0.946132	0.71951
13765	0.946131	0.376476
13766	1.41492	0.340429
13767	0.946132	0.633488
13768	0.946132	0.497825
13769	0.946131	0.553575
13770	0.946132	2.1
13771	0.946132	2.1
13772	0.946132	2.1
13773	1.35028	2.1
13774	0.946132	2.1
13775	0.946132	2.1
13776	0.946132	2.1
13777	0.946132	1.22278
13778	0.946132	2.01342
13779	0.946132	1.55195
13780	1.32461	1.4882
13781	0.946131	2.02154
13782	0.946132	1.5472
13783	0.946132	1.99328
13784	0.946131	2.1
13785	0.946131	2.1
13786	0.946131	2.1
13787	0.946132	2.1
13788	0.946129	2.1
13789	0.94613	2.1
13790	1.40369	2.1
13791	0.946132	0.244759
13792	0.946131	0.190606
13793	0.946131	0.271465
13794	0.979598	0.171438
13795	0.946131	0.17678
13796	0.946131	0.227768
13797	0.946132	0.252104
13798	0.946131	2.1
13799	1.41897	2.0839
13800	0.946132	2.1
13801	0.946132	2.1

13802	0.94613	1.28407
13803	0.946131	2.1
13804	0.946131	2.08659
13805	0.946131	2.1
13806	0.94613	2.1
13807	0.946132	2.1
13808	0.946132	2.1
13809	0.946129	2.1
13810	0.946131	2.1
13811	1.37791	2.1
13812	0.946127	2.1
13813	0.946131	2.1
13814	0.946131	2.1
13815	0.94613	2.1
13816	0.946128	2.1
13817	0.946129	2.1
13818	1.39384	2.1
13819	0.810945	1.94779
13820	0.810944	1.33676
13821	0.810958	1.56447
13822	2.70852	1.86088
13823	1.35168	1.47051
13824	1.08132	1.70446
13825	1.08132	1.02399
13826	1.0813	1.34577
13827	1.3335	2.1
13828	0.946132	2.1
13829	0.94613	1.92998
13830	0.946132	2.1
13831	0.946131	2.1
13832	0.94613	2.1
13833	0.946131	2.1
13834	0.946132	0.995929
13835	0.946131	0.445468
13836	0.94613	0.663944
13837	1.41192	0.657705
13838	0.946132	0.452981
13839	0.946131	0.593606
13840	0.94613	0.499211
13841	0.946133	0.565723
13842	0.946133	0.533855
13843	0.946132	0.525986
13844	1.39428	0.46013
13845	0.946132	0.433141
13846	0.946132	0.419683
13847	0.946133	0.476491
13848	0.94613	0.760465
13849	0.946129	2.1
13850	0.94613	0.924747
13851	0.946128	0.985767
13852	1.40684	1.87102
13853	0.94613	0.89874
13854	0.94613	2.05271
13855	0.946131	0.749009
13856	0.946131	2.1
13857	0.946131	1.24048
13858	0.946131	0.980417
13859	1.39021	2.1

13860	0.946131	1.23722
13861	0.946131	2.1
13862	0.946131	1.57241
13863	0.946131	1.98814
13864	0.946131	1.14587
13865	0.946131	1.92972
13866	0.946131	2.00185
13867	0.946131	0.987461
13868	0.946131	1.27964
13869	0.810946	1.75459
13870	0.810946	1.38139
13871	2.69264	2.1
13872	1.35168	2.1
13873	1.0813	2.1
13874	1.08132	1.737
13875	1.08132	2.1
13876	0.810958	2.1
13877	1.16687	1.34737
13878	0.946131	1.818
13879	0.946131	1.38859
13880	1.34632	2.1
13881	0.946131	2.1
13882	1.34632	2.1
13883	0.946131	2.1
13884	0.946128	0.990641
13885	0.946133	1.19145
13886	0.946133	0.755788
13887	0.946131	0.823999
13888	0.946131	0.850045
13889	1.3564	0.707531
13890	0.94613	0.716101
13891	0.946128	2.1
13892	0.946132	2.1
13893	0.94613	2.1
13894	0.94613	2.1
13895	0.946131	2.1
13896	1.35827	2.1
13897	0.94613	2.1
13898	0.946133	0.434185
13899	0.946132	0.688819
13900	0.946132	0.455885
13901	0.946133	0.675648
13902	0.946133	0.568174
13903	0.946132	0.546925
13904	0.946133	0.647421
13905	0.946132	2.00301
13906	0.946132	1.98386
13907	0.946132	0.818411
13908	0.946131	1.93762
13909	0.946132	1.23973
13910	0.946132	1.20792
13911	0.946132	1.2053
13912	0.94613	0.663866
13913	0.94613	0.515269
13914	0.946128	0.667723
13915	0.946132	0.512836
13916	0.946132	0.711704
13917	0.94613	0.585149

13918	0.946131	0.571502
13919	0.946126	0.375307
13920	0.946131	0.294146
13921	0.946127	0.403063
13922	0.946129	0.285226
13923	0.94613	0.384659
13924	0.946128	0.327841
13925	0.946128	0.325042
13926	0.946131	0.281064
13927	0.946127	0.251637
13928	0.946127	0.299376
13929	0.946131	0.249514
13930	0.946131	0.284872
13931	0.946128	0.278682
13932	0.946129	0.283039
13933	0.94613	0.294642
13934	0.946131	0.2755
13935	0.946129	0.308924
13936	0.946132	0.265955
13937	0.946132	0.301676
13938	0.946131	0.293046
13939	0.94613	0.292884
13940	0.94613	0.331696
13941	0.946131	0.313038
13942	0.946129	0.365978
13943	0.946132	0.309487
13944	0.946132	0.330709
13945	1.32827	0.319671
13946	0.946131	0.332516
13947	0.946132	2.1
13948	0.946132	1.89817
13949	0.946131	1.43189
13950	0.946131	1.50573
13951	0.946132	2.1
13952	0.946131	2.1
13953	0.946131	2.1
13954	0.946132	0.211209
13955	0.946132	0.690532
13956	0.946131	0.287427
13957	1.38356	0.271152
13958	0.946133	0.637971
13959	0.946131	0.377778
13960	0.946131	0.524289
13961	0.94613	2.1
13962	0.946131	2.1
13963	0.946131	2.1
13964	0.946128	2.1
13965	0.946133	2.1
13966	0.946131	2.1
13967	1.36918	2.1
13968	0.946131	0.580886
13969	0.94613	1.40931
13970	0.946129	0.92189
13971	0.946133	2.1
13972	0.946132	0.795553
13973	1.3771	1.51588
13974	0.946131	0.772009
13975	1.30029	1.02388

13976	0.94613	0.699434
13977	0.946129	1.30622
13978	0.946132	1.32879
13979	0.946132	0.520229
13980	0.946131	0.916866
13981	0.94613	0.807216
13982	0.946133	0.0996193
13983	0.946132	0.118416
13984	0.946132	0.1009
13985	0.946132	0.0897935
13986	0.946132	0.095344
13987	0.946132	0.106143
13988	1.21174	0.0947312
13989	0.810946	2.1
13990	0.810945	2.1
13991	2.63045	2.1
13992	1.35169	2.1
13993	1.08132	2.1
13994	1.08132	2.1
13995	1.08132	2.1
13996	0.810946	2.1
13997	1.1796	2.1
13998	0.946131	2.1
13999	0.946132	2.1
14000	1.31522	2.1
14001	0.946132	2.1
14002	1.31522	2.1
14003	0.946132	2.1
14004	0.946131	2.1
14005	1.40615	2.1
14006	0.946129	2.1
14007	0.946128	2.1
14008	0.946133	2.1
14009	0.946131	2.1
14010	0.94613	2.1
14011	0.810945	2.1
14012	0.810944	2.1
14013	2.80479	2.1
14014	1.35169	2.1
14015	1.08132	2.1
14016	1.08132	2.1
14017	1.08132	2.1
14018	0.810945	2.1
14019	1.35562	2.1
14020	0.94613	2.1
14021	1.35561	2.1
14022	0.946133	2.1
14023	0.946131	2.1
14024	1.40239	2.1
14025	0.946131	2.1
14026	0.946125	0.406401
14027	0.946131	0.612411
14028	0.946127	0.290953
14029	0.946129	0.410616
14030	0.946128	0.5665
14031	0.946129	0.571751
14032	0.946128	0.374222
14033	0.946119	2.1

14034	0.946113	2.1
14035	0.94612	2.1
14036	0.946119	2.1
14037	1.41915	2.1
14038	0.946107	2.1
14039	0.946132	2.1
14040	0.810946	1.18786
14041	0.810943	1.91056
14042	0.810934	1.57049
14043	2.31411	1.95717
14044	1.35171	1.77633
14045	1.08132	1.31006
14046	1.08132	1.94469
14047	1.08133	1.22527
14048	0.946127	1.77834
14049	0.94613	2.1
14050	0.94613	2.1
14051	0.946131	2.1
14052	0.946126	2.1
14053	0.946128	2.1
14054	0.946128	2.1
14055	0.810944	2.1
14056	0.810947	1.75324
14057	0.81096	2.1
14058	2.78019	2.1
14059	1.35168	2.1
14060	1.08132	2.1
14061	1.08132	1.78473
14062	1.08131	2.1
14063	0.946133	0.239247
14064	0.946132	0.267023
14065	0.946132	0.243291
14066	0.946133	0.221189
14067	0.946132	0.256779
14068	1.3495	0.192946
14069	0.946133	0.261853
14070	0.946133	2.1
14071	0.946132	2.1
14072	0.946132	2.1
14073	1.39529	2.1
14074	0.946133	2.1
14075	0.946132	2.1
14076	0.946133	2.1
14077	0.946133	2.1
14078	0.946132	2.1
14079	0.946132	1.83134
14080	0.946132	2.1
14081	0.946132	1.74146
14082	0.946133	2.1
14083	0.946132	2.1
14084	0.810946	2.1
14085	0.810945	2.1
14086	2.67861	2.1
14087	1.35169	2.1
14088	1.08132	2.1
14089	1.08132	2.1
14090	1.08132	2.1
14091	0.810946	2.1

14092	1.24616	2.1
14093	0.946132	2.1
14094	0.946132	2.1
14095	1.33931	2.1
14096	0.946132	2.1
14097	1.33931	2.1
14098	0.946133	2.1
14099	0.946133	0.427957
14100	0.946133	0.373881
14101	0.946132	0.380435
14102	0.946133	0.430309
14103	1.3855	0.351318
14104	1.3855	0.350369
14105	0.946133	0.433683
14106	0.946128	0.844699
14107	0.946132	2.06659
14108	0.946128	1.29357
14109	0.94613	2.1
14110	0.946131	2.04374
14111	1.40225	2.1
14112	0.94613	1.78606
14113	1.40548	0.099535
14114	0.946118	0.129287
14115	0.946114	0.114486
14116	0.946112	0.0916144
14117	0.946117	0.100755
14118	0.946128	0.106344
14119	0.946103	0.11172
14120	0.94613	2.1
14121	0.946132	2.1
14122	0.946131	2.1
14123	0.946132	2.1
14124	1.37811	2.1
14125	0.946131	2.1
14126	0.946131	2.1
14127	0.946132	2.1
14128	0.946133	2.1
14129	0.946133	2.1
14130	0.946132	2.1
14131	0.946133	2.1
14132	0.946132	2.1
14133	0.946132	2.1
14134	0.946118	1.23674
14135	0.946113	2.1
14136	0.946118	2.1
14137	0.946119	1.98644
14138	0.946117	2.1
14139	0.946105	2.1
14140	0.946131	2.1
14141	0.946118	0.137151
14142	0.946118	0.136891
14143	0.946117	0.138653
14144	0.946113	0.106323
14145	0.94612	0.109019
14146	1.41377	0.118399
14147	0.946106	0.129445
14148	0.946132	0.718019
14149	1.28436	0.561413

14150	0.946131	0.565366
14151	0.946131	0.747249
14152	0.946131	0.38572
14153	0.94613	0.77095
14154	0.946131	0.624237
14155	0.810947	0.374187
14156	0.810946	0.431065
14157	2.68675	0.288529
14158	1.35168	0.308663
14159	1.08132	0.374184
14160	1.08132	0.341798
14161	1.0813	0.392047
14162	0.81096	0.34194
14163	0.946132	0.287666
14164	1.34338	0.265687
14165	0.946132	0.27138
14166	1.34338	0.318029
14167	0.946132	0.328628
14168	1.34337	0.289621
14169	0.946132	0.288189
14170	0.946127	1.94609
14171	0.946131	1.75883
14172	0.946127	1.71665
14173	0.946131	1.54115
14174	0.946131	1.82125
14175	0.946128	1.62013
14176	1.30281	1.95223
14177	0.946132	0.597495
14178	0.946132	0.558652
14179	0.946132	0.544892
14180	0.946131	0.522699
14181	0.946132	0.603865
14182	1.37973	0.363585
14183	0.946132	0.461319
14184	0.946131	0.741805
14185	1.41798	0.652638
14186	0.946132	0.592038
14187	0.946132	0.668711
14188	0.946129	0.798245
14189	0.946131	0.66668
14190	0.946131	0.789993
14191	0.946118	0.671995
14192	1.41776	0.608386
14193	0.946113	0.619025
14194	0.946119	0.582649
14195	0.946117	0.724063
14196	1.41778	0.563618
14197	0.946105	0.727725
14198	0.946131	0.797133
14199	1.41798	0.848331
14200	0.946132	0.893076
14201	0.946132	0.756085
14202	0.94613	0.827329
14203	1.41799	0.714654
14204	0.946131	0.727652
14205	0.946133	2.1
14206	0.946132	2.1
14207	0.946132	0.925582

14208	0.946132	2.1
14209	0.946133	1.32201
14210	0.946132	1.73787
14211	0.946132	2.09852
14212	0.946131	1.91542
14213	0.946131	2.1
14214	0.94613	0.695755
14215	0.946129	1.96461
14216	0.946132	1.03665
14217	0.94613	0.970171
14218	0.946131	1.7794
14219	0.94612	1.18329
14220	0.946126	2.1
14221	0.946119	1.01048
14222	0.94612	1.33855
14223	1.40766	1.77248
14224	0.946132	0.913909
14225	0.946107	1.99586
14226	0.946128	2.1
14227	1.31213	2.1
14228	0.946129	2.1
14229	0.94613	2.1
14230	0.94613	2.1
14231	0.94613	2.1
14232	0.94613	2.1
14233	0.946127	1.24795
14234	0.946131	1.93972
14235	0.94613	2.05483
14236	0.94613	1.36425
14237	1.36653	1.86417
14238	0.946128	1.42477
14239	0.946128	1.81923
14240	0.946127	2.1
14241	0.94613	2.1
14242	0.946131	2.1
14243	0.94613	2.1
14244	0.946128	2.1
14245	0.946128	2.1
14246	0.946129	2.1
14247	0.946133	2.1
14248	1.40081	2.1
14249	0.946132	2.1
14250	0.946132	2.1
14251	0.946133	2.1
14252	0.946132	2.1
14253	0.946133	2.1
14254	0.946128	2.1
14255	1.32282	2.1
14256	0.946129	2.1
14257	0.94613	2.1
14258	0.946131	2.1
14259	0.946131	2.1
14260	0.946131	2.1
14261	0.810945	1.42184
14262	0.810943	2.1
14263	0.810933	2.02776
14264	2.44097	2.1
14265	1.3517	2.07156

14266	1.08132	1.42954
14267	1.08132	2.1
14268	1.08133	1.59997
14269	0.946131	2.1
14270	0.94613	2.07523
14271	0.946133	2.1
14272	0.946133	2.06993
14273	0.946128	1.9004
14274	0.946131	2.1
14275	1.27676	2.1
14276	0.946126	0.659416
14277	0.946126	0.708698
14278	0.946131	0.601747
14279	0.946127	0.701419
14280	0.94613	0.427444
14281	0.946128	0.581036
14282	0.946128	0.499641
14283	0.946132	0.265944
14284	0.946133	0.272269
14285	0.946132	0.273423
14286	0.946132	0.254819
14287	0.946132	0.254945
14288	0.946133	0.293979
14289	0.946132	0.264286
14290	0.946128	0.278918
14291	0.946131	0.28573
14292	0.94613	0.289411
14293	0.946129	0.265069
14294	0.94613	0.262007
14295	0.94613	0.313756
14296	0.94613	0.277196
14297	0.946132	0.234327
14298	0.946128	0.247185
14299	0.94613	0.254933
14300	0.946131	0.231786
14301	0.94613	0.267077
14302	0.94613	0.25378
14303	0.946131	0.231323
14304	0.94612	0.251718
14305	0.946119	0.260352
14306	0.946119	0.251847
14307	0.946124	0.244566
14308	0.946119	0.251352
14309	0.946132	0.267345
14310	0.946107	0.248051
14311	0.946128	2.1
14312	0.946133	2.1
14313	0.946131	2.1
14314	0.94613	2.1
14315	0.946131	2.1
14316	1.37869	2.1
14317	0.946131	2.1
14318	1.35703	2.1
14319	0.946132	2.1
14320	0.946132	2.1
14321	0.946132	2.1
14322	0.946132	2.1
14323	0.946132	2.1

14324	0.946133	2.1
14325	0.946133	2.1
14326	0.946133	0.887679
14327	0.946132	2.1
14328	1.37017	2.1
14329	0.946133	1.08739
14330	0.946132	2.1
14331	0.946133	2.1
14332	0.946114	0.0834402
14333	0.946119	0.0937871
14334	0.946119	0.117998
14335	0.94612	0.097472
14336	0.946119	0.10819
14337	0.946132	0.097371
14338	0.946107	0.101402
14339	0.946131	0.118382
14340	0.946131	0.127399
14341	0.946126	0.170489
14342	0.946127	0.143935
14343	0.94613	0.139111
14344	0.946129	0.136921
14345	0.946128	0.137886
14346	0.946131	1.05828
14347	0.946131	0.981709
14348	0.946132	0.78162
14349	0.946132	1.04775
14350	0.94613	1.26986
14351	0.946131	1.32457
14352	1.3279	1.26307
14353	0.946132	2.1
14354	1.40932	2.1
14355	0.946132	2.1
14356	0.946132	2.1
14357	0.946132	2.1
14358	0.946131	2.1
14359	0.946132	2.1
14360	0.946131	0.164329
14361	0.946131	0.162994
14362	0.946132	0.144177
14363	0.946132	0.167128
14364	0.94613	0.213679
14365	0.946131	0.189543
14366	1.26868	0.175093
14367	0.810943	2.1
14368	0.810945	2.1
14369	0.810932	2.1
14370	2.82321	2.1
14371	1.3517	2.1
14372	1.08132	2.1
14373	1.08132	2.1
14374	1.08133	2.1
14375	0.810943	0.382127
14376	0.810944	0.313611
14377	2.63079	0.480579
14378	1.35168	0.359861
14379	1.0813	0.38564
14380	1.08132	0.273288
14381	1.08132	0.649387

14382	0.810957	0.468046
14383	1.17856	0.234114
14384	0.946132	0.288172
14385	0.946128	0.34285
14386	1.3154	0.674603
14387	0.94613	0.42043
14388	1.31539	0.469359
14389	0.94613	0.605246
14390	0.94613	1.404
14391	0.94613	1.29047
14392	0.946131	1.15811
14393	0.946131	1.37808
14394	0.946129	1.4422
14395	0.94613	1.91158
14396	1.21997	1.85397
14397	0.946131	1.0463
14398	1.25083	0.878265
14399	0.946132	0.837397
14400	0.946132	1.1074
14401	0.946129	0.805556
14402	1.25083	1.09684
14403	0.946131	1.14511
14404	0.946127	1.26307
14405	1.41342	0.787558
14406	0.94613	1.12929
14407	0.946131	1.01261
14408	0.946127	0.662981
14409	0.946129	0.811113
14410	0.946129	0.871065
14411	0.946131	1.49643
14412	1.29149	1.62214
14413	0.946133	2.09106
14414	0.946133	1.16881
14415	0.946128	1.27751
14416	0.946131	0.811206
14417	0.94613	1.33654
14418	0.94613	1.70683
14419	0.94613	1.20183
14420	0.946132	2.1
14421	0.946132	0.989159
14422	0.946128	0.754726
14423	0.94613	1.53127
14424	1.41022	1.67542
14425	0.946132	0.404026
14426	0.946132	0.33547
14427	0.946128	0.372183
14428	0.94613	0.304496
14429	0.94613	0.427082
14430	0.94613	0.288113
14431	0.946131	0.423516
14432	0.946129	0.98472
14433	1.37758	1.3892
14434	0.946131	1.94969
14435	0.94613	0.767949
14436	0.946124	1.30338
14437	0.946127	0.817598
14438	0.946127	1.42874
14439	0.946129	0.985139

14440	1.38259	1.33609
14441	0.946132	1.76229
14442	0.946132	0.806649
14443	0.946126	1.36147
14444	0.946129	0.873095
14445	0.94613	1.29543
14446	0.946131	0.2304
14447	0.946132	0.239412
14448	0.946132	0.263205
14449	0.946132	0.246759
14450	0.946132	0.269275
14451	0.946132	0.245925
14452	0.946132	0.228927
14453	0.946131	0.13129
14454	0.94613	0.139139
14455	0.94613	0.123169
14456	0.946133	0.127957
14457	0.946128	0.125905
14458	1.25017	0.114693
14459	0.946131	0.132398
14460	0.946131	2.1
14461	0.946131	2.1
14462	0.946131	2.1
14463	1.3937	2.1
14464	0.946133	2.1
14465	0.946131	2.1
14466	0.94613	2.1
14467	0.946131	0.483048
14468	0.946131	0.310012
14469	0.946131	0.315897
14470	0.94613	0.288073
14471	0.946133	0.586033
14472	0.946132	0.352895
14473	0.946131	0.430139
14474	0.946132	0.317906
14475	0.946132	0.425326
14476	0.946132	0.292299
14477	0.946132	0.396209
14478	0.946131	0.372059
14479	0.946132	0.233083
14480	0.946132	0.316836
14481	0.946129	0.220635
14482	0.94613	0.549887
14483	0.94613	0.4759
14484	0.946128	0.549715
14485	0.946132	0.146119
14486	0.946131	0.470882
14487	0.94613	0.403764
14488	0.946132	1.14261
14489	0.946132	0.554267
14490	0.946132	0.671241
14491	1.41619	0.861997
14492	0.946133	0.360235
14493	0.946133	0.636577
14494	0.946132	0.557884
14495	0.946132	2.1
14496	0.946132	1.2861
14497	0.946132	2.1

14498	1.38244	2.1
14499	0.946132	0.702697
14500	0.946131	2.03278
14501	0.946132	1.69373
14502	0.810946	0.659436
14503	0.810943	0.887243
14504	0.810934	0.650185
14505	2.42741	0.487161
14506	1.35171	0.558662
14507	1.08132	0.612832
14508	1.08132	0.652186
14509	1.08133	0.650216
14510	0.810947	0.596018
14511	0.810943	0.905638
14512	2.44179	0.480604
14513	1.35171	0.569356
14514	1.08132	0.571178
14515	1.08132	0.573597
14516	1.08133	0.597168
14517	0.810934	0.570523
14518	0.946132	1.39743
14519	0.946132	1.35765
14520	0.946133	0.858988
14521	0.946133	1.3403
14522	0.946132	1.50836
14523	0.946132	1.36721
14524	1.12786	1.21921
14525	1.27806	0.725086
14526	0.946132	0.965571
14527	0.946133	0.823501
14528	0.946133	1.15547
14529	0.946132	1.4179
14530	0.946132	0.948596
14531	0.946133	1.32879
14532	0.946132	2.1
14533	0.946132	2.1
14534	0.946132	2.1
14535	1.37098	2.1
14536	0.946133	2.1
14537	0.946132	2.1
14538	1.37098	2.1
14539	0.946125	1.11884
14540	0.946129	0.982766
14541	0.946131	1.70205
14542	0.94613	0.811805
14543	0.946124	0.712857
14544	0.946127	0.828292
14545	1.39514	1.14633
14546	0.946132	2.1
14547	0.946131	2.1
14548	0.94613	2.1
14549	1.39909	2.1
14550	0.946132	0.821533
14551	0.946131	2.1
14552	0.94613	2.1
14553	0.946133	1.17134
14554	0.946133	0.45126
14555	0.946132	1.25952

14556	1.36835	0.847888
14557	0.946132	0.487004
14558	0.946133	0.822684
14559	0.946132	0.497802
14560	0.946132	1.70839
14561	0.946133	2.1
14562	0.946133	2.1
14563	0.946132	1.93029
14564	1.39047	2.1
14565	0.946132	2.1
14566	0.946132	2.1
14567	1.33174	0.0912768
14568	0.946128	0.0958916
14569	0.94613	0.0969391
14570	0.94613	0.0949668
14571	0.94613	0.0901605
14572	0.94613	0.0906392
14573	0.946131	0.0951554
14574	0.946128	0.122479
14575	1.36004	0.113683
14576	0.946129	0.113355
14577	0.94613	0.119851
14578	0.94613	0.118079
14579	1.36003	0.102829
14580	0.94613	0.11611
14581	0.946124	2.1
14582	0.946119	2.1
14583	0.946119	2.1
14584	0.946119	2.1
14585	0.94612	2.1
14586	0.946132	2.1
14587	1.29062	2.1
14588	0.946128	2.1
14589	0.946128	2.1
14590	0.946133	2.1
14591	0.94613	2.1
14592	0.946131	2.1
14593	0.946131	2.1
14594	1.30584	2.1
14595	0.946131	1.83188
14596	0.946131	2.1
14597	0.946131	2.1
14598	0.946132	2.03729
14599	1.39549	2.1
14600	0.946131	2.1
14601	0.946131	2.1
14602	0.913386	0.873657
14603	1.17466	0.799527
14604	1.00473	0.729
14605	0.946132	0.832705
14606	0.946126	1.22881
14607	0.94613	0.97819
14608	0.946129	0.894848
14609	0.946132	0.670279
14610	1.38894	0.662565
14611	0.946132	0.620728
14612	0.946132	0.630592
14613	0.946132	0.866017

14614	0.946132	0.681045
14615	0.946133	0.689363
14616	0.946128	0.642731
14617	1.23865	0.633417
14618	0.946129	0.587773
14619	0.94613	0.601879
14620	0.94613	0.688603
14621	0.94613	0.619165
14622	0.94613	0.648715
14623	0.946126	0.860058
14624	1.40248	1.00091
14625	0.946129	0.926877
14626	0.94613	0.883994
14627	0.946124	1.36526
14628	0.946127	1.03866
14629	0.946128	0.949561
14630	0.946133	0.687757
14631	1.1391	0.74104
14632	0.946132	0.732931
14633	0.946132	0.647236
14634	0.946132	1.06509
14635	0.946133	0.718522
14636	0.946132	0.73109
14637	1.17156	1.10321
14638	0.946133	1.05458
14639	1.33893	0.747566
14640	0.827864	0.834368
14641	0.995232	0.70155
14642	0.827864	1.05749
14643	0.94613	0.672626
14644	1.21387	0.64472
14645	0.946132	0.660438
14646	0.946131	0.507364
14647	0.94613	0.579542
14648	0.94613	0.557175
14649	0.94613	0.608521
14650	0.94613	0.57664
14651	0.810943	0.397945
14652	0.810945	0.318781
14653	2.42463	0.291883
14654	1.3517	0.316668
14655	1.08132	0.356963
14656	1.08132	0.383634
14657	1.08133	0.347098
14658	0.810932	0.391855
14659	0.946133	0.629562
14660	0.946132	0.47714
14661	0.946131	0.548954
14662	1.36748	0.648282
14663	0.946133	0.559968
14664	0.946131	0.486911
14665	0.946132	0.376177
14666	1.35856	0.321447
14667	0.946131	0.345506
14668	0.94613	0.29202
14669	0.94613	0.351296
14670	0.946131	0.273224
14671	0.94613	0.299738

14672	0.94613	0.350619
14673	0.946132	1.37057
14674	0.946131	2.1
14675	0.946131	2.1
14676	0.946131	2.1
14677	1.41149	2.1
14678	1.41149	2.1
14679	0.946131	2.1
14680	0.946128	2.1
14681	0.946128	2.1
14682	0.946133	2.1
14683	0.946131	2.1
14684	0.946131	2.1
14685	0.946131	2.1
14686	0.94613	2.1
14687	1.41388	0.142374
14688	0.94613	0.180011
14689	0.946132	0.130379
14690	0.946132	0.166102
14691	0.946129	0.199503
14692	0.946131	0.166601
14693	0.946131	0.181285
14694	1.02798	0.137245
14695	0.946132	0.220076
14696	1.02798	0.131685
14697	0.946133	0.220549
14698	1.14931	0.148677
14699	0.957746	0.196098
14700	0.93452	0.174768
14701	0.946129	2.1
14702	0.946134	2.1
14703	0.946133	2.1
14704	0.946131	2.1
14705	0.946131	2.1
14706	0.946131	2.1
14707	0.946131	2.1
14708	0.946133	1.22719
14709	0.946132	0.79829
14710	0.946132	1.07198
14711	0.946132	1.46169
14712	1.38773	0.849659
14713	0.946132	1.19031
14714	0.946133	0.953829
14715	0.946131	0.251452
14716	0.946131	0.293083
14717	1.41512	0.251362
14718	0.946133	0.368327
14719	0.946131	0.362079
14720	0.946131	0.344545
14721	0.946131	0.281406
14722	1.35381	0.744586
14723	0.946132	1.87706
14724	1.35381	0.643172
14725	0.946133	1.87609
14726	0.946132	0.857774
14727	0.946132	1.49291
14728	0.946133	0.965965
14729	1.3365	0.697266

14730	0.946132	1.05529
14731	1.3365	0.576642
14732	0.946129	0.901999
14733	0.94613	0.799899
14734	0.946128	0.97087
14735	0.946129	0.749258
14736	0.939895	1.29523
14737	1.14285	1.87994
14738	0.952366	1.71417
14739	0.946131	1.5483
14740	0.94613	1.59852
14741	0.946131	1.55471
14742	0.94613	1.58183
14743	0.946131	2.1
14744	0.946131	2.1
14745	0.946131	1.02273
14746	0.946129	2.1
14747	0.946133	1.07713
14748	0.946131	2.00295
14749	0.946131	2.07256
14750	0.946133	2.1
14751	0.946132	2.1
14752	0.946132	2.1
14753	0.946133	2.1
14754	0.946132	2.03637
14755	0.946132	2.1
14756	0.946133	2.1
14757	0.946133	1.9252
14758	0.946132	1.20009
14759	0.946132	1.72245
14760	0.946133	1.45686
14761	0.946132	2.1
14762	0.946132	1.87895
14763	0.946132	1.43136
14764	0.946132	0.756995
14765	0.946131	2.1
14766	0.946131	0.814327
14767	0.946131	0.857399
14768	1.38969	2.1
14769	0.946131	1.22885
14770	0.946132	2.06984
14771	0.946128	1.94367
14772	0.946133	1.46965
14773	0.946132	1.78984
14774	0.946131	1.39931
14775	0.94613	1.41894
14776	0.946131	1.70353
14777	1.28464	1.69126
14778	0.946119	0.736623
14779	1.37492	0.807758
14780	0.946125	0.899974
14781	0.946119	0.620438
14782	0.946119	1.10542
14783	0.946132	0.679104
14784	0.946107	0.845685
14785	0.810942	2.1
14786	0.810943	2.1
14787	0.709587	2.1

14788	2.64488	2.1
14789	1.21651	2.1
14790	0.946151	2.1
14791	0.979948	2.1
14792	1.0813	2.1
14793	0.946133	1.41762
14794	0.946132	1.84238
14795	0.946132	2.08862
14796	0.946133	0.957284
14797	0.946132	0.713415
14798	0.946132	0.871031
14799	1.41134	1.30507
14800	0.946133	1.82656
14801	0.946132	1.51002
14802	0.946133	2.082
14803	0.946133	1.15758
14804	0.946132	0.780336
14805	0.946133	1.35229
14806	1.31338	1.60766
14807	2.07612	1.89367
14808	0.946102	0.0780439
14809	0.752448	0.0885787
14810	1.20617	0.0834364
14811	0.94613	0.0864718
14812	0.75242	0.0838505
14813	0.946131	0.0843971
14814	0.752419	0.0757359
14815	0.946132	0.959214
14816	1.25736	0.924873
14817	1.25736	0.870981
14818	0.946132	0.894641
14819	1.08054	0.801626
14820	1.25736	0.867092
14821	0.946132	1.00722
14822	0.946128	2.1
14823	1.1896	2.09117
14824	1.18959	2.1
14825	0.946131	0.879301
14826	1.33271	1.60993
14827	0.946105	1.79914
14828	0.946156	1.62677
14829	0.741946	0.083376
14830	1.33587	0.0884751
14831	0.741947	0.0832953
14832	1.21954	0.0887214
14833	0.94613	0.0932091
14834	0.741946	0.0914365
14835	1.81347	0.0874462
14836	1.81347	0.0824088
14837	0.653322	0.0763786
14838	1.36935	0.0768019
14839	0.661882	0.0728337
14840	1.04753	0.0752276
14841	0.507464	0.0751175
14842	0.945844	1.24851
14843	1.59507	1.87749
14844	1.04043	1.14609
14845	0.946131	1.5682

14846	1.32891	1.36685
14847	0.946131	1.02916
14848	0.946131	1.07757
14849	1.28866	1.33418
14850	1.12682	0.276115
14851	0.851543	0.321259
14852	1.04072	0.294006
14853	0.946132	0.351065
14854	1.13437	0.253568
14855	1.04167	0.305395
14856	0.936652	0.304533
14857	1.258 2.1	
14858	0.946131	2.1
14859	0.946132	2.1
14860	1.36968	2.1
14861	0.946132	2.1
14862	1.36968	2.1
14863	0.946131	2.1
14864	2.30046	0.245501
14865	2.82969	0.276036
14866	0.946132	1.40409
14867	1.35022	2.1
14868	1.12356	1.29385
14869	0.94613	2.1
14870	1.35022	1.99313
14871	0.946131	1.87112
14872	0.94613	1.68484
14873	0.946131	2.1
14874	1.22401	2.1
14875	0.952456	2.1
14876	0.94613	2.1
14877	1.22401	2.1
14878	0.946131	2.1
14879	0.946131	2.1
14880	1.33047	2.1
14881	0.946132	2.1
14882	1.33047	2.1
14883	0.946131	2.1
14884	1.33047	2.1
14885	0.946132	2.1
14886	1.2107	2.1
14887	0.61957	1.5727
14888	1.80666	2.1
14889	1.53743	2.1
14890	0.619572	1.34964
14891	1.32547	2.03096
14892	1.16166	1.90608
14893	1.04074	1.90101
14894	0.557621	2.02481
14895	0.946128	0.743117
14896	1.36607	1.57509
14897	0.94613	1.27223
14898	1.36607	2.1
14899	1.39416	2.1
14900	0.94613	2.1
14901	0.94613	2.1
14902	1.23969	2.1
14903	0.946131	2.1

14904	0.946131	2.1
14905	1.13537	2.1
14906	1.05719	2.1
14907	0.806848	2.1
14908	1.30251	2.1
14909	0.851526	1.5473
14910	1.14176	2.06206
14911	0.93666	1.25835
14912	0.946114	1.80466
14913	1.15832	1.40281
14914	0.924624	1.36724
14915	0.967609	1.23745
14916	0.771764	0.20123
14917	1.26652	0.201653
14918	0.946133	0.219093
14919	1.17957	0.144382
14920	0.771735	0.192541
14921	0.946122	0.220416
14922	0.946133	0.18784
14923	0.924325	0.931087
14924	0.656739	1.00376
14925	0.637876	0.986848
14926	0.530092	0.970425
14927	0.914415	0.897738
14928	0.821311	1.27139
14929	0.630589	1.13246
14930	0.738823	1.12407
14931	0.839109	1.03115
14932	1.55029	0.0768715
14933	1.13537	0.435423
14934	0.946131	0.709079
14935	0.946128	0.538778
14936	1.30162	2.00609
14937	0.952476	0.769821
14938	1.29289	0.951292
14939	0.939783	0.945493
14940	0.94612	2.1
14941	0.946131	2.1
14942	1.07998	2.1
14943	0.836985	2.1
14944	1.1604	2.1
14945	0.94613	2.1
14946	0.837016	2.1
14947	0.712461	0.603047
14948	1.36399	0.622497
14949	1.8724	0.547945
14950	1.8724	0.486541
14951	0.623426	0.930624
14952	1.36399	0.568665
14953	1.22089	0.55982
14954	0.946132	0.558838
14955	0.946131	2.1
14956	1.32241	2.1
14957	1.32241	2.1
14958	0.946133	2.1
14959	1.40271	2.1
14960	0.946131	2.1
14961	0.946131	2.1

14962	1.1989	1.18694
14963	0.954106	1.39545
14964	0.820068	1.03374
14965	0.563139	1.28086
14966	1.06448	1.33547
14967	0.892472	1.75499
14968	0.862598	1.15036
14969	0.745851	1.77131
14970	1.13336	1.32076
14971	0.946129	2.1
14972	1.28254	2.1
14973	1.28254	2.1
14974	0.946131	2.1
14975	1.41302	2.1
14976	0.946131	2.1
14977	0.946131	2.1
14978	0.851543	1.34342
14979	1.21961	2.1
14980	0.851484	1.27105
14981	0.851543	2.08394
14982	1.15705	1.83873
14983	0.946133	1.68515
14984	0.909417	1.56286
14985	1.13808	0.602426
14986	0.946133	0.393855
14987	0.946131	0.6129
14988	1.30194	0.413196
14989	0.946131	0.489621
14990	1.30194	0.377458
14991	0.946131	0.41395
14992	0.930941	1.04661
14993	1.20185	0.803817
14994	1.02404	0.861019
14995	0.946124	1.15702
14996	1.3563	0.951241
14997	0.946127	1.23543
14998	0.946128	1.01512
14999	0.946116	2.1
15000	1.3144	1.91466
15001	1.13088	2.1
15002	0.946118	0.786571
15003	1.31438	1.50518
15004	0.946105	1.50321
15005	0.946131	1.34123
15006	1.76972	0.259048
15007	1.42091	0.185698
15008	0.946128	0.280339
15009	0.946101	0.330123
15010	0.863552	0.370043
15011	0.94613	0.22121
15012	1.21232	0.294437
15013	0.94613	0.386199
15014	1.2137	0.518467
15015	1.23224	0.717341
15016	1.86512	0.582041
15017	1.86512	0.471945
15018	0.580825	1.07609
15019	1.2137	0.756357

15020	0.946133	0.755555
15021	0.580822	0.688659
15022	0.946128	2.1
15023	1.40586	2.1
15024	0.94613	2.1
15025	1.3719	2.1
15026	1.3719	2.1
15027	1.40586	2.1
15028	0.94613	2.1
15029	0.946132	1.94041
15030	1.35025	1.85871
15031	1.1794	1.77926
15032	0.946132	0.797549
15033	1.35025	1.15206
15034	0.946131	1.16886
15035	0.946132	1.03428
15036	0.946133	2.1
15037	1.60149	2.1
15038	1.67058	2.1
15039	0.851542	2.1
15040	1.20296	2.1
15041	1.17501	2.1
15042	0.946132	2.1
15043	1.04993	2.1
15044	0.851541	1.17498
15045	1.48219	2.1
15046	0.874565	1.10019
15047	0.946129	2.1
15048	1.22049	2.04044
15049	0.946131	1.96144
15050	0.946131	1.55648
15051	0.94613	2.1
15052	0.848719	2.1
15053	1.17195	2.02846
15054	0.946129	1.23639
15055	1.35426	1.32123
15056	0.94613	1.45924
15057	0.94613	1.65953
15058	0.946133	2.1
15059	1.14342	2.1
15060	1.14342	2.1
15061	0.946133	2.1
15062	1.39009	2.1
15063	0.946132	2.1
15064	0.946132	2.1
15065	1.38819	2.1
15066	0.946133	2.1
15067	1.38819	2.1
15068	0.946131	2.1
15069	1.4116	2.1
15070	0.946131	2.1
15071	1.38819	2.1
15072	0.946127	2.1
15073	1.32244	2.1
15074	1.2176	2.1
15075	0.946131	2.1
15076	1.32244	2.1
15077	0.94613	2.1

15078	0.946129	2.1
15079	1.32415	2.1
15080	1.11239	2.1
15081	0.984099	2.1
15082	1.65532	2.1
15083	1.53082	2.1
15084	1.32068	2.1
15085	1.32068	2.1
15086	1.08172	2.1
15087	3.76293	0.106579
15088	1.36335	0.0826369
15089	0.953925	2.1
15090	0.989497	2.1
15091	0.98949	2.1
15092	1.06341	2.1
15093	1.27875	2.1
15094	1.04598	2.1
15095	0.603154	2.1
15096	0.582457	2.1
15097	0.582457	2.1
15098	0.568994	2.1
15099	1.5791	2.1
15100	1.09506	2.1
15101	1.45327	1.96216
15102	1.88406	2.1
15103	1.88404	2.1
15104	1.27389	1.8234
15105	1.14206	2.1
15106	1.00232	2.1
15107	1.00231	2.1
15108	1.68436	2.1
15109	1.4677	0.103298
15110	1.28828	0.123706
15111	1.28828	0.0961037
15112	1.46932	0.0993737
15113	1.28228	2.1
15114	1.64268	0.939728
15115	0.959473	2.1
15116	1.10101	2.1
15117	1.07976	2.1
15118	1.2873	1.14778
15119	1.70303	0.166309
15120	1.40066	0.268035
15121	1.5563	0.166005
15122	1.13727	0.233402
15123	1.03529	2.1
15124	1.11285	2.1
15125	1.03529	2.1
15126	1.28667	2.1
15127	1.32119	2.1
15128	1.44777	2.1
15129	1.44778	2.1
15130	1.61121	2.1
15131	1.09372	0.127451
15132	1.17664	0.141872
15133	1.17664	0.137196
15134	0.872931	0.118223
15135	1.02415	2.1

15136	1.28228	2.1
15137	2.14692	2.1
15138	3.90469	2.1
15139	1.00509	2.1
15140	1.28228	2.1
15141	3.32936	0.0883125
15142	1.1333	0.0868084
15143	1.11048	2.1
15144	0.979854	2.1
15145	1.65815	2.1
15146	1.00185	2.1
15147	1.84887	2.1
15148	1.59597	2.1
15149	1.78468	2.1
15150	1.87508	2.1
15151	1.82716	2.1
15152	1.33429	2.1
15153	1.34626	2.1
15154	0.797142	2.1
15155	1.00007	0.970057
15156	1.02051	1.8626
15157	1.02051	1.02213
15158	1.03373	1.65976
15159	1.05786	2.1
15160	0.887631	2.1
15161	0.992773	2.1
15162	1.28462	2.1
15163	2.35274	1.66168
15164	1.82533	1.03829
15165	1.82533	1.46176
15166	1.19496	0.797695
15167	1.28597	0.0522355
15168	1.09725	2.1
15169	1.60948	2.1
15170	1.20421	2.1
15171	1.16566	2.1
15172	1.88522	2.1
15173	1.81948	0.475713
15174	1.14042	0.344862
15175	1.21558	0.579657
15176	1.56876	0.302827
15177	1.6697	2.1
15178	1.81655	2.1
15179	1.81655	2.1
15180	1.5956	2.1
15181	0.906059	2.1
15182	1.63045	1.95487
15183	0.9533	0.982428
15184	1.1475	0.918295
15185	1.72231	1.75504
15186	1.94936	1.18519
15187	1.06826	2.1
15188	1.08576	2.1
15189	1.06826	2.1
15190	0.973559	2.1
15191	1.62737	0.0811993
15192	0.965676	0.0815684
15193	1.45055	0.0869342

15194	1.17631	0.0883727
15195	1.92535	0.119797
15196	1.9503	0.156318
15197	1.99828	0.11658
15198	1.70302	0.15092
15199	1.65139	0.153625
15200	1.26784	0.178922
15201	1.35923	0.192041
15202	1.70302	0.207963
15203	1.33033	2.1
15204	1.12481	2.1
15205	1.29452	2.1
15206	1.25399	2.1
15207	1.12481	2.1
15208	0.918358	2.1
15209	1.39332	2.1
15210	1.32368	2.1
15211	1.11712	0.862331
15212	1.07999	0.995026
15213	1.07999	0.719326
15214	1.05619	0.941111
15215	1.1766	2.1
15216	1.35735	2.1
15217	1.42456	0.122046
15218	1.089	0.151411
15219	1.11081	0.0737329
15220	2.88521	0.0764157
15221	1.09754	0.0721085
15222	1.85479	0.0713903
15223	1.27922	0.675656
15224	1.03556	0.585903
15225	1.07508	1.34515
15226	1.09795	1.83509
15227	0.967672	0.92517
15228	1.07508	1.22067
15229	1.03933	1.3375
15230	1.02975	2.1
15231	1.02975	1.30519
15232	1.01121	1.82206
15233	1.24526	0.0778208
15234	1.72368	0.0747938
15235	1.89607	0.0760859
15236	2.13512	0.0749529
15237	1.34591	0.43992
15238	1.14721	0.539659
15239	1.15539	0.91212
15240	1.90938	0.726126
15241	1.90941	1.86836
15242	1.89242	1.34045
15243	1.18907	2.1
15244	1.20408	2.1
15245	1.20409	2.1
15246	1.22655	2.00868
15247	0.701447	2.1
15248	1.57567	2.1
15249	1.38806	2.1
15250	2.09407	1.89211
15251	1.07612	1.0465

15252	0.990019	1.13365
15253	1.15564	1.48565
15254	1.01243	1.31894
15255	1.28956	0.289071
15256	1.35146	0.275907
15257	1.35145	0.281723
15258	1.22811	0.283157
15259	1.09662	2.1
15260	1.42486	2.1
15261	1.10106	0.0758548
15262	1.90814	0.0676174
15263	1.3946	0.0713995
15264	1.11926	0.0731416
15265	0.725839	2.1
15266	0.925068	2.1
15267	0.925074	2.1
15268	1.43282	2.1
15269	1.91756	0.928597
15270	1.74321	0.962539
15271	1.74321	0.827784
15272	1.63413	0.868059
15273	1.9194	0.278246
15274	1.62246	0.217314
15275	1.90456	0.181978
15276	1.06578	0.227928
15277	1.46019	0.225211
15278	1.17036	0.153304
15279	1.70233	1.20591
15280	1.53515	1.12292
15281	2.03494	2.1
15282	1.78441	2.1
15283	1.75992	2.1
15284	1.94351	2.1
15285	1.51057	0.118942
15286	0.991435	0.11335
15287	1.98068	0.143294
15288	1.64283	0.137303
15289	2.96126	0.135443
15290	2.20692	0.129533
15291	1.95054	0.106098
15292	1.21325	0.100841
15293	0.996828	0.337057
15294	1.01976	0.284129
15295	1.17129	2.1
15296	1.13664	2.1
15297	1.13664	2.1
15298	1.09627	2.1
15299	2.65123	2.1
15300	3.23062	2.1
15301	3.23062	2.1
15302	3.92038	2.1
15303	1.89019	0.344936
15304	1.79109	0.416763
15305	1.79109	0.293535
15306	1.54197	0.315599
15307	3.41824	0.922238
15308	1.93553	0.21148
15309	2.26215	0.858158

15310	2.0103	0.231065
15311	1.51569	1.08003
15312	1.01683	1.00738
15313	1.78595	2.1
15314	1.66537	2.1
15315	3.73426	2.1
15316	3.53905	2.1
15317	1.66537	2.1
15318	1.52903	2.1
15319	3.53905	2.1
15320	3.3837	2.1
15321	1.19901	0.912662
15322	1.35002	1.52121
15323	1.35002	0.636612
15324	1.11683	0.657171
15325	1.75326	1.67729
15326	0.712404	1.41595
15327	2.97768	2.1
15328	2.98949	2.1
15329	1.2832	2.1
15330	1.39518	2.1
15331	2.98949	2.1
15332	2.99339	2.1
15333	1.39517	2.1
15334	1.46124	2.1
15335	1.28534	2.1
15336	1.03391	2.1
15337	1.75366	0.987676
15338	1.26247	1.25186
15339	1.13621	1.69629
15340	2.43994	2.1
15341	1.47904	0.133343
15342	1.80085	0.154345
15343	1.80085	0.123613
15344	1.64656	0.18429
15345	2.87331	2.1
15346	2.82863	2.1
15347	2.82859	2.1
15348	2.84673	2.1
15349	1.0961	0.309038
15350	1.39448	0.430945
15351	1.21366	0.423338
15352	1.20143	0.296868
15353	1.85795	0.153726
15354	1.36768	0.139138
15355	1.36769	0.128146
15356	1.45789	0.11334
15357	1.32605	0.0822171
15358	1.27148	0.0877273
15359	1.70218	0.147541
15360	1.3063	0.228939
15361	1.45603	0.198316
15362	1.89226	0.278494
15363	1.2742	0.182191
15364	2.10112	0.141597
15365	1.10748	0.260868
15366	1.1893	0.274595
15367	1.04921	2.1

15368	1.22927	2.1
15369	1.22927	2.1
15370	1.46568	2.1
15371	0.811186	2.1
15372	0.792162	2.1
15373	0.792162	2.1
15374	0.758749	2.1
15375	1.03581	2.1
15376	0.737481	2.1
15377	2.24071	2.1
15378	1.34868	2.1
15379	1.47203	0.819866
15380	1.40967	0.88597
15381	1.40967	0.919898
15382	1.27916	0.928102
15383	1.17418	0.817819
15384	1.04921	2.1
15385	1.28291	1.18591
15386	1.3213	0.520277
15387	1.46812	0.96301
15388	2.42522	1.28541
15389	1.17879	0.755124
15390	1.28291	0.782792
15391	1.28291	0.759659
15392	1.13438	0.770061
15393	1.75004	2.1
15394	1.78247	2.1
15395	1.78247	2.1
15396	1.76421	2.1
15397	2.25975	0.0757562
15398	3.66111	0.0745895
15399	3.6611	0.0678499
15400	3.44043	0.0670743
15401	1.13339	2.1
15402	1.13322	2.1
15403	1.13323	2.1
15404	1.20761	2.1
15405	1.34337	0.752993
15406	1.90912	1.96201
15407	1.04399	1.64499
15408	2.00578	1.92682
15409	1.04973	2.1
15410	1.22615	1.51691
15411	1.93384	2.1
15412	2.62816	0.686757
15413	1.13127	2.1
15414	1.47642	1.03701
15415	2.62816	2.1
15416	3.31755	0.710967
15417	1.47642	2.1
15418	1.80454	0.90167
15419	0.964782	1.68583
15420	0.949135	2.1
15421	0.949134	1.67435
15422	0.949134	2.1
15423	1.10476	0.511116
15424	0.982858	0.162166
15425	1.10938	0.424405

15426	0.982858	0.401533
15427	1.01012	0.0646167
15428	0.974081	0.0620777
15429	1.82643	0.0625266
15430	1.29585	0.0630893
15431	1.00763	0.926028
15432	1.3294	0.779018
15433	1.3294	0.630062
15434	1.30001	0.980623
15435	1.59151	2.1
15436	1.99443	2.1
15437	1.07369	2.1
15438	1.30584	2.1
15439	1.05071	2.1
15440	0.948676	2.1
15441	1.13842	2.1
15442	1.42884	2.1
15443	1.59019	0.0856165
15444	1.38212	0.0967038
15445	1.34102	0.0881137
15446	1.02663	0.0957584
15447	0.797011	2.1
15448	0.81301	2.1
15449	0.81301	2.1
15450	0.811187	2.1
15451	0.991869	0.340048
15452	1.23868	0.330825
15453	1.67201	2.1
15454	1.61201	2.1
15455	1.61202	2.1
15456	1.57627	2.1
15457	0.94994	2.1
15458	1.77867	2.1
15459	0.854945	2.1
15460	0.973905	2.1
15461	1.64193	2.1
15462	1.68893	2.1
15463	1.68893	2.1
15464	1.70002	2.1
15465	2.08149	0.313841
15466	1.69456	0.237578
15467	1.67399	0.0522355
15468	1.83922	0.788952
15469	1.91703	2.1
15470	0.967492	2.1
15471	1.13317	2.1
15472	0.980766	2.1
15473	2.06158	0.190548
15474	0.986498	0.155441
15475	1.76289	0.183263
15476	1.17248	0.152229
15477	1.51794	0.14391
15478	1.30266	0.160612
15479	1.03165	0.339099
15480	0.947692	0.37407
15481	1.20473	0.243332
15482	0.947693	0.22789
15483	1.54357	2.1

15484	0.980841	2.1
15485	1.34124	0.469658
15486	1.39818	0.620894
15487	1.39818	0.339897
15488	1.48035	0.564288
15489	1.03932	2.1
15490	1.2065	2.1
15491	1.20651	2.1
15492	1.12717	2.1
15493	1.09483	2.1
15494	0.994293	2.1
15495	0.994293	2.1
15496	0.97356	2.1
15497	0.995252	0.742198
15498	1.66294	0.730158
15499	1.82775	0.534254
15500	1.42762	0.43127
15501	1.04713	0.337305
15502	1.05475	0.2985
15503	1.29002	0.190061
15504	1.05475	0.219156
15505	1.17585	2.1
15506	1.39223	2.1
15507	1.39223	2.1
15508	1.71255	2.1
15509	1.56166	0.783306
15510	1.37762	0.690613
15511	1.16858	1.10882
15512	1.0361	2.1
15513	1.23966	0.0745462
15514	1.10718	0.0751838
15515	1.99957	0.0669424
15516	0.658392	0.0642838
15517	1.36904	0.0718227
15518	1.12524	0.0662453
15519	1.04321	1.17578
15520	1.04627	1.16983
15521	1.91351	0.780694
15522	1.63379	0.892877
15523	1.79718	0.972056
15524	0.59634	1.05353
15525	0.958257	2.1
15526	1.06457	2.1
15527	0.958255	2.1
15528	1.03933	2.1
15529	1.14195	0.534249
15530	1.49438	0.469287
15531	1.49438	0.485435
15532	1.31259	1.09187
15533	1.23036	2.1
15534	1.2016	2.1
15535	1.2016	2.1
15536	1.17129	2.1
15537	1.05724	0.774827
15538	1.04291	0.963912
15539	1.12341	2.1
15540	2.32525	2.1
15541	3.39038	2.1

15542	2.09374	2.1
15543	1.76185	0.751852
15544	1.7934	0.856
15545	1.65809	0.858211
15546	1.54852	1.23843
15547	1.38766	2.1
15548	1.28765	2.1
15549	1.26599	2.1
15550	1.21397	2.1
15551	1.28765	2.1
15552	1.20045	2.1
15553	1.21398	2.1
15554	1.15234	2.1
15555	0.953634	0.208815
15556	1.39132	0.218697
15557	1.17285	2.1
15558	1.21408	2.1
15559	1.21408	2.1
15560	0.982848	2.1
15561	2.00101	2.1
15562	2.00973	2.1
15563	2.20314	2.1
15564	1.89318	2.1
15565	1.23267	1.22617
15566	0.947921	2.1
15567	1.18872	2.1
15568	0.740752	2.1
15569	1.92269	0.416764
15570	1.53584	0.364178
15571	1.53584	0.419601
15572	1.21067	0.322136
15573	1.40707	0.110175
15574	1.50437	0.114378
15575	1.50435	0.105878
15576	1.45446	0.111324
15577	1.47954	0.664905
15578	2.08687	0.95856
15579	2.08687	1.65083
15580	1.1233	1.97719
15581	1.04025	0.727389
15582	1.36005	1.34467
15583	1.47606	0.298168
15584	1.3407	0.306622
15585	0.758743	2.1
15586	0.716917	2.1
15587	0.716922	2.1
15588	0.672964	2.1
15589	2.40014	2.1
15590	1.96848	1.1621
15591	1.96848	2.1
15592	1.53555	0.846953
15593	1.06191	2.1
15594	1.29579	2.1
15595	1.29579	2.1
15596	1.71289	2.1
15597	1.78584	2.1
15598	2.15211	2.1
15599	2.00427	2.1

15600	3.41615	2.1
15601	1.66408	2.1
15602	1.73954	2.1
15603	2.91663	2.1
15604	3.65818	2.1
15605	1.93286	2.1
15606	1.08831	2.1
15607	1.31242	0.109819
15608	1.11712	0.112567
15609	1.26311	0.111159
15610	1.31242	0.11346
15611	1.10378	2.1
15612	0.968594	2.1
15613	1.48389	0.0807636
15614	1.49613	0.0849529
15615	1.04349	0.553035
15616	1.26409	0.437096
15617	1.26409	0.410138
15618	1.05724	0.474168
15619	1.65723	0.284299
15620	1.83116	0.290687
15621	2.06284	0.293126
15622	2.00512	0.309379
15623	1.83116	0.283826
15624	2.03139	0.284737
15625	2.20564	0.298349
15626	1.90048	0.301727
15627	0.963392	2.1
15628	1.01346	0.652109
15629	2.98092	2.1
15630	2.70295	2.1
15631	2.70295	2.1
15632	2.48828	2.1
15633	1.63661	2.1
15634	1.64158	2.1
15635	1.64158	2.1
15636	1.6488	2.1
15637	1.17506	0.230873
15638	1.09667	0.207034
15639	2.00539	0.400881
15640	1.09667	0.296814
15641	1.54946	2.1
15642	1.43577	2.1
15643	1.26103	2.1
15644	1.49808	2.1
15645	1.089	0.125532
15646	1.22586	0.124353
15647	2.35027	0.327497
15648	1.37549	0.237057
15649	3.33706	0.21838
15650	1.69894	0.193439
15651	2.00539	0.276514
15652	1.53798	0.214035
15653	1.28938	2.1
15654	1.20969	2.1
15655	1.20969	2.1
15656	1.15864	2.1
15657	1.7986	2.1

15658	1.75869	2.1
15659	1.58281	2.1
15660	1.7392	2.1
15661	1.30733	0.9344
15662	1.5419	1.2795
15663	1.50883	1.03837
15664	1.85439	0.9576
15665	1.73531	0.916585
15666	1.70225	0.832795
15667	1.22176	0.330611
15668	1.59492	0.439733
15669	1.59492	0.34771
15670	2.15542	0.402547
15671	3.24379	2.1
15672	2.64775	2.1
15673	3.16707	2.1
15674	1.93032	2.1
15675	1.50738	2.1
15676	1.24128	2.1
15677	1.24128	2.1
15678	0.994293	2.1
15679	2.90808	0.0596417
15680	2.45797	0.0582836
15681	1.85501	0.0608272
15682	2.90809	0.0595065
15683	0.892835	1.4762
15684	1.55871	1.42693
15685	1.04615	1.27545
15686	1.66643	0.689288
15687	1.70044	1.90305
15688	1.80224	2.1
15689	1.70186	0.861199
15690	3.30123	0.741003
15691	1.74789	2.1
15692	1.77446	2.1
15693	1.77901	2.1
15694	1.57995	2.1
15695	1.81518	2.1
15696	2.17716	2.1
15697	2.17716	2.1
15698	2.65123	2.1
15699	2.01899	1.36324
15700	1.77479	1.21779
15701	1.19122	0.715458
15702	1.46724	0.565774
15703	1.70944	0.0808828
15704	1.08929	0.0794392
15705	1.68454	0.0829726
15706	1.08929	0.083563
15707	1.51067	0.930351
15708	1.06729	0.775133
15709	1.37762	0.757085
15710	1.13992	0.685259
15711	1.26397	2.1
15712	1.04083	2.1
15713	1.00278	2.1
15714	1.04084	2.1
15715	1.1016	0.120399

15716	1.23477	0.120931
15717	1.23477	0.0937948
15718	1.64985	0.104907
15719	1.11117	2.1
15720	1.13006	2.1
15721	1.13006	2.1
15722	1.14721	2.1
15723	1.21946	2.1
15724	1.0186	2.1
15725	1.13178	2.1
15726	1.00133	2.1
15727	1.67046	0.433408
15728	0.986036	0.499183
15729	1.67046	0.55273
15730	1.34857	1.46447
15731	2.46016	2.1
15732	2.07161	2.1
15733	2.10732	2.1
15734	2.08371	2.1
15735	1.35602	2.1
15736	0.829065	2.1
15737	1.81667	2.1
15738	0.99406	2.1
15739	1.81667	2.1
15740	1.68073	2.1
15741	2.05736	2.1
15742	2.97311	1.58546
15743	2.14011	2.07582
15744	1.23411	2.1
15745	1.13632	0.208784
15746	1.40862	0.191361
15747	1.28055	0.183602
15748	1.14305	0.171937
15749	1.12035	0.0772997
15750	1.15483	0.0757877
15751	1.94099	0.0762114
15752	1.82126	0.0743579
15753	1.15481	0.0769893
15754	0.998957	0.0750934
15755	2.10078	0.0762874
15756	1.81617	0.0750703
15757	1.27581	2.1
15758	0.968536	2.1
15759	1.33916	2.1
15760	1.27581	2.1
15761	1.32784	2.1
15762	1.4259	2.1
15763	1.70377	2.1
15764	1.54231	2.1
15765	3.77569	2.1
15766	3.42809	2.1
15767	1.54231	2.1
15768	1.51298	2.1
15769	3.55866	2.1
15770	2.86136	2.1
15771	1.83301	0.930686
15772	1.26371	0.769725
15773	1.98301	0.949333

15774	2.0609	0.843772
15775	2.26047	0.676712
15776	2.00537	0.595189
15777	1.7913	0.589871
15778	1.48864	0.460305
15779	1.09504	2.1
15780	1.39827	2.1
15781	2.28084	2.1
15782	1.05983	2.1
15783	1.33593	0.0881399
15784	1.31508	0.0816405
15785	1.31508	0.0833329
15786	1.09599	0.0796104
15787	1.30647	2.1
15788	1.28846	2.1
15789	1.28846	2.1
15790	1.26923	2.1
15791	1.2955	2.1
15792	1.09044	2.07526
15793	2.12209	2.1
15794	1.26438	2.1
15795	0.911819	2.1
15796	1.38752	2.1
15797	2.57704	2.1
15798	2.71736	2.1
15799	1.42284	2.1
15800	1.41562	2.1
15801	2.69131	2.1
15802	2.36178	2.1
15803	1.41562	2.1
15804	1.39564	2.1
15805	1.32368	2.1
15806	0.908053	2.1
15807	0.908053	2.1
15808	0.629213	2.1
15809	1.8758	1.44503
15810	1.27362	0.934326
15811	1.3756	0.652231
15812	2.28749	0.991684
15813	1.84577	0.907108
15814	2.17765	0.895613
15815	1.38969	0.788647
15816	1.84578	0.867859
15817	1.32801	0.652155
15818	1.76377	0.662579
15819	1.22845	1.01646
15820	1.57766	0.707214
15821	1.57766	1.81436
15822	1.39067	1.41935
15823	1.15743	0.183753
15824	1.37721	0.141891
15825	1.69055	0.224032
15826	1.14858	0.278474
15827	1.38333	0.984774
15828	1.10575	0.476425
15829	1.22418	1.85257
15830	1.10574	0.992558
15831	1.91509	0.286333

15832	1.02001	0.208722
15833	1.03074	0.470175
15834	1.19122	0.295279
15835	1.67887	2.1
15836	1.67597	2.1
15837	1.84984	2.1
15838	1.77775	2.1
15839	1.73571	2.06446
15840	1.27921	2.01543
15841	2.11851	1.45085
15842	1.27921	2.06494
15843	1.42486	2.1
15844	1.12503	2.1
15845	1.12503	2.1
15846	1.30116	2.1
15847	0.568995	2.1
15848	0.557348	2.1
15849	0.557348	2.1
15850	0.53907	2.1
15851	0.633575	2.1
15852	1.33756	0.821572
15853	1.51074	2.1
15854	1.93384	0.710189
15855	0.655202	2.1
15856	0.731934	2.1
15857	0.731934	2.1
15858	1.09061	2.1
15859	0.966979	2.1
15860	1.31502	2.1
15861	1.31502	2.1
15862	1.72116	2.1
15863	1.70002	2.1
15864	1.67969	2.1
15865	1.67969	2.1
15866	1.62408	2.1
15867	1.01531	2.1
15868	1.66018	2.1
15869	1.01531	2.1
15870	0.886968	2.1
15871	1.22585	0.129926
15872	1.20674	0.123384
15873	1.20672	0.121583
15874	1.02962	0.119828
15875	0.956467	1.23624
15876	2.00551	0.808686
15877	0.553801	2.1
15878	0.617006	2.1
15879	1.26923	2.1
15880	1.23614	2.1
15881	1.23614	2.1
15882	1.16817	2.1
15883	2.14482	2.1
15884	1.72637	1.89998
15885	1.09368	2.1
15886	1.26195	2.1
15887	0.760546	2.1
15888	1.84094	2.1
15889	2.62868	2.1

15890	2.79784	2.1
15891	2.79784	2.1
15892	3.00129	2.1
15893	1.27593	0.777966
15894	1.38877	1.45751
15895	1.06486	0.446695
15896	1.2474	0.296998
15897	1.76234	0.0800206
15898	1.8685	0.0941022
15899	1.69863	0.0939884
15900	1.29666	0.0925043
15901	1.91646	2.1
15902	1.8582	2.1
15903	1.8582	2.1
15904	1.7986	2.1
15905	3.44043	0.0659515
15906	1.94764	0.064318
15907	1.94764	0.0698238
15908	1.36307	0.0672243
15909	1.32075	0.0995347
15910	1.12971	0.0924125
15911	2.06319	0.113877
15912	1.94982	0.0985948
15913	1.77255	0.115703
15914	0.979297	0.0959053
15915	1.59997	2.1
15916	1.93506	2.1
15917	1.94409	2.1
15918	1.9597	2.1
15919	0.57899	2.1
15920	0.953036	2.1
15921	0.953049	2.1
15922	1.53503	2.1
15923	1.39615	0.189
15924	1.06474	0.212346
15925	0.947564	0.254515
15926	1.45506	0.230023
15927	1.17799	0.523173
15928	1.21067	0.380737
15929	1.35945	0.320496
15930	2.05648	0.38325
15931	1.36208	0.331248
15932	2.15229	0.258377
15933	2.89288	0.247591
15934	1.95719	0.263612
15935	2.25169	0.192032
15936	1.52756	0.225242
15937	1.19322	0.460388
15938	1.23146	0.325353
15939	2.45024	0.644299
15940	0.794122	0.373139
15941	1.6343	0.383454
15942	1.34161	0.257344
15943	1.05353	0.484455
15944	1.50795	0.270931
15945	1.05353	0.228549
15946	1.20669	0.247798
15947	1.59289	2.1

15948	0.989543	2.1
15949	1.40588	2.1
15950	0.991427	2.1
15951	0.921822	1.50092
15952	1.46903	0.747176
15953	1.46903	2.1
15954	1.8214	0.550646
15955	1.39702	2.1
15956	1.25103	2.1
15957	1.25103	2.1
15958	1.10378	2.1
15959	1.71813	2.1
15960	1.39595	2.1
15961	1.386 2.1	
15962	1.13008	2.1
15963	1.31711	0.0776232
15964	1.70191	0.0794646
15965	0.893249	0.0824639
15966	1.42669	0.082421
15967	1.23943	0.730782
15968	1.1483	0.67537
15969	1.04598	2.1
15970	1.1483	0.753516
15971	1.00381	0.435532
15972	1.11433	0.39597
15973	0.973501	0.884356
15974	1.0756	0.928256
15975	1.20669	0.239539
15976	1.18508	0.248279
15977	1.18508	0.243471
15978	0.983027	0.254812
15979	1.43687	2.1
15980	1.71059	2.1
15981	1.71059	2.1
15982	1.66762	2.1
15983	2.8415	2.1
15984	2.40555	2.1
15985	1.91546	2.1
15986	1.61385	2.1
15987	2.01569	2.1
15988	1.33183	2.1
15989	1.23218	2.1
15990	1.23999	2.1
15991	1.01335	0.686639
15992	1.12088	0.800326
15993	1.25346	1.48622
15994	1.15644	2.00157
15995	1.3764	0.909681
15996	1.27558	0.725541
15997	1.2064	1.48682
15998	1.15067	2.07883
15999	1.01595	2.1
16000	1.15067	2.09772
16001	1.41902	0.661916
16002	1.30272	2.1
16003	1.30272	0.620711
16004	1.19548	2.1
16005	2.99339	2.1

16006	2.98041	2.1
16007	1.46124	2.1
16008	1.42105	2.1
16009	2.98041	2.1
16010	2.86339	2.1
16011	1.42105	2.1
16012	1.42284	2.1
16013	2.31301	0.914868
16014	1.80273	0.887268
16015	2.17765	0.966022
16016	2.31301	0.842535
16017	1.76378	0.686294
16018	2.10275	0.643158
16019	2.10275	0.688286
16020	2.26047	0.645863
16021	0.97541	2.1
16022	1.40863	2.1
16023	1.40863	2.1
16024	1.30751	2.1
16025	1.20143	0.351425
16026	1.19756	0.301932
16027	1.43707	0.2945
16028	1.28954	0.273635
16029	3.34254	0.148491
16030	1.92916	0.102175
16031	1.80089	0.132723
16032	1.1323	0.106112
16033	1.47321	0.17434
16034	1.23756	0.159249
16035	1.87291	2.08375
16036	1.53642	2.1
16037	1.53642	2.1
16038	1.5791	2.1
16039	1.0446	2.1
16040	0.992674	2.1
16041	1.12324	0.0636863
16042	1.29163	0.0618133
16043	1.29163	0.0610852
16044	1.15371	0.0610638
16045	0.996831	0.179486
16046	1.00192	0.208992
16047	1.0019	0.176738
16048	0.958937	0.203453
16049	1.07283	2.1
16050	1.25294	0.886146
16051	1.25294	0.982855
16052	1.04788	0.963912
16053	1.14496	2.1
16054	1.07652	2.1
16055	1.07652	2.1
16056	0.991406	2.1
16057	1.60976	1.85035
16058	1.37	2.1
16059	1.37	1.78392
16060	1.14195	2.1
16061	1.26593	1.45405
16062	1.75366	2.1
16063	1.57621	0.307587

16064	1.24538	0.331813
16065	0.969118	2.1
16066	1.04147	2.1
16067	0.969118	2.1
16068	1.22615	2.1
16069	0.98084	2.1
16070	1.01843	2.1
16071	1.09435	2.1
16072	1.50876	2.1
16073	1.5976	2.1
16074	1.84322	2.1
16075	1.84324	2.1
16076	1.70377	2.1
16077	1.20709	2.1
16078	2.54729	2.1
16079	0.974716	0.172498
16080	1.54468	0.263131
16081	1.39021	0.163477
16082	1.70303	0.241174
16083	1.22852	1.45886
16084	1.578	1.02108
16085	1.578	1.51529
16086	1.93852	0.768583
16087	2.2506	2.1
16088	1.39302	1.60669
16089	1.5478	2.1
16090	1.2269	2.06289
16091	1.70048	2.1
16092	1.45477	0.888265
16093	1.30929	2.1
16094	1.15069	0.908064
16095	1.00255	1.49586
16096	1.05481	0.436604
16097	1.05481	1.81891
16098	1.1032	0.467627
16099	0.993888	2.1
16100	1.19294	2.1
16101	1.01121	0.265005
16102	0.991866	0.293695
16103	2.20052	0.759528
16104	1.7409	2.1
16105	1.75142	1.62367
16106	1.46811	2.1
16107	1.7409	0.970449
16108	1.23267	2.1
16109	1.46811	2.1
16110	1.18872	2.1
16111	1.12665	2.1
16112	1.50045	2.1
16113	1.50045	2.1
16114	1.37704	2.1
16115	1.35308	0.901633
16116	1.29983	0.522083
16117	1.29983	0.916939
16118	0.970212	0.556027
16119	1.82272	2.1
16120	1.62784	2.1
16121	1.62784	2.1

16122	1.87691	2.1
16123	3.65294	2.1
16124	2.33374	2.1
16125	2.12888	2.1
16126	1.62229	2.1
16127	1.41496	1.98208
16128	1.32707	0.661644
16129	2.18871	0.574416
16130	1.4235	0.297308
16131	1.46015	0.142951
16132	1.25635	0.161431
16133	1.45501	0.264889
16134	1.3543	0.295768
16135	1.38139	1.99937
16136	1.98159	2.1
16137	2.15868	2.03005
16138	4.04308	2.1
16139	1.5362	2.1
16140	1.56328	2.1
16141	1.56327	2.1
16142	1.58163	2.1
16143	1.88987	0.235301
16144	1.08761	0.193189
16145	0.862154	0.162402
16146	1.77688	0.177266
16147	1.14238	1.5794
16148	1.11691	2.1
16149	1.01537	1.38552
16150	1.02923	2.1
16151	0.977425	0.136611
16152	1.15211	0.112504
16153	2.0815	2.1
16154	1.18621	2.1
16155	1.01485	2.1
16156	1.17054	2.1
16157	1.17054	2.1
16158	1.12665	2.1
16159	1.87213	0.0777917
16160	1.82244	0.0790575
16161	1.45039	0.0829353
16162	1.7226	0.0871338
16163	1.841	0.0780457
16164	1.79011	0.078591
16165	1.8792	0.0858868
16166	1.45053	0.0884205
16167	1.08947	2.1
16168	1.5008	2.1
16169	1.5008	2.1
16170	1.83186	2.1
16171	1.04159	0.0925943
16172	1.2489	0.0903473
16173	1.2489	0.0912277
16174	1.33592	0.0878251
16175	1.51973	0.302474
16176	0.980547	0.308566
16177	1.51974	0.334814
16178	1.15991	0.422026
16179	1.34965	2.1

16180	1.2724	2.1
16181	1.2724	2.1
16182	1.38438	2.1
16183	1.02923	1.48938
16184	1.06521	2.1
16185	1.06521	1.64573
16186	1.0752	2.1
16187	2.03139	0.280814
16188	1.92951	0.266563
16189	1.74852	0.301164
16190	1.62772	0.28409
16191	1.73655	0.266098
16192	1.76809	0.24993
16193	1.62772	0.285433
16194	1.1432	0.258446
16195	1.09235	2.1
16196	1.48081	2.1
16197	1.46712	2.1
16198	1.89321	2.1
16199	1.94548	2.1
16200	1.5636	2.1
16201	1.5636	2.1
16202	1.21124	2.1
16203	1.52314	2.1
16204	2.0219	2.1
16205	1.51113	2.1
16206	2.0219	2.1
16207	0.987247	0.472718
16208	1.18347	0.286698
16209	1.89226	0.548319
16210	1.61441	0.287639
16211	1.42884	2.1
16212	1.10961	2.1
16213	1.56991	2.1
16214	1.05337	2.1
16215	1.05337	2.1
16216	1.91547	2.1
16217	1.05931	2.1
16218	1.1729	2.1
16219	1.34526	2.1
16220	1.0593	2.1
16221	1.0856	2.1
16222	1.90854	2.1
16223	1.91674	2.1
16224	1.11345	2.1
16225	1.75067	2.1
16226	1.50022	2.1
16227	1.50022	2.1
16228	1.53619	2.1
16229	1.4748	2.1
16230	1.74431	1.8722
16231	2.02581	1.76765
16232	1.25288	1.70797
16233	1.01814	0.727553
16234	1.2881	0.533171
16235	1.2881	0.593745
16236	1.54433	0.509673
16237	1.19999	0.559483

16238	1.44056	0.390328
16239	1.02011	0.273527
16240	1.17129	0.341159
16241	1.13775	2.00794
16242	3.34815	2.06598
16243	1.60428	1.7718
16244	1.85928	1.89659
16245	1.75592	2.1
16246	1.25989	2.1
16247	1.75592	2.1
16248	1.71549	2.1
16249	1.37634	2.1
16250	1.64693	2.1
16251	1.64693	2.1
16252	1.64193	2.1
16253	1.18351	0.101181
16254	2.19398	0.0913809
16255	2.16272	1.0911
16256	1.19837	1.03145
16257	1.04973	2.1
16258	1.20149	2.1
16259	1.60574	2.1
16260	1.11164	0.759843
16261	1.87823	0.783263
16262	1.11164	0.900214
16263	0.772882	2.1
16264	1.4714	2.1
16265	2.14242	0.73261
16266	1.12336	1.97954
16267	2.8386	0.684198
16268	2.13346	2.04881
16269	1.06788	0.222106
16270	0.947662	0.208594
16271	1.05297	0.170072
16272	1.42907	0.202773
16273	2.17465	2.1
16274	2.18394	2.1
16275	1.14808	2.1
16276	1.21292	2.1
16277	1.21292	2.1
16278	1.27229	2.1
16279	1.20859	0.708987
16280	1.21073	0.84175
16281	1.21073	0.616908
16282	1.22857	0.868582
16283	1.79158	2.1
16284	1.73899	2.1
16285	1.34012	2.1
16286	1.39991	2.1
16287	1.73898	2.1
16288	1.61245	2.1
16289	1.40136	2.1
16290	1.57234	2.1
16291	1.82162	2.1
16292	3.09875	2.1
16293	1.10264	2.1
16294	1.02339	2.1
16295	1.02339	2.1

16296	1.23042	2.1
16297	2.82266	0.67599
16298	1.05698	0.522306
16299	1.96384	0.683317
16300	1.10244	0.63881
16301	0.929061	2.1
16302	1.47007	2.1
16303	0.985303	2.1
16304	1.24554	2.1
16305	1.05993	2.1
16306	2.02074	2.1
16307	2.10106	1.02567
16308	1.18171	0.917581
16309	1.68111	1.00087
16310	1.84226	0.736017
16311	1.82131	0.196733
16312	1.32371	0.181749
16313	1.41902	0.145738
16314	1.00205	0.130145
16315	1.65532	2.1
16316	1.18977	2.1
16317	1.15471	2.1
16318	0.993889	2.1
16319	2.08521	2.1
16320	2.09885	2.1
16321	2.09497	2.1
16322	2.10604	2.1
16323	0.881028	1.34294
16324	1.52828	0.900401
16325	1.40574	0.0770324
16326	1.09599	0.0753915
16327	1.45506	2.1
16328	1.50524	2.1
16329	1.50524	2.1
16330	1.54711	2.1
16331	1.41469	2.1
16332	2.40442	2.1
16333	1.40794	2.1
16334	1.89226	2.1
16335	1.31346	2.1
16336	1.51067	2.1
16337	1.31363	2.1
16338	1.11833	2.1
16339	2.3373	2.1
16340	2.19994	2.1
16341	2.19994	2.1
16342	1.09616	2.1
16343	1.63719	2.1
16344	1.52204	2.1
16345	1.52205	2.1
16346	1.40118	2.1
16347	2.5946	2.1
16348	4.07065	1.97085
16349	1.33916	2.1
16350	2.05217	2.1
16351	1.67023	2.1
16352	2.05217	1.96131
16353	1.59458	0.572331

16354	1.02247	0.520544
16355	1.68108	0.559457
16356	1.03079	0.491684
16357	1.1643	0.256195
16358	1.85795	0.225666
16359	1.70302	0.231645
16360	1.42076	0.178112
16361	1.91351	0.192169
16362	1.01148	0.179335
16363	2.52402	0.0946002
16364	0.752625	0.0905423
16365	0.886968	2.1
16366	1.28845	2.1
16367	1.28845	2.1
16368	1.27766	2.1
16369	1.82268	2.1
16370	1.87118	2.1
16371	1.87119	2.1
16372	1.91704	2.1
16373	3.13938	2.1
16374	2.97839	2.1
16375	2.97839	2.1
16376	2.87331	2.1
16377	1.73443	0.869924
16378	1.59457	0.697128
16379	2.01827	2.1
16380	2.04586	2.1
16381	2.04586	2.1
16382	2.00865	2.1
16383	1.19901	1.5478
16384	1.16661	2.1
16385	1.13215	0.269277
16386	1.96256	0.213139
16387	2.34043	0.0748846
16388	2.04154	0.0729877
16389	1.58991	0.0716614
16390	1.45893	0.0718616
16391	2.56818	0.0751302
16392	1.70532	0.0731055
16393	1.17798	0.427492
16394	1.25796	0.558498
16395	1.25797	0.565403
16396	1.10719	0.724414
16397	1.51067	2.1
16398	1.60707	2.1
16399	2.06946	1.56756
16400	3.19218	2.1
16401	2.19965	2.1
16402	1.30521	2.09206
16403	2.23684	1.09655
16404	1.43363	1.56679
16405	1.51007	2.1
16406	2.46356	2.1
16407	1.90428	2.1
16408	1.13618	2.1
16409	2.13276	0.244283
16410	0.998345	0.21709
16411	1.2861	0.258862

16412	1.20457	0.236651
16413	1.51789	2.1
16414	1.49872	2.1
16415	1.49872	2.1
16416	1.47712	2.1
16417	1.87129	0.370614
16418	0.949364	0.346424
16419	1.83142	0.362851
16420	1.55063	0.370435
16421	1.03298	0.29328
16422	0.980664	0.587338
16423	1.51696	0.254357
16424	1.34927	0.481643
16425	1.70302	0.493608
16426	1.65952	0.346549
16427	1.76658	0.274299
16428	0.986848	0.211411
16429	2.13757	1.28644
16430	1.98142	0.64359
16431	1.98142	1.03019
16432	1.65411	0.668411
16433	1.1323	0.145499
16434	1.46015	0.153617
16435	2.15376	1.90181
16436	1.64622	1.91417
16437	0.991867	0.472207
16438	1.58074	0.378727
16439	1.69368	2.1
16440	1.76403	2.1
16441	1.76403	2.1
16442	1.83318	2.1
16443	1.28418	0.572346
16444	1.16851	0.411136
16445	1.1685	0.436062
16446	1.1726	0.355412
16447	1.06455	2.1
16448	1.18773	2.1
16449	0.98511	2.1
16450	1.18774	2.1
16451	1.76476	2.1
16452	1.79825	2.1
16453	1.79825	2.1
16454	0.961895	2.1
16455	1.59589	0.078727
16456	2.25975	0.0862211
16457	2.76339	2.1
16458	3.0551	2.1
16459	1.68761	2.1
16460	1.59561	2.1
16461	2.17257	2.1
16462	1.05845	2.1
16463	1.03512	2.1
16464	2.19011	2.1
16465	1.44859	1.92751
16466	1.35897	0.803915
16467	1.35897	1.79132
16468	1.17822	1.14204
16469	1.13641	2.1

16470	1.18301	2.1
16471	1.18301	2.05944
16472	1.25173	2.1
16473	2.41297	2.1
16474	1.36463	2.1
16475	1.54711	2.1
16476	1.5735	2.1
16477	1.5735	2.1
16478	1.58713	2.1
16479	1.69044	0.0743864
16480	1.57979	0.0754078
16481	1.57979	0.0744465
16482	1.14387	0.0758342
16483	1.04199	2.1
16484	1.96491	2.1
16485	1.04199	2.1
16486	1.46892	2.1
16487	0.983724	2.1
16488	1.70579	2.1
16489	1.88522	2.1
16490	1.23914	2.02912
16491	1.79424	2.1
16492	1.52851	2.1
16493	1.04921	2.1
16494	1.11285	2.1
16495	1.25334	0.829157
16496	1.07496	0.636594
16497	1.07497	1.94373
16498	1.34852	0.729379
16499	1.27391	2.08797
16500	2.20307	2.1
16501	1.81311	2.1
16502	2.10305	2.1
16503	1.21059	0.394665
16504	1.44253	0.363769
16505	1.9194	0.214221
16506	1.21159	0.309319
16507	0.947979	2.1
16508	1.01225	0.511985
16509	0.947979	2.1
16510	1.01225	0.951282
16511	1.00255	1.89994
16512	1.01225	1.81415
16513	1.01225	2.1
16514	1.06561	1.17377
16515	3.50562	2.1
16516	3.61336	2.1
16517	3.61336	2.1
16518	3.73635	2.1
16519	1.61245	2.1
16520	1.52301	2.1
16521	1.57612	2.1
16522	1.83035	2.1
16523	1.52301	2.1
16524	1.24789	2.1
16525	1.85156	2.1
16526	1.89072	2.1
16527	0.995735	2.1

16528	1.57738	2.1
16529	1.87141	0.130624
16530	1.73388	0.163892
16531	1.61344	0.157154
16532	0.922676	0.194407
16533	1.56614	2.1
16534	0.956294	2.1
16535	1.82589	2.1
16536	1.63719	2.1
16537	1.12399	0.0861803
16538	1.50103	0.0989575
16539	1.56976	0.0915155
16540	1.03567	0.0912496
16541	1.21776	0.0751556
16542	1.89708	0.0750739
16543	1.16323	0.0738582
16544	1.63533	0.0709459
16545	2.29397	0.0755184
16546	3.01596	0.074564
16547	1.80145	0.0732183
16548	1.7666	0.0718385
16549	1.91621	0.776249
16550	1.10719	0.554275
16551	1.57828	1.11556
16552	1.17105	1.21082
16553	1.15806	2.1
16554	1.31565	2.1
16555	1.31565	2.1
16556	1.46643	2.1
16557	1.47507	2.1
16558	1.87858	2.1
16559	0.958661	2.1
16560	1.87858	2.1
16561	1.44293	0.139514
16562	1.43213	0.191134
16563	1.43213	0.138616
16564	1.10476	0.178237
16565	1.03235	1.8348
16566	1.31	0.477803
16567	1.31	1.97325
16568	1.50108	0.804018
16569	1.32215	2.1
16570	1.10611	2.1
16571	1.10611	2.1
16572	0.904978	2.1
16573	1.06486	0.440157
16574	1.18347	0.293479
16575	1.70535	2.1
16576	1.94779	2.1
16577	1.77071	2.1
16578	1.82268	2.1
16579	0.61701	2.1
16580	0.672495	2.1
16581	0.672494	2.1
16582	0.713174	2.1
16583	1.40395	2.1
16584	1.34245	2.1
16585	1.34243	2.1

16586	1.30646	2.1
16587	1.06204	0.31287
16588	0.632691	0.466899
16589	1.75766	0.469803
16590	1.14287	0.684807
16591	1.52101	0.300909
16592	1.71414	0.322421
16593	1.86411	0.284101
16594	1.6253	0.326042
16595	1.04355	0.303624
16596	1.0443	0.340756
16597	1.34852	1.91022
16598	1.56016	0.605913
16599	1.56016	0.639313
16600	1.03235	0.628291
16601	1.2966	0.122175
16602	1.82354	0.10444
16603	1.2966	0.0959089
16604	1.67687	0.0993067
16605	1.19669	1.22134
16606	1.82979	1.44402
16607	1.58972	1.2034
16608	1.09219	1.19759
16609	1.42077	0.962927
16610	1.89226	0.839129
16611	1.19547	0.623972
16612	1.10187	2.1
16613	1.10188	0.631965
16614	1.01479	2.1
16615	1.81663	2.1
16616	1.81533	2.1
16617	1.83247	2.1
16618	1.51511	2.1
16619	0.983148	2.1
16620	1.7513	2.1
16621	1.85017	2.1
16622	1.26103	2.1
16623	1.23152	2.1
16624	1.65508	2.1
16625	1.23152	2.1
16626	2.15082	2.1
16627	1.57974	2.1
16628	1.07289	2.1
16629	0.81534	2.1
16630	1.51007	2.1
16631	1.00898	0.0889933
16632	1.18959	0.092384
16633	1.32178	0.0839198
16634	1.25929	0.084571
16635	2.17892	0.29873
16636	1.22938	0.243176
16637	3.02933	0.533405
16638	2.34619	0.42389
16639	1.47679	0.364196
16640	1.0819	0.383067
16641	3.10558	2.1
16642	3.31457	2.1
16643	0.985109	2.1

16644	1.10667	2.1
16645	3.31457	2.1
16646	3.54443	2.1
16647	1.10667	2.1
16648	1.25987	2.1
16649	1.77063	1.00365
16650	1.51065	0.820969
16651	1.24688	1.74229
16652	1.37652	2.03689
16653	1.37652	1.32354
16654	1.54168	2.1
16655	1.17042	0.925635
16656	1.13092	1.17975
16657	1.13092	1.47842
16658	1.52938	2.02471
16659	1.11787	0.389863
16660	1.00613	0.530441
16661	0.781373	0.416952
16662	1.6885	0.38454
16663	2.49439	0.302097
16664	1.42657	0.366018
16665	1.77903	1.54575
16666	2.63606	2.1
16667	2.10425	2.1
16668	1.21425	1.08531
16669	1.31253	1.30118
16670	1.28418	1.51182
16671	1.28418	1.0589
16672	1.58972	1.16632
16673	0.858948	2.1
16674	1.90196	2.1
16675	1.81024	2.1
16676	1.90194	2.1
16677	1.4263	0.788568
16678	0.947921	0.673743
16679	1.22852	0.864682
16680	1.09194	0.739827
16681	1.60578	0.125292
16682	1.14721	0.127677
16683	1.06336	0.161935
16684	1.14721	0.140761
16685	1.11924	0.0760838
16686	1.53814	0.0788709
16687	1.18421	0.0944842
16688	1.8458	0.0821958
16689	1.7565	1.95404
16690	1.09344	1.30616
16691	1.09344	0.980373
16692	1.16971	1.81258
16693	1.71465	0.0915992
16694	1.19412	0.0839199
16695	1.64985	0.140932
16696	1.05417	0.122188
16697	1.2127	0.0739713
16698	1.48867	0.0735919
16699	1.27806	0.0774471
16700	1.21269	0.0723463
16701	1.37813	1.00836

16702	1.0417	1.07756
16703	1.88626	0.764705
16704	1.14977	1.0262
16705	1.78803	1.47686
16706	1.33793	1.108
16707	1.42266	1.29875
16708	1.26865	1.40092
16709	1.09518	2.1
16710	1.36844	2.1
16711	1.20455	2.1
16712	1.36844	2.1
16713	1.22297	0.999968
16714	1.11256	1.09748
16715	1.11256	0.939151
16716	1.03217	1.00893
16717	1.18422	0.0872474
16718	1.4282	0.0910623
16719	1.04159	0.101303
16720	1.4282	0.0873196
16721	0.988805	2.1
16722	1.05481	2.1
16723	1.52938	0.798649
16724	1.26593	1.22114
16725	1.53082	2.1
16726	1.09847	2.1
16727	1.75211	0.633801
16728	1.22222	1.58128
16729	1.75211	0.568247
16730	1.51592	1.38557
16731	1.63413	0.727275
16732	1.26662	0.743178
16733	1.42495	0.552322
16734	1.4356	0.621232
16735	1.29389	1.50429
16736	1.04164	2.00145
16737	1.40574	1.28434
16738	1.04164	1.07307
16739	2.22344	0.628312
16740	1.45945	0.329706
16741	0.987248	0.47515
16742	1.2474	0.316486
16743	0.974702	0.754846
16744	1.43777	0.593664
16745	1.71289	2.1
16746	1.91529	2.1
16747	1.91529	2.1
16748	2.01827	2.1
16749	1.87111	1.06936
16750	1.00864	0.614132
16751	1.26865	0.705181
16752	1.18769	0.990066
16753	1.24167	1.82626
16754	1.36133	0.689486
16755	2.28168	2.1
16756	3.98524	1.91191
16757	1.36133	1.84603
16758	1.69436	0.813354
16759	2.21926	1.23812

16760	1.70302	1.73589
16761	1.50732	0.159744
16762	1.42237	0.17201
16763	1.18659	0.179963
16764	1.50732	0.187453
16765	1.19785	0.118984
16766	1.40969	0.105659
16767	1.28339	0.139587
16768	1.40882	0.132093
16769	0.968595	0.882584
16770	1.41903	0.667913
16771	1.12567	2.1
16772	1.15898	2.1
16773	1.27241	2.1
16774	0.995796	2.1
16775	1.89983	0.0805189
16776	1.05417	0.0786489
16777	0.571999	0.0739137
16778	0.743597	0.0742623
16779	1.89353	0.0784285
16780	0.924068	0.0740977
16781	1.52851	2.1
16782	1.26969	2.1
16783	1.07763	0.321577
16784	1.82301	0.298046
16785	1.07763	0.20672
16786	1.17506	0.22325
16787	1.20045	2.1
16788	1.12486	2.1
16789	1.15234	2.1
16790	1.11598	2.1
16791	1.12486	2.1
16792	1.0491	2.1
16793	1.11598	2.1
16794	1.1047	2.1
16795	1.05902	2.1
16796	1.14361	2.1
16797	1.14361	2.1
16798	1.26851	2.1
16799	1.29389	1.09269
16800	0.988456	1.0531
16801	1.83688	0.0839772
16802	1.70302	0.1004
16803	1.49386	0.0906024
16804	1.83151	0.100391
16805	1.01346	0.590411
16806	0.983607	0.655493
16807	0.983596	2.1
16808	1.08332	0.791979
16809	2.36848	0.313231
16810	2.97066	0.270255
16811	1.12352	0.25186
16812	2.5311	0.203513
16813	1.09062	2.1
16814	0.962182	2.1
16815	0.962181	2.1
16816	1.2258	2.1
16817	0.974888	2.1

16818	1.06312	2.1
16819	1.73583	0.102679
16820	1.72399	0.100769
16821	1.724	0.128297
16822	1.50951	0.122477
16823	1.26755	2.1
16824	1.03824	2.1
16825	1.27875	2.1
16826	1.03824	2.1
16827	1.18902	2.1
16828	1.534	2.1
16829	1.11764	2.1
16830	1.534	2.1
16831	1.52008	2.1
16832	1.34406	2.1
16833	1.34406	2.1
16834	1.2187	2.1
16835	0.963571	0.828063
16836	1.19612	0.761616
16837	0.963571	1.53216
16838	1.00007	1.51409
16839	0.903333	2.1
16840	1.12724	2.1
16841	1.23771	2.1
16842	1.17683	2.1
16843	1.20059	0.712193
16844	0.772023	0.448852
16845	1.14749	0.772862
16846	1.2802	0.779928
16847	1.85091	1.17834
16848	2.11193	0.974708
16849	1.2802	0.654664
16850	1.42913	0.74101
16851	2.11193	0.885814
16852	1.27737	0.565527
16853	1.30584	2.1
16854	1.60936	2.1
16855	1.3413	0.15901
16856	1.53336	0.116678
16857	2.60679	0.128021
16858	1.3413	0.112165
16859	1.35877	1.49373
16860	1.11024	1.72104
16861	1.27839	2.1
16862	1.08171	2.1
16863	1.27841	2.1
16864	1.0577	2.1
16865	1.71255	2.1
16866	1.10611	2.1
16867	0.960106	2.1
16868	1.40921	2.1
16869	1.90705	0.44347
16870	2.79564	0.402646
16871	1.45321	0.437971
16872	1.90705	0.432949
16873	0.974718	0.294974
16874	1.53671	0.277179
16875	1.69039	0.284997

16876	1.78229	0.263494
16877	1.0101	2.1
16878	1.3167	2.1
16879	1.19699	2.1
16880	1.3212	2.1
16881	1.60811	0.354387
16882	1.43224	0.301703
16883	1.35401	0.289602
16884	1.17464	0.233772
16885	1.27696	2.1
16886	1.05025	2.1
16887	1.05025	2.1
16888	1.18076	2.1
16889	1.37224	1.33095
16890	1.13438	1.28016
16891	1.76788	1.22354
16892	1.73444	1.18732
16893	1.37704	2.1
16894	1.77042	2.1
16895	1.77042	2.1
16896	1.09778	2.1
16897	1.28667	2.1
16898	1.28228	2.1
16899	1.30646	0.770601
16900	1.68867	0.895436
16901	1.30645	1.17574
16902	0.986494	1.93426
16903	1.6253	0.296043
16904	1.48676	0.321853
16905	1.48676	0.305009
16906	1.32974	0.316587
16907	0.634347	0.0522355
16908	1.15142	0.0749204
16909	1.11395	0.0709035
16910	1.18068	0.0697417
16911	1.36904	0.0654332
16912	0.764563	0.0628569
16913	2.09213	0.0628112
16914	1.56631	2.1
16915	1.08011	2.1
16916	1.80283	2.1
16917	1.50738	2.1
16918	1.44951	0.236173
16919	1.50102	0.239063
16920	1.50102	0.208643
16921	1.39615	0.213559
16922	1.02653	0.400352
16923	2.98258	0.468494
16924	2.0815	0.0929407
16925	1.39525	0.079111
16926	1.82176	0.0802925
16927	0.977607	0.0824171
16928	1.96133	1.32955
16929	1.27189	1.09795
16930	0.826032	0.903522
16931	2.12831	0.861113
16932	1.77798	1.81501
16933	1.02969	1.44244

16934	1.06463	1.16616
16935	1.49219	1.93018
16936	1.10003	2.1
16937	1.21223	1.9525
16938	1.00513	1.1119
16939	1.43788	1.35938
16940	1.47134	0.580291
16941	0.968249	0.620542
16942	3.22585	0.155147
16943	3.36933	0.131762
16944	3.36932	0.167846
16945	3.03865	0.111431
16946	1.66295	0.173685
16947	1.65583	0.15271
16948	1.65583	0.221819
16949	1.4732	0.147939
16950	1.73704	0.0522355
16951	1.76476	0.847535
16952	2.13345	0.764924
16953	1.55816	2.04711
16954	1.55816	0.84532
16955	1.02601	2.1
16956	1.08039	1.45775
16957	1.05498	0.906487
16958	1.08039	2.1
16959	1.2873	2.1
16960	1.01121	1.8944
16961	0.987703	2.06216
16962	0.98771	1.76949
16963	0.964782	2.04974
16964	1.35719	2.1
16965	2.61092	2.1
16966	2.6607	2.1
16967	3.41693	2.1
16968	1.23125	2.1
16969	1.73772	2.1
16970	0.99845	0.585491
16971	1.5176	0.641856
16972	1.13271	0.118321
16973	1.332	0.11341
16974	1.63159	2.1
16975	1.61342	2.1
16976	1.03679	2.1
16977	1.19087	2.1
16978	1.97172	2.1
16979	2.68634	2.1
16980	1.19086	2.1
16981	1.50091	2.1
16982	2.84677	2.1
16983	2.77894	2.1
16984	2.77894	2.1
16985	2.74206	2.1
16986	1.3284	2.1
16987	1.83524	2.1
16988	1.65853	2.1
16989	1.56741	1.83393
16990	1.00509	2.1
16991	1.45246	2.1

16992	1.26193	2.1
16993	1.45244	2.1
16994	1.61963	0.328983
16995	1.68189	0.595231
16996	1.68189	0.934859
16997	1.49155	0.655021
16998	1.58054	2.1
16999	1.59169	2.1
17000	1.59169	2.1
17001	1.60497	2.1
17002	0.743595	0.0760834
17003	0.702359	0.0756868
17004	1.15317	0.0876285
17005	2.24367	0.0859194
17006	1.15252	0.0847455
17007	2.60354	0.0777011
17008	1.45158	2.1
17009	1.56004	2.1
17010	1.64527	2.1
17011	1.00371	2.1
17012	1.38287	0.476616
17013	0.893024	0.599665
17014	1.23527	0.614399
17015	1.23706	0.577438
17016	2.20993	0.49184
17017	1.54858	1.06149
17018	0.798666	0.312993
17019	1.22487	0.356126
17020	1.33158	0.290871
17021	1.85902	0.353303
17022	1.08334	2.1
17023	1.17885	0.979945
17024	1.17885	0.730852
17025	1.07283	0.912119
17026	1.30723	0.0806261
17027	1.62507	0.073985
17028	1.62507	0.0712446
17029	1.08854	0.074024
17030	1.59475	2.1
17031	1.58874	2.1
17032	1.58875	2.1
17033	1.35787	2.1
17034	1.04147	2.02577
17035	1.15564	1.54566
17036	1.78301	2.1
17037	1.90618	2.1
17038	1.71555	2.1
17039	2.13143	2.1
17040	1.10817	2.1
17041	1.032	2.1
17042	1.032	2.1
17043	0.953636	2.1
17044	1.27916	1.03093
17045	1.11938	0.935288
17046	1.11937	0.906099
17047	1.9186	1.15053
17048	1.19967	0.17556
17049	1.63489	0.181298

17050	1.00763	0.213053
17051	1.81656	0.215709
17052	1.31814	0.165239
17053	2.98183	0.182263
17054	1.20149	2.1
17055	1.29129	2.1
17056	1.29129	2.1
17057	1.37138	2.1
17058	1.23104	1.03353
17059	1.75236	1.37892
17060	1.6	0.572902
17061	1.60565	0.824754
17062	1.60565	0.748629
17063	1.40013	0.868065
17064	1.23042	2.1
17065	1.18278	2.1
17066	1.10853	2.1
17067	1.04724	2.09013
17068	1.1766	2.1
17069	1.00319	2.1
17070	1.29435	2.1
17071	1.00319	2.1
17072	1.05655	1.41497
17073	1.01606	1.4801
17074	1.80391	2.1
17075	2.7699	2.1
17076	1.52073	2.1
17077	2.06835	2.1
17078	2.00202	2.1
17079	2.04906	2.1
17080	0.968536	2.1
17081	1.02461	2.1
17082	1.59784	2.1
17083	1.78249	2.1
17084	1.81409	2.1
17085	1.79844	2.04035
17086	1.77093	1.81565
17087	2.02356	1.4171
17088	1.32449	2.1
17089	1.10003	2.1
17090	1.16915	0.732362
17091	1.05937	0.524992
17092	1.28096	2.06411
17093	1.34696	1.72211
17094	1.34695	2.1
17095	1.36457	1.78518
17096	1.06478	2.1
17097	1.06239	2.1
17098	1.06239	2.1
17099	1.09629	2.1
17100	0.798209	1.86542
17101	1.8532	2.1
17102	2.10112	0.162704
17103	2.8704	0.143896
17104	2.8704	0.149809
17105	2.92457	0.134805
17106	1.0752	1.81621
17107	1.07745	2.1

17108	1.07745	1.91008
17109	1.08911	2.1
17110	1.40182	2.1
17111	1.00133	2.1
17112	1.40182	2.1
17113	1.24509	2.1
17114	2.22243	0.0768293
17115	1.07303	0.0788059
17116	1.29736	0.0865841
17117	1.73584	0.0871471
17118	2.0815	0.569833
17119	1.07999	0.495094
17120	2.1692	2.1
17121	1.99992	2.1
17122	1.99992	2.1
17123	1.78327	2.1
17124	1.52661	0.0832489
17125	0.956003	0.0737706
17126	1.53172	0.0842215
17127	0.955991	0.0771101
17128	1.35025	0.845978
17129	1.51084	2.01039
17130	1.51084	0.918048
17131	1.67115	2.1
17132	2.00977	2.1
17133	1.63632	2.1
17134	1.63632	2.1
17135	2.66128	2.1
17136	1.13525	2.1
17137	2.37566	2.1
17138	1.25987	2.1
17139	1.4472	2.1
17140	1.4472	2.1
17141	1.66018	2.1
17142	0.994001	1.01368
17143	1.83151	0.722493
17144	1.83151	0.522536
17145	1.57621	0.568511
17146	1.40915	0.262751
17147	1.24653	0.522198
17148	1.24653	0.132449
17149	1.10609	0.678978
17150	1.61121	2.1
17151	1.74472	2.1
17152	1.74472	2.1
17153	1.0368	2.1
17154	1.04788	2.1
17155	1.28522	1.88184
17156	1.28522	2.1
17157	1.54196	1.28278
17158	1.23387	2.1
17159	0.896519	2.1
17160	1.60428	2.1
17161	1.08467	2.1
17162	1.56875	0.554796
17163	1.39072	0.261499
17164	1.39073	0.386422
17165	1.6	0.68863

17166	1.01346	1.8266
17167	1.06838	0.950113
17168	0.968463	0.934175
17169	1.06837	0.957807
17170	3.22433	0.396379
17171	2.05993	1.30978
17172	2.01836	0.355603
17173	1.66154	1.37947
17174	1.20871	1.1879
17175	1.10586	2.1
17176	1.10586	0.974913
17177	1.03853	2.1
17178	1.12076	2.1
17179	1.03899	2.1
17180	1.03899	2.1
17181	0.963973	2.1
17182	0.987132	2.1
17183	1.0894	2.1
17184	2.37823	1.9402
17185	1.75647	1.47462
17186	1.64625	2.1
17187	1.56195	2.1
17188	1.41995	2.1
17189	1.15171	2.1
17190	1.36457	2.1
17191	1.33847	1.65639
17192	1.33847	2.1
17193	0.869601	1.45364
17194	1.42255	2.1
17195	1.3868	2.1
17196	1.3868	2.1
17197	1.33962	2.1
17198	1.05481	2.1
17199	1.08074	2.1
17200	1.08074	2.1
17201	0.857557	2.1
17202	1.13537	1.49762
17203	1.09194	0.783779
17204	1.01179	1.61246
17205	1.13537	0.663264
17206	1.15645	1.86043
17207	1.56409	0.320283
17208	1.56409	0.887234
17209	1.16239	1.0783
17210	1.10746	0.443541
17211	1.47952	0.544212
17212	1.14733	1.71149
17213	3.77068	2.1
17214	1.38497	2.1
17215	1.54599	2.1
17216	1.54601	2.1
17217	1.27697	2.1
17218	1.01393	1.6903
17219	1.10558	1.77607
17220	0.995012	1.05168
17221	1.17624	1.45145
17222	1.60497	2.1
17223	1.61588	2.1

17224	1.61587	2.1
17225	1.62182	2.1
17226	1.48866	0.074281
17227	1.65977	0.0736005
17228	1.65977	0.0745719
17229	1.69044	0.0748507
17230	0.961892	2.1
17231	1.00659	2.1
17232	1.0066	2.1
17233	1.06226	2.1
17234	1.15171	2.1
17235	1.50853	0.938695
17236	1.50853	2.1
17237	1.2955	2.1
17238	1.3204	0.162283
17239	2.13295	0.144406
17240	2.75524	0.115362
17241	1.18156	0.0929931
17242	1.73729	2.1
17243	1.20356	2.1
17244	1.62483	2.1
17245	1.52008	2.1
17246	1.19964	2.1
17247	1.0577	2.1
17248	1.19964	2.1
17249	1.07156	2.1
17250	1.98241	2.1
17251	1.43687	2.08507
17252	1.27863	1.70808
17253	1.19915	1.15539
17254	0.97269	1.196
17255	1.0739	1.00883
17256	1.1427	1.9248
17257	1.22476	0.951624
17258	1.06584	1.36004
17259	1.22626	1.09337
17260	1.31388	2.1
17261	1.16932	2.1
17262	1.28819	2.1
17263	1.0499	2.1
17264	1.55894	2.1
17265	1.78709	2.1
17266	1.75986	2.1
17267	1.764 2.1	
17268	1.44801	2.1
17269	1.28186	2.1
17270	1.20797	1.95796
17271	1.06821	1.9544
17272	1.87287	0.887938
17273	2.08014	1.03454
17274	1.63225	1.08045
17275	1.39501	1.34128
17276	1.20132	0.0942233
17277	1.16182	0.096307
17278	1.08848	0.102525
17279	1.20132	0.101171
17280	1.23475	1.0838
17281	1.16661	1.50908

17282	2.31317	2.1
17283	2.50145	2.1
17284	1.04367	2.1
17285	2.23875	2.1
17286	0.953632	0.201374
17287	1.38646	0.246803
17288	1.38646	0.267965
17289	1.61963	0.224132
17290	1.62934	1.19261
17291	1.88626	1.0458
17292	1.0775	0.683219
17293	1.119	0.563716
17294	1.08133	0.462233
17295	1.48312	0.394251
17296	1.57125	0.255067
17297	1.74098	0.248667
17298	1.94372	1.0369
17299	1.22237	1.10833
17300	1.18549	0.382803
17301	1.01294	0.356284
17302	1.01294	0.555563
17303	1.2064	0.608107
17304	1.14599	1.24664
17305	1.00065	0.492187
17306	1.76357	2.1
17307	2.23468	2.1
17308	2.21735	2.1
17309	3.32769	2.1
17310	1.19647	0.866884
17311	1.35307	0.551462
17312	1.19645	0.602727
17313	1.43778	0.526394
17314	1.34412	0.392415
17315	1.1315	1.11117
17316	1.1315	0.482056
17317	1.23943	0.449898
17318	1.57829	2.1
17319	1.22537	2.1
17320	1.14357	2.1
17321	1.32772	2.07166
17322	1.4259	2.1
17323	1.34245	2.1
17324	1.47953	2.1
17325	1.70303	2.1
17326	2.21127	0.138398
17327	1.47904	0.131463
17328	1.18793	0.128427
17329	2.21126	0.14513
17330	1.1032	1.48686
17331	1.14986	0.592691
17332	1.14986	1.92983
17333	1.19611	0.736211
17334	1.91883	2.1
17335	1.83019	2.1
17336	1.83019	2.1
17337	1.82462	2.1
17338	1.27362	0.255427
17339	1.27421	0.256353

17340	1.27421	0.182848
17341	1.10736	0.227132
17342	1.3003	0.580054
17343	1.31415	1.12316
17344	1.31415	0.600775
17345	1.32201	1.26637
17346	1.21124	2.1
17347	0.81568	2.1
17348	0.81568	2.1
17349	0.551816	2.1
17350	1.67119	2.00976
17351	1.63384	2.1
17352	1.63384	2.1
17353	1.63661	2.1
17354	1.48734	2.1
17355	1.52245	2.1
17356	1.52245	2.1
17357	1.50714	2.1
17358	0.996778	0.111902
17359	1.56091	0.104758
17360	1.56091	0.110947
17361	1.90457	0.107453
17362	1.1181	2.1
17363	1.06543	2.1
17364	2.66828	2.1
17365	2.76932	2.1
17366	1.06543	2.1
17367	1.01474	2.1
17368	2.76932	2.1
17369	2.86704	2.1
17370	1.29724	0.353097
17371	1.96938	0.317461
17372	1.82462	2.1
17373	1.84553	2.1
17374	1.05556	2.1
17375	1.25363	2.1
17376	1.84552	2.1
17377	1.53208	2.1
17378	1.12826	2.1
17379	1.39075	2.1
17380	0.9702	0.915328
17381	1.16481	0.630608
17382	1.08894	0.913393
17383	1.91756	0.998715
17384	1.50865	0.68803
17385	1.06012	0.498575
17386	1.5456	0.206315
17387	1.37427	0.20894
17388	1.1211	0.130601
17389	1.5456	0.145967
17390	1.33135	0.186088
17391	1.07956	0.223626
17392	2.23255	0.194629
17393	1.21031	0.15018
17394	1.52903	2.1
17395	1.35977	2.1
17396	3.3837	2.1
17397	3.20581	2.1

17398	1.35977	2.1
17399	1.15414	2.1
17400	3.20582	2.1
17401	2.07596	2.1
17402	1.6812	0.156665
17403	1.03939	0.135216
17404	1.30047	2.1
17405	1.5586	2.1
17406	1.5586	2.1
17407	1.84214	2.1
17408	1.31825	0.5495
17409	1.31923	1.1721
17410	1.31924	0.558675
17411	1.33026	0.848796
17412	1.15818	0.817626
17413	1.17822	1.0768
17414	1.15818	1.94994
17415	1.28096	1.22369
17416	1.06848	0.574476
17417	1.89639	0.564011
17418	1.9186	0.947387
17419	1.77065	0.86386
17420	1.59103	0.791953
17421	1.56164	0.6983
17422	1.09697	0.0603512
17423	2.38719	0.0621028
17424	2.22123	0.0571578
17425	2.46059	0.0579223
17426	1.45506	0.29662
17427	1.03645	0.313067
17428	1.30347	2.1
17429	1.2627	2.1
17430	1.2627	2.1
17431	1.23036	2.1
17432	1.41469	2.1
17433	1.45257	2.1
17434	1.46907	0.098532
17435	1.70302	0.0983714
17436	1.49196	0.0816557
17437	1.36307	0.0847051
17438	1.05677	2.1
17439	0.978698	2.1
17440	0.978699	2.1
17441	2.01822	2.1
17442	1.47446	2.1
17443	1.55432	2.1
17444	1.55432	2.1
17445	1.06053	2.1
17446	1.86813	1.20009
17447	1.21892	1.56341
17448	1.53064	0.986613
17449	1.30664	1.07136
17450	1.09629	2.1
17451	1.19244	2.1
17452	1.19244	2.1
17453	1.37635	2.1
17454	1.49358	0.461598
17455	1.57702	0.279916

17456	2.09374	2.1
17457	1.88175	2.1
17458	1.88175	2.1
17459	1.51085	2.1
17460	1.10938	0.301602
17461	1.02478	0.400853
17462	1.33061	0.141812
17463	1.13272	0.115906
17464	1.75505	2.1
17465	1.78529	2.1
17466	1.6353	2.1
17467	1.59307	2.1
17468	1.60017	0.701308
17469	1.35608	0.848022
17470	1.35608	0.555684
17471	1.04713	0.561137
17472	1.01479	0.457162
17473	1.3868	0.400418
17474	1.3868	0.382816
17475	1.28418	0.662101
17476	2.15949	2.1
17477	2.90429	2.1
17478	1.51902	2.1
17479	1.54755	2.1
17480	2.90429	2.1
17481	3.49966	2.1
17482	1.70842	2.1
17483	1.97526	2.1
17484	0.949712	0.321912
17485	1.92784	0.296388
17486	1.34093	0.308255
17487	2.53426	0.358384
17488	1.92783	0.293209
17489	3.89373	0.237735
17490	2.53426	0.333617
17491	2.35961	0.343129
17492	1.68108	0.501166
17493	1.15558	0.481832
17494	1.67265	0.472621
17495	1.27608	0.448865
17496	1.42249	2.1
17497	1.39708	2.1
17498	1.39708	2.1
17499	1.39327	2.1
17500	1.97046	0.143897
17501	1.11688	0.126352
17502	1.28418	0.380148
17503	1.17302	0.636886
17504	1.17302	0.386879
17505	1.04349	0.620946
17506	0.979853	2.1
17507	1.26016	2.1
17508	1.18901	2.1
17509	1.26016	2.1
17510	1.22857	0.610393
17511	1.25848	0.808277
17512	1.25848	0.581083
17513	1.2858	0.75158

17514	1.09154	2.1
17515	2.01915	2.1
17516	1.23013	2.1
17517	1.55698	2.1
17518	1.55698	2.1
17519	1.8575	2.1
17520	2.01854	2.08671
17521	1.20686	2.01733
17522	1.20684	1.64548
17523	1.77796	1.99893
17524	1.46129	2.1
17525	1.04193	2.1
17526	1.10051	2.1
17527	0.983723	2.1
17528	1.1729	2.1
17529	0.98505	2.1
17530	0.985052	2.1
17531	1.03951	2.1
17532	1.03949	2.1
17533	1.17583	2.1
17534	1.42866	2.1
17535	1.97261	2.1
17536	1.73274	2.1
17537	1.53629	2.1
17538	1.98182	2.1
17539	1.72511	2.1
17540	1.42174	2.1
17541	1.44472	2.1
17542	1.72511	2.1
17543	1.45411	2.1
17544	1.44472	2.1
17545	1.43836	2.1
17546	0.962703	0.492476
17547	1.0171	0.623068
17548	1.53336	0.129563
17549	1.40274	0.125014
17550	1.40272	0.130734
17551	1.00213	0.123622
17552	1.50892	0.13206
17553	1.64984	0.11809
17554	1.67464	0.136636
17555	1.67481	0.132159
17556	1.08212	2.1
17557	1.28095	2.1
17558	1.46956	2.1
17559	1.70303	2.1
17560	1.2925	0.0792192
17561	1.84716	0.0716436
17562	1.48676	0.256405
17563	1.48797	0.250968
17564	1.33917	0.244066
17565	1.22691	0.233814
17566	0.528744	2.1
17567	1.13728	2.1
17568	1.13728	2.1
17569	1.99843	2.1
17570	1.17464	0.2321
17571	1.0372	0.148642

17572	2.0815	0.124735
17573	0.933462	0.11118
17574	1.54931	2.1
17575	1.5485	2.1
17576	1.4077	2.1
17577	1.29931	2.1
17578	1.14931	0.485798
17579	2.02223	0.474303
17580	1.04482	0.330256
17581	0.94971	0.417093
17582	1.54632	1.24435
17583	1.15693	1.32504
17584	1.66763	2.1
17585	1.41238	2.1
17586	1.41238	2.1
17587	1.48068	2.1
17588	1.0171	0.408461
17589	1.1211	0.595896
17590	1.1211	0.358352
17591	1.28418	0.563662
17592	0.993021	2.1
17593	1.764 2.1	
17594	0.993023	2.1
17595	1.05787	2.1
17596	1.90914	0.38099
17597	1.12229	1.26341
17598	1.02025	0.556118
17599	0.585368	1.49834
17600	1.99323	2.1
17601	2.34988	2.1
17602	2.34988	2.1
17603	2.36987	2.1
17604	1.23107	0.133177
17605	1.90664	0.163838
17606	1.53496	0.132902
17607	1.70302	0.167956
17608	1.98859	2.1
17609	1.61057	2.1
17610	1.79844	2.1
17611	2.10811	2.1
17612	1.4618	2.1
17613	1.96722	2.1
17614	1.95191	2.1
17615	2.13418	2.1
17616	1.50368	0.104938
17617	1.61417	0.0850883
17618	1.15008	0.0960589
17619	1.19685	0.0963442
17620	1.90456	0.113759
17621	1.90899	0.104895
17622	2.08864	0.112228
17623	2.10447	0.0991912
17624	1.16653	2.1
17625	1.23017	2.1
17626	1.0752	0.441941
17627	1.34412	0.369186
17628	1.06226	2.1
17629	1.09252	2.1

17630	1.09252	2.1
17631	0.88579	2.1
17632	1.56178	0.235476
17633	1.14305	0.226587
17634	1.56175	0.20638
17635	1.08211	0.17621
17636	1.32813	2.1
17637	1.32461	2.1
17638	1.32461	2.1
17639	1.32455	2.1
17640	1.42729	0.178373
17641	1.18716	0.178758
17642	1.47169	0.249863
17643	1.24687	0.228015
17644	1.09627	2.1
17645	1.04759	2.1
17646	1.04759	2.1
17647	0.987132	2.1
17648	1.11181	1.40253
17649	1.80662	2.1
17650	1.8066	2.02283
17651	1.06461	1.66339
17652	1.56091	2.1
17653	1.9842	2.1
17654	1.73525	0.203502
17655	1.40557	0.19686
17656	2.26906	0.224501
17657	1.31248	0.272953
17658	1.66832	0.187548
17659	1.32634	0.161579
17660	1.03314	0.0750281
17661	1.24682	0.0743043
17662	1.38536	0.0754876
17663	1.52661	0.0735043
17664	1.177	0.967252
17665	1.36405	0.822646
17666	1.19837	0.909104
17667	1.17701	0.415366
17668	0.993831	0.326305
17669	1.0823	0.290555
17670	1.0823	0.29494
17671	1.01121	0.301896
17672	1.49653	0.152792
17673	1.89583	0.20498
17674	0.67242	0.137844
17675	1.36047	0.189924
17676	1.00366	0.190439
17677	1.24728	0.216398
17678	1.13387	0.0973418
17679	1.60867	0.104014
17680	1.30572	0.130285
17681	1.54982	0.111203
17682	0.788592	0.0928287
17683	1.82961	0.0965884
17684	1.54982	0.119306
17685	1.82961	0.100906
17686	1.26062	0.0810078
17687	1.64002	0.080278

17688	1.12035	0.0824325
17689	1.64002	0.0896455
17690	1.1673	2.1
17691	1.75367	2.1
17692	0.964034	1.79462
17693	1.0305	1.50817
17694	1.30896	1.63641
17695	1.52776	1.54762
17696	1.52776	2.1
17697	1.17989	1.80625
17698	1.16759	2.1
17699	1.8575	2.1
17700	1.16759	2.1
17701	1.60936	2.1
17702	1.10747	0.262592
17703	1.47688	0.2679
17704	1.40794	2.1
17705	1.95729	1.30839
17706	1.95729	2.1
17707	3.18156	1.61856
17708	1.87598	0.936663
17709	1.94343	2.1
17710	1.94343	0.907318
17711	2.00551	2.1
17712	1.20067	1.0448
17713	1.70744	1.60502
17714	1.87244	1.54043
17715	1.32075	1.22991
17716	1.64955	2.1
17717	1.44221	2.1
17718	1.31109	2.1
17719	1.14206	2.1
17720	1.60452	1.65772
17721	1.12111	0.806759
17722	1.00832	2.1
17723	0.974137	2.1
17724	0.974137	2.1
17725	0.98505	2.1
17726	1.02368	2.1
17727	0.985051	2.1
17728	0.995734	0.317113
17729	1.31993	0.381708
17730	1.31993	0.327415
17731	1.18549	0.419547
17732	1.5176	0.616021
17733	1.33622	0.559375
17734	1.33621	0.579044
17735	1.02782	0.54159
17736	1.66774	2.1
17737	1.85305	2.1
17738	1.85305	2.1
17739	2.07088	2.1
17740	1.78873	2.1
17741	1.47533	2.1
17742	1.23152	2.1
17743	0.99922	2.1
17744	1.48493	2.1
17745	1.1394	2.1

17746	1.19179	2.1
17747	1.64289	2.1
17748	1.47858	2.1
17749	1.89687	2.1
17750	0.972464	2.1
17751	1.79473	2.1
17752	1.06971	2.1
17753	1.21089	2.1
17754	1.12572	2.1
17755	1.30566	2.1
17756	1.30566	2.1
17757	1.52724	2.1
17758	2.32237	0.376659
17759	2.20248	0.315064
17760	2.79564	0.425111
17761	2.32237	0.37665
17762	1.59354	0.265758
17763	3.098	0.244389
17764	3.09801	0.24431
17765	3.45132	0.222368
17766	1.24001	1.76234
17767	1.41822	0.728465
17768	1.41821	2.03902
17769	1.44859	0.854948
17770	1.34539	2.1
17771	1.06991	1.79199
17772	1.18076	2.1
17773	0.978586	2.1
17774	0.999189	0.198531
17775	2.41076	0.228816
17776	0.968475	1.89996
17777	1.02074	1.95153
17778	1.02074	1.76632
17779	1.07866	1.86392
17780	0.817702	0.170847
17781	1.5082	0.177576
17782	1.48376	0.214841
17783	1.18947	0.188457
17784	1.10638	2.1
17785	1.86766	2.1
17786	1.19785	2.1
17787	1.34141	2.1
17788	1.34141	2.1
17789	0.991985	2.1
17790	2.35516	0.696546
17791	1.24815	0.741569
17792	1.80454	2.06963
17793	1.22633	2.1
17794	0.696255	2.1
17795	1.19379	0.784483
17796	1.14721	2.1
17797	1.16153	2.1
17798	1.16153	2.1
17799	1.17261	2.1
17800	1.20814	2.1
17801	1.54791	2.1
17802	1.54791	2.1
17803	1.60477	2.1

17804	1.29931	2.1
17805	1.19098	2.1
17806	1.19098	2.1
17807	1.13341	2.1
17808	1.00503	1.79577
17809	1.25334	0.620592
17810	1.22655	2.1
17811	1.26282	2.03076
17812	1.26282	2.02769
17813	0.999489	1.35516
17814	1.54197	0.23183
17815	1.17152	0.24478
17816	1.17152	0.227189
17817	1.49627	0.2045
17818	1.36405	0.833326
17819	0.976334	0.662208
17820	1.12495	2.1
17821	1.54096	2.1
17822	1.75263	2.1
17823	1.49114	2.1
17824	0.842998	1.76171
17825	1.60244	0.855631
17826	1.48911	1.56381
17827	2.18685	0.931825
17828	1.75236	1.27575
17829	1.54143	1.58717
17830	1.40013	0.811623
17831	0.946646	0.902362
17832	1.50714	2.1
17833	1.46055	2.1
17834	1.46055	2.1
17835	1.42249	2.1
17836	2.12839	0.191223
17837	1.75211	0.170652
17838	1.76297	0.169473
17839	1.70736	0.159938
17840	1.19952	0.27598
17841	1.56475	0.229946
17842	1.74312	0.183446
17843	1.48973	0.166668
17844	1.91518	0.249008
17845	1.16534	0.267549
17846	1.53556	2.1
17847	1.56253	2.1
17848	1.56253	2.1
17849	1.58054	2.1
17850	1.27806	0.0700763
17851	1.44593	0.0700352
17852	1.44593	0.0741863
17853	1.3946	0.0718194
17854	1.34857	0.426243
17855	1.03818	1.57719
17856	1.38969	0.565053
17857	1.85548	0.64437
17858	1.03108	0.574748
17859	1.85548	0.620317
17860	1.49346	1.81174
17861	1.83192	2.1

17862	1.75291	1.43907
17863	1.35435	1.94152
17864	0.921851	0.50568
17865	1.09646	0.482311
17866	1.2183	0.473894
17867	1.36717	0.390608
17868	2.14179	0.348889
17869	1.79805	0.44821
17870	1.3428	0.452471
17871	2.30493	0.437702
17872	1.81545	0.396711
17873	1.92269	0.417723
17874	2.30492	0.478116
17875	1.3663	0.537288
17876	1.22418	1.4549
17877	0.956698	0.668153
17878	1.4706	1.6593
17879	0.953295	2.1
17880	1.47059	1.00455
17881	1.26929	2.1
17882	1.24538	0.272608
17883	1.4468	0.197956
17884	2.86762	2.1
17885	2.32911	2.1
17886	2.10864	2.1
17887	1.94548	2.1
17888	1.83134	2.1
17889	2.19519	2.1
17890	2.19853	2.1
17891	2.63855	2.1
17892	1.08213	0.180596
17893	1.46389	0.225389
17894	1.62235	0.299268
17895	0.980549	0.303242
17896	1.08195	1.75954
17897	0.99002	1.81706
17898	1.2269	2.1
17899	1.0838	2.1
17900	0.889491	2.1
17901	1.19587	1.99033
17902	1.18801	0.141001
17903	1.57125	0.142537
17904	1.47712	2.1
17905	1.45182	2.1
17906	1.45182	2.1
17907	1.42255	2.1
17908	1.17816	0.0890513
17909	1.00982	0.0925612
17910	1.21056	0.0760829
17911	1.17816	0.0742579
17912	3.80902	0.666078
17913	0.976991	0.598287
17914	1.09962	2.05254
17915	1.60976	1.74273
17916	1.87029	2.1
17917	2.33806	2.1
17918	2.12549	2.1
17919	2.21472	2.1

17920	2.16221	0.19019
17921	1.99528	0.162941
17922	1.99528	0.166157
17923	1.97969	0.150874
17924	3.60854	2.1
17925	4.00847	2.1
17926	2.70372	2.1
17927	1.253	2.1
17928	1.65739	2.1
17929	0.994294	2.1
17930	1.36203	2.1
17931	1.00965	2.1
17932	2.33374	2.1
17933	1.36209	2.1
17934	1.64983	0.10888
17935	1.36416	0.103357
17936	2.19766	0.104345
17937	1.36416	0.121578
17938	2.15871	0.649292
17939	1.81454	0.535335
17940	1.54607	0.344006
17941	1.57702	0.52227
17942	0.948748	0.0936259
17943	1.04522	0.0975459
17944	1.60994	0.0789608
17945	1.90382	0.0840677
17946	1.90382	0.0843418
17947	1.57999	0.0785186
17948	1.73905	0.675215
17949	1.26535	1.09425
17950	2.12552	0.981146
17951	1.06891	0.836378
17952	1.13999	0.91829
17953	2.29812	0.96854
17954	2.83329	0.835935
17955	2.16272	0.757491
17956	1.71277	2.1
17957	1.68401	2.1
17958	1.68401	2.1
17959	1.69368	2.1
17960	1.06472	2.1
17961	1.56017	2.1
17962	1.2765	2.1
17963	1.99541	2.1
17964	1.46041	0.0576311
17965	2.04817	0.0562103
17966	2.04817	0.0557135
17967	2.33181	0.0549065
17968	2.06353	2.1
17969	1.35449	2.1
17970	1.52045	2.1
17971	1.45437	2.1
17972	1.04405	0.0777288
17973	1.85945	0.0770115
17974	1.85945	0.0673249
17975	2.22679	0.0669302
17976	1.33339	2.1
17977	1.19376	2.1

17978	1.33266	2.1
17979	1.44244	2.1
17980	1.20455	2.1
17981	1.52852	2.1
17982	1.03882	2.1
17983	1.52852	2.1
17984	1.44801	2.04553
17985	1.00775	1.93245
17986	0.53907	2.1
17987	0.506124	2.1
17988	2.39045	2.1
17989	0.973794	2.1
17990	1.11193	2.1
17991	1.32784	2.1
17992	1.31595	2.1
17993	0.966115	2.1
17994	1.25953	2.1
17995	1.38086	2.1
17996	1.38086	2.1
17997	1.04101	2.1
17998	1.99319	0.131515
17999	1.23107	0.134869
18000	3.3606	0.141846
18001	1.23112	0.142999
18002	1.40239	2.1
18003	1.26091	2.1
18004	1.26091	2.1
18005	1.05776	2.1
18006	1.23768	0.531725
18007	1.0142	1.25591
18008	1.11435	1.08894
18009	1.01422	0.789457
18010	1.24001	0.677323
18011	1.42186	1.0081
18012	1.42186	0.741303
18013	1.47204	0.846058
18014	1.27361	2.1
18015	1.56642	2.1
18016	1.71676	2.1
18017	2.16815	2.1
18018	1.07855	1.15695
18019	1.07612	1.205
18020	1.703	0.124212
18021	1.46853	0.11338
18022	1.45448	0.109641
18023	1.27778	0.115331
18024	0.991986	2.1
18025	1.1192	2.1
18026	1.1192	2.1
18027	1.25953	2.1
18028	2.08506	2.1
18029	1.75162	0.837323
18030	1.94523	2.1
18031	1.88405	1.99841
18032	1.15506	0.0926553
18033	1.1233	0.0929929
18034	2.09736	2.1
18035	2.98303	2.1

18036	2.71184	2.1
18037	3.51614	2.1
18038	1.18769	2.0316
18039	1.24053	2.05579
18040	1.24053	2.03072
18041	1.45327	2.09646
18042	1.26865	1.24934
18043	1.1744	1.26502
18044	1.1744	0.850221
18045	1.11712	1.08396
18046	1.15841	0.323432
18047	1.02247	0.340677
18048	1.93649	2.1
18049	1.81191	2.1
18050	1.64718	2.1
18051	1.47521	2.1
18052	1.59654	2.1
18053	1.57823	2.1
18054	1.57823	2.1
18055	1.55768	2.1
18056	1.00665	0.253911
18057	1.28239	0.254814
18058	1.28239	0.246896
18059	1.44951	0.239957
18060	1.22083	2.1
18061	0.957622	2.1
18062	0.887896	2.1
18063	1.27387	2.1
18064	1.00521	0.948274
18065	1.00503	1.01844
18066	1.95844	2.1
18067	1.05042	2.1
18068	2.78662	2.1
18069	2.22399	2.1
18070	2.3356	0.0956657
18071	1.49543	0.112551
18072	1.96854	0.0889865
18073	1.21681	0.0839956
18074	2.17803	1.63528
18075	1.50998	1.92108
18076	2.35239	1.43428
18077	2.26987	1.14521
18078	1.03824	0.272046
18079	1.51396	0.358122
18080	1.51396	0.339689
18081	1.48693	0.284899
18082	1.08784	2.04635
18083	1.36865	1.86828
18084	0.712405	1.93624
18085	1.08784	2.0862
18086	2.02522	2.1
18087	1.6738	2.1
18088	1.35175	2.1
18089	1.50094	2.1
18090	1.6738	2.1
18091	1.62871	2.1
18092	1.35084	2.1
18093	1.34276	2.1

18094	1.18041	2.1
18095	1.43652	2.08699
18096	0.974196	2.1
18097	1.28037	2.1
18098	1.28037	2.1
18099	1.05071	2.1
18100	2.44737	0.0920289
18101	1.4672	0.0953208
18102	1.22883	0.0982538
18103	2.08149	0.110938
18104	1.82208	1.6897
18105	1.59826	1.69614
18106	1.59826	1.34449
18107	1.01393	1.96874
18108	1.42757	2.1
18109	0.996775	2.1
18110	1.42757	2.1
18111	1.26397	2.1
18112	1.02461	2.1
18113	1.46129	2.1
18114	1.11833	2.1
18115	1.335 2.1	
18116	2.07087	2.1
18117	1.24041	2.1
18118	1.335 2.1	
18119	1.55398	2.1
18120	1.21171	0.144155
18121	1.48589	0.133587
18122	1.32886	0.125438
18123	1.8027	0.124914
18124	1.53515	1.53559
18125	1.60428	1.54076
18126	1.00035	0.0522355
18127	1.03534	0.117271
18128	1.03535	0.72718
18129	1.09962	1.35293
18130	0.62132	1.10296
18131	1.21033	1.75804
18132	1.30326	1.42811
18133	2.6823	2.1
18134	0.991407	1.54055
18135	1.29729	1.0389
18136	1.29729	1.07569
18137	1.16858	2.1
18138	1.66137	0.217053
18139	1.23012	0.145919
18140	1.66139	0.389988
18141	1.41821	0.25723
18142	1.56043	0.609561
18143	1.06848	1.07875
18144	1.56043	1.62467
18145	1.90911	1.33825
18146	0.982847	2.1
18147	1.05863	2.1
18148	1.05863	2.1
18149	1.22352	2.1
18150	1.23019	1.29911
18151	1.60509	1.90446

18152	1.19122	2.1
18153	1.47769	2.1
18154	2.18868	2.1
18155	1.40984	1.53774
18156	3.55197	2.1
18157	3.57227	2.1
18158	3.57227	2.1
18159	3.48926	2.1
18160	1.4375	0.345243
18161	1.11314	0.302072
18162	1.62408	2.1
18163	1.74438	2.1
18164	1.72387	2.1
18165	1.50018	2.1
18166	1.1486	2.1
18167	1.18474	2.1
18168	1.18474	2.1
18169	1.2295	2.1
18170	1.28754	2.1
18171	1.36838	2.1
18172	1.36838	2.1
18173	1.21703	2.1
18174	2.74206	2.1
18175	2.70795	2.1
18176	2.70795	2.1
18177	2.55918	2.1
18178	1.35065	0.852539
18179	1.65411	0.594022
18180	1.48572	0.755994
18181	1.28393	0.706752
18182	1.25265	0.0949832
18183	1.70944	0.0798878
18184	1.31259	0.527339
18185	1.14945	0.964095
18186	1.14945	0.414563
18187	0.995725	1.04638
18188	1.00307	0.270916
18189	2.05786	0.372176
18190	1.56357	0.325881
18191	1.10907	0.376638
18192	1.27558	1.25871
18193	1.05175	0.861767
18194	1.2685	2.1
18195	1.41369	2.1
18196	1.4137	2.1
18197	1.49654	2.1
18198	1.21489	1.91766
18199	1.14605	1.4525
18200	1.22476	2.0316
18201	1.21489	1.10273
18202	2.51472	1.06481
18203	2.52091	1.05397
18204	2.52091	0.916691
18205	1.79171	0.888289
18206	1.03373	1.66407
18207	1.04037	1.92782
18208	1.04038	1.60207
18209	1.03933	1.77929

18210	1.74123	0.160223
18211	1.41902	0.148096
18212	1.89777	0.157778
18213	1.89899	0.191612
18214	1.24728	0.172748
18215	1.30394	0.194019
18216	1.30394	0.15331
18217	1.16535	0.157834
18218	1.09914	2.1
18219	1.54259	2.1
18220	1.40233	2.1
18221	1.69858	2.1
18222	1.53555	2.1
18223	1.09859	0.794731
18224	1.09859	2.05848
18225	0.725879	0.727087
18226	1.07213	2.1
18227	1.31589	2.1
18228	1.36904	2.1
18229	1.69711	2.1
18230	1.51028	0.111838
18231	0.993598	0.137135
18232	0.61563	0.107804
18233	1.26311	0.115143
18234	1.19496	2.1
18235	1.65925	2.1
18236	1.65925	2.1
18237	1.48162	2.1
18238	2.75738	1.30028
18239	1.24699	1.25789
18240	4.08729	1.68323
18241	2.35271	1.44037
18242	1.55063	0.395179
18243	1.34563	0.350057
18244	1.34563	0.367231
18245	1.08189	0.313237
18246	1.09662	0.297032
18247	2.35961	0.342007
18248	0.986952	0.24119
18249	0.813251	0.206302
18250	1.17897	0.11544
18251	1.67685	0.0959811
18252	1.00573	1.27
18253	1.08542	0.847284
18254	1.00573	2.1
18255	1.12572	2.1
18256	1.05499	1.99461
18257	1.00521	1.06338
18258	2.22679	0.0654925
18259	2.86746	0.0637767
18260	2.86746	0.0652285
18261	1.99958	0.0635169
18262	0.996517	2.1
18263	1.01169	2.1
18264	1.01169	2.1
18265	1.26346	2.1
18266	1.68274	2.1
18267	1.75505	2.1

18268	2.28748	1.65604
18269	1.36555	1.45834
18270	3.47888	0.0916122
18271	0.692788	0.0961288
18272	1.0685	0.459661
18273	1.14547	0.55166
18274	1.19017	2.1
18275	1.24976	2.1
18276	1.24976	2.1
18277	1.30682	2.1
18278	2.2153	2.1
18279	1.85457	2.1
18280	2.03267	2.1
18281	1.51042	2.1
18282	1.05619	0.639853
18283	1.05793	0.83391
18284	1.05793	0.616857
18285	1.00763	0.832623
18286	1.77677	2.1
18287	1.02524	2.1
18288	1.02524	2.1
18289	1.18803	2.1
18290	1.68669	2.1
18291	1.71162	2.1
18292	1.556	2.1
18293	1.39523	2.1
18294	0.960288	0.717453
18295	1.24201	0.925047
18296	1.24202	2.1
18297	1.3592	2.1
18298	2.14045	0.186344
18299	1.62054	0.165286
18300	2.20248	0.307219
18301	2.34699	0.242785
18302	3.45132	0.204287
18303	3.38241	0.184313
18304	3.38241	0.167942
18305	2.96127	0.155021
18306	2.02612	2.1
18307	2.09351	2.1
18308	2.09351	2.1
18309	2.17465	2.1
18310	1.07445	2.1
18311	1.14808	2.1
18312	1.39067	1.72023
18313	1.20958	1.30305
18314	1.20958	1.46434
18315	1.00382	1.28133
18316	0.986496	2.01092
18317	1.27142	2.07534
18318	1.39857	1.96285
18319	2.26482	1.83094
18320	2.24367	0.0851824
18321	1.9234	0.0842829
18322	1.59076	0.0858773
18323	1.185	0.0859948
18324	3.86179	0.0789659
18325	3.0638	0.0794365

18326	2.7574	0.0789272
18327	1.83266	0.0790876
18328	2.59418	2.1
18329	2.23048	2.1
18330	1.39564	2.1
18331	1.36763	2.1
18332	2.23048	2.1
18333	2.02522	2.1
18334	1.36763	2.1
18335	1.35175	2.1
18336	1.16181	0.0934653
18337	0.97096	0.0913359
18338	2.05665	0.180309
18339	0.483583	0.172018
18340	0.601928	0.133012
18341	3.88545	0.194981
18342	1.04909	2.1
18343	0.983143	2.1
18344	1.10469	2.1
18345	1.10516	2.1
18346	1.10517	2.1
18347	1.09951	2.1
18348	2.33985	2.1
18349	2.35026	2.1
18350	2.35025	2.1
18351	1.19462	2.1
18352	1.57627	2.1
18353	1.5493	2.1
18354	1.5493	2.1
18355	1.53556	2.1
18356	1.42763	0.497615
18357	1.24682	0.489367
18358	1.24682	0.447134
18359	1.00307	0.489411
18360	1.37392	2.1
18361	1.45408	2.1
18362	1.45408	2.1
18363	1.54249	2.1
18364	1.24474	1.27619
18365	1.07467	1.26727
18366	1.37813	1.45404
18367	1.24474	1.41323
18368	1.06341	2.1
18369	1.09194	2.1
18370	1.09194	2.1
18371	1.11117	2.1
18372	1.1658	2.1
18373	1.93707	2.1
18374	1.93707	2.1
18375	1.47729	2.1
18376	1.25669	0.599197
18377	1.07519	2.1
18378	1.65417	2.1
18379	1.50956	2.1
18380	1.37231	2.1
18381	1.27285	2.1
18382	1.23612	2.1
18383	1.1899	2.1

18384	1.08172	2.1
18385	1.20357	2.1
18386	1.5664	2.1
18387	1.7472	2.1
18388	1.48434	2.1
18389	1.23989	2.1
18390	1.58836	2.1
18391	1.38766	2.1
18392	1.23989	2.1
18393	1.26599	2.1
18394	2.56248	2.1
18395	2.08673	0.749879
18396	1.87216	0.796915
18397	1.60476	0.255544
18398	1.84355	2.1
18399	1.25971	0.715032
18400	1.44141	0.697825
18401	1.25521	0.21928
18402	1.19837	0.0783416
18403	2.31936	0.0775806
18404	1.38153	2.1
18405	2.41734	2.1
18406	2.70856	2.1
18407	3.48385	2.1
18408	1.89226	1.12408
18409	1.64617	1.46483
18410	1.89112	1.22283
18411	1.23135	0.484737
18412	1.23134	0.61373
18413	1.34927	0.551002
18414	3.60096	0.0901071
18415	1.68103	0.0993486
18416	2.25644	2.1
18417	1.91955	2.1
18418	1.91955	2.1
18419	1.47688	2.1
18420	1.67375	2.1
18421	1.50033	2.1
18422	1.5098	2.1
18423	1.46908	2.1
18424	1.69055	0.207648
18425	1.53463	0.19309
18426	1.53463	0.134318
18427	1.41943	0.123846
18428	1.19022	1.80106
18429	1.95981	1.39241
18430	1.15009	0.855754
18431	1.31612	1.01059
18432	1.31611	0.555818
18433	1.10735	0.473144
18434	1.18601	0.218385
18435	1.3499	0.241575
18436	1.3499	0.24096
18437	1.29001	0.230939
18438	1.22083	2.1
18439	1.06191	2.1
18440	1.348	0.977062
18441	1.05758	1.38514

18442	1.34799	1.87357
18443	1.18647	1.56935
18444	2.92457	0.140332
18445	1.99182	0.130681
18446	2.25313	0.138369
18447	1.30859	0.133787
18448	1.30682	2.1
18449	1.38957	2.1
18450	1.38957	2.1
18451	1.47446	2.1
18452	0.953638	1.23002
18453	1.03408	1.39208
18454	1.17885	0.541113
18455	1.3663	0.448674
18456	1.08265	0.715079
18457	1.71948	0.850305
18458	1.29501	0.862992
18459	0.955093	0.854768
18460	0.955081	0.686276
18461	1.88345	0.658722
18462	1.2243	2.1
18463	1.23169	2.1
18464	2.04726	1.49938
18465	0.842869	1.61685
18466	3.18156	2.1
18467	3.60786	1.39268
18468	3.60786	2.1
18469	2.38566	1.11366
18470	1.24526	0.078851
18471	1.59019	0.0889856
18472	1.16776	0.374811
18473	1.05042	1.08899
18474	1.17418	1.52859
18475	1.05042	2.1
18476	0.949135	2.05411
18477	0.973448	2.1
18478	0.973447	2.02764
18479	1.02958	2.1
18480	3.57768	2.07266
18481	3.04246	2.1
18482	3.04246	2.1
18483	2.47824	2.1
18484	1.35879	2.1
18485	1.10407	1.20614
18486	1.14599	0.531697
18487	1.10407	0.586439
18488	1.87794	1.37008
18489	1.53429	1.23424
18490	1.12717	2.1
18491	1.38634	2.1
18492	1.38634	2.1
18493	1.13127	2.1
18494	1.04546	2.1
18495	1.06007	2.1
18496	1.06007	2.1
18497	1.19785	2.1
18498	1.43652	2.1
18499	1.71741	1.82459

18500	1.70921	0.976999
18501	1.06994	1.03613
18502	0.960106	2.1
18503	1.48077	2.1
18504	1.48077	2.1
18505	2.5946	2.1
18506	2.03045	1.44653
18507	1.76788	1.73463
18508	2.03045	2.07825
18509	0.633098	2.08016
18510	1.06756	2.1
18511	1.38667	2.1
18512	0.966979	2.1
18513	1.38669	2.1
18514	1.07156	2.1
18515	0.986095	2.1
18516	1.32813	2.1
18517	0.986095	2.1
18518	1.98697	1.14962
18519	1.12016	1.91353
18520	1.24463	1.39481
18521	1.1967	1.78448
18522	1.30001	0.512581
18523	1.29175	1.19408
18524	1.29175	0.564108
18525	1.3003	1.26432
18526	1.5606	0.08579
18527	2.32098	0.0874098
18528	2.57888	0.0792144
18529	2.97463	0.0812187
18530	1.24555	2.1
18531	1.46101	2.1
18532	1.90723	2.1
18533	2.29126	2.1
18534	1.46101	2.1
18535	1.84089	2.1
18536	2.5519	2.1
18537	2.91663	2.1
18538	1.10476	0.141052
18539	1.44103	0.13444
18540	1.44103	0.134302
18541	1.53336	0.127645
18542	0.672964	2.1
18543	0.633884	2.1
18544	0.633884	2.1
18545	0.603154	2.1
18546	2.29663	2.1
18547	2.06892	2.1
18548	1.3685	2.1
18549	1.39067	2.1
18550	2.06892	2.1
18551	1.78208	2.1
18552	1.39067	2.1
18553	1.42174	2.1
18554	1.01335	1.67284
18555	1.08195	0.993914
18556	1.86463	1.49346
18557	1.39827	1.6464

18558	0.861515	1.9715
18559	2.10074	2.1
18560	2.09511	0.58573
18561	2.04853	0.534683
18562	2.21488	0.5401
18563	1.38287	0.679458
18564	0.974717	0.637192
18565	0.998451	0.590008
18566	1.42281	0.984662
18567	1.82595	0.917662
18568	1.50458	0.756957
18569	1.09408	0.551922
18570	1.23498	1.28187
18571	1.05654	1.24735
18572	1.17261	2.1
18573	1.18058	2.1
18574	1.18059	2.1
18575	1.18907	2.1
18576	2.38998	2.1
18577	2.77437	2.1
18578	1.04546	2.1
18579	1.16817	2.1
18580	2.77437	2.1
18581	2.97768	2.1
18582	1.16817	2.1
18583	1.2832	2.1
18584	1.75766	0.26371
18585	1.4934	0.752535
18586	1.4934	0.27383
18587	1.20324	0.996866
18588	1.07236	0.637528
18589	1.37641	0.548355
18590	1.42913	0.470788
18591	1.93373	0.606549
18592	1.42629	0.588118
18593	1.00636	0.348125
18594	0.933959	1.87732
18595	2.22873	0.79992
18596	2.22873	1.12187
18597	2.13757	0.631037
18598	1.10517	2.1
18599	1.86737	2.1
18600	0.811 2.1	
18601	1.19179	2.1
18602	1.18647	1.96162
18603	0.988453	2.06284
18604	1.12457	0.162521
18605	1.23851	0.14268
18606	0.92492	2.1
18607	1.45656	2.1
18608	1.61251	0.764209
18609	1.56696	0.784813
18610	1.90245	0.637972
18611	1.46046	0.631441
18612	1.60329	0.107677
18613	1.38247	0.101465
18614	1.38247	0.0968581
18615	1.23198	0.0948511

18616	1.09518	2.1
18617	1.28159	2.1
18618	0.977777	2.1
18619	1.80778	2.1
18620	1.80777	2.1
18621	1.16655	2.1
18622	1.3515	2.1
18623	0.956573	2.1
18624	1.08022	2.1
18625	1.03437	2.1
18626	1.03437	2.1
18627	0.975409	2.1
18628	1.47369	0.130064
18629	1.31167	0.167834
18630	1.01847	0.123924
18631	2.75562	0.124608
18632	1.13443	2.1
18633	1.32709	2.1
18634	1.01051	0.273267
18635	0.958947	0.221667
18636	1.7211	2.1
18637	1.922	2.1
18638	1.922	2.1
18639	0.991113	2.1
18640	1.00636	0.369883
18641	1.31658	0.455279
18642	1.44627	0.424306
18643	1.03824	0.311333
18644	1.21223	2.1
18645	1.01242	2.1
18646	1.01243	2.1
18647	0.956698	1.28036
18648	1.23167	2.1
18649	1.06935	2.1
18650	1.29215	0.0819497
18651	0.998971	0.0886041
18652	1.50103	0.0842718
18653	1.44836	0.0935923
18654	3.02513	2.1
18655	3.7335	2.1
18656	2.49988	2.1
18657	1.24948	2.1
18658	0.963972	2.1
18659	1.1822	2.1
18660	1.1822	2.1
18661	1.10817	2.1
18662	1.30237	2.1
18663	1.41908	2.1
18664	1.41908	2.1
18665	1.48734	2.1
18666	1.31127	0.616773
18667	1.40822	0.582523
18668	1.40822	0.473646
18669	1.22176	0.457827
18670	1.82525	2.1
18671	1.59394	2.1
18672	1.22731	0.112772
18673	0.981584	0.0997498

18674	1.1427	0.652178
18675	1.44091	0.934509
18676	1.49154	0.72785
18677	1.07526	0.691191
18678	1.07527	0.48042
18679	0.96029	0.714792
18680	2.40568	0.958993
18681	3.46486	0.842136
18682	2.417	0.943665
18683	1.52054	0.72543
18684	1.51638	0.246887
18685	1.04786	0.188155
18686	1.51404	0.241824
18687	1.3461	0.179459
18688	1.27696	0.176678
18689	0.736457	0.12816
18690	1.16817	2.1
18691	1.04263	2.1
18692	1.13449	2.1
18693	1.50524	2.1
18694	2.1182	2.1
18695	2.49001	2.1
18696	1.11764	2.1
18697	1.39292	2.1
18698	2.49001	2.1
18699	2.76339	2.1
18700	1.39292	2.1
18701	1.68761	2.1
18702	1.25173	2.08356
18703	1.34701	2.1
18704	1.02415	2.1
18705	1.34701	1.79589
18706	1.332	0.0936791
18707	0.989764	0.0888118
18708	0.963167	0.0804647
18709	1.21056	0.0735411
18710	1.67375	2.1
18711	1.17965	2.1
18712	1.09154	2.1
18713	1.52314	2.1
18714	1.54433	0.509611
18715	1.81969	0.414153
18716	1.3598	0.40468
18717	1.71041	0.294101
18718	1.70934	0.324022
18719	1.33871	0.276681
18720	1.79485	0.294229
18721	1.51404	0.228688
18722	1.30751	2.1
18723	1.20969	2.1
18724	1.20969	2.1
18725	1.12076	2.1
18726	1.49808	2.1
18727	1.50201	2.1
18728	1.50201	2.1
18729	1.31594	2.1
18730	1.2187	2.1
18731	1.11545	2.1

18732	1.227	2.1
18733	1.09645	2.1
18734	1.05902	0.0522355
18735	1.41595	0.326762
18736	1.35696	1.80572
18737	1.29446	0.644076
18738	0.857957	2.1
18739	1.21125	0.0522355
18740	2.43115	2.09636
18741	2.1151	0.366794
18742	2.0815	1.11005
18743	1.1723	1.8604
18744	1.48035	0.397035
18745	1.59971	0.55649
18746	1.33668	0.475906
18747	1.0961	0.37472
18748	1.46235	2.1
18749	0.882195	2.1
18750	2.46624	2.1
18751	2.72161	2.1
18752	2.96094	2.1
18753	2.25644	2.1
18754	1.25923	0.0880498
18755	0.976218	0.0852694
18756	1.10319	0.0762723
18757	1.82583	0.0820793
18758	0.888955	2.1
18759	1.30817	2.1
18760	1.30817	2.1
18761	1.60787	2.1
18762	0.988805	1.39563
18763	1.54168	0.731356
18764	1.19496	0.772786
18765	1.12224	1.4327
18766	1.93597	1.22223
18767	1.31113	1.16058
18768	1.17879	0.934027
18769	1.07468	0.945243
18770	2.0523	2.1
18771	2.41385	2.1
18772	2.41385	2.1
18773	3.0355	2.1
18774	1.36717	0.40021
18775	1.45535	0.287087
18776	1.45535	0.283752
18777	1.49906	0.221198
18778	1.37785	0.809989
18779	1.04413	1.72009
18780	1.06729	0.712927
18781	1.54305	0.660206
18782	1.07444	2.1
18783	1.52724	0.963845
18784	1.49151	2.1
18785	1.36814	2.1
18786	1.36814	2.1
18787	1.15541	2.1
18788	1.19379	2.1
18789	1.78657	0.6755

18790	1.6079	2.1
18791	2.59469	0.690319
18792	1.35545	0.569213
18793	1.23602	0.783265
18794	1.23452	2.02096
18795	1.56842	0.831406
18796	1.15864	2.1
18797	1.11955	2.1
18798	1.11955	2.1
18799	1.08022	2.1
18800	1.64268	1.77748
18801	1.26247	2.1
18802	1.16912	2.1
18803	2.1095	0.872624
18804	1.75549	0.0991949
18805	1.7491	0.121036
18806	1.59833	0.125885
18807	1.68338	0.140003
18808	1.14846	0.103288
18809	1.06515	0.107914
18810	1.2721	0.218277
18811	1.32639	0.181333
18812	1.32639	0.349218
18813	1.19323	0.224891
18814	1.81034	2.1
18815	1.9034	2.1
18816	2.08245	2.1
18817	1.84887	2.1
18818	1.41448	2.1
18819	1.77326	2.1
18820	1.76872	2.1
18821	1.86766	2.1
18822	1.46643	2.1
18823	1.58995	2.1
18824	1.58995	2.1
18825	1.13854	2.1
18826	1.23654	2.1
18827	1.04442	2.1
18828	1.23654	2.1
18829	1.08576	2.1
18830	0.966979	2.1
18831	1.41758	2.1
18832	1.03108	0.393945
18833	1.94412	0.461275
18834	1.32455	2.1
18835	1.32328	2.1
18836	1.32328	2.1
18837	1.34008	2.1
18838	1.60861	2.1
18839	1.69586	2.1
18840	1.69586	2.1
18841	1.78301	2.1
18842	1.7513	2.1
18843	1.67906	2.1
18844	1.67906	2.1
18845	1.60635	2.1
18846	1.68073	2.1
18847	1.57234	2.1

18848	1.57233	2.1
18849	1.47012	2.1
18850	1.22811	0.265747
18851	1.79683	0.266733
18852	1.53417	0.274397
18853	1.08732	0.292043
18854	1.8903	1.08773
18855	1.20247	1.01395
18856	1.12087	1.41873
18857	1.54699	1.67531
18858	1.43635	2.1
18859	1.02368	2.1
18860	1.43635	2.1
18861	1.23007	2.1
18862	1.035 2.1	
18863	1.07889	2.1
18864	3.0355	2.1
18865	3.78805	1.70047
18866	2.57183	1.3051
18867	1.27835	0.97538
18868	1.29813	0.269131
18869	1.42965	0.298632
18870	1.69832	0.284186
18871	2.18359	0.313404
18872	1.42964	0.279724
18873	1.65723	0.296332
18874	1.98508	0.287975
18875	2.06284	0.312601
18876	1.50705	0.746443
18877	1.51153	0.416955
18878	1.36037	0.449728
18879	1.09767	0.40175
18880	1.70048	1.4684
18881	1.15177	1.28569
18882	0.713174	2.1
18883	0.763575	2.1
18884	0.763575	2.1
18885	0.797011	2.1
18886	1.01179	0.775661
18887	1.27737	0.769539
18888	3.16531	0.185491
18889	1.0141	0.151908
18890	1.47354	0.442064
18891	1.62495	0.353425
18892	1.62495	0.366838
18893	1.39296	0.582995
18894	0.998682	2.1
18895	1.93528	2.1
18896	2.71383	2.1
18897	1.13987	2.1
18898	1.93528	2.1
18899	1.78595	2.1
18900	3.98933	2.1
18901	3.73426	2.1
18902	1.02438	2.1
18903	1.12376	2.1
18904	1.12375	2.1
18905	1.19016	2.1

18906	1.37157	0.151884
18907	1.60814	0.144765
18908	1.70303	0.199832
18909	1.01219	0.175994
18910	1.06757	1.39007
18911	1.23475	1.94551
18912	1.2295	2.1
18913	1.28014	2.1
18914	1.28014	2.1
18915	1.33339	2.1
18916	1.3201	2.1
18917	1.2679	2.1
18918	1.2679	2.1
18919	1.23504	2.1
18920	1.50108	2.1
18921	1.3715	2.1
18922	1.3715	2.1
18923	1.25075	2.1
18924	1.99222	1.0559
18925	1.4155	1.02228
18926	1.4777	2.1
18927	1.52613	2.1
18928	1.91031	2.1
18929	1.03968	2.1
18930	2.04289	0.251605
18931	0.967192	0.206079
18932	1.03043	0.164495
18933	1.65452	0.271649
18934	1.21564	2.1
18935	1.16938	2.1
18936	2.47056	2.1
18937	2.56853	2.1
18938	1.16938	2.1
18939	1.1181	2.1
18940	2.56853	2.1
18941	2.66828	2.1
18942	1.0465	2.1
18943	1.44709	2.1
18944	1.44709	2.1
18945	1.96491	2.1
18946	1.01976	0.438932
18947	0.961779	0.383254
18948	1.11764	0.423062
18949	0.961779	0.377212
18950	1.21171	0.120256
18951	1.35007	0.151027
18952	1.03102	0.545826
18953	1.42446	0.544065
18954	1.42446	0.473698
18955	1.03166	0.452659
18956	1.2951	0.209128
18957	1.33575	0.240502
18958	1.33575	0.248044
18959	1.20473	0.237364
18960	1.10049	0.752656
18961	0.99747	0.705503
18962	1.54249	2.1
18963	1.65677	2.1

18964	1.65677	2.1
18965	1.77677	2.1
18966	1.73525	0.25101
18967	1.05689	0.294589
18968	1.26051	0.21588
18969	1.16259	0.185788
18970	1.34009	2.1
18971	1.398	2.1
18972	1.398	2.1
18973	1.50437	2.1
18974	2.3245	2.1
18975	1.18237	2.1
18976	1.24509	2.1
18977	1.01595	2.1
18978	3.29047	2.1
18979	2.79168	2.1
18980	1.06561	2.1
18981	1.11077	1.31777
18982	1.11077	0.840588
18983	0.953927	1.26559
18984	1.30509	0.416813
18985	1.04685	0.431372
18986	1.04685	0.320505
18987	0.834593	0.343238
18988	1.0775	0.31384
18989	1.27362	0.385854
18990	1.71762	0.679322
18991	2.5736	0.590038
18992	2.04717	0.732444
18993	1.45521	0.619883
18994	1.33962	2.1
18995	1.27956	2.1
18996	1.27956	2.1
18997	1.20478	2.1
18998	3.65841	0.100197
18999	1.18656	0.084276
19000	1.72272	0.125432
19001	1.6812	0.104449
19002	1.09951	2.1
19003	1.08231	2.1
19004	1.08231	2.1
19005	1.06478	2.1
19006	2.16196	1.30356
19007	1.00387	0.868612
19008	1.00387	0.863476
19009	0.993999	0.67294
19010	1.04101	2.1
19011	1.27119	2.1
19012	1.27119	2.1
19013	1.51748	2.1
19014	3.00129	2.1
19015	3.27303	2.1
19016	3.27303	2.1
19017	3.60855	2.1
19018	2.22399	2.1
19019	1.71995	2.1
19020	1.54793	2.1
19021	1.227	2.1

19022	1.07624	2.1
19023	1.264 2.1	
19024	1.19323	0.999729
19025	1.21286	0.97551
19026	1.21286	0.80769
19027	1.20859	0.868528
19028	1.60578	0.167934
19029	1.27778	0.134264
19030	1.0525	2.04535
19031	1.05758	1.11497
19032	1.0525	0.884391
19033	1.17042	1.28638
19034	1.62536	0.0778452
19035	3.71662	0.0743443
19036	1.78169	0.143594
19037	1.91434	0.140348
19038	2.12331	0.153582
19039	1.33406	0.141417
19040	1.38437	2.1
19041	1.51829	2.1
19042	1.51829	2.1
19043	1.6697	2.1
19044	0.947114	2.1
19045	1.13335	2.1
19046	1.13335	2.1
19047	1.00965	2.1
19048	1.47521	2.1
19049	1.27679	2.1
19050	1.14911	2.1
19051	0.93841	2.1
19052	1.68437	2.1
19053	1.53822	2.1
19054	1.53822	2.1
19055	1.39702	2.1
19056	0.726453	2.1
19057	0.754101	2.1
19058	0.754101	2.1
19059	0.69776	2.1
19060	0.964033	2.1
19061	1.62397	1.85818
19062	1.70302	1.63677
19063	1.78637	1.41402
19064	3.68158	0.113148
19065	2.11685	0.107639
19066	1.33125	2.1
19067	1.15471	2.1
19068	1.0465	2.1
19069	1.33125	2.1
19070	1.17683	2.1
19071	1.25889	2.1
19072	1.25889	2.1
19073	1.34239	2.1
19074	1.40591	0.334946
19075	0.79348	0.243303
19076	1.04019	0.182854
19077	0.999187	0.163463
19078	1.30218	0.145439
19079	1.83339	0.14386

19080	1.88574	0.115247
19081	1.5177	0.100172
19082	1.23504	2.1
19083	1.19583	2.1
19084	1.19583	2.1
19085	1.14496	2.1
19086	1.62183	2.1
19087	1.62316	2.1
19088	1.62316	2.1
19089	1.62241	2.1
19090	1.02305	0.488333
19091	1.21136	0.407463
19092	0.993832	0.269302
19093	1.21137	0.372063
19094	1.12064	2.1
19095	1.24965	2.1
19096	1.24965	2.1
19097	1.41077	2.1
19098	1.00052	0.176908
19099	1.49842	0.148002
19100	1.49843	0.12817
19101	1.10801	0.112267
19102	1.54196	0.819674
19103	1.18041	0.987383
19104	1.73571	1.43227
19105	1.37224	1.71513
19106	1.4375	0.223524
19107	1.09529	0.205151
19108	1.44421	0.19304
19109	1.0953	0.188452
19110	1.0753	2.1
19111	0.815365	2.1
19112	0.815365	2.1
19113	0.597537	2.1
19114	1.03609	0.519806
19115	1.18952	0.496229
19116	1.54283	0.427927
19117	1.16719	0.4301
19118	1.16719	0.572508
19119	1.89226	0.518372
19120	2.55919	2.1
19121	2.37492	2.1
19122	2.37492	2.1
19123	2.07435	2.1
19124	1.36016	0.385889
19125	1.70302	0.380698
19126	1.85252	0.360187
19127	1.2111	0.496529
19128	1.62905	2.1
19129	1.49692	2.1
19130	1.49692	2.1
19131	1.40395	2.1
19132	2.19083	2.1
19133	1.30542	2.1
19134	1.58736	2.1
19135	2.16579	2.1
19136	2.0815	2.1
19137	1.17935	2.1

19138	1.53803	1.15571
19139	1.09148	0.662358
19140	1.81301	0.943872
19141	1.38333	0.46848
19142	1.04412	2.1
19143	1.44437	2.1
19144	1.4675	0.257816
19145	1.00665	0.278079
19146	1.50951	0.171237
19147	0.983105	0.14807
19148	1.80252	0.214751
19149	0.540005	0.147603
19150	1.2545	0.252425
19151	2.14514	0.218258
19152	1.32051	0.191839
19153	1.82302	0.225782
19154	2.71057	2.1
19155	2.51107	2.1
19156	1.34817	2.1
19157	1.3551	2.1
19158	2.51107	2.1
19159	2.29663	2.1
19160	1.3551	2.1
19161	1.3685	2.1
19162	1.51748	2.1
19163	1.23389	2.1
19164	0.901928	0.120611
19165	1.70302	0.149032
19166	1.66787	0.120686
19167	1.92535	0.15513
19168	1.13571	0.307174
19169	1.02836	0.344972
19170	1.55398	2.1
19171	1.54179	2.1
19172	1.89608	2.1
19173	1.07167	2.1
19174	3.3488	0.175117
19175	0.550044	0.158617
19176	0.962877	0.326104
19177	1.00065	0.355794
19178	1.00613	0.394316
19179	1.07497	0.415936
19180	1.00486	0.0522355
19181	1.00486	0.0522355
19182	0.726453	2.1
19183	1.32634	0.227883
19184	1.40008	0.328405
19185	1.40008	0.213628
19186	2.42267	0.213703
19187	1.9216	0.109532
19188	1.28066	0.0905168
19189	1.79102	0.741942
19190	1.18219	0.269372
19191	1.18219	0.679026
19192	0.709123	0.302052
19193	1.07866	2.01376
19194	1.15038	2.1
19195	1.15038	1.81415

19196	1.24687	2.078
19197	1.83613	1.74633
19198	1.99157	1.74544
19199	1.45789	0.097597
19200	1.1706	0.095481
19201	1.74746	0.0706317
19202	1.1706	0.069803
19203	1.96383	0.124787
19204	1.67727	0.116202
19205	1.67727	0.110403
19206	1.60329	0.109691
19207	1.18803	2.1
19208	1.3227	2.1
19209	1.3227	2.1
19210	1.40844	2.1
19211	1.07889	2.1
19212	1.07721	2.1
19213	1.07721	2.1
19214	1.00832	2.1
19215	0.953523	0.736389
19216	1.62875	0.69656
19217	1.07427	0.691326
19218	1.19722	0.588098
19219	1.1972	0.517022
19220	1.1599	0.512185
19221	2.97463	0.0725563
19222	1.9477	0.0715555
19223	1.95222	0.0675578
19224	1.75083	0.070388
19225	3.46787	2.1
19226	3.02896	2.1
19227	3.02896	2.1
19228	2.46145	2.1
19229	0.970962	0.0883261
19230	1.0227	0.0846895
19231	1.12328	0.0894949
19232	1.02269	0.0994055
19233	0.904978	2.1
19234	0.725887	2.1
19235	0.725887	2.1
19236	0.601592	2.1
19237	0.956294	2.1
19238	1.13179	2.1
19239	0.956294	2.1
19240	1.13179	2.1
19241	1.05556	2.1
19242	1.13179	2.1
19243	1.38859	2.1
19244	1.13179	2.1
19245	1.06659	1.70815
19246	1.05776	1.89448
19247	1.06659	1.7449
19248	1.04291	0.66927
19249	1.54699	1.01566
19250	1.42307	1.08118
19251	1.5654	1.49039
19252	1.97906	2.1
19253	1.15354	0.0965594

19254	1.15505	0.109183
19255	1.26455	0.125801
19256	2.50042	0.129871
19257	2.50042	0.148071
19258	3.22585	0.138929
19259	1.19258	0.153907
19260	1.34562	0.165134
19261	1.49515	0.203651
19262	1.66295	0.168984
19263	1.02395	2.1
19264	1.51067	2.1
19265	1.35821	1.71643
19266	2.10671	2.1
19267	1.0305	1.33366
19268	1.07855	1.27077
19269	1.20761	2.1
19270	0.98488	2.1
19271	1.4162	2.1
19272	1.14825	2.1
19273	1.29415	2.1
19274	1.6067	2.1
19275	1.23851	0.210908
19276	1.09651	0.168989
19277	1.60636	2.1
19278	1.53521	2.1
19279	1.53521	2.1
19280	1.46279	2.1
19281	1.28044	0.349879
19282	1.64147	0.320857
19283	1.18422	0.228362
19284	1.68208	0.363568
19285	1.09726	2.1
19286	1.234 2.1	
19287	1.234 2.1	
19288	1.09483	2.1
19289	1.89318	2.1
19290	1.98448	2.1
19291	1.98448	2.1
19292	2.02612	2.1
19293	1.22026	0.779348
19294	1.23527	0.650972
19295	1.09044	0.663196
19296	0.993366	0.400139
19297	1.22026	0.893316
19298	0.993368	1.00517
19299	2.61357	2.1
19300	2.48208	2.1
19301	2.48208	2.1
19302	2.62868	2.1
19303	1.85439	0.869623
19304	1.81776	0.740245
19305	1.81776	0.684949
19306	2.1587	0.721886
19307	1.8914	0.827602
19308	2.23961	0.751472
19309	1.49357	0.355958
19310	2.23961	0.698792
19311	1.78014	0.579143

19312	1.67635	0.558728
19313	1.66219	0.498024
19314	1.56036	0.490604
19315	1.14721	0.45669
19316	2.22495	0.505717
19317	1.88509	0.30228
19318	1.95249	0.251136
19319	2.11959	0.249444
19320	1.37427	0.19987
19321	1.39379	0.511855
19322	1.17614	0.413516
19323	1.39379	0.955656
19324	1.14397	0.326647
19325	1.09651	2.1
19326	1.42843	1.65939
19327	1.7069	2.1
19328	2.31293	1.85292
19329	1.56839	2.06783
19330	1.78252	0.706388
19331	1.78254	0.63273
19332	1.09142	0.789325
19333	0.99829	0.126858
19334	1.09372	0.12876
19335	1.88175	0.172671
19336	0.898452	0.159145
19337	1.06936	2.1
19338	1.4162	2.1
19339	1.17989	2.1
19340	1.47411	1.35086
19341	1.25376	1.32112
19342	1.00757	0.726268
19343	1.4185	0.307899
19344	1.02011	0.351984
19345	2.22495	0.357392
19346	1.79542	0.327434
19347	1.79542	0.256027
19348	1.19352	0.24479
19349	2.38566	2.1
19350	1.83255	1.31248
19351	1.62667	2.1
19352	1.17879	1.50842
19353	2.79168	2.1
19354	2.28162	2.1
19355	2.28162	2.1
19356	1.90905	2.1
19357	1.40117	2.1
19358	1.28268	2.1
19359	1.41096	2.1
19360	1.22978	2.1
19361	3.20034	2.1
19362	3.42052	2.1
19363	3.42052	2.1
19364	3.50562	2.1
19365	1.46279	2.1
19366	1.3864	2.1
19367	1.3864	2.1
19368	1.3201	2.1
19369	1.35561	0.073496

19370	0.974077	0.0734012
19371	0.967673	0.884182
19372	1.22482	0.751133
19373	1.02305	0.471784
19374	1.22482	0.663324
19375	0.999492	2.1
19376	1.14657	2.1
19377	1.14657	2.1
19378	1.31346	2.1
19379	1.84417	0.0888019
19380	1.31777	0.0777202
19381	1.19796	0.0708074
19382	0.691742	0.0679772
19383	1.16725	2.1
19384	1.9679	2.1
19385	1.08848	0.110923
19386	1.15356	0.103664
19387	1.58713	2.1
19388	1.58742	2.1
19389	1.58742	2.1
19390	1.49662	2.1
19391	1.32201	0.544552
19392	1.32097	0.978633
19393	1.32097	0.579449
19394	1.31825	1.13385
19395	1.12028	2.1
19396	1.15852	1.75072
19397	1.15852	2.1
19398	1.16239	1.36751
19399	1.07624	2.1
19400	1.30047	2.1
19401	1.44847	0.759291
19402	1.55292	0.729746
19403	1.21125	0.0522355
19404	1.15262	2.1
19405	1.05025	2.1
19406	1.55351	1.96462
19407	1.5329	0.104801
19408	2.26758	0.0948453
19409	1.94968	0.150997
19410	1.65747	0.139435
19411	1.42549	0.169597
19412	1.57656	0.153689
19413	2.11157	0.421513
19414	1.56531	0.503924
19415	1.69279	0.380638
19416	1.06204	0.538597
19417	0.947863	1.05267
19418	1.19854	1.29659
19419	0.947862	0.829299
19420	1.31126	0.743837
19421	1.3425	1.32032
19422	1.29976	1.07442
19423	1.05129	0.205854
19424	1.51026	0.21584
19425	1.05129	0.19296
19426	1.2951	0.203585
19427	1.7155	2.1

19428	1.72196	2.1
19429	1.72196	2.1
19430	1.77867	2.1
19431	0.964755	2.1
19432	1.14142	2.1
19433	1.03766	2.1
19434	0.986723	2.1
19435	1.28072	0.612716
19436	1.10609	0.795696
19437	1.28072	0.491816
19438	1.16776	1.23794
19439	1.61687	2.1
19440	1.60965	2.1
19441	1.60965	2.1
19442	1.59654	2.1
19443	1.26512	2.1
19444	1.23964	2.1
19445	2.07997	2.1
19446	2.60048	2.1
19447	1.23732	2.1
19448	1.3372	2.1
19449	2.62253	2.1
19450	2.47056	2.1
19451	0.92782	0.17355
19452	1.40335	0.198797
19453	1.26301	0.201301
19454	1.52668	0.196921
19455	0.99818	0.263407
19456	1.51165	0.323115
19457	1.66213	0.26941
19458	1.55246	0.316583
19459	2.31255	2.1
19460	1.34366	2.1
19461	1.90577	2.1
19462	0.969739	2.1
19463	3.4245	2.1
19464	2.8415	2.1
19465	1.97559	2.1
19466	1.82362	2.1
19467	1.48162	2.1
19468	1.23204	2.1
19469	1.23204	2.1
19470	0.95947	2.1
19471	1.05267	2.01204
19472	1.44091	1.03632
19473	1.05267	1.35844
19474	1.5795	1.84169
19475	0.738711	2.1
19476	1.28549	2.1
19477	1.15694	2.1
19478	1.72061	2.1
19479	1.69858	2.1
19480	0.954563	2.1
19481	0.859087	2.1
19482	0.788886	2.1
19483	1.08732	0.270375
19484	1.3357	0.274912
19485	1.3357	0.267791

19486	1.48676	0.26581
19487	2.15079	2.1
19488	2.12414	2.1
19489	2.12414	2.1
19490	2.04664	2.1
19491	1.98859	2.1
19492	0.992159	2.1
19493	1.48064	2.1
19494	1.2163	2.1
19495	1.26337	2.1
19496	1.63159	2.1
19497	1.00366	0.365024
19498	1.65452	0.327546
19499	1.10408	2.1
19500	1.37704	2.1
19501	1.50224	2.1
19502	1.42866	2.1
19503	1.43939	2.1
19504	1.02784	2.1
19505	1.00763	0.195283
19506	1.23746	0.190058
19507	1.23747	0.195795
19508	1.13642	0.211305
19509	1.49906	0.225468
19510	1.46506	0.173712
19511	1.46506	0.177904
19512	1.33061	0.144512
19513	1.08166	0.207361
19514	1.01052	0.18741
19515	1.02836	0.220763
19516	1.08166	0.202678
19517	1.25334	0.235572
19518	1.23749	0.235171
19519	1.37501	0.318435
19520	1.47354	0.286508
19521	1.30167	2.1
19522	1.29897	2.1
19523	1.51042	2.1
19524	1.07684	2.1
19525	1.07684	2.1
19526	0.765121	2.1
19527	1.5535	0.654516
19528	1.40602	2.1
19529	1.40603	0.688096
19530	1.25669	2.1
19531	1.08276	2.1
19532	1.60948	2.1
19533	1.26929	1.23232
19534	1.09847	2.1
19535	0.827082	0.342071
19536	1.15841	0.355096
19537	1.25063	0.282004
19538	1.14397	0.238774
19539	1.25063	0.534881
19540	1.10476	0.154558
19541	1.14872	0.117973
19542	1.3659	0.135733
19543	1.36589	0.11333

19544	1.4677	0.133492
19545	0.590354	2.1
19546	0.655202	2.1
19547	3.48926	2.1
19548	3.32709	2.1
19549	3.32709	2.1
19550	3.13938	2.1
19551	1.24458	0.305382
19552	1.29681	0.288642
19553	1.40915	0.358735
19554	1.17891	0.451766
19555	1.33171	0.0522355
19556	1.37681	0.455293
19557	1.37681	2.1
19558	1.4516	2.1
19559	2.68241	0.118714
19560	1.61031	0.0980032
19561	1.6488	2.1
19562	1.6585	2.1
19563	1.65848	2.1
19564	1.67061	2.1
19565	1.15152	2.1
19566	1.32548	2.1
19567	1.43677	2.1
19568	1.10264	2.1
19569	1.01472	2.1
19570	0.966964	2.1
19571	2.867 2.1	
19572	2.94897	2.1
19573	1.65815	2.1
19574	1.4502	2.1
19575	1.4502	2.1
19576	1.26755	2.1
19577	1.37138	2.1
19578	1.45026	2.1
19579	1.45026	2.1
19580	1.53446	2.1
19581	0.973502	1.83322
19582	1.08542	1.64596
19583	1.13387	0.109554
19584	1.71031	0.0969446
19585	1.08911	1.99019
19586	1.10748	2.1
19587	1.21823	2.1
19588	1.25005	2.1
19589	0.657014	1.90461
19590	1.38139	2.1
19591	1.01485	2.1
19592	1.26045	2.1
19593	1.12468	2.1
19594	1.26045	2.1
19595	1.89278	0.64462
19596	1.06433	0.545468
19597	1.87861	0.551718
19598	0.967544	0.447107
19599	1.50319	2.1
19600	3.47245	1.59287
19601	2.2354	0.530318

19602	1.16701	0.461647
19603	1.3592	2.1
19604	1.3305	0.776402
19605	1.3305	1.48281
19606	1.15008	0.726916
19607	1.12457	0.105088
19608	1.40707	0.115054
19609	1.01778	2.1
19610	1.35733	2.1
19611	1.01779	2.1
19612	1.12064	2.1
19613	1.30694	1.1887
19614	1.42716	1.31817
19615	1.42717	1.24504
19616	1.04321	1.31186
19617	1.19352	0.214641
19618	1.32634	0.280595
19619	1.14605	1.92546
19620	1.0417	1.65029
19621	1.19676	0.311599
19622	1.03804	0.313994
19623	2.09455	0.339896
19624	1.15338	0.354298
19625	2.21119	2.1
19626	1.26097	1.80311
19627	1.88394	0.412175
19628	1.24106	0.207904
19629	2.98143	2.1
19630	2.56248	1.01451
19631	1.88394	1.07372
19632	1.68703	0.178051
19633	1.5846	2.1
19634	1.94799	2.1
19635	1.20091	1.9611
19636	1.40393	2.1
19637	3.09031	2.1
19638	2.90243	2.1
19639	1.27227	2.1
19640	1.32592	2.1
19641	2.90247	2.1
19642	2.71057	2.1
19643	1.32594	2.1
19644	1.34817	2.1
19645	1.4092	2.1
19646	1.44859	2.1
19647	1.52008	2.1
19648	1.401	2.1
19649	1.40099	2.1
19650	1.28936	2.1
19651	1.56002	0.0641422
19652	1.08811	0.0591461
19653	1.46041	0.0631363
19654	1.56002	0.0582583
19655	1.50524	2.1
19656	1.21304	2.1
19657	1.21304	2.1
19658	0.947115	2.1
19659	1.60665	2.04775

19660	1.85179	1.60508
19661	1.85178	1.65546
19662	2.06277	0.961546
19663	2.17534	2.1
19664	1.23007	2.08743
19665	2.17533	2.1
19666	1.66635	1.813
19667	2.00869	2.1
19668	0.986724	2.1
19669	2.00475	2.1
19670	1.99394	2.1
19671	1.67244	0.215144
19672	1.39604	0.19557
19673	1.39604	0.206962
19674	0.954046	0.165583
19675	0.988863	2.03976
19676	1.9864	2.1
19677	1.9864	2.04634
19678	3.38645	2.1
19679	1.46932	0.0945549
19680	1.50847	0.0976382
19681	1.50848	0.0946893
19682	1.42705	0.0968592
19683	1.50108	2.1
19684	1.51621	0.733105
19685	1.94317	0.77165
19686	1.18382	0.466098
19687	2.05091	0.100102
19688	1.88593	0.0801528
19689	1.54299	0.0772424
19690	0.987265	0.0689869
19691	1.27766	2.1
19692	1.5178	2.1
19693	1.5178	2.1
19694	1.81518	2.1
19695	1.04626	1.09144
19696	1.31057	1.11295
19697	1.31057	1.08243
19698	1.22297	1.11243
19699	1.67844	1.00782
19700	1.62862	2.1
19701	1.62112	0.960714
19702	1.87598	2.1
19703	1.17844	2.1
19704	1.37063	2.1
19705	1.37063	2.1
19706	1.68723	2.03173
19707	1.99943	0.127854
19708	1.17631	0.098519
19709	1.4263	0.958064
19710	1.31023	2.1
19711	1.31023	0.909634
19712	1.20871	2.1
19713	1.50356	2.1
19714	1.69311	2.1
19715	1.69599	2.1
19716	1.6801	2.1
19717	1.5843	0.075179

19718	0.840233	0.0764236
19719	1.95358	0.0790062
19720	1.40216	0.0893656
19721	1.39078	2.00789
19722	1.18948	1.70614
19723	1.01606	1.33794
19724	1.18948	1.59201
19725	1.63823	2.1
19726	1.67687	2.1
19727	1.67687	2.1
19728	1.52855	2.1
19729	1.11048	2.1
19730	1.25346	2.1
19731	2.42984	1.76395
19732	3.41731	0.897052
19733	1.2258	2.1
19734	1.2966	2.1
19735	1.2966	2.1
19736	1.37392	2.1
19737	1.41758	2.1
19738	1.35504	2.1
19739	1.35504	2.1
19740	1.30347	2.1
19741	1.50877	2.1
19742	1.11193	2.1
19743	1.40984	1.91438
19744	1.54537	1.58336
19745	1.71638	2.02445
19746	1.77795	1.27467
19747	2.07593	2.1
19748	1.93387	2.1
19749	1.9339	2.1
19750	1.75669	2.1
19751	1.80299	0.96653
19752	1.31251	0.89675
19753	1.31249	0.748964
19754	1.13411	0.691416
19755	1.06313	2.1
19756	1.04921	2.1
19757	1.55766	2.1
19758	1.53728	2.1
19759	1.53729	2.1
19760	1.51789	2.1
19761	0.993021	0.541042
19762	1.02478	0.555741
19763	1.03408	0.546126
19764	1.09056	0.550288
19765	0.697755	2.1
19766	0.632901	2.1
19767	1.34072	0.252133
19768	1.80841	0.285298
19769	1.80841	0.301675
19770	1.17793	0.300833
19771	1.75032	1.72795
19772	1.13076	2.1
19773	2.11851	2.1
19774	1.73255	2.00686
19775	1.73255	2.1

19776	1.11181	1.98088
19777	1.43772	2.1
19778	1.67381	2.1
19779	1.52164	2.1
19780	1.60861	2.1
19781	1.30588	0.14364
19782	1.75241	0.145655
19783	1.33026	0.918186
19784	0.962705	0.738817
19785	0.99678	0.123726
19786	1.30572	0.111574
19787	1.41943	0.111632
19788	1.2683	0.0969123
19789	1.14145	0.0901557
19790	1.01361	0.0814833
19791	1.29943	2.1
19792	1.3483	2.1
19793	1.34832	2.1
19794	1.83488	2.1
19795	1.71065	2.1
19796	1.66178	2.1
19797	1.39076	2.1
19798	1.37456	2.1
19799	1.66126	2.1
19800	1.562 2.1	
19801	1.37456	2.1
19802	1.36065	2.1
19803	1.04026	0.463058
19804	1.51898	0.496879
19805	1.67088	0.361481
19806	1.9726	0.424351
19807	1.74852	1.33464
19808	1.95512	1.44048
19809	1.16165	0.86548
19810	1.06584	0.695911
19811	1.39327	2.1
19812	1.41394	2.1
19813	1.41394	2.1
19814	1.45506	2.1
19815	1.39523	2.1
19816	1.21119	2.1
19817	1.09005	2.1
19818	0.921311	2.1
19819	1.58164	2.1
19820	1.77574	2.1
19821	1.73184	2.1
19822	1.59423	2.1
19823	1.8214	2.06758
19824	3.10544	0.94082
19825	1.01243	1.75625
19826	1.30896	0.844446
19827	2.16956	1.03383
19828	1.29608	0.754028
19829	1.38617	1.84887
19830	1.49536	1.58789
19831	2.35239	1.23112
19832	1.24613	1.55542
19833	1.94351	2.1

19834	2.05361	2.1
19835	2.05358	2.1
19836	2.38994	2.1
19837	1.13533	2.1
19838	1.08276	2.1
19839	1.55611	0.186676
19840	1.33957	0.166259
19841	1.33957	0.144487
19842	1.69011	0.203814
19843	1.5515	0.751106
19844	1.59106	0.719545
19845	1.02958	1.957
19846	1.12266	2.1
19847	1.12266	2.03508
19848	1.24925	2.1
19849	1.80223	2.1
19850	1.20242	1.03016
19851	2.13665	0.610546
19852	1.3924	0.615328
19853	0.904894	0.984914
19854	1.82633	1.94704
19855	1.94138	0.0889744
19856	1.34009	0.073844
19857	1.15096	2.1
19858	0.994292	2.1
19859	1.10101	2.1
19860	1.15096	2.1
19861	1.50438	2.1
19862	1.67825	2.1
19863	1.67825	2.1
19864	1.91894	2.1
19865	2.04664	2.1
19866	1.92063	2.1
19867	1.72857	2.1
19868	1.89757	2.1
19869	1.61843	0.095441
19870	1.49431	0.0910777
19871	2.33925	2.1
19872	1.80683	2.1
19873	1.64257	2.1
19874	1.50108	2.1
19875	2.82561	2.1
19876	2.97847	2.1
19877	2.97848	2.1
19878	3.20034	2.1
19879	0.66055	2.1
19880	1.95919	2.1
19881	1.07976	2.1
19882	1.30237	2.1
19883	1.99943	0.101564
19884	1.31802	0.0985714
19885	1.53336	0.136667
19886	1.31802	0.107151
19887	1.27737	0.731354
19888	1.44848	0.711667
19889	2.35499	0.113727
19890	1.30041	0.109351
19891	2.35499	0.0924315

19892	1.65381	0.0723203
19893	0.996083	2.1
19894	1.28754	2.1
19895	0.996081	2.1
19896	1.44859	2.1
19897	1.24763	1.59561
19898	1.03697	1.85271
19899	0.951273	1.36293
19900	1.60018	0.985621
19901	2.0815	1.42608
19902	1.20488	1.75526
19903	2.95785	2.1
19904	2.52245	1.3258
19905	1.68455	0.103095
19906	1.12971	0.0883199
19907	1.1952	0.412081
19908	2.0815	0.526655
19909	1.3963	2.1
19910	1.4934	2.1
19911	1.47973	2.1
19912	1.25559	2.1
19913	1.1233	2.1
19914	1.15009	2.1
19915	1.15009	2.1
19916	0.977775	2.1
19917	1.16482	0.910744
19918	1.29313	0.569323
19919	1.29313	0.673064
19920	1.294	0.57758
19921	1.48693	0.309195
19922	1.47608	0.301156
19923	1.36005	1.70591
19924	1.47109	2.1
19925	1.47109	2.07589
19926	1.3515	2.1
19927	2.48828	2.1
19928	2.32378	2.1
19929	2.32378	2.1
19930	2.1692	2.1
19931	1.03217	0.850751
19932	0.953521	0.919915
19933	1.4515	0.136666
19934	3.72406	0.130463
19935	0.963395	1.47306
19936	1.01346	0.956355
19937	1.62241	2.1
19938	1.62027	2.1
19939	1.62027	2.1
19940	1.61687	2.1
19941	1.2593	0.0767291
19942	0.981586	0.0826056
19943	0.721961	2.1
19944	0.888955	2.1
19945	1.22031	0.0522355
19946	1.27566	2.1
19947	2.50066	2.1
19948	1.19814	2.1
19949	1.42278	0.0857553

19950	2.02657	0.0754631
19951	1.0756	0.681627
19952	1.10049	0.662831
19953	2.26155	2.1
19954	2.15634	2.1
19955	2.15634	2.1
19956	2.01613	2.1
19957	1.94233	1.14375
19958	1.18127	1.65622
19959	1.36865	1.67378
19960	1.75755	1.557
19961	2.01613	2.1
19962	1.78958	2.1
19963	1.97995	2.1
19964	1.79198	2.1
19965	1.20841	2.1
19966	1.6519	2.1
19967	1.80207	2.1
19968	1.66774	2.1
19969	1.17935	0.133458
19970	1.88071	0.194109
19971	1.58723	0.172069
19972	1.7361	0.156116
19973	0.794165	2.1
19974	1.21327	2.1
19975	1.21327	2.1
19976	1.34268	2.1
19977	1.34497	0.0750169
19978	0.793303	0.0760591
19979	1.12323	0.0717493
19980	1.34496	0.0714373
19981	0.928226	0.33137
19982	1.89804	0.381346
19983	2.60141	0.0522355
19984	2.60142	2.1
19985	2.60772	0.54734
19986	0.99747	1.86198
19987	1.27096	0.778123
19988	1.27096	0.628529
19989	1.03472	0.783318
19990	1.09962	0.482411
19991	1.21148	0.811425
19992	1.21148	0.80592
19993	1.35025	1.9307
19994	1.09795	2.01521
19995	1.00775	2.1
19996	1.07162	2.1
19997	1.60665	2.02551
19998	1.52984	1.95444
19999	1.61179	2.1
20000	1.70057	2.1
20001	1.76063	2.1
20002	1.14547	0.438041
20003	0.964782	0.39827
20004	1.17208	0.396752
20005	0.964769	0.350028
20006	1.04268	2.1
20007	1.57459	2.1

20008	1.57459	2.1
20009	1.32449	2.1
20010	2.07527	0.844727
20011	1.00013	0.677354
20012	0.813711	0.0522355
20013	1.05677	1.96071
20014	1.30664	1.23236
20015	1.96011	1.83558
20016	1.7151	1.04863
20017	1.61309	1.09606
20018	1.18954	0.523478
20019	1.05447	1.00514
20020	1.05446	0.37907
20021	1.11787	0.388689
20022	1.44437	2.1
20023	1.84739	1.82367
20024	1.84739	1.52393
20025	1.06203	1.95719
20026	1.78391	2.1
20027	1.89267	2.1
20028	1.9212	2.1
20029	2.02997	2.1
20030	1.32051	0.304611
20031	1.47169	0.260095
20032	1.20691	0.10272
20033	1.72271	0.0912193
20034	1.2858	0.476779
20035	1.31011	0.751863
20036	1.31011	0.466755
20037	1.34124	0.718541
20038	1.294	0.74724
20039	1.03079	0.59106
20040	1.83192	2.05617
20041	1.81979	2.1
20042	1.81979	2.1
20043	1.57829	2.1
20044	1.35435	1.82195
20045	1.19023	2.01886
20046	0.991115	2.1
20047	1.27725	2.1
20048	1.38204	2.1
20049	1.48013	2.1
20050	0.983493	2.1
20051	2.26155	2.1
20052	1.02311	0.649336
20053	1.55485	0.596393
20054	1.71033	0.592707
20055	2.07319	0.529907
20056	2.29074	0.642522
20057	1.50822	0.745201
20058	2.08147	1.42667
20059	1.1585	2.1
20060	3.74092	0.888475
20061	2.40799	2.1
20062	2.05349	1.21557
20063	1.92656	2.1
20064	1.09634	0.193868
20065	1.45502	0.302775

20066	1.17261	0.341452
20067	1.29383	0.299783
20068	1.29383	0.300345
20069	1.47689	0.292566
20070	1.85717	0.459785
20071	1.53798	0.329204
20072	1.9493	2.00552
20073	2.0371	1.25399
20074	1.8519	1.60638
20075	1.92119	1.07936
20076	1.00764	0.150665
20077	1.0302	0.141943
20078	1.0302	0.136974
20079	0.90047	0.139527
20080	1.49155	0.214215
20081	2.26403	0.144781
20082	2.06158	0.254003
20083	1.09506	0.243496
20084	1.9212	1.11033
20085	1.99586	0.926907
20086	1.99586	0.981869
20087	2.07527	0.912912
20088	1.20478	2.1
20089	1.09662	2.1
20090	1.55961	2.1
20091	1.03472	2.1
20092	1.54983	2.1
20093	2.02953	2.1
20094	1.84502	2.1
20095	2.1221	2.1
20096	1.02969	1.54327
20097	2.23523	1.91441
20098	1.2377	0.441481
20099	1.03818	0.491525
20100	1.978 2.1	
20101	1.78159	2.1
20102	1.45762	2.1
20103	1.34234	2.1
20104	2.01624	2.1
20105	2.52072	2.1
20106	1.23013	2.1
20107	2.5593	2.1
20108	3.06464	1.1512
20109	2.63697	0.753226
20110	2.63697	0.96571
20111	1.87111	0.650905
20112	1.04574	0.204653
20113	2.13428	0.179007
20114	1.61715	2.1
20115	1.44137	2.1
20116	1.44867	2.1
20117	1.18283	2.1
20118	1.50584	0.171085
20119	1.45466	0.17866
20120	1.4959	0.214003
20121	1.93489	0.21806
20122	2.60772	2.1
20123	2.68868	2.1

20124	2.68868	2.1
20125	2.82561	2.1
20126	1.49654	2.1
20127	1.38672	2.1
20128	1.36813	2.1
20129	1.98396	2.1
20130	1.93852	1.27908
20131	2.14237	0.895129
20132	1.44421	0.143361
20133	1.21749	0.127063
20134	1.09572	0.122113
20135	1.11081	0.0980722
20136	2.32363	2.1
20137	1.24375	2.1
20138	3.79242	2.1
20139	3.46787	2.1
20140	1.19854	1.41094
20141	1.35487	1.4098
20142	1.35486	1.59336
20143	1.38616	1.56881
20144	1.67265	0.40059
20145	0.909487	0.349661
20146	2.53562	2.1
20147	1.09564	2.1
20148	2.9068	2.1
20149	3.57307	0.644437
20150	2.50369	1.07983
20151	1.47348	2.03015
20152	1.24925	2.02961
20153	1.3991	2.1
20154	1.3991	2.1
20155	1.52984	2.1
20156	1.60428	2.1
20157	1.50097	2.1
20158	1.50097	2.1
20159	1.40239	2.1
20160	2.19621	2.1
20161	2.35003	2.1
20162	1.57932	0.133076
20163	1.61194	0.110211
20164	2.0815	0.08639
20165	1.18539	0.0749202
20166	1.5795	0.895591
20167	1.53064	1.08904
20168	1.89227	0.0622808
20169	3.01037	0.0589978
20170	2.08149	1.6412
20171	2.06678	1.56576
20172	1.89226	0.090655
20173	3.47888	0.0941524
20174	1.89226	0.159008
20175	3.65842	0.107638
20176	3.88546	0.178292
20177	1.89226	0.283885
20178	1.11239	2.1
20179	1.29133	0.101184
20180	0.889309	0.103926
20181	0.881934	0.121715

20182	1.88406	2.04089
20183	0.636946	1.87609
20184	0.636945	1.38269
20185	1.28073	2.1
20186	1.28075	1.91364
20187	0.942007	1.916
20188	1.68436	2.1
20189	1.4677	0.0972451
20190	0.851542	0.149023
20191	1.39063	0.116028
20192	0.851539	0.136131
20193	1.11597	0.133903
20194	1.35403	0.128778
20195	0.959473	2.1
20196	1.07976	2.1
20197	0.946129	1.08344
20198	0.946129	0.318452
20199	1.38464	0.565432
20200	1.36991	0.399781
20201	0.946131	0.481933
20202	0.94613	0.796973
20203	1.34493	0.39945
20204	1.36991	0.393242
20205	1.36991	0.30765
20206	0.946131	0.370454
20207	0.78392	0.845046
20208	0.80282	0.812298
20209	1.10351	0.836717
20210	0.80846	0.914586
20211	0.838026	0.826345
20212	1.08558	0.83295
20213	0.718203	0.90012
20214	0.501866	0.71628
20215	0.85426	0.923973
20216	1.24249	0.18004
20217	1.37052	0.260273
20218	0.851541	0.278021
20219	1.0092	0.188062
20220	0.851554	0.239356
20221	1.49826	0.216257
20222	1.13727	0.154852
20223	0.778149	0.249067
20224	1.20257	0.402855
20225	0.946133	0.395891
20226	0.778149	0.280999
20227	0.77815	0.41518
20228	0.946132	2.1
20229	1.17602	2.1
20230	0.946132	2.1
20231	1.31257	2.1
20232	1.17602	2.1
20233	1.35821	0.372915
20234	1.19993	0.301384
20235	0.946131	0.324658
20236	0.946131	0.372603
20237	1.08848	0.374458
20238	1.00509	2.1
20239	1.19629	2.1

20240	0.867593	2.1
20241	0.757921	2.1
20242	0.729116	0.101911
20243	0.89162	0.113652
20244	1.16811	0.101195
20245	1.3676	2.1
20246	1.2638	0.868139
20247	0.946132	2.07448
20248	0.946132	2.1
20249	1.2638	2.09036
20250	1.00185	2.1
20251	1.60948	2.1
20252	0.602075	2.0693
20253	0.942611	2.1
20254	0.946132	2.07023
20255	0.942611	2.1
20256	1.11822	2.1
20257	1.21558	0.290848
20258	0.607788	0.34879
20259	0.827029	0.327023
20260	1.00691	0.678178
20261	0.607789	0.341148
20262	0.607775	0.590731
20263	1.23248	0.890809
20264	0.902653	1.00243
20265	0.639143	1.03147
20266	0.851545	0.91488
20267	0.605153	0.885276
20268	1.06826	2.1
20269	1.29365	2.1
20270	1.41232	2.1
20271	0.946131	2.1
20272	1.20854	2.1
20273	0.946131	2.1
20274	0.909005	0.109099
20275	1.16672	0.0936168
20276	0.810959	0.0955022
20277	0.946131	0.16076
20278	1.40104	0.124019
20279	0.946131	0.151262
20280	1.35403	0.141303
20281	1.40104	0.137426
20282	1.92535	0.112685
20283	2.14534	0.108095
20284	1.77532	0.141372
20285	0.851541	0.199495
20286	1.05585	0.228484
20287	1.27098	0.186275
20288	0.870925	0.216258
20289	1.15547	0.220201
20290	1.47583	0.164307
20291	1.35007	0.126718
20292	0.740095	0.0775896
20293	0.644912	0.0815328
20294	0.819489	0.0750285
20295	0.983028	0.254846
20296	0.861849	0.0769693
20297	1.40511	0.0836937

20298	0.86182	0.0958541
20299	0.9705	0.0852035
20300	1.40511	0.0830348
20301	1.15539	2.1
20302	0.946119	1.45238
20303	0.736382	2.1
20304	0.736384	2.1
20305	1.41561	2.1
20306	0.785651	2.1
20307	1.34479	2.1
20308	1.38885	2.1
20309	0.94613	2.1
20310	1.29461	2.1
20311	0.94613	2.1
20312	0.776179	1.48865
20313	0.858611	2.04793
20314	0.946128	1.46576
20315	1.04073	2.09552
20316	1.16399	2.05362
20317	0.990019	2.1
20318	1.15564	1.94749
20319	1.28956	0.259807
20320	1.35145	0.266428
20321	1.09662	2.1
20322	1.8155	0.0692296
20323	0.811459	0.0643695
20324	0.731982	0.0623774
20325	0.900554	0.069619
20326	0.946127	0.766085
20327	0.817066	0.787656
20328	0.817064	0.773817
20329	1.41798	0.813348
20330	0.871606	0.791799
20331	1.30265	0.210826
20332	0.849402	1.65004
20333	0.868582	1.20486
20334	0.909137	1.33062
20335	0.807016	1.26782
20336	0.83393	0.655023
20337	0.9034	0.985181
20338	1.53515	0.993106
20339	1.17665	0.385085
20340	0.946132	0.546618
20341	0.946129	0.372073
20342	1.35977	0.360548
20343	1.34254	0.330132
20344	1.14241	0.26978
20345	0.946132	0.388717
20346	0.946132	0.267036
20347	1.27376	0.269348
20348	1.35978	0.251257
20349	1.01976	0.243761
20350	1.23498	0.882563
20351	1.35002	0.940558
20352	1.11683	1.10273
20353	0.88359	1.17173
20354	0.751374	1.15307
20355	0.791714	1.1139

20356	1.6067	2.1
20357	0.923782	2.1
20358	1.44689	2.1
20359	0.94613	2.08367
20360	0.946131	2.1
20361	1.5261	2.1
20362	1.26247	2.1
20363	1.80085	0.140483
20364	1.7693	0.128752
20365	0.900422	0.125317
20366	1.31357	0.193617
20367	0.94613	0.178607
20368	0.900424	0.171178
20369	0.900397	0.164919
20370	1.39448	0.286434
20371	1.36769	0.0940613
20372	0.713524	0.140727
20373	0.713554	0.0992914
20374	0.946124	0.130147
20375	1.24979	0.100681
20376	1.04765	0.128882
20377	0.70054	0.0777247
20378	0.776991	0.090131
20379	0.738905	0.0819674
20380	1.32605	0.0774596
20381	0.784957	0.0763291
20382	0.803955	0.0874682
20383	0.819486	0.0788826
20384	1.79306	0.161974
20385	1.18275	0.182389
20386	1.25626	0.156863
20387	0.936806	0.142898
20388	0.653124	0.199495
20389	0.946132	0.188768
20390	0.653151	0.184086
20391	1.10748	0.25359
20392	1.95244	2.1
20393	1.40967	1.67049
20394	1.27916	1.49977
20395	0.946132	0.521765
20396	0.946132	0.702093
20397	1.30555	0.766789
20398	1.38651	0.725669
20399	0.946132	0.687309
20400	1.23571	0.760762
20401	1.21261	1.17955
20402	1.34225	1.0327
20403	0.946132	1.40334
20404	0.734059	0.570471
20405	0.734084	1.01718
20406	0.78438	0.382666
20407	1.13748	0.643831
20408	0.946131	0.880091
20409	0.78438	0.383786
20410	0.784392	0.6907
20411	0.626495	2.1
20412	0.694635	2.1
20413	0.946125	2.1

20414	0.694632	2.1
20415	1.38521	2.1
20416	0.795469	2.1
20417	0.850877	2.1
20418	1.37098	2.1
20419	0.946131	2.1
20420	0.79547	2.1
20421	0.946132	0.846724
20422	1.30448	1.21442
20423	0.628361	0.712383
20424	0.686964	0.74516
20425	0.493261	0.775846
20426	1.90912	1.73862
20427	0.946127	1.18482
20428	1.36604	0.658638
20429	0.671687	0.799382
20430	0.946128	0.90707
20431	0.671699	0.725159
20432	1.04973	2.1
20433	0.982858	0.226339
20434	0.974081	0.06151
20435	1.69993	0.060433
20436	1.00763	0.692303
20437	0.755566	2.1
20438	0.946129	2.1
20439	1.04184	2.1
20440	1.13509	2.1
20441	0.755566	2.1
20442	1.114 2.1	
20443	0.810946	2.1
20444	0.786259	2.1
20445	1.43455	2.1
20446	1.05071	2.1
20447	0.746095	2.03247
20448	0.82737	2.1
20449	0.946126	1.83393
20450	1.04071	2.1
20451	1.0852	2.1
20452	1.59019	0.0823455
20453	0.621972	0.102194
20454	1.14475	0.103396
20455	0.691062	0.114386
20456	1.20057	0.0998889
20457	0.846868	0.105246
20458	1.26062	0.0855792
20459	0.795093	0.0936642
20460	1.20056	0.0932849
20461	0.795065	0.105138
20462	1.18203	0.0909874
20463	1.20057	0.0931037
20464	0.991869	0.323607
20465	0.841088	2.1
20466	0.946131	2.1
20467	0.869097	2.1
20468	0.946131	2.1
20469	0.841089	2.1
20470	1.77867	2.1
20471	1.99296	0.163116

20472	1.91703	2.1
20473	0.651334	0.197494
20474	1.31467	0.202026
20475	0.651332	0.182592
20476	0.651332	0.19319
20477	1.03079	0.205256
20478	0.808519	0.144417
20479	1.41417	0.118641
20480	1.03165	0.287205
20481	1.43577	2.1
20482	1.39053	2.1
20483	1.07006	1.99626
20484	0.946132	2.1
20485	0.946133	2.1
20486	1.07006	2.1
20487	1.12717	2.1
20488	0.994293	2.1
20489	0.946132	2.1
20490	1.31912	2.1
20491	0.946131	2.1
20492	1.40571	2.1
20493	1.31912	2.1
20494	1.12292	0.292959
20495	1.07755	0.370806
20496	0.851539	0.321835
20497	0.946131	0.269093
20498	0.929834	0.222789
20499	1.83919	0.478164
20500	1.04713	0.316342
20501	0.929726	0.220531
20502	1.50985	0.235944
20503	0.946127	0.230874
20504	1.0227	0.235001
20505	1.2582	0.227043
20506	1.1391	0.679944
20507	0.780831	0.805916
20508	0.78086	0.702395
20509	0.946133	0.79596
20510	0.780831	0.667219
20511	1.37762	0.726039
20512	0.946116	1.28348
20513	1.22882	2.1
20514	1.40015	0.97346
20515	1.40017	2.03042
20516	0.946105	2.06496
20517	0.553567	0.0774908
20518	1.19263	0.0713723
20519	0.946132	0.0749787
20520	0.553594	0.0710609
20521	1.19263	0.075965
20522	1.18237	0.0712439
20523	0.946133	0.0697675
20524	0.978467	0.0667063
20525	0.56262	0.0673029
20526	0.946132	0.0680379
20527	0.562633	0.0666171
20528	0.900175	0.0650843
20529	1.04627	1.06968

20530	0.549928	0.699369
20531	1.17765	0.830989
20532	1.11479	0.927291
20533	0.398261	0.80638
20534	0.927608	0.98951
20535	1.38467	0.725592
20536	1.04824	0.624793
20537	1.33893	0.856101
20538	1.55775	0.812492
20539	0.816895	0.887421
20540	1.96057	1.01724
20541	1.35798	2.1
20542	1.29262	2.1
20543	0.94613	2.1
20544	0.94613	2.1
20545	1.29261	2.1
20546	1.18067	2.1
20547	0.946131	2.1
20548	0.946132	2.1
20549	1.27809	2.1
20550	1.35799	2.1
20551	1.03933	2.1
20552	1.49438	1.35489
20553	0.946132	1.88896
20554	1.13537	2.1
20555	1.41666	1.29815
20556	1.74237	2.1
20557	0.946132	2.1
20558	1.04291	0.249464
20559	1.63802	2.1
20560	1.24714	1.22392
20561	0.842013	1.61914
20562	0.810959	2.1
20563	0.767923	0.401538
20564	1.19583	0.408209
20565	0.767921	0.359856
20566	0.767921	0.389634
20567	0.961348	0.456383
20568	2.08687	1.36807
20569	0.739729	0.593308
20570	1.04342	0.548294
20571	0.739757	0.569523
20572	0.946129	0.611417
20573	1.04341	0.76963
20574	1.38683	0.318505
20575	1.34153	0.334644
20576	0.946129	0.309241
20577	0.946131	0.299691
20578	1.02028	0.255516
20579	1.47606	0.241562
20580	1.20739	0.297827
20581	0.957684	0.252515
20582	0.946116	0.328742
20583	0.851528	0.323318
20584	0.957685	0.309058
20585	1.04504	2.1
20586	0.902938	2.1
20587	0.946132	2.1

20588	1.07566	2.1
20589	1.4558	2.1
20590	1.93286	2.1
20591	1.27241	2.1
20592	0.828671	2.1
20593	0.810933	2.1
20594	1.31335	0.0777563
20595	1.40305	0.0740696
20596	0.912698	0.096295
20597	0.810946	0.0916411
20598	1.11608	0.0894396
20599	1.26409	0.445931
20600	0.946132	0.466563
20601	1.27376	0.511565
20602	0.946132	0.483064
20603	1.03333	0.517341
20604	1.27376	0.514399
20605	0.94613	0.310482
20606	0.812384	0.295384
20607	0.687744	0.23508
20608	0.946132	0.3336
20609	0.687758	0.297863
20610	1.09667	0.252645
20611	1.56977	2.1
20612	0.851544	2.0034
20613	1.01136	2.1
20614	0.851481	1.68904
20615	0.946131	2.1
20616	0.705409	2.1
20617	1.32449	0.14326
20618	1.13508	0.130768
20619	0.94613	0.180287
20620	0.94613	0.160019
20621	0.965822	0.159109
20622	1.95301	0.1855
20623	0.851538	0.371612
20624	1.29262	0.344127
20625	0.95417	0.259484
20626	0.851541	0.39785
20627	0.94613	0.355948
20628	1.02727	0.783063
20629	1.37675	1.1899
20630	0.955646	0.674686
20631	0.95114	0.811947
20632	0.851528	0.792612
20633	1.76513	1.20597
20634	0.87155	2.1
20635	0.94613	2.1
20636	0.871519	2.1
20637	0.966631	2.1
20638	1.37791	2.1
20639	1.19122	0.517448
20640	0.818971	1.07523
20641	0.628799	0.466925
20642	0.861027	0.86952
20643	0.800034	0.114329
20644	0.91846	0.0995723
20645	0.765645	0.100947

20646	1.13709	0.714245
20647	1.29054	0.791152
20648	0.755336	0.888617
20649	0.755334	0.794639
20650	0.946133	0.910932
20651	0.755336	0.685675
20652	1.14444	0.785299
20653	0.872823	0.817705
20654	0.533645	0.767923
20655	0.946132	0.710755
20656	0.533645	0.578852
20657	1.02311	0.655278
20658	1.00278	2.1
20659	1.1016	0.0911604
20660	0.629615	0.0938389
20661	1.12628	0.11035
20662	0.946131	0.115244
20663	0.629615	0.0912125
20664	0.629641	0.107103
20665	1.67046	1.28398
20666	1.81667	2.1
20667	1.7145	2.06884
20668	0.94613	2.1
20669	1.29322	2.1
20670	1.00365	2.1
20671	0.946131	2.1
20672	1.29322	2.1
20673	1.27581	2.1
20674	1.33916	2.1
20675	1.32784	2.1
20676	1.74787	2.1
20677	2.28084	2.1
20678	2.98092	2.1
20679	0.946132	0.736302
20680	1.2435	2.1
20681	0.946132	1.59486
20682	1.4099	0.772093
20683	1.2435	1.186
20684	1.26438	2.1
20685	0.745287	2.1
20686	0.696827	2.1
20687	0.696781	2.1
20688	1.41195	2.1
20689	0.946131	2.1
20690	1.8758	0.946713
20691	0.832979	0.589516
20692	0.75054	0.406006
20693	0.810958	0.473694
20694	0.922889	0.856612
20695	0.694844	0.596462
20696	0.694814	0.727404
20697	0.946131	0.67131
20698	0.694844	0.682319
20699	1.02369	2.1
20700	1.4468	0.152514
20701	0.946125	0.382282
20702	0.93487	0.322208
20703	1.23608	0.472223

20704	1.34257	0.231775
20705	0.851525	0.328858
20706	1.03298	0.315973
20707	1.22418	0.775231
20708	0.946131	1.48372
20709	1.41833	1.0379
20710	1.23452	0.885394
20711	0.946131	0.604663
20712	1.41833	0.755632
20713	1.13077	0.311979
20714	0.942874	1.44719
20715	0.946132	1.21969
20716	0.504366	1.40878
20717	0.593647	2.1
20718	0.898829	1.4847
20719	1.27921	2.06495
20720	1.12503	2.1
20721	1.22539	2.1
20722	1.10332	0.88093
20723	0.851541	1.91643
20724	0.94613	2.00952
20725	0.945109	2.1
20726	0.607962	2.1
20727	0.946133	2.1
20728	0.607961	2.1
20729	0.661956	2.1
20730	1.35048	2.1
20731	1.66018	2.1
20732	0.956467	2.1
20733	0.851542	2.1
20734	0.890734	2.1
20735	0.802455	2.1
20736	1.35943	2.1
20737	1.04798	2.1
20738	1.26426	0.600055
20739	1.3689	0.519183
20740	0.946133	0.532882
20741	0.946133	0.620476
20742	1.09729	0.589199
20743	0.765259	2.1
20744	0.810945	2.1
20745	0.810957	2.1
20746	1.10284	1.80493
20747	1.09368	2.1
20748	0.772601	2.1
20749	0.788011	2.1
20750	0.810934	2.1
20751	0.694357	0.878912
20752	1.22641	0.975688
20753	0.946132	1.37884
20754	0.946132	0.987019
20755	0.946133	0.821939
20756	1.42754	1.466
20757	0.776414	0.738708
20758	0.806525	1.10091
20759	0.575339	0.816417
20760	0.97154	0.707928
20761	0.8217	1.01813

20762	0.838039	1.25966
20763	0.94613	0.894022
20764	0.946132	0.707965
20765	1.39575	0.521772
20766	1.39575	0.637107
20767	1.12818	0.636568
20768	0.946132	1.12531
20769	0.946133	0.886756
20770	1.06879	0.614061
20771	1.06879	0.776969
20772	0.970358	0.745836
20773	1.2474	0.412835
20774	1.01919	0.11665
20775	0.94613	0.136451
20776	1.28952	0.1399
20777	1.00809	0.119009
20778	0.849344	0.122381
20779	0.640304	0.134642
20780	1.35065	0.106891
20781	0.946132	0.105095
20782	0.640333	0.0991873
20783	0.946132	0.125998
20784	0.685489	2.1
20785	0.94613	2.1
20786	0.68546	2.1
20787	0.874264	2.1
20788	1.27676	2.1
20789	0.946129	0.281727
20790	1.09024	0.252702
20791	0.714563	0.254639
20792	1.12335	0.238188
20793	1.07931	0.231503
20794	1.17799	0.362594
20795	1.8795	0.249995
20796	2.15229	0.245008
20797	1.13859	0.529014
20798	0.946132	1.00542
20799	1.11374	0.648896
20800	1.1386	0.661917
20801	0.615713	0.759189
20802	1.19322	0.319995
20803	1.3377	0.230329
20804	0.670801	0.270276
20805	0.94613	0.762917
20806	0.66617	0.51639
20807	1.36173	0.531165
20808	0.980473	0.444334
20809	1.39132	0.199028
20810	0.864653	0.0964269
20811	1.23837	0.0858895
20812	0.810959	0.085608
20813	1.1483	0.224517
20814	1.00381	1.4276
20815	0.946128	1.54586
20816	1.39696	1.13818
20817	0.946131	0.987538
20818	0.946131	1.0742
20819	1.38613	1.13975

20820	0.973501	1.65652
20821	0.946127	1.03538
20822	1.35968	1.01647
20823	0.946131	0.894927
20824	0.946129	1.18489
20825	1.39696	1.12487
20826	1.12088	2.07318
20827	1.41122	1.73448
20828	1.14464	0.7326
20829	0.946131	1.84305
20830	0.946131	1.43853
20831	1.14464	1.64105
20832	1.33266	1.46412
20833	1.07341	0.904457
20834	0.827287	1.48229
20835	0.946132	1.21519
20836	0.827287	1.80826
20837	1.15644	1.29188
20838	1.27558	0.567471
20839	1.15067	0.588102
20840	0.946131	1.49084
20841	1.29143	0.501149
20842	1.36281	1.67506
20843	1.36281	0.684236
20844	0.946131	0.701397
20845	1.40863	2.1
20846	1.20143	0.269569
20847	1.19755	0.266924
20848	0.566153	0.116408
20849	0.946131	0.193433
20850	1.07979	0.123359
20851	1.00463	0.189218
20852	0.566153	0.261903
20853	0.768212	2.1
20854	1.32461	1.97457
20855	1.13268	2.1
20856	1.13268	1.5838
20857	0.946133	1.48296
20858	1.53642	2.07728
20859	1.5791	2.1
20860	0.726635	1.8224
20861	1.2334	1.49817
20862	1.29198	1.66514
20863	1.29198	1.09161
20864	0.946131	0.916242
20865	0.956585	2.1
20866	1.25294	2.1
20867	0.946133	2.03337
20868	1.35287	2.1
20869	1.23877	1.91177
20870	0.946131	2.00162
20871	1.35287	1.94805
20872	1.01843	2.1
20873	3.77569	2.1
20874	0.86707	2.1
20875	0.867066	2.1
20876	1.38639	2.1
20877	1.38639	2.1

20878	0.896168	2.1
20879	0.974716	0.208592
20880	1.09411	0.261478
20881	0.663171	0.237085
20882	0.663171	0.335881
20883	0.946132	0.319091
20884	0.66317	0.296071
20885	1.47104	0.184577
20886	1.13629	0.204451
20887	0.772342	0.175661
20888	0.772312	0.245974
20889	0.94613	0.22525
20890	0.77234	0.218084
20891	1.43154	2.1
20892	1.37704	2.1
20893	1.40025	2.1
20894	1.3063	2.1
20895	0.946132	2.1
20896	1.04075	2.1
20897	0.946131	2.1
20898	0.946118	1.13018
20899	0.750011	0.440007
20900	1.0971	0.66836
20901	0.889667	0.564133
20902	0.709598	0.684305
20903	2.39737	0.720238
20904	1.19974	0.142026
20905	0.94613	0.252897
20906	1.25809	0.173091
20907	1.19974	0.214211
20908	0.73005	0.313339
20909	1.50764	0.117698
20910	1.25809	0.145539
20911	0.946133	0.247415
20912	1.10357	0.176621
20913	1.25809	0.205803
20914	0.628177	0.277605
20915	1.3543	0.165788
20916	0.793711	0.216243
20917	1.18455	0.235156
20918	1.03966	0.200235
20919	0.946131	0.233509
20920	0.767453	0.166687
20921	0.612292	0.24153
20922	0.946131	0.19885
20923	1.24128	0.227194
20924	1.08761	0.155621
20925	0.762059	0.102406
20926	1.005 2.1	
20927	1.17054	2.1
20928	1.12665	2.1
20929	0.750368	2.1
20930	0.851542	2.1
20931	0.975436	2.1
20932	0.975442	2.1
20933	0.675367	2.1
20934	1.51973	0.330818
20935	0.94613	0.311416

20936	1.38954	0.334528
20937	0.946131	0.315156
20938	1.07912	0.33563
20939	1.38954	0.329115
20940	2.0219	2.1
20941	1.18347	0.271762
20942	0.946132	0.596198
20943	0.94613	0.438947
20944	1.00202	0.372654
20945	1.00201	0.383779
20946	0.946132	0.468431
20947	0.946132	0.692875
20948	1.37981	0.537242
20949	1.26874	0.459225
20950	1.26874	0.45431
20951	0.946132	0.511349
20952	2.8687	0.32587
20953	1.42884	2.1
20954	1.05188	2.1
20955	0.625307	2.1
20956	0.810956	2.1
20957	0.526656	2.1
20958	0.526684	2.1
20959	0.724122	2.1
20960	0.946132	2.1
20961	0.526684	2.1
20962	1.05337	2.1
20963	1.0593	2.1
20964	1.47981	2.1
20965	1.74431	2.03296
20966	0.885612	2.04546
20967	0.810946	1.37441
20968	0.845185	1.62759
20969	1.38622	0.670031
20970	0.723194	0.620499
20971	0.554302	0.734691
20972	0.709597	0.567277
20973	0.909507	0.599445
20974	0.912754	0.437132
20975	1.15174	0.352988
20976	0.946131	0.461554
20977	0.912753	0.45451
20978	1.15174	0.462266
20979	1.02011	0.220026
20980	0.792754	1.83399
20981	0.946129	2.09079
20982	0.792754	1.74139
20983	1.27575	1.65859
20984	0.94613	1.637
20985	1.60132	2.02489
20986	0.922455	2.01682
20987	1.18713	1.39444
20988	0.926957	1.60864
20989	1.66525	1.75454
20990	0.946132	2.1
20991	0.851541	2.1
20992	0.961191	2.1
20993	1.27534	2.1

20994	0.851554	2.1
20995	1.0499	2.1
20996	1.01776	0.107606
20997	0.97622	0.106843
20998	0.810934	0.126685
20999	2.6802	0.101407
21000	1.31485	0.101695
21001	1.09699	0.0824414
21002	0.681649	0.0810361
21003	0.790369	0.0780689
21004	0.946131	0.0955015
21005	0.838026	1.38121
21006	0.567833	0.692728
21007	0.891492	1.17446
21008	0.986984	0.528644
21009	0.946132	1.11409
21010	1.19028	0.679694
21011	0.986984	0.630426
21012	0.595623	0.857382
21013	0.946131	2.1
21014	1.36502	2.1
21015	1.08109	2.1
21016	0.946129	2.1
21017	1.36503	2.1
21018	0.773897	1.89995
21019	0.946133	1.14198
21020	0.613447	2.02736
21021	0.613449	1.61715
21022	0.946131	1.85165
21023	1.11163	1.89737
21024	0.966114	2.1
21025	0.735699	2.1
21026	0.662147	2.1
21027	1.04072	2.1
21028	0.946131	2.1
21029	0.862978	2.1
21030	1.48839	0.827578
21031	0.936141	0.731292
21032	0.946131	0.911539
21033	0.936142	1.86753
21034	1.40595	1.34331
21035	0.946131	0.991267
21036	1.05297	0.171288
21037	3.09035	2.1
21038	1.19952	2.1
21039	0.907198	2.1
21040	0.849144	2.1
21041	0.612469	0.539322
21042	0.551237	0.602858
21043	0.985256	0.692788
21044	0.551209	0.616942
21045	0.85153	0.78444
21046	0.624712	2.1
21047	0.900018	2.1
21048	0.851542	2.1
21049	0.864787	2.1
21050	1.27669	2.1
21051	0.946711	0.753755

21052	0.810947	0.816861
21053	0.841547	0.855574
21054	0.764114	0.784199
21055	1.26692	1.40637
21056	0.946132	0.849327
21057	1.37675	1.0495
21058	1.26692	0.869413
21059	1.37545	1.26845
21060	1.49637	0.192789
21061	0.709598	0.235136
21062	0.751252	0.213823
21063	1.36811	0.214175
21064	0.635625	0.21934
21065	1.31713	0.150006
21066	1.18977	2.1
21067	0.893377	1.47709
21068	0.707344	2.1
21069	0.946131	2.1
21070	1.20221	2.1
21071	1.20221	2.1
21072	0.707345	2.1
21073	1.26786	2.1
21074	0.988048	2.05259
21075	0.751293	1.97183
21076	0.632008	1.832
21077	1.51067	2.1
21078	1.13534	2.1
21079	0.946133	2.1
21080	0.946131	2.1
21081	1.25716	2.1
21082	1.3199	2.1
21083	1.09616	2.1
21084	1.36677	1.13279
21085	1.34752	1.23659
21086	0.838041	0.98837
21087	1.67023	2.1
21088	1.02247	0.481818
21089	1.03079	0.457119
21090	0.928982	0.250104
21091	1.13635	0.214801
21092	0.946132	0.26675
21093	0.92898	0.213607
21094	1.13635	0.229848
21095	1.1643	0.152129
21096	0.851541	0.294646
21097	1.39939	0.232877
21098	0.946129	0.298453
21099	1.07775	0.264144
21100	1.14503	0.275271
21101	1.78573	0.163629
21102	1.29827	0.163533
21103	0.872373	0.11484
21104	1.01162	0.124573
21105	0.83804	0.111579
21106	1.48851	0.12087
21107	1.47385	0.108142
21108	0.843858	0.139849
21109	0.938626	0.151394

21110	0.89567	0.126075
21111	1.59457	0.628766
21112	2.09666	0.161416
21113	0.775132	0.0827123
21114	0.927005	0.0957044
21115	0.854224	0.0717664
21116	0.775142	0.076357
21117	0.946106	0.0843359
21118	1.80784	2.1
21119	2.04593	2.1
21120	1.32282	2.1
21121	0.952138	2.1
21122	0.946132	2.1
21123	0.946132	2.1
21124	0.952138	2.1
21125	1.46988	2.1
21126	0.80465	0.23947
21127	1.14155	0.234923
21128	0.810931	0.230375
21129	1.66203	0.377328
21130	0.674605	0.529654
21131	0.946132	0.60421
21132	1.26706	0.652392
21133	0.674633	0.497992
21134	0.946132	0.659422
21135	0.980664	0.617226
21136	1.34927	0.54853
21137	0.758454	0.314734
21138	0.946133	0.3297
21139	0.985371	0.282092
21140	1.28632	0.306239
21141	0.946133	0.393777
21142	1.46868	0.265624
21143	1.7522	0.118096
21144	0.737431	2.1
21145	0.663679	2.1
21146	1.16541	1.69984
21147	1.3179	1.93145
21148	1.04081	1.91985
21149	0.887866	1.6364
21150	0.806961	1.21913
21151	0.929061	1.52476
21152	1.28418	0.3854
21153	1.38752	0.379321
21154	1.33038	0.394293
21155	0.946132	0.265111
21156	0.946132	0.30679
21157	1.14897	0.322033
21158	1.1685	0.321412
21159	1.18774	2.1
21160	0.961899	2.1
21161	1.48841	2.1
21162	1.79105	2.1
21163	3.51614	2.1
21164	0.682342	2.1
21165	0.783184	2.1
21166	0.946132	2.1
21167	0.868396	2.1

21168	1.21813	2.1
21169	0.848305	0.638969
21170	0.94559	0.732249
21171	0.805691	0.836507
21172	0.752217	2.1
21173	0.946132	2.1
21174	0.839123	2.1
21175	0.946131	2.1
21176	0.752187	2.1
21177	1.96491	2.1
21178	0.520997	2.1
21179	0.946132	2.1
21180	0.73446	2.1
21181	0.946133	2.1
21182	0.520995	2.1
21183	1.88522	2.1
21184	1.8361	2.1
21185	1.11285	2.1
21186	1.25334	1.15414
21187	2.00756	2.1
21188	1.44252	0.337369
21189	1.00255	0.916578
21190	1.64516	2.1
21191	1.12399	0.0844881
21192	1.13542	0.100467
21193	1.09038	0.10833
21194	0.746903	0.120953
21195	0.672241	0.0987111
21196	0.871774	0.109204
21197	1.73513	1.07438
21198	0.949995	2.00664
21199	1.17244	1.95101
21200	0.946127	1.96663
21201	1.3028	1.83674
21202	1.17244	1.77348
21203	0.774101	2.1
21204	0.946127	2.1
21205	0.939289	2.1
21206	0.946129	2.1
21207	0.774101	2.1
21208	1.87858	2.1
21209	1.44293	0.143509
21210	1.43213	0.136764
21211	0.946131	2.1
21212	0.716614	2.1
21213	0.716644	2.1
21214	1.35828	2.1
21215	0.790935	2.1
21216	0.730157	2.1
21217	1.41149	2.1
21218	0.730186	2.1
21219	0.730186	2.1
21220	0.946131	2.1
21221	0.946132	1.12541
21222	0.946132	0.908119
21223	0.990713	0.625088
21224	1.40779	0.734731
21225	0.946132	0.799192

21226	1.06486	0.444513
21227	0.946131	0.897827
21228	0.946133	0.649469
21229	1.40779	0.551708
21230	1.26874	0.629586
21231	0.946131	0.634823
21232	1.02588	0.542084
21233	0.826073	0.331181
21234	1.41233	0.315274
21235	0.861269	0.32803
21236	1.04073	0.36882
21237	1.27111	0.395089
21238	1.56016	1.48018
21239	1.60866	0.102282
21240	0.94613	0.138473
21241	0.946129	0.143601
21242	1.36376	0.109501
21243	1.22187	0.136769
21244	0.94613	0.140404
21245	1.82581	1.2207
21246	1.19705	1.05926
21247	1.7513	2.1
21248	1.85017	2.1
21249	1.72116	2.1
21250	1.12058	2.1
21251	0.899991	2.1
21252	0.616288	2.1
21253	1.07289	2.1
21254	1.7092	0.480668
21255	0.946132	0.40612
21256	0.946133	0.217533
21257	1.18717	0.272282
21258	1.40029	0.332289
21259	0.946132	0.239868
21260	0.94613	0.337002
21261	1.18547	0.335555
21262	0.960326	0.331385
21263	0.960345	0.320054
21264	0.942557	0.345089
21265	1.18601	0.26225
21266	0.946132	0.289366
21267	1.30307	0.306956
21268	1.08169	0.26958
21269	1.08169	0.281845
21270	1.31716	0.301493
21271	1.51065	0.788787
21272	0.877642	0.0838205
21273	0.617384	0.078258
21274	0.575741	0.0851312
21275	0.972448	0.0734128
21276	1.11334	0.0836027
21277	1.13092	2.1
21278	0.946127	0.493879
21279	1.31724	0.565865
21280	1.39751	0.376859
21281	1.39751	0.455335
21282	0.946128	0.458192
21283	0.946132	0.395192

21284	0.713284	0.345469
21285	1.40668	0.432485
21286	1.03547	0.356104
21287	0.713298	0.384452
21288	1.88774	0.315196
21289	0.830164	1.68709
21290	1.05211	2.1
21291	0.946119	0.946634
21292	0.946107	2.09169
21293	1.283	2.08779
21294	2.63609	2.0115
21295	1.28418	0.953496
21296	0.946128	0.958158
21297	1.10335	0.860202
21298	0.598377	1.15242
21299	1.18818	0.865077
21300	1.10335	0.840653
21301	1.58972	1.02108
21302	1.90194	2.1
21303	0.946118	2.1
21304	0.946115	2.1
21305	0.950969	2.1
21306	1.36918	2.1
21307	0.950955	2.1
21308	1.4263	2.01857
21309	1.11978	0.768749
21310	0.946132	1.1008
21311	0.946133	1.20194
21312	1.37658	0.787978
21313	1.2364	0.900271
21314	1.05496	0.750165
21315	0.946132	1.21095
21316	0.946132	0.988234
21317	1.21858	0.730921
21318	1.37658	0.921745
21319	1.09194	1.27038
21320	1.14721	0.114371
21321	1.53814	0.0787828
21322	0.546751	0.774219
21323	0.492063	1.15272
21324	1.16889	1.64802
21325	1.00349	1.64691
21326	0.863559	0.92024
21327	1.38102	1.49197
21328	0.597449	0.947211
21329	0.537748	0.73879
21330	1.16297	1.26805
21331	0.999366	1.1865
21332	0.896554	0.858099
21333	0.566962	0.0861531
21334	0.946129	0.10028
21335	0.452729	0.0720995
21336	0.696552	0.0815452
21337	0.852524	0.0875768
21338	1.57992	0.0755139
21339	1.64985	0.100207
21340	0.946128	2.1
21341	1.21847	2.1

21342	0.946129	2.1
21343	1.41131	2.1
21344	1.21847	2.1
21345	1.21269	0.0703848
21346	1.0417	1.8432
21347	1.15821	0.978796
21348	0.946132	0.902595
21349	0.946132	1.49747
21350	1.37967	0.722322
21351	0.851529	0.724835
21352	1.20455	2.1
21353	1.4282	0.0902525
21354	1.26593	2.1
21355	0.838026	0.148552
21356	0.985096	0.130935
21357	0.83804	0.142311
21358	0.822395	1.00596
21359	0.420272	0.543324
21360	0.946131	2.1
21361	1.17461	2.1
21362	0.94613	2.1
21363	1.40225	2.1
21364	1.17461	2.1
21365	1.09847	2.1
21366	1.22222	0.857038
21367	1.41798	0.628058
21368	0.791658	0.719684
21369	0.791656	0.728525
21370	0.946129	0.747261
21371	0.817064	0.715667
21372	0.946114	0.640352
21373	0.75824	0.646077
21374	0.75821	0.655384
21375	1.41778	0.611648
21376	0.791635	0.619965
21377	1.04164	1.66663
21378	0.946131	0.802967
21379	1.11172	0.666738
21380	1.30076	0.542868
21381	1.35721	0.598728
21382	1.11172	0.593422
21383	1.87218	0.315584
21384	0.987248	0.324919
21385	0.729695	0.62431
21386	0.974389	0.447674
21387	1.18002	0.431471
21388	0.822612	0.406658
21389	1.35721	0.418713
21390	0.634295	0.667503
21391	0.634326	0.678281
21392	1.13245	1.03777
21393	1.13245	0.882054
21394	0.937961	0.626871
21395	1.18769	0.7884
21396	0.854949	0.580259
21397	0.967357	0.645459
21398	0.810934	0.571369
21399	3.91082	1.85951

21400	0.946132	2.1
21401	0.946132	2.1
21402	1.05383	2.1
21403	1.05383	2.1
21404	0.986498	2.1
21405	1.50732	0.145819
21406	0.753659	0.191966
21407	1.22144	0.217533
21408	1.30463	0.181788
21409	0.753659	0.204345
21410	1.30463	0.206714
21411	0.886189	0.195577
21412	1.04321	0.188423
21413	0.909928	0.176639
21414	1.18659	0.159964
21415	1.19785	0.0993888
21416	1.40969	0.127277
21417	1.24094	0.103929
21418	0.946126	0.12467
21419	1.2929	0.102987
21420	1.24093	0.111208
21421	0.867944	0.105633
21422	1.41903	2.1
21423	2.47881	0.0768049
21424	1.12943	0.0717167
21425	0.764229	2.09444
21426	0.764256	2.1
21427	0.94613	2.09997
21428	0.94613	2.1
21429	1.1995	2.1
21430	1.83423	2.1
21431	0.634816	1.88448
21432	0.877837	2.1
21433	0.94613	1.89471
21434	1.04073	1.99353
21435	1.06485	2.02756
21436	0.94613	0.27948
21437	0.62725	0.248192
21438	0.798212	0.26572
21439	1.33241	0.210085
21440	0.62725	0.253327
21441	1.82301	0.20717
21442	1.40615	2.1
21443	0.782504	2.1
21444	0.748635	2.1
21445	0.946131	2.1
21446	0.748607	2.1
21447	0.988456	2.04482
21448	1.64833	0.0832732
21449	1.35657	0.101596
21450	1.26155	0.0998733
21451	0.915755	0.111804
21452	0.915755	0.0996013
21453	0.946132	0.105885
21454	1.49386	0.0870182
21455	1.01346	2.1
21456	0.946131	0.701991
21457	0.946132	1.79666

21458	1.38651	2.06396
21459	1.39523	1.86469
21460	0.946132	1.52209
21461	1.06312	2.1
21462	0.946131	1.84868
21463	0.946129	2.1
21464	1.39523	2.1
21465	1.37372	2.1
21466	0.946131	2.1
21467	1.2929	0.130497
21468	0.94613	0.177614
21469	1.24448	0.126186
21470	1.2929	0.143824
21471	0.862011	0.136668
21472	1.73583	0.0947761
21473	1.724	0.104011
21474	1.03824	2.1
21475	1.534	2.1
21476	0.725492	1.60189
21477	0.771967	1.73691
21478	0.61731	1.09021
21479	0.475743	1.39289
21480	0.719694	1.47902
21481	1.14878	1.015
21482	0.946117	1.5141
21483	0.94613	1.56643
21484	0.946105	1.5884
21485	0.482666	1.01629
21486	0.609172	1.04301
21487	0.946131	2.1
21488	1.39509	0.700869
21489	1.07439	1.70032
21490	0.946131	0.922024
21491	1.39509	1.04041
21492	0.963571	1.26574
21493	0.591011	0.534636
21494	1.1357	0.367226
21495	2.11193	0.927132
21496	1.60936	2.1
21497	1.34118	0.295443
21498	1.27696	0.340256
21499	0.94613	0.269135
21500	0.94613	0.255709
21501	1.04257	0.224656
21502	1.53336	0.100752
21503	1.0631	0.117757
21504	1.04072	0.179697
21505	0.670678	0.14452
21506	0.709531	0.158405
21507	0.891275	0.164254
21508	0.683583	0.194293
21509	0.902478	0.142643
21510	0.807671	0.149405
21511	1.11024	2.1
21512	1.08171	2.1
21513	1.16725	2.1
21514	0.946128	2.1
21515	1.13537	2.1

21516	1.40525	2.1
21517	1.28365	2.1
21518	1.04523	2.1
21519	0.960106	2.1
21520	0.946128	2.1
21521	1.23452	2.1
21522	0.946129	2.1
21523	1.3704	2.1
21524	1.23452	2.1
21525	1.16906	0.802433
21526	0.946131	1.32482
21527	0.867246	0.886558
21528	0.867217	1.48776
21529	0.946131	1.0444
21530	1.13438	1.26456
21531	1.28684	1.33824
21532	0.946132	1.43582
21533	0.883935	1.2054
21534	0.883938	2.0105
21535	0.946133	1.2865
21536	1.73444	1.02491
21537	1.21835	2.1
21538	1.28228	2.1
21539	1.8643	1.16846
21540	0.743379	0.346139
21541	1.34921	0.341613
21542	0.743381	0.343786
21543	0.743381	0.338885
21544	0.946131	0.360561
21545	0.915528	2.1
21546	0.982742	2.1
21547	0.975965	2.08903
21548	0.891132	0.0703457
21549	1.27533	0.0660636
21550	0.946132	0.0656028
21551	1.25215	0.0635584
21552	0.590343	0.0666596
21553	0.946133	0.0657006
21554	0.590331	0.0664898
21555	1.62375	2.1
21556	0.687769	0.588302
21557	1.14374	0.568355
21558	1.20885	0.528675
21559	0.946132	0.606739
21560	1.14374	0.675567
21561	1.09308	0.394897
21562	1.02055	0.345189
21563	0.810934	0.318146
21564	0.917609	1.0703
21565	1.13094	1.0392
21566	0.70959	0.993472
21567	1.11223	2.1
21568	0.83051	1.07632
21569	0.810946	1.34067
21570	0.810959	1.03899
21571	1.5773	0.0760501
21572	1.96133	0.926736
21573	1.0214	0.974011

21574	0.762783	1.34131
21575	0.838036	0.856221
21576	0.63591	1.41522
21577	0.967411	0.835676
21578	0.946132	1.87358
21579	0.946132	1.79556
21580	1.39699	1.423
21581	1.61642	1.77854
21582	0.860954	0.896181
21583	0.957411	1.34538
21584	0.660856	1.09217
21585	1.10003	2.1
21586	0.968249	1.93146
21587	0.910184	1.12597
21588	1.30029	0.62772
21589	0.754119	0.605667
21590	0.754121	0.860444
21591	0.94613	1.05617
21592	1.08039	2.1
21593	0.810942	0.190712
21594	1.15153	0.179907
21595	1.06968	0.168817
21596	1.38456	2.1
21597	1.04118	2.1
21598	0.838027	2.1
21599	1.04073	2.1
21600	0.754209	2.1
21601	1.332	0.101041
21602	1.26193	2.1
21603	1.15317	0.083113
21604	3.86179	0.0784262
21605	1.56005	2.1
21606	0.683035	0.460421
21607	0.931463	0.427469
21608	0.556019	0.670043
21609	0.929172	0.539845
21610	0.756874	0.388628
21611	1.23527	0.568467
21612	2.05961	0.669254
21613	0.946132	2.1
21614	1.16684	2.1
21615	1.3937	2.1
21616	1.3937	2.1
21617	0.946132	2.1
21618	1.17885	2.1
21619	1.62507	0.0740168
21620	1.58874	2.1
21621	0.819953	2.1
21622	1.16023	2.1
21623	1.34782	2.1
21624	1.16023	2.1
21625	0.819955	2.1
21626	0.79437	2.1
21627	0.794373	2.1
21628	1.20056	1.94787
21629	1.25	2.0939
21630	1.20056	2.09309
21631	1.35787	2.1

21632	0.946131	1.88875
21633	1.38585	1.72344
21634	0.946128	0.771714
21635	0.946129	1.2264
21636	1.35714	1.23908
21637	1.04147	2.04048
21638	0.946133	1.44416
21639	1.30382	1.1703
21640	0.946131	0.589803
21641	0.946131	0.982418
21642	1.38585	1.16087
21643	1.22305	1.28086
21644	1.00763	0.183985
21645	0.813199	0.248072
21646	0.810948	0.234667
21647	1.15928	0.222328
21648	0.944166	0.144113
21649	0.94613	0.185346
21650	0.946133	0.133403
21651	1.4179	0.165309
21652	0.946132	0.148786
21653	1.36965	0.114645
21654	1.05444	0.133693
21655	0.690302	0.1755
21656	0.838038	0.12919
21657	0.802821	0.642893
21658	1.40944	0.844524
21659	0.946126	1.10085
21660	0.799998	0.403637
21661	0.800035	0.712439
21662	1.1766	2.1
21663	1.00319	2.1
21664	1.01606	1.48847
21665	0.872158	2.1
21666	1.04884	2.01231
21667	0.679494	2.1
21668	0.946131	2.1
21669	1.37609	2.1
21670	0.94613	2.1
21671	0.946131	2.1
21672	1.18486	2.1
21673	0.726287	2.1
21674	0.946131	2.1
21675	0.726286	2.1
21676	1.30318	2.1
21677	1.01712	2.1
21678	0.946132	2.1
21679	1.04404	2.1
21680	1.37017	2.1
21681	1.37017	2.1
21682	0.946133	2.1
21683	0.992084	1.04679
21684	0.946131	0.936769
21685	0.529655	1.52521
21686	0.529686	1.07051
21687	0.946131	0.859622
21688	1.05937	1.26308
21689	1.14622	0.690274

21690	0.946131	0.799109
21691	0.584575	1.05043
21692	0.584575	0.719799
21693	0.946129	0.65198
21694	0.709714	2.1
21695	0.946132	2.1
21696	0.709714	2.1
21697	1.37199	2.1
21698	0.82899	2.1
21699	1.00133	2.1
21700	0.778424	0.0837065
21701	0.946132	0.100824
21702	1.24094	0.0871154
21703	1.00691	0.0939489
21704	0.64868	0.090897
21705	1.29736	0.0821714
21706	1.24767	0.524209
21707	1.28953	0.712136
21708	1.34612	0.725958
21709	0.946133	0.5556
21710	1.14187	0.979957
21711	0.946132	0.624559
21712	0.946129	0.598268
21713	1.4099	1.60069
21714	0.946131	0.622298
21715	1.34612	0.530279
21716	1.4099	0.465006
21717	0.956003	0.0716671
21718	1.40572	0.0786692
21719	3.54443	2.1
21720	0.994001	0.436975
21721	1.03679	2.1
21722	1.08467	2.1
21723	1.56875	0.339462
21724	0.968463	1.87195
21725	0.974889	2.1
21726	0.946131	2.1
21727	1.30203	2.1
21728	1.40199	2.1
21729	1.40199	2.1
21730	0.946132	2.1
21731	0.946132	2.1
21732	1.36994	2.1
21733	1.37372	2.1
21734	1.37372	2.1
21735	0.946132	2.1
21736	0.928979	0.94086
21737	0.565509	1.12028
21738	1.60196	0.682738
21739	1.16239	0.836582
21740	1.10746	0.441314
21741	1.15412	2.1
21742	1.15171	2.1
21743	0.946133	1.97406
21744	0.940877	2.1
21745	0.940878	2.1
21746	1.41432	2.1
21747	1.04687	2.1

21748	2.75524	0.116303
21749	0.946132	0.121863
21750	0.766452	0.111981
21751	0.766427	0.126478
21752	1.36004	0.0943341
21753	1.06648	0.119173
21754	1.62483	2.1
21755	1.0577	2.1
21756	1.39158	2.09128
21757	0.94255	2.1
21758	1.05029	2.1
21759	1.03735	1.92602
21760	0.931055	2.1
21761	1.03797	1.99433
21762	1.43687	1.94719
21763	1.28765	1.34855
21764	1.1811	0.97629
21765	0.946132	1.23306
21766	0.946132	1.21168
21767	1.1811	1.21254
21768	0.67805	0.791226
21769	1.50998	1.92492
21770	1.16661	2.1
21771	1.36749	2.1
21772	0.810945	0.95063
21773	1.14745	1.21982
21774	0.865588	0.675625
21775	1.36665	0.819471
21776	0.506964	1.19512
21777	0.506966	0.885955
21778	0.552786	0.843687
21779	0.946133	0.655566
21780	1.21336	0.719625
21781	0.552786	0.873347
21782	0.552757	1.31226
21783	0.653468	0.796559
21784	1.36665	1.53736
21785	1.27922	0.285196
21786	1.08133	0.37786
21787	1.05348	0.203362
21788	0.946132	0.320664
21789	1.19601	0.23568
21790	1.13276	0.229574
21791	0.7856	0.242974
21792	1.57125	0.223198
21793	1.19601	0.313647
21794	0.946131	0.769265
21795	1.34534	0.613959
21796	1.19601	0.380609
21797	0.870489	0.465371
21798	1.49404	2.1
21799	1.35943	1.13606
21800	1.10427	1.36833
21801	0.94613	1.0562
21802	0.946131	1.14034
21803	1.10427	1.20551
21804	1.2064	0.13052
21805	0.946132	0.359958

21806	1.11288	0.505072
21807	1.34268	0.271689
21808	1.34268	0.259386
21809	0.946132	0.306061
21810	0.946128	2.00544
21811	0.946128	0.54109
21812	1.30835	1.37735
21813	1.38463	0.733399
21814	0.946129	0.754687
21815	1.43778	0.748452
21816	1.23943	0.479982
21817	1.4748	2.1
21818	0.71295	2.1
21819	0.712951	2.1
21820	0.946132	2.1
21821	0.946133	2.1
21822	1.13976	2.1
21823	1.71109	2.1
21824	1.32426	2.1
21825	0.671255	2.00014
21826	1.29123	2.05373
21827	0.946132	2.01367
21828	0.946132	1.90395
21829	1.25603	1.98024
21830	2.21126	0.134946
21831	0.892889	2.1
21832	0.946129	2.1
21833	0.892888	2.1
21834	0.92872	2.1
21835	1.34352	2.1
21836	0.622784	0.528876
21837	1.02947	0.360343
21838	0.810957	0.33673
21839	1.91756	1.03457
21840	1.06012	0.901307
21841	1.09569	0.415477
21842	1.5456	0.142423
21843	1.1211	0.106304
21844	1.21031	0.139341
21845	1.03939	0.129383
21846	0.519697	0.143276
21847	0.933889	0.287278
21848	0.519726	0.235272
21849	1.16699	0.221648
21850	0.933889	0.23253
21851	0.840599	0.111484
21852	0.94613	0.211074
21853	0.840568	0.163296
21854	1.26868	0.161526
21855	1.16699	0.149331
21856	1.17822	1.80928
21857	0.922044	0.404649
21858	1.29322	0.442401
21859	0.946132	0.394298
21860	0.995178	0.482809
21861	0.922056	0.444113
21862	1.76232	0.691271
21863	1.77065	1.01166

21864	1.38149	0.765812
21865	0.959301	0.995918
21866	0.946133	0.824815
21867	0.946132	0.921549
21868	0.959301	0.853959
21869	1.3612	0.771551
21870	0.885314	0.972049
21871	0.885309	0.801124
21872	0.94613	0.920071
21873	0.8853	0.761907
21874	1.56164	0.818591
21875	1.45506	0.308289
21876	1.45257	2.1
21877	0.851541	0.108333
21878	1.29018	0.088745
21879	0.851541	0.103384
21880	1.21087	0.0895289
21881	1.24979	0.100359
21882	1.56983	0.0845086
21883	1.60151	0.0755392
21884	1.78952	2.1
21885	0.638164	2.1
21886	0.638163	2.1
21887	1.39754	2.1
21888	1.39754	2.1
21889	0.681589	2.1
21890	1.34993	2.1
21891	1.04525	2.1
21892	0.946133	2.1
21893	1.04072	2.1
21894	0.857805	2.1
21895	1.06053	2.1
21896	1.49066	2.1
21897	1.4185	0.943859
21898	0.934059	1.56069
21899	0.93406	0.95285
21900	0.94613	1.32823
21901	0.93406	1.09781
21902	1.25277	1.10272
21903	1.00792	1.87255
21904	0.609466	1.10238
21905	0.946132	1.51702
21906	0.609463	1.55526
21907	1.30664	2.0339
21908	0.810943	1.58926
21909	1.05741	1.28066
21910	1.3868	0.704925
21911	1.15558	0.394264
21912	1.13537	2.1
21913	0.946132	2.1
21914	0.946132	2.1
21915	1.08266	2.1
21916	1.54934	2.1
21917	1.26016	2.1
21918	2.01915	2.1
21919	0.60346	1.13532
21920	0.651529	1.25488
21921	1.15581	1.37814

21922	1.13037	1.06095
21923	1.04071	0.987238
21924	1.77796	1.71647
21925	0.532308	1.17102
21926	0.532308	1.05498
21927	1.15369	1.61371
21928	1.1537	1.5114
21929	0.903288	1.26944
21930	0.804766	2.1
21931	0.804739	2.1
21932	0.946128	2.1
21933	0.946131	2.1
21934	1.16745	2.1
21935	1.04193	2.1
21936	0.98505	2.1
21937	1.40724	0.584421
21938	1.14216	0.525759
21939	0.946131	0.400543
21940	0.946131	0.482878
21941	1.10941	0.503085
21942	0.81268	0.0848678
21943	0.946131	0.0912387
21944	1.28673	0.0767323
21945	1.28673	0.0823655
21946	0.812681	0.0853534
21947	1.84716	0.0702638
21948	1.84716	0.0703699
21949	0.92361	0.0805756
21950	0.94613	0.0833138
21951	0.923582	0.0735666
21952	1.26227	0.0796979
21953	0.923581	0.0787107
21954	0.747394	2.1
21955	1.32582	2.1
21956	0.747365	2.1
21957	0.97587	2.1
21958	1.32582	2.1
21959	1.04182	0.0957059
21960	0.961743	0.0990088
21961	0.946126	0.12785
21962	1.47463	0.110371
21963	1.12183	0.111452
21964	0.851552	0.109867
21965	1.33177	0.135933
21966	0.946131	0.196906
21967	1.41512	0.185891
21968	1.33178	0.154008
21969	0.946131	0.155877
21970	0.927735	0.595578
21971	0.97206	0.448431
21972	0.515566	0.528848
21973	0.946129	0.49893
21974	0.515538	0.482148
21975	1.04482	0.355118
21976	0.975535	0.369148
21977	0.522411	0.332367
21978	0.522382	0.434883
21979	0.925346	0.439993

21980	0.575817	0.41299
21981	0.946129	2.1
21982	1.14256	2.1
21983	1.32054	2.1
21984	1.40525	2.1
21985	1.14256	2.1
21986	1.59475	2.1
21987	0.773813	2.1
21988	1.4073	2.1
21989	0.773813	2.1
21990	0.773813	2.1
21991	1.4073	2.1
21992	0.993021	2.1
21993	1.50466	0.12796
21994	0.851539	0.186701
21995	1.20299	0.140033
21996	0.946131	0.164347
21997	1.54464	0.161648
21998	1.20309	0.158242
21999	2.00701	0.12558
22000	0.810946	0.186014
22001	1.07678	0.178237
22002	0.900128	0.165666
22003	1.26842	2.1
22004	1.11206	2.1
22005	0.946132	2.1
22006	1.04074	2.1
22007	0.948809	2.1
22008	0.946131	0.0930298
22009	0.929617	0.110362
22010	1.24057	0.0837193
22011	1.36485	0.0943969
22012	0.851554	0.0955956
22013	1.96823	0.0785679
22014	0.914836	0.104996
22015	0.94613	0.12584
22016	1.38911	0.093369
22017	0.914805	0.0958367
22018	0.946131	0.104011
22019	0.946131	0.140891
22020	1.04072	0.131478
22021	1.26837	0.108785
22022	0.970054	0.129176
22023	0.923958	0.12796
22024	1.16653	2.1
22025	1.34412	1.13916
22026	0.851541	0.176029
22027	1.38645	0.211754
22028	0.891637	0.177669
22029	0.851487	0.196978
22030	0.890871	0.203927
22031	1.56178	0.183719
22032	0.946128	0.0522355
22033	1.26868	0.0522355
22034	0.946131	0.0628882
22035	1.18789	0.114475
22036	1.26868	0.0620378
22037	1.26868	0.0644292

22038	0.946128	0.0644292
22039	1.18716	0.137459
22040	0.946131	0.228803
22041	0.59358	0.171134
22042	1.23923	0.246667
22043	1.00024	0.165213
22044	0.593593	0.21383
22045	0.946131	0.256061
22046	0.713645	0.20573
22047	1.23175	0.286007
22048	1.23923	0.176636
22049	0.713633	0.230835
22050	1.24687	0.15707
22051	1.80662	2.08219
22052	2.15762	2.1
22053	0.780455	2.1
22054	0.946131	2.1
22055	0.780455	2.1
22056	1.40583	2.09426
22057	1.39852	2.05925
22058	0.565619	2.1
22059	1.1428	2.1
22060	0.663168	0.316856
22061	1.02163	0.234374
22062	1.04072	0.334179
22063	0.663169	0.288426
22064	1.13511	0.306006
22065	1.85277	0.177763
22066	1.17701	0.814004
22067	0.993831	0.253767
22068	1.0823	0.255334
22069	0.795761	0.249271
22070	1.24596	0.197705
22071	0.566075	0.253078
22072	0.94613	0.276252
22073	0.56605	0.265418
22074	0.774911	0.114615
22075	0.946131	0.134403
22076	1.24417	0.100768
22077	0.774911	0.104986
22078	0.946132	0.118142
22079	1.54982	0.102824
22080	1.82961	0.0954813
22081	1.12035	0.0793285
22082	0.876835	2.1
22083	0.946131	1.73493
22084	0.876836	2.1
22085	1.12968	2.1
22086	1.23536	2.1
22087	2.15082	2.1
22088	0.58365	2.1
22089	0.946133	2.1
22090	0.583653	2.1
22091	0.992097	2.1
22092	1.12968	2.1
22093	1.0305	2.1
22094	0.82613	0.216487
22095	0.879644	0.193258

22096	1.00688	0.193306
22097	1.52776	1.99341
22098	0.851537	1.52892
22099	1.00851	1.40143
22100	0.946128	0.852208
22101	0.851552	1.15857
22102	1.4895	1.5117
22103	0.761574	2.1
22104	0.946132	2.1
22105	1.01095	2.1
22106	1.01095	2.1
22107	0.761572	2.1
22108	1.8575	2.1
22109	1.19984	0.274331
22110	1.25797	0.296067
22111	0.946132	0.267235
22112	0.946132	0.267345
22113	0.95332	0.264684
22114	0.946131	2.1
22115	0.851542	1.43086
22116	1.03172	2.1
22117	1.22889	2.09647
22118	1.0313	2.1
22119	1.15977	1.25854
22120	0.918875	0.951027
22121	0.8654	1.59816
22122	0.865374	0.926186
22123	0.946132	0.939308
22124	0.985051	2.1
22125	1.31993	0.479145
22126	1.33622	0.594979
22127	0.959992	0.547362
22128	0.926499	2.1
22129	1.03544	2.1
22130	0.946132	2.1
22131	0.946132	2.1
22132	1.40335	2.1
22133	0.772299	2.1
22134	0.851529	2.1
22135	0.857243	2.1
22136	1.38155	2.1
22137	1.05462	2.1
22138	0.832138	2.1
22139	0.946132	2.1
22140	0.832137	2.1
22141	0.959414	2.1
22142	1.36541	2.1
22143	1.91894	2.1
22144	1.04661	0.879163
22145	1.28355	1.85305
22146	0.877353	0.751119
22147	0.877353	0.870956
22148	0.94613	0.789934
22149	1.20537	0.200249
22150	1.09914	0.146931
22151	0.94613	0.179298
22152	0.499593	0.167713
22153	0.499606	0.185094

22154	1.0959	0.134392
22155	0.838027	0.143129
22156	0.883644	0.155364
22157	0.95019	0.178826
22158	1.10638	2.1
22159	0.946132	2.1
22160	1.41744	2.1
22161	1.05842	2.1
22162	0.94613	2.1
22163	1.41744	2.1
22164	0.991985	2.1
22165	3.31755	1.24214
22166	1.05556	2.1
22167	0.624075	0.972463
22168	0.814554	0.937862
22169	0.946131	1.10957
22170	0.814554	1.211
22171	1.31857	1.57712
22172	1.26282	2.1
22173	0.946132	2.1
22174	1.14302	2.1
22175	0.946132	2.1
22176	0.946132	2.1
22177	1.40372	2.1
22178	1.24688	0.184642
22179	0.89889	2.1
22180	1.17286	2.1
22181	0.71741	2.1
22182	0.491096	0.65952
22183	1.05432	0.472097
22184	0.719531	2.1
22185	1.25145	2.1
22186	0.864686	2.1
22187	0.867974	2.1
22188	1.21734	2.1
22189	0.405517	0.705315
22190	0.818878	1.24989
22191	0.502457	0.122319
22192	1.05868	0.146158
22193	0.912018	0.133168
22194	1.40591	0.295746
22195	0.957584	0.274189
22196	0.946131	0.440691
22197	1.11612	0.369443
22198	1.3365	0.365418
22199	0.946128	0.439622
22200	1.16534	0.220056
22201	1.38969	0.607923
22202	0.891597	1.42338
22203	0.640032	1.93172
22204	0.956758	1.49107
22205	1.3428	0.419927
22206	0.953295	1.91793
22207	0.946131	0.32388
22208	1.16167	0.36525
22209	0.94613	0.365518
22210	0.951675	0.35957
22211	1.16167	0.335207

22212	1.24538	0.164221
22213	1.13482	2.1
22214	1.38775	2.1
22215	0.934061	2.1
22216	0.91408	2.1
22217	1.06099	2.1
22218	0.980549	0.233226
22219	0.946132	0.257462
22220	1.10321	0.311511
22221	0.99126	0.258803
22222	0.991261	0.286092
22223	1.35732	0.277462
22224	1.1952	1.64692
22225	0.946132	1.12612
22226	0.946132	2.1
22227	1.32819	1.22046
22228	1.30171	0.97091
22229	1.29667	0.137187
22230	1.00982	0.0838462
22231	0.407589	0.580793
22232	1.18266	1.16837
22233	1.04037	0.600673
22234	0.956694	0.729886
22235	0.552639	0.591914
22236	0.810943	1.60922
22237	1.43144	0.744033
22238	0.918074	0.743698
22239	1.60976	2.1
22240	4.00847	2.1
22241	2.3995	0.141728
22242	1.36203	2.1
22243	0.919619	2.1
22244	1.30188	2.1
22245	0.681073	2.1
22246	0.681043	2.1
22247	1.04687	2.1
22248	3.32085	2.1
22249	1.80396	0.0980169
22250	0.773007	0.42952
22251	0.773034	0.350422
22252	1.22141	0.463334
22253	1.22141	0.456307
22254	0.946131	0.513351
22255	2.02071	0.308193
22256	0.951912	0.0940001
22257	1.39027	0.103339
22258	0.94613	0.112867
22259	0.829922	0.0910593
22260	0.829946	0.100199
22261	1.24902	1.22266
22262	2.8333	0.987504
22263	0.946131	0.0820796
22264	0.929725	0.0747471
22265	1.1134	0.0795368
22266	1.17056	0.0800741
22267	0.929713	0.0749602
22268	0.749096	2.1
22269	1.31961	2.1

22270	0.870191	2.1
22271	1.04071	2.1
22272	1.1877	2.1
22273	1.03882	2.1
22274	1.44801	2.04209
22275	3.55198	2.1
22276	1.11193	2.1
22277	0.663922	2.1
22278	0.663922	2.1
22279	0.946129	2.1
22280	0.946131	2.1
22281	0.97079	2.1
22282	1.36049	2.1
22283	1.06105	2.1
22284	0.92251	2.1
22285	0.94613	2.1
22286	0.922511	2.1
22287	1.04101	2.1
22288	0.810944	0.171727
22289	0.994109	0.152391
22290	0.623143	0.143522
22291	0.864244	0.177018
22292	1.14627	0.163282
22293	0.64586	0.152097
22294	0.721422	0.119752
22295	0.870885	0.109258
22296	0.855175	0.122321
22297	0.946131	0.847363
22298	1.38157	1.97334
22299	1.33266	0.661977
22300	0.946131	1.40529
22301	1.38158	0.902654
22302	0.946132	1.36787
22303	1.3268	2.1
22304	0.946132	0.948764
22305	0.946132	2.1
22306	1.38158	1.94604
22307	1.42186	1.71989
22308	1.47204	1.65753
22309	1.14665	1.75964
22310	0.946132	1.05298
22311	0.94613	2.0496
22312	1.30116	1.62684
22313	1.32819	1.22833
22314	1.25388	2.1
22315	0.887808	1.08878
22316	0.946132	2.1
22317	1.23536	1.8078
22318	1.34309	1.72856
22319	1.07612	2.1
22320	0.728982	0.112664
22321	1.3157	0.110371
22322	0.946131	0.152838
22323	0.686176	0.138149
22324	1.3035	0.132396
22325	0.686139	0.146889
22326	0.906806	0.125429
22327	0.794122	0.122399

22328	1.17173	0.12043
22329	1.8194	0.0993158
22330	0.851543	2.1
22331	1.19345	2.1
22332	0.946133	2.1
22333	0.946132	2.1
22334	1.56179	2.1
22335	0.620263	1.82969
22336	1.30226	1.71952
22337	1.2334	1.10874
22338	1.2334	0.911681
22339	0.946133	0.973
22340	1.24053	2.1
22341	1.4828	2.01196
22342	0.957622	2.1
22343	1.00521	1.4217
22344	1.49463	2.1
22345	1.39621	2.1
22346	1.09861	2.1
22347	0.946131	2.1
22348	0.946131	2.1
22349	1.09861	2.1
22350	2.16987	0.0900103
22351	0.572182	0.0934226
22352	1.15802	0.0989664
22353	0.63574	0.104007
22354	1.18897	0.0941889
22355	0.996733	0.0985322
22356	1.82402	1.60638
22357	0.969602	1.31027
22358	0.794777	1.73617
22359	1.02626	1.424
22360	1.51396	0.23959
22361	1.40574	0.301938
22362	1.38683	0.325307
22363	0.946126	0.307152
22364	0.946128	0.274337
22365	1.07138	0.215814
22366	0.946131	2.1
22367	1.41331	2.1
22368	0.94613	2.1
22369	0.94613	2.1
22370	1.27451	2.1
22371	0.974196	2.1
22372	1.2049	0.0945522
22373	1.42757	2.1
22374	1.02461	2.1
22375	2.07087	2.1
22376	1.92515	1.35592
22377	0.802138	1.3154
22378	0.946128	1.69283
22379	0.802107	1.28787
22380	1.23726	1.24694
22381	1.21997	1.22511
22382	0.767577	0.969392
22383	0.946131	1.51859
22384	0.767577	1.11826
22385	1.3279	1.10991

22386	1.23726	1.04615
22387	1.29729	2.1
22388	0.946128	2.1
22389	1.14186	2.1
22390	1.39214	2.1
22391	1.39214	2.1
22392	0.946129	2.1
22393	1.23012	0.275612
22394	0.653228	0.44172
22395	0.946133	1.09032
22396	1.15918	1.28129
22397	1.26893	0.666889
22398	0.653228	1.01199
22399	1.06848	1.48581
22400	0.615121	2.1
22401	0.841488	1.25232
22402	0.946131	2.1
22403	1.04071	2.1
22404	0.965717	2.1
22405	1.77893	1.29306
22406	1.11314	0.216545
22407	1.65411	0.79253
22408	0.85472	0.108694
22409	1.1604	0.0922994
22410	1.19141	0.0953972
22411	1.19141	0.0939822
22412	0.94613	0.104467
22413	1.70944	0.0773382
22414	1.89082	0.250724
22415	1.05175	0.78489
22416	0.815276	2.1
22417	0.843283	2.1
22418	0.946131	2.1
22419	0.843284	2.1
22420	1.38897	2.1
22421	1.01019	0.648446
22422	1.05197	0.577813
22423	0.810959	0.602033
22424	1.33037	0.332573
22425	1.3415	0.351287
22426	0.946118	0.256166
22427	0.946131	0.287623
22428	1.09803	0.29048
22429	0.930281	2.1
22430	1.3335	2.1
22431	0.784151	1.58844
22432	0.78415	2.1
22433	0.946132	2.1
22434	1.6299	0.103448
22435	0.995795	2.1
22436	1.84846	1.30003
22437	0.929873	1.56508
22438	0.889245	1.53181
22439	0.808072	1.70434
22440	0.851541	1.43105
22441	1.51956	1.32308
22442	0.94613	1.23366
22443	0.851553	1.33097

22444	0.946787	1.27883
22445	1.15544	1.52568
22446	1.30289	0.243647
22447	1.28323	0.257003
22448	0.85024	0.257797
22449	1.09662	0.279384
22450	1.67687	0.0989676
22451	0.946127	2.04282
22452	1.40496	2.00664
22453	1.22442	1.42249
22454	0.946128	1.53965
22455	1.40496	1.53075
22456	1.00573	2.1
22457	0.636837	1.50659
22458	0.988642	1.73274
22459	0.573141	1.18275
22460	0.946128	1.56917
22461	0.786538	1.783
22462	2.28748	1.30666
22463	0.682805	2.06418
22464	0.946132	2.00597
22465	0.682776	1.8702
22466	0.946132	2.02606
22467	0.79775	2.1
22468	1.05403	0.101638
22469	0.888273	0.112413
22470	0.876373	0.0961253
22471	0.576083	0.101277
22472	0.946132	0.127991
22473	0.576057	0.0896973
22474	0.576058	0.101217
22475	0.946131	0.109657
22476	1.41724	0.499173
22477	1.31678	0.771533
22478	0.946132	0.755982
22479	0.946132	0.705949
22480	1.31678	0.654285
22481	0.935738	2.1
22482	0.946132	2.1
22483	0.935765	2.1
22484	1.32949	2.1
22485	0.946131	2.1
22486	1.02525	2.1
22487	0.946128	2.1
22488	1.41207	2.1
22489	1.04675	2.1
22490	0.946131	2.1
22491	1.41207	2.1
22492	0.946131	2.1
22493	0.946132	2.1
22494	1.41125	2.1
22495	1.41125	2.1
22496	1.27087	2.1
22497	0.924071	0.28687
22498	0.608893	0.212042
22499	0.528907	0.285974
22500	1.78249	0.262026
22501	0.720094	0.298648

22502	0.781667	2.1
22503	0.781638	2.1
22504	1.40756	2.1
22505	1.40756	2.1
22506	0.797635	2.1
22507	1.11946	0.0522355
22508	1.32068	0.0522355
22509	0.88757	0.135618
22510	1.27891	0.117719
22511	0.810957	0.125588
22512	0.914656	0.148015
22513	0.640819	0.114094
22514	0.745643	0.141801
22515	0.865108	0.155915
22516	1.06883	0.1389
22517	0.806001	0.15381
22518	1.19462	2.1
22519	1.42763	0.397601
22520	1.24682	0.322181
22521	0.78392	0.235843
22522	1.02874	0.232354
22523	0.884512	0.223629
22524	1.1658	2.1
22525	0.663026	0.0775049
22526	1.21174	0.0908642
22527	0.663056	0.0999132
22528	1.15968	0.0877774
22529	1.21174	0.0889928
22530	0.993976	0.0818811
22531	0.945495	0.0887137
22532	0.671202	0.080045
22533	1.37312	2.1
22534	1.28499	2.1
22535	0.946133	2.1
22536	0.946132	2.1
22537	1.28499	2.1
22538	1.25081	2.1
22539	0.946132	2.1
22540	0.946132	2.1
22541	1.27985	2.1
22542	1.37312	2.1
22543	0.830638	2.1
22544	1.05358	2.1
22545	1.22209	2.1
22546	1.09874	2.1
22547	0.74758	2.1
22548	2.50011	1.56249
22549	0.6157	0.363379
22550	0.674634	0.443932
22551	0.946128	1.05516
22552	0.674633	0.518646
22553	0.946131	0.806
22554	1.96192	1.90358
22555	1.31612	0.40658
22556	1.10735	0.294259
22557	0.936676	0.226266
22558	0.808779	0.249812
22559	0.808808	0.273849

22560	1.00245	0.256285
22561	1.26179	0.228388
22562	1.05758	1.98405
22563	1.03408	2.1
22564	0.946132	0.128895
22565	0.914368	0.122646
22566	1.20524	0.0987051
22567	1.12743	0.113419
22568	0.851555	0.11591
22569	1.17885	0.575891
22570	0.867881	0.475738
22571	1.00122	0.453673
22572	0.867883	0.428796
22573	0.867881	0.459389
22574	1.19583	0.497508
22575	1.17743	1.55051
22576	0.878684	1.21531
22577	0.687087	1.92613
22578	0.955079	0.607883
22579	0.672977	0.651957
22580	0.735937	0.672922
22581	0.851531	0.67444
22582	0.672957	0.672786
22583	0.923974	0.641444
22584	1.2243	2.1
22585	0.808463	0.937737
22586	0.971078	1.00422
22587	1.06161	1.03856
22588	0.810947	0.99361
22589	0.886074	1.12752
22590	0.786829	1.24006
22591	0.55062	0.848014
22592	0.574458	1.54715
22593	0.946129	0.950039
22594	1.42526	1.06786
22595	1.15962	0.98057
22596	1.24526	0.0782611
22597	0.94613	0.332361
22598	0.946128	0.430834
22599	1.23279	0.558852
22600	1.38645	0.531623
22601	0.94613	0.480365
22602	1.05042	1.26747
22603	0.946128	2.1
22604	0.946129	1.31724
22605	1.24286	2.1
22606	1.39659	2.1
22607	0.94613	2.1
22608	1.10407	1.06924
22609	1.32809	1.53396
22610	0.993999	1.81428
22611	0.810959	1.35069
22612	0.983643	1.53047
22613	0.869552	1.82348
22614	0.747801	1.39185
22615	1.53429	1.10636
22616	1.13127	2.1
22617	1.38397	2.1

22618	1.08366	2.1
22619	0.946129	2.1
22620	0.946131	2.1
22621	1.08366	2.1
22622	1.06007	2.1
22623	1.79641	2.1
22624	0.758799	2.1
22625	1.28442	2.1
22626	1.06957	2.1
22627	0.946133	2.1
22628	1.28442	2.1
22629	1.76788	1.69904
22630	1.38669	2.1
22631	0.986095	2.1
22632	1.38478	1.74634
22633	0.946132	0.947375
22634	1.24344	0.789455
22635	0.622283	1.4599
22636	1.10335	0.828208
22637	1.24344	0.906293
22638	1.1967	1.25791
22639	0.946131	0.111164
22640	0.901005	0.1031
22641	1.05751	0.102591
22642	1.37771	0.0975965
22643	0.851527	0.105509
22644	1.26643	1.09137
22645	1.08992	1.40322
22646	0.946132	1.39016
22647	0.946132	0.965153
22648	1.08992	1.48139
22649	1.53534	2.1
22650	1.58825	1.20868
22651	1.0447	1.78152
22652	0.593818	1.28845
22653	0.89883	1.51025
22654	0.734088	1.63832
22655	0.998451	0.701536
22656	1.64156	0.797712
22657	1.05654	1.17104
22658	1.37641	0.42797
22659	1.74553	0.41644
22660	1.31112	0.3884
22661	0.74518	0.473008
22662	0.851539	0.370383
22663	0.851554	0.296281
22664	0.851475	0.24225
22665	0.946132	0.0632307
22666	1.28895	0.0584846
22667	1.11356	0.0605248
22668	0.946132	0.0602812
22669	0.946132	0.0642664
22670	1.84214	2.1
22671	1.23851	0.127914
22672	1.25866	0.433388
22673	1.28655	0.781528
22674	0.946131	0.706873
22675	0.946132	0.652247

22676	1.28655	0.602589
22677	1.11929	0.542278
22678	0.946132	0.733525
22679	0.946132	0.519171
22680	1.34254	0.501372
22681	1.25866	0.459067
22682	0.946132	2.1
22683	1.27451	2.1
22684	1.19803	2.1
22685	0.94613	2.1
22686	1.27451	2.1
22687	0.946131	2.1
22688	1.36523	2.1
22689	1.14599	2.1
22690	0.946129	2.1
22691	1.36523	2.1
22692	1.09518	2.1
22693	0.977777	2.1
22694	0.583237	2.1
22695	0.583267	2.1
22696	0.903872	2.1
22697	0.946129	2.1
22698	0.583258	2.1
22699	1.58179	0.122683
22700	0.946131	0.160624
22701	1.35123	0.137549
22702	0.655837	0.154904
22703	1.12018	0.157534
22704	1.35123	0.152065
22705	0.62967	0.142017
22706	0.860497	0.178662
22707	0.816639	0.158361
22708	0.746716	2.1
22709	0.810947	2.1
22710	0.810959	2.1
22711	0.653422	1.96042
22712	0.289127	1.2568
22713	0.475716	1.8757
22714	0.980651	1.26434
22715	0.946119	2.08251
22716	0.946132	2.07631
22717	1.08299	2.08867
22718	1.34539	2.08561
22719	1.28753	0.159724
22720	0.946132	0.290139
22721	0.946132	0.234094
22722	1.19719	0.238159
22723	0.946133	0.243116
22724	0.958947	0.177736
22725	1.3492	0.186017
22726	0.946132	0.304272
22727	0.946128	0.278437
22728	1.22588	0.262517
22729	0.946129	0.270183
22730	0.991113	2.1
22731	1.35966	2.1
22732	0.961 2.1	
22733	0.946129	2.1

22734	0.946131	2.1
22735	0.961001	2.1
22736	0.727096	1.74394
22737	0.810946	1.0907
22738	0.810959	1.34207
22739	1.31658	0.308129
22740	1.23167	2.1
22741	1.44836	0.0754528
22742	1.50103	0.0791707
22743	1.23351	0.089325
22744	1.35657	0.0922898
22745	0.851541	0.103821
22746	0.93668	0.0906412
22747	0.946131	0.0953518
22748	3.7335	2.1
22749	1.1822	2.1
22750	1.31127	0.554017
22751	1.3689	0.457812
22752	1.35822	0.37003
22753	0.946133	0.393558
22754	0.946132	0.466571
22755	1.14926	0.445598
22756	1.40822	0.40586
22757	1.04935	0.489902
22758	0.704112	0.450057
22759	0.704112	0.482262
22760	0.946131	0.534541
22761	0.704112	0.507614
22762	1.37721	0.108889
22763	0.85154	2.1
22764	1.06949	2.1
22765	0.851541	2.1
22766	0.946128	2.1
22767	1.1175	2.1
22768	1.44091	2.1
22769	0.96029	0.700569
22770	3.46486	0.982962
22771	0.603286	0.242567
22772	0.432283	0.144525
22773	0.851541	0.22767
22774	1.09355	0.145097
22775	0.851534	0.251451
22776	1.04263	2.1
22777	0.851541	2.1
22778	0.965604	2.1
22779	0.94613	2.1
22780	1.23953	2.1
22781	0.869615	2.1
22782	1.02415	2.1
22783	0.983032	2.1
22784	1.35385	1.48442
22785	1.59971	0.345437
22786	0.915178	2.1
22787	0.962435	2.1
22788	1.3116	2.1
22789	1.38302	2.1
22790	1.13227	2.1
22791	1.25923	0.0754478

22792	0.988805	2.1
22793	0.611486	0.884799
22794	1.1019	0.976954
22795	1.11124	1.29772
22796	0.716713	0.869695
22797	0.946131	1.21952
22798	1.22297	1.09489
22799	1.12873	1.30288
22800	0.946131	1.29905
22801	0.946132	1.33001
22802	1.3469	1.43988
22803	1.25366	1.57885
22804	1.1611	0.904896
22805	0.946132	1.18486
22806	0.946132	0.947724
22807	1.2854	1.17632
22808	1.3469	1.27314
22809	1.07468	0.98588
22810	1.79317	2.1
22811	1.07444	2.1
22812	1.69436	1.63145
22813	0.851539	1.96124
22814	0.870925	1.66947
22815	0.851541	2.00417
22816	0.870951	1.99069
22817	1.26097	2.03512
22818	1.26247	2.07821
22819	0.946132	1.69405
22820	1.24041	2.1
22821	0.946132	1.41528
22822	0.946132	2.1
22823	1.40274	2.03659
22824	0.946131	0.0998064
22825	0.797945	0.0917558
22826	1.12988	0.0952854
22827	1.00681	0.0928631
22828	0.797932	0.0937185
22829	0.977483	0.29032
22830	0.943362	0.58511
22831	0.9891	0.317546
22832	1.1703	0.353685
22833	0.663171	0.410197
22834	1.40882	0.170924
22835	1.32639	0.232068
22836	1.24448	0.167171
22837	0.946132	0.258017
22838	1.11015	0.16767
22839	1.24448	0.195858
22840	0.754768	0.195669
22841	1.13854	2.1
22842	1.23654	2.1
22843	0.714101	0.522431
22844	0.851538	0.555057
22845	0.79261	1.17544
22846	1.30598	0.701842
22847	1.05338	0.624156
22848	1.41758	2.1
22849	0.83317	0.350626

22850	1.00058	0.421353
22851	0.810958	0.417142
22852	1.63791	0.268258
22853	1.55116	2.1
22854	1.29735	1.18282
22855	0.945147	1.77655
22856	0.94515	1.20312
22857	0.94613	1.39156
22858	0.945149	1.41357
22859	1.02368	2.1
22860	1.12787	2.1
22861	1.04007	2.1
22862	3.78805	1.9478
22863	0.816891	0.169278
22864	0.943996	0.165075
22865	0.870002	0.154229
22866	0.564533	0.248706
22867	0.564536	0.326645
22868	0.946128	0.741738
22869	0.564535	0.391201
22870	0.946128	0.630656
22871	1.15177	2.1
22872	1.16806	0.899501
22873	0.999083	0.985367
22874	0.946131	1.02189
22875	0.946131	0.957211
22876	0.999084	1.14863
22877	1.28918	0.820312
22878	1.0383	0.987957
22879	0.946133	0.946026
22880	0.946132	0.961599
22881	1.0383	1.04598
22882	1.27737	1.30182
22883	1.39296	0.480708
22884	0.998682	2.1
22885	3.98933	2.1
22886	1.05328	0.152098
22887	1.23475	2.1
22888	0.900856	1.46489
22889	0.94001	0.898887
22890	0.707748	0.758105
22891	0.707751	1.16918
22892	0.946128	0.804914
22893	0.70775	0.986885
22894	0.996109	0.943235
22895	1.68551	2.1
22896	0.851539	2.1
22897	0.962688	1.53579
22898	0.94613	0.910743
22899	1.04073	1.95419
22900	1.44824	2.1
22901	0.794313	0.313872
22902	0.838031	0.318764
22903	1.11076	0.279888
22904	1.38813	2.1
22905	1.17204	2.1
22906	0.946132	2.1
22907	0.946131	2.1

22908	1.17204	2.1
22909	0.961779	0.324913
22910	1.0685	0.524438
22911	0.810947	1.67954
22912	1.2899	0.917255
22913	0.890404	0.925583
22914	0.909981	1.89815
22915	0.810946	1.17704
22916	0.890402	0.998352
22917	0.522844	1.62664
22918	0.593846	1.56908
22919	1.30226	1.04801
22920	1.30226	1.01201
22921	0.946132	1.26706
22922	1.03537	0.168319
22923	1.50035	0.140573
22924	0.940356	0.202679
22925	0.946131	0.17321
22926	1.03441	0.178793
22927	1.21171	0.12443
22928	1.35169	0.151557
22929	1.32449	0.139009
22930	0.946131	0.185484
22931	0.946131	0.168751
22932	1.06627	0.166307
22933	1.03102	0.532318
22934	0.488712	0.118751
22935	0.946131	0.157447
22936	1.23146	0.118089
22937	1.13242	0.142682
22938	0.946131	0.134567
22939	0.409199	0.10047
22940	0.429959	0.109383
22941	1.08357	0.1437
22942	0.80926	0.160985
22943	0.879791	0.128691
22944	0.946129	1.08117
22945	1.28828	1.44787
22946	0.946131	1.02527
22947	0.946131	1.55363
22948	1.35969	1.45113
22949	1.10049	1.54074
22950	0.923724	1.05097
22951	0.923695	1.89625
22952	0.94613	0.997635
22953	0.94613	1.85744
22954	1.28828	1.70997
22955	1.73525	0.199271
22956	0.656239	0.281972
22957	1.2996	0.226393
22958	0.812939	0.312989
22959	0.65624	0.260501
22960	1.2996	0.274207
22961	1.67372	2.1
22962	0.946131	2.1
22963	0.946132	2.1
22964	1.05978	2.1
22965	1.31049	2.1

22966	0.946132	2.1
22967	1.11077	2.1
22968	0.579725	0.454402
22969	0.373532	0.35435
22970	1.13537	0.482395
22971	1.20726	0.361208
22972	0.946131	0.350983
22973	1.45694	0.589961
22974	0.750367	0.0991268
22975	0.946126	0.116595
22976	0.750365	0.102664
22977	1.25698	0.0988649
22978	1.35726	0.0993396
22979	1.6812	0.101934
22980	1.78665	1.49795
22981	1.46568	2.1
22982	1.29606	2.1
22983	1.25298	2.1
22984	0.946115	2.1
22985	0.946129	2.1
22986	1.25296	2.1
22987	1.27778	0.114813
22988	1.17042	2.07847
22989	1.25858	0.0783342
22990	0.93106	0.0832927
22991	0.810959	0.0766923
22992	1.38905	2.1
22993	1.19216	2.1
22994	0.946131	2.1
22995	0.946131	2.1
22996	1.19216	2.1
22997	1.58339	2.00742
22998	0.81094	0.110941
22999	0.845129	0.12963
23000	0.918535	0.11713
23001	0.946132	2.1
23002	0.946132	2.1
23003	1.31904	2.1
23004	1.31904	2.1
23005	1.21275	2.1
23006	1.15471	2.1
23007	1.33125	2.1
23008	0.702954	0.244787
23009	0.942973	0.238512
23010	0.94613	0.312035
23011	0.641772	0.216788
23012	0.702942	0.299248
23013	0.991865	0.368181
23014	0.639952	0.329398
23015	1.17333	0.269387
23016	0.946132	0.229219
23017	0.490975	0.242689
23018	0.495934	0.375323
23019	1.19904	0.221866
23020	1.30488	0.116863
23021	1.02305	0.345184
23022	1.49842	0.126263
23023	1.54196	2.1

23024	1.37224	1.72936
23025	1.0953	0.151718
23026	1.18952	1.09397
23027	1.68851	0.463342
23028	0.946131	0.399778
23029	1.12491	0.379863
23030	0.583591	0.34784
23031	1.28727	0.418509
23032	1.12491	0.405972
23033	0.884641	0.529795
23034	0.810944	0.484898
23035	0.917973	0.491003
23036	0.846671	0.478108
23037	0.596968	0.357006
23038	0.716347	0.47399
23039	1.36255	0.354267
23040	1.11077	2.1
23041	0.946132	2.1
23042	0.946132	2.1
23043	1.26267	2.1
23044	1.26441	2.1
23045	1.85238	2.1
23046	1.36693	2.1
23047	0.616123	2.1
23048	0.481844	2.1
23049	0.709599	2.1
23050	0.815889	2.1
23051	1.1502	2.1
23052	1.63937	0.629015
23053	1.25797	0.275402
23054	1.22878	0.296047
23055	0.946132	0.27531
23056	0.946132	0.272611
23057	0.965737	0.270571
23058	1.1893	0.253959
23059	1.22877	0.272829
23060	0.735217	0.273315
23061	0.85154	0.284175
23062	0.851553	0.272069
23063	0.851473	0.278835
23064	1.58916	0.139515
23065	1.25545	0.154586
23066	0.810942	0.263151
23067	0.919314	0.286592
23068	0.928633	0.239232
23069	2.14514	0.180126
23070	1.23389	2.1
23071	0.946128	0.163437
23072	0.969504	0.12219
23073	0.851539	0.146416
23074	1.41503	0.14053
23075	0.958517	0.14131
23076	0.990615	0.122797
23077	1.7943	0.115326
23078	0.946133	0.436393
23079	1.27581	0.444611
23080	0.946132	0.441636
23081	1.36362	0.415444

23082	1.36936	0.41357
23083	1.71903	2.1
23084	0.751639	2.1
23085	0.77464	2.1
23086	0.946133	2.1
23087	1.04073	2.1
23088	1.23551	2.1
23089	0.94613	2.1
23090	1.18682	2.1
23091	0.946131	2.1
23092	0.94613	2.1
23093	1.40638	2.1
23094	0.483346	0.25242
23095	0.432527	0.178882
23096	0.946132	0.295381
23097	0.483312	0.214433
23098	0.946132	0.223463
23099	0.878798	0.276741
23100	0.810944	0.281424
23101	1.14604	0.241194
23102	0.946129	0.258683
23103	1.35594	0.209238
23104	0.538378	0.214389
23105	0.946131	0.269659
23106	0.538378	0.245316
23107	1.00065	0.540813
23108	0.962878	0.393533
23109	1.32634	0.250795
23110	1.28066	0.0881449
23111	0.785651	2.1
23112	1.20327	1.81379
23113	1.47058	1.75083
23114	0.551834	1.01605
23115	1.04072	1.80928
23116	0.506719	1.28526
23117	1.12786	1.44571
23118	1.45789	0.0805998
23119	0.681532	0.0848766
23120	1.21087	0.0750815
23121	0.94613	0.0813388
23122	0.681534	0.0785115
23123	1.21087	0.0818936
23124	1.98618	0.735399
23125	1.71742	1.00809
23126	1.07427	0.714942
23127	1.1972	0.554601
23128	0.933474	0.552041
23129	1.1745	0.625825
23130	0.94612	0.565489
23131	0.933485	0.622755
23132	1.17449	0.609831
23133	0.851541	0.0917128
23134	1.12774	0.0876478
23135	0.946129	0.0926551
23136	0.851486	0.0918698
23137	1.12517	0.0905377
23138	1.12328	0.0844571
23139	1.13179	2.1

23140	1.02438	2.1
23141	1.05776	2.1
23142	1.41273	1.10482
23143	1.1229	2.05207
23144	0.875912	1.11091
23145	0.946132	1.84015
23146	0.875912	1.46062
23147	1.42307	2.00709
23148	1.15354	0.0971808
23149	0.848382	0.0864664
23150	1.2685	1.52685
23151	0.938104	1.24726
23152	0.793166	2.01386
23153	1.07855	2.1
23154	0.98488	2.1
23155	1.4162	2.1
23156	1.09651	0.151558
23157	1.98502	0.370998
23158	1.41168	0.310572
23159	1.33879	0.311254
23160	0.946127	0.451028
23161	1.13528	0.276207
23162	0.946128	0.332371
23163	1.09726	2.1
23164	1.22026	0.778833
23165	1.09044	1.92977
23166	2.23961	0.726939
23167	1.14721	0.323879
23168	1.17614	0.90101
23169	0.615065	2.1
23170	0.946131	2.1
23171	0.778488	2.1
23172	0.94613	2.1
23173	0.615066	2.1
23174	1.78254	1.79475
23175	0.898452	0.126142
23176	1.47411	2.1
23177	0.59676	0.352727
23178	0.59676	0.250433
23179	0.94613	0.377343
23180	1.13511	0.291909
23181	0.946131	0.285969
23182	0.916275	2.09959
23183	0.946132	1.90846
23184	0.916274	1.2317
23185	1.36182	1.74781
23186	0.946131	1.81797
23187	0.857202	0.0823431
23188	0.941918	0.0748383
23189	0.943349	0.0724211
23190	1.2493	0.0689196
23191	0.946133	0.0824699
23192	1.01814	0.0708616
23193	0.927881	0.0657318
23194	1.11845	0.0620445
23195	1.2493	0.0650214
23196	1.11845	0.058444
23197	0.946129	0.066723

23198	0.967673	0.681049
23199	1.01332	0.0855815
23200	0.946133	0.0887955
23201	0.81938	0.0899072
23202	0.819377	0.0821599
23203	0.946133	0.0928874
23204	1.22474	0.381155
23205	1.15321	0.359298
23206	0.797489	0.367608
23207	0.946131	0.402374
23208	0.797461	0.387103
23209	1.35328	0.777409
23210	1.03318	1.05447
23211	0.946131	0.889344
23212	0.94613	0.92359
23213	1.03319	0.993359
23214	1.37938	0.77576
23215	1.00512	1.07681
23216	0.946133	0.849789
23217	0.946132	0.93383
23218	1.00512	0.916349
23219	1.5515	1.11729
23220	1.55351	2.1
23221	0.94261	0.101253
23222	0.828156	0.102021
23223	0.916495	0.105338
23224	3.06309	0.0903607
23225	0.619832	0.0805645
23226	0.705903	0.0876059
23227	1.2968	0.084118
23228	0.946133	0.0866862
23229	0.705904	0.0798089
23230	1.2968	0.0872611
23231	1.01971	0.0824851
23232	0.60302	0.0774099
23233	1.07242	0.744733
23234	1.26426	0.706638
23235	0.946131	0.701035
23236	0.946131	0.770827
23237	0.963109	0.749598
23238	0.947862	0.725628
23239	1.40248	0.981955
23240	0.767143	1.13559
23241	0.767114	1.03528
23242	0.946129	1.11142
23243	0.767143	0.949164
23244	1.22637	1.00006
23245	1.2281	0.985546
23246	0.590909	1.19838
23247	0.490832	0.991717
23248	0.804174	1.14863
23249	0.747368	0.933506
23250	1.05129	0.178389
23251	1.10609	0.581667
23252	1.3639	0.301967
23253	0.776117	0.348306
23254	0.752329	0.307723
23255	1.04073	0.318778

23256	0.67713	0.312019
23257	3.4245	2.1
23258	1.18526	2.1
23259	1.2161	1.14429
23260	0.876834	1.53739
23261	0.876831	1.03558
23262	0.946132	1.43374
23263	0.876834	1.21364
23264	0.954563	2.1
23265	0.924132	2.1
23266	1.25075	2.1
23267	1.01652	2.1
23268	0.92413	2.1
23269	1.37579	2.1
23270	0.992159	2.1
23271	1.26092	2.1
23272	0.929435	2.1
23273	0.844958	2.1
23274	0.79598	2.1
23275	0.852559	2.1
23276	0.946131	0.474787
23277	1.28435	0.33467
23278	0.827261	0.444194
23279	0.946128	0.467906
23280	0.827298	0.472285
23281	1.65452	0.21525
23282	1.79929	2.1
23283	0.791947	1.55905
23284	0.902072	1.07132
23285	0.882672	1.36637
23286	1.21703	2.1
23287	0.900338	2.1
23288	1.37712	2.1
23289	0.782591	2.1
23290	0.782562	2.1
23291	0.94613	2.1
23292	1.34749	2.1
23293	0.61359	2.1
23294	1.14397	0.393629
23295	1.02963	0.112804
23296	1.36589	0.105461
23297	0.946132	0.169458
23298	1.28644	0.126064
23299	0.946131	0.150624
23300	1.40104	0.143354
23301	1.28644	0.141562
23302	0.810946	0.21894
23303	1.09178	0.204388
23304	1.26956	0.186708
23305	0.810943	0.204541
23306	0.885633	0.204286
23307	1.00844	0.190204
23308	0.825671	0.214603
23309	0.792093	0.189121
23310	0.925037	0.197239
23311	0.946131	0.575956
23312	0.946131	0.199056
23313	1.18052	0.283014

23314	1.40265	0.289076
23315	0.946132	0.377493
23316	1.17891	0.308294
23317	0.734248	0.0819812
23318	0.810943	0.0876997
23319	0.731621	0.0819317
23320	0.799857	0.464937
23321	0.834507	0.57973
23322	0.810932	0.489867
23323	1.01316	0.116776
23324	1.23884	0.152127
23325	1.88438	2.1
23326	0.946129	1.87318
23327	1.38614	1.83688
23328	1.34245	1.08178
23329	0.946131	1.19388
23330	1.38614	1.1475
23331	0.946132	0.112313
23332	0.910733	0.117772
23333	1.24726	0.0894164
23334	1.13742	0.105646
23335	0.851555	0.105881
23336	1.50368	0.0904437
23337	0.94613	0.546511
23338	1.08998	1.40813
23339	1.36899	0.271339
23340	1.36898	0.625371
23341	0.946131	0.598832
23342	1.26045	2.1
23343	1.06432	0.735053
23344	1.89016	0.520586
23345	0.83514	1.36534
23346	1.36653	1.8725
23347	1.23426	1.56353
23348	0.94613	1.23079
23349	0.835112	1.78205
23350	1.14567	0.979932
23351	1.12096	0.895173
23352	0.881106	1.05794
23353	1.17787	0.357612
23354	1.29738	0.770131
23355	0.940472	0.405745
23356	1.03452	0.697561
23357	0.946131	0.541848
23358	2.7489	0.521966
23359	1.01778	2.1
23360	1.04321	0.965844
23361	1.19352	0.178584
23362	1.16912	1.37657
23363	0.946132	1.15868
23364	0.946132	1.56471
23365	1.40924	1.50786
23366	1.22927	1.70996
23367	2.09455	0.276633
23368	3.00575	2.1
23369	1.08928	0.647674
23370	1.94799	2.1
23371	1.4092	2.1

23372	0.946132	2.1
23373	1.16	2.1
23374	1.01985	2.1
23375	0.946133	2.1
23376	1.16	2.1
23377	1.15371	0.0592244
23378	1.56002	0.058621
23379	1.23007	2.06286
23380	1.9992	2.1
23381	0.652605	1.81601
23382	0.937903	2.1
23383	0.946132	1.70939
23384	0.937902	2.1
23385	1.228	2.1
23386	1.33694	1.16014
23387	1.00191	2.07332
23388	0.716814	1.13689
23389	0.94613	1.75387
23390	0.716816	1.65417
23391	1.70388	1.56398
23392	0.946129	0.524842
23393	1.09362	0.602547
23394	0.946131	0.590216
23395	1.4185	0.596572
23396	1.09362	0.520283
23397	1.31057	1.11313
23398	1.17631	0.0847555
23399	1.19404	0.098949
23400	0.94613	0.15745
23401	0.946129	0.125504
23402	0.99972	0.131592
23403	0.94613	0.133599
23404	0.835199	0.0771266
23405	1.23351	0.0850262
23406	0.582785	0.0958348
23407	0.701079	0.0851301
23408	0.946132	0.0851532
23409	2.17385	0.0748691
23410	1.18948	1.89005
23411	1.25346	2.1
23412	1.18586	0.769143
23413	0.880876	0.475614
23414	0.68632	0.789576
23415	0.946118	2.1
23416	0.966936	2.1
23417	0.946117	2.1
23418	1.41636	2.1
23419	1.03798	2.1
23420	0.729178	0.637039
23421	0.721888	0.911891
23422	0.946132	0.671522
23423	0.656286	0.811993
23424	1.13898	0.742028
23425	1.02351	0.174463
23426	1.48803	0.236815
23427	0.946131	0.188872
23428	1.04921	2.1
23429	1.02478	0.278266

23430	0.993023	1.80672
23431	1.80841	0.264036
23432	1.29215	0.272394
23433	0.904206	0.264885
23434	0.904207	0.293178
23435	0.946132	0.281655
23436	0.904207	0.278429
23437	1.07347	2.1
23438	0.555875	1.13517
23439	0.555904	1.33494
23440	1.30841	1.60622
23441	1.30841	1.74246
23442	0.91951	1.67774
23443	1.33026	0.532333
23444	1.43289	0.108351
23445	1.15703	1.58117
23446	0.886594	1.99644
23447	1.05129	1.76887
23448	1.08421	0.668301
23449	1.35946	0.685676
23450	0.882955	0.789018
23451	0.946131	0.781357
23452	0.882985	0.740223
23453	0.583501	0.654756
23454	0.532921	0.656112
23455	0.94613	1.387
23456	0.532892	1.06521
23457	1.23224	1.07491
23458	1.12786	0.980298
23459	0.946131	0.661622
23460	1.23732	0.291652
23461	1.40609	0.572011
23462	1.40609	0.214521
23463	0.946131	0.371064
23464	0.910672	1.33031
23465	1.08478	1.19124
23466	0.94613	0.62984
23467	0.94613	1.00532
23468	1.30382	1.03624
23469	3.10544	1.8261
23470	0.679509	1.85894
23471	1.15966	1.48001
23472	0.75499	1.90628
23473	1.37511	1.62776
23474	1.01776	1.45436
23475	2.35239	1.2359
23476	0.92406	2.1
23477	0.946118	2.1
23478	0.92406	2.1
23479	1.07979	2.1
23480	1.29604	2.1
23481	1.29897	2.1
23482	0.902645	0.691302
23483	0.729493	1.23532
23484	0.906844	0.962302
23485	0.739518	0.129203
23486	1.10564	0.208721
23487	0.946131	0.21803

23488	0.739519	0.155447
23489	0.739506	0.186897
23490	1.79907	0.169463
23491	1.59106	0.912864
23492	1.11067	0.681476
23493	0.795516	0.841504
23494	0.795519	0.733389
23495	0.946132	0.816987
23496	0.795506	0.708936
23497	1.80223	2.1
23498	1.95572	1.6703
23499	1.16237	0.850782
23500	1.07518	1.41922
23501	0.696199	0.832059
23502	0.94613	0.990986
23503	0.696199	1.08208
23504	1.6379	0.0739471
23505	0.946118	0.0894481
23506	0.851529	0.100362
23507	1.08716	0.076317
23508	0.973118	0.0810003
23509	0.851528	0.0872661
23510	1.15096	2.1
23511	0.65023	0.137476
23512	0.94613	0.11969
23513	0.650205	0.141627
23514	0.650205	0.109882
23515	0.811233	0.14628
23516	0.778721	1.741
23517	0.810945	0.915755
23518	0.997518	0.927737
23519	0.838028	0.129631
23520	0.952893	0.105375
23521	0.838037	0.112645
23522	1.58166	0.0982347
23523	1.44848	1.25688
23524	1.30041	0.0894435
23525	1.03697	0.579112
23526	0.946132	1.25601
23527	1.0277	1.2912
23528	0.946131	1.37321
23529	1.00501	1.1661
23530	1.0277	1.0576
23531	1.64988	2.1
23532	0.946131	1.63437
23533	0.94613	0.4983
23534	1.27702	1.47554
23535	1.55163	0.684402
23536	0.897905	0.680925
23537	1.12971	0.0856488
23538	1.35715	0.437645
23539	0.881884	0.69296
23540	0.664007	0.436658
23541	0.663978	0.564838
23542	0.94613	0.521059
23543	0.664006	0.554532
23544	1.48693	0.245476
23545	1.29289	0.277019

23546	1.32081	0.309874
23547	0.946119	0.263302
23548	0.946132	0.260919
23549	1.02972	0.235776
23550	0.946131	2.1
23551	0.946127	2.1
23552	1.23513	2.1
23553	1.38154	2.1
23554	0.94613	2.1
23555	1.25933	0.139524
23556	0.917954	0.150181
23557	0.868589	0.145128
23558	3.0957	2.1
23559	0.711391	0.0857924
23560	0.711391	0.0860406
23561	0.991303	0.083262
23562	1.37322	0.0808168
23563	0.729145	0.0852518
23564	2.55648	0.0773999
23565	1.0756	1.70319
23566	0.760299	1.16256
23567	0.763592	0.639579
23568	0.946126	0.715487
23569	0.76359	0.609826
23570	1.35862	1.07356
23571	0.946133	2.1
23572	1.00807	2.1
23573	0.946132	2.1
23574	1.39823	2.1
23575	1.07817	2.1
23576	1.44379	1.26562
23577	1.17934	0.150139
23578	1.13451	0.411484
23579	0.94613	0.644594
23580	1.03974	2.04125
23581	1.36835	0.647268
23582	1.36835	0.865555
23583	0.94613	0.956207
23584	1.27096	1.74167
23585	0.853042	2.1
23586	0.946131	2.1
23587	1.13658	2.1
23588	1.06997	2.1
23589	0.94613	2.1
23590	1.41556	0.487922
23591	1.21708	0.666973
23592	0.94613	0.688853
23593	0.946131	0.613262
23594	1.21708	0.589682
23595	0.964782	0.508999
23596	0.946119	0.611436
23597	1.23447	0.584455
23598	0.946118	0.615657
23599	1.41844	0.570287
23600	1.23446	0.606009
23601	1.1357	0.388082
23602	0.619856	0.0910667
23603	1.1509	0.0819448

23604	0.946126	0.0863338
23605	1.19262	0.0782537
23606	1.1509	0.0874886
23607	1.04268	2.1
23608	2.07527	0.817832
23609	1.38259	0.872681
23610	0.980054	1.31996
23611	0.946124	0.92364
23612	0.946127	1.11256
23613	0.980056	1.00163
23614	1.62916	1.78895
23615	0.946131	2.1
23616	1.40571	2.1
23617	0.946131	2.1
23618	1.38885	2.1
23619	1.40571	2.1
23620	1.11787	0.539279
23621	0.946129	0.502753
23622	1.28831	1.2548
23623	1.41498	0.415749
23624	1.41498	0.658601
23625	0.946131	0.600691
23626	0.850964	0.0951856
23627	0.921299	0.086757
23628	1.37358	0.0804317
23629	0.946132	0.0839423
23630	0.850964	0.0864262
23631	0.823988	0.099335
23632	0.823984	0.0908384
23633	1.36568	0.082051
23634	1.36569	0.0821289
23635	0.921275	0.0901731
23636	1.84739	2.1
23637	1.47169	0.184083
23638	0.946131	0.303228
23639	0.735844	0.291564
23640	1.11819	0.359204
23641	1.10904	0.228571
23642	0.735831	0.286082
23643	0.946131	2.1
23644	0.946132	2.1
23645	1.23487	2.1
23646	1.3281	2.1
23647	0.946131	2.1
23648	1.72271	0.0886751
23649	0.861359	0.0974966
23650	0.946131	0.156691
23651	0.861357	0.12402
23652	1.1369	0.125318
23653	1.26868	0.11864
23654	0.789173	2.02711
23655	0.789143	2.1
23656	0.946133	1.56181
23657	1.34386	1.89408
23658	0.789144	1.9017
23659	0.983493	2.1
23660	1.18944	0.645381
23661	1.05121	0.637567

23662	0.777398	0.677155
23663	0.946132	0.723735
23664	0.777423	0.76289
23665	1.09841	0.294713
23666	0.963919	0.292327
23667	0.474855	0.389529
23668	0.946131	0.381008
23669	0.474855	0.367985
23670	3.40074	1.07759
23671	1.29453	2.1
23672	1.01139	0.418751
23673	0.946133	0.804515
23674	1.35381	0.385235
23675	1.18322	0.622841
23676	0.946133	0.879166
23677	1.45502	0.157675
23678	0.970088	1.11363
23679	0.960333	1.23635
23680	0.838036	1.28712
23681	1.30927	0.288039
23682	0.828617	0.325907
23683	0.714795	0.295632
23684	0.946132	0.281982
23685	0.714824	0.289231
23686	0.946131	2.1
23687	0.851542	2.1
23688	1.38204	2.1
23689	1.24512	2.1
23690	1.04086	2.1
23691	0.723573	2.1
23692	0.946131	2.1
23693	0.982454	2.1
23694	0.982454	2.1
23695	0.723544	2.1
23696	1.17261	0.271131
23697	1.34153	0.293713
23698	1.41383	0.309382
23699	0.946132	0.259024
23700	0.946133	0.270582
23701	1.12818	0.277372
23702	1.29383	0.251209
23703	0.946133	0.438652
23704	1.0027	0.430994
23705	0.768989	0.315768
23706	0.946133	0.481647
23707	0.768977	0.449399
23708	0.946132	0.498973
23709	1.24333	0.375753
23710	0.928583	0.478451
23711	0.946132	0.512243
23712	0.928583	0.534647
23713	1.53798	0.233417
23714	2.95436	0.132014
23715	1.30649	0.148163
23716	0.809789	0.230084
23717	0.745744	0.209392
23718	0.745774	0.198038
23719	0.946128	0.193887

23720	1.00777	0.205395
23721	1.40271	0.191494
23722	0.768387	0.265613
23723	0.946132	0.238765
23724	0.922045	0.234545
23725	0.946132	2.1
23726	1.39021	2.1
23727	1.10046	2.1
23728	0.946132	2.1
23729	1.39021	2.1
23730	2.06158	0.203299
23731	0.913967	0.565682
23732	1.30107	0.783416
23733	0.946132	0.572955
23734	0.913966	0.617261
23735	1.30107	0.6041
23736	1.64117	2.1
23737	0.514872	1.36358
23738	0.514846	1.00118
23739	1.30811	1.51711
23740	1.30812	0.968909
23741	0.888989	1.08515
23742	1.99279	1.43703
23743	0.910271	0.847562
23744	0.838027	0.912927
23745	0.928444	0.91981
23746	1.2377	1.21492
23747	0.851537	1.58998
23748	0.899142	2.1
23749	0.810075	2.1
23750	1.3771	2.1
23751	1.03281	2.1
23752	0.719877	2.1
23753	0.901838	2.1
23754	1.01748	2.1
23755	1.12315	2.1
23756	0.64787	2.1
23757	1.173 2.1	
23758	0.876903	2.1
23759	0.597337	2.1
23760	1.00291	2.1
23761	0.597309	2.1
23762	1.09897	0.400558
23763	0.946132	0.863996
23764	1.1386	0.427481
23765	1.09897	0.494054
23766	0.596652	0.611171
23767	2.5593	2.1
23768	0.946133	0.670673
23769	1.29172	0.630287
23770	1.11337	0.492874
23771	0.946131	0.533699
23772	0.946131	0.469259
23773	0.96926	0.720473
23774	0.946132	0.90708
23775	0.946132	0.965774
23776	1.07118	0.616159
23777	1.29698	0.645851

23778	1.37867	0.833907
23779	0.946119	1.21792
23780	0.94612	1.49452
23781	1.33154	0.828494
23782	0.946107	1.06128
23783	2.17757	1.20119
23784	0.946131	0.177576
23785	0.68393	0.123694
23786	0.68396	0.15256
23787	1.33671	0.152684
23788	1.10733	0.157155
23789	2.31367	2.1
23790	0.989326	1.5146
23791	0.689952	2.08826
23792	0.854787	1.39453
23793	3.73635	2.1
23794	1.41489	2.06772
23795	1.25544	0.82564
23796	0.946128	1.69464
23797	0.946129	1.4724
23798	1.13536	1.79865
23799	2.10284	1.20243
23800	0.851542	2.1
23801	0.828523	2.08131
23802	0.946132	2.1
23803	0.946132	2.1
23804	1.50873	2.1
23805	0.989048	2.09557
23806	0.925055	2.04161
23807	0.838041	2.09975
23808	0.807714	0.961354
23809	0.656831	0.406449
23810	0.997603	0.812998
23811	0.946131	0.968444
23812	1.02602	0.854975
23813	0.946129	0.987742
23814	1.35469	0.85321
23815	1.25083	0.845806
23816	0.851541	0.309122
23817	0.851483	0.285052
23818	0.851539	0.314946
23819	1.19785	0.297324
23820	1.07615	0.303067
23821	0.714507	0.315452
23822	1.41461	0.318603
23823	0.714507	0.310754
23824	0.714507	0.308243
23825	0.946133	0.328683
23826	0.946131	0.884347
23827	0.972232	0.774761
23828	0.94613	1.05981
23829	1.25083	0.755163
23830	1.30165	0.816467
23831	1.05332	2.1
23832	0.574768	2.1
23833	1.94541	0.0999876
23834	0.789633	0.124685
23835	0.946127	0.143236

23836	1.33798	0.112331
23837	1.22903	0.130924
23838	0.946128	0.131266
23839	0.946128	2.1
23840	1.11882	2.1
23841	0.996487	2.1
23842	0.94613	2.1
23843	1.11883	2.1
23844	0.946127	2.1
23845	1.30082	2.1
23846	1.02608	2.1
23847	0.946128	2.1
23848	1.30081	2.1
23849	1.3507	1.31038
23850	1.19583	0.591205
23851	0.946127	1.61279
23852	0.94613	1.11835
23853	1.19583	1.33487
23854	0.477652	0.102988
23855	1.20416	0.156483
23856	0.946131	0.157111
23857	0.477652	0.114413
23858	0.477691	0.148305
23859	1.34536	0.0746484
23860	0.758971	0.155278
23861	1.25124	0.157324
23862	1.31467	0.141731
23863	0.758971	0.150541
23864	1.31467	0.156348
23865	0.946131	2.1
23866	1.14184	2.1
23867	1.35028	2.1
23868	1.35028	2.1
23869	0.946131	2.1
23870	1.13537	1.87663
23871	0.946132	1.12256
23872	0.946132	2.05342
23873	1.23031	1.69642
23874	1.27555	1.68439
23875	1.53064	1.952
23876	1.28421	1.07031
23877	1.07707	0.943225
23878	0.946125	0.968594
23879	0.946127	1.04095
23880	1.07707	1.04585
23881	0.946133	0.172899
23882	1.3495	0.146837
23883	0.946132	0.181668
23884	1.05704	0.157623
23885	1.3495	0.161699
23886	0.946132	0.120485
23887	1.37794	0.110489
23888	0.946132	0.127475
23889	1.01367	0.11915
23890	1.37794	0.118294
23891	0.928516	2.1
23892	0.946133	2.1
23893	0.928518	2.1

23894	1.14105	2.1
23895	1.26406	2.1
23896	0.797664	2.1
23897	0.946132	2.1
23898	0.797663	2.1
23899	1.34285	2.1
23900	0.929875	2.1
23901	0.946131	0.932843
23902	1.21139	0.451434
23903	1.41492	0.468193
23904	1.41492	0.397886
23905	0.946131	0.412494
23906	0.946131	2.1
23907	0.689388	2.1
23908	0.94365	2.1
23909	0.777345	2.1
23910	0.709599	2.1
23911	0.774968	2.1
23912	0.946132	2.1
23913	0.77497	2.1
23914	0.868145	2.1
23915	1.28401	2.1
23916	0.94613	0.504852
23917	1.19655	0.693346
23918	1.39428	0.408293
23919	1.39428	0.529105
23920	0.946131	0.5014
23921	1.1848	2.1
23922	1.07591	2.1
23923	0.946131	2.1
23924	0.946131	2.1
23925	0.874788	2.1
23926	1.35703	2.1
23927	1.35357	2.1
23928	0.946132	2.1
23929	1.26022	2.1
23930	0.946132	2.1
23931	0.946131	2.1
23932	0.966426	2.1
23933	0.946133	2.01655
23934	0.946131	2.1
23935	1.3484	2.1
23936	1.26441	2.1
23937	1.2079	2.1
23938	0.946133	2.1
23939	0.946132	2.1
23940	1.2079	2.1
23941	1.2736	2.1
23942	1.25774	2.1
23943	0.946133	2.1
23944	0.851555	2.1
23945	0.96172	2.1
23946	0.946128	2.1
23947	1.06879	2.1
23948	0.94613	2.1
23949	1.32608	2.1
23950	1.06879	2.1
23951	0.946132	2.1

23952	1.32608	2.1
23953	0.946131	2.1
23954	1.40488	2.1
23955	1.32608	2.1
23956	0.94613	0.941484
23957	1.19234	0.68269
23958	0.946129	0.842454
23959	0.94613	1.08528
23960	1.31649	1.25291
23961	0.912059	0.868821
23962	1.04514	0.91753
23963	0.946132	0.85628
23964	0.946132	1.3686
23965	1.34646	1.47099
23966	1.01187	0.480317
23967	1.33728	0.587572
23968	0.761974	0.324796
23969	0.761974	0.566351
23970	0.94613	0.436441
23971	1.40641	1.43294
23972	0.955752	0.812641
23973	0.736738	1.5303
23974	0.94613	0.939759
23975	0.736739	1.06183
23976	0.861471	2.1
23977	1.34556	2.1
23978	0.810957	2.1
23979	0.946131	2.1
23980	1.37811	2.1
23981	1.17949	2.1
23982	0.946132	2.1
23983	1.37811	2.1
23984	0.946131	2.1
23985	1.40447	2.1
23986	0.946132	2.1
23987	0.946132	2.1
23988	1.39826	2.1
23989	0.946131	2.1
23990	1.283	2.1
23991	0.94613	1.65494
23992	0.946131	2.1
23993	1.40447	2.1
23994	1.20541	2.1
23995	0.946131	1.48867
23996	0.946127	2.1
23997	1.33607	2.1
23998	1.41021	2.1
23999	1.01271	2.1
24000	0.946131	2.1
24001	0.920606	2.1
24002	1.13078	2.1
24003	1.60331	2.1
24004	0.946118	0.781335
24005	1.09718	1.61867
24006	0.946116	0.937585
24007	1.32662	0.800993
24008	1.0972	0.835873
24009	0.94613	0.794642

24010	1.32666	1.20057
24011	0.946128	0.686218
24012	1.31381	0.658795
24013	1.32666	0.589197
24014	0.94612	1.36271
24015	0.940748	2.04626
24016	1.03561	2.1
24017	1.35111	2.08713
24018	0.851527	1.89969
24019	0.607156	1.42239
24020	0.789158	1.99003
24021	0.946129	0.947047
24022	1.04071	1.73115
24023	1.13144	1.77995
24024	0.946133	2.1
24025	1.35663	2.1
24026	0.946132	2.1
24027	0.946133	2.1
24028	1.34237	2.1
24029	0.919455	1.99794
24030	1.01143	2.1
24031	0.946128	1.92986
24032	0.946131	2.1
24033	1.62796	2.1
24034	1.17796	1.28197
24035	1.23024	1.85008
24036	0.946132	1.03971
24037	0.85153	1.4166
24038	0.986867	1.34398
24039	0.889308	2.1
24040	0.94613	2.1
24041	0.889338	2.1
24042	0.946131	2.1
24043	0.889337	2.1
24044	1.18396	1.00469
24045	0.94613	0.861161
24046	0.946125	1.40813
24047	1.39514	1.0578
24048	1.30809	1.22358
24049	0.803322	2.01113
24050	0.925893	1.62664
24051	1.36183	1.29796
24052	0.925892	1.40843
24053	0.946133	1.33161
24054	1.38354	0.329208
24055	1.16009	0.388781
24056	0.946132	0.300917
24057	0.946132	0.28774
24058	1.01462	0.260556
24059	1.18854	0.898295
24060	0.888265	1.49963
24061	0.745095	1.01401
24062	0.709597	1.36552
24063	0.936541	1.25305
24064	1.24142	0.714371
24065	1.35059	0.625721
24066	0.946128	0.510936
24067	0.94613	0.667064

24068	1.16623	0.645636
24069	1.24945	0.301161
24070	1.33035	0.288465
24071	0.946127	0.271473
24072	0.946128	0.286656
24073	1.13343	0.303872
24074	0.637995	0.861498
24075	1.10455	0.986504
24076	1.22641	1.27073
24077	1.22641	1.58839
24078	0.946132	1.22833
24079	1.3665	0.292833
24080	1.28507	0.276237
24081	0.946127	0.288009
24082	0.946129	0.284422
24083	1.2051	0.298058
24084	0.797374	2.1
24085	0.797376	2.1
24086	1.13113	2.1
24087	1.20057	2.1
24088	1.12049	2.1
24089	0.59387	0.744406
24090	1.13245	0.782793
24091	0.966134	0.925099
24092	0.593842	0.709438
24093	0.946128	0.929972
24094	0.641455	0.748662
24095	0.916507	0.662687
24096	1.04072	1.16425
24097	0.415585	0.588523
24098	0.507244	1.05501
24099	0.678938	2.1
24100	1.24154	2.09628
24101	1.25	1.61455
24102	1.24154	1.90581
24103	1.25	1.83104
24104	0.851009	1.53958
24105	0.946131	1.74015
24106	0.851539	1.44911
24107	1.21997	1.28814
24108	1.45109	1.31725
24109	0.804877	0.800054
24110	1.00062	0.434122
24111	0.851536	0.705286
24112	0.909673	0.448221
24113	0.946129	0.726047
24114	1.20494	0.263144
24115	1.09088	0.297703
24116	0.887634	0.349862
24117	0.81027	0.199414
24118	0.900294	0.256851
24119	0.747713	0.118716
24120	0.975467	0.12221
24121	0.747712	0.127321
24122	1.39168	0.115908
24123	0.975467	0.124071
24124	0.94613	0.353019
24125	1.32827	0.331569

24126	0.946128	0.315413
24127	1.41201	0.350964
24128	1.32827	0.335052
24129	0.981121	0.197759
24130	0.936311	0.306195
24131	1.00715	0.201789
24132	1.15914	0.228458
24133	0.704409	0.231819
24134	0.713444	0.957894
24135	0.954538	0.718723
24136	0.944508	0.780895
24137	0.851165	1.00755
24138	0.946131	1.30288
24139	0.851192	0.944114
24140	0.946131	0.9524
24141	1.3279	0.892662
24142	1.33587	0.0923314
24143	0.946126	0.112924
24144	1.27512	0.103466
24145	1.33587	0.102639
24146	0.748059	0.0997877
24147	1.17451	0.17909
24148	1.04299	0.162703
24149	0.851542	0.231314
24150	0.946131	0.210453
24151	0.892109	0.195834
24152	0.946131	0.0831757
24153	0.851541	0.0861183
24154	1.2178	0.0746277
24155	1.2179	0.0809823
24156	0.946793	0.0821116
24157	0.709599	0.211418
24158	0.987426	0.165782
24159	1.39502	0.200804
24160	1.13624	0.167078
24161	0.73176	0.193583
24162	1.36697	0.116285
24163	1.33393	0.113106
24164	0.946131	0.151943
24165	0.946132	0.131634
24166	1.23845	0.132894
24167	0.50702	0.84735
24168	1.06442	0.833533
24169	0.946131	0.943162
24170	1.02363	0.969653
24171	1.13537	0.106399
24172	1.25049	0.0966213
24173	0.94613	0.116504
24174	1.14993	0.0996376
24175	1.38056	0.100354
24176	0.946126	0.0698689
24177	1.24678	0.0647432
24178	0.684521	0.0668897
24179	0.946124	0.0681151
24180	0.684518	0.0682164
24181	0.851542	0.569905
24182	0.925459	0.557652
24183	0.946131	0.582842

24184	0.851474	0.618911
24185	0.923305	0.618953
24186	1.4055	0.088916
24187	1.32077	0.104857
24188	0.946127	0.123664
24189	1.21757	0.106681
24190	0.946129	0.108776
24191	0.711214	0.162872
24192	1.30463	0.194492
24193	0.711185	0.168714
24194	0.711185	0.183206
24195	1.22716	0.184587
24196	1.29958	0.0914194
24197	1.26201	0.104573
24198	0.568927	0.113806
24199	0.568927	0.0927766
24200	0.946132	0.105869
24201	0.851542	0.185568
24202	1.44968	0.140655
24203	0.851543	0.162796
24204	1.35123	0.164783
24205	1.40427	0.157236
24206	0.593295	0.214022
24207	0.946129	0.229454
24208	1.22144	0.192933
24209	0.908278	0.233004
24210	1.22144	0.221154
24211	0.867593	0.372714
24212	1.23351	0.297857
24213	0.946131	0.380218
24214	1.05516	0.358772
24215	1.23351	0.341575
24216	1.15564	0.108294
24217	0.946131	0.169687
24218	0.766652	0.134926
24219	0.766681	0.123111
24220	0.946131	0.14589
24221	0.946131	0.235506
24222	0.897851	0.220068
24223	1.1751	0.266902
24224	1.00273	0.225901
24225	0.851554	0.230555
24226	0.883272	0.179395
24227	1.17111	0.411252
24228	0.851541	0.266377
24229	1.01665	0.304335
24230	0.851555	0.274826
24231	0.946131	0.466841
24232	0.911508	0.389531
24233	1.08054	0.510805
24234	1.33007	0.318499
24235	0.911471	0.385168
24236	1.26787	1.60909
24237	1.16641	1.36416
24238	0.946132	1.413
24239	0.946133	1.16837
24240	1.16641	1.54685
24241	0.946131	1.56381

24242	1.25037	0.217693
24243	1.34733	0.709423
24244	1.34733	0.45169
24245	0.946131	0.598135
24246	0.946131	0.128957
24247	1.41377	0.108232
24248	0.94613	0.135381
24249	1.30541	0.119724
24250	1.41377	0.120282
24251	1.3473	2.1
24252	1.11804	1.55517
24253	0.94613	2.1
24254	0.946131	2.1
24255	1.11805	2.1
24256	1.32068	2.1
24257	0.946131	2.1
24258	0.946128	2.1
24259	1.39384	2.1
24260	1.4181	2.1
24261	1.12913	2.1
24262	0.94613	2.1
24263	0.946129	2.1
24264	1.20328	2.1
24265	1.39385	2.1
24266	1.3335	2.1
24267	1.40987	2.1
24268	0.94613	2.1
24269	1.21474	2.1
24270	0.94613	2.1
24271	0.946132	0.694961
24272	1.15203	2.1
24273	1.41192	0.652481
24274	1.41192	1.68881
24275	0.946132	1.82529
24276	0.946131	2.01156
24277	1.40684	2.1
24278	1.2597	1.49174
24279	0.946131	1.646
24280	1.40684	1.07434
24281	0.946131	1.69168
24282	1.39113	1.97292
24283	1.34049	0.935065
24284	0.946131	1.23134
24285	1.39113	0.956201
24286	0.946132	1.54078
24287	1.31649	0.908683
24288	0.946132	0.866918
24289	0.946132	1.02964
24290	1.34297	1.06187
24291	0.946131	0.784929
24292	1.41766	0.767698
24293	0.954559	1.49105
24294	0.946131	0.67286
24295	0.946131	0.58345
24296	1.25447	0.406667
24297	1.35559	0.347708
24298	0.946127	0.312525
24299	0.946128	0.379879

24300	1.14874	0.385346
24301	1.33035	0.299831
24302	1.3665	0.288571
24303	0.946129	0.276443
24304	0.94613	0.281373
24305	1.20732	0.298049
24306	0.946131	2.1
24307	0.946131	1.30151
24308	1.32949	2.1
24309	1.34921	2.1
24310	0.946131	2.1
24311	0.946132	0.65642
24312	1.25479	0.618281
24313	1.38357	0.50276
24314	1.38357	0.372538
24315	0.946132	0.412236
24316	0.946131	1.14136
24317	1.31034	0.36835
24318	1.37127	0.644698
24319	1.37127	0.384884
24320	0.946131	0.529635
24321	1.23923	0.263078
24322	1.41169	0.305421
24323	0.94613	0.420526
24324	1.12703	0.222768
24325	0.946131	0.29843
24326	0.946119	2.1
24327	1.41915	2.1
24328	1.02679	2.1
24329	0.946107	2.1
24330	1.41917	2.1
24331	0.946133	2.1
24332	1.36581	2.1
24333	0.946131	2.1
24334	1.36581	2.1
24335	0.946131	2.1
24336	0.946132	2.1
24337	1.04384	2.1
24338	1.34973	2.1
24339	1.34967	2.1
24340	0.851555	2.1
24341	0.946132	0.294366
24342	1.3855	0.357825
24343	1.08603	0.286716
24344	1.08602	0.330494
24345	1.16234	0.34964
24346	0.946133	0.357859
24347	1.29348	0.431853
24348	1.20111	0.334269
24349	1.20112	0.399817
24350	1.3855	0.404388
24351	0.946128	0.929421
24352	1.30798	2.1
24353	0.946129	1.89149
24354	1.34828	1.71005
24355	1.30797	1.85669
24356	1.24932	0.0843901
24357	1.40549	0.0955321

24358	0.946117	0.113273
24359	1.19615	0.0986043
24360	0.946106	0.100988
24361	0.946131	2.1
24362	1.29926	2.1
24363	1.25144	2.1
24364	0.946131	2.1
24365	1.29925	2.1
24366	0.946132	2.1
24367	1.39826	2.1
24368	0.946133	2.1
24369	0.946133	2.1
24370	1.29926	2.1
24371	0.946119	0.148885
24372	1.33614	0.114494
24373	0.946118	0.146014
24374	1.41377	0.132294
24375	1.33612	0.134819
24376	0.946133	1.36602
24377	1.3933	1.81735
24378	1.34999	0.915753
24379	0.946133	1.30656
24380	1.3933	1.14018
24381	0.946131	1.41819
24382	1.36139	2.01138
24383	0.946132	1.10081
24384	0.946131	1.56929
24385	1.39329	1.07343
24386	1.37449	1.29485
24387	1.27925	0.644934
24388	0.919796	0.680222
24389	1.01176	0.822649
24390	0.94613	0.968869
24391	0.946133	1.52459
24392	1.27679	2.1
24393	0.946133	1.16549
24394	0.946132	1.95722
24395	1.39731	1.31176
24396	0.946119	2.1
24397	1.40765	1.37973
24398	1.1981	1.52939
24399	0.946131	1.15477
24400	1.40764	1.67675
24401	0.946127	2.1
24402	1.3557	2.1
24403	0.946127	2.1
24404	0.946128	2.1
24405	1.13485	2.1
24406	1.4008	2.1
24407	1.1587	2.1
24408	0.946131	2.1
24409	0.946131	2.1
24410	1.1587	2.1
24411	1.32082	0.280977
24412	1.29215	0.31231
24413	0.94613	0.273043
24414	0.946131	0.273663
24415	1.02366	0.25481

24416	1.27697	0.282346
24417	1.2929	0.31881
24418	0.946132	0.257264
24419	0.946132	0.254021
24420	1.01451	0.218512
24421	0.946132	2.1
24422	1.27252	2.1
24423	0.946132	2.1
24424	1.37869	2.1
24425	1.27252	2.1
24426	1.15627	2.1
24427	1.35703	2.1
24428	0.946132	2.1
24429	1.03783	2.1
24430	0.946132	2.1
24431	0.946128	2.1
24432	1.37869	2.1
24433	0.946131	2.1
24434	1.35357	2.1
24435	1.37869	2.1
24436	0.946132	2.1
24437	0.946131	2.1
24438	1.33471	2.1
24439	0.946131	2.1
24440	1.36373	2.1
24441	1.41342	0.851151
24442	1.06832	0.927664
24443	0.946128	1.3816
24444	0.94613	0.65649
24445	1.06832	1.2532
24446	1.26929	1.96335
24447	0.946131	0.936636
24448	0.946128	2.04596
24449	1.41022	1.3233
24450	1.37034	0.89747
24451	1.37759	0.93172
24452	1.07612	1.04837
24453	0.946126	0.89541
24454	0.946129	1.04832
24455	1.07612	0.967494
24456	0.946131	0.661062
24457	0.94613	0.319605
24458	1.23848	0.417168
24459	1.38897	0.348214
24460	0.946131	0.388195
24461	0.946132	0.428173
24462	0.946133	0.226476
24463	1.34369	0.246868
24464	1.40332	0.201767
24465	0.946132	0.260233
24466	0.946131	2.1
24467	1.24023	2.1
24468	1.38244	2.1
24469	1.38244	2.1
24470	0.946131	2.1
24471	0.946132	0.792011
24472	1.33468	2.1
24473	1.41619	0.961949

24474	1.41619	1.64615
24475	0.946131	1.65028
24476	1.1908	1.03348
24477	0.946128	0.91147
24478	0.946126	1.28353
24479	1.26654	1.30181
24480	1.39514	1.58645
24481	0.93608	1.7005
24482	1.04105	2.1
24483	1.39551	2.1
24484	1.52471	2.1
24485	1.04072	2.1
24486	1.33174	0.0755925
24487	0.946128	0.0751457
24488	0.94613	0.0780828
24489	1.17801	0.0718924
24490	0.94613	0.0811587
24491	0.946132	2.1
24492	0.946132	2.1
24493	1.29065	2.1
24494	1.29065	2.1
24495	0.975266	2.1
24496	0.946131	2.1
24497	1.26025	2.1
24498	0.955601	2.1
24499	0.946131	2.1
24500	1.26025	2.1
24501	0.94613	0.328335
24502	1.23146	0.304721
24503	0.946127	0.306809
24504	1.32827	0.318715
24505	1.23146	0.306698
24506	1.25008	0.659667
24507	1.05018	0.699664
24508	0.94613	0.658276
24509	0.932744	0.768754
24510	1.04207	0.70574
24511	0.946132	0.429779
24512	1.10023	0.6322
24513	1.36748	0.404128
24514	1.36748	0.548973
24515	0.946133	0.483214
24516	1.06023	0.280564
24517	1.08019	0.273313
24518	0.94613	0.343558
24519	1.06023	0.290178
24520	0.94613	0.294607
24521	1.16727	0.365543
24522	1.35856	0.379
24523	0.946132	0.415443
24524	1.16727	0.370304
24525	0.946132	0.361501
24526	0.946133	2.1
24527	0.946131	2.1
24528	1.21691	2.1
24529	1.39558	2.1
24530	0.946131	2.1
24531	1.09905	0.270769

24532	0.946132	0.622487
24533	1.3365	0.380809
24534	1.35381	0.417237
24535	0.946132	0.665575
24536	0.94613	0.266154
24537	0.946128	0.372293
24538	1.40332	0.315627
24539	1.39041	0.254099
24540	0.94613	0.26823
24541	0.946132	0.474343
24542	1.3659	0.719031
24543	1.39041	0.532746
24544	1.39041	0.642427
24545	0.946132	0.636523
24546	0.840292	1.88107
24547	0.946133	1.408
24548	0.851541	1.87656
24549	1.28464	1.61634
24550	1.28806	1.52083
24551	1.12591	0.703795
24552	1.05033	0.814287
24553	0.946132	0.741713
24554	0.946132	0.86285
24555	1.13537	0.818312
24556	1.21177	1.05934
24557	0.946132	1.20657
24558	0.946132	1.92499
24559	1.41134	1.16688
24560	1.27079	1.28678
24561	0.946131	0.127713
24562	1.35403	0.124633
24563	0.94613	0.157265
24564	0.94613	0.141403
24565	0.946131	0.139288
24566	0.946131	0.287222
24567	0.946132	0.161065
24568	0.94613	0.418461
24569	1.37052	0.223738
24570	0.946131	0.327015
24571	0.946133	0.197418
24572	0.946132	0.354761
24573	0.946133	0.186763
24574	1.24249	0.194789
24575	0.946132	0.221523
24576	1.20608	2.1
24577	0.946132	2.08186
24578	0.946131	1.65587
24579	0.946132	2.1
24580	0.946132	2.1
24581	0.946131	2.1
24582	0.946128	2.1
24583	0.946129	2.1
24584	1.41232	2.1
24585	0.94613	2.1
24586	0.946132	0.154588
24587	1.32449	0.15318
24588	0.946131	0.199684
24589	0.946131	0.186846

24590	1.32449	0.171352
24591	0.946116	0.826962
24592	1.41563	1.75306
24593	0.946118	1.18848
24594	0.946105	1.50849
24595	0.946131	0.77047
24596	1.26563	2.1
24597	1.32819	1.62072
24598	0.946131	0.63746
24599	0.946131	1.89678
24600	0.946131	0.975145
24601	0.946131	1.71925
24602	0.946133	1.22279
24603	1.30382	2.1
24604	0.946131	1.18151
24605	0.946131	1.03629
24606	1.30382	1.67815
24607	0.946133	0.0653959
24608	1.15803	0.0686528
24609	1.28895	0.0641677
24610	0.954071	0.0661652
24611	0.946132	0.0702735
24612	1.0264	0.688003
24613	0.946132	1.42793
24614	0.946132	1.07527
24615	0.946131	1.27217
24616	0.946132	1.12824
24617	1.35977	0.246738
24618	0.946132	0.454054
24619	0.946129	0.383915
24620	0.94613	0.384218
24621	0.946129	0.351449
24622	0.876631	0.537173
24623	0.946133	0.65619
24624	0.946132	0.978119
24625	0.946132	1.10972
24626	0.876606	0.934005
24627	0.946131	0.150732
24628	1.24979	0.104835
24629	0.94613	0.134556
24630	0.94613	0.110198
24631	0.94613	0.129458
24632	0.921791	0.0982498
24633	0.946131	0.0940556
24634	0.921762	0.1037
24635	0.946131	0.0937691
24636	0.946131	0.0941737
24637	0.810944	0.229311
24638	0.810945	0.296158
24639	0.810959	0.2794
24640	0.946133	0.27524
24641	1.25797	0.268015
24642	0.946132	0.278365
24643	0.946132	0.262737
24644	1.25797	0.262452
24645	1.13748	0.466436
24646	0.851512	0.728147
24647	0.94613	1.01145

24648	1.02373	0.471881
24649	0.946131	0.829913
24650	0.946122	2.1
24651	1.3852	2.1
24652	0.946124	2.1
24653	0.946118	2.1
24654	1.38521	2.1
24655	0.946131	0.66581
24656	0.946131	0.903681
24657	1.30448	0.861789
24658	0.946129	0.653184
24659	0.94613	0.645652
24660	0.946133	2.03582
24661	0.94613	2.1
24662	0.946131	1.62154
24663	0.946131	1.46761
24664	1.36503	2.1
24665	0.810947	0.0690592
24666	0.810947	0.0678279
24667	0.810934	0.0705646
24668	3.34066	0.0578364
24669	0.946133	0.75145
24670	1.4185	0.626757
24671	0.946132	0.533037
24672	0.946133	0.636329
24673	0.946132	0.632803
24674	0.94613	2.1
24675	0.946128	2.1
24676	1.03925	2.1
24677	0.944743	2.1
24678	0.947518	2.1
24679	0.946132	2.03049
24680	1.20573	2.1
24681	0.94613	2.1
24682	0.94613	2.1
24683	1.20573	2.1
24684	1.20056	0.0911425
24685	0.946132	0.101995
24686	0.946131	0.119468
24687	0.946131	0.104215
24688	0.946131	0.100894
24689	0.94613	2.1
24690	0.946128	2.1
24691	1.38302	2.1
24692	0.94613	2.1
24693	0.94613	2.1
24694	0.827866	0.19962
24695	0.913552	0.168228
24696	0.716526	0.233241
24697	0.82784	0.172233
24698	1.20634	0.215961
24699	0.946131	0.199325
24700	0.94613	0.215919
24701	1.39502	0.14747
24702	0.88146	0.203784
24703	0.946132	2.1
24704	0.946132	2.1
24705	0.946132	2.1

24706	0.946132	2.1
24707	1.39053	2.1
24708	0.946131	2.1
24709	1.40571	2.1
24710	0.946131	2.1
24711	0.946131	2.1
24712	0.946132	2.1
24713	0.94613	0.335603
24714	1.2582	0.441802
24715	0.946127	0.367531
24716	0.946129	0.407697
24717	0.946129	0.367382
24718	0.946129	0.89299
24719	0.946132	0.919584
24720	0.946131	0.772018
24721	0.946131	1.01098
24722	1.29054	0.762076
24723	0.810944	0.0775852
24724	0.810941	0.07691
24725	0.810931	0.0763364
24726	0.946132	0.74076
24727	0.946132	0.654206
24728	0.946132	1.11136
24729	0.946132	0.805187
24730	0.927608	0.730197
24731	0.946128	2.1
24732	0.946133	2.1
24733	0.946131	2.1
24734	0.94613	2.1
24735	1.38813	2.1
24736	0.94613	0.557603
24737	0.946128	1.7945
24738	0.946132	0.546363
24739	1.41666	0.889375
24740	0.94613	0.640183
24741	0.946118	0.28796
24742	1.34151	0.235668
24743	0.946116	0.323545
24744	0.946131	0.306898
24745	1.34149	0.273549
24746	0.946132	2.1
24747	1.31889	2.1
24748	0.946132	2.1
24749	0.946132	2.1
24750	1.31889	2.1
24751	0.864997	0.0842103
24752	0.928981	0.0785924
24753	0.872219	0.0782996
24754	0.946127	0.525708
24755	0.946131	0.452283
24756	1.37709	0.371038
24757	0.946131	0.539665
24758	0.946129	0.455478
24759	1.21882	0.0671235
24760	0.938168	0.0659779
24761	0.938161	0.068991
24762	1.06327	0.0649426
24763	1.33457	0.0708783

24764	0.946133	0.847513
24765	0.946132	0.834865
24766	0.946133	0.768841
24767	0.946132	1.07166
24768	1.14444	0.71066
24769	1.12628	0.110835
24770	0.946132	0.144738
24771	0.946131	0.176366
24772	1.12628	0.110251
24773	0.946131	0.130576
24774	0.946132	2.1
24775	0.946132	2.1
24776	0.946133	2.1
24777	0.946132	2.1
24778	1.29322	2.1
24779	0.946131	2.03771
24780	0.946132	2.1
24781	0.94613	2.06926
24782	0.946131	2.1
24783	1.28441	2.1
24784	0.946118	1.60765
24785	0.946119	1.69735
24786	0.946117	2.1
24787	1.28075	2.1
24788	0.946105	1.94786
24789	0.810945	2.1
24790	0.810942	2.1
24791	0.81093	2.1
24792	0.946127	2.1
24793	0.946127	2.1
24794	1.28228	2.1
24795	0.946129	2.1
24796	0.946132	2.1
24797	1.14042	2.1
24798	0.946132	2.1
24799	0.946133	2.1
24800	1.14042	2.1
24801	0.946133	0.318732
24802	0.946132	0.322066
24803	1.28632	0.245939
24804	1.28632	0.179344
24805	0.946133	0.299179
24806	0.946131	1.15026
24807	0.946131	0.96119
24808	0.946131	1.07121
24809	1.31817	1.19524
24810	0.946131	0.970452
24811	1.35048	2.1
24812	0.946132	2.1
24813	0.946132	2.1
24814	0.946132	2.1
24815	0.946131	2.1
24816	0.946131	2.1
24817	1.35943	2.1
24818	0.94613	2.1
24819	0.946132	2.1
24820	0.946131	2.1
24821	0.990713	2.1

24822	0.946132	2.1
24823	0.946132	2.1
24824	0.946133	2.1
24825	0.946132	2.1
24826	0.946133	1.02571
24827	1.01433	1.09518
24828	0.877931	1.09834
24829	0.96573	0.83113
24830	0.946131	0.837572
24831	0.946131	0.961547
24832	0.946133	0.788371
24833	1.39575	0.603147
24834	0.946131	0.659827
24835	0.94613	0.65618
24836	0.810943	0.512962
24837	0.810942	0.528667
24838	0.709589	0.417284
24839	0.924071	0.380717
24840	1.04293	0.297398
24841	1.15747	0.229849
24842	0.946131	0.387774
24843	1.04071	0.340678
24844	0.810946	0.565624
24845	0.810943	0.456731
24846	0.810959	0.441756
24847	0.946128	1.23707
24848	1.39696	1.62605
24849	0.94613	0.853396
24850	0.94613	0.892374
24851	1.39696	1.36006
24852	0.946131	0.766836
24853	0.946131	1.5033
24854	0.946131	1.38002
24855	0.946131	0.972073
24856	1.29735	1.56151
24857	0.946128	1.15774
24858	0.946133	0.701615
24859	0.94613	1.9246
24860	0.946131	1.20028
24861	1.33266	0.92613
24862	0.946131	1.80443
24863	0.94613	0.271478
24864	0.946131	1.38521
24865	1.40609	0.609626
24866	0.946131	0.712203
24867	0.946131	1.17815
24868	0.946133	1.34537
24869	0.946129	1.31746
24870	1.23339	1.55938
24871	0.946131	1.19935
24872	0.946132	1.62361
24873	0.946133	1.56867
24874	0.946132	1.98779
24875	1.29198	1.94779
24876	0.946132	1.31818
24877	0.946131	2.1
24878	1.07297	2.1
24879	0.946127	2.1

24880	0.94613	2.07705
24881	1.07297	2.1
24882	0.946125	2.1
24883	0.946126	2.1
24884	0.946126	2.1
24885	1.3864	2.1
24886	0.946119	2.1
24887	0.946128	0.288884
24888	1.09411	0.273637
24889	0.946131	0.388272
24890	0.946131	0.35085
24891	1.09411	0.319791
24892	0.946131	0.250789
24893	1.25837	0.23451
24894	0.946131	0.344472
24895	0.946131	0.319924
24896	1.25837	0.285902
24897	0.810945	2.1
24898	0.810943	2.1
24899	0.810933	2.1
24900	0.94613	0.249749
24901	0.946131	0.55795
24902	0.946132	0.316628
24903	0.946129	0.209115
24904	0.946131	0.410379
24905	0.946131	0.246548
24906	1.28911	0.231889
24907	0.946129	0.308024
24908	0.946133	0.243375
24909	0.946131	0.261447
24910	0.94613	0.391505
24911	1.38954	0.45309
24912	0.946132	0.376921
24913	0.94613	0.430695
24914	0.946131	0.398023
24915	0.946131	0.719596
24916	0.946131	0.56358
24917	0.946131	0.507564
24918	1.37981	0.513193
24919	0.946131	0.541388
24920	0.946131	1.39222
24921	1.26958	2.1
24922	0.946127	1.69422
24923	0.946129	2.06716
24924	1.26957	2.1
24925	0.94613	2.1
24926	1.16889	2.1
24927	0.946126	2.1
24928	0.946128	2.1
24929	1.16889	2.1
24930	0.946133	0.432188
24931	1.13511	0.340145
24932	0.946132	0.463559
24933	0.946133	0.368183
24934	0.946132	0.372942
24935	1.27575	1.28587
24936	0.946129	1.92633
24937	0.946131	1.54668

24938	0.94613	2.00005
24939	0.946131	1.82284
24940	0.946131	2.1
24941	0.946128	2.1
24942	1.41712	2.1
24943	1.41712	2.1
24944	0.94613	2.1
24945	0.946129	0.118431
24946	0.946132	0.104569
24947	0.946131	0.104873
24948	0.946131	0.116768
24949	0.946131	0.104229
24950	0.979045	1.47935
24951	0.946131	0.944229
24952	0.617549	1.59197
24953	0.555844	1.72282
24954	1.04071	0.976892
24955	0.946132	1.29441
24956	0.946132	1.00622
24957	0.946131	1.66124
24958	1.22618	0.949429
24959	0.946132	0.804648
24960	0.946131	2.1
24961	1.13976	2.1
24962	0.946129	2.1
24963	0.946131	2.1
24964	1.13976	2.1
24965	0.810945	0.610208
24966	0.810943	0.539607
24967	0.810932	0.744818
24968	1.25358	0.956117
24969	0.94613	0.61283
24970	0.946128	0.603092
24971	1.25358	1.57081
24972	1.07811	0.743549
24973	0.946129	2.1
24974	1.09197	2.1
24975	0.946131	2.1
24976	0.946131	2.1
24977	1.09197	2.1
24978	0.594912	2.1
24979	0.993703	2.1
24980	1.10173	2.1
24981	0.9916	2.1
24982	0.946131	2.0861
24983	0.946128	2.1
24984	0.946131	2.1
24985	0.94613	2.1
24986	0.946129	2.1
24987	0.946132	2.1
24988	1.40335	2.1
24989	0.946133	2.1
24990	0.946133	2.1
24991	1.40335	2.1
24992	0.946132	0.926347
24993	0.946132	1.20189
24994	0.946132	0.979841
24995	0.946132	1.0551

24996	1.411	1.20865
24997	0.611675	0.190926
24998	0.946129	0.285017
24999	1.31678	0.16405
25000	0.946124	0.294447
25001	0.946127	0.185385
25002	0.946128	0.294188
25003	0.946132	0.424005
25004	0.946131	0.312041
25005	0.946132	0.429185
25006	0.946132	0.315802
25007	0.946132	0.319539
25008	0.810946	0.356648
25009	0.810943	0.357768
25010	0.81096	0.319913
25011	1.39301	0.127556
25012	0.946132	0.137294
25013	0.625812	0.110519
25014	0.946132	0.126393
25015	0.625812	0.131033
25016	0.810946	1.00662
25017	0.810945	1.21678
25018	0.810931	1.89131
25019	0.810943	2.1
25020	0.810944	2.1
25021	0.810958	2.1
25022	0.946131	0.410409
25023	0.946131	0.378769
25024	0.946128	0.416503
25025	0.946129	0.394283
25026	0.94613	0.395655
25027	0.946133	0.48718
25028	0.946133	0.524523
25029	0.98537	0.373131
25030	0.985371	0.276163
25031	0.946132	0.576661
25032	0.946129	0.379646
25033	1.38752	0.321446
25034	0.946131	0.28627
25035	0.946131	0.303419
25036	1.38752	0.326887
25037	0.946119	0.322475
25038	1.33036	0.275818
25039	0.946119	0.261578
25040	0.946132	0.262217
25041	1.33035	0.280857
25042	1.3868	2.1
25043	0.946132	2.1
25044	0.946132	2.1
25045	0.946133	2.1
25046	0.946132	2.1
25047	0.810947	2.1
25048	0.810946	2.1
25049	0.81096	2.1
25050	0.810946	2.1
25051	0.810946	2.1
25052	0.810933	2.1
25053	0.946133	1.84799

25054	1.35342	2.1
25055	0.946133	1.75076
25056	0.946132	2.1
25057	1.35342	2.1
25058	0.946132	2.1
25059	0.946132	2.1
25060	1.28569	2.1
25061	0.946132	2.1
25062	0.946133	2.1
25063	0.946132	1.31625
25064	1.32547	2.08344
25065	0.946132	1.40044
25066	0.946132	1.56005
25067	1.32547	1.97657
25068	0.94613	0.218692
25069	0.946133	0.212408
25070	1.39664	0.194194
25071	1.39664	0.194642
25072	0.946131	0.230323
25073	0.946131	1.08998
25074	0.94613	2.01313
25075	0.946132	0.830867
25076	0.946131	1.27155
25077	1.41833	1.2478
25078	0.946131	0.112428
25079	0.946131	0.105073
25080	0.946131	0.122018
25081	1.26155	0.102683
25082	0.946132	0.108761
25083	0.810946	2.1
25084	0.810946	2.1
25085	0.810959	2.1
25086	0.94613	0.948931
25087	0.946132	0.684947
25088	1.40779	0.545771
25089	1.40779	0.612596
25090	0.94613	0.664931
25091	0.94613	0.155603
25092	0.946129	0.178177
25093	1.36376	0.128133
25094	1.36376	0.132443
25095	0.946131	0.150777
25096	0.946128	2.1
25097	0.946127	2.1
25098	0.946131	2.1
25099	1.40756	2.1
25100	0.94613	2.1
25101	0.946131	1.10876
25102	0.946132	1.44803
25103	0.94613	1.08881
25104	0.946131	1.01126
25105	1.31947	1.27893
25106	0.946131	2.1
25107	0.946131	2.1
25108	0.946129	2.1
25109	0.946131	2.1
25110	1.25768	2.1
25111	0.946132	0.363313

25112	0.94613	0.386633
25113	0.946131	0.351948
25114	0.946131	0.346682
25115	0.946131	0.388563
25116	0.810943	0.844756
25117	0.810942	1.00398
25118	0.81093	1.54617
25119	0.946128	0.902459
25120	0.946133	0.748865
25121	0.946131	0.921936
25122	0.946131	0.868318
25123	0.94613	0.960775
25124	0.946132	0.931636
25125	0.946131	0.790794
25126	0.946132	0.954317
25127	0.946132	0.928034
25128	0.946132	0.99486
25129	0.946119	2.1
25130	0.946119	2.1
25131	1.31523	2.1
25132	0.946132	2.1
25133	0.946107	2.1
25134	0.946133	1.61755
25135	0.946132	0.746981
25136	0.946132	0.705422
25137	1.28803	1.1744
25138	0.946132	1.05183
25139	1.37658	0.969708
25140	0.946132	0.87378
25141	0.946133	1.21273
25142	0.946132	1.01006
25143	0.946132	1.13361
25144	0.946132	0.925715
25145	0.94613	1.03927
25146	0.94613	1.2774
25147	1.15541	0.90769
25148	0.92943	1.12579
25149	0.946126	0.100537
25150	0.946129	0.113411
25151	1.33849	0.0827869
25152	1.33849	0.0926839
25153	0.946129	0.0987064
25154	0.810946	0.0904232
25155	0.810944	0.112628
25156	0.810959	0.0949041
25157	1.39027	0.118719
25158	0.946131	0.154291
25159	0.946131	0.190518
25160	1.39027	0.122479
25161	0.946131	0.140162
25162	1.40924	1.67913
25163	0.946131	1.16676
25164	0.946132	1.70115
25165	0.946132	0.966355
25166	0.946132	1.42659
25167	0.946131	2.1
25168	0.94613	2.1
25169	0.946128	2.1

25170	0.946128	2.1
25171	1.41744	2.1
25172	0.946132	0.602857
25173	0.946128	0.613271
25174	0.94613	0.87934
25175	1.37675	0.622292
25176	0.94613	0.916827
25177	0.946132	0.79534
25178	0.946132	0.566968
25179	0.946132	0.474607
25180	0.946132	0.556865
25181	0.946131	0.538625
25182	0.946132	0.78092
25183	0.946132	0.628699
25184	0.946133	1.12294
25185	1.10003	0.570674
25186	0.946132	0.88147
25187	0.946132	0.18944
25188	0.946131	0.206567
25189	0.946132	0.172801
25190	0.946131	0.197076
25191	0.946132	0.190248
25192	0.946132	0.215008
25193	0.946131	0.232103
25194	0.946132	0.192714
25195	0.946132	0.223378
25196	0.946131	0.21284
25197	1.24094	0.10105
25198	0.946126	0.131456
25199	0.946128	0.113837
25200	0.946129	0.12881
25201	0.946129	0.11912
25202	1.2929	0.126487
25203	0.946131	0.186613
25204	0.946131	0.153428
25205	0.946131	0.168104
25206	0.946131	0.162066
25207	0.810946	0.0916478
25208	0.810945	0.0990077
25209	0.810959	0.0880229
25210	0.810946	1.2804
25211	0.810943	1.26521
25212	0.810933	1.50683
25213	0.946128	1.77753
25214	1.18315	2.1
25215	0.94613	1.79589
25216	0.94613	1.74317
25217	1.18316	2.01151
25218	0.946126	0.419062
25219	1.3324	0.32382
25220	0.946127	0.444325
25221	0.94613	0.329432
25222	0.946128	0.373374
25223	0.94613	1.92341
25224	0.946128	2.1
25225	1.39523	2.1
25226	1.39523	2.1
25227	0.946131	2.1

25228	1.36541	2.1
25229	0.946132	2.1
25230	0.946132	2.1
25231	0.946132	2.1
25232	0.946132	2.1
25233	0.946131	1.78563
25234	0.94613	0.975573
25235	0.946132	1.31955
25236	0.946131	0.56636
25237	1.39509	1.20111
25238	0.946132	2.1
25239	0.946132	2.1
25240	1.29319	2.1
25241	0.946132	2.1
25242	0.946133	2.1
25243	0.946132	0.145416
25244	0.946131	0.203008
25245	0.946132	0.156367
25246	1.01196	0.145478
25247	0.946132	0.186272
25248	0.946116	2.1
25249	0.946117	1.08659
25250	1.38153	2.1
25251	1.38155	2.1
25252	0.946105	2.1
25253	0.946132	1.45383
25254	0.946131	1.49087
25255	0.946132	1.34327
25256	1.28684	1.39309
25257	0.946132	1.18623
25258	1.10185	1.49449
25259	0.94613	1.62035
25260	0.946128	2.02583
25261	0.94613	1.96215
25262	0.94613	1.5858
25263	0.810947	0.0755362
25264	0.810945	0.0760722
25265	0.810932	0.0766887
25266	0.946119	0.384622
25267	1.16717	0.309499
25268	0.946117	0.401652
25269	0.94613	0.338623
25270	1.16715	0.329473
25271	0.94612	0.483422
25272	0.946119	0.476676
25273	0.946119	0.456408
25274	0.946132	0.373213
25275	1.20882	0.390017
25276	1.07043	0.669479
25277	0.823268	0.673738
25278	0.810959	0.633437
25279	0.946132	1.43271
25280	1.14879	0.802755
25281	0.94613	1.25912
25282	0.946131	1.39034
25283	1.14879	0.917358
25284	1.23837	2.1
25285	0.94612	2.1

25286	0.946118	2.04793
25287	0.946131	2.1
25288	0.946105	2.1
25289	0.810946	0.101802
25290	0.810946	0.0927685
25291	0.810959	0.0958723
25292	0.810947	0.950962
25293	0.810947	0.445535
25294	0.810934	0.424388
25295	0.946128	2.1
25296	0.946131	2.1
25297	0.946128	2.1
25298	0.946129	2.1
25299	0.94613	2.1
25300	0.94613	2.1
25301	0.946131	2.1
25302	0.946131	1.95607
25303	0.946131	2.1
25304	0.946131	2.1
25305	0.946131	1.42753
25306	1.38585	2.1
25307	0.946128	1.06979
25308	0.94613	1.28688
25309	1.38585	1.5624
25310	0.946133	0.297283
25311	0.946133	0.298779
25312	0.946132	0.260178
25313	0.946132	0.294666
25314	0.946132	0.279615
25315	0.918871	0.149181
25316	0.810248	0.175681
25317	0.838037	0.157186
25318	0.946126	0.176915
25319	0.943647	0.153726
25320	1.13836	0.129498
25321	1.03802	0.132982
25322	0.946129	0.141366
25323	0.946133	0.977591
25324	0.946132	0.809657
25325	0.946132	1.42236
25326	1.1699	0.808007
25327	0.946132	0.708063
25328	0.946131	2.1
25329	0.946132	2.1
25330	1.37199	2.1
25331	1.37199	2.1
25332	0.946132	2.1
25333	0.946132	0.816417
25334	0.946133	0.663916
25335	0.946133	0.963944
25336	0.946132	0.709154
25337	1.38623	0.683141
25338	0.946132	2.1
25339	0.946132	2.1
25340	0.946132	2.1
25341	0.946132	2.1
25342	1.40098	2.1
25343	1.20787	0.610788

25344	1.41894	0.586085
25345	0.946133	0.418064
25346	1.01777	0.641323
25347	0.946132	2.1
25348	0.946132	2.1
25349	0.946133	2.1
25350	1.40199	2.1
25351	0.946132	2.1
25352	0.810946	0.922937
25353	0.810947	1.28362
25354	0.810934	1.03652
25355	0.946126	0.840127
25356	0.94613	1.12475
25357	0.946125	1.11508
25358	1.40247	0.921268
25359	0.946128	0.878937
25360	1.25703	1.26333
25361	0.87821	0.936275
25362	0.878238	0.906379
25363	0.946119	1.47112
25364	0.946132	1.8895
25365	0.946132	0.763169
25366	0.946132	0.821766
25367	0.946131	1.27013
25368	0.946132	0.84572
25369	1.28543	0.825499
25370	0.946115	2.1
25371	1.41638	2.1
25372	0.946116	2.1
25373	0.946104	2.1
25374	0.946129	2.1
25375	0.946132	1.21908
25376	1.41432	2.1
25377	0.946132	1.48895
25378	0.946132	1.91117
25379	0.946132	0.906743
25380	0.810947	0.171138
25381	0.810947	0.182903
25382	0.709567	0.189754
25383	0.851542	0.134315
25384	1.01217	0.11604
25385	0.697993	0.104319
25386	0.946133	0.122195
25387	0.851488	0.0997918
25388	0.946129	1.97443
25389	0.94613	2.1
25390	1.2254	1.60773
25391	0.946131	2.08219
25392	0.946131	1.72551
25393	0.700785	1.99347
25394	0.915893	1.52539
25395	0.838013	1.37687
25396	0.760188	1.18224
25397	1.19601	0.288109
25398	0.946133	0.48621
25399	0.946132	0.545729
25400	0.946132	0.381752
25401	0.946132	0.50715

25402	0.946132	0.841594
25403	0.946133	2.00082
25404	0.946132	1.14064
25405	0.946133	1.48253
25406	1.36956	1.75832
25407	0.946131	0.609459
25408	0.94613	0.175688
25409	0.946133	0.53382
25410	1.34268	0.198614
25411	0.946131	0.342271
25412	0.946132	1.88183
25413	1.25603	1.79221
25414	0.946132	1.87267
25415	0.946132	1.80176
25416	1.25603	1.95328
25417	0.810946	0.9336
25418	0.810943	0.944414
25419	0.810933	0.903508
25420	0.496545	0.439434
25421	0.805767	0.901465
25422	0.936671	0.817336
25423	0.530063	0.742764
25424	1.05954	0.773835
25425	0.810946	0.223209
25426	0.810943	0.195095
25427	0.810959	0.178372
25428	1.16699	0.154249
25429	0.946131	0.296757
25430	0.946129	0.244454
25431	0.946131	0.231803
25432	0.946131	0.217412
25433	0.810943	1.04695
25434	0.810942	1.15343
25435	0.810933	1.07105
25436	0.946132	1.02179
25437	1.04832	1.04309
25438	0.870714	1.08967
25439	0.957819	1.27306
25440	0.94613	0.934328
25441	1.82931	2.1
25442	0.946132	0.778437
25443	0.946129	0.712456
25444	0.94613	0.793831
25445	0.946131	0.763823
25446	0.94613	0.638158
25447	0.946119	0.864586
25448	0.94612	0.965994
25449	0.94612	0.767988
25450	0.946132	0.929249
25451	1.36118	0.805111
25452	0.810947	0.332947
25453	0.810947	0.333085
25454	0.810934	0.330616
25455	0.946132	2.1
25456	0.946132	2.1
25457	0.946132	2.1
25458	1.39754	2.1
25459	0.946131	2.1

25460	0.946129	2.1
25461	0.946133	2.1
25462	0.946131	2.1
25463	0.946131	2.1
25464	1.41732	2.1
25465	0.946132	0.81397
25466	0.946128	1.45153
25467	0.94613	0.946534
25468	0.94613	1.03636
25469	1.25277	1.31351
25470	0.94613	1.30172
25471	0.946128	1.76448
25472	1.21261	0.94883
25473	0.94613	1.33036
25474	0.94613	1.05686
25475	1.39407	2.1
25476	0.946133	2.1
25477	0.94613	2.1
25478	0.946131	2.1
25479	0.946131	2.1
25480	0.946119	0.935077
25481	0.946119	1.20325
25482	0.946119	1.17856
25483	1.1537	1.20083
25484	0.946106	0.890674
25485	0.946131	2.05464
25486	1.11822	2.1
25487	0.946129	2.08868
25488	0.946131	2.1
25489	1.11822	2.1
25490	1.46806	0.0709307
25491	0.498881	0.0780674
25492	0.996658	0.0815265
25493	1.2179	0.0734694
25494	0.991519	0.0770086
25495	0.946131	0.0874259
25496	0.946131	0.0958132
25497	1.28673	0.0786943
25498	0.946131	0.0852381
25499	0.946131	0.0837106
25500	1.33178	0.135135
25501	0.946132	0.173428
25502	0.946132	0.16553
25503	0.946132	0.169536
25504	0.946132	0.169358
25505	0.946128	0.391238
25506	0.972061	0.346901
25507	0.946129	0.485193
25508	0.946129	0.464097
25509	0.972059	0.422019
25510	0.946129	2.1
25511	0.94613	2.1
25512	0.94613	2.1
25513	0.94613	2.1
25514	0.94613	2.1
25515	0.946131	2.1
25516	0.946127	2.1
25517	0.946131	2.1

25518	0.946129	2.1
25519	1.4073	2.1
25520	0.946131	0.174815
25521	1.05016	0.145156
25522	0.946132	0.186491
25523	0.946131	0.181317
25524	0.946132	0.174996
25525	0.946128	0.174289
25526	1.13398	0.148448
25527	0.946131	0.194882
25528	0.946131	0.184816
25529	0.94613	0.177704
25530	0.946131	0.108596
25531	0.946131	0.131415
25532	1.38911	0.0890279
25533	1.38911	0.100388
25534	0.946132	0.10933
25535	0.946131	0.241546
25536	0.94613	0.2541
25537	0.946131	0.228193
25538	0.946131	0.243465
25539	0.946131	0.248665
25540	0.946131	0.298625
25541	1.23923	0.246613
25542	0.94613	0.319586
25543	0.946131	0.251727
25544	0.946131	0.276709
25545	1.40511	0.0845292
25546	0.946131	0.0931954
25547	0.946131	0.110601
25548	0.946132	0.0969415
25549	0.946131	0.0924371
25550	1.12968	2.1
25551	0.946132	1.53385
25552	0.946132	2.1
25553	0.946131	1.93257
25554	0.946132	1.99874
25555	0.946132	1.2975
25556	1.4172	2.1
25557	0.94613	1.02116
25558	0.94613	1.41453
25559	1.4172	1.78479
25560	0.946132	2.1
25561	0.946132	2.1
25562	1.31323	2.1
25563	0.946133	2.1
25564	0.946132	2.1
25565	0.946131	1.80168
25566	0.94613	0.526054
25567	1.36546	1.02073
25568	1.36546	0.810735
25569	0.946131	0.767406
25570	0.946132	0.446254
25571	0.946133	0.531154
25572	0.946132	0.34566
25573	1.36899	0.347344
25574	0.946133	0.389213
25575	0.946131	0.233015

25576	0.946132	0.205635
25577	1.03543	0.187808
25578	1.1412	0.192327
25579	0.941275	0.26168
25580	1.28952	0.172768
25581	0.94613	0.20705
25582	0.946129	0.218413
25583	0.946132	0.173979
25584	0.94613	0.204305
25585	0.94613	2.1
25586	0.94613	2.1
25587	0.946129	2.1
25588	0.94613	2.1
25589	1.35966	2.1
25590	0.810946	1.74578
25591	0.810942	2.0398
25592	0.810932	1.65104
25593	0.94613	1.78707
25594	1.40372	2.1
25595	0.946131	1.38844
25596	0.94613	2.1
25597	1.40372	2.1
25598	0.946133	2.1
25599	0.946131	2.1
25600	0.94613	2.1
25601	0.946131	2.1
25602	0.946131	2.1
25603	1.31453	2.1
25604	0.94613	2.1
25605	0.946128	2.1
25606	0.94613	2.1
25607	0.94613	2.1
25608	0.946131	0.14313
25609	1.12018	0.131191
25610	0.94613	0.154653
25611	0.94613	0.161523
25612	0.94613	0.14811
25613	0.946131	0.172953
25614	1.16373	0.164585
25615	0.946131	0.2145
25616	0.94613	0.171111
25617	0.946131	0.187026
25618	0.946126	0.410586
25619	0.946132	0.429425
25620	0.946128	0.395121
25621	0.946129	0.498519
25622	0.946129	0.525893
25623	0.946132	0.298989
25624	0.946133	0.357448
25625	0.946132	0.2901
25626	0.946132	0.323
25627	0.946133	0.32361
25628	0.946132	0.862091
25629	0.946129	1.24156
25630	0.94613	0.791342
25631	1.36399	0.935755
25632	0.946131	0.82593
25633	0.946132	2.1

25634	0.946132	2.1
25635	0.946133	2.1
25636	1.38302	2.1
25637	0.946133	2.1
25638	0.810943	2.1
25639	0.810944	2.1
25640	0.810945	2.1
25641	0.946132	2.1
25642	0.946131	2.1
25643	0.946132	2.1
25644	1.30188	2.1
25645	0.946132	2.1
25646	0.810946	0.758296
25647	0.72985	1.19304
25648	0.810934	0.937043
25649	0.856243	0.699905
25650	0.946129	0.667098
25651	0.946132	0.705817
25652	1.19686	0.628351
25653	0.851527	0.800631
25654	0.946131	2.1
25655	0.94613	2.1
25656	0.94613	2.1
25657	0.94613	2.1
25658	1.31961	2.1
25659	0.946131	2.1
25660	0.946131	2.1
25661	0.94613	2.1
25662	0.946131	2.1
25663	1.3605	2.1
25664	1.30116	1.99547
25665	0.946133	0.768542
25666	0.946131	1.91375
25667	0.946131	1.14679
25668	0.94613	1.73733
25669	0.946132	0.132257
25670	1.3035	0.108389
25671	0.94613	0.151344
25672	0.946131	0.133466
25673	0.946131	0.145333
25674	0.946132	2.1
25675	1.37869	2.1
25676	1.2051	2.1
25677	0.946132	2.1
25678	1.2051	2.1
25679	0.810946	2.1
25680	0.810945	2.1
25681	0.810946	2.1
25682	1.34623	2.1
25683	0.946131	2.1
25684	0.946131	2.1
25685	1.34623	2.1
25686	1.22993	2.1
25687	1.32299	0.105596
25688	0.946132	0.128154
25689	0.946132	0.146245
25690	0.946132	0.121661
25691	0.946131	0.124025

25692	0.94613	0.244039
25693	1.40574	0.208925
25694	0.946128	0.29892
25695	0.946131	0.336118
25696	1.40574	0.287441
25697	0.946132	2.1
25698	1.41331	2.1
25699	0.94613	2.1
25700	0.946131	2.1
25701	1.41331	2.1
25702	0.94613	2.1
25703	1.37609	2.1
25704	0.946129	2.1
25705	0.946131	2.1
25706	1.37609	2.1
25707	1.039	0.997353
25708	0.946131	1.79368
25709	0.944514	1.31331
25710	0.946132	1.68023
25711	0.946132	1.46562
25712	1.23726	0.981704
25713	0.946128	1.43527
25714	0.946128	1.23184
25715	0.946129	1.48466
25716	0.94613	1.30944
25717	0.946129	1.70068
25718	0.946131	2.1
25719	0.946131	1.5601
25720	1.39214	2.1
25721	0.94613	2.1
25722	0.946118	0.608414
25723	0.946118	0.251691
25724	1.41078	0.313728
25725	1.41076	0.216555
25726	0.946131	0.222266
25727	0.946131	0.774354
25728	0.946131	0.650749
25729	0.946132	0.712302
25730	0.94613	0.610728
25731	0.946131	0.580275
25732	0.946132	0.124626
25733	0.946132	0.105677
25734	0.946133	0.0950405
25735	1.28924	0.0964007
25736	0.946132	0.109028
25737	0.946133	0.48313
25738	1.09812	0.319341
25739	0.946132	0.502072
25740	0.946132	0.553902
25741	1.09812	0.549263
25742	0.946132	0.705388
25743	0.946132	0.669021
25744	0.946132	0.695621
25745	0.946132	0.682317
25746	1.15388	0.713757
25747	0.946132	0.484926
25748	0.974688	0.291245
25749	0.946132	0.455705

25750	0.946132	0.495176
25751	0.974687	0.483357
25752	1.0644	1.11749
25753	0.946133	1.6049
25754	0.946132	1.09754
25755	0.946133	1.19127
25756	1.26547	1.30813
25757	1.02905	0.976351
25758	0.911537	1.02914
25759	0.838038	1.21601
25760	0.946131	0.304564
25761	0.94613	0.333607
25762	0.946132	0.299836
25763	0.946131	0.346567
25764	1.33922	0.337752
25765	0.946132	0.179044
25766	0.946132	0.149012
25767	1.40926	0.144392
25768	1.40927	0.143216
25769	0.946132	0.151135
25770	0.946129	1.80403
25771	0.946131	1.63763
25772	0.946131	1.12907
25773	0.946131	0.903173
25774	1.40496	1.46051
25775	0.94613	0.808426
25776	0.94613	0.77948
25777	0.946127	0.769961
25778	0.946128	0.703685
25779	1.38914	0.73286
25780	2.675	0.0812516
25781	1.22266	0.0853114
25782	0.946131	0.101673
25783	0.946132	0.0982987
25784	0.946132	0.0939653
25785	1.22266	0.0965921
25786	0.946124	0.18066
25787	0.946128	0.124718
25788	0.946126	0.115073
25789	1.21428	0.110185
25790	0.946127	0.158713
25791	0.946131	0.145086
25792	0.946131	0.248471
25793	0.946132	0.154354
25794	0.946129	0.146088
25795	0.940118	0.213479
25796	0.810943	2.1
25797	0.810945	2.1
25798	0.810958	2.1
25799	0.946133	0.237088
25800	1.11539	0.21402
25801	0.946132	0.285029
25802	0.946132	0.264101
25803	0.946133	0.244092
25804	0.946133	2.1
25805	1.41195	2.1
25806	0.946133	2.1
25807	0.946132	2.1

25808	0.946133	2.1
25809	1.15968	0.0824518
25810	0.946131	0.0928395
25811	0.946131	0.10403
25812	0.946131	0.0932362
25813	0.946131	0.0905171
25814	0.94613	2.02807
25815	0.946128	2.07573
25816	0.946132	1.28006
25817	0.94613	1.94599
25818	0.94613	1.57744
25819	0.946131	1.82064
25820	1.04211	1.82204
25821	0.850155	1.14668
25822	0.946131	1.54923
25823	0.946131	1.32536
25824	0.946131	0.39608
25825	0.94613	0.310306
25826	0.946129	0.397977
25827	0.946131	0.30659
25828	0.946131	0.308975
25829	1.40193	0.188347
25830	1.19756	0.201925
25831	0.561936	0.23433
25832	0.808778	0.217746
25833	1.19756	0.211386
25834	0.946132	0.128822
25835	0.946132	0.124637
25836	1.33916	0.103748
25837	1.33916	0.107803
25838	0.946133	0.117225
25839	0.946127	0.466607
25840	0.946127	0.376203
25841	0.946131	0.439362
25842	0.946128	0.410524
25843	0.946129	0.39755
25844	0.589428	0.578443
25845	0.851501	0.365969
25846	0.868924	0.53599
25847	0.877703	0.533892
25848	0.85153	0.516174
25849	0.946132	1.08691
25850	1.25372	2.1
25851	0.946132	1.23672
25852	0.946132	1.59564
25853	1.25372	1.93042
25854	0.946132	0.776691
25855	0.946128	1.74064
25856	0.94613	0.950689
25857	0.94613	1.09746
25858	1.2778	1.51737
25859	0.946118	0.705181
25860	1.35629	0.716259
25861	0.946117	0.72325
25862	0.946131	0.689403
25863	0.946105	0.628633
25864	0.946132	0.52355
25865	0.946132	0.68342

25866	1.38646	0.683215
25867	1.38646	0.716957
25868	0.946132	0.726511
25869	0.946128	1.80738
25870	0.946128	0.609741
25871	1.39659	1.0459
25872	1.39659	0.782073
25873	0.946129	0.741894
25874	0.946132	1.41759
25875	0.946131	1.293
25876	0.946132	1.27212
25877	0.946132	1.29883
25878	1.15139	1.21541
25879	0.946133	2.1
25880	0.946132	1.5746
25881	0.946132	2.1
25882	0.946132	2.1
25883	1.36151	2.1
25884	0.946127	2.1
25885	0.946131	2.1
25886	0.946128	2.1
25887	0.94613	2.1
25888	1.38397	2.1
25889	0.946131	0.291841
25890	1.31112	0.280326
25891	0.946127	0.299015
25892	0.946128	0.372344
25893	1.31112	0.297051
25894	1.29778	0.0630464
25895	0.883417	0.0632244
25896	1.32613	0.0597391
25897	0.94613	0.0615393
25898	0.94613	0.0640594
25899	0.946132	2.1
25900	0.94613	2.1
25901	0.946131	2.1
25902	0.946131	2.1
25903	1.36523	2.1
25904	0.946132	0.174644
25905	1.01653	0.150337
25906	0.946132	0.213631
25907	0.946131	0.197604
25908	0.946131	0.179795
25909	0.94612	2.1
25910	1.08301	1.3857
25911	0.946119	2.09501
25912	0.946132	2.1
25913	1.08299	1.83604
25914	0.810942	2.09693
25915	0.810944	0.777074
25916	0.810957	0.948952
25917	0.946126	0.317623
25918	0.946132	0.360816
25919	0.946129	0.319532
25920	1.3492	0.286928
25921	0.94613	0.314841
25922	0.946131	0.251016
25923	1.24766	0.218651

25924	0.946128	0.280932
25925	0.946131	0.348375
25926	1.24766	0.288352
25927	0.946132	0.0950954
25928	0.946131	0.0905318
25929	0.946131	0.106163
25930	1.23351	0.0893389
25931	0.946132	0.093062
25932	0.946131	0.103261
25933	0.946132	0.0971842
25934	0.946131	0.11957
25935	1.35657	0.0957823
25936	0.946131	0.101556
25937	0.946132	0.554009
25938	1.3689	0.453742
25939	0.946132	0.529136
25940	0.946132	0.545895
25941	1.3689	0.503405
25942	0.946133	0.435837
25943	1.35822	0.345306
25944	0.946133	0.390155
25945	0.946132	0.419985
25946	1.35822	0.387695
25947	0.946131	0.669647
25948	0.946131	1.18873
25949	0.946131	1.1416
25950	0.946131	0.81796
25951	1.4101	1.3081
25952	0.810944	1.32338
25953	0.810939	1.38171
25954	0.810955	1.09327
25955	0.946132	2.1
25956	1.37733	2.1
25957	0.946132	2.1
25958	0.946131	2.1
25959	0.946131	2.1
25960	1.32582	2.1
25961	0.946132	1.60258
25962	0.946132	1.03884
25963	0.946132	1.70739
25964	0.946132	2.04812
25965	0.946132	1.73447
25966	0.946132	1.88995
25967	1.31401	2.1
25968	1.31401	2.1
25969	0.946131	2.1
25970	1.3469	1.22273
25971	0.946132	1.39375
25972	0.946132	1.45194
25973	0.946133	0.920627
25974	0.946132	1.07626
25975	0.946126	2.1
25976	0.946127	1.79061
25977	0.94613	2.1
25978	0.946128	1.80374
25979	1.41206	2.1
25980	0.946127	1.20285
25981	1.40109	1.74754

25982	0.946128	1.22182
25983	0.946129	1.67815
25984	1.40109	1.52266
25985	1.24448	0.16019
25986	0.94613	0.30205
25987	0.946132	0.218455
25988	0.946129	0.252492
25989	0.946131	0.24514
25990	1.11015	0.198894
25991	0.946133	0.478495
25992	0.946132	0.320476
25993	0.946133	0.345382
25994	0.946132	0.371734
25995	0.946132	2.1
25996	0.946131	2.1
25997	0.946131	2.1
25998	1.38775	2.1
25999	0.946131	2.1
26000	0.946133	0.616676
26001	0.946131	0.678269
26002	1.30598	1.04558
26003	1.30598	0.862352
26004	0.946131	0.839096
26005	0.946132	2.1
26006	0.946132	2.1
26007	0.946132	2.1
26008	1.40774	2.1
26009	0.946132	2.1
26010	0.798396	0.0752577
26011	0.946133	0.760823
26012	0.946128	1.18459
26013	0.946131	0.987813
26014	0.946131	0.997847
26015	1.28918	1.04829
26016	0.946132	0.408647
26017	1.40668	0.383582
26018	0.94613	0.393028
26019	0.94613	0.391308
26020	0.94613	0.396213
26021	0.810946	2.1
26022	0.810945	2.1
26023	0.810933	2.1
26024	0.946131	2.1
26025	1.30338	2.1
26026	0.94613	2.1
26027	1.30339	2.1
26028	0.946131	2.1
26029	0.946127	0.837762
26030	0.87161	1.17967
26031	0.946128	0.906135
26032	0.946129	1.02337
26033	0.827876	0.829368
26034	0.946131	2.1
26035	1.40796	2.1
26036	0.94613	2.1
26037	0.94613	2.1
26038	1.40796	2.1
26039	0.946132	0.684209

26040	0.946132	0.7786
26041	0.946132	0.667509
26042	0.946132	0.611155
26043	1.41724	0.572271
26044	1.34254	0.32553
26045	0.946132	0.620061
26046	0.946132	0.531734
26047	0.946132	0.5228
26048	0.946133	0.479123
26049	0.946133	1.52116
26050	0.946132	1.30279
26051	0.946132	1.28325
26052	1.26501	1.57381
26053	0.946132	1.20237
26054	0.946131	0.16038
26055	1.35169	0.15945
26056	0.946131	0.208349
26057	0.946131	0.189715
26058	1.35169	0.172747
26059	0.676844	0.125371
26060	0.946131	0.148981
26061	0.94613	0.183822
26062	1.13242	0.11689
26063	1.13242	0.129815
26064	0.946131	0.148155
26065	0.946128	1.01473
26066	1.28828	1.71154
26067	0.946131	1.1245
26068	0.946131	1.14119
26069	1.28828	1.40552
26070	0.946131	0.440594
26071	1.2996	0.373956
26072	0.946133	0.443915
26073	0.946131	0.401972
26074	0.946131	0.391331
26075	0.946132	0.992187
26076	1.40638	2.1
26077	0.946131	0.800159
26078	0.946132	2.07698
26079	1.40638	2.1
26080	2.84909	0.103583
26081	1.35726	0.108459
26082	0.946131	0.15535
26083	0.946131	0.138734
26084	0.946131	0.134056
26085	0.946131	0.131234
26086	1.39852	1.92258
26087	0.946132	1.6839
26088	0.94613	2.1
26089	0.94613	2.0072
26090	0.94613	1.90445
26091	0.946126	0.134785
26092	0.946131	0.124095
26093	0.946128	0.158099
26094	1.31666	0.128667
26095	0.946128	0.137077
26096	0.946126	0.173625
26097	0.946131	0.147407

26098	0.946129	0.201467
26099	1.32275	0.145738
26100	0.946129	0.179263
26101	0.946131	2.1
26102	0.946132	2.1
26103	1.41125	2.1
26104	0.946131	2.1
26105	0.946131	2.1
26106	0.946131	2.1
26107	0.946132	2.1
26108	1.31904	2.1
26109	0.946132	2.1
26110	0.946132	2.1
26111	0.946129	0.248413
26112	0.946132	0.252014
26113	0.94613	0.258161
26114	1.02426	0.170779
26115	0.9311	0.302225
26116	0.946128	1.46053
26117	0.946133	1.09477
26118	0.946131	1.32941
26119	1.40092	1.42463
26120	0.946131	1.20447
26121	0.946117	0.542609
26122	0.946118	1.27016
26123	0.946118	0.515338
26124	1.40017	0.678461
26125	0.946105	0.590472
26126	0.946132	0.421946
26127	1.03547	0.373688
26128	0.946132	0.36401
26129	0.946133	0.449665
26130	0.946132	0.413593
26131	0.946133	2.1
26132	0.946132	2.1
26133	0.946133	2.1
26134	0.946133	2.1
26135	1.36694	2.1
26136	0.946132	0.279801
26137	1.22878	0.27565
26138	0.946132	0.282638
26139	0.946132	0.273823
26140	1.22878	0.272526
26141	1.11014	0.199364
26142	0.913078	0.222012
26143	1.11015	0.272881
26144	0.49154	0.286926
26145	0.946127	2.1
26146	0.946131	2.1
26147	0.946128	2.1
26148	0.946129	2.1
26149	1.38752	2.1
26150	0.946132	2.1
26151	1.37277	2.1
26152	0.946132	2.1
26153	0.946131	2.1
26154	1.37277	2.1
26155	0.94613	0.429109

26156	1.16834	0.28865
26157	0.946129	0.439
26158	0.94613	0.328473
26159	0.94613	0.339274
26160	0.946127	0.431151
26161	0.946131	0.332743
26162	1.35594	0.282951
26163	0.946128	0.447319
26164	0.94613	0.352316
26165	0.946128	0.857287
26166	0.946128	0.366072
26167	1.38464	0.536885
26168	1.38463	0.413275
26169	0.94613	0.47905
26170	0.946129	0.830477
26171	0.946131	0.387867
26172	0.94613	0.394237
26173	1.39751	0.343951
26174	0.946131	0.42466
26175	1.25837	0.223565
26176	1.21134	0.213773
26177	0.700013	0.282914
26178	0.700041	0.255396
26179	1.40583	1.617
26180	0.946132	2.07028
26181	0.946132	2.07666
26182	0.946133	1.92401
26183	0.946132	1.744
26184	0.827865	1.9524
26185	0.946131	2.06942
26186	0.946131	1.60627
26187	0.946131	1.8022
26188	0.946131	1.55061
26189	0.946132	0.107575
26190	1.21087	0.0901996
26191	0.94613	0.105607
26192	0.94613	0.093229
26193	0.946131	0.10116
26194	0.94613	0.62529
26195	1.17452	0.770941
26196	0.946132	0.631887
26197	0.94613	0.699218
26198	0.94613	0.616169
26199	0.946119	0.54395
26200	1.27374	0.591106
26201	0.946119	0.545091
26202	0.946131	0.565513
26203	0.946106	0.534307
26204	0.946133	2.1
26205	0.946132	2.1
26206	0.946133	2.1
26207	0.946132	2.1
26208	1.39621	2.1
26209	1.34352	2.1
26210	0.946132	2.1
26211	0.946132	2.1
26212	0.946132	2.1
26213	0.946132	2.1

26214	0.946132	0.702493
26215	0.946128	1.85956
26216	0.94613	1.15863
26217	0.94613	1.44907
26218	1.41273	1.75568
26219	1.23536	2.08888
26220	0.946131	1.12672
26221	0.946132	2.1
26222	0.946132	1.66745
26223	0.946132	1.8844
26224	0.946132	2.1
26225	1.41131	2.1
26226	0.946131	2.1
26227	0.946131	2.1
26228	0.946131	2.1
26229	0.946133	0.579543
26230	0.946132	1.42572
26231	0.946133	0.549457
26232	1.41894	0.531211
26233	0.946132	0.402139
26234	0.946128	0.591786
26235	1.4099	0.970505
26236	0.946131	0.679985
26237	0.946131	1.59157
26238	0.946131	0.405258
26239	0.810947	1.10384
26240	0.810945	1.20031
26241	0.810933	1.64749
26242	0.946131	0.695464
26243	1.25765	1.81666
26244	0.946128	1.08658
26245	0.946129	0.669581
26246	1.25765	1.10847
26247	1.21347	2.1
26248	0.946129	2.1
26249	0.94613	2.1
26250	1.21347	2.1
26251	0.94613	2.1
26252	0.94613	0.077077
26253	0.946132	0.0706611
26254	1.2493	0.069401
26255	1.2493	0.0654363
26256	0.94613	0.0808229
26257	0.946132	0.814709
26258	0.946128	1.1486
26259	0.946131	0.796197
26260	0.94613	0.99035
26261	1.37938	0.909744
26262	0.946133	0.107054
26263	0.946132	0.0985844
26264	0.946133	0.10763
26265	0.946132	0.0909847
26266	0.946133	0.109663
26267	0.946129	0.692673
26268	1.26426	0.609361
26269	0.946131	0.680038
26270	0.946131	0.699357
26271	1.26426	0.659239

26272	0.872447	1.59756
26273	0.946132	1.24934
26274	0.946132	1.38606
26275	0.946132	1.5149
26276	0.946133	1.30576
26277	0.946117	1.15908
26278	0.946116	1.05352
26279	0.946113	0.957554
26280	0.946129	1.1513
26281	1.27211	0.88579
26282	0.94613	0.323578
26283	0.946129	0.377503
26284	1.40029	0.379422
26285	1.40028	0.430199
26286	0.94613	0.476889
26287	0.810942	2.1
26288	0.810943	2.1
26289	0.810932	2.1
26290	1.29521	2.1
26291	0.946129	2.1
26292	0.946131	2.1
26293	1.29521	2.1
26294	1.29521	2.1
26295	0.946132	0.473823
26296	0.946129	0.346667
26297	1.24596	0.333746
26298	0.946131	0.476497
26299	0.946131	0.398457
26300	0.946131	2.1
26301	1.35616	2.1
26302	0.946133	2.1
26303	0.94613	2.1
26304	1.35616	2.1
26305	0.879548	0.753481
26306	1.01272	0.790993
26307	0.946133	0.810661
26308	0.946133	0.696603
26309	0.946132	0.80405
26310	0.946132	0.923342
26311	0.918009	0.68469
26312	0.91801	1.06902
26313	0.946132	0.827602
26314	0.946132	0.981644
26315	0.946132	0.138121
26316	1.28644	0.127965
26317	0.946131	0.163529
26318	0.946131	0.149306
26319	0.946132	0.150555
26320	0.946131	0.134178
26321	1.40103	0.131496
26322	0.946131	0.168305
26323	0.946131	0.152889
26324	0.946131	0.152007
26325	0.946128	0.223387
26326	1.03327	0.190186
26327	0.946129	0.24137
26328	0.946131	0.198297
26329	0.94613	0.223247

26330	0.946132	0.384508
26331	0.946133	0.137224
26332	1.40265	0.28103
26333	1.40265	0.259971
26334	0.946133	0.26089
26335	0.946131	0.0942233
26336	0.946131	0.103667
26337	0.946131	0.115791
26338	0.856655	0.0882656
26339	0.856616	0.0954939
26340	0.946132	0.466067
26341	0.946132	0.485042
26342	0.946132	0.595487
26343	1.15844	0.418366
26344	0.946131	0.568799
26345	0.946131	2.1
26346	1.4031	2.1
26347	1.40304	2.1
26348	0.851554	2.1
26349	0.946131	0.120382
26350	0.94613	0.151173
26351	1.24417	0.0980646
26352	1.24416	0.106458
26353	0.946131	0.120262
26354	0.946132	0.508835
26355	1.20992	0.629568
26356	0.946132	0.532834
26357	0.946132	0.673612
26358	1.20992	0.646195
26359	0.946128	0.866883
26360	0.94613	0.964851
26361	0.94613	0.889599
26362	0.94613	0.909934
26363	1.23426	0.984357
26364	0.810943	0.54808
26365	0.810946	0.328925
26366	0.810933	0.399498
26367	0.810945	1.96315
26368	0.810945	0.595543
26369	0.810932	0.784421
26370	1.25104	0.987735
26371	0.946131	2.05275
26372	0.946131	0.641322
26373	1.25104	1.01287
26374	1.25104	0.705572
26375	1.25488	1.96264
26376	0.946132	1.93207
26377	0.946132	1.86181
26378	0.946132	2.1
26379	0.946131	2.1
26380	0.946131	0.111297
26381	0.946126	0.139614
26382	0.946128	0.11175
26383	1.32131	0.115237
26384	0.946128	0.126364
26385	0.946133	1.79968
26386	0.946132	0.854749
26387	0.946132	2.00918

26388	0.946133	1.7731
26389	1.41123	1.10726
26390	0.946132	0.184682
26391	0.946133	0.200093
26392	0.946132	0.262386
26393	1.32163	0.154458
26394	0.946133	0.248054
26395	0.946132	0.74922
26396	0.946132	1.48115
26397	1.38651	1.73913
26398	1.38651	1.68592
26399	0.946132	1.68575
26400	0.94613	0.292051
26401	1.3639	0.27063
26402	0.946128	0.319344
26403	0.946131	0.28915
26404	1.3639	0.279212
26405	0.946133	0.703608
26406	1.40724	0.529747
26407	0.946132	0.433351
26408	0.946133	0.544132
26409	1.40724	0.560198
26410	1.21099	1.22273
26411	0.946128	1.47702
26412	0.946127	2.00554
26413	0.946128	1.85282
26414	0.946129	1.35468
26415	0.946132	0.615929
26416	1.35946	0.578728
26417	0.946131	0.731687
26418	0.946131	0.715803
26419	1.35946	0.65672
26420	1.34794	0.669998
26421	0.517994	1.25545
26422	0.946132	0.73342
26423	0.946132	0.900705
26424	0.517965	0.966241
26425	1.23224	0.709082
26426	0.94613	1.52275
26427	0.946131	1.05722
26428	0.946131	0.894166
26429	0.946131	0.842907
26430	1.37511	0.99728
26431	0.946132	1.11827
26432	0.946133	1.71385
26433	0.946133	1.5709
26434	0.946132	1.11979
26435	0.851106	0.640411
26436	0.946129	0.669151
26437	0.946131	0.841085
26438	0.946131	1.04848
26439	0.851066	0.875699
26440	0.946131	0.212716
26441	0.946131	0.318064
26442	0.94613	0.259342
26443	1.31357	0.187887
26444	0.946131	0.277804
26445	0.946133	0.868241

26446	0.946132	1.1104
26447	0.946133	0.777566
26448	0.946132	0.978766
26449	1.38149	0.849951
26450	0.810944	0.768466
26451	0.810945	1.13472
26452	0.810958	1.75117
26453	0.946118	0.0951526
26454	0.946118	0.110158
26455	1.37835	0.080254
26456	1.37837	0.0890216
26457	0.946106	0.0951939
26458	0.946128	0.120598
26459	0.946131	0.111542
26460	1.34831	0.112716
26461	1.34831	0.0921388
26462	0.94613	0.124534
26463	0.946132	0.130293
26464	0.946132	0.184062
26465	0.909981	0.137165
26466	1.00099	0.128042
26467	0.946131	0.159601
26468	0.946132	0.736472
26469	0.946133	1.16717
26470	0.946133	0.842213
26471	0.946132	0.972253
26472	1.35328	0.96308
26473	0.946132	0.105036
26474	0.946132	0.0972748
26475	1.35386	0.105706
26476	1.35385	0.0847813
26477	0.946133	0.107026
26478	0.946121	0.08861
26479	0.946132	0.0910864
26480	0.900167	0.0763196
26481	1.01189	0.0824037
26482	0.946132	0.0913594
26483	0.946132	2.01398
26484	1.41585	1.17546
26485	0.946131	1.25227
26486	0.946131	1.15639
26487	0.94613	1.16056
26488	0.810946	2.1
26489	0.810946	1.98598
26490	0.810933	2.1
26491	1.34447	2.1
26492	0.946131	2.1
26493	0.946131	2.04616
26494	1.34447	2.1
26495	1.19886	2.1
26496	0.946132	0.154149
26497	0.946132	0.11513
26498	0.946132	0.111833
26499	1.35065	0.104701
26500	0.946132	0.141459
26501	0.946131	0.526462
26502	1.3022	0.506017
26503	0.94613	0.694662

26504	0.946131	0.641296
26505	1.3022	0.57984
26506	0.946127	0.265552
26507	1.38683	0.222553
26508	0.946127	0.311655
26509	0.946128	0.317357
26510	1.38683	0.280197
26511	0.810946	0.103435
26512	0.810947	0.0979709
26513	0.810959	0.0938282
26514	0.946132	0.0955286
26515	0.946131	0.0920089
26516	0.946132	0.0861934
26517	0.946132	0.0886505
26518	0.946132	0.0883786
26519	0.946127	1.06083
26520	1.35968	1.6502
26521	0.946131	0.983443
26522	0.946129	0.934162
26523	1.35969	1.49446
26524	0.946133	1.95377
26525	1.35862	1.49091
26526	0.946132	2.04855
26527	0.946132	1.02151
26528	1.35862	1.39581
26529	0.946132	0.900636
26530	0.946132	0.796399
26531	0.946132	0.956952
26532	0.946131	0.712898
26533	0.946132	0.78499
26534	0.946131	0.391429
26535	0.946131	0.362878
26536	0.946128	0.405013
26537	0.946129	0.326359
26538	1.41232	0.359176
26539	0.946128	0.987498
26540	1.29331	1.59929
26541	0.946131	1.05495
26542	0.946131	1.37625
26543	1.29331	1.30206
26544	0.946133	0.653831
26545	0.946132	0.699904
26546	0.946132	0.652766
26547	0.946132	0.599434
26548	1.41556	0.561536
26549	0.94612	0.607977
26550	0.946119	0.576741
26551	0.946119	0.607458
26552	0.946132	0.521663
26553	0.946107	0.526936
26554	0.946129	0.108394
26555	1.1509	0.0898041
26556	0.946126	0.108865
26557	0.946129	0.0911296
26558	0.94613	0.108681
26559	0.946131	0.0923073
26560	1.19263	0.0799756
26561	0.946132	0.0886774

26562	0.946132	0.0808729
26563	0.946132	0.086331
26564	0.94613	0.571531
26565	1.36939	0.694802
26566	0.946128	0.593967
26567	0.946131	0.691985
26568	1.36939	0.632451
26569	0.946133	0.889401
26570	0.827865	1.4643
26571	0.946132	0.928944
26572	0.946132	1.20462
26573	1.40398	1.21793
26574	0.946127	0.44352
26575	0.946127	0.527938
26576	0.94613	0.381547
26577	1.41498	0.467132
26578	0.946128	0.453468
26579	0.94613	0.105058
26580	0.946131	0.0953503
26581	0.946129	0.0862583
26582	0.94613	0.0925487
26583	0.94613	0.0901339
26584	0.946118	0.112404
26585	0.946119	0.0981039
26586	0.946116	0.0856119
26587	1.36569	0.0871106
26588	0.946105	0.0955861
26589	0.946132	0.294783
26590	1.23175	0.271004
26591	0.94613	0.29844
26592	0.946133	0.266117
26593	0.946131	0.297678
26594	1.25698	0.0906685
26595	0.946126	0.121781
26596	0.946129	0.108283
26597	0.946128	0.105633
26598	0.946129	0.104882
26599	0.810943	2.1
26600	0.810944	2.1
26601	0.810932	2.1
26602	0.946132	0.330046
26603	1.09841	0.300019
26604	0.946131	0.44783
26605	0.946132	0.411149
26606	1.09841	0.382017
26607	0.810936	0.762142
26608	0.810935	0.705479
26609	0.810934	0.99504
26610	1.23851	1.87573
26611	0.946132	0.797448
26612	0.946131	0.807732
26613	1.23851	2.1
26614	1.23851	1.20672
26615	0.946132	0.190585
26616	0.946131	0.325177
26617	0.946132	0.208711
26618	0.946132	0.244228
26619	0.946132	0.408666

26620	0.939141	1.04075
26621	0.953118	1.35005
26622	0.946126	0.955327
26623	0.946129	1.05017
26624	1.03306	1.16505
26625	0.946132	0.285588
26626	1.30927	0.2819
26627	0.946132	0.300606
26628	0.946132	0.283908
26629	1.30928	0.27999
26630	0.94613	2.1
26631	0.946131	2.1
26632	1.24511	2.1
26633	0.94613	2.1
26634	0.94613	2.1
26635	0.946133	0.281515
26636	1.34152	0.249575
26637	0.946131	0.252081
26638	0.946131	0.235861
26639	1.34152	0.251094
26640	0.946132	0.282405
26641	1.41383	0.257725
26642	0.946132	0.26385
26643	0.946132	0.244289
26644	1.41383	0.252163
26645	0.946132	0.617124
26646	0.946131	0.492471
26647	1.41392	0.397913
26648	0.946132	0.628382
26649	0.946132	0.519683
26650	0.946132	0.740047
26651	0.946132	0.665355
26652	1.24333	0.561801
26653	0.946133	0.809685
26654	0.946133	0.699721
26655	0.946124	0.25017
26656	0.94613	0.253139
26657	0.946126	0.276757
26658	1.3065	0.223917
26659	0.946127	0.268303
26660	0.810946	0.324611
26661	0.810943	0.262677
26662	0.81096	0.303731
26663	0.946131	0.215769
26664	1.40271	0.229084
26665	0.946131	0.31127
26666	0.946131	0.278047
26667	1.40271	0.255003
26668	0.946132	0.259633
26669	0.94613	0.293749
26670	0.946131	0.249447
26671	0.94613	0.270326
26672	0.946131	0.270365
26673	0.946133	0.586798
26674	1.30107	0.606017
26675	0.946132	0.584953
26676	0.946132	0.607245
26677	0.946133	0.624807

26678	0.810946	2.1
26679	0.810944	2.1
26680	0.810958	2.1
26681	0.946133	1.11812
26682	0.946131	1.03686
26683	0.946131	2.03577
26684	1.30812	1.43659
26685	0.946131	1.02246
26686	0.94613	0.836237
26687	0.975002	0.987498
26688	0.917259	1.49765
26689	1.00902	1.2253
26690	0.94613	0.784191
26691	0.946131	2.1
26692	0.946127	2.1
26693	1.398	2.1
26694	0.946129	2.1
26695	0.946129	2.1
26696	0.946133	2.1
26697	0.946132	2.1
26698	0.946132	2.1
26699	0.946132	2.1
26700	1.3033	2.1
26701	1.09897	0.33146
26702	0.946131	0.84699
26703	0.946132	0.698268
26704	0.946129	0.64108
26705	0.946131	0.744536
26706	0.946131	2.1
26707	0.946132	2.1
26708	1.39858	2.1
26709	0.946131	2.1
26710	0.946131	2.1
26711	0.946119	0.644592
26712	1.08878	0.679478
26713	0.946119	1.10861
26714	1.0888	0.679153
26715	0.946107	1.06735
26716	0.946132	0.150741
26717	1.10733	0.136082
26718	0.946132	0.180684
26719	0.946131	0.165779
26720	0.946132	0.166489
26721	0.946128	0.159008
26722	1.33671	0.139595
26723	0.94613	0.179167
26724	0.94613	0.171385
26725	0.94613	0.170905
26726	0.810943	2.1
26727	0.810944	2.1
26728	0.810945	2.1
26729	1.35117	2.1
26730	0.946129	2.1
26731	0.94613	2.1
26732	1.35117	2.1
26733	1.27174	2.1
26734	0.946132	1.99606
26735	0.946133	1.86706

26736	0.997296	1.16408
26737	0.946133	1.50214
26738	0.946132	1.28209
26739	0.810943	2.1
26740	0.810944	1.12461
26741	0.810945	2.00329
26742	0.810946	0.896323
26743	0.810943	1.63818
26744	0.810933	1.26877
26745	0.946127	1.07572
26746	0.946131	0.964985
26747	0.946128	1.06272
26748	0.94613	1.12415
26749	0.946129	1.16997
26750	0.946131	0.359496
26751	0.946131	0.307107
26752	0.946129	0.364437
26753	0.946131	0.293412
26754	0.94613	0.295654
26755	0.946133	0.357952
26756	0.946132	0.370346
26757	0.946132	0.354099
26758	0.946132	0.353011
26759	0.946133	0.372027
26760	0.946131	0.888356
26761	0.94613	0.75781
26762	0.946129	0.898207
26763	0.946131	0.786238
26764	0.946131	0.889166
26765	0.946132	0.848266
26766	0.946129	1.6206
26767	0.94613	1.09027
26768	0.946131	1.08582
26769	1.39356	1.3112
26770	0.810946	1.98407
26771	0.810947	2.1
26772	0.810934	1.9049
26773	0.946125	0.140484
26774	0.946127	0.151337
26775	1.33798	0.10666
26776	1.33797	0.114802
26777	0.946128	0.127944
26778	0.946131	0.122219
26779	0.946131	0.14878
26780	1.23146	0.102298
26781	1.23146	0.115614
26782	0.946131	0.126103
26783	0.946129	2.1
26784	0.94613	2.1
26785	0.946131	2.1
26786	0.94613	2.1
26787	1.30082	2.1
26788	0.946131	0.166962
26789	0.946132	0.170933
26790	0.946131	0.198379
26791	1.20416	0.139655
26792	0.946132	0.180924
26793	0.946132	0.153272

26794	0.946131	0.161669
26795	0.946131	0.224091
26796	1.18711	0.132531
26797	0.946132	0.179116
26798	0.946132	0.104594
26799	0.946132	0.0947288
26800	0.946133	0.0959611
26801	0.946132	0.0922118
26802	0.946133	0.0938481
26803	0.946131	0.21549
26804	0.946132	0.25505
26805	0.94613	0.209517
26806	0.946131	0.212421
26807	0.946131	0.237645
26808	0.946133	0.159888
26809	0.946132	0.186649
26810	0.946133	0.158402
26811	0.946132	0.15393
26812	0.946132	0.175693
26813	0.946132	2.1
26814	0.946132	2.1
26815	0.946132	2.1
26816	1.36422	2.1
26817	0.946132	2.1
26818	0.946131	0.767669
26819	0.946132	1.3831
26820	0.946132	0.967066
26821	0.946132	1.01828
26822	1.4185	1.3246
26823	0.946133	0.198392
26824	1.37794	0.163997
26825	0.946132	0.190234
26826	0.946133	0.172186
26827	0.946132	0.173784
26828	1.4045	2.1
26829	0.946132	2.1
26830	0.946132	2.1
26831	0.946132	2.1
26832	0.946132	2.1
26833	0.946131	2.1
26834	0.946132	2.07248
26835	1.34285	2.1
26836	1.34285	2.1
26837	0.946132	2.1
26838	0.946131	2.1
26839	0.946132	2.1
26840	1.40739	2.1
26841	1.40739	2.1
26842	0.946131	2.1
26843	0.946128	2.1
26844	0.946132	2.1
26845	0.94613	2.1
26846	0.94613	2.1
26847	1.3164	2.1
26848	0.946132	1.18307
26849	1.3484	2.1
26850	0.946132	1.01291
26851	0.946132	1.76096

26852	1.3484	1.863
26853	0.946132	2.1
26854	0.946132	2.1
26855	0.946133	2.1
26856	0.946133	2.1
26857	1.41507	2.1
26858	0.946132	2.1
26859	1.32608	2.1
26860	0.946131	2.1
26861	0.946131	2.1
26862	0.946132	2.1
26863	0.94613	0.899833
26864	1.34646	1.75654
26865	0.946129	0.94831
26866	0.94613	0.840344
26867	1.34646	1.34263
26868	0.946131	0.43743
26869	0.946131	1.58613
26870	0.94613	0.641905
26871	1.33728	0.388249
26872	0.946131	0.581757
26873	0.946127	1.4419
26874	0.946131	1.01924
26875	0.946128	1.79302
26876	0.946129	1.80311
26877	1.4064	1.37729
26878	0.946133	2.1
26879	0.946131	2.1
26880	0.946131	2.1
26881	0.946131	2.1
26882	0.94613	2.1
26883	1.34912	2.1
26884	0.946131	2.1
26885	0.946129	2.1
26886	0.946131	2.1
26887	0.94613	2.1
26888	0.946132	2.1
26889	1.40447	2.1
26890	0.946132	2.1
26891	0.946133	2.1
26892	1.40447	2.1
26893	1.33607	2.1
26894	0.946131	1.06195
26895	0.946127	2.1
26896	0.94613	2.04047
26897	0.946129	2.1
26898	0.946117	0.638601
26899	1.32664	0.968252
26900	0.946115	0.720939
26901	0.946105	1.18595
26902	0.94613	0.498545
26903	0.946118	2.06347
26904	0.946114	2.1
26905	1.35886	2.1
26906	1.35884	2.1
26907	0.946129	2.1
26908	0.946132	1.80505
26909	1.2571	2.1

26910	0.946132	1.97705
26911	0.946132	2.1
26912	1.2571	2.1
26913	0.946133	2.02931
26914	1.35663	2.1
26915	0.946132	2.1
26916	0.946132	2.1
26917	1.35663	2.1
26918	0.946133	0.788477
26919	0.946129	1.42022
26920	0.946131	1.17513
26921	0.946131	1.0096
26922	1.30881	1.60044
26923	0.946132	2.1
26924	0.94613	2.1
26925	1.34652	2.1
26926	0.94613	2.1
26927	0.94613	2.1
26928	0.946132	1.96078
26929	0.946133	1.40961
26930	1.36183	0.956461
26931	1.36183	1.07325
26932	0.946132	1.25591
26933	0.946132	0.309105
26934	1.38353	0.269629
26935	0.94613	0.278079
26936	0.94613	0.284961
26937	1.38354	0.280937
26938	0.946128	0.424822
26939	1.24887	0.364862
26940	0.94613	0.492004
26941	0.94613	0.544403
26942	1.24887	0.505532
26943	0.946129	0.834236
26944	0.946126	1.2657
26945	0.946124	0.861736
26946	0.946128	0.933857
26947	1.32056	1.11219
26948	0.946129	0.909701
26949	0.946128	1.33514
26950	0.946126	0.886143
26951	0.946129	1.03091
26952	1.20351	1.12141
26953	0.946131	1.01747
26954	0.946131	1.13891
26955	0.946127	1.30433
26956	1.25591	1.37094
26957	0.94613	0.867963
26958	0.946132	1.13973
26959	0.946133	1.13608
26960	0.946132	1.31234
26961	1.15436	1.03477
26962	0.930306	0.936179
26963	0.946132	2.1
26964	0.946132	2.1
26965	0.946132	1.82101
26966	0.946133	2.1
26967	0.946132	2.09795

26968	0.946129	1.07683
26969	0.946132	0.886032
26970	0.946126	1.22746
26971	1.13245	0.73977
26972	0.946129	0.890107
26973	0.94613	2.1
26974	0.946132	2.1
26975	0.946129	1.66509
26976	0.946131	2.1
26977	0.946131	1.98408
26978	1.41279	1.83705
26979	1.76254	2.1
26980	0.600454	1.77312
26981	0.858991	1.75787
26982	0.940722	1.86383
26983	1.28439	1.33731
26984	0.946131	1.28316
26985	0.94613	1.41039
26986	0.946131	1.79091
26987	0.946131	1.75713
26988	0.946132	0.907887
26989	1.39335	0.537932
26990	0.946132	0.823175
26991	0.946132	0.59851
26992	0.946132	0.778085
26993	1.33879	0.287016
26994	0.946131	0.447354
26995	0.94613	0.69707
26996	1.33879	0.286359
26997	0.946131	0.501266
26998	0.946126	0.470065
26999	0.946131	0.465168
27000	1.37342	0.43734
27001	1.37343	0.414404
27002	0.946128	0.570905
27003	1.39168	0.11153
27004	0.946131	0.139056
27005	0.946131	0.16363
27006	0.946131	0.133227
27007	0.946131	0.132192
27008	0.946131	0.378257
27009	1.412	0.318521
27010	0.946127	0.310607
27011	0.946128	0.332722
27012	0.946128	0.349971
27013	1.1387	0.240822
27014	0.943364	0.611555
27015	0.946132	0.445322
27016	0.946132	0.407336
27017	0.946133	0.488701
27018	1.21085	0.397649
27019	0.88324	0.899545
27020	0.946129	0.732627
27021	0.946131	0.810926
27022	0.946131	0.694444
27023	1.33587	0.0969296
27024	0.946125	0.129126
27025	0.946129	0.110038

27026	0.946126	0.123316
27027	0.946127	0.113767
27028	1.27512	0.117887
27029	0.946132	0.173471
27030	0.946132	0.16674
27031	0.946133	0.152898
27032	0.946132	0.137081
27033	0.946128	0.205121
27034	1.30498	0.192123
27035	0.946131	0.276639
27036	0.94613	0.251492
27037	1.30497	0.225874
27038	0.946131	0.184155
27039	1.25626	0.168464
27040	0.946131	0.233223
27041	0.946131	0.2203
27042	1.25626	0.202034
27043	0.946131	0.0867998
27044	0.94613	0.091764
27045	1.35316	0.0784535
27046	0.946131	0.0832487
27047	0.946131	0.0828592
27048	0.94613	0.426719
27049	1.28727	0.391816
27050	0.946128	0.322999
27051	0.946131	0.370259
27052	0.94613	0.375177
27053	0.946131	0.196994
27054	1.39502	0.177779
27055	0.946132	0.265096
27056	0.946132	0.217538
27057	0.946132	0.239817
27058	1.36173	0.284368
27059	0.946131	0.694039
27060	0.946126	0.536517
27061	0.946127	0.589832
27062	0.946128	0.491105
27063	0.946129	0.875737
27064	0.946133	0.764918
27065	0.946131	0.893821
27066	0.946131	0.989575
27067	0.946131	0.945532
27068	0.946128	0.0719178
27069	0.946132	0.069161
27070	1.24678	0.0671439
27071	0.94613	0.0692533
27072	0.94613	0.0694829
27073	0.946129	0.0701874
27074	0.94613	0.0686639
27075	1.25215	0.0662584
27076	0.946126	0.0685538
27077	0.946129	0.0693938
27078	0.946129	0.626105
27079	1.29689	0.609911
27080	0.946131	0.63561
27081	0.946131	0.593206
27082	0.946131	0.550543
27083	0.946128	0.193316

27084	0.94613	0.208396
27085	0.946131	0.178835
27086	0.94613	0.197246
27087	0.946131	0.198015
27088	0.946127	0.217121
27089	0.946128	0.249859
27090	0.94613	0.201568
27091	0.946129	0.228854
27092	0.946129	0.226925
27093	0.946133	0.0924393
27094	0.946132	0.104216
27095	0.946132	0.136881
27096	1.29957	0.099408
27097	0.946133	0.107856
27098	0.946132	0.110228
27099	0.946133	0.104146
27100	0.946132	0.133396
27101	1.26201	0.103557
27102	0.946132	0.112181
27103	0.946133	0.160988
27104	1.40427	0.144765
27105	0.946132	0.184732
27106	0.946132	0.178313
27107	0.946133	0.173025
27108	0.946132	0.15219
27109	1.35123	0.143552
27110	0.946132	0.176435
27111	0.946132	0.178159
27112	0.946131	0.166922
27113	0.946128	0.240498
27114	0.946129	0.271686
27115	0.946131	0.221625
27116	0.94613	0.258282
27117	0.946131	0.247201
27118	0.946127	0.494487
27119	1.23351	0.450279
27120	0.946131	0.500627
27121	0.946129	0.456228
27122	0.94613	0.439238
27123	0.946131	0.175119
27124	0.946131	0.227415
27125	0.94613	0.182032
27126	1.18119	0.166163
27127	0.946131	0.19664
27128	0.946132	0.31693
27129	1.30563	0.243334
27130	0.946133	0.346372
27131	0.946132	0.258438
27132	0.946133	0.295587
27133	0.946132	0.435628
27134	0.946132	0.29002
27135	0.946133	0.791207
27136	1.20256	0.382037
27137	0.946133	0.485053
27138	0.94613	0.61592
27139	1.33007	0.544006
27140	0.946128	0.683593
27141	0.946132	0.529017

27142	0.946131	0.586114
27143	0.946131	2.1
27144	0.946131	2.1
27145	0.946128	2.1
27146	0.946129	2.1
27147	0.94613	2.1
27148	0.810942	2.1
27149	0.810942	2.1
27150	0.81093	2.1
27151	0.946132	2.1
27152	1.39823	2.1
27153	0.946132	2.1
27154	0.946131	2.1
27155	0.946132	2.1
27156	0.946131	2.1
27157	0.946131	2.1
27158	0.946131	2.1
27159	1.37713	2.1
27160	0.946131	2.1
27161	0.946133	0.976369
27162	0.946131	1.62334
27163	1.40594	2.02745
27164	1.40594	2.01337
27165	0.94613	1.98214
27166	0.946133	2.1
27167	0.946131	2.1
27168	1.38155	2.1
27169	1.38155	2.1
27170	0.946131	2.1
27171	0.946132	0.786162
27172	0.946133	1.59251
27173	0.946132	0.974916
27174	0.946132	0.928467
27175	1.33694	1.47071
27176	0.946131	1.80666
27177	1.228 2.1	
27178	0.946129	1.93053
27179	0.946131	2.0632
27180	1.228 2.1	
27181	0.946132	1.02668
27182	0.946132	1.41162
27183	0.946131	1.6482
27184	0.946132	1.1121
27185	1.35943	1.62792
27186	0.946133	0.13768
27187	0.946133	0.133437
27188	1.02377	0.156702
27189	1.02377	0.128433
27190	0.946132	0.13241
27191	0.94613	0.365113
27192	0.946131	0.377131
27193	0.946132	0.412431
27194	1.29322	0.34296
27195	0.946131	0.404358
27196	0.946131	0.686033
27197	0.946131	0.334368
27198	0.946132	0.697447
27199	1.41492	0.307157

27200	0.946131	0.607192
27201	0.946132	2.1
27202	0.946131	2.1
27203	0.946132	2.1
27204	1.35028	2.1
27205	0.946132	2.1
27206	0.946132	2.02774
27207	0.946132	1.88225
27208	0.946131	1.83793
27209	1.32461	1.99089
27210	0.946132	1.71579
27211	0.946131	2.1
27212	0.946132	2.1
27213	1.40369	2.1
27214	0.94613	2.1
27215	0.946131	2.1
27216	0.946132	0.18547
27217	0.979597	0.171682
27218	0.946131	0.206444
27219	0.946132	0.178744
27220	0.946132	0.199072
27221	0.946131	2.1
27222	0.946131	1.16953
27223	0.94613	2.1
27224	0.946131	2.1
27225	1.41897	2.07343
27226	1.37791	2.1
27227	0.94613	2.1
27228	0.946129	2.1
27229	0.946131	2.1
27230	0.946131	2.1
27231	1.39384	2.1
27232	0.946131	2.1
27233	0.946128	2.1
27234	0.946129	2.1
27235	0.946129	2.1
27236	0.810945	1.29031
27237	0.810944	1.98592
27238	0.810958	1.49503
27239	0.946128	2.05211
27240	0.946132	2.1
27241	0.94613	2.1
27242	1.3335	2.1
27243	0.946131	2.1
27244	0.94613	0.424079
27245	0.946129	0.652132
27246	0.946132	0.317672
27247	1.41192	0.591979
27248	0.94613	0.336507
27249	0.946132	0.631898
27250	0.946133	0.51928
27251	0.946132	0.448737
27252	1.39428	0.435111
27253	0.946133	0.439092
27254	0.94613	1.54339
27255	0.946128	1.64342
27256	0.946132	0.966093
27257	0.94613	1.36422

27258	1.40684	1.61879
27259	0.946131	2.1
27260	0.946131	1.98775
27261	0.946131	1.79104
27262	0.946131	1.45281
27263	1.39021	2.05555
27264	0.946131	1.05347
27265	1.31649	1.79473
27266	0.946131	0.888539
27267	0.946131	0.868443
27268	1.31649	1.30147
27269	0.810946	2.1
27270	0.810946	1.50006
27271	0.810958	2.1
27272	1.16687	2.1
27273	0.946131	2.1
27274	0.946131	1.61352
27275	1.16687	2.1
27276	1.16687	2.1
27277	0.946128	0.863682
27278	1.3564	0.909128
27279	0.946131	0.90083
27280	0.946131	0.918506
27281	0.94613	0.744858
27282	0.946128	2.1
27283	1.35828	2.1
27284	0.94613	2.1
27285	0.946131	2.1
27286	0.94613	2.1
27287	0.946133	0.510244
27288	1.36477	0.652813
27289	0.946132	0.67647
27290	0.946132	0.698824
27291	1.36477	0.692935
27292	0.946132	0.916532
27293	1.31857	1.65027
27294	0.946132	1.16642
27295	0.946132	1.22041
27296	1.31857	1.25535
27297	0.94613	0.712109
27298	1.35059	0.538768
27299	0.946128	0.523394
27300	0.94613	0.603421
27301	1.35059	0.566554
27302	0.946126	0.387935
27303	1.35559	0.307144
27304	0.946127	0.304687
27305	0.946128	0.318567
27306	1.35559	0.311558
27307	0.946131	0.293022
27308	1.33035	0.263341
27309	0.946127	0.265997
27310	0.946128	0.255558
27311	1.33035	0.265559
27312	0.94613	0.304397
27313	1.3665	0.281252
27314	0.946129	0.281393
27315	0.946131	0.283042

27316	1.3665	0.283476
27317	0.94613	0.334719
27318	1.32828	0.302439
27319	0.946129	0.303121
27320	0.94613	0.31791
27321	0.946131	0.324277
27322	0.946131	2.1
27323	0.946131	1.22057
27324	1.32949	2.1
27325	1.32949	2.1
27326	0.946131	2.1
27327	0.946131	1.1902
27328	0.94613	0.263206
27329	0.946133	0.633873
27330	1.38357	0.360763
27331	0.946131	0.549784
27332	0.946131	2.1
27333	0.946128	2.1
27334	1.36919	2.1
27335	0.946131	2.1
27336	0.94613	2.1
27337	0.946131	0.808541
27338	1.3771	2.00172
27339	0.946129	1.50582
27340	0.94613	1.59486
27341	0.946131	0.769556
27342	0.946131	1.0996
27343	0.94613	0.77101
27344	0.946129	0.584806
27345	1.30029	0.81003
27346	0.94613	0.735187
27347	1.21174	0.0891479
27348	0.946132	0.103096
27349	0.946132	0.117956
27350	0.946132	0.103166
27351	0.946133	0.0999081
27352	0.810946	2.1
27353	0.810945	2.1
27354	0.810946	2.1
27355	1.1796	2.1
27356	0.946131	2.1
27357	0.946132	2.1
27358	1.1796	2.1
27359	1.1796	2.1
27360	0.946131	2.1
27361	0.946131	2.1
27362	0.946133	2.1
27363	0.946131	2.1
27364	1.40615	2.1
27365	0.810945	2.1
27366	0.810944	2.1
27367	0.810945	2.1
27368	1.40239	2.1
27369	0.94613	2.1
27370	1.35561	2.1
27371	0.946133	2.1
27372	1.35562	2.1
27373	1.41169	0.284994

27374	0.946131	0.472218
27375	0.946127	0.690347
27376	1.41169	0.30452
27377	0.946128	0.522982
27378	0.946119	2.1
27379	0.946119	2.1
27380	0.946119	2.1
27381	0.946107	2.1
27382	1.41917	2.1
27383	0.810946	1.01144
27384	0.810943	1.09138
27385	0.810934	1.56776
27386	0.946127	2.07693
27387	1.1995	2.1
27388	0.946126	2.0848
27389	0.946128	2.1
27390	1.1995	2.1
27391	0.810944	2.1
27392	0.810947	2.1
27393	0.81096	2.1
27394	0.946133	0.317229
27395	1.3495	0.211794
27396	0.946132	0.265449
27397	0.946132	0.222322
27398	0.946133	0.24403
27399	0.946132	2.1
27400	0.946132	2.1
27401	0.946133	2.1
27402	1.39529	2.1
27403	0.946133	2.1
27404	0.946133	2.04255
27405	1.28525	2.1
27406	0.946132	2.0014
27407	0.946133	2.1
27408	1.28525	2.1
27409	0.810946	2.1
27410	0.810945	2.1
27411	0.810946	2.1
27412	1.33931	2.1
27413	0.946132	2.1
27414	0.946132	2.1
27415	1.33931	2.1
27416	1.24616	2.1
27417	0.946133	0.398764
27418	0.946133	0.430826
27419	0.946132	0.363558
27420	0.946132	0.395892
27421	0.946133	0.388521
27422	0.946128	2.05737
27423	1.40225	2.1
27424	0.946128	2.04355
27425	0.94613	2.1
27426	0.94613	1.82805
27427	0.946113	0.115704
27428	0.946118	0.102052
27429	0.946114	0.126738
27430	1.40551	0.10529
27431	0.946103	0.109375

27432	0.94613	2.1
27433	0.946132	2.1
27434	0.946129	2.1
27435	0.946131	2.1
27436	1.37811	2.1
27437	0.946132	2.1
27438	1.39826	2.1
27439	0.946133	2.1
27440	0.946132	2.1
27441	1.39826	2.1
27442	0.946118	2.1
27443	1.28298	2.1
27444	0.946117	2.1
27445	0.946105	2.1
27446	1.283	2.1
27447	0.946118	0.107429
27448	1.41375	0.111409
27449	0.946117	0.145768
27450	0.946131	0.123616
27451	0.946106	0.124712
27452	0.946132	0.73947
27453	0.946129	0.589082
27454	1.28436	0.56086
27455	0.946131	0.749875
27456	0.946131	0.632608
27457	0.810947	0.386791
27458	0.810946	0.434632
27459	0.81096	0.393493
27460	0.946132	0.313806
27461	1.34338	0.298507
27462	0.961435	0.259563
27463	0.961435	0.279713
27464	0.946132	0.305427
27465	1.3028	1.62518
27466	0.946131	1.52658
27467	0.946127	1.74328
27468	0.946128	1.91951
27469	0.946129	1.57522
27470	0.946132	0.628828
27471	1.37973	0.502664
27472	0.946132	0.637245
27473	0.946132	0.524201
27474	0.946132	0.491829
27475	0.946131	0.810975
27476	0.94613	0.737258
27477	0.946129	0.81237
27478	0.946131	0.741763
27479	1.41798	0.7164
27480	0.946118	0.730142
27481	0.946118	0.649702
27482	0.946117	0.731705
27483	0.946131	0.667551
27484	0.946105	0.655422
27485	0.946131	0.87076
27486	0.946131	0.782956
27487	0.94613	0.867042
27488	0.946131	0.785355
27489	0.946131	0.735529

27490	0.946133	1.07471
27491	1.40274	2.02617
27492	0.946133	0.803152
27493	0.946132	1.62577
27494	1.40274	1.67396
27495	0.946131	1.22085
27496	1.39731	1.84859
27497	0.946132	0.897799
27498	0.94613	1.04193
27499	1.39731	1.50412
27500	0.94612	1.84038
27501	0.94612	1.37027
27502	0.946119	1.34388
27503	0.946132	1.39729
27504	1.40764	1.74748
27505	0.946128	2.1
27506	0.946132	2.1
27507	0.94613	2.1
27508	0.94613	2.1
27509	1.31213	2.1
27510	0.946127	1.29933
27511	0.94613	2.04344
27512	0.946127	1.32355
27513	0.946128	1.34472
27514	1.36653	1.84457
27515	0.946127	2.1
27516	1.3557	2.1
27517	0.946128	2.1
27518	0.946128	2.1
27519	1.3557	2.1
27520	0.946133	2.1
27521	0.946132	2.1
27522	0.946133	2.1
27523	0.946132	2.1
27524	1.40081	2.1
27525	0.946128	2.1
27526	0.946133	2.1
27527	0.946131	2.1
27528	0.946131	2.1
27529	1.32282	2.1
27530	0.810945	1.37348
27531	0.810943	1.40194
27532	0.810933	2.02558
27533	1.27676	2.1
27534	0.94613	1.89476
27535	0.946128	2.1
27536	0.946131	2.1
27537	0.946131	2.1
27538	0.946131	0.504366
27539	0.946127	0.45484
27540	1.41328	0.61234
27541	1.41327	0.5768
27542	0.946128	0.55274
27543	0.946132	0.261053
27544	1.32083	0.253593
27545	0.946132	0.277203
27546	0.946133	0.267234
27547	1.32082	0.260036

27548	0.946128	0.271445
27549	1.29215	0.265727
27550	0.94613	0.291352
27551	0.94613	0.273898
27552	1.29215	0.26969
27553	0.946132	0.250751
27554	1.27696	0.230009
27555	0.94613	0.248016
27556	0.94613	0.25975
27557	1.27696	0.243386
27558	0.94612	0.25088
27559	1.29289	0.240937
27560	0.946119	0.263221
27561	0.946132	0.261401
27562	1.29287	0.249565
27563	0.946128	2.1
27564	1.37869	2.1
27565	0.946131	2.1
27566	0.94613	2.1
27567	0.946131	2.1
27568	0.946133	2.1
27569	0.946132	2.1
27570	0.946132	2.1
27571	1.35703	2.1
27572	0.946133	2.1
27573	0.946132	1.25345
27574	0.946132	2.1
27575	0.946133	0.898356
27576	1.37017	2.1
27577	0.946133	2.0894
27578	0.946119	0.109572
27579	0.94612	0.118055
27580	1.38583	0.0880565
27581	1.38585	0.0953071
27582	0.946107	0.10361
27583	0.946126	0.143519
27584	0.946127	0.170209
27585	1.22904	0.118171
27586	1.22904	0.118235
27587	0.946128	0.135903
27588	1.3279	0.752341
27589	0.946131	1.27165
27590	0.94613	1.06245
27591	0.946131	1.23617
27592	0.946131	1.06457
27593	0.946132	2.1
27594	0.946132	2.1
27595	0.946132	2.1
27596	0.946131	2.1
27597	1.40932	2.1
27598	1.26868	0.12753
27599	0.946131	0.212106
27600	0.94613	0.176038
27601	0.946131	0.178141
27602	0.946131	0.168772
27603	0.810943	2.1
27604	0.810945	2.1
27605	0.810932	2.1

27606	0.810943	0.741351
27607	0.810944	0.430557
27608	0.810957	0.343355
27609	1.31539	0.511931
27610	0.946132	0.849141
27611	0.946128	0.277221
27612	1.3154	0.600665
27613	1.17856	0.383035
27614	1.21997	1.16548
27615	0.94613	1.51834
27616	0.946129	1.33545
27617	0.94613	1.73783
27618	0.94613	1.60346
27619	0.946131	1.00909
27620	0.946131	0.836957
27621	0.946129	1.03428
27622	0.94613	1.01341
27623	0.946131	1.12491
27624	0.946127	0.802494
27625	0.946131	0.621296
27626	0.946127	1.54411
27627	0.946129	0.933546
27628	1.41342	0.762196
27629	0.946131	0.690675
27630	0.946131	1.33588
27631	0.946128	1.51443
27632	0.946131	0.801577
27633	1.29149	1.3312
27634	1.41022	1.94489
27635	0.94613	0.77272
27636	0.946128	1.84846
27637	0.94613	1.60503
27638	0.94613	1.90573
27639	0.946128	0.354385
27640	0.94613	0.406384
27641	1.27864	0.290063
27642	1.27864	0.282412
27643	0.946131	0.419116
27644	0.946129	0.748557
27645	0.946125	1.29643
27646	0.946124	1.05384
27647	0.946127	0.878338
27648	1.37759	1.2083
27649	0.946129	0.792628
27650	0.94613	1.29447
27651	0.946126	0.919337
27652	0.946129	0.921453
27653	1.38259	1.14317
27654	0.946131	0.252164
27655	1.34118	0.227315
27656	0.946132	0.243754
27657	0.946132	0.247587
27658	1.34118	0.243049
27659	0.946131	0.135537
27660	1.25017	0.127254
27661	0.946128	0.129028
27662	0.946131	0.128403
27663	0.946131	0.123249

27664	0.946131	2.1
27665	0.946128	2.1
27666	0.946133	2.1
27667	1.3937	2.1
27668	0.94613	2.1
27669	0.946131	0.666999
27670	0.94613	0.405659
27671	1.40404	0.426213
27672	1.40404	0.314491
27673	0.946131	0.431469
27674	0.946132	0.486437
27675	0.946132	0.316927
27676	1.38897	0.311355
27677	1.38897	0.205347
27678	0.946132	0.339921
27679	0.94613	0.350412
27680	0.946128	0.435387
27681	1.40332	0.272437
27682	1.40331	0.426726
27683	0.94613	0.472057
27684	0.946132	0.422287
27685	0.946132	0.668764
27686	0.946133	0.563666
27687	1.41619	0.648341
27688	0.946132	0.541159
27689	0.946132	0.711946
27690	0.946132	2.1
27691	0.946132	1.26207
27692	1.38244	2.03348
27693	0.946132	1.61217
27694	0.810946	0.979145
27695	0.810943	0.683623
27696	0.810934	0.618789
27697	0.810947	0.879232
27698	0.810943	0.578724
27699	0.810934	0.597357
27700	1.12786	1.04487
27701	0.946132	1.7287
27702	0.946132	1.44065
27703	0.946132	1.22297
27704	0.946133	1.20009
27705	0.946132	1.34574
27706	0.946132	1.42228
27707	0.946132	1.15981
27708	1.27806	1.41955
27709	0.946133	1.11484
27710	0.946132	2.1
27711	0.946132	2.1
27712	0.946133	2.1
27713	0.946132	2.1
27714	0.946133	2.1
27715	1.39514	1.30882
27716	0.946129	0.928293
27717	0.946124	1.22335
27718	0.946127	0.789842
27719	0.946127	1.22083
27720	0.94613	1.79689
27721	0.946129	2.1

27722	0.946132	2.1
27723	1.3991	2.1
27724	0.94613	2.1
27725	0.946132	0.445176
27726	0.946133	0.876211
27727	0.946132	0.851215
27728	1.36835	0.616912
27729	0.946132	0.518376
27730	0.946132	2.0322
27731	0.946132	2.1
27732	0.946133	2.05867
27733	0.946132	2.1
27734	1.39047	2.1
27735	0.946132	0.0918971
27736	0.946128	0.094319
27737	0.94613	0.103702
27738	1.33174	0.0864206
27739	0.946131	0.10402
27740	0.946128	0.122853
27741	0.946131	0.112389
27742	0.94613	0.134521
27743	0.94613	0.111672
27744	0.94613	0.137272
27745	0.946119	2.1
27746	0.946119	2.1
27747	1.29064	2.1
27748	0.946132	2.1
27749	0.946107	2.1
27750	0.946133	2.1
27751	0.94613	2.1
27752	1.30584	2.1
27753	0.946131	2.1
27754	0.946131	2.1
27755	0.946131	2.1
27756	0.946132	2.1
27757	0.94613	2.1
27758	0.946131	2.1
27759	1.39549	2.1
27760	0.913386	1.1171
27761	0.978873	0.97398
27762	0.946126	0.93444
27763	0.94613	1.18138
27764	1.00473	0.811499
27765	0.946132	0.695531
27766	0.946133	0.696515
27767	0.946132	0.672876
27768	0.946132	0.782838
27769	1.38894	0.62374
27770	0.946128	0.663112
27771	0.946131	0.637385
27772	0.94613	0.649388
27773	0.94613	0.673212
27774	1.23865	0.601335
27775	0.946126	1.15721
27776	0.946129	1.20126
27777	0.946124	0.920373
27778	0.946127	1.11247
27779	1.40248	0.930745

27780	0.946133	0.740509
27781	0.946132	0.804333
27782	0.946132	0.686446
27783	0.946133	0.81437
27784	1.1391	0.684451
27785	0.827862	0.6919
27786	0.946133	0.737954
27787	0.946131	0.909717
27788	0.946131	0.761643
27789	0.94613	0.727098
27790	0.946128	0.55942
27791	0.946132	0.640025
27792	0.94613	0.711337
27793	1.21388	0.505187
27794	0.94613	0.679316
27795	0.810943	0.389581
27796	0.810945	0.458036
27797	0.810932	0.441407
27798	0.946131	0.534439
27799	0.946131	0.557916
27800	0.946133	0.629167
27801	1.36748	0.421096
27802	0.946132	0.38358
27803	0.946128	0.325233
27804	0.946131	0.328819
27805	0.94613	0.35325
27806	1.35856	0.262485
27807	0.94613	0.343475
27808	0.946132	2.1
27809	0.946131	2.1
27810	0.946133	2.1
27811	0.946132	2.1
27812	0.946131	2.1
27813	0.946133	2.1
27814	0.946131	2.1
27815	1.39558	2.1
27816	1.39558	2.1
27817	0.94613	2.1
27818	0.946131	0.165854
27819	0.94613	0.208335
27820	0.946129	0.171208
27821	1.41388	0.154981
27822	0.946131	0.188153
27823	0.946133	0.148264
27824	0.946132	0.239209
27825	0.946132	0.159596
27826	0.946133	0.159515
27827	0.93452	0.217309
27828	0.946129	2.1
27829	1.38897	2.1
27830	0.946131	2.1
27831	0.946131	2.1
27832	1.38897	2.1
27833	0.946133	1.30443
27834	0.946132	1.08424
27835	0.946133	1.19171
27836	0.946132	0.711773
27837	1.38773	0.910147

27838	1.41512	0.203494
27839	0.946131	0.379567
27840	0.946129	0.306668
27841	0.946133	0.276705
27842	0.946131	0.318834
27843	0.946132	0.769575
27844	0.946132	2.04773
27845	0.946132	0.852981
27846	0.946133	0.957828
27847	0.946133	2.03875
27848	0.946126	0.659517
27849	0.946132	0.981957
27850	0.946129	0.743493
27851	0.946129	0.78534
27852	0.946129	0.994866
27853	0.939895	1.52549
27854	1.03386	1.92545
27855	0.94613	1.49554
27856	0.946131	1.30963
27857	1.03386	1.57659
27858	0.946131	1.04727
27859	1.39309	2.1
27860	0.946133	0.920369
27861	0.946131	1.95918
27862	1.39309	2.03936
27863	0.946133	2.1
27864	1.24853	2.1
27865	0.946132	2.1
27866	0.946132	2.1
27867	1.24853	2.1
27868	0.946133	1.75715
27869	1.39699	0.857192
27870	0.946132	1.496
27871	0.946132	2.1
27872	1.39699	1.17815
27873	0.946132	2.1
27874	0.946131	0.906576
27875	0.946133	2.09715
27876	0.946131	1.67466
27877	1.38969	2.03133
27878	1.28464	1.80611
27879	0.946133	1.75631
27880	0.94613	1.87721
27881	0.946131	1.6763
27882	0.946131	1.65105
27883	0.946119	0.738753
27884	0.94612	0.831507
27885	0.946119	0.73322
27886	0.946132	0.807401
27887	1.3749	0.685682
27888	0.810942	2.1
27889	0.810943	2.1
27890	0.810957	2.1
27891	1.41134	1.79034
27892	0.946132	0.840206
27893	0.946132	1.62496
27894	0.946132	0.950091
27895	0.946132	1.59886

27896	1.31338	1.87348
27897	0.946132	1.01656
27898	0.946132	1.8412
27899	0.946133	1.29766
27900	0.946132	1.67638
27901	0.946103	0.0763359
27902	0.752448	0.0839649
27903	0.946132	0.0771978
27904	0.946131	0.0819538
27905	1.20617	0.0821398
27906	0.946132	0.910256
27907	1.08054	0.795342
27908	1.25736	0.923261
27909	0.946132	0.916408
27910	0.946131	0.895539
27911	0.946128	1.43758
27912	1.33275	0.859969
27913	1.1896	1.47079
27914	0.946131	1.53389
27915	1.18962	1.57103
27916	1.21955	0.0841838
27917	0.946131	0.0988407
27918	1.33587	0.0846002
27919	0.741946	0.086468
27920	1.07387	0.0768844
27921	0.945844	0.809292
27922	1.3292	1.7711
27923	1.59472	1.35019
27924	0.946131	0.940517
27925	1.04043	1.15069
27926	1.13529	0.243353
27927	0.946132	0.291937
27928	1.24893	0.240409
27929	1.12675	0.283693
27930	0.936652	0.286307
27931	1.36968	2.1
27932	0.946131	2.1
27933	0.946132	2.1
27934	1.36968	2.1
27935	1.258 2.1	
27936	3.17458	0.153747
27937	0.946132	1.12837
27938	1.35022	2.1
27939	1.35022	2.1
27940	0.94613	1.24813
27941	1.12356	1.77305
27942	0.946131	2.1
27943	1.22401	2.1
27944	1.22401	2.1
27945	0.94613	2.1
27946	0.952455	2.1
27947	1.2107	2.1
27948	0.946132	2.1
27949	1.33047	2.1
27950	0.946131	2.1
27951	0.946132	2.1
27952	0.61957	1.62354
27953	0.8636	2.1

27954	0.946132	1.57324
27955	1.19294	1.97224
27956	0.946128	2.1
27957	1.36607	2.1
27958	0.94613	1.32563
27959	1.39416	2.1
27960	1.36607	2.1
27961	1.21474	2.1
27962	0.946131	2.1
27963	0.946131	2.1
27964	1.16288	2.1
27965	1.08542	2.1
27966	0.851526	0.922924
27967	1.30374	1.8027
27968	1.01708	1.74775
27969	0.946114	0.918619
27970	0.96976	1.31037
27971	0.946132	0.131152
27972	1.49997	0.164421
27973	0.771735	0.150441
27974	0.771764	0.182661
27975	0.946133	0.200987
27976	0.700863	1.02526
27977	1.42222	1.06748
27978	0.946131	2.1
27979	0.946128	0.707107
27980	1.42509	1.68776
27981	1.14298	0.772236
27982	0.837014	2.1
27983	0.946131	2.1
27984	1.29426	2.1
27985	0.836985	2.1
27986	0.946129	2.1
27987	1.22089	0.490814
27988	0.946132	0.892792
27989	0.712486	0.552282
27990	0.6234	0.50591
27991	0.946131	2.1
27992	1.40271	2.1
27993	1.32241	2.1
27994	0.946133	2.1
27995	1.32241	2.1
27996	0.769373	1.17013
27997	0.946129	2.1
27998	1.41302	2.1
27999	1.28254	2.1
28000	0.946131	2.1
28001	1.28254	2.1
28002	0.851543	1.25887
28003	1.23056	2.1
28004	1.24356	2.1
28005	0.851543	1.34535
28006	0.909417	1.57697
28007	1.13808	0.488003
28008	0.946133	0.515705
28009	0.946131	0.341946
28010	1.13808	0.818482
28011	1.13808	0.484776

28012	0.930941	1.10938
28013	1.37808	0.941331
28014	1.17573	0.77232
28015	0.946124	1.14334
28016	1.02404	0.841157
28017	0.946116	1.63144
28018	1.3144	0.772381
28019	1.3144	1.64756
28020	0.946118	1.80794
28021	1.13089	1.35987
28022	0.946128	0.268283
28023	0.946101	0.204601
28024	1.2606	0.28341
28025	1.26055	0.233696
28026	0.580823	0.673075
28027	0.946132	0.974429
28028	0.580825	0.690855
28029	1.23224	0.720503
28030	0.946128	2.1
28031	1.3719	2.1
28032	0.94613	2.1
28033	1.3719	2.1
28034	0.94613	2.1
28035	0.946132	1.08839
28036	1.35025	0.754849
28037	1.35025	0.939309
28038	0.946132	1.39877
28039	1.1794	1.0755
28040	0.946133	2.1
28041	1.25598	2.1
28042	1.29249	2.1
28043	1.38154	2.1
28044	0.851541	1.50058
28045	1.31508	2.1
28046	1.37558	2.1
28047	0.946129	1.36805
28048	0.867663	1.5712
28049	0.94613	1.24995
28050	1.35426	1.1102
28051	0.84872	1.05472
28052	0.946129	0.980437
28053	1.17195	1.70359
28054	0.946133	2.1
28055	1.39009	2.1
28056	1.14342	2.1
28057	0.946133	2.1
28058	1.14343	2.1
28059	1.38819	2.1
28060	0.946133	2.1
28061	1.4116	2.1
28062	0.946131	2.1
28063	0.946131	2.1
28064	0.946127	2.1
28065	1.32244	2.1
28066	1.32244	2.1
28067	0.946131	2.1
28068	1.2176	2.1
28069	1.75823	2.1

28070	1.11239	2.1
28071	2.17979	2.1
28072	1.65533	2.1
28073	3.41356	2.1
28074	1.94841	2.1
28075	1.91767	2.1
28076	1.76726	2.1
28077	1.70302	2.1
28078	3.76137	2.1
28079	1.94841	2.1
28080	1.33962	2.1
28081	1.31365	2.1
28082	1.70302	2.1
28083	3.2123	2.1
28084	1.76726	2.1
28085	1.70302	2.1
28086	1.47581	2.1
28087	1.7457	2.1
28088	3.22764	2.1
28089	1.20478	2.1
28090	1.70302	2.1
28091	1.27956	2.1
28092	1.18569	2.1
28093	1.63979	2.1
28094	1.51949	2.1
28095	1.53082	2.1
28096	1.92911	2.1
28097	1.32068	2.1
28098	1.74454	2.1
28099	3.27536	2.1
28100	1.08172	2.1
28101	1.74454	2.1
28102	1.52603	2.1
28103	1.52603	2.1
28104	1.32068	2.1
28105	1.32068	2.1
28106	1.89226	2.1
28107	2.37364	2.1
28108	1.89226	2.1
28109	2.42769	2.1
28110	4.31995	2.1
28111	2.37365	2.1
28112	1.5362	2.1
28113	2.4277	2.1
28114	1.56328	2.1
28115	3.9639	2.1
28116	1.89225	2.1
28117	2.42768	2.1
28118	1.89213	2.1
28119	1.89225	2.1
28120	2.42756	2.1
28121	2.42768	2.1
28122	2.42768	2.1
28123	1.58163	2.1
28124	1.56315	2.1
28125	1.56327	2.1
28126	2.42757	2.1
28127	2.42768	2.1

28128	1.01641	2.1
28129	2.15005	2.1
28130	1.00099	2.1
28131	2.15114	2.1
28132	3.15104	2.1
28133	0.994813	2.1
28134	2.15114	2.1
28135	2.17159	2.1
28136	2.17159	2.1
28137	1.00099	2.1
28138	1.00099	2.1
28139	1.29608	2.1
28140	1.29608	2.1
28141	0.942035	1.69941
28142	0.942035	1.23291
28143	1.29198	1.74937
28144	0.942033	2.1
28145	0.946132	2.1
28146	0.946132	1.49665
28147	0.946132	1.24999
28148	0.942033	1.53741
28149	0.946133	1.01663
28150	1.29198	1.41935
28151	1.99985	2.1
28152	1.14207	2.1
28153	1.83584	2.1
28154	1.00232	2.1
28155	3.00217	2.1
28156	1.99867	0.100999
28157	2.0815	0.12194
28158	1.56806	0.101111
28159	1.28828	0.118259
28160	3.58519	0.115827
28161	1.89226	0.13255
28162	1.89226	0.156259
28163	1.89226	0.135629
28164	1.89226	0.161547
28165	1.89226	0.134248
28166	1.89226	0.162235
28167	1.89226	0.114326
28168	1.89226	0.14124
28169	2.23193	0.117677
28170	2.48978	0.160097
28171	4.38204	0.119917
28172	1.85293	0.667995
28173	1.64279	0.365707
28174	1.76268	0.605168
28175	1.54214	0.278119
28176	3.39507	0.459146
28177	1.6428	0.388258
28178	1.17302	0.421697
28179	1.17302	0.741505
28180	1.28419	0.47712
28181	1.54214	0.343119
28182	1.65331	0.454724
28183	1.76268	0.668155
28184	1.54214	0.411379
28185	1.65324	0.573608

28186	1.42491	0.269859
28187	3.19538	0.396426
28188	0.651043	0.522497
28189	0.651043	0.572413
28190	0.647925	0.438711
28191	0.647925	0.568927
28192	0.521747	0.461604
28193	0.58651	0.476375
28194	0.77107	0.435794
28195	0.712457	0.438973
28196	0.712457	0.382601
28197	0.521747	0.42572
28198	0.58651	0.393954
28199	0.77107	0.42755
28200	1.2873	2.1
28201	1.48751	2.1
28202	1.59659	2.1
28203	1.77596	1.36244
28204	3.06326	2.1
28205	1.07976	2.1
28206	1.59659	2.08384
28207	1.30237	2.1
28208	1.77723	2.1
28209	2.89897	2.1
28210	1.77723	2.1
28211	1.77596	1.43153
28212	1.82614	2.09418
28213	1.59659	2.02671
28214	2.0055	1.91375
28215	1.77596	1.9491
28216	2.64523	0.645244
28217	2.73982	0.35334
28218	2.50057	0.608781
28219	2.61606	0.340523
28220	5.26129	0.459648
28221	2.73982	0.366105
28222	2.68987	0.406177
28223	2.61607	0.407113
28224	2.57611	0.39028
28225	5.30593	0.325836
28226	1.89226	0.435988
28227	1.89226	0.718688
28228	1.89226	0.622235
28229	2.02178	0.620762
28230	3.78452	0.659572
28231	1.89226	0.424256
28232	1.89226	0.40936
28233	1.89226	1.00019
28234	1.89226	0.3059
28235	1.89226	1.08672
28236	1.89226	0.296588
28237	2.35204	2.1
28238	1.50108	2.1
28239	1.3715	2.1
28240	2.18469	2.1
28241	3.68577	2.1
28242	2.62514	2.1
28243	2.1847	2.1

28244	2.0487	2.1
28245	2.45213	2.1
28246	4.63682	2.1
28247	1.25075	2.1
28248	2.18469	2.1
28249	1.3715	2.1
28250	1.3715	2.1
28251	2.0487	2.1
28252	2.0487	2.1
28253	1.28667	2.1
28254	1.11285	2.1
28255	1.03529	2.1
28256	1.03529	2.1
28257	1.30711	2.1
28258	1.30711	2.1
28259	2.35053	2.1
28260	1.89225	2.1
28261	1.89225	2.1
28262	2.51216	2.1
28263	4.24278	2.1
28264	1.89225	2.1
28265	1.89225	2.1
28266	1.89225	2.1
28267	1.89225	2.1
28268	3.7845	2.1
28269	1.92025	2.1
28270	1.32119	2.1
28271	1.44777	2.1
28272	1.33834	2.1
28273	1.92025	2.1
28274	1.81082	2.1
28275	1.89226	2.1
28276	2.67272	2.1
28277	2.51218	2.1
28278	2.51218	2.1
28279	1.89226	2.1
28280	1.89226	2.1
28281	1.89226	2.1
28282	1.89226	2.1
28283	1.89226	2.1
28284	1.89226	2.1
28285	3.78452	2.1
28286	1.92027	2.1
28287	1.44778	2.1
28288	1.61121	2.1
28289	2.01555	2.1
28290	3.53147	2.1
28291	1.90727	0.32153
28292	2.29851	0.40259
28293	2.71643	0.408778
28294	2.17695	0.305383
28295	4.47547	0.306101
28296	1.90728	0.360993
28297	1.56339	0.277197
28298	1.58143	0.275703
28299	2.17696	0.34407
28300	3.74035	0.335937
28301	1.91368	0.29966

28302	2.17696	0.363934
28303	2.39986	0.368798
28304	1.75904	0.286848
28305	3.936	0.29028
28306	1.18793	0.131955
28307	2.74744	0.162221
28308	1.38716	0.168949
28309	2.57465	0.15543
28310	3.93537	0.160337
28311	1.3148	0.15747
28312	2.74744	0.189474
28313	2.70805	0.147853
28314	1.38716	0.148782
28315	2.70805	0.211488
28316	1.38716	0.188108
28317	1.20608	2.1
28318	0.975871	2.1
28319	0.946132	2.1
28320	0.946132	2.07658
28321	0.946132	2.07597
28322	0.946132	2.1
28323	0.975871	2.1
28324	0.975871	2.1
28325	1.20608	2.1
28326	0.946132	2.1
28327	0.946133	2.1
28328	0.975871	2.1
28329	2.8198	0.804519
28330	2.29967	1.43182
28331	2.09542	2.1
28332	2.63784	0.702861
28333	4.93751	1.48964
28334	2.09542	2.1
28335	1.39523	2.1
28336	1.21119	2.1
28337	1.39523	2.1
28338	2.11563	2.1
28339	2.29967	1.34687
28340	1.88584	1.92766
28341	2.63784	0.625389
28342	2.11262	1.0206
28343	1.91132	0.889994
28344	4.02153	1.07706
28345	1.88584	2.1
28346	1.21119	2.1
28347	1.12606	2.1
28348	1.91132	0.876611
28349	3.1225	2.1
28350	2.18077	1.81506
28351	1.78167	1.7564
28352	2.2947	0.823674
28353	1.93139	1.32083
28354	4.07637	0.905113
28355	1.5709	1.20646
28356	1.03235	1.85156
28357	2.05933	1.66037
28358	1.78168	1.00372
28359	1.31	0.692456

28360	1.03235	0.716272
28361	2.2947	1.06593
28362	2.31785	1.87746
28363	2.40627	1.72975
28364	2.08288	0.926726
28365	4.37758	1.91095
28366	1.95737	1.53442
28367	1.31	1.81518
28368	2.08289	0.70777
28369	1.50108	0.860744
28370	3.39289	0.823059
28371	2.49676	2.0769
28372	2.24781	1.85444
28373	2.5276	0.998913
28374	2.7352	2.1
28375	5.02436	1.86993
28376	2.49676	1.72115
28377	2.43549	2.1
28378	2.6398	2.1
28379	2.7352	2.1
28380	5.13656	2.1
28381	1.89226	2.1
28382	2.7352	2.1
28383	1.89226	1.46036
28384	2.6398	2.1
28385	1.89226	1.76644
28386	2.6398	2.1
28387	2.16843	0.0522355
28388	1.00185	0.0522355
28389	1.90039	2.1
28390	3.1547	0.0522355
28391	2.54064	0.0522355
28392	2.46752	2.1
28393	1.90039	2.1
28394	2.26044	2.1
28395	4.36791	0.47025
28396	1.65815	2.1
28397	2.48652	2.1
28398	1.65815	2.1
28399	1.9004	2.1
28400	2.21711	2.1
28401	2.5034	2.1
28402	2.37167	2.1
28403	2.59965	2.1
28404	2.62812	2.1
28405	2.40308	2.1
28406	4.99979	2.1
28407	2.50137	2.1
28408	2.59965	2.1
28409	2.62812	2.1
28410	2.5214	2.1
28411	5.12948	2.1
28412	2.62814	2.1
28413	2.4031	2.1
28414	2.43509	2.1
28415	2.65066	2.1
28416	5.06323	2.1
28417	2.62814	2.1

28418	2.52142	2.1
28419	2.53008	2.1
28420	2.65066	2.1
28421	5.15822	2.1
28422	2.0815	2.1
28423	1.92067	2.1
28424	1.59611	2.1
28425	1.89226	2.1
28426	1.62453	2.1
28427	1.92067	2.1
28428	1.89226	2.1
28429	1.89226	2.1
28430	1.89226	2.1
28431	1.89226	2.1
28432	3.78453	2.1
28433	0.946131	2.1
28434	0.946131	2.1
28435	0.929501	2.1
28436	0.910761	2.1
28437	0.9461	2.1
28438	0.9461	2.1
28439	0.910792	2.1
28440	0.910792	2.1
28441	0.946131	2.1
28442	0.929501	2.1
28443	0.910761	2.1
28444	0.946131	2.1
28445	2.0815	2.1
28446	1.81661	2.1
28447	2.1341	2.1
28448	2.0815	2.1
28449	1.86921	2.1
28450	1.81661	2.1
28451	1.89226	2.1
28452	1.89226	2.1
28453	1.89226	2.1
28454	1.89226	2.1
28455	1.89226	2.1
28456	1.89226	2.1
28457	0.946131	2.1
28458	0.946131	2.1
28459	0.949104	2.1
28460	0.9295	2.1
28461	0.946132	2.1
28462	0.946132	2.1
28463	0.929499	2.1
28464	0.929499	2.1
28465	0.946131	2.1
28466	0.949105	2.1
28467	0.946131	2.1
28468	0.929501	2.1
28469	1.66104	2.1
28470	2.34044	2.1
28471	2.08889	2.1
28472	1.33264	2.1
28473	3.67308	2.1
28474	2.0889	2.1
28475	1.08011	2.1

28476	1.08011	2.1
28477	1.33264	2.1
28478	1.87598	2.1
28479	2.12851	2.1
28480	2.27472	2.1
28481	1.89226	1.18706
28482	2.27703	1.50334
28483	1.89226	0.801895
28484	4.16699	0.82937
28485	1.89226	1.82869
28486	1.89226	1.33292
28487	1.89226	1.33279
28488	1.89226	0.991471
28489	3.78452	1.69195
28490	1.00007	1.50372
28491	1.32316	0.961589
28492	1.29417	0.862455
28493	1.29417	1.41955
28494	1.02051	0.786691
28495	1.02051	1.20062
28496	2.2846	1.68636
28497	1.89226	0.960305
28498	2.27703	0.855081
28499	2.27703	2.1
28500	1.89226	0.616967
28501	1.89226	2.1
28502	1.89226	1.72685
28503	1.89226	1.61299
28504	1.89226	0.956086
28505	1.89226	0.670311
28506	3.78452	0.89898
28507	1.02051	2.07939
28508	1.32316	1.59012
28509	1.03373	0.934387
28510	1.34066	0.935947
28511	2.35689	1.80888
28512	1.703	2.1
28513	1.89224	2.1
28514	1.52266	2.1
28515	1.89224	2.1
28516	1.23344	2.1
28517	1.53282	2.1
28518	1.89223	2.1
28519	1.89223	1.75478
28520	1.89223	2.1
28521	1.89223	2.1
28522	3.78447	2.1
28523	0.796845	2.1
28524	0.796845	2.1
28525	0.812146	2.1
28526	0.812146	2.1
28527	1.08006	2.1
28528	1.02679	2.1
28529	0.551538	2.1
28530	0.528929	2.1
28531	0.551529	2.1
28532	1.08005	2.1
28533	0.528935	2.1

28534	1.0268	2.1
28535	1.5001	2.1
28536	1.70302	2.1
28537	2.0726	2.1
28538	1.70302	2.1
28539	1.99601	2.1
28540	1.70303	2.08258
28541	1.89226	2.1
28542	1.89226	2.1
28543	1.89226	2.1
28544	1.89226	2.1
28545	1.89226	2.1
28546	1.89226	1.60539
28547	1.17285	2.1
28548	2.16015	2.1
28549	2.23563	2.1
28550	2.23563	2.1
28551	1.10309	2.1
28552	1.10309	2.1
28553	0.946128	2.1
28554	0.946128	2.1
28555	1.21847	0.59743
28556	1.2884	2.1
28557	0.94613	2.1
28558	0.94613	2.1
28559	1.21847	2.1
28560	1.21847	0.542025
28561	0.946131	2.1
28562	1.21847	2.1
28563	0.94613	2.1
28564	1.2884	2.1
28565	1.83922	0.679011
28566	2.5768	2.1
28567	1.83488	2.1
28568	1.82846	0.0522355
28569	2.43693	2.1
28570	2.43693	1.74284
28571	2.36134	2.1
28572	1.81656	2.1
28573	1.86454	2.1
28574	1.6697	2.1
28575	2.55618	2.1
28576	2.36134	2.1
28577	2.55618	2.1
28578	1.9323	2.1
28579	1.9302	2.1
28580	1.81656	2.1
28581	2.66983	2.1
28582	2.55618	2.1
28583	1.89226	0.804914
28584	1.70302	0.751013
28585	1.94935	0.923059
28586	1.70302	0.758266
28587	1.95569	0.788263
28588	1.70302	0.52715
28589	2.82465	2.1
28590	2.41707	2.1
28591	2.24481	2.1

28592	2.68958	2.1
28593	5.10665	2.1
28594	1.41232	2.1
28595	1.34479	2.1
28596	0.946129	2.1
28597	0.946129	2.1
28598	0.94613	2.1
28599	0.94613	2.1
28600	1.41232	2.1
28601	1.41232	2.1
28602	1.34479	2.1
28603	0.94613	2.1
28604	0.946131	2.1
28605	1.41232	2.1
28606	1.86859	0.0905679
28607	1.89226	0.0995832
28608	1.89226	0.112474
28609	1.89226	0.0898852
28610	1.45875	0.108335
28611	1.45875	0.0847872
28612	2.1769	0.566444
28613	2.50259	0.524436
28614	1.94955	0.417583
28615	2.25959	0.632829
28616	4.45214	0.663079
28617	1.89226	0.568015
28618	1.89226	0.61109
28619	1.89226	0.488907
28620	1.89226	0.594115
28621	3.78452	0.479358
28622	1.89226	0.662142
28623	1.89226	0.820128
28624	1.89226	0.596722
28625	1.89226	0.752632
28626	3.78452	1.09395
28627	2.50259	0.570738
28628	2.49445	0.690957
28629	2.25959	0.550439
28630	2.20843	0.573523
28631	4.71102	0.520699
28632	1.94955	0.427386
28633	2.25959	0.454879
28634	1.24689	0.339189
28635	1.79906	0.417161
28636	3.74861	0.378385
28637	1.89226	0.508364
28638	1.89226	0.524675
28639	1.89226	0.400931
28640	1.89226	0.47699
28641	3.78452	0.590387
28642	1.89226	0.532851
28643	1.89226	0.586045
28644	1.89226	0.457644
28645	1.89226	0.473207
28646	3.78452	0.44686
28647	2.25959	0.432732
28648	2.20843	0.461107
28649	1.79906	0.403288

28650	1.98391	0.499798
28651	4.00748	0.472595
28652	1.89226	2.1
28653	1.89226	2.1
28654	1.89226	2.1
28655	1.89226	2.1
28656	3.78452	2.1
28657	1.89226	2.1
28658	1.89226	2.1
28659	1.89226	2.1
28660	1.89226	2.1
28661	3.78452	2.1
28662	1.64718	2.1
28663	1.23085	2.1
28664	1.46028	2.1
28665	1.39564	2.1
28666	1.68372	2.1
28667	1.64718	2.1
28668	1.64718	2.1
28669	1.64981	2.1
28670	1.70303	2.1
28671	1.6991	2.1
28672	3.3199	2.1
28673	1.89226	2.1
28674	1.89226	2.1
28675	1.89226	2.1
28676	1.89226	2.1
28677	3.78452	2.1
28678	1.89226	2.1
28679	1.89226	2.1
28680	1.89226	2.1
28681	1.89226	2.1
28682	3.78452	2.1
28683	0.666701	2.1
28684	0.65896	2.1
28685	0.920797	2.1
28686	0.895441	2.1
28687	0.775871	2.1
28688	0.687493	2.1
28689	0.777393	2.1
28690	0.777393	2.1
28691	0.677856	2.1
28692	0.90095	2.1
28693	0.621435	2.1
28694	0.858124	2.1
28695	1.70303	2.1
28696	1.70731	2.1
28697	1.79663	2.1
28698	1.68783	2.1
28699	3.17337	2.1
28700	1.22724	2.1
28701	2.0021	2.1
28702	1.90427	2.1
28703	1.08698	2.1
28704	3.08907	2.1
28705	2.41689	2.1
28706	2.00209	2.1
28707	1.90427	2.1

28708	2.35245	2.1
28709	4.35454	2.1
28710	1.90427	2.1
28711	0.956294	2.1
28712	0.956294	2.1
28713	1.08698	2.1
28714	1.81488	2.1
28715	1.94556	2.1
28716	1.90427	2.1
28717	2.35245	2.1
28718	2.28979	2.1
28719	1.81488	2.1
28720	4.16733	2.1
28721	1.6272	2.1
28722	1.70302	2.1
28723	1.70303	2.1
28724	1.70302	2.1
28725	1.91167	2.1
28726	1.91167	2.1
28727	1.49063	2.1
28728	1.89226	2.1
28729	1.89226	2.1
28730	1.89226	2.1
28731	1.6272	2.1
28732	1.6272	2.1
28733	2.25988	0.955169
28734	1.89226	0.42827
28735	1.89226	0.93589
28736	2.23152	0.746244
28737	4.12379	0.647765
28738	2.25988	0.760393
28739	1.16361	0.937558
28740	1.15853	1.88467
28741	2.23153	0.606573
28742	3.39513	1.01244
28743	1.89226	0.888649
28744	2.23153	0.62248
28745	2.21022	1.03401
28746	2.23153	0.769939
28747	1.87095	0.582009
28748	1.89226	0.404486
28749	1.16239	1.36258
28750	2.23153	0.479567
28751	1.15853	1.05583
28752	1.15853	0.549553
28753	2.21022	1.11507
28754	2.21022	0.570409
28755	1.78381	0.12307
28756	1.70302	0.14981
28757	1.15453	0.130836
28758	1.35007	0.158625
28759	1.82021	0.123074
28760	2.12851	0.144673
28761	1.89226	0.157456
28762	1.89226	0.209692
28763	1.89226	0.174941
28764	1.89226	0.226359
28765	1.89226	0.148687

28766	1.89226	0.199393
28767	1.89258	0.123941
28768	1.089	0.151458
28769	1.22586	0.122804
28770	1.79877	0.148833
28771	2.88777	0.130044
28772	1.91564	0.119646
28773	1.79877	0.150717
28774	1.96356	0.135668
28775	1.70302	0.159217
28776	2.37828	0.122976
28777	2.04859	0.147525
28778	1.89226	0.157827
28779	1.89226	0.202585
28780	1.89226	0.150702
28781	1.89226	0.21732
28782	3.78453	0.168143
28783	1.89226	0.148775
28784	1.89226	0.196633
28785	1.89226	0.150462
28786	1.89226	0.20503
28787	1.89226	0.14652
28788	1.89226	0.19663
28789	2.28593	2.1
28790	1.89226	1.38314
28791	2.2731	2.06265
28792	1.89226	0.77242
28793	4.17819	0.990221
28794	1.89226	1.96617
28795	1.89226	1.87989
28796	1.89226	1.16146
28797	1.89226	0.99604
28798	3.78452	1.47158
28799	1.03933	1.7413
28800	1.34355	0.956027
28801	1.35048	0.879258
28802	1.35048	1.71137
28803	1.02975	0.743005
28804	1.02975	1.30898
28805	2.25105	2.01256
28806	1.89226	1.20047
28807	2.27311	1.33345
28808	2.27311	2.1
28809	1.89226	0.803442
28810	1.89226	2.1
28811	1.89226	1.90085
28812	1.89226	1.64294
28813	1.89226	1.20418
28814	1.89226	0.575022
28815	3.78452	0.999753
28816	1.02975	2.1
28817	1.34355	1.61271
28818	1.01121	1.06958
28819	1.33079	0.881615
28820	2.35476	1.85919
28821	1.24526	0.0786061
28822	2.36406	0.0929933
28823	2.81021	0.0797294

28824	1.7237	0.0826896
28825	4.08776	0.0811023
28826	1.89226	2.1
28827	1.89226	1.0168
28828	1.89226	2.1
28829	1.89226	0.857473
28830	3.78453	2.1
28831	1.79028	2.1
28832	1.89238	1.01857
28833	1.65533	2.1
28834	1.77821	1.59287
28835	3.5477	2.1
28836	1.89238	0.941287
28837	1.7465	0.339465
28838	1.77821	1.5626
28839	1.63557	0.417686
28840	3.52795	0.690873
28841	1.89226	0.929232
28842	1.89226	0.330919
28843	1.89226	0.869308
28844	1.89226	0.360702
28845	3.78452	0.618361
28846	1.89226	2.1
28847	1.89226	0.936785
28848	1.89226	2.1
28849	1.89226	1.22383
28850	3.78452	2.1
28851	1.65532	2.1
28852	1.77821	1.57358
28853	1.53278	2.1
28854	1.67571	1.26431
28855	3.33103	2.1
28856	1.77821	1.55492
28857	1.63557	0.424049
28858	1.67571	1.25608
28859	1.52615	0.392713
28860	3.31128	0.689938
28861	1.89226	0.954492
28862	1.89226	0.344745
28863	1.89226	1.25266
28864	1.89226	0.411231
28865	3.78452	0.687734
28866	2.83129	1.89463
28867	1.89226	0.351569
28868	1.89226	0.816604
28869	2.6491	1.04478
28870	4.54136	0.720126
28871	2.83129	2.09709
28872	1.47278	2.1
28873	1.3909	2.1
28874	2.6491	1.06282
28875	4.12188	2.1
28876	1.89225	1.8539
28877	2.65393	2.1
28878	1.89225	1.75981
28879	2.69591	2.1
28880	4.58816	2.1
28881	1.91703	2.1

28882	2.00209	2.1
28883	2.65393	2.1
28884	2.65393	2.1
28885	2.69591	2.1
28886	2.00209	2.1
28887	1.89226	1.98836
28888	2.69593	2.1
28889	1.93967	2.1
28890	1.89226	2.0221
28891	2.74334	2.1
28892	2.69593	1.84254
28893	2.74335	2.1
28894	2.0021	2.1
28895	2.76367	2.1
28896	2.69593	2.1
28897	2.06984	2.1
28898	2.0021	2.1
28899	2.24481	2.1
28900	2.58921	2.1
28901	2.68958	2.1
28902	2.68958	2.1
28903	2.11984	2.1
28904	2.11984	2.1
28905	2.11984	2.1
28906	2.58921	2.1
28907	2.46488	2.1
28908	1.98339	2.1
28909	4.5726	2.1
28910	2.7777	2.1
28911	2.58921	2.1
28912	2.46488	2.1
28913	2.70401	2.1
28914	5.29322	2.1
28915	2.17741	2.1
28916	2.54197	2.1
28917	2.54018	2.1
28918	2.18608	2.1
28919	4.72805	2.1
28920	2.17742	2.1
28921	1.58054	2.1
28922	1.59169	2.1
28923	2.18608	2.1
28924	3.76662	2.1
28925	2.54018	2.1
28926	2.18608	2.1
28927	2.19104	2.1
28928	2.5392	2.1
28929	4.72528	2.1
28930	1.60497	2.1
28931	2.18608	2.1
28932	1.59169	2.1
28933	1.59169	2.1
28934	2.19105	2.1
28935	2.19105	2.1
28936	2.58661	1.0305
28937	1.06203	2.1
28938	2.7043	2.1
28939	2.21397	2.029

28940	1.55236	2.1
28941	1.06203	1.88381
28942	1.89226	1.27396
28943	1.89226	1.66948
28944	1.89226	0.759823
28945	1.89226	1.66994
28946	3.78453	1.37922
28947	1.08195	1.48658
28948	0.990019	2.1
28949	1.31976	1.65757
28950	1.4539	0.857883
28951	2.40171	1.86339
28952	1.89226	1.02382
28953	1.89226	1.97286
28954	1.89226	1.01006
28955	1.89226	0.674871
28956	3.78453	1.1684
28957	1.89226	1.43873
28958	1.89226	1.28006
28959	1.89226	0.886993
28960	1.89226	1.84837
28961	3.78453	1.1904
28962	1.43895	1.76384
28963	1.31976	1.8271
28964	1.70066	0.814267
28965	1.33783	2.1
28966	1.43895	1.97942
28967	1.07612	2.1
28968	0.65988	1.72114
28969	0.850329	1.34363
28970	0.919598	0.962109
28971	0.726952	1.51694
28972	0.788106	0.791076
28973	0.788106	2.1
28974	0.789175	1.90383
28975	0.789175	0.636925
28976	0.850329	1.01717
28977	0.919598	0.965343
28978	0.659879	1.55514
28979	0.726952	1.32596
28980	1.34389	2.1
28981	1.41279	1.545
28982	1.15564	2.1
28983	1.15564	2.1
28984	1.55998	1.2828
28985	1.55998	1.72819
28986	1.89226	1.27436
28987	1.89226	1.7051
28988	1.89226	2.1
28989	1.89226	1.06949
28990	1.89226	1.96905
28991	1.89226	1.05891
28992	1.55998	2.1
28993	1.41279	1.80491
28994	1.72901	1.33688
28995	1.62414	1.99954
28996	3.1418	1.66495
28997	1.89226	1.21052

28998	1.89226	1.99962
28999	1.89226	1.3762
29000	1.89226	0.888285
29001	3.78452	1.64172
29002	1.89226	0.748526
29003	1.89226	2.06143
29004	1.89226	1.20609
29005	1.89226	1.79556
29006	1.89226	0.893874
29007	1.89226	1.13828
29008	1.62414	1.77633
29009	1.01243	1.95482
29010	1.70932	2.02059
29011	1.41279	1.34494
29012	1.30896	1.47558
29013	1.01243	1.11319
29014	1.9719	2.1
29015	1.10725	2.1
29016	1.12411	2.1
29017	1.98691	2.1
29018	3.09601	2.1
29019	1.97189	2.1
29020	2.47932	2.1
29021	2.49092	2.1
29022	1.98691	2.1
29023	4.46282	2.1
29024	1.1486	2.1
29025	1.98691	2.1
29026	1.12411	2.1
29027	1.12411	2.1
29028	2.01602	2.1
29029	2.01602	2.1
29030	2.49093	2.1
29031	1.98691	2.1
29032	2.01601	2.1
29033	2.51651	2.1
29034	4.50694	2.1
29035	1.93147	0.286297
29036	2.51593	0.268827
29037	1.35146	0.252993
29038	1.35146	0.25693
29039	2.48665	0.271043
29040	1.90664	0.239297
29041	1.63979	2.1
29042	1.7457	2.1
29043	1.66046	2.1
29044	1.56558	2.1
29045	3.31128	2.1
29046	0.819895	2.1
29047	0.782792	2.1
29048	0.60239	2.1
29049	0.54831	2.1
29050	0.688347	2.1
29051	0.688347	2.1
29052	0.679857	2.1
29053	0.679857	2.1
29054	0.782792	2.1
29055	0.54831	2.1

29056	0.60239	2.1
29057	0.819895	2.1
29058	1.66046	2.1
29059	1.42485	2.1
29060	1.56558	2.1
29061	1.56558	2.1
29062	1.52995	2.1
29063	1.52995	2.1
29064	2.58101	0.420098
29065	2.09803	0.794802
29066	2.408	0.275722
29067	1.86893	1.06884
29068	4.44994	0.596418
29069	2.36062	0.705373
29070	1.25104	2.1
29071	1.25104	1.56261
29072	1.39703	2.1
29073	1.86893	1.22649
29074	2.01492	2.1
29075	2.408	0.30057
29076	1.86893	0.732302
29077	2.22575	0.328027
29078	1.70677	0.687648
29079	4.09468	0.599404
29080	0.737605	2.1
29081	0.737605	0.708217
29082	0.741302	0.794111
29083	0.741302	2.1
29084	0.55189	2.1
29085	0.625517	2.1
29086	0.934468	0.699924
29087	0.853389	0.67464
29088	0.853389	2.1
29089	0.55189	2.0693
29090	0.625517	2.1
29091	0.934467	2.1
29092	2.35053	2.1
29093	1.09615	2.1
29094	1.25998	2.1
29095	2.51216	2.1
29096	3.61051	2.1
29097	1.33636	2.1
29098	1.25609	2.1
29099	0.698455	2.1
29100	0.629995	2.1
29101	0.986092	2.1
29102	0.986092	2.1
29103	0.980263	2.1
29104	0.980263	2.1
29105	1.33636	2.1
29106	0.698455	2.1
29107	0.629995	2.1
29108	1.25609	2.1
29109	1.89226	0.890104
29110	1.89226	0.933332
29111	1.89226	0.99348
29112	1.89226	0.918163
29113	3.78452	0.889083

29114	1.74321	0.860584
29115	1.89226	0.872702
29116	1.91755	0.919709
29117	1.91755	0.861001
29118	1.89226	0.916288
29119	1.89226	0.862595
29120	0.946132	0.864885
29121	0.946132	0.822021
29122	0.949798	0.746207
29123	1.08054	0.856235
29124	1.08054	0.795222
29125	1.08054	0.854532
29126	0.946132	0.826777
29127	0.946132	0.846453
29128	0.946132	0.826374
29129	0.949798	0.739953
29130	1.08054	0.801629
29131	0.946131	0.921815
29132	1.89226	0.951601
29133	1.89226	0.836995
29134	1.89226	0.809393
29135	1.89226	0.817175
29136	1.89226	0.890877
29137	1.89226	0.901304
29138	2.39378	0.316458
29139	2.29234	0.160477
29140	2.29234	0.214913
29141	2.7933	0.194878
29142	1.18422	0.211852
29143	1.68518	0.252014
29144	2.79329	0.194537
29145	1.89226	0.160654
29146	2.29233	0.141136
29147	1.89226	0.161569
29148	4.18459	0.13989
29149	0.857046	0.121471
29150	0.857037	0.177097
29151	0.94613	0.126716
29152	0.94613	0.158561
29153	0.851539	0.147545
29154	0.851539	0.137571
29155	1.0315	0.128334
29156	0.952867	0.133014
29157	0.851486	0.124341
29158	0.952869	0.117547
29159	1.03156	0.141796
29160	0.851528	0.1636
29161	1.61981	2.1
29162	1.93096	2.1
29163	2.00776	2.1
29164	1.62218	2.1
29165	3.62757	2.1
29166	2.00776	2.1
29167	1.68541	2.1
29168	1.83215	2.1
29169	1.62218	2.1
29170	2.21773	2.1
29171	2.00776	2.1

29172	1.89188	0.121556
29173	1.70303	0.134516
29174	1.89226	0.141147
29175	1.72465	0.124219
29176	1.89226	0.141088
29177	1.70552	0.130464
29178	2.48659	0.149363
29179	2.0815	0.178266
29180	1.89226	0.202248
29181	2.08111	0.162731
29182	1.89226	0.191229
29183	2.08111	0.165763
29184	2.35332	0.375521
29185	1.83284	0.196429
29186	2.28483	0.257128
29187	2.71955	0.226565
29188	4.63815	0.233174
29189	1.89227	2.1
29190	1.89227	2.1
29191	1.89227	2.1
29192	1.89227	2.1
29193	3.78453	2.1
29194	1.33506	2.1
29195	1.17129	2.1
29196	1.33506	2.1
29197	1.38415	2.1
29198	1.13664	2.1
29199	1.18573	2.1
29200	1.89226	2.1
29201	2.68785	2.1
29202	1.89226	2.1
29203	2.6692	2.1
29204	4.58011	2.1
29205	1.89226	2.1
29206	1.89226	2.1
29207	1.89226	2.1
29208	1.89226	2.1
29209	3.78453	2.1
29210	1.33506	2.1
29211	1.13664	2.1
29212	1.27575	2.1
29213	1.09627	2.1
29214	2.43133	2.1
29215	1.89226	2.1
29216	2.65349	2.1
29217	2.66919	2.1
29218	2.66919	2.1
29219	1.89226	2.1
29220	1.89226	2.1
29221	1.89226	2.1
29222	1.89226	2.1
29223	1.89226	2.1
29224	1.89226	2.1
29225	3.78453	2.1
29226	1.16834	2.1
29227	1.16834	2.1
29228	0.946132	2.1
29229	0.946132	2.1

29230	1.26978	2.1
29231	1.16834	2.1
29232	0.94613	2.1
29233	0.94613	2.1
29234	0.94613	2.1
29235	1.26978	2.1
29236	0.94613	2.1
29237	1.16834	2.1
29238	2.14698	2.1
29239	1.89226	2.1
29240	1.89226	2.1
29241	1.89226	2.1
29242	2.40719	2.1
29243	2.40719	2.1
29244	1.89226	2.1
29245	1.89226	2.1
29246	1.89226	2.1
29247	1.89226	2.1
29248	3.78453	2.1
29249	1.26977	2.1
29250	1.26977	2.1
29251	0.946133	2.1
29252	0.946133	2.1
29253	1.37467	2.1
29254	1.26978	2.1
29255	0.946131	2.1
29256	0.946131	2.1
29257	1.37467	2.1
29258	0.94613	2.1
29259	0.946131	2.1
29260	1.26978	2.1
29261	1.2036	2.1
29262	1.34912	2.1
29263	0.946131	2.1
29264	0.946131	2.1
29265	1.34912	2.1
29266	1.34912	2.1
29267	0.946129	2.1
29268	0.946129	2.1
29269	1.34912	2.1
29270	0.94613	2.1
29271	0.946131	2.1
29272	1.2036	2.1
29273	2.47164	0.515468
29274	1.89019	0.328851
29275	1.79109	0.49077
29276	2.41632	0.349553
29277	4.26273	0.503322
29278	1.54197	0.397778
29279	2.41632	0.275012
29280	1.79109	0.350274
29281	1.79109	0.418543
29282	2.20929	0.303133
29283	2.20929	0.394338
29284	1.70302	0.982952
29285	1.48104	1.1481
29286	1.99365	1.18154
29287	1.32897	0.976181

29288	1.70302	1.17791
29289	1.14034	0.964247
29290	1.70302	0.963606
29291	1.99365	1.01293
29292	1.99297	1.0533
29293	2.36337	0.834822
29294	4.04804	0.898477
29295	1.51569	0.871395
29296	2.49093	0.879598
29297	1.6026	0.96517
29298	1.43229	0.754455
29299	2.0815	0.981135
29300	1.83402	0.779834
29301	2.54665	2.1
29302	2.34044	2.1
29303	2.08889	2.1
29304	2.37653	2.1
29305	4.71697	2.1
29306	2.54665	2.1
29307	2.45212	2.1
29308	2.31012	2.1
29309	2.37653	2.1
29310	4.82865	2.1
29311	2.08889	2.1
29312	2.37653	2.1
29313	2.23366	2.1
29314	1.87598	2.1
29315	4.25251	2.1
29316	2.31012	2.1
29317	2.37653	2.1
29318	2.23367	2.1
29319	2.17384	2.1
29320	4.55037	2.1
29321	0.946131	2.1
29322	0.946131	2.1
29323	1.30338	2.1
29324	1.23186	2.1
29325	0.94613	2.1
29326	0.94613	2.1
29327	1.23187	2.1
29328	1.23187	2.1
29329	0.946131	2.1
29330	1.23186	2.1
29331	1.30338	2.1
29332	0.946131	2.1
29333	1.89226	2.1
29334	2.46373	2.1
29335	2.34725	2.1
29336	2.46373	2.1
29337	1.77579	2.1
29338	1.89226	2.1
29339	2.61901	2.1
29340	2.45899	2.1
29341	2.64067	2.1
29342	2.52708	2.1
29343	5.14609	2.1
29344	2.41795	2.1
29345	2.0815	2.1

29346	2.52708	2.1
29347	1.92277	2.1
29348	4.31368	2.1
29349	2.64067	2.1
29350	2.52708	2.1
29351	2.58638	2.1
29352	2.51039	2.1
29353	5.11346	2.1
29354	2.52708	2.1
29355	2.13642	2.1
29356	2.51039	2.1
29357	2.16633	2.1
29358	4.69341	2.1
29359	1.61028	1.2758
29360	1.65712	0.712341
29361	1.35002	0.521686
29362	1.35002	1.31972
29363	1.88637	0.682937
29364	1.88637	1.83515
29365	2.57663	0.994383
29366	2.24227	2.1
29367	2.48354	0.932214
29368	2.14317	2.1
29369	4.7198	2.1
29370	2.24227	2.1
29371	1.77353	2.1
29372	2.14317	2.1
29373	1.69956	2.1
29374	3.9167	2.1
29375	2.48353	0.909411
29376	2.14317	2.1
29377	2.39282	1.24313
29378	1.79824	2.1
29379	4.53599	2.1
29380	2.14317	2.1
29381	1.69956	2.1
29382	1.91427	2.1
29383	1.6347	2.1
29384	3.77787	2.1
29385	1.89226	0.852512
29386	1.89226	1.01341
29387	1.89226	1.0801
29388	1.89226	1.00156
29389	3.78453	1.36079
29390	1.89226	0.797613
29391	1.89226	0.76969
29392	1.89226	0.878322
29393	1.89226	0.914012
29394	3.78452	0.634628
29395	1.89226	0.801873
29396	1.89652	1.51137
29397	1.89226	0.679031
29398	1.89226	0.947431
29399	1.19352	0.956055
29400	1.19352	1.74406
29401	1.89226	0.959487
29402	1.89653	0.701804
29403	1.89226	1.30081

29404	1.89226	1.09964
29405	1.89226	0.84547
29406	1.89226	0.898926
29407	1.89226	1.30911
29408	1.75326	0.830113
29409	1.89226	2.01242
29410	1.12948	1.56517
29411	1.89226	0.662663
29412	1.12948	0.596154
29413	2.61064	2.1
29414	1.89226	2.1
29415	1.89226	2.1
29416	1.89226	2.1
29417	2.6085	2.1
29418	2.6085	2.1
29419	2.6085	2.1
29420	1.89226	2.1
29421	1.89226	2.1
29422	1.89226	2.1
29423	2.62161	2.1
29424	2.62161	2.1
29425	2.16535	2.1
29426	1.44603	2.1
29427	2.79784	2.1
29428	1.84756	2.1
29429	4.01292	2.1
29430	2.65262	2.1
29431	1.03391	2.07574
29432	2.20239	2.1
29433	1.74416	2.1
29434	1.49213	2.1
29435	1.03391	2.1
29436	2.29066	2.1
29437	2.49174	2.1
29438	2.56502	2.1
29439	2.33802	2.1
29440	4.85568	2.1
29441	2.39663	2.1
29442	2.49174	2.1
29443	2.56502	2.1
29444	2.45929	2.1
29445	4.96165	2.1
29446	2.56502	2.1
29447	2.33802	2.1
29448	2.37168	2.1
29449	2.59967	2.1
29450	4.9367	2.1
29451	2.56502	2.1
29452	2.45929	2.1
29453	2.50138	2.1
29454	2.59967	2.1
29455	5.0664	2.1
29456	2.27009	0.930572
29457	1.26247	2.09441
29458	1.86812	1.88192
29459	2.837	1.28814
29460	4.13822	1.86841
29461	1.89226	0.60944

29462	1.89226	2.1
29463	1.89226	1.0902
29464	1.89226	1.47788
29465	3.78453	1.45837
29466	1.75835	2.1
29467	1.72866	2.1
29468	1.65313	0.972064
29469	1.64822	2.1
29470	3.3818	2.1
29471	1.72866	2.1
29472	1.58401	2.1
29473	1.64822	2.1
29474	1.52441	2.1
29475	3.25307	2.1
29476	1.89226	2.1
29477	1.89226	2.1
29478	1.89226	1.53588
29479	1.89226	2.1
29480	3.78452	2.1
29481	1.89226	1.19555
29482	1.89226	1.56011
29483	1.89226	0.620527
29484	1.89226	2.1
29485	3.78453	1.20405
29486	1.65313	1.1432
29487	1.64822	2.1
29488	1.55779	2.1
29489	1.57968	2.1
29490	3.23281	2.1
29491	1.64822	2.1
29492	1.52441	2.1
29493	1.57967	2.1
29494	1.474 2.1	
29495	3.10408	2.1
29496	1.89226	1.59601
29497	1.89226	2.1
29498	1.89226	2.1
29499	1.89226	2.1
29500	3.78452	2.1
29501	1.86067	1.20422
29502	1.58407	2.03622
29503	2.07965	0.831054
29504	1.83123	1.3987
29505	3.66372	1.02244
29506	1.89226	0.900778
29507	1.89226	2.04396
29508	1.89226	1.02775
29509	1.89226	1.42395
29510	3.78452	1.26957
29511	1.89226	0.867288
29512	1.89226	1.77412
29513	1.89226	0.771289
29514	1.89226	2.1
29515	1.89226	0.692341
29516	1.89226	2.07184
29517	1.83123	0.886726
29518	1.03472	1.88401
29519	1.91068	1.56549

29520	1.58406	1.00837
29521	1.36133	1.07158
29522	1.03472	0.814103
29523	2.07965	1.00073
29524	1.83123	1.52781
29525	2.28385	1.02061
29526	2.09114	1.25842
29527	4.1708	1.4901
29528	1.89226	1.27772
29529	1.89226	1.7207
29530	1.89226	0.827514
29531	1.89226	1.49418
29532	3.78452	1.01607
29533	1.89226	0.918641
29534	1.89226	1.98371
29535	1.89226	0.978818
29536	1.89226	1.77508
29537	3.78452	1.18633
29538	1.83123	0.863725
29539	1.36133	2.00471
29540	2.09114	0.843839
29541	1.69436	1.49664
29542	3.45248	1.06369
29543	1.89226	2.1
29544	1.89226	1.20339
29545	1.89226	2.1
29546	1.89226	0.799112
29547	3.78453	2.1
29548	1.53278	2.1
29549	1.67571	1.24171
29550	1.40182	2.1
29551	1.55686	1.58134
29552	3.07752	2.1
29553	1.67571	1.26367
29554	1.52615	0.323876
29555	1.55686	1.51728
29556	1.38963	0.250638
29557	3.06534	0.673456
29558	1.89226	1.26296
29559	1.89226	0.410254
29560	1.89226	0.813978
29561	1.89226	0.262163
29562	3.78452	0.635352
29563	1.89226	2.1
29564	1.89226	1.06377
29565	1.89226	2.1
29566	1.89226	1.3681
29567	3.78453	2.1
29568	1.40182	2.1
29569	1.55686	1.501
29570	1.24509	2.1
29571	1.38426	1.46475
29572	2.78608	2.1
29573	1.55686	1.50437
29574	1.38963	0.386282
29575	1.2064	1.36706
29576	1.2064	0.464805
29577	1.38426	1.4365

29578	1.56749	0.60952
29579	0.810946	0.829244
29580	0.810946	0.345635
29581	0.810945	0.884422
29582	0.810945	1.49583
29583	0.810945	0.778437
29584	0.810945	0.728354
29585	1.89226	0.427077
29586	1.35169	0.573845
29587	1.08132	1.53673
29588	1.08132	0.245114
29589	1.08132	0.670243
29590	1.28418	0.525927
29591	1.59382	0.527825
29592	1.78289	0.668937
29593	1.51021	0.396229
29594	3.10403	0.473803
29595	1.76897	0.974197
29596	1.59382	0.47504
29597	1.78289	0.697569
29598	1.94302	0.732112
29599	3.53684	0.782424
29600	1.89226	0.464652
29601	1.89226	0.595563
29602	1.89226	0.698768
29603	1.89226	0.38631
29604	3.78453	0.466422
29605	1.89227	0.515427
29606	1.89227	0.330396
29607	1.89227	0.330641
29608	1.89227	0.406032
29609	1.89227	0.395436
29610	1.89227	0.468831
29611	1.78286	0.669464
29612	1.51019	0.376222
29613	1.79979	0.486976
29614	2.02511	0.510277
29615	3.5353	0.538435
29616	1.78286	0.691638
29617	1.94299	0.69847
29618	2.17831	0.912533
29619	2.02511	0.511795
29620	3.9681	0.589604
29621	1.89224	0.665096
29622	1.89224	0.40755
29623	1.89224	0.485551
29624	1.89224	0.623838
29625	3.78448	0.582115
29626	1.89224	0.483161
29627	1.89224	0.333162
29628	1.89224	0.360851
29629	1.89224	0.432489
29630	1.89224	0.392546
29631	1.89224	0.456033
29632	1.83198	0.422882
29633	1.0961	0.231519
29634	1.39448	0.379155
29635	1.99568	0.335269

29636	3.09178	0.373756
29637	1.25503	0.325009
29638	1.99568	0.294282
29639	2.3661	0.433661
29640	1.70302	0.28458
29641	3.55833	0.270885
29642	2.69899	0.164663
29643	1.85796	0.149119
29644	2.11407	0.143253
29645	1.36769	0.133512
29646	3.97203	0.126726
29647	1.89226	0.209348
29648	1.89226	0.185033
29649	1.89226	0.177748
29650	1.89226	0.139862
29651	3.78452	0.208765
29652	1.89225	0.148224
29653	2.63355	0.150637
29654	2.09529	0.142986
29655	2.09529	0.156221
29656	1.89225	0.162088
29657	1.89225	0.165004
29658	1.89226	0.227601
29659	1.89226	0.256915
29660	1.89226	0.199223
29661	1.89226	0.319666
29662	1.89226	0.187585
29663	1.89226	0.289761
29664	2.12204	0.188522
29665	1.89226	0.250013
29666	2.05798	0.180006
29667	2.01954	0.18923
29668	1.70302	0.211976
29669	1.3865	0.254258
29670	1.28326	0.273518
29671	1.33326	0.558871
29672	1.19052	0.186273
29673	1.25369	0.526493
29674	2.53694	0.478741
29675	1.33327	0.546239
29676	1.27339	0.776123
29677	1.25369	0.49528
29678	1.19733	1.33635
29679	2.52708	0.60876
29680	1.19052	0.212938
29681	1.25369	0.526617
29682	1.10938	0.199462
29683	1.17614	0.587153
29684	2.36307	0.305783
29685	1.25369	0.447806
29686	1.19733	1.54578
29687	1.17614	0.59743
29688	1.11868	1.17714
29689	2.37237	0.765174
29690	1.10747	0.25078
29691	1.47688	0.256195
29692	1.97732	0.258235
29693	1.62206	0.266339

29694	3.09894	0.257951
29695	1.89226	0.260698
29696	1.89226	0.282575
29697	1.89226	0.25754
29698	1.89226	0.271146
29699	1.89226	0.264062
29700	1.89226	0.271298
29701	1.62206	0.256652
29702	1.70302	0.272292
29703	1.22962	0.268182
29704	1.1893	0.268946
29705	2.15177	0.258512
29706	2.08121	0.255729
29707	1.97732	0.265231
29708	2.08121	0.263275
29709	1.62206	0.259844
29710	1.62206	0.269765
29711	2.41464	0.259898
29712	2.41464	0.264666
29713	1.89226	0.267062
29714	1.89226	0.284727
29715	1.89226	0.27612
29716	1.89226	0.284689
29717	3.78452	0.278709
29718	0.946133	0.2481
29719	0.946133	0.276311
29720	0.946132	0.272883
29721	0.946132	0.275729
29722	0.946132	0.281568
29723	0.946132	0.280051
29724	0.946133	0.274612
29725	0.946133	0.275529
29726	0.946132	0.275737
29727	0.946132	0.308089
29728	0.946132	0.282357
29729	0.946132	0.250859
29730	1.83157	0.691498
29731	2.19989	0.697724
29732	2.20664	0.614759
29733	1.81487	0.630685
29734	4.03146	0.731422
29735	2.41055	2.1
29736	2.19989	1.46963
29737	2.20665	1.32995
29738	2.38023	1.34611
29739	4.61044	1.12243
29740	2.19989	1.66328
29741	2.22558	1.6615
29742	2.38023	1.42537
29743	2.07065	0.896539
29744	4.42547	1.20228
29745	2.19988	0.808535
29746	1.70303	0.357903
29747	1.89939	0.707109
29748	1.86357	0.66293
29749	3.78536	0.591874
29750	1.04921	2.1
29751	1.30711	2.1

29752	1.53076	2.1
29753	1.22927	2.1
29754	2.57997	2.1
29755	1.53076	2.1
29756	1.46568	2.1
29757	1.56056	2.1
29758	1.22927	2.1
29759	1.86206	2.1
29760	1.53076	2.1
29761	1.18353	2.1
29762	2.28593	2.00502
29763	1.16979	2.1
29764	2.2731	1.6341
29765	3.45571	2.1
29766	0.854776	1.4378
29767	0.854776	2.1
29768	0.855641	2.1
29769	0.855641	2.1
29770	1.12552	1.85501
29771	1.13655	2.1
29772	0.57216	2.1
29773	0.584893	2.1
29774	0.57216	2.1
29775	1.12552	2.1
29776	0.584893	2.1
29777	1.13655	2.1
29778	1.54462	2.1
29779	2.76309	2.1
29780	1.34869	2.1
29781	2.48573	2.1
29782	4.03035	2.1
29783	1.35328	1.01572
29784	1.0383	0.873682
29785	1.03318	1.43543
29786	0.704834	1.31743
29787	1.0257	1.32739
29788	1.0257	0.922716
29789	1.03241	0.761078
29790	1.03241	1.44053
29791	1.35328	1.12273
29792	1.03319	1.46428
29793	1.0383	1.28091
29794	0.704835	1.21575
29795	2.27126	0.307522
29796	2.77291	1.23812
29797	1.81771	0.665141
29798	2.6111	0.536376
29799	4.88236	0.6877
29800	2.77291	0.916105
29801	1.89227	0.679228
29802	1.89227	0.588812
29803	1.89227	1.4983
29804	2.6111	0.496446
29805	2.6111	1.61395
29806	2.22061	0.630874
29807	2.41522	0.585493
29808	2.41522	0.437366
29809	2.6111	0.887215

29810	1.75765	0.407102
29811	1.95353	1.34606
29812	2.27502	0.784098
29813	1.89227	1.45914
29814	1.89227	0.78792
29815	2.8189	0.60311
29816	4.71116	0.688725
29817	1.89226	0.626845
29818	1.89226	1.39297
29819	1.89226	0.85268
29820	1.89226	0.723473
29821	1.89226	1.16854
29822	1.89226	0.937483
29823	1.89226	1.4401
29824	1.89226	1.36925
29825	1.89226	1.21852
29826	1.89226	0.966911
29827	3.78453	1.40261
29828	1.89226	1.34411
29829	2.57079	0.724974
29830	2.5932	1.01467
29831	1.89226	0.962401
29832	4.48546	0.888366
29833	0.589396	1.02039
29834	0.641454	0.790387
29835	0.814062	1.23455
29836	0.726518	1.04337
29837	0.675126	1.1669
29838	0.675125	1.03832
29839	0.692847	1.21773
29840	0.692847	1.02507
29841	0.641454	0.872298
29842	0.814062	1.13526
29843	0.726518	1.23304
29844	0.589396	1.04743
29845	1.89226	1.28552
29846	1.89226	0.977609
29847	1.89226	1.26693
29848	1.89226	1.45006
29849	1.89226	1.08744
29850	1.89226	1.39459
29851	1.16906	0.590034
29852	1.2966	0.941494
29853	0.946132	0.915857
29854	0.946132	1.40349
29855	1.16906	0.937764
29856	1.16906	0.665942
29857	0.946132	0.972691
29858	0.946132	1.11595
29859	1.16906	1.21112
29860	0.946131	1.10884
29861	1.2966	0.976471
29862	0.946132	1.44495
29863	0.641455	1.04887
29864	0.567191	0.78935
29865	0.807594	1.2888
29866	0.814062	1.09991
29867	0.673735	1.30751

29868	0.673734	0.899378
29869	0.707519	1.05768
29870	0.707519	1.30151
29871	0.807594	1.22583
29872	0.56719	1.44395
29873	0.641455	1.23481
29874	0.814063	1.47294
29875	1.89225	2.1
29876	1.89225	2.1
29877	1.89225	2.1
29878	1.89225	2.1
29879	3.7845	2.1
29880	1.89225	2.1
29881	1.89225	2.1
29882	1.89225	2.1
29883	1.89225	2.1
29884	1.89225	2.1
29885	1.89225	2.1
29886	1.89226	2.1
29887	1.89226	2.1
29888	1.89226	2.1
29889	1.89226	2.1
29890	3.78452	2.1
29891	1.89226	2.1
29892	1.89226	2.1
29893	1.89226	2.1
29894	1.89226	2.1
29895	1.89226	2.1
29896	1.89226	2.1
29897	1.89226	2.1
29898	1.89226	2.1
29899	1.89226	2.1
29900	1.89226	2.1
29901	3.78452	2.1
29902	1.89226	2.1
29903	1.38928	2.1
29904	1.89226	2.1
29905	1.51378	2.1
29906	1.89061	2.1
29907	1.89226	2.1
29908	1.89226	2.1
29909	1.89226	1.57313
29910	1.89226	1.54848
29911	1.89226	2.1
29912	3.78453	2.06552
29913	1.04572	2.1
29914	0.994213	2.1
29915	0.912466	1.66389
29916	0.912466	1.91904
29917	0.947743	1.80872
29918	1.00388	2.1
29919	0.900913	1.55883
29920	0.900913	2.1
29921	0.900915	2.08407
29922	0.946131	2.07792
29923	0.946131	2.07679
29924	0.999196	2.07608
29925	1.89226	1.45827

29926	1.89226	2.1
29927	1.89226	2.07713
29928	1.89226	1.68554
29929	3.78453	2.00364
29930	0.946161	2.1
29931	1.1354	2.1
29932	0.850847	2.1
29933	0.850847	2.1
29934	1.01445	2.1
29935	1.02106	2.1
29936	0.946131	2.1
29937	0.946131	2.07875
29938	1.13537	2.1
29939	1.01444	2.1
29940	0.850877	2.1
29941	0.946131	2.1
29942	1.80183	2.1
29943	1.89226	1.76908
29944	1.89226	1.72505
29945	1.89226	2.1
29946	1.79606	1.7006
29947	1.79606	2.1
29948	1.24001	1.67625
29949	1.41822	1.36027
29950	1.95665	0.823318
29951	1.77342	1.27918
29952	3.19666	1.14287
29953	1.95665	1.10388
29954	1.44859	1.55698
29955	1.41821	1.46579
29956	1.41821	1.44063
29957	1.95543	1.10884
29958	1.95543	1.2798
29959	1.95665	0.997157
29960	1.77342	1.35652
29961	2.33611	1.18395
29962	2.49694	0.782355
29963	4.29276	1.33197
29964	1.95665	1.31908
29965	1.95544	1.27588
29966	2.4728	0.686669
29967	2.49693	1.15222
29968	4.42945	1.04612
29969	2.24937	2.1
29970	2.49053	2.1
29971	2.16323	2.1
29972	2.36377	2.1
29973	4.61314	2.1
29974	2.49053	2.1
29975	2.58639	2.1
29976	2.36377	2.1
29977	2.47395	2.1
29978	4.95016	2.1
29979	2.45054	2.1
29980	2.36377	2.1
29981	1.92911	2.1
29982	2.181	2.1
29983	4.29288	2.1

29984	2.36377	2.1
29985	2.47395	2.1
29986	2.181 2.1	
29987	2.29881	2.1
29988	4.66258	2.1
29989	1.57885	2.1
29990	1.13339	2.1
29991	1.48594	2.1
29992	1.13322	2.1
29993	2.71207	2.1
29994	1.48596	2.1
29995	1.20761	2.1
29996	1.20761	2.1
29997	1.13323	2.1
29998	1.46568	2.1
29999	1.3913	2.1
30000	1.89226	0.535912
30001	1.89226	1.10663
30002	1.89226	0.645292
30003	1.89226	0.914977
30004	1.89226	0.509251
30005	1.89226	0.688591
30006	1.11712	0.927703
30007	0.946129	1.09391
30008	0.686934	0.693326
30009	0.946133	0.700943
30010	0.9461	0.917343
30011	1.10729	1.99163
30012	0.686963	1.13459
30013	0.686963	0.547383
30014	1.10945	0.991243
30015	0.80397	1.98629
30016	0.946131	0.705341
30017	0.946131	1.18176
30018	1.89226	2.1
30019	1.89226	1.28088
30020	1.89226	0.945651
30021	1.89226	1.15487
30022	3.78452	1.16768
30023	1.22615	2.1
30024	1.3439	2.1
30025	1.47868	2.1
30026	1.55998	1.07437
30027	2.78613	2.1
30028	1.04973	2.1
30029	1.47868	2.1
30030	1.20149	2.1
30031	1.61721	2.1
30032	2.68017	2.1
30033	1.89226	2.05547
30034	1.89226	2.08277
30035	1.89226	2.1
30036	1.89226	0.715512
30037	3.78452	1.99951
30038	0.946133	0.699237
30039	0.946133	1.54243
30040	0.946128	1.92611
30041	0.946128	1.93953

30042	0.946131	0.667853
30043	0.946131	1.24676
30044	0.946131	1.89433
30045	0.946131	0.787576
30046	0.94613	1.53355
30047	0.94613	1.09856
30048	0.946131	1.35044
30049	0.946131	1.05502
30050	0.798126	2.03431
30051	0.798126	1.78098
30052	0.805719	2.1
30053	0.805719	2.1
30054	0.779992	2.1
30055	0.864507	1.77822
30056	0.739338	2.09801
30057	0.808607	2.1
30058	0.808606	2.1
30059	0.864506	2.1
30060	0.739339	2.1
30061	0.779993	2.1
30062	0.946128	2.1
30063	0.946128	1.93735
30064	1.18959	0.941191
30065	1.00304	2.1
30066	1.00304	2.1
30067	1.00304	1.6636
30068	0.94613	1.80531
30069	0.94613	0.964567
30070	0.94613	1.94276
30071	1.18959	1.85319
30072	1.00304	1.70918
30073	0.94613	1.79601
30074	2.19163	2.1
30075	1.89226	1.66987
30076	2.16541	2.1
30077	1.89226	1.03435
30078	4.08389	1.40203
30079	1.89226	2.1
30080	1.89226	1.44736
30081	1.89226	1.65421
30082	1.89226	1.00738
30083	3.78452	1.76355
30084	0.964782	2.02096
30085	1.30151	0.96497
30086	1.30318	0.948179
30087	1.30318	2.09118
30088	0.949134	0.779589
30089	0.949134	1.77997
30090	2.15005	2.1
30091	1.89226	1.20571
30092	2.16541	1.59954
30093	2.16541	2.1
30094	1.89226	0.86746
30095	1.89226	2.1
30096	1.89226	1.8822
30097	1.89226	1.75561
30098	1.89226	1.496
30099	1.89226	0.688005

30100	3.78452	1.20132
30101	0.949134	2.1
30102	1.30151	1.68913
30103	0.949134	1.42031
30104	1.32143	0.972552
30105	2.25064	2.0543
30106	0.982859	0.189054
30107	1.28326	0.376366
30108	1.19052	0.233311
30109	1.19052	0.41504
30110	1.10476	0.201858
30111	1.10476	0.408699
30112	1.37265	0.669298
30113	1.38247	0.826749
30114	1.36636	0.987661
30115	1.3294	0.62967
30116	2.71187	0.726414
30117	1.89226	0.860679
30118	1.89226	0.516748
30119	1.89226	0.65348
30120	1.89226	0.731038
30121	1.89226	0.660822
30122	1.89226	0.777864
30123	1.36636	1.22292
30124	1.3294	0.576718
30125	1.30001	0.758181
30126	1.36416	0.990766
30127	2.69356	0.870296
30128	1.89226	1.03005
30129	1.89226	0.555233
30130	1.89226	0.687069
30131	1.89226	0.814746
30132	1.89226	0.612847
30133	1.89226	0.725443
30134	2.83701	0.666683
30135	1.89226	0.672685
30136	1.89226	0.977795
30137	2.79312	0.501989
30138	4.68539	0.598454
30139	1.44288	2.1
30140	0.948677	2.1
30141	1.84076	2.1
30142	1.42884	2.1
30143	2.78943	2.1
30144	1.89226	1.77214
30145	1.89226	2.1
30146	1.89226	2.1
30147	1.89226	1.78406
30148	3.78452	2.1
30149	0.909402	1.61218
30150	0.909402	1.86089
30151	1.3063	2.1
30152	1.56757	2.1
30153	1.00038	1.49331
30154	1.5235	2.1
30155	0.946127	2.1
30156	0.946127	1.54742
30157	0.946128	2.1

30158	1.26957	2.1
30159	1.00038	2.1
30160	0.946129	2.1
30161	1.17256	2.1
30162	2.2846	1.81372
30163	1.1844	2.1
30164	2.28904	1.06966
30165	3.469 2.1	
30166	0.867886	1.07475
30167	0.867886	2.1
30168	0.867277	2.1
30169	0.867277	2.08569
30170	1.14296	1.3259
30171	1.14452	1.97158
30172	0.591766	2.1
30173	0.592199	2.1
30174	0.591766	2.1
30175	1.14296	2.1
30176	0.5922	2.1
30177	1.14452	2.1
30178	1.27408	0.283485
30179	1.56339	0.336913
30180	1.52522	0.337817
30181	1.34447	0.273179
30182	2.90786	0.283933
30183	1.27408	0.314966
30184	1.01121	0.267993
30185	0.991868	0.285625
30186	1.34447	0.327817
30187	2.35569	0.303064
30188	0.944818	0.377898
30189	0.883008	0.356555
30190	0.66957	0.305643
30191	0.616883	0.301309
30192	0.755679	0.298772
30193	0.755679	0.353748
30194	0.791912	0.415137
30195	0.701943	0.274139
30196	0.901541	0.333799
30197	0.634847	0.354628
30198	0.82781	0.34609
30199	0.655405	0.345708
30200	1.73819	2.1
30201	1.70303	2.1
30202	1.70303	2.1
30203	1.70303	2.1
30204	1.94516	2.1
30205	1.94517	2.1
30206	0.94994	2.1
30207	2.07469	2.1
30208	1.08506	2.1
30209	2.0815	2.1
30210	3.12902	2.1
30211	0.946131	2.1
30212	0.946131	2.1
30213	0.946131	2.1
30214	0.946131	2.08444
30215	0.946128	2.08302

30216	0.946128	2.1
30217	0.946133	2.1
30218	0.946133	2.1
30219	0.946131	2.1
30220	0.946131	2.1
30221	0.94613	2.1
30222	0.94613	2.1
30223	2.51796	2.1
30224	1.64193	2.1
30225	2.51172	2.1
30226	1.68893	2.1
30227	4.20689	2.1
30228	2.51172	2.1
30229	1.70002	2.1
30230	1.70002	2.1
30231	1.68893	2.1
30232	2.48689	2.1
30233	2.4758	2.1
30234	2.27963	0.0884533
30235	2.21582	0.0759031
30236	2.60486	0.0751138
30237	2.5105	0.0884182
30238	4.79013	0.0817643
30239	2.21582	0.0757193
30240	2.02069	0.0883663
30241	2.5105	0.0886116
30242	2.28615	0.0732973
30243	4.53119	0.0795356
30244	2.60486	0.0756282
30245	2.5105	0.0919855
30246	2.55791	0.0953052
30247	2.47141	0.0745839
30248	5.06842	0.0823884
30249	2.5105	0.0922243
30250	2.28615	0.0756283
30251	2.47141	0.0752049
30252	2.23337	0.0903007
30253	4.74387	0.0833779
30254	2.06984	2.1
30255	0.967499	2.1
30256	2.12435	2.1
30257	2.0021	2.1
30258	1.08975	2.1
30259	0.967499	2.1
30260	1.89226	0.260007
30261	1.89226	0.24853
30262	1.89226	0.272737
30263	1.89226	0.260522
30264	1.89226	0.238531
30265	1.89226	0.23192
30266	1.89226	1.61986
30267	1.89226	2.1
30268	1.89226	2.1
30269	1.89226	1.95975
30270	3.78452	2.1
30271	1.89227	2.1
30272	1.89227	2.1
30273	1.89227	2.1

30274	1.89227	2.1
30275	3.78453	2.1
30276	0.55882	2.1
30277	0.696462	2.1
30278	1.07003	2.1
30279	0.937179	2.1
30280	0.818077	2.1
30281	0.818077	2.1
30282	0.815565	2.1
30283	0.815565	2.1
30284	0.696462	2.1
30285	1.07003	2.1
30286	0.93718	2.1
30287	0.55882	2.1
30288	2.53857	2.03138
30289	1.87436	2.1
30290	2.27541	2.1
30291	2.27541	2.1
30292	2.14005	2.1
30293	2.14005	2.1
30294	1.89226	2.1
30295	1.89226	1.84698
30296	1.89226	1.01992
30297	1.89226	2.1
30298	3.78452	2.07781
30299	1.89227	2.1
30300	1.89227	2.1
30301	1.89227	2.1
30302	1.89227	2.1
30303	3.78453	2.1
30304	1.39292	2.1
30305	2.14006	2.1
30306	2.41083	2.1
30307	1.68761	2.1
30308	3.80376	2.1
30309	2.53857	1.8913
30310	2.14005	2.1
30311	2.41083	2.1
30312	2.82043	2.1
30313	4.9494	2.1
30314	1.90439	0.339682
30315	1.51835	0.346542
30316	1.20473	0.292777
30317	0.947693	0.358303
30318	2.85208	0.351452
30319	2.586	2.1
30320	1.46262	2.1
30321	2.14009	2.1
30322	2.78101	2.1
30323	4.72609	2.1
30324	1.89224	2.1
30325	2.78101	2.1
30326	2.586	2.1
30327	2.586	2.1
30328	1.89224	2.1
30329	1.89224	2.1
30330	0.994293	2.1
30331	1.19	2.1

30332	1.09483	2.1
30333	1.09483	2.1
30334	1.06827	2.1
30335	1.06827	2.1
30336	1.89226	2.1
30337	1.89226	2.1
30338	1.89226	2.1
30339	1.89226	2.1
30340	3.78452	2.1
30341	0.946132	2.1
30342	0.946132	2.1
30343	1.31912	2.1
30344	1.3572	2.1
30345	0.946131	2.1
30346	0.946131	2.1
30347	1.31912	2.1
30348	1.31912	2.1
30349	0.946131	2.1
30350	1.31912	2.1
30351	1.35721	2.1
30352	0.946131	2.1
30353	2.24577	0.473353
30354	1.09408	0.360118
30355	1.60239	0.420479
30356	1.09408	0.40256
30357	2.39404	0.453102
30358	2.07382	0.353737
30359	1.70303	0.298355
30360	1.3216	0.376836
30361	1.23104	0.300006
30362	1.05475	0.332368
30363	2.60408	0.320622
30364	1.89226	0.44152
30365	1.89226	0.437646
30366	1.89226	0.359564
30367	1.89226	0.449399
30368	3.78452	0.453135
30369	1.89226	0.279215
30370	2.3845	0.273076
30371	2.6875	0.271892
30372	1.89226	0.262911
30373	2.6875	0.270236
30374	1.89226	0.266098
30375	1.46759	2.1
30376	1.17585	2.1
30377	1.85189	2.1
30378	1.39223	2.1
30379	2.85981	2.1
30380	1.85189	2.1
30381	1.71255	2.1
30382	1.71255	2.1
30383	1.39223	2.1
30384	2.28511	2.1
30385	1.96479	2.1
30386	1.89227	0.750111
30387	1.89227	0.874768
30388	1.89227	1.02905
30389	1.89227	0.69869

30390	1.89227	1.2684
30391	1.89227	0.78461
30392	1.89226	0.790973
30393	1.89226	0.830889
30394	1.89226	1.02731
30395	1.89226	0.686806
30396	1.89226	1.27846
30397	1.89226	0.756164
30398	1.89226	0.763849
30399	1.89226	0.920058
30400	1.89226	0.97313
30401	1.89226	0.673918
30402	1.89226	1.31486
30403	1.89226	0.779964
30404	1.89226	0.678276
30405	2.77788	0.720185
30406	2.21062	0.8032
30407	2.21062	0.627517
30408	1.89226	0.939675
30409	1.89226	0.674309
30410	0.868548	0.667858
30411	0.610104	0.743206
30412	0.796678	0.65866
30413	0.865021	0.617509
30414	1.0883	0.645429
30415	0.904938	0.720191
30416	2.21062	0.692299
30417	1.49548	0.661845
30418	1.2068	0.818545
30419	1.54764	0.663945
30420	0.790005	0.62455
30421	1.89226	1.04355
30422	2.67457	2.1
30423	1.89226	1.1775
30424	2.56352	2.1
30425	4.56683	2.1
30426	2.67456	2.1
30427	2.20011	2.1
30428	2.56351	2.1
30429	2.11083	2.1
30430	4.78539	2.1
30431	2.56348	2.1
30432	2.1108	2.1
30433	2.45763	2.1
30434	2.00986	2.1
30435	4.56843	2.1
30436	0.946133	0.0753496
30437	0.946133	0.0804674
30438	0.946132	0.0719725
30439	0.946132	0.0768257
30440	0.946132	0.0746321
30441	0.946132	0.0759193
30442	0.946132	0.0755861
30443	0.946132	0.0778019
30444	0.946132	0.0745473
30445	0.946132	0.0732046
30446	0.946133	0.0777021
30447	0.946133	0.079231

30448	1.45927	0.847225
30449	1.25133	1.17071
30450	1.00082	1.07373
30451	1.41689	1.13953
30452	1.0432	0.914135
30453	1.45927	1.07457
30454	1.41689	0.985173
30455	1.29579	1.36614
30456	1.34874	1.14971
30457	1.25133	1.14955
30458	2.76563	1.47888
30459	0.675555	1.26059
30460	0.674371	1.18728
30461	0.625664	1.0906
30462	0.523133	1.11724
30463	0.676509	1.18866
30464	0.676509	1.38458
30465	0.62471	1.17098
30466	0.62471	1.01476
30467	0.675556	1.03543
30468	0.523132	0.951722
30469	0.674373	0.964561
30470	0.625663	1.02865
30471	1.89226	0.763629
30472	1.89226	0.760152
30473	1.89226	0.751918
30474	1.89226	0.775942
30475	3.78453	0.985637
30476	0.851517	1.17702
30477	0.946133	0.811795
30478	0.946132	0.891593
30479	0.946132	1.10963
30480	0.946133	0.699782
30481	0.851517	1.33963
30482	0.946132	0.839382
30483	0.946132	0.741229
30484	0.851516	0.827602
30485	0.851516	0.722238
30486	0.946133	1.05356
30487	0.946133	0.824779
30488	0.569995	0.816117
30489	0.816895	1.03871
30490	1.33893	1.08656
30491	1.18795	0.907225
30492	1.18795	0.842917
30493	0.569995	1.08618
30494	0.816895	0.933271
30495	1.33893	1.09422
30496	0.859572	1.21662
30497	0.859572	1.08416
30498	1.04936	0.902801
30499	1.04936	0.768033
30500	1.89226	2.1
30501	1.89226	2.1
30502	2.55618	2.1
30503	2.55618	2.1
30504	2.66983	2.1
30505	2.00591	2.1

30506	1.89226	0.559532
30507	2.64586	0.909969
30508	2.83331	1.64035
30509	2.83331	0.542185
30510	1.89226	1.61403
30511	1.89226	0.478205
30512	2.64586	1.44748
30513	2.0815	2.1
30514	1.99985	1.95592
30515	1.94005	2.1
30516	2.83332	1.63489
30517	2.74859	2.1
30518	1.98061	0.73439
30519	1.68356	2.1
30520	1.83879	0.86335
30521	1.55987	2.1
30522	3.54048	2.04287
30523	1.68356	2.1
30524	1.20969	2.1
30525	1.20969	2.1
30526	1.30751	2.1
30527	1.55987	2.1
30528	1.65769	2.1
30529	1.83879	0.905151
30530	1.55987	2.1
30531	1.68443	0.739287
30532	1.4449	2.1
30533	3.2443	1.96197
30534	0.660368	2.1
30535	0.660368	2.1
30536	0.666922	2.1
30537	0.666922	2.1
30538	0.560377	2.1
30539	0.604842	2.1
30540	0.779935	2.1
30541	0.722448	2.1
30542	0.722446	2.1
30543	0.560377	2.1
30544	0.604844	2.1
30545	0.779934	2.1
30546	1.89227	2.1
30547	1.89227	2.1
30548	1.89227	2.1
30549	1.89227	2.1
30550	3.78453	2.1
30551	1.42896	2.1
30552	1.23037	2.1
30553	1.42896	2.1
30554	1.474	2.1
30555	1.2016	2.1
30556	1.24665	2.1
30557	1.89226	2.1
30558	2.7188	2.1
30559	1.89226	2.1
30560	2.70702	2.1
30561	4.61106	2.1
30562	1.89226	2.1
30563	1.89226	2.1

30564	1.89226	2.1
30565	1.89226	2.1
30566	3.78453	2.1
30567	0.644603	2.1
30568	0.644603	2.1
30569	0.648272	2.1
30570	0.648272	2.1
30571	0.585644	2.1
30572	0.600802	2.1
30573	0.692073	2.1
30574	0.714478	2.1
30575	0.585644	2.1
30576	0.692073	2.1
30577	0.714478	2.1
30578	0.600802	2.1
30579	1.35351	2.1
30580	1.35351	2.1
30581	0.946131	2.1
30582	0.946131	2.1
30583	1.34392	2.1
30584	1.35351	2.1
30585	0.946132	2.1
30586	0.946132	2.1
30587	0.946132	2.1
30588	1.34392	2.1
30589	0.946132	2.1
30590	1.35351	2.1
30591	1.87609	2.1
30592	1.79155	2.1
30593	1.79155	2.1
30594	1.89226	2.1
30595	1.89226	2.1
30596	1.99211	2.1
30597	1.89226	2.1
30598	1.71706	2.1
30599	1.71706	2.1
30600	1.79155	2.1
30601	1.89226	2.1
30602	1.96675	2.1
30603	1.46869	2.1
30604	1.89226	2.1
30605	1.89226	2.1
30606	1.89226	2.1
30607	1.5001	2.1
30608	1.5001	1.93624
30609	1.89226	2.09436
30610	1.89226	2.1
30611	1.89226	2.1
30612	1.89226	2.1
30613	1.89226	2.1
30614	1.89226	1.30311
30615	1.21408	2.1
30616	2.23563	2.1
30617	2.34021	2.1
30618	2.34021	2.1
30619	1.17285	2.1
30620	1.17285	2.1
30621	1.89226	2.1

30622	1.89226	2.1
30623	1.47937	2.1
30624	1.89226	2.1
30625	1.14822	2.1
30626	1.46869	1.54684
30627	1.89226	1.84174
30628	1.89226	2.1
30629	1.89226	2.1
30630	1.89226	2.02775
30631	1.89226	2.1
30632	1.89226	1.08393
30633	1.25716	2.1
30634	2.34021	2.1
30635	2.4542	2.1
30636	2.4542	2.1
30637	1.21408	2.1
30638	1.21408	2.1
30639	1.66824	2.1
30640	2.44883	1.80821
30641	1.93003	2.1
30642	2.24795	2.0945
30643	3.88907	2.1
30644	1.89319	2.1
30645	2.24795	2.1
30646	2.82724	2.1
30647	2.31318	2.1
30648	2.30868	2.1
30649	1.93003	2.0084
30650	1.89226	0.941846
30651	1.42589	2.1
30652	1.42589	1.44954
30653	1.80501	2.1
30654	1.89226	1.33769
30655	2.27137	2.1
30656	1.21067	0.348401
30657	2.39166	0.523116
30658	1.73577	0.42781
30659	1.73577	0.420153
30660	1.53584	0.409102
30661	1.53584	0.362518
30662	1.35025	1.10753
30663	2.0599	0.850828
30664	1.51084	0.869914
30665	2.27218	0.470856
30666	3.57074	0.926664
30667	2.0599	0.733231
30668	2.51818	0.957026
30669	2.27218	0.382417
30670	2.66532	0.84497
30671	4.72522	0.656326
30672	1.67115	0.959291
30673	2.27218	0.761327
30674	2.39605	0.917443
30675	2.39605	0.760474
30676	1.51084	0.90343
30677	1.51084	0.932953
30678	2.27218	0.654971
30679	2.66532	1.2069

30680	2.39605	0.539939
30681	2.76829	0.787855
30682	5.06137	0.720682
30683	2.08684	0.770524
30684	2.36033	1.10756
30685	1.89224	0.700086
30686	1.89224	0.999199
30687	4.25257	0.770294
30688	2.41473	0.302592
30689	1.00307	0.301843
30690	2.01365	0.323214
30691	1.00307	0.387522
30692	3.05113	0.256349
30693	2.04056	0.346352
30694	1.11099	2.1
30695	2.25103	2.1
30696	2.22227	2.1
30697	2.22227	2.01098
30698	1.14431	2.1
30699	1.14431	2.08495
30700	1.07485	2.1
30701	2.22229	2.06853
30702	2.19163	2.1
30703	2.19163	2.1
30704	1.111 2.1	
30705	1.111 2.1	
30706	1.0547	1.46481
30707	1.19886	2.1
30708	0.946131	2.1
30709	0.946131	2.08235
30710	0.946131	2.08571
30711	0.946131	1.51639
30712	1.19886	2.1
30713	1.19886	2.1
30714	1.0547	2.1
30715	0.946131	2.1
30716	0.946132	2.1
30717	1.19886	2.1
30718	1.89417	1.53075
30719	2.64424	0.341997
30720	1.76585	1.11499
30721	2.47695	0.626895
30722	4.37112	0.761566
30723	1.89226	1.69575
30724	1.89226	0.700501
30725	1.89226	2.06644
30726	1.89226	0.633775
30727	3.78453	0.844992
30728	0.930281	1.21055
30729	1.0547	2.1
30730	0.946131	2.1
30731	0.946131	1.9898
30732	0.930309	1.9374
30733	0.930309	1.26722
30734	1.07052	2.1
30735	1.07052	2.1
30736	0.93028	2.1
30737	0.946131	2.1

30738	1.0547	2.1
30739	0.946131	2.1
30740	1.00015	0.313683
30741	1.00015	0.842324
30742	1.01918	1.01674
30743	1.01918	0.560263
30744	1.17437	0.573724
30745	1.23847	0.300899
30746	0.882926	1.04534
30747	0.780859	0.838649
30748	0.780859	0.634817
30749	1.17438	0.661756
30750	0.882925	0.690244
30751	1.23848	0.690858
30752	1.89226	1.88169
30753	1.89226	0.590148
30754	1.89226	0.722121
30755	1.89226	1.33208
30756	1.89226	0.707185
30757	1.89226	1.52846
30758	1.22083	2.1
30759	1.29579	2.1
30760	1.26236	2.1
30761	1.06191	2.1
30762	1.42128	2.1
30763	1.22083	2.1
30764	1.42128	2.1
30765	1.45806	2.1
30766	1.43502	2.1
30767	1.29579	2.1
30768	1.5605	2.1
30769	1.42128	2.1
30770	1.02813	2.1
30771	1.12121	2.1
30772	1.40557	2.1
30773	1.35298	2.1
30774	1.1314	2.1
30775	1.28679	2.1
30776	1.1715	2.1
30777	1.1715	2.1
30778	0.975656	2.1
30779	1.24908	2.1
30780	0.98956	2.1
30781	1.2681	2.1
30782	0.946132	2.1
30783	0.946132	2.1
30784	1.31889	2.1
30785	1.31889	2.1
30786	1.16546	2.1
30787	1.31889	2.1
30788	0.946133	2.1
30789	0.946133	2.1
30790	0.946133	2.1
30791	1.31889	2.1
30792	0.946132	2.1
30793	1.16546	2.1
30794	2.51518	0.102358
30795	2.37584	0.115947

30796	1.11712	0.101547
30797	1.31242	0.122711
30798	3.68826	0.111043
30799	2.51518	0.105885
30800	1.26311	0.122969
30801	2.48977	0.10652
30802	2.48977	0.118835
30803	1.31242	0.103328
30804	1.31242	0.121427
30805	1.89226	0.44408
30806	1.89226	0.637087
30807	1.89226	0.370033
30808	1.89226	0.635024
30809	3.78452	0.505371
30810	2.04737	0.345307
30811	1.77122	0.629955
30812	1.94498	0.360473
30813	1.65174	0.564082
30814	3.71621	0.484046
30815	1.41077	1.57005
30816	1.29729	1.08265
30817	1.77123	0.571179
30818	1.77123	0.884272
30819	1.65175	0.565719
30820	1.29129	0.581753
30821	1.89226	0.562496
30822	1.89226	1.62964
30823	1.89226	1.80413
30824	1.89226	0.562176
30825	1.89226	1.57818
30826	1.89226	0.622281
30827	1.89226	0.37497
30828	1.89226	0.637543
30829	1.89226	0.294304
30830	1.89226	0.551408
30831	3.78451	0.460883
30832	1.94498	0.362514
30833	1.65174	0.577086
30834	1.7968	0.362537
30835	1.49831	0.541175
30836	3.44329	0.484982
30837	1.49831	0.543082
30838	1.29729	1.13495
30839	1.49831	1.5443
30840	1.65175	0.551415
30841	1.16858	1.13386
30842	1.32201	0.600318
30843	1.89226	0.511858
30844	1.89226	1.33383
30845	1.89226	0.544933
30846	1.89226	1.44348
30847	3.78452	0.652417
30848	1.89226	0.0938399
30849	1.89226	0.100189
30850	1.89226	0.10372
30851	1.89226	0.0945825
30852	1.89226	0.1018
30853	1.89226	0.0927965

30854	1.65324	0.619742
30855	1.26409	0.280139
30856	1.42491	0.276697
30857	1.42491	0.623699
30858	1.52268	0.309382
30859	1.52268	0.642413
30860	1.52268	1.00259
30861	1.26409	0.402822
30862	1.32697	0.574523
30863	1.05724	0.248668
30864	2.57991	0.48967
30865	1.89226	0.294243
30866	1.96548	0.319633
30867	2.0178	0.346038
30868	1.96548	0.306705
30869	1.94458	0.312444
30870	1.89226	0.272385
30871	1.99366	0.294124
30872	2.0178	0.327287
30873	1.89226	0.342797
30874	2.0178	0.313907
30875	1.75999	0.310731
30876	1.89226	0.279897
30877	0.946126	1.97604
30878	0.946126	2.1
30879	1.28228	2.1
30880	1.19444	2.1
30881	0.946127	2.1
30882	0.946127	1.98506
30883	1.19444	2.1
30884	1.19444	2.1
30885	0.946128	2.1
30886	1.19444	2.1
30887	1.28228	2.1
30888	0.946128	2.1
30889	1.89226	2.1
30890	1.89226	2.1
30891	1.89226	2.1
30892	1.89226	1.34077
30893	3.78452	2.1
30894	2.14104	2.1
30895	1.89226	2.1
30896	2.19948	2.1
30897	1.89226	2.1
30898	1.89226	2.1
30899	2.19948	2.1
30900	1.89226	2.1
30901	2.38888	2.1
30902	2.23932	2.1
30903	2.38888	2.1
30904	1.74269	2.1
30905	1.89226	2.1
30906	1.89226	2.1
30907	1.89226	1.20702
30908	1.89226	2.1
30909	1.89226	2.1
30910	3.78452	2.1
30911	0.946132	2.1

30912	0.946132	2.1
30913	1.07052	2.1
30914	1.07052	2.1
30915	0.946132	2.1
30916	0.946132	2.00724
30917	1.05132	2.1
30918	1.07052	2.1
30919	1.05132	2.1
30920	0.946132	2.1
30921	1.07052	2.1
30922	0.946133	2.1
30923	2.40171	2.1
30924	1.89226	2.1
30925	1.89226	2.1
30926	1.89226	2.09254
30927	2.42723	2.1
30928	2.42723	2.1
30929	1.89226	2.1
30930	1.89226	1.20861
30931	1.89226	2.1
30932	1.89226	1.48437
30933	3.78452	1.85839
30934	1.63661	2.1
30935	2.3532	1.09779
30936	2.33287	0.883692
30937	2.33287	2.1
30938	1.64158	0.809941
30939	1.64158	2.1
30940	2.38918	2.1
30941	1.89226	2.1
30942	1.89226	2.1
30943	1.89226	2.1
30944	2.40171	2.1
30945	2.40171	2.1
30946	1.89226	2.1
30947	1.89226	1.73666
30948	1.89226	2.1
30949	1.89226	1.44568
30950	1.89226	2.1
30951	1.89226	1.81295
30952	1.64158	2.1
30953	2.3532	1.35878
30954	1.6488	2.1
30955	2.37364	1.61413
30956	4.002 2.1	
30957	2.54752	0.513534
30958	1.42445	0.538689
30959	1.86697	0.555765
30960	1.86697	0.510871
30961	2.06666	0.556289
30962	2.06666	0.51882
30963	2.50872	0.39039
30964	2.06666	0.454185
30965	1.90439	0.418082
30966	1.51835	0.426795
30967	3.97105	0.407846
30968	0.755161	0.456758
30969	0.755161	0.437271

30970	0.793994	0.424825
30971	0.793994	0.455313
30972	0.712225	0.44288
30973	0.515826	0.449472
30974	1.03333	0.453005
30975	0.759175	0.439154
30976	0.759173	0.424115
30977	0.515827	0.361373
30978	0.712226	0.405005
30979	1.03333	0.430177
30980	1.69633	0.312395
30981	1.89226	0.314577
30982	1.62477	0.272725
30983	1.89226	0.290145
30984	1.62477	0.314945
30985	1.89226	0.328347
30986	1.89226	0.407577
30987	1.89226	0.513004
30988	1.89226	0.499825
30989	1.89226	0.483582
30990	3.78452	0.482378
30991	2.28622	0.313942
30992	1.09667	0.257117
30993	1.17506	0.243812
30994	2.24411	0.269889
30995	1.17506	0.339164
30996	2.24411	0.384602
30997	2.24741	2.1
30998	1.70303	2.1
30999	2.49168	2.1
31000	1.57936	2.1
31001	2.53729	2.1
31002	1.70303	2.1
31003	2.24741	2.1
31004	2.47557	2.1
31005	1.89227	2.1
31006	1.89227	2.1
31007	4.36784	2.1
31008	2.27016	0.13062
31009	1.10476	0.170258
31010	1.44103	0.137993
31011	1.10476	0.195728
31012	2.26791	0.137771
31013	1.93165	0.182129
31014	2.27016	0.126211
31015	1.89226	0.19211
31016	1.89226	0.146229
31017	2.4065	0.16842
31018	4.29876	0.165344
31019	2.27016	0.123393
31020	1.44103	0.15851
31021	1.53336	0.127988
31022	2.4065	0.163625
31023	3.84753	0.136078
31024	2.7542	0.360035
31025	1.89226	0.404842
31026	2.35027	0.391648
31027	1.89226	0.394319

31028	2.35027	0.37772
31029	1.89226	0.381514
31030	2.3301	2.1
31031	2.36071	1.26585
31032	2.07629	2.1
31033	2.01105	2.1
31034	4.37176	2.1
31035	1.86512	2.1
31036	2.01105	2.1
31037	2.01105	2.1
31038	2.3301	2.1
31039	1.57459	2.1
31040	1.89364	2.1
31041	2.52671	2.1
31042	2.01105	2.1
31043	1.78251	2.1
31044	1.70302	1.03731
31045	3.98362	2.1
31046	1.227 2.1	
31047	2.01105	2.1
31048	1.45869	2.1
31049	1.41713	2.1
31050	1.70302	2.1
31051	1.64994	2.1
31052	2.00989	2.1
31053	1.28938	2.1
31054	1.8769	2.1
31055	1.20969	2.1
31056	3.21958	2.1
31057	1.8769	2.1
31058	1.15864	2.1
31059	1.15864	2.1
31060	1.20969	2.1
31061	1.77353	2.1
31062	1.82458	2.1
31063	1.7986	2.1
31064	2.48717	2.1
31065	2.50161	2.1
31066	1.75869	2.1
31067	4.30021	2.1
31068	2.50161	2.1
31069	1.75251	2.1
31070	1.81678	2.1
31071	1.75869	2.1
31072	2.5597	2.1
31073	2.50161	2.1
31074	1.91334	0.627404
31075	1.85439	0.80841
31076	1.91334	0.676297
31077	1.70303	0.792015
31078	1.70303	0.584201
31079	1.49692	0.635526
31080	2.39986	0.30508
31081	1.89226	0.414757
31082	1.89226	0.396109
31083	2.50756	0.318215
31084	4.39982	0.341981
31085	2.39986	0.386175

31086	1.70303	0.307234
31087	1.75564	0.296826
31088	1.75904	0.359287
31089	2.50272	0.339634
31090	2.50756	0.381166
31091	1.89226	0.390944
31092	2.50756	0.31985
31093	2.47857	0.35578
31094	2.50756	0.421732
31095	1.86327	0.313452
31096	1.89226	0.366817
31097	1.22241	0.402774
31098	1.20827	0.421911
31099	0.772416	0.319484
31100	0.555208	0.362948
31101	1.004	0.318932
31102	1.004	0.381317
31103	0.93504	0.479082
31104	0.744364	0.311311
31105	1.2089	0.387323
31106	0.654935	0.398149
31107	0.773927	0.376169
31108	1.03618	0.403384
31109	1.89227	0.0764699
31110	1.89227	0.0802898
31111	1.89227	0.0675564
31112	1.89227	0.0694581
31113	3.78453	0.0723632
31114	1.89226	0.0716244
31115	1.89226	0.0778261
31116	1.89226	0.0653193
31117	1.89226	0.067975
31118	3.78452	0.0704422
31119	1.82643	0.0635621
31120	2.7084	0.0648508
31121	2.12654	0.0621645
31122	2.65614	0.0653135
31123	1.82643	0.0648352
31124	2.35603	0.0672685
31125	1.29385	0.0617382
31126	0.946133	0.0641378
31127	1.05492	0.0625136
31128	0.946132	0.0642622
31129	1.05492	0.0638905
31130	1.29385	0.0617979
31131	0.946133	0.0625449
31132	0.946133	0.0652733
31133	1.29385	0.0605379
31134	1.29385	0.0601672
31135	0.946132	0.0668939
31136	0.946132	0.0649904
31137	0.946132	0.0615291
31138	1.27329	0.0599521
31139	1.29385	0.0610067
31140	0.946129	0.0635905
31141	1.29385	0.0616265
31142	1.27328	0.0598439
31143	0.946131	0.0609754

31144	0.946131	0.0619268
31145	1.27328	0.0593688
31146	1.27328	0.0593418
31147	0.94613	0.0644253
31148	0.94613	0.0627494
31149	1.89226	1.50837
31150	1.89226	1.59636
31151	1.89226	0.840213
31152	1.89226	1.15163
31153	3.78453	1.25985
31154	1.89226	1.33293
31155	1.89226	1.59439
31156	1.89226	0.794039
31157	1.89226	1.35603
31158	3.78453	1.05551
31159	1.21488	1.57411
31160	1.14605	2.00206
31161	1.62974	1.29628
31162	1.52399	1.14774
31163	2.84463	1.63189
31164	0.577078	1.20138
31165	0.81251	1.8706
31166	0.821003	1.38894
31167	0.543413	1.72621
31168	0.683929	1.24739
31169	0.722433	2.05802
31170	0.730674	1.6725
31171	0.730674	1.10565
31172	0.857766	1.34932
31173	0.862119	1.2094
31174	0.600938	1.64126
31175	0.596094	1.42968
31176	1.89226	0.995767
31177	1.89226	1.42688
31178	1.89226	0.779438
31179	1.89226	1.21922
31180	3.78453	1.07334
31181	1.89226	0.919792
31182	1.89226	1.73057
31183	1.89226	0.886674
31184	1.89226	1.13532
31185	3.78453	1.25964
31186	1.62974	1.67435
31187	1.56958	1.81601
31188	2.2362	1.00323
31189	2.11424	1.35235
31190	3.86595	1.36019
31191	2.2362	1.09694
31192	1.85414	1.83934
31193	2.28211	0.806102
31194	1.78367	0.920819
31195	2.2362	1.55137
31196	1.73256	1.76572
31197	0.851539	1.36167
31198	0.851539	0.730247
31199	1.14454	1.01788
31200	0.938988	1.75317
31201	0.851485	1.36446

31202	0.920593	0.767666
31203	0.851541	0.420019
31204	0.851541	0.822541
31205	1.04073	0.66361
31206	1.16342	0.722398
31207	0.825315	0.902169
31208	0.825316	0.767423
31209	1.89226	1.69409
31210	1.89226	2.1
31211	1.89226	2.1
31212	1.89226	1.78946
31213	3.78453	1.9848
31214	2.57016	2.1
31215	1.74789	2.1
31216	2.57016	2.1
31217	2.522	2.1
31218	1.61727	2.1
31219	1.56911	2.1
31220	1.28075	2.1
31221	1.406	2.1
31222	0.946131	1.88277
31223	0.946131	1.71957
31224	1.28075	1.93874
31225	1.00333	2.1
31226	0.94613	2.1
31227	0.94613	1.93018
31228	1.041	2.02152
31229	1.041	2.01424
31230	0.943484	1.88776
31231	1.27717	2.00718
31232	1.89226	2.1
31233	1.89226	1.65208
31234	1.89226	1.6765
31235	1.89226	2.1
31236	1.89226	1.72323
31237	1.89226	2.1
31238	2.57016	2.1
31239	1.61727	2.1
31240	2.74196	2.1
31241	1.59094	2.1
31242	4.16109	2.1
31243	1.89226	1.67638
31244	2.0815	2.1
31245	2.2034	2.1
31246	1.82493	1.55768
31247	1.87847	2.1
31248	2.16142	2.1
31249	1.89226	2.1
31250	1.89226	2.1
31251	1.89226	2.1
31252	1.89226	2.1
31253	3.78453	2.1
31254	0.985252	2.1
31255	0.985252	2.1
31256	0.946132	2.1
31257	0.946132	2.1
31258	1.0655	2.1
31259	0.985255	2.1

31260	0.94613	2.1
31261	0.94613	2.1
31262	0.94613	2.1
31263	1.0655	2.1
31264	0.94613	2.1
31265	0.985255	2.1
31266	1.89226	2.1
31267	1.89226	2.1
31268	1.89226	2.1
31269	1.89226	2.1
31270	3.78453	2.1
31271	1.06549	2.1
31272	1.06549	2.1
31273	0.946133	2.1
31274	0.946133	2.1
31275	1.16834	2.1
31276	1.0655	2.1
31277	0.946131	2.1
31278	0.946131	2.1
31279	0.946131	2.1
31280	1.16834	2.1
31281	0.94613	2.1
31282	1.0655	2.1
31283	0.966631	2.1
31284	1.07349	2.1
31285	0.946131	2.1
31286	0.946131	2.1
31287	1.07349	2.1
31288	1.07349	2.1
31289	0.946129	2.1
31290	0.946129	2.1
31291	1.07349	2.1
31292	0.946131	2.1
31293	0.94613	2.1
31294	0.966631	2.1
31295	2.38055	0.818246
31296	1.89226	1.39016
31297	1.89226	1.21855
31298	1.46724	0.45918
31299	3.35949	0.915665
31300	1.42249	2.1
31301	2.05499	2.1
31302	1.39708	2.1
31303	2.09033	2.1
31304	3.45207	2.1
31305	2.05499	2.1
31306	2.57425	2.1
31307	2.51287	2.1
31308	2.47753	1.93951
31309	2.09033	2.1
31310	2.05499	1.58538
31311	1.39327	2.1
31312	2.09033	2.1
31313	2.16217	2.1
31314	2.16217	2.1
31315	1.39708	2.1
31316	1.39708	2.1
31317	2.16217	2.1

31318	2.57426	2.1
31319	2.73006	2.1
31320	2.73006	2.1
31321	2.09033	2.1
31322	2.09033	2.09466
31323	1.89226	0.137542
31324	1.89226	0.0929618
31325	1.89226	0.105415
31326	1.89226	0.126202
31327	3.78452	0.130057
31328	0.838749	0.0789214
31329	0.838749	0.0801216
31330	0.85824	0.0916258
31331	0.85824	0.0989191
31332	0.544644	0.0939763
31333	0.854718	0.084719
31334	0.842271	0.0858936
31335	1.19141	0.0973654
31336	0.842272	0.0866067
31337	0.544643	0.0772222
31338	0.854718	0.0871988
31339	1.19141	0.0825468
31340	1.34214	0.0885575
31341	1.34214	0.0819427
31342	0.893236	0.116412
31343	0.893236	0.104146
31344	0.893265	0.107015
31345	1.28924	0.0899554
31346	0.946133	0.0905667
31347	0.946133	0.109948
31348	0.946132	0.104861
31349	0.893265	0.110221
31350	0.946133	0.104615
31351	1.28924	0.100186
31352	2.7013	0.100798
31353	1.89226	0.120799
31354	1.9216	0.129444
31355	1.9216	0.0928872
31356	1.89226	0.128957
31357	1.89226	0.0926348
31358	1.26397	2.1
31359	1.52995	2.1
31360	1.30116	2.1
31361	1.04084	2.1
31362	2.57079	2.1
31363	1.47013	2.1
31364	2.17459	2.1
31365	2.10668	2.1
31366	1.38328	2.1
31367	3.55787	2.1
31368	2.55006	2.1
31369	2.17459	2.1
31370	2.10668	2.1
31371	2.49168	2.1
31372	4.66626	2.1
31373	0.613622	2.1
31374	0.691639	2.1
31375	1.00105	2.1

31376	1.05334	2.1
31377	0.839874	2.1
31378	0.839874	2.1
31379	0.852814	2.1
31380	0.852814	2.1
31381	0.613622	2.1
31382	1.00105	2.1
31383	1.05334	2.1
31384	0.691639	2.1
31385	1.00105	2.1
31386	1.05334	2.1
31387	1.20844	2.1
31388	1.24584	2.1
31389	1.12717	2.1
31390	1.12717	2.1
31391	1.13462	2.1
31392	1.13462	2.1
31393	1.20845	2.1
31394	1.00105	2.1
31395	1.05334	2.1
31396	1.24584	2.1
31397	2.22558	1.53527
31398	1.85981	1.21614
31399	2.18943	1.2081
31400	1.89142	0.614887
31401	4.0854	1.10191
31402	2.02641	2.04286
31403	1.85981	1.10661
31404	2.18943	1.28077
31405	1.67346	1.17387
31406	3.88622	0.999084
31407	1.85981	0.422372
31408	1.10736	0.360316
31409	1.27421	0.452007
31410	1.67346	0.431171
31411	1.27421	0.432609
31412	1.67346	0.930285
31413	1.85981	0.506721
31414	1.27362	0.543586
31415	1.89142	0.821443
31416	1.27421	0.434093
31417	3.13343	0.410393
31418	1.81893	0.134943
31419	1.64985	0.164243
31420	1.23477	0.104047
31421	2.25405	0.176204
31422	3.48882	0.172235
31423	2.23933	0.102471
31424	1.89226	0.135106
31425	1.89226	0.135498
31426	2.78053	0.129883
31427	1.89226	0.110795
31428	2.78053	0.104326
31429	0.946131	0.147503
31430	0.946131	0.142073
31431	0.946132	0.170771
31432	0.946132	0.224509
31433	0.946131	0.230286

31434	0.946131	0.198638
31435	0.946132	0.139636
31436	0.946132	0.211105
31437	0.946131	0.165666
31438	0.946131	0.192406
31439	0.946132	0.138933
31440	0.946132	0.191449
31441	1.89226	2.1
31442	2.48596	2.1
31443	1.89226	2.1
31444	2.51506	2.1
31445	4.40732	2.1
31446	2.48596	2.1
31447	1.63823	2.1
31448	2.51507	2.1
31449	1.67687	2.1
31450	4.1533	2.1
31451	1.89226	2.1
31452	2.51506	2.1
31453	1.92044	2.1
31454	1.89226	2.1
31455	2.54325	2.1
31456	2.51506	2.1
31457	2.51507	2.1
31458	1.5704	2.1
31459	1.70505	2.1
31460	1.67687	2.1
31461	2.54325	2.1
31462	2.51507	2.1
31463	2.0815	2.1
31464	1.70303	2.1
31465	1.70303	2.1
31466	1.80354	2.1
31467	1.98172	2.1
31468	2.09868	2.1
31469	1.27944	2.1
31470	1.79028	2.1
31471	1.13179	2.1
31472	1.65533	2.1
31473	2.93477	2.1
31474	1.89226	2.1
31475	1.89226	2.1
31476	1.89226	2.1
31477	1.89226	2.1
31478	1.89226	2.1
31479	1.89226	2.1
31480	1.98171	2.1
31481	1.89226	2.1
31482	1.89226	2.1
31483	1.89226	2.1
31484	1.65798	2.1
31485	1.65797	2.1
31486	1.00133	2.1
31487	1.65532	2.1
31488	1.53278	2.1
31489	1.53278	2.1
31490	1.13179	2.1
31491	1.13179	2.1

31492	0.810946	2.1
31493	0.810946	2.1
31494	0.810944	2.1
31495	0.810944	2.1
31496	0.810946	2.1
31497	0.810946	2.1
31498	1.89226	2.1
31499	1.35169	2.1
31500	1.08132	2.1
31501	1.08132	2.1
31502	1.08132	2.1
31503	1.99528	0.209124
31504	1.70303	0.235894
31505	2.08152	0.203841
31506	1.70303	0.287884
31507	2.33877	0.191622
31508	1.93967	0.248964
31509	1.89226	0.229394
31510	1.89226	0.35197
31511	1.89226	0.256989
31512	1.89226	0.30227
31513	3.78452	0.328306
31514	1.26259	0.219694
31515	2.17205	0.280376
31516	2.32468	0.239345
31517	2.32468	0.235799
31518	1.28055	0.240165
31519	1.28055	0.236369
31520	0.946131	0.240215
31521	0.946131	0.195279
31522	0.997642	0.214393
31523	0.997642	0.169248
31524	0.946131	0.222136
31525	0.946131	0.256764
31526	0.989846	0.195112
31527	0.997642	0.166257
31528	0.946132	0.190742
31529	0.989846	0.18425
31530	0.997641	0.180381
31531	0.946131	0.195783
31532	1.89226	0.254559
31533	1.89226	0.293234
31534	1.89226	0.222771
31535	1.89226	0.333864
31536	3.78452	0.229964
31537	1.28055	0.193198
31538	2.17205	0.271733
31539	1.14305	0.212232
31540	1.92737	0.251493
31541	3.3151	0.250609
31542	0.954908	0.0852733
31543	0.9705	0.077888
31544	0.946131	0.0836725
31545	0.946131	0.0939767
31546	0.946131	0.0932928
31547	0.946131	0.0857139
31548	0.9705	0.0835624
31549	0.9705	0.0767963

31550	0.954908	0.0833912
31551	0.946131	0.083696
31552	0.946131	0.0849209
31553	0.9705	0.0850111
31554	0.835187	0.0863323
31555	0.954896	0.0776024
31556	0.946118	0.0843459
31557	0.946118	0.094115
31558	0.835186	0.093338
31559	0.835186	0.0857925
31560	1.06583	0.0826346
31561	1.06583	0.0758443
31562	0.835198	0.0868037
31563	0.946131	0.0866971
31564	0.954883	0.0872287
31565	0.946106	0.0868945
31566	1.89227	2.01093
31567	1.89227	2.1
31568	1.89227	2.1
31569	1.89227	2.1
31570	1.89227	2.1
31571	1.89227	1.74151
31572	0.885265	2.1
31573	0.885265	2.1
31574	0.871467	2.1
31575	0.871467	2.1
31576	1.11883	2.1
31577	0.787875	2.1
31578	0.996487	2.1
31579	0.637907	2.1
31580	0.996487	2.1
31581	1.11883	2.1
31582	0.787875	2.1
31583	0.637906	2.1
31584	1.89226	2.1
31585	2.39258	2.1
31586	2.39258	2.1
31587	2.54729	2.1
31588	1.89226	2.1
31589	2.04697	2.1
31590	1.89226	2.1
31591	2.0815	2.1
31592	2.0815	2.1
31593	2.48371	2.1
31594	2.0815	2.1
31595	2.51469	2.1
31596	1.00035	0.0522355
31597	1.55755	0.129286
31598	1.03534	0.166515
31599	1.61333	0.190674
31600	2.59289	0.118404
31601	1.55755	0.129286
31602	1.85217	0.725511
31603	1.61333	0.193485
31604	1.92875	0.661815
31605	3.48629	0.291223
31606	1.09962	0.406898
31607	1.61334	0.296912

31608	1.70868	0.547208
31609	1.70868	0.391052
31610	1.03535	0.528772
31611	1.03535	0.336199
31612	1.61334	0.195495
31613	1.92876	0.659136
31614	1.70868	0.220407
31615	2.05274	0.758371
31616	3.63744	0.342971
31617	1.82233	0.498574
31618	2.05768	0.790126
31619	1.89226	0.866922
31620	1.65406	0.526302
31621	1.89226	0.803837
31622	1.65406	0.543026
31623	2.26047	0.578412
31624	2.0815	0.933723
31625	1.89226	0.911482
31626	2.01156	0.604522
31627	1.89226	0.839513
31628	2.01156	0.595193
31629	1.89226	1.9182
31630	1.89226	2.1
31631	1.89226	2.1
31632	1.89226	1.6139
31633	3.78453	1.75689
31634	2.70055	2.1
31635	2.28367	2.1
31636	2.56456	2.1
31637	2.33565	2.1
31638	4.84823	2.1
31639	2.28367	2.1
31640	1.5791	2.1
31641	1.96966	2.1
31642	1.09506	2.1
31643	3.37873	2.1
31644	1.89226	2.1
31645	1.89226	1.49472
31646	1.89226	1.56482
31647	1.89226	2.1
31648	3.78452	2.1
31649	0.94612	2.1
31650	0.94612	2.1
31651	0.946119	1.81219
31652	0.946119	1.57766
31653	0.946119	1.50688
31654	0.946119	2.1
31655	0.946119	2.1
31656	0.946119	1.75003
31657	0.946132	2.1
31658	0.946132	2.1
31659	0.946107	2.1
31660	0.946107	2.1
31661	2.56452	2.1
31662	1.57307	2.1
31663	2.10299	2.1
31664	2.1115	2.1
31665	2.52196	2.1

31666	2.52196	2.1
31667	1.89226	2.1
31668	1.89226	0.89651
31669	1.89226	2.1
31670	1.89226	2.1
31671	3.78452	2.1
31672	2.37665	2.1
31673	1.89226	2.1
31674	2.28084	2.1
31675	1.89226	2.1
31676	4.26891	2.1
31677	0.946129	0.918869
31678	0.946129	2.1
31679	0.946133	2.1
31680	0.946133	2.1
31681	0.946131	2.1
31682	0.946131	0.891539
31683	0.946131	2.1
31684	0.946131	2.1
31685	0.946131	2.1
31686	0.946131	2.1
31687	0.946131	2.1
31688	0.946131	2.1
31689	1.7207	0.0837309
31690	1.65815	0.0923667
31691	1.33593	0.0967093
31692	1.31508	0.0775974
31693	2.99408	0.0835389
31694	0.676047	0.0747682
31695	0.676047	0.0788513
31696	0.684362	0.093325
31697	0.684362	0.084285
31698	0.547994	0.0847831
31699	0.702871	0.0774073
31700	0.829076	0.0937299
31701	0.657538	0.0819627
31702	0.657538	0.0827907
31703	0.547994	0.0791692
31704	0.829077	0.0841867
31705	0.702871	0.083011
31706	1.9518	2.1
31707	1.30648	2.1
31708	1.28846	2.1
31709	1.92033	2.1
31710	3.22681	2.1
31711	1.89226	2.1
31712	1.89226	2.1
31713	1.89226	2.1
31714	1.89226	2.1
31715	3.78453	2.1
31716	0.946128	2.1
31717	0.946128	2.1
31718	1.10113	2.1
31719	1.09587	2.1
31720	0.946131	2.1
31721	0.946131	2.1
31722	1.09587	2.1
31723	1.09587	2.1

31724	0.946131	2.1
31725	1.09587	2.1
31726	1.10112	2.1
31727	0.94613	2.1
31728	1.26923	2.1
31729	1.92033	2.1
31730	1.28846	2.1
31731	1.28846	2.1
31732	1.89452	2.1
31733	1.89452	2.1
31734	1.89226	2.1
31735	1.89226	2.1
31736	1.89226	2.1
31737	1.89226	2.1
31738	3.78453	2.1
31739	1.89226	2.1
31740	2.19174	2.1
31741	2.15616	2.1
31742	2.19174	2.1
31743	1.85669	2.1
31744	1.89226	2.1
31745	1.89226	2.1
31746	1.89226	2.1
31747	1.89226	2.1
31748	1.89226	2.1
31749	3.78452	2.1
31750	1.89226	2.1
31751	1.89226	2.1
31752	1.89226	2.1
31753	1.89226	2.1
31754	3.78452	2.1
31755	1.51223	2.1
31756	1.41562	2.1
31757	1.5139	2.1
31758	1.42284	2.1
31759	1.6033	2.1
31760	1.51223	2.1
31761	1.51223	2.1
31762	1.25953	2.1
31763	1.38085	2.1
31764	1.6033	2.1
31765	2.89309	2.1
31766	0.946131	2.1
31767	0.946131	2.1
31768	0.946131	2.1
31769	0.946131	2.1
31770	0.946132	2.1
31771	0.946132	2.1
31772	0.946129	2.1
31773	0.946129	2.1
31774	0.946131	2.1
31775	0.946131	2.1
31776	0.94613	2.1
31777	0.94613	2.1
31778	1.89226	2.1
31779	1.89226	2.1
31780	1.89226	2.1
31781	1.89226	2.1

31782	3.78452	2.1
31783	0.707806	2.1
31784	0.697816	2.1
31785	0.823593	2.1
31786	0.801649	2.1
31787	0.74182	2.1
31788	0.74182	2.1
31789	0.757645	2.1
31790	0.757645	2.1
31791	0.823592	2.1
31792	0.697818	2.1
31793	0.707807	2.1
31794	0.801647	2.1
31795	1.52534	2.1
31796	1.6033	2.1
31797	1.64719	2.1
31798	1.38085	2.1
31799	1.64719	2.1
31800	1.38085	2.1
31801	2.67041	2.1
31802	1.89226	0.694134
31803	1.89226	1.57636
31804	1.89226	1.00209
31805	2.487	1.45154
31806	2.487	1.29937
31807	2.70097	2.1
31808	1.70303	2.1
31809	1.71163	2.1
31810	2.487	1.64134
31811	4.46723	2.1
31812	1.14984	1.52881
31813	1.2435	2.1
31814	0.778	2.1
31815	0.697616	2.1
31816	0.967211	2.1
31817	0.967211	1.16875
31818	0.973907	2.1
31819	0.973907	2.1
31820	0.697616	2.1
31821	1.14984	2.1
31822	0.778	2.1
31823	1.2435	2.1
31824	2.4158	2.1
31825	1.26438	2.1
31826	1.676	2.1
31827	2.77505	2.1
31828	4.0918	2.1
31829	2.58258	2.1
31830	1.89226	2.1
31831	1.89226	2.1
31832	1.89226	2.1
31833	2.47032	2.1
31834	2.47032	2.1
31835	1.23516	2.1
31836	1.18734	2.1
31837	0.946131	2.1
31838	0.946131	2.1
31839	1.18734	2.1

31840	1.18734	2.1
31841	0.94613	2.1
31842	0.94613	2.1
31843	1.18734	2.1
31844	0.946131	2.1
31845	0.946131	2.1
31846	1.23516	2.1
31847	2.8239	2.1
31848	1.39361	2.1
31849	1.12342	2.1
31850	2.57275	2.1
31851	3.96637	2.1
31852	0.937929	0.741723
31853	0.937929	0.678926
31854	1.39734	0.54548
31855	1.39734	0.578349
31856	1.38914	0.598214
31857	0.94613	0.691304
31858	0.937902	0.713265
31859	0.94613	0.380219
31860	0.94613	0.600054
31861	0.94613	0.634571
31862	1.38914	0.646431
31863	0.937902	0.613397
31864	1.89226	0.556397
31865	2.17765	0.886484
31866	1.84577	0.819038
31867	1.84577	0.651608
31868	1.89226	0.740834
31869	1.89226	0.585706
31870	2.11262	0.820714
31871	1.57766	0.995108
31872	2.22689	2.05817
31873	1.91132	0.401979
31874	2.19624	1.48776
31875	1.79689	0.411923
31876	1.99656	0.382747
31877	1.57766	0.839152
31878	1.39067	1.91964
31879	1.73802	0.709971
31880	3.31568	0.889769
31881	1.89226	0.499924
31882	1.89226	0.360665
31883	1.89226	0.423604
31884	1.89226	0.576715
31885	1.89226	0.27688
31886	1.89226	0.449998
31887	1.84936	0.625005
31888	2.0815	0.263181
31889	2.0815	0.393281
31890	2.47217	0.513926
31891	1.04528	0.210447
31892	1.62774	0.481229
31893	1.89226	0.432134
31894	1.89226	0.29701
31895	1.89226	0.378777
31896	1.89226	0.460806
31897	1.89226	0.287524

31898	1.89226	0.394889
31899	1.69055	0.189874
31900	2.68518	0.2934
31901	1.69055	0.172767
31902	2.25055	0.338711
31903	2.39587	0.137919
31904	3.01801	0.294306
31905	1.89226	0.307675
31906	1.89226	0.333933
31907	1.89226	0.21675
31908	1.89226	0.19038
31909	3.78452	0.274201
31910	1.24499	0.864765
31911	1.70302	0.935518
31912	1.41207	2.1
31913	1.10574	0.623561
31914	2.66056	0.938589
31915	1.70303	1.17668
31916	2.39605	1.04163
31917	2.72674	0.9491
31918	2.46904	0.761876
31919	1.84557	1.13222
31920	1.67115	1.02536
31921	2.46904	0.57498
31922	2.76829	1.38242
31923	2.83666	0.41906
31924	2.83666	0.793243
31925	2.39605	0.731362
31926	2.39605	1.38869
31927	1.07323	0.344547
31928	1.07323	1.68394
31929	1.06715	1.43114
31930	1.18052	0.857544
31931	1.30067	0.679398
31932	1.26542	0.738424
31933	0.914516	0.988745
31934	0.839633	1.22161
31935	0.936958	0.917995
31936	1.26751	0.870283
31937	0.847764	0.848556
31938	1.35395	0.939674
31939	2.69069	0.608781
31940	1.03074	0.459339
31941	1.74098	0.243914
31942	1.97397	0.4359
31943	3.71495	0.434516
31944	2.44554	2.1
31945	1.89226	2.1
31946	1.89226	2.1
31947	1.89226	2.1
31948	2.40645	2.1
31949	2.40645	2.1
31950	1.89226	2.1
31951	1.89226	1.20294
31952	1.89226	2.1
31953	1.89226	0.998914
31954	3.78452	2.1
31955	1.82117	2.1

31956	2.55786	0.956893
31957	2.51431	1.99226
31958	2.51431	2.1
31959	1.85054	1.7888
31960	1.84375	2.1
31961	2.38467	2.1
31962	1.89226	2.1
31963	1.89226	2.1
31964	1.89226	2.1
31965	2.44554	2.1
31966	2.44554	2.1
31967	1.89226	2.1
31968	1.89226	1.65779
31969	1.89226	2.1
31970	1.89226	1.57204
31971	1.89226	2.1
31972	1.89226	1.68725
31973	2.02397	2.1
31974	2.55786	1.59607
31975	1.77775	2.1
31976	2.61404	1.82633
31977	4.33561	2.1
31978	1.89226	1.20644
31979	1.89226	1.17624
31980	1.89226	0.870099
31981	1.89226	1.03027
31982	3.78453	1.12871
31983	1.89226	1.27871
31984	2.80184	1.49778
31985	1.89226	1.08076
31986	1.89226	1.35441
31987	2.03045	1.46978
31988	2.03045	1.68265
31989	2.52263	1.21797
31990	1.27921	1.96143
31991	1.58429	2.02319
31992	1.73571	1.85081
31993	2.37121	1.41746
31994	2.52263	1.28328
31995	0.764974	2.1
31996	0.650577	2.1
31997	0.712428	2.1
31998	0.562516	2.1
31999	0.67264	2.1
32000	0.67264	2.1
32001	0.654851	2.1
32002	0.654851	2.1
32003	0.562515	2.1
32004	0.650579	2.1
32005	0.712427	2.1
32006	0.764976	2.1
32007	1.89227	2.1
32008	1.89227	2.1
32009	1.89227	2.1
32010	1.89227	2.1
32011	3.78453	2.1
32012	1.89226	2.1
32013	1.89226	2.1

32014	1.89226	2.1
32015	1.89226	2.1
32016	3.78453	2.1
32017	1.49248	2.1
32018	1.24959	2.1
32019	1.28211	2.1
32020	1.26432	2.1
32021	1.51027	2.1
32022	1.49248	2.1
32023	1.49248	2.1
32024	1.19017	2.1
32025	1.24977	2.1
32026	1.51027	2.1
32027	2.74225	2.1
32028	1.89226	2.1
32029	1.89226	2.1
32030	1.89226	2.1
32031	1.89226	2.1
32032	3.78453	2.1
32033	1.89226	2.1
32034	1.89226	2.1
32035	1.89226	2.1
32036	1.89226	2.1
32037	3.78453	2.1
32038	0.624796	2.1
32039	0.618472	2.1
32040	0.770147	2.1
32041	0.755132	2.1
32042	0.681445	2.1
32043	0.681445	2.1
32044	0.69216	2.1
32045	0.69216	2.1
32046	0.618472	2.1
32047	0.770147	2.1
32048	0.624796	2.1
32049	0.755132	2.1
32050	1.54029	2.1
32051	1.51027	2.1
32052	1.54029	2.1
32053	1.30682	2.1
32054	1.24977	2.1
32055	1.24977	2.1
32056	0.994293	2.1
32057	2.17159	2.1
32058	2.21149	2.1
32059	2.21149	2.1
32060	0.994814	2.1
32061	0.994814	2.1
32062	0.990481	2.1
32063	2.21149	2.1
32064	2.2671	2.1
32065	2.2671	2.1
32066	0.994293	2.1
32067	0.994293	2.1
32068	2.41579	2.04802
32069	1.09847	2.1
32070	1.70302	0.955777
32071	2.45069	2.1

32072	3.61169	2.07687
32073	2.48689	2.1
32074	1.67969	2.1
32075	1.67969	2.1
32076	1.70002	2.1
32077	2.43826	2.1
32078	2.45859	2.1
32079	2.43827	2.1
32080	1.62408	2.1
32081	1.62408	2.1
32082	1.67969	2.1
32083	2.36729	2.1
32084	2.4229	2.1
32085	2.47199	2.1
32086	1.0823	2.1
32087	1.0607	2.1
32088	2.44276	2.1
32089	3.52506	2.1
32090	2.47199	2.1
32091	1.89226	2.1
32092	1.89226	2.1
32093	2.44277	2.1
32094	4.33503	2.1
32095	1.89226	2.1
32096	1.89226	2.1
32097	1.89226	2.1
32098	1.89226	2.1
32099	3.78453	2.1
32100	1.89226	2.1
32101	1.89226	2.1
32102	1.89226	2.1
32103	1.89226	2.1
32104	1.89226	2.1
32105	1.89226	2.1
32106	1.02986	2.1
32107	2.44276	2.1
32108	1.0607	2.1
32109	1.0607	2.1
32110	2.40055	2.1
32111	2.40055	2.1
32112	1.89226	2.1
32113	2.44277	2.1
32114	2.40056	2.1
32115	2.44277	2.1
32116	1.85005	2.1
32117	1.89226	2.1
32118	1.89226	2.1
32119	1.89226	2.1
32120	1.89226	2.1
32121	1.89226	2.1
32122	3.78453	2.1
32123	1.89226	2.1
32124	1.89226	2.1
32125	1.89226	2.1
32126	1.89226	2.1
32127	3.78452	2.1
32128	2.79502	0.364079
32129	2.63448	0.545172

32130	2.68986	0.336685
32131	2.53868	0.522349
32132	5.32434	0.439036
32133	2.63449	0.458171
32134	2.39282	1.5424
32135	2.53869	0.527589
32136	2.3085	0.823088
32137	4.94299	0.620837
32138	2.68986	0.351613
32139	2.53868	0.513666
32140	2.5761	0.303928
32141	2.43404	0.501344
32142	5.1239	0.43165
32143	2.53869	0.466533
32144	2.3085	0.814877
32145	2.43405	0.466674
32146	2.22575	0.771748
32147	4.74255	0.586577
32148	1.89226	2.1
32149	1.89226	2.1
32150	1.89226	2.1
32151	1.89226	2.1
32152	3.78453	2.1
32153	1.32391	2.1
32154	1.44951	2.1
32155	1.44951	2.1
32156	1.89226	2.1
32157	1.89226	2.1
32158	2.20203	2.1
32159	0.507656	2.1
32160	0.578945	2.1
32161	1.05568	2.1
32162	0.959007	2.1
32163	0.76264	2.1
32164	0.76264	2.1
32165	0.775313	2.1
32166	0.775313	2.1
32167	0.578945	2.1
32168	1.05568	2.1
32169	0.959007	2.1
32170	0.507656	2.1
32171	1.89226	0.137903
32172	2.4065	0.170574
32173	2.36561	0.133946
32174	2.4065	0.181474
32175	1.85138	0.128546
32176	1.89226	0.173783
32177	1.40274	0.125328
32178	2.4065	0.165813
32179	1.53336	0.137256
32180	1.53336	0.130736
32181	2.36562	0.146222
32182	2.36562	0.139782
32183	0.946115	0.165102
32184	0.946115	0.130566
32185	1.06496	0.121704
32186	1.18279	0.147728
32187	0.946117	0.15595

32188	0.946117	0.172689
32189	1.06496	0.132569
32190	1.06496	0.121398
32191	0.94613	0.139182
32192	1.06497	0.131737
32193	0.946105	0.132791
32194	1.18278	0.141399
32195	1.00213	0.123573
32196	2.36559	0.152188
32197	1.40272	0.130456
32198	1.40272	0.126123
32199	2.12992	0.140364
32200	2.12992	0.134898
32201	0.95646	2.1
32202	2.07201	1.25946
32203	1.01808	2.1
32204	2.14876	0.860933
32205	3.09009	2.1
32206	1.89226	2.1
32207	1.89226	0.841459
32208	1.89226	2.1
32209	1.89226	2.00452
32210	3.78453	2.05575
32211	1.89227	2.1
32212	1.89227	2.048
32213	1.89227	2.1
32214	1.89227	2.1
32215	1.89227	2.1
32216	1.89227	2.1
32217	2.71886	2.1
32218	1.60497	2.1
32219	1.43935	2.1
32220	2.55151	2.1
32221	4.15648	2.1
32222	2.71886	2.1
32223	1.89226	2.1
32224	1.89226	2.1
32225	2.55151	2.1
32226	4.44377	2.1
32227	1.89226	2.08987
32228	1.89226	2.02981
32229	1.89226	2.1
32230	1.89226	0.877071
32231	3.78453	2.09489
32232	1.89226	2.1
32233	1.89226	2.05935
32234	1.89226	2.1
32235	1.89226	2.1
32236	3.78453	2.1
32237	1.58942	0.584994
32238	1.92622	0.674342
32239	2.52852	0.716816
32240	2.19457	0.544579
32241	4.12079	0.553175
32242	1.92649	0.564567
32243	1.44426	0.384409
32244	1.87529	0.410423
32245	2.19457	0.542008

32246	3.63883	0.516932
32247	1.87529	0.407695
32248	2.19457	0.516506
32249	2.7378	0.540089
32250	2.29852	0.391936
32251	4.49309	0.397701
32252	1.35976	2.1
32253	1.89452	2.1
32254	1.39616	2.1
32255	1.1423	2.1
32256	1.8996	2.1
32257	1.52068	2.1
32258	1.89226	2.1
32259	1.89226	2.1
32260	1.89226	2.1
32261	1.89226	2.1
32262	1.89226	2.1
32263	1.89226	2.1
32264	1.89226	2.1
32265	2.15617	2.1
32266	2.0953	2.1
32267	2.15617	2.1
32268	1.83139	2.1
32269	1.89226	2.1
32270	1.16817	2.1
32271	1.8996	2.1
32272	1.23614	2.1
32273	1.48338	2.1
32274	1.80218	2.1
32275	2.16263	2.1
32276	1.89226	2.1
32277	1.89226	2.1
32278	1.89226	2.1
32279	1.89226	2.1
32280	1.89226	2.1
32281	1.89226	2.1
32282	1.89226	2.1
32283	2.0953	2.1
32284	2.03074	2.1
32285	2.0953	2.1
32286	1.8277	2.1
32287	1.89226	2.1
32288	1.74455	2.1
32289	2.181	2.1
32290	1.74455	2.1
32291	1.92911	2.1
32292	1.98195	2.1
32293	2.16651	2.1
32294	2.181	2.1
32295	2.29881	2.1
32296	1.98195	2.1
32297	1.80729	2.1
32298	4.28075	2.1
32299	1.74455	2.1
32300	1.98195	2.1
32301	1.52603	2.1
32302	1.76603	2.1
32303	3.51058	2.1

32304	1.76603	2.1
32305	1.94506	2.1
32306	1.76603	2.1
32307	1.98195	2.1
32308	1.86795	2.1
32309	2.08387	2.1
32310	0.984305	2.1
32311	1.9	2.1
32312	2.86619	2.1
32313	1.86189	2.1
32314	3.76189	2.1
32315	1.4621	2.1
32316	1.89226	2.1
32317	2.81374	2.1
32318	1.89226	2.1
32319	3.35437	2.1
32320	0.946133	2.0413
32321	0.946133	2.1
32322	0.946132	1.918
32323	0.946132	2.1
32324	0.946132	2.1
32325	0.946132	2.04912
32326	0.946133	2.1
32327	0.946133	2.05366
32328	0.946132	2.1
32329	0.946132	1.90377
32330	0.946132	2.04983
32331	0.946132	2.1
32332	1.64268	1.47115
32333	1.93673	2.1
32334	2.25284	2.1
32335	1.97548	2.08812
32336	3.89552	2.1
32337	1.86206	2.1
32338	1.93673	2.1
32339	2.25284	2.1
32340	2.20075	2.1
32341	4.1149	2.1
32342	2.25284	2.1
32343	2.24781	2.00017
32344	1.97548	1.38675
32345	1.97548	2.1
32346	2.49676	1.62047
32347	2.49676	2.1
32348	2.25284	2.0827
32349	2.43549	2.1
32350	2.44467	2.1
32351	2.20075	2.1
32352	2.49676	2.1
32353	2.25284	2.1
32354	2.57928	2.1
32355	2.32606	2.1
32356	2.59522	2.1
32357	2.39022	2.1
32358	4.9695	2.1
32359	2.32606	2.1
32360	1.77677	2.1
32361	2.39022	2.1

32362	2.28669	2.1
32363	4.16699	2.1
32364	2.59522	2.1
32365	2.39022	2.1
32366	2.61901	2.1
32367	2.45899	2.1
32368	5.00923	2.1
32369	2.39022	2.1
32370	2.11575	2.1
32371	2.41795	2.1
32372	2.0815	2.1
32373	4.67085	2.1
32374	1.89226	2.1
32375	2.24723	2.1
32376	1.95405	2.1
32377	1.89226	2.1
32378	2.30902	2.1
32379	2.24723	2.1
32380	1.89226	2.1
32381	1.89226	2.1
32382	1.89226	2.1
32383	1.89226	2.1
32384	3.78453	2.1
32385	0.946131	2.1
32386	0.946131	2.1
32387	1.14643	2.1
32388	1.10673	2.1
32389	1.10673	2.1
32390	1.10673	2.1
32391	0.946133	2.1
32392	0.946133	2.1
32393	0.946131	2.1
32394	1.14643	2.1
32395	1.10673	2.1
32396	0.946131	2.1
32397	1.89226	2.1
32398	2.30902	2.1
32399	1.96635	2.1
32400	1.89226	2.1
32401	2.38311	2.1
32402	2.30902	2.1
32403	1.89226	2.1
32404	1.89226	2.1
32405	1.89226	2.1
32406	1.89226	2.1
32407	1.89226	2.1
32408	1.89226	2.1
32409	0.946131	2.1
32410	0.946131	2.1
32411	1.19156	2.1
32412	1.14643	2.1
32413	1.14643	2.1
32414	1.14643	2.1
32415	0.946133	2.1
32416	0.946133	2.1
32417	0.946131	2.1
32418	1.19156	2.1
32419	0.946131	2.1

32420	1.14643	2.1
32421	1.89226	1.04177
32422	1.89226	0.767694
32423	1.89226	0.675554
32424	1.89226	1.02473
32425	1.89226	0.765607
32426	1.89226	1.37516
32427	1.89226	0.831627
32428	1.89226	0.804017
32429	1.89226	0.770519
32430	1.9645	0.877243
32431	3.78452	1.02206
32432	2.39489	2.1
32433	2.15715	2.1
32434	2.07821	2.1
32435	2.33541	2.1
32436	4.49256	2.1
32437	2.39489	2.1
32438	2.34898	2.1
32439	2.30729	2.1
32440	2.33541	2.1
32441	4.6844	2.1
32442	2.07821	2.1
32443	2.33541	2.1
32444	2.26415	2.1
32445	1.99632	2.1
32446	4.33174	2.1
32447	2.30729	2.1
32448	2.33542	2.1
32449	2.26416	2.1
32450	2.26618	2.1
32451	4.60159	2.1
32452	1.19837	1.34108
32453	2.79151	0.522955
32454	2.25636	0.959399
32455	2.25636	0.751556
32456	1.94072	0.854233
32457	1.94072	0.789101
32458	2.53725	0.140294
32459	1.16699	0.137998
32460	1.69864	0.1106
32461	2.0815	0.137737
32462	3.58273	0.131764
32463	1.91646	2.1
32464	2.51686	2.1
32465	2.49872	2.1
32466	1.85819	2.1
32467	4.41518	2.1
32468	0.929098	2.1
32469	0.8993	2.1
32470	1.24359	2.1
32471	1.24936	2.1
32472	1.06832	2.1
32473	1.06832	2.1
32474	1.08034	2.1
32475	1.08034	2.1
32476	0.899298	2.1
32477	1.24359	2.1

32478	1.24936	2.1
32479	0.929097	2.1
32480	2.0815	0.147133
32481	1.89226	0.0961743
32482	2.0815	0.122822
32483	1.82811	0.107323
32484	1.94982	0.125187
32485	1.71246	0.107719
32486	2.3256	2.1
32487	1.89226	2.1
32488	1.89226	2.1
32489	1.89226	2.1
32490	2.38467	2.1
32491	2.38467	2.1
32492	1.89226	2.1
32493	1.89226	2.04242
32494	1.89226	2.1
32495	1.89226	1.83731
32496	1.89226	2.1
32497	1.89226	1.89845
32498	1.89763	2.1
32499	2.61405	2.1
32500	2.15035	2.1
32501	2.68796	2.1
32502	1.42219	2.1
32503	1.77775	2.09518
32504	2.26306	2.1
32505	1.89226	2.1
32506	1.89226	2.1
32507	1.89226	2.1
32508	2.3256	2.1
32509	2.3256	2.1
32510	1.89226	2.1
32511	1.89226	2.1
32512	1.89226	2.1
32513	1.89226	1.98373
32514	1.89226	2.1
32515	1.89226	1.80723
32516	1.92887	2.1
32517	2.41915	2.1
32518	2.50142	2.1
32519	2.50142	2.1
32520	1.90388	2.1
32521	1.89763	2.1
32522	1.89224	2.1
32523	1.89224	2.1
32524	1.89224	2.1
32525	1.89224	2.1
32526	3.78448	2.1
32527	2.25315	2.1
32528	1.05843	2.1
32529	2.38995	2.1
32530	1.31541	2.1
32531	3.56856	2.1
32532	0.517552	2.1
32533	0.68548	2.1
32534	1.27675	2.1
32535	1.13076	2.1

32536	0.913611	2.1
32537	0.913612	2.1
32538	0.902633	2.1
32539	0.902633	2.1
32540	0.68547	2.1
32541	1.27673	2.1
32542	1.13078	2.1
32543	0.517559	2.1
32544	1.89226	2.1
32545	1.89226	2.1
32546	1.89226	2.1
32547	1.89226	2.1
32548	3.78453	2.1
32549	0.962936	2.1
32550	0.962936	2.1
32551	0.996141	2.1
32552	0.996141	2.1
32553	0.830609	2.1
32554	0.657714	2.1
32555	1.30136	2.1
32556	1.19499	2.1
32557	1.30136	2.1
32558	0.830609	2.1
32559	1.19499	2.1
32560	0.657713	2.1
32561	1.60057	0.253114
32562	1.39615	0.265115
32563	1.06474	0.260098
32564	1.39615	0.312769
32565	1.64655	0.225569
32566	2.04424	0.266351
32567	1.89226	0.277377
32568	1.89226	0.340308
32569	1.89226	0.316454
32570	1.89226	0.317211
32571	1.89226	0.306697
32572	1.89226	0.310597
32573	2.56322	0.239432
32574	1.89226	0.30568
32575	1.89226	0.276609
32576	1.89226	0.274795
32577	2.18047	0.263085
32578	2.18047	0.262348
32579	1.89226	0.637916
32580	1.89226	0.667161
32581	1.89226	0.782918
32582	1.65573	0.49691
32583	1.89226	0.799239
32584	1.65573	0.510432
32585	1.05353	0.378219
32586	1.83158	0.440817
32587	1.50282	0.342053
32588	1.20669	0.29906
32589	2.88511	0.306808
32590	1.44807	2.1
32591	1.93592	2.1
32592	1.80703	2.1
32593	1.27806	2.1

32594	3.21398	2.1
32595	2.0815	2.1
32596	1.87465	2.1
32597	1.87465	2.1
32598	1.50566	2.1
32599	2.0815	2.1
32600	1.69263	2.1
32601	1.89226	2.1
32602	1.89226	2.1
32603	1.89226	2.1
32604	1.89226	2.1
32605	1.89226	2.1
32606	1.89226	2.1
32607	1.62632	2.1
32608	0.994294	2.1
32609	0.994294	2.1
32610	1.27806	2.1
32611	1.81921	2.1
32612	2.10776	2.1
32613	2.0815	2.1
32614	1.70303	2.1
32615	1.70303	2.1
32616	1.85749	2.1
32617	1.90451	2.1
32618	2.07724	2.1
32619	1.89226	2.1
32620	1.89226	2.1
32621	1.89226	2.1
32622	1.89226	2.1
32623	1.89226	2.1
32624	1.89226	2.1
32625	2.69773	2.1
32626	1.88129	2.1
32627	2.55196	2.1
32628	2.26469	2.1
32629	4.94367	2.1
32630	1.88129	2.1
32631	1.25889	2.1
32632	1.26614	2.1
32633	1.05914	2.1
32634	2.26469	2.1
32635	1.88129	2.1
32636	2.55196	2.1
32637	2.0588	2.1
32638	2.6536	2.1
32639	2.15287	2.1
32640	4.70484	2.1
32641	0.8516	2.1
32642	0.8516	2.1
32643	0.849002	2.1
32644	0.849002	2.1
32645	0.629445	2.1
32646	0.671197	2.1
32647	1.0294	2.1
32648	1.07644	2.1
32649	1.07644	2.1
32650	0.671197	2.1
32651	0.629446	2.1

32652	1.0294	2.1
32653	2.02115	1.83859
32654	1.18382	1.89624
32655	2.29107	0.62148
32656	1.46903	1.32049
32657	3.47488	0.892199
32658	2.60763	1.09374
32659	2.29107	1.3898
32660	1.82141	1.29987
32661	1.82141	1.89108
32662	1.46903	1.72483
32663	2.29107	1.70731
32664	1.64007	2.1
32665	2.2024	2.1
32666	1.44044	2.1
32667	2.02634	2.1
32668	3.6664	2.1
32669	1.25565	2.1
32670	2.02635	2.1
32671	1.87228	2.1
32672	1.87228	2.1
32673	1.44045	2.1
32674	1.44045	2.1
32675	1.89226	0.0873462
32676	1.78503	0.0942132
32677	1.78503	0.0998806
32678	1.40048	0.0862355
32679	2.0815	0.0979945
32680	1.62972	0.0850147
32681	1.27875	2.1
32682	1.1483	0.623901
32683	1.40014	0.673772
32684	1.40014	2.1
32685	1.04598	0.696905
32686	1.04598	2.1
32687	2.63443	1.18653
32688	2.26444	1.87798
32689	2.79392	0.671776
32690	2.4863	1.88411
32691	5.12073	1.49151
32692	1.83158	0.505034
32693	1.18508	0.346915
32694	1.20669	0.351759
32695	1.81488	0.426323
32696	1.20669	0.361187
32697	1.81488	0.420515
32698	1.08134	0.317427
32699	1.70303	0.392983
32700	1.24027	0.425873
32701	1.30359	0.305494
32702	1.89939	0.528581
32703	1.70303	0.432633
32704	1.89226	2.1
32705	2.69027	2.1
32706	1.89226	2.1
32707	2.2078	2.093
32708	1.89226	2.1
32709	2.2078	2.09331

32710	1.89226	2.1
32711	2.78977	2.1
32712	1.89226	2.1
32713	2.69027	2.1
32714	1.89226	2.1
32715	2.69027	2.1
32716	1.89226	2.1
32717	1.89226	2.04091
32718	1.89226	1.52847
32719	1.89226	2.1
32720	3.78452	2.00856
32721	1.43687	2.06664
32722	1.71059	2.1
32723	2.53014	2.0774
32724	2.6521	2.1
32725	4.24073	2.1
32726	2.6521	2.1
32727	1.66762	2.1
32728	2.50299	2.1
32729	2.50299	2.1
32730	1.71059	2.1
32731	1.71059	2.1
32732	1.89226	2.1
32733	1.89226	2.1
32734	1.89226	2.1
32735	1.89226	1.95039
32736	1.89226	2.1
32737	1.89226	1.9741
32738	2.10662	0.598071
32739	2.77417	0.578388
32740	2.78873	0.902363
32741	2.11661	0.561969
32742	4.89078	0.583577
32743	2.10662	0.704682
32744	1.22857	0.418316
32745	1.25849	0.762991
32746	2.11661	0.627821
32747	3.34518	0.673907
32748	2.78873	0.737819
32749	2.11661	0.523626
32750	2.12475	0.607876
32751	2.79029	0.606706
32752	4.9069	0.616803
32753	1.2858	0.659797
32754	2.11661	0.57041
32755	1.25849	0.614093
32756	1.25849	0.624266
32757	2.12475	0.589144
32758	2.12475	0.620061
32759	1.13537	2.1
32760	1.20714	2.1
32761	0.946129	2.1
32762	0.946129	2.1
32763	0.946131	2.1
32764	0.946131	2.1
32765	1.29521	2.1
32766	1.20713	2.1
32767	1.13537	2.1

32768	0.946131	2.1
32769	0.94613	2.1
32770	1.29521	2.1
32771	1.00533	2.1
32772	0.876055	2.1
32773	0.946128	2.1
32774	0.946128	2.1
32775	0.913908	2.1
32776	0.913908	2.1
32777	1.17081	2.1
32778	0.896413	2.1
32779	0.955905	2.1
32780	0.893715	2.1
32781	1.20327	2.1
32782	0.998544	2.1
32783	1.89226	1.56076
32784	1.89226	1.75837
32785	1.89226	0.748268
32786	1.89226	1.24046
32787	3.78452	0.918625
32788	0.540975	1.23325
32789	0.726949	2.04562
32790	0.712198	1.86542
32791	0.506675	1.14408
32792	0.611916	1.20003
32793	0.611916	2.1
32794	0.621708	1.70842
32795	0.621708	1.09909
32796	0.726951	1.31746
32797	0.712196	1.14972
32798	0.506674	1.54471
32799	0.540976	1.35962
32800	1.89226	1.17376
32801	1.89226	2.05318
32802	1.89226	1.1068
32803	1.89226	1.20017
32804	3.78453	1.54495
32805	1.89226	1.00026
32806	1.89226	1.16216
32807	1.89226	0.75704
32808	1.89226	1.82767
32809	1.89226	0.794412
32810	1.89226	2.03845
32811	0.919594	1.11067
32812	0.726949	1.66326
32813	0.898318	1.20026
32814	0.712198	1.60404
32815	0.817559	0.898084
32816	0.817559	2.05267
32817	0.814234	0.901218
32818	0.814234	1.69327
32819	0.919597	0.963033
32820	0.898316	0.814023
32821	0.726951	1.6784
32822	0.712196	1.35976
32823	1.79663	0.892702
32824	1.12088	1.93797
32825	1.54699	1.16213

32826	1.17475	1.22926
32827	1.79663	1.79361
32828	1.42439	1.86201
32829	2.28928	1.04533
32830	1.23077	1.45514
32831	1.65458	0.925159
32832	2.66533	1.66667
32833	3.94385	1.2941
32834	1.89226	2.1
32835	1.89226	1.44951
32836	1.89226	2.1
32837	1.89226	1.02189
32838	3.78452	2.1
32839	1.38426	1.56788
32840	1.15067	1.98778
32841	1.38426	2.1
32842	1.01595	1.93416
32843	1.24509	2.1
32844	1.01595	2.1
32845	2.62097	2.1
32846	1.89226	1.06646
32847	2.72561	2.1
32848	2.72561	1.7074
32849	1.89226	2.1
32850	1.89226	2.1
32851	1.90103	0.307768
32852	1.41902	0.691735
32853	1.77249	0.363958
32854	1.30272	0.89368
32855	3.20375	0.581608
32856	1.77249	0.365783
32857	1.19548	0.680482
32858	1.19548	0.526524
32859	1.30272	0.624504
32860	1.65815	0.36376
32861	1.76539	0.63541
32862	2.62161	2.1
32863	1.89226	2.1
32864	1.89226	2.1
32865	1.89226	2.1
32866	2.6446	2.1
32867	2.6446	2.1
32868	2.64459	2.1
32869	1.89226	2.1
32870	1.89226	2.1
32871	1.89226	2.1
32872	2.58257	2.1
32873	2.58257	2.1
32874	1.89226	0.566114
32875	1.89226	0.981214
32876	2.31301	0.945517
32877	2.76309	0.652967
32878	1.89226	0.871099
32879	2.26047	0.625001
32880	1.89226	0.56999
32881	2.31301	0.965133
32882	2.17765	0.876068
32883	2.17765	0.649642

32884	1.89226	0.814244
32885	1.89226	0.599768
32886	2.18516	2.1
32887	1.85196	2.1
32888	1.78595	2.1
32889	2.14606	2.1
32890	3.99802	2.1
32891	2.18516	2.1
32892	2.21189	2.1
32893	2.18937	2.1
32894	2.14606	2.1
32895	4.35796	2.1
32896	1.60735	2.1
32897	2.14606	2.1
32898	2.15714	2.1
32899	1.70302	2.1
32900	3.70055	2.1
32901	2.18937	2.1
32902	2.14606	2.1
32903	2.0927	2.1
32904	2.162	2.1
32905	4.30806	2.1
32906	2.17326	2.1
32907	2.58517	2.1
32908	2.55318	2.1
32909	2.1665	2.1
32910	4.75167	2.1
32911	2.17326	2.1
32912	1.53556	2.1
32913	1.56253	2.1
32914	2.1665	2.1
32915	3.70206	2.1
32916	2.55317	2.1
32917	2.1665	2.1
32918	2.17741	2.1
32919	2.54197	2.1
32920	4.70847	2.1
32921	2.17742	2.1
32922	2.1665	2.1
32923	2.18452	2.1
32924	1.58054	2.1
32925	1.56253	2.1
32926	1.56253	2.1
32927	1.71051	0.28715
32928	1.89807	0.276185
32929	1.19755	0.237749
32930	1.19755	0.239708
32931	2.25632	0.294748
32932	2.25632	0.281115
32933	0.876046	0.158796
32934	1.27293	0.155766
32935	0.946132	0.209411
32936	0.946132	0.187014
32937	0.946131	0.174374
32938	0.869312	0.162029
32939	1.12767	0.182922
32940	0.946132	0.192918
32941	1.22225	0.19335

32942	1.37968	0.156545
32943	0.851555	0.217222
32944	0.851555	0.177366
32945	0.946133	1.22998
32946	0.946133	2.01797
32947	0.946132	1.37617
32948	0.946132	0.979858
32949	0.946133	1.19088
32950	0.946133	1.29319
32951	0.946132	1.89781
32952	0.946132	1.31349
32953	0.946132	1.82084
32954	0.946132	1.37444
32955	0.946133	1.33237
32956	0.946133	1.61422
32957	1.35152	2.1
32958	0.948936	2.1
32959	1.90166	2.1
32960	1.70303	2.1
32961	2.90858	2.1
32962	1.89226	2.1
32963	1.89226	2.1
32964	1.89226	1.84775
32965	1.89226	2.1
32966	3.78452	2.01097
32967	1.90166	2.1
32968	1.70303	2.1
32969	2.52454	1.32714
32970	2.13919	2.1
32971	3.99529	2.1
32972	1.89226	0.943357
32973	1.89226	1.54222
32974	1.89226	1.94666
32975	1.89226	2.1
32976	3.78452	2.1
32977	1.89226	2.1
32978	1.89226	2.1
32979	1.89226	2.1
32980	1.89226	2.1
32981	1.89226	0.816444
32982	1.89226	1.49276
32983	2.13918	2.1
32984	0.992674	2.1
32985	2.3397	2.1
32986	1.8023	2.1
32987	1.47769	2.1
32988	0.992674	2.1
32989	1.89226	0.361035
32990	1.89226	0.356796
32991	1.89226	0.480157
32992	1.89226	0.266829
32993	3.78452	0.352098
32994	1.89226	0.359539
32995	2.54752	0.190183
32996	2.63974	0.273839
32997	1.89226	0.263259
32998	4.53201	0.26693
32999	0.498412	0.268554

33000	0.500955	0.23226
33001	0.57955	0.34431
33002	0.563873	0.284504
33003	0.529107	0.275671
33004	0.529107	0.233541
33005	0.53572	0.356216
33006	0.53572	0.259276
33007	0.500956	0.270811
33008	0.579548	0.270819
33009	0.563871	0.272227
33010	0.498414	0.272421
33011	1.89223	0.416365
33012	1.89223	0.258972
33013	1.89223	0.321577
33014	1.89223	0.372692
33015	1.89223	0.315636
33016	1.89223	0.363359
33017	1.28752	0.190973
33018	1.31985	0.248555
33019	0.946119	0.247963
33020	0.946119	0.336791
33021	1.28752	0.249308
33022	1.28752	0.197373
33023	0.946119	0.24148
33024	0.946119	0.388359
33025	1.28753	0.239779
33026	0.946132	0.236092
33027	1.31984	0.25616
33028	0.946107	0.260282
33029	0.500948	0.246037
33030	0.479465	0.222652
33031	0.562335	0.308007
33032	0.579542	0.258389
33033	0.528436	0.252476
33034	0.528436	0.225281
33035	0.530571	0.243254
33036	0.530571	0.314387
33037	0.56234	0.251314
33038	0.479473	0.253662
33039	0.500943	0.25583
33040	0.579533	0.2554
33041	1.89226	1.99426
33042	1.89226	2.1
33043	1.89226	1.79074
33044	1.89226	2.1
33045	3.78452	2.1
33046	1.50767	1.7558
33047	1.14496	2.1
33048	1.50767	2.1
33049	1.58539	1.87358
33050	1.07653	2.1
33051	1.15425	2.1
33052	1.89226	2.1
33053	2.43826	2.1
33054	2.43826	2.1
33055	2.48688	2.1
33056	1.89226	2.1
33057	1.94088	2.1

33058	1.89226	1.55783
33059	1.89226	2.1
33060	1.89226	1.68719
33061	1.89226	2.1
33062	3.78452	2.1
33063	1.50767	1.52797
33064	1.07653	2.1
33065	1.41077	1.08771
33066	0.991407	2.1
33067	2.49907	2.1
33068	1.21913	2.1
33069	1.21913	2.1
33070	0.946128	2.1
33071	0.946128	2.1
33072	1.21913	2.1
33073	1.18364	2.1
33074	0.946131	2.1
33075	0.946131	2.1
33076	1.18364	2.1
33077	0.946129	2.1
33078	1.21913	2.1
33079	0.94613	2.1
33080	1.89226	0.618143
33081	1.89226	1.70691
33082	1.89226	0.623222
33083	1.89226	2.1
33084	3.78452	0.73107
33085	1.93823	0.620838
33086	1.60976	1.74955
33087	1.93823	1.99738
33088	2.20045	0.59546
33089	1.36999	1.83791
33090	1.63222	0.677158
33091	1.38302	1.83721
33092	1.38302	2.1
33093	0.946132	2.1
33094	0.946132	2.1
33095	1.27258	2.1
33096	1.38302	2.1
33097	0.946133	1.90156
33098	0.946133	2.08244
33099	0.946133	2.1
33100	1.27258	2.1
33101	0.946132	2.1
33102	1.38302	2.1
33103	1.89226	0.56357
33104	1.89226	1.93257
33105	1.89226	0.613941
33106	1.89226	1.80714
33107	3.78452	0.766655
33108	1.93823	0.561367
33109	1.36999	1.63496
33110	1.70423	0.553595
33111	1.14195	1.81761
33112	3.08018	0.700818
33113	1.27258	1.93446
33114	1.27258	2.1
33115	0.946132	2.1

33116	0.946132	2.1
33117	1.17233	2.1
33118	1.27258	2.1
33119	0.946132	1.91853
33120	0.946132	1.65919
33121	1.17233	2.1
33122	0.946132	2.1
33123	0.946132	2.1
33124	1.27258	2.1
33125	2.70575	2.1
33126	1.89226	0.847886
33127	2.71429	2.1
33128	2.71429	1.69163
33129	1.89226	1.49902
33130	1.89226	0.856829
33131	0.946133	0.772925
33132	0.946133	2.04125
33133	1.28713	2.1
33134	1.28713	2.1
33135	0.946131	2.03422
33136	0.946131	0.761416
33137	1.28713	2.1
33138	1.36503	1.33659
33139	0.946131	1.95663
33140	1.36503	1.86137
33141	1.28713	2.00905
33142	0.946131	2.1
33143	1.89226	2.06055
33144	1.89226	1.31717
33145	1.89226	2.03595
33146	1.89226	1.08014
33147	1.89226	1.86851
33148	1.89226	1.07181
33149	1.22615	2.09568
33150	1.04147	2.1
33151	1.3439	2.1
33152	1.3439	2.1
33153	0.969118	2.1
33154	0.969118	1.90824
33155	0.980841	2.1
33156	0.992505	2.1
33157	1.34289	2.1
33158	1.70303	2.1
33159	2.56081	2.1
33160	1.89225	2.1
33161	2.66785	2.1
33162	2.79506	2.1
33163	2.79506	2.1
33164	1.89225	2.1
33165	1.89225	2.1
33166	0.978778	2.1
33167	0.978778	2.1
33168	0.993303	2.1
33169	0.993303	2.1
33170	0.603538	2.1
33171	0.638158	2.1
33172	1.33393	2.1
33173	1.39753	2.1

33174	1.33391	2.1
33175	0.603535	2.1
33176	0.638164	2.1
33177	1.39754	2.1
33178	1.33393	2.1
33179	1.33393	2.1
33180	0.946132	2.1
33181	0.946132	2.1
33182	1.33393	2.1
33183	1.27365	2.1
33184	0.946132	2.1
33185	0.946132	2.1
33186	0.946132	2.1
33187	1.27365	2.1
33188	1.33393	2.1
33189	0.946131	2.1
33190	1.89226	2.1
33191	1.89226	2.1
33192	1.89226	2.1
33193	1.89226	2.1
33194	3.78452	2.1
33195	1.89226	2.1
33196	1.89226	2.1
33197	1.89226	2.1
33198	1.89226	2.1
33199	3.78453	2.1
33200	0.979364	2.1
33201	0.979364	2.1
33202	0.89618	2.1
33203	0.89618	2.1
33204	0.896178	2.1
33205	0.929411	2.1
33206	0.946133	2.1
33207	0.946133	2.1
33208	0.946131	2.1
33209	0.89618	2.1
33210	0.946131	2.1
33211	0.929413	2.1
33212	1.89226	2.1
33213	1.89226	2.1
33214	1.89226	2.1
33215	1.89226	2.1
33216	1.89226	2.1
33217	1.89226	2.1
33218	1.89225	2.1
33219	1.89225	2.1
33220	1.89225	2.1
33221	1.89225	2.1
33222	3.7845	2.1
33223	1.89225	2.1
33224	1.89225	2.1
33225	1.89225	2.1
33226	1.89225	2.1
33227	1.89225	2.1
33228	1.89225	2.1
33229	0.946128	0.419097
33230	0.946128	0.297274
33231	0.946132	0.385286

33232	0.946132	0.267696
33233	0.94613	0.388277
33234	0.94613	0.435409
33235	0.946129	0.28304
33236	0.946129	0.232933
33237	0.94613	0.342852
33238	0.94613	0.347114
33239	0.94613	0.320306
33240	0.94613	0.309851
33241	1.89227	1.07132
33242	1.89227	1.09979
33243	1.89227	0.892678
33244	1.89227	0.779545
33245	3.78453	0.912837
33246	1.89226	1.08511
33247	2.43717	1.47628
33248	2.63259	0.958775
33249	1.89226	0.715182
33250	4.52485	1.15517
33251	0.616808	0.926661
33252	0.792277	1.16355
33253	1.04986	0.869855
33254	0.72303	1.10197
33255	0.702675	0.559491
33256	0.76862	1.59456
33257	0.809154	1.31213
33258	0.809154	0.852589
33259	0.808757	1.08094
33260	1.07789	0.702415
33261	0.772568	0.873148
33262	0.598878	1.22839
33263	1.89226	1.09101
33264	1.89226	1.03252
33265	1.89226	0.881491
33266	1.89226	0.841318
33267	3.78453	1.0321
33268	1.36428	1.59687
33269	1.31629	0.986234
33270	0.946132	1.08193
33271	0.946132	0.96698
33272	1.36428	0.772552
33273	1.36428	1.63168
33274	0.946132	1.06382
33275	0.946132	1.34486
33276	1.36428	1.48363
33277	0.946132	0.849019
33278	1.31629	1.24155
33279	0.946132	0.73226
33280	1.578	1.17378
33281	2.85336	0.721035
33282	2.11368	0.633189
33283	2.14375	0.930728
33284	1.93852	0.963934
33285	1.93852	1.259
33286	2.34806	0.496994
33287	1.42913	0.274616
33288	1.61539	0.572308
33289	2.45322	0.371178

33290	3.88235	0.463684
33291	2.34806	0.419716
33292	1.89226	0.329797
33293	1.89226	0.553746
33294	2.45322	0.36334
33295	4.34548	0.337988
33296	1.89226	0.35037
33297	1.89226	0.247171
33298	1.89226	0.360234
33299	1.89226	0.33255
33300	3.78452	0.371925
33301	1.89226	0.307223
33302	1.89226	0.245906
33303	1.89226	0.368538
33304	1.89226	0.340437
33305	3.78452	0.291574
33306	1.25972	0.413921
33307	2.45322	0.292584
33308	2.58823	0.536607
33309	1.79398	0.338281
33310	4.2472	0.332002
33311	1.89226	0.486546
33312	2.45322	0.288766
33313	2.58823	0.362891
33314	2.45322	0.315037
33315	2.02727	0.360498
33316	1.89226	0.313531
33317	1.89226	0.283889
33318	1.89226	0.270718
33319	1.89226	0.42219
33320	1.89226	0.259458
33321	3.78452	0.245277
33322	1.89226	0.289326
33323	1.89226	0.256281
33324	1.89226	0.241923
33325	1.89226	0.176839
33326	1.89226	0.435103
33327	1.89226	0.313413
33328	1.70048	1.40761
33329	2.1572	0.312063
33330	1.45477	0.867736
33331	1.93973	0.466153
33332	3.64021	0.73512
33333	1.17614	0.907368
33334	1.93973	0.450007
33335	1.69661	1.08753
33336	1.69661	0.459598
33337	1.45477	1.16923
33338	1.45477	0.508331
33339	1.94343	1.57726
33340	2.53782	1.21198
33341	2.59903	1.33686
33342	2.59903	0.805401
33343	1.87598	1.31099
33344	1.87598	0.90164
33345	0.946131	0.765487
33346	0.946131	1.17179
33347	1.26891	1.68004

33348	1.26891	0.464279
33349	0.94613	1.27221
33350	0.94613	1.02262
33351	1.26891	1.2525
33352	1.29952	0.860647
33353	1.29952	0.635875
33354	0.946131	0.49413
33355	1.26891	0.917907
33356	0.946131	0.933822
33357	2.00551	1.54359
33358	2.59903	1.39185
33359	2.65811	1.44575
33360	2.65811	0.944721
33361	1.94343	1.24787
33362	1.94343	0.975713
33363	2.65811	0.813145
33364	1.89226	1.51456
33365	1.89226	0.723631
33366	1.89226	1.22528
33367	2.59903	0.896759
33368	2.59903	1.63506
33369	1.89227	2.1
33370	1.89227	2.1
33371	1.89227	2.1
33372	1.89227	2.1
33373	3.78453	2.1
33374	1.15471	2.1
33375	0.993889	2.1
33376	1.78919	2.1
33377	1.77325	2.1
33378	2.78308	2.1
33379	1.89226	2.1
33380	1.89226	2.1
33381	1.89226	2.1
33382	1.89226	2.1
33383	3.78453	2.1
33384	1.78919	2.1
33385	1.77325	2.1
33386	2.45969	2.1
33387	2.70835	2.1
33388	4.49753	2.1
33389	0.939314	2.02046
33390	0.939315	2.1
33391	1.01133	2.1
33392	1.01133	2.1
33393	0.596471	2.1
33394	1.20813	2.1
33395	0.886619	2.1
33396	1.35417	2.1
33397	1.35417	2.1
33398	1.20813	2.1
33399	0.886621	2.1
33400	0.59647	2.1
33401	0.946131	2.1
33402	0.946131	2.08346
33403	0.94613	2.1
33404	0.94613	2.08616
33405	0.946133	2.08273

33406	0.946133	2.01209
33407	0.946128	2.1
33408	0.946128	2.1
33409	0.946131	2.1
33410	0.946131	2.1
33411	0.946131	2.08263
33412	0.946131	2.1
33413	1.03939	2.1
33414	1.03939	2.1
33415	0.946132	0.917242
33416	0.946132	0.982438
33417	0.946132	1.09051
33418	0.946132	2.1
33419	1.23851	0.918663
33420	1.03939	2.1
33421	1.03939	1.474
33422	0.946132	1.53131
33423	0.946133	1.49411
33424	1.23851	1.46493
33425	0.902503	2.1
33426	0.946129	2.1
33427	0.946132	0.920248
33428	0.946132	1.24594
33429	0.902472	1.27715
33430	0.902472	2.1
33431	1.08736	0.930146
33432	0.994093	2.1
33433	0.946133	1.23319
33434	0.902504	1.36211
33435	1.03939	1.37473
33436	0.946132	1.40978
33437	1.35306	0.951239
33438	1.29982	0.621559
33439	1.7216	0.620523
33440	1.89183	0.581017
33441	3.07466	0.627965
33442	0.485098	0.638083
33443	0.582401	0.664159
33444	0.649908	0.795096
33445	0.8608	0.675333
33446	0.587739	0.526384
33447	0.58774	0.72073
33448	0.644567	0.649051
33449	0.644567	1.06369
33450	0.58241	0.767391
33451	0.86081	0.551846
33452	0.649898	0.90947
33453	0.485093	0.661011
33454	2.25936	2.1
33455	2.71441	2.1
33456	2.63824	2.1
33457	2.20652	2.1
33458	4.92092	2.1
33459	2.25936	2.1
33460	1.57627	2.1
33461	1.5493	2.1
33462	2.20652	2.1
33463	3.78279	2.1

33464	2.63824	2.1
33465	2.20652	2.1
33466	2.17326	2.1
33467	2.58517	2.1
33468	4.79169	2.1
33469	2.17326	2.1
33470	2.20652	2.1
33471	2.19278	2.1
33472	1.53556	2.1
33473	1.5493	2.1
33474	1.5493	2.1
33475	1.97465	2.1
33476	2.13925	2.1
33477	2.04483	2.1
33478	1.62784	2.1
33479	3.76709	2.1
33480	1.89226	2.1
33481	1.89226	2.1
33482	1.89226	2.1
33483	1.89226	2.1
33484	1.89226	2.1
33485	1.89226	2.1
33486	0.724037	2.1
33487	0.81392	2.1
33488	0.96796	2.1
33489	1.02242	2.1
33490	0.882091	2.1
33491	0.882091	2.1
33492	0.899789	2.1
33493	0.899789	2.1
33494	0.724036	2.1
33495	0.96796	2.1
33496	1.02242	2.1
33497	0.81392	2.1
33498	1.13537	2.1
33499	1.63054	2.1
33500	0.946131	2.1
33501	0.946131	2.1
33502	0.946131	2.1
33503	0.946131	2.1
33504	1.258	2.1
33505	1.63054	2.1
33506	1.22632	2.1
33507	1.22632	2.1
33508	0.946131	2.1
33509	1.258	2.1
33510	1.89226	2.1
33511	1.89226	2.1
33512	1.89226	2.1
33513	1.89226	2.1
33514	1.89226	2.1
33515	1.89226	2.1
33516	0.809739	2.1
33517	0.862674	2.1
33518	1.1995	2.1
33519	1.33899	2.1
33520	1.06324	2.1
33521	1.06324	2.1

33522	0.981932	2.1
33523	1.19563	2.1
33524	0.809727	2.1
33525	1.14092	2.1
33526	1.33902	2.1
33527	0.863494	2.1
33528	2.23742	2.1
33529	1.84902	2.1
33530	1.88088	2.1
33531	2.20392	2.1
33532	4.08644	2.1
33533	1.3592	2.1
33534	1.84902	2.1
33535	1.88088	2.1
33536	1.3305	2.08446
33537	1.88088	2.1
33538	1.3305	2.1
33539	1.84902	2.1
33540	1.15008	0.443014
33541	1.3305	1.67161
33542	1.70539	2.1
33543	2.9991	2.1
33544	1.84901	2.1
33545	2.0264	2.1
33546	2.20391	2.1
33547	1.70539	2.1
33548	3.87541	2.1
33549	1.03852	1.78552
33550	1.38247	0.711633
33551	1.36635	0.819233
33552	1.01537	1.26616
33553	2.39784	1.07998
33554	2.0815	1.2371
33555	1.91744	0.933998
33556	1.91744	0.928219
33557	2.06496	2.1
33558	1.89227	0.872263
33559	2.03474	2.1
33560	1.89226	0.729519
33561	1.89226	0.930324
33562	1.89226	0.697499
33563	1.89226	1.32905
33564	1.89226	0.68491
33565	1.89226	1.28412
33566	1.36635	0.950806
33567	1.02923	1.60489
33568	1.02923	0.871951
33569	1.01537	1.58469
33570	1.36416	0.798437
33571	1.3503	1.84805
33572	1.89226	1.26734
33573	1.89359	1.97778
33574	1.89359	1.10862
33575	1.91744	2.1
33576	1.89226	1.04095
33577	1.91611	2.1
33578	0.946128	1.50686
33579	0.946128	1.08957

33580	0.946133	0.821957
33581	0.946133	0.685511
33582	0.94613	0.690093
33583	0.94613	1.00269
33584	0.946131	1.64541
33585	0.946131	0.74137
33586	0.946131	0.891407
33587	0.946131	0.873217
33588	0.946131	0.940823
33589	0.946131	0.92451
33590	2.689	2.1
33591	2.0815	2.1
33592	2.72786	2.1
33593	2.48295	2.1
33594	5.02397	2.1
33595	2.72786	2.1
33596	1.86424	2.1
33597	2.7188	2.1
33598	2.08981	2.1
33599	4.81768	2.1
33600	2.08982	2.1
33601	0.986987	2.1
33602	2.08982	2.1
33603	1.87249	2.1
33604	1.01964	2.1
33605	0.906115	2.1
33606	1.44247	2.1
33607	1.43872	2.1
33608	1.17054	2.1
33609	1.17054	2.1
33610	1.43872	2.1
33611	1.26045	2.1
33612	2.0815	0.0895549
33613	2.0815	0.0955296
33614	2.0815	0.105235
33615	1.81468	0.0865997
33616	1.89065	0.10211
33617	1.55625	0.0841431
33618	1.89062	0.0901006
33619	1.89224	0.0991847
33620	1.89224	0.107414
33621	2.27073	0.0885134
33622	1.86856	0.104655
33623	2.24232	0.0857437
33624	1.21419	2.1
33625	2.79716	2.1
33626	2.50005	2.1
33627	2.50005	2.1
33628	1.5008	2.1
33629	1.5008	2.1
33630	1.60139	0.0890504
33631	1.7103	0.0976736
33632	1.04159	0.100953
33633	1.2489	0.0828927
33634	2.75189	0.0897227
33635	0.732812	0.0826277
33636	0.732812	0.085906
33637	0.751986	0.0982974

33638	0.751986	0.0965509
33639	0.860346	0.085435
33640	0.667961	0.0968811
33641	0.85515	0.0993882
33642	0.624452	0.0879831
33643	0.62445	0.0901456
33644	0.667962	0.0878967
33645	0.855148	0.0926158
33646	0.860348	0.0907259
33647	1.89226	0.455637
33648	2.50872	0.481452
33649	2.77909	0.458409
33650	1.89226	0.474875
33651	2.77909	0.461154
33652	1.89226	0.478501
33653	2.15825	0.378324
33654	2.50872	0.379985
33655	1.90439	0.327885
33656	1.90439	0.407526
33657	2.77909	0.337302
33658	2.77909	0.432435
33659	1.12468	2.1
33660	1.43872	2.1
33661	1.64926	2.1
33662	1.2724	2.1
33663	2.77395	2.1
33664	1.64926	2.1
33665	1.38438	2.1
33666	1.47244	2.1
33667	1.2724	2.1
33668	1.8493	2.1
33669	1.64926	2.1
33670	1.36416	0.884182
33671	1.0652	1.71583
33672	1.0652	0.965203
33673	1.02923	1.73218
33674	1.37496	0.873753
33675	1.33898	1.95652
33676	1.89227	1.4064
33677	1.90433	2.1
33678	1.90433	1.40907
33679	1.89359	2.1
33680	1.89227	1.31257
33681	1.88153	2.1
33682	0.946129	1.6688
33683	0.946129	1.35587
33684	0.946133	0.906438
33685	0.946133	0.755831
33686	0.946131	0.760694
33687	0.946131	1.2314
33688	0.946131	1.75844
33689	0.946131	0.805928
33690	0.946131	1.00982
33691	0.946131	0.997952
33692	0.946131	1.09417
33693	0.946131	1.05303
33694	1.37496	0.911003
33695	1.07519	1.83775

33696	1.07519	0.992569
33697	1.0652	1.83036
33698	1.39725	0.90075
33699	1.38726	2.01705
33700	1.89227	1.57865
33701	1.9052	2.1
33702	1.9052	1.55523
33703	1.90433	2.1
33704	1.89227	1.4302
33705	1.8914	2.1
33706	0.946129	1.79312
33707	0.946129	1.46493
33708	0.946133	0.963844
33709	0.946133	0.772257
33710	0.946131	0.779242
33711	0.946131	1.37536
33712	0.946131	1.86692
33713	0.946131	0.835946
33714	0.946131	1.07105
33715	0.946131	1.08938
33716	0.946131	1.16365
33717	0.946131	1.14172
33718	1.91316	0.289492
33719	1.89226	0.331338
33720	1.89226	0.326648
33721	1.89226	0.319655
33722	1.99366	0.296613
33723	1.99366	0.288112
33724	1.93083	0.258457
33725	1.89226	0.324397
33726	1.51379	0.299041
33727	1.89226	0.310338
33728	1.53051	0.274447
33729	1.91316	0.287166
33730	2.16731	2.1
33731	1.21373	2.1
33732	1.47804	2.1
33733	2.37601	2.1
33734	3.64535	2.1
33735	1.7211	2.1
33736	2.37601	2.1
33737	1.47804	2.1
33738	1.47804	2.1
33739	2.56843	2.1
33740	2.56843	2.1
33741	2.58731	2.1
33742	1.59094	2.1
33743	1.59094	2.1
33744	1.76816	2.1
33745	2.41707	2.1
33746	2.5943	2.1
33747	0.718346	2.1
33748	0.795468	2.1
33749	1.20854	2.1
33750	1.1224	2.1
33751	0.961203	2.1
33752	0.961203	2.1
33753	0.95667	2.1

33754	0.95667	2.1
33755	0.718346	2.1
33756	1.1224	2.1
33757	0.795469	2.1
33758	1.20854	2.1
33759	1.84076	2.1
33760	1.42884	2.1
33761	2.24274	2.1
33762	1.85415	2.1
33763	3.69491	2.1
33764	1.89226	2.1
33765	1.89226	1.38719
33766	1.89226	1.57685
33767	1.89226	2.1
33768	3.78452	2.00071
33769	2.24273	2.1
33770	2.24412	2.1
33771	1.85415	2.1
33772	1.85415	2.1
33773	2.68473	2.1
33774	2.68473	2.1
33775	1.89226	1.22813
33776	1.89226	2.1
33777	1.89226	2.1
33778	1.89226	1.06923
33779	1.89226	2.1
33780	1.89226	1.07277
33781	1.89226	1.06994
33782	1.89226	2.1
33783	1.89226	2.1
33784	1.89226	1.35339
33785	3.78451	2.01615
33786	1.85415	2.1
33787	1.10961	2.1
33788	2.24411	2.1
33789	1.51067	2.1
33790	3.35373	2.1
33791	1.70302	2.1
33792	1.89226	2.1
33793	1.76399	2.1
33794	1.43577	2.1
33795	2.08149	2.1
33796	1.70302	2.1
33797	1.89226	2.1
33798	1.89226	2.1
33799	1.89226	2.1
33800	1.89226	2.1
33801	1.89226	1.94151
33802	1.89226	2.1
33803	1.0593	2.1
33804	1.17289	2.1
33805	1.5379	2.1
33806	1.46757	2.1
33807	2.71079	2.1
33808	0.931235	2.1
33809	1.1151	2.1
33810	0.975236	2.1
33811	0.975236	2.1

33812	1.31147	2.1
33813	0.762882	2.1
33814	1.13152	2.1
33815	0.505799	2.1
33816	1.31149	2.1
33817	1.07629	2.1
33818	0.884817	2.1
33819	0.505793	2.1
33820	2.69027	2.1
33821	1.60441	2.1
33822	2.69027	2.1
33823	1.64771	2.1
33824	2.78977	2.1
33825	1.72731	2.1
33826	0.77878	0.0522355
33827	0.843226	0.0522355
33828	0.845997	0.0522355
33829	0.926091	0.0522355
33830	0.77878	0.0522355
33831	0.926093	0.0522355
33832	1.3922	1.40391
33833	1.3922	1.45149
33834	0.872156	2.01513
33835	0.872156	1.93526
33836	0.872157	2.09075
33837	0.946132	1.36703
33838	0.946129	1.10528
33839	1.31822	2.1
33840	0.94613	1.81982
33841	0.94613	1.9056
33842	1.31823	1.71467
33843	0.872157	1.90433
33844	1.81534	0.597611
33845	1.89226	0.587963
33846	1.59602	0.683773
33847	1.59602	0.772329
33848	1.89226	0.405575
33849	1.89226	0.496284
33850	1.89227	0.555778
33851	1.89227	0.577808
33852	1.89227	0.612406
33853	1.89227	0.527565
33854	3.78453	0.522048
33855	1.89226	0.576996
33856	1.89226	0.438976
33857	1.89226	0.526929
33858	1.89226	0.512
33859	3.78453	0.587744
33860	2.30348	0.341894
33861	1.4185	0.369036
33862	1.82551	0.425138
33863	1.02011	0.331981
33864	3.24401	0.321183
33865	2.46702	0.300696
33866	1.17129	0.337162
33867	1.82551	0.295351
33868	1.82551	0.313113
33869	1.73525	0.294345

33870	1.73525	0.348597
33871	1.89227	0.538221
33872	1.89227	0.42763
33873	1.89227	0.427957
33874	1.89227	0.449588
33875	1.89227	0.517918
33876	1.89227	0.5345
33877	2.80034	1.10385
33878	2.45767	2.1
33879	2.67994	0.907655
33880	2.35395	2.1
33881	5.1376	2.1
33882	2.45766	2.1
33883	1.8769	2.1
33884	2.00989	2.1
33885	2.00989	2.1
33886	2.35395	2.1
33887	2.35395	2.1
33888	2.57662	1.1288
33889	2.35395	2.1
33890	2.57662	2.1
33891	2.67993	1.58724
33892	2.24227	2.1
33893	2.34558	1.86476
33894	1.02874	2.1
33895	1.02874	2.1
33896	1.03084	2.1
33897	1.03084	2.1
33898	0.938448	2.1
33899	0.886762	2.1
33900	1.17698	2.1
33901	1.12114	2.1
33902	1.12113	2.1
33903	0.886764	2.1
33904	0.93845	2.1
33905	1.17697	2.1
33906	1.91969	2.1
33907	2.5508	2.1
33908	1.88273	2.06267
33909	2.76609	2.1
33910	4.68578	2.1
33911	2.83423	2.1
33912	1.89226	2.1
33913	2.76609	2.1
33914	1.89226	2.1
33915	4.65835	2.1
33916	1.56685	0.671476
33917	1.11712	0.600326
33918	1.07999	1.07715
33919	1.51448	0.73261
33920	2.6316	0.785172
33921	1.56685	0.527621
33922	1.67652	0.837745
33923	1.5955	1.0196
33924	1.51448	0.664353
33925	3.19099	0.773079
33926	1.89226	0.86989
33927	1.89226	0.454909

33928	1.89226	0.68311
33929	1.89226	0.742589
33930	3.78453	0.764018
33931	1.89226	0.747922
33932	1.89226	0.552763
33933	1.89226	0.867942
33934	1.89226	0.873434
33935	1.89226	0.535115
33936	1.89226	0.51823
33937	1.0562	0.830682
33938	1.51448	0.63399
33939	1.07999	0.73225
33940	1.07999	0.6299
33941	1.46441	0.767868
33942	1.46441	0.682952
33943	1.5955	1.0549
33944	1.51448	0.62143
33945	1.46441	0.713658
33946	1.52909	0.811334
33947	3.04357	0.778328
33948	1.89226	0.635747
33949	1.89226	0.721796
33950	1.89226	0.830747
33951	1.89226	0.552206
33952	3.78453	0.611981
33953	1.89227	0.648631
33954	1.89227	0.388984
33955	1.89227	0.828378
33956	1.89227	0.64442
33957	3.78453	0.702851
33958	1.89994	0.477632
33959	1.70303	0.552774
33960	1.68576	0.331296
33961	1.89608	0.52933
33962	3.8237	0.464056
33963	1.89994	0.431966
33964	2.31116	0.836643
33965	1.9386	0.766173
33966	1.88653	1.07444
33967	3.74885	0.805075
33968	2.31117	0.90668
33969	2.20665	1.22627
33970	2.09617	0.952556
33971	2.41055	2.04109
33972	2.09617	1.25497
33973	2.41055	2.07004
33974	1.10777	0.512251
33975	1.0523	0.514689
33976	0.912098	0.501536
33977	0.757533	0.540191
33978	0.89287	0.619492
33979	1.07571	0.323749
33980	0.77485	0.52519
33981	0.98645	0.378883
33982	0.989171	0.425468
33983	0.875089	0.387149
33984	0.979686	0.52979
33985	0.979686	0.695178

33986	2.52442	2.1
33987	1.49998	2.1
33988	2.51692	2.1
33989	1.56957	2.1
33990	4.09399	2.1
33991	1.01298	2.1
33992	1.01298	2.1
33993	1.03079	2.1
33994	1.03079	2.1
33995	0.820964	2.1
33996	0.784786	2.1
33997	1.25898	2.1
33998	1.25846	2.1
33999	1.25898	2.1
34000	0.820964	2.1
34001	1.25846	2.1
34002	0.784786	2.1
34003	1.89226	0.105787
34004	1.89226	0.112057
34005	2.6297	0.0932252
34006	1.89226	0.0946245
34007	3.78452	0.109305
34008	1.89226	0.094147
34009	1.89226	0.0903791
34010	1.89226	0.0857693
34011	1.89226	0.105705
34012	1.89226	0.0850906
34013	1.89226	0.107343
34014	2.19397	0.0786626
34015	1.89226	0.0905027
34016	2.34112	0.0828465
34017	2.34112	0.0733675
34018	1.89226	0.0868276
34019	1.89226	0.0852385
34020	2.23366	2.1
34021	1.87598	2.1
34022	1.64716	2.1
34023	2.06048	2.1
34024	3.93645	2.1
34025	2.23367	2.1
34026	2.17384	2.1
34027	2.04818	2.1
34028	2.06048	2.1
34029	4.23432	2.1
34030	1.9855	2.1
34031	2.06048	2.1
34032	1.93163	2.1
34033	1.50737	2.1
34034	3.56785	2.1
34035	2.04818	2.1
34036	2.06048	2.1
34037	2.27465	2.1
34038	1.92264	2.1
34039	3.98312	2.1
34040	2.08149	0.643214
34041	1.89226	1.5896
34042	1.89226	1.22827
34043	1.33153	0.613446

34044	1.68038	1.76731
34045	1.18244	0.809906
34046	1.89226	0.950925
34047	1.89226	1.63532
34048	1.89226	1.48048
34049	1.89226	0.807947
34050	3.78452	1.02262
34051	2.69069	0.602628
34052	1.89226	0.892582
34053	1.89226	0.898001
34054	1.97397	0.618645
34055	1.89226	0.864482
34056	1.97397	0.584283
34057	1.89226	1.64434
34058	2.38056	0.809975
34059	1.89226	0.814118
34060	1.89226	1.2244
34061	3.78452	1.1031
34062	0.904435	2.1
34063	0.904435	2.1
34064	0.925315	2.1
34065	0.925315	2.1
34066	1.13312	2.1
34067	1.08109	2.1
34068	0.70697	2.1
34069	0.696633	2.1
34070	0.70697	2.1
34071	1.13312	2.1
34072	0.696633	2.1
34073	1.08109	2.1
34074	1.45506	2.1
34075	2.26623	2.1
34076	2.38363	2.1
34077	2.38363	2.1
34078	1.41394	2.1
34079	1.41394	2.1
34080	0.946131	1.61498
34081	0.946131	2.1
34082	1.13311	2.1
34083	1.13311	2.1
34084	0.946127	1.33465
34085	0.946127	2.1
34086	1.13312	2.1
34087	1.19182	2.1
34088	1.19181	2.1
34089	0.946129	2.1
34090	1.13312	2.1
34091	0.94613	2.1
34092	1.89226	0.741973
34093	1.89226	1.51019
34094	1.89226	0.802511
34095	1.89226	1.3522
34096	1.89226	0.837355
34097	1.89226	1.46856
34098	1.05168	2.03601
34099	0.696527	1.83356
34100	0.907442	0.951001
34101	0.907442	1.07992

34102	0.953145	0.896728
34103	0.782285	2.1
34104	0.773928	1.97085
34105	0.773928	0.974788
34106	0.696538	1.04342
34107	0.851554	1.09326
34108	1.04071	1.16839
34109	1.12335	1.19088
34110	1.89226	0.735741
34111	1.89226	0.520732
34112	1.89226	0.517142
34113	1.89226	0.420611
34114	3.78453	0.454947
34115	1.89226	0.65525
34116	1.89226	0.522008
34117	1.89226	0.525815
34118	1.89226	0.472188
34119	3.78453	0.600783
34120	2.50912	0.492367
34121	1.89226	0.447123
34122	1.89226	0.457309
34123	2.63195	0.39424
34124	4.40139	0.399207
34125	2.50912	0.490321
34126	1.3598	0.389492
34127	1.70303	0.391096
34128	2.36874	0.305833
34129	3.82055	0.442273
34130	1.89227	0.507528
34131	1.89227	0.424421
34132	1.89227	0.402529
34133	1.89227	0.312287
34134	3.78453	0.484265
34135	1.89227	0.517032
34136	1.89227	0.456897
34137	1.89227	0.431115
34138	1.89227	0.340998
34139	3.78453	0.378406
34140	1.89226	0.438891
34141	2.67088	0.295993
34142	2.63195	0.263053
34143	2.63195	0.378624
34144	1.89226	0.313227
34145	1.89226	0.448382
34146	1.30202	0.326448
34147	1.28304	0.342057
34148	0.809902	0.307128
34149	0.888801	0.315709
34150	1.13262	0.211818
34151	0.968276	0.281224
34152	1.0733	0.341173
34153	1.0733	0.330267
34154	1.35419	0.294271
34155	0.801222	0.230291
34156	0.899732	0.385186
34157	1.23967	0.269608
34158	1.31595	2.1
34159	0.869501	2.1

34160	2.65473	2.1
34161	1.88782	2.1
34162	3.20377	2.1
34163	1.89227	2.1
34164	1.89227	2.1
34165	1.89227	2.00588
34166	1.89227	2.1
34167	3.78453	2.06947
34168	2.65473	2.1
34169	1.89226	2.1
34170	1.88781	2.1
34171	1.88782	2.1
34172	2.0815	2.1
34173	2.0815	2.1
34174	1.89226	1.53036
34175	1.89226	1.93251
34176	1.89226	1.80092
34177	1.89226	1.75101
34178	1.89226	1.61909
34179	1.89226	1.56774
34180	1.89226	1.59746
34181	1.89226	1.73125
34182	1.89226	2.00948
34183	1.89226	1.92693
34184	1.89226	1.79114
34185	1.89226	1.6724
34186	2.39057	1.65694
34187	1.89226	0.856722
34188	1.89226	0.731722
34189	1.89226	1.87191
34190	2.14243	0.689944
34191	2.14243	1.94413
34192	1.89226	0.53485
34193	1.89226	1.17635
34194	1.89226	0.665072
34195	1.89226	0.754306
34196	1.89226	0.772291
34197	1.89226	1.17822
34198	1.89226	0.583684
34199	1.89226	1.37941
34200	1.89226	0.634756
34201	1.89226	0.790379
34202	1.89226	0.702711
34203	1.89226	1.32865
34204	2.56323	0.209033
34205	1.36324	0.243503
34206	1.05296	0.213045
34207	2.18048	0.25206
34208	3.54372	0.214505
34209	0.946128	1.60987
34210	0.946128	2.1
34211	1.08733	2.1
34212	1.08733	2.1
34213	0.946131	2.1
34214	0.946131	1.86684
34215	1.09197	2.1
34216	1.08732	2.1
34217	0.946131	2.1

34218	1.09197	2.1
34219	1.08732	2.1
34220	0.946131	2.1
34221	1.35111	1.09003
34222	1.51327	1.07306
34223	1.64493	1.04586
34224	1.31058	1.1346
34225	2.99605	1.26386
34226	1.8124	1.0288
34227	1.51327	1.1738
34228	1.64493	1.19514
34229	1.31358	1.04686
34230	1.8124	1.152
34231	1.48105	0.992642
34232	1.64493	1.03293
34233	1.31058	1.107
34234	1.22297	1.02604
34235	1.70101	1.03076
34236	2.8679	0.939153
34237	1.70101	1.0872
34238	1.99297	0.989766
34239	1.64493	1.122
34240	1.64493	0.954751
34241	1.99297	1.10941
34242	1.8124	0.895586
34243	2.0815	2.1
34244	1.80258	2.1
34245	1.80258	2.1
34246	1.83506	2.1
34247	2.0815	2.1
34248	2.1212	2.1
34249	2.0815	2.1
34250	1.82129	2.1
34251	1.82129	2.1
34252	1.80445	2.1
34253	2.0815	2.1
34254	2.06092	2.1
34255	1.89224	2.1
34256	1.703 2.1	
34257	1.97839	2.1
34258	1.703 2.1	
34259	1.97402	2.1
34260	1.703 2.1	
34261	1.89226	0.696091
34262	1.89226	0.866592
34263	1.89226	0.689227
34264	2.75351	0.771838
34265	3.78453	0.78823
34266	1.89226	0.697958
34267	3.02887	1.08307
34268	2.03149	0.655744
34269	2.03149	0.721127
34270	1.89226	0.834344
34271	1.89226	0.996639
34272	2.17979	2.1
34273	1.65533	2.1
34274	2.57212	2.1
34275	2.20346	2.1

34276	4.22745	2.1
34277	1.89227	2.1
34278	2.20347	2.1
34279	1.89227	2.1
34280	2.57212	2.1
34281	1.89227	2.1
34282	2.57212	2.1
34283	2.05274	0.669615
34284	2.16783	1.88379
34285	2.24429	0.632015
34286	2.38404	2.1
34287	4.41212	0.810864
34288	2.16783	1.58245
34289	2.05967	2.1
34290	2.38404	2.1
34291	2.24695	2.1
34292	4.41478	2.1
34293	2.24429	0.64554
34294	2.38404	2.1
34295	2.51819	0.685551
34296	2.60723	1.89602
34297	4.85152	1.08945
34298	2.38404	1.8821
34299	2.24695	2.1
34300	2.60723	1.64288
34301	2.45247	2.1
34302	4.85418	2.1
34303	1.89227	2.1
34304	1.89227	2.1
34305	1.89227	2.1
34306	1.89227	2.1
34307	3.78453	2.1
34308	1.89226	2.1
34309	1.89226	2.1
34310	1.89226	2.1
34311	1.89226	2.1
34312	3.78453	2.1
34313	0.618473	2.1
34314	0.619945	2.1
34315	0.797692	2.1
34316	0.770147	2.1
34317	0.68852	2.1
34318	0.68852	2.1
34319	0.701573	2.1
34320	0.701573	2.1
34321	0.619945	2.1
34322	0.797691	2.1
34323	0.618473	2.1
34324	0.770147	2.1
34325	0.797692	2.1
34326	0.770148	2.1
34327	0.694787	2.1
34328	0.653411	2.1
34329	0.722102	2.1
34330	0.722102	2.1
34331	0.729001	2.1
34332	0.729001	2.1
34333	0.797692	2.1

34334	0.694787	2.1
34335	0.770148	2.1
34336	0.653412	2.1
34337	1.89227	2.1
34338	1.89227	2.1
34339	1.89227	2.1
34340	1.89227	2.1
34341	3.78453	2.1
34342	1.89226	2.1
34343	1.89226	2.1
34344	1.89226	2.1
34345	1.89226	2.1
34346	3.78453	2.1
34347	1.23989	2.1
34348	1.59538	2.1
34349	1.66647	2.1
34350	1.26599	2.1
34351	2.90636	2.1
34352	1.66647	2.1
34353	1.38957	2.1
34354	1.47446	2.1
34355	1.40337	2.1
34356	1.66647	2.1
34357	1.59538	2.1
34358	2.80619	2.1
34359	2.0815	2.1
34360	2.0815	2.1
34361	2.0815	2.1
34362	2.73721	2.1
34363	2.74192	2.1
34364	2.73721	2.1
34365	2.0815	2.1
34366	2.0815	2.1
34367	2.0815	2.1
34368	2.689	2.1
34369	2.69166	2.1
34370	2.62369	2.1
34371	2.19717	2.1
34372	2.60405	2.1
34373	2.20566	2.1
34374	4.80122	2.1
34375	2.19717	2.1
34376	1.37392	2.1
34377	2.20566	2.1
34378	1.45408	2.1
34379	3.65125	2.1
34380	2.60405	2.1
34381	2.20566	2.1
34382	2.57466	2.1
34383	2.21715	2.1
34384	4.8212	2.1
34385	2.20566	2.1
34386	1.54249	2.1
34387	1.54249	2.1
34388	1.45408	2.1
34389	2.21715	2.1
34390	2.12874	2.1
34391	1.60647	1.62536

34392	1.31346	2.1
34393	1.82649	1.4717
34394	1.51067	2.1
34395	3.13995	2.1
34396	1.89226	1.20029
34397	1.89226	2.1
34398	1.89226	1.47547
34399	1.89226	2.1
34400	3.78452	2.1
34401	1.89226	2.1
34402	2.6096	2.1
34403	2.8067	2.1
34404	2.8067	2.1
34405	1.89226	2.1
34406	1.89226	2.1
34407	1.82649	1.21423
34408	1.3596	2.1
34409	2.17708	1.4843
34410	1.70302	2.1
34411	3.40243	2.1
34412	1.89226	1.21621
34413	1.89226	2.1
34414	1.89226	0.996019
34415	1.89226	2.1
34416	3.78452	1.78023
34417	2.27067	2.1
34418	2.43549	2.1
34419	2.63979	2.1
34420	2.41828	2.1
34421	2.27067	2.1
34422	2.04916	2.1
34423	1.30047	2.1
34424	2.04916	2.1
34425	2.27068	2.1
34426	1.5586	2.1
34427	3.57115	2.1
34428	2.27068	2.1
34429	1.84214	2.1
34430	1.80224	2.1
34431	1.5586	2.1
34432	2.51432	2.1
34433	2.27068	2.1
34434	1.63719	2.1
34435	2.15714	2.1
34436	2.07821	2.1
34437	1.52204	2.1
34438	3.67919	2.1
34439	2.07821	2.1
34440	1.40118	2.1
34441	1.40118	2.1
34442	1.52204	2.1
34443	1.99632	2.1
34444	2.11718	2.1
34445	0.946131	1.54713
34446	0.946131	1.42679
34447	1.06957	2.05231
34448	1.06957	2.1
34449	0.946132	2.07514

34450	0.946132	1.05102
34451	1.411	1.66024
34452	1.06957	2.1
34453	0.946132	1.59749
34454	1.411	1.7679
34455	1.06957	1.89693
34456	0.946132	1.81064
34457	1.99875	0.695101
34458	2.57079	0.659482
34459	2.59319	1.00344
34460	1.9105	0.541339
34461	4.59194	0.717988
34462	1.99875	0.667882
34463	1.02247	0.440515
34464	1.07017	0.502207
34465	1.15841	0.503678
34466	1.91051	0.490787
34467	1.99875	0.50705
34468	2.59319	0.931304
34469	1.9105	0.568382
34470	2.33812	0.680003
34471	2.59319	0.658211
34472	1.59457	0.745588
34473	1.59457	0.727842
34474	2.50906	0.618527
34475	2.0467	0.483409
34476	2.03357	0.545073
34477	2.53176	0.480825
34478	4.54263	0.516933
34479	1.294	0.655444
34480	1.66636	0.557514
34481	2.03357	0.519002
34482	1.03079	0.489092
34483	3.32757	0.539303
34484	1.68108	0.513885
34485	2.53176	0.481742
34486	2.36417	0.450522
34487	2.71666	0.449997
34488	1.68108	0.645342
34489	2.03357	0.601653
34490	1.89226	0.311015
34491	2.59302	0.362346
34492	2.2727	0.309549
34493	2.2727	0.284611
34494	1.89226	0.412459
34495	1.89226	0.336624
34496	1.89226	0.475897
34497	1.89226	0.41714
34498	1.89226	0.374825
34499	1.89226	0.270563
34500	3.78452	0.466273
34501	1.89225	0.504203
34502	1.89225	0.205415
34503	1.89225	0.285154
34504	1.89225	0.341378
34505	1.89225	0.307212
34506	1.89225	0.367135
34507	0.934114	0.361834

34508	0.934114	0.248657
34509	0.944544	0.337533
34510	0.944544	0.32433
34511	0.721493	0.229486
34512	0.58215	0.203857
34513	1.2965	0.330993
34514	1.13634	0.216229
34515	1.13635	0.217008
34516	0.582148	0.20899
34517	1.29651	0.316111
34518	0.721491	0.234338
34519	1.89226	0.313996
34520	1.89226	0.260509
34521	1.89226	0.242973
34522	1.89226	0.20425
34523	3.78452	0.205274
34524	0.946131	0.420154
34525	0.946131	0.432873
34526	0.946131	0.377842
34527	0.946131	0.434948
34528	0.946131	0.379306
34529	0.946131	0.404732
34530	0.946131	0.425865
34531	0.946131	0.491841
34532	0.946131	0.402906
34533	0.946131	0.398735
34534	0.946131	0.413877
34535	0.946131	0.413797
34536	1.89226	0.13233
34537	1.89226	0.141621
34538	2.78602	0.12412
34539	1.89226	0.167337
34540	3.78453	0.129107
34541	1.52601	2.1
34542	1.76601	2.1
34543	1.27839	2.1
34544	1.56095	2.1
34545	3.0444	2.1
34546	1.76601	2.1
34547	1.86793	2.1
34548	1.56095	2.1
34549	1.68152	2.1
34550	3.44753	2.1
34551	1.27841	2.1
34552	1.56097	2.1
34553	1.0577	2.1
34554	1.36861	2.1
34555	2.64702	2.1
34556	1.56097	2.1
34557	1.68154	2.1
34558	1.36861	2.1
34559	1.53296	2.1
34560	3.05015	2.1
34561	1.89226	2.1
34562	1.89226	2.1
34563	1.89226	2.1
34564	1.89226	2.1
34565	3.78453	2.1

34566	0.983075	2.1
34567	0.788361	2.1
34568	0.724726	2.1
34569	0.724726	2.1
34570	0.983027	2.1
34571	0.724734	2.1
34572	0.788364	2.1
34573	0.788364	2.1
34574	0.788335	2.1
34575	0.94613	2.1
34576	0.724757	2.1
34577	0.946131	2.1
34578	1.15789	2.1
34579	2.11135	2.1
34580	2.25826	2.1
34581	1.28845	2.1
34582	3.41615	2.1
34583	1.89226	2.1
34584	1.89226	2.1
34585	1.89226	2.1
34586	1.89226	2.1
34587	3.78453	2.1
34588	1.57667	2.1
34589	1.70002	2.1
34590	1.70002	2.1
34591	1.89226	2.1
34592	1.89226	2.1
34593	2.05244	2.1
34594	2.25826	2.1
34595	1.42214	2.1
34596	1.43675	2.1
34597	1.28845	2.1
34598	2.40656	2.1
34599	2.25826	2.1
34600	1.58066	2.1
34601	1.74247	2.1
34602	1.85097	2.1
34603	1.61155	2.1
34604	3.43163	2.1
34605	0.785612	2.1
34606	0.789638	2.1
34607	0.882561	2.1
34608	0.763529	2.1
34609	0.686921	2.1
34610	0.86048	2.1
34611	0.871812	2.1
34612	0.871812	2.1
34613	0.830183	2.1
34614	0.882572	2.1
34615	0.902315	2.1
34616	0.785602	2.1
34617	1.01972	0.0807659
34618	0.936657	0.073154
34619	0.946131	0.084256
34620	0.946131	0.0937368
34621	0.927018	0.0923992
34622	0.927018	0.0805358
34623	1.1376	0.0841204

34624	0.953663	0.0725995
34625	1.1257	0.0804992
34626	1.04073	0.0812689
34627	1.00358	0.082733
34628	0.851528	0.0801012
34629	1.73576	0.451208
34630	2.00244	0.668521
34631	1.17798	0.490719
34632	1.25796	0.472219
34633	2.99373	0.434708
34634	2.00244	0.645253
34635	1.10719	0.540019
34636	2.03236	0.709603
34637	2.03236	0.605761
34638	1.25797	0.660699
34639	1.25797	0.536546
34640	1.89226	2.1
34641	1.89226	2.1
34642	1.89226	2.1
34643	1.89226	2.1
34644	3.78452	2.1
34645	2.3174	2.1
34646	1.50005	2.1
34647	1.90428	2.1
34648	2.64564	2.1
34649	4.22168	2.1
34650	1.89226	2.1
34651	1.89226	2.1
34652	1.89226	2.1
34653	1.89226	2.1
34654	1.89226	2.1
34655	1.89226	2.1
34656	1.32749	0.232825
34657	1.70302	0.294211
34658	2.08149	0.260462
34659	1.70302	0.28175
34660	2.19634	0.244918
34661	1.93083	0.257685
34662	1.89226	1.24647
34663	1.89226	1.74925
34664	1.89226	0.749025
34665	1.89226	1.93892
34666	3.78453	1.00702
34667	1.89226	1.27659
34668	1.89226	1.69987
34669	1.89226	0.915592
34670	1.89226	1.34319
34671	3.78452	1.53537
34672	2.17499	1.02786
34673	1.79663	1.73394
34674	2.1538	0.726912
34675	1.818	1.03721
34676	2.17499	1.46946
34677	1.83919	2.04853
34678	0.898316	1.81053
34679	1.0769	1.18804
34680	0.968276	1.86528
34681	0.773495	1.35105

34682	0.921126	1.60186
34683	0.921126	1.84262
34684	0.92927	1.59662
34685	0.92927	1.53775
34686	1.0769	1.46041
34687	0.968276	1.19694
34688	0.898316	1.829
34689	0.773495	1.3773
34690	1.89226	0.925835
34691	1.89226	2.00349
34692	1.89226	1.14022
34693	1.89226	1.08762
34694	3.78453	1.71613
34695	1.89226	1.2127
34696	1.89226	1.91491
34697	1.89226	0.791471
34698	1.89226	1.40952
34699	3.78452	1.05796
34700	2.17499	1.67001
34701	2.1538	2.05808
34702	2.53574	1.06718
34703	2.54157	0.919455
34704	4.71073	1.618
34705	1.0769	1.38279
34706	1.26787	1.05346
34707	1.16641	1.22797
34708	0.968276	1.771
34709	1.11625	1.28084
34710	1.11625	1.50041
34711	1.11989	1.65878
34712	1.11989	0.811395
34713	1.26787	1.15103
34714	1.16641	1.91601
34715	1.0769	1.89097
34716	0.968277	1.56044
34717	1.89226	0.483524
34718	1.89226	0.471584
34719	1.89226	0.458537
34720	1.89226	0.46536
34721	3.78452	0.482051
34722	1.91739	0.428959
34723	2.20045	0.429715
34724	1.79005	0.422701
34725	2.33802	0.41224
34726	4.25539	0.408228
34727	2.52754	0.394743
34728	1.89226	0.413013
34729	2.69841	0.39156
34730	2.69841	0.418584
34731	1.89226	0.380853
34732	1.89226	0.410351
34733	1.89226	0.477367
34734	1.89226	0.508926
34735	1.89226	0.459605
34736	1.89226	0.502675
34737	3.78452	0.462113
34738	1.93544	0.460149
34739	2.33802	0.443386

34740	1.55063	0.400845
34741	2.1769	0.415754
34742	3.88865	0.458772
34743	2.20929	0.312681
34744	2.55162	0.240023
34745	2.27807	0.349689
34746	1.48831	0.239549
34747	4.48736	0.248697
34748	2.27807	0.351081
34749	2.72723	0.238879
34750	2.55162	0.280319
34751	2.55162	0.340877
34752	2.43157	0.249144
34753	2.43157	0.327047
34754	2.27807	0.280716
34755	1.51612	0.197593
34756	1.96048	0.209412
34757	1.73022	0.202627
34758	1.70302	0.280648
34759	1.61016	0.248831
34760	2.27807	0.282088
34761	2.08048	0.19052
34762	1.98522	0.204919
34763	2.43157	0.24934
34764	1.83798	0.20528
34765	2.27807	0.248573
34766	1.70302	0.383959
34767	2.68507	0.30937
34768	2.23856	0.41834
34769	2.52174	0.264409
34770	2.35331	0.462731
34771	2.65099	0.323631
34772	1.70303	0.40167
34773	0.986848	0.213283
34774	2.41566	0.243789
34775	1.70303	0.222901
34776	1.88139	0.245636
34777	1.64955	0.237643
34778	1.89226	2.05777
34779	2.34978	1.70525
34780	1.89226	2.02307
34781	2.68773	1.36914
34782	1.89226	2.0986
34783	2.68773	1.5987
34784	1.89226	2.03894
34785	1.89226	1.37531
34786	1.89226	1.42961
34787	1.89226	1.96171
34788	3.78453	1.87577
34789	1.00825	1.11085
34790	1.09847	1.33284
34791	0.939987	1.19394
34792	0.783516	1.73194
34793	0.838041	1.41991
34794	0.838041	1.79833
34795	1.49693	1.86915
34796	1.26602	1.40494
34797	1.10875	1.20827

34798	1.05187	2.03133
34799	1.28743	1.46921
34800	0.946132	1.16443
34801	0.946132	1.39715
34802	0.946132	2.0731
34803	0.946132	1.89901
34804	0.946132	1.09165
34805	1.31823	2.08969
34806	0.946132	1.3651
34807	0.946132	1.91575
34808	0.946131	1.97112
34809	0.946131	2.1
34810	0.946132	1.72893
34811	1.31823	1.94068
34812	1.51688	0.308687
34813	1.79978	0.478182
34814	1.1685	0.329856
34815	1.1685	0.389959
34816	1.85857	0.372034
34817	1.51018	0.420895
34818	0.946116	0.318501
34819	0.946116	0.387392
34820	0.94612	0.273398
34821	0.94612	0.302232
34822	0.946118	0.276798
34823	0.946118	0.349337
34824	0.946118	0.2938
34825	0.946118	0.402957
34826	0.946131	0.299961
34827	0.946131	0.350346
34828	0.946105	0.334365
34829	0.946105	0.341862
34830	1.89224	2.1
34831	1.89224	2.1
34832	1.89224	2.1
34833	1.89224	2.1
34834	1.89224	2.1
34835	1.89224	2.1
34836	1.38678	2.1
34837	1.3349	2.1
34838	0.946119	2.1
34839	0.946119	2.1
34840	1.38678	2.1
34841	1.38678	2.1
34842	0.946119	2.1
34843	0.946119	2.1
34844	1.38676	2.1
34845	0.946107	2.1
34846	0.946132	2.1
34847	1.33491	2.1
34848	1.06455	2.1
34849	1.03932	2.1
34850	1.20649	2.1
34851	1.18773	2.1
34852	2.27105	2.1
34853	1.89226	2.1
34854	1.89226	2.1
34855	1.89226	2.1

34856	1.89226	2.1
34857	1.89226	2.1
34858	1.89226	2.1
34859	1.36918	2.1
34860	1.18774	2.1
34861	1.31144	2.1
34862	1.20651	2.1
34863	1.31144	2.1
34864	1.20651	2.1
34865	2.29066	2.1
34866	1.76476	2.1
34867	1.79825	2.1
34868	2.33801	2.1
34869	4.08891	2.1
34870	1.79825	2.1
34871	2.33801	2.1
34872	2.37168	2.1
34873	1.49325	2.1
34874	4.16993	2.1
34875	1.13641	2.1
34876	1.43213	0.85886
34877	1.44085	1.15081
34878	1.18301	2.00437
34879	2.61513	1.70538
34880	1.89226	1.75904
34881	2.23875	2.1
34882	2.23875	1.53255
34883	2.10287	2.1
34884	1.89226	1.54002
34885	1.75639	2.1
34886	1.89226	0.885981
34887	1.89226	1.64317
34888	1.89226	0.904698
34889	1.89226	1.90589
34890	1.89226	0.865074
34891	1.89226	2.02562
34892	1.44085	1.31394
34893	1.25173	1.92015
34894	1.25173	1.02877
34895	1.18301	2.1
34896	1.4617	0.968219
34897	1.39298	2.1
34898	1.89227	1.65453
34899	2.23875	2.1
34900	2.42504	2.1
34901	1.89227	1.21148
34902	4.13101	2.09419
34903	1.89226	0.884978
34904	1.89226	1.13675
34905	1.89226	0.818477
34906	1.89226	1.75071
34907	1.89226	0.813725
34908	1.89226	1.88524
34909	1.89226	2.04078
34910	2.41297	2.1
34911	2.70685	1.74125
34912	2.70685	2.1
34913	1.89226	1.7274

34914	1.89226	2.1
34915	1.89226	2.05418
34916	1.89226	2.1
34917	1.89226	2.1
34918	1.89226	2.1
34919	1.89226	2.1
34920	1.89226	2.1
34921	1.89226	1.78549
34922	1.89226	2.1
34923	1.89226	1.93725
34924	1.89226	2.1
34925	3.78453	2.1
34926	1.89226	2.1
34927	1.89226	2.1
34928	1.89226	2.1
34929	1.89226	2.1
34930	1.89226	2.1
34931	1.89226	2.1
34932	1.69044	0.075294
34933	1.57979	0.0826922
34934	2.27963	0.0829376
34935	2.21582	0.0747527
34936	3.85942	0.0778371
34937	2.21582	0.0745694
34938	1.30722	0.0807324
34939	2.02069	0.0811097
34940	2.02069	0.0764246
34941	1.57979	0.0819928
34942	1.57979	0.0752731
34943	1.67825	2.1
34944	1.89226	2.1
34945	1.89226	2.1
34946	1.89226	2.1
34947	1.91894	2.1
34948	1.91894	2.1
34949	0.946133	2.1
34950	0.946133	2.1
34951	0.946132	2.1
34952	0.946132	2.1
34953	0.946132	2.1
34954	0.946132	2.1
34955	0.946133	2.1
34956	0.946133	2.1
34957	0.946133	2.1
34958	0.946133	2.1
34959	0.946132	2.1
34960	0.946132	2.1
34961	0.942609	1.74127
34962	0.942609	1.88171
34963	1.329 2.1	
34964	1.329 2.1	
34965	1.32547	2.0453
34966	0.94261	2.1
34967	0.946132	1.85091
34968	0.946132	2.1
34969	0.946132	1.93194
34970	1.32547	2.1
34971	0.946132	2.1

34972	0.94261	2.1
34973	1.55831	1.8427
34974	1.07496	1.96009
34975	1.80327	0.737505
34976	1.34851	0.855369
34977	2.87824	0.999019
34978	2.14346	0.567771
34979	2.77937	0.539764
34980	2.79173	0.725057
34981	2.17129	0.483946
34982	4.95066	0.520818
34983	2.14346	0.621145
34984	1.34124	0.313214
34985	1.39818	0.548264
34986	2.1713	0.527385
34987	3.51253	0.534005
34988	2.79173	0.626605
34989	2.21882	0.449127
34990	2.17129	0.49226
34991	2.17129	0.514468
34992	2.81448	0.52535
34993	2.81448	0.540819
34994	1.10941	0.494439
34995	1.08565	0.543268
34996	0.699089	0.432467
34997	0.740176	0.496527
34998	0.908594	0.409382
34999	0.908594	0.459913
35000	0.91723	0.607965
35001	0.91723	0.45558
35002	1.10941	0.488239
35003	0.740176	0.458394
35004	0.699089	0.470094
35005	1.08565	0.441678
35006	1.89227	0.442878
35007	1.89227	0.420471
35008	1.89227	0.321109
35009	1.89227	0.278925
35010	3.78453	0.400461
35011	2.40032	0.540231
35012	2.19526	0.383171
35013	1.12907	0.270188
35014	1.44253	0.440471
35015	3.32433	0.356345
35016	2.19526	0.34813
35017	2.71093	0.409039
35018	1.29826	0.383454
35019	2.0815	0.217078
35020	4.18911	0.38993
35021	1.89226	0.374149
35022	1.89226	0.350093
35023	1.89226	0.229232
35024	1.89226	0.256669
35025	3.78452	0.276302
35026	2.30822	0.319678
35027	1.89226	0.278412
35028	2.79329	0.190514
35029	2.79329	0.172394

35030	1.89226	0.390961
35031	1.89226	0.332923
35032	0.946131	1.54802
35033	0.946131	0.794539
35034	1.38414	1.98526
35035	1.38414	0.536735
35036	0.94613	1.82308
35037	0.94613	1.58485
35038	1.38415	0.91737
35039	1.41833	0.634491
35040	0.946132	0.99956
35041	1.41833	0.890914
35042	1.38414	0.946593
35043	0.946131	1.21381
35044	1.89226	1.17709
35045	2.6536	2.1
35046	1.89226	0.81416
35047	2.72238	2.1
35048	4.61465	2.1
35049	0.946132	2.1
35050	0.946132	1.32677
35051	1.38657	1.14088
35052	1.38657	2.1
35053	1.38657	2.1
35054	1.36119	2.1
35055	0.946131	0.845045
35056	0.946131	1.48872
35057	0.946132	2.05441
35058	1.38657	1.99869
35059	0.946132	1.51239
35060	1.36119	2.07427
35061	1.89226	2.1
35062	2.4717	2.1
35063	2.5026	2.1
35064	2.4717	2.1
35065	1.92316	2.1
35066	1.89226	2.1
35067	1.89226	2.1
35068	1.89226	1.39906
35069	1.89226	2.1
35070	1.89226	0.724471
35071	3.78453	2.1
35072	0.946128	2.1
35073	0.946128	0.706588
35074	1.3419	1.30066
35075	1.3419	2.1
35076	0.946131	1.0209
35077	0.946131	0.931523
35078	1.36607	2.1
35079	1.3419	2.1
35080	0.946131	2.1
35081	1.36607	2.1
35082	1.3419	2.1
35083	0.94613	2.1
35084	1.89226	2.1
35085	2.50259	2.1
35086	2.53563	2.1
35087	2.50259	2.1

35088	1.92529	2.1
35089	1.89226	2.1
35090	0.946132	0.821899
35091	0.946132	2.1
35092	0.946131	1.2936
35093	0.946131	2.1
35094	0.946132	1.1304
35095	0.946132	1.52381
35096	0.946131	2.1
35097	0.946131	2.1
35098	0.946131	2.1
35099	0.946131	2.1
35100	0.946131	2.1
35101	0.946131	2.1
35102	2.0815	2.1
35103	1.86506	2.1
35104	1.86506	2.1
35105	1.82567	2.1
35106	2.0815	2.1
35107	2.03335	2.1
35108	2.0815	2.1
35109	2.0815	2.1
35110	2.0815	2.1
35111	1.88668	2.1
35112	1.83221	2.1
35113	1.57275	2.1
35114	2.34061	0.141331
35115	1.89226	0.188735
35116	2.23254	0.134179
35117	1.89226	0.138546
35118	2.23254	0.193085
35119	1.89226	0.199242
35120	1.8619	0.173461
35121	1.89226	0.246914
35122	2.34061	0.165337
35123	1.89226	0.168802
35124	2.34061	0.236931
35125	1.89226	0.238869
35126	1.89227	0.235478
35127	1.89227	0.167948
35128	1.89227	0.237255
35129	1.89227	0.244448
35130	1.89227	0.164528
35131	1.89227	0.179745
35132	1.89227	0.183353
35133	1.89227	0.138382
35134	1.89227	0.189203
35135	1.89227	0.193226
35136	1.89227	0.1414
35137	1.89227	0.151666
35138	1.06365	0.170238
35139	0.503305	0.147453
35140	0.794912	0.127225
35141	1.42752	0.133935
35142	1.34826	0.147827
35143	1.07163	0.180477
35144	0.533113	0.187963
35145	0.761277	0.151744

35146	0.848403	0.158859
35147	0.876191	0.125961
35148	0.963372	0.192602
35149	0.963372	0.174108
35150	1.55571	0.114306
35151	2.76574	0.14766
35152	2.92453	0.118713
35153	1.70301	0.137081
35154	2.33089	0.131498
35155	1.06905	0.155256
35156	2.28979	2.1
35157	1.81488	2.1
35158	1.63722	2.1
35159	2.22627	2.1
35160	4.04115	2.1
35161	1.98246	2.1
35162	2.15715	2.1
35163	2.22627	2.1
35164	2.22627	2.1
35165	1.63719	2.1
35166	1.63719	2.1
35167	2.1644	0.0900113
35168	1.903	0.0997198
35169	1.23639	0.0882085
35170	1.42704	0.110501
35171	3.61546	0.0959132
35172	1.89226	0.107688
35173	1.89226	0.143089
35174	1.89226	0.122173
35175	1.89226	0.130014
35176	3.78451	0.127242
35177	2.61343	0.112239
35178	1.89226	0.119844
35179	2.08149	0.12009
35180	1.74357	0.099933
35181	2.08149	0.129034
35182	1.74357	0.111779
35183	1.63483	0.0826185
35184	1.8631	0.0783988
35185	1.8631	0.0901696
35186	1.70301	0.0754566
35187	2.0815	0.088353
35188	1.94319	0.0767412
35189	2.0815	0.0833611
35190	1.89226	0.0786197
35191	1.89226	0.0921809
35192	2.05236	0.0760736
35193	2.23014	0.0894556
35194	2.41882	0.075535
35195	2.14993	2.06271
35196	1.87217	1.81708
35197	2.34488	2.1
35198	2.5254	1.50182
35199	4.39757	1.57979
35200	0.949995	2.1
35201	0.630972	2.1
35202	0.936084	2.1
35203	1.17244	2.1

35204	0.922394	2.1
35205	0.922394	2.1
35206	0.88102	2.1
35207	0.88102	2.1
35208	0.949998	2.1
35209	1.17244	2.1
35210	0.936081	2.1
35211	0.630973	2.1
35212	1.89226	2.07316
35213	1.89226	1.84588
35214	1.89226	2.02138
35215	1.89226	2.06476
35216	3.78452	2.1
35217	1.89226	1.31177
35218	1.89226	1.59248
35219	1.89226	1.23734
35220	1.89226	1.61844
35221	1.89226	1.22983
35222	1.89226	1.61468
35223	2.34488	1.74729
35224	2.5254	1.34416
35225	1.89226	1.40809
35226	1.89226	1.70871
35227	4.41766	1.89429
35228	1.89226	1.75541
35229	1.89226	1.95654
35230	1.89226	1.78205
35231	1.89226	1.79162
35232	3.78452	1.50427
35233	1.89226	1.19676
35234	1.89226	1.61839
35235	1.89226	1.19968
35236	1.89226	1.43934
35237	1.89226	1.18059
35238	1.89226	1.42335
35239	1.5605	2.1
35240	1.15806	2.1
35241	1.31565	2.1
35242	1.65642	2.1
35243	2.87615	2.1
35244	1.46643	2.1
35245	1.65642	2.1
35246	1.31565	2.1
35247	1.31565	2.1
35248	1.74246	2.1
35249	1.74246	2.1
35250	1.89226	2.1
35251	1.89226	2.1
35252	1.89226	2.1
35253	1.89226	2.1
35254	1.89226	2.07807
35255	1.89226	2.1
35256	1.15099	2.1
35257	1.15099	2.1
35258	0.939316	2.1
35259	0.939316	2.1
35260	0.946127	2.1
35261	0.946127	2.1

35262	1.14418	2.1
35263	0.939289	2.1
35264	1.14417	2.1
35265	0.946129	2.1
35266	0.939287	2.1
35267	0.94613	2.1
35268	1.89226	2.02813
35269	1.89226	2.1
35270	1.89226	2.1
35271	1.89226	1.83689
35272	3.78452	2.1
35273	1.93164	0.178213
35274	2.64898	0.138058
35275	1.43213	0.152918
35276	1.43213	0.196491
35277	2.63206	0.142962
35278	2.13254	0.18716
35279	1.89226	2.1
35280	1.89226	2.1
35281	1.89226	2.1
35282	1.89226	2.1
35283	3.78452	2.1
35284	2.71655	2.1
35285	1.43323	2.1
35286	1.28413	2.1
35287	2.53614	2.1
35288	3.96937	2.1
35289	1.89226	2.1
35290	1.89226	2.1
35291	1.89226	2.1
35292	1.89226	2.1
35293	1.89226	2.1
35294	1.89226	2.1
35295	1.33356	2.1
35296	2.82297	2.1
35297	2.771	2.1
35298	2.771	2.1
35299	1.46037	2.1
35300	1.46037	2.1
35301	2.56034	2.1
35302	1.18474	2.1
35303	1.15113	2.1
35304	2.53724	2.1
35305	3.72198	2.1
35306	2.56034	2.1
35307	1.89226	2.1
35308	1.89226	2.1
35309	2.53725	2.1
35310	4.42951	2.1
35311	1.89226	2.1
35312	1.89226	2.1
35313	1.89226	2.1
35314	1.89226	2.1
35315	3.78453	2.1
35316	1.89226	2.1
35317	1.89226	2.1
35318	1.89226	2.1
35319	1.89226	1.80609

35320	3.78452	2.1
35321	1.1229	2.1
35322	2.53724	2.1
35323	1.15113	2.1
35324	1.15113	2.1
35325	2.51559	2.1
35326	2.51559	2.1
35327	1.89226	2.1
35328	2.53724	2.1
35329	2.51559	2.1
35330	2.53724	2.1
35331	1.87061	2.1
35332	1.89226	2.1
35333	1.89226	2.1
35334	1.89226	2.1
35335	1.89226	2.1
35336	1.89226	2.1
35337	3.78453	2.1
35338	1.89226	2.1
35339	1.89226	2.1
35340	1.89226	2.1
35341	1.89226	2.1
35342	3.78452	2.1
35343	1.98143	1.11396
35344	2.81559	0.511174
35345	1.65411	0.646007
35346	2.26675	0.911034
35347	4.24818	0.733414
35348	0.810656	0.72178
35349	0.810656	2.1
35350	0.821888	2.1
35351	0.821888	1.2824
35352	1.1235	0.838726
35353	1.07439	1.40596
35354	0.535693	2.1
35355	0.509042	2.1
35356	0.535693	2.1
35357	1.1235	2.1
35358	0.509043	2.1
35359	1.07439	2.1
35360	1.10378	2.1
35361	2.247	1.40944
35362	2.27472	2.1
35363	2.27472	1.51199
35364	1.07139	2.1
35365	1.07139	1.8931
35366	2.08081	2.1
35367	1.40395	2.1
35368	1.34245	2.1
35369	2.00239	2.1
35370	3.40634	2.1
35371	1.89226	2.1
35372	1.89226	2.1
35373	1.89226	2.1
35374	1.89226	2.1
35375	3.78453	2.1
35376	0.946128	2.1
35377	0.946128	2.1

35378	1.13785	2.1
35379	1.11496	2.1
35380	0.946131	2.1
35381	0.946131	2.1
35382	1.11495	2.1
35383	1.11495	2.1
35384	0.94613	2.1
35385	1.11495	2.1
35386	1.13785	2.1
35387	0.946131	2.1
35388	0.975888	2.1
35389	1.00118	2.1
35390	0.671218	2.1
35391	0.653229	2.1
35392	0.825372	2.1
35393	0.825372	2.1
35394	0.829038	2.1
35395	0.829038	2.1
35396	0.975901	2.1
35397	0.653238	2.1
35398	0.671209	2.1
35399	1.00117	2.1
35400	1.89224	2.1
35401	1.89224	2.1
35402	1.89224	2.1
35403	1.89224	2.1
35404	3.78448	2.1
35405	0.946115	2.1
35406	0.946115	2.1
35407	1.11494	2.1
35408	1.10111	2.1
35409	0.946118	2.1
35410	0.946118	2.1
35411	1.10111	2.1
35412	1.10111	2.1
35413	1.10112	2.1
35414	0.94613	2.1
35415	0.946105	2.1
35416	1.11492	2.1
35417	1.04028	0.459623
35418	2.27126	0.357515
35419	2.0815	0.378633
35420	1.82249	0.590099
35421	1.33723	0.404069
35422	1.18684	0.55331
35423	0.812616	0.355161
35424	0.812616	0.347947
35425	0.997777	0.330063
35426	1.08122	0.369019
35427	0.94613	0.33322
35428	0.94613	0.364921
35429	0.812648	0.347383
35430	0.932779	0.337965
35431	0.812648	0.319278
35432	0.94613	0.350568
35433	0.849335	0.322786
35434	0.94613	0.323985
35435	1.80327	0.94825

35436	1.56016	0.880383
35437	1.57731	1.67725
35438	1.34851	1.59287
35439	2.03207	0.968461
35440	1.80327	1.72116
35441	2.03207	1.49839
35442	1.56016	1.4645
35443	2.18076	1.00696
35444	1.78167	1.03397
35445	3.81374	1.52113
35446	1.37597	1.64572
35447	1.60058	1.94355
35448	1.70302	0.956641
35449	1.85448	1.14103
35450	3.15028	1.1528
35451	1.60058	0.982014
35452	1.4054	1.90655
35453	1.66202	0.982591
35454	1.70302	1.66936
35455	3.09979	1.29493
35456	1.70302	1.44653
35457	1.85448	1.37335
35458	2.21317	1.08813
35459	2.0961	1.09119
35460	4.01757	1.45282
35461	1.66202	1.20002
35462	1.70302	1.71412
35463	2.09611	0.866138
35464	2.00892	1.42785
35465	3.6468	1.15108
35466	1.2966	0.120997
35467	2.25495	0.100606
35468	1.2966	0.132393
35469	2.21117	0.104555
35470	1.67687	0.127809
35471	2.6167	0.101314
35472	1.89227	0.176915
35473	1.89227	0.126014
35474	1.89227	0.132838
35475	1.89227	0.1952
35476	3.78453	0.151606
35477	2.44375	0.124782
35478	1.89227	0.16898
35479	2.81853	0.157438
35480	2.81853	0.143872
35481	1.89227	0.146278
35482	1.89227	0.1133
35483	1.89226	0.918325
35484	1.75853	1.09582
35485	2.37636	0.897576
35486	2.37636	1.10651
35487	1.89226	0.816597
35488	1.89226	0.994344
35489	1.65814	0.401615
35490	1.10187	0.657495
35491	1.10187	0.529369
35492	1.19547	0.62748
35493	1.56263	0.336947

35494	1.65624	0.642805
35495	1.56264	0.37474
35496	1.01479	0.658528
35497	1.01479	0.575367
35498	1.10187	0.636617
35499	1.47561	0.297854
35500	1.5627	0.662724
35501	1.89225	2.1
35502	1.89225	2.1
35503	1.89225	2.1
35504	1.89225	2.1
35505	3.7845	2.1
35506	2.54386	2.1
35507	1.91546	2.1
35508	2.46008	2.1
35509	1.82815	2.1
35510	4.37554	2.1
35511	1.91546	2.1
35512	1.04909	2.1
35513	1.82815	2.1
35514	0.983141	2.1
35515	2.8986	2.1
35516	1.89225	2.1
35517	1.89225	2.1
35518	1.89225	2.1
35519	1.89225	2.1
35520	3.78449	2.1
35521	1.89226	2.1
35522	1.89226	2.1
35523	1.89226	2.1
35524	1.89226	2.1
35525	1.89226	2.1
35526	1.89226	2.1
35527	2.46009	2.1
35528	1.7513	2.1
35529	1.82817	2.1
35530	1.82817	2.1
35531	2.38127	2.1
35532	2.38127	2.1
35533	2.55006	2.1
35534	2.64569	2.1
35535	2.60128	2.1
35536	2.49168	2.1
35537	5.13737	2.1
35538	2.51605	2.1
35539	2.64569	2.1
35540	2.60128	2.1
35541	2.48891	2.1
35542	5.1346	2.1
35543	2.60128	2.1
35544	2.49168	2.1
35545	2.41689	2.1
35546	2.55248	2.1
35547	5.04416	2.1
35548	2.60128	2.1
35549	2.48891	2.1
35550	2.45922	2.1
35551	2.55249	2.1

35552	5.04139	2.1
35553	1.89227	1.37293
35554	1.89227	1.80678
35555	1.89227	1.68879
35556	1.89227	1.57083
35557	1.89227	1.50535
35558	1.89227	1.41011
35559	0.96063	2.1
35560	0.96063	2.1
35561	0.958806	2.1
35562	0.958806	2.1
35563	1.28892	2.1
35564	0.749042	2.1
35565	1.17409	2.1
35566	0.630516	2.1
35567	1.17409	2.1
35568	1.28892	2.1
35569	0.630515	2.1
35570	0.749042	2.1
35571	0.92511	1.53429
35572	0.92511	1.58751
35573	1.19511	1.62561
35574	1.19511	1.81199
35575	0.946132	1.74306
35576	1.17409	1.54706
35577	0.925081	1.44278
35578	0.946129	1.77027
35579	0.946131	1.21808
35580	0.946131	1.49295
35581	1.17409	1.57087
35582	0.925083	1.38661
35583	1.89226	0.73207
35584	1.89226	0.88624
35585	1.89226	0.824323
35586	1.89226	0.791953
35587	1.89226	0.813616
35588	1.89226	0.783621
35589	1.89226	0.83437
35590	1.89226	0.960826
35591	1.89226	0.919588
35592	1.89226	0.871392
35593	3.78453	0.880306
35594	0.946132	1.99763
35595	0.946132	1.80584
35596	1.28892	2.1
35597	1.28892	2.1
35598	0.946132	2.1
35599	1.28892	2.1
35600	0.946133	1.95599
35601	1.17409	1.77417
35602	0.946132	1.74641
35603	0.946132	2.1
35604	1.17409	2.00608
35605	1.28892	2.1
35606	0.625229	2.1
35607	0.890321	2.1
35608	1.21636	2.05889
35609	1.13022	2.1

35610	1.07094	2.09727
35611	1.03541	2.1
35612	0.942207	2.1
35613	0.942207	2.07437
35614	0.898766	2.1
35615	1.26626	2.1
35616	1.0853	2.1
35617	0.600358	2.1
35618	0.719012	2.1
35619	0.719012	2.1
35620	1.17957	2.1
35621	1.17957	2.1
35622	0.719012	2.1
35623	0.952456	2.1
35624	0.946129	2.1
35625	0.946129	2.1
35626	0.946131	2.1
35627	0.952454	2.1
35628	0.946131	2.1
35629	0.719011	2.1
35630	0.94613	2.1
35631	0.94613	2.1
35632	0.94613	2.1
35633	0.94613	2.1
35634	0.946131	2.1
35635	0.946131	2.1
35636	0.946129	2.1
35637	0.946129	2.1
35638	0.94613	2.1
35639	0.94613	2.1
35640	0.94613	2.1
35641	0.94613	2.1
35642	1.1587	2.1
35643	0.973158	2.1
35644	0.750023	2.1
35645	0.536443	2.1
35646	0.84054	2.1
35647	0.84054	2.1
35648	0.854602	2.1
35649	0.854602	2.1
35650	1.1587	2.1
35651	0.750023	2.1
35652	0.973158	2.1
35653	0.536443	2.1
35654	1.89227	0.720908
35655	1.89227	1.8999
35656	1.89227	1.20974
35657	1.89227	0.840324
35658	3.78453	1.01636
35659	1.89226	2.06613
35660	1.89226	1.32881
35661	1.89226	0.972351
35662	1.89226	2.1
35663	3.78453	1.58853
35664	1.7632	1.34776
35665	1.61149	2.1
35666	1.98749	2.1
35667	2.11615	2.1

35668	3.75068	2.1
35669	1.7632	1.91759
35670	1.61149	1.56646
35671	1.97733	0.793935
35672	2.11615	2.1
35673	3.74052	1.46101
35674	1.89226	1.07159
35675	1.89226	0.93872
35676	1.89226	0.728017
35677	1.89226	1.72416
35678	3.78453	0.989244
35679	1.89226	0.853848
35680	1.89226	2.1
35681	1.89226	1.73441
35682	1.89226	1.2374
35683	3.78453	1.7635
35684	1.98749	2.1
35685	2.11615	1.93688
35686	2.43151	1.28043
35687	2.27541	2.1
35688	4.41899	2.1
35689	1.97733	0.835006
35690	2.11615	2.1
35691	2.43151	1.76174
35692	2.33986	0.799321
35693	4.40883	1.53235
35694	2.17892	0.276809
35695	2.80057	0.590011
35696	1.88349	0.51791
35697	2.63062	0.37329
35698	4.80954	0.471469
35699	1.89226	0.788891
35700	1.89226	0.412494
35701	1.89226	0.609012
35702	1.89226	0.367493
35703	3.78452	0.502189
35704	2.0155	0.679508
35705	1.97652	0.412898
35706	1.8247	0.60585
35707	1.80645	0.249137
35708	3.80122	0.466412
35709	1.97652	0.435494
35710	1.79681	0.445495
35711	1.80645	0.366028
35712	1.59244	0.419503
35713	3.56896	0.366146
35714	1.89226	0.441219
35715	1.89226	0.674081
35716	1.89226	0.409251
35717	1.89226	0.424669
35718	3.78452	0.385173
35719	1.89223	0.608274
35720	1.89223	0.432298
35721	1.89223	0.655729
35722	1.89223	0.330434
35723	3.78447	0.475938
35724	1.82467	0.605828
35725	1.80643	0.343897

35726	1.55159	0.579274
35727	1.53969	0.323961
35728	3.36437	0.416256
35729	1.80643	0.397067
35730	1.59242	0.481085
35731	1.5397	0.383351
35732	1.40538	0.410094
35733	3.13211	0.374186
35734	1.89224	0.472296
35735	1.89224	0.49282
35736	1.89224	0.384686
35737	1.89224	0.480935
35738	3.78447	0.41714
35739	1.37427	0.202902
35740	2.37086	0.262566
35741	2.37972	0.2057
35742	1.94703	0.259248
35743	2.19299	0.233934
35744	1.91813	0.285652
35745	1.07566	2.1
35746	1.12954	2.1
35747	0.946131	2.1
35748	0.946131	2.1
35749	1.12954	2.1
35750	1.12954	2.1
35751	0.946131	2.1
35752	0.946131	2.1
35753	1.12954	2.1
35754	0.946131	2.1
35755	0.946131	2.1
35756	1.07566	2.1
35757	2.25908	2.1
35758	1.89226	2.1
35759	1.89226	2.1
35760	1.89226	2.1
35761	2.37567	2.1
35762	2.37567	2.1
35763	1.37597	1.55144
35764	1.70302	0.816591
35765	1.24162	1.39817
35766	1.12219	1.77506
35767	1.37072	0.979468
35768	1.70302	2.01662
35769	1.37072	2.05947
35770	1.70302	1.2005
35771	1.54168	1.2161
35772	2.21318	1.04707
35773	3.39238	1.33946
35774	1.89226	0.118526
35775	1.89226	0.112921
35776	1.89226	0.0953112
35777	1.89226	0.0947435
35778	3.78452	0.0956289
35779	1.89227	0.120116
35780	1.89227	0.113704
35781	1.89227	0.0948719
35782	1.89227	0.0997406
35783	3.78453	0.112438

35784	1.89226	0.103424
35785	1.89226	0.105327
35786	1.89226	0.0905533
35787	1.89226	0.0916871
35788	3.78453	0.0947107
35789	1.89226	0.102475
35790	2.21654	0.0914821
35791	2.72007	0.0878351
35792	2.72007	0.10038
35793	1.89226	0.0892387
35794	1.89226	0.10565
35795	0.946128	0.0849771
35796	0.946128	0.0879815
35797	0.946132	0.0825145
35798	0.946132	0.0855307
35799	0.94613	0.0821986
35800	0.94613	0.0869462
35801	0.946131	0.082383
35802	0.946131	0.0898947
35803	0.94613	0.0805704
35804	0.94613	0.0833755
35805	0.94613	0.0954255
35806	0.94613	0.0895616
35807	1.89226	0.091927
35808	1.89226	0.093203
35809	1.89226	0.080442
35810	1.89226	0.0813835
35811	3.78453	0.0840118
35812	1.33457	0.0814712
35813	0.946131	0.0810868
35814	1.08045	0.0820298
35815	0.946132	0.0800669
35816	1.08045	0.0842919
35817	1.08045	0.0798353
35818	0.946132	0.0852349
35819	0.946132	0.08024
35820	1.33457	0.0783696
35821	1.08045	0.0760311
35822	0.946132	0.0866587
35823	0.946132	0.0825868
35824	1.53347	1.08448
35825	1.13092	2.06814
35826	1.52938	1.56664
35827	1.93568	1.48831
35828	3.06285	1.77413
35829	1.89226	0.418657
35830	2.72959	0.528962
35831	2.82996	0.535334
35832	2.82996	0.406219
35833	1.89226	0.578098
35834	1.89226	0.419377
35835	2.7296	0.530916
35836	2.57663	1.1515
35837	2.48354	0.49354
35838	2.48354	0.896972
35839	2.82996	0.498375
35840	2.82996	0.927911
35841	2.63449	0.529829

35842	2.7296	0.503356
35843	2.39282	0.573957
35844	2.39282	1.41766
35845	2.48354	0.99011
35846	2.72521	0.907821
35847	1.89224	2.1
35848	1.89224	1.527
35849	1.89224	0.799688
35850	1.89224	1.80907
35851	3.78447	1.03656
35852	1.89226	1.58569
35853	2.10425	2.1
35854	2.5142	2.1
35855	2.5142	2.1
35856	1.89226	2.1
35857	1.89226	1.54118
35858	0.946133	0.899897
35859	0.946133	1.25501
35860	0.946128	2.09471
35861	0.946128	2.1
35862	0.946131	1.76218
35863	0.946131	0.858153
35864	0.94613	0.553073
35865	0.94613	1.97774
35866	0.946131	1.31553
35867	0.946131	1.43252
35868	0.946131	1.23585
35869	0.946131	1.29257
35870	1.89226	0.724315
35871	1.89226	0.953458
35872	1.89226	0.787883
35873	1.89226	0.959857
35874	1.89226	0.760104
35875	1.89226	0.911329
35876	1.94446	0.798479
35877	2.50167	1.24208
35878	1.28418	0.901559
35879	1.94446	0.960944
35880	2.05205	0.983924
35881	2.05205	1.06232
35882	1.27835	2.1
35883	1.08341	2.1
35884	0.946132	2.1
35885	0.946132	2.1
35886	0.851542	2.1
35887	0.851542	2.1
35888	0.977356	2.1
35889	1.18368	2.1
35890	1.18378	2.1
35891	0.851555	2.1
35892	0.977346	2.1
35893	0.851476	2.1
35894	1.07369	2.1
35895	2.5813	2.1
35896	1.95053	2.1
35897	1.95053	2.1
35898	1.90196	2.1
35899	1.90196	2.1

35900	1.89226	2.1
35901	1.89226	2.1
35902	1.89226	2.1
35903	1.89226	2.1
35904	3.78452	2.1
35905	1.89224	2.1
35906	1.89224	2.1
35907	1.89224	2.1
35908	1.89224	2.1
35909	1.89224	2.1
35910	1.89224	2.1
35911	1.97004	0.575763
35912	1.8072	1.24659
35913	1.5866	1.13551
35914	1.79478	0.643511
35915	3.60198	0.90014
35916	1.23267	1.81496
35917	1.8072	0.841999
35918	1.58661	0.743786
35919	0.947921	1.51661
35920	2.75512	1.20514
35921	1.89226	0.840597
35922	1.89226	1.88647
35923	1.89226	0.75122
35924	1.89226	1.21401
35925	1.89226	0.733693
35926	1.89226	1.62853
35927	1.89226	1.24382
35928	1.89226	0.592409
35929	1.89226	0.661188
35930	1.89226	1.08447
35931	3.78452	0.812336
35932	1.4263	1.40924
35933	1.79478	0.69431
35934	1.67651	0.785796
35935	1.79478	1.41317
35936	1.46834	0.576702
35937	1.5866	0.885373
35938	1.89226	0.7116
35939	1.89226	0.973941
35940	1.89226	0.611902
35941	1.89226	0.900229
35942	1.89226	0.663464
35943	1.89226	1.38992
35944	2.23956	1.30224
35945	1.81847	1.43764
35946	2.10991	0.899948
35947	2.75317	1.02924
35948	4.34947	1.18918
35949	1.60578	0.15273
35950	1.94974	0.149497
35951	1.68062	0.196337
35952	1.14721	0.133376
35953	3.2864	0.146525
35954	1.3946	0.0810848
35955	1.692	0.0742298
35956	2.0154	0.0874692
35957	2.0154	0.076319

35958	1.11926	0.0859559
35959	1.11926	0.0748725
35960	1.90347	0.0868109
35961	1.83626	0.0748884
35962	1.84583	0.0747463
35963	1.89434	0.0895669
35964	3.79781	0.0800449
35965	1.89226	1.17351
35966	1.89226	0.972133
35967	1.89226	0.776776
35968	1.89226	1.15812
35969	3.78452	0.940059
35970	1.20986	1.30032
35971	1.20986	1.25385
35972	0.912635	1.12553
35973	0.912635	1.44889
35974	0.912667	0.896204
35975	1.17637	1.53555
35976	0.946125	1.35512
35977	0.946125	1.42971
35978	0.946128	1.51996
35979	0.912664	1.09784
35980	0.946127	1.69662
35981	1.17637	1.2302
35982	1.89226	0.095603
35983	1.65573	0.102969
35984	1.65573	0.117026
35985	1.89226	0.0885915
35986	1.89226	0.11402
35987	2.12879	0.085911
35988	1.89226	0.113982
35989	1.89226	0.115872
35990	1.89226	0.145773
35991	1.89226	0.103393
35992	1.89226	0.143307
35993	1.89226	0.101155
35994	2.40928	0.0843071
35995	1.89226	0.110298
35996	1.95959	0.0969795
35997	1.70302	0.0937607
35998	3.47338	0.100893
35999	1.89226	0.112797
36000	1.89226	0.112729
36001	1.89226	0.0975173
36002	1.89226	0.139808
36003	3.78452	0.103932
36004	1.64985	0.172373
36005	2.47846	0.24131
36006	2.25405	0.172501
36007	1.80768	0.152491
36008	4.06173	0.160077
36009	1.98846	0.140421
36010	0.912729	0.168025
36011	1.05417	0.109971
36012	1.70303	0.167213
36013	2.82107	0.165684
36014	2.47846	0.247227
36015	1.70303	0.212848

36016	2.86918	0.292926
36017	2.05973	0.265851
36018	2.51808	0.223238
36019	1.80768	0.196544
36020	0.946131	0.201606
36021	0.946131	0.181425
36022	0.946132	0.200382
36023	0.946132	0.301348
36024	0.946131	0.316628
36025	0.946131	0.25203
36026	0.946133	0.211879
36027	0.946133	0.183029
36028	0.946131	0.243833
36029	0.946131	0.268952
36030	0.946132	0.234355
36031	0.946132	0.252425
36032	0.946133	0.149466
36033	0.946133	0.145239
36034	0.946132	0.179421
36035	0.946132	0.191185
36036	0.946133	0.19357
36037	0.946133	0.181727
36038	0.946132	0.133757
36039	0.946132	0.206641
36040	0.946132	0.158456
36041	0.946132	0.18093
36042	0.946133	0.147672
36043	0.946133	0.180025
36044	1.89227	0.308359
36045	1.89227	0.320116
36046	1.89227	0.360013
36047	1.89227	0.257956
36048	1.89227	0.408266
36049	1.89227	0.316724
36050	1.1656	2.1
36051	1.21847	2.1
36052	0.836006	2.1
36053	0.806007	2.1
36054	1.00653	2.1
36055	1.00653	2.1
36056	1.01794	2.1
36057	1.01794	2.1
36058	1.1656	2.1
36059	0.806007	2.1
36060	0.836006	2.1
36061	1.21847	2.1
36062	2.82263	2.1
36063	2.3312	2.1
36064	2.25936	2.1
36065	2.71441	2.1
36066	5.0456	2.1
36067	2.25936	2.1
36068	1.61201	2.1
36069	1.57627	2.1
36070	1.61201	2.1
36071	2.29545	2.1
36072	2.3312	2.1
36073	1.21269	0.0738286

36074	1.48867	0.0815444
36075	1.68264	0.078477
36076	1.9898	0.0736257
36077	3.17131	0.0758111
36078	2.0487	2.1
36079	2.45212	2.1
36080	2.31012	2.1
36081	1.94095	2.1
36082	4.39307	2.1
36083	1.02435	2.1
36084	0.970469	2.1
36085	0.625375	2.1
36086	0.567166	2.1
36087	0.796854	2.1
36088	0.796854	2.1
36089	0.79466	2.1
36090	0.79466	2.1
36091	0.970471	2.1
36092	0.567165	2.1
36093	0.625374	2.1
36094	1.02435	2.1
36095	2.31012	2.1
36096	1.94095	2.1
36097	1.4825	2.1
36098	2.17384	2.1
36099	4.11479	2.1
36100	0.946187	2.1
36101	0.803763	2.1
36102	0.581357	2.1
36103	0.510356	2.1
36104	0.745632	2.1
36105	0.745632	2.1
36106	0.734566	2.1
36107	0.734563	2.1
36108	0.896142	2.1
36109	0.510366	2.1
36110	0.946173	2.1
36111	0.552965	2.1
36112	1.89226	0.92191
36113	1.89226	1.32394
36114	1.89226	1.4612
36115	1.89226	1.58595
36116	1.89226	0.953653
36117	1.89226	1.35913
36118	1.50743	1.30699
36119	1.0417	1.93055
36120	1.50743	1.71318
36121	1.62974	1.01446
36122	1.14605	0.989165
36123	1.14605	1.89351
36124	1.89226	0.827609
36125	1.89226	1.55615
36126	1.89226	0.704336
36127	1.89226	1.3391
36128	1.89226	0.753599
36129	1.89226	1.47835
36130	1.89226	1.46479
36131	1.89226	1.25408

36132	1.89226	0.703635
36133	1.89226	1.50399
36134	3.78453	1.04708
36135	1.77186	1.37426
36136	1.35668	1.67995
36137	1.70254	0.886658
36138	1.38756	1.62226
36139	1.77186	1.51072
36140	1.37814	1.97891
36141	0.734857	1.69765
36142	1.00549	1.44723
36143	0.835261	1.02129
36144	1.08357	1.68749
36145	0.97665	1.28357
36146	0.795559	2.03797
36147	0.891385	1.44416
36148	0.891385	0.717062
36149	0.874231	1.03769
36150	1.08356	1.22724
36151	0.734863	1.26636
36152	0.79449	1.45439
36153	1.89226	1.0172
36154	1.89226	0.603473
36155	2.42671	0.713042
36156	1.98783	1.1524
36157	4.31897	0.737731
36158	0.579854	0.768495
36159	0.965582	0.857075
36160	0.634403	0.932613
36161	1.17796	0.739974
36162	1.00592	0.750529
36163	0.797259	1.89697
36164	0.905246	1.05271
36165	0.905246	0.661196
36166	0.695715	0.76367
36167	0.637106	1.0778
36168	1.24374	0.893139
36169	0.95617	1.02045
36170	2.0815	0.725718
36171	1.60521	1.17699
36172	1.29556	1.05282
36173	2.32205	1.43915
36174	1.28445	0.656013
36175	2.0815	1.00503
36176	2.3085	0.816302
36177	2.21147	2.1
36178	2.3085	2.1
36179	2.39282	1.4184
36180	1.98899	2.1
36181	2.0733	1.73958
36182	2.3275	2.1
36183	1.63471	2.1
36184	1.98899	2.1
36185	1.56801	2.1
36186	3.62369	2.1
36187	2.3085	0.767432
36188	1.98899	2.1
36189	2.22575	0.86198

36190	1.90502	2.1
36191	4.21353	2.0815
36192	1.90502	2.1
36193	1.56801	2.1
36194	1.90502	2.1
36195	1.98899	2.1
36196	1.49733	2.1
36197	1.58129	2.1
36198	1.89226	0.929507
36199	2.6491	0.900679
36200	2.49266	2.07486
36201	2.6491	0.891052
36202	1.73582	0.865747
36203	1.89226	0.493537
36204	1.31854	2.1
36205	2.6491	0.901623
36206	1.3909	2.1
36207	1.3909	1.46081
36208	2.49266	2.1
36209	2.49266	1.69395
36210	1.89226	0.992726
36211	2.49266	0.817461
36212	2.38634	1.98128
36213	2.49266	0.822991
36214	1.78595	0.838207
36215	1.89226	0.509648
36216	1.23042	2.1
36217	2.49266	0.779451
36218	1.31854	2.1
36219	1.31854	0.971128
36220	2.38635	2.1
36221	2.38635	1.02746
36222	2.83487	2.1
36223	1.89226	2.1
36224	2.594	2.1
36225	2.594	2.1
36226	1.89226	2.1
36227	1.89226	2.1
36228	1.89226	2.1
36229	1.89226	2.1
36230	1.89226	2.1
36231	1.89226	2.1
36232	3.78452	2.1
36233	1.09518	2.1
36234	1.28158	2.1
36235	1.36844	2.1
36236	1.59972	2.1
36237	2.65003	2.1
36238	1.89226	2.1
36239	1.89226	2.02936
36240	1.89226	2.1
36241	1.89226	2.1
36242	3.78452	2.1
36243	1.36844	2.1
36244	1.59972	2.1
36245	1.67352	2.1
36246	1.94291	2.1
36247	3.31135	2.1

36248	1.52938	1.86903
36249	1.93568	1.74812
36250	2.36221	0.828018
36251	1.94723	2.01739
36252	3.89158	1.51492
36253	1.94724	1.07916
36254	1.26593	2.07696
36255	1.75367	1.81753
36256	2.4322	1.27139
36257	3.7009	1.87706
36258	2.36221	0.986727
36259	2.43219	1.74399
36260	1.94723	1.2436
36261	1.94723	1.44364
36262	2.8202	1.38391
36263	2.8202	1.61796
36264	2.51604	2.1
36265	2.18533	2.1
36266	2.17228	2.1
36267	2.4889	2.1
36268	4.67423	2.1
36269	2.17228	2.1
36270	1.62183	2.1
36271	1.62316	2.1
36272	1.62183	2.1
36273	2.18666	2.1
36274	2.18533	2.1
36275	2.17227	2.1
36276	2.4889	2.1
36277	2.45922	2.1
36278	2.15686	2.1
36279	4.64576	2.1
36280	2.17228	2.1
36281	1.62316	2.1
36282	1.62241	2.1
36283	2.15686	2.1
36284	3.78002	2.1
36285	1.89226	0.888305
36286	1.89226	0.871797
36287	1.89226	0.786608
36288	1.89226	0.728387
36289	3.78453	1.12353
36290	1.89226	0.836814
36291	1.89226	0.78359
36292	1.89226	0.686089
36293	1.89226	0.5293
36294	3.78452	0.63959
36295	1.89226	0.853504
36296	1.89226	0.615117
36297	1.89226	0.793815
36298	1.89226	0.751959
36299	1.89226	0.675913
36300	1.89226	0.684941
36301	1.89226	0.842743
36302	1.89226	0.925861
36303	2.53413	0.541786
36304	1.89226	0.538916
36305	3.78453	0.59071

36306	1.38048	0.644844
36307	1.38048	0.681746
36308	0.490332	0.613867
36309	0.490332	0.594043
36310	0.924679	0.381988
36311	0.946131	1.25791
36312	0.946131	0.990283
36313	0.490332	0.286676
36314	0.924678	0.409537
36315	0.490332	0.35095
36316	0.946131	0.765794
36317	0.946131	0.607706
36318	1.89227	0.500878
36319	1.89227	0.740929
36320	1.89227	0.585041
36321	1.89227	0.865414
36322	1.89227	0.498469
36323	1.89227	0.652291
36324	1.30803	2.1
36325	2.55151	2.1
36326	1.43935	2.1
36327	1.43935	2.1
36328	2.34922	2.1
36329	2.34922	2.1
36330	0.946131	1.8738
36331	0.946131	2.1
36332	1.27575	2.1
36333	1.17461	2.1
36334	0.94613	2.1
36335	0.94613	1.97983
36336	1.17461	2.1
36337	1.17461	2.1
36338	0.946131	2.1
36339	1.17461	2.1
36340	0.946132	2.1
36341	1.27575	2.1
36342	1.89226	2.1
36343	1.89226	0.830223
36344	1.89226	1.98287
36345	1.89226	2.1
36346	1.89226	1.67742
36347	1.89226	2.1
36348	0.946133	2.1
36349	0.946133	2.0959
36350	0.946132	2.1
36351	0.946132	2.1
36352	0.946133	2.1
36353	0.946133	1.57285
36354	0.946132	2.1
36355	0.946132	2.1
36356	0.946132	2.1
36357	0.946132	2.1
36358	0.946133	2.1
36359	0.946133	2.1
36360	1.07043	2.1
36361	1.17461	2.1
36362	0.654016	2.1
36363	0.5829	2.1

36364	0.870481	2.1
36365	0.870481	2.1
36366	0.887027	2.1
36367	0.887027	2.1
36368	1.07043	2.1
36369	0.5829	2.1
36370	0.654016	2.1
36371	1.17461	2.1
36372	1.89226	2.1
36373	1.89226	0.795895
36374	1.89226	2.04382
36375	1.89226	1.61139
36376	3.78453	2.00904
36377	1.89226	2.1
36378	1.89226	2.1
36379	1.89226	2.1
36380	1.89226	1.83949
36381	3.78453	2.1
36382	1.26929	1.70205
36383	2.19313	2.1
36384	1.09847	2.1
36385	1.69916	2.1
36386	2.96844	2.1
36387	1.84124	2.1
36388	2.24937	2.1
36389	1.69915	2.1
36390	1.75691	2.1
36391	3.94852	2.1
36392	1.53082	2.1
36393	2.04422	2.1
36394	1.53082	2.1
36395	1.69915	2.1
36396	1.92911	2.1
36397	2.09744	2.1
36398	1.39263	0.712541
36399	1.0171	0.415836
36400	1.1211	0.549853
36401	1.46482	0.594265
36402	2.48192	0.602717
36403	1.39263	0.707302
36404	1.57962	0.8016
36405	1.65238	1.04038
36406	1.46482	0.584755
36407	3.04443	0.707335
36408	1.89226	0.797908
36409	1.89226	0.421061
36410	1.89226	0.565571
36411	1.89226	0.662327
36412	3.78453	0.670157
36413	1.89227	0.410082
36414	1.89227	0.453914
36415	1.89227	0.575039
36416	1.89227	0.327823
36417	3.78453	0.376619
36418	1.28418	0.535996
36419	1.46482	0.580781
36420	1.1211	0.474228
36421	1.1211	0.70035

36422	1.59382	0.425906
36423	1.59382	0.685118
36424	1.76897	0.950122
36425	1.46482	0.577062
36426	1.59382	0.717536
36427	1.46482	0.792402
36428	1.78138	0.684954
36429	1.65238	0.754692
36430	1.89226	0.558782
36431	1.89226	0.617359
36432	1.89226	0.465905
36433	1.89226	0.75269
36434	1.89226	0.425342
36435	1.89226	0.769161
36436	1.89226	0.562177
36437	1.89226	0.310384
36438	1.89226	0.350285
36439	1.89226	0.449656
36440	1.89226	0.413667
36441	1.89226	0.498239
36442	1.96832	1.10796
36443	2.40858	0.946427
36444	1.75211	0.621175
36445	2.20797	2.1
36446	4.1607	0.844765
36447	1.75211	0.635498
36448	2.20797	2.1
36449	1.51592	1.33303
36450	2.02064	0.879689
36451	3.77275	1.40587
36452	2.71793	0.549206
36453	2.67099	0.453614
36454	2.55105	0.486222
36455	2.71297	0.403791
36456	5.26898	0.475217
36457	2.36417	0.556044
36458	2.67098	0.467665
36459	2.55104	0.49267
36460	1.83418	0.395702
36461	4.91522	0.469411
36462	2.55105	0.481901
36463	2.71297	0.419547
36464	2.58032	0.45491
36465	2.28922	0.342448
36466	5.13137	0.450047
36467	2.55104	0.487818
36468	2.23787	0.400578
36469	1.67265	0.420799
36470	2.28922	0.375132
36471	4.22369	0.419458
36472	0.851542	0.781173
36473	0.851542	0.768774
36474	0.981419	0.701019
36475	0.854774	0.757156
36476	0.949431	0.714695
36477	0.753763	0.76119
36478	0.946131	0.772508
36479	0.946131	0.780367

36480	1.04073	0.762086
36481	0.981433	0.657134
36482	0.851528	0.822953
36483	0.854828	0.750521
36484	1.89226	0.890789
36485	1.89226	0.761743
36486	1.89226	0.753579
36487	1.89226	0.760094
36488	1.89226	0.863217
36489	1.89226	0.871672
36490	0.814227	0.68038
36491	0.814227	0.684807
36492	0.946117	0.633906
36493	1.07285	0.635653
36494	0.879132	0.60947
36495	0.923293	0.625019
36496	0.946119	0.687221
36497	0.946119	0.690879
36498	0.946131	0.666974
36499	0.814236	0.535641
36500	0.946106	0.714446
36501	0.870405	0.66737
36502	1.89224	0.798282
36503	1.89224	0.671533
36504	1.89224	0.675362
36505	1.89224	0.682241
36506	1.89224	0.775923
36507	1.89224	0.783712
36508	1.29388	1.1983
36509	1.04164	1.90399
36510	1.64834	1.66112
36511	1.82955	1.0608
36512	2.94222	1.71996
36513	1.4934	0.639659
36514	2.41522	0.516476
36515	2.20387	0.45415
36516	2.20387	0.996249
36517	1.75765	0.532242
36518	1.75765	0.849911
36519	2.41522	0.469203
36520	2.77303	2.1
36521	2.20387	1.10844
36522	2.58627	0.756204
36523	5.00149	1.10523
36524	1.22222	0.68674
36525	2.20387	0.620283
36526	1.96831	0.523461
36527	1.96831	1.07495
36528	1.4934	0.591813
36529	1.4934	1.04722
36530	1.96832	1.26139
36531	2.58627	0.761983
36532	2.40858	0.643749
36533	2.40858	1.91055
36534	2.20387	0.775911
36535	2.20387	1.92818
36536	1.5605	2.1
36537	1.53775	2.1

36538	1.55398	2.1
36539	1.45806	2.1
36540	1.65642	2.1
36541	1.5605	2.1
36542	0.768876	2.1
36543	0.79033	2.1
36544	0.828211	2.1
36545	0.871233	2.1
36546	0.803843	2.1
36547	0.803843	2.1
36548	0.814698	2.1
36549	0.814698	2.1
36550	0.79033	2.1
36551	0.871233	2.1
36552	0.768876	2.1
36553	0.828212	2.1
36554	1.80252	2.1
36555	2.09715	0.683698
36556	1.9394	1.34488
36557	1.50853	1.40567
36558	3.60567	1.48586
36559	1.96416	0.643189
36560	2.09715	0.70787
36561	1.88484	1.23664
36562	1.57916	0.584454
36563	3.67631	0.736355
36564	1.89226	0.680068
36565	1.89226	0.306989
36566	1.89226	0.591231
36567	1.89226	0.707156
36568	3.78453	0.547898
36569	1.89226	0.657494
36570	1.89226	0.760889
36571	1.89226	2.02411
36572	1.89226	0.367158
36573	1.89226	1.28398
36574	1.89226	0.26194
36575	1.5852	1.18729
36576	1.50853	1.18904
36577	1.2955	2.1
36578	1.44472	0.682549
36579	2.95325	1.11414
36580	1.53065	1.09178
36581	1.57916	0.495494
36582	1.44472	0.681484
36583	1.78861	0.730451
36584	1.23527	0.754232
36585	1.23527	0.710561
36586	1.89227	0.491811
36587	1.89227	0.554862
36588	1.89227	0.753387
36589	1.89227	0.582813
36590	3.78453	0.568201
36591	1.89226	0.687453
36592	1.89226	0.698044
36593	1.89226	1.79827
36594	1.89226	0.676197
36595	1.89226	0.865833

36596	1.89226	0.228871
36597	1.89227	0.964436
36598	1.89227	0.717613
36599	1.89227	0.717036
36600	1.89227	0.69658
36601	3.78453	0.691696
36602	0.943708	0.710555
36603	0.752219	0.656117
36604	0.946132	0.675665
36605	0.946132	0.716147
36606	0.946133	0.556272
36607	0.946133	1.04686
36608	0.752218	0.913174
36609	0.752218	0.736205
36610	0.752218	0.715186
36611	0.946132	0.613036
36612	0.946133	0.855355
36613	0.943708	0.732614
36614	0.943992	2.1
36615	0.943992	1.28193
36616	1.23507	0.844587
36617	1.48209	1.79336
36618	1.0384	2.1
36619	1.47953	1.7627
36620	0.946128	0.8218
36621	0.946128	1.1179
36622	0.946129	1.73033
36623	1.23293	1.65546
36624	1.0384	1.47344
36625	0.946129	1.43032
36626	1.89226	1.37999
36627	2.46586	1.72705
36628	2.80218	0.804248
36629	2.80218	2.1
36630	1.89226	0.802456
36631	1.89226	2.1
36632	1.32455	2.1
36633	1.89128	2.1
36634	1.32328	2.1
36635	1.97299	2.1
36636	3.29754	2.1
36637	1.89128	2.1
36638	2.4255	2.1
36639	1.973	2.1
36640	2.63807	2.1
36641	4.3985	2.1
36642	1.34008	2.1
36643	1.97299	2.1
36644	2.10766	2.1
36645	2.10766	2.1
36646	1.32328	2.1
36647	1.32328	2.1
36648	2.41055	2.1
36649	2.37491	2.1
36650	2.40754	2.1
36651	2.38023	2.1
36652	4.78546	2.1
36653	2.23743	2.1

36654	2.37491	2.1
36655	2.40754	2.1
36656	2.20392	2.1
36657	4.61234	2.1
36658	2.37491	2.1
36659	2.02641	2.1
36660	2.20392	2.1
36661	2.18943	2.1
36662	4.40132	2.1
36663	2.37491	2.1
36664	2.22558	2.1
36665	2.38023	2.1
36666	2.18943	2.1
36667	4.60049	2.1
36668	1.89226	0.20634
36669	1.89226	0.311149
36670	1.89226	0.259138
36671	1.89226	0.253763
36672	1.89226	0.232188
36673	1.89226	0.228396
36674	1.03599	0.156324
36675	0.706698	0.146455
36676	1.41141	0.182032
36677	1.17573	0.162715
36678	1.11957	0.137104
36679	0.765034	0.139493
36680	1.05913	0.130683
36681	1.45054	0.185337
36682	1.04847	0.142168
36683	1.04847	0.156307
36684	1.15323	0.16493
36685	1.05825	0.141391
36686	1.89226	0.184699
36687	1.89226	0.194308
36688	1.89226	0.204329
36689	1.89226	0.16902
36690	1.89226	0.213175
36691	1.89226	0.180868
36692	2.22575	0.332318
36693	1.70677	0.715162
36694	1.90157	0.289191
36695	1.5534	0.830169
36696	3.77914	0.596049
36697	1.70678	0.67305
36698	0.968594	2.1
36699	0.968594	1.47793
36700	1.10378	2.1
36701	1.5534	0.941651
36702	1.68859	2.1
36703	2.31429	0.248821
36704	1.5534	0.938844
36705	1.90103	0.338332
36706	1.41902	0.68422
36707	3.45443	0.599031
36708	2.53709	0.0814464
36709	1.209	0.0852105
36710	1.05417	0.077983
36711	2.6513	0.0889513

36712	3.70547	0.0871882
36713	1.02675	0.0749275
36714	1.16384	0.0819568
36715	2.22062	0.0800902
36716	2.48862	0.0758286
36717	3.38446	0.0762753
36718	1.89226	0.0831274
36719	1.89226	0.100758
36720	1.89226	0.0984345
36721	1.89226	0.0841523
36722	1.89226	0.103206
36723	1.89226	0.086507
36724	1.89226	0.915518
36725	1.89226	1.64833
36726	1.89226	1.69246
36727	1.89226	1.12333
36728	1.89226	1.47436
36729	1.89226	1.05838
36730	1.89226	0.832692
36731	1.89226	1.3381
36732	1.89226	1.42023
36733	1.89226	0.971019
36734	1.89226	1.33218
36735	1.89226	0.939271
36736	1.89227	0.995515
36737	1.89227	1.53569
36738	1.75032	1.57144
36739	1.75032	1.13149
36740	1.89227	1.42438
36741	1.89227	1.06204
36742	1.89226	0.381142
36743	1.89226	0.477427
36744	1.89226	0.433135
36745	1.89226	0.477405
36746	1.89226	0.468138
36747	1.89226	0.442755
36748	0.946132	0.295772
36749	0.946132	0.295846
36750	0.798215	0.278885
36751	0.848163	0.27236
36752	0.848163	0.29856
36753	0.946131	0.311279
36754	0.946132	0.28181
36755	0.798216	0.262618
36756	0.996105	0.30067
36757	0.996105	0.266498
36758	0.79819	0.279917
36759	0.79819	0.323663
36760	1.07763	0.30331
36761	2.28622	0.296766
36762	2.16109	0.290011
36763	1.17506	0.298572
36764	3.36385	0.294136
36765	1.89226	2.1
36766	1.6663	2.1
36767	1.6663	2.1
36768	1.71706	2.1
36769	1.89226	2.1

36770	1.94302	2.1
36771	1.89226	2.1
36772	1.62801	2.1
36773	1.62801	2.1
36774	1.6663	2.1
36775	1.89226	2.1
36776	1.93055	2.1
36777	1.48734	2.1
36778	1.99609	2.09691
36779	1.52245	2.1
36780	2.04137	2.1
36781	3.51854	2.1
36782	1.99609	2.1
36783	2.28563	2.1
36784	2.04136	2.1
36785	2.35019	1.42496
36786	4.34628	2.1
36787	1.50714	2.1
36788	2.04137	2.1
36789	2.04945	2.1
36790	2.04945	2.1
36791	1.52245	2.1
36792	1.52245	2.1
36793	2.04136	2.1
36794	2.35019	1.5319
36795	2.04945	2.1
36796	2.38681	2.1
36797	4.39964	2.1
36798	2.50924	0.0522355
36799	1.38305	2.1
36800	2.56918	2.1
36801	1.44535	2.1
36802	3.95222	2.1
36803	1.89226	2.1
36804	1.89226	2.1
36805	1.89226	2.1
36806	1.89226	2.1
36807	3.78453	2.1
36808	1.40615	2.1
36809	1.38851	2.1
36810	0.748607	2.1
36811	0.724468	2.1
36812	1.06362	2.1
36813	1.06362	2.1
36814	1.067	2.1
36815	1.067	2.1
36816	1.40615	2.1
36817	0.748607	2.1
36818	0.724468	2.1
36819	1.38851	2.1
36820	2.56918	2.1
36821	1.53377	2.1
36822	1.53706	2.1
36823	1.44535	2.1
36824	2.66088	2.1
36825	2.56918	2.1
36826	1.89226	2.1
36827	1.89226	2.1

36828	1.89226	2.1
36829	1.89226	2.1
36830	3.78453	2.1
36831	1.40239	2.1
36832	1.68466	1.69802
36833	1.26091	2.1
36834	1.54641	1.35365
36835	2.94881	2.1
36836	1.68466	1.78894
36837	1.65324	0.544407
36838	1.54641	1.41475
36839	1.52268	0.466192
36840	3.19965	0.738653
36841	1.05776	2.1
36842	1.54641	1.50013
36843	1.36595	2.1
36844	1.36595	1.69966
36845	1.26091	2.1
36846	1.26091	1.87244
36847	1.54641	1.64515
36848	1.32697	0.477487
36849	1.52268	0.565206
36850	1.52268	1.80128
36851	1.36595	0.566341
36852	1.36595	1.89679
36853	1.18647	1.4197
36854	0.988454	2.1
36855	1.4867	1.70706
36856	1.63892	1.50659
36857	2.67317	1.98916
36858	1.4867	1.94628
36859	1.63892	1.59905
36860	2.09605	0.777098
36861	1.97645	1.78606
36862	3.58275	1.34567
36863	1.29389	1.8399
36864	1.97645	1.45253
36865	1.4867	1.29439
36866	1.4867	2.05164
36867	1.82955	1.01249
36868	1.82955	1.96024
36869	1.13454	1.02028
36870	0.983594	2.1
36871	1.16809	2.1
36872	1.06836	1.50592
36873	1.08332	1.53732
36874	0.983594	1.16735
36875	1.48751	2.02017
36876	1.48047	1.57727
36877	1.77596	1.4148
36878	1.75506	1.55674
36879	3.25643	1.62798
36880	1.48047	1.46126
36881	1.25334	1.98019
36882	1.75505	1.26289
36883	1.55831	0.758713
36884	3.03878	1.59851
36885	1.77596	1.4876

36886	1.75506	1.93318
36887	2.00551	1.87815
36888	2.00083	0.924059
36889	3.77679	1.83326
36890	1.75506	1.66144
36891	1.80328	0.813956
36892	1.55831	1.66911
36893	1.55831	1.8742
36894	2.00083	0.869372
36895	2.00083	1.20016
36896	2.58627	0.737456
36897	2.79047	2.1
36898	2.40858	2.08069
36899	2.64748	1.79988
36900	5.23375	2.1
36901	1.26755	2.1
36902	1.71717	2.1
36903	1.03824	2.1
36904	1.48901	2.1
36905	2.75656	2.1
36906	1.89226	2.1
36907	1.89226	2.1
36908	1.89226	2.09116
36909	1.89226	2.1
36910	1.89226	2.00561
36911	1.89226	2.1
36912	1.36541	2.1
36913	1.22156	2.1
36914	0.946132	2.1
36915	0.946132	2.1
36916	1.36541	2.1
36917	1.36541	2.1
36918	0.946132	2.1
36919	0.946132	2.1
36920	1.36541	2.1
36921	0.946133	2.1
36922	0.946132	2.1
36923	1.22156	2.1
36924	1.18901	2.1
36925	1.61149	2.1
36926	1.98748	2.1
36927	1.534 2.1	
36928	3.1765	2.1
36929	1.89226	2.1
36930	1.89226	2.1
36931	1.89226	1.65099
36932	1.89226	2.1
36933	3.78452	2.1
36934	1.98748	2.1
36935	1.534 2.1	
36936	2.27541	2.1
36937	1.98748	2.1
36938	1.87436	2.1
36939	1.87436	2.1
36940	2.2988	2.1
36941	2.2604	2.1
36942	2.20831	2.1
36943	2.02802	2.1

36944	4.32683	2.1
36945	2.26041	2.1
36946	1.98957	2.1
36947	2.02803	2.1
36948	1.75021	2.1
36949	4.0176	2.1
36950	2.34607	2.1
36951	2.02803	2.1
36952	1.86795	2.1
36953	1.81448	2.1
36954	3.89597	2.1
36955	2.02803	2.1
36956	1.75021	2.1
36957	1.81448	2.1
36958	1.57887	2.1
36959	3.6069	2.1
36960	0.946131	0.369399
36961	0.946131	2.1
36962	1.1235	1.54589
36963	1.1235	1.76197
36964	0.946131	1.20196
36965	0.946131	0.798281
36966	1.1235	1.84435
36967	1.13736	2.0059
36968	1.13736	0.739232
36969	0.946131	1.0137
36970	1.1235	1.38442
36971	0.946131	1.17853
36972	1.89226	1.87758
36973	1.89226	2.0768
36974	1.89226	0.9558
36975	1.89226	0.834383
36976	3.78452	0.767257
36977	0.563266	2.07242
36978	0.563266	1.05821
36979	0.565606	0.660071
36980	0.565606	1.91374
36981	0.647086	1.60932
36982	0.624219	1.42577
36983	0.500035	1.0526
36984	0.481785	1.05783
36985	0.500034	0.645507
36986	0.647087	0.70647
36987	0.624219	1.30732
36988	0.481785	1.00923
36989	0.915788	0.367312
36990	0.915788	0.439926
36991	1.02732	0.672504
36992	1.02732	0.548857
36993	0.660831	0.593226
36994	1.11155	0.379172
36995	0.713295	0.392138
36996	1.26874	0.643749
36997	0.713295	0.524814
36998	0.660831	0.51453
36999	1.11155	0.520791
37000	1.26874	0.513197
37001	1.89226	0.698499

37002	1.85091	0.539593
37003	2.11193	0.71529
37004	1.85091	0.603218
37005	2.15328	0.584744
37006	1.89226	0.474893
37007	0.946131	0.362944
37008	0.946131	0.478661
37009	1.18275	0.386218
37010	1.05596	0.542387
37011	0.946127	0.418286
37012	0.946127	0.219642
37013	1.18275	0.547658
37014	1.18275	0.539226
37015	0.946128	0.563686
37016	1.18275	0.360605
37017	0.946129	0.482946
37018	1.05596	0.339808
37019	1.60936	2.1
37020	2.35846	2.1
37021	2.06424	2.1
37022	2.57651	2.1
37023	4.42269	2.1
37024	1.89226	2.1
37025	1.89226	2.1
37026	1.89226	2.1
37027	1.89226	2.1
37028	3.78453	2.1
37029	1.89226	2.1
37030	1.89226	2.1
37031	1.89226	2.1
37032	1.89226	2.1
37033	1.89226	2.1
37034	1.89226	2.1
37035	1.95053	2.1
37036	2.06424	2.1
37037	1.95053	2.1
37038	1.30584	2.1
37039	2.5	2.1
37040	1.8553	2.1
37041	2.49999	2.1
37042	2.57651	2.1
37043	2.49999	2.1
37044	2.06423	2.1
37045	2.80738	2.1
37046	2.37162	2.1
37047	1.89226	2.1
37048	1.89226	2.1
37049	1.89226	2.1
37050	1.89226	2.1
37051	1.89226	2.1
37052	1.89226	2.1
37053	1.49295	0.289318
37054	1.03824	0.211719
37055	1.51396	0.3591
37056	2.08514	0.290073
37057	3.12338	0.290087
37058	1.49798	0.287189
37059	2.32017	0.192549

37060	2.68236	0.379926
37061	2.08514	0.297997
37062	4.4053	0.246961
37063	1.89226	0.219058
37064	1.89226	0.203835
37065	1.89226	0.302178
37066	1.89226	0.245884
37067	3.78452	0.268174
37068	1.89226	0.297219
37069	1.89226	0.219316
37070	1.89226	0.288895
37071	1.89226	0.254959
37072	1.89226	0.244393
37073	1.89226	0.217021
37074	1.51396	0.271647
37075	2.08514	0.20674
37076	2.55393	0.341991
37077	2.02901	0.28016
37078	4.11415	0.243119
37079	1.89226	0.22493
37080	1.89226	0.191246
37081	1.89226	0.27892
37082	1.89226	0.27619
37083	3.78451	0.206506
37084	1.89226	0.211404
37085	1.89226	0.289423
37086	1.89226	0.187828
37087	1.89226	0.183205
37088	1.89226	0.299211
37089	1.89226	0.289891
37090	0.946131	0.182014
37091	0.946131	0.201353
37092	0.946132	0.302016
37093	0.946132	0.211321
37094	0.946132	0.230152
37095	0.946132	0.196531
37096	0.946132	0.238194
37097	0.946132	0.281025
37098	0.946132	0.204846
37099	0.946132	0.229579
37100	0.946132	0.245147
37101	0.946132	0.218793
37102	1.89226	2.1
37103	1.89226	1.25788
37104	1.89226	2.1
37105	1.89226	1.035
37106	3.78452	2.1
37107	1.28367	2.1
37108	1.82707	1.23318
37109	1.11025	2.1
37110	1.6365	0.906295
37111	2.92016	2.1
37112	1.82707	1.37243
37113	1.8247	0.54511
37114	1.8247	1.55091
37115	2.01549	0.614501
37116	1.6365	0.905249
37117	1.82729	0.592051

37118	1.89226	1.45321
37119	1.89226	0.550915
37120	1.89226	0.358782
37121	1.89226	1.33985
37122	1.89226	0.361745
37123	1.89226	1.35832
37124	1.63648	1.82849
37125	1.82467	0.447168
37126	1.35877	1.32754
37127	1.55159	0.491161
37128	3.18345	0.755498
37129	1.89223	1.53737
37130	1.89223	0.446954
37131	1.89223	1.40728
37132	1.89223	0.585813
37133	3.78447	0.774521
37134	1.59972	2.1
37135	1.28037	2.1
37136	1.63702	2.1
37137	1.28158	2.1
37138	1.63581	2.1
37139	1.28037	2.1
37140	1.89226	2.1
37141	1.89226	2.1
37142	1.89226	2.1
37143	1.89226	2.1
37144	1.89226	2.1
37145	1.89226	2.1
37146	1.89226	2.1
37147	1.89226	1.99648
37148	1.89226	2.1
37149	1.89226	2.1
37150	3.78451	2.1
37151	1.28037	2.1
37152	1.05071	2.1
37153	1.63581	2.1
37154	1.44287	2.1
37155	2.72324	2.1
37156	1.59972	2.1
37157	1.63581	2.1
37158	1.94291	2.1
37159	2.00118	2.1
37160	3.57872	2.1
37161	1.89226	2.08338
37162	1.89226	2.1
37163	1.89226	2.1
37164	1.89226	1.88141
37165	3.78452	2.1
37166	0.946131	1.65331
37167	0.946131	1.93899
37168	0.946127	2.1
37169	0.946127	2.1
37170	0.94613	1.4621
37171	0.94613	2.1
37172	0.946128	2.1
37173	0.946128	1.76581
37174	0.946128	2.1
37175	0.946128	2.1

37176	0.946129	2.1
37177	0.946129	2.1
37178	0.865056	2.1
37179	0.865056	2.1
37180	0.873224	2.1
37181	0.873224	2.1
37182	0.721437	2.1
37183	0.920378	2.1
37184	0.817903	2.1
37185	1.00059	2.1
37186	1.00059	2.1
37187	0.920376	2.1
37188	0.817904	2.1
37189	0.721437	2.1
37190	3.09157	2.1
37191	1.71255	2.1
37192	2.0815	2.1
37193	1.9918	2.1
37194	2.51364	2.1
37195	2.39396	2.1
37196	2.0815	2.1
37197	1.05051	2.1
37198	2.56724	2.1
37199	1.63991	2.1
37200	3.47929	2.1
37201	1.89226	0.275636
37202	2.2973	0.496388
37203	1.90705	0.473289
37204	1.90705	0.328644
37205	1.89226	0.411424
37206	1.89226	0.2756
37207	1.89226	0.289198
37208	1.90705	0.487441
37209	1.45321	0.49517
37210	1.45321	0.34445
37211	1.89226	0.41948
37212	1.89226	0.295289
37213	0.901146	2.1
37214	0.901146	2.1
37215	1.15227	2.1
37216	1.21279	2.1
37217	0.991265	2.1
37218	1.15003	2.1
37219	0.946132	2.1
37220	0.946132	2.1
37221	0.946132	2.1
37222	1.09997	2.1
37223	0.991266	2.1
37224	0.946132	2.1
37225	1.89226	2.1
37226	1.89226	2.1
37227	1.89226	2.1
37228	1.89226	2.1
37229	1.89226	2.1
37230	1.89226	2.1
37231	1.62229	2.1
37232	1.06053	2.1
37233	1.19698	2.1

37234	1.70106	2.1
37235	2.81928	2.1
37236	0.946132	2.1
37237	0.946132	2.1
37238	1.17527	2.1
37239	1.09997	2.1
37240	1.09997	2.1
37241	1.09997	2.1
37242	0.946133	2.1
37243	0.946133	2.1
37244	1.17527	2.1
37245	0.946132	2.1
37246	0.946133	2.1
37247	1.09997	2.1
37248	1.89226	2.1
37249	1.89226	2.1
37250	1.89226	2.1
37251	1.89226	2.1
37252	1.89226	2.1
37253	1.89226	2.1
37254	0.905416	2.1
37255	0.850527	2.1
37256	0.660601	2.1
37257	0.598494	2.1
37258	0.750137	2.1
37259	0.750137	2.1
37260	0.753774	2.1
37261	0.753774	2.1
37262	0.660599	2.1
37263	0.905418	2.1
37264	0.598493	2.1
37265	0.850529	2.1
37266	2.43509	2.1
37267	2.65066	2.1
37268	2.66602	2.1
37269	2.46339	2.1
37270	5.10111	2.1
37271	2.53008	2.1
37272	2.65066	2.1
37273	2.66602	2.1
37274	2.52852	2.1
37275	5.1961	2.1
37276	2.66602	2.1
37277	2.46339	2.1
37278	2.47932	2.1
37279	2.67393	2.1
37280	5.14535	2.1
37281	2.66602	2.1
37282	2.52853	2.1
37283	2.51686	2.1
37284	2.67393	2.1
37285	5.18288	2.1
37286	1.89226	0.699879
37287	1.89226	0.400408
37288	1.89226	0.613856
37289	1.89226	0.377371
37290	3.78452	0.500883
37291	1.55161	0.619655

37292	1.53972	0.397461
37293	1.32259	0.613873
37294	1.35082	0.284748
37295	2.8623	0.480252
37296	1.53972	0.428113
37297	1.4054	0.447521
37298	1.35083	0.376741
37299	1.24913	0.402454
37300	2.78885	0.384785
37301	1.89226	0.426997
37302	1.89226	0.676667
37303	1.89226	0.441672
37304	1.89226	0.401028
37305	3.78452	0.385412
37306	1.89226	0.575302
37307	1.89226	0.440235
37308	1.89226	0.667021
37309	1.89226	0.435823
37310	1.89226	0.613418
37311	1.89226	0.398743
37312	1.14599	0.573481
37313	1.35082	0.360871
37314	1.14599	0.47871
37315	1.32259	0.633371
37316	1.19993	0.234183
37317	1.37652	0.643161
37318	1.35083	0.410072
37319	1.24913	0.51464
37320	1.19993	0.428081
37321	1.11787	0.377476
37322	2.44906	0.379176
37323	1.89226	0.50454
37324	1.89226	0.509576
37325	1.89226	0.433001
37326	1.89226	0.405179
37327	3.78452	0.407117
37328	1.51697	0.198787
37329	2.57264	0.28117
37330	1.30203	0.18234
37331	2.41701	0.187697
37332	3.93398	0.236254
37333	1.17464	0.131867
37334	2.41701	0.181642
37335	2.32462	0.164234
37336	2.32462	0.169294
37337	1.30203	0.165694
37338	1.30203	0.169404
37339	2.08149	2.1
37340	1.9839	1.72338
37341	1.71398	2.1
37342	1.99182	0.54086
37343	3.71121	1.05876
37344	2.16061	1.51301
37345	2.01399	0.378108
37346	1.99182	0.597876
37347	1.83895	0.520252
37348	4.00581	0.533127
37349	1.90444	2.1

37350	1.99182	0.587289
37351	1.71717	2.1
37352	1.805	0.648203
37353	3.70899	1.4097
37354	1.99182	0.624205
37355	1.83896	0.433581
37356	1.805	0.903059
37357	1.65243	0.453564
37358	3.64425	0.521764
37359	1.18076	2.1
37360	1.27697	2.1
37361	1.05025	2.1
37362	1.27697	2.1
37363	1.25	2.1
37364	1.47671	2.1
37365	1.89226	2.1
37366	1.89226	1.19565
37367	1.89226	2.1
37368	1.89226	2.1
37369	3.78452	2.1
37370	2.64124	2.1
37371	1.89226	2.1
37372	1.89226	2.1
37373	1.89226	2.1
37374	2.46904	2.1
37375	2.46904	2.1
37376	1.89226	2.1
37377	1.89226	2.1
37378	2.7408	2.1
37379	2.82858	1.70464
37380	4.72084	2.1
37381	1.87875	2.1
37382	1.37704	2.1
37383	1.77042	2.1
37384	2.27801	2.1
37385	3.64916	2.1
37386	1.77042	2.1
37387	2.27801	2.1
37388	2.58523	2.1
37389	1.90178	2.1
37390	4.35565	2.1
37391	1.28667	2.1
37392	1.30711	2.1
37393	1.53077	2.1
37394	1.55635	2.1
37395	2.81743	2.1
37396	1.55635	2.1
37397	1.28228	2.1
37398	1.64268	1.39933
37399	1.93673	2.1
37400	3.19903	2.1
37401	1.53077	2.1
37402	1.93673	2.1
37403	1.55635	2.1
37404	1.55635	2.1
37405	1.86206	2.1
37406	1.86206	2.1
37407	1.64892	2.1

37408	1.60532	2.1
37409	1.85282	2.1
37410	1.96716	2.1
37411	3.61608	2.1
37412	1.60531	2.1
37413	1.32414	2.1
37414	1.96716	2.1
37415	1.75823	2.1
37416	3.2913	2.1
37417	1.91219	2.1
37418	1.96716	2.1
37419	2.0815	2.1
37420	2.51396	2.1
37421	4.30843	2.1
37422	1.96716	2.1
37423	2.17979	2.1
37424	1.75823	2.1
37425	1.75823	2.1
37426	2.28541	2.1
37427	2.28541	2.1
37428	1.89226	1.12751
37429	1.89226	1.53574
37430	1.89226	1.12306
37431	1.89226	1.01229
37432	3.78453	1.19804
37433	1.89226	1.2964
37434	1.89226	1.27365
37435	1.89226	1.11698
37436	1.89226	1.32548
37437	1.89226	1.20557
37438	1.89226	1.40662
37439	1.61016	1.14492
37440	1.45303	1.4559
37441	1.62812	1.21598
37442	1.86379	1.21784
37443	3.23828	1.22417
37444	1.61016	1.38553
37445	1.78716	1.56781
37446	2.0815	0.936946
37447	1.6774	1.42413
37448	3.54454	1.40918
37449	1.89226	1.32327
37450	1.89226	1.23643
37451	1.89226	0.897591
37452	1.89226	1.31585
37453	3.78453	1.16802
37454	1.89226	1.37794
37455	1.89226	1.37138
37456	1.89226	1.22215
37457	1.89226	1.64281
37458	1.89226	0.897103
37459	1.89226	1.14042
37460	0.807591	1.39458
37461	0.814059	1.21597
37462	1.00217	1.48064
37463	0.931896	1.27478
37464	0.850586	1.30053
37465	0.850586	1.57296

37466	0.8889	1.12558
37467	0.8889	1.36715
37468	0.807592	0.971733
37469	1.00217	1.43065
37470	0.814061	1.2827
37471	0.931894	1.62525
37472	2.0815	1.12475
37473	1.6774	1.72591
37474	2.00435	1.18833
37475	2.36104	1.16139
37476	3.86809	1.2096
37477	1.00532	0.346167
37478	1.00532	0.351917
37479	1.00183	0.357691
37480	1.00183	0.342008
37481	1.26377	0.348074
37482	1.34921	0.354007
37483	0.664872	0.353712
37484	0.743381	0.360837
37485	0.664872	0.342954
37486	1.26377	0.348406
37487	1.34921	0.352902
37488	0.743381	0.3488
37489	1.26826	0.0522355
37490	0.946131	1.98337
37491	1.02404	1.85195
37492	0.94613	2.1
37493	1.30281	1.92436
37494	0.930941	2.1
37495	0.930941	1.95414
37496	1.318	1.96211
37497	1.58161	1.87754
37498	0.94613	1.91038
37499	0.94613	1.64833
37500	1.56338	1.8098
37501	1.02404	1.77996
37502	2.81374	1.83394
37503	1.89226	2.1
37504	1.89226	1.99986
37505	1.89226	2.02425
37506	3.78453	2.1
37507	0.946133	2.07588
37508	0.946133	1.79046
37509	0.946132	1.75405
37510	0.946132	1.94384
37511	0.946132	1.94067
37512	0.946132	1.8022
37513	0.946133	2.01729
37514	0.946133	1.79297
37515	0.946132	2.00645
37516	0.946132	1.72306
37517	0.946133	2.08901
37518	0.946133	1.81735
37519	1.89226	1.44289
37520	1.89226	1.96635
37521	1.89226	1.59223
37522	1.89226	1.74322
37523	3.78452	1.82621

37524	1.89226	1.97622
37525	1.89226	1.89038
37526	1.89226	2.09981
37527	1.89226	1.6183
37528	1.89226	2.1
37529	1.89226	1.63144
37530	0.946133	1.62286
37531	0.946133	1.95469
37532	0.946132	1.53527
37533	0.946132	1.87418
37534	0.946132	1.87261
37535	0.946132	1.79298
37536	0.946133	1.89009
37537	0.946133	1.6386
37538	0.946132	1.90639
37539	0.946132	1.6558
37540	0.946132	1.98114
37541	0.946132	1.70201
37542	2.56953	2.1
37543	2.64148	2.1
37544	2.48631	2.1
37545	2.62213	2.1
37546	5.19166	2.1
37547	2.64148	2.1
37548	2.57929	2.1
37549	2.62213	2.1
37550	2.59522	2.1
37551	5.20142	2.1
37552	2.48631	2.1
37553	2.62213	2.1
37554	2.41176	2.1
37555	2.59262	2.1
37556	5.03389	2.1
37557	2.62213	2.1
37558	2.59522	2.1
37559	2.59262	2.1
37560	2.61901	2.1
37561	5.24114	2.1
37562	0.946132	0.0752782
37563	0.946132	0.0720104
37564	0.946133	0.0716702
37565	0.946133	0.074003
37566	0.946132	0.0717632
37567	0.946132	0.0747347
37568	0.946133	0.0717246
37569	0.946133	0.0758172
37570	0.946132	0.069521
37571	0.946132	0.0701021
37572	0.946133	0.0795036
37573	0.946133	0.0762688
37574	1.5681	0.0702977
37575	1.01248	0.0668071
37576	1.10749	0.0677502
37577	0.570913	0.0679272
37578	1.15589	0.067003
37579	1.15588	0.0703079
37580	0.977549	0.0680128
37581	0.977549	0.0710798

37582	1.01249	0.0669051
37583	0.654445	0.0657453
37584	1.36795	0.0719351
37585	1.10748	0.069136
37586	1.73883	0.0522355
37587	1.87638	0.0522355
37588	1.85219	0.0522355
37589	1.93113	0.910002
37590	3.7407	0.0522355
37591	1.87638	0.0522355
37592	1.73883	2.1
37593	1.93113	0.874948
37594	1.80426	2.1
37595	3.66996	1.4017
37596	2.02912	0.25298
37597	1.44951	0.276944
37598	1.50103	0.254012
37599	2.02248	0.294743
37600	3.47199	0.254614
37601	1.89226	0.289204
37602	1.89226	0.317988
37603	1.89226	0.309217
37604	1.89226	0.326208
37605	3.78453	0.313682
37606	2.57881	0.271192
37607	1.89226	0.308471
37608	1.89226	0.295018
37609	1.89226	0.293002
37610	2.6428	0.279246
37611	2.6428	0.278822
37612	1.01379	0.269939
37613	0.985937	0.264418
37614	0.769295	0.262701
37615	0.715547	0.242736
37616	0.853042	0.282624
37617	0.853042	0.281558
37618	0.864117	0.279793
37619	0.864114	0.222151
37620	0.715556	0.292687
37621	1.03047	0.271278
37622	0.731721	0.262259
37623	0.985921	0.28069
37624	1.89227	0.295128
37625	1.89227	0.314686
37626	1.89227	0.296866
37627	1.89227	0.349777
37628	3.78453	0.301913
37629	0.946129	0.298966
37630	0.946129	0.281987
37631	1.28161	0.248368
37632	1.3214	0.27768
37633	0.94613	0.300166
37634	0.94613	0.296652
37635	1.28161	0.264649
37636	1.28161	0.233726
37637	1.28161	0.263204
37638	0.94613	0.285407
37639	0.946131	0.294941

37640	1.3214	0.274846
37641	0.946132	0.500487
37642	0.946132	0.476602
37643	0.706045	0.36406
37644	1.16718	0.380577
37645	0.706017	0.3445
37646	0.706017	0.537594
37647	1.4073	0.389275
37648	1.4073	0.317755
37649	0.94613	0.434849
37650	1.16719	0.436677
37651	0.706046	0.465175
37652	0.946131	0.40792
37653	1.89226	0.529412
37654	1.89226	0.457437
37655	1.89226	0.309037
37656	1.89226	0.569643
37657	1.89226	0.337614
37658	1.89226	0.566142
37659	0.946119	0.479389
37660	0.946119	0.452849
37661	0.946115	0.474144
37662	0.946115	0.386112
37663	0.946117	0.475331
37664	0.946117	0.510717
37665	0.946117	0.439791
37666	0.946117	0.405454
37667	0.94613	0.426443
37668	0.94613	0.474256
37669	0.946105	0.469767
37670	0.946105	0.425599
37671	0.946132	0.731211
37672	0.946132	0.693277
37673	0.946131	0.771306
37674	0.946131	0.664688
37675	0.946132	0.71116
37676	0.946132	0.78418
37677	0.946132	0.685582
37678	0.827865	0.688209
37679	0.946132	0.690316
37680	0.946132	0.719528
37681	0.946132	0.699011
37682	0.827865	0.70462
37683	1.89226	1.22886
37684	1.89226	1.02032
37685	1.89226	1.38151
37686	1.89226	0.930421
37687	1.89226	1.67438
37688	1.89226	1.10149
37689	0.93094	0.870613
37690	0.93094	0.987416
37691	1.04649	0.892889
37692	0.614468	0.724776
37693	0.950358	0.785393
37694	0.799012	0.742105
37695	0.682742	0.902467
37696	0.682742	0.907681
37697	1.04073	1.30578

37698	1.15596	1.13936
37699	0.614478	1.18401
37700	0.851524	0.946352
37701	2.22575	0.818336
37702	1.90502	2.1
37703	2.10893	0.76556
37704	1.80258	2.1
37705	4.01395	1.94002
37706	1.90502	2.1
37707	1.40863	2.1
37708	1.49733	2.1
37709	1.49733	2.1
37710	1.80258	2.1
37711	1.80258	2.1
37712	2.10893	0.725119
37713	1.68356	2.1
37714	1.80258	2.1
37715	1.80258	1.28469
37716	1.98061	2.1
37717	1.98061	1.21232
37718	1.80258	2.1
37719	1.40863	2.1
37720	1.68356	2.1
37721	1.30751	2.1
37722	3.11009	2.1
37723	1.35374	1.40597
37724	1.35374	1.61863
37725	0.746096	1.14132
37726	0.746096	1.12844
37727	1.1537	1.19013
37728	0.746095	1.47245
37729	0.946132	1.21704
37730	0.946132	0.96614
37731	0.946132	0.965055
37732	0.746095	1.08785
37733	0.946131	0.913745
37734	1.1537	0.999237
37735	2.15247	2.1
37736	1.70303	1.04758
37737	1.41207	2.1
37738	1.43052	2.01449
37739	3.22793	2.1
37740	0.940246	2.1
37741	0.728442	1.94395
37742	0.650037	2.1
37743	0.539874	2.1
37744	0.694756	2.1
37745	0.694756	2.00127
37746	0.776346	2.1
37747	0.656772	2.1
37748	0.733146	2.1
37749	0.563769	2.1
37750	0.66316	2.1
37751	0.899692	2.1
37752	1.21223	1.92011
37753	1.22418	2.1
37754	1.43052	2.1
37755	1.43052	1.25317

37756	1.24353	2.1
37757	1.46181	1.37024
37758	1.89226	0.277441
37759	2.39894	0.173967
37760	1.89226	0.326931
37761	2.37417	0.180087
37762	1.89226	0.315578
37763	2.37417	0.178125
37764	1.12767	0.154629
37765	1.19947	0.225954
37766	0.94613	0.284432
37767	0.94613	0.178367
37768	1.12766	0.144753
37769	0.946128	0.154904
37770	1.19947	0.187346
37771	0.946132	0.299693
37772	1.19947	0.245304
37773	1.19947	0.165894
37774	0.94613	0.26062
37775	0.94613	0.173593
37776	2.22621	0.0522355
37777	1.14533	2.1
37778	1.1131	2.1
37779	0.88238	2.1
37780	0.868521	1.09741
37781	1.01153	2.1
37782	1.01153	2.1
37783	1.00232	2.1
37784	1.00232	2.1
37785	1.14533	2.1
37786	0.882378	2.1
37787	0.868519	2.1
37788	1.11311	2.1
37789	0.910183	1.38043
37790	1.07811	0.998861
37791	0.94613	0.576974
37792	0.94613	0.668537
37793	0.910212	0.684087
37794	0.910212	1.49087
37795	1.11403	0.58192
37796	1.11403	1.65072
37797	0.910183	0.789834
37798	0.94613	0.870025
37799	0.94613	0.974532
37800	1.07811	0.980838
37801	1.87228	0.80047
37802	2.81189	2.1
37803	1.6197	1.84022
37804	2.56716	1.51119
37805	4.43944	1.76876
37806	0.946132	0.973768
37807	0.946132	1.78596
37808	0.946132	0.618181
37809	0.946132	0.692184
37810	0.946131	0.752877
37811	0.946131	1.78621
37812	0.946132	0.635228
37813	0.946132	1.1009

37814	0.946132	0.798572
37815	0.946132	0.822973
37816	0.946132	0.943793
37817	0.946132	1.0199
37818	1.28724	2.05779
37819	2.56716	1.23936
37820	2.3014	1.07697
37821	2.3014	2.1
37822	1.6197	1.31536
37823	1.6197	2.1
37824	0.946132	1.59673
37825	0.946132	1.38593
37826	0.946132	0.648599
37827	0.946132	0.7708
37828	0.946132	0.810318
37829	0.946132	1.68421
37830	0.946133	1.96481
37831	0.946133	0.65462
37832	0.946133	0.915846
37833	0.946133	0.997409
37834	0.946132	0.977242
37835	0.946132	0.998397
37836	1.08039	2.1
37837	1.24445	1.60323
37838	1.2873	1.76853
37839	1.48751	1.88815
37840	2.53175	1.93483
37841	0.946125	0.733761
37842	0.946125	2.1
37843	1.12552	1.76869
37844	1.12552	2.1
37845	0.946124	1.43757
37846	0.946124	1.56554
37847	1.12552	2.1
37848	1.11114	2.1
37849	1.11113	1.47508
37850	0.946118	1.64584
37851	1.12552	1.84282
37852	0.946131	1.71597
37853	1.89225	0.963717
37854	1.89225	1.07299
37855	1.89225	1.7192
37856	1.89225	2.1
37857	1.89225	0.722625
37858	1.89225	1.91394
37859	0.987703	0.920821
37860	1.33078	1.42936
37861	1.31565	1.05043
37862	1.31565	1.68322
37863	1.01121	1.04613
37864	1.01121	1.80612
37865	2.19163	2.1
37866	1.89226	1.61613
37867	1.89226	2.1
37868	1.89226	1.36912
37869	2.22229	2.04832
37870	2.22229	1.20011
37871	1.89226	1.98919

37872	1.89226	0.689331
37873	1.89226	0.652077
37874	1.89226	1.89943
37875	1.89226	0.676747
37876	1.89226	1.81429
37877	0.57141	1.86237
37878	0.57141	0.771914
37879	0.574036	1.00085
37880	0.574036	2.1
37881	0.651591	1.65735
37882	0.657827	1.08738
37883	0.482391	1.19035
37884	0.493855	1.44011
37885	0.482391	0.739289
37886	0.651591	0.860155
37887	0.493855	1.34499
37888	0.657828	1.34599
37889	1.88428	2.1
37890	2.03975	2.1
37891	1.97316	2.1
37892	1.8266	2.1
37893	3.86635	2.1
37894	2.23053	2.1
37895	1.42255	2.1
37896	1.3868	2.1
37897	1.8266	2.1
37898	3.24915	2.1
37899	1.97316	2.1
37900	1.8266	2.1
37901	1.77128	2.1
37902	1.91767	2.1
37903	3.74427	2.1
37904	1.77128	2.1
37905	1.8266	2.1
37906	1.77942	2.1
37907	1.33962	2.1
37908	1.3868	2.1
37909	1.3868	2.1
37910	0.944111	2.1
37911	0.944111	2.1
37912	1.26036	2.1
37913	1.16968	2.1
37914	1.03853	2.1
37915	1.16646	2.1
37916	0.946129	2.1
37917	0.946129	2.1
37918	0.94613	2.1
37919	1.25768	2.1
37920	1.03853	2.1
37921	0.946131	2.1
37922	1.82441	0.15037
37923	1.33061	0.138977
37924	1.13272	0.121304
37925	1.60249	0.132073
37926	2.95712	0.140852
37927	1.82441	0.157708
37928	2.08757	0.149488
37929	1.81292	0.127242

37930	1.60249	0.133349
37931	3.63733	0.136336
37932	1.81292	0.12044
37933	1.332	0.108038
37934	1.60249	0.102817
37935	1.60249	0.118947
37936	1.5344	0.104046
37937	1.5344	0.124054
37938	1.10107	2.1
37939	2.51559	2.1
37940	1.1229	2.1
37941	1.1229	2.1
37942	2.49456	2.1
37943	2.49456	2.1
37944	1.89226	2.1
37945	2.51559	2.1
37946	2.49457	2.1
37947	2.51559	2.1
37948	1.87124	2.1
37949	1.89226	2.1
37950	1.89226	2.1
37951	1.89226	2.1
37952	1.89226	2.1
37953	1.89226	2.1
37954	1.89226	2.1
37955	1.89226	2.1
37956	1.89226	2.1
37957	1.89226	2.1
37958	1.89226	2.1
37959	1.89226	2.1
37960	1.89226	2.09449
37961	1.89226	2.1
37962	1.23599	2.1
37963	1.24728	2.1
37964	0.550534	2.1
37965	0.541151	2.1
37966	0.893754	2.1
37967	0.893754	2.1
37968	0.894678	2.1
37969	0.894678	2.1
37970	1.23599	2.1
37971	0.541149	2.1
37972	0.550533	2.1
37973	1.24728	2.1
37974	0.946132	2.1
37975	0.946132	2.1
37976	1.23599	2.1
37977	1.24728	2.1
37978	0.946132	2.1
37979	0.946132	2.1
37980	1.23599	2.1
37981	1.23599	2.1
37982	1.23599	2.1
37983	0.946132	2.1
37984	0.946132	2.1
37985	1.24728	2.1
37986	1.89226	2.1
37987	1.89226	1.94349

37988	1.89226	2.1
37989	1.89226	2.1
37990	1.89226	2.1
37991	1.89226	2.1
37992	0.946128	1.85596
37993	0.946128	2.1
37994	0.946132	2.1
37995	0.946132	2.1
37996	0.94613	2.1
37997	0.94613	2.1
37998	0.946129	2.1
37999	0.946129	2.1
38000	0.94613	2.1
38001	0.94613	2.1
38002	0.94613	2.1
38003	0.94613	2.1
38004	2.58696	0.386797
38005	2.57073	0.468069
38006	2.12585	0.386423
38007	2.07607	0.494265
38008	4.69658	0.468175
38009	1.89226	0.466125
38010	1.89226	0.507943
38011	1.89226	0.443179
38012	1.89226	0.529012
38013	3.78452	0.456945
38014	1.89226	0.509826
38015	1.89226	0.583504
38016	1.89226	0.534482
38017	1.89226	0.564481
38018	3.78453	0.559032
38019	2.57074	0.473703
38020	2.32635	0.562315
38021	2.07608	0.498898
38022	1.73623	0.52829
38023	4.30697	0.49382
38024	2.12582	0.361061
38025	1.51971	0.404152
38026	1.57255	0.365007
38027	2.07604	0.417629
38028	1.62233	0.351364
38029	2.12582	0.394046
38030	1.89223	0.416003
38031	1.89223	0.507972
38032	1.89223	0.481147
38033	1.89223	0.455179
38034	1.89223	0.469025
38035	1.89223	0.435973
38036	0.946119	0.536454
38037	0.946119	0.51043
38038	0.946119	0.461321
38039	0.946119	0.53093
38040	0.946119	0.538405
38041	0.946119	0.538993
38042	0.94612	0.516126
38043	0.94612	0.486741
38044	0.946132	0.514824
38045	0.946132	0.492828

38046	0.946107	0.520793
38047	0.946107	0.516205
38048	0.811456	0.442695
38049	0.811456	0.519163
38050	0.806521	0.402542
38051	0.806521	0.516981
38052	0.868104	0.515674
38053	0.579949	0.50144
38054	0.759859	0.409224
38055	1.03802	0.416554
38056	0.579957	0.466027
38057	0.759868	0.463482
38058	1.03801	0.485593
38059	0.868093	0.494048
38060	2.7408	2.1
38061	2.82858	1.59123
38062	2.57368	2.1
38063	2.52633	2.1
38064	5.3549	2.1
38065	2.7408	2.1
38066	2.31353	2.1
38067	2.14063	2.1
38068	2.52632	2.1
38069	4.83985	2.1
38070	2.57368	2.1
38071	2.52633	2.1
38072	2.3301	2.1
38073	2.36071	1.45012
38074	4.88704	2.1
38075	1.86512	2.1
38076	2.52632	2.1
38077	2.14063	2.1
38078	2.14063	2.1
38079	2.3301	2.1
38080	2.3301	2.1
38081	1.86431	2.1
38082	1.89227	2.1
38083	1.68992	2.1
38084	1.68992	2.1
38085	1.89227	2.1
38086	1.89227	2.1
38087	2.0815	2.1
38088	1.89226	2.1
38089	2.04022	2.1
38090	1.86431	2.1
38091	2.07081	2.1
38092	1.89226	2.1
38093	1.91686	1.1171
38094	1.65683	1.15356
38095	1.86871	1.98667
38096	1.65683	1.08707
38097	2.13075	1.2611
38098	1.89226	0.671855
38099	1.89226	1.17804
38100	1.89226	0.557041
38101	1.89226	0.777373
38102	1.89226	1.25279
38103	3.78452	0.808529

38104	0.851541	1.93247
38105	0.851541	1.51743
38106	0.952803	2.1
38107	0.851573	2.1
38108	0.946129	2.1
38109	0.946129	0.970787
38110	0.929184	2.01474
38111	0.757482	2.1
38112	0.836274	2.1
38113	0.851528	2.1
38114	0.952816	2.1
38115	1.04073	2.1
38116	1.70303	0.990604
38117	1.70302	0.86265
38118	1.5544	1.51857
38119	1.70302	0.866746
38120	1.74957	0.91863
38121	1.91686	0.632269
38122	1.89226	0.723862
38123	1.89226	0.881583
38124	1.89226	1.27408
38125	1.89226	0.788289
38126	1.89226	0.777959
38127	1.89226	0.446718
38128	1.89226	1.61772
38129	1.89226	2.02765
38130	2.03472	2.1
38131	1.89226	1.67552
38132	1.99827	2.02825
38133	1.85836	1.05203
38134	1.00509	2.1
38135	1.55998	2.00892
38136	1.83347	2.1
38137	1.45246	2.1
38138	3.01244	2.1
38139	1.23839	2.1
38140	0.884251	2.1
38141	0.946133	2.1
38142	0.946133	2.1
38143	0.884226	2.1
38144	0.884226	2.1
38145	0.946158	2.1
38146	0.946159	2.1
38147	1.23839	2.1
38148	0.946131	2.1
38149	0.946131	2.1
38150	0.884254	2.1
38151	1.89226	2.1
38152	1.89226	1.37427
38153	1.89226	1.93066
38154	1.89226	2.07503
38155	1.89226	2.01485
38156	1.89226	2.1
38157	2.1499	2.1
38158	1.45244	2.09725
38159	1.83345	1.97571
38160	1.83345	2.1
38161	1.87214	1.93307

38162	1.87214	2.1
38163	1.89224	2.0552
38164	1.89224	1.14476
38165	1.89224	1.7343
38166	1.89224	1.85611
38167	3.78448	2.08846
38168	1.49155	0.741646
38169	2.0815	0.942881
38170	1.77515	1.12799
38171	1.72377	2.1
38172	3.50659	0.993647
38173	1.69876	0.399081
38174	2.0815	1.61146
38175	1.9133	0.574493
38176	1.68189	0.380544
38177	2.17628	1.97896
38178	1.94335	1.72272
38179	2.65129	0.0902895
38180	1.15317	0.0862113
38181	2.57085	0.0865858
38182	1.209	0.0865418
38183	2.57085	0.086547
38184	1.209	0.0870172
38185	1.89226	0.101962
38186	1.89226	0.0898879
38187	1.89226	0.0860175
38188	1.89226	0.101671
38189	3.78452	0.096977
38190	2.48861	0.0765446
38191	1.15252	0.0822297
38192	2.60515	0.0843007
38193	2.60515	0.0768417
38194	1.16384	0.0844518
38195	1.16384	0.0764074
38196	1.59903	0.752221
38197	1.26865	0.685524
38198	1.30486	1.24348
38199	1.70302	0.920006
38200	2.98573	0.860403
38201	1.59903	0.726618
38202	1.79478	0.826395
38203	1.79478	0.757597
38204	1.97005	0.882143
38205	1.50365	0.694184
38206	1.64192	1.23501
38207	1.89226	0.617619
38208	1.89226	0.701785
38209	1.89226	0.843197
38210	1.89226	0.592021
38211	3.78453	0.595015
38212	1.89226	0.758872
38213	1.89226	0.689309
38214	1.89226	0.763801
38215	1.89226	0.608046
38216	1.89226	0.796828
38217	1.89226	0.923224
38218	0.761672	0.648852
38219	0.896406	0.752812

38220	0.603509	0.718998
38221	0.574071	0.913953
38222	0.695159	0.920631
38223	0.695159	0.532838
38224	0.762564	1.35308
38225	0.770159	0.674609
38226	0.803021	0.857832
38227	0.572529	1.06887
38228	0.696466	0.596977
38229	0.899733	0.635574
38230	1.66034	0.451719
38231	1.79478	0.847055
38232	1.56685	0.661287
38233	1.68512	1.40341
38234	1.67652	0.709359
38235	1.67652	0.889531
38236	1.89226	0.862658
38237	1.89226	0.652958
38238	1.89226	0.789707
38239	1.89226	0.758274
38240	1.89226	0.782688
38241	1.89226	0.797915
38242	0.946133	0.585594
38243	0.946133	0.704338
38244	0.946132	0.884964
38245	0.946132	0.712719
38246	0.946132	0.766236
38247	0.946132	0.491381
38248	0.946133	1.36873
38249	0.946133	0.646335
38250	0.946133	0.735602
38251	0.946133	0.744566
38252	0.946132	0.585335
38253	0.946132	0.562315
38254	1.80424	2.1
38255	1.45158	2.1
38256	1.9098	2.1
38257	1.56003	2.1
38258	3.36138	2.1
38259	1.90982	2.1
38260	1.71606	2.1
38261	2.15314	2.1
38262	1.70302	2.1
38263	3.89051	2.1
38264	1.96416	0.681265
38265	1.23706	0.726459
38266	1.00053	0.580458
38267	1.38287	0.530096
38268	1.57916	0.545812
38269	1.9615	0.626498
38270	0.827865	0.527303
38271	0.946132	0.492313
38272	0.946132	0.443991
38273	0.946132	0.562949
38274	0.946132	0.643212
38275	0.946132	0.527578
38276	0.946131	0.666077
38277	0.946131	0.538962

38278	0.827865	0.727314
38279	0.946132	0.461344
38280	0.946132	0.420544
38281	0.946132	0.485748
38282	1.89227	2.1
38283	1.89227	2.1
38284	1.89227	2.1
38285	1.89227	2.1
38286	3.78453	2.1
38287	2.3819	2.1
38288	1.58089	2.1
38289	1.58089	2.1
38290	1.6287	2.1
38291	2.33368	2.1
38292	2.3815	2.1
38293	0.946133	2.1
38294	0.946133	2.1
38295	0.946132	2.1
38296	0.946132	2.1
38297	0.946132	2.1
38298	0.946132	2.1
38299	0.946132	2.1
38300	0.946132	2.1
38301	0.946133	2.1
38302	0.946133	2.1
38303	0.946132	2.1
38304	0.946132	2.1
38305	1.89226	2.1
38306	1.89226	2.1
38307	1.89226	2.1
38308	1.89226	2.1
38309	3.78453	2.1
38310	2.7874	2.1
38311	2.33368	2.1
38312	2.71897	2.1
38313	2.27495	2.1
38314	5.05266	2.1
38315	0.970991	2.1
38316	0.970991	2.1
38317	0.956929	2.1
38318	0.956929	2.1
38319	0.732928	2.1
38320	0.790444	2.1
38321	1.16684	2.1
38322	1.13748	2.1
38323	1.13748	2.1
38324	0.732928	2.1
38325	0.790444	2.1
38326	1.16684	2.1
38327	0.946132	2.1
38328	0.946132	2.1
38329	0.946131	2.1
38330	0.946131	2.1
38331	0.946132	2.1
38332	0.946132	2.1
38333	0.946132	2.1
38334	0.946132	2.1
38335	0.946132	2.1

38336	0.946132	2.1
38337	0.946132	2.1
38338	0.946132	2.1
38339	1.10747	0.38759
38340	2.30331	0.441108
38341	1.47954	0.372549
38342	2.63789	0.349912
38343	3.78285	0.3881
38344	1.65215	0.35308
38345	2.63789	0.338143
38346	2.82465	0.395667
38347	2.82465	0.384558
38348	1.47954	0.341358
38349	1.47954	0.338561
38350	1.13456	1.73107
38351	1.08334	2.1
38352	1.20674	0.862656
38353	1.17885	1.80799
38354	2.29008	1.55775
38355	1.20674	1.44484
38356	1.17885	2.05342
38357	1.28649	0.966516
38358	1.28516	1.61727
38359	2.4919	1.53088
38360	1.89226	2.02058
38361	1.89226	2.1
38362	1.89226	2.1
38363	1.89226	1.99361
38364	1.89226	2.1
38365	1.89226	1.93306
38366	2.50288	1.36039
38367	2.02115	1.85139
38368	2.7717	1.29693
38369	2.29107	0.814412
38370	4.79395	1.90754
38371	1.27339	0.76214
38372	1.10817	2.1
38373	1.19733	1.39115
38374	1.032	2.1
38375	2.30539	2.1
38376	1.19733	1.57406
38377	0.953637	2.1
38378	0.953637	1.7169
38379	1.032	2.1
38380	1.11868	1.22736
38381	1.19704	2.1
38382	2.01024	0.87748
38383	1.11938	1.02812
38384	1.9186	1.26356
38385	2.76298	0.882917
38386	3.92884	1.07817
38387	0.851541	0.256048
38388	0.851541	0.232643
38389	0.957577	0.217376
38390	0.848952	0.221962
38391	1.08043	0.261321
38392	0.851541	0.26842
38393	0.851484	0.226776

38394	0.957578	0.195377
38395	1.20045	0.250209
38396	0.726289	0.186428
38397	0.946131	0.230329
38398	0.946131	0.260423
38399	1.50524	2.1
38400	2.38363	2.1
38401	2.49018	2.1
38402	2.49018	2.1
38403	1.45506	2.1
38404	1.45506	2.1
38405	2.49017	2.1
38406	1.89226	2.1
38407	1.89226	2.1
38408	1.89226	2.1
38409	2.38363	2.1
38410	2.38363	2.1
38411	1.54711	2.1
38412	2.49018	2.1
38413	2.56975	2.1
38414	2.56975	2.1
38415	1.50524	2.1
38416	1.50524	2.1
38417	2.56975	2.1
38418	1.89226	2.1
38419	1.89226	2.1
38420	1.89226	2.1
38421	2.49017	2.1
38422	2.49017	2.1
38423	1.89226	0.189091
38424	1.89226	0.20902
38425	1.89226	0.276798
38426	1.89226	0.160442
38427	1.89226	0.28205
38428	1.89226	0.156119
38429	1.89226	0.240019
38430	1.89226	0.209958
38431	1.89226	0.388442
38432	1.89226	0.181293
38433	1.89226	0.392176
38434	1.89226	0.185079
38435	1.89226	0.179221
38436	1.89226	0.169832
38437	2.8358	0.1479
38438	1.89226	0.330668
38439	3.78453	0.152954
38440	1.60565	0.367251
38441	1.89226	1.35407
38442	1.89226	1.02102
38443	1.40013	0.705027
38444	1.89226	1.18527
38445	1.40013	0.883995
38446	1.1766	2.1
38447	1.29435	2.1
38448	1.35735	2.1
38449	1.53509	2.1
38450	2.65169	2.1
38451	0.69701	2.1

38452	0.69701	2.1
38453	0.711303	2.1
38454	0.711303	2.1
38455	0.761138	2.1
38456	0.612149	2.1
38457	0.647174	2.1
38458	0.767548	2.1
38459	0.767548	2.1
38460	0.761139	2.1
38461	0.612149	2.1
38462	0.647174	2.1
38463	1.29764	1.23328
38464	1.29579	1.42526
38465	1.34875	1.30599
38466	1.06717	1.50347
38467	1.29764	1.21288
38468	1.01606	1.25968
38469	0.756635	1.18353
38470	0.648821	1.20111
38471	0.675557	1.34606
38472	0.674374	1.25325
38473	0.74214	1.38331
38474	0.74214	1.26425
38475	0.688868	1.14251
38476	0.688868	1.21224
38477	0.675557	1.04492
38478	0.756634	1.12137
38479	0.648821	1.01028
38480	0.674374	1.08815
38481	1.51327	1.32348
38482	1.48105	1.12284
38483	1.29764	1.34181
38484	1.29764	1.23557
38485	1.48105	1.31272
38486	1.05655	1.11873
38487	1.13537	2.1
38488	1.07326	2.1
38489	0.94613	2.1
38490	0.94613	2.1
38491	0.946132	2.1
38492	0.946132	2.1
38493	1.13537	2.1
38494	1.08927	2.1
38495	1.13537	2.1
38496	0.946131	2.1
38497	0.946131	2.1
38498	0.95947	2.1
38499	1.46892	2.1
38500	1.89226	2.1
38501	1.89226	2.1
38502	1.89226	2.1
38503	1.88031	2.1
38504	1.88031	2.1
38505	1.89226	2.1
38506	1.89226	2.1
38507	1.89226	2.1
38508	1.89226	2.1
38509	3.78453	2.1

38510	2.18254	2.1
38511	1.89226	2.1
38512	2.50005	2.1
38513	1.89226	2.1
38514	4.39231	2.1
38515	1.88031	2.1
38516	2.0815	2.1
38517	2.0815	2.1
38518	1.92078	2.1
38519	2.0815	2.1
38520	1.9218	2.1
38521	1.89227	2.1
38522	1.89227	2.1
38523	1.89227	2.1
38524	1.89227	2.1
38525	1.89227	2.1
38526	1.89227	2.1
38527	1.98693	2.1
38528	1.82857	2.1
38529	2.32	2.1
38530	2.36972	2.1
38531	4.14857	2.1
38532	1.82857	2.1
38533	1.58688	2.1
38534	2.36971	2.1
38535	2.19093	2.1
38536	4.0195	2.1
38537	0.946132	2.1
38538	0.946132	2.1
38539	1.18486	2.1
38540	1.18486	2.1
38541	1.18486	2.1
38542	0.946132	2.1
38543	0.946132	2.1
38544	1.16	2.1
38545	0.946133	2.1
38546	0.946133	2.1
38547	1.16	2.1
38548	1.18486	2.1
38549	1.07798	1.4471
38550	1.08101	1.20946
38551	0.910786	2.1
38552	0.910786	1.87205
38553	0.851537	1.70683
38554	0.901377	1.21939
38555	1.14652	1.73816
38556	1.04072	2.1
38557	0.946129	1.77647
38558	0.946129	1.8223
38559	1.0019	1.56561
38560	1.04263	1.62617
38561	1.89226	2.1
38562	1.89226	2.1
38563	1.89226	2.1
38564	1.89226	2.1
38565	3.78452	2.1
38566	1.89226	2.1
38567	2.30146	2.1

38568	1.89226	2.1
38569	2.18412	2.1
38570	4.19372	2.1
38571	2.30146	2.1
38572	1.39962	2.1
38573	2.18411	2.1
38574	1.28765	2.1
38575	3.5891	2.1
38576	1.89226	2.1
38577	1.89226	2.1
38578	1.89226	2.1
38579	1.89226	2.1
38580	1.89226	2.1
38581	1.89226	2.1
38582	1.89226	2.1
38583	1.89226	2.1
38584	1.89226	2.1
38585	1.89226	2.1
38586	3.78452	2.1
38587	2.18412	2.1
38588	1.20045	2.1
38589	1.20045	2.1
38590	1.28765	2.1
38591	2.08808	2.1
38592	2.17528	2.1
38593	0.946131	2.1
38594	0.946131	2.1
38595	0.946128	2.1
38596	0.946128	2.1
38597	0.946127	2.1
38598	0.946127	2.1
38599	0.946131	2.1
38600	0.946131	2.1
38601	0.946129	2.1
38602	0.946129	2.1
38603	0.94613	2.1
38604	0.94613	2.1
38605	1.89226	0.659251
38606	1.89226	1.05903
38607	1.89226	0.990533
38608	1.89226	0.702585
38609	3.78452	0.608771
38610	1.89227	0.678534
38611	1.89227	1.3863
38612	1.89227	0.617111
38613	1.89227	0.967168
38614	1.89227	0.677111
38615	1.89227	1.11952
38616	1.89226	1.18567
38617	1.89226	0.733456
38618	1.89226	0.7359
38619	2.29245	0.761945
38620	3.78452	1.03373
38621	1.89226	0.99261
38622	1.89226	0.720968
38623	1.89226	0.717966
38624	1.89226	0.880709
38625	1.89226	0.567744

38626	1.89226	0.629599
38627	1.28095	1.45514
38628	1.81846	1.72623
38629	2.10991	0.905892
38630	1.34695	1.44137
38631	3.39086	1.53779
38632	2.10991	1.09493
38633	1.36457	1.5149
38634	1.67421	1.76198
38635	1.34695	1.53219
38636	2.43717	1.23266
38637	2.10991	1.27777
38638	2.69541	2.1
38639	1.48572	2.1
38640	2.63553	2.1
38641	1.45385	2.1
38642	4.14926	2.1
38643	2.63553	2.1
38644	1.44207	2.1
38645	1.44207	2.1
38646	1.45385	2.1
38647	2.588 2.1	
38648	2.59978	2.1
38649	1.89227	2.1
38650	1.89227	2.1
38651	1.89227	2.1
38652	1.89227	2.1
38653	3.78453	2.1
38654	1.89226	2.1
38655	1.89226	2.1
38656	1.89226	2.1
38657	1.89226	2.1
38658	3.78453	2.1
38659	2.15929	2.1
38660	1.38276	2.1
38661	1.43617	2.1
38662	1.35614	2.1
38663	2.23932	2.1
38664	2.15929	2.1
38665	2.15929	2.1
38666	2.25168	2.1
38667	2.3558	2.1
38668	2.23932	2.1
38669	4.51509	2.1
38670	1.89226	2.1
38671	1.89226	2.1
38672	1.89226	2.1
38673	1.89226	2.1
38674	3.78453	2.1
38675	1.89226	2.1
38676	1.89226	2.1
38677	1.89226	2.1
38678	1.89226	2.1
38679	1.89226	2.1
38680	1.89226	2.1
38681	2.23932	2.1
38682	1.43854	2.1
38683	1.49173	2.1

38684	1.38276	2.1
38685	2.34829	2.1
38686	2.23932	2.1
38687	2.3558	2.1
38688	2.34829	2.1
38689	2.40685	2.1
38690	2.23932	2.1
38691	2.52332	2.1
38692	2.3558	2.1
38693	1.89226	0.161024
38694	1.87361	0.189163
38695	2.25082	0.148629
38696	1.87361	0.211613
38697	2.26947	0.145971
38698	1.89226	0.198168
38699	1.89226	0.199558
38700	1.89226	0.252613
38701	1.89226	0.191558
38702	1.89226	0.304019
38703	1.89226	0.180979
38704	1.89226	0.279861
38705	2.41314	0.180061
38706	1.89226	0.222564
38707	1.89226	0.174345
38708	1.89226	0.264419
38709	2.12204	0.15954
38710	2.12204	0.240464
38711	1.89226	0.155563
38712	2.25082	0.176855
38713	2.28189	0.140223
38714	2.25082	0.211213
38715	1.92333	0.13352
38716	1.89226	0.197083
38717	1.89226	0.201248
38718	1.89226	0.22665
38719	1.89226	0.1857
38720	1.89226	0.290619
38721	1.89226	0.174595
38722	1.89226	0.260113
38723	2.42521	0.171747
38724	1.89226	0.200992
38725	1.89226	0.158804
38726	1.89226	0.236136
38727	2.41314	0.150257
38728	2.41314	0.213714
38729	1.39725	1.03466
38730	1.07745	1.93568
38731	1.07745	0.976933
38732	1.07519	1.95445
38733	1.41336	0.886117
38734	1.41111	2.06383
38735	1.89226	1.69163
38736	1.92039	2.1
38737	1.92039	1.51197
38738	1.9052	2.1
38739	1.89226	1.40165
38740	1.87708	2.1
38741	0.946128	1.88425

38742	0.946128	1.43209
38743	0.946133	0.931473
38744	0.946133	0.80494
38745	0.94613	0.810392
38746	0.94613	1.34226
38747	0.946131	1.95385
38748	0.946131	0.832164
38749	0.946131	1.0546
38750	0.946131	1.03157
38751	0.946131	1.11731
38752	0.946131	1.12481
38753	1.41336	0.95711
38754	1.08911	1.99186
38755	1.08911	1.10182
38756	1.07745	2.02335
38757	1.42381	0.981737
38758	1.41215	2.1
38759	1.89227	1.8098
38760	1.95833	2.1
38761	1.95833	1.65181
38762	1.92039	2.1
38763	1.89227	1.52121
38764	1.85433	2.1
38765	0.946129	1.97331
38766	0.946129	1.53313
38767	0.946133	1.07998
38768	0.946133	0.824889
38769	0.946131	0.825425
38770	0.946131	1.42563
38771	0.946131	2.00865
38772	0.946131	0.916849
38773	0.946131	1.19566
38774	0.946131	1.16217
38775	0.946131	1.26445
38776	0.946131	1.24297
38777	1.41943	2.1
38778	2.74398	2.1
38779	1.18238	2.1
38780	2.53897	2.1
38781	3.9584	2.1
38782	2.74398	2.1
38783	1.89226	2.1
38784	1.89226	2.1
38785	1.89226	2.1
38786	2.53897	2.1
38787	2.53897	2.1
38788	1.89227	0.878487
38789	1.89227	0.680456
38790	1.89227	0.872187
38791	1.89227	0.631154
38792	3.78453	0.699561
38793	1.89226	0.658961
38794	2.08134	0.551347
38795	2.00632	0.538859
38796	2.00632	0.571537
38797	1.89226	0.596084
38798	1.89226	0.641176
38799	1.89227	0.744263

38800	1.89227	0.609935
38801	1.89227	0.666776
38802	1.89227	0.533652
38803	3.78453	0.672013
38804	1.89227	0.821393
38805	1.89227	0.535285
38806	1.89227	0.532298
38807	1.89227	0.620938
38808	1.89227	0.65944
38809	1.89227	0.842859
38810	2.50912	0.506512
38811	1.89226	0.578248
38812	1.89226	0.440799
38813	2.31261	0.525896
38814	2.31261	0.538985
38815	1.89226	0.610321
38816	2.50912	0.504304
38817	1.19999	0.453807
38818	1.44056	0.391245
38819	1.24405	0.448271
38820	2.50912	0.494098
38821	2.31261	0.533831
38822	2.69224	0.702728
38823	2.28373	0.578043
38824	1.94788	1.09266
38825	2.4106	0.617411
38826	4.69434	0.859733
38827	2.69223	0.468713
38828	2.63783	0.706649
38829	2.34737	1.03762
38830	2.4106	0.672221
38831	5.04843	0.726948
38832	1.89226	2.1
38833	1.99753	2.1
38834	1.87366	2.1
38835	1.99753	2.1
38836	1.76839	2.1
38837	1.89226	1.99807
38838	1.89226	2.1
38839	1.89226	0.862529
38840	1.89226	2.05796
38841	1.89226	2.1
38842	3.78452	2.1
38843	0.946132	2.1
38844	0.946132	2.1
38845	1.02744	2.1
38846	1.02744	2.1
38847	0.946132	2.1
38848	0.946132	2.1
38849	1.00989	2.1
38850	1.02744	2.1
38851	1.00989	2.1
38852	0.946133	2.1
38853	1.02744	2.1
38854	0.946132	2.1
38855	1.70302	2.1
38856	1.87366	2.1
38857	1.71618	2.1

38858	1.87366	2.1
38859	1.73105	2.1
38860	1.89226	1.84264
38861	1.89226	1.82096
38862	1.89226	2.1
38863	1.89226	2.1
38864	1.89226	2.06362
38865	1.89226	2.1
38866	1.89226	0.844385
38867	1.96624	2.1
38868	1.89226	2.1
38869	1.89226	2.1
38870	1.89226	2.1
38871	2.01977	2.1
38872	2.01977	2.1
38873	1.1032	1.22438
38874	1.15114	2.1
38875	1.14986	0.472869
38876	1.19242	1.9893
38877	2.301	1.55436
38878	1.89226	1.29663
38879	1.89226	2.1
38880	1.89226	0.512103
38881	1.89226	1.42066
38882	3.78453	1.01034
38883	0.946132	2.1
38884	0.946132	1.57765
38885	0.946132	0.879674
38886	0.946132	2.1
38887	0.946133	2.1
38888	0.946133	2.1
38889	0.946132	1.41801
38890	0.946132	1.16097
38891	0.946132	2.1
38892	0.946132	2.03823
38893	0.946133	2.06146
38894	0.946133	2.09663
38895	1.19243	1.5114
38896	0.953928	2.1
38897	1.18671	2.1
38898	1.15114	1.42728
38899	0.9895	2.1
38900	0.953928	1.77215
38901	1.19611	0.592013
38902	1.19242	2.1
38903	1.24693	0.554917
38904	1.24693	1.61692
38905	1.14986	0.733806
38906	1.14986	2.06989
38907	1.89225	0.611836
38908	1.89225	2.09688
38909	1.89225	2.1
38910	1.89225	0.892704
38911	1.89225	1.25187
38912	1.89225	0.889583
38913	1.89225	1.3182
38914	1.89225	2.1
38915	1.89225	1.98652

38916	1.89225	2.1
38917	1.89225	0.79785
38918	1.89225	2.1
38919	0.561532	2.1
38920	0.561532	1.36933
38921	0.566382	1.12679
38922	0.566382	2.1
38923	0.494747	2.1
38924	0.531705	2.1
38925	0.596208	1.49589
38926	0.623464	0.902625
38927	0.623469	2.1
38928	0.531708	2.1
38929	0.596205	2.1
38930	0.494743	2.1
38931	1.25742	2.1
38932	1.18783	2.1
38933	0.946131	2.1
38934	0.946131	2.1
38935	1.25742	2.1
38936	1.25742	2.1
38937	0.946131	2.1
38938	0.946131	2.1
38939	1.25742	2.1
38940	0.946131	2.1
38941	0.946131	2.1
38942	1.18783	2.1
38943	1.35048	2.1
38944	1.25742	2.1
38945	0.946132	2.1
38946	0.946132	2.1
38947	1.35048	2.1
38948	1.35048	2.1
38949	0.946132	2.1
38950	0.946132	2.1
38951	1.35044	2.1
38952	0.946106	2.1
38953	1.25745	2.1
38954	0.946158	2.1
38955	1.13525	2.1
38956	2.70095	2.1
38957	2.51483	2.1
38958	2.51483	2.1
38959	1.21593	2.1
38960	1.21593	2.1
38961	1.93142	0.4383
38962	2.8053	0.204032
38963	1.559	0.410805
38964	2.60359	0.200544
38965	4.53501	0.311729
38966	2.80531	0.293155
38967	1.89226	0.209907
38968	2.60359	0.255046
38969	1.89226	0.463509
38970	4.49586	0.204645
38971	1.22938	0.364544
38972	2.60359	0.167263
38973	2.37434	0.510901

38974	2.37434	0.249521
38975	1.55901	0.549413
38976	1.55901	0.18043
38977	1.18717	0.482116
38978	1.18717	0.16204
38979	0.946132	0.231108
38980	0.946132	0.29421
38981	0.946133	0.319843
38982	0.946133	0.465109
38983	1.3018	0.243692
38984	1.18717	0.50185
38985	1.18714	0.311645
38986	0.946107	0.324552
38987	1.30183	0.268251
38988	0.946158	0.214409
38989	1.89226	2.1
38990	2.80196	2.1
38991	2.67272	2.1
38992	2.67272	2.1
38993	1.89226	2.1
38994	1.89226	2.1
38995	1.89226	2.1
38996	1.89226	2.1
38997	1.89226	2.1
38998	1.89226	2.1
38999	1.89226	2.1
39000	1.89226	2.1
39001	1.74471	2.1
39002	2.01555	2.1
39003	1.61121	2.1
39004	1.61121	2.1
39005	2.13878	2.1
39006	2.13878	2.1
39007	1.89226	2.1
39008	1.89226	2.1
39009	1.89226	2.1
39010	1.89226	2.1
39011	1.89226	2.1
39012	1.89226	2.1
39013	1.09766	2.1
39014	1.04263	2.1
39015	0.881794	2.1
39016	0.894188	2.1
39017	0.795402	2.1
39018	0.99524	2.1
39019	1.00647	2.1
39020	1.00647	2.1
39021	1.15401	2.1
39022	0.881789	2.1
39023	0.850526	2.1
39024	1.04263	2.1
39025	1.4054	1.02251
39026	1.04788	2.1
39027	1.70302	1.04466
39028	1.41375	2.1
39029	2.82706	1.49655
39030	1.70302	0.980915
39031	1.38776	2.00178

39032	1.59457	1.45973
39033	1.41375	2.09357
39034	1.77261	0.919464
39035	1.70302	2.1
39036	1.89226	2.1
39037	1.89226	2.1
39038	1.89226	2.1
39039	1.89226	2.1
39040	3.78453	2.1
39041	2.18042	2.1
39042	1.74738	2.1
39043	2.08433	2.1
39044	1.66347	2.1
39045	3.83171	2.1
39046	1.74738	2.1
39047	1.1181	2.1
39048	1.66347	2.1
39049	1.06543	2.1
39050	2.81281	2.1
39051	1.89226	2.1
39052	1.89226	2.1
39053	1.89226	2.1
39054	1.89226	2.1
39055	3.78453	2.1
39056	1.89227	2.1
39057	1.89227	2.1
39058	1.89227	2.1
39059	1.89227	2.1
39060	3.78453	2.1
39061	2.08433	2.1
39062	1.66347	2.1
39063	1.97092	2.1
39064	1.57431	2.1
39065	3.65864	2.1
39066	1.66347	2.1
39067	1.01474	2.1
39068	1.01474	2.1
39069	1.06544	2.1
39070	1.57431	2.1
39071	1.625	2.1
39072	1.89226	2.1
39073	1.89226	2.1
39074	1.89226	2.1
39075	1.89226	2.1
39076	1.89226	2.1
39077	1.89226	2.1
39078	1.08467	2.1
39079	1.87228	2.1
39080	1.73305	2.1
39081	1.73305	2.1
39082	1.25565	2.1
39083	1.25565	2.1
39084	0.695361	0.472414
39085	0.799996	0.646053
39086	1.13751	0.774893
39087	1.40945	0.825098
39088	1.40944	0.936298
39089	0.799999	0.301134

39090	1.13751	0.66897
39091	0.695363	0.358766
39092	1.01059	0.552458
39093	1.01059	0.61324
39094	0.926908	0.980679
39095	0.926909	0.396482
39096	1.05481	2.1
39097	1.78069	1.684
39098	1.08074	2.1
39099	1.83284	1.9919
39100	2.86143	2.1
39101	1.78069	1.99604
39102	2.22044	1.43733
39103	1.83284	2.01015
39104	2.28696	0.954427
39105	4.06766	1.47446
39106	1.07196	2.1
39107	1.64955	2.1
39108	2.02665	2.1
39109	2.0284	2.1
39110	1.08074	2.1
39111	1.08074	2.1
39112	1.64955	2.1
39113	2.28696	1.24995
39114	1.92033	1.85016
39115	2.1004	1.49957
39116	3.74988	1.74958
39117	2.83787	0.573002
39118	1.70303	1.11943
39119	1.70303	0.527749
39120	1.83195	0.571675
39121	2.64075	0.558369
39122	2.70231	0.983723
39123	1.2087	1.44733
39124	1.52909	0.713689
39125	1.43063	0.707802
39126	1.10586	1.26569
39127	2.63494	0.894854
39128	1.89227	0.850627
39129	2.10732	2.1
39130	2.10732	0.992497
39131	2.2365	2.1
39132	1.89227	0.923952
39133	2.02145	2.1
39134	1.89226	0.728048
39135	1.89226	1.10427
39136	1.89226	0.720532
39137	1.89226	1.05049
39138	1.89226	0.678427
39139	1.89226	1.34762
39140	0.51926	1.26029
39141	0.552927	1.30522
39142	0.691235	0.812158
39143	0.715314	0.681234
39144	0.619684	0.702306
39145	0.619684	1.49304
39146	0.624477	1.74287
39147	0.624477	0.708346

39148	0.519262	0.88388
39149	0.691234	0.920993
39150	0.715313	0.929036
39151	0.552928	0.910726
39152	1.89226	0.99094
39153	1.87321	2.1
39154	1.87321	1.40554
39155	1.89658	2.1
39156	2.0815	1.01746
39157	2.14789	2.1
39158	0.946128	1.35236
39159	0.946128	1.14617
39160	0.946133	0.68679
39161	0.946133	0.660958
39162	0.94613	0.639111
39163	0.94613	1.05164
39164	0.946131	0.73859
39165	0.946131	1.23233
39166	0.946131	0.763202
39167	0.946131	0.772075
39168	0.946131	0.877269
39169	0.946131	0.860539
39170	1.99297	1.01403
39171	2.19514	0.719555
39172	2.46153	0.88653
39173	2.187	0.881416
39174	4.45451	0.897252
39175	2.0815	0.807463
39176	2.33402	0.747415
39177	2.46154	0.915325
39178	2.83046	0.613561
39179	4.94496	0.764717
39180	2.46153	0.837001
39181	2.187	0.791808
39182	2.27351	0.901953
39183	2.63356	0.669999
39184	4.73504	0.802681
39185	2.46154	0.864758
39186	2.83447	0.553579
39187	2.74513	0.729005
39188	2.5731	0.819021
39189	2.63357	0.688265
39190	2.46154	0.767133
39191	2.80398	2.1
39192	2.60406	2.1
39193	2.73988	2.1
39194	2.57466	2.1
39195	5.34394	2.1
39196	1.89226	0.996068
39197	1.89226	0.754671
39198	1.89226	0.601746
39199	1.89226	1.10307
39200	1.89226	0.668197
39201	1.89226	1.46025
39202	1.89224	1.17861
39203	1.89224	0.795237
39204	1.89224	0.690912
39205	1.89224	1.35718

39206	1.89224	0.782467
39207	1.89224	1.57064
39208	1.40245	1.05411
39209	1.40246	1.62145
39210	0.946117	1.17467
39211	0.946117	1.54201
39212	1.17635	0.890806
39213	1.40246	1.67318
39214	0.946112	0.833324
39215	0.946112	1.45948
39216	0.946127	1.18675
39217	1.17637	0.889576
39218	0.946102	1.53916
39219	1.40244	1.076
39220	2.53269	2.1
39221	1.70302	2.1
39222	1.56194	2.1
39223	2.17516	2.1
39224	4.19807	2.1
39225	1.89226	2.1
39226	1.89226	0.801203
39227	1.89226	2.00322
39228	1.89226	1.71575
39229	3.78453	1.91642
39230	2.57276	2.1
39231	1.89226	1.92206
39232	1.89226	2.1
39233	1.89226	2.1
39234	2.32525	2.1
39235	2.32525	2.1
39236	1.15171	2.1
39237	2.17517	2.04746
39238	1.41995	2.1
39239	1.41995	2.1
39240	1.80252	2.1
39241	1.80252	2.1
39242	1.89226	1.88805
39243	1.89226	1.84639
39244	1.89226	2.1
39245	1.89226	0.720283
39246	3.78453	1.90646
39247	0.946133	2.1
39248	0.946133	2.1
39249	1.16263	2.1
39250	1.04687	2.1
39251	0.946133	2.1
39252	0.946133	1.7612
39253	1.04687	2.1
39254	1.04687	2.1
39255	0.946133	2.1
39256	1.04687	2.1
39257	0.946132	2.1
39258	1.16263	2.1
39259	0.682281	1.4448
39260	0.669233	1.79828
39261	1.21859	1.78342
39262	1.31629	1.26276
39263	0.916158	1.10593

39264	0.916158	1.94476
39265	0.971658	1.37018
39266	0.971658	1.48031
39267	0.669234	1.4886
39268	1.31629	1.27532
39269	0.682283	1.49245
39270	1.21858	1.5652
39271	0.669232	1.29613
39272	0.623495	1.61616
39273	1.36428	1.45476
39274	1.31629	0.999012
39275	0.946475	0.915908
39276	0.946476	1.82871
39277	0.99331	1.33333
39278	0.99331	1.35711
39279	1.36428	1.36372
39280	0.623497	1.11866
39281	0.669234	1.35157
39282	1.31629	1.19769
39283	2.02069	0.0881536
39284	1.46253	0.0736085
39285	2.51479	0.0731024
39286	1.87136	0.0807103
39287	3.89203	0.077771
39288	1.46253	0.0720479
39289	1.38536	0.0793563
39290	1.38536	0.0796685
39291	1.05579	0.0770541
39292	1.73281	0.0784478
39293	1.46916	0.0742679
39294	2.51479	0.0756077
39295	1.87136	0.090229
39296	2.23338	0.0908135
39297	2.15603	0.0754084
39298	4.55146	0.0802241
39299	0.822553	0.0739525
39300	0.822563	0.0763454
39301	0.842935	0.0895193
39302	0.842935	0.0847946
39303	0.744203	0.0847261
39304	0.675346	0.0774659
39305	0.874795	0.0901162
39306	1.00453	0.077002
39307	1.00454	0.0783216
39308	0.782417	0.0814983
39309	0.675337	0.0819725
39310	0.858007	0.0792753
39311	0.946132	1.16144
39312	1.24847	0.841
39313	0.946132	1.06412
39314	1.16806	1.20937
39315	1.24847	0.703341
39316	1.24847	1.49663
39317	0.946133	0.668062
39318	0.946133	1.35099
39319	0.946133	0.959079
39320	0.946133	0.739772
39321	1.16805	1.20316

39322	1.24847	0.839352
39323	1.2364	1.13943
39324	0.946129	0.927693
39325	0.946133	1.16673
39326	1.24847	0.947871
39327	1.2364	0.820952
39328	1.2364	1.32036
39329	0.946131	0.700762
39330	0.946131	1.55611
39331	0.946131	1.05351
39332	0.946131	0.845579
39333	1.2364	1.35512
39334	1.24847	0.899617
39335	1.89226	1.10235
39336	1.89226	1.06686
39337	1.89226	0.82156
39338	1.89226	0.856122
39339	1.89226	1.1247
39340	1.89226	1.22242
39341	1.89226	1.03866
39342	1.89226	0.897544
39343	1.89226	0.790765
39344	1.89226	1.0724
39345	1.89226	0.867002
39346	1.89226	1.18436
39347	1.69014	0.997813
39348	1.09194	1.11549
39349	1.13537	1.23689
39350	1.13537	1.15812
39351	1.58481	0.788998
39352	1.58481	0.908036
39353	1.58147	0.883463
39354	1.13537	0.950779
39355	1.69015	1.03764
39356	1.01179	0.767841
39357	1.69015	0.981677
39358	1.01179	1.12774
39359	0.946132	1.63619
39360	0.946132	1.14334
39361	1.28543	0.78188
39362	1.07341	1.76062
39363	1.07341	1.55587
39364	1.07341	0.607487
39365	0.946132	0.93586
39366	0.946132	0.820047
39367	0.946133	0.846747
39368	1.28543	0.851753
39369	1.07341	0.906427
39370	0.946132	1.06245
39371	1.89226	0.936935
39372	1.89226	0.73537
39373	1.89226	0.643895
39374	1.89226	1.58205
39375	1.89226	0.649464
39376	1.89226	2.03313
39377	0.805744	1.85464
39378	0.988659	0.991973
39379	0.782044	0.686985

39380	0.578223	1.05533
39381	0.77823	0.717061
39382	0.778231	2.1
39383	0.788653	0.617947
39384	0.788653	1.31864
39385	0.988662	0.994563
39386	0.782042	0.95086
39387	0.805745	1.10579
39388	0.578222	0.991038
39389	1.89226	0.943686
39390	1.89226	0.906603
39391	1.89226	0.634428
39392	1.89226	1.64146
39393	1.89226	0.577703
39394	1.89226	1.957
39395	1.97732	1.24861
39396	1.97819	0.851831
39397	1.56408	0.705759
39398	1.56408	1.60582
39399	2.33986	0.813877
39400	2.33986	1.90801
39401	1.84516	2.1
39402	2.21152	2.1
39403	1.90702	2.1
39404	1.54599	2.1
39405	3.75751	2.1
39406	1.89224	2.1
39407	1.89224	2.1
39408	1.89224	2.1
39409	1.89224	1.90391
39410	1.89224	2.1
39411	1.89224	1.60589
39412	1.47672	2.1
39413	1.54601	2.1
39414	1.27697	2.1
39415	1.54601	2.1
39416	1.638 2.1	
39417	1.90705	2.1
39418	0.946132	1.25887
39419	0.946132	2.1
39420	0.946131	2.1
39421	0.946131	2.1
39422	0.946131	2.1
39423	0.946131	1.32478
39424	0.946132	2.1
39425	0.946132	2.1
39426	0.946132	2.1
39427	0.946132	2.1
39428	0.946131	2.1
39429	0.946131	2.1
39430	0.946128	2.1
39431	0.946128	2.1
39432	1.32062	2.1
39433	1.4164	2.1
39434	0.946129	2.1
39435	0.946129	2.1
39436	1.32062	2.1
39437	1.32062	2.1

39438	1.32062	2.1
39439	0.94613	2.1
39440	1.41639	2.1
39441	0.946129	2.1
39442	1.98979	0.0745513
39443	1.65977	0.0840736
39444	2.21772	0.0817993
39445	2.21772	0.0768907
39446	1.48866	0.0824682
39447	1.48866	0.0747998
39448	0.973129	0.073781
39449	0.973129	0.0757421
39450	0.980954	0.0843032
39451	0.980954	0.0833815
39452	0.845218	0.0766169
39453	1.13981	0.0822687
39454	0.829886	0.0845458
39455	1.10886	0.0766129
39456	1.10886	0.0774316
39457	1.13982	0.0797895
39458	0.845219	0.0797696
39459	0.829886	0.0777682
39460	0.946132	0.820921
39461	0.946132	1.48747
39462	1.3352	2.1
39463	1.41432	1.96697
39464	0.946133	2.00895
39465	0.946133	0.641287
39466	1.3352	1.48957
39467	1.3352	2.1
39468	1.3352	1.63776
39469	0.946132	1.69777
39470	1.41432	1.65769
39471	0.946133	1.66627
39472	2.70097	2.1
39473	1.88175	2.1
39474	1.51482	2.1
39475	1.88175	2.1
39476	2.37784	2.1
39477	2.82864	2.07132
39478	1.89227	0.210045
39479	1.89227	0.177247
39480	1.89227	0.152302
39481	1.89227	0.140146
39482	3.78453	0.140909
39483	1.89226	0.163221
39484	2.13295	0.129397
39485	2.43203	0.121023
39486	2.43203	0.151809
39487	1.89226	0.13061
39488	1.89226	0.177668
39489	0.946133	0.125002
39490	0.946133	0.134053
39491	0.946132	0.112261
39492	0.946132	0.125102
39493	0.946132	0.109921
39494	0.946132	0.123779
39495	0.946133	0.113131

39496	0.946133	0.141939
39497	0.946132	0.109075
39498	0.946132	0.119422
39499	0.946132	0.151367
39500	0.946132	0.137896
39501	1.54987	2.1
39502	1.24128	2.1
39503	1.24128	2.1
39504	1.50738	2.1
39505	1.75702	2.1
39506	2.02312	2.1
39507	1.89289	2.1
39508	1.92264	2.1
39509	1.72994	2.1
39510	1.75702	2.1
39511	3.67966	2.1
39512	1.75702	2.1
39513	0.994291	2.1
39514	0.994291	2.1
39515	1.24128	2.1
39516	1.4964	2.1
39517	1.74339	2.1
39518	1.72993	2.1
39519	1.49641	2.1
39520	1.49641	2.1
39521	1.75702	2.1
39522	1.3939	2.1
39523	1.65452	2.1
39524	1.19964	2.1
39525	1.53296	2.1
39526	1.19964	2.1
39527	1.36861	2.1
39528	1.42624	2.1
39529	1.59521	2.1
39530	1.19964	2.1
39531	1.42624	2.1
39532	1.07156	2.1
39533	1.36226	2.1
39534	2.5619	2.1
39535	2.14058	1.13346
39536	1.93569	1.94595
39537	2.36221	1.28674
39538	2.5753	1.22525
39539	4.50279	1.73701
39540	2.14057	1.5647
39541	2.20831	1.92388
39542	2.64725	0.997252
39543	2.57529	1.53837
39544	4.78782	1.46418
39545	2.64725	1.18392
39546	2.57529	1.70305
39547	1.89226	1.00687
39548	1.89226	0.832558
39549	4.53951	1.37257
39550	2.0815	2.1
39551	1.86137	2.1
39552	1.86137	2.1
39553	1.86159	2.1

39554	2.0815	2.1
39555	2.10235	2.1
39556	1.33552	2.1
39557	2.32417	2.1
39558	1.18872	2.1
39559	1.93101	2.1
39560	3.26653	2.1
39561	1.89226	2.1
39562	1.89226	2.1
39563	1.89226	2.1
39564	1.89226	2.1
39565	1.89226	2.1
39566	1.89226	2.1
39567	0.955191	2.1
39568	1.03372	2.1
39569	0.946131	2.1
39570	0.946131	2.1
39571	0.930654	2.1
39572	0.930654	2.1
39573	1.04233	2.1
39574	1.05247	2.1
39575	0.955341	2.1
39576	0.946131	2.1
39577	1.02375	2.1
39578	1.13537	2.1
39579	1.1549	2.1
39580	1.93101	2.1
39581	1.91108	2.1
39582	1.52487	2.1
39583	1.3076	2.1
39584	1.06985	2.1
39585	1.89226	2.1
39586	1.89226	2.1
39587	1.89226	2.1
39588	1.89226	2.1
39589	1.89226	2.1
39590	1.89226	2.1
39591	1.58713	2.1
39592	2.6845	2.1
39593	1.58742	2.1
39594	2.74201	2.1
39595	4.27191	2.1
39596	2.68449	2.1
39597	1.89226	2.1
39598	2.74201	2.1
39599	1.89226	2.1
39600	4.57675	2.1
39601	1.57523	2.1
39602	2.74201	2.1
39603	2.8146	2.1
39604	2.8146	2.1
39605	1.58742	2.1
39606	1.58742	2.1
39607	0.946131	1.82231
39608	0.946131	2.1
39609	1.371	2.1
39610	1.371	2.1
39611	0.946127	2.1

39612	0.946127	1.45861
39613	1.37101	2.1
39614	1.4073	2.1
39615	0.94613	2.1
39616	1.4073	2.1
39617	1.371	2.1
39618	0.946129	2.1
39619	1.44801	1.95362
39620	1.06821	1.73246
39621	2.00542	1.82341
39622	2.27703	1.9127
39623	3.34524	1.62929
39624	1.70301	0.365551
39625	1.89226	0.640799
39626	1.65809	0.424059
39627	1.89226	0.593019
39628	1.62758	0.440886
39629	1.85744	0.580046
39630	1.89226	0.348506
39631	1.89226	0.536004
39632	1.89226	0.483383
39633	1.89226	0.4593
39634	3.78452	0.473676
39635	1.89226	0.542961
39636	1.89226	0.417305
39637	1.89226	0.456071
39638	1.89226	0.623875
39639	3.78452	0.5058
39640	0.851541	0.649149
39641	0.851541	0.626916
39642	0.91193	0.523124
39643	0.824837	0.562362
39644	0.854559	0.535051
39645	0.851534	0.800619
39646	0.81851	0.634137
39647	0.717219	0.399955
39648	0.871743	0.619001
39649	0.837985	0.552019
39650	0.82902	0.540414
39651	0.82902	0.639591
39652	1.89226	0.580918
39653	1.92659	0.576792
39654	2.03235	0.591197
39655	1.89226	0.697402
39656	2.03235	0.48215
39657	1.89226	0.574183
39658	0.585557	0.7644
39659	0.585557	0.651076
39660	1.22612	0.64768
39661	1.09436	0.757287
39662	0.851537	0.863037
39663	0.526987	0.688945
39664	0.983879	0.592501
39665	1.04072	0.702717
39666	0.585526	0.656256
39667	0.792325	0.658891
39668	0.946129	0.699344
39669	0.946129	0.710504

39670	1.68152	2.1
39671	1.81445	2.1
39672	1.68152	2.1
39673	1.86792	2.1
39674	1.65888	2.1
39675	1.84529	2.1
39676	1.81445	2.1
39677	1.57885	2.1
39678	1.65889	2.1
39679	1.48594	2.1
39680	3.23773	2.1
39681	1.68154	2.1
39682	1.65891	2.1
39683	1.53296	2.1
39684	1.56299	2.1
39685	3.24453	2.1
39686	1.56299	2.1
39687	1.48596	2.1
39688	1.56299	2.1
39689	1.65891	2.1
39690	1.46568	2.1
39691	1.5616	2.1
39692	2.68785	2.1
39693	2.08831	2.1
39694	2.66919	2.1
39695	2.1024	2.1
39696	4.79025	2.1
39697	2.08832	2.1
39698	1.15478	2.1
39699	1.15478	2.1
39700	1.09062	2.1
39701	2.10241	2.1
39702	2.03825	2.1
39703	2.65348	2.1
39704	2.1024	2.1
39705	2.12313	2.1
39706	2.12313	2.1
39707	2.66919	2.1
39708	2.66919	2.1
39709	0.813169	2.1
39710	0.813169	2.1
39711	0.825788	2.1
39712	0.825788	2.1
39713	0.612902	2.1
39714	0.577387	2.1
39715	1.0512	2.1
39716	1.06157	2.1
39717	1.06157	2.1
39718	0.612902	2.1
39719	0.577387	2.1
39720	1.0512	2.1
39721	1.27227	2.1
39722	1.91724	2.1
39723	1.32591	2.1
39724	1.99837	2.1
39725	3.24315	2.1
39726	1.89223	2.1
39727	1.89223	2.1

39728	1.89223	2.1
39729	1.89223	1.32139
39730	3.78446	2.1
39731	1.89224	2.1
39732	1.89224	0.969981
39733	1.89224	1.65562
39734	1.89224	1.62849
39735	3.78447	1.59987
39736	1.91724	2.1
39737	2.321	2.1
39738	1.99838	2.1
39739	2.4149	1.60265
39740	4.33214	2.1
39741	1.34817	2.1
39742	1.9984	2.1
39743	2.06238	2.1
39744	2.06238	2.1
39745	1.32593	2.1
39746	1.32593	2.1
39747	1.89226	2.1
39748	1.89226	1.28536
39749	1.89226	2.1
39750	1.89226	2.1
39751	1.89226	2.1
39752	1.89226	2.1
39753	1.89226	1.62002
39754	1.89226	1.752
39755	1.89226	2.1
39756	1.89226	0.905925
39757	3.78452	1.69223
39758	1.9984	2.1
39759	2.41494	1.71777
39760	2.06239	2.1
39761	2.5194	2.1
39762	4.47732	2.1
39763	1.19901	0.991572
39764	1.16661	2.1
39765	1.56859	1.8962
39766	1.61029	1.20467
39767	2.7676	1.79512
39768	1.56859	1.93099
39769	1.88637	0.780466
39770	1.61029	0.674383
39771	1.61029	1.84242
39772	1.856	0.669073
39773	1.856	1.81831
39774	1.856	0.782917
39775	1.57916	1.95301
39776	1.56859	0.876418
39777	1.56859	2.1
39778	1.57916	1.36648
39779	1.23475	2.1
39780	0.890924	0.423676
39781	1.07608	0.408976
39782	0.72877	0.515239
39783	0.910106	0.497858
39784	1.00196	0.405165
39785	1.09519	0.781028

39786	0.839967	0.458791
39787	0.719043	0.422095
39788	0.893859	0.450621
39789	0.942408	0.784515
39790	0.873497	0.537893
39791	0.873497	0.615397
39792	1.53046	0.32491
39793	1.89994	0.492961
39794	1.049	0.304097
39795	1.049	0.284429
39796	1.52512	0.264446
39797	1.89994	0.429749
39798	1.65573	0.804192
39799	1.89226	0.673343
39800	1.89226	0.701363
39801	2.73329	1.10349
39802	3.548	0.957939
39803	1.65573	0.692482
39804	2.42671	0.846869
39805	2.73329	0.647829
39806	1.89226	0.610083
39807	2.73329	1.11832
39808	1.89226	0.999026
39809	0.941078	0.520704
39810	1.01599	0.572908
39811	0.818936	0.445881
39812	0.651554	0.519214
39813	0.904729	0.558232
39814	0.665756	0.552598
39815	0.843423	0.630718
39816	0.918649	0.689282
39817	0.855806	0.786596
39818	0.814384	0.332958
39819	0.844437	0.643553
39820	0.844437	0.592872
39821	2.22558	1.06702
39822	1.95785	0.690593
39823	2.07065	0.830461
39824	1.89142	0.743509
39825	2.07065	1.68228
39826	1.89142	1.33073
39827	0.640813	0.730251
39828	0.951654	0.856641
39829	0.797442	0.530954
39830	0.535364	0.628482
39831	0.525269	0.688197
39832	0.620874	0.634959
39833	0.916953	0.808352
39834	0.969374	0.764105
39835	0.788925	1.10811
39836	0.69596	0.30245
39837	0.743154	0.709155
39838	0.743154	0.769607
39839	1.89226	1.63282
39840	1.89226	1.36204
39841	1.89226	0.802742
39842	1.89226	2.00836
39843	3.78452	1.03491

39844	1.32541	1.41373
39845	1.13722	1.11769
39846	0.91103	2.1
39847	0.881123	1.79094
39848	1.03363	1.88972
39849	1.03364	1.54899
39850	1.1505	1.45884
39851	1.1505	1.83842
39852	1.32539	1.6464
39853	1.07662	2.06897
39854	0.881121	1.67346
39855	0.962303	1.47197
39856	1.89226	0.93876
39857	1.89226	2.1
39858	1.89226	1.74246
39859	1.89226	1.06933
39860	3.78453	1.66298
39861	1.89226	0.787547
39862	1.89226	1.78864
39863	1.89226	1.20249
39864	1.89226	1.20038
39865	1.89226	1.26313
39866	1.89226	1.36696
39867	1.27606	1.44449
39868	1.07664	1.19005
39869	0.905895	2.03136
39870	0.696099	2.1
39871	0.984902	1.99813
39872	0.984905	1.41977
39873	1.01878	1.95869
39874	1.01878	0.830565
39875	1.27604	1.76078
39876	1.06044	1.92339
39877	1.13192	1.7554
39878	0.696111	1.74158
39879	1.89226	0.511976
39880	1.89226	0.135787
39881	1.89226	0.144473
39882	1.89226	0.513148
39883	1.89226	0.16345
39884	1.89226	0.509289
39885	1.52615	0.481054
39886	1.01294	0.1282
39887	1.01294	0.627404
39888	1.18549	0.207488
39889	1.38963	0.343389
39890	1.56218	0.174504
39891	1.89226	0.39037
39892	2.40801	0.34653
39893	2.58101	0.494982
39894	2.58101	0.380741
39895	1.89226	0.446699
39896	1.89226	0.31688
39897	2.24071	2.1
39898	2.79317	1.18118
39899	1.99136	2.1
39900	2.6167	1.09633
39901	4.85741	2.1

39902	2.79317	1.22483
39903	1.89226	0.448984
39904	1.89226	1.94916
39905	1.89226	0.64409
39906	2.6167	1.27063
39907	2.6167	0.570826
39908	1.08354	1.25472
39909	1.08354	2.1
39910	1.10222	2.1
39911	1.10222	1.50709
39912	1.2275	1.3784
39913	1.30835	1.19439
39914	0.995678	2.1
39915	0.877412	2.1
39916	0.877411	2.1
39917	1.2275	2.1
39918	0.995678	2.1
39919	1.30835	2.1
39920	1.05793	0.758089
39921	1.46441	0.623645
39922	1.0562	0.686255
39923	1.0562	0.674489
39924	1.41787	0.705731
39925	1.41787	0.70499
39926	1.41787	0.685141
39927	1.52909	0.847108
39928	1.46441	0.639821
39929	1.46441	0.847465
39930	1.43063	0.610373
39931	1.43063	0.820415
39932	1.89226	0.863034
39933	1.89226	0.562292
39934	1.89226	0.640139
39935	1.89226	0.859001
39936	3.78453	0.743134
39937	1.89227	0.753026
39938	1.89227	0.622398
39939	1.89227	0.636553
39940	1.89227	0.664406
39941	1.89227	0.608329
39942	1.89227	0.669175
39943	1.00763	0.658034
39944	1.41787	0.69334
39945	1.05793	0.646793
39946	1.05793	0.742983
39947	1.37265	0.647163
39948	1.37265	0.746652
39949	1.37265	0.679966
39950	1.43063	0.755772
39951	1.41787	0.657873
39952	1.41787	0.957304
39953	1.38247	0.609414
39954	1.38247	0.889206
39955	0.946132	0.764381
39956	0.946132	0.68961
39957	0.946132	0.638756
39958	0.946132	0.490481
39959	0.946131	0.471651

39960	0.946131	0.685351
39961	0.946132	0.789347
39962	0.946132	0.659861
39963	0.946132	0.642245
39964	0.946132	0.623439
39965	0.946132	0.641162
39966	0.946132	0.613755
39967	1.89226	0.687272
39968	1.89226	0.598374
39969	1.89226	0.612326
39970	1.89226	0.73349
39971	1.89226	0.586622
39972	1.89226	0.724988
39973	1.19645	0.874194
39974	1.89183	0.540835
39975	1.91106	0.707274
39976	1.35306	0.583304
39977	1.75446	0.728887
39978	1.19645	0.582687
39979	2.37576	0.351618
39980	1.90139	0.662795
39981	2.1845	1.93509
39982	1.86568	0.632209
39983	4.24145	0.676658
39984	2.29435	0.572193
39985	1.40602	1.59833
39986	1.40602	0.535972
39987	1.55351	0.679038
39988	1.86568	0.688681
39989	2.01317	2.01916
39990	2.18452	1.47
39991	1.8657	0.269006
39992	2.01399	0.52003
39993	1.70141	1.22442
39994	3.87969	0.52105
39995	0.774999	2.0836
39996	0.774999	0.498939
39997	0.778721	0.672737
39998	0.778721	1.48234
39999	0.628348	1.1903
40000	0.703016	0.750172
40001	0.932848	0.544669
40002	0.850704	0.581469
40003	0.850704	0.698388
40004	0.628348	0.721988
40005	0.703015	0.577102
40006	0.932848	0.586079
40007	1.65243	0.459873
40008	1.34412	0.403082
40009	1.45569	0.554109
40010	1.1315	0.663255
40011	2.78393	0.44022
40012	1.48901	2.1
40013	1.805 0.658787	
40014	1.48901	0.670996
40015	1.71717	2.1
40016	1.60635	0.590796
40017	1.83451	2.1

40018	1.805	0.861039
40019	1.65243	0.449687
40020	1.60635	0.692334
40021	1.45569	0.453916
40022	3.25879	0.53485
40023	1.27875	2.1
40024	1.60635	0.649885
40025	1.27875	0.737514
40026	1.48901	2.1
40027	1.40014	0.643085
40028	1.6104	2.1
40029	0.715199	0.492989
40030	0.715199	0.748168
40031	0.712714	0.758375
40032	0.712714	0.398732
40033	0.727843	0.408005
40034	0.619713	0.440063
40035	0.803177	0.698548
40036	0.70007	0.666751
40037	0.700069	0.536224
40038	0.619713	0.515799
40039	0.803177	0.535869
40040	0.727844	0.507615
40041	1.14357	2.1
40042	2.3506	2.09972
40043	1.14357	2.1
40044	2.68773	2.04338
40045	1.57829	2.1
40046	2.68773	2.03939
40047	2.00551	2.1
40048	2.65811	1.28789
40049	2.07203	2.05892
40050	2.72117	0.38551
40051	4.72667	1.27954
40052	0.946131	0.688604
40053	0.946131	1.7255
40054	1.32905	1.7609
40055	1.32905	0.612423
40056	0.94613	1.1327
40057	0.94613	1.35607
40058	1.32905	1.26977
40059	1.36058	1.12869
40060	1.36058	0.604559
40061	0.946131	0.512497
40062	1.32905	0.992657
40063	0.946131	1.29554
40064	1.21649	0.41888
40065	1.21649	1.9525
40066	1.21459	1.72895
40067	1.21459	1.79085
40068	1.39508	1.0545
40069	1.36057	0.798251
40070	1.03601	1.45188
40071	1.07438	1.87999
40072	1.07439	1.08428
40073	1.39509	1.08527
40074	1.036	1.17603
40075	1.36056	0.990488

40076	2.79015	0.786341
40077	2.72114	2.00805
40078	1.89225	0.781417
40079	1.89225	1.79027
40080	1.89225	1.36996
40081	2.72114	0.672935
40082	0.876258	2.1
40083	0.892889	2.1
40084	1.34352	2.1
40085	1.27985	2.1
40086	1.07462	2.1
40087	1.07462	2.1
40088	1.09812	2.1
40089	1.09812	2.1
40090	0.892888	2.1
40091	1.34352	2.1
40092	0.876256	2.1
40093	1.27985	2.1
40094	1.39725	1.46423
40095	1.30029	0.664743
40096	1.31415	0.798592
40097	1.41336	1.15426
40098	2.71366	0.938673
40099	1.89226	0.681731
40100	1.89226	0.836096
40101	1.89226	1.24826
40102	1.89226	0.645767
40103	3.78453	0.74431
40104	0.946133	0.85469
40105	0.946133	0.705535
40106	1.38709	0.531786
40107	1.39437	0.744327
40108	0.946132	0.527687
40109	0.946132	0.663398
40110	1.39437	0.93443
40111	1.39437	0.590778
40112	0.946132	0.645934
40113	1.39437	0.597514
40114	1.38709	0.638785
40115	0.946132	0.638782
40116	0.706681	1.32709
40117	0.711908	1.18089
40118	0.657077	0.697455
40119	0.661003	0.910937
40120	0.684188	0.702551
40121	0.684188	1.08737
40122	0.683496	1.62095
40123	0.683496	0.749074
40124	0.711908	0.901179
40125	0.661003	0.943587
40126	0.657076	0.933219
40127	0.706681	0.929426
40128	0.946132	1.2173
40129	0.946132	0.842126
40130	0.946132	0.72741
40131	0.946132	0.624947
40132	0.946132	0.629197
40133	0.946132	0.848333

40134	0.946133	0.756866
40135	0.946133	1.23918
40136	0.946133	0.770849
40137	0.946133	0.755293
40138	0.946132	0.814458
40139	0.946132	0.809867
40140	2.79029	0.528239
40141	1.89227	0.911976
40142	2.78873	0.647963
40143	2.78873	0.633661
40144	1.89383	0.705682
40145	1.89227	0.668266
40146	1.43669	2.1
40147	2.11984	2.1
40148	2.24481	2.1
40149	2.24481	2.1
40150	1.20311	2.1
40151	1.20311	2.1
40152	0.49703	2.1
40153	0.601553	2.1
40154	1.05992	2.1
40155	0.991694	2.1
40156	0.787558	2.1
40157	0.787558	2.1
40158	0.805689	2.1
40159	0.805689	2.1
40160	0.991694	2.1
40161	0.49703	2.1
40162	1.05992	2.1
40163	0.601553	2.1
40164	2.45905	2.1
40165	1.89226	1.92493
40166	1.89226	2.1
40167	1.89226	1.81298
40168	2.47395	2.1
40169	2.47395	2.1
40170	1.89226	2.1
40171	1.89226	1.43478
40172	1.89226	1.97265
40173	1.89226	1.0756
40174	3.78452	1.74032
40175	1.38427	2.1
40176	2.31203	0.961406
40177	2.28743	0.858284
40178	2.28743	2.1
40179	1.63384	0.793931
40180	1.63384	2.07696
40181	2.42723	2.1
40182	1.89226	2.1
40183	1.89226	2.1
40184	1.89226	2.0396
40185	2.45905	2.1
40186	2.45905	1.749
40187	0.946131	0.673305
40188	0.946131	2.0904
40189	0.946131	2.1
40190	0.946131	1.39683
40191	0.94613	1.19255

40192	0.94613	1.51347
40193	0.946132	2.1
40194	0.946132	1.49309
40195	0.946131	1.28717
40196	0.946131	1.55338
40197	0.946131	1.62417
40198	0.946131	2.05847
40199	0.989441	2.1
40200	0.989441	1.52284
40201	0.993918	2.08373
40202	0.993918	1.76817
40203	1.16644	1.64139
40204	1.15601	0.991673
40205	0.818308	2.1
40206	0.816922	2.1
40207	0.818307	1.84206
40208	1.16644	1.9562
40209	1.15601	1.8919
40210	0.816922	1.42044
40211	0.996778	0.120224
40212	2.44375	0.105542
40213	0.996778	0.141337
40214	1.92547	0.10746
40215	1.56091	0.146669
40216	2.4896	0.107814
40217	1.56091	0.144275
40218	2.44375	0.105144
40219	1.90456	0.139046
40220	1.90456	0.146001
40221	2.81853	0.110736
40222	2.47487	0.110434
40223	1.32143	1.67067
40224	1.43294	1.95642
40225	1.37444	0.868488
40226	1.51691	1.6521
40227	2.80738	1.5663
40228	1.89226	1.31892
40229	1.89226	1.98748
40230	1.89226	0.939685
40231	1.89226	1.35638
40232	3.78453	1.26002
40233	1.89226	1.28852
40234	1.89226	2.1
40235	1.89226	1.05184
40236	1.89226	2.1
40237	3.78453	1.40058
40238	1.43294	1.2882
40239	1.3268	2.1
40240	1.51691	1.23763
40241	1.44091	2.1
40242	2.87385	1.89162
40243	1.37444	1.92662
40244	1.51691	1.95756
40245	1.4714	0.845904
40246	1.65434	1.38414
40247	3.02878	1.48412
40248	1.89226	1.97139
40249	1.89226	1.69845

40250	1.89226	1.12814
40251	1.89226	1.39528
40252	3.78452	1.50031
40253	1.89226	1.2613
40254	1.89226	2.1
40255	1.89226	1.07928
40256	1.89226	2.1
40257	3.78453	1.73112
40258	1.51691	1.4695
40259	1.44091	2.1
40260	1.65434	1.10946
40261	1.60647	2.1
40262	3.09526	1.78875
40263	1.05013	2.1
40264	1.05013	2.1
40265	0.94613	2.1
40266	0.94613	2.1
40267	1.04959	2.1
40268	1.05014	2.1
40269	0.946129	2.1
40270	0.946129	2.1
40271	0.946131	2.1
40272	1.04958	2.1
40273	0.946131	2.1
40274	1.05013	2.1
40275	1.04959	2.1
40276	1.04959	2.1
40277	0.946131	2.1
40278	0.946131	2.1
40279	1.04765	2.1
40280	1.04959	2.1
40281	0.94613	2.1
40282	0.94613	2.1
40283	0.946131	2.1
40284	1.04765	2.1
40285	0.946131	2.1
40286	1.04959	2.1
40287	1.85744	2.1
40288	1.89226	2.1
40289	1.89226	2.1
40290	1.89226	2.1
40291	1.94839	2.1
40292	1.94839	2.1
40293	0.97419	2.1
40294	0.822054	2.1
40295	0.946123	2.1
40296	0.946123	2.1
40297	1.04202	2.1
40298	0.84898	2.1
40299	0.933307	2.1
40300	0.933307	2.1
40301	0.933285	2.1
40302	1.05018	2.1
40303	0.851528	2.1
40304	0.876787	2.1
40305	1.87528	0.414736
40306	1.21137	0.284105
40307	1.57893	0.326659

40308	1.21137	0.430845
40309	1.81182	0.322374
40310	1.44426	0.408794
40311	0.993833	0.275289
40312	1.21137	0.34067
40313	1.57893	0.387534
40314	1.31774	0.269557
40315	2.5291	0.279642
40316	1.87528	0.41926
40317	1.57893	0.288125
40318	1.90728	0.305788
40319	2.29851	0.407563
40320	3.87744	0.391122
40321	1.90728	0.333041
40322	1.31774	0.281475
40323	1.56339	0.289187
40324	1.31774	0.358474
40325	1.82458	0.282821
40326	1.57893	0.339877
40327	1.89226	0.84298
40328	1.89226	0.978802
40329	1.89226	0.939778
40330	1.89226	0.968185
40331	1.89226	0.957919
40332	1.89226	0.987384
40333	1.89226	0.9307
40334	1.89226	1.04445
40335	1.89226	1.10241
40336	1.89226	0.970192
40337	3.78452	1.02612
40338	1.89226	0.975693
40339	2.17251	1.02743
40340	2.45575	0.939865
40341	2.45575	0.988868
40342	1.89226	0.918889
40343	1.89226	0.971141
40344	1.08625	1.0563
40345	1.22788	0.944956
40346	0.54447	0.891169
40347	0.794861	0.978573
40348	0.967729	1.057
40349	0.967729	1.06101
40350	0.913386	0.936823
40351	0.913386	0.901199
40352	1.08625	1.02694
40353	0.544469	0.946631
40354	0.794862	1.01667
40355	1.22788	0.990965
40356	0.946128	0.995821
40357	0.946128	1.05587
40358	0.946132	0.961544
40359	0.946132	0.931615
40360	0.94613	0.95987
40361	0.94613	0.982072
40362	0.94613	1.06552
40363	0.94613	0.901076
40364	0.94613	0.986155
40365	0.94613	0.98347

40366	0.94613	0.978369
40367	0.94613	0.985436
40368	0.946127	0.928433
40369	0.946127	0.913478
40370	1.08625	0.900815
40371	0.958779	0.995565
40372	1.08625	0.930802
40373	1.08625	0.970355
40374	0.946128	0.896551
40375	0.946128	0.914646
40376	0.946129	0.93908
40377	0.958777	0.944917
40378	1.08625	0.92299
40379	0.946129	0.926105
40380	2.16338	0.187697
40381	1.94002	0.176017
40382	1.98836	0.181632
40383	2.60614	0.189721
40384	1.5456	0.164682
40385	2.16338	0.16577
40386	1.19952	0.221225
40387	2.34061	0.218484
40388	1.21031	0.194181
40389	2.1711	0.170694
40390	3.54013	0.209604
40391	1.13624	2.1
40392	1.70302	2.1
40393	1.8267	2.1
40394	1.11545	2.1
40395	2.85455	2.1
40396	1.82671	2.1
40397	1.91767	2.1
40398	1.81563	2.1
40399	1.91767	2.1
40400	1.71362	2.1
40401	1.83277	2.1
40402	1.66064	2.1
40403	0.996776	2.1
40404	0.996776	2.1
40405	1.11545	2.1
40406	1.54278	2.1
40407	1.66145	2.1
40408	1.66064	2.1
40409	1.7457	2.1
40410	1.81563	2.1
40411	1.81563	2.1
40412	1.54278	2.1
40413	1.54278	2.1
40414	2.71672	2.1
40415	2.70402	2.1
40416	2.64996	2.1
40417	2.69443	2.1
40418	5.39845	2.1
40419	2.71672	2.1
40420	2.54198	2.1
40421	2.54019	2.1
40422	2.69443	2.1
40423	5.23641	2.1

40424	2.64996	2.1
40425	2.69443	2.1
40426	2.68213	2.1
40427	2.61243	2.1
40428	5.30686	2.1
40429	2.54019	2.1
40430	2.69443	2.1
40431	2.68213	2.1
40432	2.5392	2.1
40433	5.23363	2.1
40434	1.89226	2.1
40435	2.34725	2.1
40436	2.22414	2.1
40437	2.34725	2.1
40438	1.76914	2.1
40439	1.89226	2.1
40440	0.946131	2.1
40441	0.946131	2.1
40442	1.11207	2.1
40443	1.03798	2.1
40444	0.94613	2.1
40445	0.94613	2.1
40446	1.03798	2.1
40447	1.03798	2.1
40448	0.946131	2.1
40449	1.03798	2.1
40450	0.946131	2.1
40451	1.11207	2.1
40452	1.89226	0.296569
40453	1.89226	0.276728
40454	1.89226	0.335238
40455	1.89226	0.227923
40456	1.89226	0.358508
40457	1.89226	0.251336
40458	1.89226	0.298527
40459	1.89226	0.315199
40460	1.89226	0.379422
40461	1.89226	0.249248
40462	1.89226	0.394577
40463	1.89226	0.264273
40464	1.89226	0.198622
40465	1.89226	0.195358
40466	1.89226	0.228512
40467	1.89226	0.169985
40468	1.89226	0.23905
40469	1.89226	0.182998
40470	1.44085	1.31548
40471	1.31825	0.662952
40472	1.31923	0.844374
40473	1.31825	1.11676
40474	1.43311	0.768595
40475	1.43213	0.93967
40476	1.89226	0.674997
40477	1.89226	0.89327
40478	1.89226	0.654182
40479	1.89226	0.931811
40480	1.89226	0.631371
40481	1.89226	1.03571

40482	0.946133	0.76694
40483	0.946133	0.660805
40484	1.38969	0.528504
40485	1.39587	0.64874
40486	0.946132	0.520352
40487	0.946132	0.630729
40488	1.39587	0.791526
40489	1.39587	0.565675
40490	0.946132	0.60119
40491	1.39587	0.579521
40492	1.38969	0.610125
40493	0.946132	0.61212
40494	1.44085	1.50863
40495	1.31923	0.614924
40496	1.33026	0.793075
40497	1.4617	1.05745
40498	2.78093	0.935329
40499	1.89226	0.646413
40500	1.89226	0.828182
40501	1.89226	0.631942
40502	1.89226	0.915506
40503	1.89226	0.576397
40504	1.89226	1.01324
40505	0.946133	0.714412
40506	0.946133	0.629235
40507	1.39587	0.581285
40508	1.40724	0.463813
40509	0.946132	0.456109
40510	0.946132	0.609885
40511	1.40724	0.728767
40512	1.40724	0.574587
40513	0.946132	0.580159
40514	1.40724	0.587244
40515	0.946133	0.673222
40516	1.39587	0.617045
40517	1.15818	1.54288
40518	1.81846	1.4867
40519	1.52782	0.98597
40520	1.52782	1.90898
40521	1.28095	0.823715
40522	1.28095	1.69617
40523	2.56709	2.09436
40524	1.4349	0.479317
40525	1.4349	0.689263
40526	1.75471	0.746464
40527	2.29909	0.685923
40528	2.61889	0.851757
40529	2.5671	2.1
40530	2.82858	1.45967
40531	2.57368	2.1
40532	2.29909	0.716147
40533	5.12767	1.81571
40534	2.29909	1.61455
40535	1.09148	0.391255
40536	1.09148	0.690314
40537	1.4349	0.65109
40538	1.99776	0.651583
40539	2.34119	0.729298

40540	2.57368	2.1
40541	1.99777	0.691189
40542	1.99777	1.27423
40543	2.29909	1.71805
40544	2.36071	1.25915
40545	2.66203	2.03077
40546	1.89226	0.76027
40547	1.89226	0.889453
40548	1.89226	0.83927
40549	1.89226	0.761872
40550	3.78453	0.756012
40551	0.946128	0.908784
40552	0.946128	1.29447
40553	0.946133	0.843865
40554	0.946133	0.922731
40555	0.946131	0.946681
40556	0.946131	1.02941
40557	0.946131	1.14236
40558	0.946131	0.953757
40559	0.94613	1.20052
40560	0.94613	0.986357
40561	0.946131	0.930246
40562	0.946131	1.04423
40563	1.89226	0.917933
40564	1.89226	1.02429
40565	1.89226	0.783365
40566	1.89226	1.02348
40567	3.78453	1.03523
40568	1.89226	0.948177
40569	1.89226	0.853536
40570	1.89226	0.750434
40571	1.89226	0.885355
40572	3.78453	0.916682
40573	1.89226	0.88402
40574	2.61682	1.25502
40575	1.89226	1.16193
40576	1.89226	1.19103
40577	1.89226	0.887747
40578	1.89226	1.06354
40579	1.89226	0.534822
40580	1.89226	0.529818
40581	1.89226	0.61959
40582	1.89226	0.645218
40583	3.78452	0.725808
40584	1.89226	0.526991
40585	1.89226	0.865573
40586	1.89226	0.545582
40587	1.89226	0.660185
40588	1.89226	0.686442
40589	1.89226	0.857718
40590	1.89227	0.543012
40591	1.89227	0.458025
40592	1.89227	0.732544
40593	1.89227	0.636856
40594	1.89227	0.554044
40595	1.89227	0.536646
40596	0.80447	0.916743
40597	0.534241	0.943665

40598	0.995175	0.978321
40599	0.92204	0.722718
40600	0.922042	0.547435
40601	0.534241	0.554332
40602	0.804468	0.888473
40603	0.995176	0.456252
40604	0.71543	0.736151
40605	0.71543	0.586676
40606	0.813988	0.548948
40607	0.813988	0.464765
40608	0.922045	0.427585
40609	0.942899	0.579806
40610	0.946132	0.490211
40611	0.946132	0.455634
40612	1.05169	0.332885
40613	0.936661	0.535995
40614	0.922045	0.500451
40615	0.946133	0.417652
40616	1.01665	0.420983
40617	1.13008	0.354372
40618	0.851529	0.437391
40619	0.851529	0.474534
40620	1.41908	2.1
40621	1.77723	2.1
40622	1.90485	2.1
40623	1.90485	2.1
40624	1.30237	2.1
40625	1.30237	2.1
40626	1.77723	2.1
40627	2.0055	1.54056
40628	1.90485	1.95862
40629	2.17158	1.96843
40630	3.91035	2.05421
40631	0.850906	1.74356
40632	0.850906	2.1
40633	0.85668	2.1
40634	0.85668	2.1
40635	0.998045	2.1
40636	0.952425	2.01536
40637	0.74367	2.1
40638	0.709541	2.1
40639	0.74367	2.1
40640	0.998045	2.1
40641	0.709541	2.1
40642	0.952425	2.1
40643	1.90485	2.1
40644	2.28563	2.1
40645	2.26282	2.1
40646	2.17158	1.65204
40647	1.99609	2.1
40648	1.90485	1.07723
40649	2.00098	0.287517
40650	1.90462	0.283911
40651	1.60057	0.297663
40652	1.79224	0.349548
40653	3.69686	0.290563
40654	2.65654	0.330232
40655	1.79224	0.330406

40656	2.22332	0.332187
40657	2.22332	0.338062
40658	2.24163	0.327143
40659	2.24163	0.328557
40660	1.89226	0.347877
40661	1.89226	0.338249
40662	1.89226	0.33623
40663	1.89226	0.393283
40664	3.78452	0.350147
40665	1.89226	0.295702
40666	1.89226	0.356673
40667	1.89226	0.331953
40668	1.89226	0.350883
40669	1.89226	0.354603
40670	1.89226	0.3542
40671	2.24163	0.363466
40672	1.03645	0.364866
40673	1.79224	0.374179
40674	1.79224	0.358864
40675	1.54283	0.379713
40676	1.54283	0.363388
40677	0.946132	0.36698
40678	0.946132	0.35815
40679	0.946129	0.369135
40680	0.946129	0.361613
40681	0.94613	0.369646
40682	0.94613	0.364767
40683	0.946131	0.373751
40684	0.946131	0.364171
40685	0.94613	0.37866
40686	0.94613	0.382577
40687	0.946131	0.353487
40688	0.946131	0.34971
40689	1.89226	2.1
40690	1.89226	2.1
40691	1.89226	2.1
40692	1.89226	2.1
40693	3.78453	2.1
40694	1.52441	2.1
40695	1.30347	2.1
40696	1.52441	2.1
40697	1.58401	2.1
40698	1.2627	2.1
40699	1.3223	2.1
40700	1.89226	2.1
40701	2.72787	2.1
40702	2.72787	2.1
40703	2.74762	2.1
40704	1.89226	2.1
40705	1.91202	2.1
40706	1.89227	2.1
40707	1.89227	2.1
40708	1.89227	2.1
40709	1.89227	2.1
40710	3.78453	2.1
40711	0.56414	2.1
40712	0.561557	2.1
40713	0.527284	2.1

40714	0.541143	2.1
40715	0.527276	2.1
40716	0.631685	2.1
40717	1.52441	2.1
40718	1.00232	2.1
40719	0.784495	2.1
40720	0.842304	2.1
40721	0.721569	2.1
40722	1.36393	2.1
40723	1.36393	2.1
40724	0.946132	2.1
40725	0.946132	2.1
40726	1.36393	2.1
40727	1.3594	2.1
40728	0.946132	2.1
40729	0.946132	2.1
40730	0.946107	2.1
40731	1.35936	2.1
40732	1.36397	2.1
40733	0.946158	2.1
40734	1.89226	2.1
40735	1.89226	2.1
40736	1.89226	2.1
40737	1.89226	2.1
40738	3.78453	2.1
40739	2.20802	2.1
40740	1.05677	2.1
40741	2.30479	2.1
40742	0.978699	2.1
40743	3.18672	2.1
40744	0.94613	2.1
40745	0.94613	2.1
40746	0.669866	2.1
40747	0.669866	2.1
40748	0.681588	2.1
40749	0.669865	2.1
40750	0.946133	2.1
40751	0.946133	2.1
40752	0.94613	2.1
40753	0.681589	2.1
40754	0.946131	2.1
40755	0.669867	2.1
40756	2.73514	2.1
40757	1.78301	2.1
40758	1.90618	2.1
40759	2.83464	2.1
40760	4.64132	2.1
40761	2.73514	2.1
40762	1.89226	2.1
40763	1.89226	2.1
40764	2.83464	2.1
40765	4.6274	2.1
40766	2.03915	2.1
40767	2.26616	2.1
40768	2.23549	2.1
40769	2.01704	2.1
40770	4.2832	2.1
40771	2.03916	2.1

40772	1.55766	2.1
40773	1.53728	2.1
40774	2.01704	2.1
40775	3.5747	2.1
40776	2.23551	2.1
40777	2.01705	2.1
40778	1.99823	2.1
40779	2.21189	2.1
40780	4.22894	2.1
40781	1.51788	2.1
40782	2.01705	2.1
40783	1.53729	2.1
40784	1.53729	2.1
40785	1.99823	2.1
40786	1.99823	2.1
40787	2.588 2.1	
40788	1.45581	2.1
40789	1.45581	2.1
40790	1.44207	2.1
40791	2.54949	2.1
40792	2.53574	2.1
40793	0.98641	2.1
40794	0.98641	2.1
40795	1.00371	2.1
40796	1.00371	2.1
40797	0.749992	2.1
40798	0.727904	2.1
40799	1.26221	2.1
40800	1.27474	2.1
40801	1.26221	2.1
40802	0.749993	2.1
40803	0.727904	2.1
40804	1.27474	2.1
40805	1.23105	0.831653
40806	1.35435	1.75755
40807	1.89226	1.25256
40808	1.89226	1.14884
40809	1.89226	1.59157
40810	1.7043	1.32548
40811	1.89226	1.86035
40812	1.89226	1.18818
40813	1.89226	0.832388
40814	1.89226	1.78922
40815	3.78453	1.40042
40816	1.89226	0.69416
40817	1.89226	1.74724
40818	1.89226	1.00487
40819	1.89226	1.06539
40820	1.89226	1.31169
40821	1.89226	1.39575
40822	0.946131	0.836456
40823	1.40945	0.725145
40824	0.94613	0.822753
40825	0.94613	1.42853
40826	0.946129	1.45888
40827	0.946129	1.00535
40828	0.946132	1.61327
40829	1.40945	0.68019

40830	0.94613	0.967747
40831	0.94613	0.915382
40832	0.946131	0.952831
40833	0.94613	0.913896
40834	0.946133	0.996554
40835	0.946133	0.894515
40836	0.946132	1.26144
40837	0.946132	1.66544
40838	0.946132	1.64129
40839	0.946132	1.02771
40840	0.946133	1.28217
40841	0.946133	0.904871
40842	0.946132	1.21274
40843	0.946132	1.16979
40844	0.946133	1.05337
40845	0.946133	1.05679
40846	1.89226	1.01813
40847	1.89226	1.88363
40848	1.89226	1.35791
40849	1.89226	1.23311
40850	1.89226	1.64524
40851	1.89226	1.44704
40852	1.89226	2.1
40853	1.89226	2.1
40854	1.89226	2.1
40855	1.89226	2.1
40856	1.89226	2.1
40857	1.89226	2.1
40858	1.89226	2.1
40859	1.89226	2.1
40860	1.89226	2.1
40861	1.89226	2.1
40862	3.78453	2.1
40863	1.66647	2.1
40864	1.15399	2.1
40865	1.25443	2.1
40866	1.26599	2.1
40867	1.58697	2.1
40868	1.66647	2.1
40869	1.66647	2.1
40870	1.47446	2.1
40871	1.55433	2.1
40872	1.74241	2.1
40873	3.2208	2.1
40874	1.89227	2.1
40875	1.89227	2.1
40876	1.89227	2.1
40877	1.89227	2.1
40878	3.78453	2.1
40879	0.946132	2.1
40880	0.946132	2.1
40881	0.946132	2.1
40882	0.946132	2.1
40883	0.946132	2.1
40884	0.946132	2.1
40885	0.946133	2.1
40886	0.946133	2.1
40887	0.946132	2.1

40888	0.946132	2.1
40889	0.946133	2.1
40890	0.946133	2.1
40891	0.625067	2.1
40892	0.61907	2.1
40893	0.896913	2.1
40894	0.946199	2.1
40895	0.806287	2.1
40896	0.677685	2.1
40897	0.750657	2.1
40898	0.750657	2.1
40899	0.646004	2.1
40900	0.890236	2.1
40901	0.61049	2.1
40902	0.888979	2.1
40903	1.6223	2.1
40904	1.98034	2.1
40905	1.75003	2.1
40906	1.55433	2.1
40907	1.75003	2.1
40908	1.55433	2.1
40909	1.88602	2.1
40910	2.08149	2.1
40911	2.07991	2.1
40912	1.88473	2.1
40913	2.08149	2.09519
40914	1.88602	2.1
40915	1.88458	2.1
40916	2.08149	2.1
40917	1.7069	2.1
40918	1.56284	2.1
40919	2.08149	2.1
40920	1.88458	2.1
40921	1.5955	2.1
40922	1.89226	2.1
40923	1.91975	2.1
40924	1.62299	2.1
40925	1.89226	1.97725
40926	1.5955	2.1
40927	1.89226	1.72745
40928	1.89226	1.961
40929	1.89226	1.55507
40930	1.89226	2.1
40931	1.89226	1.06056
40932	1.89226	1.91124
40933	1.89226	2.0638
40934	1.89226	2.1
40935	1.89226	2.03507
40936	1.89226	2.1
40937	1.89226	1.64119
40938	1.89226	2.1
40939	1.62299	2.1
40940	1.89226	2.1
40941	1.71752	2.1
40942	1.44824	2.1
40943	1.89226	2.1
40944	1.62299	2.1
40945	1.89226	0.749978

40946	2.8317	0.770064
40947	1.89226	0.781948
40948	2.64055	0.524157
40949	4.53281	0.76487
40950	2.8317	0.905858
40951	1.83151	0.499231
40952	2.01001	0.728881
40953	2.01001	0.87106
40954	2.64055	0.518078
40955	2.64055	0.642162
40956	0.946132	0.461015
40957	0.946132	0.553666
40958	1.16167	0.537394
40959	1.16167	0.609649
40960	1.16167	0.564911
40961	1.32027	0.428584
40962	0.946131	0.448007
40963	0.946131	0.637512
40964	0.94613	0.474797
40965	1.16167	0.398011
40966	0.946131	0.49421
40967	1.32027	0.480774
40968	2.32335	0.641379
40969	1.83151	0.475014
40970	2.38525	0.556536
40971	2.64055	0.5208
40972	1.57621	0.450907
40973	1.83151	0.440897
40974	2.25677	2.1
40975	1.88158	2.1
40976	1.86856	2.1
40977	2.0815	2.1
40978	1.70303	2.1
40979	1.88158	2.1
40980	2.50381	2.1
40981	1.89227	2.1
40982	2.25677	2.1
40983	2.25677	2.1
40984	1.89227	2.1
40985	1.89227	2.1
40986	1.89226	0.352531
40987	1.50899	0.358514
40988	2.13659	0.346404
40989	1.50899	0.457354
40990	2.51986	0.335057
40991	1.89226	0.422817
40992	0.946131	0.417166
40993	0.946131	0.317796
40994	1.06829	0.334578
40995	0.895375	0.260264
40996	0.94613	0.329621
40997	0.94613	0.430938
40998	1.33922	0.308854
40999	0.895376	0.253445
41000	0.946131	0.304846
41001	1.33922	0.30411
41002	0.946131	0.301214
41003	1.06829	0.325114

41004	0.841467	0.478513
41005	0.578428	0.509553
41006	1.18079	0.463808
41007	0.920237	0.497185
41008	0.772317	0.43324
41009	0.856555	0.531798
41010	0.910271	0.478258
41011	0.910271	0.553286
41012	0.975752	0.472823
41013	0.536929	0.439465
41014	0.81952	0.541005
41015	1.15252	0.465475
41016	1.89226	2.1
41017	2.51431	2.1
41018	1.89226	2.1
41019	1.89226	2.1
41020	2.78815	2.1
41021	2.78815	2.1
41022	2.0815	2.1
41023	2.0516	2.1
41024	2.0815	2.1
41025	3.06699	2.1
41026	2.76576	2.1
41027	2.8002	2.1
41028	1.94112	2.1
41029	1.89226	2.1
41030	2.20358	2.1
41031	1.89226	2.1
41032	4.09584	2.1
41033	1.09154	2.1
41034	2.62647	2.1
41035	2.20358	2.1
41036	2.20358	2.1
41037	1.52314	2.1
41038	1.52314	2.1
41039	1.31323	2.1
41040	1.31323	2.1
41041	0.946132	2.1
41042	0.946132	2.1
41043	0.946132	2.1
41044	0.946132	2.1
41045	1.31323	2.1
41046	1.10179	2.1
41047	1.31323	2.1
41048	0.946132	2.1
41049	1.10179	2.1
41050	0.946133	2.1
41051	1.89226	0.762595
41052	1.89226	1.0615
41053	1.89226	1.00893
41054	1.89226	1.09739
41055	1.89226	0.755826
41056	1.89226	0.928426
41057	1.35126	1.42197
41058	1.35126	2.01649
41059	0.903285	1.13523
41060	0.903285	1.36142
41061	1.30841	1.374

41062	0.903313	1.83388
41063	0.946132	1.21444
41064	0.946132	1.03714
41065	0.946132	1.27687
41066	0.903312	1.26338
41067	1.30841	1.20019
41068	0.946133	1.10381
41069	2.62097	2.1
41070	2.72561	1.72109
41071	2.39016	2.1
41072	2.51062	1.89576
41073	5.11577	2.1
41074	2.51062	1.8855
41075	2.58286	0.476449
41076	2.40107	1.40386
41077	2.40107	0.611938
41078	2.72561	1.46632
41079	2.72561	0.625594
41080	2.39016	2.1
41081	2.51062	1.92385
41082	2.20242	2.1
41083	2.34107	1.34868
41084	4.73124	2.1
41085	2.51062	1.92775
41086	2.40107	0.444816
41087	2.34107	1.35295
41088	2.25197	0.545842
41089	4.74215	0.698651
41090	2.75218	2.1
41091	1.89226	2.1
41092	2.3349	2.1
41093	2.3349	2.1
41094	1.89226	2.1
41095	1.89226	2.1
41096	1.46129	2.1
41097	0.996308	2.1
41098	1.88649	2.1
41099	1.70302	2.1
41100	2.95419	2.1
41101	1.89227	2.1
41102	1.89227	1.80948
41103	1.89227	1.95399
41104	1.89227	2.1
41105	3.78453	2.1
41106	0.646558	2.1
41107	0.646557	2.1
41108	0.60697	2.1
41109	0.60697	2.1
41110	0.587917	2.1
41111	0.519747	2.1
41112	0.586443	2.1
41113	0.733782	2.1
41114	0.733793	2.1
41115	0.587924	2.1
41116	0.519739	2.1
41117	0.586436	2.1
41118	2.1642	2.1
41119	1.42867	2.1

41120	2.28847	2.04546
41121	1.68299	2.1
41122	3.71714	2.1
41123	1.89226	2.07277
41124	1.89226	2.1
41125	1.89226	2.01554
41126	1.89226	2.1
41127	3.78453	2.1
41128	0.946131	2.1
41129	0.946131	2.1
41130	1.24994	2.1
41131	1.24994	2.1
41132	1.11582	2.1
41133	1.24994	2.1
41134	0.946133	2.1
41135	0.946133	2.1
41136	0.946131	2.1
41137	1.24994	2.1
41138	0.94613	2.1
41139	1.11582	2.1
41140	2.28847	2.1
41141	1.53629	2.1
41142	1.57498	2.1
41143	1.47417	2.1
41144	2.42221	2.07979
41145	2.28847	2.1
41146	1.89226	2.09407
41147	1.89226	2.1
41148	1.89226	1.86934
41149	1.89226	2.1
41150	3.78453	2.1
41151	1.89226	2.1
41152	2.49988	2.1
41153	2.71233	2.1
41154	2.71233	2.1
41155	1.89226	2.1
41156	1.89226	2.1
41157	0.924534	2.1
41158	0.924534	2.1
41159	0.990395	2.1
41160	0.990395	2.1
41161	0.946131	2.1
41162	0.946131	2.1
41163	0.924505	2.1
41164	0.968797	2.1
41165	0.924505	2.1
41166	0.946131	2.1
41167	0.968797	2.1
41168	0.946131	2.1
41169	0.924533	2.1
41170	0.924533	2.1
41171	0.948264	2.1
41172	0.945345	2.1
41173	0.851541	2.1
41174	0.851541	2.1
41175	0.958934	2.1
41176	1.02617	2.1
41177	0.936668	2.1

41178	1.04829	2.1
41179	0.924505	2.1
41180	0.946131	2.1
41181	2.28431	0.550351
41182	1.48035	0.349037
41183	1.59971	0.463573
41184	1.48035	0.424618
41185	2.33818	0.447021
41186	2.21882	0.435598
41187	2.28431	0.490333
41188	1.89226	0.414588
41189	1.89226	0.568975
41190	2.3968	0.335619
41191	4.28906	0.386892
41192	2.28431	0.513949
41193	1.70303	0.304847
41194	1.5428	0.295397
41195	1.59971	0.326887
41196	2.31152	0.443053
41197	2.3968	0.453968
41198	1.99823	2.1
41199	2.21189	2.1
41200	2.18937	2.1
41201	1.979	2.1
41202	4.19089	2.1
41203	0.999114	2.1
41204	0.9895	2.1
41205	0.758942	2.1
41206	0.749358	2.1
41207	0.874236	2.1
41208	0.874236	2.1
41209	0.874236	2.1
41210	0.874236	2.1
41211	0.9895	2.1
41212	0.749358	2.1
41213	0.758942	2.1
41214	0.999114	2.1
41215	2.18937	2.1
41216	1.979	2.1
41217	1.95416	2.1
41218	2.162	2.1
41219	4.14099	2.1
41220	1.95416	2.1
41221	1.49871	2.1
41222	1.47712	2.1
41223	1.49871	2.1
41224	1.9574	2.1
41225	1.979	2.1
41226	1.68508	0.123145
41227	1.64984	0.150826
41228	2.67711	0.108783
41229	2.76407	0.158747
41230	4.41391	0.149846
41231	1.84227	0.123522
41232	2.76407	0.1559
41233	2.76407	0.168917
41234	1.85007	0.127006
41235	2.72769	0.166305

41236	1.80812	0.122886
41237	1.94516	2.1
41238	1.89226	2.1
41239	1.89226	2.1
41240	1.89226	2.1
41241	1.98853	2.1
41242	1.98852	2.1
41243	1.28095	2.1
41244	2.0815	2.1
41245	2.29123	2.1
41246	2.06833	2.1
41247	1.08212	2.1
41248	0.976848	2.1
41249	1.89226	2.1
41250	1.89226	2.1
41251	1.89226	2.1
41252	1.89226	2.1
41253	1.89226	2.1
41254	1.89226	2.1
41255	1.13537	2.1
41256	1.18233	2.1
41257	0.946132	2.1
41258	0.946132	2.1
41259	0.946132	2.1
41260	0.946132	2.1
41261	0.994264	2.1
41262	1.18233	2.1
41263	1.13537	2.1
41264	0.946131	2.1
41265	0.946132	2.1
41266	0.994264	2.1
41267	0.953204	2.1
41268	0.953204	2.1
41269	0.966515	2.1
41270	0.966515	2.1
41271	1.30583	2.1
41272	1.14561	2.1
41273	0.640475	2.1
41274	0.774103	2.1
41275	1.30584	2.1
41276	0.774101	2.1
41277	0.640474	2.1
41278	1.14562	2.1
41279	0.946131	2.1
41280	0.946131	2.1
41281	0.946131	2.1
41282	0.946131	2.03474
41283	0.946128	2.03814
41284	0.946128	2.1
41285	0.946133	2.1
41286	0.946133	2.1
41287	0.94613	2.1
41288	0.94613	2.1
41289	0.946131	2.1
41290	0.946131	2.1
41291	0.946132	0.0840345
41292	0.946132	0.0796311
41293	0.946131	0.0911877

41294	0.946131	0.0970274
41295	0.946131	0.0976914
41296	0.946131	0.0896285
41297	0.946132	0.0804494
41298	0.946132	0.09377
41299	0.946131	0.088541
41300	0.946131	0.0907537
41301	0.946132	0.0892624
41302	0.946132	0.0851148
41303	0.946131	0.0872275
41304	0.946131	0.0828601
41305	0.946131	0.0954607
41306	0.946131	0.102398
41307	0.94613	0.10219
41308	0.94613	0.0914497
41309	0.946132	0.0831795
41310	0.946132	0.0976256
41311	0.946131	0.0918843
41312	0.946131	0.0931656
41313	0.946131	0.0900128
41314	0.946131	0.0925377
41315	2.57882	0.243276
41316	1.48676	0.270768
41317	1.48797	0.245276
41318	2.64281	0.272388
41319	4.12957	0.247471
41320	2.56323	0.22716
41321	2.64281	0.262724
41322	2.51807	0.232854
41323	1.36324	0.263027
41324	1.48797	0.277122
41325	1.48797	0.267652
41326	1.04367	2.1
41327	2.42504	2.1
41328	2.65164	2.1
41329	1.49479	2.1
41330	3.91983	2.1
41331	2.15622	1.08648
41332	1.70539	2.1
41333	2.02369	2.1
41334	2.39039	1.37489
41335	4.17992	2.1
41336	0.966979	2.1
41337	1.7054	2.1
41338	2.0237	2.05022
41339	1.31502	2.1
41340	2.99067	2.1
41341	2.02369	2.1
41342	2.65637	1.44893
41343	2.39039	1.0603
41344	2.39039	2.1
41345	2.29332	1.02763
41346	2.29332	2.1
41347	2.0237	1.84664
41348	1.72116	2.1
41349	1.58465	2.03027
41350	1.31502	2.1
41351	2.29332	1.73724

41352	2.0237	2.1
41353	1.0372	0.107333
41354	2.32462	0.160996
41355	2.2291	0.129822
41356	2.2291	0.151143
41357	1.17464	0.118701
41358	1.17464	0.133303
41359	2.68289	0.111127
41360	1.07805	0.113616
41361	1.70302	0.102428
41362	2.06653	0.115713
41363	3.5779	0.112541
41364	1.89226	0.200565
41365	1.89226	0.302729
41366	1.89226	0.259892
41367	1.89226	0.163495
41368	3.78453	0.217952
41369	1.89226	0.129942
41370	1.89226	0.155651
41371	1.89226	0.151463
41372	1.89226	0.139268
41373	1.89226	0.144074
41374	1.89226	0.131115
41375	1.89225	0.134365
41376	1.89225	0.139313
41377	1.89225	0.148034
41378	1.89225	0.130007
41379	1.89225	0.151294
41380	1.89225	0.135459
41381	0.94613	0.173485
41382	0.94613	0.206438
41383	0.94613	0.294668
41384	0.94613	0.237279
41385	0.946128	0.192633
41386	0.946128	0.220346
41387	0.946132	0.309042
41388	0.946132	0.169314
41389	0.94613	0.204163
41390	0.94613	0.229735
41391	0.94613	0.216134
41392	0.94613	0.186977
41393	1.29417	1.5501
41394	1.32732	2.1
41395	1.32316	0.8758
41396	1.34973	2.1
41397	2.65048	1.62326
41398	1.89226	1.21028
41399	1.89226	2.1
41400	1.89226	0.868494
41401	1.89226	1.81205
41402	3.78453	1.20722
41403	1.89226	1.34384
41404	1.89226	2.1
41405	1.89226	0.916493
41406	1.89226	2.1
41407	3.78453	1.90187
41408	1.32732	1.51583
41409	1.13006	2.1

41410	1.13358	2.1
41411	1.11117	2.1
41412	1.34973	1.33546
41413	1.32732	2.1
41414	1.32316	1.4539
41415	1.34973	2.1
41416	1.34066	0.915078
41417	1.36601	1.91159
41418	2.68918	1.60442
41419	1.89226	1.40866
41420	1.89226	2.03826
41421	1.89226	0.674365
41422	1.89226	1.96013
41423	3.78453	1.44637
41424	1.89226	1.05109
41425	1.89226	2.1
41426	1.89226	2.06837
41427	1.89226	2.1
41428	1.89226	0.944273
41429	1.89226	2.1
41430	0.624134	2.1
41431	0.624134	1.83916
41432	0.624334	0.906276
41433	0.624334	2.1
41434	0.56503	2.1
41435	0.573605	2.1
41436	0.674864	1.17268
41437	0.683007	1.2714
41438	0.683007	2.1
41439	0.573605	2.06399
41440	0.565029	2.1
41441	0.674864	2.1
41442	2.16633	2.1
41443	1.40846	2.1
41444	2.13977	2.1
41445	1.40771	2.1
41446	3.57404	2.1
41447	2.13977	2.1
41448	1.29932	2.1
41449	1.29932	2.1
41450	1.40771	2.1
41451	1.98957	2.1
41452	2.09796	2.1
41453	2.6044	0.490593
41454	1.85548	0.590044
41455	1.89226	0.410176
41456	1.89226	0.665086
41457	3.74774	0.56244
41458	1.89226	0.39174
41459	1.89226	0.663118
41460	1.89226	0.452816
41461	1.89226	0.553893
41462	3.78453	0.508768
41463	1.89226	2.1
41464	2.64107	2.1
41465	1.89226	2.1
41466	2.78977	2.1
41467	1.89226	2.1

41468	2.78977	2.1
41469	1.89226	2.1
41470	1.89226	2.1
41471	1.89226	2.1
41472	1.89226	2.1
41473	1.89226	2.1
41474	1.89226	2.1
41475	2.43173	2.1
41476	1.41238	2.1
41477	2.0815	2.1
41478	1.98048	2.1
41479	1.62014	2.1
41480	1.50086	2.1
41481	0.946129	0.500078
41482	0.946129	0.418206
41483	1.27636	0.48131
41484	1.1984	0.318529
41485	0.946131	0.288782
41486	0.946131	0.386131
41487	1.27636	0.511068
41488	1.27636	0.386552
41489	0.946131	0.399389
41490	1.27636	0.331796
41491	0.946131	0.43179
41492	1.1984	0.376523
41493	1.99569	0.328395
41494	2.55272	0.502107
41495	1.77403	0.295707
41496	1.70303	0.316589
41497	3.05772	0.405642
41498	2.55121	0.384612
41499	0.946128	0.371459
41500	0.946128	0.451458
41501	1.38752	0.455025
41502	1.27636	0.293244
41503	0.94613	0.26578
41504	0.94613	0.366444
41505	1.38752	0.468774
41506	1.38752	0.374959
41507	0.946131	0.361385
41508	1.38752	0.37227
41509	0.946131	0.438388
41510	1.27636	0.353232
41511	2.77504	0.447889
41512	1.99569	0.300329
41513	2.29794	0.346818
41514	1.99569	0.311617
41515	2.85497	0.331236
41516	2.55272	0.305772
41517	1.53284	2.1
41518	1.89226	2.1
41519	1.89226	2.1
41520	1.89226	2.1
41521	1.54763	2.1
41522	1.54763	2.1
41523	0.946131	1.84437
41524	0.946131	2.1
41525	0.946127	2.1

41526	0.946127	2.1
41527	0.946127	2.1
41528	0.946127	1.58656
41529	0.946131	2.1
41530	0.946131	2.1
41531	0.94613	2.1
41532	0.94613	2.1
41533	0.946129	2.1
41534	0.946129	2.1
41535	0.751754	2.1
41536	0.751754	2.1
41537	0.771562	2.1
41538	0.771561	2.1
41539	1.0268	2.1
41540	0.95482	2.1
41541	0.528937	2.1
41542	0.496512	2.1
41543	0.528936	2.1
41544	1.0268	2.1
41545	0.954821	2.1
41546	0.49651	2.1
41547	2.64075	0.52466
41548	1.35181	1.01035
41549	1.35181	0.443135
41550	1.83823	0.62839
41551	2.28373	0.610813
41552	2.88566	1.1763
41553	0.493018	0.962655
41554	0.675903	0.869458
41555	1.14187	0.567415
41556	0.973937	0.521865
41557	0.821166	0.595309
41558	0.821166	1.13515
41559	0.828674	1.8279
41560	0.828674	0.498322
41561	0.973937	0.714392
41562	0.493018	0.739012
41563	0.675903	0.65947
41564	1.14187	0.722604
41565	1.89226	2.1
41566	2.11667	2.1
41567	1.91184	2.1
41568	1.89226	2.1
41569	2.13625	2.1
41570	2.11667	2.1
41571	1.89226	2.1
41572	1.89226	2.1
41573	1.89226	2.1
41574	1.89226	2.1
41575	3.78453	2.1
41576	0.907729	2.1
41577	0.907729	2.1
41578	1.04459	2.1
41579	0.890955	2.1
41580	0.988181	2.1
41581	0.841856	2.1
41582	0.946133	2.1
41583	0.946133	2.1

41584	1.04072	2.1
41585	1.1064	2.1
41586	0.89099	2.1
41587	0.851541	2.1
41588	1.89225	2.1
41589	2.13623	2.1
41590	1.91044	2.1
41591	1.89225	2.1
41592	2.15442	2.1
41593	2.13623	2.1
41594	1.89225	2.1
41595	1.89225	2.1
41596	1.89225	2.1
41597	1.89225	2.1
41598	1.89225	2.1
41599	1.89225	2.1
41600	0.907723	2.1
41601	0.907723	2.1
41602	1.07536	2.1
41603	1.07814	2.1
41604	0.998499	2.1
41605	1.03575	2.1
41606	0.946126	2.1
41607	0.946126	2.1
41608	1.04072	2.1
41609	1.14025	2.1
41610	0.851528	2.1
41611	0.898284	2.1
41612	1.89226	0.165539
41613	1.89226	0.277444
41614	1.89226	0.208616
41615	1.89226	0.207664
41616	1.89226	0.193942
41617	1.89226	0.19345
41618	2.53008	2.1
41619	1.94534	2.1
41620	1.98702	2.1
41621	1.98858	2.1
41622	2.52852	2.1
41623	2.53008	2.1
41624	1.03267	2.1
41625	1.12591	2.1
41626	1.2959	2.1
41627	1.28992	2.1
41628	1.10851	2.1
41629	1.33078	2.1
41630	1.10661	2.1
41631	1.10661	2.1
41632	0.982208	2.1
41633	1.28992	2.1
41634	1.03267	2.1
41635	1.23262	2.1
41636	2.2712	2.1
41637	1.89226	2.1
41638	1.95954	2.1
41639	1.95954	2.1
41640	1.89226	2.1
41641	1.89226	2.1

41642	1.89226	2.1
41643	1.89226	2.1
41644	1.89226	2.1
41645	1.89226	2.1
41646	3.78453	2.1
41647	1.33887	2.1
41648	1.25395	2.1
41649	0.88327	2.1
41650	0.676665	2.1
41651	0.84881	2.1
41652	0.987503	2.1
41653	1.13243	2.1
41654	1.13243	2.1
41655	1.15338	2.1
41656	0.849966	2.1
41657	0.793074	2.1
41658	1.11957	2.1
41659	0.946129	2.1
41660	0.946129	2.1
41661	1.28254	2.1
41662	1.1356	2.1
41663	1.1356	2.1
41664	1.1356	2.1
41665	0.946131	2.1
41666	0.946131	2.1
41667	0.946131	2.1
41668	1.28254	2.1
41669	1.1356	2.1
41670	0.946131	2.1
41671	1.89227	2.1
41672	1.89227	2.1
41673	1.89227	2.1
41674	1.89227	2.1
41675	3.78453	2.1
41676	0.936287	0.0890331
41677	1.01399	0.0791955
41678	0.959415	0.0961855
41679	0.959415	0.106281
41680	0.910786	0.105035
41681	1.38301	0.0877575
41682	1.0286	0.090479
41683	0.535742	0.0794621
41684	0.505202	0.0944406
41685	0.937685	0.0915891
41686	1.11066	0.0916578
41687	1.46907	0.0874587
41688	1.00546	0.11144
41689	1.01524	0.109055
41690	0.94613	0.14441
41691	0.94613	0.133127
41692	1.05018	0.138048
41693	0.914025	0.103934
41694	0.913512	0.128536
41695	0.851542	0.145233
41696	1.05385	0.1281
41697	1.05494	0.107764
41698	0.91404	0.132786
41699	0.91404	0.122955

41700	2.03253	0.672097
41701	1.82325	0.359171
41702	1.93938	0.633335
41703	1.73756	0.272126
41704	3.77009	0.436796
41705	1.82325	0.352209
41706	1.47562	0.56793
41707	1.73756	0.325298
41708	1.3868	0.432806
41709	3.21317	0.349567
41710	1.93938	0.642806
41711	1.73756	0.397212
41712	1.85293	0.615296
41713	1.64279	0.262178
41714	3.59049	0.462735
41715	1.73756	0.403962
41716	1.3868	0.506777
41717	1.64279	0.335917
41718	1.28419	0.525442
41719	3.02174	0.428731
41720	1.35048	1.79363
41721	1.37999	2.03883
41722	1.34355	0.948746
41723	1.37854	2.04032
41724	2.72354	1.48755
41725	1.89226	1.14827
41726	1.89226	2.02795
41727	1.89226	0.881116
41728	1.89226	1.31485
41729	3.78452	1.03634
41730	1.89226	1.29292
41731	1.89226	2.1
41732	1.89226	0.822108
41733	1.89226	2.1
41734	3.78453	1.82519
41735	1.37999	1.35375
41736	1.18059	2.1
41737	1.17117	2.09899
41738	1.17262	2.1
41739	1.37855	1.25315
41740	1.37999	2.1
41741	1.34355	1.5717
41742	1.37854	2.1
41743	1.33079	0.957669
41744	1.3741	1.92327
41745	2.71765	1.50702
41746	1.89226	1.4054
41747	1.89226	1.87437
41748	1.89226	0.811194
41749	1.89226	1.93566
41750	3.78453	1.52248
41751	1.89226	1.02987
41752	1.89226	2.1
41753	1.89226	1.11287
41754	1.89226	2.02773
41755	3.78453	1.63714
41756	0.640305	2.1
41757	0.640305	1.463

41758	0.643507	0.772495
41759	0.643507	2.1
41760	0.590294	2.1
41761	0.594539	2.1
41762	0.689273	1.04344
41763	0.687048	1.51307
41764	0.687048	2.00077
41765	0.594538	1.28788
41766	0.590294	2.07839
41767	0.689273	2.1
41768	2.01399	0.411315
41769	1.70141	0.451737
41770	1.83896	0.654118
41771	1.52482	0.769991
41772	3.53881	0.484613
41773	1.70141	0.486812
41774	1.0752	0.892989
41775	1.0752	0.491215
41776	1.25669	0.611813
41777	1.52482	0.563547
41778	1.70632	1.18679
41779	1.83896	0.480756
41780	1.52482	0.438101
41781	1.65244	0.489267
41782	1.34412	0.759664
41783	3.17725	0.491707
41784	1.26782	0.169044
41785	2.90353	0.177255
41786	2.54203	0.156256
41787	2.41979	0.208776
41788	1.77007	0.152272
41789	1.703	0.191697
41790	0.946118	0.255833
41791	0.946118	0.217836
41792	1.38643	0.222012
41793	1.38644	0.177819
41794	0.946117	0.251482
41795	0.946117	0.267171
41796	1.38644	0.177934
41797	1.15545	0.207048
41798	0.946131	0.229401
41799	1.15546	0.20682
41800	1.38642	0.222019
41801	0.946105	0.243553
41802	2.42256	0.459552
41803	2.63056	0.389889
41804	2.43174	0.396331
41805	2.25035	0.359859
41806	4.8543	0.366095
41807	2.58031	0.455448
41808	2.63056	0.400458
41809	2.43174	0.38839
41810	2.32127	0.320308
41811	5.01205	0.427769
41812	2.43174	0.389633
41813	2.25035	0.339314
41814	2.11465	0.310616
41815	2.13042	0.269437

41816	4.54639	0.344308
41817	2.43174	0.382982
41818	2.32127	0.306315
41819	1.92997	0.287296
41820	2.13041	0.278634
41821	4.36171	0.295035
41822	1.89227	2.1
41823	1.89227	2.1
41824	1.89227	2.1
41825	1.89227	2.1
41826	3.78453	2.1
41827	0.568724	2.1
41828	0.568724	2.1
41829	0.57678	2.1
41830	0.57678	2.1
41831	0.523796	2.1
41832	0.548137	2.1
41833	0.597368	2.1
41834	0.637877	2.1
41835	0.597367	2.1
41836	0.523796	2.1
41837	0.637877	2.1
41838	0.548137	2.1
41839	1.32674	2.1
41840	1.32674	2.1
41841	0.946132	2.1
41842	0.946132	2.1
41843	1.3195	2.1
41844	1.32674	2.1
41845	0.946132	2.1
41846	0.946132	2.1
41847	0.946132	2.1
41848	1.3195	2.1
41849	1.32674	2.1
41850	0.946133	2.1
41851	1.89227	2.1
41852	1.89227	2.1
41853	1.89227	2.1
41854	1.89227	2.1
41855	3.78453	2.1
41856	1.19473	2.1
41857	0.987132	2.1
41858	0.987132	2.1
41859	1.04759	2.1
41860	1.0894	2.1
41861	1.14986	2.1
41862	1.3195	2.1
41863	1.3195	2.1
41864	0.946132	2.1
41865	0.946132	2.1
41866	1.31185	2.1
41867	1.3195	2.1
41868	0.946132	2.1
41869	0.946132	2.1
41870	1.31185	2.1
41871	0.946133	2.1
41872	0.946132	2.1
41873	1.3195	2.1

41874	1.11256	0.964698
41875	1.70101	0.939493
41876	1.22297	0.971752
41877	1.22297	0.963177
41878	1.7252	1.00886
41879	1.7252	0.99195
41880	1.99298	0.95042
41881	1.70101	0.987093
41882	1.7252	0.995195
41883	2.18701	0.853596
41884	3.71818	0.891333
41885	1.03218	0.913244
41886	1.7252	0.867018
41887	1.11256	0.929181
41888	1.11256	0.895516
41889	1.7457	0.945386
41890	1.7457	0.908942
41891	1.7457	0.913719
41892	2.187	0.766949
41893	1.7252	0.87446
41894	1.7252	0.877232
41895	2.27351	0.872102
41896	2.27351	0.875823
41897	1.95544	0.948
41898	1.35897	1.71085
41899	1.44859	1.27563
41900	1.44859	1.4154
41901	1.80709	1.06629
41902	1.80709	1.3887
41903	1.80709	0.859991
41904	1.17822	1.58925
41905	1.35897	1.26334
41906	1.35897	1.59603
41907	1.52782	0.840695
41908	1.52782	1.38364
41909	1.11978	1.34864
41910	0.903546	0.98175
41911	0.977719	1.46055
41912	1.2364	1.04053
41913	1.03812	0.874981
41914	1.03812	1.62619
41915	1.05938	0.762914
41916	1.05938	1.49784
41917	1.11978	1.109
41918	1.2364	0.804042
41919	0.97772	1.2694
41920	0.903546	1.11407
41921	1.80709	1.48879
41922	1.81847	1.21024
41923	1.96029	1.54026
41924	1.52782	1.32896
41925	2.23956	1.05828
41926	1.80709	1.18284
41927	1.89226	0.427783
41928	2.32962	0.284624
41929	2.5992	0.287894
41930	1.89226	0.306928
41931	2.5992	0.412059

41932	1.89226	0.4197
41933	2.55791	0.0964944
41934	2.47141	0.077611
41935	2.13515	0.0758594
41936	2.10939	0.0956606
41937	4.66731	0.0866518
41938	2.47141	0.0781836
41939	2.23337	0.0936626
41940	2.10939	0.0961906
41941	2.03438	0.0772812
41942	4.34276	0.0835985
41943	2.13512	0.0816622
41944	1.71027	0.100535
41945	1.71027	0.0985073
41946	2.10936	0.0859251
41947	1.60137	0.097295
41948	2.00046	0.0829213
41949	1.71027	0.102176
41950	2.03435	0.0799045
41951	1.71027	0.0943142
41952	2.10936	0.0864377
41953	1.72067	0.0928048
41954	2.11976	0.083822
41955	1.177	0.460956
41956	1.36405	0.854873
41957	2.25635	0.914001
41958	2.60151	0.468582
41959	3.6204	0.739761
41960	0.658869	0.306809
41961	0.781697	0.324806
41962	0.637037	0.262279
41963	0.541148	0.262364
41964	0.654682	0.255138
41965	0.654682	0.315472
41966	0.641224	0.364881
41967	0.641224	0.243651
41968	0.637039	0.305522
41969	0.781696	0.29548
41970	0.658867	0.30721
41971	0.541149	0.300293
41972	1.89226	0.345513
41973	1.59152	0.22571
41974	1.89226	0.257838
41975	1.82125	0.236968
41976	1.89226	0.309976
41977	1.82125	0.281163
41978	2.18967	0.111246
41979	1.89227	0.155841
41980	2.72752	0.143332
41981	2.72752	0.137445
41982	1.89227	0.125514
41983	1.89227	0.105885
41984	2.18967	0.107719
41985	1.92547	0.152798
41986	1.92547	0.128553
41987	2.72752	0.112315
41988	1.30572	0.12561
41989	2.10777	0.10909

41990	2.39663	2.1
41991	1.92293	2.1
41992	1.96889	2.1
41993	2.45928	2.1
41994	4.36552	2.1
41995	1.02605	2.1
41996	1.92293	2.1
41997	1.9689	2.1
41998	1.04679	2.1
41999	2.99495	2.1
42000	1.96889	2.1
42001	2.45928	2.1
42002	2.50138	2.1
42003	1.99782	2.1
42004	4.47027	2.1
42005	1.9689	2.1
42006	1.03449	2.1
42007	1.07572	2.1
42008	1.04679	2.1
42009	1.99783	2.1
42010	1.9689	2.1
42011	1.72162	0.782924
42012	1.89186	0.593158
42013	2.29771	0.657016
42014	1.80149	0.480531
42015	4.01933	0.601185
42016	1.16482	0.817685
42017	1.97003	0.528455
42018	1.72162	0.495079
42019	1.72162	0.593193
42020	1.29313	0.613975
42021	1.29313	0.769823
42022	2.29771	0.703944
42023	2.84159	0.441742
42024	2.19622	0.51931
42025	1.97366	0.51292
42026	2.50906	0.572645
42027	2.29771	0.587435
42028	1.29313	0.76268
42029	2.4483	0.518123
42030	2.14223	0.511423
42031	2.14228	0.584278
42032	1.294	0.586263
42033	1.294	0.685376
42034	1.21495	0.0846301
42035	1.70303	0.102427
42036	1.7924	0.0871033
42037	1.64002	0.0918479
42038	3.11963	0.0878097
42039	2.81021	0.0830068
42040	1.89226	0.108338
42041	1.89226	0.0901974
42042	1.89226	0.0932452
42043	2.36406	0.0917547
42044	2.36406	0.0947895
42045	1.89226	0.09309
42046	1.89226	0.126878
42047	1.89226	0.10073

42048	1.89226	0.107785
42049	1.89226	0.102903
42050	1.89226	0.110693
42051	2.29976	0.0832649
42052	1.64002	0.0951966
42053	1.8959	0.098986
42054	1.90873	0.0853659
42055	1.85594	0.0999664
42056	1.85594	0.0891934
42057	1.89226	0.0964078
42058	1.89226	0.129521
42059	1.89226	0.102004
42060	1.89226	0.111224
42061	3.78453	0.106505
42062	1.89226	0.823811
42063	2.52823	2.1
42064	2.83441	1.42785
42065	2.83441	2.01137
42066	1.89226	1.18236
42067	1.89226	1.5168
42068	1.04522	1.59323
42069	1.04522	1.39996
42070	1.12831	1.62605
42071	1.18364	2.1
42072	0.640525	1.55301
42073	0.924882	1.59734
42074	1.27877	1.73336
42075	1.43363	0.828662
42076	0.93474	1.61679
42077	1.58926	1.36425
42078	1.29573	1.67571
42079	0.664247	1.76375
42080	2.02191	2.1
42081	1.89226	2.1
42082	1.89226	2.1
42083	1.89226	2.1
42084	2.58639	2.1
42085	2.58639	2.1
42086	1.16759	2.1
42087	2.11828	2.1
42088	1.60936	2.1
42089	2.35846	2.1
42090	3.52605	2.1
42091	1.89226	2.1
42092	1.89226	2.1
42093	1.89226	2.1
42094	1.89226	2.1
42095	3.78453	2.1
42096	2.82765	0.278681
42097	1.89809	0.254786
42098	2.15971	0.292155
42099	1.89809	0.257131
42100	2.29233	0.26293
42101	2.03071	0.224357
42102	1.90664	0.281912
42103	2.15971	0.280264
42104	1.28956	0.240148
42105	1.28956	0.242573

42106	2.64414	0.264671
42107	2.02706	0.233107
42108	2.82765	0.243602
42109	1.89226	0.268086
42110	2.39968	0.246206
42111	2.39968	0.257378
42112	1.89226	0.266116
42113	1.89226	0.270982
42114	0.978643	2.1
42115	0.703968	2.1
42116	0.946129	2.1
42117	0.946129	2.1
42118	0.703939	2.1
42119	0.703939	2.09611
42120	0.946159	2.1
42121	0.946159	2.1
42122	0.978642	2.1
42123	0.94613	2.1
42124	0.94613	2.1
42125	0.703968	2.1
42126	1.89226	2.1
42127	2.17309	1.09113
42128	2.17309	2.02042
42129	1.47475	1.71942
42130	1.89226	1.93662
42131	1.19393	1.23298
42132	1.89226	2.08732
42133	1.89226	2.1
42134	1.89226	2.1
42135	1.89226	1.87593
42136	3.78452	2.1
42137	1.17426	2.1
42138	0.978644	2.1
42139	0.946129	2.1
42140	0.946129	2.1
42141	0.946131	2.1
42142	0.946131	2.1
42143	0.978643	2.1
42144	0.978642	2.1
42145	1.17426	2.1
42146	0.946131	2.1
42147	0.978642	2.1
42148	0.94613	2.1
42149	1.89226	2.1
42150	1.89226	1.09059
42151	1.89226	1.94765
42152	2.17309	1.20812
42153	2.56346	1.87695
42154	2.94389	1.46157
42155	0.946131	2.1
42156	0.946131	1.89459
42157	0.946128	2.1
42158	0.946128	2.09919
42159	0.946131	2.08556
42160	0.946131	1.88783
42161	0.946128	2.1
42162	0.946128	2.1
42163	0.94613	2.08401

42164	0.94613	2.07277
42165	0.946129	2.07243
42166	0.946129	2.05426
42167	1.89226	0.7713
42168	1.89226	0.716197
42169	1.89226	0.906367
42170	1.89226	0.862329
42171	3.78453	0.632
42172	1.89226	0.899899
42173	1.89226	0.722175
42174	1.89226	1.09254
42175	1.89226	1.00185
42176	1.89226	0.727811
42177	1.89226	0.687498
42178	1.89226	0.754869
42179	1.30733	0.881979
42180	1.89226	1.12116
42181	1.92403	1.02286
42182	3.38635	0.702231
42183	1.89226	0.749288
42184	3.02887	1.14522
42185	2.0815	0.602938
42186	1.88422	0.766396
42187	2.0815	0.909984
42188	1.88422	1.5347
42189	1.89226	1.239
42190	1.70221	0.885057
42191	1.89226	1.66885
42192	1.20068	1.24677
42193	1.89226	0.905726
42194	1.20068	0.738323
42195	1.01393	1.07343
42196	1.20067	0.876956
42197	1.89226	1.55112
42198	2.44958	1.50515
42199	1.01393	0.717266
42200	1.89226	0.694036
42201	1.89226	2.1
42202	1.89226	1.52199
42203	1.89226	1.96493
42204	1.89226	2.1
42205	1.89226	2.04578
42206	1.89226	2.1
42207	1.89226	2.1
42208	1.89226	0.537282
42209	1.89226	0.617864
42210	1.89226	1.66028
42211	1.89226	0.672615
42212	1.89226	1.79284
42213	1.89226	2.1
42214	1.89226	1.3466
42215	1.89226	1.88931
42216	1.89226	2.1
42217	1.89226	1.95437
42218	1.89226	2.1
42219	1.91161	2.1
42220	2.45782	0.956264
42221	1.39653	2.1

42222	2.44652	1.21145
42223	4.35812	2.1
42224	2.73092	0.920496
42225	1.89226	0.576903
42226	1.89226	1.73655
42227	1.89226	0.553685
42228	2.44652	1.31909
42229	2.44652	0.505738
42230	1.89226	2.06123
42231	1.89226	0.650185
42232	1.89226	0.713876
42233	1.89226	1.5299
42234	1.89226	0.712217
42235	1.89226	1.60431
42236	2.64924	2.1
42237	1.31109	2.1
42238	1.31109	2.1
42239	1.49958	2.1
42240	1.95039	2.1
42241	2.14451	2.1
42242	0.825157	2.1
42243	0.634856	2.1
42244	0.816836	2.1
42245	0.816836	2.1
42246	0.556743	2.1
42247	0.639142	2.1
42248	0.985106	2.1
42249	0.795045	2.1
42250	0.967461	2.1
42251	0.552494	2.1
42252	0.676829	2.1
42253	0.98803	2.1
42254	2.28564	2.1
42255	2.34084	1.27654
42256	2.3502	1.33009
42257	2.42908	2.06882
42258	4.69104	1.51302
42259	2.34084	1.63503
42260	2.18077	1.98555
42261	2.42908	1.95735
42262	2.2947	0.940909
42263	4.63554	1.78249
42264	2.3502	1.44233
42265	2.42908	2.1
42266	2.38681	2.1
42267	2.50438	1.01426
42268	4.85458	2.1
42269	2.42908	2.1
42270	2.2947	1.30097
42271	2.50438	0.96201
42272	2.40627	1.95856
42273	4.79908	1.09825
42274	1.89226	0.739832
42275	2.20711	1.18042
42276	1.89226	0.740213
42277	1.89226	1.34001
42278	2.02693	0.784816
42279	2.02693	1.52034

42280	1.2269	1.90855
42281	2.20711	1.11223
42282	2.0815	1.03208
42283	1.11506	2.1
42284	3.22109	1.67335
42285	0.545802	1.50719
42286	0.547489	1.84789
42287	0.85914	1.13677
42288	1.11641	1.17564
42289	0.899974	1.24919
42290	0.849893	1.95128
42291	0.747885	1.54445
42292	0.747885	1.02923
42293	0.557184	1.80247
42294	0.887039	1.79358
42295	1.08948	1.68572
42296	0.59815	1.75492
42297	0.974139	2.1
42298	1.33385	2.1
42299	1.02368	2.1
42300	1.02368	2.1
42301	1.00833	2.1
42302	1.00833	2.1
42303	1.89226	0.507598
42304	1.89226	0.370128
42305	1.89226	0.513376
42306	1.89226	0.278752
42307	3.78453	0.353337
42308	1.74651	0.510182
42309	1.44605	0.353323
42310	1.63557	0.5213
42311	1.31993	0.129095
42312	3.08162	0.328956
42313	1.89226	0.510005
42314	1.89226	0.382968
42315	1.89226	0.48998
42316	1.89226	0.120115
42317	3.78452	0.299734
42318	1.63557	0.530101
42319	1.31992	0.179203
42320	1.52615	0.49463
42321	1.18549	0.118626
42322	2.82106	0.246428
42323	1.89226	0.307049
42324	2.58101	0.388019
42325	2.73797	0.512148
42326	2.73797	0.366474
42327	1.89226	0.516548
42328	1.89226	0.345125
42329	1.91762	0.565258
42330	1.44354	0.536298
42331	1.33622	0.532888
42332	1.33622	0.521364
42333	2.20248	0.597296
42334	2.20248	0.572185
42335	1.11712	2.1
42336	1.11712	2.1
42337	1.11421	2.1

42338	1.11421	2.1
42339	0.833871	2.1
42340	0.926528	2.1
42341	1.3048	2.1
42342	1.40335	2.1
42343	0.926527	2.1
42344	1.40335	2.1
42345	1.3048	2.1
42346	0.833871	2.1
42347	0.879657	2.1
42348	0.937695	2.1
42349	0.946129	2.1
42350	0.946129	2.1
42351	0.851541	2.1
42352	0.851541	2.1
42353	1.1126	2.1
42354	1.04519	2.1
42355	0.851486	2.1
42356	0.980501	2.1
42357	0.898251	2.1
42358	0.851528	2.1
42359	1.37756	2.1
42360	2.73081	2.1
42361	2.44311	2.1
42362	2.44311	2.1
42363	1.66428	2.1
42364	1.66428	2.1
42365	1.89226	2.1
42366	1.89226	1.50555
42367	1.89226	2.1
42368	1.89226	1.45005
42369	3.78452	2.1
42370	1.65781	2.1
42371	1.89837	1.58641
42372	1.46713	2.1
42373	1.98027	1.35719
42374	3.63808	2.1
42375	2.29054	1.58684
42376	2.26369	0.534074
42377	1.98027	1.30793
42378	2.15617	0.34192
42379	4.24396	0.746563
42380	1.89226	1.58218
42381	1.89226	0.529339
42382	1.89226	1.41824
42383	1.89226	0.576365
42384	3.78452	0.784957
42385	0.946131	1.32074
42386	0.946131	2.1
42387	0.946128	2.1
42388	0.946128	1.64725
42389	0.946129	1.31831
42390	0.946129	1.34513
42391	0.94613	2.1
42392	0.94613	2.1
42393	0.94613	2.1
42394	0.94613	2.1
42395	0.94613	2.1

42396	0.94613	2.1
42397	0.812186	1.43337
42398	0.812186	2.1
42399	0.819781	1.44931
42400	0.819781	2.1
42401	0.913532	1.24189
42402	0.990134	1.26551
42403	0.733564	2.1
42404	0.641833	2.1
42405	0.641832	2.1
42406	0.913533	2.1
42407	0.733564	2.1
42408	0.990134	2.1
42409	0.994262	0.441336
42410	0.994262	1.5798
42411	0.997354	1.10024
42412	0.997354	0.469852
42413	1.00775	0.355629
42414	1.07808	0.434173
42415	0.913533	1.30653
42416	0.990135	1.29606
42417	0.913532	0.72901
42418	1.00775	0.727678
42419	0.990134	0.715306
42420	1.07808	0.713777
42421	0.946131	1.58214
42422	0.946131	0.549603
42423	0.946128	1.21816
42424	0.946128	0.550877
42425	0.946128	0.483689
42426	0.946128	0.465834
42427	0.94613	1.3803
42428	0.94613	1.41236
42429	0.946129	0.797638
42430	0.946129	0.795106
42431	0.946129	0.702269
42432	0.946129	0.71437
42433	0.816232	2.1
42434	0.760038	2.1
42435	0.845999	2.1
42436	0.845999	2.1
42437	1.0631	2.1
42438	1.28131	2.1
42439	0.474065	2.1
42440	0.591905	2.1
42441	0.601756	2.1
42442	1.23903	2.1
42443	0.988592	2.1
42444	0.49051	2.1
42445	2.57021	2.1
42446	1.89226	1.8274
42447	2.68172	2.1
42448	1.89226	1.02468
42449	4.46247	1.56312
42450	1.89226	2.06973
42451	1.89226	1.63434
42452	1.89226	1.34789
42453	1.89226	1.29613

42454	3.78452	1.46361
42455	1.12573	2.1
42456	1.65504	1.236
42457	1.38848	1.63341
42458	1.38848	2.04834
42459	1.30567	0.979035
42460	1.30567	1.73625
42461	2.82413	2.1
42462	1.89226	1.10914
42463	2.68172	1.96278
42464	2.68172	2.1
42465	1.89226	1.50056
42466	1.89226	2.1
42467	1.89226	1.42136
42468	1.89226	1.61459
42469	1.89226	1.90115
42470	1.89226	0.947346
42471	3.78452	1.44562
42472	1.30567	2.1
42473	1.65504	1.52634
42474	1.52724	1.87967
42475	1.89249	1.69755
42476	3.18228	1.94308
42477	2.57386	0.238489
42478	1.89226	0.419777
42479	1.89226	0.429769
42480	2.66753	0.264172
42481	1.89226	0.367603
42482	2.66753	0.256786
42483	1.89226	0.23388
42484	1.89226	0.475353
42485	2.2973	0.466461
42486	3.12479	0.291776
42487	1.89226	0.415813
42488	2.57385	0.274093
42489	0.489293	1.98423
42490	0.672698	2.1
42491	0.59038	2.1
42492	0.738357	2.1
42493	0.62269	2.1
42494	0.62269	2.07496
42495	0.640388	2.1
42496	0.640388	2.1
42497	0.59038	2.1
42498	0.489293	2.1
42499	0.738357	2.1
42500	0.672698	2.1
42501	1.89226	2.06628
42502	1.89226	1.04056
42503	1.89226	2.1
42504	1.89226	0.706337
42505	3.78452	1.48371
42506	2.82858	1.53823
42507	1.89226	0.761756
42508	2.5671	2.1
42509	1.89226	2.1
42510	2.5671	2.0382
42511	1.89226	1.21026

42512	2.06581	0.0760992
42513	2.47308	0.0949544
42514	1.89433	0.0907339
42515	1.67571	0.077399
42516	3.87274	0.0846139
42517	2.55791	0.0953217
42518	2.10276	0.0753672
42519	2.47308	0.0948922
42520	2.47308	0.0877195
42521	1.83415	0.080032
42522	1.90922	0.079793
42523	1.18421	0.0947642
42524	2.03277	0.0803321
42525	1.18421	0.0989007
42526	2.01275	0.0844633
42527	1.42818	0.0967068
42528	2.25673	0.081376
42529	0.882904	0.0793687
42530	0.958492	0.0795583
42531	0.905756	0.0953977
42532	0.905756	0.097774
42533	1.06352	0.0817131
42534	0.797654	0.0979156
42535	1.11319	0.0807076
42536	0.716795	0.0975088
42537	0.820721	0.0903177
42538	0.731963	0.0898361
42539	1.13143	0.0851521
42540	1.10528	0.0870853
42541	1.89226	0.254841
42542	1.89226	0.195415
42543	1.89226	0.238924
42544	1.89226	0.275208
42545	1.89226	0.164699
42546	1.89226	0.205428
42547	1.01241	0.290085
42548	0.703929	0.335325
42549	0.946133	0.274831
42550	0.946133	0.262125
42551	1.04072	0.284995
42552	1.163	0.193526
42553	0.639952	0.347883
42554	0.851543	0.198755
42555	0.946132	0.16931
42556	0.920761	0.230414
42557	0.711038	0.321816
42558	0.711038	0.339954
42559	1.89226	0.218914
42560	1.89226	0.156756
42561	1.89226	0.187041
42562	1.89226	0.231551
42563	1.89226	0.144545
42564	1.89226	0.186513
42565	1.89226	0.145378
42566	1.89226	0.164442
42567	2.41075	0.131681
42568	1.89226	0.114074
42569	3.78451	0.155509

42570	0.968476	1.61933
42571	1.20674	0.9217
42572	1.13456	0.916587
42573	1.13456	1.92716
42574	1.02074	0.776693
42575	1.02074	1.55249
42576	1.02074	2.1
42577	1.20674	1.57023
42578	1.07866	1.126
42579	1.28649	0.987463
42580	2.2854	1.66332
42581	1.94221	0.412925
42582	1.36717	0.368591
42583	1.45535	0.396559
42584	1.93771	0.333425
42585	3.39756	0.409796
42586	1.94222	0.464597
42587	2.25035	0.360991
42588	2.41806	0.404529
42589	2.42256	0.373824
42590	1.93772	0.417312
42591	1.94222	0.395626
42592	1.49906	0.277967
42593	1.93771	0.308345
42594	1.45535	0.344803
42595	1.45535	0.368476
42596	1.91484	0.242082
42597	1.91484	0.257597
42598	1.91484	0.291318
42599	2.25035	0.342065
42600	1.93772	0.352251
42601	1.93772	0.364173
42602	2.11465	0.262368
42603	2.11465	0.264071
42604	2.61735	0.330224
42605	1.5082	0.233941
42606	2.36481	0.393698
42607	1.58597	0.3379
42608	2.36481	0.311036
42609	1.46653	0.244347
42610	2.36482	0.420266
42611	1.08133	0.458896
42612	1.5082	0.225146
42613	2.01868	0.456203
42614	3.52688	0.443217
42615	0.94613	0.304261
42616	0.94613	0.303511
42617	0.946131	0.406722
42618	0.946131	0.487961
42619	0.946129	0.483384
42620	0.946129	0.344298
42621	0.946132	0.315392
42622	0.946132	0.403258
42623	0.94613	0.386592
42624	0.94613	0.378119
42625	0.946131	0.363522
42626	0.946131	0.35498
42627	2.53725	0.196981

42628	1.89227	0.252321
42629	1.89227	0.241716
42630	2.27073	0.23804
42631	1.89227	0.210219
42632	2.27073	0.20463
42633	1.89227	0.316274
42634	1.89227	0.481817
42635	1.89227	0.509973
42636	1.89227	0.3826
42637	1.89227	0.441355
42638	1.89227	0.326361
42639	1.86766	2.1
42640	2.83487	2.1
42641	2.84318	2.1
42642	2.59401	2.1
42643	2.11684	2.1
42644	1.86766	2.1
42645	1.10638	2.1
42646	2.11684	2.1
42647	1.29111	2.1
42648	2.37439	2.1
42649	3.40796	2.1
42650	2.56843	2.1
42651	1.89226	2.1
42652	1.89226	2.1
42653	2.71932	2.1
42654	4.46069	2.1
42655	1.89226	2.1
42656	1.89226	2.1
42657	1.89226	2.1
42658	1.89226	2.1
42659	3.78452	2.1
42660	0.6416	2.1
42661	0.697585	2.1
42662	0.670706	2.1
42663	0.598927	2.1
42664	0.644313	2.1
42665	0.644313	2.1
42666	0.652199	2.1
42667	0.652199	2.1
42668	0.697587	2.1
42669	0.670705	2.1
42670	0.598926	2.1
42671	0.641601	2.1
42672	1.89226	2.1
42673	1.89226	2.1
42674	1.89226	2.1
42675	1.89226	2.1
42676	1.89226	2.1
42677	1.89226	2.1
42678	0.697585	2.1
42679	0.730617	2.1
42680	0.712082	2.1
42681	0.670706	2.1
42682	0.69854	2.1
42683	0.69854	2.1
42684	0.702783	2.1
42685	0.702783	2.1

42686	0.730618	2.1
42687	0.712081	2.1
42688	0.697586	2.1
42689	0.670705	2.1
42690	1.89226	1.2806
42691	1.89226	1.48802
42692	1.89226	0.848276
42693	1.89226	2.08978
42694	1.89226	0.798578
42695	1.89226	2.1
42696	2.0815	1.82108
42697	1.12076	2.09793
42698	1.38662	1.0858
42699	2.01244	2.1
42700	3.41508	1.90366
42701	1.89226	2.1
42702	2.54324	2.1
42703	1.91981	2.1
42704	1.89226	2.1
42705	2.57079	2.1
42706	2.54324	2.1
42707	2.54325	2.1
42708	1.94779	2.1
42709	1.76879	2.1
42710	1.73811	2.1
42711	2.5708	2.1
42712	2.54325	2.1
42713	1.89226	2.1
42714	2.57079	2.1
42715	1.91894	2.1
42716	1.89226	2.1
42717	2.59747	2.1
42718	2.57079	2.09164
42719	2.5708	2.1
42720	1.82268	2.1
42721	1.79738	2.1
42722	1.77071	2.1
42723	2.59747	2.1
42724	2.5708	2.1
42725	1.89226	0.45822
42726	1.89226	0.595971
42727	1.89226	0.340407
42728	1.89226	0.641695
42729	3.78451	0.505132
42730	2.21582	0.382784
42731	1.94412	0.58594
42732	2.13428	0.317277
42733	1.86292	0.543155
42734	4.07839	0.475681
42735	1.94412	0.49969
42736	1.5854	1.91371
42737	1.86293	0.558955
42738	1.50767	1.62731
42739	3.45179	0.72821
42740	1.89226	0.519564
42741	1.89226	2.06719
42742	1.89226	0.582004
42743	1.89226	1.64994

42744	3.78452	0.705057
42745	1.89226	0.403071
42746	1.89226	0.774553
42747	1.89226	0.31109
42748	1.89226	0.582135
42749	3.78452	0.488626
42750	2.13428	0.382954
42751	1.86293	0.520229
42752	2.04737	0.253337
42753	1.77122	0.57567
42754	3.9055	0.462263
42755	1.86293	0.415259
42756	1.50767	1.61232
42757	1.41077	0.566179
42758	1.41077	0.876015
42759	1.77123	0.569721
42760	1.86813	0.889121
42761	0.810943	0.582465
42762	0.810943	1.9031
42763	0.810945	0.56433
42764	0.810945	0.559033
42765	0.810958	0.687174
42766	0.810958	0.671503
42767	1.89226	1.62681
42768	1.35168	0.734354
42769	1.08132	0.578576
42770	1.08132	1.62771
42771	1.0813	0.690375
42772	0.745979	2.1
42773	0.745979	2.1
42774	0.778781	2.1
42775	0.778781	2.1
42776	0.59549	2.1
42777	0.649658	2.1
42778	0.994784	2.1
42779	0.875102	2.1
42780	0.875102	2.1
42781	0.59549	2.1
42782	0.649658	2.1
42783	0.994784	2.1
42784	1.57887	2.1
42785	1.75021	2.1
42786	1.13341	2.1
42787	1.13341	2.1
42788	1.19098	2.1
42789	1.63644	2.1
42790	2.5761	0.287179
42791	2.43404	0.500357
42792	2.44813	0.291793
42793	2.31751	0.517767
42794	4.88218	0.389653
42795	2.43405	0.396008
42796	2.22575	0.799502
42797	2.31751	0.486058
42798	2.10893	0.74714
42799	4.54298	0.60695
42800	2.44813	0.304422
42801	2.31751	0.509749

42802	2.30908	0.245217
42803	2.19815	0.496232
42804	4.64628	0.38656
42805	2.31751	0.396876
42806	2.10893	0.746841
42807	2.19815	0.43874
42808	1.98061	1.35173
42809	4.30708	0.585297
42810	0.94613	2.1
42811	0.94613	1.56083
42812	1.40372	1.73339
42813	1.40372	2.1
42814	1.40372	2.1
42815	1.37167	2.1
42816	0.946132	1.10123
42817	0.946132	2.00365
42818	0.946131	2.1
42819	1.40372	2.1
42820	0.94613	2.1
42821	1.37167	2.1
42822	2.80744	2.1
42823	2.06984	2.1
42824	2.83418	2.1
42825	2.74335	2.1
42826	2.16067	2.1
42827	2.06984	2.1
42828	1.077	0.30529
42829	0.809116	0.25154
42830	0.600419	0.352979
42831	0.790277	0.264556
42832	0.927647	0.238091
42833	0.758598	0.243411
42834	0.85749	0.311609
42835	0.85749	0.334067
42836	1.02417	0.26419
42837	0.600411	0.287126
42838	0.751701	0.2696
42839	1.07702	0.26632
42840	1.89226	2.1
42841	1.89226	2.1
42842	1.89226	2.1
42843	1.89226	2.1
42844	1.89226	2.1
42845	1.89226	2.1
42846	1.89226	2.1
42847	1.89226	1.91399
42848	1.89226	1.90129
42849	1.89226	2.1
42850	3.78453	2.1
42851	1.89226	2.1
42852	1.89226	2.1
42853	1.89226	2.1
42854	1.89226	2.1
42855	1.89226	2.1
42856	1.89226	2.1
42857	0.946131	2.1
42858	0.946131	2.1
42859	0.946131	2.06194

42860	0.946131	1.85354
42861	0.946133	2.1
42862	0.946133	1.82327
42863	0.946128	2.05377
42864	0.946128	2.1
42865	0.946131	2.1
42866	0.946131	2.1
42867	0.94613	2.1
42868	0.94613	2.1
42869	2.17159	2.08483
42870	2.00083	1.71337
42871	2.21305	2.09358
42872	2.00551	1.35382
42873	2.20837	1.88206
42874	2.00083	1.29042
42875	2.20837	1.46555
42876	1.80328	1.07695
42877	2.22962	1.90001
42878	2.00083	1.33516
42879	2.03207	0.999203
42880	1.80328	0.906125
42881	2.28564	2.1
42882	2.20837	1.42255
42883	2.30406	2.0399
42884	2.17159	1.81173
42885	2.34084	1.65364
42886	2.20837	1.67562
42887	1.09523	0.816185
42888	1.09523	1.71386
42889	1.09934	1.72242
42890	1.09934	2.08468
42891	1.01603	0.815735
42892	1.09038	1.72665
42893	1.10419	0.939874
42894	1.17042	2.1
42895	1.17042	1.72084
42896	1.09038	1.40092
42897	1.10419	1.12129
42898	1.01603	1.75544
42899	2.60152	0.485019
42900	0.976334	0.671722
42901	2.22344	0.712846
42902	2.22344	0.518159
42903	1.36405	0.778085
42904	1.36405	0.319338
42905	1.89226	1.74164
42906	1.70302	2.03498
42907	1.69019	2.1
42908	1.70302	1.67837
42909	1.73933	2.1
42910	1.70303	0.882685
42911	1.89226	2.09763
42912	1.89226	0.689219
42913	1.89226	1.00614
42914	1.89226	1.62923
42915	3.78452	1.13435
42916	1.89226	1.21691
42917	1.59036	1.56227

42918	1.65683	2.1
42919	1.59036	1.29208
42920	1.95873	1.77131
42921	1.89226	0.746969
42922	0.946131	0.66509
42923	0.946131	0.762791
42924	0.946127	1.55082
42925	0.946127	1.18349
42926	0.946127	1.16821
42927	0.946127	0.517129
42928	0.946131	1.43308
42929	0.946131	0.783
42930	0.946129	0.879605
42931	0.946129	0.885674
42932	0.94613	0.904394
42933	0.94613	0.884381
42934	0.851541	2.1
42935	0.851541	1.8785
42936	0.851483	2.1
42937	1.15713	2.1
42938	0.851539	2.1
42939	0.851539	1.15345
42940	0.942127	2.1
42941	1.14455	2.1
42942	0.838683	2.1
42943	0.946131	2.1
42944	0.957503	2.1
42945	0.957504	2.1
42946	1.89226	0.746689
42947	1.89226	0.388826
42948	1.89226	0.471887
42949	1.89226	1.39526
42950	3.78452	0.472486
42951	1.89226	0.179451
42952	1.89226	0.373911
42953	1.89226	0.418389
42954	1.89226	0.371264
42955	1.89226	0.193729
42956	1.89226	0.15682
42957	1.89226	0.343664
42958	1.89226	0.242748
42959	1.89226	0.308137
42960	1.89226	0.337823
42961	3.78453	0.299211
42962	1.89226	0.367165
42963	1.89226	2.1
42964	1.89226	1.56687
42965	1.89226	1.02931
42966	3.78453	1.42441
42967	1.22575	0.577336
42968	1.05438	0.431501
42969	0.946131	0.594262
42970	0.946131	0.509572
42971	0.946129	1.21304
42972	1.22575	0.365749
42973	1.05438	0.548035
42974	0.946132	0.446835
42975	1.05438	0.610599

42976	1.05438	0.786831
42977	0.946131	0.480587
42978	0.946131	0.611625
42979	2.4515	0.585676
42980	1.13726	0.193561
42981	2.10875	0.267683
42982	1.49515	0.220552
42983	2.10875	0.263882
42984	1.49515	0.163886
42985	0.831477	0.199395
42986	0.747575	0.249143
42987	1.22575	0.526134
42988	1.28174	0.256416
42989	1.28174	0.360507
42990	0.831476	0.21751
42991	1.22575	0.408911
42992	0.747575	0.185002
42993	1.03561	0.325147
42994	1.03561	0.444532
42995	1.02162	0.262026
42996	1.02162	0.243433
42997	1.89227	1.32267
42998	2.56348	0.667828
42999	1.89227	2.1
43000	2.45151	0.525175
43001	1.89227	2.1
43002	2.45151	0.706408
43003	1.40013	0.707806
43004	1.89226	1.58423
43005	1.89226	1.03728
43006	0.946643	0.60223
43007	1.89226	1.32113
43008	0.946643	0.921642
43009	1.89226	0.133968
43010	1.89226	0.191521
43011	1.89226	0.157503
43012	1.89226	0.182065
43013	3.78453	0.185561
43014	1.89226	0.23243
43015	1.89226	0.262571
43016	1.89226	0.21522
43017	1.89226	0.348251
43018	3.78453	0.2167
43019	0.946131	0.30208
43020	0.946131	0.257624
43021	0.946128	0.191477
43022	0.946128	0.213832
43023	0.94613	0.269619
43024	0.94613	0.303956
43025	0.946129	0.225405
43026	0.946129	0.195817
43027	0.94613	0.239044
43028	0.94613	0.175862
43029	0.94613	0.234957
43030	0.94613	0.282092
43031	1.89226	0.1304
43032	1.89226	0.14553
43033	1.17244	0.133266

43034	1.89226	0.145954
43035	0.975291	0.14061
43036	1.57408	0.152675
43037	0.946132	0.258503
43038	0.946132	0.162401
43039	0.946131	0.250977
43040	0.946131	0.180538
43041	0.946132	0.226451
43042	0.946132	0.246375
43043	0.946131	0.187817
43044	0.946131	0.191212
43045	0.946131	0.229118
43046	0.946131	0.219149
43047	0.946132	0.207865
43048	0.946132	0.230349
43049	2.74744	0.199946
43050	1.89226	0.344997
43051	2.70805	0.222615
43052	1.89226	0.260277
43053	2.70805	0.263151
43054	1.89226	0.327104
43055	2.74744	0.172711
43056	1.89226	0.264501
43057	2.57465	0.265764
43058	1.89226	0.349496
43059	4.6397	0.273617
43060	1.89226	0.312859
43061	1.89226	0.47157
43062	1.89226	0.449569
43063	1.89226	0.736426
43064	3.78452	0.427681
43065	1.89226	0.364995
43066	1.89226	0.613128
43067	1.89226	0.343828
43068	1.89226	0.273699
43069	1.89226	0.745499
43070	1.89226	0.681407
43071	2.16206	0.194918
43072	2.67861	0.188801
43073	3.11996	0.200579
43074	1.70303	0.165856
43075	2.89015	0.2156
43076	1.486 0.196377	
43077	2.12839	0.207838
43078	2.83631	0.272274
43079	3.08096	0.210052
43080	1.84157	0.181526
43081	3.08096	0.294166
43082	1.84157	0.241193
43083	1.89225	0.871727
43084	1.89225	0.578912
43085	1.89225	0.709005
43086	1.89225	0.918901
43087	1.89225	0.674687
43088	1.89225	0.866693
43089	1.89226	0.532631
43090	1.89226	0.577472
43091	1.89226	0.926191

43092	1.89226	0.475966
43093	3.78452	0.489146
43094	1.89226	0.39567
43095	2.49041	0.386784
43096	1.89226	0.395953
43097	1.89226	0.25098
43098	2.27073	0.248827
43099	2.27073	0.367082
43100	1.09904	0.241501
43101	0.677147	0.237817
43102	1.3365	0.224605
43103	0.95759	0.281539
43104	0.957591	0.244411
43105	0.677148	0.270182
43106	1.09904	0.251577
43107	1.3365	0.265003
43108	0.996015	0.263123
43109	0.996015	0.24726
43110	1.01764	0.300954
43111	1.01764	0.231987
43112	2.77349	0.336844
43113	2.72723	0.225821
43114	2.43156	0.34701
43115	2.4564	0.222835
43116	5.20505	0.315762
43117	2.77349	0.31673
43118	2.68508	0.206442
43119	2.33761	0.280928
43120	2.4564	0.217043
43121	5.1111	0.24354
43122	2.43156	0.248185
43123	2.70208	0.197588
43124	1.85779	0.284287
43125	2.10695	0.185124
43126	4.77572	0.199042
43127	2.33761	0.231126
43128	2.70208	0.194087
43129	1.85779	0.252302
43130	1.61597	0.171809
43131	4.19542	0.212914
43132	0.946132	1.24387
43133	0.946132	1.62346
43134	0.746729	1.46836
43135	0.746729	1.87793
43136	0.946132	1.92144
43137	0.915958	1.57838
43138	0.946133	1.22518
43139	0.74673	1.99105
43140	0.946133	1.55469
43141	0.946133	1.55642
43142	0.915958	1.58511
43143	0.746729	1.65037
43144	1.50743	2.1
43145	2.45501	1.50925
43146	2.29118	2.1
43147	2.29118	1.67727
43148	1.75482	2.1
43149	1.75482	1.72392

43150	2.45501	1.717
43151	2.76927	0.505069
43152	2.29118	1.63483
43153	2.64523	0.514486
43154	5.10024	0.799621
43155	1.25559	2.1
43156	2.29118	1.64483
43157	2.0815	2.1
43158	2.26387	1.65313
43159	1.35668	2.1
43160	1.47667	1.72957
43161	2.28481	1.36144
43162	2.64523	0.582215
43163	2.50057	1.40506
43164	2.50057	0.588739
43165	2.29118	1.50762
43166	2.29118	0.614796
43167	0.946129	0.388392
43168	0.946129	0.364616
43169	1.23929	0.332553
43170	1.15246	0.330693
43171	0.946131	0.347082
43172	0.946131	0.412703
43173	1.15246	0.359693
43174	1.15246	0.309863
43175	0.946131	0.350056
43176	1.15246	0.392424
43177	0.946131	0.335139
43178	1.23928	0.377342
43179	0.946128	0.427601
43180	0.946128	0.415391
43181	1.15246	0.336938
43182	0.942814	0.443319
43183	0.946131	0.360316
43184	0.946131	0.438587
43185	0.961347	0.392984
43186	0.942811	0.351792
43187	0.946131	0.357194
43188	0.961347	0.318275
43189	0.946131	0.298218
43190	1.15246	0.369002
43191	2.32334	0.411319
43192	1.57621	0.274532
43193	1.90335	0.28694
43194	1.24538	0.301946
43195	3.47956	0.258145
43196	0.965762	0.206288
43197	0.965762	0.252533
43198	1.00245	0.221658
43199	1.00245	0.235405
43200	0.92786	0.25736
43201	0.705297	0.176808
43202	0.856414	0.21865
43203	1.28968	0.224284
43204	1.00306	0.214613
43205	0.705303	0.208637
43206	1.28967	0.226923
43207	0.975478	0.251586

43208	3.05308	2.1
43209	1.76816	2.1
43210	1.76816	2.1
43211	1.69129	2.1
43212	2.58731	2.1
43213	2.51043	2.1
43214	1.89226	0.239826
43215	2.71464	0.282404
43216	2.34627	0.266764
43217	1.89226	0.289305
43218	2.34627	0.228272
43219	1.89226	0.255933
43220	2.34627	0.216497
43221	2.71464	0.221464
43222	1.98252	0.194291
43223	1.98252	0.260527
43224	1.51026	0.194479
43225	2.34627	0.229262
43226	2.38698	1.07531
43227	2.35142	2.02982
43228	2.60343	1.09738
43229	2.6636	1.16439
43230	4.99041	1.19942
43231	1.07811	1.70031
43232	1.1952	2.1
43233	1.17571	1.57351
43234	1.30171	0.963498
43235	1.18322	0.894413
43236	1.18322	2.1
43237	1.18769	1.59913
43238	1.18769	1.02215
43239	1.1952	1.87283
43240	1.30171	1.659
43241	1.17571	1.59338
43242	1.07811	1.46011
43243	1.89226	1.23122
43244	2.6636	1.58121
43245	1.89226	0.709331
43246	2.60343	1.28776
43247	1.89226	1.21491
43248	2.60343	2.1
43249	1.5344	0.0964157
43250	1.4516	0.0938783
43251	1.17816	0.0864201
43252	1.21056	0.0894291
43253	2.71257	0.092859
43254	1.89226	1.60543
43255	1.89226	0.736872
43256	1.89226	0.95221
43257	1.89226	1.09279
43258	3.78452	0.886773
43259	1.89226	1.04128
43260	1.89226	0.546159
43261	1.89226	0.741646
43262	1.89226	0.653381
43263	1.65573	0.875975
43264	1.65573	0.714299
43265	1.89226	0.895639

43266	1.89226	1.14911
43267	1.89226	1.75393
43268	2.64212	0.72519
43269	3.78452	1.24255
43270	1.89226	0.697492
43271	1.89226	2.1
43272	1.89226	0.74024
43273	1.89226	2.1
43274	3.78452	0.874303
43275	2.7367	0.542936
43276	2.07947	2.1
43277	2.47319	0.744787
43278	1.85057	1.9167
43279	4.55267	1.41862
43280	2.07948	2.1
43281	1.3294	2.1
43282	1.85057	2.09888
43283	1.09962	2.1
43284	3.1791	2.1
43285	1.89226	2.1
43286	1.89226	2.1
43287	1.89226	2.1
43288	1.89226	2.1
43289	3.78453	2.1
43290	0.94613	2.1
43291	0.94613	0.642288
43292	0.94613	0.712336
43293	0.94613	2.1
43294	0.946128	2.1
43295	0.946128	1.99385
43296	0.946132	0.636662
43297	0.946132	0.648069
43298	0.94613	1.05995
43299	0.94613	1.30059
43300	0.946131	1.15063
43301	0.946131	1.21449
43302	1.00875	2.03937
43303	1.00875	0.596003
43304	1.01676	2.1
43305	1.01676	0.61202
43306	0.925282	2.07608
43307	0.804877	1.86981
43308	1.2366	0.648099
43309	1.10023	0.653475
43310	0.804878	1.00694
43311	1.10023	1.0745
43312	1.2366	0.823135
43313	0.925284	0.78307
43314	1.89226	2.1
43315	1.89226	2.1
43316	1.89226	2.1
43317	1.89226	2.1
43318	3.78453	2.1
43319	1.89226	2.1
43320	1.89226	2.1
43321	1.89226	2.1
43322	1.89226	2.1
43323	1.89226	2.1

43324	1.89226	2.1
43325	1.09766	2.1
43326	1.14842	2.1
43327	1.02909	2.1
43328	1.07659	2.1
43329	1.16718	2.1
43330	0.954189	2.1
43331	1.06653	2.1
43332	1.06653	2.1
43333	1.20738	2.1
43334	1.02909	2.1
43335	1.09767	2.1
43336	0.815951	2.1
43337	2.00793	2.1
43338	1.14048	2.1
43339	1.19086	2.1
43340	1.13723	2.1
43341	2.00793	2.1
43342	2.11772	2.1
43343	1.89226	2.1
43344	1.89226	2.1
43345	1.89226	2.1
43346	1.89226	2.1
43347	1.89226	2.1
43348	1.89226	2.1
43349	1.89226	2.1
43350	1.89226	2.1
43351	1.89226	2.1
43352	1.89226	2.1
43353	1.89226	2.1
43354	1.89226	2.1
43355	1.1779	2.1
43356	1.26166	2.1
43357	1.0875	2.1
43358	1.00397	2.1
43359	1.13286	2.1
43360	1.13286	2.1
43361	1.13277	2.1
43362	1.13277	2.1
43363	1.26166	2.1
43364	1.08749	2.1
43365	1.1779	2.1
43366	1.00396	2.1
43367	1.0875	2.1
43368	1.00397	2.1
43369	0.685749	2.1
43370	0.595432	2.1
43371	0.83976	2.1
43372	0.83976	2.1
43373	0.843169	2.1
43374	0.843169	2.1
43375	1.0875	2.1
43376	0.685749	2.1
43377	1.00397	2.1
43378	0.595432	2.1
43379	1.89226	2.1
43380	1.86443	2.1
43381	2.4988	2.1

43382	2.0815	2.1
43383	2.28173	2.1
43384	1.86443	2.1
43385	1.89226	2.1
43386	1.89226	2.1
43387	1.89226	2.1
43388	1.89226	2.1
43389	1.89226	2.1
43390	1.89226	2.1
43391	0.946131	2.1
43392	0.946131	2.1
43393	0.969491	2.1
43394	0.949105	2.1
43395	0.949103	2.1
43396	0.949103	2.1
43397	0.946133	2.1
43398	0.946133	2.1
43399	0.946131	2.1
43400	0.969492	2.1
43401	0.946131	2.1
43402	0.949105	2.1
43403	1.89226	2.1
43404	2.07428	2.1
43405	1.93464	2.1
43406	1.89226	2.1
43407	2.11667	2.1
43408	2.07428	2.1
43409	0.946132	2.1
43410	0.946132	2.1
43411	0.946131	2.1
43412	0.946131	2.1
43413	0.946132	2.1
43414	0.946132	2.1
43415	0.946132	2.1
43416	0.946132	2.1
43417	0.946132	2.1
43418	0.946132	2.1
43419	0.946132	2.1
43420	0.946132	2.1
43421	1.97992	2.1
43422	1.89226	2.1
43423	1.89226	2.1
43424	1.93898	2.1
43425	1.93898	2.1
43426	1.89226	2.1
43427	2.12475	0.553091
43428	2.79029	0.596298
43429	2.7803	0.847166
43430	2.13052	0.518341
43431	4.92081	0.55757
43432	1.06238	0.732117
43433	1.06526	0.564571
43434	0.642901	0.517081
43435	0.655055	0.623595
43436	0.856391	0.533457
43437	0.856391	0.517553
43438	0.86104	1.05827
43439	0.86104	0.512214

43440	1.06526	0.583821
43441	0.655055	0.558521
43442	0.642901	0.551267
43443	1.06238	0.53896
43444	2.7803	0.706809
43445	2.13053	0.410892
43446	2.14346	0.575315
43447	2.77937	0.574335
43448	4.9099	0.584113
43449	2.14346	0.639768
43450	1.31011	0.42324
43451	1.34124	0.568379
43452	1.31011	0.61755
43453	2.16166	0.51144
43454	2.13053	0.525254
43455	1.89226	2.1
43456	2.58176	2.1
43457	2.01422	2.1
43458	1.89226	2.1
43459	2.70373	2.1
43460	2.58176	2.1
43461	1.89226	2.1
43462	1.89226	2.1
43463	1.89226	2.1
43464	1.89226	2.1
43465	1.89226	2.1
43466	1.89226	2.1
43467	0.946128	2.1
43468	0.946128	2.1
43469	1.38521	2.1
43470	1.38521	2.1
43471	1.38521	2.1
43472	1.35186	2.1
43473	0.946131	2.1
43474	0.946131	2.1
43475	0.94613	2.1
43476	1.38521	2.1
43477	0.946131	2.1
43478	1.35186	2.1
43479	0.946133	2.1
43480	0.946133	2.1
43481	0.946132	2.1
43482	0.946132	2.1
43483	0.946132	2.1
43484	0.946132	2.1
43485	0.946132	2.1
43486	0.946132	2.1
43487	0.946132	2.1
43488	0.946132	2.1
43489	0.946133	2.1
43490	0.946133	2.1
43491	2.33374	2.1
43492	1.89226	2.1
43493	1.89226	2.1
43494	1.89226	2.1
43495	2.60377	2.1
43496	2.60377	2.1
43497	2.6771	0.110158

43498	1.36416	0.127415
43499	1.64983	0.130629
43500	1.64983	0.104106
43501	2.44131	0.131558
43502	2.44131	0.115137
43503	2.58321	0.45648
43504	1.89226	0.489513
43505	2.44283	0.331282
43506	2.44283	0.412214
43507	1.89226	0.609203
43508	1.89226	0.765962
43509	2.58321	0.580043
43510	2.44283	0.427637
43511	1.49358	0.311193
43512	1.57702	0.440144
43513	3.93641	0.38094
43514	0.951913	0.107377
43515	0.81556	0.101602
43516	0.946132	0.125079
43517	0.946132	0.120015
43518	1.04072	0.124358
43519	1.12182	0.123289
43520	0.856745	0.10382
43521	0.851542	0.10631
43522	0.923163	0.1096
43523	0.923163	0.12982
43524	1.04304	0.119663
43525	0.85098	0.10006
43526	2.70125	1.05476
43527	2.37734	1.03452
43528	2.63495	0.874518
43529	2.30539	1.94303
43530	5.01229	1.63447
43531	1.89226	1.38367
43532	1.89226	1.46672
43533	1.89226	1.11481
43534	1.89226	1.80231
43535	3.78452	0.874676
43536	1.89226	1.68975
43537	1.89226	2.1
43538	1.89226	1.6997
43539	1.89226	2.1
43540	3.78452	2.1
43541	2.37734	1.19757
43542	1.79346	2.1
43543	2.53594	1.64341
43544	1.70302	1.70565
43545	4.27682	1.61683
43546	2.63495	0.749524
43547	2.30539	1.23155
43548	2.49445	0.900801
43549	2.12089	0.780881
43550	4.75584	0.793118
43551	1.89226	0.904989
43552	1.89226	1.43086
43553	1.89226	0.899631
43554	1.89226	1.43125
43555	3.78452	1.80398

43556	1.89226	1.34654
43557	1.89226	1.52864
43558	1.89226	1.43687
43559	1.89226	1.56916
43560	1.89226	1.01988
43561	1.89226	1.08352
43562	2.53594	1.04028
43563	1.60017	1.01918
43564	1.57079	1.22938
43565	1.70302	0.87701
43566	2.15772	1.05879
43567	2.33299	0.778156
43568	2.09114	2.1
43569	2.34898	2.1
43570	2.30729	2.1
43571	2.06556	2.1
43572	4.41454	2.1
43573	2.09114	2.1
43574	1.59654	2.1
43575	1.57823	2.1
43576	2.06556	2.1
43577	3.6621	2.1
43578	2.30729	2.1
43579	2.06556	2.1
43580	2.03917	2.1
43581	2.26617	2.1
43582	4.33173	2.1
43583	2.03917	2.1
43584	2.06556	2.1
43585	2.045 2.1	
43586	1.55767	2.1
43587	1.57823	2.1
43588	1.57823	2.1
43589	1.89225	1.40088
43590	1.09726	2.1
43591	1.61196	2.1
43592	1.28597	2.1
43593	1.28597	0.0522355
43594	1.45633	2.1
43595	1.45633	2.1
43596	1.9544	0.0522355
43597	1.61196	2.1
43598	1.45633	2.1
43599	1.53735	2.1
43600	3.14931	2.1
43601	1.89226	2.1
43602	1.89226	2.1
43603	1.89226	2.1
43604	1.89226	2.1
43605	3.78453	2.1
43606	0.810946	2.1
43607	0.810946	2.1
43608	0.810947	2.1
43609	0.810947	2.1
43610	0.810933	2.1
43611	0.810933	2.1
43612	1.89226	2.1
43613	1.3517	2.1

43614	1.08132	2.1
43615	1.08132	2.1
43616	1.08133	2.1
43617	0.851539	1.97523
43618	0.851539	2.1
43619	0.972053	2.1
43620	1.05632	2.1
43621	0.94613	2.1
43622	0.94613	1.46029
43623	1.0279	2.1
43624	0.94991	2.1
43625	1.0279	2.1
43626	0.946131	2.1
43627	0.865643	2.1
43628	0.94613	2.1
43629	1.89226	1.82397
43630	1.89226	2.1
43631	1.89226	2.1
43632	1.89226	1.67728
43633	3.78452	2.1
43634	1.03882	2.1
43635	1.91121	2.1
43636	1.41833	2.1
43637	2.34547	2.1
43638	3.32954	2.1
43639	2.3979	1.89807
43640	1.89226	2.1
43641	1.89226	2.1
43642	1.89226	2.06984
43643	2.0558	2.1
43644	2.0558	1.26149
43645	1.89226	2.1
43646	1.89226	0.943394
43647	1.89226	1.64835
43648	1.89226	2.1
43649	3.78452	1.89325
43650	1.05816	2.1
43651	1.05816	2.1
43652	0.842045	2.1
43653	1.05252	2.1
43654	1.36057	2.1
43655	1.14339	2.1
43656	0.873397	2.1
43657	0.585073	2.1
43658	0.873388	2.1
43659	1.15129	2.1
43660	0.69143	2.1
43661	1.1434	2.1
43662	1.93338	2.1
43663	1.85883	2.1
43664	1.51714	2.1
43665	1.85883	2.1
43666	1.54795	2.1
43667	1.89226	2.1
43668	1.89226	2.1
43669	1.89226	2.1
43670	1.89226	2.1
43671	1.89226	2.1

43672	3.78452	2.1
43673	0.844726	2.1
43674	0.844726	2.1
43675	0.960524	2.1
43676	1.16288	2.1
43677	0.851541	2.1
43678	0.851541	2.1
43679	0.929147	2.1
43680	1.15533	2.1
43681	0.844697	2.1
43682	0.946131	2.1
43683	0.851632	2.1
43684	0.946131	2.1
43685	1.89226	2.1
43686	1.70303	2.1
43687	1.74006	2.1
43688	1.70303	2.1
43689	1.99471	2.1
43690	1.93338	2.1
43691	0.946131	2.08744
43692	0.946131	2.1
43693	0.946131	2.1
43694	0.946131	2.1
43695	0.94613	2.1
43696	0.94613	2.1
43697	0.946132	2.1
43698	0.946132	2.1
43699	0.946131	2.1
43700	0.946131	2.1
43701	0.946131	2.1
43702	0.946131	2.1
43703	1.58158	2.1
43704	1.89226	2.1
43705	1.89226	2.1
43706	1.89226	2.1
43707	1.68939	2.1
43708	1.68939	2.1
43709	0.946132	0.0816667
43710	0.946132	0.0913242
43711	1.12988	0.0812287
43712	1.30783	0.0740846
43713	1.30783	0.0823606
43714	0.946133	0.0837865
43715	0.946132	0.0825818
43716	1.12988	0.0810347
43717	1.30783	0.0813878
43718	1.30783	0.0736199
43719	0.946133	0.0823731
43720	0.946133	0.0920575
43721	1.89227	0.0916851
43722	1.89227	0.0907115
43723	1.89227	0.0866845
43724	1.89227	0.106335
43725	1.89227	0.0822424
43726	1.89227	0.101806
43727	1.89226	0.0846318
43728	1.89226	0.0837204
43729	1.89226	0.0827844

43730	1.89226	0.0948173
43731	1.89226	0.0774636
43732	1.89226	0.0888756
43733	0.946131	0.0841012
43734	0.946131	0.0759201
43735	1.30783	0.0734287
43736	1.37715	0.0689407
43737	1.37715	0.074991
43738	0.946133	0.0767441
43739	1.30783	0.0741345
43740	0.946129	0.0764673
43741	1.37715	0.0752757
43742	1.37715	0.0684926
43743	0.946131	0.0749488
43744	0.946131	0.0848531
43745	1.17056	0.0862673
43746	0.591757	0.102183
43747	0.522025	0.086496
43748	0.929727	0.0769174
43749	0.929728	0.0777189
43750	1.17056	0.0821567
43751	0.591756	0.0838388
43752	0.522025	0.0831284
43753	0.889054	0.0841889
43754	0.889054	0.078939
43755	0.803535	0.0952148
43756	0.803535	0.0870727
43757	1.49814	2.1
43758	2.37439	2.1
43759	2.63923	2.1
43760	2.63923	2.1
43761	1.29111	2.1
43762	1.29111	2.1
43763	0.946132	2.1
43764	0.946132	2.1
43765	1.18719	2.1
43766	1.18719	2.1
43767	0.94613	2.1
43768	0.94613	1.82659
43769	1.1872	2.1
43770	1.31961	2.1
43771	0.946131	2.1
43772	1.31961	2.1
43773	1.1872	2.1
43774	0.94613	2.1
43775	2.49445	0.598562
43776	2.12089	0.651812
43777	2.20843	0.534477
43778	1.82805	0.73751
43779	4.32932	0.642703
43780	1.89226	0.77781
43781	1.89226	1.07112
43782	1.89226	0.68411
43783	1.89226	0.678891
43784	3.78452	0.613219
43785	0.94613	0.731718
43786	0.94613	0.689072
43787	0.946128	0.638313

43788	0.946128	0.833674
43789	0.946131	0.795439
43790	0.946131	0.829827
43791	0.946127	0.849309
43792	0.946127	0.638198
43793	0.946129	0.732189
43794	0.946129	0.667828
43795	0.946129	0.746286
43796	0.946129	0.759606
43797	1.82804	0.682973
43798	1.60017	0.773464
43799	1.87679	0.822961
43800	2.12088	0.562917
43801	1.35608	0.852484
43802	1.60017	0.683737
43803	2.20843	0.426663
43804	1.3216	0.473474
43805	1.44426	0.453165
43806	1.82805	0.435832
43807	1.80399	0.453714
43808	2.20843	0.459277
43809	1.89226	0.544134
43810	1.89226	0.514371
43811	1.89226	0.70305
43812	1.89226	0.55856
43813	1.89226	0.590398
43814	1.89226	0.510016
43815	1.89226	0.670816
43816	1.89226	0.492602
43817	1.89226	0.565311
43818	1.89226	0.555462
43819	1.89226	0.677905
43820	1.89226	0.483477
43821	0.627705	0.427013
43822	0.634941	0.568077
43823	0.629317	0.428287
43824	0.783422	0.365589
43825	0.629328	0.460395
43826	0.448764	0.472378
43827	1.35608	0.471449
43828	1.00805	0.491461
43829	0.814295	0.356749
43830	0.598375	0.563888
43831	1.04461	0.462361
43832	1.09795	1.70671
43833	1.00775	1.68587
43834	1.61202	1.94977
43835	1.63419	1.47232
43836	2.64194	1.41031
43837	1.44801	1.66051
43838	1.61201	1.70667
43839	2.35614	1.74007
43840	2.27703	1.72467
43841	3.88904	1.80289
43842	2.35615	1.5836
43843	1.63419	1.24994
43844	2.29852	1.38912
43845	1.63419	1.31888

43846	2.27634	1.43681
43847	1.61202	1.58153
43848	0.973793	2.1
43849	2.2671	2.1
43850	2.32981	2.1
43851	2.32981	2.1
43852	0.990481	2.1
43853	0.990481	2.1
43854	1.50877	2.1
43855	1.11193	2.1
43856	2.29667	2.1
43857	1.94158	2.1
43858	3.45035	2.1
43859	2.29667	2.1
43860	1.89227	2.1
43861	1.94158	2.1
43862	1.94158	2.1
43863	1.89227	2.1
43864	1.89227	2.1
43865	2.51379	2.1
43866	1.30026	2.1
43867	1.84502	2.1
43868	2.72099	2.1
43869	4.35882	2.1
43870	1.89226	2.1
43871	2.721	2.1
43872	2.5138	2.1
43873	2.5138	2.1
43874	1.89226	2.1
43875	1.89226	2.1
43876	1.89226	0.173225
43877	1.89226	0.1877
43878	2.59101	0.153543
43879	1.89226	0.230499
43880	3.78453	0.161522
43881	0.851541	0.179618
43882	0.851541	0.155437
43883	1.42502	0.161255
43884	0.948679	0.137133
43885	0.946132	0.177851
43886	0.946132	0.19025
43887	0.853617	0.159576
43888	0.853618	0.131591
43889	0.851475	0.175344
43890	0.948666	0.187755
43891	1.166	0.172266
43892	0.851554	0.181376
43893	2.51818	0.684607
43894	2.60723	1.908
43895	2.66532	0.685234
43896	2.76315	1.91728
43897	5.27255	0.833905
43898	2.60723	1.23747
43899	2.45247	2.1
43900	2.76315	1.63688
43901	2.55197	2.1
43902	5.1592	2.1
43903	1.23768	0.635703

43904	1.11433	0.76606
43905	1.32037	1.35319
43906	1.32037	0.704227
43907	1.0142	0.909979
43908	1.0142	0.634175
43909	0.886711	0.95172
43910	1.16806	0.900775
43911	0.999083	1.59475
43912	0.710926	1.19937
43913	0.937757	1.33847
43914	0.937757	1.07033
43915	0.941224	0.702247
43916	0.941224	1.59297
43917	1.16805	1.09438
43918	0.999084	1.64167
43919	0.886709	1.38371
43920	0.710928	1.24106
43921	2.29332	1.34162
43922	1.54904	2.1
43923	2.0815	2.1
43924	2.44199	1.83485
43925	4.00418	2.1
43926	1.53873	0.246896
43927	1.53422	0.54293
43928	1.39661	0.301614
43929	1.42387	0.519707
43930	2.9626	0.441928
43931	1.53423	0.48838
43932	1.44091	0.701843
43933	1.42387	0.464474
43934	1.35215	1.46536
43935	2.86479	0.616251
43936	1.39661	0.298559
43937	1.42387	0.518207
43938	1.28325	0.283707
43939	1.33326	0.565347
43940	2.70713	0.392615
43941	1.42387	0.454066
43942	1.35215	1.49702
43943	1.33327	0.578408
43944	1.27339	0.772694
43945	2.69726	0.687488
43946	2.58442	2.1
43947	1.1468	2.1
43948	1.18775	2.1
43949	2.60047	2.1
43950	3.74728	2.1
43951	2.58442	2.1
43952	1.89226	2.1
43953	1.89226	2.1
43954	2.60048	2.1
43955	4.49274	2.1
43956	1.89226	2.1
43957	1.89226	1.71021
43958	1.89226	2.1
43959	1.89226	1.95015
43960	3.78453	2.1
43961	1.89226	2.1

43962	1.89226	1.55665
43963	1.89226	2.1
43964	1.89226	0.756585
43965	3.78452	2.1
43966	1.22262	2.1
43967	2.60047	2.1
43968	1.18775	2.1
43969	1.18775	2.1
43970	2.61433	2.1
43971	2.61433	2.1
43972	1.89226	2.1
43973	2.60048	2.1
43974	2.61434	2.1
43975	2.60048	2.1
43976	1.90612	2.1
43977	1.89226	2.1
43978	1.89226	2.1
43979	1.89226	1.66903
43980	1.89226	2.1
43981	1.89226	1.48854
43982	3.78453	2.1
43983	1.89226	2.1
43984	1.89226	1.80347
43985	1.89226	2.1
43986	1.89226	1.33549
43987	3.78452	2.1
43988	2.12556	2.1
43989	0.991113	2.1
43990	1.27725	2.1
43991	2.31081	2.1
43992	3.40281	2.1
43993	1.89226	2.1
43994	2.31082	2.1
43995	2.12556	2.1
43996	2.12556	2.1
43997	1.89226	2.1
43998	1.89226	2.1
43999	1.22544	2.1
44000	1.12649	2.1
44001	0.775254	2.1
44002	0.643133	2.1
44003	0.917924	2.1
44004	0.934955	2.1
44005	0.975579	2.1
44006	0.975579	2.1
44007	1.23113	2.1
44008	0.685272	2.1
44009	0.651719	2.1
44010	1.1791	2.1
44011	1.89226	2.1
44012	2.5138	2.1
44013	2.31082	2.1
44014	2.31082	2.1
44015	1.89226	2.1
44016	1.89226	2.1
44017	1.89226	2.1
44018	1.89226	2.1
44019	1.89226	2.1

44020	1.89226	2.1
44021	3.78453	2.1
44022	0.922224	2.1
44023	0.922224	2.1
44024	1.23555	2.1
44025	1.19715	2.1
44026	1.01445	2.1
44027	1.16061	2.1
44028	0.946132	2.1
44029	0.946132	2.1
44030	0.946131	2.1
44031	1.2051	2.1
44032	1.13537	2.1
44033	1.01445	2.1
44034	0.946133	2.1
44035	0.946133	2.1
44036	1.20648	2.09075
44037	1.20648	2.1
44038	1.06454	2.1
44039	1.20648	2.1
44040	0.946133	2.1
44041	0.946133	2.00033
44042	1.20649	2.1
44043	0.946133	2.1
44044	1.06454	2.1
44045	0.946132	2.1
44046	0.946132	2.1
44047	0.946132	2.1
44048	0.946132	2.1
44049	0.946132	2.1
44050	0.946132	2.1
44051	0.946132	2.1
44052	0.946133	2.1
44053	0.946133	2.07201
44054	0.946133	2.1
44055	0.946133	2.1
44056	0.946132	2.1
44057	0.946132	2.1
44058	1.33078	1.5757
44059	1.37409	2.04556
44060	1.31565	0.708867
44061	1.37114	1.94431
44062	2.68973	1.65348
44063	1.89225	1.6981
44064	1.89225	2.02061
44065	1.89225	0.705434
44066	1.89225	1.5219
44067	3.7845	1.15276
44068	1.89225	1.15595
44069	1.89225	2.1
44070	1.89225	0.985682
44071	1.89225	2.1
44072	3.7845	1.51278
44073	1.37409	1.2426
44074	1.20408	2.1
44075	1.18612	2.05813
44076	1.18907	2.1
44077	1.37114	0.979396

44078	1.37409	2.1
44079	1.31566	1.83773
44080	1.37115	2.1
44081	1.30318	0.670017
44082	1.37433	1.48369
44083	2.68998	1.33571
44084	1.89226	1.85836
44085	1.89226	1.78797
44086	1.89226	0.756714
44087	1.89226	1.51651
44088	3.78453	1.24905
44089	1.89226	1.12469
44090	1.89226	2.1
44091	1.89226	2.04097
44092	1.89226	2.1
44093	1.89226	0.925578
44094	1.89226	2.1
44095	0.647001	2.1
44096	0.647001	1.731
44097	0.651851	0.809195
44098	0.651851	2.1
44099	0.602046	2.1
44100	0.613277	2.1
44101	0.685576	1.03308
44102	0.687164	1.16113
44103	0.687164	1.90481
44104	0.613276	1.7304
44105	0.602046	1.91991
44106	0.685576	1.94957
44107	0.946128	0.864111
44108	0.946128	0.514307
44109	1.15307	1.34163
44110	1.19317	0.88233
44111	0.94613	1.07534
44112	0.94613	0.253235
44113	1.15307	1.14541
44114	1.15307	1.23591
44115	0.946131	0.919009
44116	1.15307	0.846355
44117	0.946131	0.775891
44118	1.19317	0.691883
44119	1.18278	2.1
44120	2.38635	0.720995
44121	1.23042	1.94183
44122	1.23042	0.935392
44123	2.30614	1.91213
44124	2.30614	0.986611
44125	0.946129	0.750127
44126	0.946129	0.629914
44127	1.12994	0.972808
44128	1.15307	0.800647
44129	0.946131	0.948761
44130	0.946131	0.289826
44131	1.12994	0.778712
44132	1.12994	0.833753
44133	1.12994	0.8502
44134	0.946131	0.881195
44135	0.946131	0.717204

44136	1.15307	0.664329
44137	2.25988	1.01414
44138	2.30614	0.529841
44139	2.28696	1.41442
44140	1.16361	1.87585
44141	1.18278	1.65944
44142	1.18278	0.792626
44143	2.3771	2.1
44144	1.90121	2.1
44145	1.81191	2.1
44146	2.46904	2.1
44147	4.37025	2.1
44148	1.15676	2.1
44149	1.23452	2.1
44150	0.823592	2.1
44151	0.737606	2.1
44152	0.988113	2.1
44153	0.988113	2.1
44154	0.984014	2.1
44155	0.984014	2.1
44156	1.15676	2.1
44157	0.737605	2.1
44158	0.823592	2.1
44159	1.23452	2.1
44160	1.30318	1.95099
44161	1.37433	2.1
44162	1.30151	0.967837
44163	1.39067	1.79274
44164	2.67584	1.41896
44165	1.89226	1.53921
44166	1.89226	2.1
44167	1.89226	0.930428
44168	1.89226	1.29685
44169	3.78453	1.12454
44170	1.89226	0.871525
44171	1.89226	2.1
44172	1.89226	0.75079
44173	1.89226	2.01726
44174	1.89226	0.929296
44175	1.89226	2.1
44176	0.656819	2.1
44177	0.656819	0.84201
44178	0.661755	0.927338
44179	0.661755	2.1
44180	0.613277	2.1
44181	0.63141	2.1
44182	0.687164	0.911525
44183	0.695336	1.32919
44184	0.695337	1.775
44185	0.63141	1.64918
44186	0.687164	2.04889
44187	0.613276	1.95388
44188	1.30151	1.71111
44189	1.39067	2.1
44190	1.32143	0.946958
44191	1.43294	1.79524
44192	2.73445	1.69851
44193	1.89226	1.63033

44194	1.89226	1.77653
44195	1.89226	0.818844
44196	1.89226	1.85763
44197	3.78452	1.39873
44198	1.89226	1.21572
44199	1.89226	1.09907
44200	1.89226	0.88446
44201	1.89226	2.1
44202	1.89226	2.1
44203	1.89226	2.1
44204	1.43294	1.19838
44205	1.26282	2.1
44206	1.3268	0.868258
44207	1.3268	2.1
44208	1.39067	1.03513
44209	1.39067	2.1
44210	1.5511	0.252033
44211	1.00665	0.27307
44212	1.28239	0.253044
44213	1.88631	0.268135
44214	2.89296	0.253911
44215	1.89227	0.280806
44216	1.89227	0.286403
44217	1.89227	0.313451
44218	1.89227	0.302384
44219	1.89227	0.276763
44220	1.89227	0.265242
44221	2.08848	0.269121
44222	1.89226	0.272086
44223	1.89226	0.264336
44224	2.37947	0.297679
44225	4.27173	0.276515
44226	1.01456	0.263097
44227	0.943157	0.260502
44228	0.641198	0.263743
44229	0.724757	0.257493
44230	0.830923	0.275277
44231	0.830923	0.272197
44232	0.836991	0.263636
44233	0.836991	0.238873
44234	1.01456	0.267561
44235	0.724757	0.300687
44236	0.641197	0.261272
44237	0.943157	0.274245
44238	1.89227	0.282803
44239	1.89227	0.303384
44240	1.89227	0.295261
44241	1.89227	0.31116
44242	3.78453	0.295856
44243	0.946129	0.28015
44244	0.946129	0.281192
44245	1.28941	0.287496
44246	1.18974	0.277982
44247	0.94613	0.289917
44248	0.94613	0.282995
44249	1.28941	0.27259
44250	1.28941	0.265208
44251	1.28941	0.271946

44252	0.946131	0.287166
44253	0.94613	0.242251
44254	1.18974	0.278949
44255	0.710636	2.1
44256	0.780249	2.1
44257	0.579033	2.1
44258	0.478812	2.1
44259	0.621822	2.1
44260	0.621822	2.1
44261	0.63724	2.1
44262	0.63724	2.1
44263	0.780251	2.1
44264	0.579032	2.1
44265	0.710637	2.1
44266	0.478811	2.1
44267	1.05499	1.87579
44268	1.00521	1.92663
44269	1.24445	1.4756
44270	1.23082	0.944706
44271	2.28581	1.84024
44272	1.48751	1.7268
44273	1.23083	1.44326
44274	1.4941	1.85956
44275	1.24445	1.27155
44276	1.48047	1.21682
44277	1.23083	1.13654
44278	0.621185	1.47534
44279	0.621185	1.75343
44280	0.620899	1.12605
44281	0.620899	2.1
44282	0.502515	1.44262
44283	0.626672	1.2248
44284	0.615412	0.987416
44285	0.740234	1.84331
44286	0.740233	0.99555
44287	0.626672	0.889847
44288	0.615413	1.17651
44289	0.502516	1.38649
44290	1.95844	2.1
44291	2.6562	2.1
44292	1.95844	2.1
44293	2.20883	2.1
44294	2.38225	2.1
44295	2.63264	2.1
44296	1.89226	2.1
44297	1.89226	2.1
44298	1.89226	2.1
44299	1.89226	2.1
44300	3.78453	2.1
44301	1.95844	2.1
44302	2.38225	2.1
44303	1.71082	2.1
44304	2.14647	2.1
44305	4.1049	2.1
44306	0.946131	2.1
44307	0.946131	2.1
44308	0.946131	2.1
44309	0.946131	2.1

44310	0.946131	2.1
44311	0.946131	2.1
44312	0.946131	2.1
44313	0.946131	2.1
44314	0.946131	2.1
44315	0.946131	2.1
44316	0.946131	2.1
44317	0.946131	2.1
44318	2.12597	2.1
44319	1.2295	2.1
44320	1.28014	2.1
44321	2.19723	2.1
44322	3.40611	2.1
44323	2.12597	2.1
44324	2.79242	2.1
44325	2.73375	2.1
44326	2.66249	2.1
44327	2.19723	2.1
44328	2.12597	2.1
44329	1.13716	2.1
44330	1.09861	2.1
44331	0.666692	2.1
44332	0.640071	2.1
44333	0.891617	2.1
44334	0.891617	2.1
44335	0.885614	2.1
44336	0.885614	2.1
44337	1.13716	2.1
44338	0.666692	2.1
44339	0.640071	2.1
44340	1.09861	2.1
44341	1.89226	0.113683
44342	1.70303	0.105035
44343	1.89226	0.0890831
44344	2.64598	0.113336
44345	1.81091	0.0853666
44346	2.53222	0.113989
44347	2.32422	0.0977051
44348	1.89226	0.12315
44349	2.78336	0.120031
44350	2.78336	0.118063
44351	1.89226	0.0953057
44352	1.89226	0.0976424
44353	0.946132	0.10281
44354	0.946132	0.0927164
44355	0.946131	0.102406
44356	0.946131	0.125164
44357	0.946132	0.128465
44358	0.946132	0.115419
44359	0.946131	0.0953324
44360	0.946131	0.112182
44361	0.946131	0.107888
44362	0.946131	0.111608
44363	0.946131	0.11029
44364	0.946131	0.103399
44365	0.946131	0.117438
44366	0.946131	0.143164
44367	0.946132	0.128586

44368	0.946132	0.161809
44369	0.946132	0.163656
44370	0.946132	0.148684
44371	0.946131	0.123924
44372	0.946131	0.113882
44373	0.946131	0.132628
44374	0.946131	0.145856
44375	0.946131	0.13986
44376	0.946131	0.12573
44377	2.24323	1.1171
44378	1.89226	1.7288
44379	2.75021	1.29825
44380	2.75021	1.49527
44381	1.89226	0.964948
44382	1.89226	1.15775
44383	2.30908	0.234311
44384	2.19815	0.4952
44385	2.1609	0.273833
44386	1.83796	0.546749
44387	4.35904	0.289768
44388	2.19815	0.373358
44389	2.02268	0.748657
44390	2.17869	0.758747
44391	1.9559	0.521403
44392	2.0815	0.738906
44393	1.83424	0.511878
44394	2.1609	0.311659
44395	1.88877	0.545753
44396	1.98413	0.262539
44397	1.87355	0.511064
44398	4.03445	0.446337
44399	1.87355	0.471997
44400	2.02268	0.851729
44401	1.87355	0.82098
44402	2.10488	0.500074
44403	1.68443	0.752125
44404	1.89448	0.578846
44405	0.946131	0.239776
44406	0.946131	0.257499
44407	1.40574	0.319513
44408	1.12373	0.304109
44409	0.946129	0.283059
44410	0.946129	0.22497
44411	1.40574	0.31091
44412	1.40574	0.265404
44413	0.946131	0.279804
44414	1.40574	0.311775
44415	1.12373	0.28481
44416	0.946131	0.238707
44417	2.14277	0.361694
44418	2.81148	0.376484
44419	1.42762	0.29571
44420	1.42762	0.305913
44421	2.96261	0.325085
44422	2.24746	0.318802
44423	1.89226	0.741042
44424	1.89226	0.823323
44425	1.89226	0.770244

44426	1.89226	0.988498
44427	3.78453	1.09795
44428	1.89226	0.62259
44429	1.89226	0.794512
44430	1.89226	0.789908
44431	1.89226	0.934863
44432	3.78452	0.692489
44433	0.68433	0.762002
44434	0.946127	1.03086
44435	0.543921	1.1556
44436	0.946131	0.84441
44437	0.946131	0.922528
44438	0.946131	1.98606
44439	0.543918	0.941254
44440	0.543918	1.04299
44441	0.684332	0.977563
44442	0.54392	2.0139
44443	0.946129	0.673453
44444	0.946129	1.0036
44445	1.89226	0.863136
44446	1.08784	1.11231
44447	1.19352	0.5699
44448	1.89226	0.998375
44449	1.19352	1.91004
44450	1.89226	1.62449
44451	0.635711	0.723541
44452	0.946132	0.992477
44453	0.946132	1.22658
44454	0.56474	0.864714
44455	0.750045	0.998333
44456	1.25657	2.03306
44457	0.635682	0.890051
44458	0.635682	0.66871
44459	0.891954	0.837366
44460	1.32751	1.91313
44461	0.564766	0.724637
44462	0.564766	1.22552
44463	0.858345	0.918409
44464	0.946128	1.16399
44465	0.635712	0.948075
44466	0.946133	0.871095
44467	0.977198	1.07332
44468	0.944521	2.03216
44469	0.635682	1.13784
44470	0.635682	0.543202
44471	1.02924	1.01292
44472	0.705625	2.08204
44473	0.851528	0.576708
44474	0.851528	1.5418
44475	2.18903	2.1
44476	2.10182	2.1
44477	2.10182	2.1
44478	1.89226	2.1
44479	1.89226	2.1
44480	1.64983	2.1
44481	1.89226	2.1
44482	1.83867	2.1
44483	1.83867	2.1

44484	1.89164	2.1
44485	2.0815	2.1
44486	2.17638	2.1
44487	2.57957	0.957141
44488	2.3506	1.48654
44489	2.69056	0.743625
44490	2.49439	1.38845
44491	5.04116	0.891796
44492	2.3506	0.947266
44493	1.8911	1.6596
44494	2.49439	1.02705
44495	2.06689	2.06029
44496	4.41748	1.5939
44497	2.69056	0.938304
44498	2.49439	1.50933
44499	2.77227	0.734909
44500	2.63443	1.27571
44501	5.32499	1.52298
44502	2.49439	1.05684
44503	2.06689	2.01097
44504	2.63443	0.739727
44505	2.26444	2.03316
44506	4.70131	1.33353
44507	2.46327	2.1
44508	2.54347	2.1
44509	2.52869	2.1
44510	2.16534	2.1
44511	2.82661	2.1
44512	2.46327	2.1
44513	0.946131	2.1
44514	0.946131	2.1
44515	1.27174	2.1
44516	1.27174	2.1
44517	1.27174	2.1
44518	0.94613	2.1
44519	0.946128	2.1
44520	1.4133	2.1
44521	0.946129	2.1
44522	0.946129	2.1
44523	1.27174	2.1
44524	1.4133	2.1
44525	1.42757	2.1
44526	1.7457	2.1
44527	1.66047	2.1
44528	1.7457	2.1
44529	1.45754	2.1
44530	1.54278	2.1
44531	1.42757	2.1
44532	1.52996	2.1
44533	1.66047	2.1
44534	1.66047	2.1
44535	1.26397	2.1
44536	1.26397	2.1
44537	0.968536	2.1
44538	1.02461	2.1
44539	1.57575	2.1
44540	1.62391	2.1
44541	2.59245	2.1

44542	0.946131	2.1
44543	0.946131	2.1
44544	0.946132	2.1
44545	0.946132	2.1
44546	0.946132	2.1
44547	0.946132	1.92934
44548	0.946132	2.1
44549	0.946132	2.1
44550	0.946132	2.1
44551	0.946132	2.1
44552	0.946132	2.1
44553	0.946132	2.1
44554	2.23765	2.1
44555	1.62391	2.1
44556	2.22154	2.1
44557	1.57575	2.1
44558	2.2697	2.1
44559	1.62391	2.1
44560	1.89226	2.06204
44561	1.89226	2.1
44562	1.89226	2.1
44563	1.89226	2.1
44564	1.89226	2.1
44565	1.89226	1.66355
44566	1.89226	2.01073
44567	1.89226	2.1
44568	1.89226	2.1
44569	1.89226	2.1
44570	1.89226	2.1
44571	1.89226	1.56275
44572	2.2697	2.1
44573	1.46129	2.1
44574	2.25873	2.1
44575	1.62391	2.1
44576	2.09611	2.1
44577	1.46129	2.1
44578	1.89226	2.1
44579	2.07088	2.1
44580	1.89226	2.1
44581	2.31884	2.1
44582	4.21111	2.1
44583	1.89226	2.1
44584	2.31884	2.1
44585	2.11713	2.1
44586	1.89226	2.1
44587	2.54371	2.1
44588	2.31884	2.1
44589	2.54371	2.1
44590	1.24041	2.1
44591	2.55208	2.1
44592	2.31884	2.1
44593	1.23712	2.1
44594	1.00388	2.1
44595	2.72769	0.153269
44596	2.57507	0.203177
44597	1.89226	0.137172
44598	1.89226	0.198466
44599	4.46733	0.158149

44600	1.89226	0.205691
44601	2.57506	0.204177
44602	1.89226	0.150945
44603	2.30076	0.183876
44604	1.89226	0.141217
44605	2.30076	0.210894
44606	1.21171	0.122817
44607	1.48589	0.152383
44608	2.09166	0.121444
44609	1.78381	0.147596
44610	3.2697	0.137553
44611	2.09166	0.120359
44612	1.70302	0.148342
44613	1.48589	0.143555
44614	1.18027	0.117598
44615	2.26571	0.143406
44616	1.79969	0.124714
44617	1.89226	0.144659
44618	1.89226	0.204095
44619	1.89226	0.213096
44620	1.89226	0.157564
44621	1.89226	0.211573
44622	1.89226	0.164284
44623	0.946131	0.153906
44624	0.946131	0.240916
44625	0.946128	0.218416
44626	0.946128	0.168161
44627	0.946129	0.217467
44628	0.946129	0.219463
44629	0.94613	0.151584
44630	0.94613	0.15334
44631	0.94613	0.181144
44632	0.94613	0.1875
44633	0.94613	0.184674
44634	0.94613	0.18029
44635	1.89226	1.49189
44636	1.89226	1.5769
44637	1.89226	1.88593
44638	1.89226	1.26968
44639	1.89226	1.82665
44640	1.89226	1.22581
44641	1.89226	1.51273
44642	1.89226	1.48669
44643	1.89226	1.77216
44644	1.89226	1.24452
44645	1.89226	1.73051
44646	1.89226	1.21791
44647	1.89226	1.29951
44648	1.89226	1.36297
44649	1.89226	1.7092
44650	1.89226	1.08037
44651	1.89226	1.69058
44652	1.89226	1.05987
44653	1.79103	0.829364
44654	0.968249	1.99553
44655	1.44807	1.32795
44656	2.17984	0.620687
44657	3.2391	1.7153

44658	1.29735	1.3361
44659	1.08992	1.32047
44660	0.945147	1.66223
44661	0.724033	2.03003
44662	1.00723	1.85679
44663	1.00723	1.44574
44664	1.01416	0.723573
44665	1.01416	1.5819
44666	1.29735	1.36435
44667	0.94515	1.73385
44668	0.724034	1.54277
44669	1.08992	1.77371
44670	1.39214	1.23088
44671	1.39214	2.1
44672	0.946129	1.64517
44673	0.946129	2.1
44674	1.33728	2.1
44675	1.39214	2.1
44676	0.94613	1.286
44677	0.94613	1.418
44678	0.94613	2.00789
44679	1.33728	2.1
44680	0.94613	2.1
44681	1.39214	2.1
44682	2.78428	2.1
44683	2.28373	2.1
44684	2.67456	2.1
44685	2.20011	2.1
44686	4.95829	2.1
44687	1.90165	0.181997
44688	2.24206	0.245524
44689	1.66137	0.224264
44690	2.07108	0.369949
44691	3.90343	0.158723
44692	1.89224	0.319046
44693	1.89224	0.222899
44694	1.89224	0.214622
44695	1.89224	0.204441
44696	3.78448	0.230972
44697	2.82159	0.310749
44698	1.89226	0.242754
44699	2.50138	0.533132
44700	1.89226	0.231728
44701	4.39365	0.255859
44702	1.66139	0.189605
44703	1.86399	0.423679
44704	1.56005	0.221003
44705	1.90324	0.290837
44706	3.56463	0.263734
44707	0.946133	0.183218
44708	0.946133	0.319339
44709	0.946132	0.209112
44710	0.946132	0.319147
44711	0.946133	0.229912
44712	0.946133	0.308583
44713	0.946132	0.24567
44714	0.946132	0.299833
44715	0.946133	0.169484

44716	0.946133	0.199977
44717	0.946132	0.176863
44718	0.946132	0.221727
44719	1.5176	0.658546
44720	1.97964	0.544132
44721	2.32889	0.593997
44722	1.91762	0.590774
44723	3.84649	0.570772
44724	2.3376	0.588617
44725	1.97963	0.563871
44726	2.32889	0.639614
44727	2.58303	0.521126
44728	4.66649	0.610448
44729	2.32889	0.571948
44730	2.20248	0.525654
44731	1.91762	0.518605
44732	1.91762	0.521769
44733	2.5615	0.59187
44734	2.5615	0.594371
44735	2.32889	0.616739
44736	2.58303	0.505701
44737	2.71793	0.550741
44738	2.56149	0.50867
44739	5.04681	0.526986
44740	1.89226	0.629243
44741	2.53785	1.626
44742	1.89226	0.689462
44743	2.60895	0.596346
44744	1.89226	0.83825
44745	2.60895	0.708405
44746	0.493248	0.714942
44747	1.30448	1.13381
44748	1.26893	1.55626
44749	0.653226	1.49508
44750	0.653227	0.970795
44751	0.493247	1.98711
44752	1.26893	1.89574
44753	1.30448	0.703013
44754	0.832232	1.84221
44755	0.832233	1.90415
44756	0.929944	0.569773
44757	0.929944	0.953686
44758	1.89226	2.1
44759	1.47937	2.1
44760	1.50143	2.1
44761	1.47937	2.1
44762	1.91432	2.1
44763	1.89226	1.30672
44764	1.89226	1.60292
44765	1.89226	2.1
44766	1.89226	2.1
44767	1.89226	1.79371
44768	1.89226	2.1
44769	1.89226	0.899364
44770	1.33419	2.1
44771	2.4542	2.1
44772	2.59834	2.1
44773	2.59834	2.1

44774	1.25716	2.1
44775	1.25716	2.1
44776	1.70303	2.03078
44777	1.50143	2.1
44778	1.60878	2.1
44779	1.50143	2.0536
44780	1.93599	2.1
44781	1.89226	1.03136
44782	0.946131	0.859627
44783	0.946131	1.16392
44784	0.946127	2.1
44785	0.946127	2.1
44786	0.946127	2.04366
44787	0.946127	0.72592
44788	0.946131	2.1
44789	0.946131	1.20068
44790	0.94613	1.47
44791	0.94613	1.46733
44792	0.946129	1.66796
44793	0.946129	1.60592
44794	1.43912	2.1
44795	2.59834	2.1
44796	2.78093	2.1
44797	2.78093	2.1
44798	1.33419	2.1
44799	1.33419	2.1
44800	1.89226	2.1
44801	2.26322	1.46399
44802	2.55682	2.1
44803	2.55682	2.0582
44804	1.89226	2.1
44805	1.89226	2.1
44806	2.58032	0.459566
44807	2.28922	0.350806
44808	1.88424	0.382396
44809	2.32127	0.333795
44810	4.46456	0.369812
44811	1.67265	0.455605
44812	2.28922	0.384754
44813	1.88424	0.328868
44814	1.11314	0.315972
44815	3.55689	0.362766
44816	1.43751	0.290318
44817	2.32127	0.319287
44818	1.92998	0.246044
44819	2.37671	0.272687
44820	1.43751	0.407622
44821	1.88424	0.413625
44822	2.36729	2.1
44823	1.56714	2.1
44824	1.56714	2.1
44825	1.62408	2.1
44826	2.28373	2.1
44827	2.34067	2.1
44828	0.937268	2.1
44829	0.937268	2.1
44830	0.94636	2.1
44831	0.94636	2.1

44832	0.76004	2.1
44833	0.783572	2.1
44834	1.14187	2.1
44835	1.10006	2.1
44836	1.10006	2.1
44837	0.76004	2.1
44838	0.783572	2.1
44839	1.14187	2.1
44840	1.28753	2.1
44841	1.36838	2.1
44842	1.62617	2.1
44843	1.82857	2.1
44844	3.1161	2.1
44845	1.82856	2.1
44846	1.17996	2.1
44847	1.53417	2.1
44848	1.56618	2.1
44849	1.36838	2.1
44850	1.36838	2.1
44851	1.35065	0.851618
44852	1.42659	0.416698
44853	2.26675	0.641922
44854	2.26675	0.605205
44855	1.62137	0.578315
44856	2.53747	0.652253
44857	1.89226	0.148863
44858	1.89226	0.0994144
44859	1.89226	0.112154
44860	1.89226	0.129253
44861	3.78452	0.129766
44862	1.36569	0.0854218
44863	1.36569	0.0778841
44864	0.946131	0.0964167
44865	0.946131	0.110743
44866	1.16041	0.0971076
44867	1.36569	0.0825255
44868	0.946129	0.0939717
44869	0.946129	0.111991
44870	1.1604	0.0878657
44871	0.94613	0.0939432
44872	0.946131	0.0945661
44873	1.36569	0.0918603
44874	2.73139	0.0767761
44875	1.25265	0.0917535
44876	1.25265	0.0981717
44877	1.64799	0.0847848
44878	2.32081	0.0945565
44879	2.71615	0.0771643
44880	0.946133	0.0935299
44881	0.946133	0.0992696
44882	0.946132	0.128448
44883	0.946132	0.11469
44884	0.946132	0.0986476
44885	0.946132	0.115875
44886	0.946133	0.100248
44887	0.946133	0.120911
44888	0.946132	0.106134
44889	0.946132	0.110656

44890	0.946133	0.108495
44891	0.946133	0.105834
44892	0.946131	0.0979047
44893	0.946131	0.108262
44894	0.94613	0.160971
44895	0.94613	0.123907
44896	0.946132	0.133217
44897	0.946132	0.106613
44898	0.946129	0.121936
44899	0.946129	0.157932
44900	0.946131	0.118459
44901	0.946131	0.107786
44902	0.946131	0.111533
44903	0.946131	0.119156
44904	1.89226	0.554652
44905	2.48503	0.936105
44906	2.64586	1.45913
44907	2.64586	0.545243
44908	1.89226	1.38557
44909	1.89226	0.437105
44910	2.48504	1.4224
44911	1.99985	2.1
44912	1.83584	1.89148
44913	1.83584	2.1
44914	2.64586	1.60704
44915	2.64586	2.1
44916	1.89225	0.545551
44917	2.33095	0.936086
44918	2.48501	1.36315
44919	2.48501	0.523781
44920	1.89225	1.30816
44921	1.89225	0.448437
44922	2.33096	1.18051
44923	1.83583	2.1
44924	1.68436	1.77286
44925	1.68436	2.1
44926	2.48502	1.43448
44927	2.48502	2.1
44928	1.14807	2.1
44929	1.76545	2.09143
44930	1.21292	2.1
44931	1.83936	2.1
44932	2.97837	2.1
44933	1.89226	2.1
44934	1.89226	2.1
44935	1.89226	2.1
44936	1.89226	1.2496
44937	3.78451	2.1
44938	1.89226	2.1
44939	1.89226	1.10466
44940	1.89226	1.47933
44941	1.89226	1.24196
44942	3.78452	1.48072
44943	1.76545	2.0954
44944	2.12608	2.1
44945	1.83937	2.1
44946	2.22529	1.23724
44947	3.99074	2.1

44948	0.780222	2.01317
44949	0.780222	2.1
44950	0.784871	2.1
44951	0.784871	2.1
44952	0.95863	2.1
44953	0.919681	2.1
44954	0.636145	2.1
44955	0.606461	2.1
44956	0.636144	2.1
44957	0.958631	2.1
44958	0.60646	2.1
44959	0.919683	2.1
44960	1.89226	2.1
44961	1.89226	1.28302
44962	1.89226	2.1
44963	1.89226	2.1
44964	3.78452	2.1
44965	1.89226	1.52595
44966	1.89226	1.43868
44967	1.89226	2.1
44968	1.89226	0.891437
44969	3.78452	1.37173
44970	1.03789	1.66523
44971	1.03789	2.1
44972	1.04231	2.1
44973	1.04231	2.1
44974	1.16052	2.1
44975	1.11265	0.864999
44976	0.958634	2.1
44977	0.919685	2.1
44978	0.958634	2.1
44979	1.16052	2.1
44980	0.919685	2.09451
44981	1.11265	2.1
44982	1.43837	2.1
44983	2.57085	2.1
44984	1.39332	2.1
44985	2.58228	2.1
44986	3.96417	2.1
44987	1.89226	2.1
44988	1.89226	2.1
44989	1.89226	2.1
44990	1.89226	2.1
44991	1.89226	2.1
44992	1.89226	2.1
44993	1.89226	2.1
44994	1.89226	0.91696
44995	1.89226	1.97906
44996	1.89226	2.09212
44997	3.78452	1.96672
44998	2.57085	2.1
44999	1.89226	2.1
45000	2.58229	2.1
45001	1.89226	2.08679
45002	4.46312	2.1
45003	1.32368	2.1
45004	2.58228	2.1
45005	2.55352	2.1

45006	2.55352	2.1
45007	1.39332	2.1
45008	1.39332	2.1
45009	1.89226	2.1
45010	1.89226	2.1
45011	1.89226	2.1
45012	1.89226	2.1
45013	1.89226	2.1
45014	1.89226	2.1
45015	1.89226	2.06826
45016	1.89226	2.1
45017	1.89226	2.1
45018	1.89226	1.96997
45019	1.89226	2.1
45020	1.89226	1.30011
45021	2.55353	2.1
45022	1.89226	2.09832
45023	2.58228	2.1
45024	1.89226	2.1
45025	1.89226	2.1
45026	2.58228	2.1
45027	1.40574	1.33104
45028	1.05175	1.09001
45029	1.71613	1.38868
45030	1.99961	1.04808
45031	3.12187	1.27858
45032	1.71613	1.37541
45033	2.50733	1.089
45034	1.99961	1.5041
45035	1.99961	1.36133
45036	2.25746	1.03984
45037	2.25746	1.06822
45038	2.25746	0.963952
45039	1.89526	1.01025
45040	1.71613	1.30187
45041	1.71613	0.765274
45042	1.89526	1.04042
45043	1.27558	0.777779
45044	1.07536	2.1
45045	1.07536	2.1
45046	1.07034	2.1
45047	1.07034	2.1
45048	0.766879	2.1
45049	0.81527	2.1
45050	1.38896	2.1
45051	1.33043	2.1
45052	0.815264	2.1
45053	1.38895	2.1
45054	0.766884	2.1
45055	1.33044	2.1
45056	1.89225	2.1
45057	1.89225	2.1
45058	1.89225	2.1
45059	1.89225	2.1
45060	1.89225	2.1
45061	1.89225	2.1
45062	1.64059	2.1
45063	1.89225	2.1

45064	1.565	2.1
45065	1.565	2.1
45066	1.89225	2.1
45067	1.89225	2.1
45068	1.89226	2.1
45069	1.89226	2.1
45070	1.89226	2.1
45071	1.89226	2.1
45072	1.89226	2.1
45073	1.89226	2.1
45074	1.67906	2.1
45075	1.89226	2.1
45076	1.6406	2.1
45077	1.6406	2.1
45078	1.89226	2.1
45079	1.89226	2.1
45080	2.18106	1.39316
45081	2.20831	1.96114
45082	2.64725	1.00359
45083	2.63102	1.29543
45084	4.82831	1.43411
45085	2.18106	1.24296
45086	2.09605	2.1
45087	2.5601	1.33342
45088	2.63102	1.34187
45089	4.74116	1.73448
45090	2.64725	1.19068
45091	1.89226	1.27562
45092	1.87603	1.89105
45093	2.63102	1.20778
45094	1.89226	1.46002
45095	2.64725	0.94699
45096	0.946132	1.31904
45097	1.28005	1.15847
45098	0.946132	1.80793
45099	1.31551	1.01511
45100	1.28005	1.4013
45101	1.28005	1.76234
45102	0.946132	1.00956
45103	0.946132	1.28554
45104	0.946132	1.04041
45105	0.946132	1.21699
45106	1.28005	1.36066
45107	1.31551	1.50458
45108	2.0815	2.1
45109	1.59318	2.1
45110	2.0815	2.1
45111	1.40161	2.1
45112	2.49782	2.1
45113	1.68553	2.1
45114	0.819885	2.1
45115	0.935082	2.1
45116	0.867363	2.1
45117	0.867363	2.1
45118	0.715431	2.1
45119	0.902777	2.1
45120	0.738933	2.1
45121	0.980053	2.1

45122	0.936716	2.1
45123	0.917651	2.1
45124	0.775853	2.1
45125	0.74037	2.1
45126	2.78977	2.1
45127	2.38239	2.1
45128	2.78977	2.1
45129	1.96468	2.1
45130	2.64107	2.1
45131	1.80733	2.1
45132	1.80141	2.1
45133	2.28512	2.1
45134	2.28512	2.1
45135	1.8519	2.1
45136	2.64107	2.1
45137	2.20785	2.1
45138	0.946133	0.588785
45139	0.946133	0.538986
45140	0.946132	0.573432
45141	0.946132	0.532666
45142	0.946132	0.571384
45143	0.946132	0.578708
45144	0.946132	0.529882
45145	0.946132	0.514112
45146	0.946132	0.573821
45147	0.946132	0.572511
45148	0.946133	0.557429
45149	0.946133	0.580627
45150	1.89226	0.375034
45151	1.89226	0.430881
45152	1.89226	0.284705
45153	1.89226	0.395991
45154	1.89226	0.366061
45155	1.89226	0.481063
45156	1.89226	0.414443
45157	1.89226	0.473586
45158	1.89226	0.439605
45159	1.89226	0.505423
45160	3.78453	0.487138
45161	1.89226	0.577404
45162	1.89226	0.529078
45163	1.89226	0.407722
45164	1.89226	0.577109
45165	1.89226	0.485012
45166	1.89226	0.567555
45167	2.12925	0.109528
45168	2.51397	0.141498
45169	2.22488	0.107571
45170	1.86113	0.136149
45171	4.3751	0.131027
45172	2.82754	0.105248
45173	2.51397	0.139557
45174	2.22489	0.103575
45175	2.47291	0.140072
45176	4.98688	0.110202
45177	1.89226	0.143895
45178	1.89226	0.199273
45179	1.89226	0.148338

45180	1.89226	0.188445
45181	3.78453	0.18153
45182	1.89226	0.145832
45183	1.89226	0.189251
45184	1.89226	0.141862
45185	1.89226	0.192013
45186	3.78452	0.149948
45187	0.90848	0.126153
45188	1.11244	0.105862
45189	0.930566	0.110009
45190	0.73385	0.127412
45191	0.921298	0.125424
45192	0.921298	0.131564
45193	0.924994	0.104962
45194	0.924994	0.106759
45195	0.908478	0.115007
45196	0.733851	0.108248
45197	1.11244	0.11027
45198	0.930567	0.114603
45199	2.22489	0.103603
45200	2.0815	0.126534
45201	2.0815	0.129908
45202	2.30674	0.110018
45203	1.99867	0.124197
45204	2.33358	0.105326
45205	1.89226	0.139065
45206	1.89226	0.182462
45207	1.89226	0.142624
45208	1.89226	0.167789
45209	1.89226	0.142671
45210	1.89226	0.166099
45211	0.946131	0.19119
45212	0.946131	0.154894
45213	0.946128	0.178148
45214	0.946128	0.136908
45215	0.946129	0.17591
45216	0.946129	0.191128
45217	0.94613	0.131046
45218	0.94613	0.1365
45219	0.94613	0.15487
45220	0.94613	0.138485
45221	0.94613	0.156256
45222	0.94613	0.166078
45223	2.28904	2.02678
45224	1.89226	1.02363
45225	2.2846	1.22323
45226	2.2846	2.1
45227	1.89226	0.700036
45228	1.89226	2.1
45229	1.89226	1.09485
45230	1.89226	1.13424
45231	1.89226	2.01686
45232	1.89226	1.97003
45233	1.89226	0.795957
45234	1.89226	1.54616
45235	1.04037	0.917036
45236	1.34066	1.45409
45237	1.34955	0.895402

45238	1.34955	1.70345
45239	1.03373	1.06324
45240	1.03373	2.02579
45241	0.946131	0.528322
45242	0.946131	2.1
45243	1.14452	1.60269
45244	1.14452	1.93611
45245	0.946131	1.10943
45246	0.946131	1.03635
45247	1.14296	1.42281
45248	1.14452	2.1
45249	1.14296	0.816174
45250	0.946131	1.08061
45251	1.14452	1.39141
45252	0.946132	1.15093
45253	1.89226	1.02458
45254	1.89226	1.71655
45255	1.89226	1.40578
45256	1.89226	2.02125
45257	1.89226	0.70065
45258	1.89226	1.85624
45259	0.597454	1.01214
45260	0.597454	1.49948
45261	0.597972	0.876993
45262	0.597972	2.1
45263	0.675238	1.67251
45264	0.674776	1.11073
45265	0.519667	1.08051
45266	0.520188	1.22425
45267	0.519667	0.761279
45268	0.675238	0.86635
45269	0.520188	1.22407
45270	0.674777	1.24008
45271	1.89226	0.320958
45272	1.82125	0.219396
45273	1.89226	0.263335
45274	1.84314	0.220523
45275	1.89226	0.406463
45276	1.84314	0.340613
45277	1.89226	0.244237
45278	1.84314	0.25363
45279	1.89226	0.241718
45280	1.65794	0.210835
45281	1.89226	0.254817
45282	1.65794	0.222617
45283	1.348	1.78652
45284	1.43485	2.06753
45285	1.79929	0.984503
45286	1.75309	1.58756
45287	3.14728	1.62028
45288	1.348	1.27738
45289	1.63892	1.72135
45290	1.59157	2.01273
45291	1.18647	1.79978
45292	1.75309	1.33456
45293	1.348	1.60703
45294	1.79929	1.47192
45295	1.75309	2.09831

45296	2.18106	1.23592
45297	2.20831	1.26801
45298	3.98034	1.64384
45299	2.18106	1.09944
45300	1.63892	1.81843
45301	1.75309	1.11149
45302	1.75309	1.90494
45303	2.09605	1.02319
45304	2.09605	1.95394
45305	2.66076	0.382814
45306	1.70302	0.242598
45307	2.41575	0.318462
45308	1.70302	0.295237
45309	3.08598	0.284108
45310	2.3661	0.267077
45311	1.71053	0.273392
45312	2.41575	0.377996
45313	1.20143	0.227982
45314	1.20143	0.247298
45315	2.2123	0.260216
45316	1.66739	0.260744
45317	2.25633	0.276046
45318	2.68302	0.330785
45319	1.71051	0.236076
45320	1.71051	0.261901
45321	2.74192	0.264198
45322	2.1961	0.236673
45323	1.32553	0.790808
45324	2.34875	0.46999
45325	2.19139	0.954811
45326	2.19139	0.369393
45327	1.56172	0.93482
45328	1.56172	0.33824
45329	1.89226	1.71033
45330	1.89226	0.48562
45331	1.89226	1.03462
45332	1.89226	0.677536
45333	3.78453	0.795807
45334	2.667 2.1	
45335	1.26097	0.915593
45336	1.26097	1.93106
45337	1.5683	2.09693
45338	2.42949	1.88069
45339	2.73682	2.1
45340	1.13213	0.732512
45341	2.19139	0.399896
45342	2.0815	0.856095
45343	1.77615	0.2769
45344	1.37767	0.850582
45345	1.19297	0.241797
45346	0.946132	0.486695
45347	0.946132	0.95353
45348	0.946131	1.58582
45349	0.946131	0.656554
45350	0.946131	0.585535
45351	0.946131	0.378909
45352	0.946132	0.938144
45353	0.946132	1.70052

45354	0.946132	0.828022
45355	0.946132	0.824015
45356	0.946131	0.760284
45357	0.946131	0.785479
45358	2.02064	0.986011
45359	2.3606	2.1
45360	1.47478	2.1
45361	2.22997	2.03025
45362	4.25061	2.1
45363	2.36059	2.1
45364	2.56952	2.1
45365	2.22997	2.01972
45366	2.48631	2.1
45367	4.79949	2.1
45368	1.84261	2.1
45369	2.22997	2.1
45370	1.65706	1.44499
45371	1.78837	2.1
45372	3.88703	2.06366
45373	2.22997	2.1
45374	2.48631	2.1
45375	2.19528	2.1
45376	2.41175	2.1
45377	4.64172	2.1
45378	1.6466	2.1
45379	1.89226	2.1
45380	1.35972	2.1
45381	1.35972	2.1
45382	1.89226	2.1
45383	1.89226	2.1
45384	1.89226	2.1
45385	1.89226	2.1
45386	1.89226	2.1
45387	1.89226	2.1
45388	3.78453	2.1
45389	2.44797	2.1
45390	1.07214	2.1
45391	1.31589	2.1
45392	1.1211	2.1
45393	2.44797	2.1
45394	2.25318	2.1
45395	1.95954	2.1
45396	1.89226	2.1
45397	1.6466	2.1
45398	1.6466	2.1
45399	1.89226	2.1
45400	1.89226	2.1
45401	0.946133	2.1
45402	0.946133	2.1
45403	0.946132	2.1
45404	0.946132	2.1
45405	0.946132	2.1
45406	0.946132	2.1
45407	0.946133	2.1
45408	0.946133	2.1
45409	0.946132	2.1
45410	0.946132	2.1
45411	0.946133	2.1

45412	0.946133	2.1
45413	2.44797	2.1
45414	1.36904	2.1
45415	1.70303	2.1
45416	2.37586	2.1
45417	4.00507	2.1
45418	0.631553	0.125541
45419	1.24489	0.106064
45420	1.11597	0.109373
45421	0.496799	0.130683
45422	0.872272	0.127079
45423	0.872272	0.124099
45424	0.869415	0.10503
45425	0.869415	0.106203
45426	0.4968	0.121131
45427	0.631553	0.119532
45428	1.11597	0.113744
45429	1.24489	0.120722
45430	1.84253	2.1
45431	2.17384	2.1
45432	2.04818	2.1
45433	1.65925	2.1
45434	3.83309	2.1
45435	2.04818	2.1
45436	1.65925	2.1
45437	1.48162	2.1
45438	1.92264	2.1
45439	3.58189	2.1
45440	2.3305	0.367447
45441	1.89226	0.401416
45442	2.52753	0.382869
45443	2.52753	0.408376
45444	1.89226	0.373447
45445	1.89226	0.401538
45446	1.89226	0.405553
45447	1.89226	0.520573
45448	1.89226	0.473532
45449	1.89226	0.490951
45450	1.89226	0.440853
45451	1.89226	0.488914
45452	1.34563	0.369436
45453	2.1769	0.431139
45454	1.94955	0.387607
45455	1.94955	0.488947
45456	1.55063	0.398048
45457	1.55063	0.488316
45458	2.09455	0.334489
45459	1.89226	0.391197
45460	2.3305	0.360871
45461	2.3305	0.377101
45462	1.89226	0.351966
45463	1.89226	0.372437
45464	1.89226	0.379348
45465	1.89226	0.404028
45466	1.89226	0.455126
45467	1.89226	0.384115
45468	1.89226	0.396914
45469	1.89226	0.452597

45470	0.773392	0.327841
45471	0.612765	0.665538
45472	0.79504	0.409481
45473	0.575323	0.538711
45474	1.0819	0.33519
45475	1.06106	0.404205
45476	1.04675	0.347203
45477	0.86858	0.422698
45478	0.796873	0.38273
45479	0.557469	0.390945
45480	0.807663	0.411159
45481	2.2024	2.1
45482	2.34106	1.379
45483	2.02634	2.1
45484	2.20125	2.08786
45485	4.36739	2.1
45486	2.34106	1.46003
45487	2.25195	0.500166
45488	2.20125	2.09208
45489	2.13132	0.517673
45490	4.47237	0.773357
45491	2.02635	2.1
45492	2.22465	2.07537
45493	1.68504	2.1
45494	2.0815	1.57386
45495	3.92673	2.1
45496	2.22465	2.09041
45497	2.13133	0.487366
45498	2.0815	1.59483
45499	2.23579	0.591369
45500	4.44862	0.743065
45501	1.89226	0.935782
45502	1.89226	1.31335
45503	1.89226	1.49728
45504	1.89226	1.01172
45505	1.89226	1.41315
45506	1.89226	0.966987
45507	1.93967	0.199804
45508	1.70302	0.258712
45509	1.21883	0.223521
45510	1.70302	0.30315
45511	1.19889	0.202592
45512	1.70302	0.28264
45513	0.946131	0.347597
45514	0.946131	0.285201
45515	0.946131	0.250645
45516	0.946131	0.303051
45517	0.94613	0.312711
45518	0.94613	0.370485
45519	0.946131	0.243314
45520	0.946131	0.280525
45521	0.946131	0.25747
45522	0.946131	0.269177
45523	0.946131	0.281062
45524	0.946131	0.253047
45525	0.883128	0.261254
45526	0.883128	0.316719
45527	0.827519	0.244865

45528	0.82752	0.294271
45529	1.16234	0.286644
45530	1.19063	0.311532
45531	0.631294	0.256852
45532	0.548311	0.248324
45533	0.631293	0.241597
45534	1.16234	0.263833
45535	1.19063	0.281996
45536	0.54831	0.316375
45537	1.89226	0.127614
45538	1.89226	0.188249
45539	1.89226	0.198889
45540	1.89226	0.133855
45541	3.78453	0.15135
45542	2.33328	0.100926
45543	1.87991	0.119239
45544	1.87991	0.131984
45545	2.82938	0.0983204
45546	1.84457	0.129023
45547	2.79403	0.0952929
45548	2.82937	0.0974312
45549	2.70881	0.114366
45550	1.8799	0.122893
45551	1.82739	0.0873021
45552	4.58874	0.0973662
45553	1.89226	0.19541
45554	1.89226	0.118685
45555	1.89226	0.138386
45556	1.89226	0.176369
45557	3.78452	0.167585
45558	1.17897	0.119503
45559	1.82739	0.0902061
45560	1.17897	0.110012
45561	1.76199	0.0976612
45562	1.23885	0.112981
45563	1.80989	0.0953622
45564	0.946131	0.129311
45565	0.946131	0.122885
45566	0.946131	0.158685
45567	0.946131	0.15316
45568	0.946129	0.164525
45569	0.946129	0.117623
45570	0.946133	0.139955
45571	0.946133	0.159403
45572	0.946131	0.136094
45573	0.946131	0.122052
45574	0.946131	0.130792
45575	0.946131	0.130692
45576	0.946126	0.558927
45577	0.946126	2.1
45578	1.24887	2.1
45579	1.24887	2.1
45580	0.946127	1.67352
45581	0.946127	0.726951
45582	1.24887	2.1
45583	1.28511	1.46515
45584	1.2851	1.39226
45585	0.946128	1.49672

45586	1.24887	1.68904
45587	0.946128	1.80467
45588	1.89226	1.95333
45589	1.89226	1.6222
45590	1.89226	1.5663
45591	1.89226	0.78476
45592	3.78452	1.34544
45593	0.592142	1.73905
45594	0.592142	1.53264
45595	0.60496	1.90856
45596	0.60496	2.06863
45597	0.694237	1.86354
45598	0.608714	1.03859
45599	0.562863	1.54377
45600	0.502864	1.92242
45601	0.562863	1.65561
45602	0.694237	1.69385
45603	0.608714	1.94684
45604	0.502864	1.65069
45605	1.89226	0.6368
45606	1.89226	2.1
45607	1.89226	1.24335
45608	1.89226	1.48201
45609	3.78453	1.51094
45610	1.55779	2.1
45611	1.57968	2.1
45612	1.47186	1.04745
45613	1.51725	2.1
45614	3.05154	2.1
45615	1.57967	2.1
45616	1.474	2.1
45617	1.51725	2.1
45618	1.42895	2.1
45619	3.00863	2.1
45620	1.89226	2.1
45621	1.89226	2.1
45622	1.89226	1.54203
45623	1.89226	2.1
45624	3.78452	2.1
45625	1.89226	1.13218
45626	1.89226	1.57081
45627	1.89226	0.628342
45628	1.89226	2.1
45629	3.78453	1.18348
45630	1.47186	1.00996
45631	1.51725	2.1
45632	1.38859	2.1
45633	1.45332	2.1
45634	2.92519	2.1
45635	1.51725	2.1
45636	1.42895	2.1
45637	1.45332	2.1
45638	1.38414	2.1
45639	2.88228	2.1
45640	1.89226	1.61655
45641	1.89226	2.1
45642	1.89226	2.1
45643	1.89226	2.1

45644	3.78452	2.1
45645	1.34497	0.0716243
45646	1.36747	0.0631312
45647	1.18919	0.0664705
45648	2.08149	0.0667343
45649	1.23556	0.0628862
45650	2.13702	0.0639685
45651	1.10106	0.0731277
45652	2.08001	0.0658531
45653	1.34497	0.0710519
45654	2.31605	0.0713916
45655	1.34497	0.064246
45656	2.31605	0.0667672
45657	2.31605	0.0680054
45658	2.0815	0.0677159
45659	1.9419	0.0598101
45660	2.46665	0.0583306
45661	4.52922	0.061669
45662	1.20349	0.0604105
45663	2.08149	0.0673263
45664	1.36948	0.0596623
45665	1.34737	0.0632276
45666	2.08149	0.0605698
45667	2.08149	0.0635175
45668	0.946132	0.0747539
45669	0.946132	0.0792097
45670	1.37715	0.0711837
45671	0.823199	0.0713929
45672	1.21598	0.0730598
45673	0.946132	0.0745045
45674	1.37715	0.0723407
45675	0.946132	0.0739852
45676	1.21598	0.0740301
45677	0.823198	0.0715178
45678	0.946133	0.0725495
45679	0.946133	0.0792072
45680	1.89226	0.0820708
45681	1.89226	0.0891277
45682	1.89226	0.07846
45683	1.89226	0.0765064
45684	3.78453	0.0813155
45685	1.89227	0.0857833
45686	1.89227	0.0798184
45687	1.89227	0.0740306
45688	1.89227	0.0764303
45689	3.78453	0.0794851
45690	0.946132	0.0783803
45691	0.946132	0.0723652
45692	1.21599	0.0719787
45693	1.21599	0.0691743
45694	0.966749	0.0726015
45695	0.946133	0.0738293
45696	1.21598	0.0733224
45697	0.946132	0.0740367
45698	0.966748	0.0726505
45699	1.21598	0.0708441
45700	0.946133	0.0728671
45701	0.946133	0.0756851

45702	0.946133	0.0672292
45703	0.946133	0.0766159
45704	1.1134	0.0699274
45705	1.11555	0.0636946
45706	1.11555	0.0687978
45707	0.946132	0.068473
45708	0.946132	0.0698589
45709	1.1134	0.0694189
45710	1.11555	0.0670998
45711	1.11555	0.0641142
45712	0.946132	0.0769528
45713	0.946132	0.070976
45714	0.946133	0.0660293
45715	0.946133	0.0717702
45716	1.11555	0.0679379
45717	0.978467	0.0639654
45718	0.978468	0.0672729
45719	0.946133	0.0672166
45720	1.11555	0.0673764
45721	0.946132	0.0676142
45722	0.978468	0.0657715
45723	0.978468	0.0637869
45724	0.946132	0.0680435
45725	0.946132	0.0717937
45726	0.851542	2.09437
45727	0.851542	2.03221
45728	1.14168	2.1
45729	1.27897	2.07893
45730	1.02817	2.1
45731	1.13536	2.1
45732	0.946131	1.98058
45733	0.861207	1.94478
45734	0.94613	1.8441
45735	0.94613	2.1
45736	0.868751	2.05607
45737	1.15181	2.1
45738	0.946132	1.18798
45739	0.946132	1.17197
45740	0.946128	1.25328
45741	0.946128	0.881645
45742	0.94613	1.95607
45743	0.94613	1.26822
45744	0.946131	1.04956
45745	0.946131	0.85948
45746	0.94613	0.78874
45747	0.94613	1.18247
45748	0.94613	1.03055
45749	0.94613	1.54607
45750	2.53273	0.0973037
45751	1.89226	0.133207
45752	1.89226	0.115581
45753	1.89226	0.124123
45754	1.89226	0.102948
45755	1.89226	0.113503
45756	1.89226	2.1
45757	1.89226	2.1
45758	1.89226	2.1
45759	1.89226	2.1

45760	3.78452	2.1
45761	1.89226	2.1
45762	2.54394	2.1
45763	1.89226	2.1
45764	2.42221	2.1
45765	4.43621	2.1
45766	2.54394	2.1
45767	1.62743	2.1
45768	2.42221	2.1
45769	1.5437	2.1
45770	4.08764	2.1
45771	1.89226	2.1
45772	1.89226	2.1
45773	1.89226	2.1
45774	1.89226	2.1
45775	3.78452	2.1
45776	1.89226	2.1
45777	1.89226	2.1
45778	1.89226	2.1
45779	1.89226	2.1
45780	3.78452	2.1
45781	1.89226	2.1
45782	2.30146	2.1
45783	2.42221	2.1
45784	1.89226	2.1
45785	1.89226	2.1
45786	2.42221	2.1
45787	2.30146	2.1
45788	2.42221	2.1
45789	1.39962	2.1
45790	1.39962	2.1
45791	1.5437	2.1
45792	2.44553	2.1
45793	1.89226	2.1
45794	1.89226	2.1
45795	1.89226	2.1
45796	1.89226	2.1
45797	3.78452	2.1
45798	1.62196	2.1
45799	2.00695	2.1
45800	1.95288	2.1
45801	1.70303	2.1
45802	3.53316	2.1
45803	1.80703	2.1
45804	2.00695	2.1
45805	1.92991	2.1
45806	2.00695	2.1
45807	1.85888	2.1
45808	1.93592	2.1
45809	1.89226	2.1
45810	1.89226	2.1
45811	1.89226	2.1
45812	1.89226	2.1
45813	1.89226	2.1
45814	1.89226	2.1
45815	1.89226	2.1
45816	1.89226	2.1
45817	1.89226	2.1

45818	1.89226	2.1
45819	3.78453	2.1
45820	1.95288	2.1
45821	1.50524	2.1
45822	1.83564	2.1
45823	1.70303	2.1
45824	2.00749	2.1
45825	1.84671	2.1
45826	1.62632	2.1
45827	1.82498	2.1
45828	1.92991	2.1
45829	1.92991	2.1
45830	1.47928	2.1
45831	1.47751	2.1
45832	0.946128	2.1
45833	0.946128	2.1
45834	0.946132	2.1
45835	0.946132	2.1
45836	0.94613	2.1
45837	0.94613	2.1
45838	0.946129	2.1
45839	0.946129	2.1
45840	0.94613	2.1
45841	0.94613	2.1
45842	0.94613	2.1
45843	0.94613	2.1
45844	1.89226	2.1
45845	1.89226	2.1
45846	1.89226	2.1
45847	1.89226	2.1
45848	1.89226	2.1
45849	1.89226	2.1
45850	2.27351	0.841577
45851	2.74068	0.622138
45852	2.72278	0.722197
45853	2.63356	0.78714
45854	2.36273	0.707269
45855	2.27351	0.770083
45856	2.36273	0.76384
45857	2.83112	0.562151
45858	2.81212	0.664465
45859	2.74068	0.719577
45860	2.43416	0.652011
45861	2.36273	0.706039
45862	1.89227	0.696915
45863	2.83112	0.550419
45864	2.74069	0.677245
45865	2.74069	0.520532
45866	1.89227	0.737169
45867	1.89227	0.609728
45868	2.40887	2.1
45869	1.4494	2.1
45870	1.52164	2.1
45871	2.47534	2.1
45872	3.93051	2.1
45873	1.89226	2.1
45874	2.47534	2.1
45875	2.40887	2.1

45876	2.40887	2.1
45877	1.89226	2.1
45878	1.89226	2.1
45879	1.60861	2.1
45880	2.47534	2.1
45881	1.52164	2.1
45882	1.52164	2.1
45883	2.55301	2.1
45884	2.55301	2.1
45885	0.946128	2.1
45886	0.946128	2.1
45887	1.23767	2.1
45888	1.2765	2.1
45889	1.23767	2.1
45890	1.23767	2.1
45891	0.946131	2.1
45892	0.946131	2.1
45893	0.946131	2.1
45894	1.2765	2.1
45895	1.23767	2.1
45896	0.946131	2.1
45897	1.89226	2.1
45898	1.87511	2.1
45899	1.87511	2.1
45900	1.95852	2.1
45901	2.0815	2.1
45902	2.22169	2.1
45903	1.89226	2.1
45904	1.89226	2.1
45905	1.89226	2.1
45906	1.89226	2.1
45907	1.89226	2.1
45908	1.89226	2.1
45909	2.0815	2.1
45910	1.80067	2.1
45911	1.80067	2.1
45912	2.05518	2.1
45913	1.89226	2.1
45914	2.16509	2.1
45915	1.87153	2.1
45916	2.65897	2.1
45917	1.58724	2.1
45918	2.38924	2.1
45919	4.26077	2.1
45920	1.89226	2.1
45921	1.89226	2.1
45922	1.89226	2.1
45923	1.89226	2.1
45924	1.89226	2.1
45925	1.89226	2.1
45926	1.89226	0.834521
45927	2.21022	0.491016
45928	2.19769	0.917544
45929	2.21022	0.702271
45930	1.87973	0.576319
45931	1.89226	0.397221
45932	1.18376	1.11497
45933	2.21022	0.36461

45934	1.16239	0.998228
45935	1.16239	0.447891
45936	2.19769	1.00126
45937	2.19769	0.455239
45938	0.946128	0.664525
45939	0.946128	0.570604
45940	1.09362	0.835695
45941	1.09884	0.585962
45942	0.94613	0.665404
45943	0.94613	0.374319
45944	1.09362	0.75417
45945	1.09362	0.600927
45946	0.946131	0.702954
45947	1.09362	0.709303
45948	0.946131	0.563232
45949	1.09884	0.462393
45950	1.19323	0.994394
45951	2.19769	0.333137
45952	1.18376	0.87621
45953	1.18376	0.498652
45954	2.18724	0.879227
45955	2.18724	0.555795
45956	2.0815	2.1
45957	0.922714	2.1
45958	2.0815	2.1
45959	1.84882	2.1
45960	1.20787	2.1
45961	1.08321	2.1
45962	2.6238	0.175389
45963	1.89226	0.246728
45964	1.89226	0.25763
45965	2.4866	0.181755
45966	1.89226	0.23598
45967	2.4866	0.183791
45968	2.66753	0.215432
45969	1.89226	0.328049
45970	1.89226	0.362226
45971	2.6238	0.227467
45972	1.89226	0.316765
45973	2.6238	0.230097
45974	0.946129	1.58418
45975	0.946129	2.1
45976	1.04676	2.1
45977	1.04676	2.1
45978	0.946131	2.1
45979	0.946131	1.95999
45980	1.08732	2.1
45981	1.04676	2.1
45982	1.08732	2.1
45983	0.946131	2.1
45984	1.04676	2.1
45985	0.946131	2.1
45986	1.20958	1.52239
45987	1.73802	0.682832
45988	1.39067	0.799468
45989	1.39067	0.635953
45990	1.50969	0.905219
45991	1.50969	0.959367

45992	1.00382	1.11846
45993	1.50969	0.379196
45994	1.20958	0.947855
45995	1.20958	0.491653
45996	1.32039	1.22081
45997	1.32039	0.700823
45998	2.0815	2.1
45999	2.51396	2.1
46000	2.19826	2.1
46001	2.54175	2.1
46002	4.79599	2.1
46003	2.54174	2.1
46004	2.17978	2.1
46005	2.54174	2.1
46006	2.2854	2.1
46007	2.57212	2.1
46008	2.31578	2.1
46009	2.27103	2.1
46010	2.54175	2.1
46011	2.4255	2.1
46012	2.82251	2.1
46013	4.96725	2.1
46014	2.60614	0.179686
46015	2.75374	0.169859
46016	1.94002	0.162466
46017	2.08392	0.177403
46018	4.69006	0.165342
46019	2.75373	0.167523
46020	2.6875	0.194366
46021	2.08392	0.156778
46022	1.99944	0.190099
46023	4.77142	0.188253
46024	1.1211	0.108723
46025	2.08392	0.139271
46026	1.17787	0.130765
46027	1.94002	0.117535
46028	1.32178	0.126331
46029	2.08392	0.12577
46030	2.08392	0.121125
46031	1.99944	0.129122
46032	1.32178	0.108444
46033	1.25929	0.115336
46034	3.34321	0.115754
46035	1.22266	0.0907228
46036	0.779473	0.0869502
46037	0.94613	0.100627
46038	0.94613	0.0973821
46039	1.04072	0.0968056
46040	1.1407	0.0941398
46041	1.10042	0.0940048
46042	0.851542	0.0978721
46043	0.942046	0.093432
46044	0.942046	0.0990001
46045	1.04114	0.0972142
46046	0.826602	0.0939987
46047	2.37468	2.1
46048	1.89226	2.1
46049	1.89226	2.1

46050	1.89226	2.1
46051	2.27917	2.1
46052	2.27917	2.1
46053	1.13959	2.1
46054	1.09451	2.1
46055	0.946131	2.1
46056	0.946131	2.1
46057	1.09451	2.1
46058	1.09451	2.1
46059	0.94613	2.1
46060	0.94613	2.1
46061	1.09451	2.1
46062	0.946131	2.1
46063	0.946131	2.1
46064	1.13959	2.1
46065	2.52454	0.803149
46066	2.12221	2.1
46067	2.67624	0.90226
46068	2.33865	1.38842
46069	4.86319	1.07928
46070	1.89226	1.21042
46071	1.89226	2.1
46072	1.89226	1.27634
46073	1.89226	2.1
46074	3.78452	2.1
46075	1.89226	2.1
46076	1.89226	2.1
46077	1.89226	2.1
46078	1.89226	2.1
46079	3.78452	2.1
46080	2.12221	2.1
46081	1.47769	2.1
46082	2.33865	2.1
46083	1.62898	2.1
46084	3.81634	2.1
46085	2.67624	1.9163
46086	2.37734	1.23667
46087	2.33865	2.1
46088	2.33865	1.69848
46089	2.70125	2.1
46090	2.70125	2.09571
46091	1.89226	1.58043
46092	1.89226	1.90408
46093	1.89226	2.1
46094	1.89226	2.1
46095	1.89226	0.916852
46096	1.89226	1.09334
46097	1.89226	2.01715
46098	1.89226	2.1
46099	1.89226	2.1
46100	1.89226	2.1
46101	1.89226	2.1
46102	1.89226	2.1
46103	2.37733	1.49108
46104	1.95927	2.1
46105	1.82516	1.14724
46106	1.82516	2.1
46107	2.33865	2.1

46108	2.33865	2.1
46109	2.57466	2.1
46110	2.21715	2.1
46111	2.55653	2.1
46112	2.24775	2.1
46113	4.77367	2.1
46114	0.937095	2.1
46115	0.937095	2.1
46116	0.958028	2.1
46117	0.958028	2.1
46118	0.828386	2.1
46119	0.771244	2.1
46120	1.10858	2.1
46121	1.12388	2.1
46122	1.12388	2.1
46123	0.828386	2.1
46124	0.771244	2.1
46125	1.10858	2.1
46126	2.55653	2.1
46127	2.32606	2.1
46128	2.24775	2.1
46129	2.24775	2.1
46130	2.57928	2.1
46131	2.57928	2.1
46132	0.990481	2.1
46133	0.990481	2.1
46134	1.00094	2.1
46135	1.00094	2.1
46136	0.888385	2.1
46137	0.828386	2.1
46138	1.16303	2.1
46139	1.12388	2.1
46140	1.16303	2.1
46141	0.888385	2.1
46142	1.12388	2.1
46143	0.828387	2.1
46144	0.962524	2.1
46145	0.962524	2.1
46146	0.7976	2.1
46147	0.7976	2.1
46148	0.797628	2.1
46149	0.814 2.1	
46150	0.946125	2.1
46151	0.946125	2.1
46152	0.946118	2.1
46153	0.797623	2.1
46154	0.814006	2.1
46155	0.946131	2.1
46156	1.8978	0.0522355
46157	2.23892	0.0522355
46158	1.71469	0.0522355
46159	4.13672	0.0522355
46160	2.64136	0.0522355
46161	2.23892	0.233413
46162	2.37578	0.294567
46163	1.77489	1.79515
46164	4.6147	0.288153
46165	1.02284	2.1

46166	0.898796	0.479974
46167	0.948554	0.684354
46168	0.948554	1.96052
46169	0.878797	1.326
46170	0.796197	2.0275
46171	1.09145	0.561289
46172	0.963109	0.570181
46173	1.12114	0.959641
46174	0.782739	0.865197
46175	1.11084	0.701059
46176	0.850742	0.701059
46177	2.48024	2.1
46178	2.405 2.1	
46179	2.24585	2.1
46180	2.34795	2.1
46181	4.75295	2.1
46182	1.93794	2.1
46183	2.405 2.1	
46184	2.24585	2.1
46185	1.67594	1.39764
46186	4.08094	2.1
46187	1.89226	2.1
46188	1.89226	0.617276
46189	1.89226	1.32637
46190	1.89226	0.82277
46191	3.78452	1.03179
46192	1.89226	2.1
46193	1.89226	1.57126
46194	1.89226	2.1
46195	1.89226	0.852464
46196	3.78452	1.99081
46197	2.24585	2.1
46198	2.34795	2.1
46199	2.21155	2.1
46200	2.06626	2.1
46201	4.4142	2.1
46202	2.24585	2.1
46203	1.41498	1.268
46204	1.41498	2.1
46205	1.67594	2.1
46206	2.06625	2.09257
46207	2.32721	2.1
46208	1.89226	1.20387
46209	1.89226	0.810949
46210	1.89226	2.03895
46211	1.89226	0.710569
46212	1.89226	1.94225
46213	1.89226	0.637412
46214	1.89226	2.1
46215	1.89226	0.831333
46216	1.89226	2.1
46217	1.89226	1.44012
46218	3.78452	1.97652
46219	1.89226	0.182238
46220	1.89226	0.245135
46221	1.89226	0.310938
46222	1.89226	0.158626
46223	3.78452	0.164815

46224	1.89226	0.207318
46225	2.53655	0.122936
46226	1.89226	0.130026
46227	1.89226	0.132976
46228	1.89226	0.188489
46229	1.89226	0.189794
46230	1.89227	2.1
46231	1.89227	2.1
46232	1.89227	2.1
46233	1.89227	2.1
46234	1.89227	2.1
46235	1.89227	2.1
46236	0.91339	2.1
46237	0.91339	2.1
46238	1.2158	2.1
46239	1.20629	2.1
46240	1.00473	2.1
46241	1.15749	2.1
46242	0.946132	2.1
46243	0.946132	2.1
46244	0.946133	2.1
46245	1.17513	2.1
46246	1.00473	2.1
46247	0.946132	2.1
46248	1.89226	2.1
46249	1.89226	2.1
46250	1.89226	2.1
46251	1.89226	2.1
46252	1.89226	2.1
46253	1.89226	2.1
46254	1.89226	2.1
46255	2.6066	2.1
46256	2.35026	2.1
46257	2.35026	2.1
46258	1.89226	2.1
46259	1.89226	2.1
46260	2.04056	0.365563
46261	2.77366	0.3564
46262	1.24682	0.262036
46263	1.24682	0.363331
46264	2.93651	0.295099
46265	2.14277	0.353323
46266	2.68524	1.46278
46267	2.81847	1.39922
46268	1.89226	0.931054
46269	1.89226	1.37619
46270	4.57751	1.12681
46271	2.68524	1.23331
46272	1.89226	1.54373
46273	1.89226	1.5736
46274	2.50732	1.3278
46275	1.89226	1.44586
46276	2.50732	1.49368
46277	1.89226	1.03823
46278	1.89226	1.51044
46279	1.89226	1.33955
46280	1.89226	1.59374
46281	1.89226	1.08799

46282	1.89226	1.49808
46283	1.89226	1.55523
46284	1.89226	1.26165
46285	1.89226	1.32131
46286	1.89226	1.68753
46287	1.89226	0.909302
46288	1.89226	1.1275
46289	1.61016	1.12697
46290	1.07467	1.30791
46291	1.24474	1.00027
46292	1.24474	1.56555
46293	1.45303	1.11875
46294	1.45303	1.58421
46295	1.61016	1.36177
46296	1.24474	1.80571
46297	1.61016	1.42972
46298	1.77186	1.22954
46299	1.37813	1.20798
46300	1.37813	1.39939
46301	1.73305	2.1
46302	2.0815	1.63727
46303	1.97658	2.1
46304	1.75791	2.02558
46305	1.87228	2.1
46306	1.66515	2.1
46307	2.0815	1.71487
46308	1.93938	0.604486
46309	2.03253	0.432444
46310	2.2142	2.04861
46311	1.97658	0.442772
46312	2.15324	2.04037
46313	1.73305	2.1
46314	1.97658	1.66395
46315	1.60428	2.1
46316	1.8589	2.02066
46317	3.58085	2.1
46318	1.97657	1.59092
46319	1.93938	0.571298
46320	1.51316	1.94796
46321	1.85293	0.577984
46322	3.82951	0.770334
46323	1.70303	0.218151
46324	1.96087	0.27771
46325	1.42308	0.261974
46326	1.96087	0.280962
46327	1.25023	0.226555
46328	1.70303	0.248236
46329	0.946132	0.290866
46330	0.946132	0.272582
46331	0.946131	0.249379
46332	0.946131	0.27963
46333	0.946132	0.31309
46334	0.946132	0.305922
46335	0.946132	0.246159
46336	0.946132	0.265848
46337	0.946132	0.298808
46338	0.946132	0.266629
46339	0.946132	0.275763

46340	0.946132	0.300639
46341	1.89226	0.317826
46342	1.89226	0.356744
46343	1.89226	0.330742
46344	1.89226	0.350064
46345	3.78452	0.333952
46346	1.89226	0.267609
46347	1.89226	0.331837
46348	1.89226	0.316127
46349	1.89226	0.33184
46350	1.89226	0.260477
46351	1.89226	0.285818
46352	1.89226	0.307007
46353	1.89226	0.331455
46354	1.89226	0.337033
46355	1.89226	0.326753
46356	1.89226	0.315368
46357	1.89226	0.30409
46358	1.89226	0.236742
46359	1.89226	0.304029
46360	2.48255	0.249084
46361	1.89226	0.280727
46362	3.78452	0.250581
46363	1.89226	2.1
46364	2.42758	2.1
46365	1.9209	2.1
46366	1.89226	2.1
46367	2.45622	2.1
46368	2.42758	2.1
46369	2.42758	2.1
46370	1.49987	2.1
46371	1.61028	2.1
46372	1.58164	2.1
46373	2.45622	2.1
46374	2.42758	2.1
46375	0.94613	2.1
46376	0.94613	2.1
46377	1.24298	2.1
46378	1.24298	2.1
46379	1.24298	2.1
46380	1.22811	2.1
46381	0.946131	2.04554
46382	0.946131	2.1
46383	0.94613	2.1
46384	1.24298	2.1
46385	0.94613	2.1
46386	1.22811	2.1
46387	2.48596	2.1
46388	2.45622	2.1
46389	1.63823	2.1
46390	1.4304	2.1
46391	1.45739	2.1
46392	2.45622	2.1
46393	2.4169	2.1
46394	2.55249	2.1
46395	2.51125	2.1
46396	2.35245	2.1
46397	4.90494	2.1

46398	2.45922	2.1
46399	2.55249	2.1
46400	2.51126	2.1
46401	2.43226	2.1
46402	4.98474	2.1
46403	2.51125	2.1
46404	2.35245	2.1
46405	2.28979	2.1
46406	2.47228	2.1
46407	4.82473	2.1
46408	2.51126	2.1
46409	2.43226	2.1
46410	2.40668	2.1
46411	2.47228	2.1
46412	4.90453	2.1
46413	2.8045	2.1
46414	1.93707	2.1
46415	2.14087	2.1
46416	2.14087	2.1
46417	2.61595	2.1
46418	2.61595	1.91245
46419	1.89226	2.1
46420	1.89226	2.1
46421	1.89226	2.1
46422	1.89226	0.953673
46423	1.89226	2.1
46424	1.89226	0.791769
46425	1.22368	1.60688
46426	1.32339	2.1
46427	0.957125	2.1
46428	0.750074	2.1
46429	1.08438	2.1
46430	1.08438	1.69367
46431	1.13094	2.1
46432	0.970797	2.1
46433	1.17568	2.1
46434	0.764392	2.1
46435	0.944286	2.1
46436	1.27523	2.1
46437	1.89226	1.84083
46438	1.89226	2.09992
46439	1.89226	2.1
46440	1.89226	0.835111
46441	3.78453	2.08484
46442	2.8239	2.1
46443	1.89227	2.1
46444	2.57276	2.1
46445	2.57276	2.1
46446	1.89227	2.1
46447	1.89227	2.1
46448	1.18139	2.1
46449	1.85195	2.1
46450	1.78595	2.1
46451	1.08172	2.1
46452	2.93367	2.1
46453	1.60735	2.1
46454	1.20356	2.1
46455	0.967034	2.1

46456	1.08172	2.1
46457	1.67324	2.1
46458	1.77293	2.1
46459	1.89226	2.1
46460	1.89226	2.1
46461	1.8213	2.1
46462	1.82129	2.1
46463	2.14171	2.1
46464	2.40192	2.1
46465	0.810943	2.1
46466	0.810943	2.1
46467	0.798469	2.1
46468	0.788336	2.1
46469	0.804015	2.1
46470	0.810945	2.1
46471	1.947 2.1	
46472	1.36506	2.1
46473	1.08686	2.1
46474	1.07208	2.1
46475	1.08132	2.1
46476	1.13537	0.357811
46477	1.14754	0.801111
46478	0.946131	0.603476
46479	0.946131	0.969912
46480	0.94613	0.574838
46481	0.94613	0.376544
46482	1.25104	1.04692
46483	1.14754	0.749289
46484	1.13537	0.660959
46485	0.946131	0.66771
46486	0.946131	0.701149
46487	1.25104	0.690832
46488	0.96542	0.274809
46489	0.922284	0.690001
46490	0.946131	0.96855
46491	0.946131	0.631199
46492	0.946131	0.62376
46493	0.946131	0.297243
46494	1.13537	0.987875
46495	0.924824	0.728914
46496	0.859476	0.624438
46497	0.842305	0.635745
46498	1.25996	0.641502
46499	1.04996	0.650507
46500	1.71007	0.0848249
46501	2.73283	0.0835564
46502	2.73283	0.0919633
46503	1.89226	0.0812629
46504	1.89226	0.0903797
46505	1.13262	0.0764439
46506	0.946131	0.0860655
46507	0.946131	0.0926614
46508	0.946131	0.0988668
46509	0.946131	0.116243
46510	0.946132	0.117522
46511	0.946132	0.102048
46512	0.946131	0.0876106
46513	0.946131	0.105069

46514	0.946131	0.0990704
46515	0.946131	0.101248
46516	0.946132	0.100135
46517	0.946132	0.0938253
46518	0.946131	0.0877226
46519	1.36642	0.0814158
46520	0.946125	0.0899181
46521	0.946125	0.0995015
46522	0.946128	0.100147
46523	0.946128	0.0907425
46524	0.946126	0.0835144
46525	0.946128	0.0900198
46526	0.946128	0.0925333
46527	0.946128	0.093076
46528	1.36641	0.0890832
46529	0.946128	0.0908138
46530	1.89226	0.0927623
46531	1.89226	0.124263
46532	1.89226	0.103626
46533	1.89226	0.102844
46534	1.89226	0.106213
46535	1.89226	0.105405
46536	2.69784	2.1
46537	2.51651	2.1
46538	2.56999	2.1
46539	2.74623	2.1
46540	5.26783	2.1
46541	2.69784	2.1
46542	2.48718	2.1
46543	2.50161	2.1
46544	2.74623	2.1
46545	5.19945	2.1
46546	1.89226	2.1
46547	1.89226	2.1
46548	1.89226	2.1
46549	1.89226	2.1
46550	3.78453	2.1
46551	0.874264	2.1
46552	1.09879	2.1
46553	0.946131	2.1
46554	0.946131	2.1
46555	1.17062	2.1
46556	1.17062	2.1
46557	0.874293	2.1
46558	0.874293	2.1
46559	1.09879	2.1
46560	0.94613	2.1
46561	0.946131	2.1
46562	0.874265	2.1
46563	1.89226	2.1
46564	1.89226	2.1
46565	1.89226	2.1
46566	1.89226	2.1
46567	3.78453	2.1
46568	0.977382	2.1
46569	1.16424	2.1
46570	0.946133	2.1
46571	0.946133	2.1

46572	1.33012	2.1
46573	1.25589	2.1
46574	0.888498	2.1
46575	0.888498	2.1
46576	1.25375	2.1
46577	0.946131	2.1
46578	0.946131	2.1
46579	0.977384	2.1
46580	1.89226	2.1
46581	2.19757	2.1
46582	2.62906	2.1
46583	1.89226	2.1
46584	1.89226	2.1
46585	2.62906	2.1
46586	1.27944	1.18145
46587	1.27731	1.86141
46588	0.615701	1.66407
46589	0.615701	1.21694
46590	0.615673	0.29791
46591	0.946133	1.52576
46592	0.948264	0.708671
46593	0.946132	1.13316
46594	0.94752	0.729961
46595	0.61706	0.430119
46596	0.946132	0.870961
46597	0.946132	0.867499
46598	2.76604	2.1
46599	1.66728	2.1
46600	1.66728	2.1
46601	1.83036	2.1
46602	2.54515	2.1
46603	2.70823	2.1
46604	2.54515	2.1
46605	1.49958	2.1
46606	1.49958	2.1
46607	1.66728	2.1
46608	2.34465	2.1
46609	2.51235	2.1
46610	1.21128	2.1
46611	1.21128	2.1
46612	0.946131	2.1
46613	0.946131	1.90473
46614	0.946131	1.84537
46615	0.946131	2.1
46616	1.31401	2.09002
46617	1.21128	2.1
46618	1.21128	2.1
46619	0.946131	2.1
46620	0.946131	2.1
46621	1.31401	2.1
46622	1.67374	2.1
46623	2.29586	2.1
46624	1.50033	2.1
46625	2.16598	2.1
46626	3.83972	2.1
46627	1.89226	2.1
46628	1.89226	1.94427
46629	1.89226	2.05343

46630	1.89226	2.1
46631	3.78452	2.1
46632	1.13537	2.1
46633	1.02188	2.1
46634	0.946132	2.1
46635	0.946132	2.08702
46636	0.946132	1.98211
46637	0.946132	2.1
46638	1.21128	2.1
46639	1.02188	2.1
46640	1.04672	2.1
46641	1.04672	2.1
46642	1.21128	2.1
46643	0.946132	2.1
46644	0.815411	2.1
46645	0.870464	2.1
46646	0.885437	2.1
46647	0.885437	2.1
46648	1.06395	2.1
46649	1.00483	2.1
46650	0.757125	2.1
46651	0.673956	2.1
46652	0.684461	2.1
46653	1.06979	2.1
46654	0.731403	2.1
46655	1.0559	2.1
46656	1.89226	2.1
46657	1.89226	2.1
46658	1.89226	2.1
46659	1.89226	2.1
46660	1.89226	2.06622
46661	1.89226	2.1
46662	1.76897	1.01644
46663	1.76932	1.00774
46664	1.97455	1.72465
46665	1.94302	0.767623
46666	3.71233	0.92703
46667	1.83347	2.1
46668	1.76932	1.02858
46669	1.97455	1.75482
46670	1.76932	1.81815
46671	1.76522	1.80851
46672	1.55999	1.9092
46673	1.89227	1.0267
46674	1.89227	1.23216
46675	1.89227	1.8373
46676	1.89227	0.877792
46677	3.78453	1.00462
46678	1.89226	1.00808
46679	1.89226	0.607768
46680	1.89226	0.735693
46681	1.89226	0.847238
46682	3.78452	0.877552
46683	1.97452	1.54308
46684	1.94299	0.720129
46685	2.17831	0.93128
46686	2.23195	0.920825
46687	4.17495	1.03164

46688	2.23196	0.969362
46689	2.1499	2.01443
46690	1.97452	1.54788
46691	1.97452	1.34484
46692	2.09088	1.70255
46693	1.83345	1.58328
46694	0.94612	1.09809
46695	0.94612	1.42025
46696	0.946119	0.899962
46697	0.946119	0.849906
46698	0.946119	0.850603
46699	0.946119	1.07163
46700	0.94612	0.955774
46701	0.94612	1.42656
46702	0.946132	0.98913
46703	0.946132	0.937814
46704	0.946107	1.03715
46705	0.946107	1.01935
46706	1.89224	0.685875
46707	1.89224	0.80947
46708	1.89224	0.881552
46709	1.89224	0.616371
46710	3.78447	0.658296
46711	1.70302	0.537395
46712	1.056	0.498971
46713	1.21829	0.551392
46714	1.89906	0.497454
46715	3.07892	0.512971
46716	2.20248	0.534037
46717	1.56498	0.500922
46718	1.46539	0.49112
46719	1.48787	0.481938
46720	2.37625	0.548746
46721	2.37625	0.540495
46722	1.14104	0.482866
46723	1.00675	0.488931
46724	0.700689	0.471388
46725	0.624389	0.494737
46726	0.782149	0.387786
46727	0.940197	0.465974
46728	0.803437	0.459616
46729	0.803437	0.493602
46730	0.995423	0.455045
46731	0.700699	0.398266
46732	0.593895	0.535624
46733	1.00673	0.469715
46734	1.18097	0.48634
46735	1.15839	0.474058
46736	0.995407	0.481722
46737	0.865932	0.468443
46738	1.17299	0.428381
46739	0.957054	0.475076
46740	1.08016	0.479523
46741	1.08016	0.517174
46742	0.995425	0.490342
46743	1.24161	0.413097
46744	0.796771	0.438056
46745	1.15837	0.51473

46746	2.57264	0.174597
46747	1.89226	0.209109
46748	2.41701	0.15641
46749	1.89226	0.152246
46750	4.30928	0.162141
46751	1.69055	0.179408
46752	2.39587	0.169207
46753	1.53463	0.148802
46754	2.2053	0.118631
46755	3.89585	0.173346
46756	0.946129	0.165843
46757	0.946129	0.145953
46758	0.94613	0.160731
46759	0.94613	0.17124
46760	0.946132	0.145361
46761	0.946132	0.216617
46762	0.946126	0.178195
46763	0.946126	0.160232
46764	0.94613	0.132428
46765	0.94613	0.157463
46766	0.946129	0.178244
46767	0.946129	0.197179
46768	2.41701	0.150249
46769	1.89226	0.116578
46770	1.89226	0.118361
46771	1.89226	0.137951
46772	2.32462	0.13211
46773	2.32462	0.157803
46774	1.41942	0.0957601
46775	2.2053	0.114137
46776	2.02097	0.104252
46777	2.02097	0.114032
46778	1.53463	0.0950534
46779	1.53463	0.100126
46780	1.89226	0.109016
46781	1.89226	0.143786
46782	1.89226	0.118982
46783	1.89226	0.13504
46784	1.89226	0.118568
46785	1.89226	0.128854
46786	1.89226	1.13694
46787	1.89226	1.94655
46788	1.89226	1.89424
46789	1.89226	1.33277
46790	1.89226	1.71982
46791	1.89226	1.21733
46792	1.02816	1.21335
46793	1.02816	1.53381
46794	0.595141	1.50465
46795	0.595141	1.75211
46796	0.946132	1.38528
46797	0.595113	1.82187
46798	0.677174	1.72533
46799	0.946132	1.1786
46800	0.946132	1.4841
46801	0.946132	1.47528
46802	0.595113	1.4736
46803	0.677174	1.58203

46804	1.89226	1.22071
46805	1.89226	2.01722
46806	1.89226	1.70363
46807	1.89226	1.30441
46808	1.89226	1.8615
46809	1.89226	1.43081
46810	0.946133	1.19194
46811	0.946133	0.935369
46812	0.946132	1.67911
46813	0.946132	1.77524
46814	0.946132	1.75847
46815	0.946132	1.20051
46816	0.946132	1.45539
46817	0.946132	0.942247
46818	0.946132	1.36388
46819	0.946132	1.50938
46820	0.946133	1.24483
46821	0.946133	1.23063
46822	0.946131	1.43441
46823	0.946131	1.37366
46824	0.946131	1.96974
46825	0.946131	1.97797
46826	0.946128	2.03466
46827	0.946128	1.43757
46828	0.946133	1.9999
46829	0.946133	1.33361
46830	0.94613	1.71401
46831	0.94613	1.86355
46832	0.946131	1.74656
46833	0.946131	1.5986
46834	2.0264	1.09164
46835	1.67345	1.19272
46836	2.0264	0.881162
46837	1.70539	0.738537
46838	1.31611	0.955422
46839	1.31611	0.86458
46840	1.89226	0.468897
46841	1.89226	0.466609
46842	1.89226	0.607043
46843	1.89226	0.416053
46844	3.78452	0.421402
46845	1.89226	0.580447
46846	1.89226	0.525597
46847	1.89226	0.521543
46848	1.89226	0.616307
46849	1.89226	0.548776
46850	1.89226	0.650499
46851	1.02645	0.258586
46852	0.810942	0.305518
46853	0.882781	0.32967
46854	0.882781	0.312438
46855	2.80386	0.273704
46856	1.56719	0.307231
46857	1.22499	0.267018
46858	1.08132	0.331801
46859	1.36865	0.287097
46860	0.810957	0.31445
46861	0.810957	0.297644

46862	0.946132	0.405955
46863	0.946132	0.456394
46864	0.946129	0.316415
46865	0.946129	0.360486
46866	0.946131	0.31594
46867	0.946131	0.33773
46868	0.946131	0.445666
46869	0.946131	0.389168
46870	0.94613	0.410395
46871	0.94613	0.398249
46872	0.946131	0.507008
46873	0.946131	0.399467
46874	1.89226	0.452007
46875	1.89226	0.34467
46876	1.89226	0.525835
46877	1.89226	0.346686
46878	3.78453	0.351294
46879	1.30462	0.302332
46880	1.79906	0.358794
46881	1.01299	0.390134
46882	1.44185	0.321359
46883	1.3499	0.337853
46884	1.79906	0.317404
46885	1.89226	0.346987
46886	1.89226	0.416576
46887	1.89226	0.322416
46888	1.89226	0.366519
46889	1.89226	0.402989
46890	1.89226	0.459117
46891	1.89226	0.399261
46892	1.89226	0.387707
46893	1.89226	0.428877
46894	1.89226	0.402337
46895	1.89226	0.404077
46896	1.89226	0.380858
46897	1.3499	0.306458
46898	1.79905	0.336275
46899	1.29001	0.332991
46900	1.36005	0.35145
46901	1.88294	0.328171
46902	1.79905	0.328568
46903	1.89226	0.308965
46904	2.75374	0.270329
46905	2.60614	0.301035
46906	1.89226	0.298491
46907	2.60614	0.268739
46908	1.89226	0.269888
46909	0.946131	0.278947
46910	0.946131	0.283483
46911	1.37687	0.298787
46912	1.37687	0.305776
46913	0.946131	0.297419
46914	1.34375	0.318507
46915	1.37686	0.275891
46916	0.946128	0.265263
46917	1.37687	0.270917
46918	1.34375	0.290817
46919	0.94613	0.300248

46920	0.94613	0.281602
46921	1.44247	2.1
46922	1.12665	2.1
46923	1.50044	2.1
46924	1.73651	2.1
46925	2.94291	2.1
46926	1.73652	2.1
46927	1.64926	2.1
46928	1.44247	2.1
46929	1.44247	2.1
46930	1.64926	2.1
46931	1.43872	2.1
46932	1.50044	2.1
46933	1.73651	2.1
46934	2.00955	2.1
46935	1.87875	2.1
46936	3.50999	2.1
46937	1.64926	2.1
46938	1.73652	2.1
46939	2.00955	2.1
46940	1.8493	2.1
46941	3.65881	2.1
46942	1.89226	0.144256
46943	2.28188	0.167622
46944	1.99182	0.136857
46945	2.28188	0.199583
46946	1.6022	0.125387
46947	1.89226	0.185018
46948	1.89226	0.194834
46949	1.89226	0.217647
46950	1.89226	0.181902
46951	1.89226	0.279515
46952	1.89226	0.168958
46953	1.89226	0.246356
46954	1.89226	0.164806
46955	1.89226	0.178643
46956	2.19578	0.14361
46957	1.89226	0.230674
46958	2.81422	0.148737
46959	2.42521	0.202096
46960	1.89224	0.138152
46961	1.99179	0.155985
46962	1.454	0.138726
46963	1.99179	0.173715
46964	1.35444	0.124251
46965	1.89224	0.161322
46966	1.89224	0.173951
46967	1.89224	0.201589
46968	1.89224	0.172859
46969	1.89224	0.249426
46970	1.89224	0.160144
46971	1.89224	0.220418
46972	1.703	0.155363
46973	2.19575	0.170771
46974	1.9911	0.146688
46975	2.19575	0.20453
46976	1.67811	0.129905
46977	1.89223	0.183205

46978	1.69586	2.1
46979	2.55301	2.1
46980	1.60861	2.1
46981	1.60861	2.1
46982	2.65233	2.1
46983	2.65233	2.1
46984	0.946129	2.1
46985	0.946129	2.1
46986	1.2765	2.1
46987	1.32617	2.1
46988	1.2765	2.1
46989	1.2765	2.1
46990	0.946131	2.1
46991	0.946131	2.1
46992	0.946131	2.1
46993	1.32616	2.1
46994	0.946131	2.1
46995	1.2765	2.1
46996	1.36757	2.1
46997	1.32617	2.1
46998	0.891503	2.1
46999	0.847932	2.1
47000	1.10722	2.1
47001	1.10722	2.1
47002	1.10829	2.1
47003	1.10829	2.1
47004	1.36757	2.1
47005	0.891504	2.1
47006	0.847932	2.1
47007	1.32617	2.1
47008	0.946129	2.1
47009	0.946129	2.1
47010	1.36757	2.1
47011	1.32617	2.1
47012	1.32616	2.1
47013	1.32616	2.1
47014	0.946131	2.1
47015	0.946131	2.1
47016	1.36757	2.1
47017	0.946131	2.1
47018	0.946131	2.1
47019	1.32616	2.1
47020	1.89226	0.166976
47021	1.89226	0.127607
47022	1.89226	0.12384
47023	1.89226	0.182328
47024	3.78452	0.145575
47025	2.36336	0.0964169
47026	1.89226	0.126962
47027	2.67831	0.121423
47028	2.67831	0.106772
47029	1.89226	0.11723
47030	1.89226	0.0919966
47031	1.89227	0.12841
47032	1.89227	0.194592
47033	1.89227	0.183442
47034	1.89227	0.133113
47035	3.78453	0.157077

47036	1.89226	0.119916
47037	1.89226	0.183855
47038	1.89226	0.178673
47039	1.89226	0.135859
47040	3.78452	0.151261
47041	2.36336	0.0982382
47042	2.0815	0.131604
47043	2.0815	0.12191
47044	2.41048	0.100035
47045	1.66267	0.123065
47046	2.13452	0.0971579
47047	1.89226	0.185063
47048	1.89226	0.135048
47049	1.89226	0.178191
47050	1.89226	0.128664
47051	1.89226	0.188629
47052	1.89226	0.143319
47053	1.89226	0.376374
47054	1.89226	0.483472
47055	1.89226	0.348556
47056	1.89226	0.488145
47057	1.89226	0.328997
47058	1.89226	0.47169
47059	0.946128	0.559081
47060	0.946128	0.544416
47061	1.00122	0.450872
47062	1.00122	0.438962
47063	0.94613	0.588883
47064	1.01618	0.499218
47065	0.946131	0.510935
47066	1.00122	0.425039
47067	1.00122	0.424004
47068	1.01618	0.481634
47069	0.946131	0.559802
47070	0.946131	0.500906
47071	1.89226	1.24565
47072	1.89226	0.819376
47073	1.89226	0.772017
47074	1.89226	1.52097
47075	3.78452	0.892994
47076	1.89226	0.966267
47077	1.89226	1.92103
47078	1.89226	1.9376
47079	1.89226	1.12717
47080	1.89226	1.76191
47081	1.89226	1.12633
47082	1.89226	0.682076
47083	1.89226	1.42014
47084	1.89226	1.16389
47085	1.89226	0.784589
47086	1.89226	1.16382
47087	1.89226	0.795119
47088	1.89226	0.800632
47089	1.89226	1.76601
47090	1.89226	1.54138
47091	1.89226	0.943593
47092	1.89226	1.49172
47093	1.89226	1.01135

47094	2.71279	0.8856
47095	1.4155	0.768252
47096	2.28488	0.707888
47097	1.06122	0.823636
47098	3.70038	0.709813
47099	1.89226	0.914822
47100	1.89226	0.836125
47101	1.89226	0.790814
47102	1.89226	0.786248
47103	3.78452	1.08341
47104	0.851542	0.746238
47105	0.851542	0.710809
47106	0.851484	0.740159
47107	0.865567	0.865312
47108	0.981616	0.746276
47109	0.864767	0.794057
47110	0.851542	0.78846
47111	0.851542	0.731079
47112	0.851477	0.775208
47113	0.851477	0.816598
47114	0.865635	0.750777
47115	0.816472	0.716209
47116	1.68443	0.710621
47117	1.44489	2.1
47118	1.54924	0.962038
47119	1.34389	2.1
47120	3.02832	2.09564
47121	1.44489	2.1
47122	1.03899	2.1
47123	1.03899	2.1
47124	1.12075	2.1
47125	1.34389	2.1
47126	1.42566	2.1
47127	1.54924	0.943962
47128	1.34389	2.1
47129	1.44091	0.811599
47130	1.25866	2.1
47131	2.7848	1.92221
47132	0.573141	2.1
47133	0.573141	2.1
47134	0.575681	2.1
47135	0.575681	2.1
47136	0.481985	2.1
47137	0.519492	2.1
47138	0.671948	2.1
47139	0.629331	2.1
47140	0.629329	2.1
47141	0.481986	2.1
47142	0.519494	2.1
47143	0.671947	2.1
47144	1.89226	0.668802
47145	1.89226	1.05636
47146	1.89226	0.816097
47147	2.31925	0.812328
47148	3.78452	0.989229
47149	1.30916	2.1
47150	1.17426	2.1
47151	0.946129	2.1

47152	0.946129	2.1
47153	0.94613	2.1
47154	0.94613	2.1
47155	1.17426	2.1
47156	1.17426	2.1
47157	1.30916	2.1
47158	0.94613	2.1
47159	1.17426	2.1
47160	0.94613	2.1
47161	2.56346	2.08949
47162	1.89226	1.11812
47163	1.89226	1.9055
47164	1.89226	1.26762
47165	2.6074	1.87856
47166	2.6074	1.27495
47167	1.89226	2.09494
47168	1.89226	2.09546
47169	1.89226	2.1
47170	1.89226	1.88208
47171	3.78452	2.1
47172	1.33064	2.09961
47173	1.84121	2.1
47174	0.946129	2.1
47175	0.946129	2.1
47176	0.946131	2.1
47177	0.946131	2.1
47178	1.30916	2.1
47179	1.8412	2.1
47180	1.33064	2.1
47181	0.94613	2.1
47182	1.30916	2.1
47183	0.946131	2.1
47184	1.19283	1.12973
47185	1.19283	0.999975
47186	0.946131	2.09732
47187	0.946131	1.88633
47188	0.946132	1.93605
47189	0.946132	1.09736
47190	1.3037	2.09772
47191	1.19283	1.0303
47192	1.19283	1.55959
47193	0.946131	1.55783
47194	0.946131	1.49468
47195	1.3037	1.60858
47196	1.89226	2.1
47197	1.89226	1.88733
47198	1.89226	2.06225
47199	1.89226	2.09711
47200	1.89226	2.07687
47201	1.89226	2.1
47202	2.17708	1.26882
47203	1.70302	2.1
47204	2.28188	1.25087
47205	2.13621	2.1
47206	4.28253	1.92386
47207	1.89226	0.989205
47208	1.89226	2.1
47209	1.89226	1.28884

47210	1.89226	2.1
47211	3.78452	1.90149
47212	0.946132	2.1
47213	0.946132	2.1
47214	0.946132	2.03956
47215	0.946132	2.1
47216	0.946131	2.1
47217	0.946131	2.1
47218	0.946132	2.04577
47219	0.946132	1.93662
47220	0.946132	2.1
47221	0.946132	2.1
47222	0.946132	2.1
47223	0.946132	2.1
47224	1.74933	2.1
47225	1.11833	2.1
47226	1.90074	2.1
47227	1.669	2.09855
47228	1.335	2.1
47229	1.11833	2.01228
47230	2.28188	1.10055
47231	2.31804	2.1
47232	2.42758	1.26624
47233	2.14895	2.1
47234	4.43083	1.98912
47235	1.89226	1.11852
47236	1.89226	2.1
47237	1.89226	1.04761
47238	1.89226	2.1
47239	3.78452	1.5015
47240	1.89226	1.9711
47241	1.89226	2.1
47242	1.89226	2.1
47243	1.89226	2.1
47244	1.89226	1.94816
47245	1.89226	2.1
47246	0.864133	2.1
47247	0.864133	2.1
47248	0.967939	2.02024
47249	0.793739	2.1
47250	0.650797	2.1
47251	0.757546	2.1
47252	1.08941	2.0071
47253	1.10136	2.02888
47254	1.10138	2.1
47255	0.796445	2.1
47256	0.866907	2.1
47257	0.650788	2.1
47258	2.63062	0.530752
47259	1.89226	0.496069
47260	1.89226	0.45457
47261	1.89226	0.654665
47262	2.46557	0.327104
47263	2.46557	0.696929
47264	1.16777	0.568896
47265	1.38859	2.1
47266	1.05042	0.895419
47267	1.28909	1.33933

47268	2.45686	1.31709
47269	1.89227	0.599272
47270	1.89227	0.652633
47271	1.89227	0.431351
47272	1.89227	1.512
47273	3.78453	0.587177
47274	2.15114	2.1
47275	1.89226	1.72947
47276	2.15005	1.82703
47277	2.15005	2.1
47278	1.89226	0.983278
47279	1.89226	2.1
47280	1.89226	1.90268
47281	1.89226	1.02205
47282	1.89226	2.01418
47283	1.89226	2.1
47284	1.89226	0.810849
47285	1.89226	1.87059
47286	0.973447	1.64716
47287	1.32143	1.76498
47288	1.37444	1.69081
47289	1.37444	1.78499
47290	0.949134	1.85078
47291	0.949134	2.03562
47292	0.946131	0.812206
47293	0.946131	2.1
47294	1.07557	1.80636
47295	1.07557	2.1
47296	0.946131	1.50395
47297	0.946131	1.68455
47298	1.07557	2.1
47299	1.08579	2.1
47300	1.08579	2.0495
47301	0.946131	2.07234
47302	1.07557	2.09011
47303	0.946131	2.08157
47304	0.946131	0.634964
47305	0.946131	2.1
47306	0.946131	1.3673
47307	0.946131	1.57007
47308	0.94613	1.01698
47309	0.94613	1.27381
47310	0.946131	1.65956
47311	0.946131	1.83299
47312	0.946131	1.29486
47313	0.946131	1.2561
47314	0.946131	1.37444
47315	0.946131	1.54169
47316	0.606115	1.56499
47317	0.606115	1.58196
47318	0.616308	1.83576
47319	0.616308	2.1
47320	0.68722	1.02439
47321	0.735699	1.77276
47322	0.514789	1.47602
47323	0.486724	1.87903
47324	0.514788	1.22831
47325	0.735699	1.24286

47326	0.486723	1.73747
47327	0.687221	1.59264
47328	2.33761	0.670742
47329	2.51749	0.505637
47330	2.68092	0.566866
47331	2.58304	0.531447
47332	5.01853	0.534782
47333	2.50906	0.606232
47334	2.51749	0.529463
47335	2.68091	0.586181
47336	2.53176	0.462454
47337	5.18997	0.560333
47338	2.68092	0.572188
47339	2.58304	0.515243
47340	2.71793	0.567932
47341	2.67098	0.458665
47342	5.39885	0.537822
47343	2.68091	0.594326
47344	2.53175	0.463685
47345	2.36417	0.526682
47346	2.67098	0.473268
47347	5.04508	0.50162
47348	2.76309	2.1
47349	1.89226	1.40564
47350	1.89226	2.1
47351	1.89226	1.83538
47352	2.48573	2.1
47353	2.48573	1.63976
47354	1.35879	1.90319
47355	1.55161	0.541587
47356	1.10407	1.1994
47357	1.32259	0.579412
47358	2.68138	0.826977
47359	1.89226	1.96666
47360	1.89226	0.537563
47361	1.89226	1.35573
47362	1.89226	0.584126
47363	3.78452	0.751349
47364	1.19612	1.58361
47365	1.24694	2.1
47366	1.24844	0.579205
47367	1.29747	1.86464
47368	2.49358	1.49881
47369	1.89226	1.64857
47370	1.89226	2.05358
47371	1.89226	0.513704
47372	1.89226	1.87591
47373	3.78453	1.35543
47374	1.89226	1.2702
47375	1.89226	2.1
47376	1.89226	2.1
47377	1.89226	2.1
47378	1.89226	1.18834
47379	1.89226	2.1
47380	0.587463	2.1
47381	0.587463	2.02021
47382	0.581977	1.1635
47383	0.581977	2.1

47384	0.531709	2.1
47385	0.545972	2.1
47386	0.623469	1.50682
47387	0.648733	1.50714
47388	0.648733	2.1
47389	0.545971	2.1
47390	0.623469	2.1
47391	0.531709	2.1
47392	1.29417	0.660816
47393	1.29747	2.1
47394	1.27829	2.1
47395	1.24844	0.97977
47396	1.32732	1.39583
47397	1.29747	0.888458
47398	1.89226	0.773202
47399	1.89226	2.1
47400	1.89226	0.52714
47401	1.89226	0.92667
47402	1.89226	1.12537
47403	1.89226	2.1
47404	1.89226	2.1
47405	1.89226	2.1
47406	1.89226	0.888491
47407	1.89226	1.92914
47408	1.89226	1.86672
47409	1.89226	0.867415
47410	0.60346	2.1
47411	0.60346	1.30814
47412	0.60086	0.742438
47413	0.60086	2.1
47414	0.545971	2.1
47415	0.555587	2.1
47416	0.648733	1.21849
47417	0.663661	1.19234
47418	0.663661	1.9945
47419	0.555587	1.49639
47420	0.648733	2.1
47421	0.545971	2.1
47422	1.34066	1.41752
47423	1.36601	2.1
47424	1.34955	0.608423
47425	1.37623	2.01234
47426	2.71556	1.54725
47427	1.89226	1.1565
47428	1.89226	2.1
47429	1.89226	0.608356
47430	1.89226	1.39762
47431	3.78452	1.0394
47432	0.946132	2.1
47433	0.946132	1.32238
47434	0.946132	0.691869
47435	0.946132	2.1
47436	0.946132	2.1
47437	0.946132	2.1
47438	0.946131	0.975505
47439	0.946131	0.966131
47440	0.946132	1.88584
47441	0.946132	1.70609

47442	0.946132	1.88843
47443	0.946132	1.87753
47444	0.631351	0.755471
47445	0.631351	2.1
47446	0.632421	1.08535
47447	0.632421	2.1
47448	0.573604	2.1
47449	0.580765	2.1
47450	0.683006	0.780131
47451	0.688118	1.34342
47452	0.688118	2.01477
47453	0.580765	2.06305
47454	0.683007	2.1
47455	0.573604	2.1
47456	1.34955	1.60608
47457	1.37624	2.1
47458	1.35048	0.638135
47459	1.37999	1.94267
47460	2.72954	1.35193
47461	0.946131	1.34622
47462	0.946131	1.06395
47463	0.946131	1.90432
47464	0.946131	1.9974
47465	0.94613	2.1
47466	0.94613	1.22281
47467	0.946133	0.970801
47468	0.946133	1.33914
47469	0.946131	1.41235
47470	0.946131	0.98095
47471	0.946132	1.22687
47472	0.946132	1.13251
47473	1.89226	1.25812
47474	1.89226	1.29211
47475	1.89226	1.32486
47476	1.89226	2.1
47477	1.89226	2.1
47478	1.89226	2.1
47479	0.532189	0.820871
47480	0.531141	2.1
47481	0.497784	2.1
47482	0.502536	2.1
47483	1.37999	1.31713
47484	0.92433	2.01921
47485	0.670081	1.74904
47486	0.727155	2.1
47487	0.786438	0.909664
47488	0.589798	2.06916
47489	0.497784	2.1
47490	1.89226	2.1
47491	2.72301	2.1
47492	2.4158	1.87256
47493	2.4158	2.1
47494	1.89226	1.84432
47495	1.89226	2.1
47496	1.89226	2.1
47497	1.89226	2.1
47498	1.89226	2.1
47499	1.89226	2.1

47500	1.89226	2.1
47501	1.89226	2.1
47502	1.36919	2.1
47503	1.12717	2.1
47504	1.38634	2.1
47505	1.56541	2.1
47506	2.75552	2.1
47507	1.89226	2.1
47508	1.89226	2.1
47509	1.89226	2.1
47510	1.89226	2.1
47511	1.89226	2.1
47512	1.89226	2.1
47513	0.907036	2.1
47514	0.782706	2.1
47515	0.838808	2.1
47516	0.693168	2.1
47517	0.794747	2.1
47518	0.794747	2.1
47519	0.805458	2.1
47520	0.805458	2.1
47521	0.907036	2.1
47522	0.838808	2.1
47523	0.693169	2.1
47524	0.782707	2.1
47525	0.94613	2.1
47526	0.94613	2.1
47527	1.188	2.1
47528	1.28421	2.1
47529	1.18801	2.1
47530	1.18801	2.1
47531	0.946129	2.1
47532	0.946129	2.1
47533	1.28421	2.1
47534	0.94613	2.1
47535	1.188	2.1
47536	0.94613	2.1
47537	1.89226	2.1
47538	1.89226	2.1
47539	1.89226	2.1
47540	1.89226	2.1
47541	1.89226	2.1
47542	1.89226	2.1
47543	1.16817	2.1
47544	1.06007	2.1
47545	1.19785	2.1
47546	1.2832	2.1
47547	2.36602	2.1
47548	0.960106	2.1
47549	2.0073	2.1
47550	1.51754	2.1
47551	2.56883	2.1
47552	3.52484	2.1
47553	1.06756	2.1
47554	1.57913	1.81888
47555	1.87532	1.33183
47556	1.38666	2.1
47557	2.94287	2.1

47558	2.15622	0.981581
47559	1.38668	2.1
47560	1.87534	2.1
47561	1.87534	2.1
47562	1.70539	1.76356
47563	1.70539	2.1
47564	0.986095	2.1
47565	1.36226	2.1
47566	1.3331	2.1
47567	1.3331	2.1
47568	1.07156	2.1
47569	1.07156	2.1
47570	0.682081	1.19698
47571	0.68748	1.00862
47572	0.650003	0.657425
47573	0.645873	0.828979
47574	0.666344	0.663365
47575	0.666344	0.949373
47576	0.66161	1.55535
47577	0.66161	0.702098
47578	0.68748	0.825187
47579	0.645873	0.831717
47580	0.650004	0.833284
47581	0.682082	0.82655
47582	0.946132	0.994135
47583	0.946132	0.784322
47584	0.946132	0.689306
47585	0.946132	0.538216
47586	0.946132	0.540779
47587	0.946132	0.777484
47588	0.946133	0.717689
47589	0.946133	1.00487
47590	0.946132	0.721897
47591	0.946132	0.71512
47592	0.946133	0.758618
47593	0.946133	0.744234
47594	1.89227	0.940249
47595	2.79312	0.499565
47596	2.77591	0.693388
47597	2.79312	0.702004
47598	1.87505	0.5993
47599	1.89227	0.627157
47600	0.68748	1.26413
47601	0.698626	1.04212
47602	0.645873	0.68854
47603	0.650148	0.827009
47604	0.670561	0.696999
47605	0.670561	1.01106
47606	0.667067	1.5247
47607	0.667067	0.692333
47608	0.698627	0.862082
47609	0.650148	0.899601
47610	0.68748	0.89044
47611	0.645873	0.898377
47612	0.946132	1.06912
47613	0.946132	0.795827
47614	0.946132	0.730493
47615	0.946132	0.615154

47616	0.946132	0.612726
47617	0.946132	0.798382
47618	0.946133	0.752171
47619	0.946133	1.10528
47620	0.946133	0.764341
47621	0.946133	0.764171
47622	0.946132	0.809146
47623	0.946132	0.787531
47624	0.946133	0.789579
47625	0.946133	0.651227
47626	1.38709	0.734844
47627	1.38795	0.51154
47628	0.946132	0.51721
47629	0.946132	0.651173
47630	1.38709	0.853028
47631	1.38709	0.587157
47632	1.38709	0.683644
47633	0.946133	0.684078
47634	0.946132	0.686252
47635	1.38795	0.639778
47636	1.70301	0.115855
47637	2.07659	0.0998908
47638	1.89226	0.112414
47639	1.89226	0.114961
47640	1.76332	0.0976193
47641	1.76331	0.101153
47642	1.89226	0.145409
47643	1.89226	0.119303
47644	1.89226	0.143039
47645	1.89226	0.144746
47646	1.89226	0.117018
47647	1.89226	0.120772
47648	1.89226	0.134578
47649	1.89226	0.141623
47650	1.89226	0.109755
47651	1.89226	0.113975
47652	3.78452	0.131761
47653	1.89226	0.105174
47654	2.12492	0.097162
47655	1.89226	0.111983
47656	1.89226	0.107364
47657	2.07659	0.0972059
47658	2.07659	0.0936961
47659	0.94613	0.0915702
47660	0.94613	0.101393
47661	1.17498	0.0891894
47662	1.32446	0.0846748
47663	1.32446	0.089349
47664	0.94613	0.0930023
47665	0.946127	0.0960327
47666	1.17498	0.0897327
47667	1.32446	0.0901125
47668	1.32446	0.0815912
47669	0.946128	0.10406
47670	0.946128	0.0937517
47671	1.89226	2.1
47672	1.86472	2.1
47673	2.11049	2.1

47674	1.89226	2.1
47675	2.08295	2.1
47676	1.86472	2.1
47677	1.89226	2.1
47678	2.08295	2.1
47679	2.14023	2.1
47680	1.89226	2.1
47681	2.33091	2.1
47682	2.08295	2.1
47683	0.809215	2.1
47684	0.809215	1.57795
47685	0.810915	2.1
47686	0.810915	2.1
47687	1.09581	2.08591
47688	1.08271	2.1
47689	0.520996	2.1
47690	0.537426	2.1
47691	0.520997	2.1
47692	1.08271	2.1
47693	0.537426	2.1
47694	1.09581	2.1
47695	0.79674	2.1
47696	0.79674	2.1
47697	0.799279	2.1
47698	0.799279	2.1
47699	1.07502	2.1
47700	1.0827	2.03006
47701	0.508206	2.1
47702	0.520996	2.1
47703	0.508206	2.1
47704	1.07502	2.1
47705	0.520996	2.1
47706	1.0827	2.1
47707	2.15714	2.1
47708	1.70302	2.1
47709	1.78732	2.1
47710	2.02219	2.1
47711	3.98971	2.1
47712	2.0927	2.1
47713	2.162	2.1
47714	2.11563	2.1
47715	2.02219	2.1
47716	4.18419	2.1
47717	1.62483	2.1
47718	2.02219	2.1
47719	1.93617	2.1
47720	1.52008	2.1
47721	3.54227	2.1
47722	2.11563	2.1
47723	2.02219	2.1
47724	2.29218	2.1
47725	2.03975	2.1
47726	4.06194	2.1
47727	2.007	2.1
47728	1.89226	2.1
47729	1.89226	2.1
47730	1.89226	2.1
47731	2.06776	2.1

47732	2.06776	2.1
47733	0.946129	2.1
47734	0.946129	2.1
47735	1.0035	2.1
47736	1.0035	2.1
47737	0.946131	2.1
47738	0.946131	2.1
47739	0.968797	2.1
47740	1.0035	2.1
47741	0.968771	2.1
47742	0.946105	2.1
47743	1.00353	2.1
47744	0.946157	2.1
47745	1.79103	1.80692
47746	2.53286	0.630249
47747	2.60781	1.91634
47748	2.219	0.868528
47749	2.17985	1.45861
47750	1.79103	0.622575
47751	2.219	1.45481
47752	2.66359	1.43302
47753	2.70084	1.9422
47754	2.38698	1.20604
47755	2.53285	0.960687
47756	2.219	0.789784
47757	1.33179	1.35608
47758	0.946128	0.916617
47759	0.946133	1.12271
47760	1.26643	1.69423
47761	1.3318	1.00744
47762	1.3318	1.61026
47763	0.946131	0.813885
47764	0.946131	1.30531
47765	0.946131	0.825636
47766	0.946131	0.720937
47767	1.33179	0.986807
47768	1.26643	1.73896
47769	2.28979	2.1
47770	2.47227	2.1
47771	2.43451	2.1
47772	2.22627	2.1
47773	4.69855	2.1
47774	2.40667	2.1
47775	2.47228	2.1
47776	2.43451	2.1
47777	2.38069	2.1
47778	4.85296	2.1
47779	2.43451	2.1
47780	2.15715	2.1
47781	2.15715	2.1
47782	2.22627	2.1
47783	2.39489	2.1
47784	2.46402	2.1
47785	2.43451	2.1
47786	2.34899	2.1
47787	2.38069	2.1
47788	2.38069	2.1
47789	2.3949	2.1

47790	2.3949	2.1
47791	2.85425	0.826531
47792	1.70302	0.506407
47793	1.70302	0.483767
47794	1.52254	0.525259
47795	2.34181	0.528077
47796	2.08499	0.595453
47797	1.89226	0.514673
47798	1.89226	0.538435
47799	1.89226	0.401258
47800	1.89226	0.474097
47801	1.89226	0.425123
47802	1.89226	0.578504
47803	2.34181	0.641428
47804	1.38287	0.634724
47805	1.38287	0.588006
47806	1.84132	0.54504
47807	1.96416	0.540327
47808	2.4907	0.571801
47809	0.931432	0.716278
47810	0.946135	0.461058
47811	0.946133	0.423807
47812	0.946133	0.409764
47813	0.931464	0.515239
47814	0.931464	0.713795
47815	1.15573	0.422058
47816	0.96378	0.956404
47817	0.931434	0.545276
47818	0.946131	0.410184
47819	0.946131	0.432187
47820	1.13808	0.411862
47821	0.946132	0.639847
47822	0.946132	0.509147
47823	0.946132	0.5978
47824	0.946132	0.426614
47825	0.946133	0.604739
47826	0.946133	0.438504
47827	0.946132	0.656546
47828	0.946132	0.560765
47829	0.946133	0.504733
47830	0.946133	0.640881
47831	0.946132	0.446364
47832	0.946132	0.351255
47833	1.68074	2.1
47834	2.29459	2.1
47835	2.23361	2.1
47836	1.57234	2.1
47837	3.86693	2.1
47838	2.61243	2.1
47839	2.29458	2.1
47840	2.2336	2.1
47841	2.58234	2.1
47842	4.87692	2.1
47843	0.73506	2.1
47844	0.786166	2.1
47845	1.08729	2.1
47846	1.11679	2.1
47847	0.93134	2.1

47848	0.93134	2.1
47849	0.942113	2.1
47850	0.942113	2.1
47851	0.735065	2.1
47852	1.08729	2.1
47853	1.11679	2.1
47854	0.78616	2.1
47855	1.08729	2.1
47856	1.11679	2.1
47857	1.27502	2.1
47858	1.29116	2.1
47859	1.19259	2.1
47860	1.19259	2.1
47861	1.19923	2.1
47862	1.19923	2.1
47863	1.27503	2.1
47864	1.08729	2.1
47865	1.11679	2.1
47866	1.29115	2.1
47867	0.974715	0.785371
47868	1.43779	0.5907
47869	1.93915	0.625883
47870	1.53908	0.640879
47871	2.91387	0.625119
47872	1.53908	0.607648
47873	0.99845	0.617208
47874	1.5176	0.759745
47875	1.97963	0.568291
47876	3.05668	0.633092
47877	1.93915	0.611426
47878	1.97963	0.585571
47879	1.53908	0.595203
47880	1.53908	0.526875
47881	2.3376	0.739069
47882	2.3376	0.604
47883	1.89226	2.06597
47884	2.59747	2.1
47885	1.91802	2.1
47886	1.89226	2.1
47887	2.62322	2.1
47888	2.59747	2.03516
47889	2.59747	2.1
47890	1.87119	2.1
47891	1.84844	2.1
47892	1.82268	2.1
47893	2.62323	2.1
47894	2.59747	2.1
47895	0.94613	2.1
47896	0.94613	2.00131
47897	1.32697	2.06028
47898	1.32697	2.1
47899	1.32697	2.1
47900	1.31161	2.1
47901	0.946132	2.1
47902	0.946132	1.63469
47903	0.94613	2.1
47904	1.32697	2.1
47905	0.946131	2.1

47906	1.31161	2.1
47907	2.62323	2.1
47908	1.91704	2.1
47909	1.90191	2.1
47910	1.87119	2.1
47911	2.65395	2.1
47912	2.62323	2.1
47913	1.89226	2.1
47914	1.89226	2.1
47915	2.15958	2.1
47916	2.15958	2.1
47917	2.46558	2.1
47918	2.19826	2.1
47919	2.46558	2.1
47920	1.89226	2.1
47921	1.89226	2.1
47922	1.89226	2.1
47923	2.61064	2.1
47924	2.61064	2.1
47925	1.81759	0.685621
47926	1.89526	0.71056
47927	2.32219	0.937194
47928	1.881	0.603679
47929	1.81759	0.797085
47930	1.3764	0.570538
47931	1.81759	0.69976
47932	2.57079	0.659007
47933	2.32219	1.0936
47934	2.32219	0.629364
47935	1.99875	0.782645
47936	1.99875	0.542953
47937	0.536182	0.507412
47938	0.579206	0.397716
47939	0.908796	0.68497
47940	0.999375	0.560677
47941	0.732091	0.586289
47942	0.732091	0.405635
47943	0.755911	0.498047
47944	0.755911	0.617214
47945	0.999376	0.543314
47946	0.579206	0.579744
47947	0.908796	0.564187
47948	0.536182	0.554486
47949	0.94613	0.357675
47950	0.94613	0.34303
47951	1.31112	0.397299
47952	1.18275	0.330473
47953	0.946126	0.362018
47954	0.946126	0.220556
47955	1.31112	0.409089
47956	1.31112	0.352391
47957	0.946128	0.3452
47958	1.31112	0.371968
47959	0.946128	0.349843
47960	1.18275	0.243539
47961	2.64617	0.648025
47962	1.27737	0.475406
47963	1.83076	0.593232

47964	1.27737	0.528469
47965	3.03764	0.632549
47966	2.3655	0.428838
47967	1.12991	0.0590952
47968	1.22375	0.0555588
47969	1.35931	0.0567481
47970	1.04971	0.0619529
47971	1.0419	0.0584629
47972	1.15237	0.0580745
47973	1.19593	0.0579222
47974	1.30306	0.0573677
47975	1.12937	0.0552676
47976	1.31934	0.0553684
47977	1.15203	0.0616413
47978	1.15203	0.0592041
47979	2.65227	0.0558972
47980	1.46041	0.0619668
47981	2.22712	0.0566894
47982	2.04816	0.0600229
47983	2.22712	0.0580157
47984	2.04816	0.0621364
47985	1.89226	0.161338
47986	1.89226	0.584283
47987	1.89226	0.367012
47988	1.89226	0.372832
47989	3.78452	0.431663
47990	1.24653	0.475363
47991	1.66554	0.569651
47992	1.53538	0.320164
47993	1.53538	0.658064
47994	1.40915	0.359208
47995	1.40915	0.664038
47996	1.66555	0.56245
47997	1.75835	2.1
47998	1.53538	0.753726
47999	1.65313	0.979705
48000	3.31868	0.976427
48001	1.89226	0.574132
48002	1.89226	0.608784
48003	1.89226	0.354252
48004	1.89226	1.12118
48005	3.78453	0.555949
48006	1.89226	0.287668
48007	1.89226	0.40158
48008	1.89226	0.247675
48009	1.89226	0.647531
48010	3.78452	0.266928
48011	0.658778	1.23454
48012	0.658778	0.387638
48013	0.661956	0.385307
48014	0.661956	0.619455
48015	0.703822	0.606204
48016	0.767689	0.915736
48017	0.623266	0.328196
48018	0.553046	0.601485
48019	0.703804	0.602916
48020	0.55303	0.61205
48021	0.623282	0.493758

48022	0.767711	0.519238
48023	1.53538	0.738725
48024	1.65313	1.13283
48025	1.40765	0.598208
48026	1.55779	2.1
48027	3.06078	0.724699
48028	1.89227	0.370547
48029	1.89227	1.20935
48030	1.89227	0.625085
48031	1.89227	0.62048
48032	3.78453	0.671058
48033	1.10517	2.1
48034	2.16532	2.1
48035	1.94374	2.1
48036	1.94001	2.1
48037	1.37756	2.1
48038	1.37756	2.1
48039	1.7453	0.16125
48040	1.83284	0.183342
48041	2.28483	0.241883
48042	1.77804	0.149765
48043	1.7453	0.278357
48044	1.23851	0.16168
48045	1.7453	0.20355
48046	2.54752	0.174716
48047	2.28483	0.216408
48048	2.28483	0.160334
48049	2.0189	0.225973
48050	2.0189	0.173888
48051	1.12457	0.150487
48052	2.0189	0.141616
48053	1.7453	0.17507
48054	1.7453	0.132001
48055	1.40707	0.183163
48056	1.40707	0.144573
48057	2.41176	2.1
48058	2.59262	2.1
48059	2.34085	2.1
48060	2.55827	2.1
48061	4.97003	2.1
48062	2.59262	2.1
48063	2.61901	2.1
48064	2.55827	2.1
48065	2.64067	2.1
48066	5.17728	2.1
48067	2.34084	2.1
48068	2.55827	2.1
48069	2.24937	2.1
48070	2.49053	2.1
48071	4.80764	2.1
48072	2.55826	2.1
48073	2.64067	2.1
48074	2.49053	2.1
48075	2.58639	2.1
48076	5.14465	2.1
48077	2.27918	0.102906
48078	2.44132	0.136955
48079	1.89226	0.102272

48080	1.89226	0.11426
48081	4.33359	0.104924
48082	1.89226	0.112058
48083	2.44132	0.138472
48084	2.6771	0.144044
48085	2.6771	0.118538
48086	1.89226	0.144924
48087	1.89226	0.120659
48088	1.60329	0.100301
48089	1.38246	0.115802
48090	1.98182	0.0974981
48091	2.2217	0.120551
48092	3.60417	0.112874
48093	2.05362	0.0946786
48094	1.02663	0.100433
48095	1.43255	0.105483
48096	1.52072	0.0906763
48097	1.70302	0.105477
48098	1.7685	0.0948511
48099	0.946131	0.117901
48100	0.946131	0.100909
48101	0.946131	0.106287
48102	0.946131	0.139298
48103	0.946131	0.140244
48104	0.946131	0.126431
48105	0.946131	0.101095
48106	0.946131	0.108135
48107	0.946131	0.120367
48108	0.946131	0.123496
48109	0.946131	0.118668
48110	0.946131	0.119984
48111	1.89226	0.121435
48112	1.89226	0.153097
48113	1.89226	0.155979
48114	1.89226	0.120016
48115	1.89226	0.158403
48116	1.89226	0.128399
48117	1.92645	0.677531
48118	2.83045	0.644
48119	1.92225	0.762368
48120	2.01873	0.523817
48121	2.51732	0.795373
48122	2.51732	0.567133
48123	2.51732	0.501162
48124	2.23857	0.474202
48125	1.92645	0.607452
48126	1.92645	0.39621
48127	2.23857	0.712374
48128	1.50865	0.367778
48129	2.02687	2.1
48130	2.39604	2.1
48131	2.29198	2.1
48132	2.73046	2.1
48133	4.68803	2.1
48134	1.21148	0.505139
48135	1.87957	0.617819
48136	2.03555	0.391196
48137	2.03555	0.489457

48138	1.09962	0.38322
48139	1.09962	0.618404
48140	1.92558	0.419083
48141	2.05274	0.74283
48142	2.2443	0.327858
48143	2.24429	0.687709
48144	1.70869	0.322751
48145	1.70868	0.83554
48146	0.818397	0.435489
48147	0.818397	0.773047
48148	0.827911	0.537421
48149	0.827908	0.69591
48150	1.00418	0.62476
48151	1.02054	0.581349
48152	0.692018	0.620663
48153	0.620899	0.566991
48154	0.65823	0.348567
48155	1.00418	0.470401
48156	0.620899	0.536006
48157	1.0262	0.384843
48158	1.92558	0.69488
48159	2.51818	0.851022
48160	1.94816	0.536355
48161	2.01985	0.767224
48162	1.85389	0.477332
48163	1.92558	0.856368
48164	0.903844	2.1
48165	0.903844	2.1
48166	0.946148	2.1
48167	0.946151	2.1
48168	0.946118	2.1
48169	0.903876	2.1
48170	0.946118	2.1
48171	1.10235	2.1
48172	0.946131	2.1
48173	0.946131	2.1
48174	1.10234	2.1
48175	0.903863	2.1
48176	1.63471	2.1
48177	1.08022	2.1
48178	1.56801	2.1
48179	1.03437	2.1
48180	2.66908	2.1
48181	1.56801	2.1
48182	0.97541	2.1
48183	0.97541	2.1
48184	1.03437	2.1
48185	1.49733	2.1
48186	1.55629	2.1
48187	1.89226	0.158019
48188	1.89226	0.254991
48189	1.89226	0.197067
48190	1.89226	0.231687
48191	3.78452	0.252441
48192	0.946131	0.185805
48193	1.16373	0.176107
48194	0.946131	0.199558
48195	0.734637	0.166162

48196	0.946132	0.189064
48197	0.946132	0.195438
48198	1.16373	0.155032
48199	0.734636	0.154851
48200	0.946093	0.18606
48201	0.946093	0.179508
48202	0.734675	0.169411
48203	0.734675	0.18022
48204	1.98413	0.239324
48205	1.87355	0.511719
48206	1.72774	0.240583
48207	1.67865	0.554438
48208	3.60128	0.290061
48209	1.87355	0.416121
48210	1.54924	0.808202
48211	1.68443	0.721173
48212	1.68443	0.471643
48213	1.67866	0.723937
48214	1.67866	0.490628
48215	1.72774	0.266047
48216	1.53422	0.541897
48217	1.67865	0.434586
48218	1.67865	0.23631
48219	1.53873	0.491903
48220	1.53873	0.311003
48221	1.67866	0.402596
48222	1.54924	0.915319
48223	1.53423	0.499612
48224	1.44091	0.815491
48225	3.11957	0.634597
48226	1.89226	2.07789
48227	1.89226	1.43321
48228	1.65573	1.71325
48229	1.65573	1.85806
48230	1.89226	1.79378
48231	1.89226	1.95641
48232	1.89226	1.97515
48233	1.89614	1.25264
48234	1.7508	1.45754
48235	1.72666	1.57369
48236	1.89226	1.55502
48237	1.89226	1.76566
48238	1.89223	1.83559
48239	1.89223	1.28227
48240	1.89223	1.3206
48241	1.89223	1.58126
48242	1.89223	1.40426
48243	1.89223	1.80494
48244	1.89224	1.74473
48245	1.89224	1.00902
48246	1.89224	1.30645
48247	1.89224	1.30497
48248	1.89224	1.45071
48249	1.89224	1.53454
48250	2.09773	2.1
48251	2.06623	2.1
48252	2.43268	2.1
48253	2.33713	2.1

48254	1.703	2.1
48255	1.61372	2.1
48256	0.646929	1.11199
48257	0.689776	2.09098
48258	1.05897	2.1
48259	0.956364	2.1
48260	0.905594	2.1
48261	0.904315	1.18061
48262	0.727014	2.1
48263	0.850757	2.1
48264	0.798445	2.1
48265	0.654101	2.1
48266	0.717539	2.1
48267	1.0478	2.1
48268	1.89223	1.04986
48269	1.89223	0.800169
48270	1.89223	2.07159
48271	1.6557	0.493145
48272	3.54794	0.844662
48273	0.946118	0.929655
48274	0.946118	2.1
48275	0.946118	2.1
48276	0.946118	1.83234
48277	0.946117	1.82381
48278	0.946117	0.781237
48279	0.946119	2.1
48280	0.946119	2.1
48281	0.946105	1.85435
48282	0.946105	1.93187
48283	0.946131	1.85483
48284	0.946131	1.85546
48285	1.47671	2.1
48286	1.70303	2.1
48287	1.90705	2.1
48288	1.98945	2.1
48289	1.3454	2.1
48290	1.40353	2.1
48291	1.89226	2.07581
48292	1.89226	1.31662
48293	1.89226	2.1
48294	1.89226	0.880061
48295	1.89226	2.1
48296	1.89226	0.743262
48297	2.39438	0.213498
48298	2.69842	0.187154
48299	2.45177	0.263873
48300	2.28563	0.165836
48301	4.84615	0.197754
48302	1.28421	2.1
48303	1.35966	2.1
48304	0.961001	2.1
48305	0.860549	2.1
48306	1.1039	2.1
48307	1.1039	2.1
48308	1.11631	2.1
48309	1.11631	2.1
48310	1.35966	2.1
48311	0.961001	2.1

48312	0.860549	2.1
48313	1.28421	2.1
48314	1.89226	1.36126
48315	1.89226	1.89685
48316	1.89226	2.02346
48317	1.89226	1.14964
48318	3.78452	1.38854
48319	1.48702	0.996584
48320	1.40656	1.10681
48321	0.626442	1.60679
48322	0.626442	1.42781
48323	0.626442	1.77091
48324	0.946132	1.08915
48325	1.05489	0.800697
48326	1.05012	1.89004
48327	0.946131	1.37795
48328	0.946131	1.41157
48329	0.626442	1.44423
48330	1.05012	1.33612
48331	1.89226	1.89083
48332	1.97212	1.18802
48333	1.89226	1.14581
48334	1.89226	1.48144
48335	3.78453	1.55311
48336	1.89226	1.97495
48337	1.89226	1.15032
48338	1.89226	1.25223
48339	1.89226	1.72362
48340	3.78452	1.66647
48341	1.89226	1.09546
48342	1.89226	1.44466
48343	1.89226	1.70023
48344	1.89226	0.903277
48345	1.89226	1.73151
48346	1.89226	0.928969
48347	0.946161	0.781834
48348	0.946161	1.10836
48349	0.932156	1.45777
48350	0.932156	1.19387
48351	0.932184	1.39647
48352	0.946132	0.662254
48353	0.946133	1.44166
48354	0.946133	0.896112
48355	0.946132	1.02149
48356	0.946132	1.08034
48357	0.932185	1.05159
48358	0.946133	0.922697
48359	0.946132	1.08988
48360	0.946132	0.914474
48361	0.946132	1.98873
48362	0.946132	1.47832
48363	0.946132	1.4086
48364	0.946132	1.06764
48365	0.946133	1.14477
48366	0.946133	1.64925
48367	0.946132	1.33218
48368	0.946132	1.50936
48369	0.946133	1.09605

48370	0.946133	1.22463
48371	1.79397	0.364133
48372	1.00636	0.22773
48373	1.31658	0.482315
48374	2.02924	0.323021
48375	3.0356	0.349076
48376	1.89226	0.328785
48377	1.89226	0.20789
48378	1.89226	0.289069
48379	1.89226	0.242004
48380	1.89226	0.265325
48381	1.89226	0.220165
48382	0.946131	0.247752
48383	0.946131	0.280814
48384	1.24766	0.347033
48385	1.03691	0.296322
48386	0.946129	0.285233
48387	0.946129	0.210965
48388	1.24766	0.344095
48389	1.24766	0.300273
48390	0.946131	0.284119
48391	1.24766	0.331463
48392	1.03691	0.291172
48393	0.946131	0.248455
48394	1.44824	0.301944
48395	2.02924	0.233658
48396	1.7065	0.333151
48397	1.38604	0.476059
48398	2.32017	0.295408
48399	2.32017	0.287509
48400	1.89226	0.237678
48401	1.89226	0.251608
48402	1.89226	0.190071
48403	1.89226	0.16061
48404	1.89226	0.346249
48405	1.89226	0.289178
48406	0.478349	0.690879
48407	0.612092	1.56339
48408	0.606115	1.98185
48409	0.506213	2.1
48410	0.550677	1.8831
48411	0.550677	0.683921
48412	0.567628	1.87083
48413	0.567628	2.1
48414	0.506213	1.61095
48415	0.478349	1.66281
48416	0.606116	1.58273
48417	0.612092	1.78145
48418	1.52226	2.1
48419	1.54241	2.1
48420	1.23167	2.1
48421	1.23167	2.1
48422	1.75555	2.1
48423	1.75555	2.1
48424	1.75557	2.1
48425	1.54243	2.1
48426	1.9943	2.1
48427	1.85871	2.1

48428	3.53673	2.1
48429	1.85871	2.1
48430	1.06936	2.1
48431	1.88926	2.1
48432	1.54243	2.1
48433	1.41619	2.1
48434	1.06936	2.1
48435	1.28649	1.54627
48436	1.28516	2.07715
48437	1.38986	0.686316
48438	1.42399	1.47496
48439	2.67502	1.35626
48440	1.42399	0.895951
48441	1.07283	2.1
48442	1.46528	2.1
48443	1.28517	1.02111
48444	1.25294	1.85785
48445	1.07283	0.8908
48446	1.38986	1.82413
48447	1.42399	1.99144
48448	1.52885	0.921843
48449	1.60058	1.17811
48450	2.99043	1.30884
48451	0.715316	2.00299
48452	0.715316	1.45444
48453	0.719126	0.629852
48454	0.719126	2.1
48455	0.722447	1.83502
48456	0.626469	1.90038
48457	0.711995	0.923455
48458	0.800291	1.02243
48459	0.80029	1.37125
48460	0.722447	1.17373
48461	0.711995	1.48663
48462	0.62647	1.52724
48463	2.05632	0.082381
48464	1.70303	0.0917568
48465	2.3923	0.0973853
48466	2.61277	0.0831692
48467	1.44836	0.099496
48468	1.58184	0.0858694
48469	1.89226	0.0934343
48470	1.89226	0.125817
48471	1.89226	0.10427
48472	1.89226	0.108956
48473	3.78453	0.110122
48474	1.44091	0.699044
48475	1.1822	2.1
48476	1.25866	2.1
48477	1.25866	1.40208
48478	1.35215	2.1
48479	1.35215	1.32565
48480	1.27338	0.779129
48481	1.1822	2.1
48482	1.27338	2.1
48483	1.35215	1.44612
48484	1.10817	2.1
48485	1.18694	1.91323

48486	0.946132	0.579932
48487	0.946132	0.528957
48488	0.946132	0.47086
48489	0.946132	0.425634
48490	0.946133	0.468981
48491	0.946133	0.567003
48492	0.946132	0.52119
48493	0.946132	0.413791
48494	0.946132	0.513477
48495	0.946132	0.503329
48496	0.946133	0.496623
48497	0.946133	0.504888
48498	2.6875	0.164651
48499	2.40558	0.161192
48500	2.1994	0.167667
48501	1.70303	0.164296
48502	4.68305	0.166643
48503	0.915523	0.153182
48504	0.915523	0.165165
48505	0.861057	0.146498
48506	0.946796	0.16218
48507	0.838652	0.169638
48508	0.654182	0.139507
48509	1.14044	0.147434
48510	0.80252	0.156597
48511	0.836435	0.154069
48512	0.608659	0.15215
48513	1.22198	0.158423
48514	0.863472	0.170763
48515	2.1994	0.111313
48516	0.981586	0.106325
48517	1.59619	0.113936
48518	1.70303	0.111499
48519	1.24816	0.10918
48520	1.38523	0.102567
48521	2.71141	2.1
48522	1.70302	2.1
48523	2.56527	2.1
48524	1.88649	2.1
48525	2.41279	2.1
48526	1.70302	2.1
48527	1.89226	1.38745
48528	1.89226	2.1
48529	1.89226	2.1
48530	1.89226	1.23054
48531	3.78453	2.03508
48532	1.89226	1.43988
48533	1.89226	2.1
48534	1.89226	2.1
48535	1.89226	2.06816
48536	1.89226	2.0941
48537	1.89226	1.39285
48538	2.18867	2.1
48539	0.983723	2.1
48540	2.42263	2.1
48541	1.8014	2.1
48542	1.62992	2.1
48543	0.983723	2.1

48544	2.71141	2.1
48545	1.89226	2.1
48546	2.31157	2.1
48547	2.31157	2.1
48548	1.89226	2.1
48549	1.89226	2.1
48550	1.89227	1.84284
48551	1.89227	1.0056
48552	1.89227	0.98948
48553	1.89227	1.74008
48554	3.78453	1.23825
48555	1.89226	1.13681
48556	1.89226	2.04139
48557	1.89226	1.93492
48558	1.89226	1.18406
48559	1.89226	1.84699
48560	1.89226	1.13913
48561	1.89226	1.33072
48562	1.89226	1.1531
48563	1.89226	1.88039
48564	1.89226	0.861867
48565	1.89226	1.22608
48566	1.89226	0.648098
48567	1.1427	1.66321
48568	1.14956	1.98967
48569	1.70302	1.16723
48570	1.81267	1.68739
48571	2.88983	1.64048
48572	1.89226	0.852099
48573	1.89226	1.67089
48574	1.89226	0.863483
48575	1.89226	1.11106
48576	3.78453	1.19181
48577	1.89226	1.28579
48578	1.89226	1.43057
48579	1.89226	0.720538
48580	1.89226	1.19377
48581	3.78452	0.99373
48582	0.884197	1.79056
48583	1.1125	1.25384
48584	1.13273	1.2736
48585	0.844557	1.51552
48586	0.981137	1.0754
48587	1.13971	1.61163
48588	0.980432	1.55883
48589	0.980432	1.10396
48590	1.15859	1.18285
48591	1.12206	0.873562
48592	0.898619	1.74296
48593	0.94426	1.34877
48594	2.21016	1.11025
48595	1.44091	1.96208
48596	1.98408	1.18235
48597	1.4218	1.04331
48598	2.21016	1.7425
48599	1.64788	1.76956
48600	1.07526	0.436583
48601	1.91531	2.1

48602	1.49155	1.67293
48603	1.58551	0.641124
48604	1.49155	0.764699
48605	1.58551	1.54099
48606	1.98979	0.0746527
48607	2.21773	0.0870824
48608	2.30533	0.0841459
48609	2.55895	0.0736049
48610	4.52305	0.0784649
48611	2.55896	0.0742895
48612	2.27963	0.0883012
48613	2.60486	0.0866816
48614	2.60486	0.0780876
48615	2.21773	0.0870292
48616	2.21773	0.0759359
48617	2.30533	0.0868113
48618	2.55895	0.0738413
48619	2.29539	0.0736678
48620	2.47308	0.0900594
48621	4.7784	0.0791846
48622	2.55896	0.0745199
48623	2.55792	0.0941927
48624	2.55792	0.0909551
48625	2.60486	0.0788898
48626	2.47308	0.0891136
48627	2.52003	0.0760633
48628	0.946133	1.50673
48629	0.946133	1.63647
48630	1.20851	1.20793
48631	1.20851	1.13163
48632	0.967411	1.16746
48633	1.20851	0.953212
48634	0.946132	1.83353
48635	0.946132	1.41888
48636	0.946132	1.51097
48637	1.20851	1.19504
48638	0.967412	1.16613
48639	0.946133	1.43678
48640	1.89226	1.53253
48641	1.89226	1.07706
48642	1.89226	1.09388
48643	1.89226	1.09246
48644	1.89226	1.54415
48645	1.89226	1.5927
48646	1.89226	1.44687
48647	2.41701	1.40724
48648	2.71724	1.05931
48649	2.71724	1.36409
48650	1.89226	1.69642
48651	1.89226	1.91657
48652	0.946131	1.64766
48653	0.946131	1.92133
48654	0.946128	1.39272
48655	0.946128	1.47768
48656	0.94613	0.941071
48657	0.94613	1.1269
48658	0.94613	1.71385
48659	0.94613	1.78429

48660	0.94613	1.48702
48661	0.94613	1.07362
48662	0.94613	1.24348
48663	0.94613	1.81826
48664	0.851541	0.269536
48665	0.851541	0.313691
48666	0.851481	0.276771
48667	0.997219	0.299158
48668	0.997112	0.230272
48669	0.992853	0.198841
48670	0.85154	0.235247
48671	0.85154	0.289781
48672	0.894824	0.195929
48673	0.894824	0.281728
48674	0.94613	0.329494
48675	0.906485	0.211113
48676	0.802947	2.1
48677	0.845556	2.1
48678	0.582339	2.1
48679	0.519756	2.1
48680	0.714508	2.1
48681	0.714508	2.1
48682	0.628932	2.1
48683	0.708518	2.1
48684	0.801358	2.1
48685	0.476016	2.1
48686	0.569477	2.1
48687	0.878553	2.1
48688	1.89226	2.1
48689	1.89226	2.1
48690	1.89226	2.1
48691	1.89226	2.1
48692	1.89226	2.1
48693	1.89226	2.1
48694	0.946128	2.1
48695	0.946128	2.1
48696	1.01537	2.1
48697	0.9662	2.1
48698	0.946131	2.1
48699	0.946131	2.1
48700	0.966198	2.1
48701	0.966198	2.1
48702	0.94613	2.1
48703	0.966198	2.1
48704	0.946131	2.1
48705	1.01537	2.1
48706	1.89226	2.1
48707	1.89226	2.1
48708	1.91883	2.1
48709	1.91883	2.1
48710	2.15905	2.1
48711	2.13249	2.1
48712	1.07953	2.1
48713	1.2107	2.1
48714	0.946132	2.1
48715	0.946132	2.1
48716	1.2107	2.1
48717	1.2107	2.1

48718	0.946132	2.1
48719	0.946132	2.1
48720	1.2107	2.1
48721	0.946133	2.1
48722	0.946132	2.1
48723	1.07953	2.1
48724	2.46752	2.1
48725	2.53733	2.1
48726	2.26044	2.1
48727	2.33222	0.864408
48728	4.79974	2.1
48729	2.53733	2.1
48730	2.37576	0.31408
48731	2.33222	1.14008
48732	2.1845	1.40093
48733	4.70798	1.01349
48734	2.48652	2.1
48735	2.33224	0.865861
48736	2.08149	2.1
48737	1.9839	2.08994
48738	4.45623	2.1
48739	2.33223	0.935125
48740	2.18452	0.902949
48741	2.16061	2.02137
48742	2.01399	0.474574
48743	4.34623	0.712176
48744	1.4617	0.98375
48745	1.34701	1.90892
48746	1.34701	0.996602
48747	1.25173	2.06481
48748	1.5117	0.939016
48749	1.41642	2.1
48750	2.65164	2.1
48751	1.89227	1.22058
48752	1.89227	1.94114
48753	1.89227	2.1
48754	2.42504	1.74219
48755	2.42504	2.1
48756	1.89226	0.859351
48757	1.89226	1.16286
48758	1.89226	1.94203
48759	1.89226	0.747858
48760	3.78452	0.929384
48761	1.5117	1.0428
48762	1.34701	1.4875
48763	1.47001	2.1
48764	1.57961	0.848285
48765	2.92663	1.1521
48766	1.89226	1.51396
48767	1.89226	0.737498
48768	1.89226	0.846136
48769	1.89226	1.039
48770	3.78452	0.991838
48771	0.412773	0.0861111
48772	0.570839	0.0906843
48773	0.50721	0.0858495
48774	0.513638	0.0998003
48775	0.518805	0.0851694

48776	0.41278	0.0842358
48777	1.5344	0.0972597
48778	0.939282	0.0948167
48779	0.720051	0.0853447
48780	0.691767	0.0959353
48781	0.761152	0.0956976
48782	1.89226	1.4258
48783	1.89226	2.1
48784	1.89226	1.779
48785	1.89226	2.1
48786	1.89226	1.59991
48787	1.89226	2.1
48788	1.24618	2.1
48789	2.10585	2.1
48790	1.08134	2.1
48791	1.93032	2.1
48792	3.1765	2.1
48793	1.98474	2.1
48794	2.57777	2.1
48795	1.93031	2.1
48796	2.19041	2.1
48797	3.99251	2.1
48798	1.89226	1.74189
48799	1.89226	2.1
48800	1.89226	2.1
48801	1.89226	1.71408
48802	3.78452	2.1
48803	1.67374	2.1
48804	2.19042	2.1
48805	1.67374	2.1
48806	2.09769	2.1
48807	2.29586	2.1
48808	2.7198	2.1
48809	1.89226	2.1
48810	1.89226	1.89267
48811	1.89226	1.97019
48812	1.89226	2.1
48813	1.89226	1.78066
48814	1.89226	2.1
48815	0.946132	0.549703
48816	0.946132	0.489341
48817	0.947763	0.490334
48818	0.909304	0.519467
48819	0.946127	0.453333
48820	0.947831	0.376307
48821	0.907673	0.410435
48822	0.907673	0.529077
48823	0.94613	0.435229
48824	0.947834	0.401867
48825	0.907671	0.591844
48826	0.94613	0.424382
48827	0.906519	0.366354
48828	0.906519	0.321425
48829	0.946097	0.335624
48830	0.946097	0.374601
48831	0.947834	0.34467
48832	0.906485	0.294522
48833	0.946131	0.314933

48834	0.946131	0.379287
48835	0.94613	0.404198
48836	0.906485	0.259382
48837	0.946131	0.397744
48838	0.947835	0.373816
48839	2.58638	2.1
48840	2.51039	2.1
48841	2.47395	2.1
48842	2.41539	2.1
48843	5.00178	2.1
48844	2.51039	2.1
48845	2.16633	2.1
48846	2.4154	2.1
48847	2.13977	2.1
48848	4.58173	2.1
48849	2.47395	2.1
48850	2.41539	2.1
48851	2.2988	2.1
48852	2.2604	2.1
48853	4.7142	2.1
48854	2.4154	2.1
48855	2.13977	2.1
48856	2.26041	2.1
48857	1.98957	2.1
48858	4.40497	2.1
48859	2.15721	0.477426
48860	2.31843	0.288715
48861	1.93973	0.584892
48862	2.13243	0.148705
48863	4.25816	0.293576
48864	2.31844	0.394007
48865	2.30908	0.343529
48866	2.13243	0.244282
48867	2.1609	0.356378
48868	4.47934	0.33473
48869	1.93973	0.582729
48870	2.13243	0.230337
48871	1.69661	0.519941
48872	1.9339	0.198497
48873	3.87363	0.326138
48874	2.13243	0.312268
48875	2.1609	0.376932
48876	1.9339	0.330215
48877	1.98414	0.362746
48878	4.0948	0.313711
48879	1.88857	0.0522355
48880	2.34107	0.0522355
48881	0.502431	0.0522355
48882	0.71416	0.0522355
48883	0.50243	0.0522355
48884	0.944284	0.0522355
48885	1.88856	2.1
48886	2.34107	2.1
48887	2.39662	2.1
48888	1.92293	2.1
48889	4.28519	2.1
48890	1.00486	2.1
48891	1.92293	2.1

48892	1.88857	2.1
48893	1.88857	2.1
48894	1.02605	2.1
48895	1.02605	2.1
48896	2.53728	2.1
48897	1.70303	2.1
48898	2.03734	2.1
48899	1.3624	2.1
48900	2.54706	2.1
48901	1.70303	2.1
48902	2.54706	2.1
48903	1.50876	2.1
48904	2.74067	2.1
48905	1.85391	2.1
48906	2.29667	2.1
48907	1.50876	2.1
48908	0.946133	2.1
48909	0.946133	2.1
48910	1.24191	2.1
48911	1.24191	2.1
48912	0.946132	2.1
48913	1.24191	2.1
48914	1.23779	2.1
48915	0.946132	2.1
48916	0.946132	2.1
48917	0.946132	2.1
48918	1.23779	2.1
48919	1.24191	2.1
48920	0.946131	2.1
48921	0.946131	2.1
48922	1.14833	2.1
48923	1.14833	2.1
48924	1.14834	2.1
48925	0.946132	2.1
48926	0.94613	2.1
48927	1.24191	2.1
48928	0.946131	2.1
48929	0.946131	2.1
48930	1.24191	2.1
48931	1.14833	2.1
48932	2.08149	2.03948
48933	2.31116	2.1
48934	1.9701	1.30678
48935	2.18328	2.1
48936	4.49563	1.72684
48937	2.08149	1.85067
48938	2.14059	2.1
48939	1.72377	2.1
48940	2.18328	2.1
48941	3.88931	2.1
48942	2.14059	2.1
48943	2.40755	2.1
48944	2.31145	2.1
48945	2.23743	2.1
48946	2.31145	2.1
48947	2.23743	2.1
48948	2.31117	2.1
48949	2.40754	2.1

48950	2.41055	2.1
48951	2.31145	2.1
48952	2.41055	1.83176
48953	2.31145	2.1
48954	2.5615	0.523533
48955	2.37625	0.474034
48956	2.20248	0.465192
48957	2.20248	0.472649
48958	2.65967	0.534235
48959	2.65967	0.547002
48960	2.71793	0.533051
48961	2.65966	0.461231
48962	2.56149	0.425721
48963	2.56149	0.461699
48964	2.71296	0.503642
48965	2.71296	0.535301
48966	1.31528	0.47082
48967	1.32983	0.451658
48968	1.21128	0.472664
48969	1.18812	0.456997
48970	1.24232	0.420629
48971	1.24232	0.450234
48972	1.26109	0.461947
48973	1.26109	0.510502
48974	1.21128	0.480567
48975	1.31528	0.402194
48976	1.18812	0.430529
48977	1.32983	0.505986
48978	2.63056	0.417962
48979	2.58032	0.451993
48980	2.65966	0.338249
48981	2.65966	0.443478
48982	2.58032	0.492627
48983	2.71296	0.506069
48984	1.92039	2.1
48985	1.06226	2.1
48986	1.09252	2.1
48987	1.96052	2.1
48988	3.01291	2.1
48989	1.92039	2.1
48990	2.46338	2.1
48991	2.47522	2.1
48992	2.43508	2.1
48993	1.96052	2.1
48994	1.92039	2.1
48995	0.985949	2.1
48996	0.980261	2.1
48997	0.553625	2.1
48998	0.546262	2.1
48999	0.765671	2.1
49000	0.765671	2.1
49001	0.766539	2.1
49002	0.766539	2.1
49003	0.985949	2.1
49004	0.553625	2.1
49005	0.546261	2.1
49006	0.980261	2.1
49007	1.96052	2.1

49008	2.46338	2.1
49009	1.97189	2.1
49010	1.96052	2.1
49011	2.47932	2.1
49012	2.47932	2.1
49013	0.964608	0.0522355
49014	0.691524	0.0522355
49015	1.25462	0.0522355
49016	0.691524	0.0522355
49017	1.89226	0.0522355
49018	1.89226	0.0522355
49019	1.44894	0.0522355
49020	2.77655	0.0522355
49021	1.4237	0.0522355
49022	2.77701	0.0522355
49023	2.77701	0.0522355
49024	1.38847	2.1
49025	1.46031	1.61086
49026	1.65504	1.21153
49027	1.75396	1.21808
49028	3.11535	1.37395
49029	1.89226	1.51734
49030	1.89226	1.73183
49031	1.89226	1.27196
49032	1.89226	0.959283
49033	3.78452	1.57257
49034	1.89226	1.38591
49035	1.89226	1.87041
49036	1.89226	0.789875
49037	1.89226	1.53573
49038	3.78452	0.972386
49039	1.46031	1.21662
49040	1.34851	1.85462
49041	1.75395	1.07701
49042	1.62287	1.2525
49043	3.08318	1.64262
49044	1.65504	1.48484
49045	1.75396	1.71797
49046	1.89249	1.48038
49047	1.9857	1.13814
49048	3.64073	1.69088
49049	1.89226	1.58764
49050	1.89226	1.42864
49051	1.89226	1.01758
49052	1.89226	1.23962
49053	3.78452	1.10917
49054	1.89226	1.0528
49055	1.89226	1.85964
49056	1.89226	1.14233
49057	1.89226	1.40282
49058	3.78452	1.69696
49059	1.75395	1.50117
49060	1.62287	1.59002
49061	1.98569	0.973926
49062	1.86067	1.20934
49063	3.60856	1.1485
49064	1.35697	1.30553
49065	2.02064	1.04311

49066	1.89989	0.763774
49067	1.49571	2.1
49068	1.66753	0.869905
49069	1.36431	2.1
49070	0.744004	2.1
49071	0.743997	1.31114
49072	0.717972	0.77895
49073	0.717972	1.37892
49074	0.997982	2.1
49075	0.807796	1.56157
49076	0.632235	0.829297
49077	0.488575	1.95004
49078	0.849275	1.51014
49079	0.488581	1.62548
49080	0.724729	1.57482
49081	0.997967	1.60832
49082	2.5276	1.37579
49083	1.70791	1.38741
49084	1.82872	0.827457
49085	1.56362	2.08869
49086	2.5276	1.03128
49087	2.24781	2.1
49088	1.37591	2.1
49089	1.23217	1.16807
49090	1.00442	1.67661
49091	0.945282	0.924581
49092	1.15446	1.11072
49093	1.09123	2.1
49094	1.21506	1.32379
49095	1.21506	1.06065
49096	1.39452	1.21637
49097	1.13108	1.69072
49098	1.27876	1.09766
49099	0.929006	0.936264
49100	1.46505	0.220149
49101	1.91484	0.238817
49102	1.49906	0.249734
49103	1.49906	0.268845
49104	1.90196	0.191992
49105	1.90196	0.201168
49106	1.90196	0.223893
49107	2.11465	0.252375
49108	1.91484	0.269675
49109	1.91484	0.279856
49110	2.04016	0.205646
49111	2.04016	0.207441
49112	0.912201	0.195204
49113	0.950978	0.180321
49114	0.665307	0.173107
49115	0.732528	0.192337
49116	0.829512	0.151488
49117	0.829512	0.191283
49118	0.815217	0.1668
49119	0.815217	0.188588
49120	0.912202	0.170861
49121	0.665305	0.154203
49122	0.732527	0.218532
49123	0.95098	0.178243

49124	1.90196	0.208521
49125	2.08757	0.167608
49126	1.9626	0.192167
49127	2.04016	0.172196
49128	1.82441	0.190712
49129	1.90196	0.173638
49130	1.13048	2.1
49131	0.821162	2.1
49132	0.946131	2.1
49133	0.946131	2.1
49134	1.04567	2.1
49135	0.882347	2.1
49136	0.897276	2.1
49137	0.897276	2.1
49138	0.863109	2.1
49139	1.01055	2.1
49140	0.940305	2.1
49141	0.942166	2.1
49142	2.23933	0.0855038
49143	0.976217	0.0820302
49144	2.78053	0.0904509
49145	2.78053	0.0898324
49146	0.976217	0.103773
49147	1.65984	0.0865426
49148	1.54168	2.04943
49149	2.01197	1.50237
49150	1.69136	1.9286
49151	2.13284	0.889496
49152	3.67452	1.54062
49153	0.988805	2.1
49154	1.78069	1.37262
49155	1.69136	1.36582
49156	1.69136	2.1
49157	1.05481	1.28277
49158	1.05481	2.1
49159	1.69136	2.1
49160	2.13284	1.21584
49161	1.78069	1.72956
49162	2.22044	1.13457
49163	3.91353	1.64499
49164	2.69379	0.859733
49165	2.32219	0.953993
49166	2.25746	1.47146
49167	2.25746	0.818179
49168	2.32219	0.985765
49169	1.89526	0.76688
49170	1.4556	2.1
49171	1.85028	2.1
49172	2.4909	0.769917
49173	2.03799	2.05327
49174	2.73495	1.01995
49175	1.85028	1.50478
49176	2.71938	0.796403
49177	1.85028	2.1
49178	3.36051	1.99885
49179	2.73495	1.41483
49180	2.47584	1.62719
49181	1.85028	0.983202

49182	2.47584	1.18642
49183	1.04413	2.1
49184	2.1461	2.1
49185	1.74586	1.81436
49186	1.44437	2.1
49187	1.04413	1.70022
49188	1.89226	2.1
49189	1.89226	1.2049
49190	1.89226	1.27182
49191	1.89226	1.22701
49192	3.78452	1.30191
49193	1.52724	2.1
49194	1.89249	2.03847
49195	1.68143	2.1
49196	2.02433	1.1588
49197	3.55157	2.07487
49198	1.07444	2.1
49199	1.76545	2.0272
49200	1.68142	2.1
49201	1.68142	2.1
49202	1.14807	2.1
49203	1.14807	2.1
49204	1.89226	2.1
49205	1.89226	2.05104
49206	1.89226	2.1
49207	1.89226	1.01822
49208	1.89226	2.1
49209	1.89226	0.795751
49210	1.89226	2.1
49211	1.89226	1.37135
49212	1.89226	2.06528
49213	1.89226	0.883549
49214	3.78452	1.269
49215	1.68143	2.1
49216	2.02433	1.30094
49217	1.76545	2.03398
49218	2.12608	2.01821
49219	3.78978	2.02616
49220	2.62871	1.71199
49221	1.49149	2.1
49222	1.36814	2.1
49223	2.44761	1.08151
49224	3.9391	2.1
49225	1.89226	2.1
49226	1.89226	0.647398
49227	1.89226	1.09683
49228	1.89226	1.78989
49229	3.78452	1.04059
49230	0.851542	2.1
49231	0.851542	1.44283
49232	1.00897	2.1
49233	0.931224	2.1
49234	0.838952	2.1
49235	0.838952	1.18697
49236	0.956848	2.1
49237	0.942474	2.1
49238	0.946132	2.1
49239	0.838952	2.1

49240	0.878743	2.1
49241	0.946132	2.1
49242	1.15541	2.1
49243	2.44761	0.78654
49244	1.36814	2.1
49245	1.36814	1.35898
49246	2.19439	2.1
49247	2.19439	1.84864
49248	1.89226	0.779265
49249	1.89226	1.40572
49250	1.89226	2.1
49251	1.89226	0.738587
49252	3.78452	1.26641
49253	1.89226	2.1
49254	1.67791	2.1
49255	1.5713	2.1
49256	1.67791	2.1
49257	1.78566	2.1
49258	1.89226	1.51809
49259	1.89226	0.664195
49260	1.62911	1.73638
49261	2.24602	1.63046
49262	1.89226	1.54268
49263	1.98287	1.01561
49264	1.62911	0.95479
49265	1.89226	1.36248
49266	1.89226	1.5894
49267	1.89226	0.92238
49268	1.89226	1.9668
49269	3.78452	1.17844
49270	1.26688	2.1
49271	1.11804	1.95295
49272	0.852467	0.966457
49273	0.693314	2.1
49274	0.977459	1.56046
49275	0.977459	2.1
49276	0.982741	0.914892
49277	0.982741	1.81475
49278	1.26689	2.02933
49279	0.852464	2.02853
49280	0.693312	2.1
49281	1.11804	1.97436
49282	0.946131	2.05814
49283	0.946131	0.825361
49284	1.1794	0.824111
49285	1.1794	1.91532
49286	0.991434	1.93765
49287	1.1794	0.965251
49288	0.946131	0.810042
49289	0.946131	0.812484
49290	0.946131	1.50996
49291	1.1794	1.36079
49292	0.946131	1.39018
49293	0.991434	1.11326
49294	1.89226	0.798598
49295	1.89226	2.1
49296	1.89226	0.858983
49297	1.89226	1.17667

49298	3.78452	1.14952
49299	2.53377	2.00523
49300	1.53443	1.8817
49301	2.0815	0.874253
49302	2.82979	2.1
49303	4.35462	1.87588
49304	1.96192	0.765025
49305	1.23602	1.80065
49306	2.16506	0.883282
49307	1.56841	1.44612
49308	3.40108	1.22919
49309	1.89226	1.07239
49310	1.89226	1.77201
49311	1.89226	0.928779
49312	1.89226	1.85483
49313	3.78452	1.17949
49314	0.946129	2.1
49315	0.946129	1.62326
49316	1.13067	0.897953
49317	1.12952	1.9898
49318	0.946129	2.1
49319	1.13089	1.80737
49320	0.944978	0.882105
49321	0.944978	1.54529
49322	0.946131	2.05766
49323	1.13089	2.00648
49324	0.944977	2.1
49325	0.946131	1.95959
49326	0.710208	2.1
49327	0.710208	2.1
49328	0.718894	2.1
49329	0.718894	2.1
49330	0.559773	2.1
49331	0.579321	2.1
49332	0.886767	2.1
49333	0.849781	2.1
49334	0.849781	2.1
49335	0.559774	2.1
49336	0.579321	2.1
49337	0.886766	2.1
49338	1.63471	2.1
49339	1.69956	2.1
49340	1.08022	2.1
49341	1.08022	2.1
49342	1.11955	2.1
49343	1.67403	2.1
49344	1.03766	2.1
49345	2.02825	2.1
49346	2.02794	2.1
49347	2.0348	2.1
49348	1.07196	2.1
49349	1.07196	2.1
49350	1.9276	2.01946
49351	2.1004	1.9513
49352	2.1306	2.08813
49353	2.1306	1.80542
49354	1.92178	2.1
49355	1.92032	1.52674

49356	0.986724	2.1
49357	2.03064	2.1
49358	2.01385	2.1
49359	2.02404	2.1
49360	1.03766	2.1
49361	1.03766	2.1
49362	1.92027	2.1
49363	2.1306	2.1
49364	2.15388	2.1
49365	2.15388	2.06727
49366	1.92614	2.1
49367	1.9276	1.85944
49368	1.09578	2.1
49369	0.963018	2.06617
49370	0.782956	1.09881
49371	0.529693	2.06577
49372	0.6994	1.10165
49373	0.862532	2.1
49374	0.898605	2.03364
49375	0.898605	1.21744
49376	0.776756	1.84952
49377	1.14805	1.91157
49378	0.966521	1.821
49379	0.617676	1.40444
49380	2.67185	1.34465
49381	2.099	2.1
49382	2.80548	1.27138
49383	2.2727	2.1
49384	4.94455	2.1
49385	1.89226	1.35256
49386	1.89226	2.1
49387	1.89226	1.4069
49388	1.89226	2.1
49389	3.78453	2.1
49390	1.89226	2.1
49391	1.89226	2.1
49392	1.89226	2.1
49393	1.89226	2.1
49394	1.89226	2.1
49395	1.89226	2.1
49396	0.884801	2.1
49397	0.884801	2.1
49398	0.895199	2.1
49399	0.895199	2.1
49400	0.622774	2.1
49401	0.730501	2.1
49402	1.0495	2.1
49403	1.13635	2.1
49404	1.13635	2.1
49405	0.730503	2.1
49406	1.0495	2.1
49407	0.622775	2.1
49408	1.89226	1.04286
49409	1.89226	2.1
49410	1.89226	1.6758
49411	1.89226	2.1
49412	1.89226	1.22291
49413	1.89226	2.1

49414	1.89226	2.1
49415	1.89226	2.1
49416	1.89226	2.1
49417	1.89226	2.1
49418	1.89226	2.1
49419	1.89226	2.1
49420	2.2727	2.1
49421	1.89586	2.1
49422	1.66913	2.1
49423	1.46101	2.1
49424	2.48082	2.1
49425	2.2727	2.1
49426	1.89226	1.45266
49427	1.89226	2.07143
49428	1.84641	2.1
49429	1.89226	1.78136
49430	1.30868	1.99096
49431	1.34118	1.00229
49432	1.89226	0.800475
49433	1.89226	0.892723
49434	1.89226	0.987363
49435	1.89226	0.800147
49436	1.89226	0.854773
49437	1.89226	0.67605
49438	1.89226	0.90647
49439	1.89226	0.474211
49440	1.89226	0.742085
49441	1.89226	0.892473
49442	3.78452	0.7883
49443	0.946132	1.31035
49444	0.946132	1.10681
49445	0.946131	1.89552
49446	0.946131	2.05891
49447	0.946132	1.99144
49448	0.946132	0.89677
49449	0.946132	2.00749
49450	0.946132	1.41224
49451	0.946132	1.6798
49452	0.946132	1.62174
49453	0.946132	1.85806
49454	0.946132	1.76645
49455	1.89227	1.12449
49456	1.84641	1.13577
49457	2.29517	1.6747
49458	1.84641	1.02172
49459	2.34102	1.13779
49460	1.89227	0.847092
49461	1.89226	0.699908
49462	1.89226	0.712263
49463	1.89226	0.831924
49464	1.89226	0.661037
49465	1.89226	0.713132
49466	1.89226	0.48136
49467	1.89226	0.569258
49468	1.89226	0.719801
49469	1.89226	0.777447
49470	1.89226	0.60563
49471	1.89226	0.664295

49472	1.89226	0.415936
49473	1.89226	1.207
49474	1.89226	0.920369
49475	1.89226	1.4129
49476	1.89226	0.832722
49477	1.89226	1.04896
49478	1.89226	0.827272
49479	0.851544	0.102493
49480	0.757542	0.123906
49481	1.13421	0.0944354
49482	1.04072	0.102454
49483	0.946132	0.0994854
49484	0.946132	0.101188
49485	0.841686	0.104901
49486	0.834428	0.0982432
49487	1.13649	0.0967642
49488	0.937687	0.101377
49489	0.841713	0.10628
49490	0.841713	0.119793
49491	1.89226	0.411802
49492	1.89226	0.48867
49493	1.89226	0.51546
49494	1.89226	0.34588
49495	1.89226	0.524366
49496	1.89226	0.353903
49497	2.31988	1.14334
49498	2.00082	1.69003
49499	2.4594	0.869517
49500	2.17638	1.70574
49501	4.46022	1.09565
49502	2.00082	0.931515
49503	1.41829	1.94146
49504	1.61207	1.24516
49505	1.43652	2.1
49506	2.17637	0.884727
49507	2.00082	2.1
49508	2.4594	0.857018
49509	2.17638	2.00681
49510	2.57957	0.862075
49511	2.3506	1.58625
49512	4.81	1.23122
49513	2.3506	1.03874
49514	1.57368	2.07574
49515	1.89111	0.713241
49516	1.89111	2.00839
49517	2.17638	0.75692
49518	2.17638	2.05505
49519	1.32813	2.1
49520	1.7237	2.1
49521	1.32461	2.1
49522	1.81656	2.1
49523	3.14468	2.1
49524	1.55132	2.1
49525	2.0815	2.1
49526	1.81656	2.1
49527	2.19826	2.1
49528	3.65818	2.1
49529	0.775951	2.1

49530	0.775951	2.1
49531	0.794601	2.1
49532	0.794601	2.1
49533	0.945641	2.1
49534	0.90828	2.1
49535	0.662302	2.1
49536	0.662273	2.1
49537	0.662274	2.1
49538	0.945639	2.1
49539	0.662304	2.1
49540	0.908279	2.1
49541	1.03062	2.1
49542	1.03062	2.1
49543	1.05054	2.1
49544	1.05054	2.1
49545	1.13551	2.1
49546	1.21275	2.1
49547	0.945641	2.1
49548	0.90828	2.1
49549	0.945641	2.1
49550	1.21275	2.1
49551	0.90828	2.1
49552	1.13552	2.1
49553	1.89226	2.1
49554	1.79883	2.1
49555	1.95341	2.1
49556	1.89226	2.1
49557	1.85998	2.1
49558	1.79883	2.1
49559	1.89226	2.1
49560	1.89226	2.1
49561	1.89226	2.1
49562	1.89226	2.1
49563	3.78453	2.1
49564	0.851541	2.1
49565	0.851541	2.1
49566	0.970398	2.1
49567	0.954365	2.1
49568	0.855356	2.1
49569	0.855357	2.1
49570	0.946133	2.1
49571	0.946133	2.1
49572	1.04072	2.1
49573	0.970397	2.1
49574	0.950003	2.1
49575	0.851538	2.1
49576	1.89226	2.1
49577	1.85998	2.1
49578	1.95296	2.1
49579	1.89226	2.1
49580	1.92067	2.1
49581	1.85998	2.1
49582	0.946133	2.1
49583	0.946133	2.1
49584	0.946132	2.1
49585	0.946132	2.1
49586	0.946132	2.1
49587	0.946132	2.1

49588	0.946132	2.1
49589	0.946132	2.1
49590	0.946132	2.1
49591	0.946132	2.1
49592	0.946133	2.1
49593	0.946133	2.1
49594	1.82152	2.1
49595	1.89226	2.1
49596	1.89226	2.1
49597	2.11152	2.1
49598	1.7472	2.1
49599	1.89226	2.1
49600	2.02687	2.1
49601	2.29199	2.1
49602	1.2734	2.1
49603	1.41077	2.1
49604	1.47381	2.1
49605	2.29199	2.1
49606	1.46889	2.1
49607	2.29199	2.1
49608	1.86766	2.1
49609	2.59401	2.1
49610	4.15965	2.1
49611	1.58995	2.1
49612	1.74246	2.1
49613	1.46643	2.1
49614	1.46643	2.1
49615	1.85097	2.1
49616	1.85097	2.1
49617	1.58995	2.1
49618	1.66584	2.1
49619	1.89214	2.1
49620	1.91787	2.1
49621	3.48208	2.1
49622	1.18474	2.1
49623	1.8144	2.1
49624	1.1486	2.1
49625	1.1486	2.1
49626	2.13071	2.1
49627	2.1257	2.1
49628	2.0815	2.1
49629	2.51651	2.1
49630	1.73681	2.1
49631	2.01601	2.1
49632	2.21406	2.1
49633	2.56998	2.1
49634	1.19995	2.1
49635	1.06256	2.1
49636	0.620677	2.1
49637	0.598084	2.1
49638	0.831426	2.1
49639	0.946056	2.1
49640	0.827692	2.1
49641	0.827692	2.1
49642	1.08958	2.1
49643	0.630132	2.1
49644	0.577547	2.1
49645	1.06104	2.1

49646	2.0815	2.1
49647	2.66249	2.1
49648	2.78966	2.1
49649	2.47108	2.1
49650	2.33858	2.1
49651	2.0815	2.1
49652	1.53735	2.1
49653	1.42278	2.1
49654	1.23654	2.1
49655	1.34678	2.1
49656	2.76955	2.1
49657	1.89226	2.1
49658	1.89226	2.1
49659	1.89226	2.1
49660	1.89226	2.1
49661	3.78452	2.1
49662	1.23654	2.1
49663	1.34678	2.1
49664	1.19	2.1
49665	1.08576	2.1
49666	2.43254	2.1
49667	1.38776	2.1
49668	1.29366	2.1
49669	0.946131	2.1
49670	0.946131	2.1
49671	0.946131	2.1
49672	0.946131	2.1
49673	1.38775	2.1
49674	1.38775	2.1
49675	1.29366	2.1
49676	0.946132	2.1
49677	0.946131	2.1
49678	1.38775	2.1
49679	0.946131	2.1
49680	0.946131	2.1
49681	0.946129	2.1
49682	0.946129	2.1
49683	0.94613	2.1
49684	0.94613	2.1
49685	0.946131	2.1
49686	0.946131	2.1
49687	0.946131	2.1
49688	0.946131	2.1
49689	0.94613	2.1
49690	0.94613	2.1
49691	1.89226	0.387806
49692	1.89226	0.983031
49693	1.89226	0.556813
49694	1.89226	0.631143
49695	1.89226	0.627038
49696	1.89226	0.925022
49697	1.89226	0.519048
49698	1.89226	0.99574
49699	1.89226	0.520823
49700	1.89226	0.63665
49701	1.89226	0.588099
49702	1.89226	0.898525
49703	1.89226	0.548019

49704	1.89226	0.984978
49705	1.89226	0.585636
49706	1.89226	0.803254
49707	1.89226	0.566401
49708	1.89226	1.04201
49709	0.92338	2.09256
49710	0.92338	0.773931
49711	0.94428	0.739815
49712	0.94428	0.596312
49713	1.19528	0.8109
49714	1.30598	1.98989
49715	0.714073	0.638301
49716	0.561679	1.29799
49717	1.19528	0.837347
49718	0.561678	0.846134
49719	0.714072	1.07217
49720	1.30598	1.03865
49721	2.61197	1.37459
49722	1.89226	0.861419
49723	1.89226	0.84914
49724	1.89226	1.80647
49725	2.39056	0.733675
49726	2.39056	1.83385
49727	0.946132	1.00326
49728	0.946132	0.778596
49729	0.946132	0.462113
49730	0.946132	0.587502
49731	0.946131	0.608096
49732	0.946131	1.01829
49733	0.946132	0.846779
49734	0.946132	0.556553
49735	0.946132	0.688031
49736	0.946132	0.657639
49737	0.946132	0.716022
49738	0.946132	0.75515
49739	1.89224	2.1
49740	1.89224	2.1
49741	1.89224	2.1
49742	1.89224	2.1
49743	3.78448	2.1
49744	1.97089	2.1
49745	1.57428	2.1
49746	1.84991	2.1
49747	1.49078	2.1
49748	3.42419	2.1
49749	0.621927	2.1
49750	0.621927	2.1
49751	0.630824	2.1
49752	0.630824	2.1
49753	0.483483	2.1
49754	0.507361	2.1
49755	0.787142	2.1
49756	0.745391	2.1
49757	0.745381	2.1
49758	0.483476	2.1
49759	0.507368	2.1
49760	0.787152	2.1
49761	1.89224	2.1

49762	1.89224	2.1
49763	1.89224	2.1
49764	1.89224	2.1
49765	1.89224	2.1
49766	1.89224	2.1
49767	1.89227	2.1
49768	1.89227	2.1
49769	1.89227	2.1
49770	1.89227	2.1
49771	1.89227	2.1
49772	1.89227	2.1
49773	1.84994	2.1
49774	1.41758	2.1
49775	1.4908	2.1
49776	1.4908	2.1
49777	1.74391	2.1
49778	1.74391	2.1
49779	1.89226	2.1
49780	1.89226	2.1
49781	1.89226	2.1
49782	1.89226	2.1
49783	3.78452	2.1
49784	1.01323	2.1
49785	1.01323	2.1
49786	1.01445	2.1
49787	1.01445	2.1
49788	0.839531	2.1
49789	0.875652	2.1
49790	1.19063	2.1
49791	1.15203	2.1
49792	1.15203	2.1
49793	0.839531	2.1
49794	0.875652	2.1
49795	1.19063	2.1
49796	1.89226	2.1
49797	2.75587	2.1
49798	2.81512	2.1
49799	2.81512	2.1
49800	1.89226	2.1
49801	1.89226	2.1
49802	1.89226	2.1
49803	1.89226	2.1
49804	1.89226	2.1
49805	1.89226	2.1
49806	3.78452	2.1
49807	0.974831	2.1
49808	0.974831	2.1
49809	0.976681	2.1
49810	0.976681	2.1
49811	0.803179	2.1
49812	0.83953	2.1
49813	1.11198	2.1
49814	1.15203	2.1
49815	1.11198	2.1
49816	0.803179	2.1
49817	1.15203	2.1
49818	0.83953	2.1
49819	1.37794	2.1

49820	1.37794	2.1
49821	0.946128	2.1
49822	0.946128	2.1
49823	1.3477	2.1
49824	1.37793	2.1
49825	0.946131	2.1
49826	0.946131	2.1
49827	0.946129	2.1
49828	1.3477	2.1
49829	1.37793	2.1
49830	0.946129	2.1
49831	2.38577	1.09741
49832	1.20247	2.1
49833	1.75182	2.05761
49834	2.82546	1.22067
49835	4.1376	2.02402
49836	2.82546	1.94722
49837	1.89226	0.787675
49838	2.38577	1.38912
49839	2.38577	1.39229
49840	1.89226	1.61874
49841	1.89226	1.3109
49842	1.035 2.1	
49843	2.15429	2.1
49844	0.970998	2.1
49845	2.0815	2.1
49846	2.89672	2.1
49847	0.946131	0.326328
49848	0.946131	0.311365
49849	0.853249	0.308802
49850	1.02715	0.337694
49851	0.851537	0.31032
49852	0.851537	0.320592
49853	1.02663	0.295563
49854	1.13139	0.293591
49855	0.946128	0.290833
49856	0.932039	0.333117
49857	0.843985	0.281168
49858	0.946129	0.292039
49859	1.89226	0.291059
49860	1.86408	0.311203
49861	1.96548	0.343866
49862	1.86408	0.309645
49863	1.99366	0.306297
49864	1.89226	0.274647
49865	2.70702	2.1
49866	2.08981	2.1
49867	2.08693	2.1
49868	2.08693	2.1
49869	2.7188	2.1
49870	2.7188	2.1
49871	2.08982	2.1
49872	1.01964	2.1
49873	2.08693	2.1
49874	1.04615	2.1
49875	3.13597	2.1
49876	2.70702	2.1
49877	2.08693	2.1

49878	2.68784	2.1
49879	2.08831	2.1
49880	4.77477	2.1
49881	0.778233	2.1
49882	0.778233	2.1
49883	0.789001	2.1
49884	0.789001	2.1
49885	0.545307	2.1
49886	0.523075	2.1
49887	1.04347	2.1
49888	1.04416	2.1
49889	1.04416	2.1
49890	0.545308	2.1
49891	1.04347	2.1
49892	0.523075	2.1
49893	1.89226	0.594943
49894	1.89226	0.566251
49895	1.89226	0.521143
49896	1.89226	0.390084
49897	3.78453	0.444591
49898	1.39237	0.61077
49899	1.17933	1.43385
49900	0.755796	0.830777
49901	0.755796	1.15319
49902	0.755767	0.290968
49903	0.94613	1.05406
49904	1.15918	0.485635
49905	0.94613	0.706116
49906	1.11632	0.514084
49907	0.92595	0.322021
49908	0.94613	0.635987
49909	0.94613	0.602162
49910	1.89226	0.576429
49911	1.89226	0.467299
49912	1.89226	0.380323
49913	1.89226	0.393318
49914	3.78452	0.439035
49915	0.950067	0.385309
49916	0.75071	0.421249
49917	0.906516	0.35556
49918	0.906516	0.347248
49919	0.81586	0.273273
49920	0.851541	0.482869
49921	0.962101	0.299477
49922	0.851476	0.499332
49923	0.962105	0.28531
49924	0.815808	0.288912
49925	0.851529	0.498866
49926	0.851529	0.396738
49927	1.05032	0.368467
49928	0.972326	0.408353
49929	0.906515	0.328005
49930	0.906515	0.318572
49931	1.12245	0.236728
49932	1.04072	0.320944
49933	0.815857	0.292506
49934	0.851539	0.447686
49935	1.04451	0.214996

49936	0.815853	0.383117
49937	0.919795	0.402275
49938	0.919795	0.399581
49939	1.5855	1.77631
49940	2.14058	2.1
49941	1.9153	2.1
49942	1.79922	2.1
49943	3.72608	2.1
49944	1.58551	0.879078
49945	1.24201	0.902799
49946	0.960287	1.15725
49947	1.79922	2.1
49948	2.82752	2.1
49949	0.679602	2.1
49950	0.621009	1.66721
49951	0.899614	2.1
49952	0.940441	2.1
49953	0.679602	2.1
49954	0.94044	2.1
49955	0.621009	2.1
49956	0.899615	2.1
49957	0.794027	2.1
49958	0.794027	2.1
49959	0.785189	2.1
49960	0.785189	0.678406
49961	2.14058	2.1
49962	1.79922	2.1
49963	2.23742	2.1
49964	2.23742	2.1
49965	1.78404	2.1
49966	1.88088	2.1
49967	1.15177	2.1
49968	2.0815	1.39854
49969	1.93765	2.1
49970	1.78342	1.36059
49971	1.39511	2.1
49972	1.28406	1.44696
49973	1.97719	1.3936
49974	2.50057	0.581933
49975	2.35349	1.08814
49976	2.35349	0.55583
49977	2.31686	1.26403
49978	2.32857	0.601383
49979	1.93765	1.22904
49980	2.35349	0.574286
49981	1.70048	1.18725
49982	2.15721	0.379678
49983	4.05397	0.73548
49984	0.850388	0.810819
49985	0.850388	2.1
49986	0.840019	2.1
49987	0.840019	2.04408
49988	1.13736	1.64093
49989	1.13852	1.32376
49990	0.573661	2.1
49991	0.551891	2.1
49992	0.573661	2.1
49993	1.13852	2.1

49994	0.551892	2.1
49995	1.13736	2.1
49996	0.860203	1.06457
49997	0.860203	2.1
49998	0.855758	2.1
49999	0.855758	1.98801
50000	1.1423	1.3698
50001	1.13852	1.86258
50002	0.58628	2.1
50003	0.573661	2.1
50004	0.586281	2.1
50005	1.1423	2.1
50006	0.573661	2.1
50007	1.13852	2.1
50008	1.28918	1.03752
50009	0.999086	0.895621
50010	1.0383	1.54346
50011	0.736019	1.26255
50012	1.00954	1.3185
50013	1.00954	0.960399
50014	1.01566	0.721351
50015	1.01566	1.49091
50016	1.28918	1.08864
50017	1.0383	1.56238
50018	0.999086	1.31688
50019	0.736019	1.21228
50020	2.20797	2.1
50021	2.64748	1.92396
50022	2.50375	1.10161
50023	2.50375	2.1
50024	2.40858	1.52511
50025	2.40858	2.1
50026	2.64748	1.95486
50027	2.74744	2.1
50028	2.50375	2.1
50029	2.6521	2.1
50030	5.29958	2.1
50031	2.20797	2.1
50032	2.50375	2.09364
50033	2.02064	0.837602
50034	2.3606	2.1
50035	4.52439	2.03147
50036	2.36059	2.1
50037	2.6521	2.1
50038	2.56952	2.1
50039	2.56952	2.1
50040	2.50375	2.1
50041	2.50375	2.1
50042	2.49439	0.308197
50043	1.89226	0.361408
50044	2.81337	0.377659
50045	1.89226	0.385191
50046	4.38665	0.36899
50047	1.89226	0.37959
50048	1.89226	0.446274
50049	1.89226	0.52554
50050	1.89226	0.500567
50051	3.78452	0.439551

50052	1.89226	0.364252
50053	1.89226	0.385111
50054	1.89226	0.391913
50055	1.89226	0.431054
50056	3.78452	0.39182
50057	1.93948	0.611728
50058	1.98874	0.415384
50059	1.62494	0.565653
50060	2.14218	0.692413
50061	1.62494	0.358856
50062	2.14218	0.416949
50063	1.47354	0.445237
50064	1.98874	0.371714
50065	2.01865	0.443494
50066	1.62495	0.368018
50067	2.01865	0.486661
50068	1.62495	0.365331
50069	1.89226	0.619904
50070	1.89226	2.1
50071	1.89226	0.909145
50072	1.89226	1.50707
50073	3.78453	1.39177
50074	2.05257	2.1
50075	1.94441	2.1
50076	1.88487	0.824739
50077	1.82441	2.1
50078	3.82928	2.1
50079	1.94441	2.1
50080	1.74391	2.1
50081	1.82441	2.1
50082	1.65636	2.1
50083	3.60077	2.1
50084	1.89226	2.1
50085	1.89226	2.1
50086	1.89226	1.5556
50087	1.89226	2.1
50088	3.78452	2.1
50089	1.89226	0.986312
50090	1.89226	1.56866
50091	1.89226	0.613822
50092	1.89226	2.1
50093	3.78453	1.07894
50094	1.88487	0.934178
50095	1.82441	2.1
50096	1.75835	2.1
50097	1.72866	2.1
50098	3.61354	2.1
50099	1.82441	2.1
50100	1.65636	2.1
50101	1.72866	2.1
50102	1.58401	2.1
50103	3.38503	2.1
50104	1.89226	1.58376
50105	1.89226	2.1
50106	1.89226	2.1
50107	1.89226	2.1
50108	3.78452	2.1
50109	2.27432	2.1

50110	1.33339	2.1
50111	1.38905	2.1
50112	2.34471	2.1
50113	3.66337	2.1
50114	1.89226	2.1
50115	2.34471	2.1
50116	2.27432	2.1
50117	2.27432	2.1
50118	1.89226	2.1
50119	1.89226	2.1
50120	1.20443	2.1
50121	1.17235	2.1
50122	0.724695	2.1
50123	0.694522	2.1
50124	0.949994	2.1
50125	0.949993	2.1
50126	0.948955	2.1
50127	0.948955	2.1
50128	1.20443	2.1
50129	0.7247	2.1
50130	0.694517	2.1
50131	1.17234	2.1
50132	1.89225	2.1
50133	2.40885	2.1
50134	2.3447	2.1
50135	2.3447	2.1
50136	1.89225	2.1
50137	1.89225	2.1
50138	1.37138	2.1
50139	1.82597	2.1
50140	1.45027	2.1
50141	1.92772	2.1
50142	3.27624	2.1
50143	1.89226	2.06855
50144	1.89226	2.1
50145	1.89226	2.1
50146	1.89226	1.4935
50147	3.78452	2.1
50148	1.89226	2.1
50149	1.89226	0.85781
50150	1.89226	1.37574
50151	1.89226	1.09784
50152	3.78452	1.59815
50153	1.82597	2.1
50154	2.02312	0.74577
50155	1.92772	2.1
50156	2.15888	2.1
50157	3.98485	1.29357
50158	1.53446	2.1
50159	1.92772	2.1
50160	2.03975	2.1
50161	2.03975	2.1
50162	1.45027	2.1
50163	1.45027	2.1
50164	1.89226	2.1
50165	1.89226	1.58722
50166	1.89226	2.1
50167	1.89226	2.1

50168	1.89226	2.1
50169	1.89226	2.1
50170	1.89226	1.44404
50171	1.89226	2.06015
50172	1.89226	2.1
50173	1.89226	0.999689
50174	3.78452	1.6412
50175	1.92772	2.1
50176	2.15888	2.1
50177	2.03975	2.1
50178	2.3111	2.1
50179	4.19863	2.1
50180	1.89226	2.1
50181	1.89226	2.1
50182	1.89226	2.1
50183	1.89226	2.1
50184	3.78452	2.1
50185	1.78376	2.1
50186	1.3201	2.1
50187	1.78376	2.1
50188	1.86102	2.1
50189	1.2679	2.1
50190	1.34517	2.1
50191	1.89226	2.1
50192	2.52442	2.1
50193	1.89226	2.1
50194	2.51691	2.1
50195	4.41667	2.1
50196	1.89226	2.07613
50197	1.89226	2.1
50198	1.89226	2.07818
50199	1.89226	2.1
50200	3.78452	2.1
50201	1.78376	2.07477
50202	1.2679	2.1
50203	1.71839	1.83525
50204	1.23504	2.1
50205	3.0188	2.1
50206	1.89226	2.1
50207	2.51795	2.1
50208	2.51691	2.1
50209	2.51691	2.1
50210	1.89226	2.1
50211	1.89226	2.1
50212	1.89226	0.808429
50213	1.89226	1.0977
50214	1.89226	0.93052
50215	1.89226	0.881732
50216	3.78452	0.851735
50217	1.89227	0.820999
50218	1.89227	0.876371
50219	3.26088	1.03634
50220	2.79543	0.893106
50221	2.20733	0.877617
50222	1.89227	0.831287
50223	1.89226	0.997082
50224	1.89226	0.850647
50225	1.89226	0.77768

50226	1.89226	0.855699
50227	1.89226	0.879618
50228	1.89226	1.03284
50229	1.89227	0.808566
50230	2.20733	0.834615
50231	1.46973	1.0523
50232	1.89227	0.805997
50233	1.7848	1.02385
50234	2.20733	0.781532
50235	1.89226	2.1
50236	2.55682	2.1
50237	2.81593	2.1
50238	2.81593	2.1
50239	1.89226	2.1
50240	1.89226	2.1
50241	1.0607	2.04104
50242	1.0607	2.1
50243	1.12617	2.1
50244	0.986957	2.1
50245	0.699328	1.84408
50246	0.785567	1.53488
50247	1.28855	2.1
50248	1.41913	2.1
50249	0.766737	1.06371
50250	1.34711	2.1
50251	1.24643	2.1
50252	0.687289	1.43034
50253	3.11063	0.319409
50254	1.89226	0.390675
50255	1.96536	0.357225
50256	1.89226	0.368253
50257	1.96536	0.334863
50258	1.89226	0.346059
50259	1.89226	0.652696
50260	1.89226	0.441041
50261	1.89226	0.542575
50262	1.89226	0.506425
50263	3.78452	0.644847
50264	1.89226	0.381733
50265	1.96256	0.235548
50266	1.89226	0.268995
50267	1.89226	0.380271
50268	2.49192	0.361471
50269	2.49192	0.253254
50270	1.85716	0.335136
50271	2.56871	0.339049
50272	1.03043	0.262679
50273	1.03043	0.267972
50274	2.48125	0.218054
50275	1.65452	0.327053
50276	1.89226	2.1
50277	2.09622	2.1
50278	2.09622	2.1
50279	2.08508	2.1
50280	1.89226	2.1
50281	1.88112	2.1
50282	1.04811	2.1
50283	1.04811	2.1

50284	0.946131	2.1
50285	0.946131	2.1
50286	1.05014	2.1
50287	1.04811	2.1
50288	0.94613	2.1
50289	0.94613	2.1
50290	0.946131	2.1
50291	1.05013	2.1
50292	1.04811	2.1
50293	0.946131	2.1
50294	1.398	2.1
50295	2.10766	2.1
50296	2.3018	2.1
50297	2.3018	2.1
50298	1.34008	2.1
50299	1.34008	2.1
50300	1.1509	2.1
50301	1.1509	2.1
50302	0.946132	2.1
50303	0.946132	2.1
50304	0.946132	2.1
50305	0.946132	2.1
50306	1.05383	2.1
50307	1.1509	2.1
50308	1.1509	2.1
50309	0.946133	2.1
50310	0.946132	2.1
50311	1.05383	2.1
50312	0.931141	2.1
50313	0.931141	2.1
50314	0.971946	2.1
50315	0.971946	2.1
50316	1.28569	2.1
50317	1.1509	2.1
50318	0.699	2.1
50319	0.752185	2.1
50320	1.28568	2.1
50321	0.752186	2.1
50322	0.699001	2.1
50323	1.1509	2.1
50324	2.30181	2.1
50325	1.89226	2.1
50326	1.89226	2.1
50327	1.89226	2.1
50328	2.57137	2.1
50329	2.57137	2.1
50330	2.0815	2.1
50331	1.21962	2.1
50332	1.55173	2.1
50333	2.57849	2.1
50334	3.79001	2.1
50335	1.38779	2.1
50336	1.2923	2.1
50337	1.03974	2.1
50338	1.28832	2.1
50339	1.33566	2.1
50340	1.21116	2.1
50341	1.22343	2.1

50342	1.22343	2.1
50343	1.42288	2.1
50344	1.20138	2.1
50345	1.26025	2.1
50346	1.00644	2.1
50347	1.26066	2.1
50348	1.14271	2.1
50349	0.886635	2.1
50350	0.795277	2.1
50351	1.02966	2.1
50352	1.02967	2.1
50353	1.05216	2.1
50354	1.05216	2.1
50355	1.26068	2.1
50356	0.790042	2.1
50357	0.795269	2.1
50358	1.20135	2.1
50359	1.89226	0.617266
50360	1.89226	0.532541
50361	1.89226	0.639707
50362	1.89226	0.461966
50363	1.89226	0.689923
50364	1.89226	0.52365
50365	1.89227	0.498112
50366	1.89227	0.456245
50367	1.89227	0.556533
50368	1.89227	0.377034
50369	1.89227	0.592844
50370	1.89227	0.413514
50371	0.563873	0.353475
50372	0.50988	0.296026
50373	0.598693	0.453569
50374	0.558819	0.369888
50375	0.564855	0.355651
50376	0.564855	0.305315
50377	0.557838	0.43734
50378	0.557838	0.370144
50379	0.563873	0.379037
50380	0.598694	0.375851
50381	0.558819	0.349265
50382	0.50988	0.355733
50383	0.598693	0.439241
50384	0.558819	0.355502
50385	0.572737	0.53141
50386	0.534248	0.457076
50387	0.566788	0.445442
50388	0.566788	0.37903
50389	0.566153	0.51315
50390	0.566153	0.454381
50391	0.598694	0.457438
50392	0.572737	0.457872
50393	0.534248	0.424107
50394	0.558819	0.418122
50395	1.85217	0.725511
50396	1.93111	1.24456
50397	1.92875	0.673577
50398	2.02391	2.1
50399	3.85986	2.1

50400	1.93112	1.23356
50401	1.80425	2.1
50402	2.02392	2.1
50403	1.90981	2.1
50404	3.84092	2.1
50405	1.92876	0.626764
50406	2.02393	2.1
50407	2.05274	0.727138
50408	2.16783	1.87294
50409	4.09659	1.19764
50410	2.02393	2.1
50411	1.90982	2.1
50412	2.16784	1.87452
50413	2.05967	2.1
50414	4.07766	2.1
50415	1.89226	1.20006
50416	1.89226	1.06503
50417	1.89226	1.22329
50418	1.89226	1.1535
50419	3.78453	0.937804
50420	1.89226	0.934926
50421	1.89226	1.30007
50422	1.89226	1.6568
50423	2.44829	0.865699
50424	3.78453	1.5713
50425	1.89226	1.17219
50426	1.89226	1.33324
50427	1.89226	1.01283
50428	2.60452	0.954256
50429	3.78453	1.65107
50430	1.58723	0.142692
50431	1.88071	0.214606
50432	2.97376	0.142682
50433	2.13255	0.187225
50434	4.43982	0.17061
50435	2.20641	0.245425
50436	1.33575	0.319532
50437	2.15824	0.280684
50438	2.15824	0.271886
50439	1.2951	0.280396
50440	1.2951	0.277231
50441	0.825383	0.339013
50442	0.825383	0.302898
50443	0.856104	0.28402
50444	0.856104	0.285395
50445	0.952196	0.323332
50446	0.602363	0.288604
50447	0.667877	0.325035
50448	1.07913	0.28599
50449	0.602363	0.322721
50450	0.667876	0.325186
50451	1.07913	0.33356
50452	0.952197	0.316144
50453	2.57657	0.919639
50454	1.44437	2.1
50455	2.65378	2.06622
50456	2.25076	1.48657
50457	1.84739	1.91747

50458	1.44437	1.15288
50459	2.0189	0.189443
50460	2.54752	0.182005
50461	2.63975	0.254274
50462	2.31049	0.1459
50463	4.65865	0.183781
50464	1.40707	0.144201
50465	1.89605	0.138867
50466	2.0189	0.163575
50467	2.0189	0.128875
50468	1.50438	0.168342
50469	1.50438	0.135083
50470	2.63971	0.232191
50471	2.38246	0.147383
50472	2.13402	0.179878
50473	2.57503	0.166756
50474	4.77373	0.180898
50475	1.50436	0.140334
50476	1.96803	0.136844
50477	2.13402	0.174256
50478	1.45446	0.11779
50479	3.63838	0.137921
50480	2.30793	0.280956
50481	2.11032	0.245831
50482	1.24082	0.249032
50483	1.05689	0.273559
50484	3.36482	0.28149
50485	1.89226	0.381998
50486	1.89226	0.432061
50487	1.89226	0.433974
50488	1.89226	0.38844
50489	3.78453	0.393168
50490	0.812967	0.285493
50491	0.812967	0.302379
50492	1.00181	0.282035
50493	1.08141	0.270041
50494	0.946132	0.253959
50495	0.812938	0.30154
50496	0.932815	0.331376
50497	1.05577	0.270322
50498	0.812938	0.285096
50499	0.932812	0.306996
50500	0.946133	0.315592
50501	0.946133	0.292477
50502	2.53897	2.1
50503	1.89226	2.1
50504	1.89226	2.1
50505	1.89226	2.1
50506	2.3245	2.1
50507	2.3245	2.1
50508	1.00255	0.962731
50509	1.06561	2.1
50510	1.05481	0.534641
50511	1.11077	2.00863
50512	2.12042	1.32332
50513	1.89226	1.29917
50514	1.89226	2.1
50515	1.89226	0.578365

50516	1.89226	1.73291
50517	3.78453	1.0149
50518	1.89226	1.08939
50519	2.77314	2.1
50520	2.81276	0.874012
50521	2.81276	2.1
50522	1.89226	1.10006
50523	1.89226	2.1
50524	1.11077	2.1
50525	1.15114	1.81739
50526	1.10274	0.802913
50527	1.1032	1.21122
50528	1.05481	1.35666
50529	1.1032	0.932002
50530	1.89226	1.06207
50531	1.89226	1.86612
50532	1.89226	0.45992
50533	1.89226	1.92244
50534	3.78452	1.07708
50535	0.721957	0.550923
50536	0.721957	0.557549
50537	0.946103	0.565914
50538	0.946103	0.458481
50539	0.814237	0.480393
50540	0.721929	0.44106
50541	0.946132	0.573702
50542	0.946132	0.592216
50543	0.721928	0.608984
50544	0.946131	0.627508
50545	0.946132	0.642593
50546	0.814237	0.623729
50547	1.89226	0.71812
50548	1.89226	0.56443
50549	1.89226	0.5701
50550	1.89226	0.582691
50551	1.89226	0.684086
50552	1.89226	0.694947
50553	1.37196	0.528759
50554	2.83556	0.415535
50555	1.5165	0.483944
50556	1.5165	0.542624
50557	2.74923	0.441453
50558	2.74923	0.517272
50559	0.946128	0.537536
50560	0.946128	0.575425
50561	0.827866	0.452415
50562	0.946132	0.508798
50563	0.94613	0.432707
50564	0.94613	0.455008
50565	0.94613	0.584812
50566	0.94613	0.587717
50567	0.94613	0.598573
50568	0.827864	0.631536
50569	0.94613	0.588913
50570	0.94613	0.688417
50571	1.36208	0.40741
50572	2.74923	0.319549
50573	1.37196	0.365013

50574	1.37196	0.399501
50575	2.53256	0.330227
50576	2.53256	0.380848
50577	1.07705	2.1
50578	1.07705	2.1
50579	1.08253	2.1
50580	1.08253	2.1
50581	0.762985	2.1
50582	0.781638	2.1
50583	1.37794	2.1
50584	1.40756	2.1
50585	1.37794	2.1
50586	0.762986	2.1
50587	0.781638	2.1
50588	1.40756	2.1
50589	2.69541	2.1
50590	2.75587	2.1
50591	1.48572	2.1
50592	1.48572	2.1
50593	1.52597	2.1
50594	2.73566	2.1
50595	2.0554	1.36116
50596	1.00387	0.844227
50597	2.01001	0.720869
50598	0.994002	0.716013
50599	3.01389	0.614607
50600	1.89226	2.1
50601	2.41579	2.1
50602	2.12209	2.1
50603	2.12209	2.1
50604	1.89226	2.1
50605	1.89226	2.1
50606	1.89226	2.1
50607	1.89226	2.1
50608	1.89226	2.1
50609	1.89226	2.1
50610	1.89226	2.1
50611	1.89226	2.1
50612	0.88209	2.1
50613	0.899471	2.1
50614	0.651501	2.1
50615	0.533531	2.1
50616	0.724105	2.1
50617	0.711243	2.1
50618	0.692622	2.1
50619	0.692622	2.1
50620	0.890668	2.1
50621	0.738066	2.1
50622	0.778223	2.1
50623	0.531121	2.1
50624	1.89226	2.1
50625	2.77504	2.1
50626	2.4158	2.1
50627	2.4158	2.1
50628	1.89226	2.1
50629	1.89226	2.1
50630	1.89226	2.1
50631	1.89226	2.1

50632	1.89226	2.1
50633	1.89226	2.1
50634	1.89226	2.1
50635	1.89226	2.1
50636	1.70303	2.1
50637	1.39832	2.1
50638	1.51749	2.1
50639	1.68783	2.1
50640	3.24844	2.1
50641	1.89226	2.1
50642	2.38311	2.1
50643	1.98321	2.1
50644	1.89226	2.1
50645	2.47406	2.1
50646	2.38311	2.1
50647	1.89226	2.1
50648	1.89226	2.1
50649	1.89226	2.1
50650	1.89226	2.1
50651	1.89226	2.1
50652	1.89226	2.1
50653	0.946131	2.1
50654	0.946131	2.1
50655	1.25167	2.1
50656	1.19156	2.1
50657	1.19156	2.1
50658	1.19156	2.1
50659	0.946133	2.1
50660	0.946133	2.1
50661	0.946131	2.1
50662	1.25167	2.1
50663	0.946131	2.1
50664	1.19156	2.1
50665	1.89226	2.1
50666	2.47406	2.1
50667	1.99996	2.1
50668	1.89226	2.1
50669	2.58176	2.1
50670	2.47406	2.1
50671	1.89226	2.1
50672	1.89226	2.1
50673	1.89226	2.1
50674	1.89226	2.1
50675	1.89226	2.1
50676	1.89226	2.1
50677	0.946131	2.1
50678	0.946131	2.1
50679	1.32241	2.1
50680	1.25167	2.1
50681	1.25167	2.1
50682	1.25167	2.1
50683	0.946133	2.1
50684	0.946133	2.1
50685	0.946131	2.1
50686	1.32241	2.1
50687	0.946131	2.1
50688	1.25167	2.1
50689	1.65151	2.1

50690	1.92471	2.1
50691	1.81834	2.1
50692	2.13272	2.1
50693	3.74306	2.1
50694	1.92472	2.1
50695	1.9943	2.1
50696	2.13273	2.1
50697	2.24475	2.1
50698	4.16947	2.1
50699	1.81834	2.1
50700	2.13272	2.1
50701	2.02687	2.1
50702	2.39605	2.1
50703	4.21439	2.1
50704	2.13273	2.1
50705	2.24475	2.1
50706	2.39605	2.1
50707	2.54903	2.1
50708	4.6408	2.1
50709	1.10534	2.1
50710	1.22993	2.1
50711	0.946132	2.1
50712	0.946132	2.1
50713	0.946132	2.1
50714	0.946132	2.1
50715	1.22993	2.1
50716	1.22993	2.1
50717	1.10534	2.1
50718	0.946131	2.1
50719	0.946132	2.1
50720	1.22993	2.1
50721	1.47221	2.1
50722	2.14647	2.1
50723	1.95567	2.1
50724	1.95567	2.1
50725	1.71082	2.1
50726	1.71082	2.1
50727	0.946131	2.1
50728	0.946131	2.1
50729	0.946131	2.1
50730	0.946131	2.1
50731	0.946132	2.1
50732	0.946132	2.1
50733	0.946131	2.1
50734	0.946131	2.1
50735	0.946131	2.1
50736	0.946131	2.1
50737	0.946132	2.1
50738	0.946132	2.1
50739	0.998086	2.1
50740	1.09429	2.1
50741	0.946125	2.1
50742	0.946125	2.1
50743	0.90732	2.1
50744	0.90732	2.1
50745	1.14414	2.1
50746	1.16553	2.1
50747	1.05018	2.1

50748	0.907332	2.1
50749	0.99482	2.1
50750	0.851529	2.1
50751	0.80534	2.1
50752	0.80534	2.1
50753	0.812206	2.1
50754	0.812206	2.1
50755	0.895134	2.1
50756	0.977828	2.1
50757	0.736099	2.1
50758	0.639718	2.1
50759	0.639722	2.1
50760	0.89514	2.1
50761	0.736095	2.1
50762	0.977821	2.1
50763	0.946125	2.1
50764	0.946125	2.1
50765	0.946125	2.1
50766	0.946125	2.1
50767	0.946125	2.1
50768	0.946125	2.1
50769	0.946124	2.1
50770	0.946124	2.1
50771	0.946131	2.1
50772	0.946131	2.1
50773	0.946118	2.1
50774	0.946118	2.1
50775	2.22622	0.0522355
50776	2.43347	0.0522355
50777	2.34108	0.0522355
50778	1.24587	2.1
50779	1.21674	2.1
50780	1.14533	2.1
50781	1.11311	2.1
50782	1.17874	2.1
50783	1.17874	2.1
50784	1.18024	2.1
50785	1.18024	2.1
50786	1.14533	2.1
50787	1.24587	2.1
50788	1.21674	2.1
50789	1.11311	2.1
50790	2.43347	2.1
50791	2.34107	2.1
50792	2.39663	2.1
50793	2.49174	2.1
50794	4.8301	2.1
50795	1.86206	2.1
50796	1.41058	2.1
50797	1.70302	2.1
50798	2.42083	2.1
50799	3.68129	2.1
50800	1.70302	2.1
50801	2.43549	2.1
50802	2.20075	2.1
50803	2.50358	2.1
50804	2.04916	2.1
50805	2.33113	2.1

50806	1.07624	2.1
50807	2.12621	2.1
50808	1.57297	2.1
50809	1.60383	2.1
50810	1.30047	2.1
50811	1.30047	2.1
50812	2.33379	1.87011
50813	2.56149	1.20079
50814	2.36734	1.13203
50815	2.62796	1.13753
50816	4.92883	1.24386
50817	2.5615	1.25463
50818	2.57957	1.30567
50819	2.62796	1.07066
50820	2.69056	1.03205
50821	5.25206	1.50121
50822	2.39322	1.26896
50823	2.62796	1.63953
50824	2.6849	1.66038
50825	2.6849	1.5805
50826	2.36734	1.71452
50827	2.36734	1.59182
50828	2.62796	1.49771
50829	2.69056	1.57844
50830	2.6849	0.862461
50831	2.77227	0.794839
50832	5.37546	0.987577
50833	1.89226	0.0807392
50834	1.89226	0.102769
50835	1.89226	0.0893566
50836	1.89226	0.0864012
50837	1.89226	0.0904814
50838	1.89226	0.0870278
50839	1.06974	0.071334
50840	1.06974	0.0715626
50841	1.02299	0.0783588
50842	1.02299	0.0830067
50843	1.35299	0.082119
50844	1.26227	0.0727115
50845	0.646252	0.0795632
50846	0.830464	0.0720047
50847	0.830463	0.0755892
50848	1.35299	0.0762443
50849	1.26227	0.0752128
50850	0.646252	0.0773335
50851	2.52454	0.074378
50852	1.89226	0.094034
50853	2.70598	0.085226
50854	1.89226	0.0764296
50855	4.59824	0.0816919
50856	1.89226	0.0875103
50857	1.89226	0.116031
50858	1.89226	0.101636
50859	1.89226	0.0955063
50860	1.89226	0.10381
50861	1.89226	0.0968159
50862	1.89226	0.0814244
50863	1.89226	0.105212

50864	1.89226	0.0902747
50865	1.89226	0.0891164
50866	1.89226	0.0904038
50867	1.89226	0.0878395
50868	1.7666	0.0728802
50869	2.70598	0.0850944
50870	2.65378	0.0790295
50871	2.65378	0.0782235
50872	1.66093	0.0775702
50873	1.66093	0.0754804
50874	2.70598	0.087274
50875	1.89226	0.0795011
50876	1.89226	0.0926097
50877	1.89226	0.0763146
50878	2.65377	0.0910183
50879	2.65377	0.0767322
50880	0.946132	0.0883727
50881	0.946132	0.0929015
50882	0.946131	0.105475
50883	0.946131	0.117373
50884	0.946131	0.116102
50885	0.946131	0.0949215
50886	0.946132	0.106731
50887	0.946132	0.0885644
50888	0.946132	0.0993113
50889	0.946132	0.100049
50890	0.946131	0.098199
50891	0.946131	0.0995165
50892	1.69858	2.1
50893	2.18238	2.1
50894	2.506	2.1
50895	1.93586	2.1
50896	4.20458	2.1
50897	2.21772	2.1
50898	2.18238	2.1
50899	2.506	2.1
50900	2.59215	2.1
50901	4.72372	2.1
50902	2.50596	2.1
50903	1.93584	2.1
50904	2.16728	2.1
50905	2.7679	2.1
50906	4.67325	2.1
50907	1.53296	2.1
50908	1.56299	2.1
50909	1.42624	2.1
50910	1.52961	2.1
50911	2.98923	2.1
50912	1.56299	2.1
50913	1.46568	2.1
50914	1.52961	2.1
50915	1.51916	2.1
50916	3.08215	2.1
50917	1.36226	2.1
50918	1.52961	2.1
50919	1.36226	2.1
50920	1.42624	2.1
50921	1.55369	2.1

50922	1.61768	2.1
50923	1.52961	2.1
50924	1.51916	2.1
50925	1.55369	2.1
50926	1.64892	2.1
50927	3.07285	2.1
50928	2.13405	0.171348
50929	2.57507	0.163286
50930	2.39438	0.221052
50931	1.93408	0.142085
50932	4.52843	0.165273
50933	2.13405	0.165771
50934	1.27778	0.121615
50935	1.2545	0.138219
50936	1.45448	0.141395
50937	1.93408	0.133104
50938	2.13405	0.138619
50939	2.39438	0.205115
50940	1.93407	0.142392
50941	1.60578	0.166378
50942	1.87985	0.159489
50943	4.00016	0.170786
50944	1.0525	1.98915
50945	1.05758	1.99697
50946	1.43484	0.915554
50947	1.38506	1.6322
50948	2.48734	1.73544
50949	1.20455	2.1
50950	1.67352	2.1
50951	1.52852	2.1
50952	2.05164	2.1
50953	3.20203	2.1
50954	1.89226	1.97384
50955	1.89226	2.1
50956	1.89226	2.1
50957	1.89226	2.02819
50958	3.78452	2.1
50959	1.89226	2.1
50960	1.89226	2.1
50961	1.89226	2.1
50962	1.89226	2.1
50963	1.89226	2.1
50964	1.89226	1.78987
50965	2.05165	2.1
50966	1.94291	2.1
50967	2.09398	2.1
50968	1.67352	2.1
50969	2.36337	2.1
50970	1.94291	2.1
50971	1.52851	2.1
50972	2.52049	2.1
50973	2.43433	2.1
50974	2.05164	2.1
50975	1.9112	2.1
50976	1.52851	2.1
50977	1.89226	2.1
50978	1.89226	1.43511
50979	1.89226	1.97938

50980	1.89226	2.1
50981	3.78451	2.1
50982	1.89226	1.60755
50983	1.89226	2.1
50984	1.89226	2.1
50985	1.89226	1.28913
50986	3.78452	2.1
50987	2.5205	2.1
50988	2.36337	2.1
50989	2.4605	2.1
50990	2.05165	2.1
50991	2.77222	2.1
50992	2.36337	2.1
50993	1.89226	2.1
50994	1.89226	2.1
50995	1.89226	2.1
50996	1.89226	2.1
50997	1.89226	2.1
50998	1.89226	2.1
50999	1.89226	2.1
51000	1.89226	2.1
51001	1.89226	2.1
51002	1.89226	2.1
51003	3.78452	2.1
51004	1.38905	2.1
51005	1.26166	2.1
51006	1.19216	2.1
51007	1.0875	2.1
51008	1.24393	2.1
51009	1.24393	2.1
51010	1.23261	2.1
51011	1.23261	2.1
51012	1.38905	2.1
51013	1.19216	2.1
51014	1.26166	2.1
51015	1.0875	2.1
51016	1.16233	2.1
51017	1.06028	2.1
51018	0.777395	2.1
51019	0.705508	2.1
51020	0.940018	2.1
51021	0.936465	2.1
51022	0.954216	2.1
51023	0.954216	2.1
51024	1.16125	2.1
51025	0.685505	2.1
51026	1.1157	2.1
51027	0.703534	2.1
51028	1.89226	2.1
51029	1.89226	2.1
51030	1.89226	2.1
51031	1.89226	2.1
51032	1.89226	2.1
51033	1.89226	2.1
51034	1.89226	2.1
51035	1.89226	2.1
51036	1.89226	2.1
51037	1.89226	2.1

51038	1.89226	2.1
51039	1.89226	2.1
51040	2.38433	2.1
51041	1.43456	2.1
51042	1.93881	2.1
51043	2.36952	2.1
51044	4.32313	2.1
51045	1.8493	2.1
51046	1.5927	2.1
51047	1.45535	2.1
51048	1.38438	2.1
51049	1.89657	2.1
51050	1.8493	2.1
51051	2.0815	2.1
51052	1.6697	2.1
51053	2.0657	2.1
51054	1.5927	2.1
51055	2.59749	2.1
51056	2.0815	2.1
51057	2.76597	2.1
51058	1.89226	2.1
51059	1.89226	2.1
51060	1.89226	2.1
51061	2.6126	2.1
51062	2.6126	2.1
51063	2.62513	2.1
51064	1.89226	2.1
51065	1.89226	2.1
51066	1.89226	2.1
51067	2.76598	2.1
51068	2.76598	2.1
51069	2.54665	2.1
51070	2.6126	2.1
51071	2.76598	2.1
51072	2.76598	2.1
51073	2.34044	2.1
51074	2.34044	2.1
51075	2.62514	2.1
51076	2.76598	2.1
51077	2.54666	2.1
51078	2.45212	2.1
51079	5.2181	2.1
51080	2.14063	2.1
51081	1.47521	2.1
51082	1.27679	2.1
51083	1.47521	2.1
51084	2.11511	2.1
51085	2.31353	2.1
51086	0.93256	2.1
51087	1.07032	2.1
51088	0.638396	2.1
51089	0.521342	2.1
51090	0.790674	2.1
51091	0.790674	2.1
51092	0.80098	2.1
51093	0.800981	2.1
51094	0.932535	2.1
51095	0.521328	2.1

51096	1.07034	2.1
51097	0.638414	2.1
51098	1.89226	2.1
51099	1.89226	1.2642
51100	1.89226	1.31529
51101	1.89226	2.05501
51102	3.78453	1.53618
51103	1.89226	1.97181
51104	1.9842	2.1
51105	1.89226	1.80208
51106	1.89226	2.1
51107	2.79705	1.92552
51108	2.79705	2.1
51109	0.469955	2.1
51110	0.791665	2.1
51111	0.989115	1.42094
51112	0.763235	1.88719
51113	0.776356	1.60163
51114	0.805461	2.1
51115	0.776759	1.61207
51116	0.776759	2.1
51117	0.799151	1.93196
51118	0.940869	1.99695
51119	0.78449	1.79799
51120	0.489635	1.81887
51121	1.89226	1.19938
51122	1.89226	1.81341
51123	1.89226	1.23171
51124	1.89226	1.96827
51125	1.89226	1.14536
51126	1.89226	1.98532
51127	2.46058	1.63823
51128	2.28991	1.94827
51129	1.8525	1.71406
51130	1.87697	2.06469
51131	2.28991	1.63407
51132	1.62397	2.00118
51133	0.946131	0.12104
51134	0.974033	0.111152
51135	1.31667	0.121584
51136	0.946131	0.130826
51137	0.885497	0.129035
51138	0.885497	0.124482
51139	1.0468	0.112784
51140	1.04678	0.116898
51141	1.31667	0.120245
51142	0.974019	0.113885
51143	0.946131	0.119437
51144	0.946131	0.116512
51145	1.89226	0.133258
51146	1.89226	0.208308
51147	1.89226	0.181796
51148	1.89226	0.166029
51149	3.78452	0.19067
51150	0.946125	0.211104
51151	0.946125	0.183064
51152	0.946131	0.208288
51153	0.946131	0.239531

51154	0.946128	0.234371
51155	0.946128	0.214859
51156	0.946128	0.172276
51157	0.946128	0.193827
51158	0.946128	0.199957
51159	0.946128	0.205116
51160	0.946128	0.213951
51161	0.946128	0.207097
51162	0.946131	0.143946
51163	0.98453	0.130301
51164	1.32276	0.16097
51165	0.946131	0.167678
51166	0.946132	0.158818
51167	0.946132	0.151394
51168	0.98453	0.138504
51169	1.32276	0.14136
51170	1.32276	0.153029
51171	1.32276	0.129786
51172	0.946132	0.146926
51173	0.946132	0.13015
51174	1.89227	2.1
51175	1.89227	2.1
51176	1.89227	2.1
51177	1.89227	2.1
51178	1.89227	2.1
51179	1.89227	2.1
51180	1.89226	2.1
51181	1.89226	2.1
51182	1.89226	2.1
51183	1.89226	2.1
51184	1.89226	2.1
51185	1.89226	2.1
51186	1.05556	2.1
51187	1.38859	2.1
51188	1.46996	2.1
51189	1.25364	2.1
51190	2.52552	2.1
51191	0.694296	2.1
51192	0.73498	2.1
51193	0.561881	2.1
51194	0.512189	2.1
51195	0.621304	2.1
51196	0.621304	2.1
51197	0.625865	2.1
51198	0.625865	2.1
51199	0.734979	2.1
51200	0.561881	2.1
51201	0.51219	2.1
51202	0.694296	2.1
51203	0.946126	2.1
51204	0.946126	2.1
51205	0.946126	2.1
51206	0.946126	2.1
51207	0.946126	2.1
51208	0.946126	2.1
51209	0.946126	2.1
51210	0.946126	2.1
51211	0.946132	2.1

51212	0.946132	2.1
51213	0.94612	2.1
51214	0.94612	2.1
51215	1.89225	2.1
51216	1.89225	2.1
51217	1.89225	2.1
51218	1.89225	2.1
51219	1.89225	2.1
51220	1.89225	2.1
51221	0.626814	2.1
51222	0.632155	2.1
51223	0.746234	2.1
51224	0.734974	2.1
51225	0.68202	2.1
51226	0.68202	2.1
51227	0.685108	2.1
51228	0.685108	2.1
51229	0.746239	2.1
51230	0.632159	2.1
51231	0.734969	2.1
51232	0.62681	2.1
51233	0.746235	2.1
51234	0.734975	2.1
51235	0.595081	2.1
51236	0.561877	2.1
51237	0.648584	2.1
51238	0.648584	2.1
51239	0.659528	2.1
51240	0.659528	2.1
51241	0.746239	2.1
51242	0.595085	2.1
51243	0.734969	2.1
51244	0.561874	2.1
51245	1.89249	2.1
51246	2.2137	2.1
51247	1.33125	2.1
51248	1.06499	2.1
51249	2.12571	2.1
51250	1.78918	2.1
51251	0.946131	2.1
51252	0.946131	2.1
51253	0.94613	2.1
51254	0.94613	2.1
51255	0.946132	2.1
51256	0.946132	2.1
51257	0.946129	2.1
51258	0.946129	2.1
51259	0.946131	2.1
51260	0.946131	2.1
51261	0.946131	2.1
51262	0.946131	2.1
51263	1.89227	2.1
51264	1.89227	2.1
51265	1.89227	2.1
51266	1.89227	2.1
51267	3.78453	2.1
51268	1.89226	2.1
51269	1.89226	2.1

51270	1.89226	2.1
51271	1.89226	2.1
51272	1.89226	2.1
51273	1.89226	2.1
51274	2.01388	2.1
51275	1.38757	2.1
51276	1.24229	2.1
51277	1.3309	2.1
51278	1.88011	2.1
51279	2.01388	2.1
51280	2.01388	2.1
51281	2.01555	2.1
51282	2.13879	2.1
51283	1.80766	2.1
51284	4.15267	2.1
51285	0.946133	2.1
51286	0.946133	2.1
51287	0.946132	2.1
51288	0.946132	2.1
51289	0.946132	2.1
51290	0.946132	2.1
51291	0.946133	2.1
51292	0.946133	2.1
51293	0.946132	2.1
51294	0.946132	2.1
51295	0.946132	2.1
51296	0.946132	2.1
51297	1.89226	2.1
51298	1.89226	2.1
51299	1.89226	2.1
51300	1.89226	2.1
51301	1.89226	2.1
51302	1.89226	2.1
51303	0.676745	2.1
51304	0.68382	2.1
51305	1.07048	2.1
51306	1.1327	2.1
51307	0.929324	2.1
51308	0.871438	2.1
51309	0.867362	2.1
51310	0.867362	2.1
51311	1.10666	2.1
51312	0.695036	2.1
51313	0.744098	2.1
51314	1.10144	2.1
51315	2.25168	2.1
51316	2.22499	2.1
51317	2.15929	2.1
51318	2.13879	2.1
51319	2.15929	2.1
51320	2.13879	2.1
51321	2.15314	2.1
51322	1.70302	2.1
51323	2.24694	2.1
51324	1.65245	2.1
51325	3.82722	2.1
51326	1.65245	2.1
51327	1.00371	2.1

51328	1.7437	2.1
51329	1.65748	2.1
51330	1.10459	2.1
51331	1.00371	2.1
51332	2.24694	2.1
51333	1.65246	2.1
51334	2.69773	2.1
51335	1.88129	2.1
51336	4.12824	2.1
51337	1.65246	2.1
51338	1.10459	2.1
51339	1.88129	2.1
51340	1.05914	2.1
51341	2.76496	2.1
51342	1.89226	2.1
51343	1.89226	2.1
51344	1.89226	1.94575
51345	1.89226	2.1
51346	3.78452	2.1
51347	0.718577	2.1
51348	0.718577	1.80362
51349	0.725249	2.1
51350	0.725249	2.00733
51351	0.597916	2.1
51352	0.61752	2.1
51353	0.826306	1.79403
51354	0.859194	1.82551
51355	0.826306	2.1
51356	0.597916	2.1
51357	0.859194	2.1
51358	0.61752	2.1
51359	1.25898	2.1
51360	1.25898	2.1
51361	0.946128	2.1
51362	0.946128	2.1
51363	1.25897	2.1
51364	1.25585	2.1
51365	0.946131	2.1
51366	0.946131	2.1
51367	0.946129	2.1
51368	1.25586	2.1
51369	1.25898	2.1
51370	0.946129	2.1
51371	1.89226	1.74473
51372	1.89226	2.1
51373	1.89226	1.9503
51374	1.89226	2.1
51375	3.78452	2.1
51376	0.693258	2.1
51377	0.693258	1.54277
51378	0.697356	1.82279
51379	0.697356	2.1
51380	0.572479	2.1
51381	0.597916	2.1
51382	0.792698	1.52841
51383	0.826306	1.77457
51384	0.572478	2.1
51385	0.792697	2.1

51386	0.826307	2.1
51387	0.597917	2.1
51388	1.25586	2.1
51389	1.25586	2.1
51390	0.946128	2.1
51391	0.946128	2.1
51392	1.25585	2.1
51393	1.24344	2.1
51394	0.946131	2.1
51395	0.946131	2.1
51396	0.946129	2.1
51397	1.24344	2.1
51398	0.94613	2.1
51399	1.25586	2.1
51400	1.65151	2.1
51401	1.81835	2.1
51402	1.12064	2.1
51403	1.12064	2.1
51404	1.24965	2.1
51405	1.81835	2.1
51406	1.24965	2.1
51407	1.81835	2.1
51408	1.41077	2.1
51409	2.02687	2.1
51410	3.22911	2.1
51411	1.89122	1.11844
51412	1.38776	1.91117
51413	2.37014	0.932051
51414	1.80818	2.00061
51415	3.6994	1.62703
51416	2.15466	0.93621
51417	2.00083	1.7711
51418	1.80818	1.39891
51419	1.80818	0.95364
51420	2.31988	1.5008
51421	2.31988	0.952009
51422	1.80818	0.810262
51423	1.18041	2.1
51424	2.00082	1.10229
51425	1.43652	2.1
51426	3.18123	1.70742
51427	1.89226	0.933082
51428	1.89226	1.33164
51429	1.89226	1.01799
51430	1.89226	0.922649
51431	3.78453	1.01989
51432	1.89226	1.36617
51433	1.89226	1.38339
51434	1.89226	1.01065
51435	1.89226	1.06217
51436	3.78452	1.43432
51437	0.80759	1.20193
51438	0.686119	1.38417
51439	0.972147	1.26636
51440	1.00217	1.39121
51441	0.821334	1.62367
51442	0.821334	1.37222
51443	0.866957	1.22969

51444	0.866957	1.17936
51445	0.972145	1.4515
51446	0.686121	1.934
51447	0.807592	1.51952
51448	1.00217	1.90188
51449	0.972146	1.22774
51450	1.00217	1.30762
51451	1.26654	1.47578
51452	1.1908	1.27705
51453	1.05501	1.30643
51454	1.05501	1.87999
51455	1.10794	1.20261
51456	1.10794	0.848689
51457	0.972146	0.875198
51458	1.26654	1.3423
51459	1.00217	1.22769
51460	1.1908	1.65144
51461	1.89226	1.1938
51462	1.89226	0.858138
51463	1.89226	0.933973
51464	1.89226	1.49409
51465	1.89226	0.774102
51466	1.89226	1.07039
51467	0.972147	1.31141
51468	0.867854	1.41398
51469	1.26132	1.09516
51470	1.26654	1.37386
51471	1.0424	1.50691
51472	1.0424	1.73182
51473	1.092	0.826277
51474	1.092	1.31857
51475	1.26132	1.37
51476	0.867854	2.03833
51477	0.972147	1.58302
51478	1.26654	1.96037
51479	1.4375	0.318744
51480	1.92998	0.280595
51481	1.66953	0.190927
51482	1.0953	0.20067
51483	3.10703	0.226959
51484	1.89226	2.1
51485	1.89226	2.1
51486	1.89226	2.1
51487	1.89226	2.1
51488	3.78452	2.1
51489	2.74034	2.1
51490	2.08808	2.1
51491	2.63859	2.1
51492	2.00343	2.1
51493	4.72667	2.1
51494	0.799825	2.1
51495	0.799825	2.1
51496	0.80211	2.1
51497	0.80211	2.1
51498	0.562428	2.1
51499	0.600223	2.1
51500	1.04404	2.1
51501	1.00171	2.1

51502	1.00171	2.1
51503	0.56243	2.1
51504	0.600225	2.1
51505	1.04404	2.1
51506	0.94613	2.1
51507	0.94613	2.1
51508	0.946128	2.1
51509	0.946128	2.1
51510	0.946127	2.1
51511	0.946127	2.1
51512	0.946131	2.1
51513	0.946131	2.1
51514	0.946129	2.1
51515	0.946129	2.1
51516	0.946129	2.1
51517	0.946129	2.1
51518	1.89226	2.1
51519	1.89226	2.1
51520	1.89226	2.1
51521	1.89226	2.1
51522	3.78452	2.1
51523	2.63859	2.1
51524	2.00343	2.1
51525	2.54388	2.1
51526	1.91548	2.1
51527	4.55407	2.1
51528	1.91548	2.1
51529	2.00342	2.1
51530	1.04909	2.1
51531	1.04909	2.1
51532	1.12486	2.1
51533	1.99124	2.1
51534	0.810942	2.1
51535	0.810942	2.1
51536	0.810942	2.1
51537	0.810942	2.1
51538	1.89226	2.1
51539	1.35169	2.1
51540	1.08131	2.1
51541	1.08132	2.1
51542	1.08132	2.1
51543	0.810944	2.1
51544	0.810944	2.1
51545	1.89226	0.430312
51546	1.89226	0.58311
51547	1.89226	0.384435
51548	1.89226	0.645683
51549	3.78452	0.48662
51550	1.7968	0.357247
51551	1.49831	0.567933
51552	1.59244	0.363915
51553	1.3395	0.517621
51554	3.09074	0.464598
51555	1.49831	0.535865
51556	1.16858	1.29018
51557	1.33951	0.460444
51558	1.0361	0.946061
51559	2.53442	0.644405

51560	1.89226	0.56386
51561	1.89226	0.541497
51562	1.89226	0.576635
51563	1.89226	1.45693
51564	1.89226	1.1303
51565	1.89226	1.00205
51566	1.89223	0.391342
51567	1.89223	0.554846
51568	1.89223	0.487247
51569	1.89223	0.582581
51570	1.89223	0.362943
51571	1.89223	0.528834
51572	1.59241	0.3731
51573	1.18952	0.53821
51574	1.33949	0.515271
51575	1.33949	0.396897
51576	1.40538	0.517696
51577	1.40538	0.38959
51578	1.89226	0.314359
51579	1.89226	0.448262
51580	1.89226	0.359899
51581	1.89226	0.424055
51582	1.89226	0.409276
51583	1.89226	0.475487
51584	1.89226	2.1
51585	1.89226	2.1
51586	1.89226	2.1
51587	1.89226	2.1
51588	3.78452	2.1
51589	1.89226	2.1
51590	1.89226	2.1
51591	1.89226	2.1
51592	1.89226	2.1
51593	3.78452	2.1
51594	2.01867	2.1
51595	1.46955	2.1
51596	1.58476	2.1
51597	1.38189	2.1
51598	2.22153	2.1
51599	2.01867	2.1
51600	2.01867	2.1
51601	2.52881	2.1
51602	2.21732	2.1
51603	2.21732	2.1
51604	2.22154	2.1
51605	2.22154	2.1
51606	1.89226	2.1
51607	1.89226	2.1
51608	1.89226	2.1
51609	1.89226	2.1
51610	1.89226	2.1
51611	1.89226	2.1
51612	1.89226	2.1
51613	1.89226	2.1
51614	1.89226	2.1
51615	1.89226	2.1
51616	1.89226	2.1
51617	1.89226	2.1

51618	2.22153	2.1
51619	1.63361	2.1
51620	1.77336	2.1
51621	1.46955	2.1
51622	2.52534	2.1
51623	2.22153	2.1
51624	2.34431	2.1
51625	1.62905	2.1
51626	1.49693	2.1
51627	2.19197	2.1
51628	3.82103	2.1
51629	1.89226	2.1
51630	1.89226	2.1
51631	1.89226	2.1
51632	1.89226	2.1
51633	3.78453	2.1
51634	0.946128	2.1
51635	0.946128	2.1
51636	1.22372	2.1
51637	1.17331	2.1
51638	0.946131	2.1
51639	0.946131	2.1
51640	1.17331	2.1
51641	1.17331	2.1
51642	0.946131	2.1
51643	1.17331	2.1
51644	1.22372	2.1
51645	0.94613	2.1
51646	1.04041	2.1
51647	1.09599	2.1
51648	0.748462	2.1
51649	0.701976	2.1
51650	0.896701	2.1
51651	0.896701	2.1
51652	0.901262	2.1
51653	0.901262	2.1
51654	1.04041	2.1
51655	0.701977	2.1
51656	0.748463	2.1
51657	1.09599	2.1
51658	1.89226	2.1
51659	1.89226	2.1
51660	1.89226	2.1
51661	1.89226	2.1
51662	3.78453	2.1
51663	0.946128	2.1
51664	0.946128	2.1
51665	1.17331	2.1
51666	1.13785	2.1
51667	0.94613	2.1
51668	0.94613	2.1
51669	1.13785	2.1
51670	1.13785	2.1
51671	1.13785	2.1
51672	0.94613	2.1
51673	0.94613	2.1
51674	1.17331	2.1
51675	2.50381	2.1

51676	1.89226	2.1
51677	1.89226	2.1
51678	2.73387	2.1
51679	4.39607	2.1
51680	1.80045	2.1
51681	1.99777	0.681777
51682	2.45134	1.14994
51683	2.12463	1.34836
51684	1.70302	1.18503
51685	1.42655	2.09965
51686	2.20231	2.1
51687	1.70302	0.912536
51688	1.93128	1.8905
51689	1.66768	1.28098
51690	1.24499	0.680837
51691	1.70302	0.664241
51692	2.55572	0.268978
51693	1.22811	0.277446
51694	1.82584	0.298266
51695	1.22811	0.272482
51696	2.62764	0.270269
51697	1.93147	0.250015
51698	2.14513	0.261049
51699	1.89226	0.362589
51700	2.66481	0.265428
51701	1.89226	0.265047
51702	2.66481	0.383367
51703	1.89226	0.377026
51704	2.11649	2.1
51705	1.89226	2.1
51706	1.89226	2.1
51707	1.89226	2.1
51708	2.15426	2.1
51709	2.15426	2.1
51710	2.06776	2.1
51711	1.89226	2.1
51712	1.89226	2.1
51713	1.89226	2.1
51714	2.11649	2.1
51715	2.11649	2.1
51716	1.89226	2.1
51717	1.89226	2.1
51718	1.89226	2.1
51719	1.89226	2.1
51720	1.89226	2.1
51721	1.89226	2.1
51722	0.998134	2.1
51723	0.918904	2.1
51724	0.894708	2.1
51725	0.758742	2.1
51726	0.864276	2.1
51727	0.864276	2.1
51728	0.8926	2.1
51729	0.8926	2.1
51730	0.998134	2.1
51731	0.894709	2.1
51732	0.758742	2.1
51733	0.918904	2.1

51734	1.89226	2.1
51735	1.89226	2.1
51736	1.89226	2.1
51737	1.89226	2.1
51738	1.89226	2.1
51739	1.89226	2.1
51740	1.89226	2.1
51741	1.89226	2.1
51742	1.89226	2.1
51743	1.89226	2.1
51744	1.89226	2.1
51745	1.89226	2.1
51746	1.99627	2.1
51747	1.61047	2.1
51748	2.0815	2.1
51749	2.07569	2.1
51750	3.75063	2.1
51751	0.848077	2.1
51752	1.07748	2.1
51753	0.802498	2.1
51754	0.626808	2.1
51755	0.875552	2.1
51756	0.737527	2.1
51757	0.835602	2.1
51758	0.835602	2.1
51759	0.983245	2.1
51760	0.781108	2.1
51761	0.872306	2.1
51762	0.601497	2.1
51763	2.74745	2.1
51764	2.73988	2.1
51765	2.6521	2.1
51766	2.67792	2.1
51767	5.39198	2.1
51768	2.73988	2.1
51769	2.57466	2.1
51770	2.67791	2.1
51771	2.55653	2.1
51772	5.29641	2.1
51773	2.6521	2.1
51774	2.64148	2.1
51775	2.67792	2.1
51776	2.67792	2.1
51777	2.56953	2.1
51778	2.56953	2.1
51779	2.64148	2.1
51780	2.55653	2.1
51781	2.57929	2.1
51782	2.57929	2.1
51783	2.67792	2.1
51784	2.67792	2.1
51785	1.89226	0.517428
51786	1.89226	0.294285
51787	1.89226	0.598383
51788	1.89226	0.172392
51789	3.78453	0.301728
51790	1.68703	0.51385
51791	2.50385	0.185193

51792	1.45958	0.314174
51793	2.0815	0.169037
51794	3.93806	0.253126
51795	2.50385	0.169815
51796	2.57414	0.250512
51797	2.0815	0.213079
51798	2.39921	0.341191
51799	4.91181	0.174501
51800	1.89226	0.444158
51801	1.89226	0.226365
51802	1.89226	0.198095
51803	1.89226	0.22112
51804	3.78453	0.265616
51805	1.89226	0.62244
51806	1.89226	0.183656
51807	1.89226	0.29779
51808	1.89226	0.362204
51809	1.89226	0.239963
51810	1.89226	0.497687
51811	1.23013	0.246993
51812	2.0815	0.18556
51813	1.90167	0.39492
51814	1.71243	0.241067
51815	1.45887	0.363753
51816	1.31369	0.195729
51817	2.0815	0.251898
51818	2.39921	0.357226
51819	1.7115	0.200376
51820	2.2421	0.258623
51821	4.09829	0.288135
51822	1.89227	0.218055
51823	1.89227	0.242446
51824	1.89227	0.294535
51825	1.89227	0.244426
51826	3.78453	0.191385
51827	2.39697	0.085229
51828	1.85594	0.0967311
51829	1.91161	0.0808901
51830	2.49867	0.102458
51831	4.35461	0.0912352
51832	1.12035	0.0794294
51833	1.85594	0.0935713
51834	1.91161	0.0780071
51835	1.15483	0.0829554
51836	3.01077	0.082159
51837	1.89226	0.0850248
51838	1.89226	0.11042
51839	1.89226	0.0943675
51840	1.89226	0.0975555
51841	1.89226	0.0945887
51842	1.89226	0.098624
51843	1.89226	0.104464
51844	1.89226	0.113871
51845	1.89226	0.122451
51846	1.89226	0.0983188
51847	1.89226	0.124087
51848	1.89226	0.101956
51849	0.936516	0.0908422

51850	1.03496	0.0801587
51851	1.22607	0.103019
51852	1.28058	0.0838743
51853	1.06317	0.0992502
51854	1.06317	0.0929472
51855	1.19735	0.0791022
51856	1.0813	0.0830145
51857	0.858168	0.0904091
51858	1.32278	0.0885647
51859	0.957674	0.0864497
51860	1.25178	0.0876388
51861	1.92659	0.0787258
51862	0.998957	0.0830891
51863	0.998957	0.0929039
51864	1.15481	0.0794103
51865	1.6031	0.0890257
51866	1.74459	0.0779592
51867	0.946119	0.0862489
51868	0.946119	0.096987
51869	0.946118	0.0923139
51870	0.946118	0.110457
51871	0.946118	0.11
51872	0.946118	0.0974575
51873	0.946119	0.0838276
51874	0.946119	0.0905611
51875	0.946131	0.0952916
51876	0.946131	0.0955612
51877	0.946106	0.0973226
51878	0.946106	0.097747
51879	0.946118	0.0968575
51880	0.946118	0.115988
51881	0.946119	0.104859
51882	0.946119	0.124986
51883	0.946118	0.125162
51884	0.946118	0.115806
51885	0.946119	0.103216
51886	0.946119	0.0963379
51887	0.946131	0.105274
51888	0.946131	0.106394
51889	0.946106	0.108785
51890	0.946106	0.108796
51891	2.47164	0.521618
51892	2.83689	0.352149
51893	2.73872	0.543375
51894	2.41632	0.355824
51895	5.21036	0.371049
51896	0.946133	0.44543
51897	0.946133	0.541858
51898	1.41845	0.368723
51899	1.36936	0.520069
51900	1.41845	0.365664
51901	1.41845	0.345368
51902	0.946132	0.538409
51903	0.946132	0.55091
51904	0.946132	0.468278
51905	1.36936	0.402786
51906	1.41845	0.446209
51907	0.946133	0.504819

51908	1.36936	0.246359
51909	1.27581	0.396969
51910	1.20816	0.300487
51911	1.10464	0.460767
51912	1.24454	0.276434
51913	1.24454	0.263716
51914	1.23943	0.411838
51915	1.23943	0.44655
51916	1.10465	0.386209
51917	1.27581	0.366906
51918	1.36936	0.379222
51919	1.20816	0.35403
51920	0.946131	2.1
51921	0.946131	2.1
51922	1.37277	2.1
51923	1.37277	2.1
51924	1.37277	2.1
51925	1.27186	2.1
51926	0.946131	2.1
51927	0.946131	2.1
51928	0.946131	2.1
51929	1.37277	2.1
51930	0.946131	2.1
51931	1.27186	2.1
51932	2.74554	2.1
51933	1.23712	2.1
51934	2.80992	2.1
51935	2.54371	2.1
51936	1.50333	2.1
51937	1.23712	2.1
51938	2.77314	2.1
51939	2.33316	2.1
51940	2.28748	2.1
51941	2.28748	2.1
51942	2.81275	2.1
51943	2.81275	2.1
51944	2.28749	2.1
51945	1.45887	2.1
51946	2.33317	2.1
51947	1.50022	2.1
51948	3.79204	2.1
51949	2.37364	2.1
51950	2.33317	2.1
51951	1.5362	2.1
51952	1.5362	2.1
51953	1.50022	2.1
51954	2.33317	2.1
51955	1.89226	0.526428
51956	1.89226	0.519108
51957	1.89226	0.475224
51958	1.89226	0.542213
51959	1.89226	0.50071
51960	1.89226	0.578572
51961	0.946128	0.350888
51962	0.946128	0.389206
51963	1.0644	0.306629
51964	0.946133	0.352919
51965	1.0644	0.296558

51966	0.94613	0.323968
51967	0.946131	0.409678
51968	0.946131	0.345454
51969	1.0644	0.336908
51970	1.0644	0.349625
51971	0.946131	0.361544
51972	0.946131	0.344496
51973	1.89226	0.747089
51974	1.89226	0.428976
51975	1.89226	0.608613
51976	1.89226	0.252308
51977	3.78452	0.455701
51978	1.14599	0.743717
51979	1.19993	0.370784
51980	1.00065	0.602565
51981	1.07497	0.255512
51982	2.22096	0.520469
51983	0.546551	0.436682
51984	0.546551	0.453806
51985	0.54987	0.415222
51986	0.54987	0.598991
51987	0.503067	0.394364
51988	0.558937	0.393373
51989	0.599966	0.355034
51990	0.537484	0.402891
51991	0.503067	0.320647
51992	0.537485	0.324422
51993	0.599966	0.373337
51994	0.558936	0.345006
51995	0.946132	0.406552
51996	0.946132	0.493223
51997	0.94613	0.386005
51998	0.94613	0.567081
51999	0.946131	0.42981
52000	0.946131	0.429332
52001	0.946131	0.413483
52002	0.946131	0.417893
52003	0.946131	0.353232
52004	0.946131	0.347981
52005	0.946131	0.406722
52006	0.946131	0.449175
52007	2.19104	2.1
52008	2.5392	2.1
52009	2.53302	2.1
52010	2.19226	2.1
52011	4.73146	2.1
52012	1.09552	2.1
52013	1.09613	2.1
52014	0.802484	2.1
52015	0.807941	2.1
52016	0.95052	2.1
52017	0.95052	2.1
52018	0.952944	2.1
52019	0.952944	2.1
52020	1.09613	2.1
52021	0.807941	2.1
52022	0.802484	2.1
52023	1.09552	2.1

52024	2.533	2.1
52025	2.18531	2.1
52026	2.19224	2.1
52027	2.19224	2.1
52028	2.51603	2.1
52029	2.51603	2.1
52030	1.09266	2.1
52031	1.09612	2.1
52032	0.807935	2.1
52033	0.81091	2.1
52034	0.951897	2.1
52035	0.951897	2.1
52036	0.955134	2.1
52037	0.955134	2.1
52038	0.810915	2.1
52039	1.09266	2.1
52040	1.09611	2.1
52041	0.80793	2.1
52042	2.12741	0.636101
52043	2.79312	0.60789
52044	2.77591	0.970313
52045	2.10714	0.587395
52046	4.90026	0.552971
52047	2.12741	0.635892
52048	1.20859	0.431935
52049	1.21073	0.952984
52050	2.10714	0.648769
52051	3.31573	0.713838
52052	1.38708	0.772925
52053	1.38795	0.566849
52054	1.05331	0.637926
52055	1.05357	0.568523
52056	1.22049	0.587822
52057	1.22049	0.616942
52058	1.22078	1.11733
52059	1.22078	0.49658
52060	1.05331	0.686896
52061	1.38709	0.689614
52062	1.38795	0.586513
52063	1.05357	0.621169
52064	2.10662	0.812809
52065	2.10714	0.541082
52066	2.12499	0.572592
52067	1.22857	0.760487
52068	1.21073	0.619953
52069	1.21073	0.422522
52070	1.82014	0.154194
52071	2.08757	0.152834
52072	1.81292	0.12901
52073	1.67485	0.134566
52074	3.63306	0.140782
52075	1.82013	0.150669
52076	1.21748	0.134335
52077	1.29891	0.15153
52078	1.4442	0.145542
52079	1.67484	0.135079
52080	1.82013	0.134547
52081	1.81292	0.122234

52082	1.4516	0.103951
52083	1.39633	0.114688
52084	1.67485	0.109822
52085	1.5344	0.112182
52086	1.81292	0.109874
52087	1.67484	0.110694
52088	1.00982	0.103726
52089	0.994235	0.114111
52090	1.21748	0.110825
52091	1.45159	0.103525
52092	1.67484	0.106717
52093	1.55216	2.1
52094	1.34407	2.1
52095	1.34407	2.1
52096	1.52008	2.1
52097	1.81783	2.1
52098	1.99384	2.1
52099	1.90817	2.1
52100	2.03975	2.1
52101	1.97317	2.1
52102	1.81783	2.1
52103	3.85758	2.1
52104	0.599049	2.1
52105	0.660673	2.1
52106	0.785063	2.1
52107	0.924278	2.1
52108	0.760416	2.1
52109	0.760416	2.1
52110	0.719088	2.1
52111	0.694532	2.1
52112	0.522441	2.1
52113	0.796789	2.1
52114	0.655207	2.1
52115	0.886155	2.1
52116	1.97317	2.1
52117	1.81783	2.1
52118	1.65098	2.1
52119	1.91767	2.1
52120	3.7355	2.1
52121	1.15038	1.10828
52122	1.28649	1.60809
52123	1.38986	1.21232
52124	1.38986	1.86079
52125	1.07866	1.31025
52126	1.07866	1.94298
52127	0.664498	0.631794
52128	0.664498	2.04852
52129	0.675122	1.49898
52130	0.675122	2.1
52131	0.69493	0.895968
52132	0.764429	1.46342
52133	0.623438	1.22269
52134	0.575191	1.73432
52135	0.764429	1.02028
52136	0.623438	0.969837
52137	0.575191	1.69219
52138	0.69493	1.59491
52139	1.89226	0.562814

52140	1.89226	0.398925
52141	1.89226	0.50138
52142	1.89226	0.437659
52143	3.78453	0.372551
52144	2.50075	0.531509
52145	2.15276	0.416465
52146	2.24902	0.506394
52147	2.30422	0.385577
52148	4.40178	0.363691
52149	1.70424	0.545441
52150	1.34494	0.555635
52151	2.15276	0.39468
52152	2.15276	0.544186
52153	2.0793	0.379881
52154	1.63078	0.471514
52155	1.89226	0.370235
52156	1.89226	0.543538
52157	1.89226	0.659734
52158	1.89226	0.37297
52159	1.89226	0.579961
52160	1.89226	0.461778
52161	1.89226	0.512482
52162	1.89226	0.458898
52163	1.89226	0.462319
52164	1.89226	0.34196
52165	3.78452	0.385905
52166	2.24902	0.521674
52167	1.91497	0.376192
52168	1.97135	0.552785
52169	1.73957	0.366768
52170	3.98859	0.371704
52171	1.73957	0.336112
52172	1.34494	0.521573
52173	1.73957	0.651074
52174	1.69006	0.34368
52175	1.3126	0.551612
52176	1.34848	0.464417
52177	0.946132	0.360382
52178	0.946132	0.567484
52179	0.946132	0.405719
52180	0.946132	0.606402
52181	0.946132	0.574335
52182	0.946132	0.545631
52183	0.946131	0.374176
52184	0.946131	0.334018
52185	0.946132	0.421666
52186	0.946132	0.430032
52187	0.946132	0.473754
52188	0.946132	0.484024
52189	1.89226	2.04394
52190	1.89226	1.44715
52191	1.89226	1.8839
52192	1.89226	1.62125
52193	3.78453	1.70308
52194	1.89226	1.81407
52195	2.82624	1.38928
52196	1.89226	1.64223
52197	2.02536	1.45736

52198	1.89226	1.64104
52199	2.02536	1.37323
52200	2.25027	0.0868248
52201	1.1706	0.0853936
52202	2.02733	0.0874667
52203	2.02733	0.0913171
52204	1.61462	0.0835847
52205	1.69832	0.0862583
52206	1.89226	0.11779
52207	1.89226	0.105664
52208	1.89226	0.0989046
52209	1.89226	0.0956765
52210	3.78452	0.0951674
52211	0.946131	0.0795322
52212	0.946131	0.0835622
52213	1.18182	0.0763606
52214	1.18182	0.0725777
52215	0.973819	0.0752779
52216	0.94613	0.0786141
52217	1.18182	0.0761687
52218	0.94613	0.0823267
52219	0.973819	0.0793884
52220	1.18182	0.0776802
52221	0.94613	0.088366
52222	0.94613	0.0810014
52223	2.23944	2.1
52224	1.52903	2.1
52225	1.35977	2.1
52226	2.04847	2.1
52227	3.5775	2.1
52228	2.23944	2.1
52229	2.34795	2.1
52230	2.34795	2.1
52231	2.48025	2.1
52232	2.04847	2.1
52233	2.18077	2.1
52234	1.89226	2.1
52235	1.89226	1.64162
52236	1.89226	2.1
52237	1.89226	2.1
52238	3.78452	2.1
52239	0.946127	2.1
52240	0.946127	2.1
52241	0.946131	2.1
52242	0.946131	2.1
52243	0.946128	2.1
52244	0.946128	2.1
52245	0.94613	2.1
52246	0.94613	2.1
52247	0.946129	2.1
52248	0.946129	2.1
52249	0.946129	2.1
52250	0.946129	2.1
52251	1.26956	2.1
52252	2.04847	2.1
52253	1.49576	2.1
52254	1.22377	2.1
52255	1.91172	2.1

52256	1.50198	2.1
52257	1.48568	2.1
52258	2.34795	2.1
52259	2.04847	2.1
52260	2.04847	2.1
52261	2.21155	2.1
52262	2.21155	2.1
52263	1.89226	2.1
52264	1.89226	2.1
52265	1.89226	2.1
52266	1.89226	1.75412
52267	1.89226	2.1
52268	1.89226	1.38046
52269	0.810943	2.1
52270	0.810943	2.1
52271	0.810944	2.1
52272	0.810944	2.1
52273	0.810957	2.1
52274	0.810957	2.1
52275	1.89226	2.1
52276	1.35168	2.1
52277	1.08132	2.1
52278	1.08132	2.1
52279	1.0813	2.1
52280	0.946132	0.121707
52281	1.33855	0.158472
52282	0.946131	0.11791
52283	1.38204	0.175921
52284	0.946131	0.176472
52285	0.946131	0.163193
52286	1.38204	0.125535
52287	1.38204	0.123403
52288	0.946131	0.140944
52289	0.946131	0.150684
52290	1.33855	0.126132
52291	1.38204	0.151653
52292	1.89226	0.19196
52293	2.76407	0.184602
52294	1.89226	0.133591
52295	2.72769	0.170519
52296	1.89226	0.124876
52297	2.72769	0.18528
52298	2.03914	0.117427
52299	1.67727	0.136984
52300	1.94831	0.13906
52301	1.81591	0.11084
52302	2.32098	0.140553
52303	2.32098	0.119949
52304	0.801647	0.0998145
52305	1.11086	0.119752
52306	0.838634	0.130743
52307	1.16049	0.111214
52308	0.977891	0.130371
52309	0.977891	0.127249
52310	0.984246	0.0995833
52311	0.984246	0.11123
52312	1.11086	0.116442
52313	1.16049	0.120192

52314	0.801647	0.118487
52315	0.838634	0.117668
52316	0.946132	0.131284
52317	0.946132	0.175082
52318	0.946131	0.12805
52319	0.946131	0.185748
52320	0.946131	0.186739
52321	0.946131	0.173174
52322	0.946132	0.131235
52323	0.946132	0.128005
52324	0.946131	0.146442
52325	0.946131	0.151135
52326	0.946131	0.144467
52327	0.946131	0.155347
52328	0.946131	0.138819
52329	0.946131	0.202452
52330	0.946131	0.189285
52331	0.946131	0.141644
52332	0.946131	0.190722
52333	0.946131	0.189092
52334	0.946131	0.135621
52335	0.946131	0.143638
52336	0.946131	0.159573
52337	0.946131	0.165848
52338	0.946131	0.17015
52339	0.946131	0.168555
52340	2.0815	2.1
52341	1.20787	2.1
52342	1.92277	2.1
52343	1.3227	2.1
52344	3.29401	2.1
52345	2.16633	2.1
52346	2.13642	2.1
52347	1.40846	2.1
52348	1.40846	2.1
52349	1.3227	2.1
52350	2.08058	2.1
52351	0.938106	2.1
52352	0.790258	2.1
52353	0.742975	2.1
52354	0.742975	2.1
52355	0.874389	2.1
52356	1.17364	2.1
52357	0.525948	2.1
52358	0.52513	2.1
52359	0.553732	2.1
52360	0.876996	2.1
52361	0.530954	2.1
52362	1.095	2.1
52363	1.00833	2.1
52364	1.70608	2.1
52365	1.33385	2.1
52366	1.33385	2.1
52367	1.07722	2.1
52368	1.07722	2.1
52369	0.953523	0.857978
52370	1.7457	0.802593
52371	1.03218	0.854828

52372	1.03218	0.820875
52373	1.79675	0.861661
52374	1.79675	0.827304
52375	1.13676	0.825331
52376	1.18136	0.71053
52377	0.87285	0.805923
52378	0.898375	0.833342
52379	1.03191	0.798363
52380	1.03191	0.700091
52381	1.0223	0.809608
52382	1.0223	0.84063
52383	1.18136	0.784678
52384	0.898374	0.757719
52385	0.87285	0.781076
52386	1.13676	0.781658
52387	2.36273	0.746932
52388	1.46965	0.682997
52389	1.79675	0.715292
52390	1.79675	0.61049
52391	2.43417	0.757439
52392	2.43417	0.675194
52393	1.4714	1.97951
52394	1.65434	1.73486
52395	1.62322	0.940799
52396	1.86068	1.5369
52397	3.27756	1.48756
52398	1.89226	2.1
52399	1.89226	1.73523
52400	1.89226	0.976748
52401	1.89226	1.11869
52402	3.78453	1.2124
52403	1.89226	1.26359
52404	1.89226	2.08542
52405	1.89226	0.960087
52406	1.89226	2.09882
52407	3.78453	1.36992
52408	1.65434	1.29715
52409	1.60647	2.1
52410	1.86068	1.12515
52411	1.82649	2.09343
52412	3.48083	1.79366
52413	1.62322	1.95312
52414	1.86068	1.67619
52415	1.84368	0.910804
52416	2.18862	1.24008
52417	3.81184	1.38397
52418	1.89226	1.98954
52419	1.89226	1.61135
52420	1.89226	0.86892
52421	1.89226	1.26904
52422	3.78453	1.4013
52423	1.89226	1.33007
52424	1.89226	2.06214
52425	1.89226	1.06302
52426	1.89226	1.97527
52427	3.78453	1.72587
52428	1.86068	1.1672
52429	1.82649	2.05475

52430	2.18862	1.05294
52431	2.1128	2.02938
52432	4.01511	1.35342
52433	1.74351	2.1
52434	1.13854	2.1
52435	1.40233	2.1
52436	1.922 2.1	
52437	3.14584	2.1
52438	1.922 2.1	
52439	2.00775	2.1
52440	1.7435	2.1
52441	2.09224	2.1
52442	2.00775	2.1
52443	2.09295	2.1
52444	1.69858	2.1
52445	1.922 2.1	
52446	1.40233	2.1
52447	1.40233	2.1
52448	2.18238	2.1
52449	2.18238	2.1
52450	1.00387	2.1
52451	1.10886	2.1
52452	0.961001	2.1
52453	1.09119	2.1
52454	1.02868	2.1
52455	1.02868	2.1
52456	1.04118	2.1
52457	1.04118	2.1
52458	1.10886	2.1
52459	1.09119	2.1
52460	0.960999	2.1
52461	1.00388	2.1
52462	0.885958	0.594688
52463	0.885958	0.544669
52464	0.875826	0.548867
52465	0.875826	0.710752
52466	0.913965	0.718131
52467	0.598609	0.573372
52468	1.16317	0.563924
52469	0.868115	0.537245
52470	0.868115	0.598792
52471	0.598609	0.583925
52472	1.16317	0.59793
52473	0.913965	0.615515
52474	0.946131	0.611151
52475	0.946131	0.580943
52476	0.946131	0.64786
52477	0.946131	0.584565
52478	0.946131	0.632599
52479	0.946131	0.621958
52480	0.946131	0.585661
52481	0.946131	0.57514
52482	0.946131	0.611757
52483	0.946131	0.617746
52484	0.946131	0.602668
52485	0.946131	0.603354
52486	0.946133	0.0962976
52487	0.946133	0.0950576

52488	1.06246	0.0946958
52489	1.06246	0.0970276
52490	0.973851	0.0972219
52491	0.946133	0.0981218
52492	1.06246	0.0878152
52493	0.946132	0.103267
52494	0.97385	0.0966895
52495	1.06246	0.0928461
52496	0.946133	0.0949751
52497	0.946133	0.100798
52498	1.89226	0.124897
52499	1.89226	0.109119
52500	1.89226	0.11347
52501	1.89226	0.120549
52502	1.89226	0.109256
52503	1.89226	0.114056
52504	0.946131	0.108656
52505	0.946131	0.108224
52506	0.946127	0.107686
52507	0.946127	0.107711
52508	0.946131	0.122082
52509	0.946131	0.0999936
52510	0.946127	0.107694
52511	0.946127	0.112708
52512	0.946128	0.10468
52513	0.946128	0.1009
52514	0.946129	0.111229
52515	0.946129	0.114946
52516	0.946132	0.0861991
52517	0.946132	0.089693
52518	1.32446	0.0819606
52519	0.794912	0.0814404
52520	1.32429	0.0844711
52521	0.946131	0.0864326
52522	1.32446	0.0824246
52523	0.94613	0.0859754
52524	1.32429	0.0857266
52525	0.794915	0.0772733
52526	0.94613	0.0853605
52527	0.94613	0.0916966
52528	1.89226	0.0844155
52529	2.36365	0.0792371
52530	2.64857	0.0847732
52531	1.89226	0.0830643
52532	2.64857	0.0762731
52533	1.89226	0.0819909
52534	1.18463	2.1
52535	1.27174	2.1
52536	0.946129	2.1
52537	0.946129	2.1
52538	0.946131	2.1
52539	0.946131	2.1
52540	1.27174	2.1
52541	1.27174	2.1
52542	1.18463	2.1
52543	0.94613	2.1
52544	0.946131	2.1
52545	1.27174	2.1

52546	2.57997	2.1
52547	1.89226	2.1
52548	1.89226	2.1
52549	1.89226	2.1
52550	2.41915	2.1
52551	2.41915	2.1
52552	1.89226	2.1
52553	1.89226	2.1
52554	1.89226	2.1
52555	1.89226	2.1
52556	3.78452	2.1
52557	1.08807	2.1
52558	1.18463	2.1
52559	0.946129	2.1
52560	0.946129	2.1
52561	0.946131	2.1
52562	0.946131	2.1
52563	1.18463	2.1
52564	1.18463	2.1
52565	0.946131	2.1
52566	1.08807	2.1
52567	1.18463	2.1
52568	0.94613	2.1
52569	1.07017	2.1
52570	1.07017	2.1
52571	0.946131	2.1
52572	0.946131	2.1
52573	0.946132	2.1
52574	0.946132	2.1
52575	1.20957	2.1
52576	1.07017	2.1
52577	1.07017	2.1
52578	0.946132	2.1
52579	0.946131	2.1
52580	1.20957	2.1
52581	1.89226	2.1
52582	1.89226	2.1
52583	1.89226	2.1
52584	1.89226	2.1
52585	1.89226	2.1
52586	1.89226	2.1
52587	1.16181	0.0913491
52588	1.0227	0.0953496
52589	1.0227	0.10257
52590	0.970959	0.0910508
52591	1.24295	0.0978137
52592	1.19121	0.088327
52593	1.12076	2.1
52594	2.53614	2.1
52595	1.28413	2.1
52596	1.28413	2.1
52597	2.36423	2.1
52598	2.36423	2.1
52599	1.89226	2.1
52600	1.89226	2.1
52601	1.89226	2.1
52602	1.89226	2.1
52603	3.78452	2.1

52604	1.22557	2.1
52605	2.771	2.1
52606	2.73012	2.1
52607	2.73012	2.1
52608	1.33356	2.1
52609	1.33356	2.1
52610	0.998682	2.1
52611	2.36423	2.1
52612	1.12076	2.1
52613	1.12076	2.1
52614	2.13757	2.1
52615	2.13757	2.1
52616	1.89226	2.1
52617	1.89226	2.1
52618	1.89226	2.1
52619	1.89226	2.1
52620	1.89226	2.1
52621	1.89226	2.1
52622	1.13987	2.1
52623	2.73011	2.1
52624	2.70875	2.1
52625	2.70875	2.1
52626	1.22557	2.1
52627	1.22557	2.1
52628	1.89227	2.1
52629	2.79243	2.1
52630	2.6625	2.1
52631	2.6625	2.1
52632	1.89227	2.1
52633	1.89227	2.1
52634	1.89226	2.1
52635	2.55971	2.1
52636	1.89226	2.1
52637	1.89226	2.1
52638	2.68704	2.1
52639	2.68704	2.1
52640	2.22043	1.46292
52641	2.38975	1.52151
52642	2.28696	1.10388
52643	2.48682	0.905154
52644	4.67671	1.07581
52645	2.38976	1.08643
52646	2.31988	1.52085
52647	2.48683	0.978515
52648	2.4594	1.26103
52649	4.84915	1.34801
52650	2.28696	1.43839
52651	2.48682	1.36429
52652	2.33379	1.40526
52653	2.56149	0.829637
52654	4.84845	1.33168
52655	2.48683	1.46317
52656	2.4594	1.34758
52657	2.5615	0.870061
52658	2.57957	1.11553
52659	5.0209	1.02202
52660	1.06659	1.46472
52661	1.04291	0.644116

52662	1.36595	0.611239
52663	1.36595	1.61578
52664	1.02761	2.04649
52665	1.32697	0.608494
52666	0.946132	1.37609
52667	0.946132	1.26015
52668	1.36956	2.01521
52669	1.1229	2.1
52670	1.1229	1.23398
52671	1.1229	2.1
52672	0.946132	0.839866
52673	0.946132	1.87629
52674	0.946132	1.5641
52675	1.36956	1.63786
52676	1.1229	1.59348
52677	0.946133	1.55337
52678	0.946133	0.989251
52679	0.946133	0.902309
52680	0.946128	1.86878
52681	0.946128	1.62981
52682	0.946131	1.68524
52683	0.946131	1.30133
52684	0.946131	0.646915
52685	0.946131	1.83655
52686	0.94613	1.5512
52687	0.94613	1.46237
52688	0.946131	1.36499
52689	0.946131	1.30486
52690	0.966427	1.21211
52691	0.802265	1.79374
52692	0.803714	1.7458
52693	0.808476	1.43866
52694	1.94371	1.76813
52695	1.36851	1.83749
52696	0.853864	1.98405
52697	1.26635	1.39354
52698	1.09085	1.27825
52699	0.857653	1.81791
52700	0.630243	1.49912
52701	1.20132	0.0933272
52702	1.24295	0.100961
52703	1.24295	0.108183
52704	1.16182	0.0951886
52705	1.28465	0.104472
52706	1.20351	0.092461
52707	0.600658	0.0949622
52708	0.642323	0.107266
52709	0.54424	0.111814
52710	0.576779	0.100306
52711	0.590985	0.112338
52712	0.590985	0.111452
52713	0.586452	0.0937401
52714	0.586452	0.101476
52715	0.642323	0.104344
52716	0.576779	0.102312
52717	0.600658	0.106353
52718	0.54424	0.104749
52719	1.28463	0.0946519

52720	1.24294	0.101092
52721	1.12328	0.0896592
52722	1.15505	0.103174
52723	2.39798	0.0969541
52724	1.98431	0.151794
52725	2.18735	0.147956
52726	1.98431	0.174071
52727	1.70303	0.122289
52728	1.70302	0.219454
52729	1.37788	0.14416
52730	1.89226	0.220813
52731	2.37417	0.176763
52732	1.89226	0.218865
52733	2.18735	0.155757
52734	1.89226	0.27947
52735	2.18735	0.205983
52736	1.89226	0.183614
52737	1.89226	0.539335
52738	1.89226	0.25924
52739	1.89226	0.366561
52740	3.78452	0.40014
52741	1.8613	0.358465
52742	2.03576	0.533839
52743	1.61652	0.224875
52744	1.83082	0.717678
52745	3.65228	0.347204
52746	2.03576	0.51525
52747	2.05257	2.0929
52748	1.83082	0.694335
52749	1.88487	0.813934
52750	3.92064	0.830827
52751	1.89226	0.517931
52752	1.89226	0.623667
52753	1.89226	0.331115
52754	1.89226	0.901795
52755	3.78453	0.538316
52756	0.946131	0.563823
52757	0.946131	0.230792
52758	0.94613	0.143395
52759	0.94613	0.445513
52760	0.946129	0.354909
52761	0.946129	0.55096
52762	0.946132	0.447177
52763	0.946132	0.14712
52764	0.94613	0.291801
52765	0.94613	0.270815
52766	0.946131	0.209031
52767	0.946131	0.266676
52768	0.804708	1.00467
52769	0.804708	0.325154
52770	0.815274	0.565998
52771	0.815274	0.290797
52772	0.83277	0.553527
52773	0.915407	0.834032
52774	0.80826	0.403931
52775	0.704574	0.262016
52776	0.832771	0.517759
52777	0.704573	0.528092

52778	0.808259	0.524743
52779	0.915409	0.521174
52780	1.83082	0.719271
52781	1.88487	0.912149
52782	1.66555	0.55838
52783	1.75835	2.1
52784	3.55042	0.694337
52785	0.810947	0.330619
52786	0.810947	0.617814
52787	0.810947	0.34562
52788	0.810947	0.524839
52789	0.810934	0.563728
52790	0.810934	0.569298
52791	1.89227	0.978499
52792	1.3517	0.651368
52793	1.08132	0.578722
52794	1.08132	0.617457
52795	1.08133	0.58005
52796	1.89226	1.25563
52797	1.70303	1.49457
52798	1.89226	2.0152
52799	2.24712	2.1
52800	2.07363	2.1
52801	1.74617	1.42794
52802	1.89226	2.05698
52803	1.89226	1.27296
52804	1.89226	1.02306
52805	1.89226	1.99265
52806	3.78452	1.24468
52807	1.50108	1.9804
52808	1.43894	1.8968
52809	1.8322	0.918967
52810	1.40967	2.1
52811	1.50108	1.9673
52812	1.07854	2.1
52813	1.89226	0.967929
52814	1.89226	2.02648
52815	1.89226	1.73517
52816	1.89226	0.775628
52817	3.78452	1.62559
52818	1.89226	2.08496
52819	1.89226	1.20046
52820	1.89226	1.13717
52821	1.89226	2.04419
52822	3.78453	1.25087
52823	1.54457	2.06281
52824	1.35097	1.59386
52825	1.70754	0.949154
52826	1.29629	2.1
52827	1.54457	1.87849
52828	1.0305	2.1
52829	0.731763	2.06729
52830	1.00043	1.72945
52831	1.13883	1.18838
52832	0.939026	1.75316
52833	1.04003	1.20012
52834	0.853868	2.1
52835	0.927188	1.95327

52836	0.927188	0.75888
52837	0.861565	1.37818
52838	1.13882	1.64589
52839	0.731769	1.66692
52840	0.89319	1.82706
52841	1.46568	2.1
52842	1.20761	2.1
52843	1.51916	2.1
52844	1.36261	2.1
52845	2.72677	2.1
52846	1.51916	2.1
52847	1.60532	2.1
52848	1.36261	2.1
52849	1.36261	2.1
52850	1.64892	2.1
52851	1.64892	2.1
52852	1.3626	2.1
52853	0.98488	2.1
52854	1.60532	2.1
52855	1.32414	2.1
52856	2.68675	2.1
52857	1.78711	2.1
52858	1.14825	2.1
52859	2.16535	2.1
52860	1.6067	2.1
52861	3.3136	2.1
52862	1.89226	2.1
52863	1.89226	2.1
52864	1.89226	2.1
52865	1.89226	2.1
52866	3.78452	2.1
52867	2.34552	2.1
52868	1.53521	2.1
52869	1.53521	2.1
52870	1.60636	2.1
52871	1.92797	2.1
52872	1.99589	2.1
52873	1.34771	2.1
52874	1.34771	2.1
52875	0.946128	2.1
52876	0.946128	2.1
52877	1.3477	2.1
52878	1.31776	2.1
52879	0.946131	2.1
52880	0.946131	2.1
52881	0.94613	2.1
52882	1.31776	2.1
52883	0.946129	2.1
52884	1.3477	2.1
52885	1.89226	2.1
52886	1.89226	2.1
52887	1.89226	2.1
52888	1.89226	2.1
52889	3.78452	2.1
52890	0.92843	2.1
52891	0.672114	2.0529
52892	0.89514	2.1
52893	0.89514	2.1

52894	0.720104	2.1
52895	0.755753	2.1
52896	0.789158	2.1
52897	0.967302	2.1
52898	1.06565	2.1
52899	0.7397	2.1
52900	0.758893	2.1
52901	0.94672	2.1
52902	1.89226	2.1
52903	2.588	2.1
52904	2.588	2.1
52905	2.63552	2.1
52906	1.89226	2.1
52907	1.93978	2.1
52908	1.49347	0.162253
52909	2.67757	0.239338
52910	1.80061	0.163157
52911	2.27056	0.187639
52912	4.07117	0.158638
52913	1.1016	0.125364
52914	2.27057	0.16581
52915	1.49348	0.18824
52916	1.81893	0.199844
52917	1.49348	0.129275
52918	1.81893	0.121974
52919	1.81893	0.179727
52920	2.82339	0.27919
52921	2.27057	0.180572
52922	2.25405	0.169325
52923	4.52462	0.154924
52924	1.89226	0.190194
52925	1.89226	0.268423
52926	1.89226	0.309384
52927	1.89226	0.206602
52928	1.89226	0.304197
52929	1.89226	0.229653
52930	1.89226	0.147964
52931	1.89226	0.23516
52932	1.89226	0.148896
52933	1.89226	0.169805
52934	3.78453	0.14513
52935	1.89226	0.257439
52936	1.89226	0.303458
52937	1.89226	0.174101
52938	1.89226	0.36399
52939	3.78452	0.338712
52940	1.89226	0.294027
52941	1.89226	0.566503
52942	1.89226	0.306922
52943	1.89226	0.369398
52944	3.78451	0.308391
52945	1.45633	2.1
52946	1.34678	2.1
52947	1.34678	2.1
52948	1.53735	2.1
52949	1.234	2.1
52950	1.42457	2.1
52951	1.89226	2.1

52952	1.89226	2.1
52953	1.89226	2.1
52954	1.89226	2.1
52955	1.89226	2.1
52956	1.89226	2.1
52957	1.09483	2.1
52958	1.34678	2.1
52959	1.234	2.1
52960	1.234	2.1
52961	1.19	2.1
52962	1.19	2.1
52963	0.946131	2.1
52964	0.946131	2.1
52965	0.946131	2.1
52966	0.946131	2.1
52967	0.946131	2.1
52968	0.946131	2.1
52969	0.946132	2.1
52970	0.946132	2.1
52971	0.946132	2.1
52972	0.946132	2.1
52973	0.946131	2.1
52974	0.946131	2.1
52975	2.82263	2.1
52976	1.89226	2.1
52977	2.71441	2.1
52978	2.71441	2.1
52979	1.89226	2.1
52980	1.89226	2.1
52981	1.1412	1.11933
52982	1.1412	2.1
52983	1.14626	2.1
52984	1.14626	2.1
52985	1.28511	1.43212
52986	1.34086	2.1
52987	0.992242	2.1
52988	0.946593	2.1
52989	0.992242	2.1
52990	1.34086	2.1
52991	0.946594	2.1
52992	1.28511	2.1
52993	1.18954	1.71757
52994	1.18954	2.1
52995	1.21477	2.1
52996	1.21477	2.1
52997	1.41207	2.1
52998	1.34086	2.01305
52999	1.01306	2.1
53000	0.992242	2.1
53001	1.01306	2.1
53002	1.41207	2.1
53003	0.992242	2.1
53004	1.34086	2.1
53005	1.09044	1.92521
53006	1.44472	0.57327
53007	1.2955	1.21812
53008	1.2955	0.756538
53009	1.22025	1.26693

53010	1.22025	0.892606
53011	0.810944	0.653893
53012	0.810944	1.71241
53013	0.810946	0.746999
53014	0.810946	0.316493
53015	0.810959	0.709496
53016	0.810959	0.640473
53017	1.89226	0.586773
53018	1.35167	0.704683
53019	1.08132	1.71454
53020	1.08132	0.339498
53021	1.0813	0.692418
53022	1.41894	0.643024
53023	1.28953	0.480081
53024	0.946132	0.501615
53025	0.946132	1.15562
53026	0.946133	0.849064
53027	0.946133	0.496193
53028	1.41894	0.685349
53029	1.41894	1.02617
53030	1.28953	0.596607
53031	0.946132	0.777454
53032	0.946132	0.675669
53033	1.41894	0.534262
53034	1.89226	2.1
53035	2.15444	2.1
53036	1.93239	2.1
53037	1.89226	2.1
53038	2.19457	2.1
53039	2.15444	2.1
53040	1.89226	2.1
53041	1.89226	2.1
53042	1.89226	2.1
53043	1.89226	2.1
53044	1.89226	2.1
53045	1.89226	2.1
53046	0.938507	2.1
53047	0.938507	2.1
53048	1.07871	2.1
53049	1.15801	2.1
53050	1.03236	2.1
53051	1.14766	2.1
53052	0.946133	2.1
53053	0.946133	2.1
53054	0.946131	2.1
53055	1.07009	2.1
53056	0.946131	2.1
53057	1.03237	2.1
53058	1.89226	2.1
53059	2.19457	2.1
53060	1.94492	2.1
53061	1.89226	2.1
53062	2.24723	2.1
53063	2.19457	2.1
53064	0.946132	2.1
53065	0.946132	2.1
53066	0.946131	2.1
53067	0.946131	2.1

53068	0.946132	2.1
53069	0.946132	2.1
53070	0.946132	2.1
53071	0.946132	2.1
53072	0.946132	2.1
53073	0.946132	2.1
53074	0.946132	2.1
53075	0.946132	2.1
53076	2.21345	2.1
53077	1.89226	2.1
53078	1.89226	2.1
53079	2.14017	2.1
53080	2.14017	2.1
53081	1.89226	2.1
53082	1.89226	0.520933
53083	1.89226	0.802185
53084	1.89226	0.579266
53085	1.85439	0.755062
53086	2.23961	0.487546
53087	2.19479	0.577015
53088	1.2916	0.532231
53089	1.2916	0.508741
53090	0.946128	0.51273
53091	0.946128	0.520472
53092	1.2916	0.452174
53093	0.946131	0.513331
53094	0.946126	0.734378
53095	1.1198	0.414855
53096	1.2916	0.447126
53097	1.1198	0.593732
53098	0.946128	0.645871
53099	0.946128	0.572314
53100	1.89226	0.654173
53101	2.71262	0.580744
53102	1.89226	0.597401
53103	2.49959	0.610822
53104	4.39185	0.704574
53105	2.71262	0.576686
53106	1.34592	0.563429
53107	2.49959	0.597786
53108	1.14721	0.531158
53109	3.84551	0.49015
53110	0.946131	0.559127
53111	0.946131	0.573144
53112	1.11247	0.52927
53113	1.11247	0.51256
53114	1.24979	0.516223
53115	1.11247	0.507336
53116	0.946129	0.585223
53117	0.946129	0.555491
53118	0.946131	0.481041
53119	1.11247	0.351714
53120	0.946131	0.425894
53121	1.24979	0.462105
53122	0.946131	2.1
53123	0.946131	2.1
53124	0.94613	2.1
53125	0.94613	2.1

53126	0.946132	2.1
53127	0.946132	2.1
53128	0.946129	2.1
53129	0.946129	2.1
53130	0.946131	2.1
53131	0.946131	2.1
53132	0.946131	2.1
53133	0.946131	2.1
53134	2.77816	2.1
53135	1.89226	2.1
53136	1.89226	2.1
53137	1.89226	2.1
53138	3.78453	2.1
53139	1.89226	2.1
53140	1.89226	2.1
53141	1.89226	2.1
53142	1.89226	2.1
53143	1.89226	2.1
53144	1.89226	2.1
53145	1.89226	2.1
53146	1.89226	2.1
53147	1.89226	2.1
53148	1.89226	2.1
53149	3.78452	2.1
53150	1.55698	2.1
53151	1.89226	2.1
53152	1.89226	2.1
53153	1.89226	2.1
53154	1.8575	2.1
53155	1.8575	2.1
53156	1.89226	2.1
53157	1.89226	2.1
53158	1.89226	2.1
53159	1.89226	2.1
53160	1.89226	2.1
53161	1.89226	2.1
53162	1.89227	2.1
53163	1.89227	2.1
53164	1.89227	2.1
53165	1.89227	2.1
53166	1.89227	2.1
53167	1.89227	2.1
53168	1.35509	2.1
53169	2.06238	2.1
53170	2.12244	2.1
53171	2.12244	2.1
53172	1.34817	2.1
53173	1.34817	2.1
53174	0.946126	1.12102
53175	0.946126	2.1
53176	0.946131	2.1
53177	0.946131	2.1
53178	0.946127	2.1
53179	0.946127	1.27957
53180	0.94613	2.1
53181	0.94613	2.1
53182	0.946128	2.1
53183	0.946128	2.1

53184	0.946128	2.1
53185	0.946128	2.1
53186	1.89226	2.1
53187	1.89226	0.97243
53188	1.89226	1.54719
53189	1.89226	2.06126
53190	1.89226	0.99373
53191	1.89226	1.9446
53192	1.16742	2.1
53193	1.16742	1.63572
53194	1.18122	2.1
53195	1.18122	2.1
53196	1.31745	2.1
53197	1.2597	0.942861
53198	1.03119	2.1
53199	1.06122	2.1
53200	1.06122	2.1
53201	1.31745	2.1
53202	1.03119	2.1
53203	1.2597	2.1
53204	1.36849	2.1
53205	2.12244	2.1
53206	2.20092	2.1
53207	2.20092	2.1
53208	1.35509	2.1
53209	1.35509	2.1
53210	1.89226	2.1
53211	1.89226	2.1
53212	1.89226	2.1
53213	1.89226	2.1
53214	1.89226	2.1
53215	1.89226	1.8963
53216	1.89226	2.1
53217	1.89226	0.87617
53218	1.89226	1.69343
53219	1.89226	1.29131
53220	3.78452	1.73696
53221	1.21731	2.1
53222	1.21731	1.22962
53223	1.23412	2.1
53224	1.23412	2.1
53225	1.39021	2.1
53226	1.31745	1.36057
53227	1.06122	2.1
53228	1.10046	2.1
53229	1.10046	2.1
53230	1.39021	2.1
53231	1.06122	2.1
53232	1.31745	2.1
53233	2.16503	0.803858
53234	1.78252	1.12164
53235	1.77293	0.953643
53236	1.56839	1.89292
53237	2.36957	0.550421
53238	2.16503	1.95541
53239	1.89223	0.844012
53240	1.89223	2.1
53241	1.89223	0.730208

53242	1.89223	1.10384
53243	3.78447	1.28562
53244	2.3696	1.12011
53245	1.78254	1.46232
53246	2.63298	0.630909
53247	2.07312	1.57004
53248	4.44272	1.34432
53249	1.89226	1.14782
53250	1.89226	1.41209
53251	1.89226	1.00725
53252	1.89226	2.0195
53253	1.89226	0.609443
53254	1.89226	2.03962
53255	1.89226	0.623671
53256	1.89226	0.436973
53257	1.89226	0.624069
53258	1.89226	0.251273
53259	3.78452	0.463161
53260	2.26369	0.624901
53261	2.2168	0.378904
53262	2.15617	0.527317
53263	2.10864	0.243875
53264	4.37234	0.412861
53265	2.2168	0.384978
53266	1.94499	0.423699
53267	1.94499	0.519158
53268	2.04737	0.434278
53269	2.10864	0.348735
53270	2.21103	0.411428
53271	1.89226	0.577457
53272	1.89226	0.42026
53273	1.89226	0.444747
53274	1.89226	0.440113
53275	1.89226	0.511255
53276	1.89226	0.486735
53277	1.89226	0.587881
53278	1.89226	0.423679
53279	1.89226	0.472797
53280	1.89226	0.643477
53281	1.89226	0.327815
53282	1.89226	0.650621
53283	1.02995	0.208577
53284	1.02995	0.615675
53285	1.03212	0.419612
53286	1.03212	0.564589
53287	0.988256	0.376028
53288	1.05432	0.334661
53289	1.00775	0.546362
53290	1.07809	0.569091
53291	1.00775	0.346178
53292	0.988257	0.429276
53293	1.07808	0.46957
53294	1.05432	0.47519
53295	2.10864	0.444925
53296	1.94499	0.461727
53297	1.97652	0.380032
53298	1.79681	0.412074
53299	3.9215	0.369715

53300	0.810943	0.401223
53301	0.810943	0.651039
53302	0.810943	0.412862
53303	0.810943	0.436075
53304	0.810958	0.334995
53305	0.810958	0.348888
53306	1.89226	0.459247
53307	1.35168	0.387727
53308	1.08132	0.427237
53309	1.08132	0.474589
53310	1.0813	0.416651
53311	0.657403	0.173042
53312	1.35403	0.163078
53313	1.24038	0.155174
53314	0.499144	0.156728
53315	1.24039	0.178596
53316	1.35403	0.16483
53317	0.657402	0.155217
53318	0.499144	0.158214
53319	0.915415	0.175188
53320	0.915415	0.178751
53321	0.937755	0.159382
53322	0.937755	0.161364
53323	1.72526	2.1
53324	1.17989	1.76698
53325	1.96035	2.04028
53326	1.47412	2.1
53327	3.14024	2.1
53328	1.89226	1.17728
53329	1.89226	1.86749
53330	1.89226	1.28314
53331	1.89226	0.855031
53332	3.78452	1.53554
53333	0.946131	1.34847
53334	0.946131	1.26506
53335	1.25764	1.4596
53336	1.25764	2.03645
53337	1.07087	1.16188
53338	1.25765	1.10235
53339	0.946128	1.06902
53340	0.946128	0.660902
53341	0.94613	0.912427
53342	1.25765	1.08098
53343	1.07086	1.33521
53344	0.946129	1.27817
53345	1.96035	1.89044
53346	1.32668	2.1
53347	2.29301	2.1
53348	1.70303	1.4785
53349	3.56428	2.1
53350	1.89226	1.19855
53351	1.89226	1.23956
53352	1.89226	0.64696
53353	1.89226	1.65939
53354	3.78452	0.60774
53355	0.897738	0.448443
53356	0.897738	0.456334
53357	0.946102	0.418398

53358	0.946102	0.322267
53359	1.11247	0.377423
53360	0.89771	0.317394
53361	0.94613	0.446532
53362	0.94613	0.475397
53363	0.897709	0.478852
53364	0.946131	0.509812
53365	0.946131	0.554035
53366	1.11247	0.467582
53367	1.89226	2.1
53368	1.89226	1.86132
53369	1.89226	2.08179
53370	1.89226	2.05238
53371	1.89226	2.07678
53372	1.89226	2.06377
53373	2.15429	2.1
53374	1.89226	2.1
53375	1.89226	2.1
53376	1.89226	2.1
53377	2.27074	2.1
53378	2.27074	2.1
53379	0.993311	1.27779
53380	0.993311	1.60934
53381	0.904322	2.1
53382	0.904322	1.84491
53383	0.803324	2.06206
53384	1.36183	1.28149
53385	0.916274	2.1
53386	0.535808	1.44955
53387	0.535808	1.65841
53388	0.803322	1.72107
53389	0.916276	1.62159
53390	1.36183	1.76436
53391	1.89226	2.1
53392	1.89226	1.62966
53393	1.89226	2.06806
53394	1.89226	2.1
53395	1.89226	2.1
53396	1.89226	2.1
53397	2.11955	2.1
53398	2.62097	2.1
53399	1.85542	2.1
53400	2.39016	2.1
53401	4.50971	2.1
53402	2.39016	2.1
53403	2.20242	2.1
53404	2.39016	2.1
53405	1.64008	2.1
53406	1.85542	2.1
53407	1.64008	2.1
53408	1.79667	2.1
53409	1.28267	2.1
53410	1.28267	2.1
53411	1.40117	2.1
53412	1.74514	2.1
53413	1.83994	2.1
53414	0.575914	2.1
53415	0.625292	2.1

53416	0.949144	2.1
53417	0.882782	2.1
53418	0.773697	2.1
53419	0.773697	2.1
53420	0.786497	2.1
53421	0.786494	2.1
53422	0.605477	2.1
53423	0.949148	2.1
53424	0.625291	2.1
53425	0.862374	2.1
53426	1.89226	2.1
53427	2.44918	2.1
53428	2.4616	2.1
53429	2.44918	2.1
53430	1.90468	2.1
53431	1.89226	2.1
53432	1.89226	2.1
53433	1.89226	1.1552
53434	1.89226	2.1
53435	1.89226	0.736646
53436	3.78453	2.1
53437	0.946128	2.1
53438	0.946128	0.701463
53439	1.23631	1.31114
53440	1.23631	2.1
53441	0.946131	1.08087
53442	0.946131	1.09911
53443	1.3147	2.1
53444	1.23631	2.1
53445	1.3147	2.07747
53446	0.946131	2.1
53447	1.23631	2.04724
53448	0.94613	2.0136
53449	1.89226	2.1
53450	2.4616	2.1
53451	2.4717	2.1
53452	2.4616	2.1
53453	1.90237	2.1
53454	1.89226	2.1
53455	0.946132	0.760786
53456	0.946132	2.1
53457	0.946131	2.1
53458	0.946131	1.15923
53459	0.946132	0.87359
53460	0.946132	1.58155
53461	0.946131	2.1
53462	0.946131	2.1
53463	0.946131	2.1
53464	0.946131	2.1
53465	0.946131	2.1
53466	0.946131	2.1
53467	0.946128	2.1
53468	0.946128	1.49362
53469	1.3147	0.858378
53470	1.3147	2.1
53471	0.94613	0.83652
53472	0.94613	1.35322
53473	1.3419	2.1

53474	1.3147	2.1
53475	1.3419	2.1
53476	0.94613	2.1
53477	1.3147	1.78286
53478	0.94613	1.98911
53479	1.89226	2.1
53480	1.89226	2.1
53481	1.89226	2.1
53482	1.89226	2.1
53483	3.78452	2.1
53484	2.3333	1.99911
53485	1.3864	2.1
53486	1.3864	2.1
53487	1.4628	2.1
53488	1.95763	2.1
53489	2.03403	2.1
53490	1.294	2.1
53491	1.294	2.1
53492	0.946128	2.1
53493	0.946128	2.1
53494	1.294	2.1
53495	1.27474	2.1
53496	0.946131	2.1
53497	0.946131	2.1
53498	0.946129	2.1
53499	1.27474	2.1
53500	1.294	2.1
53501	0.94613	2.1
53502	1.89226	2.1
53503	1.89226	2.1
53504	1.89226	2.1
53505	1.89226	2.1
53506	1.89226	2.1
53507	1.89226	2.1
53508	0.808088	2.1
53509	0.808088	2.1
53510	0.815623	2.1
53511	0.815623	2.1
53512	0.660053	2.1
53513	0.693199	2.1
53514	0.930512	2.07328
53515	0.978817	2.1
53516	0.660053	2.1
53517	0.930513	2.1
53518	0.693198	2.1
53519	0.978817	2.1
53520	1.27474	2.1
53521	1.27474	2.1
53522	0.946128	2.1
53523	0.946128	2.1
53524	1.26221	2.1
53525	1.27474	2.1
53526	0.946131	2.1
53527	0.946131	2.1
53528	0.94613	2.1
53529	1.26221	2.1
53530	0.946129	2.1
53531	1.27474	2.1

53532	0.90207	0.0648052
53533	0.90207	0.0679976
53534	0.895719	0.0673384
53535	0.895719	0.0727425
53536	0.927855	0.0632374
53537	1.2493	0.0663235
53538	0.85723	0.0667008
53539	0.548484	0.0652151
53540	0.548485	0.0658086
53541	0.927855	0.0625361
53542	0.85723	0.0736838
53543	1.2493	0.0689907
53544	1.41319	0.760649
53545	1.22482	0.515646
53546	1.46629	0.610421
53547	1.92622	0.724511
53548	3.15104	0.717047
53549	1.02305	0.453642
53550	1.22482	0.530288
53551	1.80336	0.596166
53552	1.44426	0.410836
53553	2.66908	0.441891
53554	1.3268	1.98903
53555	0.999489	2.1
53556	1.44091	1.75814
53557	1.14657	2.1
53558	2.4404	2.1
53559	1.89226	1.28691
53560	1.89226	2.1
53561	1.89226	1.75346
53562	1.89226	2.1
53563	3.78452	2.1
53564	1.89226	2.1
53565	2.28605	2.1
53566	2.43157	2.1
53567	2.43157	2.1
53568	1.89226	2.1
53569	1.89226	2.1
53570	1.60647	1.96358
53571	1.44091	1.54708
53572	1.31346	2.1
53573	1.31346	2.1
53574	1.14657	2.1
53575	1.44091	2.1
53576	1.89226	1.52584
53577	1.89226	2.1
53578	1.89226	1.443
53579	1.89226	2.1
53580	3.78452	2.07669
53581	0.946132	2.1
53582	0.946132	2.1
53583	1.3048	2.1
53584	1.3048	2.1
53585	1.3048	2.1
53586	1.21578	2.1
53587	0.946132	2.1
53588	0.946132	2.1
53589	1.3048	2.1

53590	0.946132	2.1
53591	0.946132	2.1
53592	1.21578	2.1
53593	1.89227	0.0987245
53594	1.89227	0.0982094
53595	1.89227	0.0809074
53596	1.89227	0.0907991
53597	3.78453	0.0942744
53598	1.6671	0.0760354
53599	1.87338	0.083608
53600	1.6671	0.0872776
53601	2.02098	0.0940735
53602	1.38328	0.0747927
53603	1.73715	0.0814805
53604	1.89226	0.0775799
53605	1.89226	0.0864462
53606	1.89226	0.0702327
53607	1.89226	0.074623
53608	3.78453	0.0770308
53609	1.6671	0.0744416
53610	1.38328	0.0723643
53611	1.35562	0.0667618
53612	1.01012	0.0723209
53613	2.67722	0.0726284
53614	0.819377	0.0714997
53615	0.647925	0.0709914
53616	1.17802	0.0673599
53617	1.33174	0.0706961
53618	0.98543	0.0687691
53619	0.98543	0.0741078
53620	0.994237	0.0687437
53621	0.994237	0.0699194
53622	1.17802	0.0684707
53623	0.647926	0.0670455
53624	1.33174	0.0714476
53625	0.819377	0.0711757
53626	1.27806	0.0791358
53627	1.68264	0.0730482
53628	1.44593	0.0716321
53629	1.878	0.0805878
53630	3.15606	0.0751708
53631	1.878	0.0817586
53632	1.9898	0.0737129
53633	1.878	0.0833184
53634	1.68264	0.0760209
53635	2.30533	0.0826462
53636	2.10997	0.0749983
53637	0.814587	0.0738726
53638	0.814596	0.0732206
53639	0.82775	0.0807656
53640	0.82775	0.0837392
53641	1.0107	0.0829684
53642	0.91549	0.0748286
53643	0.741058	0.0805197
53644	0.714748	0.0741838
53645	0.714758	0.0780709
53646	1.03225	0.0772267
53647	0.704864	0.0762332

53648	0.915488	0.0781004
53649	1.90347	0.0841488
53650	2.06584	0.072997
53651	1.878	0.085899
53652	1.50236	0.0760175
53653	2.05749	0.0758199
53654	2.30533	0.0852955
53655	2.24475	2.1
53656	1.85871	2.1
53657	2.13711	2.1
53658	2.13711	2.1
53659	1.9943	2.1
53660	1.9943	2.1
53661	1.85871	2.1
53662	1.7871	2.1
53663	1.41619	2.1
53664	1.41619	2.1
53665	2.13711	2.1
53666	2.13711	2.1
53667	2.24475	2.1
53668	2.13711	2.1
53669	2.54902	2.1
53670	2.46327	2.1
53671	4.70802	2.1
53672	1.06913	2.1
53673	1.06913	2.1
53674	1.0821	2.1
53675	1.0821	2.1
53676	0.893552	2.1
53677	1.08267	2.1
53678	1.06855	2.1
53679	1.23163	2.1
53680	1.23163	2.1
53681	1.08267	2.1
53682	0.893551	2.1
53683	1.06855	2.1
53684	1.67046	0.861619
53685	2.4106	0.503695
53686	2.05835	0.991323
53687	2.4106	1.14925
53688	1.59562	0.580466
53689	1.94788	0.554204
53690	2.34737	0.800556
53691	2.4106	0.523447
53692	2.05834	1.07393
53693	1.99655	1.01741
53694	4.40715	0.77317
53695	1.67046	0.681638
53696	2.05835	0.49249
53697	1.588	0.933416
53698	1.34857	1.35396
53699	3.40692	0.706042
53700	1.73802	0.682976
53701	1.89668	0.863527
53702	1.99655	1.69715
53703	1.99655	0.554408
53704	1.79981	0.753568
53705	2.05834	0.482455

53706	1.89226	1.136
53707	1.89226	0.886691
53708	1.89226	0.593977
53709	1.89226	1.68511
53710	1.89226	0.635609
53711	1.89226	2.0825
53712	1.89226	0.954862
53713	1.89226	2.01617
53714	1.89226	1.36598
53715	1.89226	0.667024
53716	3.78453	1.40822
53717	0.719473	1.97217
53718	0.916103	1.45827
53719	1.02744	1.06383
53720	0.850328	1.80957
53721	0.888094	0.966757
53722	0.888094	2.1
53723	0.878337	1.91602
53724	0.878337	0.630135
53725	0.916103	1.19595
53726	1.02744	1.27599
53727	0.719473	1.63323
53728	0.850328	1.75404
53729	1.02744	1.21115
53730	0.850329	1.85395
53731	1.0875	1.1212
53732	0.919597	1.32243
53733	0.975841	0.775725
53734	0.975841	2.1
53735	0.971196	0.769988
53736	0.971196	1.74673
53737	1.02744	1.38711
53738	1.0875	1.05874
53739	0.850328	1.72168
53740	0.919598	1.54824
53741	1.89226	1.62373
53742	1.89226	1.33344
53743	1.89226	0.751444
53744	1.89226	1.42178
53745	3.78453	1.24244
53746	1.89226	1.27098
53747	1.89226	0.891732
53748	1.89226	0.640395
53749	1.89226	1.7692
53750	1.89226	0.600171
53751	1.89226	1.97675
53752	1.11103	1.43215
53753	0.916103	1.97196
53754	1.21177	1.20023
53755	1.02744	1.25935
53756	1.07188	0.839196
53757	1.07188	2.1
53758	1.06659	0.828906
53759	1.06659	1.85632
53760	1.11103	1.69461
53761	1.21177	1.4252
53762	0.916104	1.73761
53763	1.02744	1.81351

53764	2.54158	0.975393
53765	2.42354	1.28492
53766	2.17499	0.726325
53767	2.17499	2.03178
53768	2.42354	1.49323
53769	2.05488	2.1
53770	1.89226	0.137337
53771	1.89226	0.184117
53772	1.89226	0.147333
53773	1.89226	0.189297
53774	3.78453	0.148655
53775	1.89226	0.128132
53776	1.89226	0.174784
53777	1.89226	0.136552
53778	1.89226	0.165481
53779	1.89226	0.145738
53780	1.89226	0.177699
53781	1.89226	0.215553
53782	1.89226	0.258519
53783	1.89226	0.238842
53784	1.89226	0.177352
53785	1.89226	0.310888
53786	1.89226	0.240998
53787	1.89226	0.224675
53788	1.89226	0.287855
53789	1.89226	0.231595
53790	1.89226	0.303109
53791	3.78452	0.27606
53792	0.802544	0.153349
53793	0.946132	0.119219
53794	0.946132	0.127449
53795	0.787038	0.157967
53796	0.787039	0.143109
53797	0.787039	0.148036
53798	0.961637	0.130175
53799	0.961637	0.131403
53800	0.787038	0.141336
53801	0.802544	0.14448
53802	0.946132	0.13574
53803	0.946132	0.137411
53804	1.89226	0.116528
53805	1.60509	0.149088
53806	1.89226	0.122448
53807	1.89226	0.134357
53808	1.3905	0.133146
53809	1.3905	0.147421
53810	0.946132	0.249614
53811	0.946132	0.14434
53812	0.946131	0.1974
53813	0.946131	0.213743
53814	0.946132	0.220114
53815	0.946132	0.2182
53816	0.946131	0.190317
53817	0.946131	0.18731
53818	0.946131	0.20047
53819	0.946131	0.203969
53820	0.946132	0.198888
53821	0.946132	0.193036

53822	0.946131	0.225333
53823	0.946131	0.247504
53824	0.946131	0.274751
53825	0.946131	0.171904
53826	0.946132	0.234237
53827	0.946132	0.245956
53828	0.946131	0.193709
53829	0.946131	0.191127
53830	0.946131	0.21704
53831	0.946131	0.171206
53832	0.946132	0.201425
53833	0.946132	0.196052
53834	1.42382	1.59465
53835	1.32201	0.67781
53836	1.32096	0.815496
53837	1.42866	1.26603
53838	2.75067	0.964311
53839	1.89226	1.40729
53840	1.89226	0.555621
53841	1.89226	0.758099
53842	1.89226	0.868819
53843	1.89226	0.689333
53844	1.89226	0.788531
53845	1.89226	1.05982
53846	2.79029	0.545201
53847	2.7803	0.646592
53848	2.79029	0.68433
53849	1.88227	0.58558
53850	1.89226	0.635437
53851	0.716065	1.03011
53852	0.714333	1.56482
53853	0.660483	0.70854
53854	0.659126	0.895577
53855	0.687538	0.703869
53856	0.687538	0.966919
53857	0.685921	1.83148
53858	0.685921	0.75265
53859	0.659126	0.840728
53860	0.716065	0.867549
53861	0.660483	0.9166
53862	0.714333	0.915688
53863	1.89226	0.979633
53864	1.89226	0.531685
53865	1.89226	0.732369
53866	1.89226	0.846166
53867	3.78453	0.817166
53868	0.946133	0.621398
53869	0.946133	0.741415
53870	1.39015	0.607517
53871	1.38969	0.444176
53872	0.946132	0.454701
53873	0.946132	0.619417
53874	1.38969	0.769933
53875	1.38969	0.611449
53876	1.38969	0.605169
53877	0.946132	0.608974
53878	0.946133	0.710944
53879	1.39015	0.625127

53880	2.72157	0.42922
53881	1.22176	0.370483
53882	1.59492	0.382756
53883	1.22176	0.471762
53884	2.40743	0.373504
53885	2.03426	0.461226
53886	0.946131	0.533483
53887	0.946131	0.451399
53888	1.36079	0.41042
53889	1.01713	0.448796
53890	0.94613	0.44219
53891	0.94613	0.534261
53892	1.36079	0.432802
53893	1.36079	0.403582
53894	0.946131	0.476435
53895	1.36079	0.472452
53896	1.01713	0.464376
53897	0.946131	0.473598
53898	1.89226	0.510373
53899	1.89226	0.442788
53900	1.89226	0.40567
53901	1.89226	0.575196
53902	3.78453	0.495947
53903	1.89226	0.417455
53904	1.89226	0.378601
53905	1.89226	0.362435
53906	1.89226	0.487189
53907	1.89226	0.334244
53908	1.89226	0.455043
53909	0.946131	0.518154
53910	0.946131	0.444391
53911	0.946131	0.433178
53912	1.36079	0.362745
53913	0.94613	0.433356
53914	0.94613	0.521057
53915	0.946133	0.433631
53916	0.946133	0.287087
53917	0.946131	0.448122
53918	0.946131	0.464757
53919	0.946132	0.381575
53920	1.36079	0.423478
53921	1.89226	0.412875
53922	1.89226	0.560301
53923	1.89226	0.436044
53924	1.89226	0.500945
53925	1.89226	0.439815
53926	1.89226	0.505205
53927	1.89227	0.428534
53928	1.89227	0.336461
53929	1.89227	0.36109
53930	1.89227	0.480236
53931	1.89227	0.322722
53932	1.89227	0.430223
53933	1.60428	2.1
53934	2.2367	1.82146
53935	1.50097	2.1
53936	1.80125	1.0349
53937	3.40553	2.1

53938	1.89095	1.97243
53939	1.85293	0.580488
53940	1.80125	1.16991
53941	1.76268	0.549656
53942	3.65418	0.771932
53943	0.803177	1.83584
53944	0.803177	2.1
53945	0.798644	2.1
53946	0.798644	1.75736
53947	0.84233	1.65404
53948	0.900624	1.68743
53949	0.750483	2.1
53950	0.701197	2.1
53951	0.701197	2.1
53952	0.84233	2.1
53953	0.750483	2.1
53954	0.900625	2.1
53955	0.860925	0.561694
53956	0.860925	1.95018
53957	0.862743	1.61965
53958	0.862743	0.620031
53959	0.881338	0.588551
53960	0.826621	0.51158
53961	0.84233	1.74236
53962	0.900625	1.7705
53963	0.84233	0.728741
53964	0.826621	0.734532
53965	0.900624	0.761624
53966	0.881339	0.731086
53967	1.37938	0.965564
53968	1.03319	0.934073
53969	1.00512	1.23603
53970	0.639582	1.28241
53971	1.00463	1.23416
53972	1.00463	0.890912
53973	1.01434	0.837312
53974	1.01434	1.2913
53975	1.37938	1.07908
53976	1.00512	1.28455
53977	1.03319	1.17624
53978	0.639582	1.17289
53979	0.728713	0.141868
53980	0.728713	0.141519
53981	0.73425	0.435315
53982	0.73425	0.616778
53983	0.525122	2.09704
53984	0.605622	0.110983
53985	0.948901	0.110983
53986	0.857342	0.110983
53987	0.85734	0.102391
53988	0.525123	0.628025
53989	0.948899	0.102391
53990	0.605624	0.102391
53991	1.75449	0.629534
53992	1.4378	0.652916
53993	1.93916	0.74681
53994	2.22512	0.52608
53995	3.69364	0.674365

53996	1.75448	0.798092
53997	1.89186	0.549079
53998	2.29771	0.614275
53999	2.22512	0.551568
54000	4.05219	0.59677
54001	1.93916	0.737787
54002	2.51749	0.498742
54003	2.62357	0.580705
54004	2.22512	0.536854
54005	2.33761	0.683687
54006	1.93916	0.598644
54007	2.29771	0.651307
54008	2.51749	0.519538
54009	2.22512	0.518657
54010	2.22512	0.490099
54011	2.50905	0.68301
54012	2.50905	0.610928
54013	0.87382	0.115539
54014	1.33662	0.159108
54015	1.20756	0.143906
54016	0.890467	0.202564
54017	1.08859	0.198298
54018	1.08859	0.180652
54019	1.13084	0.116688
54020	1.08793	0.144013
54021	1.34722	0.15952
54022	1.27184	0.169294
54023	0.917678	0.161836
54024	0.97821	0.159711
54025	1.28396	0.138212
54026	1.70302	0.211254
54027	2.30076	0.18826
54028	1.20417	0.142795
54029	2.57506	0.190277
54030	2.2656	0.15769
54031	1.03642	0.847844
54032	1.03642	0.298213
54033	1.07029	0.297445
54034	1.07029	0.376444
54035	1.31531	0.703589
54036	1.23279	0.381527
54037	0.941743	0.362087
54038	0.791399	0.636382
54039	0.791399	0.38638
54040	1.23279	0.389277
54041	0.941744	0.416001
54042	1.31531	0.428701
54043	0.937354	0.922386
54044	0.937354	0.269614
54045	0.954794	0.330461
54046	0.954794	0.382633
54047	1.13563	0.382354
54048	1.23279	0.715016
54049	0.791398	0.367629
54050	0.659359	0.624137
54051	1.13563	0.389353
54052	0.659359	0.394766
54053	0.791399	0.396009

54054	1.23279	0.405382
54055	2.29852	0.912067
54056	1.89227	0.851001
54057	2.14485	0.775674
54058	2.14485	1.0119
54059	1.89227	0.815509
54060	1.89227	1.04969
54061	0.947863	0.851563
54062	1.19854	0.993113
54063	2.16841	0.961829
54064	1.76597	0.865047
54065	2.96451	0.830953
54066	1.89227	0.785683
54067	1.89227	0.773909
54068	1.89227	0.718025
54069	1.89227	0.939833
54070	1.89227	0.725557
54071	1.89227	0.941581
54072	2.46489	2.1
54073	1.98339	2.1
54074	1.81667	2.1
54075	2.36914	2.1
54076	4.35253	2.1
54077	2.46488	2.1
54078	2.70401	2.1
54079	2.64996	2.1
54080	2.36914	2.1
54081	5.07315	2.1
54082	1.81667	2.1
54083	2.29459	2.1
54084	2.36914	2.1
54085	2.36914	2.1
54086	1.68073	2.1
54087	1.68073	2.1
54088	2.64996	2.1
54089	2.29458	2.1
54090	2.29458	2.1
54091	2.36914	2.1
54092	2.61243	2.1
54093	2.68698	2.1
54094	1.89226	1.16722
54095	1.89226	1.00673
54096	1.89226	1.37796
54097	1.89226	1.03293
54098	1.89226	1.41525
54099	1.89226	1.02434
54100	1.89224	1.33197
54101	1.89224	0.977178
54102	1.65571	1.22118
54103	1.65571	0.957523
54104	1.89224	1.33782
54105	1.89224	1.05784
54106	1.89224	1.15791
54107	1.89224	1.10056
54108	1.89224	1.32502
54109	1.89224	0.912215
54110	1.89224	1.47059
54111	1.89224	0.982607

54112	1.98252	0.222903
54113	2.20641	0.238669
54114	1.05129	0.207051
54115	1.2951	0.270905
54116	3.27762	0.221979
54117	1.85598	1.83546
54118	1.88635	1.11066
54119	2.12346	0.941562
54120	2.10838	1.87098
54121	3.97943	1.28177
54122	2.10838	1.23451
54123	1.87532	1.95841
54124	1.85597	0.746803
54125	1.85597	2.1
54126	1.87532	1.28135
54127	1.57914	2.1
54128	2.12348	1.17986
54129	2.35141	1.65678
54130	2.37191	1.25212
54131	2.10841	1.38902
54132	2.38698	0.987093
54133	2.12348	1.35671
54134	1.07811	1.65645
54135	0.937672	2.1
54136	1.17571	1.58663
54137	1.05421	0.810343
54138	1.07087	1.0973
54139	1.07087	2.1
54140	1.06145	1.75742
54141	1.06145	0.975581
54142	1.17571	1.77485
54143	1.07811	1.68088
54144	1.05421	1.63716
54145	0.937672	1.66599
54146	1.67334	2.1
54147	2.76609	2.1
54148	2.74144	2.1
54149	2.74144	2.1
54150	1.71157	2.1
54151	1.71157	2.1
54152	2.76609	2.1
54153	1.89226	2.1
54154	1.89226	2.1
54155	1.89226	2.1
54156	2.74143	2.1
54157	2.74143	2.1
54158	1.10393	2.1
54159	1.10393	2.1
54160	1.10788	2.1
54161	1.10788	2.1
54162	1.38302	2.1
54163	1.37072	2.1
54164	0.836672	2.1
54165	0.841089	2.1
54166	1.38302	2.1
54167	0.84109	2.1
54168	0.836671	2.1
54169	1.37072	2.1

54170	1.38302	2.1
54171	1.38302	2.1
54172	0.946131	2.1
54173	0.946131	2.1
54174	0.946128	2.1
54175	0.946128	2.1
54176	1.38302	2.1
54177	1.37072	2.1
54178	1.38302	2.1
54179	0.946131	2.1
54180	0.94613	2.1
54181	1.37072	2.1
54182	1.28072	0.625072
54183	1.55779	2.1
54184	1.28072	1.14021
54185	1.40765	0.720376
54186	1.47186	1.26219
54187	1.59879	1.04512
54188	1.89227	0.654858
54189	1.89227	0.632977
54190	1.89227	0.472927
54191	1.89227	1.15383
54192	1.89227	0.472513
54193	1.89227	0.944598
54194	2.80057	0.549056
54195	1.89226	0.521068
54196	1.89226	0.494369
54197	1.89226	0.640098
54198	2.63061	0.281327
54199	2.63061	0.690587
54200	1.28072	0.584594
54201	1.38859	2.1
54202	1.47186	1.19892
54203	1.47186	0.762331
54204	1.16777	1.31755
54205	1.16777	0.839094
54206	1.89226	0.602046
54207	1.89226	0.628154
54208	1.89226	0.558545
54209	1.89226	1.17249
54210	1.89226	0.461403
54211	1.89226	1.46301
54212	1.83221	2.1
54213	2.0815	2.1
54214	2.0815	2.1
54215	2.08153	2.1
54216	1.86703	2.1
54217	1.87402	2.1
54218	1.86703	2.1
54219	2.08508	2.1
54220	2.08508	2.1
54221	2.29001	2.1
54222	1.89226	2.1
54223	2.08048	2.1
54224	2.7278	0.283664
54225	1.10909	0.298869
54226	1.50472	0.327331
54227	1.10909	0.320295

54228	2.69881	0.261404
54229	2.30319	0.300251
54230	1.23204	2.1
54231	1.92264	2.1
54232	1.48162	2.1
54233	1.48162	2.1
54234	1.72994	2.1
54235	1.72994	2.1
54236	0.864968	2.1
54237	0.696952	2.1
54238	0.61602	2.1
54239	0.479736	2.1
54240	0.66444	2.1
54241	0.66444	2.1
54242	0.680264	2.1
54243	0.680264	2.1
54244	0.696952	2.1
54245	0.479736	2.1
54246	0.61602	2.1
54247	0.864968	2.1
54248	1.89226	0.651786
54249	2.43219	1.71442
54250	1.89226	1.16519
54251	2.82019	1.20509
54252	1.89226	1.48546
54253	2.82019	1.27395
54254	1.89226	0.739713
54255	2.83701	1.87817
54256	2.2701	1.17892
54257	2.2701	1.02769
54258	1.89226	1.24312
54259	1.89226	1.22431
54260	1.89226	1.36469
54261	1.89226	0.73845
54262	1.89226	0.856727
54263	1.89226	1.15007
54264	1.89226	1.21195
54265	1.89226	1.17154
54266	1.5795	1.47819
54267	1.44091	1.47057
54268	1.05267	1.68962
54269	1.05267	1.24105
54270	1.98408	1.49624
54271	1.98408	1.2008
54272	2.20624	2.1
54273	1.08831	2.1
54274	2.49053	2.1
54275	1.42486	2.1
54276	3.57883	2.1
54277	1.89226	2.1
54278	1.89226	2.1
54279	1.89226	2.1
54280	1.89226	2.1
54281	3.78453	2.1
54282	0.946132	2.1
54283	0.946132	2.1
54284	0.853591	2.1
54285	0.642207	2.1

54286	0.94613	2.1
54287	0.94613	2.1
54288	0.642209	2.1
54289	0.642209	2.1
54290	0.946131	2.1
54291	0.853591	2.1
54292	0.642207	2.1
54293	0.946132	2.1
54294	1.0506	2.1
54295	1.0506	2.1
54296	1.0879	2.1
54297	0.960424	2.1
54298	0.708687	2.1
54299	0.782927	2.1
54300	1.40041	2.1
54301	1.2518	2.1
54302	0.767748	2.1
54303	1.31801	2.1
54304	1.21412	2.1
54305	0.69461	2.1
54306	1.89226	2.1
54307	1.89226	2.1
54308	1.89226	2.1
54309	1.89226	2.1
54310	1.89226	2.1
54311	1.89226	2.1
54312	0.922223	2.1
54313	0.922223	2.1
54314	1.04548	2.1
54315	0.768229	2.1
54316	0.948441	2.1
54317	0.806483	2.1
54318	0.85362	2.1
54319	0.85362	2.1
54320	1.05942	2.1
54321	0.922187	2.1
54322	0.851554	2.1
54323	0.768264	2.1
54324	2.16728	2.1
54325	0.95455	2.1
54326	1.21372	2.1
54327	0.98227	2.1
54328	2.16728	2.1
54329	1.93584	2.1
54330	2.08849	0.261015
54331	1.08732	0.283438
54332	1.3357	0.265563
54333	2.37948	0.277547
54334	3.4668	0.267885
54335	2.57882	0.250048
54336	2.37948	0.266953
54337	2.53055	0.253362
54338	1.48676	0.286831
54339	1.3357	0.287923
54340	1.3357	0.277373
54341	1.89226	2.1
54342	1.89226	2.1
54343	2.17471	2.1

54344	1.89226	2.1
54345	2.60085	2.1
54346	2.26306	2.1
54347	0.946131	1.23872
54348	0.946131	2.1
54349	0.946131	1.78322
54350	0.946131	2.1
54351	0.94613	1.93166
54352	0.94613	1.85127
54353	0.946132	2.1
54354	0.946132	2.1
54355	0.946131	2.1
54356	0.946131	2.1
54357	0.946131	2.1
54358	0.946131	2.1
54359	1.89226	2.1
54360	2.17471	2.1
54361	2.08196	2.1
54362	2.17471	2.1
54363	1.79952	2.1
54364	1.89226	2.1
54365	1.89226	2.1
54366	1.89226	2.1
54367	1.89226	2.1
54368	1.89226	2.08209
54369	1.89226	2.1
54370	1.89226	1.96495
54371	1.86016	2.1
54372	1.89226	2.1
54373	1.89226	2.1
54374	1.89226	2.1
54375	1.84826	2.1
54376	1.84826	2.1
54377	2.50136	2.1
54378	1.99781	2.1
54379	1.99729	2.1
54380	2.5214	2.1
54381	4.49865	2.1
54382	0.51724	2.1
54383	0.496076	2.1
54384	0.998906	2.1
54385	0.998645	2.1
54386	0.742249	2.1
54387	0.742249	2.1
54388	0.752734	2.1
54389	0.752734	2.1
54390	0.998639	2.1
54391	0.496073	2.1
54392	0.517243	2.1
54393	0.998913	2.1
54394	0.998653	2.1
54395	0.994293	2.1
54396	1.26071	2.1
54397	1.26504	2.1
54398	1.12529	2.1
54399	1.12529	2.1
54400	1.12971	2.1
54401	1.12971	2.1

54402	1.26504	2.1
54403	0.994292	2.1
54404	0.998651	2.1
54405	1.26071	2.1
54406	1.89226	2.1
54407	1.89226	2.1
54408	1.89226	2.1
54409	1.89226	2.1
54410	3.78452	2.1
54411	1.89226	2.1
54412	1.89226	2.1
54413	1.89226	2.1
54414	1.89226	2.1
54415	3.78452	2.1
54416	0.675872	2.1
54417	0.675641	2.1
54418	0.943072	2.1
54419	0.898317	2.1
54420	0.775717	2.1
54421	0.775717	2.1
54422	0.79824	2.1
54423	0.79824	2.1
54424	0.675642	2.1
54425	0.94307	2.1
54426	0.675874	2.1
54427	0.898315	2.1
54428	0.943071	2.1
54429	0.898316	2.1
54430	0.998133	2.1
54431	0.918903	2.1
54432	0.922369	2.1
54433	0.922369	2.1
54434	0.939606	2.1
54435	0.939606	2.1
54436	0.943071	2.1
54437	0.998133	2.1
54438	0.898316	2.1
54439	0.918903	2.1
54440	0.946131	2.1
54441	0.946131	2.1
54442	0.946131	2.1
54443	0.946131	2.1
54444	0.946132	2.1
54445	0.946132	2.1
54446	0.946129	2.1
54447	0.946129	2.1
54448	0.94613	2.1
54449	0.94613	2.1
54450	0.946131	2.1
54451	0.946131	2.1
54452	1.89226	2.1
54453	1.89226	2.1
54454	1.89226	2.1
54455	1.89226	2.1
54456	1.89226	2.1
54457	1.89226	2.1
54458	1.35129	2.1
54459	1.88614	2.1

54460	2.01867	2.1
54461	1.38189	2.1
54462	3.36995	2.1
54463	0.943071	2.1
54464	1.00934	2.1
54465	0.998134	2.1
54466	1.10866	2.1
54467	0.992617	2.1
54468	0.992617	2.1
54469	1.01485	2.1
54470	1.01485	2.1
54471	1.00934	2.1
54472	1.10866	2.1
54473	0.943072	2.1
54474	0.998134	2.1
54475	2.67272	2.1
54476	1.61859	2.1
54477	1.70303	2.1
54478	2.52175	2.1
54479	4.5326	2.1
54480	2.00954	2.1
54481	2.27801	2.1
54482	1.87875	2.1
54483	1.87875	2.1
54484	2.37837	2.1
54485	2.37837	2.1
54486	2.00955	2.1
54487	1.78	2.1
54488	2.21813	2.1
54489	1.8493	2.1
54490	2.37838	2.1
54491	2.00955	2.1
54492	2.37838	2.1
54493	2.27801	2.1
54494	2.58523	2.1
54495	2.71597	2.1
54496	4.9636	2.1
54497	2.37838	2.1
54498	2.71597	2.1
54499	1.72709	2.1
54500	2.0815	2.1
54501	2.59749	2.1
54502	2.78034	2.1
54503	0.94613	0.746467
54504	0.94613	0.784819
54505	0.946128	0.645006
54506	0.946128	0.592728
54507	0.94613	0.602484
54508	0.94613	0.659866
54509	0.946128	0.781118
54510	0.946128	0.538942
54511	0.946129	0.724248
54512	0.946129	0.705326
54513	0.946129	0.670539
54514	0.946129	0.68421
54515	1.89226	0.893473
54516	1.89226	0.815595
54517	1.89226	0.890571

54518	1.89226	0.71757
54519	3.78452	0.736832
54520	0.946133	0.581545
54521	0.946133	0.610195
54522	0.946132	0.419826
54523	0.946132	0.45546
54524	0.946132	0.495865
54525	0.946132	0.473179
54526	0.946133	0.595478
54527	0.946133	0.558507
54528	0.946132	0.537304
54529	0.946132	0.280779
54530	0.946132	0.471658
54531	0.946132	0.49859
54532	1.89226	0.644096
54533	2.10495	0.435152
54534	1.89226	0.41934
54535	2.30932	0.412192
54536	1.89226	0.743383
54537	2.30932	0.742238
54538	2.30932	0.465866
54539	1.00366	0.235069
54540	2.10496	0.303351
54541	1.24728	0.360605
54542	3.31298	0.367913
54543	1.87557	2.1
54544	1.07167	2.1
54545	2.03045	2.1
54546	1.25185	2.1
54547	3.10212	2.1
54548	1.89226	2.1
54549	1.89226	2.1
54550	1.89226	2.1
54551	1.89226	2.1
54552	3.78453	2.1
54553	0.851541	2.1
54554	0.851541	2.1
54555	1.12236	2.1
54556	1.05783	2.1
54557	0.870663	2.1
54558	0.951856	2.1
54559	0.946132	2.1
54560	0.946132	2.1
54561	1.04073	2.1
54562	1.1296	2.1
54563	0.776062	2.1
54564	0.851527	2.1
54565	0.859395	2.1
54566	0.859395	2.1
54567	0.870165	2.1
54568	0.870165	2.1
54569	0.625924	2.1
54570	0.714333	2.1
54571	1.0821	2.1
54572	1.01523	2.1
54573	1.0821	2.1
54574	0.714333	2.1
54575	1.01523	2.1

54576	0.625924	2.1
54577	1.89226	2.1
54578	1.89226	2.1
54579	1.89226	2.1
54580	1.89226	2.1
54581	3.78453	2.1
54582	1.89226	2.1
54583	1.89226	2.1
54584	2.23164	2.1
54585	2.67802	2.1
54586	2.03663	2.1
54587	1.89226	2.1
54588	2.42221	2.1
54589	1.61933	2.1
54590	2.80896	1.97901
54591	1.87043	2.1
54592	4.29262	2.1
54593	1.89226	1.64094
54594	1.89226	2.1
54595	1.89226	2.1
54596	1.89226	2.1
54597	1.89226	1.86895
54598	1.89226	2.1
54599	2.80892	1.90651
54600	1.87041	2.1
54601	2.67182	1.50929
54602	2.30891	2.1
54603	5.07665	2.1
54604	1.89224	1.78092
54605	1.89224	2.1
54606	1.89224	1.50044
54607	1.89224	2.1
54608	3.78448	2.1
54609	0.946118	2.1
54610	0.946118	2.1
54611	0.946118	2.1
54612	0.946118	2.1
54613	0.946115	2.1
54614	0.946115	2.1
54615	0.94612	2.1
54616	0.94612	2.1
54617	0.94613	2.1
54618	0.94613	2.1
54619	0.946105	2.1
54620	0.946105	2.1
54621	2.3089	2.1
54622	1.02783	2.1
54623	1.98533	2.1
54624	1.76762	2.1
54625	1.24553	2.1
54626	1.02783	2.1
54627	2.2049	0.225053
54628	2.34673	0.218695
54629	1.00763	0.212509
54630	1.23746	0.257213
54631	3.44236	0.223845
54632	1.89226	0.300679
54633	1.89226	0.387748

54634	1.89226	0.326344
54635	1.89226	0.370894
54636	3.78452	0.377938
54637	1.89226	0.392697
54638	1.89226	0.366385
54639	1.89226	0.373923
54640	1.89226	0.416993
54641	3.78453	0.383036
54642	0.891618	0.270081
54643	0.891618	0.258269
54644	0.902158	0.219901
54645	0.902158	0.240878
54646	1.15396	0.266028
54647	0.620408	0.24643
54648	1.17337	0.220027
54649	0.618733	0.260472
54650	0.620408	0.24994
54651	0.618733	0.236847
54652	1.17337	0.253595
54653	1.15396	0.263462
54654	0.69978	0.256495
54655	0.69978	0.248422
54656	0.946102	0.252778
54657	0.946102	0.261773
54658	0.831702	0.273824
54659	0.946129	0.275511
54660	0.699752	0.253134
54661	0.946132	0.233464
54662	0.831703	0.261645
54663	0.699751	0.233088
54664	0.946131	0.239265
54665	0.946131	0.254001
54666	0.831675	0.28483
54667	0.831675	0.279577
54668	0.946163	0.276155
54669	0.864397	0.282024
54670	0.853471	0.314113
54671	0.962623	0.268111
54672	0.748555	0.273523
54673	0.851543	0.277114
54674	0.864714	0.284443
54675	0.760411	0.28074
54676	0.946133	0.280426
54677	0.946133	0.282867
54678	1.89226	0.502302
54679	1.89226	0.758525
54680	1.89226	0.662769
54681	1.89226	0.61587
54682	3.78452	0.666588
54683	1.89226	0.704629
54684	1.89226	0.60747
54685	1.89226	0.592209
54686	1.89226	0.837771
54687	3.78452	0.641496
54688	0.946132	0.804766
54689	0.946132	0.874093
54690	0.946132	0.686398
54691	0.946132	0.727784

54692	0.946132	0.669943
54693	0.946132	1.01242
54694	0.946133	0.591873
54695	0.946133	0.84886
54696	0.946133	0.712371
54697	0.946133	0.679382
54698	0.946132	0.752321
54699	0.946132	0.879227
54700	1.89226	0.764619
54701	2.39715	0.764177
54702	1.89226	0.900452
54703	1.89226	1.0014
54704	1.89226	0.626507
54705	1.89226	0.69254
54706	0.94613	0.822902
54707	0.94613	0.765663
54708	0.94613	0.692241
54709	0.94613	0.660681
54710	0.946131	0.696124
54711	0.946131	0.926263
54712	0.946129	0.762988
54713	0.946129	0.647547
54714	0.94613	0.656885
54715	0.94613	0.714528
54716	0.94613	0.756413
54717	0.94613	0.769583
54718	2.54204	2.1
54719	2.3148	2.1
54720	2.55328	2.1
54721	2.39646	2.1
54722	4.71126	2.1
54723	1.62611	2.1
54724	2.3148	2.1
54725	2.16986	2.1
54726	1.43745	2.1
54727	3.75225	2.1
54728	1.89226	2.1
54729	1.89226	2.1
54730	1.89226	2.1
54731	1.89226	2.1
54732	3.78453	2.1
54733	1.89226	2.1
54734	1.89226	2.1
54735	1.89226	2.1
54736	1.89226	2.1
54737	3.78453	2.1
54738	2.12125	2.1
54739	2.39642	2.1
54740	2.2941	2.1
54741	1.9933	2.1
54742	4.38972	2.1
54743	1.73784	2.1
54744	1.26892	2.1
54745	1.26892	2.1
54746	1.43743	2.1
54747	1.9933	2.1
54748	2.1618	2.1
54749	0.946118	2.1

54750	0.946118	2.1
54751	0.946118	2.1
54752	0.946118	2.1
54753	0.946119	2.1
54754	0.946119	2.1
54755	0.946118	2.1
54756	0.946118	2.1
54757	0.946131	2.1
54758	0.946131	2.1
54759	0.946106	2.1
54760	0.946106	2.1
54761	1.89224	2.1
54762	1.89224	2.1
54763	1.89224	2.1
54764	1.89224	2.1
54765	3.78448	2.1
54766	2.45922	2.1
54767	2.15686	2.1
54768	2.14259	2.1
54769	2.43225	2.1
54770	4.58911	2.1
54771	1.62027	2.1
54772	2.15686	2.1
54773	1.62241	2.1
54774	1.62241	2.1
54775	2.1426	2.1
54776	2.1426	2.1
54777	2.14259	2.1
54778	2.43225	2.1
54779	2.40667	2.1
54780	2.1281	2.1
54781	4.56035	2.1
54782	1.0713	2.1
54783	1.06405	2.1
54784	0.810136	2.1
54785	0.808433	2.1
54786	0.938508	2.1
54787	0.938508	2.1
54788	0.941223	2.1
54789	0.941223	2.1
54790	1.06405	2.1
54791	0.808433	2.1
54792	0.810135	2.1
54793	1.0713	2.1
54794	1.89226	0.521948
54795	1.89226	0.360282
54796	1.89226	0.485604
54797	1.89226	0.403497
54798	3.78453	0.414723
54799	1.89227	0.400773
54800	1.89227	0.356548
54801	1.89227	0.465898
54802	1.89227	0.303109
54803	3.78453	0.377927
54804	1.19	0.32327
54805	1.12469	0.231633
54806	1.01052	0.277806
54807	1.08166	0.2549

54808	2.20051	0.257769
54809	0.594999	0.327035
54810	0.540832	0.274585
54811	0.514182	0.36805
54812	0.567857	0.298579
54813	0.554259	0.288937
54814	0.554259	0.263127
54815	0.55443	0.327999
54816	0.55443	0.352244
54817	0.540832	0.301746
54818	0.514182	0.284519
54819	0.567857	0.294212
54820	0.594999	0.290971
54821	1.38674	0.33785
54822	0.946132	0.400924
54823	1.36361	0.352611
54824	0.946132	0.406118
54825	1.36362	0.345162
54826	1.36362	0.277189
54827	0.946131	0.400406
54828	0.946131	0.439356
54829	1.38674	0.359907
54830	1.36361	0.316184
54831	0.946132	0.392117
54832	0.946132	0.378941
54833	1.89226	0.38457
54834	2.68508	0.266518
54835	2.77349	0.300629
54836	1.89226	0.33374
54837	2.77349	0.309693
54838	1.89226	0.333596
54839	2.15315	0.245934
54840	1.96254	0.298047
54841	1.51377	0.296233
54842	1.89226	0.285546
54843	1.47799	0.256967
54844	1.89226	0.242004
54845	2.49439	0.29654
54846	1.89226	0.349031
54847	2.15315	0.343504
54848	1.89226	0.326221
54849	2.15315	0.324249
54850	1.89226	0.273302
54851	1.89226	0.353722
54852	1.89226	0.365238
54853	1.89226	0.349699
54854	1.89226	0.336418
54855	1.89226	0.395009
54856	1.89226	0.373879
54857	1.89226	0.334832
54858	1.89226	0.329324
54859	1.89226	0.302316
54860	1.89226	0.333257
54861	1.89226	0.290068
54862	1.89226	0.318558
54863	1.375	0.322449
54864	2.01865	0.346532
54865	2.12047	0.314343

54866	1.47354	0.304061
54867	2.12047	0.358498
54868	1.47354	0.34196
54869	0.687499	0.252963
54870	1.06023	0.258271
54871	1.08019	0.255546
54872	0.604229	0.235327
54873	1.08018	0.266287
54874	1.06023	0.270859
54875	0.687502	0.233848
54876	0.60423	0.237423
54877	0.80646	0.239796
54878	0.806461	0.263656
54879	0.858001	0.269479
54880	0.858001	0.239013
54881	2.26414	2.1
54882	1.87342	2.1
54883	1.87342	2.1
54884	1.79667	2.1
54885	2.44045	2.1
54886	2.31924	2.1
54887	2.26414	2.1
54888	2.26616	2.1
54889	2.2355	2.1
54890	2.21858	2.1
54891	4.48474	2.1
54892	2.18516	2.1
54893	1.87344	2.1
54894	2.21859	2.1
54895	2.66233	2.1
54896	1.85196	2.1
54897	2.22236	2.1
54898	2.23551	2.1
54899	2.21859	2.1
54900	2.18516	2.1
54901	2.21189	2.1
54902	4.43049	2.1
54903	1.17171	2.1
54904	2.38924	2.1
54905	2.0815	2.1
54906	2.33348	2.1
54907	1.42851	2.1
54908	1.5959	2.1
54909	1.89226	2.1
54910	1.89226	2.1
54911	1.89226	2.1
54912	1.89226	2.1
54913	3.78452	2.1
54914	2.75425	2.1
54915	1.34366	2.1
54916	1.34366	2.1
54917	1.56512	2.1
54918	2.54186	2.1
54919	2.76332	2.1
54920	1.07751	2.1
54921	2.0815	2.1
54922	1.97259	2.1
54923	1.76455	2.1

54924	1.30191	2.1
54925	1.16461	2.1
54926	0.946131	2.1
54927	0.946131	2.1
54928	0.946129	2.1
54929	0.946129	2.1
54930	0.946131	2.1
54931	0.946131	2.1
54932	0.94613	2.1
54933	0.94613	2.1
54934	0.94613	2.1
54935	0.94613	2.1
54936	0.946131	2.1
54937	0.946131	2.1
54938	1.89226	0.394836
54939	1.89226	0.303701
54940	1.89226	0.277628
54941	1.89226	0.23141
54942	3.78453	0.238002
54943	1.89226	0.416564
54944	1.89226	0.333172
54945	1.89226	0.308116
54946	1.89226	0.233452
54947	3.78453	0.379794
54948	0.946132	0.295543
54949	0.946132	0.330737
54950	1.30327	0.247693
54951	1.33544	0.300766
54952	1.33544	0.251712
54953	1.33544	0.212073
54954	0.946132	0.273016
54955	0.946132	0.235616
54956	0.946132	0.225979
54957	1.30327	0.198201
54958	1.33544	0.281465
54959	0.946132	0.303875
54960	1.30327	0.22233
54961	1.33544	0.245369
54962	0.709426	0.20863
54963	0.790129	0.224162
54964	1.05886	0.169532
54965	1.05886	0.219404
54966	1.03454	0.279058
54967	1.03454	0.22321
54968	1.30327	0.207658
54969	0.709426	0.17427
54970	1.33544	0.283424
54971	0.790128	0.216943
54972	0.946132	0.208052
54973	0.946132	0.237963
54974	0.827866	0.170012
54975	0.946133	0.210604
54976	0.946132	0.161166
54977	0.946132	0.192575
54978	0.946133	0.187535
54979	0.946133	0.256505
54980	0.946132	0.159344
54981	0.827866	0.208875

54982	0.946133	0.267036
54983	0.946133	0.234294
54984	1.89227	0.29141
54985	1.89227	0.182497
54986	1.89227	0.220778
54987	1.89227	0.220507
54988	1.89227	0.210939
54989	1.89227	0.212783
54990	0.946133	0.210951
54991	0.946133	0.227374
54992	1.21602	0.177983
54993	1.30327	0.217916
54994	1.30327	0.177919
54995	1.30327	0.165322
54996	0.946132	0.175747
54997	0.946132	0.200555
54998	0.946132	0.172407
54999	1.21602	0.150735
55000	0.946133	0.219872
55001	1.30327	0.192733
55002	1.04227	0.153292
55003	1.11706	0.186448
55004	0.968413	0.136833
55005	0.945482	0.176777
55006	1.3204	0.165072
55007	1.23215	0.164373
55008	0.810791	0.167227
55009	1.38978	0.139127
55010	1.07728	0.190328
55011	1.11704	0.197112
55012	0.608052	0.169183
55013	1.89226	0.205626
55014	1.89226	0.128034
55015	1.89226	0.150675
55016	1.89226	0.16462
55017	3.78453	0.166489
55018	1.89226	0.113954
55019	1.89226	0.121386
55020	1.89226	0.146771
55021	1.89226	0.0991722
55022	3.78451	0.11544
55023	2.58476	0.117932
55024	1.56598	0.0918004
55025	1.55045	0.104356
55026	1.5124	0.101328
55027	2.62281	0.100537
55028	2.58476	0.0982411
55029	2.58477	0.124227
55030	1.89226	0.136893
55031	1.89226	0.153114
55032	1.89226	0.117689
55033	2.62282	0.150795
55034	2.62282	0.116992
55035	1.89224	0.152745
55036	1.89224	0.169789
55037	1.89224	0.21431
55038	1.89224	0.131946
55039	3.78448	0.15046

55040	1.89223	0.148523
55041	1.89223	0.102543
55042	1.89223	0.122253
55043	1.89223	0.116923
55044	1.89223	0.125069
55045	1.89223	0.120557
55046	2.62278	0.122195
55047	1.45053	0.0929016
55048	1.49678	0.0992236
55049	1.56596	0.103098
55050	2.5536	0.0970453
55051	2.62278	0.101137
55052	1.31139	0.125962
55053	1.2768	0.105704
55054	0.946118	0.171515
55055	0.946118	0.137696
55056	1.2768	0.137267
55057	1.2768	0.11749
55058	0.946116	0.162981
55059	0.946116	0.125323
55060	1.27682	0.125655
55061	0.94613	0.142926
55062	0.946105	0.133181
55063	1.31138	0.127258
55064	1.38506	1.53432
55065	1.53348	1.90106
55066	1.17042	1.47054
55067	1.17042	1.78143
55068	1.73669	1.50789
55069	1.73669	1.77854
55070	1.73669	1.33381
55071	1.79928	0.894716
55072	1.38506	1.21718
55073	1.38506	1.76095
55074	1.79928	1.73135
55075	1.43484	1.81715
55076	1.73669	1.2003
55077	1.93568	1.68585
55078	1.53347	1.08108
55079	1.53347	1.6829
55080	2.14058	1.15773
55081	2.14058	1.77578
55082	0.89964	0.889545
55083	1.10415	1.97883
55084	0.868346	1.25103
55085	1.07029	1.83441
55086	0.98687	1.36913
55087	0.98687	2.1
55088	0.985627	0.673921
55089	0.985627	1.76976
55090	1.07029	1.55529
55091	1.10415	1.35308
55092	0.868344	1.65518
55093	0.899642	1.71332
55094	2.40667	2.1
55095	2.1281	2.1
55096	2.11176	2.1
55097	2.38069	2.1

55098	4.50878	2.1
55099	2.11176	2.1
55100	1.61687	2.1
55101	1.60965	2.1
55102	1.61687	2.1
55103	2.12088	2.1
55104	2.1281	2.1
55105	2.11176	2.1
55106	2.34898	2.1
55107	2.34898	2.1
55108	2.38069	2.1
55109	2.09114	2.1
55110	2.12285	2.1
55111	1.05588	2.1
55112	1.04557	2.1
55113	0.804824	2.1
55114	0.798269	2.1
55115	0.92615	2.1
55116	0.92615	2.1
55117	0.927999	2.1
55118	0.927999	2.1
55119	1.04557	2.1
55120	0.798269	2.1
55121	1.05588	2.1
55122	0.804824	2.1
55123	1.43548	0.358354
55124	1.53873	0.414379
55125	1.25063	0.367319
55126	1.39662	0.385453
55127	2.78937	0.335066
55128	1.25063	0.371428
55129	1.39662	0.404521
55130	1.10476	0.206147
55131	1.28326	0.372664
55132	2.53389	0.363354
55133	1.51147	0.113357
55134	1.86114	0.142121
55135	1.86114	0.135855
55136	2.12926	0.117878
55137	1.3659	0.130137
55138	1.63401	0.11239
55139	1.89226	0.13614
55140	1.89226	0.190253
55141	1.89226	0.165048
55142	1.89226	0.142376
55143	1.89226	0.179133
55144	1.89226	0.16708
55145	1.89226	0.199916
55146	1.89226	0.243162
55147	2.46286	0.178727
55148	1.89226	0.281583
55149	3.78452	0.179357
55150	1.89226	0.248499
55151	1.89226	0.385364
55152	1.89226	0.318672
55153	1.89226	0.315947
55154	1.89226	0.258326
55155	1.89226	0.260492

55156	1.89226	0.217386
55157	1.89226	0.246147
55158	2.28992	0.19902
55159	1.89226	0.294157
55160	3.78452	0.199864
55161	1.13537	0.181883
55162	0.886595	0.386241
55163	0.946131	0.426228
55164	0.946131	0.217808
55165	0.94613	0.387941
55166	0.94613	0.195238
55167	1.25069	0.439782
55168	0.841855	0.407004
55169	1.27436	0.230109
55170	1.06196	0.229364
55171	1.09758	0.163428
55172	0.830306	0.206868
55173	0.826931	0.582449
55174	0.826933	0.169656
55175	0.764631	0.183065
55176	0.764631	0.319734
55177	1.00427	0.484753
55178	0.788034	0.245103
55179	0.726852	0.191105
55180	0.604206	0.382547
55181	0.828475	0.260228
55182	0.604198	0.244015
55183	0.8332	0.240932
55184	1.00428	0.246937
55185	1.89226	0.371103
55186	1.89226	0.218097
55187	1.89226	0.165991
55188	1.89226	0.305581
55189	1.89226	0.255068
55190	1.89226	0.340996
55191	0.768789	0.545936
55192	0.849132	0.0522355
55193	0.666423	0.542656
55194	0.688993	0.48854
55195	0.868671	0.0522355
55196	0.89117	0.539916
55197	0.705632	1.30306
55198	1.80425	2.1
55199	1.73883	2.1
55200	1.45159	2.1
55201	1.45159	2.1
55202	1.37681	2.1
55203	1.73883	2.1
55204	1.39679	0.0801463
55205	2.08149	0.108936
55206	1.89226	0.0939112
55207	2.32763	0.0835442
55208	1.89226	0.0901856
55209	2.31274	0.081927
55210	1.89226	0.0937674
55211	1.89226	0.145668
55212	1.89226	0.11601
55213	1.89226	0.0991914

55214	1.89226	0.125499
55215	1.89226	0.106664
55216	1.89226	0.0956523
55217	1.89226	0.127032
55218	1.89226	0.106927
55219	1.89226	0.102609
55220	1.89226	0.107887
55221	1.89226	0.103478
55222	1.95181	0.0824138
55223	1.89226	0.102276
55224	1.89226	0.0909664
55225	1.89226	0.0834904
55226	3.78451	0.0830319
55227	0.946131	0.091706
55228	0.946131	0.0870309
55229	0.946125	0.100129
55230	0.946125	0.110672
55231	0.946127	0.112807
55232	0.946127	0.103305
55233	0.946128	0.103918
55234	0.946128	0.0880287
55235	0.946128	0.0976116
55236	0.946128	0.102755
55237	0.946128	0.091032
55238	0.946128	0.100836
55239	1.89226	0.104374
55240	1.89226	0.15236
55241	1.89226	0.123421
55242	1.89226	0.117814
55243	1.89226	0.127189
55244	1.89226	0.121499
55245	1.89226	0.676037
55246	1.89226	0.644255
55247	1.89226	0.556779
55248	1.89226	0.523045
55249	3.78453	0.593136
55250	1.89226	0.700018
55251	1.89226	0.617136
55252	1.89226	0.607225
55253	1.89226	0.589462
55254	3.78452	0.605729
55255	1.21387	0.586271
55256	0.8164	0.521566
55257	0.946133	0.56059
55258	0.946133	0.624867
55259	0.946102	0.482353
55260	0.946102	0.671056
55261	0.816431	0.729684
55262	0.816431	0.597762
55263	0.816401	0.561838
55264	0.94613	0.506619
55265	0.946131	0.847359
55266	1.21388	0.599299
55267	0.946132	0.483522
55268	0.946132	0.511153
55269	0.946132	0.54838
55270	0.946132	0.557156
55271	0.946132	0.461251

55272	0.946132	0.535771
55273	0.946132	0.620426
55274	0.946132	0.512303
55275	0.946131	0.451248
55276	0.946131	0.520786
55277	0.946132	0.627063
55278	0.946132	0.545194
55279	1.89226	0.612697
55280	1.89226	0.447715
55281	1.89226	0.42848
55282	1.89226	0.622241
55283	1.89226	0.481683
55284	1.89226	0.789796
55285	1.89226	0.678297
55286	1.89226	0.638324
55287	2.15871	0.504549
55288	2.15871	0.670597
55289	1.81454	0.410421
55290	1.54809	0.727276
55291	1.89226	0.853931
55292	1.89226	1.19227
55293	1.89226	0.902457
55294	1.89226	0.796564
55295	3.78453	1.01684
55296	1.89226	0.992545
55297	1.89226	0.733354
55298	1.89226	0.710517
55299	1.89226	0.944825
55300	1.89226	1.24825
55301	1.89226	1.04511
55302	2.29205	0.9836
55303	1.42633	0.904161
55304	1.69015	1.11822
55305	1.69015	1.04451
55306	2.10364	0.666279
55307	2.05344	0.697997
55308	2.29205	0.79357
55309	1.58147	0.908317
55310	2.16529	0.694132
55311	1.56339	0.611472
55312	2.29205	1.16809
55313	1.69015	1.02595
55314	1.89226	1.03566
55315	1.89226	0.901322
55316	1.89226	0.765358
55317	1.89226	0.914565
55318	3.78453	0.806087
55319	1.89226	0.878679
55320	1.89226	1.12985
55321	1.89226	0.965282
55322	1.89226	0.738858
55323	3.78452	1.11623
55324	2.29205	1.13968
55325	2.85335	0.582414
55326	2.55409	0.917614
55327	2.04345	0.68528
55328	2.76338	0.909907
55329	2.29205	0.746663

55330	2.29205	0.914064
55331	2.16529	1.03144
55332	2.72418	0.914461
55333	2.76338	0.539008
55334	5.01623	0.904854
55335	1.89227	0.195264
55336	1.89227	0.172834
55337	1.89227	0.128176
55338	1.89227	0.141277
55339	3.78453	0.149082
55340	2.39588	0.138522
55341	2.14918	0.124886
55342	2.14918	0.130654
55343	2.37907	0.176777
55344	2.20531	0.0957856
55345	2.4352	0.130965
55346	1.89226	0.156287
55347	2.64327	0.160994
55348	2.36175	0.127136
55349	1.89226	0.122595
55350	4.25402	0.154737
55351	1.89226	0.128679
55352	1.89226	0.10602
55353	1.89226	0.108719
55354	1.89226	0.12426
55355	1.89226	0.11055
55356	1.89226	0.127885
55357	1.05395	0.0902718
55358	1.05395	0.0983414
55359	1.03114	0.0995972
55360	1.03114	0.106783
55361	0.936689	0.0927266
55362	1.07459	0.113548
55363	1.10265	0.0950958
55364	1.01049	0.0984676
55365	0.93669	0.0898606
55366	1.01049	0.0950596
55367	1.07459	0.117357
55368	1.10265	0.0953843
55369	1.18088	0.107879
55370	0.776509	0.105702
55371	0.946132	0.100649
55372	0.946132	0.109743
55373	1.04225	0.10333
55374	0.830392	0.130587
55375	0.931352	0.106133
55376	0.931352	0.110667
55377	1.13006	0.0969445
55378	1.04074	0.100051
55379	1.0628	0.111534
55380	0.851529	0.115997
55381	2.40032	2.1
55382	1.89226	2.1
55383	1.89226	2.1
55384	1.89226	2.1
55385	2.38918	2.1
55386	2.38918	2.1
55387	1.89226	2.1

55388	1.89226	1.78933
55389	1.89226	2.1
55390	1.89226	1.52167
55391	1.89226	2.1
55392	1.89226	1.78464
55393	1.6585	2.1
55394	2.37364	1.92399
55395	2.39622	2.1
55396	2.39622	1.85072
55397	1.6488	2.1
55398	1.6488	1.87818
55399	2.44603	2.1
55400	1.89224	2.1
55401	1.89224	2.1
55402	1.89224	2.1
55403	2.40029	2.1
55404	2.40029	2.1
55405	1.89224	2.1
55406	1.89224	1.48566
55407	1.89224	2.1
55408	1.89224	1.33134
55409	1.89224	2.1
55410	1.89224	1.38108
55411	1.01946	1.61806
55412	1.01946	2.1
55413	1.02497	2.1
55414	1.02497	1.96935
55415	1.21519	1.43544
55416	1.19809	1.26302
55417	0.835303	2.1
55418	0.82924	2.1
55419	0.835314	2.1
55420	1.2152	2.08972
55421	0.829228	1.99683
55422	1.19808	2.04464
55423	1.91027	2.1
55424	1.05809	2.1
55425	1.00659	2.1
55426	2.06727	2.1
55427	3.11819	2.1
55428	2.37166	2.1
55429	2.06726	2.1
55430	1.66994	2.1
55431	1.67623	2.1
55432	2.40308	2.1
55433	2.40308	2.1
55434	0.960194	2.1
55435	0.939664	2.1
55436	0.531131	2.1
55437	0.503298	2.1
55438	0.729927	2.1
55439	0.729927	2.1
55440	0.733565	2.1
55441	0.733565	2.1
55442	0.960195	2.1
55443	0.531132	2.1
55444	0.503297	2.1
55445	0.939663	2.1

55446	1.20154	2.1
55447	1.21754	2.1
55448	0.960195	2.1
55449	0.939665	2.1
55450	1.07745	2.1
55451	1.07745	2.1
55452	1.07976	2.1
55453	1.07976	2.1
55454	0.960194	2.1
55455	1.21754	2.1
55456	0.939662	2.1
55457	1.20155	2.1
55458	1.89224	2.1
55459	1.89224	2.1
55460	2.08609	2.1
55461	2.08609	2.1
55462	2.09527	2.1
55463	1.90142	2.1
55464	1.65815	2.1
55465	1.71398	2.1
55466	2.2001	2.1
55467	2.23791	2.1
55468	1.4502	2.1
55469	1.4502	2.1
55470	0.793587	2.1
55471	0.793587	2.1
55472	0.792408	2.1
55473	0.792408	2.1
55474	0.952221	2.1
55475	0.858585	2.05173
55476	0.725101	2.1
55477	0.633774	2.1
55478	0.633775	2.1
55479	0.858585	2.1
55480	0.952221	2.1
55481	0.725101	2.1
55482	1.5735	2.1
55483	2.56975	2.1
55484	2.63166	2.1
55485	2.63166	2.1
55486	1.54711	2.1
55487	1.54711	2.1
55488	0.946131	2.06202
55489	0.946131	2.1
55490	1.28487	2.1
55491	1.28487	2.1
55492	0.946127	1.85598
55493	0.946127	2.1
55494	1.28488	2.1
55495	1.31583	2.1
55496	1.31583	2.1
55497	0.94613	2.1
55498	1.28487	2.1
55499	0.946129	2.1
55500	1.0596	2.1
55501	1.0596	2.1
55502	1.06939	2.1
55503	1.06939	2.1

55504	1.34225	2.1
55505	1.31583	2.1
55506	0.793563	2.1
55507	0.786748	2.1
55508	0.793563	2.1
55509	1.34225	2.1
55510	0.786748	2.1
55511	1.31583	2.1
55512	0.946131	2.1
55513	0.946131	2.1
55514	1.31582	2.1
55515	1.31582	2.1
55516	0.946127	2.1
55517	0.946127	2.1
55518	1.34225	2.1
55519	1.31583	2.1
55520	1.34225	2.1
55521	0.946129	2.1
55522	1.31583	2.1
55523	0.946129	2.1
55524	1.89226	2.1
55525	1.89226	2.1
55526	1.89226	2.1
55527	1.89226	2.1
55528	1.89226	2.1
55529	1.89226	2.1
55530	1.89226	2.1
55531	1.89226	2.1
55532	1.89226	2.1
55533	1.89226	2.1
55534	1.89226	2.1
55535	1.89226	2.1
55536	1.26992	2.1
55537	1.75003	2.1
55538	1.79883	2.1
55539	1.26732	2.1
55540	3.06875	2.1
55541	0.875017	2.1
55542	0.899414	2.1
55543	0.811147	2.1
55544	0.85053	2.1
55545	0.851513	2.1
55546	0.851513	2.1
55547	0.859048	2.1
55548	0.859048	2.1
55549	0.899413	2.1
55550	0.85053	2.1
55551	0.811147	2.1
55552	0.875016	2.1
55553	1.89227	2.1
55554	1.89227	2.1
55555	1.89227	2.1
55556	1.89227	2.1
55557	1.89227	2.1
55558	1.89227	2.1
55559	1.89226	2.1
55560	1.89226	2.1
55561	1.89226	2.1

55562	1.89226	2.1
55563	1.89226	2.1
55564	1.89226	2.1
55565	0.633661	2.1
55566	0.642005	2.1
55567	0.93285	2.1
55568	0.899413	2.1
55569	0.764487	2.1
55570	0.764487	2.1
55571	0.776931	2.1
55572	0.776931	2.1
55573	0.642004	2.1
55574	0.93285	2.1
55575	0.899414	2.1
55576	0.633661	2.1
55577	0.932851	2.1
55578	0.899414	2.1
55579	0.905419	2.1
55580	0.85053	2.1
55581	0.886336	2.1
55582	0.886336	2.1
55583	0.897045	2.1
55584	0.897045	2.1
55585	0.93285	2.1
55586	0.905419	2.1
55587	0.899414	2.1
55588	0.850531	2.1
55589	2.39322	2.08918
55590	2.74149	0.799721
55591	2.70944	2.1
55592	2.6849	0.859789
55593	2.41776	1.91452
55594	2.39322	0.598908
55595	2.41776	2.04044
55596	2.80992	1.6278
55597	2.77256	2.1
55598	2.74149	1.21621
55599	2.44882	2.05027
55600	2.41776	0.73548
55601	2.80992	1.83983
55602	1.89226	1.5722
55603	2.74149	1.62287
55604	2.74149	1.84032
55605	1.89226	0.975268
55606	1.89226	1.26196
55607	1.89226	0.130008
55608	1.89226	0.115765
55609	1.89226	0.157534
55610	1.89226	0.1015
55611	1.89226	0.15415
55612	1.89226	0.103151
55613	2.38796	0.0830866
55614	2.0815	0.114868
55615	2.0815	0.101691
55616	2.49452	0.087731
55617	1.55994	0.104243
55618	2.14721	0.086577
55619	1.89227	0.150303

55620	1.89227	0.123215
55621	1.89227	0.171183
55622	1.89227	0.112721
55623	1.89227	0.170015
55624	1.89227	0.116917
55625	1.13387	0.108883
55626	2.27494	0.0976835
55627	1.13387	0.129397
55628	2.19489	0.0972978
55629	1.60867	0.123299
55630	2.6994	0.0944919
55631	1.89226	0.148326
55632	1.89226	0.118975
55633	1.89226	0.113139
55634	1.89226	0.180969
55635	3.78453	0.127201
55636	1.42381	1.12048
55637	1.10747	2.05197
55638	1.10747	0.993546
55639	1.08911	2.05764
55640	1.42866	0.932943
55641	1.4103	2.1
55642	1.89226	1.89925
55643	1.88181	2.1
55644	1.88181	1.71477
55645	1.76249	2.1
55646	2.0815	1.56264
55647	1.97539	2.1
55648	0.946128	2.01665
55649	0.946128	1.57124
55650	0.946133	1.02585
55651	0.946133	0.773505
55652	0.94613	0.778904
55653	0.94613	1.45907
55654	0.946131	2.05764
55655	0.946131	0.886164
55656	0.946131	1.13499
55657	0.946131	1.12765
55658	0.946131	1.21353
55659	0.946131	1.20013
55660	1.42866	1.00523
55661	1.43213	0.965319
55662	1.10748	1.67681
55663	1.10748	2.1
55664	1.13641	2.09774
55665	1.43213	2.08949
55666	2.0815	1.98033
55667	2.10287	2.1
55668	2.10287	1.71044
55669	2.0921	2.1
55670	1.89227	1.65662
55671	1.83938	2.1
55672	0.810944	0.908555
55673	0.810944	0.787275
55674	0.810945	0.804218
55675	0.810945	1.51498
55676	0.810933	1.02955
55677	0.810933	1.06265

55678	1.89226	2.04559
55679	1.3517	1.36937
55680	1.08132	1.02549
55681	1.08132	1.72342
55682	1.08133	1.05269
55683	2.33097	1.09109
55684	1.53822	2.1
55685	1.53822	1.6289
55686	1.68437	2.1
55687	2.17996	1.60158
55688	2.32612	2.1
55689	0.99189	0.951589
55690	1.18948	0.41335
55691	1.19756	0.422133
55692	1.19756	1.33066
55693	0.978851	1.09435
55694	0.926995	0.747064
55695	1.26924	0.271536
55696	1.34642	0.473252
55697	1.3228	0.590481
55698	0.990899	0.590063
55699	1.0627	0.601239
55700	1.33473	0.597261
55701	1.96196	0.882397
55702	1.39702	2.1
55703	1.39702	1.47055
55704	1.53822	2.1
55705	2.17372	1.3062
55706	2.31011	2.1
55707	1.89226	0.648682
55708	1.89226	0.375357
55709	1.89226	0.500148
55710	1.89226	0.341627
55711	3.78453	0.406864
55712	2.38475	0.579745
55713	1.73957	0.391654
55714	1.88452	0.504231
55715	1.58695	0.242354
55716	3.6241	0.427761
55717	1.73958	0.374261
55718	1.3126	0.548353
55719	1.58695	0.255869
55720	1.14946	0.565672
55721	2.88903	0.443722
55722	1.89226	0.361735
55723	1.89226	0.594453
55724	1.89226	0.267242
55725	1.89226	0.562256
55726	3.78453	0.465984
55727	1.89225	0.500863
55728	1.89225	0.383339
55729	1.89225	0.472376
55730	1.89225	0.35548
55731	3.7845	0.33655
55732	1.88451	0.513409
55733	1.58694	0.250752
55734	1.74649	0.535868
55735	1.44604	0.357714

55736	3.33055	0.324062
55737	1.58694	0.294493
55738	0.995727	0.532525
55739	0.995727	0.493368
55740	1.14945	0.482081
55741	1.44604	0.313166
55742	1.59976	0.516007
55743	1.89225	0.321559
55744	1.89225	0.52511
55745	1.89225	0.553572
55746	1.89225	0.381517
55747	1.89225	0.554467
55748	1.89225	0.37183
55749	2.01197	1.51352
55750	2.0961	1.42782
55751	2.13283	1.04845
55752	2.25399	0.8304
55753	4.22894	1.26693
55754	2.09611	1.04869
55755	2.37014	1.06253
55756	2.29031	1.4177
55757	2.10084	1.65128
55758	2.25399	0.810972
55759	2.09611	1.6202
55760	2.13283	1.43772
55761	2.25399	1.86688
55762	2.22044	1.1724
55763	2.38975	0.886672
55764	4.52258	1.33454
55765	2.38976	0.706793
55766	2.15466	1.17487
55767	2.31988	0.566034
55768	2.31988	1.52446
55769	2.25399	0.740168
55770	2.25399	1.98974
55771	2.58286	0.584631
55772	2.29193	0.290599
55773	2.40107	0.622636
55774	2.13815	0.20379
55775	4.72101	0.359292
55776	2.29193	0.3301
55777	1.90104	0.477811
55778	2.13815	0.245288
55779	1.77249	0.45404
55780	4.03919	0.346837
55781	2.25197	0.601553
55782	2.13815	0.324404
55783	2.00625	0.524204
55784	2.00625	0.345279
55785	2.40107	0.488691
55786	2.40107	0.358105
55787	2.13815	0.324186
55788	1.77249	0.461202
55789	2.00625	0.340814
55790	1.65815	0.48168
55791	3.7963	0.3625
55792	2.43417	0.681196
55793	2.06653	0.572134

55794	1.64587	0.617718
55795	1.65284	0.527461
55796	2.46898	0.690261
55797	2.46898	0.607455
55798	2.46895	0.597558
55799	1.89016	0.459246
55800	1.87861	0.516883
55801	1.87861	0.404087
55802	2.47161	0.609241
55803	2.47161	0.501529
55804	0.946132	0.744436
55805	0.946132	0.733425
55806	0.863311	0.876276
55807	1.34768	0.83982
55808	0.851542	0.863172
55809	0.851542	0.635455
55810	1.30451	0.805399
55811	1.45304	0.846692
55812	0.946132	0.75222
55813	1.20992	0.79463
55814	0.855162	0.864191
55815	0.946132	0.778008
55816	1.89226	0.658976
55817	1.89226	0.872038
55818	1.89226	0.853422
55819	1.89226	0.637131
55820	1.89226	0.889486
55821	1.89226	0.678981
55822	0.946132	0.538047
55823	0.946132	0.408015
55824	0.946133	0.586325
55825	0.946133	0.516079
55826	0.946132	0.568594
55827	0.946132	0.386335
55828	0.946132	0.418037
55829	0.946132	0.524082
55830	0.946132	0.584615
55831	0.946132	0.478133
55832	0.946133	0.475137
55833	0.946133	0.50544
55834	2.35574	0.735204
55835	1.89226	0.51291
55836	1.89226	1.02181
55837	1.89226	0.496291
55838	2.67456	0.77154
55839	2.67456	0.202328
55840	0.583505	0.369822
55841	0.761972	0.466195
55842	1.17787	0.666608
55843	1.33728	0.32525
55844	0.965128	0.639574
55845	0.965128	0.389805
55846	0.974714	0.529921
55847	0.974715	0.647767
55848	1.17787	0.443261
55849	0.583506	0.653703
55850	1.33728	0.292265
55851	0.761974	0.48768

55852	1.89226	0.636187
55853	1.89226	0.447518
55854	1.89226	0.365323
55855	1.89226	0.309088
55856	1.89226	0.391737
55857	1.89226	0.578758
55858	1.89226	0.423027
55859	1.89226	0.252924
55860	1.89226	0.461898
55861	1.89226	0.428729
55862	3.78453	0.387189
55863	0.946132	0.336261
55864	0.946132	0.409173
55865	0.946132	0.504831
55866	0.946132	0.299085
55867	0.946133	0.517424
55868	0.946133	0.360963
55869	0.946132	0.533427
55870	0.946132	0.462003
55871	0.946133	0.399712
55872	0.946133	0.60054
55873	0.946132	0.339942
55874	0.946132	0.500521
55875	1.01779	2.1
55876	1.50685	2.1
55877	1.12064	2.1
55878	1.65151	2.1
55879	2.6275	2.1
55880	1.35733	2.1
55881	1.53507	2.1
55882	1.50683	2.1
55883	1.73626	2.1
55884	3.09359	2.1
55885	1.73626	2.1
55886	1.52226	2.1
55887	1.75555	2.1
55888	1.75555	2.1
55889	1.53507	2.1
55890	1.53507	2.1
55891	1.65151	2.1
55892	1.73628	2.1
55893	1.69528	2.1
55894	1.50685	2.1
55895	1.92471	2.1
55896	1.73628	2.1
55897	1.73629	2.1
55898	1.75558	2.1
55899	1.9943	2.1
55900	1.9943	2.1
55901	1.92471	2.1
55902	1.73629	2.1
55903	2.5601	1.06764
55904	1.97645	1.77241
55905	2.09605	0.936613
55906	2.09605	1.652
55907	2.45853	1.10137
55908	2.45853	1.88494
55909	2.33824	1.49299

55910	1.97646	1.42182
55911	2.45853	0.90674
55912	1.94984	1.77061
55913	2.33824	1.50984
55914	1.82955	2.09441
55915	1.22926	1.80308
55916	0.946128	1.71124
55917	0.946133	1.87185
55918	1.28005	1.30097
55919	1.22927	1.31864
55920	1.22926	1.56844
55921	0.946131	1.44703
55922	0.946131	1.32715
55923	0.946131	1.33829
55924	0.946131	1.25177
55925	1.28005	1.39984
55926	1.22926	1.36435
55927	2.35349	0.595147
55928	2.61606	0.389628
55929	2.48209	0.587044
55930	2.48209	0.339752
55931	2.50057	0.582221
55932	2.50057	0.343501
55933	2.61607	0.416282
55934	2.57611	0.388209
55935	2.4821	0.392646
55936	2.44814	0.355907
55937	5.06421	0.340344
55938	2.15721	0.445886
55939	2.48209	0.349631
55940	2.31844	0.603164
55941	2.31844	0.272015
55942	2.35349	0.600034
55943	2.35349	0.249207
55944	2.44814	0.363014
55945	2.30908	0.34815
55946	2.44814	0.357462
55947	2.31844	0.351002
55948	2.4821	0.381537
55949	2.31844	0.358587
55950	0.91766	0.33658
55951	0.91766	0.349962
55952	0.912463	0.322743
55953	0.912463	0.35982
55954	1.16525	0.349707
55955	1.26377	0.349215
55956	0.576692	0.349271
55957	0.664871	0.355886
55958	1.16525	0.325809
55959	0.576692	0.336997
55960	0.664871	0.356543
55961	1.26377	0.354659
55962	1.72901	1.82381
55963	1.62414	2.1
55964	1.88971	2.04317
55965	1.8411	1.2754
55966	3.57011	2.0013
55967	1.89226	1.93256

55968	1.89226	1.24954
55969	1.89226	0.795119
55970	1.89226	1.51662
55971	3.78452	0.85789
55972	1.89226	0.942581
55973	1.89226	2.1
55974	1.89226	1.34682
55975	1.89226	1.03818
55976	3.78452	2.0035
55977	1.62414	1.96918
55978	1.30896	2.00936
55979	1.8411	1.0131
55980	1.52776	1.83649
55981	3.15006	1.2668
55982	1.51448	1.91734
55983	1.8411	1.72693
55984	2.02311	1.53883
55985	1.99309	0.886609
55986	3.86421	1.23433
55987	1.89226	0.839767
55988	1.89226	2.04322
55989	1.89226	1.38532
55990	1.89226	0.93973
55991	3.78452	1.89405
55992	1.89226	1.96836
55993	1.89226	1.34235
55994	1.89226	1.10144
55995	1.89226	1.96658
55996	3.78452	1.44063
55997	1.8411	1.43058
55998	1.52776	2.1
55999	1.99309	2.1
56000	1.72526	1.78165
56001	3.56636	2.1
56002	1.89226	2.00453
56003	1.89226	1.41022
56004	1.89226	1.52743
56005	1.89226	2.06322
56006	3.78453	1.60829
56007	1.0424	2.1
56008	1.12935	2.1
56009	0.929383	2.09256
56010	0.929383	2.09862
56011	1.24941	2.1
56012	1.12686	2.1
56013	0.946132	2.1
56014	0.946132	2.05767
56015	1.12944	2.05309
56016	0.851554	2.00637
56017	1.05017	2.05308
56018	0.929315	2.06309
56019	0.898029	2.03629
56020	0.973994	2.1
56021	0.946131	1.4558
56022	0.946131	1.73256
56023	1.0221	1.75779
56024	1.0221	2.1
56025	0.898028	1.37479

56026	0.898028	2.05843
56027	0.973994	2.04495
56028	0.946131	2.09467
56029	0.946131	2.03513
56030	0.898029	2.03963
56031	1.89226	1.50839
56032	1.89226	1.80784
56033	1.89226	1.34886
56034	1.89226	1.74206
56035	1.89226	1.4037
56036	1.89226	1.78239
56037	1.13537	1.75898
56038	0.879308	2.1
56039	0.946131	1.48158
56040	0.946131	1.54214
56041	1.13537	1.59946
56042	0.85187	2.1
56043	0.94613	1.77127
56044	0.94613	1.31286
56045	1.05777	1.83375
56046	1.05777	1.98097
56047	0.799318	1.85748
56048	0.776452	1.89527
56049	0.946116	2.08001
56050	0.946116	2.1
56051	1.09531	2.1
56052	1.09531	2.1
56053	0.946118	2.1
56054	0.946118	2.1
56055	1.09135	2.1
56056	1.09531	2.1
56057	1.09134	2.1
56058	0.946105	2.1
56059	1.09532	2.1
56060	0.946131	2.1
56061	0.946129	2.1
56062	0.946129	2.1
56063	1.09137	2.1
56064	1.09137	2.1
56065	0.946131	2.1
56066	0.946131	2.1
56067	1.07713	2.1
56068	1.09137	2.1
56069	0.946131	2.1
56070	1.07713	2.1
56071	1.09137	2.1
56072	0.946131	2.1
56073	0.938916	2.1
56074	0.938916	2.1
56075	0.936995	2.1
56076	1.00447	2.1
56077	0.979819	2.1
56078	1.16821	2.1
56079	1.01263	2.1
56080	0.719167	2.1
56081	1.04536	2.1
56082	1.18901	2.1
56083	0.758057	2.1

56084	0.924111	2.1
56085	1.00532	2.1
56086	1.00532	2.1
56087	0.9925	2.1
56088	0.9925	2.1
56089	1.29322	2.1
56090	1.01985	2.1
56091	1.00365	2.1
56092	0.704601	2.1
56093	1.00365	2.1
56094	1.29322	2.1
56095	1.01985	2.1
56096	0.704602	2.1
56097	2.20011	2.1
56098	1.401	2.1
56099	1.401	2.1
56100	1.52008	2.1
56101	2.11084	2.1
56102	2.22991	2.1
56103	0.854068	2.1
56104	0.854068	2.1
56105	0.851357	2.1
56106	0.851357	2.1
56107	0.644681	2.1
56108	0.700493	2.1
56109	1.0554	2.1
56110	1.00493	2.1
56111	1.00495	2.1
56112	0.64469	2.1
56113	0.700483	2.1
56114	1.05539	2.1
56115	2.46665	0.0572101
56116	2.22713	0.0649594
56117	1.85877	0.0595403
56118	1.8967	0.0582959
56119	1.56002	0.0628066
56120	2.22713	0.0627453
56121	1.89225	2.1
56122	1.89225	2.1
56123	1.89225	2.1
56124	1.89225	2.1
56125	1.89225	2.1
56126	1.89225	2.1
56127	1.89225	2.1
56128	1.89225	2.1
56129	1.89225	2.1
56130	1.89225	2.1
56131	1.89225	2.1
56132	1.89225	2.1
56133	1.284	2.1
56134	1.86569	2.1
56135	1.95398	2.1
56136	1.31091	2.1
56137	3.23798	2.1
56138	0.932844	2.1
56139	0.976992	2.1
56140	0.960128	2.1
56141	0.905413	2.1

56142	0.938531	2.1
56143	0.938531	2.1
56144	0.943874	2.1
56145	0.943874	2.1
56146	0.976985	2.1
56147	0.960122	2.1
56148	0.93285	2.1
56149	0.905419	2.1
56150	1.89227	2.1
56151	1.89227	2.1
56152	1.89227	2.1
56153	1.89227	2.1
56154	3.78453	2.1
56155	1.89226	2.1
56156	1.89226	2.1
56157	1.89226	2.1
56158	1.89226	2.1
56159	1.89226	2.1
56160	1.89226	2.1
56161	0.65546	2.1
56162	0.665451	2.1
56163	1.00694	2.1
56164	0.976998	2.1
56165	0.816261	2.1
56166	0.816261	2.1
56167	0.826189	2.1
56168	0.826189	2.1
56169	1.00694	2.1
56170	0.665451	2.1
56171	0.976998	2.1
56172	0.65546	2.1
56173	2.01555	2.1
56174	2.01388	2.1
56175	1.92027	2.1
56176	1.92027	2.1
56177	2.01388	2.1
56178	1.954 2.1	
56179	2.75466	2.1
56180	1.89226	2.1
56181	1.89226	2.1
56182	2.54931	2.1
56183	4.44158	2.1
56184	2.47917	2.1
56185	1.92772	2.1
56186	1.80682	2.1
56187	2.54931	2.1
56188	4.47702	2.1
56189	0.946132	2.1
56190	0.946132	2.1
56191	1.17602	2.1
56192	1.27466	2.1
56193	0.946132	2.1
56194	0.946132	2.1
56195	1.17602	2.1
56196	1.17602	2.1
56197	0.946132	2.1
56198	1.17602	2.1
56199	0.946131	2.1

56200	1.27466	2.1
56201	2.35204	2.1
56202	2.54931	2.1
56203	2.40783	2.1
56204	1.50108	2.1
56205	1.64256	2.1
56206	1.64256	2.1
56207	1.89226	2.1
56208	1.89226	0.984417
56209	2.31012	1.71603
56210	2.17534	1.86022
56211	4.0676	1.70471
56212	2.17533	1.82771
56213	1.89226	0.866541
56214	2.17533	1.08889
56215	1.89226	0.989664
56216	1.66635	1.74908
56217	1.38328	1.70228
56218	1.92027	2.1
56219	2.17597	1.86047
56220	2.14619	2.1
56221	2.15388	1.79167
56222	1.91087	2.1
56223	1.92027	0.882078
56224	1.08622	0.921915
56225	1.08622	2.1
56226	0.971184	2.1
56227	0.972761	2.1
56228	1.14845	1.42178
56229	1.16321	2.1
56230	0.894769	2.1
56231	0.894967	2.1
56232	0.894775	2.1
56233	1.28563	2.1
56234	1.14844	2.1
56235	1.0167	2.1
56236	1.89226	0.925679
56237	1.89226	2.01871
56238	1.89226	1.87413
56239	1.89226	1.02307
56240	1.89226	1.8783
56241	1.89226	1.09999
56242	0.937933	1.57891
56243	0.937933	1.74605
56244	1.26192	2.1
56245	1.26192	2.1
56246	0.937903	1.41967
56247	1.25372	2.1
56248	0.946132	1.57028
56249	0.946132	2.1
56250	0.946133	2.05143
56251	1.25372	2.1
56252	0.946132	2.07976
56253	0.937902	2.08488
56254	0.946132	0.96495
56255	0.946132	0.80672
56256	0.946128	1.87957
56257	0.946128	1.80929

56258	0.94613	1.73293
56259	0.94613	0.959023
56260	0.94613	0.729147
56261	0.94613	1.61644
56262	0.946131	1.21448
56263	0.946131	1.31847
56264	0.94613	1.18823
56265	0.94613	1.15338
56266	0.946132	1.16162
56267	0.946132	0.960193
56268	1.2778	1.98065
56269	1.00191	2.1
56270	1.00191	1.09761
56271	1.00191	2.1
56272	0.94613	1.95573
56273	0.94613	1.08255
56274	0.946131	1.50601
56275	1.2778	1.66266
56276	1.00191	1.3935
56277	0.94613	1.34271
56278	1.50847	0.0952065
56279	2.0815	0.116869
56280	1.56806	0.0969965
56281	2.0815	0.110271
56282	1.52043	0.0967119
56283	1.9224	0.110038
56284	1.06882	0.0924279
56285	0.69567	0.113187
56286	0.773112	0.0927244
56287	1.09473	0.106192
56288	0.8941	0.109522
56289	0.8941	0.113118
56290	0.937064	0.0937783
56291	0.981131	0.0935473
56292	0.763762	0.102756
56293	0.722535	0.101977
56294	1.2153	0.102085
56295	1.07279	0.103511
56296	1.89226	0.128924
56297	1.89226	0.149037
56298	1.89226	0.141292
56299	1.89226	0.120044
56300	1.89226	0.159267
56301	1.89226	0.138923
56302	1.89226	0.127594
56303	1.89226	0.159592
56304	1.89226	0.133121
56305	1.89226	0.164786
56306	3.78453	0.152126
56307	2.51518	0.120886
56308	1.89226	0.130705
56309	1.89226	0.126945
56310	1.89226	0.110247
56311	2.37584	0.13872
56312	2.37584	0.124879
56313	1.25759	0.128474
56314	0.946131	0.138183
56315	0.946131	0.115916

56316	1.24489	0.151267
56317	0.946131	0.137301
56318	0.946131	0.141517
56319	1.25759	0.123476
56320	1.25759	0.119948
56321	1.25759	0.12997
56322	1.24489	0.128285
56323	0.946131	0.129341
56324	0.946131	0.131204
56325	1.08028	0.604655
56326	1.09362	0.697989
56327	0.596616	0.660552
56328	0.606432	0.74958
56329	0.844265	0.758923
56330	0.844265	0.292152
56331	0.855786	1.01798
56332	0.855786	0.627542
56333	1.08028	0.773416
56334	0.606433	0.602164
56335	0.596616	0.559493
56336	1.09362	0.557273
56337	2.837	0.763374
56338	2.16055	0.432992
56339	2.12741	0.710706
56340	2.79312	0.652462
56341	4.95367	0.704019
56342	2.12741	0.738387
56343	1.21286	0.462047
56344	1.20859	0.828513
56345	1.21286	0.91548
56346	2.15628	0.630157
56347	2.16056	0.428944
56348	1.89226	2.1
56349	1.89226	2.1
56350	1.89226	2.1
56351	1.89226	2.1
56352	3.78453	2.1
56353	0.946102	2.1
56354	0.946102	2.1
56355	0.850039	2.1
56356	0.850039	2.1
56357	0.914891	2.1
56358	0.85001	2.1
56359	0.946131	2.1
56360	0.946131	2.1
56361	0.94613	2.1
56362	0.91489	2.1
56363	0.850011	2.1
56364	0.946131	2.1
56365	0.71107	2.1
56366	0.784813	2.1
56367	1.28453	2.1
56368	1.20328	2.1
56369	0.992128	2.1
56370	0.992128	2.1
56371	0.995965	2.1
56372	0.995965	2.1
56373	0.784813	2.1

56374	1.28453	2.1
56375	0.711071	2.1
56376	1.20328	2.1
56377	1.89226	2.1
56378	1.89226	2.1
56379	1.89226	2.1
56380	1.89226	2.1
56381	3.78453	2.1
56382	0.946132	2.1
56383	0.946132	2.1
56384	0.914889	2.1
56385	0.914889	2.1
56386	0.985255	2.1
56387	0.914891	2.1
56388	0.946131	2.1
56389	0.946131	2.1
56390	0.946131	2.1
56391	0.985255	2.1
56392	0.94613	2.1
56393	0.91489	2.1
56394	0.784813	2.1
56395	0.871551	2.1
56396	1.37791	2.1
56397	1.28453	2.1
56398	1.07644	2.1
56399	1.07644	2.1
56400	1.07964	2.1
56401	1.07964	2.1
56402	0.871551	2.1
56403	1.37791	2.1
56404	0.784813	2.1
56405	1.28453	2.1
56406	1.73075	1.25818
56407	2.10582	0.91368
56408	1.89319	0.85856
56409	1.38766	1.70153
56410	3.27777	0.886815
56411	1.08715	1.46776
56412	1.54185	1.31214
56413	1.73074	1.22371
56414	1.73074	1.77903
56415	1.18948	1.0157
56416	1.18948	1.3907
56417	1.89319	0.823607
56418	1.41689	1.55585
56419	1.11619	1.00341
56420	1.38766	1.29226
56421	1.6052	0.883796
56422	1.89319	1.14271
56423	0.647894	1.33938
56424	0.594738	1.50332
56425	0.708445	1.29192
56426	0.770926	1.10084
56427	0.738326	1.30336
56428	0.738326	1.72831
56429	0.680494	1.27682
56430	0.680494	0.954979
56431	0.708443	1.04884

56432	0.647895	1.07685
56433	0.770925	1.10665
56434	0.59474	0.947478
56435	1.89111	0.895006
56436	1.06994	2.1
56437	2.06689	1.52146
56438	1.37063	2.1
56439	3.13683	2.04838
56440	2.06689	1.08726
56441	1.39295	2.05577
56442	1.56818	1.54519
56443	1.37063	2.1
56444	2.26444	1.14356
56445	2.06689	2.1
56446	1.49808	2.1
56447	1.50201	2.1
56448	2.57784	2.1
56449	2.58615	2.1
56450	4.08424	2.1
56451	0.946133	1.68182
56452	0.946133	1.52754
56453	0.946132	2.1
56454	0.946132	2.1
56455	0.946132	2.1
56456	0.946132	1.63734
56457	0.946132	1.90491
56458	0.946132	1.53798
56459	0.946133	1.59522
56460	0.946133	1.97675
56461	0.946132	1.92611
56462	0.946132	2.1
56463	1.89226	1.64786
56464	1.89226	2.1
56465	1.89226	2.1
56466	1.89226	1.98469
56467	3.78452	2.1
56468	0.977201	2.1
56469	0.977201	2.1
56470	0.980489	2.1
56471	0.980489	2.1
56472	0.657973	2.1
56473	1.20669	2.1
56474	0.751002	2.1
56475	1.29307	2.1
56476	1.29308	2.1
56477	1.20669	2.1
56478	0.751003	2.1
56479	0.657972	2.1
56480	0.946131	2.1
56481	0.946131	2.1
56482	1.29308	2.1
56483	1.29308	2.1
56484	0.946132	2.1
56485	1.29308	2.1
56486	0.946132	2.06672
56487	1.28892	2.1
56488	0.946132	2.0614
56489	0.946132	2.1

56490	1.28892	2.1
56491	1.29308	2.1
56492	0.946132	1.07261
56493	0.946132	0.87263
56494	0.946131	1.29471
56495	0.946131	1.12703
56496	0.946132	1.30206
56497	0.946132	1.23758
56498	0.946132	0.986757
56499	0.946132	0.875553
56500	0.946132	0.937182
56501	0.946132	1.11798
56502	0.946132	1.04497
56503	0.946132	1.3821
56504	1.89226	1.31989
56505	1.89226	1.48986
56506	1.89226	1.75151
56507	1.89226	1.21247
56508	1.89226	1.67693
56509	1.89226	1.12013
56510	0.94613	2.1
56511	0.94613	2.1
56512	1.20669	2.1
56513	1.20669	2.1
56514	1.20669	2.1
56515	0.946132	2.1
56516	0.946129	2.1
56517	1.29307	2.1
56518	0.946131	2.1
56519	0.946131	2.1
56520	1.29308	2.1
56521	1.20669	2.1
56522	1.89226	0.214901
56523	1.89226	0.132207
56524	1.89226	0.151688
56525	1.89226	0.17378
56526	3.78453	0.185567
56527	0.946132	0.103254
56528	0.946132	0.117528
56529	0.946126	0.123835
56530	0.946126	0.152785
56531	0.946129	0.126581
56532	0.946129	0.109005
56533	0.946129	0.124172
56534	0.946129	0.15281
56535	0.946128	0.118834
56536	0.946128	0.121973
56537	0.946129	0.123696
56538	0.946129	0.125468
56539	0.725277	0.0978947
56540	0.588155	0.0929221
56541	1.19403	0.122843
56542	1.27681	0.10152
56543	0.918903	0.101002
56544	0.918903	0.095052
56545	0.946068	0.115211
56546	0.946068	0.0981383
56547	1.19404	0.103974

56548	0.588153	0.102168
56549	0.725275	0.101438
56550	1.27682	0.100169
56551	1.19404	0.126766
56552	1.27682	0.104819
56553	0.946131	0.158264
56554	0.946131	0.135195
56555	1.19404	0.136494
56556	1.19404	0.117352
56557	0.946129	0.12619
56558	0.946129	0.143919
56559	1.19404	0.127519
56560	0.94613	0.127997
56561	1.27682	0.117032
56562	0.946131	0.126229
56563	1.89226	0.141855
56564	1.89226	0.183154
56565	1.89226	0.200043
56566	1.89226	0.145626
56567	1.89226	0.182684
56568	1.89226	0.129992
56569	1.4263	1.44345
56570	1.67651	0.600568
56571	1.5955	0.766534
56572	1.31022	1.53883
56573	2.98674	0.968135
56574	1.18824	2.1
56575	1.28802	2.1
56576	0.946132	0.931695
56577	0.946132	0.910777
56578	0.946132	0.920753
56579	0.946132	2.1
56580	1.28802	0.827519
56581	1.28802	2.1
56582	1.18824	1.27257
56583	0.946132	1.38855
56584	0.946132	1.52222
56585	1.28802	1.37076
56586	1.89226	0.64117
56587	1.89226	0.962028
56588	1.89226	1.49358
56589	1.89226	0.581658
56590	3.78452	0.759767
56591	1.5955	0.822941
56592	1.52909	0.659579
56593	1.31022	0.780498
56594	1.31022	1.52324
56595	1.2087	1.34666
56596	1.52909	1.34995
56597	1.11825	2.1
56598	1.18824	2.1
56599	0.946132	1.03369
56600	0.946132	0.903367
56601	0.946132	0.953546
56602	0.946132	2.1
56603	1.18824	0.861364
56604	1.18824	2.1
56605	0.946132	0.997825

56606	1.11825	1.32162
56607	1.18824	1.44627
56608	0.946133	1.52186
56609	0.810943	1.20012
56610	0.810943	0.932857
56611	0.810945	0.669052
56612	0.810945	0.991629
56613	1.89226	0.687797
56614	1.35168	0.784957
56615	1.08132	0.641569
56616	1.0813	0.860542
56617	1.08132	1.12777
56618	0.810959	0.803346
56619	0.810959	0.839931
56620	1.89226	0.451774
56621	1.89226	0.227227
56622	1.89226	0.276229
56623	1.89226	0.365346
56624	3.78453	0.400797
56625	1.89226	0.171589
56626	1.89226	0.203225
56627	1.89226	0.215744
56628	1.89226	0.133076
56629	1.89226	0.215342
56630	1.89226	0.134097
56631	0.946131	0.129418
56632	0.946131	0.134388
56633	0.946126	0.216358
56634	0.946126	0.163005
56635	0.946128	0.209556
56636	0.946128	0.1384
56637	0.946128	0.213044
56638	0.946128	0.161911
56639	0.946128	0.168992
56640	0.946128	0.168842
56641	0.946128	0.176255
56642	0.946128	0.164702
56643	1.89226	0.384606
56644	1.89226	0.168623
56645	1.89226	0.273163
56646	1.89226	0.264894
56647	1.89226	0.241198
56648	1.89226	0.235235
56649	1.89226	0.229877
56650	1.89226	0.324171
56651	1.89226	0.541731
56652	1.89226	0.213268
56653	3.78453	0.217846
56654	1.07176	0.134901
56655	1.07176	0.161437
56656	0.946126	0.207299
56657	0.946126	0.177921
56658	1.07176	0.171558
56659	0.946129	0.124722
56660	1.27474	0.18104
56661	0.94613	0.209764
56662	1.27474	0.163965
56663	1.07176	0.104983

56664	0.94613	0.145111
56665	0.94613	0.143344
56666	0.723486	0.115954
56667	0.94613	0.122309
56668	0.946126	0.16027
56669	0.946126	0.135924
56670	0.946129	0.149251
56671	0.723484	0.118153
56672	1.07176	0.152071
56673	0.946129	0.110554
56674	1.32398	0.142461
56675	1.19835	0.118856
56676	0.723471	0.126511
56677	0.723471	0.131232
56678	0.810946	0.404078
56679	0.810946	0.233006
56680	0.810946	0.400659
56681	0.810946	0.283099
56682	0.810933	0.151363
56683	0.810933	0.286823
56684	1.89226	0.132498
56685	1.3517	0.285333
56686	1.08132	0.179334
56687	1.08132	0.270723
56688	1.08133	0.264518
56689	2.39923	2.1
56690	1.89226	2.1
56691	1.89226	2.1
56692	1.89226	2.1
56693	2.44606	2.1
56694	2.44606	2.1
56695	1.89226	2.1
56696	1.89226	1.9605
56697	1.89226	2.1
56698	1.89226	1.74862
56699	1.89226	2.1
56700	1.89226	1.83272
56701	1.70302	2.1
56702	2.43041	2.04278
56703	2.25275	2.1
56704	2.4747	1.72607
56705	1.52079	2.1
56706	1.67063	1.96066
56707	2.40644	2.1
56708	1.89226	2.1
56709	1.89226	2.1
56710	1.89226	2.1
56711	2.39923	2.1
56712	2.39923	2.1
56713	0.946131	0.825168
56714	0.946131	2.1
56715	0.946131	2.1
56716	0.946131	1.701
56717	0.94613	1.40241
56718	0.94613	1.36329
56719	0.946131	2.1
56720	0.946131	2.1
56721	0.946131	1.96556

56722	0.946131	2.02523
56723	0.946131	2.03314
56724	0.946131	2.04112
56725	1.04938	2.1
56726	1.04938	1.8141
56727	1.111	2.1
56728	1.06978	2.1
56729	1.2748	1.83677
56730	1.25472	1.23289
56731	0.88782	2.1
56732	0.882987	2.1
56733	0.973779	2.1
56734	1.31997	2.1
56735	0.913905	2.1
56736	1.31312	2.09685
56737	2.6536	2.1
56738	2.15287	2.1
56739	2.72238	2.1
56740	2.22887	2.1
56741	4.87525	2.1
56742	2.15288	2.1
56743	1.40719	2.1
56744	1.41839	2.1
56745	1.34239	2.1
56746	2.22887	2.1
56747	2.15288	2.1
56748	2.72237	2.1
56749	2.22887	2.1
56750	2.77314	2.1
56751	2.28748	2.1
56752	5.00985	2.1
56753	0.922802	2.1
56754	0.922802	2.1
56755	0.92107	2.1
56756	0.92107	2.1
56757	0.703593	2.1
56758	0.729436	2.1
56759	1.11444	2.1
56760	1.14374	2.1
56761	1.14374	2.1
56762	0.729436	2.1
56763	0.703593	2.1
56764	1.11444	2.1
56765	1.89226	1.8559
56766	1.89226	2.1
56767	1.89226	1.34044
56768	1.89226	2.1
56769	1.89226	1.52256
56770	1.89226	2.1
56771	0.489927	2.1
56772	0.63008	2.1
56773	0.697385	2.1
56774	0.55524	1.91516
56775	0.592141	2.1
56776	0.592141	2.1
56777	0.593179	1.9021
56778	0.593179	2.1
56779	0.63008	2.1

56780	0.697385	2.1
56781	0.55524	2.1
56782	0.489927	2.1
56783	0.946133	2.0861
56784	0.946133	1.64939
56785	0.946132	1.27725
56786	0.946132	0.837892
56787	0.946133	1.04137
56788	0.946133	2.1
56789	0.946132	1.7395
56790	0.946132	0.925586
56791	0.946132	1.30328
56792	0.946132	1.15927
56793	0.946132	0.968288
56794	0.946132	1.07197
56795	1.89226	2.1
56796	1.89226	1.52854
56797	1.89226	1.16751
56798	1.89226	2.1
56799	1.89226	1.17274
56800	1.89226	2.1
56801	0.805746	2.1
56802	0.63008	2.1
56803	0.881598	1.79795
56804	0.697385	2.1
56805	0.749417	2.1
56806	0.749417	2.1
56807	0.753714	1.56371
56808	0.753714	2.1
56809	0.881598	2.1
56810	0.805746	2.1
56811	0.63008	2.1
56812	0.697385	2.1
56813	0.881599	2.03369
56814	0.697385	2.1
56815	0.805746	1.54356
56816	0.626731	1.03656
56817	0.755479	1.31277
56818	0.755479	2.1
56819	0.752851	1.01057
56820	0.752851	2.09547
56821	0.881598	2.00205
56822	0.805747	1.95485
56823	0.697385	2.03417
56824	0.626731	1.98216
56825	2.65348	2.1
56826	2.12314	2.1
56827	2.63899	2.1
56828	2.13898	2.1
56829	4.76213	2.1
56830	0.763518	2.1
56831	0.765889	2.1
56832	0.84086	2.1
56833	0.84086	2.1
56834	0.632078	2.1
56835	0.597565	2.1
56836	1.08813	2.1
56837	0.985643	2.1

56838	0.982464	2.1
56839	0.66404	2.1
56840	1.0358	2.1
56841	0.598023	2.1
56842	1.29293	2.1
56843	1.29628	2.1
56844	1.19266	2.1
56845	1.19266	2.1
56846	1.07109	2.1
56847	1.13093	2.1
56848	1.34467	2.1
56849	1.35251	2.1
56850	1.34369	2.1
56851	1.12525	2.1
56852	1.28746	2.1
56853	1.13511	2.1
56854	2.19717	2.1
56855	2.16547	2.1
56856	1.37392	2.1
56857	1.37392	2.1
56858	1.2966	2.1
56859	2.11985	2.1
56860	1.89226	2.1
56861	1.89226	2.1
56862	1.89226	2.1
56863	1.89226	2.1
56864	1.89226	2.1
56865	1.89226	2.1
56866	1.65637	2.1
56867	1.41758	2.1
56868	1.65637	2.1
56869	1.74391	2.1
56870	1.35504	2.1
56871	1.44258	2.1
56872	1.40774	2.1
56873	1.40774	2.1
56874	0.946131	2.1
56875	0.946131	2.1
56876	1.40774	2.1
56877	1.38882	2.1
56878	0.946132	2.1
56879	0.946132	2.1
56880	0.946132	2.1
56881	1.38882	2.1
56882	0.946132	2.1
56883	1.40774	2.1
56884	1.89227	2.1
56885	1.89227	2.1
56886	1.89227	2.1
56887	1.89227	2.1
56888	3.78453	2.1
56889	0.603148	2.1
56890	0.600177	2.1
56891	0.558615	2.1
56892	0.580714	2.1
56893	0.558606	2.1
56894	0.67883	2.1
56895	1.65637	2.1

56896	1.07933	2.1
56897	0.842649	2.1
56898	0.905168	2.1
56899	0.774334	2.1
56900	1.38882	2.1
56901	1.38882	2.1
56902	0.946132	2.1
56903	0.946132	2.1
56904	1.38882	2.1
56905	1.37381	2.1
56906	0.946132	2.1
56907	0.946132	2.1
56908	0.946132	2.1
56909	1.37381	2.1
56910	0.946133	2.1
56911	1.38882	2.1
56912	2.3771	2.1
56913	1.9339	2.1
56914	1.70782	2.1
56915	1.9339	2.1
56916	2.42693	2.1
56917	2.83279	2.1
56918	2.34901	0.631454
56919	1.45833	0.652537
56920	1.86695	0.59112
56921	1.03101	0.67997
56922	3.32528	0.612062
56923	1.89226	0.388831
56924	1.89226	0.378625
56925	1.89226	0.282416
56926	1.89226	0.243135
56927	3.78453	0.344179
56928	2.71753	0.237709
56929	1.89226	0.367582
56930	2.76228	0.189192
56931	2.76228	0.250148
56932	1.89226	0.392176
56933	1.89226	0.447462
56934	0.946131	0.27274
56935	0.946131	0.26307
56936	0.946132	0.271523
56937	0.946132	0.296779
56938	0.946132	0.332284
56939	0.946132	0.218987
56940	0.946132	0.348202
56941	0.946132	0.230575
56942	0.946132	0.207208
56943	0.946132	0.246027
56944	0.946132	0.343757
56945	0.946132	0.294445
56946	1.89227	0.322103
56947	1.89227	0.167727
56948	1.89227	0.206261
56949	1.89227	0.286101
56950	1.89227	0.163841
56951	1.89227	0.225918
56952	2.71753	0.253213
56953	2.37907	0.136632

56954	2.37907	0.183798
56955	2.76228	0.274578
56956	2.39588	0.115641
56957	2.77909	0.186162
56958	1.6571	0.861252
56959	1.11682	1.42746
56960	1.47132	0.950954
56961	1.93763	0.487056
56962	3.12842	1.23746
56963	1.93762	0.673229
56964	2.12345	1.03812
56965	1.65709	0.579397
56966	1.65709	1.56807
56967	2.12345	0.980722
56968	1.88635	1.81296
56969	1.47134	1.68039
56970	2.219	0.66127
56971	2.25734	1.93148
56972	1.93765	0.785846
56973	1.79103	1.20523
56974	1.47134	0.5841
56975	1.06174	0.912788
56976	1.19349	1.72462
56977	1.1095	1.52839
56978	0.968827	0.844
56979	1.07892	0.754414
56980	1.07892	2.1
56981	1.0834	0.804673
56982	1.0834	1.06081
56983	1.1095	1.20038
56984	1.19349	1.00744
56985	0.968825	1.15122
56986	1.06174	1.10123
56987	2.40627	2.05592
56988	2.08288	1.20107
56989	2.5444	0.824695
56990	2.29285	1.44069
56991	4.62729	0.940848
56992	2.08289	0.856341
56993	1.65703	1.00992
56994	1.71105	2.1
56995	1.50108	1.54645
56996	2.29286	1.51294
56997	2.08289	1.62068
56998	2.5444	0.994461
56999	2.29285	2.1
57000	2.71429	1.72953
57001	2.50288	0.7861
57002	5.04728	1.94052
57003	2.50288	0.793972
57004	2.00927	1.76018
57005	2.02116	1.04478
57006	2.02116	1.95648
57007	2.29286	0.955433
57008	2.29286	1.50156
57009	1.89226	1.37679
57010	1.89226	1.81735
57011	1.89226	0.659223

57012	1.89226	2.1
57013	3.78453	1.29433
57014	1.17418	1.30025
57015	1.29741	2.1
57016	1.04921	2.1
57017	1.19352	2.1
57018	2.3677	2.1
57019	1.29741	2.1
57020	1.27575	2.1
57021	1.19352	2.1
57022	1.19473	2.1
57023	2.46927	2.1
57024	1.89226	2.1
57025	1.89226	2.1
57026	1.89226	1.93731
57027	1.89226	2.1
57028	1.89226	1.98943
57029	1.89226	2.1
57030	0.561332	2.1
57031	0.561332	2.1
57032	0.567597	2.1
57033	0.567597	2.1
57034	0.597365	2.1
57035	0.544699	2.1
57036	0.596761	2.1
57037	0.531564	2.1
57038	0.5447	2.1
57039	0.531563	2.1
57040	0.596759	2.1
57041	0.597366	2.1
57042	0.946131	2.1
57043	0.946131	2.1
57044	0.946131	1.5395
57045	0.946131	2.1
57046	0.946129	2.1
57047	0.946129	2.1
57048	0.946133	2.1
57049	0.946133	1.56688
57050	0.946131	2.1
57051	0.946131	2.1
57052	0.946131	2.1
57053	0.946131	2.1
57054	1.10938	0.234724
57055	1.17614	0.580322
57056	1.02478	0.26532
57057	1.09056	0.549005
57058	2.19994	0.521138
57059	1.17614	0.584846
57060	1.11868	1.14128
57061	1.03408	0.553633
57062	1.03408	1.34099
57063	1.09056	0.55025
57064	1.17516	1.56025
57065	1.89226	0.572914
57066	1.89226	0.486192
57067	1.89226	0.582854
57068	1.89226	0.434597
57069	1.89226	0.637854

57070	1.89226	0.494109
57071	1.89226	0.430533
57072	1.89226	0.513516
57073	1.89226	0.514315
57074	1.89226	0.365425
57075	1.89226	0.589803
57076	1.89226	0.35871
57077	0.579551	0.342964
57078	0.563873	0.282694
57079	0.611341	0.418549
57080	0.598693	0.345336
57081	0.589856	0.335037
57082	0.589856	0.29423
57083	0.588387	0.402573
57084	0.588387	0.352678
57085	0.57955	0.353356
57086	0.611341	0.34854
57087	0.563873	0.336114
57088	0.598693	0.332905
57089	0.611341	0.410713
57090	0.598693	0.337904
57091	0.586048	0.403659
57092	0.572737	0.44594
57093	0.591824	0.40215
57094	0.591824	0.353478
57095	0.592254	0.453958
57096	0.592254	0.421136
57097	0.611341	0.407868
57098	0.586048	0.396569
57099	0.598693	0.393868
57100	0.572737	0.394254
57101	0.946119	0.414057
57102	0.946119	0.555816
57103	0.946119	0.443519
57104	0.946119	0.569804
57105	0.94612	0.438972
57106	0.94612	0.397964
57107	0.946119	0.549555
57108	0.946119	0.604345
57109	0.946132	0.488179
57110	0.946132	0.482287
57111	0.946107	0.511089
57112	0.946107	0.514922
57113	1.89224	0.448006
57114	1.89224	0.383729
57115	1.89224	0.484119
57116	1.89224	0.331447
57117	1.89224	0.544503
57118	1.89224	0.387727
57119	0.562335	0.310518
57120	0.579543	0.260416
57121	0.59499	0.35446
57122	0.611333	0.300419
57123	0.586619	0.292999
57124	0.586619	0.270355
57125	0.587049	0.342402
57126	0.587049	0.3155
57127	0.594999	0.320457

57128	0.562342	0.319727
57129	0.579535	0.309536
57130	0.611325	0.305231
57131	0.59499	0.348939
57132	0.611333	0.295626
57133	0.567849	0.345463
57134	0.58604	0.389451
57135	0.590978	0.342423
57136	0.590978	0.306322
57137	0.590052	0.395292
57138	0.590052	0.358546
57139	0.567857	0.369733
57140	0.594998	0.365533
57141	0.611325	0.360441
57142	0.586032	0.352099
57143	1.69661	0.555883
57144	1.9339	0.292726
57145	1.39379	0.511876
57146	1.69107	0.150008
57147	3.32768	0.322176
57148	1.9339	0.37075
57149	1.98414	0.370286
57150	1.69107	0.217284
57151	1.72774	0.353889
57152	3.66164	0.335725
57153	1.39379	0.625561
57154	1.69107	0.203212
57155	1.14397	0.434219
57156	1.43548	0.175139
57157	2.82927	0.356599
57158	1.69107	0.281991
57159	1.72774	0.374858
57160	1.53873	0.30976
57161	1.53873	0.402749
57162	1.43548	0.289404
57163	1.62449	0.374881
57164	2.58431	0.275928
57165	1.34072	0.264854
57166	1.80841	0.300481
57167	1.34072	0.27951
57168	2.51501	0.256559
57169	2.04732	0.24742
57170	1.89226	0.294896
57171	1.89226	0.295422
57172	1.89226	0.321426
57173	1.89226	0.312408
57174	1.89226	0.286233
57175	1.89226	0.272347
57176	0.946131	0.281877
57177	0.946131	0.299632
57178	1.3639	0.318344
57179	1.1516	0.299532
57180	0.946129	0.299759
57181	0.946129	0.315589
57182	1.36391	0.294649
57183	1.36391	0.2956
57184	0.946131	0.317426
57185	1.3639	0.306246

57186	1.1516	0.282662
57187	0.946131	0.288796
57188	0.946131	0.319343
57189	0.946131	0.291224
57190	0.94613	0.321662
57191	0.94613	0.27818
57192	0.946128	0.329977
57193	0.946128	0.313627
57194	0.946133	0.280997
57195	0.946133	0.289456
57196	0.946131	0.299815
57197	0.946131	0.345167
57198	0.946131	0.271283
57199	0.946131	0.29455
57200	1.89226	1.06549
57201	2.63634	1.75067
57202	1.89226	1.12457
57203	1.83902	1.22213
57204	3.73128	1.09546
57205	1.47059	1.57541
57206	2.02928	2.1
57207	1.47059	2.1
57208	1.65706	2.1
57209	1.98628	2.1
57210	2.17274	2.1
57211	2.43618	2.1
57212	2.41176	2.1
57213	1.98627	2.1
57214	2.34084	2.1
57215	4.39803	2.1
57216	1.47059	1.51133
57217	1.82453	2.1
57218	1.98627	2.1
57219	1.98627	2.1
57220	1.26929	2.1
57221	1.26929	2.1
57222	1.98627	2.1
57223	2.34084	2.1
57224	1.47264	2.1
57225	2.24937	2.1
57226	4.23564	2.1
57227	1.33026	0.743204
57228	1.51171	0.974013
57229	1.51171	0.700392
57230	1.4617	1.07139
57231	1.35382	0.695666
57232	1.30382	1.46813
57233	1.89226	0.631581
57234	1.89226	0.733035
57235	1.89226	0.587503
57236	1.89226	0.881161
57237	1.89226	0.548098
57238	1.89226	0.951391
57239	1.39263	0.747495
57240	0.962704	0.429137
57241	1.0171	0.589613
57242	0.962704	0.664836
57243	1.40822	0.515115

57244	1.35382	0.59758
57245	0.755853	1.20596
57246	0.789808	0.830679
57247	0.696315	0.745946
57248	0.676912	0.614859
57249	0.729751	0.577325
57250	0.729751	0.802393
57251	0.722417	1.52527
57252	0.722417	0.661091
57253	0.696315	0.795992
57254	0.789808	0.774793
57255	0.755853	0.790901
57256	0.676912	0.789533
57257	0.946132	0.729643
57258	0.946132	0.796363
57259	0.946132	0.632093
57260	0.946132	0.471186
57261	0.946131	0.460102
57262	0.946131	0.723276
57263	0.946132	0.585384
57264	0.946132	0.799326
57265	0.946132	0.585233
57266	0.946132	0.621444
57267	0.946132	0.655462
57268	0.946132	0.651636
57269	0.946133	0.60589
57270	0.946133	0.448756
57271	0.946132	0.380085
57272	0.946132	0.51064
57273	0.946132	0.35575
57274	0.946132	0.473908
57275	0.946133	0.44556
57276	0.946133	0.637617
57277	0.946132	0.524504
57278	0.946132	0.503851
57279	0.946132	0.494397
57280	0.946132	0.55759
57281	1.89227	1.7306
57282	2.53014	1.77676
57283	1.89227	2.06412
57284	1.98241	1.50888
57285	1.89227	2.08977
57286	1.98241	1.57699
57287	1.89226	1.31425
57288	1.89226	2.09602
57289	1.89226	1.67683
57290	1.89226	1.61343
57291	1.89226	2.06261
57292	1.89226	2.02049
57293	1.89226	1.23835
57294	1.89226	1.99397
57295	1.89226	1.51528
57296	1.89226	1.51011
57297	3.78452	1.59063
57298	2.45304	1.15774
57299	1.89226	1.8315
57300	1.89226	1.50477
57301	1.89226	1.45856

57302	1.89226	1.3037
57303	1.89226	1.28341
57304	1.89227	1.75541
57305	1.89227	1.61415
57306	1.89227	2.00789
57307	1.89227	1.31826
57308	1.89227	2.02933
57309	1.89227	1.36184
57310	1.89226	1.43088
57311	1.89226	2.0658
57312	1.89226	1.6447
57313	1.89226	1.68505
57314	1.89226	1.94521
57315	1.89226	1.96443
57316	2.09871	0.619191
57317	2.71891	0.698908
57318	1.31126	0.615909
57319	1.31126	0.706432
57320	2.55341	0.616874
57321	1.76597	0.81517
57322	1.89226	0.759666
57323	1.89226	0.75272
57324	1.89226	0.643569
57325	1.89226	0.918273
57326	1.89226	0.649536
57327	1.89226	0.923812
57328	0.946128	0.752349
57329	0.946128	0.638928
57330	0.946133	0.610124
57331	0.946133	0.560462
57332	0.946131	0.604764
57333	0.946131	0.711779
57334	0.94613	0.570415
57335	0.94613	0.651085
57336	0.946131	0.638942
57337	0.946131	0.632542
57338	0.946131	0.633555
57339	0.946131	0.643985
57340	2.71891	0.573967
57341	1.89226	0.573977
57342	2.09871	0.506652
57343	2.09871	0.717092
57344	1.89226	0.477215
57345	1.89226	0.698533
57346	1.89227	0.72371
57347	1.89227	0.713671
57348	1.89227	0.641885
57349	1.89227	0.86884
57350	1.89227	0.644065
57351	1.89227	0.869635
57352	0.946129	0.715063
57353	0.946129	0.636966
57354	0.946133	0.5861
57355	0.946133	0.535358
57356	0.946131	0.583272
57357	0.946131	0.686531
57358	0.946131	0.545491
57359	0.946131	0.648934

57360	0.946131	0.611649
57361	0.946131	0.607027
57362	0.946131	0.616059
57363	0.946131	0.61192
57364	2.32462	0.124812
57365	1.89226	0.0912796
57366	1.89226	0.0899561
57367	1.89226	0.102518
57368	2.2291	0.110546
57369	2.2291	0.129644
57370	0.847669	0.0844362
57371	0.847669	0.0907274
57372	0.796966	0.0878914
57373	0.796966	0.100067
57374	0.833552	0.0816877
57375	1.01049	0.0898964
57376	0.709709	0.0831759
57377	0.634147	0.0835732
57378	0.634148	0.0787193
57379	0.833549	0.090284
57380	0.709711	0.0896284
57381	1.01049	0.0997103
57382	1.89226	0.0855596
57383	1.89226	0.118647
57384	1.89226	0.0957084
57385	1.89226	0.114732
57386	1.89226	0.0889891
57387	1.89226	0.102265
57388	1.03611	0.0831016
57389	0.936655	0.0909302
57390	0.946132	0.0899684
57391	0.946132	0.105457
57392	0.941918	0.0838833
57393	0.941918	0.0895953
57394	1.00308	0.0947123
57395	1.04117	0.0896398
57396	1.1406	0.0766758
57397	1.04074	0.0847401
57398	0.851529	0.0900792
57399	1.00311	0.110242
57400	1.01814	0.0729718
57401	1.6671	0.087033
57402	1.35561	0.0740208
57403	1.35561	0.0844579
57404	1.2683	0.0708302
57405	1.2683	0.0749971
57406	0.946128	0.0874315
57407	0.946128	0.0740044
57408	0.946129	0.0800269
57409	0.946129	0.0943266
57410	0.946132	0.087904
57411	0.946132	0.0806372
57412	0.946126	0.0813474
57413	0.946126	0.0859559
57414	0.946129	0.0716839
57415	0.946129	0.0794354
57416	0.946129	0.0837041
57417	0.946129	0.0952645

57418	2.23338	0.0941703
57419	1.96	0.0773889
57420	2.03438	0.0771599
57421	1.87835	0.0882401
57422	4.11172	0.0861226
57423	1.96	0.0769078
57424	1.52661	0.0846352
57425	1.87834	0.0880292
57426	1.53175	0.0739986
57427	3.40495	0.0774379
57428	2.03435	0.0797841
57429	1.65813	0.0901859
57430	1.65813	0.0949801
57431	1.87832	0.0821488
57432	1.72067	0.0920855
57433	1.94086	0.0780772
57434	1.65813	0.0904523
57435	1.40572	0.0749082
57436	1.87832	0.0845779
57437	1.87832	0.0789097
57438	1.31154	0.0793086
57439	1.53173	0.0828395
57440	1.86662	2.1
57441	2.0815	2.1
57442	2.0815	2.1
57443	2.0815	2.1
57444	1.87246	2.1
57445	1.87362	2.1
57446	1.0273	2.1
57447	0.996347	2.1
57448	0.946129	2.1
57449	0.946129	2.1
57450	1.04427	2.1
57451	1.04196	2.1
57452	0.910674	2.1
57453	0.910674	2.1
57454	1.04072	2.1
57455	1.10931	2.1
57456	1.03132	2.1
57457	0.933698	2.1
57458	2.63789	0.439446
57459	1.89226	0.539072
57460	2.3033	0.429769
57461	2.3033	0.53626
57462	1.89226	0.41155
57463	1.89226	0.542046
57464	1.89226	0.585803
57465	1.89226	0.631019
57466	1.89226	0.558682
57467	1.89226	0.599023
57468	3.78452	0.626464
57469	1.51898	0.536972
57470	1.36006	0.621477
57471	2.03143	0.664425
57472	2.03143	0.6151
57473	1.04026	0.589598
57474	1.04026	0.573092
57475	0.94613	0.420232

57476	0.94613	0.466298
57477	1.31894	0.380867
57478	1.31894	0.435218
57479	0.94613	0.373341
57480	0.94613	0.485113
57481	1.31894	0.420271
57482	1.41232	0.464536
57483	0.946129	0.424392
57484	1.41232	0.437705
57485	1.31895	0.426878
57486	0.946129	0.423435
57487	1.89226	0.476679
57488	1.89226	0.551504
57489	1.89226	0.43994
57490	1.89226	0.559381
57491	1.89226	0.406572
57492	1.89226	0.556544
57493	1.89804	0.445092
57494	2.03143	0.473104
57495	2.46286	0.540657
57496	2.46287	0.462078
57497	1.51898	0.473895
57498	1.51898	0.418345
57499	1.89226	2.1
57500	1.89226	2.1
57501	1.89226	2.1
57502	1.89226	2.1
57503	3.78452	2.1
57504	1.3864	2.1
57505	1.3864	2.1
57506	0.946132	2.1
57507	0.946132	2.1
57508	1.32905	2.1
57509	1.3864	2.1
57510	0.946132	2.1
57511	0.946132	2.1
57512	0.946132	2.1
57513	1.32905	2.1
57514	0.946133	2.1
57515	1.3864	2.1
57516	2.77279	2.1
57517	1.67883	2.1
57518	1.67883	2.1
57519	1.73415	2.1
57520	2.65811	2.1
57521	2.71343	2.1
57522	0.946131	2.1
57523	0.946131	2.1
57524	0.946128	2.1
57525	0.946128	2.1
57526	0.946127	2.1
57527	0.946127	2.1
57528	0.946131	2.1
57529	0.946131	2.1
57530	0.946129	2.1
57531	0.946129	2.1
57532	0.94613	2.1
57533	0.94613	2.1

57534	1.89226	2.1
57535	1.89226	2.1
57536	1.89226	2.1
57537	1.89226	2.1
57538	3.78452	2.1
57539	1.89226	2.1
57540	2.54394	2.1
57541	2.54394	2.1
57542	2.65811	2.1
57543	1.89226	2.1
57544	2.00643	2.1
57545	2.54394	2.1
57546	2.6581	2.1
57547	1.62743	2.1
57548	1.62743	2.1
57549	1.67883	2.1
57550	2.59533	2.1
57551	1.89226	2.1
57552	1.89226	2.1
57553	1.89226	2.1
57554	1.89226	2.1
57555	1.89226	2.1
57556	1.89226	2.1
57557	1.89226	1.29336
57558	1.89226	1.14926
57559	1.89226	1.81449
57560	1.89226	0.909021
57561	1.89226	1.63466
57562	1.89226	0.873057
57563	1.89226	1.30943
57564	1.89226	1.13354
57565	1.89226	1.76038
57566	1.89226	1.01693
57567	1.89226	1.56757
57568	1.89226	0.929823
57569	1.89226	0.892431
57570	1.89226	0.703309
57571	1.89226	1.28504
57572	1.89226	0.656665
57573	1.89226	1.1494
57574	1.89226	0.647926
57575	2.68537	0.347054
57576	2.22575	0.501322
57577	2.47464	0.224816
57578	1.79093	0.377263
57579	4.70039	0.322659
57580	2.81217	0.591778
57581	2.47464	0.298841
57582	2.58286	0.53751
57583	2.29193	0.174818
57584	5.0575	0.332089
57585	2.47464	0.312436
57586	2.20365	0.451192
57587	2.29193	0.272374
57588	1.90104	0.47766
57589	4.37568	0.32827
57590	1.89226	0.63556
57591	2.16957	2.09749

57592	2.52823	1.04753
57593	2.52823	2.00679
57594	1.89226	0.856067
57595	1.89226	1.40695
57596	1.36226	2.1
57597	1.55369	2.1
57598	1.33309	2.1
57599	1.62593	2.1
57600	2.98819	2.1
57601	0.802281	2.1
57602	0.802281	2.1
57603	0.835143	2.1
57604	0.835143	2.1
57605	0.824458	2.1
57606	0.92641	2.1
57607	0.776847	2.1
57608	0.812967	2.1
57609	0.812967	2.1
57610	0.926411	2.1
57611	0.776847	2.1
57612	0.824458	2.1
57613	1.33309	2.1
57614	1.62593	2.1
57615	1.32813	2.1
57616	1.7237	2.1
57617	2.95406	2.1
57618	0.814544	2.1
57619	0.875312	2.1
57620	0.910502	2.1
57621	0.910502	2.1
57622	0.886633	2.1
57623	0.975739	2.1
57624	0.792623	2.1
57625	0.840283	2.1
57626	0.796372	2.1
57627	1.06159	2.1
57628	0.81948	2.1
57629	0.933833	2.1
57630	2.63997	2.1
57631	1.29694	2.1
57632	1.28141	2.1
57633	2.62623	2.1
57634	3.92317	2.1
57635	2.63998	2.1
57636	1.89226	2.1
57637	1.89226	2.1
57638	2.62623	2.1
57639	4.5185	2.1
57640	1.89226	2.1
57641	1.89226	2.1
57642	1.89226	2.1
57643	1.89226	1.5696
57644	3.78453	2.1
57645	1.89226	2.1
57646	1.89226	2.01963
57647	1.89226	2.1
57648	1.89226	2.05444
57649	1.89226	2.1

57650	1.89226	2.1
57651	1.25468	2.1
57652	2.62623	2.1
57653	1.28141	2.1
57654	1.28141	2.1
57655	2.60665	2.1
57656	2.60665	2.1
57657	1.89226	2.1
57658	2.62623	2.1
57659	2.60666	2.1
57660	2.62623	2.1
57661	1.87269	2.1
57662	1.89226	2.1
57663	1.89226	2.1
57664	1.89226	2.09026
57665	1.89226	2.1
57666	1.89226	2.03442
57667	3.78453	2.1
57668	1.89226	2.1
57669	1.89226	1.56702
57670	1.89226	2.1
57671	1.89226	2.1
57672	1.89226	2.1
57673	1.89226	1.80972
57674	2.30147	1.29695
57675	2.27703	1.76747
57676	1.89227	1.17401
57677	1.89227	1.58182
57678	4.1693	1.50062
57679	1.38617	1.25033
57680	1.24613	1.37647
57681	2.59886	1.13842
57682	2.6238	1.40895
57683	3.86993	1.14709
57684	0.946133	1.41024
57685	0.946133	0.972284
57686	0.946132	0.974874
57687	0.946132	1.73434
57688	0.946133	1.70372
57689	0.946133	1.54085
57690	0.946132	0.954925
57691	0.946132	1.09271
57692	0.946133	1.26057
57693	0.946133	1.21201
57694	0.946132	1.10701
57695	0.946132	1.12822
57696	2.21773	2.1
57697	2.59216	2.1
57698	1.68541	2.1
57699	1.68541	2.1
57700	1.84814	2.1
57701	2.38046	2.1
57702	1.89966	2.1
57703	1.70303	2.1
57704	1.2028	2.1
57705	1.29897	2.1
57706	2.02873	2.1
57707	2.19093	2.1

57708	2.75218	2.1
57709	1.70303	2.1
57710	3.10254	2.1
57711	2.24783	2.1
57712	2.56839	2.1
57713	1.70303	2.1
57714	2.56839	2.1
57715	1.08276	2.1
57716	2.24061	2.1
57717	1.67491	2.1
57718	1.60948	2.1
57719	1.08276	2.1
57720	1.89226	0.884861
57721	1.89226	1.02834
57722	1.89226	0.916916
57723	1.89226	0.87033
57724	3.78453	0.734612
57725	1.89226	0.812677
57726	1.89226	0.995125
57727	1.89226	0.847993
57728	1.89226	0.869107
57729	3.78452	1.3112
57730	1.89226	0.983672
57731	2.7535	0.671217
57732	1.89226	1.18056
57733	1.89226	1.01272
57734	1.89226	0.798477
57735	1.89226	0.797652
57736	1.89226	1.01734
57737	1.89226	0.860466
57738	1.89226	0.660401
57739	1.89226	1.01247
57740	1.89226	0.820151
57741	1.89226	1.35354
57742	1.89227	1.00721
57743	1.89227	0.889706
57744	1.89227	0.903748
57745	1.89227	0.833655
57746	3.78453	1.44071
57747	1.89226	0.929802
57748	2.53412	0.576907
57749	1.89226	1.50101
57750	1.89226	0.71563
57751	1.89226	0.837698
57752	1.89226	0.667072
57753	2.21126	0.204432
57754	1.89226	0.244119
57755	2.57464	0.244149
57756	1.89226	0.248861
57757	2.57464	0.1924
57758	1.89226	0.205215
57759	1.89226	0.294019
57760	1.89226	0.329169
57761	1.89226	0.708703
57762	1.89226	0.305045
57763	1.89226	0.705803
57764	1.89226	0.275395
57765	1.1418	0.182489

57766	0.653021	0.133202
57767	1.37282	0.175513
57768	0.931386	0.19256
57769	1.34568	0.235338
57770	1.17669	0.167768
57771	0.666595	0.158636
57772	0.786895	0.197548
57773	0.933982	0.19582
57774	1.05351	0.211691
57775	1.00792	0.155874
57776	1.00792	0.166042
57777	1.89226	2.1
57778	1.89226	2.1
57779	1.89226	2.1
57780	1.89226	2.1
57781	3.78452	2.1
57782	1.89226	2.1
57783	1.89226	2.1
57784	1.89226	2.1
57785	1.89226	2.1
57786	1.89226	2.1
57787	1.89226	2.1
57788	1.65642	2.1
57789	1.30248	2.1
57790	1.38888	2.1
57791	1.25987	2.1
57792	1.70611	2.1
57793	1.65642	2.1
57794	1.65642	2.1
57795	1.81407	2.1
57796	2.19428	2.1
57797	1.92555	2.1
57798	3.8507	2.1
57799	1.89226	2.1
57800	1.89226	2.1
57801	1.89226	2.1
57802	1.89226	2.1
57803	3.78452	2.1
57804	1.89226	2.1
57805	1.89226	2.1
57806	1.89226	2.1
57807	1.89226	2.1
57808	1.89226	2.1
57809	1.89226	2.1
57810	0.705495	2.1
57811	0.809317	2.1
57812	1.1655	2.1
57813	1.09605	2.1
57814	0.983375	2.1
57815	0.797301	2.1
57816	0.906633	2.1
57817	0.906633	2.1
57818	0.85085	2.1
57819	1.16548	2.1
57820	0.705501	2.1
57821	1.00314	2.1
57822	2.19428	2.1
57823	2.31862	2.1

57824	2.27408	2.1
57825	2.45893	2.1
57826	4.46836	2.1
57827	1.89226	0.746337
57828	1.89226	0.972499
57829	1.89226	0.966462
57830	1.89226	0.736298
57831	1.89226	1.20024
57832	1.89226	0.92301
57833	2.0815	0.754794
57834	2.22136	0.828729
57835	1.5515	0.777284
57836	1.86323	0.665581
57837	2.74986	1.0108
57838	3.30238	0.772588
57839	1.89224	0.749771
57840	1.89224	0.927256
57841	1.89224	1.00316
57842	1.89224	0.713185
57843	1.89224	1.2609
57844	1.89224	0.850927
57845	1.89224	0.736066
57846	1.89224	1.05111
57847	1.89224	0.85493
57848	1.89224	0.684421
57849	1.89224	1.18879
57850	1.89224	0.865245
57851	1.17846	0.671082
57852	0.810935	0.694618
57853	0.93347	1.08376
57854	0.93347	0.694426
57855	0.810932	0.832331
57856	0.810932	0.725915
57857	2.22133	0.739721
57858	1.71919	0.735175
57859	1.32629	0.650062
57860	1.0813	0.83748
57861	1.57137	0.729568
57862	2.21149	2.1
57863	1.89226	1.85201
57864	1.89226	2.1
57865	1.89226	1.52477
57866	2.17159	2.1
57867	2.17159	1.67849
57868	0.946131	0.596738
57869	0.946131	2.1
57870	0.946131	1.54883
57871	0.946131	1.97754
57872	0.94613	1.07283
57873	0.94613	1.68102
57874	0.946132	1.96483
57875	0.946132	1.76125
57876	0.946131	1.2808
57877	0.946131	1.1302
57878	0.946131	1.56966
57879	0.946131	1.46282
57880	1.12266	1.5419
57881	1.4714	1.68963

57882	1.62322	1.42303
57883	1.62322	1.60034
57884	1.02958	1.67883
57885	1.02958	1.99587
57886	2.2671	2.1
57887	1.89226	1.93739
57888	1.89226	2.1
57889	1.89226	1.71965
57890	2.21149	2.1
57891	2.21149	1.95672
57892	1.89226	1.71113
57893	1.89226	1.50862
57894	1.89226	1.54225
57895	1.89226	1.24683
57896	1.89226	1.8753
57897	1.89226	1.77509
57898	0.736914	1.67994
57899	0.736914	1.70966
57900	0.822839	1.75085
57901	0.822836	2.1
57902	0.791298	0.96012
57903	1.00003	1.74669
57904	0.640254	1.72601
57905	0.575379	1.9012
57906	0.609001	1.30762
57907	1.00005	1.26608
57908	0.575371	1.77782
57909	0.831908	1.3753
57910	1.89226	2.1
57911	1.89226	2.1
57912	1.89226	2.1
57913	1.89226	2.1
57914	1.89226	2.1
57915	1.89226	2.1
57916	1.89226	2.1
57917	1.89226	2.1
57918	1.89226	2.1
57919	1.89226	2.1
57920	1.89226	2.1
57921	1.89226	2.1
57922	0.730617	2.1
57923	0.71052	2.1
57924	0.7329	2.1
57925	0.712083	2.1
57926	0.701051	2.1
57927	0.701051	2.1
57928	0.721552	2.1
57929	0.721552	2.1
57930	0.710521	2.1
57931	0.732898	2.1
57932	0.730619	2.1
57933	0.712081	2.1
57934	0.7329	2.1
57935	0.712082	2.1
57936	0.559601	2.1
57937	0.495993	2.1
57938	0.603718	2.1
57939	0.603718	2.1

57940	0.625174	2.1
57941	0.625174	2.1
57942	0.732899	2.1
57943	0.559601	2.1
57944	0.712082	2.1
57945	0.495993	2.1
57946	0.94613	2.1
57947	0.94613	2.1
57948	0.94613	2.1
57949	0.94613	2.1
57950	0.946131	2.1
57951	0.946131	2.1
57952	0.946129	2.1
57953	0.946129	2.1
57954	0.94613	2.1
57955	0.94613	2.1
57956	0.94613	2.1
57957	0.94613	2.1
57958	1.89226	2.1
57959	1.89226	2.1
57960	1.89226	2.1
57961	1.89226	2.1
57962	1.89226	2.1
57963	1.89226	2.1
57964	1.42104	2.1
57965	1.4658	2.1
57966	1.51223	2.1
57967	1.42283	2.1
57968	2.93327	2.1
57969	0.732899	2.1
57970	0.756115	2.1
57971	0.629763	2.1
57972	0.559601	2.1
57973	0.646134	2.1
57974	0.646134	2.1
57975	0.669582	2.1
57976	0.669582	2.1
57977	0.756115	2.1
57978	0.629763	2.1
57979	0.732899	2.1
57980	0.559601	2.1
57981	2.566 2.1	
57982	1.20242	1.42381
57983	2.64084	2.1
57984	2.18297	1.86714
57985	1.66029	1.98913
57986	1.20242	1.31111
57987	0.946133	1.18072
57988	0.946133	1.38206
57989	0.946129	2.00546
57990	0.946129	1.91289
57991	0.946131	1.79233
57992	0.946131	1.09831
57993	0.946131	0.514783
57994	0.946131	1.75551
57995	0.946131	0.883239
57996	0.946131	1.39658
57997	0.946131	1.25796

57998	0.946131	1.33794
57999	1.89226	0.082813
58000	1.89226	0.106778
58001	1.89226	0.0954683
58002	1.89226	0.0894454
58003	3.78453	0.0945113
58004	1.03073	0.0718256
58005	1.03073	0.0726245
58006	1.04066	0.0799406
58007	1.04066	0.0877654
58008	1.20802	0.0856357
58009	1.32689	0.0724511
58010	0.883301	0.0792977
58011	0.744506	0.0716068
58012	1.20802	0.0782931
58013	0.744506	0.0774548
58014	0.883301	0.0749877
58015	1.32689	0.0767812
58016	1.20802	0.0806809
58017	1.20802	0.0740788
58018	0.946131	0.0889691
58019	0.946131	0.0984188
58020	0.94613	0.097543
58021	0.94613	0.0805931
58022	1.20802	0.0884786
58023	1.32689	0.0775675
58024	1.20802	0.0831731
58025	0.946131	0.085069
58026	0.946131	0.0794309
58027	1.32689	0.0830472
58028	1.89226	0.0908407
58029	1.89226	0.122314
58030	1.89226	0.108193
58031	1.89226	0.103307
58032	1.89226	0.107012
58033	1.89226	0.101366
58034	0.946119	0.101791
58035	0.946119	0.091527
58036	0.946118	0.111848
58037	0.946118	0.130501
58038	0.946118	0.129307
58039	0.946118	0.0992266
58040	0.946119	0.0909343
58041	0.946119	0.10928
58042	0.946131	0.108288
58043	0.946131	0.108369
58044	0.946106	0.107635
58045	0.946106	0.108346
58046	1.3939	2.1
58047	1.49641	2.1
58048	1.15096	2.1
58049	1.10101	2.1
58050	2.59742	2.1
58051	1.22106	2.1
58052	2.60663	2.1
58053	1.25467	2.1
58054	1.25467	2.1
58055	2.58377	2.1

58056	2.58377	2.1
58057	1.89225	2.1
58058	2.60664	2.1
58059	2.58377	2.1
58060	2.60664	2.1
58061	1.86938	2.1
58062	1.89225	2.1
58063	1.89225	2.1
58064	1.89225	1.96123
58065	1.89225	2.1
58066	1.89225	2.1
58067	1.89225	2.1
58068	1.89225	2.1
58069	0.946121	1.90652
58070	0.946121	2.1
58071	0.946126	2.1
58072	0.946126	1.98988
58073	0.946124	1.63474
58074	0.946124	1.56723
58075	0.946123	2.1
58076	0.946123	2.1
58077	0.946117	2.1
58078	0.946117	2.1
58079	0.94613	2.1
58080	0.94613	2.1
58081	1.28017	2.1
58082	1.29189	2.1
58083	0.610534	2.1
58084	0.592373	2.1
58085	0.943764	2.1
58086	0.943764	2.1
58087	0.940501	2.1
58088	0.940501	2.1
58089	1.28017	2.1
58090	0.592371	2.1
58091	0.610533	2.1
58092	1.29189	2.1
58093	0.946133	2.1
58094	0.946133	2.1
58095	1.28017	2.1
58096	1.29189	2.1
58097	0.946132	2.1
58098	0.946132	2.1
58099	1.28017	2.1
58100	1.28017	2.1
58101	1.28017	2.1
58102	0.946132	2.1
58103	0.946133	2.1
58104	1.2919	2.1
58105	1.89226	2.1
58106	1.89226	2.1
58107	1.89226	2.1
58108	1.89226	2.1
58109	1.89226	2.1
58110	1.89226	2.09351
58111	1.89226	2.1
58112	1.89226	2.1
58113	1.89226	2.1

58114	1.89226	2.1
58115	3.78452	2.1
58116	1.89226	2.1
58117	2.08196	2.1
58118	1.97854	2.1
58119	2.08196	2.1
58120	1.78884	2.1
58121	1.89226	2.1
58122	1.89226	2.1
58123	1.89226	2.1
58124	1.89226	2.1
58125	1.89226	2.1
58126	1.89226	2.1
58127	1.89226	2.1
58128	1.83688	2.1
58129	1.89226	2.1
58130	1.89226	2.1
58131	1.89226	2.1
58132	1.86015	2.1
58133	1.86015	2.1
58134	1.89226	2.1
58135	1.97854	2.1
58136	1.85883	2.1
58137	1.97854	2.1
58138	1.77255	2.1
58139	1.89226	2.1
58140	0.946131	1.75793
58141	0.946131	2.1
58142	0.946131	2.1
58143	0.946131	2.1
58144	0.94613	2.1
58145	0.94613	2.08497
58146	0.946132	2.1
58147	0.946132	2.1
58148	0.946131	2.1
58149	0.946131	2.1
58150	0.946131	2.1
58151	0.946131	2.1
58152	0.918412	2.1
58153	0.918412	2.1
58154	0.94893	2.1
58155	0.750211	2.1
58156	0.851541	2.1
58157	0.851541	2.1
58158	0.970566	2.1
58159	0.826567	2.1
58160	0.970554	2.1
58161	1.04071	2.1
58162	0.826632	2.1
58163	0.851554	2.1
58164	1.89226	0.141619
58165	1.89226	0.165633
58166	1.89226	0.135468
58167	1.89226	0.131947
58168	3.78453	0.1533
58169	0.946131	0.131024
58170	0.946131	0.193537
58171	0.811232	0.161227

58172	0.811232	0.141677
58173	0.94613	0.177393
58174	0.94613	0.176831
58175	0.959701	0.170328
58176	0.811233	0.199133
58177	0.94613	0.129262
58178	0.811233	0.14585
58179	0.959702	0.150847
58180	0.946131	0.185447
58181	1.89226	0.125942
58182	1.89226	0.149879
58183	1.89226	0.142252
58184	1.89226	0.134727
58185	1.89226	0.120057
58186	1.89226	0.121035
58187	1.89227	0.119644
58188	1.89227	0.150568
58189	1.89227	0.131757
58190	1.89227	0.137741
58191	1.89227	0.130511
58192	1.89227	0.137917
58193	1.89226	0.133179
58194	1.89226	0.126062
58195	1.89226	0.108125
58196	1.89226	0.10368
58197	3.78453	0.109067
58198	1.89226	0.112991
58199	1.89226	0.101633
58200	1.89226	0.103765
58201	1.89226	0.121419
58202	1.89226	0.0988713
58203	1.89226	0.11607
58204	1.89226	2.1
58205	2.36204	2.1
58206	2.39853	2.1
58207	2.36204	2.1
58208	1.92876	2.1
58209	1.89226	2.1
58210	1.89226	2.1
58211	1.89226	1.09477
58212	1.89226	2.1
58213	1.89226	0.614079
58214	3.78453	2.00637
58215	0.946128	2.1
58216	0.946128	0.630523
58217	1.13182	0.940572
58218	1.13182	2.08664
58219	0.94613	0.903037
58220	0.94613	1.08067
58221	1.1749	2.1
58222	1.13182	2.1
58223	1.1749	2.03536
58224	0.94613	1.77045
58225	1.13182	1.79828
58226	0.94613	1.87858
58227	1.89226	2.1
58228	2.39853	2.1
58229	2.44918	2.1

58230	2.39853	2.1
58231	1.94291	2.1
58232	1.89226	2.1
58233	0.946132	0.71548
58234	0.946132	2.1
58235	0.946131	2.1
58236	0.946131	1.25707
58237	0.946132	0.990452
58238	0.946132	1.52362
58239	0.946131	2.1
58240	0.946131	2.1
58241	0.946131	2.08714
58242	0.946131	2.1
58243	0.946132	1.96397
58244	0.946132	2.1
58245	0.946128	2.1
58246	0.946128	1.69519
58247	1.1749	0.9946
58248	1.1749	2.1
58249	0.946131	0.992016
58250	0.946131	1.09212
58251	1.23631	2.1
58252	1.1749	2.1
58253	1.23631	2.0702
58254	0.94613	2.02948
58255	1.1749	1.74912
58256	0.946131	1.04182
58257	1.89226	0.651492
58258	1.89226	2.1
58259	1.89226	1.45526
58260	1.89226	1.45072
58261	3.78453	1.69367
58262	1.38859	2.1
58263	1.45333	2.1
58264	1.28909	1.30083
58265	1.38091	2.1
58266	2.74242	2.1
58267	1.45332	2.1
58268	1.38414	2.1
58269	1.38091	2.1
58270	1.33506	2.1
58271	2.78838	2.1
58272	1.89226	2.1
58273	1.89226	2.1
58274	1.89226	1.50676
58275	1.89226	2.1
58276	3.78452	2.1
58277	1.89226	1.66548
58278	1.89226	1.54985
58279	1.89226	0.750699
58280	1.89226	2.1
58281	1.89226	0.784638
58282	1.89226	2.1
58283	1.17418	1.59211
58284	1.38091	2.1
58285	1.17418	2.1
58286	1.28909	2.03991
58287	1.29741	2.1

58288	1.41232	2.1
58289	1.38091	2.1
58290	1.33506	2.1
58291	1.27575	2.1
58292	1.27575	2.1
58293	1.29741	2.1
58294	1.35671	2.1
58295	1.89226	1.60052
58296	1.89226	2.1
58297	1.89226	2.1
58298	1.89226	2.1
58299	3.78452	2.1
58300	1.92737	0.236
58301	2.40223	0.301128
58302	1.56178	0.230984
58303	1.97605	0.311987
58304	3.90343	0.231491
58305	1.89226	0.260768
58306	1.89226	0.38499
58307	1.89226	0.303688
58308	1.89226	0.359023
58309	3.78452	0.367365
58310	1.89226	0.386663
58311	1.89226	0.44044
58312	1.89226	0.418225
58313	1.89226	0.46628
58314	1.89226	0.360987
58315	1.89226	0.407878
58316	1.13641	0.356551
58317	1.13641	0.317284
58318	1.12763	0.300075
58319	1.12763	0.375888
58320	1.29348	0.376062
58321	1.06293	0.391976
58322	1.20111	0.296275
58323	0.988028	0.318833
58324	0.988028	0.336733
58325	1.06293	0.351334
58326	1.29348	0.339814
58327	1.20111	0.317979
58328	1.56176	0.210508
58329	1.46387	0.284731
58330	1.97603	0.2543
58331	1.97603	0.234307
58332	1.08211	0.251686
58333	1.08211	0.225153
58334	1.89224	0.269785
58335	1.89224	0.321638
58336	1.89224	0.295933
58337	1.89224	0.339882
58338	1.89224	0.253072
58339	1.89224	0.295238
58340	1.89224	0.342396
58341	1.89224	0.41276
58342	1.89224	0.326039
58343	1.89224	0.420472
58344	3.78448	0.33738
58345	0.820185	0.256894

58346	0.818947	0.354006
58347	0.911041	0.359442
58348	0.695265	0.355138
58349	1.46388	0.28891
58350	1.23607	0.306517
58351	0.927068	0.304764
58352	1.02691	0.352215
58353	1.12918	0.259574
58354	0.846843	0.290926
58355	0.911039	0.321872
58356	1.29129	2.1
58357	1.45547	2.1
58358	1.67327	2.1
58359	1.66698	2.1
58360	1.20149	2.1
58361	1.20149	2.1
58362	0.946133	0.807585
58363	0.946133	2.02849
58364	0.946129	2.1
58365	0.946129	2.1
58366	0.946131	1.91443
58367	0.946131	0.801955
58368	0.946131	2.1
58369	0.946131	1.68454
58370	0.946131	2.01202
58371	0.946131	1.97569
58372	0.946131	1.87688
58373	0.946131	1.9898
58374	1.89226	1.64616
58375	1.89226	1.53209
58376	1.89226	2.0126
58377	1.89226	0.824367
58378	3.78452	1.30999
58379	0.86875	1.42111
58380	0.86875	2.05651
58381	0.899002	2.1
58382	0.966245	2.1
58383	0.849364	0.922736
58384	1.01918	2.03425
58385	0.764737	2.1
58386	0.89249	2.0389
58387	0.764624	1.97843
58388	0.967869	2.06652
58389	0.808606	1.91527
58390	0.864506	1.67078
58391	0.773929	2.1
58392	0.773929	2.1
58393	0.781991	2.1
58394	0.836559	2.1
58395	0.983761	2.1
58396	0.834687	2.1
58397	0.680545	2.1
58398	0.640799	2.1
58399	0.70286	2.1
58400	0.935844	2.1
58401	0.629479	2.1
58402	0.823655	2.1
58403	0.946133	0.927572

58404	0.946133	2.03593
58405	0.946128	2.1
58406	0.946128	2.1
58407	0.94613	2.1
58408	0.94613	0.885973
58409	0.946131	2.1
58410	0.946131	1.91041
58411	0.946131	2.1
58412	0.946131	2.1
58413	0.946131	2.1
58414	0.946131	2.1
58415	1.89226	2.1
58416	1.89226	0.874432
58417	1.89226	1.52696
58418	1.89226	1.33039
58419	1.89226	1.06187
58420	1.89226	1.2162
58421	0.931638	2.1
58422	0.931638	1.14839
58423	1.03559	2.1
58424	0.977442	1.86078
58425	0.826487	1.37182
58426	0.993902	1.23491
58427	1.04922	2.1
58428	0.894324	2.1
58429	0.912984	2.04286
58430	1.01156	2.1
58431	0.944692	1.70568
58432	1.15456	1.79312
58433	1.89226	0.135494
58434	1.89226	0.169428
58435	1.89226	0.215451
58436	1.89226	0.121271
58437	3.78453	0.129653
58438	1.89226	0.133115
58439	1.89226	0.11938
58440	2.64262	0.135985
58441	2.64262	0.106982
58442	1.78052	0.0990768
58443	1.03015	0.114171
58444	0.999721	0.111308
58445	0.659011	0.100399
58446	1.15564	0.163511
58447	1.41388	0.119347
58448	1.01583	0.120221
58449	1.01583	0.105481
58450	1.05706	0.16178
58451	1.05706	0.120004
58452	1.15564	0.117407
58453	0.65901	0.112764
58454	1.41388	0.119615
58455	0.999721	0.116326
58456	1.89226	2.1
58457	1.89226	2.1
58458	1.89226	2.1
58459	1.89226	2.1
58460	3.78453	2.1
58461	2.39022	2.1

58462	1.90734	2.1
58463	2.27634	2.1
58464	1.82666	2.1
58465	4.18368	2.1
58466	1.90734	2.1
58467	1.21564	2.1
58468	1.82666	2.1
58469	1.16938	2.1
58470	3.07672	2.1
58471	1.89226	2.1
58472	1.89226	2.1
58473	1.89226	2.1
58474	1.89226	2.1
58475	1.89226	2.1
58476	1.89226	2.1
58477	1.89227	2.1
58478	1.89227	2.1
58479	1.89227	2.1
58480	1.89227	2.1
58481	3.78453	2.1
58482	2.27634	2.1
58483	1.82666	2.1
58484	2.18042	2.1
58485	1.74738	2.1
58486	4.02372	2.1
58487	1.74738	2.1
58488	1.82666	2.1
58489	1.1181	2.1
58490	1.1181	2.1
58491	1.16938	2.1
58492	1.79865	2.1
58493	1.89226	2.1
58494	1.89226	2.1
58495	1.89226	2.1
58496	1.89226	2.1
58497	1.89226	2.1
58498	1.89226	2.1
58499	1.89226	1.13087
58500	1.89226	0.740333
58501	1.89226	0.897571
58502	1.89226	0.869987
58503	1.89226	1.21963
58504	1.89226	0.862617
58505	0.790736	0.904808
58506	1.08265	0.814393
58507	0.938909	1.11308
58508	0.638683	0.888083
58509	0.858588	0.972507
58510	0.858588	0.769749
58511	0.862743	1.15905
58512	0.862743	0.975882
58513	1.08265	1.06287
58514	0.938911	0.742608
58515	0.790735	1.06411
58516	0.638685	0.782224
58517	1.89226	0.744863
58518	1.89226	1.06782
58519	1.89226	0.935618

58520	1.89226	0.757685
58521	1.89226	1.20183
58522	1.89226	1.01263
58523	1.89226	0.816638
58524	1.89226	1.10369
58525	1.89226	0.793154
58526	1.89226	0.781037
58527	1.89226	1.02073
58528	1.89226	1.04245
58529	1.08265	0.732132
58530	1.36209	0.705879
58531	1.25233	0.770695
58532	0.938909	0.860619
58533	1.14195	0.716483
58534	1.14195	0.64252
58535	1.15904	1.11103
58536	1.15904	0.809691
58537	1.36209	0.943518
58538	1.25233	0.608808
58539	1.08265	0.927328
58540	0.938911	0.67713
58541	1.22077	0.70392
58542	0.915244	0.748577
58543	0.967835	0.939899
58544	0.742495	0.90977
58545	0.78648	0.882841
58546	0.985041	0.724673
58547	0.989098	1.19018
58548	0.989098	0.839165
58549	1.03206	0.770018
58550	0.967897	1.02518
58551	0.915404	1.00668
58552	0.706105	0.78723
58553	2.69662	0.0911257
58554	1.89226	0.102951
58555	2.35499	0.0826592
58556	1.89226	0.0724577
58557	4.24725	0.0903353
58558	1.89227	0.0945172
58559	1.89227	0.0986226
58560	1.89227	0.078234
58561	1.89227	0.0824585
58562	3.78453	0.0926264
58563	2.35499	0.0817226
58564	1.70302	0.064712
58565	1.70302	0.0682535
58566	1.703	0.0714413
58567	1.97374	0.0698084
58568	1.97372	0.0736552
58569	0.946133	0.0778397
58570	0.946133	0.0677217
58571	0.946132	0.0715297
58572	0.946132	0.081334
58573	0.946133	0.0749655
58574	0.946133	0.074578
58575	0.946132	0.0747468
58576	0.946132	0.074355
58577	0.946133	0.0662215

58578	0.946133	0.0704024
58579	0.946132	0.0766478
58580	0.946132	0.0794029
58581	0.6794	2.1
58582	0.6794	2.1
58583	0.670812	2.1
58584	0.62182	2.1
58585	0.648326	2.1
58586	0.792154	2.1
58587	0.719167	2.1
58588	0.494515	2.1
58589	0.649421	2.1
58590	0.804869	2.1
58591	0.485585	2.1
58592	0.627666	2.1
58593	1.79347	2.1
58594	1.03968	1.38217
58595	1.70303	1.67843
58596	0.933265	1.33484
58597	2.62068	1.16598
58598	1.89226	2.1
58599	1.89226	2.1
58600	1.89226	2.1
58601	1.89226	1.11461
58602	3.78452	2.1
58603	1.89226	1.37254
58604	2.24604	2.1
58605	2.0554	1.92897
58606	2.0554	1.57321
58607	1.89226	2.1
58608	1.89226	1.98625
58609	1.89226	2.09676
58610	1.89226	0.514314
58611	1.89226	0.698469
58612	1.89226	1.54969
58613	1.89226	0.70702
58614	1.89226	1.66151
58615	2.0815	1.80716
58616	2.64424	0.333551
58617	2.80807	0.518401
58618	3.232	1.14046
58619	1.89417	0.554713
58620	2.18012	1.23651
58621	0.946131	1.80947
58622	0.946131	0.539654
58623	0.946131	1.96089
58624	0.946131	0.743894
58625	0.94613	0.713077
58626	0.94613	0.601633
58627	0.946132	1.87022
58628	0.946132	2.04431
58629	0.946131	0.878232
58630	0.946131	0.925074
58631	0.946131	0.853052
58632	0.946131	0.856045
58633	1.89227	0.12055
58634	1.89227	0.156377
58635	1.89227	0.181838

58636	1.89227	0.116544
58637	3.78453	0.126676
58638	2.70881	0.116483
58639	2.68687	0.0810924
58640	2.03046	0.0894534
58641	1.9682	0.109095
58642	4.67701	0.103745
58643	1.08518	0.0774744
58644	1.08518	0.0833495
58645	1.09033	0.10914
58646	1.09033	0.0947481
58647	0.842273	0.0779876
58648	1.19141	0.0902552
58649	1.34343	0.0836613
58650	0.9841	0.105596
58651	0.9841	0.0927599
58652	0.842273	0.0963612
58653	1.34344	0.0952886
58654	1.19141	0.091763
58655	0.946133	0.114587
58656	0.946133	0.102289
58657	0.946132	0.17207
58658	0.946132	0.13401
58659	0.946132	0.12412
58660	0.946132	0.101916
58661	0.946132	0.171198
58662	0.946132	0.132016
58663	0.946132	0.108946
58664	0.946132	0.127679
58665	0.946133	0.140868
58666	0.946133	0.127987
58667	1.89227	0.167206
58668	1.89227	0.106193
58669	1.89227	0.148861
58670	1.89227	0.119246
58671	1.89227	0.147073
58672	1.89227	0.11736
58673	2.03046	0.0919063
58674	1.9682	0.100533
58675	1.23886	0.119073
58676	1.12971	0.0866632
58677	3.20706	0.0918706
58678	1.89226	0.542382
58679	1.89226	0.874551
58680	1.89226	0.503272
58681	1.89226	0.726589
58682	3.78452	0.651435
58683	2.45569	0.393739
58684	1.89226	0.505564
58685	2.22922	0.414166
58686	2.0815	0.610404
58687	1.85074	0.302395
58688	1.70303	0.536202
58689	2.72856	0.390497
58690	1.89226	0.644831
58691	1.89226	0.368742
58692	1.89226	0.560829
58693	3.78452	0.523961

58694	1.89226	0.576463
58695	1.89226	0.76256
58696	1.89226	0.501591
58697	1.89226	0.76648
58698	3.78452	0.627493
58699	0.946131	0.762312
58700	0.946131	0.494934
58701	0.946131	0.664017
58702	0.946131	0.450613
58703	0.94613	0.636521
58704	0.94613	0.697036
58705	0.946132	0.479103
58706	0.946132	0.513381
58707	0.946131	0.577542
58708	0.946131	0.590633
58709	0.946131	0.588679
58710	0.946131	0.583623
58711	2.0595	0.290549
58712	2.58581	0.309439
58713	1.48694	0.20059
58714	1.48694	0.224902
58715	2.60157	0.256164
58716	2.02901	0.231154
58717	1.89226	0.233472
58718	1.89226	0.324121
58719	1.89226	0.198234
58720	1.89226	0.211438
58721	1.89226	0.293694
58722	1.89226	0.315695
58723	2.04728	0.299876
58724	2.64161	0.286611
58725	1.47606	0.218308
58726	1.47606	0.249202
58727	2.63069	0.25893
58728	2.05947	0.237937
58729	1.89224	0.280595
58730	1.89224	0.285194
58731	1.89224	0.312403
58732	1.89224	0.278898
58733	1.89224	0.277713
58734	1.89224	0.236748
58735	0.946114	0.236846
58736	0.946114	0.257478
58737	0.946118	0.302566
58738	0.946118	0.228786
58739	0.946114	0.311652
58740	0.946114	0.277343
58741	0.946118	0.285094
58742	0.946118	0.267286
58743	0.946128	0.314425
58744	0.946128	0.328358
58745	0.946103	0.249181
58746	0.946103	0.24703
58747	1.1233	2.02107
58748	1.15009	2.1
58749	2.36036	2.1
58750	2.38225	2.1
58751	3.50554	2.1

58752	2.38224	2.1
58753	0.977775	2.1
58754	2.20473	2.1
58755	2.20473	2.1
58756	1.15009	2.1
58757	1.15009	2.1
58758	0.946119	1.79517
58759	0.946119	1.81944
58760	1.19111	1.93478
58761	1.19111	1.81245
58762	0.946118	1.86489
58763	1.19111	2.03989
58764	1.18016	1.76907
58765	0.946118	0.997444
58766	0.94613	0.636586
58767	0.94613	1.67868
58768	1.18015	1.72047
58769	1.19109	1.76035
58770	0.946118	2.1
58771	0.946118	2.1
58772	1.10235	2.1
58773	1.10235	2.1
58774	0.946118	2.1
58775	1.10235	2.1
58776	0.946115	1.88317
58777	1.1911	2.07413
58778	0.946129	1.75747
58779	0.946129	2.1
58780	1.19109	2.1
58781	1.10234	2.1
58782	1.89226	2.1
58783	2.23932	2.1
58784	2.11568	2.1
58785	2.23932	2.1
58786	1.76862	2.1
58787	1.89226	2.1
58788	1.89226	2.1
58789	1.89226	2.1
58790	1.89226	2.1
58791	1.89226	2.1
58792	1.89226	2.1
58793	1.89226	1.19575
58794	2.07838	2.1
58795	1.89226	2.1
58796	1.89226	2.1
58797	1.89226	2.1
58798	2.10264	2.1
58799	2.10264	2.1
58800	1.89226	2.1
58801	2.11568	2.1
58802	1.99753	2.1
58803	2.11568	2.1
58804	1.77411	2.1
58805	1.89226	2.08041
58806	0.946128	0.881449
58807	0.946128	2.01132
58808	0.946133	2.1
58809	0.946133	2.1

58810	0.94613	2.1
58811	0.94613	0.843639
58812	0.946131	2.1
58813	0.946131	2.01114
58814	0.946131	2.1
58815	0.946131	2.1
58816	0.946131	2.1
58817	0.946131	2.1
58818	0.946132	2.1
58819	0.946132	2.1
58820	1.03919	2.1
58821	1.03919	2.1
58822	0.946131	2.1
58823	0.946131	2.0233
58824	1.02744	2.1
58825	1.03919	2.1
58826	1.02744	2.1
58827	0.946132	2.1
58828	1.03919	2.1
58829	0.946132	2.1
58830	1.64834	1.73443
58831	1.82955	1.46889
58832	2.33824	1.07726
58833	2.19301	1.74354
58834	3.98658	1.40399
58835	1.40574	1.78563
58836	2.19301	1.32368
58837	1.64834	1.47424
58838	1.64834	1.5579
58839	1.99961	1.09291
58840	1.99961	1.32458
58841	2.33824	1.19952
58842	2.19301	1.7124
58843	2.68524	1.45195
58844	2.81847	1.29819
58845	5.02349	1.50801
58846	1.99961	1.73137
58847	2.19301	1.23132
58848	2.68524	1.23875
58849	2.50732	1.45334
58850	4.68485	1.31718
58851	1.89226	2.097
58852	1.89226	1.05469
58853	1.89226	1.07071
58854	1.89226	2.1
58855	3.78453	1.51216
58856	1.75142	2.09106
58857	2.47026	2.1
58858	1.46811	2.1
58859	2.26652	2.1
58860	4.01794	2.1
58861	1.89226	1.22395
58862	1.89226	2.1
58863	1.89226	1.59099
58864	1.89226	2.1
58865	1.89226	1.44321
58866	1.89226	2.1
58867	1.89226	1.36392

58868	1.89226	2.1
58869	1.89226	1.52323
58870	1.89226	2.1
58871	1.89226	1.27959
58872	1.89226	2.1
58873	0.812082	2.1
58874	0.878759	2.1
58875	0.882782	2.1
58876	0.882782	2.1
58877	1.05572	2.1
58878	1.04037	2.1
58879	0.73849	2.1
58880	0.626857	2.1
58881	0.638677	2.1
58882	1.05581	2.1
58883	0.715695	2.1
58884	1.10492	2.1
58885	1.17058	2.1
58886	1.07863	2.1
58887	1.21113	2.1
58888	1.21113	2.1
58889	1.2811	2.1
58890	1.37319	2.1
58891	1.01788	2.1
58892	0.999492	2.1
58893	1.04352	2.1
58894	1.32112	2.1
58895	1.10492	2.1
58896	1.34699	2.1
58897	1.89226	1.48411
58898	1.89226	2.1
58899	1.89226	1.88188
58900	1.89226	2.1
58901	1.89226	1.62063
58902	1.89226	2.1
58903	1.19813	0.0522355
58904	1.19813	0.0522355
58905	2.43432	0.0522355
58906	1.21716	0.0522355
58907	0.946124	0.0522355
58908	1.21715	0.0522355
58909	0.946118	0.0522355
58910	2.56627	2.1
58911	1.89226	2.1
58912	1.89226	2.1
58913	1.89226	2.1
58914	2.43434	2.1
58915	2.43434	2.1
58916	1.89227	0.0971001
58917	1.89227	0.106521
58918	1.89227	0.104711
58919	1.89227	0.0986036
58920	1.89227	0.103121
58921	1.89227	0.0969448
58922	1.89226	0.108464
58923	1.89226	0.135031
58924	1.89226	0.135394
58925	1.89226	0.0970994

58926	3.78453	0.115644
58927	1.89226	0.0923969
58928	2.0764	0.0773304
58929	1.89226	0.0871522
58930	2.81674	0.084814
58931	1.89226	0.0831385
58932	2.81674	0.0786961
58933	0.946132	0.100621
58934	0.946132	0.0917725
58935	0.946131	0.129612
58936	0.946131	0.109779
58937	0.946132	0.0974818
58938	0.946132	0.104967
58939	0.946132	0.133997
58940	0.946132	0.0986768
58941	0.946132	0.105658
58942	0.946132	0.109707
58943	0.946132	0.107735
58944	0.946132	0.105103
58945	0.578684	1.24689
58946	0.578684	2.08922
58947	0.589487	1.387
58948	0.589487	2.1
58949	0.625461	1.87254
58950	0.5378	1.84643
58951	0.608714	2.1
58952	0.54271	1.35279
58953	0.608714	1.37775
58954	0.625461	1.0307
58955	0.54271	1.59149
58956	0.537801	1.64057
58957	0.946128	1.04434
58958	0.946128	1.77569
58959	0.946133	0.984531
58960	0.946133	2.1
58961	0.946131	1.23818
58962	0.946131	1.01272
58963	0.946131	0.992988
58964	0.946131	1.30353
58965	0.94613	1.20505
58966	0.94613	0.920851
58967	0.946131	1.21071
58968	0.946131	1.3454
58969	1.21743	1.6963
58970	1.25092	1.99796
58971	1.38847	1.04608
58972	1.46031	1.07601
58973	2.67774	1.49357
58974	0.946128	0.98074
58975	0.946128	1.82292
58976	0.946132	1.84999
58977	0.946132	1.13415
58978	0.94613	1.52498
58979	0.94613	1.02041
58980	0.94613	1.33611
58981	0.94613	1.10319
58982	0.94613	1.02334
58983	0.94613	0.929973

58984	0.94613	1.42727
58985	0.94613	1.09097
58986	1.89226	0.960446
58987	1.89226	1.10983
58988	1.89226	0.695085
58989	1.89226	1.98594
58990	1.89226	1.9886
58991	1.89226	1.67776
58992	0.539566	1.01142
58993	0.534046	2.1
58994	0.577914	1.63023
58995	0.471623	1.5957
58996	1.46031	0.842451
58997	0.945865	1.09483
58998	0.770603	0.857577
58999	0.737201	1.89808
59000	0.714829	1.12716
59001	0.536085	1.39253
59002	0.471617	1.25969
59003	1.89226	1.8673
59004	1.89226	0.893755
59005	1.89226	0.889165
59006	1.89226	1.22325
59007	1.89226	1.22823
59008	1.89226	1.87878
59009	1.89225	1.37602
59010	1.89225	0.716267
59011	1.65572	0.736161
59012	1.65572	0.939267
59013	1.89225	0.942592
59014	1.89225	1.47304
59015	1.89226	1.26602
59016	1.89226	0.998308
59017	1.89226	1.01794
59018	1.89226	0.541014
59019	3.78453	1.32865
59020	1.89226	1.64379
59021	1.89226	0.720428
59022	1.89226	0.748654
59023	1.89226	1.07795
59024	1.89226	0.998854
59025	1.89226	1.76058
59026	1.89225	1.1855
59027	1.89225	0.564533
59028	1.89225	0.617123
59029	1.89225	0.811612
59030	1.89225	0.734288
59031	1.89225	1.26459
59032	0.763592	0.726156
59033	0.763592	1.12095
59034	0.987417	0.845225
59035	0.987417	0.877867
59036	0.763592	0.637758
59037	0.804881	0.56428
59038	0.946126	0.87472
59039	0.946126	0.676341
59040	0.804879	0.541685
59041	0.94613	0.781422

59042	0.946129	0.94536
59043	0.76359	0.653153
59044	1.46055	2.1
59045	2.04945	2.1
59046	2.04587	2.1
59047	2.04587	2.1
59048	1.50714	2.1
59049	1.50714	2.1
59050	2.04587	2.1
59051	2.38681	2.1
59052	2.42082	2.1
59053	2.42082	2.1
59054	2.04945	2.1
59055	2.04945	1.9165
59056	0.872964	2.1
59057	0.872964	2.1
59058	0.884806	2.1
59059	0.884806	2.1
59060	1.0275	2.1
59061	1.02293	2.1
59062	0.711243	2.1
59063	0.730273	2.1
59064	0.711244	2.1
59065	1.0275	2.1
59066	0.730273	2.1
59067	1.02293	2.1
59068	2.04587	2.1
59069	2.47753	2.1
59070	2.42994	2.1
59071	2.42082	2.01706
59072	2.05499	2.1
59073	2.04587	1.4651
59074	0.946132	2.1
59075	0.946132	2.1
59076	1.13078	2.1
59077	1.07817	2.1
59078	0.946132	2.1
59079	0.946132	2.1
59080	1.07817	2.1
59081	1.07817	2.1
59082	0.946132	2.1
59083	1.07817	2.1
59084	1.13078	2.1
59085	0.946132	2.1
59086	1.89226	2.1
59087	1.89226	2.1
59088	1.89226	2.1
59089	1.89226	2.1
59090	1.89226	2.1
59091	1.89226	2.1
59092	1.89226	2.1
59093	1.89226	2.1
59094	1.89226	2.1
59095	1.89226	2.1
59096	1.89226	2.1
59097	1.89226	2.1
59098	1.89226	2.1
59099	1.89226	2.1

59100	1.8173	2.1
59101	2.66885	2.1
59102	2.7438	2.1
59103	2.7438	2.1
59104	1.89226	0.759601
59105	1.89226	0.693485
59106	1.89226	0.687965
59107	1.89226	0.976874
59108	3.78453	0.75165
59109	1.89226	0.594083
59110	1.89226	0.773782
59111	1.89226	0.665187
59112	1.89226	1.13451
59113	1.89226	0.504057
59114	1.89226	0.741292
59115	1.89226	0.641048
59116	1.97385	1.01402
59117	1.7531	0.811931
59118	1.89226	1.30846
59119	1.7531	0.542463
59120	1.89226	0.782424
59121	0.946131	1.07867
59122	0.840904	0.758974
59123	0.684333	0.801319
59124	0.94613	0.864566
59125	0.972337	0.90366
59126	0.946278	1.78677
59127	0.684304	0.672097
59128	0.684304	0.930262
59129	0.999838	0.777743
59130	0.759605	1.77135
59131	0.851528	0.71749
59132	0.851528	1.16653
59133	1.89226	0.673347
59134	2.23818	1.24647
59135	1.79771	0.828886
59136	1.89226	1.16855
59137	1.79771	0.605466
59138	1.89226	0.816493
59139	2.79647	2.1
59140	2.01613	2.1
59141	1.46826	2.1
59142	2.54504	2.1
59143	4.56117	2.1
59144	1.89226	2.1
59145	1.89226	2.1
59146	1.89226	2.1
59147	1.89226	2.1
59148	1.89226	2.1
59149	1.89226	2.1
59150	1.89226	2.1
59151	2.66884	2.1
59152	2.56022	2.1
59153	2.66884	2.1
59154	1.78364	2.1
59155	1.89226	2.1
59156	1.14282	2.1
59157	1.24068	2.1

59158	0.880748	2.1
59159	0.834916	2.1
59160	1.05539	2.1
59161	1.05539	2.1
59162	1.04312	2.1
59163	1.04313	2.1
59164	1.2015	2.1
59165	0.83492	2.1
59166	0.789149	2.1
59167	1.24066	2.1
59168	1.89226	2.1
59169	1.89226	2.1
59170	1.89226	2.1
59171	1.89226	2.1
59172	3.78453	2.1
59173	0.946128	2.1
59174	0.946128	2.1
59175	1.28011	2.1
59176	1.22372	2.1
59177	0.946131	2.1
59178	0.946131	2.1
59179	1.22372	2.1
59180	1.22372	2.1
59181	1.22372	2.1
59182	0.94613	2.1
59183	0.946131	2.1
59184	1.28011	2.1
59185	2.28604	2.1
59186	1.34268	2.1
59187	2.43157	2.1
59188	1.50172	2.1
59189	3.77425	2.1
59190	2.6096	2.1
59191	2.43157	2.1
59192	1.66774	2.1
59193	1.66774	2.1
59194	1.50172	2.1
59195	2.43157	2.1
59196	2.16067	2.1
59197	1.08975	2.1
59198	2.19336	2.1
59199	2.06984	2.1
59200	1.21327	2.1
59201	1.08975	2.1
59202	2.28604	2.1
59203	1.21327	2.1
59204	2.29008	2.1
59205	2.16067	2.1
59206	1.34268	2.1
59207	1.21327	2.1
59208	1.89226	0.450749
59209	1.89226	0.41149
59210	1.89226	0.445732
59211	1.89226	0.4528
59212	3.78452	0.41868
59213	1.89804	0.410122
59214	2.21656	0.347669
59215	1.70537	0.465138

59216	2.50023	0.376179
59217	3.8982	0.382895
59218	1.03136	0.389872
59219	1.89486	0.335194
59220	0.949364	0.389321
59221	1.82283	0.35537
59222	2.84422	0.383867
59223	1.89226	0.35844
59224	1.89226	0.369573
59225	1.89226	0.41869
59226	1.89226	0.396203
59227	3.78452	0.40681
59228	0.946132	0.474961
59229	0.946132	0.458471
59230	0.946129	0.495181
59231	0.946129	0.421475
59232	0.94613	0.421282
59233	0.94613	0.538929
59234	0.946131	0.442894
59235	0.946131	0.473133
59236	0.946131	0.483529
59237	0.946131	0.471023
59238	0.946131	0.348433
59239	0.946131	0.534334
59240	1.09466	0.441068
59241	1.09466	0.343728
59242	0.866512	0.433366
59243	1.08352	0.40628
59244	1.19189	0.386503
59245	1.21883	0.436347
59246	0.971139	0.436909
59247	0.873213	0.383831
59248	0.873202	0.384539
59249	1.00855	0.432467
59250	0.923735	0.42487
59251	1.21885	0.378335
59252	1.89225	0.0522355
59253	2.40019	0.0522355
59254	1.89225	0.119144
59255	1.89225	0.116054
59256	1.89225	0.109909
59257	1.89225	0.182441
59258	1.89225	0.119144
59259	1.89225	0.149158
59260	2.05088	0.110484
59261	1.89225	0.0796466
59262	1.89225	0.0952231
59263	1.89225	0.130631
59264	2.03956	0.110484
59265	2.03956	0.116054
59266	1.89226	2.1
59267	2.40021	0.905166
59268	2.38473	0.485611
59269	2.40021	1.61742
59270	1.87679	0.650126
59271	1.89226	1.0025
59272	1.89226	1.77551
59273	1.89226	0.413482

59274	1.89226	1.17455
59275	1.89226	0.436979
59276	1.89226	0.739944
59277	1.89226	0.326003
59278	2.0692	1.03949
59279	1.89226	0.576938
59280	1.89226	1.66792
59281	1.89226	0.470471
59282	2.05089	1.49813
59283	2.05089	0.39086
59284	1.89226	0.876248
59285	1.89226	2.1
59286	1.89226	0.968245
59287	1.89226	2.1
59288	3.78452	1.53321
59289	1.89226	0.614411
59290	2.50051	2.1
59291	1.89226	0.959049
59292	2.30076	2.1
59293	4.39277	1.9288
59294	2.50052	2.1
59295	1.70377	2.1
59296	2.30076	2.1
59297	1.54231	2.1
59298	4.04283	2.1
59299	1.89226	2.1
59300	1.89226	2.1
59301	1.89226	2.1
59302	1.89226	2.1
59303	1.89226	2.1
59304	1.89226	2.1
59305	1.89226	0.653813
59306	1.89226	2.1
59307	1.89226	0.793865
59308	1.89226	2.1
59309	3.78452	1.76073
59310	2.07948	2.1
59311	2.30076	2.1
59312	1.3294	2.1
59313	1.3294	2.1
59314	1.54232	2.1
59315	2.29239	2.1
59316	0.810947	2.1
59317	0.810947	2.1
59318	0.810946	2.1
59319	0.810946	2.1
59320	1.89226	2.1
59321	1.35169	2.1
59322	1.08132	2.1
59323	1.08132	2.1
59324	1.08132	2.1
59325	0.810946	2.1
59326	0.810946	2.1
59327	1.34851	1.0975
59328	0.997471	2.00406
59329	1.62287	0.864893
59330	1.27096	1.53099
59331	2.62034	1.12739

59332	1.89226	0.978001
59333	1.89226	2.02726
59334	1.89226	1.06215
59335	1.89226	1.81127
59336	3.78452	1.27697
59337	0.946128	2.1
59338	0.946128	1.41489
59339	1.29331	0.794675
59340	1.29331	1.83953
59341	1.29331	1.27947
59342	1.10699	2.1
59343	0.946131	0.771899
59344	0.946131	1.16657
59345	0.94613	1.45042
59346	1.29331	1.45067
59347	1.10699	1.26888
59348	0.946131	1.20397
59349	1.62287	0.974474
59350	1.27096	1.72162
59351	1.86067	0.922818
59352	1.58407	1.63626
59353	3.20694	1.48389
59354	1.89226	1.23066
59355	1.89226	1.74527
59356	1.89226	0.91094
59357	1.89226	1.66702
59358	3.78452	1.17915
59359	2.62761	0.703783
59360	2.55849	0.907399
59361	2.12261	1.30699
59362	2.22939	0.764569
59363	4.78788	0.826619
59364	0.772522	1.42371
59365	0.839588	1.19236
59366	1.31126	0.65298
59367	1.08787	0.665721
59368	1.01318	0.707399
59369	1.01318	1.5709
59370	0.900815	1.82735
59371	1.10799	0.470782
59372	0.772511	0.878297
59373	1.03473	0.938012
59374	1.31126	0.946188
59375	0.841464	1.06376
59376	1.89227	0.666249
59377	1.89227	1.20802
59378	1.89227	0.711013
59379	1.89227	1.22995
59380	1.89227	0.677128
59381	1.89227	1.22781
59382	1.89226	0.753018
59383	1.89226	0.816028
59384	1.89226	0.67501
59385	1.89226	0.917882
59386	1.89226	0.597984
59387	1.89226	0.840951
59388	2.12261	1.04376
59389	2.22939	0.634422

59390	1.97004	0.680517
59391	1.8072	1.04477
59392	4.03658	0.773413
59393	1.5847	1.53225
59394	1.8072	0.719484
59395	2.12262	0.914996
59396	2.12262	1.42477
59397	1.23267	0.798011
59398	1.23267	1.41352
59399	1.89227	0.719832
59400	1.89227	1.47911
59401	1.89227	0.742385
59402	1.89227	1.3029
59403	1.89227	0.707808
59404	1.89227	1.38613
59405	1.89226	0.637184
59406	1.89226	0.834508
59407	1.89226	1.05145
59408	1.89226	0.59234
59409	3.78452	0.716714
59410	2.49093	2.1
59411	2.67393	2.1
59412	2.47932	2.1
59413	2.47932	2.1
59414	2.67994	2.1
59415	2.67994	2.1
59416	2.49873	2.1
59417	2.67393	2.1
59418	2.67993	2.1
59419	2.69807	2.1
59420	2.49873	2.1
59421	2.51686	2.1
59422	2.49093	2.1
59423	2.67994	2.1
59424	2.69784	2.1
59425	2.51651	2.1
59426	5.18877	2.1
59427	1.24359	2.1
59428	1.24936	2.1
59429	1.33997	2.1
59430	1.34892	2.1
59431	1.28805	2.1
59432	1.28805	2.1
59433	1.2955	2.1
59434	1.2955	2.1
59435	1.34892	2.1
59436	1.24359	2.1
59437	1.24936	2.1
59438	1.33997	2.1
59439	1.15506	1.58781
59440	1.15506	2.1
59441	0.946128	2.1
59442	0.946128	2.1
59443	0.946131	2.1
59444	1.15506	1.64737
59445	1.06997	2.1
59446	0.946128	2.1
59447	1.06997	1.9527

59448	1.15506	2.02936
59449	0.946129	2.05907
59450	0.946129	2.09276
59451	1.70608	2.1
59452	2.13994	2.1
59453	1.33384	2.1
59454	1.43635	2.1
59455	3.14243	2.1
59456	2.13994	2.1
59457	2.31012	2.1
59458	1.43635	2.1
59459	1.23007	2.1
59460	3.74646	2.1
59461	2.46361	2.1
59462	1.89226	1.84729
59463	1.89226	2.1
59464	1.89226	1.69567
59465	2.39045	2.1
59466	2.39045	2.03497
59467	1.89226	2.1
59468	1.89226	1.48509
59469	1.89226	1.85009
59470	1.89226	1.0181
59471	3.78452	1.76719
59472	1.52984	2.1
59473	2.25682	0.937942
59474	2.20831	0.803474
59475	2.20831	2.08981
59476	1.61178	0.705609
59477	1.61178	1.92602
59478	2.47395	2.1
59479	1.89226	2.1
59480	1.89226	2.1
59481	1.89226	1.91379
59482	2.46362	2.1
59483	2.46362	1.8593
59484	0.946131	0.692261
59485	0.946131	2.1
59486	0.946131	1.94162
59487	0.946131	1.36906
59488	0.94613	1.20711
59489	0.94613	1.6615
59490	0.946132	2.1
59491	0.946132	1.61335
59492	0.946131	1.1348
59493	0.946131	1.42015
59494	0.946131	1.73237
59495	0.946131	2.02066
59496	0.982398	2.08715
59497	0.982398	1.50076
59498	0.931703	1.89221
59499	0.97549	1.69431
59500	1.11509	1.61067
59501	1.10017	1.01181
59502	0.774576	2.1
59503	0.789859	2.09991
59504	0.716502	1.6104
59505	1.12793	1.74819

59506	1.14398	1.79111
59507	0.817011	1.27118
59508	1.82499	2.1
59509	1.50524	2.1
59510	1.21304	2.1
59511	1.54191	2.1
59512	3.04715	2.1
59513	1.54191	2.1
59514	1.49823	2.1
59515	1.82499	2.1
59516	1.82499	2.1
59517	1.21147	2.1
59518	1.48977	2.1
59519	1.89226	2.1
59520	1.89226	2.1
59521	1.89226	2.1
59522	1.89226	2.1
59523	3.78452	2.1
59524	1.89226	2.1
59525	1.89226	2.1
59526	1.89226	2.1
59527	1.89226	2.1
59528	1.89226	2.1
59529	1.89226	2.1
59530	0.566674	2.1
59531	0.770957	2.1
59532	0.60652	2.1
59533	0.473557	2.1
59534	0.604441	2.1
59535	0.604441	2.1
59536	0.640073	2.1
59537	0.640073	2.1
59538	0.473557	2.1
59539	0.566674	2.1
59540	0.60652	2.1
59541	0.770956	2.1
59542	1.36203	2.1
59543	1.13335	2.1
59544	1.54191	2.1
59545	1.54191	2.1
59546	1.00965	2.1
59547	1.00965	2.1
59548	1.89226	2.1
59549	1.89226	2.1
59550	1.89226	2.1
59551	1.89226	2.1
59552	1.89226	2.1
59553	1.89226	2.1
59554	0.946131	2.1
59555	0.946131	2.1
59556	0.946131	2.1
59557	0.946131	2.1
59558	0.946132	2.1
59559	0.946132	2.1
59560	0.946131	2.1
59561	0.946131	2.1
59562	0.946132	2.1
59563	0.946132	2.1

59564	0.946131	2.1
59565	0.946131	2.1
59566	0.946132	0.10144
59567	0.946132	0.111271
59568	0.946133	0.0933383
59569	0.946133	0.106599
59570	0.946132	0.0944432
59571	0.946132	0.100838
59572	0.946132	0.097096
59573	0.946132	0.114369
59574	0.946132	0.0968288
59575	0.946132	0.0988212
59576	0.946133	0.11254
59577	0.946133	0.106005
59578	1.89226	0.129971
59579	1.89226	0.120015
59580	1.89226	0.108335
59581	1.89226	0.099416
59582	3.78453	0.100809
59583	1.89226	0.119185
59584	1.89226	0.0939303
59585	1.89226	0.0968491
59586	1.89226	0.109791
59587	1.89226	0.10239
59588	1.89226	0.121394
59589	0.946132	0.0890931
59590	0.946132	0.0953412
59591	0.946131	0.0824942
59592	0.946131	0.0906465
59593	0.946132	0.085696
59594	0.946132	0.0895524
59595	0.946132	0.0859156
59596	0.946132	0.0985574
59597	0.946132	0.0865115
59598	0.946132	0.0870117
59599	0.946132	0.10407
59600	0.946132	0.095111
59601	0.946129	0.0866828
59602	0.946129	0.0937254
59603	0.946128	0.0796352
59604	0.946128	0.0862135
59605	0.946132	0.081737
59606	0.946132	0.0861773
59607	0.946126	0.0895153
59608	0.946126	0.092597
59609	0.946129	0.0822045
59610	0.946129	0.0843706
59611	0.946129	0.0910989
59612	0.946129	0.0988416
59613	1.20674	0.122685
59614	1.79877	0.146531
59615	1.22586	0.132674
59616	1.22586	0.122607
59617	1.7919	0.139212
59618	1.7919	0.130044
59619	2.27622	0.11883
59620	1.79877	0.148396
59621	1.7919	0.120064

59622	2.27102	0.148447
59623	4.06979	0.12603
59624	1.89226	0.148527
59625	1.89226	0.216241
59626	1.89226	0.149564
59627	1.89226	0.201688
59628	3.78452	0.192893
59629	1.89226	0.14662
59630	1.89226	0.178514
59631	1.89226	0.139908
59632	1.89226	0.199429
59633	3.78453	0.151375
59634	1.13259	0.118998
59635	1.79187	0.139245
59636	1.32741	0.125764
59637	1.18784	0.121313
59638	1.703	0.132286
59639	1.49575	0.126843
59640	1.46201	0.117233
59641	2.27099	0.148786
59642	1.79187	0.129319
59643	1.79187	0.119432
59644	2.12923	0.140076
59645	2.12923	0.130434
59646	1.89224	0.149383
59647	1.89224	0.194723
59648	1.89224	0.142181
59649	1.89224	0.195342
59650	3.78447	0.154069
59651	0.946119	0.201217
59652	0.946119	0.154099
59653	0.946118	0.177203
59654	0.946118	0.139267
59655	0.946118	0.178675
59656	0.946118	0.194303
59657	0.946119	0.140006
59658	0.946119	0.132067
59659	0.946131	0.15646
59660	0.946131	0.145615
59661	0.946106	0.160261
59662	0.946106	0.166431
59663	2.07526	0.536474
59664	1.89226	0.426846
59665	1.89226	0.711546
59666	2.13445	0.621493
59667	4.02671	0.4527
59668	1.89226	0.417933
59669	1.89226	0.476227
59670	1.89226	0.560575
59671	1.89226	0.261378
59672	1.89226	0.685288
59673	1.89226	0.399858
59674	2.13446	0.585819
59675	1.00013	0.544489
59676	1.1475	0.896164
59677	2.20046	0.590115
59678	3.20059	0.699786
59679	0.946133	0.485111

59680	0.946133	0.580027
59681	1.06722	0.561333
59682	1.10023	0.44777
59683	0.946131	0.493142
59684	0.946131	0.296905
59685	1.10023	0.635885
59686	1.10023	0.486458
59687	0.946131	0.586432
59688	1.10023	0.426456
59689	0.94613	0.554718
59690	1.06723	0.445207
59691	1.89226	0.353763
59692	1.89226	0.342966
59693	1.89226	0.536258
59694	1.89226	0.429647
59695	3.78452	0.464888
59696	1.89226	0.593384
59697	1.89226	0.454943
59698	1.89226	0.604508
59699	1.89226	0.521724
59700	1.89226	0.519995
59701	1.89226	0.430527
59702	1.89226	2.1
59703	1.89226	0.23969
59704	1.89226	0.501129
59705	1.89226	0.249422
59706	1.89226	2.1
59707	0.828588	0.0522355
59708	0.828588	0.0522355
59709	0.841316	0.0522355
59710	0.841316	1.14794
59711	0.528387	2.1
59712	0.565893	0.0522355
59713	1.10401	0.0522355
59714	1.10401	1.14794
59715	0.528387	2.1
59716	1.89226	2.1
59717	1.36047	0.501129
59718	1.33973	2.1
59719	1.33973	2.1
59720	1.89226	0.0522355
59721	2.28951	0.269244
59722	1.89226	0.942461
59723	2.80796	1.79302
59724	2.01584	1.59703
59725	2.01584	1.13015
59726	1.89226	1.50254
59727	1.89226	1.0878
59728	1.89226	0.778447
59729	1.89226	1.44432
59730	1.89226	1.23115
59731	1.89226	0.900109
59732	1.89226	1.25887
59733	1.89226	0.9457
59734	2.15223	0.916939
59735	1.30664	1.84178
59736	1.96011	1.65122
59737	2.76516	0.976654

59738	4.11234	1.6639
59739	1.89226	0.728832
59740	1.89226	1.39668
59741	1.89226	1.13816
59742	1.89226	0.847409
59743	1.89226	1.18839
59744	1.89226	0.895063
59745	2.81143	2.1
59746	2.58517	2.1
59747	2.55318	2.1
59748	2.7542	2.1
59749	5.33937	2.1
59750	2.7777	2.1
59751	2.7542	2.1
59752	2.71672	2.1
59753	2.70401	2.1
59754	5.45821	2.1
59755	2.55318	2.1
59756	2.7542	2.1
59757	2.71672	2.1
59758	2.54198	2.1
59759	5.29618	2.1
59760	1.95416	2.1
59761	2.162 2.1	
59762	2.11563	2.1
59763	1.91813	2.1
59764	4.08013	2.1
59765	1.95417	2.1
59766	1.47712	2.1
59767	1.45182	2.1
59768	1.91813	2.1
59769	3.39525	2.1
59770	2.11562	2.1
59771	1.91813	2.1
59772	1.50979	2.1
59773	2.03974	2.1
59774	3.95787	2.1
59775	1.85605	2.1
59776	1.91813	2.1
59777	1.88885	2.1
59778	1.42255	2.1
59779	1.45182	2.1
59780	1.45182	2.1
59781	0.94613	0.556659
59782	0.94613	0.412498
59783	0.946128	0.324599
59784	0.946128	0.665926
59785	0.946127	0.539859
59786	0.946127	0.546513
59787	0.946131	0.375517
59788	0.946131	0.375443
59789	0.946129	0.453662
59790	0.946129	0.462265
59791	0.946128	0.484727
59792	0.946128	0.481265
59793	0.543052	0.55609
59794	0.54608	0.390457
59795	0.602293	0.36194

59796	0.535325	0.391832
59797	1.05446	0.544546
59798	0.850496	0.489962
59799	0.699838	0.415904
59800	0.679751	0.53909
59801	0.713797	0.464041
59802	0.509794	0.420251
59803	0.602304	0.412981
59804	1.40017	0.529224
59805	1.40017	1.04783
59806	0.94613	0.378835
59807	0.94613	1.22236
59808	1.40017	1.06672
59809	1.33997	0.864001
59810	0.946131	0.449314
59811	0.946131	0.496036
59812	1.33997	0.626574
59813	0.946131	0.630854
59814	0.946131	0.635162
59815	1.40017	0.630558
59816	1.89226	0.109087
59817	1.89226	0.135017
59818	1.89226	0.142652
59819	1.89226	0.0982761
59820	3.78452	0.112556
59821	0.946133	0.090047
59822	0.946133	0.0989945
59823	0.946132	0.137457
59824	0.946132	0.109017
59825	0.946132	0.096098
59826	0.946132	0.109388
59827	0.946133	0.136792
59828	0.946133	0.100881
59829	0.946132	0.104876
59830	0.946132	0.112205
59831	0.946132	0.108588
59832	0.946132	0.108094
59833	1.89224	0.145373
59834	1.89224	0.102584
59835	1.89224	0.119899
59836	1.89224	0.116673
59837	1.89224	0.126834
59838	1.89224	0.124525
59839	0.94612	0.0921771
59840	0.94612	0.101805
59841	0.946119	0.130866
59842	0.946119	0.110811
59843	0.946119	0.0986216
59844	0.946119	0.112499
59845	0.94612	0.105857
59846	0.94612	0.12878
59847	0.946132	0.103844
59848	0.946132	0.100742
59849	0.946107	0.0992319
59850	0.946107	0.107354
59851	2.63939	2.1
59852	1.2862	2.1
59853	1.29891	2.1

59854	2.64419	2.1
59855	3.93039	2.1
59856	2.6394	2.1
59857	1.89226	2.1
59858	1.89226	2.1
59859	2.64419	2.1
59860	4.53646	2.1
59861	1.89226	2.1
59862	1.89226	2.1
59863	1.89226	2.1
59864	1.89226	1.53022
59865	3.78453	2.1
59866	1.89226	2.1
59867	1.89226	1.80662
59868	1.89226	2.1
59869	1.89226	1.96026
59870	1.89226	2.1
59871	1.89226	2.1
59872	1.31998	2.1
59873	1.32209	2.1
59874	0.649456	2.1
59875	0.648474	2.1
59876	0.984995	2.1
59877	0.984995	2.1
59878	0.985573	2.1
59879	0.985573	2.1
59880	1.31999	2.1
59881	0.648472	2.1
59882	0.649455	2.1
59883	1.32209	2.1
59884	0.946133	2.1
59885	0.946133	2.1
59886	1.31999	2.1
59887	1.3221	2.1
59888	0.946132	2.1
59889	0.946132	2.1
59890	1.31999	2.1
59891	1.31999	2.1
59892	1.31999	2.1
59893	0.946132	2.1
59894	0.946133	2.1
59895	1.3221	2.1
59896	0.946132	1.61723
59897	0.946132	2.1
59898	0.946131	2.1
59899	0.946131	1.9644
59900	0.946132	1.53485
59901	0.946132	2.00432
59902	0.946131	2.1
59903	0.946131	2.1
59904	0.946131	2.1
59905	0.946131	2.1
59906	0.946132	2.1
59907	0.946132	2.1
59908	0.946128	2.1
59909	0.946128	2.07537
59910	0.946132	1.57008
59911	0.946132	2.1

59912	0.946131	1.61971
59913	0.946131	1.64068
59914	0.94613	2.1
59915	0.94613	2.1
59916	0.94613	2.1
59917	0.94613	2.1
59918	0.946131	2.1
59919	0.946131	2.1
59920	1.89226	0.278271
59921	1.89226	0.577291
59922	1.89226	0.553281
59923	1.89226	0.498885
59924	1.89226	0.399566
59925	1.89226	0.365986
59926	1.89226	0.447015
59927	1.89226	0.795057
59928	1.89226	0.602516
59929	1.89226	0.753659
59930	3.78453	0.805677
59931	0.946133	0.497645
59932	0.946133	0.49289
59933	0.946132	0.436486
59934	0.946132	0.408214
59935	0.946133	0.561847
59936	0.946133	0.560151
59937	0.946132	0.519495
59938	0.946132	0.330895
59939	0.946132	0.488082
59940	0.946132	0.589909
59941	0.946132	0.439882
59942	0.946132	0.683905
59943	2.23638	0.321745
59944	1.89226	0.369611
59945	2.66014	0.538934
59946	2.66014	0.587968
59947	1.89226	0.548715
59948	1.89226	0.416039
59949	1.11819	0.272773
59950	1.33007	0.269817
59951	0.660255	0.293959
59952	0.91151	0.285788
59953	0.911511	0.327087
59954	1.33007	0.318638
59955	1.11819	0.229811
59956	0.660255	0.261468
59957	0.985327	0.259747
59958	0.985327	0.333473
59959	1.005	0.262074
59960	1.005	0.307753
59961	1.89226	2.1
59962	1.89226	2.1
59963	1.89226	2.1
59964	1.89226	2.1
59965	3.78453	2.1
59966	1.59533	2.1
59967	2.68571	2.1
59968	1.28291	2.1
59969	2.46974	2.1

59970	4.06506	2.1
59971	2.68571	2.1
59972	1.89226	2.1
59973	1.89226	2.1
59974	1.89226	2.1
59975	2.46973	2.1
59976	2.46973	2.1
59977	1.89226	2.1
59978	1.89226	2.1
59979	1.89226	2.1
59980	1.89226	2.1
59981	1.89226	2.1
59982	1.89226	2.1
59983	1.89226	2.1
59984	1.89226	2.1
59985	1.89226	2.1
59986	1.89226	2.1
59987	1.89226	2.1
59988	1.89226	2.1
59989	0.883594	2.1
59990	0.883594	2.1
59991	0.876484	2.1
59992	0.876484	2.1
59993	1.10442	2.1
59994	1.23487	2.1
59995	0.641457	2.1
59996	0.52521	2.1
59997	1.10442	2.1
59998	0.52521	2.1
59999	0.641457	2.1
60000	1.23487	2.1
60001	0.946131	2.1
60002	0.946131	2.1
60003	0.946131	2.1
60004	0.946131	2.1
60005	0.946131	2.1
60006	0.946131	2.1
60007	0.946131	2.1
60008	0.946131	2.1
60009	0.946131	2.1
60010	0.946131	2.1
60011	0.946131	2.1
60012	0.946131	2.1
60013	2.04016	0.226762
60014	2.13042	0.250338
60015	2.11465	0.277265
60016	2.11465	0.30989
60017	1.95908	0.202274
60018	1.95908	0.214449
60019	1.66953	0.217286
60020	2.13041	0.258604
60021	1.95908	0.190177
60022	2.21952	0.218424
60023	1.66953	0.294805
60024	1.92997	0.323011
60025	2.04016	0.213159
60026	1.95908	0.195804
60027	1.82014	0.158582

60028	2.08757	0.171252
60029	3.86029	0.173791
60030	0.834764	0.200835
60031	0.722102	0.168426
60032	0.979537	0.200981
60033	0.910065	0.170768
60034	0.840053	0.161436
60035	0.840052	0.223045
60036	0.861587	0.171524
60037	0.861587	0.205809
60038	0.910067	0.168167
60039	0.722101	0.149705
60040	0.834763	0.22687
60041	0.979539	0.199417
60042	1.89226	0.14496
60043	1.89226	0.149277
60044	1.89226	0.164161
60045	1.89226	0.129407
60046	1.89226	0.169427
60047	1.89226	0.13682
60048	1.89226	0.151808
60049	1.89226	0.170377
60050	1.89226	0.219362
60051	1.89226	0.123549
60052	3.78453	0.159175
60053	1.89226	0.112038
60054	1.89226	0.117554
60055	1.89226	0.130035
60056	1.89226	0.105025
60057	1.89226	0.131459
60058	1.89226	0.108027
60059	1.20691	0.099608
60060	2.27379	0.0978646
60061	2.58476	0.114107
60062	1.5124	0.0879285
60063	3.79167	0.0995831
60064	1.89226	0.16649
60065	2.2738	0.108407
60066	2.58477	0.119767
60067	1.89226	0.134138
60068	4.47703	0.132744
60069	0.952225	1.45737
60070	0.952225	1.82522
60071	0.909865	1.75624
60072	0.909865	1.97858
60073	0.909895	2.06585
60074	0.946132	1.53863
60075	0.946132	1.80257
60076	0.915958	1.46213
60077	0.946132	1.76904
60078	0.946132	1.73714
60079	0.909894	1.72182
60080	0.915958	1.85236
60081	0.983493	2.1
60082	2.40055	2.1
60083	1.02986	2.1
60084	1.02986	2.1
60085	2.34217	2.1

60086	2.34217	2.1
60087	1.89226	2.1
60088	2.40056	2.1
60089	2.34217	2.1
60090	2.40056	2.1
60091	1.83388	2.1
60092	1.89226	2.1
60093	1.89226	2.1
60094	1.89226	2.1
60095	1.89226	2.1
60096	1.89226	2.1
60097	1.89226	2.1
60098	1.89226	2.1
60099	1.89226	2.1
60100	1.89226	2.1
60101	1.89226	2.1
60102	1.89226	2.1
60103	1.89226	2.1
60104	1.89226	2.1
60105	0.946133	2.1
60106	0.946133	2.1
60107	1.17109	2.1
60108	1.13078	2.1
60109	0.946132	2.1
60110	0.946132	2.1
60111	1.13078	2.1
60112	1.13078	2.1
60113	1.13078	2.1
60114	0.946133	2.1
60115	0.946132	2.1
60116	1.17109	2.1
60117	1.89226	2.1
60118	1.89226	2.1
60119	1.89226	2.1
60120	1.89226	2.1
60121	1.89226	2.1
60122	1.89226	2.1
60123	2.33963	1.02408
60124	1.89227	0.689383
60125	1.74565	0.756727
60126	1.74565	0.833642
60127	1.89227	0.612636
60128	1.89227	0.71623
60129	1.89227	0.838613
60130	1.89227	0.713073
60131	1.89227	1.21412
60132	1.89227	0.776365
60133	3.78453	0.756688
60134	1.31398	0.647426
60135	1.10392	0.681114
60136	0.796878	0.662143
60137	0.524356	0.621941
60138	0.870614	0.628684
60139	0.945739	0.602602
60140	0.89208	0.696893
60141	0.89208	0.815276
60142	1.23425	0.622061
60143	0.774658	0.772093

60144	1.03182	0.629626
60145	0.557289	0.581691
60146	2.33963	0.920793
60147	1.89226	0.600903
60148	1.89226	0.928504
60149	2.77245	0.68133
60150	4.23189	0.814309
60151	1.89226	0.961818
60152	1.89226	0.692786
60153	1.89226	1.02603
60154	1.89226	0.951563
60155	1.89226	0.712074
60156	1.89226	0.68766
60157	1.89226	0.443726
60158	1.89226	0.502204
60159	1.89226	0.420458
60160	1.89226	0.609076
60161	1.89226	0.39987
60162	1.89226	0.586264
60163	1.89226	0.408003
60164	1.89226	0.426067
60165	1.89226	0.365006
60166	1.89226	0.538362
60167	1.89226	0.329826
60168	1.89226	0.493454
60169	0.946128	0.366773
60170	0.946128	0.273009
60171	0.963918	0.325415
60172	1.37543	0.233959
60173	0.946129	0.312545
60174	0.946129	0.386994
60175	1.37542	0.257407
60176	1.37542	0.229471
60177	0.94613	0.321254
60178	1.37542	0.323483
60179	0.963916	0.324429
60180	0.94613	0.325219
60181	0.946132	0.442437
60182	0.946132	0.337369
60183	0.946131	0.385415
60184	0.946131	0.284674
60185	0.946131	0.386883
60186	0.946131	0.457085
60187	0.946132	0.324321
60188	0.946132	0.255119
60189	0.946131	0.360155
60190	0.946131	0.371445
60191	0.946131	0.348245
60192	0.946131	0.334313
60193	1.89226	0.460739
60194	1.89226	0.511955
60195	1.89226	0.374074
60196	1.89226	0.580086
60197	3.78452	0.466971
60198	0.946127	0.513038
60199	0.946127	0.373879
60200	0.946131	0.427146
60201	0.946131	0.324862

60202	0.946128	0.429354
60203	0.946128	0.499318
60204	0.94613	0.306315
60205	0.94613	0.381402
60206	0.946129	0.390215
60207	0.946129	0.407853
60208	0.946129	0.380969
60209	0.946129	0.403881
60210	1.89226	0.704743
60211	1.89226	0.816284
60212	1.89226	1.64664
60213	1.89226	0.457744
60214	3.78453	0.497247
60215	1.89226	0.627324
60216	1.89226	0.181882
60217	1.89226	0.381394
60218	1.89226	0.440931
60219	3.78453	0.595872
60220	0.946131	0.314289
60221	0.946131	0.318182
60222	0.946132	0.951204
60223	0.946132	0.56353
60224	0.946132	0.817434
60225	0.946132	0.469933
60226	0.946132	0.83314
60227	0.946132	0.577442
60228	0.946132	0.616486
60229	0.946132	0.734924
60230	0.946132	0.79451
60231	0.946132	0.574989
60232	0.548169	0.22843
60233	0.736603	0.214828
60234	1.18322	0.284455
60235	1.01139	0.231323
60236	1.01139	0.231256
60237	0.548168	0.201994
60238	0.736603	0.230518
60239	1.18322	0.201719
60240	0.861589	0.219666
60241	0.861589	0.292561
60242	0.869798	0.216321
60243	0.869798	0.221643
60244	1.16037	0.310359
60245	0.942012	0.212275
60246	0.942012	0.250161
60247	0.62356	0.243131
60248	0.891932	0.286573
60249	0.900255	0.290061
60250	2.02279	0.220761
60251	1.48677	0.240518
60252	1.54724	0.243285
60253	0.831456	0.236798
60254	1.19775	0.249737
60255	1.89226	1.14981
60256	2.76291	1.26557
60257	1.89226	1.46484
60258	1.89226	1.06405
60259	3.78452	1.33426

60260	1.89226	0.854509
60261	1.89226	1.23201
60262	1.89226	1.39464
60263	1.89226	0.912197
60264	1.89226	1.38324
60265	1.89226	0.907705
60266	1.89226	1.37815
60267	1.89226	0.947186
60268	1.89226	1.16731
60269	1.89226	1.13484
60270	3.78452	1.03655
60271	1.89226	0.801765
60272	1.89226	1.25737
60273	1.89226	1.28714
60274	1.89226	0.893377
60275	3.78453	1.15543
60276	0.946128	0.898476
60277	0.946128	1.0605
60278	0.946129	1.29333
60279	0.946129	1.16961
60280	0.946132	0.81757
60281	0.946132	1.20562
60282	0.946126	1.21809
60283	0.946126	0.902546
60284	0.946129	1.13585
60285	0.946129	1.08704
60286	0.946129	1.05417
60287	0.946129	0.979032
60288	2.80495	1.181
60289	1.89225	0.930834
60290	1.89225	1.28255
60291	1.87794	1.28908
60292	1.89225	1.0471
60293	1.87794	1.0106
60294	2.25195	0.622125
60295	2.00624	0.41191
60296	2.13132	0.570206
60297	1.90239	0.204588
60298	4.13756	0.345872
60299	0.804661	0.460738
60300	0.804663	0.334386
60301	0.889591	0.293351
60302	0.889591	0.510785
60303	0.808324	0.445234
60304	0.761765	0.469806
60305	1.02822	0.342842
60306	0.879624	0.314157
60307	0.879617	0.373043
60308	0.800864	0.355933
60309	0.978023	0.397087
60310	0.808329	0.364124
60311	2.13133	0.594093
60312	2.00558	0.202987
60313	2.09316	0.183905
60314	2.09316	0.625259
60315	2.03253	0.247039
60316	2.03253	0.736552
60317	0.84577	0.422106

60318	0.845767	0.409635
60319	0.843917	0.278574
60320	0.843917	0.588851
60321	0.76177	0.40347
60322	0.719345	0.455421
60323	0.934435	0.309814
60324	0.884197	0.305444
60325	0.934439	0.303185
60326	0.75627	0.316703
60327	0.849424	0.342274
60328	0.76177	0.374538
60329	2.61855	0.278299
60330	1.17793	0.28498
60331	1.42965	0.319714
60332	1.17793	0.284103
60333	2.50057	0.279514
60334	2.24885	0.267408
60335	0.946133	0.300586
60336	0.946133	0.291559
60337	1.30928	0.312507
60338	1.12443	0.286376
60339	0.946132	0.290192
60340	0.946132	0.279927
60341	1.30928	0.27154
60342	1.30928	0.28552
60343	0.946132	0.292764
60344	1.30928	0.282112
60345	1.12443	0.268886
60346	0.946133	0.278282
60347	1.89226	0.274969
60348	1.89226	0.324656
60349	1.89226	0.323316
60350	1.89226	0.330736
60351	3.78452	0.325078
60352	1.89226	0.340609
60353	1.89226	0.303182
60354	1.89226	0.278841
60355	1.89226	0.305854
60356	1.89226	0.305294
60357	1.89226	0.273403
60358	1.89226	0.302951
60359	1.89226	0.308868
60360	1.89226	0.308597
60361	1.89226	0.360773
60362	3.78452	0.306483
60363	0.94613	0.307562
60364	0.94613	0.325271
60365	0.946131	0.341522
60366	0.946131	0.319086
60367	0.946128	0.319702
60368	0.946128	0.316824
60369	0.946133	0.295897
60370	0.946133	0.306745
60371	0.946131	0.304762
60372	0.946131	0.343219
60373	0.946131	0.312142
60374	0.946131	0.279207
60375	1.89226	2.1

60376	1.89226	2.1
60377	1.89226	2.1
60378	1.89226	2.1
60379	3.78453	2.1
60380	1.0465	2.1
60381	2.73926	2.1
60382	2.03721	2.1
60383	2.03721	2.1
60384	1.44709	2.1
60385	1.44709	2.1
60386	0.946131	2.1
60387	0.946131	2.1
60388	0.946131	2.1
60389	0.946131	2.1
60390	0.946132	2.1
60391	0.946132	2.1
60392	0.94613	2.1
60393	0.94613	2.1
60394	0.946131	2.1
60395	0.946131	2.1
60396	0.946131	2.1
60397	0.946131	2.1
60398	0.946132	2.1
60399	0.946132	2.1
60400	0.946131	2.1
60401	0.946131	2.1
60402	0.946132	2.1
60403	0.946132	2.1
60404	0.946132	2.1
60405	0.946132	2.1
60406	0.946132	2.1
60407	0.946132	2.1
60408	0.946132	2.1
60409	0.946132	2.1
60410	1.89226	2.1
60411	1.89226	2.1
60412	1.89226	2.1
60413	1.89226	2.1
60414	3.78452	2.1
60415	1.61969	0.311809
60416	1.79374	0.250127
60417	1.29382	0.244816
60418	1.29382	0.288467
60419	2.05759	0.251936
60420	2.05759	0.293766
60421	1.89226	0.284119
60422	1.89226	0.269295
60423	1.89226	0.24495
60424	1.89226	0.293952
60425	1.89226	0.244646
60426	1.89226	0.28279
60427	1.89226	0.617796
60428	1.89226	0.669849
60429	1.89226	0.635223
60430	1.89226	0.641505
60431	1.89226	0.582117
60432	1.89226	0.590246
60433	1.89226	1.08552

60434	1.89226	0.761387
60435	1.89226	0.87828
60436	1.89226	0.944705
60437	3.78453	1.07469
60438	0.946131	0.721808
60439	0.946131	0.747253
60440	0.946131	0.642433
60441	0.946131	0.719257
60442	0.946131	0.659303
60443	0.946131	0.627959
60444	0.946131	0.744673
60445	0.946131	0.810214
60446	0.946131	0.701464
60447	0.946131	0.730122
60448	0.946131	0.725022
60449	0.946131	0.676343
60450	2.45177	0.235436
60451	2.68507	0.196971
60452	2.3376	0.269213
60453	2.22086	0.178383
60454	4.78937	0.219236
60455	2.45178	0.220599
60456	2.35552	0.160222
60457	1.68062	0.205852
60458	1.81902	0.171355
60459	4.13239	0.188388
60460	2.3376	0.221178
60461	1.55612	0.156923
60462	1.69811	0.167827
60463	2.31527	0.193825
60464	1.79553	0.164541
60465	2.3376	0.192847
60466	1.55613	0.148595
60467	1.06336	0.164821
60468	1.97516	0.172478
60469	1.88966	0.150127
60470	1.06336	0.207711
60471	1.68062	0.147014
60472	1.89226	0.906837
60473	1.91634	0.778469
60474	2.03709	1.19238
60475	1.91634	0.819821
60476	1.83185	0.865085
60477	1.70303	0.504923
60478	1.89226	0.697442
60479	1.89226	0.706253
60480	1.89226	1.00559
60481	1.89226	0.705766
60482	1.89226	0.715284
60483	1.89226	0.405366
60484	1.89226	1.26714
60485	2.03472	1.44726
60486	2.22183	2.07998
60487	2.03472	0.998991
60488	2.07936	1.50862
60489	1.89226	0.75263
60490	1.89226	0.889146
60491	1.8519	0.664286

60492	1.92119	0.987055
60493	1.8519	0.746945
60494	1.96156	0.73018
60495	1.89226	0.472346
60496	1.89226	0.657159
60497	1.89226	0.667118
60498	1.89226	0.913095
60499	1.89226	0.68488
60500	1.89226	0.631082
60501	1.89226	0.372582
60502	1.89226	1.04381
60503	2.22183	0.994834
60504	2.39541	1.63212
60505	2.22183	0.865632
60506	2.06585	1.02798
60507	1.89226	0.715119
60508	1.47001	1.99629
60509	1.57962	0.808747
60510	1.65238	1.03508
60511	1.60502	1.22915
60512	3.18464	1.21537
60513	1.02415	2.1
60514	1.60503	1.41563
60515	1.47001	2.06228
60516	1.47001	2.1
60517	1.28228	2.1
60518	1.28228	2.1
60519	0.946133	2.1
60520	0.946133	1.824
60521	0.946132	1.26
60522	0.946132	0.964864
60523	0.946132	0.963396
60524	0.946132	1.75631
60525	0.946133	1.50083
60526	0.946133	2.1
60527	0.946132	1.53828
60528	0.946132	1.30624
60529	0.946133	1.66031
60530	0.946133	1.75655
60531	1.89226	0.805811
60532	1.89226	0.908338
60533	1.89226	1.21453
60534	1.89226	0.661641
60535	3.78452	0.803398
60536	1.76897	0.998016
60537	1.60502	1.07503
60538	1.65238	0.847249
60539	1.65238	1.71842
60540	1.76932	0.820113
60541	1.76932	1.57648
60542	1.55998	2.1
60543	1.60503	1.14577
60544	1.76932	1.86988
60545	1.60503	2.05315
60546	1.44657	1.90964
60547	1.28228	2.07219
60548	0.946133	1.49985
60549	0.946133	1.99811

60550	0.946132	1.39709
60551	0.946132	0.885133
60552	0.946132	0.869874
60553	0.946132	1.3515
60554	0.946133	1.15948
60555	0.946133	2.02222
60556	0.946132	1.46754
60557	0.946132	1.46111
60558	0.946132	1.66347
60559	0.946132	1.49632
60560	0.946128	1.10534
60561	0.946128	0.814785
60562	0.946133	0.606772
60563	0.946133	0.760177
60564	0.94613	0.616308
60565	0.94613	0.824753
60566	0.946131	1.10512
60567	0.946131	0.818666
60568	0.946131	0.799091
60569	0.946131	0.782465
60570	0.946131	0.836584
60571	0.946131	0.837476
60572	1.85779	0.198975
60573	1.00763	0.164223
60574	1.74874	0.180089
60575	1.74873	0.168446
60576	1.14467	0.215334
60577	1.14466	0.191416
60578	1.85779	0.177377
60579	1.61597	0.154314
60580	1.00053	0.182403
60581	1.03018	0.147633
60582	2.65819	0.155424
60583	1.89226	0.45569
60584	1.89226	0.330789
60585	1.89226	0.490406
60586	1.89226	0.424802
60587	3.78451	0.454375
60588	1.89226	0.472538
60589	1.89226	0.222149
60590	1.89226	0.306666
60591	1.89226	0.330269
60592	1.89226	0.28963
60593	1.89226	0.312115
60594	1.11983	0.204718
60595	0.810945	0.23165
60596	0.913906	0.196799
60597	0.913906	0.221327
60598	2.23303	0.163892
60599	1.66057	0.171452
60600	1.28724	0.176595
60601	1.08132	0.187653
60602	1.49316	0.203644
60603	0.810958	0.208929
60604	0.810958	0.203435
60605	0.827861	0.313498
60606	0.946126	0.345147
60607	0.946132	0.434166

60608	0.946132	0.288938
60609	0.827863	0.325222
60610	0.827863	0.316903
60611	0.946129	0.363312
60612	0.946129	0.318973
60613	0.827863	0.329731
60614	0.946128	0.339235
60615	0.946129	0.275845
60616	0.946129	0.326029
60617	0.974718	0.176479
60618	1.53671	0.298039
60619	2.80542	0.194065
60620	2.27259	0.255853
60621	3.8093	0.227747
60622	1.89226	0.296117
60623	1.89226	0.49822
60624	1.89226	0.268092
60625	1.89226	0.45898
60626	3.78453	0.399116
60627	0.946132	0.28285
60628	0.946132	0.427542
60629	0.946131	0.417733
60630	0.946131	0.309123
60631	0.946132	0.419542
60632	0.946132	0.46519
60633	0.946131	0.267405
60634	0.946131	0.273003
60635	0.946132	0.376442
60636	0.946132	0.414773
60637	0.946131	0.35333
60638	0.946131	0.354131
60639	2.80542	0.217675
60640	1.89226	0.278632
60641	2.27259	0.233929
60642	2.27259	0.342978
60643	1.89226	0.168797
60644	1.89226	0.27974
60645	1.89226	0.272865
60646	1.89226	0.480489
60647	1.89226	0.296785
60648	1.89226	0.470826
60649	1.89226	0.296344
60650	1.89226	0.476411
60651	0.946132	0.482211
60652	0.946132	0.296685
60653	0.946131	0.45549
60654	0.946131	0.325772
60655	0.946132	0.459513
60656	0.946132	0.456838
60657	0.946131	0.283492
60658	0.946131	0.303725
60659	0.946131	0.363995
60660	0.946131	0.383057
60661	0.946131	0.327607
60662	0.946131	0.363023
60663	2.68213	2.1
60664	2.58234	2.1
60665	2.58234	2.1

60666	2.61243	2.1
60667	2.67035	2.1
60668	2.70044	2.1
60669	2.68213	2.1
60670	2.53921	2.1
60671	2.53303	2.1
60672	2.67035	2.1
60673	5.20956	2.1
60674	2.67033	2.1
60675	2.58232	2.1
60676	2.55004	2.1
60677	2.64567	2.1
60678	5.228	2.1
60679	2.53301	2.1
60680	2.64567	2.1
60681	2.64567	2.1
60682	2.67033	2.1
60683	2.51603	2.1
60684	2.54069	2.1
60685	0.907497	2.07771
60686	0.907497	2.1
60687	0.926641	2.1
60688	0.926641	2.1
60689	1.14989	2.1
60690	1.10046	2.1
60691	0.695334	2.1
60692	0.684247	2.1
60693	0.695333	2.1
60694	1.14989	2.1
60695	0.684246	2.1
60696	1.10046	2.1
60697	0.946126	0.905497
60698	0.946126	2.1
60699	0.946131	2.1
60700	0.946131	2.1
60701	0.946127	2.1
60702	0.946127	1.13341
60703	0.94613	2.1
60704	0.94613	2.1
60705	0.946128	2.1
60706	0.946128	2.1
60707	0.946128	2.1
60708	0.946128	2.1
60709	1.89226	1.87762
60710	1.89226	1.31788
60711	1.89226	2.1
60712	1.89226	0.864429
60713	1.89226	2.1
60714	1.89226	0.736738
60715	1.42174	2.1
60716	2.29978	2.1
60717	2.41233	2.1
60718	2.41233	2.1
60719	1.39067	2.1
60720	1.39067	2.1
60721	1.89226	2.1
60722	1.89226	2.1
60723	1.89226	2.1

60724	1.89226	1.86388
60725	3.78452	2.1
60726	1.89226	1.99831
60727	1.89226	1.90096
60728	1.89226	2.1
60729	1.89226	1.65412
60730	1.89226	2.1
60731	1.89226	1.08708
60732	2.41234	2.1
60733	1.89226	1.92808
60734	2.29979	2.1
60735	1.89226	2.1
60736	1.89226	2.1
60737	2.29979	2.1
60738	1.09506	0.249779
60739	2.82921	0.300251
60740	2.53251	0.257697
60741	2.53251	0.295605
60742	1.42901	0.26065
60743	1.42901	0.299302
60744	0.946132	0.315146
60745	0.946132	0.308124
60746	0.946128	0.277682
60747	0.946128	0.319334
60748	0.94613	0.333044
60749	0.94613	0.334501
60750	0.946131	0.30871
60751	0.946131	0.29723
60752	0.94613	0.315646
60753	0.94613	0.297804
60754	0.94613	0.324425
60755	0.94613	0.335735
60756	1.89226	0.346364
60757	1.89226	0.365034
60758	1.89226	0.346513
60759	1.89226	0.355655
60760	3.78452	0.346183
60761	2.53251	0.282138
60762	1.89226	0.344395
60763	2.82921	0.286641
60764	1.89226	0.30868
60765	1.89226	0.358104
60766	2.82921	0.360214
60767	1.89226	0.330404
60768	1.89226	0.315187
60769	1.89226	0.336104
60770	1.89226	0.337444
60771	1.89226	0.318847
60772	1.89226	0.320844
60773	2.53251	0.253078
60774	1.89226	0.308384
60775	2.06158	0.236763
60776	1.89226	0.26861
60777	4.42477	0.235341
60778	1.23296	2.1
60779	2.55352	2.1
60780	2.47476	2.1
60781	2.47476	2.1

60782	1.32368	2.1
60783	1.32368	2.1
60784	1.89226	2.1
60785	1.89226	2.1
60786	1.89226	2.1
60787	1.89226	2.1
60788	1.89226	2.1
60789	1.89226	2.1
60790	1.89226	2.09654
60791	1.89226	2.1
60792	1.89226	2.1
60793	1.89226	1.98388
60794	1.89226	2.1
60795	1.89226	1.09867
60796	2.47476	2.1
60797	1.89226	2.1
60798	1.89226	2.1
60799	1.89226	2.1
60800	2.55353	2.1
60801	2.55353	2.1
60802	1.05983	2.1
60803	2.47476	2.1
60804	2.37664	2.1
60805	2.37664	2.1
60806	1.23296	2.1
60807	1.23296	2.1
60808	0.946127	1.84625
60809	0.946127	2.1
60810	0.946131	2.1
60811	0.946131	2.1
60812	0.946127	2.1
60813	0.946127	1.83921
60814	0.946131	2.1
60815	0.946131	2.1
60816	0.946129	2.1
60817	0.946129	2.1
60818	0.946128	2.1
60819	0.946128	2.1
60820	1.89226	2.1
60821	1.89226	2.1
60822	1.89226	2.1
60823	1.89226	0.884822
60824	3.78452	2.1
60825	2.37665	2.1
60826	1.89226	2.1
60827	1.89226	2.1
60828	1.89226	2.1
60829	2.47476	2.1
60830	2.47476	2.1
60831	1.89226	0.769819
60832	1.92119	0.576678
60833	1.99586	0.886925
60834	1.92119	0.731587
60835	1.96693	0.608548
60836	1.89226	0.453716
60837	1.89226	0.646505
60838	1.89226	0.577299
60839	1.89226	0.807149

60840	1.89226	0.625595
60841	1.89226	0.591831
60842	1.89226	0.332806
60843	1.89226	0.887646
60844	2.39541	0.888677
60845	2.57183	1.3314
60846	2.39541	0.789086
60847	2.06868	0.810228
60848	1.89226	0.608396
60849	0.946133	0.582746
60850	0.946133	0.614723
60851	0.997928	0.598593
60852	1.03763	0.67708
60853	0.946131	0.702497
60854	0.946131	0.301051
60855	1.03763	0.655689
60856	1.03763	0.550974
60857	1.03763	0.666576
60858	0.946131	0.653149
60859	0.94613	0.61239
60860	0.99793	0.482236
60861	1.89226	0.383459
60862	1.89226	0.626406
60863	1.89226	0.672798
60864	1.89226	0.289187
60865	1.89226	0.798495
60866	1.89226	0.369963
60867	0.946131	0.567466
60868	0.946131	0.627469
60869	1.36939	0.735368
60870	1.28592	0.859671
60871	0.946129	0.796468
60872	0.946129	0.282297
60873	1.36939	0.677809
60874	1.36939	0.789254
60875	0.946131	0.612377
60876	1.36939	0.622872
60877	0.946131	0.698002
60878	1.28592	0.53418
60879	1.89226	0.540711
60880	1.89226	0.674814
60881	1.89226	0.599925
60882	1.89226	0.588221
60883	3.78453	0.611002
60884	1.89226	0.677212
60885	1.89226	0.68205
60886	1.89226	0.593221
60887	1.89226	0.683988
60888	3.78452	0.63552
60889	1.91005	0.739452
60890	1.89226	0.793912
60891	2.60215	0.609988
60892	2.60215	0.715465
60893	1.89226	0.656695
60894	1.89226	0.844372
60895	0.946132	0.652898
60896	0.946132	0.614129
60897	0.946132	0.661143

60898	0.946132	0.590823
60899	0.946132	0.647354
60900	0.946132	0.677107
60901	0.946132	0.611306
60902	0.946132	0.571707
60903	0.946132	0.619835
60904	0.946132	0.620527
60905	0.946131	0.599961
60906	0.946131	0.618801
60907	1.89226	0.647545
60908	1.89226	0.699392
60909	1.89226	0.881965
60910	1.89226	0.679034
60911	1.89226	0.636298
60912	1.89226	0.841327
60913	0.780483	0.747331
60914	0.78928	1.00171
60915	0.818566	0.747859
60916	0.460387	0.976081
60917	1.82793	0.608936
60918	1.32039	0.785991
60919	0.613889	0.676824
60920	1.0592	0.787658
60921	1.48698	0.840939
60922	0.818555	0.764984
60923	1.11515	0.825579
60924	1.2802	0.782421
60925	2.20046	0.449035
60926	1.1475	0.671606
60927	1.1475	0.462062
60928	2.27062	0.619491
60929	2.27062	0.460642
60930	1.89226	0.626045
60931	2.20046	0.399637
60932	2.27062	0.555991
60933	2.20046	0.493695
60934	1.96242	0.468135
60935	1.89226	0.430077
60936	1.89226	0.41004
60937	1.89226	0.338029
60938	1.89226	0.527886
60939	1.89226	0.396628
60940	3.78452	0.37308
60941	1.89226	0.454037
60942	1.89226	0.340894
60943	1.89226	0.590273
60944	1.89226	0.424521
60945	3.78452	0.462715
60946	1.17403	0.431285
60947	1.13531	0.578644
60948	0.6401	0.439597
60949	0.714566	0.552908
60950	0.915987	0.486778
60951	0.915987	0.267031
60952	0.933891	0.689238
60953	0.933891	0.491787
60954	1.17403	0.588491
60955	0.714566	0.447858

60956	0.6401	0.536081
60957	1.13531	0.44008
60958	0.946133	0.413771
60959	0.946133	0.508848
60960	1.17403	0.371699
60961	1.13531	0.435346
60962	0.946131	0.397833
60963	0.946131	0.318394
60964	1.17403	0.565861
60965	1.17403	0.354616
60966	1.17403	0.453735
60967	0.94613	0.44056
60968	0.946131	0.50451
60969	1.13531	0.398099
60970	1.89226	0.341931
60971	1.89226	0.346284
60972	1.89226	0.326537
60973	1.89226	0.23379
60974	1.89226	0.490823
60975	1.89226	0.344308
60976	0.946131	0.343959
60977	0.946131	0.342733
60978	0.946131	0.362218
60979	0.946131	0.413571
60980	0.946129	0.38554
60981	0.946129	0.2714
60982	0.946133	0.554948
60983	0.946133	0.30003
60984	0.946131	0.3538
60985	0.946131	0.36248
60986	0.946131	0.297211
60987	0.946131	0.332128
60988	2.29413	2.1
60989	1.99332	2.1
60990	1.84774	2.1
60991	2.21582	2.1
60992	4.20915	2.1
60993	0.517359	2.1
60994	0.634469	2.1
60995	0.996661	2.1
60996	0.92387	2.1
60997	0.768098	2.1
60998	0.768098	2.1
60999	0.790242	2.1
61000	0.790242	2.1
61001	0.923871	2.1
61002	0.517359	2.1
61003	0.634469	2.1
61004	0.996661	2.1
61005	1.89226	2.1
61006	1.89226	2.1
61007	1.89226	2.1
61008	1.89226	2.1
61009	3.78453	2.1
61010	0.946132	2.1
61011	0.946132	2.1
61012	0.946131	2.1
61013	0.946131	2.1

61014	0.946132	2.1
61015	0.946132	2.1
61016	0.946131	2.1
61017	0.946131	2.1
61018	0.946132	2.1
61019	0.946132	2.1
61020	0.946131	2.1
61021	0.946131	2.1
61022	1.62708	2.1
61023	2.21582	2.1
61024	2.13925	2.1
61025	2.21582	2.1
61026	1.77117	2.1
61027	1.84774	2.1
61028	0.946132	2.1
61029	0.946132	2.1
61030	0.946131	2.1
61031	0.946131	2.1
61032	0.946132	2.1
61033	0.946132	2.1
61034	0.946131	2.1
61035	0.946131	2.1
61036	0.946131	2.1
61037	0.946131	2.1
61038	0.946131	2.1
61039	0.946131	2.1
61040	1.89226	1.26053
61041	1.89226	0.742285
61042	1.89226	0.72995
61043	1.89226	0.928956
61044	3.78453	1.03079
61045	1.89226	0.738267
61046	1.89226	1.14365
61047	1.89226	1.09498
61048	1.89226	1.49966
61049	1.89226	0.690957
61050	1.89226	0.931389
61051	1.89226	0.739422
61052	1.89226	1.4181
61053	1.89226	0.682469
61054	1.89226	1.1785
61055	1.89226	0.74686
61056	1.89226	1.50145
61057	0.914369	0.955479
61058	0.914369	1.03614
61059	0.810945	1.82701
61060	0.810945	0.992427
61061	2.26581	2.06938
61062	1.66199	1.72165
61063	1.49503	1.07044
61064	1.28816	2.00154
61065	1.08133	1.23274
61066	0.810933	1.22833
61067	1.1212	1.23793
61068	1.89227	0.627436
61069	1.89227	0.612944
61070	1.89227	0.534499
61071	1.89227	0.709723

61072	3.78453	0.572535
61073	1.89226	0.54597
61074	1.89226	0.721231
61075	1.89226	0.668022
61076	1.89226	0.603217
61077	3.78453	0.65531
61078	1.89226	0.728072
61079	1.89226	0.652042
61080	1.89226	0.605216
61081	1.89226	0.837591
61082	3.78453	0.652116
61083	1.89226	0.62182
61084	1.89226	0.898599
61085	1.89226	0.713532
61086	2.25318	0.702108
61087	3.78453	0.824814
61088	1.89226	0.690552
61089	1.89226	0.864313
61090	1.89226	0.673998
61091	1.9637	0.685149
61092	3.78453	0.779972
61093	1.89226	0.709851
61094	1.89226	0.702025
61095	1.89226	0.599114
61096	1.89226	0.773958
61097	3.78453	0.651522
61098	1.89226	0.708062
61099	1.89226	1.02327
61100	1.89226	0.775439
61101	2.53384	0.770956
61102	3.78453	0.886847
61103	1.55405	0.687549
61104	1.03818	1.28182
61105	1.03818	0.581947
61106	1.34857	0.705037
61107	1.46338	0.593107
61108	1.77377	1.16716
61109	1.86273	0.574529
61110	1.73802	0.668369
61111	1.50969	1.49732
61112	1.46337	0.991543
61113	3.20139	0.711321
61114	1.2377	1.05421
61115	1.46337	0.645228
61116	1.27408	0.738024
61117	1.32039	1.41012
61118	1.50969	0.893481
61119	1.50969	0.603
61120	1.89226	1.60183
61121	1.89226	2.1
61122	1.89226	2.1
61123	1.89226	2.07779
61124	1.89226	2.1
61125	1.89226	0.832738
61126	1.89169	2.1
61127	1.89226	2.1
61128	1.89226	2.1
61129	1.89226	2.1

61130	1.96624	2.1
61131	1.96624	2.1
61132	2.62871	2.1
61133	1.6201	2.1
61134	1.49149	2.1
61135	1.6201	2.1
61136	2.62559	2.1
61137	2.75419	1.18592
61138	0.946129	0.685621
61139	0.946129	1.54114
61140	0.946133	2.1
61141	0.946133	1.95855
61142	0.946131	1.80136
61143	0.946131	0.646613
61144	0.946131	2.1
61145	0.946131	1.6239
61146	0.946131	1.49294
61147	0.946131	1.51528
61148	0.946131	1.64977
61149	0.946131	1.43825
61150	0.851543	2.1
61151	0.851543	2.1
61152	1.04043	2.1
61153	0.997939	2.1
61154	0.946132	2.1
61155	0.946132	1.32838
61156	0.894185	2.1
61157	0.893901	2.1
61158	0.998267	2.1
61159	1.04074	2.1
61160	0.85127	2.1
61161	0.85153	2.1
61162	1.89226	2.1
61163	1.89226	1.92546
61164	1.89226	2.1
61165	1.89226	2.1
61166	1.89226	2.1
61167	1.89226	2.1
61168	0.928691	2.1
61169	0.928691	2.1
61170	0.935332	2.1
61171	0.935332	2.1
61172	1.398	2.1
61173	1.14418	2.1
61174	0.479331	2.1
61175	0.719848	2.1
61176	1.398	2.1
61177	0.719848	2.1
61178	1.14417	2.1
61179	0.47933	2.1
61180	2.28835	2.1
61181	1.89226	2.1
61182	1.89226	2.1
61183	1.89226	2.1
61184	2.796	2.1
61185	2.796	2.1
61186	0.946131	2.1
61187	0.946131	2.1

61188	0.946131	2.1
61189	0.946131	1.63121
61190	0.946128	1.64829
61191	0.946128	2.1
61192	0.946133	1.93414
61193	0.946133	2.1
61194	0.946131	2.1
61195	0.946131	2.1
61196	0.94613	2.1
61197	0.94613	2.1
61198	0.946131	2.1
61199	0.946131	2.1
61200	0.946131	2.1
61201	0.946131	1.73323
61202	0.946131	1.75101
61203	0.946131	2.1
61204	0.946131	2.1
61205	0.946131	2.06478
61206	0.946131	2.1
61207	0.946131	2.1
61208	0.946131	2.1
61209	0.946131	2.1
61210	1.89226	2.1
61211	1.89226	1.36804
61212	1.89226	1.83237
61213	1.89226	2.1
61214	3.78452	2.1
61215	2.05207	0.557629
61216	1.70302	0.70972
61217	1.33794	1.50058
61218	1.98194	0.763755
61219	3.36873	0.810463
61220	2.62761	0.686415
61221	1.98194	0.768945
61222	2.28009	1.18883
61223	2.28009	0.584679
61224	2.22939	1.12998
61225	2.22939	0.619067
61226	1.89226	0.781714
61227	1.89226	0.576245
61228	1.89226	0.671316
61229	1.89226	0.592778
61230	1.89226	0.73277
61231	1.89226	0.708044
61232	1.89227	0.77955
61233	1.89227	0.692126
61234	1.89227	1.47044
61235	1.89227	0.958211
61236	1.89227	0.601785
61237	1.89227	0.353804
61238	0.888355	0.57749
61239	0.990973	0.91081
61240	0.71133	0.773916
61241	0.634324	1.04364
61242	0.806209	0.943682
61243	0.806209	0.364659
61244	0.819087	1.43098
61245	0.819087	0.924772

61246	0.888355	1.0003
61247	0.634324	1.07467
61248	0.71133	0.774425
61249	0.990972	0.798855
61250	1.11469	0.988138
61251	0.985023	0.852345
61252	0.888354	0.630239
61253	0.990972	1.0621
61254	0.994754	0.853275
61255	0.994754	0.682876
61256	1.0083	1.13615
61257	1.0083	0.651646
61258	0.888354	0.685458
61259	0.985024	0.850131
61260	0.990971	0.659455
61261	1.11469	0.607872
61262	0.946132	0.885764
61263	0.946132	0.639575
61264	0.946132	0.697368
61265	0.946132	0.668477
61266	0.946132	0.661147
61267	0.946132	0.608153
61268	0.946133	1.24267
61269	0.946133	0.716644
61270	0.946133	0.774375
61271	0.946133	0.708377
61272	0.946132	0.619401
61273	0.946132	0.692898
61274	0.810944	0.711689
61275	0.810944	0.592036
61276	0.810947	0.833577
61277	0.810947	0.352574
61278	1.89227	1.30285
61279	1.35168	0.756315
61280	1.08132	0.719437
61281	1.08131	0.811326
61282	1.08132	0.54581
61283	0.810959	0.569148
61284	0.810959	0.600634
61285	1.70303	2.1
61286	2.03975	2.1
61287	1.99324	2.1
61288	2.17615	2.1
61289	1.40549	2.1
61290	1.53446	2.1
61291	0.946133	1.68434
61292	0.946133	2.09973
61293	0.946129	2.1
61294	0.946129	2.1
61295	0.946131	2.1
61296	0.946131	1.0733
61297	0.946131	2.1
61298	0.946131	2.1
61299	0.946131	2.1
61300	0.946131	2.1
61301	0.946131	2.1
61302	0.946131	2.1
61303	1.89226	2.1

61304	1.89226	1.35231
61305	1.89226	2.1
61306	1.89226	1.36789
61307	1.89226	2.1
61308	1.89226	1.58021
61309	2.17615	2.1
61310	2.3111	2.1
61311	2.50288	2.1
61312	2.50288	2.1
61313	2.03975	2.1
61314	2.03975	2.1
61315	1.76401	2.1
61316	1.95852	2.1
61317	2.32134	2.1
61318	2.12306	2.1
61319	1.8513	2.1
61320	1.70303	2.1
61321	1.89226	2.00747
61322	1.89226	2.1
61323	1.89226	2.1
61324	1.89226	1.36738
61325	3.78452	2.1
61326	1.89226	2.1
61327	1.89226	0.753807
61328	1.89226	1.35555
61329	1.89226	2.1
61330	3.78452	1.94541
61331	1.2243	2.1
61332	1.2243	2.1
61333	1.24189	2.1
61334	1.24189	2.1
61335	1.37811	2.1
61336	1.25144	1.83799
61337	1.08807	2.1
61338	1.17949	2.1
61339	1.17949	2.1
61340	1.37811	2.1
61341	1.08807	2.1
61342	1.25144	2.1
61343	1.89226	2.1
61344	1.89226	2.1
61345	1.89226	2.1
61346	1.89226	2.1
61347	1.89226	2.1
61348	1.89226	2.1
61349	1.89227	2.1
61350	1.89227	2.1
61351	1.89227	2.1
61352	1.89227	2.1
61353	1.89227	2.1
61354	1.89227	2.1
61355	1.89224	2.1
61356	1.89224	2.1
61357	1.89224	2.1
61358	1.89224	2.1
61359	3.78447	2.1
61360	1.89224	2.1
61361	1.89224	2.1

61362	1.89224	2.1
61363	1.89224	2.1
61364	1.89224	2.1
61365	1.89224	2.1
61366	1.89224	2.1
61367	1.89224	2.1
61368	1.89224	2.1
61369	1.89224	2.1
61370	3.78448	2.1
61371	1.89224	2.1
61372	1.703 2.1	
61373	1.89224	2.1
61374	1.72991	2.1
61375	1.07643	2.1
61376	1.36657	2.1
61377	1.44472	2.1
61378	2.41233	2.1
61379	2.51628	2.1
61380	2.51628	2.1
61381	1.42174	2.1
61382	1.42174	2.1
61383	1.89226	2.1
61384	1.89226	1.70071
61385	1.89226	2.1
61386	1.89226	2.1
61387	1.89226	2.1
61388	1.89226	2.1
61389	1.89226	1.87888
61390	1.89226	2.04407
61391	1.89226	2.1
61392	1.89226	0.81826
61393	3.78452	1.94743
61394	2.51628	2.1
61395	1.89226	2.1
61396	1.89226	2.1
61397	1.89226	2.1
61398	2.41234	2.1
61399	2.41234	2.1
61400	0.996254	2.1
61401	0.996254	2.1
61402	1.01153	2.1
61403	1.01153	2.1
61404	1.28542	2.1
61405	1.25814	2.1
61406	0.719184	2.1
61407	0.72236	2.1
61408	0.719183	2.1
61409	1.28542	2.1
61410	0.722359	2.1
61411	1.25814	2.1
61412	0.946126	1.7092
61413	0.946126	2.1
61414	0.946131	2.1
61415	0.946131	2.1
61416	0.946127	2.1
61417	0.946127	1.68503
61418	0.94613	2.1
61419	0.94613	2.1

61420	0.946128	2.1
61421	0.946128	2.1
61422	0.946128	2.1
61423	0.946128	2.1
61424	1.89226	2.1
61425	1.89226	0.962254
61426	1.89226	1.99278
61427	1.89226	2.1
61428	1.89226	1.45434
61429	1.89226	2.07679
61430	0.946132	2.1
61431	0.946132	2.04516
61432	1.25814	2.1
61433	1.25814	2.1
61434	0.946131	2.1
61435	0.946131	1.4404
61436	1.28543	2.1
61437	1.25814	2.1
61438	1.28543	2.1
61439	0.946132	2.1
61440	1.25814	2.1
61441	0.946132	2.1
61442	1.89226	1.26228
61443	1.89226	0.69817
61444	1.89226	1.16352
61445	1.89226	1.00269
61446	1.89226	0.885094
61447	1.89226	0.770938
61448	1.89226	1.15119
61449	2.27719	0.633489
61450	1.89226	0.698957
61451	1.89226	0.888969
61452	3.78452	0.850637
61453	1.89225	1.30696
61454	1.89225	0.75377
61455	1.89225	0.919356
61456	1.89225	1.07065
61457	3.78451	1.0411
61458	1.89226	0.620167
61459	1.89226	0.757636
61460	1.89226	0.913872
61461	1.89226	0.480843
61462	3.78452	0.651277
61463	0.946132	0.396778
61464	0.946132	0.647343
61465	0.946132	0.772908
61466	0.946132	0.639165
61467	0.946131	0.574261
61468	0.946131	0.446099
61469	0.946132	0.75148
61470	0.946132	0.474983
61471	0.946132	0.524905
61472	0.946132	0.575239
61473	0.946132	0.545333
61474	0.946132	0.430309
61475	0.946126	0.690003
61476	0.946126	0.557671
61477	0.946132	1.24355

61478	0.946132	0.859022
61479	0.946128	0.643755
61480	0.946128	0.806606
61481	0.946129	0.753057
61482	0.946129	1.04264
61483	0.946129	0.871576
61484	0.946129	0.94138
61485	0.946129	0.754968
61486	0.946129	0.772808
61487	1.39858	2.1
61488	1.39858	2.1
61489	0.94613	2.1
61490	0.94613	2.1
61491	0.946132	2.1
61492	0.946132	2.1
61493	1.39858	2.1
61494	1.25002	2.1
61495	1.39858	2.1
61496	0.946131	2.1
61497	1.25002	2.1
61498	0.946131	2.1
61499	2.0815	2.1
61500	1.89226	2.1
61501	1.89226	2.1
61502	2.05298	2.1
61503	2.5593	2.1
61504	2.77667	2.1
61505	0.946133	2.1
61506	0.946133	2.1
61507	0.946132	2.1
61508	0.946132	2.1
61509	0.946132	2.1
61510	0.946132	2.1
61511	0.946133	2.1
61512	0.946133	2.1
61513	0.946132	2.1
61514	0.946132	2.1
61515	0.946132	2.1
61516	0.946132	2.1
61517	1.38908	2.1
61518	1.38908	2.1
61519	0.946132	2.1
61520	0.946132	2.1
61521	0.946132	2.1
61522	0.946132	2.1
61523	1.38908	2.1
61524	1.27965	2.1
61525	1.38908	2.1
61526	0.946132	2.1
61527	0.946133	2.1
61528	1.27965	2.1
61529	0.946133	2.1
61530	0.946133	2.1
61531	0.946132	2.1
61532	0.946132	2.1
61533	0.946132	2.1
61534	0.946132	2.1
61535	0.946133	2.1

61536	0.946133	2.1
61537	0.946132	2.1
61538	0.946132	2.1
61539	0.946133	2.1
61540	0.946133	2.1
61541	1.89227	1.06379
61542	1.89227	1.2436
61543	1.89227	0.874564
61544	1.89227	0.634488
61545	3.78453	0.913541
61546	1.33154	0.941343
61547	1.33154	1.10646
61548	0.946132	1.08197
61549	0.946132	0.8626
61550	1.19095	0.746357
61551	1.33154	1.16152
61552	0.946133	1.04427
61553	0.946133	1.1694
61554	0.946133	1.5068
61555	1.19095	0.814601
61556	0.946132	1.37418
61557	1.33154	0.941133
61558	1.0888	0.804259
61559	1.04745	0.814477
61560	0.946132	0.780578
61561	0.946132	0.816231
61562	1.04745	0.623643
61563	1.04745	1.08898
61564	0.946132	1.25327
61565	0.946132	0.641847
61566	1.04745	1.15482
61567	0.946133	0.669084
61568	0.946132	1.14374
61569	1.0888	0.812302
61570	1.89226	1.45932
61571	1.89226	0.634526
61572	1.89226	0.604858
61573	1.89226	0.782349
61574	1.89226	0.726475
61575	1.89226	1.01694
61576	1.89226	1.34666
61577	1.87592	0.598396
61578	1.87592	0.680127
61579	2.3819	0.837481
61580	1.89226	1.01201
61581	2.39825	1.2003
61582	1.89226	0.671301
61583	2.0949	1.01146
61584	1.88741	0.559341
61585	1.89226	0.584907
61586	1.89226	0.64304
61587	1.88741	1.03991
61588	1.07508	1.14081
61589	1.63419	1.34619
61590	1.63419	1.17715
61591	1.09795	1.53144
61592	1.56137	1.09972
61593	1.02513	1.75064

61594	0.967672	0.879259
61595	1.56137	1.01284
61596	1.56137	0.868924
61597	1.07508	1.07125
61598	1.41319	0.820069
61599	0.926902	1.19316
61600	1.56137	1.08245
61601	1.63419	1.16232
61602	2.29852	1.39826
61603	2.14485	0.958393
61604	3.77904	0.950723
61605	1.41319	0.774425
61606	1.92622	0.738818
61607	1.56137	0.7046
61608	1.56137	1.06734
61609	2.14485	0.958482
61610	1.92622	0.83811
61611	1.89227	2.1
61612	1.89227	2.1
61613	1.89227	2.1
61614	1.89227	2.1
61615	3.78453	2.1
61616	2.56618	2.1
61617	2.08612	2.1
61618	2.48048	2.1
61619	1.99644	2.1
61620	4.5666	2.1
61621	2.08612	2.1
61622	1.30659	2.1
61623	1.99644	2.1
61624	1.25404	2.1
61625	3.34016	2.1
61626	1.89226	2.1
61627	1.89226	2.1
61628	1.89226	2.1
61629	1.89226	2.1
61630	1.89226	2.1
61631	1.89226	2.1
61632	1.89227	2.1
61633	1.89227	2.1
61634	1.89227	2.1
61635	1.89227	2.1
61636	3.78453	2.1
61637	2.48048	2.1
61638	1.99644	2.1
61639	2.39022	2.1
61640	1.90734	2.1
61641	4.38782	2.1
61642	1.90734	2.1
61643	1.99644	2.1
61644	1.21564	2.1
61645	1.21564	2.1
61646	1.25404	2.1
61647	1.94574	2.1
61648	0.810946	2.1
61649	0.810946	2.1
61650	0.810947	2.1
61651	0.810947	2.1

61652	1.89226	2.1
61653	1.3517	2.1
61654	1.08133	2.1
61655	1.08132	2.1
61656	1.08132	2.1
61657	0.810934	2.1
61658	0.810934	2.1
61659	1.89226	2.1
61660	1.89226	0.675987
61661	1.89226	1.03898
61662	1.89226	1.85611
61663	3.78453	1.70996
61664	1.89226	0.339419
61665	1.89226	0.215349
61666	1.89226	1.01085
61667	1.89226	0.324282
61668	1.89226	0.777569
61669	1.89226	0.20678
61670	0.946131	0.444421
61671	0.946131	0.31127
61672	0.94613	0.677182
61673	0.94613	0.580022
61674	0.946128	0.459934
61675	0.946128	0.320823
61676	0.946133	0.215745
61677	0.946133	0.777655
61678	0.946131	0.476285
61679	0.946131	0.458226
61680	0.946131	0.603901
61681	0.946131	0.448419
61682	1.89226	2.01879
61683	1.89226	0.584317
61684	1.89226	0.974275
61685	1.89226	0.885529
61686	1.89226	0.831717
61687	1.89226	0.73402
61688	2.56348	0.664517
61689	1.89226	1.36066
61690	1.89226	2.1
61691	2.53234	0.969314
61692	4.45574	0.769918
61693	2.53234	0.350453
61694	1.65583	0.145445
61695	2.56348	0.298031
61696	2.56348	0.340024
61697	1.66295	0.166818
61698	1.65583	0.162664
61699	0.736602	0.229358
61700	0.827916	0.260676
61701	1.26617	0.311023
61702	1.18322	0.152234
61703	0.7366	0.236199
61704	1.18321	0.227273
61705	1.26617	0.240863
61706	0.827918	0.19215
61707	0.99929	0.251743
61708	0.99929	0.310496
61709	1.00348	0.21909

61710	1.00348	0.232662
61711	1.26617	0.556625
61712	1.18322	0.505124
61713	0.946131	1.92579
61714	0.946131	0.661974
61715	0.946129	0.643287
61716	1.18321	0.421063
61717	0.946133	1.60095
61718	1.26617	1.08586
61719	1.26617	1.0866
61720	1.26617	0.280533
61721	0.946131	1.33851
61722	0.946131	0.848508
61723	2.23267	0.183961
61724	1.89226	0.216456
61725	2.07099	0.203793
61726	1.89226	0.222609
61727	2.07099	0.201722
61728	1.89226	0.206405
61729	0.946132	0.324329
61730	1.35403	0.191124
61731	1.24038	0.20437
61732	0.946131	0.210014
61733	0.946131	0.270511
61734	0.946131	0.281236
61735	1.24039	0.215881
61736	1.35403	0.213562
61737	1.24038	0.26382
61738	1.24038	0.287059
61739	0.946131	0.231965
61740	0.946131	0.352685
61741	1.89226	0.354624
61742	1.89226	0.545861
61743	1.89226	0.317902
61744	1.89226	0.2922
61745	1.89226	0.650594
61746	1.89226	0.621969
61747	1.89226	0.226453
61748	1.89226	0.527965
61749	1.89226	0.359976
61750	1.89226	0.431605
61751	3.78452	0.501103
61752	1.56971	0.252487
61753	2.80086	0.335523
61754	2.59806	0.252235
61755	1.93489	0.239312
61756	2.59806	0.394621
61757	1.93489	0.316383
61758	0.827467	0.223245
61759	1.26652	0.228994
61760	1.13679	0.23391
61761	0.639035	0.23316
61762	1.11547	0.302946
61763	1.30656	0.266661
61764	0.869062	0.206089
61765	0.696608	0.228793
61766	1.07205	0.246959
61767	0.953656	0.317366

61768	0.970744	0.21653
61769	0.970744	0.247408
61770	2.38681	2.1
61771	2.50438	1.18478
61772	2.42082	1.53731
61773	2.59106	2.1
61774	4.92521	1.78767
61775	2.50438	1.06661
61776	2.40627	2.1
61777	2.59106	2.1
61778	2.5444	0.973774
61779	5.04879	2.05498
61780	2.42082	2.1
61781	2.70575	2.1
61782	2.64778	2.1
61783	2.59106	1.43654
61784	2.47753	2.1
61785	2.42082	0.941101
61786	2.70575	2.1
61787	2.5444	1.28096
61788	2.76095	2.1
61789	2.59106	1.46732
61790	2.71429	2.05056
61791	2.5444	1.25974
61792	1.89226	2.1
61793	2.38473	2.1
61794	2.36689	2.1
61795	2.38473	2.1
61796	1.87442	2.1
61797	1.89226	2.1
61798	1.89226	1.8755
61799	1.89226	0.659481
61800	1.89226	0.940447
61801	1.89226	0.667671
61802	1.89226	1.31469
61803	1.89226	0.693993
61804	2.14952	1.16263
61805	1.89226	0.463475
61806	1.89226	0.979258
61807	1.89226	0.403036
61808	2.0692	1.77658
61809	2.0692	0.73114
61810	1.89226	2.1
61811	2.36689	2.1
61812	2.36204	2.1
61813	2.36689	2.1
61814	1.88742	2.1
61815	1.89226	2.1
61816	0.946132	0.62042
61817	0.946132	2.1
61818	0.946131	2.1
61819	0.946131	1.44408
61820	0.946132	0.948357
61821	0.946132	1.5656
61822	0.946131	2.1
61823	0.946131	1.6842
61824	0.946132	1.54164
61825	0.946132	1.85308

61826	0.946131	1.31451
61827	0.946131	2.1
61828	0.946128	2.1
61829	0.946128	1.75557
61830	1.07476	0.97037
61831	1.07476	2.06517
61832	0.946131	1.15012
61833	0.946131	1.63406
61834	1.13182	2.1
61835	1.07476	2.08516
61836	1.13182	1.426
61837	0.946131	1.3656
61838	1.07476	1.411
61839	0.94613	0.800988
61840	1.89227	0.441167
61841	1.89227	0.531706
61842	1.89227	0.663927
61843	1.89227	0.6066
61844	3.78453	0.51204
61845	1.89226	0.468883
61846	1.89226	0.526014
61847	1.89226	0.479241
61848	1.89226	0.751952
61849	3.78452	0.500567
61850	1.89226	0.520912
61851	1.89226	0.618832
61852	1.89226	0.857337
61853	1.89226	0.825779
61854	3.78452	0.56262
61855	1.89226	0.526757
61856	1.89226	0.632598
61857	1.89226	0.567501
61858	1.89226	0.58771
61859	1.89226	0.650674
61860	1.89226	0.645877
61861	2.58344	0.921614
61862	2.00267	0.44364
61863	2.14218	0.81215
61864	2.22674	1.17429
61865	2.14218	0.37553
61866	2.22674	0.465071
61867	2.83531	1.23547
61868	1.56043	0.606952
61869	2.22674	1.14244
61870	1.90912	1.55894
61871	2.22674	0.431643
61872	1.90912	0.553331
61873	0.780217	0.645513
61874	1.11337	0.699731
61875	1.00133	1.0915
61876	0.804472	0.776097
61877	0.804472	0.930082
61878	0.780217	0.556836
61879	1.00133	0.437184
61880	1.11337	0.91109
61881	0.856668	0.878814
61882	0.856668	1.4782
61883	0.924883	0.54724

61884	0.924883	0.492844
61885	1.93107	2.1
61886	1.68657	2.1
61887	1.7237	2.1
61888	1.89226	2.1
61889	1.53436	2.1
61890	1.68657	2.1
61891	1.89226	2.1
61892	1.89226	2.1
61893	1.89226	2.1
61894	1.89226	2.1
61895	3.78453	2.1
61896	0.946131	2.1
61897	0.946131	2.1
61898	0.855092	2.1
61899	0.83953	2.1
61900	0.946131	2.1
61901	0.946131	2.1
61902	0.839531	2.1
61903	0.839531	2.1
61904	0.946131	2.1
61905	0.855093	2.1
61906	0.839531	2.1
61907	0.946131	2.1
61908	1.89226	2.1
61909	1.70302	2.1
61910	2.21419	2.1
61911	1.93107	2.1
61912	1.97872	2.1
61913	1.70302	2.1
61914	0.946133	2.1
61915	0.946133	2.1
61916	0.946132	2.1
61917	0.946132	2.1
61918	0.946132	2.1
61919	0.946132	2.1
61920	0.946132	2.1
61921	0.946132	2.1
61922	0.946133	2.1
61923	0.946133	2.1
61924	0.946132	2.1
61925	0.946132	2.1
61926	1.90081	2.1
61927	1.89226	2.1
61928	1.51379	2.1
61929	1.36813	2.1
61930	1.71019	2.1
61931	1.89226	2.1
61932	1.89227	1.12929
61933	1.89227	0.712579
61934	1.89227	0.709153
61935	1.89227	0.952662
61936	1.89227	1.15082
61937	1.89227	1.44094
61938	1.36428	1.07139
61939	1.37869	0.930172
61940	0.946132	0.785515
61941	0.946132	1.01939

61942	1.37869	0.724355
61943	1.37869	1.67156
61944	0.946133	1.08049
61945	0.946133	1.24264
61946	1.37869	0.723771
61947	0.946132	0.887437
61948	1.36428	1.16426
61949	0.946133	0.616944
61950	0.94612	0.945614
61951	0.94612	0.8631
61952	0.946119	0.896224
61953	0.946119	0.979947
61954	0.946119	0.64831
61955	0.946119	1.30437
61956	0.94612	1.06365
61957	0.94612	1.30842
61958	0.946132	0.882992
61959	0.946132	0.666474
61960	0.946107	0.698784
61961	0.946107	1.11742
61962	1.89225	0.0522355
61963	1.89225	1.84192
61964	1.63564	0.0522355
61965	1.42278	2.1
61966	1.42278	2.1
61967	1.57534	0.0522355
61968	1.53735	2.1
61969	1.68991	0.0522355
61970	1.57535	0.0522355
61971	1.04442	2.1
61972	1.04442	2.1
61973	1.22031	0.0522355
61974	1.42278	2.1
61975	1.59868	0.0522355
61976	0.946132	2.1
61977	0.946132	2.1
61978	0.946131	2.1
61979	0.946131	2.1
61980	0.946131	2.1
61981	0.946131	2.1
61982	0.946132	2.1
61983	0.946132	2.1
61984	0.946132	2.1
61985	0.946132	2.1
61986	0.946131	2.1
61987	0.946131	2.1
61988	1.89226	2.1
61989	1.89226	2.1
61990	1.89226	2.1
61991	1.89226	2.1
61992	1.89226	2.1
61993	1.89226	2.1
61994	2.03143	0.727679
61995	1.47111	1.25674
61996	2.28685	1.21408
61997	2.28685	1.04587
61998	1.36006	0.830019
61999	1.36006	0.802451

62000	1.89226	0.668326
62001	1.89226	1.01756
62002	1.89226	0.928084
62003	1.89226	0.989497
62004	1.89226	0.661545
62005	1.89226	0.747941
62006	1.89226	0.964208
62007	1.89226	2.1
62008	1.89226	1.9965
62009	1.89226	2.1
62010	1.89226	1.01533
62011	1.89226	1.57631
62012	0.902821	1.97921
62013	0.902821	1.56969
62014	0.889218	2.1
62015	0.889218	2.1
62016	0.675759	2.1
62017	1.05649	2.1
62018	0.735554	1.92397
62019	1.14342	1.8398
62020	1.14343	1.12638
62021	1.05649	2.1
62022	0.735554	1.99638
62023	0.675758	2.1
62024	2.46286	0.565981
62025	2.28685	0.709871
62026	2.67843	0.748214
62027	2.67843	0.632402
62028	2.03143	0.634248
62029	2.03143	0.570244
62030	1.89226	0.601432
62031	1.89226	0.651442
62032	1.89226	0.611865
62033	1.89226	0.617526
62034	3.78452	0.679697
62035	0.94613	0.952723
62036	0.94613	0.902624
62037	0.946128	1.21559
62038	0.946128	0.776248
62039	0.94613	2.1
62040	0.94613	0.796804
62041	0.946128	0.618987
62042	0.946128	0.848787
62043	0.946129	0.500095
62044	0.946129	0.9178
62045	0.946129	0.595844
62046	0.946129	1.87805
62047	2.67843	0.702495
62048	2.11297	1.3272
62049	2.52453	1.26694
62050	2.52453	1.32709
62051	2.28685	0.863179
62052	2.28685	0.791191
62053	1.89226	0.466066
62054	1.89226	0.753031
62055	1.89226	0.408713
62056	1.89226	0.745337
62057	3.78451	0.55493

62058	2.39524	0.387253
62059	2.10188	0.710115
62060	2.29839	0.398663
62061	2.02242	0.589955
62062	4.40028	0.503899
62063	1.71839	1.98483
62064	1.65261	1.77302
62065	2.10189	0.593062
62066	2.10189	1.1304
62067	2.02243	0.587806
62068	1.63893	0.590303
62069	0.946132	2.06651
62070	0.946132	0.616628
62071	0.946129	0.606602
62072	0.946129	2.1
62073	0.94613	2.1
62074	0.94613	1.8201
62075	0.946131	0.5913
62076	0.946131	0.603727
62077	0.946131	0.754833
62078	0.946131	0.774697
62079	0.946131	0.821888
62080	0.946131	0.884374
62081	1.89226	0.409659
62082	1.89226	0.853817
62083	1.89226	0.317399
62084	1.89226	0.600879
62085	3.78452	0.496518
62086	2.2984	0.391975
62087	2.02242	0.629141
62088	2.21582	0.225743
62089	1.94412	0.590396
62090	4.24251	0.49968
62091	1.94412	0.570412
62092	1.5854	1.47327
62093	2.02243	1.74671
62094	2.02243	0.598042
62095	1.57431	0.586666
62096	1.65261	1.75536
62097	0.810943	0.602462
62098	0.810943	2.05525
62099	0.810945	1.71065
62100	0.810945	1.93118
62101	1.89226	0.574196
62102	1.3517	0.72034
62103	1.08133	0.770298
62104	1.08132	1.8046
62105	1.08132	0.572977
62106	0.810933	0.766428
62107	0.810933	0.790454
62108	1.89226	0.148197
62109	1.89226	0.227207
62110	1.89226	0.19903
62111	1.89226	0.177788
62112	1.89226	0.18631
62113	1.89226	0.167271
62114	1.89226	0.198146
62115	1.89226	0.314886

62116	1.89226	0.232422
62117	1.89226	0.279871
62118	3.78452	0.292984
62119	1.89226	0.181769
62120	1.89226	0.267376
62121	1.89226	0.226007
62122	1.89226	0.206655
62123	1.89226	0.215652
62124	1.89226	0.197454
62125	0.946128	0.263699
62126	0.946128	0.203596
62127	0.946132	0.194245
62128	0.946132	0.237599
62129	0.94613	0.270213
62130	0.94613	0.286587
62131	0.946129	0.217861
62132	0.946129	0.190701
62133	0.94613	0.192229
62134	0.94613	0.221574
62135	0.94613	0.208191
62136	0.94613	0.226422
62137	1.89226	0.154822
62138	1.89226	0.242009
62139	1.89226	0.187766
62140	1.89226	0.184763
62141	1.89226	0.223086
62142	1.89226	0.222239
62143	2.81478	2.1
62144	1.89226	2.1
62145	2.65557	2.1
62146	1.89226	2.1
62147	4.54783	2.1
62148	1.89226	2.1
62149	1.89226	2.1
62150	1.89226	2.1
62151	1.89226	2.1
62152	3.78452	2.1
62153	2.65557	2.1
62154	1.89226	2.1
62155	1.89226	2.1
62156	1.89226	2.1
62157	2.57997	2.1
62158	2.57997	2.1
62159	0.946131	2.1
62160	0.946131	2.1
62161	0.946128	2.1
62162	0.946128	2.1
62163	0.94613	2.1
62164	0.94613	2.1
62165	0.946129	2.1
62166	0.946129	2.1
62167	0.94613	2.1
62168	0.94613	2.1
62169	0.94613	2.1
62170	0.94613	2.1
62171	1.89226	1.01663
62172	2.27703	1.56285
62173	2.35615	1.59501

62174	2.35615	1.12145
62175	1.89226	1.56378
62176	1.89226	1.08644
62177	0.946132	0.973363
62178	1.17807	1.6038
62179	1.14926	0.927417
62180	0.946132	1.10902
62181	0.946133	1.10911
62182	0.946133	1.21159
62183	1.14926	1.03209
62184	1.14926	1.01847
62185	0.946132	1.16319
62186	0.946132	1.03302
62187	1.17807	1.25984
62188	1.14926	1.01947
62189	2.16841	0.990172
62190	1.35487	1.17367
62191	1.19854	1.03904
62192	1.19854	1.05848
62193	2.46078	1.03376
62194	2.46078	1.07202
62195	0.69308	1.07579
62196	1.3119	1.26788
62197	0.677433	1.2621
62198	1.23039	1.13401
62199	0.978178	1.25658
62200	0.978178	1.26953
62201	0.945294	1.12433
62202	0.945294	1.14532
62203	1.3119	1.16993
62204	1.23039	1.10608
62205	0.693081	1.12619
62206	0.677432	1.12902
62207	1.89226	0.919097
62208	1.89226	1.63796
62209	1.89226	1.4783
62210	1.89226	0.996878
62211	1.89226	1.42426
62212	1.89226	0.987007
62213	0.946132	0.891012
62214	0.946132	1.52278
62215	0.946132	0.802689
62216	0.946132	1.00887
62217	0.946133	1.00649
62218	0.946133	1.15863
62219	0.946132	0.911274
62220	0.946132	0.844953
62221	0.946133	0.990846
62222	0.946133	0.905189
62223	0.946132	1.03449
62224	0.946132	0.933199
62225	2.60637	1.72564
62226	1.89226	2.1
62227	1.89226	2.1
62228	1.89226	1.26988
62229	3.78452	2.1
62230	1.89226	0.884214
62231	1.8396	1.4198

62232	1.89226	1.60327
62233	1.89226	0.897001
62234	1.60993	1.97241
62235	1.60993	0.938677
62236	0.946131	1.09949
62237	0.946131	1.41084
62238	0.946128	2.0791
62239	0.946128	1.6734
62240	0.946131	1.08506
62241	0.946131	1.51134
62242	0.946127	2.1
62243	0.946127	1.33158
62244	0.946129	1.65837
62245	0.946129	1.6439
62246	0.94613	1.53974
62247	0.94613	1.54419
62248	1.89226	2.1
62249	1.89226	1.37923
62250	1.89226	2.01661
62251	1.89226	1.92334
62252	1.89226	1.98313
62253	1.89226	1.89117
62254	1.89226	0.859832
62255	1.89226	1.24051
62256	1.89226	1.56354
62257	1.89226	0.818337
62258	1.89226	1.74422
62259	1.89226	0.791316
62260	0.946131	1.10905
62261	0.946131	1.49147
62262	0.946128	2.02611
62263	0.946128	1.51932
62264	0.946131	1.08952
62265	0.946131	1.39343
62266	0.946127	1.47895
62267	0.946127	2.02723
62268	0.94613	1.59978
62269	0.94613	1.60717
62270	0.946129	1.51534
62271	0.946129	1.55409
62272	0.946133	2.1
62273	0.946133	2.1
62274	1.28153	2.1
62275	1.26781	2.1
62276	0.946132	2.1
62277	0.946132	2.1
62278	1.28153	2.1
62279	1.28153	2.1
62280	0.946132	2.1
62281	1.28153	2.1
62282	0.946133	2.1
62283	1.26781	2.1
62284	1.89226	2.1
62285	1.89226	0.828853
62286	1.89226	2.1
62287	1.89226	1.94092
62288	1.89226	2.1
62289	1.89226	1.17196

62290	2.5844	2.1
62291	1.09563	2.1
62292	1.1468	2.1
62293	1.09563	2.1
62294	2.6142	2.1
62295	2.56304	2.1
62296	0.946126	2.1
62297	0.946126	2.1
62298	1.2922	2.1
62299	1.28152	2.1
62300	0.946126	2.1
62301	0.946126	2.1
62302	1.2922	2.1
62303	1.2922	2.1
62304	1.29221	2.1
62305	0.946132	2.1
62306	0.946119	2.1
62307	1.28151	2.1
62308	1.89225	2.1
62309	1.89225	1.3559
62310	1.89225	2.1
62311	1.89225	1.94798
62312	1.89225	2.1
62313	1.89225	1.26571
62314	0.946121	1.04937
62315	0.946121	2.1
62316	0.946126	1.10546
62317	0.946126	2.1
62318	0.946124	0.922653
62319	0.946124	2.08655
62320	0.946123	2.1
62321	0.946123	2.1
62322	0.94613	2.1
62323	0.94613	2.1
62324	0.946117	2.1
62325	0.946117	2.1
62326	1.89226	0.915873
62327	1.89226	0.9944
62328	1.89226	0.790547
62329	1.89226	2.05569
62330	1.89226	0.6239
62331	1.89226	2.1
62332	1.89226	1.41745
62333	1.89226	1.31854
62334	1.89226	0.726467
62335	1.89226	2.05908
62336	1.89226	0.579082
62337	1.89226	2.1
62338	1.89226	1.5462
62339	2.81281	1.94212
62340	2.50369	0.894183
62341	2.50369	2.1
62342	1.89226	0.943032
62343	1.89226	2.1
62344	1.89226	2.1
62345	1.89226	2.1
62346	1.89226	1.54271
62347	1.89226	2.1

62348	1.89226	1.58096
62349	1.89226	2.1
62350	1.89226	2.1
62351	1.89226	2.1
62352	1.89226	2.1
62353	1.89226	2.1
62354	1.89226	2.1
62355	1.89226	2.1
62356	0.907036	2.1
62357	1.09714	2.1
62358	0.994609	2.1
62359	0.838808	2.1
62360	0.976536	2.1
62361	0.976536	2.1
62362	0.959412	2.1
62363	0.959412	2.1
62364	1.09714	2.1
62365	0.994609	2.1
62366	0.907036	2.1
62367	0.838808	2.1
62368	0.99461	2.1
62369	0.838808	2.1
62370	0.738212	2.1
62371	0.565634	2.1
62372	0.775921	2.1
62373	0.775921	2.1
62374	0.784322	2.1
62375	0.784322	2.1
62376	0.994609	2.1
62377	0.738212	2.1
62378	0.838808	2.1
62379	0.565635	2.1
62380	1.89226	2.1
62381	1.89226	2.1
62382	1.89226	1.28653
62383	1.89226	2.1
62384	1.89226	1.01189
62385	1.89226	2.1
62386	1.89226	2.1
62387	1.89226	2.1
62388	1.89226	2.1
62389	1.89226	2.1
62390	1.89226	2.1
62391	1.89226	2.1
62392	2.19428	2.1
62393	1.98922	2.1
62394	2.27322	2.1
62395	2.45893	2.1
62396	4.4675	2.1
62397	0.99461	2.1
62398	1.13661	2.1
62399	0.902272	2.1
62400	0.738212	2.1
62401	0.931874	2.1
62402	0.931874	2.1
62403	0.942953	2.09947
62404	0.942953	2.1
62405	1.13658	2.1

62406	0.902248	2.1
62407	0.994637	2.1
62408	0.738233	2.1
62409	1.89226	1.47507
62410	1.89226	1.29055
62411	1.89226	1.69421
62412	1.89226	1.58096
62413	1.89226	1.49136
62414	1.89226	1.36836
62415	1.89227	1.65678
62416	2.58246	1.10795
62417	1.89227	1.32105
62418	1.89227	1.48482
62419	3.78453	1.25436
62420	1.89226	1.91541
62421	1.89226	1.41212
62422	1.65573	1.67506
62423	1.65573	1.74004
62424	1.89226	1.69569
62425	1.89226	1.77904
62426	1.89226	1.16664
62427	1.89226	1.24049
62428	1.89226	1.245
62429	1.89226	1.23136
62430	1.89226	1.2016
62431	1.89226	1.16794
62432	1.89226	1.24337
62433	1.89226	1.20796
62434	1.89226	1.22497
62435	1.89226	1.25988
62436	1.89226	1.21022
62437	1.89226	1.24822
62438	0.946132	1.73524
62439	0.946132	1.62161
62440	1.01023	1.1829
62441	1.13651	1.52428
62442	1.01023	1.14458
62443	0.946133	1.41838
62444	1.0644	1.45336
62445	1.0644	1.70031
62446	0.946132	1.72477
62447	0.946132	1.52869
62448	1.13651	1.3575
62449	0.946132	1.41979
62450	1.89226	0.801809
62451	1.89226	1.59411
62452	1.89226	1.17291
62453	1.89226	0.68562
62454	3.78451	0.896051
62455	1.00934	0.561593
62456	0.741561	0.491529
62457	0.946131	0.776537
62458	0.946131	0.914959
62459	0.946131	1.16991
62460	0.741561	0.318827
62461	1.00934	0.418222
62462	0.946131	0.985996
62463	0.741586	0.661887

62464	0.741586	0.660052
62465	0.946106	0.738623
62466	0.946106	0.535986
62467	1.14895	0.778558
62468	0.957449	0.915803
62469	0.934806	1.30577
62470	0.934806	1.20607
62471	1.13536	0.640299
62472	1.02829	1.57549
62473	0.946129	1.68215
62474	0.946129	0.753068
62475	0.946127	1.0951
62476	0.946127	1.18133
62477	0.946127	1.0465
62478	1.0283	1.15911
62479	1.89226	1.51518
62480	1.89226	0.85729
62481	1.89226	0.821603
62482	1.89226	1.33116
62483	3.78452	1.13842
62484	1.89226	0.618134
62485	1.89226	0.988771
62486	1.89226	0.892507
62487	1.89226	0.565318
62488	3.78453	0.68199
62489	1.89226	0.988833
62490	2.69068	0.602742
62491	1.89226	0.571851
62492	1.89226	0.883478
62493	3.78452	0.819888
62494	1.89226	0.857257
62495	1.89226	1.49232
62496	1.89226	1.45456
62497	1.89226	0.992504
62498	1.89226	1.29341
62499	1.89226	0.883743
62500	1.89226	2.1
62501	1.89226	2.1
62502	1.89226	2.1
62503	1.89226	2.1
62504	1.89226	2.1
62505	1.89226	2.1
62506	1.89226	2.1
62507	1.89226	2.1
62508	1.89226	2.1
62509	1.89226	2.1
62510	1.89226	2.1
62511	1.89226	2.1
62512	0.985109	2.1
62513	1.31144	2.1
62514	1.45864	2.1
62515	1.10667	2.1
62516	2.44375	2.1
62517	0.655718	2.1
62518	0.729316	2.1
62519	0.684594	2.1
62520	0.782707	2.1
62521	0.700849	2.1

62522	0.700849	2.1
62523	0.71306	2.1
62524	0.71306	2.1
62525	0.729318	2.1
62526	0.782705	2.1
62527	0.684592	2.1
62528	0.655719	2.1
62529	0.946128	2.1
62530	0.946128	2.1
62531	0.946133	2.1
62532	0.946133	2.1
62533	0.946131	2.1
62534	0.946131	2.1
62535	0.946131	2.1
62536	0.946131	2.1
62537	0.94613	2.1
62538	0.94613	2.1
62539	0.946131	2.1
62540	0.946131	2.1
62541	1.89226	2.1
62542	1.89226	2.1
62543	1.89226	2.1
62544	1.89226	2.1
62545	1.89226	2.1
62546	1.89226	2.1
62547	0.553334	2.1
62548	0.629936	2.1
62549	0.828211	2.1
62550	0.729319	2.1
62551	0.674025	2.1
62552	0.674025	2.1
62553	0.68523	2.1
62554	0.68523	2.1
62555	0.828211	2.1
62556	0.629935	2.1
62557	0.72932	2.1
62558	0.553334	2.1
62559	0.828208	2.1
62560	0.729316	2.1
62561	0.907037	2.1
62562	0.782707	2.1
62563	0.799048	2.1
62564	0.799048	2.1
62565	0.811867	2.1
62566	0.811867	2.1
62567	0.828209	2.1
62568	0.907035	2.1
62569	0.729318	2.1
62570	0.782706	2.1
62571	2.32981	2.1
62572	1.89226	1.96564
62573	1.89226	2.1
62574	1.89226	1.74573
62575	2.2671	2.1
62576	2.2671	2.09329
62577	1.89226	1.77297
62578	1.89226	1.89781
62579	1.89226	1.77173

62580	1.89226	1.38217
62581	1.89226	1.89551
62582	1.89226	1.75799
62583	1.3991	1.70124
62584	1.81793	1.64489
62585	2.07226	1.40062
62586	2.07226	1.37411
62587	1.24924	1.84876
62588	1.24924	1.97075
62589	0.946131	0.776471
62590	0.946131	2.1
62591	1.16491	2.1
62592	1.16491	2.1
62593	0.946131	1.93046
62594	0.946131	1.1225
62595	1.16491	2.1
62596	1.19523	2.1
62597	0.946132	2.1
62598	1.19523	2.03516
62599	1.16491	2.1
62600	0.946131	2.1
62601	1.89226	2.1
62602	1.89226	1.3536
62603	1.89226	1.95337
62604	1.89226	0.740905
62605	3.78452	1.12805
62606	0.901204	1.45811
62607	0.901204	1.98137
62608	0.902504	1.68666
62609	0.902504	1.93451
62610	1.10416	1.57242
62611	1.03613	0.991966
62612	0.764922	2.01119
62613	0.699552	1.98403
62614	0.764921	1.41389
62615	1.10416	1.54802
62616	0.699551	1.70267
62617	1.03613	1.28209
62618	1.89226	0.822323
62619	1.89226	0.998842
62620	1.89226	1.00613
62621	1.89226	0.86519
62622	3.78453	0.960177
62623	1.89226	0.972119
62624	1.89226	1.09564
62625	1.89226	0.965613
62626	1.89226	1.2365
62627	1.89226	0.976725
62628	1.89226	1.25005
62629	0.946131	1.01757
62630	0.946131	1.0136
62631	0.946131	0.90482
62632	0.946131	0.871134
62633	0.946131	0.903359
62634	0.946131	1.0228
62635	0.946131	1.02733
62636	0.946131	0.829067
62637	0.946131	0.90161

62638	0.946131	0.90023
62639	0.946131	0.893357
62640	0.946131	0.914973
62641	1.89226	0.968159
62642	1.89226	1.10219
62643	1.89226	0.998226
62644	1.89226	1.14375
62645	1.89226	0.997987
62646	1.89226	1.1453
62647	0.946127	1.02178
62648	0.946127	1.01811
62649	1.22788	0.969387
62650	1.35469	1.01348
62651	1.35469	1.04848
62652	1.35469	1.02452
62653	0.946128	1.02335
62654	0.946128	0.93421
62655	0.946129	0.988083
62656	1.22788	1.01477
62657	1.35469	1.00705
62658	0.946129	0.972256
62659	1.22788	1.02413
62660	1.35469	1.07432
62661	1.02602	0.89824
62662	0.794861	0.999638
62663	1.15301	1.06352
62664	1.15301	1.08301
62665	1.10089	0.887208
62666	1.10089	0.995734
62667	0.794861	0.96398
62668	1.22788	1.05093
62669	1.02602	1.03516
62670	1.35469	1.03812
62671	1.89226	0.414895
62672	1.89226	0.619647
62673	1.89226	1.05441
62674	1.89226	0.498609
62675	3.78452	0.583188
62676	1.13536	1.24515
62677	0.942524	0.538421
62678	0.946131	0.563497
62679	0.946131	0.458655
62680	0.946127	0.596306
62681	0.946127	1.24355
62682	1.26282	0.453688
62683	0.917471	1.20716
62684	1.21023	0.68831
62685	1.00851	0.687646
62686	1.17955	0.651709
62687	0.883746	0.629962
62688	1.89226	0.544192
62689	1.89226	1.18723
62690	1.89226	0.661531
62691	1.89226	0.790714
62692	1.89226	0.675692
62693	1.89226	0.954703
62694	1.89226	0.474078
62695	1.89226	0.716229

62696	1.89226	0.513451
62697	1.89226	0.580849
62698	1.89226	0.530982
62699	1.89226	0.706677
62700	0.94613	0.784783
62701	0.94613	0.574109
62702	0.94613	0.476588
62703	0.94613	0.541392
62704	0.946128	0.557051
62705	0.946128	0.807933
62706	0.946131	1.00002
62707	0.946131	0.439253
62708	0.94613	0.602905
62709	0.94613	0.644942
62710	0.94613	0.611713
62711	0.94613	0.586891
62712	0.883758	1.15311
62713	1.03041	0.819452
62714	0.946131	0.656768
62715	0.946131	0.514588
62716	0.851537	0.625968
62717	0.851537	1.12907
62718	1.23942	0.479527
62719	1.14906	1.37404
62720	0.851484	0.626162
62721	0.985518	0.597135
62722	1.0156	0.679523
62723	0.851526	0.69005
62724	1.89226	0.57481
62725	1.89226	1.43824
62726	1.89226	0.666736
62727	1.89226	0.835472
62728	1.89226	0.692123
62729	1.89226	1.01162
62730	1.89226	0.53772
62731	1.89226	0.738832
62732	1.89226	0.569371
62733	1.89226	0.678563
62734	1.89226	0.545277
62735	1.89226	0.732432
62736	1.89227	0.390059
62737	1.89227	0.444884
62738	1.89227	0.433964
62739	1.89227	0.541334
62740	3.78453	0.42704
62741	1.89226	0.44706
62742	1.89226	0.443572
62743	1.89226	0.506802
62744	1.89226	0.648047
62745	3.78452	0.516825
62746	0.946132	0.553094
62747	0.946132	0.582311
62748	0.946128	0.722016
62749	0.946128	0.576396
62750	0.94613	0.517737
62751	0.94613	0.581905
62752	0.94613	0.437464
62753	0.94613	0.618589

62754	0.946131	0.552219
62755	0.946131	0.750387
62756	0.94613	0.475874
62757	0.94613	0.513737
62758	1.89226	0.559208
62759	1.89226	0.500125
62760	1.89226	0.560536
62761	1.89226	0.464737
62762	1.89226	0.589299
62763	1.89226	0.473113
62764	1.98874	0.374091
62765	2.33455	0.409607
62766	2.01865	0.50751
62767	2.07024	0.504118
62768	4.32329	0.514739
62769	1.98874	0.419866
62770	2.00266	0.458648
62771	2.14218	0.675897
62772	2.07024	0.811947
62773	3.9914	0.454589
62774	1.00133	0.570819
62775	0.804471	0.462338
62776	1.03512	0.663802
62777	0.995176	0.609217
62778	0.80447	1.08063
62779	0.995178	0.480979
62780	1.00133	0.517567
62781	1.03512	0.959226
62782	0.880587	0.933585
62783	0.880587	0.748613
62784	0.959002	0.424798
62785	0.959002	0.471189
62786	2.33455	0.418239
62787	1.99036	0.432523
62788	2.07024	0.512789
62789	2.58645	0.531796
62790	4.32491	0.394259
62791	1.89226	0.58941
62792	1.89226	0.54191
62793	1.89226	0.61588
62794	1.89226	0.475552
62795	3.78453	0.47084
62796	1.89226	0.726764
62797	1.89226	0.559325
62798	1.89226	0.650049
62799	1.89226	0.624835
62800	3.78453	0.725169
62801	1.89226	0.492687
62802	1.89226	0.401459
62803	1.89226	0.426468
62804	1.89226	0.472376
62805	1.89226	0.424726
62806	1.89226	0.474754
62807	1.89226	0.475116
62808	2.1523	0.248212
62809	2.53257	0.307539
62810	2.53257	0.290948
62811	1.89226	0.466077

62812	1.89226	0.44663
62813	0.946133	0.43098
62814	0.946133	0.508453
62815	0.946132	0.358219
62816	0.946132	0.389451
62817	0.946132	0.352402
62818	0.946132	0.371782
62819	0.946132	0.517104
62820	0.946132	0.381053
62821	0.946133	0.45536
62822	0.946133	0.465707
62823	0.946132	0.53411
62824	0.946132	0.45892
62825	0.946132	0.536344
62826	0.946132	0.617438
62827	0.946132	0.473831
62828	0.946132	0.551427
62829	0.946132	0.473255
62830	0.946132	0.49385
62831	0.946132	0.620661
62832	0.946132	0.554515
62833	0.946132	0.543269
62834	0.946132	0.545013
62835	0.946131	0.556047
62836	0.946131	0.550798
62837	1.62772	0.307298
62838	1.70303	0.346237
62839	1.70303	0.342827
62840	1.70303	0.32796
62841	1.90116	0.325906
62842	1.90116	0.305946
62843	1.89226	0.316157
62844	1.89226	0.372836
62845	1.89226	0.376939
62846	1.89226	0.378199
62847	1.89226	0.327251
62848	1.89226	0.333731
62849	1.89226	0.392538
62850	1.89226	0.408784
62851	1.89226	0.369622
62852	1.89226	0.410773
62853	3.78452	0.402364
62854	1.89226	0.386217
62855	1.89226	0.378614
62856	1.89226	0.372597
62857	1.89226	0.384802
62858	1.89226	0.368856
62859	1.89226	0.384643
62860	0.946132	0.341297
62861	0.946132	0.337884
62862	0.946129	0.348197
62863	0.946129	0.35259
62864	0.94613	0.352882
62865	0.94613	0.355523
62866	0.946131	0.341075
62867	0.946131	0.328547
62868	0.94613	0.330515
62869	0.94613	0.342134

62870	0.946131	0.297384
62871	0.946131	0.362425
62872	1.89226	0.361556
62873	1.89226	0.375481
62874	1.89226	0.403123
62875	1.89226	0.402355
62876	1.89226	0.352671
62877	1.89226	0.351741
62878	1.89226	0.58024
62879	1.89226	0.845833
62880	1.89226	0.81475
62881	1.89226	0.575353
62882	3.78452	0.682978
62883	1.89226	0.700604
62884	1.89226	0.855251
62885	1.89226	0.641359
62886	1.89226	0.892778
62887	1.89226	0.664201
62888	1.89226	0.9542
62889	1.30165	0.759292
62890	0.946127	0.839149
62891	1.09659	0.692637
62892	0.946131	0.724779
62893	1.09659	0.697017
62894	1.09659	1.19165
62895	0.946128	1.01012
62896	0.946128	0.600535
62897	1.30165	0.724505
62898	1.09659	1.04255
62899	0.946129	0.75915
62900	0.946129	0.952828
62901	0.946128	0.824911
62902	0.946128	0.860031
62903	0.946133	0.69569
62904	0.946133	0.759835
62905	0.946131	0.674103
62906	0.946131	1.04845
62907	0.946131	0.853371
62908	0.946131	0.619852
62909	0.94613	0.690046
62910	0.94613	0.651621
62911	0.946131	0.640768
62912	0.946131	0.789268
62913	0.946133	0.877191
62914	0.946133	0.908143
62915	0.946132	0.742343
62916	0.946132	0.777948
62917	0.946132	0.719329
62918	0.946132	0.965739
62919	0.946132	0.907078
62920	0.946132	0.714564
62921	0.946132	0.777803
62922	0.946132	0.782329
62923	0.946133	0.819626
62924	0.946133	0.861712
62925	1.30165	1.17434
62926	0.972228	1.06944
62927	1.09659	0.920164

62928	0.656268	1.27748
62929	1.06217	0.904366
62930	1.06217	1.65467
62931	1.00665	0.7129
62932	1.00665	1.15265
62933	0.97223	0.760061
62934	0.656267	0.7243
62935	1.30165	0.804803
62936	1.09659	0.986796
62937	0.946132	0.949704
62938	0.946132	1.24798
62939	1.19895	1.77733
62940	1.19895	2.1
62941	0.94613	1.85467
62942	0.94613	0.76995
62943	1.39356	1.42217
62944	1.19895	2.1
62945	0.94613	1.23435
62946	1.39356	1.33694
62947	1.19895	1.52781
62948	0.946131	1.2687
62949	1.89226	0.824546
62950	1.89226	1.70094
62951	1.89226	1.93787
62952	1.89226	0.800576
62953	1.89226	1.97592
62954	1.89226	0.828442
62955	0.946132	0.73655
62956	0.946132	0.697564
62957	0.946128	0.996057
62958	0.946128	1.10957
62959	0.94613	1.00129
62960	0.94613	0.574952
62961	0.94613	0.745215
62962	0.94613	1.01237
62963	0.946131	0.782381
62964	0.946131	0.77215
62965	0.94613	0.814141
62966	0.94613	0.847083
62967	1.89226	0.136478
62968	1.89226	0.149985
62969	1.89226	0.186556
62970	1.89226	0.116862
62971	1.89226	0.188452
62972	1.89226	0.115424
62973	1.29881	0.0989701
62974	1.09985	0.0958039
62975	0.946126	0.142699
62976	0.946126	0.112533
62977	0.851539	0.141987
62978	1.20421	0.113659
62979	0.851483	0.0983548
62980	0.999426	0.12397
62981	1.47172	0.113061
62982	0.999413	0.101104
62983	0.851552	0.112834
62984	0.851552	0.112962
62985	1.89226	0.175775

62986	1.89226	0.165795
62987	1.89226	0.240795
62988	1.89226	0.145693
62989	1.89226	0.237044
62990	1.89226	0.143589
62991	1.89226	0.149349
62992	1.89226	0.157849
62993	1.89226	0.205155
62994	1.89226	0.131548
62995	1.89226	0.208027
62996	1.89226	0.128241
62997	1.33915	2.1
62998	1.99297	2.1
62999	2.05216	2.1
63000	2.60163	2.1
63001	4.04513	2.1
63002	1.89226	1.16934
63003	1.89226	2.02898
63004	1.89226	2.1
63005	1.89226	1.31908
63006	3.78452	1.82246
63007	0.946132	1.48098
63008	0.946132	1.33076
63009	0.946133	2.0818
63010	0.946133	2.1
63011	0.946132	2.0923
63012	0.946132	1.00606
63013	0.946133	2.06721
63014	0.946133	1.41465
63015	0.946132	1.87662
63016	0.946132	1.67515
63017	0.946132	1.94117
63018	0.946132	1.93553
63019	1.89226	1.18549
63020	1.89226	0.830249
63021	1.89226	0.921385
63022	1.89226	1.26666
63023	3.78452	0.999417
63024	1.89227	0.866142
63025	1.89227	1.20308
63026	1.89227	1.27243
63027	1.89227	0.824903
63028	1.89227	1.38809
63029	1.89227	0.851185
63030	2.29301	2.1
63031	1.70303	2.08782
63032	2.46749	2.1
63033	2.30728	2.1
63034	4.53937	1.79212
63035	1.89226	0.835145
63036	1.89226	2.1
63037	1.89226	2.07861
63038	1.89226	0.93504
63039	3.78452	2.1
63040	1.89226	0.694041
63041	1.89226	1.71155
63042	1.89226	0.917701
63043	1.89226	1.70791

63044	1.89226	0.74781
63045	1.89226	1.1763
63046	2.30728	2.1
63047	1.00757	1.20319
63048	2.0833	2.1
63049	1.65318	1.42584
63050	1.417	1.32119
63051	1.00757	1.54746
63052	2.46749	2.1
63053	2.0975	2.1
63054	2.79653	2.1
63055	2.44785	1.90084
63056	4.91534	2.1
63057	1.89226	2.1
63058	1.89226	1.59022
63059	1.89226	0.731681
63060	1.89226	2.08292
63061	3.78452	0.86574
63062	1.89226	1.01142
63063	1.89226	1.49836
63064	1.89226	2.1
63065	1.89226	1.43207
63066	1.89226	2.06616
63067	1.89226	1.39591
63068	0.970559	1.32621
63069	0.970559	2.1
63070	0.979305	2.1
63071	0.979305	2.1
63072	0.708502	1.2433
63073	0.901117	2.1
63074	1.04875	2.1
63075	1.22392	1.13629
63076	1.22392	2.0328
63077	0.901117	2.1
63078	1.04875	2.1
63079	0.7085	2.06983
63080	1.89226	1.17622
63081	1.89226	0.747551
63082	1.89226	0.885037
63083	1.89226	0.959079
63084	3.78452	1.23676
63085	1.89226	1.01521
63086	1.89226	0.718583
63087	1.89226	0.929842
63088	1.89226	0.82782
63089	3.78452	0.803618
63090	1.89226	0.462607
63091	1.89226	0.307077
63092	1.89226	0.50549
63093	1.89226	0.439859
63094	3.78453	0.458899
63095	1.89226	0.607782
63096	1.89226	0.645339
63097	1.89226	0.492199
63098	1.89226	0.463772
63099	1.89226	0.482857
63100	1.89226	0.395628
63101	1.89226	0.660741

63102	2.35441	0.648783
63103	1.89226	0.897422
63104	2.30931	0.439552
63105	4.24667	0.528107
63106	1.11612	0.504037
63107	0.946131	0.501717
63108	1.1772	0.515552
63109	0.946131	0.605732
63110	1.17721	0.651684
63111	1.11612	0.511594
63112	0.94613	0.574318
63113	0.94613	0.589282
63114	1.11612	0.566113
63115	1.11612	0.494311
63116	0.946131	0.48554
63117	0.946131	0.492554
63118	1.11612	0.260542
63119	0.582675	0.259844
63120	1.17721	0.290409
63121	0.651969	0.239189
63122	0.651969	0.255485
63123	0.582674	0.231196
63124	1.17721	0.261232
63125	1.11612	0.23542
63126	0.877955	0.283146
63127	0.877955	0.274315
63128	0.881925	0.235447
63129	0.881925	0.254388
63130	1.17721	0.365146
63131	0.651969	0.291282
63132	1.15466	0.323866
63133	0.623642	0.31989
63134	0.623642	0.318894
63135	0.651969	0.278676
63136	1.17721	0.349848
63137	1.15466	0.34404
63138	0.904714	0.379512
63139	0.904714	0.300496
63140	0.901914	0.38472
63141	0.901914	0.317526
63142	1.89226	1.06326
63143	1.89226	0.942874
63144	1.89226	0.725951
63145	1.89226	1.25032
63146	1.89226	0.628664
63147	1.89226	1.77729
63148	1.89226	1.08107
63149	1.89226	0.692881
63150	1.89226	0.643632
63151	1.89226	1.89477
63152	1.89226	0.608922
63153	1.89226	1.96992
63154	2.33986	1.61547
63155	1.97819	0.926307
63156	2.39166	0.579085
63157	2.70141	1.69226
63158	4.73151	0.923827
63159	0.989095	1.02384

63160	1.19583	0.705307
63161	0.851194	0.739468
63162	0.581197	0.817211
63163	0.872704	0.555821
63164	0.872704	1.19894
63165	0.904322	0.76172
63166	0.904322	0.896907
63167	1.19583	0.618393
63168	0.851193	0.668659
63169	0.989095	0.801241
63170	0.581197	0.709008
63171	1.89226	0.831753
63172	1.89226	0.970326
63173	1.89226	0.826565
63174	1.89226	1.37652
63175	1.89226	0.736639
63176	1.89226	1.73524
63177	1.89226	1.08704
63178	1.89226	0.669814
63179	1.89226	0.647911
63180	1.89226	1.817
63181	1.89226	0.556497
63182	1.89226	1.99219
63183	2.39166	0.840729
63184	1.70239	1.10047
63185	2.13665	0.772589
63186	2.82684	0.805756
63187	4.52831	0.894884
63188	1.89226	0.14551
63189	1.89226	0.268877
63190	1.89226	0.205088
63191	1.89226	0.234186
63192	3.78453	0.246033
63193	1.89226	0.146032
63194	1.89226	0.175414
63195	1.89226	0.21748
63196	1.89226	0.126738
63197	1.89226	0.23154
63198	1.89226	0.126727
63199	1.89226	0.199781
63200	1.89226	0.220959
63201	1.89226	0.282246
63202	1.89226	0.165498
63203	1.89226	0.29128
63204	1.89226	0.162263
63205	1.89226	0.284479
63206	1.89226	0.3129
63207	1.89226	0.520069
63208	1.89226	0.210813
63209	1.89226	0.539501
63210	1.89226	0.211327
63211	1.89226	0.218699
63212	1.89226	0.297946
63213	1.89226	0.389496
63214	1.89226	0.18007
63215	1.89226	0.457036
63216	1.89226	0.195868
63217	1.5169	0.402321

63218	1.61969	0.270465
63219	1.17262	0.271827
63220	1.17262	0.334887
63221	1.88868	0.293349
63222	1.88868	0.36605
63223	1.79981	0.480734
63224	1.88868	0.30506
63225	1.5169	0.331622
63226	1.5169	0.414468
63227	2.12891	0.355993
63228	2.12891	0.448423
63229	1.89226	0.410402
63230	1.89226	0.287361
63231	1.89226	0.298559
63232	1.89226	0.352228
63233	3.78452	0.353076
63234	0.946132	0.266088
63235	0.946132	0.329074
63236	0.946132	0.248938
63237	0.946132	0.267629
63238	0.946132	0.246277
63239	0.946132	0.304866
63240	0.946133	0.258259
63241	0.946133	0.34342
63242	0.946133	0.262409
63243	0.946133	0.288166
63244	0.946132	0.293553
63245	0.946132	0.28542
63246	1.88868	0.383748
63247	2.05759	0.263894
63248	1.61969	0.275184
63249	1.61969	0.340653
63250	2.29747	0.281483
63251	2.29747	0.346373
63252	2.12891	0.488833
63253	2.29747	0.300211
63254	1.88868	0.327871
63255	1.88868	0.38733
63256	2.50894	0.342076
63257	2.50894	0.413115
63258	1.89226	0.294716
63259	1.89226	0.355535
63260	1.89226	0.259908
63261	1.89226	0.387245
63262	1.89226	0.263901
63263	1.89226	0.394962
63264	0.946132	0.323859
63265	0.946132	0.266582
63266	0.946132	0.273255
63267	0.946132	0.251981
63268	0.946131	0.246441
63269	0.946131	0.337529
63270	0.946132	0.257959
63271	0.946132	0.34119
63272	0.946132	0.264796
63273	0.946132	0.295244
63274	0.946132	0.281337
63275	0.946132	0.318128

63276	2.42758	1.15192
63277	2.14895	2.1
63278	2.56975	1.11731
63279	2.28142	2.1
63280	4.71869	1.60523
63281	1.89226	1.07092
63282	1.89226	2.1
63283	1.89226	1.13409
63284	1.89226	2.1
63285	3.78452	1.82223
63286	1.89226	1.69745
63287	1.89226	2.1
63288	1.89226	1.54194
63289	1.89226	2.1
63290	1.89226	1.55757
63291	1.89226	2.1
63292	0.960916	2.1
63293	0.960916	1.53038
63294	0.978118	1.82731
63295	0.875674	2.1
63296	0.775661	2.1
63297	0.773613	2.1
63298	1.07631	1.63711
63299	1.14266	1.98735
63300	1.16927	2.1
63301	0.898612	2.1
63302	1.04757	2.1
63303	0.757536	2.1
63304	2.56975	1.04572
63305	2.28142	2.1
63306	2.64384	1.15639
63307	2.40621	2.09972
63308	4.97596	1.71826
63309	1.89226	1.05984
63310	1.89226	2.1
63311	1.89226	0.783219
63312	1.89226	2.09904
63313	3.78452	1.21747
63314	1.89226	1.43776
63315	1.89226	2.1
63316	1.89226	1.93684
63317	1.89226	2.1
63318	1.89226	1.60214
63319	1.89226	2.1
63320	1.03328	2.1
63321	1.03328	1.95801
63322	1.04321	1.33618
63323	1.15424	2.1
63324	0.936183	2.1
63325	0.944491	2.1
63326	1.14266	1.53496
63327	1.20517	1.66851
63328	1.23323	2.1
63329	0.961268	2.1
63330	1.11215	2.1
63331	0.827247	2.1
63332	1.89226	2.1
63333	1.89226	0.738846

63334	1.89226	2.1
63335	1.89226	0.756856
63336	3.78453	1.74568
63337	2.14647	2.1
63338	2.20589	1.178
63339	1.95567	2.1
63340	2.03224	0.752979
63341	4.17871	2.1
63342	2.06035	1.26016
63343	1.88452	0.477992
63344	1.88452	1.05038
63345	2.26997	0.359343
63346	2.03224	0.756065
63347	2.42272	0.55424
63348	1.89226	0.787813
63349	1.89226	0.364197
63350	1.89226	0.191863
63351	1.89226	0.775687
63352	1.89226	0.272327
63353	1.89226	1.03648
63354	1.89225	2.1
63355	1.89225	1.2117
63356	1.89225	1.14262
63357	1.89225	2.1
63358	1.89225	0.820025
63359	1.89225	2.1
63360	0.952418	1.66364
63361	0.952418	2.1
63362	0.95883	1.19978
63363	0.95883	2.1
63364	0.946182	1.03216
63365	1.01611	1.28993
63366	0.895134	2.1
63367	0.977828	2.1
63368	0.89514	2.1
63369	0.946189	2.1
63370	0.977821	2.1
63371	1.01611	2.1
63372	2.03223	1.64752
63373	1.88451	0.41773
63374	1.89236	1.08596
63375	1.74649	0.364413
63376	3.77687	0.64444
63377	0.81094	1.03024
63378	0.81094	0.467423
63379	0.810939	1.10639
63380	0.810939	0.837399
63381	0.810945	0.675267
63382	0.810945	0.692971
63383	1.89225	0.300519
63384	1.35167	0.5083
63385	1.08131	1.17023
63386	1.08131	0.293434
63387	1.0813	0.644802
63388	1.89226	0.0998104
63389	1.89226	0.110484
63390	1.89226	0.117961
63391	1.89226	0.0997896

63392	1.89226	0.115328
63393	1.89226	0.0972116
63394	1.01842	0.0811171
63395	0.936656	0.0787295
63396	0.946131	0.0921108
63397	0.946131	0.087275
63398	1.11157	0.0779574
63399	1.04281	0.0874434
63400	0.925803	0.0811496
63401	0.925803	0.0938221
63402	0.851553	0.0813486
63403	1.11158	0.0843972
63404	1.05017	0.088951
63405	0.925798	0.0854313
63406	1.89226	0.104759
63407	1.89226	0.127966
63408	1.89226	0.13669
63409	1.89226	0.1057
63410	1.89226	0.131018
63411	1.89226	0.099738
63412	1.23504	0.0875951
63413	1.23504	0.0771447
63414	0.946131	0.0983792
63415	0.946131	0.0909699
63416	1.37358	0.0903842
63417	1.23504	0.0814347
63418	0.946129	0.0880948
63419	0.946129	0.102224
63420	1.37358	0.0857304
63421	0.946131	0.0876698
63422	0.94613	0.0869003
63423	1.23504	0.0910669
63424	1.00261	0.075169
63425	1.00261	0.0756369
63426	1.02952	0.0916362
63427	1.02952	0.0824605
63428	0.850964	0.0818129
63429	0.658553	0.0783401
63430	1.37358	0.0868804
63431	1.23504	0.0764249
63432	1.37358	0.0807801
63433	0.850965	0.0851269
63434	1.23504	0.0804262
63435	0.658553	0.0792457
63436	0.946133	0.0959484
63437	0.946133	0.0867159
63438	0.946132	0.130869
63439	0.946132	0.103398
63440	0.946132	0.0908733
63441	0.946132	0.101423
63442	0.946133	0.12142
63443	0.946133	0.0994938
63444	0.946133	0.105437
63445	0.946133	0.104708
63446	0.946132	0.0999217
63447	0.946132	0.0994213
63448	1.89226	0.296106
63449	1.89226	0.316177

63450	1.89226	0.249334
63451	1.89226	0.275968
63452	3.78452	0.313783
63453	1.89226	0.319974
63454	1.89226	0.328206
63455	1.89226	0.269933
63456	1.89226	0.293414
63457	3.78452	0.27499
63458	1.89227	0.278431
63459	1.89227	0.280332
63460	1.89227	0.245158
63461	1.89227	0.243196
63462	1.89227	0.250857
63463	1.89227	0.276499
63464	0.94613	0.190685
63465	0.94613	0.194349
63466	0.946127	0.194705
63467	0.946127	0.19648
63468	0.94613	0.190483
63469	0.94613	0.206198
63470	0.946127	0.176622
63471	0.946127	0.236871
63472	0.946128	0.180462
63473	0.946128	0.169163
63474	0.946128	0.217086
63475	0.946128	0.205903
63476	0.946132	0.217273
63477	0.946132	0.219142
63478	0.946128	0.205028
63479	0.946128	0.205239
63480	0.94613	0.201699
63481	0.94613	0.23398
63482	0.946131	0.189715
63483	0.946131	0.207615
63484	0.94613	0.191732
63485	0.94613	0.18452
63486	0.94613	0.21828
63487	0.94613	0.24067
63488	1.89226	2.1
63489	1.89226	1.71115
63490	1.89226	1.89078
63491	1.89226	2.1
63492	3.78453	2.1
63493	2.72844	2.1
63494	1.89226	2.1
63495	2.26536	2.1
63496	2.26536	2.1
63497	1.89226	2.1
63498	1.89226	2.1
63499	1.89226	2.1
63500	1.89226	1.34499
63501	1.89226	1.72685
63502	1.89226	1.89585
63503	1.89226	1.71134
63504	1.89226	1.85529
63505	1.89226	1.87315
63506	1.89226	2.1
63507	1.89226	1.96672

63508	1.89226	2.1
63509	1.89226	1.69624
63510	1.89226	2.1
63511	2.72844	2.1
63512	2.26536	2.1
63513	2.28367	2.1
63514	1.5791	2.1
63515	4.54903	2.1
63516	0.810945	1.88135
63517	0.810945	1.46533
63518	0.810943	1.42404
63519	0.810943	1.98326
63520	1.89226	2.1
63521	1.3517	1.70193
63522	1.08133	1.92979
63523	1.08132	1.89432
63524	1.08132	1.7731
63525	0.810933	1.99845
63526	0.810933	1.80369
63527	1.89227	2.1
63528	1.89227	2.1
63529	1.89227	2.1
63530	1.89227	2.1
63531	3.78453	2.1
63532	2.71897	2.1
63533	2.27495	2.1
63534	2.648	2.1
63535	2.20832	2.1
63536	4.92729	2.1
63537	2.27495	2.1
63538	1.38455	2.1
63539	1.38455	2.1
63540	1.46586	2.1
63541	2.20831	2.1
63542	2.28962	2.1
63543	0.946133	2.1
63544	0.946133	2.1
63545	0.946132	2.1
63546	0.946132	2.1
63547	0.946132	2.1
63548	0.946132	2.1
63549	0.946132	2.1
63550	0.946132	2.1
63551	0.946132	2.1
63552	0.946132	2.1
63553	0.946133	2.1
63554	0.946133	2.1
63555	1.89226	2.1
63556	1.89226	2.1
63557	1.89226	2.1
63558	1.89226	2.1
63559	1.89226	2.1
63560	1.89226	2.1
63561	2.648	2.1
63562	2.08612	2.1
63563	2.08612	2.1
63564	2.20831	2.1
63565	2.56618	2.1

63566	2.68837	2.1
63567	2.08612	2.1
63568	2.20831	2.1
63569	1.30659	2.1
63570	1.30659	2.1
63571	1.38455	2.1
63572	2.16408	2.1
63573	0.810946	2.1
63574	0.810946	2.1
63575	0.810946	2.1
63576	0.810946	2.1
63577	1.89226	2.1
63578	1.3517	2.1
63579	1.08133	2.1
63580	1.08132	2.1
63581	1.08132	2.1
63582	0.810934	2.1
63583	0.810934	2.1
63584	1.89226	1.41371
63585	1.89226	1.26689
63586	1.89226	0.879299
63587	1.89226	1.81975
63588	3.78452	1.13773
63589	1.89226	0.948351
63590	1.89226	1.98413
63591	1.89226	1.85504
63592	1.89226	1.14925
63593	3.78453	1.691
63594	1.08733	1.81146
63595	0.856615	2.1
63596	1.25964	1.31565
63597	1.12378	1.31183
63598	1.23551	1.05903
63599	0.969894	2.1
63600	0.985977	1.91977
63601	0.985977	1.07582
63602	1.16169	1.42266
63603	1.21072	1.58269
63604	0.765175	1.55887
63605	0.863419	1.51475
63606	2.39934	1.75365
63607	2.44427	1.40994
63608	2.64003	0.921314
63609	2.39077	1.89309
63610	2.39934	1.60561
63611	2.10194	2.1
63612	1.89226	0.917296
63613	1.89226	1.94751
63614	1.89226	1.3409
63615	1.89226	0.744721
63616	3.78452	1.43906
63617	0.946132	1.53602
63618	0.946132	1.79388
63619	0.946131	0.983949
63620	0.946131	0.999826
63621	0.946132	0.972171
63622	0.946132	2.1
63623	0.946132	1.58207

63624	0.946132	1.05171
63625	0.946132	1.28468
63626	0.946132	1.42218
63627	0.946132	1.2793
63628	0.946132	1.58389
63629	1.19967	1.83167
63630	0.946128	1.49547
63631	0.946133	1.04465
63632	1.32002	1.45129
63633	1.19967	1.75975
63634	1.19967	1.71796
63635	0.94613	1.82978
63636	0.94613	0.72419
63637	0.946131	1.20007
63638	0.946131	1.3038
63639	1.19967	1.47017
63640	1.32001	1.48978
63641	1.89226	0.518072
63642	1.89226	0.673907
63643	1.89226	0.426181
63644	1.89226	1.04344
63645	3.78451	0.588664
63646	2.6402	0.426865
63647	2.28552	0.664817
63648	2.50958	0.413113
63649	2.18948	0.628351
63650	4.79509	0.522442
63651	2.28552	0.572099
63652	1.86102	2.1
63653	2.18949	0.613955
63654	1.78376	2.1
63655	4.06928	0.938156
63656	1.89226	0.586763
63657	1.89226	2.1
63658	1.89226	0.654189
63659	1.89226	2.1
63660	3.78452	1.12856
63661	1.89226	0.425142
63662	1.89226	1.10505
63663	1.89226	0.292003
63664	1.89226	0.695369
63665	3.78452	0.533156
63666	2.50958	0.404521
63667	2.18948	0.56544
63668	2.39524	0.31036
63669	2.10188	0.686254
63670	4.61146	0.488582
63671	2.18949	0.482155
63672	1.78376	2.09196
63673	1.71839	0.614066
63674	1.71839	1.52231
63675	2.10189	0.620392
63676	2.16726	1.62248
63677	0.810943	0.632884
63678	0.810943	2.1
63679	0.810945	0.629464
63680	0.810945	0.613186
63681	0.810959	0.804646

63682	0.810959	0.885052
63683	1.89226	2.09393
63684	1.35168	1.06942
63685	1.08132	0.633411
63686	1.08132	2.04813
63687	1.0813	0.853544
63688	1.89226	0.77515
63689	1.89226	1.42712
63690	1.89226	0.939304
63691	1.89226	1.04804
63692	1.89226	1.12436
63693	1.89226	1.38009
63694	1.28421	1.36643
63695	0.992041	0.819468
63696	1.07707	1.85263
63697	0.789746	1.40824
63698	1.03815	1.64426
63699	1.03815	1.45162
63700	1.03581	1.4895
63701	1.03581	1.23171
63702	1.28421	1.35569
63703	1.07707	1.6639
63704	0.789748	1.45231
63705	0.992038	1.20578
63706	1.89226	0.771138
63707	1.89226	1.49188
63708	1.89226	1.07156
63709	1.89226	0.936964
63710	1.89226	1.16798
63711	1.89226	1.08684
63712	1.89226	0.749777
63713	1.89226	1.16537
63714	1.89226	0.842251
63715	1.89226	0.938028
63716	1.89226	0.977979
63717	1.89226	1.21714
63718	0.92236	1.83952
63719	0.655971	1.5254
63720	0.916772	1.69151
63721	0.913834	1.26768
63722	1.18074	1.51514
63723	0.922346	1.79517
63724	2.15414	0.937545
63725	1.53528	1.48166
63726	1.22748	1.61232
63727	1.57442	1.03848
63728	0.874683	1.50145
63729	1.25935	2.1
63730	2.61433	2.1
63731	1.22262	2.1
63732	1.22262	2.1
63733	2.62813	2.1
63734	2.62813	2.1
63735	1.89226	2.1
63736	2.61434	2.1
63737	2.62814	2.1
63738	2.61434	2.1
63739	1.90607	2.1

63740	1.89226	2.1
63741	1.89226	2.1
63742	1.89226	1.85709
63743	1.89226	2.1
63744	1.89226	2.08529
63745	1.89226	2.1
63746	1.89226	2.1
63747	1.89226	0.853711
63748	1.89226	1.72018
63749	1.89226	2.1
63750	1.89226	2.1
63751	1.89226	2.1
63752	1.89226	2.06391
63753	1.31969	2.1
63754	1.31406	2.1
63755	0.629677	2.1
63756	0.643104	2.1
63757	0.976621	2.1
63758	0.976621	2.1
63759	0.98055	2.1
63760	0.98055	2.1
63761	1.3197	2.1
63762	0.643102	2.1
63763	0.629675	2.1
63764	1.31407	2.1
63765	0.946133	2.1
63766	0.946133	2.1
63767	1.3197	2.1
63768	1.31407	2.1
63769	0.946132	2.1
63770	0.946132	2.1
63771	1.3197	2.1
63772	1.3197	2.1
63773	1.3197	2.1
63774	0.946133	2.1
63775	0.946132	2.1
63776	1.31407	2.1
63777	1.89226	2.1
63778	1.89226	1.49624
63779	1.89226	2.1
63780	1.89226	2.1
63781	1.89226	2.1
63782	1.89226	1.73515
63783	1.89226	2.1
63784	1.89226	1.23289
63785	1.89226	2.1
63786	1.89226	2.04171
63787	1.89226	2.1
63788	1.89226	1.32417
63789	1.89226	0.274914
63790	1.89226	0.214377
63791	1.89226	0.188916
63792	1.89226	0.184783
63793	3.78452	0.18804
63794	1.89226	0.219211
63795	1.89226	0.164648
63796	1.89226	0.167297
63797	1.89226	0.190471

63798	1.89226	0.191979
63799	1.89226	0.225829
63800	0.946131	0.141019
63801	0.946131	0.145686
63802	0.946128	0.138321
63803	0.946128	0.140896
63804	0.94613	0.130229
63805	0.94613	0.137921
63806	0.94613	0.124091
63807	0.94613	0.170089
63808	0.94613	0.142877
63809	0.94613	0.137475
63810	0.94613	0.162478
63811	0.94613	0.147532
63812	1.89226	0.135215
63813	1.89226	0.148023
63814	1.89226	0.137136
63815	1.89226	0.119684
63816	1.89226	0.127313
63817	1.89226	0.148427
63818	0.924614	0.116999
63819	0.914275	0.111978
63820	0.906003	0.11402
63821	0.62479	0.10451
63822	2.02733	0.112112
63823	1.48332	0.105837
63824	0.833106	0.106163
63825	1.19364	0.10602
63826	1.57482	0.116255
63827	1.18103	0.108772
63828	0.905988	0.108601
63829	1.89226	2.1
63830	1.89226	2.1
63831	1.89226	2.1
63832	1.89226	2.1
63833	3.78452	2.1
63834	1.89226	2.1
63835	1.89226	2.1
63836	1.89226	2.1
63837	1.89226	2.1
63838	1.89226	2.1
63839	1.89226	2.1
63840	0.816805	2.1
63841	0.928516	2.1
63842	1.4045	2.1
63843	1.26268	2.1
63844	1.0881	2.1
63845	1.0881	2.1
63846	1.10309	2.1
63847	1.10309	2.1
63848	0.928518	2.1
63849	1.4045	2.1
63850	0.816807	2.1
63851	1.26267	2.1
63852	1.89226	2.1
63853	2.52535	2.1
63854	1.89226	2.1
63855	1.89226	2.1

63856	2.809	2.1
63857	2.809	2.1
63858	1.89226	2.1
63859	1.89226	2.1
63860	1.89226	2.1
63861	1.89226	2.1
63862	3.78452	2.1
63863	0.946127	2.1
63864	0.946127	2.1
63865	0.946131	2.1
63866	0.946131	2.1
63867	0.94613	2.1
63868	0.94613	2.1
63869	0.946128	2.1
63870	0.946128	2.1
63871	0.946129	2.1
63872	0.946129	2.1
63873	0.946129	2.1
63874	0.946129	2.1
63875	1.89224	0.60226
63876	1.89224	2.1
63877	1.89224	0.801718
63878	1.89224	1.66259
63879	3.78448	1.30469
63880	2.39244	1.69838
63881	2.27036	2.1
63882	2.25945	0.747789
63883	2.27398	2.1
63884	4.52981	2.08439
63885	2.27036	2.1
63886	1.97088	2.1
63887	1.86302	2.1
63888	1.84991	2.1
63889	4.12027	2.1
63890	1.89224	2.1
63891	1.89224	2.1
63892	1.89224	1.72818
63893	1.89224	2.1
63894	3.78447	2.1
63895	1.89226	0.837842
63896	1.89226	1.57384
63897	1.89226	0.612702
63898	1.89226	2.1
63899	3.78453	1.09808
63900	2.25948	0.803859
63901	2.33611	2.1
63902	2.05257	2.05531
63903	1.94441	2.1
63904	4.20389	2.1
63905	1.92514	2.1
63906	1.84993	2.1
63907	1.94441	2.1
63908	1.74391	2.1
63909	3.79434	2.1
63910	1.89226	1.63358
63911	1.89226	2.1
63912	1.89226	2.1
63913	1.89226	2.1

63914	3.78452	2.1
63915	15.1381	0.452622
63916	15.1381	0.45769
63917	15.1381	0.444205
63918	15.1381	0.79313
63919	30.2762	0.43777
63920	15.1381	0.409327
63921	15.1381	0.604001
63922	15.1381	0.471491
63923	15.1381	0.566349
63924	30.2762	0.555125
63925	15.1381	1.64908
63926	15.1381	1.82909
63927	15.1381	1.33962
63928	15.1381	1.98963
63929	30.2762	1.81001
63930	15.1381	0.378432
63931	15.1381	0.481513
63932	15.1381	0.452585
63933	15.1381	0.456072
63934	30.2762	0.382573
63935	1.89225	1.05928
63936	1.89225	2.1
63937	1.89225	1.56977
63938	1.89225	2.1
63939	3.78449	1.95422
63940	1.39909	1.97248
63941	1.39909	2.1
63942	0.946123	2.1
63943	0.946123	1.87483
63944	1.3291	2.1
63945	1.39908	2.1
63946	0.946126	1.98051
63947	0.946126	1.4307
63948	0.946118	1.92012
63949	1.32909	2.1
63950	0.94613	2.1
63951	1.3991	2.1
63952	3.07801	2.1
63953	1.84322	2.1
63954	1.84322	2.1
63955	1.68788	2.1
63956	2.6582	2.1
63957	2.50287	2.1
63958	1.89225	2.1
63959	1.89225	2.1
63960	1.89225	2.1
63961	1.89225	2.1
63962	3.7845	2.1
63963	1.89226	1.08722
63964	1.89226	2.1
63965	1.89226	1.01656
63966	1.89226	2.1
63967	3.78452	2.1
63968	1.89226	1.09151
63969	2.50051	2.1
63970	2.50051	0.890477
63971	2.65822	2.1

63972	1.89226	1.14586
63973	2.04997	2.1
63974	2.50052	2.1
63975	2.65822	2.1
63976	1.70377	2.1
63977	1.70377	2.1
63978	1.84324	2.1
63979	2.63998	2.1
63980	0.810947	2.1
63981	0.810947	2.1
63982	0.810946	2.1
63983	0.810946	2.1
63984	1.89226	2.1
63985	1.35169	2.1
63986	1.08132	2.1
63987	1.08132	2.1
63988	1.08132	2.1
63989	0.810946	2.1
63990	0.810946	2.1
63991	1.89226	2.1
63992	1.89226	2.1
63993	1.89226	2.1
63994	1.89226	2.1
63995	3.78453	2.1
63996	0.946164	2.1
63997	0.946164	2.1
63998	0.929842	2.1
63999	0.929842	2.1
64000	0.946132	2.1
64001	0.946132	2.1
64002	1.04101	2.1
64003	0.929874	2.1
64004	0.946132	2.1
64005	0.929874	2.1
64006	1.04101	2.1
64007	0.946132	2.1
64008	1.89226	2.1
64009	1.89226	2.1
64010	1.89226	2.1
64011	1.89226	2.1
64012	3.78453	2.1
64013	1.89226	2.1
64014	1.89226	2.1
64015	1.89226	2.1
64016	1.89226	2.1
64017	1.89226	2.1
64018	1.89226	2.1
64019	1.89226	2.1
64020	1.89226	2.1
64021	1.89226	2.1
64022	1.89226	2.1
64023	1.89226	2.1
64024	1.89226	2.1
64025	0.946131	2.1
64026	0.946131	2.1
64027	0.946131	2.1
64028	0.946131	2.1
64029	0.946132	2.09554

64030	0.946132	2.01618
64031	0.946131	2.1
64032	0.946131	2.1
64033	0.946131	2.1
64034	0.946131	2.1
64035	0.946132	2.1
64036	0.946132	2.1
64037	1.89226	0.726493
64038	1.89226	0.534451
64039	1.89226	0.451547
64040	1.89226	0.563308
64041	3.78453	0.544501
64042	2.73497	0.390622
64043	1.93823	0.621897
64044	1.93823	0.435123
64045	2.20046	0.617248
64046	2.42279	0.448726
64047	2.68501	0.892697
64048	1.89226	0.429853
64049	1.89226	0.62035
64050	1.89226	0.781329
64051	1.89226	0.417695
64052	1.89226	0.689609
64053	1.89226	0.384639
64054	1.89226	0.474325
64055	1.89226	0.402142
64056	1.89226	0.426808
64057	1.89226	0.596283
64058	1.89226	0.414644
64059	1.89226	0.462003
64060	2.82984	0.493115
64061	2.15276	0.418811
64062	2.15276	0.628492
64063	2.42278	0.345549
64064	2.50074	0.419067
64065	2.77077	0.384138
64066	2.42279	0.359854
64067	1.93823	0.575155
64068	1.70423	0.347468
64069	1.70423	0.559858
64070	2.15276	0.355226
64071	2.38676	0.591155
64072	0.810946	0.430025
64073	0.810946	0.651083
64074	0.810944	0.385225
64075	0.810944	0.383074
64076	0.810946	0.45452
64077	0.810946	0.465695
64078	1.89226	0.573612
64079	1.35169	0.471582
64080	1.08132	0.39926
64081	1.08132	0.593156
64082	1.08132	0.484094
64083	1.89226	2.1
64084	1.89226	2.1
64085	1.89226	2.1
64086	1.89226	2.1
64087	3.78452	2.1

64088	1.89226	2.1
64089	1.89226	2.1
64090	1.89226	2.1
64091	1.89226	2.1
64092	3.78453	2.1
64093	1.04144	2.1
64094	1.04144	2.1
64095	0.938972	2.1
64096	0.938972	2.1
64097	0.969661	2.1
64098	1.03285	2.1
64099	0.946133	2.1
64100	0.946133	2.1
64101	0.946131	2.1
64102	0.969663	2.1
64103	0.946131	2.1
64104	1.03284	2.1
64105	0.946131	2.1
64106	0.946131	2.1
64107	0.946128	2.1
64108	0.946128	2.1
64109	0.946127	2.1
64110	0.946127	2.1
64111	0.946131	2.1
64112	0.946131	2.1
64113	0.94613	2.1
64114	0.94613	2.1
64115	0.946129	2.1
64116	0.946129	2.1
64117	1.89226	2.1
64118	1.89226	2.1
64119	1.89226	2.1
64120	1.89226	2.1
64121	3.78452	2.1
64122	1.89226	2.1
64123	1.89226	2.1
64124	1.89226	2.1
64125	1.89226	2.1
64126	3.78453	2.1
64127	1.89226	2.1
64128	1.89226	2.1
64129	1.85882	2.1
64130	1.85882	2.1
64131	1.93932	2.1
64132	1.97276	2.1
64133	0.94613	2.1
64134	0.94613	2.1
64135	0.946128	2.1
64136	0.946128	2.1
64137	0.946127	2.1
64138	0.946127	2.1
64139	0.946131	2.1
64140	0.946131	2.1
64141	0.946129	2.1
64142	0.946129	2.1
64143	0.946129	2.1
64144	0.946129	2.1
64145	1.89225	2.1

64146	1.89225	2.1
64147	1.89225	2.1
64148	1.89225	2.1
64149	3.7845	2.1
64150	1.0086	2.1
64151	1.09028	2.1
64152	0.946125	2.1
64153	0.946125	2.1
64154	0.916906	2.1
64155	0.916906	2.1
64156	1.13051	2.1
64157	1.1505	2.1
64158	1.11557	2.1
64159	1.04071	2.1
64160	0.851542	2.1
64161	0.991196	2.1
64162	1.89225	2.1
64163	1.89225	2.1
64164	1.89225	2.1
64165	1.89225	2.1
64166	3.7845	2.1
64167	1.89225	2.1
64168	1.89225	2.1
64169	1.89225	2.1
64170	1.89225	2.1
64171	1.89225	2.1
64172	1.89225	2.1
64173	1.89226	2.1
64174	1.89226	2.1
64175	1.89226	2.1
64176	1.89226	2.1
64177	3.78452	2.1
64178	0.890962	2.1
64179	0.952547	2.1
64180	0.946132	2.1
64181	0.946132	2.1
64182	0.851543	2.1
64183	0.851543	2.1
64184	1.11558	2.1
64185	1.06304	2.1
64186	0.994315	2.1
64187	1.04072	2.1
64188	0.900943	2.1
64189	0.851542	2.1
64190	1.89226	2.1
64191	1.89226	2.1
64192	1.89226	2.1
64193	1.89226	2.1
64194	1.89226	2.1
64195	1.89226	2.1
64196	1.89226	2.1
64197	1.89226	2.1
64198	1.89226	2.1
64199	1.89226	2.1
64200	3.78452	2.1
64201	1.89226	2.1
64202	1.89226	2.1
64203	1.89226	2.1

64204	1.89226	2.1
64205	3.78452	2.1
64206	0.782015	2.1
64207	0.929718	2.1
64208	0.946132	2.1
64209	0.946132	2.1
64210	0.782016	2.1
64211	0.782016	2.1
64212	1.06706	2.1
64213	1.11785	2.1
64214	0.782015	2.1
64215	0.946133	2.1
64216	0.878931	2.1
64217	0.946132	2.1
64218	1.89226	2.1
64219	1.89226	2.1
64220	1.89226	2.1
64221	1.89226	2.1
64222	1.89226	2.1
64223	1.89226	2.1
64224	0.946131	2.1
64225	0.946131	2.1
64226	0.946129	2.1
64227	0.946129	2.1
64228	0.946131	2.1
64229	0.946131	2.1
64230	0.94613	2.1
64231	0.94613	2.1
64232	0.946131	2.1
64233	0.946131	2.1
64234	0.94613	2.1
64235	0.94613	2.1
64236	0.827866	2.1
64237	0.946131	2.1
64238	0.946129	2.1
64239	0.946129	2.1
64240	0.94613	2.1
64241	0.94613	2.1
64242	0.94613	2.1
64243	0.94613	2.1
64244	0.827865	2.1
64245	0.94613	2.1
64246	0.94613	2.1
64247	0.94613	2.1
64248	1.89226	2.1
64249	1.89226	2.1
64250	1.89226	2.1
64251	1.89226	2.1
64252	3.78452	2.1
64253	1.89226	2.1
64254	1.89226	2.1
64255	1.89226	2.1
64256	1.89226	2.1
64257	1.89226	2.1
64258	1.89226	2.1
64259	1.89226	2.1
64260	1.89226	2.1
64261	1.89226	2.1

64262	1.89226	2.1
64263	3.78453	2.1
64264	1.89226	2.1
64265	1.89226	2.1
64266	1.89226	2.1
64267	1.89226	2.1
64268	3.78452	2.1
64269	0.744377	2.1
64270	0.541919	2.1
64271	0.652022	2.1
64272	0.544198	2.1
64273	0.764633	2.1
64274	0.673254	2.1
64275	1.89226	2.1
64276	1.31149	2.1
64277	1.05423	2.1
64278	1.02189	2.1
64279	0.870602	2.1
64280	1.89226	2.1
64281	1.89226	2.1
64282	1.89226	2.1
64283	1.89226	2.1
64284	1.89226	2.1
64285	1.89226	2.1
64286	1.89226	2.1
64287	1.89226	2.1
64288	1.89226	2.1
64289	1.89226	2.1
64290	1.89226	2.1
64291	1.89226	2.1
64292	1.89226	2.1
64293	1.89226	2.1
64294	1.89226	2.1
64295	1.89226	2.1
64296	1.89226	2.1
64297	1.89226	2.1
64298	0.815314	2.1
64299	0.815313	2.1
64300	0.714029	2.1
64301	0.782951	2.1
64302	0.810944	2.1
64303	0.782954	2.1
64304	1.89226	2.1
64305	1.30999	2.1
64306	1.08132	2.1
64307	1.03962	2.1
64308	0.95209	2.1
64309	1.89226	2.1
64310	1.89226	2.1
64311	1.89226	2.1
64312	1.89226	2.1
64313	1.89226	2.1
64314	1.89226	2.1
64315	1.89226	2.1
64316	1.89226	2.1
64317	1.89226	2.1
64318	1.89226	2.1
64319	1.89226	2.1

64320	1.89226	2.1
64321	1.89226	2.1
64322	1.89226	2.1
64323	1.89226	2.1
64324	1.89226	2.1
64325	1.89226	2.1
64326	1.89226	2.1
64327	0.871558	2.1
64328	0.713078	2.1
64329	0.756858	2.1
64330	0.703674	2.1
64331	0.838025	2.1
64332	0.862196	2.1
64333	1.89226	2.1
64334	1.33194	2.1
64335	1.10834	2.1
64336	1.06163	2.1
64337	1.01785	2.1
64338	1.89226	2.1
64339	1.89226	2.1
64340	1.89226	2.1
64341	1.89226	2.1
64342	3.78452	2.1
64343	1.89226	2.1
64344	1.89226	2.1
64345	1.89226	2.1
64346	1.89226	2.1
64347	1.89226	2.1
64348	1.89226	2.1
64349	1.89226	2.1
64350	1.89226	2.1
64351	1.89226	2.1
64352	1.89226	2.1
64353	1.89226	2.1
64354	1.89226	2.1
64355	0.958406	2.1
64356	1.1383	2.1
64357	0.865636	2.1
64358	1.10093	2.1
64359	0.838024	2.1
64360	0.975154	2.1
64361	1.89226	2.1
64362	1.38553	2.1
64363	1.10834	2.1
64364	1.11522	2.1
64365	1.15271	2.1
64366	1.89227	2.1
64367	1.89227	2.1
64368	1.89227	2.1
64369	1.89227	2.1
64370	3.78453	2.1
64371	0.946132	2.1
64372	0.946132	2.1
64373	0.946132	2.1
64374	0.946132	2.1
64375	0.946132	2.1
64376	0.946132	2.1
64377	0.946133	2.1

64378	0.946133	2.1
64379	0.946132	2.1
64380	0.946132	2.1
64381	0.946133	2.1
64382	0.946133	2.1
64383	0.719271	2.1
64384	0.774968	2.1
64385	1.28401	2.1
64386	1.17415	2.1
64387	0.960999	2.1
64388	0.960999	2.1
64389	0.988115	2.1
64390	0.988115	2.1
64391	0.774968	2.1
64392	1.28401	2.1
64393	0.719271	2.1
64394	1.17415	2.1
64395	2.77811	2.1
64396	2.34829	2.1
64397	2.56802	2.1
64398	2.52333	2.1
64399	2.56802	2.1
64400	2.52333	2.1
64401	1.89227	2.1
64402	1.89227	2.1
64403	1.89227	2.1
64404	1.89227	2.1
64405	3.78453	2.1
64406	1.89226	2.1
64407	1.89226	2.1
64408	1.89226	2.1
64409	1.89226	2.1
64410	1.89226	2.1
64411	1.89226	2.1
64412	1.89226	2.1
64413	2.77811	2.1
64414	1.89226	2.1
64415	2.56802	2.1
64416	1.89226	2.1
64417	2.56802	2.1
64418	1.89226	0.466577
64419	1.89226	1.43416
64420	1.89226	0.41471
64421	1.89226	0.754646
64422	3.78452	0.55277
64423	2.41802	0.603638
64424	1.95764	2.1
64425	1.95764	0.679132
64426	2.27727	2.04723
64427	2.39311	0.644513
64428	2.73961	1.96432
64429	0.946132	2.1
64430	0.946132	0.64468
64431	0.94613	0.671102
64432	0.94613	2.1
64433	0.946131	2.1
64434	0.946131	2.1
64435	0.946131	0.690152

64436	0.946131	0.653121
64437	0.946131	0.955457
64438	0.946131	1.06701
64439	0.946131	0.779083
64440	0.946131	0.863415
64441	1.89226	0.515651
64442	1.89226	0.695591
64443	1.89226	0.800459
64444	1.89226	0.476819
64445	1.89226	0.992073
64446	1.89226	0.507935
64447	2.78856	0.410823
64448	2.28552	0.678721
64449	2.28552	0.469404
64450	2.3931	0.601085
64451	2.6402	0.412679
64452	2.74778	0.574689
64453	2.39311	0.552742
64454	1.95764	2.1
64455	2.28552	0.610466
64456	1.86103	2.08426
64457	4.24316	0.91476
64458	1.89226	0.61762
64459	1.89226	2.1
64460	1.89226	0.655141
64461	1.89226	2.1
64462	1.89226	0.674008
64463	1.89226	2.1
64464	1.47412	1.77355
64465	2.18862	1.48805
64466	2.07226	1.02057
64467	2.33645	1.17956
64468	4.26088	1.24474
64469	1.89226	0.928142
64470	1.89226	1.52083
64471	1.89226	1.04161
64472	1.89226	1.70353
64473	1.89226	1.31183
64474	1.89226	2.00934
64475	1.89226	1.20823
64476	1.89226	2.05273
64477	1.89226	0.540625
64478	1.89226	2.04842
64479	3.78453	1.22583
64480	2.18862	1.20357
64481	2.1128	1.94091
64482	2.33645	0.981328
64483	2.28188	2.01611
64484	4.47051	1.67038
64485	2.07226	1.99781
64486	2.33645	1.44442
64487	2.20831	0.891943
64488	2.4646	1.00822
64489	4.53686	1.15594
64490	0.946131	0.786201
64491	0.946131	2.1
64492	0.946131	2.1
64493	0.946131	1.20899

64494	0.94613	1.99736
64495	0.94613	1.11986
64496	0.946133	1.83973
64497	0.946133	1.95328
64498	0.946132	0.836591
64499	0.946132	0.772884
64500	0.946131	1.66655
64501	0.946131	1.3532
64502	1.89226	1.61423
64503	1.89226	1.99963
64504	1.89226	0.914401
64505	1.89226	1.80797
64506	3.78453	1.42796
64507	2.33646	1.16677
64508	2.28189	1.96471
64509	2.4646	0.888975
64510	2.42758	1.93286
64511	4.74648	1.27174
64512	1.89226	2.1
64513	1.89226	2.1
64514	1.89226	2.1
64515	1.89226	2.1
64516	1.89226	2.1
64517	1.89226	2.1
64518	1.89226	2.1
64519	1.89226	2.1
64520	1.89226	2.1
64521	1.89226	2.1
64522	1.89226	2.1
64523	1.89226	2.1
64524	1.89226	2.1
64525	1.89226	2.1
64526	1.89226	2.1
64527	1.89226	2.1
64528	3.78453	2.1
64529	1.89226	2.1
64530	1.89226	2.1
64531	1.89226	2.1
64532	1.89226	2.1
64533	1.89226	2.1
64534	1.89226	2.1
64535	1.89226	2.1
64536	1.89226	2.1
64537	1.89226	2.1
64538	1.89226	2.1
64539	3.78452	2.1
64540	0.877238	2.1
64541	0.877238	2.1
64542	1.24746	2.1
64543	1.04764	2.1
64544	0.964969	2.1
64545	1.1521	2.1
64546	0.946132	2.1
64547	0.946132	2.1
64548	0.946132	2.1
64549	1.12659	2.1
64550	0.964969	2.1
64551	0.946131	2.1

64552	1.89226	2.1
64553	1.89226	2.1
64554	1.89226	2.1
64555	1.89226	2.1
64556	1.89226	2.1
64557	1.89226	2.1
64558	1.89226	2.1
64559	1.89226	2.1
64560	1.89226	2.1
64561	1.89226	2.1
64562	3.78453	2.1
64563	0.868144	2.1
64564	0.992762	2.1
64565	0.946131	2.1
64566	0.946131	2.1
64567	1.07072	2.1
64568	1.07072	2.1
64569	0.868171	2.1
64570	0.868171	2.1
64571	0.992762	2.1
64572	0.946131	2.1
64573	0.946131	2.1
64574	0.868144	2.1
64575	0.946132	2.1
64576	0.946132	2.1
64577	0.946131	2.1
64578	0.946131	2.1
64579	0.946132	2.1
64580	0.946132	2.1
64581	0.946132	2.1
64582	0.946132	2.1
64583	0.946132	2.1
64584	0.946132	2.1
64585	0.946132	2.1
64586	0.946132	2.1
64587	1.89227	2.1
64588	1.89227	2.1
64589	1.89227	2.1
64590	1.89227	2.1
64591	1.89227	2.1
64592	1.89227	2.1
64593	1.89226	2.1
64594	1.89226	2.1
64595	1.89226	2.1
64596	1.89226	2.1
64597	1.89226	2.1
64598	1.89226	2.1
64599	1.98552	2.1
64600	2.22298	2.1
64601	2.22298	2.1
64602	1.89226	2.1
64603	1.89226	2.1
64604	1.54525	2.1
64605	0.810946	2.1
64606	0.810946	2.1
64607	0.810947	2.1
64608	0.810947	2.1
64609	0.81096	2.1

64610	0.81096	2.1
64611	1.89226	2.1
64612	1.35168	2.1
64613	1.08132	2.1
64614	1.08132	2.1
64615	1.0813	2.1
64616	1.89226	2.1
64617	1.89226	1.56821
64618	1.89226	2.1
64619	1.89226	1.24924
64620	3.78452	2.1
64621	1.7753	2.1
64622	2.39703	1.59004
64623	1.90577	2.1
64624	2.03445	1.70235
64625	3.82335	2.1
64626	2.39703	1.50526
64627	2.47949	0.517756
64628	2.26051	1.67342
64629	2.36891	0.406181
64630	4.76594	0.780703
64631	1.89226	1.51895
64632	1.89226	0.545506
64633	1.89226	1.31526
64634	1.89226	0.41968
64635	3.78452	0.712747
64636	1.89226	2.1
64637	1.89226	1.64311
64638	1.89226	2.1
64639	1.89226	1.54646
64640	1.89226	2.1
64641	1.89226	1.60697
64642	0.883745	1.49398
64643	0.973143	2.1
64644	0.988173	2.1
64645	0.988173	1.41558
64646	0.941927	1.17509
64647	1.02971	1.50635
64648	0.854065	2.1
64649	0.82702	2.1
64650	0.847835	2.1
64651	0.981126	2.1
64652	1.02996	2.1
64653	1.10704	2.1
64654	2.28031	1.86117
64655	2.26369	0.589043
64656	2.26369	1.83274
64657	2.36891	0.625761
64658	2.30788	1.09699
64659	2.42455	0.616108
64660	1.89226	1.73497
64661	1.89226	0.588648
64662	1.89226	0.542917
64663	1.89226	1.58654
64664	1.89226	0.568375
64665	1.89226	1.57479
64666	2.71406	2.1
64667	1.83244	2.1

64668	1.83244	2.1
64669	2.07567	2.1
64670	2.52044	2.1
64671	2.76367	2.1
64672	1.89226	2.1
64673	1.89226	2.1
64674	1.89226	2.1
64675	1.89226	2.1
64676	3.78453	2.1
64677	1.89226	2.1
64678	1.89226	2.1
64679	1.89226	2.1
64680	1.89226	2.1
64681	1.89226	2.1
64682	1.89226	2.1
64683	2.70714	2.1
64684	2.3148	2.1
64685	2.3148	2.1
64686	2.52044	2.1
64687	2.54204	2.1
64688	2.74768	2.1
64689	2.52044	2.1
64690	2.3148	2.1
64691	1.83244	2.1
64692	1.83244	2.1
64693	1.62611	2.1
64694	2.3148	2.1
64695	0.946131	2.1
64696	0.946131	2.1
64697	0.946131	2.1
64698	0.946131	2.1
64699	0.946131	2.1
64700	0.946131	2.1
64701	0.946131	2.1
64702	0.946131	2.1
64703	0.946131	2.1
64704	0.946131	2.1
64705	0.946131	2.1
64706	0.946131	2.1
64707	1.89226	2.1
64708	1.89226	2.1
64709	1.89226	2.1
64710	1.89226	2.1
64711	3.78453	2.1
64712	1.89226	2.1
64713	1.89226	2.1
64714	1.89226	2.1
64715	1.89226	2.1
64716	1.89226	2.1
64717	1.89226	2.1
64718	1.89227	2.1
64719	1.89227	2.1
64720	1.89227	2.1
64721	1.89227	2.1
64722	3.78453	2.1
64723	1.89226	2.1
64724	1.89226	2.1
64725	1.89226	2.1

64726	1.89226	2.1
64727	3.78453	2.1
64728	0.915781	2.1
64729	0.863524	2.1
64730	0.717056	2.1
64731	0.830182	2.1
64732	0.865094	2.1
64733	0.918003	2.1
64734	1.89226	2.1
64735	1.29933	2.1
64736	1.11215	2.1
64737	1.05227	2.1
64738	0.966269	2.1
64739	1.89226	2.1
64740	1.89226	2.1
64741	1.89226	2.1
64742	1.89226	2.1
64743	1.89226	2.1
64744	1.89226	2.1
64745	1.89226	2.1
64746	1.89226	2.1
64747	1.89226	2.1
64748	1.89226	2.1
64749	1.89226	2.1
64750	1.89226	2.1
64751	1.89226	2.1
64752	1.89226	2.1
64753	1.89226	2.1
64754	1.89226	2.1
64755	1.89226	2.1
64756	1.89226	2.1
64757	1.01725	2.1
64758	0.997048	2.1
64759	0.818657	2.1
64760	0.960724	2.1
64761	0.838013	2.1
64762	1.04859	2.1
64763	1.89226	2.1
64764	1.41754	2.1
64765	1.17625	2.1
64766	1.0793	2.1
64767	1.18822	2.1
64768	2.16402	2.1
64769	1.89452	2.1
64770	1.50084	2.1
64771	2.09288	2.1
64772	3.9874	2.1
64773	2.16402	2.1
64774	2.13925	2.1
64775	2.04483	2.1
64776	2.09288	2.1
64777	4.23212	2.1
64778	1.89226	2.1
64779	1.89226	2.1
64780	1.89226	2.1
64781	1.89226	2.1
64782	3.78452	2.1
64783	1.89226	2.1

64784	1.89226	2.1
64785	1.89226	2.1
64786	1.89226	2.1
64787	1.89226	2.1
64788	1.89226	2.1
64789	1.9824	2.1
64790	2.09288	2.1
64791	1.69889	2.1
64792	1.6817	2.1
64793	2.00695	2.1
64794	2.00695	2.1
64795	1.93592	2.1
64796	2.09288	2.1
64797	2.00695	2.1
64798	2.09288	2.1
64799	1.9589	2.1
64800	2.04483	2.1
64801	0.946128	2.1
64802	0.946128	2.1
64803	0.946132	2.1
64804	0.946132	2.1
64805	0.94613	2.1
64806	0.94613	2.1
64807	0.946129	2.1
64808	0.946129	2.1
64809	0.94613	2.1
64810	0.94613	2.1
64811	0.94613	2.1
64812	0.94613	2.1
64813	0.946131	2.1
64814	0.946131	2.1
64815	0.946131	2.1
64816	0.946131	2.1
64817	0.946131	2.1
64818	0.946131	2.1
64819	0.946131	2.1
64820	0.946131	2.1
64821	0.946131	2.1
64822	0.946131	2.1
64823	0.946131	2.1
64824	0.946131	2.1
64825	1.89226	1.15548
64826	2.48083	2.1
64827	2.6968	0.976742
64828	2.6968	2.1
64829	1.89226	1.04134
64830	1.89226	2.1
64831	1.89226	1.14963
64832	1.89226	2.1
64833	1.89226	1.18671
64834	1.89226	2.1
64835	3.78453	1.86375
64836	1.89226	2.1
64837	1.89226	2.1
64838	1.89226	2.00696
64839	1.89226	2.1
64840	1.89226	1.95663
64841	1.89226	2.1

64842	1.1017	2.1
64843	1.1017	2.01062
64844	1.10605	2.1
64845	1.01647	2.1
64846	0.868255	2.1
64847	0.883424	2.1
64848	1.24624	2.1
64849	1.35473	2.01673
64850	0.942231	2.1
64851	1.31464	2.1
64852	0.910064	2.1
64853	1.27147	2.1
64854	1.89226	1.14962
64855	1.89226	2.1
64856	1.89226	0.735386
64857	1.89226	2.1
64858	3.78453	1.81422
64859	1.89226	2.097
64860	1.89226	2.1
64861	1.89226	2.06517
64862	1.89226	2.1
64863	1.89226	1.99266
64864	1.89226	2.1
64865	1.89226	2.1
64866	1.89226	2.1
64867	1.89226	2.1
64868	1.89226	2.1
64869	3.78453	2.1
64870	1.89226	2.1
64871	2.70015	2.1
64872	1.89226	2.1
64873	2.61064	2.1
64874	4.59241	2.1
64875	2.70015	2.1
64876	2.3372	2.1
64877	2.61064	2.1
64878	1.85184	2.1
64879	4.55198	2.1
64880	1.89226	2.1
64881	1.89226	2.1
64882	1.89226	2.1
64883	1.89226	2.1
64884	1.89226	2.1
64885	1.89226	2.1
64886	1.89227	2.1
64887	1.89227	2.1
64888	1.89227	2.1
64889	1.89227	2.1
64890	3.78453	2.1
64891	1.89226	2.1
64892	2.52194	2.1
64893	2.61064	2.1
64894	2.61064	2.1
64895	1.89226	2.1
64896	1.89226	2.1
64897	1.095	2.1
64898	1.095	2.1
64899	1.09189	2.1

64900	1.09189	2.1
64901	0.875334	2.1
64902	0.925919	2.1
64903	1.26097	2.1
64904	1.30532	2.1
64905	1.26097	2.1
64906	0.875334	2.1
64907	1.30532	2.1
64908	0.92592	2.1
64909	1.89226	2.1
64910	1.89226	2.1
64911	1.89226	2.1
64912	1.89226	2.1
64913	1.89226	2.1
64914	1.89226	2.1
64915	1.89226	2.1
64916	1.89226	2.1
64917	1.89226	2.1
64918	1.89226	2.1
64919	3.78453	2.1
64920	1.26097	2.1
64921	1.26097	2.1
64922	0.946132	2.1
64923	0.946132	2.1
64924	1.22176	2.1
64925	1.26097	2.1
64926	0.946132	2.1
64927	0.946132	2.1
64928	0.946132	2.1
64929	1.22176	2.1
64930	1.26097	2.1
64931	0.946132	2.1
64932	2.52194	2.1
64933	1.67381	2.1
64934	1.67381	2.1
64935	1.75067	2.1
64936	2.44352	2.1
64937	2.52038	2.1
64938	1.89226	2.1
64939	1.89226	2.1
64940	1.89226	2.1
64941	1.89226	2.1
64942	1.89226	2.1
64943	1.89226	2.1
64944	1.89227	2.1
64945	1.89227	2.1
64946	1.89227	2.1
64947	1.89227	2.1
64948	1.89227	2.1
64949	1.89227	2.1
64950	1.22176	2.1
64951	1.22176	2.1
64952	0.946132	2.1
64953	0.946132	2.1
64954	1.19095	2.1
64955	1.22176	2.1
64956	0.946133	2.1
64957	0.946133	2.1

64958	1.19095	2.1
64959	0.946132	2.1
64960	0.946133	2.1
64961	1.22176	2.1
64962	2.44352	2.1
64963	1.6287	2.1
64964	1.6287	2.1
64965	1.67381	2.1
64966	2.3819	2.1
64967	2.427	2.1
64968	1.89226	2.1
64969	1.89226	2.1
64970	1.89226	2.1
64971	1.89226	2.1
64972	1.89226	2.1
64973	1.89226	2.1
64974	1.89226	1.66871
64975	1.89226	2.1
64976	1.89226	1.5548
64977	1.89226	2.1
64978	3.78453	2.1
64979	1.89226	1.78017
64980	1.89226	2.1
64981	1.89226	1.85406
64982	1.89226	2.1
64983	1.89226	1.33809
64984	1.89226	2.1
64985	1.89226	2.1
64986	1.89226	2.1
64987	1.89226	2.1
64988	1.89226	2.1
64989	1.89226	2.1
64990	1.89226	2.1
64991	0.851542	2.1
64992	0.851542	2.1
64993	1.1736	2.1
64994	1.29261	2.1
64995	0.852708	2.1
64996	1.18843	2.1
64997	0.946132	2.1
64998	0.946132	2.1
64999	0.946132	2.1
65000	1.07901	2.1
65001	1.04208	2.1
65002	0.936669	2.1
65003	1.89227	1.10658
65004	1.89227	2.1
65005	1.89227	1.2636
65006	1.89227	2.1
65007	3.78453	2.00167
65008	1.89226	1.63719
65009	1.89226	2.1
65010	1.89226	1.34155
65011	1.89226	2.1
65012	3.78453	2.07324
65013	1.89226	2.1
65014	1.89226	2.1
65015	1.89226	2.1

65016	1.89226	2.1
65017	1.89226	2.1
65018	1.89226	2.1
65019	0.946133	2.1
65020	0.946133	2.1
65021	1.28525	2.0122
65022	1.28525	2.1
65023	1.07901	2.1
65024	1.28525	2.1
65025	0.946132	2.1
65026	0.946132	1.88975
65027	0.946132	2.1
65028	1.28525	2.1
65029	0.946133	2.1
65030	1.07901	2.1
65031	1.89226	2.1
65032	1.89226	2.1
65033	1.89226	2.1
65034	1.89226	2.1
65035	1.89226	2.1
65036	1.89226	2.1
65037	1.89226	2.1
65038	1.89226	2.1
65039	1.89226	2.1
65040	1.89226	2.1
65041	1.89226	2.1
65042	1.89226	2.1
65043	1.24419	2.1
65044	1.09093	2.1
65045	1.22722	2.1
65046	1.18154	2.1
65047	1.27455	2.1
65048	1.08473	2.1
65049	1.15541	2.1
65050	1.15541	2.1
65051	1.29607	2.1
65052	1.23814	2.1
65053	1.13024	2.1
65054	1.12096	2.1
65055	1.18858	2.1
65056	1.13181	2.1
65057	0.988718	2.1
65058	0.802498	2.1
65059	0.994034	2.1
65060	1.15773	2.1
65061	1.0029	2.1
65062	1.0029	2.1
65063	1.23814	2.1
65064	0.997523	2.1
65065	1.11498	2.1
65066	0.899532	2.1
65067	1.89226	2.1
65068	1.89226	2.1
65069	1.89226	2.1
65070	1.89226	2.1
65071	1.89226	2.1
65072	1.89226	2.1
65073	1.89226	2.1

65074	1.89226	2.1
65075	1.89226	2.1
65076	1.89226	2.1
65077	1.89226	2.1
65078	1.89226	2.1
65079	0.993139	2.1
65080	0.834108	2.1
65081	1.01147	2.1
65082	1.01358	2.1
65083	1.20067	2.1
65084	0.993156	2.1
65085	2.4158	2.1
65086	1.69956	2.1
65087	1.3544	2.1
65088	1.60095	2.1
65089	1.11219	2.1
65090	1.89226	2.1
65091	1.89226	2.1
65092	1.89226	1.80621
65093	1.89226	2.1
65094	3.78452	2.1
65095	1.89226	2.1
65096	1.89226	2.1
65097	1.89226	2.1
65098	1.89226	2.1
65099	1.89226	2.1
65100	1.89226	2.1
65101	1.89226	2.1
65102	2.83014	2.1
65103	2.32404	2.1
65104	2.32404	2.1
65105	1.89226	2.1
65106	1.89226	2.1
65107	1.37966	2.1
65108	1.13294	2.1
65109	0.881723	2.1
65110	0.582304	2.1
65111	0.977944	2.1
65112	0.977948	2.1
65113	1.0264	2.1
65114	1.0264	2.1
65115	1.37967	2.1
65116	1.03896	2.1
65117	1.19108	2.1
65118	0.582298	2.1
65119	1.89226	1.48887
65120	1.89226	2.1
65121	1.89226	2.05211
65122	1.89226	2.1
65123	3.78452	2.1
65124	0.946131	2.1
65125	0.946131	2.1
65126	0.946131	2.1
65127	0.946131	2.07528
65128	0.946132	2.1
65129	0.946132	2.1
65130	0.946129	2.1
65131	0.946129	2.01133

65132	0.946131	2.1
65133	0.946131	2.1
65134	0.94613	2.1
65135	0.94613	2.1
65136	1.89226	2.1
65137	1.89226	1.64214
65138	1.89226	2.1
65139	1.89226	2.1
65140	3.78453	2.1
65141	1.89226	2.1
65142	1.89226	2.1
65143	1.89226	2.1
65144	1.89226	2.1
65145	1.89226	2.1
65146	1.89226	2.1
65147	2.65216	2.1
65148	1.93528	2.1
65149	1.78595	2.1
65150	2.48544	2.1
65151	4.42072	2.1
65152	1.89226	2.1
65153	1.89226	2.1
65154	1.89226	2.1
65155	1.89226	2.1
65156	1.89226	2.1
65157	1.89226	2.0638
65158	1.89226	2.1
65159	1.89226	2.1
65160	1.89226	2.1
65161	1.89226	2.1
65162	3.78452	2.1
65163	1.66537	2.1
65164	2.48544	2.1
65165	1.78595	2.1
65166	1.78595	2.1
65167	2.36197	2.1
65168	2.36197	2.1
65169	2.80975	2.1
65170	2.48544	2.1
65171	2.36198	2.1
65172	2.65135	2.1
65173	5.13679	2.1
65174	1.89226	2.1
65175	1.89226	2.1
65176	1.89226	2.1
65177	1.89226	2.1
65178	1.89226	2.1
65179	1.89226	1.98721
65180	0.946127	2.1
65181	0.946127	2.1
65182	0.946131	2.1
65183	0.946131	2.1
65184	0.946128	2.1
65185	0.946128	2.1
65186	0.94613	2.1
65187	0.94613	2.1
65188	0.946129	2.1
65189	0.946129	2.1

65190	0.946129	2.1
65191	0.946129	2.1
65192	1.11972	2.1
65193	1.18099	2.1
65194	0.832687	2.1
65195	0.764517	2.1
65196	0.974426	2.1
65197	0.974426	2.1
65198	0.971078	2.1
65199	0.971078	2.1
65200	1.11972	2.1
65201	0.764515	2.1
65202	0.832686	2.1
65203	1.18099	2.1
65204	2.36198	2.1
65205	2.48025	2.1
65206	2.48025	2.1
65207	2.65135	2.1
65208	2.23944	2.1
65209	2.41055	2.1
65210	0.946132	1.30647
65211	0.946132	2.1
65212	0.946131	2.1
65213	0.946131	2.1
65214	0.946131	2.1
65215	0.946131	1.56365
65216	0.946132	2.1
65217	0.946132	2.1
65218	0.946131	2.1
65219	0.946131	2.1
65220	0.946132	2.1
65221	0.946132	2.1
65222	0.810942	2.1
65223	0.810942	2.1
65224	0.810944	2.1
65225	0.810944	2.1
65226	1.89226	2.1
65227	1.3517	2.1
65228	1.08132	2.1
65229	1.08133	2.1
65230	1.08132	2.1
65231	0.810932	2.1
65232	0.810932	2.1
65233	1.89249	1.89353
65234	1.9857	1.57334
65235	2.02433	1.30759
65236	2.15807	1.30508
65237	4.01003	1.38291
65238	1.89226	1.27248
65239	1.89226	1.73079
65240	1.89226	1.43091
65241	1.89226	0.982409
65242	3.78452	1.67969
65243	1.89226	1.57526
65244	1.89226	1.53792
65245	1.89226	0.815931
65246	1.89226	1.37821
65247	3.78452	0.991479

65248	1.98569	1.33295
65249	1.86067	1.57339
65250	2.15807	1.16306
65251	2.07965	1.16192
65252	4.06534	1.73096
65253	2.02433	1.49268
65254	2.15807	1.84408
65255	2.12608	1.98246
65256	2.3025	1.05165
65257	4.32683	1.80073
65258	1.89226	1.60954
65259	1.89226	1.36449
65260	1.89226	0.976599
65261	1.89226	1.14038
65262	3.78452	1.11075
65263	1.89226	1.06642
65264	1.89226	1.74786
65265	1.89226	1.0982
65266	1.89226	1.09778
65267	3.78452	1.60436
65268	2.15807	1.6388
65269	2.07965	1.49612
65270	2.3025	0.97804
65271	2.28384	1.2405
65272	4.38215	1.04484
65273	1.89226	0.682688
65274	1.89226	0.275408
65275	1.89226	0.537342
65276	1.89226	0.271928
65277	3.78453	0.414965
65278	2.16905	0.369812
65279	2.56369	0.271474
65280	1.88394	0.49902
65281	2.41938	0.167352
65282	4.44763	0.302028
65283	2.56369	0.355972
65284	2.80663	0.409025
65285	2.41938	0.18972
65286	2.69045	0.26277
65287	5.25414	0.273418
65288	1.89226	0.359895
65289	1.89226	0.369535
65290	1.89226	0.40368
65291	1.89226	0.289722
65292	3.78453	0.302873
65293	1.89226	0.470436
65294	1.89226	0.325215
65295	1.89226	0.654745
65296	1.89226	0.363504
65297	1.89226	0.627298
65298	1.89226	0.249176
65299	1.01988	0.207615
65300	1.01988	0.230319
65301	1.03333	0.401288
65302	1.03333	0.316662
65303	1.13811	0.278797
65304	1.20969	0.249735
65305	0.941974	0.435368

65306	0.843515	0.22358
65307	0.843514	0.175641
65308	1.13811	0.215833
65309	0.941973	0.239526
65310	1.20969	0.205013
65311	2.41938	0.165124
65312	2.57414	0.29048
65313	2.57414	0.431751
65314	2.69045	0.438204
65315	2.27623	0.169547
65316	2.39253	0.430156
65317	1.89227	0.411734
65318	1.89227	0.27173
65319	1.89227	0.256621
65320	1.89227	0.326971
65321	1.89227	0.199333
65322	1.89227	0.264867
65323	2.28385	1.35963
65324	2.09114	1.44602
65325	2.46968	0.814232
65326	2.32438	1.31737
65327	4.56082	0.885448
65328	1.89226	0.913786
65329	1.89226	1.69269
65330	1.89226	1.0045
65331	1.89226	1.30943
65332	3.78452	1.41736
65333	1.89226	0.992995
65334	1.89226	1.72747
65335	1.89226	0.912886
65336	1.89226	1.36786
65337	3.78452	1.07685
65338	2.09114	0.846309
65339	1.5249	1.70766
65340	2.55684	0.843504
65341	1.86899	1.42206
65342	3.96011	1.27719
65343	2.46968	1.02888
65344	2.32439	1.57046
65345	2.62768	0.977227
65346	2.506	1.26384
65347	4.97568	1.71974
65348	1.89226	1.30523
65349	1.89226	1.3653
65350	1.89226	0.68287
65351	1.89226	1.33022
65352	3.78452	0.88215
65353	1.89226	0.893753
65354	1.89226	1.33281
65355	1.89226	1.06691
65356	1.89226	2.05333
65357	1.89226	0.679211
65358	1.89226	2.02814
65359	2.55684	1.06283
65360	2.16506	1.25238
65361	1.86901	1.50348
65362	1.86899	1.05375
65363	2.75664	1.14844

65364	2.75662	0.85865
65365	1.89224	0.186921
65366	1.89224	0.384107
65367	1.89224	0.150608
65368	1.89224	0.554291
65369	3.78447	0.19937
65370	2.24206	0.216864
65371	2.38862	0.646549
65372	2.07107	0.339594
65373	2.23097	0.533143
65374	4.47303	0.501067
65375	2.38863	0.654606
65376	2.25945	0.735039
65377	2.25945	0.54288
65378	2.39244	1.67969
65379	2.23098	0.535881
65380	2.36397	1.86774
65381	1.89224	0.285216
65382	1.89224	0.78927
65383	1.89224	0.599771
65384	1.89224	0.531858
65385	1.89224	0.584578
65386	1.89224	0.543195
65387	1.89226	0.188283
65388	1.89226	0.485342
65389	1.89226	0.458744
65390	1.89226	0.252694
65391	1.89226	0.334021
65392	1.89226	0.324835
65393	0.920975	0.785272
65394	0.920979	0.382786
65395	1.03015	0.497683
65396	1.03015	0.193667
65397	0.992407	0.487168
65398	1.08759	0.694844
65399	0.854346	0.360361
65400	0.87241	0.198024
65401	0.872397	0.461205
65402	1.04334	0.454727
65403	1.00965	0.495865
65404	1.0876	0.454196
65405	2.23101	0.646961
65406	2.25948	0.792231
65407	2.03577	0.513171
65408	2.05257	2.0181
65409	4.29524	0.667794
65410	0.810947	0.298903
65411	0.810947	0.601152
65412	0.810947	0.477934
65413	0.810947	0.329161
65414	0.810934	0.537889
65415	0.810934	0.549733
65416	1.89227	0.830379
65417	1.3517	0.631119
65418	1.08132	0.553962
65419	1.08132	0.611411
65420	1.08133	0.556985
65421	1.89226	1.13314

65422	1.89226	1.55459
65423	1.89226	0.916373
65424	1.89226	2.08376
65425	1.89226	0.654246
65426	1.89226	2.1
65427	1.89226	1.29866
65428	1.89226	1.43745
65429	1.89226	0.9362
65430	1.89226	2.1
65431	1.89226	0.87556
65432	1.89226	2.1
65433	0.879432	1.73147
65434	0.879432	1.05851
65435	0.900223	0.630833
65436	0.900223	1.86383
65437	0.54571	1.67437
65438	0.743092	1.31396
65439	1.03656	1.73533
65440	1.19234	0.812185
65441	1.19234	1.27538
65442	0.743092	1.45667
65443	1.03656	1.51085
65444	0.545711	1.27526
65445	1.89226	0.774614
65446	2.38467	1.60812
65447	2.69293	0.757398
65448	2.69293	1.82219
65449	1.89226	0.674272
65450	1.89226	1.49525
65451	1.89226	0.956676
65452	1.89226	1.5346
65453	1.89226	0.803473
65454	1.89226	1.58927
65455	3.78452	1.22111
65456	0.946129	2.1
65457	0.946129	1.4848
65458	0.946133	0.728278
65459	0.946133	2.00523
65460	0.946131	1.06402
65461	0.946131	2.0982
65462	0.946131	0.70478
65463	0.946131	1.16933
65464	0.946131	1.27498
65465	0.946131	1.28578
65466	0.946131	1.14229
65467	0.946131	1.07614
65468	1.04846	0.636847
65469	1.04846	1.62426
65470	1.05594	0.694978
65471	1.05594	1.82116
65472	0.743092	1.48478
65473	0.912061	1.14236
65474	1.19234	0.740591
65475	1.34647	0.867368
65476	0.912061	0.760308
65477	1.34646	1.04185
65478	1.19234	0.909078
65479	0.743093	0.911098

65480	1.89226	0.910422
65481	1.89226	1.5774
65482	1.89226	0.615549
65483	1.89226	1.01403
65484	3.78452	0.959135
65485	0.946128	2.1
65486	0.946128	1.06405
65487	0.946133	1.41191
65488	0.946133	0.784764
65489	0.94613	2.1
65490	0.94613	1.78829
65491	0.946131	1.24182
65492	0.946131	0.855482
65493	0.946131	1.60081
65494	0.946131	1.59025
65495	0.946131	1.08382
65496	0.946131	1.1088
65497	1.89226	1.17969
65498	1.89226	2.06061
65499	1.89226	0.856878
65500	1.89226	1.23853
65501	3.78452	1.46358
65502	1.89226	1.10454
65503	1.89226	1.21219
65504	1.89226	0.937648
65505	1.89226	2.0513
65506	1.89226	0.546282
65507	1.89226	2.05869
65508	1.89226	1.05853
65509	1.89226	1.39432
65510	1.89226	0.86614
65511	1.89226	2.09152
65512	1.89226	0.75784
65513	1.89226	2.1
65514	0.946132	1.83961
65515	0.946132	1.09916
65516	1.17758	0.770958
65517	1.17759	2.0246
65518	1.04514	1.77407
65519	1.17758	1.04531
65520	0.946133	0.739274
65521	0.946133	0.900308
65522	0.946107	1.08486
65523	1.17755	1.10653
65524	1.04517	1.17203
65525	0.946158	1.07415
65526	1.89226	2.1
65527	2.10171	2.1
65528	1.95064	2.1
65529	2.10171	2.1
65530	1.7412	2.1
65531	1.89226	2.1
65532	1.89226	2.1
65533	1.89226	2.1
65534	1.89226	2.1
65535	1.89226	2.1
65536	1.89226	2.1
65537	1.89226	2.1

65538	1.89226	2.1
65539	1.89226	0.808641
65540	1.89226	2.1
65541	1.89226	1.72766
65542	3.78453	2.00707
65543	0.946133	2.1
65544	0.946133	1.55182
65545	0.946132	2.1
65546	0.946132	2.1
65547	0.946133	2.1
65548	0.946133	1.7523
65549	0.946132	2.1
65550	0.946132	2.1
65551	0.946132	2.1
65552	0.946132	2.1
65553	0.946132	2.1
65554	0.946132	2.1
65555	1.70303	2.1
65556	1.95064	2.1
65557	1.74039	2.1
65558	1.95064	2.1
65559	1.69368	2.1
65560	1.89226	2.1
65561	1.89226	2.1
65562	1.89226	2.1
65563	1.89226	2.1
65564	1.89226	2.07956
65565	3.78453	2.1
65566	0.946132	0.754435
65567	0.946132	2.07139
65568	0.946132	2.1
65569	0.946132	1.78242
65570	0.946133	1.53002
65571	0.946133	0.80433
65572	0.946132	2.1
65573	0.946132	2.09164
65574	0.946107	2.00941
65575	0.946107	2.00167
65576	0.946158	1.9223
65577	0.946158	2.01986
65578	0.946133	2.1
65579	0.946133	2.07584
65580	0.946132	2.1
65581	0.946132	2.1
65582	0.946133	2.1
65583	0.946133	1.47389
65584	0.946132	2.1
65585	0.946132	2.1
65586	0.946107	2.1
65587	0.946107	2.1
65588	0.946158	2.1
65589	0.946158	2.1
65590	1.89226	2.04262
65591	1.89226	2.05171
65592	1.89226	1.34982
65593	1.89226	2.1
65594	1.89226	1.24064
65595	1.89226	2.1

65596	1.89226	2.1
65597	1.89226	2.1
65598	1.89226	2.1
65599	1.89226	2.1
65600	1.89226	1.91989
65601	1.89226	2.1
65602	2.5198	2.1
65603	2.45893	2.1
65604	2.27322	2.1
65605	2.27322	2.1
65606	2.63576	2.1
65607	2.63576	2.1
65608	1.24045	2.1
65609	1.11906	2.1
65610	1.13578	2.1
65611	0.916205	1.99715
65612	1.12539	2.1
65613	1.08404	2.1
65614	1.08484	2.01597
65615	1.08484	2.1
65616	1.22836	2.1
65617	0.919363	2.1
65618	1.16507	2.1
65619	0.924864	2.1
65620	1.89226	2.04359
65621	1.89226	1.63548
65622	1.89226	0.960636
65623	1.89226	2.1
65624	1.89226	0.876694
65625	1.89226	2.1
65626	1.89226	2.1
65627	1.89226	2.1
65628	1.89226	2.0286
65629	1.89226	2.1
65630	1.89226	1.84018
65631	1.89226	2.1
65632	2.6946	2.1
65633	2.63576	2.1
65634	2.5198	2.1
65635	2.5198	2.1
65636	2.8362	2.1
65637	2.8362	2.1
65638	1.3265	2.1
65639	1.24045	2.1
65640	0.983223	1.56587
65641	0.954429	2.1
65642	1.06046	2.1
65643	1.10594	2.1
65644	1.1915	1.48789
65645	1.1915	2.1
65646	1.31357	2.1
65647	1.09005	2.1
65648	1.29145	2.1
65649	0.995825	2.1
65650	1.89226	2.1
65651	1.89226	2.1
65652	1.89226	2.1
65653	1.89226	2.1

65654	3.78452	2.1
65655	1.89226	2.1
65656	1.89226	2.1
65657	1.89226	2.1
65658	1.89226	2.1
65659	3.78453	2.1
65660	1.91802	2.1
65661	2.27409	2.1
65662	2.48048	2.1
65663	2.22264	2.1
65664	1.91802	2.1
65665	1.66018	2.1
65666	1.13704	2.1
65667	1.24023	2.1
65668	1.22947	2.1
65669	1.31788	2.1
65670	1.23859	2.1
65671	1.23859	2.1
65672	1.23111	2.1
65673	1.23111	2.1
65674	1.24024	2.1
65675	1.31788	2.1
65676	1.13704	2.1
65677	1.22947	2.1
65678	1.89226	2.1
65679	1.89226	2.1
65680	1.89226	2.1
65681	1.89226	2.1
65682	1.89226	2.1
65683	1.89226	2.1
65684	1.89226	2.1
65685	1.89226	2.1
65686	1.89226	2.1
65687	1.89226	2.1
65688	3.78453	2.1
65689	1.05568	2.1
65690	0.959008	2.1
65691	1.32068	2.1
65692	1.24024	2.1
65693	1.15206	2.1
65694	1.15206	2.1
65695	1.14386	2.1
65696	1.14386	2.1
65697	1.05568	2.1
65698	1.32068	2.1
65699	0.959008	2.1
65700	1.24024	2.1
65701	1.32068	2.1
65702	1.24023	2.1
65703	1.31788	2.1
65704	1.4181	2.1
65705	1.31436	2.1
65706	1.31436	2.1
65707	1.3242	2.1
65708	1.3242	2.1
65709	1.32068	2.1
65710	1.4181	2.1
65711	1.24024	2.1

65712	1.31788	2.1
65713	1.89226	0.87334
65714	1.89226	0.357
65715	1.89226	0.613757
65716	1.89226	0.616864
65717	1.89226	0.610608
65718	1.89226	0.661336
65719	1.02925	0.256757
65720	1.16678	0.433277
65721	0.946132	0.313444
65722	0.946132	0.7365
65723	0.935677	0.507577
65724	0.935677	0.292638
65725	1.18902	0.628675
65726	1.21562	0.571027
65727	1.13433	0.391158
65728	1.04071	0.44204
65729	0.851553	0.478222
65730	1.06075	0.402567
65731	1.89226	0.499133
65732	1.89226	0.338473
65733	1.89226	0.335616
65734	1.89226	0.240203
65735	1.89226	0.33564
65736	1.89226	0.53956
65737	1.89226	0.636351
65738	1.89226	0.221969
65739	1.89226	0.389375
65740	1.89226	0.373491
65741	3.78453	0.316606
65742	0.946128	0.533243
65743	0.946128	0.428704
65744	0.946133	0.40623
65745	0.946133	0.281285
65746	0.946131	0.336298
65747	0.946131	0.389518
65748	0.94613	0.517322
65749	0.94613	0.403702
65750	0.946131	0.451073
65751	0.946131	0.393209
65752	0.946131	0.237095
65753	0.946131	0.267938
65754	0.946132	0.585045
65755	0.946132	0.343399
65756	0.946132	0.632105
65757	0.946132	0.302582
65758	0.946132	0.489256
65759	0.946132	0.220623
65760	0.946131	0.52039
65761	0.946131	0.609176
65762	0.946132	0.41639
65763	0.946132	0.591923
65764	0.946132	0.303519
65765	0.946132	0.440121
65766	0.946131	1.76984
65767	0.946131	1.06993
65768	0.946131	2.07441
65769	0.946131	0.7888

65770	0.946131	0.87821
65771	0.946131	1.64372
65772	0.946131	0.864444
65773	0.946131	0.74286
65774	0.946131	1.01869
65775	0.946131	1.035
65776	0.946131	1.13629
65777	0.946131	1.12102
65778	1.89226	0.971863
65779	1.89226	1.20011
65780	1.89226	0.791827
65781	1.89226	2.03048
65782	1.89226	0.710995
65783	1.89226	2.1
65784	1.89226	0.941389
65785	1.89226	0.924145
65786	1.89226	0.95176
65787	1.89226	1.79817
65788	1.89226	0.728659
65789	1.89226	2.03574
65790	1.89226	1.05843
65791	1.89226	0.938143
65792	1.89226	0.713712
65793	1.89226	1.9622
65794	1.89226	0.582313
65795	1.89226	2.1
65796	1.89226	1.4397
65797	1.89226	1.94517
65798	1.89226	0.790215
65799	1.89226	2.06564
65800	3.78452	1.42716
65801	0.946131	1.7745
65802	0.946131	0.79107
65803	1.17195	0.773617
65804	1.17195	2.05124
65805	0.958489	1.95666
65806	1.17195	1.29669
65807	0.946129	0.783518
65808	0.946129	1.20745
65809	0.946131	1.09062
65810	1.17195	1.12911
65811	0.958487	1.20478
65812	0.94613	1.18705
65813	2.69656	2.00727
65814	2.17516	1.88548
65815	2.41885	2.1
65816	2.41885	1.25367
65817	2.46476	2.1
65818	2.46476	0.879716
65819	2.69657	1.75452
65820	2.30233	0.604892
65821	2.30233	1.13735
65822	2.59476	0.777954
65823	2.46477	1.19787
65824	2.7572	0.829722
65825	1.89226	1.29563
65826	1.89226	0.452197
65827	1.89226	0.461779

65828	1.89226	0.578749
65829	1.89226	0.436945
65830	1.89226	0.640626
65831	1.89226	1.20327
65832	1.89226	0.766631
65833	1.89226	1.6865
65834	1.89226	0.394149
65835	3.78452	0.828445
65836	2.46477	1.64812
65837	2.17516	1.82788
65838	1.80252	2.1
65839	2.09715	0.663007
65840	4.27231	1.82651
65841	2.46477	1.66882
65842	2.09715	0.689155
65843	2.30233	0.823234
65844	2.30233	0.766306
65845	1.96416	0.439142
65846	2.09715	0.466676
65847	1.89227	0.674758
65848	1.89227	0.315034
65849	1.89227	0.498677
65850	1.89227	0.442062
65851	1.89227	0.620491
65852	1.89227	0.71341
65853	1.89226	1.58238
65854	1.89226	0.336104
65855	1.89226	0.623751
65856	1.89226	0.671235
65857	3.78452	0.642758
65858	0.946132	2.1
65859	0.946132	2.1
65860	0.946132	2.1
65861	0.946132	2.1
65862	0.946132	2.1
65863	0.946132	2.1
65864	0.946132	2.1
65865	0.946132	2.1
65866	0.946132	2.1
65867	0.946132	2.1
65868	0.946131	2.1
65869	0.946131	2.1
65870	1.89226	2.1
65871	1.89226	2.1
65872	1.89226	2.1
65873	1.89226	2.1
65874	3.78453	2.1
65875	1.89226	2.1
65876	1.89226	2.1
65877	1.89226	2.1
65878	1.89226	2.1
65879	1.89226	2.1
65880	1.89226	2.1
65881	1.89226	2.1
65882	1.63707	2.1
65883	1.63707	2.1
65884	1.38859	2.1
65885	1.89226	2.1

65886	1.64377	2.1
65887	0.94613	2.1
65888	0.94613	2.1
65889	0.94613	2.1
65890	0.94613	2.1
65891	0.946131	2.1
65892	0.946131	2.1
65893	0.946129	2.1
65894	0.946129	2.1
65895	0.94613	2.1
65896	0.94613	2.1
65897	0.94613	2.1
65898	0.94613	2.1
65899	2.15888	2.1
65900	1.99309	2.0629
65901	2.16841	2.1
65902	2.16841	2.04646
65903	2.02311	0.822688
65904	2.02311	0.775917
65905	1.89226	0.8511
65906	1.89226	1.14078
65907	1.89226	2.1
65908	1.89226	0.941336
65909	1.89226	1.65709
65910	1.89226	0.696784
65911	1.89226	1.34712
65912	1.89226	1.99019
65913	1.89226	2.09895
65914	1.89226	1.25095
65915	1.89226	2.1
65916	1.89226	1.3564
65917	1.99309	2.1
65918	1.96035	2.1
65919	1.72526	2.1
65920	1.72526	1.60856
65921	2.16841	2.1
65922	2.16841	2.1
65923	2.15888	2.1
65924	2.16841	2.1
65925	2.3111	2.1
65926	2.35938	2.1
65927	4.51826	2.1
65928	1.89226	1.66201
65929	1.89226	0.838856
65930	1.89226	0.734147
65931	1.89226	0.326308
65932	3.78452	1.71769
65933	1.89226	1.31768
65934	1.89226	1.93867
65935	1.89226	1.88845
65936	1.89226	0.595954
65937	3.78452	1.85201
65938	2.16841	2.1
65939	1.96035	2.1
65940	2.35938	2.1
65941	2.18493	2.1
65942	4.31973	2.1
65943	1.89226	2.1

65944	1.89226	2.1
65945	1.89226	2.1
65946	1.89226	2.1
65947	3.78453	2.1
65948	1.89226	2.1
65949	1.89226	2.1
65950	1.89226	2.1
65951	1.89226	2.1
65952	3.78452	2.1
65953	0.946158	2.1
65954	0.946158	2.1
65955	0.818509	2.1
65956	0.818509	2.1
65957	0.933975	2.1
65958	0.818539	2.1
65959	0.946128	2.1
65960	0.946128	2.1
65961	0.94613	2.1
65962	0.933974	2.1
65963	0.818537	2.1
65964	0.946129	2.1
65965	0.946131	2.1
65966	0.946131	2.1
65967	0.946131	2.1
65968	0.946131	2.1
65969	0.946132	2.1
65970	0.946132	2.1
65971	0.946129	2.1
65972	0.946129	2.1
65973	0.946131	2.1
65974	0.946131	2.1
65975	0.94613	2.1
65976	0.94613	2.1
65977	1.89226	2.1
65978	1.89226	2.1
65979	1.89226	2.1
65980	1.89226	2.1
65981	3.78453	2.1
65982	1.89226	2.1
65983	1.89226	2.1
65984	1.89226	2.1
65985	1.89226	2.1
65986	3.78452	2.1
65987	1.89226	2.1
65988	1.8706	2.1
65989	1.8706	2.1
65990	1.68112	2.1
65991	2.0815	2.1
65992	1.88715	2.1
65993	0.94613	2.1
65994	0.94613	2.1
65995	0.94613	2.1
65996	0.94613	2.1
65997	0.946131	2.1
65998	0.946131	2.1
65999	0.946129	2.1
66000	0.946129	2.1
66001	0.94613	2.1

66002	0.94613	2.1
66003	0.94613	2.1
66004	0.94613	2.1
66005	1.76401	2.1
66006	2.60983	2.1
66007	2.50461	2.1
66008	2.35897	2.1
66009	1.90965	2.1
66010	1.76401	2.1
66011	1.89226	2.1
66012	1.89226	1.15685
66013	1.89226	2.1
66014	1.89226	2.1
66015	3.78452	2.1
66016	1.89226	1.13418
66017	1.89226	2.1
66018	1.89226	2.1
66019	1.89226	0.768697
66020	1.89226	2.1
66021	1.89226	0.816137
66022	1.90965	2.1
66023	2.83834	2.1
66024	2.7538	2.1
66025	2.60983	2.1
66026	2.05361	2.1
66027	1.90965	2.1
66028	1.89226	1.91742
66029	1.89226	2.1
66030	1.89226	2.1
66031	1.89226	1.24947
66032	1.89226	2.1
66033	1.89226	1.29699
66034	1.89226	1.42933
66035	1.89226	2.1
66036	1.89226	2.1
66037	1.89226	0.774301
66038	3.78452	1.99946
66039	2.83834	2.1
66040	1.89226	2.1
66041	2.60983	2.1
66042	2.60983	2.1
66043	1.89226	2.1
66044	1.89226	2.1
66045	1.89226	1.82272
66046	1.89226	1.77682
66047	1.89226	2.1
66048	1.89226	1.37955
66049	1.89226	2.1
66050	1.89226	1.23839
66051	1.89226	1.13854
66052	1.89226	1.91305
66053	1.89226	2.1
66054	1.89226	1.14422
66055	1.89226	2.05772
66056	1.89226	1.25353
66057	2.44785	2.1
66058	1.80223	2.1
66059	2.80894	2.1

66060	2.18296	1.46341
66061	4.63081	2.1
66062	1.89226	1.43198
66063	1.89226	2.07111
66064	1.89226	2.1
66065	1.89226	1.09469
66066	1.89226	2.1
66067	1.89226	1.15214
66068	0.946131	0.935914
66069	0.946131	1.31411
66070	0.946127	1.48024
66071	0.946127	2.1
66072	0.946131	1.22914
66073	0.946131	2.1
66074	0.946128	2.1
66075	0.946128	1.39073
66076	0.94613	1.61102
66077	0.94613	1.61663
66078	0.946129	1.66587
66079	0.946129	1.56022
66080	1.89226	1.00933
66081	1.89226	2.1
66082	1.89226	2.1
66083	1.89226	1.25935
66084	3.78452	2.04982
66085	1.89226	2.1
66086	1.89226	2.1
66087	1.89226	2.1
66088	1.89226	2.1
66089	3.78453	2.1
66090	2.41083	2.1
66091	2.93937	2.1
66092	1.68761	2.1
66093	1.68761	2.1
66094	1.67248	2.1
66095	2.3957	2.1
66096	1.89226	2.1
66097	1.89226	1.03517
66098	1.89226	0.88935
66099	1.89226	2.1
66100	1.89226	0.889693
66101	1.89226	2.1
66102	1.89227	2.1
66103	1.89227	2.1
66104	1.89227	2.1
66105	1.89227	2.1
66106	1.89227	2.1
66107	1.89227	2.1
66108	1.89224	0.663419
66109	1.89224	0.48991
66110	1.89224	0.88006
66111	1.89224	0.682072
66112	3.78448	0.581673
66113	1.89224	0.896779
66114	1.89224	0.628601
66115	1.89224	0.871523
66116	1.89224	0.493493
66117	1.89224	1.11304

66118	1.89224	0.761506
66119	1.11152	0.569252
66120	1.29346	0.843385
66121	0.978596	0.790654
66122	0.918324	1.04308
66123	1.06821	1.04577
66124	1.06821	0.489284
66125	0.985654	1.37423
66126	1.15278	0.950249
66127	1.07693	0.985461
66128	0.902432	1.17273
66129	0.968939	1.17418
66130	1.34644	0.771271
66131	0.946132	0.582734
66132	0.946132	0.696375
66133	0.946132	0.753726
66134	0.946132	0.58661
66135	0.946131	0.638094
66136	0.946131	0.466742
66137	0.946132	0.915023
66138	0.946132	0.582828
66139	0.946132	0.679066
66140	0.946132	0.6397
66141	0.946132	0.503703
66142	0.946132	0.544623
66143	0.946133	0.745509
66144	0.946133	0.535429
66145	0.946132	0.70392
66146	0.946132	0.922067
66147	0.946132	0.7794
66148	0.946132	0.363688
66149	0.946133	1.40274
66150	0.946133	0.590671
66151	0.946132	0.840466
66152	0.946132	0.716686
66153	0.946132	0.578623
66154	0.946132	0.543693
66155	1.89224	0.708925
66156	1.89224	2.06107
66157	1.89224	0.860189
66158	1.89224	1.47598
66159	1.89224	0.890808
66160	1.89224	1.9106
66161	1.89224	0.863217
66162	1.89224	2.1
66163	1.89224	1.331
66164	1.89224	2.02591
66165	1.89224	1.20672
66166	1.89224	2.1
66167	1.89226	0.812485
66168	1.89226	2.1
66169	1.89226	2.05117
66170	1.89226	0.919792
66171	3.78453	1.6356
66172	1.11056	2.1
66173	1.21595	2.1
66174	1.12763	2.1
66175	1.12763	1.84306

66176	1.23339	2.1
66177	1.35697	2.1
66178	1.03581	1.97973
66179	0.95954	2.1
66180	0.89761	2.1
66181	1.26602	2.1
66182	1.12749	2.1
66183	1.45307	2.1
66184	2.71775	2.1
66185	1.89226	2.1
66186	1.89226	2.1
66187	1.89226	2.1
66188	2.47025	2.1
66189	2.47025	2.1
66190	0.94613	2.1
66191	0.94613	2.1
66192	0.94613	0.923361
66193	0.94613	1.51065
66194	0.946128	1.34298
66195	0.946128	2.1
66196	0.946132	2.1
66197	0.946132	1.22388
66198	0.94613	1.89419
66199	0.94613	1.87499
66200	0.94613	1.99682
66201	0.94613	2.00453
66202	1.89226	0.828212
66203	1.89226	1.86089
66204	1.89226	2.06363
66205	1.89226	1.04997
66206	1.89226	1.93293
66207	1.89226	1.19836
66208	1.89226	0.959399
66209	1.89226	1.83353
66210	1.89226	1.71094
66211	1.89226	0.968249
66212	1.89226	1.65719
66213	1.89226	1.20085
66214	0.946132	1.17642
66215	0.946132	1.42697
66216	0.946133	2.1
66217	0.946133	2.1
66218	0.946133	2.1
66219	0.946133	1.05194
66220	0.946132	0.94391
66221	0.946132	2.1
66222	0.946133	2.07368
66223	0.946133	2.05385
66224	0.946132	1.98735
66225	0.946132	2.00634
66226	1.89226	0.902675
66227	1.89226	1.84985
66228	1.89226	1.91469
66229	1.89226	1.23196
66230	1.89226	1.90753
66231	1.89226	1.24318
66232	0.946131	1.32104
66233	0.946131	0.948613

66234	0.946127	1.64927
66235	0.946127	1.88037
66236	0.946131	1.25736
66237	0.946131	2.1
66238	0.946127	1.97094
66239	0.946127	1.20348
66240	0.94613	1.47446
66241	0.94613	1.49575
66242	0.946129	1.4928
66243	0.946129	1.49816
66244	1.94291	2.1
66245	2.00118	2.1
66246	2.36337	2.1
66247	2.41217	2.1
66248	4.35508	2.1
66249	1.89226	2.1
66250	1.89226	1.49526
66251	1.89226	1.92005
66252	1.89226	2.1
66253	3.78452	2.09891
66254	1.89226	1.5745
66255	1.89226	2.1
66256	1.89226	2.1
66257	1.89226	1.50704
66258	3.78452	2.1
66259	1.06209	2.1
66260	1.06209	2.1
66261	1.05987	2.1
66262	1.05987	2.1
66263	0.920378	2.1
66264	1.12137	2.1
66265	1.00059	2.1
66266	1.20608	2.1
66267	1.20608	2.1
66268	1.12137	2.1
66269	1.00059	2.1
66270	0.920376	2.1
66271	2.77222	2.1
66272	2.41216	2.1
66273	2.77002	2.1
66274	2.36337	2.1
66275	2.81882	2.1
66276	2.41216	2.1
66277	1.89226	1.42597
66278	1.89226	2.1
66279	1.89226	2.1
66280	1.89226	1.93106
66281	1.89226	2.1
66282	1.89226	1.23097
66283	1.89226	2.1
66284	1.89226	1.1703
66285	1.89226	1.42641
66286	1.89226	2.1
66287	3.78451	1.83253
66288	2.41216	2.1
66289	2.24273	2.1
66290	2.81882	2.1
66291	2.68473	2.1

66292	5.09689	2.1
66293	1.89226	2.1
66294	1.89226	2.1
66295	1.89226	2.1
66296	1.89226	2.1
66297	3.78453	2.1
66298	2.17614	2.1
66299	2.65326	2.1
66300	1.93101	2.1
66301	2.58552	2.1
66302	4.58427	2.1
66303	2.65326	2.1
66304	1.89226	2.1
66305	1.89226	2.1
66306	1.89226	2.1
66307	2.58552	2.1
66308	2.58552	2.1
66309	1.89226	2.1
66310	1.89226	2.1
66311	1.89226	2.1
66312	1.89226	2.1
66313	3.78453	2.1
66314	1.89226	2.1
66315	1.89226	2.1
66316	1.89226	2.1
66317	1.89226	2.1
66318	3.78453	2.1
66319	1.93101	2.1
66320	2.58552	2.1
66321	1.46507	2.1
66322	2.52893	2.1
66323	4.45994	2.1
66324	1.26446	2.1
66325	1.26446	2.1
66326	0.946132	2.1
66327	0.946132	2.1
66328	0.94613	2.1
66329	0.94613	2.1
66330	1.29276	2.1
66331	1.26446	2.1
66332	1.26446	2.1
66333	0.94613	2.1
66334	0.94613	2.1
66335	1.29276	2.1
66336	1.89226	2.1
66337	1.89226	2.1
66338	1.89226	2.1
66339	1.89226	2.07767
66340	3.78453	2.1
66341	1.89226	2.1
66342	1.89226	2.1
66343	1.89226	2.1
66344	1.89226	2.07673
66345	3.78453	2.1
66346	1.83378	2.1
66347	2.52893	2.1
66348	1.75592	2.1
66349	2.49844	2.1

66350	4.28485	2.1
66351	1.24922	2.1
66352	1.24922	2.1
66353	0.946133	2.1
66354	0.946133	2.1
66355	0.946131	2.1
66356	0.946131	2.1
66357	1.26446	2.1
66358	1.24922	2.1
66359	1.24922	2.1
66360	0.946131	2.1
66361	1.26446	2.1
66362	0.94613	2.1
66363	0.946132	2.1
66364	0.946132	2.1
66365	0.946132	2.08197
66366	0.946132	2.05747
66367	0.946132	2.00876
66368	0.946132	2.1
66369	0.946132	2.1
66370	0.946132	2.1
66371	0.946132	2.1
66372	0.946132	2.1
66373	0.946131	2.1
66374	0.946131	2.1
66375	1.89226	2.1
66376	1.89226	2.1
66377	1.89226	2.1
66378	1.89226	2.1
66379	1.89226	2.1
66380	1.89226	2.1
66381	1.0465	2.1
66382	1.0465	2.1
66383	1.06047	2.1
66384	1.06047	2.1
66385	1.25687	2.1
66386	1.24922	2.1
66387	0.87796	2.1
66388	0.857749	2.1
66389	0.857749	2.1
66390	1.25687	2.1
66391	0.877959	2.1
66392	1.24922	2.1
66393	2.49843	2.1
66394	1.89226	2.1
66395	2.51374	2.1
66396	1.89226	2.1
66397	4.406 2.1	
66398	1.89226	2.1
66399	1.89226	2.06168
66400	1.89226	2.1
66401	1.89226	2.1
66402	1.89226	2.1
66403	1.89226	2.1
66404	1.89226	0.946295
66405	1.89226	2.09386
66406	1.89226	2.09672
66407	1.89226	1.081

66408	1.89226	2.08991
66409	1.89226	1.15551
66410	1.89226	2.0899
66411	1.89226	1.13938
66412	1.89226	2.0783
66413	1.89226	1.11559
66414	1.89226	2.09819
66415	1.89226	1.14262
66416	1.03835	2.1
66417	1.03835	2.06395
66418	1.15388	2.1
66419	0.94267	2.1
66420	0.736433	1.85301
66421	0.849239	2.1
66422	1.15014	2.1
66423	1.39058	2.1
66424	0.838639	2.1
66425	1.39056	2.1
66426	1.09399	2.1
66427	0.736444	2.1
66428	1.89226	0.885751
66429	1.89226	1.84493
66430	1.89226	1.9366
66431	1.89226	0.920661
66432	1.89226	1.85502
66433	1.89226	0.963332
66434	0.94613	0.98874
66435	0.94613	0.948987
66436	0.946127	2.0585
66437	0.946127	1.82637
66438	0.946131	1.9187
66439	0.946131	0.953193
66440	0.946126	2.01333
66441	0.946126	0.997201
66442	0.946128	1.60416
66443	0.946128	1.59973
66444	0.946128	1.27544
66445	0.946128	1.46227
66446	1.89226	1.63262
66447	1.89226	1.05158
66448	1.89226	0.730856
66449	1.89226	1.95452
66450	3.78453	1.02983
66451	0.946133	1.38317
66452	0.946133	0.70657
66453	0.946128	1.53364
66454	0.946128	1.25144
66455	0.946131	1.35554
66456	0.946131	1.29453
66457	0.946131	0.571061
66458	0.946131	1.27885
66459	0.946131	1.07567
66460	0.946131	1.04858
66461	0.94613	1.1838
66462	0.94613	1.21734
66463	1.89226	0.813431
66464	1.89226	1.88568
66465	1.89226	1.42626

66466	1.89226	0.99873
66467	1.89226	1.42778
66468	1.89226	1.16453
66469	1.89226	0.798513
66470	1.89226	1.42459
66471	1.89226	1.06189
66472	1.89226	0.998043
66473	1.89226	1.14702
66474	1.89226	1.27278
66475	1.89226	0.814292
66476	1.89226	1.64955
66477	1.89226	1.07332
66478	1.89226	1.23494
66479	1.89226	1.14812
66480	1.89226	1.42473
66481	1.89226	0.886733
66482	2.67388	2.08954
66483	2.23684	1.65215
66484	2.23684	1.29583
66485	1.89226	1.69651
66486	1.89226	1.43118
66487	1.89226	2.1
66488	1.89226	2.07128
66489	1.89226	2.1
66490	1.89226	2.1
66491	1.89226	2.1
66492	1.89226	2.1
66493	1.72197	2.1
66494	2.51374	2.1
66495	2.58004	2.1
66496	2.58004	2.1
66497	1.7155	2.1
66498	1.7155	2.1
66499	2.51374	2.1
66500	1.89226	2.1
66501	1.89226	2.1
66502	1.89226	2.1
66503	2.58003	2.1
66504	2.58003	2.1
66505	0.946132	2.1
66506	0.946132	2.1
66507	0.946132	2.1
66508	0.946132	1.96641
66509	0.946132	1.88871
66510	0.946132	2.1
66511	0.946132	1.97862
66512	0.946132	2.1
66513	0.946131	2.1
66514	0.946131	2.1
66515	0.946132	2.1
66516	0.946132	2.1
66517	1.89226	2.1
66518	1.89226	2.1
66519	1.89226	2.1
66520	1.89226	2.1
66521	3.78453	2.1
66522	1.08262	2.1
66523	1.08262	2.1

66524	1.09674	2.1
66525	1.09674	2.1
66526	1.34652	2.1
66527	1.29002	2.1
66528	0.860984	2.1
66529	0.889338	2.1
66530	1.34652	2.1
66531	0.889338	2.1
66532	0.860983	2.1
66533	1.29002	2.1
66534	1.34652	2.1
66535	1.34652	2.1
66536	0.946133	2.1
66537	0.946133	2.1
66538	0.946131	2.1
66539	0.946131	2.1
66540	1.34652	2.1
66541	1.29002	2.1
66542	1.34652	2.1
66543	0.946131	2.1
66544	0.94613	2.1
66545	1.29002	2.1
66546	1.89226	2.1
66547	1.89226	1.96716
66548	1.89226	2.1
66549	1.89226	2.1
66550	1.89226	2.04171
66551	1.89226	2.1
66552	1.89226	2.1
66553	1.89226	2.1
66554	1.89226	2.1
66555	1.89226	2.1
66556	1.89226	2.1
66557	1.89226	2.1
66558	0.946131	2.1
66559	0.946131	2.1
66560	0.946131	2.1
66561	0.946131	1.71667
66562	0.946131	1.71777
66563	0.946131	2.1
66564	0.946131	2.1
66565	0.946131	2.03267
66566	0.946131	2.1
66567	0.946131	2.1
66568	0.946131	2.1
66569	0.946131	2.1
66570	1.89226	2.1
66571	1.89226	2.1
66572	1.89226	2.04267
66573	1.89226	2.1
66574	1.89226	2.06393
66575	1.89226	2.1
66576	1.04103	2.1
66577	1.13849	2.1
66578	0.943014	2.1
66579	0.943014	2.1
66580	0.943591	2.1
66581	0.943591	2.1

66582	1.03757	2.1
66583	1.04098	2.1
66584	1.03761	2.1
66585	0.946131	2.1
66586	0.943041	2.1
66587	0.946131	2.1
66588	0.946128	2.1
66589	0.946128	2.1
66590	0.946133	2.1
66591	0.946133	2.1
66592	0.946131	2.1
66593	0.946131	2.1
66594	0.946131	2.1
66595	0.946131	2.1
66596	0.946131	2.1
66597	0.946131	2.1
66598	0.94613	2.1
66599	0.94613	2.1
66600	0.946132	2.1
66601	0.946132	2.1
66602	0.946132	2.1
66603	0.946132	1.74468
66604	0.946132	1.75806
66605	0.946132	2.1
66606	0.946132	2.1
66607	0.946132	2.1
66608	0.946132	2.1
66609	0.946132	2.1
66610	0.946131	2.1
66611	0.946131	2.1
66612	1.89226	0.855265
66613	1.89226	1.67066
66614	1.89226	0.926347
66615	1.89226	0.926114
66616	3.78452	1.218
66617	0.946132	1.09869
66618	0.946132	1.23819
66619	0.946131	0.928383
66620	0.946131	0.879429
66621	0.946132	0.91787
66622	0.946132	1.87835
66623	0.946132	1.11255
66624	0.946132	1.1024
66625	0.946132	1.04769
66626	0.946132	1.07849
66627	0.946132	1.14245
66628	0.946132	1.22912
66629	0.935482	1.44754
66630	1.08999	1.3651
66631	1.24315	1.21007
66632	1.12559	1.38883
66633	1.21343	1.14132
66634	0.889397	2.03845
66635	1.01265	0.777538
66636	1.01265	1.52873
66637	1.07332	1.01001
66638	1.34292	1.29268
66639	0.865649	1.3505

66640	0.840024	1.62593
66641	1.15433	1.33824
66642	1.02858	1.57247
66643	1.34082	1.25896
66644	1.08357	1.27717
66645	1.10938	0.909268
66646	1.33349	1.94455
66647	1.12232	1.56498
66648	1.12232	0.813435
66649	1.21357	1.24878
66650	1.3408	1.31304
66651	1.02859	1.44667
66652	1.24211	1.5801
66653	1.89226	1.14563
66654	1.89226	0.687235
66655	1.89226	0.850016
66656	1.89226	1.68002
66657	1.89226	0.673706
66658	1.89226	1.21578
66659	1.89226	0.798239
66660	1.89226	0.985584
66661	1.89226	0.80548
66662	1.89226	1.44053
66663	1.89226	1.0824
66664	1.89226	1.41708
66665	2.0815	1.32583
66666	2.79029	1.19798
66667	2.36793	1.07332
66668	2.58428	1.79886
66669	2.38161	0.762285
66670	2.59922	1.4302
66671	1.89226	2.04489
66672	1.89226	1.36465
66673	1.89226	1.73351
66674	1.89226	1.8256
66675	1.89226	1.70254
66676	1.89226	1.84163
66677	1.89226	1.42331
66678	1.89226	0.636981
66679	1.89226	0.830212
66680	1.89226	0.976751
66681	3.78452	0.890538
66682	1.89226	1.75849
66683	1.89226	0.907281
66684	1.89226	1.43263
66685	1.89226	1.40771
66686	1.89226	1.3643
66687	1.89226	1.40515
66688	0.946132	1.02203
66689	0.946132	1.66128
66690	0.94613	2.07604
66691	0.94613	1.51111
66692	0.946131	1.8122
66693	0.946131	1.01809
66694	0.946131	1.63524
66695	0.946131	2.1
66696	0.946131	1.59768
66697	0.946131	1.71091

66698	0.946131	1.71209
66699	0.946131	1.64672
66700	1.85178	1.62887
66701	1.89226	0.925324
66702	2.0815	0.74023
66703	2.27054	1.27165
66704	1.70303	0.616865
66705	1.5217	0.736387
66706	1.89226	1.79595
66707	1.89226	0.859664
66708	1.89226	1.24134
66709	1.89226	1.04952
66710	1.89226	1.68333
66711	1.89226	1.29175
66712	2.58823	0.448252
66713	2.02924	0.303827
66714	1.79398	0.28132
66715	1.79398	0.212165
66716	2.76708	0.472502
66717	2.76708	0.375321
66718	0.946133	0.329794
66719	0.946133	0.393074
66720	1.29411	0.355437
66721	1.38353	0.291388
66722	0.946131	0.284769
66723	0.946131	0.296131
66724	1.38354	0.427617
66725	1.38354	0.335591
66726	0.946131	0.313718
66727	1.38354	0.332807
66728	0.94613	0.407234
66729	1.29411	0.321486
66730	1.89226	0.28416
66731	1.89226	0.185237
66732	1.89226	0.303252
66733	1.89226	0.268953
66734	3.78452	0.291838
66735	0.946131	0.202304
66736	0.946131	0.279859
66737	0.946131	0.287521
66738	0.946131	0.231358
66739	0.946129	0.233428
66740	0.946129	0.186968
66741	0.946133	0.319562
66742	0.946133	0.277708
66743	0.946131	0.323456
66744	0.946131	0.227451
66745	0.946131	0.181467
66746	0.946131	0.248899
66747	1.89226	0.211953
66748	1.89226	0.215581
66749	1.89226	0.279826
66750	1.89226	0.248712
66751	3.78452	0.199806
66752	0.94613	0.241669
66753	0.94613	0.238053
66754	0.946131	0.214664
66755	0.946131	0.241182

66756	0.946128	0.230084
66757	0.946128	0.201641
66758	0.946133	0.25944
66759	0.946133	0.237018
66760	0.946131	0.26921
66761	0.946131	0.206688
66762	0.946131	0.264763
66763	0.946131	0.193018
66764	0.946129	0.705315
66765	0.946129	0.628516
66766	1.13486	0.639077
66767	0.859753	0.742579
66768	0.851541	0.731257
66769	0.851541	0.577773
66770	1.34346	0.662692
66771	1.26212	0.691723
66772	0.851553	0.580224
66773	1.12403	0.620866
66774	0.851474	0.462332
66775	0.956163	0.638879
66776	0.946132	0.396632
66777	0.946132	0.364311
66778	0.946133	0.446449
66779	0.946133	0.311008
66780	0.946132	0.448079
66781	0.946132	0.285292
66782	0.946133	0.382691
66783	0.946133	0.376169
66784	0.946133	0.430526
66785	0.946133	0.369099
66786	0.946132	0.287259
66787	0.946132	0.376515
66788	1.89226	0.329414
66789	1.89226	0.448624
66790	1.89226	0.50402
66791	1.89226	0.394129
66792	3.78453	0.366472
66793	0.946128	0.683048
66794	0.946128	0.66193
66795	0.946132	0.719215
66796	0.946132	0.724925
66797	0.94613	0.715086
66798	0.94613	0.630046
66799	0.94613	0.767286
66800	0.94613	0.648952
66801	0.946131	0.724217
66802	0.946131	0.695761
66803	0.94613	0.677104
66804	0.94613	0.68807
66805	0.946132	0.338781
66806	0.946132	0.276913
66807	0.946133	0.346391
66808	0.946133	0.259621
66809	0.946132	0.326225
66810	0.946132	0.258115
66811	0.946133	0.285469
66812	0.946133	0.281497
66813	0.946132	0.354757

66814	0.946132	0.304205
66815	0.946132	0.287555
66816	0.946132	0.250503
66817	1.89226	0.359807
66818	1.89226	0.305622
66819	1.89226	0.470583
66820	1.89226	0.259754
66821	1.89226	0.490451
66822	1.89226	0.27973
66823	1.89226	0.598344
66824	1.89226	0.466502
66825	1.89226	0.459673
66826	1.89226	0.451879
66827	1.89226	0.489289
66828	1.89226	0.4985
66829	1.89226	2.1
66830	1.89226	2.1
66831	1.89226	2.1
66832	1.89226	2.1
66833	3.78452	2.1
66834	1.07652	2.1
66835	1.07652	2.1
66836	0.928749	2.1
66837	0.928749	2.1
66838	0.946132	2.1
66839	0.946132	2.1
66840	1.05914	2.1
66841	0.928746	2.1
66842	1.05914	2.1
66843	0.946131	2.1
66844	0.94613	2.1
66845	0.928747	2.1
66846	1.89226	2.1
66847	1.89226	2.1
66848	1.89226	2.1
66849	1.89226	2.1
66850	1.89226	2.1
66851	1.89226	2.1
66852	1.89227	2.1
66853	1.89227	2.1
66854	1.89227	2.0687
66855	1.89227	2.1
66856	1.89227	2.09493
66857	1.89227	2.1
66858	1.89226	2.1
66859	1.89226	2.1
66860	1.89226	2.1
66861	1.89226	2.1
66862	1.89226	2.1
66863	1.89226	2.1
66864	2.11828	2.1
66865	1.89226	2.1
66866	2.35845	2.1
66867	1.89226	2.1
66868	4.25071	2.1
66869	1.89226	2.1
66870	1.89226	2.1
66871	1.89226	2.1

66872	1.89226	2.1
66873	3.78453	2.1
66874	1.89226	2.1
66875	1.89226	2.1
66876	1.89226	2.1
66877	1.89226	2.1
66878	1.89226	2.08684
66879	1.89226	2.1
66880	1.89226	0.80401
66881	1.89226	1.49037
66882	1.65573	1.32996
66883	1.65573	0.88337
66884	1.89226	1.32245
66885	1.89226	0.892177
66886	1.89226	0.705847
66887	1.89226	1.27288
66888	1.89226	1.09038
66889	1.89226	0.8088
66890	1.89226	1.14105
66891	1.89226	0.848926
66892	1.89226	0.761179
66893	1.89226	1.32705
66894	1.89226	1.24387
66895	1.89226	0.845713
66896	1.89226	1.26251
66897	1.89226	0.865178
66898	1.89226	0.688796
66899	1.89226	1.29225
66900	1.89226	1.06089
66901	1.89226	0.771994
66902	1.89226	1.09941
66903	1.89226	0.804938
66904	0.946128	0.876273
66905	0.946128	0.76933
66906	0.946125	1.18969
66907	0.946125	1.18135
66908	0.94613	0.832225
66909	0.94613	1.63775
66910	0.946124	1.33277
66911	0.946124	0.81417
66912	0.946127	0.987231
66913	0.946127	1.00173
66914	0.946127	1.03446
66915	0.946127	1.07702
66916	1.89226	0.72323
66917	1.89226	1.02366
66918	1.89226	0.711065
66919	1.89226	0.646162
66920	3.78452	0.74628
66921	1.89226	0.951745
66922	1.89226	0.987753
66923	1.89226	0.760891
66924	1.89226	0.98518
66925	3.78453	0.931316
66926	1.05926	1.28997
66927	0.891358	2.02072
66928	0.946129	1.09865
66929	0.946129	1.42689

66930	0.946101	1.09933
66931	0.946101	2.1
66932	0.891386	1.16988
66933	0.891386	1.2353
66934	0.891357	1.31165
66935	0.946129	1.19784
66936	0.94613	1.27477
66937	1.05926	1.26489
66938	0.946126	0.834775
66939	0.946126	1.0104
66940	0.94613	0.823526
66941	0.94613	1.05561
66942	0.946131	0.915333
66943	0.946131	1.08542
66944	0.946125	1.0458
66945	0.946125	0.64806
66946	0.946127	0.844718
66947	0.946127	0.812964
66948	0.946128	1.01503
66949	0.946128	0.933539
66950	1.89226	0.795838
66951	1.89226	0.684318
66952	1.89226	0.612881
66953	1.89226	0.952916
66954	1.89226	0.660944
66955	1.89226	1.11011
66956	1.89226	0.850793
66957	1.89226	1.14422
66958	1.89226	1.01281
66959	1.89226	1.11026
66960	1.89226	0.844377
66961	1.89226	1.03786
66962	1.16109	1.55086
66963	1.23243	2.01683
66964	0.659819	1.2077
66965	0.659819	1.6073
66966	0.697965	0.991961
66967	1.00087	2.09522
66968	0.891386	1.50192
66969	0.891386	1.23294
66970	0.946129	1.49548
66971	0.65979	1.11984
66972	0.891357	1.42007
66973	0.946129	1.29755
66974	1.89225	0.719632
66975	1.89225	0.978921
66976	1.89225	1.16846
66977	1.89225	0.65027
66978	1.89225	1.51359
66979	1.89225	0.834559
66980	1.79981	0.488175
66981	2.02514	0.506019
66982	2.33247	0.666843
66983	2.12891	0.378423
66984	4.15405	0.44114
66985	2.48284	0.857097
66986	2.02514	0.506924
66987	2.33247	0.649096

66988	2.02514	0.724755
66989	2.48567	0.643845
66990	2.17834	0.710697
66991	1.89226	0.482779
66992	1.89226	0.614114
66993	1.89226	0.70422
66994	1.89226	0.43369
66995	3.78453	0.517628
66996	1.89227	0.481095
66997	1.89227	0.311932
66998	1.89227	0.338308
66999	1.89227	0.439633
67000	1.89227	0.369832
67001	1.89227	0.464358
67002	2.50894	0.360088
67003	2.70118	0.619279
67004	2.12891	0.431638
67005	2.12891	0.500013
67006	2.7125	0.448926
67007	2.33247	0.500063
67008	1.89226	0.666203
67009	1.89226	0.440112
67010	1.89226	0.479661
67011	1.89226	0.608882
67012	1.89226	0.457245
67013	1.89226	0.586788
67014	0.946133	0.434022
67015	0.946133	0.484748
67016	0.946132	0.325959
67017	0.946132	0.377954
67018	0.946132	0.313136
67019	0.946132	0.471031
67020	0.946133	0.346988
67021	0.946133	0.500851
67022	0.946132	0.381411
67023	0.946132	0.405127
67024	0.946132	0.414799
67025	0.946132	0.39987
67026	1.90098	0.334856
67027	1.36463	0.363582
67028	1.56656	0.366669
67029	1.36463	0.373285
67030	2.04386	0.308381
67031	1.89227	0.346884
67032	1.89226	0.347602
67033	1.89226	0.359991
67034	1.89226	0.37363
67035	1.89226	0.386876
67036	1.89226	0.330273
67037	1.89226	0.348303
67038	1.89226	0.402827
67039	1.89226	0.415786
67040	1.89226	0.42371
67041	1.89226	0.451645
67042	1.89226	0.382256
67043	1.89226	0.425613
67044	1.89226	0.395982
67045	1.89226	0.410953

67046	1.89226	0.429754
67047	1.89226	0.442219
67048	1.89226	0.38449
67049	1.89226	0.411575
67050	1.89226	0.315623
67051	1.70303	0.342092
67052	2.1292	0.363345
67053	1.70303	0.340675
67054	2.32302	0.313558
67055	1.90098	0.314338
67056	1.89226	0.322048
67057	1.89226	0.34984
67058	1.89226	0.366505
67059	1.89226	0.36063
67060	1.89226	0.324676
67061	1.89226	0.322507
67062	1.89226	0.383069
67063	1.89226	0.398912
67064	1.89226	0.410491
67065	1.89226	0.41149
67066	1.89226	0.371993
67067	1.89226	0.379234
67068	1.89226	0.368078
67069	1.89226	0.39889
67070	1.89226	0.407287
67071	1.89226	0.404871
67072	1.89226	0.364253
67073	1.89226	0.366936
67074	1.89226	0.722021
67075	1.89226	0.987545
67076	1.89226	0.948117
67077	1.89226	0.747225
67078	3.78453	0.706648
67079	1.89226	0.923226
67080	1.89226	1.26112
67081	1.89226	0.801294
67082	1.89226	1.3817
67083	1.89226	0.945813
67084	1.89226	1.76073
67085	1.89226	0.981403
67086	1.89226	0.765526
67087	1.89226	0.7409
67088	1.89226	0.855018
67089	3.78452	0.931264
67090	1.89224	0.947108
67091	1.89224	0.716774
67092	1.89224	0.791219
67093	1.89224	1.02473
67094	1.89224	0.73089
67095	1.89224	0.884083
67096	1.89224	0.925487
67097	1.89224	1.48967
67098	1.89224	1.10725
67099	1.89224	1.95242
67100	1.89224	0.785795
67101	1.89224	1.3769
67102	1.31307	1.80555
67103	1.31307	1.86697

67104	0.888948	1.14149
67105	0.888948	1.01611
67106	1.2559	1.22251
67107	0.888979	1.85778
67108	0.946114	1.43599
67109	0.946114	0.963051
67110	0.946129	0.889167
67111	0.888989	1.12244
67112	0.946104	0.801678
67113	1.25588	0.956791
67114	1.89224	0.709433
67115	1.89224	1.07222
67116	1.89224	0.750187
67117	1.89224	1.02002
67118	1.89224	0.854484
67119	1.89224	1.30139
67120	1.79375	0.278089
67121	1.47689	0.246639
67122	1.97732	0.2499
67123	2.26687	0.271634
67124	3.74375	0.265762
67125	2.05759	0.317422
67126	2.26686	0.261369
67127	1.79374	0.251496
67128	1.79374	0.300757
67129	2.49889	0.26164
67130	2.49889	0.307068
67131	1.89226	0.294924
67132	1.89226	0.275531
67133	1.89226	0.259748
67134	1.89226	0.296265
67135	3.78452	0.29662
67136	1.89227	0.27199
67137	1.89227	0.269725
67138	1.89227	0.258424
67139	1.89227	0.277592
67140	1.89227	0.253465
67141	1.89227	0.248864
67142	1.97732	0.256064
67143	2.26687	0.271994
67144	2.6607	0.277473
67145	2.41464	0.265282
67146	4.68151	0.253186
67147	1.89226	0.262455
67148	1.89226	0.314852
67149	1.89226	0.307566
67150	1.89226	0.287489
67151	3.78452	0.267869
67152	1.89226	0.27214
67153	1.89226	0.283757
67154	1.89226	0.271667
67155	1.89226	0.306711
67156	1.89226	0.265086
67157	1.89226	0.290752
67158	1.89226	0.706479
67159	1.89226	1.01325
67160	1.89226	0.805603
67161	1.89226	0.731876

67162	3.78452	0.737528
67163	1.89226	0.788966
67164	1.89226	1.20387
67165	1.89226	0.80848
67166	1.89226	0.983861
67167	1.89226	0.842945
67168	1.89226	1.14076
67169	1.89226	1.06112
67170	1.89226	0.751548
67171	1.89226	0.68794
67172	1.89226	1.05156
67173	1.89226	0.732422
67174	1.89226	1.12849
67175	1.89226	0.822225
67176	1.89226	0.745384
67177	1.89226	0.661385
67178	1.89226	0.986442
67179	1.89226	0.710214
67180	1.89226	1.1797
67181	1.89226	0.806247
67182	2.45282	1.08687
67183	1.89226	1.1163
67184	1.89226	1.20132
67185	1.89226	0.787259
67186	1.89226	0.950482
67187	1.89226	0.76466
67188	1.89226	1.05365
67189	1.89226	0.741572
67190	1.89226	0.816585
67191	1.89226	1.18043
67192	1.89226	1.58462
67193	1.87308	0.263776
67194	1.88631	0.275419
67195	1.63908	0.27136
67196	1.70302	0.274913
67197	2.08745	0.2624
67198	2.16916	0.262503
67199	2.41019	0.275929
67200	2.733	0.274393
67201	2.08121	0.268266
67202	2.08121	0.277307
67203	2.74362	0.261715
67204	2.41464	0.258488
67205	1.89226	0.287577
67206	1.89226	0.295059
67207	1.89226	0.281675
67208	1.89226	0.290633
67209	1.89226	0.296054
67210	1.89226	0.303274
67211	0.946133	0.291816
67212	0.946133	0.275529
67213	0.946132	0.304968
67214	0.946132	0.287856
67215	0.946132	0.294779
67216	0.946132	0.297936
67217	0.946133	0.284336
67218	0.946133	0.286383
67219	0.946132	0.288906

67220	0.946132	0.324231
67221	0.946133	0.264838
67222	0.946133	0.296029
67223	2.02912	0.286854
67224	2.57016	0.271468
67225	1.88631	0.286331
67226	1.88631	0.290982
67227	2.55301	0.256017
67228	2.4102	0.269753
67229	1.89226	0.296354
67230	1.89226	0.317884
67231	1.89226	0.313972
67232	1.89226	0.31915
67233	3.78452	0.305983
67234	1.89227	0.295705
67235	1.89227	0.319675
67236	1.89227	0.31026
67237	1.89227	0.317828
67238	1.89227	0.299712
67239	1.89227	0.303423
67240	1.89226	2.1
67241	1.89226	2.08349
67242	1.89226	1.94921
67243	1.89226	2.1
67244	3.78452	2.1
67245	1.2515	1.99318
67246	1.34788	2.1
67247	0.946132	2.1
67248	0.946132	2.1
67249	0.973043	2.1
67250	1.16766	2.1
67251	0.919222	1.75879
67252	1.2159	2.1
67253	0.946132	2.1
67254	0.946132	2.1
67255	1.33746	2.1
67256	1.13537	2.1
67257	1.89226	2.07375
67258	1.89226	2.1
67259	1.89226	2.1
67260	1.89226	2.1
67261	1.89226	2.1
67262	1.89226	2.1
67263	1.89226	1.93615
67264	1.89226	2.1
67265	1.89226	2.1
67266	1.89226	1.97359
67267	3.78452	2.03678
67268	1.89227	2.1
67269	1.89227	1.55953
67270	1.89227	2.1
67271	1.89227	1.86769
67272	1.89227	2.1
67273	1.89227	1.84975
67274	0.946132	2.01576
67275	0.946132	1.97207
67276	0.946129	2.1
67277	0.946129	2.1

67278	0.946131	2.06247
67279	0.946131	2.1
67280	0.946131	2.1
67281	0.946131	1.97873
67282	0.946131	2.08974
67283	0.946131	2.1
67284	0.94613	2.08952
67285	0.94613	2.07239
67286	1.89226	0.959065
67287	1.89226	0.919582
67288	1.89226	0.939455
67289	1.89226	0.790693
67290	3.78453	0.855249
67291	0.946132	1.38792
67292	0.946132	0.893372
67293	0.946132	1.06058
67294	0.946132	0.926787
67295	0.946132	0.720733
67296	0.946132	1.40472
67297	0.946132	0.928368
67298	0.946132	0.981169
67299	0.946131	1.40362
67300	0.946131	0.819793
67301	0.946132	1.06714
67302	0.946132	0.886607
67303	1.89226	1.07647
67304	1.89226	0.691415
67305	1.89226	0.652948
67306	1.89226	0.8022
67307	1.89226	0.894424
67308	1.89226	1.19199
67309	1.89226	1.14038
67310	1.89226	0.783353
67311	1.89226	0.699886
67312	1.89226	0.781115
67313	3.78453	0.808113
67314	0.96614	0.977901
67315	0.96614	0.896605
67316	0.946131	0.6429
67317	0.946131	0.787374
67318	0.946131	0.838776
67319	0.96614	0.79064
67320	1.37736	0.967354
67321	0.946131	1.08785
67322	1.37736	1.26923
67323	0.96614	0.75575
67324	0.946131	1.15131
67325	0.946131	0.725396
67326	0.946129	1.22178
67327	0.946129	0.834506
67328	0.946128	0.922523
67329	0.946128	1.13509
67330	0.946132	0.667639
67331	0.946132	1.433
67332	0.946126	0.948035
67333	0.946126	0.819559
67334	0.946129	0.695442
67335	0.946129	0.778123

67336	0.946129	0.699062
67337	0.946129	0.927679
67338	1.89226	0.274487
67339	1.65723	0.299782
67340	1.83117	0.323347
67341	1.65723	0.299867
67342	2.0662	0.281052
67343	1.89226	0.261573
67344	1.89226	0.28799
67345	1.89226	0.313106
67346	1.89226	0.335819
67347	1.89226	0.319613
67348	1.89226	0.283622
67349	1.89226	0.268321
67350	1.89226	0.321586
67351	1.89226	0.353233
67352	1.89226	0.370933
67353	1.89226	0.363017
67354	1.89226	0.315633
67355	1.89226	0.302832
67356	1.89226	0.30446
67357	1.89226	0.321674
67358	1.89226	0.35736
67359	1.89226	0.34144
67360	1.89226	0.304698
67361	1.89226	0.287591
67362	1.89226	0.283166
67363	1.83117	0.306833
67364	1.92807	0.320396
67365	1.83117	0.304048
67366	1.98916	0.277733
67367	1.89226	0.262937
67368	1.89226	0.277074
67369	1.89226	0.322277
67370	1.89226	0.30489
67371	1.89226	0.32245
67372	3.78453	0.299442
67373	1.89226	0.335522
67374	1.89226	0.363188
67375	1.89226	0.373611
67376	1.89226	0.37411
67377	1.89226	0.316611
67378	1.89226	0.318003
67379	1.89226	0.304932
67380	1.89226	0.343711
67381	1.89226	0.361454
67382	1.89226	0.350739
67383	1.89226	0.307268
67384	1.89226	0.294331
67385	1.88037	0.314777
67386	1.89227	0.334803
67387	1.89227	0.358178
67388	1.89227	0.319415
67389	1.98599	0.336753
67390	1.98599	0.298364
67391	1.89226	0.325371
67392	1.89226	0.359514
67393	1.89226	0.381953

67394	1.89226	0.366352
67395	1.89226	0.338038
67396	1.89226	0.31882
67397	1.89226	0.383408
67398	1.89226	0.39379
67399	1.89226	0.382999
67400	1.89226	0.426083
67401	3.78452	0.384545
67402	1.89226	0.369723
67403	1.89226	0.394261
67404	1.89226	0.380838
67405	1.89226	0.37622
67406	1.89226	0.377769
67407	1.89226	0.375
67408	1.90116	0.311126
67409	1.89227	0.340031
67410	1.89226	0.355221
67411	1.89227	0.322438
67412	1.88036	0.336508
67413	1.88037	0.300373
67414	1.89226	0.327022
67415	1.89226	0.370094
67416	1.89226	0.378714
67417	1.89226	0.361311
67418	1.89226	0.340379
67419	1.89226	0.316605
67420	0.946132	0.401303
67421	0.946132	0.394141
67422	0.946128	0.405717
67423	0.946128	0.399362
67424	0.94613	0.40264
67425	0.94613	0.412069
67426	0.94613	0.392116
67427	0.94613	0.389443
67428	0.946131	0.400482
67429	0.946131	0.404845
67430	0.94613	0.369319
67431	0.94613	0.420742
67432	1.89226	0.388259
67433	1.89226	0.387056
67434	1.89226	0.376277
67435	1.89226	0.389275
67436	1.89226	0.377684
67437	1.89226	0.390351
67438	1.89226	0.716219
67439	1.89226	1.31104
67440	2.35239	1.28763
67441	2.27873	0.82482
67442	1.89226	1.21336
67443	1.83301	0.82039
67444	2.68449	0.920155
67445	1.89226	1.85515
67446	1.89226	0.983822
67447	1.89226	1.11511
67448	3.78453	1.03058
67449	0.946132	1.70916
67450	0.946132	0.989185
67451	0.946129	0.884933

67452	0.946129	1.62626
67453	0.946131	1.62702
67454	0.946131	1.24058
67455	0.94613	0.981403
67456	0.94613	1.12163
67457	0.946131	1.25859
67458	0.946131	1.05068
67459	0.946131	1.11829
67460	0.946131	1.34408
67461	1.89226	0.713768
67462	1.89226	1.07723
67463	1.89226	1.26596
67464	1.89226	0.756816
67465	1.89226	1.2081
67466	1.89226	0.757268
67467	1.70303	0.730745
67468	1.89226	1.21123
67469	1.89226	0.705148
67470	1.89226	0.852982
67471	3.78453	0.78352
67472	1.89227	1.06912
67473	1.89227	1.18098
67474	1.89227	0.798092
67475	1.89227	1.53748
67476	3.78453	1.14079
67477	1.89226	0.95655
67478	1.89226	1.64717
67479	1.89226	1.03495
67480	1.89226	1.36897
67481	3.78452	1.25415
67482	1.89226	1.94535
67483	1.89226	2.09613
67484	1.89226	2.09626
67485	1.89226	1.91552
67486	3.78452	1.87258
67487	0.946132	1.59503
67488	0.946132	1.92671
67489	0.946129	2.1
67490	0.946129	2.08223
67491	0.94613	2.0864
67492	0.94613	1.81266
67493	0.946131	2.07806
67494	0.946131	1.88761
67495	0.946131	1.90141
67496	0.946131	2.03268
67497	0.946131	1.9717
67498	0.946131	1.74859
67499	1.89226	2.06128
67500	1.89226	1.71041
67501	1.89226	1.85082
67502	1.89226	2.02706
67503	3.78452	2.04374
67504	1.89226	1.42051
67505	1.89226	2.02472
67506	1.89226	2.1
67507	1.89226	1.50728
67508	1.89226	2.07343
67509	1.89226	1.46093

67510	1.89227	2.00409
67511	2.48308	1.38496
67512	1.89227	1.50929
67513	1.89227	1.89717
67514	3.78453	2.01353
67515	1.89226	1.66979
67516	1.89226	1.94004
67517	1.89226	2.0577
67518	1.89226	1.59678
67519	1.89226	1.98992
67520	1.89226	1.4789
67521	1.89226	1.42322
67522	1.89226	1.51822
67523	1.89226	1.75308
67524	1.89226	1.41279
67525	3.78453	1.8176
67526	0.946131	1.64663
67527	0.946131	1.83925
67528	0.946131	1.76955
67529	0.946131	1.98636
67530	0.946131	1.80515
67531	0.946131	1.80223
67532	0.946131	1.85779
67533	0.946131	1.81364
67534	0.946131	1.76125
67535	0.946131	1.93073
67536	0.946131	1.85641
67537	0.946131	1.68402
67538	1.23719	1.8909
67539	1.28439	1.59605
67540	0.94613	1.35795
67541	0.94613	1.5212
67542	1.28439	1.66219
67543	1.6431	1.92902
67544	0.946129	1.51097
67545	0.946129	1.32297
67546	1.28439	1.25972
67547	0.946131	1.19214
67548	1.21038	1.17415
67549	1.21038	1.24386
67550	1.89226	1.65564
67551	1.89226	1.30462
67552	1.89226	1.42397
67553	1.89226	1.49592
67554	1.89226	1.33907
67555	1.89226	1.42443
67556	1.89226	1.62294
67557	1.89226	1.70944
67558	1.89226	1.89615
67559	1.89226	1.48393
67560	1.89226	1.7751
67561	1.89226	1.3715
67562	1.89226	1.01089
67563	1.89226	0.567647
67564	1.89226	0.603586
67565	1.89226	0.742397
67566	1.89226	0.722798
67567	1.89226	1.04961

67568	0.946128	0.592868
67569	0.946128	0.738425
67570	0.946125	0.424331
67571	0.946125	0.597292
67572	0.94613	0.498301
67573	0.94613	0.611544
67574	0.946124	0.651733
67575	0.946124	0.766766
67576	0.946127	0.517804
67577	0.946127	0.447931
67578	0.946127	0.649023
67579	0.946127	0.745162
67580	1.89226	0.397749
67581	2.78671	0.467192
67582	2.59302	0.431154
67583	2.59302	0.422951
67584	1.89226	0.554836
67585	1.89226	0.480536
67586	1.89226	0.531522
67587	1.89226	0.582513
67588	1.89226	0.491811
67589	1.89226	0.489375
67590	1.89226	0.641532
67591	1.89226	0.579166
67592	1.89225	0.467485
67593	1.89225	0.300765
67594	1.89225	0.572687
67595	1.89225	0.448112
67596	1.89225	0.589012
67597	1.89225	0.422788
67598	0.979536	0.403799
67599	0.816597	0.417832
67600	1.15446	0.386551
67601	1.07423	0.297
67602	1.08452	0.394306
67603	0.603522	0.343109
67604	1.70302	0.3231
67605	1.29344	0.310162
67606	1.21806	0.331823
67607	0.723758	0.303949
67608	1.37562	0.411934
67609	1.89226	0.262125
67610	1.92807	0.308935
67611	1.90843	0.305382
67612	1.92807	0.310361
67613	1.87263	0.266395
67614	1.89226	0.273135
67615	0.946132	0.308099
67616	0.946132	0.301838
67617	0.946131	0.298048
67618	0.946131	0.307871
67619	0.946132	0.321457
67620	0.946132	0.3183
67621	0.946132	0.275101
67622	0.946132	0.2882
67623	0.946132	0.299897
67624	0.946132	0.34321
67625	0.946132	0.297866

67626	0.946132	0.320018
67627	1.89226	0.324374
67628	1.89226	0.367053
67629	1.89226	0.345634
67630	1.89226	0.365069
67631	3.78452	0.347496
67632	1.89226	0.307386
67633	1.89226	0.353787
67634	1.89226	0.355612
67635	1.89226	0.349692
67636	1.89226	0.304647
67637	1.89226	0.300704
67638	1.70303	0.256564
67639	1.90843	0.294229
67640	1.77002	0.28736
67641	1.90843	0.29624
67642	1.75709	0.250677
67643	1.89226	0.256869
67644	1.89226	0.272217
67645	1.89226	0.319938
67646	1.89226	0.289579
67647	1.89226	0.324111
67648	3.78453	0.28924
67649	1.89226	0.332803
67650	1.89226	0.358689
67651	1.89226	0.330412
67652	1.89226	0.372544
67653	3.78452	0.354363
67654	0.946131	0.305084
67655	0.946131	0.31628
67656	0.946131	0.305174
67657	0.946131	0.32689
67658	0.946129	0.337474
67659	0.946129	0.334806
67660	0.946133	0.300152
67661	0.946133	0.29936
67662	0.946131	0.347731
67663	0.946131	0.310439
67664	0.946131	0.288836
67665	0.946131	0.33612
67666	1.89226	0.397935
67667	2.10275	0.697053
67668	1.76377	0.661213
67669	1.76377	0.50021
67670	1.89226	0.588786
67671	1.89226	0.427823
67672	1.89226	0.392695
67673	1.89226	0.713442
67674	2.10275	0.680605
67675	2.55889	0.46709
67676	1.89226	0.622512
67677	2.30274	0.434847
67678	1.89226	0.569253
67679	1.89226	0.950054
67680	1.89226	0.912441
67681	1.89226	0.612805
67682	1.89226	0.836374
67683	1.89226	0.590425

67684	1.89226	0.527046
67685	1.89226	0.946069
67686	1.89226	0.856972
67687	1.89226	0.619683
67688	1.89226	0.789002
67689	1.89226	0.593204
67690	1.89226	0.381529
67691	1.89226	0.665618
67692	1.89226	0.633713
67693	1.89226	0.446912
67694	1.89226	0.577261
67695	1.89226	0.416359
67696	1.89226	0.385115
67697	1.89226	0.685507
67698	1.89226	0.648759
67699	1.89226	0.463228
67700	1.89226	0.6031
67701	1.89226	0.440184
67702	1.89226	0.572888
67703	1.89226	0.904414
67704	1.89226	0.853409
67705	1.89226	0.624353
67706	1.89226	0.810546
67707	1.89226	0.610869
67708	1.89226	0.778885
67709	1.89226	0.878505
67710	1.89226	0.555105
67711	1.89226	0.628885
67712	1.89226	0.557953
67713	1.89226	0.801625
67714	0.946131	0.350048
67715	0.976399	0.297209
67716	1.10124	0.25991
67717	0.946131	0.583479
67718	0.887633	0.545488
67719	0.887633	0.406995
67720	1.16933	0.359656
67721	1.23163	0.294333
67722	0.946131	0.411873
67723	0.946131	0.386324
67724	0.976399	0.358038
67725	1.14992	0.359367
67726	1.89226	0.452497
67727	1.89226	0.864659
67728	1.89226	0.462455
67729	1.89226	0.570133
67730	3.78452	0.486772
67731	1.89226	0.328585
67732	1.89226	0.605227
67733	1.89226	0.313158
67734	1.89226	0.429734
67735	3.78453	0.356494
67736	0.946132	0.233872
67737	0.946132	0.454034
67738	0.946131	0.337881
67739	0.946131	0.477555
67740	0.946131	0.476676
67741	0.946131	0.380316

67742	0.946132	0.222928
67743	0.946132	0.257251
67744	0.946131	0.320667
67745	0.946131	0.345085
67746	0.946132	0.314667
67747	0.946132	0.262207
67748	0.946132	0.557268
67749	0.946132	0.355238
67750	0.946131	0.414611
67751	0.946131	0.648048
67752	0.946131	0.650387
67753	0.946131	0.525888
67754	0.946132	0.434563
67755	0.946132	0.344588
67756	0.946131	0.482072
67757	0.946131	0.511636
67758	0.946132	0.480109
67759	0.946132	0.450092
67760	1.89226	0.579026
67761	1.89226	0.690831
67762	1.89226	0.419131
67763	1.89226	0.746345
67764	3.78452	0.665716
67765	1.89226	0.754282
67766	1.89226	0.686066
67767	1.89226	0.619018
67768	1.89226	0.541518
67769	3.78453	0.58521
67770	1.89226	0.525684
67771	1.89226	0.721189
67772	1.89226	0.6313
67773	1.89226	0.757748
67774	1.89226	0.513985
67775	1.89226	0.592425
67776	1.89225	0.482482
67777	1.89225	0.57782
67778	1.89225	0.552291
67779	1.89225	0.700139
67780	1.89225	0.445645
67781	1.89225	0.521662
67782	1.89226	0.530772
67783	1.89226	0.618518
67784	1.89226	0.578707
67785	1.89226	0.674089
67786	1.89226	0.474677
67787	1.89226	0.529114
67788	1.89226	0.767308
67789	1.89226	0.552595
67790	1.89226	0.465919
67791	1.89226	0.44366
67792	3.78453	0.493727
67793	1.39491	0.50478
67794	1.39491	0.502599
67795	0.924649	0.516996
67796	0.924649	0.519336
67797	0.946131	0.538803
67798	1.37343	0.422389
67799	0.924678	0.299221

67800	0.946131	0.837484
67801	0.924678	0.480569
67802	1.37343	0.420323
67803	0.946131	0.647112
67804	0.946131	0.605578
67805	1.89226	0.543406
67806	1.89226	0.404885
67807	1.89226	0.473507
67808	1.89226	0.515331
67809	1.89226	0.411197
67810	1.89226	0.450333
67811	0.946126	0.129267
67812	0.975461	0.121039
67813	0.946132	0.116061
67814	1.12674	0.169061
67815	0.946129	0.168565
67816	0.946129	0.147405
67817	1.12673	0.129165
67818	1.12673	0.120917
67819	0.946129	0.14357
67820	0.946129	0.155595
67821	0.975464	0.14495
67822	1.12673	0.145073
67823	1.89226	0.150587
67824	1.89226	0.144584
67825	1.89226	0.188023
67826	1.89226	0.139334
67827	1.89226	0.1775
67828	1.89226	0.141293
67829	0.946126	0.149934
67830	0.946126	0.183576
67831	0.946131	0.155037
67832	0.946131	0.257662
67833	0.946129	0.256808
67834	0.946129	0.219685
67835	0.946128	0.17102
67836	0.946128	0.149809
67837	0.946129	0.203692
67838	0.946129	0.225105
67839	0.946128	0.197669
67840	0.946128	0.196677
67841	1.89226	0.135079
67842	1.89226	0.223305
67843	1.89226	0.137425
67844	1.89226	0.158345
67845	3.78451	0.140285
67846	1.89226	0.180123
67847	1.89226	0.183715
67848	1.89226	0.132682
67849	1.89226	0.214558
67850	3.78452	0.192934
67851	0.946125	0.171874
67852	0.946125	0.215076
67853	0.946131	0.189728
67854	0.946131	0.289096
67855	0.946128	0.291305
67856	0.946128	0.258941
67857	0.946128	0.212539

67858	0.946128	0.172126
67859	0.946128	0.22057
67860	0.946128	0.242724
67861	0.946128	0.202999
67862	0.946128	0.225881
67863	1.89226	0.303447
67864	1.89226	0.389642
67865	1.89226	0.389966
67866	1.89226	0.336238
67867	3.78452	0.345507
67868	1.89226	0.321702
67869	1.89226	0.387765
67870	1.89226	0.357961
67871	1.89226	0.385096
67872	1.89226	0.345568
67873	1.89226	0.375622
67874	1.12492	0.402625
67875	1.41201	0.407349
67876	1.12082	0.365435
67877	0.771416	0.369594
67878	1.10725	0.365874
67879	1.10725	0.374767
67880	1.07618	0.489703
67881	1.07618	0.307069
67882	1.12492	0.391245
67883	0.771415	0.416373
67884	1.12082	0.373866
67885	1.41201	0.392613
67886	2.82401	0.33792
67887	1.89226	0.425921
67888	1.89226	0.33691
67889	1.89226	0.371091
67890	2.24983	0.369355
67891	2.24983	0.400578
67892	0.946133	0.403377
67893	0.946133	0.389502
67894	0.946129	0.350639
67895	0.946129	0.366558
67896	0.946131	0.384591
67897	0.946131	0.417096
67898	0.946131	0.417738
67899	0.946131	0.312388
67900	0.946131	0.369468
67901	0.946131	0.386335
67902	0.946131	0.373073
67903	0.946131	0.393758
67904	0.946132	0.396619
67905	0.946132	0.394337
67906	0.946133	0.377776
67907	0.946133	0.372431
67908	0.946132	0.385769
67909	0.946132	0.396368
67910	0.946133	0.375324
67911	0.946133	0.381672
67912	0.946132	0.39103
67913	0.946132	0.376471
67914	0.946133	0.385702
67915	0.946133	0.374445

67916	1.89227	0.460488
67917	1.89227	0.603529
67918	1.89227	0.619891
67919	1.89227	0.410874
67920	1.89227	0.622329
67921	1.89227	0.416248
67922	1.42982	0.212123
67923	1.19944	0.156409
67924	0.936311	0.316999
67925	0.936311	0.228025
67926	0.946132	0.241256
67927	1.02993	0.227005
67928	1.41513	0.249352
67929	0.946133	0.304431
67930	0.946133	0.244037
67931	0.946133	0.280546
67932	1.02993	0.263701
67933	1.18181	0.213349
67934	1.89226	0.505459
67935	1.89226	0.684278
67936	1.89226	0.765862
67937	1.89226	0.396868
67938	3.78453	0.537365
67939	1.89226	0.272639
67940	1.89226	0.372497
67941	1.89226	0.384531
67942	1.89226	0.273118
67943	1.89226	0.37085
67944	1.89226	0.261074
67945	1.89226	0.266839
67946	1.89226	0.322858
67947	1.89226	0.368173
67948	1.89226	0.255003
67949	1.89226	0.361744
67950	1.89226	0.250038
67951	0.946131	0.383818
67952	0.946131	0.497819
67953	0.946132	0.66072
67954	0.946132	0.582037
67955	0.946132	0.426975
67956	0.946132	0.542258
67957	0.946132	0.692178
67958	0.946132	0.404
67959	0.946132	0.484157
67960	0.946132	0.52045
67961	0.946132	0.491848
67962	0.946132	0.419943
67963	1.89226	1.04839
67964	1.89226	1.10628
67965	1.89226	1.31845
67966	1.89226	0.905104
67967	1.89226	1.31539
67968	1.89226	0.899362
67969	1.89226	1.27385
67970	2.22749	0.693708
67971	1.89226	0.808686
67972	1.89226	0.914836
67973	3.78453	0.928619

67974	1.89226	1.08417
67975	1.89226	1.1864
67976	1.89226	1.3491
67977	1.89226	0.851739
67978	3.78453	0.989845
67979	1.89226	0.851431
67980	1.89226	0.907878
67981	1.89226	1.06621
67982	1.89226	0.722956
67983	1.89226	1.09638
67984	1.89226	0.749637
67985	0.94613	0.68478
67986	0.94613	0.985779
67987	0.94613	1.24591
67988	0.94613	0.915297
67989	0.946131	0.850504
67990	0.946131	0.713724
67991	0.946129	1.17083
67992	0.946129	0.84518
67993	0.94613	0.876029
67994	0.94613	0.911583
67995	0.94613	0.857203
67996	0.94613	0.808449
67997	1.89226	0.165912
67998	1.89226	0.17727
67999	1.89226	0.185072
68000	1.89226	0.154372
68001	1.89226	0.186866
68002	1.89226	0.157657
68003	1.89226	0.182577
68004	1.89226	0.249291
68005	1.89226	0.254871
68006	1.89226	0.14673
68007	3.78452	0.19696
68008	1.89225	0.126516
68009	1.89225	0.135293
68010	1.89225	0.141998
68011	1.89225	0.117242
68012	1.89225	0.147111
68013	1.89225	0.124535
68014	1.89226	0.111936
68015	1.89226	0.113715
68016	1.89226	0.129573
68017	1.89226	0.107347
68018	1.89226	0.129598
68019	1.89226	0.109746
68020	0.946132	0.139899
68021	0.946132	0.172624
68022	0.946132	0.235187
68023	0.946132	0.17649
68024	0.946131	0.161203
68025	0.946131	0.141097
68026	0.946132	0.247554
68027	0.946132	0.146893
68028	0.946132	0.163821
68029	0.946132	0.172918
68030	0.946132	0.16859
68031	0.946132	0.16308

68032	1.89226	0.281623
68033	1.89226	0.377372
68034	1.89226	0.277349
68035	1.89226	0.435374
68036	1.89226	0.264672
68037	1.89226	0.413724
68038	1.89226	0.32847
68039	1.89226	0.266594
68040	1.89226	0.261893
68041	1.89226	0.380698
68042	1.89226	0.210006
68043	1.89226	0.360135
68044	1.89226	0.189952
68045	1.89226	0.254255
68046	1.89226	0.207236
68047	1.89226	0.283817
68048	1.89226	0.173781
68049	1.89226	0.247908
68050	1.89226	0.280318
68051	1.89226	0.379523
68052	1.89226	0.255294
68053	1.89226	0.41367
68054	3.78452	0.301784
68055	1.89226	0.251528
68056	1.89226	0.30263
68057	1.89226	0.239953
68058	1.89226	0.397575
[truncated: 361,193 more chars]
