# Supplementary material for: On the Use of Bone Remodelling Models to Estimate the Density Distribution of Bones. Uniqueness of the Solution
Source: PLoS One. 2016 Feb 9;11(2):e0148603. doi: 10.1371/journal.pone.0148603 (PMC4747586; doi:10.1371/journal.pone.0148603)
Supplement: S2 File — (PDF) [file pone.0148603.s002.pdf]

N° Elem	Volume (mm3)	Density (g/cm3)
1	1.32415	2.1
2	1.75823	2.1
3	0.984099	2.1
4	1.46442	2.1
5	1.53082	2.1
6	1.32068	2.1
7	1.91404	0.342191
8	1.17036	0.317048
9	0.929209	0.281291
10	1.27254	0.257104
11	2.0926	0.262738
12	2.20407	0.248796
13	1.21488	0.282896
14	1.26024	0.250749
15	0.794454	0.27327
16	1.95087	0.20767
17	0.953925	2.1
18	0.98949	2.1
19	1.27875	2.1
20	1.04598	2.1
21	0.594005	2.1
22	0.578861	2.1
23	1.09506	2.1
24	1.45327	2.02433
25	2.58396	1.72924
26	1.58581	2.1
27	1.5858	2.1
28	0.636946	1.89436
29	0.94202	2.1
30	0.946119	1.69293
31	0.636953	2.08916
32	0.946107	2.09967
33	1.09505	2.1
34	1.27389	1.6347
35	1.00232	2.1
36	1.00231	2.1
37	1.83583	2.1
38	1.00231	2.1
39	1.28828	0.288389
40	1.81696	0.266305
41	1.28828	0.257202
42	1.28828	0.243436
43	1.0044	0.33154
44	0.85148	0.345494
45	0.851539	0.359934
46	1.57767	0.271944
47	1.52227	0.261898
48	0.730657	0.268387
49	0.94613	0.33814
50	1.28228	2.1
51	1.64268	1.91584
52	1.10101	2.1
53	1.3939	2.1
54	0.959471	2.1
55	1.10101	2.1

56	1.2873	2.1
57	1.59659	2.1
58	1.80841	0.552308
59	1.80841	0.887003
60	0.946129	0.502798
61	1.36991	0.462423
62	1.32262	0.931846
63	1.32261	0.709642
64	1.38464	0.807106
65	0.962877	0.57738
66	0.962878	0.60844
67	1.82129	0.64026
68	1.82129	0.430624
69	0.94613	0.640169
70	1.39751	0.576523
71	0.946131	0.730036
72	1.34493	0.481379
73	1.39751	0.626501
74	0.837577	0.750838
75	0.823478	0.686223
76	1.10889	1.10949
77	1.01686	0.734279
78	1.1942	1.15685
79	0.836129	0.737609
80	0.937165	0.686859
81	0.969438	0.816212
82	0.895386	0.726226
83	1.30554	1.17053
84	1.15798	0.754198
85	1.22578	1.30576
86	1.15255	0.804552
87	0.9765	0.705004
88	2.02178	1.18754
89	1.63755	1.1242
90	0.727713	0.840092
91	0.810945	0.803869
92	1.00559	1.23667
93	0.879098	0.905204
94	1.07852	1.25794
95	0.657764	0.8159
96	0.829406	0.75108
97	0.720201	0.482867
98	0.946131	0.582903
99	1.11827	0.526612
100	1.50754	0.45617
101	1.64835	0.364188
102	1.71903	0.589185
103	0.851554	0.508957
104	1.75626	0.362715
105	2.1646	0.444767
106	1.5563	0.456366
107	1.5563	0.331181
108	2.10876	0.43806
109	1.05438	0.556949
110	0.946133	0.661423
111	1.05438	0.800344
112	1.20256	0.484798
113	1.47879	0.467123

114	1.47879	1.05916
115	0.77815	0.608499
116	0.947114	2.1
117	1.31257	2.1
118	1.09235	2.1
119	0.946132	2.1
120	1.54246	2.1
121	1.54246	2.1
122	1.09235	2.1
123	0.946131	2.1
124	1.29507	1.58168
125	1.0353	2.1
126	1.03529	2.1
127	1.28667	2.1
128	1.44777	2.1
129	1.61121	2.1
130	1.22176	0.699995
131	0.946129	0.688492
132	1.08848	0.623805
133	0.946131	0.690555
134	1.61201	0.675163
135	1.61201	0.632095
136	1.19993	0.680804
137	1.35822	0.685732
138	1.1539	0.314742
139	1.00765	0.266515
140	1.28228	2.1
141	2.65339	2.1
142	1.28228	2.1
143	1.55998	2.1
144	1.00509	2.1
145	0.93845	2.1
146	1.27674	2.1
147	0.810958	2.1
148	1.91381	2.1
149	1.1861	2.1
150	1.24886	2.1
151	2.02842	2.1
152	0.991436	0.235337
153	1.34588	0.236789
154	2.02729	0.229085
155	0.72904	0.282068
156	1.06139	0.279357
157	0.835368	0.289733
158	1.63819	0.220887
159	1.10745	0.240598
160	1.79925	0.231332
161	1.00085	0.273859
162	0.946133	1.93303
163	1.41123	1.70058
164	1.8327	1.89232
165	1.8327	2.1
166	0.946132	1.09779
167	1.41123	1.98352
168	1.3676	2.07197
169	1.11048	2.1
170	1.11048	2.1
171	1.65815	2.1

172	1.9004	2.1
173	1.88691	2.1
174	1.82232	2.1
175	1.46591	2.1
176	1.09115	2.1
177	1.02051	1.09331
178	1.03373	1.16249
179	1.10307	2.1
180	1.05556	2.1
181	1.82533	1.02009
182	1.19496	0.935793
183	1.28597	2.1
184	1.20421	2.1
185	2.23644	2.1
186	1.08378	2.1
187	0.983724	2.1
188	0.602075	2.06348
189	1.45876	2.1
190	1.45875	2.1
191	0.602103	1.86394
192	1.11822	2.1
193	0.946133	2.1
194	0.602103	2.1
195	1.20421	2.08898
196	1.26371	1.03561
197	1.55179	0.769903
198	1.14042	0.634543
199	1.56876	0.67031
200	2.27496	0.890208
201	1.13748	0.777847
202	1.02884	0.91032
203	1.25124	0.868608
204	0.82703	0.794687
205	0.607775	0.815472
206	1.49242	0.862649
207	1.35674	0.8681
208	1.6697	2.1
209	1.81655	2.1
210	0.900108	1.1319
211	0.851537	1.00444
212	0.703076	1.1867
213	0.639143	1.05749
214	1.42358	1.15347
215	1.4976	1.27471
216	0.86284	1.07904
217	1.2965	1.1238
218	1.27835	1.75121
219	1.00012	1.02094
220	1.74332	1.28244
221	1.08576	2.1
222	1.19 2.1	
223	1.08576	2.1
224	1.20854	2.1
225	0.946131	2.1
226	0.946131	2.1
227	1.75985	2.1
228	1.75985	2.1
229	1.41232	2.1

230	1.29365	2.1
231	0.973559	2.1
232	1.23698	0.234117
233	1.45055	0.251109
234	1.26947	0.268327
235	0.856218	0.292454
236	1.75303	0.218421
237	1.14938	0.252063
238	0.984711	0.22572
239	2.05771	0.244169
240	0.625171	0.230265
241	0.965675	0.220758
242	1.17631	0.233438
243	1.4677	0.265172
244	1.35403	0.348861
245	0.975149	0.373281
246	0.946131	0.38838
247	1.80893	0.283626
248	1.80893	0.27015
249	0.975149	0.287797
250	0.946132	0.368261
251	1.9503	0.359067
252	2.80207	0.314126
253	1.76411	0.354135
254	2.77469	0.306693
255	1.9503	0.299162
256	1.26784	0.35176
257	2.31093	0.447505
258	0.851541	0.520177
259	1.39521	0.378827
260	1.45613	0.474384
261	0.851541	0.534911
262	0.996013	0.387527
263	1.07989	0.508831
264	0.946131	0.519823
265	1.08213	0.434965
266	1.74289	0.397599
267	1.75838	0.41224
268	2.11163	0.494446
269	1.23773	2.1
270	1.25398	2.1
271	1.0304	2.1
272	1.32368	2.1
273	1.11712	0.897304
274	1.07999	0.854679
275	1.1766	2.1
276	1.35735	2.1
277	1.089 0.306437	
278	1.81193	0.347426
279	1.11081	0.200036
280	1.78069	0.207651
281	0.717512	0.213925
282	0.810946	0.202507
283	0.703901	0.207536
284	1.09028	0.196403
285	0.958031	0.203306
286	0.892585	0.202648
287	0.873431	0.204395

288	0.997755	0.197978
289	1.85479	0.199881
290	0.983029	0.619675
291	1.27922	0.588034
292	1.64346	0.918054
293	1.27922	0.773526
294	1.09795	1.53482
295	1.07508	1.07425
296	1.02975	1.29949
297	1.01121	1.50002
298	1.72369	0.216372
299	1.12035	0.202762
300	2.13511	0.218495
301	0.9705	0.221106
302	0.946131	0.245072
303	0.86182	0.221059
304	1.51379	0.21202
305	1.51379	0.206084
306	0.946132	0.222114
307	0.861849	0.221923
308	1.34591	0.796715
309	1.14721	0.612473
310	1.15539	2.1
311	1.90938	1.96625
312	2.19436	1.28798
313	1.41563	2.1
314	0.785641	2.1
315	0.736384	2.04273
316	1.46489	2.1
317	1.46488	2.1
318	0.736372	2.1
319	0.946132	2.1
320	1.90941	1.1461
321	1.18907	2.09291
322	1.20409	2.1
323	0.973559	2.1
324	1.29461	2.1
325	0.946129	2.1
326	0.94613	2.1
327	1.78751	2.1
328	1.78751	2.1
329	1.38885	2.1
330	1.34479	2.1
331	1.07283	1.21781
332	1.6869	2.1
333	1.52544	2.1
334	1.07797	2.1
335	1.13819	1.02838
336	0.776179	2.1
337	1.61092	1.14447
338	1.36155	2.1
339	0.77618	2.1
340	1.2933	1.08929
341	1.15946	2.1
342	0.69857	1.98064
343	1.87607	2.1
344	1.07612	2.1
345	1.31976	1.10686

346	1.01243	1.58284
347	1.41279	1.18329
348	1.35146	0.383281
349	1.90664	0.376275
350	1.35145	0.404781
351	1.22811	0.38395
352	1.93147	0.371956
353	1.42486	2.1
354	1.56558	2.1
355	1.0476	0.188696
356	2.31605	0.178924
357	1.89367	0.186875
358	0.804706	0.180345
359	0.783919	0.169459
360	1.3116	0.162401
361	1.03891	0.162396
362	0.960473	0.170503
363	1.08462	0.172094
364	0.785994	0.170625
365	1.1318	0.162442
366	1.29576	0.160189
367	1.3946	0.208627
368	1.11924	0.200713
369	0.834322	2.1
370	1.0256	2.1
371	1.74321	1.06484
372	1.41798	1.04397
373	0.871608	1.05767
374	1.47253	1.06502
375	1.47252	1.06161
376	0.817064	1.00864
377	0.817064	1.02933
378	0.946129	1.01665
379	1.63413	1.00743
380	1.9194	0.355787
381	1.93562	0.329496
382	2.06307	0.363096
383	1.46021	0.302123
384	0.955002	1.54437
385	0.833409	1.68432
386	0.838039	1.43839
387	1.48702	1.58274
388	1.1697	1.48412
389	1.23549	1.62587
390	1.18373	1.51424
391	2.02254	1.55401
392	1.68867	1.64117
393	0.875481	1.26098
394	0.821635	1.55648
395	0.858878	1.13101
396	1.30245	1.30533
397	1.07476	1.23483
398	1.11907	1.32744
399	1.10453	1.25167
400	1.70233	1.34099
401	1.70233	1.22475
402	2.6558	1.46758
403	1.7818	2.1

404	1.87059	2.1
405	1.34412	0.292029
406	1.98068	0.341633
407	2.56369	0.322797
408	1.49512	0.261974
409	1.35977	0.581194
410	1.34254	0.692857
411	1.75618	0.627552
412	1.75618	0.550622
413	0.946129	0.801099
414	0.946129	0.676394
415	1.17665	0.668925
416	1.01976	0.626266
417	1.27376	0.493929
418	1.35978	0.56999
419	1.68741	0.517271
420	1.68741	0.465839
421	0.946132	0.670124
422	0.946133	0.576113
423	1.14241	0.566886
424	0.996831	0.514497
425	0.996828	0.565382
426	1.12774	0.66176
427	1.13664	2.1
428	1.09627	2.1
429	2.8814	2.1
430	3.49751	2.1
431	1.79109	0.756408
432	1.54197	0.65292
433	2.75153	0.753139
434	1.93019	0.664957
435	1.51569	1.13849
436	1.55145	1.30545
437	1.78595	2.1
438	3.74452	2.1
439	1.66537	2.1
440	3.55161	2.1
441	1.35002	0.774815
442	1.19901	0.825675
443	1.61028	1.56223
444	1.35002	0.846875
445	1.65712	0.802086
446	1.10585	0.765449
447	0.961347	0.746272
448	0.810944	0.746247
449	1.16148	1.25675
450	1.08066	0.970087
451	0.934577	0.742245
452	1.05164	1.03366
453	0.79216	0.728988
454	2.99194	2.1
455	1.2832	2.1
456	2.97842	2.1
457	1.39517	2.1
458	1.03391	2.1
459	1.74416	2.1
460	1.05071	2.1
461	0.923782	2.09421

462	1.55369	2.1
463	1.86444	2.1
464	1.01617	2.0014
465	1.27174	2.1
466	1.20573	2.1
467	1.01616	2.1
468	1.13621	1.92219
469	1.75366	1.85997
470	2.27009	1.36182
471	1.6813	1.64393
472	1.47904	0.295397
473	2.62714	0.357527
474	1.80085	0.332557
475	1.80085	0.278944
476	2.74104	0.39989
477	1.37052	0.400589
478	0.946131	0.498438
479	1.37052	0.436848
480	1.31357	0.429611
481	1.7837	0.358753
482	1.7837	0.500122
483	0.900397	0.396924
484	1.74487	0.410528
485	2.88672	2.1
486	2.88249	2.1
487	1.0961	0.500345
488	1.20143	0.474732
489	1.66739	0.466451
490	1.85795	0.302003
491	1.45789	0.229395
492	2.11407	0.267462
493	0.713524	0.265212
494	1.58391	0.267355
495	1.58391	0.222788
496	1.04765	0.252205
497	1.24979	0.274214
498	0.713551	0.225227
499	0.946127	0.287919
500	1.36768	0.256731
501	1.14433	0.205555
502	1.66923	0.210513
503	0.699479	0.229622
504	0.70351	0.218003
505	0.758416	0.212704
506	1.07678	0.204515
507	0.909874	0.209063
508	0.940218	0.215561
509	0.876604	0.224876
510	1.27148	0.219185
511	2.42348	0.219081
512	1.19837	0.204852
513	0.764764	0.226803
514	0.829259	0.219683
515	0.740584	0.212471
516	1.2772	0.202503
517	0.964465	0.208974
518	1.06231	0.207743
519	1.06924	0.21593

520	1.17566	0.367018
521	1.3063	0.37591
522	2.51253	0.466559
523	2.42267	0.434384
524	1.89226	0.433788
525	1.46492	0.452442
526	0.946132	0.471833
527	0.653151	0.450637
528	0.653124	0.511337
529	1.53995	0.339945
530	1.53995	0.324821
531	0.936806	0.424139
532	1.25626	0.39548
533	1.1893	0.351915
534	1.62206	0.367283
535	1.04921	2.1
536	1.22927	2.1
537	0.801765	2.1
538	0.774917	2.1
539	0.844623	2.1
540	0.737481	2.1
541	3.24379	2.1
542	2.48573	2.1
543	1.40967	1.16805
544	1.47203	1.27021
545	2.07659	1.00931
546	1.40967	1.30908
547	2.06638	1.07122
548	1.17418	1.81149
549	1.04921	2.1
550	1.74593	2.01962
551	1.74593	1.46862
552	0.946132	1.10353
553	1.38651	1.93955
554	1.20761	0.836239
555	1.20761	1.02319
556	1.30555	0.967129
557	1.46812	1.09969
558	2.41602	1.29199
559	1.46812	1.4314
560	1.46812	1.00445
561	0.734059	0.998712
562	0.946132	1.5295
563	1.21261	1.25753
564	1.34225	1.3114
565	0.734084	1.15829
566	1.82077	1.21878
567	1.82077	1.22379
568	1.13751	0.845207
569	0.946131	1.07122
570	1.13751	0.980299
571	1.13748	0.976677
572	0.784392	0.925403
573	1.4906	0.954307
574	1.4906	0.832052
575	1.17879	1.05659
576	1.28291	1.0366
577	0.626495	2.1

578	1.45334	2.1
579	1.45334	2.1
580	0.626494	2.1
581	1.3852	2.1
582	0.946116	2.1
583	0.626499	2.1
584	1.47203	1.0239
585	1.42639	2.1
586	1.42639	2.1
587	0.795469	2.1
588	0.795469	2.1
589	0.946132	2.1
590	0.850877	2.1
591	1.37098	2.1
592	1.87194	2.1
593	1.76988	2.1
594	3.16295	0.204492
595	3.26725	0.185642
596	1.13322	2.1
597	1.20761	2.1
598	0.686933	0.798017
599	0.493248	0.708358
600	1.2053	1.29813
601	0.947149	0.754171
602	1.27591	0.808397
603	1.75732	1.31351
604	0.493261	0.82413
605	1.34337	1.41651
606	2.73207	0.723737
607	1.19857	0.799945
608	0.671688	0.690663
609	0.946134	1.38425
610	1.36603	0.723294
611	2.07817	0.803795
612	1.82573	1.16265
613	0.671699	0.880958
614	1.47772	0.697377
615	0.975349	1.29097
616	1.22615	2.1
617	1.47868	2.1
618	2.29351	1.29412
619	1.47642	2.1
620	2.94443	1.20005
621	1.80454	2.1
622	0.949135	1.67105
623	0.949134	1.71928
624	1.10476	0.255441
625	0.982857	0.320491
626	1.10938	0.338641
627	1.19052	0.566382
628	1.01012	0.168134
629	1.35562	0.16812
630	0.691742	0.163945
631	2.39596	0.165321
632	2.35604	0.167378
633	0.974081	0.160013
634	3.22444	0.156952
635	1.3294	0.759895

636	1.37265	0.888875
637	1.3294	0.928951
638	1.51113	2.1
639	1.43643	2.1
640	1.72726	2.1
641	1.634	2.1
642	0.755566	2.1
643	1.29319	2.1
644	0.755567	2.1
645	0.946131	2.1
646	1.29319	2.1
647	0.973563	2.1
648	0.904398	2.1
649	0.904406	2.1
650	1.1538	2.1
651	1.10437	2.1
652	0.935418	2.1
653	1.16649	2.1
654	0.975667	2.1
655	1.17952	2.1
656	1.95053	2.1
657	0.948676	2.1
658	1.44287	2.1
659	0.746095	1.94839
660	1.51224	2.1
661	1.27354	2.1
662	0.746066	1.84559
663	1.20573	2.1
664	1.12442	2.1
665	0.671487	2.1
666	1.04355	2.1
667	0.948675	2.1
668	0.967673	1.00026
669	1.12931	0.232114
670	1.38212	0.237778
671	2.40113	0.228454
672	1.08054	0.239461
673	1.04072	0.284886
674	0.691062	0.248179
675	1.58052	0.240192
676	1.33502	0.231861
677	0.946131	0.260044
678	0.691062	0.267851
679	1.02663	0.235817
680	1.80569	0.230208
681	1.26062	0.213395
682	1.18203	0.221558
683	0.946131	0.26619
684	0.795065	0.236443
685	1.58753	0.2225
686	1.58753	0.213601
687	0.946131	0.238212
688	0.795093	0.225347
689	0.805057	2.1
690	0.812415	2.1
691	1.23868	0.437027
692	1.34448	0.464633
693	1.67201	2.1

694	1.61202	2.1
695	1.41103	2.1
696	1.41103	2.1
697	0.841088	2.1
698	1.38302	2.1
699	0.841089	2.1
700	0.869097	2.1
701	1.38302	2.1
702	0.949941	2.1
703	0.94994	2.1
704	1.88608	2.1
705	0.854942	2.1
706	1.68893	2.1
707	1.70002	2.1
708	2.02107	0.427854
709	2.04982	0.413091
710	3.11063	0.511919
711	2.03805	0.437337
712	1.67638	2.1
713	0.967492	2.1
714	2.00209	2.1
715	0.967491	2.1
716	0.870742	2.1
717	1.30266	0.309173
718	0.651334	0.316397
719	1.69413	0.27732
720	1.69413	0.321865
721	0.946132	0.327365
722	1.03079	0.321444
723	1.31468	0.29582
724	0.946132	0.345007
725	0.919055	0.278371
726	1.16815	0.289434
727	2.79004	0.285783
728	1.2136	0.263445
729	2.62935	0.273909
730	0.947692	0.63702
731	1.51835	0.610782
732	0.947693	0.452027
733	0.947692	0.493493
734	0.980841	2.1
735	1.43577	2.1
736	0.980841	2.1
737	1.67858	2.1
738	1.34124	0.729448
739	1.39818	0.618325
740	1.2065	2.1
741	0.946133	2.1
742	1.2079	2.1
743	1.65229	2.1
744	1.65229	2.1
745	0.946132	2.1
746	1.2079	2.1
747	1.39053	2.1
748	1.20651	2.1
749	1.36919	2.1
750	1.12717	2.1
751	1.09483	2.1

752	0.994294	2.1
753	0.97356	2.1
754	1.06827	2.1
755	1.40571	2.1
756	1.29259	2.1
757	0.946131	2.1
758	1.7787	2.1
759	1.7787	2.1
760	1.29259	2.1
761	0.946132	2.1
762	1.09408	1.00176
763	1.03824	0.702838
764	0.851541	0.547353
765	1.23608	0.571299
766	0.851539	0.516903
767	1.5021	0.668731
768	1.54839	0.673967
769	1.12373	0.523616
770	1.22617	0.543065
771	1.42762	0.74522
772	2.24746	0.744462
773	1.05475	0.574495
774	1.46845	0.587974
775	1.05475	0.33145
776	0.929726	0.439391
777	1.52072	0.466703
778	1.82488	0.438987
779	1.02271	0.427775
780	1.19225	0.455425
781	1.43071	0.424258
782	0.94613	0.466554
783	1.05475	0.376417
784	1.39223	2.1
785	1.71255	2.1
786	0.946132	0.865678
787	1.10531	0.902459
788	1.46355	0.883094
789	1.46355	0.848943
790	0.78086	0.984728
791	1.10531	0.897319
792	1.1391	0.873232
793	1.56166	0.959022
794	2.21062	0.901702
795	1.0361	1.80055
796	1.73577	2.1
797	1.73577	2.04376
798	0.946116	2.1
799	1.28174	2.1
800	0.946118	1.84358
801	1.22883	2.1
802	1.28172	2.1
803	1.03609	1.85005
804	0.553567	0.181341
805	1.60581	0.179536
806	1.60581	0.174575
807	0.553594	0.178053
808	0.966748	0.18676
809	0.966748	0.180087

810	0.946133	0.192338
811	1.05602	0.180293
812	2.38525	0.187656
813	1.14503	0.169485
814	1.24678	0.170136
815	0.56262	0.168203
816	1.24678	0.173526
817	0.978467	0.172946
818	1.66262	0.167408
819	1.66262	0.164556
820	0.562633	0.175923
821	1.09522	0.172806
822	2.49356	0.171503
823	0.592547	0.173457
824	1.04321	1.10569
825	1.25133	1.25117
826	1.70508	1.17425
827	1.81043	0.943427
828	0.494934	1.04275
829	0.601047	0.999116
830	0.993428	0.990678
831	0.946573	1.12318
832	1.35039	0.948477
833	0.816895	1.22017
834	1.5063	0.901933
835	0.816895	0.844916
836	0.946132	0.844349
837	1.9067	1.16418
838	2.14504	1.19506
839	0.816895	0.831467
840	2.29624	0.851499
841	2.76933	0.878672
842	1.254	1.32869
843	0.946128	2.1
844	1.38813	2.1
845	1.79998	2.1
846	1.79998	2.1
847	0.94613	2.1
848	1.38813	2.1
849	1.35798	2.1
850	1.27809	2.1
851	1.35798	2.1
852	1.68994	2.1
853	1.68994	2.1
854	0.946132	2.1
855	0.946132	2.1
856	1.18067	2.1
857	0.958256	2.1
858	0.958255	2.1
859	0.958257	2.1
860	0.958255	2.1
861	1.06457	2.1
862	1.14195	1.86561
863	1.70423	0.818869
864	1.14195	1.87372
865	1.64285	2.1
866	2.02057	2.1
867	0.946132	2.1

868	1.44186	2.1
869	0.946133	2.1
870	1.16366	2.1
871	1.17233	2.1
872	1.31259	0.820005
873	1.2016	2.1
874	1.17129	2.1
875	1.05724	0.543278
876	1.32697	0.941115
877	1.05724	0.752856
878	1.04292	0.988972
879	3.39037	2.1
880	2.57275	2.1
881	0.629212	2.1
882	2.32525	2.1
883	1.76949	0.737645
884	1.65809	0.862831
885	1.27665	2.1
886	1.19861	2.1
887	1.20045	2.1
888	1.1635	2.1
889	0.953633	0.472445
890	0.953634	0.590582
891	1.21408	2.1
892	1.25716	2.1
893	2.01791	2.1
894	1.89318	2.1
895	0.947921	1.28294
896	0.939049	2.1
897	0.947921	1.66137
898	0.740753	2.1
899	0.916104	1.86612
900	1.16941	2.1
901	1.72038	2.1
902	2.04441	2.1
903	0.973182	2.1
904	1.11751	1.92137
905	0.611089	2.1
906	1.53584	0.683817
907	0.767923	0.695181
908	1.38925	0.700635
909	1.38926	0.691958
910	0.946131	0.708341
911	0.961347	0.702907
912	1.19583	0.700709
913	0.946131	0.704349
914	1.3663	0.720328
915	1.21067	0.680419
916	1.00166	0.304739
917	1.40707	0.335041
918	1.50435	0.333131
919	1.47954	0.854447
920	1.1233	2.08497
921	2.36036	1.28776
922	1.04024	0.842224
923	0.739729	0.855104
924	1.45533	0.812995
925	1.45532	0.803907

926	0.946118	0.84613
927	1.15163	0.675535
928	1.15165	0.867862
929	0.739748	0.826119
930	1.04025	0.985244
931	1.47608	0.462088
932	0.946131	0.531601
933	1.02028	0.484719
934	0.946129	0.493018
935	1.78223	0.423816
936	1.78223	0.404363
937	1.34153	0.447568
938	1.38683	0.419038
939	1.3407	0.428881
940	1.3407	0.408807
941	2.04729	0.416336
942	0.851529	0.511257
943	1.26671	0.458451
944	0.946116	0.505675
945	1.4869	0.400836
946	1.29553	0.387855
947	1.03645	0.459755
948	1.20731	0.412579
949	0.73665	2.1
950	0.693596	2.1
951	2.11152	1.725
952	1.73627	1.43277
953	1.06191	2.1
954	1.52226	2.1
955	1.76869	2.1
956	1.1881	2.1
957	1.32349	2.1
958	1.51041	2.1
959	1.61591	2.1
960	0.946132	2.1
961	0.946132	2.1
962	1.13537	2.1
963	3.0048	2.1
964	0.985109	2.1
965	1.68899	2.1
966	3.23261	2.1
967	1.08831	2.1
968	2.20623	2.1
969	0.91749	2.1
970	1.15515	2.1
971	0.550438	2.1
972	1.66294	2.1
973	1.08257	2.1
974	0.87851	2.1
975	2.09046	2.1
976	0.738711	2.1
977	1.31242	0.263809
978	1.26311	0.268053
979	1.67652	0.199724
980	0.968594	2.1
981	0.968594	2.1
982	0.981099	0.208816
983	1.32418	0.22678

984	2.67175	0.223935
985	2.09678	0.211989
986	1.2511	0.214302
987	0.912698	0.247955
988	0.810946	0.26238
989	0.810959	0.252562
990	1.67479	0.227569
991	1.65677	0.232777
992	1.28469	0.232201
993	1.48817	0.243994
994	1.08131	0.252799
995	1.04349	0.73759
996	1.42492	0.737406
997	1.05724	0.719431
998	1.65723	0.373613
999	1.95313	0.393921
1000	1.9293	0.378349
1001	2.02729	0.403518
1002	0.963393	1.02731
1003	0.963392	0.93129
1004	2.90171	2.1
1005	2.65447	2.1
1006	1.64158	2.1
1007	1.6488	2.1
1008	1.15991	0.687832
1009	0.946132	0.751113
1010	1.58199	0.718534
1011	1.58199	0.740057
1012	1.03333	0.751257
1013	1.25436	0.727077
1014	1.25436	0.754718
1015	0.946132	0.756869
1016	1.3771	0.558403
1017	0.687745	0.546978
1018	1.37709	0.504145
1019	0.812386	0.579252
1020	1.50172	0.573998
1021	1.50172	0.468247
1022	0.687758	0.551161
1023	1.17506	0.483839
1024	2.00539	0.547918
1025	2.2441	0.622063
1026	1.09667	0.496154
1027	1.43577	2.1
1028	2.24741	2.1
1029	1.26103	2.1
1030	0.851544	2.02996
1031	1.5543	2.1
1032	1.59756	2.1
1033	0.851543	2.07827
1034	1.44673	2.1
1035	1.31947	2.1
1036	1.1237	2.1
1037	1.26103	2.1
1038	1.089	0.304793
1039	0.946128	0.42251
1040	0.965824	0.409152
1041	0.94613	0.444886

1042	1.51344	0.327463
1043	1.51344	0.313958
1044	1.13508	0.368049
1045	1.32449	0.361842
1046	1.089	0.361529
1047	3.33706	0.438062
1048	2.7542	0.518185
1049	1.27256	0.619336
1050	0.954172	0.567963
1051	1.55527	0.537682
1052	1.05765	0.69917
1053	1.57326	0.636662
1054	1.38251	0.454769
1055	0.94613	0.600702
1056	2.38829	0.462363
1057	2.00539	0.526182
1058	1.53798	0.51537
1059	1.20969	2.1
1060	1.15864	2.1
1061	1.7986	2.1
1062	1.74533	2.1
1063	0.944827	1.37715
1064	0.946131	0.973648
1065	0.851543	0.921336
1066	1.52816	1.0529
1067	1.5314	1.32368
1068	1.39266	0.845064
1069	0.851528	0.82175
1070	1.39589	0.814567
1071	1.7348	1.03786
1072	1.85439	1.43765
1073	1.86367	1.21222
1074	1.90905	1.09557
1075	1.22176	0.697661
1076	1.59492	0.695451
1077	2.97929	2.1
1078	2.1905	2.1
1079	1.24128	2.1
1080	0.994293	2.1
1081	2.49767	0.159911
1082	2.37105	0.163021
1083	1.10834	0.949985
1084	1.53214	0.850662
1085	1.417	1.57316
1086	2.13599	0.774893
1087	1.61727	2.1
1088	1.75004	2.1
1089	1.96301	2.1
1090	0.966631	2.1
1091	1.37791	2.1
1092	1.47302	2.1
1093	1.47302	2.1
1094	0.871519	2.1
1095	0.94613	2.1
1096	0.87155	2.1
1097	2.38731	2.1
1098	2.12399	1.5051
1099	1.74865	1.49511

1100	1.46724	1.1042
1101	2.38056	1.02585
1102	1.46724	1.11576
1103	0.818887	1.10612
1104	0.810944	1.55413
1105	0.805227	1.0502
1106	1.06558	1.25647
1107	0.841487	1.10236
1108	1.08456	1.31035
1109	0.903083	1.25175
1110	1.08929	0.229992
1111	1.68454	0.236742
1112	1.08929	0.229802
1113	1.07658	0.270351
1114	0.904439	0.296973
1115	1.58501	0.218254
1116	1.19729	0.243894
1117	1.05427	0.23324
1118	1.47764	0.253971
1119	0.810959	0.244972
1120	1.60946	1.00023
1121	2.28887	0.985637
1122	0.946133	1.0062
1123	1.14444	0.94173
1124	1.67964	0.878294
1125	1.67964	0.901697
1126	0.755334	1.05318
1127	1.14444	0.999621
1128	1.29054	0.942646
1129	1.37762	0.936493
1130	0.946132	0.993279
1131	0.533645	0.871958
1132	1.48361	0.835338
1133	1.48361	0.895663
1134	0.533645	0.968425
1135	0.872823	1.00851
1136	1.14444	0.889121
1137	1.37762	0.908647
1138	2.10101	0.901906
1139	1.02311	0.927506
1140	1.04083	2.1
1141	1.04084	2.1
1142	1.04084	2.1
1143	1.30116	2.1
1144	1.23477	0.236579
1145	1.81893	0.295202
1146	1.23477	0.249402
1147	1.23477	0.273216
1148	1.11967	0.245309
1149	0.946131	0.290409
1150	1.11966	0.287157
1151	1.12628	0.244389
1152	0.629641	0.294772
1153	1.6163	0.277901
1154	1.6163	0.220126
1155	1.11117	2.1
1156	1.13006	2.1
1157	1.15149	2.1

1158	1.13179	2.1
1159	0.585368	0.788774
1160	0.986036	0.906598
1161	1.94788	0.711768
1162	1.34857	0.773853
1163	2.31806	2.1
1164	2.06363	2.1
1165	1.00423	2.1
1166	0.829065	2.1
1167	0.551816	2.1
1168	0.99406	2.1
1169	1.98339	2.1
1170	1.68073	2.1
1171	2.558	2.1
1172	0.711814	1.19541
1173	2.97316	1.69717
1174	2.41579	2.1
1175	1.15248	0.523632
1176	1.14305	0.504684
1177	1.15483	0.213832
1178	1.90981	0.217924
1179	0.998957	0.210776
1180	1.83742	0.219454
1181	1.27581	2.1
1182	0.94613	2.1
1183	1.6315	2.1
1184	1.6315	2.1
1185	0.946132	2.1
1186	1.28441	2.1
1187	1.28441	2.1
1188	1.00365	2.1
1189	0.968536	2.1
1190	1.57575	2.1
1191	1.27581	2.1
1192	1.99297	2.1
1193	1.32784	2.1
1194	1.4259	2.1
1195	2.27952	2.1
1196	1.5685	2.1
1197	1.54231	2.1
1198	3.54638	2.1
1199	1.3294	2.1
1200	2.98237	2.1
1201	2.01633	1.14606
1202	2.0985	1.18031
1203	2.26047	0.882191
1204	1.7913	0.835587
1205	1.09504	2.1
1206	1.74126	2.1
1207	1.05983	2.1
1208	2.37664	2.1
1209	2.28084	2.1
1210	2.19948	2.1
1211	1.33593	0.224414
1212	1.31508	0.224166
1213	1.30647	2.1
1214	1.28846	2.1
1215	1.2955	1.92819

1216	1.09044	1.63546
1217	1.4099	1.49435
1218	1.14984	1.76187
1219	0.946132	1.08268
1220	1.70727	1.54613
1221	1.70727	2.1
1222	1.14984	1.30879
1223	0.946132	1.46345
1224	0.911818	2.1
1225	2.12209	2.1
1226	2.4158	2.1
1227	1.20867	2.1
1228	2.84085	2.1
1229	1.42284	2.1
1230	2.45689	2.1
1231	1.41562	2.1
1232	0.918358	2.1
1233	1.11222	2.1
1234	0.999083	2.1
1235	0.696781	2.1
1236	1.30869	2.1
1237	1.66132	2.1
1238	0.696807	2.1
1239	0.745315	2.1
1240	1.23296	2.1
1241	0.780548	2.1
1242	1.93242	1.09112
1243	1.27362	1.13153
1244	2.77828	1.40447
1245	1.02653	0.875798
1246	0.808576	0.91097
1247	0.744012	0.981596
1248	0.605174	0.809841
1249	1.16501	0.734271
1250	0.803432	0.756999
1251	0.961016	0.751939
1252	1.10425	0.858661
1253	1.3756	0.928995
1254	2.17765	1.03252
1255	1.84578	0.93487
1256	1.32801	0.813669
1257	1.76377	0.821438
1258	0.946131	0.776247
1259	1.3022	0.855952
1260	0.694814	0.886368
1261	1.53028	0.785183
1262	1.53028	0.786041
1263	1.3022	0.835016
1264	0.922889	0.847926
1265	1.57766	1.98121
1266	2.06911	1.41476
1267	1.57766	1.36569
1268	1.37721	0.280342
1269	1.72174	0.336877
1270	1.4468	0.329853
1271	1.37721	0.230387
1272	1.73972	0.694317
1273	1.62324	0.706142

1274	0.946125	0.631553
1275	1.34259	0.483888
1276	1.04187	0.431559
1277	1.05019	0.529357
1278	1.2361	0.680829
1279	1.69055	0.461159
1280	2.25056	0.566417
1281	1.10574	0.894621
1282	1.21632	0.975543
1283	1.10574	1.01631
1284	1.46181	1.29668
1285	0.946131	0.86237
1286	1.74111	1.22369
1287	1.74111	0.969667
1288	0.94613	0.990042
1289	1.26891	0.944703
1290	1.26891	0.867334
1291	1.23452	1.19565
1292	1.00255	0.932098
1293	1.72286	0.647011
1294	1.12233	0.523436
1295	1.30682	0.758251
1296	1.97397	0.792486
1297	1.67425	2.1
1298	1.77775	2.1
1299	1.73571	1.54025
1300	0.426265	1.8692
1301	1.31817	1.37101
1302	1.81042	2.00278
1303	1.85456	1.97743
1304	0.466532	1.36576
1305	1.28025	1.3088
1306	0.964467	1.28084
1307	1.27921	1.97319
1308	2.11851	1.33704
1309	2.37121	1.59315
1310	1.49357	0.709739
1311	1.42486	2.1
1312	1.00278	2.1
1313	1.00278	2.1
1314	1.12503	2.1
1315	0.568697	2.1
1316	0.555211	2.1
1317	0.982272	1.33808
1318	1.57558	1.20522
1319	0.851539	1.90149
1320	1.07307	2.1
1321	1.58292	2.08281
1322	1.50731	2.1
1323	0.851541	1.1091
1324	1.00304	2.04358
1325	1.36003	2.1
1326	1.13127	2.1
1327	0.672234	2.1
1328	0.763044	2.1
1329	0.966979	2.1
1330	1.31502	2.1
1331	1.67969	2.1

1332	1.62408	2.1
1333	0.661955	2.1
1334	1.35048	2.1
1335	1.40447	2.1
1336	1.40447	2.1
1337	0.607961	2.1
1338	0.946131	2.1
1339	0.607963	2.1
1340	1.01531	2.1
1341	1.01531	2.1
1342	1.91802	2.1
1343	0.777749	2.1
1344	1.22585	0.334913
1345	1.20672	0.318817
1346	1.03166	0.547592
1347	0.553805	2.1
1348	2.00551	2.1
1349	2.07203	1.43075
1350	0.589479	2.1
1351	1.22352	2.1
1352	0.984227	2.1
1353	0.802455	2.1
1354	1.40942	2.1
1355	1.6662	2.1
1356	0.802485	2.1
1357	0.936669	2.1
1358	1.31127	0.850901
1359	0.946132	0.81139
1360	1.09729	0.741272
1361	0.946133	0.81798
1362	1.68703	0.771976
1363	1.68703	0.740079
1364	1.3689	0.793228
1365	1.26426	0.806963
1366	1.26923	2.1
1367	1.23614	2.1
1368	0.828371	2.1
1369	2.04556	2.1
1370	0.772648	2.1
1371	0.791059	2.1
1372	0.809958	2.1
1373	1.70012	2.1
1374	0.267032	2.1
1375	1.67604	2.1
1376	0.803823	2.1
1377	0.267305	2.1
1378	0.80246	2.1
1379	0.874582	2.1
1380	1.00188	2.1
1381	0.709588	2.1
1382	1.50679	1.99473
1383	1.22456	1.98023
1384	0.870622	2.09908
1385	1.05754	1.9918
1386	1.1689	2.1
1387	1.54556	2.03935
1388	1.89226	2.06581
1389	0.995311	2.1

1390	1.26195	2.1
1391	1.9	2.1
1392	0.845055	2.1
1393	0.737836	2.1
1394	0.823707	2.1
1395	0.626587	2.1
1396	1.16109	2.1
1397	0.85517	2.1
1398	0.932036	2.1
1399	1.08321	2.1
1400	1.43863	2.1
1401	2.72297	2.1
1402	2.90798	2.1
1403	1.47818	1.15814
1404	1.47819	1.13421
1405	0.694357	1.06441
1406	0.694382	1.11027
1407	0.946132	0.886185
1408	0.694382	1.00818
1409	1.22641	0.958601
1410	1.31155	1.4024
1411	2.45282	1.20135
1412	0.787843	0.978529
1413	0.808984	0.851574
1414	1.14232	1.20967
1415	0.795037	1.15959
1416	0.887382	1.00512
1417	1.14493	1.0268
1418	0.78393	0.916682
1419	1.09129	1.08452
1420	0.791482	1.01843
1421	1.1137	1.26677
1422	1.11819	1.16984
1423	0.960173	1.02853
1424	1.09447	1.23499
1425	0.874365	1.02687
1426	1.2474	0.576432
1427	1.75038	0.725437
1428	1.75038	0.806886
1429	0.94613	0.945656
1430	1.30076	0.751605
1431	1.12817	1.08838
1432	0.94613	0.875751
1433	1.30076	0.865922
1434	1.51841	0.802245
1435	1.51841	0.863846
1436	0.946132	1.08118
1437	1.39575	0.838249
1438	0.970358	1.25474
1439	0.946133	0.981988
1440	1.39575	0.950656
1441	1.06486	0.670878
1442	1.06486	0.661209
1443	1.38622	0.712084
1444	1.04521	0.260099
1445	1.00809	0.319872
1446	1.28952	0.337329
1447	0.849316	0.305961

1448	0.946132	0.320247
1449	0.849344	0.383205
1450	1.53631	0.335452
1451	1.53631	0.349274
1452	1.77843	0.250772
1453	1.69863	0.266299
1454	1.91646	2.1
1455	1.8582	2.1
1456	2.8349	0.177772
1457	1.36307	0.173662
1458	1.23886	0.241471
1459	1.12971	0.251215
1460	1.59662	0.249681
1461	1.59662	0.23684
1462	0.640304	0.33188
1463	0.640333	0.227367
1464	0.886276	0.349109
1465	0.886276	0.325694
1466	1.35065	0.297281
1467	2.09002	0.265281
1468	1.35543	0.27979
1469	1.94688	2.1
1470	1.97221	2.1
1471	0.719563	2.1
1472	1.20358	2.1
1473	0.874264	2.1
1474	1.27676	2.1
1475	1.46557	2.1
1476	1.46557	2.1
1477	0.68546	2.1
1478	0.94613	2.1
1479	0.685489	2.1
1480	1.39615	0.321733
1481	1.05404	0.264747
1482	1.06474	0.319695
1483	1.00713	0.32987
1484	0.714535	0.350551
1485	0.714563	0.368537
1486	1.41646	0.27665
1487	1.52582	0.29046
1488	0.721694	0.364026
1489	0.93665	0.345903
1490	1.21067	0.682823
1491	1.21067	0.678459
1492	1.73577	0.7
1493	1.17799	0.696773
1494	3.21432	0.646675
1495	2.41452	0.626769
1496	1.36208	0.557805
1497	2.53256	0.621687
1498	0.834593	0.517675
1499	2.5853	0.570153
1500	1.77259	0.517649
1501	0.615724	0.789513
1502	1.11374	1.02751
1503	0.615726	0.736526
1504	0.946129	1.13224
1505	0.615714	1.08721

1506	1.75489	1.03756
1507	1.75489	0.963499
1508	1.23146	0.779161
1509	2.2772	0.816956
1510	0.74463	0.787423
1511	1.3546	0.71983
1512	1.80252	0.60146
1513	2.38304	0.710789
1514	1.19151	0.810562
1515	1.1231	0.851322
1516	1.82345	0.778719
1517	1.79805	0.710092
1518	0.761337	0.976766
1519	0.946127	0.823652
1520	0.557726	0.854724
1521	1.05353	0.55521
1522	1.05353	0.532944
1523	1.68599	0.604025
1524	1.05353	0.535531
1525	1.40588	2.1
1526	1.09373	2.1
1527	1.18382	0.998632
1528	1.46903	0.954061
1529	1.25103	2.1
1530	1.10378	2.1
1531	1.58941	2.1
1532	1.29639	2.1
1533	1.14874	0.236381
1534	0.899817	0.249507
1535	0.666898	0.242026
1536	1.76519	0.223722
1537	1.17289	0.228791
1538	1.03526	0.229015
1539	1.97708	0.233496
1540	1.31711	0.21084
1541	0.992517	0.220745
1542	1.70193	0.222886
1543	1.13852	0.243096
1544	1.23943	0.497233
1545	1.40014	1.05061
1546	1.04598	1.90666
1547	1.1483	1.21891
1548	1.11433	0.930581
1549	1.32037	0.741983
1550	1.00381	0.871406
1551	1.11435	0.721085
1552	0.973502	0.963174
1553	0.946128	1.38959
1554	1.83697	1.14667
1555	1.83697	2.00991
1556	1.31721	1.42502
1557	1.38614	1.20636
1558	1.39696	1.10926
1559	1.31721	1.45681
1560	1.0756	1.57806
1561	1.08542	0.990859
1562	0.946127	1.66926
1563	1.81052	0.938523

1564	1.81052	1.97112
1565	1.24315	1.59441
1566	1.39696	0.909025
1567	1.35969	1.20648
1568	1.24315	1.20829
1569	1.0756	0.79586
1570	1.20669	0.515494
1571	1.18508	0.535409
1572	1.71059	2.1
1573	1.66762	2.1
1574	2.50853	2.1
1575	1.8236	2.1
1576	1.78872	2.1
1577	1.41721	2.1
1578	1.01335	1.30477
1579	1.42439	0.988471
1580	0.946129	1.11929
1581	1.33266	1.15988
1582	1.79776	1.30345
1583	1.79776	1.57647
1584	0.946131	0.908268
1585	1.33266	1.25788
1586	1.41123	1.20424
1587	1.25346	2.1
1588	0.946132	0.896878
1589	0.827287	1.49427
1590	1.57878	1.15096
1591	1.57878	1.12527
1592	0.827287	1.00236
1593	1.07341	1.00554
1594	1.33266	1.09297
1595	1.25346	1.13662
1596	1.61149	1.91486
1597	1.15644	1.36833
1598	1.27558	0.967297
1599	1.3764	0.809225
1600	1.89526	1.04939
1601	1.37641	0.802881
1602	1.2064	0.903672
1603	1.38426	2.06677
1604	1.01595	2.1
1605	1.15067	2.1
1606	1.82276	1.92607
1607	1.82276	0.885666
1608	0.946131	0.867473
1609	1.40609	0.864924
1610	0.946132	1.72189
1611	1.29143	1.00715
1612	1.40609	1.0044
1613	1.30272	1.61082
1614	1.19548	1.36292
1615	2.95451	2.1
1616	1.46124	2.1
1617	2.93215	2.1
1618	1.42105	2.1
1619	1.75477	1.16084
1620	2.31301	1.19539
1621	2.10275	0.850242

1622	1.80099	0.912323
1623	0.97541	2.1
1624	1.49733	2.1
1625	0.97541	2.1
1626	1.30751	2.1
1627	1.28614	0.260507
1628	1.74759	0.295884
1629	1.19756	0.423969
1630	1.71053	0.428862
1631	1.19757	0.518283
1632	1.28954	0.383977
1633	2.02703	0.393877
1634	2.29453	0.350744
1635	1.28265	0.291604
1636	0.793267	0.372263
1637	1.19974	0.473052
1638	0.509551	0.342681
1639	1.04072	0.460733
1640	1.35302	0.314269
1641	1.53133	0.485385
1642	0.566153	0.406381
1643	1.09634	0.378762
1644	1.12792	0.421931
1645	1.68908	2.1
1646	1.68908	2.1
1647	0.768212	1.87736
1648	0.768213	2.1
1649	0.946132	2.09696
1650	0.768212	1.8837
1651	1.32461	1.87903
1652	1.87291	1.92006
1653	2.64921	1.93934
1654	1.53642	2.1
1655	2.26537	2.1
1656	1.53642	2.1
1657	1.79874	2.06333
1658	1.79874	2.06369
1659	0.726635	1.44843
1660	0.726636	2.03483
1661	0.946128	1.52744
1662	0.726634	1.51351
1663	1.23339	1.31435
1664	0.992674	2.1
1665	1.8023	2.1
1666	1.12324	0.175403
1667	1.29163	0.167708
1668	0.996831	0.529977
1669	1.0019	0.509928
1670	0.993831	0.52515
1671	1.07283	1.29125
1672	1.04788	2.1
1673	1.44489	1.09867
1674	1.04788	1.92392
1675	1.07652	2.1
1676	0.991406	2.1
1677	1.37	2.1
1678	1.14195	2.1
1679	1.47904	0.302865

1680	1.26593	1.46305
1681	1.75366	1.34446
1682	1.57621	0.620404
1683	1.24538	0.437036
1684	0.969118	2.1
1685	0.946133	1.11449
1686	1.69387	2.0687
1687	1.69387	2.1
1688	0.94613	1.55965
1689	1.28713	2.1
1690	1.28713	1.9455
1691	1.23877	1.94879
1692	0.969118	2.02338
1693	1.22615	2.1
1694	0.98084	2.1
1695	1.01843	2.1
1696	1.50876	2.1
1697	1.85391	2.1
1698	1.84322	2.1
1699	1.70377	2.1
1700	1.78919	2.1
1701	2.66787	2.1
1702	1.41549	2.1
1703	1.41549	2.1
1704	0.86707	2.1
1705	0.896172	2.1
1706	0.946126	2.1
1707	0.867074	2.1
1708	0.946118	2.1
1709	1.54468	0.462367
1710	1.54468	0.46607
1711	2.27259	0.515054
1712	0.946131	0.645724
1713	1.25837	0.60451
1714	0.663171	0.671388
1715	1.68931	0.5151
1716	1.68931	0.502153
1717	1.25837	0.600718
1718	1.09411	0.593961
1719	1.73571	0.419232
1720	2.34894	0.486162
1721	0.946128	0.544267
1722	1.30497	0.499694
1723	0.772312	0.582928
1724	1.66895	0.425711
1725	1.66895	0.375679
1726	1.30497	0.482002
1727	1.13629	0.453406
1728	1.22852	0.823853
1729	1.578	0.822548
1730	2.07781	2.00956
1731	1.5478	1.98811
1732	1.70048	1.74195
1733	1.42329	1.58039
1734	1.05481	1.49038
1735	1.1032	1.09629
1736	1.19267	2.1
1737	1.77324	2.1

1738	1.01121	0.423359
1739	0.991867	0.405442
1740	2.0744	1.17915
1741	1.75142	2.1
1742	1.35418	1.20464
1743	1.40041	2.1
1744	1.50045	2.1
1745	1.37704	2.1
1746	1.50045	2.1
1747	1.87875	2.1
1748	1.35308	0.75245
1749	1.29983	0.746099
1750	1.62784	2.1
1751	1.44807	2.1
1752	1.00965	2.1
1753	2.69264	2.1
1754	1.04075	2.1
1755	0.946131	2.1
1756	0.946132	2.1
1757	1.52704	2.1
1758	1.83246	2.1
1759	1.56757	2.1
1760	1.16687	2.1
1761	2.04857	2.1
1762	1.32707	1.24898
1763	1.25519	0.381711
1764	1.32101	0.583861
1765	1.25055	0.972567
1766	0.827853	0.820611
1767	1.11835	0.540798
1768	1.07281	1.22368
1769	1.54302	0.847149
1770	0.724064	0.9723
1771	1.32709	1.95178
1772	3.44123	1.06833
1773	2.46889	0.56584
1774	0.730076	0.383148
1775	1.25809	0.561271
1776	0.730077	0.425298
1777	0.946129	0.524095
1778	1.72777	0.339142
1779	1.72777	0.54386
1780	0.73005	0.465332
1781	1.7522	0.316722
1782	2.51618	0.389282
1783	0.628177	0.382801
1784	1.1068	0.56748
1785	0.747435	0.407202
1786	0.946133	0.533966
1787	1.84668	0.377336
1788	1.8563	0.529942
1789	0.628177	0.489424
1790	1.45501	0.403743
1791	2.19809	0.564604
1792	1.45501	0.443017
1793	1.48974	0.405905
1794	1.7709	1.90955
1795	2.63288	1.80825

1796	1.5362	2.1
1797	1.56327	2.1
1798	3.03945	0.280567
1799	0.899979	0.323175
1800	0.869386	0.325635
1801	1.85864	0.301094
1802	1.78028	0.312879
1803	1.28719	0.330358
1804	1.09532	0.282586
1805	0.881898	0.330894
1806	1.28911	0.327958
1807	1.39644	0.339036
1808	0.688829	0.321757
1809	2.14611	0.296345
1810	1.80313	0.29648
1811	0.68883	0.328991
1812	0.946131	0.356379
1813	1.25334	0.263463
1814	1.88987	0.306729
1815	1.94432	0.30815
1816	1.37592	0.290793
1817	1.11691	1.36153
1818	1.02923	1.50594
1819	0.45824	0.221146
1820	0.646512	0.30869
1821	2.46292	0.259459
1822	0.493451	0.272952
1823	2.00826	2.1
1824	2.49784	2.1
1825	1.17054	2.1
1826	1.01485	2.1
1827	1.26045	2.1
1828	1.17054	2.1
1829	1.44247	2.1
1830	2.02687	0.22958
1831	1.66365	0.248866
1832	1.64656	0.22926
1833	1.56597	0.256128
1834	1.21419	2.1
1835	1.5008	2.1
1836	1.10711	2.1
1837	1.44287	2.1
1838	1.44287	2.1
1839	0.750368	2.1
1840	1.25876	2.1
1841	0.82542	2.1
1842	1.04074	2.1
1843	1.25874	2.1
1844	1.04159	0.242421
1845	1.2489	0.242563
1846	0.980547	0.628653
1847	1.62235	0.588191
1848	1.15991	0.617025
1849	0.980547	0.533182
1850	0.94613	0.636957
1851	1.54662	0.594988
1852	1.54661	0.601436
1853	1.07912	0.651756

1854	1.1032	0.600681
1855	1.1032	0.611627
1856	0.94613	0.626025
1857	1.12468	2.1
1858	1.2724	2.1
1859	1.06521	1.59953
1860	1.0752	1.7056
1861	1.94936	0.378181
1862	1.62772	0.397646
1863	1.94733	0.356463
1864	1.42901	0.399826
1865	1.33022	2.1
1866	1.70839	2.1
1867	1.75048	2.1
1868	1.41201	2.1
1869	1.52314	2.1
1870	1.44039	2.1
1871	1.51113	2.1
1872	2.58638	2.1
1873	0.987247	0.493535
1874	1.38622	0.707612
1875	0.858359	0.525194
1876	1.19967	0.588768
1877	1.06414	0.498842
1878	0.946132	0.714959
1879	0.94613	0.503568
1880	0.687079	0.800596
1881	0.946132	0.691839
1882	0.822611	0.691323
1883	1.70242	0.534574
1884	1.70242	0.666824
1885	0.946132	0.804655
1886	1.11155	0.597536
1887	0.946132	0.864305
1888	1.11155	0.756066
1889	1.37981	0.784119
1890	1.18348	0.548199
1891	2.44179	0.666928
1892	2.00403	0.786155
1893	1.10961	2.1
1894	1.85415	2.1
1895	1.44825	2.1
1896	0.932272	2.1
1897	0.810942	2.1
1898	1.00189	2.1
1899	1.11779	2.1
1900	1.3359	2.1
1901	0.965279	2.1
1902	0.833779	2.1
1903	1.05337	2.1
1904	0.526656	2.1
1905	1.44599	2.1
1906	1.44599	2.1
1907	1.24853	2.1
1908	0.946132	2.1
1909	1.24853	2.1
1910	0.724122	2.1
1911	1.91547	2.1

1912	2.49705	2.1
1913	1.05931	2.1
1914	1.1729	2.1
1915	1.34526	2.1
1916	1.5379	2.1
1917	1.90855	2.1
1918	2.08597	2.1
1919	1.68165	2.1
1920	1.45887	2.1
1921	1.50022	2.1
1922	1.4748	2.1
1923	2.63645	2.0199
1924	1.74431	2.1
1925	1.25288	2.0369
1926	1.25289	1.91656
1927	0.970025	1.82171
1928	0.747542	2.09973
1929	0.810959	1.69701
1930	1.15369	1.92559
1931	0.977472	1.80892
1932	1.12695	1.9391
1933	0.996762	1.81478
1934	1.28249	0.878418
1935	0.946132	0.871287
1936	0.528621	0.911068
1937	1.59186	0.813789
1938	1.44072	0.82184
1939	0.633488	0.847855
1940	0.598506	0.901189
1941	1.51618	0.906973
1942	1.19999	0.793696
1943	1.46511	0.793393
1944	1.44056	0.7691
1945	0.912754	0.687535
1946	1.4725	0.625045
1947	1.4725	0.579872
1948	0.912753	0.678016
1949	1.23351	0.697061
1950	1.23351	0.718965
1951	0.946131	0.765612
1952	1.17129	0.568457
1953	1.82551	0.538583
1954	1.27575	1.97321
1955	0.946129	2.05727
1956	0.946129	2.1
1957	0.946128	2.0704
1958	0.792754	2.0499
1959	0.94613	1.76102
1960	0.792752	1.72552
1961	2.08054	2.1
1962	1.20091	2.1
1963	2.1669	2.095
1964	1.24383	1.82771
1965	0.904669	1.95192
1966	0.83804	1.59036
1967	1.90486	1.91862
1968	1.29162	1.74077
1969	1.37383	1.9892

1970	1.86533	1.75014
1971	1.76472	2.02083
1972	1.60428	1.73101
1973	2.43993	1.94559
1974	1.37833	2.1
1975	1.18632	2.1
1976	0.946132	2.1
1977	1.27544	2.1
1978	1.05085	2.1
1979	0.961178	2.1
1980	0.859844	2.1
1981	1.0499	2.1
1982	1.75592	2.1
1983	2.08668	2.1
1984	1.75592	2.1
1985	1.4402	2.1
1986	1.72285	2.1
1987	0.83312	0.294387
1988	1.07295	0.274579
1989	1.37607	0.307386
1990	1.32097	0.271368
1991	1.11332	0.325611
1992	1.18568	0.269933
1993	0.877482	0.288125
1994	1.4458	0.238916
1995	2.34112	0.21704
1996	2.68021	0.268882
1997	0.681677	0.199772
1998	0.946131	0.234569
1999	0.681649	0.211414
2000	1.83115	0.212757
2001	1.68029	0.223072
2002	1.05482	0.198954
2003	1.31485	0.221895
2004	2.08247	1.32779
2005	1.33153	1.30525
2006	0.935062	1.39433
2007	0.806958	1.73619
2008	0.827683	1.25181
2009	1.01041	1.52439
2010	0.741832	1.25491
2011	1.15668	1.58861
2012	0.908649	1.3957
2013	0.59561	0.75225
2014	1.19028	1.07204
2015	0.59561	1.07356
2016	0.946132	1.09623
2017	0.595623	1.2859
2018	1.58164	1.10686
2019	1.58164	1.16403
2020	0.946131	1.70259
2021	1.55201	2.1
2022	1.55201	2.1
2023	0.946127	2.1
2024	1.13312	2.1
2025	1.13312	2.1
2026	1.08108	2.1
2027	1.04973	2.1

2028	1.20149	2.1
2029	1.86902	1.14099
2030	0.613447	2.1
2031	1.22618	1.21605
2032	1.38663	1.39956
2033	1.38662	2.1
2034	0.613447	1.28281
2035	1.22617	1.84607
2036	0.773899	1.75581
2037	1.23515	1.53617
2038	1.87823	1.22781
2039	1.70303	0.864483
2040	1.4714	2.1
2041	1.74067	2.1
2042	0.735699	2.1
2043	1.73461	2.1
2044	1.47224	2.1
2045	1.22827	2.1
2046	1.1634	2.1
2047	1.36471	2.1
2048	0.735701	2.1
2049	2.8386	0.878065
2050	2.39056	1.96172
2051	2.44128	1.0118
2052	1.54102	2.1
2053	1.54102	1.94537
2054	0.936141	1.25033
2055	1.40594	2.1
2056	1.07121	0.987172
2057	0.936142	1.57995
2058	1.07121	1.25245
2059	1.22691	0.308375
2060	1.42907	0.304406
2061	2.18048	0.284209
2062	0.947662	0.288533
2063	2.18394	2.1
2064	1.21292	2.1
2065	2.18394	2.1
2066	2.19064	2.1
2067	1.27229	2.1
2068	1.20859	0.858256
2069	1.21073	0.817547
2070	1.73899	2.1
2071	1.3164	2.1
2072	1.61245	2.1
2073	1.41444	2.1
2074	2.55116	2.1
2075	1.26193	2.1
2076	1.89553	2.1
2077	0.916438	2.1
2078	1.26113	2.1
2079	0.810934	2.1
2080	2.00419	2.1
2081	1.27872	2.1
2082	1.35306	2.1
2083	1.96749	2.1
2084	1.07955	2.1
2085	1.31854	2.1

2086	1.29505	0.86397
2087	1.29504	0.849871
2088	0.612469	0.892494
2089	0.990053	0.936935
2090	1.04072	0.850894
2091	0.985266	0.843613
2092	0.990039	0.835726
2093	1.06892	1.00124
2094	1.57158	0.775875
2095	1.895	0.870721
2096	1.05698	0.802388
2097	0.925053	2.1
2098	0.624712	2.1
2099	1.42465	2.1
2100	1.49014	2.1
2101	0.687198	2.1
2102	1.22058	2.1
2103	0.851488	2.1
2104	0.588128	2.1
2105	1.12452	2.1
2106	1.02783	2.1
2107	1.7083	2.1
2108	1.06977	0.874296
2109	0.818569	0.787971
2110	1.14386	1.12851
2111	0.983791	0.858553
2112	1.12214	1.18378
2113	1.0915	0.84248
2114	0.810934	0.813512
2115	0.764114	0.906064
2116	1.87956	0.878484
2117	1.87956	1.08767
2118	0.764141	0.919966
2119	1.37675	0.825651
2120	0.764141	0.891119
2121	0.946132	0.860294
2122	1.9637	1.24018
2123	2.10107	0.829741
2124	1.67814	0.960955
2125	3.02886	0.950621
2126	0.908982	0.440385
2127	0.621727	0.492484
2128	0.525963	0.466563
2129	0.946128	0.520298
2130	1.60979	0.441034
2131	1.45596	0.481305
2132	0.556172	0.493061
2133	2.06779	0.386362
2134	2.17911	0.437709
2135	1.00205	0.323452
2136	1.30685	0.372564
2137	1.16534	0.40097
2138	1.65532	2.1
2139	2.20346	2.1
2140	0.993889	2.1
2141	1.09328	2.1
2142	2.09372	2.1
2143	2.10036	2.1

2144	1.33727	1.0919
2145	2.53383	1.12042
2146	0.893379	1.2665
2147	1.52828	1.09151
2148	1.09599	0.220563
2149	1.09599	0.216771
2150	1.50524	2.1
2151	1.54711	2.1
2152	1.41469	2.1
2153	1.99459	1.82148
2154	1.79805	1.82123
2155	1.79805	2.1
2156	0.707344	2.1
2157	1.30318	1.73912
2158	0.707344	2.1
2159	0.946129	2.1
2160	1.30318	2.1
2161	2.40442	2.1
2162	1.89226	2.1
2163	1.40794	2.00546
2164	0.898721	1.95131
2165	0.791921	1.27802
2166	0.810933	1.80819
2167	1.10904	1.48904
2168	0.991102	1.95549
2169	0.808502	1.21985
2170	1.1398	1.66177
2171	1.31346	2.1
2172	1.11833	2.1
2173	1.669	2.1
2174	1.25715	2.1
2175	1.3199	2.1
2176	1.63092	2.1
2177	1.63092	2.1
2178	0.946131	2.1
2179	0.946131	2.1
2180	1.13534	2.1
2181	0.979853	2.1
2182	2.19994	2.1
2183	0.725844	2.1
2184	2.19994	2.1
2185	2.35055	2.1
2186	1.52204	2.1
2187	1.40118	2.1
2188	3.42285	1.87796
2189	2.05217	2.1
2190	1.50319	2.1
2191	0.947516	1.27944
2192	0.903572	1.25094
2193	0.76996	1.31678
2194	1.90959	1.96024
2195	1.18897	1.76259
2196	1.24166	1.65333
2197	2.26785	1.492
2198	2.05217	2.1
2199	2.73306	2.1
2200	1.42729	0.430374
2201	1.387	0.430077

2202	1.59458	0.757719
2203	1.91051	0.772664
2204	1.68108	0.727889
2205	2.03357	0.748506
2206	0.928982	0.446635
2207	1.55687	0.472789
2208	1.55687	0.315924
2209	0.92898	0.403005
2210	1.3495	0.501259
2211	1.3495	0.407847
2212	0.946133	0.509016
2213	1.85795	0.356714
2214	2.2727	0.454167
2215	1.20324	0.883165
2216	1.07784	0.545041
2217	0.957792	0.409535
2218	1.2745	0.408531
2219	1.4659	0.45025
2220	0.946129	0.566204
2221	0.95778	0.553678
2222	0.851553	0.637841
2223	1.74103	0.457644
2224	2.28994	0.486636
2225	2.08213	0.411259
2226	2.1556	0.438125
2227	2.20372	0.38159
2228	0.838027	0.296976
2229	1.11373	0.266147
2230	1.2909	0.221001
2231	0.998424	0.273703
2232	1.40621	0.220148
2233	1.19478	0.241492
2234	1.11783	0.26609
2235	1.6695	0.225483
2236	1.7498	0.252436
2237	0.477652	0.264747
2238	1.77291	0.272792
2239	0.590448	0.254247
2240	1.07668	0.254278
2241	0.810946	0.349418
2242	1.1946	0.306446
2243	2.03914	0.270573
2244	1.25585	0.327355
2245	2.02622	0.269723
2246	1.39091	0.279613
2247	1.31871	0.31218
2248	1.15789	2.1
2249	1.15755	2.1
2250	1.82268	2.1
2251	1.87119	2.1
2252	3.10636	2.1
2253	2.96285	2.1
2254	1.73443	0.853993
2255	2.33812	0.858224
2256	1.59457	0.777882
2257	1.73443	1.02467
2258	2.00665	2.1
2259	1.99841	2.1

2260	1.19901	1.90825
2261	1.16661	1.99899
2262	2.15883	0.409519
2263	1.20951	0.40561
2264	2.49192	0.498289
2265	0.65112	0.389006
2266	2.45317	0.217737
2267	1.73091	0.208974
2268	2.0795	0.214274
2269	1.45891	0.19597
2270	1.6531	0.200379
2271	1.78304	0.191765
2272	0.775132	0.244085
2273	1.35051	0.21659
2274	1.0461	0.215189
2275	1.37837	0.212991
2276	1.00259	0.221842
2277	1.25796	0.7
2278	1.10719	0.734098
2279	1.51067	2.1
2280	2.65632	1.47484
2281	1.30521	2.1
2282	2.456	2.07865
2283	3.19218	2.00166
2284	2.67387	1.58085
2285	0.988862	2.1
2286	1.917	2.1
2287	0.946132	2.1
2288	1.16202	2.1
2289	1.53871	2.1
2290	1.53871	2.1
2291	0.946132	2.1
2292	1.16202	2.1
2293	1.32282	2.1
2294	2.46356	2.1
2295	2.32404	2.1
2296	1.37562	0.336479
2297	0.985546	0.36254
2298	1.28983	0.304895
2299	1.05566	0.3317
2300	0.895312	0.346851
2301	1.53325	0.314498
2302	1.04342	0.348883
2303	1.80881	0.328366
2304	0.876865	0.315751
2305	0.5689	0.321281
2306	1.09506	0.34375
2307	1.51789	2.1
2308	1.49872	2.1
2309	0.949364	0.5452
2310	2.01233	0.602391
2311	1.55063	0.565749
2312	1.57783	0.754074
2313	1.57783	0.755462
2314	0.674605	0.704285
2315	0.98537	0.702175
2316	0.674633	0.688295
2317	0.98537	0.667769

2318	1.26706	0.738355
2319	0.980664	0.796967
2320	1.03298	0.700048
2321	1.84936	0.766165
2322	1.51696	0.698444
2323	1.97074	0.619124
2324	1.03298	0.58538
2325	1.51324	0.541493
2326	1.51324	0.553682
2327	0.758454	0.506764
2328	1.28632	0.478226
2329	0.758482	0.398253
2330	0.758482	0.446698
2331	0.985372	0.539988
2332	1.14105	0.347297
2333	1.59787	0.665022
2334	2.46244	0.816794
2335	1.70367	0.486273
2336	2.13757	1.2397
2337	1.98142	1.25091
2338	1.35878	0.298439
2339	2.39947	0.380053
2340	1.24554	0.311166
2341	1.7522	0.324762
2342	1.49693	1.9199
2343	1.49652	2.0366
2344	1.6211	1.90131
2345	1.45938	2.01098
2346	0.737431	2.02666
2347	1.18643	2.01718
2348	1.04072	2.08197
2349	0.9459	1.91149
2350	0.66364	2.04201
2351	0.985109	1.72099
2352	0.638368	1.94892
2353	0.83804	1.60497
2354	0.998024	1.88047
2355	0.914471	1.57896
2356	1.14281	1.92814
2357	0.833972	1.72073
2358	0.991865	0.642747
2359	1.42208	0.649278
2360	1.70718	2.1
2361	1.79888	2.1
2362	1.16851	0.694796
2363	1.16851	0.665938
2364	1.51021	0.711281
2365	0.946132	0.501545
2366	1.14897	0.408247
2367	0.946132	0.521276
2368	1.77177	0.561181
2369	1.77177	0.469802
2370	1.33038	0.479099
2371	1.38752	0.492435
2372	1.1726	0.541492
2373	1.51688	0.570877
2374	1.06455	2.1
2375	0.98511	2.1

2376	1.31144	2.1
2377	1.79825	2.1
2378	1.79825	2.1
2379	1.73044	2.1
2380	1.59589	0.232658
2381	1.59589	0.229742
2382	2.78009	2.1
2383	1.68761	2.1
2384	3.05511	2.1
2385	2.25318	2.1
2386	0.578998	2.1
2387	1.03512	2.1
2388	2.26156	2.1
2389	1.44859	0.904958
2390	1.35897	1.04206
2391	1.18301	1.82633
2392	1.25173	1.77488
2393	1.98851	2.1
2394	2.70685	2.1
2395	0.682342	2.1
2396	1.52491	2.1
2397	1.37119	2.1
2398	0.682313	2.1
2399	1.35342	2.1
2400	0.947555	2.1
2401	0.614107	2.1
2402	1.23007	2.1
2403	1.5735	2.1
2404	1.58713	2.1
2405	1.57979	0.217889
2406	1.30723	0.216546
2407	0.935658	0.91646
2408	0.8952	0.871569
2409	1.27352	1.09954
2410	1.0985	1.00924
2411	1.07163	0.880521
2412	1.15823	1.01656
2413	0.836451	0.861947
2414	2.08051	0.789618
2415	1.92601	1.12541
2416	2.0644	0.84306
2417	1.37259	2.1
2418	1.37259	2.1
2419	0.752217	2.1
2420	1.28569	2.1
2421	0.752186	2.1
2422	0.839124	2.1
2423	1.28568	2.1
2424	1.04199	2.1
2425	1.04199	2.1
2426	2.59268	2.1
2427	1.04199	2.1
2428	1.5098	2.1
2429	1.5098	2.1
2430	0.520997	2.1
2431	1.29634	2.1
2432	0.520996	2.1
2433	0.73446	2.1

2434	1.29634	2.1
2435	0.983724	2.1
2436	1.23914	2.1
2437	2.65095	2.03676
2438	1.52851	2.1
2439	2.399	2.1
2440	1.68137	2.1
2441	1.11285	2.1
2442	1.04921	2.1
2443	1.30711	2.1
2444	1.04921	2.1
2445	1.07496	1.04881
2446	1.55831	0.804591
2447	1.07497	0.757169
2448	1.07497	0.909
2449	1.27391	2.1
2450	2.5615	1.98067
2451	1.2739	2.1
2452	1.75253	1.89072
2453	1.12907	0.464801
2454	1.9194	0.54408
2455	1.96617	0.418962
2456	1.01225	1.95697
2457	0.947979	1.89234
2458	1.01225	1.10875
2459	1.06561	1.92596
2460	1.01225	1.58902
2461	3.56876	2.1
2462	3.68767	2.1
2463	1.52301	2.1
2464	1.61013	2.1
2465	1.43726	2.1
2466	2.04275	2.1
2467	0.995734	2.1
2468	1.16199	2.1
2469	1.73704	0.299846
2470	1.04019	0.343677
2471	0.956294	2.1
2472	1.81488	2.1
2473	1.63719	2.1
2474	1.50103	0.211452
2475	1.75289	0.235904
2476	1.31491	0.226008
2477	1.03567	0.22045
2478	0.672257	0.223998
2479	1.04072	0.286448
2480	0.746903	0.258384
2481	1.43317	0.238083
2482	1.39986	0.218238
2483	1.06385	0.255079
2484	1.1624	0.266058
2485	1.61667	0.213923
2486	1.27258	0.208396
2487	2.559	0.21465
2488	1.66093	0.206021
2489	1.10719	0.734713
2490	2.03235	0.75592
2491	1.17105	0.99314

2492	1.10719	0.770177
2493	1.3028	2.05368
2494	0.946131	1.86289
2495	0.946127	1.86283
2496	1.52912	2.1
2497	1.52912	2.06256
2498	0.946128	1.48466
2499	0.949997	1.7571
2500	1.31565	2.1
2501	1.46643	2.1
2502	1.5482	2.1
2503	1.47102	2.1
2504	1.47102	2.1
2505	0.774101	2.1
2506	1.30583	2.1
2507	0.774103	2.1
2508	0.939287	2.1
2509	1.30584	2.1
2510	0.958661	2.1
2511	2.28835	2.1
2512	1.43213	0.334278
2513	2.13255	0.408391
2514	1.43213	0.323754
2515	1.10476	0.319814
2516	1.93165	0.393244
2517	1.03235	0.969728
2518	1.31	0.965411
2519	1.22082	2.1
2520	1.35827	2.1
2521	0.790936	2.1
2522	0.716644	2.1
2523	1.43257	2.1
2524	1.43257	2.1
2525	0.716615	2.1
2526	0.946131	2.1
2527	0.730157	2.1
2528	1.47212	2.1
2529	1.47212	2.1
2530	0.946131	2.1
2531	0.790792	2.1
2532	1.41149	2.1
2533	0.790791	2.1
2534	1.01006	2.1
2535	1.53045	0.781224
2536	1.53045	0.877068
2537	0.946132	1.07203
2538	1.40779	0.82304
2539	1.06879	1.20682
2540	0.990713	0.948447
2541	1.06879	1.00103
2542	1.06486	0.67387
2543	1.18347	0.659842
2544	1.38622	0.699326
2545	1.18347	0.568673
2546	1.7304	0.649768
2547	1.7304	0.752797
2548	0.946131	0.892653
2549	1.26874	0.746108

2550	1.13337	1.07725
2551	1.13337	0.853126
2552	1.40779	0.858537
2553	1.695 2.1	
2554	1.77071	2.1
2555	0.649313	2.1
2556	0.692829	2.1
2557	1.40395	2.1
2558	1.34243	2.1
2559	0.813292	0.480651
2560	0.632691	0.460202
2561	1.75766	0.681977
2562	2.25727	0.846182
2563	1.74206	0.413816
2564	0.826073	0.524266
2565	1.30022	0.537778
2566	1.45641	0.460291
2567	0.946131	0.51265
2568	0.752245	0.472731
2569	0.960952	0.548529
2570	0.743477	0.446634
2571	1.04355	0.446847
2572	1.6253	0.415403
2573	0.960577	0.425961
2574	1.34852	0.931439
2575	1.03235	0.996201
2576	1.78168	0.795283
2577	1.2966	0.296114
2578	2.21116	0.268649
2579	1.6395	0.284569
2580	1.6395	0.271221
2581	0.94613	0.361945
2582	1.22187	0.324302
2583	0.962737	0.370491
2584	0.962735	0.333541
2585	1.36376	0.343922
2586	1.2966	0.240877
2587	1.2966	0.247098
2588	1.19669	0.942533
2589	2.37636	1.00279
2590	1.08894	1.08609
2591	1.62551	1.14123
2592	2.51472	1.12721
2593	1.89226	0.994118
2594	0.980038	1.17244
2595	1.10187	1.09814
2596	1.01479	0.967049
2597	1.81663	2.1
2598	1.83247	2.1
2599	0.921851	0.785954
2600	0.983148	2.1
2601	1.82816	2.1
2602	1.26103	2.1
2603	2.34818	2.1
2604	1.23152	2.1
2605	2.15738	2.1
2606	1.23152	2.1
2607	1.51042	2.1

2608	0.842995	2.1
2609	1.22257	2.1
2610	0.810959	2.1
2611	1.68242	2.1
2612	0.95701	2.1
2613	1.12389	2.1
2614	1.95255	2.1
2615	1.57974	2.1
2616	1.94632	2.1
2617	0.81534	2.1
2618	1.13996	2.1
2619	1.18958	0.225583
2620	1.25929	0.210288
2621	3.02933	0.827797
2622	2.37434	0.396651
2623	1.10609	0.825334
2624	1.64132	0.718913
2625	1.64132	0.744102
2626	0.946132	0.564958
2627	1.40029	0.62058
2628	1.08946	0.482895
2629	1.08946	0.523471
2630	1.18717	0.370696
2631	2.71421	0.370371
2632	1.08189	0.472627
2633	0.94613	0.461474
2634	1.37109	0.494898
2635	1.17925	0.362862
2636	0.851541	0.491009
2637	0.942512	0.479943
2638	1.18541	0.420598
2639	0.851526	0.500642
2640	1.0819	0.419803
2641	1.86009	0.522509
2642	0.946132	0.403752
2643	1.67409	0.295767
2644	1.67409	0.44779
2645	0.946131	0.448183
2646	1.31716	0.410898
2647	1.30307	0.368439
2648	0.946131	0.462465
2649	1.18601	0.373467
2650	3.15646	2.1
2651	0.985109	2.1
2652	3.37048	2.1
2653	1.10667	2.1
2654	1.77065	1.22108
2655	1.45837	1.08384
2656	1.77063	1.07611
2657	2.58104	1.0251
2658	1.37652	1.5886
2659	1.54168	1.55804
2660	1.00224	0.204118
2661	1.05851	0.203724
2662	1.39093	0.194734
2663	1.43358	0.204215
2664	0.575741	0.197403
2665	0.518161	0.191719

2666	1.04071	0.206437
2667	1.13092	1.17784
2668	1.17042	1.51432
2669	1.53347	1.29347
2670	1.52938	1.20749
2671	1.00613	0.569165
2672	1.81618	0.851196
2673	1.81618	0.543987
2674	0.946127	0.829983
2675	1.36479	0.828943
2676	0.94613	0.565023
2677	1.31724	0.758373
2678	1.3648	0.760173
2679	1.00613	0.567684
2680	0.781375	0.370539
2681	1.13431	0.438894
2682	1.03547	0.472369
2683	1.40668	0.498686
2684	0.94613	0.500541
2685	0.713285	0.530517
2686	1.72885	0.572998
2687	1.72885	0.511772
2688	0.713298	0.498888
2689	3.30075	0.381654
2690	2.81337	0.464398
2691	1.39296	0.606463
2692	0.830164	1.25427
2693	1.50493	2.1
2694	1.50494	2.1
2695	0.830136	1.2101
2696	1.28298	2.1
2697	1.0521	2.08748
2698	0.830147	2.1
2699	2.0799	1.34782
2700	1.52115	1.17256
2701	2.5142	1.26871
2702	1.31253	0.837208
2703	1.94446	0.97411
2704	1.18817	0.956222
2705	0.598348	0.980099
2706	1.69315	0.943208
2707	1.69315	1.05455
2708	0.598377	0.868051
2709	0.598347	0.872968
2710	0.94613	0.855343
2711	1.28418	0.991124
2712	2.05205	1.07307
2713	1.28418	1.07126
2714	1.0737	2.1
2715	1.81024	2.1
2716	2.73834	2.1
2717	1.71369	2.1
2718	1.71368	2.1
2719	0.946118	2.1
2720	1.29063	2.1
2721	1.36917	2.1
2722	0.94613	2.1
2723	1.29062	2.1

2724	0.947921	1.90482
2725	1.58661	0.950112
2726	1.37658	1.05868
2727	1.2364	1.03768
2728	1.66685	0.803393
2729	1.66685	1.16432
2730	0.946133	0.781797
2731	0.946133	0.90809
2732	1.11978	1.01406
2733	1.09194	0.842121
2734	1.21858	1.24866
2735	1.37658	0.989709
2736	1.64903	0.814441
2737	1.64903	1.41909
2738	0.946132	0.815716
2739	0.946132	0.912401
2740	1.05496	0.900724
2741	1.22851	0.811443
2742	1.22852	1.28111
2743	1.58482	0.747224
2744	1.60578	0.366063
2745	1.06336	0.347043
2746	1.68062	0.361763
2747	1.14721	0.310733
2748	1.11924	0.2101
2749	1.18421	0.224877
2750	2.01276	0.222057
2751	1.23502	1.71144
2752	1.34926	1.55275
2753	0.546751	1.33519
2754	0.790446	1.71569
2755	1.04072	1.30967
2756	0.555868	1.29628
2757	0.930305	1.2747
2758	1.09344	1.38064
2759	1.18351	1.63056
2760	1.97215	1.36746
2761	1.07416	1.66935
2762	1.33204	1.28292
2763	1.22038	1.28164
2764	0.597449	1.05884
2765	0.821422	1.41903
2766	1.04071	1.20296
2767	0.608026	0.983236
2768	0.929427	1.14639
2769	1.64929	0.202976
2770	1.68541	0.193625
2771	0.496093	0.258213
2772	1.3385	0.220597
2773	0.790798	0.219202
2774	1.22971	0.217649
2775	0.898853	0.222134
2776	2.14361	0.211962
2777	2.10813	0.210305
2778	1.05417	0.245575
2779	1.80768	0.354458
2780	1.09725	2.1
2781	1.41131	2.1

2782	1.1656	2.1
2783	0.946129	2.1
2784	1.68365	2.1
2785	1.68365	2.1
2786	1.1656	2.1
2787	0.94613	2.1
2788	1.21269	0.197954
2789	1.48867	0.210254
2790	1.27806	0.201101
2791	1.68264	0.206583
2792	1.37813	1.30163
2793	1.50743	1.0532
2794	1.04121	1.05608
2795	1.12434	0.813874
2796	1.39553	0.809451
2797	1.44721	1.25434
2798	0.946132	0.855074
2799	1.23943	0.871971
2800	1.05292	0.913002
2801	1.28607	0.815254
2802	1.16915	0.772237
2803	1.76306	1.03108
2804	1.42266	1.01195
2805	1.36844	2.1
2806	1.36844	2.1
2807	1.67352	2.1
2808	1.11256	1.17933
2809	1.03217	1.13863
2810	1.18422	0.236903
2811	1.04159	0.254709
2812	1.60139	0.235116
2813	1.04159	0.239641
2814	0.988805	2.1
2815	1.05481	2.1
2816	1.52938	1.48535
2817	1.94724	1.26612
2818	0.896336	0.354866
2819	0.911484	0.376412
2820	1.18315	0.261773
2821	1.10515	0.304183
2822	1.04067	0.26542
2823	1.12758	0.34089
2824	0.904106	0.340113
2825	1.6901	0.261077
2826	1.68671	0.271308
2827	1.99908	0.294561
2828	0.772426	0.854934
2829	0.810945	1.3089
2830	0.667562	1.18782
2831	0.496617	0.742863
2832	0.847689	1.0436
2833	0.914378	1.23135
2834	0.934003	0.817684
2835	1.07459	0.960139
2836	1.40225	2.1
2837	1.07043	2.1
2838	0.94613	2.1
2839	1.63072	2.1

2840	1.63072	2.1
2841	1.07043	2.1
2842	0.946131	2.1
2843	1.53082	2.1
2844	1.69916	2.1
2845	1.75211	0.8132
2846	1.96831	1.65501
2847	1.75211	0.933866
2848	0.946127	0.946355
2849	0.817065	0.981885
2850	1.41798	0.984573
2851	1.41798	0.917264
2852	0.791656	0.956208
2853	0.791656	0.978494
2854	1.41798	0.95552
2855	1.58331	0.944928
2856	1.41776	0.872628
2857	0.791647	0.8655
2858	1.4512	0.851971
2859	1.45119	0.859321
2860	0.75821	0.889583
2861	0.758248	0.884548
2862	0.946104	0.870466
2863	1.36482	0.875206
2864	1.29389	1.7031
2865	1.04164	1.38214
2866	1.40574	1.37761
2867	1.64833	1.277
2868	1.71183	0.537223
2869	1.71183	0.63389
2870	0.946131	0.838523
2871	1.35721	0.680036
2872	0.946131	0.96705
2873	0.946131	0.847558
2874	1.30076	0.849538
2875	1.2474	0.587596
2876	2.85225	0.759026
2877	2.71441	0.596633
2878	1.2474	0.50292
2879	1.38622	0.719548
2880	0.858359	0.51699
2881	1.3682	0.560305
2882	1.57364	0.50467
2883	0.729695	0.725704
2884	0.729723	0.485723
2885	0.822611	0.828836
2886	0.946133	0.728424
2887	0.729723	0.57823
2888	0.974704	0.828067
2889	0.974702	0.811632
2890	1.81603	2.1
2891	1.96348	2.1
2892	1.43612	1.04608
2893	1.43612	1.11138
2894	0.634295	0.981012
2895	0.93796	1.25114
2896	0.946133	1.05849
2897	0.634326	0.88649

2898	0.946133	0.987626
2899	1.2654	0.826426
2900	1.26865	1.09262
2901	2.2649	0.990538
2902	1.00864	1.04013
2903	1.09689	0.800614
2904	0.832021	0.817961
2905	1.53229	0.785786
2906	1.1145	0.797155
2907	0.929583	0.823122
2908	1.613	0.780513
2909	0.644729	0.810201
2910	1.03471	1.16311
2911	2.7739	2.1
2912	1.36133	1.15731
2913	2.50215	2.1
2914	2.52195	2.01434
2915	1.0465	2.1
2916	1.42673	2.1
2917	1.42673	2.1
2918	0.946132	2.1
2919	1.31904	2.1
2920	0.986499	2.1
2921	0.946132	2.1
2922	1.31904	2.1
2923	0.966978	2.1
2924	1.42237	0.37515
2925	2.60925	0.440339
2926	0.753659	0.489642
2927	1.77241	0.39968
2928	1.77241	0.454429
2929	0.946129	0.509369
2930	0.75366	0.396769
2931	1.22144	0.490912
2932	0.94613	0.509152
2933	1.50732	0.393741
2934	1.18514	0.434786
2935	0.902187	0.470747
2936	1.89858	0.35119
2937	1.24294	0.403562
2938	1.67211	0.428447
2939	1.41786	0.37036
2940	0.810959	0.469819
2941	1.50732	0.401402
2942	2.44288	0.460753
2943	1.40969	0.271555
2944	2.06654	0.304074
2945	1.40969	0.277442
2946	1.40882	0.344546
2947	1.94063	0.365321
2948	0.867921	0.285211
2949	1.29289	0.301122
2950	0.867918	0.287196
2951	0.946128	0.311596
2952	0.867944	0.311777
2953	1.66589	0.299418
2954	1.66589	0.272228
2955	0.968595	2.1

2956	1.5534	1.65744
2957	1.27793	2.1
2958	1.13433	2.1
2959	1.3439	0.206179
2960	2.53709	0.221666
2961	0.571998	0.196884
2962	0.64837	0.207397
2963	1.99755	0.201183
2964	1.99854	0.206752
2965	0.764229	2.03563
2966	1.61842	2.1
2967	1.61842	2.1
2968	1.18315	2.1
2969	1.1995	2.1
2970	1.18315	2.1
2971	0.764256	2.1
2972	1.52364	2.1
2973	2.36631	2.1
2974	0.641912	1.69154
2975	1.7181	2.1
2976	1.459 2.1	
2977	0.634845	1.57458
2978	1.18315	2.09557
2979	1.17615	2.01146
2980	0.571367	2.02318
2981	1.3324	0.548244
2982	0.798214	0.557123
2983	0.946128	0.580828
2984	0.627252	0.525355
2985	1.50337	0.544193
2986	1.50337	0.480127
2987	0.62725	0.583999
2988	1.07763	0.48437
2989	1.07763	0.536745
2990	2.16109	0.601902
2991	1.07763	0.499295
2992	1.12486	2.1
2993	1.11827	2.1
2994	1.0491	2.1
2995	1.09034	2.1
2996	1.09431	2.1
2997	0.946131	2.1
2998	0.748607	2.1
2999	1.44002	2.1
3000	1.44002	2.1
3001	0.748635	2.1
3002	0.782505	2.1
3003	1.40615	2.1
3004	1.19539	2.1
3005	1.29389	1.65259
3006	1.4867	1.12998
3007	1.50103	0.207931
3008	1.63025	0.210056
3009	2.72109	0.247183
3010	0.915755	0.213291
3011	0.946132	0.271918
3012	0.915755	0.260145
3013	1.70236	0.225661

3014	1.70236	0.204823
3015	1.26155	0.247549
3016	1.35657	0.259661
3017	1.83151	0.208565
3018	1.83151	0.210769
3019	2.52309	0.242485
3020	0.983607	2.1
3021	1.06838	0.896161
3022	0.983609	1.10578
3023	0.983596	1.03626
3024	2.74384	0.368583
3025	1.6261	0.320641
3026	1.15477	2.1
3027	1.02136	2.1
3028	1.83561	2.1
3029	1.83561	2.1
3030	0.946131	2.0389
3031	1.39523	2.1
3032	1.29313	1.04006
3033	1.29313	2.02362
3034	1.38651	1.90871
3035	1.06313	2.1
3036	0.974888	2.1
3037	1.0894	2.1
3038	0.974888	2.1
3039	1.82282	2.1
3040	1.82282	2.1
3041	0.946131	2.1
3042	1.37372	2.1
3043	1.32374	2.04424
3044	1.32374	2.1
3045	1.39523	2.1
3046	1.40882	0.352122
3047	0.861998	0.351544
3048	1.24448	0.375367
3049	0.861999	0.346914
3050	0.946129	0.406572
3051	0.862011	0.3964
3052	1.67537	0.379184
3053	1.67537	0.333837
3054	1.72399	0.259871
3055	2.58579	0.278485
3056	1.72399	0.258677
3057	1.50951	0.299346
3058	2.48897	0.336886
3059	1.26755	2.1
3060	1.03824	2.1
3061	1.27875	2.1
3062	1.48901	2.1
3063	1.18902	2.1
3064	1.11764	2.1
3065	1.87436	2.1
3066	0.95345	1.87018
3067	0.724191	1.52403
3068	0.86668	1.50195
3069	0.811523	1.62735
3070	1.04074	1.21691
3071	0.843097	1.38459

3072	0.833434	1.57017
3073	0.965727	1.40765
3074	0.85346	1.53354
3075	1.00185	1.01364
3076	1.00184	1.36299
3077	0.980651	1.56879
3078	0.980664	1.35982
3079	0.475737	1.16237
3080	0.719696	1.18588
3081	0.946109	1.14363
3082	0.946116	1.23333
3083	0.719693	1.13759
3084	0.946117	1.23055
3085	1.14879	1.45803
3086	0.719683	1.39125
3087	0.900977	1.1697
3088	0.972364	1.28825
3089	1.22961	1.25367
3090	0.405384	1.18525
3091	1.34406	2.1
3092	1.2187	2.1
3093	0.963571	0.718837
3094	0.946131	0.712675
3095	1.57246	0.891956
3096	1.57246	1.91583
3097	0.946131	0.912307
3098	1.1235	1.13017
3099	1.1235	1.07353
3100	1.07439	1.20802
3101	1.19612	1.16346
3102	1.24844	1.07399
3103	1.00007	1.33937
3104	1.02429	2.1
3105	1.10459	2.1
3106	1.06724	0.655861
3107	0.67519	0.833815
3108	0.49762	0.678113
3109	1.43435	0.581194
3110	0.833315	0.760712
3111	0.58135	0.730802
3112	1.14749	0.934923
3113	1.85091	1.16227
3114	1.2802	0.893276
3115	1.27737	1.02256
3116	2.3655	1.00144
3117	1.30584	2.1
3118	2.06424	2.1
3119	0.946131	0.3776
3120	1.04257	0.364027
3121	0.94613	0.38621
3122	1.67201	0.447757
3123	1.67201	0.382227
3124	1.27696	0.373385
3125	1.34118	0.339155
3126	1.3413	0.347051
3127	2.36238	0.310224
3128	0.6036	0.400886
3129	1.11745	0.407755

3130	1.46126	0.352066
3131	1.39612	0.312403
3132	0.670678	0.403543
3133	1.03402	0.418934
3134	1.11271	0.396169
3135	0.838028	0.331761
3136	0.807019	0.407177
3137	1.27787	0.284591
3138	0.994705	0.32615
3139	1.20014	0.291443
3140	0.964793	0.383753
3141	0.863868	0.359232
3142	1.3413	0.30292
3143	1.79549	0.317982
3144	1.35877	2.1
3145	1.63648	1.97072
3146	1.08171	2.1
3147	1.27839	2.1
3148	1.52601	2.1
3149	1.27841	2.1
3150	1.71255	2.1
3151	2.16307	2.1
3152	1.91592	2.1
3153	1.78508	2.1
3154	0.946128	2.1
3155	1.41199	2.1
3156	0.946128	2.1
3157	0.901088	2.1
3158	1.55244	2.1
3159	1.59475	2.1
3160	1.40921	2.1
3161	0.960106	2.1
3162	1.40921	2.1
3163	2.0073	2.1
3164	2.2973	0.698625
3165	1.90705	0.703528
3166	1.53671	0.574318
3167	1.86035	0.562882
3168	1.33747	2.00515
3169	1.14007	2.1
3170	1.3212	2.1
3171	1.66867	0.498121
3172	1.30203	0.425162
3173	1.27696	2.1
3174	1.05025	2.1
3175	1.05025	2.1
3176	1.3704	2.1
3177	1.15677	2.1
3178	0.946129	2.1
3179	1.65879	2.1
3180	1.65879	2.1
3181	1.15676	2.1
3182	0.94613	2.1
3183	0.867217	1.05206
3184	1.28684	1.01526
3185	1.58866	1.0405
3186	1.58866	0.897152
3187	0.867246	1.3257

3188	1.28684	0.970444
3189	1.16906	1.03462
3190	1.13438	1.03747
3191	1.37224	1.05785
3192	1.61519	1.12298
3193	0.883939	1.34642
3194	1.40092	1.12271
3195	1.80383	1.49157
3196	1.80383	1.29243
3197	0.883935	1.37362
3198	1.40092	1.072
3199	1.28684	1.33207
3200	1.76788	0.979487
3201	2.57368	1.06927
3202	1.37224	1.49588
3203	1.65411	1.01322
3204	1.77042	2.1
3205	1.77042	2.1
3206	1.90446	2.1
3207	1.28667	2.1
3208	1.55635	2.1
3209	1.30646	0.721172
3210	2.31836	0.937357
3211	1.30645	0.77166
3212	1.86429	1.102
3213	1.48676	0.41413
3214	0.743379	0.52749
3215	1.41848	0.519908
3216	1.41848	0.486505
3217	0.946131	0.548372
3218	0.812649	0.494971
3219	1.34921	0.512229
3220	0.812649	0.471937
3221	0.949366	0.425211
3222	1.32974	0.426025
3223	0.794204	0.147815
3224	0.634352	2.1
3225	0.838025	2.08062
3226	1.08802	1.97043
3227	0.838038	1.91823
3228	1.18314	2.1
3229	1.12495	2.1
3230	1.22774	2.07383
3231	1.04343	2.04639
3232	0.921111	0.182281
3233	2.1609	0.177894
3234	1.09987	0.174237
3235	2.5043	0.169777
3236	1.05492	0.168236
3237	0.590344	0.172972
3238	1.05492	0.169647
3239	1.25215	0.169011
3240	1.71674	0.163123
3241	1.71674	0.1632
3242	0.590331	0.171089
3243	1.17078	0.166789
3244	0.625542	0.170439
3245	0.724673	2.1

3246	1.08011	2.1
3247	1.87598	2.1
3248	1.50738	2.1
3249	1.44951	0.327424
3250	1.50102	0.322293
3251	1.8042	0.745373
3252	0.687769	0.866542
3253	1.66483	0.760992
3254	1.66483	0.812329
3255	0.946132	0.861143
3256	0.687798	0.76547
3257	1.20885	0.861423
3258	0.687799	0.838613
3259	0.886063	0.635887
3260	0.798751	0.642975
3261	0.584813	0.588375
3262	1.49483	0.461049
3263	0.888005	0.501201
3264	1.03589	0.514072
3265	1.70435	0.54748
3266	0.710414	1.98732
3267	0.917609	1.24593
3268	0.810947	1.25278
3269	0.81096	1.24982
3270	1.48658	1.31007
3271	1.53653	1.30679
3272	1.19329	1.31054
3273	0.946153	1.28736
3274	1.50798	1.3076
3275	1.85017	2.1
3276	1.19768	2.1
3277	0.864304	1.61824
3278	0.792906	1.26365
3279	0.989025	1.28445
3280	1.19768	1.3878
3281	1.54548	1.35164
3282	1.18798	1.51264
3283	1.31875	1.22596
3284	1.05725	1.32656
3285	2.17365	0.219889
3286	2.48106	0.205013
3287	1.87451	0.230206
3288	1.27189	1.20638
3289	2.79399	1.63952
3290	0.859565	1.22943
3291	0.926174	1.17541
3292	0.604744	1.13938
3293	1.41832	0.883835
3294	1.05494	1.02024
3295	0.874749	1.06367
3296	1.50067	0.962859
3297	0.917817	1.32124
3298	0.63591	1.29761
3299	1.72849	1.2686
3300	1.72849	1.03709
3301	0.635943	1.14068
3302	1.39699	1.3037
3303	0.967411	1.68523

3304	0.635943	1.20227
3305	1.39908	1.14052
3306	1.26971	1.27477
3307	1.02969	1.15403
3308	1.15326	1.78902
3309	2.30741	1.36818
3310	0.93545	1.17568
3311	0.802747	1.00242
3312	1.15466	1.21224
3313	0.859164	1.39765
3314	1.02616	1.23615
3315	1.23149	1.12907
3316	0.83804	1.06174
3317	1.13266	1.52685
3318	1.64142	1.52901
3319	1.21223	2.1
3320	1.43051	2.1
3321	1.32418	0.894212
3322	0.621319	0.993168
3323	1.47134	1.36027
3324	1.79103	0.813014
3325	3.25951	0.334891
3326	3.16764	0.298048
3327	1.65583	0.338332
3328	1.4732	0.396092
3329	1.76476	2.1
3330	1.80114	1.15023
3331	0.754121	1.6028
3332	0.94613	0.900163
3333	0.754119	1.60755
3334	1.45636	1.58205
3335	1.45636	0.830181
3336	1.30029	1.05923
3337	0.910183	1.02915
3338	1.29099	1.48158
3339	1.08039	2.03906
3340	1.05498	1.81564
3341	1.24445	1.57264
3342	1.2873	2.1
3343	0.987703	1.6724
3344	0.964782	1.49989
3345	1.73645	0.343062
3346	0.871279	0.421115
3347	0.884744	0.456957
3348	1.85193	0.333493
3349	1.68681	0.38399
3350	1.33689	0.415599
3351	1.23493	0.348093
3352	0.892482	0.4512
3353	2.95185	0.375456
3354	2.10496	0.377644
3355	1.87334	2.1
3356	0.94411	2.1
3357	0.754196	2.1
3358	1.43122	2.1
3359	1.58684	2.1
3360	0.838027	2.1
3361	1.1536	2.1

3362	1.24878	2.1
3363	1.23125	2.1
3364	2.86862	2.1
3365	1.57636	2.1
3366	0.998451	0.840954
3367	1.5176	0.812466
3368	1.13271	0.248998
3369	1.60248	0.247936
3370	1.22083	2.1
3371	1.79996	2.1
3372	1.19087	2.1
3373	2.00342	2.1
3374	1.3715	2.1
3375	2.79227	2.1
3376	2.7873	2.1
3377	0.987265	0.175533
3378	1.62715	2.07128
3379	1.70117	1.59013
3380	1.27806	0.18997
3381	1.45246	2.1
3382	1.45244	2.1
3383	1.87214	2.1
3384	0.995796	2.1
3385	1.68189	0.732199
3386	1.49155	0.921854
3387	1.58054	2.1
3388	1.59169	2.1
3389	0.736364	0.215522
3390	0.70236	0.214185
3391	2.24367	0.22769
3392	2.57085	0.231844
3393	1.70952	0.221707
3394	2.60516	0.224901
3395	1.45158	2.1
3396	1.00371	2.1
3397	1.65748	2.1
3398	1.23706	0.605081
3399	1.55834	0.804551
3400	1.72671	0.780458
3401	0.597656	0.728042
3402	1.39137	0.791821
3403	0.855578	0.564845
3404	1.48398	0.749788
3405	0.745117	0.700873
3406	1.23706	0.742172
3407	1.57916	0.589579
3408	0.893025	0.570695
3409	3.22433	0.733423
3410	2.47324	0.879437
3411	1.63852	2.1
3412	1.63852	2.1
3413	0.946132	2.1
3414	1.19095	2.1
3415	0.946133	2.1
3416	1.16684	2.1
3417	1.19095	2.1
3418	1.06698	0.493518
3419	1.48692	0.455063

3420	1.08334	1.03756
3421	1.07283	2.1
3422	1.28517	0.983796
3423	1.2746	0.210376
3424	1.30723	0.213186
3425	2.02069	0.216469
3426	1.05579	0.210598
3427	1.59475	2.1
3428	2.40113	2.1
3429	1.58875	2.1
3430	1.6881	2.1
3431	1.6881	2.1
3432	0.819953	2.1
3433	0.819954	2.1
3434	0.946131	2.1
3435	0.946131	2.1
3436	1.34782	2.1
3437	1.65619	2.1
3438	1.65619	2.1
3439	0.79437	2.1
3440	1.25	2.1
3441	0.94613	2.1
3442	0.946131	2.09135
3443	0.794371	2.1
3444	1.58875	2.1
3445	2.5	2.1
3446	1.04147	2.0447
3447	0.946131	0.908799
3448	1.79686	1.86195
3449	1.79686	1.9848
3450	1.25144	0.91668
3451	1.35714	1.30469
3452	1.38585	1.29298
3453	1.25144	1.71394
3454	1.15564	1.69511
3455	1.34389	1.47111
3456	0.946133	1.0752
3457	1.74353	1.52594
3458	1.74353	2.01454
3459	1.14553	1.02381
3460	1.38585	1.03081
3461	1.30382	1.10577
3462	1.14553	1.52165
3463	1.15564	1.45677
3464	1.90618	2.1
3465	2.11056	2.1
3466	1.032	2.1
3467	0.953636	2.1
3468	1.23131	1.32183
3469	1.39763	1.3177
3470	2.01024	1.11057
3471	1.9186	1.12846
3472	1.83658	0.453664
3473	1.81656	0.440142
3474	2.2049	0.491029
3475	2.16081	0.463971
3476	1.12219	0.492484
3477	0.599771	0.540567

3478	1.61401	0.436911
3479	1.05912	0.488657
3480	0.951648	0.492065
3481	1.89328	0.439768
3482	0.913682	0.529734
3483	1.00763	0.451278
3484	1.29129	2.1
3485	1.37138	2.1
3486	1.41986	0.354908
3487	1.41986	0.354939
3488	0.944166	0.450999
3489	1.41789	0.409769
3490	0.944167	0.471207
3491	0.944166	0.396129
3492	0.946132	0.382498
3493	1.98713	0.282516
3494	1.23626	0.28364
3495	0.932791	0.374488
3496	0.810251	0.27681
3497	0.986748	0.292269
3498	0.898155	0.366969
3499	0.92624	0.287497
3500	1.1349	0.277222
3501	0.934572	0.327003
3502	1.23104	1.42109
3503	1.23104	1.41169
3504	0.799994	0.878562
3505	0.94613	1.16796
3506	0.802822	0.740843
3507	1.40944	1.29882
3508	0.800035	1.03237
3509	1.41223	1.12337
3510	1.41223	1.12457
3511	1.6	0.975988
3512	1.60565	0.974849
3513	1.23042	2.1
3514	1.0645	2.00057
3515	1.00319	2.1
3516	1.29435	2.1
3517	1.2243	2.1
3518	1.2243	2.1
3519	1.05655	1.54593
3520	1.29764	1.68091
3521	2.55252	2.1
3522	1.15676	2.1
3523	0.855295	2.1
3524	0.810959	2.1
3525	1.65857	2.1
3526	1.00849	2.1
3527	1.16021	2.1
3528	1.75869	2.1
3529	1.13775	2.1
3530	1.20091	2.1
3531	1.61582	2.1
3532	1.88031	2.1
3533	0.968536	2.1
3534	0.946131	2.1
3535	1.61481	2.1

3536	1.61481	2.1
3537	1.09547	2.1
3538	1.18486	2.1
3539	1.37609	2.1
3540	1.09546	2.1
3541	1.02461	2.1
3542	1.83223	2.1
3543	1.74002	2.1
3544	1.70939	1.7303
3545	1.7094	2.1
3546	0.726287	2.06615
3547	1.01712	1.65131
3548	1.30318	2.1
3549	0.946131	2.1
3550	0.87155	2.1
3551	1.77093	1.8054
3552	2.02356	1.8016
3553	1.32449	2.1
3554	1.10003	2.1
3555	1.5161	2.1
3556	1.5161	2.1
3557	0.946132	2.1
3558	1.09206	2.1
3559	0.946132	2.1
3560	1.04404	2.1
3561	1.09206	2.1
3562	0.635628	0.984738
3563	1.14623	0.796894
3564	1.73197	0.864245
3565	1.73197	1.06078
3566	0.529655	0.848524
3567	1.14623	0.893913
3568	0.992085	0.934541
3569	1.16915	1.26136
3570	2.29245	0.789649
3571	0.584573	0.917368
3572	0.956756	0.768981
3573	1.52471	0.790755
3574	1.51409	1.24004
3575	0.584575	0.766269
3576	0.94613	0.86083
3577	1.14622	0.777479
3578	1.28096	1.03063
3579	1.34695	1.15731
3580	1.04193	2.1
3581	1.05378	2.1
3582	0.798209	2.1
3583	1.32007	1.84787
3584	1.64327	0.228758
3585	2.64674	1.35752
3586	2.38936	0.420088
3587	2.88475	0.403319
3588	1.07745	1.78894
3589	1.08911	1.86087
3590	1.49127	2.1
3591	1.49127	2.1
3592	0.709714	2.1
3593	1.37199	2.1

3594	0.82899	2.1
3595	0.709714	2.1
3596	0.946132	2.1
3597	1.00133	2.1
3598	1.40182	2.1
3599	1.53278	2.1
3600	1.40182	2.1
3601	1.31764	0.23838
3602	1.00691	0.242443
3603	1.24094	0.254787
3604	0.64868	0.241805
3605	0.946133	0.261126
3606	0.64868	0.261756
3607	1.71762	0.250898
3608	1.71762	0.236197
3609	1.41662	0.225957
3610	0.975477	0.217656
3611	1.73584	0.232878
3612	2.48188	0.240188
3613	2.2045	0.78276
3614	2.08134	0.774411
3615	0.993366	0.602168
3616	0.993368	0.851477
3617	1.14187	0.725128
3618	0.946132	0.753668
3619	0.946133	0.838886
3620	1.68951	0.816226
3621	1.68951	0.869218
3622	1.34612	0.782249
3623	1.28953	0.740695
3624	1.34611	0.907574
3625	1.31892	0.855682
3626	0.946131	0.832652
3627	1.80988	0.811566
3628	1.80988	1.51219
3629	1.31892	0.863241
3630	0.946131	0.858803
3631	2.13214	2.1
3632	1.94691	2.1
3633	1.52661	0.202758
3634	1.53175	0.209082
3635	0.956005	0.199901
3636	0.955991	0.200867
3637	1.40572	0.217766
3638	0.955992	0.202584
3639	1.51084	1.06965
3640	1.67115	1.17156
3641	1.73143	2.1
3642	1.55234	2.1
3643	1.69377	2.1
3644	2.51483	2.1
3645	1.25987	2.1
3646	1.4472	2.1
3647	1.83151	0.842376
3648	2.01001	1.20229
3649	0.993998	0.87877
3650	1.83151	0.79014
3651	1.40915	0.549819

3652	1.24653	0.661547
3653	1.74472	2.1
3654	1.74472	2.1
3655	1.74244	2.1
3656	1.04788	1.85997
3657	1.28522	1.52925
3658	1.03785	2.1
3659	0.896519	2.1
3660	1.60428	2.1
3661	1.73305	2.1
3662	1.39072	0.675859
3663	2.27501	0.917693
3664	1.39073	0.836383
3665	1.39073	0.791255
3666	1.06838	1.82746
3667	0.968462	0.989963
3668	1.06837	1.39162
3669	1.13455	1.26301
3670	1.23527	0.764331
3671	2.71686	0.734266
3672	1.86881	0.757995
3673	1.10586	1.14785
3674	1.03852	1.25318
3675	1.03899	2.1
3676	0.963973	2.1
3677	1.00319	2.1
3678	1.00319	2.1
3679	0.987132	2.1
3680	0.974889	2.1
3681	0.987131	2.1
3682	1.7677	2.1
3683	1.7677	2.1
3684	0.946131	2.1
3685	1.31185	2.1
3686	0.946132	2.1
3687	1.30203	2.1
3688	1.31185	2.1
3689	1.82958	2.1
3690	1.82958	2.1
3691	0.946132	2.1
3692	1.40199	2.1
3693	0.946133	2.1
3694	1.36994	2.1
3695	1.40199	2.1
3696	0.97489	2.1
3697	1.05939	1.67216
3698	1.01922	1.64996
3699	0.810941	1.40189
3700	1.05372	1.93375
3701	0.754065	1.97039
3702	0.926131	1.69619
3703	1.35905	1.66632
3704	0.810931	1.57404
3705	1.75647	1.14131
3706	1.68265	2.1
3707	1.41995	2.1
3708	1.36457	1.21343
3709	0.933392	1.17285

3710	1.42255	2.1
3711	1.3868	2.1
3712	1.08074	2.1
3713	1.07196	2.1
3714	1.13537	0.849897
3715	1.01179	0.770159
3716	1.60196	0.729664
3717	1.72798	0.729833
3718	1.3715	0.738682
3719	1.56409	0.986532
3720	1.56409	0.799202
3721	1.97819	1.02157
3722	1.47954	0.84617
3723	0.798655	0.57773
3724	1.47952	0.792081
3725	2.30327	0.629799
3726	1.49111	2.1
3727	1.96013	1.82443
3728	1.54599	2.1
3729	2.06632	2.1
3730	1.54601	2.1
3731	1.10557	0.768546
3732	1.17624	0.716082
3733	1.60497	2.1
3734	1.61587	2.1
3735	1.65977	0.213065
3736	1.69044	0.216586
3737	1.00659	2.1
3738	1.06226	2.1
3739	1.15171	2.1
3740	1.50853	2.1
3741	1.80252	2.1
3742	1.41432	2.1
3743	1.04687	2.1
3744	0.940878	2.1
3745	1.52031	2.1
3746	1.52031	2.1
3747	0.940877	2.1
3748	0.946132	2.1
3749	1.50853	2.07092
3750	0.963973	2.1
3751	1.70562	0.304703
3752	2.43203	0.294789
3753	1.36004	0.273931
3754	1.06648	0.279016
3755	1.66009	0.236928
3756	1.66009	0.266087
3757	0.766427	0.258391
3758	0.766452	0.246204
3759	0.946132	0.281928
3760	1.98012	0.233739
3761	1.20357	2.1
3762	1.20356	2.1
3763	1.67324	2.1
3764	1.52008	2.1
3765	1.19964	2.1
3766	1.36861	2.1
3767	1.19964	2.1

3768	2.85415	2.1
3769	1.43687	2.1
3770	0.935852	2.1
3771	1.09972	2.1
3772	0.810933	2.00281
3773	2.25974	2.1
3774	1.74149	2.1
3775	1.36984	2.1
3776	1.87602	2.1
3777	0.890748	2.1
3778	1.20587	1.91463
3779	0.810956	1.74769
3780	2.03174	2.00422
3781	1.34403	2.03529
3782	1.58825	1.96828
3783	1.63096	1.93435
3784	2.53735	2.1
3785	1.98241	2.04124
3786	2.53014	2.1
3787	1.1775	1.12361
3788	1.06538	1.00595
3789	0.946132	1.04397
3790	1.4101	1.52197
3791	1.75162	1.85511
3792	1.75162	0.990232
3793	0.946132	1.04344
3794	1.4101	0.990185
3795	1.28765	1.01894
3796	1.1427	0.947591
3797	1.1427	0.836344
3798	1.3109	2.1
3799	1.02721	1.43891
3800	0.846447	1.56506
3801	0.862076	2.1
3802	0.644098	1.60846
3803	0.581308	1.61924
3804	1.02306	1.49085
3805	0.322641	1.07459
3806	0.589346	1.08405
3807	2.25572	1.38796
3808	1.31372	2.1
3809	1.18873	2.1
3810	1.78225	2.1
3811	1.764 2.1	
3812	1.17503	2.1
3813	1.06821	2.1
3814	1.06821	1.79081
3815	1.7777	1.93754
3816	1.9096	0.900663
3817	1.70239	1.00446
3818	1.16182	0.242564
3819	1.20132	0.260934
3820	1.23475	2.1
3821	1.56859	1.25689
3822	2.46263	2.1
3823	0.528744	2.1
3824	2.93337	2.1
3825	2.42504	2.1

3826	1.38646	0.489912
3827	1.61963	0.583914
3828	1.88016	0.748489
3829	0.622283	0.885535
3830	1.03243	0.782142
3831	0.710307	1.07225
3832	0.798124	0.861349
3833	0.931073	0.898411
3834	0.892862	1.29001
3835	1.06791	0.787824
3836	0.552787	0.952359
3837	0.946132	0.786468
3838	1.36665	0.869518
3839	1.36665	1.17724
3840	0.506966	0.806104
3841	0.506964	1.15095
3842	1.36665	0.808999
3843	0.653469	0.80219
3844	1.36665	0.701908
3845	1.21339	0.753676
3846	1.21339	1.00105
3847	0.552757	0.750808
3848	0.552786	0.987731
3849	1.21336	0.724081
3850	1.70744	1.38771
3851	1.0775	0.619218
3852	1.59527	0.910347
3853	1.48312	0.907669
3854	2.01868	0.766755
3855	1.18947	0.488542
3856	1.48312	0.908365
3857	0.785626	0.509322
3858	1.19601	0.553882
3859	0.785626	0.576424
3860	0.946132	0.54521
3861	0.7856	0.628301
3862	1.59087	0.573947
3863	1.51158	0.579834
3864	1.74098	0.587661
3865	2.39202	0.540022
3866	0.870489	0.683486
3867	1.34534	0.797141
3868	0.870488	0.579107
3869	0.946133	0.817516
3870	0.870489	0.915305
3871	1.67086	0.839808
3872	1.67086	0.854255
3873	2.11311	1.81507
3874	1.98765	1.21521
3875	0.946133	0.939314
3876	1.30881	1.56896
3877	1.72211	1.9147
3878	1.72211	1.14083
3879	0.94613	1.40938
3880	1.30881	1.17727
3881	1.35943	1.00882
3882	1.01294	0.477537
3883	1.01294	0.229039

3884	1.38963	0.873301
3885	1.01294	0.529936
3886	1.60056	0.49204
3887	1.60056	0.584128
3888	0.946132	0.797834
3889	1.204	0.576336
3890	0.946132	0.461502
3891	1.11288	0.416606
3892	1.204	0.499226
3893	1.14599	0.927109
3894	1.00065	0.897943
3895	1.74685	0.940281
3896	1.74685	1.82354
3897	0.946128	0.925254
3898	1.38463	0.821252
3899	1.2275	1.99944
3900	1.2275	1.05592
3901	1.30835	1.1022
3902	2.04407	2.1
3903	2.50425	2.1
3904	1.19647	0.768054
3905	1.19647	0.776278
3906	1.19645	0.813158
3907	1.75446	0.762672
3908	1.1315	0.76112
3909	1.1315	0.771358
3910	1.45569	0.703146
3911	1.1315	0.731983
3912	1.14357	2.1
3913	1.25793	2.1
3914	1.14357	2.1
3915	2.08181	2.1
3916	1.4714	2.1
3917	0.71295	2.06564
3918	1.68285	2.1
3919	1.68285	2.1
3920	1.25603	2.1
3921	1.13976	2.1
3922	1.25603	2.1
3923	0.71295	2.1
3924	1.61095	2.1
3925	2.51207	2.1
3926	1.63762	2.1
3927	2.74451	2.1
3928	0.671255	1.66117
3929	1.87601	2.02577
3930	1.87601	2.08133
3931	0.671226	1.65519
3932	1.25603	1.9518
3933	1.29123	1.97848
3934	0.671226	1.95152
3935	1.63723	2.1
3936	0.872928	0.268853
3937	1.47904	0.32708
3938	1.18793	0.308051
3939	2.57464	0.328553
3940	1.14986	1.61423
3941	1.19611	1.61555

3942	0.96029	0.759281
3943	1.7961	2.1
3944	0.928721	2.1
3945	1.34352	2.1
3946	1.37935	2.1
3947	1.37935	2.1
3948	0.892888	2.1
3949	0.946131	2.1
3950	0.892888	2.1
3951	1.75422	2.1
3952	1.05556	2.1
3953	1.27362	0.854546
3954	1.27421	0.544163
3955	1.3003	1.00375
3956	1.31415	0.971587
3957	1.01431	2.1
3958	0.673951	2.1
3959	1.63384	1.98494
3960	1.63661	2.1
3961	1.52245	2.1
3962	1.50714	2.1
3963	0.996778	0.228511
3964	1.56091	0.253188
3965	1.06543	2.1
3966	2.66689	2.1
3967	1.01474	2.1
3968	2.76422	2.1
3969	1.45321	0.702389
3970	0.974716	0.573235
3971	0.810943	0.582345
3972	0.909982	0.705366
3973	0.937793	0.627002
3974	1.05138	0.592368
3975	1.25044	0.599264
3976	0.92095	0.588298
3977	0.830414	0.661379
3978	2.1402	0.635423
3979	1.75938	2.1
3980	1.05556	2.1
3981	1.78558	2.1
3982	1.379 2.1	
3983	0.970214	0.764148
3984	0.9702	0.758718
3985	1.08894	1.13773
3986	2.17251	1.09779
3987	1.51569	1.14662
3988	1.06012	1.10074
3989	0.98966	1.11584
3990	1.55926	0.849431
3991	1.92645	0.995349
3992	1.37427	0.285047
3993	2.16338	0.323283
3994	1.5456	0.261746
3995	1.5456	0.239676
3996	1.94002	0.258199
3997	1.33135	0.29221
3998	1.31948	0.336958
3999	2.23255	0.305003

4000	2.34061	0.294039
4001	1.52903	2.1
4002	3.38327	2.1
4003	1.35977	2.1
4004	2.22414	2.1
4005	1.6812	0.375065
4006	2.33397	0.403108
4007	1.16699	0.473147
4008	0.946131	0.462574
4009	1.58115	0.474088
4010	1.58115	0.438256
4011	0.519726	0.600384
4012	0.946131	0.513873
4013	0.519697	0.492275
4014	1.26868	0.391507
4015	1.16699	0.403019
4016	1.5951	0.364727
4017	1.5951	0.340279
4018	0.840568	0.456823
4019	0.946131	0.40739
4020	0.840598	0.393105
4021	1.30047	2.1
4022	1.5586	2.1
4023	1.31825	1.00646
4024	1.31924	0.979898
4025	1.15818	1.33646
4026	1.52782	0.886786
4027	1.15818	1.0161
4028	1.15818	1.03534
4029	0.99518	0.673153
4030	0.94613	0.618369
4031	0.922045	0.695762
4032	1.29322	0.67983
4033	1.29321	0.544895
4034	1.29321	0.67929
4035	0.922056	0.692588
4036	1.06848	0.7
4037	1.84409	0.698258
4038	1.9186	1.22137
4039	2.72244	1.04733
4040	0.946132	0.94652
4041	1.36122	1.06484
4042	1.79658	1.06203
4043	1.79658	0.949053
4044	0.946133	1.16623
4045	1.36122	1.01291
4046	1.38149	0.949909
4047	1.59106	0.950104
4048	0.946116	0.965695
4049	1.29052	0.995109
4050	1.76641	0.957685
4051	1.76641	0.934193
4052	0.885309	1.15265
4053	1.29054	1.01247
4054	1.36118	0.958773
4055	1.59103	0.998369
4056	2.27816	0.898422
4057	1.56164	0.950699

4058	1.41114	0.173474
4059	2.04128	0.162175
4060	0.986723	2.1
4061	1.03645	0.433698
4062	1.79224	0.386221
4063	1.2627	2.1
4064	1.23036	2.1
4065	1.45257	2.1
4066	1.41469	2.1
4067	1.41469	2.1
4068	2.60636	2.1
4069	1.45789	0.217868
4070	0.851541	0.221619
4071	1.57877	0.222274
4072	1.53134	0.196871
4073	0.851479	0.213668
4074	1.08982	0.232534
4075	0.716284	0.202886
4076	0.946131	0.235946
4077	1.73193	0.21048
4078	2.57309	0.224564
4079	1.36307	0.192484
4080	2.42175	0.199308
4081	1.72948	0.201001
4082	0.978698	2.1
4083	0.978699	2.1
4084	2.08398	2.1
4085	0.978698	2.1
4086	1.44096	2.1
4087	1.44096	2.1
4088	0.638164	2.1
4089	0.681588	2.1
4090	0.946133	2.1
4091	0.638165	2.1
4092	0.94613	2.1
4093	1.55432	2.1
4094	0.901147	2.1
4095	0.85777	2.1
4096	1.36073	2.1
4097	1.44004	2.1
4098	0.946133	2.1
4099	1.09982	2.1
4100	1.27562	2.1
4101	1.55432	2.1
4102	1.62229	2.1
4103	1.0101	2.1
4104	2.28459	1.92822
4105	2.50554	1.65313
4106	0.946131	1.06005
4107	1.25276	1.87037
4108	1.73721	1.95882
4109	1.73721	1.21469
4110	0.93406	1.54933
4111	1.25277	1.33344
4112	1.4185	1.18432
4113	1.53064	1.20467
4114	0.946133	1.22735
4115	0.609462	1.94701

4116	1.65123	1.938
4117	1.65123	1.48823
4118	0.609466	1.82217
4119	1.00792	1.55443
4120	1.25277	1.36233
4121	1.53064	1.61188
4122	2.15223	1.19983
4123	1.30664	1.56171
4124	1.10674	2.1
4125	1.22862	2.1
4126	1.49358	0.660106
4127	2.0207	0.504159
4128	2.09374	2.1
4129	1.88175	2.1
4130	1.10938	0.380332
4131	1.02478	0.690907
4132	1.13272	0.26332
4133	1.13272	0.247977
4134	1.03935	1.49492
4135	0.810945	1.65323
4136	0.527573	1.14701
4137	0.925011	1.39741
4138	1.03193	1.27523
4139	1.38587	1.37014
4140	0.703469	1.30105
4141	0.912282	1.48861
4142	1.75505	2.1
4143	1.78529	2.1
4144	1.60017	0.976672
4145	1.35608	0.780277
4146	1.01479	0.989681
4147	1.47561	0.759226
4148	1.01479	0.975946
4149	1.28418	0.732297
4150	2.62605	2.1
4151	1.71233	2.1
4152	3.22222	2.1
4153	1.97526	2.1
4154	0.949712	0.688491
4155	1.78986	0.676423
4156	2.72879	0.626133
4157	2.66428	0.661119
4158	1.68108	0.720178
4159	1.67265	0.704738
4160	2.24877	0.718152
4161	1.15558	0.746829
4162	1.39708	2.1
4163	1.39327	2.1
4164	1.69788	0.350223
4165	1.1273	0.327073
4166	1.17302	0.664317
4167	1.04349	0.54259
4168	0.979853	2.1
4169	1.19094	2.1
4170	1.39408	2.1
4171	1.61857	2.1
4172	1.66523	2.1
4173	0.946132	2.1

4174	0.838554	2.1
4175	1.05151	2.1
4176	1.18901	2.1
4177	1.18901	2.1
4178	1.61149	2.1
4179	1.22857	0.827199
4180	1.25848	0.822456
4181	1.09154	2.1
4182	2.20358	2.1
4183	1.23013	2.1
4184	1.55698	2.1
4185	1.55828	1.6761
4186	1.52391	1.92792
4187	0.60346	1.39157
4188	1.06336	1.97525
4189	0.870714	1.20042
4190	0.543109	1.39654
4191	1.10367	1.20791
4192	1.20686	1.37599
4193	1.20684	1.89389
4194	2.5118	1.29393
4195	1.20684	1.95588
4196	1.52468	1.51361
4197	1.52469	1.72498
4198	0.532308	1.42141
4199	0.903301	1.86227
4200	0.946119	1.26329
4201	0.532315	1.33717
4202	0.946107	1.34247
4203	1.46129	2.1
4204	0.804766	2.1
4205	1.4809	2.1
4206	1.4809	2.1
4207	1.11822	2.1
4208	1.16744	2.1
4209	1.11822	2.1
4210	0.804737	2.1
4211	1.04193	2.1
4212	0.983723	2.1
4213	1.8014	2.1
4214	1.1729	2.1
4215	1.03951	2.1
4216	1.03951	2.1
4217	1.17583	2.1
4218	1.42866	2.1
4219	1.78503	2.1
4220	1.17822	0.967955
4221	1.89121	2.1
4222	1.44472	2.1
4223	1.62143	2.1
4224	1.43837	2.1
4225	1.01121	0.423189
4226	0.962703	0.899391
4227	0.946129	0.738138
4228	1.10941	0.614258
4229	0.946131	0.758454
4230	1.60326	0.854238
4231	1.60326	0.659226

4232	1.14215	0.728861
4233	1.40724	0.765837
4234	0.962703	0.788205
4235	1.53336	0.344601
4236	1.40272	0.328635
4237	1.64984	0.296382
4238	1.67345	0.31409
4239	1.08212	2.1
4240	1.40905	2.1
4241	0.946132	0.211845
4242	0.946132	0.208785
4243	0.81268	0.23945
4244	0.946131	0.211328
4245	0.812681	0.2234
4246	0.946131	0.223065
4247	0.946132	0.226868
4248	0.997755	0.206247
4249	1.94585	0.19691
4250	1.2925	0.199724
4251	2.52454	0.207372
4252	1.69182	0.201281
4253	1.69182	0.196713
4254	0.92361	0.227114
4255	1.35316	0.20918
4256	1.26227	0.214665
4257	0.946131	0.21786
4258	1.35316	0.206406
4259	1.48676	0.343716
4260	1.33917	0.339909
4261	0.757292	2.1
4262	1.02415	2.1
4263	1.48495	2.1
4264	0.97587	2.1
4265	0.946132	2.1
4266	0.747365	2.1
4267	1.55432	2.1
4268	1.55432	2.1
4269	0.946132	2.1
4270	0.747394	2.1
4271	1.17464	0.31258
4272	1.0372	0.315069
4273	2.16553	0.232153
4274	2.00616	0.290298
4275	1.12184	0.291232
4276	1.33177	0.298155
4277	0.936674	0.276541
4278	0.946129	0.30175
4279	0.851552	0.326382
4280	1.60205	0.293521
4281	1.77391	0.286431
4282	1.03327	0.393306
4283	1.41512	0.420604
4284	1.03327	0.357331
4285	0.946133	0.426161
4286	0.946131	0.444112
4287	1.80077	0.433009
4288	1.80077	0.412092
4289	1.54848	2.1

4290	1.29931	2.1
4291	1.04482	0.714021
4292	2.08069	0.723806
4293	0.946127	0.703987
4294	0.515539	0.731489
4295	0.515566	0.757504
4296	1.38423	0.70668
4297	1.38423	0.706101
4298	0.972058	0.730989
4299	0.927737	0.737597
4300	0.94971	0.657474
4301	2.19682	0.687045
4302	0.946132	0.723387
4303	1.09841	0.688753
4304	0.522382	0.735255
4305	1.5962	0.645658
4306	1.72791	0.637492
4307	1.07428	0.702254
4308	1.05215	0.697422
4309	1.54632	1.26301
4310	1.15693	1.33606
4311	1.41238	2.1
4312	1.41238	2.1
4313	1.77966	2.1
4314	1.77966	2.1
4315	0.946129	2.1
4316	1.40525	2.1
4317	0.946131	2.1
4318	0.94613	2.1
4319	1.32054	2.1
4320	1.41238	2.1
4321	1.94026	2.1
4322	1.0171	0.759324
4323	1.1211	0.71671
4324	0.993022	2.1
4325	0.773813	2.1
4326	1.4073	2.1
4327	1.4073	2.1
4328	0.94613	2.1
4329	0.787615	2.1
4330	0.94613	2.1
4331	0.787615	2.1
4332	1.764	2.1
4333	1.90964	2.1
4334	1.05787	2.1
4335	1.403	0.766713
4336	0.744189	0.816715
4337	2.32834	2.1
4338	2.34986	2.1
4339	1.17106	1.09853
4340	1.94826	0.381854
4341	2.40606	0.340747
4342	1.26387	0.400209
4343	0.954013	0.411397
4344	0.946131	0.447593
4345	1.71	0.318527
4346	1.51926	0.305775
4347	0.953997	0.316831

4348	0.851554	0.414536
4349	1.96502	0.339606
4350	1.72954	0.395867
4351	2.89117	0.350743
4352	0.971958	0.407145
4353	0.985575	0.451682
4354	1.41943	0.326796
4355	1.20021	0.374044
4356	1.29599	0.401512
4357	1.15687	0.342322
4358	0.810958	0.44425
4359	1.12919	0.204375
4360	1.98859	2.1
4361	1.82044	2.1
4362	1.90488	2.1
4363	0.851542	2.1
4364	1.02973	2.1
4365	1.39286	2.1
4366	1.44873	2.1
4367	0.946132	2.1
4368	1.11826	2.1
4369	1.26842	2.1
4370	2.09461	2.1
4371	1.76952	0.211167
4372	1.6473	0.196502
4373	0.946131	0.281901
4374	1.36486	0.239203
4375	1.04243	0.24723
4376	1.05017	0.249084
4377	1.24059	0.245565
4378	2.11646	0.205612
4379	1.50368	0.242213
4380	2.38796	0.222684
4381	1.50368	0.228716
4382	1.71844	0.230213
4383	1.71844	0.216728
4384	0.914836	0.315036
4385	1.24416	0.269362
4386	0.914805	0.27975
4387	1.24416	0.267068
4388	1.38911	0.262187
4389	1.46859	0.288127
4390	1.35348	0.290279
4391	0.946131	0.352515
4392	1.1226	0.306551
4393	0.857078	0.379469
4394	0.9746	0.330265
4395	1.37624	0.329964
4396	1.15131	0.235058
4397	1.139	0.243618
4398	1.90456	0.242022
4399	2.09692	0.282824
4400	1.16655	2.1
4401	1.23017	2.1
4402	2.14592	2.1
4403	1.23017	2.1
4404	1.0752	1.21845
4405	1.52482	0.750801

4406	1.09252	2.1
4407	1.10725	2.1
4408	1.14305	0.437523
4409	0.851541	0.497022
4410	1.48073	0.44532
4411	1.45607	0.349216
4412	0.946131	0.520928
4413	0.932823	0.353579
4414	1.45828	0.475616
4415	0.851528	0.473703
4416	1.14305	0.431518
4417	1.92737	0.542915
4418	1.08211	0.5195
4419	1.69831	0.147815
4420	1.69831	0.147815
4421	0.946131	0.147815
4422	1.26868	0.147815
4423	1.23377	0.147815
4424	1.23377	0.147815
4425	0.94723	0.147815
4426	1.64322	0.174675
4427	1.64322	0.165444
4428	0.946131	0.581078
4429	1.32068	0.180221
4430	0.946129	0.180221
4431	1.18789	0.61352
4432	1.32068	0.187335
4433	1.32813	2.1
4434	1.32461	2.1
4435	1.42729	0.363545
4436	2.47846	0.445351
4437	1.00024	0.525484
4438	1.23923	0.437731
4439	0.946131	0.530178
4440	0.712304	0.424187
4441	1.77501	0.544914
4442	1.77501	0.373012
4443	0.593593	0.628463
4444	1.23923	0.552048
4445	1.23175	0.482618
4446	0.94613	0.585811
4447	0.713646	0.46775
4448	1.75735	0.579772
4449	1.75735	0.397658
4450	0.713633	0.678323
4451	1.37157	0.44384
4452	1.47169	0.413036
4453	2.21808	0.524791
4454	1.47169	0.501965
4455	1.04759	2.1
4456	0.987132	2.1
4457	1.11181	2.08689
4458	2.61682	1.55538
4459	1.11181	2.08299
4460	1.06461	1.24725
4461	1.69733	2.1
4462	2.79705	2.1
4463	1.08245	2.1

4464	1.40583	2.00268
4465	1.39852	1.88848
4466	2.0239	1.80456
4467	2.0239	1.91441
4468	0.780455	2.1
4469	0.946132	2.04636
4470	0.780455	2.04999
4471	1.01885	2.1
4472	0.564217	2.1
4473	0.733461	2.1
4474	0.537126	2.1
4475	0.683838	2.1
4476	0.779662	2.1
4477	0.634141	2.1
4478	0.685337	2.1
4479	1.58848	1.68047
4480	1.17129	0.402515
4481	0.663168	0.554887
4482	1.55587	0.490706
4483	1.31342	0.450738
4484	0.596868	0.54856
4485	1.1666	0.584129
4486	0.867261	0.602875
4487	0.946132	0.639024
4488	1.28843	0.457225
4489	1.31248	0.543854
4490	2.59921	0.503014
4491	1.32634	0.3748
4492	1.35564	0.212265
4493	1.52661	0.22457
4494	1.12323	0.181313
4495	1.17701	1.1805
4496	1.36405	1.15296
4497	1.19837	1.1373
4498	2.25636	1.08072
4499	1.0823	0.408464
4500	1.31774	0.506885
4501	1.0823	0.479744
4502	1.01121	0.365135
4503	1.27408	0.446308
4504	0.946131	0.575701
4505	0.566074	0.513522
4506	0.79576	0.54411
4507	1.24596	0.554282
4508	1.47567	0.564252
4509	1.47567	0.414034
4510	0.56605	0.544592
4511	1.6567	0.454489
4512	0.967792	0.424544
4513	1.00366	0.536707
4514	1.00366	0.50593
4515	1.56409	0.257543
4516	1.56409	0.239995
4517	0.774911	0.335079
4518	1.09483	0.302532
4519	0.774912	0.2992
4520	1.09483	0.30013
4521	1.24417	0.292978

4522	1.13387	0.247296
4523	1.13387	0.252535
4524	1.30572	0.311329
4525	2.18966	0.281399
4526	0.98575	0.232418
4527	1.54982	0.284556
4528	2.48833	0.255376
4529	1.54982	0.25406
4530	1.28954	0.443026
4531	1.64002	0.232087
4532	1.64002	0.217057
4533	1.85594	0.216327
4534	1.12968	2.1
4535	1.23536	1.67408
4536	1.4882	1.88033
4537	1.4882	2.1
4538	0.876836	1.80285
4539	0.946131	2.07313
4540	0.876835	2.1
4541	1.43166	2.1
4542	2.25936	2.1
4543	1.0305	1.79733
4544	0.992095	2.1
4545	1.12968	2.01816
4546	1.53813	2.08197
4547	1.53812	2.1
4548	0.583653	2.1
4549	0.94613	2.1
4550	0.583651	2.1
4551	0.964033	2.07083
4552	0.964034	2.1
4553	1.54457	1.64891
4554	3.54109	0.40558
4555	0.99974	0.465832
4556	0.78814	0.50748
4557	1.65729	0.395485
4558	1.1283	0.444419
4559	1.09407	0.464988
4560	1.55091	0.40313
4561	0.870773	0.492377
4562	1.19967	0.423609
4563	1.56521	0.427199
4564	1.30896	1.20571
4565	1.17989	2.02917
4566	1.72526	1.81962
4567	0.851537	1.35303
4568	1.58944	1.72117
4569	1.56915	1.80473
4570	0.891039	1.29139
4571	1.4172	1.36605
4572	0.963818	1.22535
4573	0.851474	1.54957
4574	1.17989	1.98232
4575	1.56261	2.1
4576	1.56261	2.1
4577	0.761574	2.1
4578	1.31323	2.1
4579	0.761573	2.1

4580	0.946133	2.1
4581	1.31323	2.1
4582	1.16759	2.1
4583	1.16759	2.1
4584	2.11829	2.1
4585	1.16759	2.1
4586	1.10748	0.401781
4587	1.47688	0.372862
4588	0.946133	0.353184
4589	0.953319	0.354449
4590	0.946132	0.377902
4591	1.51168	0.368109
4592	1.51168	0.335437
4593	1.25797	0.337311
4594	1.19984	0.294824
4595	1.95729	2.07924
4596	2.34853	2.09666
4597	1.94343	1.58702
4598	2.00551	1.64631
4599	1.20067	0.885647
4600	1.87244	0.925386
4601	1.4178	1.95876
4602	1.33538	2.1
4603	0.946131	1.99233
4604	1.22895	1.56768
4605	1.12433	2.1
4606	0.956757	2.1
4607	0.91826	2.1
4608	1.44221	2.1
4609	1.14206	2.1
4610	1.80514	1.02287
4611	0.890304	1.31302
4612	1.1362	0.940367
4613	1.32819	0.985791
4614	1.46311	1.74146
4615	0.8654	0.992605
4616	1.1699	1.15631
4617	1.00267	1.17325
4618	1.02882	1.05242
4619	1.00832	2.1
4620	0.974137	2.1
4621	1.02368	2.1
4622	0.974138	2.1
4623	0.995734	0.666094
4624	1.44605	0.546707
4625	1.18549	0.498411
4626	1.33621	0.807166
4627	1.5176	0.811743
4628	1.91762	0.80017
4629	1.33621	0.795398
4630	1.6638	0.791723
4631	1.66774	2.1
4632	1.11833	2.1
4633	0.926499	2.1
4634	1.51229	2.1
4635	1.51229	2.1
4636	0.926528	2.1
4637	1.40335	2.1

4638	1.03544	2.1
4639	0.926527	2.1
4640	1.85305	2.1
4641	1.5812	2.1
4642	1.15529	2.1
4643	1.31086	2.1
4644	0.999206	2.1
4645	1.46714	2.1
4646	1.73087	2.1
4647	0.772299	2.1
4648	1.24341	2.1
4649	0.997867	2.1
4650	0.772311	2.1
4651	0.936657	2.1
4652	1.35986	2.1
4653	0.959414	2.1
4654	1.36541	2.1
4655	1.49268	2.1
4656	1.49268	2.1
4657	0.832137	2.1
4658	0.946132	2.1
4659	0.832138	2.1
4660	1.64523	2.1
4661	1.11764	2.1
4662	1.05776	2.1
4663	0.972465	2.1
4664	0.972464	2.1
4665	1.98425	2.1
4666	0.969822	2.1
4667	1.30566	2.1
4668	1.52724	2.1
4669	2.32237	0.666002
4670	2.30274	0.699669
4671	2.27761	0.509088
4672	3.33012	0.501461
4673	1.24001	0.834364
4674	1.41821	0.977842
4675	0.978584	2.03522
4676	1.18076	2.1
4677	1.07645	2.1
4678	0.877351	0.720406
4679	0.946132	1.04987
4680	0.877353	0.89243
4681	1.58444	2.0455
4682	1.49614	1.43305
4683	1.28355	1.17176
4684	1.13491	1.07608
4685	1.56875	0.781803
4686	0.499592	0.419976
4687	0.946131	0.321907
4688	1.20538	0.340717
4689	1.09913	0.347235
4690	1.8049	0.377781
4691	1.8049	0.328671
4692	0.499606	0.413816
4693	2.41076	0.37348
4694	0.860202	0.325506
4695	0.906798	0.35036

4696	1.8626	0.4349
4697	1.33986	0.339372
4698	1.2992	0.459102
4699	1.58702	0.349963
4700	1.05939	0.38621
4701	1.02074	1.75347
4702	1.07866	1.71397
4703	1.5082	0.49072
4704	1.5522	0.412342
4705	1.65903	0.562917
4706	1.86766	2.1
4707	2.11684	2.1
4708	0.946132	2.1
4709	1.6585	2.1
4710	1.6585	2.1
4711	0.94613	2.1
4712	1.1872	2.1
4713	1.1872	2.1
4714	1.05842	2.1
4715	1.20455	2.1
4716	1.34141	2.1
4717	1.34141	2.1
4718	1.42416	2.1
4719	1.75818	1.56386
4720	2.63715	1.01483
4721	1.80454	2.03871
4722	2.26198	2.1
4723	0.696255	2.1
4724	0.624075	1.65404
4725	1.50905	0.92018
4726	1.50905	1.72616
4727	0.624075	1.74632
4728	1.31857	0.881087
4729	0.946131	1.4169
4730	0.624075	1.10573
4731	0.914628	1.64742
4732	1.14721	2.1
4733	1.16153	2.1
4734	1.47664	2.1
4735	1.54791	2.1
4736	1.19098	2.1
4737	1.13341	2.1
4738	1.00503	0.778699
4739	1.00503	1.01762
4740	1.22655	1.93969
4741	0.999489	2.1
4742	1.3268	1.6821
4743	0.946132	2.1
4744	1.60061	2.1
4745	1.60061	2.1
4746	1.08034	2.1
4747	1.40372	2.1
4748	1.14302	2.1
4749	1.08034	2.1
4750	0.999491	2.1
4751	1.17152	0.594599
4752	1.17152	0.426794
4753	1.17152	0.461165

4754	2.05423	0.502544
4755	1.40984	1.82199
4756	1.2489	2.1
4757	0.885089	2.1
4758	0.810931	2.1
4759	1.84128	2.1
4760	1.11206	2.1
4761	1.21926	2.1
4762	1.99084	2.1
4763	0.976334	1.02499
4764	0.599363	0.922216
4765	1.02621	0.780647
4766	0.462959	0.888082
4767	1.42612	0.733402
4768	0.299682	0.789301
4769	1.51379	2.1
4770	0.719531	1.95337
4771	1.3702	2.1
4772	1.33293	2.1
4773	0.851539	2.1
4774	0.62998	2.1
4775	0.851543	2.1
4776	0.629966	2.1
4777	1.43132	2.1
4778	1.13328	1.12658
4779	1.93212	0.931931
4780	1.62345	1.56579
4781	1.38511	1.68671
4782	1.40013	1.23778
4783	1.04132	1.23318
4784	0.83473	1.46694
4785	0.868732	1.46745
4786	0.810595	1.7037
4787	0.729919	1.39814
4788	0.992428	1.58612
4789	0.577107	1.27341
4790	1.18464	1.59724
4791	0.791199	1.50869
4792	0.810943	0.35814
4793	0.860723	0.364137
4794	1.33348	0.277459
4795	1.57467	0.300684
4796	0.981032	0.354519
4797	0.731569	0.286846
4798	0.770312	0.370457
4799	1.46055	2.1
4800	1.42249	2.1
4801	1.75999	0.388886
4802	1.6868	0.32622
4803	1.17042	0.904436
4804	1.01485	2.1
4805	1.19952	0.397268
4806	1.40592	0.501213
4807	1.19952	0.436724
4808	2.20145	0.427785
4809	1.33649	0.563937
4810	1.11612	0.835508
4811	0.957588	0.709809

4812	0.946126	0.818981
4813	1.50648	0.565823
4814	1.50648	0.740833
4815	0.946128	0.68928
4816	2.10671	0.394532
4817	1.91518	0.487776
4818	2.23223	0.558038
4819	1.53556	2.1
4820	1.56253	2.1
4821	1.27806	0.203967
4822	1.44593	0.210606
4823	1.03818	0.783489
4824	1.03818	0.783636
4825	1.38969	0.911977
4826	1.85548	0.784727
4827	2.6044	0.847844
4828	1.85548	0.768808
4829	1.49346	2.07635
4830	1.83192	1.92578
4831	0.834043	1.90918
4832	0.810943	1.46508
4833	0.887113	1.4684
4834	0.893827	1.60112
4835	0.991061	1.59746
4836	0.91939	1.66157
4837	0.793307	1.62299
4838	1.75291	1.85597
4839	1.35435	1.67346
4840	1.16989	0.763371
4841	1.36717	0.725436
4842	2.12276	0.68841
4843	2.30493	0.620023
4844	2.47857	0.69118
4845	1.92269	0.693206
4846	1.66426	0.701492
4847	0.956698	1.00464
4848	0.956698	1.43479
4849	0.857957	2.03651
4850	1.4706	1.553
4851	1.65706	2.1
4852	1.47059	1.96831
4853	1.04713	0.580494
4854	0.946131	0.616935
4855	1.47374	0.687637
4856	1.47374	0.602421
4857	0.951675	0.561365
4858	1.2582	0.688698
4859	1.2582	0.61027
4860	0.94613	0.672442
4861	1.4468	0.375139
4862	1.90335	0.504512
4863	1.14886	2.1
4864	2.59689	2.1
4865	1.02747	2.1
4866	0.946131	2.1
4867	0.934061	2.1
4868	1.63979	2.1
4869	1.65206	2.1

4870	1.48	2.1
4871	1.27467	2.1
4872	2.20449	2.1
4873	1.1333	0.210012
4874	1.95334	2.1
4875	2.20984	2.1
4876	1.46389	0.480296
4877	1.4639	0.510606
4878	1.4639	0.58706
4879	0.946132	0.585668
4880	1.5144	0.503229
4881	1.5144	0.567691
4882	0.946132	0.599011
4883	1.35732	0.506177
4884	1.10321	0.575766
4885	0.946132	0.588047
4886	0.980549	0.525205
4887	1.32819	1.98383
4888	1.30171	1.16627
4889	1.68377	1.02583
4890	1.68377	2.1
4891	0.946132	0.950659
4892	0.946132	1.46145
4893	1.1952	1.20369
4894	0.990019	1.23632
4895	1.08195	1.46565
4896	1.2269	2.02128
4897	1.19219	1.86969
4898	1.01485	2.1
4899	1.57125	0.382409
4900	2.10696	0.354759
4901	1.29666	0.249989
4902	1.57125	0.389129
4903	1.47712	2.1
4904	1.45182	2.1
4905	1.17816	0.203251
4906	1.4516	0.218522
4907	1.17816	0.186196
4908	1.17816	0.183118
4909	0.596954	1.04133
4910	1.65133	0.913192
4911	0.583859	0.863053
4912	1.04072	1.10098
4913	1.28795	0.910196
4914	1.59423	0.905991
4915	0.483559	0.928005
4916	1.74088	0.870108
4917	1.09545	1.01651
4918	1.02519	1.18088
4919	2.47579	0.959481
4920	1.54732	1.03255
4921	2.17663	0.938989
4922	1.75006	0.973893
4923	1.32354	1.01597
4924	1.09962	2.1
4925	1.85057	2.1
4926	1.91046	2.1
4927	2.17037	2.1

4928	1.99528	0.487208
4929	1.97969	0.451402
4930	3.82765	2.1
4931	1.85767	2.1
4932	2.77042	2.1
4933	1.37236	0.286249
4934	2.23254	0.284403
4935	1.10089	0.264433
4936	0.994294	2.1
4937	1.81921	2.1
4938	1.00073	2.1
4939	0.681043	2.1
4940	0.946131	2.1
4941	0.681073	2.1
4942	1.51804	2.1
4943	1.60653	2.1
4944	1.30188	2.1
4945	0.919622	2.1
4946	1.00965	2.1
4947	1.93821	2.1
4948	2.60377	2.1
4949	1.36416	0.246277
4950	1.36416	0.265464
4951	1.36416	0.286247
4952	2.44132	0.267558
4953	2.15871	0.844105
4954	2.22171	0.773069
4955	1.70747	0.715683
4956	1.49515	0.684197
4957	0.773007	0.710491
4958	0.946125	0.725371
4959	0.946131	0.723901
4960	0.773034	0.652348
4961	1.15844	0.716409
4962	1.98104	0.616751
4963	2.44283	0.700626
4964	1.05417	0.244881
4965	1.1596	0.278431
4966	0.829921	0.255932
4967	0.946131	0.294399
4968	0.951911	0.292256
4969	1.39027	0.24457
4970	0.829946	0.296094
4971	1.51223	0.27182
4972	1.51223	0.220706
4973	1.65984	0.227139
4974	1.90382	0.232436
4975	1.93228	1.16584
4976	2.47924	1.09565
4977	1.73904	1.25104
4978	1.40547	0.860253
4979	2.37589	0.957003
4980	0.662605	1.21087
4981	2.33293	1.09689
4982	1.72249	2.1
4983	1.68478	2.1
4984	1.00514	0.730532
4985	1.4537	2.1

4986	1.92698	2.1
4987	1.46041	0.155751
4988	2.04817	0.153801
4989	1.65954	2.1
4990	1.42342	2.1
4991	1.04405	0.22528
4992	1.17056	0.183339
4993	1.11339	0.173567
4994	0.946133	0.196177
4995	0.929724	0.190147
4996	1.35424	0.180566
4997	1.35424	0.182371
4998	0.929713	0.20508
4999	1.85945	0.195469
5000	1.38905	2.1
5001	1.39056	2.1
5002	1.52852	2.1
5003	1.06472	2.1
5004	0.749096	2.09882
5005	1.46076	2.1
5006	1.29263	2.1
5007	0.94613	2.1
5008	0.69097	2.1
5009	0.982284	2.1
5010	0.67419	2.1
5011	1.52852	2.1
5012	1.91121	2.1
5013	1.00775	1.78128
5014	1.61201	1.95259
5015	0.529987	2.1
5016	0.506125	2.1
5017	1.44696	2.1
5018	2.32981	2.1
5019	1.32784	2.1
5020	1.94158	2.1
5021	1.31595	2.1
5022	0.663922	2.1
5023	1.44663	2.1
5024	1.44663	2.1
5025	1.13976	2.1
5026	0.970788	2.1
5027	1.13976	2.1
5028	0.663921	2.1
5029	0.966114	2.1
5030	1.05498	1.04537
5031	1.38086	2.1
5032	0.946127	2.1
5033	0.922513	2.1
5034	1.49903	2.1
5035	1.49903	2.1
5036	0.92251	2.1
5037	1.06105	2.1
5038	1.36049	2.1
5039	1.38086	2.1
5040	1.52534	2.1
5041	1.04101	2.1
5042	1.30861	0.313752
5043	0.949112	0.344451

5044	0.894275	0.414674
5045	1.10232	0.298503
5046	0.830889	0.328674
5047	1.26554	0.33653
5048	0.948347	0.319808
5049	0.81096	0.350944
5050	1.18338	0.366879
5051	0.883764	0.435028
5052	1.54793	0.306124
5053	1.02867	0.367706
5054	1.8573	0.356387
5055	1.06065	0.3297
5056	0.64842	0.362621
5057	1.99319	0.387477
5058	1.9476	0.412384
5059	2.51436	0.154219
5060	1.68337	0.322244
5061	1.92321	0.340841
5062	0.810942	0.243131
5063	0.921001	0.275484
5064	0.99758	0.321301
5065	0.960785	0.279175
5066	1.01565	0.327895
5067	0.908624	0.27652
5068	0.912835	0.282598
5069	1.18351	0.278966
5070	1.40239	2.1
5071	1.26091	2.1
5072	0.947979	2.07299
5073	0.946131	1.72997
5074	1.81959	1.72167
5075	1.81959	1.04662
5076	0.94613	1.85058
5077	1.38415	1.01326
5078	1.38414	1.42649
5079	1.33266	1.58261
5080	0.946132	2.1
5081	1.76225	2.08305
5082	1.76225	2.1
5083	1.27599	2.1
5084	1.38158	1.73053
5085	1.3268	2.1
5086	1.27599	2.1
5087	0.947979	2.04892
5088	1.0142	0.741948
5089	1.01422	0.737163
5090	1.11435	0.79112
5091	1.42186	0.922447
5092	1.24001	1.05362
5093	1.77342	0.993663
5094	1.42186	1.19277
5095	1.99817	0.938906
5096	1.27361	2.1
5097	1.68914	2.1
5098	1.30116	2.1
5099	1.32819	1.22524
5100	1.68322	1.22009
5101	1.68322	2.1

5102	0.94613	1.00219
5103	0.94613	1.65834
5104	1.14666	1.261
5105	1.07612	1.20876
5106	1.31151	2.1
5107	1.22095	1.35249
5108	1.57256	1.51477
5109	1.6679	2.1
5110	0.946132	1.19165
5111	0.946133	1.80205
5112	1.13537	1.78318
5113	1.07855	1.42949
5114	1.07855	2.1
5115	1.43895	1.14898
5116	1.38481	0.248687
5117	1.72385	0.244481
5118	0.550441	0.245156
5119	0.79807	0.251795
5120	1.11627	0.285769
5121	1.30349	0.269936
5122	0.94613	0.285662
5123	0.686176	0.287742
5124	1.11627	0.259975
5125	1.73363	0.258138
5126	1.73363	0.2399
5127	0.686139	0.306496
5128	1.24932	0.242833
5129	0.899582	0.235399
5130	2.00472	0.219374
5131	1.17121	0.241342
5132	1.2874	0.228801
5133	2.0194	0.236205
5134	0.88601	0.267073
5135	1.77409	0.277104
5136	1.73293	0.241317
5137	2.74045	0.268276
5138	1.83787	0.24355
5139	1.45448	0.337472
5140	1.27778	0.307419
5141	1.1192	2.1
5142	1.25953	2.1
5143	1.93611	2.1
5144	1.92104	2.02208
5145	1.15506	0.240407
5146	1.15506	0.249675
5147	1.23943	0.787295
5148	0.851543	2.1
5149	1.6061	2.1
5150	1.80058	2.1
5151	0.869471	2.1
5152	1.3931	2.1
5153	1.06454	2.1
5154	0.93667	2.1
5155	2.16916	2.1
5156	3.10246	2.1
5157	1.91539	1.91262
5158	1.91539	1.64653
5159	0.620263	1.36799

5160	0.620265	1.88621
5161	0.946132	1.4046
5162	0.620265	1.34411
5163	1.30226	1.24533
5164	1.87292	1.91635
5165	1.18769	2.02666
5166	2.60452	1.74182
5167	1.26574	2.03191
5168	2.46679	1.55053
5169	1.36459	2.038
5170	1.26865	0.995082
5171	1.1744	0.886667
5172	1.15841	0.733091
5173	1.02247	0.616514
5174	1.93649	2.1
5175	1.64718	2.1
5176	1.59654	2.1
5177	1.57823	2.1
5178	1.00665	0.343188
5179	1.28239	0.332973
5180	0.887895	2.1
5181	1.22083	2.1
5182	1.42127	2.1
5183	1.07374	2.1
5184	1.00503	1.26417
5185	1.23082	0.947302
5186	2.78663	2.1
5187	2.20883	2.1
5188	2.54588	2.1
5189	0.946128	2.1
5190	1.13716	2.1
5191	1.58724	2.1
5192	1.58724	2.1
5193	0.946131	2.1
5194	1.13716	2.1
5195	1.39621	2.1
5196	1.02438	2.1
5197	1.49543	0.27614
5198	2.78336	0.251084
5199	1.27148	0.226469
5200	1.0906	0.231375
5201	1.04072	0.264895
5202	0.63574	0.231912
5203	1.45506	0.220599
5204	1.5863	0.229295
5205	0.928352	0.251271
5206	0.647687	0.253808
5207	2.2375	1.96782
5208	1.50998	1.65768
5209	2.75021	1.64623
5210	1.91912	1.55182
5211	1.99872	1.59381
5212	0.918127	1.7797
5213	1.03031	1.42836
5214	0.951169	1.27288
5215	1.27975	1.45469
5216	1.12735	1.54598
5217	1.15741	1.43832

5218	1.24998	1.43353
5219	1.65953	0.772406
5220	0.967555	0.957982
5221	0.967542	0.747374
5222	1.03824	0.505078
5223	1.48693	0.512692
5224	1.48693	0.415797
5225	2.02901	0.443732
5226	0.946127	0.512743
5227	1.07139	0.489275
5228	0.946126	0.470178
5229	1.84644	0.460507
5230	1.84644	0.439602
5231	1.38683	0.440153
5232	1.40574	0.412977
5233	1.36866	0.797663
5234	0.649325	0.783714
5235	1.93631	2.1
5236	1.50145	2.1
5237	1.6287	2.1
5238	1.31302	2.1
5239	1.18041	2.1
5240	1.18041	2.1
5241	0.974196	2.1
5242	0.946131	2.1
5243	1.74169	2.1
5244	1.74169	2.1
5245	1.23163	2.1
5246	1.27451	2.1
5247	1.41331	2.1
5248	1.23163	2.1
5249	1.28037	2.1
5250	1.28158	2.1
5251	1.28037	2.1
5252	2.28015	0.233584
5253	2.06561	0.213698
5254	2.61343	0.263971
5255	1.11712	0.252877
5256	1.63689	0.894478
5257	1.01393	0.795554
5258	0.996775	2.1
5259	1.54277	2.1
5260	1.26397	2.1
5261	1.46129	2.1
5262	1.62391	2.1
5263	1.08848	0.264635
5264	1.11833	2.1
5265	1.24041	2.1
5266	2.31884	2.1
5267	1.335	2.1
5268	1.48589	0.336735
5269	1.77131	0.299221
5270	1.8422	1.46324
5271	2.47453	1.77659
5272	1.23726	1.53984
5273	1.21996	1.56282
5274	1.65512	1.70541
5275	1.65512	1.67422

5276	0.802107	1.69818
5277	0.946129	1.36838
5278	0.802137	1.41462
5279	1.3279	1.33022
5280	1.23726	1.38465
5281	1.79759	1.42928
5282	1.79759	1.35346
5283	0.767577	1.55455
5284	0.946131	1.24579
5285	0.767577	1.26484
5286	1.03534	0.306115
5287	1.09962	0.752002
5288	0.929217	1.15198
5289	1.70126	1.1807
5290	0.991407	2.1
5291	1.41077	2.00696
5292	0.991406	2.1
5293	1.62966	2.1
5294	1.62966	2.1
5295	0.946128	2.1
5296	1.18364	2.1
5297	0.946131	2.1
5298	1.14186	2.1
5299	1.18364	2.1
5300	1.16858	1.8758
5301	1.66137	0.591264
5302	1.90165	0.294761
5303	1.66139	0.290304
5304	1.26893	0.700816
5305	1.15918	0.7
5306	0.653228	0.7
5307	0.946133	0.7
5308	1.77488	1.18966
5309	1.77489	0.877664
5310	0.653228	0.7
5311	1.56043	0.702052
5312	1.60894	0.795985
5313	1.56043	0.68124
5314	1.06848	1.10866
5315	1.04307	2.1
5316	1.14188	2.1
5317	1.09195	2.1
5318	0.615121	2.1
5319	1.50867	2.1
5320	1.27152	1.91576
5321	0.615094	1.62669
5322	1.07297	2.1
5323	1.1372	2.1
5324	0.553608	2.1
5325	1.23019	1.9622
5326	0.992675	2.1
5327	1.40984	1.34657
5328	2.55682	2.1
5329	3.66069	2.1
5330	3.58726	2.1
5331	1.4375	0.473978
5332	1.88424	0.614503
5333	1.56714	2.1

5334	1.6721	2.1
5335	1.18474	2.1
5336	1.2295	2.1
5337	1.28754	2.1
5338	1.36838	2.1
5339	2.78661	2.1
5340	2.64055	2.1
5341	1.35065	1.18518
5342	2.26675	0.847833
5343	1.35065	1.00193
5344	1.21558	0.880161
5345	1.49709	0.221226
5346	1.4971	0.205829
5347	0.85472	0.262832
5348	0.854718	0.206729
5349	0.946129	0.286327
5350	0.854717	0.260842
5351	1.1604	0.266304
5352	1.25265	0.237993
5353	2.32081	0.242043
5354	1.15371	0.165186
5355	1.14945	0.821472
5356	0.995725	0.80522
5357	1.00307	0.543527
5358	1.10907	0.502398
5359	2.30315	0.48994
5360	1.10908	0.527439
5361	1.27558	0.966632
5362	1.71613	1.21498
5363	1.32977	2.1
5364	0.815276	2.1
5365	1.41697	2.1
5366	1.41697	2.1
5367	0.815276	2.1
5368	1.38897	2.1
5369	0.946131	2.1
5370	0.815275	2.1
5371	1.44684	2.1
5372	1.21489	1.32045
5373	1.22476	1.28274
5374	0.870366	1.00132
5375	0.862572	0.944524
5376	0.835435	0.909048
5377	1.6993	0.890858
5378	1.23618	0.884294
5379	1.18624	0.898547
5380	1.53058	0.919821
5381	2.16108	1.02088
5382	2.21589	0.979367
5383	1.04037	1.46607
5384	1.03933	1.49326
5385	1.57753	0.403076
5386	1.87644	0.450099
5387	1.24728	0.505254
5388	1.30394	0.424026
5389	1.3538	2.1
5390	1.69858	2.1
5391	1.1726	0.585144

5392	0.94612	0.428746
5393	1.09805	0.367545
5394	0.946118	0.454729
5395	1.72576	0.470471
5396	1.72575	0.40235
5397	1.34152	0.410771
5398	1.33034	0.408111
5399	0.784151	1.34763
5400	0.946131	1.99608
5401	0.784151	1.53979
5402	1.47963	2.1
5403	1.47963	2.1
5404	1.3335	2.1
5405	0.930281	2.1
5406	1.29435	1.24371
5407	0.883308	1.12871
5408	1.31589	2.1
5409	1.67846	2.1
5410	0.993598	0.318508
5411	2.23193	0.289627
5412	0.9936	0.277997
5413	0.615629	0.272173
5414	1.65925	2.1
5415	2.05992	2.1
5416	1.65925	2.1
5417	4.08735	1.28692
5418	2.72856	1.38133
5419	0.869602	2.05665
5420	3.42833	1.01697
5421	1.14397	0.72013
5422	1.34563	0.571859
5423	1.08189	0.513369
5424	0.782128	1.70005
5425	1.0022	1.91763
5426	0.900427	1.7419
5427	1.51499	1.84068
5428	1.05588	1.76731
5429	1.13739	1.97065
5430	1.37004	1.77867
5431	1.14603	1.90993
5432	0.851541	1.31492
5433	0.946799	1.76449
5434	1.14854	1.70086
5435	1.33793	1.41141
5436	0.94613	1.41267
5437	1.24336	1.5062
5438	0.851473	1.49087
5439	2.08792	1.55392
5440	1.01869	1.70799
5441	0.986953	0.532406
5442	0.896238	0.592594
5443	0.906574	0.607717
5444	0.852145	0.554088
5445	2.14191	0.504351
5446	1.15979	0.532844
5447	1.18327	0.505363
5448	2.32742	0.538077
5449	2.35961	0.554012

5450	2.38126	0.629354
5451	1.13632	0.547166
5452	1.17897	0.278093
5453	1.76199	0.249925
5454	1.00573	1.41199
5455	0.946127	0.876886
5456	1.7077	1.37361
5457	1.7077	2.1
5458	0.946127	1.22111
5459	1.24887	2.1
5460	1.24887	1.55852
5461	1.22441	1.57762
5462	1.08542	1.56377
5463	1.21743	1.46032
5464	1.12572	2.1
5465	1.05499	0.965705
5466	1.00521	0.930339
5467	2.86191	0.179247
5468	2.27972	0.173925
5469	1.01169	2.1
5470	1.03373	2.1
5471	1.39319	2.1
5472	0.636837	1.85423
5473	1.63908	1.69436
5474	1.45713	1.62388
5475	1.04072	1.69256
5476	1.25025	1.66829
5477	1.38914	1.59994
5478	0.636807	1.54124
5479	1.50211	2.1
5480	1.36555	1.78645
5481	2.77545	1.33494
5482	0.682805	2.06704
5483	1.50267	1.75357
5484	1.50267	1.933
5485	0.797749	2.09702
5486	1.38773	1.68303
5487	1.38773	1.97047
5488	0.682776	1.93692
5489	2.01245	0.209305
5490	0.925918	0.283636
5491	0.75689	0.223958
5492	1.25823	0.204883
5493	1.16505	0.243782
5494	0.838779	0.226456
5495	1.14722	0.217781
5496	0.756849	0.230684
5497	1.52298	0.255186
5498	2.11034	0.239184
5499	1.92174	0.230928
5500	1.92174	0.224056
5501	0.576083	0.322917
5502	1.23146	0.28201
5503	1.26637	0.254798
5504	1.23146	0.280342
5505	1.26637	0.270937
5506	0.946133	0.958277
5507	1.37034	0.919648

5508	1.84145	0.943349
5509	1.84145	0.827879
5510	0.946132	1.01153
5511	1.37034	0.935163
5512	1.41724	0.939824
5513	1.0685	0.817867
5514	1.14547	0.840434
5515	1.24976	2.1
5516	1.30682	2.1
5517	2.12095	2.1
5518	1.46392	2.1
5519	1.46392	2.1
5520	0.935738	2.1
5521	1.32949	2.1
5522	1.07017	2.1
5523	0.935766	2.1
5524	1.07017	2.1
5525	1.78095	2.1
5526	1.05619	0.819296
5527	1.05793	0.814938
5528	1.77677	2.1
5529	2.04766	2.1
5530	1.02524	2.1
5531	1.18803	2.1
5532	1.70047	2.1
5533	1.556 2.1	
5534	1.24201	1.02596
5535	1.3592	2.1
5536	2.11824	0.428013
5537	2.20248	0.603968
5538	3.43878	0.436194
5539	3.12466	0.378267
5540	2.09351	2.1
5541	0.946128	1.95396
5542	1.51269	2.1
5543	1.51269	2.1
5544	0.946131	2.1
5545	1.01306	2.1
5546	1.04676	2.1
5547	1.01306	2.1
5548	1.07444	2.1
5549	2.17465	2.1
5550	1.14808	2.1
5551	1.39067	1.10099
5552	1.20958	1.06046
5553	0.662832	0.717659
5554	1.93512	0.751892
5555	1.15471	2.1
5556	1.75119	2.1
5557	1.75118	2.1
5558	0.946131	2.1
5559	1.28606	2.1
5560	1.27087	2.1
5561	0.946131	2.1
5562	1.28606	2.1
5563	1.58916	0.30736
5564	0.927822	0.42872
5565	2.24367	0.247021

5566	1.8734	0.245812
5567	3.51668	0.230315
5568	2.68986	0.230219
5569	2.32395	2.1
5570	1.39564	2.1
5571	2.10862	2.1
5572	1.36763	2.1
5573	0.970963	0.226213
5574	0.97096	0.24048
5575	1.11897	0.43518
5576	1.26885	0.553941
5577	1.11605	0.447636
5578	2.06612	0.390174
5579	1.717	0.415843
5580	0.59502	0.544284
5581	0.825755	0.35275
5582	0.709624	0.586761
5583	1.65741	0.387619
5584	0.307486	0.378893
5585	1.4785	0.423614
5586	0.983143	2.1
5587	1.08247	2.1
5588	1.42353	2.1
5589	1.42353	2.1
5590	0.781667	2.1
5591	0.797634	2.1
5592	0.946131	2.1
5593	0.781638	2.1
5594	0.946131	2.1
5595	0.98315	2.1
5596	1.083	2.1
5597	1.61975	0.147815
5598	1.11946	0.147815
5599	1.18977	0.329058
5600	0.996484	0.356657
5601	2.24343	0.229166
5602	1.4787	0.262452
5603	1.66127	0.249117
5604	1.86028	0.303563
5605	1.09128	0.302548
5606	1.03115	0.36255
5607	0.838025	0.364959
5608	1.3736	0.242514
5609	1.44009	0.264055
5610	0.92917	0.260175
5611	1.03889	0.356133
5612	0.956974	0.309381
5613	3.23468	0.274326
5614	0.953922	0.384783
5615	0.957104	0.393948
5616	1.66231	0.261643
5617	1.26778	0.295524
5618	1.47615	0.27887
5619	1.28621	0.365986
5620	1.08043	0.347415
5621	2.35026	2.1
5622	2.35025	2.1
5623	2.60659	2.1

5624	1.57627	2.1
5625	1.5493	2.1
5626	1.24682	0.710781
5627	2.14277	0.735287
5628	1.24682	0.898931
5629	1.00307	0.655522
5630	2.04056	0.673245
5631	1.45408	2.1
5632	1.54249	2.1
5633	1.24474	1.1902
5634	1.37813	1.25409
5635	0.924158	0.314134
5636	0.916623	0.330751
5637	1.35344	0.308218
5638	1.00859	0.326576
5639	1.34786	0.316088
5640	0.952457	0.301383
5641	0.85404	0.307974
5642	1.06341	2.1
5643	1.09194	2.1
5644	1.93707	2.1
5645	2.14087	2.1
5646	1.93707	2.1
5647	1.07519	1.10811
5648	1.07519	0.935561
5649	1.62518	2.1
5650	1.3592	2.1
5651	1.13183	2.1
5652	1.20357	2.1
5653	1.4858	2.1
5654	1.23694	2.1
5655	1.44011	2.1
5656	1.23989	2.1
5657	2.21604	1.37149
5658	1.85574	0.644045
5659	1.55649	1.29194
5660	1.48861	0.521489
5661	1.15968	0.224163
5662	0.946132	0.255909
5663	0.663056	0.22174
5664	1.70837	0.213253
5665	1.70837	0.218588
5666	0.946131	0.231496
5667	0.663026	0.21713
5668	2.09604	0.220405
5669	0.81094	0.234987
5670	1.06559	0.217997
5671	0.810956	0.217677
5672	1.15474	0.201944
5673	0.990051	0.211552
5674	0.894971	0.206969
5675	1.32536	0.211788
5676	0.946133	2.1
5677	1.33125	2.1
5678	1.75823	2.1
5679	1.75823	2.1
5680	0.946133	2.1
5681	1.33125	2.1

5682	1.37312	2.1
5683	1.27985	2.1
5684	1.37312	2.1
5685	1.70684	2.1
5686	1.70684	2.1
5687	0.946132	2.1
5688	0.946132	2.1
5689	1.25081	2.1
5690	0.956293	2.1
5691	0.956294	2.1
5692	1.36035	2.1
5693	1.41944	2.1
5694	0.830638	2.1
5695	0.747568	2.1
5696	0.888498	2.1
5697	1.04071	2.1
5698	1.17128	2.1
5699	1.92339	2.1
5700	2.91208	2.1
5701	1.82208	0.877666
5702	1.89653	1.30852
5703	1.82588	1.25871
5704	1.26703	0.753799
5705	1.26703	1.04974
5706	0.6157	0.718608
5707	1.26706	1.10592
5708	0.61567	0.701926
5709	0.615671	0.70797
5710	1.26706	0.75303
5711	1.89158	0.972452
5712	1.23134	0.712136
5713	2.49497	0.282885
5714	1.68103	0.233204
5715	2.0554	2.1
5716	1.72647	2.1
5717	1.67375	2.1
5718	1.65037	2.1
5719	1.04349	0.571786
5720	1.69055	0.344357
5721	1.53463	0.268762
5722	1.19022	2.05755
5723	1.89226	1.56971
5724	1.15009	0.903921
5725	1.7054	1.02398
5726	1.31612	0.549573
5727	1.31611	0.612348
5728	1.67345	0.978796
5729	1.06577	0.568839
5730	1.40193	0.435599
5731	1.72411	0.408158
5732	1.46123	0.442344
5733	0.808808	0.547529
5734	0.897714	0.447597
5735	0.851554	0.588872
5736	1.3499	0.375013
5737	1.29001	0.377447
5738	1.22083	2.1
5739	1.06191	2.1

5740	1.05758	1.22309
5741	1.348	1.54704
5742	1.43485	1.20207
5743	1.34799	1.46398
5744	1.36307	0.180858
5745	2.28188	0.395458
5746	1.7926	0.372912
5747	1.38957	2.1
5748	1.47446	2.1
5749	0.953638	2.1
5750	1.11868	1.73351
5751	0.953636	2.1
5752	1.03408	2.04543
5753	1.52443	0.256322
5754	1.38539	0.247145
5755	0.946132	0.319887
5756	1.12747	0.270989
5757	1.04393	0.336083
5758	1.05017	0.297141
5759	1.20529	0.296798
5760	1.3663	0.725916
5761	1.89226	0.717418
5762	1.17885	0.75125
5763	0.867881	0.701538
5764	1.32917	0.697202
5765	1.32917	0.698369
5766	0.946127	0.706822
5767	1.19583	0.701947
5768	1.00122	0.700794
5769	0.946128	0.703213
5770	1.71405	0.770302
5771	0.871815	1.56725
5772	1.16435	2.1
5773	0.686619	1.9679
5774	1.63102	2.1
5775	1.06589	1.87045
5776	1.05375	2.1
5777	1.9023	2.05002
5778	1.27394	0.923477
5779	1.88345	0.890656
5780	2.05636	0.91051
5781	0.672977	0.893006
5782	1.41274	0.891137
5783	1.65606	0.89492
5784	0.869329	0.894936
5785	1.2207	0.89629
5786	1.35631	0.902986
5787	0.908776	0.886752
5788	0.955081	0.860657
5789	1.2243	2.1
5790	1.23169	2.1
5791	1.52228	2.1
5792	1.23169	2.1
5793	0.990892	0.890649
5794	1.08376	0.755253
5795	1.79348	1.16638
5796	1.50043	0.820568
5797	1.32599	1.33687

5798	1.37924	0.827535
5799	0.838014	0.750948
5800	0.87737	1.01601
5801	0.947086	0.827179
5802	1.27199	1.02159
5803	1.04918	0.810732
5804	1.04851	1.15478
5805	1.1699	0.932118
5806	0.810934	0.969216
5807	1.83602	1.38795
5808	2.21909	1.35859
5809	0.619441	0.941092
5810	2.03755	0.858944
5811	1.83472	1.18718
5812	1.20416	1.11784
5813	1.26691	0.812821
5814	0.655795	0.961878
5815	0.94613	0.900295
5816	2.35169	1.10728
5817	3.54705	2.04655
5818	2.60741	2.05215
5819	1.59019	0.212864
5820	1.24526	0.206417
5821	1.59019	0.218885
5822	2.36406	0.211849
5823	1.16776	0.920063
5824	1.67311	1.00678
5825	1.67311	0.921626
5826	0.94613	0.816561
5827	1.38645	0.946525
5828	1.13563	0.562045
5829	1.13563	0.816871
5830	1.23279	0.734078
5831	1.05042	1.43695
5832	1.17418	1.0341
5833	1.28909	2.1
5834	0.973448	1.96543
5835	1.02958	1.88452
5836	3.3005	2.1
5837	2.86325	2.1
5838	1.35879	2.0034
5839	1.69332	2.1
5840	1.69332	2.1
5841	0.946128	1.96378
5842	1.39659	1.72882
5843	1.12035	2.1
5844	1.12035	2.1
5845	1.24286	2.1
5846	1.10407	1.89642
5847	1.14599	1.36506
5848	1.32259	0.912141
5849	0.788592	0.225921
5850	2.4588	2.1
5851	0.909581	1.71183
5852	1.35656	1.40039
5853	0.986895	1.60786
5854	2.30602	1.43483
5855	1.46873	1.64266

5856	1.55584	1.39088
5857	2.1547	1.44158
5858	1.87794	1.78788
5859	0.808286	1.74516
5860	0.928464	1.59817
5861	0.810959	1.59232
5862	1.50962	1.29539
5863	1.1053	1.50212
5864	1.0712	1.45748
5865	1.40504	1.44819
5866	1.87794	1.30844
5867	2.80496	1.38987
5868	1.41479	1.67058
5869	1.38634	2.1
5870	1.38634	2.1
5871	1.67762	2.1
5872	0.946131	2.1
5873	1.188 2.1	
5874	1.62585	2.1
5875	1.62585	2.1
5876	0.946129	2.1
5877	1.18801	2.1
5878	1.38397	2.1
5879	1.04546	2.1
5880	1.16817	2.1
5881	1.06007	2.1
5882	1.19785	2.1
5883	1.43652	2.01372
5884	1.06994	2.1
5885	1.89111	1.48698
5886	1.07144	2.1
5887	1.51754	2.1
5888	2.0873	2.1
5889	0.758799	2.03625
5890	1.59518	2.1
5891	1.59518	2.1
5892	0.946132	2.06313
5893	0.758771	2.1
5894	1.06957	2.08719
5895	0.758771	2.1
5896	1.33916	2.1
5897	2.03045	1.15832
5898	2.80184	1.33404
5899	1.76788	1.56132
5900	1.27442	1.61393
5901	1.06756	2.1
5902	0.966979	2.1
5903	1.7054	2.1
5904	1.07156	2.1
5905	0.986096	2.1
5906	1.32813	2.1
5907	1.3331	2.1
5908	2.08369	0.743134
5909	2.23818	1.16942
5910	1.10335	0.821639
5911	0.622314	0.971167
5912	1.72451	0.775127
5913	1.72451	1.09293

5914	0.622283	0.760223
5915	0.622314	0.759104
5916	0.946131	0.756888
5917	1.31254	1.33903
5918	1.15704	1.4335
5919	1.24463	0.794703
5920	2.2067	1.00346
5921	1.30001	0.921579
5922	1.29175	0.965072
5923	1.1233	0.228527
5924	1.8689	0.234239
5925	1.29494	0.233719
5926	1.11895	0.217408
5927	0.851539	0.241886
5928	0.851474	0.23724
5929	1.58352	0.219114
5930	1.58228	0.20649
5931	0.851527	0.255539
5932	2.6389	0.211918
5933	1.77548	0.285609
5934	1.24555	2.1
5935	2.0512	2.1
5936	1.46101	2.1
5937	2.60635	2.1
5938	1.10476	0.386713
5939	1.44103	0.371901
5940	0.652391	2.1
5941	0.618322	2.1
5942	2.22915	2.1
5943	1.39067	2.1
5944	1.99737	2.1
5945	1.42174	2.1
5946	0.946132	0.860464
5947	1.29735	1.24319
5948	1.61765	1.92607
5949	1.61765	0.981038
5950	0.946132	1.37436
5951	1.29735	0.947765
5952	1.26643	0.940084
5953	1.01335	1.11076
5954	1.01335	0.734749
5955	0.947669	2.1
5956	2.17167	2.04402
5957	2.59733	1.73444
5958	1.50954	1.83302
5959	1.02638	1.36213
5960	0.555785	1.92329
5961	1.91101	1.88896
5962	2.09809	1.66825
5963	0.641974	1.77602
5964	1.2022	1.64492
5965	1.36359	1.53162
5966	1.8128	1.83494
5967	2.34348	0.493194
5968	1.66343	0.552909
5969	0.974717	0.861726
5970	1.53908	0.808369
5971	1.27737	1.1362

5972	1.09408	0.986821
5973	2.07382	0.972665
5974	1.10466	1.27372
5975	1.05654	1.74941
5976	1.23498	1.33751
5977	1.48104	1.5173
5978	1.23498	1.44721
5979	1.17261	2.1
5980	1.18059	2.1
5981	2.43005	2.1
5982	1.04546	2.1
5983	2.81222	2.1
5984	1.16817	2.1
5985	1.75766	0.725541
5986	1.47021	0.790997
5987	1.07236	0.648269
5988	1.81759	0.864452
5989	1.42913	0.804315
5990	1.00636	0.879001
5991	1.00636	0.604242
5992	1.79397	0.695719
5993	0.946131	0.664754
5994	1.03691	0.730192
5995	0.851539	0.634448
5996	1.46001	0.857096
5997	1.37878	0.868998
5998	0.933258	0.667894
5999	1.32423	0.724781
6000	1.32614	0.159413
6001	1.11356	0.171743
6002	1.32614	0.161613
6003	1.28895	0.162266
6004	1.66895	0.1555
6005	1.66895	0.155084
6006	0.946132	0.167072
6007	1.29576	0.154859
6008	1.1318	0.156553
6009	1.37621	1.37327
6010	1.94071	1.27768
6011	1.10517	2.1
6012	1.97098	2.1
6013	0.811 2.1	
6014	0.956127	2.1
6015	1.18647	1.7185
6016	0.988455	1.29899
6017	1.12457	0.366101
6018	1.7453	0.408907
6019	0.92492	2.1
6020	1.24353	2.1
6021	1.84536	0.942294
6022	1.67662	0.879917
6023	1.38247	0.263893
6024	1.02663	0.246029
6025	0.946132	0.917181
6026	1.41723	0.868056
6027	1.72976	0.913813
6028	1.72976	0.771299
6029	0.946131	1.01551

6030	1.41723	0.916332
6031	1.25866	0.903293
6032	1.34254	0.702112
6033	1.25866	0.831767
6034	1.65507	0.757763
6035	1.65507	0.666427
6036	0.946132	0.933653
6037	0.946132	0.812621
6038	1.11929	0.803017
6039	0.961779	0.75719
6040	0.961779	0.751324
6041	0.946132	2.1
6042	1.69361	2.1
6043	1.69361	2.1
6044	0.94613	2.1
6045	1.36523	2.1
6046	1.36523	2.1
6047	1.19802	2.1
6048	0.974196	2.1
6049	1.09518	2.1
6050	0.946131	2.1
6051	1.7161	2.1
6052	1.7161	2.1
6053	0.94613	2.1
6054	1.297 2.1	
6055	1.297 2.1	
6056	1.14599	2.1
6057	0.974197	2.1
6058	0.974197	2.1
6059	1.80778	2.1
6060	2.20473	2.1
6061	1.80777	2.1
6062	1.3515	2.1
6063	0.583237	2.1
6064	1.39359	2.1
6065	1.39359	2.1
6066	1.07296	2.1
6067	0.946114	2.1
6068	1.07297	2.1
6069	0.903862	2.1
6070	0.956572	2.1
6071	1.03437	2.1
6072	0.975409	2.1
6073	1.31167	0.397223
6074	2.70246	0.352405
6075	1.12018	0.38394
6076	0.655836	0.404591
6077	0.655837	0.398563
6078	1.81557	0.297029
6079	1.81557	0.286205
6080	0.655836	0.391775
6081	0.946131	0.425539
6082	1.01847	0.333345
6083	1.61334	0.31816
6084	0.810946	0.430167
6085	0.8311	0.441937
6086	1.2466	0.323877
6087	1.20286	0.359796

6088	0.97508	0.431804
6089	0.870805	0.339461
6090	0.760087	0.449985
6091	0.853389	2.1
6092	0.820894	2.1
6093	0.928291	2.1
6094	1.06922	2.08853
6095	1.32829	2.1
6096	1.12173	2.1
6097	1.16615	2.1
6098	0.957748	2.1
6099	0.612491	1.78633
6100	0.548141	2.09171
6101	0.979614	2.1
6102	0.658184	2.06044
6103	0.678456	1.62456
6104	0.529544	1.70983
6105	0.739013	2.07546
6106	0.694037	1.9495
6107	0.475716	1.64562
6108	1.58794	1.59469
6109	1.58794	1.34453
6110	0.475743	1.41675
6111	1.08301	1.89821
6112	0.980664	2.00508
6113	0.475737	1.73497
6114	1.32709	1.98999
6115	1.59396	2.1
6116	1.19719	0.521959
6117	1.34921	0.534995
6118	1.69061	0.51228
6119	1.69061	0.419443
6120	0.946132	0.584454
6121	1.34921	0.548336
6122	1.28753	0.540558
6123	0.95895	0.446812
6124	1.01051	0.48861
6125	1.12468	0.51381
6126	1.22588	0.582113
6127	1.34254	0.555374
6128	1.74561	0.534917
6129	1.74561	0.464553
6130	0.946128	0.610861
6131	1.34253	0.568245
6132	1.3492	0.570266
6133	1.01051	0.451736
6134	1.922	2.1
6135	1.922	2.1
6136	2.12556	2.1
6137	0.946131	2.1
6138	1.06278	2.1
6139	1.47631	2.1
6140	1.47631	2.1
6141	0.946129	2.1
6142	1.06278	2.1
6143	1.35966	2.1
6144	0.991985	2.1
6145	0.738507	1.60464

6146	0.536935	1.87191
6147	0.799004	1.68597
6148	1.04281	1.52276
6149	0.879197	1.39908
6150	1.06538	1.5073
6151	0.715942	1.61317
6152	1.00636	0.687629
6153	1.03824	0.484258
6154	1.70147	0.571776
6155	1.21223	2.1
6156	1.01243	1.36471
6157	1.01242	2.1
6158	1.06935	2.1
6159	1.54241	2.1
6160	0.998971	0.199053
6161	1.60312	0.225238
6162	1.44836	0.202056
6163	2.05632	0.235504
6164	1.44836	0.197867
6165	0.973765	0.203648
6166	0.946131	0.253313
6167	0.851541	0.231478
6168	1.73854	0.214294
6169	1.91752	0.199676
6170	1.49622	0.227543
6171	1.23351	0.239576
6172	3.33289	2.1
6173	1.86606	2.1
6174	2.71233	2.1
6175	0.788816	2.1
6176	0.963972	2.1
6177	1.25866	2.1
6178	1.10817	2.1
6179	1.41908	2.1
6180	1.48734	2.1
6181	1.40822	0.737981
6182	1.40822	0.732941
6183	2.09871	0.789838
6184	0.946133	0.73053
6185	1.14926	0.685031
6186	0.946133	0.734264
6187	1.78098	0.708649
6188	1.78098	0.694983
6189	1.35822	0.721061
6190	1.3689	0.724407
6191	1.22176	0.69879
6192	2.03426	0.716421
6193	0.946131	0.753263
6194	1.01713	0.714194
6195	0.704112	0.757025
6196	1.36237	0.721676
6197	1.36237	0.707664
6198	1.01713	0.748506
6199	1.04936	0.74817
6200	1.87377	2.1
6201	1.60501	2.1
6202	0.981584	0.227337
6203	1.69435	0.227293

6204	0.85154	2.1
6205	1.47841	2.1
6206	1.4952	2.1
6207	1.31941	2.1
6208	1.04023	2.1
6209	1.1995	2.1
6210	0.914791	2.1
6211	1.1427	1.25897
6212	1.64787	1.06881
6213	1.07526	0.751248
6214	1.07527	0.643467
6215	1.07527	0.707063
6216	1.58551	0.870895
6217	2.77362	1.23431
6218	2.17974	0.960155
6219	2.71723	1.11349
6220	1.29868	0.436381
6221	1.3594	0.411522
6222	1.04786	0.377398
6223	0.527876	0.398278
6224	1.4085	0.420006
6225	1.48538	0.311111
6226	0.76829	0.357492
6227	1.18514	0.41467
6228	0.617954	0.308314
6229	0.864161	0.368531
6230	1.18835	0.328079
6231	1.16817	2.1
6232	1.04263	2.1
6233	1.50524	2.1
6234	1.85355	2.1
6235	1.23963	2.1
6236	1.06279	2.1
6237	0.94613	2.1
6238	1.35229	2.1
6239	1.15947	2.1
6240	0.965593	2.1
6241	0.851553	2.1
6242	2.21296	2.1
6243	1.11764	2.1
6244	2.46399	2.1
6245	1.39292	2.1
6246	1.34701	1.83741
6247	1.34701	2.1
6248	1.47001	2.1
6249	0.963166	0.208423
6250	0.893445	0.197969
6251	1.21056	0.195332
6252	1.67375	2.1
6253	2.0977	2.1
6254	1.09154	2.1
6255	1.09154	2.1
6256	1.57122	1.02731
6257	1.60976	0.731217
6258	1.69533	0.661138
6259	1.6951	0.592653
6260	1.5831	0.532281
6261	1.20969	2.1

6262	1.12076	2.1
6263	1.49808	2.1
6264	1.50201	2.1
6265	1.00278	2.1
6266	1.22811	0.374118
6267	1.11545	2.1
6268	1.09646	2.1
6269	1.03107	0.147815
6270	1.44293	0.370987
6271	1.66752	1.19208
6272	1.11023	1.17883
6273	2.57831	1.53846
6274	2.20191	1.13265
6275	2.18051	1.70042
6276	1.48035	0.64597
6277	1.0961	0.546405
6278	1.63241	0.54455
6279	1.0961	0.675312
6280	1.20959	2.1
6281	2.86136	2.1
6282	1.64933	2.1
6283	1.47217	2.1
6284	0.915178	2.1
6285	1.19074	2.1
6286	0.897276	2.1
6287	0.915177	2.1
6288	0.987037	2.1
6289	1.04288	2.1
6290	2.39323	2.1
6291	0.976218	0.20398
6292	2.23933	0.227765
6293	0.976215	0.206083
6294	1.07384	0.21632
6295	1.07652	2.1
6296	1.55678	2.1
6297	1.54168	2.1
6298	1.69136	1.6749
6299	1.64391	1.38519
6300	1.76477	1.23513
6301	0.611486	1.34455
6302	0.683421	1.13827
6303	0.946131	1.18655
6304	1.1019	1.23301
6305	1.12946	1.12045
6306	1.54226	1.58362
6307	1.19496	1.32431
6308	1.96199	1.21632
6309	2.0775	1.45365
6310	1.22297	1.27693
6311	1.3469	0.96228
6312	1.25366	1.06384
6313	1.65443	1.07331
6314	1.65443	1.06216
6315	0.946132	1.24256
6316	0.946132	1.26758
6317	1.12873	1.18104
6318	1.07468	1.01256
6319	1.2854	0.833629

6320	1.3469	0.979191
6321	1.68616	0.953836
6322	1.68616	0.876751
6323	0.946132	1.1823
6324	0.946132	1.06814
6325	1.16109	1.01936
6326	1.17879	0.931134
6327	1.17879	0.931621
6328	1.45304	1.0831
6329	2.21058	2.1
6330	2.81552	1.98414
6331	1.45535	0.619567
6332	1.49906	0.506344
6333	1.04413	2.1
6334	1.74586	2.01512
6335	1.34526	2.1
6336	1.17402	1.06673
6337	1.74564	0.961349
6338	1.52724	2.1
6339	1.68142	2.1
6340	1.09651	2.1
6341	1.49149	2.1
6342	1.36814	2.1
6343	1.46783	1.22742
6344	2.18116	1.39577
6345	1.23602	1.14112
6346	1.74538	0.960719
6347	0.851539	2.1
6348	1.45775	1.29994
6349	1.28919	2.1
6350	0.851483	2.1
6351	1.26102	1.26781
6352	0.869889	2.1
6353	0.85151	2.016
6354	1.23451	1.01976
6355	1.23602	1.20002
6356	1.11955	2.1
6357	1.08022	2.1
6358	0.977776	2.1
6359	1.27896	2.08634
6360	1.64268	1.88426
6361	1.97548	2.1
6362	1.72527	1.50496
6363	0.946132	2.1
6364	1.69702	1.47015
6365	1.69702	2.1
6366	1.13635	2.1
6367	1.40274	1.22504
6368	1.24041	2.1
6369	1.13635	2.06726
6370	1.78427	0.253376
6371	1.68338	0.297215
6372	1.00681	0.221789
6373	1.12988	0.212014
6374	0.946132	0.228357
6375	0.957543	0.219717
6376	1.40515	0.224397
6377	1.40515	0.214142

6378	0.797932	0.243166
6379	1.30187	0.248704
6380	1.14846	0.242816
6381	0.665137	0.591259
6382	1.09575	0.716395
6383	0.596894	0.664224
6384	1.04072	0.772723
6385	0.663171	0.799493
6386	1.47218	0.748169
6387	1.32889	0.708107
6388	1.32639	0.472098
6389	1.94887	0.502
6390	1.32639	0.477847
6391	1.19323	0.598646
6392	2.19794	0.667594
6393	0.754756	0.431631
6394	1.11015	0.471882
6395	0.754755	0.429619
6396	0.946133	0.511174
6397	0.754768	0.497143
6398	1.59986	0.478866
6399	1.59986	0.42135
6400	1.84082	2.1
6401	1.79044	2.1
6402	1.75922	2.1
6403	1.86766	2.1
6404	1.58995	2.1
6405	1.58995	2.1
6406	1.74351	2.1
6407	1.04442	2.1
6408	1.42278	2.1
6409	1.08576	2.1
6410	0.994294	2.1
6411	1.44884	1.38148
6412	1.71036	1.11378
6413	0.714101	0.852728
6414	1.17541	1.23009
6415	1.00872	0.745801
6416	0.714072	0.900803
6417	0.936677	0.867657
6418	0.966979	2.1
6419	1.4908	2.1
6420	1.03108	0.733909
6421	0.949712	0.658691
6422	0.810946	0.714405
6423	0.88209	0.715493
6424	0.646702	0.690569
6425	0.972663	0.677326
6426	0.862301	0.678114
6427	0.868143	0.646147
6428	1.11094	0.685992
6429	1.35809	0.683413
6430	1.32455	2.1
6431	1.32328	2.1
6432	1.69586	2.1
6433	1.78301	2.1
6434	1.67906	2.1
6435	1.60635	2.1

6436	1.57234	2.1
6437	1.47012	2.1
6438	1.22811	0.337317
6439	1.08732	0.388607
6440	2.08849	0.356872
6441	1.08732	0.370069
6442	1.30635	0.186388
6443	0.77315	0.183693
6444	1.25732	0.173682
6445	2.43845	2.01172
6446	2.38577	1.20007
6447	0.946132	0.935601
6448	1.19288	1.75872
6449	1.54508	2.09389
6450	1.54508	1.23205
6451	0.94515	1.70235
6452	1.19288	1.3376
6453	1.29735	1.12268
6454	1.12087	0.881472
6455	1.54699	1.07539
6456	1.43635	2.1
6457	1.33385	2.1
6458	1.43635	2.1
6459	1.02368	2.1
6460	1.035 2.1	
6461	1.00128	2.1
6462	1.04009	2.1
6463	1.14052	2.1
6464	0.443552	2.1
6465	0.984351	2.1
6466	0.591443	2.1
6467	0.929561	2.1
6468	1.52079	2.1
6469	1.035 2.1	
6470	3.5282	1.72516
6471	1.88288	1.70642
6472	2.73877	1.20335
6473	0.906059	2.1
6474	1.23107	0.339915
6475	0.906712	0.379111
6476	0.841322	0.426614
6477	1.53005	0.330518
6478	1.10007	0.382864
6479	1.38033	0.373121
6480	1.03442	0.342628
6481	0.787223	0.363885
6482	1.42589	0.366587
6483	2.35665	0.364504
6484	1.17793	0.378638
6485	1.81356	0.398802
6486	1.42964	0.366481
6487	1.95641	0.395551
6488	1.39139	0.7
6489	1.39139	0.701356
6490	0.564533	0.7
6491	1.20016	0.700862
6492	0.755765	0.589591
6493	0.755766	0.62523

6494	1.20016	0.7
6495	1.91011	0.7
6496	1.4695	0.616212
6497	1.70048	2.1
6498	1.93765	1.66075
6499	0.741307	2.1
6500	0.780371	2.1
6501	0.946133	0.797097
6502	1.28918	1.07603
6503	1.5111	1.29541
6504	1.5111	0.832655
6505	0.946131	1.08348
6506	1.28918	0.846389
6507	1.16805	0.802924
6508	1.01179	0.785041
6509	0.946132	0.83314
6510	1.35328	1.13061
6511	1.69633	1.27702
6512	1.69633	0.87384
6513	0.946133	1.13575
6514	1.35328	0.88962
6515	1.28918	0.841686
6516	1.01179	0.937969
6517	1.58147	0.909181
6518	1.27737	0.84037
6519	2.17223	0.296956
6520	0.697168	0.285845
6521	1.62495	0.454906
6522	1.62495	0.430475
6523	1.93948	0.674473
6524	0.601592	2.1
6525	1.93528	2.1
6526	2.13757	2.1
6527	1.67561	2.1
6528	2.70875	2.1
6529	1.93528	2.1
6530	3.98475	2.1
6531	1.12376	2.1
6532	1.19016	2.1
6533	1.67323	0.439216
6534	1.74019	0.404485
6535	1.84719	0.45907
6536	1.00764	0.398889
6537	1.23475	2.1
6538	1.06757	2.1
6539	1.57916	1.70508
6540	1.06758	2.1
6541	1.28014	2.1
6542	1.33339	2.1
6543	1.2679	2.1
6544	1.23504	2.1
6545	1.50108	2.1
6546	1.3715	2.1
6547	0.58831	0.982473
6548	0.865971	0.974833
6549	0.813704	1.11786
6550	0.658856	1.01857
6551	0.495869	1.11757

6552	1.13845	1.03554
6553	0.780804	1.0539
6554	0.642023	1.0833
6555	0.707748	0.962583
6556	1.64476	0.928507
6557	1.64476	1.01476
6558	0.99611	0.974754
6559	1.35639	0.9136
6560	1.3564	0.985037
6561	0.946129	0.96854
6562	1.99222	0.959576
6563	1.4777	2.1
6564	1.03968	2.1
6565	1.82516	2.1
6566	0.851539	2.1
6567	1.63693	2.1
6568	1.59416	2.1
6569	0.878566	2.1
6570	1.40796	2.1
6571	1.13172	1.94619
6572	0.851527	1.27558
6573	1.14366	2.1
6574	0.823822	0.578482
6575	0.882001	0.592527
6576	1.09756	0.340792
6577	1.11857	0.43432
6578	1.0934	0.421305
6579	0.960104	0.565224
6580	0.982001	0.667624
6581	1.74319	0.495956
6582	1.03043	0.491273
6583	0.967194	0.453891
6584	1.16938	2.1
6585	2.48377	2.1
6586	1.1181	2.1
6587	2.57349	2.1
6588	1.0465	2.1
6589	1.44709	2.1
6590	0.946132	2.1
6591	1.29302	2.1
6592	1.73502	2.1
6593	1.73502	2.1
6594	0.946132	2.1
6595	1.29302	2.1
6596	1.38813	2.1
6597	1.03933	2.1
6598	1.01976	0.67722
6599	1.11764	0.767411
6600	0.961779	0.857291
6601	1.0685	0.770177
6602	1.08426	1.20123
6603	0.99695	1.31742
6604	2.25593	1.4182
6605	1.47992	1.56862
6606	1.88989	1.14687
6607	1.65813	1.1487
6608	1.20278	1.22924
6609	0.981842	1.2761

6610	0.802655	1.78136
6611	0.953489	1.82921
6612	0.961719	1.75075
6613	0.975614	1.62427
6614	0.991617	1.29419
6615	0.940748	1.37387
6616	2.25019	1.80458
6617	2.30712	1.2688
6618	1.98823	1.66863
6619	0.522844	1.28868
6620	0.946122	1.70002
6621	0.946132	1.39328
6622	0.593846	1.25921
6623	1.26501	1.24719
6624	1.21171	0.332257
6625	0.851542	0.440502
6626	0.940327	0.464109
6627	0.940356	0.468427
6628	1.52117	0.366866
6629	1.58072	0.326328
6630	1.35169	0.389206
6631	1.13889	0.381112
6632	1.35007	0.297055
6633	1.35007	0.303763
6634	1.78381	0.340468
6635	0.946132	0.440137
6636	1.06627	0.41822
6637	0.946131	0.466547
6638	1.73005	0.338034
6639	1.73005	0.326941
6640	1.32449	0.381805
6641	1.35169	0.381373
6642	1.42446	0.809389
6643	1.86697	0.786616
6644	1.13413	0.779262
6645	1.42446	0.687066
6646	1.33575	0.481372
6647	1.20473	0.498165
6648	1.87517	0.257592
6649	1.87517	0.2533
6650	0.488712	0.389353
6651	1.13242	0.335707
6652	0.488713	0.349747
6653	0.488713	0.341465
6654	1.23146	0.331128
6655	1.57932	0.260484
6656	1.01698	0.276987
6657	1.42085	0.276213
6658	0.246725	0.253916
6659	0.996283	0.260835
6660	1.36965	0.271211
6661	0.86361	0.379412
6662	0.810945	0.264615
6663	1.72938	0.27738
6664	1.21649	0.370531
6665	1.1865	0.26923
6666	1.62387	0.287219
6667	1.05302	0.325806

6668	1.10049	0.829461
6669	0.946129	1.94298
6670	1.70184	0.994484
6671	1.70184	2.03842
6672	1.12538	1.9202
6673	1.35969	0.946639
6674	1.28828	1.52418
6675	1.12538	1.23089
6676	0.99747	1.35093
6677	1.34851	0.908786
6678	0.923724	2.1
6679	1.47154	1.04246
6680	1.47154	2.02834
6681	1.10698	2.1
6682	1.28828	0.984279
6683	1.10698	1.85581
6684	0.923695	1.54429
6685	0.99747	0.896512
6686	1.65677	2.1
6687	1.77677	2.1
6688	1.05689	0.548481
6689	2.11031	0.51866
6690	1.07367	0.426856
6691	0.656239	0.529303
6692	1.4563	0.491028
6693	1.4563	0.456641
6694	0.656238	0.515271
6695	0.946133	0.522295
6696	0.812937	0.581517
6697	0.946131	0.591651
6698	1.05689	0.440304
6699	1.34009	2.1
6700	1.398 2.1	
6701	3.29047	2.1
6702	2.53897	2.1
6703	1.24509	2.1
6704	3.06162	2.1
6705	1.01595	2.1
6706	1.52661	2.1
6707	1.5266	2.1
6708	0.946131	2.1
6709	1.31049	2.1
6710	1.16225	2.1
6711	1.05978	2.1
6712	1.16225	2.1
6713	1.06561	1.47459
6714	0.953927	2.1
6715	1.15114	1.42322
6716	1.1807	0.775808
6717	0.507259	0.695984
6718	1.47709	0.680872
6719	1.36192	0.645888
6720	0.487293	0.677183
6721	1.65569	0.740673
6722	0.679725	0.580902
6723	0.721928	0.675485
6724	1.03931	0.674587
6725	1.0775	0.763822

6726	1.0775	0.813998
6727	2.30207	0.934283
6728	1.01814	0.89804
6729	2.33963	0.854077
6730	2.42589	0.917569
6731	1.33962	2.1
6732	1.27956	2.1
6733	2.14624	0.246236
6734	1.6812	0.27509
6735	1.25698	0.253781
6736	1.35725	0.276341
6737	1.86387	0.249583
6738	1.86387	0.241506
6739	0.750365	0.296946
6740	0.946128	0.273168
6741	0.750365	0.244824
6742	1.72271	0.241478
6743	1.72272	0.303796
6744	2.53736	0.338893
6745	1.07327	2.1
6746	1.05376	2.1
6747	1.00387	1.10346
6748	2.0554	1.10409
6749	1.00387	1.04359
6750	1.00387	1.00928
6751	1.27119	2.1
6752	1.51748	2.1
6753	3.15271	2.1
6754	3.45801	2.1
6755	1.99871	2.1
6756	1.41186	2.1
6757	1.07624	2.1
6758	1.5954	2.1
6759	1.19323	0.954016
6760	1.21286	0.892799
6761	0.946114	2.1
6762	1.38395	2.1
6763	1.73389	2.1
6764	1.7339	2.1
6765	0.946115	2.1
6766	1.38397	2.1
6767	1.29604	2.1
6768	1.04544	2.1
6769	1.60578	0.350612
6770	1.93407	0.350697
6771	1.0525	0.913149
6772	1.0525	1.1784
6773	1.0525	1.37816
6774	1.38506	1.36209
6775	2.34737	0.217575
6776	1.3531	0.200804
6777	1.2925	0.195986
6778	0.871466	0.223707
6779	1.26623	0.208385
6780	0.700631	0.212891
6781	1.88774	0.200995
6782	1.14167	0.207024
6783	1.18606	0.202974

6784	2.1238	0.206435
6785	1.42762	0.987881
6786	1.67791	0.427416
6787	1.47118	0.408914
6788	0.946128	2.1
6789	1.31641	2.1
6790	1.75933	2.1
6791	1.75933	2.1
6792	0.946131	2.1
6793	1.31641	2.1
6794	1.38905	2.1
6795	1.38437	2.1
6796	1.51829	2.1
6797	0.947114	2.1
6798	1.13335	2.1
6799	1.47521	2.1
6800	1.14911	2.1
6801	1.53822	2.1
6802	1.39702	2.1
6803	0.739166	2.1
6804	0.706056	2.1
6805	0.964033	2.04649
6806	1.58993	1.8872
6807	2.51892	1.63708
6808	0.595905	0.349745
6809	1.18155	0.282954
6810	1.73364	0.26846
6811	0.952735	0.291883
6812	2.10381	0.266312
6813	1.12115	0.269977
6814	1.27826	0.280083
6815	2.74021	0.25951
6816	1.78416	2.1
6817	1.78416	2.1
6818	0.946132	2.1
6819	1.41126	2.1
6820	1.21275	2.1
6821	0.946133	2.1
6822	1.41126	2.1
6823	1.33125	2.1
6824	1.33125	2.1
6825	1.78918	2.1
6826	1.0465	2.1
6827	1.99715	2.1
6828	1.17683	2.1
6829	1.25889	2.1
6830	0.860834	0.442807
6831	0.946131	0.394727
6832	1.05008	0.352499
6833	0.970444	0.443106
6834	1.19401	0.410203
6835	1.25989	0.342744
6836	0.702942	0.497113
6837	1.40591	0.558706
6838	1.72168	0.528251
6839	0.446352	0.568717
6840	1.04072	0.426981
6841	0.711038	0.569474

6842	0.980737	0.533132
6843	1.05099	0.500086
6844	1.26003	0.394095
6845	0.495934	0.60391
6846	1.03114	0.374812
6847	1.09911	0.429083
6848	1.24825	0.397379
6849	2.19827	0.363633
6850	2.0953	0.269765
6851	2.30437	0.307444
6852	0.775923	0.288712
6853	2.2283	0.241692
6854	1.19583	2.1
6855	1.14496	2.1
6856	1.62183	2.1
6857	1.62316	2.1
6858	1.02305	0.745897
6859	1.21136	0.64283
6860	1.44426	0.720169
6861	1.21137	0.523757
6862	1.20143	0.558383
6863	1.15009	0.800236
6864	1.24965	2.1
6865	1.41077	2.1
6866	1.00052	0.382177
6867	1.79553	0.378498
6868	1.06336	0.311751
6869	1.00053	0.344604
6870	1.18041	2.1
6871	1.80818	1.28698
6872	1.73571	1.15029
6873	1.94429	1.26177
6874	1.4375	0.375317
6875	1.44421	0.324188
6876	1.66953	0.431097
6877	1.09529	0.374026
6878	0.952395	2.1
6879	0.717337	2.1
6880	1.03609	1.79264
6881	1.33949	0.80714
6882	1.2774	0.616706
6883	2.24983	0.537181
6884	1.28727	0.645977
6885	0.583592	0.54359
6886	0.583591	0.698285
6887	1.82859	0.646983
6888	1.82859	0.580202
6889	0.583593	0.662674
6890	0.946128	0.569782
6891	0.692233	0.681795
6892	1.12226	0.697224
6893	2.57454	0.719251
6894	0.810944	0.697025
6895	0.810945	0.676525
6896	1.12226	0.684396
6897	1.45867	0.682599
6898	1.22403	0.624161
6899	1.15262	0.702596

6900	1.08132	0.687662
6901	0.846671	0.661596
6902	0.480954	0.689454
6903	0.917975	0.658623
6904	0.917975	0.713807
6905	0.720957	0.615291
6906	0.795998	0.599303
6907	0.69316	0.71542
6908	0.641306	0.659338
6909	0.690817	0.605953
6910	0.961899	2.1
6911	2.49579	2.1
6912	2.31106	2.1
6913	1.70482	0.513305
6914	1.93156	0.567063
6915	1.23868	0.481108
6916	1.73878	0.611666
6917	1.26267	2.1
6918	1.26441	2.1
6919	1.58095	2.1
6920	1.58095	2.1
6921	0.946132	2.1
6922	0.946132	2.1
6923	1.11077	2.1
6924	1.62905	2.1
6925	1.49692	2.1
6926	3.49966	2.1
6927	2.39213	2.1
6928	2.12603	2.1
6929	0.946129	2.1
6930	0.461661	2.1
6931	1.52841	2.1
6932	1.39091	2.1
6933	0.550679	2.1
6934	0.509894	2.1
6935	1.49508	2.1
6936	2.05862	2.1
6937	2.47851	2.1
6938	1.07213	2.1
6939	1.09148	0.893103
6940	1.99776	0.941305
6941	1.38333	0.935854
6942	1.04412	2.1
6943	1.04412	2.1
6944	1.1893	0.377553
6945	0.946132	0.3619
6946	0.965737	0.365944
6947	0.946132	0.383089
6948	1.54061	0.365693
6949	1.54061	0.334866
6950	1.22878	0.348289
6951	1.25797	0.305502
6952	1.00665	0.353092
6953	1.00665	0.35632
6954	1.80369	0.354943
6955	0.946128	0.355862
6956	1.04424	0.366359
6957	0.85154	0.385496

6958	1.39322	0.351286
6959	1.35464	0.339802
6960	0.939857	0.338815
6961	1.28272	0.318088
6962	1.19323	0.617707
6963	1.03498	0.381712
6964	2.22029	0.425844
6965	0.540005	0.377468
6966	0.970382	0.423686
6967	2.14514	0.466964
6968	1.31542	0.466272
6969	1.05575	0.553685
6970	0.810943	0.595532
6971	1.31542	0.439941
6972	1.70493	0.48077
6973	1.22582	0.524918
6974	1.31681	0.568464
6975	1.40775	0.471674
6976	0.928633	0.642415
6977	1.2545	0.460088
6978	2.66481	0.507555
6979	1.32051	0.504424
6980	1.32051	0.550005
6981	2.66305	2.1
6982	1.3551	2.1
6983	2.45325	2.1
6984	1.3685	2.1
6985	1.51748	2.1
6986	1.78941	2.1
6987	1.02963	0.285388
6988	1.28643	0.340656
6989	0.86131	0.362229
6990	0.851539	0.393435
6991	1.46585	0.292299
6992	1.38822	0.27428
6993	0.996402	0.303614
6994	0.851553	0.35609
6995	1.73156	0.3377
6996	1.91695	0.301707
6997	1.92535	0.350912
6998	2.57287	0.311615
6999	1.77743	0.304978
7000	1.02836	0.679924
7001	1.36362	0.690857
7002	1.36936	0.596528
7003	1.78684	0.557169
7004	1.78685	0.607583
7005	0.946132	0.736896
7006	1.27581	0.712238
7007	0.946132	0.768559
7008	1.02836	0.531316
7009	1.55398	2.1
7010	1.07167	2.1
7011	1.87557	2.1
7012	0.751639	2.1
7013	1.50884	2.1
7014	1.33793	2.1
7015	0.751664	2.1

7016	1.37277	2.1
7017	0.979515	2.1
7018	0.676508	2.1
7019	1.09578	2.1
7020	0.953925	2.1
7021	0.94613	2.1
7022	1.64707	2.1
7023	1.64707	2.1
7024	1.16658	2.1
7025	1.40638	2.1
7026	1.18682	2.1
7027	1.16658	2.1
7028	0.543765	0.480353
7029	1.57462	0.462918
7030	1.57462	0.399253
7031	1.00083	0.469312
7032	1.16834	0.540333
7033	1.16834	0.507049
7034	0.889626	0.511343
7035	1.25944	0.51337
7036	0.824061	0.538437
7037	1.9397	0.379646
7038	1.21893	0.429498
7039	1.91364	0.413294
7040	1.33672	0.503844
7041	0.928069	0.605359
7042	1.41484	0.5137
7043	0.538379	0.471872
7044	1.41484	0.479659
7045	1.35594	0.526238
7046	2.11414	0.497046
7047	2.11414	0.382675
7048	0.538378	0.494604
7049	1.38532	0.381496
7050	0.962877	0.804652
7051	1.07497	0.606824
7052	1.00613	0.463639
7053	0.962878	0.597827
7054	0.711447	2.1
7055	1.40008	0.539515
7056	2.51674	0.599771
7057	1.40008	0.538798
7058	1.7263	0.409393
7059	1.40008	0.595041
7060	1.9216	0.256237
7061	2.7013	0.262978
7062	2.58584	0.238219
7063	0.979296	0.261666
7064	1.46459	0.674604
7065	0.899254	0.558465
7066	1.15038	1.76128
7067	1.24687	1.48746
7068	1.16738	1.83783
7069	0.942323	2.1
7070	1.65573	1.5239
7071	0.844791	2.06118
7072	0.54674	1.63719
7073	0.795811	1.87175

7074	1.08131	1.89969
7075	1.03553	1.87163
7076	0.758334	1.96531
7077	0.708665	1.62719
7078	1.22626	1.63402
7079	0.835439	1.62885
7080	0.861131	1.47456
7081	0.68296	1.74803
7082	1.65224	1.6293
7083	2.82624	1.74414
7084	1.0151	1.27707
7085	1.15905	1.43637
7086	1.71076	1.34194
7087	1.4529	1.20001
7088	0.494895	1.89007
7089	0.946132	1.4338
7090	0.613132	1.3358
7091	0.863631	1.35984
7092	1.78391	2.1
7093	1.1706	0.199872
7094	2.02734	0.216127
7095	1.1706	0.174724
7096	0.681532	0.191659
7097	1.50316	0.185473
7098	1.50316	0.178247
7099	0.681534	0.186844
7100	0.973819	0.196704
7101	0.97382	0.190428
7102	0.94613	0.201257
7103	1.1706	0.18162
7104	1.67727	0.300173
7105	1.60329	0.274725
7106	1.3227	2.1
7107	1.40846	2.1
7108	1.07889	2.1
7109	1.07721	2.1
7110	0.953523	0.994989
7111	1.79675	0.979351
7112	2.09859	0.999228
7113	1.21309	1.97637
7114	1.54567	0.909552
7115	1.19722	0.820544
7116	1.82793	0.796272
7117	1.1599	0.76615
7118	1.73621	0.73426
7119	0.933474	0.800257
7120	1.51477	0.774372
7121	1.51477	0.806377
7122	0.93347	0.799525
7123	1.27374	0.773337
7124	1.27376	0.803681
7125	0.946105	0.806522
7126	1.1972	0.766422
7127	0.851541	0.205503
7128	1.36971	0.19617
7129	1.27832	0.1942
7130	0.853489	0.201151
7131	0.973847	0.205933

7132	0.976308	0.205387
7133	0.851528	0.215944
7134	2.72654	0.196197
7135	1.77867	0.184193
7136	3.23032	2.1
7137	2.78208	2.1
7138	1.0227	0.240547
7139	1.02269	0.217727
7140	1.02269	0.221092
7141	1.24293	0.246936
7142	0.831695	2.1
7143	0.67513	2.1
7144	0.956294	2.1
7145	1.08623	2.1
7146	1.05556	2.1
7147	1.38859	2.1
7148	1.13179	2.1
7149	1.02438	2.1
7150	1.06659	2.1
7151	1.36595	2.05314
7152	1.06659	2.1
7153	1.06659	0.996551
7154	0.946131	1.14757
7155	0.875912	2.06607
7156	1.65971	2.1
7157	1.65971	1.33292
7158	0.875912	2.0533
7159	1.1229	1.75898
7160	1.41273	1.62162
7161	1.54699	1.31949
7162	1.93655	1.2797
7163	1.42307	1.09956
7164	1.97907	1.24955
7165	0.996774	2.1
7166	1.15505	0.267565
7167	1.28463	0.250164
7168	1.86353	0.298595
7169	2.71398	0.313598
7170	1.56787	0.320648
7171	1.66295	0.33052
7172	1.73432	1.80953
7173	1.05019	0.223634
7174	1.42117	0.22606
7175	0.888299	0.219545
7176	1.38127	0.23073
7177	1.72993	0.220641
7178	0.664129	0.223347
7179	0.99302	0.937085
7180	0.993023	0.935419
7181	1.13491	2.1
7182	0.876316	1.59863
7183	1.2588	2.1
7184	0.895613	1.99552
7185	2.07526	2.1
7186	1.23369	1.83914
7187	1.25146	2.1
7188	2.15282	2.04708
7189	1.22239	1.57848

7190	1.10962	2.1
7191	1.77402	1.59945
7192	1.0305	2.1
7193	1.50108	1.29084
7194	1.20761	2.1
7195	1.3626	2.1
7196	1.14825	2.1
7197	1.78711	2.1
7198	1.17933	2.1
7199	1.14824	2.1
7200	1.09651	0.468036
7201	1.23851	0.430261
7202	1.83284	0.493907
7203	1.23851	0.357888
7204	1.53521	2.1
7205	1.46279	2.1
7206	1.9233	0.484237
7207	1.2966	0.461267
7208	2.11443	0.416687
7209	1.30265	0.486277
7210	1.13528	0.593811
7211	0.946131	0.74995
7212	1.41169	0.67115
7213	1.33879	0.583215
7214	0.946128	0.619988
7215	1.80435	0.639977
7216	1.80435	0.483785
7217	1.234	2.1
7218	1.45633	2.1
7219	1.234	2.1
7220	1.98448	2.1
7221	2.02612	2.1
7222	1.23527	0.859079
7223	1.44472	0.894949
7224	0.993366	1.05187
7225	1.22025	0.987435
7226	0.993368	0.864902
7227	2.43669	2.1
7228	2.56707	2.1
7229	1.80348	1.35786
7230	1.74406	1.04814
7231	1.90937	1.20706
7232	1.49357	0.644651
7233	2.5832	0.782949
7234	1.82274	0.802976
7235	1.6957	0.73601
7236	2.22495	0.739191
7237	2.49959	0.749477
7238	2.01183	0.428031
7239	1.37427	0.345877
7240	1.15069	1.36914
7241	1.39379	1.14823
7242	1.69661	0.827082
7243	1.39379	0.901756
7244	1.39654	2.1
7245	1.93505	2.1
7246	1.5525	2.1
7247	1.5525	2.1

7248	0.615065	2.1
7249	1.38908	2.1
7250	0.615065	2.1
7251	0.77849	2.1
7252	1.38908	2.1
7253	1.56839	1.14373
7254	1.09142	1.2679
7255	2.07313	1.03484
7256	0.998288	0.300088
7257	1.31481	0.308414
7258	1.88175	0.322788
7259	2.23267	0.379007
7260	1.06935	2.1
7261	1.06936	2.1
7262	1.17989	1.30581
7263	1.00757	0.985565
7264	1.65318	1.00195
7265	1.4185	0.617908
7266	2.22495	0.66397
7267	1.02011	0.475777
7268	0.59676	0.581713
7269	1.43606	0.539349
7270	1.43606	0.490457
7271	0.89771	0.544909
7272	1.13511	0.608357
7273	0.59676	0.419838
7274	0.897709	0.556898
7275	1.79542	0.535056
7276	2.38566	2.05815
7277	1.63838	1.08477
7278	1.63837	1.37467
7279	0.916275	1.90427
7280	1.36183	1.1421
7281	1.19282	2.01525
7282	0.916275	1.56595
7283	1.19283	1.77053
7284	2.01582	2.1
7285	1.00215	0.307854
7286	2.60643	2.1
7287	2.15971	2.1
7288	1.28267	2.1
7289	1.29954	2.1
7290	3.40327	2.1
7291	3.49128	2.1
7292	1.3864	2.1
7293	1.3201	2.1
7294	1.45666	0.18673
7295	1.55798	0.182478
7296	0.857202	0.188333
7297	1.24374	0.190283
7298	1.04157	0.192778
7299	0.85723	0.188059
7300	1.02722	0.188613
7301	1.01361	0.195317
7302	0.974077	0.174588
7303	1.01814	0.181866
7304	0.974077	0.171693
7305	1.43987	0.167834

7306	1.43986	0.163913
7307	0.927881	0.164471
7308	0.946132	0.169255
7309	0.927849	0.166826
7310	0.927852	0.163273
7311	1.2493	0.167493
7312	1.22482	0.838216
7313	1.41319	0.938312
7314	1.22482	0.767487
7315	0.999492	2.1
7316	1.14657	2.1
7317	1.3617	0.194305
7318	0.983269	0.20271
7319	1.33174	0.210663
7320	1.62816	0.213097
7321	1.62817	0.202201
7322	0.81938	0.197725
7323	1.33174	0.20062
7324	1.01332	0.211604
7325	0.874788	0.177626
7326	1.16725	2.1
7327	1.63991	2.1
7328	1.08848	0.290996
7329	1.15354	0.260377
7330	1.12665	2.1
7331	1.58742	2.1
7332	1.57523	2.1
7333	1.32201	1.01824
7334	1.32097	0.98461
7335	0.851542	0.707628
7336	0.877188	0.68341
7337	0.797489	0.707548
7338	1.44721	0.673051
7339	1.61761	0.683517
7340	0.943799	0.696815
7341	1.36079	0.693251
7342	1.16361	1.74266
7343	1.15852	1.25894
7344	1.07624	2.1
7345	1.07624	2.1
7346	0.946132	0.870376
7347	1.37938	1.16049
7348	1.78653	1.26433
7349	1.78653	0.919224
7350	0.946131	1.18661
7351	1.37938	0.943957
7352	1.35328	0.883798
7353	1.44847	0.886929
7354	0.946133	0.914236
7355	1.38149	1.13377
7356	1.81474	1.18936
7357	1.81474	0.950305
7358	0.946133	1.20733
7359	1.38149	0.989899
7360	1.37938	0.925161
7361	1.44847	0.988163
7362	1.93665	0.873356
7363	1.5515	0.92176

7364	1.05025	2.1
7365	1.05025	2.1
7366	1.71468	1.97685
7367	1.04759	2.1
7368	1.04646	0.226441
7369	1.00739	0.23041
7370	1.37086	0.213314
7371	1.12547	0.228817
7372	1.03853	0.225526
7373	1.34824	0.208987
7374	0.799786	0.220244
7375	2.10105	0.226073
7376	2.72007	0.215626
7377	0.589918	0.196064
7378	0.589918	0.203475
7379	1.1509	0.200144
7380	0.619832	0.191555
7381	1.1509	0.205789
7382	0.705903	0.203011
7383	1.53703	0.195444
7384	1.53703	0.200199
7385	0.705904	0.199495
7386	0.946133	0.204156
7387	0.946132	0.198923
7388	0.946132	0.211984
7389	0.588467	0.185669
7390	1.1078	0.196195
7391	0.408061	0.18954
7392	1.38703	0.207292
7393	1.68328	0.338278
7394	1.87031	0.379435
7395	1.86268	0.375565
7396	1.27472	0.419692
7397	0.947862	1.05995
7398	1.19854	1.19416
7399	0.946129	0.936459
7400	0.963111	0.837701
7401	0.946131	0.950888
7402	1.39055	0.891587
7403	1.39055	0.846537
7404	1.26426	0.906877
7405	1.07242	0.921174
7406	1.31126	0.840886
7407	1.76597	0.955185
7408	0.94613	1.20967
7409	1.27214	1.10916
7410	1.90751	1.09459
7411	1.90751	1.05538
7412	0.767114	1.20372
7413	1.27214	1.19492
7414	1.40248	1.13798
7415	1.41479	1.24825
7416	2.22625	1.25588
7417	0.913373	1.23398
7418	1.0158	1.04736
7419	1.61259	0.999693
7420	1.69051	0.938484
7421	0.560949	1.20464

7422	0.898559	1.20907
7423	1.28011	1.12696
7424	1.51026	0.433996
7425	1.98252	0.496564
7426	1.05129	0.451892
7427	1.2951	0.461099
7428	1.46041	0.157523
7429	1.7155	2.1
7430	1.72196	2.1
7431	0.933885	2.1
7432	0.986723	2.1
7433	1.28072	0.730207
7434	1.40765	1.07206
7435	1.28072	0.94087
7436	1.61687	2.1
7437	1.60965	2.1
7438	1.21424	2.1
7439	2.07297	2.1
7440	1.19943	2.1
7441	2.38784	2.1
7442	1.10278	0.443977
7443	1.43678	0.455754
7444	1.1142	0.446522
7445	1.18034	0.391521
7446	0.94613	0.458568
7447	0.752356	0.422776
7448	0.752329	0.446923
7449	1.46634	0.380589
7450	1.31789	0.35824
7451	0.958159	0.434833
7452	1.22752	0.377947
7453	1.50472	0.405072
7454	1.98974	2.1
7455	2.54186	2.1
7456	0.76512	2.1
7457	2.08777	2.1
7458	1.7753	2.1
7459	3.07372	2.1
7460	1.9756	2.1
7461	1.48162	2.1
7462	1.23204	2.1
7463	0.946131	1.0012
7464	1.13505	1.83986
7465	1.47432	2.034
7466	1.47432	1.1328
7467	0.876831	1.48204
7468	1.13505	1.29111
7469	1.2161	1.1328
7470	1.05267	1.21714
7471	1.05267	1.08261
7472	1.5795	1.08911
7473	0.98186	2.1
7474	1.56735	2.1
7475	0.859099	2.1
7476	1.69858	2.1
7477	1.93586	2.1
7478	1.09233	2.1
7479	1.08732	0.356746

7480	1.3357	0.360703
7481	1.84826	2.1
7482	0.924132	1.74858
7483	1.39188	2.1
7484	1.43685	2.1
7485	0.851541	2.09994
7486	1.16251	2.1
7487	0.946131	2.1
7488	1.05685	2.1
7489	2.13781	2.1
7490	1.98859	2.1
7491	1.99731	2.1
7492	1.51379	2.1
7493	1.48796	2.1
7494	0.851542	2.1
7495	0.802558	2.1
7496	1.09244	2.1
7497	1.33891	2.1
7498	0.844958	2.1
7499	0.710864	2.1
7500	1.26087	2.1
7501	1.03679	2.1
7502	1.51026	0.427511
7503	1.05248	0.814706
7504	0.827258	0.761129
7505	1.05248	0.77698
7506	1.28435	0.807921
7507	1.50953	0.839701
7508	1.50953	0.553838
7509	0.827298	0.785896
7510	1.00366	0.523178
7511	2.10495	0.649094
7512	1.17885	2.1
7513	1.25185	2.1
7514	1.02784	2.1
7515	1.76764	2.1
7516	1.23746	0.449738
7517	1.24082	0.457663
7518	1.46506	0.421592
7519	1.33061	0.328779
7520	0.768407	0.902389
7521	0.972058	0.745201
7522	1.16536	1.03963
7523	1.13325	0.819004
7524	0.978906	1.30838
7525	1.02291	0.801726
7526	0.838015	0.74251
7527	1.01052	0.509455
7528	1.08166	0.560031
7529	1.42607	0.331904
7530	1.47354	0.385785
7531	1.21703	2.1
7532	1.29897	2.1
7533	1.83293	2.1
7534	1.29897	2.1
7535	1.24688	0.395698
7536	0.782563	2.1
7537	0.946129	2.1

7538	0.782591	2.1
7539	1.49487	2.1
7540	1.49487	2.1
7541	1.37713	2.1
7542	0.900337	2.1
7543	1.31284	2.1
7544	0.924468	2.1
7545	1.40602	1.81755
7546	1.25669	1.81435
7547	0.986901	2.1
7548	0.958565	2.1
7549	1.37266	2.1
7550	1.64146	2.1
7551	0.49345	2.1
7552	1.08276	2.1
7553	1.08276	2.1
7554	1.26929	2.06382
7555	1.09847	2.1
7556	0.827081	0.636193
7557	1.07236	0.758341
7558	1.25063	0.605794
7559	1.43548	0.362808
7560	1.25063	0.308895
7561	1.3659	0.308927
7562	1.72441	0.29426
7563	1.3659	0.275053
7564	1.4677	0.307694
7565	1.86114	0.28942
7566	1.40104	0.350843
7567	0.962675	0.376054
7568	0.946131	0.403174
7569	1.74134	0.292872
7570	1.74134	0.277879
7571	0.962676	0.300673
7572	0.946131	0.365036
7573	1.05548	0.452185
7574	0.902187	0.497025
7575	2.14178	0.372384
7576	1.74998	0.424917
7577	1.38541	0.452286
7578	1.64468	0.377
7579	0.949317	0.486416
7580	2.06654	0.413601
7581	0.839626	0.463617
7582	0.810945	0.489052
7583	1.29203	0.352299
7584	1.18091	0.415726
7585	1.06872	0.464433
7586	1.11956	0.380503
7587	0.926911	0.505189
7588	0.901716	0.47905
7589	0.909146	0.511067
7590	1.33776	0.39541
7591	1.02048	0.448928
7592	1.08365	0.481299
7593	1.21042	0.407911
7594	0.865634	0.507183
7595	0.590354	2.1

7596	0.61173	2.1
7597	3.46825	2.1
7598	3.28404	2.1
7599	1.56004	0.297092
7600	1.69344	0.375607
7601	1.7151	0.668637
7602	0.946131	0.338708
7603	1.40265	0.32618
7604	1.13537	0.676914
7605	0.965708	0.39445
7606	1.13537	0.504246
7607	1.29681	0.304806
7608	1.40915	0.469439
7609	1.61652	0.743849
7610	1.33171	0.822296
7611	1.37681	2.1
7612	0.81094	0.221828
7613	0.598606	0.210236
7614	0.723111	0.223187
7615	1.04562	0.203125
7616	0.979052	0.21222
7617	0.798185	0.207393
7618	0.880117	0.221003
7619	0.979341	0.742917
7620	0.800229	0.724467
7621	1.39126	0.740007
7622	1.13005	0.744886
7623	0.913877	0.76413
7624	1.26959	0.736131
7625	0.699741	0.75948
7626	1.19496	1.38097
7627	1.13392	0.272038
7628	1.11913	0.248036
7629	1.06866	0.246268
7630	0.615546	0.282394
7631	0.831006	0.295455
7632	1.53286	0.252002
7633	0.690977	0.279733
7634	1.01956	0.277803
7635	2.33818	0.225218
7636	1.6585	2.1
7637	1.67061	2.1
7638	1.41414	2.1
7639	1.16756	2.1
7640	1.00898	0.226504
7641	0.966964	2.1
7642	2.85443	2.1
7643	0.966977	2.1
7644	2.08427	2.1
7645	2.94901	2.1
7646	1.10735	0.498769
7647	1.65815	2.1
7648	1.4502	2.1
7649	1.45026	2.1
7650	1.53446	2.1
7651	0.946129	0.962971
7652	1.81075	2.06909
7653	1.81075	1.00925

7654	0.946131	1.37694
7655	1.37075	2.02322
7656	1.37075	1.00955
7657	1.34245	1.47478
7658	0.973502	1.04596
7659	1.08542	1.82511
7660	1.5814	0.237539
7661	1.43836	0.220258
7662	0.946132	0.300725
7663	1.13747	0.25753
7664	1.04429	0.294654
7665	1.05017	0.275411
7666	1.24732	0.272681
7667	1.13387	0.249034
7668	2.19489	0.249333
7669	1.10748	1.93034
7670	1.25006	1.97922
7671	1.58834	1.8818
7672	1.58834	0.794095
7673	0.94613	2.06814
7674	1.16548	1.74391
7675	0.946132	0.803005
7676	1.08998	0.910743
7677	1.16548	0.87577
7678	0.995732	0.780564
7679	0.657014	2.1
7680	0.957173	1.97425
7681	1.01485	2.1
7682	1.12468	2.1
7683	1.43871	2.1
7684	1.23716	2.1
7685	1.89277	0.969636
7686	2.06653	0.87852
7687	0.967544	0.752479
7688	1.89016	0.802873
7689	2.22165	1.71875
7690	0.83514	1.59202
7691	1.76565	2.03954
7692	1.76565	2.1
7693	0.94613	1.53388
7694	0.835112	2.1
7695	1.23426	1.59035
7696	1.36653	1.80498
7697	0.895892	1.19372
7698	0.859278	1.14704
7699	0.791461	1.19662
7700	1.86249	1.32103
7701	1.14607	1.21571
7702	1.19904	1.27611
7703	1.84898	1.23739
7704	1.03452	0.941572
7705	0.946131	0.742262
7706	0.940472	0.633918
7707	1.53478	1.01116
7708	1.84175	0.898711
7709	1.55687	0.871245
7710	1.17787	0.70023
7711	1.60589	0.641567

7712	2.35574	0.426442
7713	1.3305	0.679398
7714	1.15008	1.0946
7715	1.12457	0.302134
7716	1.12457	0.332707
7717	1.01778	2.1
7718	1.35733	2.1
7719	1.50683	2.1
7720	1.12064	2.1
7721	1.42716	0.774272
7722	1.42717	0.913204
7723	1.45927	1.09222
7724	1.04321	1.26308
7725	1.32634	0.533879
7726	2.27022	0.482577
7727	1.19352	0.411837
7728	1.32634	0.39627
7729	1.40924	1.36192
7730	1.22927	1.08877
7731	1.69237	1.26018
7732	1.69237	1.84021
7733	0.946132	0.9899
7734	0.946133	1.28042
7735	1.16912	1.23094
7736	1.0417	1.13092
7737	1.14605	1.34978
7738	1.1233	2.1
7739	1.96882	1.96414
7740	1.03804	0.428615
7741	1.15338	0.428779
7742	1.15338	0.431647
7743	2.33051	0.488327
7744	1.81507	1.3282
7745	2.42949	1.95091
7746	0.725879	1.01538
7747	1.88394	1.07895
7748	2.2699	0.492212
7749	2.78952	1.91358
7750	1.88394	0.672231
7751	1.79606	2.1
7752	1.20091	2.1
7753	1.9614	2.09228
7754	1.19901	1.21649
7755	3.07915	2.1
7756	1.32592	2.1
7757	2.86461	2.1
7758	1.34817	2.1
7759	1.44859	2.1
7760	2.03969	2.1
7761	0.946132	2.1
7762	1.50709	2.1
7763	1.50709	2.1
7764	0.946132	2.1
7765	1.29322	2.1
7766	1.29322	2.1
7767	1.01985	2.1
7768	0.968537	2.1
7769	1.401	2.1

7770	1.28936	2.1
7771	1.56002	0.161621
7772	1.92548	0.160025
7773	1.46041	0.161744
7774	2.22712	0.158002
7775	1.56001	0.16057
7776	1.50524	2.1
7777	1.21304	2.1
7778	1.60665	1.53193
7779	1.85178	1.20046
7780	2.17534	2.07618
7781	2.31012	2.1
7782	2.17533	1.47226
7783	0.986724	2.1
7784	2.01895	2.1
7785	2.00433	2.1
7786	1.45752	0.44535
7787	1.12695	0.410599
7788	0.652605	1.97282
7789	1.5133	2.1
7790	1.5133	2.1
7791	0.652603	1.79727
7792	1.228	2.1
7793	0.946132	2.1
7794	0.652604	2.1
7795	0.946132	1.24867
7796	0.716815	2.05893
7797	1.62203	1.51553
7798	1.62203	2.08133
7799	0.716814	2.0512
7800	1.00191	1.75022
7801	1.33694	1.47096
7802	1.38216	1.99878
7803	2.53338	1.88439
7804	1.46932	0.24308
7805	1.50848	0.238479
7806	1.50108	1.09208
7807	1.18382	0.890352
7808	2.02115	0.796178
7809	0.921823	0.739094
7810	2.08356	0.209728
7811	1.543	0.183169
7812	1.00763	0.740493
7813	1.4185	0.772097
7814	1.08028	0.69848
7815	0.946131	0.83361
7816	1.56599	0.749484
7817	1.56599	0.845862
7818	1.08028	0.774796
7819	0.946131	0.657485
7820	1.37518	2.1
7821	1.63453	2.1
7822	1.04627	1.22246
7823	1.04626	1.21357
7824	1.35111	1.28938
7825	1.22297	1.24566
7826	1.64499	1.36536
7827	1.87598	1.13103

7828	1.06994	2.1
7829	1.37063	2.1
7830	1.99943	0.269174
7831	2.38807	0.264918
7832	0.99972	0.339925
7833	1.41388	0.354052
7834	1.66179	0.327084
7835	1.66179	0.275945
7836	0.946129	0.38273
7837	1.41388	0.353852
7838	1.19404	0.350561
7839	1.31023	1.09234
7840	1.20871	1.1301
7841	1.69731	2.1
7842	1.67971	2.1
7843	0.998971	0.197865
7844	1.32988	0.219273
7845	0.701079	0.223428
7846	0.946131	0.235953
7847	0.582785	0.222025
7848	1.48592	0.206684
7849	1.48592	0.194283
7850	1.23351	0.213217
7851	0.835199	0.221778
7852	0.92427	0.195391
7853	1.68262	0.198867
7854	2.46702	0.220117
7855	1.08715	1.88778
7856	1.01606	1.74568
7857	1.29579	1.66066
7858	1.01606	1.97774
7859	1.63823	2.1
7860	1.67687	2.1
7861	1.11048	1.86355
7862	1.39477	2.1
7863	0.991114	2.1
7864	2.90978	0.863302
7865	0.906811	0.933502
7866	0.872849	0.726186
7867	1.17091	0.840722
7868	0.685763	0.811998
7869	1.6386	1.1406
7870	1.06907	0.829217
7871	1.05516	0.907205
7872	1.9189	0.906655
7873	1.04615	0.741101
7874	1.2966	2.1
7875	1.37392	2.1
7876	1.35504	2.1
7877	1.30347	2.1
7878	1.50877	2.1
7879	1.11193	2.1
7880	1.40984	2.0759
7881	1.67682	2.09155
7882	1.00371	2.1
7883	1.15412	2.1
7884	1.41638	2.1
7885	1.03796	2.1

7886	0.946117	2.1
7887	1.50823	2.1
7888	1.50822	2.1
7889	0.966923	2.1
7890	0.946131	2.1
7891	2.07593	2.1
7892	1.9339	2.1
7893	1.1817	0.853543
7894	0.729178	0.856146
7895	1.5079	0.831576
7896	1.26991	0.904267
7897	0.994049	0.863579
7898	1.17452	0.824076
7899	1.05711	0.872467
7900	1.04071	0.857858
7901	1.80299	0.85766
7902	1.60417	0.833703
7903	0.943405	0.396099
7904	1.01129	0.320233
7905	1.42972	0.347896
7906	1.15904	0.361628
7907	0.680409	0.326284
7908	1.2816	0.270663
7909	0.806176	0.32026
7910	1.18954	0.309729
7911	1.26055	0.371256
7912	1.06313	2.1
7913	1.19352	2.1
7914	1.55766	2.1
7915	1.53729	2.1
7916	0.993021	0.576728
7917	1.09056	0.864537
7918	1.03408	2.1
7919	0.993023	0.855798
7920	0.659901	2.1
7921	0.632905	2.1
7922	1.34072	0.364448
7923	1.17793	0.414059
7924	2.24885	0.393096
7925	0.946132	0.391631
7926	1.12443	0.383589
7927	0.904207	0.407358
7928	1.51237	0.354209
7929	1.51237	0.352473
7930	1.12443	0.365294
7931	1.29215	0.336554
7932	1.6943	1.69924
7933	2.43858	1.82195
7934	2.0589	1.31176
7935	1.67204	2.02484
7936	1.67204	1.60615
7937	0.555875	1.57878
7938	0.91951	2.1
7939	0.946131	1.29731
7940	0.555904	1.52862
7941	0.946131	1.50711
7942	1.6943	2.1
7943	1.04744	1.40704

7944	1.50938	2.1
7945	1.60861	2.1
7946	1.92406	0.367172
7947	1.18716	0.373924
7948	0.962705	0.867743
7949	1.35382	1.00144
7950	1.43632	0.263307
7951	1.09386	0.306254
7952	1.92547	0.281615
7953	0.994164	0.265938
7954	0.903974	2.00949
7955	0.928829	1.82932
7956	0.810958	1.57356
7957	1.92853	1.84374
7958	1.598	1.78118
7959	1.28967	1.9131
7960	1.44993	1.75445
7961	0.946132	0.955343
7962	0.882984	0.901995
7963	0.882955	0.983345
7964	1.56071	0.825352
7965	1.56071	0.845178
7966	1.35946	0.916499
7967	1.08421	0.946362
7968	1.41943	0.233097
7969	1.26265	0.204681
7970	1.03235	0.792715
7971	1.50778	2.1
7972	1.70387	2.1
7973	1.65608	2.1
7974	1.39076	2.1
7975	1.6104	2.1
7976	1.37456	2.1
7977	1.51898	0.70297
7978	1.9726	0.545285
7979	2.13757	1.56636
7980	1.74856	1.66032
7981	0.635949	0.928506
7982	2.46448	1.06913
7983	1.23224	1.03724
7984	1.12786	1.15007
7985	1.82721	1.02176
7986	1.82721	0.915026
7987	0.532892	1.48861
7988	0.946131	1.19037
7989	0.53292	1.12895
7990	1.41394	2.1
7991	1.45506	2.1
7992	1.39523	2.1
7993	1.09006	2.1
7994	1.80264	0.275775
7995	1.80264	0.85917
7996	0.946131	0.425305
7997	1.34268	0.394964
7998	0.946132	0.807938
7999	1.23732	0.491306
8000	1.34268	0.535253
8001	1.2064	0.916166

8002	1.58164	2.1
8003	1.73853	2.1
8004	1.01243	1.0331
8005	0.910672	1.33261
8006	1.47793	1.30457
8007	1.47793	1.98109
8008	0.910702	1.3007
8009	1.30382	1.04786
8010	1.08478	1.02697
8011	0.910702	1.4929
8012	2.60709	1.14858
8013	1.01243	1.09066
8014	1.85516	1.28944
8015	2.52823	0.856631
8016	1.24001	0.830531
8017	1.89793	1.35611
8018	1.73584	0.231268
8019	1.24613	1.94945
8020	1.37074	1.78588
8021	1.23763	1.53205
8022	1.04072	1.81964
8023	0.75499	1.37752
8024	1.72329	1.66745
8025	1.46195	1.54962
8026	0.946132	1.3962
8027	0.747438	1.86496
8028	1.24613	1.4638
8029	2.59886	1.34067
8030	1.87279	2.1
8031	1.07977	2.1
8032	1.29606	2.1
8033	1.45178	2.1
8034	1.45177	2.1
8035	0.92406	2.1
8036	0.946131	2.1
8037	0.924047	2.1
8038	2.04529	2.1
8039	1.04544	2.1
8040	1.08276	2.1
8041	1.67491	2.1
8042	0.961826	0.863107
8043	0.810942	0.908408
8044	0.826622	1.55703
8045	0.914378	1.36507
8046	0.89022	0.87895
8047	0.922383	1.07548
8048	0.988165	0.82169
8049	1.31357	0.341508
8050	0.946131	0.474428
8051	1.31357	0.416351
8052	1.10564	0.40236
8053	1.6797	0.359233
8054	1.6797	0.46841
8055	0.739506	0.429751
8056	1.33957	0.406357
8057	1.33957	0.403276
8058	1.33957	0.356936
8059	2.34223	0.424811

8060	1.5515	0.976371
8061	2.22137	0.896717
8062	0.94612	0.847425
8063	1.13908	0.921782
8064	1.45423	0.847245
8065	1.45422	0.926599
8066	0.795519	1.01475
8067	1.1391	0.892458
8068	1.11065	0.873912
8069	1.12266	1.87132
8070	1.24925	1.97291
8071	1.20242	1.28045
8072	2.18297	1.43924
8073	3.00107	1.10784
8074	2.58297	0.774224
8075	0.884783	1.49939
8076	0.851538	0.852402
8077	0.713587	1.32133
8078	1.47949	0.882236
8079	1.66673	1.49318
8080	0.696199	1.33994
8081	0.9383	0.952638
8082	1.29149	0.965544
8083	1.32266	1.08741
8084	2.11428	0.211639
8085	2.17439	0.20107
8086	1.40489	0.207278
8087	1.21071	0.192489
8088	0.946118	0.256026
8089	1.0746	0.228506
8090	0.973119	0.234254
8091	1.31333	0.22842
8092	1.08723	0.228467
8093	0.994292	2.1
8094	1.4964	2.1
8095	1.15096	2.1
8096	1.10101	2.1
8097	1.50438	2.1
8098	1.67825	2.1
8099	2.02103	2.1
8100	2.01978	2.1
8101	1.50931	0.281371
8102	1.50931	0.257621
8103	0.65023	0.246231
8104	1.34831	0.260126
8105	0.811233	0.274923
8106	1.34831	0.260794
8107	0.94613	0.280947
8108	1.00754	1.19526
8109	0.714132	1.69291
8110	1.28877	1.36807
8111	1.39905	1.31924
8112	1.03282	1.26694
8113	0.997769	1.19036
8114	1.11743	1.21703
8115	1.86863	1.16376
8116	3.30723	1.33112
8117	1.66635	1.82643

8118	1.35609	0.224548
8119	1.38212	0.240536
8120	1.95597	2.1
8121	1.64257	2.1
8122	2.93316	2.1
8123	3.13417	2.1
8124	0.733947	2.1
8125	1.15259	2.1
8126	1.07976	2.1
8127	1.30237	2.1
8128	1.99943	0.28929
8129	0.907431	0.304573
8130	0.878161	0.336289
8131	1.07544	0.233324
8132	1.03401	0.279577
8133	0.991975	0.236691
8134	1.03635	0.278634
8135	0.788549	0.267508
8136	1.44984	0.26145
8137	1.84007	0.303375
8138	2.31127	0.284986
8139	1.27362	2.1
8140	1.27737	0.984021
8141	1.87783	0.854647
8142	2.35499	0.220517
8143	2.69662	0.260009
8144	2.24065	0.193182
8145	0.996082	2.1
8146	0.996083	2.1
8147	1.44859	2.1
8148	1.03968	2.06183
8149	1.60018	1.13326
8150	1.73082	1.63508
8151	0.946132	1.26235
8152	1.49742	1.76151
8153	1.49742	0.915528
8154	1.00501	1.13487
8155	1.41585	1.93715
8156	1.41585	1.20508
8157	0.946131	1.50377
8158	1.03697	1.37975
8159	2.95786	2.1
8160	2.32185	1.86762
8161	1.65783	0.890336
8162	1.772	1.6676
8163	0.946131	0.919513
8164	1.40404	0.694459
8165	1.13537	1.76888
8166	1.04559	1.03657
8167	1.16091	1.07291
8168	2.63751	1.91195
8169	1.68455	0.237804
8170	1.9682	0.261366
8171	2.16834	0.717272
8172	2.45569	0.75445
8173	0.946128	0.703708
8174	1.36428	0.757949
8175	0.663978	0.786151

8176	1.58218	0.714847
8177	1.58219	0.715011
8178	1.36428	0.743364
8179	0.881886	0.757262
8180	1.73849	2.1
8181	1.35668	2.1
8182	1.1233	2.1
8183	1.15009	2.1
8184	1.16482	0.752802
8185	1.29313	0.764947
8186	1.47608	0.416149
8187	2.05949	0.427535
8188	0.946119	0.384263
8189	1.02974	0.375133
8190	0.946119	0.38082
8191	1.66758	0.380721
8192	1.66757	0.349203
8193	1.32082	0.349993
8194	1.29287	0.307557
8195	1.36005	1.81154
8196	1.47109	2.1
8197	2.45958	2.1
8198	2.2975	2.1
8199	0.953521	1.0791
8200	0.953521	0.993804
8201	1.67054	2.1
8202	1.67054	2.1
8203	0.946131	2.1
8204	1.38154	2.1
8205	1.13326	2.1
8206	1.13326	2.1
8207	1.23513	2.1
8208	1.50998	2.1
8209	2.47982	0.30421
8210	1.45091	0.292698
8211	0.958953	0.358988
8212	0.79239	0.361984
8213	2.11929	0.345863
8214	1.26511	0.413149
8215	1.27733	0.312234
8216	2.18127	0.328578
8217	1.30169	0.340754
8218	1.19259	0.340373
8219	0.963395	1.07879
8220	0.999947	1.69074
8221	1.62241	2.1
8222	1.62027	2.1
8223	0.981586	0.195557
8224	0.981585	0.180157
8225	0.721961	2.1
8226	0.891321	2.1
8227	1.04442	2.1
8228	1.66554	2.1
8229	2.43434	2.1
8230	1.73944	0.217775
8231	1.88032	0.214838
8232	0.711391	0.236435
8233	1.40837	0.223032

8234	0.946133	0.236533
8235	0.92252	0.232495
8236	1.13964	0.227369
8237	1.83394	0.225321
8238	2.81674	0.221847
8239	1.10049	1.41814
8240	1.25092	0.876727
8241	0.760299	1.04993
8242	1.48017	1.16998
8243	1.48017	1.25038
8244	0.760271	0.98392
8245	1.35861	1.11375
8246	0.946129	1.04112
8247	0.760269	0.992276
8248	2.26155	2.1
8249	2.15634	2.1
8250	1.39824	2.1
8251	1.07817	2.1
8252	0.946132	2.1
8253	1.53028	2.1
8254	1.53028	2.1
8255	1.00807	2.1
8256	0.946133	2.1
8257	2.06126	0.721223
8258	1.97385	1.13403
8259	1.75754	0.704751
8260	2.01613	2.1
8261	1.97995	2.1
8262	1.47696	2.1
8263	1.50172	2.1
8264	1.5177	0.229841
8265	1.44146	0.37161
8266	1.8807	0.356821
8267	2.07068	0.402192
8268	1.84267	0.383783
8269	1.08975	2.1
8270	1.21327	2.1
8271	1.10106	0.194948
8272	1.34496	0.188513
8273	2.08452	0.612063
8274	1.70537	0.439696
8275	2.62464	1.31344
8276	1.5726	1.12753
8277	1.5726	2.1
8278	0.94613	2.1
8279	1.15038	2.1
8280	0.946132	1.00512
8281	1.03974	1.76492
8282	1.15038	1.74765
8283	0.99747	0.991169
8284	1.03472	1.35518
8285	1.58407	0.930912
8286	1.21148	0.844112
8287	1.35025	1.00786
8288	1.3428	0.650868
8289	1.00775	2.1
8290	1.00775	2.0525
8291	1.3879	2.07361

8292	1.39958	2.1
8293	0.853042	2.1
8294	1.06997	2.05976
8295	0.853042	2.1
8296	0.853042	2.1
8297	1.12491	2.1
8298	1.17879	2.1
8299	1.07162	1.99812
8300	1.61179	1.88856
8301	1.76016	2.1
8302	0.946132	0.910616
8303	1.23449	0.86689
8304	1.70392	0.875901
8305	1.70392	0.808303
8306	0.94613	0.923517
8307	1.23449	0.870523
8308	1.41556	0.872228
8309	1.14547	0.848864
8310	1.1721	0.728931
8311	0.96478	0.807321
8312	1.41843	0.807237
8313	1.2358	0.765301
8314	1.70678	0.756067
8315	1.70678	0.75771
8316	0.946118	0.87487
8317	1.23582	0.848632
8318	0.946105	0.887821
8319	0.964769	0.678079
8320	1.1357	0.69474
8321	0.964767	0.682177
8322	0.619856	0.20127
8323	1.72366	0.20119
8324	1.72367	0.191892
8325	0.619831	0.196447
8326	1.19262	0.20621
8327	0.619829	0.181758
8328	0.946129	0.212255
8329	1.57459	2.1
8330	1.86512	2.1
8331	0.93841	2.1
8332	1.57459	2.1
8333	1.10476	0.315488
8334	1.00013	0.942975
8335	2.13446	0.91846
8336	0.759781	2.1
8337	1.96011	1.2739
8338	0.946129	0.951744
8339	1.32056	1.46575
8340	1.75702	1.05446
8341	1.75702	1.60575
8342	0.946124	1.31715
8343	1.32056	1.14038
8344	1.38258	1.00074
8345	1.96011	1.30765
8346	2.31099	1.23225
8347	0.97356	2.1
8348	1.38885	2.1
8349	1.3771	2.1

8350	0.946131	2.1
8351	1.84843	2.1
8352	1.84843	2.1
8353	1.3771	2.1
8354	0.946131	2.1
8355	1.00278	2.1
8356	1.05447	0.824998
8357	1.05446	0.838594
8358	1.24913	0.584152
8359	1.05447	0.819438
8360	1.80882	0.829462
8361	1.80882	2.07872
8362	0.946129	1.83236
8363	1.33997	2.05147
8364	0.946131	0.804077
8365	1.28831	0.967838
8366	1.33997	0.917377
8367	1.44391	0.213108
8368	1.44391	0.209861
8369	0.850964	0.238023
8370	0.850963	0.223377
8371	0.946132	0.245585
8372	0.921299	0.239991
8373	1.37358	0.224183
8374	1.46297	0.208322
8375	1.46298	0.197069
8376	0.823988	0.244719
8377	0.921287	0.229999
8378	0.94612	0.268562
8379	0.823995	0.238776
8380	0.946107	0.22885
8381	1.44437	2.1
8382	1.06203	2.1
8383	2.21397	2.1
8384	0.637677	2.1
8385	1.8892	2.1
8386	2.075	2.1
8387	1.32051	0.465623
8388	2.23637	0.573785
8389	1.10904	0.663917
8390	1.11819	0.605783
8391	0.94613	0.676628
8392	0.735845	0.562471
8393	1.4914	0.720468
8394	1.4914	0.466847
8395	0.735831	0.764562
8396	1.61683	2.1
8397	1.61683	2.1
8398	0.946131	2.1
8399	1.3281	2.1
8400	1.10441	2.1
8401	1.10442	2.1
8402	1.23487	2.1
8403	1.09914	2.1
8404	1.20691	0.252883
8405	2.27379	0.285437
8406	1.1369	0.319792
8407	1.26868	0.333313

8408	1.54422	0.309539
8409	1.54422	0.289312
8410	0.861357	0.375043
8411	0.94613	0.33533
8412	0.861359	0.321268
8413	1.2858	0.767646
8414	1.31011	0.78824
8415	1.294	0.748044
8416	1.03079	0.800709
8417	1.81979	2.03917
8418	1.57829	2.1
8419	1.19023	1.89863
8420	1.19022	2.05527
8421	1.46458	1.87361
8422	1.46458	2.1
8423	0.789173	2.1
8424	1.34386	2.1
8425	0.909896	2.05894
8426	0.946131	1.94271
8427	0.909893	1.96239
8428	1.27725	2.1
8429	1.68451	2.1
8430	2.26155	2.1
8431	2.34217	2.1
8432	1.55485	0.879661
8433	0.90854	0.838198
8434	0.858256	0.830772
8435	1.47042	0.797308
8436	1.62771	0.798406
8437	0.777398	0.85619
8438	0.982525	0.826371
8439	1.23865	0.819297
8440	2.09928	0.819285
8441	0.946128	0.688061
8442	0.474856	0.685266
8443	0.474855	0.703543
8444	1.58747	0.557536
8445	1.58747	0.56802
8446	0.963917	0.655313
8447	1.09841	0.657103
8448	2.23904	1.50578
8449	2.60055	0.943209
8450	1.02599	2.1
8451	2.17837	1.89724
8452	2.07122	2.1
8453	2.7309	1.01604
8454	2.08482	2.09691
8455	1.18322	0.754605
8456	1.35381	1.14842
8457	1.01139	0.903377
8458	0.946133	1.10787
8459	1.59089	1.17312
8460	1.59089	0.462208
8461	0.946133	0.943961
8462	1.09634	0.380125
8463	2.02279	0.534865
8464	1.03108	0.745419
8465	0.808978	1.29309

8466	1.07317	1.39903
8467	0.831695	1.34629
8468	1.22583	1.42159
8469	1.10215	1.27679
8470	1.07508	1.47895
8471	1.22204	1.33327
8472	1.04679	1.84964
8473	1.92535	0.304906
8474	1.11081	0.205689
8475	0.946131	0.389927
8476	0.714824	0.39511
8477	0.714795	0.40183
8478	1.4231	0.365464
8479	1.4231	0.350845
8480	0.828617	0.421902
8481	1.30928	0.345533
8482	1.49894	2.1
8483	1.55702	2.1
8484	0.946131	2.1
8485	1.10689	2.1
8486	0.946227	2.1
8487	0.946131	2.1
8488	1.21754	2.1
8489	1.504	2.1
8490	1.504	2.1
8491	0.723573	2.1
8492	1.24512	2.1
8493	0.723544	2.1
8494	0.946131	2.1
8495	1.24512	2.1
8496	1.29383	0.507293
8497	1.29383	0.445166
8498	1.61969	0.470481
8499	0.946132	0.378611
8500	1.12818	0.339979
8501	0.946132	0.425484
8502	1.80922	0.41083
8503	1.80922	0.33015
8504	1.41383	0.358285
8505	1.34153	0.326008
8506	1.47689	0.407494
8507	1.79375	0.41344
8508	1.24333	0.738568
8509	0.768989	0.697738
8510	1.24333	0.627052
8511	1.0027	0.815258
8512	1.47705	0.79073
8513	1.47705	0.550621
8514	0.768977	0.693477
8515	1.28436	0.818811
8516	0.928583	0.838884
8517	1.28436	0.656902
8518	1.24333	0.87068
8519	1.5991	0.872738
8520	1.5991	0.485538
8521	0.928583	0.828895
8522	1.85717	0.556759
8523	2.48665	0.657643

8524	1.18423	0.2264
8525	1.9493	1.24213
8526	1.8519	1.09922
8527	1.68802	2.1
8528	1.0302	0.38686
8529	0.900473	0.364246
8530	1.99663	0.415519
8531	2.61301	0.368308
8532	0.745772	0.498969
8533	0.946131	0.408922
8534	1.48881	0.412304
8535	1.48881	0.378902
8536	0.745745	0.46403
8537	0.809786	0.46192
8538	1.3065	0.448976
8539	0.946132	0.547815
8540	0.768357	0.579779
8541	0.768387	0.585462
8542	1.76897	0.433437
8543	1.76896	0.415508
8544	1.40271	0.500655
8545	1.00777	0.512471
8546	1.37427	0.314977
8547	1.1967	1.28003
8548	0.946132	1.56995
8549	1.59397	2.1
8550	1.59397	2.1
8551	0.946131	2.09614
8552	1.14989	2.1
8553	1.14989	2.1
8554	1.10046	2.1
8555	1.09506	0.335727
8556	2.53251	0.366692
8557	1.9212	1.0065
8558	1.99586	0.960228
8559	0.913967	0.763132
8560	1.55028	0.745942
8561	1.55028	0.805337
8562	0.913966	0.769607
8563	1.16318	0.724923
8564	1.16318	0.743624
8565	0.946132	0.733013
8566	1.20478	2.1
8567	1.09662	2.1
8568	1.03472	2.1
8569	1.84774	2.1
8570	1.7984	2.1
8571	2.1221	2.1
8572	1.68223	1.89746
8573	1.68224	1.76997
8574	0.514872	1.51463
8575	0.888991	1.73656
8576	0.946127	1.13592
8577	0.514845	1.54437
8578	0.946129	1.31837
8579	1.10127	1.47144
8580	2.61623	1.2112
8581	0.983581	0.862389

8582	0.869663	0.764905
8583	1.1818	1.20231
8584	1.10406	0.830959
8585	1.07133	1.30397
8586	1.09405	0.822699
8587	0.838014	0.782891
8588	0.842998	1.19022
8589	1.03818	0.96626
8590	1.46338	0.702979
8591	1.903	2.1
8592	1.23942	2.1
8593	0.965933	2.1
8594	0.810075	2.03099
8595	1.40571	2.1
8596	1.66219	2.1
8597	0.810047	2.1
8598	0.936678	2.1
8599	1.45808	2.1
8600	1.6384	2.1
8601	1.54699	2.1
8602	0.719877	2.1
8603	1.33256	2.1
8604	0.753547	2.1
8605	1.04073	2.1
8606	1.25822	2.1
8607	0.851542	2.1
8608	0.714102	2.1
8609	1.45886	2.1
8610	1.80171	2.1
8611	0.597337	2.1
8612	0.957713	2.1
8613	1.3033	2.1
8614	0.596613	0.643438
8615	1.1386	0.90128
8616	0.596612	0.78198
8617	0.946133	0.982934
8618	0.596652	1.02386
8619	1.64092	0.92259
8620	1.64092	0.887173
8621	1.991	2.1
8622	1.23013	2.1
8623	2.77816	2.1
8624	2.74107	0.861615
8625	2.07681	0.831759
8626	1.47764	0.42913
8627	1.72677	0.463566
8628	1.52161	2.1
8629	1.28526	2.1
8630	1.90502	2.1
8631	1.87359	0.412118
8632	1.93489	0.474275
8633	2.70895	2.1
8634	2.83141	2.1
8635	1.41766	0.678094
8636	1.11337	0.673023
8637	1.41765	0.941622
8638	1.29172	0.680771
8639	1.76324	0.681778

8640	1.76324	0.84667
8641	0.946131	0.679128
8642	1.52428	2.1
8643	1.71459	2.1
8644	1.93852	0.794978
8645	1.07119	1.04541
8646	1.29698	0.89485
8647	1.42203	0.77452
8648	1.42203	0.988067
8649	0.946132	0.795475
8650	0.946132	0.833148
8651	0.96926	0.899941
8652	0.769913	1.35017
8653	0.946119	1.1893
8654	1.37867	0.978416
8655	1.94028	0.945252
8656	0.94612	0.83691
8657	0.946132	1.09246
8658	1.37865	0.92782
8659	2.14234	1.00933
8660	3.06459	1.09556
8661	1.44421	0.269096
8662	1.33924	0.226076
8663	1.33671	0.377766
8664	1.10733	0.374134
8665	0.68396	0.434219
8666	1.76008	0.320568
8667	1.76008	0.310209
8668	0.68393	0.32576
8669	0.946131	0.393419
8670	3.79242	2.1
8671	2.46293	2.1
8672	3.56947	2.1
8673	1.35487	1.4779
8674	1.38616	1.71506
8675	0.954048	0.401659
8676	1.36663	0.558091
8677	0.909487	0.622786
8678	0.887288	1.66641
8679	0.810947	1.16531
8680	0.810934	1.48837
8681	1.07046	1.66917
8682	1.13979	1.83242
8683	0.927838	1.2209
8684	0.919995	1.54372
8685	1.61447	2.1
8686	2.56305	2.1
8687	0.943414	1.40663
8688	1.17016	1.89986
8689	1.7238	2.07511
8690	1.89334	1.72905
8691	0.946128	1.00151
8692	1.25184	1.50594
8693	1.55639	1.72946
8694	3.12098	1.12364
8695	3.57307	0.952616
8696	2.81281	1.94784
8697	0.851542	1.96099

8698	1.27689	1.83048
8699	1.52568	2.03628
8700	0.839689	2.04415
8701	1.36471	1.91629
8702	0.827865	2.1
8703	0.93667	2.06099
8704	0.900861	2.00554
8705	0.943152	2.03663
8706	1.00667	2.06082
8707	1.18141	2.09415
8708	1.15298	2.09754
8709	1.20335	2.05845
8710	1.00713	2.06246
8711	0.597537	2.1
8712	1.0577	2.1
8713	0.967342	1.12296
8714	0.838027	1.43025
8715	0.893587	0.965614
8716	1.06436	1.22966
8717	0.850048	0.972009
8718	1.22026	1.31935
8719	0.988077	1.14152
8720	1.18769	0.7617
8721	1.3991	2.00399
8722	1.52984	2.1
8723	1.35469	1.18839
8724	1.25083	1.07851
8725	1.6594	1.29517
8726	1.6594	1.15163
8727	0.946129	1.02274
8728	1.02602	1.01969
8729	0.94613	0.97111
8730	1.29724	0.626233
8731	1.14501	0.573208
8732	1.13078	0.477452
8733	1.48381	0.478934
8734	1.45921	0.442065
8735	0.851539	0.589046
8736	0.890836	0.597969
8737	0.946131	0.657995
8738	1.60428	2.1
8739	1.50097	2.1
8740	0.714507	0.442624
8741	1.51396	0.453754
8742	1.51396	0.421397
8743	0.946132	0.457656
8744	0.813862	0.436194
8745	1.41461	0.436449
8746	0.813862	0.420308
8747	0.921311	2.1
8748	1.25084	1.03247
8749	1.30165	0.915481
8750	1.60636	1.13218
8751	1.60636	0.974996
8752	0.94613	0.90164
8753	0.972232	0.906157
8754	0.946131	0.870811
8755	2.35003	2.1

8756	0.92546	1.8384
8757	1.00713	2.1
8758	0.810947	2.08588
8759	0.810934	2.1
8760	1.056	2.1
8761	0.926811	2.1
8762	0.766408	2.1
8763	1.34292	2.1
8764	1.57932	0.292395
8765	2.67595	0.267212
8766	1.77737	0.261593
8767	1.77738	0.252023
8768	0.789633	0.3598
8769	1.22903	0.316812
8770	0.789663	0.33675
8771	0.789662	0.325817
8772	1.33797	0.326113
8773	0.946128	2.1
8774	1.47351	2.1
8775	1.47351	2.1
8776	0.94613	2.1
8777	1.30082	2.1
8778	1.30082	2.1
8779	0.996486	2.1
8780	0.946127	2.02151
8781	1.72122	2.1
8782	1.72122	2.1
8783	0.94613	2.09017
8784	1.36653	2.1
8785	1.36653	2.1
8786	1.02608	2.1
8787	0.709123	0.861424
8788	0.946127	0.938809
8789	1.41342	0.974416
8790	1.818	1.05639
8791	1.818	1.03196
8792	0.946127	0.750076
8793	1.41342	0.941382
8794	1.35071	0.911869
8795	1.8993	0.903499
8796	1.4748	2.1
8797	1.18711	0.279122
8798	0.946131	0.38784
8799	1.18711	0.328319
8800	1.20416	0.300258
8801	1.91358	0.373741
8802	1.91358	0.266778
8803	0.477691	0.331208
8804	2.16798	0.215117
8805	2.22307	0.214155
8806	0.758971	0.276755
8807	1.80694	0.275139
8808	1.80694	0.239659
8809	0.946132	0.289449
8810	0.75897	0.275697
8811	1.25124	0.262461
8812	0.946131	0.306691
8813	1.76837	2.1

8814	1.76837	2.1
8815	0.946131	2.1
8816	1.36422	2.1
8817	0.946131	2.1
8818	1.14184	2.1
8819	1.36422	2.1
8820	0.793303	0.206407
8821	1.79584	0.207335
8822	1.35333	1.74463
8823	1.19967	1.24459
8824	1.55088	1.36146
8825	1.64246	1.92921
8826	0.946132	1.04786
8827	0.886293	1.59553
8828	1.25018	1.59084
8829	1.5795	1.33746
8830	2.15414	1.15484
8831	0.94613	0.852544
8832	1.37758	1.29303
8833	1.71567	0.89502
8834	1.71567	1.51666
8835	0.946125	1.03572
8836	1.37759	0.905245
8837	1.28421	0.856126
8838	0.933959	1.14778
8839	0.946133	0.328666
8840	1.7813	0.348099
8841	1.7813	0.27549
8842	1.05704	0.305543
8843	1.37794	0.369541
8844	1.37794	0.325783
8845	0.946132	0.367641
8846	0.946132	0.252203
8847	1.68197	0.254612
8848	1.68197	0.226576
8849	1.01367	0.241337
8850	1.25017	0.266775
8851	1.25017	0.24671
8852	0.946132	0.271809
8853	0.838953	2.1
8854	1.4045	2.1
8855	1.56388	2.1
8856	1.3182	2.1
8857	0.928518	2.1
8858	1.04073	2.1
8859	0.835676	2.1
8860	1.47507	2.1
8861	1.47506	2.1
8862	0.797664	2.1
8863	1.34285	2.1
8864	0.929875	2.1
8865	0.797663	2.1
8866	0.946133	2.1
8867	1.83628	0.744995
8868	1.83627	0.76271
8869	0.946131	0.77745
8870	1.36748	0.733518
8871	0.946133	0.733112

8872	1.21139	0.746254
8873	1.36748	0.631139
8874	1.06795	2.1
8875	1.08192	2.1
8876	0.827866	2.1
8877	1.02548	2.1
8878	0.95006	2.1
8879	1.53933	2.1
8880	0.586511	2.1
8881	0.868145	2.1
8882	1.28401	2.1
8883	1.37719	2.1
8884	1.37719	2.1
8885	0.77497	2.1
8886	0.946132	2.1
8887	0.774968	2.1
8888	1.7053	0.614376
8889	1.7053	0.889974
8890	0.94613	1.18669
8891	1.25715	0.865668
8892	0.946132	0.745963
8893	1.19655	0.807147
8894	1.25716	0.799272
8895	0.851542	2.1
8896	0.80043	2.1
8897	1.27239	2.1
8898	1.02719	2.1
8899	0.946131	2.1
8900	0.981318	2.1
8901	1.06628	2.1
8902	1.26022	2.1
8903	0.946132	2.1
8904	0.946132	2.1
8905	1.76447	2.1
8906	1.76447	2.1
8907	1.35357	2.1
8908	1.35703	2.1
8909	0.946131	2.1
8910	1.50538	2.1
8911	1.50538	2.1
8912	1.10311	2.1
8913	1.3484	1.92799
8914	1.10312	2.1
8915	0.966428	2.1
8916	0.946133	2.1
8917	1.4008	2.1
8918	1.71908	2.1
8919	1.71908	2.1
8920	0.946133	2.1
8921	1.40081	2.1
8922	1.26441	2.1
8923	0.851543	2.1
8924	0.961732	2.1
8925	1.54576	2.1
8926	1.35364	2.1
8927	0.946133	2.1
8928	1.02913	2.1
8929	1.2735	2.1

8930	1.32608	2.1
8931	0.967643	2.1
8932	0.94613	2.1
8933	1.44873	2.1
8934	1.44873	2.1
8935	0.967641	2.1
8936	0.946131	2.1
8937	1.40488	2.1
8938	1.24272	2.1
8939	0.946131	2.1
8940	1.78483	2.1
8941	1.78483	2.1
8942	1.24272	2.1
8943	0.946131	2.1
8944	0.94613	1.53557
8945	1.5627	0.867253
8946	1.5627	1.63429
8947	1.03656	1.52333
8948	1.31649	0.809847
8949	1.19233	1.11864
8950	1.03656	1.05256
8951	0.912059	1.63818
8952	1.47954	0.904303
8953	1.47954	1.80356
8954	0.912061	1.59629
8955	1.34647	0.857133
8956	1.04514	1.32224
8957	0.912061	1.05504
8958	0.761972	0.483691
8959	0.946132	0.469466
8960	0.761974	0.560216
8961	1.70225	0.786983
8962	1.70225	0.692215
8963	1.33728	0.599177
8964	1.01188	0.543156
8965	0.94613	1.03533
8966	0.746361	1.22338
8967	1.6369	1.33296
8968	1.62454	1.25226
8969	0.736738	0.977546
8970	0.958487	1.01243
8971	1.40641	1.14214
8972	1.2284	2.1
8973	0.92609	2.1
8974	0.595102	2.1
8975	1.80871	2.1
8976	1.1516	2.1
8977	0.962474	2.1
8978	2.24024	2.1
8979	0.946131	2.1
8980	1.73689	2.1
8981	1.73689	2.1
8982	0.946132	2.1
8983	1.30492	2.1
8984	1.30492	2.1
8985	1.17949	2.1
8986	0.946131	1.99408
8987	1.8566	2.1

8988	1.8566	2.1
8989	1.22392	2.04102
8990	1.39826	2.1
8991	1.40447	2.1
8992	1.22392	2.1
8993	0.946131	1.54079
8994	1.74134	2.1
8995	1.74134	2.1
8996	1.09148	1.48921
8997	1.40447	2.1
8998	1.283	2.1
8999	1.09148	2.1
9000	1.33607	2.1
9001	1.41022	1.90841
9002	1.80016	2.07265
9003	1.80015	2.1
9004	0.946127	1.66258
9005	0.946129	2.1
9006	1.20541	2.1
9007	1.13078	2.1
9008	1.33607	2.1
9009	1.54624	2.1
9010	1.85553	2.1
9011	0.920606	2.1
9012	0.946131	2.1
9013	1.01271	2.1
9014	1.32664	1.05821
9015	0.954692	1.08311
9016	0.946116	0.953869
9017	1.47771	0.951738
9018	1.4777	2.00818
9019	0.95468	1.01073
9020	0.94613	0.983969
9021	1.31381	0.922549
9022	1.14005	0.812278
9023	0.946128	0.916278
9024	1.69433	0.76331
9025	1.69433	1.05484
9026	1.14005	0.872324
9027	0.94613	0.78452
9028	1.521	2.1
9029	1.411	2.1
9030	0.94612	2.1
9031	1.35115	2.1
9032	1.04127	1.75752
9033	1.05017	2.1
9034	1.03564	2.1
9035	0.607156	1.34112
9036	1.54549	2.1
9037	1.36715	2.1
9038	0.607127	1.29441
9039	1.2571	2.1
9040	0.998701	1.70698
9041	0.546436	1.79246
9042	0.946133	2.1
9043	1.75286	2.1
9044	1.75286	2.1
9045	1.12206	2.05677

9046	1.34237	2.1
9047	1.35663	2.1
9048	1.12206	2.1
9049	0.919455	2.0654
9050	1.66517	2.1
9051	1.99822	2.1
9052	1.228 2.1	
9053	1.35662	2.1
9054	1.228 2.1	
9055	1.01143	2.1
9056	0.851543	1.04443
9057	0.986856	1.91606
9058	1.42762	1.24722
9059	1.23095	2.1
9060	0.946132	1.76769
9061	1.00659	1.37826
9062	1.1779	1.20029
9063	1.40025	2.1
9064	1.40025	2.1
9065	0.889308	2.1
9066	1.34652	2.1
9067	0.943042	2.1
9068	0.943041	2.1
9069	1.34652	2.1
9070	1.39514	1.04959
9071	1.30809	0.946745
9072	1.75711	0.907051
9073	1.7571	1.48021
9074	0.946125	0.791938
9075	0.946127	0.987796
9076	1.18396	1.02251
9077	1.4844	1.05414
9078	1.4844	1.2857
9079	0.803322	1.47848
9080	0.946133	0.994526
9081	0.803324	1.97233
9082	0.803324	1.38919
9083	1.36183	1.40468
9084	0.946131	0.435563
9085	1.01462	0.419128
9086	0.946132	0.441119
9087	1.59749	0.538795
9088	1.59749	0.467195
9089	1.16009	0.459042
9090	1.38354	0.458948
9091	0.851539	1.02897
9092	1.36005	1.53448
9093	1.5532	1.18274
9094	1.80402	1.63635
9095	0.851537	1.45922
9096	0.902632	1.29266
9097	1.44437	1.1617
9098	0.633098	2.03492
9099	1.00307	0.752221
9100	0.94613	0.887311
9101	1.16623	0.804121
9102	0.946128	0.897245
9103	1.64588	0.896476

9104	1.64588	0.83071
9105	1.35059	0.871387
9106	1.24142	0.881493
9107	0.946131	0.462373
9108	1.13343	0.390542
9109	0.946127	0.496964
9110	1.63367	0.46736
9111	1.63367	0.380039
9112	1.33035	0.429263
9113	1.24945	0.423519
9114	1.78733	1.22376
9115	1.70856	1.48618
9116	0.637995	1.1457
9117	0.712454	1.18327
9118	0.946132	1.13388
9119	0.637964	1.09905
9120	1.13021	1.16969
9121	0.94613	0.39563
9122	1.20509	0.377955
9123	0.946127	0.443071
9124	1.70545	0.406687
9125	1.70545	0.325649
9126	1.28508	0.372906
9127	1.3665	0.346893
9128	1.58612	2.1
9129	1.51845	2.1
9130	0.797374	2.1
9131	1.20057	2.1
9132	0.946132	2.1
9133	0.946132	2.1
9134	0.899819	2.1
9135	1.50471	1.02067
9136	1.50472	1.17343
9137	0.59387	1.11516
9138	0.593844	0.960745
9139	0.946126	1.00778
9140	0.966137	1.0203
9141	1.13245	0.96857
9142	1.20801	1.09593
9143	0.946131	1.28934
9144	1.47644	1.14367
9145	0.733223	1.11365
9146	0.563609	1.03524
9147	1.69218	1.13187
9148	1.6173	1.28925
9149	1.8126	2.00027
9150	1.8126	2.1
9151	0.678938	2.0151
9152	0.678935	2.1
9153	0.946133	2.09329
9154	0.946132	1.95712
9155	0.678936	2.1
9156	1.21997	1.70962
9157	1.28439	1.73807
9158	1.65282	1.95421
9159	1.86735	1.96119
9160	0.851539	1.72864
9161	0.94613	1.39797

9162	0.87688	1.44952
9163	0.804877	0.795406
9164	1.40747	0.910475
9165	1.41312	0.721139
9166	0.885349	0.761644
9167	1.25405	0.896865
9168	1.34463	0.762469
9169	0.804878	0.777987
9170	0.810299	0.510285
9171	1.04072	0.665614
9172	1.25601	0.607556
9173	1.04943	0.52003
9174	0.900294	0.635226
9175	1.38777	0.599686
9176	1.33131	0.461558
9177	1.39168	0.263082
9178	0.946132	0.31507
9179	0.747712	0.28739
9180	1.61944	0.253715
9181	1.61944	0.238921
9182	0.946132	0.31799
9183	0.747712	0.306688
9184	1.41201	0.489426
9185	1.12082	0.445844
9186	0.946128	0.529687
9187	1.79415	0.473812
9188	1.79415	0.458286
9189	1.12082	0.456539
9190	0.94613	0.460536
9191	0.711721	0.483546
9192	1.12316	0.532606
9193	0.707038	0.502226
9194	1.04072	0.553371
9195	0.704409	0.57496
9196	1.49036	0.552878
9197	1.27689	0.505309
9198	0.793044	1.02556
9199	0.810886	1.20317
9200	0.885957	1.02192
9201	1.38254	1.0505
9202	1.35991	0.989335
9203	1.01375	1.11552
9204	1.03169	1.10362
9205	0.946131	1.04002
9206	1.3279	1.20066
9207	1.42284	1.11538
9208	1.42284	1.0457
9209	0.851192	1.33736
9210	0.946131	1.24269
9211	0.851165	1.12422
9212	0.748062	0.270048
9213	1.27511	0.286217
9214	0.74806	0.257662
9215	0.94613	0.29437
9216	0.748059	0.285955
9217	1.86293	0.283226
9218	1.86293	0.258058
9219	0.851542	0.525785

9220	1.38186	0.478585
9221	0.851542	0.564196
9222	1.67869	0.378783
9223	1.66579	0.351178
9224	1.25626	0.450711
9225	1.23046	0.422702
9226	1.46023	0.207447
9227	1.27083	0.19902
9228	0.946131	0.232653
9229	0.999304	0.220946
9230	0.946803	0.224887
9231	0.851554	0.223142
9232	0.999227	0.2165
9233	0.750097	0.340502
9234	1.91815	0.316695
9235	0.946132	0.341982
9236	0.768117	0.314032
9237	1.78003	0.313192
9238	1.99572	0.290895
9239	0.650453	0.347187
9240	0.946132	0.349837
9241	1.23845	0.353906
9242	0.946131	0.360607
9243	1.75477	0.278469
9244	1.75477	0.281651
9245	1.33393	0.3089
9246	1.36697	0.312715
9247	0.749018	1.00917
9248	1.02366	1.11504
9249	1.43924	1.06384
9250	1.00018	1.01703
9251	1.06438	0.918232
9252	0.946127	0.948949
9253	0.74906	0.961297
9254	0.946131	0.954943
9255	1.26488	0.235484
9256	0.946131	0.288487
9257	0.94613	0.260195
9258	1.49051	0.233588
9259	1.59837	0.229964
9260	0.907797	0.253345
9261	1.04455	0.263549
9262	1.25214	0.171635
9263	0.68452	0.175837
9264	1.25215	0.174828
9265	1.24677	0.172262
9266	1.8144	0.166642
9267	1.81441	0.165386
9268	0.684518	0.176715
9269	0.851542	0.819327
9270	1.52275	0.80433
9271	1.45223	0.806029
9272	1.0269	0.801553
9273	1.29689	0.818055
9274	1.32822	0.838569
9275	0.851553	0.85421
9276	1.21756	0.279315
9277	0.946131	0.294993

9278	0.946127	0.277849
9279	1.78014	0.247772
9280	1.78014	0.219306
9281	1.32076	0.259026
9282	1.40551	0.269768
9283	0.711214	0.456728
9284	1.82057	0.411807
9285	1.82057	0.353285
9286	0.946129	0.480389
9287	1.22716	0.359331
9288	1.30463	0.461506
9289	0.94613	0.458991
9290	0.568927	0.254849
9291	0.946132	0.274682
9292	0.568927	0.264328
9293	1.99266	0.217134
9294	1.99266	0.210142
9295	1.26201	0.261114
9296	1.29957	0.26431
9297	1.21614	0.41162
9298	0.851484	0.414167
9299	0.851543	0.447391
9300	1.85815	0.313656
9301	1.81937	0.300203
9302	0.697705	0.29876
9303	0.946132	0.4228
9304	0.593295	0.516389
9305	1.53642	0.479824
9306	1.53642	0.435762
9307	0.908276	0.544089
9308	0.593296	0.434032
9309	0.593295	0.464799
9310	0.94613	0.522915
9311	0.867593	0.638039
9312	1.42107	0.54691
9313	1.42107	0.533283
9314	0.867621	0.612136
9315	1.05516	0.621884
9316	0.867624	0.52977
9317	0.946129	0.658858
9318	0.766681	0.35898
9319	1.18119	0.382232
9320	1.57018	0.33364
9321	1.57018	0.293438
9322	0.766652	0.399375
9323	1.18119	0.372979
9324	1.15564	0.36807
9325	0.946131	0.554633
9326	1.239	0.484691
9327	0.851541	0.578172
9328	0.851487	0.471683
9329	1.40021	0.555085
9330	1.38373	0.441562
9331	0.851554	0.598867
9332	1.12266	0.448076
9333	0.946131	0.67539
9334	1.08234	0.597129
9335	1.2882	0.574481

9336	1.49963	0.837257
9337	1.46188	0.510861
9338	0.851555	0.548224
9339	1.33007	0.745183
9340	1.08054	0.73749
9341	0.946129	0.804875
9342	0.91151	0.65754
9343	1.49914	0.782277
9344	1.49914	0.620217
9345	0.911471	0.828316
9346	0.946132	0.980758
9347	1.35943	1.50878
9348	1.68117	1.22915
9349	1.68117	1.8367
9350	0.946132	1.29518
9351	1.35943	1.0882
9352	1.26787	0.947987
9353	1.81612	0.982979
9354	1.81612	0.642468
9355	0.946131	0.604223
9356	1.41492	0.457317
9357	0.946132	1.01738
9358	1.25037	0.852834
9359	1.41492	0.831275
9360	1.30541	0.281086
9361	1.23646	0.336174
9362	0.94613	0.31452
9363	1.77304	0.25811
9364	1.77304	0.248605
9365	1.23646	0.29887
9366	0.946132	0.308998
9367	0.946131	2.1
9368	1.26689	2.1
9369	1.66806	2.1
9370	1.66806	2.1
9371	0.94613	1.85673
9372	1.26689	2.1
9373	1.3473	2.1
9374	1.39384	2.1
9375	1.4181	2.1
9376	1.86581	2.1
9377	1.86581	2.1
9378	0.946128	2.1
9379	0.946129	2.1
9380	1.32068	2.1
9381	1.20328	2.1
9382	1.39384	2.1
9383	1.651	2.1
9384	1.651	2.1
9385	0.946129	2.1
9386	0.946131	2.1
9387	1.12913	2.1
9388	1.21474	1.94881
9389	0.946132	2.1
9390	0.94613	2.09363
9391	1.79724	2.1
9392	1.79724	2.1
9393	1.40987	2.1

9394	1.3335	2.1
9395	1.65642	1.39192
9396	1.65642	2.1
9397	0.946132	2.1
9398	1.19063	2.1
9399	0.946133	0.937454
9400	1.15203	2.1
9401	1.19063	2.1
9402	0.946131	0.95308
9403	1.77815	1.62176
9404	1.77815	2.1
9405	0.946131	1.27385
9406	1.31745	2.1
9407	1.31745	1.41631
9408	1.2597	1.58521
9409	0.946131	0.851397
9410	1.85184	1.07658
9411	1.85184	2.1
9412	0.946128	1.23114
9413	1.40684	2.1
9414	1.40684	1.05141
9415	1.34049	1.19407
9416	0.946132	1.25338
9417	1.71333	1.10524
9418	1.71333	1.72084
9419	1.1848	1.26334
9420	1.34297	1.00735
9421	1.31649	1.04689
9422	1.1848	1.33785
9423	1.36604	0.685119
9424	0.954559	0.680947
9425	1.36604	0.707274
9426	1.41766	0.680632
9427	1.83756	0.710302
9428	1.83756	1.17049
9429	0.946131	0.712708
9430	0.946127	0.681891
9431	1.14874	0.551165
9432	0.946127	0.695037
9433	1.66393	0.688077
9434	1.66393	0.596212
9435	1.35559	0.659852
9436	1.25447	0.661503
9437	0.94613	0.416787
9438	1.20732	0.373307
9439	0.946129	0.466454
9440	1.75073	0.43378
9441	1.75073	0.33639
9442	1.3665	0.38562
9443	1.33035	0.361407
9444	1.73256	2.1
9445	1.73256	2.1
9446	0.946131	1.98371
9447	1.34921	2.04858
9448	1.19462	2.1
9449	1.19462	2.1
9450	1.32949	2.1
9451	1.75754	0.725666

9452	1.75754	0.551258
9453	0.946132	0.746446
9454	1.3201	0.720038
9455	0.946132	0.726443
9456	1.25479	0.536749
9457	1.3201	0.709905
9458	1.8087	0.899455
9459	1.8087	0.414958
9460	0.946131	0.584066
9461	1.38357	0.688978
9462	0.946133	0.890692
9463	1.31034	0.70235
9464	1.38357	0.802109
9465	1.12702	0.575814
9466	0.946131	0.745382
9467	1.23923	0.686153
9468	1.4117	0.609549
9469	0.946131	0.645432
9470	1.70479	0.652399
9471	1.70479	0.468736
9472	0.946119	2.1
9473	1.55309	2.1
9474	1.55309	2.1
9475	0.946119	2.1
9476	1.08006	2.1
9477	1.08005	2.1
9478	1.02681	2.1
9479	1.36581	2.1
9480	1.93075	2.1
9481	0.946133	2.1
9482	0.772538	2.1
9483	0.946131	2.1
9484	0.946131	2.1
9485	1.33748	2.1
9486	1.44991	2.1
9487	1.31183	2.1
9488	0.946132	2.1
9489	0.944104	2.1
9490	0.915236	2.1
9491	1.05017	2.1
9492	0.944148	2.1
9493	0.946132	0.65365
9494	1.60171	0.66177
9495	1.60171	0.568574
9496	0.946131	0.671383
9497	1.16234	0.571089
9498	1.3855	0.673994
9499	0.946132	0.671379
9500	0.946133	0.677949
9501	1.73285	0.692456
9502	1.73285	0.638215
9503	0.946133	0.689956
9504	1.3855	0.64188
9505	1.29348	0.695335
9506	0.946132	0.695974
9507	1.34828	2.1
9508	1.20943	1.93591
9509	0.946129	1.19589

9510	1.71013	2.1
9511	1.71013	2.1
9512	1.20943	1.9308
9513	0.94613	1.92679
9514	1.19613	0.260083
9515	0.946118	0.276351
9516	0.946117	0.260091
9517	1.70868	0.231714
9518	1.70869	0.213294
9519	1.40551	0.244209
9520	1.2493	0.253719
9521	0.946131	2.1
9522	1.73123	2.1
9523	1.73123	2.1
9524	0.946132	2.1
9525	1.37811	2.1
9526	1.37811	2.1
9527	1.25144	2.1
9528	0.946132	2.1
9529	1.75139	2.1
9530	1.75139	2.1
9531	1.23374	2.1
9532	1.29926	2.1
9533	1.39827	2.1
9534	1.23374	2.1
9535	1.41375	0.297983
9536	1.25697	0.361571
9537	0.946118	0.356347
9538	1.80377	0.26777
9539	1.80377	0.25546
9540	1.25699	0.326335
9541	0.946106	0.337712
9542	0.946133	1.33597
9543	1.81806	1.14487
9544	1.81806	1.85584
9545	0.946133	1.64921
9546	1.37089	1.88909
9547	1.37089	1.16445
9548	1.34999	1.70006
9549	0.946131	1.84993
9550	1.80855	1.10885
9551	1.80855	2.1
9552	1.31184	1.79892
9553	1.3933	1.24174
9554	1.36139	1.30621
9555	1.31184	1.44138
9556	1.01175	1.13012
9557	0.94613	0.860423
9558	0.919796	1.28348
9559	1.50484	0.796172
9560	1.80584	1.40508
9561	1.53512	0.915632
9562	1.14539	0.874374
9563	0.946133	2.1
9564	1.72797	1.26209
9565	1.72797	2.1
9566	1.2111	2.1
9567	1.39731	1.20367

9568	1.27679	1.84804
9569	1.21111	1.68808
9570	0.946119	0.813655
9571	1.67673	1.13458
9572	1.67673	2.1
9573	0.946118	1.16383
9574	1.21519	1.69647
9575	1.2152	1.3936
9576	1.19808	1.70468
9577	0.946127	2.1
9578	1.54442	2.1
9579	1.54442	2.1
9580	1.04806	2.1
9581	1.13485	2.1
9582	1.3557	2.1
9583	1.04805	2.1
9584	0.946129	2.1
9585	1.32282	2.1
9586	1.77749	2.1
9587	1.77749	2.1
9588	0.946131	2.1
9589	1.32282	2.1
9590	1.4008	2.1
9591	0.946128	0.397948
9592	1.02366	0.385087
9593	0.94613	0.391158
9594	1.66684	0.36695
9595	1.66684	0.345302
9596	1.29215	0.358825
9597	1.32082	0.314791
9598	0.946133	0.371413
9599	1.01451	0.367844
9600	0.946132	0.370275
9601	1.62374	0.411178
9602	1.62374	0.36501
9603	1.2929	0.356936
9604	1.27697	0.318683
9605	1.37869	2.1
9606	1.17215	2.1
9607	0.946132	2.1
9608	1.70508	2.1
9609	1.70508	2.1
9610	1.17215	2.1
9611	0.946132	2.1
9612	1.03784	2.1
9613	0.946132	2.1
9614	0.946132	2.1
9615	1.56718	2.1
9616	1.56717	2.1
9617	1.35703	2.1
9618	1.15628	2.1
9619	1.35356	2.1
9620	1.28976	2.1
9621	0.946131	2.1
9622	1.78612	2.1
9623	1.78612	2.1
9624	1.28976	2.1
9625	0.94613	2.1

9626	0.946132	2.1
9627	1.36373	2.1
9628	1.92382	2.1
9629	0.946131	2.1
9630	1.92382	2.1
9631	1.33471	2.1
9632	0.946132	2.1
9633	0.94613	0.773333
9634	1.29149	0.970838
9635	1.75878	0.844019
9636	1.75878	1.4017
9637	0.946128	0.915042
9638	1.29149	0.78016
9639	1.41342	0.794998
9640	1.41022	2.1
9641	1.37034	1.20559
9642	1.83443	1.38914
9643	1.83443	2.1
9644	0.946128	1.1285
9645	0.946131	1.49682
9646	1.26928	1.42493
9647	0.946128	0.884252
9648	1.38259	1.40111
9649	1.81405	0.96712
9650	1.81405	1.58628
9651	0.946126	1.13069
9652	1.38259	0.988521
9653	1.37759	0.912119
9654	1.68131	0.365937
9655	1.68131	0.647426
9656	0.946131	0.434856
9657	1.38896	0.347702
9658	1.17438	0.923449
9659	1.17438	0.536956
9660	1.23848	0.643566
9661	1.80088	0.534796
9662	1.80088	0.334969
9663	0.946132	0.375335
9664	1.40332	0.525018
9665	1.28185	0.53923
9666	1.28185	0.314619
9667	1.34369	0.315583
9668	1.71939	2.1
9669	1.71939	2.1
9670	0.946131	2.1
9671	1.28308	2.1
9672	0.946133	2.1
9673	1.24024	2.1
9674	1.28309	2.1
9675	1.8525	1.91603
9676	1.8525	2.1
9677	0.946132	2.1
9678	1.38244	2.1
9679	0.946132	1.01424
9680	1.33469	2.01968
9681	1.38244	2.04602
9682	1.26654	1.02014
9683	1.39514	0.969744

9684	1.71556	0.954757
9685	1.71556	1.50572
9686	0.946126	0.851074
9687	0.946129	1.08657
9688	1.1908	1.03447
9689	1.49995	2.1
9690	1.63875	2.1
9691	0.93608	2.1
9692	0.937208	2.1
9693	0.943708	2.1
9694	0.936134	2.1
9695	1.14341	2.1
9696	1.17802	0.180015
9697	1.3542	0.187578
9698	1.73981	0.189204
9699	1.73981	0.180187
9700	0.94613	0.180213
9701	1.3542	0.180122
9702	1.33174	0.188837
9703	1.59452	2.1
9704	1.59452	2.1
9705	0.946132	2.1
9706	1.25 2.1	
9707	0.975266	2.1
9708	0.946132	2.1
9709	1.25 2.1	
9710	0.946131	2.1
9711	1.48685	2.1
9712	1.48685	2.1
9713	0.946132	2.1
9714	1.17273	2.1
9715	1.17273	2.1
9716	0.955602	2.1
9717	1.32827	0.424719
9718	1.11166	0.411316
9719	0.946127	0.46077
9720	1.6136	0.396012
9721	1.6136	0.388652
9722	1.11166	0.422999
9723	0.946129	0.42436
9724	0.851541	0.850549
9725	1.22107	0.862513
9726	1.64594	0.820876
9727	1.5271	0.830026
9728	0.94613	0.898432
9729	1.22112	0.867473
9730	1.25002	0.837156
9731	1.65795	0.754993
9732	1.65795	1.16965
9733	0.946132	1.02766
9734	1.2366	0.907374
9735	0.946132	0.696725
9736	1.10023	0.842791
9737	1.2366	0.785557
9738	1.35856	0.420891
9739	0.946132	0.403306
9740	1.35856	0.369368
9741	1.08019	0.398052

9742	1.49261	0.357266
9743	1.49261	0.385887
9744	0.94613	0.456868
9745	1.29322	0.573744
9746	0.946132	0.524831
9747	1.29322	0.460792
9748	1.35856	0.531653
9749	1.70565	0.431436
9750	1.70565	0.507066
9751	0.946132	0.576542
9752	1.66635	2.1
9753	1.66635	2.1
9754	0.946133	2.1
9755	1.39558	2.1
9756	1.14793	2.1
9757	1.14793	2.1
9758	1.21691	2.1
9759	1.35381	0.637927
9760	1.3365	1.102
9761	1.09905	0.841652
9762	0.946132	0.996522
9763	1.74418	1.03982
9764	1.74418	0.523892
9765	0.946132	0.785628
9766	1.84759	0.859633
9767	1.84759	0.666198
9768	0.94613	0.774008
9769	1.3904	0.853406
9770	1.34522	0.361528
9771	1.34522	0.564484
9772	1.40331	0.383072
9773	1.86047	0.881703
9774	1.86047	2.07692
9775	0.946132	1.18662
9776	1.41619	2.0467
9777	0.946132	0.746782
9778	1.3659	0.931253
9779	1.41619	0.957562
9780	1.28464	1.82488
9781	1.14496	1.33406
9782	1.57806	1.42152
9783	1.7753	1.67391
9784	0.851541	1.44785
9785	0.946131	1.53891
9786	0.872786	1.50077
9787	0.85062	0.784997
9788	1.52635	0.892238
9789	1.63785	0.792801
9790	1.69972	0.964557
9791	0.946132	0.988225
9792	1.37493	0.807311
9793	1.23854	0.810706
9794	1.41134	1.64619
9795	1.27079	1.38598
9796	1.736	1.20268
9797	1.736	2.0381
9798	0.946132	0.903766
9799	0.946133	1.32009

9800	1.21177	1.47966
9801	0.946131	0.379007
9802	0.946129	0.344181
9803	0.94613	0.37958
9804	0.946131	0.297196
9805	0.946131	0.310758
9806	1.35403	0.315981
9807	0.946131	0.378511
9808	1.37052	0.557935
9809	0.946132	0.905249
9810	0.94613	0.689506
9811	0.946133	0.573199
9812	0.946131	0.615909
9813	0.946131	0.68875
9814	0.946131	0.618822
9815	1.24249	0.524224
9816	0.946132	0.524564
9817	0.946133	0.473758
9818	0.946132	0.536217
9819	0.946132	0.485526
9820	0.946132	0.519293
9821	0.946132	0.503083
9822	0.946132	2.1
9823	0.946132	1.75833
9824	0.946131	2.1
9825	0.946132	2.1
9826	0.946132	2.1
9827	0.946132	1.9738
9828	1.20608	2.1
9829	1.41232	2.1
9830	0.946128	2.1
9831	0.946129	2.1
9832	0.94613	2.1
9833	0.94613	2.1
9834	0.94613	2.1
9835	0.94613	2.1
9836	0.946132	0.449757
9837	0.946131	0.446213
9838	0.946131	0.470628
9839	0.946132	0.319305
9840	0.946132	0.362752
9841	0.946131	0.437836
9842	0.946132	0.398678
9843	0.946116	0.902436
9844	0.94612	1.41589
9845	0.946118	1.10801
9846	0.946124	2.1
9847	0.946118	1.08621
9848	1.41561	1.5118
9849	0.946131	1.06404
9850	0.945846	1.94729
9851	1.03508	2.07516
9852	0.77579	1.50476
9853	0.658908	2.0942
9854	1.17474	2.09359
9855	0.94303	2.1
9856	0.849189	2.1
9857	0.94298	2.1

9858	1.03963	2.07922
9859	0.946131	1.3759
9860	0.946131	1.24313
9861	0.946131	1.99795
9862	0.946131	1.04367
9863	0.946131	0.812843
9864	0.946131	1.13281
9865	1.32819	1.2913
9866	0.946133	1.04366
9867	0.946129	1.96135
9868	0.946128	1.50449
9869	0.94613	1.43589
9870	0.946131	2.05785
9871	0.946131	1.03645
9872	0.946131	1.40018
9873	0.954072	0.160318
9874	1.28895	0.160076
9875	0.946133	0.169686
9876	1.15803	0.172288
9877	1.29689	0.160764
9878	1.29689	0.166041
9879	0.946132	0.173085
9880	0.946132	1.26237
9881	0.946132	1.27797
9882	0.946132	1.16985
9883	0.946132	1.35152
9884	0.946132	1.53161
9885	0.946131	1.55451
9886	1.0264	1.54843
9887	0.946126	0.690204
9888	0.946132	0.664506
9889	0.946131	0.569799
9890	0.94613	0.668618
9891	0.946129	0.770841
9892	0.94613	0.708482
9893	1.35977	0.681225
9894	0.946132	0.704004
9895	0.946133	0.701211
9896	0.876632	0.720958
9897	0.946132	0.7
9898	1.01566	0.722856
9899	1.01566	0.701046
9900	0.876606	0.711258
9901	0.946131	0.265602
9902	0.946128	0.250526
9903	0.946128	0.271747
9904	0.94613	0.257765
9905	0.94613	0.281546
9906	1.24979	0.236681
9907	0.94613	0.282334
9908	0.946131	0.230835
9909	0.946131	0.267657
9910	0.921762	0.24169
9911	0.9705	0.217264
9912	0.9705	0.229328
9913	0.946131	0.257356
9914	0.921791	0.250839
9915	0.810944	0.595155

9916	0.810945	0.61945
9917	0.810959	0.575347
9918	2.42267	0.433819
9919	1.35168	0.529315
9920	1.08132	0.572315
9921	1.08132	0.481002
9922	1.08131	0.55122
9923	0.946133	0.347962
9924	0.946132	0.341566
9925	0.946132	0.370967
9926	0.946133	0.306217
9927	0.946133	0.363279
9928	0.946132	0.396366
9929	0.946133	0.360519
9930	0.946131	0.941656
9931	0.946131	1.10909
9932	0.94613	0.884341
9933	0.851514	0.896553
9934	0.946131	0.981389
9935	0.965268	1.03642
9936	0.851513	0.919566
9937	0.946122	2.1
9938	0.946123	2.1
9939	0.946126	2.1
9940	0.946124	2.1
9941	0.946124	2.1
9942	0.946118	2.1
9943	0.94613	2.1
9944	0.946131	0.689822
9945	1.30448	0.6823
9946	0.946132	0.699925
9947	0.946129	0.679618
9948	0.946131	0.822249
9949	0.946131	0.679151
9950	0.94613	0.696786
9951	0.946133	1.06692
9952	0.946128	2.1
9953	0.946128	1.74393
9954	0.94613	1.21431
9955	1.36503	2.1
9956	0.946131	1.61776
9957	0.946131	2.1
9958	0.810947	0.173369
9959	0.810947	0.173332
9960	2.48233	0.168238
9961	1.3517	0.168816
9962	1.08132	0.173215
9963	1.08132	0.169929
9964	1.08133	0.170337
9965	0.810934	0.173606
9966	1.48874	0.157625
9967	2.48233	0.158609
9968	2.2369	0.163049
9969	0.946133	0.808664
9970	0.946132	0.692748
9971	0.946132	0.87376
9972	0.946133	0.856227
9973	0.946133	0.865815

9974	1.41851	0.802934
9975	0.946132	0.746665
9976	1.03925	2.1
9977	0.946131	2.1
9978	0.94613	2.1
9979	0.946128	2.1
9980	0.946133	2.1
9981	0.944743	2.1
9982	1.13703	2.1
9983	0.946132	2.01002
9984	0.946128	2.1
9985	0.946128	2.1
9986	0.946131	2.1
9987	0.94613	2.1
9988	0.94613	2.1
9989	0.94613	2.1
9990	0.946131	0.264124
9991	0.946132	0.299323
9992	0.946131	0.26733
9993	0.946132	0.240837
9994	0.946132	0.24857
9995	0.946131	0.269118
9996	1.20057	0.251988
9997	0.946129	2.1
9998	0.94613	2.1
9999	0.94613	2.09078
10000	0.946128	2.1
10001	0.946132	2.1
10002	0.94613	2.1
10003	1.38302	2.1
10004	0.856567	0.331575
10005	1.14319	0.333409
10006	0.716526	0.321558
10007	1.20631	0.318655
10008	0.626936	0.292493
10009	0.946131	0.325498
10010	0.94613	0.361207
10011	1.39502	0.302331
10012	0.881445	0.27989
10013	0.946128	0.316856
10014	0.94613	0.291238
10015	0.881447	0.310286
10016	0.946117	0.286192
10017	0.946116	0.288561
10018	0.88146	0.357588
10019	0.946132	2.1
10020	1.39053	2.1
10021	0.946132	2.1
10022	0.946132	2.1
10023	0.946132	2.1
10024	0.946132	2.1
10025	0.946131	2.1
10026	0.946131	2.1
10027	0.946131	2.1
10028	0.946131	2.1
10029	0.946132	2.1
10030	0.946132	2.1
10031	1.40571	2.1

10032	0.946132	2.1
10033	0.94613	0.661414
10034	0.946127	0.730987
10035	0.946128	0.684836
10036	0.946131	0.668004
10037	0.946127	0.679462
10038	1.25819	0.639196
10039	0.946129	0.650231
10040	0.946129	0.915528
10041	1.29054	0.937746
10042	0.94613	0.904681
10043	0.94613	0.914836
10044	0.946131	1.18059
10045	0.946131	0.979807
10046	0.94613	0.935588
10047	0.810944	0.18487
10048	0.810941	0.188004
10049	1.61558	0.178239
10050	1.3517	0.179545
10051	1.08131	0.186043
10052	1.08132	0.186503
10053	1.08133	0.180338
10054	0.810931	0.186435
10055	1.02036	1.19738
10056	0.946132	0.992225
10057	1.02036	0.976899
10058	0.946132	0.858148
10059	1.1576	0.939345
10060	0.964656	1.03364
10061	0.927608	0.851046
10062	0.946128	2.1
10063	1.38813	2.1
10064	0.946129	2.1
10065	0.946131	2.1
10066	0.946131	2.1
10067	0.94613	2.1
10068	0.946131	2.1
10069	0.946131	2.1
10070	0.94613	0.837223
10071	0.94613	2.1
10072	1.41665	1.99494
10073	0.946132	0.847704
10074	0.946131	1.64003
10075	0.94613	0.959432
10076	0.946118	0.464674
10077	0.946118	0.442304
10078	0.946116	0.460751
10079	0.946126	0.351576
10080	0.94612	0.395297
10081	0.946131	0.452982
10082	0.946105	0.394798
10083	0.946132	2.1
10084	0.946132	2.1
10085	0.946132	2.1
10086	0.946131	2.1
10087	0.946132	2.1
10088	0.946132	2.1
10089	0.946132	2.1

10090	0.864997	0.228021
10091	0.786573	0.229947
10092	0.978561	0.228599
10093	1.47385	0.209952
10094	1.68652	0.214132
10095	1.27803	0.21044
10096	1.19771	0.22622
10097	1.27595	0.222388
10098	0.946127	0.765761
10099	1.3771	0.692596
10100	0.946127	0.672711
10101	0.946131	0.83664
10102	0.946129	0.638975
10103	0.946129	0.786245
10104	0.946129	0.709035
10105	0.95697	0.16894
10106	1.48133	0.175938
10107	1.24499	0.169269
10108	1.5295	0.17559
10109	0.938161	0.169927
10110	0.808717	0.168944
10111	1.3542	0.175927
10112	0.946133	0.924744
10113	1.14444	0.895713
10114	0.946132	0.878908
10115	0.946132	0.911533
10116	0.946133	1.13507
10117	0.946132	0.987682
10118	0.946132	0.922146
10119	0.946131	0.303237
10120	0.946132	0.414948
10121	0.946131	0.304641
10122	0.946132	0.29536
10123	0.946131	0.375705
10124	0.946132	0.276365
10125	0.946132	0.356284
10126	0.946132	2.1
10127	0.946133	2.1
10128	0.946133	2.1
10129	0.946132	2.1
10130	1.29322	2.1
10131	0.946132	2.1
10132	0.946132	2.1
10133	0.946131	2.02281
10134	0.946132	2.1
10135	0.946131	2.1
10136	0.946132	2.00195
10137	1.28441	2.1
10138	0.946131	2.07172
10139	0.946131	2.1
10140	0.946113	2.1
10141	0.946118	2.1
10142	0.946118	1.49199
10143	1.28073	2.1
10144	0.946117	1.78066
10145	0.946131	2.1
10146	0.946105	2.07731
10147	0.810945	2.06475

10148	0.810942	2.1
10149	2.75904	2.1
10150	1.3517	2.1
10151	1.08131	2.1
10152	1.08133	2.1
10153	1.08132	2.1
10154	0.81093	2.1
10155	0.946127	2.09941
10156	1.37952	2.1
10157	1.86965	2.1
10158	0.946127	2.1
10159	1.86965	2.1
10160	1.28228	2.1
10161	1.37952	2.1
10162	0.946128	2.1
10163	0.946132	2.1
10164	1.14042	2.1
10165	0.946132	2.1
10166	1.14042	2.1
10167	1.09974	2.1
10168	1.09974	2.1
10169	0.946132	2.1
10170	0.946132	0.539065
10171	0.946132	0.472789
10172	0.946133	0.535079
10173	0.946132	0.452469
10174	0.946132	0.544683
10175	0.946132	0.454583
10176	0.946133	0.567139
10177	1.31817	1.3184
10178	0.946131	1.12355
10179	0.946131	1.27769
10180	0.946131	1.23088
10181	0.946131	1.01113
10182	0.946131	1.14725
10183	0.946131	1.14106
10184	0.946132	2.1
10185	0.946132	2.1
10186	0.946132	2.1
10187	0.946132	2.1
10188	0.946132	2.1
10189	0.946132	2.1
10190	1.35048	2.1
10191	0.946131	2.08793
10192	0.946131	2.1
10193	0.94613	2.1
10194	0.946133	2.1
10195	0.946133	2.1
10196	1.35943	2.1
10197	0.946131	2.1
10198	0.946133	2.1
10199	0.946132	2.1
10200	0.946132	2.1
10201	0.946133	2.1
10202	0.946133	2.1
10203	0.946133	2.07588
10204	0.990712	2.1
10205	0.965725	1.49197

10206	0.946129	0.990404
10207	0.946133	1.16808
10208	1.21721	1.09292
10209	0.877931	0.899128
10210	0.946131	1.15016
10211	0.946131	0.971646
10212	0.946132	0.80588
10213	0.946131	0.740855
10214	0.946131	0.93833
10215	0.946133	0.787177
10216	0.946128	1.08692
10217	0.946131	0.887736
10218	1.39575	0.890987
10219	0.810943	0.744324
10220	0.810942	0.756535
10221	2.58794	0.637515
10222	1.21652	0.607676
10223	0.946154	0.702992
10224	0.979952	0.696782
10225	1.0813	0.697144
10226	0.810959	0.700182
10227	0.924071	0.6366
10228	1.16453	0.524685
10229	1.28586	0.517672
10230	0.946131	0.666251
10231	1.1227	0.498547
10232	1.16462	0.587797
10233	0.851553	0.705546
10234	0.709578	0.677766
10235	0.810943	0.878714
10236	0.810959	0.771852
10237	1.82615	0.630958
10238	1.21651	0.667181
10239	1.08132	0.692009
10240	0.946155	0.710115
10241	0.979937	0.803667
10242	0.946128	1.17564
10243	0.946132	2.0223
10244	0.946132	0.978491
10245	0.94613	1.6412
10246	0.94613	1.52821
10247	0.94613	0.969589
10248	0.94613	1.45532
10249	0.946131	1.3477
10250	1.29735	1.6419
10251	0.946131	2.08556
10252	0.946131	0.979872
10253	0.946131	1.22704
10254	0.946131	0.924047
10255	0.946131	1.51871
10256	0.946128	1.81139
10257	1.33266	1.11636
10258	0.94613	1.18807
10259	0.946131	1.5521
10260	0.94613	0.858937
10261	0.946131	1.10679
10262	0.946131	1.06329
10263	0.946131	0.866391

10264	0.946131	1.98647
10265	0.946131	0.769636
10266	1.40608	0.877912
10267	0.946131	1.82095
10268	0.946131	0.939354
10269	0.946131	1.02789
10270	0.946131	1.17931
10271	0.946131	1.64656
10272	0.946131	1.23365
10273	1.2334	1.27588
10274	0.946129	1.33816
10275	0.946131	1.28047
10276	0.946131	1.56276
10277	0.946133	1.78062
10278	0.946133	2.08474
10279	0.946132	1.31477
10280	1.29198	1.84687
10281	0.946132	1.62947
10282	0.946132	1.6661
10283	0.946132	1.84743
10284	0.946131	2.1
10285	0.946128	2.1
10286	0.946128	2.1
10287	0.946131	2.1
10288	0.946127	2.1
10289	0.94613	2.1
10290	0.946129	2.1
10291	0.946123	2.1
10292	0.946126	2.1
10293	0.946125	2.1
10294	1.38639	2.1
10295	0.946126	2.1
10296	0.946132	2.1
10297	0.946119	2.1
10298	0.946128	0.67613
10299	0.946132	0.655109
10300	0.946131	0.693384
10301	0.94613	0.523423
10302	0.94613	0.573185
10303	0.946131	0.642304
10304	0.94613	0.608259
10305	0.946131	0.642913
10306	0.946131	0.619719
10307	0.946131	0.666976
10308	0.946131	0.446706
10309	0.946131	0.516096
10310	0.946131	0.620007
10311	0.946131	0.559902
10312	0.810945	2.1
10313	0.810943	2.1
10314	0.810933	2.1
10315	2.24688	2.1
10316	1.3517	2.1
10317	1.08132	2.1
10318	1.08132	2.1
10319	1.08133	2.1
10320	1.25809	0.539942
10321	0.946131	0.850226

10322	1.25809	0.539215
10323	0.946129	0.806996
10324	0.94613	0.556101
10325	0.946131	0.794474
10326	0.946131	0.636927
10327	0.946131	0.376434
10328	0.946131	0.366943
10329	0.946129	0.386761
10330	1.28911	0.355531
10331	0.946131	0.348596
10332	0.946131	0.380331
10333	0.946131	0.399676
10334	0.94613	0.697352
10335	0.946131	0.713012
10336	0.94613	0.65169
10337	0.946128	0.700171
10338	0.946132	0.647867
10339	1.38954	0.679479
10340	0.946131	0.681417
10341	0.946131	0.543496
10342	0.946131	0.739928
10343	0.946131	0.813663
10344	1.37981	0.671169
10345	0.946131	0.927089
10346	0.946131	0.775339
10347	0.946131	0.798451
10348	0.946131	1.64528
10349	0.946127	2.1
10350	0.946127	2.1
10351	0.946131	1.87333
10352	0.946127	2.1
10353	0.946129	2.08186
10354	0.946128	2.08362
10355	0.94613	2.1
10356	0.946126	2.1
10357	0.946127	2.1
10358	0.946131	2.1
10359	0.946126	1.98947
10360	0.946128	2.1
10361	0.946128	2.1
10362	0.946133	0.688426
10363	0.946132	0.618038
10364	0.946132	0.642183
10365	0.946132	0.635067
10366	0.946132	0.69719
10367	1.13511	0.532105
10368	0.946132	0.591014
10369	0.946132	1.73756
10370	0.946129	1.77546
10371	0.94613	1.55522
10372	0.946131	1.85645
10373	0.946131	1.86056
10374	0.94613	2.08513
10375	1.27575	2.04006
10376	0.946129	2.1
10377	0.946131	2.1
10378	0.946131	2.1
10379	0.946128	2.1

10380	0.946133	2.1
10381	0.946131	2.1
10382	0.94613	2.1
10383	1.15581	0.229642
10384	0.946132	0.21782
10385	0.946131	0.250596
10386	0.94613	0.236748
10387	1.15581	0.231786
10388	0.94613	0.233623
10389	0.946131	0.265417
10390	0.611407	1.09391
10391	0.946131	0.936732
10392	1.37335	1.04151
10393	1.15086	1.14448
10394	0.617549	0.979165
10395	0.851553	1.04161
10396	1.14592	1.13103
10397	1.22618	1.2619
10398	0.946132	0.929992
10399	0.946132	1.30959
10400	0.946132	0.966358
10401	0.946131	0.985637
10402	0.946132	1.09184
10403	0.946132	1.06254
10404	0.946131	2.00863
10405	0.94613	2.1
10406	0.946131	2.1
10407	0.946133	2.1
10408	0.946129	2.1
10409	0.946131	2.1
10410	0.946131	2.1
10411	0.810945	0.814147
10412	0.810943	1.07863
10413	2.50715	0.976829
10414	1.3517	0.915696
10415	1.08133	0.888778
10416	1.08132	1.04066
10417	1.08132	0.716919
10418	0.810932	0.835163
10419	1.07811	1.11422
10420	0.94613	0.867551
10421	0.946128	1.17872
10422	1.25358	0.775028
10423	0.946131	0.928716
10424	1.25358	0.862168
10425	0.94613	0.881357
10426	0.946129	2.1
10427	1.09197	2.1
10428	0.946131	2.1
10429	1.09197	2.1
10430	1.09532	2.1
10431	1.09532	2.1
10432	0.946131	2.1
10433	1.58788	2.1
10434	1.34255	2.1
10435	0.594912	2.1
10436	0.58894	2.1
10437	0.946129	2.1

10438	1.16679	2.1
10439	1.04071	2.1
10440	0.535418	2.1
10441	0.946131	1.9668
10442	0.946128	2.1
10443	0.946131	1.86794
10444	1.20221	2.1
10445	0.946128	2.1
10446	0.94613	2.1
10447	1.20221	2.1
10448	0.946132	2.1
10449	0.946133	2.1
10450	0.946132	2.1
10451	0.946132	2.1
10452	0.946133	2.1
10453	0.946133	2.1
10454	0.946132	2.1
10455	0.946132	1.15719
10456	0.946132	1.32212
10457	0.946133	1.57285
10458	0.946132	1.20333
10459	1.411	1.41967
10460	0.946132	1.28678
10461	0.946133	1.3867
10462	1.05575	0.46348
10463	0.563734	0.46624
10464	1.05575	0.472261
10465	0.773833	0.390102
10466	0.751686	0.420632
10467	0.75884	0.471113
10468	0.815612	0.40631
10469	0.793465	0.4413
10470	0.768563	0.512032
10471	0.946129	0.439497
10472	0.946125	0.372623
10473	0.946126	0.46183
10474	0.94613	0.405942
10475	0.946124	0.488108
10476	1.31678	0.353014
10477	0.946128	0.418901
10478	0.946132	0.661731
10479	1.29914	0.561215
10480	0.946132	0.573939
10481	0.946132	0.579612
10482	0.946132	0.674197
10483	1.29914	0.457583
10484	0.946132	0.586431
10485	0.810946	0.636848
10486	0.810943	0.624455
10487	2.21082	0.474891
10488	1.35168	0.493872
10489	1.08132	0.548087
10490	1.08132	0.562145
10491	1.0813	0.613059
10492	0.81096	0.60521
10493	0.625813	0.266256
10494	0.946132	0.289487
10495	0.946131	0.340778

10496	0.946132	0.306409
10497	0.946131	0.301027
10498	1.39301	0.323769
10499	0.625812	0.305458
10500	0.810946	1.3694
10501	0.810945	2.1
10502	0.810931	1.84475
10503	2.70043	2.06676
10504	1.3517	1.90556
10505	1.08131	1.43853
10506	1.08131	2.08467
10507	1.08133	1.54501
10508	0.810943	2.1
10509	0.810944	2.09473
10510	0.810958	2.1
10511	2.44802	2.1
10512	1.35168	2.1
10513	1.08132	2.1
10514	1.08132	2.1
10515	1.0813	2.1
10516	0.946131	0.642218
10517	0.946129	0.68518
10518	0.946128	0.61342
10519	0.946131	0.616434
10520	1.3492	0.603718
10521	1.3492	0.638021
10522	0.94613	0.615977
10523	0.946132	0.716336
10524	0.946132	0.705496
10525	0.946133	0.704729
10526	0.946133	0.700667
10527	0.946132	0.716277
10528	0.946132	0.700303
10529	0.946132	0.714817
10530	0.946129	0.590844
10531	0.946133	0.482799
10532	0.946131	0.597217
10533	0.94613	0.53852
10534	0.946131	0.666161
10535	0.946131	0.548161
10536	0.946131	0.552749
10537	0.946119	0.476538
10538	0.946119	0.402619
10539	0.946119	0.48805
10540	0.946125	0.425412
10541	0.94612	0.537253
10542	0.946132	0.448793
10543	0.946107	0.450238
10544	0.946133	2.1
10545	0.946132	2.1
10546	0.946132	2.1
10547	0.946132	2.1
10548	0.946132	2.1
10549	0.946133	2.1
10550	1.3868	2.1
10551	0.810947	2.1
10552	0.810946	2.1
10553	0.81096	2.1

10554	2.66094	2.1
10555	1.35168	2.1
10556	1.08132	2.1
10557	1.08132	2.1
10558	1.0813	2.1
10559	0.810946	2.1
10560	0.810946	2.1
10561	0.810933	2.1
10562	2.75737	2.1
10563	1.3517	2.1
10564	1.08132	2.1
10565	1.08132	2.1
10566	1.08133	2.1
10567	0.946133	2.1
10568	0.946132	2.1
10569	0.946132	1.52725
10570	0.946132	2.1
10571	0.946133	1.50285
10572	0.946132	2.1
10573	0.946133	2.1
10574	0.946133	2.1
10575	0.946133	2.1
10576	0.946132	2.1
10577	0.946132	2.1
10578	0.946133	2.1
10579	0.946132	2.1
10580	1.28569	2.1
10581	0.946132	1.46734
10582	0.946133	2.1
10583	0.946133	2.1
10584	0.946132	1.77096
10585	0.946132	2.1
10586	0.946132	1.89466
10587	0.946133	1.88642
10588	0.946132	0.426733
10589	0.946131	0.408722
10590	0.94613	0.371263
10591	0.946133	0.358406
10592	0.946128	0.414272
10593	0.946131	0.348523
10594	0.946131	0.433499
10595	0.946131	1.55089
10596	0.94613	1.57552
10597	0.946131	1.97264
10598	0.94613	1.63772
10599	1.41833	1.2003
10600	0.946131	1.14967
10601	0.946131	1.63782
10602	1.26155	0.251649
10603	0.946131	0.32256
10604	0.946131	0.285356
10605	0.946132	0.244058
10606	0.946132	0.264903
10607	0.946131	0.266437
10608	0.946132	0.263438
10609	0.810946	2.04324
10610	0.810946	2.1
10611	0.810959	2.1

10612	2.47938	2.1
10613	1.35168	2.1
10614	1.08132	2.1
10615	1.08132	2.1
10616	1.08131	2.1
10617	0.946131	0.776937
10618	0.94613	0.726538
10619	0.94613	0.90438
10620	0.946132	0.706703
10621	0.946128	1.04556
10622	0.94613	0.858049
10623	0.94613	0.885565
10624	0.94613	0.317102
10625	0.946131	0.32539
10626	0.94613	0.431993
10627	0.946129	0.352119
10628	0.946132	0.384268
10629	0.94613	0.359457
10630	0.946131	0.371484
10631	0.946132	2.1
10632	0.946131	2.1
10633	0.946128	2.1
10634	1.40756	2.1
10635	0.946131	2.1
10636	0.946129	2.1
10637	0.94613	2.1
10638	0.946131	1.3156
10639	0.946131	1.31414
10640	0.946131	1.61577
10641	0.946132	1.39324
10642	1.31947	1.38541
10643	0.946131	1.36997
10644	0.946131	1.42729
10645	0.946131	2.1
10646	1.25768	2.1
10647	0.946132	2.1
10648	0.946132	2.1
10649	0.946129	2.1
10650	0.946131	2.1
10651	0.94613	2.1
10652	0.946132	0.526455
10653	0.946129	0.469459
10654	0.946129	0.622866
10655	0.94613	0.548609
10656	1.31716	0.519967
10657	1.31716	0.511552
10658	0.946131	0.627399
10659	0.810943	0.996107
10660	0.810942	1.87402
10661	0.81093	1.4116
10662	2.37052	1.95858
10663	1.3517	1.32523
10664	1.08132	0.952057
10665	1.08132	1.68578
10666	1.08133	1.05743
10667	0.946128	0.914422
10668	1.10335	0.8017
10669	0.94613	0.797669

10670	0.946131	0.936311
10671	0.946131	0.807764
10672	1.10335	0.92357
10673	0.94613	0.931542
10674	0.946132	1.03927
10675	1.18818	0.939778
10676	0.946132	0.929523
10677	0.946132	1.05045
10678	0.946132	0.921377
10679	1.18818	1.05533
10680	0.946132	1.05658
10681	0.946125	2.1
10682	0.94612	2.1
10683	0.946119	2.1
10684	0.946119	2.1
10685	0.946119	2.1
10686	0.946132	2.1
10687	1.31522	2.1
10688	1.28803	1.73231
10689	0.946132	0.979027
10690	0.946132	1.98987
10691	0.946133	1.92461
10692	0.946133	0.945357
10693	0.946133	1.63576
10694	0.946132	1.31091
10695	0.946133	0.833424
10696	0.946132	0.967701
10697	0.946132	0.881193
10698	0.946132	0.78637
10699	0.946133	0.771814
10700	0.946132	0.767475
10701	1.37658	0.823053
10702	1.02239	1.44685
10703	0.946128	1.04222
10704	0.946132	1.25849
10705	1.02239	1.13062
10706	0.94613	0.964474
10707	0.96283	1.25484
10708	0.92943	1.02387
10709	0.946132	0.213375
10710	0.946131	0.237417
10711	0.946126	0.287899
10712	0.946129	0.246239
10713	0.946128	0.254027
10714	0.946128	0.239429
10715	0.946129	0.248059
10716	0.810946	0.292471
10717	0.810944	0.247019
10718	2.22493	0.209477
10719	1.21651	0.214263
10720	1.0813	0.239823
10721	0.946154	0.231181
10722	0.979951	0.229206
10723	0.70959	0.235054
10724	0.946132	0.340314
10725	0.946131	0.43768
10726	0.946131	0.334071
10727	0.946133	0.328637

10728	0.946131	0.429165
10729	0.946132	0.335682
10730	0.946132	0.394895
10731	0.946132	1.51059
10732	0.946131	1.26249
10733	0.946132	1.6828
10734	0.946132	1.03502
10735	0.946132	0.916157
10736	0.946132	1.08626
10737	1.40924	1.31712
10738	0.946131	2.1
10739	0.946128	2.1
10740	0.946127	2.1
10741	0.94613	2.1
10742	1.41743	2.1
10743	0.946128	2.1
10744	0.946129	2.1
10745	1.37675	0.774248
10746	0.946128	0.798022
10747	0.94613	0.768256
10748	0.94613	0.888454
10749	0.94613	0.768737
10750	0.946129	0.796095
10751	0.94613	0.826047
10752	0.946132	0.616249
10753	0.946132	0.73991
10754	0.946132	0.818074
10755	1.35721	0.616982
10756	0.946132	0.937512
10757	0.946132	0.808942
10758	1.35721	0.773612
10759	0.946133	0.911557
10760	0.946133	0.838089
10761	0.946132	0.871408
10762	1.10003	0.798299
10763	0.946133	0.787559
10764	0.946132	0.838235
10765	0.946132	0.836909
10766	0.946132	0.493599
10767	0.946131	0.401317
10768	0.946131	0.448785
10769	0.946131	0.517547
10770	1.0616	0.408751
10771	1.0616	0.427324
10772	0.946132	0.509993
10773	0.946132	0.527977
10774	0.946131	0.44278
10775	0.946131	0.48511
10776	0.946131	0.555013
10777	1.09402	0.450703
10778	1.09402	0.463401
10779	0.946131	0.55708
10780	0.946132	0.317576
10781	0.946126	0.314682
10782	1.24093	0.306207
10783	0.946129	0.361291
10784	0.946129	0.330118
10785	0.946128	0.334794

10786	0.946129	0.331142
10787	0.946131	0.403374
10788	0.946131	0.406787
10789	1.2929	0.387376
10790	0.946131	0.471631
10791	0.946131	0.425445
10792	0.946131	0.437819
10793	0.946131	0.424461
10794	0.810946	0.256466
10795	0.810945	0.221232
10796	0.810959	0.239994
10797	2.41233	0.218801
10798	1.35168	0.221494
10799	1.08132	0.23245
10800	1.08132	0.223981
10801	1.0813	0.239039
10802	0.810946	1.38881
10803	0.810943	1.89401
10804	0.750103	1.66846
10805	2.22778	1.94353
10806	1.27059	1.80415
10807	1.00021	1.55751
10808	1.02049	1.90335
10809	1.08133	1.53963
10810	0.946128	1.67711
10811	0.946132	2.1
10812	0.946132	2.09999
10813	0.94613	1.92306
10814	0.94613	2.1
10815	0.94613	1.97738
10816	0.946131	1.97405
10817	0.946126	0.731304
10818	0.946131	0.704877
10819	0.946127	0.783988
10820	1.3324	0.66479
10821	0.946127	0.679305
10822	0.946128	0.74151
10823	0.946128	0.813875
10824	0.94613	2.1
10825	0.946131	2.1
10826	0.94613	2.1
10827	0.946128	2.1
10828	0.946133	2.03822
10829	0.946131	2.1
10830	0.946131	2.1
10831	0.946133	2.1
10832	0.946132	2.1
10833	0.946133	2.1
10834	0.946133	2.1
10835	0.946132	2.1
10836	0.946132	2.1
10837	1.36541	2.1
10838	0.946131	0.841096
10839	0.94613	1.9899
10840	0.946131	0.997114
10841	0.94613	0.985918
10842	1.39509	1.40448
10843	0.946131	0.852082

10844	0.946131	1.49794
10845	0.946133	2.1
10846	0.946133	2.1
10847	0.946132	2.1
10848	0.946132	2.1
10849	0.946133	2.1
10850	0.946132	2.1
10851	1.29319	2.1
10852	1.01196	0.345159
10853	0.946131	0.44402
10854	0.946132	0.329477
10855	0.946132	0.382604
10856	0.946132	0.44675
10857	0.946132	0.41429
10858	0.946132	0.402915
10859	0.946112	2.1
10860	0.946118	2.08057
10861	0.946116	1.90971
10862	0.946117	1.92877
10863	0.946118	2.1
10864	0.94613	2.1
10865	0.946105	2.1
10866	1.28684	1.00685
10867	0.946131	1.06847
10868	0.946132	1.03799
10869	0.946132	1.20083
10870	0.946132	1.26143
10871	0.946132	1.08726
10872	0.946132	1.21874
10873	0.94613	1.87916
10874	0.94613	1.92631
10875	0.946128	1.66349
10876	0.946131	1.62356
10877	0.946131	1.79712
10878	0.94613	1.66362
10879	1.10185	1.9192
10880	0.810947	0.183728
10881	0.810945	0.185952
10882	1.57348	0.178906
10883	1.3517	0.179416
10884	1.08131	0.185053
10885	1.08132	0.186165
10886	1.08133	0.17771
10887	0.810932	0.185151
10888	0.946119	0.648488
10889	0.946116	0.560073
10890	0.946117	0.640103
10891	0.946121	0.470455
10892	0.946118	0.518323
10893	0.94613	0.579714
10894	0.946105	0.519777
10895	0.94612	0.790417
10896	0.946122	0.588816
10897	0.946119	0.693125
10898	0.946119	0.789249
10899	1.20884	0.5854
10900	0.946132	0.750724
10901	0.946107	0.684129

10902	2.67849	1.19055
10903	1.17709	1.16101
10904	0.605418	1.31842
10905	0.286984	1.20215
10906	0.172654	1.23355
10907	0.810946	1.00281
10908	0.940878	0.967472
10909	0.941235	0.959834
10910	1.26696	0.982098
10911	1.56837	0.998175
10912	1.13737	0.986674
10913	1.34115	0.988992
10914	1.24889	0.958227
10915	0.946132	1.25491
10916	0.946129	1.08644
10917	0.946128	1.2155
10918	0.94613	1.16253
10919	0.94613	1.32265
10920	0.946131	1.2531
10921	0.94613	1.20405
10922	0.946116	2.1
10923	0.94612	1.99246
10924	0.946118	2.1
10925	0.946123	2.1
10926	0.946118	2.1
10927	0.946131	2.05591
10928	1.23836	2.1
10929	0.810946	0.273341
10930	0.810946	0.266983
10931	0.810959	0.272711
10932	2.80677	0.244407
10933	1.35168	0.257576
10934	1.08132	0.242193
10935	1.08132	0.267955
10936	1.08131	0.267191
10937	0.810947	0.66668
10938	0.810947	0.762314
10939	2.41574	0.753747
10940	1.21653	0.74925
10941	1.08133	0.731344
10942	0.946153	0.751368
10943	0.979949	0.710912
10944	0.709567	0.706554
10945	0.94613	2.1
10946	0.946131	2.1
10947	0.946128	2.1
10948	1.34782	2.1
10949	0.946128	2.1
10950	0.946129	2.1
10951	1.34782	2.1
10952	0.946132	2.03936
10953	0.946132	2.06344
10954	0.94613	2.1
10955	1.16023	2.1
10956	0.946131	2.06628
10957	0.946131	2.1
10958	1.16023	2.1
10959	0.946131	1.00202

10960	0.946127	1.94185
10961	0.946127	1.56884
10962	0.946131	1.36738
10963	0.946128	2.08893
10964	0.94613	1.12311
10965	0.946129	1.3543
10966	0.946133	0.598196
10967	0.946132	0.539769
10968	0.946132	0.566479
10969	0.946133	0.625013
10970	1.20045	0.541158
10971	1.20045	0.542306
10972	0.946132	0.632537
10973	0.918871	0.425623
10974	0.971961	0.365091
10975	1.37324	0.275609
10976	1.78255	0.276452
10977	1.25155	0.368675
10978	1.42172	0.27972
10979	1.40554	0.33695
10980	1.08862	0.361847
10981	1.03803	0.33384
10982	0.946132	0.34714
10983	0.946126	0.399205
10984	0.943647	0.373897
10985	0.948611	0.388549
10986	0.946129	0.361879
10987	0.946129	0.366446
10988	1.1699	0.926938
10989	0.946132	0.807455
10990	0.946133	0.992054
10991	0.946133	0.822309
10992	0.946132	0.844288
10993	0.946133	0.880931
10994	0.946132	0.907823
10995	0.946132	2.1
10996	0.946131	2.1
10997	0.946131	2.1
10998	0.946132	2.1
10999	0.946131	2.1
11000	0.946131	2.1
11001	0.946132	2.1
11002	0.946132	0.915756
11003	1.38623	0.873564
11004	0.946132	0.848141
11005	0.946132	0.840666
11006	0.946133	0.884499
11007	0.946132	0.818345
11008	0.946133	0.966056
11009	0.946132	2.1
11010	1.40098	2.1
11011	0.946131	2.1
11012	0.946131	2.1
11013	0.946132	2.1
11014	0.946132	2.1
11015	0.946132	2.1
11016	1.01777	0.749597
11017	0.946132	0.662218

11018	0.946133	0.813377
11019	1.68068	0.736166
11020	1.68068	0.831609
11021	1.41894	0.729366
11022	1.20787	0.663882
11023	0.946132	0.709891
11024	0.946133	2.1
11025	0.946133	2.1
11026	0.946132	2.1
11027	1.40199	2.1
11028	0.946133	2.1
11029	0.946132	2.1
11030	0.946132	2.1
11031	0.810946	1.13505
11032	0.810947	0.958237
11033	2.37827	1.32558
11034	1.3517	1.17817
11035	1.08132	1.1938
11036	1.08132	1.19127
11037	1.08133	1.11076
11038	0.810934	1.11603
11039	0.946124	1.51929
11040	0.94613	1.20221
11041	1.40248	1.10973
11042	0.946131	1.0871
11043	0.946125	0.898355
11044	0.946127	1.17399
11045	0.946128	0.974313
11046	1.25703	1.69191
11047	0.87821	1.18461
11048	1.18912	1.68426
11049	0.946119	1.08128
11050	0.87825	1.21424
11051	1.1891	1.29291
11052	0.946107	1.40378
11053	0.946132	1.60317
11054	1.28543	1.36734
11055	0.946132	1.37963
11056	0.946132	1.20871
11057	0.946131	0.845338
11058	0.946132	0.82985
11059	0.946132	0.914729
11060	0.946115	2.1
11061	0.946119	2.1
11062	0.946116	2.1
11063	0.946123	2.1
11064	0.946118	2.1
11065	1.41636	2.1
11066	0.946129	2.1
11067	0.946132	0.989896
11068	0.946132	1.83401
11069	0.946132	1.20045
11070	0.946131	2.1
11071	0.946131	1.53316
11072	1.41432	1.93246
11073	0.946132	1.27731
11074	0.810947	0.336805
11075	0.810947	0.343071

11076	2.44433	0.271777
11077	1.21654	0.268834
11078	0.946154	0.332392
11079	0.97995	0.316182
11080	1.08133	0.278187
11081	0.810934	0.317992
11082	0.851542	0.269238
11083	0.835698	0.284977
11084	1.22216	0.256766
11085	0.946133	0.2628
11086	1.01217	0.25817
11087	1.09996	0.272654
11088	0.851529	0.282018
11089	0.946132	1.67312
11090	0.946132	1.96803
11091	0.946129	2.06226
11092	0.94613	1.70685
11093	0.946131	2.00758
11094	0.946131	1.88539
11095	1.2254	2.06587
11096	0.800897	1.29108
11097	0.838029	1.92042
11098	0.838013	1.50858
11099	0.93679	1.22706
11100	1.20217	1.30898
11101	1.16923	1.38835
11102	0.94948	1.34306
11103	0.958118	1.50851
11104	0.36751	1.43697
11105	0.800535	1.71199
11106	0.272087	1.11137
11107	0.428084	1.25437
11108	0.986335	1.70139
11109	0.946132	0.737172
11110	0.946133	0.848096
11111	1.19601	0.750565
11112	0.946132	0.775671
11113	0.946132	0.801641
11114	0.946133	0.875376
11115	0.946133	0.732086
11116	0.946132	1.30237
11117	1.36956	1.94761
11118	0.946132	2.1
11119	0.946132	1.25658
11120	0.946132	1.99354
11121	0.946133	1.41851
11122	0.946132	1.63812
11123	0.946132	0.262126
11124	0.946131	0.860728
11125	0.946131	0.324076
11126	1.34268	0.275038
11127	0.946133	0.657723
11128	0.946132	0.358096
11129	0.946131	0.502994
11130	0.946132	1.77629
11131	0.946131	1.90985
11132	0.946131	1.95666
11133	0.946132	1.93911

11134	0.946132	1.828
11135	0.946132	1.95404
11136	0.946132	1.83777
11137	0.810946	1.09967
11138	0.810943	1.0113
11139	2.51472	1.1086
11140	1.3517	1.07305
11141	1.08132	1.00709
11142	1.08132	1.10156
11143	1.08133	1.07141
11144	0.810933	1.07735
11145	0.902316	0.981758
11146	1.04517	1.0391
11147	0.974924	0.84268
11148	0.530063	1.13923
11149	1.1272	1.06536
11150	0.68207	1.03222
11151	0.477061	1.12628
11152	0.810946	0.397102
11153	0.810943	0.51772
11154	0.810959	0.444722
11155	1.99211	0.392921
11156	1.35168	0.394294
11157	1.08132	0.408965
11158	1.08132	0.412597
11159	1.08131	0.477506
11160	0.94613	0.500466
11161	0.946131	0.492503
11162	0.946132	0.440333
11163	0.946132	0.499103
11164	0.946129	0.619773
11165	0.946131	0.519893
11166	1.16699	0.499736
11167	0.810943	1.22479
11168	0.810942	1.14354
11169	1.6943	1.50566
11170	1.27059	1.52637
11171	1.00021	1.48818
11172	1.02049	1.3226
11173	1.08133	1.23054
11174	0.750103	1.2031
11175	0.957813	1.2705
11176	0.946128	1.34817
11177	0.946132	1.12654
11178	1.04832	1.43288
11179	0.870714	1.11367
11180	0.94613	1.17977
11181	0.94613	1.23487
11182	1.86374	1.84224
11183	2.0815	1.20917
11184	1.15918	0.7
11185	0.946129	0.7
11186	1.15918	0.7
11187	0.946131	0.7
11188	0.946131	0.7
11189	0.946131	0.7
11190	0.94613	0.7
11191	0.946119	0.90756

11192	1.3612	0.970973
11193	0.946125	0.943567
11194	0.946119	0.910827
11195	0.94612	1.14455
11196	0.946132	0.960955
11197	0.946107	0.94127
11198	0.810947	0.446031
11199	0.810947	0.520462
11200	2.50398	0.424012
11201	1.3517	0.418006
11202	1.08132	0.42354
11203	1.08133	0.482914
11204	1.08132	0.400282
11205	0.810934	0.461892
11206	0.946132	2.1
11207	0.946132	2.1
11208	0.946132	2.1
11209	1.39754	2.1
11210	0.946132	2.1
11211	0.946132	2.1
11212	0.946131	2.1
11213	0.946129	2.1
11214	1.41732	2.1
11215	0.946129	2.1
11216	0.946131	2.1
11217	0.946131	2.1
11218	0.946131	2.1
11219	0.946131	2.1
11220	0.946132	1.07852
11221	1.25276	1.45077
11222	0.946131	1.69882
11223	0.94613	1.09381
11224	0.94613	1.36309
11225	0.94613	1.13675
11226	0.946131	1.21284
11227	1.21261	1.14382
11228	0.94613	1.25249
11229	0.94613	1.60285
11230	0.946128	1.08953
11231	0.946131	1.35496
11232	0.94613	1.3092
11233	0.946129	1.42516
11234	0.946128	2.1
11235	0.946133	2.1
11236	0.946132	2.1
11237	0.946131	2.1
11238	0.94613	2.1
11239	0.946131	2.1
11240	1.39407	2.1
11241	0.946116	1.26966
11242	0.946119	1.27352
11243	0.946119	1.08492
11244	1.15369	1.28297
11245	0.946119	1.00003
11246	0.946131	1.15251
11247	0.946106	1.14878
11248	0.946131	1.94856
11249	0.946131	2.1

11250	0.946131	2.1
11251	0.946133	2.1
11252	0.946129	2.1
11253	0.946131	2.1
11254	0.946131	2.1
11255	1.19564	0.196352
11256	0.498881	0.22058
11257	1.33964	0.200077
11258	0.498878	0.210628
11259	0.923583	0.201128
11260	0.831259	0.210238
11261	0.553735	0.200143
11262	0.946132	0.207353
11263	0.946132	0.217872
11264	0.946131	0.250312
11265	0.946131	0.219144
11266	0.946132	0.226919
11267	0.946131	0.230871
11268	1.28673	0.217617
11269	0.946132	0.42207
11270	0.946132	0.440074
11271	1.33178	0.415432
11272	0.946132	0.449752
11273	0.946132	0.436745
11274	0.946131	0.454464
11275	0.946131	0.425568
11276	0.946128	0.741461
11277	0.946132	0.713037
11278	0.946129	0.749784
11279	0.946131	0.636201
11280	0.946131	0.67427
11281	0.946129	0.713095
11282	0.94613	0.693238
11283	0.946131	2.1
11284	0.946131	2.1
11285	0.946129	2.1
11286	1.40525	2.1
11287	0.94613	2.1
11288	0.94613	2.1
11289	1.40525	2.1
11290	0.946131	2.1
11291	0.946126	2.1
11292	0.946127	2.1
11293	0.946127	2.1
11294	1.4073	2.1
11295	0.946129	2.1
11296	0.946129	2.1
11297	0.946131	0.46641
11298	0.946131	0.415802
11299	0.946132	0.487696
11300	0.946131	0.366909
11301	0.946131	0.393267
11302	1.05016	0.39783
11303	0.946132	0.483023
11304	0.946128	0.473364
11305	0.946132	0.42639
11306	0.946131	0.491192
11307	0.946129	0.376429

11308	0.94613	0.399194
11309	1.13398	0.406023
11310	0.94613	0.505708
11311	0.946132	0.224928
11312	0.946132	0.261699
11313	0.946131	0.329667
11314	0.946131	0.275712
11315	0.946132	0.276063
11316	0.946131	0.262051
11317	0.946132	0.277514
11318	0.946131	0.591561
11319	0.94613	0.478947
11320	0.946131	0.54918
11321	0.94613	0.617824
11322	1.38645	0.496665
11323	1.38645	0.517902
11324	0.946131	0.574122
11325	0.946131	0.695742
11326	0.946131	0.578146
11327	0.94613	0.727828
11328	1.23923	0.551935
11329	0.94613	0.531428
11330	0.946131	0.696253
11331	0.946131	0.773955
11332	0.946132	0.2484
11333	0.946131	0.278756
11334	0.946131	0.249064
11335	0.946132	0.225289
11336	0.946132	0.236595
11337	0.946132	0.24987
11338	1.40511	0.237967
11339	0.946132	2.1
11340	0.946132	1.67676
11341	0.946132	2.07403
11342	0.946132	1.68617
11343	0.946132	1.53804
11344	0.946131	2.00902
11345	1.12968	2.032
11346	0.946132	1.26002
11347	0.946128	1.9146
11348	0.946128	1.75435
11349	0.94613	1.41694
11350	0.94613	2.01655
11351	0.94613	1.34575
11352	0.94613	1.61873
11353	0.946133	2.1
11354	0.946133	2.1
11355	0.946132	2.1
11356	0.946132	2.1
11357	0.946132	2.1
11358	0.946133	2.1
11359	1.31323	2.1
11360	0.94613	1.8304
11361	0.946131	0.943905
11362	0.946131	0.94768
11363	0.94613	0.903311
11364	0.946131	1.8141
11365	0.946131	1.11469

11366	0.946131	1.06866
11367	0.946132	0.680933
11368	0.946132	0.471187
11369	0.946132	0.818283
11370	1.36899	0.631105
11371	0.946132	0.571985
11372	0.946132	0.484343
11373	0.946133	0.551625
11374	1.03543	0.432444
11375	0.946131	0.426107
11376	0.946131	0.343724
11377	0.946132	0.352808
11378	0.94613	0.416576
11379	0.950987	0.334861
11380	0.941275	0.454376
11381	0.946131	0.473894
11382	0.94613	0.504021
11383	1.28952	0.503426
11384	0.946132	0.465399
11385	0.94613	0.556803
11386	0.946131	0.553847
11387	0.946131	0.48328
11388	0.94613	2.1
11389	1.35966	2.1
11390	0.946131	2.1
11391	0.946131	2.1
11392	0.946129	2.1
11393	0.94613	2.1
11394	0.94613	2.1
11395	0.810946	2.1
11396	0.810942	0.998783
11397	0.810932	1.82697
11398	2.6655	1.65096
11399	1.3517	1.38126
11400	1.08132	2.1
11401	1.08131	0.955807
11402	1.08133	1.57084
11403	0.94613	2.1
11404	0.946131	2.1
11405	0.94613	1.70413
11406	0.946128	2.1
11407	0.946131	1.80281
11408	0.94613	2.1
11409	0.94613	2.1
11410	0.946128	2.1
11411	0.946128	2.1
11412	0.946133	2.1
11413	1.25375	2.1
11414	0.94613	2.1
11415	0.946131	2.1
11416	1.25375	2.1
11417	0.94613	2.1
11418	0.94613	2.1
11419	0.946132	2.1
11420	0.946132	2.1
11421	0.946128	2.1
11422	0.94613	2.1
11423	1.31453	2.1

11424	0.946131	0.437986
11425	0.946129	0.350845
11426	0.94613	0.4369
11427	0.94613	0.324012
11428	0.94613	0.339842
11429	1.12018	0.329211
11430	0.94613	0.433393
11431	0.946131	0.530428
11432	0.946129	0.435578
11433	0.946131	0.524853
11434	1.16373	0.411229
11435	0.94613	0.40208
11436	0.94613	0.500428
11437	0.946131	0.549193
11438	1.36811	0.713523
11439	0.946132	0.76936
11440	1.36811	0.649104
11441	0.946129	0.739608
11442	0.94613	0.777777
11443	0.946129	0.751364
11444	0.946129	0.723872
11445	0.946132	0.644489
11446	0.946133	0.574784
11447	0.946132	0.622149
11448	0.946133	0.65708
11449	1.35732	0.586079
11450	1.35732	0.597057
11451	0.946133	0.634873
11452	1.36399	0.828926
11453	0.946129	0.948995
11454	0.94613	0.84703
11455	0.946131	0.960763
11456	0.94613	1.17197
11457	0.946131	0.920214
11458	0.946131	0.994895
11459	0.946133	2.1
11460	0.946132	2.1
11461	0.946132	2.1
11462	1.38302	2.1
11463	0.946133	2.1
11464	0.946132	2.1
11465	0.946133	2.1
11466	0.810943	2.1
11467	0.810944	2.1
11468	0.810945	2.1
11469	2.80542	2.1
11470	1.35169	2.1
11471	1.08132	2.1
11472	1.08132	2.1
11473	1.08132	2.1
11474	1.30189	2.1
11475	0.946131	2.1
11476	0.946132	2.1
11477	0.946131	2.1
11478	0.946131	2.1
11479	0.946131	2.1
11480	0.946132	2.1
11481	0.810946	0.922114

11482	0.810947	0.854737
11483	2.33293	0.901349
11484	1.24357	0.878086
11485	1.08132	0.882006
11486	0.973183	0.901103
11487	1.00023	0.905283
11488	0.810934	0.895167
11489	1.06584	0.849691
11490	0.946129	0.881362
11491	0.84731	0.848729
11492	1.16646	0.884789
11493	0.946126	0.905328
11494	1.2831	0.861614
11495	0.851527	0.873319
11496	0.946131	2.04476
11497	0.946128	2.1
11498	0.946128	2.1
11499	0.94613	2.1
11500	1.31961	2.1
11501	0.94613	2.1
11502	0.94613	2.1
11503	0.946131	2.1
11504	1.3605	2.1
11505	0.946133	2.1
11506	0.946132	2.1
11507	0.94613	2.1
11508	0.946131	2.1
11509	0.946131	2.1
11510	0.946128	1.71574
11511	0.946133	1.29848
11512	0.946132	2.1
11513	0.946131	1.17335
11514	0.946131	0.927094
11515	0.946131	1.29854
11516	1.30116	1.56793
11517	0.946132	0.259332
11518	0.946129	0.262227
11519	0.94613	0.258046
11520	1.3035	0.252669
11521	0.946131	0.231942
11522	0.94613	0.258879
11523	0.946131	0.299536
11524	0.946132	2.1
11525	1.2051	2.1
11526	1.2051	2.1
11527	0.946132	2.1
11528	1.37869	2.1
11529	0.946132	2.1
11530	0.946131	2.1
11531	0.810946	2.1
11532	0.810945	2.1
11533	2.69247	2.1
11534	1.35169	2.1
11535	1.08132	2.1
11536	1.08132	2.1
11537	1.08132	2.1
11538	0.810946	2.1
11539	1.22993	2.1

11540	0.946131	2.1
11541	0.946131	2.1
11542	1.34623	2.1
11543	0.946131	2.1
11544	1.34623	2.1
11545	0.946131	2.1
11546	0.946131	0.290574
11547	0.946132	0.363182
11548	0.946132	0.30637
11549	0.946132	0.271132
11550	0.946131	0.280974
11551	0.946132	0.33742
11552	1.32299	0.26384
11553	0.94613	0.380509
11554	0.946131	0.415168
11555	0.946128	0.375168
11556	0.946132	0.358723
11557	0.946133	0.387475
11558	0.946131	0.414563
11559	0.946131	0.371731
11560	0.946132	2.1
11561	0.946129	2.1
11562	0.946128	2.1
11563	0.94613	2.1
11564	0.94613	2.1
11565	0.946131	2.1
11566	0.94613	2.1
11567	0.94613	2.1
11568	0.94613	2.1
11569	0.946131	2.1
11570	0.946132	2.1
11571	0.946129	2.1
11572	0.946131	2.1
11573	0.946131	2.1
11574	0.946132	1.51063
11575	0.946131	1.54904
11576	1.13731	1.40721
11577	0.94775	1.65025
11578	0.944514	1.7797
11579	0.946132	1.94578
11580	1.039	1.92802
11581	0.946131	1.4299
11582	0.946128	1.36992
11583	0.94613	1.23967
11584	0.946131	1.44526
11585	0.946128	1.54379
11586	0.946129	1.75662
11587	1.23726	1.72114
11588	0.946132	2.1
11589	0.946132	2.05191
11590	0.946129	2.1
11591	1.39214	2.1
11592	0.946131	2.05382
11593	0.946131	2.1
11594	0.94613	2.1
11595	0.946125	0.736351
11596	0.946119	0.365157
11597	0.946118	0.417676

11598	0.946118	0.354292
11599	0.94612	0.753866
11600	0.946106	0.45081
11601	0.946131	0.4395
11602	1.26893	0.700466
11603	0.946131	0.697373
11604	1.26893	0.70158
11605	0.94613	0.69321
11606	0.946131	0.700706
11607	0.946131	0.695882
11608	0.946131	0.7
11609	0.946133	0.240294
11610	0.946133	0.25423
11611	0.946132	0.304415
11612	1.28924	0.239131
11613	0.946133	0.317084
11614	0.946132	0.278293
11615	0.946132	0.283509
11616	0.946133	0.788241
11617	0.946132	0.826311
11618	0.946132	0.779874
11619	0.946133	0.718979
11620	0.946133	0.749191
11621	0.946132	0.799849
11622	0.946133	0.759214
11623	0.946132	0.984087
11624	0.946133	0.833714
11625	0.946133	0.977726
11626	0.946132	0.992581
11627	1.15388	0.939854
11628	0.946132	0.981549
11629	0.946133	0.961923
11630	0.946132	0.782288
11631	0.946133	0.747361
11632	0.946132	0.77667
11633	0.946133	0.649449
11634	0.946133	0.703171
11635	0.946132	0.760017
11636	0.946132	0.69378
11637	1.0644	1.19135
11638	1.12486	1.58974
11639	1.08674	1.67159
11640	0.946132	1.25342
11641	0.946132	1.52336
11642	0.946133	1.33378
11643	0.946132	1.33625
11644	0.808978	1.13799
11645	0.911537	1.40765
11646	0.932832	1.30109
11647	1.14604	1.58946
11648	1.56953	1.42333
11649	1.25866	1.25733
11650	1.18488	1.48825
11651	1.341	1.20013
11652	0.946131	0.654988
11653	0.946131	0.551359
11654	0.946131	0.641862
11655	0.94613	0.671632

11656	1.33922	0.547564
11657	0.946131	0.671839
11658	0.946131	0.637365
11659	0.946132	0.369865
11660	0.946133	0.339508
11661	0.946132	0.39528
11662	0.946132	0.341927
11663	0.946132	0.416591
11664	0.946133	0.367643
11665	0.946132	0.380685
11666	0.946129	0.91554
11667	0.946133	2.1
11668	0.946133	1.04322
11669	0.946131	1.17238
11670	1.40496	1.73483
11671	0.946131	0.947437
11672	0.946131	1.91511
11673	0.94613	1.06406
11674	0.946127	0.89217
11675	0.946127	1.02281
11676	0.94613	1.08025
11677	1.38913	0.959049
11678	0.946128	1.03162
11679	0.946128	0.995162
11680	1.98863	0.21235
11681	2.59915	0.26371
11682	1.40338	0.246941
11683	0.946131	0.273581
11684	1.22266	0.259246
11685	1.40338	0.253024
11686	0.946132	0.265112
11687	0.946132	0.271787
11688	1.22266	0.246571
11689	0.94613	0.273307
11690	0.94613	0.29335
11691	0.946124	0.40484
11692	1.21428	0.273876
11693	0.946126	0.420909
11694	0.946127	0.371188
11695	0.946127	0.344178
11696	1.03411	0.347034
11697	0.946131	0.49191
11698	1.03411	0.344763
11699	0.946129	0.5079
11700	1.14258	0.355231
11701	0.952143	0.460566
11702	0.940118	0.411857
11703	0.810943	2.1
11704	0.810945	2.1
11705	0.810958	2.1
11706	2.82603	2.1
11707	1.35168	2.1
11708	1.08132	2.1
11709	1.08132	2.1
11710	1.0813	2.1
11711	0.946133	0.360652
11712	0.946132	0.341759
11713	0.946132	0.374412

11714	0.946132	0.321825
11715	0.946132	0.315381
11716	1.11539	0.318245
11717	0.946133	0.375941
11718	0.946133	2.1
11719	0.946132	2.1
11720	0.946133	2.1
11721	0.946133	2.1
11722	0.946132	2.1
11723	1.41195	2.1
11724	0.946133	2.1
11725	0.946131	0.23902
11726	0.946131	0.26721
11727	0.946131	0.239038
11728	0.946131	0.223309
11729	0.946131	0.234851
11730	0.946131	0.244959
11731	1.15968	0.23527
11732	0.946131	1.42673
11733	0.94613	1.46029
11734	0.94613	2.03498
11735	1.34386	1.5662
11736	0.946132	1.83681
11737	0.94613	1.98897
11738	1.34386	1.76533
11739	0.946131	1.32993
11740	0.935202	1.32434
11741	0.946131	1.85688
11742	1.25054	1.43202
11743	0.850155	1.6717
11744	0.946131	1.72247
11745	0.935205	1.5915
11746	0.946131	0.649663
11747	1.40193	0.559459
11748	0.946132	0.580665
11749	0.946132	0.580808
11750	0.946129	0.66775
11751	1.40193	0.497298
11752	0.946131	0.602156
11753	0.808779	0.535543
11754	0.561937	0.424082
11755	1.40193	0.441913
11756	1.40193	0.404487
11757	0.561936	0.48738
11758	0.561937	0.379754
11759	1.40193	0.427133
11760	0.946132	0.254266
11761	0.946132	0.265454
11762	0.946132	0.327379
11763	0.946132	0.27207
11764	0.946133	0.326197
11765	0.946132	0.287254
11766	0.946133	0.296911
11767	0.946127	0.707665
11768	0.946131	0.705087
11769	0.946131	0.683784
11770	0.946127	0.701931
11771	1.19583	0.692657

11772	1.19583	0.689321
11773	0.946129	0.699518
11774	0.589428	0.716122
11775	1.08737	0.689673
11776	1.10961	0.705204
11777	0.872901	0.705332
11778	0.541327	0.687131
11779	0.541312	0.704467
11780	0.85153	0.705414
11781	0.946132	1.42246
11782	0.946131	2.1
11783	0.946131	2.1
11784	0.946132	1.66846
11785	0.946132	2.1
11786	0.946132	1.87753
11787	0.946132	1.91316
11788	0.946132	1.20021
11789	1.2778	1.7997
11790	0.946131	2.04313
11791	0.946131	1.22168
11792	0.94613	1.75063
11793	0.94613	1.27003
11794	0.94613	1.4071
11795	0.946118	0.91478
11796	0.946112	0.938235
11797	0.946118	0.878196
11798	0.946119	0.89651
11799	0.946117	0.896834
11800	1.35631	0.851994
11801	0.946105	0.851503
11802	0.946133	1.05317
11803	0.946133	1.88132
11804	0.946132	1.2047
11805	0.946132	1.86794
11806	0.946133	0.791714
11807	0.946132	1.02798
11808	0.946132	0.993293
11809	0.94613	1.94875
11810	0.946131	0.929172
11811	0.946128	0.935549
11812	0.946128	0.886321
11813	0.94613	1.83274
11814	0.946129	1.16512
11815	0.946129	1.0489
11816	0.946132	1.24552
11817	1.15139	1.28627
11818	0.946132	1.2258
11819	0.946132	1.32786
11820	0.946132	1.3467
11821	0.946132	1.39017
11822	0.946132	1.28284
11823	0.946133	2.1
11824	1.36151	2.1
11825	0.946132	2.1
11826	0.946132	2.1
11827	0.946132	1.92024
11828	0.946132	2.1
11829	0.946133	2.1

11830	0.946127	2.1
11831	1.38397	2.1
11832	0.94613	2.1
11833	0.946131	2.1
11834	0.946128	2.1
11835	0.94613	2.1
11836	0.946129	2.1
11837	0.946131	0.487474
11838	0.946127	0.514857
11839	0.946127	0.47419
11840	0.946132	0.549527
11841	0.946131	0.57812
11842	0.946128	0.49106
11843	0.946129	0.515235
11844	1.0268	0.156064
11845	1.23823	0.15846
11846	1.2171	0.164254
11847	0.883446	0.164436
11848	1.23827	0.158525
11849	1.32614	0.157191
11850	0.94613	0.167788
11851	0.946132	2.1
11852	0.946129	2.1
11853	0.946129	2.1
11854	0.94613	2.1
11855	1.36523	2.1
11856	0.946131	2.1
11857	0.94613	2.1
11858	0.946132	0.494881
11859	0.946131	0.425163
11860	0.946132	0.51095
11861	0.946131	0.379267
11862	0.946131	0.396647
11863	1.01653	0.394614
11864	0.946131	0.53516
11865	0.94612	2.01747
11866	0.946121	1.7858
11867	0.946119	1.96599
11868	0.946119	1.87101
11869	0.946119	2.09983
11870	0.946132	2.00586
11871	0.946107	1.99562
11872	0.810942	1.06206
11873	0.810944	0.927504
11874	2.60324	1.12136
11875	1.21651	1.06404
11876	1.0813	1.03552
11877	0.946153	0.926121
11878	0.979948	1.99188
11879	0.709588	1.16111
11880	1.3492	0.565133
11881	0.946132	0.593608
11882	0.946131	0.528263
11883	0.94613	0.594432
11884	0.946129	0.676913
11885	0.946129	0.594126
11886	0.94613	0.62741
11887	0.946131	0.395535

11888	0.94613	0.442595
11889	0.946128	0.386573
11890	0.946133	0.445364
11891	0.946133	0.48352
11892	0.946131	0.431638
11893	0.946131	0.421322
11894	1.23351	0.218583
11895	0.946131	0.270956
11896	0.946131	0.244138
11897	0.946132	0.210281
11898	0.946132	0.230795
11899	0.946131	0.231302
11900	0.946132	0.237102
11901	1.35657	0.233771
11902	0.946132	0.308402
11903	0.946131	0.26533
11904	0.946132	0.224622
11905	0.946132	0.24638
11906	0.946131	0.248972
11907	0.946131	0.25497
11908	0.946132	0.774905
11909	0.946132	0.734284
11910	0.946132	0.775051
11911	0.946131	0.719871
11912	0.946131	0.755941
11913	0.946132	0.732087
11914	0.946132	0.729005
11915	0.946133	0.717316
11916	0.946132	0.684428
11917	0.946133	0.717619
11918	0.946132	0.666065
11919	0.946132	0.706326
11920	0.946132	0.697804
11921	0.946133	0.688565
11922	0.946131	0.935153
11923	1.4101	1.3001
11924	0.946131	1.85498
11925	0.946131	0.819891
11926	0.946131	1.03114
11927	0.946131	0.82176
11928	0.946131	1.30816
11929	0.810944	1.26182
11930	0.810939	1.20133
11931	0.709586	1.05215
11932	2.71261	1.07486
11933	1.21651	1.14224
11934	0.946151	1.01409
11935	0.979943	1.15795
11936	1.0813	1.31981
11937	0.946132	2.1
11938	0.946131	2.1
11939	0.946132	2.1
11940	0.946131	2.1
11941	0.946131	2.1
11942	1.37733	2.1
11943	0.946131	2.1
11944	0.946133	2.05209
11945	0.946132	1.28516

11946	0.946132	2.06793
11947	0.946133	2.1
11948	0.946133	1.92834
11949	0.946132	1.70837
11950	1.32582	2.04971
11951	0.946132	2.1
11952	0.946132	2.1
11953	0.946132	2.03145
11954	0.946132	2.1
11955	0.946132	1.87808
11956	0.946132	2.1
11957	0.946131	2.1
11958	0.946133	1.31956
11959	0.946132	1.12139
11960	0.946132	1.12888
11961	0.946132	1.06414
11962	0.946132	1.1313
11963	0.946133	1.08322
11964	1.3469	1.08462
11965	0.946126	1.032
11966	0.94613	2.1
11967	0.946131	1.59953
11968	0.946127	1.25654
11969	1.41206	2.1
11970	0.946128	1.76967
11971	0.946128	2.1
11972	0.946127	2.1
11973	0.94613	1.74625
11974	0.946131	0.989917
11975	0.94613	2.1
11976	0.946128	1.07315
11977	0.946129	1.61505
11978	0.946129	1.5277
11979	0.946131	0.513682
11980	0.94613	0.497651
11981	1.24448	0.478912
11982	0.946129	0.625963
11983	0.946131	0.544705
11984	0.94613	0.564748
11985	0.946131	0.536621
11986	0.946132	0.614989
11987	0.946133	0.607972
11988	1.11015	0.57388
11989	0.946133	0.792735
11990	0.946132	0.701201
11991	0.946132	0.722099
11992	0.946132	0.656598
11993	1.38775	2.1
11994	0.946131	2.1
11995	0.946131	2.1
11996	0.946132	2.1
11997	0.946132	2.1
11998	0.946131	2.1
11999	0.946131	2.1
12000	0.946129	1.45437
12001	0.946129	1.96669
12002	0.946133	1.05237
12003	0.946131	1.95373

12004	0.946131	0.846215
12005	0.946131	1.19493
12006	0.946131	1.10346
12007	0.946132	2.1
12008	0.946133	2.1
12009	0.946132	2.1
12010	1.40774	2.1
12011	0.946132	2.1
12012	0.946133	2.1
12013	0.946132	2.1
12014	0.610968	0.183029
12015	0.688233	0.184709
12016	0.715226	0.186954
12017	0.792409	0.18559
12018	1.13418	0.179394
12019	0.956667	0.184648
12020	0.757321	0.185451
12021	0.968845	0.178388
12022	0.595679	0.181152
12023	0.946133	0.94106
12024	1.28917	1.06278
12025	0.946131	1.24657
12026	0.94613	0.827366
12027	0.946131	1.01526
12028	0.946131	0.896752
12029	0.946131	1.07489
12030	0.946132	0.544182
12031	0.946128	0.563731
12032	0.94613	0.652676
12033	1.40668	0.579267
12034	0.946131	0.608928
12035	0.946131	0.610513
12036	0.94613	0.612986
12037	0.810946	2.1
12038	0.810945	2.1
12039	2.78163	2.1
12040	1.3517	2.1
12041	1.08132	2.1
12042	1.08133	2.1
12043	1.08132	2.1
12044	0.810933	2.1
12045	0.946131	2.1
12046	1.39081	2.1
12047	0.94613	2.1
12048	1.30339	2.1
12049	1.30339	2.1
12050	1.39081	2.1
12051	0.946131	2.1
12052	0.946127	0.961273
12053	0.821631	0.996062
12054	0.946131	0.895801
12055	0.946131	0.964778
12056	0.946128	0.893949
12057	0.996109	0.936594
12058	0.827876	0.926669
12059	0.946131	2.1
12060	0.946129	2.1
12061	0.946129	2.1

12062	0.94613	2.1
12063	0.94613	2.1
12064	0.94613	2.1
12065	0.94613	2.1
12066	0.946132	0.805908
12067	1.41723	0.864775
12068	0.946132	0.843446
12069	0.946133	0.759326
12070	0.946132	0.969435
12071	0.946132	0.88512
12072	0.946133	0.884198
12073	0.946133	0.808374
12074	0.946132	0.759677
12075	0.946133	0.67237
12076	0.946133	0.773971
12077	0.946132	0.894782
12078	0.946132	0.836937
12079	1.34254	0.806173
12080	1.26501	1.08509
12081	0.946132	1.15436
12082	0.946133	1.15393
12083	0.946133	1.32902
12084	0.946132	1.25941
12085	0.946133	1.15374
12086	0.946132	1.3828
12087	0.946131	0.458198
12088	0.946131	0.45888
12089	0.946131	0.47688
12090	0.946131	0.336083
12091	0.946131	0.368413
12092	0.946131	0.436897
12093	0.946131	0.4024
12094	0.878843	0.307021
12095	0.82561	0.344593
12096	0.586974	0.30172
12097	1.05343	0.345995
12098	0.80631	0.260833
12099	0.782663	0.308716
12100	0.789405	0.274662
12101	0.902491	0.26204
12102	1.05344	0.304996
12103	0.94613	0.301939
12104	0.946131	0.350104
12105	0.946131	0.466521
12106	0.94613	0.408367
12107	0.946131	0.356806
12108	0.946131	0.371584
12109	0.946131	0.351033
12110	0.946128	1.75804
12111	0.946133	1.97265
12112	0.946133	0.934372
12113	0.94613	1.89012
12114	0.946131	1.12048
12115	0.946131	1.17894
12116	0.946131	1.31025
12117	0.946131	0.691184
12118	0.946131	0.670689
12119	0.94613	0.648959

12120	0.946128	0.649691
12121	0.946133	0.692037
12122	1.2996	0.584457
12123	0.946131	0.628811
12124	0.946132	2.1
12125	0.946132	2.1
12126	0.946132	1.52128
12127	0.946132	2.1
12128	0.946131	1.86107
12129	0.946132	2.1
12130	0.946132	2.1
12131	1.99211	0.33277
12132	2.55024	0.298491
12133	0.946131	0.344802
12134	0.946131	0.344403
12135	0.946131	0.329272
12136	0.946131	0.347515
12137	0.946131	0.404981
12138	0.946131	0.360948
12139	1.35726	0.34902
12140	0.946128	2.1
12141	0.946132	1.5674
12142	0.946131	1.68809
12143	0.94613	1.61803
12144	0.94613	1.65545
12145	0.94613	2.0102
12146	1.39852	1.99792
12147	1.31666	0.305354
12148	0.946131	0.430688
12149	0.946128	0.342595
12150	0.94613	0.288325
12151	0.946128	0.322982
12152	0.946128	0.327199
12153	0.946128	0.32801
12154	1.32275	0.394081
12155	0.946131	0.53692
12156	0.946129	0.431878
12157	0.946128	0.41941
12158	0.946129	0.437791
12159	0.946128	0.421924
12160	0.946129	0.411
12161	0.946132	2.1
12162	0.946131	2.1
12163	0.946131	2.1
12164	0.946132	2.1
12165	0.94613	2.1
12166	0.946131	2.1
12167	1.41125	2.1
12168	0.946132	2.1
12169	0.946132	2.1
12170	0.946131	2.1
12171	0.946132	2.1
12172	0.946132	2.1
12173	0.946132	2.1
12174	1.31904	2.1
12175	1.02425	0.433308
12176	0.946132	0.384852
12177	0.94613	0.341272

12178	0.946131	0.460949
12179	1.1534	0.389176
12180	0.961161	0.336263
12181	0.9311	0.466849
12182	1.40092	1.08155
12183	0.946133	1.10906
12184	0.946132	1.1203
12185	0.946131	1.26318
12186	0.946131	1.07879
12187	0.94613	1.12161
12188	0.946131	1.27039
12189	0.946113	1.97406
12190	0.946119	0.81058
12191	0.946117	1.8548
12192	1.40015	1.70201
12193	0.946118	0.818083
12194	0.946131	1.23567
12195	0.946105	0.87398
12196	0.946132	0.598633
12197	0.946133	0.5649
12198	0.946132	0.682532
12199	1.03547	0.586722
12200	0.946133	0.547029
12201	0.946133	0.625274
12202	0.946132	0.595001
12203	0.946133	2.1
12204	1.36694	2.1
12205	0.946133	2.1
12206	0.946132	2.1
12207	0.946133	2.1
12208	0.946133	2.1
12209	0.946132	2.1
12210	0.946132	0.364717
12211	0.946133	0.359498
12212	0.946132	0.389123
12213	0.946133	0.321231
12214	0.946133	0.365573
12215	0.946132	0.408149
12216	0.946132	0.368308
12217	0.491552	0.468032
12218	0.913078	0.584688
12219	0.946132	0.684558
12220	0.491552	0.436708
12221	0.946132	0.639757
12222	0.49154	0.629653
12223	1.53168	0.596521
12224	1.53168	0.488713
12225	0.946127	2.1
12226	1.38752	2.1
12227	0.946129	2.1
12228	0.94613	2.1
12229	0.946128	2.1
12230	0.946129	2.1
12231	0.946129	2.1
12232	0.946132	2.1
12233	0.946132	2.1
12234	0.946132	2.1
12235	0.946132	2.1

12236	0.946132	2.1
12237	0.946131	2.1
12238	0.946132	2.1
12239	0.94613	0.663054
12240	0.94613	0.575927
12241	0.94613	0.583287
12242	0.946131	0.587242
12243	0.946129	0.662805
12244	1.16834	0.463701
12245	0.94613	0.576886
12246	0.946127	0.6838
12247	1.35594	0.565061
12248	0.946131	0.594237
12249	0.946128	0.703198
12250	0.946127	0.463664
12251	0.946129	0.65078
12252	0.94613	0.605961
12253	0.94613	0.933101
12254	0.946131	0.572978
12255	0.946128	0.517374
12256	0.946128	0.473307
12257	0.946131	0.901241
12258	0.94613	0.686187
12259	0.94613	0.79888
12260	0.946132	0.492569
12261	0.946132	0.692934
12262	0.946129	0.614152
12263	1.39751	0.534077
12264	0.94613	0.742209
12265	0.946131	0.520879
12266	0.946131	0.68425
12267	0.946132	0.606595
12268	0.700041	0.582569
12269	0.700013	0.641193
12270	1.7697	0.455291
12271	1.7697	0.441568
12272	1.21134	0.529549
12273	0.946131	0.535155
12274	1.25837	0.528385
12275	0.946133	2.0338
12276	0.946132	1.56673
12277	0.946132	1.41262
12278	0.946132	1.65237
12279	0.946132	1.98672
12280	0.946133	1.95191
12281	1.40583	1.8681
12282	0.946131	1.24411
12283	0.946131	1.59783
12284	0.946131	1.24919
12285	0.946131	1.99729
12286	0.946131	1.66657
12287	0.827865	1.58484
12288	0.946131	1.55122
12289	0.946132	0.222132
12290	0.946129	0.209747
12291	0.946129	0.225513
12292	0.946131	0.219068
12293	0.94613	0.232356

12294	1.21087	0.20833
12295	0.946131	0.227028
12296	0.94613	0.816797
12297	0.946131	0.84149
12298	0.94613	0.777987
12299	0.946128	0.815452
12300	0.946132	0.779022
12301	1.17452	0.790219
12302	0.94613	0.786582
12303	0.946119	0.772879
12304	0.946114	0.782103
12305	0.946119	0.743231
12306	0.946119	0.771438
12307	0.946119	0.744426
12308	1.27376	0.754044
12309	0.946106	0.751171
12310	0.946133	2.1
12311	1.39621	2.1
12312	0.946132	2.1
12313	0.946132	2.1
12314	0.946133	2.1
12315	0.946132	2.1
12316	0.946132	2.1
12317	0.946132	2.1
12318	0.946132	2.1
12319	0.946131	2.1
12320	0.946131	2.1
12321	0.946132	2.1
12322	0.946132	2.1
12323	1.34352	2.1
12324	0.946132	1.51331
12325	1.41272	1.80164
12326	0.946131	2.06809
12327	0.94613	1.2975
12328	0.94613	1.67015
12329	0.94613	1.31844
12330	0.94613	1.63929
12331	0.946132	2.06086
12332	0.946131	1.44204
12333	0.946132	2.1
12334	0.946132	1.34136
12335	0.946132	1.20029
12336	0.946132	1.74477
12337	1.23536	1.8306
12338	0.946132	2.1
12339	0.946131	2.1
12340	0.946131	2.1
12341	0.946132	2.1
12342	0.946132	2.1
12343	1.41131	2.1
12344	0.946131	2.1
12345	1.41894	0.838281
12346	0.946132	0.774386
12347	0.946133	0.764775
12348	0.946132	0.818064
12349	0.946132	0.87425
12350	0.946133	0.788048
12351	0.946132	0.760648

12352	0.946128	0.524342
12353	0.946133	0.943112
12354	0.946131	0.849727
12355	0.946129	1.78784
12356	0.94613	0.83064
12357	1.4099	0.984596
12358	0.946131	0.762958
12359	0.810947	2.1
12360	0.810945	1.08182
12361	0.810933	1.78804
12362	2.62883	1.85781
12363	1.3517	1.6662
12364	1.08132	2.1
12365	1.08132	1.01352
12366	1.08133	1.54182
12367	0.946131	0.924561
12368	0.946126	1.45402
12369	0.946127	0.954613
12370	0.94613	0.977829
12371	0.946128	1.13056
12372	0.946129	0.848826
12373	0.946129	0.934079
12374	1.21347	2.00713
12375	0.946129	2.1
12376	0.94613	2.00602
12377	1.21347	2.1
12378	1.33064	2.1
12379	0.94613	2.1
12380	1.33064	2.1
12381	0.94613	0.165572
12382	0.946129	0.184681
12383	0.94613	0.180799
12384	0.946132	0.182805
12385	0.946126	0.18089
12386	0.946129	0.180743
12387	0.94613	0.184031
12388	0.946132	0.889908
12389	1.37938	1.02049
12390	0.946131	1.09567
12391	0.94613	0.871778
12392	0.946131	1.16736
12393	0.94613	0.920354
12394	0.94613	0.9635
12395	0.946133	0.230879
12396	0.946133	0.216999
12397	1.2968	0.216651
12398	0.946132	0.230488
12399	0.946133	0.233392
12400	1.2968	0.21045
12401	0.946133	0.231524
12402	1.43643	0.227549
12403	0.946129	0.876893
12404	0.946133	0.81728
12405	0.946131	0.880502
12406	0.94613	0.787901
12407	0.946131	0.846622
12408	0.946131	0.821613
12409	0.946131	0.812257

12410	0.872447	1.24794
12411	0.946132	1.20091
12412	0.872447	1.14655
12413	0.827867	1.22899
12414	0.946132	1.31915
12415	0.827867	1.38988
12416	0.872447	1.36606
12417	0.946117	1.02162
12418	1.27213	1.01303
12419	0.946124	0.959229
12420	0.946119	1.02655
12421	0.946113	1.22844
12422	0.946129	1.11373
12423	0.946104	1.04586
12424	0.94613	0.821889
12425	0.946131	0.917293
12426	0.94613	0.823328
12427	0.946129	0.913412
12428	0.946132	0.397164
12429	0.946131	0.791348
12430	0.94613	0.738166
12431	0.810942	2.1
12432	0.810943	2.1
12433	2.80212	2.1
12434	1.3517	2.1
12435	1.08133	2.1
12436	1.08132	2.1
12437	1.08132	2.1
12438	0.810932	2.1
12439	1.29521	2.1
12440	0.946129	2.1
12441	0.946131	2.1
12442	1.40106	2.1
12443	0.94613	2.1
12444	1.40106	2.1
12445	0.946131	2.1
12446	0.946132	0.771829
12447	1.24596	0.64146
12448	0.94613	0.686094
12449	0.946131	0.745519
12450	0.946131	0.573597
12451	0.946131	0.741204
12452	0.946131	0.708359
12453	0.946131	2.1
12454	0.946131	2.1
12455	0.946131	2.1
12456	0.946128	2.1
12457	0.946133	2.1
12458	0.94613	2.1
12459	0.946131	2.1
12460	0.879548	0.725798
12461	1.21527	0.707283
12462	0.967506	0.715256
12463	0.946132	0.793258
12464	0.946133	0.704376
12465	0.967511	0.856944
12466	0.946132	0.802292
12467	0.946132	0.874992

12468	0.946132	0.749781
12469	0.974253	0.762061
12470	0.974253	0.903064
12471	0.91801	0.770791
12472	0.918009	0.783336
12473	0.946132	0.845982
12474	0.946132	0.40036
12475	0.946131	0.366727
12476	0.946131	0.410473
12477	0.946133	0.305868
12478	0.946132	0.330939
12479	1.28644	0.340003
12480	0.946132	0.388641
12481	0.946131	0.412076
12482	0.946131	0.370431
12483	0.946131	0.421932
12484	0.946131	0.311617
12485	0.946131	0.329836
12486	1.40103	0.337093
12487	0.946131	0.393327
12488	0.946128	0.580963
12489	0.946132	0.500791
12490	0.946129	0.625177
12491	1.03327	0.468938
12492	0.946129	0.467377
12493	0.946129	0.570277
12494	0.94613	0.624365
12495	0.946134	0.565336
12496	0.946133	0.593596
12497	0.946132	0.39067
12498	0.946133	0.579855
12499	0.946132	0.398162
12500	0.946132	0.428937
12501	0.946133	0.297791
12502	0.856654	0.239807
12503	0.946131	0.288908
12504	0.946131	0.240789
12505	0.946132	0.245354
12506	0.856616	0.268472
12507	1.03565	0.22325
12508	1.03565	0.259256
12509	1.15844	0.721181
12510	0.946132	0.72388
12511	0.946132	0.708142
12512	0.946132	0.71938
12513	0.946132	0.706475
12514	0.946132	0.714653
12515	0.946131	0.722219
12516	1.4582	2.1
12517	1.35067	2.1
12518	0.946131	2.1
12519	1.04106	2.1
12520	0.938748	2.1
12521	0.943015	2.1
12522	1.05017	2.1
12523	0.938793	2.1
12524	0.946131	0.242353
12525	0.946132	0.277286

12526	0.946131	0.378
12527	0.94613	0.303422
12528	0.946133	0.303006
12529	0.946131	0.291067
12530	0.946131	0.302141
12531	0.946132	0.828832
12532	0.946133	0.980354
12533	0.946132	0.823469
12534	0.946133	0.990321
12535	0.946133	0.95245
12536	0.946132	0.921561
12537	0.946133	0.969114
12538	0.946128	1.10967
12539	0.946132	1.27084
12540	0.946132	1.13363
12541	0.94613	1.1402
12542	1.23426	1.20037
12543	0.94613	1.18442
12544	0.946131	1.20513
12545	0.810943	0.662249
12546	0.810946	0.73574
12547	2.60388	0.739026
12548	1.3517	0.709262
12549	1.08133	0.675604
12550	1.08132	0.729691
12551	1.08132	0.609213
12552	0.810933	0.537491
12553	0.810945	0.97474
12554	0.810945	0.791791
12555	2.72377	1.23118
12556	1.3517	1.05247
12557	1.08133	0.991593
12558	1.08132	0.888877
12559	1.08132	1.88133
12560	0.810932	1.06696
12561	1.25104	0.690897
12562	0.946131	0.950865
12563	0.946131	0.788428
12564	1.36188	1.9252
12565	0.946131	0.988573
12566	1.36188	1.13898
12567	0.946132	1.15301
12568	0.946132	1.9849
12569	0.946132	2.0125
12570	0.946132	2.09334
12571	0.946132	2.03339
12572	0.946132	1.86537
12573	0.946132	2.1
12574	1.25488	2.1
12575	1.32132	0.275135
12576	0.946126	0.337847
12577	0.946127	0.24922
12578	0.946128	0.324312
12579	0.946128	0.347412
12580	0.946128	0.309841
12581	0.946128	0.328103
12582	0.946133	2.1
12583	1.41123	1.33155

12584	0.946134	1.49247
12585	0.946133	2.07994
12586	0.946132	1.04952
12587	0.946133	1.5797
12588	0.946132	1.43986
12589	1.32163	0.369386
12590	0.946133	0.402057
12591	0.946133	0.367566
12592	0.946132	0.397338
12593	0.946132	0.324669
12594	0.946132	0.35206
12595	0.946133	0.409058
12596	0.946133	2.03927
12597	0.946133	2.1
12598	0.946132	1.92624
12599	0.946132	2.1
12600	0.946132	1.03063
12601	0.946133	1.98779
12602	0.946132	1.9465
12603	0.94613	0.449975
12604	0.946131	0.427701
12605	0.946128	0.448172
12606	0.946133	0.360861
12607	0.946133	0.388282
12608	0.946131	0.448665
12609	0.946131	0.396638
12610	0.946133	0.916113
12611	0.946132	0.68361
12612	0.946132	0.921337
12613	0.946134	0.765436
12614	0.946133	0.997047
12615	0.946133	0.788662
12616	0.946132	0.835095
12617	0.94613	1.70378
12618	0.946128	1.89492
12619	0.946127	1.45545
12620	0.94613	1.40126
12621	0.946131	1.64048
12622	0.946128	1.57165
12623	1.21099	1.81945
12624	0.946132	0.87042
12625	0.946131	0.836561
12626	0.946131	0.890597
12627	0.946133	0.760991
12628	0.946133	0.790555
12629	0.946131	0.836333
12630	0.946132	0.800078
12631	1.34794	1.02198
12632	0.517994	1.01155
12633	0.517965	0.924663
12634	0.946132	1.06992
12635	0.9198	1.51192
12636	0.946132	1.16465
12637	0.9198	1.2205
12638	0.946132	1.05645
12639	0.94613	1.07815
12640	0.946131	0.854327
12641	0.946131	1.06869

12642	0.946131	1.30205
12643	0.946131	1.08218
12644	1.23224	0.995341
12645	0.946132	1.50273
12646	0.946132	1.58234
12647	0.946133	1.34578
12648	0.946132	1.12226
12649	0.946132	1.25902
12650	0.946133	1.34814
12651	1.37511	1.43146
12652	0.946133	0.771561
12653	0.946129	0.78198
12654	0.851104	0.810368
12655	0.946131	0.774754
12656	1.0412	0.870117
12657	1.0412	0.809221
12658	0.851066	0.789629
12659	1.31357	0.500408
12660	0.946131	0.723613
12661	0.94613	0.556917
12662	0.946131	0.588553
12663	0.94613	0.522724
12664	0.946131	0.586946
12665	0.946131	0.509913
12666	0.946133	0.891073
12667	1.38149	0.999271
12668	0.946132	1.00131
12669	0.946132	0.888849
12670	0.946133	1.20236
12671	0.946132	0.944239
12672	0.946133	0.948375
12673	0.810944	1.04023
12674	0.810945	2.02855
12675	0.810958	1.61294
12676	2.65782	1.75498
12677	1.35168	1.43868
12678	1.08132	1.05318
12679	1.08132	1.67653
12680	1.0813	1.03696
12681	0.946113	0.211713
12682	0.946119	0.233176
12683	0.946118	0.281514
12684	0.946118	0.241007
12685	0.946119	0.245356
12686	0.946131	0.233676
12687	0.946106	0.243579
12688	0.946132	0.201567
12689	0.946132	0.255516
12690	0.946128	0.237677
12691	0.946131	0.247404
12692	0.94613	0.247787
12693	0.94613	0.237833
12694	0.94613	0.26042
12695	1.00099	0.302753
12696	0.946132	0.39507
12697	1.17876	0.291545
12698	0.982282	0.342614
12699	0.909981	0.418742

12700	0.946132	0.373302
12701	0.946131	0.37163
12702	0.946132	0.87566
12703	1.35328	1.03336
12704	0.946132	1.19695
12705	0.946132	0.837348
12706	0.946133	1.10231
12707	0.946132	0.875022
12708	0.946133	1.0222
12709	0.946133	0.1959
12710	0.946133	0.237677
12711	0.946132	0.212351
12712	0.946132	0.230605
12713	0.946132	0.224471
12714	0.946132	0.221135
12715	0.946133	0.237143
12716	0.900195	0.187821
12717	0.946132	0.186881
12718	0.900167	0.194659
12719	1.41963	0.204258
12720	0.900195	0.186861
12721	0.946133	0.190648
12722	1.35385	0.208632
12723	0.946132	1.20233
12724	0.946129	1.76049
12725	0.946129	1.58067
12726	0.946131	1.20005
12727	0.946131	1.60714
12728	1.41585	1.29671
12729	0.94613	1.79725
12730	0.810946	2.1
12731	0.810946	1.92012
12732	2.68895	2.1
12733	1.3517	2.1
12734	1.08133	2.1
12735	1.08132	2.02457
12736	1.08132	2.1
12737	0.810933	2.1
12738	1.19886	1.95521
12739	0.946131	2.1
12740	0.946131	2.04128
12741	1.34447	2.1
12742	0.946131	2.1
12743	1.34447	2.1
12744	0.946131	2.1
12745	0.946133	0.256835
12746	0.946133	0.282472
12747	0.946132	0.355608
12748	1.35065	0.260674
12749	0.946132	0.382815
12750	0.946133	0.329369
12751	0.946132	0.317756
12752	0.946131	0.827965
12753	0.946131	0.807618
12754	0.94613	0.848064
12755	0.946131	0.729937
12756	0.946131	0.749029
12757	0.946131	0.790468

12758	0.946131	0.763329
12759	0.946127	0.422069
12760	0.946131	0.434703
12761	0.946127	0.418215
12762	0.94613	0.357237
12763	0.946131	0.392105
12764	0.946128	0.434815
12765	0.946129	0.383689
12766	0.810946	0.266389
12767	0.810947	0.281405
12768	0.810959	0.267747
12769	2.4391	0.247642
12770	1.35168	0.250641
12771	1.08132	0.24838
12772	1.08132	0.263736
12773	1.08131	0.272958
12774	0.946132	0.225396
12775	0.946132	0.230358
12776	0.946132	0.25454
12777	1.40837	0.23622
12778	0.946132	0.25587
12779	0.946132	0.250233
12780	1.40837	0.237341
12781	0.946127	1.4029
12782	0.946132	1.99952
12783	0.946131	0.910175
12784	0.946127	1.71318
12785	0.946131	1.31721
12786	0.946129	1.02053
12787	0.94613	1.39318
12788	0.946133	1.66083
12789	0.946132	1.48684
12790	0.946132	1.46615
12791	0.946132	1.26624
12792	0.946132	1.54416
12793	0.946132	1.10937
12794	0.946133	1.95082
12795	0.946132	0.786489
12796	1.24344	0.707589
12797	0.946132	0.705131
12798	0.946132	0.844092
12799	0.946132	0.726311
12800	1.24344	0.754875
12801	0.946132	0.823624
12802	0.946131	0.608191
12803	0.946128	0.552
12804	0.946128	0.550503
12805	0.946131	0.613023
12806	1.41232	0.530375
12807	0.946129	0.622661
12808	0.94613	0.529785
12809	0.946128	1.97714
12810	0.946133	1.67353
12811	0.946133	0.947847
12812	0.946131	2.01491
12813	0.946131	1.09494
12814	0.946131	1.37841
12815	0.94613	1.3181

12816	0.946133	0.825078
12817	1.41556	0.828745
12818	0.946133	0.796168
12819	0.946133	0.77282
12820	0.946132	0.923358
12821	0.946132	0.851896
12822	0.946132	0.881301
12823	0.94612	0.783088
12824	1.41843	0.788603
12825	0.946114	0.790274
12826	0.94612	0.743542
12827	0.946119	0.875521
12828	1.41845	0.777059
12829	0.946107	0.846242
12830	0.946129	0.227164
12831	0.946129	0.211867
12832	0.94613	0.231171
12833	0.946132	0.225694
12834	0.946126	0.237246
12835	1.1509	0.208115
12836	0.94613	0.226767
12837	0.946131	0.201284
12838	0.946132	0.192391
12839	0.946132	0.202591
12840	0.946132	0.199943
12841	0.946132	0.206977
12842	1.19263	0.187458
12843	0.946132	0.205825
12844	0.94613	0.764543
12845	0.946131	0.899833
12846	0.946128	0.761717
12847	0.946133	0.923837
12848	0.946133	0.867144
12849	0.946131	0.815662
12850	0.946131	0.890091
12851	0.946133	1.10868
12852	1.22848	1.30032
12853	1.00336	1.30648
12854	0.827866	1.14264
12855	0.946132	1.40596
12856	0.827865	1.21734
12857	0.946133	1.20054
12858	0.946131	0.850089
12859	0.94613	0.617132
12860	0.946127	0.808632
12861	1.41498	0.80793
12862	0.94613	0.715177
12863	0.946128	0.769444
12864	0.946128	0.73958
12865	0.94613	0.211572
12866	0.94613	0.229868
12867	0.94613	0.259567
12868	1.37358	0.232387
12869	0.946129	0.274476
12870	0.94613	0.261646
12871	1.37357	0.239898
12872	0.946111	0.216849
12873	0.946118	0.230892

12874	0.946118	0.27182
12875	1.36568	0.218897
12876	0.946116	0.286475
12877	0.94613	0.24549
12878	0.946105	0.244125
12879	0.946132	0.714344
12880	0.946131	0.612176
12881	0.94613	0.781367
12882	1.23175	0.577727
12883	0.946131	0.55851
12884	0.946131	0.729428
12885	0.946131	0.770579
12886	0.946132	0.280074
12887	0.946126	0.292677
12888	0.946128	0.273964
12889	0.946129	0.292283
12890	0.946129	0.327207
12891	0.946128	0.293924
12892	1.25698	0.282086
12893	0.810943	2.1
12894	0.810944	2.1
12895	2.81171	2.1
12896	1.3517	2.1
12897	1.08132	2.1
12898	1.08133	2.1
12899	1.08132	2.1
12900	0.810932	2.1
12901	0.946132	0.706726
12902	0.946131	0.692404
12903	0.946131	0.71592
12904	0.946132	0.553815
12905	0.946132	0.611893
12906	0.946132	0.684224
12907	0.946131	0.650898
12908	0.810936	1.00113
12909	0.810935	1.83032
12910	2.80914	1.42787
12911	1.35167	1.25522
12912	1.0813	1.18423
12913	1.0813	1.81367
12914	1.0813	0.924085
12915	0.810934	1.1743
12916	1.23851	2.08295
12917	0.946132	1.0756
12918	0.946131	2.07854
12919	1.40459	1.0201
12920	0.946132	1.48474
12921	1.40459	1.30621
12922	0.946132	1.34972
12923	1.19974	0.464145
12924	0.946131	0.682594
12925	1.19974	0.45822
12926	0.946132	0.660549
12927	0.946132	0.480412
12928	0.946132	0.656307
12929	0.946132	0.527353
12930	0.939141	1.0585
12931	1.14375	1.29631

12932	1.03306	1.29724
12933	0.946132	1.12381
12934	0.946126	1.32657
12935	0.946129	1.20011
12936	0.94613	1.15739
12937	0.946132	0.394063
12938	0.946131	0.392418
12939	0.946132	0.415568
12940	0.946132	0.344531
12941	0.946132	0.370431
12942	0.946132	0.444424
12943	0.946132	0.384383
12944	0.946131	2.1
12945	0.94613	2.1
12946	0.94613	2.1
12947	0.946131	2.1
12948	0.946129	2.1
12949	0.94613	2.1
12950	1.24512	2.1
12951	0.946133	0.398904
12952	0.946129	0.346055
12953	0.946131	0.436285
12954	0.946132	0.343079
12955	0.946131	0.43925
12956	0.946131	0.407355
12957	0.946131	0.384032
12958	0.946132	0.367284
12959	0.946133	0.336329
12960	0.946132	0.381145
12961	0.946132	0.30821
12962	0.946133	0.405409
12963	0.946132	0.37468
12964	0.946132	0.36664
12965	0.946132	0.832927
12966	1.41392	0.715416
12967	0.946132	0.71585
12968	0.946132	0.878061
12969	0.946132	0.630498
12970	0.946132	0.840928
12971	0.946132	0.771419
12972	0.946132	0.987882
12973	1.24333	0.907251
12974	0.946132	0.854202
12975	0.946133	1.07277
12976	0.946132	0.725096
12977	0.946132	1.05328
12978	0.946133	0.888512
12979	1.30649	0.498627
12980	0.94613	0.525475
12981	0.94613	0.489146
12982	0.946128	0.501892
12983	0.946126	0.546251
12984	0.946127	0.523815
12985	0.946127	0.53454
12986	0.709578	0.616671
12987	0.810943	0.600589
12988	2.53303	0.460902
12989	1.21651	0.467972

12990	0.979951	0.507094
12991	1.08132	0.48994
12992	0.946141	0.611851
12993	0.81096	0.567872
12994	0.946131	0.560613
12995	0.946131	0.590395
12996	0.946131	0.577649
12997	0.946131	0.438141
12998	0.946131	0.475125
12999	0.946131	0.577205
13000	0.946131	0.52675
13001	0.946132	0.384883
13002	0.946129	0.360775
13003	0.946129	0.38233
13004	0.94613	0.388157
13005	1.20634	0.364354
13006	1.20634	0.352765
13007	0.946131	0.410416
13008	0.946133	0.751131
13009	0.946132	0.719608
13010	0.946132	0.705995
13011	0.946132	0.759137
13012	0.946132	0.701999
13013	1.30107	0.744924
13014	0.946133	0.75009
13015	0.810946	2.1
13016	0.810944	2.1
13017	2.73936	2.1
13018	1.35168	2.1
13019	1.0813	2.1
13020	1.08132	2.1
13021	1.08132	2.1
13022	0.810958	2.1
13023	0.946129	1.74761
13024	0.946129	1.99611
13025	0.946133	1.0976
13026	1.30812	1.74658
13027	0.946131	1.18547
13028	0.946131	1.42964
13029	0.946131	1.38048
13030	1.00901	1.1471
13031	0.94613	1.41149
13032	0.94613	0.945081
13033	1.17001	1.22618
13034	0.917259	0.948297
13035	0.94613	1.06666
13036	0.94613	1.08045
13037	0.946126	2.1
13038	0.946127	2.1
13039	0.946131	2.1
13040	0.946127	2.1
13041	0.946131	2.1
13042	0.946129	2.1
13043	1.398	2.1
13044	0.946133	2.1
13045	1.3033	2.1
13046	0.946132	2.1
13047	0.946132	2.1

13048	0.946132	2.1
13049	0.946132	2.1
13050	0.946133	2.1
13051	0.946131	0.892557
13052	0.946131	0.956382
13053	1.09897	0.827991
13054	0.946129	1.05938
13055	0.946131	0.990809
13056	0.94613	1.04293
13057	0.94613	0.916017
13058	0.946132	2.1
13059	0.946131	2.1
13060	0.946131	2.1
13061	0.946132	2.1
13062	0.94613	2.1
13063	0.946131	2.1
13064	1.39858	2.1
13065	1.07117	0.82481
13066	1.08878	0.912158
13067	0.946119	0.914792
13068	1.08878	1.04775
13069	0.946119	0.766985
13070	0.946132	0.871069
13071	1.07116	0.832128
13072	0.946132	0.438372
13073	0.946132	0.406705
13074	0.946132	0.457815
13075	0.946132	0.332833
13076	0.946132	0.361534
13077	1.10733	0.383872
13078	0.946132	0.422077
13079	0.946128	0.457512
13080	0.946132	0.40446
13081	0.94613	0.478352
13082	0.946129	0.34029
13083	0.94613	0.372927
13084	1.3367	0.381048
13085	0.94613	0.448318
13086	0.810943	2.1
13087	0.810944	2.1
13088	2.70234	2.1
13089	1.21653	2.1
13090	1.08132	2.1
13091	0.946155	2.1
13092	0.979951	2.1
13093	0.709578	2.1
13094	1.27174	2.1
13095	0.946129	2.1
13096	0.94613	2.1
13097	1.35117	2.1
13098	0.94613	2.1
13099	1.35117	2.1
13100	0.94613	2.1
13101	0.946133	1.19686
13102	0.946133	1.16771
13103	0.946132	1.54324
13104	0.946133	1.1084
13105	0.946132	1.95749

13106	0.946133	1.50132
13107	0.997296	1.48758
13108	0.810943	1.33969
13109	0.810944	1.69005
13110	0.810945	2.1
13111	2.78833	2.1
13112	1.35169	2.1
13113	1.08132	1.45339
13114	1.08132	2.1
13115	1.08132	2.1
13116	0.810946	1.85964
13117	0.810943	1.00387
13118	0.810933	1.29547
13119	2.70049	1.59537
13120	1.3517	1.17348
13121	1.08132	1.62123
13122	1.08132	0.840886
13123	1.08133	1.04193
13124	0.946127	1.23904
13125	1.35469	1.20128
13126	0.94613	1.18944
13127	0.946131	1.2608
13128	0.946128	1.11466
13129	1.35469	1.32262
13130	0.946129	1.36231
13131	0.946131	0.623805
13132	1.2722	0.566894
13133	0.946132	0.574587
13134	0.946132	0.57177
13135	0.946129	0.652414
13136	1.2722	0.503055
13137	0.94613	0.579388
13138	0.946133	0.517339
13139	0.946132	0.493338
13140	0.946132	0.542299
13141	0.946132	0.533459
13142	1.41461	0.49489
13143	1.41461	0.494177
13144	0.946133	0.576985
13145	0.946131	0.980637
13146	1.30165	0.843753
13147	0.946132	0.821744
13148	0.946132	1.01028
13149	0.946129	0.863754
13150	1.30165	0.972893
13151	0.946131	1.01587
13152	0.946132	1.08086
13153	0.946129	1.40689
13154	0.94613	1.08094
13155	1.39356	1.56651
13156	0.946131	1.14072
13157	0.946131	1.16648
13158	0.94613	1.44839
13159	0.810946	1.75897
13160	0.810947	1.93473
13161	0.810934	2.01438
13162	2.35003	2.1
13163	1.3517	2.1

13164	1.08132	2.1
13165	1.08132	2.04636
13166	1.08133	2.1
13167	0.946131	0.265006
13168	0.946131	0.293322
13169	0.946125	0.384216
13170	0.946127	0.319117
13171	0.946129	0.335282
13172	0.946128	0.308248
13173	0.946128	0.315717
13174	0.946131	0.258298
13175	0.946131	0.304765
13176	0.946131	0.394696
13177	0.946131	0.336244
13178	0.946132	0.303682
13179	0.946132	0.308705
13180	0.946131	0.304237
13181	0.946129	2.02996
13182	0.946132	2.1
13183	0.946132	2.1
13184	0.94613	2.08359
13185	1.30082	2.1
13186	0.94613	2.1
13187	0.946131	2.1
13188	1.20416	0.360448
13189	0.946132	0.535284
13190	0.946131	0.450462
13191	0.946132	0.424999
13192	0.946131	0.372911
13193	0.946131	0.433966
13194	0.946132	0.372
13195	1.18711	0.360721
13196	0.946131	0.541372
13197	0.946131	0.45402
13198	0.946132	0.417755
13199	0.946131	0.368993
13200	0.946131	0.438948
13201	0.946132	0.409354
13202	0.946133	0.23435
13203	0.946133	0.250011
13204	0.946132	0.261796
13205	1.30212	0.24776
13206	0.946133	0.283007
13207	0.946132	0.257796
13208	1.30212	0.24803
13209	0.946131	0.328131
13210	0.946131	0.303357
13211	0.946131	0.351113
13212	0.946132	0.330991
13213	1.31467	0.321636
13214	1.31467	0.30845
13215	0.946131	0.383948
13216	0.946133	0.279659
13217	0.946132	0.259354
13218	0.946132	0.297172
13219	0.946132	0.276523
13220	1.25124	0.274432
13221	1.25124	0.263061

13222	0.946132	0.322961
13223	0.946132	2.1
13224	0.946132	2.1
13225	0.946132	2.1
13226	1.36422	2.1
13227	0.946132	2.1
13228	0.946131	2.1
13229	0.946132	2.1
13230	0.946131	0.998686
13231	1.41851	1.31335
13232	0.946132	1.61929
13233	0.946132	0.975633
13234	0.946132	1.21685
13235	0.946132	1.00641
13236	0.946132	1.19376
13237	0.946133	0.350441
13238	0.946132	0.377489
13239	0.946132	0.349687
13240	0.946132	0.336324
13241	0.946132	0.361837
13242	1.37794	0.318671
13243	0.946132	0.349653
13244	0.946132	2.1
13245	0.946132	2.1
13246	0.946131	2.1
13247	0.946131	2.1
13248	0.946132	2.1
13249	0.946132	2.1
13250	1.4045	2.1
13251	0.946133	2.1
13252	0.946132	2.1
13253	0.946131	2.1
13254	0.946132	2.1
13255	0.946131	2.1
13256	0.946131	2.1
13257	0.946132	2.1
13258	0.946132	2.1
13259	0.946132	2.1
13260	0.946131	2.1
13261	0.946132	2.1
13262	0.946131	2.1
13263	0.946131	2.1
13264	0.946131	2.1
13265	0.946128	2.1
13266	1.31641	2.1
13267	0.946129	2.1
13268	0.94613	2.1
13269	0.94613	2.1
13270	0.94613	2.1
13271	0.94613	2.1
13272	0.946132	2.1
13273	0.946131	2.1
13274	0.946131	1.16268
13275	0.946132	2.1
13276	0.946132	1.3682
13277	0.946132	1.76628
13278	0.946132	2.00195
13279	0.946132	2.1

13280	1.41507	2.1
13281	0.946132	2.1
13282	0.946132	2.1
13283	0.946133	2.1
13284	0.946133	2.1
13285	0.946132	2.1
13286	0.946132	2.1
13287	0.946131	2.1
13288	0.946131	2.1
13289	0.946133	2.1
13290	0.946132	2.1
13291	1.32608	2.1
13292	0.946132	2.1
13293	0.94613	1.35721
13294	0.94613	1.89844
13295	0.94613	0.892199
13296	0.946131	1.54778
13297	0.946129	1.21725
13298	0.94613	0.923594
13299	0.94613	1.20798
13300	1.33728	0.963426
13301	0.946131	0.923451
13302	0.94613	0.728834
13303	0.946132	0.960157
13304	0.946132	1.13976
13305	0.946131	0.846868
13306	0.946131	0.88931
13307	0.946127	2.08296
13308	1.40641	1.44659
13309	0.946129	1.68885
13310	0.94613	1.96494
13311	0.946128	1.1434
13312	0.946129	1.56711
13313	0.946129	1.40958
13314	0.946129	2.1
13315	0.946128	2.1
13316	0.946133	2.1
13317	1.37467	2.1
13318	0.946131	2.1
13319	0.946131	2.1
13320	1.37467	2.1
13321	0.946131	2.1
13322	0.946131	2.1
13323	0.946132	2.1
13324	0.946132	2.1
13325	0.946129	2.1
13326	0.946131	2.1
13327	1.34912	2.1
13328	0.946132	2.08174
13329	0.946133	2.1
13330	0.946133	2.1
13331	0.946132	2.1
13332	0.946132	2.1
13333	0.946133	2.1
13334	0.946132	2.1
13335	0.946127	2.1
13336	0.946131	1.61722
13337	0.946132	2.1

13338	0.946131	1.63211
13339	0.946127	1.41419
13340	0.94613	2.07357
13341	1.33607	2.1
13342	0.946117	0.717397
13343	0.946117	0.835652
13344	0.946115	0.951193
13345	0.946124	1.5746
13346	0.946119	0.737119
13347	1.32662	0.86276
13348	0.94613	0.752181
13349	0.946119	2.1
13350	0.946114	2.1
13351	0.946118	2.1
13352	0.946114	2.1
13353	0.946118	2.1
13354	0.946104	2.1
13355	0.946129	2.1
13356	0.946132	1.44338
13357	0.946133	2.1
13358	0.946133	2.1
13359	0.946133	1.79838
13360	0.946132	2.1
13361	0.946132	2.1
13362	0.946133	2.1
13363	0.946133	2.0149
13364	0.946132	2.1
13365	0.946132	2.1
13366	0.946133	2.1
13367	0.946132	2.1
13368	0.946132	2.1
13369	0.946132	2.1
13370	0.946133	1.29518
13371	1.30881	1.63822
13372	0.946132	2.02258
13373	0.946131	1.14231
13374	0.946131	1.25992
13375	0.946131	1.06324
13376	0.946131	1.45042
13377	0.946128	2.1
13378	0.946128	2.1
13379	0.946132	2.1
13380	0.94613	2.1
13381	0.94613	2.1
13382	0.94613	2.1
13383	1.34652	2.1
13384	0.946133	1.08776
13385	0.946133	1.01748
13386	0.946132	1.53744
13387	0.946133	1.02009
13388	0.946132	1.80289
13389	0.946132	1.18082
13390	0.946132	1.33021
13391	0.946132	0.421474
13392	0.946128	0.397623
13393	0.94613	0.419476
13394	0.946131	0.424267
13395	0.94613	0.500874

13396	0.94613	0.371804
13397	0.94613	0.414528
13398	0.946128	0.73271
13399	0.946132	0.827603
13400	0.94613	0.719748
13401	0.94613	0.764571
13402	0.946131	0.74285
13403	0.94613	0.767495
13404	0.94613	0.759564
13405	0.946129	0.949093
13406	1.32056	1.20974
13407	0.946131	1.38092
13408	0.94613	0.957246
13409	0.946124	1.194
13410	0.946128	0.991286
13411	0.946127	1.06522
13412	0.946129	1.00781
13413	1.20351	1.25994
13414	0.946132	1.3344
13415	0.946132	1.03874
13416	0.946126	1.27131
13417	0.946129	1.09177
13418	0.946129	1.11043
13419	0.946127	1.33772
13420	0.946127	1.64713
13421	0.946131	1.08865
13422	1.25592	1.39019
13423	0.946127	1.11956
13424	0.946129	1.22403
13425	0.94613	1.28503
13426	1.02332	1.55835
13427	0.946133	1.14279
13428	0.946132	1.17994
13429	1.02332	1.27434
13430	0.946132	0.973238
13431	0.96196	1.23273
13432	0.930306	1.08172
13433	0.946133	1.97936
13434	0.946133	1.95603
13435	0.946132	2.1
13436	1.20057	2.06341
13437	0.946132	2.0709
13438	0.946133	2.1
13439	1.20057	2.1
13440	0.946128	1.31808
13441	0.946129	0.94787
13442	0.946129	1.10438
13443	1.13245	0.951135
13444	0.946126	0.852511
13445	0.946128	1.03793
13446	0.946129	0.942209
13447	0.946131	1.6206
13448	0.946131	1.84446
13449	0.94613	2.08085
13450	1.25	1.82926
13451	0.946129	2.03569
13452	0.946131	2.1
13453	1.25	2.1

13454	0.540425	1.90565
13455	0.748493	1.99645
13456	1.46266	2.1
13457	1.20046	2.1
13458	0.600454	1.87996
13459	1.41283	2.09204
13460	0.582567	1.95832
13461	0.946131	1.61296
13462	0.946131	1.50907
13463	0.946132	1.54195
13464	0.946132	1.56427
13465	0.94613	1.38682
13466	0.946131	1.96776
13467	1.28439	1.9152
13468	0.946132	0.862744
13469	0.946131	0.85182
13470	0.946131	0.959627
13471	0.946132	0.829631
13472	0.946132	0.983411
13473	1.39335	0.808147
13474	0.946132	0.868672
13475	0.946131	0.713501
13476	0.946131	0.86163
13477	0.94613	0.66782
13478	0.946132	0.678915
13479	0.946131	0.787281
13480	0.946131	0.757662
13481	0.946131	0.739512
13482	0.946131	0.716667
13483	0.94613	0.710038
13484	0.946126	0.710662
13485	0.946131	0.665945
13486	0.946125	0.728647
13487	0.946127	0.674846
13488	0.946128	0.717265
13489	0.946131	0.312137
13490	0.946131	0.394431
13491	0.946131	0.326155
13492	0.946131	0.285543
13493	0.946131	0.300986
13494	0.946131	0.356112
13495	1.39168	0.287235
13496	0.946131	0.552303
13497	0.946126	0.456396
13498	0.946127	0.618518
13499	0.946131	0.482972
13500	0.94613	0.564465
13501	1.412	0.499374
13502	0.946128	0.530817
13503	0.948901	0.703922
13504	0.943364	0.733072
13505	1.03768	0.699481
13506	0.946132	0.871144
13507	0.946133	0.83988
13508	1.03767	0.867714
13509	0.946132	0.763294
13510	1.00902	0.956441
13511	0.88324	0.931825

13512	0.971584	0.826445
13513	0.946132	0.976991
13514	0.946129	1.12797
13515	0.946131	1.0663
13516	0.971572	1.08837
13517	0.946131	0.304772
13518	0.946125	0.304053
13519	1.33587	0.289246
13520	0.946126	0.356092
13521	0.946127	0.311507
13522	0.946126	0.315329
13523	0.946128	0.316148
13524	0.946132	0.355865
13525	0.946132	0.356156
13526	1.27512	0.312275
13527	0.946133	0.438595
13528	0.946132	0.369975
13529	0.946133	0.368046
13530	0.946133	0.391307
13531	0.946128	0.552206
13532	0.946133	0.548419
13533	0.946131	0.569562
13534	0.94613	0.371775
13535	0.946131	0.446167
13536	0.94613	0.525529
13537	0.946131	0.478437
13538	0.946131	0.510859
13539	0.946131	0.493276
13540	0.946131	0.533238
13541	0.946131	0.334532
13542	0.946131	0.409633
13543	0.946131	0.486214
13544	0.946131	0.439979
13545	0.94613	0.209454
13546	0.946131	0.215624
13547	0.946131	0.243214
13548	0.94613	0.217293
13549	0.946132	0.228115
13550	0.946131	0.22979
13551	1.35316	0.21807
13552	0.94613	0.656049
13553	0.94613	0.562183
13554	0.946128	0.679152
13555	0.946132	0.59039
13556	0.946132	0.670591
13557	1.28727	0.625641
13558	0.94613	0.629798
13559	0.946131	0.337205
13560	0.946132	0.333204
13561	0.946132	0.329948
13562	1.39502	0.322859
13563	0.946132	0.304574
13564	0.946132	0.332878
13565	0.946132	0.387275
13566	0.946125	0.806341
13567	0.946131	0.783763
13568	0.94613	0.667467
13569	0.946129	0.815265

13570	0.946126	0.945834
13571	0.946127	0.885305
13572	1.36173	0.858215
13573	0.946129	0.96343
13574	1.02363	0.893506
13575	0.94613	0.892012
13576	0.946131	0.974848
13577	0.946131	0.851739
13578	1.02363	0.959977
13579	0.946131	0.950982
13580	0.946128	0.170448
13581	1.24678	0.168866
13582	0.94613	0.173733
13583	0.94613	0.175324
13584	0.94613	0.16548
13585	0.946131	0.169478
13586	0.94613	0.182056
13587	0.946129	0.169434
13588	1.25215	0.169224
13589	0.946132	0.172442
13590	0.946126	0.173987
13591	0.946129	0.165956
13592	0.94613	0.167853
13593	0.946129	0.17911
13594	0.946129	0.827416
13595	0.946133	0.844111
13596	0.946133	0.792172
13597	0.946131	0.810817
13598	0.946131	0.809258
13599	1.29689	0.758708
13600	0.946131	0.77459
13601	0.946128	0.507959
13602	0.946132	0.380578
13603	0.946132	0.45999
13604	0.94613	0.534518
13605	1.22716	0.397709
13606	1.22716	0.436329
13607	0.946131	0.509177
13608	0.946127	0.546229
13609	0.946131	0.453199
13610	0.946131	0.514139
13611	0.946128	0.571167
13612	1.30463	0.465717
13613	1.30463	0.483572
13614	0.946129	0.555479
13615	1.29958	0.2665
13616	0.946132	0.347631
13617	0.946132	0.276732
13618	0.946132	0.237316
13619	0.946132	0.261609
13620	0.946132	0.26723
13621	0.946133	0.266263
13622	1.26201	0.249358
13623	0.946133	0.357502
13624	0.946132	0.290807
13625	0.946132	0.246776
13626	0.946132	0.267631
13627	0.946133	0.279048

13628	0.946132	0.271269
13629	0.946133	0.46902
13630	0.946132	0.404646
13631	0.946132	0.485149
13632	0.946133	0.351111
13633	0.946132	0.377244
13634	1.40427	0.377654
13635	0.946133	0.464777
13636	0.946132	0.475896
13637	0.946132	0.394318
13638	0.946132	0.485494
13639	0.946132	0.348912
13640	0.946132	0.367661
13641	1.35123	0.360858
13642	0.946131	0.461762
13643	0.946128	0.576812
13644	0.946132	0.493696
13645	0.946132	0.535806
13646	0.946129	0.607621
13647	1.22144	0.498501
13648	1.22144	0.505735
13649	0.946131	0.582617
13650	0.946127	0.729132
13651	0.946132	0.715651
13652	0.946131	0.686513
13653	0.946127	0.695976
13654	0.946131	0.720125
13655	1.23351	0.626889
13656	0.94613	0.661602
13657	1.18119	0.388699
13658	0.946131	0.460338
13659	0.946132	0.368432
13660	0.946132	0.422373
13661	0.94613	0.49012
13662	0.946131	0.437319
13663	0.946131	0.449472
13664	0.946132	0.641666
13665	0.946132	0.597987
13666	0.946133	0.691878
13667	1.30563	0.554201
13668	0.946132	0.572529
13669	0.946132	0.63869
13670	0.946133	0.712913
13671	1.20256	0.779745
13672	0.946132	1.39329
13673	0.946133	1.05033
13674	0.946132	0.765782
13675	0.946132	0.903566
13676	0.946132	1.16665
13677	0.946133	0.903755
13678	0.94613	0.993626
13679	0.94613	0.912734
13680	0.946128	1.13396
13681	1.33007	0.886087
13682	0.946129	0.896534
13683	0.94613	1.04513
13684	0.946131	1.11149
13685	0.946127	2.1

13686	0.946127	2.1
13687	0.946131	2.1
13688	1.30136	2.1
13689	0.946128	2.1
13690	0.946129	2.1
13691	1.30136	2.1
13692	0.810942	2.1
13693	0.810942	2.1
13694	0.81093	2.1
13695	2.3208	2.1
13696	1.3517	2.1
13697	1.08132	2.1
13698	1.08132	2.1
13699	1.08133	2.1
13700	0.946132	2.1
13701	0.946132	2.1
13702	0.946132	2.1
13703	0.946132	2.1
13704	0.946132	2.1
13705	1.39823	2.1
13706	0.946132	2.1
13707	1.37713	2.1
13708	0.946131	2.1
13709	0.946131	2.1
13710	0.946131	2.1
13711	0.946131	2.1
13712	0.946131	2.1
13713	0.946131	2.1
13714	0.946128	2.1
13715	0.946128	2.1
13716	0.946133	1.91147
13717	0.946131	2.1
13718	0.946131	1.21692
13719	0.946131	2.1
13720	0.94613	2.1
13721	0.946129	2.1
13722	0.946129	2.1
13723	0.946133	2.1
13724	0.946131	2.1
13725	0.946131	2.1
13726	0.946131	2.1
13727	0.946131	2.1
13728	0.946132	1.09454
13729	1.33694	1.63907
13730	0.946133	2.01234
13731	0.946133	1.06704
13732	0.946132	1.55538
13733	0.946132	1.11915
13734	0.946133	1.32052
13735	0.946131	1.7869
13736	0.94613	2.1
13737	0.946131	2.1
13738	0.946133	1.99059
13739	0.946129	2.1
13740	0.946131	2.1
13741	0.946131	2.1
13742	0.946132	1.36874
13743	1.35943	1.59145

13744	0.946132	2.02705
13745	0.946132	1.10708
13746	0.946131	1.22103
13747	0.946132	1.02179
13748	0.946132	1.55311
13749	0.946132	0.287241
13750	0.946132	0.333106
13751	0.946133	0.259543
13752	0.946133	0.301788
13753	0.946132	0.289298
13754	0.946132	0.284097
13755	0.946132	0.325768
13756	1.29322	0.547965
13757	0.946131	0.591815
13758	0.946132	0.453175
13759	0.946129	0.642737
13760	0.946131	0.503888
13761	0.946131	0.4588
13762	0.946131	0.64252
13763	0.946132	0.760457
13764	0.946132	1.09801
13765	0.946131	0.659154
13766	1.41492	0.525077
13767	0.946132	0.930714
13768	0.946132	0.822769
13769	0.946131	0.873206
13770	0.946132	2.1
13771	0.946132	2.1
13772	0.946132	2.1
13773	1.35028	2.1
13774	0.946132	2.1
13775	0.946132	2.1
13776	0.946132	2.1
13777	0.946132	1.38015
13778	0.946132	1.98007
13779	0.946132	1.64372
13780	1.32461	1.57532
13781	0.946131	1.99746
13782	0.946132	1.64506
13783	0.946132	1.96757
13784	0.946131	2.1
13785	0.946131	2.1
13786	0.946131	2.1
13787	0.946132	2.1
13788	0.946129	2.1
13789	0.94613	2.1
13790	1.40369	2.1
13791	0.946132	0.547745
13792	0.946131	0.461673
13793	0.946131	0.576878
13794	0.979598	0.432642
13795	0.946131	0.431517
13796	0.946131	0.52879
13797	0.946132	0.566766
13798	0.946131	2.1
13799	1.41897	2.1
13800	0.946132	2.1
13801	0.946132	2.1

13802	0.94613	1.54445
13803	0.946131	2.1
13804	0.946131	2.1
13805	0.946131	2.1
13806	0.94613	2.1
13807	0.946132	2.1
13808	0.946132	2.1
13809	0.946129	2.1
13810	0.946131	2.1
13811	1.37791	2.1
13812	0.946127	2.1
13813	0.946131	2.1
13814	0.946131	2.1
13815	0.94613	2.1
13816	0.946128	2.1
13817	0.946129	2.1
13818	1.39384	2.1
13819	0.810945	1.71466
13820	0.810944	1.29238
13821	0.810958	1.42268
13822	2.70852	1.6835
13823	1.35168	1.41606
13824	1.08132	1.52919
13825	1.08132	1.10522
13826	1.0813	1.32898
13827	1.3335	2.1
13828	0.946132	2.1
13829	0.94613	2.07556
13830	0.946132	2.1
13831	0.946131	2.1
13832	0.94613	2.1
13833	0.946131	2.1
13834	0.946132	1.47652
13835	0.946131	0.757271
13836	0.94613	0.941745
13837	1.41192	0.999219
13838	0.946132	0.734242
13839	0.946131	0.852493
13840	0.94613	0.794315
13841	0.946133	0.767452
13842	0.946133	0.740962
13843	0.946132	0.746163
13844	1.39428	0.680131
13845	0.946132	0.716404
13846	0.946132	0.589204
13847	0.946133	0.720823
13848	0.94613	0.908769
13849	0.946129	2.1
13850	0.94613	1.06133
13851	0.946128	1.05643
13852	1.40684	1.72663
13853	0.94613	1.01334
13854	0.94613	1.92447
13855	0.946131	1.03476
13856	0.946131	2.1
13857	0.946131	1.48689
13858	0.946131	1.20993
13859	1.39021	2.1

13860	0.946131	1.46553
13861	0.946131	2.1
13862	0.946131	1.36432
13863	0.946131	1.76698
13864	0.946131	1.10122
13865	0.946131	1.67881
13866	0.946131	1.73212
13867	0.946131	1.04321
13868	0.946131	1.27048
13869	0.810946	2.01936
13870	0.810946	1.8545
13871	2.69264	2.1
13872	1.35168	2.1
13873	1.0813	2.1
13874	1.08132	2.04013
13875	1.08132	2.1
13876	0.810958	2.1
13877	1.16687	1.82099
13878	0.946131	2.07034
13879	0.946131	1.85516
13880	1.34632	2.1
13881	0.946131	2.1
13882	1.34632	2.1
13883	0.946131	2.1
13884	0.946128	0.925836
13885	0.946133	0.941908
13886	0.946133	0.856073
13887	0.946131	0.914477
13888	0.946131	0.865338
13889	1.3564	0.875388
13890	0.94613	0.871957
13891	0.946128	2.1
13892	0.946132	2.1
13893	0.94613	2.1
13894	0.94613	2.1
13895	0.946131	2.1
13896	1.35827	2.1
13897	0.94613	2.1
13898	0.946133	0.851038
13899	0.946132	1.02934
13900	0.946132	0.862326
13901	0.946133	1.02044
13902	0.946133	0.949679
13903	0.946132	0.952631
13904	0.946133	0.99902
13905	0.946132	1.78247
13906	0.946132	1.72077
13907	0.946132	0.902933
13908	0.946131	1.72171
13909	0.946132	1.17225
13910	0.946132	1.12398
13911	0.946132	1.16387
13912	0.94613	0.861185
13913	0.94613	0.792515
13914	0.946128	0.863717
13915	0.946132	0.789194
13916	0.946132	0.889574
13917	0.94613	0.816497

13918	0.946131	0.811551
13919	0.946126	0.677848
13920	0.946131	0.562197
13921	0.946127	0.684134
13922	0.946129	0.571671
13923	0.94613	0.693078
13924	0.946128	0.621931
13925	0.946128	0.61017
13926	0.946131	0.456445
13927	0.946127	0.37579
13928	0.946127	0.482273
13929	0.946131	0.365116
13930	0.946131	0.476812
13931	0.946128	0.435559
13932	0.946129	0.422231
13933	0.94613	0.425274
13934	0.946131	0.37257
13935	0.946129	0.454515
13936	0.946132	0.339409
13937	0.946132	0.443056
13938	0.946131	0.422618
13939	0.94613	0.405141
13940	0.94613	0.466052
13941	0.946131	0.409809
13942	0.946129	0.529676
13943	0.946132	0.419042
13944	0.946132	0.460235
13945	1.32827	0.426801
13946	0.946131	0.46018
13947	0.946132	2.1
13948	0.946132	2.1
13949	0.946131	1.98881
13950	0.946131	1.98664
13951	0.946132	2.1
13952	0.946131	2.1
13953	0.946131	2.1
13954	0.946132	0.370962
13955	0.946132	0.921393
13956	0.946131	0.562786
13957	1.38356	0.507071
13958	0.946133	0.890347
13959	0.946131	0.706929
13960	0.946131	0.825629
13961	0.94613	2.1
13962	0.946131	2.1
13963	0.946131	2.1
13964	0.946128	2.1
13965	0.946133	2.1
13966	0.946131	2.1
13967	1.36918	2.1
13968	0.946131	0.916922
13969	0.94613	1.63999
13970	0.946129	1.16427
13971	0.946133	2.1
13972	0.946132	1.11549
13973	1.3771	1.63292
13974	0.946131	1.09354
13975	1.30029	0.982827

13976	0.94613	0.804446
13977	0.946129	1.15654
13978	0.946132	1.10534
13979	0.946132	0.706481
13980	0.946131	0.935138
13981	0.94613	0.836292
13982	0.946133	0.257293
13983	0.946132	0.299783
13984	0.946132	0.260314
13985	0.946132	0.236783
13986	0.946132	0.248511
13987	0.946132	0.271991
13988	1.21174	0.245302
13989	0.810946	2.1
13990	0.810945	2.1
13991	2.63045	2.1
13992	1.35169	2.1
13993	1.08132	2.1
13994	1.08132	2.1
13995	1.08132	2.1
13996	0.810946	2.1
13997	1.1796	2.1
13998	0.946131	2.1
13999	0.946132	2.1
14000	1.31522	2.1
14001	0.946132	2.1
14002	1.31522	2.1
14003	0.946132	2.1
14004	0.946131	2.1
14005	1.40615	2.1
14006	0.946129	2.1
14007	0.946128	2.1
14008	0.946133	2.1
14009	0.946131	2.1
14010	0.94613	2.1
14011	0.810945	2.1
14012	0.810944	2.1
14013	2.80479	2.1
14014	1.35169	2.1
14015	1.08132	2.1
14016	1.08132	2.1
14017	1.08132	2.1
14018	0.810945	2.1
14019	1.35562	2.1
14020	0.94613	2.1
14021	1.35561	2.1
14022	0.946133	2.1
14023	0.946131	2.1
14024	1.40239	2.1
14025	0.946131	2.1
14026	0.946125	0.718202
14027	0.946131	0.859839
14028	0.946127	0.618764
14029	0.946129	0.715367
14030	0.946128	0.821002
14031	0.946129	0.831228
14032	0.946128	0.730891
14033	0.946119	2.1

14034	0.946113	2.1
14035	0.94612	2.1
14036	0.946119	2.1
14037	1.41915	2.1
14038	0.946107	2.1
14039	0.946132	2.1
14040	0.810946	1.3163
14041	0.810943	1.91995
14042	0.810934	1.59332
14043	2.31411	1.95153
14044	1.35171	1.80017
14045	1.08132	1.43176
14046	1.08132	1.92694
14047	1.08133	1.40059
14048	0.946127	1.87657
14049	0.94613	2.1
14050	0.94613	2.1
14051	0.946131	2.1
14052	0.946126	2.1
14053	0.946128	2.1
14054	0.946128	2.1
14055	0.810944	2.1
14056	0.810947	1.97334
14057	0.81096	2.1
14058	2.78019	2.1
14059	1.35168	2.1
14060	1.08132	2.1
14061	1.08132	2.03928
14062	1.08131	2.1
14063	0.946133	0.471192
14064	0.946132	0.504782
14065	0.946132	0.483811
14066	0.946133	0.442096
14067	0.946132	0.504119
14068	1.3495	0.405276
14069	0.946133	0.496279
14070	0.946133	2.1
14071	0.946132	2.1
14072	0.946132	2.1
14073	1.39529	2.1
14074	0.946133	2.1
14075	0.946132	2.1
14076	0.946133	2.1
14077	0.946133	2.1
14078	0.946132	2.1
14079	0.946132	2.05483
14080	0.946132	2.1
14081	0.946132	1.97357
14082	0.946133	2.1
14083	0.946132	2.1
14084	0.810946	2.1
14085	0.810945	2.1
14086	2.67861	2.1
14087	1.35169	2.1
14088	1.08132	2.1
14089	1.08132	2.1
14090	1.08132	2.1
14091	0.810946	2.1

14092	1.24616	2.1
14093	0.946132	2.1
14094	0.946132	2.1
14095	1.33931	2.1
14096	0.946132	2.1
14097	1.33931	2.1
14098	0.946133	2.1
14099	0.946133	0.69545
14100	0.946133	0.682153
14101	0.946132	0.697188
14102	0.946133	0.697864
14103	1.3855	0.680335
14104	1.3855	0.678717
14105	0.946133	0.698661
14106	0.946128	1.15121
14107	0.946132	2.0987
14108	0.946128	1.55657
14109	0.94613	2.1
14110	0.946131	2.06107
14111	1.40225	2.1
14112	0.94613	1.91123
14113	1.40548	0.252454
14114	0.946118	0.317802
14115	0.946114	0.285944
14116	0.946112	0.234448
14117	0.946117	0.254329
14118	0.946128	0.267421
14119	0.946103	0.281059
14120	0.94613	2.1
14121	0.946132	2.1
14122	0.946131	2.1
14123	0.946132	2.1
14124	1.37811	2.1
14125	0.946131	2.1
14126	0.946131	2.1
14127	0.946132	2.1
14128	0.946133	2.1
14129	0.946133	2.1
14130	0.946132	2.1
14131	0.946133	2.1
14132	0.946132	2.1
14133	0.946132	2.1
14134	0.946118	1.47966
14135	0.946113	2.1
14136	0.946118	2.1
14137	0.946119	2.06933
14138	0.946117	2.1
14139	0.946105	2.1
14140	0.946131	2.1
14141	0.946118	0.335334
14142	0.946118	0.333026
14143	0.946117	0.338126
14144	0.946113	0.262907
14145	0.94612	0.272013
14146	1.41377	0.291353
14147	0.946106	0.318549
14148	0.946132	1.03634
14149	1.28436	0.904438

14150	0.946131	0.901205
14151	0.946131	1.04898
14152	0.946131	0.716656
14153	0.94613	1.08147
14154	0.946131	0.934005
14155	0.810947	0.666544
14156	0.810946	0.720786
14157	2.68675	0.560501
14158	1.35168	0.58262
14159	1.08132	0.657341
14160	1.08132	0.619709
14161	1.0813	0.68197
14162	0.81096	0.613224
14163	0.946132	0.562554
14164	1.34338	0.527634
14165	0.946132	0.536531
14166	1.34338	0.597499
14167	0.946132	0.610083
14168	1.34337	0.566376
14169	0.946132	0.551771
14170	0.946127	1.80559
14171	0.946131	1.61551
14172	0.946127	1.61552
14173	0.946131	1.61865
14174	0.946131	1.75648
14175	0.946128	1.54602
14176	1.30281	1.84846
14177	0.946132	0.835654
14178	0.946132	0.797103
14179	0.946132	0.789511
14180	0.946131	0.785768
14181	0.946132	0.83441
14182	1.37973	0.665182
14183	0.946132	0.732948
14184	0.946131	0.953268
14185	1.41798	0.922856
14186	0.946132	0.90562
14187	0.946132	0.916104
14188	0.946129	0.990188
14189	0.946131	0.91621
14190	0.946131	0.98859
14191	0.946118	0.897704
14192	1.41776	0.878085
14193	0.946113	0.885433
14194	0.946119	0.858949
14195	0.946117	0.933796
14196	1.41778	0.849337
14197	0.946105	0.936482
14198	0.946131	1.00608
14199	1.41798	1.06022
14200	0.946132	1.09477
14201	0.946132	0.986407
14202	0.94613	1.01808
14203	1.41799	0.966256
14204	0.946131	0.968091
14205	0.946133	2.07381
14206	0.946132	2.1
14207	0.946132	1.05919

14208	0.946132	2.1
14209	0.946133	1.40128
14210	0.946132	1.62627
14211	0.946132	1.97028
14212	0.946131	1.70901
14213	0.946131	2.1
14214	0.94613	0.952445
14215	0.946129	1.89602
14216	0.946132	1.23122
14217	0.94613	1.12216
14218	0.946131	1.76917
14219	0.94612	1.23707
14220	0.946126	2.1
14221	0.946119	1.12935
14222	0.94612	1.36666
14223	1.40766	1.71163
14224	0.946132	1.08248
14225	0.946107	1.92539
14226	0.946128	2.1
14227	1.31213	2.1
14228	0.946129	2.1
14229	0.94613	2.1
14230	0.94613	2.1
14231	0.94613	2.1
14232	0.94613	2.1
14233	0.946127	1.5683
14234	0.946131	1.92805
14235	0.94613	2.02579
14236	0.94613	1.63364
14237	1.36653	1.88654
14238	0.946128	1.66271
14239	0.946128	1.86765
14240	0.946127	2.1
14241	0.94613	2.1
14242	0.946131	2.1
14243	0.94613	2.1
14244	0.946128	2.1
14245	0.946128	2.1
14246	0.946129	2.1
14247	0.946133	2.1
14248	1.40081	2.1
14249	0.946132	2.1
14250	0.946132	2.1
14251	0.946133	2.1
14252	0.946132	2.1
14253	0.946133	2.1
14254	0.946128	2.1
14255	1.32282	2.1
14256	0.946129	2.1
14257	0.94613	2.1
14258	0.946131	2.1
14259	0.946131	2.1
14260	0.946131	2.1
14261	0.810945	1.40172
14262	0.810943	2.1
14263	0.810933	1.97102
14264	2.44097	2.1
14265	1.3517	2.01177

14266	1.08132	1.45758
14267	1.08132	2.1
14268	1.08133	1.63272
14269	0.946131	2.1
14270	0.94613	2.1
14271	0.946133	2.1
14272	0.946133	2.1
14273	0.946128	2.07332
14274	0.946131	2.1
14275	1.27676	2.1
14276	0.946126	0.847192
14277	0.946126	0.906704
14278	0.946131	0.801507
14279	0.946127	0.903345
14280	0.94613	0.639102
14281	0.946128	0.822787
14282	0.946128	0.777305
14283	0.946132	0.346686
14284	0.946133	0.350233
14285	0.946132	0.366523
14286	0.946132	0.310077
14287	0.946132	0.348674
14288	0.946133	0.393489
14289	0.946132	0.344226
14290	0.946128	0.374222
14291	0.946131	0.377168
14292	0.94613	0.395924
14293	0.946129	0.324324
14294	0.94613	0.358155
14295	0.94613	0.421322
14296	0.94613	0.366146
14297	0.946132	0.324933
14298	0.946128	0.319913
14299	0.94613	0.347354
14300	0.946131	0.305365
14301	0.94613	0.38107
14302	0.94613	0.352177
14303	0.946131	0.320378
14304	0.94612	0.32987
14305	0.946119	0.329014
14306	0.946119	0.347616
14307	0.946124	0.304113
14308	0.946119	0.346334
14309	0.946132	0.363673
14310	0.946107	0.323255
14311	0.946128	2.1
14312	0.946133	2.1
14313	0.946131	2.1
14314	0.94613	2.1
14315	0.946131	2.1
14316	1.37869	2.1
14317	0.946131	2.1
14318	1.35703	2.1
14319	0.946132	2.1
14320	0.946132	2.1
14321	0.946132	2.1
14322	0.946132	2.1
14323	0.946132	2.1

14324	0.946133	2.1
14325	0.946133	2.1
14326	0.946133	1.74616
14327	0.946132	2.1
14328	1.37017	2.1
14329	0.946133	1.83657
14330	0.946132	2.1
14331	0.946133	2.1
14332	0.946114	0.222727
14333	0.946119	0.247082
14334	0.946119	0.304076
14335	0.94612	0.256299
14336	0.946119	0.280744
14337	0.946132	0.255148
14338	0.946107	0.263572
14339	0.946131	0.299136
14340	0.946131	0.323956
14341	0.946126	0.416798
14342	0.946127	0.366068
14343	0.94613	0.347534
14344	0.946129	0.3455
14345	0.946128	0.34199
14346	0.946131	1.25091
14347	0.946131	1.20824
14348	0.946132	1.07439
14349	0.946132	1.26196
14350	0.94613	1.41235
14351	0.946131	1.48135
14352	1.3279	1.4245
14353	0.946132	2.1
14354	1.40932	2.1
14355	0.946132	2.1
14356	0.946132	2.1
14357	0.946132	2.1
14358	0.946131	2.1
14359	0.946132	2.1
14360	0.946131	0.416909
14361	0.946131	0.413589
14362	0.946132	0.380162
14363	0.946132	0.42005
14364	0.94613	0.494229
14365	0.946131	0.44843
14366	1.26868	0.427849
14367	0.810943	2.1
14368	0.810945	2.1
14369	0.810932	2.1
14370	2.82321	2.1
14371	1.3517	2.1
14372	1.08132	2.1
14373	1.08132	2.1
14374	1.08133	2.1
14375	0.810943	0.660274
14376	0.810944	0.495874
14377	2.63079	0.750557
14378	1.35168	0.596507
14379	1.0813	0.614816
14380	1.08132	0.450217
14381	1.08132	0.928797

14382	0.810957	0.680483
14383	1.17856	0.375765
14384	0.946132	0.532907
14385	0.946128	0.488863
14386	1.3154	0.937293
14387	0.94613	0.597329
14388	1.31539	0.692074
14389	0.94613	0.860258
14390	0.94613	1.55951
14391	0.94613	1.50294
14392	0.946131	1.39726
14393	0.946131	1.58969
14394	0.946129	1.57934
14395	0.94613	1.94468
14396	1.21997	1.90608
14397	0.946131	1.18889
14398	1.25083	1.03219
14399	0.946132	1.00849
14400	0.946132	1.21598
14401	0.946129	0.991543
14402	1.25083	1.23474
14403	0.946131	1.23111
14404	0.946127	1.1618
14405	1.41342	0.868008
14406	0.94613	1.12428
14407	0.946131	0.977121
14408	0.946127	0.768649
14409	0.946129	0.845713
14410	0.946129	0.919537
14411	0.946131	1.31019
14412	1.29149	1.36322
14413	0.946133	1.84146
14414	0.946133	1.05289
14415	0.946128	1.12367
14416	0.946131	0.846955
14417	0.94613	1.22597
14418	0.94613	1.8029
14419	0.94613	1.24595
14420	0.946132	2.08441
14421	0.946132	1.15355
14422	0.946128	0.976328
14423	0.94613	1.62174
14424	1.41022	1.77396
14425	0.946132	0.7
14426	0.946132	0.609763
14427	0.946128	0.672289
14428	0.94613	0.551116
14429	0.94613	0.7
14430	0.94613	0.536451
14431	0.946131	0.7
14432	0.946129	0.887502
14433	1.37758	1.19519
14434	0.946131	1.57353
14435	0.94613	0.829524
14436	0.946124	1.10086
14437	0.946127	0.855595
14438	0.946127	1.18302
14439	0.946129	0.904579

14440	1.38259	1.18946
14441	0.946132	1.4444
14442	0.946132	0.88767
14443	0.946126	1.16585
14444	0.946129	0.919953
14445	0.94613	1.10195
14446	0.946131	0.342121
14447	0.946132	0.329008
14448	0.946132	0.365133
14449	0.946132	0.340882
14450	0.946132	0.398056
14451	0.946132	0.357509
14452	0.946132	0.339562
14453	0.946131	0.267633
14454	0.94613	0.279879
14455	0.94613	0.255253
14456	0.946133	0.265222
14457	0.946128	0.257485
14458	1.25017	0.249401
14459	0.946131	0.273797
14460	0.946131	2.1
14461	0.946131	2.1
14462	0.946131	2.1
14463	1.3937	2.1
14464	0.946133	2.1
14465	0.946131	2.1
14466	0.94613	2.1
14467	0.946131	0.795706
14468	0.946131	0.473912
14469	0.946131	0.533012
14470	0.94613	0.453396
14471	0.946133	0.897645
14472	0.946132	0.562451
14473	0.946131	0.612538
14474	0.946132	0.485049
14475	0.946132	0.630232
14476	0.946132	0.395569
14477	0.946132	0.486308
14478	0.946131	0.480271
14479	0.946132	0.326407
14480	0.946132	0.42763
14481	0.946129	0.518481
14482	0.94613	0.861241
14483	0.94613	0.79715
14484	0.946128	0.860565
14485	0.946132	0.262478
14486	0.946131	0.771652
14487	0.94613	0.746716
14488	0.946132	1.75408
14489	0.946132	0.862446
14490	0.946132	0.99787
14491	1.41619	1.4678
14492	0.946133	0.760336
14493	0.946133	0.990129
14494	0.946132	0.873552
14495	0.946132	2.1
14496	0.946132	1.74971
14497	0.946132	2.1

14498	1.38244	2.1
14499	0.946132	1.14669
14500	0.946131	2.1
14501	0.946132	2.02518
14502	0.810946	0.866223
14503	0.810943	1.02428
14504	0.810934	0.861877
14505	2.42741	0.753057
14506	1.35171	0.804746
14507	1.08132	0.836596
14508	1.08132	0.861318
14509	1.08133	0.868173
14510	0.810947	0.832383
14511	0.810943	0.996307
14512	2.44179	0.756325
14513	1.35171	0.803957
14514	1.08132	0.809727
14515	1.08132	0.806646
14516	1.08133	0.830765
14517	0.810934	0.813655
14518	0.946132	1.33739
14519	0.946132	1.29334
14520	0.946133	1.02875
14521	0.946133	1.28613
14522	0.946132	1.51258
14523	0.946132	1.32544
14524	1.12786	1.26952
14525	1.27806	0.929457
14526	0.946132	1.06172
14527	0.946133	0.977016
14528	0.946133	1.14351
14529	0.946132	1.31556
14530	0.946132	1.05131
14531	0.946133	1.23758
14532	0.946132	2.1
14533	0.946132	2.1
14534	0.946132	2.1
14535	1.37098	2.1
14536	0.946133	2.08383
14537	0.946132	2.1
14538	1.37098	2.1
14539	0.946125	0.982371
14540	0.946129	0.900304
14541	0.946131	1.33322
14542	0.94613	0.845716
14543	0.946124	0.802781
14544	0.946127	0.845442
14545	1.39514	0.978833
14546	0.946132	2.1
14547	0.946131	2.1
14548	0.94613	2.1
14549	1.39909	2.1
14550	0.946132	1.19237
14551	0.946131	2.1
14552	0.94613	2.1
14553	0.946133	1.44314
14554	0.946133	0.783902
14555	0.946132	1.52572

14556	1.36835	1.21249
14557	0.946132	0.810788
14558	0.946133	1.12349
14559	0.946132	0.817583
14560	0.946132	1.93538
14561	0.946133	2.1
14562	0.946133	2.1
14563	0.946132	2.05528
14564	1.39047	2.1
14565	0.946132	2.1
14566	0.946132	2.1
14567	1.33174	0.215326
14568	0.946128	0.221805
14569	0.94613	0.226745
14570	0.94613	0.220866
14571	0.94613	0.212877
14572	0.94613	0.213398
14573	0.946131	0.220385
14574	0.946128	0.269918
14575	1.36004	0.25583
14576	0.946129	0.258703
14577	0.94613	0.267186
14578	0.94613	0.262436
14579	1.36003	0.237008
14580	0.94613	0.260741
14581	0.946124	2.1
14582	0.946119	2.1
14583	0.946119	2.1
14584	0.946119	2.1
14585	0.94612	2.1
14586	0.946132	2.1
14587	1.29062	2.1
14588	0.946128	2.1
14589	0.946128	2.1
14590	0.946133	2.1
14591	0.94613	2.1
14592	0.946131	2.1
14593	0.946131	2.1
14594	1.30584	2.1
14595	0.946131	1.97727
14596	0.946131	2.1
14597	0.946131	2.1
14598	0.946132	2.06919
14599	1.39549	2.1
14600	0.946131	2.1
14601	0.946131	2.1
14602	0.913386	1.02367
14603	1.17466	0.971752
14604	1.00473	0.934438
14605	0.946132	0.998079
14606	0.946126	1.23063
14607	0.94613	1.10517
14608	0.946129	1.03403
14609	0.946132	0.826226
14610	1.38894	0.83956
14611	0.946132	0.820239
14612	0.946132	0.810944
14613	0.946132	0.903687

14614	0.946132	0.832636
14615	0.946133	0.844727
14616	0.946128	0.821455
14617	1.23865	0.823896
14618	0.946129	0.804728
14619	0.94613	0.801176
14620	0.94613	0.845391
14621	0.94613	0.808986
14622	0.94613	0.832965
14623	0.946126	1.0042
14624	1.40248	1.11484
14625	0.946129	1.07065
14626	0.94613	1.02677
14627	0.946124	1.2786
14628	0.946127	1.1057
14629	0.946128	1.06779
14630	0.946133	0.831117
14631	1.1391	0.883899
14632	0.946132	0.871163
14633	0.946132	0.821794
14634	0.946132	0.995736
14635	0.946133	0.853402
14636	0.946132	0.860298
14637	1.17156	1.0358
14638	0.946133	0.947573
14639	1.33893	0.872718
14640	0.827864	0.874288
14641	0.995232	0.853043
14642	0.827864	0.980554
14643	0.94613	0.807195
14644	1.21387	0.766607
14645	0.946132	0.769115
14646	0.946131	0.734877
14647	0.94613	0.75334
14648	0.94613	0.743709
14649	0.94613	0.755501
14650	0.94613	0.758926
14651	0.810943	0.660962
14652	0.810945	0.544103
14653	2.42463	0.532445
14654	1.3517	0.550987
14655	1.08132	0.609114
14656	1.08132	0.655752
14657	1.08133	0.582936
14658	0.810932	0.665758
14659	0.946133	0.80458
14660	0.946132	0.755236
14661	0.946131	0.777495
14662	1.36748	0.773901
14663	0.946133	0.769089
14664	0.946131	0.758868
14665	0.946132	0.713663
14666	1.35856	0.44461
14667	0.946131	0.482949
14668	0.94613	0.39201
14669	0.94613	0.497916
14670	0.946131	0.398662
14671	0.94613	0.402298

14672	0.94613	0.513116
14673	0.946132	1.80543
14674	0.946131	2.1
14675	0.946131	2.1
14676	0.946131	2.1
14677	1.41149	2.1
14678	1.41149	2.1
14679	0.946131	2.1
14680	0.946128	2.1
14681	0.946128	2.1
14682	0.946133	2.1
14683	0.946131	2.1
14684	0.946131	2.1
14685	0.946131	2.1
14686	0.94613	2.1
14687	1.41388	0.367725
14688	0.94613	0.435207
14689	0.946132	0.345066
14690	0.946132	0.413207
14691	0.946129	0.464806
14692	0.946131	0.409215
14693	0.946131	0.438999
14694	1.02798	0.366533
14695	0.946132	0.513438
14696	1.02798	0.359614
14697	0.946133	0.50444
14698	1.14931	0.379697
14699	0.957746	0.47436
14700	0.93452	0.428451
14701	0.946129	2.1
14702	0.946134	2.1
14703	0.946133	2.1
14704	0.946131	2.1
14705	0.946131	2.1
14706	0.946131	2.1
14707	0.946131	2.1
14708	0.946133	1.20193
14709	0.946132	1.0355
14710	0.946132	1.17001
14711	0.946132	1.34728
14712	1.38773	1.04348
14713	0.946132	1.19704
14714	0.946133	1.10715
14715	0.946131	0.571581
14716	0.946131	0.596276
14717	1.41512	0.569003
14718	0.946133	0.671743
14719	0.946131	0.667931
14720	0.946131	0.672114
14721	0.946131	0.60306
14722	1.35381	1.07073
14723	0.946132	2.07831
14724	1.35381	1.05547
14725	0.946133	2.00498
14726	0.946132	1.15524
14727	0.946132	1.86798
14728	0.946133	1.24379
14729	1.3365	1.04372

14730	0.946132	1.3392
14731	1.3365	0.981472
14732	0.946129	1.18023
14733	0.94613	1.16046
14734	0.946128	1.27513
14735	0.946129	1.03577
14736	0.939895	1.47389
14737	1.14285	1.94602
14738	0.952366	1.82746
14739	0.946131	1.68193
14740	0.94613	1.75406
14741	0.946131	1.68223
14742	0.94613	1.704
14743	0.946131	2.1
14744	0.946131	2.1
14745	0.946131	1.30378
14746	0.946129	2.1
14747	0.946133	1.35111
14748	0.946131	2.07973
14749	0.946131	2.1
14750	0.946133	2.1
14751	0.946132	2.1
14752	0.946132	2.1
14753	0.946133	2.1
14754	0.946132	2.04437
14755	0.946132	2.1
14756	0.946133	2.1
14757	0.946133	1.89684
14758	0.946132	1.30567
14759	0.946132	1.71045
14760	0.946133	1.57515
14761	0.946132	2.0266
14762	0.946132	1.90062
14763	0.946132	1.47344
14764	0.946132	1.0261
14765	0.946131	2.1
14766	0.946131	1.10019
14767	0.946131	1.10617
14768	1.38969	2.1
14769	0.946131	1.48852
14770	0.946132	2.06192
14771	0.946128	1.82917
14772	0.946133	1.40908
14773	0.946132	1.56572
14774	0.946131	1.3691
14775	0.94613	1.46088
14776	0.946131	1.61824
14777	1.28464	1.59164
14778	0.946119	0.804704
14779	1.37492	0.854057
14780	0.946125	0.871967
14781	0.946119	0.770133
14782	0.946119	0.946638
14783	0.946132	0.787328
14784	0.946107	0.846763
14785	0.810942	2.1
14786	0.810943	2.1
14787	0.709587	2.1

14788	2.64488	2.1
14789	1.21651	2.1
14790	0.946151	2.1
14791	0.979948	2.1
14792	1.0813	2.1
14793	0.946133	1.34642
14794	0.946132	1.59056
14795	0.946132	1.87221
14796	0.946133	1.02864
14797	0.946132	0.853021
14798	0.946132	0.968651
14799	1.41134	1.27872
14800	0.946133	1.71702
14801	0.946132	1.37342
14802	0.946133	1.87787
14803	0.946133	1.1705
14804	0.946132	0.980492
14805	0.946133	1.31039
14806	1.31338	1.47722
14807	2.07612	1.33181
14808	0.946102	0.211925
14809	0.752448	0.23524
14810	1.20617	0.223255
14811	0.94613	0.230053
14812	0.75242	0.221476
14813	0.946131	0.225606
14814	0.752419	0.203102
14815	0.946132	1.08252
14816	1.25736	1.10875
14817	1.25736	1.07263
14818	0.946132	1.02339
14819	1.08054	1.01944
14820	1.25736	1.04899
14821	0.946132	1.06799
14822	0.946128	2.1
14823	1.1896	2.07039
14824	1.18959	2.1
14825	0.946131	1.07517
14826	1.33271	1.70461
14827	0.946105	1.83204
14828	0.946156	1.7523
14829	0.741946	0.232113
14830	1.33587	0.246621
14831	0.741947	0.233265
14832	1.21954	0.24573
14833	0.94613	0.25632
14834	0.741946	0.252723
14835	1.81347	0.242741
14836	1.81347	0.22952
14837	0.653322	0.216119
14838	1.36935	0.216876
14839	0.661882	0.205891
14840	1.04753	0.214014
14841	0.507464	0.212794
14842	0.945844	1.05745
14843	1.59507	1.56392
14844	1.04043	1.00353
14845	0.946131	1.47181

14846	1.32891	1.22507
14847	0.946131	0.99477
14848	0.946131	0.976901
14849	1.28866	1.28037
14850	1.12682	0.362982
14851	0.851543	0.391328
14852	1.04072	0.375654
14853	0.946132	0.442908
14854	1.13437	0.344751
14855	1.04167	0.400585
14856	0.936652	0.398824
14857	1.258 2.1	
14858	0.946131	2.1
14859	0.946132	2.1
14860	1.36968	2.1
14861	0.946132	2.1
14862	1.36968	2.1
14863	0.946131	2.1
14864	2.30046	0.519404
14865	2.82969	0.550508
14866	0.946132	1.51039
14867	1.35022	2.1
14868	1.12356	1.43673
14869	0.94613	2.1
14870	1.35022	1.97731
14871	0.946131	1.9048
14872	0.94613	1.7667
14873	0.946131	2.1
14874	1.22401	2.1
14875	0.952456	2.1
14876	0.94613	2.1
14877	1.22401	2.1
14878	0.946131	2.1
14879	0.946131	2.1
14880	1.33047	2.1
14881	0.946132	2.1
14882	1.33047	2.1
14883	0.946131	2.1
14884	1.33047	2.1
14885	0.946132	2.1
14886	1.2107	2.1
14887	0.61957	1.75665
14888	1.80666	2.1
14889	1.53743	2.1
14890	0.619572	1.56508
14891	1.32547	2.05642
14892	1.16166	1.94693
14893	1.04074	1.93935
14894	0.557621	2.00454
14895	0.946128	1.20732
14896	1.36607	1.81943
14897	0.94613	1.63031
14898	1.36607	2.1
14899	1.39416	2.1
14900	0.94613	2.1
14901	0.94613	2.1
14902	1.23969	2.1
14903	0.946131	2.1

14904	0.946131	2.1
14905	1.13537	2.1
14906	1.05719	2.1
14907	0.806848	2.1
14908	1.30251	2.1
14909	0.851526	1.25857
14910	1.14176	1.82252
14911	0.93666	1.12588
14912	0.946114	1.64705
14913	1.15832	1.30338
14914	0.924624	1.258
14915	0.967609	1.10646
14916	0.771764	0.463804
14917	1.26652	0.470169
14918	0.946133	0.488849
14919	1.17957	0.371328
14920	0.771735	0.449383
14921	0.946122	0.500999
14922	0.946133	0.446307
14923	0.924325	1.17631
14924	0.656739	1.24209
14925	0.637876	1.22846
14926	0.530092	1.2051
14927	0.914415	1.15455
14928	0.821311	1.44044
14929	0.630589	1.34647
14930	0.738823	1.32677
14931	0.839109	1.26102
14932	1.55029	0.199388
14933	1.13537	0.809397
14934	0.946131	1.00452
14935	0.946128	0.916165
14936	1.30162	2.03787
14937	0.952476	1.05013
14938	1.29289	1.21578
14939	0.939783	1.24732
14940	0.94612	2.1
14941	0.946131	2.1
14942	1.07998	2.1
14943	0.836985	2.1
14944	1.1604	2.1
14945	0.94613	2.1
14946	0.837016	2.1
14947	0.712461	0.840441
14948	1.36399	0.85517
14949	1.8724	0.788994
14950	1.8724	0.755055
14951	0.623426	1.01387
14952	1.36399	0.830886
14953	1.22089	0.812307
14954	0.946132	0.810689
14955	0.946131	2.1
14956	1.32241	2.1
14957	1.32241	2.1
14958	0.946133	2.1
14959	1.40271	2.1
14960	0.946131	2.1
14961	0.946131	2.1

14962	1.1989	1.31339
14963	0.954106	1.49407
14964	0.820068	1.2031
14965	0.563139	1.40462
14966	1.06448	1.43346
14967	0.892472	1.75884
14968	0.862598	1.30942
14969	0.745851	1.77492
14970	1.13336	1.43931
14971	0.946129	2.1
14972	1.28254	2.1
14973	1.28254	2.1
14974	0.946131	2.1
14975	1.41302	2.1
14976	0.946131	2.1
14977	0.946131	2.1
14978	0.851543	1.53259
14979	1.21961	2.1
14980	0.851484	1.45743
14981	0.851543	2.08237
14982	1.15705	1.87462
14983	0.946133	1.77951
14984	0.909417	1.67956
14985	1.13808	0.75522
14986	0.946133	0.621513
14987	0.946131	0.760725
14988	1.30194	0.567229
14989	0.946131	0.707623
14990	1.30194	0.550027
14991	0.946131	0.599478
14992	0.930941	1.17896
14993	1.20185	0.985946
14994	1.02404	1.03847
14995	0.946124	1.24397
14996	1.3563	1.12493
14997	0.946127	1.30851
14998	0.946128	1.15534
14999	0.946116	2.1
15000	1.3144	1.91632
15001	1.13088	2.1
15002	0.946118	1.03731
15003	1.31438	1.66571
15004	0.946105	1.6817
15005	0.946131	1.62359
15006	1.76972	0.47589
15007	1.42091	0.364653
15008	0.946128	0.49498
15009	0.946101	0.567394
15010	0.863552	0.601424
15011	0.94613	0.423118
15012	1.21232	0.522821
15013	0.94613	0.618917
15014	1.2137	0.784187
15015	1.23224	0.928652
15016	1.86512	0.829042
15017	1.86512	0.737551
15018	0.580825	1.13115
15019	1.2137	0.944151

15020	0.946133	0.943455
15021	0.580822	0.916115
15022	0.946128	2.1
15023	1.40586	2.1
15024	0.94613	2.1
15025	1.3719	2.1
15026	1.3719	2.1
15027	1.40586	2.1
15028	0.94613	2.1
15029	0.946132	1.68575
15030	1.35025	1.65534
15031	1.1794	1.54834
15032	0.946132	0.925911
15033	1.35025	1.08681
15034	0.946131	1.08782
15035	0.946132	1.07499
15036	0.946133	2.1
15037	1.60149	2.1
15038	1.67058	2.1
15039	0.851542	2.1
15040	1.20296	2.1
15041	1.17501	2.1
15042	0.946132	2.1
15043	1.04993	2.1
15044	0.851541	1.23348
15045	1.48219	2.1
15046	0.874565	1.22026
15047	0.946129	2.1
15048	1.22049	2.00118
15049	0.946131	1.93838
15050	0.946131	1.60569
15051	0.94613	1.97144
15052	0.848719	2.06803
15053	1.17195	1.83944
15054	0.946129	1.21692
15055	1.35426	1.24835
15056	0.94613	1.32461
15057	0.94613	1.5175
15058	0.946133	2.1
15059	1.14342	2.1
15060	1.14342	2.1
15061	0.946133	2.1
15062	1.39009	2.1
15063	0.946132	2.1
15064	0.946132	2.1
15065	1.38819	2.1
15066	0.946133	2.1
15067	1.38819	2.1
15068	0.946131	2.1
15069	1.4116	2.1
15070	0.946131	2.1
15071	1.38819	2.1
15072	0.946127	2.1
15073	1.32244	2.1
15074	1.2176	2.1
15075	0.946131	2.1
15076	1.32244	2.1
15077	0.94613	2.1

15078	0.946129	2.1
15079	1.32415	2.1
15080	1.11239	2.1
15081	0.984099	2.1
15082	1.65532	2.1
15083	1.53082	2.1
15084	1.32068	2.1
15085	1.32068	2.1
15086	1.08172	2.1
15087	3.76293	0.247928
15088	1.36335	0.20643
15089	0.953925	2.1
15090	0.989497	2.1
15091	0.98949	2.1
15092	1.06341	2.1
15093	1.27875	2.1
15094	1.04598	2.1
15095	0.603154	2.1
15096	0.582457	2.1
15097	0.582457	2.1
15098	0.568994	2.1
15099	1.5791	2.1
15100	1.09506	2.1
15101	1.45327	1.9649
15102	1.88406	2.1
15103	1.88404	2.1
15104	1.27389	1.85942
15105	1.14206	2.1
15106	1.00232	2.1
15107	1.00231	2.1
15108	1.68436	2.1
15109	1.4677	0.256972
15110	1.28828	0.306055
15111	1.28828	0.235158
15112	1.46932	0.243803
15113	1.28228	2.1
15114	1.64268	1.16087
15115	0.959473	2.1
15116	1.10101	2.1
15117	1.07976	2.1
15118	1.2873	1.39155
15119	1.70303	0.461508
15120	1.40066	0.485154
15121	1.5563	0.363767
15122	1.13727	0.381286
15123	1.03529	2.1
15124	1.11285	2.1
15125	1.03529	2.1
15126	1.28667	2.1
15127	1.32119	2.1
15128	1.44777	2.1
15129	1.44778	2.1
15130	1.61121	2.1
15131	1.09372	0.297082
15132	1.17664	0.315215
15133	1.17664	0.30681
15134	0.872931	0.269939
15135	1.02415	2.1

15136	1.28228	2.1
15137	2.14692	2.1
15138	3.90469	2.1
15139	1.00509	2.1
15140	1.28228	2.1
15141	3.32936	0.227546
15142	1.1333	0.219529
15143	1.11048	2.1
15144	0.979854	2.1
15145	1.65815	2.1
15146	1.00185	2.1
15147	1.84887	2.1
15148	1.59597	2.1
15149	1.78468	2.1
15150	1.87508	2.1
15151	1.82716	2.1
15152	1.33429	2.1
15153	1.34626	2.1
15154	0.797142	2.1
15155	1.00007	1.2041
15156	1.02051	1.73978
15157	1.02051	1.26723
15158	1.03373	1.5853
15159	1.05786	2.1
15160	0.887631	2.1
15161	0.992773	2.1
15162	1.28462	2.1
15163	2.35274	1.62916
15164	1.82533	1.17241
15165	1.82533	1.50117
15166	1.19496	0.971696
15167	1.28597	0.147815
15168	1.09725	2.1
15169	1.60948	2.1
15170	1.20421	2.1
15171	1.16566	2.1
15172	1.88522	2.1
15173	1.81948	0.768366
15174	1.14042	0.68546
15175	1.21558	0.93468
15176	1.56876	0.679568
15177	1.6697	2.1
15178	1.81655	2.1
15179	1.81655	2.1
15180	1.5956	2.1
15181	0.906059	2.1
15182	1.63045	1.87899
15183	0.9533	1.10775
15184	1.1475	1.03893
15185	1.72231	1.68709
15186	1.94936	1.2901
15187	1.06826	2.1
15188	1.08576	2.1
15189	1.06826	2.1
15190	0.973559	2.1
15191	1.62737	0.221398
15192	0.965676	0.220585
15193	1.45055	0.237341

15194	1.17631	0.239677
15195	1.92535	0.296528
15196	1.9503	0.381468
15197	1.99828	0.288233
15198	1.70302	0.369837
15199	1.65139	0.381492
15200	1.26784	0.440897
15201	1.35923	0.457332
15202	1.70302	0.489121
15203	1.33033	2.1
15204	1.12481	2.1
15205	1.29452	2.1
15206	1.25399	2.1
15207	1.12481	2.1
15208	0.918358	2.1
15209	1.39332	2.1
15210	1.32368	2.1
15211	1.11712	0.921757
15212	1.07999	0.936031
15213	1.07999	0.837475
15214	1.05619	0.899728
15215	1.1766	2.1
15216	1.35735	2.1
15217	1.42456	0.297297
15218	1.089	0.357305
15219	1.11081	0.19993
15220	2.88521	0.204374
15221	1.09754	0.196684
15222	1.85479	0.196914
15223	1.27922	0.964439
15224	1.03556	0.913011
15225	1.07508	1.34096
15226	1.09795	1.72753
15227	0.967672	1.01845
15228	1.07508	1.25512
15229	1.03933	1.46401
15230	1.02975	2.09972
15231	1.02975	1.55419
15232	1.01121	1.81847
15233	1.24526	0.208423
15234	1.72368	0.20159
15235	1.89607	0.203486
15236	2.13512	0.200091
15237	1.34591	0.742442
15238	1.14721	0.79316
15239	1.15539	1.22745
15240	1.90938	1.011
15241	1.90941	1.96327
15242	1.89242	1.34013
15243	1.18907	2.1
15244	1.20408	2.1
15245	1.20409	2.1
15246	1.22655	2.1
15247	0.701447	2.1
15248	1.57567	2.1
15249	1.38806	2.1
15250	2.09407	2.08023
15251	1.07612	1.08729

15252	0.990019	1.15944
15253	1.15564	1.4153
15254	1.01243	1.28652
15255	1.28956	0.421831
15256	1.35146	0.380279
15257	1.35145	0.398359
15258	1.22811	0.38703
15259	1.09662	2.1
15260	1.42486	2.1
15261	1.10106	0.192176
15262	1.90814	0.178882
15263	1.3946	0.194527
15264	1.11926	0.196046
15265	0.725839	2.1
15266	0.925068	2.1
15267	0.925074	2.1
15268	1.43282	2.1
15269	1.91756	1.12921
15270	1.74321	1.14519
15271	1.74321	1.05953
15272	1.63413	1.07859
15273	1.9194	0.517156
15274	1.62246	0.415778
15275	1.90456	0.374749
15276	1.06578	0.444073
15277	1.46019	0.426122
15278	1.17036	0.316503
15279	1.70233	1.41988
15280	1.53515	1.37181
15281	2.03494	2.1
15282	1.78441	2.1
15283	1.75992	2.1
15284	1.94351	2.1
15285	1.51057	0.289189
15286	0.991435	0.274254
15287	1.98068	0.339375
15288	1.64283	0.32383
15289	2.96126	0.320848
15290	2.20692	0.305349
15291	1.95054	0.262394
15292	1.21325	0.247841
15293	0.996828	0.66316
15294	1.01976	0.603842
15295	1.17129	2.1
15296	1.13664	2.1
15297	1.13664	2.1
15298	1.09627	2.1
15299	2.65123	2.1
15300	3.23062	2.1
15301	3.23062	2.1
15302	3.92038	2.1
15303	1.89019	0.65833
15304	1.79109	0.723206
15305	1.79109	0.595094
15306	1.54197	0.615218
15307	3.41824	1.17445
15308	1.93553	0.480491
15309	2.26215	1.11233

15310	2.0103	0.471718
15311	1.51569	1.29798
15312	1.01683	1.25546
15313	1.78595	2.1
15314	1.66537	2.1
15315	3.73426	2.1
15316	3.53905	2.1
15317	1.66537	2.1
15318	1.52903	2.1
15319	3.53905	2.1
15320	3.3837	2.1
15321	1.19901	0.915823
15322	1.35002	1.37716
15323	1.35002	0.757752
15324	1.11683	0.762558
15325	1.75326	1.15552
15326	0.712404	1.15043
15327	2.97768	2.1
15328	2.98949	2.1
15329	1.2832	2.1
15330	1.39518	2.1
15331	2.98949	2.1
15332	2.99339	2.1
15333	1.39517	2.1
15334	1.46124	2.1
15335	1.28534	2.1
15336	1.03391	2.1
15337	1.75366	1.17313
15338	1.26247	1.3666
15339	1.13621	1.76522
15340	2.43994	2.1
15341	1.47904	0.364632
15342	1.80085	0.378492
15343	1.80085	0.356687
15344	1.64656	0.41793
15345	2.87331	2.1
15346	2.82863	2.1
15347	2.82859	2.1
15348	2.84673	2.1
15349	1.0961	0.466758
15350	1.39448	0.593336
15351	1.21366	0.59227
15352	1.20143	0.434118
15353	1.85795	0.318562
15354	1.36768	0.291351
15355	1.36769	0.272162
15356	1.45789	0.256626
15357	1.32605	0.217866
15358	1.27148	0.22932
15359	1.70218	0.339093
15360	1.3063	0.482028
15361	1.45603	0.428194
15362	1.89226	0.556815
15363	1.2742	0.405993
15364	2.10112	0.325987
15365	1.10748	0.381978
15366	1.1893	0.383814
15367	1.04921	2.1

15368	1.22927	2.1
15369	1.22927	2.1
15370	1.46568	2.1
15371	0.811186	2.1
15372	0.792162	2.1
15373	0.792162	2.1
15374	0.758749	2.1
15375	1.03581	2.1
15376	0.737481	2.1
15377	2.24071	2.1
15378	1.34868	2.1
15379	1.47203	0.941592
15380	1.40967	0.990783
15381	1.40967	1.03203
15382	1.27916	1.0614
15383	1.17418	1.14526
15384	1.04921	2.1
15385	1.28291	1.42037
15386	1.3213	0.935855
15387	1.46812	1.21444
15388	2.42522	1.52829
15389	1.17879	0.881661
15390	1.28291	0.899873
15391	1.28291	0.885073
15392	1.13438	0.913354
15393	1.75004	2.1
15394	1.78247	2.1
15395	1.78247	2.1
15396	1.76421	2.1
15397	2.25975	0.196687
15398	3.66111	0.18903
15399	3.6611	0.174797
15400	3.44043	0.171971
15401	1.13339	2.1
15402	1.13322	2.1
15403	1.13323	2.1
15404	1.20761	2.1
15405	1.34337	0.69596
15406	1.90912	1.31008
15407	1.04399	0.993096
15408	2.00578	1.2661
15409	1.04973	2.1
15410	1.22615	1.74716
15411	1.93384	2.1
15412	2.62816	0.900208
15413	1.13127	2.1
15414	1.47642	1.48474
15415	2.62816	2.1
15416	3.31755	0.900721
15417	1.47642	2.1
15418	1.80454	1.28727
15419	0.964782	1.76181
15420	0.949135	2.1
15421	0.949134	1.78637
15422	0.949134	2.1
15423	1.10476	0.779729
15424	0.982858	0.2466
15425	1.10938	0.664455

15426	0.982858	0.537788
15427	1.01012	0.170044
15428	0.974081	0.166876
15429	1.82643	0.165348
15430	1.29585	0.165387
15431	1.00763	0.88504
15432	1.3294	0.84873
15433	1.3294	0.768102
15434	1.30001	0.998537
15435	1.59151	2.1
15436	1.99443	2.1
15437	1.07369	2.1
15438	1.30584	2.1
15439	1.05071	2.1
15440	0.948676	2.1
15441	1.13842	2.1
15442	1.42884	2.1
15443	1.59019	0.224204
15444	1.38212	0.249332
15445	1.34102	0.229361
15446	1.02663	0.24575
15447	0.797011	2.1
15448	0.81301	2.1
15449	0.81301	2.1
15450	0.811187	2.1
15451	0.991869	0.47086
15452	1.23868	0.463522
15453	1.67201	2.1
15454	1.61201	2.1
15455	1.61202	2.1
15456	1.57627	2.1
15457	0.94994	2.1
15458	1.77867	2.1
15459	0.854945	2.1
15460	0.973905	2.1
15461	1.64193	2.1
15462	1.68893	2.1
15463	1.68893	2.1
15464	1.70002	2.1
15465	2.08149	0.599759
15466	1.69456	0.514621
15467	1.67399	0.147815
15468	1.83922	1.21591
15469	1.91703	2.1
15470	0.967492	2.1
15471	1.13317	2.1
15472	0.980766	2.1
15473	2.06158	0.317706
15474	0.986498	0.287174
15475	1.76289	0.315221
15476	1.17248	0.289157
15477	1.51794	0.274276
15478	1.30266	0.291364
15479	1.03165	0.594562
15480	0.947692	0.636711
15481	1.20473	0.501886
15482	0.947693	0.477101
15483	1.54357	2.1

15484	0.980841	2.1
15485	1.34124	0.658559
15486	1.39818	0.809464
15487	1.39818	0.543767
15488	1.48035	0.765895
15489	1.03932	2.1
15490	1.2065	2.1
15491	1.20651	2.1
15492	1.12717	2.1
15493	1.09483	2.1
15494	0.994293	2.1
15495	0.994293	2.1
15496	0.97356	2.1
15497	0.995252	1.02013
15498	1.66294	1.00296
15499	1.82775	0.826185
15500	1.42762	0.724307
15501	1.04713	0.596976
15502	1.05475	0.562431
15503	1.29002	0.332426
15504	1.05475	0.380673
15505	1.17585	2.1
15506	1.39223	2.1
15507	1.39223	2.1
15508	1.71255	2.1
15509	1.56166	0.936542
15510	1.37762	0.882809
15511	1.16858	1.8813
15512	1.0361	2.1
15513	1.23966	0.185071
15514	1.10718	0.186597
15515	1.99957	0.171511
15516	0.658392	0.167748
15517	1.36904	0.180646
15518	1.12524	0.170604
15519	1.04321	1.29422
15520	1.04627	1.27838
15521	1.91351	0.87394
15522	1.63379	1.00881
15523	1.79718	1.08396
15524	0.59634	1.16909
15525	0.958257	2.1
15526	1.06457	2.1
15527	0.958255	2.1
15528	1.03933	2.1
15529	1.14195	0.82406
15530	1.49438	0.808257
15531	1.49438	0.797849
15532	1.31259	1.88463
15533	1.23036	2.1
15534	1.2016	2.1
15535	1.2016	2.1
15536	1.17129	2.1
15537	1.05724	0.934625
15538	1.04291	1.14797
15539	1.12341	2.1
15540	2.32525	2.1
15541	3.39038	2.1

15542	2.09374	2.1
15543	1.76185	0.727682
15544	1.7934	0.73791
15545	1.65809	0.730744
15546	1.54852	0.929326
15547	1.38766	2.1
15548	1.28765	2.1
15549	1.26599	2.1
15550	1.21397	2.1
15551	1.28765	2.1
15552	1.20045	2.1
15553	1.21398	2.1
15554	1.15234	2.1
15555	0.953634	0.447778
15556	1.39132	0.543903
15557	1.17285	2.1
15558	1.21408	2.1
15559	1.21408	2.1
15560	0.982848	2.1
15561	2.00101	2.1
15562	2.00973	2.1
15563	2.20314	2.1
15564	1.89318	2.1
15565	1.23267	1.431
15566	0.947921	2.1
15567	1.18872	2.1
15568	0.740752	2.1
15569	1.92269	0.699799
15570	1.53584	0.667761
15571	1.53584	0.69725
15572	1.21067	0.640984
15573	1.40707	0.301798
15574	1.50437	0.310282
15575	1.50435	0.290668
15576	1.45446	0.303486
15577	1.47954	0.881428
15578	2.08687	1.10871
15579	2.08687	1.67174
15580	1.1233	1.81598
15581	1.04025	0.922637
15582	1.36005	1.42547
15583	1.47606	0.43216
15584	1.3407	0.428275
15585	0.758743	2.1
15586	0.716917	2.1
15587	0.716922	2.1
15588	0.672964	2.1
15589	2.40014	2.1
15590	1.96848	1.48316
15591	1.96848	2.1
15592	1.53555	1.20168
15593	1.06191	2.1
15594	1.29579	2.1
15595	1.29579	2.1
15596	1.71289	2.1
15597	1.78584	2.1
15598	2.15211	2.1
15599	2.00427	2.1

15600	3.41615	2.1
15601	1.66408	2.1
15602	1.73954	2.1
15603	2.91663	2.1
15604	3.65818	2.1
15605	1.93286	2.1
15606	1.08831	2.1
15607	1.31242	0.259733
15608	1.11712	0.266948
15609	1.26311	0.265007
15610	1.31242	0.2682
15611	1.10378	2.1
15612	0.968594	2.1
15613	1.48389	0.224148
15614	1.49613	0.236029
15615	1.04349	0.746747
15616	1.26409	0.73559
15617	1.26409	0.724498
15618	1.05724	0.609625
15619	1.65723	0.37843
15620	1.83116	0.390566
15621	2.06284	0.394369
15622	2.00512	0.419093
15623	1.83116	0.38419
15624	2.03139	0.38677
15625	2.20564	0.407179
15626	1.90048	0.415734
15627	0.963392	2.1
15628	1.01346	0.930024
15629	2.98092	2.1
15630	2.70295	2.1
15631	2.70295	2.1
15632	2.48828	2.1
15633	1.63661	2.1
15634	1.64158	2.1
15635	1.64158	2.1
15636	1.6488	2.1
15637	1.17506	0.504797
15638	1.09667	0.468774
15639	2.00539	0.723487
15640	1.09667	0.592153
15641	1.54946	2.1
15642	1.43577	2.1
15643	1.26103	2.1
15644	1.49808	2.1
15645	1.089	0.303375
15646	1.22586	0.300461
15647	2.35027	0.611876
15648	1.37549	0.504777
15649	3.33706	0.470955
15650	1.69894	0.43105
15651	2.00539	0.564735
15652	1.53798	0.477018
15653	1.28938	2.1
15654	1.20969	2.1
15655	1.20969	2.1
15656	1.15864	2.1
15657	1.7986	2.1

15658	1.75869	2.1
15659	1.58281	2.1
15660	1.7392	2.1
15661	1.30733	0.915142
15662	1.5419	1.37974
15663	1.50883	1.12293
15664	1.85439	1.08249
15665	1.73531	1.07855
15666	1.70225	0.97442
15667	1.22176	0.663238
15668	1.59492	0.69805
15669	1.59492	0.675565
15670	2.15542	0.690183
15671	3.24379	2.1
15672	2.64775	2.1
15673	3.16707	2.1
15674	1.93032	2.1
15675	1.50738	2.1
15676	1.24128	2.1
15677	1.24128	2.1
15678	0.994293	2.1
15679	2.90808	0.159356
15680	2.45797	0.157174
15681	1.85501	0.161163
15682	2.90809	0.15896
15683	0.892835	1.11716
15684	1.55871	1.46367
15685	1.04615	1.063
15686	1.66643	0.804937
15687	1.70044	1.64138
15688	1.80224	2.1
15689	1.70186	0.854901
15690	3.30123	0.848335
15691	1.74789	2.1
15692	1.77446	2.1
15693	1.77901	2.1
15694	1.57995	2.1
15695	1.81518	2.1
15696	2.17716	2.1
15697	2.17716	2.1
15698	2.65123	2.1
15699	2.01899	1.58523
15700	1.77479	1.46723
15701	1.19122	1.10825
15702	1.46724	0.94761
15703	1.70944	0.215098
15704	1.08929	0.212541
15705	1.68454	0.215786
15706	1.08929	0.219266
15707	1.51067	1.06607
15708	1.06729	0.954593
15709	1.37762	0.9253
15710	1.13992	0.880148
15711	1.26397	2.1
15712	1.04083	2.1
15713	1.00278	2.1
15714	1.04084	2.1
15715	1.1016	0.295323

15716	1.23477	0.307646
15717	1.23477	0.232245
15718	1.64985	0.258445
15719	1.11117	2.1
15720	1.13006	2.1
15721	1.13006	2.1
15722	1.14721	2.1
15723	1.21946	2.1
15724	1.0186	2.1
15725	1.13178	2.1
15726	1.00133	2.1
15727	1.67046	0.698213
15728	0.986036	0.711981
15729	1.67046	0.71668
15730	1.34857	1.27077
15731	2.46016	2.1
15732	2.07161	2.1
15733	2.10732	2.1
15734	2.08371	2.1
15735	1.35602	2.1
15736	0.829065	2.1
15737	1.81667	2.1
15738	0.99406	2.1
15739	1.81667	2.1
15740	1.68073	2.1
15741	2.05736	2.1
15742	2.97311	1.82997
15743	2.14011	2.1
15744	1.23411	2.1
15745	1.13632	0.490359
15746	1.40862	0.436678
15747	1.28055	0.440623
15748	1.14305	0.396584
15749	1.12035	0.203434
15750	1.15483	0.199533
15751	1.94099	0.201439
15752	1.82126	0.197826
15753	1.15481	0.200922
15754	0.998957	0.19604
15755	2.10078	0.200377
15756	1.81617	0.197347
15757	1.27581	2.1
15758	0.968536	2.1
15759	1.33916	2.1
15760	1.27581	2.1
15761	1.32784	2.1
15762	1.4259	2.1
15763	1.70377	2.1
15764	1.54231	2.1
15765	3.77569	2.1
15766	3.42809	2.1
15767	1.54231	2.1
15768	1.51298	2.1
15769	3.55866	2.1
15770	2.86136	2.1
15771	1.83301	1.14253
15772	1.26371	1.02717
15773	1.98301	1.13674

15774	2.0609	1.05549
15775	2.26047	0.875215
15776	2.00537	0.822831
15777	1.7913	0.829839
15778	1.48864	0.770824
15779	1.09504	2.1
15780	1.39827	2.1
15781	2.28084	2.1
15782	1.05983	2.1
15783	1.33593	0.230678
15784	1.31508	0.21613
15785	1.31508	0.220804
15786	1.09599	0.212708
15787	1.30647	2.1
15788	1.28846	2.1
15789	1.28846	2.1
15790	1.26923	2.1
15791	1.2955	2.1
15792	1.09044	2.09314
15793	2.12209	2.1
15794	1.26438	2.1
15795	0.911819	2.1
15796	1.38752	2.1
15797	2.57704	2.1
15798	2.71736	2.1
15799	1.42284	2.1
15800	1.41562	2.1
15801	2.69131	2.1
15802	2.36178	2.1
15803	1.41562	2.1
15804	1.39564	2.1
15805	1.32368	2.1
15806	0.908053	2.1
15807	0.908053	2.1
15808	0.629213	2.1
15809	1.8758	1.51471
15810	1.27362	1.20334
15811	1.3756	0.891445
15812	2.28749	1.13585
15813	1.84577	1.05266
15814	2.17765	1.04992
15815	1.38969	0.928867
15816	1.84578	1.0154
15817	1.32801	0.824052
15818	1.76377	0.838141
15819	1.22845	1.43142
15820	1.57766	1.01292
15821	1.57766	1.97842
15822	1.39067	1.55715
15823	1.15743	0.345204
15824	1.37721	0.294039
15825	1.69055	0.419349
15826	1.14858	0.432215
15827	1.38333	1.13746
15828	1.10575	0.769174
15829	1.22418	1.93264
15830	1.10574	1.22218
15831	1.91509	0.592949

15832	1.02001	0.527086
15833	1.03074	0.857624
15834	1.19122	0.659711
15835	1.67887	2.1
15836	1.67597	2.1
15837	1.84984	2.1
15838	1.77775	2.1
15839	1.73571	1.86226
15840	1.27921	1.83921
15841	2.11851	1.36939
15842	1.27921	1.86452
15843	1.42486	2.1
15844	1.12503	2.1
15845	1.12503	2.1
15846	1.30116	2.1
15847	0.568995	2.1
15848	0.557348	2.1
15849	0.557348	2.1
15850	0.53907	2.1
15851	0.633575	2.1
15852	1.33756	1.04836
15853	1.51074	2.1
15854	1.93384	0.928639
15855	0.655202	2.1
15856	0.731934	2.1
15857	0.731934	2.1
15858	1.09061	2.1
15859	0.966979	2.1
15860	1.31502	2.1
15861	1.31502	2.1
15862	1.72116	2.1
15863	1.70002	2.1
15864	1.67969	2.1
15865	1.67969	2.1
15866	1.62408	2.1
15867	1.01531	2.1
15868	1.66018	2.1
15869	1.01531	2.1
15870	0.886968	2.1
15871	1.22585	0.314406
15872	1.20674	0.300351
15873	1.20672	0.297805
15874	1.02962	0.293406
15875	0.956467	1.50077
15876	2.00551	1.09952
15877	0.553801	2.1
15878	0.617006	2.1
15879	1.26923	2.1
15880	1.23614	2.1
15881	1.23614	2.1
15882	1.16817	2.1
15883	2.14482	2.1
15884	1.72637	1.95851
15885	1.09368	2.1
15886	1.26195	2.1
15887	0.760546	2.1
15888	1.84094	2.1
15889	2.62868	2.1

15890	2.79784	2.1
15891	2.79784	2.1
15892	3.00129	2.1
15893	1.27593	0.989759
15894	1.38877	1.41093
15895	1.06486	0.720224
15896	1.2474	0.549886
15897	1.76234	0.218272
15898	1.8685	0.244245
15899	1.69863	0.244815
15900	1.29666	0.243167
15901	1.91646	2.1
15902	1.8582	2.1
15903	1.8582	2.1
15904	1.7986	2.1
15905	3.44043	0.167773
15906	1.94764	0.165587
15907	1.94764	0.175072
15908	1.36307	0.172217
15909	1.32075	0.254771
15910	1.12971	0.236084
15911	2.06319	0.280745
15912	1.94982	0.247302
15913	1.77255	0.292994
15914	0.979297	0.242617
15915	1.59997	2.1
15916	1.93506	2.1
15917	1.94409	2.1
15918	1.9597	2.1
15919	0.57899	2.1
15920	0.953036	2.1
15921	0.953049	2.1
15922	1.53503	2.1
15923	1.39615	0.284626
15924	1.06474	0.309338
15925	0.947564	0.339591
15926	1.45506	0.309048
15927	1.17799	0.699658
15928	1.21067	0.696246
15929	1.35945	0.597006
15930	2.05648	0.67011
15931	1.36208	0.629458
15932	2.15229	0.540564
15933	2.89288	0.513029
15934	1.95719	0.530527
15935	2.25169	0.443596
15936	1.52756	0.486515
15937	1.19322	0.875786
15938	1.23146	0.70318
15939	2.45024	1.03111
15940	0.794122	0.747182
15941	1.6343	0.717173
15942	1.34161	0.581934
15943	1.05353	0.896051
15944	1.50795	0.600754
15945	1.05353	0.528509
15946	1.20669	0.573724
15947	1.59289	2.1

15948	0.989543	2.1
15949	1.40588	2.1
15950	0.991427	2.1
15951	0.921822	1.30576
15952	1.46903	0.852391
15953	1.46903	1.88325
15954	1.8214	0.777804
15955	1.39702	2.1
15956	1.25103	2.1
15957	1.25103	2.1
15958	1.10378	2.1
15959	1.71813	2.1
15960	1.39595	2.1
15961	1.386 2.1	
15962	1.13008	2.1
15963	1.31711	0.2154
15964	1.70191	0.218418
15965	0.893249	0.227768
15966	1.42669	0.226779
15967	1.23943	1.12111
15968	1.1483	0.96546
15969	1.04598	2.1
15970	1.1483	1.22664
15971	1.00381	0.687957
15972	1.11433	0.674395
15973	0.973501	1.00568
15974	1.0756	0.988265
15975	1.20669	0.561365
15976	1.18508	0.56023
15977	1.18508	0.562706
15978	0.983027	0.548596
15979	1.43687	2.1
15980	1.71059	2.1
15981	1.71059	2.1
15982	1.66762	2.1
15983	2.8415	2.1
15984	2.40555	2.1
15985	1.91546	2.1
15986	1.61385	2.1
15987	2.01569	2.1
15988	1.33183	2.1
15989	1.23218	2.1
15990	1.23999	2.1
15991	1.01335	0.817209
15992	1.12088	0.890203
15993	1.25346	1.39643
15994	1.15644	1.85392
15995	1.3764	1.02668
15996	1.27558	0.924649
15997	1.2064	2.01597
15998	1.15067	2.1
15999	1.01595	2.1
16000	1.15067	2.1
16001	1.41902	1.25823
16002	1.30272	2.1
16003	1.30272	1.04456
16004	1.19548	2.1
16005	2.99339	2.1

16006	2.98041	2.1
16007	1.46124	2.1
16008	1.42105	2.1
16009	2.98041	2.1
16010	2.86339	2.1
16011	1.42105	2.1
16012	1.42284	2.1
16013	2.31301	1.08643
16014	1.80273	1.07393
16015	2.17765	1.11605
16016	2.31301	1.01586
16017	1.76378	0.857696
16018	2.10275	0.825863
16019	2.10275	0.864567
16020	2.26047	0.845446
16021	0.97541	2.1
16022	1.40863	2.1
16023	1.40863	2.1
16024	1.30751	2.1
16025	1.20143	0.508174
16026	1.19756	0.42911
16027	1.43707	0.433146
16028	1.28954	0.379897
16029	3.34254	0.364687
16030	1.92916	0.259956
16031	1.80089	0.342164
16032	1.1323	0.272266
16033	1.47321	0.411907
16034	1.23756	0.373634
16035	1.87291	2.06523
16036	1.53642	2.1
16037	1.53642	2.1
16038	1.5791	2.1
16039	1.0446	2.1
16040	0.992674	2.1
16041	1.12324	0.170656
16042	1.29163	0.165058
16043	1.29163	0.162925
16044	1.15371	0.161861
16045	0.996831	0.446351
16046	1.00192	0.492394
16047	1.0019	0.440578
16048	0.958937	0.483099
16049	1.07283	2.1
16050	1.25294	1.10311
16051	1.25294	1.14037
16052	1.04788	1.1454
16053	1.14496	2.1
16054	1.07652	2.1
16055	1.07652	2.1
16056	0.991406	2.1
16057	1.60976	2.1
16058	1.37	2.1
16059	1.37	2.1
16060	1.14195	2.1
16061	1.26593	1.43309
16062	1.75366	2.06194
16063	1.57621	0.595336

16064	1.24538	0.620188
16065	0.969118	2.1
16066	1.04147	2.03564
16067	0.969118	2.1
16068	1.22615	2.1
16069	0.98084	2.1
16070	1.01843	2.1
16071	1.09435	2.1
16072	1.50876	2.1
16073	1.5976	2.1
16074	1.84322	2.1
16075	1.84324	2.1
16076	1.70377	2.1
16077	1.20709	2.1
16078	2.54729	2.1
16079	0.974716	0.400962
16080	1.54468	0.538258
16081	1.39021	0.37098
16082	1.70303	0.501332
16083	1.22852	1.23155
16084	1.578	0.93741
16085	1.578	1.30925
16086	1.93852	0.842346
16087	2.2506	2.1
16088	1.39302	1.8824
16089	1.5478	2.1
16090	1.2269	2.1
16091	1.70048	2.1
16092	1.45477	1.29867
16093	1.30929	2.1
16094	1.15069	1.25673
16095	1.00255	1.53043
16096	1.05481	0.748205
16097	1.05481	1.7761
16098	1.1032	0.737095
16099	0.993888	2.1
16100	1.19294	2.1
16101	1.01121	0.357405
16102	0.991866	0.414189
16103	2.20052	1.00649
16104	1.7409	2.1
16105	1.75142	1.94063
16106	1.46811	2.1
16107	1.7409	1.17826
16108	1.23267	2.1
16109	1.46811	2.1
16110	1.18872	2.1
16111	1.12665	2.1
16112	1.50045	2.1
16113	1.50045	2.1
16114	1.37704	2.1
16115	1.35308	0.832012
16116	1.29983	0.721891
16117	1.29983	0.809994
16118	0.970212	0.730842
16119	1.82272	2.1
16120	1.62784	2.1
16121	1.62784	2.1

16122	1.87691	2.1
16123	3.65294	2.1
16124	2.33374	2.1
16125	2.12888	2.1
16126	1.62229	2.1
16127	1.41496	2.0323
16128	1.32707	0.953009
16129	2.18871	0.897817
16130	1.4235	0.554715
16131	1.46015	0.390414
16132	1.25635	0.402499
16133	1.45501	0.616235
16134	1.3543	0.595258
16135	1.38139	1.9756
16136	1.98159	2.1
16137	2.15868	1.98825
16138	4.04308	2.1
16139	1.5362	2.1
16140	1.56328	2.1
16141	1.56327	2.1
16142	1.58163	2.1
16143	1.88987	0.35609
16144	1.08761	0.310534
16145	0.862154	0.292219
16146	1.77688	0.302914
16147	1.14238	1.68237
16148	1.11691	2.1
16149	1.01537	1.61902
16150	1.02923	2.1
16151	0.977425	0.320263
16152	1.15211	0.268458
16153	2.0815	2.1
16154	1.18621	2.1
16155	1.01485	2.1
16156	1.17054	2.1
16157	1.17054	2.1
16158	1.12665	2.1
16159	1.87213	0.215355
16160	1.82244	0.216374
16161	1.45039	0.228321
16162	1.7226	0.238111
16163	1.841	0.215061
16164	1.79011	0.214822
16165	1.8792	0.235066
16166	1.45053	0.240873
16167	1.08947	2.1
16168	1.5008	2.1
16169	1.5008	2.1
16170	1.83186	2.1
16171	1.04159	0.239169
16172	1.2489	0.235281
16173	1.2489	0.238219
16174	1.33592	0.230764
16175	1.51973	0.585698
16176	0.980547	0.60351
16177	1.51974	0.616221
16178	1.15991	0.678048
16179	1.34965	2.1

16180	1.2724	2.1
16181	1.2724	2.1
16182	1.38438	2.1
16183	1.02923	1.68766
16184	1.06521	2.1
16185	1.06521	1.79557
16186	1.0752	2.1
16187	2.03139	0.38581
16188	1.92951	0.370274
16189	1.74852	0.414755
16190	1.62772	0.3982
16191	1.73655	0.374652
16192	1.76809	0.355018
16193	1.62772	0.399648
16194	1.1432	0.376161
16195	1.09235	2.1
16196	1.48081	2.1
16197	1.46712	2.1
16198	1.89321	2.1
16199	1.94548	2.1
16200	1.5636	2.1
16201	1.5636	2.1
16202	1.21124	2.1
16203	1.52314	2.1
16204	2.0219	2.1
16205	1.51113	2.1
16206	2.0219	2.1
16207	0.987247	0.72859
16208	1.18347	0.537839
16209	1.89226	0.793187
16210	1.61441	0.530932
16211	1.42884	2.1
16212	1.10961	2.1
16213	1.56991	2.1
16214	1.05337	2.1
16215	1.05337	2.1
16216	1.91547	2.1
16217	1.05931	2.1
16218	1.1729	2.1
16219	1.34526	2.1
16220	1.0593	2.1
16221	1.0856	2.1
16222	1.90854	2.1
16223	1.91674	2.1
16224	1.11345	2.1
16225	1.75067	2.1
16226	1.50022	2.1
16227	1.50022	2.1
16228	1.53619	2.1
16229	1.4748	2.1
16230	1.74431	2.00619
16231	2.02581	1.9376
16232	1.25288	1.89595
16233	1.01814	0.9162
16234	1.2881	0.782876
16235	1.2881	0.812218
16236	1.54433	0.756763
16237	1.19999	0.780196

16238	1.44056	0.696967
16239	1.02011	0.520365
16240	1.17129	0.60451
16241	1.13775	2.1
16242	3.34815	2.1
16243	1.60428	1.89248
16244	1.85928	2.03408
16245	1.75592	2.1
16246	1.25989	2.1
16247	1.75592	2.1
16248	1.71549	2.1
16249	1.37634	2.1
16250	1.64693	2.1
16251	1.64693	2.1
16252	1.64193	2.1
16253	1.18351	0.240224
16254	2.19398	0.227922
16255	2.16272	1.31725
16256	1.19837	1.29668
16257	1.04973	2.1
16258	1.20149	2.1
16259	1.60574	2.1
16260	1.11164	1.00526
16261	1.87823	0.956917
16262	1.11164	1.06315
16263	0.772882	2.1
16264	1.4714	2.1
16265	2.14242	0.973602
16266	1.12336	1.95015
16267	2.8386	0.906164
16268	2.13346	2.04108
16269	1.06788	0.325158
16270	0.947662	0.304862
16271	1.05297	0.26819
16272	1.42907	0.299773
16273	2.17465	2.1
16274	2.18394	2.1
16275	1.14808	2.1
16276	1.21292	2.1
16277	1.21292	2.1
16278	1.27229	2.1
16279	1.20859	0.861825
16280	1.21073	0.887316
16281	1.21073	0.779847
16282	1.22857	0.876618
16283	1.79158	2.1
16284	1.73899	2.1
16285	1.34012	2.1
16286	1.39991	2.1
16287	1.73898	2.1
16288	1.61245	2.1
16289	1.40136	2.1
16290	1.57234	2.1
16291	1.82162	2.1
16292	3.09875	2.1
16293	1.10264	2.1
16294	1.02339	2.1
16295	1.02339	2.1

16296	1.23042	2.1
16297	2.82266	0.850387
16298	1.05698	0.758952
16299	1.96384	0.880627
16300	1.10244	0.841174
16301	0.929061	2.1
16302	1.47007	2.1
16303	0.985303	2.1
16304	1.24554	2.1
16305	1.05993	2.1
16306	2.02074	2.1
16307	2.10106	0.91368
16308	1.18171	0.888234
16309	1.68111	0.928783
16310	1.84226	0.854748
16311	1.82131	0.469737
16312	1.32371	0.432524
16313	1.41902	0.372409
16314	1.00205	0.334568
16315	1.65532	2.1
16316	1.18977	2.1
16317	1.15471	2.1
16318	0.993889	2.1
16319	2.08521	2.1
16320	2.09885	2.1
16321	2.09497	2.1
16322	2.10604	2.1
16323	0.881028	1.0996
16324	1.52828	0.923515
16325	1.40574	0.208465
16326	1.09599	0.203566
16327	1.45506	2.1
16328	1.50524	2.1
16329	1.50524	2.1
16330	1.54711	2.1
16331	1.41469	2.1
16332	2.40442	2.1
16333	1.40794	2.1
16334	1.89226	2.1
16335	1.31346	2.1
16336	1.51067	2.1
16337	1.31363	2.1
16338	1.11833	2.1
16339	2.3373	2.1
16340	2.19994	2.1
16341	2.19994	2.1
16342	1.09616	2.1
16343	1.63719	2.1
16344	1.52204	2.1
16345	1.52205	2.1
16346	1.40118	2.1
16347	2.5946	2.1
16348	4.07065	1.94233
16349	1.33916	2.1
16350	2.05217	2.1
16351	1.67023	2.1
16352	2.05217	1.97229
16353	1.59458	0.809311

16354	1.02247	0.7759
16355	1.68108	0.74408
16356	1.03079	0.729157
16357	1.1643	0.477165
16358	1.85795	0.449532
16359	1.70302	0.5017
16360	1.42076	0.421947
16361	1.91351	0.444542
16362	1.01148	0.423745
16363	2.52402	0.222008
16364	0.752625	0.215309
16365	0.886968	2.1
16366	1.28845	2.1
16367	1.28845	2.1
16368	1.27766	2.1
16369	1.82268	2.1
16370	1.87118	2.1
16371	1.87119	2.1
16372	1.91704	2.1
16373	3.13938	2.1
16374	2.97839	2.1
16375	2.97839	2.1
16376	2.87331	2.1
16377	1.73443	0.965713
16378	1.59457	0.869232
16379	2.01827	2.1
16380	2.04586	2.1
16381	2.04586	2.1
16382	2.00865	2.1
16383	1.19901	1.39691
16384	1.16661	2.1
16385	1.13215	0.559883
16386	1.96256	0.483144
16387	2.34043	0.199868
16388	2.04154	0.196632
16389	1.58991	0.193646
16390	1.45893	0.19368
16391	2.56818	0.198384
16392	1.70532	0.194269
16393	1.17798	0.698721
16394	1.25796	0.709225
16395	1.25797	0.711278
16396	1.10719	0.7685
16397	1.51067	2.1
16398	1.60707	2.1
16399	2.06946	1.55988
16400	3.19218	2.1
16401	2.19965	2.1
16402	1.30521	2.09505
16403	2.23684	1.27643
16404	1.43363	1.58199
16405	1.51007	2.1
16406	2.46356	2.1
16407	1.90428	2.1
16408	1.13618	2.1
16409	2.13276	0.354042
16410	0.998345	0.323744
16411	1.2861	0.376003

16412	1.20457	0.353709
16413	1.51789	2.1
16414	1.49872	2.1
16415	1.49872	2.1
16416	1.47712	2.1
16417	1.87129	0.568459
16418	0.949364	0.547518
16419	1.83142	0.564206
16420	1.55063	0.526715
16421	1.03298	0.616053
16422	0.980664	0.762585
16423	1.51696	0.510451
16424	1.34927	0.70169
16425	1.70302	0.820228
16426	1.65952	0.682931
16427	1.76658	0.596063
16428	0.986848	0.496599
16429	2.13757	1.33123
16430	1.98142	0.899197
16431	1.98142	1.17892
16432	1.65411	0.915982
16433	1.1323	0.389976
16434	1.46015	0.384483
16435	2.15376	2.02859
16436	1.64622	2.02739
16437	0.991867	0.672249
16438	1.58074	0.577732
16439	1.69368	2.1
16440	1.76403	2.1
16441	1.76403	2.1
16442	1.83318	2.1
16443	1.28418	0.821311
16444	1.16851	0.689005
16445	1.1685	0.656272
16446	1.1726	0.564872
16447	1.06455	2.1
16448	1.18773	2.1
16449	0.98511	2.1
16450	1.18774	2.1
16451	1.76476	2.1
16452	1.79825	2.1
16453	1.79825	2.1
16454	0.961895	2.1
16455	1.59589	0.202333
16456	2.25975	0.214948
16457	2.76339	2.1
16458	3.0551	2.1
16459	1.68761	2.1
16460	1.59561	2.1
16461	2.17257	2.1
16462	1.05845	2.1
16463	1.03512	2.1
16464	2.19011	2.1
16465	1.44859	1.52457
16466	1.35897	0.78248
16467	1.35897	1.37963
16468	1.17822	0.918004
16469	1.13641	2.1

16470	1.18301	2.1
16471	1.18301	2.01773
16472	1.25173	2.1
16473	2.41297	2.1
16474	1.36463	2.1
16475	1.54711	2.1
16476	1.5735	2.1
16477	1.5735	2.1
16478	1.58713	2.1
16479	1.69044	0.21043
16480	1.57979	0.214912
16481	1.57979	0.21168
16482	1.14387	0.216291
16483	1.04199	2.1
16484	1.96491	2.1
16485	1.04199	2.1
16486	1.46892	2.1
16487	0.983724	2.1
16488	1.70579	2.1
16489	1.88522	2.1
16490	1.23914	2.04536
16491	1.79424	2.1
16492	1.52851	2.1
16493	1.04921	2.1
16494	1.11285	2.1
16495	1.25334	0.883902
16496	1.07496	0.812041
16497	1.07497	1.75373
16498	1.34852	0.861267
16499	1.27391	2.08478
16500	2.20307	2.1
16501	1.81311	2.1
16502	2.10305	2.1
16503	1.21059	0.65016
16504	1.44253	0.692672
16505	1.9194	0.436897
16506	1.21159	0.582339
16507	0.947979	2.1
16508	1.01225	0.849349
16509	0.947979	2.1
16510	1.01225	1.56374
16511	1.00255	1.93602
16512	1.01225	1.87931
16513	1.01225	2.1
16514	1.06561	1.62213
16515	3.50562	2.1
16516	3.61336	2.1
16517	3.61336	2.1
16518	3.73635	2.1
16519	1.61245	2.1
16520	1.52301	2.1
16521	1.57612	2.1
16522	1.83035	2.1
16523	1.52301	2.1
16524	1.24789	2.1
16525	1.85156	2.1
16526	1.89072	2.1
16527	0.995735	2.1

16528	1.57738	2.1
16529	1.87141	0.281615
16530	1.73388	0.325436
16531	1.61344	0.323222
16532	0.922676	0.370215
16533	1.56614	2.1
16534	0.956294	2.1
16535	1.82589	2.1
16536	1.63719	2.1
16537	1.12399	0.215547
16538	1.50103	0.245119
16539	1.56976	0.220705
16540	1.03567	0.223886
16541	1.21776	0.202921
16542	1.89708	0.202524
16543	1.16323	0.199325
16544	1.63533	0.193875
16545	2.29397	0.20241
16546	3.01596	0.200224
16547	1.80145	0.196811
16548	1.7666	0.194133
16549	1.91621	0.771648
16550	1.10719	0.710369
16551	1.57828	0.961403
16552	1.17105	1.03598
16553	1.15806	2.1
16554	1.31565	2.1
16555	1.31565	2.1
16556	1.46643	2.1
16557	1.47507	2.1
16558	1.87858	2.1
16559	0.958661	2.1
16560	1.87858	2.1
16561	1.44293	0.328792
16562	1.43213	0.42321
16563	1.43213	0.325673
16564	1.10476	0.40016
16565	1.03235	1.6061
16566	1.31	0.758435
16567	1.31	1.72287
16568	1.50108	0.867558
16569	1.32215	2.1
16570	1.10611	2.1
16571	1.10611	2.1
16572	0.904978	2.1
16573	1.06486	0.707434
16574	1.18347	0.546511
16575	1.70535	2.1
16576	1.94779	2.1
16577	1.77071	2.1
16578	1.82268	2.1
16579	0.61701	2.1
16580	0.672495	2.1
16581	0.672494	2.1
16582	0.713174	2.1
16583	1.40395	2.1
16584	1.34245	2.1
16585	1.34243	2.1

16586	1.30646	2.1
16587	1.06204	0.45743
16588	0.632691	0.666976
16589	1.75766	0.700004
16590	1.14287	0.843798
16591	1.52101	0.408735
16592	1.71414	0.445514
16593	1.86411	0.388969
16594	1.6253	0.44969
16595	1.04355	0.416249
16596	1.0443	0.483656
16597	1.34852	1.64329
16598	1.56016	0.777192
16599	1.56016	0.790979
16600	1.03235	0.796697
16601	1.2966	0.315604
16602	1.82354	0.266207
16603	1.2966	0.240402
16604	1.67687	0.249692
16605	1.19669	1.02485
16606	1.82979	1.08424
16607	1.58972	1.15165
16608	1.09219	1.09704
16609	1.42077	1.10338
16610	1.89226	1.00442
16611	1.19547	0.924996
16612	1.10187	2.1
16613	1.10188	0.893692
16614	1.01479	2.1
16615	1.81663	2.1
16616	1.81533	2.1
16617	1.83247	2.1
16618	1.51511	2.1
16619	0.983148	2.1
16620	1.7513	2.1
16621	1.85017	2.1
16622	1.26103	2.07975
16623	1.23152	2.1
16624	1.65508	2.1
16625	1.23152	2.1
16626	2.15082	2.1
16627	1.57974	2.1
16628	1.07289	2.1
16629	0.81534	2.1
16630	1.51007	2.1
16631	1.00898	0.21344
16632	1.18959	0.210954
16633	1.32178	0.198155
16634	1.25929	0.196442
16635	2.17892	0.476193
16636	1.22938	0.376326
16637	3.02933	0.723207
16638	2.34619	0.583551
16639	1.47679	0.534335
16640	1.0819	0.591048
16641	3.10558	2.1
16642	3.31457	2.1
16643	0.985109	2.1

16644	1.10667	2.1
16645	3.31457	2.1
16646	3.54443	2.1
16647	1.10667	2.1
16648	1.25987	2.1
16649	1.77063	1.11383
16650	1.51065	0.994144
16651	1.24688	1.76739
16652	1.37652	2.06872
16653	1.37652	1.45172
16654	1.54168	2.1
16655	1.17042	0.973706
16656	1.13092	1.18561
16657	1.13092	1.3072
16658	1.52938	1.8917
16659	1.11787	0.569239
16660	1.00613	0.825654
16661	0.781373	0.511294
16662	1.6885	0.465448
16663	2.49439	0.406244
16664	1.42657	0.460521
16665	1.77903	1.4323
16666	2.63606	2.1
16667	2.10425	2.1
16668	1.21425	1.24746
16669	1.31253	0.999801
16670	1.28418	1.02826
16671	1.28418	1.09103
16672	1.58972	1.1211
16673	0.858948	2.1
16674	1.90196	2.1
16675	1.81024	2.1
16676	1.90194	2.1
16677	1.4263	0.966804
16678	0.947921	0.944532
16679	1.22852	0.81652
16680	1.09194	0.767734
16681	1.60578	0.33092
16682	1.14721	0.3365
16683	1.06336	0.40731
16684	1.14721	0.363729
16685	1.11924	0.202952
16686	1.53814	0.209168
16687	1.18421	0.247932
16688	1.8458	0.218846
16689	1.7565	1.87176
16690	1.09344	1.40987
16691	1.09344	1.14202
16692	1.16971	1.72878
16693	1.71465	0.228679
16694	1.19412	0.212567
16695	1.64985	0.34976
16696	1.05417	0.331538
16697	1.2127	0.206368
16698	1.48867	0.205924
16699	1.27806	0.211889
16700	1.21269	0.200227
16701	1.37813	0.984591

16702	1.0417	1.00568
16703	1.88626	0.786529
16704	1.14977	0.922444
16705	1.78803	1.57633
16706	1.33793	1.10625
16707	1.42266	1.2784
16708	1.26865	1.13818
16709	1.09518	2.1
16710	1.36844	2.1
16711	1.20455	2.1
16712	1.36844	2.1
16713	1.22297	1.20147
16714	1.11256	1.25585
16715	1.11256	1.16537
16716	1.03217	1.21384
16717	1.18422	0.227398
16718	1.4282	0.236973
16719	1.04159	0.265625
16720	1.4282	0.22949
16721	0.988805	2.1
16722	1.05481	2.1
16723	1.52938	0.999782
16724	1.26593	1.23404
16725	1.53082	2.1
16726	1.09847	2.1
16727	1.75211	0.887421
16728	1.22222	1.65157
16729	1.75211	0.833038
16730	1.51592	1.6301
16731	1.63413	0.975801
16732	1.26662	0.979678
16733	1.42495	0.845009
16734	1.4356	0.886655
16735	1.29389	1.32499
16736	1.04164	1.77847
16737	1.40574	1.304
16738	1.04164	1.14648
16739	2.22344	0.851573
16740	1.45945	0.573014
16741	0.987248	0.737425
16742	1.2474	0.571731
16743	0.974702	0.871804
16744	1.43777	0.783036
16745	1.71289	2.1
16746	1.91529	2.1
16747	1.91529	2.1
16748	2.01827	2.1
16749	1.87111	1.18874
16750	1.00864	0.80684
16751	1.26865	0.877096
16752	1.18769	1.19697
16753	1.24167	1.81492
16754	1.36133	0.908591
16755	2.28168	2.1
16756	3.98524	2.03172
16757	1.36133	1.73386
16758	1.69436	0.929835
16759	2.21926	1.45213

16760	1.70302	2.00518
16761	1.50732	0.399873
16762	1.42237	0.426384
16763	1.18659	0.43859
16764	1.50732	0.45228
16765	1.19785	0.309904
16766	1.40969	0.288356
16767	1.28339	0.368773
16768	1.40882	0.365293
16769	0.968595	1.73801
16770	1.41903	1.19926
16771	1.12567	2.1
16772	1.15898	2.1
16773	1.27241	2.1
16774	0.995796	2.1
16775	1.89983	0.213775
16776	1.05417	0.215711
16777	0.571999	0.205509
16778	0.743597	0.205144
16779	1.89353	0.214467
16780	0.924068	0.202118
16781	1.52851	2.1
16782	1.26969	2.1
16783	1.07763	0.651447
16784	1.82301	0.606232
16785	1.07763	0.476382
16786	1.17506	0.497777
16787	1.20045	2.1
16788	1.12486	2.1
16789	1.15234	2.1
16790	1.11598	2.1
16791	1.12486	2.1
16792	1.0491	2.1
16793	1.11598	2.1
16794	1.1047	2.1
16795	1.05902	2.1
16796	1.14361	2.1
16797	1.14361	2.1
16798	1.26851	2.1
16799	1.29389	1.12606
16800	0.988456	1.0766
16801	1.83688	0.210944
16802	1.70302	0.248129
16803	1.49386	0.221744
16804	1.83151	0.244451
16805	1.01346	0.909697
16806	0.983607	0.946173
16807	0.983596	2.1
16808	1.08332	1.01192
16809	2.36848	0.389682
16810	2.97066	0.337873
16811	1.12352	0.329201
16812	2.5311	0.286185
16813	1.09062	2.1
16814	0.962182	2.1
16815	0.962181	2.1
16816	1.2258	2.1
16817	0.974888	2.1

16818	1.06312	2.1
16819	1.73583	0.280049
16820	1.72399	0.278448
16821	1.724	0.343847
16822	1.50951	0.337231
16823	1.26755	2.1
16824	1.03824	2.1
16825	1.27875	2.1
16826	1.03824	2.1
16827	1.18902	2.1
16828	1.534	2.1
16829	1.11764	2.1
16830	1.534	2.1
16831	1.52008	2.1
16832	1.34406	2.1
16833	1.34406	2.1
16834	1.2187	2.1
16835	0.963571	0.980911
16836	1.19612	0.909446
16837	0.963571	1.54438
16838	1.00007	1.44098
16839	0.903333	2.1
16840	1.12724	2.1
16841	1.23771	2.1
16842	1.17683	2.1
16843	1.20059	0.923792
16844	0.772023	0.729997
16845	1.14749	0.964741
16846	1.2802	0.948712
16847	1.85091	1.3255
16848	2.11193	1.13709
16849	1.2802	0.916468
16850	1.42913	0.916149
16851	2.11193	1.07662
16852	1.27737	0.947619
16853	1.30584	2.1
16854	1.60936	2.1
16855	1.3413	0.397679
16856	1.53336	0.311786
16857	2.60679	0.337111
16858	1.3413	0.297264
16859	1.35877	1.9761
16860	1.11024	2.02586
16861	1.27839	2.1
16862	1.08171	2.1
16863	1.27841	2.1
16864	1.0577	2.1
16865	1.71255	2.1
16866	1.10611	2.1
16867	0.960106	2.1
16868	1.40921	2.1
16869	1.90705	0.701704
16870	2.79564	0.688548
16871	1.45321	0.706511
16872	1.90705	0.699719
16873	0.974718	0.577382
16874	1.53671	0.552922
16875	1.69039	0.557536

16876	1.78229	0.5204
16877	1.0101	2.1
16878	1.3167	2.1
16879	1.19699	2.1
16880	1.3212	2.1
16881	1.60811	0.632981
16882	1.43224	0.540008
16883	1.35401	0.525159
16884	1.17464	0.436871
16885	1.27696	2.1
16886	1.05025	2.1
16887	1.05025	2.1
16888	1.18076	2.1
16889	1.37224	1.22856
16890	1.13438	1.19841
16891	1.76788	1.19073
16892	1.73444	1.17293
16893	1.37704	2.1
16894	1.77042	2.1
16895	1.77042	2.1
16896	1.09778	2.1
16897	1.28667	2.1
16898	1.28228	2.1
16899	1.30646	0.701091
16900	1.68867	0.862078
16901	1.30645	1.01627
16902	0.986494	1.47764
16903	1.6253	0.407608
16904	1.48676	0.444774
16905	1.48676	0.426069
16906	1.32974	0.443974
16907	0.634347	0.147815
16908	1.15142	0.185432
16909	1.11395	0.179405
16910	1.18068	0.17683
16911	1.36904	0.169315
16912	0.764563	0.164867
16913	2.09213	0.16479
16914	1.56631	2.1
16915	1.08011	2.1
16916	1.80283	2.1
16917	1.50738	2.1
16918	1.44951	0.322204
16919	1.50102	0.33059
16920	1.50102	0.296128
16921	1.39615	0.306468
16922	1.02653	0.666993
16923	2.98258	0.713888
16924	2.0815	0.236798
16925	1.39525	0.205402
16926	1.82176	0.204821
16927	0.977607	0.208619
16928	1.96133	1.46256
16929	1.27189	1.23415
16930	0.826032	1.0754
16931	2.12831	1.05592
16932	1.77798	1.72065
16933	1.02969	1.38686

16934	1.06463	1.26948
16935	1.49219	1.75817
16936	1.10003	2.1
16937	1.21223	1.97853
16938	1.00513	0.992686
16939	1.43788	1.24675
16940	1.47134	0.803384
16941	0.968249	0.815521
16942	3.22585	0.358867
16943	3.36933	0.322851
16944	3.36932	0.380499
16945	3.03865	0.285147
16946	1.66295	0.376292
16947	1.65583	0.335498
16948	1.65583	0.464838
16949	1.4732	0.354908
16950	1.73704	0.147815
16951	1.76476	1.16961
16952	2.13345	0.989343
16953	1.55816	2.05509
16954	1.55816	1.07435
16955	1.02601	2.1
16956	1.08039	1.45577
16957	1.05498	1.04727
16958	1.08039	2.1
16959	1.2873	2.1
16960	1.01121	1.87028
16961	0.987703	2.02645
16962	0.98771	1.78655
16963	0.964782	2.02994
16964	1.35719	2.1
16965	2.61092	2.1
16966	2.6607	2.1
16967	3.41693	2.1
16968	1.23125	2.1
16969	1.73772	2.1
16970	0.99845	0.803859
16971	1.5176	0.843073
16972	1.13271	0.262855
16973	1.332	0.257068
16974	1.63159	2.1
16975	1.61342	2.1
16976	1.03679	2.1
16977	1.19087	2.1
16978	1.97172	2.1
16979	2.68634	2.1
16980	1.19086	2.1
16981	1.50091	2.1
16982	2.84677	2.1
16983	2.77894	2.1
16984	2.77894	2.1
16985	2.74206	2.1
16986	1.3284	2.1
16987	1.83524	2.07498
16988	1.65853	2.1
16989	1.56741	1.80522
16990	1.00509	2.1
16991	1.45246	2.1

16992	1.26193	2.1
16993	1.45244	2.1
16994	1.61963	0.620951
16995	1.68189	0.789702
16996	1.68189	1.03067
16997	1.49155	0.715781
16998	1.58054	2.1
16999	1.59169	2.1
17000	1.59169	2.1
17001	1.60497	2.1
17002	0.743595	0.211236
17003	0.702359	0.210214
17004	1.15317	0.232885
17005	2.24367	0.238632
17006	1.15252	0.232738
17007	2.60354	0.21553
17008	1.45158	2.1
17009	1.56004	2.1
17010	1.64527	2.1
17011	1.00371	2.1
17012	1.38287	0.632683
17013	0.893024	0.753
17014	1.23527	0.761561
17015	1.23706	0.718904
17016	2.20993	0.7322
17017	1.54858	0.889415
17018	0.798666	0.481452
17019	1.22487	0.521604
17020	1.33158	0.429624
17021	1.85902	0.496893
17022	1.08334	2.1
17023	1.17885	1.13729
17024	1.17885	0.945801
17025	1.07283	1.06078
17026	1.30723	0.224016
17027	1.62507	0.208204
17028	1.62507	0.201995
17029	1.08854	0.209795
17030	1.59475	2.1
17031	1.58874	2.1
17032	1.58875	2.1
17033	1.35787	2.1
17034	1.04147	1.94862
17035	1.15564	1.52733
17036	1.78301	2.1
17037	1.90618	2.1
17038	1.71555	2.1
17039	2.13143	2.1
17040	1.10817	2.1
17041	1.032	2.1
17042	1.032	2.1
17043	0.953636	2.1
17044	1.27916	1.13409
17045	1.11938	1.10499
17046	1.11937	1.10752
17047	1.9186	1.25713
17048	1.19967	0.427958
17049	1.63489	0.436939

17050	1.00763	0.47858
17051	1.81656	0.489982
17052	1.31814	0.414798
17053	2.98183	0.442631
17054	1.20149	2.1
17055	1.29129	2.1
17056	1.29129	2.1
17057	1.37138	2.1
17058	1.23104	1.30725
17059	1.75236	1.60694
17060	1.6	0.915092
17061	1.60565	1.14475
17062	1.60565	1.07477
17063	1.40013	1.19535
17064	1.23042	2.1
17065	1.18278	2.1
17066	1.10853	2.1
17067	1.04724	2.06017
17068	1.1766	2.1
17069	1.00319	2.1
17070	1.29435	2.1
17071	1.00319	2.1
17072	1.05655	1.63444
17073	1.01606	1.71264
17074	1.80391	2.1
17075	2.7699	2.1
17076	1.52073	2.1
17077	2.06835	2.1
17078	2.00202	2.1
17079	2.04906	2.1
17080	0.968536	2.1
17081	1.02461	2.1
17082	1.59784	2.1
17083	1.78249	2.1
17084	1.81409	2.1
17085	1.79844	2.1
17086	1.77093	1.87544
17087	2.02356	1.57507
17088	1.32449	2.1
17089	1.10003	2.1
17090	1.16915	0.835009
17091	1.05937	0.783175
17092	1.28096	1.76266
17093	1.34696	1.4105
17094	1.34695	1.8882
17095	1.36457	1.5157
17096	1.06478	2.1
17097	1.06239	2.1
17098	1.06239	2.1
17099	1.09629	2.1
17100	0.798209	1.93139
17101	1.8532	2.1
17102	2.10112	0.375228
17103	2.8704	0.334381
17104	2.8704	0.353162
17105	2.92457	0.319443
17106	1.0752	1.90187
17107	1.07745	2.1

17108	1.07745	1.961
17109	1.08911	2.1
17110	1.40182	2.1
17111	1.00133	2.1
17112	1.40182	2.1
17113	1.24509	2.1
17114	2.22243	0.21624
17115	1.07303	0.225112
17116	1.29736	0.239993
17117	1.73584	0.244343
17118	2.0815	0.78775
17119	1.07999	0.756426
17120	2.1692	2.1
17121	1.99992	2.1
17122	1.99992	2.1
17123	1.78327	2.1
17124	1.52661	0.226197
17125	0.956003	0.202968
17126	1.53172	0.227547
17127	0.955991	0.210034
17128	1.35025	1.06747
17129	1.51084	2.1
17130	1.51084	1.17295
17131	1.67115	2.1
17132	2.00977	2.1
17133	1.63632	2.1
17134	1.63632	2.1
17135	2.66128	2.1
17136	1.13525	2.1
17137	2.37566	2.1
17138	1.25987	2.1
17139	1.4472	2.1
17140	1.4472	2.1
17141	1.66018	2.1
17142	0.994001	1.3206
17143	1.83151	0.954894
17144	1.83151	0.7678
17145	1.57621	0.805872
17146	1.40915	0.434677
17147	1.24653	0.808625
17148	1.24653	0.281426
17149	1.10609	0.899019
17150	1.61121	2.1
17151	1.74472	2.1
17152	1.74472	2.1
17153	1.0368	2.1
17154	1.04788	2.1
17155	1.28522	1.98502
17156	1.28522	2.1
17157	1.54196	1.49139
17158	1.23387	2.1
17159	0.896519	2.1
17160	1.60428	2.1
17161	1.08467	2.1
17162	1.56875	0.948593
17163	1.39072	0.629205
17164	1.39073	0.721456
17165	1.6	1.01563

17166	1.01346	1.83055
17167	1.06838	1.07644
17168	0.968463	1.12387
17169	1.06837	1.09393
17170	3.22433	0.698182
17171	2.05993	1.13949
17172	2.01836	0.642453
17173	1.66154	1.20669
17174	1.20871	1.31975
17175	1.10586	2.1
17176	1.10586	1.23144
17177	1.03853	2.1
17178	1.12076	2.1
17179	1.03899	2.1
17180	1.03899	2.1
17181	0.963973	2.1
17182	0.987132	2.1
17183	1.0894	2.1
17184	2.37823	1.86919
17185	1.75647	1.52263
17186	1.64625	2.1
17187	1.56195	2.1
17188	1.41995	2.1
17189	1.15171	2.1
17190	1.36457	2.07612
17191	1.33847	1.51355
17192	1.33847	2.01059
17193	0.869601	1.41528
17194	1.42255	2.1
17195	1.3868	2.1
17196	1.3868	2.1
17197	1.33962	2.1
17198	1.05481	2.1
17199	1.08074	2.1
17200	1.08074	2.1
17201	0.857557	2.1
17202	1.13537	1.22112
17203	1.09194	0.746895
17204	1.01179	1.28866
17205	1.13537	0.749461
17206	1.15645	1.60561
17207	1.56409	0.532503
17208	1.56409	0.863558
17209	1.16239	0.967603
17210	1.10746	0.685392
17211	1.47952	0.795452
17212	1.14733	1.86472
17213	3.77068	2.1
17214	1.38497	2.1
17215	1.54599	2.1
17216	1.54601	2.1
17217	1.27697	2.1
17218	1.01393	1.17702
17219	1.10558	1.27702
17220	0.995012	0.817004
17221	1.17624	1.05881
17222	1.60497	2.1
17223	1.61588	2.1

17224	1.61587	2.1
17225	1.62182	2.1
17226	1.48866	0.208472
17227	1.65977	0.207667
17228	1.65977	0.211012
17229	1.69044	0.211617
17230	0.961892	2.1
17231	1.00659	2.1
17232	1.0066	2.1
17233	1.06226	2.1
17234	1.15171	2.1
17235	1.50853	1.33377
17236	1.50853	2.1
17237	1.2955	2.1
17238	1.3204	0.33797
17239	2.13295	0.312889
17240	2.75524	0.261832
17241	1.18156	0.223613
17242	1.73729	2.1
17243	1.20356	2.1
17244	1.62483	2.1
17245	1.52008	2.1
17246	1.19964	2.1
17247	1.0577	2.1
17248	1.19964	2.1
17249	1.07156	2.1
17250	1.98241	2.1
17251	1.43687	2.1
17252	1.27863	1.62692
17253	1.19915	1.15726
17254	0.97269	1.18791
17255	1.0739	1.03604
17256	1.1427	1.64246
17257	1.22476	0.958291
17258	1.06584	1.39789
17259	1.22626	1.25611
17260	1.31388	2.1
17261	1.16932	2.1
17262	1.28819	2.1
17263	1.0499	2.1
17264	1.55894	2.1
17265	1.78709	2.1
17266	1.75986	2.1
17267	1.764 2.1	
17268	1.44801	2.1
17269	1.28186	2.09226
17270	1.20797	1.97752
17271	1.06821	1.91527
17272	1.87287	0.796803
17273	2.08014	0.861846
17274	1.63225	0.89056
17275	1.39501	1.03494
17276	1.20132	0.238534
17277	1.16182	0.245711
17278	1.08848	0.256308
17279	1.20132	0.256079
17280	1.23475	1.22524
17281	1.16661	1.51227

17282	2.31317	2.1
17283	2.50145	2.1
17284	1.04367	2.1
17285	2.23875	2.1
17286	0.953632	0.493628
17287	1.38646	0.509704
17288	1.38646	0.557204
17289	1.61963	0.476943
17290	1.62934	1.09458
17291	1.88626	0.965266
17292	1.0775	1.01834
17293	1.119	0.912017
17294	1.08133	0.825999
17295	1.48312	0.747089
17296	1.57125	0.568659
17297	1.74098	0.556603
17298	1.94372	1.15119
17299	1.22237	1.20178
17300	1.18549	0.600609
17301	1.01294	0.578128
17302	1.01294	0.861881
17303	1.2064	0.906996
17304	1.14599	1.82513
17305	1.00065	0.907931
17306	1.76357	2.1
17307	2.23468	2.1
17308	2.21735	2.1
17309	3.32769	2.1
17310	1.19647	0.840194
17311	1.35307	0.740225
17312	1.19645	0.762867
17313	1.43778	0.745832
17314	1.34412	0.73374
17315	1.1315	1.25182
17316	1.1315	0.755817
17317	1.23943	0.700864
17318	1.57829	2.1
17319	1.22537	2.1
17320	1.14357	2.1
17321	1.32772	2.1
17322	1.4259	2.1
17323	1.34245	2.1
17324	1.47953	2.1
17325	1.70303	2.1
17326	2.21127	0.318599
17327	1.47904	0.30439
17328	1.18793	0.323294
17329	2.21126	0.342247
17330	1.1032	1.48385
17331	1.14986	0.82589
17332	1.14986	1.86997
17333	1.19611	0.87432
17334	1.91883	2.1
17335	1.83019	2.1
17336	1.83019	2.1
17337	1.82462	2.1
17338	1.27362	0.529517
17339	1.27421	0.570601

17340	1.27421	0.448293
17341	1.10736	0.491427
17342	1.3003	0.818684
17343	1.31415	1.06616
17344	1.31415	0.846095
17345	1.32201	1.11522
17346	1.21124	2.1
17347	0.81568	2.1
17348	0.81568	2.1
17349	0.551816	2.1
17350	1.67119	2.054
17351	1.63384	2.1
17352	1.63384	2.1
17353	1.63661	2.1
17354	1.48734	2.1
17355	1.52245	2.1
17356	1.52245	2.1
17357	1.50714	2.1
17358	0.996778	0.268508
17359	1.56091	0.251549
17360	1.56091	0.26818
17361	1.90457	0.248801
17362	1.1181	2.1
17363	1.06543	2.1
17364	2.66828	2.1
17365	2.76932	2.1
17366	1.06543	2.1
17367	1.01474	2.1
17368	2.76932	2.1
17369	2.86704	2.1
17370	1.29724	0.632059
17371	1.96938	0.604966
17372	1.82462	2.1
17373	1.84553	2.1
17374	1.05556	2.1
17375	1.25363	2.1
17376	1.84552	2.1
17377	1.53208	2.1
17378	1.12826	2.1
17379	1.39075	2.1
17380	0.9702	0.893566
17381	1.16481	0.77665
17382	1.08894	1.09898
17383	1.91756	1.1847
17384	1.50865	0.99139
17385	1.06012	0.880751
17386	1.5456	0.325988
17387	1.37427	0.336139
17388	1.1211	0.258449
17389	1.5456	0.277434
17390	1.33135	0.319574
17391	1.07956	0.356214
17392	2.23255	0.339077
17393	1.21031	0.305655
17394	1.52903	2.1
17395	1.35977	2.1
17396	3.3837	2.1
17397	3.20581	2.1

17398	1.35977	2.1
17399	1.15414	2.1
17400	3.20582	2.1
17401	2.07596	2.1
17402	1.6812	0.401732
17403	1.03939	0.361083
17404	1.30047	2.1
17405	1.5586	2.1
17406	1.5586	2.1
17407	1.84214	2.1
17408	1.31825	0.858898
17409	1.31923	1.12623
17410	1.31924	0.857699
17411	1.33026	1.0634
17412	1.15818	0.843174
17413	1.17822	0.93059
17414	1.15818	1.54308
17415	1.28096	1.10806
17416	1.06848	0.699572
17417	1.89639	0.7
17418	1.9186	1.06864
17419	1.77065	1.02735
17420	1.59103	0.925331
17421	1.56164	0.881454
17422	1.09697	0.16474
17423	2.38719	0.166881
17424	2.22123	0.157952
17425	2.46059	0.158484
17426	1.45506	0.379349
17427	1.03645	0.400855
17428	1.30347	2.1
17429	1.2627	2.1
17430	1.2627	2.1
17431	1.23036	2.1
17432	1.41469	2.1
17433	1.45257	2.1
17434	1.46907	0.226284
17435	1.70302	0.229118
17436	1.49196	0.196067
17437	1.36307	0.203117
17438	1.05677	2.1
17439	0.978698	2.1
17440	0.978699	2.1
17441	2.01822	2.1
17442	1.47446	2.1
17443	1.55432	2.1
17444	1.55432	2.1
17445	1.06053	2.1
17446	1.86813	1.34691
17447	1.21892	1.67023
17448	1.53064	1.07398
17449	1.30664	1.18506
17450	1.09629	2.1
17451	1.19244	2.1
17452	1.19244	2.1
17453	1.37635	2.1
17454	1.49358	0.694661
17455	1.57702	0.515433

17456	2.09374	2.1
17457	1.88175	2.1
17458	1.88175	2.1
17459	1.51085	2.1
17460	1.10938	0.372112
17461	1.02478	0.577641
17462	1.33061	0.311833
17463	1.13272	0.268136
17464	1.75505	2.1
17465	1.78529	2.1
17466	1.6353	2.1
17467	1.59307	2.1
17468	1.60017	0.951877
17469	1.35608	1.0649
17470	1.35608	0.805449
17471	1.04713	0.808959
17472	1.01479	0.751746
17473	1.3868	0.621795
17474	1.3868	0.614849
17475	1.28418	0.940182
17476	2.15949	2.1
17477	2.90429	2.1
17478	1.51902	2.1
17479	1.54755	2.1
17480	2.90429	2.1
17481	3.49966	2.1
17482	1.70842	2.1
17483	1.97526	2.1
17484	0.949712	0.642842
17485	1.92784	0.583552
17486	1.34093	0.669723
17487	2.53426	0.652966
17488	1.92783	0.604781
17489	3.89373	0.50776
17490	2.53426	0.668593
17491	2.35961	0.637098
17492	1.68108	0.733037
17493	1.15558	0.724869
17494	1.67265	0.716468
17495	1.27608	0.713184
17496	1.42249	2.1
17497	1.39708	2.1
17498	1.39708	2.1
17499	1.39327	2.1
17500	1.97046	0.376519
17501	1.11688	0.330974
17502	1.28418	0.59479
17503	1.17302	0.889231
17504	1.17302	0.567138
17505	1.04349	0.889813
17506	0.979853	2.1
17507	1.26016	2.1
17508	1.18901	2.1
17509	1.26016	2.1
17510	1.22857	0.771086
17511	1.25848	0.870337
17512	1.25848	0.753359
17513	1.2858	0.847521

17514	1.09154	2.1
17515	2.01915	2.1
17516	1.23013	2.1
17517	1.55698	2.1
17518	1.55698	2.1
17519	1.8575	2.1
17520	2.01854	1.98599
17521	1.20686	1.89231
17522	1.20684	1.65036
17523	1.77796	1.90352
17524	1.46129	2.1
17525	1.04193	2.1
17526	1.10051	2.1
17527	0.983723	2.1
17528	1.1729	2.1
17529	0.98505	2.1
17530	0.985052	2.1
17531	1.03951	2.1
17532	1.03949	2.1
17533	1.17583	2.1
17534	1.42866	2.1
17535	1.97261	2.1
17536	1.73274	2.1
17537	1.53629	2.1
17538	1.98182	2.1
17539	1.72511	2.1
17540	1.42174	2.1
17541	1.44472	2.1
17542	1.72511	2.1
17543	1.45411	2.1
17544	1.44472	2.1
17545	1.43836	2.1
17546	0.962703	0.724313
17547	1.0171	0.884818
17548	1.53336	0.312971
17549	1.40274	0.303359
17550	1.40272	0.320255
17551	1.00213	0.303753
17552	1.50892	0.306923
17553	1.64984	0.281248
17554	1.67464	0.314357
17555	1.67481	0.305934
17556	1.08212	2.1
17557	1.28095	2.1
17558	1.46956	2.1
17559	1.70303	2.1
17560	1.2925	0.213456
17561	1.84716	0.196847
17562	1.48676	0.352072
17563	1.48797	0.342795
17564	1.33917	0.344035
17565	1.22691	0.332252
17566	0.528744	2.1
17567	1.13728	2.1
17568	1.13728	2.1
17569	1.99843	2.1
17570	1.17464	0.43415
17571	1.0372	0.312333

17572	2.0815	0.272329
17573	0.933462	0.261058
17574	1.54931	2.1
17575	1.5485	2.1
17576	1.4077	2.1
17577	1.29931	2.1
17578	1.14931	0.716761
17579	2.02223	0.716273
17580	1.04482	0.630988
17581	0.94971	0.686894
17582	1.54632	1.24909
17583	1.15693	1.28792
17584	1.66763	2.1
17585	1.41238	2.1
17586	1.41238	2.1
17587	1.48068	2.1
17588	1.0171	0.619012
17589	1.1211	0.868079
17590	1.1211	0.599296
17591	1.28418	0.824062
17592	0.993021	2.1
17593	1.764 2.1	
17594	0.993023	2.1
17595	1.05787	2.1
17596	1.90914	0.69893
17597	1.12229	1.16568
17598	1.02025	0.77689
17599	0.585368	1.34152
17600	1.99323	2.1
17601	2.34988	2.1
17602	2.34988	2.1
17603	2.36987	2.1
17604	1.23107	0.331183
17605	1.90664	0.402294
17606	1.53496	0.331799
17607	1.70302	0.41349
17608	1.98859	2.1
17609	1.61057	2.1
17610	1.79844	2.1
17611	2.10811	2.1
17612	1.4618	2.1
17613	1.96722	2.1
17614	1.95191	2.1
17615	2.13418	2.1
17616	1.50368	0.268057
17617	1.61417	0.220329
17618	1.15008	0.241798
17619	1.19685	0.244335
17620	1.90456	0.268371
17621	1.90899	0.25424
17622	2.08864	0.275691
17623	2.10447	0.239637
17624	1.16653	2.1
17625	1.23017	2.1
17626	1.0752	0.777183
17627	1.34412	0.72053
17628	1.06226	2.1
17629	1.09252	2.1

17630	1.09252	2.1
17631	0.88579	2.1
17632	1.56178	0.534254
17633	1.14305	0.53212
17634	1.56175	0.488021
17635	1.08211	0.422953
17636	1.32813	2.1
17637	1.32461	2.1
17638	1.32461	2.1
17639	1.32455	2.1
17640	1.42729	0.469606
17641	1.18716	0.448727
17642	1.47169	0.590913
17643	1.24687	0.521895
17644	1.09627	2.1
17645	1.04759	2.1
17646	1.04759	2.1
17647	0.987132	2.1
17648	1.11181	1.44077
17649	1.80662	2.0137
17650	1.8066	1.93783
17651	1.06461	1.65983
17652	1.56091	2.1
17653	1.9842	2.1
17654	1.73525	0.440424
17655	1.40557	0.426256
17656	2.26906	0.470342
17657	1.31248	0.548692
17658	1.66832	0.417696
17659	1.32634	0.374915
17660	1.03314	0.209406
17661	1.24682	0.206733
17662	1.38536	0.210079
17663	1.52661	0.202578
17664	1.177	1.18779
17665	1.36405	1.06065
17666	1.19837	1.11742
17667	1.17701	0.783187
17668	0.993831	0.538891
17669	1.0823	0.489569
17670	1.0823	0.45336
17671	1.01121	0.449986
17672	1.49653	0.377307
17673	1.89583	0.461025
17674	0.67242	0.349381
17675	1.36047	0.434913
17676	1.00366	0.446958
17677	1.24728	0.497693
17678	1.13387	0.243533
17679	1.60867	0.262387
17680	1.30572	0.330403
17681	1.54982	0.277485
17682	0.788592	0.226381
17683	1.82961	0.236579
17684	1.54982	0.30188
17685	1.82961	0.253527
17686	1.26062	0.213746
17687	1.64002	0.21093

17688	1.12035	0.21498
17689	1.64002	0.231232
17690	1.1673	2.1
17691	1.75367	2.1
17692	0.964034	1.76813
17693	1.0305	1.53946
17694	1.30896	1.4831
17695	1.52776	1.41398
17696	1.52776	2.1
17697	1.17989	1.72705
17698	1.16759	2.1
17699	1.8575	2.1
17700	1.16759	2.1
17701	1.60936	2.1
17702	1.10747	0.379772
17703	1.47688	0.393533
17704	1.40794	2.1
17705	1.95729	1.41275
17706	1.95729	2.1
17707	3.18156	1.57492
17708	1.87598	1.30555
17709	1.94343	2.1
17710	1.94343	1.29746
17711	2.00551	2.1
17712	1.20067	1.08443
17713	1.70744	1.18056
17714	1.87244	1.33118
17715	1.32075	1.23194
17716	1.64955	2.1
17717	1.44221	2.1
17718	1.31109	2.1
17719	1.14206	2.1
17720	1.60452	1.654
17721	1.12111	0.942167
17722	1.00832	2.1
17723	0.974137	2.1
17724	0.974137	2.1
17725	0.98505	2.1
17726	1.02368	2.1
17727	0.985051	2.1
17728	0.995734	0.480079
17729	1.31993	0.594252
17730	1.31993	0.485762
17731	1.18549	0.592067
17732	1.5176	0.806541
17733	1.33622	0.780639
17734	1.33621	0.792043
17735	1.02782	0.776834
17736	1.66774	2.1
17737	1.85305	2.1
17738	1.85305	2.1
17739	2.07088	2.1
17740	1.78873	2.1
17741	1.47533	2.1
17742	1.23152	2.1
17743	0.99922	2.1
17744	1.48493	2.1
17745	1.1394	2.1

17746	1.19179	2.1
17747	1.64289	2.1
17748	1.47858	2.1
17749	1.89687	2.1
17750	0.972464	2.1
17751	1.79473	2.1
17752	1.06971	2.1
17753	1.21089	2.1
17754	1.12572	2.1
17755	1.30566	2.1
17756	1.30566	2.1
17757	1.52724	2.1
17758	2.32237	0.667444
17759	2.20248	0.626307
17760	2.79564	0.697445
17761	2.32237	0.675152
17762	1.59354	0.516996
17763	3.098	0.503007
17764	3.09801	0.503904
17765	3.45132	0.468647
17766	1.24001	1.43248
17767	1.41822	0.788884
17768	1.41821	1.66611
17769	1.44859	0.799904
17770	1.34539	2.1
17771	1.06991	1.85715
17772	1.18076	2.1
17773	0.978586	2.1
17774	0.999189	0.381279
17775	2.41076	0.453427
17776	0.968475	1.83997
17777	1.02074	1.88671
17778	1.02074	1.78918
17779	1.07866	1.86257
17780	0.817702	0.422313
17781	1.5082	0.441434
17782	1.48376	0.487028
17783	1.18947	0.478712
17784	1.10638	2.1
17785	1.86766	2.1
17786	1.19785	2.1
17787	1.34141	2.1
17788	1.34141	2.1
17789	0.991985	2.1
17790	2.35516	0.848283
17791	1.24815	0.925186
17792	1.80454	2.1
17793	1.22633	2.1
17794	0.696255	2.1
17795	1.19379	0.987394
17796	1.14721	2.1
17797	1.16153	2.1
17798	1.16153	2.1
17799	1.17261	2.1
17800	1.20814	2.1
17801	1.54791	2.1
17802	1.54791	2.1
17803	1.60477	2.1

17804	1.29931	2.1
17805	1.19098	2.1
17806	1.19098	2.1
17807	1.13341	2.1
17808	1.00503	1.57095
17809	1.25334	0.797005
17810	1.22655	2.1
17811	1.26282	2.1
17812	1.26282	2.0943
17813	0.999489	1.72864
17814	1.54197	0.51057
17815	1.17152	0.526436
17816	1.17152	0.506385
17817	1.49627	0.472196
17818	1.36405	1.05233
17819	0.976334	0.933145
17820	1.12495	2.1
17821	1.54096	2.1
17822	1.75263	2.1
17823	1.49114	2.1
17824	0.842998	1.51263
17825	1.60244	0.915542
17826	1.48911	1.33021
17827	2.18685	0.918302
17828	1.75236	1.50855
17829	1.54143	1.77738
17830	1.40013	1.14184
17831	0.946646	1.261
17832	1.50714	2.1
17833	1.46055	2.1
17834	1.46055	2.1
17835	1.42249	2.1
17836	2.12839	0.437104
17837	1.75211	0.397898
17838	1.76297	0.400396
17839	1.70736	0.372687
17840	1.19952	0.40002
17841	1.56475	0.359934
17842	1.74312	0.455309
17843	1.48973	0.396749
17844	1.91518	0.545928
17845	1.16534	0.556326
17846	1.53556	2.1
17847	1.56253	2.1
17848	1.56253	2.1
17849	1.58054	2.1
17850	1.27806	0.191725
17851	1.44593	0.192878
17852	1.44593	0.199677
17853	1.3946	0.195382
17854	1.34857	0.726932
17855	1.03818	1.39585
17856	1.38969	0.777974
17857	1.85548	0.806284
17858	1.03108	0.752127
17859	1.85548	0.785232
17860	1.49346	1.95716
17861	1.83192	2.1

17862	1.75291	1.65839
17863	1.35435	2.05172
17864	0.921851	0.766367
17865	1.09646	0.752278
17866	1.2183	0.745738
17867	1.36717	0.68809
17868	2.14179	0.668469
17869	1.79805	0.702006
17870	1.3428	0.691772
17871	2.30493	0.680158
17872	1.81545	0.698568
17873	1.92269	0.692191
17874	2.30492	0.683388
17875	1.3663	0.714602
17876	1.22418	1.60623
17877	0.956698	0.956093
17878	1.4706	1.9419
17879	0.953295	2.1
17880	1.47059	1.40419
17881	1.26929	2.1
17882	1.24538	0.506284
17883	1.4468	0.41285
17884	2.86762	2.1
17885	2.32911	2.1
17886	2.10864	2.1
17887	1.94548	2.1
17888	1.83134	2.1
17889	2.19519	2.1
17890	2.19853	2.1
17891	2.63855	2.1
17892	1.08213	0.440394
17893	1.46389	0.510928
17894	1.62235	0.582157
17895	0.980549	0.599053
17896	1.08195	1.63172
17897	0.99002	1.6206
17898	1.2269	2.1
17899	1.0838	2.1
17900	0.889491	2.1
17901	1.19587	2.0732
17902	1.18801	0.365252
17903	1.57125	0.375047
17904	1.47712	2.1
17905	1.45182	2.1
17906	1.45182	2.1
17907	1.42255	2.1
17908	1.17816	0.210347
17909	1.00982	0.216808
17910	1.21056	0.187581
17911	1.17816	0.184531
17912	3.80902	0.892881
17913	0.976991	0.866383
17914	1.09962	2.1
17915	1.60976	2.1
17916	1.87029	2.1
17917	2.33806	2.1
17918	2.12549	2.1
17919	2.21472	2.1

17920	2.16221	0.438246
17921	1.99528	0.372823
17922	1.99528	0.39134
17923	1.97969	0.353056
17924	3.60854	2.1
17925	4.00847	2.1
17926	2.70372	2.1
17927	1.253 2.1	
17928	1.65739	2.1
17929	0.994294	2.1
17930	1.36203	2.1
17931	1.00965	2.1
17932	2.33374	2.1
17933	1.36209	2.1
17934	1.64983	0.263756
17935	1.36416	0.253569
17936	2.19766	0.263977
17937	1.36416	0.302224
17938	2.15871	0.849129
17939	1.81454	0.793029
17940	1.54607	0.606885
17941	1.57702	0.731242
17942	0.948748	0.238683
17943	1.04522	0.253182
17944	1.60994	0.210993
17945	1.90382	0.219784
17946	1.90382	0.222559
17947	1.57999	0.211984
17948	1.73905	0.873445
17949	1.26535	1.18658
17950	2.12552	1.15643
17951	1.06891	1.03978
17952	1.13999	0.9675
17953	2.29812	1.18762
17954	2.83329	1.03585
17955	2.16272	0.964935
17956	1.71277	2.1
17957	1.68401	2.1
17958	1.68401	2.1
17959	1.69368	2.1
17960	1.06472	2.1
17961	1.56017	2.1
17962	1.2765	2.1
17963	1.99541	2.1
17964	1.46041	0.155807
17965	2.04817	0.153539
17966	2.04817	0.153585
17967	2.33181	0.151672
17968	2.06353	2.1
17969	1.35449	2.1
17970	1.52045	2.1
17971	1.45437	2.1
17972	1.04405	0.202957
17973	1.85945	0.197607
17974	1.85945	0.178731
17975	2.22679	0.175292
17976	1.33339	2.1
17977	1.19376	2.1

17978	1.33266	2.1
17979	1.44244	2.1
17980	1.20455	2.1
17981	1.52852	2.1
17982	1.03882	2.1
17983	1.52852	2.1
17984	1.44801	1.9736
17985	1.00775	1.82718
17986	0.53907	2.1
17987	0.506124	2.1
17988	2.39045	2.1
17989	0.973794	2.1
17990	1.11193	2.1
17991	1.32784	2.1
17992	1.31595	2.1
17993	0.966115	2.1
17994	1.25953	2.1
17995	1.38086	2.1
17996	1.38086	2.1
17997	1.04101	2.1
17998	1.99319	0.330285
17999	1.23107	0.332788
18000	3.3606	0.354174
18001	1.23112	0.353992
18002	1.40239	2.1
18003	1.26091	2.1
18004	1.26091	2.1
18005	1.05776	2.1
18006	1.23768	0.712303
18007	1.0142	1.04255
18008	1.11435	0.885989
18009	1.01422	0.793593
18010	1.24001	0.799113
18011	1.42186	0.928693
18012	1.42186	0.864899
18013	1.47204	0.915895
18014	1.27361	2.1
18015	1.56642	2.1
18016	1.71676	2.1
18017	2.16815	2.1
18018	1.07855	1.20112
18019	1.07612	1.21216
18020	1.703	0.26498
18021	1.46853	0.263883
18022	1.45448	0.298678
18023	1.27778	0.311326
18024	0.991986	2.1
18025	1.1192	2.1
18026	1.1192	2.1
18027	1.25953	2.1
18028	2.08506	2.1
18029	1.75162	0.895177
18030	1.94523	2.1
18031	1.88405	1.93946
18032	1.15506	0.23322
18033	1.1233	0.235841
18034	2.09736	2.1
18035	2.98303	2.1

18036	2.71184	2.1
18037	3.51614	2.1
18038	1.18769	1.92728
18039	1.24053	1.95992
18040	1.24053	1.97919
18041	1.45327	2.05694
18042	1.26865	1.12944
18043	1.1744	1.04466
18044	1.1744	0.95168
18045	1.11712	0.956511
18046	1.15841	0.560476
18047	1.02247	0.618072
18048	1.93649	2.1
18049	1.81191	2.1
18050	1.64718	2.1
18051	1.47521	2.1
18052	1.59654	2.1
18053	1.57823	2.1
18054	1.57823	2.1
18055	1.55768	2.1
18056	1.00665	0.335414
18057	1.28239	0.351477
18058	1.28239	0.324431
18059	1.44951	0.336039
18060	1.22083	2.1
18061	0.957622	2.1
18062	0.887896	2.1
18063	1.27387	2.1
18064	1.00521	0.978966
18065	1.00503	1.00092
18066	1.95844	2.1
18067	1.05042	2.1
18068	2.78662	2.1
18069	2.22399	2.1
18070	2.3356	0.245194
18071	1.49543	0.283649
18072	1.96854	0.230114
18073	1.21681	0.218579
18074	2.17803	1.76654
18075	1.50998	1.88617
18076	2.35239	1.59645
18077	2.26987	1.32744
18078	1.03824	0.49078
18079	1.51396	0.552943
18080	1.51396	0.492873
18081	1.48693	0.415401
18082	1.08784	1.3752
18083	1.36865	1.28267
18084	0.712405	1.48737
18085	1.08784	1.4896
18086	2.02522	2.1
18087	1.6738	2.1
18088	1.35175	2.1
18089	1.50094	2.1
18090	1.6738	2.1
18091	1.62871	2.1
18092	1.35084	2.1
18093	1.34276	2.1

18094	1.18041	2.1
18095	1.43652	2.1
18096	0.974196	2.1
18097	1.28037	2.1
18098	1.28037	2.1
18099	1.05071	2.1
18100	2.44737	0.217659
18101	1.4672	0.230272
18102	1.22883	0.236568
18103	2.08149	0.269929
18104	1.82208	1.28251
18105	1.59826	1.33812
18106	1.59826	1.20641
18107	1.01393	1.43695
18108	1.42757	2.1
18109	0.996775	2.1
18110	1.42757	2.1
18111	1.26397	2.1
18112	1.02461	2.1
18113	1.46129	2.1
18114	1.11833	2.1
18115	1.335 2.1	
18116	2.07087	2.1
18117	1.24041	2.1
18118	1.335 2.1	
18119	1.55398	2.1
18120	1.21171	0.33474
18121	1.48589	0.315323
18122	1.32886	0.298011
18123	1.8027	0.299716
18124	1.53515	1.72158
18125	1.60428	1.77008
18126	1.00035	0.147815
18127	1.03534	0.288096
18128	1.03535	0.958405
18129	1.09962	1.81206
18130	0.62132	1.13376
18131	1.21033	1.75667
18132	1.30326	1.35276
18133	2.6823	2.06061
18134	0.991407	2.04055
18135	1.29729	1.80125
18136	1.29729	1.84096
18137	1.16858	2.1
18138	1.66137	0.378236
18139	1.23012	0.26544
18140	1.66139	0.586712
18141	1.41821	0.421309
18142	1.56043	0.697667
18143	1.06848	0.863735
18144	1.56043	1.13788
18145	1.90911	0.97235
18146	0.982847	2.1
18147	1.05863	2.1
18148	1.05863	2.1
18149	1.22352	2.1
18150	1.23019	1.40232
18151	1.60509	1.84249

18152	1.19122	2.1
18153	1.47769	2.1
18154	2.18868	2.1
18155	1.40984	1.70501
18156	3.55197	2.1
18157	3.57227	2.1
18158	3.57227	2.1
18159	3.48926	2.1
18160	1.4375	0.591811
18161	1.11314	0.55222
18162	1.62408	2.1
18163	1.74438	2.1
18164	1.72387	2.1
18165	1.50018	2.1
18166	1.1486	2.1
18167	1.18474	2.1
18168	1.18474	2.1
18169	1.2295	2.1
18170	1.28754	2.1
18171	1.36838	2.1
18172	1.36838	2.1
18173	1.21703	2.1
18174	2.74206	2.1
18175	2.70795	2.1
18176	2.70795	2.1
18177	2.55918	2.1
18178	1.35065	1.03604
18179	1.65411	0.84033
18180	1.48572	0.988357
18181	1.28393	0.916767
18182	1.25265	0.25611
18183	1.70944	0.217082
18184	1.31259	0.806525
18185	1.14945	1.80521
18186	1.14945	0.770219
18187	0.995725	1.78225
18188	1.00307	0.503758
18189	2.05786	0.560175
18190	1.56357	0.47424
18191	1.10907	0.544123
18192	1.27558	1.23264
18193	1.05175	1.0288
18194	1.2685	2.1
18195	1.41369	2.1
18196	1.4137	2.1
18197	1.49654	2.1
18198	1.21489	1.58825
18199	1.14605	1.25816
18200	1.22476	1.71331
18201	1.21489	1.04363
18202	2.51472	1.13939
18203	2.52091	1.10888
18204	2.52091	1.08303
18205	1.79171	1.02987
18206	1.03373	1.66641
18207	1.04037	1.87246
18208	1.04038	1.66136
18209	1.03933	1.70542

18210	1.74123	0.394703
18211	1.41902	0.377898
18212	1.89777	0.392888
18213	1.89899	0.440489
18214	1.24728	0.431612
18215	1.30394	0.453545
18216	1.30394	0.402349
18217	1.16535	0.40049
18218	1.09914	2.1
18219	1.54259	2.1
18220	1.40233	2.1
18221	1.69858	2.1
18222	1.53555	2.1
18223	1.09859	1.09232
18224	1.09859	2.1
18225	0.725879	1.03113
18226	1.07213	2.1
18227	1.31589	2.1
18228	1.36904	2.1
18229	1.69711	2.1
18230	1.51028	0.274318
18231	0.993598	0.335292
18232	0.61563	0.26054
18233	1.26311	0.274628
18234	1.19496	2.1
18235	1.65925	2.1
18236	1.65925	2.1
18237	1.48162	2.1
18238	2.75738	1.22997
18239	1.24699	1.27111
18240	4.08729	1.60608
18241	2.35271	1.41991
18242	1.55063	0.632135
18243	1.34563	0.483035
18244	1.34563	0.546167
18245	1.08189	0.426939
18246	1.09662	0.633253
18247	2.35961	0.643825
18248	0.986952	0.544522
18249	0.813251	0.460154
18250	1.17897	0.30085
18251	1.67685	0.246204
18252	1.00573	1.30577
18253	1.08542	1.02038
18254	1.00573	2.1
18255	1.12572	2.1
18256	1.05499	1.82735
18257	1.00521	1.0704
18258	2.22679	0.17264
18259	2.86746	0.166348
18260	2.86746	0.16808
18261	1.99958	0.165846
18262	0.996517	2.1
18263	1.01169	2.1
18264	1.01169	2.1
18265	1.26346	2.1
18266	1.68274	2.1
18267	1.75505	2.1

18268	2.28748	1.63656
18269	1.36555	1.42874
18270	3.47888	0.220609
18271	0.692788	0.230131
18272	1.0685	0.80038
18273	1.14547	0.871585
18274	1.19017	2.1
18275	1.24976	2.1
18276	1.24976	2.1
18277	1.30682	2.1
18278	2.2153	2.1
18279	1.85457	2.1
18280	2.03267	2.1
18281	1.51042	2.1
18282	1.05619	0.76387
18283	1.05793	0.848446
18284	1.05793	0.715088
18285	1.00763	0.840202
18286	1.77677	2.1
18287	1.02524	2.1
18288	1.02524	2.1
18289	1.18803	2.1
18290	1.68669	2.1
18291	1.71162	2.1
18292	1.556	2.1
18293	1.39523	2.1
18294	0.960288	0.877746
18295	1.24201	1.05835
18296	1.24202	2.1
18297	1.3592	2.1
18298	2.14045	0.420875
18299	1.62054	0.387231
18300	2.20248	0.606097
18301	2.34699	0.538487
18302	3.45132	0.439192
18303	3.38241	0.401225
18304	3.38241	0.377199
18305	2.96127	0.349433
18306	2.02612	2.1
18307	2.09351	2.1
18308	2.09351	2.1
18309	2.17465	2.1
18310	1.07445	2.1
18311	1.14808	2.1
18312	1.39067	1.77725
18313	1.20958	1.24798
18314	1.20958	1.49666
18315	1.00382	1.11121
18316	0.986496	1.47631
18317	1.27142	1.42306
18318	1.39857	1.2111
18319	2.26482	1.22437
18320	2.24367	0.233374
18321	1.9234	0.23068
18322	1.59076	0.234468
18323	1.185	0.237096
18324	3.86179	0.2204
18325	3.0638	0.222985

18326	2.7574	0.220593
18327	1.83266	0.223199
18328	2.59418	2.1
18329	2.23048	2.1
18330	1.39564	2.1
18331	1.36763	2.1
18332	2.23048	2.1
18333	2.02522	2.1
18334	1.36763	2.1
18335	1.35175	2.1
18336	1.16181	0.237619
18337	0.97096	0.23673
18338	2.05665	0.417476
18339	0.483583	0.400336
18340	0.601928	0.338042
18341	3.88545	0.439314
18342	1.04909	2.1
18343	0.983143	2.1
18344	1.10469	2.1
18345	1.10516	2.1
18346	1.10517	2.1
18347	1.09951	2.1
18348	2.33985	2.1
18349	2.35026	2.1
18350	2.35025	2.1
18351	1.19462	2.1
18352	1.57627	2.1
18353	1.5493	2.1
18354	1.5493	2.1
18355	1.53556	2.1
18356	1.42763	0.75253
18357	1.24682	0.743641
18358	1.24682	0.669509
18359	1.00307	0.711007
18360	1.37392	2.1
18361	1.45408	2.1
18362	1.45408	2.1
18363	1.54249	2.1
18364	1.24474	1.1539
18365	1.07467	1.14183
18366	1.37813	1.22266
18367	1.24474	1.21547
18368	1.06341	2.1
18369	1.09194	2.1
18370	1.09194	2.1
18371	1.11117	2.1
18372	1.1658	2.1
18373	1.93707	2.1
18374	1.93707	2.1
18375	1.47729	2.1
18376	1.25669	0.925815
18377	1.07519	2.1
18378	1.65417	2.1
18379	1.50956	2.1
18380	1.37231	2.1
18381	1.27285	2.1
18382	1.23612	2.1
18383	1.1899	2.1

18384	1.08172	2.1
18385	1.20357	2.1
18386	1.5664	2.1
18387	1.7472	2.1
18388	1.48434	2.1
18389	1.23989	2.1
18390	1.58836	2.1
18391	1.38766	2.1
18392	1.23989	2.1
18393	1.26599	2.1
18394	2.56248	2.1
18395	2.08673	1.02563
18396	1.87216	1.05159
18397	1.60476	0.479479
18398	1.84355	2.1
18399	1.25971	0.998277
18400	1.44141	0.971045
18401	1.25521	0.424171
18402	1.19837	0.209076
18403	2.31936	0.208126
18404	1.38153	2.1
18405	2.41734	2.1
18406	2.70856	2.1
18407	3.48385	2.1
18408	1.89226	0.828072
18409	1.64617	1.35537
18410	1.89112	1.02992
18411	1.23135	0.726662
18412	1.23134	0.720266
18413	1.34927	0.710409
18414	3.60096	0.242231
18415	1.68103	0.263935
18416	2.25644	2.1
18417	1.91955	2.1
18418	1.91955	2.1
18419	1.47688	2.1
18420	1.67375	2.1
18421	1.50033	2.1
18422	1.5098	2.1
18423	1.46908	2.1
18424	1.69055	0.363098
18425	1.53463	0.353062
18426	1.53463	0.289587
18427	1.41943	0.267217
18428	1.19022	1.8612
18429	1.95981	1.5993
18430	1.15009	1.25852
18431	1.31612	1.30223
18432	1.31611	0.912033
18433	1.10735	0.814976
18434	1.18601	0.348492
18435	1.3499	0.377708
18436	1.3499	0.392922
18437	1.29001	0.383201
18438	1.22083	2.1
18439	1.06191	2.1
18440	1.348	1.04094
18441	1.05758	1.30378

18442	1.34799	1.64721
18443	1.18647	1.40403
18444	2.92457	0.338595
18445	1.99182	0.315091
18446	2.25313	0.340403
18447	1.30859	0.325679
18448	1.30682	2.1
18449	1.38957	2.1
18450	1.38957	2.1
18451	1.47446	2.1
18452	0.953638	1.74499
18453	1.03408	1.87015
18454	1.17885	0.71764
18455	1.3663	0.708716
18456	1.08265	0.740442
18457	1.71948	0.786889
18458	1.29501	1.00312
18459	0.955093	0.964894
18460	0.955081	0.887084
18461	1.88345	0.863055
18462	1.2243	2.1
18463	1.23169	2.1
18464	2.04726	1.04864
18465	0.842869	1.15766
18466	3.18156	2.1
18467	3.60786	1.41202
18468	3.60786	2.07534
18469	2.38566	1.24211
18470	1.24526	0.209983
18471	1.59019	0.231863
18472	1.16776	0.780728
18473	1.05042	1.44541
18474	1.17418	1.8741
18475	1.05042	2.1
18476	0.949135	2.04914
18477	0.973448	2.1
18478	0.973447	2.04396
18479	1.02958	2.1
18480	3.57768	2.1
18481	3.04246	2.1
18482	3.04246	2.1
18483	2.47824	2.1
18484	1.35879	2.1
18485	1.10407	1.77755
18486	1.14599	0.913002
18487	1.10407	0.922111
18488	1.87794	1.43849
18489	1.53429	1.36351
18490	1.12717	2.1
18491	1.38634	2.1
18492	1.38634	2.1
18493	1.13127	2.1
18494	1.04546	2.1
18495	1.06007	2.1
18496	1.06007	2.1
18497	1.19785	2.1
18498	1.43652	2.1
18499	1.71741	1.91802

18500	1.70921	1.21263
18501	1.06994	1.43921
18502	0.960106	2.1
18503	1.48077	2.1
18504	1.48077	2.1
18505	2.5946	2.1
18506	2.03045	1.37407
18507	1.76788	1.59037
18508	2.03045	1.95126
18509	0.633098	1.96334
18510	1.06756	2.1
18511	1.38667	2.1
18512	0.966979	2.1
18513	1.38669	2.1
18514	1.07156	2.1
18515	0.986095	2.1
18516	1.32813	2.1
18517	0.986095	2.1
18518	1.98697	0.882246
18519	1.12016	1.1985
18520	1.24463	1.01611
18521	1.1967	1.1577
18522	1.30001	0.746843
18523	1.29175	1.0636
18524	1.29175	0.79455
18525	1.3003	1.08372
18526	1.5606	0.212498
18527	2.32098	0.210261
18528	2.57888	0.196966
18529	2.97463	0.197243
18530	1.24555	2.1
18531	1.46101	2.1
18532	1.90723	2.1
18533	2.29126	2.1
18534	1.46101	2.1
18535	1.84089	2.1
18536	2.5519	2.1
18537	2.91663	2.1
18538	1.10476	0.329974
18539	1.44103	0.315529
18540	1.44103	0.319001
18541	1.53336	0.304222
18542	0.672964	2.1
18543	0.633884	2.1
18544	0.633884	2.1
18545	0.603154	2.1
18546	2.29663	2.1
18547	2.06892	2.1
18548	1.3685	2.1
18549	1.39067	2.1
18550	2.06892	2.1
18551	1.78208	2.1
18552	1.39067	2.1
18553	1.42174	2.1
18554	1.01335	1.52578
18555	1.08195	1.00253
18556	1.86463	1.60658
18557	1.39827	1.75792

18558	0.861515	2.00095
18559	2.10074	2.1
18560	2.09511	0.750731
18561	2.04853	0.738091
18562	2.21488	0.689772
18563	1.38287	0.771662
18564	0.974717	0.818757
18565	0.998451	0.796111
18566	1.42281	1.19811
18567	1.82595	1.13261
18568	1.50458	1.02318
18569	1.09408	0.941795
18570	1.23498	1.49647
18571	1.05654	1.46952
18572	1.17261	2.1
18573	1.18058	2.1
18574	1.18059	2.1
18575	1.18907	2.1
18576	2.38998	2.1
18577	2.77437	2.1
18578	1.04546	2.1
18579	1.16817	2.1
18580	2.77437	2.1
18581	2.97768	2.1
18582	1.16817	2.1
18583	1.2832	2.1
18584	1.75766	0.521671
18585	1.4934	0.880495
18586	1.4934	0.550151
18587	1.20324	0.957694
18588	1.07236	0.852693
18589	1.37641	0.804748
18590	1.42913	0.797122
18591	1.93373	0.839348
18592	1.42629	0.777137
18593	1.00636	0.598439
18594	0.933959	1.811
18595	2.22873	1.01475
18596	2.22873	1.24444
18597	2.13757	0.885038
18598	1.10517	2.1
18599	1.86737	2.1
18600	0.811 2.1	
18601	1.19179	2.1
18602	1.18647	1.73196
18603	0.988453	1.85076
18604	1.12457	0.411764
18605	1.23851	0.374041
18606	0.92492	2.1
18607	1.45656	2.1
18608	1.61251	0.992469
18609	1.56696	0.993417
18610	1.90245	0.891222
18611	1.46046	0.883927
18612	1.60329	0.268127
18613	1.38247	0.256418
18614	1.38247	0.245781
18615	1.23198	0.244598

18616	1.09518	2.1
18617	1.28159	2.1
18618	0.977777	2.1
18619	1.80778	2.1
18620	1.80777	2.1
18621	1.16655	2.1
18622	1.3515	2.1
18623	0.956573	2.1
18624	1.08022	2.1
18625	1.03437	2.1
18626	1.03437	2.1
18627	0.975409	2.1
18628	1.47369	0.324218
18629	1.31167	0.413101
18630	1.01847	0.310021
18631	2.75562	0.310213
18632	1.13443	2.1
18633	1.32709	2.1
18634	1.01051	0.582301
18635	0.958947	0.516481
18636	1.7211	2.1
18637	1.922 2.1	
18638	1.922 2.1	
18639	0.991113	2.1
18640	1.00636	0.675762
18641	1.31658	0.716594
18642	1.44627	0.628234
18643	1.03824	0.497334
18644	1.21223	2.1
18645	1.01242	2.1
18646	1.01243	2.1
18647	0.956698	1.43486
18648	1.23167	2.1
18649	1.06935	2.1
18650	1.29215	0.210931
18651	0.998971	0.225543
18652	1.50103	0.214234
18653	1.44836	0.235554
18654	3.02513	2.1
18655	3.7335	2.1
18656	2.49988	2.1
18657	1.24948	2.1
18658	0.963972	2.1
18659	1.1822	2.1
18660	1.1822	2.1
18661	1.10817	2.1
18662	1.30237	2.1
18663	1.41908	2.1
18664	1.41908	2.1
18665	1.48734	2.1
18666	1.31127	0.781473
18667	1.40822	0.764614
18668	1.40822	0.708169
18669	1.22176	0.706806
18670	1.82525	2.1
18671	1.59394	2.1
18672	1.22731	0.228689
18673	0.981584	0.219957

18674	1.1427	0.778258
18675	1.44091	0.944066
18676	1.49154	0.808937
18677	1.07526	0.824024
18678	1.07527	0.730264
18679	0.96029	0.925306
18680	2.40568	1.15094
18681	3.46486	1.04648
18682	2.417	1.13405
18683	1.52054	0.944462
18684	1.51638	0.482396
18685	1.04786	0.391337
18686	1.51404	0.474066
18687	1.3461	0.378877
18688	1.27696	0.367482
18689	0.736457	0.28842
18690	1.16817	2.1
18691	1.04263	2.1
18692	1.13449	2.1
18693	1.50524	2.1
18694	2.1182	2.1
18695	2.49001	2.1
18696	1.11764	2.1
18697	1.39292	2.1
18698	2.49001	2.1
18699	2.76339	2.1
18700	1.39292	2.1
18701	1.68761	2.1
18702	1.25173	2.03376
18703	1.34701	2.1
18704	1.02415	2.1
18705	1.34701	1.86039
18706	1.332	0.220681
18707	0.989764	0.212419
18708	0.963167	0.195681
18709	1.21056	0.183672
18710	1.67375	2.1
18711	1.17965	2.1
18712	1.09154	2.1
18713	1.52314	2.1
18714	1.54433	0.745299
18715	1.81969	0.717938
18716	1.3598	0.693671
18717	1.71041	0.574796
18718	1.70934	0.619636
18719	1.33871	0.556783
18720	1.79485	0.556989
18721	1.51404	0.464593
18722	1.30751	2.1
18723	1.20969	2.1
18724	1.20969	2.1
18725	1.12076	2.1
18726	1.49808	2.1
18727	1.50201	2.1
18728	1.50201	2.1
18729	1.31594	2.1
18730	1.2187	2.1
18731	1.11545	2.1

18732	1.227	2.1
18733	1.09645	2.1
18734	1.05902	0.147815
18735	1.41595	0.735824
18736	1.35696	1.85458
18737	1.29446	0.89752
18738	0.857957	2.1
18739	1.21125	0.147815
18740	2.43115	1.95995
18741	2.1151	0.680587
18742	2.0815	1.11697
18743	1.1723	1.64567
18744	1.48035	0.559086
18745	1.59971	0.767653
18746	1.33668	0.646506
18747	1.0961	0.536581
18748	1.46235	2.1
18749	0.882195	2.1
18750	2.46624	2.1
18751	2.72161	2.1
18752	2.96094	2.1
18753	2.25644	2.1
18754	1.25923	0.225651
18755	0.976218	0.226176
18756	1.10319	0.206014
18757	1.82583	0.216633
18758	0.888955	2.1
18759	1.30817	2.1
18760	1.30817	2.1
18761	1.60787	2.1
18762	0.988805	1.6307
18763	1.54168	1.01084
18764	1.19496	0.951859
18765	1.12224	1.49211
18766	1.93597	1.28422
18767	1.31113	1.26674
18768	1.17879	1.01587
18769	1.07468	0.993578
18770	2.0523	2.1
18771	2.41385	2.1
18772	2.41385	2.1
18773	3.0355	2.06614
18774	1.36717	0.679694
18775	1.45535	0.573379
18776	1.45535	0.557062
18777	1.49906	0.463356
18778	1.37785	1.1694
18779	1.04413	1.99879
18780	1.06729	0.918432
18781	1.54305	0.870123
18782	1.07444	2.1
18783	1.52724	1.34543
18784	1.49151	2.1
18785	1.36814	2.1
18786	1.36814	2.1
18787	1.15541	2.1
18788	1.19379	2.1
18789	1.78657	0.884211

18790	1.6079	2.06175
18791	2.59469	0.881287
18792	1.35545	0.793543
18793	1.23602	0.931546
18794	1.23452	2.02648
18795	1.56842	1.01573
18796	1.15864	2.1
18797	1.11955	2.1
18798	1.11955	2.1
18799	1.08022	2.1
18800	1.64268	1.83813
18801	1.26247	2.1
18802	1.16912	2.1
18803	2.1095	0.978575
18804	1.75549	0.241729
18805	1.7491	0.273232
18806	1.59833	0.287093
18807	1.68338	0.30641
18808	1.14846	0.24793
18809	1.06515	0.253292
18810	1.2721	0.521396
18811	1.32639	0.471216
18812	1.32639	0.714969
18813	1.19323	0.559635
18814	1.81034	2.1
18815	1.9034	2.1
18816	2.08245	2.1
18817	1.84887	2.1
18818	1.41448	2.1
18819	1.77326	2.1
18820	1.76872	2.1
18821	1.86766	2.1
18822	1.46643	2.1
18823	1.58995	2.1
18824	1.58995	2.1
18825	1.13854	2.1
18826	1.23654	2.1
18827	1.04442	2.1
18828	1.23654	2.1
18829	1.08576	2.1
18830	0.966979	2.1
18831	1.41758	2.1
18832	1.03108	0.696102
18833	1.94412	0.709383
18834	1.32455	2.1
18835	1.32328	2.1
18836	1.32328	2.1
18837	1.34008	2.1
18838	1.60861	2.1
18839	1.69586	2.1
18840	1.69586	2.1
18841	1.78301	2.1
18842	1.7513	2.1
18843	1.67906	2.1
18844	1.67906	2.1
18845	1.60635	2.1
18846	1.68073	2.1
18847	1.57234	2.1

18848	1.57233	2.1
18849	1.47012	2.1
18850	1.22811	0.341171
18851	1.79683	0.364068
18852	1.53417	0.364248
18853	1.08732	0.394061
18854	1.8903	1.20165
18855	1.20247	1.20077
18856	1.12087	1.27067
18857	1.54699	1.55614
18858	1.43635	2.1
18859	1.02368	2.1
18860	1.43635	2.1
18861	1.23007	2.1
18862	1.035	2.1
18863	1.07889	2.1
18864	3.0355	2.1
18865	3.78805	1.68708
18866	2.57183	1.36859
18867	1.27835	1.18678
18868	1.29813	0.357228
18869	1.42965	0.401113
18870	1.69832	0.38451
18871	2.18359	0.42193
18872	1.42964	0.370622
18873	1.65723	0.389287
18874	1.98508	0.386486
18875	2.06284	0.419772
18876	1.50705	0.7
18877	1.51153	0.7
18878	1.36037	0.7
18879	1.09767	0.7
18880	1.70048	1.9113
18881	1.15177	1.6735
18882	0.713174	2.1
18883	0.763575	2.1
18884	0.763575	2.1
18885	0.797011	2.1
18886	1.01179	0.7897
18887	1.27737	0.801972
18888	3.16531	0.31241
18889	1.0141	0.277118
18890	1.47354	0.538783
18891	1.62495	0.421088
18892	1.62495	0.511917
18893	1.39296	0.678836
18894	0.998682	2.1
18895	1.93528	2.1
18896	2.71383	2.1
18897	1.13987	2.1
18898	1.93528	2.1
18899	1.78595	2.1
18900	3.98933	2.1
18901	3.73426	2.1
18902	1.02438	2.1
18903	1.12376	2.1
18904	1.12375	2.1
18905	1.19016	2.1

18906	1.37157	0.384073
18907	1.60814	0.376052
18908	1.70303	0.464698
18909	1.01219	0.423509
18910	1.06757	1.70213
18911	1.23475	1.96487
18912	1.2295	2.1
18913	1.28014	2.1
18914	1.28014	2.1
18915	1.33339	2.1
18916	1.3201	2.1
18917	1.2679	2.1
18918	1.2679	2.1
18919	1.23504	2.1
18920	1.50108	2.1
18921	1.3715	2.1
18922	1.3715	2.1
18923	1.25075	2.1
18924	1.99222	1.04732
18925	1.4155	1.0189
18926	1.4777	2.1
18927	1.52613	2.1
18928	1.91031	2.1
18929	1.03968	2.1
18930	2.04289	0.537495
18931	0.967192	0.471234
18932	1.03043	0.405612
18933	1.65452	0.577921
18934	1.21564	2.1
18935	1.16938	2.1
18936	2.47056	2.1
18937	2.56853	2.1
18938	1.16938	2.1
18939	1.1181	2.1
18940	2.56853	2.1
18941	2.66828	2.1
18942	1.0465	2.1
18943	1.44709	2.1
18944	1.44709	2.1
18945	1.96491	2.1
18946	1.01976	0.773168
18947	0.961779	0.727473
18948	1.11764	0.758474
18949	0.961779	0.718729
18950	1.21171	0.292432
18951	1.35007	0.353042
18952	1.03102	0.778842
18953	1.42446	0.779183
18954	1.42446	0.702302
18955	1.03166	0.687187
18956	1.2951	0.474954
18957	1.33575	0.505892
18958	1.33575	0.513938
18959	1.20473	0.492494
18960	1.10049	0.892815
18961	0.99747	0.881495
18962	1.54249	2.1
18963	1.65677	2.1

18964	1.65677	2.1
18965	1.77677	2.1
18966	1.73525	0.501034
18967	1.05689	0.566811
18968	1.26051	0.458555
18969	1.16259	0.415803
18970	1.34009	2.1
18971	1.398 2.1	
18972	1.398 2.1	
18973	1.50437	2.1
18974	2.3245	2.1
18975	1.18237	2.1
18976	1.24509	2.1
18977	1.01595	2.1
18978	3.29047	2.1
18979	2.79168	2.1
18980	1.06561	2.1
18981	1.11077	1.70264
18982	1.11077	1.25442
18983	0.953927	1.55239
18984	1.30509	0.728729
18985	1.04685	0.742426
18986	1.04685	0.620588
18987	0.834593	0.638412
18988	1.0775	0.614263
18989	1.27362	0.730508
18990	1.71762	0.891569
18991	2.5736	0.819886
18992	2.04717	0.923712
18993	1.45521	0.842882
18994	1.33962	2.1
18995	1.27956	2.1
18996	1.27956	2.1
18997	1.20478	2.1
18998	3.65841	0.269811
18999	1.18656	0.234583
19000	1.72272	0.33545
19001	1.6812	0.28683
19002	1.09951	2.1
19003	1.08231	2.1
19004	1.08231	2.1
19005	1.06478	2.1
19006	2.16196	1.4987
19007	1.00387	1.09665
19008	1.00387	1.04992
19009	0.993999	0.917806
19010	1.04101	2.1
19011	1.27119	2.1
19012	1.27119	2.1
19013	1.51748	2.1
19014	3.00129	2.1
19015	3.27303	2.1
19016	3.27303	2.1
19017	3.60855	2.1
19018	2.22399	2.1
19019	1.71995	2.1
19020	1.54793	2.1
19021	1.227 2.1	

19022	1.07624	2.1
19023	1.264 2.1	
19024	1.19323	1.06041
19025	1.21286	0.989075
19026	1.21286	0.936639
19027	1.20859	0.915427
19028	1.60578	0.414961
19029	1.27778	0.353549
19030	1.0525	1.82275
19031	1.05758	1.1395
19032	1.0525	0.949931
19033	1.17042	1.24765
19034	1.62536	0.207091
19035	3.71662	0.202891
19036	1.78169	0.349149
19037	1.91434	0.337936
19038	2.12331	0.374336
19039	1.33406	0.344217
19040	1.38437	2.1
19041	1.51829	2.1
19042	1.51829	2.1
19043	1.6697	2.1
19044	0.947114	2.1
19045	1.13335	2.1
19046	1.13335	2.1
19047	1.00965	2.1
19048	1.47521	2.1
19049	1.27679	2.1
19050	1.14911	2.1
19051	0.93841	2.1
19052	1.68437	2.1
19053	1.53822	2.1
19054	1.53822	2.1
19055	1.39702	2.1
19056	0.726453	2.1
19057	0.754101	2.1
19058	0.754101	2.1
19059	0.69776	2.1
19060	0.964033	2.1
19061	1.62397	1.83924
19062	1.70302	1.67043
19063	1.78637	1.46321
19064	3.68158	0.265203
19065	2.11685	0.253749
19066	1.33125	2.1
19067	1.15471	2.1
19068	1.0465	2.1
19069	1.33125	2.1
19070	1.17683	2.1
19071	1.25889	2.1
19072	1.25889	2.1
19073	1.34239	2.1
19074	1.40591	0.466575
19075	0.79348	0.418239
19076	1.04019	0.341879
19077	0.999187	0.34476
19078	1.30218	0.311852
19079	1.83339	0.311866

19080	1.88574	0.256775
19081	1.5177	0.232996
19082	1.23504	2.1
19083	1.19583	2.1
19084	1.19583	2.1
19085	1.14496	2.1
19086	1.62183	2.1
19087	1.62316	2.1
19088	1.62316	2.1
19089	1.62241	2.1
19090	1.02305	0.731773
19091	1.21136	0.703513
19092	0.993832	0.496368
19093	1.21137	0.644298
19094	1.12064	2.1
19095	1.24965	2.1
19096	1.24965	2.1
19097	1.41077	2.1
19098	1.00052	0.436927
19099	1.49842	0.378415
19100	1.49843	0.339061
19101	1.10801	0.305578
19102	1.54196	1.03265
19103	1.18041	1.26623
19104	1.73571	1.32373
19105	1.37224	1.48815
19106	1.4375	0.43762
19107	1.09529	0.405452
19108	1.44421	0.380312
19109	1.0953	0.392391
19110	1.0753	2.1
19111	0.815365	2.1
19112	0.815365	2.1
19113	0.597537	2.1
19114	1.03609	0.812079
19115	1.18952	0.820171
19116	1.54283	0.544719
19117	1.16719	0.572992
19118	1.16719	0.729705
19119	1.89226	0.702459
19120	2.55919	2.1
19121	2.37492	2.1
19122	2.37492	2.1
19123	2.07435	2.1
19124	1.36016	0.576052
19125	1.70302	0.572674
19126	1.85252	0.491314
19127	1.2111	0.698
19128	1.62905	2.1
19129	1.49692	2.1
19130	1.49692	2.1
19131	1.40395	2.1
19132	2.19083	2.1
19133	1.30542	2.1
19134	1.58736	2.1
19135	2.16579	2.1
19136	2.0815	2.1
19137	1.17935	2.1

19138	1.53803	1.34001
19139	1.09148	0.938962
19140	1.81301	1.20005
19141	1.38333	0.808442
19142	1.04412	2.1
19143	1.44437	2.1
19144	1.4675	0.357685
19145	1.00665	0.381996
19146	1.50951	0.434562
19147	0.983105	0.398945
19148	1.80252	0.516576
19149	0.540005	0.397197
19150	1.2545	0.55638
19151	2.14514	0.501952
19152	1.32051	0.464812
19153	1.82302	0.522046
19154	2.71057	2.1
19155	2.51107	2.1
19156	1.34817	2.1
19157	1.3551	2.1
19158	2.51107	2.1
19159	2.29663	2.1
19160	1.3551	2.1
19161	1.3685	2.1
19162	1.51748	2.1
19163	1.23389	2.1
19164	0.901928	0.297059
19165	1.70302	0.362241
19166	1.66787	0.298457
19167	1.92535	0.377162
19168	1.13571	0.624005
19169	1.02836	0.659435
19170	1.55398	2.1
19171	1.54179	2.1
19172	1.89608	2.1
19173	1.07167	2.1
19174	3.3488	0.404195
19175	0.550044	0.377261
19176	0.962877	0.530904
19177	1.00065	0.583003
19178	1.00613	0.70723
19179	1.07497	0.715529
19180	1.00486	0.147815
19181	1.00486	0.147815
19182	0.726453	2.1
19183	1.32634	0.489722
19184	1.40008	0.604647
19185	1.40008	0.486257
19186	2.42267	0.450583
19187	1.9216	0.283229
19188	1.28066	0.235188
19189	1.79102	1.01149
19190	1.18219	0.510944
19191	1.18219	0.945515
19192	0.709123	0.529675
19193	1.07866	1.9398
19194	1.15038	2.09185
19195	1.15038	1.77723

19196	1.24687	2.08357
19197	1.83613	1.75168
19198	1.99157	1.6696
19199	1.45789	0.217106
19200	1.1706	0.218855
19201	1.74746	0.173043
19202	1.1706	0.17361
19203	1.96383	0.299571
19204	1.67727	0.284077
19205	1.67727	0.271836
19206	1.60329	0.272544
19207	1.18803	2.1
19208	1.3227	2.1
19209	1.3227	2.1
19210	1.40844	2.1
19211	1.07889	2.1
19212	1.07721	2.1
19213	1.07721	2.1
19214	1.00832	2.1
19215	0.953523	0.976122
19216	1.62875	0.968999
19217	1.07427	0.794871
19218	1.19722	0.772476
19219	1.1972	0.732931
19220	1.1599	0.738407
19221	2.97463	0.181361
19222	1.9477	0.17697
19223	1.95222	0.168128
19224	1.75083	0.174188
19225	3.46787	2.1
19226	3.02896	2.1
19227	3.02896	2.1
19228	2.46145	2.1
19229	0.970962	0.228523
19230	1.0227	0.218819
19231	1.12328	0.229401
19232	1.02269	0.251435
19233	0.904978	2.1
19234	0.725887	2.1
19235	0.725887	2.1
19236	0.601592	2.1
19237	0.956294	2.1
19238	1.13179	2.1
19239	0.956294	2.1
19240	1.13179	2.1
19241	1.05556	2.1
19242	1.13179	2.1
19243	1.38859	2.1
19244	1.13179	2.1
19245	1.06659	2.08156
19246	1.05776	2.0936
19247	1.06659	2.1
19248	1.04291	0.983444
19249	1.54699	1.01974
19250	1.42307	1.10225
19251	1.5654	1.35514
19252	1.97906	2.0375
19253	1.15354	0.244763

19254	1.15505	0.272795
19255	1.26455	0.303971
19256	2.50042	0.321049
19257	2.50042	0.335287
19258	3.22585	0.340499
19259	1.19258	0.346223
19260	1.34562	0.331913
19261	1.49515	0.371641
19262	1.66295	0.341672
19263	1.02395	2.1
19264	1.51067	2.1
19265	1.35821	1.68875
19266	2.10671	2.1
19267	1.0305	1.37204
19268	1.07855	1.31158
19269	1.20761	2.1
19270	0.98488	2.1
19271	1.4162	2.1
19272	1.14825	2.1
19273	1.29415	2.1
19274	1.6067	2.1
19275	1.23851	0.493938
19276	1.09651	0.433016
19277	1.60636	2.1
19278	1.53521	2.1
19279	1.53521	2.1
19280	1.46279	2.1
19281	1.28044	0.548333
19282	1.64147	0.560152
19283	1.18422	0.45396
19284	1.68208	0.578635
19285	1.09726	2.1
19286	1.234	2.1
19287	1.234	2.1
19288	1.09483	2.1
19289	1.89318	2.1
19290	1.98448	2.1
19291	1.98448	2.1
19292	2.02612	2.1
19293	1.22026	1.00883
19294	1.23527	0.897293
19295	1.09044	0.982249
19296	0.993366	0.732301
19297	1.22026	0.97159
19298	0.993368	0.85946
19299	2.61357	2.1
19300	2.48208	2.1
19301	2.48208	2.1
19302	2.62868	2.1
19303	1.85439	1.02845
19304	1.81776	0.944254
19305	1.81776	0.896159
19306	2.1587	0.912753
19307	1.8914	0.992794
19308	2.23961	0.931526
19309	1.49357	0.660709
19310	2.23961	0.86464
19311	1.78014	0.817733

19312	1.67635	0.801234
19313	1.66219	0.735216
19314	1.56036	0.719283
19315	1.14721	0.714869
19316	2.22495	0.764195
19317	1.88509	0.421933
19318	1.95249	0.372643
19319	2.11959	0.368476
19320	1.37427	0.324717
19321	1.39379	0.915561
19322	1.17614	0.860465
19323	1.39379	1.4772
19324	1.14397	0.688377
19325	1.09651	2.1
19326	1.42843	1.98745
19327	1.7069	2.1
19328	2.31293	2.04436
19329	1.56839	1.99557
19330	1.78252	0.882592
19331	1.78254	0.836058
19332	1.09142	0.957175
19333	0.99829	0.304399
19334	1.09372	0.302428
19335	1.88175	0.44584
19336	0.898452	0.379819
19337	1.06936	2.1
19338	1.4162	2.1
19339	1.17989	1.87222
19340	1.47411	1.30316
19341	1.25376	1.60149
19342	1.00757	0.935266
19343	1.4185	0.565077
19344	1.02011	0.594289
19345	2.22495	0.62258
19346	1.79542	0.587048
19347	1.79542	0.518377
19348	1.19352	0.494117
19349	2.38566	2.1
19350	1.83255	1.40782
19351	1.62667	2.1
19352	1.17879	1.57781
19353	2.79168	2.1
19354	2.28162	2.1
19355	2.28162	2.1
19356	1.90905	2.1
19357	1.40117	2.1
19358	1.28268	2.1
19359	1.41096	2.1
19360	1.22978	2.1
19361	3.20034	2.1
19362	3.42052	2.1
19363	3.42052	2.1
19364	3.50562	2.1
19365	1.46279	2.1
19366	1.3864	2.1
19367	1.3864	2.1
19368	1.3201	2.1
19369	1.35561	0.187818

19370	0.974077	0.189385
19371	0.967673	0.982425
19372	1.22482	0.908286
19373	1.02305	0.751619
19374	1.22482	0.828162
19375	0.999492	2.1
19376	1.14657	2.1
19377	1.14657	2.1
19378	1.31346	2.1
19379	1.84417	0.207601
19380	1.31777	0.194036
19381	1.19796	0.180496
19382	0.691742	0.177755
19383	1.16725	2.1
19384	1.9679	2.1
19385	1.08848	0.275663
19386	1.15356	0.26164
19387	1.58713	2.1
19388	1.58742	2.1
19389	1.58742	2.1
19390	1.49662	2.1
19391	1.32201	0.853309
19392	1.32097	1.06786
19393	1.32097	0.855621
19394	1.31825	1.08855
19395	1.12028	2.1
19396	1.15852	1.81861
19397	1.15852	2.05316
19398	1.16239	1.39231
19399	1.07624	2.1
19400	1.30047	2.1
19401	1.44847	0.858928
19402	1.55292	0.856593
19403	1.21125	0.147815
19404	1.15262	2.1
19405	1.05025	2.1
19406	1.55351	2.1
19407	1.5329	0.237734
19408	2.26758	0.22286
19409	1.94968	0.341047
19410	1.65747	0.3195
19411	1.42549	0.376232
19412	1.57656	0.345103
19413	2.11157	0.564238
19414	1.56531	0.694157
19415	1.69279	0.487371
19416	1.06204	0.732768
19417	0.947863	1.09795
19418	1.19854	1.37203
19419	0.947862	0.952984
19420	1.31126	0.877364
19421	1.3425	1.4054
19422	1.29976	1.22537
19423	1.05129	0.475309
19424	1.51026	0.497682
19425	1.05129	0.455908
19426	1.2951	0.468854
19427	1.7155	2.1

19428	1.72196	2.1
19429	1.72196	2.1
19430	1.77867	2.1
19431	0.964755	2.1
19432	1.14142	2.1
19433	1.03766	2.1
19434	0.986723	2.1
19435	1.28072	0.908265
19436	1.10609	1.09172
19437	1.28072	0.807403
19438	1.16776	1.56068
19439	1.61687	2.1
19440	1.60965	2.1
19441	1.60965	2.1
19442	1.59654	2.1
19443	1.26512	2.1
19444	1.23964	2.1
19445	2.07997	2.1
19446	2.60048	2.1
19447	1.23732	2.1
19448	1.3372	2.1
19449	2.62253	2.1
19450	2.47056	2.1
19451	0.92782	0.418623
19452	1.40335	0.454649
19453	1.26301	0.454182
19454	1.52668	0.445491
19455	0.99818	0.401785
19456	1.51165	0.46254
19457	1.66213	0.381898
19458	1.55246	0.436468
19459	2.31255	2.1
19460	1.34366	2.1
19461	1.90577	2.1
19462	0.969739	2.1
19463	3.4245	2.1
19464	2.8415	2.1
19465	1.97559	2.1
19466	1.82362	2.1
19467	1.48162	2.1
19468	1.23204	2.1
19469	1.23204	2.1
19470	0.95947	2.1
19471	1.05267	1.77806
19472	1.44091	1.00768
19473	1.05267	1.21257
19474	1.5795	1.66239
19475	0.738711	2.1
19476	1.28549	2.1
19477	1.15694	2.1
19478	1.72061	2.1
19479	1.69858	2.1
19480	0.954563	2.1
19481	0.859087	2.1
19482	0.788886	2.1
19483	1.08732	0.358319
19484	1.3357	0.36427
19485	1.3357	0.358783

19486	1.48676	0.363347
19487	2.15079	2.1
19488	2.12414	2.1
19489	2.12414	2.1
19490	2.04664	2.1
19491	1.98859	2.1
19492	0.992159	2.1
19493	1.48064	2.1
19494	1.2163	2.1
19495	1.26337	2.1
19496	1.63159	2.1
19497	1.00366	0.697751
19498	1.65452	0.650922
19499	1.10408	2.1
19500	1.37704	2.1
19501	1.50224	2.1
19502	1.42866	2.1
19503	1.43939	2.1
19504	1.02784	2.1
19505	1.00763	0.448052
19506	1.23746	0.437294
19507	1.23747	0.443628
19508	1.13642	0.453072
19509	1.49906	0.457311
19510	1.46506	0.379553
19511	1.46506	0.376507
19512	1.33061	0.321715
19513	1.08166	0.493194
19514	1.01052	0.460829
19515	1.02836	0.519528
19516	1.08166	0.48461
19517	1.25334	0.332665
19518	1.23749	0.328808
19519	1.37501	0.403348
19520	1.47354	0.369279
19521	1.30167	2.1
19522	1.29897	2.1
19523	1.51042	2.1
19524	1.07684	2.1
19525	1.07684	2.1
19526	0.765121	2.1
19527	1.5535	1.18219
19528	1.40602	2.1
19529	1.40603	1.33873
19530	1.25669	2.1
19531	1.08276	2.1
19532	1.60948	2.1
19533	1.26929	1.59777
19534	1.09847	2.1
19535	0.827082	0.580574
19536	1.15841	0.609037
19537	1.25063	0.444171
19538	1.14397	0.375714
19539	1.25063	0.850953
19540	1.10476	0.275856
19541	1.14872	0.291122
19542	1.3659	0.331157
19543	1.36589	0.281328

19544	1.4677	0.327878
19545	0.590354	2.1
19546	0.655202	2.1
19547	3.48926	2.1
19548	3.32709	2.1
19549	3.32709	2.1
19550	3.13938	2.1
19551	1.24458	0.463479
19552	1.29681	0.484379
19553	1.40915	0.583887
19554	1.17891	0.731484
19555	1.33171	0.147815
19556	1.37681	0.787783
19557	1.37681	2.1
19558	1.4516	2.1
19559	2.68241	0.250962
19560	1.61031	0.233523
19561	1.6488	2.1
19562	1.6585	2.1
19563	1.65848	2.1
19564	1.67061	2.1
19565	1.15152	2.1
19566	1.32548	2.1
19567	1.43677	2.1
19568	1.10264	2.1
19569	1.01472	2.1
19570	0.966964	2.1
19571	2.867	2.1
19572	2.94897	2.1
19573	1.65815	2.1
19574	1.4502	2.1
19575	1.4502	2.1
19576	1.26755	2.1
19577	1.37138	2.1
19578	1.45026	2.1
19579	1.45026	2.1
19580	1.53446	2.1
19581	0.973502	1.76406
19582	1.08542	1.62079
19583	1.13387	0.28069
19584	1.71031	0.247524
19585	1.08911	2.00734
19586	1.10748	2.1
19587	1.21823	2.1
19588	1.25005	2.1
19589	0.657014	1.90489
19590	1.38139	2.1
19591	1.01485	2.1
19592	1.26045	2.1
19593	1.12468	2.1
19594	1.26045	2.1
19595	1.89278	0.909403
19596	1.06433	0.861851
19597	1.87861	0.844479
19598	0.967544	0.772682
19599	1.50319	2.1
19600	3.47245	1.68735
19601	2.2354	0.700314

19602	1.16701	0.551555
19603	1.3592	2.1
19604	1.3305	0.830211
19605	1.3305	1.57718
19606	1.15008	0.700749
19607	1.12457	0.291192
19608	1.40707	0.312555
19609	1.01778	2.1
19610	1.35733	2.1
19611	1.01779	2.1
19612	1.12064	2.1
19613	1.30694	0.87722
19614	1.42716	1.05169
19615	1.42717	1.12243
19616	1.04321	1.21871
19617	1.19352	0.456058
19618	1.32634	0.550647
19619	1.14605	1.5522
19620	1.0417	1.35649
19621	1.19676	0.435572
19622	1.03804	0.44038
19623	2.09455	0.484713
19624	1.15338	0.504739
19625	2.21119	2.1
19626	1.26097	1.96552
19627	1.88394	0.817012
19628	1.24106	0.469689
19629	2.98143	2.1
19630	2.56248	1.25205
19631	1.88394	1.27923
19632	1.68703	0.401845
19633	1.5846	2.1
19634	1.94799	2.1
19635	1.20091	2.0395
19636	1.40393	2.1
19637	3.09031	2.1
19638	2.90243	2.1
19639	1.27227	2.1
19640	1.32592	2.1
19641	2.90247	2.1
19642	2.71057	2.1
19643	1.32594	2.1
19644	1.34817	2.1
19645	1.4092	2.1
19646	1.44859	2.1
19647	1.52008	2.1
19648	1.401	2.1
19649	1.40099	2.1
19650	1.28936	2.1
19651	1.56002	0.167397
19652	1.08811	0.15876
19653	1.46041	0.165351
19654	1.56002	0.156878
19655	1.50524	2.1
19656	1.21304	2.1
19657	1.21304	2.1
19658	0.947115	2.1
19659	1.60665	2.01241

19660	1.85179	1.66037
19661	1.85178	1.67571
19662	2.06277	1.1687
19663	2.17534	2.1
19664	1.23007	2.05334
19665	2.17533	2.07644
19666	1.66635	1.84131
19667	2.00869	2.1
19668	0.986724	2.1
19669	2.00475	2.1
19670	1.99394	2.1
19671	1.67244	0.468139
19672	1.39604	0.435534
19673	1.39604	0.451152
19674	0.954046	0.382986
19675	0.988863	2.0258
19676	1.9864	2.1
19677	1.9864	2.01688
19678	3.38645	2.1
19679	1.46932	0.229354
19680	1.50847	0.2385
19681	1.50848	0.229357
19682	1.42705	0.235927
19683	1.50108	1.89894
19684	1.51621	0.830904
19685	1.94317	0.853152
19686	1.18382	0.728669
19687	2.05091	0.234038
19688	1.88593	0.201606
19689	1.54299	0.192826
19690	0.987265	0.180811
19691	1.27766	2.1
19692	1.5178	2.1
19693	1.5178	2.1
19694	1.81518	2.1
19695	1.04626	1.26115
19696	1.31057	1.27625
19697	1.31057	1.26914
19698	1.22297	1.26944
19699	1.67844	1.24013
19700	1.62862	2.1
19701	1.62112	1.27763
19702	1.87598	2.1
19703	1.17844	2.1
19704	1.37063	2.1
19705	1.37063	2.1
19706	1.68723	2.09247
19707	1.99943	0.336753
19708	1.17631	0.26829
19709	1.4263	1.09812
19710	1.31023	2.1
19711	1.31023	1.14033
19712	1.20871	2.1
19713	1.50356	2.1
19714	1.69311	2.1
19715	1.69599	2.1
19716	1.6801	2.1
19717	1.5843	0.196258

19718	0.840233	0.19822
19719	1.95358	0.202033
19720	1.40216	0.22451
19721	1.39078	2.094
19722	1.18948	1.8977
19723	1.01606	1.57087
19724	1.18948	1.84026
19725	1.63823	2.1
19726	1.67687	2.1
19727	1.67687	2.1
19728	1.52855	2.1
19729	1.11048	2.09684
19730	1.25346	2.1
19731	2.42984	1.48328
19732	3.41731	0.891989
19733	1.2258	2.1
19734	1.2966	2.1
19735	1.2966	2.1
19736	1.37392	2.1
19737	1.41758	2.1
19738	1.35504	2.1
19739	1.35504	2.1
19740	1.30347	2.1
19741	1.50877	2.1
19742	1.11193	2.1
19743	1.40984	2.00693
19744	1.54537	1.80811
19745	1.71638	2.0813
19746	1.77795	1.46196
19747	2.07593	2.1
19748	1.93387	2.1
19749	1.9339	2.1
19750	1.75669	2.1
19751	1.80299	0.931638
19752	1.31251	0.901724
19753	1.31249	0.899143
19754	1.13411	0.851172
19755	1.06313	2.1
19756	1.04921	2.1
19757	1.55766	2.1
19758	1.53728	2.1
19759	1.53729	2.1
19760	1.51789	2.1
19761	0.993021	0.85516
19762	1.02478	0.868464
19763	1.03408	0.860366
19764	1.09056	0.865922
19765	0.697755	2.1
19766	0.632901	2.1
19767	1.34072	0.359048
19768	1.80841	0.404602
19769	1.80841	0.405643
19770	1.17793	0.418068
19771	1.75032	1.64923
19772	1.13076	2.01162
19773	2.11851	2.09068
19774	1.73255	1.86264
19775	1.73255	2.1

19776	1.11181	1.84764
19777	1.43772	2.1
19778	1.67381	2.1
19779	1.52164	2.1
19780	1.60861	2.1
19781	1.30588	0.362609
19782	1.75241	0.359171
19783	1.33026	1.07612
19784	0.962705	1.00147
19785	0.99678	0.321951
19786	1.30572	0.278569
19787	1.41943	0.250006
19788	1.2683	0.227875
19789	1.14145	0.214425
19790	1.01361	0.203865
19791	1.29943	2.1
19792	1.3483	2.1
19793	1.34832	2.1
19794	1.83488	2.1
19795	1.71065	2.1
19796	1.66178	2.1
19797	1.39076	2.1
19798	1.37456	2.1
19799	1.66126	2.1
19800	1.562 2.1	
19801	1.37456	2.1
19802	1.36065	2.1
19803	1.04026	0.712071
19804	1.51898	0.712413
19805	1.67088	0.514397
19806	1.9726	0.597148
19807	1.74852	1.57065
19808	1.95512	1.66482
19809	1.16165	1.04726
19810	1.06584	0.947051
19811	1.39327	2.1
19812	1.41394	2.1
19813	1.41394	2.1
19814	1.45506	2.1
19815	1.39523	2.1
19816	1.21119	2.1
19817	1.09005	2.1
19818	0.921311	2.1
19819	1.58164	2.1
19820	1.77574	2.1
19821	1.73184	2.1
19822	1.59423	2.1
19823	1.8214	1.83571
19824	3.10544	0.966542
19825	1.01243	1.54123
19826	1.30896	0.998323
19827	2.16956	1.04283
19828	1.29608	0.873709
19829	1.38617	1.82637
19830	1.49536	1.68042
19831	2.35239	1.35804
19832	1.24613	1.557
19833	1.94351	2.1

19834	2.05361	2.1
19835	2.05358	2.1
19836	2.38994	2.1
19837	1.13533	2.1
19838	1.08276	2.1
19839	1.55611	0.412484
19840	1.33957	0.392076
19841	1.33957	0.437325
19842	1.69011	0.483816
19843	1.5515	0.883487
19844	1.59106	0.879721
19845	1.02958	1.99558
19846	1.12266	2.1
19847	1.12266	2.06044
19848	1.24925	2.1
19849	1.80223	2.1
19850	1.20242	1.23826
19851	2.13665	0.783174
19852	1.3924	0.776592
19853	0.904894	1.04465
19854	1.82633	1.91666
19855	1.94138	0.23053
19856	1.34009	0.197312
19857	1.15096	2.1
19858	0.994292	2.1
19859	1.10101	2.1
19860	1.15096	2.1
19861	1.50438	2.1
19862	1.67825	2.1
19863	1.67825	2.1
19864	1.91894	2.1
19865	2.04664	2.1
19866	1.92063	2.1
19867	1.72857	2.1
19868	1.89757	2.1
19869	1.61843	0.243625
19870	1.49431	0.235833
19871	2.33925	2.1
19872	1.80683	2.1
19873	1.64257	2.1
19874	1.50108	2.1
19875	2.82561	2.1
19876	2.97847	2.1
19877	2.97848	2.1
19878	3.20034	2.1
19879	0.66055	2.1
19880	1.95919	2.1
19881	1.07976	2.1
19882	1.30237	2.1
19883	1.99943	0.272447
19884	1.31802	0.264458
19885	1.53336	0.354241
19886	1.31802	0.288385
19887	1.27737	0.818353
19888	1.44848	0.828521
19889	2.35499	0.25451
19890	1.30041	0.249517
19891	2.35499	0.220947

19892	1.65381	0.187838
19893	0.996083	2.1
19894	1.28754	2.1
19895	0.996081	2.1
19896	1.44859	2.1
19897	1.24763	1.89692
19898	1.03697	2.0946
19899	0.951273	1.63617
19900	1.60018	1.258
19901	2.0815	1.84049
19902	1.20488	1.96058
19903	2.95785	2.1
19904	2.52245	1.62843
19905	1.68455	0.270432
19906	1.12971	0.230356
19907	1.1952	0.710085
19908	2.0815	0.740012
19909	1.3963	2.1
19910	1.4934	2.1
19911	1.47973	2.1
19912	1.25559	2.1
19913	1.1233	2.1
19914	1.15009	2.1
19915	1.15009	2.1
19916	0.977775	2.1
19917	1.16482	0.967798
19918	1.29313	0.757388
19919	1.29313	0.808275
19920	1.294	0.759157
19921	1.48693	0.457327
19922	1.47608	0.424557
19923	1.36005	1.69083
19924	1.47109	2.1
19925	1.47109	2.04868
19926	1.3515	2.1
19927	2.48828	2.1
19928	2.32378	2.1
19929	2.32378	2.1
19930	2.1692	2.1
19931	1.03217	1.09157
19932	0.953521	1.13565
19933	1.4515	0.334916
19934	3.72406	0.317759
19935	0.963395	1.55344
19936	1.01346	1.08934
19937	1.62241	2.1
19938	1.62027	2.1
19939	1.62027	2.1
19940	1.61687	2.1
19941	1.2593	0.183503
19942	0.981586	0.18967
19943	0.721961	2.1
19944	0.888955	2.1
19945	1.22031	0.147815
19946	1.27566	2.1
19947	2.50066	2.1
19948	1.19814	2.1
19949	1.42278	0.236983

19950	2.02657	0.211758
19951	1.0756	0.887035
19952	1.10049	0.878854
19953	2.26155	2.1
19954	2.15634	2.1
19955	2.15634	2.1
19956	2.01613	2.1
19957	1.94233	0.942272
19958	1.18127	1.14298
19959	1.36865	1.09397
19960	1.75755	1.05372
19961	2.01613	2.1
19962	1.78958	2.1
19963	1.97995	2.1
19964	1.79198	2.1
19965	1.20841	2.1
19966	1.6519	2.1
19967	1.80207	2.1
19968	1.66774	2.1
19969	1.17935	0.32035
19970	1.88071	0.432217
19971	1.58723	0.379312
19972	1.7361	0.353496
19973	0.794165	2.1
19974	1.21327	2.1
19975	1.21327	2.1
19976	1.34268	2.1
19977	1.34497	0.195271
19978	0.793303	0.195985
19979	1.12323	0.188247
19980	1.34496	0.188081
19981	0.928226	0.467951
19982	1.89804	0.538286
19983	2.60141	0.147815
19984	2.60142	2.1
19985	2.60772	0.863776
19986	0.99747	1.79268
19987	1.27096	0.923793
19988	1.27096	0.831322
19989	1.03472	0.913762
19990	1.09962	0.849454
19991	1.21148	1.10367
19992	1.21148	1.00997
19993	1.35025	2.1
19994	1.09795	1.89403
19995	1.00775	2.03446
19996	1.07162	2.1
19997	1.60665	1.99837
19998	1.52984	2.00181
19999	1.61179	2.1
20000	1.70057	2.1
20001	1.76063	2.1
20002	1.14547	0.768346
20003	0.964782	0.725755
20004	1.17208	0.725329
20005	0.964769	0.677154
20006	1.04268	2.1
20007	1.57459	2.1

20008	1.57459	2.1
20009	1.32449	2.1
20010	2.07527	0.946172
20011	1.00013	0.896522
20012	0.813711	0.147815
20013	1.05677	1.96434
20014	1.30664	1.33448
20015	1.96011	1.75605
20016	1.7151	1.13499
20017	1.61309	1.21769
20018	1.18954	0.824203
20019	1.05447	1.83144
20020	1.05446	0.640135
20021	1.11787	0.589177
20022	1.44437	2.1
20023	1.84739	2.03456
20024	1.84739	1.82087
20025	1.06203	2.1
20026	1.78391	2.1
20027	1.89267	2.1
20028	1.9212	2.1
20029	2.02997	2.1
20030	1.32051	0.650902
20031	1.47169	0.572866
20032	1.20691	0.27875
20033	1.72271	0.251396
20034	1.2858	0.679458
20035	1.31011	0.842455
20036	1.31011	0.66008
20037	1.34124	0.836896
20038	1.294	0.85972
20039	1.03079	0.758871
20040	1.83192	2.1
20041	1.81979	2.1
20042	1.81979	2.1
20043	1.57829	2.1
20044	1.35435	1.96524
20045	1.19023	2.0966
20046	0.991115	2.1
20047	1.27725	2.1
20048	1.38204	2.1
20049	1.48013	2.1
20050	0.983493	2.1
20051	2.26155	2.1
20052	1.02311	0.859677
20053	1.55485	0.818323
20054	1.71033	0.817837
20055	2.07319	0.772408
20056	2.29074	0.82923
20057	1.50822	0.931045
20058	2.08147	1.76361
20059	1.1585	2.1
20060	3.74092	1.04772
20061	2.40799	2.1
20062	2.05349	1.62745
20063	1.92656	2.1
20064	1.09634	0.508455
20065	1.45502	0.614994

20066	1.17261	0.535292
20067	1.29383	0.467325
20068	1.29383	0.458898
20069	1.47689	0.434989
20070	1.85717	0.791885
20071	1.53798	0.646362
20072	1.9493	1.90349
20073	2.0371	1.28057
20074	1.8519	1.52117
20075	1.92119	1.1325
20076	1.00764	0.376257
20077	1.0302	0.3612
20078	1.0302	0.352467
20079	0.90047	0.35884
20080	1.49155	0.490156
20081	2.26403	0.368572
20082	2.06158	0.375067
20083	1.09506	0.361702
20084	1.9212	1.1497
20085	1.99586	1.01835
20086	1.99586	1.07186
20087	2.07527	0.988888
20088	1.20478	2.1
20089	1.09662	2.1
20090	1.55961	2.1
20091	1.03472	2.1
20092	1.54983	2.1
20093	2.02953	2.1
20094	1.84502	2.1
20095	2.1221	2.1
20096	1.02969	1.60107
20097	2.23523	1.96527
20098	1.2377	0.69818
20099	1.03818	0.701511
20100	1.978 2.1	
20101	1.78159	2.1
20102	1.45762	2.1
20103	1.34234	2.1
20104	2.01624	2.1
20105	2.52072	2.1
20106	1.23013	2.1
20107	2.5593	2.1
20108	3.06464	1.19406
20109	2.63697	0.879191
20110	2.63697	1.08571
20111	1.87111	0.827085
20112	1.04574	0.462325
20113	2.13428	0.398471
20114	1.61715	2.1
20115	1.44137	2.1
20116	1.44867	2.1
20117	1.18283	2.1
20118	1.50584	0.421499
20119	1.45466	0.429857
20120	1.4959	0.476501
20121	1.93489	0.486028
20122	2.60772	2.1
20123	2.68868	2.1

20124	2.68868	2.1
20125	2.82561	2.1
20126	1.49654	2.1
20127	1.38672	2.1
20128	1.36813	2.1
20129	1.98396	2.1
20130	1.93852	1.17548
20131	2.14237	0.918414
20132	1.44421	0.306545
20133	1.21749	0.280685
20134	1.09572	0.265872
20135	1.11081	0.228745
20136	2.32363	2.1
20137	1.24375	2.1
20138	3.79242	2.1
20139	3.46787	2.1
20140	1.19854	1.45075
20141	1.35487	1.48275
20142	1.35486	1.61477
20143	1.38616	1.61934
20144	1.67265	0.676445
20145	0.909487	0.588755
20146	2.53562	2.1
20147	1.09564	2.1
20148	2.9068	2.06359
20149	3.57307	0.842744
20150	2.50369	1.15989
20151	1.47348	1.91589
20152	1.24925	2.0546
20153	1.3991	2.1
20154	1.3991	2.1
20155	1.52984	2.1
20156	1.60428	2.1
20157	1.50097	2.1
20158	1.50097	2.1
20159	1.40239	2.1
20160	2.19621	2.1
20161	2.35003	2.1
20162	1.57932	0.327344
20163	1.61194	0.269353
20164	2.0815	0.236836
20165	1.18539	0.207951
20166	1.5795	0.935655
20167	1.53064	1.07383
20168	1.89227	0.166177
20169	3.01037	0.160113
20170	2.08149	1.61522
20171	2.06678	1.51223
20172	1.89226	0.226013
20173	3.47888	0.232474
20174	1.89226	0.4077
20175	3.65842	0.293615
20176	3.88546	0.414759
20177	1.89226	0.565934
20178	1.11239	2.1
20179	1.29133	0.243595
20180	0.889309	0.24462
20181	0.881934	0.27345

20182	1.88406	2.0583
20183	0.636946	1.90833
20184	0.636945	1.599
20185	1.28073	2.1
20186	1.28075	1.98225
20187	0.942007	1.99033
20188	1.68436	2.1
20189	1.4677	0.241875
20190	0.851542	0.363349
20191	1.39063	0.286979
20192	0.851539	0.338455
20193	1.11597	0.329912
20194	1.35403	0.317077
20195	0.959473	2.1
20196	1.07976	2.1
20197	0.946129	1.04411
20198	0.946129	0.575946
20199	1.38464	0.881815
20200	1.36991	0.703972
20201	0.946131	0.812544
20202	0.94613	0.787128
20203	1.34493	0.677926
20204	1.36991	0.669334
20205	1.36991	0.418595
20206	0.946131	0.688345
20207	0.78392	0.776844
20208	0.80282	0.776117
20209	1.10351	0.752927
20210	0.80846	0.839775
20211	0.838026	0.785197
20212	1.08558	0.805264
20213	0.718203	0.832868
20214	0.501866	0.792919
20215	0.85426	0.85392
20216	1.24249	0.452895
20217	1.37052	0.56521
20218	0.851541	0.572828
20219	1.0092	0.44287
20220	0.851554	0.510582
20221	1.49826	0.415064
20222	1.13727	0.350242
20223	0.778149	0.514622
20224	1.20257	0.700792
20225	0.946133	0.70219
20226	0.778149	0.540093
20227	0.77815	0.553604
20228	0.946132	2.1
20229	1.17602	2.1
20230	0.946132	2.1
20231	1.31257	2.1
20232	1.17602	2.1
20233	1.35821	0.68209
20234	1.19993	0.626016
20235	0.946131	0.631349
20236	0.946131	0.681561
20237	1.08848	0.671408
20238	1.00509	2.1
20239	1.19629	2.1

20240	0.867593	2.1
20241	0.757921	2.1
20242	0.729116	0.261165
20243	0.89162	0.28737
20244	1.16811	0.257755
20245	1.3676	2.1
20246	1.2638	1.08312
20247	0.946132	2.05511
20248	0.946132	2.08922
20249	1.2638	2.06698
20250	1.00185	2.1
20251	1.60948	2.1
20252	0.602075	2.06942
20253	0.942611	2.1
20254	0.946132	2.07425
20255	0.942611	2.1
20256	1.11822	2.1
20257	1.21558	0.635477
20258	0.607788	0.719006
20259	0.827029	0.693785
20260	1.00691	0.906925
20261	0.607789	0.697009
20262	0.607775	0.843681
20263	1.23248	1.06859
20264	0.902653	1.21163
20265	0.639143	1.23249
20266	0.851545	1.11338
20267	0.605153	1.1051
20268	1.06826	2.1
20269	1.29365	2.1
20270	1.41232	2.1
20271	0.946131	2.1
20272	1.20854	2.1
20273	0.946131	2.1
20274	0.909005	0.291444
20275	1.16672	0.252632
20276	0.810959	0.259215
20277	0.946131	0.391254
20278	1.40104	0.306841
20279	0.946131	0.3703
20280	1.35403	0.346866
20281	1.40104	0.337559
20282	1.92535	0.281345
20283	2.14534	0.269104
20284	1.77532	0.349166
20285	0.851541	0.468428
20286	1.05585	0.522864
20287	1.27098	0.450124
20288	0.870925	0.503482
20289	1.15547	0.507464
20290	1.47583	0.404042
20291	1.35007	0.303039
20292	0.740095	0.208004
20293	0.644912	0.214187
20294	0.819489	0.203106
20295	0.983028	0.561987
20296	0.861849	0.207609
20297	1.40511	0.21976

20298	0.86182	0.24575
20299	0.9705	0.222086
20300	1.40511	0.217151
20301	1.15539	2.1
20302	0.946119	1.74513
20303	0.736382	2.1
20304	0.736384	2.1
20305	1.41561	2.1
20306	0.785651	2.1
20307	1.34479	2.1
20308	1.38885	2.1
20309	0.94613	2.1
20310	1.29461	2.1
20311	0.94613	2.1
20312	0.776179	1.78404
20313	0.858611	2.09514
20314	0.946128	1.74289
20315	1.04073	2.1
20316	1.16399	2.04976
20317	0.990019	2.1
20318	1.15564	1.73327
20319	1.28956	0.356394
20320	1.35145	0.351456
20321	1.09662	2.1
20322	1.8155	0.18108
20323	0.811459	0.17267
20324	0.731982	0.16639
20325	0.900554	0.182035
20326	0.946127	0.984117
20327	0.817066	1.00343
20328	0.817064	0.987189
20329	1.41798	1.02945
20330	0.871606	1.01594
20331	1.30265	0.406825
20332	0.849402	1.77518
20333	0.868582	1.45213
20334	0.909137	1.56225
20335	0.807016	1.46476
20336	0.83393	1.0116
20337	0.9034	1.2526
20338	1.53515	1.27273
20339	1.17665	0.70498
20340	0.946132	0.837942
20341	0.946129	0.696536
20342	1.35977	0.688243
20343	1.34254	0.663588
20344	1.14241	0.574917
20345	0.946132	0.708138
20346	0.946132	0.574285
20347	1.27376	0.580252
20348	1.35978	0.560862
20349	1.01976	0.544972
20350	1.23498	1.15387
20351	1.35002	0.995451
20352	1.11683	0.99692
20353	0.88359	0.721481
20354	0.751374	0.749114
20355	0.791714	0.736814

20356	1.6067	2.1
20357	0.923782	2.1
20358	1.44689	2.1
20359	0.94613	2.1
20360	0.946131	2.1
20361	1.5261	2.1
20362	1.26247	2.04435
20363	1.80085	0.325565
20364	1.7693	0.304584
20365	0.900422	0.36269
20366	1.31357	0.49887
20367	0.94613	0.470073
20368	0.900424	0.45152
20369	0.900397	0.440482
20370	1.39448	0.445712
20371	1.36769	0.217844
20372	0.713524	0.299497
20373	0.713554	0.233277
20374	0.946124	0.279726
20375	1.24979	0.233219
20376	1.04765	0.281111
20377	0.70054	0.207073
20378	0.776991	0.231618
20379	0.738905	0.21744
20380	1.32605	0.20583
20381	0.784957	0.20717
20382	0.803955	0.228845
20383	0.819486	0.21054
20384	1.79306	0.36171
20385	1.18275	0.401649
20386	1.25626	0.364286
20387	0.936806	0.332383
20388	0.653124	0.438189
20389	0.946132	0.418697
20390	0.653151	0.411431
20391	1.10748	0.335058
20392	1.95244	2.1
20393	1.40967	1.50455
20394	1.27916	1.46109
20395	0.946132	0.838448
20396	0.946132	1.00507
20397	1.30555	1.03997
20398	1.38651	1.12474
20399	0.946132	0.959077
20400	1.23571	1.07596
20401	1.21261	1.37231
20402	1.34225	1.23291
20403	0.946132	1.47357
20404	0.734059	0.962972
20405	0.734084	1.19405
20406	0.78438	0.760191
20407	1.13748	0.954437
20408	0.946131	1.05858
20409	0.78438	0.76412
20410	0.784392	0.920863
20411	0.626495	2.1
20412	0.694635	2.1
20413	0.946125	2.1

20414	0.694632	2.1
20415	1.38521	2.1
20416	0.795469	2.1
20417	0.850877	2.1
20418	1.37098	2.1
20419	0.946131	2.1
20420	0.79547	2.1
20421	0.946132	0.732767
20422	1.30448	0.755916
20423	0.628361	0.724387
20424	0.686964	0.700262
20425	0.493261	0.692256
20426	1.90912	1.19789
20427	0.946127	0.904234
20428	1.36604	0.696414
20429	0.671687	0.707893
20430	0.946128	0.775438
20431	0.671699	0.718273
20432	1.04973	2.1
20433	0.982858	0.296493
20434	0.974081	0.166578
20435	1.69993	0.161948
20436	1.00763	0.804618
20437	0.755566	2.1
20438	0.946129	2.1
20439	1.04184	2.1
20440	1.13509	2.1
20441	0.755566	2.1
20442	1.114 2.1	
20443	0.810946	2.1
20444	0.786259	2.1
20445	1.43455	2.1
20446	1.05071	2.1
20447	0.746095	2.04815
20448	0.82737	2.1
20449	0.946126	1.96055
20450	1.04071	2.1
20451	1.0852	2.1
20452	1.59019	0.218717
20453	0.621972	0.260818
20454	1.14475	0.264019
20455	0.691062	0.285055
20456	1.20057	0.255775
20457	0.846868	0.26828
20458	1.26062	0.224543
20459	0.795093	0.242422
20460	1.20056	0.242395
20461	0.795065	0.266705
20462	1.18203	0.236957
20463	1.20057	0.242159
20464	0.991869	0.437927
20465	0.841088	2.1
20466	0.946131	2.1
20467	0.869097	2.1
20468	0.946131	2.1
20469	0.841089	2.1
20470	1.77867	2.1
20471	1.99296	0.387359

20472	1.91703	2.1
20473	0.651334	0.323272
20474	1.31467	0.324083
20475	0.651332	0.312484
20476	0.651332	0.317438
20477	1.03079	0.338398
20478	0.808519	0.275933
20479	1.41417	0.250421
20480	1.03165	0.539465
20481	1.43577	2.1
20482	1.39053	2.1
20483	1.07006	2.1
20484	0.946132	2.1
20485	0.946133	2.1
20486	1.07006	2.1
20487	1.12717	2.1
20488	0.994293	2.1
20489	0.946132	2.1
20490	1.31912	2.1
20491	0.946131	2.1
20492	1.40571	2.1
20493	1.31912	2.1
20494	1.12292	0.552622
20495	1.07755	0.665134
20496	0.851539	0.556767
20497	0.946131	0.478327
20498	0.929834	0.47695
20499	1.83919	0.812819
20500	1.04713	0.581635
20501	0.929726	0.401673
20502	1.50985	0.430409
20503	0.946127	0.420517
20504	1.0227	0.442446
20505	1.2582	0.425706
20506	1.1391	0.845454
20507	0.780831	0.940722
20508	0.78086	0.866759
20509	0.946133	0.92234
20510	0.780831	0.858589
20511	1.37762	0.912878
20512	0.946116	1.88984
20513	1.22882	2.1
20514	1.40015	1.72765
20515	1.40017	2.1
20516	0.946105	2.1
20517	0.553567	0.189509
20518	1.19263	0.179822
20519	0.946132	0.185353
20520	0.553594	0.17923
20521	1.19263	0.186942
20522	1.18237	0.180591
20523	0.946133	0.177346
20524	0.978467	0.170954
20525	0.56262	0.173111
20526	0.946132	0.173251
20527	0.562633	0.170771
20528	0.900175	0.168531
20529	1.04627	1.18988

20530	0.549928	0.864504
20531	1.17765	0.984116
20532	1.11479	1.00879
20533	0.398261	0.931016
20534	0.927608	1.04372
20535	1.38467	0.847574
20536	1.04824	0.80199
20537	1.33893	0.984412
20538	1.55775	0.866337
20539	0.816895	0.874579
20540	1.96057	1.04877
20541	1.35798	2.1
20542	1.29262	2.1
20543	0.94613	2.1
20544	0.94613	2.1
20545	1.29261	2.1
20546	1.18067	2.1
20547	0.946131	2.1
20548	0.946132	2.1
20549	1.27809	2.1
20550	1.35799	2.1
20551	1.03933	2.1
20552	1.49438	1.92693
20553	0.946132	2.1
20554	1.13537	2.1
20555	1.41666	2.00772
20556	1.74237	2.1
20557	0.946132	2.1
20558	1.04291	0.476796
20559	1.63802	2.1
20560	1.24714	1.60274
20561	0.842013	1.9214
20562	0.810959	2.1
20563	0.767923	0.691367
20564	1.19583	0.697382
20565	0.767921	0.682952
20566	0.767921	0.697751
20567	0.961348	0.702537
20568	2.08687	1.3934
20569	0.739729	0.830896
20570	1.04342	0.811721
20571	0.739757	0.814937
20572	0.946129	0.841811
20573	1.04341	0.94494
20574	1.38683	0.463822
20575	1.34153	0.458664
20576	0.946129	0.464242
20577	0.946131	0.44
20578	1.02028	0.403258
20579	1.47606	0.367519
20580	1.20739	0.436278
20581	0.957684	0.387789
20582	0.946116	0.466613
20583	0.851528	0.461405
20584	0.957685	0.443105
20585	1.04504	2.1
20586	0.902938	2.1
20587	0.946132	2.1

20588	1.07566	2.1
20589	1.4558	2.1
20590	1.93286	2.1
20591	1.27241	2.1
20592	0.828671	2.1
20593	0.810933	2.1
20594	1.31335	0.215954
20595	1.40305	0.206849
20596	0.912698	0.263937
20597	0.810946	0.251906
20598	1.11608	0.246117
20599	1.26409	0.601423
20600	0.946132	0.716928
20601	1.27376	0.742698
20602	0.946132	0.725414
20603	1.03333	0.748588
20604	1.27376	0.745741
20605	0.94613	0.598068
20606	0.812384	0.577758
20607	0.687744	0.495766
20608	0.946132	0.62777
20609	0.687758	0.577685
20610	1.09667	0.531573
20611	1.56977	2.1
20612	0.851544	2.00561
20613	1.01136	2.1
20614	0.851481	1.80878
20615	0.946131	2.1
20616	0.705409	2.1
20617	1.32449	0.341374
20618	1.13508	0.31286
20619	0.94613	0.408222
20620	0.94613	0.37039
20621	0.965822	0.371029
20622	1.95301	0.422161
20623	0.851538	0.673131
20624	1.29262	0.630961
20625	0.95417	0.531947
20626	0.851541	0.699865
20627	0.94613	0.640773
20628	1.02727	0.879811
20629	1.37675	1.05534
20630	0.955646	0.83675
20631	0.95114	0.859274
20632	0.851528	0.865447
20633	1.76513	1.25154
20634	0.87155	2.1
20635	0.94613	2.1
20636	0.871519	2.1
20637	0.966631	2.1
20638	1.37791	2.1
20639	1.19122	0.945209
20640	0.818971	1.29989
20641	0.628799	0.836707
20642	0.861027	1.16697
20643	0.800034	0.299889
20644	0.91846	0.263388
20645	0.765645	0.267883

20646	1.13709	0.923485
20647	1.29054	0.948759
20648	0.755336	1.02053
20649	0.755334	0.955855
20650	0.946133	1.03708
20651	0.755336	0.888461
20652	1.14444	0.952246
20653	0.872823	0.964206
20654	0.533645	0.936798
20655	0.946132	0.89052
20656	0.533645	0.814205
20657	1.02311	0.869637
20658	1.00278	2.1
20659	1.1016	0.22517
20660	0.629615	0.237837
20661	1.12628	0.280466
20662	0.946131	0.290064
20663	0.629615	0.23126
20664	0.629641	0.269995
20665	1.67046	0.996329
20666	1.81667	2.1
20667	1.7145	2.1
20668	0.94613	2.1
20669	1.29322	2.1
20670	1.00365	2.1
20671	0.946131	2.1
20672	1.29322	2.1
20673	1.27581	2.1
20674	1.33916	2.1
20675	1.32784	2.1
20676	1.74787	2.1
20677	2.28084	2.1
20678	2.98092	2.1
20679	0.946132	1.01923
20680	1.2435	2.1
20681	0.946132	1.84253
20682	1.4099	1.08635
20683	1.2435	1.45707
20684	1.26438	2.1
20685	0.745287	2.1
20686	0.696827	2.1
20687	0.696781	2.1
20688	1.41195	2.1
20689	0.946131	2.1
20690	1.8758	1.14392
20691	0.832979	0.859468
20692	0.75054	0.726352
20693	0.810958	0.773672
20694	0.922889	0.947324
20695	0.694844	0.790357
20696	0.694814	0.867689
20697	0.946131	0.83432
20698	0.694844	0.829791
20699	1.02369	2.1
20700	1.4468	0.288993
20701	0.946125	0.687494
20702	0.93487	0.563124
20703	1.23608	0.707823

20704	1.34257	0.470028
20705	0.851525	0.596607
20706	1.03298	0.45814
20707	1.22418	1.04916
20708	0.946131	1.44988
20709	1.41833	1.12328
20710	1.23452	1.01338
20711	0.946131	0.869096
20712	1.41833	0.981313
20713	1.13077	0.690234
20714	0.942874	1.37742
20715	0.946132	1.25495
20716	0.504366	1.33918
20717	0.593647	2.05511
20718	0.898829	1.3933
20719	1.27921	1.86329
20720	1.12503	2.1
20721	1.22539	2.1
20722	1.10332	1.13419
20723	0.851541	2.00049
20724	0.94613	2.05766
20725	0.945109	2.1
20726	0.607962	2.1
20727	0.946133	2.1
20728	0.607961	2.1
20729	0.661956	2.1
20730	1.35048	2.1
20731	1.66018	2.1
20732	0.956467	2.1
20733	0.851542	2.1
20734	0.890734	2.1
20735	0.802455	2.1
20736	1.35943	2.1
20737	1.04798	2.1
20738	1.26426	0.79008
20739	1.3689	0.736263
20740	0.946133	0.747941
20741	0.946133	0.797406
20742	1.09729	0.778382
20743	0.765259	2.1
20744	0.810945	2.1
20745	0.810957	2.1
20746	1.10284	1.90206
20747	1.09368	2.1
20748	0.772601	2.1
20749	0.788011	2.1
20750	0.810934	2.1
20751	0.694357	1.00714
20752	1.22641	1.10087
20753	0.946132	1.23953
20754	0.946132	0.897174
20755	0.946133	0.929939
20756	1.42754	1.45221
20757	0.776414	0.912982
20758	0.806525	1.00039
20759	0.575339	1.03166
20760	0.97154	0.897399
20761	0.8217	1.05214

20762	0.838039	1.18745
20763	0.94613	1.06102
20764	0.946132	0.923805
20765	1.39575	0.793394
20766	1.39575	0.871945
20767	1.12818	0.895551
20768	0.946132	1.21728
20769	0.946133	1.05766
20770	1.06879	0.872809
20771	1.06879	0.976691
20772	0.970358	0.971005
20773	1.2474	0.669699
20774	1.01919	0.302896
20775	0.94613	0.37435
20776	1.28952	0.354457
20777	1.00809	0.316322
20778	0.849344	0.327675
20779	0.640304	0.346977
20780	1.35065	0.274488
20781	0.946132	0.273053
20782	0.640333	0.253271
20783	0.946132	0.323423
20784	0.685489	2.1
20785	0.94613	2.1
20786	0.68546	2.1
20787	0.874264	2.1
20788	1.27676	2.1
20789	0.946129	0.350147
20790	1.09024	0.334124
20791	0.714563	0.353815
20792	1.12335	0.329537
20793	1.07931	0.31836
20794	1.17799	0.678272
20795	1.8795	0.528831
20796	2.15229	0.524001
20797	1.13859	0.903732
20798	0.946132	1.20752
20799	1.11374	1.03441
20800	1.1386	1.01141
20801	0.615713	1.04315
20802	1.19322	0.681203
20803	1.3377	0.546303
20804	0.670801	0.613188
20805	0.94613	1.0092
20806	0.66617	0.856284
20807	1.36173	0.863934
20808	0.980473	0.809855
20809	1.39132	0.451738
20810	0.864653	0.262498
20811	1.23837	0.23568
20812	0.810959	0.233827
20813	1.1483	0.546562
20814	1.00381	1.16523
20815	0.946128	1.53466
20816	1.39696	1.22354
20817	0.946131	1.04027
20818	0.946131	1.16171
20819	1.38613	1.22227

20820	0.973501	1.62532
20821	0.946127	1.18777
20822	1.35968	1.12103
20823	0.946131	0.958564
20824	0.946129	1.2082
20825	1.39696	1.20028
20826	1.12088	1.82403
20827	1.41122	1.60606
20828	1.14464	0.901037
20829	0.946131	1.70861
20830	0.946131	1.37984
20831	1.14464	1.5111
20832	1.33266	1.24868
20833	1.07341	0.964961
20834	0.827287	1.3537
20835	0.946132	1.08419
20836	0.827287	1.53503
20837	1.15644	1.23922
20838	1.27558	0.809874
20839	1.15067	0.907964
20840	0.946131	2.00513
20841	1.29143	0.84912
20842	1.36281	2.09123
20843	1.36281	0.997347
20844	0.946131	1.00735
20845	1.40863	2.1
20846	1.20143	0.39882
20847	1.19755	0.37315
20848	0.566153	0.332009
20849	0.946131	0.463468
20850	1.07979	0.333456
20851	1.00463	0.458927
20852	0.566153	0.571232
20853	0.768212	2.1
20854	1.32461	1.99528
20855	1.13268	2.1
20856	1.13268	1.73004
20857	0.946133	1.64283
20858	1.53642	2.06926
20859	1.5791	2.1
20860	0.726635	1.84571
20861	1.2334	1.6243
20862	1.29198	1.78043
20863	1.29198	1.31092
20864	0.946131	1.19062
20865	0.956585	2.1
20866	1.25294	2.1
20867	0.946133	2.0043
20868	1.35287	2.1
20869	1.23877	1.99531
20870	0.946131	1.99158
20871	1.35287	2.00304
20872	1.01843	2.1
20873	3.77569	2.1
20874	0.86707	2.1
20875	0.867066	2.1
20876	1.38639	2.1
20877	1.38639	2.1

20878	0.896168	2.1
20879	0.974716	0.447056
20880	1.09411	0.553351
20881	0.663171	0.5053
20882	0.663171	0.617216
20883	0.946132	0.603488
20884	0.66317	0.580674
20885	1.47104	0.398389
20886	1.13629	0.457141
20887	0.772342	0.390603
20888	0.772312	0.51222
20889	0.94613	0.485065
20890	0.77234	0.476216
20891	1.43154	2.1
20892	1.37704	2.1
20893	1.40025	2.1
20894	1.3063	2.1
20895	0.946132	2.1
20896	1.04075	2.1
20897	0.946131	2.1
20898	0.946118	1.4319
20899	0.750011	0.782147
20900	1.0971	0.951106
20901	0.889667	0.860249
20902	0.709598	0.972727
20903	2.39737	0.994803
20904	1.19974	0.377767
20905	0.94613	0.547479
20906	1.25809	0.424339
20907	1.19974	0.501167
20908	0.73005	0.640942
20909	1.50764	0.3098
20910	1.25809	0.377711
20911	0.946133	0.554562
20912	1.10357	0.440263
20913	1.25809	0.491727
20914	0.628177	0.605981
20915	1.3543	0.39158
20916	0.793711	0.311253
20917	1.18455	0.332568
20918	1.03966	0.296547
20919	0.946131	0.337913
20920	0.767453	0.290659
20921	0.612292	0.354717
20922	0.946131	0.322127
20923	1.24128	0.342005
20924	1.08761	0.285773
20925	0.762059	0.243007
20926	1.005 2.1	
20927	1.17054	2.1
20928	1.12665	2.1
20929	0.750368	2.1
20930	0.851542	2.1
20931	0.975436	2.1
20932	0.975442	2.1
20933	0.675367	2.1
20934	1.51973	0.612498
20935	0.94613	0.598597

20936	1.38954	0.604414
20937	0.946131	0.60241
20938	1.07912	0.61996
20939	1.38954	0.611969
20940	2.0219	2.1
20941	1.18347	0.504014
20942	0.946132	0.824365
20943	0.94613	0.714643
20944	1.00202	0.624979
20945	1.00201	0.654959
20946	0.946132	0.736938
20947	0.946132	0.890628
20948	1.37981	0.788495
20949	1.26874	0.72521
20950	1.26874	0.730259
20951	0.946132	0.785377
20952	2.8687	0.566764
20953	1.42884	2.1
20954	1.05188	2.1
20955	0.625307	2.1
20956	0.810956	2.1
20957	0.526656	2.1
20958	0.526684	2.1
20959	0.724122	2.1
20960	0.946132	2.1
20961	0.526684	2.1
20962	1.05337	2.1
20963	1.0593	2.1
20964	1.47981	2.1
20965	1.74431	2.1
20966	0.885612	2.05563
20967	0.810946	1.61161
20968	0.845185	1.82191
20969	1.38622	0.864768
20970	0.723194	0.844251
20971	0.554302	0.930353
20972	0.709597	0.795271
20973	0.909507	0.823811
20974	0.912754	0.690736
20975	1.15174	0.589309
20976	0.946131	0.706506
20977	0.912753	0.69623
20978	1.15174	0.70261
20979	1.02011	0.463731
20980	0.792754	1.96712
20981	0.946129	2.08287
20982	0.792754	1.9071
20983	1.27575	1.86804
20984	0.94613	1.85016
20985	1.60132	2.1
20986	0.922455	2.027
20987	1.18713	1.62939
20988	0.926957	1.80546
20989	1.66525	1.95506
20990	0.946132	2.1
20991	0.851541	2.1
20992	0.961191	2.1
20993	1.27534	2.1

20994	0.851554	2.1
20995	1.0499	2.1
20996	1.01776	0.256473
20997	0.97622	0.250626
20998	0.810934	0.282154
20999	2.6802	0.239771
21000	1.31485	0.239959
21001	1.09699	0.209611
21002	0.681649	0.203469
21003	0.790369	0.19834
21004	0.946131	0.228116
21005	0.838026	1.53019
21006	0.567833	1.02986
21007	0.891492	1.37722
21008	0.986984	0.896955
21009	0.946132	1.27432
21010	1.19028	1.01928
21011	0.986984	0.988204
21012	0.595623	1.10657
21013	0.946131	2.1
21014	1.36502	2.1
21015	1.08109	2.1
21016	0.946129	2.1
21017	1.36503	2.1
21018	0.773897	1.97582
21019	0.946133	1.30442
21020	0.613447	2.04697
21021	0.613449	1.73922
21022	0.946131	1.93569
21023	1.11163	1.87114
21024	0.966114	2.1
21025	0.735699	2.1
21026	0.662147	2.1
21027	1.04072	2.1
21028	0.946131	2.1
21029	0.862978	2.1
21030	1.48839	1.01204
21031	0.936141	0.988184
21032	0.946131	1.20187
21033	0.936142	1.99538
21034	1.40595	1.656
21035	0.946131	1.22804
21036	1.05297	0.275296
21037	3.09035	2.1
21038	1.19952	2.1
21039	0.907198	2.1
21040	0.849144	2.1
21041	0.612469	0.788849
21042	0.551237	0.800648
21043	0.985256	0.865812
21044	0.551209	0.804322
21045	0.85153	0.909046
21046	0.624712	2.1
21047	0.900018	2.1
21048	0.851542	2.1
21049	0.864787	2.1
21050	1.27669	2.1
21051	0.946711	0.805211

21052	0.810947	0.817019
21053	0.841547	0.848625
21054	0.764114	0.857406
21055	1.26692	1.09596
21056	0.946132	0.8613
21057	1.37675	0.876889
21058	1.26692	0.88074
21059	1.37545	1.07529
21060	1.49637	0.448135
21061	0.709598	0.532009
21062	0.751252	0.490937
21063	1.36811	0.489848
21064	0.635625	0.506272
21065	1.31713	0.381751
21066	1.18977	2.1
21067	0.893377	1.19244
21068	0.707344	2.1
21069	0.946131	2.1
21070	1.20221	2.1
21071	1.20221	2.1
21072	0.707345	2.1
21073	1.26786	2.1
21074	0.988048	2.00071
21075	0.751293	1.93068
21076	0.632008	1.79763
21077	1.51067	2.1
21078	1.13534	2.1
21079	0.946133	2.1
21080	0.946131	2.1
21081	1.25716	2.1
21082	1.3199	2.1
21083	1.09616	2.1
21084	1.36677	1.32384
21085	1.34752	1.49666
21086	0.838041	1.22972
21087	1.67023	2.1
21088	1.02247	0.751961
21089	1.03079	0.719669
21090	0.928982	0.491604
21091	1.13635	0.41007
21092	0.946132	0.510664
21093	0.92898	0.427833
21094	1.13635	0.463583
21095	1.1643	0.320301
21096	0.851541	0.58332
21097	1.39939	0.503377
21098	0.946129	0.588474
21099	1.07775	0.549448
21100	1.14503	0.563346
21101	1.78573	0.396618
21102	1.29827	0.393773
21103	0.872373	0.284562
21104	1.01162	0.303261
21105	0.83804	0.277408
21106	1.48851	0.276398
21107	1.47385	0.255825
21108	0.843858	0.340808
21109	0.938626	0.360015

21110	0.89567	0.316363
21111	1.59457	0.828891
21112	2.09666	0.389154
21113	0.775132	0.214856
21114	0.927005	0.245369
21115	0.854224	0.193232
21116	0.775142	0.199904
21117	0.946106	0.219855
21118	1.80784	2.1
21119	2.04593	2.1
21120	1.32282	2.1
21121	0.952138	2.1
21122	0.946132	2.1
21123	0.946132	2.1
21124	0.952138	2.1
21125	1.46988	2.1
21126	0.80465	0.346906
21127	1.14155	0.350621
21128	0.810931	0.347395
21129	1.66203	0.53969
21130	0.674605	0.709221
21131	0.946132	0.712946
21132	1.26706	0.763551
21133	0.674633	0.70875
21134	0.946132	0.73116
21135	0.980664	0.751116
21136	1.34927	0.700938
21137	0.758454	0.587543
21138	0.946133	0.571206
21139	0.985371	0.51059
21140	1.28632	0.517214
21141	0.946133	0.693137
21142	1.46868	0.589079
21143	1.7522	0.301693
21144	0.737431	2.1
21145	0.663679	2.1
21146	1.16541	1.85474
21147	1.3179	2.00658
21148	1.04081	2.00638
21149	0.887866	1.74804
21150	0.806961	1.44463
21151	0.929061	1.69868
21152	1.28418	0.639093
21153	1.38752	0.575108
21154	1.33038	0.548882
21155	0.946132	0.417584
21156	0.946132	0.486041
21157	1.14897	0.497584
21158	1.1685	0.514371
21159	1.18774	2.1
21160	0.961899	2.1
21161	1.48841	2.1
21162	1.79105	2.1
21163	3.51614	2.1
21164	0.682342	2.1
21165	0.783184	2.1
21166	0.946132	2.1
21167	0.868396	2.1

21168	1.21813	2.1
21169	0.848305	0.835934
21170	0.94559	0.859751
21171	0.805691	0.893984
21172	0.752217	2.1
21173	0.946132	2.1
21174	0.839123	2.1
21175	0.946131	2.1
21176	0.752187	2.1
21177	1.96491	2.1
21178	0.520997	2.1
21179	0.946132	2.1
21180	0.73446	2.1
21181	0.946133	2.1
21182	0.520995	2.1
21183	1.88522	2.1
21184	1.8361	2.1
21185	1.11285	2.1
21186	1.25334	1.16275
21187	2.00756	2.1
21188	1.44252	0.610594
21189	1.00255	1.1455
21190	1.64516	2.1
21191	1.12399	0.210317
21192	1.13542	0.246264
21193	1.09038	0.268789
21194	0.746903	0.298501
21195	0.672241	0.236961
21196	0.871774	0.26998
21197	1.73513	0.956118
21198	0.949995	1.9023
21199	1.17244	1.87836
21200	0.946127	1.84268
21201	1.3028	1.80056
21202	1.17244	1.76
21203	0.774101	2.1
21204	0.946127	2.1
21205	0.939289	2.1
21206	0.946129	2.1
21207	0.774101	2.1
21208	1.87858	2.1
21209	1.44293	0.329004
21210	1.43213	0.31665
21211	0.946131	2.1
21212	0.716614	2.1
21213	0.716644	2.1
21214	1.35828	2.1
21215	0.790935	2.1
21216	0.730157	2.1
21217	1.41149	2.1
21218	0.730186	2.1
21219	0.730186	2.1
21220	0.946131	2.1
21221	0.946132	1.21485
21222	0.946132	1.06109
21223	0.990713	0.881409
21224	1.40779	0.941801
21225	0.946132	1.00638

21226	1.06486	0.706442
21227	0.946131	1.04841
21228	0.946133	0.869718
21229	1.40779	0.815074
21230	1.26874	0.858453
21231	0.946131	0.863517
21232	1.02588	0.75525
21233	0.826073	0.481833
21234	1.41233	0.44225
21235	0.861269	0.475968
21236	1.04073	0.528912
21237	1.27111	0.560471
21238	1.56016	1.32076
21239	1.60866	0.257938
21240	0.94613	0.353823
21241	0.946129	0.362943
21242	1.36376	0.275641
21243	1.22187	0.351079
21244	0.94613	0.361784
21245	1.82581	1.0173
21246	1.19705	1.17131
21247	1.7513	2.1
21248	1.85017	2.1
21249	1.72116	2.1
21250	1.12058	2.1
21251	0.899991	2.1
21252	0.616288	2.1
21253	1.07289	2.1
21254	1.7092	0.690391
21255	0.946132	0.560563
21256	0.946133	0.347725
21257	1.18717	0.381705
21258	1.40029	0.583366
21259	0.946132	0.401263
21260	0.94613	0.490015
21261	1.18547	0.486337
21262	0.960326	0.482275
21263	0.960345	0.457762
21264	0.942557	0.505967
21265	1.18601	0.355669
21266	0.946132	0.430354
21267	1.30307	0.447763
21268	1.08169	0.415597
21269	1.08169	0.411395
21270	1.31716	0.453755
21271	1.51065	0.987721
21272	0.877642	0.201885
21273	0.617384	0.192244
21274	0.575741	0.203192
21275	0.972448	0.184358
21276	1.11334	0.201625
21277	1.13092	1.95547
21278	0.946127	0.757366
21279	1.31724	0.821727
21280	1.39751	0.490506
21281	1.39751	0.76598
21282	0.946128	0.759933
21283	0.946132	0.496627

21284	0.713284	0.459287
21285	1.40668	0.565766
21286	1.03547	0.444422
21287	0.713298	0.486171
21288	1.88774	0.398576
21289	0.830164	1.71185
21290	1.05211	2.1
21291	0.946119	1.26851
21292	0.946107	2.07216
21293	1.283 2.1	
21294	2.63609	1.96241
21295	1.28418	0.86697
21296	0.946128	0.946393
21297	1.10335	0.86755
21298	0.598377	0.980341
21299	1.18818	0.865898
21300	1.10335	0.863762
21301	1.58972	1.07114
21302	1.90194	2.1
21303	0.946118	2.1
21304	0.946115	2.1
21305	0.950969	2.1
21306	1.36918	2.1
21307	0.950955	2.1
21308	1.4263	1.93209
21309	1.11978	0.780959
21310	0.946132	0.908932
21311	0.946133	0.959544
21312	1.37658	0.778382
21313	1.2364	0.83854
21314	1.05496	0.846871
21315	0.946132	0.96506
21316	0.946132	0.904129
21317	1.21858	0.813822
21318	1.37658	0.902558
21319	1.09194	1.02884
21320	1.14721	0.308814
21321	1.53814	0.209279
21322	0.546751	0.987829
21323	0.492063	1.24217
21324	1.16889	1.55774
21325	1.00349	1.57948
21326	0.863559	1.10493
21327	1.38102	1.53347
21328	0.597449	1.04056
21329	0.537748	0.930098
21330	1.16297	1.31345
21331	0.999366	1.27771
21332	0.896554	1.0193
21333	0.566962	0.219612
21334	0.946129	0.2533
21335	0.452729	0.191428
21336	0.696552	0.207241
21337	0.852524	0.22237
21338	1.57992	0.193463
21339	1.64985	0.246782
21340	0.946128	2.1
21341	1.21847	2.1

21342	0.946129	2.1
21343	1.41131	2.1
21344	1.21847	2.1
21345	1.21269	0.195596
21346	1.0417	1.48057
21347	1.15821	1.09895
21348	0.946132	0.883789
21349	0.946132	1.18701
21350	1.37967	0.875019
21351	0.851529	0.887638
21352	1.20455	2.1
21353	1.4282	0.234707
21354	1.26593	1.99719
21355	0.838026	0.373897
21356	0.985096	0.341542
21357	0.83804	0.36462
21358	0.822395	0.952116
21359	0.420272	0.73689
21360	0.946131	2.1
21361	1.17461	2.1
21362	0.94613	2.1
21363	1.40225	2.1
21364	1.17461	2.1
21365	1.09847	2.1
21366	1.22222	0.980445
21367	1.41798	0.913532
21368	0.791658	0.950494
21369	0.791656	0.95061
21370	0.946129	0.972724
21371	0.817064	0.95531
21372	0.946114	0.890868
21373	0.75824	0.890918
21374	0.75821	0.894842
21375	1.41778	0.878214
21376	0.791635	0.87969
21377	1.04164	1.45904
21378	0.946131	0.9708
21379	1.11172	0.878483
21380	1.30076	0.819403
21381	1.35721	0.832439
21382	1.11172	0.845114
21383	1.87218	0.562385
21384	0.987248	0.573844
21385	0.729695	0.837002
21386	0.974389	0.71655
21387	1.18002	0.693479
21388	0.822612	0.676707
21389	1.35721	0.684518
21390	0.634295	0.84367
21391	0.634326	0.847997
21392	1.13245	1.12688
21393	1.13245	1.02584
21394	0.937961	0.833785
21395	1.18769	0.983925
21396	0.854949	0.781099
21397	0.967357	0.821293
21398	0.810934	0.77271
21399	3.91082	2.06415

21400	0.946132	2.1
21401	0.946132	2.1
21402	1.05383	2.1
21403	1.05383	2.1
21404	0.986498	2.1
21405	1.50732	0.366925
21406	0.753659	0.460358
21407	1.22144	0.501921
21408	1.30463	0.443934
21409	0.753659	0.48228
21410	1.30463	0.485954
21411	0.886189	0.470774
21412	1.04321	0.457631
21413	0.909928	0.436023
21414	1.18659	0.399369
21415	1.19785	0.268339
21416	1.40969	0.341942
21417	1.24094	0.283455
21418	0.946126	0.341706
21419	1.2929	0.283656
21420	1.24093	0.305108
21421	0.867944	0.287482
21422	1.41903	2.1
21423	2.47881	0.206084
21424	1.12943	0.196698
21425	0.764229	2.07404
21426	0.764256	2.1
21427	0.94613	2.0796
21428	0.94613	2.1
21429	1.1995	2.1
21430	1.83423	2.1
21431	0.634816	1.89834
21432	0.877837	2.1
21433	0.94613	1.92108
21434	1.04073	1.99754
21435	1.06485	2.02835
21436	0.94613	0.578887
21437	0.62725	0.543874
21438	0.798212	0.57168
21439	1.33241	0.483867
21440	0.62725	0.549548
21441	1.82301	0.482927
21442	1.40615	2.1
21443	0.782504	2.1
21444	0.748635	2.1
21445	0.946131	2.1
21446	0.748607	2.1
21447	0.988456	1.79635
21448	1.64833	0.208291
21449	1.35657	0.25179
21450	1.26155	0.249119
21451	0.915755	0.277075
21452	0.915755	0.24708
21453	0.946132	0.264394
21454	1.49386	0.213295
21455	1.01346	2.08007
21456	0.946131	1.02583
21457	0.946132	2.01528

21458	1.38651	2.1
21459	1.39523	2.05043
21460	0.946132	1.90776
21461	1.06312	2.1
21462	0.946131	2.02999
21463	0.946129	2.1
21464	1.39523	2.1
21465	1.37372	2.1
21466	0.946131	2.1
21467	1.2929	0.353239
21468	0.94613	0.450293
21469	1.24448	0.342829
21470	1.2929	0.388037
21471	0.862011	0.365677
21472	1.73583	0.259995
21473	1.724	0.287614
21474	1.03824	2.1
21475	1.534	2.1
21476	0.725492	1.64758
21477	0.771967	1.75955
21478	0.61731	1.25986
21479	0.475743	1.52802
21480	0.719694	1.47777
21481	1.14878	1.20009
21482	0.946117	1.54489
21483	0.94613	1.57185
21484	0.946105	1.60025
21485	0.482666	1.1914
21486	0.609172	1.21181
21487	0.946131	2.1
21488	1.39509	0.926414
21489	1.07439	1.78439
21490	0.946131	1.05813
21491	1.39509	1.21711
21492	0.963571	1.29526
21493	0.591011	0.79788
21494	1.1357	0.632644
21495	2.11193	1.12354
21496	1.60936	2.1
21497	1.34118	0.422788
21498	1.27696	0.443614
21499	0.94613	0.359608
21500	0.94613	0.360969
21501	1.04257	0.33045
21502	1.53336	0.271811
21503	1.0631	0.315002
21504	1.04072	0.430619
21505	0.670678	0.375032
21506	0.709531	0.397127
21507	0.891275	0.409101
21508	0.683583	0.452457
21509	0.902478	0.374855
21510	0.807671	0.387416
21511	1.11024	2.1
21512	1.08171	2.1
21513	1.16725	2.1
21514	0.946128	2.1
21515	1.13537	2.1

21516	1.40525	2.1
21517	1.28365	2.1
21518	1.04523	2.1
21519	0.960106	2.1
21520	0.946128	2.1
21521	1.23452	2.1
21522	0.946129	2.1
21523	1.3704	2.1
21524	1.23452	2.1
21525	1.16906	0.910346
21526	0.946131	1.21411
21527	0.867246	0.954434
21528	0.867217	1.32676
21529	0.946131	1.00338
21530	1.13438	1.14736
21531	1.28684	1.22366
21532	0.946132	1.32587
21533	0.883935	1.12414
21534	0.883938	1.79762
21535	0.946133	1.20555
21536	1.73444	1.0572
21537	1.21835	2.1
21538	1.28228	2.1
21539	1.8643	0.996747
21540	0.743379	0.505278
21541	1.34921	0.497423
21542	0.743381	0.502546
21543	0.743381	0.489581
21544	0.946131	0.534429
21545	0.915528	2.08372
21546	0.982742	2.06895
21547	0.975965	2.06715
21548	0.891132	0.178252
21549	1.27533	0.170591
21550	0.946132	0.169803
21551	1.25215	0.165969
21552	0.590343	0.17155
21553	0.946133	0.169512
21554	0.590331	0.171072
21555	1.62375	2.1
21556	0.687769	0.843785
21557	1.14374	0.836975
21558	1.20885	0.799403
21559	0.946132	0.858193
21560	1.14374	0.902566
21561	1.09308	0.585605
21562	1.02055	0.532715
21563	0.810934	0.493515
21564	0.917609	1.29347
21565	1.13094	1.30705
21566	0.70959	1.25154
21567	1.11223	2.03698
21568	0.83051	1.28525
21569	0.810946	1.48107
21570	0.810959	1.2453
21571	1.5773	0.197615
21572	1.96133	1.10864
21573	1.0214	1.12511

21574	0.762783	1.35404
21575	0.838036	1.02296
21576	0.63591	1.44501
21577	0.967411	1.06768
21578	0.946132	1.84955
21579	0.946132	1.80278
21580	1.39699	1.4926
21581	1.61642	1.69506
21582	0.860954	1.05956
21583	0.957411	1.23545
21584	0.660856	1.25434
21585	1.10003	2.1
21586	0.968249	1.66399
21587	0.910184	1.17668
21588	1.30029	0.815851
21589	0.754119	0.868042
21590	0.754121	1.03552
21591	0.94613	1.05431
21592	1.08039	2.1
21593	0.810942	0.457978
21594	1.15153	0.44158
21595	1.06968	0.416977
21596	1.38456	2.1
21597	1.04118	2.1
21598	0.838027	2.1
21599	1.04073	2.1
21600	0.754209	2.1
21601	1.332	0.235537
21602	1.26193	2.1
21603	1.15317	0.226766
21604	3.86179	0.219031
21605	1.56005	2.1
21606	0.683035	0.659626
21607	0.931463	0.650179
21608	0.556019	0.779521
21609	0.929172	0.754273
21610	0.756874	0.571652
21611	1.23527	0.757046
21612	2.05961	0.780081
21613	0.946132	2.1
21614	1.16684	2.1
21615	1.3937	2.1
21616	1.3937	2.1
21617	0.946132	2.1
21618	1.17885	2.1
21619	1.62507	0.210385
21620	1.58874	2.1
21621	0.819953	2.1
21622	1.16023	2.1
21623	1.34782	2.1
21624	1.16023	2.1
21625	0.819955	2.1
21626	0.79437	2.1
21627	0.794373	2.1
21628	1.20056	2.05231
21629	1.25	2.1
21630	1.20056	2.1
21631	1.35787	2.1

21632	0.946131	1.7237
21633	1.38585	1.42354
21634	0.946128	0.953746
21635	0.946129	1.23445
21636	1.35714	1.32607
21637	1.04147	1.89762
21638	0.946133	1.32905
21639	1.30382	1.08234
21640	0.946131	0.814795
21641	0.946131	1.04457
21642	1.38585	1.22669
21643	1.22305	1.34196
21644	1.00763	0.439944
21645	0.813199	0.53148
21646	0.810948	0.512893
21647	1.15928	0.497704
21648	0.944166	0.372944
21649	0.94613	0.445117
21650	0.946133	0.339998
21651	1.4179	0.413266
21652	0.946132	0.371664
21653	1.36965	0.273448
21654	1.05444	0.350236
21655	0.690302	0.414607
21656	0.838038	0.34039
21657	0.802821	1.00605
21658	1.40944	1.10104
21659	0.946126	1.2953
21660	0.799998	0.781199
21661	0.800035	0.975249
21662	1.1766	2.1
21663	1.00319	2.1
21664	1.01606	1.6959
21665	0.872158	2.1
21666	1.04884	2.1
21667	0.679494	2.1
21668	0.946131	2.1
21669	1.37609	2.1
21670	0.94613	2.1
21671	0.946131	2.1
21672	1.18486	2.1
21673	0.726287	2.1
21674	0.946131	2.09692
21675	0.726286	2.1
21676	1.30318	2.1
21677	1.01712	2.1
21678	0.946132	2.1
21679	1.04404	2.1
21680	1.37017	2.1
21681	1.37017	2.1
21682	0.946133	2.1
21683	0.992084	1.10126
21684	0.946131	0.915581
21685	0.529655	1.3331
21686	0.529686	1.19277
21687	0.946131	0.941633
21688	1.05937	1.24148
21689	1.14622	0.823938

21690	0.946131	0.832922
21691	0.584575	0.911839
21692	0.584575	0.761509
21693	0.946129	0.764718
21694	0.709714	2.1
21695	0.946132	2.1
21696	0.709714	2.1
21697	1.37199	2.1
21698	0.82899	2.1
21699	1.00133	2.1
21700	0.778424	0.232668
21701	0.946132	0.274736
21702	1.24094	0.243229
21703	1.00691	0.257014
21704	0.64868	0.247657
21705	1.29736	0.228609
21706	1.24767	0.770279
21707	1.28953	0.756433
21708	1.34612	0.817264
21709	0.946133	0.753147
21710	1.14187	0.858821
21711	0.946132	0.756864
21712	0.946129	0.810395
21713	1.4099	1.63535
21714	0.946131	0.887775
21715	1.34612	0.661651
21716	1.4099	0.810033
21717	0.956003	0.19958
21718	1.40572	0.211521
21719	3.54443	2.1
21720	0.994001	0.777355
21721	1.03679	2.1
21722	1.08467	2.1
21723	1.56875	0.693005
21724	0.968463	1.81385
21725	0.974889	2.1
21726	0.946131	2.1
21727	1.30203	2.1
21728	1.40199	2.1
21729	1.40199	2.1
21730	0.946132	2.1
21731	0.946132	2.1
21732	1.36994	2.1
21733	1.37372	2.1
21734	1.37372	2.1
21735	0.946132	2.1
21736	0.928979	1.08168
21737	0.565509	1.21875
21738	1.60196	0.728891
21739	1.16239	0.874611
21740	1.10746	0.686091
21741	1.15412	2.1
21742	1.15171	2.1
21743	0.946133	2.05761
21744	0.940877	2.1
21745	0.940878	2.1
21746	1.41432	2.1
21747	1.04687	2.1

21748	2.75524	0.265818
21749	0.946132	0.270369
21750	0.766452	0.254316
21751	0.766427	0.275919
21752	1.36004	0.224566
21753	1.06648	0.267693
21754	1.62483	2.1
21755	1.0577	2.1
21756	1.39158	2.09598
21757	0.94255	2.1
21758	1.05029	2.1
21759	1.03735	1.97289
21760	0.931055	2.08279
21761	1.03797	2.00798
21762	1.43687	2.04478
21763	1.28765	1.20884
21764	1.1811	1.01636
21765	0.946132	1.13833
21766	0.946132	1.1116
21767	1.1811	1.11703
21768	0.67805	1.03646
21769	1.50998	1.97008
21770	1.16661	2.1
21771	1.36749	2.1
21772	0.810945	0.894152
21773	1.14745	1.09743
21774	0.865588	0.768012
21775	1.36665	0.773776
21776	0.506964	0.84889
21777	0.506966	0.78945
21778	0.552786	0.758912
21779	0.946133	0.713132
21780	1.21336	0.736986
21781	0.552786	0.739001
21782	0.552757	1.08183
21783	0.653468	0.741774
21784	1.36665	1.33325
21785	1.27922	0.589145
21786	1.08133	0.783792
21787	1.05348	0.502572
21788	0.946132	0.631961
21789	1.19601	0.541957
21790	1.13276	0.535192
21791	0.7856	0.542935
21792	1.57125	0.533581
21793	1.19601	0.665977
21794	0.946131	0.973428
21795	1.34534	0.940726
21796	1.19601	0.737285
21797	0.870489	0.779616
21798	1.49404	2.01537
21799	1.35943	1.09507
21800	1.10427	1.358
21801	0.94613	1.08851
21802	0.946131	1.18289
21803	1.10427	1.19541
21804	1.2064	0.310371
21805	0.946132	0.602284

21806	1.11288	0.787236
21807	1.34268	0.421358
21808	1.34268	0.390311
21809	0.946132	0.423976
21810	0.946128	2.1
21811	0.946128	0.909393
21812	1.30835	1.85103
21813	1.38463	1.04335
21814	0.946129	1.07591
21815	1.43778	0.838611
21816	1.23943	0.772564
21817	1.4748	2.1
21818	0.71295	2.1
21819	0.712951	2.1
21820	0.946132	2.1
21821	0.946133	2.1
21822	1.13976	2.1
21823	1.71109	2.1
21824	1.32426	2.1
21825	0.671255	1.9558
21826	1.29123	2.06127
21827	0.946132	1.97207
21828	0.946132	1.913
21829	1.25603	1.97409
21830	2.21126	0.311144
21831	0.892889	2.1
21832	0.946129	2.1
21833	0.892888	2.1
21834	0.92872	2.1
21835	1.34352	2.1
21836	0.622784	0.728763
21837	1.02947	0.658068
21838	0.810957	0.628045
21839	1.91756	1.19805
21840	1.06012	1.16691
21841	1.09569	0.795667
21842	1.5456	0.265629
21843	1.1211	0.227993
21844	1.21031	0.283385
21845	1.03939	0.349575
21846	0.519697	0.390751
21847	0.933889	0.601881
21848	0.519726	0.540686
21849	1.16699	0.513543
21850	0.933889	0.539298
21851	0.840599	0.312414
21852	0.94613	0.486067
21853	0.840568	0.416938
21854	1.26868	0.412448
21855	1.16699	0.394598
21856	1.17822	1.38813
21857	0.922044	0.630631
21858	1.29322	0.637535
21859	0.946132	0.532798
21860	0.995178	0.687185
21861	0.922056	0.684147
21862	1.76232	0.7
21863	1.77065	1.17939

21864	1.38149	0.915537
21865	0.959301	1.08533
21866	0.946133	0.95987
21867	0.946132	1.01258
21868	0.959301	0.96726
21869	1.3612	0.930503
21870	0.885314	1.07851
21871	0.885309	0.956414
21872	0.94613	1.03563
21873	0.8853	0.940788
21874	1.56164	0.968328
21875	1.45506	0.394149
21876	1.45257	2.1
21877	0.851541	0.242538
21878	1.29018	0.209589
21879	0.851541	0.23322
21880	1.21087	0.212399
21881	1.24979	0.229428
21882	1.56983	0.206332
21883	1.60151	0.18746
21884	1.78952	2.1
21885	0.638164	2.1
21886	0.638163	2.1
21887	1.39754	2.1
21888	1.39754	2.1
21889	0.681589	2.1
21890	1.34993	2.1
21891	1.04525	2.1
21892	0.946133	2.1
21893	1.04072	2.1
21894	0.857805	2.1
21895	1.06053	2.1
21896	1.49066	2.09184
21897	1.4185	1.08058
21898	0.934059	1.46703
21899	0.93406	1.10913
21900	0.94613	1.27858
21901	0.93406	1.20008
21902	1.25277	1.26153
21903	1.00792	1.84198
21904	0.609466	1.2872
21905	0.946132	1.52314
21906	0.609463	1.59827
21907	1.30664	1.87532
21908	0.810943	1.71953
21909	1.05741	1.47102
21910	1.3868	0.987798
21911	1.15558	0.682358
21912	1.13537	2.1
21913	0.946132	2.1
21914	0.946132	2.1
21915	1.08266	2.1
21916	1.54934	2.1
21917	1.26016	2.1
21918	2.01915	2.1
21919	0.60346	1.26089
21920	0.651529	1.34435
21921	1.15581	1.42142

21922	1.13037	1.20168
21923	1.04071	1.16397
21924	1.77796	1.66157
21925	0.532308	1.24759
21926	0.532308	1.20178
21927	1.15369	1.49934
21928	1.1537	1.44177
21929	0.903288	1.38927
21930	0.804766	2.1
21931	0.804739	2.1
21932	0.946128	2.1
21933	0.946131	2.1
21934	1.16745	2.1
21935	1.04193	2.1
21936	0.98505	2.1
21937	1.40724	0.852554
21938	1.14216	0.771609
21939	0.946131	0.592659
21940	0.946131	0.706256
21941	1.10941	0.72692
21942	0.81268	0.225408
21943	0.946131	0.237802
21944	1.28673	0.210304
21945	1.28673	0.220322
21946	0.812681	0.227049
21947	1.84716	0.191905
21948	1.84716	0.192231
21949	0.92361	0.216889
21950	0.94613	0.223175
21951	0.923582	0.203283
21952	1.26227	0.215171
21953	0.923581	0.213997
21954	0.747394	2.1
21955	1.32582	2.1
21956	0.747365	2.08646
21957	0.97587	2.1
21958	1.32582	2.1
21959	1.04182	0.230703
21960	0.961743	0.265205
21961	0.946126	0.339116
21962	1.47463	0.292524
21963	1.12183	0.29984
21964	0.851552	0.293775
21965	1.33177	0.373885
21966	0.946131	0.480413
21967	1.41512	0.449096
21968	1.33178	0.411842
21969	0.946131	0.402826
21970	0.927735	0.764564
21971	0.97206	0.707887
21972	0.515566	0.739626
21973	0.946129	0.728286
21974	0.515538	0.714451
21975	1.04482	0.654155
21976	0.975535	0.673762
21977	0.522411	0.635709
21978	0.522382	0.703581
21979	0.925346	0.704638

21980	0.575817	0.683419
21981	0.946129	2.1
21982	1.14256	2.1
21983	1.32054	2.1
21984	1.40525	2.1
21985	1.14256	2.1
21986	1.59475	2.1
21987	0.773813	2.1
21988	1.4073	2.1
21989	0.773813	2.1
21990	0.773813	2.1
21991	1.4073	2.1
21992	0.993021	2.1
21993	1.50466	0.320856
21994	0.851539	0.445803
21995	1.20299	0.34683
21996	0.946131	0.402792
21997	1.54464	0.396729
21998	1.20309	0.387322
21999	2.00701	0.315314
22000	0.810946	0.453546
22001	1.07678	0.437002
22002	0.900128	0.411411
22003	1.26842	2.1
22004	1.11206	2.1
22005	0.946132	2.1
22006	1.04074	2.1
22007	0.948809	2.1
22008	0.946131	0.238402
22009	0.929617	0.280013
22010	1.24057	0.21762
22011	1.36485	0.242363
22012	0.851554	0.246525
22013	1.96823	0.204005
22014	0.914836	0.266629
22015	0.94613	0.31775
22016	1.38911	0.235131
22017	0.914805	0.240423
22018	0.946131	0.265682
22019	0.946131	0.365684
22020	1.04072	0.337349
22021	1.26837	0.272509
22022	0.970054	0.338937
22023	0.923958	0.334545
22024	1.16653	2.1
22025	1.34412	1.37541
22026	0.851541	0.41874
22027	1.38645	0.4971
22028	0.891637	0.425837
22029	0.851487	0.465266
22030	0.890871	0.47226
22031	1.56178	0.432137
22032	0.946128	0.147815
22033	1.26868	0.147815
22034	0.946131	0.183154
22035	1.18789	0.328879
22036	1.26868	0.180221
22037	1.26868	0.187335

22038	0.946128	0.187335
22039	1.18716	0.351057
22040	0.946131	0.522
22041	0.59358	0.433863
22042	1.23923	0.541125
22043	1.00024	0.423841
22044	0.593593	0.496262
22045	0.946131	0.562565
22046	0.713645	0.500483
22047	1.23175	0.597691
22048	1.23923	0.443843
22049	0.713633	0.530022
22050	1.24687	0.395711
22051	1.80662	1.99527
22052	2.15762	2.1
22053	0.780455	2.1
22054	0.946131	2.1
22055	0.780455	2.1
22056	1.40583	2.07482
22057	1.39852	2.04963
22058	0.565619	2.1
22059	1.1428	2.1
22060	0.663168	0.587046
22061	1.02163	0.480225
22062	1.04072	0.601524
22063	0.663169	0.553917
22064	1.13511	0.569991
22065	1.85277	0.401031
22066	1.17701	1.05332
22067	0.993831	0.40328
22068	1.0823	0.360827
22069	0.795761	0.536399
22070	1.24596	0.457657
22071	0.566075	0.538918
22072	0.94613	0.568592
22073	0.56605	0.54224
22074	0.774911	0.292524
22075	0.946131	0.337205
22076	1.24417	0.252444
22077	0.774911	0.263827
22078	0.946132	0.300369
22079	1.54982	0.25025
22080	1.82961	0.233354
22081	1.12035	0.209078
22082	0.876835	2.1
22083	0.946131	1.8768
22084	0.876836	2.1
22085	1.12968	2.09497
22086	1.23536	2.1
22087	2.15082	2.1
22088	0.58365	2.1
22089	0.946133	2.1
22090	0.583653	2.1
22091	0.992097	2.1
22092	1.12968	2.1
22093	1.0305	2.1
22094	0.82613	0.497197
22095	0.879644	0.465987

22096	1.00688	0.462909
22097	1.52776	1.85473
22098	0.851537	1.4031
22099	1.00851	1.23301
22100	0.946128	0.978677
22101	0.851552	1.2107
22102	1.4895	1.45326
22103	0.761574	2.1
22104	0.946132	2.1
22105	1.01095	2.1
22106	1.01095	2.1
22107	0.761572	2.1
22108	1.8575	2.1
22109	1.19984	0.372918
22110	1.25797	0.387891
22111	0.946132	0.352503
22112	0.946132	0.355683
22113	0.95332	0.34691
22114	0.946131	2.1
22115	0.851542	1.87665
22116	1.03172	2.1
22117	1.22889	2.1
22118	1.0313	2.1
22119	1.15977	1.45605
22120	0.918875	1.04815
22121	0.8654	1.54278
22122	0.865374	1.09271
22123	0.946132	1.10614
22124	0.985051	2.1
22125	1.31993	0.686792
22126	1.33622	0.810891
22127	0.959992	0.787079
22128	0.926499	2.1
22129	1.03544	2.1
22130	0.946132	2.1
22131	0.946132	2.1
22132	1.40335	2.1
22133	0.772299	2.1
22134	0.851529	2.1
22135	0.857243	2.1
22136	1.38155	2.1
22137	1.05462	2.1
22138	0.832138	2.1
22139	0.946132	2.1
22140	0.832137	2.1
22141	0.959414	2.1
22142	1.36541	2.1
22143	1.91894	2.1
22144	1.04661	1.1278
22145	1.28355	1.9514
22146	0.877353	1.03825
22147	0.877353	1.12872
22148	0.94613	1.07626
22149	1.20537	0.394002
22150	1.09914	0.304246
22151	0.94613	0.344296
22152	0.499593	0.350084
22153	0.499606	0.34544

22154	1.0959	0.297714
22155	0.838027	0.311245
22156	0.883644	0.328202
22157	0.95019	0.450573
22158	1.10638	2.1
22159	0.946132	2.1
22160	1.41744	2.1
22161	1.05842	2.1
22162	0.94613	2.1
22163	1.41744	2.1
22164	0.991985	2.1
22165	3.31755	1.20838
22166	1.05556	2.1
22167	0.624075	1.1193
22168	0.814554	1.04766
22169	0.946131	1.13604
22170	0.814554	1.21847
22171	1.31857	1.45725
22172	1.26282	2.1
22173	0.946132	2.1
22174	1.14302	2.1
22175	0.946132	2.1
22176	0.946132	2.1
22177	1.40372	2.1
22178	1.24688	0.440432
22179	0.89889	2.1
22180	1.17286	2.1
22181	0.71741	2.1
22182	0.491096	0.885799
22183	1.05432	0.748259
22184	0.719531	2.1
22185	1.25145	2.1
22186	0.864686	2.1
22187	0.867974	2.1
22188	1.21734	2.1
22189	0.405517	1.0956
22190	0.818878	1.45593
22191	0.502457	0.305558
22192	1.05868	0.357891
22193	0.912018	0.32414
22194	1.40591	0.418284
22195	0.957584	0.634878
22196	0.946131	0.799406
22197	1.11612	0.729412
22198	1.3365	0.714639
22199	0.946128	0.796855
22200	1.16534	0.491185
22201	1.38969	0.782557
22202	0.891597	1.62698
22203	0.640032	1.9351
22204	0.956758	1.66972
22205	1.3428	0.648769
22206	0.953295	2.02838
22207	0.946131	0.620925
22208	1.16167	0.635887
22209	0.94613	0.656909
22210	0.951675	0.632018
22211	1.16167	0.636101

22212	1.24538	0.337179
22213	1.13482	2.1
22214	1.38775	2.1
22215	0.934061	2.1
22216	0.91408	2.1
22217	1.06099	2.1
22218	0.980549	0.521427
22219	0.946132	0.550546
22220	1.10321	0.597393
22221	0.99126	0.55352
22222	0.991261	0.575859
22223	1.35732	0.566679
22224	1.1952	1.65045
22225	0.946132	1.16094
22226	0.946132	2.02811
22227	1.32819	1.21802
22228	1.30171	1.08929
22229	1.29667	0.362931
22230	1.00982	0.203376
22231	0.407589	0.851211
22232	1.18266	1.17879
22233	1.04037	0.864531
22234	0.956694	0.954588
22235	0.552639	0.872694
22236	0.810943	1.47491
22237	1.43144	0.955183
22238	0.918074	0.937705
22239	1.60976	2.1
22240	4.00847	2.1
22241	2.3995	0.272076
22242	1.36203	2.1
22243	0.919619	2.1
22244	1.30188	2.1
22245	0.681073	2.1
22246	0.681043	2.1
22247	1.04687	2.1
22248	3.32085	2.1
22249	1.80396	0.248444
22250	0.773007	0.70971
22251	0.773034	0.628124
22252	1.22141	0.716794
22253	1.22141	0.710474
22254	0.946131	0.725948
22255	2.02071	0.542482
22256	0.951912	0.245014
22257	1.39027	0.270503
22258	0.94613	0.294132
22259	0.829922	0.243943
22260	0.829946	0.26266
22261	1.24902	1.30501
22262	2.8333	1.18444
22263	0.946131	0.202313
22264	0.929725	0.189745
22265	1.1134	0.199302
22266	1.17056	0.20244
22267	0.929713	0.190351
22268	0.749096	2.1
22269	1.31961	2.1

22270	0.870191	2.1
22271	1.04071	2.1
22272	1.1877	2.1
22273	1.03882	2.1
22274	1.44801	1.97528
22275	3.55198	2.1
22276	1.11193	2.1
22277	0.663922	2.1
22278	0.663922	2.1
22279	0.946129	2.1
22280	0.946131	2.1
22281	0.97079	2.1
22282	1.36049	2.1
22283	1.06105	2.1
22284	0.92251	2.1
22285	0.94613	2.1
22286	0.922511	2.1
22287	1.04101	2.1
22288	0.810944	0.414377
22289	0.994109	0.371063
22290	0.623143	0.352523
22291	0.864244	0.436205
22292	1.14627	0.399309
22293	0.64586	0.377269
22294	0.721422	0.273632
22295	0.870885	0.251586
22296	0.855175	0.275951
22297	0.946131	1.02243
22298	1.38157	2.04091
22299	1.33266	0.884921
22300	0.946131	1.48262
22301	1.38158	1.3291
22302	0.946132	1.82938
22303	1.3268	2.1
22304	0.946132	1.51022
22305	0.946132	2.1
22306	1.38158	2.1
22307	1.42186	1.44745
22308	1.47204	1.43777
22309	1.14665	1.80206
22310	0.946132	1.16485
22311	0.94613	1.96807
22312	1.30116	1.58552
22313	1.32819	1.27594
22314	1.25388	2.1
22315	0.887808	1.27984
22316	0.946132	2.1
22317	1.23536	1.80199
22318	1.34309	1.77074
22319	1.07612	2.1
22320	0.728982	0.25298
22321	1.3157	0.244011
22322	0.946131	0.292308
22323	0.686176	0.274599
22324	1.3035	0.27426
22325	0.686139	0.284974
22326	0.906806	0.26006
22327	0.794122	0.245275

22328	1.17173	0.253072
22329	1.8194	0.23722
22330	0.851543	2.1
22331	1.19345	2.1
22332	0.946133	2.1
22333	0.946132	2.1
22334	1.56179	2.1
22335	0.620263	1.78603
22336	1.30226	1.71847
22337	1.2334	1.28157
22338	1.2334	1.17115
22339	0.946133	1.1631
22340	1.24053	2.02695
22341	1.4828	1.95039
22342	0.957622	2.1
22343	1.00521	1.35738
22344	1.49463	2.1
22345	1.39621	2.1
22346	1.09861	2.1
22347	0.946131	2.1
22348	0.946131	2.1
22349	1.09861	2.1
22350	2.16987	0.230232
22351	0.572182	0.24166
22352	1.15802	0.255106
22353	0.63574	0.263472
22354	1.18897	0.244201
22355	0.996733	0.253445
22356	1.82402	1.73879
22357	0.969602	1.48375
22358	0.794777	1.76602
22359	1.02626	1.56016
22360	1.51396	0.399839
22361	1.40574	0.456254
22362	1.38683	0.45874
22363	0.946126	0.463716
22364	0.946128	0.41442
22365	1.07138	0.368852
22366	0.946131	2.1
22367	1.41331	2.1
22368	0.94613	2.1
22369	0.94613	2.1
22370	1.27451	2.1
22371	0.974196	2.1
22372	1.2049	0.227471
22373	1.42757	2.1
22374	1.02461	2.1
22375	2.07087	2.1
22376	1.92515	1.60693
22377	0.802138	1.57333
22378	0.946128	1.78267
22379	0.802107	1.48532
22380	1.23726	1.46655
22381	1.21997	1.45146
22382	0.767577	1.24065
22383	0.946131	1.64907
22384	0.767577	1.32056
22385	1.3279	1.32744

22386	1.23726	1.29133
22387	1.29729	2.1
22388	0.946128	2.1
22389	1.14186	2.1
22390	1.39214	2.1
22391	1.39214	2.1
22392	0.946129	2.1
22393	1.23012	0.466891
22394	0.653228	0.7
22395	0.946133	0.700666
22396	1.15918	0.852439
22397	1.26893	0.7
22398	0.653228	0.7
22399	1.06848	1.10918
22400	0.615121	2.1
22401	0.841488	1.38291
22402	0.946131	2.1
22403	1.04071	2.1
22404	0.965717	2.1
22405	1.77893	1.37338
22406	1.11314	0.444912
22407	1.65411	1.00068
22408	0.85472	0.288438
22409	1.1604	0.246884
22410	1.19141	0.254536
22411	1.19141	0.248019
22412	0.94613	0.276895
22413	1.70944	0.207767
22414	1.89082	0.404757
22415	1.05175	0.961387
22416	0.815276	2.1
22417	0.843283	2.1
22418	0.946131	2.1
22419	0.843284	2.1
22420	1.38897	2.1
22421	1.01019	0.923941
22422	1.05197	0.904988
22423	0.810959	0.88611
22424	1.33037	0.481776
22425	1.3415	0.476496
22426	0.946118	0.375303
22427	0.946131	0.423315
22428	1.09803	0.428375
22429	0.930281	2.1
22430	1.3335	2.1
22431	0.784151	1.91456
22432	0.78415	2.1
22433	0.946132	2.1
22434	1.6299	0.255174
22435	0.995795	2.1
22436	1.84846	1.29921
22437	0.929873	1.70899
22438	0.889245	1.65909
22439	0.808072	1.79637
22440	0.851541	1.47541
22441	1.51956	1.47516
22442	0.94613	1.39883
22443	0.851553	1.43695

22444	0.946787	1.43582
22445	1.15544	1.64054
22446	1.30289	0.524742
22447	1.28323	0.552253
22448	0.85024	0.55196
22449	1.09662	0.587079
22450	1.67687	0.250363
22451	0.946127	1.97827
22452	1.40496	1.88244
22453	1.22442	1.62041
22454	0.946128	1.57371
22455	1.40496	1.60452
22456	1.00573	2.1
22457	0.636837	1.58177
22458	0.988642	1.70007
22459	0.573141	1.32781
22460	0.946128	1.58636
22461	0.786538	1.74816
22462	2.28748	1.33859
22463	0.682805	1.94709
22464	0.946132	1.89427
22465	0.682776	1.74639
22466	0.946132	1.92116
22467	0.79775	2.1
22468	1.05403	0.253135
22469	0.888273	0.279729
22470	0.876373	0.235948
22471	0.576083	0.248487
22472	0.946132	0.315292
22473	0.576057	0.217133
22474	0.576058	0.237878
22475	0.946131	0.272413
22476	1.41724	0.831789
22477	1.31678	1.0002
22478	0.946132	0.974157
22479	0.946132	0.947934
22480	1.31678	0.92688
22481	0.935738	2.1
22482	0.946132	2.1
22483	0.935765	2.1
22484	1.32949	2.1
22485	0.946131	2.1
22486	1.02525	2.1
22487	0.946128	2.1
22488	1.41207	2.1
22489	1.04675	2.1
22490	0.946131	2.1
22491	1.41207	2.1
22492	0.946131	2.1
22493	0.946132	2.1
22494	1.41125	2.1
22495	1.41125	2.1
22496	1.27087	2.1
22497	0.924071	0.568887
22498	0.608893	0.466832
22499	0.528907	0.567805
22500	1.78249	0.53403
22501	0.720094	0.580593

22502	0.781667	2.1
22503	0.781638	2.1
22504	1.40756	2.1
22505	1.40756	2.1
22506	0.797635	2.1
22507	1.11946	0.147815
22508	1.32068	0.147815
22509	0.88757	0.353818
22510	1.27891	0.313641
22511	0.810957	0.334107
22512	0.914656	0.370673
22513	0.640819	0.306268
22514	0.745643	0.368024
22515	0.865108	0.395936
22516	1.06883	0.365865
22517	0.806001	0.402979
22518	1.19462	2.1
22519	1.42763	0.711491
22520	1.24682	0.601884
22521	0.78392	0.328598
22522	1.02874	0.33288
22523	0.884512	0.327165
22524	1.1658	2.1
22525	0.663026	0.209233
22526	1.21174	0.237942
22527	0.663056	0.255739
22528	1.15968	0.231054
22529	1.21174	0.23301
22530	0.993976	0.218028
22531	0.945495	0.231962
22532	0.671202	0.213292
22533	1.37312	2.1
22534	1.28499	2.1
22535	0.946133	2.1
22536	0.946132	2.1
22537	1.28499	2.1
22538	1.25081	2.1
22539	0.946132	2.1
22540	0.946132	2.1
22541	1.27985	2.1
22542	1.37312	2.1
22543	0.830638	2.1
22544	1.05358	2.1
22545	1.22209	2.1
22546	1.09874	2.1
22547	0.74758	2.1
22548	2.50011	1.34249
22549	0.6157	0.706554
22550	0.674634	0.705571
22551	0.946128	0.863238
22552	0.674633	0.711784
22553	0.946131	0.792612
22554	1.96192	2.02885
22555	1.31612	0.627919
22556	1.10735	0.561838
22557	0.936676	0.500107
22558	0.808779	0.532666
22559	0.808808	0.561591

22560	1.00245	0.539843
22561	1.26179	0.496036
22562	1.05758	1.74988
22563	1.03408	2.1
22564	0.946132	0.333032
22565	0.914368	0.315185
22566	1.20524	0.254387
22567	1.12743	0.293943
22568	0.851555	0.302206
22569	1.17885	0.711135
22570	0.867881	0.700523
22571	1.00122	0.699731
22572	0.867883	0.699906
22573	0.867881	0.7
22574	1.19583	0.701179
22575	1.17743	1.68823
22576	0.878684	1.48059
22577	0.687087	1.95556
22578	0.955079	0.862532
22579	0.672977	0.879591
22580	0.735937	0.909
22581	0.851531	0.886808
22582	0.672957	0.901156
22583	0.923974	0.877782
22584	1.2243	2.1
22585	0.808463	0.869802
22586	0.971078	0.889431
22587	1.06161	0.894322
22588	0.810947	0.911154
22589	0.886074	0.929716
22590	0.786829	0.967558
22591	0.55062	0.88181
22592	0.574458	1.14024
22593	0.946129	0.891957
22594	1.42526	0.896214
22595	1.15962	0.907475
22596	1.24526	0.209679
22597	0.94613	0.588844
22598	0.946128	0.766413
22599	1.23279	0.817126
22600	1.38645	0.829084
22601	0.94613	0.736472
22602	1.05042	1.59255
22603	0.946128	2.1
22604	0.946129	1.87586
22605	1.24286	2.1
22606	1.39659	2.1
22607	0.94613	2.1
22608	1.10407	1.72377
22609	1.32809	1.62542
22610	0.993999	1.75857
22611	0.810959	1.491
22612	0.983643	1.63516
22613	0.869552	1.78865
22614	0.747801	1.54196
22615	1.53429	1.2924
22616	1.13127	2.1
22617	1.38397	2.1

22618	1.08366	2.1
22619	0.946129	2.1
22620	0.946131	2.1
22621	1.08366	2.1
22622	1.06007	2.1
22623	1.79641	2.1
22624	0.758799	2.1
22625	1.28442	2.1
22626	1.06957	2.1
22627	0.946133	2.08161
22628	1.28442	2.1
22629	1.76788	1.53298
22630	1.38669	2.1
22631	0.986095	2.1
22632	1.38478	1.07935
22633	0.946132	0.833134
22634	1.24344	0.734856
22635	0.622283	1.00383
22636	1.10335	0.756463
22637	1.24344	0.792548
22638	1.1967	0.84941
22639	0.946131	0.246552
22640	0.901005	0.234767
22641	1.05751	0.237401
22642	1.37771	0.234761
22643	0.851527	0.237404
22644	1.26643	1.07193
22645	1.08992	1.32446
22646	0.946132	1.30932
22647	0.946132	0.980172
22648	1.08992	1.34981
22649	1.53534	2.1
22650	1.58825	1.37141
22651	1.0447	1.79771
22652	0.593818	1.43858
22653	0.89883	1.57562
22654	0.734088	1.69156
22655	0.998451	0.86452
22656	1.64156	1.05281
22657	1.05654	1.38634
22658	1.37641	0.676571
22659	1.74553	0.696278
22660	1.31112	0.73348
22661	0.74518	0.84761
22662	0.851539	0.70618
22663	0.851554	0.601408
22664	0.851475	0.577573
22665	0.946132	0.167746
22666	1.28895	0.16031
22667	1.11356	0.162513
22668	0.946132	0.162289
22669	0.946132	0.16973
22670	1.84214	2.1
22671	1.23851	0.345481
22672	1.25866	0.778619
22673	1.28655	1.01067
22674	0.946131	0.946915
22675	0.946132	0.922119

22676	1.28655	0.90056
22677	1.11929	0.839482
22678	0.946132	0.961616
22679	0.946132	0.830133
22680	1.34254	0.824701
22681	1.25866	0.799526
22682	0.946132	2.1
22683	1.27451	2.1
22684	1.19803	2.1
22685	0.94613	2.1
22686	1.27451	2.1
22687	0.946131	2.1
22688	1.36523	2.1
22689	1.14599	2.1
22690	0.946129	2.1
22691	1.36523	2.1
22692	1.09518	2.1
22693	0.977777	2.1
22694	0.583237	2.1
22695	0.583267	2.1
22696	0.903872	2.1
22697	0.946129	2.1
22698	0.583258	2.1
22699	1.58179	0.30757
22700	0.946131	0.39919
22701	1.35123	0.343489
22702	0.655837	0.38702
22703	1.12018	0.391996
22704	1.35123	0.378665
22705	0.62967	0.364768
22706	0.860497	0.439687
22707	0.816639	0.395026
22708	0.746716	2.1
22709	0.810947	2.1
22710	0.810959	2.1
22711	0.653422	1.94283
22712	0.289127	1.45104
22713	0.475716	1.81794
22714	0.980651	1.42991
22715	0.946119	2.01694
22716	0.946132	2.03158
22717	1.08299	2.04212
22718	1.34539	2.08628
22719	1.28753	0.408515
22720	0.946132	0.597112
22721	0.946132	0.525622
22722	1.19719	0.530802
22723	0.946133	0.534209
22724	0.958947	0.445617
22725	1.3492	0.453003
22726	0.946132	0.613698
22727	0.946128	0.582774
22728	1.22588	0.563554
22729	0.946129	0.568477
22730	0.991113	2.1
22731	1.35966	2.1
22732	0.961 2.1	
22733	0.946129	2.1

22734	0.946131	2.1
22735	0.961001	2.1
22736	0.727096	1.8467
22737	0.810946	1.34013
22738	0.810959	1.56492
22739	1.31658	0.54486
22740	1.23167	2.1
22741	1.44836	0.195616
22742	1.50103	0.201864
22743	1.23351	0.224482
22744	1.35657	0.231972
22745	0.851541	0.259405
22746	0.93668	0.227307
22747	0.946131	0.240166
22748	3.7335	2.1
22749	1.1822	2.1
22750	1.31127	0.741304
22751	1.3689	0.719372
22752	1.35822	0.693402
22753	0.946133	0.691019
22754	0.946132	0.723159
22755	1.14926	0.712837
22756	1.40822	0.699609
22757	1.04935	0.732747
22758	0.704112	0.706767
22759	0.704112	0.72113
22760	0.946131	0.745923
22761	0.704112	0.730262
22762	1.37721	0.225313
22763	0.85154	2.1
22764	1.06949	2.1
22765	0.851541	2.1
22766	0.946128	2.1
22767	1.1175	2.1
22768	1.44091	1.89971
22769	0.96029	0.829479
22770	3.46486	1.15617
22771	0.603286	0.467593
22772	0.432283	0.323031
22773	0.851541	0.44576
22774	1.09355	0.314994
22775	0.851534	0.482555
22776	1.04263	2.1
22777	0.851541	2.1
22778	0.965604	2.1
22779	0.94613	2.1
22780	1.23953	2.1
22781	0.869615	2.1
22782	1.02415	2.1
22783	0.983032	2.1
22784	1.35385	1.39868
22785	1.59971	0.505799
22786	0.915178	2.1
22787	0.962435	2.1
22788	1.3116	2.1
22789	1.38302	2.1
22790	1.13227	2.1
22791	1.25923	0.20146

22792	0.988805	2.1
22793	0.611486	1.00714
22794	1.1019	1.13663
22795	1.11124	1.28506
22796	0.716713	1.02502
22797	0.946131	1.21967
22798	1.22297	1.26524
22799	1.12873	1.20154
22800	0.946131	1.22858
22801	0.946132	1.21628
22802	1.3469	1.27368
22803	1.25366	1.36042
22804	1.1611	0.982791
22805	0.946132	1.12297
22806	0.946132	1.00469
22807	1.2854	1.07509
22808	1.3469	1.15436
22809	1.07468	0.978211
22810	1.79317	2.1
22811	1.07444	2.1
22812	1.69436	1.44298
22813	0.851539	2.08682
22814	0.870925	1.9498
22815	0.851541	2.08069
22816	0.870951	2.09705
22817	1.26097	2.07562
22818	1.26247	1.98922
22819	0.946132	1.87742
22820	1.24041	2.1
22821	0.946132	1.62878
22822	0.946132	2.1
22823	1.40274	2.09747
22824	0.946131	0.234352
22825	0.797945	0.223246
22826	1.12988	0.230949
22827	1.00681	0.224189
22828	0.797932	0.225609
22829	0.977483	0.635829
22830	0.943362	0.871723
22831	0.9891	0.652896
22832	1.1703	0.716234
22833	0.663171	0.736842
22834	1.40882	0.447052
22835	1.32639	0.557074
22836	1.24448	0.433128
22837	0.946132	0.554405
22838	1.11015	0.429942
22839	1.24448	0.485104
22840	0.754768	0.470041
22841	1.13854	2.1
22842	1.23654	2.1
22843	0.714101	0.764079
22844	0.851538	0.834087
22845	0.79261	1.10492
22846	1.30598	0.94877
22847	1.05338	0.829287
22848	1.41758	2.1
22849	0.83317	0.657331

22850	1.00058	0.692726
22851	0.810958	0.688593
22852	1.63791	0.344338
22853	1.55116	2.07112
22854	1.29735	1.08477
22855	0.945147	1.62439
22856	0.94515	1.13561
22857	0.94613	1.31648
22858	0.945149	1.25492
22859	1.02368	2.1
22860	1.12787	2.1
22861	1.04007	2.1
22862	3.78805	1.8744
22863	0.816891	0.421465
22864	0.943996	0.40989
22865	0.870002	0.385013
22866	0.564533	0.653657
22867	0.564536	0.620505
22868	0.946128	0.702626
22869	0.564535	0.7
22870	0.946128	0.7
22871	1.15177	2.1
22872	1.16806	0.835843
22873	0.999083	0.955384
22874	0.946131	0.882008
22875	0.946131	0.883783
22876	0.999084	0.952024
22877	1.28918	0.841019
22878	1.0383	0.997282
22879	0.946133	0.889299
22880	0.946132	0.911438
22881	1.0383	0.908133
22882	1.27737	1.16102
22883	1.39296	0.570625
22884	0.998682	2.1
22885	3.98933	2.1
22886	1.05328	0.383595
22887	1.23475	2.1
22888	0.900856	1.15778
22889	0.94001	0.961957
22890	0.707748	0.908427
22891	0.707751	1.0447
22892	0.946128	0.912937
22893	0.70775	0.971363
22894	0.996109	0.957724
22895	1.68551	2.1
22896	0.851539	2.1
22897	0.962688	1.80767
22898	0.94613	1.23415
22899	1.04073	2.03457
22900	1.44824	2.1
22901	0.794313	0.604219
22902	0.838031	0.609951
22903	1.11076	0.561187
22904	1.38813	2.1
22905	1.17204	2.1
22906	0.946132	2.1
22907	0.946131	2.1

22908	1.17204	2.1
22909	0.961779	0.653811
22910	1.0685	0.847361
22911	0.810947	1.57503
22912	1.2899	1.08068
22913	0.890404	1.06432
22914	0.909981	1.84837
22915	0.810946	1.27736
22916	0.890402	1.18883
22917	0.522844	1.54526
22918	0.593846	1.52682
22919	1.30226	1.16232
22920	1.30226	1.19001
22921	0.946132	1.26414
22922	1.03537	0.391196
22923	1.50035	0.332064
22924	0.940356	0.449037
22925	0.946131	0.396265
22926	1.03441	0.409793
22927	1.21171	0.297692
22928	1.35169	0.359016
22929	1.32449	0.32787
22930	0.946131	0.415784
22931	0.946131	0.386306
22932	1.06627	0.384737
22933	1.03102	0.775405
22934	0.488712	0.288672
22935	0.946131	0.37616
22936	1.23146	0.284775
22937	1.13242	0.349224
22938	0.946131	0.33173
22939	0.409199	0.25194
22940	0.429959	0.250664
22941	1.08357	0.368189
22942	0.80926	0.399752
22943	0.879791	0.345284
22944	0.946129	1.19406
22945	1.28828	1.42773
22946	0.946131	1.0595
22947	0.946131	1.51988
22948	1.35969	1.42551
22949	1.10049	1.50076
22950	0.923724	1.26792
22951	0.923695	1.92131
22952	0.94613	1.16974
22953	0.94613	1.89339
22954	1.28828	1.73718
22955	1.73525	0.430618
22956	0.656239	0.548157
22957	1.2996	0.468824
22958	0.812939	0.585958
22959	0.65624	0.527305
22960	1.2996	0.535512
22961	1.67372	2.1
22962	0.946131	2.1
22963	0.946132	2.1
22964	1.05978	2.1
22965	1.31049	2.1

22966	0.946132	2.1
22967	1.11077	2.1
22968	0.579725	0.75212
22969	0.373532	0.645556
22970	1.13537	0.763512
22971	1.20726	0.660215
22972	0.946131	0.652786
22973	1.45694	0.822627
22974	0.750367	0.270467
22975	0.946126	0.314895
22976	0.750365	0.280605
22977	1.25698	0.270696
22978	1.35726	0.272453
22979	1.6812	0.281487
22980	1.78665	1.66927
22981	1.46568	2.1
22982	1.29606	2.1
22983	1.25298	2.1
22984	0.946115	2.1
22985	0.946129	2.1
22986	1.25296	2.1
22987	1.27778	0.309983
22988	1.17042	1.85835
22989	1.25858	0.210806
22990	0.93106	0.221251
22991	0.810959	0.20659
22992	1.38905	2.1
22993	1.19216	2.1
22994	0.946131	2.1
22995	0.946131	2.1
22996	1.19216	2.1
22997	1.58339	1.87721
22998	0.81094	0.268293
22999	0.845129	0.311331
23000	0.918535	0.281217
23001	0.946132	2.1
23002	0.946132	2.1
23003	1.31904	2.1
23004	1.31904	2.1
23005	1.21275	2.1
23006	1.15471	2.1
23007	1.33125	2.1
23008	0.702954	0.388286
23009	0.942973	0.391298
23010	0.94613	0.462341
23011	0.641772	0.343107
23012	0.702942	0.432066
23013	0.991865	0.523112
23014	0.639952	0.520226
23015	1.17333	0.426689
23016	0.946132	0.407982
23017	0.490975	0.404066
23018	0.495934	0.534195
23019	1.19904	0.42225
23020	1.30488	0.26597
23021	1.02305	0.640676
23022	1.49842	0.334823
23023	1.54196	2.1

23024	1.37224	1.47182
23025	1.0953	0.334491
23026	1.18952	1.7945
23027	1.68851	0.574137
23028	0.946131	0.610187
23029	1.12491	0.508399
23030	0.583591	0.468804
23031	1.28727	0.600256
23032	1.12491	0.569957
23033	0.884641	0.694789
23034	0.810944	0.688684
23035	0.917973	0.678313
23036	0.846671	0.664472
23037	0.596968	0.514958
23038	0.716347	0.61102
23039	1.36255	0.503203
23040	1.11077	2.1
23041	0.946132	2.1
23042	0.946132	2.1
23043	1.26267	2.1
23044	1.26441	2.1
23045	1.85238	2.1
23046	1.36693	2.1
23047	0.616123	2.1
23048	0.481844	2.1
23049	0.709599	2.1
23050	0.815889	2.1
23051	1.1502	2.1
23052	1.63937	0.919295
23053	1.25797	0.371919
23054	1.22878	0.389705
23055	0.946132	0.358766
23056	0.946132	0.36247
23057	0.965737	0.3463
23058	1.1893	0.336935
23059	1.22877	0.357663
23060	0.735217	0.348978
23061	0.85154	0.370434
23062	0.851553	0.357107
23063	0.851473	0.351631
23064	1.58916	0.370344
23065	1.25545	0.397769
23066	0.810942	0.571514
23067	0.919314	0.598471
23068	0.928633	0.536948
23069	2.14514	0.439828
23070	1.23389	2.1
23071	0.946128	0.393384
23072	0.969504	0.301014
23073	0.851539	0.356061
23074	1.41503	0.342651
23075	0.958517	0.34426
23076	0.990615	0.302996
23077	1.7943	0.287286
23078	0.946133	0.741464
23079	1.27581	0.752083
23080	0.946132	0.746308
23081	1.36362	0.721228

23082	1.36936	0.721967
23083	1.71903	2.1
23084	0.751639	2.1
23085	0.77464	2.1
23086	0.946133	2.1
23087	1.04073	2.1
23088	1.23551	2.1
23089	0.94613	2.1
23090	1.18682	2.1
23091	0.946131	2.1
23092	0.94613	2.1
23093	1.40638	2.1
23094	0.483346	0.530577
23095	0.432527	0.403704
23096	0.946132	0.567269
23097	0.483312	0.469752
23098	0.946132	0.471684
23099	0.878798	0.54964
23100	0.810944	0.552801
23101	1.14604	0.497654
23102	0.946129	0.535265
23103	1.35594	0.461514
23104	0.538378	0.465222
23105	0.946131	0.547504
23106	0.538378	0.500776
23107	1.00065	0.864214
23108	0.962878	0.430545
23109	1.32634	0.523208
23110	1.28066	0.232017
23111	0.785651	2.1
23112	1.20327	1.87121
23113	1.47058	1.64855
23114	0.551834	1.18398
23115	1.04072	1.75184
23116	0.506719	1.34378
23117	1.12786	1.4419
23118	1.45789	0.191745
23119	0.681532	0.201742
23120	1.21087	0.184723
23121	0.94613	0.194358
23122	0.681534	0.191359
23123	1.21087	0.195553
23124	1.98618	1.00014
23125	1.71742	1.14647
23126	1.07427	0.847292
23127	1.1972	0.762765
23128	0.933474	0.770884
23129	1.1745	0.808405
23130	0.94612	0.772006
23131	0.933485	0.800037
23132	1.17449	0.794046
23133	0.851541	0.210131
23134	1.12774	0.198823
23135	0.946129	0.205324
23136	0.851486	0.207995
23137	1.12517	0.203289
23138	1.12328	0.217313
23139	1.13179	2.1

23140	1.02438	2.1
23141	1.05776	2.1
23142	1.41273	1.17839
23143	1.1229	1.99385
23144	0.875912	1.24187
23145	0.946132	1.76273
23146	0.875912	1.44389
23147	1.42307	1.80793
23148	1.15354	0.245421
23149	0.848382	0.238988
23150	1.2685	1.6202
23151	0.938104	1.48213
23152	0.793166	1.99721
23153	1.07855	2.1
23154	0.98488	2.1
23155	1.4162	2.1
23156	1.09651	0.401339
23157	1.98502	0.583631
23158	1.41168	0.607952
23159	1.33879	0.652185
23160	0.946127	0.743422
23161	1.13528	0.583284
23162	0.946128	0.631335
23163	1.09726	2.1
23164	1.22026	0.890123
23165	1.09044	1.88356
23166	2.23961	0.904989
23167	1.14721	0.617447
23168	1.17614	1.22015
23169	0.615065	2.1
23170	0.946131	2.1
23171	0.778488	2.1
23172	0.94613	2.1
23173	0.615066	2.1
23174	1.78254	1.63003
23175	0.898452	0.308724
23176	1.47411	1.87604
23177	0.59676	0.62575
23178	0.59676	0.495758
23179	0.94613	0.641924
23180	1.13511	0.556052
23181	0.946131	0.537203
23182	0.916275	2.02899
23183	0.946132	1.82395
23184	0.916274	1.34553
23185	1.36182	1.69617
23186	0.946131	1.7705
23187	0.857202	0.202382
23188	0.941918	0.190406
23189	0.943349	0.18519
23190	1.2493	0.179521
23191	0.946133	0.202178
23192	1.01814	0.184945
23193	0.927881	0.17196
23194	1.11845	0.165912
23195	1.2493	0.172986
23196	1.11845	0.159829
23197	0.946129	0.1741

23198	0.967673	0.839315
23199	1.01332	0.203225
23200	0.946133	0.209103
23201	0.81938	0.209486
23202	0.819377	0.199227
23203	0.946133	0.216922
23204	1.22474	0.680131
23205	1.15321	0.682065
23206	0.797489	0.684345
23207	0.946131	0.695602
23208	0.797461	0.688674
23209	1.35328	0.869343
23210	1.03318	1.07093
23211	0.946131	0.915824
23212	0.94613	0.946458
23213	1.03319	0.931025
23214	1.37938	0.899985
23215	1.00512	1.10984
23216	0.946133	0.945962
23217	0.946132	0.985503
23218	1.00512	0.957583
23219	1.5515	1.11295
23220	1.55351	2.1
23221	0.94261	0.233344
23222	0.828156	0.233224
23223	0.916495	0.239128
23224	3.06309	0.215272
23225	0.619832	0.196945
23226	0.705903	0.20871
23227	1.2968	0.203713
23228	0.946133	0.206522
23229	0.705904	0.196266
23230	1.2968	0.208466
23231	1.01971	0.200028
23232	0.60302	0.19114
23233	1.07242	0.904406
23234	1.26426	0.842007
23235	0.946131	0.847459
23236	0.946131	0.908169
23237	0.963109	0.890628
23238	0.947862	0.852438
23239	1.40248	1.13227
23240	0.767143	1.21726
23241	0.767114	1.17843
23242	0.946129	1.2042
23243	0.767143	1.12767
23244	1.22637	1.20024
23245	1.2281	1.13483
23246	0.590909	1.2404
23247	0.490832	1.14826
23248	0.804174	1.22423
23249	0.747368	1.09152
23250	1.05129	0.432719
23251	1.10609	0.869244
23252	1.3639	0.417762
23253	0.776117	0.4642
23254	0.752329	0.423742
23255	1.04073	0.438305

23256	0.67713	0.4282
23257	3.4245	2.1
23258	1.18526	2.1
23259	1.2161	1.05955
23260	0.876834	1.40914
23261	0.876831	1.05974
23262	0.946132	1.30104
23263	0.876834	1.1623
23264	0.954563	2.1
23265	0.924132	2.1
23266	1.25075	2.1
23267	1.01652	2.1
23268	0.92413	2.1
23269	1.37579	2.1
23270	0.992159	2.1
23271	1.26092	2.1
23272	0.929435	2.1
23273	0.844958	2.1
23274	0.79598	2.1
23275	0.852559	2.1
23276	0.946131	0.814425
23277	1.28435	0.666834
23278	0.827261	0.775254
23279	0.946128	0.805718
23280	0.827298	0.783843
23281	1.65452	0.484964
23282	1.79929	2.1
23283	0.791947	0.943931
23284	0.902072	0.920978
23285	0.882672	0.925982
23286	1.21703	2.1
23287	0.900338	2.1
23288	1.37712	2.1
23289	0.782591	2.1
23290	0.782562	2.1
23291	0.94613	2.1
23292	1.34749	2.1
23293	0.61359	2.1
23294	1.14397	0.593648
23295	1.02963	0.278962
23296	1.36589	0.26283
23297	0.946132	0.405255
23298	1.28644	0.310252
23299	0.946131	0.367011
23300	1.40104	0.349646
23301	1.28644	0.345016
23302	0.810946	0.501766
23303	1.09178	0.482391
23304	1.26956	0.450966
23305	0.810943	0.483815
23306	0.885633	0.484679
23307	1.00844	0.460199
23308	0.825671	0.498773
23309	0.792093	0.467879
23310	0.925037	0.472604
23311	0.946131	0.88649
23312	0.946131	0.31276
23313	1.18052	0.51094

23314	1.40265	0.517164
23315	0.946132	0.619671
23316	1.17891	0.521769
23317	0.734248	0.217601
23318	0.810943	0.225909
23319	0.731621	0.217761
23320	0.799857	0.736652
23321	0.834507	0.777617
23322	0.810932	0.73769
23323	1.01316	0.249804
23324	1.23884	0.290233
23325	1.88438	2.1
23326	0.946129	1.69767
23327	1.38614	1.71734
23328	1.34245	1.20019
23329	0.946131	1.15932
23330	1.38614	1.21057
23331	0.946132	0.289677
23332	0.910733	0.301202
23333	1.24726	0.232455
23334	1.13742	0.27334
23335	0.851555	0.275183
23336	1.50368	0.22627
23337	0.94613	0.822722
23338	1.08998	2.06912
23339	1.36899	0.59722
23340	1.36898	0.901964
23341	0.946131	0.872469
23342	1.26045	2.1
23343	1.06432	0.977584
23344	1.89016	0.829439
23345	0.83514	1.58461
23346	1.36653	1.89263
23347	1.23426	1.70215
23348	0.94613	1.49441
23349	0.835112	1.85006
23350	1.14567	1.22084
23351	1.12096	1.18214
23352	0.881106	1.31337
23353	1.17787	0.565204
23354	1.29738	1.00456
23355	0.940472	0.668498
23356	1.03452	0.889665
23357	0.946131	0.808675
23358	2.7489	0.728976
23359	1.01778	2.1
23360	1.04321	0.982206
23361	1.19352	0.404599
23362	1.16912	1.27316
23363	0.946132	1.0923
23364	0.946132	1.39223
23365	1.40924	1.32508
23366	1.22927	1.47056
23367	2.09455	0.395622
23368	3.00575	2.1
23369	1.08928	1.00233
23370	1.94799	2.1
23371	1.4092	2.1

23372	0.946132	2.1
23373	1.16	2.1
23374	1.01985	2.1
23375	0.946133	2.1
23376	1.16	2.1
23377	1.15371	0.158488
23378	1.56002	0.157371
23379	1.23007	2.05558
23380	1.9992	2.1
23381	0.652605	1.89755
23382	0.937903	2.1
23383	0.946132	1.8624
23384	0.937902	2.1
23385	1.228	2.1
23386	1.33694	1.29186
23387	1.00191	2.02368
23388	0.716814	1.32704
23389	0.94613	1.7194
23390	0.716816	1.66335
23391	1.70388	1.36357
23392	0.946129	0.690058
23393	1.09362	0.7093
23394	0.946131	0.710628
23395	1.4185	0.758967
23396	1.09362	0.664994
23397	1.31057	1.28308
23398	1.17631	0.232959
23399	1.19404	0.270237
23400	0.94613	0.393585
23401	0.946129	0.332848
23402	0.99972	0.345528
23403	0.94613	0.350684
23404	0.835199	0.202685
23405	1.23351	0.217607
23406	0.582785	0.242122
23407	0.701079	0.217482
23408	0.946132	0.216386
23409	2.17385	0.192566
23410	1.18948	1.98208
23411	1.25346	2.07408
23412	1.18586	0.805095
23413	0.880876	0.728229
23414	0.68632	0.766618
23415	0.946118	2.1
23416	0.966936	2.1
23417	0.946117	2.1
23418	1.41636	2.1
23419	1.03798	2.1
23420	0.729178	0.810915
23421	0.721888	0.901711
23422	0.946132	0.816498
23423	0.656286	0.864454
23424	1.13898	0.848422
23425	1.02351	0.351525
23426	1.48803	0.409829
23427	0.946131	0.382092
23428	1.04921	2.1
23429	1.02478	0.625414

23430	0.993023	2.1
23431	1.80841	0.350814
23432	1.29215	0.366801
23433	0.904206	0.346139
23434	0.904207	0.387278
23435	0.946132	0.375599
23436	0.904207	0.359391
23437	1.07347	2.03282
23438	0.555875	1.23695
23439	0.555904	1.37871
23440	1.30841	1.50374
23441	1.30841	1.63331
23442	0.91951	1.64336
23443	1.33026	0.837089
23444	1.43289	0.265899
23445	1.15703	1.73035
23446	0.886594	1.97233
23447	1.05129	1.87144
23448	1.08421	0.850865
23449	1.35946	0.838031
23450	0.882955	0.895925
23451	0.946131	0.90838
23452	0.882985	0.886421
23453	0.583501	0.900988
23454	0.532921	0.896084
23455	0.94613	1.37898
23456	0.532892	1.15722
23457	1.23224	1.18751
23458	1.12786	1.14842
23459	0.946131	0.862286
23460	1.23732	0.482227
23461	1.40609	0.881045
23462	1.40609	0.393048
23463	0.946131	0.560379
23464	0.910672	1.27763
23465	1.08478	1.12108
23466	0.94613	0.815008
23467	0.94613	1.08044
23468	1.30382	1.12813
23469	3.10544	1.58834
23470	0.679509	1.78411
23471	1.15966	1.5746
23472	0.75499	1.79143
23473	1.37511	1.6511
23474	1.01776	1.54525
23475	2.35239	1.37566
23476	0.92406	2.1
23477	0.946118	2.1
23478	0.92406	2.1
23479	1.07979	2.1
23480	1.29604	2.1
23481	1.29897	2.1
23482	0.902645	0.769858
23483	0.729493	0.955782
23484	0.906844	0.844035
23485	0.739518	0.340221
23486	1.10564	0.476313
23487	0.946131	0.478849

23488	0.739519	0.408612
23489	0.739506	0.443431
23490	1.79907	0.391892
23491	1.59106	1.02087
23492	1.11067	0.832387
23493	0.795516	0.951617
23494	0.795519	0.862545
23495	0.946132	0.915421
23496	0.795506	0.867473
23497	1.80223	2.09859
23498	1.95572	1.31815
23499	1.16237	0.874322
23500	1.07518	1.2766
23501	0.696199	0.933284
23502	0.94613	0.954237
23503	0.696199	1.01688
23504	1.6379	0.19634
23505	0.946118	0.232847
23506	0.851529	0.258752
23507	1.08716	0.204924
23508	0.973118	0.211215
23509	0.851528	0.228636
23510	1.15096	2.1
23511	0.65023	0.295294
23512	0.94613	0.265621
23513	0.650205	0.294138
23514	0.650205	0.250751
23515	0.811233	0.310959
23516	0.778721	1.66754
23517	0.810945	1.09125
23518	0.997518	1.10879
23519	0.838028	0.336554
23520	0.952893	0.280428
23521	0.838037	0.297907
23522	1.58166	0.264205
23523	1.44848	1.16459
23524	1.30041	0.216282
23525	1.03697	1.02146
23526	0.946132	1.39272
23527	1.0277	1.60072
23528	0.946131	1.53325
23529	1.00501	1.4085
23530	1.0277	1.32071
23531	1.64988	2.1
23532	0.946131	1.98676
23533	0.94613	0.910844
23534	1.27702	1.78773
23535	1.55163	1.0261
23536	0.897905	1.05748
23537	1.12971	0.225131
23538	1.35715	0.709568
23539	0.881884	0.836364
23540	0.664007	0.718604
23541	0.663978	0.765052
23542	0.94613	0.742225
23543	0.664006	0.750585
23544	1.48693	0.38771
23545	1.29289	0.385055

23546	1.32081	0.407053
23547	0.946119	0.354208
23548	0.946132	0.350288
23549	1.02972	0.314581
23550	0.946131	2.1
23551	0.946127	2.1
23552	1.23513	2.1
23553	1.38154	2.1
23554	0.94613	2.1
23555	1.25933	0.372252
23556	0.917954	0.391654
23557	0.868589	0.407587
23558	3.0957	2.1
23559	0.711391	0.237842
23560	0.711391	0.237687
23561	0.991303	0.232336
23562	1.37322	0.225298
23563	0.729145	0.237792
23564	2.55648	0.218938
23565	1.0756	1.61654
23566	0.760299	1.25448
23567	0.763592	0.864808
23568	0.946126	0.944768
23569	0.76359	0.851773
23570	1.35862	1.24022
23571	0.946133	2.1
23572	1.00807	2.1
23573	0.946132	2.1
23574	1.39823	2.1
23575	1.07817	2.1
23576	1.44379	0.832436
23577	1.17934	0.345033
23578	1.13451	0.572962
23579	0.94613	0.967499
23580	1.03974	2.1
23581	1.36835	0.97152
23582	1.36835	1.58611
23583	0.94613	1.74693
23584	1.27096	1.62539
23585	0.853042	2.1
23586	0.946131	2.1
23587	1.13658	2.1
23588	1.06997	2.1
23589	0.94613	2.1
23590	1.41556	0.809678
23591	1.21708	0.914407
23592	0.94613	0.91719
23593	0.946131	0.885668
23594	1.21708	0.875339
23595	0.964782	0.82701
23596	0.946119	0.866854
23597	1.23447	0.865088
23598	0.946118	0.869828
23599	1.41844	0.852504
23600	1.23446	0.874715
23601	1.1357	0.728392
23602	0.619856	0.213681
23603	1.1509	0.199479

23604	0.946126	0.205815
23605	1.19262	0.193034
23606	1.1509	0.208129
23607	1.04268	2.1
23608	2.07527	0.958791
23609	1.38259	0.96123
23610	0.980054	1.27062
23611	0.946124	1.00156
23612	0.946127	1.09959
23613	0.980056	1.03937
23614	1.62916	1.69655
23615	0.946131	2.1
23616	1.40571	2.1
23617	0.946131	2.1
23618	1.38885	2.1
23619	1.40571	2.1
23620	1.11787	0.835618
23621	0.946129	0.831474
23622	1.28831	1.85253
23623	1.41498	0.809663
23624	1.41498	0.973788
23625	0.946131	0.914879
23626	0.850964	0.256698
23627	0.921299	0.237084
23628	1.37358	0.220151
23629	0.946132	0.226786
23630	0.850964	0.237583
23631	0.823988	0.267206
23632	0.823984	0.246183
23633	1.36568	0.224051
23634	1.36569	0.220863
23635	0.921275	0.246281
23636	1.84739	2.1
23637	1.47169	0.449802
23638	0.946131	0.644165
23639	0.735844	0.62096
23640	1.11819	0.709226
23641	1.10904	0.507419
23642	0.735831	0.606917
23643	0.946131	2.1
23644	0.946132	2.1
23645	1.23487	2.1
23646	1.3281	2.1
23647	0.946131	2.1
23648	1.72271	0.245289
23649	0.861359	0.271287
23650	0.946131	0.400335
23651	0.861357	0.335596
23652	1.1369	0.338474
23653	1.26868	0.325023
23654	0.789173	2.03277
23655	0.789143	2.1
23656	0.946133	1.75881
23657	1.34386	1.98777
23658	0.789144	1.99249
23659	0.983493	2.1
23660	1.18944	0.833464
23661	1.05121	0.833729

23662	0.777398	0.85471
23663	0.946132	0.868587
23664	0.777423	0.895411
23665	1.09841	0.595128
23666	0.963919	0.578331
23667	0.474855	0.668812
23668	0.946131	0.657541
23669	0.474855	0.658408
23670	3.40074	1.21292
23671	1.29453	2.1
23672	1.01139	0.809239
23673	0.946133	1.1103
23674	1.35381	0.728216
23675	1.18322	1.00571
23676	0.946133	1.15873
23677	1.45502	0.377717
23678	0.970088	1.24751
23679	0.960333	1.288
23680	0.838036	1.37812
23681	1.30927	0.380246
23682	0.828617	0.420277
23683	0.714795	0.393376
23684	0.946132	0.378498
23685	0.714824	0.380321
23686	0.946131	2.1
23687	0.851542	2.1
23688	1.38204	2.1
23689	1.24512	2.1
23690	1.04086	2.1
23691	0.723573	2.1
23692	0.946131	2.1
23693	0.982454	2.1
23694	0.982454	2.1
23695	0.723544	2.1
23696	1.17261	0.421289
23697	1.34153	0.420391
23698	1.41383	0.416075
23699	0.946132	0.34978
23700	0.946133	0.378255
23701	1.12818	0.387485
23702	1.29383	0.375413
23703	0.946133	0.758975
23704	1.0027	0.742501
23705	0.768989	0.621601
23706	0.946133	0.799568
23707	0.768977	0.749276
23708	0.946132	0.831779
23709	1.24333	0.69832
23710	0.928583	0.808195
23711	0.946132	0.848535
23712	0.928583	0.844741
23713	1.53798	0.508124
23714	2.95436	0.345305
23715	1.30649	0.37454
23716	0.809789	0.504417
23717	0.745744	0.486033
23718	0.745774	0.461819
23719	0.946128	0.456507

23720	1.00777	0.460622
23721	1.40271	0.420769
23722	0.768387	0.549046
23723	0.946132	0.506367
23724	0.922045	0.508155
23725	0.946132	2.1
23726	1.39021	2.1
23727	1.10046	2.1
23728	0.946132	2.1
23729	1.39021	2.1
23730	2.06158	0.327505
23731	0.913967	0.737792
23732	1.30107	0.807929
23733	0.946132	0.737304
23734	0.913966	0.748064
23735	1.30107	0.745143
23736	1.64117	2.1
23737	0.514872	1.4477
23738	0.514846	1.16164
23739	1.30811	1.65187
23740	1.30812	1.17723
23741	0.888989	1.25936
23742	1.99279	1.58451
23743	0.910271	0.84237
23744	0.838027	0.85873
23745	0.928444	0.869191
23746	1.2377	0.98368
23747	0.851537	1.87082
23748	0.899142	2.1
23749	0.810075	2.1
23750	1.3771	2.1
23751	1.03281	2.1
23752	0.719877	2.1
23753	0.901838	2.1
23754	1.01748	2.1
23755	1.12315	2.1
23756	0.64787	2.1
23757	1.173 2.1	
23758	0.876903	2.1
23759	0.597337	2.1
23760	1.00291	2.1
23761	0.597309	2.1
23762	1.09897	0.768855
23763	0.946132	1.06901
23764	1.1386	0.799437
23765	1.09897	0.872671
23766	0.596652	0.946678
23767	2.5593	2.1
23768	0.946133	0.687411
23769	1.29172	0.676804
23770	1.11337	0.675251
23771	0.946131	0.684751
23772	0.946131	0.663607
23773	0.96926	0.828615
23774	0.946132	0.847106
23775	0.946132	0.914741
23776	1.07118	0.770844
23777	1.29698	0.782934

23778	1.37867	0.937235
23779	0.946119	1.06823
23780	0.94612	1.27375
23781	1.33154	0.916078
23782	0.946107	1.02225
23783	2.17757	1.16887
23784	0.946131	0.43098
23785	0.68393	0.308683
23786	0.68396	0.377966
23787	1.33671	0.376895
23788	1.10733	0.38643
23789	2.31367	2.1
23790	0.989326	1.62162
23791	0.689952	2.04649
23792	0.854787	1.49815
23793	3.73635	2.1
23794	1.41489	2.0866
23795	1.25544	1.00149
23796	0.946128	1.69084
23797	0.946129	1.55642
23798	1.13536	1.79138
23799	2.10284	1.17213
23800	0.851542	2.09656
23801	0.828523	2.07222
23802	0.946132	2.1
23803	0.946132	2.1
23804	1.50873	2.1
23805	0.989048	2.06221
23806	0.925055	2.01486
23807	0.838041	2.0626
23808	0.807714	1.17084
23809	0.656831	0.762887
23810	0.997603	1.07385
23811	0.946131	1.12115
23812	1.02602	1.02497
23813	0.946129	1.12419
23814	1.35469	1.02086
23815	1.25083	1.01202
23816	0.851541	0.606442
23817	0.851483	0.576972
23818	0.851539	0.612311
23819	1.19785	0.585709
23820	1.07615	0.597196
23821	0.714507	0.442748
23822	1.41461	0.448958
23823	0.714507	0.436944
23824	0.714507	0.433465
23825	0.946133	0.466967
23826	0.946131	1.00515
23827	0.972232	0.902293
23828	0.94613	1.0456
23829	1.25083	0.906077
23830	1.30165	0.911643
23831	1.05332	2.1
23832	0.574768	2.1
23833	1.94541	0.239401
23834	0.789633	0.31051
23835	0.946127	0.357934

23836	1.33798	0.276529
23837	1.22903	0.331848
23838	0.946128	0.334368
23839	0.946128	2.1
23840	1.11882	2.1
23841	0.996487	2.1
23842	0.94613	2.1
23843	1.11883	2.1
23844	0.946127	2.1
23845	1.30082	2.1
23846	1.02608	2.06967
23847	0.946128	2.1
23848	1.30081	2.1
23849	1.3507	1.2001
23850	1.19583	0.746776
23851	0.946127	1.40573
23852	0.94613	1.01162
23853	1.19583	1.1936
23854	0.477652	0.260696
23855	1.20416	0.373926
23856	0.946131	0.369459
23857	0.477652	0.288601
23858	0.477691	0.348479
23859	1.34536	0.208491
23860	0.758971	0.287638
23861	1.25124	0.284003
23862	1.31467	0.274919
23863	0.758971	0.278258
23864	1.31467	0.292456
23865	0.946131	2.1
23866	1.14184	2.1
23867	1.35028	2.1
23868	1.35028	2.1
23869	0.946131	2.1
23870	1.13537	1.77605
23871	0.946132	1.16801
23872	0.946132	1.91358
23873	1.23031	1.55567
23874	1.27555	1.54709
23875	1.53064	1.73253
23876	1.28421	0.938785
23877	1.07707	0.969199
23878	0.946125	0.906557
23879	0.946127	0.960579
23880	1.07707	0.939224
23881	0.946133	0.353194
23882	1.3495	0.301087
23883	0.946132	0.362998
23884	1.05704	0.329841
23885	1.3495	0.336631
23886	0.946132	0.257536
23887	1.37794	0.235098
23888	0.946132	0.262202
23889	1.01367	0.25584
23890	1.37794	0.251984
23891	0.928516	2.1
23892	0.946133	2.1
23893	0.928518	2.1

23894	1.14105	2.1
23895	1.26406	2.1
23896	0.797664	2.1
23897	0.946132	2.1
23898	0.797663	2.1
23899	1.34285	2.1
23900	0.929875	2.1
23901	0.946131	0.823007
23902	1.21139	0.744669
23903	1.41492	0.737666
23904	1.41492	0.682293
23905	0.946131	0.72438
23906	0.946131	2.1
23907	0.689388	2.1
23908	0.94365	2.1
23909	0.777345	2.1
23910	0.709599	2.1
23911	0.774968	2.1
23912	0.946132	2.1
23913	0.77497	2.1
23914	0.868145	2.1
23915	1.28401	2.1
23916	0.94613	0.778483
23917	1.19655	1.0706
23918	1.39428	0.615879
23919	1.39428	0.804924
23920	0.946131	0.798157
23921	1.1848	2.1
23922	1.07591	2.1
23923	0.946131	2.1
23924	0.946131	2.1
23925	0.874788	2.1
23926	1.35703	2.1
23927	1.35357	2.1
23928	0.946132	2.1
23929	1.26022	2.1
23930	0.946132	2.1
23931	0.946131	2.1
23932	0.966426	2.1
23933	0.946133	2.1
23934	0.946131	2.1
23935	1.3484	2.1
23936	1.26441	2.1
23937	1.2079	2.1
23938	0.946133	2.1
23939	0.946132	2.1
23940	1.2079	2.1
23941	1.2736	2.1
23942	1.25774	2.1
23943	0.946133	2.1
23944	0.851555	2.1
23945	0.96172	2.1
23946	0.946128	2.1
23947	1.06879	2.1
23948	0.94613	2.1
23949	1.32608	2.1
23950	1.06879	2.1
23951	0.946132	2.1

23952	1.32608	2.1
23953	0.946131	2.1
23954	1.40488	2.1
23955	1.32608	2.1
23956	0.94613	1.07607
23957	1.19234	0.862896
23958	0.946129	0.901421
23959	0.94613	1.09983
23960	1.31649	1.20106
23961	0.912059	0.999242
23962	1.04514	1.00994
23963	0.946132	0.933569
23964	0.946132	1.31274
23965	1.34646	1.39279
23966	1.01187	0.573267
23967	1.33728	0.816841
23968	0.761974	0.461979
23969	0.761974	0.756692
23970	0.94613	0.606958
23971	1.40641	1.35829
23972	0.955752	0.924805
23973	0.736738	1.40179
23974	0.94613	0.999558
23975	0.736739	1.08207
23976	0.861471	2.1
23977	1.34556	2.1
23978	0.810957	2.1
23979	0.946131	2.1
23980	1.37811	2.1
23981	1.17949	2.1
23982	0.946132	2.1
23983	1.37811	2.1
23984	0.946131	2.1
23985	1.40447	2.1
23986	0.946132	2.1
23987	0.946132	2.1
23988	1.39826	2.1
23989	0.946131	2.1
23990	1.283 2.1	
23991	0.94613	1.94783
23992	0.946131	2.1
23993	1.40447	2.1
23994	1.20541	2.1
23995	0.946131	1.75776
23996	0.946127	2.1
23997	1.33607	2.1
23998	1.41021	2.1
23999	1.01271	2.1
24000	0.946131	2.1
24001	0.920606	2.1
24002	1.13078	2.1
24003	1.60331	2.1
24004	0.946118	0.899993
24005	1.09718	1.67358
24006	0.946116	1.07388
24007	1.32662	0.948407
24008	1.0972	0.989614
24009	0.94613	0.843711

24010	1.32666	1.03399
24011	0.946128	0.800317
24012	1.31381	0.795242
24013	1.32666	0.767446
24014	0.94612	1.71856
24015	0.940748	2.1
24016	1.03561	2.1
24017	1.35111	2.1
24018	0.851527	2.06223
24019	0.607156	1.37763
24020	0.789158	1.90176
24021	0.946129	1.18865
24022	1.04071	1.70195
24023	1.13144	1.80892
24024	0.946133	2.1
24025	1.35663	2.1
24026	0.946132	2.1
24027	0.946133	2.1
24028	1.34237	2.1
24029	0.919455	2.04904
24030	1.01143	2.1
24031	0.946128	2.0198
24032	0.946131	2.1
24033	1.62796	2.1
24034	1.17796	1.15998
24035	1.23024	1.72476
24036	0.946132	1.13128
24037	0.85153	1.35982
24038	0.986867	1.2378
24039	0.889308	2.1
24040	0.94613	2.1
24041	0.889338	2.1
24042	0.946131	2.1
24043	0.889337	2.1
24044	1.18396	0.951268
24045	0.94613	0.854692
24046	0.946125	1.20059
24047	1.39514	0.939363
24048	1.30809	1.08899
24049	0.803322	1.93637
24050	0.925893	1.60148
24051	1.36183	1.38183
24052	0.925892	1.46925
24053	0.946133	1.40879
24054	1.38354	0.482673
24055	1.16009	0.496078
24056	0.946132	0.423089
24057	0.946132	0.429472
24058	1.01462	0.410428
24059	1.18854	1.03772
24060	0.888265	1.45834
24061	0.745095	1.08997
24062	0.709597	1.31274
24063	0.936541	1.22684
24064	1.24142	0.904584
24065	1.35059	0.836982
24066	0.946128	0.810077
24067	0.94613	0.872193

24068	1.16623	0.861871
24069	1.24945	0.480855
24070	1.33035	0.417806
24071	0.946127	0.4029
24072	0.946128	0.446652
24073	1.13343	0.455837
24074	0.637995	1.01695
24075	1.10455	1.14088
24076	1.22641	1.22399
24077	1.22641	1.4524
24078	0.946132	1.21555
24079	1.3665	0.404957
24080	1.28507	0.369794
24081	0.946127	0.379594
24082	0.946129	0.39125
24083	1.2051	0.397996
24084	0.797374	2.1
24085	0.797376	2.1
24086	1.13113	2.1
24087	1.20057	2.1
24088	1.12049	2.1
24089	0.59387	0.902207
24090	1.13245	0.959539
24091	0.966134	1.05084
24092	0.593842	0.897462
24093	0.946128	1.03445
24094	0.641455	1.04945
24095	0.916507	0.943159
24096	1.04072	1.27611
24097	0.415585	0.934816
24098	0.507244	1.2079
24099	0.678938	2.1
24100	1.24154	2.1
24101	1.25	1.82491
24102	1.24154	2.01175
24103	1.25	1.99512
24104	0.851009	1.79016
24105	0.946131	1.80137
24106	0.851539	1.62479
24107	1.21997	1.49829
24108	1.45109	1.54437
24109	0.804877	0.948594
24110	1.00062	0.742441
24111	0.851536	0.904474
24112	0.909673	0.746016
24113	0.946129	0.917033
24114	1.20494	0.557185
24115	1.09088	0.6298
24116	0.887634	0.669562
24117	0.81027	0.497144
24118	0.900294	0.56712
24119	0.747713	0.295801
24120	0.975467	0.303832
24121	0.747712	0.311285
24122	1.39168	0.290446
24123	0.975467	0.308962
24124	0.94613	0.479905
24125	1.32827	0.437524

24126	0.946128	0.417448
24127	1.41201	0.491678
24128	1.32827	0.449452
24129	0.981121	0.487364
24130	0.936311	0.625868
24131	1.00715	0.495775
24132	1.15914	0.54557
24133	0.704409	0.529772
24134	0.713444	1.20418
24135	0.954538	1.05811
24136	0.944508	1.07617
24137	0.851165	1.21705
24138	0.946131	1.45398
24139	0.851192	1.17743
24140	0.946131	1.19128
24141	1.3279	1.14977
24142	1.33587	0.254319
24143	0.946126	0.311181
24144	1.27512	0.280317
24145	1.33587	0.281952
24146	0.748059	0.271234
24147	1.17451	0.409711
24148	1.04299	0.365831
24149	0.851542	0.488641
24150	0.946131	0.457056
24151	0.892109	0.435388
24152	0.946131	0.223889
24153	0.851541	0.23146
24154	1.2178	0.208624
24155	1.2179	0.219708
24156	0.946793	0.222318
24157	0.709599	0.33537
24158	0.987426	0.300563
24159	1.39502	0.32326
24160	1.13624	0.298661
24161	0.73176	0.319434
24162	1.36697	0.290996
24163	1.33393	0.282663
24164	0.946131	0.362863
24165	0.946132	0.31921
24166	1.23845	0.325778
24167	0.50702	0.947401
24168	1.06442	0.902974
24169	0.946131	0.951443
24170	1.02363	0.945568
24171	1.13537	0.26883
24172	1.25049	0.248265
24173	0.94613	0.29157
24174	1.14993	0.253405
24175	1.38056	0.256496
24176	0.946126	0.177501
24177	1.24678	0.167777
24178	0.684521	0.172397
24179	0.946124	0.173612
24180	0.684518	0.173898
24181	0.851542	0.805907
24182	0.925459	0.799943
24183	0.946131	0.810806

24184	0.851474	0.834068
24185	0.923305	0.833078
24186	1.4055	0.229355
24187	1.32077	0.263566
24188	0.946127	0.304461
24189	1.21757	0.266718
24190	0.946129	0.272122
24191	0.711214	0.401026
24192	1.30463	0.467177
24193	0.711185	0.415865
24194	0.711185	0.447145
24195	1.22716	0.445607
24196	1.29958	0.221216
24197	1.26201	0.261295
24198	0.568927	0.282757
24199	0.568927	0.219233
24200	0.946132	0.26156
24201	0.851542	0.445802
24202	1.44968	0.349156
24203	0.851543	0.401795
24204	1.35123	0.405764
24205	1.40427	0.386532
24206	0.593295	0.492268
24207	0.946129	0.512658
24208	1.22144	0.46166
24209	0.908278	0.517069
24210	1.22144	0.50203
24211	0.867593	0.636136
24212	1.23351	0.541861
24213	0.946131	0.640351
24214	1.05516	0.615694
24215	1.23351	0.598302
24216	1.15564	0.291875
24217	0.946131	0.41443
24218	0.766652	0.352245
24219	0.766681	0.325759
24220	0.946131	0.374522
24221	0.946131	0.536369
24222	0.897851	0.510321
24223	1.1751	0.574183
24224	1.00273	0.511036
24225	0.851554	0.524319
24226	0.883272	0.476346
24227	1.17111	0.742178
24228	0.851541	0.617988
24229	1.01665	0.652442
24230	0.851555	0.582061
24231	0.946131	0.786282
24232	0.911508	0.723598
24233	1.08054	0.823293
24234	1.33007	0.634557
24235	0.911471	0.716329
24236	1.26787	1.40885
24237	1.16641	1.29659
24238	0.946132	1.30302
24239	0.946133	1.16788
24240	1.16641	1.39632
24241	0.946131	1.91085

24242	1.25037	0.381131
24243	1.34733	1.03131
24244	1.34733	0.852835
24245	0.946131	0.873458
24246	0.946131	0.316609
24247	1.41377	0.269532
24248	0.94613	0.33281
24249	1.30541	0.294686
24250	1.41377	0.29654
24251	1.3473	2.1
24252	1.11804	1.84139
24253	0.94613	2.1
24254	0.946131	2.1
24255	1.11805	2.1
24256	1.32068	2.1
24257	0.946131	2.1
24258	0.946128	2.1
24259	1.39384	2.1
24260	1.4181	2.1
24261	1.12913	2.1
24262	0.94613	2.1
24263	0.946129	2.1
24264	1.20328	2.1
24265	1.39385	2.1
24266	1.3335	2.1
24267	1.40987	2.1
24268	0.94613	2.1
24269	1.21474	2.1
24270	0.94613	2.1
24271	0.946132	1.33463
24272	1.15203	2.1
24273	1.41192	1.00009
24274	1.41192	2.1
24275	0.946132	2.1
24276	0.946131	1.95515
24277	1.40684	2.03548
24278	1.2597	1.71481
24279	0.946131	1.60868
24280	1.40684	1.27653
24281	0.946131	1.56286
24282	1.39113	1.75652
24283	1.34049	1.12404
24284	0.946131	1.17057
24285	1.39113	1.04967
24286	0.946132	1.41545
24287	1.31649	0.977332
24288	0.946132	0.904632
24289	0.946132	1.05027
24290	1.34297	1.10406
24291	0.946131	0.712571
24292	1.41766	0.685579
24293	0.954559	1.06575
24294	0.946131	0.700584
24295	0.946131	0.678661
24296	1.25447	0.704531
24297	1.35559	0.609612
24298	0.946127	0.574482
24299	0.946128	0.671307

24300	1.14874	0.658149
24301	1.33035	0.442149
24302	1.3665	0.392469
24303	0.946129	0.379464
24304	0.94613	0.405004
24305	1.20732	0.419391
24306	0.946131	2.1
24307	0.946131	1.83185
24308	1.32949	2.1
24309	1.34921	2.1
24310	0.946131	2.1
24311	0.946132	0.75185
24312	1.25479	0.74128
24313	1.38357	0.740094
24314	1.38357	0.533602
24315	0.946132	0.705082
24316	0.946131	0.984604
24317	1.31034	0.704178
24318	1.37127	0.923718
24319	1.37127	0.60092
24320	0.946131	0.806259
24321	1.23923	0.563911
24322	1.41169	0.64175
24323	0.94613	0.729465
24324	1.12703	0.534027
24325	0.946131	0.604338
24326	0.946119	2.1
24327	1.41915	2.1
24328	1.02679	2.1
24329	0.946107	2.1
24330	1.41917	2.1
24331	0.946133	2.1
24332	1.36581	2.1
24333	0.946131	2.1
24334	1.36581	2.1
24335	0.946131	2.1
24336	0.946132	2.1
24337	1.04384	2.1
24338	1.34973	2.1
24339	1.34967	2.1
24340	0.851555	2.1
24341	0.946132	0.615251
24342	1.3855	0.680504
24343	1.08603	0.612434
24344	1.08602	0.659766
24345	1.16234	0.663096
24346	0.946133	0.659955
24347	1.29348	0.698665
24348	1.20111	0.64982
24349	1.20112	0.689593
24350	1.3855	0.687858
24351	0.946128	1.20433
24352	1.30798	2.1
24353	0.946129	1.96945
24354	1.34828	1.83099
24355	1.30797	1.94094
24356	1.24932	0.221494
24357	1.40549	0.243966

24358	0.946117	0.282959
24359	1.19615	0.250006
24360	0.946106	0.255882
24361	0.946131	2.1
24362	1.29926	2.1
24363	1.25144	2.1
24364	0.946131	2.1
24365	1.29925	2.1
24366	0.946132	2.1
24367	1.39826	2.1
24368	0.946133	2.1
24369	0.946133	2.1
24370	1.29926	2.1
24371	0.946119	0.358644
24372	1.33614	0.28487
24373	0.946118	0.35366
24374	1.41377	0.321744
24375	1.33612	0.32817
24376	0.946133	1.43872
24377	1.3933	1.73721
24378	1.34999	1.0942
24379	0.946133	1.34283
24380	1.3933	1.28191
24381	0.946131	1.51786
24382	1.36139	2.00251
24383	0.946132	1.20509
24384	0.946131	1.54099
24385	1.39329	1.22744
24386	1.37449	1.19745
24387	1.27925	0.790202
24388	0.919796	0.833435
24389	1.01176	0.94986
24390	0.94613	0.923118
24391	0.946133	1.6034
24392	1.27679	2.1
24393	0.946133	1.25039
24394	0.946132	1.92756
24395	1.39731	1.48343
24396	0.946119	2.0864
24397	1.40765	1.41977
24398	1.1981	1.61331
24399	0.946131	1.22438
24400	1.40764	1.68842
24401	0.946127	2.1
24402	1.3557	2.1
24403	0.946127	2.1
24404	0.946128	2.1
24405	1.13485	2.1
24406	1.4008	2.1
24407	1.1587	2.1
24408	0.946131	2.1
24409	0.946131	2.1
24410	1.1587	2.1
24411	1.32082	0.384392
24412	1.29215	0.410132
24413	0.94613	0.365031
24414	0.946131	0.364812
24415	1.02366	0.331887

24416	1.27697	0.399922
24417	1.2929	0.418673
24418	0.946132	0.349677
24419	0.946132	0.348319
24420	1.01451	0.31117
24421	0.946132	2.1
24422	1.27252	2.1
24423	0.946132	2.1
24424	1.37869	2.1
24425	1.27252	2.1
24426	1.15627	2.1
24427	1.35703	2.1
24428	0.946132	2.1
24429	1.03783	2.1
24430	0.946132	2.1
24431	0.946128	2.1
24432	1.37869	2.1
24433	0.946131	2.1
24434	1.35357	2.1
24435	1.37869	2.1
24436	0.946132	2.1
24437	0.946131	2.1
24438	1.33471	2.1
24439	0.946131	2.1
24440	1.36373	2.1
24441	1.41342	0.866811
24442	1.06832	0.896065
24443	0.946128	1.23501
24444	0.94613	0.772004
24445	1.06832	1.12302
24446	1.26929	2.05021
24447	0.946131	1.13749
24448	0.946128	2.06447
24449	1.41022	1.49589
24450	1.37034	1.22729
24451	1.37759	0.902489
24452	1.07612	1.07556
24453	0.946126	0.932739
24454	0.946129	0.98909
24455	1.07612	0.959338
24456	0.946131	0.960068
24457	0.94613	0.461437
24458	1.23848	0.698808
24459	1.38897	0.58667
24460	0.946131	0.671121
24461	0.946132	0.675225
24462	0.946133	0.325148
24463	1.34369	0.323067
24464	1.40332	0.308024
24465	0.946132	0.376043
24466	0.946131	2.1
24467	1.24023	2.1
24468	1.38244	2.1
24469	1.38244	2.1
24470	0.946131	2.1
24471	0.946132	1.31461
24472	1.33468	2.1
24473	1.41619	1.55057

24474	1.41619	2.00688
24475	0.946131	2.02149
24476	1.1908	1.0096
24477	0.946128	0.931507
24478	0.946126	1.12516
24479	1.26654	1.12722
24480	1.39514	1.32703
24481	0.93608	2.05918
24482	1.04105	2.1
24483	1.39551	2.1
24484	1.52471	2.1
24485	1.04072	2.1
24486	1.33174	0.185922
24487	0.946128	0.186185
24488	0.94613	0.190625
24489	1.17801	0.180764
24490	0.94613	0.196359
24491	0.946132	2.1
24492	0.946132	2.1
24493	1.29065	2.1
24494	1.29065	2.1
24495	0.975266	2.1
24496	0.946131	2.1
24497	1.26025	2.1
24498	0.955601	2.1
24499	0.946131	2.1
24500	1.26025	2.1
24501	0.94613	0.423621
24502	1.23146	0.394189
24503	0.946127	0.401501
24504	1.32827	0.430147
24505	1.23146	0.399358
24506	1.25008	0.837003
24507	1.05018	0.870588
24508	0.94613	0.847838
24509	0.932744	0.899533
24510	1.04207	0.868963
24511	0.946132	0.755534
24512	1.10023	0.921909
24513	1.36748	0.733658
24514	1.36748	0.8207
24515	0.946133	0.794561
24516	1.06023	0.395104
24517	1.08019	0.401629
24518	0.94613	0.484448
24519	1.06023	0.404778
24520	0.94613	0.400854
24521	1.16727	0.499516
24522	1.35856	0.5297
24523	0.946132	0.630587
24524	1.16727	0.532332
24525	0.946132	0.511955
24526	0.946133	2.1
24527	0.946131	2.1
24528	1.21691	2.1
24529	1.39558	2.1
24530	0.946131	2.1
24531	1.09905	0.611614

24532	0.946132	1.00673
24533	1.3365	0.776416
24534	1.35381	0.816075
24535	0.946132	1.04349
24536	0.94613	0.453601
24537	0.946128	0.767269
24538	1.40332	0.541396
24539	1.39041	0.569929
24540	0.94613	0.498379
24541	0.946132	0.826018
24542	1.3659	1.16338
24543	1.39041	0.870682
24544	1.39041	0.961306
24545	0.946132	0.950817
24546	0.840292	1.73673
24547	0.946133	1.46271
24548	0.851541	1.77343
24549	1.28464	1.55236
24550	1.28806	1.47439
24551	1.12591	0.789914
24552	1.05033	0.87653
24553	0.946132	0.821032
24554	0.946132	0.850667
24555	1.13537	0.837814
24556	1.21177	1.12358
24557	0.946132	1.17777
24558	0.946132	1.70569
24559	1.41134	1.15437
24560	1.27079	1.26636
24561	0.946131	0.314192
24562	1.35403	0.307019
24563	0.94613	0.384101
24564	0.94613	0.347815
24565	0.946131	0.341361
24566	0.946131	0.592294
24567	0.946132	0.43134
24568	0.94613	0.763046
24569	1.37052	0.534777
24570	0.946131	0.623489
24571	0.946133	0.480657
24572	0.946132	0.685517
24573	0.946133	0.496806
24574	1.24249	0.478417
24575	0.946132	0.496721
24576	1.20608	2.1
24577	0.946132	2.023
24578	0.946131	1.71255
24579	0.946132	2.09606
24580	0.946132	2.1
24581	0.946131	2.1
24582	0.946128	2.1
24583	0.946129	2.1
24584	1.41232	2.1
24585	0.94613	2.1
24586	0.946132	0.366339
24587	1.32449	0.361053
24588	0.946131	0.445003
24589	0.946131	0.424776

24590	1.32449	0.395131
24591	0.946116	1.10167
24592	1.41563	1.84362
24593	0.946118	1.53566
24594	0.946105	1.57216
24595	0.946131	1.08112
24596	1.26563	2.1
24597	1.32819	1.52917
24598	0.946131	0.848638
24599	0.946131	1.79914
24600	0.946131	1.08523
24601	0.946131	1.59161
24602	0.946133	1.23234
24603	1.30382	2.05501
24604	0.946131	1.20074
24605	0.946131	1.11315
24606	1.30382	1.4816
24607	0.946133	0.172331
24608	1.15803	0.178002
24609	1.28895	0.169915
24610	0.954071	0.173745
24611	0.946132	0.182031
24612	1.0264	1.09366
24613	0.946132	1.55698
24614	0.946132	1.28313
24615	0.946131	1.4485
24616	0.946132	1.32863
24617	1.35977	0.556042
24618	0.946132	0.764609
24619	0.946129	0.708969
24620	0.94613	0.705088
24621	0.946129	0.66937
24622	0.876631	0.703996
24623	0.946133	0.700384
24624	0.946132	0.717038
24625	0.946132	0.703615
24626	0.876606	0.701689
24627	0.946131	0.314391
24628	1.24979	0.242102
24629	0.94613	0.287191
24630	0.94613	0.248924
24631	0.94613	0.278651
24632	0.921791	0.251973
24633	0.946131	0.245206
24634	0.921762	0.265325
24635	0.946131	0.245196
24636	0.946131	0.244738
24637	0.810944	0.492749
24638	0.810945	0.578321
24639	0.810959	0.564151
24640	0.946133	0.36914
24641	1.25797	0.342835
24642	0.946132	0.351525
24643	0.946132	0.356717
24644	1.25797	0.34441
24645	1.13748	0.826653
24646	0.851512	0.949194
24647	0.94613	1.12134

24648	1.02373	0.821234
24649	0.946131	0.989071
24650	0.946122	2.1
24651	1.3852	2.1
24652	0.946124	2.1
24653	0.946118	2.1
24654	1.38521	2.1
24655	0.946131	0.696445
24656	0.946131	0.689833
24657	1.30448	0.710844
24658	0.946129	0.698957
24659	0.94613	0.681584
24660	0.946133	2.05107
24661	0.94613	2.1
24662	0.946131	1.83775
24663	0.946131	1.69598
24664	1.36503	2.1
24665	0.810947	0.177152
24666	0.810947	0.175632
24667	0.810934	0.180324
24668	3.34066	0.158158
24669	0.946133	0.866342
24670	1.4185	0.762135
24671	0.946132	0.693493
24672	0.946133	0.801319
24673	0.946132	0.761008
24674	0.94613	2.1
24675	0.946128	2.1
24676	1.03925	2.1
24677	0.944743	2.1
24678	0.947518	2.1
24679	0.946132	2.07675
24680	1.20573	2.1
24681	0.94613	2.1
24682	0.94613	2.1
24683	1.20573	2.1
24684	1.20056	0.239127
24685	0.946132	0.261809
24686	0.946131	0.298445
24687	0.946131	0.26574
24688	0.946131	0.259513
24689	0.94613	2.1
24690	0.946128	2.1
24691	1.38302	2.1
24692	0.94613	2.1
24693	0.94613	2.1
24694	0.827866	0.321575
24695	0.913552	0.29886
24696	0.716526	0.355398
24697	0.82784	0.296085
24698	1.20634	0.341886
24699	0.946131	0.322532
24700	0.94613	0.336723
24701	1.39502	0.281414
24702	0.88146	0.334362
24703	0.946132	2.1
24704	0.946132	2.1
24705	0.946132	2.1

24706	0.946132	2.1
24707	1.39053	2.1
24708	0.946131	2.1
24709	1.40571	2.1
24710	0.946131	2.1
24711	0.946131	2.1
24712	0.946132	2.1
24713	0.94613	0.640393
24714	1.2582	0.701012
24715	0.946127	0.663509
24716	0.946129	0.678349
24717	0.946129	0.657051
24718	0.946129	0.962955
24719	0.946132	1.00971
24720	0.946131	0.925451
24721	0.946131	1.0538
24722	1.29054	0.920143
24723	0.810944	0.190871
24724	0.810941	0.18886
24725	0.810931	0.187519
24726	0.946132	0.871061
24727	0.946132	0.833899
24728	0.946132	1.05309
24729	0.946132	0.869257
24730	0.927608	0.847827
24731	0.946128	2.1
24732	0.946133	2.1
24733	0.946131	2.1
24734	0.94613	2.1
24735	1.38813	2.1
24736	0.94613	0.842589
24737	0.946128	2.1
24738	0.946132	0.836048
24739	1.41666	1.56132
24740	0.94613	0.934452
24741	0.946118	0.411777
24742	1.34151	0.346954
24743	0.946116	0.443191
24744	0.946131	0.43237
24745	1.34149	0.400615
24746	0.946132	2.1
24747	1.31889	2.1
24748	0.946132	2.1
24749	0.946132	2.1
24750	1.31889	2.1
24751	0.864997	0.233472
24752	0.928981	0.220201
24753	0.872219	0.217231
24754	0.946127	0.824729
24755	0.946131	0.754751
24756	1.37709	0.678631
24757	0.946131	0.832274
24758	0.946129	0.754089
24759	1.21882	0.171893
24760	0.938168	0.170712
24761	0.938161	0.175361
24762	1.06327	0.168582
24763	1.33457	0.178672

24764	0.946133	0.958901
24765	0.946132	0.966813
24766	0.946133	0.932148
24767	0.946132	1.11014
24768	1.14444	0.891186
24769	1.12628	0.281886
24770	0.946132	0.368485
24771	0.946131	0.42349
24772	1.12628	0.279122
24773	0.946131	0.32594
24774	0.946132	2.1
24775	0.946132	2.1
24776	0.946133	2.1
24777	0.946132	2.1
24778	1.29322	2.1
24779	0.946131	2.03018
24780	0.946132	2.1
24781	0.94613	2.05257
24782	0.946131	2.1
24783	1.28441	2.1
24784	0.946118	1.75808
24785	0.946119	1.82527
24786	0.946117	2.1
24787	1.28075	2.1
24788	0.946105	1.99995
24789	0.810945	2.1
24790	0.810942	2.1
24791	0.81093	2.1
24792	0.946127	2.1
24793	0.946127	2.1
24794	1.28228	2.1
24795	0.946129	2.1
24796	0.946132	2.1
24797	1.14042	2.1
24798	0.946132	2.1
24799	0.946133	2.1
24800	1.14042	2.1
24801	0.946133	0.562014
24802	0.946132	0.551886
24803	1.28632	0.468078
24804	1.28632	0.358711
24805	0.946133	0.537235
24806	0.946131	1.17306
24807	0.946131	1.06277
24808	0.946131	1.11664
24809	1.31817	1.21902
24810	0.946131	1.06226
24811	1.35048	2.1
24812	0.946132	2.1
24813	0.946132	2.1
24814	0.946132	2.1
24815	0.946131	2.1
24816	0.946131	2.1
24817	1.35943	2.1
24818	0.94613	2.1
24819	0.946132	2.1
24820	0.946131	2.1
24821	0.990713	2.1

24822	0.946132	2.1
24823	0.946132	2.1
24824	0.946133	2.1
24825	0.946132	2.1
24826	0.946133	1.00068
24827	1.01433	1.13301
24828	0.877931	1.06311
24829	0.96573	0.979274
24830	0.946131	0.972525
24831	0.946131	1.09133
24832	0.946133	0.968616
24833	1.39575	0.85735
24834	0.946131	0.883745
24835	0.94613	0.895473
24836	0.810943	0.78479
24837	0.810942	0.791366
24838	0.709589	0.687725
24839	0.924071	0.666556
24840	1.04293	0.582418
24841	1.15747	0.49399
24842	0.946131	0.672271
24843	1.04071	0.621891
24844	0.810946	0.868428
24845	0.810943	0.796925
24846	0.810959	0.761189
24847	0.946128	1.29821
24848	1.39696	1.58297
24849	0.94613	1.01177
24850	0.94613	1.02054
24851	1.39696	1.38455
24852	0.946131	0.879499
24853	0.946131	1.35281
24854	0.946131	1.30851
24855	0.946131	0.979426
24856	1.29735	1.40218
24857	0.946128	1.20751
24858	0.946133	0.87946
24859	0.94613	1.75216
24860	0.946131	1.16485
24861	1.33266	1.02642
24862	0.946131	2.09922
24863	0.94613	0.618082
24864	0.946131	1.98224
24865	1.40609	0.93354
24866	0.946131	1.02593
24867	0.946131	1.37107
24868	0.946133	1.4165
24869	0.946129	1.47951
24870	1.23339	1.63864
24871	0.946131	1.36024
24872	0.946132	1.74851
24873	0.946133	1.68023
24874	0.946132	2.00044
24875	1.29198	1.97261
24876	0.946132	1.52855
24877	0.946131	2.1
24878	1.07297	2.1
24879	0.946127	2.1

24880	0.94613	2.02197
24881	1.07297	2.1
24882	0.946125	2.1
24883	0.946126	2.1
24884	0.946126	2.1
24885	1.3864	2.1
24886	0.946119	2.1
24887	0.946128	0.591765
24888	1.09411	0.567789
24889	0.946131	0.664568
24890	0.946131	0.638012
24891	1.09411	0.608001
24892	0.946131	0.531017
24893	1.25837	0.508519
24894	0.946131	0.625435
24895	0.946131	0.607622
24896	1.25837	0.564737
24897	0.810945	2.1
24898	0.810943	2.1
24899	0.810933	2.1
24900	0.94613	0.537499
24901	0.946131	0.854653
24902	0.946132	0.624301
24903	0.946129	0.500373
24904	0.946131	0.752868
24905	0.946131	0.346164
24906	1.28911	0.346608
24907	0.946129	0.426165
24908	0.946133	0.347711
24909	0.946131	0.362971
24910	0.94613	0.66947
24911	1.38954	0.703012
24912	0.946132	0.660563
24913	0.94613	0.690431
24914	0.946131	0.669834
24915	0.946131	0.910797
24916	0.946131	0.810961
24917	0.946131	0.768686
24918	1.37981	0.773565
24919	0.946131	0.799493
24920	0.946131	1.65366
24921	1.26958	2.1
24922	0.946127	1.85278
24923	0.946129	2.06547
24924	1.26957	2.1
24925	0.94613	2.1
24926	1.16889	2.1
24927	0.946126	2.1
24928	0.946128	2.1
24929	1.16889	2.1
24930	0.946133	0.697375
24931	1.13511	0.593839
24932	0.946132	0.716444
24933	0.946133	0.629121
24934	0.946132	0.624043
24935	1.27575	1.54771
24936	0.946129	1.91018
24937	0.946131	1.67

24938	0.94613	2.00885
24939	0.946131	1.88594
24940	0.946131	2.1
24941	0.946128	2.1
24942	1.41712	2.1
24943	1.41712	2.1
24944	0.94613	2.1
24945	0.946129	0.264966
24946	0.946132	0.241001
24947	0.946131	0.24184
24948	0.946131	0.266264
24949	0.946131	0.242019
24950	0.979045	1.53978
24951	0.946131	1.03779
24952	0.617549	1.45588
24953	0.555844	1.79293
24954	1.04071	1.10248
24955	0.946132	1.34014
24956	0.946132	1.03851
24957	0.946131	1.5744
24958	1.22618	1.10151
24959	0.946132	1.00882
24960	0.946131	2.1
24961	1.13976	2.1
24962	0.946129	2.1
24963	0.946131	2.1
24964	1.13976	2.1
24965	0.810945	0.761935
24966	0.810943	0.771307
24967	0.810932	0.817797
24968	1.25358	1.01405
24969	0.94613	0.802019
24970	0.946128	0.819749
24971	1.25358	1.29426
24972	1.07811	0.918737
24973	0.946129	2.1
24974	1.09197	2.1
24975	0.946131	2.1
24976	0.946131	2.1
24977	1.09197	2.1
24978	0.594912	2.1
24979	0.993703	2.1
24980	1.10173	2.1
24981	0.9916	2.1
24982	0.946131	2.03284
24983	0.946128	2.1
24984	0.946131	2.1
24985	0.94613	2.1
24986	0.946129	2.1
24987	0.946132	2.1
24988	1.40335	2.1
24989	0.946133	2.1
24990	0.946133	2.1
24991	1.40335	2.1
24992	0.946132	1.20206
24993	0.946132	1.50943
24994	0.946132	1.23804
24995	0.946132	1.32835

24996	1.411	1.4805
24997	0.611675	0.482989
24998	0.946129	0.530081
24999	1.31678	0.350784
25000	0.946124	0.542446
25001	0.946127	0.380236
25002	0.946128	0.543586
25003	0.946132	0.712744
25004	0.946131	0.592554
25005	0.946132	0.716495
25006	0.946132	0.610258
25007	0.946132	0.594117
25008	0.810946	0.660678
25009	0.810943	0.66248
25010	0.81096	0.600129
25011	1.39301	0.314276
25012	0.946132	0.331591
25013	0.625812	0.273988
25014	0.946132	0.306362
25015	0.625812	0.31727
25016	0.810946	1.2485
25017	0.810945	1.39249
25018	0.810931	1.83989
25019	0.810943	2.1
25020	0.810944	2.1
25021	0.810958	2.1
25022	0.946131	0.643573
25023	0.946131	0.59617
25024	0.946128	0.651087
25025	0.946129	0.622475
25026	0.94613	0.630435
25027	0.946133	0.706269
25028	0.946133	0.707136
25029	0.98537	0.704827
25030	0.985371	0.580719
25031	0.946132	0.717096
25032	0.946129	0.665599
25033	1.38752	0.544412
25034	0.946131	0.491919
25035	0.946131	0.547493
25036	1.38752	0.54594
25037	0.946119	0.537186
25038	1.33036	0.440038
25039	0.946119	0.412128
25040	0.946132	0.443457
25041	1.33035	0.441964
25042	1.3868	2.1
25043	0.946132	2.1
25044	0.946132	2.1
25045	0.946133	2.1
25046	0.946132	2.1
25047	0.810947	2.1
25048	0.810946	2.1
25049	0.81096	2.1
25050	0.810946	2.1
25051	0.810946	2.1
25052	0.810933	2.1
25053	0.946133	2.05065

25054	1.35342	2.1
25055	0.946133	2.01282
25056	0.946132	2.1
25057	1.35342	2.1
25058	0.946132	2.1
25059	0.946132	2.1
25060	1.28569	2.1
25061	0.946132	2.1
25062	0.946133	2.1
25063	0.946132	1.53278
25064	1.32547	2.1
25065	0.946132	1.6031
25066	0.946132	1.71082
25067	1.32547	2.01006
25068	0.94613	0.389584
25069	0.946133	0.394705
25070	1.39664	0.382968
25071	1.39664	0.382973
25072	0.946131	0.397525
25073	0.946131	1.21778
25074	0.94613	2.03258
25075	0.946132	1.02049
25076	0.946131	1.23605
25077	1.41833	1.30652
25078	0.946131	0.279613
25079	0.946131	0.259824
25080	0.946131	0.305083
25081	1.26155	0.255919
25082	0.946132	0.270368
25083	0.810946	2.1
25084	0.810946	2.06999
25085	0.810959	2.1
25086	0.94613	1.07796
25087	0.946132	0.90036
25088	1.40779	0.810717
25089	1.40779	0.853749
25090	0.94613	0.892641
25091	0.94613	0.39168
25092	0.946129	0.430604
25093	1.36376	0.329336
25094	1.36376	0.335769
25095	0.946131	0.377246
25096	0.946128	2.1
25097	0.946127	2.1
25098	0.946131	2.1
25099	1.40756	2.1
25100	0.94613	2.1
25101	0.946131	1.34096
25102	0.946132	1.59247
25103	0.94613	1.3286
25104	0.946131	1.29518
25105	1.31947	1.46087
25106	0.946131	2.1
25107	0.946131	2.1
25108	0.946129	2.1
25109	0.946131	2.1
25110	1.25768	2.1
25111	0.946132	0.560836

25112	0.94613	0.625205
25113	0.946131	0.536097
25114	0.946131	0.524094
25115	0.946131	0.618459
25116	0.810943	0.907473
25117	0.810942	1.05047
25118	0.81093	1.37296
25119	0.946128	0.903307
25120	0.946133	0.811408
25121	0.946131	0.917093
25122	0.946131	0.866886
25123	0.94613	0.923028
25124	0.946132	1.01657
25125	0.946131	0.931176
25126	0.946132	1.02213
25127	0.946132	1.01198
25128	0.946132	1.06238
25129	0.946119	2.1
25130	0.946119	2.1
25131	1.31523	2.1
25132	0.946132	2.1
25133	0.946107	2.1
25134	0.946133	1.85197
25135	0.946132	0.978769
25136	0.946132	0.972435
25137	1.28803	1.46242
25138	0.946132	1.31145
25139	1.37658	0.853719
25140	0.946132	0.799453
25141	0.946133	0.969145
25142	0.946132	0.830018
25143	0.946132	0.945809
25144	0.946132	1.00892
25145	0.94613	1.13891
25146	0.94613	1.21385
25147	1.15541	1.04307
25148	0.92943	1.13154
25149	0.946126	0.254969
25150	0.946129	0.287131
25151	1.33849	0.213929
25152	1.33849	0.234247
25153	0.946129	0.250383
25154	0.810946	0.22685
25155	0.810944	0.282267
25156	0.810959	0.237812
25157	1.39027	0.310913
25158	0.946131	0.404449
25159	0.946131	0.465008
25160	1.39027	0.317082
25161	0.946131	0.359877
25162	1.40924	1.42634
25163	0.946131	1.12013
25164	0.946132	1.47321
25165	0.946132	1.00358
25166	0.946132	1.26816
25167	0.946131	2.1
25168	0.94613	2.1
25169	0.946128	2.1

25170	0.946128	2.1
25171	1.41744	2.1
25172	0.946132	0.767506
25173	0.946128	0.763879
25174	0.94613	0.840223
25175	1.37675	0.774914
25176	0.94613	0.891877
25177	0.946132	0.959882
25178	0.946132	0.813063
25179	0.946132	0.736345
25180	0.946132	0.806326
25181	0.946131	0.798283
25182	0.946132	0.854988
25183	0.946132	0.810154
25184	0.946133	1.07847
25185	1.10003	0.787709
25186	0.946132	0.935538
25187	0.946132	0.455673
25188	0.946131	0.484249
25189	0.946132	0.427457
25190	0.946131	0.470929
25191	0.946132	0.460281
25192	0.946132	0.503506
25193	0.946131	0.525158
25194	0.946132	0.466055
25195	0.946132	0.511708
25196	0.946131	0.497214
25197	1.24094	0.279036
25198	0.946126	0.352899
25199	0.946128	0.313296
25200	0.946129	0.346771
25201	0.946129	0.320611
25202	1.2929	0.351905
25203	0.946131	0.462688
25204	0.946131	0.407115
25205	0.946131	0.441011
25206	0.946131	0.420162
25207	0.810946	0.243118
25208	0.810945	0.256934
25209	0.810959	0.235348
25210	0.810946	1.41618
25211	0.810943	1.44784
25212	0.810933	1.62113
25213	0.946128	1.81274
25214	1.18315	2.1
25215	0.94613	1.82991
25216	0.94613	1.81667
25217	1.18316	2.03281
25218	0.946126	0.743327
25219	1.3324	0.654884
25220	0.946127	0.763911
25221	0.94613	0.65467
25222	0.946128	0.700204
25223	0.94613	2.0482
25224	0.946128	2.1
25225	1.39523	2.1
25226	1.39523	2.1
25227	0.946131	2.1

25228	1.36541	2.1
25229	0.946132	2.1
25230	0.946132	2.1
25231	0.946132	2.1
25232	0.946132	2.1
25233	0.946131	1.64995
25234	0.94613	1.09932
25235	0.946132	1.29584
25236	0.946131	0.850691
25237	1.39509	1.30076
25238	0.946132	2.1
25239	0.946132	2.1
25240	1.29319	2.1
25241	0.946132	2.1
25242	0.946133	2.1
25243	0.946132	0.368013
25244	0.946131	0.470484
25245	0.946132	0.391783
25246	1.01196	0.366643
25247	0.946132	0.445715
25248	0.946116	2.1
25249	0.946117	1.74492
25250	1.38153	2.1
25251	1.38155	2.1
25252	0.946105	2.1
25253	0.946132	1.2695
25254	0.946131	1.36787
25255	0.946132	1.21
25256	1.28684	1.25859
25257	0.946132	1.10435
25258	1.10185	1.63098
25259	0.94613	1.67708
25260	0.946128	1.91925
25261	0.94613	1.88993
25262	0.94613	1.66415
25263	0.810947	0.186997
25264	0.810945	0.187554
25265	0.810932	0.188455
25266	0.946119	0.5595
25267	1.16717	0.465395
25268	0.946117	0.574231
25269	0.94613	0.503856
25270	1.16715	0.489797
25271	0.94612	0.741474
25272	0.946119	0.727498
25273	0.946119	0.684351
25274	0.946132	0.573559
25275	1.20882	0.608337
25276	1.07043	0.95881
25277	0.823268	0.974601
25278	0.810959	0.935411
25279	0.946132	1.41302
25280	1.14879	1.00388
25281	0.94613	1.31599
25282	0.946131	1.45197
25283	1.14879	1.07378
25284	1.23837	2.1
25285	0.94612	2.08897

25286	0.946118	1.9709
25287	0.946131	2.1
25288	0.946105	2.1
25289	0.810946	0.273832
25290	0.810946	0.251625
25291	0.810959	0.258464
25292	0.810947	0.849271
25293	0.810947	0.60956
25294	0.810934	0.582387
25295	0.946128	2.1
25296	0.946131	2.1
25297	0.946128	2.1
25298	0.946129	2.1
25299	0.94613	2.1
25300	0.94613	2.1
25301	0.946131	2.1
25302	0.946131	2.02156
25303	0.946131	2.1
25304	0.946131	2.1
25305	0.946131	1.36961
25306	1.38585	1.9425
25307	0.946128	1.18669
25308	0.94613	1.26917
25309	1.38585	1.40041
25310	0.946133	0.59186
25311	0.946133	0.599256
25312	0.946132	0.550099
25313	0.946132	0.590621
25314	0.946132	0.575098
25315	0.918871	0.365782
25316	0.810248	0.421542
25317	0.838037	0.38772
25318	0.946126	0.422527
25319	0.943647	0.402016
25320	1.13836	0.325155
25321	1.03802	0.326931
25322	0.946129	0.350747
25323	0.946133	1.03226
25324	0.946132	0.865256
25325	0.946132	1.14479
25326	1.1699	0.90207
25327	0.946132	0.872614
25328	0.946131	2.1
25329	0.946132	2.1
25330	1.37199	2.1
25331	1.37199	2.1
25332	0.946132	2.1
25333	0.946132	0.9348
25334	0.946133	0.852392
25335	0.946133	1.03629
25336	0.946132	0.868122
25337	1.38623	0.8688
25338	0.946132	2.1
25339	0.946132	2.1
25340	0.946132	2.1
25341	0.946132	2.1
25342	1.40098	2.1
25343	1.20787	0.718878

25344	1.41894	0.744159
25345	0.946133	0.621802
25346	1.01777	0.763473
25347	0.946132	2.1
25348	0.946132	2.1
25349	0.946133	2.1
25350	1.40199	2.1
25351	0.946132	2.1
25352	0.810946	1.04605
25353	0.810947	1.23085
25354	0.810934	1.11972
25355	0.946126	0.993526
25356	0.94613	1.02574
25357	0.946125	1.0874
25358	1.40247	1.02786
25359	0.946128	0.996515
25360	1.25703	1.2899
25361	0.87821	1.07372
25362	0.878238	1.08277
25363	0.946119	1.38088
25364	0.946132	1.6674
25365	0.946132	0.840554
25366	0.946132	0.814841
25367	0.946131	1.0925
25368	0.946132	0.845672
25369	1.28543	0.880551
25370	0.946115	2.1
25371	1.41638	2.1
25372	0.946116	2.1
25373	0.946104	2.1
25374	0.946129	2.1
25375	0.946132	1.53207
25376	1.41432	2.1
25377	0.946132	1.82071
25378	0.946132	1.94974
25379	0.946132	1.39601
25380	0.810947	0.352793
25381	0.810947	0.368855
25382	0.709567	0.379772
25383	0.851542	0.288425
25384	1.01217	0.259575
25385	0.697993	0.244604
25386	0.946133	0.267218
25387	0.851488	0.235451
25388	0.946129	1.9249
25389	0.94613	2.07476
25390	1.2254	1.76368
25391	0.946131	2.04177
25392	0.946131	1.81863
25393	0.700785	1.95138
25394	0.915893	1.59641
25395	0.838013	1.5031
25396	0.760188	1.36179
25397	1.19601	0.629244
25398	0.946133	0.793621
25399	0.946132	0.830591
25400	0.946132	0.721449
25401	0.946132	0.800007

25402	0.946132	1.09034
25403	0.946133	1.89402
25404	0.946132	1.23456
25405	0.946133	1.44015
25406	1.36956	1.64286
25407	0.946131	0.872515
25408	0.94613	0.288243
25409	0.946133	0.865319
25410	1.34268	0.336641
25411	0.946131	0.520643
25412	0.946132	1.84792
25413	1.25603	1.90896
25414	0.946132	1.85123
25415	0.946132	1.80761
25416	1.25603	1.97489
25417	0.810946	1.06712
25418	0.810943	1.06476
25419	0.810933	1.07362
25420	0.496545	0.831527
25421	0.805767	1.14144
25422	0.936671	1.08864
25423	0.530063	1.01532
25424	1.05954	1.03475
25425	0.810946	0.505767
25426	0.810943	0.47589
25427	0.810959	0.437392
25428	1.16699	0.412526
25429	0.946131	0.607195
25430	0.946129	0.546423
25431	0.946131	0.519933
25432	0.946131	0.506945
25433	0.810943	1.18446
25434	0.810942	1.24931
25435	0.810933	1.18753
25436	0.946132	1.18184
25437	1.04832	1.1997
25438	0.870714	1.26233
25439	0.957819	1.41153
25440	0.94613	1.1389
25441	1.82931	2.1
25442	0.946132	0.7
25443	0.946129	0.7
25444	0.94613	0.7
25445	0.946131	0.7
25446	0.94613	0.7
25447	0.946119	0.931164
25448	0.94612	1.02327
25449	0.94612	0.910122
25450	0.946132	0.992017
25451	1.36118	0.936065
25452	0.810947	0.435475
25453	0.810947	0.440528
25454	0.810934	0.417928
25455	0.946132	2.1
25456	0.946132	2.1
25457	0.946132	2.1
25458	1.39754	2.1
25459	0.946131	2.1

25460	0.946129	2.1
25461	0.946133	2.1
25462	0.946131	2.1
25463	0.946131	2.1
25464	1.41732	2.1
25465	0.946132	0.983193
25466	0.946128	1.3399
25467	0.94613	1.05667
25468	0.94613	1.10632
25469	1.25277	1.22668
25470	0.94613	1.38267
25471	0.946128	1.73541
25472	1.21261	1.1693
25473	0.94613	1.40247
25474	0.94613	1.24086
25475	1.39407	2.1
25476	0.946133	2.1
25477	0.94613	2.1
25478	0.946131	2.1
25479	0.946131	2.1
25480	0.946119	1.08093
25481	0.946119	1.24539
25482	0.946119	1.24393
25483	1.1537	1.27879
25484	0.946106	1.06928
25485	0.946131	2.06073
25486	1.11822	2.1
25487	0.946129	2.08245
25488	0.946131	2.1
25489	1.11822	2.1
25490	1.46806	0.194628
25491	0.498881	0.21024
25492	0.996658	0.217101
25493	1.2179	0.199625
25494	0.991519	0.206844
25495	0.946131	0.232039
25496	0.946131	0.250352
25497	1.28673	0.213741
25498	0.946131	0.227479
25499	0.946131	0.223794
25500	1.33178	0.368591
25501	0.946132	0.444686
25502	0.946132	0.429955
25503	0.946132	0.435438
25504	0.946132	0.433721
25505	0.946128	0.689292
25506	0.972061	0.655585
25507	0.946129	0.720509
25508	0.946129	0.71323
25509	0.972059	0.690304
25510	0.946129	2.1
25511	0.94613	2.1
25512	0.94613	2.1
25513	0.94613	2.1
25514	0.94613	2.1
25515	0.946131	2.1
25516	0.946127	2.1
25517	0.946131	2.1

25518	0.946129	2.1
25519	1.4073	2.1
25520	0.946131	0.42216
25521	1.05016	0.363193
25522	0.946132	0.453899
25523	0.946131	0.43896
25524	0.946132	0.429085
25525	0.946128	0.432235
25526	1.13398	0.373686
25527	0.946131	0.474981
25528	0.946131	0.451667
25529	0.94613	0.439101
25530	0.946131	0.277381
25531	0.946131	0.330709
25532	1.38911	0.231574
25533	1.38911	0.254961
25534	0.946132	0.278055
25535	0.946131	0.535997
25536	0.94613	0.565149
25537	0.946131	0.52208
25538	0.946131	0.546141
25539	0.946131	0.5561
25540	0.946131	0.636332
25541	1.23923	0.553897
25542	0.94613	0.668763
25543	0.946131	0.554063
25544	0.946131	0.586894
25545	1.40511	0.223556
25546	0.946131	0.240987
25547	0.946131	0.279067
25548	0.946132	0.249298
25549	0.946131	0.239797
25550	1.12968	2.06399
25551	0.946132	1.67759
25552	0.946132	2.1
25553	0.946131	1.98282
25554	0.946132	2.00974
25555	0.946132	1.35603
25556	1.4172	2.02076
25557	0.94613	1.20993
25558	0.94613	1.4313
25559	1.4172	1.60958
25560	0.946132	2.1
25561	0.946132	2.1
25562	1.31323	2.1
25563	0.946133	2.1
25564	0.946132	2.1
25565	0.946131	2.07764
25566	0.94613	0.924521
25567	1.36546	1.51902
25568	1.36546	1.1489
25569	0.946131	1.07994
25570	0.946132	0.753262
25571	0.946133	0.806887
25572	0.946132	0.493682
25573	1.36899	0.489006
25574	0.946133	0.563391
25575	0.946131	0.371674

25576	0.946132	0.361497
25577	1.03543	0.369294
25578	1.1412	0.391373
25579	0.941275	0.390248
25580	1.28952	0.437424
25581	0.94613	0.494623
25582	0.946129	0.511476
25583	0.946132	0.451527
25584	0.94613	0.494682
25585	0.94613	2.1
25586	0.94613	2.1
25587	0.946129	2.1
25588	0.94613	2.1
25589	1.35966	2.1
25590	0.810946	1.80627
25591	0.810942	1.9561
25592	0.810932	1.69082
25593	0.94613	1.97256
25594	1.40372	2.1
25595	0.946131	1.64411
25596	0.94613	2.1
25597	1.40372	2.1
25598	0.946133	2.1
25599	0.946131	2.1
25600	0.94613	2.1
25601	0.946131	2.1
25602	0.946131	2.1
25603	1.31453	2.1
25604	0.94613	2.1
25605	0.946128	2.1
25606	0.94613	2.1
25607	0.94613	2.1
25608	0.946131	0.355669
25609	1.12018	0.327719
25610	0.94613	0.391472
25611	0.94613	0.401144
25612	0.94613	0.365787
25613	0.946131	0.43645
25614	1.16373	0.409185
25615	0.946131	0.5025
25616	0.94613	0.418446
25617	0.946131	0.448568
25618	0.946126	0.704029
25619	0.946132	0.759521
25620	0.946128	0.719761
25621	0.946129	0.777109
25622	0.946129	0.816841
25623	0.946132	0.597239
25624	0.946133	0.644598
25625	0.946132	0.590432
25626	0.946132	0.617866
25627	0.946133	0.620344
25628	0.946132	0.966391
25629	0.946129	1.14436
25630	0.94613	0.931603
25631	1.36399	1.00764
25632	0.946131	0.956079
25633	0.946132	2.1

25634	0.946132	2.1
25635	0.946133	2.1
25636	1.38302	2.1
25637	0.946133	2.1
25638	0.810943	2.1
25639	0.810944	2.1
25640	0.810945	2.1
25641	0.946132	2.1
25642	0.946131	2.1
25643	0.946132	2.1
25644	1.30188	2.1
25645	0.946132	2.1
25646	0.810946	0.872147
25647	0.72985	1.06024
25648	0.810934	0.958093
25649	0.856243	0.878656
25650	0.946129	0.839376
25651	0.946132	0.866593
25652	1.19686	0.830538
25653	0.851527	0.924182
25654	0.946131	2.1
25655	0.94613	2.1
25656	0.94613	2.1
25657	0.94613	2.1
25658	1.31961	2.1
25659	0.946131	2.1
25660	0.946131	2.1
25661	0.94613	2.1
25662	0.946131	2.1
25663	1.3605	2.1
25664	1.30116	1.87553
25665	0.946133	0.964979
25666	0.946131	1.84253
25667	0.946131	1.2608
25668	0.94613	1.65085
25669	0.946132	0.279142
25670	1.3035	0.241225
25671	0.94613	0.281959
25672	0.946131	0.266001
25673	0.946131	0.279755
25674	0.946132	2.1
25675	1.37869	2.1
25676	1.2051	2.1
25677	0.946132	2.1
25678	1.2051	2.1
25679	0.810946	2.1
25680	0.810945	2.1
25681	0.810946	2.1
25682	1.34623	2.1
25683	0.946131	2.1
25684	0.946131	2.1
25685	1.34623	2.1
25686	1.22993	2.1
25687	1.32299	0.270263
25688	0.946132	0.325362
25689	0.946132	0.360691
25690	0.946132	0.305494
25691	0.946131	0.314494

25692	0.94613	0.36996
25693	1.40574	0.330501
25694	0.946128	0.405159
25695	0.946131	0.451638
25696	1.40574	0.416019
25697	0.946132	2.1
25698	1.41331	2.1
25699	0.94613	2.1
25700	0.946131	2.1
25701	1.41331	2.1
25702	0.94613	2.1
25703	1.37609	2.1
25704	0.946129	2.1
25705	0.946131	2.1
25706	1.37609	2.1
25707	1.039	1.30279
25708	0.946131	1.83186
25709	0.944514	1.48662
25710	0.946132	1.80461
25711	0.946132	1.62943
25712	1.23726	1.24311
25713	0.946128	1.57394
25714	0.946128	1.39745
25715	0.946129	1.65006
25716	0.94613	1.48736
25717	0.946129	2.07293
25718	0.946131	2.1
25719	0.946131	2.04461
25720	1.39214	2.1
25721	0.94613	2.1
25722	0.946118	0.907758
25723	0.946118	0.436455
25724	1.41078	0.585026
25725	1.41076	0.417219
25726	0.946131	0.462442
25727	0.946131	0.701023
25728	0.946131	0.697588
25729	0.946132	0.7
25730	0.94613	0.700471
25731	0.946131	0.697276
25732	0.946132	0.332262
25733	0.946132	0.283266
25734	0.946133	0.256147
25735	1.28924	0.258102
25736	0.946132	0.29353
25737	0.946133	0.745373
25738	1.09812	0.614234
25739	0.946132	0.812217
25740	0.946132	0.822129
25741	1.09812	0.83876
25742	0.946132	0.980541
25743	0.946132	0.978967
25744	0.946132	0.970417
25745	0.946132	0.965968
25746	1.15388	0.992047
25747	0.946132	0.721286
25748	0.974688	0.541612
25749	0.946132	0.747317

25750	0.946132	0.742331
25751	0.974687	0.759334
25752	1.0644	1.20824
25753	0.946133	1.55078
25754	0.946132	1.22841
25755	0.946133	1.24692
25756	1.26547	1.38477
25757	1.02905	1.13157
25758	0.911537	1.16636
25759	0.838038	1.26129
25760	0.946131	0.604582
25761	0.94613	0.642187
25762	0.946132	0.604133
25763	0.946131	0.655481
25764	1.33922	0.639291
25765	0.946132	0.427947
25766	0.946132	0.383733
25767	1.40926	0.367528
25768	1.40927	0.364772
25769	0.946132	0.382177
25770	0.946129	1.63583
25771	0.946131	1.52279
25772	0.946131	1.2044
25773	0.946131	1.02211
25774	1.40496	1.59482
25775	0.94613	1.03447
25776	0.94613	1.03455
25777	0.946127	1.00591
25778	0.946128	0.965347
25779	1.38914	0.989337
25780	2.675	0.202748
25781	1.22266	0.23552
25782	0.946131	0.274418
25783	0.946132	0.267647
25784	0.946132	0.255213
25785	1.22266	0.264469
25786	0.946124	0.430584
25787	0.946128	0.325903
25788	0.946126	0.307481
25789	1.21428	0.287357
25790	0.946127	0.402414
25791	0.946131	0.365128
25792	0.946131	0.52547
25793	0.946132	0.385011
25794	0.946129	0.382375
25795	0.940118	0.487374
25796	0.810943	2.1
25797	0.810945	2.1
25798	0.810958	2.1
25799	0.946133	0.332624
25800	1.11539	0.322748
25801	0.946132	0.388295
25802	0.946132	0.360875
25803	0.946133	0.335483
25804	0.946133	2.1
25805	1.41195	2.1
25806	0.946133	2.1
25807	0.946132	2.1

25808	0.946133	2.1
25809	1.15968	0.221463
25810	0.946131	0.242455
25811	0.946131	0.267124
25812	0.946131	0.243556
25813	0.946131	0.237481
25814	0.94613	1.95196
25815	0.946128	2.02429
25816	0.946132	1.42833
25817	0.94613	1.91167
25818	0.94613	1.64839
25819	0.946131	1.79715
25820	1.04211	1.83579
25821	0.850155	1.31879
25822	0.946131	1.6316
25823	0.946131	1.47043
25824	0.946131	0.701978
25825	0.94613	0.602703
25826	0.946129	0.705768
25827	0.946131	0.612815
25828	0.946131	0.597502
25829	1.40193	0.434427
25830	1.19756	0.456981
25831	0.561936	0.516805
25832	0.808778	0.48842
25833	1.19756	0.470713
25834	0.946132	0.332062
25835	0.946132	0.323403
25836	1.33916	0.273326
25837	1.33916	0.279571
25838	0.946133	0.304868
25839	0.946127	0.707396
25840	0.946127	0.68502
25841	0.946131	0.70436
25842	0.946128	0.698615
25843	0.946129	0.693694
25844	0.589428	0.713963
25845	0.851501	0.683239
25846	0.868924	0.712918
25847	0.877703	0.704964
25848	0.85153	0.704835
25849	0.946132	1.38056
25850	1.25372	2.1
25851	0.946132	1.52515
25852	0.946132	1.75403
25853	1.25372	1.98777
25854	0.946132	1.05749
25855	0.946128	1.66095
25856	0.94613	1.15609
25857	0.94613	1.24053
25858	1.2778	1.45894
25859	0.946118	0.892043
25860	1.35629	0.906798
25861	0.946117	0.894985
25862	0.946131	0.883891
25863	0.946105	0.853018
25864	0.946132	0.819231
25865	0.946132	1.0161

25866	1.38646	0.989272
25867	1.38646	1.02822
25868	0.946132	1.01939
25869	0.946128	2.08113
25870	0.946128	0.922339
25871	1.39659	1.66717
25872	1.39659	1.22913
25873	0.946129	1.06442
25874	0.946132	1.43692
25875	0.946131	1.37632
25876	0.946132	1.36016
25877	0.946132	1.34849
25878	1.15139	1.35139
25879	0.946133	2.1
25880	0.946132	1.94295
25881	0.946132	2.1
25882	0.946132	2.1
25883	1.36151	2.1
25884	0.946127	2.1
25885	0.946131	2.1
25886	0.946128	2.1
25887	0.94613	2.1
25888	1.38397	2.1
25889	0.946131	0.531684
25890	1.31112	0.5362
25891	0.946127	0.503679
25892	0.946128	0.597767
25893	1.31112	0.551886
25894	1.29778	0.167797
25895	0.883417	0.167818
25896	1.32613	0.161304
25897	0.94613	0.166142
25898	0.94613	0.170265
25899	0.946132	2.1
25900	0.94613	2.1
25901	0.946131	2.1
25902	0.946131	2.1
25903	1.36523	2.1
25904	0.946132	0.435298
25905	1.01653	0.379631
25906	0.946132	0.492468
25907	0.946131	0.47188
25908	0.946131	0.439709
25909	0.94612	2.06125
25910	1.08301	1.59907
25911	0.946119	2.02858
25912	0.946132	2.1
25913	1.08299	1.87808
25914	0.810942	2.1
25915	0.810944	1.05854
25916	0.810957	1.22486
25917	0.946126	0.62895
25918	0.946132	0.678062
25919	0.946129	0.631174
25920	1.3492	0.595631
25921	0.94613	0.627697
25922	0.946131	0.441132
25923	1.24766	0.403063

25924	0.946128	0.426103
25925	0.946131	0.510787
25926	1.24766	0.486645
25927	0.946132	0.24137
25928	0.946131	0.229405
25929	0.946131	0.26807
25930	1.23351	0.225799
25931	0.946132	0.235729
25932	0.946131	0.258987
25933	0.946132	0.243537
25934	0.946131	0.298785
25935	1.35657	0.239151
25936	0.946131	0.25409
25937	0.946132	0.761798
25938	1.3689	0.722049
25939	0.946132	0.738537
25940	0.946132	0.743271
25941	1.3689	0.72377
25942	0.946133	0.708985
25943	1.35822	0.676378
25944	0.946133	0.686606
25945	0.946132	0.701412
25946	1.35822	0.68899
25947	0.946131	0.773301
25948	0.946131	1.06986
25949	0.946131	1.0306
25950	0.946131	0.852256
25951	1.4101	1.17298
25952	0.810944	1.35883
25953	0.810939	1.4202
25954	0.810955	1.2016
25955	0.946132	2.1
25956	1.37733	2.1
25957	0.946132	2.1
25958	0.946131	2.1
25959	0.946131	2.1
25960	1.32582	2.1
25961	0.946132	1.72573
25962	0.946132	1.28236
25963	0.946132	1.80382
25964	0.946132	2.03005
25965	0.946132	1.90041
25966	0.946132	2.03577
25967	1.31401	2.1
25968	1.31401	2.1
25969	0.946131	2.1
25970	1.3469	1.12202
25971	0.946132	1.31363
25972	0.946132	1.28932
25973	0.946133	1.02362
25974	0.946132	1.07985
25975	0.946126	2.1
25976	0.946127	1.90398
25977	0.94613	2.1
25978	0.946128	1.87826
25979	1.41206	2.1
25980	0.946127	1.29085
25981	1.40109	1.69627

25982	0.946128	1.28282
25983	0.946129	1.68418
25984	1.40109	1.48898
25985	1.24448	0.430935
25986	0.94613	0.60744
25987	0.946132	0.509762
25988	0.946129	0.57192
25989	0.946131	0.539744
25990	1.11015	0.503999
25991	0.946133	0.781964
25992	0.946132	0.636394
25993	0.946133	0.68681
25994	0.946132	0.679694
25995	0.946132	2.1
25996	0.946131	2.1
25997	0.946131	2.1
25998	1.38775	2.1
25999	0.946131	2.1
26000	0.946133	0.872009
26001	0.946131	0.977805
26002	1.30598	1.29835
26003	1.30598	1.18153
26004	0.946131	1.09776
26005	0.946132	2.1
26006	0.946132	2.1
26007	0.946132	2.1
26008	1.40774	2.1
26009	0.946132	2.1
26010	0.798396	0.186344
26011	0.946133	0.791794
26012	0.946128	1.03267
26013	0.946131	0.881575
26014	0.946131	0.900867
26015	1.28918	0.929574
26016	0.946132	0.559418
26017	1.40668	0.51888
26018	0.94613	0.550068
26019	0.94613	0.507092
26020	0.94613	0.509846
26021	0.810946	2.1
26022	0.810945	2.1
26023	0.810933	2.1
26024	0.946131	2.1
26025	1.30338	2.1
26026	0.94613	2.1
26027	1.30339	2.1
26028	0.946131	2.1
26029	0.946127	0.912015
26030	0.87161	0.982362
26031	0.946128	0.914685
26032	0.946129	0.946116
26033	0.827876	0.930269
26034	0.946131	2.1
26035	1.40796	2.1
26036	0.94613	2.1
26037	0.94613	2.1
26038	1.40796	2.1
26039	0.946132	0.915835

26040	0.946132	0.991648
26041	0.946132	0.910299
26042	0.946132	0.889803
26043	1.41724	0.874649
26044	1.34254	0.66221
26045	0.946132	0.89069
26046	0.946132	0.832278
26047	0.946132	0.830646
26048	0.946133	0.794458
26049	0.946133	1.40991
26050	0.946132	1.28926
26051	0.946132	1.22389
26052	1.26501	1.49289
26053	0.946132	1.20001
26054	0.946131	0.376647
26055	1.35169	0.374079
26056	0.946131	0.460473
26057	0.946131	0.426651
26058	1.35169	0.396432
26059	0.676844	0.298176
26060	0.946131	0.363473
26061	0.94613	0.448699
26062	1.13242	0.296599
26063	1.13242	0.305676
26064	0.946131	0.357567
26065	0.946128	1.16751
26066	1.28828	1.62816
26067	0.946131	1.16818
26068	0.946131	1.16822
26069	1.28828	1.32829
26070	0.946131	0.708546
26071	1.2996	0.636935
26072	0.946133	0.710964
26073	0.946131	0.670239
26074	0.946131	0.65415
26075	0.946132	1.24355
26076	1.40638	2.1
26077	0.946131	1.07737
26078	0.946132	2.1
26079	1.40638	2.1
26080	2.84909	0.286911
26081	1.35726	0.301189
26082	0.946131	0.39809
26083	0.946131	0.369302
26084	0.946131	0.354482
26085	0.946131	0.352293
26086	1.39852	1.88877
26087	0.946132	1.77403
26088	0.94613	2.09594
26089	0.94613	2.0199
26090	0.94613	1.92075
26091	0.946126	0.330523
26092	0.946131	0.306394
26093	0.946128	0.383346
26094	1.31666	0.318273
26095	0.946128	0.335485
26096	0.946126	0.41458
26097	0.946131	0.377771

26098	0.946129	0.464052
26099	1.32275	0.365722
26100	0.946129	0.432717
26101	0.946131	2.1
26102	0.946132	2.1
26103	1.41125	2.1
26104	0.946131	2.1
26105	0.946131	2.1
26106	0.946131	2.1
26107	0.946132	2.1
26108	1.31904	2.1
26109	0.946132	2.1
26110	0.946132	2.1
26111	0.946129	0.404117
26112	0.946132	0.387606
26113	0.94613	0.398969
26114	1.02426	0.331122
26115	0.9311	0.439286
26116	0.946128	1.31646
26117	0.946133	1.1296
26118	0.946131	1.2292
26119	1.40092	1.32851
26120	0.946131	1.16811
26121	0.946117	0.828499
26122	0.946118	1.8478
26123	0.946118	0.807273
26124	1.40017	1.25626
26125	0.946105	0.870662
26126	0.946132	0.56876
26127	1.03547	0.503139
26128	0.946132	0.487513
26129	0.946133	0.628079
26130	0.946132	0.550232
26131	0.946133	2.1
26132	0.946132	2.1
26133	0.946133	2.1
26134	0.946133	2.1
26135	1.36694	2.1
26136	0.946132	0.36983
26137	1.22878	0.354531
26138	0.946132	0.365754
26139	0.946132	0.37224
26140	1.22878	0.355789
26141	1.11014	0.494891
26142	0.913078	0.528393
26143	1.11015	0.59987
26144	0.49154	0.597552
26145	0.946127	2.1
26146	0.946131	2.1
26147	0.946128	2.1
26148	0.946129	2.1
26149	1.38752	2.1
26150	0.946132	2.1
26151	1.37277	2.1
26152	0.946132	2.1
26153	0.946131	2.1
26154	1.37277	2.1
26155	0.94613	0.701714

26156	1.16834	0.546177
26157	0.946129	0.709108
26158	0.94613	0.605461
26159	0.94613	0.594696
26160	0.946127	0.724375
26161	0.946131	0.610712
26162	1.35594	0.557012
26163	0.946128	0.735493
26164	0.94613	0.626912
26165	0.946128	0.969867
26166	0.946128	0.667198
26167	1.38464	0.871024
26168	1.38463	0.676192
26169	0.94613	0.802526
26170	0.946129	0.776479
26171	0.946131	0.64093
26172	0.94613	0.678694
26173	1.39751	0.451541
26174	0.946131	0.715784
26175	1.25837	0.490948
26176	1.21134	0.452748
26177	0.700013	0.561028
26178	0.700041	0.533689
26179	1.40583	1.63443
26180	0.946132	2.04687
26181	0.946132	2.03259
26182	0.946133	1.9448
26183	0.946132	1.78607
26184	0.827865	1.89829
26185	0.946131	2.02172
26186	0.946131	1.62114
26187	0.946131	1.75841
26188	0.946131	1.61964
26189	0.946132	0.239826
26190	1.21087	0.213844
26191	0.94613	0.233355
26192	0.94613	0.217352
26193	0.946131	0.226297
26194	0.94613	0.78668
26195	1.17452	0.823598
26196	0.946132	0.786853
26197	0.94613	0.803211
26198	0.94613	0.786129
26199	0.946119	0.75288
26200	1.27374	0.772566
26201	0.946119	0.75216
26202	0.946131	0.762523
26203	0.946106	0.750015
26204	0.946133	2.1
26205	0.946132	2.1
26206	0.946133	2.1
26207	0.946132	2.1
26208	1.39621	2.1
26209	1.34352	2.1
26210	0.946132	2.1
26211	0.946132	2.1
26212	0.946132	2.1
26213	0.946132	2.1

26214	0.946132	0.894826
26215	0.946128	1.68064
26216	0.94613	1.1431
26217	0.94613	1.31899
26218	1.41273	1.56587
26219	1.23536	2.01243
26220	0.946131	1.26751
26221	0.946132	2.06341
26222	0.946132	1.71045
26223	0.946132	1.84697
26224	0.946132	2.1
26225	1.41131	2.1
26226	0.946131	2.1
26227	0.946131	2.1
26228	0.946131	2.1
26229	0.946133	0.760503
26230	0.946132	1.10198
26231	0.946133	0.739981
26232	1.41894	0.749108
26233	0.946132	0.61788
26234	0.946128	0.886739
26235	1.4099	1.13384
26236	0.946131	0.940611
26237	0.946131	1.25417
26238	0.946131	0.717754
26239	0.810947	1.36476
26240	0.810945	1.3372
26241	0.810933	1.71877
26242	0.946131	0.833841
26243	1.25765	1.41435
26244	0.946128	0.991548
26245	0.946129	0.828245
26246	1.25765	0.9628
26247	1.21347	2.1
26248	0.946129	2.1
26249	0.94613	2.1
26250	1.21347	2.1
26251	0.94613	2.1
26252	0.94613	0.192088
26253	0.946132	0.180616
26254	1.2493	0.178967
26255	1.2493	0.174194
26256	0.94613	0.200531
26257	0.946132	0.860796
26258	0.946128	1.06659
26259	0.946131	0.881249
26260	0.94613	0.948549
26261	1.37938	0.93306
26262	0.946133	0.242678
26263	0.946132	0.229252
26264	0.946133	0.241889
26265	0.946132	0.215843
26266	0.946133	0.244955
26267	0.946129	0.854119
26268	1.26426	0.794878
26269	0.946131	0.823545
26270	0.946131	0.834721
26271	1.26426	0.807405

26272	0.872447	1.56045
26273	0.946132	1.34404
26274	0.946132	1.47046
26275	0.946132	1.53819
26276	0.946133	1.40301
26277	0.946117	1.16999
26278	0.946116	1.13218
26279	0.946113	1.07204
26280	0.946129	1.19712
26281	1.27211	1.03028
26282	0.94613	0.584926
26283	0.946129	0.730496
26284	1.40029	0.680087
26285	1.40028	0.771322
26286	0.94613	0.764127
26287	0.810942	2.1
26288	0.810943	2.1
26289	0.810932	2.1
26290	1.29521	2.1
26291	0.946129	2.1
26292	0.946131	2.1
26293	1.29521	2.1
26294	1.29521	2.1
26295	0.946132	0.796613
26296	0.946129	0.657663
26297	1.24596	0.644536
26298	0.946131	0.797128
26299	0.946131	0.698293
26300	0.946131	2.1
26301	1.35616	2.1
26302	0.946133	2.1
26303	0.94613	2.1
26304	1.35616	2.1
26305	0.879548	0.753502
26306	1.01272	0.712704
26307	0.946133	0.77017
26308	0.946133	0.705499
26309	0.946132	0.717597
26310	0.946132	0.868639
26311	0.918009	0.75789
26312	0.91801	0.895741
26313	0.946132	0.788639
26314	0.946132	0.831364
26315	0.946132	0.335512
26316	1.28644	0.31422
26317	0.946131	0.393017
26318	0.946131	0.361455
26319	0.946132	0.364048
26320	0.946131	0.328215
26321	1.40103	0.3221
26322	0.946131	0.401151
26323	0.946131	0.369862
26324	0.946131	0.366811
26325	0.946128	0.527575
26326	1.03327	0.469751
26327	0.946129	0.556727
26328	0.946131	0.477011
26329	0.94613	0.512646

26330	0.946132	0.533493
26331	0.946133	0.24328
26332	1.40265	0.461172
26333	1.40265	0.440369
26334	0.946133	0.37027
26335	0.946131	0.242777
26336	0.946131	0.266778
26337	0.946131	0.289834
26338	0.856655	0.235365
26339	0.856616	0.248998
26340	0.946132	0.718416
26341	0.946132	0.719008
26342	0.946132	0.74714
26343	1.15844	0.706667
26344	0.946131	0.737811
26345	0.946131	2.1
26346	1.4031	2.1
26347	1.40304	2.1
26348	0.851554	2.1
26349	0.946131	0.307656
26350	0.94613	0.378841
26351	1.24417	0.253127
26352	1.24416	0.270027
26353	0.946131	0.30514
26354	0.946132	0.908749
26355	1.20992	0.974602
26356	0.946132	0.947749
26357	0.946132	1.00471
26358	1.20992	1.00572
26359	0.946128	1.14927
26360	0.94613	1.23908
26361	0.94613	1.16839
26362	0.94613	1.20441
26363	1.23426	1.26194
26364	0.810943	0.720459
26365	0.810946	0.462915
26366	0.810933	0.554765
26367	0.810945	2.0759
26368	0.810945	0.954805
26369	0.810932	1.07739
26370	1.25104	1.2117
26371	0.946131	2.1
26372	0.946131	0.937888
26373	1.25104	1.24451
26374	1.25104	0.978486
26375	1.25488	2.09806
26376	0.946132	1.93209
26377	0.946132	1.93304
26378	0.946132	2.1
26379	0.946131	2.1
26380	0.946131	0.297979
26381	0.946126	0.362178
26382	0.946128	0.298534
26383	1.32131	0.306041
26384	0.946128	0.333362
26385	0.946133	1.88687
26386	0.946132	1.0533
26387	0.946132	1.99841

26388	0.946133	1.76597
26389	1.41123	1.29385
26390	0.946132	0.373157
26391	0.946133	0.389824
26392	0.946132	0.466553
26393	1.32163	0.329022
26394	0.946133	0.448405
26395	0.946132	1.06813
26396	0.946132	1.86587
26397	1.38651	2.01064
26398	1.38651	1.99188
26399	0.946132	1.99316
26400	0.94613	0.408747
26401	1.3639	0.365779
26402	0.946128	0.432771
26403	0.946131	0.40408
26404	1.3639	0.382412
26405	0.946133	0.98222
26406	1.40724	0.801429
26407	0.946132	0.692241
26408	0.946133	0.848612
26409	1.40724	0.835656
26410	1.21099	1.4095
26411	0.946128	1.55814
26412	0.946127	1.90749
26413	0.946128	1.80276
26414	0.946129	1.49013
26415	0.946132	0.800617
26416	1.35946	0.776503
26417	0.946131	0.842997
26418	0.946131	0.834742
26419	1.35946	0.799771
26420	1.34794	0.93647
26421	0.517994	1.33479
26422	0.946132	0.99831
26423	0.946132	1.08749
26424	0.517965	1.18019
26425	1.23224	0.912683
26426	0.94613	1.42958
26427	0.946131	1.11629
26428	0.946131	1.04253
26429	0.946131	1.00934
26430	1.37511	1.17222
26431	0.946132	1.21616
26432	0.946133	1.57586
26433	0.946133	1.51029
26434	0.946132	1.21452
26435	0.851106	0.770276
26436	0.946129	0.754156
26437	0.946131	0.854066
26438	0.946131	0.779562
26439	0.851066	0.770869
26440	0.946131	0.504518
26441	0.946131	0.629486
26442	0.94613	0.60722
26443	1.31357	0.495251
26444	0.946131	0.585527
26445	0.946133	0.898905

26446	0.946132	1.07568
26447	0.946133	0.896078
26448	0.946132	0.975359
26449	1.38149	0.939356
26450	0.810944	0.888808
26451	0.810945	1.08662
26452	0.810958	1.56737
26453	0.946118	0.245756
26454	0.946118	0.28173
26455	1.37835	0.212441
26456	1.37837	0.229961
26457	0.946106	0.246004
26458	0.946128	0.267811
26459	0.946131	0.251048
26460	1.34831	0.251369
26461	1.34831	0.222097
26462	0.94613	0.277892
26463	0.946132	0.338293
26464	0.946132	0.434134
26465	0.909981	0.355511
26466	1.00099	0.332921
26467	0.946131	0.397829
26468	0.946132	0.809464
26469	0.946133	1.03888
26470	0.946133	0.85673
26471	0.946132	0.900073
26472	1.35328	0.908913
26473	0.946132	0.241509
26474	0.946132	0.227724
26475	1.35386	0.240992
26476	1.35385	0.210083
26477	0.946133	0.247015
26478	0.946121	0.213453
26479	0.946132	0.217945
26480	0.900167	0.19388
26481	1.01189	0.203466
26482	0.946132	0.218852
26483	0.946132	1.94527
26484	1.41585	1.43815
26485	0.946131	1.44425
26486	0.946131	1.39012
26487	0.94613	1.36885
26488	0.810946	2.1
26489	0.810946	2.1
26490	0.810933	2.1
26491	1.34447	2.1
26492	0.946131	2.1
26493	0.946131	2.1
26494	1.34447	2.1
26495	1.19886	2.1
26496	0.946132	0.386467
26497	0.946132	0.301562
26498	0.946132	0.292916
26499	1.35065	0.275416
26500	0.946132	0.361857
26501	0.946131	0.760166
26502	1.3022	0.74536
26503	0.94613	0.817863

26504	0.946131	0.791592
26505	1.3022	0.759834
26506	0.946127	0.39105
26507	1.38683	0.342082
26508	0.946127	0.427621
26509	0.946128	0.441209
26510	1.38683	0.412219
26511	0.810946	0.28299
26512	0.810947	0.268767
26513	0.810959	0.255743
26514	0.946132	0.261015
26515	0.946131	0.251057
26516	0.946132	0.23859
26517	0.946132	0.241417
26518	0.946132	0.244653
26519	0.946127	1.20084
26520	1.35968	1.60627
26521	0.946131	1.09007
26522	0.946129	1.01607
26523	1.35969	1.39994
26524	0.946133	1.90348
26525	1.35862	1.70135
26526	0.946132	1.99661
26527	0.946132	1.20272
26528	1.35862	1.55839
26529	0.946132	0.816853
26530	0.946132	0.720425
26531	0.946132	0.841564
26532	0.946131	0.730888
26533	0.946132	0.761899
26534	0.946131	0.587911
26535	0.946131	0.530988
26536	0.946128	0.604493
26537	0.946129	0.506585
26538	1.41232	0.534473
26539	0.946128	1.15805
26540	1.29331	1.59396
26541	0.946131	1.19434
26542	0.946131	1.39817
26543	1.29331	1.29906
26544	0.946133	0.887397
26545	0.946132	0.925809
26546	0.946132	0.889242
26547	0.946132	0.871786
26548	1.41556	0.856821
26549	0.94612	0.858886
26550	0.946119	0.855931
26551	0.946119	0.859682
26552	0.946132	0.815347
26553	0.946107	0.82262
26554	0.946129	0.244436
26555	1.1509	0.215859
26556	0.946126	0.243377
26557	0.946129	0.216112
26558	0.94613	0.242299
26559	0.946131	0.215993
26560	1.19263	0.196539
26561	0.946132	0.208203

26562	0.946132	0.19675
26563	0.946132	0.203999
26564	0.94613	0.819007
26565	1.36939	0.940068
26566	0.946128	0.900141
26567	0.946131	0.916107
26568	1.36939	0.899372
26569	0.946133	1.03728
26570	0.827865	1.37069
26571	0.946132	1.08314
26572	0.946132	1.19746
26573	1.40398	1.23706
26574	0.946127	0.734652
26575	0.946127	0.801805
26576	0.94613	0.6415
26577	1.41498	0.769645
26578	0.946128	0.740191
26579	0.94613	0.282054
26580	0.946131	0.257152
26581	0.946129	0.235676
26582	0.94613	0.248079
26583	0.94613	0.245646
26584	0.946118	0.298759
26585	0.946119	0.263931
26586	0.946116	0.232109
26587	1.36569	0.235633
26588	0.946105	0.260329
26589	0.946132	0.645466
26590	1.23175	0.585653
26591	0.94613	0.657135
26592	0.946133	0.574748
26593	0.946131	0.61178
26594	1.25698	0.251014
26595	0.946126	0.326923
26596	0.946129	0.294843
26597	0.946128	0.284627
26598	0.946129	0.286562
26599	0.810943	2.1
26600	0.810944	2.1
26601	0.810932	2.1
26602	0.946132	0.625242
26603	1.09841	0.604116
26604	0.946131	0.698317
26605	0.946132	0.678749
26606	1.09841	0.658107
26607	0.810936	0.953512
26608	0.810935	0.978207
26609	0.810934	1.17646
26610	1.23851	1.97958
26611	0.946132	1.02301
26612	0.946131	1.07437
26613	1.23851	2.1
26614	1.23851	1.50069
26615	0.946132	0.470923
26616	0.946131	0.683407
26617	0.946132	0.498627
26618	0.946132	0.580948
26619	0.946132	0.783554

26620	0.939141	1.08403
26621	0.953118	1.33637
26622	0.946126	1.0838
26623	0.946129	1.14243
26624	1.03306	1.20984
26625	0.946132	0.382124
26626	1.30927	0.368966
26627	0.946132	0.400475
26628	0.946132	0.386255
26629	1.30928	0.369842
26630	0.94613	2.1
26631	0.946131	2.1
26632	1.24511	2.1
26633	0.94613	2.1
26634	0.94613	2.1
26635	0.946133	0.441225
26636	1.34152	0.369623
26637	0.946131	0.359285
26638	0.946131	0.373348
26639	1.34152	0.370502
26640	0.946132	0.410343
26641	1.41383	0.349864
26642	0.946132	0.350455
26643	0.946132	0.352705
26644	1.41383	0.345129
26645	0.946132	0.879574
26646	0.946131	0.782361
26647	1.41392	0.706021
26648	0.946132	0.885108
26649	0.946132	0.795315
26650	0.946132	1.00708
26651	0.946132	0.930701
26652	1.24333	0.873211
26653	0.946133	1.06357
26654	0.946133	0.954495
26655	0.946124	0.532511
26656	0.94613	0.551095
26657	0.946126	0.565477
26658	1.3065	0.500026
26659	0.946127	0.562407
26660	0.810946	0.632346
26661	0.810943	0.554862
26662	0.81096	0.614465
26663	0.946131	0.479758
26664	1.40271	0.499724
26665	0.946131	0.592178
26666	0.946131	0.55826
26667	1.40271	0.531716
26668	0.946132	0.372008
26669	0.94613	0.411946
26670	0.946131	0.363726
26671	0.94613	0.380575
26672	0.946131	0.388845
26673	0.946133	0.730947
26674	1.30107	0.72578
26675	0.946132	0.729538
26676	0.946132	0.731029
26677	0.946133	0.742165

26678	0.810946	2.1
26679	0.810944	2.1
26680	0.810958	2.1
26681	0.946133	1.25134
26682	0.946131	1.23733
26683	0.946131	2.02677
26684	1.30812	1.56962
26685	0.946131	1.26231
26686	0.94613	1.01203
26687	0.975002	1.08099
26688	0.917259	1.39607
26689	1.00902	1.26566
26690	0.94613	0.999047
26691	0.946131	2.1
26692	0.946127	2.1
26693	1.398	2.1
26694	0.946129	2.1
26695	0.946129	2.1
26696	0.946133	2.1
26697	0.946132	2.1
26698	0.946132	2.1
26699	0.946132	2.1
26700	1.3033	2.1
26701	1.09897	0.698316
26702	0.946131	1.05113
26703	0.946132	0.966504
26704	0.946129	0.942339
26705	0.946131	0.982642
26706	0.946131	2.1
26707	0.946132	2.1
26708	1.39858	2.1
26709	0.946131	2.1
26710	0.946131	2.1
26711	0.946119	0.7855
26712	1.08878	0.801216
26713	0.946119	1.0086
26714	1.0888	0.799663
26715	0.946107	0.984717
26716	0.946132	0.364789
26717	1.10733	0.334315
26718	0.946132	0.430119
26719	0.946131	0.399856
26720	0.946132	0.400595
26721	0.946128	0.384201
26722	1.33671	0.343828
26723	0.94613	0.432631
26724	0.94613	0.414549
26725	0.94613	0.413138
26726	0.810943	2.1
26727	0.810944	2.1
26728	0.810945	2.1
26729	1.35117	2.1
26730	0.946129	2.1
26731	0.94613	2.1
26732	1.35117	2.1
26733	1.27174	2.1
26734	0.946132	1.94598
26735	0.946133	1.80994

26736	0.997296	1.29246
26737	0.946133	1.53607
26738	0.946132	1.40217
26739	0.810943	2.1
26740	0.810944	1.41887
26741	0.810945	2.1
26742	0.810946	1.01602
26743	0.810943	1.4376
26744	0.810933	1.20308
26745	0.946127	1.22232
26746	0.946131	1.1347
26747	0.946128	1.20966
26748	0.94613	1.28319
26749	0.946129	1.35062
26750	0.946131	0.673801
26751	0.946131	0.602482
26752	0.946129	0.679
26753	0.946131	0.590785
26754	0.94613	0.588312
26755	0.946133	0.526841
26756	0.946132	0.565469
26757	0.946132	0.511177
26758	0.946132	0.517095
26759	0.946133	0.556881
26760	0.946131	0.98718
26761	0.94613	0.873463
26762	0.946129	0.998919
26763	0.946131	0.918653
26764	0.946131	0.990359
26765	0.946132	1.10262
26766	0.946129	1.70088
26767	0.94613	1.38493
26768	0.946131	1.31329
26769	1.39356	1.50344
26770	0.810946	2.01842
26771	0.810947	2.1
26772	0.810934	1.96916
26773	0.946125	0.346887
26774	0.946127	0.381891
26775	1.33798	0.27036
26776	1.33797	0.284696
26777	0.946128	0.318652
26778	0.946131	0.302666
26779	0.946131	0.375384
26780	1.23146	0.256697
26781	1.23146	0.280806
26782	0.946131	0.309662
26783	0.946129	2.0621
26784	0.94613	2.1
26785	0.946131	2.07918
26786	0.94613	2.1
26787	1.30082	2.1
26788	0.946131	0.395547
26789	0.946132	0.42838
26790	0.946131	0.473542
26791	1.20416	0.352828
26792	0.946132	0.429399
26793	0.946132	0.385207

26794	0.946131	0.419885
26795	0.946131	0.507786
26796	1.18711	0.34613
26797	0.946132	0.427461
26798	0.946132	0.28557
26799	0.946132	0.260029
26800	0.946133	0.262485
26801	0.946132	0.250451
26802	0.946133	0.258619
26803	0.946131	0.34375
26804	0.946132	0.369665
26805	0.94613	0.332618
26806	0.946131	0.327167
26807	0.946131	0.367331
26808	0.946133	0.296508
26809	0.946132	0.309343
26810	0.946133	0.284486
26811	0.946132	0.278904
26812	0.946132	0.309166
26813	0.946132	2.1
26814	0.946132	2.1
26815	0.946132	2.1
26816	1.36422	2.1
26817	0.946132	2.1
26818	0.946131	0.881064
26819	0.946132	1.23217
26820	0.946132	0.963765
26821	0.946132	0.995339
26822	1.4185	1.19696
26823	0.946133	0.377916
26824	1.37794	0.340341
26825	0.946132	0.361555
26826	0.946133	0.348499
26827	0.946132	0.343238
26828	1.4045	2.1
26829	0.946132	2.1
26830	0.946132	2.1
26831	0.946132	2.1
26832	0.946132	2.1
26833	0.946131	2.1
26834	0.946132	2.1
26835	1.34285	2.1
26836	1.34285	2.1
26837	0.946132	2.1
26838	0.946131	2.1
26839	0.946132	2.1
26840	1.40739	2.1
26841	1.40739	2.1
26842	0.946131	2.1
26843	0.946128	2.1
26844	0.946132	2.1
26845	0.94613	2.1
26846	0.94613	2.1
26847	1.3164	2.1
26848	0.946132	1.29663
26849	1.3484	2.1
26850	0.946132	1.19039
26851	0.946132	1.78177

26852	1.3484	1.9013
26853	0.946132	2.1
26854	0.946132	2.1
26855	0.946133	2.1
26856	0.946133	2.1
26857	1.41507	2.1
26858	0.946132	2.1
26859	1.32608	2.1
26860	0.946131	2.1
26861	0.946131	2.1
26862	0.946132	2.1
26863	0.94613	0.996515
26864	1.34646	1.58156
26865	0.946129	0.998479
26866	0.94613	0.921641
26867	1.34646	1.21848
26868	0.946131	0.674434
26869	0.946131	1.6771
26870	0.94613	0.91615
26871	1.33728	0.649993
26872	0.946131	0.887982
26873	0.946127	1.50786
26874	0.946131	1.08493
26875	0.946128	1.71002
26876	0.946129	1.70361
26877	1.4064	1.34628
26878	0.946133	2.1
26879	0.946131	2.1
26880	0.946131	2.1
26881	0.946131	2.1
26882	0.94613	2.1
26883	1.34912	2.1
26884	0.946131	2.1
26885	0.946129	2.1
26886	0.946131	2.1
26887	0.94613	2.1
26888	0.946132	2.1
26889	1.40447	2.1
26890	0.946132	2.1
26891	0.946133	2.1
26892	1.40447	2.1
26893	1.33607	2.1
26894	0.946131	1.42819
26895	0.946127	2.1
26896	0.94613	2.09885
26897	0.946129	2.1
26898	0.946117	0.779483
26899	1.32664	0.914525
26900	0.946115	0.8347
26901	0.946105	0.949117
26902	0.94613	0.71659
26903	0.946118	2.1
26904	0.946114	2.1
26905	1.35886	2.1
26906	1.35884	2.1
26907	0.946129	2.1
26908	0.946132	1.81742
26909	1.2571	2.1

26910	0.946132	1.99427
26911	0.946132	2.1
26912	1.2571	2.1
26913	0.946133	2.05996
26914	1.35663	2.1
26915	0.946132	2.1
26916	0.946132	2.1
26917	1.35663	2.1
26918	0.946133	0.897717
26919	0.946129	1.33174
26920	0.946131	1.15676
26921	0.946131	1.0464
26922	1.30881	1.41128
26923	0.946132	2.1
26924	0.94613	2.1
26925	1.34652	2.1
26926	0.94613	2.1
26927	0.94613	2.1
26928	0.946132	1.87105
26929	0.946133	1.42633
26930	1.36183	1.12788
26931	1.36183	1.18383
26932	0.946132	1.32612
26933	0.946132	0.47661
26934	1.38353	0.417517
26935	0.94613	0.405606
26936	0.94613	0.420782
26937	1.38354	0.418313
26938	0.946128	0.720682
26939	1.24887	0.696722
26940	0.94613	0.805481
26941	0.94613	0.825678
26942	1.24887	0.82285
26943	0.946129	0.907686
26944	0.946126	1.17923
26945	0.946124	0.946184
26946	0.946128	0.978132
26947	1.32056	1.06408
26948	0.946129	0.985133
26949	0.946128	1.26732
26950	0.946126	1.01031
26951	0.946129	1.05755
26952	1.20351	1.14258
26953	0.946131	1.22014
26954	0.946131	1.26507
26955	0.946127	1.45946
26956	1.25591	1.48659
26957	0.94613	1.11579
26958	0.946132	1.12477
26959	0.946133	1.20527
26960	0.946132	1.21628
26961	1.15436	1.13535
26962	0.930306	1.05458
26963	0.946132	2.1
26964	0.946132	2.1
26965	0.946132	1.90946
26966	0.946133	2.1
26967	0.946132	2.1

26968	0.946129	1.03486
26969	0.946132	0.979937
26970	0.946126	1.15278
26971	1.13245	0.901611
26972	0.946129	0.982226
26973	0.94613	2.08109
26974	0.946132	2.09771
26975	0.946129	1.78247
26976	0.946131	2.1
26977	0.946131	2.00683
26978	1.41279	1.91771
26979	1.76254	2.1
26980	0.600454	1.92138
26981	0.858991	1.93151
26982	0.940722	1.99033
26983	1.28439	1.57003
26984	0.946131	1.44123
26985	0.94613	1.54839
26986	0.946131	1.86936
26987	0.946131	1.8428
26988	0.946132	1.06777
26989	1.39335	0.80939
26990	0.946132	1.00845
26991	0.946132	0.831521
26992	0.946132	0.976121
26993	1.33879	0.62323
26994	0.946131	0.77575
26995	0.94613	0.87358
26996	1.33879	0.620419
26997	0.946131	0.755815
26998	0.946126	0.712047
26999	0.946131	0.708245
27000	1.37342	0.710537
27001	1.37343	0.70373
27002	0.946128	0.732112
27003	1.39168	0.284622
27004	0.946131	0.345469
27005	0.946131	0.39308
27006	0.946131	0.329771
27007	0.946131	0.330628
27008	0.946131	0.579447
27009	1.412	0.457643
27010	0.946127	0.436689
27011	0.946128	0.498994
27012	0.946128	0.512872
27013	1.1387	0.577677
27014	0.943364	0.877496
27015	0.946132	0.765229
27016	0.946132	0.76428
27017	0.946133	0.793448
27018	1.21085	0.774971
27019	0.88324	1.11742
27020	0.946129	0.993503
27021	0.946131	1.04848
27022	0.946131	0.978065
27023	1.33587	0.268817
27024	0.946125	0.348253
27025	0.946129	0.303128

27026	0.946126	0.334531
27027	0.946127	0.306649
27028	1.27512	0.323035
27029	0.946132	0.434722
27030	0.946132	0.422041
27031	0.946133	0.403874
27032	0.946132	0.36328
27033	0.946128	0.45626
27034	1.30498	0.432
27035	0.946131	0.551717
27036	0.94613	0.520611
27037	1.30497	0.482308
27038	0.946131	0.417057
27039	1.25626	0.384989
27040	0.946131	0.494679
27041	0.946131	0.476347
27042	1.25626	0.441903
27043	0.946131	0.23278
27044	0.94613	0.243669
27045	1.35316	0.214259
27046	0.946131	0.224403
27047	0.946131	0.224466
27048	0.94613	0.68045
27049	1.28727	0.599749
27050	0.946128	0.507339
27051	0.946131	0.622605
27052	0.94613	0.630467
27053	0.946131	0.325996
27054	1.39502	0.309677
27055	0.946132	0.388459
27056	0.946132	0.341498
27057	0.946132	0.36146
27058	1.36173	0.631003
27059	0.946131	0.937922
27060	0.946126	0.846307
27061	0.946127	0.875904
27062	0.946128	0.805905
27063	0.946129	0.919087
27064	0.946133	0.861199
27065	0.946131	0.922227
27066	0.946131	0.932004
27067	0.946131	0.954383
27068	0.946128	0.181826
27069	0.946132	0.176704
27070	1.24678	0.173124
27071	0.94613	0.176123
27072	0.94613	0.176886
27073	0.946129	0.178541
27074	0.94613	0.175953
27075	1.25215	0.17157
27076	0.946126	0.174815
27077	0.946129	0.17669
27078	0.946129	0.820218
27079	1.29689	0.818346
27080	0.946131	0.82205
27081	0.946131	0.805613
27082	0.946131	0.783984
27083	0.946128	0.457479

27084	0.94613	0.48853
27085	0.946131	0.435591
27086	0.94613	0.469727
27087	0.946131	0.473416
27088	0.946127	0.499647
27089	0.946128	0.546228
27090	0.94613	0.480065
27091	0.946129	0.520461
27092	0.946129	0.519112
27093	0.946133	0.231903
27094	0.946132	0.259145
27095	0.946132	0.336783
27096	1.29957	0.24427
27097	0.946133	0.26681
27098	0.946132	0.273021
27099	0.946133	0.258796
27100	0.946132	0.331838
27101	1.26201	0.253555
27102	0.946132	0.276784
27103	0.946133	0.392238
27104	1.40427	0.356521
27105	0.946132	0.440386
27106	0.946132	0.427669
27107	0.946133	0.416493
27108	0.946132	0.380949
27109	1.35123	0.354844
27110	0.946132	0.43518
27111	0.946132	0.432036
27112	0.946131	0.406525
27113	0.946128	0.531284
27114	0.946129	0.570094
27115	0.946131	0.507025
27116	0.94613	0.553972
27117	0.946131	0.541248
27118	0.946127	0.736652
27119	1.23351	0.686463
27120	0.946131	0.741107
27121	0.946129	0.696969
27122	0.94613	0.681445
27123	0.946131	0.424565
27124	0.946131	0.507073
27125	0.94613	0.437922
27126	1.18119	0.409409
27127	0.946131	0.465919
27128	0.946132	0.624611
27129	1.30563	0.546592
27130	0.946133	0.6578
27131	0.946132	0.561077
27132	0.946133	0.606285
27133	0.946132	0.788549
27134	0.946132	0.628933
27135	0.946133	1.1279
27136	1.20256	0.714803
27137	0.946133	0.847106
27138	0.94613	0.961452
27139	1.33007	0.888033
27140	0.946128	0.988858
27141	0.946132	0.859243

27142	0.946131	0.889426
27143	0.946131	2.1
27144	0.946131	2.1
27145	0.946128	2.1
27146	0.946129	2.1
27147	0.94613	2.1
27148	0.810942	2.1
27149	0.810942	2.1
27150	0.81093	2.1
27151	0.946132	2.1
27152	1.39823	2.1
27153	0.946132	2.1
27154	0.946131	2.1
27155	0.946132	2.1
27156	0.946131	2.1
27157	0.946131	2.1
27158	0.946131	2.1
27159	1.37713	2.1
27160	0.946131	2.1
27161	0.946133	1.27123
27162	0.946131	1.90515
27163	1.40594	2.1
27164	1.40594	2.1
27165	0.94613	2.1
27166	0.946133	2.1
27167	0.946131	2.1
27168	1.38155	2.1
27169	1.38155	2.1
27170	0.946131	2.1
27171	0.946132	0.978516
27172	0.946133	1.48236
27173	0.946132	1.07781
27174	0.946132	1.1028
27175	1.33694	1.32834
27176	0.946131	1.9105
27177	1.228 2.1	
27178	0.946129	1.98697
27179	0.946131	2.08395
27180	1.228 2.1	
27181	0.946132	1.02574
27182	0.946132	1.32639
27183	0.946131	1.4655
27184	0.946132	1.09931
27185	1.35943	1.45432
27186	0.946133	0.29098
27187	0.946133	0.285182
27188	1.02377	0.329079
27189	1.02377	0.288961
27190	0.946132	0.288555
27191	0.94613	0.521303
27192	0.946131	0.550692
27193	0.946132	0.637733
27194	1.29322	0.457181
27195	0.946131	0.614064
27196	0.946131	0.964668
27197	0.946131	0.525726
27198	0.946132	1.06117
27199	1.41492	0.659379

27200	0.946131	0.883322
27201	0.946132	2.1
27202	0.946131	2.1
27203	0.946132	2.1
27204	1.35028	2.1
27205	0.946132	2.1
27206	0.946132	2.00716
27207	0.946132	1.87614
27208	0.946131	1.82114
27209	1.32461	1.97602
27210	0.946132	1.75088
27211	0.946131	2.1
27212	0.946132	2.1
27213	1.40369	2.1
27214	0.94613	2.1
27215	0.946131	2.1
27216	0.946132	0.473631
27217	0.979597	0.43337
27218	0.946131	0.504378
27219	0.946132	0.442758
27220	0.946132	0.475053
27221	0.946131	2.1
27222	0.946131	1.46839
27223	0.94613	2.1
27224	0.946131	2.1
27225	1.41897	2.1
27226	1.37791	2.1
27227	0.94613	2.1
27228	0.946129	2.1
27229	0.946131	2.1
27230	0.946131	2.1
27231	1.39384	2.1
27232	0.946131	2.1
27233	0.946128	2.1
27234	0.946129	2.1
27235	0.946129	2.1
27236	0.810945	1.30308
27237	0.810944	1.78188
27238	0.810958	1.37106
27239	0.946128	2.1
27240	0.946132	2.1
27241	0.94613	2.1
27242	1.3335	2.1
27243	0.946131	2.1
27244	0.94613	0.719678
27245	0.946129	0.937935
27246	0.946132	0.607423
27247	1.41192	0.851157
27248	0.94613	0.696446
27249	0.946132	0.754789
27250	0.946133	0.734842
27251	0.946132	0.730912
27252	1.39428	0.653601
27253	0.946133	0.713042
27254	0.94613	1.44872
27255	0.946128	1.47758
27256	0.946132	1.10523
27257	0.94613	1.25245

27258	1.40684	1.63028
27259	0.946131	2.07431
27260	0.946131	1.95744
27261	0.946131	1.91188
27262	0.946131	1.57519
27263	1.39021	2.08917
27264	0.946131	1.13992
27265	1.31649	1.62616
27266	0.946131	0.996117
27267	0.946131	0.978032
27268	1.31649	1.2729
27269	0.810946	2.1
27270	0.810946	1.90632
27271	0.810958	2.1
27272	1.16687	2.1
27273	0.946131	2.1
27274	0.946131	1.98936
27275	1.16687	2.1
27276	1.16687	2.1
27277	0.946128	0.892781
27278	1.3564	0.915955
27279	0.946131	0.893642
27280	0.946131	0.898591
27281	0.94613	0.873437
27282	0.946128	2.1
27283	1.35828	2.1
27284	0.94613	2.1
27285	0.946131	2.1
27286	0.94613	2.1
27287	0.946133	0.906028
27288	1.36477	1.02088
27289	0.946132	1.01691
27290	0.946132	1.01413
27291	1.36477	1.0174
27292	0.946132	1.05461
27293	1.31857	1.49321
27294	0.946132	1.16423
27295	0.946132	1.14015
27296	1.31857	1.14827
27297	0.94613	0.889462
27298	1.35059	0.800128
27299	0.946128	0.795069
27300	0.94613	0.827336
27301	1.35059	0.809127
27302	0.946126	0.698257
27303	1.35559	0.586779
27304	0.946127	0.577178
27305	0.946128	0.625239
27306	1.35559	0.597603
27307	0.946131	0.479129
27308	1.33035	0.394889
27309	0.946127	0.38959
27310	0.946128	0.420253
27311	1.33035	0.404641
27312	0.94613	0.441065
27313	1.3665	0.381285
27314	0.946129	0.380115
27315	0.946131	0.408114

27316	1.3665	0.38828
27317	0.94613	0.478569
27318	1.32828	0.408843
27319	0.946129	0.401823
27320	0.94613	0.44152
27321	0.946131	0.438339
27322	0.946131	2.1
27323	0.946131	1.85973
27324	1.32949	2.1
27325	1.32949	2.1
27326	0.946131	2.1
27327	0.946131	1.05319
27328	0.94613	0.54921
27329	0.946133	0.909794
27330	1.38357	0.648338
27331	0.946131	0.824441
27332	0.946131	2.1
27333	0.946128	2.1
27334	1.36919	2.1
27335	0.946131	2.1
27336	0.94613	2.1
27337	0.946131	1.12446
27338	1.3771	2.02625
27339	0.946129	1.73701
27340	0.94613	1.64488
27341	0.946131	1.10298
27342	0.946131	1.04667
27343	0.94613	0.818087
27344	0.946129	0.754492
27345	1.30029	0.889099
27346	0.94613	0.802017
27347	1.21174	0.235126
27348	0.946132	0.266075
27349	0.946132	0.297512
27350	0.946132	0.265448
27351	0.946133	0.25834
27352	0.810946	2.1
27353	0.810945	2.1
27354	0.810946	2.1
27355	1.1796	2.1
27356	0.946131	2.1
27357	0.946132	2.1
27358	1.1796	2.1
27359	1.1796	2.1
27360	0.946131	2.1
27361	0.946131	2.1
27362	0.946133	2.1
27363	0.946131	2.1
27364	1.40615	2.1
27365	0.810945	2.1
27366	0.810944	2.1
27367	0.810945	2.1
27368	1.40239	2.1
27369	0.94613	2.1
27370	1.35561	2.1
27371	0.946133	2.1
27372	1.35562	2.1
27373	1.41169	0.62401

27374	0.946131	0.789381
27375	0.946127	0.894979
27376	1.41169	0.641862
27377	0.946128	0.780266
27378	0.946119	2.1
27379	0.946119	2.1
27380	0.946119	2.1
27381	0.946107	2.1
27382	1.41917	2.1
27383	0.810946	1.23507
27384	0.810943	1.29585
27385	0.810934	1.57143
27386	0.946127	2.06718
27387	1.1995	2.1
27388	0.946126	2.06888
27389	0.946128	2.08338
27390	1.1995	2.1
27391	0.810944	2.1
27392	0.810947	2.1
27393	0.81096	2.1
27394	0.946133	0.573442
27395	1.3495	0.431237
27396	0.946132	0.512494
27397	0.946132	0.450019
27398	0.946133	0.484364
27399	0.946132	2.1
27400	0.946132	2.1
27401	0.946133	2.1
27402	1.39529	2.1
27403	0.946133	2.1
27404	0.946133	2.1
27405	1.28525	2.1
27406	0.946132	2.1
27407	0.946133	2.1
27408	1.28525	2.1
27409	0.810946	2.1
27410	0.810945	2.1
27411	0.810946	2.1
27412	1.33931	2.1
27413	0.946132	2.1
27414	0.946132	2.1
27415	1.33931	2.1
27416	1.24616	2.1
27417	0.946133	0.678391
27418	0.946133	0.699987
27419	0.946132	0.670525
27420	0.946132	0.690968
27421	0.946133	0.694152
27422	0.946128	2.06631
27423	1.40225	2.1
27424	0.946128	2.09123
27425	0.94613	2.1
27426	0.94613	1.93692
27427	0.946113	0.28887
27428	0.946118	0.257257
27429	0.946114	0.313304
27430	1.40551	0.264554
27431	0.946103	0.274251

27432	0.94613	2.1
27433	0.946132	2.1
27434	0.946129	2.1
27435	0.946131	2.1
27436	1.37811	2.1
27437	0.946132	2.1
27438	1.39826	2.1
27439	0.946133	2.1
27440	0.946132	2.1
27441	1.39826	2.1
27442	0.946118	2.1
27443	1.28298	2.1
27444	0.946117	2.1
27445	0.946105	2.1
27446	1.283	2.1
27447	0.946118	0.270645
27448	1.41375	0.277417
27449	0.946117	0.35304
27450	0.946131	0.305236
27451	0.946106	0.306078
27452	0.946132	1.04366
27453	0.946129	0.914684
27454	1.28436	0.9024
27455	0.946131	1.04781
27456	0.946131	0.937925
27457	0.810947	0.681611
27458	0.810946	0.723818
27459	0.81096	0.668832
27460	0.946132	0.590975
27461	1.34338	0.568247
27462	0.961435	0.519137
27463	0.961435	0.551875
27464	0.946132	0.575887
27465	1.3028	1.65923
27466	0.946131	1.53404
27467	0.946127	1.60237
27468	0.946128	1.78186
27469	0.946129	1.57039
27470	0.946132	0.854073
27471	1.37973	0.761071
27472	0.946132	0.857907
27473	0.946132	0.780991
27474	0.946132	0.748514
27475	0.946131	0.991946
27476	0.94613	0.957035
27477	0.946129	0.994451
27478	0.946131	0.953609
27479	1.41798	0.948894
27480	0.946118	0.935669
27481	0.946118	0.896566
27482	0.946117	0.937105
27483	0.946131	0.895523
27484	0.946105	0.898215
27485	0.946131	1.04705
27486	0.946131	0.997159
27487	0.94613	1.04406
27488	0.946131	1.00146
27489	0.946131	0.970526

27490	0.946133	1.17644
27491	1.40274	1.98335
27492	0.946133	0.979533
27493	0.946132	1.54118
27494	1.40274	1.68733
27495	0.946131	1.35048
27496	1.39731	1.84927
27497	0.946132	1.0836
27498	0.94613	1.15353
27499	1.39731	1.56519
27500	0.94612	1.74837
27501	0.94612	1.40356
27502	0.946119	1.37141
27503	0.946132	1.39963
27504	1.40764	1.74337
27505	0.946128	2.1
27506	0.946132	2.1
27507	0.94613	2.1
27508	0.94613	2.1
27509	1.31213	2.1
27510	0.946127	1.55535
27511	0.94613	2.01739
27512	0.946127	1.56588
27513	0.946128	1.62002
27514	1.36653	1.8775
27515	0.946127	2.1
27516	1.3557	2.1
27517	0.946128	2.1
27518	0.946128	2.1
27519	1.3557	2.1
27520	0.946133	2.1
27521	0.946132	2.1
27522	0.946133	2.1
27523	0.946132	2.1
27524	1.40081	2.1
27525	0.946128	2.1
27526	0.946133	2.1
27527	0.946131	2.1
27528	0.946131	2.1
27529	1.32282	2.1
27530	0.810945	1.41617
27531	0.810943	1.50382
27532	0.810933	1.96847
27533	1.27676	2.1
27534	0.94613	2.06819
27535	0.946128	2.1
27536	0.946131	2.1
27537	0.946131	2.1
27538	0.946131	0.745111
27539	0.946127	0.749462
27540	1.41328	0.81366
27541	1.41327	0.820355
27542	0.946128	0.772162
27543	0.946132	0.352607
27544	1.32083	0.322176
27545	0.946132	0.351984
27546	0.946133	0.361013
27547	1.32082	0.348039

27548	0.946128	0.367401
27549	1.29215	0.342604
27550	0.94613	0.38143
27551	0.94613	0.376247
27552	1.29215	0.36154
27553	0.946132	0.363144
27554	1.27696	0.313883
27555	0.94613	0.318846
27556	0.94613	0.354914
27557	1.27696	0.339422
27558	0.94612	0.341183
27559	1.29289	0.308191
27560	0.946119	0.327442
27561	0.946132	0.349573
27562	1.29287	0.336787
27563	0.946128	2.1
27564	1.37869	2.1
27565	0.946131	2.1
27566	0.94613	2.1
27567	0.946131	2.1
27568	0.946133	2.1
27569	0.946132	2.1
27570	0.946132	2.1
27571	1.35703	2.1
27572	0.946133	2.1
27573	0.946132	1.91891
27574	0.946132	2.1
27575	0.946133	1.72582
27576	1.37017	2.1
27577	0.946133	2.1
27578	0.946119	0.284435
27579	0.94612	0.304327
27580	1.38583	0.23486
27581	1.38585	0.248657
27582	0.946107	0.270592
27583	0.946126	0.359852
27584	0.946127	0.416892
27585	1.22904	0.298685
27586	1.22904	0.295543
27587	0.946128	0.336984
27588	1.3279	1.07871
27589	0.946131	1.41884
27590	0.94613	1.24811
27591	0.946131	1.40607
27592	0.946131	1.26067
27593	0.946132	2.1
27594	0.946132	2.1
27595	0.946132	2.1
27596	0.946131	2.1
27597	1.40932	2.1
27598	1.26868	0.349363
27599	0.946131	0.489483
27600	0.94613	0.438535
27601	0.946131	0.43629
27602	0.946131	0.421412
27603	0.810943	2.1
27604	0.810945	2.1
27605	0.810932	2.1

27606	0.810943	0.999317
27607	0.810944	0.626475
27608	0.810957	0.620219
27609	1.31539	0.697107
27610	0.946132	1.04811
27611	0.946128	0.432686
27612	1.3154	0.879479
27613	1.17856	0.540955
27614	1.21997	1.40468
27615	0.94613	1.63233
27616	0.946129	1.49554
27617	0.94613	1.84197
27618	0.94613	1.73247
27619	0.946131	1.14654
27620	0.946131	1.00753
27621	0.946129	1.15734
27622	0.94613	1.15553
27623	0.946131	1.2509
27624	0.946127	0.886644
27625	0.946131	0.773474
27626	0.946127	1.32646
27627	0.946129	0.887373
27628	1.41342	0.862674
27629	0.946131	0.809628
27630	0.946131	1.18021
27631	0.946128	1.38046
27632	0.946131	0.851541
27633	1.29149	1.19703
27634	1.41022	1.9541
27635	0.94613	0.988289
27636	0.946128	1.85726
27637	0.94613	1.65263
27638	0.94613	1.88824
27639	0.946128	0.647723
27640	0.94613	0.698329
27641	1.27864	0.546832
27642	1.27864	0.505042
27643	0.946131	0.7
27644	0.946129	0.793464
27645	0.946125	1.10472
27646	0.946124	0.914827
27647	0.946127	0.865206
27648	1.37759	1.03581
27649	0.946129	0.845444
27650	0.94613	1.14288
27651	0.946126	0.899681
27652	0.946129	0.920437
27653	1.38259	1.03266
27654	0.946131	0.384788
27655	1.34118	0.338001
27656	0.946132	0.33247
27657	0.946132	0.35858
27658	1.34118	0.354851
27659	0.946131	0.27302
27660	1.25017	0.267732
27661	0.946128	0.258825
27662	0.946131	0.267204
27663	0.946131	0.254352

27664	0.946131	2.1
27665	0.946128	2.1
27666	0.946133	2.1
27667	1.3937	2.1
27668	0.94613	2.1
27669	0.946131	0.959027
27670	0.94613	0.599048
27671	1.40404	0.712236
27672	1.40404	0.509861
27673	0.946131	0.717987
27674	0.946132	0.795497
27675	0.946132	0.400454
27676	1.38897	0.403944
27677	1.38897	0.291821
27678	0.946132	0.500811
27679	0.94613	0.552554
27680	0.946128	0.7867
27681	1.40332	0.487909
27682	1.40331	0.722347
27683	0.94613	0.791216
27684	0.946132	0.79634
27685	0.946132	1.0018
27686	0.946133	0.873858
27687	1.41619	1.07858
27688	0.946132	0.865289
27689	0.946132	1.18728
27690	0.946132	2.1
27691	0.946132	1.75233
27692	1.38244	2.1
27693	0.946132	2.00325
27694	0.810946	1.05766
27695	0.810943	0.882466
27696	0.810934	0.851776
27697	0.810947	0.97604
27698	0.810943	0.808248
27699	0.810934	0.824567
27700	1.12786	1.15086
27701	0.946132	1.64928
27702	0.946132	1.37494
27703	0.946132	1.283
27704	0.946133	1.24738
27705	0.946132	1.23374
27706	0.946132	1.31908
27707	0.946132	1.11721
27708	1.27806	1.32222
27709	0.946133	1.11092
27710	0.946132	2.07704
27711	0.946132	2.1
27712	0.946133	2.1
27713	0.946132	2.1
27714	0.946133	2.1
27715	1.39514	1.09405
27716	0.946129	0.872249
27717	0.946124	1.04921
27718	0.946127	0.824474
27719	0.946127	1.02508
27720	0.94613	2.04955
27721	0.946129	2.1

27722	0.946132	2.1
27723	1.3991	2.1
27724	0.94613	2.1
27725	0.946132	0.776272
27726	0.946133	1.29251
27727	0.946132	1.01672
27728	1.36835	0.917016
27729	0.946132	0.834471
27730	0.946132	2.09448
27731	0.946132	2.1
27732	0.946133	2.1
27733	0.946132	2.1
27734	1.39047	2.1
27735	0.946132	0.214893
27736	0.946128	0.219535
27737	0.94613	0.231077
27738	1.33174	0.206607
27739	0.946131	0.233314
27740	0.946128	0.270595
27741	0.946131	0.253764
27742	0.94613	0.288806
27743	0.94613	0.251154
27744	0.94613	0.293038
27745	0.946119	2.1
27746	0.946119	2.1
27747	1.29064	2.1
27748	0.946132	2.1
27749	0.946107	2.1
27750	0.946133	2.1
27751	0.94613	2.1
27752	1.30584	2.1
27753	0.946131	2.1
27754	0.946131	2.1
27755	0.946131	2.1
27756	0.946132	2.1
27757	0.94613	2.1
27758	0.946131	2.1
27759	1.39549	2.1
27760	0.913386	1.16013
27761	0.978873	1.09335
27762	0.946126	1.07192
27763	0.94613	1.2138
27764	1.00473	0.976643
27765	0.946132	0.835742
27766	0.946133	0.85333
27767	0.946132	0.837232
27768	0.946132	0.871505
27769	1.38894	0.819697
27770	0.946128	0.83255
27771	0.946131	0.825948
27772	0.94613	0.834622
27773	0.94613	0.833966
27774	1.23865	0.810214
27775	0.946126	1.1395
27776	0.946129	1.2073
27777	0.946124	1.04914
27778	0.946127	1.14256
27779	1.40248	1.06592

27780	0.946133	0.847776
27781	0.946132	0.908822
27782	0.946132	0.840603
27783	0.946133	0.888597
27784	1.1391	0.84636
27785	0.827862	0.825393
27786	0.946133	0.815275
27787	0.946131	0.897927
27788	0.946131	0.823621
27789	0.94613	0.821445
27790	0.946128	0.754225
27791	0.946132	0.769875
27792	0.94613	0.795962
27793	1.21388	0.73394
27794	0.94613	0.784347
27795	0.810943	0.654961
27796	0.810945	0.707745
27797	0.810932	0.704801
27798	0.946131	0.777295
27799	0.946131	0.766982
27800	0.946133	0.779133
27801	1.36748	0.740879
27802	0.946132	0.718528
27803	0.946128	0.435301
27804	0.946131	0.449364
27805	0.94613	0.511006
27806	1.35856	0.368875
27807	0.94613	0.480431
27808	0.946132	2.1
27809	0.946131	2.1
27810	0.946133	2.1
27811	0.946132	2.1
27812	0.946131	2.1
27813	0.946133	2.1
27814	0.946131	2.1
27815	1.39558	2.1
27816	1.39558	2.1
27817	0.94613	2.1
27818	0.946131	0.409377
27819	0.94613	0.480076
27820	0.946129	0.417361
27821	1.41388	0.38942
27822	0.946131	0.448808
27823	0.946133	0.382085
27824	0.946132	0.53553
27825	0.946132	0.406842
27826	0.946133	0.409176
27827	0.93452	0.508933
27828	0.946129	2.1
27829	1.38897	2.1
27830	0.946131	2.1
27831	0.946131	2.1
27832	1.38897	2.1
27833	0.946133	1.22594
27834	0.946132	1.187
27835	0.946133	1.15376
27836	0.946132	0.937842
27837	1.38773	1.06293

27838	1.41512	0.502348
27839	0.946131	0.678267
27840	0.946129	0.61089
27841	0.946133	0.604028
27842	0.946131	0.626384
27843	0.946132	1.04306
27844	0.946132	2.1
27845	0.946132	1.17004
27846	0.946133	1.22716
27847	0.946133	2.1
27848	0.946126	0.969819
27849	0.946132	1.24777
27850	0.946129	1.084
27851	0.946129	1.1095
27852	0.946129	1.27073
27853	0.939895	1.58419
27854	1.03386	1.97995
27855	0.94613	1.59487
27856	0.946131	1.47621
27857	1.03386	1.7236
27858	0.946131	1.38815
27859	1.39309	2.1
27860	0.946133	1.26722
27861	0.946131	2.06976
27862	1.39309	2.1
27863	0.946133	2.1
27864	1.24853	2.1
27865	0.946132	2.1
27866	0.946132	2.07834
27867	1.24853	2.1
27868	0.946133	1.72932
27869	1.39699	1.0816
27870	0.946132	1.54437
27871	0.946132	2.1
27872	1.39699	1.2748
27873	0.946132	2.1
27874	0.946131	1.16513
27875	0.946133	2.1
27876	0.946131	1.73444
27877	1.38969	2.06129
27878	1.28464	1.65807
27879	0.946133	1.67881
27880	0.94613	1.77393
27881	0.946131	1.61838
27882	0.946131	1.56958
27883	0.946119	0.796418
27884	0.94612	0.855649
27885	0.946119	0.812148
27886	0.946132	0.828188
27887	1.3749	0.80359
27888	0.810942	2.1
27889	0.810943	2.1
27890	0.810957	2.1
27891	1.41134	1.58099
27892	0.946132	0.951526
27893	0.946132	1.49467
27894	0.946132	1.03413
27895	0.946132	1.46337

27896	1.31338	1.70669
27897	0.946132	1.12322
27898	0.946132	1.71179
27899	0.946133	1.28859
27900	0.946132	1.52898
27901	0.946103	0.205132
27902	0.752448	0.222852
27903	0.946132	0.211453
27904	0.946131	0.219573
27905	1.20617	0.218118
27906	0.946132	1.03514
27907	1.08054	0.992494
27908	1.25736	1.11613
27909	0.946132	1.03575
27910	0.946131	1.04539
27911	0.946128	1.68685
27912	1.33275	1.05753
27913	1.1896	1.64968
27914	0.946131	1.695
27915	1.18962	1.69871
27916	1.21955	0.233841
27917	0.946131	0.271033
27918	1.33587	0.237127
27919	0.741946	0.23755
27920	1.07387	0.216741
27921	0.945844	0.861799
27922	1.3292	1.54952
27923	1.59472	1.16294
27924	0.946131	0.967313
27925	1.04043	1.01866
27926	1.13529	0.338603
27927	0.946132	0.393577
27928	1.24893	0.32488
27929	1.12675	0.372443
27930	0.936652	0.362261
27931	1.36968	2.1
27932	0.946131	2.1
27933	0.946132	2.1
27934	1.36968	2.1
27935	1.258 2.1	
27936	3.17458	0.375664
27937	0.946132	1.37204
27938	1.35022	2.1
27939	1.35022	2.1
27940	0.94613	1.46822
27941	1.12356	1.81189
27942	0.946131	2.1
27943	1.22401	2.1
27944	1.22401	2.1
27945	0.94613	2.1
27946	0.952455	2.1
27947	1.2107	2.1
27948	0.946132	2.1
27949	1.33047	2.1
27950	0.946131	2.1
27951	0.946132	2.1
27952	0.61957	1.72359
27953	0.8636	2.1

27954	0.946132	1.7138
27955	1.19294	1.98784
27956	0.946128	2.1
27957	1.36607	2.1
27958	0.94613	1.63714
27959	1.39416	2.1
27960	1.36607	2.1
27961	1.21474	2.1
27962	0.946131	2.1
27963	0.946131	2.1
27964	1.16288	2.1
27965	1.08542	2.1
27966	0.851526	0.929031
27967	1.30374	1.62112
27968	1.01708	1.46644
27969	0.946114	0.999999
27970	0.96976	1.15598
27971	0.946132	0.346111
27972	1.49997	0.408303
27973	0.771735	0.396829
27974	0.771764	0.440551
27975	0.946133	0.477669
27976	0.700863	1.26339
27977	1.42222	1.30722
27978	0.946131	2.1
27979	0.946128	1.00373
27980	1.42509	1.78862
27981	1.14298	1.04966
27982	0.837014	2.1
27983	0.946131	2.1
27984	1.29426	2.1
27985	0.836985	2.1
27986	0.946129	2.1
27987	1.22089	0.761127
27988	0.946132	0.986567
27989	0.712486	0.804289
27990	0.6234	0.817875
27991	0.946131	2.1
27992	1.40271	2.1
27993	1.32241	2.1
27994	0.946133	2.1
27995	1.32241	2.1
27996	0.769373	1.23198
27997	0.946129	2.1
27998	1.41302	2.1
27999	1.28254	2.1
28000	0.946131	2.1
28001	1.28254	2.1
28002	0.851543	1.42321
28003	1.23056	2.09588
28004	1.24356	2.1
28005	0.851543	1.52803
28006	0.909417	1.7041
28007	1.13808	0.665332
28008	0.946133	0.701531
28009	0.946131	0.465275
28010	1.13808	0.784835
28011	1.13808	0.698918

28012	0.930941	1.20245
28013	1.37808	1.11128
28014	1.17573	0.965612
28015	0.946124	1.23452
28016	1.02404	1.01758
28017	0.946116	1.84065
28018	1.3144	1.01011
28019	1.3144	1.78406
28020	0.946118	1.89746
28021	1.13089	1.60576
28022	0.946128	0.483987
28023	0.946101	0.412644
28024	1.2606	0.498282
28025	1.26055	0.413378
28026	0.580823	0.900454
28027	0.946132	1.07868
28028	0.580825	0.9151
28029	1.23224	0.933367
28030	0.946128	2.1
28031	1.3719	2.1
28032	0.94613	2.1
28033	1.3719	2.1
28034	0.94613	2.1
28035	0.946132	1.17835
28036	1.35025	0.894192
28037	1.35025	1.01636
28038	0.946132	1.32873
28039	1.1794	1.08187
28040	0.946133	2.1
28041	1.25598	2.1
28042	1.29249	2.1
28043	1.38154	2.1
28044	0.851541	1.44969
28045	1.31508	2.1
28046	1.37558	2.09498
28047	0.946129	1.5045
28048	0.867663	1.60259
28049	0.94613	1.27174
28050	1.35426	1.14985
28051	0.84872	1.11195
28052	0.946129	1.08527
28053	1.17195	1.5358
28054	0.946133	2.1
28055	1.39009	2.1
28056	1.14342	2.1
28057	0.946133	2.1
28058	1.14343	2.1
28059	1.38819	2.1
28060	0.946133	2.1
28061	1.4116	2.1
28062	0.946131	2.1
28063	0.946131	2.1
28064	0.946127	2.1
28065	1.32244	2.1
28066	1.32244	2.1
28067	0.946131	2.1
28068	1.2176	2.1
28069	1.75823	2.1

28070	1.11239	2.1
28071	2.17979	2.1
28072	1.65533	2.1
28073	3.41356	2.1
28074	1.94841	2.1
28075	1.91767	2.1
28076	1.76726	2.1
28077	1.70302	2.1
28078	3.76137	2.1
28079	1.94841	2.1
28080	1.33962	2.1
28081	1.31365	2.1
28082	1.70302	2.1
28083	3.2123	2.1
28084	1.76726	2.1
28085	1.70302	2.1
28086	1.47581	2.1
28087	1.7457	2.1
28088	3.22764	2.1
28089	1.20478	2.1
28090	1.70302	2.1
28091	1.27956	2.1
28092	1.18569	2.1
28093	1.63979	2.1
28094	1.51949	2.1
28095	1.53082	2.1
28096	1.92911	2.1
28097	1.32068	2.1
28098	1.74454	2.1
28099	3.27536	2.1
28100	1.08172	2.1
28101	1.74454	2.1
28102	1.52603	2.1
28103	1.52603	2.1
28104	1.32068	2.1
28105	1.32068	2.1
28106	1.89226	2.1
28107	2.37364	2.1
28108	1.89226	2.1
28109	2.42769	2.1
28110	4.31995	2.1
28111	2.37365	2.1
28112	1.5362	2.1
28113	2.4277	2.1
28114	1.56328	2.1
28115	3.9639	2.1
28116	1.89225	2.1
28117	2.42768	2.1
28118	1.89213	2.1
28119	1.89225	2.1
28120	2.42756	2.1
28121	2.42768	2.1
28122	2.42768	2.1
28123	1.58163	2.1
28124	1.56315	2.1
28125	1.56327	2.1
28126	2.42757	2.1
28127	2.42768	2.1

28128	1.01641	2.1
28129	2.15005	2.1
28130	1.00099	2.1
28131	2.15114	2.1
28132	3.15104	2.1
28133	0.994813	2.1
28134	2.15114	2.1
28135	2.17159	2.1
28136	2.17159	2.1
28137	1.00099	2.1
28138	1.00099	2.1
28139	1.29608	2.1
28140	1.29608	2.1
28141	0.942035	1.79383
28142	0.942035	1.4589
28143	1.29198	1.83717
28144	0.942033	2.1
28145	0.946132	2.1
28146	0.946132	1.68103
28147	0.946132	1.55039
28148	0.942033	1.70839
28149	0.946133	1.29288
28150	1.29198	1.67943
28151	1.99985	2.1
28152	1.14207	2.1
28153	1.83584	2.1
28154	1.00232	2.1
28155	3.00217	2.1
28156	1.99867	0.25162
28157	2.0815	0.300818
28158	1.56806	0.250687
28159	1.28828	0.291509
28160	3.58519	0.285789
28161	1.89226	0.322188
28162	1.89226	0.380141
28163	1.89226	0.331303
28164	1.89226	0.391404
28165	1.89226	0.326836
28166	1.89226	0.391767
28167	1.89226	0.279942
28168	1.89226	0.343664
28169	2.23193	0.289308
28170	2.48978	0.385618
28171	4.38204	0.294438
28172	1.85293	0.8943
28173	1.64279	0.573932
28174	1.76268	0.907533
28175	1.54214	0.402384
28176	3.39507	0.742486
28177	1.6428	0.674722
28178	1.17302	0.600152
28179	1.17302	0.818597
28180	1.28419	0.722238
28181	1.54214	0.457133
28182	1.65331	0.611847
28183	1.76268	0.921619
28184	1.54214	0.734122
28185	1.65324	0.879014

28186	1.42491	0.43587
28187	3.19538	0.649588
28188	0.651043	0.782191
28189	0.651043	0.77212
28190	0.647925	0.533159
28191	0.647925	0.75917
28192	0.521747	0.691068
28193	0.58651	0.721831
28194	0.77107	0.711461
28195	0.712457	0.731289
28196	0.712457	0.48314
28197	0.521747	0.611806
28198	0.58651	0.646684
28199	0.77107	0.696559
28200	1.2873	2.0865
28201	1.48751	1.98895
28202	1.59659	2.1
28203	1.77596	1.32388
28204	3.06326	2.04863
28205	1.07976	2.1
28206	1.59659	2.0846
28207	1.30237	2.1
28208	1.77723	2.1
28209	2.89897	2.1
28210	1.77723	2.1
28211	1.77596	1.37056
28212	1.82614	2.05476
28213	1.59659	2.00913
28214	2.0055	1.85476
28215	1.77596	1.92408
28216	2.64523	0.929659
28217	2.73982	0.532664
28218	2.50057	0.92104
28219	2.61606	0.506618
28220	5.26129	0.680303
28221	2.73982	0.639267
28222	2.68987	0.67915
28223	2.61607	0.610855
28224	2.57611	0.427356
28225	5.30593	0.48921
28226	1.89226	0.699433
28227	1.89226	0.743416
28228	1.89226	0.720537
28229	2.02178	0.717797
28230	3.78452	0.726829
28231	1.89226	0.778513
28232	1.89226	0.739204
28233	1.89226	1.24968
28234	1.89226	0.632593
28235	1.89226	1.3195
28236	1.89226	0.624882
28237	2.35204	2.1
28238	1.50108	2.1
28239	1.3715	2.1
28240	2.18469	2.1
28241	3.68577	2.1
28242	2.62514	2.1
28243	2.1847	2.1

28244	2.0487	2.1
28245	2.45213	2.1
28246	4.63682	2.1
28247	1.25075	2.1
28248	2.18469	2.1
28249	1.3715	2.1
28250	1.3715	2.1
28251	2.0487	2.1
28252	2.0487	2.1
28253	1.28667	2.1
28254	1.11285	2.1
28255	1.03529	2.1
28256	1.03529	2.1
28257	1.30711	2.1
28258	1.30711	2.1
28259	2.35053	2.1
28260	1.89225	2.1
28261	1.89225	2.1
28262	2.51216	2.1
28263	4.24278	2.1
28264	1.89225	2.1
28265	1.89225	2.1
28266	1.89225	2.1
28267	1.89225	2.1
28268	3.7845	2.1
28269	1.92025	2.1
28270	1.32119	2.1
28271	1.44777	2.1
28272	1.33834	2.1
28273	1.92025	2.1
28274	1.81082	2.1
28275	1.89226	2.1
28276	2.67272	2.1
28277	2.51218	2.1
28278	2.51218	2.1
28279	1.89226	2.1
28280	1.89226	2.1
28281	1.89226	2.1
28282	1.89226	2.1
28283	1.89226	2.1
28284	1.89226	2.1
28285	3.78452	2.1
28286	1.92027	2.1
28287	1.44778	2.1
28288	1.61121	2.1
28289	2.01555	2.1
28290	3.53147	2.1
28291	1.90727	0.636081
28292	2.29851	0.685168
28293	2.71643	0.697186
28294	2.17695	0.611865
28295	4.47547	0.622908
28296	1.90728	0.647148
28297	1.56339	0.479308
28298	1.58143	0.505373
28299	2.17696	0.633031
28300	3.74035	0.609364
28301	1.91368	0.507051

28302	2.17696	0.638037
28303	2.39986	0.661676
28304	1.75904	0.495361
28305	3.936	0.511318
28306	1.18793	0.333125
28307	2.74744	0.381182
28308	1.38716	0.39811
28309	2.57465	0.366563
28310	3.93537	0.385182
28311	1.3148	0.368803
28312	2.74744	0.437056
28313	2.70805	0.36107
28314	1.38716	0.370066
28315	2.70805	0.456444
28316	1.38716	0.427973
28317	1.20608	2.1
28318	0.975871	2.1
28319	0.946132	2.1
28320	0.946132	2.05129
28321	0.946132	2.05376
28322	0.946132	2.1
28323	0.975871	2.1
28324	0.975871	2.1
28325	1.20608	2.1
28326	0.946132	2.1
28327	0.946133	2.1
28328	0.975871	2.1
28329	2.8198	1.11536
28330	2.29967	1.61367
28331	2.09542	2.1
28332	2.63784	0.970916
28333	4.93751	1.6487
28334	2.09542	2.1
28335	1.39523	2.1
28336	1.21119	2.1
28337	1.39523	2.1
28338	2.11563	2.1
28339	2.29967	1.68813
28340	1.88584	1.99092
28341	2.63784	0.895408
28342	2.11262	1.16509
28343	1.91132	1.12256
28344	4.02153	1.18691
28345	1.88584	2.1
28346	1.21119	2.1
28347	1.12606	2.1
28348	1.91132	1.19619
28349	3.1225	2.1
28350	2.18077	1.61756
28351	1.78167	1.57534
28352	2.2947	0.926482
28353	1.93139	1.21476
28354	4.07637	0.986579
28355	1.5709	1.02883
28356	1.03235	1.62312
28357	2.05933	1.44014
28358	1.78168	1.04149
28359	1.31	0.855385

28360	1.03235	0.841509
28361	2.2947	1.1232
28362	2.31785	1.65128
28363	2.40627	1.60226
28364	2.08288	0.977885
28365	4.37758	1.66352
28366	1.95737	1.31824
28367	1.31	1.57205
28368	2.08289	0.831629
28369	1.50108	0.912504
28370	3.39289	0.896025
28371	2.49676	2.1
28372	2.24781	1.88451
28373	2.5276	1.20069
28374	2.7352	2.1
28375	5.02436	1.9317
28376	2.49676	2.00127
28377	2.43549	2.1
28378	2.6398	2.1
28379	2.7352	2.1
28380	5.13656	2.1
28381	1.89226	2.1
28382	2.7352	2.1
28383	1.89226	1.83891
28384	2.6398	2.1
28385	1.89226	2.01032
28386	2.6398	2.1
28387	2.16843	0.147815
28388	1.00185	0.147815
28389	1.90039	2.1
28390	3.1547	0.147815
28391	2.54064	0.147815
28392	2.46752	2.1
28393	1.90039	2.1
28394	2.26044	2.1
28395	4.36791	0.821306
28396	1.65815	2.1
28397	2.48652	2.1
28398	1.65815	2.1
28399	1.9004	2.1
28400	2.21711	2.1
28401	2.5034	2.1
28402	2.37167	2.1
28403	2.59965	2.1
28404	2.62812	2.1
28405	2.40308	2.1
28406	4.99979	2.1
28407	2.50137	2.1
28408	2.59965	2.1
28409	2.62812	2.1
28410	2.5214	2.1
28411	5.12948	2.1
28412	2.62814	2.1
28413	2.4031	2.1
28414	2.43509	2.1
28415	2.65066	2.1
28416	5.06323	2.1
28417	2.62814	2.1

28418	2.52142	2.1
28419	2.53008	2.1
28420	2.65066	2.1
28421	5.15822	2.1
28422	2.0815	2.1
28423	1.92067	2.1
28424	1.59611	2.1
28425	1.89226	2.1
28426	1.62453	2.1
28427	1.92067	2.1
28428	1.89226	2.1
28429	1.89226	2.1
28430	1.89226	2.1
28431	1.89226	2.1
28432	3.78453	2.1
28433	0.946131	2.1
28434	0.946131	2.1
28435	0.929501	2.1
28436	0.910761	2.1
28437	0.9461	2.1
28438	0.9461	2.1
28439	0.910792	2.1
28440	0.910792	2.1
28441	0.946131	2.1
28442	0.929501	2.1
28443	0.910761	2.1
28444	0.946131	2.1
28445	2.0815	2.1
28446	1.81661	2.1
28447	2.1341	2.1
28448	2.0815	2.1
28449	1.86921	2.1
28450	1.81661	2.1
28451	1.89226	2.1
28452	1.89226	2.1
28453	1.89226	2.1
28454	1.89226	2.1
28455	1.89226	2.1
28456	1.89226	2.1
28457	0.946131	2.1
28458	0.946131	2.1
28459	0.949104	2.1
28460	0.9295	2.1
28461	0.946132	2.1
28462	0.946132	2.1
28463	0.929499	2.1
28464	0.929499	2.1
28465	0.946131	2.1
28466	0.949105	2.1
28467	0.946131	2.1
28468	0.929501	2.1
28469	1.66104	2.1
28470	2.34044	2.1
28471	2.08889	2.1
28472	1.33264	2.1
28473	3.67308	2.1
28474	2.0889	2.1
28475	1.08011	2.1

28476	1.08011	2.1
28477	1.33264	2.1
28478	1.87598	2.1
28479	2.12851	2.1
28480	2.27472	2.1
28481	1.89226	1.32181
28482	2.27703	1.49656
28483	1.89226	0.944806
28484	4.16699	1.01055
28485	1.89226	1.70102
28486	1.89226	1.40405
28487	1.89226	1.24424
28488	1.89226	1.08525
28489	3.78452	1.57995
28490	1.00007	1.50626
28491	1.32316	1.04987
28492	1.29417	0.956041
28493	1.29417	1.43273
28494	1.02051	0.926304
28495	1.02051	1.30142
28496	2.2846	1.62655
28497	1.89226	1.14813
28498	2.27703	1.08217
28499	2.27703	2.1
28500	1.89226	0.877075
28501	1.89226	2.1
28502	1.89226	1.63488
28503	1.89226	1.6664
28504	1.89226	1.01764
28505	1.89226	0.880143
28506	3.78452	1.07632
28507	1.02051	2.00951
28508	1.32316	1.61376
28509	1.03373	1.00155
28510	1.34066	1.08866
28511	2.35689	1.71676
28512	1.703	2.1
28513	1.89224	2.1
28514	1.52266	2.1
28515	1.89224	2.1
28516	1.23344	2.1
28517	1.53282	2.1
28518	1.89223	2.1
28519	1.89223	1.9784
28520	1.89223	2.1
28521	1.89223	2.1
28522	3.78447	2.1
28523	0.796845	2.1
28524	0.796845	2.1
28525	0.812146	2.1
28526	0.812146	2.1
28527	1.08006	2.1
28528	1.02679	2.1
28529	0.551538	2.1
28530	0.528929	2.1
28531	0.551529	2.1
28532	1.08005	2.1
28533	0.528935	2.1

28534	1.0268	2.1
28535	1.5001	2.1
28536	1.70302	2.1
28537	2.0726	2.1
28538	1.70302	2.1
28539	1.99601	2.1
28540	1.70303	2.1
28541	1.89226	2.1
28542	1.89226	2.1
28543	1.89226	2.1
28544	1.89226	2.1
28545	1.89226	2.1
28546	1.89226	1.94387
28547	1.17285	2.1
28548	2.16015	2.1
28549	2.23563	2.1
28550	2.23563	2.1
28551	1.10309	2.1
28552	1.10309	2.1
28553	0.946128	2.1
28554	0.946128	2.1
28555	1.21847	0.880112
28556	1.2884	2.1
28557	0.94613	2.1
28558	0.94613	2.1
28559	1.21847	2.1
28560	1.21847	0.88017
28561	0.946131	2.1
28562	1.21847	2.1
28563	0.94613	2.1
28564	1.2884	2.1
28565	1.83922	0.992529
28566	2.5768	2.1
28567	1.83488	2.1
28568	1.82846	0.147815
28569	2.43693	2.1
28570	2.43693	1.8436
28571	2.36134	2.1
28572	1.81656	2.1
28573	1.86454	2.1
28574	1.6697	2.1
28575	2.55618	2.1
28576	2.36134	2.1
28577	2.55618	2.1
28578	1.9323	2.1
28579	1.9302	2.1
28580	1.81656	2.1
28581	2.66983	2.1
28582	2.55618	2.1
28583	1.89226	0.995075
28584	1.70302	1.01203
28585	1.94935	1.12219
28586	1.70302	0.998137
28587	1.95569	1.00648
28588	1.70302	0.860911
28589	2.82465	2.1
28590	2.41707	2.1
28591	2.24481	2.1

28592	2.68958	2.1
28593	5.10665	2.1
28594	1.41232	2.1
28595	1.34479	2.1
28596	0.946129	2.1
28597	0.946129	2.1
28598	0.94613	2.1
28599	0.94613	2.1
28600	1.41232	2.1
28601	1.41232	2.1
28602	1.34479	2.1
28603	0.94613	2.1
28604	0.946131	2.1
28605	1.41232	2.1
28606	1.86859	0.246026
28607	1.89226	0.269724
28608	1.89226	0.300678
28609	1.89226	0.247521
28610	1.45875	0.288275
28611	1.45875	0.230975
28612	2.1769	0.700622
28613	2.50259	0.720617
28614	1.94955	0.7
28615	2.25959	0.7
28616	4.45214	0.700604
28617	1.89226	0.733913
28618	1.89226	0.762496
28619	1.89226	0.702847
28620	1.89226	0.725784
28621	3.78452	0.709511
28622	1.89226	0.815291
28623	1.89226	0.883331
28624	1.89226	0.741646
28625	1.89226	0.873375
28626	3.78452	1.09124
28627	2.50259	0.76057
28628	2.49445	0.867189
28629	2.25959	0.7
28630	2.20843	0.780159
28631	4.71102	0.741319
28632	1.94955	0.700537
28633	2.25959	0.7
28634	1.24689	0.526204
28635	1.79906	0.7
28636	3.74861	0.665359
28637	1.89226	0.704141
28638	1.89226	0.71215
28639	1.89226	0.699033
28640	1.89226	0.701294
28641	3.78452	0.710414
28642	1.89226	0.725026
28643	1.89226	0.774298
28644	1.89226	0.701184
28645	1.89226	0.719554
28646	3.78452	0.706753
28647	2.25959	0.7
28648	2.20843	0.727816
28649	1.79906	0.7

28650	1.98391	0.705292
28651	4.00748	0.702742
28652	1.89226	2.1
28653	1.89226	2.1
28654	1.89226	2.1
28655	1.89226	2.1
28656	3.78452	2.1
28657	1.89226	2.1
28658	1.89226	2.1
28659	1.89226	2.1
28660	1.89226	2.1
28661	3.78452	2.1
28662	1.64718	2.1
28663	1.23085	2.1
28664	1.46028	2.1
28665	1.39564	2.1
28666	1.68372	2.1
28667	1.64718	2.1
28668	1.64718	2.1
28669	1.64981	2.1
28670	1.70303	2.1
28671	1.6991	2.1
28672	3.3199	2.1
28673	1.89226	2.1
28674	1.89226	2.1
28675	1.89226	2.1
28676	1.89226	2.1
28677	3.78452	2.1
28678	1.89226	2.1
28679	1.89226	2.1
28680	1.89226	2.1
28681	1.89226	2.1
28682	3.78452	2.1
28683	0.666701	2.1
28684	0.65896	2.1
28685	0.920797	2.1
28686	0.895441	2.1
28687	0.775871	2.1
28688	0.687493	2.1
28689	0.777393	2.1
28690	0.777393	2.1
28691	0.677856	2.1
28692	0.90095	2.1
28693	0.621435	2.1
28694	0.858124	2.1
28695	1.70303	2.1
28696	1.70731	2.1
28697	1.79663	2.1
28698	1.68783	2.1
28699	3.17337	2.1
28700	1.22724	2.1
28701	2.0021	2.1
28702	1.90427	2.1
28703	1.08698	2.1
28704	3.08907	2.1
28705	2.41689	2.1
28706	2.00209	2.1
28707	1.90427	2.1

28708	2.35245	2.1
28709	4.35454	2.1
28710	1.90427	2.1
28711	0.956294	2.1
28712	0.956294	2.1
28713	1.08698	2.1
28714	1.81488	2.1
28715	1.94556	2.1
28716	1.90427	2.1
28717	2.35245	2.1
28718	2.28979	2.1
28719	1.81488	2.1
28720	4.16733	2.1
28721	1.6272	2.1
28722	1.70302	2.1
28723	1.70303	2.1
28724	1.70302	2.1
28725	1.91167	2.1
28726	1.91167	2.1
28727	1.49063	2.1
28728	1.89226	2.1
28729	1.89226	2.1
28730	1.89226	2.1
28731	1.6272	2.1
28732	1.6272	2.1
28733	2.25988	0.963219
28734	1.89226	0.642499
28735	1.89226	0.924433
28736	2.23152	0.893775
28737	4.12379	0.819492
28738	2.25988	0.951174
28739	1.16361	1.14299
28740	1.15853	1.71295
28741	2.23153	0.853125
28742	3.39513	1.12369
28743	1.89226	0.893207
28744	2.23153	0.787408
28745	2.21022	0.972177
28746	2.23153	0.862674
28747	1.87095	0.776898
28748	1.89226	0.649455
28749	1.16239	1.27009
28750	2.23153	0.704133
28751	1.15853	1.14639
28752	1.15853	0.814091
28753	2.21022	1.15383
28754	2.21022	0.805729
28755	1.78381	0.299845
28756	1.70302	0.352564
28757	1.15453	0.313633
28758	1.35007	0.370327
28759	1.82021	0.297982
28760	2.12851	0.344069
28761	1.89226	0.370171
28762	1.89226	0.461761
28763	1.89226	0.402003
28764	1.89226	0.489448
28765	1.89226	0.350987

28766	1.89226	0.446737
28767	1.89258	0.302923
28768	1.089	0.359004
28769	1.22586	0.297299
28770	1.79877	0.352244
28771	2.88777	0.314289
28772	1.91564	0.293346
28773	1.79877	0.355477
28774	1.96356	0.325033
28775	1.70302	0.374164
28776	2.37828	0.299479
28777	2.04859	0.352504
28778	1.89226	0.372151
28779	1.89226	0.454831
28780	1.89226	0.35587
28781	1.89226	0.478625
28782	3.78453	0.393999
28783	1.89226	0.352565
28784	1.89226	0.446605
28785	1.89226	0.358055
28786	1.89226	0.461854
28787	1.89226	0.349434
28788	1.89226	0.446764
28789	2.28593	2.1
28790	1.89226	1.50403
28791	2.2731	2.01812
28792	1.89226	0.94053
28793	4.17819	1.16402
28794	1.89226	1.86452
28795	1.89226	1.80394
28796	1.89226	1.14758
28797	1.89226	1.12004
28798	3.78452	1.47156
28799	1.03933	1.72899
28800	1.34355	1.08263
28801	1.35048	1.02172
28802	1.35048	1.67717
28803	1.02975	0.940729
28804	1.02975	1.44518
28805	2.25105	1.97205
28806	1.89226	1.26076
28807	2.27311	1.47789
28808	2.27311	2.1
28809	1.89226	1.0348
28810	1.89226	2.1
28811	1.89226	1.77366
28812	1.89226	1.72055
28813	1.89226	1.20049
28814	1.89226	0.865704
28815	3.78452	1.14047
28816	1.02975	2.1
28817	1.34355	1.67378
28818	1.01121	1.10723
28819	1.33079	1.0704
28820	2.35476	1.78798
28821	1.24526	0.210072
28822	2.36406	0.239643
28823	2.81021	0.210169

28824	1.7237	0.218714
28825	4.08776	0.215003
28826	1.89226	2.1
28827	1.89226	1.81356
28828	1.89226	2.1
28829	1.89226	1.59291
28830	3.78453	2.1
28831	1.79028	2.1
28832	1.89238	1.84673
28833	1.65533	2.1
28834	1.77821	2.1
28835	3.5477	2.1
28836	1.89238	1.80094
28837	1.7465	0.79716
28838	1.77821	2.1
28839	1.63557	0.764946
28840	3.52795	0.994531
28841	1.89226	1.77399
28842	1.89226	0.682032
28843	1.89226	1.56842
28844	1.89226	0.724143
28845	3.78452	0.920515
28846	1.89226	2.1
28847	1.89226	1.69975
28848	1.89226	2.1
28849	1.89226	1.91
28850	3.78452	2.1
28851	1.65532	2.1
28852	1.77821	2.09888
28853	1.53278	2.1
28854	1.67571	1.93511
28855	3.33103	2.1
28856	1.77821	2.1
28857	1.63557	0.813386
28858	1.67571	1.92029
28859	1.52615	0.836934
28860	3.31128	0.99498
28861	1.89226	1.67072
28862	1.89226	0.754607
28863	1.89226	1.92964
28864	1.89226	0.730407
28865	3.78452	0.991117
28866	2.83129	1.9699
28867	1.89226	0.736625
28868	1.89226	1.03906
28869	2.6491	1.26247
28870	4.54136	1.02207
28871	2.83129	2.1
28872	1.47278	2.1
28873	1.3909	2.1
28874	2.6491	1.48782
28875	4.12188	2.1
28876	1.89225	2.05616
28877	2.65393	2.1
28878	1.89225	2.00993
28879	2.69591	2.1
28880	4.58816	2.1
28881	1.91703	2.1

28882	2.00209	2.1
28883	2.65393	2.1
28884	2.65393	2.1
28885	2.69591	2.1
28886	2.00209	2.1
28887	1.89226	2.1
28888	2.69593	2.1
28889	1.93967	2.1
28890	1.89226	2.1
28891	2.74334	2.1
28892	2.69593	2.00945
28893	2.74335	2.1
28894	2.0021	2.1
28895	2.76367	2.1
28896	2.69593	2.1
28897	2.06984	2.1
28898	2.0021	2.1
28899	2.24481	2.1
28900	2.58921	2.1
28901	2.68958	2.1
28902	2.68958	2.1
28903	2.11984	2.1
28904	2.11984	2.1
28905	2.11984	2.1
28906	2.58921	2.1
28907	2.46488	2.1
28908	1.98339	2.1
28909	4.5726	2.1
28910	2.7777	2.1
28911	2.58921	2.1
28912	2.46488	2.1
28913	2.70401	2.1
28914	5.29322	2.1
28915	2.17741	2.1
28916	2.54197	2.1
28917	2.54018	2.1
28918	2.18608	2.1
28919	4.72805	2.1
28920	2.17742	2.1
28921	1.58054	2.1
28922	1.59169	2.1
28923	2.18608	2.1
28924	3.76662	2.1
28925	2.54018	2.1
28926	2.18608	2.1
28927	2.19104	2.1
28928	2.5392	2.1
28929	4.72528	2.1
28930	1.60497	2.1
28931	2.18608	2.1
28932	1.59169	2.1
28933	1.59169	2.1
28934	2.19105	2.1
28935	2.19105	2.1
28936	2.58661	1.27323
28937	1.06203	2.1
28938	2.7043	2.1
28939	2.21397	2.08914

28940	1.55236	2.1
28941	1.06203	2.04499
28942	1.89226	1.28015
28943	1.89226	1.55708
28944	1.89226	0.868728
28945	1.89226	1.47935
28946	3.78453	1.31391
28947	1.08195	1.44738
28948	0.990019	2.04973
28949	1.31976	1.48772
28950	1.4539	0.974121
28951	2.40171	1.68065
28952	1.89226	1.09982
28953	1.89226	1.78583
28954	1.89226	1.04252
28955	1.89226	0.846229
28956	3.78453	1.17001
28957	1.89226	1.42772
28958	1.89226	1.27937
28959	1.89226	0.958946
28960	1.89226	1.67548
28961	3.78453	1.21545
28962	1.43895	1.64093
28963	1.31976	1.65218
28964	1.70066	0.943648
28965	1.33783	2.00562
28966	1.43895	1.78648
28967	1.07612	2.1
28968	0.65988	1.59036
28969	0.850329	1.33986
28970	0.919598	1.04346
28971	0.726952	1.40872
28972	0.788106	0.907792
28973	0.788106	1.97221
28974	0.789175	1.7397
28975	0.789175	0.824279
28976	0.850329	1.05048
28977	0.919598	1.01021
28978	0.659879	1.48705
28979	0.726952	1.31813
28980	1.34389	2.1
28981	1.41279	1.4151
28982	1.15564	2.01562
28983	1.15564	1.99146
28984	1.55998	1.30735
28985	1.55998	1.62713
28986	1.89226	1.25835
28987	1.89226	1.51406
28988	1.89226	2.05698
28989	1.89226	1.11704
28990	1.89226	1.8222
28991	1.89226	1.09862
28992	1.55998	2.1
28993	1.41279	1.64464
28994	1.72901	1.42656
28995	1.62414	1.8777
28996	3.1418	1.63239
28997	1.89226	1.28363

28998	1.89226	1.81547
28999	1.89226	1.43814
29000	1.89226	1.01198
29001	3.78452	1.53032
29002	1.89226	0.925423
29003	1.89226	1.83998
29004	1.89226	1.21075
29005	1.89226	1.62708
29006	1.89226	1.00402
29007	1.89226	1.20849
29008	1.62414	1.71087
29009	1.01243	1.74267
29010	1.70932	1.82069
29011	1.41279	1.33556
29012	1.30896	1.34949
29013	1.01243	1.15696
29014	1.9719	2.1
29015	1.10725	2.1
29016	1.12411	2.1
29017	1.98691	2.1
29018	3.09601	2.1
29019	1.97189	2.1
29020	2.47932	2.1
29021	2.49092	2.1
29022	1.98691	2.1
29023	4.46282	2.1
29024	1.1486	2.1
29025	1.98691	2.1
29026	1.12411	2.1
29027	1.12411	2.1
29028	2.01602	2.1
29029	2.01602	2.1
29030	2.49093	2.1
29031	1.98691	2.1
29032	2.01601	2.1
29033	2.51651	2.1
29034	4.50694	2.1
29035	1.93147	0.384805
29036	2.51593	0.383441
29037	1.35146	0.337469
29038	1.35146	0.335454
29039	2.48665	0.345101
29040	1.90664	0.327139
29041	1.63979	2.1
29042	1.7457	2.1
29043	1.66046	2.1
29044	1.56558	2.1
29045	3.31128	2.1
29046	0.819895	2.1
29047	0.782792	2.1
29048	0.60239	2.1
29049	0.54831	2.1
29050	0.688347	2.1
29051	0.688347	2.1
29052	0.679857	2.1
29053	0.679857	2.1
29054	0.782792	2.1
29055	0.54831	2.1

29056	0.60239	2.1
29057	0.819895	2.1
29058	1.66046	2.1
29059	1.42485	2.1
29060	1.56558	2.1
29061	1.56558	2.1
29062	1.52995	2.1
29063	1.52995	2.1
29064	2.58101	0.777948
29065	2.09803	1.55026
29066	2.408	0.614455
29067	1.86893	1.90509
29068	4.44994	0.86671
29069	2.36062	1.55427
29070	1.25104	2.1
29071	1.25104	2.06102
29072	1.39703	2.1
29073	1.86893	1.96578
29074	2.01492	2.1
29075	2.408	0.619626
29076	1.86893	1.54908
29077	2.22575	0.717595
29078	1.70677	1.32369
29079	4.09468	0.855606
29080	0.737605	2.1
29081	0.737605	1.55992
29082	0.741302	1.87652
29083	0.741302	2.1
29084	0.55189	2.1
29085	0.625517	2.1
29086	0.934468	1.46526
29087	0.853389	1.24157
29088	0.853389	2.1
29089	0.55189	2.1
29090	0.625517	2.1
29091	0.934467	2.1
29092	2.35053	2.1
29093	1.09615	2.1
29094	1.25998	2.1
29095	2.51216	2.1
29096	3.61051	2.1
29097	1.33636	2.1
29098	1.25609	2.1
29099	0.698455	2.1
29100	0.629995	2.1
29101	0.986092	2.1
29102	0.986092	2.1
29103	0.980263	2.1
29104	0.980263	2.1
29105	1.33636	2.1
29106	0.698455	2.1
29107	0.629995	2.1
29108	1.25609	2.1
29109	1.89226	1.03967
29110	1.89226	1.08635
29111	1.89226	1.11127
29112	1.89226	1.08483
29113	3.78452	1.06216

29114	1.74321	1.05795
29115	1.89226	1.08945
29116	1.91755	1.11569
29117	1.91755	1.05495
29118	1.89226	1.12873
29119	1.89226	1.06534
29120	0.946132	1.03325
29121	0.946132	0.998049
29122	0.949798	0.965589
29123	1.08054	1.06369
29124	1.08054	1.01688
29125	1.08054	1.06702
29126	0.946132	1.0004
29127	0.946132	1.00081
29128	0.946132	1.00775
29129	0.949798	0.974709
29130	1.08054	1.00928
29131	0.946131	1.03757
29132	1.89226	1.07435
29133	1.89226	1.02998
29134	1.89226	1.00394
29135	1.89226	1.0104
29136	1.89226	1.03973
29137	1.89226	1.04727
29138	2.39378	0.570665
29139	2.29234	0.34688
29140	2.29234	0.404181
29141	2.7933	0.382299
29142	1.18422	0.428143
29143	1.68518	0.466966
29144	2.79329	0.376064
29145	1.89226	0.339353
29146	2.29233	0.310865
29147	1.89226	0.332076
29148	4.18459	0.31053
29149	0.857046	0.27177
29150	0.857037	0.35922
29151	0.94613	0.28299
29152	0.94613	0.338819
29153	0.851539	0.314938
29154	0.851539	0.297582
29155	1.0315	0.283528
29156	0.952867	0.293596
29157	0.851486	0.273155
29158	0.952869	0.267932
29159	1.03156	0.312542
29160	0.851528	0.334138
29161	1.61981	2.1
29162	1.93096	2.1
29163	2.00776	2.1
29164	1.62218	2.1
29165	3.62757	2.1
29166	2.00776	2.1
29167	1.68541	2.1
29168	1.83215	2.1
29169	1.62218	2.1
29170	2.21773	2.1
29171	2.00776	2.1

29172	1.89188	0.305865
29173	1.70303	0.330677
29174	1.89226	0.352698
29175	1.72465	0.310804
29176	1.89226	0.348799
29177	1.70552	0.323645
29178	2.48659	0.371162
29179	2.0815	0.427254
29180	1.89226	0.467951
29181	2.08111	0.398378
29182	1.89226	0.441696
29183	2.08111	0.400617
29184	2.35332	0.700034
29185	1.83284	0.474544
29186	2.28483	0.55349
29187	2.71955	0.516744
29188	4.63815	0.520633
29189	1.89227	2.1
29190	1.89227	2.1
29191	1.89227	2.1
29192	1.89227	2.1
29193	3.78453	2.1
29194	1.33506	2.1
29195	1.17129	2.1
29196	1.33506	2.1
29197	1.38415	2.1
29198	1.13664	2.1
29199	1.18573	2.1
29200	1.89226	2.1
29201	2.68785	2.1
29202	1.89226	2.1
29203	2.6692	2.1
29204	4.58011	2.1
29205	1.89226	2.1
29206	1.89226	2.1
29207	1.89226	2.1
29208	1.89226	2.1
29209	3.78453	2.1
29210	1.33506	2.1
29211	1.13664	2.1
29212	1.27575	2.1
29213	1.09627	2.1
29214	2.43133	2.1
29215	1.89226	2.1
29216	2.65349	2.1
29217	2.66919	2.1
29218	2.66919	2.1
29219	1.89226	2.1
29220	1.89226	2.1
29221	1.89226	2.1
29222	1.89226	2.1
29223	1.89226	2.1
29224	1.89226	2.1
29225	3.78453	2.1
29226	1.16834	2.1
29227	1.16834	2.1
29228	0.946132	2.1
29229	0.946132	2.1

29230	1.26978	2.1
29231	1.16834	2.1
29232	0.94613	2.1
29233	0.94613	2.1
29234	0.94613	2.1
29235	1.26978	2.1
29236	0.94613	2.1
29237	1.16834	2.1
29238	2.14698	2.1
29239	1.89226	2.1
29240	1.89226	2.1
29241	1.89226	2.1
29242	2.40719	2.1
29243	2.40719	2.1
29244	1.89226	2.1
29245	1.89226	2.1
29246	1.89226	2.1
29247	1.89226	2.1
29248	3.78453	2.1
29249	1.26977	2.1
29250	1.26977	2.1
29251	0.946133	2.1
29252	0.946133	2.1
29253	1.37467	2.1
29254	1.26978	2.1
29255	0.946131	2.1
29256	0.946131	2.1
29257	1.37467	2.1
29258	0.94613	2.1
29259	0.946131	2.1
29260	1.26978	2.1
29261	1.2036	2.1
29262	1.34912	2.1
29263	0.946131	2.1
29264	0.946131	2.1
29265	1.34912	2.1
29266	1.34912	2.1
29267	0.946129	2.1
29268	0.946129	2.1
29269	1.34912	2.1
29270	0.94613	2.1
29271	0.946131	2.1
29272	1.2036	2.1
29273	2.47164	0.807187
29274	1.89019	0.652077
29275	1.79109	0.796115
29276	2.41632	0.66293
29277	4.26273	0.794739
29278	1.54197	0.704468
29279	2.41632	0.571177
29280	1.79109	0.655568
29281	1.79109	0.724076
29282	2.20929	0.606173
29283	2.20929	0.704246
29284	1.70302	1.2064
29285	1.48104	1.35895
29286	1.99365	1.37568
29287	1.32897	1.2311

29288	1.70302	1.3704
29289	1.14034	1.21869
29290	1.70302	1.18094
29291	1.99365	1.24912
29292	1.99297	1.25407
29293	2.36337	1.09264
29294	4.04804	1.12917
29295	1.51569	1.11142
29296	2.49093	1.13529
29297	1.6026	1.19902
29298	1.43229	1.04253
29299	2.0815	1.20214
29300	1.83402	1.04416
29301	2.54665	2.1
29302	2.34044	2.1
29303	2.08889	2.1
29304	2.37653	2.1
29305	4.71697	2.1
29306	2.54665	2.1
29307	2.45212	2.1
29308	2.31012	2.1
29309	2.37653	2.1
29310	4.82865	2.1
29311	2.08889	2.1
29312	2.37653	2.1
29313	2.23366	2.1
29314	1.87598	2.1
29315	4.25251	2.1
29316	2.31012	2.1
29317	2.37653	2.1
29318	2.23367	2.1
29319	2.17384	2.1
29320	4.55037	2.1
29321	0.946131	2.1
29322	0.946131	2.1
29323	1.30338	2.1
29324	1.23186	2.1
29325	0.94613	2.1
29326	0.94613	2.1
29327	1.23187	2.1
29328	1.23187	2.1
29329	0.946131	2.1
29330	1.23186	2.1
29331	1.30338	2.1
29332	0.946131	2.1
29333	1.89226	2.1
29334	2.46373	2.1
29335	2.34725	2.1
29336	2.46373	2.1
29337	1.77579	2.1
29338	1.89226	2.1
29339	2.61901	2.1
29340	2.45899	2.1
29341	2.64067	2.1
29342	2.52708	2.1
29343	5.14609	2.1
29344	2.41795	2.1
29345	2.0815	2.1

29346	2.52708	2.1
29347	1.92277	2.1
29348	4.31368	2.1
29349	2.64067	2.1
29350	2.52708	2.1
29351	2.58638	2.1
29352	2.51039	2.1
29353	5.11346	2.1
29354	2.52708	2.1
29355	2.13642	2.1
29356	2.51039	2.1
29357	2.16633	2.1
29358	4.69341	2.1
29359	1.61028	1.25995
29360	1.65712	0.811957
29361	1.35002	0.747577
29362	1.35002	1.28413
29363	1.88637	0.801177
29364	1.88637	1.5935
29365	2.57663	1.74193
29366	2.24227	2.1
29367	2.48354	1.7179
29368	2.14317	2.1
29369	4.7198	2.1
29370	2.24227	2.1
29371	1.77353	2.1
29372	2.14317	2.1
29373	1.69956	2.1
29374	3.9167	2.1
29375	2.48353	1.61852
29376	2.14317	2.1
29377	2.39282	1.84734
29378	1.79824	2.1
29379	4.53599	2.1
29380	2.14317	2.1
29381	1.69956	2.1
29382	1.91427	2.1
29383	1.6347	2.1
29384	3.77787	2.1
29385	1.89226	0.703933
29386	1.89226	0.713956
29387	1.89226	0.700448
29388	1.89226	0.708256
29389	3.78453	0.70707
29390	1.89226	0.704602
29391	1.89226	0.718354
29392	1.89226	0.701165
29393	1.89226	0.744551
29394	3.78452	0.707851
29395	1.89226	0.721068
29396	1.89652	1.12691
29397	1.89226	0.764556
29398	1.89226	0.78649
29399	1.19352	0.743235
29400	1.19352	0.772057
29401	1.89226	0.716307
29402	1.89653	0.726746
29403	1.89226	0.769278

29404	1.89226	0.735206
29405	1.89226	0.719708
29406	1.89226	0.710508
29407	1.89226	0.77545
29408	1.75326	0.768547
29409	1.89226	1.27151
29410	1.12948	1.28867
29411	1.89226	0.7
29412	1.12948	0.700451
29413	2.61064	2.1
29414	1.89226	2.1
29415	1.89226	2.1
29416	1.89226	2.1
29417	2.6085	2.1
29418	2.6085	2.1
29419	2.6085	2.1
29420	1.89226	2.1
29421	1.89226	2.1
29422	1.89226	2.1
29423	2.62161	2.1
29424	2.62161	2.1
29425	2.16535	2.1
29426	1.44603	2.1
29427	2.79784	2.1
29428	1.84756	2.1
29429	4.01292	2.1
29430	2.65262	2.1
29431	1.03391	2.07496
29432	2.20239	2.1
29433	1.74416	2.1
29434	1.49213	2.1
29435	1.03391	2.1
29436	2.29066	2.1
29437	2.49174	2.1
29438	2.56502	2.1
29439	2.33802	2.1
29440	4.85568	2.1
29441	2.39663	2.1
29442	2.49174	2.1
29443	2.56502	2.1
29444	2.45929	2.1
29445	4.96165	2.1
29446	2.56502	2.1
29447	2.33802	2.1
29448	2.37168	2.1
29449	2.59967	2.1
29450	4.9367	2.1
29451	2.56502	2.1
29452	2.45929	2.1
29453	2.50138	2.1
29454	2.59967	2.1
29455	5.0664	2.1
29456	2.27009	1.11105
29457	1.26247	1.98214
29458	1.86812	1.76434
29459	2.837	1.29383
29460	4.13822	1.68667
29461	1.89226	0.911918

29462	1.89226	2.1
29463	1.89226	1.55159
29464	1.89226	1.84779
29465	3.78453	1.82732
29466	1.75835	2.1
29467	1.72866	2.1
29468	1.65313	1.515
29469	1.64822	2.1
29470	3.3818	2.1
29471	1.72866	2.1
29472	1.58401	2.1
29473	1.64822	2.1
29474	1.52441	2.1
29475	3.25307	2.1
29476	1.89226	2.1
29477	1.89226	2.1
29478	1.89226	1.88478
29479	1.89226	2.1
29480	3.78452	2.1
29481	1.89226	1.6429
29482	1.89226	1.88781
29483	1.89226	0.935435
29484	1.89226	2.1
29485	3.78453	1.65617
29486	1.65313	1.66807
29487	1.64822	2.1
29488	1.55779	2.1
29489	1.57968	2.1
29490	3.23281	2.1
29491	1.64822	2.1
29492	1.52441	2.1
29493	1.57967	2.1
29494	1.474	2.1
29495	3.10408	2.1
29496	1.89226	1.90872
29497	1.89226	2.1
29498	1.89226	2.1
29499	1.89226	2.1
29500	3.78452	2.1
29501	1.86067	1.22391
29502	1.58407	1.90132
29503	2.07965	0.922663
29504	1.83123	1.30232
29505	3.66372	1.0591
29506	1.89226	1.00404
29507	1.89226	1.88671
29508	1.89226	1.00159
29509	1.89226	1.34354
29510	3.78452	1.25847
29511	1.89226	0.9943
29512	1.89226	1.61008
29513	1.89226	0.931231
29514	1.89226	2.09772
29515	1.89226	0.881007
29516	1.89226	2.01183
29517	1.83123	0.938359
29518	1.03472	1.71997
29519	1.91068	1.45994

29520	1.58406	1.13633
29521	1.36133	1.11271
29522	1.03472	0.961692
29523	2.07965	1.09629
29524	1.83123	1.43867
29525	2.28385	0.987901
29526	2.09114	1.20914
29527	4.1708	1.39286
29528	1.89226	1.24042
29529	1.89226	1.5971
29530	1.89226	0.903732
29531	1.89226	1.31269
29532	3.78452	1.01104
29533	1.89226	1.0417
29534	1.89226	1.82182
29535	1.89226	0.959813
29536	1.89226	1.66432
29537	3.78452	1.19406
29538	1.83123	0.989013
29539	1.36133	1.86795
29540	2.09114	0.907593
29541	1.69436	1.32672
29542	3.45248	1.07671
29543	1.89226	2.1
29544	1.89226	1.92678
29545	1.89226	2.1
29546	1.89226	1.44356
29547	3.78453	2.1
29548	1.53278	2.1
29549	1.67571	1.95027
29550	1.40182	2.1
29551	1.55686	2.07909
29552	3.07752	2.1
29553	1.67571	1.93733
29554	1.52615	0.789429
29555	1.55686	2.07675
29556	1.38963	0.626444
29557	3.06534	0.980937
29558	1.89226	1.94666
29559	1.89226	0.789892
29560	1.89226	1.41493
29561	1.89226	0.63417
29562	3.78452	0.935642
29563	1.89226	2.1
29564	1.89226	1.72545
29565	1.89226	2.1
29566	1.89226	1.95243
29567	3.78453	2.1
29568	1.40182	2.1
29569	1.55686	2.04601
29570	1.24509	2.1
29571	1.38426	1.99403
29572	2.78608	2.1
29573	1.55686	2.05919
29574	1.38963	0.833031
29575	1.2064	1.94819
29576	1.2064	0.895574
29577	1.38426	1.97953

29578	1.56749	0.926134
29579	0.810946	1.41776
29580	0.810946	0.813022
29581	0.810945	1.53431
29582	0.810945	1.9772
29583	0.810945	1.24428
29584	0.810945	1.05589
29585	1.89226	0.857525
29586	1.35169	0.942785
29587	1.08132	2.03094
29588	1.08132	0.619426
29589	1.08132	0.981322
29590	1.28418	0.800788
29591	1.59382	0.840995
29592	1.78289	0.919574
29593	1.51021	0.709836
29594	3.10403	0.794789
29595	1.76897	1.12617
29596	1.59382	0.833673
29597	1.78289	0.940458
29598	1.94302	0.996149
29599	3.53684	1.00166
29600	1.89226	0.828448
29601	1.89226	0.927162
29602	1.89226	0.983798
29603	1.89226	0.753553
29604	3.78453	0.843517
29605	1.89227	0.836206
29606	1.89227	0.606896
29607	1.89227	0.649444
29608	1.89227	0.728671
29609	1.89227	0.701213
29610	1.89227	0.768684
29611	1.78286	0.888068
29612	1.51019	0.688455
29613	1.79979	0.78471
29614	2.02511	0.835807
29615	3.5353	0.827218
29616	1.78286	0.903541
29617	1.94299	0.95139
29618	2.17831	1.07528
29619	2.02511	0.847526
29620	3.9681	0.889899
29621	1.89224	0.940286
29622	1.89224	0.764957
29623	1.89224	0.833798
29624	1.89224	0.912433
29625	3.78448	0.890484
29626	1.89224	0.822049
29627	1.89224	0.609486
29628	1.89224	0.678475
29629	1.89224	0.769638
29630	1.89224	0.683886
29631	1.89224	0.769249
29632	1.83198	0.584512
29633	1.0961	0.383811
29634	1.39448	0.520232
29635	1.99568	0.493664

29636	3.09178	0.523478
29637	1.25503	0.460156
29638	1.99568	0.446601
29639	2.3661	0.593234
29640	1.70302	0.423962
29641	3.55833	0.425387
29642	2.69899	0.337817
29643	1.85796	0.314138
29644	2.11407	0.308919
29645	1.36769	0.280507
29646	3.97203	0.274665
29647	1.89226	0.4141
29648	1.89226	0.375111
29649	1.89226	0.366492
29650	1.89226	0.302487
29651	3.78452	0.414929
29652	1.89225	0.314474
29653	2.63355	0.323263
29654	2.09529	0.309206
29655	2.09529	0.330216
29656	1.89225	0.335456
29657	1.89225	0.3436
29658	1.89226	0.490485
29659	1.89226	0.537249
29660	1.89226	0.437397
29661	1.89226	0.609695
29662	1.89226	0.421019
29663	1.89226	0.579
29664	2.12204	0.420997
29665	1.89226	0.529638
29666	2.05798	0.405645
29667	2.01954	0.414934
29668	1.70302	0.472556
29669	1.3865	0.534104
29670	1.28326	0.396327
29671	1.33326	0.86717
29672	1.19052	0.388418
29673	1.25369	0.837224
29674	2.53694	0.808612
29675	1.33327	0.873436
29676	1.27339	1.43039
29677	1.25369	0.841859
29678	1.19733	1.96025
29679	2.52708	0.919423
29680	1.19052	0.425136
29681	1.25369	0.836805
29682	1.10938	0.350978
29683	1.17614	0.899728
29684	2.36307	0.558284
29685	1.25369	0.838367
29686	1.19733	2.04973
29687	1.17614	0.909826
29688	1.11868	1.77046
29689	2.37237	1.29035
29690	1.10747	0.335844
29691	1.47688	0.35861
29692	1.97732	0.38904
29693	1.62206	0.369027

29694	3.09894	0.354236
29695	1.89226	0.352517
29696	1.89226	0.381071
29697	1.89226	0.349584
29698	1.89226	0.361545
29699	1.89226	0.359125
29700	1.89226	0.356898
29701	1.62206	0.344631
29702	1.70302	0.365497
29703	1.22962	0.354915
29704	1.1893	0.353218
29705	2.15177	0.354694
29706	2.08121	0.341882
29707	1.97732	0.387578
29708	2.08121	0.358252
29709	1.62206	0.355489
29710	1.62206	0.366992
29711	2.41464	0.36787
29712	2.41464	0.367261
29713	1.89226	0.356281
29714	1.89226	0.370416
29715	1.89226	0.366758
29716	1.89226	0.393118
29717	3.78452	0.369574
29718	0.946133	0.326469
29719	0.946133	0.360947
29720	0.946132	0.359366
29721	0.946132	0.361948
29722	0.946132	0.378646
29723	0.946132	0.364125
29724	0.946133	0.363416
29725	0.946133	0.356301
29726	0.946132	0.37044
29727	0.946132	0.417479
29728	0.946132	0.366024
29729	0.946132	0.317126
29730	1.83157	1.12079
29731	2.19989	1.0346
29732	2.20664	0.960375
29733	1.81487	0.988928
29734	4.03146	1.11575
29735	2.41055	2.1
29736	2.19989	1.75421
29737	2.20665	1.66553
29738	2.38023	1.57161
29739	4.61044	1.43031
29740	2.19989	1.92415
29741	2.22558	1.79588
29742	2.38023	1.75306
29743	2.07065	1.18426
29744	4.42547	1.46001
29745	2.19988	1.13794
29746	1.70303	0.766436
29747	1.89939	1.08257
29748	1.86357	0.980737
29749	3.78536	0.98442
29750	1.04921	2.1
29751	1.30711	2.1

29752	1.53076	2.1
29753	1.22927	2.1
29754	2.57997	2.1
29755	1.53076	2.1
29756	1.46568	2.1
29757	1.56056	2.1
29758	1.22927	2.1
29759	1.86206	2.1
29760	1.53076	2.1
29761	1.18353	2.1
29762	2.28593	2.1
29763	1.16979	2.1
29764	2.2731	1.83023
29765	3.45571	2.1
29766	0.854776	1.79932
29767	0.854776	2.1
29768	0.855641	2.1
29769	0.855641	2.1
29770	1.12552	1.96841
29771	1.13655	2.1
29772	0.57216	2.1
29773	0.584893	2.1
29774	0.57216	2.1
29775	1.12552	2.1
29776	0.584893	2.1
29777	1.13655	2.1
29778	1.54462	2.1
29779	2.76309	2.1
29780	1.34869	2.1
29781	2.48573	2.1
29782	4.03035	2.1
29783	1.35328	0.969083
29784	1.0383	0.906403
29785	1.03318	1.35267
29786	0.704834	1.24038
29787	1.0257	1.28726
29788	1.0257	0.974449
29789	1.03241	0.890722
29790	1.03241	1.26151
29791	1.35328	1.13424
29792	1.03319	1.29
29793	1.0383	1.16139
29794	0.704835	1.13274
29795	2.27126	0.518752
29796	2.77291	1.4014
29797	1.81771	0.893603
29798	2.6111	0.847668
29799	4.88236	0.917765
29800	2.77291	1.27248
29801	1.89227	0.977185
29802	1.89227	0.885234
29803	1.89227	1.80714
29804	2.6111	0.854055
29805	2.6111	1.85394
29806	2.22061	0.87614
29807	2.41522	0.858023
29808	2.41522	0.752388
29809	2.6111	1.09043

29810	1.75765	0.726076
29811	1.95353	1.53188
29812	2.27502	1.0289
29813	1.89227	1.55804
29814	1.89227	0.981057
29815	2.8189	0.902371
29816	4.71116	0.930836
29817	1.89226	0.886278
29818	1.89226	1.40887
29819	1.89226	1.00571
29820	1.89226	0.95911
29821	1.89226	1.24108
29822	1.89226	1.1064
29823	1.89226	1.27208
29824	1.89226	1.21317
29825	1.89226	1.19875
29826	1.89226	0.978879
29827	3.78453	1.24313
29828	1.89226	1.21845
29829	2.57079	0.867781
29830	2.5932	1.01881
29831	1.89226	0.98884
29832	4.48546	0.962876
29833	0.589396	0.991856
29834	0.641454	0.889602
29835	0.814062	1.13553
29836	0.726518	1.00161
29837	0.675126	1.07477
29838	0.675125	0.949483
29839	0.692847	1.11179
29840	0.692847	0.994142
29841	0.641454	0.933147
29842	0.814062	1.0209
29843	0.726518	1.10892
29844	0.589396	1.01673
29845	1.89226	1.22755
29846	1.89226	0.986828
29847	1.89226	1.19547
29848	1.89226	1.26921
29849	1.89226	1.07969
29850	1.89226	1.22889
29851	1.16906	0.813098
29852	1.2966	0.978035
29853	0.946132	0.972991
29854	0.946132	1.28897
29855	1.16906	0.982729
29856	1.16906	0.838805
29857	0.946132	0.989263
29858	0.946132	1.09434
29859	1.16906	1.10006
29860	0.946131	1.02452
29861	1.2966	1.02431
29862	0.946132	1.29448
29863	0.641455	1.0022
29864	0.567191	0.906222
29865	0.807594	1.20843
29866	0.814062	1.04944
29867	0.673735	1.19778

29868	0.673734	0.946493
29869	0.707519	1.00848
29870	0.707519	1.20108
29871	0.807594	1.12347
29872	0.56719	1.26546
29873	0.641455	1.13268
29874	0.814063	1.28516
29875	1.89225	2.1
29876	1.89225	2.1
29877	1.89225	2.1
29878	1.89225	2.1
29879	3.7845	2.1
29880	1.89225	2.1
29881	1.89225	2.1
29882	1.89225	2.1
29883	1.89225	2.1
29884	1.89225	2.1
29885	1.89225	2.1
29886	1.89226	2.1
29887	1.89226	2.1
29888	1.89226	2.1
29889	1.89226	2.1
29890	3.78452	2.1
29891	1.89226	2.1
29892	1.89226	2.1
29893	1.89226	2.1
29894	1.89226	2.1
29895	1.89226	2.1
29896	1.89226	2.1
29897	1.89226	2.1
29898	1.89226	2.1
29899	1.89226	2.1
29900	1.89226	2.1
29901	3.78452	2.1
29902	1.89226	2.1
29903	1.38928	2.1
29904	1.89226	2.1
29905	1.51378	2.1
29906	1.89061	2.1
29907	1.89226	2.1
29908	1.89226	2.1
29909	1.89226	1.74563
29910	1.89226	1.64808
29911	1.89226	2.1
29912	3.78453	2.09001
29913	1.04572	2.1
29914	0.994213	2.1
29915	0.912466	1.79597
29916	0.912466	1.9925
29917	0.947743	1.94666
29918	1.00388	2.1
29919	0.900913	1.73944
29920	0.900913	2.1
29921	0.900915	2.1
29922	0.946131	2.09505
29923	0.946131	2.09526
29924	0.999196	2.09612
29925	1.89226	1.55503

29926	1.89226	2.1
29927	1.89226	2.07939
29928	1.89226	1.81292
29929	3.78453	2.00509
29930	0.946161	2.1
29931	1.1354	2.1
29932	0.850847	2.1
29933	0.850847	2.1
29934	1.01445	2.1
29935	1.02106	2.1
29936	0.946131	2.1
29937	0.946131	2.08885
29938	1.13537	2.1
29939	1.01444	2.1
29940	0.850877	2.1
29941	0.946131	2.1
29942	1.80183	2.1
29943	1.89226	1.92185
29944	1.89226	1.85315
29945	1.89226	2.1
29946	1.79606	1.83059
29947	1.79606	2.1
29948	1.24001	1.34491
29949	1.41822	1.09914
29950	1.95665	0.777102
29951	1.77342	1.11426
29952	3.19666	0.980072
29953	1.95665	0.958907
29954	1.44859	1.20966
29955	1.41821	1.17308
29956	1.41821	1.15723
29957	1.95543	0.944798
29958	1.95543	1.03791
29959	1.95665	0.85677
29960	1.77342	1.15502
29961	2.33611	1.01437
29962	2.49694	0.774542
29963	4.29276	1.09879
29964	1.95665	1.10109
29965	1.95544	1.03721
29966	2.4728	0.734511
29967	2.49693	0.955921
29968	4.42945	0.885421
29969	2.24937	2.1
29970	2.49053	2.1
29971	2.16323	2.1
29972	2.36377	2.1
29973	4.61314	2.1
29974	2.49053	2.1
29975	2.58639	2.1
29976	2.36377	2.1
29977	2.47395	2.1
29978	4.95016	2.1
29979	2.45054	2.1
29980	2.36377	2.1
29981	1.92911	2.1
29982	2.181	2.1
29983	4.29288	2.1

29984	2.36377	2.1
29985	2.47395	2.1
29986	2.181	2.1
29987	2.29881	2.1
29988	4.66258	2.1
29989	1.57885	2.1
29990	1.13339	2.1
29991	1.48594	2.1
29992	1.13322	2.1
29993	2.71207	2.1
29994	1.48596	2.1
29995	1.20761	2.1
29996	1.20761	2.1
29997	1.13323	2.1
29998	1.46568	2.1
29999	1.3913	2.1
30000	1.89226	0.692029
30001	1.89226	0.824268
30002	1.89226	0.7
30003	1.89226	0.729333
30004	1.89226	0.686144
30005	1.89226	0.700874
30006	1.11712	0.748294
30007	0.946129	0.798478
30008	0.686934	0.689964
30009	0.946133	0.684207
30010	0.9461	0.735622
30011	1.10729	1.29317
30012	0.686963	0.833209
30013	0.686963	0.688221
30014	1.10945	0.779752
30015	0.80397	1.26297
30016	0.946131	0.692064
30017	0.946131	0.841272
30018	1.89226	2.1
30019	1.89226	1.2576
30020	1.89226	1.14058
30021	1.89226	1.25547
30022	3.78452	1.27536
30023	1.22615	2.1
30024	1.3439	2.1
30025	1.47868	2.1
30026	1.55998	1.24684
30027	2.78613	2.1
30028	1.04973	2.1
30029	1.47868	2.1
30030	1.20149	2.1
30031	1.61721	2.1
30032	2.68017	2.1
30033	1.89226	2.06187
30034	1.89226	2.09667
30035	1.89226	2.1
30036	1.89226	1.05459
30037	3.78452	2.04914
30038	0.946133	0.952804
30039	0.946133	1.57558
30040	0.946128	1.93692
30041	0.946128	1.87195

30042	0.946131	0.92812
30043	0.946131	1.2933
30044	0.946131	1.91472
30045	0.946131	1.06904
30046	0.94613	1.52052
30047	0.94613	1.26573
30048	0.946131	1.40372
30049	0.946131	1.24438
30050	0.798126	2.02678
30051	0.798126	1.75249
30052	0.805719	2.1
30053	0.805719	2.1
30054	0.779992	2.1
30055	0.864507	1.73715
30056	0.739338	2.07796
30057	0.808607	2.1
30058	0.808606	2.1
30059	0.864506	2.08946
30060	0.739339	2.1
30061	0.779993	2.1
30062	0.946128	2.1
30063	0.946128	2.02078
30064	1.18959	1.15589
30065	1.00304	2.1
30066	1.00304	2.1
30067	1.00304	1.79072
30068	0.94613	1.94494
30069	0.94613	1.17504
30070	0.94613	2.00003
30071	1.18959	1.93934
30072	1.00304	1.84571
30073	0.94613	1.91078
30074	2.19163	2.1
30075	1.89226	1.7557
30076	2.16541	2.1
30077	1.89226	1.1911
30078	4.08389	1.58634
30079	1.89226	2.09625
30080	1.89226	1.53906
30081	1.89226	1.60364
30082	1.89226	1.18222
30083	3.78452	1.67756
30084	0.964782	1.99723
30085	1.30151	1.14329
30086	1.30318	1.11307
30087	1.30318	2.02347
30088	0.949134	1.01728
30089	0.949134	1.84611
30090	2.15005	2.1
30091	1.89226	1.32241
30092	2.16541	1.70857
30093	2.16541	2.1
30094	1.89226	1.1014
30095	1.89226	2.1
30096	1.89226	1.8421
30097	1.89226	1.83894
30098	1.89226	1.40339
30099	1.89226	0.911737

30100	3.78452	1.27737
30101	0.949134	2.1
30102	1.30151	1.78749
30103	0.949134	1.35881
30104	1.32143	1.18525
30105	2.25064	1.96784
30106	0.982859	0.271238
30107	1.28326	0.473227
30108	1.19052	0.324352
30109	1.19052	0.587718
30110	1.10476	0.286191
30111	1.10476	0.534853
30112	1.37265	0.840771
30113	1.38247	0.973993
30114	1.36636	1.08689
30115	1.3294	0.840448
30116	2.71187	0.904911
30117	1.89226	0.986515
30118	1.89226	0.76732
30119	1.89226	0.855277
30120	1.89226	0.937682
30121	1.89226	0.859836
30122	1.89226	0.951952
30123	1.36636	1.18172
30124	1.3294	0.828373
30125	1.30001	0.941676
30126	1.36416	1.10987
30127	2.69356	1.01759
30128	1.89226	1.12144
30129	1.89226	0.785995
30130	1.89226	0.904406
30131	1.89226	1.03159
30132	1.89226	0.844238
30133	1.89226	0.960154
30134	2.83701	0.792419
30135	1.89226	0.843646
30136	1.89226	0.997495
30137	2.79312	0.704264
30138	4.68539	0.769485
30139	1.44288	2.1
30140	0.948677	2.1
30141	1.84076	2.1
30142	1.42884	2.1
30143	2.78943	2.1
30144	1.89226	1.92556
30145	1.89226	2.1
30146	1.89226	2.1
30147	1.89226	1.92034
30148	3.78452	2.1
30149	0.909402	1.77478
30150	0.909402	1.92207
30151	1.3063	2.1
30152	1.56757	2.1
30153	1.00038	1.68357
30154	1.5235	2.1
30155	0.946127	2.1
30156	0.946127	1.76247
30157	0.946128	2.1

30158	1.26957	2.1
30159	1.00038	2.1
30160	0.946129	2.1
30161	1.17256	2.1
30162	2.2846	1.97882
30163	1.1844	2.1
30164	2.28904	1.41141
30165	3.469 2.1	
30166	0.867886	1.52574
30167	0.867886	2.1
30168	0.867277	2.1
30169	0.867277	2.1
30170	1.14296	1.56821
30171	1.14452	2.02884
30172	0.591766	2.1
30173	0.592199	2.1
30174	0.591766	2.1
30175	1.14296	2.1
30176	0.5922	2.1
30177	1.14452	2.1
30178	1.27408	0.41237
30179	1.56339	0.529391
30180	1.52522	0.555602
30181	1.34447	0.419788
30182	2.90786	0.441621
30183	1.27408	0.451709
30184	1.01121	0.357062
30185	0.991868	0.400249
30186	1.34447	0.48482
30187	2.35569	0.429916
30188	0.944818	0.588439
30189	0.883008	0.565881
30190	0.66957	0.436679
30191	0.616883	0.433143
30192	0.755679	0.445454
30193	0.755679	0.534838
30194	0.791912	0.649872
30195	0.701943	0.399308
30196	0.901541	0.51133
30197	0.634847	0.531514
30198	0.82781	0.508904
30199	0.655405	0.505747
30200	1.73819	2.1
30201	1.70303	2.1
30202	1.70303	2.1
30203	1.70303	2.1
30204	1.94516	2.1
30205	1.94517	2.1
30206	0.94994	2.1
30207	2.07469	2.1
30208	1.08506	2.1
30209	2.0815	2.1
30210	3.12902	2.1
30211	0.946131	2.1
30212	0.946131	2.1
30213	0.946131	2.1
30214	0.946131	2.07493
30215	0.946128	2.08132

30216	0.946128	2.1
30217	0.946133	2.1
30218	0.946133	2.1
30219	0.946131	2.1
30220	0.946131	2.1
30221	0.94613	2.1
30222	0.94613	2.1
30223	2.51796	2.1
30224	1.64193	2.1
30225	2.51172	2.1
30226	1.68893	2.1
30227	4.20689	2.1
30228	2.51172	2.1
30229	1.70002	2.1
30230	1.70002	2.1
30231	1.68893	2.1
30232	2.48689	2.1
30233	2.4758	2.1
30234	2.27963	0.240493
30235	2.21582	0.212485
30236	2.60486	0.209039
30237	2.5105	0.240876
30238	4.79013	0.225198
30239	2.21582	0.212012
30240	2.02069	0.241574
30241	2.5105	0.241528
30242	2.28615	0.205725
30243	4.53119	0.220841
30244	2.60486	0.210062
30245	2.5105	0.249258
30246	2.55791	0.256274
30247	2.47141	0.206891
30248	5.06842	0.225523
30249	2.5105	0.25008
30250	2.28615	0.210149
30251	2.47141	0.209229
30252	2.23337	0.244681
30253	4.74387	0.228189
30254	2.06984	2.1
30255	0.967499	2.1
30256	2.12435	2.1
30257	2.0021	2.1
30258	1.08975	2.1
30259	0.967499	2.1
30260	1.89226	0.381857
30261	1.89226	0.362835
30262	1.89226	0.388657
30263	1.89226	0.383018
30264	1.89226	0.351971
30265	1.89226	0.353022
30266	1.89226	1.9437
30267	1.89226	2.1
30268	1.89226	2.1
30269	1.89226	2.08344
30270	3.78452	2.1
30271	1.89227	2.1
30272	1.89227	2.1
30273	1.89227	2.1

30274	1.89227	2.1
30275	3.78453	2.1
30276	0.55882	2.1
30277	0.696462	2.1
30278	1.07003	2.1
30279	0.937179	2.1
30280	0.818077	2.1
30281	0.818077	2.1
30282	0.815565	2.1
30283	0.815565	2.1
30284	0.696462	2.1
30285	1.07003	2.1
30286	0.93718	2.1
30287	0.55882	2.1
30288	2.53857	2.1
30289	1.87436	2.1
30290	2.27541	2.1
30291	2.27541	2.1
30292	2.14005	2.1
30293	2.14005	2.1
30294	1.89226	2.1
30295	1.89226	2.01464
30296	1.89226	1.47795
30297	1.89226	2.1
30298	3.78452	2.1
30299	1.89227	2.1
30300	1.89227	2.1
30301	1.89227	2.1
30302	1.89227	2.1
30303	3.78453	2.1
30304	1.39292	2.1
30305	2.14006	2.1
30306	2.41083	2.1
30307	1.68761	2.1
30308	3.80376	2.1
30309	2.53857	2.04808
30310	2.14005	2.1
30311	2.41083	2.1
30312	2.82043	2.1
30313	4.9494	2.1
30314	1.90439	0.609242
30315	1.51835	0.597979
30316	1.20473	0.557299
30317	0.947693	0.623486
30318	2.85208	0.616814
30319	2.586	2.1
30320	1.46262	2.1
30321	2.14009	2.1
30322	2.78101	2.1
30323	4.72609	2.1
30324	1.89224	2.1
30325	2.78101	2.1
30326	2.586	2.1
30327	2.586	2.1
30328	1.89224	2.1
30329	1.89224	2.1
30330	0.994293	2.1
30331	1.19	2.1

30332	1.09483	2.1
30333	1.09483	2.1
30334	1.06827	2.1
30335	1.06827	2.1
30336	1.89226	2.1
30337	1.89226	2.1
30338	1.89226	2.1
30339	1.89226	2.1
30340	3.78452	2.1
30341	0.946132	2.1
30342	0.946132	2.1
30343	1.31912	2.1
30344	1.3572	2.1
30345	0.946131	2.1
30346	0.946131	2.1
30347	1.31912	2.1
30348	1.31912	2.1
30349	0.946131	2.1
30350	1.31912	2.1
30351	1.35721	2.1
30352	0.946131	2.1
30353	2.24577	0.78535
30354	1.09408	0.732149
30355	1.60239	0.770825
30356	1.09408	0.731042
30357	2.39404	0.793535
30358	2.07382	0.698962
30359	1.70303	0.529089
30360	1.3216	0.640781
30361	1.23104	0.494955
30362	1.05475	0.601399
30363	2.60408	0.563903
30364	1.89226	0.694111
30365	1.89226	0.711344
30366	1.89226	0.637536
30367	1.89226	0.706487
30368	3.78452	0.706698
30369	1.89226	0.472006
30370	2.3845	0.488992
30371	2.6875	0.489629
30372	1.89226	0.48
30373	2.6875	0.453211
30374	1.89226	0.452399
30375	1.46759	2.1
30376	1.17585	2.1
30377	1.85189	2.1
30378	1.39223	2.1
30379	2.85981	2.1
30380	1.85189	2.1
30381	1.71255	2.1
30382	1.71255	2.1
30383	1.39223	2.1
30384	2.28511	2.1
30385	1.96479	2.1
30386	1.89227	0.883657
30387	1.89227	0.955111
30388	1.89227	1.01537
30389	1.89227	0.860977

30390	1.89227	1.14857
30391	1.89227	0.891838
30392	1.89226	0.918979
30393	1.89226	0.941271
30394	1.89226	1.03968
30395	1.89226	0.867313
30396	1.89226	1.17922
30397	1.89226	0.900294
30398	1.89226	0.879437
30399	1.89226	0.943069
30400	1.89226	0.974366
30401	1.89226	0.844062
30402	1.89226	1.15276
30403	1.89226	0.887143
30404	1.89226	0.846926
30405	2.77788	0.87216
30406	2.21062	0.907212
30407	2.21062	0.824946
30408	1.89226	0.954349
30409	1.89226	0.844091
30410	0.868548	0.867289
30411	0.610104	0.910552
30412	0.796678	0.85366
30413	0.865021	0.833689
30414	1.0883	0.850187
30415	0.904938	0.888943
30416	2.21062	0.872495
30417	1.49548	0.853864
30418	1.2068	0.942461
30419	1.54764	0.851108
30420	0.790005	0.837856
30421	1.89226	1.7706
30422	2.67457	2.1
30423	1.89226	1.91093
30424	2.56352	2.1
30425	4.56683	2.1
30426	2.67456	2.1
30427	2.20011	2.1
30428	2.56351	2.1
30429	2.11083	2.1
30430	4.78539	2.1
30431	2.56348	2.1
30432	2.1108	2.1
30433	2.45763	2.1
30434	2.00986	2.1
30435	4.56843	2.1
30436	0.946133	0.186607
30437	0.946133	0.196178
30438	0.946132	0.180374
30439	0.946132	0.189103
30440	0.946132	0.186603
30441	0.946132	0.188155
30442	0.946132	0.187627
30443	0.946132	0.190198
30444	0.946132	0.185693
30445	0.946132	0.181833
30446	0.946133	0.189888
30447	0.946133	0.194695

30448	1.45927	1.00832
30449	1.25133	1.31433
30450	1.00082	1.15709
30451	1.41689	1.25621
30452	1.0432	1.08073
30453	1.45927	1.22089
30454	1.41689	1.19693
30455	1.29579	1.53468
30456	1.34874	1.35065
30457	1.25133	1.34062
30458	2.76563	1.61941
30459	0.675555	1.42838
30460	0.674371	1.37958
30461	0.625664	1.26104
30462	0.523133	1.28769
30463	0.676509	1.35224
30464	0.676509	1.49946
30465	0.62471	1.37441
30466	0.62471	1.19728
30467	0.675556	1.22453
30468	0.523132	1.15946
30469	0.674373	1.17182
30470	0.625663	1.21282
30471	1.89226	0.842828
30472	1.89226	0.858466
30473	1.89226	0.839237
30474	1.89226	0.872577
30475	3.78453	0.911765
30476	0.851517	1.1739
30477	0.946133	0.909335
30478	0.946132	0.929614
30479	0.946132	1.05449
30480	0.946133	0.86451
30481	0.851517	1.16148
30482	0.946132	0.899372
30483	0.946132	0.880811
30484	0.851516	0.94683
30485	0.851516	0.872077
30486	0.946133	0.989907
30487	0.946133	0.896792
30488	0.569995	0.971782
30489	0.816895	1.08346
30490	1.33893	1.13904
30491	1.18795	1.06161
30492	1.18795	0.996186
30493	0.569995	1.03052
30494	0.816895	0.99302
30495	1.33893	1.21185
30496	0.859572	1.22844
30497	0.859572	1.20201
30498	1.04936	1.02064
30499	1.04936	0.899907
30500	1.89226	2.1
30501	1.89226	2.1
30502	2.55618	2.1
30503	2.55618	2.1
30504	2.66983	2.1
30505	2.00591	2.1

30506	1.89226	0.84302
30507	2.64586	1.8366
30508	2.83331	2.08487
30509	2.83331	0.820786
30510	1.89226	2.09183
30511	1.89226	0.81811
30512	2.64586	2.06395
30513	2.0815	2.1
30514	1.99985	2.1
30515	1.94005	2.1
30516	2.83332	2.1
30517	2.74859	2.1
30518	1.98061	1.41001
30519	1.68356	2.1
30520	1.83879	1.64935
30521	1.55987	2.1
30522	3.54048	2.1
30523	1.68356	2.1
30524	1.20969	2.1
30525	1.20969	2.1
30526	1.30751	2.1
30527	1.55987	2.1
30528	1.65769	2.1
30529	1.83879	1.69063
30530	1.55987	2.1
30531	1.68443	1.38154
30532	1.4449	2.1
30533	3.2443	2.1
30534	0.660368	2.1
30535	0.660368	2.1
30536	0.666922	2.1
30537	0.666922	2.1
30538	0.560377	2.1
30539	0.604842	2.1
30540	0.779935	2.1
30541	0.722448	2.1
30542	0.722446	2.1
30543	0.560377	2.1
30544	0.604844	2.1
30545	0.779934	2.1
30546	1.89227	2.1
30547	1.89227	2.1
30548	1.89227	2.1
30549	1.89227	2.1
30550	3.78453	2.1
30551	1.42896	2.1
30552	1.23037	2.1
30553	1.42896	2.1
30554	1.474	2.1
30555	1.2016	2.1
30556	1.24665	2.1
30557	1.89226	2.1
30558	2.7188	2.1
30559	1.89226	2.1
30560	2.70702	2.1
30561	4.61106	2.1
30562	1.89226	2.1
30563	1.89226	2.1

30564	1.89226	2.1
30565	1.89226	2.1
30566	3.78453	2.1
30567	0.644603	2.1
30568	0.644603	2.1
30569	0.648272	2.1
30570	0.648272	2.1
30571	0.585644	2.1
30572	0.600802	2.1
30573	0.692073	2.1
30574	0.714478	2.1
30575	0.585644	2.1
30576	0.692073	2.1
30577	0.714478	2.1
30578	0.600802	2.1
30579	1.35351	2.1
30580	1.35351	2.1
30581	0.946131	2.1
30582	0.946131	2.1
30583	1.34392	2.1
30584	1.35351	2.1
30585	0.946132	2.1
30586	0.946132	2.1
30587	0.946132	2.1
30588	1.34392	2.1
30589	0.946132	2.1
30590	1.35351	2.1
30591	1.87609	2.1
30592	1.79155	2.1
30593	1.79155	2.1
30594	1.89226	2.1
30595	1.89226	2.1
30596	1.99211	2.1
30597	1.89226	2.1
30598	1.71706	2.1
30599	1.71706	2.1
30600	1.79155	2.1
30601	1.89226	2.1
30602	1.96675	2.1
30603	1.46869	2.1
30604	1.89226	2.1
30605	1.89226	2.1
30606	1.89226	2.1
30607	1.5001	2.1
30608	1.5001	2.0644
30609	1.89226	2.1
30610	1.89226	2.1
30611	1.89226	2.1
30612	1.89226	2.1
30613	1.89226	2.1
30614	1.89226	1.72991
30615	1.21408	2.1
30616	2.23563	2.1
30617	2.34021	2.1
30618	2.34021	2.1
30619	1.17285	2.1
30620	1.17285	2.1
30621	1.89226	2.1

30622	1.89226	2.1
30623	1.47937	2.1
30624	1.89226	2.1
30625	1.14822	2.1
30626	1.46869	1.84853
30627	1.89226	2.03079
30628	1.89226	2.1
30629	1.89226	2.1
30630	1.89226	2.1
30631	1.89226	2.1
30632	1.89226	1.52213
30633	1.25716	2.1
30634	2.34021	2.1
30635	2.4542	2.1
30636	2.4542	2.1
30637	1.21408	2.1
30638	1.21408	2.1
30639	1.66824	2.1
30640	2.44883	1.91823
30641	1.93003	2.1
30642	2.24795	2.1
30643	3.88907	2.1
30644	1.89319	2.1
30645	2.24795	2.1
30646	2.82724	2.1
30647	2.31318	2.1
30648	2.30868	2.1
30649	1.93003	2.09396
30650	1.89226	1.24975
30651	1.42589	2.1
30652	1.42589	1.79083
30653	1.80501	2.1
30654	1.89226	1.71983
30655	2.27137	2.1
30656	1.21067	0.673545
30657	2.39166	0.70151
30658	1.73577	0.699001
30659	1.73577	0.699709
30660	1.53584	0.695937
30661	1.53584	0.693718
30662	1.35025	1.2345
30663	2.0599	1.0879
30664	1.51084	0.952782
30665	2.27218	0.799089
30666	3.57074	1.02395
30667	2.0599	0.976365
30668	2.51818	1.10625
30669	2.27218	0.704194
30670	2.66532	0.98676
30671	4.72522	0.886316
30672	1.67115	1.02157
30673	2.27218	1.00888
30674	2.39605	0.926504
30675	2.39605	0.957375
30676	1.51084	1.02767
30677	1.51084	1.16674
30678	2.27218	0.902441
30679	2.66532	1.29708

30680	2.39605	0.798482
30681	2.76829	0.931796
30682	5.06137	0.938619
30683	2.08684	0.956913
30684	2.36033	1.14469
30685	1.89224	0.898102
30686	1.89224	1.12962
30687	4.25257	0.938084
30688	2.41473	0.46343
30689	1.00307	0.498952
30690	2.01365	0.477352
30691	1.00307	0.547852
30692	3.05113	0.436494
30693	2.04056	0.544044
30694	1.11099	2.1
30695	2.25103	2.1
30696	2.22227	2.1
30697	2.22227	2.1
30698	1.14431	2.1
30699	1.14431	2.1
30700	1.07485	2.1
30701	2.22229	2.1
30702	2.19163	2.1
30703	2.19163	2.1
30704	1.111	2.1
30705	1.111	2.1
30706	1.0547	1.80534
30707	1.19886	2.1
30708	0.946131	2.1
30709	0.946131	2.1
30710	0.946131	2.1
30711	0.946131	1.84277
30712	1.19886	2.1
30713	1.19886	2.1
30714	1.0547	2.1
30715	0.946131	2.1
30716	0.946132	2.1
30717	1.19886	2.1
30718	1.89417	1.87167
30719	2.64424	0.684878
30720	1.76585	1.47386
30721	2.47695	0.93511
30722	4.37112	1.0549
30723	1.89226	1.95627
30724	1.89226	1.01718
30725	1.89226	2.1
30726	1.89226	0.945635
30727	3.78453	1.17151
30728	0.930281	1.53876
30729	1.0547	2.1
30730	0.946131	2.1
30731	0.946131	2.1
30732	0.930309	2.0811
30733	0.930309	1.59211
30734	1.07052	2.1
30735	1.07052	2.1
30736	0.93028	2.1
30737	0.946131	2.1

30738	1.0547	2.1
30739	0.946131	2.1
30740	1.00015	0.60194
30741	1.00015	1.13517
30742	1.01918	1.41664
30743	1.01918	0.90858
30744	1.17437	0.915584
30745	1.23847	0.580847
30746	0.882926	1.4323
30747	0.780859	1.13385
30748	0.780859	0.933714
30749	1.17438	0.958283
30750	0.882925	0.988976
30751	1.23848	0.989013
30752	1.89226	2.06526
30753	1.89226	0.921364
30754	1.89226	1.03348
30755	1.89226	1.6717
30756	1.89226	1.01262
30757	1.89226	1.8617
30758	1.22083	2.1
30759	1.29579	2.1
30760	1.26236	2.1
30761	1.06191	2.1
30762	1.42128	2.1
30763	1.22083	2.1
30764	1.42128	2.1
30765	1.45806	2.1
30766	1.43502	2.1
30767	1.29579	2.1
30768	1.5605	2.1
30769	1.42128	2.1
30770	1.02813	2.1
30771	1.12121	2.1
30772	1.40557	2.1
30773	1.35298	2.1
30774	1.1314	2.1
30775	1.28679	2.1
30776	1.1715	2.1
30777	1.1715	2.1
30778	0.975656	2.1
30779	1.24908	2.1
30780	0.98956	2.1
30781	1.2681	2.1
30782	0.946132	2.1
30783	0.946132	2.1
30784	1.31889	2.1
30785	1.31889	2.1
30786	1.16546	2.1
30787	1.31889	2.1
30788	0.946133	2.1
30789	0.946133	2.1
30790	0.946133	2.1
30791	1.31889	2.1
30792	0.946132	2.1
30793	1.16546	2.1
30794	2.51518	0.246158
30795	2.37584	0.281148

30796	1.11712	0.247317
30797	1.31242	0.297837
30798	3.68826	0.270582
30799	2.51518	0.25742
30800	1.26311	0.301776
30801	2.48977	0.260636
30802	2.48977	0.289385
30803	1.31242	0.252989
30804	1.31242	0.294089
30805	1.89226	0.738382
30806	1.89226	0.846986
30807	1.89226	0.515241
30808	1.89226	0.844234
30809	3.78452	0.782513
30810	2.04737	0.505389
30811	1.77122	0.84946
30812	1.94498	0.468875
30813	1.65174	0.852878
30814	3.71621	0.772265
30815	1.41077	2.02882
30816	1.29729	1.87792
30817	1.77123	0.862374
30818	1.77123	1.6088
30819	1.65175	0.852999
30820	1.29129	0.865227
30821	1.89226	0.847498
30822	1.89226	2.0361
30823	1.89226	2.1
30824	1.89226	0.844834
30825	1.89226	2.05067
30826	1.89226	0.924714
30827	1.89226	0.572893
30828	1.89226	0.843457
30829	1.89226	0.447276
30830	1.89226	0.830876
30831	3.78451	0.73073
30832	1.94498	0.51899
30833	1.65174	0.840529
30834	1.7968	0.498242
30835	1.49831	0.829301
30836	3.44329	0.785339
30837	1.49831	0.832883
30838	1.29729	1.8499
30839	1.49831	2.04395
30840	1.65175	0.838725
30841	1.16858	1.83
30842	1.32201	0.894538
30843	1.89226	0.836719
30844	1.89226	1.88929
30845	1.89226	0.833017
30846	1.89226	1.98179
30847	3.78452	0.961189
30848	1.89226	0.256096
30849	1.89226	0.27553
30850	1.89226	0.282477
30851	1.89226	0.26277
30852	1.89226	0.274548
30853	1.89226	0.254558

30854	1.65324	0.881966
30855	1.26409	0.437912
30856	1.42491	0.453306
30857	1.42491	0.893234
30858	1.52268	0.537109
30859	1.52268	0.900315
30860	1.52268	0.998596
30861	1.26409	0.739554
30862	1.32697	0.885135
30863	1.05724	0.445526
30864	2.57991	0.815435
30865	1.89226	0.402187
30866	1.96548	0.440669
30867	2.0178	0.47084
30868	1.96548	0.439987
30869	1.94458	0.406206
30870	1.89226	0.380984
30871	1.99366	0.401712
30872	2.0178	0.45525
30873	1.89226	0.473357
30874	2.0178	0.450034
30875	1.75999	0.40964
30876	1.89226	0.39263
30877	0.946126	2.06666
30878	0.946126	2.1
30879	1.28228	2.1
30880	1.19444	2.1
30881	0.946127	2.1
30882	0.946127	2.0706
30883	1.19444	2.1
30884	1.19444	2.1
30885	0.946128	2.1
30886	1.19444	2.1
30887	1.28228	2.1
30888	0.946128	2.1
30889	1.89226	2.1
30890	1.89226	2.1
30891	1.89226	2.1
30892	1.89226	1.72995
30893	3.78452	2.1
30894	2.14104	2.1
30895	1.89226	2.1
30896	2.19948	2.1
30897	1.89226	2.1
30898	1.89226	2.1
30899	2.19948	2.1
30900	1.89226	2.1
30901	2.38888	2.1
30902	2.23932	2.1
30903	2.38888	2.1
30904	1.74269	2.1
30905	1.89226	2.1
30906	1.89226	2.1
30907	1.89226	1.64123
30908	1.89226	2.1
30909	1.89226	2.1
30910	3.78452	2.1
30911	0.946132	2.1

30912	0.946132	2.1
30913	1.07052	2.1
30914	1.07052	2.1
30915	0.946132	2.1
30916	0.946132	2.08995
30917	1.05132	2.1
30918	1.07052	2.1
30919	1.05132	2.1
30920	0.946132	2.1
30921	1.07052	2.1
30922	0.946133	2.1
30923	2.40171	2.1
30924	1.89226	2.1
30925	1.89226	2.1
30926	1.89226	2.1
30927	2.42723	2.1
30928	2.42723	2.1
30929	1.89226	2.1
30930	1.89226	1.27897
30931	1.89226	2.1
30932	1.89226	1.4391
30933	3.78452	1.84745
30934	1.63661	2.1
30935	2.3532	1.18715
30936	2.33287	1.1262
30937	2.33287	2.1
30938	1.64158	1.07114
30939	1.64158	2.1
30940	2.38918	2.1
30941	1.89226	2.1
30942	1.89226	2.1
30943	1.89226	2.1
30944	2.40171	2.1
30945	2.40171	2.1
30946	1.89226	2.1
30947	1.89226	1.69724
30948	1.89226	2.1
30949	1.89226	1.58124
30950	1.89226	2.1
30951	1.89226	1.80703
30952	1.64158	2.1
30953	2.3532	1.43502
30954	1.6488	2.1
30955	2.37364	1.61714
30956	4.002 2.0966	
30957	2.54752	0.744017
30958	1.42445	0.774562
30959	1.86697	0.781908
30960	1.86697	0.745708
30961	2.06666	0.781544
30962	2.06666	0.752779
30963	2.50872	0.649382
30964	2.06666	0.702043
30965	1.90439	0.682446
30966	1.51835	0.670061
30967	3.97105	0.656368
30968	0.755161	0.699263
30969	0.755161	0.691614

30970	0.793994	0.670018
30971	0.793994	0.689305
30972	0.712225	0.684023
30973	0.515826	0.683864
30974	1.03333	0.700921
30975	0.759175	0.681174
30976	0.759173	0.666918
30977	0.515827	0.616176
30978	0.712226	0.653273
30979	1.03333	0.674563
30980	1.69633	0.610907
30981	1.89226	0.604957
30982	1.62477	0.558902
30983	1.89226	0.58048
30984	1.62477	0.610682
30985	1.89226	0.62464
30986	1.89226	0.754837
30987	1.89226	0.813545
30988	1.89226	0.809655
30989	1.89226	0.79145
30990	3.78452	0.795003
30991	2.28622	0.629813
30992	1.09667	0.545219
30993	1.17506	0.531647
30994	2.24411	0.570787
30995	1.17506	0.636444
30996	2.24411	0.685905
30997	2.24741	2.1
30998	1.70303	2.1
30999	2.49168	2.1
31000	1.57936	2.1
31001	2.53729	2.1
31002	1.70303	2.1
31003	2.24741	2.1
31004	2.47557	2.05173
31005	1.89227	2.09333
31006	1.89227	2.1
31007	4.36784	2.1
31008	2.27016	0.308389
31009	1.10476	0.388241
31010	1.44103	0.324413
31011	1.10476	0.435724
31012	2.26791	0.32412
31013	1.93165	0.411612
31014	2.27016	0.30617
31015	1.89226	0.438161
31016	1.89226	0.347634
31017	2.4065	0.390403
31018	4.29876	0.384176
31019	2.27016	0.298023
31020	1.44103	0.370944
31021	1.53336	0.30591
31022	2.4065	0.379676
31023	3.84753	0.3251
31024	2.7542	0.669669
31025	1.89226	0.71091
31026	2.35027	0.702875
31027	1.89226	0.70314

31028	2.35027	0.677445
31029	1.89226	0.67944
31030	2.3301	2.1
31031	2.36071	1.4785
31032	2.07629	2.1
31033	2.01105	2.1
31034	4.37176	2.1
31035	1.86512	2.1
31036	2.01105	2.1
31037	2.01105	2.1
31038	2.3301	2.1
31039	1.57459	2.1
31040	1.89364	2.1
31041	2.52671	2.1
31042	2.01105	2.1
31043	1.78251	2.1
31044	1.70302	1.39822
31045	3.98362	2.1
31046	1.227 2.1	
31047	2.01105	2.1
31048	1.45869	2.1
31049	1.41713	2.1
31050	1.70302	2.1
31051	1.64994	2.1
31052	2.00989	2.1
31053	1.28938	2.1
31054	1.8769	2.1
31055	1.20969	2.1
31056	3.21958	2.1
31057	1.8769	2.1
31058	1.15864	2.1
31059	1.15864	2.1
31060	1.20969	2.1
31061	1.77353	2.1
31062	1.82458	2.1
31063	1.7986	2.1
31064	2.48717	2.1
31065	2.50161	2.1
31066	1.75869	2.1
31067	4.30021	2.1
31068	2.50161	2.1
31069	1.75251	2.1
31070	1.81678	2.1
31071	1.75869	2.1
31072	2.5597	2.1
31073	2.50161	2.1
31074	1.91334	0.8154
31075	1.85439	0.885418
31076	1.91334	0.843933
31077	1.70303	0.86438
31078	1.70303	0.782911
31079	1.49692	0.774155
31080	2.39986	0.631176
31081	1.89226	0.683961
31082	1.89226	0.69007
31083	2.50756	0.627013
31084	4.39982	0.652823
31085	2.39986	0.661123

31086	1.70303	0.516096
31087	1.75564	0.50444
31088	1.75904	0.624045
31089	2.50272	0.582527
31090	2.50756	0.649216
31091	1.89226	0.68708
31092	2.50756	0.623539
31093	2.47857	0.659961
31094	2.50756	0.684982
31095	1.86327	0.649489
31096	1.89226	0.676239
31097	1.22241	0.666451
31098	1.20827	0.680805
31099	0.772416	0.515471
31100	0.555208	0.58194
31101	1.004	0.518522
31102	1.004	0.643855
31103	0.93504	0.70139
31104	0.744364	0.52692
31105	1.2089	0.645243
31106	0.654935	0.651331
31107	0.773927	0.629296
31108	1.03618	0.652417
31109	1.89227	0.188402
31110	1.89227	0.195203
31111	1.89227	0.173243
31112	1.89227	0.177233
31113	3.78453	0.181461
31114	1.89226	0.180317
31115	1.89226	0.19187
31116	1.89226	0.170493
31117	1.89226	0.174982
31118	3.78452	0.179565
31119	1.82643	0.166575
31120	2.7084	0.168407
31121	2.12654	0.164578
31122	2.65614	0.170096
31123	1.82643	0.168494
31124	2.35603	0.172257
31125	1.29385	0.163499
31126	0.946133	0.167769
31127	1.05492	0.16437
31128	0.946132	0.167885
31129	1.05492	0.166933
31130	1.29385	0.163609
31131	0.946133	0.164831
31132	0.946133	0.16901
31133	1.29385	0.161246
31134	1.29385	0.160554
31135	0.946132	0.171819
31136	0.946132	0.169019
31137	0.946132	0.16343
31138	1.27329	0.161012
31139	1.29385	0.162311
31140	0.946129	0.16705
31141	1.29385	0.163548
31142	1.27328	0.161176
31143	0.946131	0.162356

31144	0.946131	0.16396
31145	1.27328	0.15995
31146	1.27328	0.160121
31147	0.94613	0.16862
31148	0.94613	0.165335
31149	1.89226	1.31589
31150	1.89226	1.35648
31151	1.89226	0.871485
31152	1.89226	1.04851
31153	3.78453	1.16748
31154	1.89226	1.22265
31155	1.89226	1.35301
31156	1.89226	0.851152
31157	1.89226	1.18493
31158	3.78453	1.00569
31159	1.21488	1.34043
31160	1.14605	1.63911
31161	1.62974	1.15676
31162	1.52399	1.04581
31163	2.84463	1.36104
31164	0.577078	1.09897
31165	0.81251	1.5122
31166	0.821003	1.21229
31167	0.543413	1.39492
31168	0.683929	1.12722
31169	0.722433	1.69386
31170	0.730674	1.39651
31171	0.730674	1.02726
31172	0.857766	1.19102
31173	0.862119	1.08879
31174	0.600938	1.37742
31175	0.596094	1.25469
31176	1.89226	0.971817
31177	1.89226	1.23161
31178	1.89226	0.81776
31179	1.89226	1.05261
31180	3.78453	0.969434
31181	1.89226	0.926667
31182	1.89226	1.40104
31183	1.89226	0.898761
31184	1.89226	0.994623
31185	3.78453	1.1209
31186	1.62974	1.37825
31187	1.56958	1.45772
31188	2.2362	0.932016
31189	2.11424	1.11503
31190	3.86595	1.17791
31191	2.2362	0.991753
31192	1.85414	1.48622
31193	2.28211	0.827207
31194	1.78367	0.890556
31195	2.2362	1.29865
31196	1.73256	1.42842
31197	0.851539	1.02877
31198	0.851539	0.791683
31199	1.14454	0.95517
31200	0.938988	1.24085
31201	0.851485	1.05512

31202	0.920593	0.827845
31203	0.851541	0.69801
31204	0.851541	0.882704
31205	1.04073	0.780956
31206	1.16342	0.809833
31207	0.825315	0.906946
31208	0.825316	0.811703
31209	1.89226	1.77262
31210	1.89226	2.1
31211	1.89226	2.1
31212	1.89226	1.89079
31213	3.78453	1.99664
31214	2.57016	2.1
31215	1.74789	2.1
31216	2.57016	2.1
31217	2.522	2.1
31218	1.61727	2.1
31219	1.56911	2.1
31220	1.28075	2.1
31221	1.406	2.1
31222	0.946131	1.94691
31223	0.946131	1.87148
31224	1.28075	2.00429
31225	1.00333	2.1
31226	0.94613	2.1
31227	0.94613	1.98876
31228	1.041	2.05665
31229	1.041	2.04135
31230	0.943484	1.96098
31231	1.27717	2.0428
31232	1.89226	2.1
31233	1.89226	1.78288
31234	1.89226	1.75816
31235	1.89226	2.1
31236	1.89226	1.79954
31237	1.89226	2.1
31238	2.57016	2.1
31239	1.61727	2.1
31240	2.74196	2.1
31241	1.59094	2.1
31242	4.16109	2.1
31243	1.89226	1.80306
31244	2.0815	2.1
31245	2.2034	2.1
31246	1.82493	1.74664
31247	1.87847	2.1
31248	2.16142	2.1
31249	1.89226	2.1
31250	1.89226	2.1
31251	1.89226	2.1
31252	1.89226	2.1
31253	3.78453	2.1
31254	0.985252	2.1
31255	0.985252	2.1
31256	0.946132	2.1
31257	0.946132	2.1
31258	1.0655	2.1
31259	0.985255	2.1

31260	0.94613	2.1
31261	0.94613	2.1
31262	0.94613	2.1
31263	1.0655	2.1
31264	0.94613	2.1
31265	0.985255	2.1
31266	1.89226	2.1
31267	1.89226	2.1
31268	1.89226	2.1
31269	1.89226	2.1
31270	3.78453	2.1
31271	1.06549	2.1
31272	1.06549	2.1
31273	0.946133	2.1
31274	0.946133	2.1
31275	1.16834	2.1
31276	1.0655	2.1
31277	0.946131	2.1
31278	0.946131	2.1
31279	0.946131	2.1
31280	1.16834	2.1
31281	0.94613	2.1
31282	1.0655	2.1
31283	0.966631	2.1
31284	1.07349	2.1
31285	0.946131	2.1
31286	0.946131	2.1
31287	1.07349	2.1
31288	1.07349	2.1
31289	0.946129	2.1
31290	0.946129	2.1
31291	1.07349	2.1
31292	0.946131	2.1
31293	0.94613	2.1
31294	0.966631	2.1
31295	2.38055	1.10613
31296	1.89226	1.55375
31297	1.89226	1.34312
31298	1.46724	0.835241
31299	3.35949	1.16066
31300	1.42249	2.1
31301	2.05499	2.1
31302	1.39708	2.1
31303	2.09033	2.1
31304	3.45207	2.1
31305	2.05499	2.1
31306	2.57425	2.1
31307	2.51287	2.1
31308	2.47753	2.01679
31309	2.09033	2.1
31310	2.05499	1.81773
31311	1.39327	2.1
31312	2.09033	2.1
31313	2.16217	2.1
31314	2.16217	2.1
31315	1.39708	2.1
31316	1.39708	2.1
31317	2.16217	2.1

31318	2.57426	2.1
31319	2.73006	2.1
31320	2.73006	2.1
31321	2.09033	2.1
31322	2.09033	2.1
31323	1.89226	0.358267
31324	1.89226	0.248461
31325	1.89226	0.280729
31326	1.89226	0.328827
31327	3.78452	0.339694
31328	0.838749	0.207629
31329	0.838749	0.212041
31330	0.85824	0.242545
31331	0.85824	0.263581
31332	0.544644	0.24904
31333	0.854718	0.225429
31334	0.842271	0.22989
31335	1.19141	0.25918
31336	0.842272	0.231194
31337	0.544643	0.205614
31338	0.854718	0.234234
31339	1.19141	0.217972
31340	1.34214	0.237024
31341	1.34214	0.219032
31342	0.893236	0.307978
31343	0.893236	0.278065
31344	0.893265	0.284103
31345	1.28924	0.24293
31346	0.946133	0.242998
31347	0.946133	0.290213
31348	0.946132	0.278867
31349	0.893265	0.295191
31350	0.946133	0.280968
31351	1.28924	0.26852
31352	2.7013	0.266835
31353	1.89226	0.314858
31354	1.9216	0.337746
31355	1.9216	0.244705
31356	1.89226	0.336507
31357	1.89226	0.244057
31358	1.26397	2.1
31359	1.52995	2.1
31360	1.30116	2.1
31361	1.04084	2.1
31362	2.57079	2.1
31363	1.47013	2.1
31364	2.17459	2.1
31365	2.10668	2.1
31366	1.38328	2.1
31367	3.55787	2.1
31368	2.55006	2.1
31369	2.17459	2.1
31370	2.10668	2.1
31371	2.49168	2.1
31372	4.66626	2.1
31373	0.613622	2.1
31374	0.691639	2.1
31375	1.00105	2.1

31376	1.05334	2.1
31377	0.839874	2.1
31378	0.839874	2.1
31379	0.852814	2.1
31380	0.852814	2.1
31381	0.613622	2.1
31382	1.00105	2.1
31383	1.05334	2.1
31384	0.691639	2.1
31385	1.00105	2.1
31386	1.05334	2.1
31387	1.20844	2.1
31388	1.24584	2.1
31389	1.12717	2.1
31390	1.12717	2.1
31391	1.13462	2.1
31392	1.13462	2.1
31393	1.20845	2.1
31394	1.00105	2.1
31395	1.05334	2.1
31396	1.24584	2.1
31397	2.22558	1.72628
31398	1.85981	1.61269
31399	2.18943	1.57156
31400	1.89142	0.870643
31401	4.0854	1.38033
31402	2.02641	2.1
31403	1.85981	1.40545
31404	2.18943	1.53417
31405	1.67346	1.4774
31406	3.88622	1.35177
31407	1.85981	0.792928
31408	1.10736	0.737723
31409	1.27421	0.832241
31410	1.67346	0.840941
31411	1.27421	0.796896
31412	1.67346	1.15548
31413	1.85981	0.929279
31414	1.27362	0.901527
31415	1.89142	1.06384
31416	1.27421	0.87863
31417	3.13343	0.821713
31418	1.81893	0.326802
31419	1.64985	0.384464
31420	1.23477	0.269572
31421	2.25405	0.424008
31422	3.48882	0.40759
31423	2.23933	0.258299
31424	1.89226	0.347196
31425	1.89226	0.348926
31426	2.78053	0.335165
31427	1.89226	0.282009
31428	2.78053	0.264877
31429	0.946131	0.374683
31430	0.946131	0.360887
31431	0.946132	0.438499
31432	0.946132	0.511654
31433	0.946131	0.518781

31434	0.946131	0.480525
31435	0.946132	0.362636
31436	0.946132	0.485653
31437	0.946131	0.412211
31438	0.946131	0.479183
31439	0.946132	0.350387
31440	0.946132	0.468976
31441	1.89226	2.1
31442	2.48596	2.1
31443	1.89226	2.1
31444	2.51506	2.1
31445	4.40732	2.1
31446	2.48596	2.1
31447	1.63823	2.1
31448	2.51507	2.1
31449	1.67687	2.1
31450	4.1533	2.1
31451	1.89226	2.1
31452	2.51506	2.1
31453	1.92044	2.1
31454	1.89226	2.1
31455	2.54325	2.1
31456	2.51506	2.1
31457	2.51507	2.1
31458	1.5704	2.1
31459	1.70505	2.1
31460	1.67687	2.1
31461	2.54325	2.1
31462	2.51507	2.1
31463	2.0815	2.1
31464	1.70303	2.1
31465	1.70303	2.1
31466	1.80354	2.1
31467	1.98172	2.1
31468	2.09868	2.1
31469	1.27944	2.1
31470	1.79028	2.1
31471	1.13179	2.1
31472	1.65533	2.1
31473	2.93477	2.1
31474	1.89226	2.1
31475	1.89226	2.1
31476	1.89226	2.1
31477	1.89226	2.1
31478	1.89226	2.1
31479	1.89226	2.1
31480	1.98171	2.1
31481	1.89226	2.1
31482	1.89226	2.1
31483	1.89226	2.1
31484	1.65798	2.1
31485	1.65797	2.1
31486	1.00133	2.1
31487	1.65532	2.1
31488	1.53278	2.1
31489	1.53278	2.1
31490	1.13179	2.1
31491	1.13179	2.1

31492	0.810946	2.1
31493	0.810946	2.1
31494	0.810944	2.1
31495	0.810944	2.1
31496	0.810946	2.1
31497	0.810946	2.1
31498	1.89226	2.1
31499	1.35169	2.1
31500	1.08132	2.1
31501	1.08132	2.1
31502	1.08132	2.1
31503	1.99528	0.472869
31504	1.70303	0.531785
31505	2.08152	0.452292
31506	1.70303	0.591255
31507	2.33877	0.442126
31508	1.93967	0.548825
31509	1.89226	0.524087
31510	1.89226	0.651275
31511	1.89226	0.553051
31512	1.89226	0.624382
31513	3.78452	0.635642
31514	1.26259	0.503207
31515	2.17205	0.605785
31516	2.32468	0.563209
31517	2.32468	0.528542
31518	1.28055	0.565036
31519	1.28055	0.532632
31520	0.946131	0.536847
31521	0.946131	0.449176
31522	0.997642	0.494902
31523	0.997642	0.392346
31524	0.946131	0.506932
31525	0.946131	0.556223
31526	0.989846	0.450368
31527	0.997642	0.392307
31528	0.946132	0.453027
31529	0.989846	0.445071
31530	0.997641	0.440175
31531	0.946131	0.46021
31532	1.89226	0.555454
31533	1.89226	0.609709
31534	1.89226	0.506324
31535	1.89226	0.646142
31536	3.78452	0.534487
31537	1.28055	0.453497
31538	2.17205	0.5899
31539	1.14305	0.489107
31540	1.92737	0.567131
31541	3.3151	0.551428
31542	0.954908	0.222293
31543	0.9705	0.207895
31544	0.946131	0.218586
31545	0.946131	0.241458
31546	0.946131	0.238915
31547	0.946131	0.2232
31548	0.9705	0.217778
31549	0.9705	0.203925

31550	0.954908	0.21657
31551	0.946131	0.217564
31552	0.946131	0.221771
31553	0.9705	0.222041
31554	0.835187	0.222584
31555	0.954896	0.205454
31556	0.946118	0.218123
31557	0.946118	0.240526
31558	0.835186	0.237855
31559	0.835186	0.221664
31560	1.06583	0.213319
31561	1.06583	0.198995
31562	0.835198	0.223622
31563	0.946131	0.221975
31564	0.954883	0.225421
31565	0.946106	0.224487
31566	1.89227	2.02979
31567	1.89227	2.1
31568	1.89227	2.1
31569	1.89227	2.1
31570	1.89227	2.1
31571	1.89227	1.88456
31572	0.885265	2.1
31573	0.885265	2.1
31574	0.871467	2.1
31575	0.871467	2.1
31576	1.11883	2.1
31577	0.787875	2.1
31578	0.996487	2.1
31579	0.637907	2.1
31580	0.996487	2.1
31581	1.11883	2.1
31582	0.787875	2.1
31583	0.637906	2.1
31584	1.89226	2.1
31585	2.39258	2.1
31586	2.39258	2.1
31587	2.54729	2.1
31588	1.89226	2.1
31589	2.04697	2.1
31590	1.89226	2.1
31591	2.0815	2.1
31592	2.0815	2.1
31593	2.48371	2.1
31594	2.0815	2.1
31595	2.51469	2.1
31596	1.00035	0.147815
31597	1.55755	0.295771
31598	1.03534	0.373346
31599	1.61333	0.373342
31600	2.59289	0.254534
31601	1.55755	0.295771
31602	1.85217	1.01887
31603	1.61333	0.503782
31604	1.92875	0.943846
31605	3.48629	0.728043
31606	1.09962	0.673565
31607	1.61334	0.525617

31608	1.70868	0.767843
31609	1.70868	0.63526
31610	1.03535	0.800321
31611	1.03535	0.581384
31612	1.61334	0.429053
31613	1.92876	0.946239
31614	1.70868	0.455869
31615	2.05274	1.05897
31616	3.63744	0.648516
31617	1.82233	0.793823
31618	2.05768	0.972271
31619	1.89226	1.0248
31620	1.65406	0.832276
31621	1.89226	0.964207
31622	1.65406	0.827853
31623	2.26047	0.816573
31624	2.0815	1.06353
31625	1.89226	1.04042
31626	2.01156	0.845259
31627	1.89226	0.977951
31628	2.01156	0.832898
31629	1.89226	1.94384
31630	1.89226	2.1
31631	1.89226	2.1
31632	1.89226	1.72828
31633	3.78453	1.83116
31634	2.70055	2.1
31635	2.28367	2.1
31636	2.56456	2.1
31637	2.33565	2.1
31638	4.84823	2.1
31639	2.28367	2.1
31640	1.5791	2.1
31641	1.96966	2.1
31642	1.09506	2.1
31643	3.37873	2.1
31644	1.89226	2.1
31645	1.89226	1.66955
31646	1.89226	1.70942
31647	1.89226	2.1
31648	3.78452	2.1
31649	0.94612	2.1
31650	0.94612	2.1
31651	0.946119	1.85885
31652	0.946119	1.73111
31653	0.946119	1.70059
31654	0.946119	2.1
31655	0.946119	2.1
31656	0.946119	1.82677
31657	0.946132	2.1
31658	0.946132	2.1
31659	0.946107	2.1
31660	0.946107	2.1
31661	2.56452	2.1
31662	1.57307	2.1
31663	2.10299	2.1
31664	2.1115	2.1
31665	2.52196	2.1

31666	2.52196	2.1
31667	1.89226	2.1
31668	1.89226	1.25807
31669	1.89226	2.1
31670	1.89226	2.1
31671	3.78452	2.1
31672	2.37665	2.1
31673	1.89226	2.1
31674	2.28084	2.1
31675	1.89226	2.1
31676	4.26891	2.1
31677	0.946129	1.27387
31678	0.946129	2.1
31679	0.946133	2.1
31680	0.946133	2.1
31681	0.946131	2.1
31682	0.946131	1.24116
31683	0.946131	2.1
31684	0.946131	2.1
31685	0.946131	2.1
31686	0.946131	2.1
31687	0.946131	2.1
31688	0.946131	2.1
31689	1.7207	0.222966
31690	1.65815	0.246916
31691	1.33593	0.255944
31692	1.31508	0.208356
31693	2.99408	0.223058
31694	0.676047	0.201317
31695	0.676047	0.211668
31696	0.684362	0.248453
31697	0.684362	0.226944
31698	0.547994	0.227187
31699	0.702871	0.210457
31700	0.829076	0.249918
31701	0.657538	0.22163
31702	0.657538	0.222579
31703	0.547994	0.211868
31704	0.829077	0.226531
31705	0.702871	0.222147
31706	1.9518	2.1
31707	1.30648	2.1
31708	1.28846	2.1
31709	1.92033	2.1
31710	3.22681	2.1
31711	1.89226	2.1
31712	1.89226	2.1
31713	1.89226	2.1
31714	1.89226	2.1
31715	3.78453	2.1
31716	0.946128	2.1
31717	0.946128	2.1
31718	1.10113	2.1
31719	1.09587	2.1
31720	0.946131	2.1
31721	0.946131	2.1
31722	1.09587	2.1
31723	1.09587	2.1

31724	0.946131	2.1
31725	1.09587	2.1
31726	1.10112	2.1
31727	0.94613	2.1
31728	1.26923	2.1
31729	1.92033	2.1
31730	1.28846	2.1
31731	1.28846	2.1
31732	1.89452	2.1
31733	1.89452	2.1
31734	1.89226	2.1
31735	1.89226	2.1
31736	1.89226	2.1
31737	1.89226	2.1
31738	3.78453	2.1
31739	1.89226	2.1
31740	2.19174	2.1
31741	2.15616	2.1
31742	2.19174	2.1
31743	1.85669	2.1
31744	1.89226	2.1
31745	1.89226	2.1
31746	1.89226	2.1
31747	1.89226	2.1
31748	1.89226	2.1
31749	3.78452	2.1
31750	1.89226	2.1
31751	1.89226	2.1
31752	1.89226	2.1
31753	1.89226	2.1
31754	3.78452	2.1
31755	1.51223	2.1
31756	1.41562	2.1
31757	1.5139	2.1
31758	1.42284	2.1
31759	1.6033	2.1
31760	1.51223	2.1
31761	1.51223	2.1
31762	1.25953	2.1
31763	1.38085	2.1
31764	1.6033	2.1
31765	2.89309	2.1
31766	0.946131	2.1
31767	0.946131	2.1
31768	0.946131	2.1
31769	0.946131	2.1
31770	0.946132	2.1
31771	0.946132	2.1
31772	0.946129	2.1
31773	0.946129	2.1
31774	0.946131	2.1
31775	0.946131	2.1
31776	0.94613	2.1
31777	0.94613	2.1
31778	1.89226	2.1
31779	1.89226	2.1
31780	1.89226	2.1
31781	1.89226	2.1

31782	3.78452	2.1
31783	0.707806	2.1
31784	0.697816	2.1
31785	0.823593	2.1
31786	0.801649	2.1
31787	0.74182	2.1
31788	0.74182	2.1
31789	0.757645	2.1
31790	0.757645	2.1
31791	0.823592	2.1
31792	0.697818	2.1
31793	0.707807	2.1
31794	0.801647	2.1
31795	1.52534	2.1
31796	1.6033	2.1
31797	1.64719	2.1
31798	1.38085	2.1
31799	1.64719	2.1
31800	1.38085	2.1
31801	2.67041	2.1
31802	1.89226	1.00136
31803	1.89226	1.80666
31804	1.89226	1.43738
31805	2.487	1.70743
31806	2.487	1.65794
31807	2.70097	2.1
31808	1.70303	2.1
31809	1.71163	2.1
31810	2.487	1.87973
31811	4.46723	2.1
31812	1.14984	1.76892
31813	1.2435	2.1
31814	0.778	2.1
31815	0.697616	2.1
31816	0.967211	2.1
31817	0.967211	1.54055
31818	0.973907	2.1
31819	0.973907	2.1
31820	0.697616	2.1
31821	1.14984	2.1
31822	0.778	2.1
31823	1.2435	2.1
31824	2.4158	2.1
31825	1.26438	2.1
31826	1.676	2.1
31827	2.77505	2.1
31828	4.0918	2.1
31829	2.58258	2.1
31830	1.89226	2.1
31831	1.89226	2.1
31832	1.89226	2.1
31833	2.47032	2.1
31834	2.47032	2.1
31835	1.23516	2.1
31836	1.18734	2.1
31837	0.946131	2.1
31838	0.946131	2.1
31839	1.18734	2.1

31840	1.18734	2.1
31841	0.94613	2.1
31842	0.94613	2.1
31843	1.18734	2.1
31844	0.946131	2.1
31845	0.946131	2.1
31846	1.23516	2.1
31847	2.8239	2.1
31848	1.39361	2.1
31849	1.12342	2.1
31850	2.57275	2.1
31851	3.96637	2.1
31852	0.937929	0.990574
31853	0.937929	0.939332
31854	1.39734	0.861327
31855	1.39734	0.861208
31856	1.38914	0.912546
31857	0.94613	0.952631
31858	0.937902	0.958795
31859	0.94613	0.696073
31860	0.94613	0.872424
31861	0.94613	0.898129
31862	1.38914	0.918928
31863	0.937902	0.891471
31864	1.89226	0.777127
31865	2.17765	0.983235
31866	1.84577	0.934239
31867	1.84577	0.827797
31868	1.89226	0.88041
31869	1.89226	0.790397
31870	2.11262	1.01326
31871	1.57766	1.14557
31872	2.22689	1.97473
31873	1.91132	0.749141
31874	2.19624	1.6148
31875	1.79689	0.616426
31876	1.99656	0.670298
31877	1.57766	1.02615
31878	1.39067	1.7619
31879	1.73802	0.862782
31880	3.31568	1.02405
31881	1.89226	0.705107
31882	1.89226	0.651289
31883	1.89226	0.70018
31884	1.89226	0.720545
31885	1.89226	0.520611
31886	1.89226	0.704712
31887	1.84936	0.775802
31888	2.0815	0.479549
31889	2.0815	0.700226
31890	2.47217	0.714456
31891	1.04528	0.417278
31892	1.62774	0.70848
31893	1.89226	0.700646
31894	1.89226	0.547669
31895	1.89226	0.6581
31896	1.89226	0.704323
31897	1.89226	0.514179

31898	1.89226	0.697517
31899	1.69055	0.362233
31900	2.68518	0.483561
31901	1.69055	0.342972
31902	2.25055	0.548158
31903	2.39587	0.289648
31904	3.01801	0.497961
31905	1.89226	0.533984
31906	1.89226	0.578983
31907	1.89226	0.4066
31908	1.89226	0.375415
31909	3.78452	0.500195
31910	1.24499	1.11093
31911	1.70302	1.20332
31912	1.41207	2.1
31913	1.10574	0.928449
31914	2.66056	1.20184
31915	1.70303	1.17322
31916	2.39605	1.21364
31917	2.72674	0.978492
31918	2.46904	0.97718
31919	1.84557	1.16885
31920	1.67115	1.21591
31921	2.46904	0.818913
31922	2.76829	1.48457
31923	2.83666	0.746033
31924	2.83666	0.999171
31925	2.39605	0.934295
31926	2.39605	1.40763
31927	1.07323	0.652951
31928	1.07323	1.73773
31929	1.06715	1.15526
31930	1.18052	1.08547
31931	1.30067	0.921706
31932	1.26542	0.952861
31933	0.914516	1.15708
31934	0.839633	1.32814
31935	0.936958	0.993617
31936	1.26751	0.938308
31937	0.847764	1.06332
31938	1.35395	1.07533
31939	2.69069	0.937958
31940	1.03074	0.83622
31941	1.74098	0.571413
31942	1.97397	0.78396
31943	3.71495	0.777313
31944	2.44554	2.1
31945	1.89226	2.1
31946	1.89226	2.1
31947	1.89226	2.1
31948	2.40645	2.1
31949	2.40645	2.1
31950	1.89226	2.1
31951	1.89226	1.41635
31952	1.89226	2.1
31953	1.89226	1.3339
31954	3.78452	2.1
31955	1.82117	2.1

31956	2.55786	1.30096
31957	2.51431	2.0514
31958	2.51431	2.1
31959	1.85054	1.97948
31960	1.84375	2.1
31961	2.38467	2.1
31962	1.89226	2.1
31963	1.89226	2.1
31964	1.89226	2.1
31965	2.44554	2.1
31966	2.44554	2.1
31967	1.89226	2.1
31968	1.89226	1.86494
31969	1.89226	2.1
31970	1.89226	1.83132
31971	1.89226	2.1
31972	1.89226	1.88749
31973	2.02397	2.1
31974	2.55786	1.8396
31975	1.77775	2.1
31976	2.61404	2.00141
31977	4.33561	2.1
31978	1.89226	1.17084
31979	1.89226	1.14636
31980	1.89226	0.992158
31981	1.89226	1.08093
31982	3.78453	1.14566
31983	1.89226	1.26396
31984	2.80184	1.37623
31985	1.89226	1.1471
31986	1.89226	1.26171
31987	2.03045	1.40129
31988	2.03045	1.49826
31989	2.52263	1.17226
31990	1.27921	1.73313
31991	1.58429	1.78978
31992	1.73571	1.59194
31993	2.37121	1.32151
31994	2.52263	1.20102
31995	0.764974	2.1
31996	0.650577	2.1
31997	0.712428	2.1
31998	0.562516	2.1
31999	0.67264	2.1
32000	0.67264	2.1
32001	0.654851	2.1
32002	0.654851	2.1
32003	0.562515	2.1
32004	0.650579	2.1
32005	0.712427	2.1
32006	0.764976	2.1
32007	1.89227	2.1
32008	1.89227	2.1
32009	1.89227	2.1
32010	1.89227	2.1
32011	3.78453	2.1
32012	1.89226	2.1
32013	1.89226	2.1

32014	1.89226	2.1
32015	1.89226	2.1
32016	3.78453	2.1
32017	1.49248	2.1
32018	1.24959	2.1
32019	1.28211	2.1
32020	1.26432	2.1
32021	1.51027	2.1
32022	1.49248	2.1
32023	1.49248	2.1
32024	1.19017	2.1
32025	1.24977	2.1
32026	1.51027	2.1
32027	2.74225	2.1
32028	1.89226	2.1
32029	1.89226	2.1
32030	1.89226	2.1
32031	1.89226	2.1
32032	3.78453	2.1
32033	1.89226	2.1
32034	1.89226	2.1
32035	1.89226	2.1
32036	1.89226	2.1
32037	3.78453	2.1
32038	0.624796	2.1
32039	0.618472	2.1
32040	0.770147	2.1
32041	0.755132	2.1
32042	0.681445	2.1
32043	0.681445	2.1
32044	0.69216	2.1
32045	0.69216	2.1
32046	0.618472	2.1
32047	0.770147	2.1
32048	0.624796	2.1
32049	0.755132	2.1
32050	1.54029	2.1
32051	1.51027	2.1
32052	1.54029	2.1
32053	1.30682	2.1
32054	1.24977	2.1
32055	1.24977	2.1
32056	0.994293	2.1
32057	2.17159	2.1
32058	2.21149	2.1
32059	2.21149	2.1
32060	0.994814	2.1
32061	0.994814	2.1
32062	0.990481	2.1
32063	2.21149	2.1
32064	2.2671	2.1
32065	2.2671	2.1
32066	0.994293	2.1
32067	0.994293	2.1
32068	2.41579	2.1
32069	1.09847	2.1
32070	1.70302	1.21291
32071	2.45069	2.1

32072	3.61169	2.1
32073	2.48689	2.1
32074	1.67969	2.1
32075	1.67969	2.1
32076	1.70002	2.1
32077	2.43826	2.1
32078	2.45859	2.1
32079	2.43827	2.1
32080	1.62408	2.1
32081	1.62408	2.1
32082	1.67969	2.1
32083	2.36729	2.1
32084	2.4229	2.1
32085	2.47199	2.1
32086	1.0823	2.1
32087	1.0607	2.1
32088	2.44276	2.1
32089	3.52506	2.1
32090	2.47199	2.1
32091	1.89226	2.1
32092	1.89226	2.1
32093	2.44277	2.1
32094	4.33503	2.1
32095	1.89226	2.1
32096	1.89226	2.1
32097	1.89226	2.1
32098	1.89226	2.1
32099	3.78453	2.1
32100	1.89226	2.1
32101	1.89226	2.1
32102	1.89226	2.1
32103	1.89226	2.1
32104	1.89226	2.1
32105	1.89226	2.1
32106	1.02986	2.1
32107	2.44276	2.1
32108	1.0607	2.1
32109	1.0607	2.1
32110	2.40055	2.1
32111	2.40055	2.1
32112	1.89226	2.1
32113	2.44277	2.1
32114	2.40056	2.1
32115	2.44277	2.1
32116	1.85005	2.1
32117	1.89226	2.1
32118	1.89226	2.1
32119	1.89226	2.1
32120	1.89226	2.1
32121	1.89226	2.1
32122	3.78453	2.1
32123	1.89226	2.1
32124	1.89226	2.1
32125	1.89226	2.1
32126	1.89226	2.1
32127	3.78452	2.1
32128	2.79502	0.479746
32129	2.63448	0.813385

32130	2.68986	0.456135
32131	2.53868	0.821194
32132	5.32434	0.60646
32133	2.63449	0.810051
32134	2.39282	1.98701
32135	2.53869	0.831449
32136	2.3085	1.50324
32137	4.94299	0.928056
32138	2.68986	0.492149
32139	2.53868	0.813922
32140	2.5761	0.378093
32141	2.43404	0.805707
32142	5.1239	0.681249
32143	2.53869	0.817436
32144	2.3085	1.50001
32145	2.43405	0.811239
32146	2.22575	1.5326
32147	4.74255	0.88423
32148	1.89226	2.1
32149	1.89226	2.1
32150	1.89226	2.1
32151	1.89226	2.1
32152	3.78453	2.1
32153	1.32391	2.1
32154	1.44951	2.1
32155	1.44951	2.1
32156	1.89226	2.1
32157	1.89226	2.1
32158	2.20203	2.1
32159	0.507656	2.1
32160	0.578945	2.1
32161	1.05568	2.1
32162	0.959007	2.1
32163	0.76264	2.1
32164	0.76264	2.1
32165	0.775313	2.1
32166	0.775313	2.1
32167	0.578945	2.1
32168	1.05568	2.1
32169	0.959007	2.1
32170	0.507656	2.1
32171	1.89226	0.331396
32172	2.4065	0.400823
32173	2.36561	0.324204
32174	2.4065	0.421982
32175	1.85138	0.314386
32176	1.89226	0.407816
32177	1.40274	0.303891
32178	2.4065	0.390425
32179	1.53336	0.330097
32180	1.53336	0.317303
32181	2.36562	0.348256
32182	2.36562	0.335921
32183	0.946115	0.394644
32184	0.946115	0.318475
32185	1.06496	0.30009
32186	1.18279	0.357651
32187	0.946117	0.374635

32188	0.946117	0.410776
32189	1.06496	0.321932
32190	1.06496	0.300343
32191	0.94613	0.338644
32192	1.06497	0.322471
32193	0.946105	0.325072
32194	1.18278	0.344405
32195	1.00213	0.302638
32196	2.36559	0.367093
32197	1.40272	0.318075
32198	1.40272	0.30865
32199	2.12992	0.339572
32200	2.12992	0.32827
32201	0.95646	2.1
32202	2.07201	1.56465
32203	1.01808	2.1
32204	2.14876	1.13348
32205	3.09009	2.1
32206	1.89226	2.1
32207	1.89226	1.18835
32208	1.89226	2.1
32209	1.89226	2.1
32210	3.78453	2.1
32211	1.89227	2.1
32212	1.89227	2.1
32213	1.89227	2.1
32214	1.89227	2.1
32215	1.89227	2.1
32216	1.89227	2.1
32217	2.71886	2.1
32218	1.60497	2.1
32219	1.43935	2.1
32220	2.55151	2.1
32221	4.15648	2.1
32222	2.71886	2.1
32223	1.89226	2.1
32224	1.89226	2.1
32225	2.55151	2.1
32226	4.44377	2.1
32227	1.89226	2.1
32228	1.89226	2.1
32229	1.89226	2.1
32230	1.89226	1.2177
32231	3.78453	2.1
32232	1.89226	2.1
32233	1.89226	2.1
32234	1.89226	2.1
32235	1.89226	2.1
32236	3.78453	2.1
32237	1.58942	0.770591
32238	1.92622	0.828545
32239	2.52852	0.847315
32240	2.19457	0.760509
32241	4.12079	0.759459
32242	1.92649	0.754501
32243	1.44426	0.69897
32244	1.87529	0.715133
32245	2.19457	0.756109

32246	3.63883	0.746886
32247	1.87529	0.69972
32248	2.19457	0.737231
32249	2.7378	0.739225
32250	2.29852	0.694779
32251	4.49309	0.697745
32252	1.35976	2.1
32253	1.89452	2.1
32254	1.39616	2.1
32255	1.1423	2.1
32256	1.8996	2.1
32257	1.52068	2.1
32258	1.89226	2.1
32259	1.89226	2.1
32260	1.89226	2.1
32261	1.89226	2.1
32262	1.89226	2.1
32263	1.89226	2.1
32264	1.89226	2.1
32265	2.15617	2.1
32266	2.0953	2.1
32267	2.15617	2.1
32268	1.83139	2.1
32269	1.89226	2.1
32270	1.16817	2.1
32271	1.8996	2.1
32272	1.23614	2.1
32273	1.48338	2.1
32274	1.80218	2.1
32275	2.16263	2.1
32276	1.89226	2.1
32277	1.89226	2.1
32278	1.89226	2.1
32279	1.89226	2.1
32280	1.89226	2.1
32281	1.89226	2.1
32282	1.89226	2.1
32283	2.0953	2.1
32284	2.03074	2.1
32285	2.0953	2.1
32286	1.8277	2.1
32287	1.89226	2.1
32288	1.74455	2.1
32289	2.181	2.1
32290	1.74455	2.1
32291	1.92911	2.1
32292	1.98195	2.1
32293	2.16651	2.1
32294	2.181	2.1
32295	2.29881	2.1
32296	1.98195	2.1
32297	1.80729	2.1
32298	4.28075	2.1
32299	1.74455	2.1
32300	1.98195	2.1
32301	1.52603	2.1
32302	1.76603	2.1
32303	3.51058	2.1

32304	1.76603	2.1
32305	1.94506	2.1
32306	1.76603	2.1
32307	1.98195	2.1
32308	1.86795	2.1
32309	2.08387	2.1
32310	0.984305	2.1
32311	1.9	2.0948
32312	2.86619	2.1
32313	1.86189	2.08385
32314	3.76189	2.1
32315	1.4621	2.1
32316	1.89226	2.1
32317	2.81374	2.1
32318	1.89226	2.1
32319	3.35437	2.1
32320	0.946133	2.02388
32321	0.946133	2.09861
32322	0.946132	1.87553
32323	0.946132	2.01035
32324	0.946132	2.00936
32325	0.946132	1.98204
32326	0.946133	2.09709
32327	0.946133	2.037
32328	0.946132	2.06741
32329	0.946132	1.84586
32330	0.946132	1.9922
32331	0.946132	2.1
32332	1.64268	1.71499
32333	1.93673	2.1
32334	2.25284	2.1
32335	1.97548	2.09688
32336	3.89552	2.1
32337	1.86206	2.1
32338	1.93673	2.1
32339	2.25284	2.1
32340	2.20075	2.1
32341	4.1149	2.1
32342	2.25284	2.1
32343	2.24781	2.02208
32344	1.97548	1.5952
32345	1.97548	2.1
32346	2.49676	1.75109
32347	2.49676	2.1
32348	2.25284	2.1
32349	2.43549	2.1
32350	2.44467	2.1
32351	2.20075	2.1
32352	2.49676	2.1
32353	2.25284	2.1
32354	2.57928	2.1
32355	2.32606	2.1
32356	2.59522	2.1
32357	2.39022	2.1
32358	4.9695	2.1
32359	2.32606	2.1
32360	1.77677	2.1
32361	2.39022	2.1

32362	2.28669	2.1
32363	4.16699	2.1
32364	2.59522	2.1
32365	2.39022	2.1
32366	2.61901	2.1
32367	2.45899	2.1
32368	5.00923	2.1
32369	2.39022	2.1
32370	2.11575	2.1
32371	2.41795	2.1
32372	2.0815	2.1
32373	4.67085	2.1
32374	1.89226	2.1
32375	2.24723	2.1
32376	1.95405	2.1
32377	1.89226	2.1
32378	2.30902	2.1
32379	2.24723	2.1
32380	1.89226	2.1
32381	1.89226	2.1
32382	1.89226	2.1
32383	1.89226	2.1
32384	3.78453	2.1
32385	0.946131	2.1
32386	0.946131	2.1
32387	1.14643	2.1
32388	1.10673	2.1
32389	1.10673	2.1
32390	1.10673	2.1
32391	0.946133	2.1
32392	0.946133	2.1
32393	0.946131	2.1
32394	1.14643	2.1
32395	1.10673	2.1
32396	0.946131	2.1
32397	1.89226	2.1
32398	2.30902	2.1
32399	1.96635	2.1
32400	1.89226	2.1
32401	2.38311	2.1
32402	2.30902	2.1
32403	1.89226	2.1
32404	1.89226	2.1
32405	1.89226	2.1
32406	1.89226	2.1
32407	1.89226	2.1
32408	1.89226	2.1
32409	0.946131	2.1
32410	0.946131	2.1
32411	1.19156	2.1
32412	1.14643	2.1
32413	1.14643	2.1
32414	1.14643	2.1
32415	0.946133	2.1
32416	0.946133	2.1
32417	0.946131	2.1
32418	1.19156	2.1
32419	0.946131	2.1

32420	1.14643	2.1
32421	1.89226	0.983513
32422	1.89226	0.898873
32423	1.89226	0.871182
32424	1.89226	1.03265
32425	1.89226	0.863712
32426	1.89226	1.1588
32427	1.89226	0.89582
32428	1.89226	0.926256
32429	1.89226	0.87591
32430	1.9645	0.957341
32431	3.78452	1.0261
32432	2.39489	2.1
32433	2.15715	2.1
32434	2.07821	2.1
32435	2.33541	2.1
32436	4.49256	2.1
32437	2.39489	2.1
32438	2.34898	2.1
32439	2.30729	2.1
32440	2.33541	2.1
32441	4.6844	2.1
32442	2.07821	2.1
32443	2.33541	2.1
32444	2.26415	2.1
32445	1.99632	2.1
32446	4.33174	2.1
32447	2.30729	2.1
32448	2.33542	2.1
32449	2.26416	2.1
32450	2.26618	2.1
32451	4.60159	2.1
32452	1.19837	1.37331
32453	2.79151	0.796947
32454	2.25636	1.11286
32455	2.25636	0.97009
32456	1.94072	1.04654
32457	1.94072	0.997957
32458	2.53725	0.353286
32459	1.16699	0.354613
32460	1.69864	0.30615
32461	2.0815	0.364264
32462	3.58273	0.345088
32463	1.91646	2.1
32464	2.51686	2.1
32465	2.49872	2.1
32466	1.85819	2.1
32467	4.41518	2.1
32468	0.929098	2.1
32469	0.8993	2.1
32470	1.24359	2.1
32471	1.24936	2.1
32472	1.06832	2.1
32473	1.06832	2.1
32474	1.08034	2.1
32475	1.08034	2.1
32476	0.899298	2.1
32477	1.24359	2.1

32478	1.24936	2.1
32479	0.929097	2.1
32480	2.0815	0.378614
32481	1.89226	0.250087
32482	2.0815	0.326014
32483	1.82811	0.283382
32484	1.94982	0.324941
32485	1.71246	0.277905
32486	2.3256	2.1
32487	1.89226	2.1
32488	1.89226	2.1
32489	1.89226	2.1
32490	2.38467	2.1
32491	2.38467	2.1
32492	1.89226	2.1
32493	1.89226	2.1
32494	1.89226	2.1
32495	1.89226	2.01833
32496	1.89226	2.1
32497	1.89226	2.04298
32498	1.89763	2.1
32499	2.61405	2.1
32500	2.15035	2.1
32501	2.68796	2.1
32502	1.42219	2.1
32503	1.77775	2.1
32504	2.26306	2.1
32505	1.89226	2.1
32506	1.89226	2.1
32507	1.89226	2.1
32508	2.3256	2.1
32509	2.3256	2.1
32510	1.89226	2.1
32511	1.89226	2.1
32512	1.89226	2.1
32513	1.89226	2.09237
32514	1.89226	2.1
32515	1.89226	2.01649
32516	1.92887	2.1
32517	2.41915	2.1
32518	2.50142	2.1
32519	2.50142	2.1
32520	1.90388	2.1
32521	1.89763	2.1
32522	1.89224	2.1
32523	1.89224	2.1
32524	1.89224	2.1
32525	1.89224	2.1
32526	3.78448	2.1
32527	2.25315	2.1
32528	1.05843	2.1
32529	2.38995	2.1
32530	1.31541	2.1
32531	3.56856	2.1
32532	0.517552	2.1
32533	0.68548	2.1
32534	1.27675	2.1
32535	1.13076	2.1

32536	0.913611	2.1
32537	0.913612	2.1
32538	0.902633	2.1
32539	0.902633	2.1
32540	0.68547	2.1
32541	1.27673	2.1
32542	1.13078	2.1
32543	0.517559	2.1
32544	1.89226	2.1
32545	1.89226	2.1
32546	1.89226	2.1
32547	1.89226	2.1
32548	3.78453	2.1
32549	0.962936	2.1
32550	0.962936	2.1
32551	0.996141	2.1
32552	0.996141	2.1
32553	0.830609	2.1
32554	0.657714	2.1
32555	1.30136	2.1
32556	1.19499	2.1
32557	1.30136	2.1
32558	0.830609	2.1
32559	1.19499	2.1
32560	0.657713	2.1
32561	1.60057	0.345324
32562	1.39615	0.3501
32563	1.06474	0.358759
32564	1.39615	0.396904
32565	1.64655	0.311613
32566	2.04424	0.338963
32567	1.89226	0.361968
32568	1.89226	0.435856
32569	1.89226	0.422628
32570	1.89226	0.415688
32571	1.89226	0.386289
32572	1.89226	0.387953
32573	2.56322	0.324424
32574	1.89226	0.385249
32575	1.89226	0.379056
32576	1.89226	0.364105
32577	2.18047	0.348042
32578	2.18047	0.33685
32579	1.89226	0.926066
32580	1.89226	0.944324
32581	1.89226	1.00779
32582	1.65573	0.832554
32583	1.89226	1.01534
32584	1.65573	0.842683
32585	1.05353	0.794747
32586	1.83158	0.822939
32587	1.50282	0.646603
32588	1.20669	0.643877
32589	2.88511	0.676776
32590	1.44807	2.1
32591	1.93592	2.1
32592	1.80703	2.1
32593	1.27806	2.1

32594	3.21398	2.1
32595	2.0815	2.1
32596	1.87465	2.1
32597	1.87465	2.1
32598	1.50566	2.1
32599	2.0815	2.1
32600	1.69263	2.1
32601	1.89226	2.1
32602	1.89226	2.1
32603	1.89226	2.1
32604	1.89226	2.1
32605	1.89226	2.1
32606	1.89226	2.1
32607	1.62632	2.1
32608	0.994294	2.1
32609	0.994294	2.1
32610	1.27806	2.1
32611	1.81921	2.1
32612	2.10776	2.1
32613	2.0815	2.1
32614	1.70303	2.1
32615	1.70303	2.1
32616	1.85749	2.1
32617	1.90451	2.1
32618	2.07724	2.1
32619	1.89226	2.1
32620	1.89226	2.1
32621	1.89226	2.1
32622	1.89226	2.1
32623	1.89226	2.1
32624	1.89226	2.1
32625	2.69773	2.1
32626	1.88129	2.1
32627	2.55196	2.1
32628	2.26469	2.1
32629	4.94367	2.1
32630	1.88129	2.1
32631	1.25889	2.1
32632	1.26614	2.1
32633	1.05914	2.1
32634	2.26469	2.1
32635	1.88129	2.1
32636	2.55196	2.1
32637	2.0588	2.1
32638	2.6536	2.1
32639	2.15287	2.1
32640	4.70484	2.1
32641	0.8516	2.1
32642	0.8516	2.1
32643	0.849002	2.1
32644	0.849002	2.1
32645	0.629445	2.1
32646	0.671197	2.1
32647	1.0294	2.1
32648	1.07644	2.1
32649	1.07644	2.1
32650	0.671197	2.1
32651	0.629446	2.1

32652	1.0294	2.1
32653	2.02115	1.559
32654	1.18382	1.65263
32655	2.29107	0.792715
32656	1.46903	1.16512
32657	3.47488	0.936093
32658	2.60763	1.05606
32659	2.29107	1.29225
32660	1.82141	1.14635
32661	1.82141	1.61336
32662	1.46903	1.48873
32663	2.29107	1.48535
32664	1.64007	2.1
32665	2.2024	2.1
32666	1.44044	2.1
32667	2.02634	2.1
32668	3.6664	2.1
32669	1.25565	2.1
32670	2.02635	2.1
32671	1.87228	2.1
32672	1.87228	2.1
32673	1.44045	2.1
32674	1.44045	2.1
32675	1.89226	0.239376
32676	1.78503	0.258726
32677	1.78503	0.27159
32678	1.40048	0.24009
32679	2.0815	0.264214
32680	1.62972	0.233696
32681	1.27875	2.1
32682	1.1483	0.948843
32683	1.40014	0.992743
32684	1.40014	2.1
32685	1.04598	1.13084
32686	1.04598	2.1
32687	2.63443	1.25687
32688	2.26444	1.80944
32689	2.79392	0.887465
32690	2.4863	1.70284
32691	5.12073	1.48292
32692	1.83158	0.878957
32693	1.18508	0.679231
32694	1.20669	0.704953
32695	1.81488	0.775052
32696	1.20669	0.705032
32697	1.81488	0.781291
32698	1.08134	0.662028
32699	1.70303	0.758531
32700	1.24027	0.776949
32701	1.30359	0.656104
32702	1.89939	0.888087
32703	1.70303	0.799474
32704	1.89226	2.1
32705	2.69027	2.1
32706	1.89226	2.1
32707	2.2078	2.1
32708	1.89226	2.1
32709	2.2078	2.1

32710	1.89226	2.1
32711	2.78977	2.1
32712	1.89226	2.1
32713	2.69027	2.1
32714	1.89226	2.1
32715	2.69027	2.1
32716	1.89226	2.07722
32717	1.89226	2.05322
32718	1.89226	1.6933
32719	1.89226	2.1
32720	3.78452	1.9817
32721	1.43687	2.1
32722	1.71059	2.1
32723	2.53014	2.0881
32724	2.6521	2.1
32725	4.24073	2.1
32726	2.6521	2.1
32727	1.66762	2.1
32728	2.50299	2.1
32729	2.50299	2.1
32730	1.71059	2.1
32731	1.71059	2.1
32732	1.89226	2.08921
32733	1.89226	2.1
32734	1.89226	2.1
32735	1.89226	1.98238
32736	1.89226	2.1
32737	1.89226	2.00052
32738	2.10662	0.772985
32739	2.77417	0.770443
32740	2.78873	0.935501
32741	2.11661	0.751382
32742	4.89078	0.765483
32743	2.10662	0.806565
32744	1.22857	0.631395
32745	1.25849	0.843433
32746	2.11661	0.803786
32747	3.34518	0.804521
32748	2.78873	0.878263
32749	2.11661	0.704027
32750	2.12475	0.810701
32751	2.79029	0.832873
32752	4.9069	0.814341
32753	1.2858	0.80276
32754	2.11661	0.751353
32755	1.25849	0.788594
32756	1.25849	0.780665
32757	2.12475	0.771504
32758	2.12475	0.777829
32759	1.13537	2.1
32760	1.20714	2.1
32761	0.946129	2.1
32762	0.946129	2.1
32763	0.946131	2.1
32764	0.946131	2.1
32765	1.29521	2.1
32766	1.20713	2.1
32767	1.13537	2.1

32768	0.946131	2.1
32769	0.94613	2.1
32770	1.29521	2.1
32771	1.00533	2.1
32772	0.876055	2.1
32773	0.946128	2.1
32774	0.946128	2.1
32775	0.913908	2.1
32776	0.913908	2.1
32777	1.17081	2.1
32778	0.896413	2.1
32779	0.955905	2.1
32780	0.893715	2.1
32781	1.20327	2.1
32782	0.998544	2.1
32783	1.89226	1.42474
32784	1.89226	1.55962
32785	1.89226	0.844103
32786	1.89226	1.19029
32787	3.78452	0.99058
32788	0.540975	1.2217
32789	0.726949	1.82059
32790	0.712198	1.58197
32791	0.506675	1.12878
32792	0.611916	1.15466
32793	0.611916	2.01527
32794	0.621708	1.5289
32795	0.621708	1.07129
32796	0.726951	1.2689
32797	0.712196	1.15467
32798	0.506674	1.44089
32799	0.540976	1.34476
32800	1.89226	1.17544
32801	1.89226	1.82918
32802	1.89226	1.12884
32803	1.89226	1.13086
32804	3.78453	1.37737
32805	1.89226	1.05302
32806	1.89226	1.1325
32807	1.89226	0.852124
32808	1.89226	1.62517
32809	1.89226	0.878822
32810	1.89226	1.82778
32811	0.919594	1.13991
32812	0.726949	1.47853
32813	0.898318	1.16184
32814	0.712198	1.41553
32815	0.817559	0.933154
32816	0.817559	1.83295
32817	0.814234	0.961347
32818	0.814234	1.54931
32819	0.919597	1.02309
32820	0.898316	0.886597
32821	0.726951	1.53358
32822	0.712196	1.33871
32823	1.79663	0.928496
32824	1.12088	1.69388
32825	1.54699	1.12335

32826	1.17475	1.20698
32827	1.79663	1.58806
32828	1.42439	1.66187
32829	2.28928	1.13338
32830	1.23077	1.36391
32831	1.65458	0.988182
32832	2.66533	1.38703
32833	3.94385	1.24883
32834	1.89226	2.1
32835	1.89226	2.0117
32836	1.89226	2.1
32837	1.89226	1.76689
32838	3.78452	2.1
32839	1.38426	2.05361
32840	1.15067	2.1
32841	1.38426	2.1
32842	1.01595	2.1
32843	1.24509	2.1
32844	1.01595	2.1
32845	2.62097	2.1
32846	1.89226	1.80691
32847	2.72561	2.1
32848	2.72561	2.1
32849	1.89226	2.1
32850	1.89226	2.1
32851	1.90103	0.584886
32852	1.41902	1.21917
32853	1.77249	0.654364
32854	1.30272	1.60968
32855	3.20375	0.843921
32856	1.77249	0.644998
32857	1.19548	1.23687
32858	1.19548	0.805136
32859	1.30272	0.966984
32860	1.65815	0.634414
32861	1.76539	0.916426
32862	2.62161	2.1
32863	1.89226	2.1
32864	1.89226	2.1
32865	1.89226	2.1
32866	2.6446	2.1
32867	2.6446	2.1
32868	2.64459	2.1
32869	1.89226	2.1
32870	1.89226	2.1
32871	1.89226	2.1
32872	2.58257	2.1
32873	2.58257	2.1
32874	1.89226	0.794603
32875	1.89226	1.0757
32876	2.31301	1.05004
32877	2.76309	0.856493
32878	1.89226	0.984816
32879	2.26047	0.832259
32880	1.89226	0.786783
32881	2.31301	1.05197
32882	2.17765	0.983743
32883	2.17765	0.837129

32884	1.89226	0.935103
32885	1.89226	0.806016
32886	2.18516	2.1
32887	1.85196	2.1
32888	1.78595	2.1
32889	2.14606	2.1
32890	3.99802	2.1
32891	2.18516	2.1
32892	2.21189	2.1
32893	2.18937	2.1
32894	2.14606	2.1
32895	4.35796	2.1
32896	1.60735	2.1
32897	2.14606	2.1
32898	2.15714	2.1
32899	1.70302	2.1
32900	3.70055	2.1
32901	2.18937	2.1
32902	2.14606	2.1
32903	2.0927	2.1
32904	2.162 2.1	
32905	4.30806	2.1
32906	2.17326	2.1
32907	2.58517	2.1
32908	2.55318	2.1
32909	2.1665	2.1
32910	4.75167	2.1
32911	2.17326	2.1
32912	1.53556	2.1
32913	1.56253	2.1
32914	2.1665	2.1
32915	3.70206	2.1
32916	2.55317	2.1
32917	2.1665	2.1
32918	2.17741	2.1
32919	2.54197	2.1
32920	4.70847	2.1
32921	2.17742	2.1
32922	2.1665	2.1
32923	2.18452	2.1
32924	1.58054	2.1
32925	1.56253	2.1
32926	1.56253	2.1
32927	1.71051	0.427189
32928	1.89807	0.376402
32929	1.19755	0.338534
32930	1.19755	0.338899
32931	2.25632	0.40846
32932	2.25632	0.390228
32933	0.876046	0.409925
32934	1.27293	0.40163
32935	0.946132	0.496686
32936	0.946132	0.477249
32937	0.946131	0.420578
32938	0.869312	0.406483
32939	1.12767	0.466881
32940	0.946132	0.47681
32941	1.22225	0.479286

32942	1.37968	0.390525
32943	0.851555	0.537462
32944	0.851555	0.433807
32945	0.946133	1.43683
32946	0.946133	1.99589
32947	0.946132	1.55172
32948	0.946132	1.20357
32949	0.946133	1.39364
32950	0.946133	1.50304
32951	0.946132	1.90416
32952	0.946132	1.51109
32953	0.946132	1.85382
32954	0.946132	1.55372
32955	0.946133	1.49044
32956	0.946133	1.69711
32957	1.35152	2.1
32958	0.948936	2.1
32959	1.90166	2.1
32960	1.70303	2.1
32961	2.90858	2.1
32962	1.89226	2.1
32963	1.89226	2.1
32964	1.89226	1.77346
32965	1.89226	2.1
32966	3.78452	1.92288
32967	1.90166	2.1
32968	1.70303	2.1
32969	2.52454	1.48867
32970	2.13919	2.1
32971	3.99529	2.09106
32972	1.89226	1.03067
32973	1.89226	1.66657
32974	1.89226	1.94084
32975	1.89226	2.1
32976	3.78452	2.1
32977	1.89226	2.07045
32978	1.89226	2.1
32979	1.89226	2.1
32980	1.89226	2.1
32981	1.89226	1.06719
32982	1.89226	1.73403
32983	2.13918	2.1
32984	0.992674	2.1
32985	2.3397	2.1
32986	1.8023	2.1
32987	1.47769	2.1
32988	0.992674	2.1
32989	1.89226	0.677831
32990	1.89226	0.674066
32991	1.89226	0.778243
32992	1.89226	0.571915
32993	3.78452	0.675078
32994	1.89226	0.671954
32995	2.54752	0.464413
32996	2.63974	0.573651
32997	1.89226	0.564513
32998	4.53201	0.567278
32999	0.498412	0.57606

33000	0.500955	0.529474
33001	0.57955	0.661321
33002	0.563873	0.602791
33003	0.529107	0.590628
33004	0.529107	0.532453
33005	0.53572	0.675592
33006	0.53572	0.563708
33007	0.500956	0.578777
33008	0.579548	0.577452
33009	0.563871	0.584835
33010	0.498414	0.586063
33011	1.89223	0.727561
33012	1.89223	0.558632
33013	1.89223	0.639818
33014	1.89223	0.682378
33015	1.89223	0.631094
33016	1.89223	0.671993
33017	1.28752	0.46304
33018	1.31985	0.546188
33019	0.946119	0.544078
33020	0.946119	0.645074
33021	1.28752	0.546908
33022	1.28752	0.47366
33023	0.946119	0.535244
33024	0.946119	0.693332
33025	1.28753	0.535246
33026	0.946132	0.528783
33027	1.31984	0.559027
33028	0.946107	0.564494
33029	0.500948	0.545338
33030	0.479465	0.51381
33031	0.562335	0.626303
33032	0.579542	0.568015
33033	0.528436	0.558612
33034	0.528436	0.51897
33035	0.530571	0.540726
33036	0.530571	0.635745
33037	0.56234	0.554725
33038	0.479473	0.5585
33039	0.500943	0.565596
33040	0.579533	0.563177
33041	1.89226	2.1
33042	1.89226	2.1
33043	1.89226	2.1
33044	1.89226	2.1
33045	3.78452	2.1
33046	1.50767	2.1
33047	1.14496	2.1
33048	1.50767	2.1
33049	1.58539	2.1
33050	1.07653	2.1
33051	1.15425	2.1
33052	1.89226	2.1
33053	2.43826	2.1
33054	2.43826	2.1
33055	2.48688	2.1
33056	1.89226	2.1
33057	1.94088	2.1

33058	1.89226	2.07765
33059	1.89226	2.1
33060	1.89226	2.1
33061	1.89226	2.1
33062	3.78452	2.1
33063	1.50767	2.06873
33064	1.07653	2.1
33065	1.41077	1.82624
33066	0.991407	2.1
33067	2.49907	2.1
33068	1.21913	2.1
33069	1.21913	2.1
33070	0.946128	2.1
33071	0.946128	2.1
33072	1.21913	2.1
33073	1.18364	2.1
33074	0.946131	2.1
33075	0.946131	2.1
33076	1.18364	2.1
33077	0.946129	2.1
33078	1.21913	2.1
33079	0.94613	2.1
33080	1.89226	0.903644
33081	1.89226	2.1
33082	1.89226	0.917673
33083	1.89226	2.1
33084	3.78452	1.22471
33085	1.93823	0.914021
33086	1.60976	2.1
33087	1.93823	2.1
33088	2.20045	0.891487
33089	1.36999	2.1
33090	1.63222	1.06715
33091	1.38302	2.1
33092	1.38302	2.1
33093	0.946132	2.1
33094	0.946132	2.1
33095	1.27258	2.1
33096	1.38302	2.1
33097	0.946133	2.1
33098	0.946133	2.1
33099	0.946133	2.1
33100	1.27258	2.1
33101	0.946132	2.1
33102	1.38302	2.1
33103	1.89226	0.849521
33104	1.89226	2.1
33105	1.89226	0.904578
33106	1.89226	2.1
33107	3.78452	1.52513
33108	1.93823	0.847607
33109	1.36999	2.1
33110	1.70423	0.85908
33111	1.14195	2.1
33112	3.08018	1.0808
33113	1.27258	2.1
33114	1.27258	2.1
33115	0.946132	2.1

33116	0.946132	2.1
33117	1.17233	2.1
33118	1.27258	2.1
33119	0.946132	2.1
33120	0.946132	2.1
33121	1.17233	2.1
33122	0.946132	2.1
33123	0.946132	2.1
33124	1.27258	2.1
33125	2.70575	2.1
33126	1.89226	1.015
33127	2.71429	2.00452
33128	2.71429	1.64661
33129	1.89226	1.51433
33130	1.89226	1.06934
33131	0.946133	1.06488
33132	0.946133	2.04806
33133	1.28713	2.1
33134	1.28713	2.1
33135	0.946131	2.03529
33136	0.946131	1.04922
33137	1.28713	2.1
33138	1.36503	1.69517
33139	0.946131	2.00432
33140	1.36503	1.96886
33141	1.28713	2.06183
33142	0.946131	2.1
33143	1.89226	2.05161
33144	1.89226	1.29242
33145	1.89226	1.94142
33146	1.89226	1.24773
33147	1.89226	1.70826
33148	1.89226	1.19843
33149	1.22615	2.08148
33150	1.04147	2.1
33151	1.3439	2.1
33152	1.3439	2.1
33153	0.969118	2.1
33154	0.969118	1.85151
33155	0.980841	2.1
33156	0.992505	2.1
33157	1.34289	2.1
33158	1.70303	2.1
33159	2.56081	2.1
33160	1.89225	2.1
33161	2.66785	2.1
33162	2.79506	2.1
33163	2.79506	2.1
33164	1.89225	2.1
33165	1.89225	2.1
33166	0.978778	2.1
33167	0.978778	2.1
33168	0.993303	2.1
33169	0.993303	2.1
33170	0.603538	2.1
33171	0.638158	2.1
33172	1.33393	2.1
33173	1.39753	2.1

33174	1.33391	2.1
33175	0.603535	2.1
33176	0.638164	2.1
33177	1.39754	2.1
33178	1.33393	2.1
33179	1.33393	2.1
33180	0.946132	2.1
33181	0.946132	2.1
33182	1.33393	2.1
33183	1.27365	2.1
33184	0.946132	2.1
33185	0.946132	2.1
33186	0.946132	2.1
33187	1.27365	2.1
33188	1.33393	2.1
33189	0.946131	2.1
33190	1.89226	2.1
33191	1.89226	2.1
33192	1.89226	2.1
33193	1.89226	2.1
33194	3.78452	2.1
33195	1.89226	2.1
33196	1.89226	2.1
33197	1.89226	2.1
33198	1.89226	2.1
33199	3.78453	2.1
33200	0.979364	2.1
33201	0.979364	2.1
33202	0.89618	2.1
33203	0.89618	2.1
33204	0.896178	2.1
33205	0.929411	2.1
33206	0.946133	2.1
33207	0.946133	2.1
33208	0.946131	2.1
33209	0.89618	2.1
33210	0.946131	2.1
33211	0.929413	2.1
33212	1.89226	2.1
33213	1.89226	2.1
33214	1.89226	2.1
33215	1.89226	2.1
33216	1.89226	2.1
33217	1.89226	2.1
33218	1.89225	2.1
33219	1.89225	2.1
33220	1.89225	2.1
33221	1.89225	2.1
33222	3.7845	2.1
33223	1.89225	2.1
33224	1.89225	2.1
33225	1.89225	2.1
33226	1.89225	2.1
33227	1.89225	2.1
33228	1.89225	2.1
33229	0.946128	0.688556
33230	0.946128	0.591393
33231	0.946132	0.659223

33232	0.946132	0.55109
33233	0.94613	0.66122
33234	0.94613	0.695495
33235	0.946129	0.573689
33236	0.946129	0.507334
33237	0.94613	0.632882
33238	0.94613	0.63465
33239	0.94613	0.612372
33240	0.94613	0.607657
33241	1.89227	0.898223
33242	1.89227	0.930561
33243	1.89227	0.820621
33244	1.89227	0.805058
33245	3.78453	0.836579
33246	1.89226	0.922913
33247	2.43717	1.24106
33248	2.63259	0.923127
33249	1.89226	0.814868
33250	4.52485	0.971632
33251	0.616808	0.859726
33252	0.792277	1.01825
33253	1.04986	0.808226
33254	0.72303	0.951488
33255	0.702675	0.735967
33256	0.76862	1.23941
33257	0.809154	1.07501
33258	0.809154	0.830597
33259	0.808757	0.919756
33260	1.07789	0.76238
33261	0.772568	0.841093
33262	0.598878	1.04706
33263	1.89226	0.91692
33264	1.89226	0.936816
33265	1.89226	0.844668
33266	1.89226	0.816851
33267	3.78453	0.876363
33268	1.36428	1.36761
33269	1.31629	0.963324
33270	0.946132	0.972722
33271	0.946132	0.911815
33272	1.36428	0.853509
33273	1.36428	1.38585
33274	0.946132	0.970806
33275	0.946132	1.10012
33276	1.36428	1.22451
33277	0.946132	0.883787
33278	1.31629	1.04245
33279	0.946132	0.850102
33280	1.578	1.0327
33281	2.85336	0.772566
33282	2.11368	0.744167
33283	2.14375	0.863498
33284	1.93852	0.852313
33285	1.93852	1.11757
33286	2.34806	0.691546
33287	1.42913	0.526212
33288	1.61539	0.740174
33289	2.45322	0.611964

33290	3.88235	0.660277
33291	2.34806	0.609727
33292	1.89226	0.521014
33293	1.89226	0.750969
33294	2.45322	0.543946
33295	4.34548	0.545149
33296	1.89226	0.515682
33297	1.89226	0.39872
33298	1.89226	0.521135
33299	1.89226	0.481155
33300	3.78452	0.511528
33301	1.89226	0.519634
33302	1.89226	0.428063
33303	1.89226	0.590371
33304	1.89226	0.542338
33305	3.78452	0.508278
33306	1.25972	0.5858
33307	2.45322	0.502624
33308	2.58823	0.714967
33309	1.79398	0.534174
33310	4.2472	0.554518
33311	1.89226	0.651282
33312	2.45322	0.451891
33313	2.58823	0.554823
33314	2.45322	0.506126
33315	2.02727	0.539178
33316	1.89226	0.492259
33317	1.89226	0.431873
33318	1.89226	0.412471
33319	1.89226	0.543121
33320	1.89226	0.39405
33321	3.78452	0.402299
33322	1.89226	0.482491
33323	1.89226	0.411754
33324	1.89226	0.430497
33325	1.89226	0.335731
33326	1.89226	0.562483
33327	1.89226	0.448144
33328	1.70048	1.86012
33329	2.1572	0.70712
33330	1.45477	1.22253
33331	1.93973	0.873777
33332	3.64021	1.03515
33333	1.17614	1.24648
33334	1.93973	0.857666
33335	1.69661	1.58471
33336	1.69661	0.866369
33337	1.45477	1.58909
33338	1.45477	0.888158
33339	1.94343	1.50626
33340	2.53782	1.33186
33341	2.59903	1.20538
33342	2.59903	0.977341
33343	1.87598	1.31176
33344	1.87598	1.09788
33345	0.946131	0.870722
33346	0.946131	1.20113
33347	1.26891	1.62855

33348	1.26891	0.777063
33349	0.94613	1.32296
33350	0.94613	1.11809
33351	1.26891	1.31352
33352	1.29952	0.981485
33353	1.29952	0.879741
33354	0.946131	0.814368
33355	1.26891	1.11812
33356	0.946131	1.13282
33357	2.00551	1.53404
33358	2.59903	1.4544
33359	2.65811	1.30729
33360	2.65811	1.08195
33361	1.94343	1.28035
33362	1.94343	1.17852
33363	2.65811	0.90082
33364	1.89226	1.52156
33365	1.89226	0.900968
33366	1.89226	1.25426
33367	2.59903	1.05858
33368	2.59903	1.58061
33369	1.89227	2.1
33370	1.89227	2.1
33371	1.89227	2.1
33372	1.89227	2.1
33373	3.78453	2.1
33374	1.15471	2.1
33375	0.993889	2.1
33376	1.78919	2.1
33377	1.77325	2.1
33378	2.78308	2.1
33379	1.89226	2.1
33380	1.89226	2.1
33381	1.89226	2.1
33382	1.89226	2.1
33383	3.78453	2.1
33384	1.78919	2.1
33385	1.77325	2.1
33386	2.45969	2.1
33387	2.70835	2.1
33388	4.49753	2.1
33389	0.939314	2.1
33390	0.939315	2.1
33391	1.01133	2.1
33392	1.01133	2.1
33393	0.596471	2.1
33394	1.20813	2.1
33395	0.886619	2.1
33396	1.35417	2.1
33397	1.35417	2.1
33398	1.20813	2.1
33399	0.886621	2.1
33400	0.59647	2.1
33401	0.946131	2.1
33402	0.946131	2.1
33403	0.94613	2.1
33404	0.94613	2.1
33405	0.946133	2.1

33406	0.946133	2.0658
33407	0.946128	2.1
33408	0.946128	2.1
33409	0.946131	2.1
33410	0.946131	2.1
33411	0.946131	2.1
33412	0.946131	2.1
33413	1.03939	2.1
33414	1.03939	2.1
33415	0.946132	1.13079
33416	0.946132	1.24508
33417	0.946132	1.37366
33418	0.946132	2.1
33419	1.23851	1.17003
33420	1.03939	2.1
33421	1.03939	1.75475
33422	0.946132	1.75945
33423	0.946133	1.65498
33424	1.23851	1.66665
33425	0.902503	2.1
33426	0.946129	2.1
33427	0.946132	1.16444
33428	0.946132	1.6028
33429	0.902472	1.57607
33430	0.902472	2.1
33431	1.08736	1.20118
33432	0.994093	2.1
33433	0.946133	1.60334
33434	0.902504	1.69328
33435	1.03939	1.68935
33436	0.946132	1.67057
33437	1.35306	0.845648
33438	1.29982	0.761656
33439	1.7216	0.744742
33440	1.89183	0.744878
33441	3.07466	0.752326
33442	0.485098	0.76396
33443	0.582401	0.7796
33444	0.649908	0.796488
33445	0.8608	0.765276
33446	0.587739	0.730218
33447	0.58774	0.796474
33448	0.644567	0.76295
33449	0.644567	0.917715
33450	0.58241	0.787241
33451	0.86081	0.730947
33452	0.649898	0.854737
33453	0.485093	0.77317
33454	2.25936	2.1
33455	2.71441	2.1
33456	2.63824	2.1
33457	2.20652	2.1
33458	4.92092	2.1
33459	2.25936	2.1
33460	1.57627	2.1
33461	1.5493	2.1
33462	2.20652	2.1
33463	3.78279	2.1

33464	2.63824	2.1
33465	2.20652	2.1
33466	2.17326	2.1
33467	2.58517	2.1
33468	4.79169	2.1
33469	2.17326	2.1
33470	2.20652	2.1
33471	2.19278	2.1
33472	1.53556	2.1
33473	1.5493	2.1
33474	1.5493	2.1
33475	1.97465	2.1
33476	2.13925	2.1
33477	2.04483	2.1
33478	1.62784	2.1
33479	3.76709	2.1
33480	1.89226	2.1
33481	1.89226	2.1
33482	1.89226	2.1
33483	1.89226	2.1
33484	1.89226	2.1
33485	1.89226	2.1
33486	0.724037	2.1
33487	0.81392	2.1
33488	0.96796	2.1
33489	1.02242	2.1
33490	0.882091	2.1
33491	0.882091	2.1
33492	0.899789	2.1
33493	0.899789	2.1
33494	0.724036	2.1
33495	0.96796	2.1
33496	1.02242	2.1
33497	0.81392	2.1
33498	1.13537	2.1
33499	1.63054	2.1
33500	0.946131	2.1
33501	0.946131	2.1
33502	0.946131	2.1
33503	0.946131	2.1
33504	1.258	2.1
33505	1.63054	2.1
33506	1.22632	2.1
33507	1.22632	2.1
33508	0.946131	2.1
33509	1.258	2.1
33510	1.89226	2.1
33511	1.89226	2.1
33512	1.89226	2.1
33513	1.89226	2.1
33514	1.89226	2.1
33515	1.89226	2.1
33516	0.809739	2.1
33517	0.862674	2.1
33518	1.1995	2.1
33519	1.33899	2.1
33520	1.06324	2.1
33521	1.06324	2.1

33522	0.981932	2.1
33523	1.19563	2.1
33524	0.809727	2.1
33525	1.14092	2.1
33526	1.33902	2.1
33527	0.863494	2.1
33528	2.23742	2.1
33529	1.84902	2.1
33530	1.88088	2.1
33531	2.20392	2.1
33532	4.08644	2.1
33533	1.3592	2.1
33534	1.84902	2.1
33535	1.88088	2.1
33536	1.3305	2.1
33537	1.88088	2.1
33538	1.3305	2.1
33539	1.84902	2.1
33540	1.15008	0.650135
33541	1.3305	1.94657
33542	1.70539	2.1
33543	2.9991	2.1
33544	1.84901	2.1
33545	2.0264	2.1
33546	2.20391	2.1
33547	1.70539	2.1
33548	3.87541	2.1
33549	1.03852	1.76068
33550	1.38247	0.950246
33551	1.36635	1.04139
33552	1.01537	1.40385
33553	2.39784	1.20252
33554	2.0815	1.42645
33555	1.91744	1.43323
33556	1.91744	1.20674
33557	2.06496	2.1
33558	1.89227	1.20235
33559	2.03474	2.1
33560	1.89226	0.960709
33561	1.89226	1.19883
33562	1.89226	0.960717
33563	1.89226	1.46652
33564	1.89226	0.953158
33565	1.89226	1.42484
33566	1.36635	1.12058
33567	1.02923	1.68519
33568	1.02923	1.1244
33569	1.01537	1.66009
33570	1.36416	1.05271
33571	1.3503	1.83382
33572	1.89226	1.515
33573	1.89359	2.01138
33574	1.89359	1.36773
33575	1.91744	2.1
33576	1.89226	1.32026
33577	1.91611	2.1
33578	0.946128	1.63049
33579	0.946128	1.33053

33580	0.946133	1.05213
33581	0.946133	0.964065
33582	0.94613	0.973975
33583	0.94613	1.2764
33584	0.946131	1.72358
33585	0.946131	1.01334
33586	0.946131	1.16813
33587	0.946131	1.15773
33588	0.946131	1.18628
33589	0.946131	1.16263
33590	2.689 2.1	
33591	2.0815	2.1
33592	2.72786	2.1
33593	2.48295	2.1
33594	5.02397	2.1
33595	2.72786	2.1
33596	1.86424	2.1
33597	2.7188	2.1
33598	2.08981	2.1
33599	4.81768	2.1
33600	2.08982	2.1
33601	0.986987	2.1
33602	2.08982	2.1
33603	1.87249	2.1
33604	1.01964	2.1
33605	0.906115	2.1
33606	1.44247	2.1
33607	1.43872	2.1
33608	1.17054	2.1
33609	1.17054	2.1
33610	1.43872	2.1
33611	1.26045	2.1
33612	2.0815	0.244897
33613	2.0815	0.26081
33614	2.0815	0.286147
33615	1.81468	0.24122
33616	1.89065	0.275589
33617	1.55625	0.231302
33618	1.89062	0.24513
33619	1.89224	0.269889
33620	1.89224	0.29038
33621	2.27073	0.244185
33622	1.86856	0.281168
33623	2.24232	0.23451
33624	1.21419	2.1
33625	2.79716	2.1
33626	2.50005	2.1
33627	2.50005	2.1
33628	1.5008	2.1
33629	1.5008	2.1
33630	1.60139	0.235327
33631	1.7103	0.258403
33632	1.04159	0.264486
33633	1.2489	0.218737
33634	2.75189	0.236567
33635	0.732812	0.219155
33636	0.732812	0.228653
33637	0.751986	0.259428

33638	0.751986	0.256733
33639	0.860346	0.228672
33640	0.667961	0.257056
33641	0.85515	0.263279
33642	0.624452	0.236048
33643	0.62445	0.23942
33644	0.667962	0.232412
33645	0.855148	0.246673
33646	0.860348	0.240568
33647	1.89226	0.709297
33648	2.50872	0.718888
33649	2.77909	0.702277
33650	1.89226	0.719772
33651	2.77909	0.70861
33652	1.89226	0.725224
33653	2.15825	0.656324
33654	2.50872	0.643064
33655	1.90439	0.599443
33656	1.90439	0.675988
33657	2.77909	0.606038
33658	2.77909	0.692222
33659	1.12468	2.1
33660	1.43872	2.1
33661	1.64926	2.1
33662	1.2724	2.1
33663	2.77395	2.1
33664	1.64926	2.1
33665	1.38438	2.1
33666	1.47244	2.1
33667	1.2724	2.1
33668	1.8493	2.1
33669	1.64926	2.1
33670	1.36416	1.12292
33671	1.0652	1.78411
33672	1.0652	1.18912
33673	1.02923	1.77602
33674	1.37496	1.11094
33675	1.33898	1.93029
33676	1.89227	1.62179
33677	1.90433	2.1
33678	1.90433	1.59383
33679	1.89359	2.1
33680	1.89227	1.52832
33681	1.88153	2.1
33682	0.946129	1.74173
33683	0.946129	1.53569
33684	0.946133	1.11232
33685	0.946133	1.02815
33686	0.946131	1.04194
33687	0.946131	1.48982
33688	0.946131	1.81265
33689	0.946131	1.06387
33690	0.946131	1.24631
33691	0.946131	1.24443
33692	0.946131	1.25009
33693	0.946131	1.24638
33694	1.37496	1.15355
33695	1.07519	1.87212

33696	1.07519	1.21603
33697	1.0652	1.86806
33698	1.39725	1.15154
33699	1.38726	1.9873
33700	1.89227	1.72893
33701	1.9052	2.1
33702	1.9052	1.69932
33703	1.90433	2.1
33704	1.89227	1.61834
33705	1.8914	2.1
33706	0.946129	1.83156
33707	0.946129	1.62411
33708	0.946133	1.16629
33709	0.946133	1.04947
33710	0.946131	1.06562
33711	0.946131	1.59221
33712	0.946131	1.89146
33713	0.946131	1.1056
33714	0.946131	1.29847
33715	0.946131	1.31526
33716	0.946131	1.30989
33717	0.946131	1.31402
33718	1.91316	0.398577
33719	1.89226	0.462914
33720	1.89226	0.453687
33721	1.89226	0.444793
33722	1.99366	0.404363
33723	1.99366	0.395421
33724	1.93083	0.365376
33725	1.89226	0.455549
33726	1.51379	0.420477
33727	1.89226	0.429168
33728	1.53051	0.386885
33729	1.91316	0.396095
33730	2.16731	2.1
33731	1.21373	2.1
33732	1.47804	2.1
33733	2.37601	2.1
33734	3.64535	2.1
33735	1.7211	2.1
33736	2.37601	2.1
33737	1.47804	2.1
33738	1.47804	2.1
33739	2.56843	2.1
33740	2.56843	2.1
33741	2.58731	2.1
33742	1.59094	2.1
33743	1.59094	2.1
33744	1.76816	2.1
33745	2.41707	2.1
33746	2.5943	2.1
33747	0.718346	2.1
33748	0.795468	2.1
33749	1.20854	2.1
33750	1.1224	2.1
33751	0.961203	2.1
33752	0.961203	2.1
33753	0.95667	2.1

33754	0.95667	2.1
33755	0.718346	2.1
33756	1.1224	2.1
33757	0.795469	2.1
33758	1.20854	2.1
33759	1.84076	2.1
33760	1.42884	2.1
33761	2.24274	2.1
33762	1.85415	2.1
33763	3.69491	2.1
33764	1.89226	2.1
33765	1.89226	1.66589
33766	1.89226	1.82133
33767	1.89226	2.1
33768	3.78452	2.04739
33769	2.24273	2.1
33770	2.24412	2.1
33771	1.85415	2.1
33772	1.85415	2.1
33773	2.68473	2.1
33774	2.68473	2.1
33775	1.89226	1.56074
33776	1.89226	2.1
33777	1.89226	2.1
33778	1.89226	1.39974
33779	1.89226	2.1
33780	1.89226	1.38623
33781	1.89226	1.37743
33782	1.89226	2.1
33783	1.89226	2.1
33784	1.89226	1.57442
33785	3.78451	2.04795
33786	1.85415	2.1
33787	1.10961	2.1
33788	2.24411	2.1
33789	1.51067	2.1
33790	3.35373	2.1
33791	1.70302	2.1
33792	1.89226	2.1
33793	1.76399	2.1
33794	1.43577	2.1
33795	2.08149	2.1
33796	1.70302	2.1
33797	1.89226	2.07884
33798	1.89226	2.1
33799	1.89226	2.08917
33800	1.89226	2.1
33801	1.89226	1.92392
33802	1.89226	2.1
33803	1.0593	2.1
33804	1.17289	2.1
33805	1.5379	2.1
33806	1.46757	2.1
33807	2.71079	2.1
33808	0.931235	2.1
33809	1.1151	2.1
33810	0.975236	2.1
33811	0.975236	2.1

33812	1.31147	2.1
33813	0.762882	2.1
33814	1.13152	2.1
33815	0.505799	2.1
33816	1.31149	2.1
33817	1.07629	2.1
33818	0.884817	2.1
33819	0.505793	2.1
33820	2.69027	2.1
33821	1.60441	2.1
33822	2.69027	2.1
33823	1.64771	2.1
33824	2.78977	2.1
33825	1.72731	2.1
33826	0.77878	0.147815
33827	0.843226	0.147815
33828	0.845997	0.147815
33829	0.926091	0.147815
33830	0.77878	0.147815
33831	0.926093	0.147815
33832	1.3922	1.5994
33833	1.3922	1.67627
33834	0.872156	1.99925
33835	0.872156	1.97712
33836	0.872157	2.09259
33837	0.946132	1.60817
33838	0.946129	1.33643
33839	1.31822	2.08236
33840	0.94613	1.8955
33841	0.94613	1.95601
33842	1.31823	1.84367
33843	0.872157	1.97418
33844	1.81534	0.80473
33845	1.89226	0.801781
33846	1.59602	0.876878
33847	1.59602	0.940448
33848	1.89226	0.725425
33849	1.89226	0.752933
33850	1.89227	0.786179
33851	1.89227	0.807653
33852	1.89227	0.830118
33853	1.89227	0.760375
33854	3.78453	0.754001
33855	1.89226	0.806862
33856	1.89226	0.674538
33857	1.89226	0.760119
33858	1.89226	0.761499
33859	3.78453	0.811949
33860	2.30348	0.578483
33861	1.4185	0.621206
33862	1.82551	0.679747
33863	1.02011	0.574939
33864	3.24401	0.56648
33865	2.46702	0.545213
33866	1.17129	0.596841
33867	1.82551	0.538937
33868	1.82551	0.568556
33869	1.73525	0.535858

33870	1.73525	0.607277
33871	1.89227	0.781609
33872	1.89227	0.673867
33873	1.89227	0.675252
33874	1.89227	0.70036
33875	1.89227	0.76079
33876	1.89227	0.77809
33877	2.80034	1.84435
33878	2.45767	2.1
33879	2.67994	1.7466
33880	2.35395	2.1
33881	5.1376	2.1
33882	2.45766	2.1
33883	1.8769	2.1
33884	2.00989	2.1
33885	2.00989	2.1
33886	2.35395	2.1
33887	2.35395	2.1
33888	2.57662	1.8323
33889	2.35395	2.1
33890	2.57662	2.1
33891	2.67993	2.04537
33892	2.24227	2.1
33893	2.34558	2.1
33894	1.02874	2.1
33895	1.02874	2.1
33896	1.03084	2.1
33897	1.03084	2.1
33898	0.938448	2.1
33899	0.886762	2.1
33900	1.17698	2.1
33901	1.12114	2.1
33902	1.12113	2.1
33903	0.886764	2.1
33904	0.93845	2.1
33905	1.17697	2.1
33906	1.91969	2.1
33907	2.5508	2.1
33908	1.88273	1.97556
33909	2.76609	2.1
33910	4.68578	2.07099
33911	2.83423	2.1
33912	1.89226	2.1
33913	2.76609	2.1
33914	1.89226	2.1
33915	4.65835	2.1
33916	1.56685	0.777331
33917	1.11712	0.749527
33918	1.07999	0.929381
33919	1.51448	0.833043
33920	2.6316	0.827008
33921	1.56685	0.714324
33922	1.67652	0.864307
33923	1.5955	0.99914
33924	1.51448	0.804565
33925	3.19099	0.840316
33926	1.89226	0.87978
33927	1.89226	0.682065

33928	1.89226	0.819252
33929	1.89226	0.887622
33930	3.78453	0.842738
33931	1.89226	0.847548
33932	1.89226	0.696662
33933	1.89226	0.88302
33934	1.89226	0.910359
33935	1.89226	0.712136
33936	1.89226	0.732443
33937	1.0562	0.855942
33938	1.51448	0.763499
33939	1.07999	0.821517
33940	1.07999	0.76623
33941	1.46441	0.830822
33942	1.46441	0.779285
33943	1.5955	0.965023
33944	1.51448	0.753737
33945	1.46441	0.847926
33946	1.52909	0.937911
33947	3.04357	0.868399
33948	1.89226	0.765925
33949	1.89226	0.870816
33950	1.89226	0.948439
33951	1.89226	0.768835
33952	3.78453	0.796151
33953	1.89227	0.77593
33954	1.89227	0.613327
33955	1.89227	0.854711
33956	1.89227	0.802244
33957	3.78453	0.79887
33958	1.89994	0.80409
33959	1.70303	0.964106
33960	1.68576	0.624972
33961	1.89608	0.869429
33962	3.8237	0.828889
33963	1.89994	0.769284
33964	2.31116	1.15249
33965	1.9386	1.02444
33966	1.88653	1.35818
33967	3.74885	1.11628
33968	2.31117	1.19695
33969	2.20665	1.54502
33970	2.09617	1.30954
33971	2.41055	2.1
33972	2.09617	1.46405
33973	2.41055	2.1
33974	1.10777	0.8781
33975	1.0523	0.936294
33976	0.912098	0.907586
33977	0.757533	0.953794
33978	0.89287	1.07002
33979	1.07571	0.721815
33980	0.77485	0.912646
33981	0.98645	0.6763
33982	0.989171	0.722141
33983	0.875089	0.792466
33984	0.979686	0.924238
33985	0.979686	1.10389

33986	2.52442	2.1
33987	1.49998	2.1
33988	2.51692	2.1
33989	1.56957	2.1
33990	4.09399	2.1
33991	1.01298	2.1
33992	1.01298	2.1
33993	1.03079	2.1
33994	1.03079	2.1
33995	0.820964	2.1
33996	0.784786	2.1
33997	1.25898	2.1
33998	1.25846	2.1
33999	1.25898	2.1
34000	0.820964	2.1
34001	1.25846	2.1
34002	0.784786	2.1
34003	1.89226	0.245656
34004	1.89226	0.253744
34005	2.6297	0.225331
34006	1.89226	0.227342
34007	3.78452	0.247762
34008	1.89226	0.222509
34009	1.89226	0.217199
34010	1.89226	0.205774
34011	1.89226	0.241979
34012	1.89226	0.207928
34013	1.89226	0.247613
34014	2.19397	0.200956
34015	1.89226	0.217217
34016	2.34112	0.20395
34017	2.34112	0.190866
34018	1.89226	0.213542
34019	1.89226	0.210209
34020	2.23366	2.1
34021	1.87598	2.1
34022	1.64716	2.1
34023	2.06048	2.1
34024	3.93645	2.1
34025	2.23367	2.1
34026	2.17384	2.1
34027	2.04818	2.1
34028	2.06048	2.1
34029	4.23432	2.1
34030	1.9855	2.1
34031	2.06048	2.1
34032	1.93163	2.1
34033	1.50737	2.1
34034	3.56785	2.1
34035	2.04818	2.1
34036	2.06048	2.1
34037	2.27465	2.1
34038	1.92264	2.1
34039	3.98312	2.1
34040	2.08149	0.972772
34041	1.89226	1.71171
34042	1.89226	1.36826
34043	1.33153	0.986504

34044	1.68038	1.82166
34045	1.18244	1.14827
34046	1.89226	1.11668
34047	1.89226	1.68468
34048	1.89226	1.50859
34049	1.89226	1.02846
34050	3.78452	1.17417
34051	2.69069	0.93255
34052	1.89226	1.09709
34053	1.89226	1.09172
34054	1.97397	0.973149
34055	1.89226	1.05115
34056	1.97397	0.92622
34057	1.89226	1.68163
34058	2.38056	1.10014
34059	1.89226	1.02954
34060	1.89226	1.34787
34061	3.78452	1.25723
34062	0.904435	2.1
34063	0.904435	2.1
34064	0.925315	2.1
34065	0.925315	2.1
34066	1.13312	2.1
34067	1.08109	2.1
34068	0.70697	2.1
34069	0.696633	2.1
34070	0.70697	2.1
34071	1.13312	2.1
34072	0.696633	2.1
34073	1.08109	2.1
34074	1.45506	2.1
34075	2.26623	2.1
34076	2.38363	2.1
34077	2.38363	2.1
34078	1.41394	2.1
34079	1.41394	2.1
34080	0.946131	1.8899
34081	0.946131	2.1
34082	1.13311	2.1
34083	1.13311	2.1
34084	0.946127	1.67377
34085	0.946127	2.1
34086	1.13312	2.1
34087	1.19182	2.1
34088	1.19181	2.1
34089	0.946129	2.1
34090	1.13312	2.1
34091	0.94613	2.1
34092	1.89226	0.949455
34093	1.89226	1.45332
34094	1.89226	0.961497
34095	1.89226	1.36668
34096	1.89226	0.9955
34097	1.89226	1.50816
34098	1.05168	2.05456
34099	0.696527	1.95151
34100	0.907442	1.13714
34101	0.907442	1.24452

34102	0.953145	1.11913
34103	0.782285	2.1
34104	0.773928	2.0023
34105	0.773928	1.18055
34106	0.696538	1.25598
34107	0.851554	1.27737
34108	1.04071	1.34961
34109	1.12335	1.39329
34110	1.89226	0.85162
34111	1.89226	0.748592
34112	1.89226	0.735621
34113	1.89226	0.715457
34114	3.78453	0.722322
34115	1.89226	0.790049
34116	1.89226	0.746281
34117	1.89226	0.734664
34118	1.89226	0.7186
34119	3.78453	0.768147
34120	2.50912	0.733214
34121	1.89226	0.715917
34122	1.89226	0.714542
34123	2.63195	0.67948
34124	4.40139	0.687429
34125	2.50912	0.734836
34126	1.3598	0.689116
34127	1.70303	0.691324
34128	2.36874	0.588825
34129	3.82055	0.723235
34130	1.89227	0.72906
34131	1.89227	0.711391
34132	1.89227	0.70046
34133	1.89227	0.585333
34134	3.78453	0.722774
34135	1.89227	0.72879
34136	1.89227	0.711988
34137	1.89227	0.703211
34138	1.89227	0.620931
34139	3.78453	0.661199
34140	1.89226	0.708372
34141	2.67088	0.559281
34142	2.63195	0.518619
34143	2.63195	0.663568
34144	1.89226	0.587242
34145	1.89226	0.710759
34146	1.30202	0.606541
34147	1.28304	0.627425
34148	0.809902	0.587902
34149	0.888801	0.594447
34150	1.13262	0.448351
34151	0.968276	0.550481
34152	1.0733	0.629477
34153	1.0733	0.60447
34154	1.35419	0.55887
34155	0.801222	0.474707
34156	0.899732	0.672914
34157	1.23967	0.537944
34158	1.31595	2.1
34159	0.869501	2.1

34160	2.65473	2.1
34161	1.88782	2.1
34162	3.20377	2.1
34163	1.89227	2.1
34164	1.89227	2.04975
34165	1.89227	1.96393
34166	1.89227	2.1
34167	3.78453	2.01008
34168	2.65473	2.1
34169	1.89226	2.1
34170	1.88781	2.1
34171	1.88782	2.1
34172	2.0815	2.1
34173	2.0815	2.1
34174	1.89226	1.5157
34175	1.89226	1.90175
34176	1.89226	1.81495
34177	1.89226	1.69251
34178	1.89226	1.66153
34179	1.89226	1.53339
34180	1.89226	1.59248
34181	1.89226	1.83378
34182	1.89226	1.98543
34183	1.89226	1.8774
34184	1.89226	1.81241
34185	1.89226	1.67634
34186	2.39057	1.82111
34187	1.89226	1.12861
34188	1.89226	0.997442
34189	1.89226	2.02076
34190	2.14243	0.972704
34191	2.14243	2.06057
34192	1.89226	0.771744
34193	1.89226	1.33877
34194	1.89226	0.890975
34195	1.89226	0.963431
34196	1.89226	0.935804
34197	1.89226	1.17888
34198	1.89226	0.814089
34199	1.89226	1.52426
34200	1.89226	0.902889
34201	1.89226	1.0071
34202	1.89226	0.928667
34203	1.89226	1.30083
34204	2.56323	0.29474
34205	1.36324	0.336021
34206	1.05296	0.309646
34207	2.18048	0.332071
34208	3.54372	0.310623
34209	0.946128	1.98691
34210	0.946128	2.1
34211	1.08733	2.1
34212	1.08733	2.1
34213	0.946131	2.1
34214	0.946131	2.07993
34215	1.09197	2.1
34216	1.08732	2.1
34217	0.946131	2.1

34218	1.09197	2.1
34219	1.08732	2.1
34220	0.946131	2.1
34221	1.35111	1.27138
34222	1.51327	1.28523
34223	1.64493	1.25045
34224	1.31058	1.31463
34225	2.99605	1.44782
34226	1.8124	1.24134
34227	1.51327	1.37175
34228	1.64493	1.38558
34229	1.31358	1.27367
34230	1.8124	1.34695
34231	1.48105	1.22926
34232	1.64493	1.23099
34233	1.31058	1.30643
34234	1.22297	1.22471
34235	1.70101	1.23743
34236	2.8679	1.15427
34237	1.70101	1.28991
34238	1.99297	1.2008
34239	1.64493	1.31704
34240	1.64493	1.20044
34241	1.99297	1.29922
34242	1.8124	1.15527
34243	2.0815	2.1
34244	1.80258	2.1
34245	1.80258	2.1
34246	1.83506	2.1
34247	2.0815	2.1
34248	2.1212	2.1
34249	2.0815	2.1
34250	1.82129	2.1
34251	1.82129	2.1
34252	1.80445	2.1
34253	2.0815	2.1
34254	2.06092	2.1
34255	1.89224	2.1
34256	1.703	2.1
34257	1.97839	2.1
34258	1.703	2.1
34259	1.97402	2.1
34260	1.703	2.1
34261	1.89226	0.780038
34262	1.89226	0.859223
34263	1.89226	0.820792
34264	2.75351	0.817027
34265	3.78453	0.826878
34266	1.89226	0.821927
34267	3.02887	0.955034
34268	2.03149	0.801916
34269	2.03149	0.85129
34270	1.89226	0.815925
34271	1.89226	0.868511
34272	2.17979	2.1
34273	1.65533	2.1
34274	2.57212	2.1
34275	2.20346	2.1

34276	4.22745	2.1
34277	1.89227	2.1
34278	2.20347	2.1
34279	1.89227	2.1
34280	2.57212	2.1
34281	1.89227	2.1
34282	2.57212	2.1
34283	2.05274	0.968307
34284	2.16783	2.1
34285	2.24429	0.910977
34286	2.38404	2.1
34287	4.41212	1.29894
34288	2.16783	2.05317
34289	2.05967	2.1
34290	2.38404	2.1
34291	2.24695	2.1
34292	4.41478	2.1
34293	2.24429	0.939632
34294	2.38404	2.1
34295	2.51819	0.94345
34296	2.60723	2.0516
34297	4.85152	1.44912
34298	2.38404	2.1
34299	2.24695	2.1
34300	2.60723	2.00964
34301	2.45247	2.1
34302	4.85418	2.1
34303	1.89227	2.1
34304	1.89227	2.1
34305	1.89227	2.1
34306	1.89227	2.1
34307	3.78453	2.1
34308	1.89226	2.1
34309	1.89226	2.1
34310	1.89226	2.1
34311	1.89226	2.1
34312	3.78453	2.1
34313	0.618473	2.1
34314	0.619945	2.1
34315	0.797692	2.1
34316	0.770147	2.1
34317	0.68852	2.1
34318	0.68852	2.1
34319	0.701573	2.1
34320	0.701573	2.1
34321	0.619945	2.1
34322	0.797691	2.1
34323	0.618473	2.1
34324	0.770147	2.1
34325	0.797692	2.1
34326	0.770148	2.1
34327	0.694787	2.1
34328	0.653411	2.1
34329	0.722102	2.1
34330	0.722102	2.1
34331	0.729001	2.1
34332	0.729001	2.1
34333	0.797692	2.1

34334	0.694787	2.1
34335	0.770148	2.1
34336	0.653412	2.1
34337	1.89227	2.1
34338	1.89227	2.1
34339	1.89227	2.1
34340	1.89227	2.1
34341	3.78453	2.1
34342	1.89226	2.1
34343	1.89226	2.1
34344	1.89226	2.1
34345	1.89226	2.1
34346	3.78453	2.1
34347	1.23989	2.1
34348	1.59538	2.1
34349	1.66647	2.1
34350	1.26599	2.1
34351	2.90636	2.1
34352	1.66647	2.1
34353	1.38957	2.1
34354	1.47446	2.1
34355	1.40337	2.1
34356	1.66647	2.1
34357	1.59538	2.1
34358	2.80619	2.1
34359	2.0815	2.1
34360	2.0815	2.1
34361	2.0815	2.1
34362	2.73721	2.1
34363	2.74192	2.1
34364	2.73721	2.1
34365	2.0815	2.1
34366	2.0815	2.1
34367	2.0815	2.1
34368	2.689	2.1
34369	2.69166	2.1
34370	2.62369	2.1
34371	2.19717	2.1
34372	2.60405	2.1
34373	2.20566	2.1
34374	4.80122	2.1
34375	2.19717	2.1
34376	1.37392	2.1
34377	2.20566	2.1
34378	1.45408	2.1
34379	3.65125	2.1
34380	2.60405	2.1
34381	2.20566	2.1
34382	2.57466	2.1
34383	2.21715	2.1
34384	4.8212	2.1
34385	2.20566	2.1
34386	1.54249	2.1
34387	1.54249	2.1
34388	1.45408	2.1
34389	2.21715	2.1
34390	2.12874	2.1
34391	1.60647	1.82469

34392	1.31346	2.1
34393	1.82649	1.56973
34394	1.51067	2.1
34395	3.13995	2.1
34396	1.89226	1.29746
34397	1.89226	2.1
34398	1.89226	1.58643
34399	1.89226	2.1
34400	3.78452	2.1
34401	1.89226	2.1
34402	2.6096	2.1
34403	2.8067	2.1
34404	2.8067	2.1
34405	1.89226	2.1
34406	1.89226	2.1
34407	1.82649	1.34788
34408	1.3596	2.1
34409	2.17708	1.61922
34410	1.70302	2.1
34411	3.40243	2.1
34412	1.89226	1.36669
34413	1.89226	2.1
34414	1.89226	1.1354
34415	1.89226	2.1
34416	3.78452	1.9056
34417	2.27067	2.1
34418	2.43549	2.1
34419	2.63979	2.1
34420	2.41828	2.1
34421	2.27067	2.1
34422	2.04916	2.1
34423	1.30047	2.1
34424	2.04916	2.1
34425	2.27068	2.1
34426	1.5586	2.1
34427	3.57115	2.1
34428	2.27068	2.1
34429	1.84214	2.1
34430	1.80224	2.1
34431	1.5586	2.1
34432	2.51432	2.1
34433	2.27068	2.1
34434	1.63719	2.1
34435	2.15714	2.1
34436	2.07821	2.1
34437	1.52204	2.1
34438	3.67919	2.1
34439	2.07821	2.1
34440	1.40118	2.1
34441	1.40118	2.1
34442	1.52204	2.1
34443	1.99632	2.1
34444	2.11718	2.1
34445	0.946131	1.67071
34446	0.946131	1.66618
34447	1.06957	2.03146
34448	1.06957	2.1
34449	0.946132	2.05256

34450	0.946132	1.32622
34451	1.411	1.78784
34452	1.06957	2.1
34453	0.946132	1.78029
34454	1.411	1.858
34455	1.06957	1.93432
34456	0.946132	1.88726
34457	1.99875	0.871029
34458	2.57079	0.842689
34459	2.59319	1.01902
34460	1.9105	0.785526
34461	4.59194	0.869098
34462	1.99875	0.855436
34463	1.02247	0.718011
34464	1.07017	0.764637
34465	1.15841	0.767626
34466	1.91051	0.761113
34467	1.99875	0.769815
34468	2.59319	0.976379
34469	1.9105	0.799079
34470	2.33812	0.859873
34471	2.59319	0.85471
34472	1.59457	0.882175
34473	1.59457	0.884657
34474	2.50906	0.747605
34475	2.0467	0.715314
34476	2.03357	0.725638
34477	2.53176	0.715526
34478	4.54263	0.721138
34479	1.294	0.777346
34480	1.66636	0.737717
34481	2.03357	0.727526
34482	1.03079	0.721406
34483	3.32757	0.730799
34484	1.68108	0.71988
34485	2.53176	0.718514
34486	2.36417	0.708177
34487	2.71666	0.708772
34488	1.68108	0.756683
34489	2.03357	0.746584
34490	1.89226	0.622123
34491	2.59302	0.626546
34492	2.2727	0.601611
34493	2.2727	0.575055
34494	1.89226	0.68548
34495	1.89226	0.62532
34496	1.89226	0.724513
34497	1.89226	0.705571
34498	1.89226	0.690553
34499	1.89226	0.542629
34500	3.78452	0.725654
34501	1.89225	0.732422
34502	1.89225	0.433935
34503	1.89225	0.547596
34504	1.89225	0.637507
34505	1.89225	0.570486
34506	1.89225	0.664926
34507	0.934114	0.659972

34508	0.934114	0.498674
34509	0.944544	0.598373
34510	0.944544	0.58942
34511	0.721493	0.46602
34512	0.58215	0.432144
34513	1.2965	0.592134
34514	1.13634	0.492385
34515	1.13635	0.447723
34516	0.582148	0.436668
34517	1.29651	0.585607
34518	0.721491	0.498304
34519	1.89226	0.589559
34520	1.89226	0.49664
34521	1.89226	0.47815
34522	1.89226	0.418569
34523	3.78452	0.417943
34524	0.946131	0.719585
34525	0.946131	0.736622
34526	0.946131	0.672678
34527	0.946131	0.724102
34528	0.946131	0.673517
34529	0.946131	0.696833
34530	0.946131	0.729461
34531	0.946131	0.7684
34532	0.946131	0.710885
34533	0.946131	0.706002
34534	0.946131	0.70712
34535	0.946131	0.706464
34536	1.89226	0.328555
34537	1.89226	0.343156
34538	2.78602	0.306779
34539	1.89226	0.419406
34540	3.78453	0.31415
34541	1.52601	2.1
34542	1.76601	2.1
34543	1.27839	2.1
34544	1.56095	2.1
34545	3.0444	2.1
34546	1.76601	2.1
34547	1.86793	2.1
34548	1.56095	2.1
34549	1.68152	2.1
34550	3.44753	2.1
34551	1.27841	2.1
34552	1.56097	2.1
34553	1.0577	2.1
34554	1.36861	2.1
34555	2.64702	2.1
34556	1.56097	2.1
34557	1.68154	2.1
34558	1.36861	2.1
34559	1.53296	2.1
34560	3.05015	2.1
34561	1.89226	2.1
34562	1.89226	2.1
34563	1.89226	2.1
34564	1.89226	2.1
34565	3.78453	2.1

34566	0.983075	2.1
34567	0.788361	2.1
34568	0.724726	2.1
34569	0.724726	2.1
34570	0.983027	2.1
34571	0.724734	2.1
34572	0.788364	2.1
34573	0.788364	2.1
34574	0.788335	2.1
34575	0.94613	2.1
34576	0.724757	2.1
34577	0.946131	2.1
34578	1.15789	2.1
34579	2.11135	2.1
34580	2.25826	2.1
34581	1.28845	2.1
34582	3.41615	2.1
34583	1.89226	2.1
34584	1.89226	2.1
34585	1.89226	2.1
34586	1.89226	2.1
34587	3.78453	2.1
34588	1.57667	2.1
34589	1.70002	2.1
34590	1.70002	2.1
34591	1.89226	2.1
34592	1.89226	2.1
34593	2.05244	2.1
34594	2.25826	2.1
34595	1.42214	2.1
34596	1.43675	2.1
34597	1.28845	2.1
34598	2.40656	2.1
34599	2.25826	2.1
34600	1.58066	2.1
34601	1.74247	2.1
34602	1.85097	2.1
34603	1.61155	2.1
34604	3.43163	2.1
34605	0.785612	2.1
34606	0.789638	2.1
34607	0.882561	2.1
34608	0.763529	2.1
34609	0.686921	2.1
34610	0.86048	2.1
34611	0.871812	2.1
34612	0.871812	2.1
34613	0.830183	2.1
34614	0.882572	2.1
34615	0.902315	2.1
34616	0.785602	2.1
34617	1.01972	0.21365
34618	0.936657	0.199632
34619	0.946131	0.220947
34620	0.946131	0.243397
34621	0.927018	0.239754
34622	0.927018	0.213282
34623	1.1376	0.221035

34624	0.953663	0.197564
34625	1.1257	0.211752
34626	1.04073	0.213662
34627	1.00358	0.219059
34628	0.851528	0.211536
34629	1.73576	0.7
34630	2.00244	0.732251
34631	1.17798	0.695681
34632	1.25796	0.700014
34633	2.99373	0.699177
34634	2.00244	0.72305
34635	1.10719	0.709451
34636	2.03236	0.7516
34637	2.03236	0.713664
34638	1.25797	0.749755
34639	1.25797	0.702877
34640	1.89226	2.1
34641	1.89226	2.1
34642	1.89226	2.1
34643	1.89226	2.1
34644	3.78452	2.1
34645	2.3174	2.1
34646	1.50005	2.1
34647	1.90428	2.1
34648	2.64564	2.1
34649	4.22168	2.1
34650	1.89226	2.1
34651	1.89226	2.1
34652	1.89226	2.1
34653	1.89226	2.1
34654	1.89226	2.1
34655	1.89226	2.1
34656	1.32749	0.341321
34657	1.70302	0.411788
34658	2.08149	0.376549
34659	1.70302	0.384986
34660	2.19634	0.359084
34661	1.93083	0.362286
34662	1.89226	1.23814
34663	1.89226	1.54048
34664	1.89226	0.839896
34665	1.89226	1.68195
34666	3.78453	1.04671
34667	1.89226	1.27659
34668	1.89226	1.53477
34669	1.89226	0.972894
34670	1.89226	1.23989
34671	3.78452	1.36515
34672	2.17499	1.04692
34673	1.79663	1.5564
34674	2.1538	0.85417
34675	1.818	1.08909
34676	2.17499	1.40542
34677	1.83919	1.85775
34678	0.898316	1.57713
34679	1.0769	1.17925
34680	0.968276	1.67573
34681	0.773495	1.32239

34682	0.921126	1.42367
34683	0.921126	1.65295
34684	0.92927	1.44964
34685	0.92927	1.40909
34686	1.0769	1.3576
34687	0.968276	1.17785
34688	0.898316	1.60628
34689	0.773495	1.33874
34690	1.89226	0.985315
34691	1.89226	1.78737
34692	1.89226	1.13625
34693	1.89226	1.08553
34694	3.78453	1.49052
34695	1.89226	1.21436
34696	1.89226	1.6846
34697	1.89226	0.898726
34698	1.89226	1.3043
34699	3.78452	1.11935
34700	2.17499	1.53275
34701	2.1538	1.85337
34702	2.53574	1.08667
34703	2.54157	0.987626
34704	4.71073	1.43587
34705	1.0769	1.31539
34706	1.26787	1.08898
34707	1.16641	1.2301
34708	0.968276	1.59732
34709	1.11625	1.23606
34710	1.11625	1.4113
34711	1.11989	1.50764
34712	1.11989	0.883665
34713	1.26787	1.11675
34714	1.16641	1.57055
34715	1.0769	1.64427
34716	0.968277	1.39399
34717	1.89226	0.708093
34718	1.89226	0.715901
34719	1.89226	0.710694
34720	1.89226	0.7024
34721	3.78452	0.7069
34722	1.91739	0.701688
34723	2.20045	0.699847
34724	1.79005	0.698493
34725	2.33802	0.697066
34726	4.25539	0.689826
34727	2.52754	0.610709
34728	1.89226	0.682031
34729	2.69841	0.635137
34730	2.69841	0.672935
34731	1.89226	0.621197
34732	1.89226	0.664166
34733	1.89226	0.711533
34734	1.89226	0.709457
34735	1.89226	0.7024
34736	1.89226	0.706021
34737	3.78452	0.706167
34738	1.93544	0.7
34739	2.33802	0.70081

34740	1.55063	0.688735
34741	2.1769	0.699116
34742	3.88865	0.7
34743	2.20929	0.624601
34744	2.55162	0.522978
34745	2.27807	0.660839
34746	1.48831	0.523747
34747	4.48736	0.538643
34748	2.27807	0.662379
34749	2.72723	0.527782
34750	2.55162	0.576167
34751	2.55162	0.649597
34752	2.43157	0.536831
34753	2.43157	0.63532
34754	2.27807	0.578601
34755	1.51612	0.461636
34756	1.96048	0.480877
34757	1.73022	0.472427
34758	1.70302	0.575189
34759	1.61016	0.536443
34760	2.27807	0.580408
34761	2.08048	0.448153
34762	1.98522	0.477541
34763	2.43157	0.541978
34764	1.83798	0.476827
34765	2.27807	0.540038
34766	1.70302	0.712043
34767	2.68507	0.63549
34768	2.23856	0.743428
34769	2.52174	0.578852
34770	2.35331	0.779683
34771	2.65099	0.649808
34772	1.70303	0.724772
34773	0.986848	0.504348
34774	2.41566	0.547591
34775	1.70303	0.520513
34776	1.88139	0.545285
34777	1.64955	0.535201
34778	1.89226	2.00315
34779	2.34978	1.85811
34780	1.89226	2.00795
34781	2.68773	1.54429
34782	1.89226	2.08427
34783	2.68773	1.75904
34784	1.89226	1.9564
34785	1.89226	1.51194
34786	1.89226	1.47496
34787	1.89226	1.91294
34788	3.78453	1.84995
34789	1.00825	1.3356
34790	1.09847	1.56692
34791	0.939987	1.38884
34792	0.783516	1.77205
34793	0.838041	1.5633
34794	0.838041	1.87322
34795	1.49693	1.92455
34796	1.26602	1.56914
34797	1.10875	1.44008

34798	1.05187	1.99069
34799	1.28743	1.63227
34800	0.946132	1.40068
34801	0.946132	1.54365
34802	0.946132	2.01114
34803	0.946132	1.90342
34804	0.946132	1.33718
34805	1.31823	2.06941
34806	0.946132	1.50265
34807	0.946132	1.87454
34808	0.946131	1.95201
34809	0.946131	2.08829
34810	0.946132	1.78941
34811	1.31823	1.95417
34812	1.51688	0.557354
34813	1.79978	0.771115
34814	1.1685	0.611807
34815	1.1685	0.669597
34816	1.85857	0.627824
34817	1.51018	0.683136
34818	0.946116	0.580473
34819	0.946116	0.669683
34820	0.94612	0.476542
34821	0.94612	0.526859
34822	0.946118	0.475862
34823	0.946118	0.595085
34824	0.946118	0.539542
34825	0.946118	0.674509
34826	0.946131	0.546717
34827	0.946131	0.575093
34828	0.946105	0.579648
34829	0.946105	0.574559
34830	1.89224	2.1
34831	1.89224	2.1
34832	1.89224	2.1
34833	1.89224	2.1
34834	1.89224	2.1
34835	1.89224	2.1
34836	1.38678	2.1
34837	1.3349	2.1
34838	0.946119	2.1
34839	0.946119	2.1
34840	1.38678	2.1
34841	1.38678	2.1
34842	0.946119	2.1
34843	0.946119	2.1
34844	1.38676	2.1
34845	0.946107	2.1
34846	0.946132	2.1
34847	1.33491	2.1
34848	1.06455	2.1
34849	1.03932	2.1
34850	1.20649	2.1
34851	1.18773	2.1
34852	2.27105	2.1
34853	1.89226	2.1
34854	1.89226	2.1
34855	1.89226	2.1

34856	1.89226	2.1
34857	1.89226	2.1
34858	1.89226	2.1
34859	1.36918	2.1
34860	1.18774	2.1
34861	1.31144	2.1
34862	1.20651	2.1
34863	1.31144	2.1
34864	1.20651	2.1
34865	2.29066	2.1
34866	1.76476	2.1
34867	1.79825	2.1
34868	2.33801	2.1
34869	4.08891	2.1
34870	1.79825	2.1
34871	2.33801	2.1
34872	2.37168	2.1
34873	1.49325	2.1
34874	4.16993	2.1
34875	1.13641	2.1
34876	1.43213	1.14299
34877	1.44085	1.33634
34878	1.18301	1.98983
34879	2.61513	1.71402
34880	1.89226	1.82651
34881	2.23875	2.1
34882	2.23875	1.70032
34883	2.10287	2.1
34884	1.89226	1.72883
34885	1.75639	2.1
34886	1.89226	1.15282
34887	1.89226	1.79271
34888	1.89226	1.17111
34889	1.89226	1.914
34890	1.89226	1.15365
34891	1.89226	2.01999
34892	1.44085	1.42928
34893	1.25173	1.91483
34894	1.25173	1.24642
34895	1.18301	2.04864
34896	1.4617	1.22364
34897	1.39298	2.1
34898	1.89227	1.74246
34899	2.23875	2.1
34900	2.42504	2.1
34901	1.89227	1.4953
34902	4.13101	2.06782
34903	1.89226	1.16261
34904	1.89226	1.41822
34905	1.89226	1.11843
34906	1.89226	1.80438
34907	1.89226	1.11992
34908	1.89226	1.92049
34909	1.89226	2.1
34910	2.41297	2.1
34911	2.70685	1.97815
34912	2.70685	2.1
34913	1.89226	1.9538

34914	1.89226	2.1
34915	1.89226	2.1
34916	1.89226	2.1
34917	1.89226	2.1
34918	1.89226	2.1
34919	1.89226	2.1
34920	1.89226	2.1
34921	1.89226	2.0255
34922	1.89226	2.1
34923	1.89226	2.1
34924	1.89226	2.1
34925	3.78453	2.1
34926	1.89226	2.1
34927	1.89226	2.1
34928	1.89226	2.1
34929	1.89226	2.1
34930	1.89226	2.1
34931	1.89226	2.1
34932	1.69044	0.212101
34933	1.57979	0.22852
34934	2.27963	0.228846
34935	2.21582	0.210315
34936	3.85942	0.217208
34937	2.21582	0.209858
34938	1.30722	0.224674
34939	2.02069	0.225573
34940	2.02069	0.216223
34941	1.57979	0.225688
34942	1.57979	0.21244
34943	1.67825	2.1
34944	1.89226	2.1
34945	1.89226	2.1
34946	1.89226	2.1
34947	1.91894	2.1
34948	1.91894	2.1
34949	0.946133	2.1
34950	0.946133	2.1
34951	0.946132	2.1
34952	0.946132	2.1
34953	0.946132	2.1
34954	0.946132	2.1
34955	0.946133	2.1
34956	0.946133	2.1
34957	0.946133	2.1
34958	0.946133	2.1
34959	0.946132	2.1
34960	0.946132	2.1
34961	0.942609	1.89595
34962	0.942609	1.94307
34963	1.329	2.1
34964	1.329	2.1
34965	1.32547	2.05768
34966	0.94261	2.1
34967	0.946132	1.93178
34968	0.946132	2.1
34969	0.946132	1.98238
34970	1.32547	2.08949
34971	0.946132	2.1

34972	0.94261	2.1
34973	1.55831	1.56053
34974	1.07496	1.72446
34975	1.80327	0.866366
34976	1.34851	0.934045
34977	2.87824	1.0335
34978	2.14346	0.795577
34979	2.77937	0.777431
34980	2.79173	0.919795
34981	2.17129	0.687669
34982	4.95066	0.736409
34983	2.14346	0.811262
34984	1.34124	0.531348
34985	1.39818	0.767099
34986	2.1713	0.736621
34987	3.51253	0.7633
34988	2.79173	0.873919
34989	2.21882	0.66381
34990	2.17129	0.696022
34991	2.17129	0.734145
34992	2.81448	0.777034
34993	2.81448	0.812712
34994	1.10941	0.700303
34995	1.08565	0.768243
34996	0.699089	0.59094
34997	0.740176	0.711066
34998	0.908594	0.578963
34999	0.908594	0.644481
35000	0.91723	0.831765
35001	0.91723	0.628249
35002	1.10941	0.671952
35003	0.740176	0.6328
35004	0.699089	0.632029
35005	1.08565	0.621769
35006	1.89227	0.699411
35007	1.89227	0.698788
35008	1.89227	0.545751
35009	1.89227	0.45997
35010	3.78453	0.665407
35011	2.40032	0.704968
35012	2.19526	0.609331
35013	1.12907	0.465689
35014	1.44253	0.7
35015	3.32433	0.605249
35016	2.19526	0.539861
35017	2.71093	0.7
35018	1.29826	0.656971
35019	2.0815	0.440568
35020	4.18911	0.661649
35021	1.89226	0.63452
35022	1.89226	0.581228
35023	1.89226	0.399122
35024	1.89226	0.464605
35025	3.78452	0.46596
35026	2.30822	0.578634
35027	1.89226	0.45593
35028	2.79329	0.380435
35029	2.79329	0.363722

35030	1.89226	0.62951
35031	1.89226	0.54488
35032	0.946131	1.53767
35033	0.946131	1.02459
35034	1.38414	2.03867
35035	1.38414	0.858821
35036	0.94613	1.9439
35037	0.94613	1.79241
35038	1.38415	1.09275
35039	1.41833	0.879604
35040	0.946132	1.15711
35041	1.41833	1.05995
35042	1.38414	1.15081
35043	0.946131	1.27575
35044	1.89226	1.59893
35045	2.6536	2.1
35046	1.89226	1.47953
35047	2.72238	2.1
35048	4.61465	2.1
35049	0.946132	2.1
35050	0.946132	1.69437
35051	1.38657	1.59543
35052	1.38657	2.1
35053	1.38657	2.1
35054	1.36119	2.1
35055	0.946131	1.26027
35056	0.946131	1.77485
35057	0.946132	2.1
35058	1.38657	2.1
35059	0.946132	1.96885
35060	1.36119	2.1
35061	1.89226	2.1
35062	2.4717	2.1
35063	2.5026	2.1
35064	2.4717	2.1
35065	1.92316	2.1
35066	1.89226	2.1
35067	1.89226	2.1
35068	1.89226	1.88307
35069	1.89226	2.1
35070	1.89226	1.13176
35071	3.78453	2.1
35072	0.946128	2.1
35073	0.946128	1.11331
35074	1.3419	1.65633
35075	1.3419	2.1
35076	0.946131	1.43815
35077	0.946131	1.47128
35078	1.36607	2.1
35079	1.3419	2.1
35080	0.946131	2.1
35081	1.36607	2.1
35082	1.3419	2.1
35083	0.94613	2.1
35084	1.89226	2.1
35085	2.50259	2.1
35086	2.53563	2.1
35087	2.50259	2.1

35088	1.92529	2.1
35089	1.89226	2.1
35090	0.946132	1.51615
35091	0.946132	2.1
35092	0.946131	1.71733
35093	0.946131	2.1
35094	0.946132	1.54205
35095	0.946132	1.91267
35096	0.946131	2.1
35097	0.946131	2.1
35098	0.946131	2.1
35099	0.946131	2.1
35100	0.946131	2.1
35101	0.946131	2.1
35102	2.0815	2.1
35103	1.86506	2.1
35104	1.86506	2.1
35105	1.82567	2.1
35106	2.0815	2.1
35107	2.03335	2.1
35108	2.0815	2.1
35109	2.0815	2.1
35110	2.0815	2.1
35111	1.88668	2.1
35112	1.83221	2.1
35113	1.57275	2.1
35114	2.34061	0.29205
35115	1.89226	0.325183
35116	2.23254	0.277817
35117	1.89226	0.27917
35118	2.23254	0.335137
35119	1.89226	0.336879
35120	1.8619	0.333958
35121	1.89226	0.383857
35122	2.34061	0.316417
35123	1.89226	0.315869
35124	2.34061	0.384585
35125	1.89226	0.38189
35126	1.89227	0.364404
35127	1.89227	0.328111
35128	1.89227	0.378122
35129	1.89227	0.394919
35130	1.89227	0.29814
35131	1.89227	0.321408
35132	1.89227	0.316249
35133	1.89227	0.287578
35134	1.89227	0.320116
35135	1.89227	0.340177
35136	1.89227	0.270131
35137	1.89227	0.294939
35138	1.06365	0.346192
35139	0.503305	0.311812
35140	0.794912	0.283214
35141	1.42752	0.290886
35142	1.34826	0.320532
35143	1.07163	0.341928
35144	0.533113	0.348588
35145	0.761277	0.317436

35146	0.848403	0.334842
35147	0.876191	0.289543
35148	0.963372	0.364656
35149	0.963372	0.327007
35150	1.55571	0.256695
35151	2.76574	0.304122
35152	2.92453	0.262657
35153	1.70301	0.281276
35154	2.33089	0.2932
35155	1.06905	0.305178
35156	2.28979	2.1
35157	1.81488	2.1
35158	1.63722	2.1
35159	2.22627	2.1
35160	4.04115	2.1
35161	1.98246	2.1
35162	2.15715	2.1
35163	2.22627	2.1
35164	2.22627	2.1
35165	1.63719	2.1
35166	1.63719	2.1
35167	2.1644	0.223229
35168	1.903	0.248854
35169	1.23639	0.219716
35170	1.42704	0.272402
35171	3.61546	0.237384
35172	1.89226	0.267543
35173	1.89226	0.348757
35174	1.89226	0.300115
35175	1.89226	0.32297
35176	3.78451	0.313051
35177	2.61343	0.275784
35178	1.89226	0.296562
35179	2.08149	0.293217
35180	1.74357	0.246751
35181	2.08149	0.315423
35182	1.74357	0.278672
35183	1.63483	0.22041
35184	1.8631	0.210303
35185	1.8631	0.235676
35186	1.70301	0.20476
35187	2.0815	0.232598
35188	1.94319	0.208409
35189	2.0815	0.221198
35190	1.89226	0.209246
35191	1.89226	0.240307
35192	2.05236	0.20599
35193	2.23014	0.234192
35194	2.41882	0.205029
35195	2.14993	1.95576
35196	1.87217	1.71775
35197	2.34488	2.09515
35198	2.5254	1.49043
35199	4.39757	1.56871
35200	0.949995	2.1
35201	0.630972	2.08109
35202	0.936084	2.03566
35203	1.17244	2.1

35204	0.922394	2.01702
35205	0.922394	2.04851
35206	0.88102	2.1
35207	0.88102	2.1
35208	0.949998	2.1
35209	1.17244	2.1
35210	0.936081	2.1
35211	0.630973	2.1
35212	1.89226	2.0221
35213	1.89226	1.74944
35214	1.89226	1.93728
35215	1.89226	1.98485
35216	3.78452	2.08345
35217	1.89226	1.37609
35218	1.89226	1.53716
35219	1.89226	1.30414
35220	1.89226	1.59741
35221	1.89226	1.28621
35222	1.89226	1.58605
35223	2.34488	1.70768
35224	2.5254	1.35128
35225	1.89226	1.47008
35226	1.89226	1.61994
35227	4.41766	1.78724
35228	1.89226	1.6735
35229	1.89226	1.82696
35230	1.89226	1.75872
35231	1.89226	1.64765
35232	3.78452	1.49888
35233	1.89226	1.26723
35234	1.89226	1.49877
35235	1.89226	1.21923
35236	1.89226	1.42736
35237	1.89226	1.21257
35238	1.89226	1.4185
35239	1.5605	2.1
35240	1.15806	2.1
35241	1.31565	2.1
35242	1.65642	2.1
35243	2.87615	2.1
35244	1.46643	2.1
35245	1.65642	2.1
35246	1.31565	2.1
35247	1.31565	2.1
35248	1.74246	2.1
35249	1.74246	2.1
35250	1.89226	2.1
35251	1.89226	2.1
35252	1.89226	2.07923
35253	1.89226	2.1
35254	1.89226	2.07118
35255	1.89226	2.1
35256	1.15099	2.1
35257	1.15099	2.1
35258	0.939316	2.1
35259	0.939316	2.1
35260	0.946127	2.1
35261	0.946127	2.1

35262	1.14418	2.1
35263	0.939289	2.1
35264	1.14417	2.1
35265	0.946129	2.1
35266	0.939287	2.1
35267	0.94613	2.1
35268	1.89226	2.01356
35269	1.89226	2.1
35270	1.89226	2.1
35271	1.89226	1.89235
35272	3.78452	2.1
35273	1.93164	0.400719
35274	2.64898	0.321829
35275	1.43213	0.351825
35276	1.43213	0.434278
35277	2.63206	0.334251
35278	2.13254	0.418525
35279	1.89226	2.1
35280	1.89226	2.1
35281	1.89226	2.1
35282	1.89226	2.1
35283	3.78452	2.1
35284	2.71655	2.1
35285	1.43323	2.1
35286	1.28413	2.1
35287	2.53614	2.1
35288	3.96937	2.1
35289	1.89226	2.1
35290	1.89226	2.1
35291	1.89226	2.1
35292	1.89226	2.1
35293	1.89226	2.1
35294	1.89226	2.1
35295	1.33356	2.1
35296	2.82297	2.1
35297	2.771	2.1
35298	2.771	2.1
35299	1.46037	2.1
35300	1.46037	2.1
35301	2.56034	2.1
35302	1.18474	2.1
35303	1.15113	2.1
35304	2.53724	2.1
35305	3.72198	2.1
35306	2.56034	2.1
35307	1.89226	2.1
35308	1.89226	2.1
35309	2.53725	2.1
35310	4.42951	2.1
35311	1.89226	2.1
35312	1.89226	2.1
35313	1.89226	2.1
35314	1.89226	2.1
35315	3.78453	2.1
35316	1.89226	2.1
35317	1.89226	2.1
35318	1.89226	2.1
35319	1.89226	2.05395

35320	3.78452	2.1
35321	1.1229	2.1
35322	2.53724	2.1
35323	1.15113	2.1
35324	1.15113	2.1
35325	2.51559	2.1
35326	2.51559	2.1
35327	1.89226	2.1
35328	2.53724	2.1
35329	2.51559	2.1
35330	2.53724	2.1
35331	1.87061	2.1
35332	1.89226	2.1
35333	1.89226	2.1
35334	1.89226	2.1
35335	1.89226	2.1
35336	1.89226	2.1
35337	3.78453	2.1
35338	1.89226	2.1
35339	1.89226	2.1
35340	1.89226	2.1
35341	1.89226	2.1
35342	3.78452	2.1
35343	1.98143	1.21219
35344	2.81559	0.78388
35345	1.65411	0.893041
35346	2.26675	1.07629
35347	4.24818	0.95828
35348	0.810656	1.0077
35349	0.810656	2.1
35350	0.821888	2.1
35351	0.821888	1.54648
35352	1.1235	1.05734
35353	1.07439	1.62133
35354	0.535693	2.1
35355	0.509042	2.1
35356	0.535693	2.1
35357	1.1235	2.1
35358	0.509043	2.1
35359	1.07439	2.1
35360	1.10378	2.1
35361	2.247	1.68219
35362	2.27472	2.1
35363	2.27472	1.77622
35364	1.07139	2.1
35365	1.07139	2.03496
35366	2.08081	2.1
35367	1.40395	2.1
35368	1.34245	2.1
35369	2.00239	2.1
35370	3.40634	2.1
35371	1.89226	2.1
35372	1.89226	2.1
35373	1.89226	2.1
35374	1.89226	2.1
35375	3.78453	2.1
35376	0.946128	2.1
35377	0.946128	2.1

35378	1.13785	2.1
35379	1.11496	2.1
35380	0.946131	2.1
35381	0.946131	2.1
35382	1.11495	2.1
35383	1.11495	2.1
35384	0.94613	2.1
35385	1.11495	2.1
35386	1.13785	2.1
35387	0.946131	2.1
35388	0.975888	2.1
35389	1.00118	2.1
35390	0.671218	2.1
35391	0.653229	2.1
35392	0.825372	2.1
35393	0.825372	2.1
35394	0.829038	2.1
35395	0.829038	2.1
35396	0.975901	2.1
35397	0.653238	2.1
35398	0.671209	2.1
35399	1.00117	2.1
35400	1.89224	2.1
35401	1.89224	2.1
35402	1.89224	2.1
35403	1.89224	2.1
35404	3.78448	2.1
35405	0.946115	2.1
35406	0.946115	2.1
35407	1.11494	2.1
35408	1.10111	2.1
35409	0.946118	2.1
35410	0.946118	2.1
35411	1.10111	2.1
35412	1.10111	2.1
35413	1.10112	2.1
35414	0.94613	2.1
35415	0.946105	2.1
35416	1.11492	2.1
35417	1.04028	0.677306
35418	2.27126	0.548692
35419	2.0815	0.613793
35420	1.82249	0.804589
35421	1.33723	0.63165
35422	1.18684	0.755331
35423	0.812616	0.512613
35424	0.812616	0.502862
35425	0.997777	0.469966
35426	1.08122	0.530207
35427	0.94613	0.469378
35428	0.94613	0.524975
35429	0.812648	0.501435
35430	0.932779	0.467045
35431	0.812648	0.454785
35432	0.94613	0.515718
35433	0.849335	0.464067
35434	0.94613	0.464433
35435	1.80327	1.02374

35436	1.56016	0.931326
35437	1.57731	1.46951
35438	1.34851	1.41369
35439	2.03207	0.987616
35440	1.80327	1.46137
35441	2.03207	1.39862
35442	1.56016	1.35352
35443	2.18076	1.02987
35444	1.78167	1.02666
35445	3.81374	1.36458
35446	1.37597	1.57712
35447	1.60058	1.82567
35448	1.70302	1.02033
35449	1.85448	1.21401
35450	3.15028	1.21434
35451	1.60058	1.19248
35452	1.4054	1.82092
35453	1.66202	1.07077
35454	1.70302	1.57791
35455	3.09979	1.3337
35456	1.70302	1.40001
35457	1.85448	1.47931
35458	2.21317	1.10137
35459	2.0961	1.16492
35460	4.01757	1.40334
35461	1.66202	1.32792
35462	1.70302	1.64002
35463	2.09611	0.966313
35464	2.00892	1.26671
35465	3.6468	1.21376
35466	1.2966	0.313214
35467	2.25495	0.257024
35468	1.2966	0.337135
35469	2.21117	0.269179
35470	1.67687	0.325528
35471	2.6167	0.258793
35472	1.89227	0.428917
35473	1.89227	0.325306
35474	1.89227	0.343043
35475	1.89227	0.452221
35476	3.78453	0.381617
35477	2.44375	0.319018
35478	1.89227	0.41182
35479	2.81853	0.392205
35480	2.81853	0.365062
35481	1.89227	0.370094
35482	1.89227	0.293374
35483	1.89226	1.00854
35484	1.75853	1.06164
35485	2.37636	1.00549
35486	2.37636	1.11609
35487	1.89226	0.942896
35488	1.89226	1.049
35489	1.65814	0.679534
35490	1.10187	1.0053
35491	1.10187	0.80789
35492	1.19547	0.908917
35493	1.56263	0.58971

35494	1.65624	0.946988
35495	1.56264	0.609968
35496	1.01479	0.950317
35497	1.01479	0.837441
35498	1.10187	0.903605
35499	1.47561	0.533079
35500	1.5627	0.978456
35501	1.89225	2.1
35502	1.89225	2.1
35503	1.89225	2.1
35504	1.89225	2.1
35505	3.7845	2.1
35506	2.54386	2.1
35507	1.91546	2.1
35508	2.46008	2.1
35509	1.82815	2.1
35510	4.37554	2.1
35511	1.91546	2.1
35512	1.04909	2.1
35513	1.82815	2.1
35514	0.983141	2.1
35515	2.8986	2.1
35516	1.89225	2.1
35517	1.89225	2.1
35518	1.89225	2.1
35519	1.89225	2.1
35520	3.78449	2.1
35521	1.89226	2.1
35522	1.89226	2.1
35523	1.89226	2.1
35524	1.89226	2.1
35525	1.89226	2.1
35526	1.89226	2.1
35527	2.46009	2.1
35528	1.7513	2.1
35529	1.82817	2.1
35530	1.82817	2.1
35531	2.38127	2.1
35532	2.38127	2.1
35533	2.55006	2.1
35534	2.64569	2.1
35535	2.60128	2.1
35536	2.49168	2.1
35537	5.13737	2.1
35538	2.51605	2.1
35539	2.64569	2.1
35540	2.60128	2.1
35541	2.48891	2.1
35542	5.1346	2.1
35543	2.60128	2.1
35544	2.49168	2.1
35545	2.41689	2.1
35546	2.55248	2.1
35547	5.04416	2.1
35548	2.60128	2.1
35549	2.48891	2.1
35550	2.45922	2.1
35551	2.55249	2.1

35552	5.04139	2.1
35553	1.89227	1.52314
35554	1.89227	1.80446
35555	1.89227	1.7603
35556	1.89227	1.66881
35557	1.89227	1.59891
35558	1.89227	1.52654
35559	0.96063	2.1
35560	0.96063	2.1
35561	0.958806	2.1
35562	0.958806	2.1
35563	1.28892	2.1
35564	0.749042	2.1
35565	1.17409	2.1
35566	0.630516	2.1
35567	1.17409	2.1
35568	1.28892	2.1
35569	0.630515	2.1
35570	0.749042	2.1
35571	0.92511	1.6201
35572	0.92511	1.62101
35573	1.19511	1.65871
35574	1.19511	1.76208
35575	0.946132	1.74156
35576	1.17409	1.60042
35577	0.925081	1.50078
35578	0.946129	1.75796
35579	0.946131	1.3953
35580	0.946131	1.56946
35581	1.17409	1.62187
35582	0.925083	1.47663
35583	1.89226	1.02848
35584	1.89226	1.16227
35585	1.89226	1.08891
35586	1.89226	1.07957
35587	1.89226	1.09343
35588	1.89226	1.08608
35589	1.89226	1.11371
35590	1.89226	1.21988
35591	1.89226	1.19163
35592	1.89226	1.14963
35593	3.78453	1.16657
35594	0.946132	1.90385
35595	0.946132	1.76923
35596	1.28892	2.04163
35597	1.28892	2.1
35598	0.946132	2.04307
35599	1.28892	2.1
35600	0.946133	1.88707
35601	1.17409	1.73968
35602	0.946132	1.72423
35603	0.946132	2.03454
35604	1.17409	1.91107
35605	1.28892	2.1
35606	0.625229	2.1
35607	0.890321	2.1
35608	1.21636	2.1
35609	1.13022	2.1

35610	1.07094	2.1
35611	1.03541	2.1
35612	0.942207	2.1
35613	0.942207	2.1
35614	0.898766	2.1
35615	1.26626	2.1
35616	1.0853	2.1
35617	0.600358	2.1
35618	0.719012	2.1
35619	0.719012	2.1
35620	1.17957	2.1
35621	1.17957	2.1
35622	0.719012	2.1
35623	0.952456	2.1
35624	0.946129	2.1
35625	0.946129	2.1
35626	0.946131	2.1
35627	0.952454	2.1
35628	0.946131	2.1
35629	0.719011	2.1
35630	0.94613	2.1
35631	0.94613	2.1
35632	0.94613	2.1
35633	0.94613	2.1
35634	0.946131	2.1
35635	0.946131	2.1
35636	0.946129	2.1
35637	0.946129	2.1
35638	0.94613	2.1
35639	0.94613	2.1
35640	0.94613	2.1
35641	0.94613	2.1
35642	1.1587	2.1
35643	0.973158	2.1
35644	0.750023	2.1
35645	0.536443	2.1
35646	0.84054	2.1
35647	0.84054	2.1
35648	0.854602	2.1
35649	0.854602	2.1
35650	1.1587	2.1
35651	0.750023	2.1
35652	0.973158	2.1
35653	0.536443	2.1
35654	1.89227	0.935319
35655	1.89227	1.85605
35656	1.89227	1.30197
35657	1.89227	0.983979
35658	3.78453	1.15734
35659	1.89226	2.1
35660	1.89226	1.60736
35661	1.89226	1.20887
35662	1.89226	2.1
35663	3.78453	1.80167
35664	1.7632	1.66534
35665	1.61149	2.1
35666	1.98749	2.1
35667	2.11615	2.0981

35668	3.75068	2.1
35669	1.7632	1.90185
35670	1.61149	1.52621
35671	1.97733	0.968591
35672	2.11615	2.1
35673	3.74052	1.48297
35674	1.89226	1.20883
35675	1.89226	1.03382
35676	1.89226	0.877882
35677	1.89226	1.64699
35678	3.78453	1.0819
35679	1.89226	1.11612
35680	1.89226	2.1
35681	1.89226	1.95453
35682	1.89226	1.42076
35683	3.78453	1.96158
35684	1.98749	2.1
35685	2.11615	1.97295
35686	2.43151	1.48311
35687	2.27541	2.1
35688	4.41899	2.1
35689	1.97733	0.975709
35690	2.11615	2.09358
35691	2.43151	1.69199
35692	2.33986	0.979376
35693	4.40883	1.47372
35694	2.17892	0.37514
35695	2.80057	0.819067
35696	1.88349	0.728684
35697	2.63062	0.574427
35698	4.80954	0.688322
35699	1.89226	0.940656
35700	1.89226	0.745803
35701	1.89226	0.904818
35702	1.89226	0.591034
35703	3.78452	0.81394
35704	2.0155	0.95402
35705	1.97652	0.745357
35706	1.8247	0.900732
35707	1.80645	0.396459
35708	3.80122	0.759136
35709	1.97652	0.729648
35710	1.79681	0.698791
35711	1.80645	0.481612
35712	1.59244	0.629448
35713	3.56896	0.593184
35714	1.89226	0.732953
35715	1.89226	0.749552
35716	1.89226	0.691951
35717	1.89226	0.658923
35718	3.78452	0.695664
35719	1.89223	0.907246
35720	1.89223	0.745509
35721	1.89223	0.89492
35722	1.89223	0.487041
35723	3.78447	0.792596
35724	1.82467	0.903597
35725	1.80643	0.534722

35726	1.55159	0.891596
35727	1.53969	0.484514
35728	3.36437	0.608158
35729	1.80643	0.680915
35730	1.59242	0.718905
35731	1.5397	0.557111
35732	1.40538	0.507894
35733	3.13211	0.58814
35734	1.89224	0.734917
35735	1.89224	0.723699
35736	1.89224	0.553526
35737	1.89224	0.715332
35738	3.78447	0.708587
35739	1.37427	0.33147
35740	2.37086	0.377727
35741	2.37972	0.323567
35742	1.94703	0.381217
35743	2.19299	0.360087
35744	1.91813	0.420035
35745	1.07566	2.1
35746	1.12954	2.1
35747	0.946131	2.1
35748	0.946131	2.1
35749	1.12954	2.1
35750	1.12954	2.1
35751	0.946131	2.1
35752	0.946131	2.1
35753	1.12954	2.1
35754	0.946131	2.1
35755	0.946131	2.1
35756	1.07566	2.1
35757	2.25908	2.1
35758	1.89226	2.1
35759	1.89226	2.1
35760	1.89226	2.1
35761	2.37567	2.1
35762	2.37567	2.1
35763	1.37597	1.60294
35764	1.70302	0.957551
35765	1.24162	1.45835
35766	1.12219	1.7126
35767	1.37072	1.12513
35768	1.70302	1.89182
35769	1.37072	2.0331
35770	1.70302	1.2495
35771	1.54168	1.21826
35772	2.21318	1.11397
35773	3.39238	1.35923
35774	1.89226	0.259567
35775	1.89226	0.253084
35776	1.89226	0.220913
35777	1.89226	0.222052
35778	3.78452	0.223583
35779	1.89227	0.26083
35780	1.89227	0.252875
35781	1.89227	0.220163
35782	1.89227	0.230173
35783	3.78453	0.249198

35784	1.89226	0.233925
35785	1.89226	0.238354
35786	1.89226	0.212714
35787	1.89226	0.216618
35788	3.78453	0.220842
35789	1.89226	0.233861
35790	2.21654	0.216378
35791	2.72007	0.210486
35792	2.72007	0.231045
35793	1.89226	0.212128
35794	1.89226	0.239046
35795	0.946128	0.20324
35796	0.946128	0.208911
35797	0.946132	0.199817
35798	0.946132	0.205164
35799	0.94613	0.200216
35800	0.94613	0.207499
35801	0.946131	0.199634
35802	0.946131	0.21168
35803	0.94613	0.196409
35804	0.94613	0.200307
35805	0.94613	0.220724
35806	0.94613	0.2116
35807	1.89226	0.214442
35808	1.89226	0.218028
35809	1.89226	0.195066
35810	1.89226	0.197617
35811	3.78453	0.201757
35812	1.33457	0.196778
35813	0.946131	0.19646
35814	1.08045	0.198125
35815	0.946132	0.195275
35816	1.08045	0.201965
35817	1.08045	0.194863
35818	0.946132	0.203087
35819	0.946132	0.19515
35820	1.33457	0.191616
35821	1.08045	0.188176
35822	0.946132	0.20539
35823	0.946132	0.199518
35824	1.53347	1.07029
35825	1.13092	1.86473
35826	1.52938	1.42287
35827	1.93568	1.31367
35828	3.06285	1.53267
35829	1.89226	0.726531
35830	2.72959	0.822917
35831	2.82996	0.823311
35832	2.82996	0.710756
35833	1.89226	0.824217
35834	1.89226	0.718884
35835	2.7296	0.831877
35836	2.57663	1.78494
35837	2.48354	0.833195
35838	2.48354	1.58553
35839	2.82996	0.830324
35840	2.82996	1.60469
35841	2.63449	0.82987

35842	2.7296	0.826342
35843	2.39282	0.866711
35844	2.39282	1.88223
35845	2.48354	1.61465
35846	2.72521	1.6473
35847	1.89224	2.06957
35848	1.89224	1.40263
35849	1.89224	0.964821
35850	1.89224	1.61114
35851	3.78447	1.12571
35852	1.89226	1.77609
35853	2.10425	2.1
35854	2.5142	2.1
35855	2.5142	2.1
35856	1.89226	2.1
35857	1.89226	1.67023
35858	0.946133	0.954184
35859	0.946133	1.1668
35860	0.946128	2.00527
35861	0.946128	1.97878
35862	0.946131	1.53455
35863	0.946131	0.95567
35864	0.94613	0.865475
35865	0.94613	1.85287
35866	0.946131	1.26398
35867	0.946131	1.32723
35868	0.946131	1.22285
35869	0.946131	1.22689
35870	1.89226	0.877248
35871	1.89226	1.0356
35872	1.89226	0.90271
35873	1.89226	1.00556
35874	1.89226	0.898299
35875	1.89226	0.999542
35876	1.94446	0.914169
35877	2.50167	1.11763
35878	1.28418	0.945534
35879	1.94446	1.04951
35880	2.05205	1.00959
35881	2.05205	1.06219
35882	1.27835	2.1
35883	1.08341	2.1
35884	0.946132	2.1
35885	0.946132	2.1
35886	0.851542	2.1
35887	0.851542	2.1
35888	0.977356	2.1
35889	1.18368	2.1
35890	1.18378	2.1
35891	0.851555	2.1
35892	0.977346	2.1
35893	0.851476	2.1
35894	1.07369	2.1
35895	2.5813	2.1
35896	1.95053	2.1
35897	1.95053	2.1
35898	1.90196	2.1
35899	1.90196	2.1

35900	1.89226	2.1
35901	1.89226	2.1
35902	1.89226	2.1
35903	1.89226	2.1
35904	3.78452	2.1
35905	1.89224	2.1
35906	1.89224	2.1
35907	1.89224	2.1
35908	1.89224	2.1
35909	1.89224	2.1
35910	1.89224	2.1
35911	1.97004	0.75259
35912	1.8072	1.08338
35913	1.5866	1.10234
35914	1.79478	0.827463
35915	3.60198	0.950065
35916	1.23267	1.67259
35917	1.8072	0.976946
35918	1.58661	0.948174
35919	0.947921	1.5818
35920	2.75512	1.21969
35921	1.89226	0.978668
35922	1.89226	1.94717
35923	1.89226	0.999416
35924	1.89226	1.37329
35925	1.89226	0.987936
35926	1.89226	1.68
35927	1.89226	1.08765
35928	1.89226	0.79431
35929	1.89226	0.841304
35930	1.89226	1.17985
35931	3.78452	0.917552
35932	1.4263	1.26982
35933	1.79478	0.829674
35934	1.67651	0.907087
35935	1.79478	1.25653
35936	1.46834	0.811963
35937	1.5866	0.996326
35938	1.89226	0.84242
35939	1.89226	1.10331
35940	1.89226	0.854342
35941	1.89226	1.02322
35942	1.89226	0.862226
35943	1.89226	1.24251
35944	2.23956	1.05454
35945	1.81847	1.17042
35946	2.10991	0.852437
35947	2.75317	0.90255
35948	4.34947	0.955575
35949	1.60578	0.387243
35950	1.94974	0.381707
35951	1.68062	0.455732
35952	1.14721	0.350196
35953	3.2864	0.375806
35954	1.3946	0.217513
35955	1.692	0.201683
35956	2.0154	0.230678
35957	2.0154	0.206736

35958	1.11926	0.226645
35959	1.11926	0.203689
35960	1.90347	0.232083
35961	1.83626	0.20455
35962	1.84583	0.20499
35963	1.89434	0.237082
35964	3.79781	0.21543
35965	1.89226	1.1154
35966	1.89226	1.0365
35967	1.89226	0.934726
35968	1.89226	1.15543
35969	3.78452	1.02215
35970	1.20986	1.29546
35971	1.20986	1.35531
35972	0.912635	1.14129
35973	0.912635	1.34688
35974	0.912667	1.02164
35975	1.17637	1.5459
35976	0.946125	1.25832
35977	0.946125	1.33511
35978	0.946128	1.40026
35979	0.912664	1.15346
35980	0.946127	1.48856
35981	1.17637	1.25605
35982	1.89226	0.242817
35983	1.65573	0.260147
35984	1.65573	0.294158
35985	1.89226	0.22854
35986	1.89226	0.286667
35987	2.12879	0.219224
35988	1.89226	0.288537
35989	1.89226	0.293357
35990	1.89226	0.361929
35991	1.89226	0.263797
35992	1.89226	0.356083
35993	1.89226	0.257392
35994	2.40928	0.214292
35995	1.89226	0.277172
35996	1.95959	0.243802
35997	1.70302	0.234867
35998	3.47338	0.256044
35999	1.89226	0.28388
36000	1.89226	0.283865
36001	1.89226	0.246133
36002	1.89226	0.348737
36003	3.78452	0.260683
36004	1.64985	0.403421
36005	2.47846	0.527139
36006	2.25405	0.435309
36007	1.80768	0.411866
36008	4.06173	0.39798
36009	1.98846	0.346093
36010	0.912729	0.410233
36011	1.05417	0.294816
36012	1.70303	0.415961
36013	2.82107	0.411249
36014	2.47846	0.538647
36015	1.70303	0.509982

36016	2.86918	0.597074
36017	2.05973	0.576026
36018	2.51808	0.525762
36019	1.80768	0.496288
36020	0.946131	0.492771
36021	0.946131	0.461962
36022	0.946132	0.490597
36023	0.946132	0.607378
36024	0.946131	0.623818
36025	0.946131	0.56906
36026	0.946133	0.500323
36027	0.946133	0.461519
36028	0.946131	0.546107
36029	0.946131	0.589304
36030	0.946132	0.550687
36031	0.946132	0.578322
36032	0.946133	0.394824
36033	0.946133	0.382991
36034	0.946132	0.466444
36035	0.946132	0.47182
36036	0.946133	0.476343
36037	0.946133	0.462223
36038	0.946132	0.363354
36039	0.946132	0.492456
36040	0.946132	0.413686
36041	0.946132	0.473561
36042	0.946133	0.385487
36043	0.946133	0.457767
36044	1.89227	0.618845
36045	1.89227	0.634168
36046	1.89227	0.658061
36047	1.89227	0.576983
36048	1.89227	0.697258
36049	1.89227	0.638674
36050	1.1656	2.1
36051	1.21847	2.1
36052	0.836006	2.1
36053	0.806007	2.1
36054	1.00653	2.1
36055	1.00653	2.1
36056	1.01794	2.1
36057	1.01794	2.1
36058	1.1656	2.1
36059	0.806007	2.1
36060	0.836006	2.1
36061	1.21847	2.1
36062	2.82263	2.1
36063	2.3312	2.1
36064	2.25936	2.1
36065	2.71441	2.1
36066	5.0456	2.1
36067	2.25936	2.1
36068	1.61201	2.1
36069	1.57627	2.1
36070	1.61201	2.1
36071	2.29545	2.1
36072	2.3312	2.1
36073	1.21269	0.20548

36074	1.48867	0.221713
36075	1.68264	0.215893
36076	1.9898	0.20468
36077	3.17131	0.209244
36078	2.0487	2.1
36079	2.45212	2.1
36080	2.31012	2.1
36081	1.94095	2.1
36082	4.39307	2.1
36083	1.02435	2.1
36084	0.970469	2.1
36085	0.625375	2.1
36086	0.567166	2.1
36087	0.796854	2.1
36088	0.796854	2.1
36089	0.79466	2.1
36090	0.79466	2.1
36091	0.970471	2.1
36092	0.567165	2.1
36093	0.625374	2.1
36094	1.02435	2.1
36095	2.31012	2.1
36096	1.94095	2.1
36097	1.4825	2.1
36098	2.17384	2.1
36099	4.11479	2.1
36100	0.946187	2.1
36101	0.803763	2.1
36102	0.581357	2.1
36103	0.510356	2.1
36104	0.745632	2.1
36105	0.745632	2.1
36106	0.734566	2.1
36107	0.734563	2.1
36108	0.896142	2.1
36109	0.510366	2.1
36110	0.946173	2.1
36111	0.552965	2.1
36112	1.89226	0.967838
36113	1.89226	1.1766
36114	1.89226	1.26936
36115	1.89226	1.37017
36116	1.89226	0.948005
36117	1.89226	1.23793
36118	1.50743	1.16628
36119	1.0417	1.563
36120	1.50743	1.40353
36121	1.62974	0.96932
36122	1.14605	0.964274
36123	1.14605	1.5162
36124	1.89226	0.876671
36125	1.89226	1.2994
36126	1.89226	0.818805
36127	1.89226	1.23062
36128	1.89226	0.844842
36129	1.89226	1.30897
36130	1.89226	1.31464
36131	1.89226	1.14294

36132	1.89226	0.873782
36133	1.89226	1.2578
36134	3.78453	1.01919
36135	1.77186	1.21093
36136	1.35668	1.34746
36137	1.70254	0.939074
36138	1.38756	1.32647
36139	1.77186	1.2696
36140	1.37814	1.58023
36141	0.734857	1.36405
36142	1.00549	1.24004
36143	0.835261	0.984255
36144	1.08357	1.34404
36145	0.97665	1.08919
36146	0.795559	1.6321
36147	0.891385	1.24504
36148	0.891385	0.832527
36149	0.874231	0.95693
36150	1.08356	1.06393
36151	0.734863	1.1446
36152	0.79449	1.24955
36153	1.89226	0.891557
36154	1.89226	0.72589
36155	2.42671	0.768979
36156	1.98783	1.00909
36157	4.31897	0.81309
36158	0.579854	0.864554
36159	0.965582	0.891025
36160	0.634403	0.797233
36161	1.17796	0.745732
36162	1.00592	0.749361
36163	0.797259	1.70316
36164	0.905246	0.90161
36165	0.905246	0.751905
36166	0.695715	0.803445
36167	0.637106	0.861043
36168	1.24374	0.812131
36169	0.95617	0.954278
36170	2.0815	0.867467
36171	1.60521	1.18048
36172	1.29556	0.966303
36173	2.32205	1.26824
36174	1.28445	0.848921
36175	2.0815	1.04428
36176	2.3085	1.54821
36177	2.21147	2.1
36178	2.3085	2.1
36179	2.39282	1.96275
36180	1.98899	2.1
36181	2.0733	2.09943
36182	2.3275	2.1
36183	1.63471	2.1
36184	1.98899	2.1
36185	1.56801	2.1
36186	3.62369	2.1
36187	2.3085	1.48743
36188	1.98899	2.1
36189	2.22575	1.67298

36190	1.90502	2.1
36191	4.21353	2.1
36192	1.90502	2.1
36193	1.56801	2.1
36194	1.90502	2.1
36195	1.98899	2.1
36196	1.49733	2.1
36197	1.58129	2.1
36198	1.89226	1.04387
36199	2.6491	1.1249
36200	2.49266	1.98826
36201	2.6491	1.06405
36202	1.73582	1.1113
36203	1.89226	0.812228
36204	1.31854	2.1
36205	2.6491	1.20007
36206	1.3909	2.1
36207	1.3909	1.73667
36208	2.49266	2.1
36209	2.49266	1.86396
36210	1.89226	1.02871
36211	2.49266	1.05383
36212	2.38634	1.82855
36213	2.49266	1.00413
36214	1.78595	1.04241
36215	1.89226	0.81347
36216	1.23042	2.1
36217	2.49266	1.07838
36218	1.31854	2.1
36219	1.31854	1.37433
36220	2.38635	2.1
36221	2.38635	1.46105
36222	2.83487	2.1
36223	1.89226	2.1
36224	2.594	2.1
36225	2.594	2.1
36226	1.89226	2.1
36227	1.89226	2.1
36228	1.89226	2.1
36229	1.89226	2.1
36230	1.89226	2.1
36231	1.89226	2.1
36232	3.78452	2.1
36233	1.09518	2.1
36234	1.28158	2.1
36235	1.36844	2.1
36236	1.59972	2.1
36237	2.65003	2.1
36238	1.89226	2.1
36239	1.89226	2.1
36240	1.89226	2.1
36241	1.89226	2.1
36242	3.78452	2.1
36243	1.36844	2.1
36244	1.59972	2.1
36245	1.67352	2.1
36246	1.94291	2.1
36247	3.31135	2.1

36248	1.52938	1.60451
36249	1.93568	1.47305
36250	2.36221	0.903189
36251	1.94723	1.7848
36252	3.89158	1.39382
36253	1.94724	1.06461
36254	1.26593	1.91819
36255	1.75367	1.62479
36256	2.4322	1.20145
36257	3.7009	1.61229
36258	2.36221	0.97203
36259	2.43219	1.54052
36260	1.94723	1.21697
36261	1.94723	1.28374
36262	2.8202	1.26585
36263	2.8202	1.35583
36264	2.51604	2.1
36265	2.18533	2.1
36266	2.17228	2.1
36267	2.4889	2.1
36268	4.67423	2.1
36269	2.17228	2.1
36270	1.62183	2.1
36271	1.62316	2.1
36272	1.62183	2.1
36273	2.18666	2.1
36274	2.18533	2.1
36275	2.17227	2.1
36276	2.4889	2.1
36277	2.45922	2.1
36278	2.15686	2.1
36279	4.64576	2.1
36280	2.17228	2.1
36281	1.62316	2.1
36282	1.62241	2.1
36283	2.15686	2.1
36284	3.78002	2.1
36285	1.89226	0.845291
36286	1.89226	0.843481
36287	1.89226	0.766202
36288	1.89226	0.753935
36289	3.78453	0.885871
36290	1.89226	0.81758
36291	1.89226	0.814595
36292	1.89226	0.782632
36293	1.89226	0.728925
36294	3.78452	0.774286
36295	1.89226	0.823401
36296	1.89226	0.73017
36297	1.89226	0.778497
36298	1.89226	0.783626
36299	1.89226	0.738324
36300	1.89226	0.74725
36301	1.89226	0.787271
36302	1.89226	0.831122
36303	2.53413	0.726353
36304	1.89226	0.71026
36305	3.78453	0.739366

36306	1.38048	0.807999
36307	1.38048	0.818779
36308	0.490332	0.74178
36309	0.490332	0.739833
36310	0.924679	0.706176
36311	0.946131	1.16396
36312	0.946131	0.954509
36313	0.490332	0.695549
36314	0.924678	0.70925
36315	0.490332	0.709378
36316	0.946131	0.829889
36317	0.946131	0.765272
36318	1.89227	0.706967
36319	1.89227	0.766657
36320	1.89227	0.709938
36321	1.89227	0.797154
36322	1.89227	0.707201
36323	1.89227	0.762675
36324	1.30803	2.1
36325	2.55151	2.1
36326	1.43935	2.1
36327	1.43935	2.1
36328	2.34922	2.1
36329	2.34922	2.1
36330	0.946131	1.9879
36331	0.946131	2.1
36332	1.27575	2.1
36333	1.17461	2.1
36334	0.94613	2.1
36335	0.94613	2.05004
36336	1.17461	2.1
36337	1.17461	2.1
36338	0.946131	2.1
36339	1.17461	2.1
36340	0.946132	2.1
36341	1.27575	2.1
36342	1.89226	2.1
36343	1.89226	1.15813
36344	1.89226	2.08421
36345	1.89226	2.1
36346	1.89226	1.92803
36347	1.89226	2.1
36348	0.946133	2.1
36349	0.946133	2.1
36350	0.946132	2.1
36351	0.946132	2.1
36352	0.946133	2.1
36353	0.946133	1.84662
36354	0.946132	2.1
36355	0.946132	2.1
36356	0.946132	2.1
36357	0.946132	2.1
36358	0.946133	2.1
36359	0.946133	2.1
36360	1.07043	2.1
36361	1.17461	2.1
36362	0.654016	2.1
36363	0.5829	2.1

36364	0.870481	2.1
36365	0.870481	2.1
36366	0.887027	2.1
36367	0.887027	2.1
36368	1.07043	2.1
36369	0.5829	2.1
36370	0.654016	2.1
36371	1.17461	2.1
36372	1.89226	2.1
36373	1.89226	1.13831
36374	1.89226	2.09401
36375	1.89226	1.81939
36376	3.78453	2.08534
36377	1.89226	2.1
36378	1.89226	2.1
36379	1.89226	2.1
36380	1.89226	1.98426
36381	3.78453	2.1
36382	1.26929	1.98631
36383	2.19313	2.1
36384	1.09847	2.1
36385	1.69916	2.1
36386	2.96844	2.1
36387	1.84124	2.1
36388	2.24937	2.1
36389	1.69915	2.1
36390	1.75691	2.1
36391	3.94852	2.1
36392	1.53082	2.1
36393	2.04422	2.1
36394	1.53082	2.1
36395	1.69915	2.1
36396	1.92911	2.1
36397	2.09744	2.1
36398	1.39263	0.957207
36399	1.0171	0.705041
36400	1.1211	0.848663
36401	1.46482	0.919141
36402	2.48192	0.88918
36403	1.39263	0.962435
36404	1.57962	1.07055
36405	1.65238	1.22926
36406	1.46482	0.932949
36407	3.04443	0.998342
36408	1.89226	1.06526
36409	1.89226	0.785649
36410	1.89226	0.926647
36411	1.89226	1.00013
36412	3.78453	0.973547
36413	1.89227	0.701168
36414	1.89227	0.780165
36415	1.89227	0.912632
36416	1.89227	0.620401
36417	3.78453	0.69197
36418	1.28418	0.81873
36419	1.46482	0.89674
36420	1.1211	0.758398
36421	1.1211	0.943689

36422	1.59382	0.740774
36423	1.59382	0.938831
36424	1.76897	1.13387
36425	1.46482	0.90884
36426	1.59382	0.969208
36427	1.46482	1.04592
36428	1.78138	0.951417
36429	1.65238	1.01823
36430	1.89226	0.902719
36431	1.89226	0.954291
36432	1.89226	0.832694
36433	1.89226	1.01319
36434	1.89226	0.800198
36435	1.89226	1.02894
36436	1.89226	0.890368
36437	1.89226	0.600596
36438	1.89226	0.673848
36439	1.89226	0.776162
36440	1.89226	0.733475
36441	1.89226	0.813192
36442	1.96832	1.45894
36443	2.40858	1.3389
36444	1.75211	0.907468
36445	2.20797	2.1
36446	4.1607	1.22766
36447	1.75211	0.912949
36448	2.20797	2.1
36449	1.51592	1.63572
36450	2.02064	1.32961
36451	3.77275	1.71393
36452	2.71793	0.739069
36453	2.67099	0.714313
36454	2.55105	0.713652
36455	2.71297	0.698422
36456	5.26898	0.716512
36457	2.36417	0.730479
36458	2.67098	0.719537
36459	2.55104	0.71608
36460	1.83418	0.69021
36461	4.91522	0.714641
36462	2.55105	0.714815
36463	2.71297	0.701042
36464	2.58032	0.710819
36465	2.28922	0.619288
36466	5.13137	0.712032
36467	2.55104	0.717509
36468	2.23787	0.686078
36469	1.67265	0.703967
36470	2.28922	0.646197
36471	4.22369	0.705141
36472	0.851542	0.97672
36473	0.851542	0.960454
36474	0.981419	0.934631
36475	0.854774	0.990216
36476	0.949431	0.95327
36477	0.753763	0.994194
36478	0.946131	0.96237
36479	0.946131	0.968521

36480	1.04073	0.963112
36481	0.981433	0.919054
36482	0.851528	0.99281
36483	0.854828	0.969019
36484	1.89226	1.02748
36485	1.89226	0.973304
36486	1.89226	0.961638
36487	1.89226	0.968325
36488	1.89226	1.01215
36489	1.89226	1.0203
36490	0.814227	0.904846
36491	0.814227	0.903875
36492	0.946117	0.880973
36493	1.07285	0.892255
36494	0.879132	0.879692
36495	0.923293	0.889525
36496	0.946119	0.905473
36497	0.946119	0.906208
36498	0.946131	0.894741
36499	0.814236	0.837237
36500	0.946106	0.922509
36501	0.870405	0.90502
36502	1.89224	0.970895
36503	1.89224	0.903858
36504	1.89224	0.903988
36505	1.89224	0.90902
36506	1.89224	0.956012
36507	1.89224	0.962019
36508	1.29388	1.20254
36509	1.04164	1.63233
36510	1.64834	1.51966
36511	1.82955	1.10286
36512	2.94222	1.51255
36513	1.4934	0.877926
36514	2.41522	0.819028
36515	2.20387	0.768722
36516	2.20387	1.1946
36517	1.75765	0.816032
36518	1.75765	1.07736
36519	2.41522	0.818442
36520	2.77303	2.1
36521	2.20387	1.43558
36522	2.58627	1.08581
36523	5.00149	1.42225
36524	1.22222	0.90493
36525	2.20387	0.895351
36526	1.96831	0.80536
36527	1.96831	1.28611
36528	1.4934	0.842127
36529	1.4934	1.25861
36530	1.96832	1.53315
36531	2.58627	1.09391
36532	2.40858	0.943189
36533	2.40858	2.03287
36534	2.20387	1.05459
36535	2.20387	2.0655
36536	1.5605	2.1
36537	1.53775	2.1

36538	1.55398	2.1
36539	1.45806	2.1
36540	1.65642	2.1
36541	1.5605	2.1
36542	0.768876	2.1
36543	0.79033	2.1
36544	0.828211	2.1
36545	0.871233	2.1
36546	0.803843	2.1
36547	0.803843	2.1
36548	0.814698	2.1
36549	0.814698	2.1
36550	0.79033	2.1
36551	0.871233	2.1
36552	0.768876	2.1
36553	0.828212	2.1
36554	1.80252	2.1
36555	2.09715	0.979524
36556	1.9394	1.65764
36557	1.50853	1.59378
36558	3.60567	1.74393
36559	1.96416	0.90618
36560	2.09715	0.963554
36561	1.88484	1.38998
36562	1.57916	0.824893
36563	3.67631	0.973653
36564	1.89226	0.942812
36565	1.89226	0.47477
36566	1.89226	0.828597
36567	1.89226	0.872074
36568	3.78453	0.802658
36569	1.89226	0.955443
36570	1.89226	1.05032
36571	1.89226	2.06922
36572	1.89226	0.733385
36573	1.89226	1.61604
36574	1.89226	0.570483
36575	1.5852	1.3993
36576	1.50853	1.48618
36577	1.2955	2.1
36578	1.44472	0.971901
36579	2.95325	1.42709
36580	1.53065	1.21919
36581	1.57916	0.661141
36582	1.44472	0.940615
36583	1.78861	0.934319
36584	1.23527	0.95051
36585	1.23527	0.925036
36586	1.89227	0.66844
36587	1.89227	0.796696
36588	1.89227	0.960953
36589	1.89227	0.759858
36590	3.78453	0.821639
36591	1.89226	0.977565
36592	1.89226	0.994402
36593	1.89226	1.80072
36594	1.89226	0.959912
36595	1.89226	1.14706

36596	1.89226	0.570553
36597	1.89227	0.941694
36598	1.89227	0.828645
36599	1.89227	0.820895
36600	1.89227	0.834029
36601	3.78453	0.817947
36602	0.943708	0.838293
36603	0.752219	0.810061
36604	0.946132	0.799008
36605	0.946132	0.80844
36606	0.946133	0.760042
36607	0.946133	1.06947
36608	0.752218	0.917535
36609	0.752218	0.8465
36610	0.752218	0.835139
36611	0.946132	0.782712
36612	0.946133	0.906411
36613	0.943708	0.843715
36614	0.943992	2.1
36615	0.943992	1.5912
36616	1.23507	1.05542
36617	1.48209	1.8136
36618	1.0384	2.1
36619	1.47953	1.85859
36620	0.946128	1.03674
36621	0.946128	1.41083
36622	0.946129	1.81643
36623	1.23293	1.74354
36624	1.0384	1.64986
36625	0.946129	1.64512
36626	1.89226	1.46282
36627	2.46586	1.66591
36628	2.80218	1.01133
36629	2.80218	2.08818
36630	1.89226	1.01493
36631	1.89226	2.09197
36632	1.32455	2.1
36633	1.89128	2.1
36634	1.32328	2.1
36635	1.97299	2.1
36636	3.29754	2.1
36637	1.89128	2.1
36638	2.4255	2.1
36639	1.973 2.1	
36640	2.63807	2.1
36641	4.3985	2.1
36642	1.34008	2.1
36643	1.97299	2.1
36644	2.10766	2.1
36645	2.10766	2.1
36646	1.32328	2.1
36647	1.32328	2.1
36648	2.41055	2.1
36649	2.37491	2.1
36650	2.40754	2.1
36651	2.38023	2.1
36652	4.78546	2.1
36653	2.23743	2.1

36654	2.37491	2.1
36655	2.40754	2.1
36656	2.20392	2.1
36657	4.61234	2.1
36658	2.37491	2.1
36659	2.02641	2.1
36660	2.20392	2.1
36661	2.18943	2.1
36662	4.40132	2.1
36663	2.37491	2.1
36664	2.22558	2.1
36665	2.38023	2.1
36666	2.18943	2.1
36667	4.60049	2.1
36668	1.89226	0.485461
36669	1.89226	0.618992
36670	1.89226	0.56283
36671	1.89226	0.557648
36672	1.89226	0.5249
36673	1.89226	0.521366
36674	1.03599	0.399099
36675	0.706698	0.38501
36676	1.41141	0.45069
36677	1.17573	0.415852
36678	1.11957	0.365015
36679	0.765034	0.368732
36680	1.05913	0.35724
36681	1.45054	0.448113
36682	1.04847	0.373899
36683	1.04847	0.406083
36684	1.15323	0.416825
36685	1.05825	0.36781
36686	1.89226	0.459958
36687	1.89226	0.478912
36688	1.89226	0.487664
36689	1.89226	0.443617
36690	1.89226	0.495951
36691	1.89226	0.456785
36692	2.22575	0.683316
36693	1.70677	1.36927
36694	1.90157	0.608773
36695	1.5534	1.71341
36696	3.77914	0.862936
36697	1.70678	1.42376
36698	0.968594	2.1
36699	0.968594	2.03945
36700	1.10378	2.1
36701	1.5534	1.78474
36702	1.68859	2.1
36703	2.31429	0.530091
36704	1.5534	1.66115
36705	1.90103	0.66203
36706	1.41902	1.21674
36707	3.45443	0.855889
36708	2.53709	0.217681
36709	1.209	0.223388
36710	1.05417	0.2143
36711	2.6513	0.23778

36712	3.70547	0.232168
36713	1.02675	0.205955
36714	1.16384	0.220126
36715	2.22062	0.219188
36716	2.48862	0.208809
36717	3.38446	0.208737
36718	1.89226	0.226865
36719	1.89226	0.266478
36720	1.89226	0.261728
36721	1.89226	0.227936
36722	1.89226	0.269245
36723	1.89226	0.231242
36724	1.89226	1.19604
36725	1.89226	1.79718
36726	1.89226	1.80164
36727	1.89226	1.3339
36728	1.89226	1.63947
36729	1.89226	1.28991
36730	1.89226	1.09861
36731	1.89226	1.439
36732	1.89226	1.48832
36733	1.89226	1.20003
36734	1.89226	1.4186
36735	1.89226	1.17371
36736	1.89227	1.20099
36737	1.89227	1.63126
36738	1.75032	1.60856
36739	1.75032	1.29508
36740	1.89227	1.49275
36741	1.89227	1.25395
36742	1.89226	0.734707
36743	1.89226	0.796126
36744	1.89226	0.752278
36745	1.89226	0.774695
36746	1.89226	0.774017
36747	1.89226	0.770327
36748	0.946132	0.597865
36749	0.946132	0.59187
36750	0.798215	0.579281
36751	0.848163	0.568187
36752	0.848163	0.598686
36753	0.946131	0.609019
36754	0.946132	0.57603
36755	0.798216	0.563365
36756	0.996105	0.596399
36757	0.996105	0.55941
36758	0.79819	0.579225
36759	0.79819	0.622713
36760	1.07763	0.626092
36761	2.28622	0.61337
36762	2.16109	0.585738
36763	1.17506	0.603238
36764	3.36385	0.604808
36765	1.89226	2.1
36766	1.6663	2.1
36767	1.6663	2.1
36768	1.71706	2.1
36769	1.89226	2.1

36770	1.94302	2.1
36771	1.89226	2.1
36772	1.62801	2.1
36773	1.62801	2.1
36774	1.6663	2.1
36775	1.89226	2.1
36776	1.93055	2.1
36777	1.48734	2.1
36778	1.99609	2.1
36779	1.52245	2.1
36780	2.04137	2.1
36781	3.51854	2.1
36782	1.99609	2.1
36783	2.28563	2.1
36784	2.04136	2.1
36785	2.35019	1.52691
36786	4.34628	2.1
36787	1.50714	2.1
36788	2.04137	2.1
36789	2.04945	2.1
36790	2.04945	2.1
36791	1.52245	2.1
36792	1.52245	2.1
36793	2.04136	2.1
36794	2.35019	1.64446
36795	2.04945	2.1
36796	2.38681	2.1
36797	4.39964	2.1
36798	2.50924	0.147815
36799	1.38305	2.1
36800	2.56918	2.1
36801	1.44535	2.1
36802	3.95222	2.1
36803	1.89226	2.1
36804	1.89226	2.1
36805	1.89226	2.1
36806	1.89226	2.1
36807	3.78453	2.1
36808	1.40615	2.1
36809	1.38851	2.1
36810	0.748607	2.1
36811	0.724468	2.1
36812	1.06362	2.1
36813	1.06362	2.1
36814	1.067	2.1
36815	1.067	2.1
36816	1.40615	2.1
36817	0.748607	2.1
36818	0.724468	2.1
36819	1.38851	2.1
36820	2.56918	2.1
36821	1.53377	2.1
36822	1.53706	2.1
36823	1.44535	2.1
36824	2.66088	2.1
36825	2.56918	2.1
36826	1.89226	2.1
36827	1.89226	2.1

36828	1.89226	2.1
36829	1.89226	2.1
36830	3.78453	2.1
36831	1.40239	2.1
36832	1.68466	2.08157
36833	1.26091	2.1
36834	1.54641	1.92744
36835	2.94881	2.1
36836	1.68466	2.1
36837	1.65324	0.885896
36838	1.54641	1.93431
36839	1.52268	0.893157
36840	3.19965	1.05902
36841	1.05776	2.1
36842	1.54641	1.9918
36843	1.36595	2.1
36844	1.36595	2.02978
36845	1.26091	2.1
36846	1.26091	2.08792
36847	1.54641	2.02005
36848	1.32697	0.885738
36849	1.52268	0.9023
36850	1.52268	2.05368
36851	1.36595	0.90002
36852	1.36595	2.0646
36853	1.18647	1.34756
36854	0.988454	1.91115
36855	1.4867	1.5149
36856	1.63892	1.32729
36857	2.67317	1.74685
36858	1.4867	1.71377
36859	1.63892	1.38681
36860	2.09605	0.901309
36861	1.97645	1.51443
36862	3.58275	1.25292
36863	1.29389	1.62456
36864	1.97645	1.26604
36865	1.4867	1.23988
36866	1.4867	1.76785
36867	1.82955	1.06516
36868	1.82955	1.66526
36869	1.13454	1.18246
36870	0.983594	2.08495
36871	1.16809	2.1
36872	1.06836	1.61256
36873	1.08332	1.55113
36874	0.983594	1.24536
36875	1.48751	1.91225
36876	1.48047	1.48757
36877	1.77596	1.39917
36878	1.75506	1.40965
36879	3.25643	1.51345
36880	1.48047	1.36384
36881	1.25334	1.766
36882	1.75505	1.21129
36883	1.55831	0.89071
36884	3.03878	1.44683
36885	1.77596	1.4531

36886	1.75506	1.73955
36887	2.00551	1.83995
36888	2.00083	1.02491
36889	3.77679	1.66129
36890	1.75506	1.50277
36891	1.80328	0.927818
36892	1.55831	1.5167
36893	1.55831	1.66379
36894	2.00083	0.967156
36895	2.00083	1.17476
36896	2.58627	1.09693
36897	2.79047	2.1
36898	2.40858	2.1
36899	2.64748	2.03236
36900	5.23375	2.1
36901	1.26755	2.1
36902	1.71717	2.1
36903	1.03824	2.1
36904	1.48901	2.1
36905	2.75656	2.1
36906	1.89226	2.1
36907	1.89226	2.1
36908	1.89226	2.1
36909	1.89226	2.1
36910	1.89226	2.1
36911	1.89226	2.1
36912	1.36541	2.1
36913	1.22156	2.1
36914	0.946132	2.1
36915	0.946132	2.1
36916	1.36541	2.1
36917	1.36541	2.1
36918	0.946132	2.1
36919	0.946132	2.1
36920	1.36541	2.1
36921	0.946133	2.1
36922	0.946132	2.1
36923	1.22156	2.1
36924	1.18901	2.1
36925	1.61149	2.1
36926	1.98748	2.1
36927	1.534 2.1	
36928	3.1765	2.1
36929	1.89226	2.1
36930	1.89226	2.1
36931	1.89226	1.95664
36932	1.89226	2.1
36933	3.78452	2.1
36934	1.98748	2.1
36935	1.534 2.1	
36936	2.27541	2.1
36937	1.98748	2.1
36938	1.87436	2.1
36939	1.87436	2.1
36940	2.2988	2.1
36941	2.2604	2.1
36942	2.20831	2.1
36943	2.02802	2.1

36944	4.32683	2.1
36945	2.26041	2.1
36946	1.98957	2.1
36947	2.02803	2.1
36948	1.75021	2.1
36949	4.0176	2.1
36950	2.34607	2.1
36951	2.02803	2.1
36952	1.86795	2.1
36953	1.81448	2.1
36954	3.89597	2.1
36955	2.02803	2.1
36956	1.75021	2.1
36957	1.81448	2.1
36958	1.57887	2.1
36959	3.6069	2.1
36960	0.946131	0.683962
36961	0.946131	2.1
36962	1.1235	1.48658
36963	1.1235	1.54462
36964	0.946131	1.27856
36965	0.946131	0.982602
36966	1.1235	1.87835
36967	1.13736	1.99337
36968	1.13736	0.943388
36969	0.946131	1.04242
36970	1.1235	1.26982
36971	0.946131	1.21403
36972	1.89226	1.73093
36973	1.89226	1.97739
36974	1.89226	1.0134
36975	1.89226	0.964923
36976	3.78452	0.983424
36977	0.563266	1.98398
36978	0.563266	1.06881
36979	0.565606	0.875183
36980	0.565606	1.87186
36981	0.647086	1.60057
36982	0.624219	1.4654
36983	0.500035	1.13292
36984	0.481785	1.17635
36985	0.500034	0.897819
36986	0.647087	0.936084
36987	0.624219	1.42162
36988	0.481785	1.2158
36989	0.915788	0.629507
36990	0.915788	0.707175
36991	1.02732	0.879503
36992	1.02732	0.801791
36993	0.660831	0.83284
36994	1.11155	0.640339
36995	0.713295	0.661877
36996	1.26874	0.868827
36997	0.713295	0.789128
36998	0.660831	0.778664
36999	1.11155	0.778539
37000	1.26874	0.773253
37001	1.89226	0.92806

37002	1.85091	0.934952
37003	2.11193	0.995291
37004	1.85091	0.922043
37005	2.15328	0.893029
37006	1.89226	0.797194
37007	0.946131	0.692851
37008	0.946131	0.795415
37009	1.18275	0.758304
37010	1.05596	0.887279
37011	0.946127	0.797001
37012	0.946127	0.495471
37013	1.18275	0.847503
37014	1.18275	0.868305
37015	0.946128	0.872048
37016	1.18275	0.7189
37017	0.946129	0.801267
37018	1.05596	0.693225
37019	1.60936	2.1
37020	2.35846	2.1
37021	2.06424	2.1
37022	2.57651	2.1
37023	4.42269	2.1
37024	1.89226	2.1
37025	1.89226	2.1
37026	1.89226	2.1
37027	1.89226	2.1
37028	3.78453	2.1
37029	1.89226	2.1
37030	1.89226	2.1
37031	1.89226	2.1
37032	1.89226	2.1
37033	1.89226	2.1
37034	1.89226	2.1
37035	1.95053	2.1
37036	2.06424	2.1
37037	1.95053	2.1
37038	1.30584	2.1
37039	2.5	2.1
37040	1.8553	2.1
37041	2.49999	2.1
37042	2.57651	2.1
37043	2.49999	2.1
37044	2.06423	2.1
37045	2.80738	2.1
37046	2.37162	2.1
37047	1.89226	2.1
37048	1.89226	2.1
37049	1.89226	2.1
37050	1.89226	2.1
37051	1.89226	2.1
37052	1.89226	2.1
37053	1.49295	0.481974
37054	1.03824	0.357732
37055	1.51396	0.49889
37056	2.08514	0.432865
37057	3.12338	0.428773
37058	1.49798	0.434465
37059	2.32017	0.329899

37060	2.68236	0.540214
37061	2.08514	0.402528
37062	4.4053	0.406175
37063	1.89226	0.334105
37064	1.89226	0.294722
37065	1.89226	0.383603
37066	1.89226	0.33474
37067	3.78452	0.346523
37068	1.89226	0.418465
37069	1.89226	0.344257
37070	1.89226	0.384075
37071	1.89226	0.363594
37072	1.89226	0.371281
37073	1.89226	0.355455
37074	1.51396	0.400034
37075	2.08514	0.331413
37076	2.55393	0.493011
37077	2.02901	0.387752
37078	4.11415	0.384226
37079	1.89226	0.298889
37080	1.89226	0.268448
37081	1.89226	0.378878
37082	1.89226	0.347879
37083	3.78451	0.291908
37084	1.89226	0.322119
37085	1.89226	0.366262
37086	1.89226	0.287145
37087	1.89226	0.27411
37088	1.89226	0.385146
37089	1.89226	0.365165
37090	0.946131	0.437646
37091	0.946131	0.466632
37092	0.946132	0.619869
37093	0.946132	0.489611
37094	0.946132	0.506989
37095	0.946132	0.464076
37096	0.946132	0.531246
37097	0.946132	0.593082
37098	0.946132	0.464814
37099	0.946132	0.51676
37100	0.946132	0.544618
37101	0.946132	0.497088
37102	1.89226	2.1
37103	1.89226	1.85034
37104	1.89226	2.1
37105	1.89226	1.63969
37106	3.78452	2.1
37107	1.28367	2.1
37108	1.82707	1.80149
37109	1.11025	2.1
37110	1.6365	1.53842
37111	2.92016	2.1
37112	1.82707	1.88143
37113	1.8247	0.900719
37114	1.8247	1.95425
37115	2.01549	0.946131
37116	1.6365	1.51386
37117	1.82729	0.952353

37118	1.89226	1.90458
37119	1.89226	0.904046
37120	1.89226	0.865241
37121	1.89226	1.78592
37122	1.89226	0.882793
37123	1.89226	1.75089
37124	1.63648	2.06231
37125	1.82467	0.888627
37126	1.35877	1.88638
37127	1.55159	0.868379
37128	3.18345	1.08149
37129	1.89223	1.97362
37130	1.89223	0.887105
37131	1.89223	1.9334
37132	1.89223	0.893557
37133	3.78447	1.10388
37134	1.59972	2.1
37135	1.28037	2.1
37136	1.63702	2.1
37137	1.28158	2.1
37138	1.63581	2.1
37139	1.28037	2.1
37140	1.89226	2.1
37141	1.89226	2.1
37142	1.89226	2.1
37143	1.89226	2.1
37144	1.89226	2.1
37145	1.89226	2.1
37146	1.89226	2.1
37147	1.89226	2.08131
37148	1.89226	2.1
37149	1.89226	2.1
37150	3.78451	2.1
37151	1.28037	2.1
37152	1.05071	2.1
37153	1.63581	2.1
37154	1.44287	2.1
37155	2.72324	2.1
37156	1.59972	2.1
37157	1.63581	2.1
37158	1.94291	2.1
37159	2.00118	2.1
37160	3.57872	2.1
37161	1.89226	2.1
37162	1.89226	2.1
37163	1.89226	2.1
37164	1.89226	2.05093
37165	3.78452	2.1
37166	0.946131	1.88729
37167	0.946131	2.05253
37168	0.946127	2.1
37169	0.946127	2.1
37170	0.94613	1.77244
37171	0.94613	2.1
37172	0.946128	2.1
37173	0.946128	1.97152
37174	0.946128	2.1
37175	0.946128	2.1

37176	0.946129	2.1
37177	0.946129	2.1
37178	0.865056	2.1
37179	0.865056	2.1
37180	0.873224	2.1
37181	0.873224	2.1
37182	0.721437	2.1
37183	0.920378	2.1
37184	0.817903	2.1
37185	1.00059	2.1
37186	1.00059	2.1
37187	0.920376	2.1
37188	0.817904	2.1
37189	0.721437	2.1
37190	3.09157	2.1
37191	1.71255	2.1
37192	2.0815	2.1
37193	1.9918	2.1
37194	2.51364	2.1
37195	2.39396	2.1
37196	2.0815	2.1
37197	1.05051	2.1
37198	2.56724	2.1
37199	1.63991	2.1
37200	3.47929	2.1
37201	1.89226	0.574699
37202	2.2973	0.721728
37203	1.90705	0.719572
37204	1.90705	0.644589
37205	1.89226	0.691778
37206	1.89226	0.584949
37207	1.89226	0.581592
37208	1.90705	0.720307
37209	1.45321	0.726111
37210	1.45321	0.652467
37211	1.89226	0.695312
37212	1.89226	0.599422
37213	0.901146	2.1
37214	0.901146	2.1
37215	1.15227	2.1
37216	1.21279	2.1
37217	0.991265	2.1
37218	1.15003	2.1
37219	0.946132	2.1
37220	0.946132	2.1
37221	0.946132	2.1
37222	1.09997	2.1
37223	0.991266	2.1
37224	0.946132	2.1
37225	1.89226	2.1
37226	1.89226	2.1
37227	1.89226	2.1
37228	1.89226	2.1
37229	1.89226	2.1
37230	1.89226	2.1
37231	1.62229	2.1
37232	1.06053	2.1
37233	1.19698	2.1

37234	1.70106	2.1
37235	2.81928	2.1
37236	0.946132	2.1
37237	0.946132	2.1
37238	1.17527	2.1
37239	1.09997	2.1
37240	1.09997	2.1
37241	1.09997	2.1
37242	0.946133	2.1
37243	0.946133	2.1
37244	1.17527	2.1
37245	0.946132	2.1
37246	0.946133	2.1
37247	1.09997	2.1
37248	1.89226	2.1
37249	1.89226	2.1
37250	1.89226	2.1
37251	1.89226	2.1
37252	1.89226	2.1
37253	1.89226	2.1
37254	0.905416	2.1
37255	0.850527	2.1
37256	0.660601	2.1
37257	0.598494	2.1
37258	0.750137	2.1
37259	0.750137	2.1
37260	0.753774	2.1
37261	0.753774	2.1
37262	0.660599	2.1
37263	0.905418	2.1
37264	0.598493	2.1
37265	0.850529	2.1
37266	2.43509	2.1
37267	2.65066	2.1
37268	2.66602	2.1
37269	2.46339	2.1
37270	5.10111	2.1
37271	2.53008	2.1
37272	2.65066	2.1
37273	2.66602	2.1
37274	2.52852	2.1
37275	5.1961	2.1
37276	2.66602	2.1
37277	2.46339	2.1
37278	2.47932	2.1
37279	2.67393	2.1
37280	5.14535	2.1
37281	2.66602	2.1
37282	2.52853	2.1
37283	2.51686	2.1
37284	2.67393	2.1
37285	5.18288	2.1
37286	1.89226	0.920485
37287	1.89226	0.702616
37288	1.89226	0.90706
37289	1.89226	0.626552
37290	3.78452	0.812776
37291	1.55161	0.920962

37292	1.53972	0.697151
37293	1.32259	0.90541
37294	1.35082	0.448162
37295	2.8623	0.781878
37296	1.53972	0.733855
37297	1.4054	0.692618
37298	1.35083	0.517438
37299	1.24913	0.579536
37300	2.78885	0.626766
37301	1.89226	0.735979
37302	1.89226	0.742712
37303	1.89226	0.733954
37304	1.89226	0.566657
37305	3.78452	0.702503
37306	1.89226	0.893718
37307	1.89226	0.757682
37308	1.89226	0.913586
37309	1.89226	0.765429
37310	1.89226	0.911947
37311	1.89226	0.702749
37312	1.14599	0.892921
37313	1.35082	0.594808
37314	1.14599	0.798785
37315	1.32259	0.912038
37316	1.19993	0.40483
37317	1.37652	0.941151
37318	1.35083	0.695658
37319	1.24913	0.723745
37320	1.19993	0.579722
37321	1.11787	0.424103
37322	2.44906	0.604818
37323	1.89226	0.751864
37324	1.89226	0.724635
37325	1.89226	0.600377
37326	1.89226	0.631331
37327	3.78452	0.712046
37328	1.51697	0.392067
37329	2.57264	0.464177
37330	1.30203	0.343577
37331	2.41701	0.374997
37332	3.93398	0.411224
37333	1.17464	0.286457
37334	2.41701	0.364991
37335	2.32462	0.332133
37336	2.32462	0.358766
37337	1.30203	0.322188
37338	1.30203	0.341321
37339	2.08149	2.1
37340	1.9839	1.92686
37341	1.71398	2.1
37342	1.99182	0.849758
37343	3.71121	1.74663
37344	2.16061	1.83215
37345	2.01399	0.70411
37346	1.99182	0.896225
37347	1.83895	0.854251
37348	4.00581	0.852841
37349	1.90444	2.1

37350	1.99182	0.878513
37351	1.71717	2.1
37352	1.805	1.09761
37353	3.70899	1.96568
37354	1.99182	0.89877
37355	1.83896	0.758381
37356	1.805	1.36603
37357	1.65243	0.776545
37358	3.64425	0.824197
37359	1.18076	2.1
37360	1.27697	2.1
37361	1.05025	2.1
37362	1.27697	2.1
37363	1.25	2.1
37364	1.47671	2.1
37365	1.89226	2.1
37366	1.89226	1.59064
37367	1.89226	2.1
37368	1.89226	2.1
37369	3.78452	2.1
37370	2.64124	2.1
37371	1.89226	2.1
37372	1.89226	2.1
37373	1.89226	2.1
37374	2.46904	2.1
37375	2.46904	2.1
37376	1.89226	2.1
37377	1.89226	2.1
37378	2.7408	2.1
37379	2.82858	1.86425
37380	4.72084	2.1
37381	1.87875	2.1
37382	1.37704	2.1
37383	1.77042	2.1
37384	2.27801	2.1
37385	3.64916	2.1
37386	1.77042	2.1
37387	2.27801	2.1
37388	2.58523	2.1
37389	1.90178	2.1
37390	4.35565	2.1
37391	1.28667	2.1
37392	1.30711	2.1
37393	1.53077	2.1
37394	1.55635	2.1
37395	2.81743	2.1
37396	1.55635	2.1
37397	1.28228	2.1
37398	1.64268	1.69418
37399	1.93673	2.1
37400	3.19903	2.1
37401	1.53077	2.1
37402	1.93673	2.1
37403	1.55635	2.1
37404	1.55635	2.1
37405	1.86206	2.1
37406	1.86206	2.1
37407	1.64892	2.1

37408	1.60532	2.1
37409	1.85282	2.1
37410	1.96716	2.1
37411	3.61608	2.1
37412	1.60531	2.1
37413	1.32414	2.1
37414	1.96716	2.1
37415	1.75823	2.1
37416	3.2913	2.1
37417	1.91219	2.1
37418	1.96716	2.1
37419	2.0815	2.1
37420	2.51396	2.1
37421	4.30843	2.1
37422	1.96716	2.1
37423	2.17979	2.1
37424	1.75823	2.1
37425	1.75823	2.1
37426	2.28541	2.1
37427	2.28541	2.1
37428	1.89226	1.11549
37429	1.89226	1.28177
37430	1.89226	1.11354
37431	1.89226	1.00227
37432	3.78453	1.11306
37433	1.89226	1.21996
37434	1.89226	1.17367
37435	1.89226	1.14424
37436	1.89226	1.20458
37437	1.89226	1.161
37438	1.89226	1.21372
37439	1.61016	1.10383
37440	1.45303	1.21577
37441	1.62812	1.11502
37442	1.86379	1.09646
37443	3.23828	1.0948
37444	1.61016	1.22118
37445	1.78716	1.26942
37446	2.0815	0.982336
37447	1.6774	1.18553
37448	3.54454	1.21092
37449	1.89226	1.21942
37450	1.89226	1.11888
37451	1.89226	0.983533
37452	1.89226	1.16199
37453	3.78453	1.10606
37454	1.89226	1.26141
37455	1.89226	1.23083
37456	1.89226	1.20993
37457	1.89226	1.36786
37458	1.89226	1.01872
37459	1.89226	1.05539
37460	0.807591	1.24515
37461	0.814059	1.09824
37462	1.00217	1.30205
37463	0.931896	1.17446
37464	0.850586	1.20188
37465	0.850586	1.27965

37466	0.8889	1.10419
37467	0.8889	1.23325
37468	0.807592	0.985312
37469	1.00217	1.24587
37470	0.814061	1.19115
37471	0.931894	1.3606
37472	2.0815	1.06959
37473	1.6774	1.36928
37474	2.00435	1.09437
37475	2.36104	1.06931
37476	3.86809	1.0767
37477	1.00532	0.50734
37478	1.00532	0.513196
37479	1.00183	0.508533
37480	1.00183	0.50652
37481	1.26377	0.503241
37482	1.34921	0.532116
37483	0.664872	0.516643
37484	0.743381	0.513096
37485	0.664872	0.493615
37486	1.26377	0.509376
37487	1.34921	0.524332
37488	0.743381	0.505354
37489	1.26826	0.147815
37490	0.946131	1.91869
37491	1.02404	1.88502
37492	0.94613	1.99809
37493	1.30281	1.88516
37494	0.930941	1.987
37495	0.930941	1.88312
37496	1.318	1.93082
37497	1.58161	1.8976
37498	0.94613	1.8572
37499	0.94613	1.66658
37500	1.56338	1.82218
37501	1.02404	1.80605
37502	2.81374	1.90632
37503	1.89226	2.06516
37504	1.89226	1.95835
37505	1.89226	1.99616
37506	3.78453	2.05419
37507	0.946133	1.97603
37508	0.946133	1.76048
37509	0.946132	1.65499
37510	0.946132	1.78641
37511	0.946132	1.78273
37512	0.946132	1.72775
37513	0.946133	1.89149
37514	0.946133	1.76396
37515	0.946132	1.87892
37516	0.946132	1.62967
37517	0.946133	1.99301
37518	0.946133	1.73992
37519	1.89226	1.51538
37520	1.89226	1.79917
37521	1.89226	1.56538
37522	1.89226	1.66359
37523	3.78452	1.73024

37524	1.89226	1.86115
37525	1.89226	1.81538
37526	1.89226	1.97573
37527	1.89226	1.6205
37528	1.89226	1.98844
37529	1.89226	1.63614
37530	0.946133	1.5579
37531	0.946133	1.79234
37532	0.946132	1.44021
37533	0.946132	1.68835
37534	0.946132	1.68484
37535	0.946132	1.64307
37536	0.946133	1.7245
37537	0.946133	1.57064
37538	0.946132	1.73602
37539	0.946132	1.52399
37540	0.946132	1.824
37541	0.946132	1.58034
37542	2.56953	2.1
37543	2.64148	2.1
37544	2.48631	2.1
37545	2.62213	2.1
37546	5.19166	2.1
37547	2.64148	2.1
37548	2.57929	2.1
37549	2.62213	2.1
37550	2.59522	2.1
37551	5.20142	2.1
37552	2.48631	2.1
37553	2.62213	2.1
37554	2.41176	2.1
37555	2.59262	2.1
37556	5.03389	2.1
37557	2.62213	2.1
37558	2.59522	2.1
37559	2.59262	2.1
37560	2.61901	2.1
37561	5.24114	2.1
37562	0.946132	0.18685
37563	0.946132	0.181022
37564	0.946133	0.180206
37565	0.946133	0.184619
37566	0.946132	0.180967
37567	0.946132	0.185554
37568	0.946133	0.180724
37569	0.946133	0.186983
37570	0.946132	0.176971
37571	0.946132	0.177618
37572	0.946133	0.1932
37573	0.946133	0.188223
37574	1.5681	0.17756
37575	1.01248	0.171603
37576	1.10749	0.173437
37577	0.570913	0.173355
37578	1.15589	0.172061
37579	1.15588	0.178028
37580	0.977549	0.173923
37581	0.977549	0.178771

37582	1.01249	0.171775
37583	0.654445	0.169889
37584	1.36795	0.180377
37585	1.10748	0.17599
37586	1.73883	0.147815
37587	1.87638	0.147815
37588	1.85219	0.147815
37589	1.93113	1.78064
37590	3.7407	0.147815
37591	1.87638	0.147815
37592	1.73883	2.1
37593	1.93113	1.74861
37594	1.80426	2.1
37595	3.66996	2.1
37596	2.02912	0.33226
37597	1.44951	0.359957
37598	1.50103	0.347537
37599	2.02248	0.383834
37600	3.47199	0.337904
37601	1.89226	0.368129
37602	1.89226	0.404979
37603	1.89226	0.392891
37604	1.89226	0.437609
37605	3.78453	0.40347
37606	2.57881	0.353452
37607	1.89226	0.40653
37608	1.89226	0.394338
37609	1.89226	0.38011
37610	2.6428	0.369717
37611	2.6428	0.35653
37612	1.01379	0.355544
37613	0.985937	0.35562
37614	0.769295	0.347094
37615	0.715547	0.334418
37616	0.853042	0.37076
37617	0.853042	0.35894
37618	0.864117	0.371524
37619	0.864114	0.306015
37620	0.715556	0.389374
37621	1.03047	0.364566
37622	0.731721	0.347633
37623	0.985921	0.368146
37624	1.89227	0.366714
37625	1.89227	0.411761
37626	1.89227	0.386915
37627	1.89227	0.461009
37628	3.78453	0.388904
37629	0.946129	0.374515
37630	0.946129	0.366374
37631	1.28161	0.34197
37632	1.3214	0.367919
37633	0.94613	0.39808
37634	0.94613	0.382377
37635	1.28161	0.342246
37636	1.28161	0.317566
37637	1.28161	0.346079
37638	0.94613	0.381054
37639	0.946131	0.38794

37640	1.3214	0.354659
37641	0.946132	0.762117
37642	0.946132	0.70072
37643	0.706045	0.614686
37644	1.16718	0.586522
37645	0.706017	0.591883
37646	0.706017	0.814517
37647	1.4073	0.600071
37648	1.4073	0.552066
37649	0.94613	0.657122
37650	1.16719	0.662183
37651	0.706046	0.695392
37652	0.946131	0.662135
37653	1.89226	0.814339
37654	1.89226	0.738377
37655	1.89226	0.562379
37656	1.89226	0.850329
37657	1.89226	0.582194
37658	1.89226	0.843804
37659	0.946119	0.75203
37660	0.946119	0.676994
37661	0.946115	0.734028
37662	0.946115	0.578886
37663	0.946117	0.737472
37664	0.946117	0.788995
37665	0.946117	0.643983
37666	0.946117	0.578343
37667	0.94613	0.621045
37668	0.94613	0.728299
37669	0.946105	0.704765
37670	0.946105	0.633466
37671	0.946132	1.03055
37672	0.946132	0.991192
37673	0.946131	1.05069
37674	0.946131	0.963741
37675	0.946132	1.00822
37676	0.946132	1.06666
37677	0.946132	0.984258
37678	0.827865	0.987559
37679	0.946132	0.997639
37680	0.946132	1.01439
37681	0.946132	0.990952
37682	0.827865	1.00144
37683	1.89226	1.29505
37684	1.89226	1.19292
37685	1.89226	1.47036
37686	1.89226	1.09863
37687	1.89226	1.65423
37688	1.89226	1.21579
37689	0.93094	1.03005
37690	0.93094	1.10966
37691	1.04649	1.06551
37692	0.614468	0.93588
37693	0.950358	0.97147
37694	0.799012	0.938406
37695	0.682742	1.06976
37696	0.682742	1.06825
37697	1.04073	1.35377

37698	1.15596	1.24104
37699	0.614478	1.23417
37700	0.851524	1.08418
37701	2.22575	1.63227
37702	1.90502	2.1
37703	2.10893	1.39799
37704	1.80258	2.1
37705	4.01395	2.1
37706	1.90502	2.1
37707	1.40863	2.1
37708	1.49733	2.1
37709	1.49733	2.1
37710	1.80258	2.1
37711	1.80258	2.1
37712	2.10893	1.3254
37713	1.68356	2.1
37714	1.80258	2.1
37715	1.80258	1.83334
37716	1.98061	2.1
37717	1.98061	1.77801
37718	1.80258	2.1
37719	1.40863	2.1
37720	1.68356	2.1
37721	1.30751	2.1
37722	3.11009	2.1
37723	1.35374	1.39612
37724	1.35374	1.52348
37725	0.746096	1.21185
37726	0.746096	1.16791
37727	1.1537	1.27259
37728	0.746095	1.44988
37729	0.946132	1.23644
37730	0.946132	1.08807
37731	0.946132	1.03741
37732	0.746095	1.17001
37733	0.946131	1.04243
37734	1.1537	1.11034
37735	2.15247	2.1
37736	1.70303	1.3504
37737	1.41207	2.1
37738	1.43052	2.04645
37739	3.22793	2.1
37740	0.940246	2.1
37741	0.728442	1.98422
37742	0.650037	2.1
37743	0.539874	2.1
37744	0.694756	2.1
37745	0.694756	2.01627
37746	0.776346	2.1
37747	0.656772	2.1
37748	0.733146	2.1
37749	0.563769	2.1
37750	0.66316	2.1
37751	0.899692	2.1
37752	1.21223	1.96868
37753	1.22418	2.1
37754	1.43052	2.1
37755	1.43052	1.43127

37756	1.24353	2.1
37757	1.46181	1.52472
37758	1.89226	0.589694
37759	2.39894	0.438774
37760	1.89226	0.625077
37761	2.37417	0.466891
37762	1.89226	0.619039
37763	2.37417	0.457654
37764	1.12767	0.417191
37765	1.19947	0.504447
37766	0.94613	0.581463
37767	0.94613	0.460588
37768	1.12766	0.393498
37769	0.946128	0.404117
37770	1.19947	0.468931
37771	0.946132	0.60962
37772	1.19947	0.551798
37773	1.19947	0.415638
37774	0.94613	0.570541
37775	0.94613	0.435275
37776	2.22621	0.147815
37777	1.14533	2.1
37778	1.1131	2.1
37779	0.88238	2.1
37780	0.868521	1.47503
37781	1.01153	2.1
37782	1.01153	2.1
37783	1.00232	2.1
37784	1.00232	2.1
37785	1.14533	2.1
37786	0.882378	2.1
37787	0.868519	2.1
37788	1.11311	2.1
37789	0.910183	1.28857
37790	1.07811	1.06525
37791	0.94613	0.785457
37792	0.94613	0.84945
37793	0.910212	0.885958
37794	0.910212	1.35283
37795	1.11403	0.787517
37796	1.11403	1.42862
37797	0.910183	0.933985
37798	0.94613	0.955569
37799	0.94613	0.992776
37800	1.07811	0.981952
37801	1.87228	1.06274
37802	2.81189	2.1
37803	1.6197	2.0002
37804	2.56716	1.79265
37805	4.43944	1.96258
37806	0.946132	1.12646
37807	0.946132	1.86428
37808	0.946132	0.848017
37809	0.946132	0.920599
37810	0.946131	1.003
37811	0.946131	1.86006
37812	0.946132	0.867707
37813	0.946132	1.14893

37814	0.946132	1.04025
37815	0.946132	1.0732
37816	0.946132	1.14722
37817	0.946132	1.1494
37818	1.28724	2.1
37819	2.56716	1.64395
37820	2.3014	1.38774
37821	2.3014	2.1
37822	1.6197	1.64713
37823	1.6197	2.1
37824	0.946132	1.75947
37825	0.946132	1.53148
37826	0.946132	0.887502
37827	0.946132	0.990374
37828	0.946132	1.04037
37829	0.946132	1.8144
37830	0.946133	1.88979
37831	0.946133	0.89367
37832	0.946133	1.1102
37833	0.946133	1.09041
37834	0.946132	1.10911
37835	0.946132	1.14304
37836	1.08039	2.1
37837	1.24445	1.46363
37838	1.2873	1.74864
37839	1.48751	1.75013
37840	2.53175	1.84286
37841	0.946125	0.934814
37842	0.946125	2.1
37843	1.12552	1.76318
37844	1.12552	2.03795
37845	0.946124	1.4723
37846	0.946124	1.56837
37847	1.12552	2.1
37848	1.11114	2.1
37849	1.11113	1.47473
37850	0.946118	1.64133
37851	1.12552	1.80092
37852	0.946131	1.73721
37853	1.89225	1.09812
37854	1.89225	1.27672
37855	1.89225	1.70589
37856	1.89225	2.06721
37857	1.89225	0.977005
37858	1.89225	1.80401
37859	0.987703	1.05844
37860	1.33078	1.58794
37861	1.31565	1.11351
37862	1.31565	1.62886
37863	1.01121	1.16378
37864	1.01121	1.7804
37865	2.19163	2.1
37866	1.89226	1.69092
37867	1.89226	2.1
37868	1.89226	1.35625
37869	2.22229	2.06033
37870	2.22229	1.25875
37871	1.89226	1.94105

37872	1.89226	0.936262
37873	1.89226	0.927397
37874	1.89226	1.81668
37875	1.89226	0.939477
37876	1.89226	1.82743
37877	0.57141	1.81442
37878	0.57141	0.948616
37879	0.574036	1.10005
37880	0.574036	2.1
37881	0.651591	1.62454
37882	0.657827	1.20353
37883	0.482391	1.26448
37884	0.493855	1.49818
37885	0.482391	0.981225
37886	0.651591	1.05222
37887	0.493855	1.52877
37888	0.657828	1.47498
37889	1.88428	2.1
37890	2.03975	2.1
37891	1.97316	2.1
37892	1.8266	2.1
37893	3.86635	2.1
37894	2.23053	2.1
37895	1.42255	2.1
37896	1.3868	2.1
37897	1.8266	2.1
37898	3.24915	2.1
37899	1.97316	2.1
37900	1.8266	2.1
37901	1.77128	2.1
37902	1.91767	2.1
37903	3.74427	2.1
37904	1.77128	2.1
37905	1.8266	2.1
37906	1.77942	2.1
37907	1.33962	2.1
37908	1.3868	2.1
37909	1.3868	2.1
37910	0.944111	2.1
37911	0.944111	2.1
37912	1.26036	2.1
37913	1.16968	2.1
37914	1.03853	2.1
37915	1.16646	2.1
37916	0.946129	2.1
37917	0.946129	2.1
37918	0.94613	2.1
37919	1.25768	2.1
37920	1.03853	2.1
37921	0.946131	2.1
37922	1.82441	0.321087
37923	1.33061	0.306698
37924	1.13272	0.271965
37925	1.60249	0.292934
37926	2.95712	0.306596
37927	1.82441	0.331582
37928	2.08757	0.323418
37929	1.81292	0.279846

37930	1.60249	0.29343
37931	3.63733	0.298331
37932	1.81292	0.266597
37933	1.332	0.246158
37934	1.60249	0.23707
37935	1.60249	0.266783
37936	1.5344	0.237849
37937	1.5344	0.273407
37938	1.10107	2.1
37939	2.51559	2.1
37940	1.1229	2.1
37941	1.1229	2.1
37942	2.49456	2.1
37943	2.49456	2.1
37944	1.89226	2.1
37945	2.51559	2.1
37946	2.49457	2.1
37947	2.51559	2.1
37948	1.87124	2.1
37949	1.89226	2.1
37950	1.89226	2.1
37951	1.89226	2.1
37952	1.89226	2.1
37953	1.89226	2.1
37954	1.89226	2.1
37955	1.89226	2.1
37956	1.89226	2.1
37957	1.89226	2.1
37958	1.89226	2.1
37959	1.89226	2.1
37960	1.89226	2.1
37961	1.89226	2.1
37962	1.23599	2.1
37963	1.24728	2.1
37964	0.550534	2.1
37965	0.541151	2.1
37966	0.893754	2.1
37967	0.893754	2.1
37968	0.894678	2.1
37969	0.894678	2.1
37970	1.23599	2.1
37971	0.541149	2.1
37972	0.550533	2.1
37973	1.24728	2.1
37974	0.946132	2.1
37975	0.946132	2.1
37976	1.23599	2.1
37977	1.24728	2.1
37978	0.946132	2.1
37979	0.946132	2.1
37980	1.23599	2.1
37981	1.23599	2.1
37982	1.23599	2.1
37983	0.946132	2.1
37984	0.946132	2.1
37985	1.24728	2.1
37986	1.89226	2.1
37987	1.89226	2.1

37988	1.89226	2.1
37989	1.89226	2.1
37990	1.89226	2.1
37991	1.89226	2.1
37992	0.946128	2.1
37993	0.946128	2.1
37994	0.946132	2.1
37995	0.946132	2.1
37996	0.94613	2.1
37997	0.94613	2.1
37998	0.946129	2.1
37999	0.946129	2.1
38000	0.94613	2.1
38001	0.94613	2.1
38002	0.94613	2.1
38003	0.94613	2.1
38004	2.58696	0.671743
38005	2.57073	0.709286
38006	2.12585	0.672342
38007	2.07607	0.704922
38008	4.69658	0.700198
38009	1.89226	0.709569
38010	1.89226	0.711046
38011	1.89226	0.695952
38012	1.89226	0.733889
38013	3.78452	0.705691
38014	1.89226	0.711589
38015	1.89226	0.759503
38016	1.89226	0.736083
38017	1.89226	0.738756
38018	3.78453	0.739147
38019	2.57074	0.710588
38020	2.32635	0.737746
38021	2.07608	0.70678
38022	1.73623	0.734706
38023	4.30697	0.713762
38024	2.12582	0.647646
38025	1.51971	0.66546
38026	1.57255	0.640851
38027	2.07604	0.68034
38028	1.62233	0.634007
38029	2.12582	0.668982
38030	1.89223	0.682783
38031	1.89223	0.73689
38032	1.89223	0.709654
38033	1.89223	0.704806
38034	1.89223	0.709423
38035	1.89223	0.702574
38036	0.946119	0.749136
38037	0.946119	0.734362
38038	0.946119	0.701532
38039	0.946119	0.735844
38040	0.946119	0.742
38041	0.946119	0.749572
38042	0.94612	0.738756
38043	0.94612	0.711817
38044	0.946132	0.734206
38045	0.946132	0.723853

38046	0.946107	0.732869
38047	0.946107	0.731799
38048	0.811456	0.69478
38049	0.811456	0.738945
38050	0.806521	0.665108
38051	0.806521	0.733678
38052	0.868104	0.731758
38053	0.579949	0.734541
38054	0.759859	0.668013
38055	1.03802	0.67967
38056	0.579957	0.705857
38057	0.759868	0.704211
38058	1.03801	0.721196
38059	0.868093	0.724347
38060	2.7408	2.1
38061	2.82858	1.77425
38062	2.57368	2.1
38063	2.52633	2.1
38064	5.3549	2.1
38065	2.7408	2.1
38066	2.31353	2.1
38067	2.14063	2.1
38068	2.52632	2.1
38069	4.83985	2.1
38070	2.57368	2.1
38071	2.52633	2.1
38072	2.3301	2.1
38073	2.36071	1.66905
38074	4.88704	2.1
38075	1.86512	2.1
38076	2.52632	2.1
38077	2.14063	2.1
38078	2.14063	2.1
38079	2.3301	2.1
38080	2.3301	2.1
38081	1.86431	2.1
38082	1.89227	2.1
38083	1.68992	2.1
38084	1.68992	2.1
38085	1.89227	2.1
38086	1.89227	2.1
38087	2.0815	2.1
38088	1.89226	2.1
38089	2.04022	2.1
38090	1.86431	2.1
38091	2.07081	2.1
38092	1.89226	2.1
38093	1.91686	1.15605
38094	1.65683	1.42621
38095	1.86871	1.91796
38096	1.65683	1.21467
38097	2.13075	1.37414
38098	1.89226	0.934034
38099	1.89226	1.46732
38100	1.89226	0.915499
38101	1.89226	1.02627
38102	1.89226	1.44278
38103	3.78452	1.08485

38104	0.851541	1.98215
38105	0.851541	1.6568
38106	0.952803	2.1
38107	0.851573	2.1
38108	0.946129	2.1
38109	0.946129	1.31023
38110	0.929184	2.03184
38111	0.757482	2.1
38112	0.836274	2.1
38113	0.851528	2.1
38114	0.952816	2.1
38115	1.04073	2.1
38116	1.70303	1.04265
38117	1.70302	1.09701
38118	1.5544	1.48639
38119	1.70302	1.04866
38120	1.74957	1.06556
38121	1.91686	0.884593
38122	1.89226	0.958972
38123	1.89226	1.15367
38124	1.89226	1.35932
38125	1.89226	1.02829
38126	1.89226	1.02906
38127	1.89226	0.789429
38128	1.89226	1.66567
38129	1.89226	2.0526
38130	2.03472	2.1
38131	1.89226	1.81255
38132	1.99827	2.01297
38133	1.85836	1.32455
38134	1.00509	2.1
38135	1.55998	1.94128
38136	1.83347	2.1
38137	1.45246	2.1
38138	3.01244	2.1
38139	1.23839	2.1
38140	0.884251	2.1
38141	0.946133	2.1
38142	0.946133	2.1
38143	0.884226	2.1
38144	0.884226	2.1
38145	0.946158	2.1
38146	0.946159	2.1
38147	1.23839	2.1
38148	0.946131	2.1
38149	0.946131	2.1
38150	0.884254	2.1
38151	1.89226	2.1
38152	1.89226	1.48144
38153	1.89226	1.88362
38154	1.89226	2.01854
38155	1.89226	1.94373
38156	1.89226	2.0562
38157	2.1499	2.04799
38158	1.45244	2.02058
38159	1.83345	1.91019
38160	1.83345	2.1
38161	1.87214	1.85818

38162	1.87214	2.07857
38163	1.89224	1.98435
38164	1.89224	1.28698
38165	1.89224	1.7375
38166	1.89224	1.79642
38167	3.78448	1.99181
38168	1.49155	1.09542
38169	2.0815	1.28558
38170	1.77515	1.4152
38171	1.72377	2.1
38172	3.50659	1.42062
38173	1.69876	0.647237
38174	2.0815	1.81359
38175	1.9133	0.926302
38176	1.68189	0.65619
38177	2.17628	2.00083
38178	1.94335	1.79823
38179	2.65129	0.242042
38180	1.15317	0.228491
38181	2.57085	0.230593
38182	1.209	0.228947
38183	2.57085	0.231752
38184	1.209	0.232416
38185	1.89226	0.270264
38186	1.89226	0.242536
38187	1.89226	0.235661
38188	1.89226	0.273854
38189	3.78452	0.261974
38190	2.48861	0.211319
38191	1.15252	0.224093
38192	2.60515	0.230186
38193	2.60515	0.212699
38194	1.16384	0.228288
38195	1.16384	0.211111
38196	1.59903	0.813025
38197	1.26865	0.825086
38198	1.30486	1.04828
38199	1.70302	0.88032
38200	2.98573	0.880078
38201	1.59903	0.778601
38202	1.79478	0.891373
38203	1.79478	0.812553
38204	1.97005	0.848384
38205	1.50365	0.806486
38206	1.64192	1.02099
38207	1.89226	0.751674
38208	1.89226	0.8147
38209	1.89226	0.904553
38210	1.89226	0.783578
38211	3.78453	0.772519
38212	1.89226	0.864336
38213	1.89226	0.806022
38214	1.89226	0.866123
38215	1.89226	0.770215
38216	1.89226	0.864375
38217	1.89226	0.880151
38218	0.761672	0.778434
38219	0.896406	0.820838

38220	0.603509	0.821419
38221	0.574071	0.874961
38222	0.695159	0.891305
38223	0.695159	0.722056
38224	0.762564	1.06378
38225	0.770159	0.792869
38226	0.803021	0.878327
38227	0.572529	0.926974
38228	0.696466	0.759487
38229	0.899733	0.764282
38230	1.66034	0.67325
38231	1.79478	0.870389
38232	1.56685	0.803491
38233	1.68512	1.11799
38234	1.67652	0.826575
38235	1.67652	0.886909
38236	1.89226	0.881402
38237	1.89226	0.811958
38238	1.89226	0.860883
38239	1.89226	0.847548
38240	1.89226	0.862669
38241	1.89226	0.856839
38242	0.946133	0.762717
38243	0.946133	0.799069
38244	0.946132	0.859598
38245	0.946132	0.814033
38246	0.946132	0.833207
38247	0.946132	0.688682
38248	0.946133	1.08175
38249	0.946133	0.778916
38250	0.946133	0.834673
38251	0.946133	0.854924
38252	0.946132	0.745691
38253	0.946132	0.739947
38254	1.80424	2.1
38255	1.45158	2.1
38256	1.9098	2.1
38257	1.56003	2.1
38258	3.36138	2.1
38259	1.90982	2.1
38260	1.71606	2.1
38261	2.15314	2.1
38262	1.70302	2.1
38263	3.89051	2.1
38264	1.96416	0.895868
38265	1.23706	0.762111
38266	1.00053	0.732064
38267	1.38287	0.726901
38268	1.57916	0.761459
38269	1.9615	0.793851
38270	0.827865	0.714726
38271	0.946132	0.703237
38272	0.946132	0.590653
38273	0.946132	0.752481
38274	0.946132	0.756823
38275	0.946132	0.716787
38276	0.946131	0.760249
38277	0.946131	0.724426

38278	0.827865	0.787918
38279	0.946132	0.700391
38280	0.946132	0.570426
38281	0.946132	0.679136
38282	1.89227	2.1
38283	1.89227	2.1
38284	1.89227	2.1
38285	1.89227	2.1
38286	3.78453	2.1
38287	2.3819	2.1
38288	1.58089	2.1
38289	1.58089	2.1
38290	1.6287	2.1
38291	2.33368	2.1
38292	2.3815	2.1
38293	0.946133	2.1
38294	0.946133	2.1
38295	0.946132	2.1
38296	0.946132	2.1
38297	0.946132	2.1
38298	0.946132	2.1
38299	0.946132	2.1
38300	0.946132	2.1
38301	0.946133	2.1
38302	0.946133	2.1
38303	0.946132	2.1
38304	0.946132	2.1
38305	1.89226	2.1
38306	1.89226	2.1
38307	1.89226	2.1
38308	1.89226	2.1
38309	3.78453	2.1
38310	2.7874	2.1
38311	2.33368	2.1
38312	2.71897	2.1
38313	2.27495	2.1
38314	5.05266	2.1
38315	0.970991	2.1
38316	0.970991	2.1
38317	0.956929	2.1
38318	0.956929	2.1
38319	0.732928	2.1
38320	0.790444	2.1
38321	1.16684	2.1
38322	1.13748	2.1
38323	1.13748	2.1
38324	0.732928	2.1
38325	0.790444	2.1
38326	1.16684	2.1
38327	0.946132	2.1
38328	0.946132	2.1
38329	0.946131	2.1
38330	0.946131	2.1
38331	0.946132	2.1
38332	0.946132	2.1
38333	0.946132	2.1
38334	0.946132	2.1
38335	0.946132	2.1

38336	0.946132	2.1
38337	0.946132	2.1
38338	0.946132	2.1
38339	1.10747	0.57342
38340	2.30331	0.66597
38341	1.47954	0.547297
38342	2.63789	0.526941
38343	3.78285	0.573217
38344	1.65215	0.509162
38345	2.63789	0.486711
38346	2.82465	0.571216
38347	2.82465	0.56149
38348	1.47954	0.49394
38349	1.47954	0.498281
38350	1.13456	1.76002
38351	1.08334	2.1
38352	1.20674	1.05974
38353	1.17885	1.69154
38354	2.29008	1.58568
38355	1.20674	1.54342
38356	1.17885	2.00816
38357	1.28649	1.12908
38358	1.28516	1.51292
38359	2.4919	1.56709
38360	1.89226	2.00919
38361	1.89226	2.08357
38362	1.89226	2.09317
38363	1.89226	2.03966
38364	1.89226	2.05987
38365	1.89226	1.9976
38366	2.50288	1.31998
38367	2.02115	1.65859
38368	2.7717	1.25526
38369	2.29107	0.913954
38370	4.79395	1.66177
38371	1.27339	1.44075
38372	1.10817	2.1
38373	1.19733	1.95426
38374	1.032 2.1	
38375	2.30539	2.1
38376	1.19733	2.04277
38377	0.953637	2.1
38378	0.953637	2.0081
38379	1.032 2.1	
38380	1.11868	1.7734
38381	1.19704	2.1
38382	2.01024	1.00346
38383	1.11938	1.14947
38384	1.9186	1.24444
38385	2.76298	1.03211
38386	3.92884	1.14203
38387	0.851541	0.548057
38388	0.851541	0.521094
38389	0.957577	0.497914
38390	0.848952	0.507168
38391	1.08043	0.555143
38392	0.851541	0.562586
38393	0.851484	0.509672

38394	0.957578	0.468785
38395	1.20045	0.541562
38396	0.726289	0.454979
38397	0.946131	0.51501
38398	0.946131	0.554916
38399	1.50524	2.1
38400	2.38363	2.1
38401	2.49018	2.1
38402	2.49018	2.1
38403	1.45506	2.1
38404	1.45506	2.1
38405	2.49017	2.1
38406	1.89226	2.1
38407	1.89226	2.1
38408	1.89226	2.1
38409	2.38363	2.1
38410	2.38363	2.1
38411	1.54711	2.1
38412	2.49018	2.1
38413	2.56975	2.1
38414	2.56975	2.1
38415	1.50524	2.1
38416	1.50524	2.1
38417	2.56975	2.1
38418	1.89226	2.1
38419	1.89226	2.1
38420	1.89226	2.1
38421	2.49017	2.1
38422	2.49017	2.1
38423	1.89226	0.457831
38424	1.89226	0.474629
38425	1.89226	0.564882
38426	1.89226	0.400655
38427	1.89226	0.569108
38428	1.89226	0.393066
38429	1.89226	0.530047
38430	1.89226	0.501352
38431	1.89226	0.684613
38432	1.89226	0.443884
38433	1.89226	0.687755
38434	1.89226	0.447734
38435	1.89226	0.446251
38436	1.89226	0.427437
38437	2.8358	0.3876
38438	1.89226	0.620121
38439	3.78453	0.39336
38440	1.60565	0.737268
38441	1.89226	1.52491
38442	1.89226	1.21299
38443	1.40013	1.0399
38444	1.89226	1.35583
38445	1.40013	1.16336
38446	1.1766	2.1
38447	1.29435	2.1
38448	1.35735	2.1
38449	1.53509	2.1
38450	2.65169	2.1
38451	0.69701	2.1

38452	0.69701	2.1
38453	0.711303	2.1
38454	0.711303	2.1
38455	0.761138	2.1
38456	0.612149	2.1
38457	0.647174	2.1
38458	0.767548	2.1
38459	0.767548	2.1
38460	0.761139	2.1
38461	0.612149	2.1
38462	0.647174	2.1
38463	1.29764	1.44577
38464	1.29579	1.65269
38465	1.34875	1.50648
38466	1.06717	1.69617
38467	1.29764	1.42498
38468	1.01606	1.485
38469	0.756635	1.38488
38470	0.648821	1.39622
38471	0.675557	1.53598
38472	0.674374	1.45863
38473	0.74214	1.57081
38474	0.74214	1.45137
38475	0.688868	1.34613
38476	0.688868	1.42034
38477	0.675557	1.24891
38478	0.756634	1.31614
38479	0.648821	1.23799
38480	0.674374	1.29615
38481	1.51327	1.52641
38482	1.48105	1.34004
38483	1.29764	1.5411
38484	1.29764	1.44415
38485	1.48105	1.51638
38486	1.05655	1.34054
38487	1.13537	2.1
38488	1.07326	2.1
38489	0.94613	2.1
38490	0.94613	2.1
38491	0.946132	2.1
38492	0.946132	2.1
38493	1.13537	2.1
38494	1.08927	2.1
38495	1.13537	2.1
38496	0.946131	2.1
38497	0.946131	2.1
38498	0.95947	2.1
38499	1.46892	2.1
38500	1.89226	2.1
38501	1.89226	2.1
38502	1.89226	2.1
38503	1.88031	2.1
38504	1.88031	2.1
38505	1.89226	2.1
38506	1.89226	2.1
38507	1.89226	2.1
38508	1.89226	2.1
38509	3.78453	2.1

38510	2.18254	2.1
38511	1.89226	2.1
38512	2.50005	2.1
38513	1.89226	2.1
38514	4.39231	2.1
38515	1.88031	2.1
38516	2.0815	2.1
38517	2.0815	2.1
38518	1.92078	2.1
38519	2.0815	2.1
38520	1.9218	2.1
38521	1.89227	2.1
38522	1.89227	2.1
38523	1.89227	2.1
38524	1.89227	2.1
38525	1.89227	2.1
38526	1.89227	2.1
38527	1.98693	2.1
38528	1.82857	2.1
38529	2.32	2.1
38530	2.36972	2.1
38531	4.14857	2.1
38532	1.82857	2.1
38533	1.58688	2.1
38534	2.36971	2.1
38535	2.19093	2.1
38536	4.0195	2.1
38537	0.946132	2.1
38538	0.946132	2.1
38539	1.18486	2.1
38540	1.18486	2.1
38541	1.18486	2.1
38542	0.946132	2.1
38543	0.946132	2.1
38544	1.16	2.1
38545	0.946133	2.1
38546	0.946133	2.1
38547	1.16	2.1
38548	1.18486	2.1
38549	1.07798	1.58369
38550	1.08101	1.40263
38551	0.910786	2.1
38552	0.910786	1.89875
38553	0.851537	1.77204
38554	0.901377	1.40149
38555	1.14652	1.78982
38556	1.04072	2.1
38557	0.946129	1.85368
38558	0.946129	1.86538
38559	1.0019	1.65484
38560	1.04263	1.71761
38561	1.89226	2.1
38562	1.89226	2.1
38563	1.89226	2.1
38564	1.89226	2.1
38565	3.78452	2.1
38566	1.89226	2.1
38567	2.30146	2.1

38568	1.89226	2.1
38569	2.18412	2.1
38570	4.19372	2.1
38571	2.30146	2.1
38572	1.39962	2.1
38573	2.18411	2.1
38574	1.28765	2.1
38575	3.5891	2.1
38576	1.89226	2.1
38577	1.89226	2.1
38578	1.89226	2.1
38579	1.89226	2.1
38580	1.89226	2.1
38581	1.89226	2.1
38582	1.89226	2.1
38583	1.89226	2.1
38584	1.89226	2.1
38585	1.89226	2.1
38586	3.78452	2.1
38587	2.18412	2.1
38588	1.20045	2.1
38589	1.20045	2.1
38590	1.28765	2.1
38591	2.08808	2.1
38592	2.17528	2.1
38593	0.946131	2.1
38594	0.946131	2.1
38595	0.946128	2.1
38596	0.946128	2.1
38597	0.946127	2.1
38598	0.946127	2.1
38599	0.946131	2.1
38600	0.946131	2.1
38601	0.946129	2.1
38602	0.946129	2.1
38603	0.94613	2.1
38604	0.94613	2.1
38605	1.89226	0.795306
38606	1.89226	0.942605
38607	1.89226	0.881887
38608	1.89226	0.783379
38609	3.78452	0.789574
38610	1.89227	0.83851
38611	1.89227	1.22207
38612	1.89227	0.812715
38613	1.89227	0.999483
38614	1.89227	0.83397
38615	1.89227	1.08705
38616	1.89226	0.996312
38617	1.89226	0.848489
38618	1.89226	0.792456
38619	2.29245	0.864839
38620	3.78452	0.970141
38621	1.89226	0.889567
38622	1.89226	0.747091
38623	1.89226	0.794736
38624	1.89226	0.871545
38625	1.89226	0.760801

38626	1.89226	0.801888
38627	1.28095	1.18579
38628	1.81846	1.37256
38629	2.10991	0.905626
38630	1.34695	1.22502
38631	3.39086	1.25765
38632	2.10991	0.991105
38633	1.36457	1.32181
38634	1.67421	1.46097
38635	1.34695	1.29198
38636	2.43717	1.09385
38637	2.10991	1.10255
38638	2.69541	2.1
38639	1.48572	2.1
38640	2.63553	2.1
38641	1.45385	2.1
38642	4.14926	2.1
38643	2.63553	2.1
38644	1.44207	2.1
38645	1.44207	2.1
38646	1.45385	2.1
38647	2.588	2.1
38648	2.59978	2.1
38649	1.89227	2.1
38650	1.89227	2.1
38651	1.89227	2.1
38652	1.89227	2.1
38653	3.78453	2.1
38654	1.89226	2.1
38655	1.89226	2.1
38656	1.89226	2.1
38657	1.89226	2.1
38658	3.78453	2.1
38659	2.15929	2.1
38660	1.38276	2.1
38661	1.43617	2.1
38662	1.35614	2.1
38663	2.23932	2.1
38664	2.15929	2.1
38665	2.15929	2.1
38666	2.25168	2.1
38667	2.3558	2.1
38668	2.23932	2.1
38669	4.51509	2.1
38670	1.89226	2.1
38671	1.89226	2.1
38672	1.89226	2.1
38673	1.89226	2.1
38674	3.78453	2.1
38675	1.89226	2.1
38676	1.89226	2.1
38677	1.89226	2.1
38678	1.89226	2.1
38679	1.89226	2.1
38680	1.89226	2.1
38681	2.23932	2.1
38682	1.43854	2.1
38683	1.49173	2.1

38684	1.38276	2.1
38685	2.34829	2.1
38686	2.23932	2.1
38687	2.3558	2.1
38688	2.34829	2.1
38689	2.40685	2.1
38690	2.23932	2.1
38691	2.52332	2.1
38692	2.3558	2.1
38693	1.89226	0.371074
38694	1.87361	0.42752
38695	2.25082	0.345186
38696	1.87361	0.468524
38697	2.26947	0.341492
38698	1.89226	0.44663
38699	1.89226	0.448851
38700	1.89226	0.535499
38701	1.89226	0.431485
38702	1.89226	0.597781
38703	1.89226	0.414166
38704	1.89226	0.570636
38705	2.41314	0.411287
38706	1.89226	0.493921
38707	1.89226	0.394352
38708	1.89226	0.552588
38709	2.12204	0.372951
38710	2.12204	0.522326
38711	1.89226	0.365199
38712	2.25082	0.409146
38713	2.28189	0.332205
38714	2.25082	0.472836
38715	1.92333	0.320968
38716	1.89226	0.449297
38717	1.89226	0.45433
38718	1.89226	0.503647
38719	1.89226	0.425568
38720	1.89226	0.586061
38721	1.89226	0.405684
38722	1.89226	0.549103
38723	2.42521	0.398192
38724	1.89226	0.460621
38725	1.89226	0.369499
38726	1.89226	0.517915
38727	2.41314	0.357084
38728	2.41314	0.48514
38729	1.39725	1.22363
38730	1.07745	1.9426
38731	1.07745	1.22155
38732	1.07519	1.96152
38733	1.41336	1.1638
38734	1.41111	2.03866
38735	1.89226	1.80587
38736	1.92039	2.1
38737	1.92039	1.67558
38738	1.9052	2.1
38739	1.89226	1.60827
38740	1.87708	2.1
38741	0.946128	1.90093

38742	0.946128	1.61383
38743	0.946133	1.17137
38744	0.946133	1.08091
38745	0.94613	1.0956
38746	0.94613	1.57532
38747	0.946131	1.95543
38748	0.946131	1.12321
38749	0.946131	1.30002
38750	0.946131	1.29462
38751	0.946131	1.30804
38752	0.946131	1.31891
38753	1.41336	1.2121
38754	1.08911	1.99042
38755	1.08911	1.27874
38756	1.07745	2.01201
38757	1.42381	1.21944
38758	1.41215	2.07436
38759	1.89227	1.87775
38760	1.95833	2.1
38761	1.95833	1.7669
38762	1.92039	2.1
38763	1.89227	1.6958
38764	1.85433	2.1
38765	0.946129	1.96373
38766	0.946129	1.68694
38767	0.946133	1.24472
38768	0.946133	1.10471
38769	0.946131	1.11659
38770	0.946131	1.64484
38771	0.946131	1.99829
38772	0.946131	1.17902
38773	0.946131	1.38139
38774	0.946131	1.38256
38775	0.946131	1.40401
38776	0.946131	1.40849
38777	1.41943	2.1
38778	2.74398	2.1
38779	1.18238	2.1
38780	2.53897	2.1
38781	3.9584	2.1
38782	2.74398	2.1
38783	1.89226	2.1
38784	1.89226	2.1
38785	1.89226	2.1
38786	2.53897	2.1
38787	2.53897	2.1
38788	1.89227	0.952452
38789	1.89227	0.841559
38790	1.89227	0.919449
38791	1.89227	0.814171
38792	3.78453	0.851313
38793	1.89226	0.829585
38794	2.08134	0.77448
38795	2.00632	0.768944
38796	2.00632	0.792045
38797	1.89226	0.792953
38798	1.89226	0.827626
38799	1.89227	0.880504

38800	1.89227	0.810654
38801	1.89227	0.821376
38802	1.89227	0.757581
38803	3.78453	0.839642
38804	1.89227	0.890091
38805	1.89227	0.755083
38806	1.89227	0.753882
38807	1.89227	0.803318
38808	1.89227	0.803683
38809	1.89227	0.914667
38810	2.50912	0.742292
38811	1.89226	0.778656
38812	1.89226	0.72427
38813	2.31261	0.751888
38814	2.31261	0.764113
38815	1.89226	0.794619
38816	2.50912	0.744159
38817	1.19999	0.73956
38818	1.44056	0.704921
38819	1.24405	0.736895
38820	2.50912	0.747023
38821	2.31261	0.764179
38822	2.69224	0.812101
38823	2.28373	0.712534
38824	1.94788	0.851637
38825	2.4106	0.762554
38826	4.69434	0.793778
38827	2.69223	0.648187
38828	2.63783	0.868604
38829	2.34737	1.04675
38830	2.4106	0.792359
38831	5.04843	0.876225
38832	1.89226	2.1
38833	1.99753	2.1
38834	1.87366	2.1
38835	1.99753	2.1
38836	1.76839	2.1
38837	1.89226	2.08394
38838	1.89226	2.1
38839	1.89226	1.23472
38840	1.89226	2.1
38841	1.89226	2.1
38842	3.78452	2.1
38843	0.946132	2.1
38844	0.946132	2.1
38845	1.02744	2.1
38846	1.02744	2.1
38847	0.946132	2.1
38848	0.946132	2.1
38849	1.00989	2.1
38850	1.02744	2.1
38851	1.00989	2.1
38852	0.946133	2.1
38853	1.02744	2.1
38854	0.946132	2.1
38855	1.70302	2.1
38856	1.87366	2.1
38857	1.71618	2.1

38858	1.87366	2.1
38859	1.73105	2.1
38860	1.89226	2.0284
38861	1.89226	2.02775
38862	1.89226	2.1
38863	1.89226	2.1
38864	1.89226	2.1
38865	1.89226	2.1
38866	1.89226	1.21282
38867	1.96624	2.1
38868	1.89226	2.1
38869	1.89226	2.1
38870	1.89226	2.1
38871	2.01977	2.1
38872	2.01977	2.1
38873	1.1032	1.41586
38874	1.15114	2.1
38875	1.14986	0.795489
38876	1.19242	1.98148
38877	2.301	1.61458
38878	1.89226	1.28384
38879	1.89226	2.1
38880	1.89226	0.83123
38881	1.89226	1.39075
38882	3.78453	1.19204
38883	0.946132	2.1
38884	0.946132	1.70718
38885	0.946132	1.23089
38886	0.946132	2.1
38887	0.946133	2.1
38888	0.946133	2.1
38889	0.946132	1.59899
38890	0.946132	1.34625
38891	0.946132	2.1
38892	0.946132	2.1
38893	0.946133	2.1
38894	0.946133	2.1
38895	1.19243	1.74929
38896	0.953928	2.1
38897	1.18671	2.1
38898	1.15114	1.70573
38899	0.9895	2.1
38900	0.953928	1.96281
38901	1.19611	0.865267
38902	1.19242	2.1
38903	1.24693	0.900125
38904	1.24693	1.80916
38905	1.14986	0.999753
38906	1.14986	2.03125
38907	1.89225	0.891167
38908	1.89225	2.04349
38909	1.89225	2.1
38910	1.89225	1.15836
38911	1.89225	1.28155
38912	1.89225	1.05235
38913	1.89225	1.48568
38914	1.89225	2.1
38915	1.89225	2.0672

38916	1.89225	2.1
38917	1.89225	1.03944
38918	1.89225	2.1
38919	0.561532	2.1
38920	0.561532	1.68961
38921	0.566382	1.49282
38922	0.566382	2.1
38923	0.494747	2.1
38924	0.531705	2.1
38925	0.596208	1.71745
38926	0.623464	1.1897
38927	0.623469	2.1
38928	0.531708	2.1
38929	0.596205	2.1
38930	0.494743	2.1
38931	1.25742	2.1
38932	1.18783	2.1
38933	0.946131	2.1
38934	0.946131	2.1
38935	1.25742	2.1
38936	1.25742	2.1
38937	0.946131	2.1
38938	0.946131	2.1
38939	1.25742	2.1
38940	0.946131	2.1
38941	0.946131	2.1
38942	1.18783	2.1
38943	1.35048	2.1
38944	1.25742	2.1
38945	0.946132	2.1
38946	0.946132	2.1
38947	1.35048	2.1
38948	1.35048	2.1
38949	0.946132	2.1
38950	0.946132	2.1
38951	1.35044	2.1
38952	0.946106	2.1
38953	1.25745	2.1
38954	0.946158	2.1
38955	1.13525	2.1
38956	2.70095	2.1
38957	2.51483	2.1
38958	2.51483	2.1
38959	1.21593	2.1
38960	1.21593	2.1
38961	1.93142	0.70079
38962	2.8053	0.349528
38963	1.559	0.62803
38964	2.60359	0.373663
38965	4.53501	0.503804
38966	2.80531	0.438521
38967	1.89226	0.325126
38968	2.60359	0.438352
38969	1.89226	0.730571
38970	4.49586	0.316435
38971	1.22938	0.578903
38972	2.60359	0.301883
38973	2.37434	0.745837

38974	2.37434	0.4098
38975	1.55901	0.779369
38976	1.55901	0.297867
38977	1.18717	0.71645
38978	1.18717	0.328554
38979	0.946132	0.409264
38980	0.946132	0.416618
38981	0.946133	0.525203
38982	0.946133	0.691846
38983	1.3018	0.420128
38984	1.18717	0.719047
38985	1.18714	0.510947
38986	0.946107	0.525732
38987	1.30183	0.400893
38988	0.946158	0.408778
38989	1.89226	2.1
38990	2.80196	2.1
38991	2.67272	2.1
38992	2.67272	2.1
38993	1.89226	2.1
38994	1.89226	2.1
38995	1.89226	2.1
38996	1.89226	2.1
38997	1.89226	2.1
38998	1.89226	2.1
38999	1.89226	2.1
39000	1.89226	2.1
39001	1.74471	2.1
39002	2.01555	2.1
39003	1.61121	2.1
39004	1.61121	2.1
39005	2.13878	2.1
39006	2.13878	2.1
39007	1.89226	2.1
39008	1.89226	2.1
39009	1.89226	2.1
39010	1.89226	2.1
39011	1.89226	2.1
39012	1.89226	2.1
39013	1.09766	2.1
39014	1.04263	2.1
39015	0.881794	2.1
39016	0.894188	2.1
39017	0.795402	2.1
39018	0.99524	2.1
39019	1.00647	2.1
39020	1.00647	2.1
39021	1.15401	2.1
39022	0.881789	2.1
39023	0.850526	2.1
39024	1.04263	2.1
39025	1.4054	1.17342
39026	1.04788	2.1
39027	1.70302	1.11625
39028	1.41375	2.1
39029	2.82706	1.56548
39030	1.70302	1.09691
39031	1.38776	1.92219

39032	1.59457	1.52144
39033	1.41375	2.04415
39034	1.77261	1.03654
39035	1.70302	2.06527
39036	1.89226	2.1
39037	1.89226	2.1
39038	1.89226	2.1
39039	1.89226	2.1
39040	3.78453	2.1
39041	2.18042	2.1
39042	1.74738	2.1
39043	2.08433	2.1
39044	1.66347	2.1
39045	3.83171	2.1
39046	1.74738	2.1
39047	1.1181	2.1
39048	1.66347	2.1
39049	1.06543	2.1
39050	2.81281	2.1
39051	1.89226	2.1
39052	1.89226	2.1
39053	1.89226	2.1
39054	1.89226	2.1
39055	3.78453	2.1
39056	1.89227	2.1
39057	1.89227	2.1
39058	1.89227	2.1
39059	1.89227	2.1
39060	3.78453	2.1
39061	2.08433	2.1
39062	1.66347	2.1
39063	1.97092	2.1
39064	1.57431	2.1
39065	3.65864	2.1
39066	1.66347	2.1
39067	1.01474	2.1
39068	1.01474	2.1
39069	1.06544	2.1
39070	1.57431	2.1
39071	1.625 2.1	
39072	1.89226	2.1
39073	1.89226	2.1
39074	1.89226	2.1
39075	1.89226	2.1
39076	1.89226	2.1
39077	1.89226	2.1
39078	1.08467	2.1
39079	1.87228	2.1
39080	1.73305	2.1
39081	1.73305	2.1
39082	1.25565	2.1
39083	1.25565	2.1
39084	0.695361	0.835679
39085	0.799996	0.950381
39086	1.13751	1.09552
39087	1.40945	1.07842
39088	1.40944	1.1547
39089	0.799999	0.648388

39090	1.13751	0.981594
39091	0.695363	0.731061
39092	1.01059	0.853574
39093	1.01059	0.917696
39094	0.926908	1.2793
39095	0.926909	0.801697
39096	1.05481	2.1
39097	1.78069	1.82119
39098	1.08074	2.1
39099	1.83284	2.03868
39100	2.86143	2.1
39101	1.78069	1.97607
39102	2.22044	1.4619
39103	1.83284	2.0308
39104	2.28696	1.03374
39105	4.06766	1.50731
39106	1.07196	2.1
39107	1.64955	2.1
39108	2.02665	2.1
39109	2.0284	2.1
39110	1.08074	2.1
39111	1.08074	2.1
39112	1.64955	2.1
39113	2.28696	1.28278
39114	1.92033	1.88837
39115	2.1004	1.51398
39116	3.74988	1.77365
39117	2.83787	0.753971
39118	1.70303	0.886141
39119	1.70303	0.715795
39120	1.83195	0.708551
39121	2.64075	0.717763
39122	2.70231	0.786866
39123	1.2087	1.42332
39124	1.52909	0.908085
39125	1.43063	0.923272
39126	1.10586	1.34097
39127	2.63494	1.05679
39128	1.89227	1.1505
39129	2.10732	2.1
39130	2.10732	1.28499
39131	2.2365	2.1
39132	1.89227	1.22087
39133	2.02145	2.1
39134	1.89226	0.916942
39135	1.89226	1.30433
39136	1.89226	0.968656
39137	1.89226	1.21671
39138	1.89226	0.93563
39139	1.89226	1.42526
39140	0.51926	1.40316
39141	0.552927	1.37191
39142	0.691235	0.963875
39143	0.715314	0.918597
39144	0.619684	0.946335
39145	0.619684	1.60894
39146	0.624477	1.72958
39147	0.624477	0.920392

39148	0.519262	1.10415
39149	0.691234	1.09721
39150	0.715313	1.0786
39151	0.552928	1.07506
39152	1.89226	1.26855
39153	1.87321	2.1
39154	1.87321	1.66605
39155	1.89658	2.1
39156	2.0815	1.3286
39157	2.14789	2.1
39158	0.946128	1.44358
39159	0.946128	1.37866
39160	0.946133	0.925703
39161	0.946133	0.922305
39162	0.94613	0.919178
39163	0.94613	1.36541
39164	0.946131	0.967497
39165	0.946131	1.35855
39166	0.946131	1.01284
39167	0.946131	1.02545
39168	0.946131	1.09859
39169	0.946131	1.07878
39170	1.99297	1.21519
39171	2.19514	1.00092
39172	2.46153	1.10032
39173	2.187	1.12815
39174	4.45451	1.13881
39175	2.0815	1.03328
39176	2.33402	1.02538
39177	2.46154	1.12875
39178	2.83046	0.929496
39179	4.94496	1.00818
39180	2.46153	1.05007
39181	2.187	1.04975
39182	2.27351	1.10933
39183	2.63356	0.949723
39184	4.73504	1.03035
39185	2.46154	1.07638
39186	2.83447	0.87398
39187	2.74513	0.992005
39188	2.5731	1.0358
39189	2.63357	0.962603
39190	2.46154	0.990535
39191	2.80398	2.1
39192	2.60406	2.1
39193	2.73988	2.1
39194	2.57466	2.1
39195	5.34394	2.1
39196	1.89226	1.02746
39197	1.89226	0.906022
39198	1.89226	0.832731
39199	1.89226	1.08538
39200	1.89226	0.867119
39201	1.89226	1.25746
39202	1.89224	1.12223
39203	1.89224	0.933731
39204	1.89224	0.869649
39205	1.89224	1.25407

39206	1.89224	0.91412
39207	1.89224	1.41631
39208	1.40245	1.13642
39209	1.40246	1.56243
39210	0.946117	1.12898
39211	0.946117	1.33966
39212	1.17635	1.01199
39213	1.40246	1.61259
39214	0.946112	0.951129
39215	0.946112	1.32333
39216	0.946127	1.13192
39217	1.17637	1.01586
39218	0.946102	1.34552
39219	1.40244	1.14888
39220	2.53269	2.1
39221	1.70302	2.1
39222	1.56194	2.1
39223	2.17516	2.1
39224	4.19807	2.1
39225	1.89226	2.1
39226	1.89226	1.11911
39227	1.89226	2.06806
39228	1.89226	1.9188
39229	3.78453	2.02066
39230	2.57276	2.1
39231	1.89226	2.01098
39232	1.89226	2.1
39233	1.89226	2.1
39234	2.32525	2.1
39235	2.32525	2.1
39236	1.15171	2.1
39237	2.17517	2.07642
39238	1.41995	2.1
39239	1.41995	2.1
39240	1.80252	2.1
39241	1.80252	2.1
39242	1.89226	1.99783
39243	1.89226	1.98943
39244	1.89226	2.1
39245	1.89226	1.03563
39246	3.78453	2.01854
39247	0.946133	2.1
39248	0.946133	2.1
39249	1.16263	2.1
39250	1.04687	2.1
39251	0.946133	2.1
39252	0.946133	1.88727
39253	1.04687	2.1
39254	1.04687	2.1
39255	0.946133	2.1
39256	1.04687	2.1
39257	0.946132	2.1
39258	1.16263	2.1
39259	0.682281	1.28552
39260	0.669233	1.56708
39261	1.21859	1.49883
39262	1.31629	1.16404
39263	0.916158	1.07033

39264	0.916158	1.69881
39265	0.971658	1.20525
39266	0.971658	1.24807
39267	0.669234	1.24636
39268	1.31629	1.17426
39269	0.682283	1.30782
39270	1.21858	1.30325
39271	0.669232	1.22709
39272	0.623495	1.4693
39273	1.36428	1.31681
39274	1.31629	1.01204
39275	0.946475	1.0182
39276	0.946476	1.64054
39277	0.99331	1.22345
39278	0.99331	1.21392
39279	1.36428	1.26528
39280	0.623497	1.15689
39281	0.669234	1.22883
39282	1.31629	1.17013
39283	2.02069	0.24062
39284	1.46253	0.207646
39285	2.51479	0.205322
39286	1.87136	0.224331
39287	3.89203	0.215971
39288	1.46253	0.203092
39289	1.38536	0.220852
39290	1.38536	0.220399
39291	1.05579	0.216672
39292	1.73281	0.216358
39293	1.46916	0.207958
39294	2.51479	0.210429
39295	1.87136	0.245832
39296	2.23338	0.246073
39297	2.15603	0.209594
39298	4.55146	0.220993
39299	0.822553	0.205989
39300	0.822563	0.211899
39301	0.842935	0.243207
39302	0.842935	0.23265
39303	0.744203	0.232416
39304	0.675346	0.216742
39305	0.874795	0.244308
39306	1.00453	0.214963
39307	1.00454	0.214746
39308	0.782417	0.225123
39309	0.675337	0.227473
39310	0.858007	0.218206
39311	0.946132	0.943348
39312	1.24847	0.773278
39313	0.946132	0.938852
39314	1.16806	1.04041
39315	1.24847	0.758801
39316	1.24847	1.23062
39317	0.946133	0.73975
39318	0.946133	1.11928
39319	0.946133	0.823083
39320	0.946133	0.759705
39321	1.16805	0.998899

39322	1.24847	0.803373
39323	1.2364	0.926614
39324	0.946129	0.80074
39325	0.946133	0.967983
39326	1.24847	0.83599
39327	1.2364	0.775173
39328	1.2364	1.0917
39329	0.946131	0.737284
39330	0.946131	1.21391
39331	0.946131	0.863793
39332	0.946131	0.772995
39333	1.2364	1.09553
39334	1.24847	0.851299
39335	1.89226	0.897961
39336	1.89226	0.94625
39337	1.89226	0.789771
39338	1.89226	0.804156
39339	1.89226	0.931683
39340	1.89226	1.00347
39341	1.89226	0.869475
39342	1.89226	0.81683
39343	1.89226	0.757847
39344	1.89226	0.907393
39345	1.89226	0.802595
39346	1.89226	0.992922
39347	1.69014	0.873266
39348	1.09194	0.9268
39349	1.13537	1.033
39350	1.13537	0.950712
39351	1.58481	0.784776
39352	1.58481	0.807674
39353	1.58147	0.869118
39354	1.13537	0.857391
39355	1.69015	0.934861
39356	1.01179	0.806022
39357	1.69015	0.861331
39358	1.01179	0.954037
39359	0.946132	1.32385
39360	0.946132	1.06689
39361	1.28543	0.85171
39362	1.07341	1.45937
39363	1.07341	1.30181
39364	1.07341	0.786924
39365	0.946132	0.876818
39366	0.946132	0.867373
39367	0.946133	0.869909
39368	1.28543	0.86406
39369	1.07341	0.950667
39370	0.946132	1.03851
39371	1.89226	1.04265
39372	1.89226	0.849031
39373	1.89226	0.803744
39374	1.89226	1.45066
39375	1.89226	0.804357
39376	1.89226	1.82233
39377	0.805744	1.70456
39378	0.988659	1.09816
39379	0.782044	0.843172

39380	0.578223	1.07784
39381	0.77823	0.879823
39382	0.778231	1.97063
39383	0.788653	0.808793
39384	0.788653	1.27058
39385	0.988662	1.00352
39386	0.782042	0.98342
39387	0.805745	1.15552
39388	0.578222	1.06881
39389	1.89226	0.945356
39390	1.89226	0.936092
39391	1.89226	0.782521
39392	1.89226	1.4378
39393	1.89226	0.761139
39394	1.89226	1.66636
39395	1.97732	1.21136
39396	1.97819	0.899995
39397	1.56408	0.797424
39398	1.56408	1.49407
39399	2.33986	0.861318
39400	2.33986	1.73321
39401	1.84516	2.1
39402	2.21152	2.1
39403	1.90702	2.1
39404	1.54599	2.1
39405	3.75751	2.1
39406	1.89224	2.1
39407	1.89224	2.1
39408	1.89224	2.1
39409	1.89224	2.02139
39410	1.89224	2.1
39411	1.89224	1.82914
39412	1.47672	2.1
39413	1.54601	2.1
39414	1.27697	2.1
39415	1.54601	2.1
39416	1.638 2.1	
39417	1.90705	2.1
39418	0.946132	1.58308
39419	0.946132	2.1
39420	0.946131	2.1
39421	0.946131	2.1
39422	0.946131	2.1
39423	0.946131	1.63352
39424	0.946132	2.1
39425	0.946132	2.1
39426	0.946132	2.1
39427	0.946132	2.1
39428	0.946131	2.1
39429	0.946131	2.1
39430	0.946128	2.1
39431	0.946128	2.1
39432	1.32062	2.1
39433	1.4164	2.1
39434	0.946129	2.1
39435	0.946129	2.1
39436	1.32062	2.1
39437	1.32062	2.1

39438	1.32062	2.1
39439	0.94613	2.1
39440	1.41639	2.1
39441	0.946129	2.1
39442	1.98979	0.207712
39443	1.65977	0.228653
39444	2.21772	0.224708
39445	2.21772	0.214677
39446	1.48866	0.224682
39447	1.48866	0.208997
39448	0.973129	0.20733
39449	0.973129	0.212159
39450	0.980954	0.230936
39451	0.980954	0.229011
39452	0.845218	0.216275
39453	1.13981	0.22636
39454	0.829886	0.230257
39455	1.10886	0.215042
39456	1.10886	0.213739
39457	1.13982	0.221837
39458	0.845219	0.222154
39459	0.829886	0.215756
39460	0.946132	1.12438
39461	0.946132	1.75545
39462	1.3352	2.1
39463	1.41432	2.07548
39464	0.946133	2.07768
39465	0.946133	0.957717
39466	1.3352	1.7986
39467	1.3352	2.1
39468	1.3352	1.93055
39469	0.946132	1.93827
39470	1.41432	1.88297
39471	0.946133	1.89264
39472	2.70097	2.1
39473	1.88175	2.1
39474	1.51482	2.1
39475	1.88175	2.1
39476	2.37784	2.1
39477	2.82864	2.1
39478	1.89227	0.413169
39479	1.89227	0.365344
39480	1.89227	0.317547
39481	1.89227	0.302731
39482	3.78453	0.302661
39483	1.89226	0.340791
39484	2.13295	0.288895
39485	2.43203	0.273928
39486	2.43203	0.326052
39487	1.89226	0.287101
39488	1.89226	0.364116
39489	0.946133	0.273634
39490	0.946133	0.287852
39491	0.946132	0.254284
39492	0.946132	0.275298
39493	0.946132	0.253347
39494	0.946132	0.274343
39495	0.946133	0.253664

39496	0.946133	0.300796
39497	0.946132	0.250108
39498	0.946132	0.263299
39499	0.946132	0.316484
39500	0.946132	0.295901
39501	1.54987	2.1
39502	1.24128	2.1
39503	1.24128	2.1
39504	1.50738	2.1
39505	1.75702	2.1
39506	2.02312	2.1
39507	1.89289	2.1
39508	1.92264	2.1
39509	1.72994	2.1
39510	1.75702	2.1
39511	3.67966	2.1
39512	1.75702	2.1
39513	0.994291	2.1
39514	0.994291	2.1
39515	1.24128	2.1
39516	1.4964	2.1
39517	1.74339	2.1
39518	1.72993	2.1
39519	1.49641	2.1
39520	1.49641	2.1
39521	1.75702	2.1
39522	1.3939	2.1
39523	1.65452	2.1
39524	1.19964	2.1
39525	1.53296	2.1
39526	1.19964	2.1
39527	1.36861	2.1
39528	1.42624	2.1
39529	1.59521	2.1
39530	1.19964	2.1
39531	1.42624	2.1
39532	1.07156	2.1
39533	1.36226	2.1
39534	2.5619	2.1
39535	2.14058	1.0972
39536	1.93569	1.69468
39537	2.36221	1.21673
39538	2.5753	1.14568
39539	4.50279	1.45772
39540	2.14057	1.39254
39541	2.20831	1.63258
39542	2.64725	0.987638
39543	2.57529	1.36339
39544	4.78782	1.33783
39545	2.64725	1.10319
39546	2.57529	1.46037
39547	1.89226	0.970419
39548	1.89226	0.875105
39549	4.53951	1.20056
39550	2.0815	2.1
39551	1.86137	2.1
39552	1.86137	2.1
39553	1.86159	2.1

39554	2.0815	2.1
39555	2.10235	2.1
39556	1.33552	2.1
39557	2.32417	2.1
39558	1.18872	2.1
39559	1.93101	2.1
39560	3.26653	2.1
39561	1.89226	2.1
39562	1.89226	2.1
39563	1.89226	2.1
39564	1.89226	2.1
39565	1.89226	2.1
39566	1.89226	2.1
39567	0.955191	2.1
39568	1.03372	2.1
39569	0.946131	2.1
39570	0.946131	2.1
39571	0.930654	2.1
39572	0.930654	2.1
39573	1.04233	2.1
39574	1.05247	2.1
39575	0.955341	2.1
39576	0.946131	2.1
39577	1.02375	2.1
39578	1.13537	2.1
39579	1.1549	2.1
39580	1.93101	2.1
39581	1.91108	2.1
39582	1.52487	2.1
39583	1.3076	2.1
39584	1.06985	2.1
39585	1.89226	2.1
39586	1.89226	2.1
39587	1.89226	2.1
39588	1.89226	2.1
39589	1.89226	2.1
39590	1.89226	2.1
39591	1.58713	2.1
39592	2.6845	2.1
39593	1.58742	2.1
39594	2.74201	2.1
39595	4.27191	2.1
39596	2.68449	2.1
39597	1.89226	2.1
39598	2.74201	2.1
39599	1.89226	2.1
39600	4.57675	2.1
39601	1.57523	2.1
39602	2.74201	2.1
39603	2.8146	2.1
39604	2.8146	2.1
39605	1.58742	2.1
39606	1.58742	2.1
39607	0.946131	1.99192
39608	0.946131	2.1
39609	1.371	2.1
39610	1.371	2.1
39611	0.946127	2.1

39612	0.946127	1.76901
39613	1.37101	2.1
39614	1.4073	2.1
39615	0.94613	2.1
39616	1.4073	2.1
39617	1.371 2.1	
39618	0.946129	2.1
39619	1.44801	1.93829
39620	1.06821	1.71605
39621	2.00542	1.8633
39622	2.27703	1.81588
39623	3.34524	1.67421
39624	1.70301	0.68063
39625	1.89226	0.711234
39626	1.65809	0.688577
39627	1.89226	0.710815
39628	1.62758	0.694445
39629	1.85744	0.707387
39630	1.89226	0.698102
39631	1.89226	0.707973
39632	1.89226	0.704025
39633	1.89226	0.701496
39634	3.78452	0.701817
39635	1.89226	0.708211
39636	1.89226	0.698803
39637	1.89226	0.700953
39638	1.89226	0.718894
39639	3.78452	0.704362
39640	0.851541	0.71043
39641	0.851541	0.708953
39642	0.91193	0.682918
39643	0.824837	0.680821
39644	0.854559	0.680897
39645	0.851534	0.788358
39646	0.81851	0.7097
39647	0.717219	0.686662
39648	0.871743	0.69987
39649	0.837985	0.698077
39650	0.82902	0.69076
39651	0.82902	0.692688
39652	1.89226	0.706189
39653	1.92659	0.694478
39654	2.03235	0.700713
39655	1.89226	0.729404
39656	2.03235	0.690863
39657	1.89226	0.705569
39658	0.585557	0.773057
39659	0.585557	0.732887
39660	1.22612	0.707175
39661	1.09436	0.754508
39662	0.851537	0.795796
39663	0.526987	0.809381
39664	0.983879	0.702395
39665	1.04072	0.731253
39666	0.585526	0.756482
39667	0.792325	0.740193
39668	0.946129	0.746406
39669	0.946129	0.752873

39670	1.68152	2.1
39671	1.81445	2.1
39672	1.68152	2.1
39673	1.86792	2.1
39674	1.65888	2.1
39675	1.84529	2.1
39676	1.81445	2.1
39677	1.57885	2.1
39678	1.65889	2.1
39679	1.48594	2.1
39680	3.23773	2.1
39681	1.68154	2.1
39682	1.65891	2.1
39683	1.53296	2.1
39684	1.56299	2.1
39685	3.24453	2.1
39686	1.56299	2.1
39687	1.48596	2.1
39688	1.56299	2.1
39689	1.65891	2.1
39690	1.46568	2.1
39691	1.5616	2.1
39692	2.68785	2.1
39693	2.08831	2.1
39694	2.66919	2.1
39695	2.1024	2.1
39696	4.79025	2.1
39697	2.08832	2.1
39698	1.15478	2.1
39699	1.15478	2.1
39700	1.09062	2.1
39701	2.10241	2.1
39702	2.03825	2.1
39703	2.65348	2.1
39704	2.1024	2.1
39705	2.12313	2.1
39706	2.12313	2.1
39707	2.66919	2.1
39708	2.66919	2.1
39709	0.813169	2.1
39710	0.813169	2.1
39711	0.825788	2.1
39712	0.825788	2.1
39713	0.612902	2.1
39714	0.577387	2.1
39715	1.0512	2.1
39716	1.06157	2.1
39717	1.06157	2.1
39718	0.612902	2.1
39719	0.577387	2.1
39720	1.0512	2.1
39721	1.27227	2.1
39722	1.91724	2.1
39723	1.32591	2.1
39724	1.99837	2.1
39725	3.24315	2.1
39726	1.89223	2.1
39727	1.89223	2.1

39728	1.89223	2.1
39729	1.89223	1.69821
39730	3.78446	2.1
39731	1.89224	2.1
39732	1.89224	1.11018
39733	1.89224	1.73353
39734	1.89224	1.7059
39735	3.78447	1.66568
39736	1.91724	2.1
39737	2.321	2.1
39738	1.99838	2.1
39739	2.4149	1.68984
39740	4.33214	2.1
39741	1.34817	2.1
39742	1.9984	2.1
39743	2.06238	2.1
39744	2.06238	2.1
39745	1.32593	2.1
39746	1.32593	2.1
39747	1.89226	2.1
39748	1.89226	1.66621
39749	1.89226	2.1
39750	1.89226	2.1
39751	1.89226	2.1
39752	1.89226	2.1
39753	1.89226	1.7007
39754	1.89226	1.83096
39755	1.89226	2.1
39756	1.89226	1.08745
39757	3.78452	1.82242
39758	1.9984	2.1
39759	2.41494	1.81001
39760	2.06239	2.1
39761	2.5194	2.1
39762	4.47732	2.1
39763	1.19901	1.04924
39764	1.16661	2.09103
39765	1.56859	1.708
39766	1.61029	1.16932
39767	2.7676	1.61987
39768	1.56859	1.75692
39769	1.88637	0.864997
39770	1.61029	0.830058
39771	1.61029	1.67614
39772	1.856	0.837743
39773	1.856	1.67492
39774	1.856	1.00282
39775	1.57916	1.91621
39776	1.56859	1.07538
39777	1.56859	2.1
39778	1.57916	1.39861
39779	1.23475	2.1
39780	0.890924	0.713673
39781	1.07608	0.774077
39782	0.72877	0.86546
39783	0.910106	0.884929
39784	1.00196	0.882326
39785	1.09519	1.09579

39786	0.839967	0.815566
39787	0.719043	0.793954
39788	0.893859	0.716207
39789	0.942408	1.05745
39790	0.873497	0.864278
39791	0.873497	1.14926
39792	1.53046	0.658048
39793	1.89994	0.913273
39794	1.049	0.598005
39795	1.049	0.59195
39796	1.52512	0.567891
39797	1.89994	0.708429
39798	1.65573	0.764616
39799	1.89226	0.774815
39800	1.89226	0.740943
39801	2.73329	0.965341
39802	3.548	0.816707
39803	1.65573	0.741448
39804	2.42671	0.814954
39805	2.73329	0.742308
39806	1.89226	0.732164
39807	2.73329	0.999887
39808	1.89226	0.9497
39809	0.941078	0.878604
39810	1.01599	0.931632
39811	0.818936	0.818988
39812	0.651554	0.904304
39813	0.904729	0.893036
39814	0.665756	0.900827
39815	0.843423	1.07136
39816	0.918649	1.03881
39817	0.855806	1.12626
39818	0.814384	0.628989
39819	0.844437	0.98553
39820	0.844437	0.928617
39821	2.22558	1.34648
39822	1.95785	0.993007
39823	2.07065	1.1103
39824	1.89142	1.03225
39825	2.07065	1.86575
39826	1.89142	1.62574
39827	0.640813	1.06084
39828	0.951654	1.15612
39829	0.797442	0.878177
39830	0.535364	0.961725
39831	0.525269	1.00734
39832	0.620874	0.963083
39833	0.916953	1.10945
39834	0.969374	1.06936
39835	0.788925	1.42397
39836	0.69596	0.633926
39837	0.743154	0.986622
39838	0.743154	1.03878
39839	1.89226	1.52031
39840	1.89226	1.27543
39841	1.89226	0.907374
39842	1.89226	1.75524
39843	3.78452	1.09163

39844	1.32541	1.28987
39845	1.13722	1.07291
39846	0.91103	2.02251
39847	0.881123	1.62473
39848	1.03363	1.7394
39849	1.03364	1.42768
39850	1.1505	1.31541
39851	1.1505	1.63806
39852	1.32539	1.53453
39853	1.07662	1.81198
39854	0.881121	1.44673
39855	0.962303	1.36332
39856	1.89226	1.09701
39857	1.89226	2.01557
39858	1.89226	1.69245
39859	1.89226	1.1427
39860	3.78453	1.52699
39861	1.89226	0.902844
39862	1.89226	1.57149
39863	1.89226	1.20015
39864	1.89226	1.15453
39865	1.89226	1.21218
39866	1.89226	1.23254
39867	1.27606	1.28306
39868	1.07664	1.16875
39869	0.905895	1.91577
39870	0.696099	1.94946
39871	0.984902	1.85756
39872	0.984905	1.32371
39873	1.01878	1.83019
39874	1.01878	1.03174
39875	1.27604	1.61309
39876	1.06044	1.66181
39877	1.13192	1.54598
39878	0.696111	1.5475
39879	1.89226	0.825928
39880	1.89226	0.243676
39881	1.89226	0.251884
39882	1.89226	0.82618
39883	1.89226	0.261269
39884	1.89226	0.822048
39885	1.52615	0.852002
39886	1.01294	0.226603
39887	1.01294	0.893565
39888	1.18549	0.346854
39889	1.38963	0.635526
39890	1.56218	0.35341
39891	1.89226	0.68934
39892	2.40801	0.551748
39893	2.58101	0.711475
39894	2.58101	0.606252
39895	1.89226	0.688819
39896	1.89226	0.509621
39897	2.24071	2.1
39898	2.79317	1.75473
39899	1.99136	2.1
39900	2.6167	1.6827
39901	4.85741	2.1

39902	2.79317	1.77485
39903	1.89226	0.898835
39904	1.89226	2.1
39905	1.89226	0.9576
39906	2.6167	1.77811
39907	2.6167	0.929148
39908	1.08354	1.79809
39909	1.08354	2.1
39910	1.10222	2.1
39911	1.10222	1.9784
39912	1.2275	1.92291
39913	1.30835	1.75419
39914	0.995678	2.1
39915	0.877412	2.1
39916	0.877411	2.1
39917	1.2275	2.1
39918	0.995678	2.1
39919	1.30835	2.1
39920	1.05793	0.823001
39921	1.46441	0.746242
39922	1.0562	0.797703
39923	1.0562	0.779847
39924	1.41787	0.806151
39925	1.41787	0.788438
39926	1.41787	0.834892
39927	1.52909	0.938142
39928	1.46441	0.816334
39929	1.46441	0.913071
39930	1.43063	0.809568
39931	1.43063	0.909671
39932	1.89226	0.945811
39933	1.89226	0.743058
39934	1.89226	0.824561
39935	1.89226	1.00298
39936	3.78453	0.880719
39937	1.89227	0.826273
39938	1.89227	0.769809
39939	1.89227	0.782919
39940	1.89227	0.773749
39941	1.89227	0.773543
39942	1.89227	0.773862
39943	1.00763	0.78638
39944	1.41787	0.789066
39945	1.05793	0.783091
39946	1.05793	0.808044
39947	1.37265	0.784078
39948	1.37265	0.807936
39949	1.37265	0.83855
39950	1.43063	0.918447
39951	1.41787	0.832544
39952	1.41787	0.966619
39953	1.38247	0.818696
39954	1.38247	0.959
39955	0.946132	0.939685
39956	0.946132	0.917659
39957	0.946132	0.810125
39958	0.946132	0.722861
39959	0.946131	0.722675

39960	0.946131	0.908493
39961	0.946132	0.937125
39962	0.946132	0.852956
39963	0.946132	0.86268
39964	0.946132	0.854783
39965	0.946132	0.8525
39966	0.946132	0.844407
39967	1.89226	0.802014
39968	1.89226	0.738328
39969	1.89226	0.766456
39970	1.89226	0.813219
39971	1.89226	0.751836
39972	1.89226	0.81282
39973	1.19645	0.844058
39974	1.89183	0.729996
39975	1.91106	0.794846
39976	1.35306	0.750692
39977	1.75446	0.798657
39978	1.19645	0.746785
39979	2.37576	0.804994
39980	1.90139	1.24366
39981	2.1845	2.1
39982	1.86568	0.978502
39983	4.24145	1.29359
39984	2.29435	0.953672
39985	1.40602	2.02015
39986	1.40602	0.865206
39987	1.55351	1.03974
39988	1.86568	1.36285
39989	2.01317	2.1
39990	2.18452	1.63716
39991	1.8657	0.625059
39992	2.01399	0.856194
39993	1.70141	1.48929
39994	3.87969	0.857735
39995	0.774999	2.1
39996	0.774999	0.844877
39997	0.778721	1.07394
39998	0.778721	1.81696
39999	0.628348	1.64099
40000	0.703016	1.37801
40001	0.932848	0.888924
40002	0.850704	1.09148
40003	0.850704	1.15288
40004	0.628348	1.20008
40005	0.703015	0.896907
40006	0.932848	0.921733
40007	1.65243	0.754461
40008	1.34412	0.734806
40009	1.45569	0.822026
40010	1.1315	0.810812
40011	2.78393	0.754199
40012	1.48901	2.1
40013	1.805 1.11001	
40014	1.48901	1.05931
40015	1.71717	2.1
40016	1.60635	0.884076
40017	1.83451	2.1

40018	1.805	1.22799
40019	1.65243	0.754692
40020	1.60635	0.981752
40021	1.45569	0.748443
40022	3.25879	0.830603
40023	1.27875	2.1
40024	1.60635	0.945269
40025	1.27875	1.2903
40026	1.48901	2.1
40027	1.40014	0.9623
40028	1.6104	2.1
40029	0.715199	0.8179
40030	0.715199	1.03046
40031	0.712714	1.06075
40032	0.712714	0.589962
40033	0.727843	0.616927
40034	0.619713	0.665015
40035	0.803177	1.02404
40036	0.70007	0.971573
40037	0.700069	0.847406
40038	0.619713	0.803832
40039	0.803177	0.821397
40040	0.727844	0.774203
40041	1.14357	2.1
40042	2.3506	2.1
40043	1.14357	2.09905
40044	2.68773	2.1
40045	1.57829	2.1
40046	2.68773	2.1
40047	2.00551	2.1
40048	2.65811	1.44098
40049	2.07203	1.92702
40050	2.72117	0.678919
40051	4.72667	1.37456
40052	0.946131	0.835058
40053	0.946131	1.59153
40054	1.32905	1.68171
40055	1.32905	0.8379
40056	0.94613	1.14336
40057	0.94613	1.3738
40058	1.32905	1.2864
40059	1.36058	1.16973
40060	1.36058	0.893805
40061	0.946131	0.838323
40062	1.32905	1.19522
40063	0.946131	1.36329
40064	1.21649	0.722818
40065	1.21649	1.9188
40066	1.21459	1.63196
40067	1.21459	1.74886
40068	1.39508	1.16366
40069	1.36057	0.981817
40070	1.03601	1.57667
40071	1.07438	1.86738
40072	1.07439	1.14438
40073	1.39509	1.15423
40074	1.036	1.21282
40075	1.36056	1.12551

40076	2.79015	0.948793
40077	2.72114	1.8874
40078	1.89225	1.02165
40079	1.89225	1.75631
40080	1.89225	1.45019
40081	2.72114	0.851028
40082	0.876258	2.1
40083	0.892889	2.1
40084	1.34352	2.1
40085	1.27985	2.1
40086	1.07462	2.1
40087	1.07462	2.1
40088	1.09812	2.1
40089	1.09812	2.1
40090	0.892888	2.1
40091	1.34352	2.1
40092	0.876256	2.1
40093	1.27985	2.1
40094	1.39725	1.37123
40095	1.30029	0.920587
40096	1.31415	1.01047
40097	1.41336	1.24362
40098	2.71366	1.10039
40099	1.89226	0.927168
40100	1.89226	1.07801
40101	1.89226	1.26851
40102	1.89226	0.917936
40103	3.78453	1.0012
40104	0.946133	0.99161
40105	0.946133	0.926657
40106	1.38709	0.767357
40107	1.39437	0.882397
40108	0.946132	0.772872
40109	0.946132	0.897874
40110	1.39437	1.01397
40111	1.39437	0.821251
40112	0.946132	0.880407
40113	1.39437	0.863474
40114	1.38709	0.861343
40115	0.946132	0.86216
40116	0.706681	1.35053
40117	0.711908	1.25867
40118	0.657077	0.953037
40119	0.661003	1.04584
40120	0.684188	0.964385
40121	0.684188	1.22175
40122	0.683496	1.54204
40123	0.683496	0.988394
40124	0.711908	1.12376
40125	0.661003	1.12632
40126	0.657076	1.09389
40127	0.706681	1.09793
40128	0.946132	1.25631
40129	0.946132	1.10576
40130	0.946132	0.969762
40131	0.946132	0.905775
40132	0.946132	0.913603
40133	0.946132	1.0983

40134	0.946133	0.993288
40135	0.946133	1.25225
40136	0.946133	1.03123
40137	0.946133	1.02792
40138	0.946132	1.03509
40139	0.946132	1.03722
40140	2.79029	0.773528
40141	1.89227	1.01838
40142	2.78873	0.875541
40143	2.78873	0.894976
40144	1.89383	0.85551
40145	1.89227	0.903453
40146	1.43669	2.1
40147	2.11984	2.1
40148	2.24481	2.1
40149	2.24481	2.1
40150	1.20311	2.1
40151	1.20311	2.1
40152	0.49703	2.1
40153	0.601553	2.1
40154	1.05992	2.1
40155	0.991694	2.1
40156	0.787558	2.1
40157	0.787558	2.1
40158	0.805689	2.1
40159	0.805689	2.1
40160	0.991694	2.1
40161	0.49703	2.1
40162	1.05992	2.1
40163	0.601553	2.1
40164	2.45905	2.1
40165	1.89226	2.01334
40166	1.89226	2.1
40167	1.89226	2.00717
40168	2.47395	2.1
40169	2.47395	2.1
40170	1.89226	2.1
40171	1.89226	1.41456
40172	1.89226	1.96772
40173	1.89226	1.14927
40174	3.78452	1.70581
40175	1.38427	2.1
40176	2.31203	1.07857
40177	2.28743	1.0912
40178	2.28743	2.1
40179	1.63384	1.02867
40180	1.63384	2.07685
40181	2.42723	2.1
40182	1.89226	2.1
40183	1.89226	2.1
40184	1.89226	2.08802
40185	2.45905	2.1
40186	2.45905	1.99074
40187	0.946131	0.906918
40188	0.946131	2.06788
40189	0.946131	2.09866
40190	0.946131	1.43435
40191	0.94613	1.20548

40192	0.94613	1.37547
40193	0.946132	2.1
40194	0.946132	1.70031
40195	0.946131	1.47097
40196	0.946131	1.57844
40197	0.946131	1.71806
40198	0.946131	2.01221
40199	0.989441	2.1
40200	0.989441	1.53757
40201	0.993918	2.08081
40202	0.993918	1.78051
40203	1.16644	1.61329
40204	1.15601	1.16353
40205	0.818308	2.1
40206	0.816922	2.1
40207	0.818307	1.85419
40208	1.16644	1.92059
40209	1.15601	1.80164
40210	0.816922	1.53353
40211	0.996778	0.315356
40212	2.44375	0.261137
40213	0.996778	0.365841
40214	1.92547	0.268377
40215	1.56091	0.381664
40216	2.4896	0.271218
40217	1.56091	0.375991
40218	2.44375	0.260665
40219	1.90456	0.360178
40220	1.90456	0.378253
40221	2.81853	0.275744
40222	2.47487	0.277076
40223	1.32143	1.80267
40224	1.43294	1.88812
40225	1.37444	1.03859
40226	1.51691	1.59634
40227	2.80738	1.60066
40228	1.89226	1.43042
40229	1.89226	1.93095
40230	1.89226	1.0923
40231	1.89226	1.26537
40232	3.78453	1.30833
40233	1.89226	1.42032
40234	1.89226	2.1
40235	1.89226	1.11058
40236	1.89226	2.1
40237	3.78453	1.51534
40238	1.43294	1.39882
40239	1.3268	2.1
40240	1.51691	1.31615
40241	1.44091	2.1
40242	2.87385	1.89326
40243	1.37444	1.85513
40244	1.51691	1.93179
40245	1.4714	1.06132
40246	1.65434	1.35891
40247	3.02878	1.53139
40248	1.89226	1.90305
40249	1.89226	1.6544

40250	1.89226	1.19879
40251	1.89226	1.39236
40252	3.78452	1.52772
40253	1.89226	1.37068
40254	1.89226	2.1
40255	1.89226	1.18748
40256	1.89226	2.02206
40257	3.78453	1.70903
40258	1.51691	1.596
40259	1.44091	2.1
40260	1.65434	1.18984
40261	1.60647	2.1
40262	3.09526	1.80913
40263	1.05013	2.1
40264	1.05013	2.1
40265	0.94613	2.1
40266	0.94613	2.1
40267	1.04959	2.1
40268	1.05014	2.1
40269	0.946129	2.1
40270	0.946129	2.1
40271	0.946131	2.1
40272	1.04958	2.1
40273	0.946131	2.1
40274	1.05013	2.1
40275	1.04959	2.1
40276	1.04959	2.1
40277	0.946131	2.1
40278	0.946131	2.1
40279	1.04765	2.1
40280	1.04959	2.1
40281	0.94613	2.1
40282	0.94613	2.1
40283	0.946131	2.1
40284	1.04765	2.1
40285	0.946131	2.1
40286	1.04959	2.1
40287	1.85744	2.1
40288	1.89226	2.1
40289	1.89226	2.1
40290	1.89226	2.1
40291	1.94839	2.1
40292	1.94839	2.1
40293	0.97419	2.1
40294	0.822054	2.1
40295	0.946123	2.1
40296	0.946123	2.1
40297	1.04202	2.1
40298	0.84898	2.1
40299	0.933307	2.1
40300	0.933307	2.1
40301	0.933285	2.1
40302	1.05018	2.1
40303	0.851528	2.1
40304	0.876787	2.1
40305	1.87528	0.707697
40306	1.21137	0.588293
40307	1.57893	0.635774

40308	1.21137	0.706492
40309	1.81182	0.634554
40310	1.44426	0.698305
40311	0.993833	0.483385
40312	1.21137	0.605707
40313	1.57893	0.661807
40314	1.31774	0.482348
40315	2.5291	0.516317
40316	1.87528	0.697189
40317	1.57893	0.586251
40318	1.90728	0.631779
40319	2.29851	0.693776
40320	3.87744	0.686492
40321	1.90728	0.642651
40322	1.31774	0.487608
40323	1.56339	0.521979
40324	1.31774	0.637576
40325	1.82458	0.51056
40326	1.57893	0.613399
40327	1.89226	1.00198
40328	1.89226	1.11973
40329	1.89226	1.05949
40330	1.89226	1.08061
40331	1.89226	1.06109
40332	1.89226	1.08252
40333	1.89226	1.06819
40334	1.89226	1.1666
40335	1.89226	1.1869
40336	1.89226	1.11957
40337	3.78452	1.15898
40338	1.89226	1.13335
40339	2.17251	1.20396
40340	2.45575	1.12875
40341	2.45575	1.17683
40342	1.89226	1.0936
40343	1.89226	1.14281
40344	1.08625	1.20325
40345	1.22788	1.11563
40346	0.54447	1.05276
40347	0.794861	1.12455
40348	0.967729	1.19381
40349	0.967729	1.20646
40350	0.913386	1.11071
40351	0.913386	1.04947
40352	1.08625	1.17079
40353	0.544469	1.09756
40354	0.794862	1.17253
40355	1.22788	1.15208
40356	0.946128	1.13188
40357	0.946128	1.14646
40358	0.946132	1.10891
40359	0.946132	1.07429
40360	0.94613	1.10999
40361	0.94613	1.12896
40362	0.94613	1.14872
40363	0.94613	1.05467
40364	0.94613	1.10796
40365	0.94613	1.11039

40366	0.94613	1.09338
40367	0.94613	1.1037
40368	0.946127	1.13082
40369	0.946127	1.09908
40370	1.08625	1.09509
40371	0.958779	1.19527
40372	1.08625	1.14546
40373	1.08625	1.18069
40374	0.946128	1.08128
40375	0.946128	1.09693
40376	0.946129	1.1097
40377	0.958777	1.10557
40378	1.08625	1.10237
40379	0.946129	1.08393
40380	2.16338	0.328247
40381	1.94002	0.29115
40382	1.98836	0.307499
40383	2.60614	0.317145
40384	1.5456	0.288329
40385	2.16338	0.287675
40386	1.19952	0.356328
40387	2.34061	0.356876
40388	1.21031	0.329458
40389	2.1711	0.328667
40390	3.54013	0.350778
40391	1.13624	2.1
40392	1.70302	2.1
40393	1.8267	2.1
40394	1.11545	2.1
40395	2.85455	2.1
40396	1.82671	2.1
40397	1.91767	2.1
40398	1.81563	2.1
40399	1.91767	2.1
40400	1.71362	2.1
40401	1.83277	2.1
40402	1.66064	2.1
40403	0.996776	2.1
40404	0.996776	2.1
40405	1.11545	2.1
40406	1.54278	2.1
40407	1.66145	2.1
40408	1.66064	2.1
40409	1.7457	2.1
40410	1.81563	2.1
40411	1.81563	2.1
40412	1.54278	2.1
40413	1.54278	2.1
40414	2.71672	2.1
40415	2.70402	2.1
40416	2.64996	2.1
40417	2.69443	2.1
40418	5.39845	2.1
40419	2.71672	2.1
40420	2.54198	2.1
40421	2.54019	2.1
40422	2.69443	2.1
40423	5.23641	2.1

40424	2.64996	2.1
40425	2.69443	2.1
40426	2.68213	2.1
40427	2.61243	2.1
40428	5.30686	2.1
40429	2.54019	2.1
40430	2.69443	2.1
40431	2.68213	2.1
40432	2.5392	2.1
40433	5.23363	2.1
40434	1.89226	2.1
40435	2.34725	2.1
40436	2.22414	2.1
40437	2.34725	2.1
40438	1.76914	2.1
40439	1.89226	2.1
40440	0.946131	2.1
40441	0.946131	2.1
40442	1.11207	2.1
40443	1.03798	2.1
40444	0.94613	2.1
40445	0.94613	2.1
40446	1.03798	2.1
40447	1.03798	2.1
40448	0.946131	2.1
40449	1.03798	2.1
40450	0.946131	2.1
40451	1.11207	2.1
40452	1.89226	0.612559
40453	1.89226	0.58388
40454	1.89226	0.643595
40455	1.89226	0.529828
40456	1.89226	0.669182
40457	1.89226	0.566061
40458	1.89226	0.615151
40459	1.89226	0.626678
40460	1.89226	0.68859
40461	1.89226	0.554153
40462	1.89226	0.704672
40463	1.89226	0.578654
40464	1.89226	0.476919
40465	1.89226	0.467406
40466	1.89226	0.514311
40467	1.89226	0.430911
40468	1.89226	0.52988
40469	1.89226	0.456249
40470	1.44085	1.38852
40471	1.31825	0.965478
40472	1.31923	1.08163
40473	1.31825	1.24459
40474	1.43311	1.03639
40475	1.43213	1.16156
40476	1.89226	0.969221
40477	1.89226	1.15536
40478	1.89226	0.947236
40479	1.89226	1.16181
40480	1.89226	0.937393
40481	1.89226	1.22065

40482	0.946133	1.00504
40483	0.946133	0.938976
40484	1.38969	0.792556
40485	1.39587	0.898834
40486	0.946132	0.785632
40487	0.946132	0.917448
40488	1.39587	1.01454
40489	1.39587	0.859432
40490	0.946132	0.891536
40491	1.39587	0.875124
40492	1.38969	0.886267
40493	0.946132	0.888159
40494	1.44085	1.49424
40495	1.31923	0.945704
40496	1.33026	1.05439
40497	1.4617	1.24191
40498	2.78093	1.13688
40499	1.89226	0.948831
40500	1.89226	1.12551
40501	1.89226	0.939752
40502	1.89226	1.16752
40503	1.89226	0.933091
40504	1.89226	1.25166
40505	0.946133	0.974601
40506	0.946133	0.927972
40507	1.39587	0.858251
40508	1.40724	0.720844
40509	0.946132	0.725582
40510	0.946132	0.912883
40511	1.40724	0.983502
40512	1.40724	0.857761
40513	0.946132	0.881524
40514	1.40724	0.886155
40515	0.946133	0.919793
40516	1.39587	0.898044
40517	1.15818	1.22642
40518	1.81846	1.19459
40519	1.52782	0.882563
40520	1.52782	1.47317
40521	1.28095	0.848289
40522	1.28095	1.30623
40523	2.56709	2.1
40524	1.4349	0.821405
40525	1.4349	0.982052
40526	1.75471	1.02589
40527	2.29909	0.979204
40528	2.61889	1.12819
40529	2.5671	2.1
40530	2.82858	1.69704
40531	2.57368	2.1
40532	2.29909	1.01641
40533	5.12767	1.93538
40534	2.29909	1.76105
40535	1.09148	0.686178
40536	1.09148	0.97983
40537	1.4349	0.942734
40538	1.99776	0.944374
40539	2.34119	1.00419

40540	2.57368	2.1
40541	1.99777	0.985507
40542	1.99777	1.5472
40543	2.29909	1.83857
40544	2.36071	1.50282
40545	2.66203	2.04298
40546	1.89226	0.970441
40547	1.89226	1.06087
40548	1.89226	1.02933
40549	1.89226	0.967379
40550	3.78453	0.963212
40551	0.946128	1.12871
40552	0.946128	1.35867
40553	0.946133	1.04897
40554	0.946133	1.10764
40555	0.946131	1.11754
40556	0.946131	1.21459
40557	0.946131	1.24884
40558	0.946131	1.1192
40559	0.94613	1.30005
40560	0.94613	1.15137
40561	0.946131	1.09693
40562	0.946131	1.17972
40563	1.89226	1.07118
40564	1.89226	1.13125
40565	1.89226	0.977697
40566	1.89226	1.14655
40567	3.78453	1.15275
40568	1.89226	1.10519
40569	1.89226	1.03498
40570	1.89226	0.95636
40571	1.89226	1.06745
40572	3.78453	1.0865
40573	1.89226	1.04995
40574	2.61682	1.3114
40575	1.89226	1.23223
40576	1.89226	1.27071
40577	1.89226	1.05479
40578	1.89226	1.15569
40579	1.89226	0.691888
40580	1.89226	0.698659
40581	1.89226	0.68572
40582	1.89226	0.695926
40583	3.78452	0.697742
40584	1.89226	0.695154
40585	1.89226	0.699521
40586	1.89226	0.695958
40587	1.89226	0.7
40588	1.89226	0.687181
40589	1.89226	0.698504
40590	1.89227	0.699474
40591	1.89227	0.699959
40592	1.89227	0.7
40593	1.89227	0.7
40594	1.89227	0.697167
40595	1.89227	0.694893
40596	0.80447	0.695957
40597	0.534241	0.697918

40598	0.995175	0.697695
40599	0.92204	0.698496
40600	0.922042	0.699075
40601	0.534241	0.699603
40602	0.804468	0.690363
40603	0.995176	0.682985
40604	0.71543	0.692378
40605	0.71543	0.698236
40606	0.813988	0.699537
40607	0.813988	0.690737
40608	0.922045	0.688441
40609	0.942899	0.7
40610	0.946132	0.685839
40611	0.946132	0.667468
40612	1.05169	0.496781
40613	0.936661	0.7
40614	0.922045	0.697138
40615	0.946133	0.59611
40616	1.01665	0.593695
40617	1.13008	0.517843
40618	0.851529	0.697188
40619	0.851529	0.695742
40620	1.41908	2.1
40621	1.77723	2.1
40622	1.90485	2.1
40623	1.90485	2.1
40624	1.30237	2.1
40625	1.30237	2.1
40626	1.77723	2.1
40627	2.0055	1.52643
40628	1.90485	1.97837
40629	2.17158	1.94794
40630	3.91035	2.03778
40631	0.850906	1.90642
40632	0.850906	2.1
40633	0.85668	2.1
40634	0.85668	2.1
40635	0.998045	2.1
40636	0.952425	2.04158
40637	0.74367	2.1
40638	0.709541	2.1
40639	0.74367	2.1
40640	0.998045	2.1
40641	0.709541	2.1
40642	0.952425	2.1
40643	1.90485	2.1
40644	2.28563	2.1
40645	2.26282	2.1
40646	2.17158	1.71156
40647	1.99609	2.1
40648	1.90485	1.28076
40649	2.00098	0.370143
40650	1.90462	0.373548
40651	1.60057	0.388926
40652	1.79224	0.450006
40653	3.69686	0.380493
40654	2.65654	0.425227
40655	1.79224	0.438322

40656	2.22332	0.430905
40657	2.22332	0.442457
40658	2.24163	0.43213
40659	2.24163	0.434684
40660	1.89226	0.452141
40661	1.89226	0.432751
40662	1.89226	0.442024
40663	1.89226	0.549049
40664	3.78452	0.463182
40665	1.89226	0.384979
40666	1.89226	0.463028
40667	1.89226	0.417785
40668	1.89226	0.44245
40669	1.89226	0.473402
40670	1.89226	0.459854
40671	2.24163	0.461735
40672	1.03645	0.473692
40673	1.79224	0.481709
40674	1.79224	0.460254
40675	1.54283	0.48731
40676	1.54283	0.468412
40677	0.946132	0.479125
40678	0.946132	0.476147
40679	0.946129	0.477211
40680	0.946129	0.466102
40681	0.94613	0.492432
40682	0.94613	0.478844
40683	0.946131	0.485436
40684	0.946131	0.466297
40685	0.94613	0.507364
40686	0.94613	0.483858
40687	0.946131	0.453242
40688	0.946131	0.471873
40689	1.89226	2.1
40690	1.89226	2.1
40691	1.89226	2.1
40692	1.89226	2.1
40693	3.78453	2.1
40694	1.52441	2.1
40695	1.30347	2.1
40696	1.52441	2.1
40697	1.58401	2.1
40698	1.2627	2.1
40699	1.3223	2.1
40700	1.89226	2.1
40701	2.72787	2.1
40702	2.72787	2.1
40703	2.74762	2.1
40704	1.89226	2.1
40705	1.91202	2.1
40706	1.89227	2.1
40707	1.89227	2.1
40708	1.89227	2.1
40709	1.89227	2.1
40710	3.78453	2.1
40711	0.56414	2.1
40712	0.561557	2.1
40713	0.527284	2.1

40714	0.541143	2.1
40715	0.527276	2.1
40716	0.631685	2.1
40717	1.52441	2.1
40718	1.00232	2.1
40719	0.784495	2.1
40720	0.842304	2.1
40721	0.721569	2.1
40722	1.36393	2.1
40723	1.36393	2.1
40724	0.946132	2.1
40725	0.946132	2.1
40726	1.36393	2.1
40727	1.3594	2.1
40728	0.946132	2.1
40729	0.946132	2.1
40730	0.946107	2.1
40731	1.35936	2.1
40732	1.36397	2.1
40733	0.946158	2.1
40734	1.89226	2.1
40735	1.89226	2.1
40736	1.89226	2.1
40737	1.89226	2.1
40738	3.78453	2.1
40739	2.20802	2.1
40740	1.05677	2.1
40741	2.30479	2.1
40742	0.978699	2.1
40743	3.18672	2.1
40744	0.94613	2.1
40745	0.94613	2.1
40746	0.669866	2.1
40747	0.669866	2.1
40748	0.681588	2.1
40749	0.669865	2.1
40750	0.946133	2.1
40751	0.946133	2.1
40752	0.94613	2.1
40753	0.681589	2.1
40754	0.946131	2.1
40755	0.669867	2.1
40756	2.73514	2.1
40757	1.78301	2.1
40758	1.90618	2.1
40759	2.83464	2.1
40760	4.64132	2.1
40761	2.73514	2.1
40762	1.89226	2.1
40763	1.89226	2.1
40764	2.83464	2.1
40765	4.6274	2.1
40766	2.03915	2.1
40767	2.26616	2.1
40768	2.23549	2.1
40769	2.01704	2.1
40770	4.2832	2.1
40771	2.03916	2.1

40772	1.55766	2.1
40773	1.53728	2.1
40774	2.01704	2.1
40775	3.5747	2.1
40776	2.23551	2.1
40777	2.01705	2.1
40778	1.99823	2.1
40779	2.21189	2.1
40780	4.22894	2.1
40781	1.51788	2.1
40782	2.01705	2.1
40783	1.53729	2.1
40784	1.53729	2.1
40785	1.99823	2.1
40786	1.99823	2.1
40787	2.588	2.1
40788	1.45581	2.1
40789	1.45581	2.1
40790	1.44207	2.1
40791	2.54949	2.1
40792	2.53574	2.1
40793	0.98641	2.1
40794	0.98641	2.1
40795	1.00371	2.1
40796	1.00371	2.1
40797	0.749992	2.1
40798	0.727904	2.1
40799	1.26221	2.1
40800	1.27474	2.1
40801	1.26221	2.1
40802	0.749993	2.1
40803	0.727904	2.1
40804	1.27474	2.1
40805	1.23105	1.13412
40806	1.35435	1.83005
40807	1.89226	1.42237
40808	1.89226	1.38649
40809	1.89226	1.69553
40810	1.7043	1.53681
40811	1.89226	1.80552
40812	1.89226	1.35903
40813	1.89226	1.08051
40814	1.89226	1.74863
40815	3.78453	1.49535
40816	1.89226	0.929325
40817	1.89226	1.68278
40818	1.89226	1.10383
40819	1.89226	1.17328
40820	1.89226	1.31056
40821	1.89226	1.40827
40822	0.946131	1.04258
40823	1.40945	0.974458
40824	0.94613	1.01918
40825	0.94613	1.46255
40826	0.946129	1.48829
40827	0.946129	1.18456
40828	0.946132	1.65396
40829	1.40945	0.952121

40830	0.94613	1.11865
40831	0.94613	1.08321
40832	0.946131	1.14738
40833	0.94613	1.11696
40834	0.946133	1.20147
40835	0.946133	1.09362
40836	0.946132	1.30381
40837	0.946132	1.63602
40838	0.946132	1.62501
40839	0.946132	1.21642
40840	0.946133	1.33163
40841	0.946133	1.0981
40842	0.946132	1.29982
40843	0.946132	1.26449
40844	0.946133	1.21918
40845	0.946133	1.2232
40846	1.89226	1.13014
40847	1.89226	1.78708
40848	1.89226	1.3465
40849	1.89226	1.27971
40850	1.89226	1.57802
40851	1.89226	1.45465
40852	1.89226	2.1
40853	1.89226	2.1
40854	1.89226	2.1
40855	1.89226	2.1
40856	1.89226	2.1
40857	1.89226	2.1
40858	1.89226	2.1
40859	1.89226	2.1
40860	1.89226	2.1
40861	1.89226	2.1
40862	3.78453	2.1
40863	1.66647	2.1
40864	1.15399	2.1
40865	1.25443	2.1
40866	1.26599	2.1
40867	1.58697	2.1
40868	1.66647	2.1
40869	1.66647	2.1
40870	1.47446	2.1
40871	1.55433	2.1
40872	1.74241	2.1
40873	3.2208	2.1
40874	1.89227	2.1
40875	1.89227	2.1
40876	1.89227	2.1
40877	1.89227	2.1
40878	3.78453	2.1
40879	0.946132	2.1
40880	0.946132	2.1
40881	0.946132	2.1
40882	0.946132	2.1
40883	0.946132	2.1
40884	0.946132	2.1
40885	0.946133	2.1
40886	0.946133	2.1
40887	0.946132	2.1

40888	0.946132	2.1
40889	0.946133	2.1
40890	0.946133	2.1
40891	0.625067	2.1
40892	0.61907	2.1
40893	0.896913	2.1
40894	0.946199	2.1
40895	0.806287	2.1
40896	0.677685	2.1
40897	0.750657	2.1
40898	0.750657	2.1
40899	0.646004	2.1
40900	0.890236	2.1
40901	0.61049	2.1
40902	0.888979	2.1
40903	1.6223	2.1
40904	1.98034	2.1
40905	1.75003	2.1
40906	1.55433	2.1
40907	1.75003	2.1
40908	1.55433	2.1
40909	1.88602	2.09039
40910	2.08149	2.1
40911	2.07991	2.1
40912	1.88473	2.1
40913	2.08149	2.02975
40914	1.88602	2.1
40915	1.88458	2.1
40916	2.08149	2.1
40917	1.7069	2.1
40918	1.56284	2.1
40919	2.08149	2.1
40920	1.88458	2.1
40921	1.5955	2.09489
40922	1.89226	2.1
40923	1.91975	2.1
40924	1.62299	2.1
40925	1.89226	1.90199
40926	1.5955	2.1
40927	1.89226	1.57984
40928	1.89226	1.8827
40929	1.89226	1.58943
40930	1.89226	2.0273
40931	1.89226	1.25111
40932	1.89226	1.72006
40933	1.89226	1.91775
40934	1.89226	2.0763
40935	1.89226	1.9769
40936	1.89226	2.1
40937	1.89226	1.67196
40938	1.89226	2.02409
40939	1.62299	2.1
40940	1.89226	2.1
40941	1.71752	2.1
40942	1.44824	2.1
40943	1.89226	2.1
40944	1.62299	2.1
40945	1.89226	1.00106

40946	2.8317	1.03707
40947	1.89226	1.0356
40948	2.64055	0.853171
40949	4.53281	1.01494
40950	2.8317	1.17271
40951	1.83151	0.829
40952	2.01001	0.989079
40953	2.01001	1.11039
40954	2.64055	0.857512
40955	2.64055	0.905441
40956	0.946132	0.765521
40957	0.946132	0.787686
40958	1.16167	0.819581
40959	1.16167	0.841771
40960	1.16167	0.81615
40961	1.32027	0.763746
40962	0.946131	0.769674
40963	0.946131	0.853221
40964	0.94613	0.773632
40965	1.16167	0.755578
40966	0.946131	0.790562
40967	1.32027	0.785114
40968	2.32335	0.867831
40969	1.83151	0.77286
40970	2.38525	0.807911
40971	2.64055	0.802322
40972	1.57621	0.76692
40973	1.83151	0.765482
40974	2.25677	2.1
40975	1.88158	2.1
40976	1.86856	2.1
40977	2.0815	2.1
40978	1.70303	2.1
40979	1.88158	2.1
40980	2.50381	2.1
40981	1.89227	2.1
40982	2.25677	2.1
40983	2.25677	2.1
40984	1.89227	2.1
40985	1.89227	2.1
40986	1.89226	0.646851
40987	1.50899	0.670872
40988	2.13659	0.645316
40989	1.50899	0.703273
40990	2.51986	0.646539
40991	1.89226	0.694184
40992	0.946131	0.686934
40993	0.946131	0.614545
40994	1.06829	0.651407
40995	0.895375	0.564649
40996	0.94613	0.646162
40997	0.94613	0.695087
40998	1.33922	0.610198
40999	0.895376	0.572781
41000	0.946131	0.607189
41001	1.33922	0.612096
41002	0.946131	0.616554
41003	1.06829	0.633807

41004	0.841467	0.717219
41005	0.578428	0.721945
41006	1.18079	0.712799
41007	0.920237	0.720144
41008	0.772317	0.709171
41009	0.856555	0.726581
41010	0.910271	0.716524
41011	0.910271	0.734023
41012	0.975752	0.714284
41013	0.536929	0.709625
41014	0.81952	0.731125
41015	1.15252	0.714383
41016	1.89226	2.1
41017	2.51431	2.1
41018	1.89226	2.1
41019	1.89226	2.1
41020	2.78815	2.1
41021	2.78815	2.1
41022	2.0815	2.1
41023	2.0516	2.1
41024	2.0815	2.1
41025	3.06699	2.1
41026	2.76576	2.1
41027	2.8002	2.1
41028	1.94112	2.1
41029	1.89226	2.1
41030	2.20358	2.1
41031	1.89226	2.1
41032	4.09584	2.1
41033	1.09154	2.1
41034	2.62647	2.1
41035	2.20358	2.1
41036	2.20358	2.1
41037	1.52314	2.1
41038	1.52314	2.1
41039	1.31323	2.1
41040	1.31323	2.1
41041	0.946132	2.1
41042	0.946132	2.1
41043	0.946132	2.1
41044	0.946132	2.1
41045	1.31323	2.1
41046	1.10179	2.1
41047	1.31323	2.1
41048	0.946132	2.1
41049	1.10179	2.1
41050	0.946133	2.1
41051	1.89226	0.98788
41052	1.89226	1.1889
41053	1.89226	1.13527
41054	1.89226	1.22177
41055	1.89226	0.97504
41056	1.89226	1.09456
41057	1.35126	1.42368
41058	1.35126	1.91373
41059	0.903285	1.24883
41060	0.903285	1.3679
41061	1.30841	1.42681

41062	0.903313	1.75279
41063	0.946132	1.27771
41064	0.946132	1.17236
41065	0.946132	1.25404
41066	0.903312	1.31903
41067	1.30841	1.27527
41068	0.946133	1.19383
41069	2.62097	2.1
41070	2.72561	2.06976
41071	2.39016	2.1
41072	2.51062	2.1
41073	5.11577	2.1
41074	2.51062	2.1
41075	2.58286	0.848228
41076	2.40107	1.94725
41077	2.40107	0.93005
41078	2.72561	1.96402
41079	2.72561	0.935224
41080	2.39016	2.1
41081	2.51062	2.1
41082	2.20242	2.1
41083	2.34107	1.9623
41084	4.73124	2.1
41085	2.51062	2.1
41086	2.40107	0.854874
41087	2.34107	1.95151
41088	2.25197	0.910813
41089	4.74215	1.00613
41090	2.75218	2.1
41091	1.89226	2.1
41092	2.3349	2.1
41093	2.3349	2.1
41094	1.89226	2.1
41095	1.89226	2.1
41096	1.46129	2.1
41097	0.996308	2.1
41098	1.88649	2.1
41099	1.70302	2.1
41100	2.95419	2.1
41101	1.89227	2.1
41102	1.89227	1.92218
41103	1.89227	2.0085
41104	1.89227	2.1
41105	3.78453	2.1
41106	0.646558	2.1
41107	0.646557	2.1
41108	0.60697	2.1
41109	0.60697	2.1
41110	0.587917	2.1
41111	0.519747	2.1
41112	0.586443	2.1
41113	0.733782	2.1
41114	0.733793	2.1
41115	0.587924	2.1
41116	0.519739	2.1
41117	0.586436	2.1
41118	2.1642	2.1
41119	1.42867	2.1

41120	2.28847	2.1
41121	1.68299	2.1
41122	3.71714	2.1
41123	1.89226	2.1
41124	1.89226	2.1
41125	1.89226	2.1
41126	1.89226	2.1
41127	3.78453	2.1
41128	0.946131	2.1
41129	0.946131	2.1
41130	1.24994	2.1
41131	1.24994	2.1
41132	1.11582	2.1
41133	1.24994	2.1
41134	0.946133	2.1
41135	0.946133	2.1
41136	0.946131	2.1
41137	1.24994	2.1
41138	0.94613	2.1
41139	1.11582	2.1
41140	2.28847	2.1
41141	1.53629	2.1
41142	1.57498	2.1
41143	1.47417	2.1
41144	2.42221	2.1
41145	2.28847	2.1
41146	1.89226	2.1
41147	1.89226	2.1
41148	1.89226	2.08155
41149	1.89226	2.1
41150	3.78453	2.1
41151	1.89226	2.1
41152	2.49988	2.1
41153	2.71233	2.1
41154	2.71233	2.1
41155	1.89226	2.1
41156	1.89226	2.1
41157	0.924534	2.1
41158	0.924534	2.1
41159	0.990395	2.1
41160	0.990395	2.1
41161	0.946131	2.1
41162	0.946131	2.1
41163	0.924505	2.1
41164	0.968797	2.1
41165	0.924505	2.1
41166	0.946131	2.1
41167	0.968797	2.1
41168	0.946131	2.1
41169	0.924533	2.1
41170	0.924533	2.1
41171	0.948264	2.1
41172	0.945345	2.1
41173	0.851541	2.1
41174	0.851541	2.1
41175	0.958934	2.1
41176	1.02617	2.1
41177	0.936668	2.1

41178	1.04829	2.1
41179	0.924505	2.1
41180	0.946131	2.1
41181	2.28431	0.779169
41182	1.48035	0.511079
41183	1.59971	0.645441
41184	1.48035	0.638106
41185	2.33818	0.618163
41186	2.21882	0.598489
41187	2.28431	0.701591
41188	1.89226	0.642669
41189	1.89226	0.826044
41190	2.3968	0.552302
41191	4.28906	0.610142
41192	2.28431	0.722551
41193	1.70303	0.47746
41194	1.5428	0.491626
41195	1.59971	0.519442
41196	2.31152	0.611563
41197	2.3968	0.635906
41198	1.99823	2.1
41199	2.21189	2.1
41200	2.18937	2.1
41201	1.979 2.1	
41202	4.19089	2.1
41203	0.999114	2.1
41204	0.9895	2.1
41205	0.758942	2.1
41206	0.749358	2.1
41207	0.874236	2.1
41208	0.874236	2.1
41209	0.874236	2.1
41210	0.874236	2.1
41211	0.9895	2.1
41212	0.749358	2.1
41213	0.758942	2.1
41214	0.999114	2.1
41215	2.18937	2.1
41216	1.979 2.1	
41217	1.95416	2.1
41218	2.162 2.1	
41219	4.14099	2.1
41220	1.95416	2.1
41221	1.49871	2.1
41222	1.47712	2.1
41223	1.49871	2.1
41224	1.9574	2.1
41225	1.979 2.1	
41226	1.68508	0.298511
41227	1.64984	0.356563
41228	2.67711	0.271219
41229	2.76407	0.371935
41230	4.41391	0.353977
41231	1.84227	0.2974
41232	2.76407	0.365804
41233	2.76407	0.38751
41234	1.85007	0.309118
41235	2.72769	0.381505

41236	1.80812	0.301365
41237	1.94516	2.1
41238	1.89226	2.1
41239	1.89226	2.1
41240	1.89226	2.1
41241	1.98853	2.1
41242	1.98852	2.1
41243	1.28095	2.1
41244	2.0815	2.1
41245	2.29123	2.1
41246	2.06833	2.1
41247	1.08212	2.1
41248	0.976848	2.1
41249	1.89226	2.1
41250	1.89226	2.1
41251	1.89226	2.1
41252	1.89226	2.1
41253	1.89226	2.1
41254	1.89226	2.1
41255	1.13537	2.1
41256	1.18233	2.1
41257	0.946132	2.1
41258	0.946132	2.1
41259	0.946132	2.1
41260	0.946132	2.1
41261	0.994264	2.1
41262	1.18233	2.1
41263	1.13537	2.1
41264	0.946131	2.1
41265	0.946132	2.1
41266	0.994264	2.1
41267	0.953204	2.1
41268	0.953204	2.1
41269	0.966515	2.1
41270	0.966515	2.1
41271	1.30583	2.1
41272	1.14561	2.1
41273	0.640475	2.1
41274	0.774103	2.1
41275	1.30584	2.1
41276	0.774101	2.1
41277	0.640474	2.1
41278	1.14562	2.1
41279	0.946131	2.1
41280	0.946131	2.1
41281	0.946131	2.1
41282	0.946131	2.03657
41283	0.946128	2.04913
41284	0.946128	2.1
41285	0.946133	2.1
41286	0.946133	2.1
41287	0.94613	2.1
41288	0.94613	2.1
41289	0.946131	2.1
41290	0.946131	2.1
41291	0.946132	0.225648
41292	0.946132	0.214375
41293	0.946131	0.24109

41294	0.946131	0.253492
41295	0.946131	0.255882
41296	0.946131	0.238527
41297	0.946132	0.219128
41298	0.946132	0.245355
41299	0.946131	0.23546
41300	0.946131	0.241085
41301	0.946132	0.236603
41302	0.946132	0.226284
41303	0.946131	0.233551
41304	0.946131	0.223384
41305	0.946131	0.25201
41306	0.946131	0.268521
41307	0.94613	0.268459
41308	0.94613	0.243645
41309	0.946132	0.225878
41310	0.946132	0.256364
41311	0.946131	0.24376
41312	0.946131	0.246967
41313	0.946131	0.239013
41314	0.946131	0.24563
41315	2.57882	0.323528
41316	1.48676	0.361362
41317	1.48797	0.34009
41318	2.64281	0.364496
41319	4.12957	0.339469
41320	2.56323	0.313696
41321	2.64281	0.348047
41322	2.51807	0.326854
41323	1.36324	0.364188
41324	1.48797	0.372452
41325	1.48797	0.356521
41326	1.04367	2.1
41327	2.42504	2.1
41328	2.65164	2.1
41329	1.49479	2.1
41330	3.91983	2.1
41331	2.15622	1.24624
41332	1.70539	2.1
41333	2.02369	2.1
41334	2.39039	1.43094
41335	4.17992	2.04488
41336	0.966979	2.1
41337	1.7054	2.1
41338	2.0237	2.09344
41339	1.31502	2.1
41340	2.99067	2.1
41341	2.02369	2.1
41342	2.65637	1.42244
41343	2.39039	1.20762
41344	2.39039	2.1
41345	2.29332	1.2074
41346	2.29332	2.1
41347	2.0237	1.95376
41348	1.72116	2.1
41349	1.58465	2.02398
41350	1.31502	2.1
41351	2.29332	1.87248

41352	2.0237	2.1
41353	1.0372	0.250459
41354	2.32462	0.332065
41355	2.2291	0.281547
41356	2.2291	0.327755
41357	1.17464	0.260888
41358	1.17464	0.292165
41359	2.68289	0.294418
41360	1.07805	0.295539
41361	1.70302	0.27755
41362	2.06653	0.307357
41363	3.5779	0.296365
41364	1.89226	0.4821
41365	1.89226	0.593875
41366	1.89226	0.551154
41367	1.89226	0.42016
41368	3.78453	0.502408
41369	1.89226	0.344127
41370	1.89226	0.395847
41371	1.89226	0.390735
41372	1.89226	0.366546
41373	1.89226	0.375332
41374	1.89226	0.348819
41375	1.89225	0.35262
41376	1.89225	0.371095
41377	1.89225	0.381855
41378	1.89225	0.350761
41379	1.89225	0.383719
41380	1.89225	0.356637
41381	0.94613	0.442147
41382	0.94613	0.481821
41383	0.94613	0.588224
41384	0.94613	0.528253
41385	0.946128	0.47347
41386	0.946128	0.50624
41387	0.946132	0.598872
41388	0.946132	0.430903
41389	0.94613	0.482077
41390	0.94613	0.517118
41391	0.94613	0.507375
41392	0.94613	0.468726
41393	1.29417	1.68159
41394	1.32732	2.08214
41395	1.32316	1.0152
41396	1.34973	2.06656
41397	2.65048	1.63139
41398	1.89226	1.30858
41399	1.89226	2.06202
41400	1.89226	1.03033
41401	1.89226	1.64021
41402	3.78453	1.26532
41403	1.89226	1.51935
41404	1.89226	2.1
41405	1.89226	1.05482
41406	1.89226	2.1
41407	3.78453	1.98752
41408	1.32732	1.66603
41409	1.13006	2.1

41410	1.13358	2.1
41411	1.11117	2.1
41412	1.34973	1.61034
41413	1.32732	2.1
41414	1.32316	1.53956
41415	1.34973	2.1
41416	1.34066	1.10592
41417	1.36601	1.76976
41418	2.68918	1.63845
41419	1.89226	1.55365
41420	1.89226	1.96272
41421	1.89226	0.876628
41422	1.89226	1.8118
41423	3.78453	1.44347
41424	1.89226	1.22597
41425	1.89226	2.1
41426	1.89226	2.09706
41427	1.89226	2.1
41428	1.89226	1.1916
41429	1.89226	2.1
41430	0.624134	2.1
41431	0.624134	1.95953
41432	0.624334	1.24144
41433	0.624334	2.1
41434	0.56503	2.1
41435	0.573605	2.1
41436	0.674864	1.39723
41437	0.683007	1.50077
41438	0.683007	2.1
41439	0.573605	2.1
41440	0.565029	2.1
41441	0.674864	2.1
41442	2.16633	2.1
41443	1.40846	2.1
41444	2.13977	2.1
41445	1.40771	2.1
41446	3.57404	2.1
41447	2.13977	2.1
41448	1.29932	2.1
41449	1.29932	2.1
41450	1.40771	2.1
41451	1.98957	2.1
41452	2.09796	2.1
41453	2.6044	0.742405
41454	1.85548	0.763396
41455	1.89226	0.704682
41456	1.89226	0.801132
41457	3.74774	0.764285
41458	1.89226	0.688593
41459	1.89226	0.797028
41460	1.89226	0.717037
41461	1.89226	0.742386
41462	3.78453	0.723888
41463	1.89226	2.1
41464	2.64107	2.1
41465	1.89226	2.1
41466	2.78977	2.1
41467	1.89226	2.1

41468	2.78977	2.1
41469	1.89226	2.1
41470	1.89226	2.1
41471	1.89226	2.1
41472	1.89226	2.1
41473	1.89226	2.1
41474	1.89226	2.1
41475	2.43173	2.1
41476	1.41238	2.1
41477	2.0815	2.1
41478	1.98048	2.1
41479	1.62014	2.1
41480	1.50086	2.1
41481	0.946129	0.749992
41482	0.946129	0.651679
41483	1.27636	0.68748
41484	1.1984	0.509147
41485	0.946131	0.496762
41486	0.946131	0.616108
41487	1.27636	0.75102
41488	1.27636	0.612612
41489	0.946131	0.604616
41490	1.27636	0.589072
41491	0.946131	0.636569
41492	1.1984	0.609721
41493	1.99569	0.476095
41494	2.55272	0.71277
41495	1.77403	0.50464
41496	1.70303	0.496511
41497	3.05772	0.564511
41498	2.55121	0.533993
41499	0.946128	0.617322
41500	0.946128	0.713148
41501	1.38752	0.661721
41502	1.27636	0.482824
41503	0.94613	0.471946
41504	0.94613	0.601948
41505	1.38752	0.713294
41506	1.38752	0.600125
41507	0.946131	0.601723
41508	1.38752	0.59702
41509	0.946131	0.629791
41510	1.27636	0.595303
41511	2.77504	0.650015
41512	1.99569	0.444198
41513	2.29794	0.53161
41514	1.99569	0.503771
41515	2.85497	0.502635
41516	2.55272	0.475224
41517	1.53284	2.1
41518	1.89226	2.1
41519	1.89226	2.1
41520	1.89226	2.1
41521	1.54763	2.1
41522	1.54763	2.1
41523	0.946131	2.00474
41524	0.946131	2.1
41525	0.946127	2.1

41526	0.946127	2.1
41527	0.946127	2.1
41528	0.946127	1.85779
41529	0.946131	2.1
41530	0.946131	2.1
41531	0.94613	2.1
41532	0.94613	2.1
41533	0.946129	2.1
41534	0.946129	2.1
41535	0.751754	2.1
41536	0.751754	2.1
41537	0.771562	2.1
41538	0.771561	2.1
41539	1.0268	2.1
41540	0.95482	2.1
41541	0.528937	2.1
41542	0.496512	2.1
41543	0.528936	2.1
41544	1.0268	2.1
41545	0.954821	2.1
41546	0.49651	2.1
41547	2.64075	0.714216
41548	1.35181	0.892021
41549	1.35181	0.710668
41550	1.83823	0.746123
41551	2.28373	0.748325
41552	2.88566	0.862681
41553	0.493018	0.886541
41554	0.675903	0.859859
41555	1.14187	0.712222
41556	0.973937	0.700133
41557	0.821166	0.755632
41558	0.821166	0.9224
41559	0.828674	1.25063
41560	0.828674	0.710033
41561	0.973937	0.783084
41562	0.493018	0.792663
41563	0.675903	0.747502
41564	1.14187	0.733497
41565	1.89226	2.1
41566	2.11667	2.1
41567	1.91184	2.1
41568	1.89226	2.1
41569	2.13625	2.1
41570	2.11667	2.1
41571	1.89226	2.1
41572	1.89226	2.1
41573	1.89226	2.1
41574	1.89226	2.1
41575	3.78453	2.1
41576	0.907729	2.1
41577	0.907729	2.1
41578	1.04459	2.1
41579	0.890955	2.1
41580	0.988181	2.1
41581	0.841856	2.1
41582	0.946133	2.1
41583	0.946133	2.1

41584	1.04072	2.1
41585	1.1064	2.1
41586	0.89099	2.1
41587	0.851541	2.1
41588	1.89225	2.1
41589	2.13623	2.1
41590	1.91044	2.1
41591	1.89225	2.1
41592	2.15442	2.1
41593	2.13623	2.1
41594	1.89225	2.1
41595	1.89225	2.1
41596	1.89225	2.1
41597	1.89225	2.1
41598	1.89225	2.1
41599	1.89225	2.1
41600	0.907723	2.1
41601	0.907723	2.1
41602	1.07536	2.1
41603	1.07814	2.1
41604	0.998499	2.1
41605	1.03575	2.1
41606	0.946126	2.1
41607	0.946126	2.1
41608	1.04072	2.1
41609	1.14025	2.1
41610	0.851528	2.1
41611	0.898284	2.1
41612	1.89226	0.405197
41613	1.89226	0.579126
41614	1.89226	0.492941
41615	1.89226	0.488236
41616	1.89226	0.461577
41617	1.89226	0.456422
41618	2.53008	2.1
41619	1.94534	2.1
41620	1.98702	2.1
41621	1.98858	2.1
41622	2.52852	2.1
41623	2.53008	2.1
41624	1.03267	2.1
41625	1.12591	2.1
41626	1.2959	2.1
41627	1.28992	2.1
41628	1.10851	2.1
41629	1.33078	2.1
41630	1.10661	2.1
41631	1.10661	2.1
41632	0.982208	2.1
41633	1.28992	2.1
41634	1.03267	2.1
41635	1.23262	2.1
41636	2.2712	2.1
41637	1.89226	2.1
41638	1.95954	2.1
41639	1.95954	2.1
41640	1.89226	2.1
41641	1.89226	2.1

41642	1.89226	2.1
41643	1.89226	2.1
41644	1.89226	2.1
41645	1.89226	2.1
41646	3.78453	2.1
41647	1.33887	2.1
41648	1.25395	2.1
41649	0.88327	2.1
41650	0.676665	2.1
41651	0.84881	2.1
41652	0.987503	2.1
41653	1.13243	2.1
41654	1.13243	2.1
41655	1.15338	2.1
41656	0.849966	2.1
41657	0.793074	2.1
41658	1.11957	2.1
41659	0.946129	2.1
41660	0.946129	2.1
41661	1.28254	2.1
41662	1.1356	2.1
41663	1.1356	2.1
41664	1.1356	2.1
41665	0.946131	2.1
41666	0.946131	2.1
41667	0.946131	2.1
41668	1.28254	2.1
41669	1.1356	2.1
41670	0.946131	2.1
41671	1.89227	2.1
41672	1.89227	2.1
41673	1.89227	2.1
41674	1.89227	2.1
41675	3.78453	2.1
41676	0.936287	0.223244
41677	1.01399	0.203138
41678	0.959415	0.242532
41679	0.959415	0.269346
41680	0.910786	0.266218
41681	1.38301	0.220664
41682	1.0286	0.227356
41683	0.535742	0.203416
41684	0.505202	0.239741
41685	0.937685	0.230715
41686	1.11066	0.23231
41687	1.46907	0.219065
41688	1.00546	0.291256
41689	1.01524	0.283179
41690	0.94613	0.369487
41691	0.94613	0.34507
41692	1.05018	0.356484
41693	0.914025	0.272603
41694	0.913512	0.333608
41695	0.851542	0.369862
41696	1.05385	0.332824
41697	1.05494	0.280513
41698	0.91404	0.34313
41699	0.91404	0.31909

41700	2.03253	0.881367
41701	1.82325	0.584715
41702	1.93938	0.92716
41703	1.73756	0.377152
41704	3.77009	0.686514
41705	1.82325	0.590879
41706	1.47562	0.765401
41707	1.73756	0.46744
41708	1.3868	0.530975
41709	3.21317	0.422853
41710	1.93938	0.921184
41711	1.73756	0.680519
41712	1.85293	0.883767
41713	1.64279	0.372854
41714	3.59049	0.695445
41715	1.73756	0.722721
41716	1.3868	0.723994
41717	1.64279	0.439733
41718	1.28419	0.761403
41719	3.02174	0.684044
41720	1.35048	1.83915
41721	1.37999	1.98728
41722	1.34355	1.07831
41723	1.37854	1.96081
41724	2.72354	1.53559
41725	1.89226	1.29868
41726	1.89226	1.98303
41727	1.89226	1.0473
41728	1.89226	1.27932
41729	3.78452	1.14984
41730	1.89226	1.45127
41731	1.89226	2.1
41732	1.89226	0.970384
41733	1.89226	2.1
41734	3.78453	1.90235
41735	1.37999	1.49358
41736	1.18059	2.1
41737	1.17117	2.1
41738	1.17262	2.1
41739	1.37855	1.50819
41740	1.37999	2.1
41741	1.34355	1.65485
41742	1.37854	2.1
41743	1.33079	1.17612
41744	1.3741	1.80024
41745	2.71765	1.56805
41746	1.89226	1.57015
41747	1.89226	1.77467
41748	1.89226	0.990729
41749	1.89226	1.82265
41750	3.78453	1.50659
41751	1.89226	1.17355
41752	1.89226	2.1
41753	1.89226	1.21717
41754	1.89226	2.02568
41755	3.78453	1.73354
41756	0.640305	2.1
41757	0.640305	1.51003

41758	0.643507	0.988801
41759	0.643507	2.1
41760	0.590294	2.1
41761	0.594539	2.1
41762	0.689273	1.24343
41763	0.687048	1.59043
41764	0.687048	1.95416
41765	0.594538	1.51383
41766	0.590294	2.1
41767	0.689273	2.1
41768	2.01399	0.753105
41769	1.70141	0.785351
41770	1.83896	1.09453
41771	1.52482	0.912803
41772	3.53881	0.807986
41773	1.70141	0.825483
41774	1.0752	1.49002
41775	1.0752	0.813973
41776	1.25669	0.929988
41777	1.52482	0.890318
41778	1.70632	1.6445
41779	1.83896	0.789658
41780	1.52482	0.76115
41781	1.65244	0.792377
41782	1.34412	0.849135
41783	3.17725	0.792099
41784	1.26782	0.406882
41785	2.90353	0.427014
41786	2.54203	0.37687
41787	2.41979	0.490014
41788	1.77007	0.371985
41789	1.703	0.463692
41790	0.946118	0.5621
41791	0.946118	0.496774
41792	1.38643	0.520489
41793	1.38644	0.426837
41794	0.946117	0.558938
41795	0.946117	0.575309
41796	1.38644	0.427866
41797	1.15545	0.485851
41798	0.946131	0.525668
41799	1.15546	0.493981
41800	1.38642	0.514066
41801	0.946105	0.540933
41802	2.42256	0.714329
41803	2.63056	0.665824
41804	2.43174	0.673971
41805	2.25035	0.63158
41806	4.8543	0.642628
41807	2.58031	0.710733
41808	2.63056	0.672327
41809	2.43174	0.663893
41810	2.32127	0.589931
41811	5.01205	0.69687
41812	2.43174	0.660952
41813	2.25035	0.60898
41814	2.11465	0.568542
41815	2.13042	0.528712

41816	4.54639	0.612067
41817	2.43174	0.650073
41818	2.32127	0.572033
41819	1.92997	0.5386
41820	2.13041	0.533391
41821	4.36171	0.554625
41822	1.89227	2.1
41823	1.89227	2.1
41824	1.89227	2.1
41825	1.89227	2.1
41826	3.78453	2.1
41827	0.568724	2.1
41828	0.568724	2.1
41829	0.57678	2.1
41830	0.57678	2.1
41831	0.523796	2.1
41832	0.548137	2.1
41833	0.597368	2.1
41834	0.637877	2.1
41835	0.597367	2.1
41836	0.523796	2.1
41837	0.637877	2.1
41838	0.548137	2.1
41839	1.32674	2.1
41840	1.32674	2.1
41841	0.946132	2.1
41842	0.946132	2.1
41843	1.3195	2.1
41844	1.32674	2.1
41845	0.946132	2.1
41846	0.946132	2.1
41847	0.946132	2.1
41848	1.3195	2.1
41849	1.32674	2.1
41850	0.946133	2.1
41851	1.89227	2.1
41852	1.89227	2.1
41853	1.89227	2.1
41854	1.89227	2.1
41855	3.78453	2.1
41856	1.19473	2.1
41857	0.987132	2.1
41858	0.987132	2.1
41859	1.04759	2.1
41860	1.0894	2.1
41861	1.14986	2.1
41862	1.3195	2.1
41863	1.3195	2.1
41864	0.946132	2.1
41865	0.946132	2.1
41866	1.31185	2.1
41867	1.3195	2.1
41868	0.946132	2.1
41869	0.946132	2.1
41870	1.31185	2.1
41871	0.946133	2.1
41872	0.946132	2.1
41873	1.3195	2.1

41874	1.11256	1.16454
41875	1.70101	1.1853
41876	1.22297	1.18585
41877	1.22297	1.17169
41878	1.7252	1.22321
41879	1.7252	1.20084
41880	1.99298	1.16071
41881	1.70101	1.23004
41882	1.7252	1.19952
41883	2.18701	1.10293
41884	3.71818	1.11374
41885	1.03218	1.13286
41886	1.7252	1.10984
41887	1.11256	1.15529
41888	1.11256	1.11266
41889	1.7457	1.17532
41890	1.7457	1.1286
41891	1.7457	1.12576
41892	2.187 1.02746	
41893	1.7252	1.10774
41894	1.7252	1.09077
41895	2.27351	1.11361
41896	2.27351	1.09737
41897	1.95544	0.855958
41898	1.35897	1.29624
41899	1.44859	1.03622
41900	1.44859	1.14134
41901	1.80709	0.909334
41902	1.80709	1.10856
41903	1.80709	0.813328
41904	1.17822	1.22613
41905	1.35897	1.00756
41906	1.35897	1.24994
41907	1.52782	0.81188
41908	1.52782	1.11467
41909	1.11978	1.06953
41910	0.903546	0.849186
41911	0.977719	1.15557
41912	1.2364	0.896349
41913	1.03812	0.800073
41914	1.03812	1.2524
41915	1.05938	0.766994
41916	1.05938	1.20411
41917	1.11978	0.889461
41918	1.2364	0.761454
41919	0.97772	1.03656
41920	0.903546	0.928959
41921	1.80709	1.17676
41922	1.81847	0.964665
41923	1.96029	1.22152
41924	1.52782	1.05135
41925	2.23956	0.908819
41926	1.80709	0.948315
41927	1.89226	0.689166
41928	2.32962	0.53963
41929	2.5992	0.545196
41930	1.89226	0.569726
41931	2.5992	0.671619

41932	1.89226	0.680336
41933	2.55791	0.258575
41934	2.47141	0.212515
41935	2.13515	0.209759
41936	2.10939	0.255493
41937	4.66731	0.233923
41938	2.47141	0.214811
41939	2.23337	0.252457
41940	2.10939	0.257282
41941	2.03438	0.212422
41942	4.34276	0.22685
41943	2.13512	0.220255
41944	1.71027	0.265342
41945	1.71027	0.261285
41946	2.10936	0.231924
41947	1.60137	0.256113
41948	2.00046	0.222157
41949	1.71027	0.270407
41950	2.03435	0.217104
41951	1.71027	0.252548
41952	2.10936	0.233763
41953	1.72067	0.246855
41954	2.11976	0.224988
41955	1.177	0.809392
41956	1.36405	1.05053
41957	2.25635	1.10117
41958	2.60151	0.762579
41959	3.6204	0.977887
41960	0.658869	0.514851
41961	0.781697	0.543933
41962	0.637037	0.389883
41963	0.541148	0.394652
41964	0.654682	0.390012
41965	0.654682	0.496211
41966	0.641224	0.607027
41967	0.641224	0.376009
41968	0.637039	0.485492
41969	0.781696	0.47611
41970	0.658867	0.47606
41971	0.541149	0.469906
41972	1.89226	0.657459
41973	1.59152	0.500457
41974	1.89226	0.538613
41975	1.82125	0.515236
41976	1.89226	0.628898
41977	1.82125	0.598107
41978	2.18967	0.282048
41979	1.89227	0.390126
41980	2.72752	0.359712
41981	2.72752	0.349271
41982	1.89227	0.318491
41983	1.89227	0.270738
41984	2.18967	0.268342
41985	1.92547	0.382757
41986	1.92547	0.327997
41987	2.72752	0.282095
41988	1.30572	0.319648
41989	2.10777	0.274237

41990	2.39663	2.1
41991	1.92293	2.1
41992	1.96889	2.1
41993	2.45928	2.1
41994	4.36552	2.1
41995	1.02605	2.1
41996	1.92293	2.1
41997	1.9689	2.1
41998	1.04679	2.1
41999	2.99495	2.1
42000	1.96889	2.1
42001	2.45928	2.1
42002	2.50138	2.1
42003	1.99782	2.1
42004	4.47027	2.1
42005	1.9689	2.1
42006	1.03449	2.1
42007	1.07572	2.1
42008	1.04679	2.1
42009	1.99783	2.1
42010	1.9689	2.1
42011	1.72162	0.791009
42012	1.89186	0.751283
42013	2.29771	0.75477
42014	1.80149	0.712669
42015	4.01933	0.746544
42016	1.16482	0.847181
42017	1.97003	0.726727
42018	1.72162	0.717018
42019	1.72162	0.75265
42020	1.29313	0.755074
42021	1.29313	0.857379
42022	2.29771	0.770115
42023	2.84159	0.70568
42024	2.19622	0.725223
42025	1.97366	0.72129
42026	2.50906	0.732725
42027	2.29771	0.733786
42028	1.29313	0.814593
42029	2.4483	0.724502
42030	2.14223	0.721614
42031	2.14228	0.749824
42032	1.294	0.744504
42033	1.294	0.790682
42034	1.21495	0.223491
42035	1.70303	0.260113
42036	1.7924	0.225932
42037	1.64002	0.238425
42038	3.11963	0.2292
42039	2.81021	0.220298
42040	1.89226	0.274403
42041	1.89226	0.234427
42042	1.89226	0.242318
42043	2.36406	0.23734
42044	2.36406	0.245342
42045	1.89226	0.242677
42046	1.89226	0.31635
42047	1.89226	0.258592

42048	1.89226	0.275454
42049	1.89226	0.262601
42050	1.89226	0.281105
42051	2.29976	0.217172
42052	1.64002	0.245755
42053	1.8959	0.252511
42054	1.90873	0.223228
42055	1.85594	0.253695
42056	1.85594	0.23134
42057	1.89226	0.250007
42058	1.89226	0.321613
42059	1.89226	0.259827
42060	1.89226	0.282502
42061	3.78453	0.270862
42062	1.89226	0.988635
42063	2.52823	2.03758
42064	2.83441	1.37573
42065	2.83441	1.82509
42066	1.89226	1.21878
42067	1.89226	1.48239
42068	1.04522	1.44547
42069	1.04522	1.35587
42070	1.12831	1.47439
42071	1.18364	1.95711
42072	0.640525	1.41471
42073	0.924882	1.39161
42074	1.27877	1.51777
42075	1.43363	0.958895
42076	0.93474	1.39244
42077	1.58926	1.30375
42078	1.29573	1.52221
42079	0.664247	1.5895
42080	2.02191	2.1
42081	1.89226	2.1
42082	1.89226	2.1
42083	1.89226	2.1
42084	2.58639	2.1
42085	2.58639	2.1
42086	1.16759	2.1
42087	2.11828	2.1
42088	1.60936	2.1
42089	2.35846	2.1
42090	3.52605	2.1
42091	1.89226	2.1
42092	1.89226	2.1
42093	1.89226	2.1
42094	1.89226	2.1
42095	3.78453	2.1
42096	2.82765	0.411561
42097	1.89809	0.333015
42098	2.15971	0.394443
42099	1.89809	0.372304
42100	2.29233	0.346478
42101	2.03071	0.319861
42102	1.90664	0.385822
42103	2.15971	0.42479
42104	1.28956	0.336349
42105	1.28956	0.329666

42106	2.64414	0.347029
42107	2.02706	0.334444
42108	2.82765	0.343073
42109	1.89226	0.357346
42110	2.39968	0.332986
42111	2.39968	0.340111
42112	1.89226	0.356675
42113	1.89226	0.352654
42114	0.978643	2.05971
42115	0.703968	2.1
42116	0.946129	2.1
42117	0.946129	2.1
42118	0.703939	2.1
42119	0.703939	2.02688
42120	0.946159	2.1
42121	0.946159	2.1
42122	0.978642	2.1
42123	0.94613	2.1
42124	0.94613	2.1
42125	0.703968	2.1
42126	1.89226	2.04243
42127	2.17309	1.22784
42128	2.17309	1.9447
42129	1.47475	1.67309
42130	1.89226	1.8664
42131	1.19393	1.33489
42132	1.89226	2.01572
42133	1.89226	2.1
42134	1.89226	2.1
42135	1.89226	1.81187
42136	3.78452	2.1
42137	1.17426	2.08969
42138	0.978644	2.1
42139	0.946129	2.1
42140	0.946129	2.1
42141	0.946131	2.1
42142	0.946131	2.06607
42143	0.978643	2.1
42144	0.978642	2.1
42145	1.17426	2.1
42146	0.946131	2.1
42147	0.978642	2.1
42148	0.94613	2.1
42149	1.89226	2.07437
42150	1.89226	1.21791
42151	1.89226	1.85838
42152	2.17309	1.28796
42153	2.56346	1.80818
42154	2.94389	1.46705
42155	0.946131	2.04334
42156	0.946131	1.81794
42157	0.946128	2.1
42158	0.946128	2.04827
42159	0.946131	2.03346
42160	0.946131	1.81367
42161	0.946128	2.1
42162	0.946128	2.06515
42163	0.94613	2.01872

42164	0.94613	2.00704
42165	0.946129	2.00131
42166	0.946129	1.98059
42167	1.89226	0.761121
42168	1.89226	0.779
42169	1.89226	0.776668
42170	1.89226	0.856495
42171	3.78453	0.763913
42172	1.89226	0.796711
42173	1.89226	0.792306
42174	1.89226	0.91702
42175	1.89226	0.916088
42176	1.89226	0.755699
42177	1.89226	0.754046
42178	1.89226	0.80615
42179	1.30733	0.855748
42180	1.89226	0.94573
42181	1.92403	1.08723
42182	3.38635	0.81204
42183	1.89226	0.790945
42184	3.02887	1.13358
42185	2.0815	0.794676
42186	1.88422	0.815925
42187	2.0815	0.880043
42188	1.88422	1.17487
42189	1.89226	0.884687
42190	1.70221	0.885434
42191	1.89226	1.30231
42192	1.20068	1.2744
42193	1.89226	0.768838
42194	1.20068	0.757163
42195	1.01393	0.875924
42196	1.20067	0.854095
42197	1.89226	1.33214
42198	2.44958	1.14158
42199	1.01393	0.743147
42200	1.89226	0.734114
42201	1.89226	2.1
42202	1.89226	1.91293
42203	1.89226	2.1
42204	1.89226	2.1
42205	1.89226	2.1
42206	1.89226	2.1
42207	1.89226	2.1
42208	1.89226	0.936324
42209	1.89226	0.985631
42210	1.89226	1.99492
42211	1.89226	1.00072
42212	1.89226	2.04158
42213	1.89226	2.1
42214	1.89226	1.75521
42215	1.89226	2.09214
42216	1.89226	2.1
42217	1.89226	2.1
42218	1.89226	2.1
42219	1.91161	2.1
42220	2.45782	1.43397
42221	1.39653	2.1

42222	2.44652	1.64671
42223	4.35812	2.1
42224	2.73092	1.36617
42225	1.89226	0.950268
42226	1.89226	2.01113
42227	1.89226	0.93301
42228	2.44652	1.71319
42229	2.44652	0.909187
42230	1.89226	2.1
42231	1.89226	0.972057
42232	1.89226	1.02198
42233	1.89226	1.8928
42234	1.89226	1.02753
42235	1.89226	1.91631
42236	2.64924	2.1
42237	1.31109	2.1
42238	1.31109	2.1
42239	1.49958	2.1
42240	1.95039	2.1
42241	2.14451	2.1
42242	0.825157	2.1
42243	0.634856	2.1
42244	0.816836	2.1
42245	0.816836	2.1
42246	0.556743	2.1
42247	0.639142	2.1
42248	0.985106	2.1
42249	0.795045	2.1
42250	0.967461	2.1
42251	0.552494	2.1
42252	0.676829	2.1
42253	0.98803	2.1
42254	2.28564	2.1
42255	2.34084	1.27843
42256	2.3502	1.42451
42257	2.42908	1.97514
42258	4.69104	1.5446
42259	2.34084	1.52224
42260	2.18077	1.78007
42261	2.42908	1.87656
42262	2.2947	0.997606
42263	4.63554	1.59062
42264	2.3502	1.54285
42265	2.42908	2.1
42266	2.38681	2.1
42267	2.50438	1.14209
42268	4.85458	2.05034
42269	2.42908	2.04415
42270	2.2947	1.25691
42271	2.50438	1.07743
42272	2.40627	1.76288
42273	4.79908	1.1592
42274	1.89226	0.941481
42275	2.20711	1.36363
42276	1.89226	0.951185
42277	1.89226	1.35506
42278	2.02693	0.978773
42279	2.02693	1.51985

42280	1.2269	1.98392
42281	2.20711	1.33245
42282	2.0815	1.22466
42283	1.11506	2.1
42284	3.22109	1.80984
42285	0.545802	1.69543
42286	0.547489	1.97404
42287	0.85914	1.30068
42288	1.11641	1.39944
42289	0.899974	1.48276
42290	0.849893	2.02565
42291	0.747885	1.70864
42292	0.747885	1.23135
42293	0.557184	1.92697
42294	0.887039	1.87899
42295	1.08948	1.83692
42296	0.59815	1.89085
42297	0.974139	2.1
42298	1.33385	2.1
42299	1.02368	2.1
42300	1.02368	2.1
42301	1.00833	2.1
42302	1.00833	2.1
42303	1.89226	0.802598
42304	1.89226	0.521688
42305	1.89226	0.814408
42306	1.89226	0.35206
42307	3.78453	0.447878
42308	1.74651	0.859435
42309	1.44605	0.467618
42310	1.63557	0.796315
42311	1.31993	0.20751
42312	3.08162	0.479505
42313	1.89226	0.846532
42314	1.89226	0.587953
42315	1.89226	0.773891
42316	1.89226	0.225837
42317	3.78452	0.464505
42318	1.63557	0.846489
42319	1.31992	0.305948
42320	1.52615	0.862235
42321	1.18549	0.214563
42322	2.82106	0.424616
42323	1.89226	0.464661
42324	2.58101	0.571594
42325	2.73797	0.792382
42326	2.73797	0.539459
42327	1.89226	0.796117
42328	1.89226	0.514134
42329	1.91762	0.778134
42330	1.44354	0.770879
42331	1.33622	0.767993
42332	1.33622	0.762452
42333	2.20248	0.791669
42334	2.20248	0.782864
42335	1.11712	2.1
42336	1.11712	2.1
42337	1.11421	2.1

42338	1.11421	2.1
42339	0.833871	2.1
42340	0.926528	2.1
42341	1.3048	2.1
42342	1.40335	2.1
42343	0.926527	2.1
42344	1.40335	2.1
42345	1.3048	2.1
42346	0.833871	2.1
42347	0.879657	2.1
42348	0.937695	2.1
42349	0.946129	2.1
42350	0.946129	2.1
42351	0.851541	2.1
42352	0.851541	2.1
42353	1.1126	2.1
42354	1.04519	2.1
42355	0.851486	2.1
42356	0.980501	2.1
42357	0.898251	2.1
42358	0.851528	2.1
42359	1.37756	2.1
42360	2.73081	2.1
42361	2.44311	2.1
42362	2.44311	2.1
42363	1.66428	2.1
42364	1.66428	2.1
42365	1.89226	2.1
42366	1.89226	1.98139
42367	1.89226	2.1
42368	1.89226	1.97394
42369	3.78452	2.1
42370	1.65781	2.1
42371	1.89837	2.01609
42372	1.46713	2.1
42373	1.98027	1.93211
42374	3.63808	2.1
42375	2.29054	2.01218
42376	2.26369	0.907964
42377	1.98027	1.92757
42378	2.15617	0.797567
42379	4.24396	1.06249
42380	1.89226	1.97815
42381	1.89226	0.908608
42382	1.89226	1.974
42383	1.89226	0.889211
42384	3.78452	1.13814
42385	0.946131	1.89491
42386	0.946131	2.1
42387	0.946128	2.1
42388	0.946128	2.03631
42389	0.946129	1.87505
42390	0.946129	1.92971
42391	0.94613	2.1
42392	0.94613	2.1
42393	0.94613	2.1
42394	0.94613	2.1
42395	0.94613	2.1

42396	0.94613	2.1
42397	0.812186	1.93827
42398	0.812186	2.1
42399	0.819781	1.96927
42400	0.819781	2.1
42401	0.913532	1.86998
42402	0.990134	1.84874
42403	0.733564	2.1
42404	0.641833	2.1
42405	0.641832	2.1
42406	0.913533	2.1
42407	0.733564	2.1
42408	0.990134	2.1
42409	0.994262	0.903915
42410	0.994262	2.00864
42411	0.997354	1.77688
42412	0.997354	0.896879
42413	1.00775	0.790748
42414	1.07808	0.855133
42415	0.913533	1.90934
42416	0.990135	1.87224
42417	0.913532	1.04224
42418	1.00775	1.03471
42419	0.990134	1.03066
42420	1.07808	1.02616
42421	0.946131	2.04764
42422	0.946131	0.912604
42423	0.946128	1.76634
42424	0.946128	0.901405
42425	0.946128	0.825916
42426	0.946128	0.849928
42427	0.94613	1.88072
42428	0.94613	1.94679
42429	0.946129	1.16621
42430	0.946129	1.14746
42431	0.946129	1.01858
42432	0.946129	1.01301
42433	0.816232	2.1
42434	0.760038	2.1
42435	0.845999	2.1
42436	0.845999	2.1
42437	1.0631	2.1
42438	1.28131	2.1
42439	0.474065	2.1
42440	0.591905	2.1
42441	0.601756	2.1
42442	1.23903	2.1
42443	0.988592	2.1
42444	0.49051	2.1
42445	2.57021	2.1
42446	1.89226	1.8654
42447	2.68172	2.1
42448	1.89226	1.16739
42449	4.46247	1.73979
42450	1.89226	2.01834
42451	1.89226	1.49437
42452	1.89226	1.36151
42453	1.89226	1.24902

42454	3.78452	1.43078
42455	1.12573	2.1
42456	1.65504	1.22251
42457	1.38848	1.58262
42458	1.38848	1.99227
42459	1.30567	1.15911
42460	1.30567	1.77554
42461	2.82413	2.1
42462	1.89226	1.23103
42463	2.68172	2.03441
42464	2.68172	2.1
42465	1.89226	1.71365
42466	1.89226	2.1
42467	1.89226	1.44886
42468	1.89226	1.51502
42469	1.89226	1.91454
42470	1.89226	0.991222
42471	3.78452	1.37988
42472	1.30567	2.1
42473	1.65504	1.45921
42474	1.52724	1.88565
42475	1.89249	1.65489
42476	3.18228	1.90644
42477	2.57386	0.528533
42478	1.89226	0.68522
42479	1.89226	0.698771
42480	2.66753	0.577502
42481	1.89226	0.658211
42482	2.66753	0.561982
42483	1.89226	0.527945
42484	1.89226	0.717213
42485	2.2973	0.716107
42486	3.12479	0.609865
42487	1.89226	0.691621
42488	2.57385	0.584627
42489	0.489293	2.02697
42490	0.672698	2.1
42491	0.59038	2.1
42492	0.738357	2.1
42493	0.62269	2.1
42494	0.62269	2.08711
42495	0.640388	2.1
42496	0.640388	2.1
42497	0.59038	2.1
42498	0.489293	2.1
42499	0.738357	2.1
42500	0.672698	2.1
42501	1.89226	2.09238
42502	1.89226	1.47322
42503	1.89226	2.1
42504	1.89226	1.00706
42505	3.78452	1.76807
42506	2.82858	1.77109
42507	1.89226	1.05565
42508	2.5671	2.1
42509	1.89226	2.1
42510	2.5671	2.07783
42511	1.89226	1.46223

42512	2.06581	0.208405
42513	2.47308	0.252246
42514	1.89433	0.240973
42515	1.67571	0.210342
42516	3.87274	0.227366
42517	2.55791	0.254585
42518	2.10276	0.207407
42519	2.47308	0.252589
42520	2.47308	0.235545
42521	1.83415	0.220007
42522	1.90922	0.217518
42523	1.18421	0.248571
42524	2.03277	0.215181
42525	1.18421	0.259484
42526	2.01275	0.226461
42527	1.42818	0.252444
42528	2.25673	0.216997
42529	0.882904	0.213043
42530	0.958492	0.21476
42531	0.905756	0.251052
42532	0.905756	0.258444
42533	1.06352	0.220222
42534	0.797654	0.258334
42535	1.11319	0.217461
42536	0.716795	0.25523
42537	0.820721	0.23885
42538	0.731963	0.23631
42539	1.13143	0.225906
42540	1.10528	0.232862
42541	1.89226	0.385879
42542	1.89226	0.375264
42543	1.89226	0.388112
42544	1.89226	0.443412
42545	1.89226	0.308154
42546	1.89226	0.362777
42547	1.01241	0.490159
42548	0.703929	0.551251
42549	0.946133	0.420905
42550	0.946133	0.415167
42551	1.04072	0.447533
42552	1.163	0.393983
42553	0.639952	0.517817
42554	0.851543	0.376124
42555	0.946132	0.335021
42556	0.920761	0.467162
42557	0.711038	0.466374
42558	0.711038	0.529892
42559	1.89226	0.347387
42560	1.89226	0.320476
42561	1.89226	0.330609
42562	1.89226	0.392401
42563	1.89226	0.280626
42564	1.89226	0.340097
42565	1.89226	0.288569
42566	1.89226	0.331318
42567	2.41075	0.293447
42568	1.89226	0.263425
42569	3.78451	0.313204

42570	0.968476	1.67652
42571	1.20674	1.1088
42572	1.13456	1.08788
42573	1.13456	1.83777
42574	1.02074	1.00422
42575	1.02074	1.64395
42576	1.02074	2.07415
42577	1.20674	1.63701
42578	1.07866	1.16108
42579	1.28649	1.167
42580	2.2854	1.58688
42581	1.94221	0.702644
42582	1.36717	0.664737
42583	1.45535	0.679236
42584	1.93771	0.623427
42585	3.39756	0.695708
42586	1.94222	0.721143
42587	2.25035	0.637538
42588	2.41806	0.685076
42589	2.42256	0.666243
42590	1.93772	0.691104
42591	1.94222	0.679332
42592	1.49906	0.546062
42593	1.93771	0.586858
42594	1.45535	0.621862
42595	1.45535	0.649064
42596	1.91484	0.495677
42597	1.91484	0.517677
42598	1.91484	0.549217
42599	2.25035	0.616937
42600	1.93772	0.627718
42601	1.93772	0.646288
42602	2.11465	0.52232
42603	2.11465	0.528486
42604	2.61735	0.641702
42605	1.5082	0.550384
42606	2.36481	0.72045
42607	1.58597	0.678186
42608	2.36481	0.623381
42609	1.46653	0.560326
42610	2.36482	0.750149
42611	1.08133	0.824231
42612	1.5082	0.538256
42613	2.01868	0.776338
42614	3.52688	0.786597
42615	0.94613	0.629188
42616	0.94613	0.624098
42617	0.946131	0.71236
42618	0.946131	0.768616
42619	0.946129	0.771123
42620	0.946129	0.69206
42621	0.946132	0.632764
42622	0.946132	0.692897
42623	0.94613	0.69366
42624	0.94613	0.713356
42625	0.946131	0.721352
42626	0.946131	0.694995
42627	2.53725	0.478788

42628	1.89227	0.557469
42629	1.89227	0.548404
42630	2.27073	0.548634
42631	1.89227	0.498228
42632	2.27073	0.493072
42633	1.89227	0.657172
42634	1.89227	0.789899
42635	1.89227	0.798203
42636	1.89227	0.727544
42637	1.89227	0.736241
42638	1.89227	0.660922
42639	1.86766	2.1
42640	2.83487	2.1
42641	2.84318	2.1
42642	2.59401	2.1
42643	2.11684	2.1
42644	1.86766	2.1
42645	1.10638	2.1
42646	2.11684	2.1
42647	1.29111	2.1
42648	2.37439	2.1
42649	3.40796	2.1
42650	2.56843	2.1
42651	1.89226	2.1
42652	1.89226	2.1
42653	2.71932	2.1
42654	4.46069	2.1
42655	1.89226	2.1
42656	1.89226	2.1
42657	1.89226	2.1
42658	1.89226	2.1
42659	3.78452	2.1
42660	0.6416	2.1
42661	0.697585	2.1
42662	0.670706	2.1
42663	0.598927	2.1
42664	0.644313	2.1
42665	0.644313	2.1
42666	0.652199	2.1
42667	0.652199	2.1
42668	0.697587	2.1
42669	0.670705	2.1
42670	0.598926	2.1
42671	0.641601	2.1
42672	1.89226	2.1
42673	1.89226	2.1
42674	1.89226	2.1
42675	1.89226	2.1
42676	1.89226	2.1
42677	1.89226	2.1
42678	0.697585	2.1
42679	0.730617	2.1
42680	0.712082	2.1
42681	0.670706	2.1
42682	0.69854	2.1
42683	0.69854	2.1
42684	0.702783	2.1
42685	0.702783	2.1

42686	0.730618	2.1
42687	0.712081	2.1
42688	0.697586	2.1
42689	0.670705	2.1
42690	1.89226	1.45806
42691	1.89226	1.43288
42692	1.89226	1.02707
42693	1.89226	2.06041
42694	1.89226	0.989675
42695	1.89226	2.1
42696	2.0815	1.98185
42697	1.12076	2.1
42698	1.38662	1.26336
42699	2.01244	2.1
42700	3.41508	1.99416
42701	1.89226	2.1
42702	2.54324	2.1
42703	1.91981	2.1
42704	1.89226	2.1
42705	2.57079	2.1
42706	2.54324	2.1
42707	2.54325	2.1
42708	1.94779	2.1
42709	1.76879	2.1
42710	1.73811	2.1
42711	2.5708	2.1
42712	2.54325	2.1
42713	1.89226	2.1
42714	2.57079	2.1
42715	1.91894	2.1
42716	1.89226	2.1
42717	2.59747	2.1
42718	2.57079	2.1
42719	2.5708	2.1
42720	1.82268	2.1
42721	1.79738	2.1
42722	1.77071	2.1
42723	2.59747	2.1
42724	2.5708	2.1
42725	1.89226	0.737958
42726	1.89226	0.835386
42727	1.89226	0.504176
42728	1.89226	0.857808
42729	3.78451	0.784821
42730	2.21582	0.497435
42731	1.94412	0.836071
42732	2.13428	0.456874
42733	1.86292	0.822945
42734	4.07839	0.694837
42735	1.94412	0.834322
42736	1.5854	2.1
42737	1.86293	0.846847
42738	1.50767	2.1
42739	3.45179	1.24329
42740	1.89226	0.835474
42741	1.89226	2.1
42742	1.89226	0.863955
42743	1.89226	2.1

42744	3.78452	1.28516
42745	1.89226	0.657615
42746	1.89226	0.928458
42747	1.89226	0.531659
42748	1.89226	0.842829
42749	3.78452	0.77742
42750	2.13428	0.592333
42751	1.86293	0.805312
42752	2.04737	0.355607
42753	1.77122	0.845082
42754	3.9055	0.757436
42755	1.86293	0.777165
42756	1.50767	2.07271
42757	1.41077	0.852802
42758	1.41077	1.57445
42759	1.77123	0.857749
42760	1.86813	1.71504
42761	0.810943	0.867591
42762	0.810943	2.1
42763	0.810945	0.857151
42764	0.810945	0.841483
42765	0.810958	1.20905
42766	0.810958	1.07029
42767	1.89226	2.08011
42768	1.35168	1.26583
42769	1.08132	0.863106
42770	1.08132	2.06339
42771	1.0813	1.0857
42772	0.745979	2.1
42773	0.745979	2.1
42774	0.778781	2.1
42775	0.778781	2.1
42776	0.59549	2.1
42777	0.649658	2.1
42778	0.994784	2.1
42779	0.875102	2.1
42780	0.875102	2.1
42781	0.59549	2.1
42782	0.649658	2.1
42783	0.994784	2.1
42784	1.57887	2.1
42785	1.75021	2.1
42786	1.13341	2.1
42787	1.13341	2.1
42788	1.19098	2.1
42789	1.63644	2.1
42790	2.5761	0.38119
42791	2.43404	0.80226
42792	2.44813	0.370533
42793	2.31751	0.821359
42794	4.88218	0.512124
42795	2.43405	0.789225
42796	2.22575	1.57128
42797	2.31751	0.832918
42798	2.10893	1.32295
42799	4.54298	0.914598
42800	2.44813	0.396233
42801	2.31751	0.812664

42802	2.30908	0.342547
42803	2.19815	0.802883
42804	4.64628	0.593173
42805	2.31751	0.816097
42806	2.10893	1.34019
42807	2.19815	0.809188
42808	1.98061	1.94098
42809	4.30708	0.885497
42810	0.94613	2.1
42811	0.94613	1.81065
42812	1.40372	1.97185
42813	1.40372	2.1
42814	1.40372	2.1
42815	1.37167	2.1
42816	0.946132	1.44265
42817	0.946132	2.081
42818	0.946131	2.1
42819	1.40372	2.1
42820	0.94613	2.1
42821	1.37167	2.1
42822	2.80744	2.1
42823	2.06984	2.1
42824	2.83418	2.1
42825	2.74335	2.1
42826	2.16067	2.1
42827	2.06984	2.1
42828	1.077	0.613709
42829	0.809116	0.540858
42830	0.600419	0.660923
42831	0.790277	0.553036
42832	0.927647	0.518176
42833	0.758598	0.52802
42834	0.85749	0.618721
42835	0.85749	0.638541
42836	1.02417	0.559936
42837	0.600411	0.584757
42838	0.751701	0.563824
42839	1.07702	0.558132
42840	1.89226	2.1
42841	1.89226	2.1
42842	1.89226	2.1
42843	1.89226	2.1
42844	1.89226	2.1
42845	1.89226	2.1
42846	1.89226	2.1
42847	1.89226	2.06454
42848	1.89226	2.07562
42849	1.89226	2.1
42850	3.78453	2.1
42851	1.89226	2.1
42852	1.89226	2.1
42853	1.89226	2.1
42854	1.89226	2.1
42855	1.89226	2.1
42856	1.89226	2.1
42857	0.946131	2.1
42858	0.946131	2.1
42859	0.946131	2.1

42860	0.946131	2.04405
42861	0.946133	2.1
42862	0.946133	2.00435
42863	0.946128	2.1
42864	0.946128	2.1
42865	0.946131	2.1
42866	0.946131	2.1
42867	0.94613	2.1
42868	0.94613	2.1
42869	2.17159	2.02769
42870	2.00083	1.55375
42871	2.21305	1.96669
42872	2.00551	1.41286
42873	2.20837	1.65718
42874	2.00083	1.30257
42875	2.20837	1.38579
42876	1.80328	1.1306
42877	2.22962	1.69964
42878	2.00083	1.30916
42879	2.03207	1.02196
42880	1.80328	0.965465
42881	2.28564	2.09144
42882	2.20837	1.37897
42883	2.30406	1.92529
42884	2.17159	1.79556
42885	2.34084	1.50302
42886	2.20837	1.64574
42887	1.09523	0.946297
42888	1.09523	1.53133
42889	1.09934	1.57871
42890	1.09934	1.89913
42891	1.01603	0.939217
42892	1.09038	1.44703
42893	1.10419	1.10239
42894	1.17042	2.02119
42895	1.17042	1.54168
42896	1.09038	1.36043
42897	1.10419	1.20022
42898	1.01603	1.52203
42899	2.60152	0.777343
42900	0.976334	0.910394
42901	2.22344	0.935289
42902	2.22344	0.800729
42903	1.36405	0.999944
42904	1.36405	0.602684
42905	1.89226	1.76232
42906	1.70302	2.06516
42907	1.69019	2.1
42908	1.70302	1.7858
42909	1.73933	2.1
42910	1.70303	1.15517
42911	1.89226	2.1
42912	1.89226	1.01627
42913	1.89226	1.29344
42914	1.89226	1.85428
42915	3.78452	1.47282
42916	1.89226	1.30079
42917	1.59036	1.7549

42918	1.65683	2.1
42919	1.59036	1.42853
42920	1.95873	1.83516
42921	1.89226	1.02158
42922	0.946131	0.964788
42923	0.946131	1.04482
42924	0.946127	1.77428
42925	0.946127	1.4837
42926	0.946127	1.46202
42927	0.946127	0.909122
42928	0.946131	1.66453
42929	0.946131	1.05769
42930	0.946129	1.15951
42931	0.946129	1.15546
42932	0.94613	1.17481
42933	0.94613	1.17059
42934	0.851541	2.1
42935	0.851541	1.95616
42936	0.851483	2.1
42937	1.15713	2.1
42938	0.851539	2.1
42939	0.851539	1.47224
42940	0.942127	2.1
42941	1.14455	2.1
42942	0.838683	2.1
42943	0.946131	2.1
42944	0.957503	2.1
42945	0.957504	2.1
42946	1.89226	1.00987
42947	1.89226	0.738685
42948	1.89226	0.791002
42949	1.89226	1.69671
42950	3.78452	0.801901
42951	1.89226	0.472749
42952	1.89226	0.746082
42953	1.89226	0.792697
42954	1.89226	0.758188
42955	1.89226	0.481356
42956	1.89226	0.435387
42957	1.89226	0.702376
42958	1.89226	0.583808
42959	1.89226	0.653033
42960	1.89226	0.75378
42961	3.78453	0.64726
42962	1.89226	0.702422
42963	1.89226	2.1
42964	1.89226	1.77185
42965	1.89226	1.36111
42966	3.78453	1.75767
42967	1.22575	0.830847
42968	1.05438	0.721999
42969	0.946131	0.917328
42970	0.946131	0.883404
42971	0.946129	1.37166
42972	1.22575	0.619498
42973	1.05438	0.785951
42974	0.946132	0.722724
42975	1.05438	0.934598

42976	1.05438	1.10477
42977	0.946131	0.695805
42978	0.946131	0.882166
42979	2.4515	0.591972
42980	1.13726	0.357746
42981	2.10875	0.477337
42982	1.49515	0.481868
42983	2.10875	0.426749
42984	1.49515	0.361689
42985	0.831477	0.394663
42986	0.747575	0.455747
42987	1.22575	0.685012
42988	1.28174	0.523247
42989	1.28174	0.532758
42990	0.831476	0.431866
42991	1.22575	0.483796
42992	0.747575	0.42548
42993	1.03561	0.48763
42994	1.03561	0.684017
42995	1.02162	0.420293
42996	1.02162	0.427673
42997	1.89227	1.59018
42998	2.56348	1.04003
42999	1.89227	2.1
43000	2.45151	0.843514
43001	1.89227	2.1
43002	2.45151	0.935349
43003	1.40013	1.03152
43004	1.89226	1.72283
43005	1.89226	1.24457
43006	0.946643	1.02273
43007	1.89226	1.47424
43008	0.946643	1.23146
43009	1.89226	0.332605
43010	1.89226	0.448146
43011	1.89226	0.384675
43012	1.89226	0.436814
43013	3.78453	0.440954
43014	1.89226	0.520138
43015	1.89226	0.555975
43016	1.89226	0.497115
43017	1.89226	0.649934
43018	3.78453	0.498943
43019	0.946131	0.607134
43020	0.946131	0.561654
43021	0.946128	0.461569
43022	0.946128	0.493881
43023	0.94613	0.574884
43024	0.94613	0.609879
43025	0.946129	0.509262
43026	0.946129	0.468605
43027	0.94613	0.540376
43028	0.94613	0.439537
43029	0.94613	0.520637
43030	0.94613	0.587896
43031	1.89226	0.3151
43032	1.89226	0.349476
43033	1.17244	0.325817

43034	1.89226	0.350092
43035	0.975291	0.342194
43036	1.57408	0.365208
43037	0.946132	0.549386
43038	0.946132	0.419311
43039	0.946131	0.535891
43040	0.946131	0.438203
43041	0.946132	0.512292
43042	0.946132	0.533715
43043	0.946131	0.450964
43044	0.946131	0.458486
43045	0.946131	0.51984
43046	0.946131	0.505533
43047	0.946132	0.481169
43048	0.946132	0.519085
43049	2.74744	0.503942
43050	1.89226	0.654342
43051	2.70805	0.523076
43052	1.89226	0.566216
43053	2.70805	0.581873
43054	1.89226	0.64035
43055	2.74744	0.466573
43056	1.89226	0.544981
43057	2.57465	0.561781
43058	1.89226	0.650193
43059	4.6397	0.562688
43060	1.89226	0.632796
43061	1.89226	0.809339
43062	1.89226	0.808592
43063	1.89226	0.991701
43064	3.78452	0.77468
43065	1.89226	0.72865
43066	1.89226	0.884893
43067	1.89226	0.690213
43068	1.89226	0.606904
43069	1.89226	1.0007
43070	1.89226	0.94816
43071	2.16206	0.476211
43072	2.67861	0.478737
43073	3.11996	0.509282
43074	1.70303	0.469994
43075	2.89015	0.513583
43076	1.486	0.449384
43077	2.12839	0.492553
43078	2.83631	0.612047
43079	3.08096	0.514024
43080	1.84157	0.484351
43081	3.08096	0.608406
43082	1.84157	0.54264
43083	1.89225	1.14745
43084	1.89225	0.865991
43085	1.89225	0.976285
43086	1.89225	1.1576
43087	1.89225	0.955594
43088	1.89225	1.12945
43089	1.89226	0.834565
43090	1.89226	0.866191
43091	1.89226	1.18845

43092	1.89226	0.79971
43093	3.78452	0.803234
43094	1.89226	0.700329
43095	2.49041	0.704017
43096	1.89226	0.70346
43097	1.89226	0.554064
43098	2.27073	0.555103
43099	2.27073	0.689765
43100	1.09904	0.52743
43101	0.677147	0.528957
43102	1.3365	0.534874
43103	0.95759	0.676372
43104	0.957591	0.562045
43105	0.677148	0.583789
43106	1.09904	0.581747
43107	1.3365	0.58193
43108	0.996015	0.546639
43109	0.996015	0.559459
43110	1.01764	0.701734
43111	1.01764	0.543307
43112	2.77349	0.64582
43113	2.72723	0.510694
43114	2.43156	0.655817
43115	2.4564	0.502571
43116	5.20505	0.621841
43117	2.77349	0.626153
43118	2.68508	0.481222
43119	2.33761	0.586456
43120	2.4564	0.494486
43121	5.1111	0.532286
43122	2.43156	0.541346
43123	2.70208	0.459532
43124	1.85779	0.578563
43125	2.10695	0.439897
43126	4.77572	0.464262
43127	2.33761	0.51904
43128	2.70208	0.454287
43129	1.85779	0.543091
43130	1.61597	0.42502
43131	4.19542	0.49032
43132	0.946132	1.47188
43133	0.946132	1.81057
43134	0.746729	1.58219
43135	0.746729	1.89419
43136	0.946132	1.92964
43137	0.915958	1.75345
43138	0.946133	1.44866
43139	0.74673	1.95526
43140	0.946133	1.67312
43141	0.946133	1.67603
43142	0.915958	1.73487
43143	0.746729	1.78635
43144	1.50743	2.1
43145	2.45501	1.9088
43146	2.29118	2.1
43147	2.29118	1.95942
43148	1.75482	2.1
43149	1.75482	1.97125

43150	2.45501	1.98208
43151	2.76927	0.897057
43152	2.29118	1.92916
43153	2.64523	0.916919
43154	5.10024	1.11888
43155	1.25559	2.1
43156	2.29118	1.94194
43157	2.0815	2.1
43158	2.26387	1.94895
43159	1.35668	2.1
43160	1.47667	1.97739
43161	2.28481	1.75663
43162	2.64523	0.931258
43163	2.50057	1.83363
43164	2.50057	0.931777
43165	2.29118	1.86376
43166	2.29118	0.932635
43167	0.946129	0.683686
43168	0.946129	0.675587
43169	1.23929	0.642757
43170	1.15246	0.631736
43171	0.946131	0.63995
43172	0.946131	0.681481
43173	1.15246	0.675839
43174	1.15246	0.638833
43175	0.946131	0.665598
43176	1.15246	0.672684
43177	0.946131	0.66636
43178	1.23928	0.665455
43179	0.946128	0.695881
43180	0.946128	0.695547
43181	1.15246	0.65097
43182	0.942814	0.708806
43183	0.946131	0.659864
43184	0.946131	0.693699
43185	0.961347	0.689726
43186	0.942811	0.689928
43187	0.946131	0.672409
43188	0.961347	0.660443
43189	0.946131	0.658276
43190	1.15246	0.673902
43191	2.32334	0.67675
43192	1.57621	0.556177
43193	1.90335	0.585144
43194	1.24538	0.580515
43195	3.47956	0.5394
43196	0.965762	0.406175
43197	0.965762	0.465441
43198	1.00245	0.415199
43199	1.00245	0.445583
43200	0.92786	0.464044
43201	0.705297	0.369366
43202	0.856414	0.440547
43203	1.28968	0.415142
43204	1.00306	0.415377
43205	0.705303	0.400717
43206	1.28967	0.432998
43207	0.975478	0.452698

43208	3.05308	2.1
43209	1.76816	2.1
43210	1.76816	2.1
43211	1.69129	2.1
43212	2.58731	2.1
43213	2.51043	2.1
43214	1.89226	0.539518
43215	2.71464	0.583109
43216	2.34627	0.566
43217	1.89226	0.590251
43218	2.34627	0.5241
43219	1.89226	0.560989
43220	2.34627	0.500678
43221	2.71464	0.504214
43222	1.98252	0.460592
43223	1.98252	0.553032
43224	1.51026	0.46258
43225	2.34627	0.519197
43226	2.38698	1.13506
43227	2.35142	1.87273
43228	2.60343	1.06638
43229	2.6636	1.15269
43230	4.99041	1.19314
43231	1.07811	1.64724
43232	1.1952	2.1
43233	1.17571	1.45141
43234	1.30171	1.05206
43235	1.18322	1.01599
43236	1.18322	2.1
43237	1.18769	1.55808
43238	1.18769	1.11192
43239	1.1952	1.72835
43240	1.30171	1.5671
43241	1.17571	1.48436
43242	1.07811	1.40439
43243	1.89226	1.18989
43244	2.6636	1.48881
43245	1.89226	0.840945
43246	2.60343	1.21746
43247	1.89226	1.22822
43248	2.60343	1.96281
43249	1.5344	0.222479
43250	1.4516	0.220213
43251	1.17816	0.207199
43252	1.21056	0.211963
43253	2.71257	0.217103
43254	1.89226	1.46974
43255	1.89226	0.916205
43256	1.89226	1.05306
43257	1.89226	1.118
43258	3.78452	1.02737
43259	1.89226	1.10427
43260	1.89226	0.801577
43261	1.89226	0.928603
43262	1.89226	0.866179
43263	1.65573	1.00736
43264	1.65573	0.905523
43265	1.89226	1.04525

43266	1.89226	1.17475
43267	1.89226	1.64062
43268	2.64212	0.958025
43269	3.78452	1.20416
43270	1.89226	1.10069
43271	1.89226	2.1
43272	1.89226	1.21311
43273	1.89226	2.1
43274	3.78452	1.64758
43275	2.7367	0.906269
43276	2.07947	2.1
43277	2.47319	1.20322
43278	1.85057	2.1
43279	4.55267	1.98961
43280	2.07948	2.1
43281	1.3294	2.1
43282	1.85057	2.1
43283	1.09962	2.1
43284	3.1791	2.1
43285	1.89226	2.1
43286	1.89226	2.1
43287	1.89226	2.1
43288	1.89226	2.1
43289	3.78453	2.1
43290	0.94613	2.1
43291	0.94613	0.94203
43292	0.94613	1.07974
43293	0.94613	2.1
43294	0.946128	2.1
43295	0.946128	2.1
43296	0.946132	0.938058
43297	0.946132	0.948429
43298	0.94613	1.80488
43299	0.94613	1.90283
43300	0.946131	1.86453
43301	0.946131	1.87733
43302	1.00875	2.1
43303	1.00875	0.892159
43304	1.01676	2.1
43305	1.01676	0.906779
43306	0.925282	2.1
43307	0.804877	2.1
43308	1.2366	0.98058
43309	1.10023	0.97156
43310	0.804878	1.66906
43311	1.10023	1.74761
43312	1.2366	1.54965
43313	0.925284	1.46426
43314	1.89226	2.1
43315	1.89226	2.1
43316	1.89226	2.1
43317	1.89226	2.1
43318	3.78453	2.1
43319	1.89226	2.1
43320	1.89226	2.1
43321	1.89226	2.1
43322	1.89226	2.1
43323	1.89226	2.1

43324	1.89226	2.1
43325	1.09766	2.1
43326	1.14842	2.1
43327	1.02909	2.1
43328	1.07659	2.1
43329	1.16718	2.1
43330	0.954189	2.1
43331	1.06653	2.1
43332	1.06653	2.1
43333	1.20738	2.1
43334	1.02909	2.1
43335	1.09767	2.1
43336	0.815951	2.1
43337	2.00793	2.1
43338	1.14048	2.1
43339	1.19086	2.1
43340	1.13723	2.1
43341	2.00793	2.1
43342	2.11772	2.1
43343	1.89226	2.1
43344	1.89226	2.1
43345	1.89226	2.1
43346	1.89226	2.1
43347	1.89226	2.1
43348	1.89226	2.1
43349	1.89226	2.1
43350	1.89226	2.1
43351	1.89226	2.1
43352	1.89226	2.1
43353	1.89226	2.1
43354	1.89226	2.1
43355	1.1779	2.1
43356	1.26166	2.1
43357	1.0875	2.1
43358	1.00397	2.1
43359	1.13286	2.1
43360	1.13286	2.1
43361	1.13277	2.1
43362	1.13277	2.1
43363	1.26166	2.1
43364	1.08749	2.1
43365	1.1779	2.1
43366	1.00396	2.1
43367	1.0875	2.1
43368	1.00397	2.1
43369	0.685749	2.1
43370	0.595432	2.1
43371	0.83976	2.1
43372	0.83976	2.1
43373	0.843169	2.1
43374	0.843169	2.1
43375	1.0875	2.1
43376	0.685749	2.1
43377	1.00397	2.1
43378	0.595432	2.1
43379	1.89226	2.1
43380	1.86443	2.1
43381	2.4988	2.1

43382	2.0815	2.1
43383	2.28173	2.1
43384	1.86443	2.1
43385	1.89226	2.1
43386	1.89226	2.1
43387	1.89226	2.1
43388	1.89226	2.1
43389	1.89226	2.1
43390	1.89226	2.1
43391	0.946131	2.1
43392	0.946131	2.1
43393	0.969491	2.1
43394	0.949105	2.1
43395	0.949103	2.1
43396	0.949103	2.1
43397	0.946133	2.1
43398	0.946133	2.1
43399	0.946131	2.1
43400	0.969492	2.1
43401	0.946131	2.1
43402	0.949105	2.1
43403	1.89226	2.1
43404	2.07428	2.1
43405	1.93464	2.1
43406	1.89226	2.1
43407	2.11667	2.1
43408	2.07428	2.1
43409	0.946132	2.1
43410	0.946132	2.1
43411	0.946131	2.1
43412	0.946131	2.1
43413	0.946132	2.1
43414	0.946132	2.1
43415	0.946132	2.1
43416	0.946132	2.1
43417	0.946132	2.1
43418	0.946132	2.1
43419	0.946132	2.1
43420	0.946132	2.1
43421	1.97992	2.1
43422	1.89226	2.1
43423	1.89226	2.1
43424	1.93898	2.1
43425	1.93898	2.1
43426	1.89226	2.1
43427	2.12475	0.781568
43428	2.79029	0.826197
43429	2.7803	0.947328
43430	2.13052	0.731478
43431	4.92081	0.76531
43432	1.06238	0.853166
43433	1.06526	0.774347
43434	0.642901	0.694302
43435	0.655055	0.773696
43436	0.856391	0.709657
43437	0.856391	0.688364
43438	0.86104	0.916536
43439	0.86104	0.685772

43440	1.06526	0.78311
43441	0.655055	0.742472
43442	0.642901	0.723075
43443	1.06238	0.715955
43444	2.7803	0.888963
43445	2.13053	0.621745
43446	2.14346	0.810472
43447	2.77937	0.82145
43448	4.9099	0.821562
43449	2.14346	0.835169
43450	1.31011	0.609518
43451	1.34124	0.776468
43452	1.31011	0.824705
43453	2.16166	0.71141
43454	2.13053	0.725069
43455	1.89226	2.1
43456	2.58176	2.1
43457	2.01422	2.1
43458	1.89226	2.1
43459	2.70373	2.1
43460	2.58176	2.1
43461	1.89226	2.1
43462	1.89226	2.1
43463	1.89226	2.1
43464	1.89226	2.1
43465	1.89226	2.1
43466	1.89226	2.1
43467	0.946128	2.1
43468	0.946128	2.1
43469	1.38521	2.1
43470	1.38521	2.1
43471	1.38521	2.1
43472	1.35186	2.1
43473	0.946131	2.1
43474	0.946131	2.1
43475	0.94613	2.1
43476	1.38521	2.1
43477	0.946131	2.1
43478	1.35186	2.1
43479	0.946133	2.1
43480	0.946133	2.1
43481	0.946132	2.1
43482	0.946132	2.1
43483	0.946132	2.1
43484	0.946132	2.1
43485	0.946132	2.1
43486	0.946132	2.1
43487	0.946132	2.1
43488	0.946132	2.1
43489	0.946133	2.1
43490	0.946133	2.1
43491	2.33374	2.1
43492	1.89226	2.1
43493	1.89226	2.1
43494	1.89226	2.1
43495	2.60377	2.1
43496	2.60377	2.1
43497	2.6771	0.27242

43498	1.36416	0.310809
43499	1.64983	0.318248
43500	1.64983	0.262163
43501	2.44131	0.317953
43502	2.44131	0.283913
43503	2.58321	0.729428
43504	1.89226	0.721437
43505	2.44283	0.642553
43506	2.44283	0.703563
43507	1.89226	0.776501
43508	1.89226	0.821369
43509	2.58321	0.788486
43510	2.44283	0.692657
43511	1.49358	0.604905
43512	1.57702	0.69385
43513	3.93641	0.650919
43514	0.951913	0.284069
43515	0.81556	0.265828
43516	0.946132	0.332602
43517	0.946132	0.316331
43518	1.04072	0.330336
43519	1.12182	0.328095
43520	0.856745	0.270783
43521	0.851542	0.27748
43522	0.923163	0.289366
43523	0.923163	0.34936
43524	1.04304	0.315343
43525	0.85098	0.256604
43526	2.70125	1.25275
43527	2.37734	1.25223
43528	2.63495	1.01708
43529	2.30539	1.98526
43530	5.01229	1.68682
43531	1.89226	1.27429
43532	1.89226	1.56205
43533	1.89226	1.15986
43534	1.89226	1.60096
43535	3.78452	1.0328
43536	1.89226	1.71699
43537	1.89226	2.1
43538	1.89226	1.54549
43539	1.89226	2.1
43540	3.78452	2.1
43541	2.37734	1.30979
43542	1.79346	2.1
43543	2.53594	1.72929
43544	1.70302	1.91852
43545	4.27682	1.84303
43546	2.63495	0.959334
43547	2.30539	1.47015
43548	2.49445	1.03087
43549	2.12089	1.03081
43550	4.75584	1.0015
43551	1.89226	1.0693
43552	1.89226	1.32765
43553	1.89226	0.949198
43554	1.89226	1.49289
43555	3.78452	1.68316

43556	1.89226	1.30729
43557	1.89226	1.71468
43558	1.89226	1.60205
43559	1.89226	1.66541
43560	1.89226	1.19214
43561	1.89226	1.22059
43562	2.53594	1.23632
43563	1.60017	1.26318
43564	1.57079	1.37105
43565	1.70302	1.09071
43566	2.15772	1.30913
43567	2.33299	1.04157
43568	2.09114	2.1
43569	2.34898	2.1
43570	2.30729	2.1
43571	2.06556	2.1
43572	4.41454	2.1
43573	2.09114	2.1
43574	1.59654	2.1
43575	1.57823	2.1
43576	2.06556	2.1
43577	3.6621	2.1
43578	2.30729	2.1
43579	2.06556	2.1
43580	2.03917	2.1
43581	2.26617	2.1
43582	4.33173	2.1
43583	2.03917	2.1
43584	2.06556	2.1
43585	2.045 2.1	
43586	1.55767	2.1
43587	1.57823	2.1
43588	1.57823	2.1
43589	1.89225	1.7479
43590	1.09726	2.1
43591	1.61196	2.1
43592	1.28597	2.1
43593	1.28597	0.147815
43594	1.45633	2.1
43595	1.45633	2.1
43596	1.9544	0.147815
43597	1.61196	2.1
43598	1.45633	2.1
43599	1.53735	2.1
43600	3.14931	2.1
43601	1.89226	2.1
43602	1.89226	2.1
43603	1.89226	2.1
43604	1.89226	2.1
43605	3.78453	2.1
43606	0.810946	2.1
43607	0.810946	2.1
43608	0.810947	2.1
43609	0.810947	2.1
43610	0.810933	2.1
43611	0.810933	2.1
43612	1.89226	2.1
43613	1.3517	2.1

43614	1.08132	2.1
43615	1.08132	2.1
43616	1.08133	2.1
43617	0.851539	2.00875
43618	0.851539	2.1
43619	0.972053	2.1
43620	1.05632	2.1
43621	0.94613	2.1
43622	0.94613	1.7058
43623	1.0279	2.1
43624	0.94991	2.1
43625	1.0279	2.1
43626	0.946131	2.1
43627	0.865643	2.1
43628	0.94613	2.1
43629	1.89226	1.94415
43630	1.89226	2.1
43631	1.89226	2.1
43632	1.89226	1.91031
43633	3.78452	2.1
43634	1.03882	2.1
43635	1.91121	2.1
43636	1.41833	2.1
43637	2.34547	2.1
43638	3.32954	2.1
43639	2.3979	1.95189
43640	1.89226	2.1
43641	1.89226	2.1
43642	1.89226	2.05956
43643	2.0558	2.1
43644	2.0558	1.5541
43645	1.89226	2.1
43646	1.89226	1.25541
43647	1.89226	1.82142
43648	1.89226	2.1
43649	3.78452	1.98314
43650	1.05816	2.1
43651	1.05816	2.1
43652	0.842045	2.1
43653	1.05252	2.1
43654	1.36057	2.1
43655	1.14339	2.1
43656	0.873397	2.1
43657	0.585073	2.1
43658	0.873388	2.1
43659	1.15129	2.1
43660	0.69143	2.1
43661	1.1434	2.1
43662	1.93338	2.1
43663	1.85883	2.1
43664	1.51714	2.1
43665	1.85883	2.1
43666	1.54795	2.1
43667	1.89226	2.1
43668	1.89226	2.1
43669	1.89226	2.1
43670	1.89226	2.1
43671	1.89226	2.1

43672	3.78452	2.1
43673	0.844726	2.1
43674	0.844726	2.1
43675	0.960524	2.1
43676	1.16288	2.1
43677	0.851541	2.1
43678	0.851541	2.1
43679	0.929147	2.1
43680	1.15533	2.1
43681	0.844697	2.1
43682	0.946131	2.1
43683	0.851632	2.1
43684	0.946131	2.1
43685	1.89226	2.1
43686	1.70303	2.1
43687	1.74006	2.1
43688	1.70303	2.1
43689	1.99471	2.1
43690	1.93338	2.1
43691	0.946131	2.1
43692	0.946131	2.1
43693	0.946131	2.1
43694	0.946131	2.1
43695	0.94613	2.1
43696	0.94613	2.1
43697	0.946132	2.1
43698	0.946132	2.1
43699	0.946131	2.1
43700	0.946131	2.1
43701	0.946131	2.1
43702	0.946131	2.1
43703	1.58158	2.1
43704	1.89226	2.1
43705	1.89226	2.1
43706	1.89226	2.1
43707	1.68939	2.1
43708	1.68939	2.1
43709	0.946132	0.201903
43710	0.946132	0.220521
43711	1.12988	0.202313
43712	1.30783	0.187129
43713	1.30783	0.204296
43714	0.946133	0.204967
43715	0.946132	0.202699
43716	1.12988	0.201527
43717	1.30783	0.201873
43718	1.30783	0.187844
43719	0.946133	0.203135
43720	0.946133	0.219581
43721	1.89227	0.218133
43722	1.89227	0.220119
43723	1.89227	0.208715
43724	1.89227	0.24462
43725	1.89227	0.203781
43726	1.89227	0.240185
43727	1.89226	0.204737
43728	1.89226	0.204784
43729	1.89226	0.197786

43730	1.89226	0.223319
43731	1.89226	0.190882
43732	1.89226	0.215864
43733	0.946131	0.205478
43734	0.946131	0.187966
43735	1.30783	0.18522
43736	1.37715	0.173244
43737	1.37715	0.187872
43738	0.946133	0.189635
43739	1.30783	0.185681
43740	0.946129	0.188734
43741	1.37715	0.187157
43742	1.37715	0.172873
43743	0.946131	0.187106
43744	0.946131	0.205615
43745	1.17056	0.214317
43746	0.591757	0.241198
43747	0.522025	0.214627
43748	0.929727	0.197285
43749	0.929728	0.19685
43750	1.17056	0.208695
43751	0.591756	0.210854
43752	0.522025	0.210439
43753	0.889054	0.210999
43754	0.889054	0.205014
43755	0.803535	0.228472
43756	0.803535	0.215881
43757	1.49814	2.1
43758	2.37439	2.1
43759	2.63923	2.1
43760	2.63923	2.1
43761	1.29111	2.1
43762	1.29111	2.1
43763	0.946132	2.1
43764	0.946132	2.1
43765	1.18719	2.1
43766	1.18719	2.1
43767	0.94613	2.1
43768	0.94613	1.96568
43769	1.1872	2.1
43770	1.31961	2.1
43771	0.946131	2.1
43772	1.31961	2.1
43773	1.1872	2.1
43774	0.94613	2.1
43775	2.49445	0.79221
43776	2.12089	0.904449
43777	2.20843	0.762507
43778	1.82805	0.955534
43779	4.32932	0.854037
43780	1.89226	0.889586
43781	1.89226	1.25808
43782	1.89226	0.829886
43783	1.89226	0.871732
43784	3.78452	0.816959
43785	0.94613	0.936015
43786	0.94613	0.892151
43787	0.946128	0.88997

43788	0.946128	1.08716
43789	0.946131	1.03625
43790	0.946131	1.07703
43791	0.946127	1.04116
43792	0.946127	0.866868
43793	0.946129	0.936861
43794	0.946129	0.911417
43795	0.946129	0.993974
43796	0.946129	0.992345
43797	1.82804	0.901454
43798	1.60017	0.998963
43799	1.87679	1.03853
43800	2.12088	0.847715
43801	1.35608	1.06027
43802	1.60017	0.906361
43803	2.20843	0.717879
43804	1.3216	0.725402
43805	1.44426	0.716315
43806	1.82805	0.743959
43807	1.80399	0.70318
43808	2.20843	0.733782
43809	1.89226	0.755781
43810	1.89226	0.747174
43811	1.89226	0.83175
43812	1.89226	0.756469
43813	1.89226	0.79147
43814	1.89226	0.748253
43815	1.89226	0.875124
43816	1.89226	0.774432
43817	1.89226	0.768479
43818	1.89226	0.781997
43819	1.89226	0.834364
43820	1.89226	0.744291
43821	0.627705	0.712376
43822	0.634941	0.8074
43823	0.629317	0.716999
43824	0.783422	0.723963
43825	0.629328	0.734072
43826	0.448764	0.733036
43827	1.35608	0.780237
43828	1.00805	0.781797
43829	0.814295	0.721724
43830	0.598375	0.807281
43831	1.04461	0.764992
43832	1.09795	1.63357
43833	1.00775	1.6247
43834	1.61202	1.89067
43835	1.63419	1.44523
43836	2.64194	1.43589
43837	1.44801	1.72331
43838	1.61201	1.58604
43839	2.35614	1.72675
43840	2.27703	1.65055
43841	3.88904	1.75119
43842	2.35615	1.61796
43843	1.63419	1.25499
43844	2.29852	1.4186
43845	1.63419	1.3213

43846	2.27634	1.45267
43847	1.61202	1.49998
43848	0.973793	2.1
43849	2.2671	2.1
43850	2.32981	2.1
43851	2.32981	2.1
43852	0.990481	2.1
43853	0.990481	2.1
43854	1.50877	2.1
43855	1.11193	2.1
43856	2.29667	2.1
43857	1.94158	2.1
43858	3.45035	2.1
43859	2.29667	2.1
43860	1.89227	2.1
43861	1.94158	2.1
43862	1.94158	2.1
43863	1.89227	2.1
43864	1.89227	2.1
43865	2.51379	2.1
43866	1.30026	2.1
43867	1.84502	2.1
43868	2.72099	2.1
43869	4.35882	2.1
43870	1.89226	2.1
43871	2.721	2.1
43872	2.5138	2.1
43873	2.5138	2.1
43874	1.89226	2.1
43875	1.89226	2.1
43876	1.89226	0.414841
43877	1.89226	0.454471
43878	2.59101	0.374704
43879	1.89226	0.517791
43880	3.78453	0.39617
43881	0.851541	0.436158
43882	0.851541	0.381316
43883	1.42502	0.39873
43884	0.948679	0.340207
43885	0.946132	0.432451
43886	0.946132	0.456354
43887	0.853617	0.391594
43888	0.853618	0.331143
43889	0.851475	0.426672
43890	0.948666	0.449739
43891	1.166	0.418422
43892	0.851554	0.433903
43893	2.51818	0.954881
43894	2.60723	2.0442
43895	2.66532	0.896814
43896	2.76315	2.04645
43897	5.27255	1.121
43898	2.60723	1.75889
43899	2.45247	2.1
43900	2.76315	2.01523
43901	2.55197	2.1
43902	5.1592	2.1
43903	1.23768	0.735477

43904	1.11433	0.818189
43905	1.32037	1.03372
43906	1.32037	0.769942
43907	1.0142	0.860626
43908	1.0142	0.743328
43909	0.886711	0.863381
43910	1.16806	0.830292
43911	0.999083	1.37004
43912	0.710926	1.08913
43913	0.937757	1.19753
43914	0.937757	0.951404
43915	0.941224	0.782366
43916	0.941224	1.28702
43917	1.16805	1.00426
43918	0.999084	1.32049
43919	0.886709	1.14534
43920	0.710928	1.06422
43921	2.29332	1.54709
43922	1.54904	2.1
43923	2.0815	2.1
43924	2.44199	1.82335
43925	4.00418	2.1
43926	1.53873	0.350011
43927	1.53422	0.854965
43928	1.39661	0.455189
43929	1.42387	0.827916
43930	2.9626	0.746323
43931	1.53423	0.858025
43932	1.44091	1.25839
43933	1.42387	0.835894
43934	1.35215	2.02084
43935	2.86479	0.927115
43936	1.39661	0.452003
43937	1.42387	0.82584
43938	1.28325	0.389791
43939	1.33326	0.872726
43940	2.70713	0.58952
43941	1.42387	0.828024
43942	1.35215	2.0178
43943	1.33327	0.886432
43944	1.27339	1.39043
43945	2.69726	1.05513
43946	2.58442	2.1
43947	1.1468	2.1
43948	1.18775	2.1
43949	2.60047	2.1
43950	3.74728	2.1
43951	2.58442	2.1
43952	1.89226	2.1
43953	1.89226	2.1
43954	2.60048	2.1
43955	4.49274	2.1
43956	1.89226	2.1
43957	1.89226	1.97035
43958	1.89226	2.1
43959	1.89226	2.1
43960	3.78453	2.1
43961	1.89226	2.1

43962	1.89226	1.91742
43963	1.89226	2.1
43964	1.89226	1.04169
43965	3.78452	2.1
43966	1.22262	2.1
43967	2.60047	2.1
43968	1.18775	2.1
43969	1.18775	2.1
43970	2.61433	2.1
43971	2.61433	2.1
43972	1.89226	2.1
43973	2.60048	2.1
43974	2.61434	2.1
43975	2.60048	2.1
43976	1.90612	2.1
43977	1.89226	2.1
43978	1.89226	2.1
43979	1.89226	2.02199
43980	1.89226	2.1
43981	1.89226	1.84612
43982	3.78453	2.1
43983	1.89226	2.1
43984	1.89226	1.97835
43985	1.89226	2.1
43986	1.89226	1.77467
43987	3.78452	2.1
43988	2.12556	2.1
43989	0.991113	2.1
43990	1.27725	2.1
43991	2.31081	2.1
43992	3.40281	2.1
43993	1.89226	2.1
43994	2.31082	2.1
43995	2.12556	2.1
43996	2.12556	2.1
43997	1.89226	2.1
43998	1.89226	2.1
43999	1.22544	2.1
44000	1.12649	2.1
44001	0.775254	2.1
44002	0.643133	2.1
44003	0.917924	2.1
44004	0.934955	2.1
44005	0.975579	2.1
44006	0.975579	2.1
44007	1.23113	2.1
44008	0.685272	2.1
44009	0.651719	2.1
44010	1.1791	2.1
44011	1.89226	2.1
44012	2.5138	2.1
44013	2.31082	2.1
44014	2.31082	2.1
44015	1.89226	2.1
44016	1.89226	2.1
44017	1.89226	2.1
44018	1.89226	2.1
44019	1.89226	2.1

44020	1.89226	2.1
44021	3.78453	2.1
44022	0.922224	2.1
44023	0.922224	2.1
44024	1.23555	2.1
44025	1.19715	2.1
44026	1.01445	2.1
44027	1.16061	2.1
44028	0.946132	2.1
44029	0.946132	2.1
44030	0.946131	2.1
44031	1.2051	2.1
44032	1.13537	2.1
44033	1.01445	2.1
44034	0.946133	2.1
44035	0.946133	2.1
44036	1.20648	2.1
44037	1.20648	2.1
44038	1.06454	2.1
44039	1.20648	2.1
44040	0.946133	2.1
44041	0.946133	2.1
44042	1.20649	2.1
44043	0.946133	2.1
44044	1.06454	2.1
44045	0.946132	2.1
44046	0.946132	2.1
44047	0.946132	2.1
44048	0.946132	2.1
44049	0.946132	2.1
44050	0.946132	2.1
44051	0.946132	2.1
44052	0.946133	2.1
44053	0.946133	2.1
44054	0.946133	2.1
44055	0.946133	2.1
44056	0.946132	2.1
44057	0.946132	2.1
44058	1.33078	1.73538
44059	1.37409	1.99313
44060	1.31565	0.968663
44061	1.37114	1.85028
44062	2.68973	1.66028
44063	1.89225	1.67558
44064	1.89225	1.9844
44065	1.89225	0.977706
44066	1.89225	1.3506
44067	3.7845	1.2452
44068	1.89225	1.2936
44069	1.89225	2.1
44070	1.89225	1.06876
44071	1.89225	2.1
44072	3.7845	1.63195
44073	1.37409	1.36939
44074	1.20408	2.1
44075	1.18612	2.07763
44076	1.18907	2.1
44077	1.37114	1.20182

44078	1.37409	2.1
44079	1.31566	1.80458
44080	1.37115	2.1
44081	1.30318	0.985321
44082	1.37433	1.39846
44083	2.68998	1.49041
44084	1.89226	1.8456
44085	1.89226	1.67463
44086	1.89226	0.958051
44087	1.89226	1.41861
44088	3.78453	1.31514
44089	1.89226	1.23087
44090	1.89226	2.1
44091	1.89226	2.05761
44092	1.89226	2.1
44093	1.89226	1.09532
44094	1.89226	2.1
44095	0.647001	2.1
44096	0.647001	1.82616
44097	0.651851	1.03934
44098	0.651851	2.1
44099	0.602046	2.1
44100	0.613277	2.1
44101	0.685576	1.24182
44102	0.687164	1.32242
44103	0.687164	1.93949
44104	0.613276	1.85096
44105	0.602046	2.00224
44106	0.685576	2.00277
44107	0.946128	0.922254
44108	0.946128	0.746948
44109	1.15307	1.25349
44110	1.19317	1.0545
44111	0.94613	1.09699
44112	0.94613	0.493626
44113	1.15307	0.998285
44114	1.15307	1.10995
44115	0.946131	0.980417
44116	1.15307	0.986516
44117	0.946131	0.922419
44118	1.19317	0.905883
44119	1.18278	2.1
44120	2.38635	1.0104
44121	1.23042	1.95432
44122	1.23042	1.21973
44123	2.30614	1.93795
44124	2.30614	1.29922
44125	0.946129	0.853845
44126	0.946129	0.812389
44127	1.12994	1.02592
44128	1.15307	0.955011
44129	0.946131	0.989793
44130	0.946131	0.532006
44131	1.12994	0.875964
44132	1.12994	0.939804
44133	1.12994	0.935986
44134	0.946131	0.945397
44135	0.946131	0.859557

44136	1.15307	0.863517
44137	2.25988	1.14059
44138	2.30614	0.897224
44139	2.28696	1.5104
44140	1.16361	1.80225
44141	1.18278	1.67827
44142	1.18278	1.06337
44143	2.3771	2.1
44144	1.90121	2.1
44145	1.81191	2.1
44146	2.46904	2.1
44147	4.37025	2.1
44148	1.15676	2.1
44149	1.23452	2.1
44150	0.823592	2.1
44151	0.737606	2.1
44152	0.988113	2.1
44153	0.988113	2.1
44154	0.984014	2.1
44155	0.984014	2.1
44156	1.15676	2.1
44157	0.737605	2.1
44158	0.823592	2.1
44159	1.23452	2.1
44160	1.30318	1.94065
44161	1.37433	2.1
44162	1.30151	1.14064
44163	1.39067	1.72251
44164	2.67584	1.52955
44165	1.89226	1.59195
44166	1.89226	2.1
44167	1.89226	1.12905
44168	1.89226	1.25542
44169	3.78453	1.23221
44170	1.89226	1.01896
44171	1.89226	2.1
44172	1.89226	0.981619
44173	1.89226	2.04268
44174	1.89226	1.13404
44175	1.89226	2.1
44176	0.656819	2.1
44177	0.656819	1.06954
44178	0.661755	1.13059
44179	0.661755	2.1
44180	0.613277	2.1
44181	0.63141	2.1
44182	0.687164	1.13198
44183	0.695336	1.47057
44184	0.695337	1.80707
44185	0.63141	1.72562
44186	0.687164	2.06133
44187	0.613276	2.016
44188	1.30151	1.78186
44189	1.39067	2.1
44190	1.32143	1.16623
44191	1.43294	1.65147
44192	2.73445	1.69502
44193	1.89226	1.75692

44194	1.89226	1.68814
44195	1.89226	0.994098
44196	1.89226	1.71685
44197	3.78452	1.42131
44198	1.89226	1.27824
44199	1.89226	1.23575
44200	1.89226	1.11402
44201	1.89226	2.1
44202	1.89226	2.1
44203	1.89226	2.1
44204	1.43294	1.25115
44205	1.26282	2.1
44206	1.3268	1.08999
44207	1.3268	2.1
44208	1.39067	1.23154
44209	1.39067	2.1
44210	1.5511	0.332865
44211	1.00665	0.35068
44212	1.28239	0.346662
44213	1.88631	0.361529
44214	2.89296	0.335232
44215	1.89227	0.36723
44216	1.89227	0.369476
44217	1.89227	0.417742
44218	1.89227	0.412764
44219	1.89227	0.34103
44220	1.89227	0.336763
44221	2.08848	0.343118
44222	1.89226	0.35217
44223	1.89226	0.355971
44224	2.37947	0.400243
44225	4.27173	0.359982
44226	1.01456	0.352044
44227	0.943157	0.352284
44228	0.641198	0.349098
44229	0.724757	0.350027
44230	0.830923	0.364875
44231	0.830923	0.349845
44232	0.836991	0.365192
44233	0.836991	0.317837
44234	1.01456	0.367399
44235	0.724757	0.404905
44236	0.641197	0.352342
44237	0.943157	0.360334
44238	1.89227	0.35434
44239	1.89227	0.39059
44240	1.89227	0.385485
44241	1.89227	0.414513
44242	3.78453	0.382748
44243	0.946129	0.361407
44244	0.946129	0.363976
44245	1.28941	0.380969
44246	1.18974	0.371169
44247	0.94613	0.389887
44248	0.94613	0.366925
44249	1.28941	0.354292
44250	1.28941	0.339801
44251	1.28941	0.355207

44252	0.946131	0.381238
44253	0.94613	0.305267
44254	1.18974	0.360512
44255	0.710636	2.1
44256	0.780249	2.1
44257	0.579033	2.1
44258	0.478812	2.1
44259	0.621822	2.1
44260	0.621822	2.1
44261	0.63724	2.1
44262	0.63724	2.1
44263	0.780251	2.1
44264	0.579032	2.1
44265	0.710637	2.1
44266	0.478811	2.1
44267	1.05499	1.74515
44268	1.00521	1.77804
44269	1.24445	1.43813
44270	1.23082	1.02154
44271	2.28581	1.68545
44272	1.48751	1.64631
44273	1.23083	1.3974
44274	1.4941	1.69294
44275	1.24445	1.28429
44276	1.48047	1.1851
44277	1.23083	1.14407
44278	0.621185	1.30119
44279	0.621185	1.51509
44280	0.620899	1.08294
44281	0.620899	1.91725
44282	0.502515	1.29721
44283	0.626672	1.18242
44284	0.615412	1.0755
44285	0.740234	1.61845
44286	0.740233	1.02287
44287	0.626672	0.974075
44288	0.615413	1.20547
44289	0.502516	1.31461
44290	1.95844	2.1
44291	2.6562	2.1
44292	1.95844	2.1
44293	2.20883	2.1
44294	2.38225	2.1
44295	2.63264	2.1
44296	1.89226	2.1
44297	1.89226	2.1
44298	1.89226	2.1
44299	1.89226	2.1
44300	3.78453	2.1
44301	1.95844	2.1
44302	2.38225	2.1
44303	1.71082	2.1
44304	2.14647	2.1
44305	4.1049	2.1
44306	0.946131	2.1
44307	0.946131	2.1
44308	0.946131	2.1
44309	0.946131	2.1

44310	0.946131	2.1
44311	0.946131	2.1
44312	0.946131	2.1
44313	0.946131	2.1
44314	0.946131	2.1
44315	0.946131	2.1
44316	0.946131	2.1
44317	0.946131	2.1
44318	2.12597	2.1
44319	1.2295	2.1
44320	1.28014	2.1
44321	2.19723	2.1
44322	3.40611	2.1
44323	2.12597	2.1
44324	2.79242	2.1
44325	2.73375	2.1
44326	2.66249	2.1
44327	2.19723	2.1
44328	2.12597	2.1
44329	1.13716	2.1
44330	1.09861	2.1
44331	0.666692	2.1
44332	0.640071	2.1
44333	0.891617	2.1
44334	0.891617	2.1
44335	0.885614	2.1
44336	0.885614	2.1
44337	1.13716	2.1
44338	0.666692	2.1
44339	0.640071	2.1
44340	1.09861	2.1
44341	1.89226	0.28675
44342	1.70303	0.265308
44343	1.89226	0.234334
44344	2.64598	0.287365
44345	1.81091	0.223145
44346	2.53222	0.287438
44347	2.32422	0.251606
44348	1.89226	0.307617
44349	2.78336	0.302763
44350	2.78336	0.295967
44351	1.89226	0.249247
44352	1.89226	0.251298
44353	0.946132	0.265406
44354	0.946132	0.242175
44355	0.946131	0.265689
44356	0.946131	0.317795
44357	0.946132	0.326618
44358	0.946132	0.296299
44359	0.946131	0.252051
44360	0.946131	0.284187
44361	0.946131	0.278294
44362	0.946131	0.288552
44363	0.946131	0.283538
44364	0.946131	0.263935
44365	0.946131	0.299389
44366	0.946131	0.355846
44367	0.946132	0.324749

44368	0.946132	0.399219
44369	0.946132	0.401757
44370	0.946132	0.371171
44371	0.946131	0.312849
44372	0.946131	0.291379
44373	0.946131	0.332877
44374	0.946131	0.365465
44375	0.946131	0.351207
44376	0.946131	0.315179
44377	2.24323	1.28892
44378	1.89226	1.63697
44379	2.75021	1.39307
44380	2.75021	1.53435
44381	1.89226	1.17188
44382	1.89226	1.28325
44383	2.30908	0.350698
44384	2.19815	0.801266
44385	2.1609	0.376141
44386	1.83796	0.856903
44387	4.35904	0.462102
44388	2.19815	0.772127
44389	2.02268	1.53707
44390	2.17869	1.47971
44391	1.9559	0.825958
44392	2.0815	1.42078
44393	1.83424	0.825701
44394	2.1609	0.412228
44395	1.88877	0.846437
44396	1.98413	0.387644
44397	1.87355	0.817281
44398	4.03445	0.736085
44399	1.87355	0.822325
44400	2.02268	1.64725
44401	1.87355	1.49437
44402	2.10488	0.843125
44403	1.68443	1.38699
44404	1.89448	0.892298
44405	0.946131	0.40851
44406	0.946131	0.410001
44407	1.40574	0.472018
44408	1.12373	0.488982
44409	0.946129	0.447714
44410	0.946129	0.395922
44411	1.40574	0.474308
44412	1.40574	0.451485
44413	0.946131	0.416578
44414	1.40574	0.462206
44415	1.12373	0.486985
44416	0.946131	0.412406
44417	2.14277	0.574641
44418	2.81148	0.591943
44419	1.42762	0.547462
44420	1.42762	0.537368
44421	2.96261	0.581395
44422	2.24746	0.565503
44423	1.89226	0.702353
44424	1.89226	0.726125
44425	1.89226	0.7

44426	1.89226	0.790371
44427	3.78453	0.717932
44428	1.89226	0.7
44429	1.89226	0.730462
44430	1.89226	0.704221
44431	1.89226	0.796204
44432	3.78452	0.700029
44433	0.68433	0.703483
44434	0.946127	0.799084
44435	0.543921	0.758864
44436	0.946131	0.743124
44437	0.946131	0.808267
44438	0.946131	1.4681
44439	0.543918	0.764304
44440	0.543918	0.831065
44441	0.684332	0.796353
44442	0.54392	1.39296
44443	0.946129	0.700565
44444	0.946129	0.82456
44445	1.89226	0.732576
44446	1.08784	0.803285
44447	1.19352	0.723117
44448	1.89226	0.831922
44449	1.19352	1.39553
44450	1.89226	1.2701
44451	0.635711	0.700192
44452	0.946132	0.774685
44453	0.946132	0.737954
44454	0.56474	0.725237
44455	0.750045	0.790115
44456	1.25657	1.3884
44457	0.635682	0.766515
44458	0.635682	0.7
44459	0.891954	0.743397
44460	1.32751	1.2461
44461	0.564766	0.7
44462	0.564766	0.774101
44463	0.858345	0.733305
44464	0.946128	0.808084
44465	0.635712	0.772435
44466	0.946133	0.73595
44467	0.977198	0.797464
44468	0.944521	1.3846
44469	0.635682	0.819859
44470	0.635682	0.7
44471	1.02924	0.782376
44472	0.705625	1.39689
44473	0.851528	0.7
44474	0.851528	0.966777
44475	2.18903	2.1
44476	2.10182	2.1
44477	2.10182	2.1
44478	1.89226	2.1
44479	1.89226	2.1
44480	1.64983	2.1
44481	1.89226	2.1
44482	1.83867	2.1
44483	1.83867	2.1

44484	1.89164	2.1
44485	2.0815	2.1
44486	2.17638	2.1
44487	2.57957	1.11347
44488	2.3506	1.42984
44489	2.69056	0.874419
44490	2.49439	1.3171
44491	5.04116	0.977323
44492	2.3506	1.06279
44493	1.8911	1.64078
44494	2.49439	1.06565
44495	2.06689	1.98987
44496	4.41748	1.50402
44497	2.69056	1.09676
44498	2.49439	1.46782
44499	2.77227	0.896227
44500	2.63443	1.2145
44501	5.32499	1.46457
44502	2.49439	1.1748
44503	2.06689	1.951
44504	2.63443	0.895902
44505	2.26444	1.86127
44506	4.70131	1.36883
44507	2.46327	2.1
44508	2.54347	2.1
44509	2.52869	2.1
44510	2.16534	2.1
44511	2.82661	2.1
44512	2.46327	2.1
44513	0.946131	2.1
44514	0.946131	2.1
44515	1.27174	2.1
44516	1.27174	2.1
44517	1.27174	2.1
44518	0.94613	2.1
44519	0.946128	2.1
44520	1.4133	2.1
44521	0.946129	2.1
44522	0.946129	2.1
44523	1.27174	2.1
44524	1.4133	2.1
44525	1.42757	2.1
44526	1.7457	2.1
44527	1.66047	2.1
44528	1.7457	2.1
44529	1.45754	2.1
44530	1.54278	2.1
44531	1.42757	2.1
44532	1.52996	2.1
44533	1.66047	2.1
44534	1.66047	2.1
44535	1.26397	2.1
44536	1.26397	2.1
44537	0.968536	2.1
44538	1.02461	2.1
44539	1.57575	2.1
44540	1.62391	2.1
44541	2.59245	2.1

44542	0.946131	2.1
44543	0.946131	2.1
44544	0.946132	2.1
44545	0.946132	2.1
44546	0.946132	2.1
44547	0.946132	1.99193
44548	0.946132	2.1
44549	0.946132	2.1
44550	0.946132	2.1
44551	0.946132	2.1
44552	0.946132	2.1
44553	0.946132	2.1
44554	2.23765	2.1
44555	1.62391	2.1
44556	2.22154	2.1
44557	1.57575	2.1
44558	2.2697	2.1
44559	1.62391	2.1
44560	1.89226	2.06796
44561	1.89226	2.1
44562	1.89226	2.1
44563	1.89226	2.1
44564	1.89226	2.1
44565	1.89226	1.86118
44566	1.89226	2.04869
44567	1.89226	2.1
44568	1.89226	2.1
44569	1.89226	2.1
44570	1.89226	2.1
44571	1.89226	1.82214
44572	2.2697	2.1
44573	1.46129	2.1
44574	2.25873	2.1
44575	1.62391	2.1
44576	2.09611	2.1
44577	1.46129	2.1
44578	1.89226	2.1
44579	2.07088	2.1
44580	1.89226	2.1
44581	2.31884	2.1
44582	4.21111	2.1
44583	1.89226	2.1
44584	2.31884	2.1
44585	2.11713	2.1
44586	1.89226	2.1
44587	2.54371	2.1
44588	2.31884	2.1
44589	2.54371	2.1
44590	1.24041	2.1
44591	2.55208	2.1
44592	2.31884	2.1
44593	1.23712	2.1
44594	1.00388	2.1
44595	2.72769	0.364144
44596	2.57507	0.45434
44597	1.89226	0.33029
44598	1.89226	0.444315
44599	4.46733	0.374416

44600	1.89226	0.453064
44601	2.57506	0.454974
44602	1.89226	0.355417
44603	2.30076	0.416877
44604	1.89226	0.337264
44605	2.30076	0.462176
44606	1.21171	0.297157
44607	1.48589	0.359033
44608	2.09166	0.294961
44609	1.78381	0.347179
44610	3.2697	0.326125
44611	2.09166	0.292761
44612	1.70302	0.352469
44613	1.48589	0.343168
44614	1.18027	0.289312
44615	2.26571	0.341385
44616	1.79969	0.303269
44617	1.89226	0.345627
44618	1.89226	0.459411
44619	1.89226	0.472988
44620	1.89226	0.37564
44621	1.89226	0.468885
44622	1.89226	0.386793
44623	0.946131	0.363346
44624	0.946131	0.514117
44625	0.946128	0.475956
44626	0.946128	0.390985
44627	0.946129	0.475142
44628	0.946129	0.481324
44629	0.94613	0.360585
44630	0.94613	0.360937
44631	0.94613	0.413578
44632	0.94613	0.426351
44633	0.94613	0.424146
44634	0.94613	0.415076
44635	1.89226	1.58523
44636	1.89226	1.68192
44637	1.89226	1.85963
44638	1.89226	1.45419
44639	1.89226	1.80469
44640	1.89226	1.40426
44641	1.89226	1.56553
44642	1.89226	1.56823
44643	1.89226	1.74344
44644	1.89226	1.38999
44645	1.89226	1.70264
44646	1.89226	1.35721
44647	1.89226	1.42993
44648	1.89226	1.50074
44649	1.89226	1.73458
44650	1.89226	1.27704
44651	1.89226	1.70868
44652	1.89226	1.25432
44653	1.79103	0.919845
44654	0.968249	1.74679
44655	1.44807	1.27189
44656	2.17984	0.812361
44657	3.2391	1.46742

44658	1.29735	1.19436
44659	1.08992	1.20586
44660	0.945147	1.50587
44661	0.724033	1.82108
44662	1.00723	1.6317
44663	1.00723	1.29293
44664	1.01416	0.865874
44665	1.01416	1.43196
44666	1.29735	1.2728
44667	0.94515	1.42914
44668	0.724034	1.41811
44669	1.08992	1.53659
44670	1.39214	1.88059
44671	1.39214	2.1
44672	0.946129	2.07534
44673	0.946129	2.1
44674	1.33728	2.1
44675	1.39214	2.1
44676	0.94613	1.91643
44677	0.94613	1.97861
44678	0.94613	2.1
44679	1.33728	2.1
44680	0.94613	2.1
44681	1.39214	2.1
44682	2.78428	2.1
44683	2.28373	2.1
44684	2.67456	2.1
44685	2.20011	2.1
44686	4.95829	2.1
44687	1.90165	0.322541
44688	2.24206	0.392215
44689	1.66137	0.356621
44690	2.07108	0.667389
44691	3.90343	0.260144
44692	1.89224	0.489758
44693	1.89224	0.390469
44694	1.89224	0.359254
44695	1.89224	0.371942
44696	3.78448	0.400636
44697	2.82159	0.552829
44698	1.89226	0.454073
44699	2.50138	0.874597
44700	1.89226	0.402317
44701	4.39365	0.486072
44702	1.66139	0.27914
44703	1.86399	0.72904
44704	1.56005	0.412406
44705	1.90324	0.48426
44706	3.56463	0.441141
44707	0.946133	0.284077
44708	0.946133	0.4845
44709	0.946132	0.364804
44710	0.946132	0.44194
44711	0.946133	0.382104
44712	0.946133	0.453469
44713	0.946132	0.377014
44714	0.946132	0.443354
44715	0.946133	0.310444

44716	0.946133	0.368918
44717	0.946132	0.266431
44718	0.946132	0.37093
44719	1.5176	0.819727
44720	1.97964	0.762216
44721	2.32889	0.776808
44722	1.91762	0.790463
44723	3.84649	0.773692
44724	2.3376	0.764785
44725	1.97963	0.773201
44726	2.32889	0.792549
44727	2.58303	0.739256
44728	4.66649	0.784782
44729	2.32889	0.765311
44730	2.20248	0.757928
44731	1.91762	0.753746
44732	1.91762	0.756627
44733	2.5615	0.776474
44734	2.5615	0.783204
44735	2.32889	0.780573
44736	2.58303	0.735465
44737	2.71793	0.736339
44738	2.56149	0.741579
44739	5.04681	0.738391
44740	1.89226	0.697523
44741	2.53785	1.04151
44742	1.89226	0.710869
44743	2.60895	0.700726
44744	1.89226	0.682997
44745	2.60895	0.685257
44746	0.493248	0.754908
44747	1.30448	0.856069
44748	1.26893	0.922359
44749	0.653226	0.994909
44750	0.653227	0.770482
44751	0.493247	1.49927
44752	1.26893	1.23537
44753	1.30448	0.702057
44754	0.832232	1.18694
44755	0.832233	1.41636
44756	0.929944	0.7
44757	0.929944	0.71977
44758	1.89226	2.1
44759	1.47937	2.1
44760	1.50143	2.1
44761	1.47937	2.1
44762	1.91432	2.1
44763	1.89226	1.62973
44764	1.89226	1.87001
44765	1.89226	2.1
44766	1.89226	2.1
44767	1.89226	2.01576
44768	1.89226	2.1
44769	1.89226	1.25286
44770	1.33419	2.1
44771	2.4542	2.1
44772	2.59834	2.1
44773	2.59834	2.1

44774	1.25716	2.1
44775	1.25716	2.1
44776	1.70303	2.03053
44777	1.50143	2.1
44778	1.60878	2.1
44779	1.50143	2.0779
44780	1.93599	2.1
44781	1.89226	1.32615
44782	0.946131	1.19988
44783	0.946131	1.49008
44784	0.946127	2.1
44785	0.946127	2.1
44786	0.946127	2.1
44787	0.946127	1.05545
44788	0.946131	2.1
44789	0.946131	1.51435
44790	0.94613	1.83244
44791	0.94613	1.84293
44792	0.946129	1.91038
44793	0.946129	1.90957
44794	1.43912	2.1
44795	2.59834	2.1
44796	2.78093	2.1
44797	2.78093	2.1
44798	1.33419	2.1
44799	1.33419	2.1
44800	1.89226	2.1
44801	2.26322	1.79814
44802	2.55682	2.1
44803	2.55682	2.08365
44804	1.89226	2.1
44805	1.89226	2.1
44806	2.58032	0.71168
44807	2.28922	0.621858
44808	1.88424	0.655358
44809	2.32127	0.596857
44810	4.46456	0.645464
44811	1.67265	0.711159
44812	2.28922	0.648788
44813	1.88424	0.587085
44814	1.11314	0.571094
44815	3.55689	0.619379
44816	1.43751	0.535958
44817	2.32127	0.578349
44818	1.92998	0.477757
44819	2.37671	0.518305
44820	1.43751	0.668093
44821	1.88424	0.675681
44822	2.36729	2.1
44823	1.56714	2.1
44824	1.56714	2.1
44825	1.62408	2.1
44826	2.28373	2.1
44827	2.34067	2.1
44828	0.937268	2.1
44829	0.937268	2.1
44830	0.94636	2.1
44831	0.94636	2.1

44832	0.76004	2.1
44833	0.783572	2.1
44834	1.14187	2.1
44835	1.10006	2.1
44836	1.10006	2.1
44837	0.76004	2.1
44838	0.783572	2.1
44839	1.14187	2.1
44840	1.28753	2.1
44841	1.36838	2.1
44842	1.62617	2.1
44843	1.82857	2.1
44844	3.1161	2.1
44845	1.82856	2.1
44846	1.17996	2.1
44847	1.53417	2.1
44848	1.56618	2.1
44849	1.36838	2.1
44850	1.36838	2.1
44851	1.35065	1.02345
44852	1.42659	0.691914
44853	2.26675	0.870498
44854	2.26675	0.841588
44855	1.62137	0.832697
44856	2.53747	0.875282
44857	1.89226	0.380606
44858	1.89226	0.269375
44859	1.89226	0.296222
44860	1.89226	0.340482
44861	3.78452	0.340348
44862	1.36569	0.230083
44863	1.36569	0.210835
44864	0.946131	0.259602
44865	0.946131	0.292806
44866	1.16041	0.26077
44867	1.36569	0.22512
44868	0.946129	0.252886
44869	0.946129	0.296862
44870	1.1604	0.236224
44871	0.94613	0.252498
44872	0.946131	0.254319
44873	1.36569	0.246819
44874	2.73139	0.207482
44875	1.25265	0.247857
44876	1.25265	0.264436
44877	1.64799	0.231968
44878	2.32081	0.253157
44879	2.71615	0.20944
44880	0.946133	0.253659
44881	0.946133	0.268458
44882	0.946132	0.340637
44883	0.946132	0.305614
44884	0.946132	0.268378
44885	0.946132	0.308194
44886	0.946133	0.271137
44887	0.946133	0.322203
44888	0.946132	0.284872
44889	0.946132	0.297198

44890	0.946133	0.291546
44891	0.946133	0.284272
44892	0.946131	0.261091
44893	0.946131	0.287838
44894	0.94613	0.398367
44895	0.94613	0.32832
44896	0.946132	0.348521
44897	0.946132	0.285755
44898	0.946129	0.321004
44899	0.946129	0.394064
44900	0.946131	0.314611
44901	0.946131	0.290448
44902	0.946131	0.295937
44903	0.946131	0.316749
44904	1.89226	0.830128
44905	2.48503	1.78635
44906	2.64586	2.04287
44907	2.64586	0.821874
44908	1.89226	2.05089
44909	1.89226	0.81651
44910	2.48504	2.0484
44911	1.99985	2.1
44912	1.83584	2.1
44913	1.83584	2.1
44914	2.64586	2.09873
44915	2.64586	2.1
44916	1.89225	0.821941
44917	2.33095	1.70293
44918	2.48501	1.98956
44919	2.48501	0.802551
44920	1.89225	2.00092
44921	1.89225	0.798624
44922	2.33096	1.88736
44923	1.83583	2.1
44924	1.68436	2.1
44925	1.68436	2.1
44926	2.48502	2.0429
44927	2.48502	2.1
44928	1.14807	2.1
44929	1.76545	2.1
44930	1.21292	2.1
44931	1.83936	2.1
44932	2.97837	2.1
44933	1.89226	2.1
44934	1.89226	2.1
44935	1.89226	2.1
44936	1.89226	1.49273
44937	3.78451	2.1
44938	1.89226	2.1
44939	1.89226	1.13519
44940	1.89226	1.52903
44941	1.89226	1.32054
44942	3.78452	1.51348
44943	1.76545	2.1
44944	2.12608	2.1
44945	1.83937	2.1
44946	2.22529	1.31436
44947	3.99074	2.1

44948	0.780222	2.09826
44949	0.780222	2.1
44950	0.784871	2.1
44951	0.784871	2.1
44952	0.95863	2.1
44953	0.919681	2.1
44954	0.636145	2.1
44955	0.606461	2.1
44956	0.636144	2.1
44957	0.958631	2.1
44958	0.60646	2.1
44959	0.919683	2.1
44960	1.89226	2.1
44961	1.89226	1.59757
44962	1.89226	2.1
44963	1.89226	2.1
44964	3.78452	2.1
44965	1.89226	1.63767
44966	1.89226	1.49706
44967	1.89226	2.1
44968	1.89226	1.02801
44969	3.78452	1.45669
44970	1.03789	1.68044
44971	1.03789	2.1
44972	1.04231	2.1
44973	1.04231	2.1
44974	1.16052	2.1
44975	1.11265	1.12278
44976	0.958634	2.1
44977	0.919685	2.1
44978	0.958634	2.1
44979	1.16052	2.1
44980	0.919685	2.1
44981	1.11265	2.1
44982	1.43837	2.1
44983	2.57085	2.1
44984	1.39332	2.1
44985	2.58228	2.1
44986	3.96417	2.1
44987	1.89226	2.1
44988	1.89226	2.1
44989	1.89226	2.1
44990	1.89226	2.1
44991	1.89226	2.1
44992	1.89226	2.1
44993	1.89226	2.1
44994	1.89226	1.25757
44995	1.89226	2.07815
44996	1.89226	2.1
44997	3.78452	2.0744
44998	2.57085	2.1
44999	1.89226	2.1
45000	2.58229	2.1
45001	1.89226	2.1
45002	4.46312	2.1
45003	1.32368	2.1
45004	2.58228	2.1
45005	2.55352	2.1

45006	2.55352	2.1
45007	1.39332	2.1
45008	1.39332	2.1
45009	1.89226	2.1
45010	1.89226	2.1
45011	1.89226	2.1
45012	1.89226	2.1
45013	1.89226	2.1
45014	1.89226	2.1
45015	1.89226	2.1
45016	1.89226	2.1
45017	1.89226	2.1
45018	1.89226	2.08284
45019	1.89226	2.1
45020	1.89226	1.70296
45021	2.55353	2.1
45022	1.89226	2.1
45023	2.58228	2.1
45024	1.89226	2.1
45025	1.89226	2.1
45026	2.58228	2.1
45027	1.40574	1.31434
45028	1.05175	1.10258
45029	1.71613	1.28856
45030	1.99961	1.11806
45031	3.12187	1.21664
45032	1.71613	1.26362
45033	2.50733	1.09143
45034	1.99961	1.41646
45035	1.99961	1.21464
45036	2.25746	1.13914
45037	2.25746	1.05031
45038	2.25746	1.03361
45039	1.89526	1.07038
45040	1.71613	1.25792
45041	1.71613	0.940036
45042	1.89526	1.09799
45043	1.27558	0.943606
45044	1.07536	2.1
45045	1.07536	2.1
45046	1.07034	2.1
45047	1.07034	2.1
45048	0.766879	2.1
45049	0.81527	2.1
45050	1.38896	2.1
45051	1.33043	2.1
45052	0.815264	2.1
45053	1.38895	2.1
45054	0.766884	2.1
45055	1.33044	2.1
45056	1.89225	2.1
45057	1.89225	2.1
45058	1.89225	2.1
45059	1.89225	2.1
45060	1.89225	2.1
45061	1.89225	2.1
45062	1.64059	2.1
45063	1.89225	2.1

45064	1.565	2.1
45065	1.565	2.1
45066	1.89225	2.1
45067	1.89225	2.1
45068	1.89226	2.1
45069	1.89226	2.1
45070	1.89226	2.1
45071	1.89226	2.1
45072	1.89226	2.1
45073	1.89226	2.1
45074	1.67906	2.1
45075	1.89226	2.1
45076	1.6406	2.1
45077	1.6406	2.1
45078	1.89226	2.1
45079	1.89226	2.1
45080	2.18106	1.2861
45081	2.20831	1.65618
45082	2.64725	1.00116
45083	2.63102	1.19772
45084	4.82831	1.26392
45085	2.18106	1.20746
45086	2.09605	1.87501
45087	2.5601	1.23531
45088	2.63102	1.21774
45089	4.74116	1.48206
45090	2.64725	1.12023
45091	1.89226	1.18164
45092	1.87603	1.58917
45093	2.63102	1.13592
45094	1.89226	1.2853
45095	2.64725	0.946269
45096	0.946132	1.18125
45097	1.28005	1.08363
45098	0.946132	1.52267
45099	1.31551	1.01561
45100	1.28005	1.25129
45101	1.28005	1.47934
45102	0.946132	0.997311
45103	0.946132	1.20533
45104	0.946132	1.01509
45105	0.946132	1.1373
45106	1.28005	1.2408
45107	1.31551	1.30346
45108	2.0815	2.1
45109	1.59318	2.1
45110	2.0815	2.1
45111	1.40161	2.1
45112	2.49782	2.1
45113	1.68553	2.1
45114	0.819885	2.1
45115	0.935082	2.1
45116	0.867363	2.1
45117	0.867363	2.1
45118	0.715431	2.1
45119	0.902777	2.1
45120	0.738933	2.1
45121	0.980053	2.1

45122	0.936716	2.1
45123	0.917651	2.1
45124	0.775853	2.1
45125	0.74037	2.1
45126	2.78977	2.1
45127	2.38239	2.1
45128	2.78977	2.1
45129	1.96468	2.1
45130	2.64107	2.1
45131	1.80733	2.1
45132	1.80141	2.1
45133	2.28512	2.1
45134	2.28512	2.1
45135	1.8519	2.1
45136	2.64107	2.1
45137	2.20785	2.1
45138	0.946133	0.900802
45139	0.946133	0.809456
45140	0.946132	0.885453
45141	0.946132	0.839252
45142	0.946132	0.882244
45143	0.946132	0.888608
45144	0.946132	0.799162
45145	0.946132	0.826825
45146	0.946132	0.867852
45147	0.946132	0.863871
45148	0.946133	0.85198
45149	0.946133	0.877403
45150	1.89226	0.582828
45151	1.89226	0.637148
45152	1.89226	0.506756
45153	1.89226	0.609506
45154	1.89226	0.566523
45155	1.89226	0.679933
45156	1.89226	0.631662
45157	1.89226	0.726817
45158	1.89226	0.650848
45159	1.89226	0.768881
45160	3.78453	0.699655
45161	1.89226	0.872021
45162	1.89226	0.795792
45163	1.89226	0.707447
45164	1.89226	0.875793
45165	1.89226	0.782536
45166	1.89226	0.86784
45167	2.12925	0.271805
45168	2.51397	0.344861
45169	2.22488	0.266784
45170	1.86113	0.332274
45171	4.3751	0.320801
45172	2.82754	0.262845
45173	2.51397	0.339666
45174	2.22489	0.2571
45175	2.47291	0.341549
45176	4.98688	0.274033
45177	1.89226	0.346501
45178	1.89226	0.455592
45179	1.89226	0.357023

45180	1.89226	0.442051
45181	3.78453	0.425002
45182	1.89226	0.351663
45183	1.89226	0.441008
45184	1.89226	0.343868
45185	1.89226	0.450834
45186	3.78452	0.364231
45187	0.90848	0.310926
45188	1.11244	0.262914
45189	0.930566	0.271902
45190	0.73385	0.312467
45191	0.921298	0.308283
45192	0.921298	0.324974
45193	0.924994	0.259279
45194	0.924994	0.264464
45195	0.908478	0.285027
45196	0.733851	0.26811
45197	1.11244	0.273236
45198	0.930567	0.285157
45199	2.22489	0.257686
45200	2.0815	0.311602
45201	2.0815	0.320224
45202	2.30674	0.274638
45203	1.99867	0.305993
45204	2.33358	0.26132
45205	1.89226	0.337635
45206	1.89226	0.42951
45207	1.89226	0.346754
45208	1.89226	0.403489
45209	1.89226	0.347023
45210	1.89226	0.400398
45211	0.946131	0.448323
45212	0.946131	0.376583
45213	0.946128	0.420411
45214	0.946128	0.333609
45215	0.946129	0.417729
45216	0.946129	0.447908
45217	0.94613	0.318759
45218	0.94613	0.333509
45219	0.94613	0.375694
45220	0.94613	0.337485
45221	0.94613	0.376693
45222	0.94613	0.398855
45223	2.28904	1.97015
45224	1.89226	1.2118
45225	2.2846	1.35311
45226	2.2846	2.1
45227	1.89226	0.932341
45228	1.89226	2.1
45229	1.89226	1.15528
45230	1.89226	1.29163
45231	1.89226	1.94107
45232	1.89226	1.78947
45233	1.89226	1.00398
45234	1.89226	1.3221
45235	1.04037	1.02658
45236	1.34066	1.58737
45237	1.34955	1.02348

45238	1.34955	1.66341
45239	1.03373	1.18469
45240	1.03373	1.95906
45241	0.946131	0.849357
45242	0.946131	2.1
45243	1.14452	1.63681
45244	1.14452	1.77294
45245	0.946131	1.22467
45246	0.946131	1.19484
45247	1.14296	1.6453
45248	1.14452	2.1
45249	1.14296	1.01573
45250	0.946131	1.14702
45251	1.14452	1.44878
45252	0.946132	1.2571
45253	1.89226	1.07986
45254	1.89226	1.68097
45255	1.89226	1.48344
45256	1.89226	1.95778
45257	1.89226	0.93232
45258	1.89226	1.70597
45259	0.597454	1.07736
45260	0.597454	1.6096
45261	0.597972	0.996942
45262	0.597972	2.05226
45263	0.675238	1.65851
45264	0.674776	1.22252
45265	0.519667	1.16533
45266	0.520188	1.25605
45267	0.519667	0.973687
45268	0.675238	1.04437
45269	0.520188	1.40194
45270	0.674777	1.38235
45271	1.89226	0.629642
45272	1.82125	0.509839
45273	1.89226	0.557768
45274	1.84314	0.496728
45275	1.89226	0.713176
45276	1.84314	0.647938
45277	1.89226	0.54838
45278	1.84314	0.548456
45279	1.89226	0.534541
45280	1.65794	0.493963
45281	1.89226	0.554201
45282	1.65794	0.514373
45283	1.348	1.57757
45284	1.43485	1.82369
45285	1.79929	1.00729
45286	1.75309	1.41585
45287	3.14728	1.4354
45288	1.348	1.25809
45289	1.63892	1.50882
45290	1.59157	1.7592
45291	1.18647	1.58349
45292	1.75309	1.27817
45293	1.348	1.44527
45294	1.79929	1.33701
45295	1.75309	1.84991

45296	2.18106	1.19429
45297	2.20831	1.21501
45298	3.98034	1.45212
45299	2.18106	1.09018
45300	1.63892	1.57966
45301	1.75309	1.09302
45302	1.75309	1.66953
45303	2.09605	1.06334
45304	2.09605	1.72371
45305	2.66076	0.549222
45306	1.70302	0.371087
45307	2.41575	0.467466
45308	1.70302	0.447414
45309	3.08598	0.428267
45310	2.3661	0.402566
45311	1.71053	0.386514
45312	2.41575	0.530075
45313	1.20143	0.36677
45314	1.20143	0.375233
45315	2.2123	0.38295
45316	1.66739	0.396465
45317	2.25633	0.389967
45318	2.68302	0.488987
45319	1.71051	0.373266
45320	1.71051	0.377981
45321	2.74192	0.387736
45322	2.1961	0.361449
45323	1.32553	1.07967
45324	2.34875	0.860479
45325	2.19139	1.33639
45326	2.19139	0.724879
45327	1.56172	1.23995
45328	1.56172	0.675471
45329	1.89226	1.98201
45330	1.89226	0.874218
45331	1.89226	1.45842
45332	1.89226	0.986635
45333	3.78453	1.0935
45334	2.667 2.1	
45335	1.26097	1.23024
45336	1.26097	2.04898
45337	1.5683	2.1
45338	2.42949	2.01543
45339	2.73682	2.1
45340	1.13213	1.02876
45341	2.19139	0.821033
45342	2.0815	1.13983
45343	1.77615	0.612116
45344	1.37767	1.13047
45345	1.19297	0.545642
45346	0.946132	0.893326
45347	0.946132	1.32394
45348	0.946131	1.87324
45349	0.946131	0.98577
45350	0.946131	0.965192
45351	0.946131	0.77515
45352	0.946132	1.27314
45353	0.946132	1.9284

45354	0.946132	1.13125
45355	0.946132	1.1337
45356	0.946131	1.05883
45357	0.946131	1.08396
45358	2.02064	1.48072
45359	2.3606	2.1
45360	1.47478	2.1
45361	2.22997	2.1
45362	4.25061	2.1
45363	2.36059	2.1
45364	2.56952	2.1
45365	2.22997	2.1
45366	2.48631	2.1
45367	4.79949	2.1
45368	1.84261	2.1
45369	2.22997	2.1
45370	1.65706	1.78864
45371	1.78837	2.1
45372	3.88703	2.1
45373	2.22997	2.1
45374	2.48631	2.1
45375	2.19528	2.1
45376	2.41175	2.1
45377	4.64172	2.1
45378	1.6466	2.1
45379	1.89226	2.1
45380	1.35972	2.1
45381	1.35972	2.1
45382	1.89226	2.1
45383	1.89226	2.1
45384	1.89226	2.1
45385	1.89226	2.1
45386	1.89226	2.1
45387	1.89226	2.1
45388	3.78453	2.1
45389	2.44797	2.1
45390	1.07214	2.1
45391	1.31589	2.1
45392	1.1211	2.1
45393	2.44797	2.1
45394	2.25318	2.1
45395	1.95954	2.1
45396	1.89226	2.1
45397	1.6466	2.1
45398	1.6466	2.1
45399	1.89226	2.1
45400	1.89226	2.1
45401	0.946133	2.1
45402	0.946133	2.1
45403	0.946132	2.1
45404	0.946132	2.1
45405	0.946132	2.1
45406	0.946132	2.1
45407	0.946133	2.1
45408	0.946133	2.1
45409	0.946132	2.1
45410	0.946132	2.1
45411	0.946133	2.1

45412	0.946133	2.1
45413	2.44797	2.1
45414	1.36904	2.1
45415	1.70303	2.1
45416	2.37586	2.1
45417	4.00507	2.1
45418	0.631553	0.30703
45419	1.24489	0.258436
45420	1.11597	0.267287
45421	0.496799	0.320156
45422	0.872272	0.311093
45423	0.872272	0.303884
45424	0.869415	0.255863
45425	0.869415	0.258368
45426	0.4968	0.296946
45427	0.631553	0.293564
45428	1.11597	0.277845
45429	1.24489	0.294689
45430	1.84253	2.1
45431	2.17384	2.1
45432	2.04818	2.1
45433	1.65925	2.1
45434	3.83309	2.1
45435	2.04818	2.1
45436	1.65925	2.1
45437	1.48162	2.1
45438	1.92264	2.1
45439	3.58189	2.1
45440	2.3305	0.554667
45441	1.89226	0.677651
45442	2.52753	0.611248
45443	2.52753	0.655218
45444	1.89226	0.594573
45445	1.89226	0.651177
45446	1.89226	0.69824
45447	1.89226	0.706279
45448	1.89226	0.702214
45449	1.89226	0.706882
45450	1.89226	0.7
45451	1.89226	0.703994
45452	1.34563	0.584564
45453	2.1769	0.7
45454	1.94955	0.59801
45455	1.94955	0.701628
45456	1.55063	0.664498
45457	1.55063	0.702074
45458	2.09455	0.483104
45459	1.89226	0.635578
45460	2.3305	0.542858
45461	2.3305	0.577035
45462	1.89226	0.526746
45463	1.89226	0.578446
45464	1.89226	0.621765
45465	1.89226	0.699523
45466	1.89226	0.700195
45467	1.89226	0.620544
45468	1.89226	0.650106
45469	1.89226	0.7

45470	0.773392	0.434838
45471	0.612765	0.7
45472	0.79504	0.669734
45473	0.575323	0.7
45474	1.0819	0.490863
45475	1.06106	0.644441
45476	1.04675	0.493691
45477	0.86858	0.699982
45478	0.796873	0.587082
45479	0.557469	0.651936
45480	0.807663	0.664971
45481	2.2024	2.1
45482	2.34106	1.99906
45483	2.02634	2.1
45484	2.20125	2.1
45485	4.36739	2.1
45486	2.34106	2.01213
45487	2.25195	0.926719
45488	2.20125	2.1
45489	2.13132	0.868969
45490	4.47237	1.10868
45491	2.02635	2.1
45492	2.22465	2.1
45493	1.68504	2.1
45494	2.0815	2.02689
45495	3.92673	2.1
45496	2.22465	2.1
45497	2.13133	0.877032
45498	2.0815	2.0244
45499	2.23579	0.912891
45500	4.44862	1.06952
45501	1.89226	1.07083
45502	1.89226	1.31864
45503	1.89226	1.41082
45504	1.89226	1.14352
45505	1.89226	1.33685
45506	1.89226	1.10551
45507	1.93967	0.453253
45508	1.70302	0.551675
45509	1.21883	0.482127
45510	1.70302	0.599892
45511	1.19889	0.459381
45512	1.70302	0.584747
45513	0.946131	0.643137
45514	0.946131	0.578338
45515	0.946131	0.534397
45516	0.946131	0.625961
45517	0.94613	0.632735
45518	0.94613	0.660439
45519	0.946131	0.533207
45520	0.946131	0.576984
45521	0.946131	0.568076
45522	0.946131	0.576092
45523	0.946131	0.58687
45524	0.946131	0.561359
45525	0.883128	0.553683
45526	0.883128	0.639506
45527	0.827519	0.542187

45528	0.82752	0.633926
45529	1.16234	0.625419
45530	1.19063	0.629567
45531	0.631294	0.553414
45532	0.548311	0.549196
45533	0.631293	0.554339
45534	1.16234	0.574756
45535	1.19063	0.606834
45536	0.54831	0.633977
45537	1.89226	0.330376
45538	1.89226	0.445206
45539	1.89226	0.458345
45540	1.89226	0.346747
45541	3.78453	0.382165
45542	2.33328	0.258421
45543	1.87991	0.311095
45544	1.87991	0.337635
45545	2.82938	0.255004
45546	1.84457	0.329307
45547	2.79403	0.244863
45548	2.82937	0.252353
45549	2.70881	0.299824
45550	1.8799	0.32166
45551	1.82739	0.226673
45552	4.58874	0.254689
45553	1.89226	0.456206
45554	1.89226	0.311841
45555	1.89226	0.357602
45556	1.89226	0.428038
45557	3.78452	0.412662
45558	1.17897	0.312422
45559	1.82739	0.23105
45560	1.17897	0.285981
45561	1.76199	0.252653
45562	1.23885	0.29335
45563	1.80989	0.246549
45564	0.946131	0.339481
45565	0.946131	0.321794
45566	0.946131	0.402295
45567	0.946131	0.387615
45568	0.946129	0.409107
45569	0.946129	0.309039
45570	0.946133	0.362838
45571	0.946133	0.403286
45572	0.946131	0.35602
45573	0.946131	0.325787
45574	0.946131	0.34291
45575	0.946131	0.343375
45576	0.946126	0.90206
45577	0.946126	2.07845
45578	1.24887	2.06142
45579	1.24887	2.1
45580	0.946127	1.66759
45581	0.946127	0.985186
45582	1.24887	2.1
45583	1.28511	1.72425
45584	1.2851	1.52869
45585	0.946128	1.65556

45586	1.24887	1.78793
45587	0.946128	1.8017
45588	1.89226	1.83644
45589	1.89226	1.55957
45590	1.89226	1.60756
45591	1.89226	0.920124
45592	3.78452	1.31628
45593	0.592142	1.65099
45594	0.592142	1.58394
45595	0.60496	1.88452
45596	0.60496	1.95927
45597	0.694237	1.72372
45598	0.608714	1.16234
45599	0.562863	1.61908
45600	0.502864	1.89771
45601	0.562863	1.64914
45602	0.694237	1.64816
45603	0.608714	1.81662
45604	0.502864	1.61815
45605	1.89226	0.939468
45606	1.89226	2.1
45607	1.89226	1.61315
45608	1.89226	1.83893
45609	3.78453	1.84347
45610	1.55779	2.1
45611	1.57968	2.1
45612	1.47186	1.53839
45613	1.51725	2.1
45614	3.05154	2.1
45615	1.57967	2.1
45616	1.474	2.1
45617	1.51725	2.1
45618	1.42895	2.1
45619	3.00863	2.1
45620	1.89226	2.1
45621	1.89226	2.1
45622	1.89226	1.87667
45623	1.89226	2.1
45624	3.78452	2.1
45625	1.89226	1.51935
45626	1.89226	1.88726
45627	1.89226	0.941631
45628	1.89226	2.1
45629	3.78453	1.59017
45630	1.47186	1.50241
45631	1.51725	2.1
45632	1.38859	2.1
45633	1.45332	2.1
45634	2.92519	2.1
45635	1.51725	2.1
45636	1.42895	2.1
45637	1.45332	2.1
45638	1.38414	2.1
45639	2.88228	2.1
45640	1.89226	1.9127
45641	1.89226	2.1
45642	1.89226	2.1
45643	1.89226	2.1

45644	3.78452	2.1
45645	1.34497	0.183858
45646	1.36747	0.169573
45647	1.18919	0.174107
45648	2.08149	0.175277
45649	1.23556	0.16898
45650	2.13702	0.17156
45651	1.10106	0.187065
45652	2.08001	0.175858
45653	1.34497	0.182312
45654	2.31605	0.184163
45655	1.34497	0.172738
45656	2.31605	0.177751
45657	2.31605	0.176089
45658	2.0815	0.175707
45659	1.9419	0.162952
45660	2.46665	0.159758
45661	4.52922	0.16658
45662	1.20349	0.162241
45663	2.08149	0.174267
45664	1.36948	0.161086
45665	1.34737	0.166648
45666	2.08149	0.161766
45667	2.08149	0.167555
45668	0.946132	0.184742
45669	0.946132	0.195016
45670	1.37715	0.178584
45671	0.823199	0.176097
45672	1.21598	0.182858
45673	0.946132	0.184608
45674	1.37715	0.180034
45675	0.946132	0.182203
45676	1.21598	0.18372
45677	0.823198	0.175994
45678	0.946133	0.180443
45679	0.946133	0.195003
45680	1.89226	0.197507
45681	1.89226	0.212368
45682	1.89226	0.192992
45683	1.89226	0.189228
45684	3.78453	0.198249
45685	1.89227	0.205783
45686	1.89227	0.195092
45687	1.89227	0.18374
45688	1.89227	0.189612
45689	3.78453	0.193338
45690	0.946132	0.193358
45691	0.946132	0.180995
45692	1.21599	0.179579
45693	1.21599	0.174467
45694	0.966749	0.182741
45695	0.946133	0.184337
45696	1.21598	0.181149
45697	0.946132	0.181976
45698	0.966748	0.181748
45699	1.21598	0.176361
45700	0.946133	0.180384
45701	0.946133	0.188724

45702	0.946133	0.173666
45703	0.946133	0.192151
45704	1.1134	0.179393
45705	1.11555	0.166136
45706	1.11555	0.176919
45707	0.946132	0.176015
45708	0.946132	0.178629
45709	1.1134	0.178126
45710	1.11555	0.173281
45711	1.11555	0.167402
45712	0.946132	0.190892
45713	0.946132	0.180908
45714	0.946133	0.170542
45715	0.946133	0.182074
45716	1.11555	0.174152
45717	0.978467	0.165749
45718	0.978468	0.173414
45719	0.946133	0.173032
45720	1.11555	0.17264
45721	0.946132	0.172987
45722	0.978468	0.170062
45723	0.978468	0.165255
45724	0.946132	0.174214
45725	0.946132	0.182056
45726	0.851542	1.94331
45727	0.851542	1.92134
45728	1.14168	2.06788
45729	1.27897	2.01772
45730	1.02817	2.1
45731	1.13536	2.06824
45732	0.946131	1.86949
45733	0.861207	1.85553
45734	0.94613	1.72042
45735	0.94613	2.03044
45736	0.868751	1.94991
45737	1.15181	2.1
45738	0.946132	1.23693
45739	0.946132	1.20724
45740	0.946128	1.32668
45741	0.946128	1.12142
45742	0.94613	1.72058
45743	0.94613	1.32796
45744	0.946131	1.15062
45745	0.946131	1.08771
45746	0.94613	1.04525
45747	0.94613	1.26764
45748	0.94613	1.18101
45749	0.94613	1.51232
45750	2.53273	0.241818
45751	1.89226	0.33158
45752	1.89226	0.289147
45753	1.89226	0.309488
45754	1.89226	0.255682
45755	1.89226	0.282543
45756	1.89226	2.1
45757	1.89226	2.1
45758	1.89226	2.1
45759	1.89226	2.1

45760	3.78452	2.1
45761	1.89226	2.1
45762	2.54394	2.1
45763	1.89226	2.1
45764	2.42221	2.1
45765	4.43621	2.1
45766	2.54394	2.1
45767	1.62743	2.1
45768	2.42221	2.1
45769	1.5437	2.1
45770	4.08764	2.1
45771	1.89226	2.1
45772	1.89226	2.1
45773	1.89226	2.1
45774	1.89226	2.1
45775	3.78452	2.1
45776	1.89226	2.1
45777	1.89226	2.1
45778	1.89226	2.1
45779	1.89226	2.1
45780	3.78452	2.1
45781	1.89226	2.1
45782	2.30146	2.1
45783	2.42221	2.1
45784	1.89226	2.1
45785	1.89226	2.1
45786	2.42221	2.1
45787	2.30146	2.1
45788	2.42221	2.1
45789	1.39962	2.1
45790	1.39962	2.1
45791	1.5437	2.1
45792	2.44553	2.1
45793	1.89226	2.1
45794	1.89226	2.1
45795	1.89226	2.1
45796	1.89226	2.1
45797	3.78452	2.1
45798	1.62196	2.1
45799	2.00695	2.1
45800	1.95288	2.1
45801	1.70303	2.1
45802	3.53316	2.1
45803	1.80703	2.1
45804	2.00695	2.1
45805	1.92991	2.1
45806	2.00695	2.1
45807	1.85888	2.1
45808	1.93592	2.1
45809	1.89226	2.1
45810	1.89226	2.1
45811	1.89226	2.1
45812	1.89226	2.1
45813	1.89226	2.1
45814	1.89226	2.1
45815	1.89226	2.1
45816	1.89226	2.1
45817	1.89226	2.1

45818	1.89226	2.1
45819	3.78453	2.1
45820	1.95288	2.1
45821	1.50524	2.1
45822	1.83564	2.1
45823	1.70303	2.1
45824	2.00749	2.1
45825	1.84671	2.1
45826	1.62632	2.1
45827	1.82498	2.1
45828	1.92991	2.1
45829	1.92991	2.1
45830	1.47928	2.1
45831	1.47751	2.1
45832	0.946128	2.1
45833	0.946128	2.1
45834	0.946132	2.1
45835	0.946132	2.1
45836	0.94613	2.1
45837	0.94613	2.1
45838	0.946129	2.1
45839	0.946129	2.1
45840	0.94613	2.1
45841	0.94613	2.1
45842	0.94613	2.1
45843	0.94613	2.1
45844	1.89226	2.1
45845	1.89226	2.1
45846	1.89226	2.1
45847	1.89226	2.1
45848	1.89226	2.1
45849	1.89226	2.1
45850	2.27351	1.05421
45851	2.74068	0.91845
45852	2.72278	0.982651
45853	2.63356	1.00714
45854	2.36273	0.970844
45855	2.27351	0.991003
45856	2.36273	0.98758
45857	2.83112	0.870873
45858	2.81212	0.939706
45859	2.74068	0.947588
45860	2.43416	0.930115
45861	2.36273	0.935129
45862	1.89227	0.92298
45863	2.83112	0.869688
45864	2.74069	0.927821
45865	2.74069	0.841292
45866	1.89227	0.95711
45867	1.89227	0.883885
45868	2.40887	2.1
45869	1.4494	2.1
45870	1.52164	2.1
45871	2.47534	2.1
45872	3.93051	2.1
45873	1.89226	2.1
45874	2.47534	2.1
45875	2.40887	2.1

45876	2.40887	2.1
45877	1.89226	2.1
45878	1.89226	2.1
45879	1.60861	2.1
45880	2.47534	2.1
45881	1.52164	2.1
45882	1.52164	2.1
45883	2.55301	2.1
45884	2.55301	2.1
45885	0.946128	2.1
45886	0.946128	2.1
45887	1.23767	2.1
45888	1.2765	2.1
45889	1.23767	2.1
45890	1.23767	2.1
45891	0.946131	2.1
45892	0.946131	2.1
45893	0.946131	2.1
45894	1.2765	2.1
45895	1.23767	2.1
45896	0.946131	2.1
45897	1.89226	2.1
45898	1.87511	2.1
45899	1.87511	2.1
45900	1.95852	2.1
45901	2.0815	2.1
45902	2.22169	2.1
45903	1.89226	2.1
45904	1.89226	2.1
45905	1.89226	2.1
45906	1.89226	2.1
45907	1.89226	2.1
45908	1.89226	2.1
45909	2.0815	2.1
45910	1.80067	2.1
45911	1.80067	2.1
45912	2.05518	2.1
45913	1.89226	2.1
45914	2.16509	2.1
45915	1.87153	2.1
45916	2.65897	2.1
45917	1.58724	2.1
45918	2.38924	2.1
45919	4.26077	2.1
45920	1.89226	2.1
45921	1.89226	2.1
45922	1.89226	2.1
45923	1.89226	2.1
45924	1.89226	2.1
45925	1.89226	2.1
45926	1.89226	0.858323
45927	2.21022	0.674569
45928	2.19769	0.896483
45929	2.21022	0.824746
45930	1.87973	0.717384
45931	1.89226	0.62013
45932	1.18376	1.09934
45933	2.21022	0.598182

45934	1.16239	1.05569
45935	1.16239	0.676059
45936	2.19769	1.05339
45937	2.19769	0.671372
45938	0.946128	0.770855
45939	0.946128	0.712261
45940	1.09362	0.828504
45941	1.09884	0.731826
45942	0.94613	0.783526
45943	0.94613	0.551743
45944	1.09362	0.81425
45945	1.09362	0.698127
45946	0.946131	0.802829
45947	1.09362	0.805446
45948	0.946131	0.698953
45949	1.09884	0.602997
45950	1.19323	0.995941
45951	2.19769	0.550827
45952	1.18376	0.950555
45953	1.18376	0.676844
45954	2.18724	0.952168
45955	2.18724	0.713138
45956	2.0815	2.1
45957	0.922714	2.1
45958	2.0815	2.1
45959	1.84882	2.1
45960	1.20787	2.1
45961	1.08321	2.1
45962	2.6238	0.424061
45963	1.89226	0.534235
45964	1.89226	0.553353
45965	2.4866	0.442019
45966	1.89226	0.521865
45967	2.4866	0.443796
45968	2.66753	0.491791
45969	1.89226	0.620543
45970	1.89226	0.647316
45971	2.6238	0.525109
45972	1.89226	0.610812
45973	2.6238	0.521864
45974	0.946129	1.96115
45975	0.946129	2.1
45976	1.04676	2.1
45977	1.04676	2.1
45978	0.946131	2.1
45979	0.946131	2.09458
45980	1.08732	2.1
45981	1.04676	2.1
45982	1.08732	2.1
45983	0.946131	2.1
45984	1.04676	2.1
45985	0.946131	2.1
45986	1.20958	1.3094
45987	1.73802	0.822794
45988	1.39067	0.957887
45989	1.39067	0.861924
45990	1.50969	0.97907
45991	1.50969	0.969953

45992	1.00382	1.06977
45993	1.50969	0.645641
45994	1.20958	1.00336
45995	1.20958	0.748038
45996	1.32039	1.07516
45997	1.32039	0.820855
45998	2.0815	2.1
45999	2.51396	2.1
46000	2.19826	2.1
46001	2.54175	2.1
46002	4.79599	2.1
46003	2.54174	2.1
46004	2.17978	2.1
46005	2.54174	2.1
46006	2.2854	2.1
46007	2.57212	2.1
46008	2.31578	2.1
46009	2.27103	2.1
46010	2.54175	2.1
46011	2.4255	2.1
46012	2.82251	2.1
46013	4.96725	2.1
46014	2.60614	0.321128
46015	2.75374	0.301296
46016	1.94002	0.278023
46017	2.08392	0.312696
46018	4.69006	0.291438
46019	2.75373	0.303743
46020	2.6875	0.352041
46021	2.08392	0.28609
46022	1.99944	0.329668
46023	4.77142	0.332468
46024	1.1211	0.227691
46025	2.08392	0.270907
46026	1.17787	0.25917
46027	1.94002	0.245474
46028	1.32178	0.245818
46029	2.08392	0.248539
46030	2.08392	0.246193
46031	1.99944	0.253204
46032	1.32178	0.22
46033	1.25929	0.243807
46034	3.34321	0.234008
46035	1.22266	0.249452
46036	0.779473	0.239345
46037	0.94613	0.272546
46038	0.94613	0.267728
46039	1.04072	0.264426
46040	1.1407	0.258363
46041	1.10042	0.257533
46042	0.851542	0.265521
46043	0.942046	0.254455
46044	0.942046	0.270017
46045	1.04114	0.26583
46046	0.826602	0.255609
46047	2.37468	2.1
46048	1.89226	2.1
46049	1.89226	2.1

46050	1.89226	2.1
46051	2.27917	2.1
46052	2.27917	2.1
46053	1.13959	2.1
46054	1.09451	2.1
46055	0.946131	2.1
46056	0.946131	2.1
46057	1.09451	2.1
46058	1.09451	2.1
46059	0.94613	2.1
46060	0.94613	2.1
46061	1.09451	2.1
46062	0.946131	2.1
46063	0.946131	2.1
46064	1.13959	2.1
46065	2.52454	1.06431
46066	2.12221	2.1
46067	2.67624	1.12689
46068	2.33865	1.60353
46069	4.86319	1.51826
46070	1.89226	1.33632
46071	1.89226	2.1
46072	1.89226	1.295
46073	1.89226	2.1
46074	3.78452	2.1
46075	1.89226	2.1
46076	1.89226	2.1
46077	1.89226	2.1
46078	1.89226	2.1
46079	3.78452	2.1
46080	2.12221	2.1
46081	1.47769	2.1
46082	2.33865	2.1
46083	1.62898	2.1
46084	3.81634	2.1
46085	2.67624	2.03086
46086	2.37734	1.37744
46087	2.33865	2.1
46088	2.33865	1.79125
46089	2.70125	2.1
46090	2.70125	2.1
46091	1.89226	1.51873
46092	1.89226	1.88447
46093	1.89226	2.1
46094	1.89226	2.1
46095	1.89226	1.05237
46096	1.89226	1.11165
46097	1.89226	1.97941
46098	1.89226	2.1
46099	1.89226	2.1
46100	1.89226	2.1
46101	1.89226	2.1
46102	1.89226	2.1
46103	2.37733	1.49335
46104	1.95927	2.1
46105	1.82516	1.42342
46106	1.82516	2.1
46107	2.33865	2.1

46108	2.33865	2.1
46109	2.57466	2.1
46110	2.21715	2.1
46111	2.55653	2.1
46112	2.24775	2.1
46113	4.77367	2.1
46114	0.937095	2.1
46115	0.937095	2.1
46116	0.958028	2.1
46117	0.958028	2.1
46118	0.828386	2.1
46119	0.771244	2.1
46120	1.10858	2.1
46121	1.12388	2.1
46122	1.12388	2.1
46123	0.828386	2.1
46124	0.771244	2.1
46125	1.10858	2.1
46126	2.55653	2.1
46127	2.32606	2.1
46128	2.24775	2.1
46129	2.24775	2.1
46130	2.57928	2.1
46131	2.57928	2.1
46132	0.990481	2.1
46133	0.990481	2.1
46134	1.00094	2.1
46135	1.00094	2.1
46136	0.888385	2.1
46137	0.828386	2.1
46138	1.16303	2.1
46139	1.12388	2.1
46140	1.16303	2.1
46141	0.888385	2.1
46142	1.12388	2.1
46143	0.828387	2.1
46144	0.962524	2.1
46145	0.962524	2.1
46146	0.7976	2.1
46147	0.7976	2.1
46148	0.797628	2.1
46149	0.814 2.1	
46150	0.946125	2.1
46151	0.946125	2.1
46152	0.946118	2.1
46153	0.797623	2.1
46154	0.814006	2.1
46155	0.946131	2.1
46156	1.8978	0.147815
46157	2.23892	0.147815
46158	1.71469	0.147815
46159	4.13672	0.147815
46160	2.64136	0.147815
46161	2.23892	0.64767
46162	2.37578	0.780276
46163	1.77489	1.99007
46164	4.6147	0.789067
46165	1.02284	2.1

46166	0.898796	0.83357
46167	0.948554	1.04582
46168	0.948554	2.1
46169	0.878797	1.9856
46170	0.796197	2.1
46171	1.09145	0.906064
46172	0.963109	0.911426
46173	1.12114	1.57654
46174	0.782739	1.34568
46175	1.11084	1.3357
46176	0.850742	1.3357
46177	2.48024	2.1
46178	2.405	2.1
46179	2.24585	2.1
46180	2.34795	2.1
46181	4.75295	2.1
46182	1.93794	2.1
46183	2.405	2.1
46184	2.24585	2.1
46185	1.67594	1.64185
46186	4.08094	2.1
46187	1.89226	2.1
46188	1.89226	0.927722
46189	1.89226	1.61867
46190	1.89226	1.11247
46191	3.78452	1.37312
46192	1.89226	2.1
46193	1.89226	1.88382
46194	1.89226	2.1
46195	1.89226	1.15196
46196	3.78452	2.08013
46197	2.24585	2.1
46198	2.34795	2.1
46199	2.21155	2.1
46200	2.06626	2.1
46201	4.4142	2.1
46202	2.24585	2.1
46203	1.41498	1.53967
46204	1.41498	2.1
46205	1.67594	2.1
46206	2.06625	2.1
46207	2.32721	2.1
46208	1.89226	1.50914
46209	1.89226	1.09617
46210	1.89226	2.09802
46211	1.89226	1.00298
46212	1.89226	2.00801
46213	1.89226	0.935478
46214	1.89226	2.1
46215	1.89226	1.12094
46216	1.89226	2.1
46217	1.89226	1.70807
46218	3.78452	2.05411
46219	1.89226	0.438344
46220	1.89226	0.52794
46221	1.89226	0.623428
46222	1.89226	0.399522
46223	3.78452	0.407165

46224	1.89226	0.476318
46225	2.53655	0.325612
46226	1.89226	0.33958
46227	1.89226	0.348035
46228	1.89226	0.450711
46229	1.89226	0.456003
46230	1.89227	2.1
46231	1.89227	2.1
46232	1.89227	2.1
46233	1.89227	2.1
46234	1.89227	2.1
46235	1.89227	2.1
46236	0.91339	2.1
46237	0.91339	2.1
46238	1.2158	2.1
46239	1.20629	2.1
46240	1.00473	2.1
46241	1.15749	2.1
46242	0.946132	2.1
46243	0.946132	2.1
46244	0.946133	2.1
46245	1.17513	2.1
46246	1.00473	2.1
46247	0.946132	2.1
46248	1.89226	2.1
46249	1.89226	2.1
46250	1.89226	2.1
46251	1.89226	2.1
46252	1.89226	2.1
46253	1.89226	2.1
46254	1.89226	2.1
46255	2.6066	2.1
46256	2.35026	2.1
46257	2.35026	2.1
46258	1.89226	2.1
46259	1.89226	2.1
46260	2.04056	0.559515
46261	2.77366	0.542364
46262	1.24682	0.479223
46263	1.24682	0.568842
46264	2.93651	0.522719
46265	2.14277	0.570545
46266	2.68524	1.33552
46267	2.81847	1.23043
46268	1.89226	1.03153
46269	1.89226	1.19694
46270	4.57751	1.09002
46271	2.68524	1.21525
46272	1.89226	1.3891
46273	1.89226	1.3678
46274	2.50732	1.219
46275	1.89226	1.30522
46276	2.50732	1.29623
46277	1.89226	1.08034
46278	1.89226	1.26858
46279	1.89226	1.19021
46280	1.89226	1.36627
46281	1.89226	1.04981

46282	1.89226	1.32365
46283	1.89226	1.39297
46284	1.89226	1.15668
46285	1.89226	1.26453
46286	1.89226	1.40522
46287	1.89226	1.03383
46288	1.89226	1.05996
46289	1.61016	1.08714
46290	1.07467	1.15517
46291	1.24474	1.00939
46292	1.24474	1.34268
46293	1.45303	1.07359
46294	1.45303	1.34696
46295	1.61016	1.20875
46296	1.24474	1.42938
46297	1.61016	1.22415
46298	1.77186	1.113
46299	1.37813	1.0751
46300	1.37813	1.19768
46301	1.73305	2.1
46302	2.0815	2.07397
46303	1.97658	2.1
46304	1.75791	2.1
46305	1.87228	2.1
46306	1.66515	2.1
46307	2.0815	2.08044
46308	1.93938	0.925345
46309	2.03253	0.879022
46310	2.2142	2.1
46311	1.97658	0.878379
46312	2.15324	2.1
46313	1.73305	2.1
46314	1.97658	2.0477
46315	1.60428	2.1
46316	1.8589	2.1
46317	3.58085	2.1
46318	1.97657	2.00177
46319	1.93938	0.921797
46320	1.51316	2.1
46321	1.85293	0.882187
46322	3.82951	1.13299
46323	1.70303	0.316901
46324	1.96087	0.384486
46325	1.42308	0.369395
46326	1.96087	0.376452
46327	1.25023	0.334697
46328	1.70303	0.342746
46329	0.946132	0.387915
46330	0.946132	0.360443
46331	0.946131	0.350049
46332	0.946131	0.375312
46333	0.946132	0.413286
46334	0.946132	0.400554
46335	0.946132	0.342601
46336	0.946132	0.341511
46337	0.946132	0.392534
46338	0.946132	0.350718
46339	0.946132	0.365574

46340	0.946132	0.400188
46341	1.89226	0.410521
46342	1.89226	0.520493
46343	1.89226	0.443434
46344	1.89226	0.492059
46345	3.78452	0.448852
46346	1.89226	0.369239
46347	1.89226	0.45656
46348	1.89226	0.432237
46349	1.89226	0.438922
46350	1.89226	0.373207
46351	1.89226	0.378875
46352	1.89226	0.40754
46353	1.89226	0.439587
46354	1.89226	0.473726
46355	1.89226	0.439856
46356	1.89226	0.414529
46357	1.89226	0.389797
46358	1.89226	0.33879
46359	1.89226	0.418964
46360	2.48255	0.354669
46361	1.89226	0.374936
46362	3.78452	0.354308
46363	1.89226	2.1
46364	2.42758	2.1
46365	1.9209	2.1
46366	1.89226	2.1
46367	2.45622	2.1
46368	2.42758	2.1
46369	2.42758	2.1
46370	1.49987	2.1
46371	1.61028	2.1
46372	1.58164	2.1
46373	2.45622	2.1
46374	2.42758	2.1
46375	0.94613	2.1
46376	0.94613	2.1
46377	1.24298	2.1
46378	1.24298	2.1
46379	1.24298	2.1
46380	1.22811	2.1
46381	0.946131	2.1
46382	0.946131	2.1
46383	0.94613	2.1
46384	1.24298	2.1
46385	0.94613	2.1
46386	1.22811	2.1
46387	2.48596	2.1
46388	2.45622	2.1
46389	1.63823	2.1
46390	1.4304	2.1
46391	1.45739	2.1
46392	2.45622	2.1
46393	2.4169	2.1
46394	2.55249	2.1
46395	2.51125	2.1
46396	2.35245	2.1
46397	4.90494	2.1

46398	2.45922	2.1
46399	2.55249	2.1
46400	2.51126	2.1
46401	2.43226	2.1
46402	4.98474	2.1
46403	2.51125	2.1
46404	2.35245	2.1
46405	2.28979	2.1
46406	2.47228	2.1
46407	4.82473	2.1
46408	2.51126	2.1
46409	2.43226	2.1
46410	2.40668	2.1
46411	2.47228	2.1
46412	4.90453	2.1
46413	2.8045	2.1
46414	1.93707	2.1
46415	2.14087	2.1
46416	2.14087	2.1
46417	2.61595	2.1
46418	2.61595	1.97081
46419	1.89226	2.1
46420	1.89226	2.1
46421	1.89226	2.1
46422	1.89226	1.23278
46423	1.89226	2.1
46424	1.89226	1.09746
46425	1.22368	1.76064
46426	1.32339	2.1
46427	0.957125	2.1
46428	0.750074	2.1
46429	1.08438	2.1
46430	1.08438	1.83263
46431	1.13094	2.1
46432	0.970797	2.1
46433	1.17568	2.1
46434	0.764392	2.1
46435	0.944286	2.1
46436	1.27523	2.1
46437	1.89226	1.96341
46438	1.89226	2.1
46439	1.89226	2.1
46440	1.89226	1.17244
46441	3.78453	2.1
46442	2.8239	2.1
46443	1.89227	2.1
46444	2.57276	2.1
46445	2.57276	2.1
46446	1.89227	2.1
46447	1.89227	2.1
46448	1.18139	2.1
46449	1.85195	2.1
46450	1.78595	2.1
46451	1.08172	2.1
46452	2.93367	2.1
46453	1.60735	2.1
46454	1.20356	2.1
46455	0.967034	2.1

46456	1.08172	2.1
46457	1.67324	2.1
46458	1.77293	2.1
46459	1.89226	2.1
46460	1.89226	2.1
46461	1.8213	2.1
46462	1.82129	2.1
46463	2.14171	2.1
46464	2.40192	2.1
46465	0.810943	2.1
46466	0.810943	2.1
46467	0.798469	2.1
46468	0.788336	2.1
46469	0.804015	2.1
46470	0.810945	2.1
46471	1.947 2.1	
46472	1.36506	2.1
46473	1.08686	2.1
46474	1.07208	2.1
46475	1.08132	2.1
46476	1.13537	0.677573
46477	1.14754	1.05443
46478	0.946131	0.916438
46479	0.946131	1.30043
46480	0.94613	0.910981
46481	0.94613	0.685014
46482	1.25104	1.35425
46483	1.14754	1.01851
46484	1.13537	0.947832
46485	0.946131	0.955331
46486	0.946131	0.989168
46487	1.25104	0.976432
46488	0.96542	0.543829
46489	0.922284	0.966662
46490	0.946131	1.25415
46491	0.946131	0.925912
46492	0.946131	0.919767
46493	0.946131	0.573734
46494	1.13537	1.26071
46495	0.924824	1.00399
46496	0.859476	0.919399
46497	0.842305	0.931199
46498	1.25996	0.931262
46499	1.04996	0.940959
46500	1.71007	0.224136
46501	2.73283	0.222368
46502	2.73283	0.240202
46503	1.89226	0.216719
46504	1.89226	0.238176
46505	1.13262	0.208677
46506	0.946131	0.228272
46507	0.946131	0.243691
46508	0.946131	0.257066
46509	0.946131	0.295731
46510	0.946132	0.299191
46511	0.946132	0.264848
46512	0.946131	0.234722
46513	0.946131	0.269311

46514	0.946131	0.258127
46515	0.946131	0.263066
46516	0.946132	0.259806
46517	0.946132	0.244936
46518	0.946131	0.232091
46519	1.36642	0.218657
46520	0.946125	0.236399
46521	0.946125	0.258058
46522	0.946128	0.259595
46523	0.946128	0.238591
46524	0.946126	0.224522
46525	0.946128	0.236872
46526	0.946128	0.242631
46527	0.946128	0.243794
46528	1.36641	0.234695
46529	0.946128	0.240231
46530	1.89226	0.245305
46531	1.89226	0.316146
46532	1.89226	0.268522
46533	1.89226	0.26764
46534	1.89226	0.273831
46535	1.89226	0.273012
46536	2.69784	2.1
46537	2.51651	2.1
46538	2.56999	2.1
46539	2.74623	2.1
46540	5.26783	2.1
46541	2.69784	2.1
46542	2.48718	2.1
46543	2.50161	2.1
46544	2.74623	2.1
46545	5.19945	2.1
46546	1.89226	2.1
46547	1.89226	2.1
46548	1.89226	2.1
46549	1.89226	2.1
46550	3.78453	2.1
46551	0.874264	2.1
46552	1.09879	2.1
46553	0.946131	2.1
46554	0.946131	2.1
46555	1.17062	2.1
46556	1.17062	2.1
46557	0.874293	2.1
46558	0.874293	2.1
46559	1.09879	2.1
46560	0.94613	2.1
46561	0.946131	2.1
46562	0.874265	2.1
46563	1.89226	2.1
46564	1.89226	2.1
46565	1.89226	2.1
46566	1.89226	2.1
46567	3.78453	2.1
46568	0.977382	2.1
46569	1.16424	2.1
46570	0.946133	2.1
46571	0.946133	2.1

46572	1.33012	2.1
46573	1.25589	2.1
46574	0.888498	2.1
46575	0.888498	2.1
46576	1.25375	2.1
46577	0.946131	2.1
46578	0.946131	2.1
46579	0.977384	2.1
46580	1.89226	2.1
46581	2.19757	2.1
46582	2.62906	2.1
46583	1.89226	2.1
46584	1.89226	2.1
46585	2.62906	2.1
46586	1.27944	0.801061
46587	1.27731	1.3072
46588	0.615701	1.07899
46589	0.615701	0.925635
46590	0.615673	0.702982
46591	0.946133	1.08192
46592	0.948264	0.732436
46593	0.946132	0.771423
46594	0.94752	0.735496
46595	0.61706	0.707326
46596	0.946132	0.765972
46597	0.946132	0.767495
46598	2.76604	2.1
46599	1.66728	2.1
46600	1.66728	2.1
46601	1.83036	2.1
46602	2.54515	2.1
46603	2.70823	2.1
46604	2.54515	2.1
46605	1.49958	2.1
46606	1.49958	2.1
46607	1.66728	2.1
46608	2.34465	2.1
46609	2.51235	2.1
46610	1.21128	2.1
46611	1.21128	2.1
46612	0.946131	2.1
46613	0.946131	2.05472
46614	0.946131	2.03777
46615	0.946131	2.1
46616	1.31401	2.1
46617	1.21128	2.1
46618	1.21128	2.1
46619	0.946131	2.1
46620	0.946131	2.1
46621	1.31401	2.1
46622	1.67374	2.1
46623	2.29586	2.1
46624	1.50033	2.1
46625	2.16598	2.1
46626	3.83972	2.1
46627	1.89226	2.1
46628	1.89226	2.04167
46629	1.89226	2.09625

46630	1.89226	2.1
46631	3.78452	2.1
46632	1.13537	2.1
46633	1.02188	2.1
46634	0.946132	2.1
46635	0.946132	2.1
46636	0.946132	2.09811
46637	0.946132	2.1
46638	1.21128	2.1
46639	1.02188	2.1
46640	1.04672	2.1
46641	1.04672	2.1
46642	1.21128	2.1
46643	0.946132	2.1
46644	0.815411	2.1
46645	0.870464	2.1
46646	0.885437	2.1
46647	0.885437	2.1
46648	1.06395	2.1
46649	1.00483	2.1
46650	0.757125	2.1
46651	0.673956	2.1
46652	0.684461	2.1
46653	1.06979	2.1
46654	0.731403	2.1
46655	1.0559	2.1
46656	1.89226	2.1
46657	1.89226	2.1
46658	1.89226	2.1
46659	1.89226	2.1
46660	1.89226	2.1
46661	1.89226	2.1
46662	1.76897	1.15878
46663	1.76932	1.20546
46664	1.97455	1.68126
46665	1.94302	1.02586
46666	3.71233	1.13741
46667	1.83347	2.08455
46668	1.76932	1.22148
46669	1.97455	1.70122
46670	1.76932	1.79442
46671	1.76522	1.73367
46672	1.55999	1.84931
46673	1.89227	1.21885
46674	1.89227	1.35731
46675	1.89227	1.80901
46676	1.89227	1.11542
46677	3.78453	1.20669
46678	1.89226	1.20286
46679	1.89226	0.937017
46680	1.89226	1.0116
46681	1.89226	1.08929
46682	3.78452	1.10605
46683	1.97452	1.53443
46684	1.94299	0.970352
46685	2.17831	1.0906
46686	2.23195	1.11319
46687	4.17495	1.16566

46688	2.23196	1.15286
46689	2.1499	1.90346
46690	1.97452	1.52475
46691	1.97452	1.42388
46692	2.09088	1.64931
46693	1.83345	1.59382
46694	0.94612	1.25892
46695	0.94612	1.48596
46696	0.946119	1.10478
46697	0.946119	1.07031
46698	0.946119	1.07254
46699	0.946119	1.24747
46700	0.94612	1.14765
46701	0.94612	1.49738
46702	0.946132	1.17497
46703	0.946132	1.13429
46704	0.946107	1.21912
46705	0.946107	1.20584
46706	1.89224	0.957831
46707	1.89224	1.03409
46708	1.89224	1.09008
46709	1.89224	0.915598
46710	3.78447	0.945802
46711	1.70302	0.767564
46712	1.056	0.759085
46713	1.21829	0.768771
46714	1.89906	0.750451
46715	3.07892	0.759625
46716	2.20248	0.759267
46717	1.56498	0.748157
46718	1.46539	0.743174
46719	1.48787	0.746752
46720	2.37625	0.760708
46721	2.37625	0.763453
46722	1.14104	0.73792
46723	1.00675	0.738615
46724	0.700689	0.737898
46725	0.624389	0.744235
46726	0.782149	0.697512
46727	0.940197	0.73667
46728	0.803437	0.727408
46729	0.803437	0.742375
46730	0.995423	0.727785
46731	0.700699	0.700954
46732	0.593895	0.757662
46733	1.00673	0.741076
46734	1.18097	0.731584
46735	1.15839	0.728357
46736	0.995407	0.736046
46737	0.865932	0.731341
46738	1.17299	0.712228
46739	0.957054	0.735245
46740	1.08016	0.726557
46741	1.08016	0.74447
46742	0.995425	0.731244
46743	1.24161	0.701396
46744	0.796771	0.726403
46745	1.15837	0.745337

46746	2.57264	0.332454
46747	1.89226	0.392915
46748	2.41701	0.33
46749	1.89226	0.307928
46750	4.30928	0.333196
46751	1.69055	0.335296
46752	2.39587	0.322482
46753	1.53463	0.283835
46754	2.2053	0.263765
46755	3.89585	0.318776
46756	0.946129	0.328392
46757	0.946129	0.294459
46758	0.94613	0.318727
46759	0.94613	0.342511
46760	0.946132	0.302426
46761	0.946132	0.393729
46762	0.946126	0.332841
46763	0.946126	0.316639
46764	0.94613	0.275383
46765	0.94613	0.323237
46766	0.946129	0.339896
46767	0.946129	0.380099
46768	2.41701	0.319714
46769	1.89226	0.252598
46770	1.89226	0.257289
46771	1.89226	0.279994
46772	2.32462	0.289832
46773	2.32462	0.320011
46774	1.41942	0.225025
46775	2.2053	0.253758
46776	2.02097	0.2375
46777	2.02097	0.261597
46778	1.53463	0.219214
46779	1.53463	0.232243
46780	1.89226	0.240493
46781	1.89226	0.300887
46782	1.89226	0.262635
46783	1.89226	0.29698
46784	1.89226	0.247151
46785	1.89226	0.266615
46786	1.89226	1.31353
46787	1.89226	1.88089
46788	1.89226	1.82736
46789	1.89226	1.47709
46790	1.89226	1.68181
46791	1.89226	1.37632
46792	1.02816	1.43225
46793	1.02816	1.72823
46794	0.595141	1.64899
46795	0.595141	1.85549
46796	0.946132	1.59654
46797	0.595113	1.88633
46798	0.677174	1.78874
46799	0.946132	1.39285
46800	0.946132	1.64142
46801	0.946132	1.63234
46802	0.595113	1.64002
46803	0.677174	1.72366

46804	1.89226	1.26354
46805	1.89226	1.9218
46806	1.89226	1.61527
46807	1.89226	1.36159
46808	1.89226	1.75333
46809	1.89226	1.46159
46810	0.946133	1.37427
46811	0.946133	1.14978
46812	0.946132	1.62793
46813	0.946132	1.75547
46814	0.946132	1.75161
46815	0.946132	1.36434
46816	0.946132	1.46443
46817	0.946132	1.14714
46818	0.946132	1.42879
46819	0.946132	1.55854
46820	0.946133	1.3745
46821	0.946133	1.36647
46822	0.946131	1.48595
46823	0.946131	1.41067
46824	0.946131	1.85777
46825	0.946131	1.89605
46826	0.946128	1.95507
46827	0.946128	1.49128
46828	0.946133	1.89575
46829	0.946133	1.37625
46830	0.94613	1.67248
46831	0.94613	1.79457
46832	0.946131	1.70106
46833	0.946131	1.58776
46834	2.0264	1.5123
46835	1.67345	1.50001
46836	2.0264	1.17615
46837	1.70539	1.20143
46838	1.31611	1.33192
46839	1.31611	1.21462
46840	1.89226	0.768089
46841	1.89226	0.76088
46842	1.89226	0.864205
46843	1.89226	0.708411
46844	3.78452	0.712947
46845	1.89226	0.84969
46846	1.89226	0.811485
46847	1.89226	0.809937
46848	1.89226	0.863198
46849	1.89226	0.827819
46850	1.89226	0.885934
46851	1.02645	0.54258
46852	0.810942	0.593056
46853	0.882781	0.6277
46854	0.882781	0.604632
46855	2.80386	0.565058
46856	1.56719	0.604912
46857	1.22499	0.554963
46858	1.08132	0.628895
46859	1.36865	0.580272
46860	0.810957	0.603927
46861	0.810957	0.586054

46862	0.946132	0.718593
46863	0.946132	0.756501
46864	0.946129	0.615689
46865	0.946129	0.659428
46866	0.946131	0.61423
46867	0.946131	0.642227
46868	0.946131	0.744749
46869	0.946131	0.697898
46870	0.94613	0.723791
46871	0.94613	0.7109
46872	0.946131	0.803475
46873	0.946131	0.702984
46874	1.89226	0.75785
46875	1.89226	0.661973
46876	1.89226	0.826401
46877	1.89226	0.655556
46878	3.78453	0.664054
46879	1.30462	0.412055
46880	1.79906	0.598108
46881	1.01299	0.614865
46882	1.44185	0.481493
46883	1.3499	0.504305
46884	1.79906	0.481155
46885	1.89226	0.544346
46886	1.89226	0.7
46887	1.89226	0.492284
46888	1.89226	0.646764
46889	1.89226	0.664635
46890	1.89226	0.700118
46891	1.89226	0.685789
46892	1.89226	0.690742
46893	1.89226	0.7
46894	1.89226	0.686827
46895	1.89226	0.684355
46896	1.89226	0.652181
46897	1.3499	0.448416
46898	1.79905	0.547205
46899	1.29001	0.503852
46900	1.36005	0.585812
46901	1.88294	0.572124
46902	1.79905	0.531772
46903	1.89226	0.475193
46904	2.75374	0.418014
46905	2.60614	0.480601
46906	1.89226	0.477841
46907	2.60614	0.394798
46908	1.89226	0.397969
46909	0.946131	0.458551
46910	0.946131	0.455443
46911	1.37687	0.490813
46912	1.37687	0.514177
46913	0.946131	0.498507
46914	1.34375	0.530889
46915	1.37686	0.445669
46916	0.946128	0.428372
46917	1.37687	0.451131
46918	1.34375	0.477629
46919	0.94613	0.493736

46920	0.94613	0.467589
46921	1.44247	2.1
46922	1.12665	2.1
46923	1.50044	2.1
46924	1.73651	2.1
46925	2.94291	2.1
46926	1.73652	2.1
46927	1.64926	2.1
46928	1.44247	2.1
46929	1.44247	2.1
46930	1.64926	2.1
46931	1.43872	2.1
46932	1.50044	2.1
46933	1.73651	2.1
46934	2.00955	2.1
46935	1.87875	2.1
46936	3.50999	2.1
46937	1.64926	2.1
46938	1.73652	2.1
46939	2.00955	2.1
46940	1.8493	2.1
46941	3.65881	2.1
46942	1.89226	0.344999
46943	2.28188	0.398009
46944	1.99182	0.330032
46945	2.28188	0.45987
46946	1.6022	0.308333
46947	1.89226	0.434166
46948	1.89226	0.449549
46949	1.89226	0.497785
46950	1.89226	0.424199
46951	1.89226	0.57558
46952	1.89226	0.400346
46953	1.89226	0.533189
46954	1.89226	0.391287
46955	1.89226	0.427759
46956	2.19578	0.345679
46957	1.89226	0.516362
46958	2.81422	0.357491
46959	2.42521	0.472933
46960	1.89224	0.334776
46961	1.99179	0.378777
46962	1.454	0.337645
46963	1.99179	0.414067
46964	1.35444	0.308989
46965	1.89224	0.38928
46966	1.89224	0.412356
46967	1.89224	0.472942
46968	1.89224	0.411946
46969	1.89224	0.543293
46970	1.89224	0.386608
46971	1.89224	0.501854
46972	1.703	0.375056
46973	2.19575	0.409206
46974	1.9911	0.355004
46975	2.19575	0.476733
46976	1.67811	0.323996
46977	1.89223	0.441137

46978	1.69586	2.1
46979	2.55301	2.1
46980	1.60861	2.1
46981	1.60861	2.1
46982	2.65233	2.1
46983	2.65233	2.1
46984	0.946129	2.1
46985	0.946129	2.1
46986	1.2765	2.1
46987	1.32617	2.1
46988	1.2765	2.1
46989	1.2765	2.1
46990	0.946131	2.1
46991	0.946131	2.1
46992	0.946131	2.1
46993	1.32616	2.1
46994	0.946131	2.1
46995	1.2765	2.1
46996	1.36757	2.1
46997	1.32617	2.1
46998	0.891503	2.1
46999	0.847932	2.1
47000	1.10722	2.1
47001	1.10722	2.1
47002	1.10829	2.1
47003	1.10829	2.1
47004	1.36757	2.1
47005	0.891504	2.1
47006	0.847932	2.1
47007	1.32617	2.1
47008	0.946129	2.1
47009	0.946129	2.1
47010	1.36757	2.1
47011	1.32617	2.1
47012	1.32616	2.1
47013	1.32616	2.1
47014	0.946131	2.1
47015	0.946131	2.1
47016	1.36757	2.1
47017	0.946131	2.1
47018	0.946131	2.1
47019	1.32616	2.1
47020	1.89226	0.410909
47021	1.89226	0.328539
47022	1.89226	0.318547
47023	1.89226	0.437838
47024	3.78452	0.367289
47025	2.36336	0.250343
47026	1.89226	0.326882
47027	2.67831	0.311602
47028	2.67831	0.27935
47029	1.89226	0.302723
47030	1.89226	0.24216
47031	1.89227	0.331643
47032	1.89227	0.458208
47033	1.89227	0.43999
47034	1.89227	0.343519
47035	3.78453	0.394149

47036	1.89226	0.309846
47037	1.89226	0.438835
47038	1.89226	0.426922
47039	1.89226	0.34694
47040	3.78452	0.382141
47041	2.36336	0.251474
47042	2.0815	0.335897
47043	2.0815	0.313244
47044	2.41048	0.258526
47045	1.66267	0.312992
47046	2.13452	0.248587
47047	1.89226	0.441008
47048	1.89226	0.345762
47049	1.89226	0.428592
47050	1.89226	0.33353
47051	1.89226	0.444864
47052	1.89226	0.366217
47053	1.89226	0.685108
47054	1.89226	0.704328
47055	1.89226	0.698106
47056	1.89226	0.707024
47057	1.89226	0.692845
47058	1.89226	0.704453
47059	0.946128	0.704299
47060	0.946128	0.703317
47061	1.00122	0.694035
47062	1.00122	0.693287
47063	0.94613	0.707657
47064	1.01618	0.696973
47065	0.946131	0.703394
47066	1.00122	0.691695
47067	1.00122	0.697176
47068	1.01618	0.698227
47069	0.946131	0.704663
47070	0.946131	0.702719
47071	1.89226	1.35794
47072	1.89226	1.08008
47073	1.89226	1.06146
47074	1.89226	1.49934
47075	3.78452	1.15842
47076	1.89226	1.24357
47077	1.89226	1.98932
47078	1.89226	1.97284
47079	1.89226	1.36411
47080	1.89226	1.86448
47081	1.89226	1.35048
47082	1.89226	0.987576
47083	1.89226	1.39172
47084	1.89226	1.26756
47085	1.89226	1.03401
47086	1.89226	1.27712
47087	1.89226	1.03946
47088	1.89226	1.0351
47089	1.89226	1.66269
47090	1.89226	1.51855
47091	1.89226	1.11106
47092	1.89226	1.46561
47093	1.89226	1.12325

47094	2.71279	0.927435
47095	1.4155	0.940006
47096	2.28488	0.888475
47097	1.06122	0.96077
47098	3.70038	0.911843
47099	1.89226	0.948404
47100	1.89226	0.948069
47101	1.89226	0.909958
47102	1.89226	0.944343
47103	3.78452	0.98722
47104	0.851542	0.954041
47105	0.851542	0.917558
47106	0.851484	0.919432
47107	0.865567	1.00063
47108	0.981616	0.960521
47109	0.864767	0.99397
47110	0.851542	0.937205
47111	0.851542	0.922096
47112	0.851477	0.964507
47113	0.851477	0.971405
47114	0.865635	0.943712
47115	0.816472	0.937389
47116	1.68443	1.33143
47117	1.44489	2.1
47118	1.54924	1.79148
47119	1.34389	2.1
47120	3.02832	2.1
47121	1.44489	2.1
47122	1.03899	2.1
47123	1.03899	2.1
47124	1.12075	2.1
47125	1.34389	2.1
47126	1.42566	2.1
47127	1.54924	1.76771
47128	1.34389	2.1
47129	1.44091	1.52335
47130	1.25866	2.1
47131	2.7848	2.1
47132	0.573141	2.1
47133	0.573141	2.1
47134	0.575681	2.1
47135	0.575681	2.1
47136	0.481985	2.1
47137	0.519492	2.1
47138	0.671948	2.1
47139	0.629331	2.1
47140	0.629329	2.1
47141	0.481986	2.1
47142	0.519494	2.1
47143	0.671947	2.1
47144	1.89226	0.765333
47145	1.89226	0.877955
47146	1.89226	0.855635
47147	2.31925	0.801294
47148	3.78452	0.862668
47149	1.30916	2.03598
47150	1.17426	2.1
47151	0.946129	2.1

47152	0.946129	2.1
47153	0.94613	2.1
47154	0.94613	2.025
47155	1.17426	2.1
47156	1.17426	2.1
47157	1.30916	2.1
47158	0.94613	2.1
47159	1.17426	2.1
47160	0.94613	2.1
47161	2.56346	2.01081
47162	1.89226	1.22571
47163	1.89226	1.8068
47164	1.89226	1.32051
47165	2.6074	1.79753
47166	2.6074	1.33693
47167	1.89226	2.01569
47168	1.89226	2.03649
47169	1.89226	2.1
47170	1.89226	1.79729
47171	3.78452	2.06191
47172	1.33064	2.02212
47173	1.84121	2.04955
47174	0.946129	2.1
47175	0.946129	2.1
47176	0.946131	2.1
47177	0.946131	2.02536
47178	1.30916	2.1
47179	1.8412	2.1
47180	1.33064	2.1
47181	0.94613	2.1
47182	1.30916	2.1
47183	0.946131	2.1
47184	1.19283	1.22983
47185	1.19283	1.15972
47186	0.946131	2.01853
47187	0.946131	1.80479
47188	0.946132	1.85227
47189	0.946132	1.21794
47190	1.3037	2.02181
47191	1.19283	1.19446
47192	1.19283	1.52349
47193	0.946131	1.52368
47194	0.946131	1.47596
47195	1.3037	1.57028
47196	1.89226	2.1
47197	1.89226	1.80172
47198	1.89226	1.99495
47199	1.89226	2.01703
47200	1.89226	2.01312
47201	1.89226	2.02803
47202	2.17708	1.45636
47203	1.70302	2.1
47204	2.28188	1.33335
47205	2.13621	2.1
47206	4.28253	1.98648
47207	1.89226	1.15289
47208	1.89226	2.1
47209	1.89226	1.37534

47210	1.89226	2.1
47211	3.78452	1.93671
47212	0.946132	2.1
47213	0.946132	2.1
47214	0.946132	2.1
47215	0.946132	2.1
47216	0.946131	2.1
47217	0.946131	2.1
47218	0.946132	2.1
47219	0.946132	2.09254
47220	0.946132	2.1
47221	0.946132	2.1
47222	0.946132	2.1
47223	0.946132	2.1
47224	1.74933	2.1
47225	1.11833	2.1
47226	1.90074	2.1
47227	1.669	2.1
47228	1.335	2.1
47229	1.11833	2.1
47230	2.28188	1.23521
47231	2.31804	2.1
47232	2.42758	1.37306
47233	2.14895	2.1
47234	4.43083	2.01704
47235	1.89226	1.2545
47236	1.89226	2.1
47237	1.89226	1.11717
47238	1.89226	2.1
47239	3.78452	1.63653
47240	1.89226	2.09737
47241	1.89226	2.1
47242	1.89226	2.1
47243	1.89226	2.1
47244	1.89226	2.09906
47245	1.89226	2.1
47246	0.864133	2.1
47247	0.864133	2.1
47248	0.967939	2.1
47249	0.793739	2.1
47250	0.650797	2.1
47251	0.757546	2.1
47252	1.08941	2.1
47253	1.10136	2.1
47254	1.10138	2.1
47255	0.796445	2.1
47256	0.866907	2.1
47257	0.650788	2.1
47258	2.63062	0.823023
47259	1.89226	0.799611
47260	1.89226	0.761432
47261	1.89226	0.903096
47262	2.46557	0.614885
47263	2.46557	0.938029
47264	1.16777	0.886644
47265	1.38859	2.1
47266	1.05042	1.33718
47267	1.28909	1.77945

47268	2.45686	1.67762
47269	1.89227	0.892858
47270	1.89227	0.940857
47271	1.89227	0.792587
47272	1.89227	1.84296
47273	3.78453	0.888425
47274	2.15114	2.1
47275	1.89226	1.82326
47276	2.15005	1.91483
47277	2.15005	2.1
47278	1.89226	1.22175
47279	1.89226	2.1
47280	1.89226	1.83083
47281	1.89226	1.20991
47282	1.89226	1.97421
47283	1.89226	2.0979
47284	1.89226	1.04059
47285	1.89226	1.75667
47286	0.973447	1.61042
47287	1.32143	1.85537
47288	1.37444	1.60892
47289	1.37444	1.76025
47290	0.949134	1.80324
47291	0.949134	1.99673
47292	0.946131	1.04355
47293	0.946131	2.1
47294	1.07557	1.87845
47295	1.07557	2.1
47296	0.946131	1.57078
47297	0.946131	1.70419
47298	1.07557	2.1
47299	1.08579	2.1
47300	1.08579	2.03488
47301	0.946131	2.06896
47302	1.07557	2.09638
47303	0.946131	2.08836
47304	0.946131	0.91705
47305	0.946131	2.1
47306	0.946131	1.30823
47307	0.946131	1.70127
47308	0.94613	1.21187
47309	0.94613	1.3249
47310	0.946131	1.71592
47311	0.946131	1.84167
47312	0.946131	1.31796
47313	0.946131	1.25526
47314	0.946131	1.47066
47315	0.946131	1.56338
47316	0.606115	1.52182
47317	0.606115	1.73737
47318	0.616308	1.73039
47319	0.616308	2.1
47320	0.68722	1.20702
47321	0.735699	1.74147
47322	0.514789	1.51374
47323	0.486724	1.82888
47324	0.514788	1.27889
47325	0.735699	1.31737

47326	0.486723	1.77079
47327	0.687221	1.6537
47328	2.33761	0.779085
47329	2.51749	0.724609
47330	2.68092	0.731739
47331	2.58304	0.741722
47332	5.01853	0.729305
47333	2.50906	0.743353
47334	2.51749	0.734888
47335	2.68091	0.740294
47336	2.53176	0.710338
47337	5.18997	0.737234
47338	2.68092	0.733644
47339	2.58304	0.73812
47340	2.71793	0.742698
47341	2.67098	0.713789
47342	5.39885	0.738597
47343	2.68091	0.74361
47344	2.53175	0.712738
47345	2.36417	0.718897
47346	2.67098	0.718783
47347	5.04508	0.717855
47348	2.76309	2.1
47349	1.89226	1.90516
47350	1.89226	2.1
47351	1.89226	2.08511
47352	2.48573	2.1
47353	2.48573	2.00261
47354	1.35879	2.1
47355	1.55161	0.925585
47356	1.10407	1.79871
47357	1.32259	0.903161
47358	2.68138	1.19352
47359	1.89226	2.1
47360	1.89226	0.893451
47361	1.89226	1.87353
47362	1.89226	0.905561
47363	3.78452	1.0721
47364	1.19612	1.57846
47365	1.24694	2.1
47366	1.24844	0.867343
47367	1.29747	1.8035
47368	2.49358	1.53434
47369	1.89226	1.6528
47370	1.89226	1.96975
47371	1.89226	0.79819
47372	1.89226	1.80913
47373	3.78453	1.34848
47374	1.89226	1.43235
47375	1.89226	2.1
47376	1.89226	2.1
47377	1.89226	2.1
47378	1.89226	1.41423
47379	1.89226	2.1
47380	0.587463	2.1
47381	0.587463	2.08141
47382	0.581977	1.52195
47383	0.581977	2.1

47384	0.531709	2.1
47385	0.545972	2.1
47386	0.623469	1.75317
47387	0.648733	1.73556
47388	0.648733	2.1
47389	0.545971	2.1
47390	0.623469	2.1
47391	0.531709	2.1
47392	1.29417	0.942143
47393	1.29747	2.1
47394	1.27829	2.1
47395	1.24844	1.22685
47396	1.32732	1.50222
47397	1.29747	1.08715
47398	1.89226	0.932731
47399	1.89226	2.1
47400	1.89226	0.842703
47401	1.89226	1.1515
47402	1.89226	1.21923
47403	1.89226	1.99887
47404	1.89226	2.1
47405	1.89226	2.1
47406	1.89226	1.11923
47407	1.89226	2.05442
47408	1.89226	1.92263
47409	1.89226	1.14556
47410	0.60346	2.1
47411	0.60346	1.55353
47412	0.60086	1.00711
47413	0.60086	2.1
47414	0.545971	2.1
47415	0.555587	2.1
47416	0.648733	1.42913
47417	0.663661	1.40752
47418	0.663661	2.03401
47419	0.555587	1.76921
47420	0.648733	2.1
47421	0.545971	2.1
47422	1.34066	1.5837
47423	1.36601	2.1
47424	1.34955	0.908065
47425	1.37623	1.93774
47426	2.71556	1.57859
47427	1.89226	1.29501
47428	1.89226	2.1
47429	1.89226	0.919532
47430	1.89226	1.18409
47431	3.78452	1.1532
47432	0.946132	2.1
47433	0.946132	1.44536
47434	0.946132	0.941907
47435	0.946132	2.1
47436	0.946132	2.1
47437	0.946132	2.1
47438	0.946131	1.19762
47439	0.946131	1.1445
47440	0.946132	1.92013
47441	0.946132	1.86954

47442	0.946132	2.00507
47443	0.946132	1.9544
47444	0.631351	1.01303
47445	0.631351	2.1
47446	0.632421	1.34603
47447	0.632421	2.1
47448	0.573604	2.1
47449	0.580765	2.1
47450	0.683006	1.02642
47451	0.688118	1.52522
47452	0.688118	2.04476
47453	0.580765	2.075
47454	0.683007	2.1
47455	0.573604	2.1
47456	1.34955	1.61402
47457	1.37624	2.1
47458	1.35048	0.952323
47459	1.37999	1.83469
47460	2.72954	1.46273
47461	0.946131	1.15531
47462	0.946131	1.22724
47463	0.946131	1.86604
47464	0.946131	1.89795
47465	0.94613	2.1
47466	0.94613	1.29831
47467	0.946133	1.142
47468	0.946133	1.34685
47469	0.946131	1.25801
47470	0.946131	1.1164
47471	0.946132	1.28139
47472	0.946132	1.21694
47473	1.89226	1.38201
47474	1.89226	1.45397
47475	1.89226	1.49331
47476	1.89226	2.1
47477	1.89226	2.1
47478	1.89226	2.1
47479	0.532189	1.05019
47480	0.531141	2.1
47481	0.497784	2.1
47482	0.502536	2.1
47483	1.37999	1.41703
47484	0.92433	2.04544
47485	0.670081	1.89619
47486	0.727155	2.1
47487	0.786438	1.14402
47488	0.589798	2.1
47489	0.497784	2.1
47490	1.89226	2.1
47491	2.72301	2.1
47492	2.4158	2.06797
47493	2.4158	2.1
47494	1.89226	2.06338
47495	1.89226	2.1
47496	1.89226	2.1
47497	1.89226	2.1
47498	1.89226	2.1
47499	1.89226	2.1

47500	1.89226	2.1
47501	1.89226	2.1
47502	1.36919	2.1
47503	1.12717	2.1
47504	1.38634	2.1
47505	1.56541	2.1
47506	2.75552	2.1
47507	1.89226	2.1
47508	1.89226	2.1
47509	1.89226	2.1
47510	1.89226	2.1
47511	1.89226	2.1
47512	1.89226	2.1
47513	0.907036	2.1
47514	0.782706	2.1
47515	0.838808	2.1
47516	0.693168	2.1
47517	0.794747	2.1
47518	0.794747	2.1
47519	0.805458	2.1
47520	0.805458	2.1
47521	0.907036	2.1
47522	0.838808	2.1
47523	0.693169	2.1
47524	0.782707	2.1
47525	0.94613	2.1
47526	0.94613	2.1
47527	1.188	2.1
47528	1.28421	2.1
47529	1.18801	2.1
47530	1.18801	2.1
47531	0.946129	2.1
47532	0.946129	2.1
47533	1.28421	2.1
47534	0.94613	2.1
47535	1.188	2.1
47536	0.94613	2.1
47537	1.89226	2.1
47538	1.89226	2.1
47539	1.89226	2.1
47540	1.89226	2.1
47541	1.89226	2.1
47542	1.89226	2.1
47543	1.16817	2.1
47544	1.06007	2.1
47545	1.19785	2.1
47546	1.2832	2.1
47547	2.36602	2.1
47548	0.960106	2.1
47549	2.0073	2.1
47550	1.51754	2.1
47551	2.56883	2.1
47552	3.52484	2.1
47553	1.06756	2.1
47554	1.57913	1.83635
47555	1.87532	1.5568
47556	1.38666	2.1
47557	2.94287	2.1

47558	2.15622	1.21636
47559	1.38668	2.1
47560	1.87534	2.05769
47561	1.87534	2.1
47562	1.70539	1.80458
47563	1.70539	2.1
47564	0.986095	2.1
47565	1.36226	2.1
47566	1.3331	2.1
47567	1.3331	2.1
47568	1.07156	2.1
47569	1.07156	2.1
47570	0.682081	1.23721
47571	0.68748	1.13629
47572	0.650003	0.88862
47573	0.645873	0.971036
47574	0.666344	0.899796
47575	0.666344	1.09555
47576	0.66161	1.41551
47577	0.66161	0.92114
47578	0.68748	1.02524
47579	0.645873	1.01804
47580	0.650004	0.994884
47581	0.682082	0.993576
47582	0.946132	1.12477
47583	0.946132	1.03079
47584	0.946132	0.906482
47585	0.946132	0.824687
47586	0.946132	0.832995
47587	0.946132	1.01852
47588	0.946133	0.931149
47589	0.946133	1.11742
47590	0.946132	0.958612
47591	0.946132	0.961417
47592	0.946133	0.964186
47593	0.946133	0.963347
47594	1.89227	0.98948
47595	2.79312	0.679398
47596	2.77591	0.864338
47597	2.79312	0.896738
47598	1.87505	0.792694
47599	1.89227	0.847525
47600	0.68748	1.27544
47601	0.698626	1.18211
47602	0.645873	0.920272
47603	0.650148	0.981053
47604	0.670561	0.932266
47605	0.670561	1.14358
47606	0.667067	1.42809
47607	0.667067	0.929888
47608	0.698627	1.06904
47609	0.650148	1.06938
47610	0.68748	1.04871
47611	0.645873	1.04358
47612	0.946132	1.17911
47613	0.946132	1.05041
47614	0.946132	0.940831
47615	0.946132	0.885001

47616	0.946132	0.890678
47617	0.946132	1.04339
47618	0.946133	0.964855
47619	0.946133	1.17243
47620	0.946133	0.986536
47621	0.946133	0.991647
47622	0.946132	0.992587
47623	0.946132	0.994245
47624	0.946133	0.956774
47625	0.946133	0.894284
47626	1.38709	0.870489
47627	1.38795	0.737551
47628	0.946132	0.750745
47629	0.946132	0.878846
47630	1.38709	0.978566
47631	1.38709	0.804764
47632	1.38709	0.884771
47633	0.946133	0.884949
47634	0.946132	0.862122
47635	1.38795	0.846182
47636	1.70301	0.236896
47637	2.07659	0.226238
47638	1.89226	0.232823
47639	1.89226	0.250458
47640	1.76332	0.21597
47641	1.76331	0.233972
47642	1.89226	0.273265
47643	1.89226	0.250913
47644	1.89226	0.266446
47645	1.89226	0.285602
47646	1.89226	0.239735
47647	1.89226	0.259736
47648	1.89226	0.260152
47649	1.89226	0.280801
47650	1.89226	0.233073
47651	1.89226	0.240796
47652	3.78452	0.265504
47653	1.89226	0.224242
47654	2.12492	0.218709
47655	1.89226	0.227844
47656	1.89226	0.235937
47657	2.07659	0.211475
47658	2.07659	0.218268
47659	0.94613	0.214189
47660	0.94613	0.234234
47661	1.17498	0.21344
47662	1.32446	0.2012
47663	1.32446	0.213555
47664	0.94613	0.216749
47665	0.946127	0.219555
47666	1.17498	0.213091
47667	1.32446	0.213523
47668	1.32446	0.195789
47669	0.946128	0.2285
47670	0.946128	0.218616
47671	1.89226	2.1
47672	1.86472	2.1
47673	2.11049	2.1

47674	1.89226	2.1
47675	2.08295	2.1
47676	1.86472	2.1
47677	1.89226	2.1
47678	2.08295	2.1
47679	2.14023	2.1
47680	1.89226	2.1
47681	2.33091	2.1
47682	2.08295	2.1
47683	0.809215	2.1
47684	0.809215	1.96384
47685	0.810915	2.1
47686	0.810915	2.1
47687	1.09581	2.1
47688	1.08271	2.1
47689	0.520996	2.1
47690	0.537426	2.1
47691	0.520997	2.1
47692	1.08271	2.1
47693	0.537426	2.1
47694	1.09581	2.1
47695	0.79674	2.1
47696	0.79674	2.1
47697	0.799279	2.1
47698	0.799279	2.1
47699	1.07502	2.1
47700	1.0827	2.1
47701	0.508206	2.1
47702	0.520996	2.1
47703	0.508206	2.1
47704	1.07502	2.1
47705	0.520996	2.1
47706	1.0827	2.1
47707	2.15714	2.1
47708	1.70302	2.1
47709	1.78732	2.1
47710	2.02219	2.1
47711	3.98971	2.1
47712	2.0927	2.1
47713	2.162	2.1
47714	2.11563	2.1
47715	2.02219	2.1
47716	4.18419	2.1
47717	1.62483	2.1
47718	2.02219	2.1
47719	1.93617	2.1
47720	1.52008	2.1
47721	3.54227	2.1
47722	2.11563	2.1
47723	2.02219	2.1
47724	2.29218	2.1
47725	2.03975	2.1
47726	4.06194	2.1
47727	2.007	2.1
47728	1.89226	2.1
47729	1.89226	2.1
47730	1.89226	2.1
47731	2.06776	2.1

47732	2.06776	2.1
47733	0.946129	2.1
47734	0.946129	2.1
47735	1.0035	2.1
47736	1.0035	2.1
47737	0.946131	2.1
47738	0.946131	2.1
47739	0.968797	2.1
47740	1.0035	2.1
47741	0.968771	2.1
47742	0.946105	2.1
47743	1.00353	2.1
47744	0.946157	2.1
47745	1.79103	1.55249
47746	2.53286	0.790866
47747	2.60781	1.68564
47748	2.219	0.934255
47749	2.17985	1.26364
47750	1.79103	0.739468
47751	2.219	1.33165
47752	2.66359	1.26468
47753	2.70084	1.72947
47754	2.38698	1.14673
47755	2.53285	1.02829
47756	2.219	0.834166
47757	1.33179	1.2457
47758	0.946128	0.943184
47759	0.946133	1.14196
47760	1.26643	1.50182
47761	1.3318	1.00076
47762	1.3318	1.47909
47763	0.946131	0.859577
47764	0.946131	1.27806
47765	0.946131	0.897266
47766	0.946131	0.831142
47767	1.33179	1.08085
47768	1.26643	1.55142
47769	2.28979	2.1
47770	2.47227	2.1
47771	2.43451	2.1
47772	2.22627	2.1
47773	4.69855	2.1
47774	2.40667	2.1
47775	2.47228	2.1
47776	2.43451	2.1
47777	2.38069	2.1
47778	4.85296	2.1
47779	2.43451	2.1
47780	2.15715	2.1
47781	2.15715	2.1
47782	2.22627	2.1
47783	2.39489	2.1
47784	2.46402	2.1
47785	2.43451	2.1
47786	2.34899	2.1
47787	2.38069	2.1
47788	2.38069	2.1
47789	2.3949	2.1

47790	2.3949	2.1
47791	2.85425	1.03208
47792	1.70302	0.731338
47793	1.70302	0.674838
47794	1.52254	0.708264
47795	2.34181	0.780804
47796	2.08499	0.858728
47797	1.89226	0.707979
47798	1.89226	0.738529
47799	1.89226	0.510349
47800	1.89226	0.600335
47801	1.89226	0.588127
47802	1.89226	0.7712
47803	2.34181	0.903497
47804	1.38287	0.770269
47805	1.38287	0.767087
47806	1.84132	0.730659
47807	1.96416	0.765593
47808	2.4907	0.809951
47809	0.931432	0.800818
47810	0.946135	0.680354
47811	0.946133	0.633174
47812	0.946133	0.590099
47813	0.931464	0.73731
47814	0.931464	0.798794
47815	1.15573	0.629139
47816	0.96378	0.810661
47817	0.931434	0.751217
47818	0.946131	0.672332
47819	0.946131	0.656209
47820	1.13808	0.630709
47821	0.946132	0.77821
47822	0.946132	0.726271
47823	0.946132	0.800376
47824	0.946132	0.566284
47825	0.946133	0.769606
47826	0.946133	0.662821
47827	0.946132	0.774762
47828	0.946132	0.759584
47829	0.946133	0.747476
47830	0.946133	0.788791
47831	0.946132	0.611381
47832	0.946132	0.473033
47833	1.68074	2.1
47834	2.29459	2.1
47835	2.23361	2.1
47836	1.57234	2.1
47837	3.86693	2.1
47838	2.61243	2.1
47839	2.29458	2.1
47840	2.2336	2.1
47841	2.58234	2.1
47842	4.87692	2.1
47843	0.73506	2.1
47844	0.786166	2.1
47845	1.08729	2.1
47846	1.11679	2.1
47847	0.93134	2.1

47848	0.93134	2.1
47849	0.942113	2.1
47850	0.942113	2.1
47851	0.735065	2.1
47852	1.08729	2.1
47853	1.11679	2.1
47854	0.78616	2.1
47855	1.08729	2.1
47856	1.11679	2.1
47857	1.27502	2.1
47858	1.29116	2.1
47859	1.19259	2.1
47860	1.19259	2.1
47861	1.19923	2.1
47862	1.19923	2.1
47863	1.27503	2.1
47864	1.08729	2.1
47865	1.11679	2.1
47866	1.29115	2.1
47867	0.974715	0.869004
47868	1.43779	0.776548
47869	1.93915	0.782606
47870	1.53908	0.808076
47871	2.91387	0.79138
47872	1.53908	0.796033
47873	0.99845	0.81119
47874	1.5176	0.859254
47875	1.97963	0.774001
47876	3.05668	0.816256
47877	1.93915	0.77269
47878	1.97963	0.783527
47879	1.53908	0.781205
47880	1.53908	0.751765
47881	2.3376	0.814988
47882	2.3376	0.775165
47883	1.89226	2.1
47884	2.59747	2.1
47885	1.91802	2.1
47886	1.89226	2.1
47887	2.62322	2.1
47888	2.59747	2.1
47889	2.59747	2.1
47890	1.87119	2.1
47891	1.84844	2.1
47892	1.82268	2.1
47893	2.62323	2.1
47894	2.59747	2.1
47895	0.94613	2.1
47896	0.94613	2.1
47897	1.32697	2.1
47898	1.32697	2.1
47899	1.32697	2.1
47900	1.31161	2.1
47901	0.946132	2.1
47902	0.946132	1.92207
47903	0.94613	2.1
47904	1.32697	2.1
47905	0.946131	2.1

47906	1.31161	2.1
47907	2.62323	2.1
47908	1.91704	2.1
47909	1.90191	2.1
47910	1.87119	2.1
47911	2.65395	2.1
47912	2.62323	2.1
47913	1.89226	2.1
47914	1.89226	2.1
47915	2.15958	2.1
47916	2.15958	2.1
47917	2.46558	2.1
47918	2.19826	2.1
47919	2.46558	2.1
47920	1.89226	2.1
47921	1.89226	2.1
47922	1.89226	2.1
47923	2.61064	2.1
47924	2.61064	2.1
47925	1.81759	0.87775
47926	1.89526	0.888124
47927	2.32219	1.00979
47928	1.881	0.8315
47929	1.81759	0.954602
47930	1.3764	0.815077
47931	1.81759	0.881187
47932	2.57079	0.848448
47933	2.32219	1.06136
47934	2.32219	0.824563
47935	1.99875	0.938214
47936	1.99875	0.784937
47937	0.536182	0.767663
47938	0.579206	0.656782
47939	0.908796	0.875476
47940	0.999375	0.796626
47941	0.732091	0.805918
47942	0.732091	0.672046
47943	0.755911	0.762633
47944	0.755911	0.838514
47945	0.999376	0.787319
47946	0.579206	0.804994
47947	0.908796	0.805158
47948	0.536182	0.800297
47949	0.94613	0.640412
47950	0.94613	0.613617
47951	1.31112	0.691302
47952	1.18275	0.645751
47953	0.946126	0.679684
47954	0.946126	0.463634
47955	1.31112	0.695118
47956	1.31112	0.677919
47957	0.946128	0.610912
47958	1.31112	0.653305
47959	0.946128	0.624926
47960	1.18275	0.518715
47961	2.64617	0.939112
47962	1.27737	0.876863
47963	1.83076	0.958567

47964	1.27737	0.891262
47965	3.03764	0.944979
47966	2.3655	0.798944
47967	1.12991	0.159724
47968	1.22375	0.153503
47969	1.35931	0.156546
47970	1.04971	0.165295
47971	1.0419	0.159098
47972	1.15237	0.15868
47973	1.19593	0.158741
47974	1.30306	0.157317
47975	1.12937	0.153266
47976	1.31934	0.153417
47977	1.15203	0.164982
47978	1.15203	0.159815
47979	2.65227	0.153508
47980	1.46041	0.164125
47981	2.22712	0.154976
47982	2.04816	0.161086
47983	2.22712	0.156862
47984	2.04816	0.164309
47985	1.89226	0.310056
47986	1.89226	0.873853
47987	1.89226	0.675907
47988	1.89226	0.708749
47989	3.78452	0.761487
47990	1.24653	0.814933
47991	1.66554	0.871931
47992	1.53538	0.645881
47993	1.53538	0.951897
47994	1.40915	0.700622
47995	1.40915	0.962344
47996	1.66555	0.875271
47997	1.75835	2.1
47998	1.53538	1.11157
47999	1.65313	1.5221
48000	3.31868	1.42349
48001	1.89226	0.876092
48002	1.89226	0.909629
48003	1.89226	0.727176
48004	1.89226	1.58801
48005	3.78453	0.869232
48006	1.89226	0.544126
48007	1.89226	0.726421
48008	1.89226	0.476682
48009	1.89226	0.934198
48010	3.78452	0.502319
48011	0.658778	1.55068
48012	0.658778	0.795083
48013	0.661956	0.699068
48014	0.661956	0.915604
48015	0.703822	0.909288
48016	0.767689	1.36695
48017	0.623266	0.647278
48018	0.553046	0.881136
48019	0.703804	0.904735
48020	0.55303	0.906946
48021	0.623282	0.82785

48022	0.767711	0.832245
48023	1.53538	1.09018
48024	1.65313	1.65806
48025	1.40765	0.913846
48026	1.55779	2.1
48027	3.06078	1.1929
48028	1.89227	0.725017
48029	1.89227	1.66142
48030	1.89227	0.932685
48031	1.89227	0.931709
48032	3.78453	0.972256
48033	1.10517	2.1
48034	2.16532	2.1
48035	1.94374	2.1
48036	1.94001	2.1
48037	1.37756	2.1
48038	1.37756	2.1
48039	1.7453	0.419146
48040	1.83284	0.452205
48041	2.28483	0.528028
48042	1.77804	0.391889
48043	1.7453	0.574409
48044	1.23851	0.416404
48045	1.7453	0.478765
48046	2.54752	0.436123
48047	2.28483	0.50044
48048	2.28483	0.41034
48049	2.0189	0.515004
48050	2.0189	0.436216
48051	1.12457	0.388279
48052	2.0189	0.370893
48053	1.7453	0.427187
48054	1.7453	0.354527
48055	1.40707	0.439046
48056	1.40707	0.377285
48057	2.41176	2.1
48058	2.59262	2.1
48059	2.34085	2.1
48060	2.55827	2.1
48061	4.97003	2.1
48062	2.59262	2.1
48063	2.61901	2.1
48064	2.55827	2.1
48065	2.64067	2.1
48066	5.17728	2.1
48067	2.34084	2.1
48068	2.55827	2.1
48069	2.24937	2.1
48070	2.49053	2.1
48071	4.80764	2.1
48072	2.55826	2.1
48073	2.64067	2.1
48074	2.49053	2.1
48075	2.58639	2.1
48076	5.14465	2.1
48077	2.27918	0.262008
48078	2.44132	0.335989
48079	1.89226	0.261069

48080	1.89226	0.2872
48081	4.33359	0.266314
48082	1.89226	0.282352
48083	2.44132	0.338693
48084	2.6771	0.349028
48085	2.6771	0.296066
48086	1.89226	0.350537
48087	1.89226	0.301621
48088	1.60329	0.255357
48089	1.38246	0.289725
48090	1.98182	0.249118
48091	2.2217	0.298454
48092	3.60417	0.282408
48093	2.05362	0.242557
48094	1.02663	0.256607
48095	1.43255	0.26731
48096	1.52072	0.236421
48097	1.70302	0.266399
48098	1.7685	0.244587
48099	0.946131	0.295997
48100	0.946131	0.260185
48101	0.946131	0.270742
48102	0.946131	0.341791
48103	0.946131	0.342939
48104	0.946131	0.314593
48105	0.946131	0.25995
48106	0.946131	0.273733
48107	0.946131	0.300308
48108	0.946131	0.308154
48109	0.946131	0.298672
48110	0.946131	0.30214
48111	1.89226	0.301648
48112	1.89226	0.371255
48113	1.89226	0.375386
48114	1.89226	0.301338
48115	1.89226	0.379208
48116	1.89226	0.319935
48117	1.92645	0.945227
48118	2.83045	0.95154
48119	1.92225	1.01166
48120	2.01873	0.869713
48121	2.51732	1.02569
48122	2.51732	0.887554
48123	2.51732	0.838253
48124	2.23857	0.806282
48125	1.92645	0.909542
48126	1.92645	0.738059
48127	2.23857	0.967137
48128	1.50865	0.713542
48129	2.02687	2.1
48130	2.39604	2.1
48131	2.29198	2.1
48132	2.73046	2.1
48133	4.68803	2.1
48134	1.21148	0.78794
48135	1.87957	0.859021
48136	2.03555	0.615024
48137	2.03555	0.793901

48138	1.09962	0.694607
48139	1.09962	0.86163
48140	1.92558	0.741145
48141	2.05274	1.01672
48142	2.2443	0.581775
48143	2.24429	0.940723
48144	1.70869	0.673437
48145	1.70868	1.07773
48146	0.818397	0.765991
48147	0.818397	0.912054
48148	0.827911	0.742826
48149	0.827908	0.911048
48150	1.00418	0.867161
48151	1.02054	0.829895
48152	0.692018	0.83738
48153	0.620899	0.828178
48154	0.65823	0.701817
48155	1.00418	0.74447
48156	0.620899	0.850784
48157	1.0262	0.685169
48158	1.92558	0.884414
48159	2.51818	1.011
48160	1.94816	0.817437
48161	2.01985	0.960948
48162	1.85389	0.779913
48163	1.92558	0.973598
48164	0.903844	2.1
48165	0.903844	2.1
48166	0.946148	2.1
48167	0.946151	2.1
48168	0.946118	2.1
48169	0.903876	2.1
48170	0.946118	2.1
48171	1.10235	2.1
48172	0.946131	2.1
48173	0.946131	2.1
48174	1.10234	2.1
48175	0.903863	2.1
48176	1.63471	2.1
48177	1.08022	2.1
48178	1.56801	2.1
48179	1.03437	2.1
48180	2.66908	2.1
48181	1.56801	2.1
48182	0.97541	2.1
48183	0.97541	2.1
48184	1.03437	2.1
48185	1.49733	2.1
48186	1.55629	2.1
48187	1.89226	0.400346
48188	1.89226	0.550893
48189	1.89226	0.473172
48190	1.89226	0.519359
48191	3.78452	0.547993
48192	0.946131	0.450309
48193	1.16373	0.434812
48194	0.946131	0.471092
48195	0.734637	0.41321

48196	0.946132	0.454218
48197	0.946132	0.461893
48198	1.16373	0.38927
48199	0.734636	0.387994
48200	0.946093	0.449073
48201	0.946093	0.441662
48202	0.734675	0.414829
48203	0.734675	0.43453
48204	1.98413	0.366883
48205	1.87355	0.816817
48206	1.72774	0.354735
48207	1.67865	0.864056
48208	3.60128	0.475519
48209	1.87355	0.81804
48210	1.54924	1.66943
48211	1.68443	1.34407
48212	1.68443	0.829422
48213	1.67866	1.37953
48214	1.67866	0.837614
48215	1.72774	0.35948
48216	1.53422	0.849773
48217	1.67865	0.779782
48218	1.67865	0.368268
48219	1.53873	0.80288
48220	1.53873	0.422315
48221	1.67866	0.803182
48222	1.54924	1.7377
48223	1.53423	0.855377
48224	1.44091	1.50847
48225	3.11957	0.946685
48226	1.89226	1.9946
48227	1.89226	1.60057
48228	1.65573	1.76298
48229	1.65573	1.84914
48230	1.89226	1.81386
48231	1.89226	1.91366
48232	1.89226	1.91797
48233	1.89614	1.42528
48234	1.7508	1.59846
48235	1.72666	1.67571
48236	1.89226	1.64948
48237	1.89226	1.77747
48238	1.89223	1.73223
48239	1.89223	1.41248
48240	1.89223	1.42851
48241	1.89223	1.59623
48242	1.89223	1.49485
48243	1.89223	1.74615
48244	1.89224	1.67788
48245	1.89224	1.201
48246	1.89224	1.41827
48247	1.89224	1.42604
48248	1.89224	1.51381
48249	1.89224	1.56507
48250	2.09773	2.1
48251	2.06623	2.1
48252	2.43268	2.1
48253	2.33713	2.1

48254	1.703	2.1
48255	1.61372	2.1
48256	0.646929	1.3608
48257	0.689776	2.09499
48258	1.05897	2.1
48259	0.956364	2.1
48260	0.905594	2.1
48261	0.904315	1.41214
48262	0.727014	2.1
48263	0.850757	2.1
48264	0.798445	2.1
48265	0.654101	2.1
48266	0.717539	2.1
48267	1.0478	2.1
48268	1.89223	1.3206
48269	1.89223	1.09657
48270	1.89223	2.1
48271	1.6557	0.87248
48272	3.54794	1.13259
48273	0.946118	1.23585
48274	0.946118	2.1
48275	0.946118	2.1
48276	0.946118	1.99057
48277	0.946117	1.9791
48278	0.946117	1.08156
48279	0.946119	2.1
48280	0.946119	2.1
48281	0.946105	2.018
48282	0.946105	2.04931
48283	0.946131	2.0054
48284	0.946131	1.99643
48285	1.47671	2.1
48286	1.70303	2.1
48287	1.90705	2.1
48288	1.98945	2.1
48289	1.3454	2.1
48290	1.40353	2.1
48291	1.89226	2.1
48292	1.89226	1.67079
48293	1.89226	2.1
48294	1.89226	1.20047
48295	1.89226	2.1
48296	1.89226	1.03834
48297	2.39438	0.491347
48298	2.69842	0.453263
48299	2.45177	0.561193
48300	2.28563	0.413681
48301	4.84615	0.470085
48302	1.28421	2.1
48303	1.35966	2.1
48304	0.961001	2.1
48305	0.860549	2.1
48306	1.1039	2.1
48307	1.1039	2.1
48308	1.11631	2.1
48309	1.11631	2.1
48310	1.35966	2.1
48311	0.961001	2.1

48312	0.860549	2.1
48313	1.28421	2.1
48314	1.89226	1.4676
48315	1.89226	1.89818
48316	1.89226	1.9582
48317	1.89226	1.33728
48318	3.78452	1.49457
48319	1.48702	1.25697
48320	1.40656	1.35383
48321	0.626442	1.70092
48322	0.626442	1.60411
48323	0.626442	1.86812
48324	0.946132	1.3371
48325	1.05489	1.1105
48326	1.05012	1.9053
48327	0.946131	1.53677
48328	0.946131	1.57129
48329	0.626442	1.60564
48330	1.05012	1.52411
48331	1.89226	1.89481
48332	1.97212	1.42749
48333	1.89226	1.34356
48334	1.89226	1.63814
48335	3.78453	1.68462
48336	1.89226	1.89057
48337	1.89226	1.2899
48338	1.89226	1.32639
48339	1.89226	1.71437
48340	3.78452	1.67464
48341	1.89226	1.23189
48342	1.89226	1.55317
48343	1.89226	1.725
48344	1.89226	1.13326
48345	1.89226	1.75196
48346	1.89226	1.15862
48347	0.946161	1.09859
48348	0.946161	1.29247
48349	0.932156	1.55486
48350	0.932156	1.38379
48351	0.932184	1.57943
48352	0.946132	1.03197
48353	0.946133	1.55084
48354	0.946133	1.12704
48355	0.946132	1.22184
48356	0.946132	1.26563
48357	0.932185	1.27677
48358	0.946133	1.19482
48359	0.946132	1.25581
48360	0.946132	1.16116
48361	0.946132	1.92281
48362	0.946132	1.57256
48363	0.946132	1.51936
48364	0.946132	1.261
48365	0.946133	1.29175
48366	0.946133	1.67652
48367	0.946132	1.46068
48368	0.946132	1.61563
48369	0.946133	1.2648

48370	0.946133	1.36001
48371	1.79397	0.595799
48372	1.00636	0.439169
48373	1.31658	0.65829
48374	2.02924	0.524931
48375	3.0356	0.539865
48376	1.89226	0.505903
48377	1.89226	0.383112
48378	1.89226	0.44482
48379	1.89226	0.407605
48380	1.89226	0.444708
48381	1.89226	0.408354
48382	0.946131	0.498521
48383	0.946131	0.510984
48384	1.24766	0.586656
48385	1.03691	0.576771
48386	0.946129	0.54365
48387	0.946129	0.441468
48388	1.24766	0.595735
48389	1.24766	0.57732
48390	0.946131	0.497205
48391	1.24766	0.569135
48392	1.03691	0.586101
48393	0.946131	0.506665
48394	1.44824	0.484072
48395	2.02924	0.415411
48396	1.7065	0.491462
48397	1.38604	0.627309
48398	2.32017	0.440727
48399	2.32017	0.488697
48400	1.89226	0.399643
48401	1.89226	0.356098
48402	1.89226	0.337483
48403	1.89226	0.28656
48404	1.89226	0.448013
48405	1.89226	0.386611
48406	0.478349	0.992183
48407	0.612092	1.69968
48408	0.606115	2.03927
48409	0.506213	2.1
48410	0.550677	1.9741
48411	0.550677	0.992688
48412	0.567628	1.91652
48413	0.567628	2.1
48414	0.506213	1.789
48415	0.478349	1.82553
48416	0.606116	1.78863
48417	0.612092	1.89176
48418	1.52226	2.1
48419	1.54241	2.1
48420	1.23167	2.1
48421	1.23167	2.1
48422	1.75555	2.1
48423	1.75555	2.1
48424	1.75557	2.1
48425	1.54243	2.1
48426	1.9943	2.1
48427	1.85871	2.1

48428	3.53673	2.1
48429	1.85871	2.1
48430	1.06936	2.1
48431	1.88926	2.1
48432	1.54243	2.1
48433	1.41619	2.1
48434	1.06936	2.1
48435	1.28649	1.63522
48436	1.28516	2.00183
48437	1.38986	0.918644
48438	1.42399	1.42341
48439	2.67502	1.42636
48440	1.42399	1.05329
48441	1.07283	2.1
48442	1.46528	2.1
48443	1.28517	1.22872
48444	1.25294	1.78073
48445	1.07283	1.04936
48446	1.38986	1.73108
48447	1.42399	1.93479
48448	1.52885	1.03497
48449	1.60058	1.19824
48450	2.99043	1.41158
48451	0.715316	1.8529
48452	0.715316	1.51323
48453	0.719126	0.883156
48454	0.719126	2.1
48455	0.722447	1.82324
48456	0.626469	1.89128
48457	0.711995	1.12718
48458	0.800291	1.15561
48459	0.80029	1.32823
48460	0.722447	1.21006
48461	0.711995	1.498
48462	0.62647	1.55049
48463	2.05632	0.210493
48464	1.70303	0.234365
48465	2.3923	0.2455
48466	2.61277	0.213655
48467	1.44836	0.249454
48468	1.58184	0.21936
48469	1.89226	0.238727
48470	1.89226	0.312551
48471	1.89226	0.261485
48472	1.89226	0.274765
48473	3.78453	0.277557
48474	1.44091	1.27671
48475	1.1822	2.1
48476	1.25866	2.1
48477	1.25866	1.94985
48478	1.35215	2.1
48479	1.35215	1.91721
48480	1.27338	1.42376
48481	1.1822	2.1
48482	1.27338	2.1
48483	1.35215	1.99646
48484	1.10817	2.1
48485	1.18694	2.1

48486	0.946132	0.756501
48487	0.946132	0.743158
48488	0.946132	0.718164
48489	0.946132	0.708713
48490	0.946133	0.71748
48491	0.946133	0.74675
48492	0.946132	0.738842
48493	0.946132	0.709364
48494	0.946132	0.732033
48495	0.946132	0.722046
48496	0.946133	0.728478
48497	0.946133	0.735795
48498	2.6875	0.306766
48499	2.40558	0.305608
48500	2.1994	0.298434
48501	1.70303	0.323504
48502	4.68305	0.306811
48503	0.915523	0.302848
48504	0.915523	0.314299
48505	0.861057	0.276041
48506	0.946796	0.3115
48507	0.838652	0.318324
48508	0.654182	0.290469
48509	1.14044	0.27703
48510	0.80252	0.309559
48511	0.836435	0.302237
48512	0.608659	0.297031
48513	1.22198	0.30568
48514	0.863472	0.320534
48515	2.1994	0.224323
48516	0.981586	0.231139
48517	1.59619	0.233249
48518	1.70303	0.236169
48519	1.24816	0.229514
48520	1.38523	0.223672
48521	2.71141	2.1
48522	1.70302	2.1
48523	2.56527	2.1
48524	1.88649	2.1
48525	2.41279	2.1
48526	1.70302	2.1
48527	1.89226	1.63022
48528	1.89226	2.1
48529	1.89226	2.1
48530	1.89226	1.57946
48531	3.78453	2.06685
48532	1.89226	1.71943
48533	1.89226	2.1
48534	1.89226	2.1
48535	1.89226	2.08078
48536	1.89226	2.0896
48537	1.89226	1.65704
48538	2.18867	2.1
48539	0.983723	2.1
48540	2.42263	2.1
48541	1.8014	2.1
48542	1.62992	2.1
48543	0.983723	2.1

48544	2.71141	2.1
48545	1.89226	2.1
48546	2.31157	2.1
48547	2.31157	2.1
48548	1.89226	2.1
48549	1.89226	2.1
48550	1.89227	1.93394
48551	1.89227	1.31877
48552	1.89227	1.30472
48553	1.89227	1.87006
48554	3.78453	1.49398
48555	1.89226	1.42764
48556	1.89226	2.07827
48557	1.89226	1.99567
48558	1.89226	1.42961
48559	1.89226	1.93202
48560	1.89226	1.39137
48561	1.89226	1.21249
48562	1.89226	1.03397
48563	1.89226	1.56483
48564	1.89226	0.894106
48565	1.89226	1.11759
48566	1.89226	0.764746
48567	1.1427	1.40685
48568	1.14956	1.63845
48569	1.70302	1.04629
48570	1.81267	1.40218
48571	2.88983	1.38111
48572	1.89226	0.846283
48573	1.89226	1.3976
48574	1.89226	0.891636
48575	1.89226	0.974425
48576	3.78453	1.02792
48577	1.89226	1.14732
48578	1.89226	1.22001
48579	1.89226	0.772067
48580	1.89226	1.05956
48581	3.78452	0.952289
48582	0.884197	1.43825
48583	1.1125	1.10889
48584	1.13273	1.11283
48585	0.844557	1.26628
48586	0.981137	0.991528
48587	1.13971	1.31768
48588	0.980432	1.29455
48589	0.980432	1.02029
48590	1.15859	1.02947
48591	1.12206	0.845987
48592	0.898619	1.42416
48593	0.94426	1.20135
48594	2.21016	0.982508
48595	1.44091	1.65415
48596	1.98408	1.07367
48597	1.4218	0.963504
48598	2.21016	1.44769
48599	1.64788	1.45265
48600	1.07526	0.706048
48601	1.91531	2.1

48602	1.49155	2.01544
48603	1.58551	1.07367
48604	1.49155	0.932985
48605	1.58551	1.10325
48606	1.98979	0.207843
48607	2.21773	0.235526
48608	2.30533	0.228981
48609	2.55895	0.20471
48610	4.52305	0.215454
48611	2.55896	0.206999
48612	2.27963	0.239792
48613	2.60486	0.235856
48614	2.60486	0.217671
48615	2.21773	0.235073
48616	2.21773	0.210915
48617	2.30533	0.234233
48618	2.55895	0.2049
48619	2.29539	0.2039
48620	2.47308	0.241542
48621	4.7784	0.216117
48622	2.55896	0.207185
48623	2.55792	0.25257
48624	2.55792	0.245151
48625	2.60486	0.220505
48626	2.47308	0.238436
48627	2.52003	0.211005
48628	0.946133	1.63075
48629	0.946133	1.6934
48630	1.20851	1.38622
48631	1.20851	1.30965
48632	0.967411	1.33398
48633	1.20851	1.18363
48634	0.946132	1.78359
48635	0.946132	1.55875
48636	0.946132	1.58976
48637	1.20851	1.34468
48638	0.967412	1.36949
48639	0.946133	1.59062
48640	1.89226	1.55866
48641	1.89226	1.22949
48642	1.89226	1.24259
48643	1.89226	1.23149
48644	1.89226	1.54738
48645	1.89226	1.57593
48646	1.89226	1.53869
48647	2.41701	1.58747
48648	2.71724	1.28191
48649	2.71724	1.52165
48650	1.89226	1.7453
48651	1.89226	1.89453
48652	0.946131	1.64127
48653	0.946131	1.82281
48654	0.946128	1.40958
48655	0.946128	1.44075
48656	0.94613	1.12369
48657	0.94613	1.26526
48658	0.94613	1.64991
48659	0.94613	1.7278

48660	0.94613	1.51268
48661	0.94613	1.21691
48662	0.94613	1.36174
48663	0.94613	1.76237
48664	0.851541	0.519088
48665	0.851541	0.585837
48666	0.851481	0.540556
48667	0.997219	0.572035
48668	0.997112	0.459007
48669	0.992853	0.416945
48670	0.85154	0.479745
48671	0.85154	0.554124
48672	0.894824	0.410732
48673	0.894824	0.531554
48674	0.94613	0.606675
48675	0.906485	0.440638
48676	0.802947	2.1
48677	0.845556	2.1
48678	0.582339	2.1
48679	0.519756	2.1
48680	0.714508	2.1
48681	0.714508	2.1
48682	0.628932	2.1
48683	0.708518	2.1
48684	0.801358	2.1
48685	0.476016	2.1
48686	0.569477	2.1
48687	0.878553	2.1
48688	1.89226	2.1
48689	1.89226	2.1
48690	1.89226	2.1
48691	1.89226	2.1
48692	1.89226	2.1
48693	1.89226	2.1
48694	0.946128	2.1
48695	0.946128	2.1
48696	1.01537	2.1
48697	0.9662	2.1
48698	0.946131	2.1
48699	0.946131	2.1
48700	0.966198	2.1
48701	0.966198	2.1
48702	0.94613	2.1
48703	0.966198	2.1
48704	0.946131	2.1
48705	1.01537	2.1
48706	1.89226	2.1
48707	1.89226	2.1
48708	1.91883	2.1
48709	1.91883	2.1
48710	2.15905	2.1
48711	2.13249	2.1
48712	1.07953	2.1
48713	1.2107	2.1
48714	0.946132	2.1
48715	0.946132	2.1
48716	1.2107	2.1
48717	1.2107	2.1

48718	0.946132	2.1
48719	0.946132	2.1
48720	1.2107	2.1
48721	0.946133	2.1
48722	0.946132	2.1
48723	1.07953	2.1
48724	2.46752	2.1
48725	2.53733	2.1
48726	2.26044	2.1
48727	2.33222	1.28847
48728	4.79974	2.1
48729	2.53733	2.1
48730	2.37576	0.776724
48731	2.33222	1.51479
48732	2.1845	1.92555
48733	4.70798	1.60726
48734	2.48652	2.1
48735	2.33224	1.20303
48736	2.08149	2.1
48737	1.9839	2.1
48738	4.45623	2.1
48739	2.33223	1.3042
48740	2.18452	1.23737
48741	2.16061	2.1
48742	2.01399	0.81042
48743	4.34623	1.15136
48744	1.4617	1.21197
48745	1.34701	1.92543
48746	1.34701	1.22821
48747	1.25173	2.00777
48748	1.5117	1.20464
48749	1.41642	2.1
48750	2.65164	2.1
48751	1.89227	1.47581
48752	1.89227	1.95537
48753	1.89227	2.1
48754	2.42504	1.84165
48755	2.42504	2.1
48756	1.89226	1.1367
48757	1.89226	1.41289
48758	1.89226	1.93645
48759	1.89226	1.05481
48760	3.78452	1.20833
48761	1.5117	1.23736
48762	1.34701	1.62841
48763	1.47001	2.08586
48764	1.57961	1.13022
48765	2.92663	1.39372
48766	1.89226	1.63463
48767	1.89226	1.03552
48768	1.89226	1.1236
48769	1.89226	1.31238
48770	3.78452	1.24412
48771	0.412773	0.207098
48772	0.570839	0.214106
48773	0.50721	0.206486
48774	0.513638	0.230046
48775	0.518805	0.204565

48776	0.41278	0.203353
48777	1.5344	0.224728
48778	0.939282	0.221746
48779	0.720051	0.205387
48780	0.691767	0.223347
48781	0.761152	0.223306
48782	1.89226	1.75734
48783	1.89226	2.1
48784	1.89226	1.97467
48785	1.89226	2.1
48786	1.89226	1.87753
48787	1.89226	2.1
48788	1.24618	2.1
48789	2.10585	2.1
48790	1.08134	2.1
48791	1.93032	2.1
48792	3.1765	2.1
48793	1.98474	2.1
48794	2.57777	2.1
48795	1.93031	2.1
48796	2.19041	2.1
48797	3.99251	2.1
48798	1.89226	1.9417
48799	1.89226	2.1
48800	1.89226	2.1
48801	1.89226	1.92209
48802	3.78452	2.1
48803	1.67374	2.1
48804	2.19042	2.1
48805	1.67374	2.1
48806	2.09769	2.1
48807	2.29586	2.1
48808	2.7198	2.1
48809	1.89226	2.1
48810	1.89226	2.02712
48811	1.89226	2.03727
48812	1.89226	2.1
48813	1.89226	1.9991
48814	1.89226	2.1
48815	0.946132	0.761911
48816	0.946132	0.732184
48817	0.947763	0.748541
48818	0.909304	0.762742
48819	0.946127	0.724269
48820	0.947831	0.691738
48821	0.907673	0.72001
48822	0.907673	0.760114
48823	0.94613	0.718913
48824	0.947834	0.706791
48825	0.907671	0.79324
48826	0.94613	0.725531
48827	0.906519	0.656363
48828	0.906519	0.605119
48829	0.946097	0.640098
48830	0.946097	0.680897
48831	0.947834	0.635816
48832	0.906485	0.581185
48833	0.946131	0.622545

48834	0.946131	0.676199
48835	0.94613	0.698805
48836	0.906485	0.529776
48837	0.946131	0.692299
48838	0.947835	0.673068
48839	2.58638	2.1
48840	2.51039	2.1
48841	2.47395	2.1
48842	2.41539	2.1
48843	5.00178	2.1
48844	2.51039	2.1
48845	2.16633	2.1
48846	2.4154	2.1
48847	2.13977	2.1
48848	4.58173	2.1
48849	2.47395	2.1
48850	2.41539	2.1
48851	2.2988	2.1
48852	2.2604	2.1
48853	4.7142	2.1
48854	2.4154	2.1
48855	2.13977	2.1
48856	2.26041	2.1
48857	1.98957	2.1
48858	4.40497	2.1
48859	2.15721	0.776577
48860	2.31843	0.49619
48861	1.93973	0.895993
48862	2.13243	0.260694
48863	4.25816	0.507555
48864	2.31844	0.649669
48865	2.30908	0.406255
48866	2.13243	0.331343
48867	2.1609	0.398797
48868	4.47934	0.409054
48869	1.93973	0.889485
48870	2.13243	0.366587
48871	1.69661	0.840206
48872	1.9339	0.359605
48873	3.87363	0.530463
48874	2.13243	0.432931
48875	2.1609	0.504888
48876	1.9339	0.433683
48877	1.98414	0.4196
48878	4.0948	0.37077
48879	1.88857	0.147815
48880	2.34107	0.147815
48881	0.502431	0.147815
48882	0.71416	0.147815
48883	0.50243	0.147815
48884	0.944284	0.147815
48885	1.88856	2.1
48886	2.34107	2.1
48887	2.39662	2.1
48888	1.92293	2.1
48889	4.28519	2.1
48890	1.00486	2.1
48891	1.92293	2.1

48892	1.88857	2.1
48893	1.88857	2.1
48894	1.02605	2.1
48895	1.02605	2.1
48896	2.53728	2.1
48897	1.70303	2.1
48898	2.03734	2.1
48899	1.3624	2.1
48900	2.54706	2.1
48901	1.70303	2.1
48902	2.54706	2.1
48903	1.50876	2.1
48904	2.74067	2.1
48905	1.85391	2.1
48906	2.29667	2.1
48907	1.50876	2.1
48908	0.946133	2.1
48909	0.946133	2.1
48910	1.24191	2.1
48911	1.24191	2.1
48912	0.946132	2.1
48913	1.24191	2.0991
48914	1.23779	2.1
48915	0.946132	2.1
48916	0.946132	2.1
48917	0.946132	2.1
48918	1.23779	2.1
48919	1.24191	2.1
48920	0.946131	2.1
48921	0.946131	2.1
48922	1.14833	2.1
48923	1.14833	2.1
48924	1.14834	2.1
48925	0.946132	2.1
48926	0.94613	2.1
48927	1.24191	2.1
48928	0.946131	2.1
48929	0.946131	2.1
48930	1.24191	2.1
48931	1.14833	2.1
48932	2.08149	2.1
48933	2.31116	2.1
48934	1.9701	1.47041
48935	2.18328	2.1
48936	4.49563	2.01164
48937	2.08149	2.07651
48938	2.14059	2.1
48939	1.72377	2.1
48940	2.18328	2.1
48941	3.88931	2.1
48942	2.14059	2.1
48943	2.40755	2.1
48944	2.31145	2.1
48945	2.23743	2.1
48946	2.31145	2.1
48947	2.23743	2.1
48948	2.31117	2.1
48949	2.40754	2.1

48950	2.41055	2.1
48951	2.31145	2.1
48952	2.41055	2.1
48953	2.31145	2.1
48954	2.5615	0.746167
48955	2.37625	0.7345
48956	2.20248	0.731512
48957	2.20248	0.735476
48958	2.65967	0.747689
48959	2.65967	0.75604
48960	2.71793	0.732569
48961	2.65966	0.723855
48962	2.56149	0.711621
48963	2.56149	0.723419
48964	2.71296	0.731208
48965	2.71296	0.742453
48966	1.31528	0.719835
48967	1.32983	0.715945
48968	1.21128	0.725804
48969	1.18812	0.722106
48970	1.24232	0.698396
48971	1.24232	0.71691
48972	1.26109	0.715569
48973	1.26109	0.735816
48974	1.21128	0.721943
48975	1.31528	0.685351
48976	1.18812	0.711749
48977	1.32983	0.735862
48978	2.63056	0.691851
48979	2.58032	0.710108
48980	2.65966	0.624496
48981	2.65966	0.712719
48982	2.58032	0.726523
48983	2.71296	0.728617
48984	1.92039	2.1
48985	1.06226	2.1
48986	1.09252	2.1
48987	1.96052	2.1
48988	3.01291	2.1
48989	1.92039	2.1
48990	2.46338	2.1
48991	2.47522	2.1
48992	2.43508	2.1
48993	1.96052	2.1
48994	1.92039	2.1
48995	0.985949	2.1
48996	0.980261	2.1
48997	0.553625	2.1
48998	0.546262	2.1
48999	0.765671	2.1
49000	0.765671	2.1
49001	0.766539	2.1
49002	0.766539	2.1
49003	0.985949	2.1
49004	0.553625	2.1
49005	0.546261	2.1
49006	0.980261	2.1
49007	1.96052	2.1

49008	2.46338	2.1
49009	1.97189	2.1
49010	1.96052	2.1
49011	2.47932	2.1
49012	2.47932	2.1
49013	0.964608	0.147815
49014	0.691524	0.147815
49015	1.25462	0.147815
49016	0.691524	0.147815
49017	1.89226	0.147815
49018	1.89226	0.147815
49019	1.44894	0.147815
49020	2.77655	0.147815
49021	1.4237	0.147815
49022	2.77701	0.147815
49023	2.77701	0.147815
49024	1.38847	2.06168
49025	1.46031	1.57495
49026	1.65504	1.20552
49027	1.75396	1.21857
49028	3.11535	1.35154
49029	1.89226	1.42825
49030	1.89226	1.63846
49031	1.89226	1.23992
49032	1.89226	1.03633
49033	3.78452	1.45245
49034	1.89226	1.35876
49035	1.89226	1.71188
49036	1.89226	0.906399
49037	1.89226	1.38593
49038	3.78452	1.03216
49039	1.46031	1.26065
49040	1.34851	1.71398
49041	1.75395	1.06311
49042	1.62287	1.24496
49043	3.08318	1.52459
49044	1.65504	1.43758
49045	1.75396	1.63804
49046	1.89249	1.50424
49047	1.9857	1.14968
49048	3.64073	1.56463
49049	1.89226	1.50433
49050	1.89226	1.41011
49051	1.89226	1.01853
49052	1.89226	1.20336
49053	3.78452	1.11488
49054	1.89226	1.1628
49055	1.89226	1.68386
49056	1.89226	1.08711
49057	1.89226	1.31556
49058	3.78452	1.54309
49059	1.75395	1.41769
49060	1.62287	1.52875
49061	1.98569	1.01176
49062	1.86067	1.21451
49063	3.60856	1.17394
49064	1.35697	1.66838
49065	2.02064	1.50531

49066	1.89989	1.08933
49067	1.49571	2.1
49068	1.66753	1.20933
49069	1.36431	2.1
49070	0.744004	2.1
49071	0.743997	1.74117
49072	0.717972	1.07881
49073	0.717972	1.76856
49074	0.997982	2.1
49075	0.807796	1.8444
49076	0.632235	1.13511
49077	0.488575	2.07669
49078	0.849275	1.87528
49079	0.488581	1.92185
49080	0.724729	1.86074
49081	0.997967	1.87555
49082	2.5276	1.43052
49083	1.70791	1.41901
49084	1.82872	1.00121
49085	1.56362	2.02848
49086	2.5276	1.15408
49087	2.24781	2.1
49088	1.37591	2.1
49089	1.23217	1.28057
49090	1.00442	1.54298
49091	0.945282	1.02499
49092	1.15446	1.1694
49093	1.09123	2.1
49094	1.21506	1.32931
49095	1.21506	1.12673
49096	1.39452	1.25168
49097	1.13108	1.5407
49098	1.27876	1.18127
49099	0.929006	1.09106
49100	1.46505	0.441713
49101	1.91484	0.480958
49102	1.49906	0.488296
49103	1.49906	0.523896
49104	1.90196	0.401621
49105	1.90196	0.425062
49106	1.90196	0.443101
49107	2.11465	0.500233
49108	1.91484	0.508774
49109	1.91484	0.527924
49110	2.04016	0.423838
49111	2.04016	0.43234
49112	0.912201	0.398228
49113	0.950978	0.371564
49114	0.665307	0.363173
49115	0.732528	0.394438
49116	0.829512	0.333106
49117	0.829512	0.397372
49118	0.815217	0.346623
49119	0.815217	0.390593
49120	0.912202	0.356842
49121	0.665305	0.334334
49122	0.732527	0.436732
49123	0.95098	0.375788

49124	1.90196	0.414395
49125	2.08757	0.355027
49126	1.9626	0.396776
49127	2.04016	0.365584
49128	1.82441	0.38824
49129	1.90196	0.359149
49130	1.13048	2.1
49131	0.821162	2.1
49132	0.946131	2.1
49133	0.946131	2.1
49134	1.04567	2.1
49135	0.882347	2.1
49136	0.897276	2.1
49137	0.897276	2.1
49138	0.863109	2.1
49139	1.01055	2.1
49140	0.940305	2.1
49141	0.942166	2.1
49142	2.23933	0.220637
49143	0.976217	0.22119
49144	2.78053	0.236461
49145	2.78053	0.230048
49146	0.976217	0.276667
49147	1.65984	0.229923
49148	1.54168	1.96287
49149	2.01197	1.49125
49150	1.69136	1.90022
49151	2.13284	0.989728
49152	3.67452	1.53571
49153	0.988805	2.1
49154	1.78069	1.55277
49155	1.69136	1.62135
49156	1.69136	2.1
49157	1.05481	1.58779
49158	1.05481	2.1
49159	1.69136	2.1
49160	2.13284	1.24012
49161	1.78069	1.72429
49162	2.22044	1.20031
49163	3.91353	1.63367
49164	2.69379	0.964244
49165	2.32219	1.02498
49166	2.25746	1.32425
49167	2.25746	0.92423
49168	2.32219	1.05505
49169	1.89526	0.910798
49170	1.4556	2.1
49171	1.85028	2.09562
49172	2.4909	0.942256
49173	2.03799	1.97187
49174	2.73495	1.17996
49175	1.85028	1.59615
49176	2.71938	0.950639
49177	1.85028	2.1
49178	3.36051	1.88355
49179	2.73495	1.47532
49180	2.47584	1.55151
49181	1.85028	1.11997

49182	2.47584	1.40846
49183	1.04413	2.1
49184	2.1461	2.1
49185	1.74586	1.97076
49186	1.44437	2.1
49187	1.04413	1.95847
49188	1.89226	2.1
49189	1.89226	1.19264
49190	1.89226	1.31003
49191	1.89226	1.24475
49192	3.78452	1.36653
49193	1.52724	2.1
49194	1.89249	1.96118
49195	1.68143	2.1
49196	2.02433	1.21188
49197	3.55157	2.03584
49198	1.07444	2.1
49199	1.76545	2.0712
49200	1.68142	2.1
49201	1.68142	2.1
49202	1.14807	2.1
49203	1.14807	2.1
49204	1.89226	2.1
49205	1.89226	2.08458
49206	1.89226	2.1
49207	1.89226	1.42469
49208	1.89226	2.1
49209	1.89226	1.11972
49210	1.89226	2.04334
49211	1.89226	1.37616
49212	1.89226	2.0681
49213	1.89226	0.993395
49214	3.78452	1.32353
49215	1.68143	2.1
49216	2.02433	1.33557
49217	1.76545	2.04404
49218	2.12608	1.99154
49219	3.78978	2.01955
49220	2.62871	1.93849
49221	1.49149	2.1
49222	1.36814	2.1
49223	2.44761	1.42922
49224	3.9391	2.1
49225	1.89226	2.1
49226	1.89226	0.981656
49227	1.89226	1.4641
49228	1.89226	1.91155
49229	3.78452	1.50528
49230	0.851542	2.1
49231	0.851542	1.76049
49232	1.00897	2.1
49233	0.931224	2.1
49234	0.838952	2.1
49235	0.838952	1.59073
49236	0.956848	2.1
49237	0.942474	2.1
49238	0.946132	2.1
49239	0.838952	2.1

49240	0.878743	2.1
49241	0.946132	2.1
49242	1.15541	2.1
49243	2.44761	1.0903
49244	1.36814	2.1
49245	1.36814	1.69675
49246	2.19439	2.1
49247	2.19439	1.94611
49248	1.89226	1.09378
49249	1.89226	1.72251
49250	1.89226	2.1
49251	1.89226	1.03103
49252	3.78452	1.55999
49253	1.89226	2.1
49254	1.67791	2.1
49255	1.5713	2.1
49256	1.67791	2.1
49257	1.78566	2.1
49258	1.89226	1.85579
49259	1.89226	0.824655
49260	1.62911	1.57872
49261	2.24602	1.47066
49262	1.89226	1.45879
49263	1.98287	1.01963
49264	1.62911	0.995418
49265	1.89226	1.46697
49266	1.89226	1.48944
49267	1.89226	0.987778
49268	1.89226	1.95303
49269	3.78452	1.23262
49270	1.26688	2.1
49271	1.11804	2.02704
49272	0.852467	1.1722
49273	0.693314	2.1
49274	0.977459	1.70819
49275	0.977459	2.1
49276	0.982741	1.13946
49277	0.982741	1.94815
49278	1.26689	2.04461
49279	0.852464	2.04341
49280	0.693312	2.1
49281	1.11804	2.04053
49282	0.946131	1.87153
49283	0.946131	0.950918
49284	1.1794	0.929859
49285	1.1794	1.67763
49286	0.991434	1.73519
49287	1.1794	1.0121
49288	0.946131	0.881698
49289	0.946131	0.91197
49290	0.946131	1.37913
49291	1.1794	1.26885
49292	0.946131	1.29763
49293	0.991434	1.11563
49294	1.89226	0.946711
49295	1.89226	2.1
49296	1.89226	1.04412
49297	1.89226	1.21368

49298	3.78452	1.24636
49299	2.53377	2.03576
49300	1.53443	1.8879
49301	2.0815	1.07197
49302	2.82979	2.1
49303	4.35462	1.89799
49304	1.96192	0.900789
49305	1.23602	1.64907
49306	2.16506	0.941594
49307	1.56841	1.26825
49308	3.40108	1.14187
49309	1.89226	1.25004
49310	1.89226	1.62503
49311	1.89226	0.965799
49312	1.89226	1.8675
49313	3.78452	1.21542
49314	0.946129	2.1
49315	0.946129	1.87007
49316	1.13067	1.1359
49317	1.12952	2.01444
49318	0.946129	2.1
49319	1.13089	1.96789
49320	0.944978	1.12781
49321	0.944978	1.81668
49322	0.946131	2.08249
49323	1.13089	2.0467
49324	0.944977	2.1
49325	0.946131	2.06
49326	0.710208	2.1
49327	0.710208	2.1
49328	0.718894	2.1
49329	0.718894	2.1
49330	0.559773	2.1
49331	0.579321	2.1
49332	0.886767	2.1
49333	0.849781	2.1
49334	0.849781	2.1
49335	0.559774	2.1
49336	0.579321	2.1
49337	0.886766	2.1
49338	1.63471	2.1
49339	1.69956	2.1
49340	1.08022	2.1
49341	1.08022	2.1
49342	1.11955	2.1
49343	1.67403	2.1
49344	1.03766	2.1
49345	2.02825	2.1
49346	2.02794	2.1
49347	2.0348	2.1
49348	1.07196	2.1
49349	1.07196	2.1
49350	1.9276	2.03082
49351	2.1004	1.89771
49352	2.1306	2.1
49353	2.1306	1.82418
49354	1.92178	2.1
49355	1.92032	1.53907

49356	0.986724	2.1
49357	2.03064	2.1
49358	2.01385	2.1
49359	2.02404	2.1
49360	1.03766	2.1
49361	1.03766	2.1
49362	1.92027	2.1
49363	2.1306	2.1
49364	2.15388	2.1
49365	2.15388	2.05718
49366	1.92614	2.1
49367	1.9276	1.8787
49368	1.09578	2.1
49369	0.963018	2.02863
49370	0.782956	1.19262
49371	0.529693	1.92375
49372	0.6994	1.20695
49373	0.862532	2.1
49374	0.898605	1.98469
49375	0.898605	1.27625
49376	0.776756	1.78867
49377	1.14805	1.83124
49378	0.966521	1.76585
49379	0.617676	1.46118
49380	2.67185	1.62206
49381	2.099 2.1	
49382	2.80548	1.55116
49383	2.2727	2.1
49384	4.94455	2.1
49385	1.89226	1.62624
49386	1.89226	2.1
49387	1.89226	1.66165
49388	1.89226	2.1
49389	3.78453	2.1
49390	1.89226	2.1
49391	1.89226	2.1
49392	1.89226	2.1
49393	1.89226	2.1
49394	1.89226	2.1
49395	1.89226	2.1
49396	0.884801	2.1
49397	0.884801	2.1
49398	0.895199	2.1
49399	0.895199	2.1
49400	0.622774	2.1
49401	0.730501	2.1
49402	1.0495	2.1
49403	1.13635	2.1
49404	1.13635	2.1
49405	0.730503	2.1
49406	1.0495	2.1
49407	0.622775	2.1
49408	1.89226	1.35772
49409	1.89226	2.1
49410	1.89226	1.86591
49411	1.89226	2.1
49412	1.89226	1.53629
49413	1.89226	2.1

49414	1.89226	2.1
49415	1.89226	2.1
49416	1.89226	2.1
49417	1.89226	2.1
49418	1.89226	2.1
49419	1.89226	2.1
49420	2.2727	2.1
49421	1.89586	2.1
49422	1.66913	2.1
49423	1.46101	2.1
49424	2.48082	2.1
49425	2.2727	2.1
49426	1.89226	1.62064
49427	1.89226	2.0472
49428	1.84641	2.1
49429	1.89226	1.84603
49430	1.30868	1.97717
49431	1.34118	1.27576
49432	1.89226	1.04927
49433	1.89226	1.16696
49434	1.89226	1.23251
49435	1.89226	1.07506
49436	1.89226	1.10476
49437	1.89226	0.977343
49438	1.89226	1.1869
49439	1.89226	0.872941
49440	1.89226	1.03428
49441	1.89226	1.17986
49442	3.78452	1.0832
49443	0.946132	1.57436
49444	0.946132	1.34586
49445	0.946131	1.96702
49446	0.946131	2.06087
49447	0.946132	2.02549
49448	0.946132	1.20335
49449	0.946132	2.01292
49450	0.946132	1.64202
49451	0.946132	1.77355
49452	0.946132	1.79344
49453	0.946132	1.91206
49454	0.946132	1.85643
49455	1.89227	1.30599
49456	1.84641	1.41174
49457	2.29517	1.77171
49458	1.84641	1.27588
49459	2.34102	1.36038
49460	1.89227	1.12311
49461	1.89226	0.967123
49462	1.89226	1.01585
49463	1.89226	1.08978
49464	1.89226	0.971738
49465	1.89226	0.991711
49466	1.89226	0.842548
49467	1.89226	0.923788
49468	1.89226	1.03695
49469	1.89226	1.07417
49470	1.89226	0.962468
49471	1.89226	0.989312

49472	1.89226	0.792709
49473	1.89226	1.53387
49474	1.89226	1.20547
49475	1.89226	1.68321
49476	1.89226	1.15572
49477	1.89226	1.32599
49478	1.89226	1.14149
49479	0.851544	0.244893
49480	0.757542	0.278645
49481	1.13421	0.231651
49482	1.04072	0.243118
49483	0.946132	0.234248
49484	0.946132	0.242736
49485	0.841686	0.24348
49486	0.834428	0.237119
49487	1.13649	0.236553
49488	0.937687	0.242451
49489	0.841713	0.246058
49490	0.841713	0.269372
49491	1.89226	0.733952
49492	1.89226	0.78735
49493	1.89226	0.804194
49494	1.89226	0.685135
49495	1.89226	0.806648
49496	1.89226	0.690926
49497	2.31988	1.29574
49498	2.00082	1.68071
49499	2.4594	0.939514
49500	2.17638	1.54965
49501	4.46022	1.1776
49502	2.00082	1.10378
49503	1.41829	1.8805
49504	1.61207	1.38084
49505	1.43652	2.1
49506	2.17637	1.05034
49507	2.00082	2.1
49508	2.4594	1.01443
49509	2.17638	1.92702
49510	2.57957	0.963995
49511	2.3506	1.48921
49512	4.81	1.28645
49513	2.3506	1.10884
49514	1.57368	2.04091
49515	1.89111	0.921174
49516	1.89111	1.98182
49517	2.17638	0.943506
49518	2.17638	1.99872
49519	1.32813	2.1
49520	1.7237	2.1
49521	1.32461	2.1
49522	1.81656	2.1
49523	3.14468	2.1
49524	1.55132	2.1
49525	2.0815	2.1
49526	1.81656	2.1
49527	2.19826	2.1
49528	3.65818	2.1
49529	0.775951	2.1

49530	0.775951	2.1
49531	0.794601	2.1
49532	0.794601	2.1
49533	0.945641	2.1
49534	0.90828	2.1
49535	0.662302	2.1
49536	0.662273	2.1
49537	0.662274	2.1
49538	0.945639	2.1
49539	0.662304	2.1
49540	0.908279	2.1
49541	1.03062	2.1
49542	1.03062	2.1
49543	1.05054	2.1
49544	1.05054	2.1
49545	1.13551	2.1
49546	1.21275	2.1
49547	0.945641	2.1
49548	0.90828	2.1
49549	0.945641	2.1
49550	1.21275	2.1
49551	0.90828	2.1
49552	1.13552	2.1
49553	1.89226	2.1
49554	1.79883	2.1
49555	1.95341	2.1
49556	1.89226	2.1
49557	1.85998	2.1
49558	1.79883	2.1
49559	1.89226	2.1
49560	1.89226	2.1
49561	1.89226	2.1
49562	1.89226	2.1
49563	3.78453	2.1
49564	0.851541	2.1
49565	0.851541	2.1
49566	0.970398	2.1
49567	0.954365	2.1
49568	0.855356	2.1
49569	0.855357	2.1
49570	0.946133	2.1
49571	0.946133	2.1
49572	1.04072	2.1
49573	0.970397	2.1
49574	0.950003	2.1
49575	0.851538	2.1
49576	1.89226	2.1
49577	1.85998	2.1
49578	1.95296	2.1
49579	1.89226	2.1
49580	1.92067	2.1
49581	1.85998	2.1
49582	0.946133	2.1
49583	0.946133	2.1
49584	0.946132	2.1
49585	0.946132	2.1
49586	0.946132	2.1
49587	0.946132	2.1

49588	0.946132	2.1
49589	0.946132	2.1
49590	0.946132	2.1
49591	0.946132	2.1
49592	0.946133	2.1
49593	0.946133	2.1
49594	1.82152	2.1
49595	1.89226	2.1
49596	1.89226	2.1
49597	2.11152	2.1
49598	1.7472	2.1
49599	1.89226	2.1
49600	2.02687	2.1
49601	2.29199	2.1
49602	1.2734	2.1
49603	1.41077	2.1
49604	1.47381	2.1
49605	2.29199	2.1
49606	1.46889	2.1
49607	2.29199	2.1
49608	1.86766	2.1
49609	2.59401	2.1
49610	4.15965	2.1
49611	1.58995	2.1
49612	1.74246	2.1
49613	1.46643	2.1
49614	1.46643	2.1
49615	1.85097	2.1
49616	1.85097	2.1
49617	1.58995	2.1
49618	1.66584	2.1
49619	1.89214	2.1
49620	1.91787	2.1
49621	3.48208	2.1
49622	1.18474	2.1
49623	1.8144	2.1
49624	1.1486	2.1
49625	1.1486	2.1
49626	2.13071	2.1
49627	2.1257	2.1
49628	2.0815	2.1
49629	2.51651	2.1
49630	1.73681	2.1
49631	2.01601	2.1
49632	2.21406	2.1
49633	2.56998	2.1
49634	1.19995	2.1
49635	1.06256	2.1
49636	0.620677	2.1
49637	0.598084	2.1
49638	0.831426	2.1
49639	0.946056	2.1
49640	0.827692	2.1
49641	0.827692	2.1
49642	1.08958	2.1
49643	0.630132	2.1
49644	0.577547	2.1
49645	1.06104	2.1

49646	2.0815	2.1
49647	2.66249	2.1
49648	2.78966	2.1
49649	2.47108	2.1
49650	2.33858	2.1
49651	2.0815	2.1
49652	1.53735	2.1
49653	1.42278	2.1
49654	1.23654	2.1
49655	1.34678	2.1
49656	2.76955	2.1
49657	1.89226	2.1
49658	1.89226	2.1
49659	1.89226	2.1
49660	1.89226	2.1
49661	3.78452	2.1
49662	1.23654	2.1
49663	1.34678	2.1
49664	1.19 2.1	
49665	1.08576	2.1
49666	2.43254	2.1
49667	1.38776	2.1
49668	1.29366	2.1
49669	0.946131	2.1
49670	0.946131	2.1
49671	0.946131	2.1
49672	0.946131	2.1
49673	1.38775	2.1
49674	1.38775	2.1
49675	1.29366	2.1
49676	0.946132	2.1
49677	0.946131	2.1
49678	1.38775	2.1
49679	0.946131	2.1
49680	0.946131	2.1
49681	0.946129	2.1
49682	0.946129	2.1
49683	0.94613	2.1
49684	0.94613	2.1
49685	0.946131	2.1
49686	0.946131	2.1
49687	0.946131	2.1
49688	0.946131	2.1
49689	0.94613	2.1
49690	0.94613	2.1
49691	1.89226	0.701713
49692	1.89226	0.981601
49693	1.89226	0.759877
49694	1.89226	0.775988
49695	1.89226	0.780294
49696	1.89226	0.864529
49697	1.89226	0.752372
49698	1.89226	1.20569
49699	1.89226	0.813115
49700	1.89226	0.854046
49701	1.89226	0.822163
49702	1.89226	0.971039
49703	1.89226	0.738637

49704	1.89226	0.987763
49705	1.89226	0.768546
49706	1.89226	0.815123
49707	1.89226	0.790949
49708	1.89226	1.01252
49709	0.92338	2.062
49710	0.92338	0.969808
49711	0.94428	0.940332
49712	0.94428	0.82105
49713	1.19528	1.04171
49714	1.30598	1.98827
49715	0.714073	0.844583
49716	0.561679	1.27057
49717	1.19528	1.05737
49718	0.561678	1.06769
49719	0.714072	1.10466
49720	1.30598	1.12973
49721	2.61197	1.66496
49722	1.89226	1.17487
49723	1.89226	1.12056
49724	1.89226	1.96685
49725	2.39056	1.01102
49726	2.39056	1.98534
49727	0.946132	1.24175
49728	0.946132	0.975522
49729	0.946132	0.749436
49730	0.946132	0.827554
49731	0.946131	0.863349
49732	0.946131	1.24368
49733	0.946132	0.951354
49734	0.946132	0.793235
49735	0.946132	0.865844
49736	0.946132	0.892775
49737	0.946132	0.93227
49738	0.946132	0.915846
49739	1.89224	2.1
49740	1.89224	2.1
49741	1.89224	2.1
49742	1.89224	2.1
49743	3.78448	2.1
49744	1.97089	2.1
49745	1.57428	2.1
49746	1.84991	2.1
49747	1.49078	2.1
49748	3.42419	2.1
49749	0.621927	2.1
49750	0.621927	2.1
49751	0.630824	2.1
49752	0.630824	2.1
49753	0.483483	2.1
49754	0.507361	2.1
49755	0.787142	2.1
49756	0.745391	2.1
49757	0.745381	2.1
49758	0.483476	2.1
49759	0.507368	2.1
49760	0.787152	2.1
49761	1.89224	2.1

49762	1.89224	2.1
49763	1.89224	2.1
49764	1.89224	2.1
49765	1.89224	2.1
49766	1.89224	2.1
49767	1.89227	2.1
49768	1.89227	2.1
49769	1.89227	2.1
49770	1.89227	2.1
49771	1.89227	2.1
49772	1.89227	2.1
49773	1.84994	2.1
49774	1.41758	2.1
49775	1.4908	2.1
49776	1.4908	2.1
49777	1.74391	2.1
49778	1.74391	2.1
49779	1.89226	2.1
49780	1.89226	2.1
49781	1.89226	2.1
49782	1.89226	2.1
49783	3.78452	2.1
49784	1.01323	2.1
49785	1.01323	2.1
49786	1.01445	2.1
49787	1.01445	2.1
49788	0.839531	2.1
49789	0.875652	2.1
49790	1.19063	2.1
49791	1.15203	2.1
49792	1.15203	2.1
49793	0.839531	2.1
49794	0.875652	2.1
49795	1.19063	2.1
49796	1.89226	2.1
49797	2.75587	2.1
49798	2.81512	2.1
49799	2.81512	2.1
49800	1.89226	2.1
49801	1.89226	2.1
49802	1.89226	2.1
49803	1.89226	2.1
49804	1.89226	2.1
49805	1.89226	2.1
49806	3.78452	2.1
49807	0.974831	2.1
49808	0.974831	2.1
49809	0.976681	2.1
49810	0.976681	2.1
49811	0.803179	2.1
49812	0.83953	2.1
49813	1.11198	2.1
49814	1.15203	2.1
49815	1.11198	2.1
49816	0.803179	2.1
49817	1.15203	2.1
49818	0.83953	2.1
49819	1.37794	2.1

49820	1.37794	2.1
49821	0.946128	2.1
49822	0.946128	2.1
49823	1.3477	2.1
49824	1.37793	2.1
49825	0.946131	2.1
49826	0.946131	2.1
49827	0.946129	2.1
49828	1.3477	2.1
49829	1.37793	2.1
49830	0.946129	2.1
49831	2.38577	1.20912
49832	1.20247	2.05811
49833	1.75182	2.00454
49834	2.82546	1.24235
49835	4.1376	1.89994
49836	2.82546	1.75303
49837	1.89226	0.914342
49838	2.38577	1.36192
49839	2.38577	1.27263
49840	1.89226	1.48189
49841	1.89226	1.24668
49842	1.035 2.1	
49843	2.15429	2.1
49844	0.970998	2.1
49845	2.0815	2.1
49846	2.89672	2.1
49847	0.946131	0.446161
49848	0.946131	0.424868
49849	0.853249	0.424131
49850	1.02715	0.448544
49851	0.851537	0.424887
49852	0.851537	0.437669
49853	1.02663	0.403643
49854	1.13139	0.378814
49855	0.946128	0.394779
49856	0.932039	0.458556
49857	0.843985	0.385241
49858	0.946129	0.39958
49859	1.89226	0.392009
49860	1.86408	0.429111
49861	1.96548	0.463334
49862	1.86408	0.445125
49863	1.99366	0.39469
49864	1.89226	0.38404
49865	2.70702	2.1
49866	2.08981	2.1
49867	2.08693	2.1
49868	2.08693	2.1
49869	2.7188	2.1
49870	2.7188	2.1
49871	2.08982	2.1
49872	1.01964	2.1
49873	2.08693	2.1
49874	1.04615	2.1
49875	3.13597	2.1
49876	2.70702	2.1
49877	2.08693	2.1

49878	2.68784	2.1
49879	2.08831	2.1
49880	4.77477	2.1
49881	0.778233	2.1
49882	0.778233	2.1
49883	0.789001	2.1
49884	0.789001	2.1
49885	0.545307	2.1
49886	0.523075	2.1
49887	1.04347	2.1
49888	1.04416	2.1
49889	1.04416	2.1
49890	0.545308	2.1
49891	1.04347	2.1
49892	0.523075	2.1
49893	1.89226	0.7
49894	1.89226	0.7
49895	1.89226	0.7
49896	1.89226	0.602203
49897	3.78453	0.7
49898	1.39237	0.7
49899	1.17933	0.79196
49900	0.755796	0.7
49901	0.755796	0.7
49902	0.755767	0.566395
49903	0.94613	0.708606
49904	1.15918	0.7
49905	0.94613	0.7
49906	1.11632	0.7
49907	0.92595	0.633335
49908	0.94613	0.7
49909	0.94613	0.7
49910	1.89226	0.7
49911	1.89226	0.7
49912	1.89226	0.574504
49913	1.89226	0.64726
49914	3.78452	0.69145
49915	0.950067	0.694421
49916	0.75071	0.7
49917	0.906516	0.53731
49918	0.906516	0.524484
49919	0.81586	0.459354
49920	0.851541	0.7
49921	0.962101	0.496446
49922	0.851476	0.7
49923	0.962105	0.44298
49924	0.815808	0.546966
49925	0.851529	0.7
49926	0.851529	0.61938
49927	1.05032	0.6066
49928	0.972326	0.663287
49929	0.906515	0.466611
49930	0.906515	0.455278
49931	1.12245	0.465947
49932	1.04072	0.529312
49933	0.815857	0.472863
49934	0.851539	0.698237
49935	1.04451	0.418406

49936	0.815853	0.608773
49937	0.919795	0.616214
49938	0.919795	0.651724
49939	1.5855	1.87422
49940	2.14058	2.1
49941	1.9153	2.1
49942	1.79922	2.1
49943	3.72608	2.1
49944	1.58551	0.987879
49945	1.24201	1.38596
49946	0.960287	1.03491
49947	1.79922	2.1
49948	2.82752	2.1
49949	0.679602	2.1
49950	0.621009	2.1
49951	0.899614	2.1
49952	0.940441	2.1
49953	0.679602	2.1
49954	0.94044	2.1
49955	0.621009	2.1
49956	0.899615	2.1
49957	0.794027	2.1
49958	0.794027	2.1
49959	0.785189	2.1
49960	0.785189	1.26996
49961	2.14058	2.1
49962	1.79922	2.1
49963	2.23742	2.1
49964	2.23742	2.1
49965	1.78404	2.1
49966	1.88088	2.1
49967	1.15177	2.1
49968	2.0815	1.79966
49969	1.93765	2.1
49970	1.78342	1.74466
49971	1.39511	2.1
49972	1.28406	1.79349
49973	1.97719	1.79979
49974	2.50057	0.909964
49975	2.35349	1.62446
49976	2.35349	0.910721
49977	2.31686	1.71577
49978	2.32857	0.916677
49979	1.93765	1.65496
49980	2.35349	0.924085
49981	1.70048	1.72843
49982	2.15721	0.741943
49983	4.05397	1.04393
49984	0.850388	1.34797
49985	0.850388	2.1
49986	0.840019	2.1
49987	0.840019	2.1
49988	1.13736	1.83333
49989	1.13852	1.59221
49990	0.573661	2.1
49991	0.551891	2.1
49992	0.573661	2.1
49993	1.13852	2.1

49994	0.551892	2.1
49995	1.13736	2.1
49996	0.860203	1.45254
49997	0.860203	2.1
49998	0.855758	2.1
49999	0.855758	2.06871
50000	1.1423	1.60415
50001	1.13852	1.97183
50002	0.58628	2.1
50003	0.573661	2.1
50004	0.586281	2.1
50005	1.1423	2.1
50006	0.573661	2.1
50007	1.13852	2.1
50008	1.28918	0.921472
50009	0.999086	0.857093
50010	1.0383	1.36986
50011	0.736019	1.16636
50012	1.00954	1.2424
50013	1.00954	0.935063
50014	1.01566	0.83467
50015	1.01566	1.25263
50016	1.28918	1.0638
50017	1.0383	1.30789
50018	0.999086	1.13405
50019	0.736019	1.07419
50020	2.20797	2.1
50021	2.64748	2.08402
50022	2.50375	1.45761
50023	2.50375	2.1
50024	2.40858	1.80646
50025	2.40858	2.1
50026	2.64748	2.09191
50027	2.74744	2.1
50028	2.50375	2.1
50029	2.6521	2.1
50030	5.29958	2.1
50031	2.20797	2.1
50032	2.50375	2.1
50033	2.02064	1.28476
50034	2.3606	2.1
50035	4.52439	2.1
50036	2.36059	2.1
50037	2.6521	2.1
50038	2.56952	2.1
50039	2.56952	2.1
50040	2.50375	2.1
50041	2.50375	2.1
50042	2.49439	0.407896
50043	1.89226	0.478104
50044	2.81337	0.467853
50045	1.89226	0.514633
50046	4.38665	0.474065
50047	1.89226	0.526667
50048	1.89226	0.632102
50049	1.89226	0.648003
50050	1.89226	0.648757
50051	3.78452	0.611785

50052	1.89226	0.484458
50053	1.89226	0.554774
50054	1.89226	0.528604
50055	1.89226	0.597577
50056	3.78452	0.548655
50057	1.93948	0.676719
50058	1.98874	0.584606
50059	1.62494	0.663008
50060	2.14218	0.68381
50061	1.62494	0.491255
50062	2.14218	0.556538
50063	1.47354	0.544912
50064	1.98874	0.503937
50065	2.01865	0.562343
50066	1.62495	0.471653
50067	2.01865	0.602551
50068	1.62495	0.471976
50069	1.89226	0.918774
50070	1.89226	2.1
50071	1.89226	1.3984
50072	1.89226	1.86534
50073	3.78453	1.79407
50074	2.05257	2.1
50075	1.94441	2.1
50076	1.88487	1.38282
50077	1.82441	2.1
50078	3.82928	2.1
50079	1.94441	2.1
50080	1.74391	2.1
50081	1.82441	2.1
50082	1.65636	2.1
50083	3.60077	2.1
50084	1.89226	2.1
50085	1.89226	2.1
50086	1.89226	1.89668
50087	1.89226	2.1
50088	3.78452	2.1
50089	1.89226	1.47153
50090	1.89226	1.88665
50091	1.89226	0.923457
50092	1.89226	2.1
50093	3.78453	1.54483
50094	1.88487	1.49908
50095	1.82441	2.1
50096	1.75835	2.1
50097	1.72866	2.1
50098	3.61354	2.1
50099	1.82441	2.1
50100	1.65636	2.1
50101	1.72866	2.1
50102	1.58401	2.1
50103	3.38503	2.1
50104	1.89226	1.89639
50105	1.89226	2.1
50106	1.89226	2.1
50107	1.89226	2.1
50108	3.78452	2.1
50109	2.27432	2.1

50110	1.33339	2.1
50111	1.38905	2.1
50112	2.34471	2.1
50113	3.66337	2.1
50114	1.89226	2.1
50115	2.34471	2.1
50116	2.27432	2.1
50117	2.27432	2.1
50118	1.89226	2.1
50119	1.89226	2.1
50120	1.20443	2.1
50121	1.17235	2.1
50122	0.724695	2.1
50123	0.694522	2.1
50124	0.949994	2.1
50125	0.949993	2.1
50126	0.948955	2.1
50127	0.948955	2.1
50128	1.20443	2.1
50129	0.7247	2.1
50130	0.694517	2.1
50131	1.17234	2.1
50132	1.89225	2.1
50133	2.40885	2.1
50134	2.3447	2.1
50135	2.3447	2.1
50136	1.89225	2.1
50137	1.89225	2.1
50138	1.37138	2.1
50139	1.82597	2.1
50140	1.45027	2.1
50141	1.92772	2.1
50142	3.27624	2.1
50143	1.89226	2.1
50144	1.89226	2.1
50145	1.89226	2.1
50146	1.89226	1.78144
50147	3.78452	2.1
50148	1.89226	2.1
50149	1.89226	1.17163
50150	1.89226	1.6161
50151	1.89226	1.31403
50152	3.78452	1.81685
50153	1.82597	2.1
50154	2.02312	0.981327
50155	1.92772	2.1
50156	2.15888	2.1
50157	3.98485	1.33446
50158	1.53446	2.1
50159	1.92772	2.1
50160	2.03975	2.1
50161	2.03975	2.1
50162	1.45027	2.1
50163	1.45027	2.1
50164	1.89226	2.1
50165	1.89226	1.87259
50166	1.89226	2.1
50167	1.89226	2.1

50168	1.89226	2.1
50169	1.89226	2.1
50170	1.89226	1.69631
50171	1.89226	2.05669
50172	1.89226	2.1
50173	1.89226	1.26164
50174	3.78452	1.73037
50175	1.92772	2.1
50176	2.15888	2.1
50177	2.03975	2.1
50178	2.3111	2.1
50179	4.19863	2.1
50180	1.89226	2.1
50181	1.89226	2.1
50182	1.89226	2.1
50183	1.89226	2.1
50184	3.78452	2.1
50185	1.78376	2.1
50186	1.3201	2.1
50187	1.78376	2.1
50188	1.86102	2.1
50189	1.2679	2.1
50190	1.34517	2.1
50191	1.89226	2.1
50192	2.52442	2.1
50193	1.89226	2.1
50194	2.51691	2.1
50195	4.41667	2.1
50196	1.89226	2.1
50197	1.89226	2.1
50198	1.89226	2.1
50199	1.89226	2.1
50200	3.78452	2.1
50201	1.78376	2.1
50202	1.2679	2.1
50203	1.71839	2.1
50204	1.23504	2.1
50205	3.0188	2.1
50206	1.89226	2.1
50207	2.51795	2.1
50208	2.51691	2.1
50209	2.51691	2.1
50210	1.89226	2.1
50211	1.89226	2.1
50212	1.89226	0.902995
50213	1.89226	1.00249
50214	1.89226	0.982707
50215	1.89226	0.953866
50216	3.78452	0.948278
50217	1.89227	0.943377
50218	1.89227	1.02899
50219	3.26088	1.02277
50220	2.79543	0.988493
50221	2.20733	1.04286
50222	1.89227	0.99404
50223	1.89226	0.974358
50224	1.89226	0.965886
50225	1.89226	0.926461

50226	1.89226	0.971981
50227	1.89226	0.937229
50228	1.89226	0.988528
50229	1.89227	0.954935
50230	2.20733	1.00689
50231	1.46973	1.05873
50232	1.89227	0.960379
50233	1.7848	1.05705
50234	2.20733	0.961714
50235	1.89226	2.1
50236	2.55682	2.1
50237	2.81593	2.1
50238	2.81593	2.1
50239	1.89226	2.1
50240	1.89226	2.1
50241	1.0607	2.09914
50242	1.0607	2.1
50243	1.12617	2.1
50244	0.986957	2.1
50245	0.699328	2.0205
50246	0.785567	1.86403
50247	1.28855	2.1
50248	1.41913	2.1
50249	0.766737	1.3944
50250	1.34711	2.1
50251	1.24643	2.1
50252	0.687289	1.73963
50253	3.11063	0.624922
50254	1.89226	0.690627
50255	1.96536	0.665021
50256	1.89226	0.671456
50257	1.96536	0.634234
50258	1.89226	0.642298
50259	1.89226	0.909345
50260	1.89226	0.7373
50261	1.89226	0.828739
50262	1.89226	0.816041
50263	3.78452	0.901188
50264	1.89226	0.681103
50265	1.96256	0.513165
50266	1.89226	0.555671
50267	1.89226	0.70334
50268	2.49192	0.68643
50269	2.49192	0.536388
50270	1.85716	0.665256
50271	2.56871	0.664943
50272	1.03043	0.569668
50273	1.03043	0.584831
50274	2.48125	0.502406
50275	1.65452	0.651572
50276	1.89226	2.1
50277	2.09622	2.1
50278	2.09622	2.1
50279	2.08508	2.1
50280	1.89226	2.1
50281	1.88112	2.1
50282	1.04811	2.1
50283	1.04811	2.1

50284	0.946131	2.1
50285	0.946131	2.1
50286	1.05014	2.1
50287	1.04811	2.1
50288	0.94613	2.1
50289	0.94613	2.1
50290	0.946131	2.1
50291	1.05013	2.1
50292	1.04811	2.1
50293	0.946131	2.1
50294	1.398	2.1
50295	2.10766	2.1
50296	2.3018	2.1
50297	2.3018	2.1
50298	1.34008	2.1
50299	1.34008	2.1
50300	1.1509	2.1
50301	1.1509	2.1
50302	0.946132	2.1
50303	0.946132	2.1
50304	0.946132	2.1
50305	0.946132	2.1
50306	1.05383	2.1
50307	1.1509	2.1
50308	1.1509	2.1
50309	0.946133	2.1
50310	0.946132	2.1
50311	1.05383	2.1
50312	0.931141	2.1
50313	0.931141	2.1
50314	0.971946	2.1
50315	0.971946	2.1
50316	1.28569	2.1
50317	1.1509	2.1
50318	0.699	2.1
50319	0.752185	2.1
50320	1.28568	2.1
50321	0.752186	2.1
50322	0.699001	2.1
50323	1.1509	2.1
50324	2.30181	2.1
50325	1.89226	2.1
50326	1.89226	2.1
50327	1.89226	2.1
50328	2.57137	2.1
50329	2.57137	2.1
50330	2.0815	2.1
50331	1.21962	2.1
50332	1.55173	2.1
50333	2.57849	2.1
50334	3.79001	2.1
50335	1.38779	2.1
50336	1.2923	2.1
50337	1.03974	2.1
50338	1.28832	2.1
50339	1.33566	2.1
50340	1.21116	2.1
50341	1.22343	2.1

50342	1.22343	2.1
50343	1.42288	2.1
50344	1.20138	2.1
50345	1.26025	2.1
50346	1.00644	2.1
50347	1.26066	2.1
50348	1.14271	2.1
50349	0.886635	2.1
50350	0.795277	2.1
50351	1.02966	2.1
50352	1.02967	2.1
50353	1.05216	2.1
50354	1.05216	2.1
50355	1.26068	2.1
50356	0.790042	2.1
50357	0.795269	2.1
50358	1.20135	2.1
50359	1.89226	0.876471
50360	1.89226	0.84521
50361	1.89226	0.898233
50362	1.89226	0.785628
50363	1.89226	0.918007
50364	1.89226	0.826244
50365	1.89227	0.799355
50366	1.89227	0.777134
50367	1.89227	0.84634
50368	1.89227	0.704564
50369	1.89227	0.862544
50370	1.89227	0.736467
50371	0.563873	0.68268
50372	0.50988	0.621753
50373	0.598693	0.779952
50374	0.558819	0.708998
50375	0.564855	0.693167
50376	0.564855	0.632081
50377	0.557838	0.767739
50378	0.557838	0.701023
50379	0.563873	0.713338
50380	0.598694	0.70918
50381	0.558819	0.682056
50382	0.50988	0.689799
50383	0.598693	0.768954
50384	0.558819	0.695581
50385	0.572737	0.847585
50386	0.534248	0.798662
50387	0.566788	0.78774
50388	0.566788	0.715326
50389	0.566153	0.838976
50390	0.566153	0.78301
50391	0.598694	0.794803
50392	0.572737	0.791058
50393	0.534248	0.759604
50394	0.558819	0.758081
50395	1.85217	1.01887
50396	1.93111	2.1
50397	1.92875	0.981725
50398	2.02391	2.1
50399	3.85986	2.1

50400	1.93112	2.1
50401	1.80425	2.1
50402	2.02392	2.1
50403	1.90981	2.1
50404	3.84092	2.1
50405	1.92876	0.921968
50406	2.02393	2.1
50407	2.05274	1.06637
50408	2.16783	2.1
50409	4.09659	1.83204
50410	2.02393	2.1
50411	1.90982	2.1
50412	2.16784	2.1
50413	2.05967	2.1
50414	4.07766	2.1
50415	1.89226	1.29543
50416	1.89226	1.16313
50417	1.89226	1.28682
50418	1.89226	1.22888
50419	3.78453	1.10037
50420	1.89226	1.11601
50421	1.89226	1.31548
50422	1.89226	1.61709
50423	2.44829	1.08458
50424	3.78453	1.49326
50425	1.89226	1.27785
50426	1.89226	1.36225
50427	1.89226	1.19862
50428	2.60452	1.15127
50429	3.78453	1.61501
50430	1.58723	0.334198
50431	1.88071	0.468047
50432	2.97376	0.330532
50433	2.13255	0.41659
50434	4.43982	0.384305
50435	2.20641	0.518403
50436	1.33575	0.602746
50437	2.15824	0.552141
50438	2.15824	0.55299
50439	1.2951	0.552666
50440	1.2951	0.565672
50441	0.825383	0.614484
50442	0.825383	0.583123
50443	0.856104	0.554757
50444	0.856104	0.550838
50445	0.952196	0.594649
50446	0.602363	0.554128
50447	0.667877	0.607628
50448	1.07913	0.557438
50449	0.602363	0.599573
50450	0.667876	0.603343
50451	1.07913	0.608456
50452	0.952197	0.591702
50453	2.57657	1.11177
50454	1.44437	2.1
50455	2.65378	2.04237
50456	2.25076	1.66076
50457	1.84739	1.98071

50458	1.44437	1.44785
50459	2.0189	0.458658
50460	2.54752	0.448003
50461	2.63975	0.547906
50462	2.31049	0.380992
50463	4.65865	0.452022
50464	1.40707	0.377145
50465	1.89605	0.365362
50466	2.0189	0.410266
50467	2.0189	0.346627
50468	1.50438	0.418659
50469	1.50438	0.359611
50470	2.63971	0.523152
50471	2.38246	0.385787
50472	2.13402	0.440371
50473	2.57503	0.418155
50474	4.77373	0.441351
50475	1.50436	0.37013
50476	1.96803	0.363493
50477	2.13402	0.428697
50478	1.45446	0.320944
50479	3.63838	0.36542
50480	2.30793	0.54668
50481	2.11032	0.495303
50482	1.24082	0.504523
50483	1.05689	0.542167
50484	3.36482	0.544354
50485	1.89226	0.662261
50486	1.89226	0.701529
50487	1.89226	0.718458
50488	1.89226	0.660828
50489	3.78453	0.66662
50490	0.812967	0.557743
50491	0.812967	0.577746
50492	1.00181	0.548431
50493	1.08141	0.530838
50494	0.946132	0.510151
50495	0.812938	0.573101
50496	0.932815	0.610365
50497	1.05577	0.540944
50498	0.812938	0.557338
50499	0.932812	0.587393
50500	0.946133	0.596216
50501	0.946133	0.561311
50502	2.53897	2.1
50503	1.89226	2.1
50504	1.89226	2.1
50505	1.89226	2.1
50506	2.3245	2.1
50507	2.3245	2.1
50508	1.00255	1.22271
50509	1.06561	2.1
50510	1.05481	0.827088
50511	1.11077	1.99167
50512	2.12042	1.44412
50513	1.89226	1.3887
50514	1.89226	2.1
50515	1.89226	0.861051

50516	1.89226	1.71216
50517	3.78453	1.2005
50518	1.89226	1.33447
50519	2.77314	2.1
50520	2.81276	1.46602
50521	2.81276	2.1
50522	1.89226	1.54915
50523	1.89226	2.1
50524	1.11077	2.1
50525	1.15114	1.81626
50526	1.10274	1.00057
50527	1.1032	1.41034
50528	1.05481	1.47739
50529	1.1032	1.12545
50530	1.89226	1.21842
50531	1.89226	1.86159
50532	1.89226	0.763855
50533	1.89226	1.90867
50534	3.78452	1.19368
50535	0.721957	0.816385
50536	0.721957	0.821387
50537	0.946103	0.817851
50538	0.946103	0.755141
50539	0.814237	0.782168
50540	0.721929	0.748587
50541	0.946132	0.829101
50542	0.946132	0.833654
50543	0.721928	0.85049
50544	0.946131	0.860414
50545	0.946132	0.867706
50546	0.814237	0.861992
50547	1.89226	0.916265
50548	1.89226	0.827616
50549	1.89226	0.831829
50550	1.89226	0.84124
50551	1.89226	0.893035
50552	1.89226	0.900695
50553	1.37196	0.808757
50554	2.83556	0.720625
50555	1.5165	0.772539
50556	1.5165	0.821459
50557	2.74923	0.746306
50558	2.74923	0.810399
50559	0.946128	0.803087
50560	0.946128	0.828879
50561	0.827866	0.734151
50562	0.946132	0.783674
50563	0.94613	0.720286
50564	0.94613	0.746811
50565	0.94613	0.83276
50566	0.94613	0.830389
50567	0.94613	0.839271
50568	0.827864	0.856428
50569	0.94613	0.837589
50570	0.94613	0.891055
50571	1.36208	0.704507
50572	2.74923	0.616735
50573	1.37196	0.662052

50574	1.37196	0.708102
50575	2.53256	0.63146
50576	2.53256	0.695565
50577	1.07705	2.1
50578	1.07705	2.1
50579	1.08253	2.1
50580	1.08253	2.1
50581	0.762985	2.1
50582	0.781638	2.1
50583	1.37794	2.1
50584	1.40756	2.1
50585	1.37794	2.1
50586	0.762986	2.1
50587	0.781638	2.1
50588	1.40756	2.1
50589	2.69541	2.1
50590	2.75587	2.1
50591	1.48572	2.1
50592	1.48572	2.1
50593	1.52597	2.1
50594	2.73566	2.1
50595	2.0554	1.64233
50596	1.00387	1.05763
50597	2.01001	0.983364
50598	0.994002	0.952842
50599	3.01389	0.899323
50600	1.89226	2.1
50601	2.41579	2.1
50602	2.12209	2.1
50603	2.12209	2.1
50604	1.89226	2.1
50605	1.89226	2.1
50606	1.89226	2.1
50607	1.89226	2.1
50608	1.89226	2.1
50609	1.89226	2.1
50610	1.89226	2.1
50611	1.89226	2.1
50612	0.88209	2.1
50613	0.899471	2.1
50614	0.651501	2.1
50615	0.533531	2.1
50616	0.724105	2.1
50617	0.711243	2.1
50618	0.692622	2.1
50619	0.692622	2.1
50620	0.890668	2.1
50621	0.738066	2.1
50622	0.778223	2.1
50623	0.531121	2.1
50624	1.89226	2.1
50625	2.77504	2.1
50626	2.4158	2.1
50627	2.4158	2.1
50628	1.89226	2.1
50629	1.89226	2.1
50630	1.89226	2.1
50631	1.89226	2.1

50632	1.89226	2.1
50633	1.89226	2.1
50634	1.89226	2.1
50635	1.89226	2.1
50636	1.70303	2.1
50637	1.39832	2.1
50638	1.51749	2.1
50639	1.68783	2.1
50640	3.24844	2.1
50641	1.89226	2.1
50642	2.38311	2.1
50643	1.98321	2.1
50644	1.89226	2.1
50645	2.47406	2.1
50646	2.38311	2.1
50647	1.89226	2.1
50648	1.89226	2.1
50649	1.89226	2.1
50650	1.89226	2.1
50651	1.89226	2.1
50652	1.89226	2.1
50653	0.946131	2.1
50654	0.946131	2.1
50655	1.25167	2.1
50656	1.19156	2.1
50657	1.19156	2.1
50658	1.19156	2.1
50659	0.946133	2.1
50660	0.946133	2.1
50661	0.946131	2.1
50662	1.25167	2.1
50663	0.946131	2.1
50664	1.19156	2.1
50665	1.89226	2.1
50666	2.47406	2.1
50667	1.99996	2.1
50668	1.89226	2.1
50669	2.58176	2.1
50670	2.47406	2.1
50671	1.89226	2.1
50672	1.89226	2.1
50673	1.89226	2.1
50674	1.89226	2.1
50675	1.89226	2.1
50676	1.89226	2.1
50677	0.946131	2.1
50678	0.946131	2.1
50679	1.32241	2.1
50680	1.25167	2.1
50681	1.25167	2.1
50682	1.25167	2.1
50683	0.946133	2.1
50684	0.946133	2.1
50685	0.946131	2.1
50686	1.32241	2.1
50687	0.946131	2.1
50688	1.25167	2.1
50689	1.65151	2.1

50690	1.92471	2.1
50691	1.81834	2.1
50692	2.13272	2.1
50693	3.74306	2.1
50694	1.92472	2.1
50695	1.9943	2.1
50696	2.13273	2.1
50697	2.24475	2.1
50698	4.16947	2.1
50699	1.81834	2.1
50700	2.13272	2.1
50701	2.02687	2.1
50702	2.39605	2.1
50703	4.21439	2.1
50704	2.13273	2.1
50705	2.24475	2.1
50706	2.39605	2.1
50707	2.54903	2.1
50708	4.6408	2.1
50709	1.10534	2.1
50710	1.22993	2.1
50711	0.946132	2.1
50712	0.946132	2.1
50713	0.946132	2.1
50714	0.946132	2.1
50715	1.22993	2.1
50716	1.22993	2.1
50717	1.10534	2.1
50718	0.946131	2.1
50719	0.946132	2.1
50720	1.22993	2.1
50721	1.47221	2.1
50722	2.14647	2.1
50723	1.95567	2.1
50724	1.95567	2.1
50725	1.71082	2.1
50726	1.71082	2.1
50727	0.946131	2.1
50728	0.946131	2.1
50729	0.946131	2.1
50730	0.946131	2.1
50731	0.946132	2.1
50732	0.946132	2.1
50733	0.946131	2.1
50734	0.946131	2.1
50735	0.946131	2.1
50736	0.946131	2.1
50737	0.946132	2.1
50738	0.946132	2.1
50739	0.998086	2.1
50740	1.09429	2.1
50741	0.946125	2.1
50742	0.946125	2.1
50743	0.90732	2.1
50744	0.90732	2.1
50745	1.14414	2.1
50746	1.16553	2.1
50747	1.05018	2.1

50748	0.907332	2.1
50749	0.99482	2.1
50750	0.851529	2.1
50751	0.80534	2.1
50752	0.80534	2.1
50753	0.812206	2.1
50754	0.812206	2.1
50755	0.895134	2.1
50756	0.977828	2.1
50757	0.736099	2.1
50758	0.639718	2.1
50759	0.639722	2.1
50760	0.89514	2.1
50761	0.736095	2.1
50762	0.977821	2.1
50763	0.946125	2.1
50764	0.946125	2.1
50765	0.946125	2.1
50766	0.946125	2.1
50767	0.946125	2.1
50768	0.946125	2.1
50769	0.946124	2.1
50770	0.946124	2.1
50771	0.946131	2.1
50772	0.946131	2.1
50773	0.946118	2.1
50774	0.946118	2.1
50775	2.22622	0.147815
50776	2.43347	0.147815
50777	2.34108	0.147815
50778	1.24587	2.1
50779	1.21674	2.1
50780	1.14533	2.1
50781	1.11311	2.1
50782	1.17874	2.1
50783	1.17874	2.1
50784	1.18024	2.1
50785	1.18024	2.1
50786	1.14533	2.1
50787	1.24587	2.1
50788	1.21674	2.1
50789	1.11311	2.1
50790	2.43347	2.1
50791	2.34107	2.1
50792	2.39663	2.1
50793	2.49174	2.1
50794	4.8301	2.1
50795	1.86206	2.1
50796	1.41058	2.1
50797	1.70302	2.1
50798	2.42083	2.1
50799	3.68129	2.1
50800	1.70302	2.1
50801	2.43549	2.1
50802	2.20075	2.1
50803	2.50358	2.1
50804	2.04916	2.1
50805	2.33113	2.1

50806	1.07624	2.1
50807	2.12621	2.1
50808	1.57297	2.1
50809	1.60383	2.1
50810	1.30047	2.1
50811	1.30047	2.1
50812	2.33379	1.85158
50813	2.56149	1.22872
50814	2.36734	1.14936
50815	2.62796	1.14155
50816	4.92883	1.25217
50817	2.5615	1.25436
50818	2.57957	1.39595
50819	2.62796	1.09431
50820	2.69056	1.08082
50821	5.25206	1.40898
50822	2.39322	1.29893
50823	2.62796	1.54109
50824	2.6849	1.69905
50825	2.6849	1.51699
50826	2.36734	1.67558
50827	2.36734	1.49262
50828	2.62796	1.45098
50829	2.69056	1.50117
50830	2.6849	0.939129
50831	2.77227	0.956738
50832	5.37546	1.08343
50833	1.89226	0.218106
50834	1.89226	0.266788
50835	1.89226	0.236003
50836	1.89226	0.230363
50837	1.89226	0.238109
50838	1.89226	0.231359
50839	1.06974	0.195862
50840	1.06974	0.196304
50841	1.02299	0.210211
50842	1.02299	0.220171
50843	1.35299	0.217548
50844	1.26227	0.199679
50845	0.646252	0.212022
50846	0.830464	0.197926
50847	0.830463	0.203197
50848	1.35299	0.205906
50849	1.26227	0.202803
50850	0.646252	0.210107
50851	2.52454	0.205589
50852	1.89226	0.247142
50853	2.70598	0.227528
50854	1.89226	0.207657
50855	4.59824	0.21956
50856	1.89226	0.234222
50857	1.89226	0.2994
50858	1.89226	0.265665
50859	1.89226	0.252429
50860	1.89226	0.270217
50861	1.89226	0.255018
50862	1.89226	0.218573
50863	1.89226	0.27249

50864	1.89226	0.237306
50865	1.89226	0.236324
50866	1.89226	0.237312
50867	1.89226	0.233031
50868	1.7666	0.196996
50869	2.70598	0.22424
50870	2.65378	0.210468
50871	2.65378	0.211313
50872	1.66093	0.205374
50873	1.66093	0.202921
50874	2.70598	0.231591
50875	1.89226	0.213469
50876	1.89226	0.242864
50877	1.89226	0.206958
50878	2.65377	0.23999
50879	2.65377	0.209076
50880	0.946132	0.23563
50881	0.946132	0.245755
50882	0.946131	0.274094
50883	0.946131	0.303297
50884	0.946131	0.300053
50885	0.946131	0.251074
50886	0.946132	0.277317
50887	0.946132	0.236845
50888	0.946132	0.259616
50889	0.946132	0.261494
50890	0.946131	0.258048
50891	0.946131	0.26158
50892	1.69858	2.1
50893	2.18238	2.1
50894	2.506	2.1
50895	1.93586	2.1
50896	4.20458	2.1
50897	2.21772	2.1
50898	2.18238	2.1
50899	2.506	2.1
50900	2.59215	2.1
50901	4.72372	2.1
50902	2.50596	2.1
50903	1.93584	2.1
50904	2.16728	2.1
50905	2.7679	2.1
50906	4.67325	2.1
50907	1.53296	2.1
50908	1.56299	2.1
50909	1.42624	2.1
50910	1.52961	2.1
50911	2.98923	2.1
50912	1.56299	2.1
50913	1.46568	2.1
50914	1.52961	2.1
50915	1.51916	2.1
50916	3.08215	2.1
50917	1.36226	2.1
50918	1.52961	2.1
50919	1.36226	2.1
50920	1.42624	2.1
50921	1.55369	2.1

50922	1.61768	2.1
50923	1.52961	2.1
50924	1.51916	2.1
50925	1.55369	2.1
50926	1.64892	2.1
50927	3.07285	2.1
50928	2.13405	0.426486
50929	2.57507	0.412459
50930	2.39438	0.503243
50931	1.93408	0.370918
50932	4.52843	0.417435
50933	2.13405	0.41464
50934	1.27778	0.32772
50935	1.2545	0.361961
50936	1.45448	0.367904
50937	1.93408	0.351091
50938	2.13405	0.362012
50939	2.39438	0.476233
50940	1.93407	0.371571
50941	1.60578	0.413881
50942	1.87985	0.400461
50943	4.00016	0.419372
50944	1.0525	1.74849
50945	1.05758	1.7596
50946	1.43484	0.990473
50947	1.38506	1.44582
50948	2.48734	1.54339
50949	1.20455	2.1
50950	1.67352	2.1
50951	1.52852	2.1
50952	2.05164	2.1
50953	3.20203	2.1
50954	1.89226	2.05916
50955	1.89226	2.1
50956	1.89226	2.1
50957	1.89226	2.1
50958	3.78452	2.1
50959	1.89226	2.1
50960	1.89226	2.1
50961	1.89226	2.1
50962	1.89226	2.1
50963	1.89226	2.1
50964	1.89226	1.98737
50965	2.05165	2.1
50966	1.94291	2.1
50967	2.09398	2.1
50968	1.67352	2.1
50969	2.36337	2.1
50970	1.94291	2.1
50971	1.52851	2.1
50972	2.52049	2.1
50973	2.43433	2.1
50974	2.05164	2.1
50975	1.9112	2.1
50976	1.52851	2.1
50977	1.89226	2.1
50978	1.89226	1.78777
50979	1.89226	2.06765

50980	1.89226	2.1
50981	3.78451	2.1
50982	1.89226	1.87963
50983	1.89226	2.1
50984	1.89226	2.1
50985	1.89226	1.73358
50986	3.78452	2.1
50987	2.5205	2.1
50988	2.36337	2.1
50989	2.4605	2.1
50990	2.05165	2.1
50991	2.77222	2.1
50992	2.36337	2.1
50993	1.89226	2.1
50994	1.89226	2.1
50995	1.89226	2.1
50996	1.89226	2.1
50997	1.89226	2.1
50998	1.89226	2.1
50999	1.89226	2.1
51000	1.89226	2.1
51001	1.89226	2.1
51002	1.89226	2.1
51003	3.78452	2.1
51004	1.38905	2.1
51005	1.26166	2.1
51006	1.19216	2.1
51007	1.0875	2.1
51008	1.24393	2.1
51009	1.24393	2.1
51010	1.23261	2.1
51011	1.23261	2.1
51012	1.38905	2.1
51013	1.19216	2.1
51014	1.26166	2.1
51015	1.0875	2.1
51016	1.16233	2.1
51017	1.06028	2.1
51018	0.777395	2.1
51019	0.705508	2.1
51020	0.940018	2.1
51021	0.936465	2.1
51022	0.954216	2.1
51023	0.954216	2.1
51024	1.16125	2.1
51025	0.685505	2.1
51026	1.1157	2.1
51027	0.703534	2.1
51028	1.89226	2.1
51029	1.89226	2.1
51030	1.89226	2.1
51031	1.89226	2.1
51032	1.89226	2.1
51033	1.89226	2.1
51034	1.89226	2.1
51035	1.89226	2.1
51036	1.89226	2.1
51037	1.89226	2.1

51038	1.89226	2.1
51039	1.89226	2.1
51040	2.38433	2.1
51041	1.43456	2.1
51042	1.93881	2.1
51043	2.36952	2.1
51044	4.32313	2.1
51045	1.8493	2.1
51046	1.5927	2.1
51047	1.45535	2.1
51048	1.38438	2.1
51049	1.89657	2.1
51050	1.8493	2.1
51051	2.0815	2.1
51052	1.6697	2.1
51053	2.0657	2.1
51054	1.5927	2.1
51055	2.59749	2.1
51056	2.0815	2.1
51057	2.76597	2.1
51058	1.89226	2.1
51059	1.89226	2.1
51060	1.89226	2.1
51061	2.6126	2.1
51062	2.6126	2.1
51063	2.62513	2.1
51064	1.89226	2.1
51065	1.89226	2.1
51066	1.89226	2.1
51067	2.76598	2.1
51068	2.76598	2.1
51069	2.54665	2.1
51070	2.6126	2.1
51071	2.76598	2.1
51072	2.76598	2.1
51073	2.34044	2.1
51074	2.34044	2.1
51075	2.62514	2.1
51076	2.76598	2.1
51077	2.54666	2.1
51078	2.45212	2.1
51079	5.2181	2.1
51080	2.14063	2.1
51081	1.47521	2.1
51082	1.27679	2.1
51083	1.47521	2.1
51084	2.11511	2.1
51085	2.31353	2.1
51086	0.93256	2.1
51087	1.07032	2.1
51088	0.638396	2.1
51089	0.521342	2.1
51090	0.790674	2.1
51091	0.790674	2.1
51092	0.80098	2.1
51093	0.800981	2.1
51094	0.932535	2.1
51095	0.521328	2.1

51096	1.07034	2.1
51097	0.638414	2.1
51098	1.89226	2.09881
51099	1.89226	1.34783
51100	1.89226	1.50583
51101	1.89226	1.98111
51102	3.78453	1.59698
51103	1.89226	2.02439
51104	1.9842	2.1
51105	1.89226	1.85402
51106	1.89226	2.1
51107	2.79705	1.96587
51108	2.79705	2.1
51109	0.469955	2.09921
51110	0.791665	2.0213
51111	0.989115	1.42359
51112	0.763235	1.67384
51113	0.776356	1.49956
51114	0.805461	2.07244
51115	0.776759	1.572
51116	0.776759	2.1
51117	0.799151	1.81649
51118	0.940869	1.86051
51119	0.78449	1.68538
51120	0.489635	1.70892
51121	1.89226	1.36894
51122	1.89226	1.73146
51123	1.89226	1.34284
51124	1.89226	1.92538
51125	1.89226	1.30289
51126	1.89226	1.94405
51127	2.46058	1.48094
51128	2.28991	1.84674
51129	1.8525	1.63091
51130	1.87697	1.90017
51131	2.28991	1.58465
51132	1.62397	1.84946
51133	0.946131	0.296208
51134	0.974033	0.275679
51135	1.31667	0.301472
51136	0.946131	0.321527
51137	0.885497	0.31744
51138	0.885497	0.305215
51139	1.0468	0.280518
51140	1.04678	0.288469
51141	1.31667	0.297675
51142	0.974019	0.276028
51143	0.946131	0.29309
51144	0.946131	0.283816
51145	1.89226	0.330027
51146	1.89226	0.48177
51147	1.89226	0.440045
51148	1.89226	0.415581
51149	3.78452	0.45847
51150	0.946125	0.483699
51151	0.946125	0.442723
51152	0.946131	0.487087
51153	0.946131	0.524333

51154	0.946128	0.512285
51155	0.946128	0.495152
51156	0.946128	0.433057
51157	0.946128	0.467298
51158	0.946128	0.472446
51159	0.946128	0.488301
51160	0.946128	0.492531
51161	0.946128	0.471333
51162	0.946131	0.343471
51163	0.98453	0.320982
51164	1.32276	0.394937
51165	0.946131	0.404598
51166	0.946132	0.383205
51167	0.946132	0.369629
51168	0.98453	0.344972
51169	1.32276	0.344664
51170	1.32276	0.373185
51171	1.32276	0.304973
51172	0.946132	0.354167
51173	0.946132	0.306624
51174	1.89227	2.1
51175	1.89227	2.1
51176	1.89227	2.1
51177	1.89227	2.1
51178	1.89227	2.1
51179	1.89227	2.1
51180	1.89226	2.1
51181	1.89226	2.1
51182	1.89226	2.1
51183	1.89226	2.1
51184	1.89226	2.1
51185	1.89226	2.1
51186	1.05556	2.1
51187	1.38859	2.1
51188	1.46996	2.1
51189	1.25364	2.1
51190	2.52552	2.1
51191	0.694296	2.1
51192	0.73498	2.1
51193	0.561881	2.1
51194	0.512189	2.1
51195	0.621304	2.1
51196	0.621304	2.1
51197	0.625865	2.1
51198	0.625865	2.1
51199	0.734979	2.1
51200	0.561881	2.1
51201	0.51219	2.1
51202	0.694296	2.1
51203	0.946126	2.1
51204	0.946126	2.1
51205	0.946126	2.1
51206	0.946126	2.1
51207	0.946126	2.1
51208	0.946126	2.1
51209	0.946126	2.1
51210	0.946126	2.1
51211	0.946132	2.1

51212	0.946132	2.1
51213	0.94612	2.1
51214	0.94612	2.1
51215	1.89225	2.1
51216	1.89225	2.1
51217	1.89225	2.1
51218	1.89225	2.1
51219	1.89225	2.1
51220	1.89225	2.1
51221	0.626814	2.1
51222	0.632155	2.1
51223	0.746234	2.1
51224	0.734974	2.1
51225	0.68202	2.1
51226	0.68202	2.1
51227	0.685108	2.1
51228	0.685108	2.1
51229	0.746239	2.1
51230	0.632159	2.1
51231	0.734969	2.1
51232	0.62681	2.1
51233	0.746235	2.1
51234	0.734975	2.1
51235	0.595081	2.1
51236	0.561877	2.1
51237	0.648584	2.1
51238	0.648584	2.1
51239	0.659528	2.1
51240	0.659528	2.1
51241	0.746239	2.1
51242	0.595085	2.1
51243	0.734969	2.1
51244	0.561874	2.1
51245	1.89249	2.1
51246	2.2137	2.1
51247	1.33125	2.1
51248	1.06499	2.1
51249	2.12571	2.1
51250	1.78918	2.1
51251	0.946131	2.1
51252	0.946131	2.1
51253	0.94613	2.1
51254	0.94613	2.1
51255	0.946132	2.1
51256	0.946132	2.1
51257	0.946129	2.1
51258	0.946129	2.1
51259	0.946131	2.1
51260	0.946131	2.1
51261	0.946131	2.1
51262	0.946131	2.1
51263	1.89227	2.1
51264	1.89227	2.1
51265	1.89227	2.1
51266	1.89227	2.1
51267	3.78453	2.1
51268	1.89226	2.1
51269	1.89226	2.1

51270	1.89226	2.1
51271	1.89226	2.1
51272	1.89226	2.1
51273	1.89226	2.1
51274	2.01388	2.1
51275	1.38757	2.1
51276	1.24229	2.1
51277	1.3309	2.1
51278	1.88011	2.1
51279	2.01388	2.1
51280	2.01388	2.1
51281	2.01555	2.1
51282	2.13879	2.1
51283	1.80766	2.1
51284	4.15267	2.1
51285	0.946133	2.1
51286	0.946133	2.1
51287	0.946132	2.1
51288	0.946132	2.1
51289	0.946132	2.1
51290	0.946132	2.1
51291	0.946133	2.1
51292	0.946133	2.1
51293	0.946132	2.1
51294	0.946132	2.1
51295	0.946132	2.1
51296	0.946132	2.1
51297	1.89226	2.1
51298	1.89226	2.1
51299	1.89226	2.1
51300	1.89226	2.1
51301	1.89226	2.1
51302	1.89226	2.1
51303	0.676745	2.1
51304	0.68382	2.1
51305	1.07048	2.1
51306	1.1327	2.1
51307	0.929324	2.1
51308	0.871438	2.1
51309	0.867362	2.1
51310	0.867362	2.1
51311	1.10666	2.1
51312	0.695036	2.1
51313	0.744098	2.1
51314	1.10144	2.1
51315	2.25168	2.1
51316	2.22499	2.1
51317	2.15929	2.1
51318	2.13879	2.1
51319	2.15929	2.1
51320	2.13879	2.1
51321	2.15314	2.1
51322	1.70302	2.1
51323	2.24694	2.1
51324	1.65245	2.1
51325	3.82722	2.1
51326	1.65245	2.1
51327	1.00371	2.1

51328	1.7437	2.1
51329	1.65748	2.1
51330	1.10459	2.1
51331	1.00371	2.1
51332	2.24694	2.1
51333	1.65246	2.1
51334	2.69773	2.1
51335	1.88129	2.1
51336	4.12824	2.1
51337	1.65246	2.1
51338	1.10459	2.1
51339	1.88129	2.1
51340	1.05914	2.1
51341	2.76496	2.1
51342	1.89226	2.1
51343	1.89226	2.1
51344	1.89226	2.1
51345	1.89226	2.1
51346	3.78452	2.1
51347	0.718577	2.1
51348	0.718577	2.1
51349	0.725249	2.1
51350	0.725249	2.1
51351	0.597916	2.1
51352	0.61752	2.1
51353	0.826306	2.1
51354	0.859194	2.1
51355	0.826306	2.1
51356	0.597916	2.1
51357	0.859194	2.1
51358	0.61752	2.1
51359	1.25898	2.1
51360	1.25898	2.1
51361	0.946128	2.1
51362	0.946128	2.1
51363	1.25897	2.1
51364	1.25585	2.1
51365	0.946131	2.1
51366	0.946131	2.1
51367	0.946129	2.1
51368	1.25586	2.1
51369	1.25898	2.1
51370	0.946129	2.1
51371	1.89226	2.1
51372	1.89226	2.1
51373	1.89226	2.1
51374	1.89226	2.1
51375	3.78452	2.1
51376	0.693258	2.1
51377	0.693258	2.07628
51378	0.697356	2.1
51379	0.697356	2.1
51380	0.572479	2.1
51381	0.597916	2.1
51382	0.792698	2.08003
51383	0.826306	2.1
51384	0.572478	2.1
51385	0.792697	2.1

51386	0.826307	2.1
51387	0.597917	2.1
51388	1.25586	2.1
51389	1.25586	2.1
51390	0.946128	2.1
51391	0.946128	2.1
51392	1.25585	2.1
51393	1.24344	2.1
51394	0.946131	2.1
51395	0.946131	2.1
51396	0.946129	2.1
51397	1.24344	2.1
51398	0.94613	2.1
51399	1.25586	2.1
51400	1.65151	2.1
51401	1.81835	2.1
51402	1.12064	2.1
51403	1.12064	2.1
51404	1.24965	2.1
51405	1.81835	2.1
51406	1.24965	2.1
51407	1.81835	2.1
51408	1.41077	2.1
51409	2.02687	2.1
51410	3.22911	2.1
51411	1.89122	1.24797
51412	1.38776	1.89265
51413	2.37014	1.01446
51414	1.80818	1.86137
51415	3.6994	1.57052
51416	2.15466	1.07583
51417	2.00083	1.72091
51418	1.80818	1.42215
51419	1.80818	1.11665
51420	2.31988	1.36858
51421	2.31988	1.04008
51422	1.80818	1.03008
51423	1.18041	2.1
51424	2.00082	1.24996
51425	1.43652	2.1
51426	3.18123	1.80444
51427	1.89226	1.02007
51428	1.89226	1.19172
51429	1.89226	1.04974
51430	1.89226	0.994768
51431	3.78453	1.0231
51432	1.89226	1.24642
51433	1.89226	1.2513
51434	1.89226	1.07673
51435	1.89226	1.04184
51436	3.78452	1.30796
51437	0.80759	1.09599
51438	0.686119	1.23009
51439	0.972147	1.20844
51440	1.00217	1.24489
51441	0.821334	1.40057
51442	0.821334	1.20484
51443	0.866957	1.17247

51444	0.866957	1.09096
51445	0.972145	1.29606
51446	0.686121	1.65057
51447	0.807592	1.33761
51448	1.00217	1.60726
51449	0.972146	1.1273
51450	1.00217	1.15325
51451	1.26654	1.27147
51452	1.1908	1.20309
51453	1.05501	1.17718
51454	1.05501	1.48533
51455	1.10794	1.11373
51456	1.10794	0.965392
51457	0.972146	0.947835
51458	1.26654	1.19389
51459	1.00217	1.15009
51460	1.1908	1.38228
51461	1.89226	1.13206
51462	1.89226	0.987056
51463	1.89226	1.02305
51464	1.89226	1.30304
51465	1.89226	0.937634
51466	1.89226	1.06931
51467	0.972147	1.20384
51468	0.867854	1.28456
51469	1.26132	1.1003
51470	1.26654	1.21876
51471	1.0424	1.32811
51472	1.0424	1.42351
51473	1.092	0.970834
51474	1.092	1.21348
51475	1.26132	1.24425
51476	0.867854	1.78613
51477	0.972147	1.38256
51478	1.26654	1.67765
51479	1.4375	0.561982
51480	1.92998	0.513444
51481	1.66953	0.386979
51482	1.0953	0.421425
51483	3.10703	0.44552
51484	1.89226	2.1
51485	1.89226	2.1
51486	1.89226	2.1
51487	1.89226	2.1
51488	3.78452	2.1
51489	2.74034	2.1
51490	2.08808	2.1
51491	2.63859	2.1
51492	2.00343	2.1
51493	4.72667	2.1
51494	0.799825	2.1
51495	0.799825	2.1
51496	0.80211	2.1
51497	0.80211	2.1
51498	0.562428	2.1
51499	0.600223	2.1
51500	1.04404	2.1
51501	1.00171	2.1

51502	1.00171	2.1
51503	0.56243	2.1
51504	0.600225	2.1
51505	1.04404	2.1
51506	0.94613	2.1
51507	0.94613	2.1
51508	0.946128	2.1
51509	0.946128	2.1
51510	0.946127	2.1
51511	0.946127	2.1
51512	0.946131	2.1
51513	0.946131	2.1
51514	0.946129	2.1
51515	0.946129	2.1
51516	0.946129	2.1
51517	0.946129	2.1
51518	1.89226	2.1
51519	1.89226	2.1
51520	1.89226	2.1
51521	1.89226	2.1
51522	3.78452	2.1
51523	2.63859	2.1
51524	2.00343	2.1
51525	2.54388	2.1
51526	1.91548	2.1
51527	4.55407	2.1
51528	1.91548	2.1
51529	2.00342	2.1
51530	1.04909	2.1
51531	1.04909	2.1
51532	1.12486	2.1
51533	1.99124	2.1
51534	0.810942	2.1
51535	0.810942	2.1
51536	0.810942	2.1
51537	0.810942	2.1
51538	1.89226	2.1
51539	1.35169	2.1
51540	1.08131	2.1
51541	1.08132	2.1
51542	1.08132	2.1
51543	0.810944	2.1
51544	0.810944	2.1
51545	1.89226	0.733754
51546	1.89226	0.830403
51547	1.89226	0.523157
51548	1.89226	0.849716
51549	3.78452	0.774128
51550	1.7968	0.485951
51551	1.49831	0.828761
51552	1.59244	0.467516
51553	1.3395	0.809519
51554	3.09074	0.71594
51555	1.49831	0.829188
51556	1.16858	1.93327
51557	1.33951	0.812685
51558	1.0361	1.71476
51559	2.53442	0.96571

51560	1.89226	0.848736
51561	1.89226	0.829385
51562	1.89226	0.859038
51563	1.89226	1.95
51564	1.89226	1.77409
51565	1.89226	1.81059
51566	1.89223	0.638162
51567	1.89223	0.821376
51568	1.89223	0.763506
51569	1.89223	0.808683
51570	1.89223	0.488545
51571	1.89223	0.820825
51572	1.59241	0.564259
51573	1.18952	0.831063
51574	1.33949	0.809486
51575	1.33949	0.630345
51576	1.40538	0.81189
51577	1.40538	0.598287
51578	1.89226	0.43524
51579	1.89226	0.669956
51580	1.89226	0.529805
51581	1.89226	0.654121
51582	1.89226	0.590259
51583	1.89226	0.689883
51584	1.89226	2.1
51585	1.89226	2.1
51586	1.89226	2.1
51587	1.89226	2.1
51588	3.78452	2.1
51589	1.89226	2.1
51590	1.89226	2.1
51591	1.89226	2.1
51592	1.89226	2.1
51593	3.78452	2.1
51594	2.01867	2.1
51595	1.46955	2.1
51596	1.58476	2.1
51597	1.38189	2.1
51598	2.22153	2.1
51599	2.01867	2.1
51600	2.01867	2.1
51601	2.52881	2.1
51602	2.21732	2.1
51603	2.21732	2.1
51604	2.22154	2.1
51605	2.22154	2.1
51606	1.89226	2.1
51607	1.89226	2.1
51608	1.89226	2.1
51609	1.89226	2.1
51610	1.89226	2.1
51611	1.89226	2.1
51612	1.89226	2.1
51613	1.89226	2.1
51614	1.89226	2.1
51615	1.89226	2.1
51616	1.89226	2.1
51617	1.89226	2.1

51618	2.22153	2.1
51619	1.63361	2.1
51620	1.77336	2.1
51621	1.46955	2.1
51622	2.52534	2.1
51623	2.22153	2.1
51624	2.34431	2.1
51625	1.62905	2.1
51626	1.49693	2.1
51627	2.19197	2.1
51628	3.82103	2.1
51629	1.89226	2.1
51630	1.89226	2.1
51631	1.89226	2.1
51632	1.89226	2.1
51633	3.78453	2.1
51634	0.946128	2.1
51635	0.946128	2.1
51636	1.22372	2.1
51637	1.17331	2.1
51638	0.946131	2.1
51639	0.946131	2.1
51640	1.17331	2.1
51641	1.17331	2.1
51642	0.946131	2.1
51643	1.17331	2.1
51644	1.22372	2.1
51645	0.94613	2.1
51646	1.04041	2.1
51647	1.09599	2.1
51648	0.748462	2.1
51649	0.701976	2.1
51650	0.896701	2.1
51651	0.896701	2.1
51652	0.901262	2.1
51653	0.901262	2.1
51654	1.04041	2.1
51655	0.701977	2.1
51656	0.748463	2.1
51657	1.09599	2.1
51658	1.89226	2.1
51659	1.89226	2.1
51660	1.89226	2.1
51661	1.89226	2.1
51662	3.78453	2.1
51663	0.946128	2.1
51664	0.946128	2.1
51665	1.17331	2.1
51666	1.13785	2.1
51667	0.94613	2.1
51668	0.94613	2.1
51669	1.13785	2.1
51670	1.13785	2.1
51671	1.13785	2.1
51672	0.94613	2.1
51673	0.94613	2.1
51674	1.17331	2.1
51675	2.50381	2.1

51676	1.89226	2.1
51677	1.89226	2.1
51678	2.73387	2.1
51679	4.39607	2.1
51680	1.80045	2.1
51681	1.99777	0.97022
51682	2.45134	1.37731
51683	2.12463	1.52643
51684	1.70302	1.39124
51685	1.42655	2.07994
51686	2.20231	2.1
51687	1.70302	1.19907
51688	1.93128	1.95107
51689	1.66768	1.51475
51690	1.24499	0.967411
51691	1.70302	0.959119
51692	2.55572	0.369452
51693	1.22811	0.351711
51694	1.82584	0.389528
51695	1.22811	0.386299
51696	2.62764	0.34197
51697	1.93147	0.333314
51698	2.14513	0.561012
51699	1.89226	0.693678
51700	2.66481	0.569278
51701	1.89226	0.569802
51702	2.66481	0.71196
51703	1.89226	0.70715
51704	2.11649	2.1
51705	1.89226	2.1
51706	1.89226	2.1
51707	1.89226	2.1
51708	2.15426	2.1
51709	2.15426	2.1
51710	2.06776	2.1
51711	1.89226	2.1
51712	1.89226	2.1
51713	1.89226	2.1
51714	2.11649	2.1
51715	2.11649	2.1
51716	1.89226	2.1
51717	1.89226	2.1
51718	1.89226	2.1
51719	1.89226	2.1
51720	1.89226	2.1
51721	1.89226	2.1
51722	0.998134	2.1
51723	0.918904	2.1
51724	0.894708	2.1
51725	0.758742	2.1
51726	0.864276	2.1
51727	0.864276	2.1
51728	0.8926	2.1
51729	0.8926	2.1
51730	0.998134	2.1
51731	0.894709	2.1
51732	0.758742	2.1
51733	0.918904	2.1

51734	1.89226	2.1
51735	1.89226	2.1
51736	1.89226	2.1
51737	1.89226	2.1
51738	1.89226	2.1
51739	1.89226	2.1
51740	1.89226	2.1
51741	1.89226	2.1
51742	1.89226	2.1
51743	1.89226	2.1
51744	1.89226	2.1
51745	1.89226	2.1
51746	1.99627	2.1
51747	1.61047	2.1
51748	2.0815	2.1
51749	2.07569	2.1
51750	3.75063	2.1
51751	0.848077	2.1
51752	1.07748	2.1
51753	0.802498	2.1
51754	0.626808	2.1
51755	0.875552	2.1
51756	0.737527	2.1
51757	0.835602	2.1
51758	0.835602	2.1
51759	0.983245	2.1
51760	0.781108	2.1
51761	0.872306	2.1
51762	0.601497	2.1
51763	2.74745	2.1
51764	2.73988	2.1
51765	2.6521	2.1
51766	2.67792	2.1
51767	5.39198	2.1
51768	2.73988	2.1
51769	2.57466	2.1
51770	2.67791	2.1
51771	2.55653	2.1
51772	5.29641	2.1
51773	2.6521	2.1
51774	2.64148	2.1
51775	2.67792	2.1
51776	2.67792	2.1
51777	2.56953	2.1
51778	2.56953	2.1
51779	2.64148	2.1
51780	2.55653	2.1
51781	2.57929	2.1
51782	2.57929	2.1
51783	2.67792	2.1
51784	2.67792	2.1
51785	1.89226	0.78119
51786	1.89226	0.551987
51787	1.89226	0.90932
51788	1.89226	0.297741
51789	3.78453	0.561354
51790	1.68703	0.785679
51791	2.50385	0.253142

51792	1.45958	0.485043
51793	2.0815	0.306863
51794	3.93806	0.399946
51795	2.50385	0.255424
51796	2.57414	0.377081
51797	2.0815	0.346103
51798	2.39921	0.599334
51799	4.91181	0.243677
51800	1.89226	0.68661
51801	1.89226	0.370528
51802	1.89226	0.311286
51803	1.89226	0.350592
51804	3.78453	0.391017
51805	1.89226	0.91944
51806	1.89226	0.297111
51807	1.89226	0.579612
51808	1.89226	0.635072
51809	1.89226	0.434125
51810	1.89226	0.767564
51811	1.23013	0.437349
51812	2.0815	0.311569
51813	1.90167	0.616651
51814	1.71243	0.343332
51815	1.45887	0.574934
51816	1.31369	0.281153
51817	2.0815	0.35693
51818	2.39921	0.649696
51819	1.7115	0.351661
51820	2.2421	0.391263
51821	4.09829	0.424622
51822	1.89227	0.313338
51823	1.89227	0.362844
51824	1.89227	0.455584
51825	1.89227	0.398238
51826	3.78453	0.287342
51827	2.39697	0.221064
51828	1.85594	0.248216
51829	1.91161	0.212575
51830	2.49867	0.25887
51831	4.35461	0.234158
51832	1.12035	0.209812
51833	1.85594	0.239311
51834	1.91161	0.204309
51835	1.15483	0.215926
51836	3.01077	0.214689
51837	1.89226	0.222422
51838	1.89226	0.279432
51839	1.89226	0.242097
51840	1.89226	0.250873
51841	1.89226	0.242395
51842	1.89226	0.253436
51843	1.89226	0.264212
51844	1.89226	0.288967
51845	1.89226	0.306934
51846	1.89226	0.252845
51847	1.89226	0.309155
51848	1.89226	0.260629
51849	0.936516	0.232302

51850	1.03496	0.210084
51851	1.22607	0.25872
51852	1.28058	0.216596
51853	1.06317	0.250368
51854	1.06317	0.238312
51855	1.19735	0.205961
51856	1.0813	0.215078
51857	0.858168	0.231851
51858	1.32278	0.225477
51859	0.957674	0.222746
51860	1.25178	0.226348
51861	1.92659	0.205074
51862	0.998957	0.214372
51863	0.998957	0.236853
51864	1.15481	0.208792
51865	1.6031	0.226309
51866	1.74459	0.203342
51867	0.946119	0.223276
51868	0.946119	0.248342
51869	0.946118	0.236852
51870	0.946118	0.278829
51871	0.946118	0.277322
51872	0.946118	0.249517
51873	0.946119	0.217354
51874	0.946119	0.232106
51875	0.946131	0.243811
51876	0.946131	0.243456
51877	0.946106	0.249049
51878	0.946106	0.250102
51879	0.946118	0.248115
51880	0.946118	0.292074
51881	0.946119	0.264875
51882	0.946119	0.311799
51883	0.946118	0.311756
51884	0.946118	0.29155
51885	0.946119	0.260479
51886	0.946119	0.247198
51887	0.946131	0.265523
51888	0.946131	0.26816
51889	0.946106	0.274922
51890	0.946106	0.275957
51891	2.47164	0.816026
51892	2.83689	0.660576
51893	2.73872	0.822945
51894	2.41632	0.668898
51895	5.21036	0.684642
51896	0.946133	0.74736
51897	0.946133	0.816884
51898	1.41845	0.684981
51899	1.36936	0.815523
51900	1.41845	0.681651
51901	1.41845	0.659096
51902	0.946132	0.814372
51903	0.946132	0.832529
51904	0.946132	0.769908
51905	1.36936	0.723283
51906	1.41845	0.75588
51907	0.946133	0.795978

51908	1.36936	0.543766
51909	1.27581	0.711333
51910	1.20816	0.601466
51911	1.10464	0.757802
51912	1.24454	0.571779
51913	1.24454	0.561903
51914	1.23943	0.724397
51915	1.23943	0.749161
51916	1.10465	0.702704
51917	1.27581	0.682661
51918	1.36936	0.689825
51919	1.20816	0.663628
51920	0.946131	2.1
51921	0.946131	2.1
51922	1.37277	2.1
51923	1.37277	2.1
51924	1.37277	2.1
51925	1.27186	2.1
51926	0.946131	2.1
51927	0.946131	2.1
51928	0.946131	2.1
51929	1.37277	2.1
51930	0.946131	2.1
51931	1.27186	2.1
51932	2.74554	2.1
51933	1.23712	2.1
51934	2.80992	2.1
51935	2.54371	2.1
51936	1.50333	2.1
51937	1.23712	2.1
51938	2.77314	2.1
51939	2.33316	2.1
51940	2.28748	2.1
51941	2.28748	2.1
51942	2.81275	2.1
51943	2.81275	2.1
51944	2.28749	2.1
51945	1.45887	2.1
51946	2.33317	2.1
51947	1.50022	2.1
51948	3.79204	2.1
51949	2.37364	2.1
51950	2.33317	2.1
51951	1.5362	2.1
51952	1.5362	2.1
51953	1.50022	2.1
51954	2.33317	2.1
51955	1.89226	0.787988
51956	1.89226	0.784092
51957	1.89226	0.759564
51958	1.89226	0.805785
51959	1.89226	0.77464
51960	1.89226	0.824979
51961	0.946128	0.641964
51962	0.946128	0.676297
51963	1.0644	0.579331
51964	0.946133	0.634372
51965	1.0644	0.569177

51966	0.94613	0.605176
51967	0.946131	0.693056
51968	0.946131	0.641339
51969	1.0644	0.627232
51970	1.0644	0.639514
51971	0.946131	0.637032
51972	0.946131	0.618706
51973	1.89226	0.972454
51974	1.89226	0.756188
51975	1.89226	0.899088
51976	1.89226	0.408805
51977	3.78452	0.792074
51978	1.14599	0.969575
51979	1.19993	0.638124
51980	1.00065	0.913785
51981	1.07497	0.403314
51982	2.22096	0.843008
51983	0.546551	0.590102
51984	0.546551	0.745793
51985	0.54987	0.588561
51986	0.54987	0.753465
51987	0.503067	0.568892
51988	0.558937	0.56186
51989	0.599966	0.560513
51990	0.537484	0.697225
51991	0.503067	0.422134
51992	0.537485	0.411425
51993	0.599966	0.670098
51994	0.558936	0.561488
51995	0.946132	0.585135
51996	0.946132	0.719983
51997	0.94613	0.640002
51998	0.94613	0.740867
51999	0.946131	0.706027
52000	0.946131	0.699555
52001	0.946131	0.714863
52002	0.946131	0.711111
52003	0.946131	0.5347
52004	0.946131	0.636685
52005	0.946131	0.701221
52006	0.946131	0.72091
52007	2.19104	2.1
52008	2.5392	2.1
52009	2.53302	2.1
52010	2.19226	2.1
52011	4.73146	2.1
52012	1.09552	2.1
52013	1.09613	2.1
52014	0.802484	2.1
52015	0.807941	2.1
52016	0.95052	2.1
52017	0.95052	2.1
52018	0.952944	2.1
52019	0.952944	2.1
52020	1.09613	2.1
52021	0.807941	2.1
52022	0.802484	2.1
52023	1.09552	2.1

52024	2.533	2.1
52025	2.18531	2.1
52026	2.19224	2.1
52027	2.19224	2.1
52028	2.51603	2.1
52029	2.51603	2.1
52030	1.09266	2.1
52031	1.09612	2.1
52032	0.807935	2.1
52033	0.81091	2.1
52034	0.951897	2.1
52035	0.951897	2.1
52036	0.955134	2.1
52037	0.955134	2.1
52038	0.810915	2.1
52039	1.09266	2.1
52040	1.09611	2.1
52041	0.80793	2.1
52042	2.12741	0.756843
52043	2.79312	0.754159
52044	2.77591	0.943099
52045	2.10714	0.753701
52046	4.90026	0.707626
52047	2.12741	0.765019
52048	1.20859	0.630123
52049	1.21073	0.914685
52050	2.10714	0.798206
52051	3.31573	0.814955
52052	1.38708	0.880756
52053	1.38795	0.769395
52054	1.05331	0.792357
52055	1.05357	0.740035
52056	1.22049	0.758858
52057	1.22049	0.767283
52058	1.22078	0.961582
52059	1.22078	0.701334
52060	1.05331	0.834811
52061	1.38709	0.835395
52062	1.38795	0.766202
52063	1.05357	0.76756
52064	2.10662	0.871794
52065	2.10714	0.693525
52066	2.12499	0.72061
52067	1.22857	0.84204
52068	1.21073	0.772498
52069	1.21073	0.623854
52070	1.82014	0.323897
52071	2.08757	0.326082
52072	1.81292	0.279814
52073	1.67485	0.297519
52074	3.63306	0.30298
52075	1.82013	0.312987
52076	1.21748	0.297178
52077	1.29891	0.320372
52078	1.4442	0.311143
52079	1.67484	0.294111
52080	1.82013	0.293201
52081	1.81292	0.26663

52082	1.4516	0.240938
52083	1.39633	0.257318
52084	1.67485	0.249573
52085	1.5344	0.251782
52086	1.81292	0.248074
52087	1.67484	0.24771
52088	1.00982	0.240242
52089	0.994235	0.254388
52090	1.21748	0.250023
52091	1.45159	0.238293
52092	1.67484	0.243801
52093	1.55216	2.1
52094	1.34407	2.1
52095	1.34407	2.1
52096	1.52008	2.1
52097	1.81783	2.1
52098	1.99384	2.1
52099	1.90817	2.1
52100	2.03975	2.1
52101	1.97317	2.1
52102	1.81783	2.1
52103	3.85758	2.1
52104	0.599049	2.1
52105	0.660673	2.1
52106	0.785063	2.1
52107	0.924278	2.1
52108	0.760416	2.1
52109	0.760416	2.1
52110	0.719088	2.1
52111	0.694532	2.1
52112	0.522441	2.1
52113	0.796789	2.1
52114	0.655207	2.1
52115	0.886155	2.1
52116	1.97317	2.1
52117	1.81783	2.1
52118	1.65098	2.1
52119	1.91767	2.1
52120	3.7355	2.1
52121	1.15038	1.18039
52122	1.28649	1.6974
52123	1.38986	1.2474
52124	1.38986	1.79115
52125	1.07866	1.34063
52126	1.07866	1.87128
52127	0.664498	0.873068
52128	0.664498	1.94468
52129	0.675122	1.45428
52130	0.675122	2.05274
52131	0.69493	1.07751
52132	0.764429	1.45799
52133	0.623438	1.30268
52134	0.575191	1.68189
52135	0.764429	1.14535
52136	0.623438	1.08645
52137	0.575191	1.68646
52138	0.69493	1.62165
52139	1.89226	0.781841

52140	1.89226	0.572777
52141	1.89226	0.794236
52142	1.89226	0.725555
52143	3.78453	0.468756
52144	2.50075	0.758151
52145	2.15276	0.599778
52146	2.24902	0.796478
52147	2.30422	0.522906
52148	4.40178	0.514495
52149	1.70424	0.817415
52150	1.34494	0.84312
52151	2.15276	0.575823
52152	2.15276	0.819487
52153	2.0793	0.568607
52154	1.63078	0.722294
52155	1.89226	0.515673
52156	1.89226	0.815046
52157	1.89226	0.880647
52158	1.89226	0.614624
52159	1.89226	0.848333
52160	1.89226	0.771467
52161	1.89226	0.793798
52162	1.89226	0.73688
52163	1.89226	0.657912
52164	1.89226	0.437271
52165	3.78452	0.636315
52166	2.24902	0.797091
52167	1.91497	0.40992
52168	1.97135	0.848319
52169	1.73957	0.48228
52170	3.98859	0.404328
52171	1.73957	0.410342
52172	1.34494	0.800776
52173	1.73957	0.878833
52174	1.69006	0.486303
52175	1.3126	0.825512
52176	1.34848	0.633059
52177	0.946132	0.466107
52178	0.946132	0.819465
52179	0.946132	0.507464
52180	0.946132	0.859935
52181	0.946132	0.846073
52182	0.946132	0.823775
52183	0.946131	0.502685
52184	0.946131	0.457074
52185	0.946132	0.564103
52186	0.946132	0.625522
52187	0.946132	0.763087
52188	0.946132	0.771771
52189	1.89226	1.97974
52190	1.89226	1.52094
52191	1.89226	1.88138
52192	1.89226	1.59599
52193	3.78453	1.68915
52194	1.89226	1.72107
52195	2.82624	1.3681
52196	1.89226	1.63277
52197	2.02536	1.38645

52198	1.89226	1.62618
52199	2.02536	1.34963
52200	2.25027	0.195704
52201	1.1706	0.201847
52202	2.02733	0.199447
52203	2.02733	0.209052
52204	1.61462	0.194951
52205	1.69832	0.201464
52206	1.89226	0.243691
52207	1.89226	0.236439
52208	1.89226	0.223458
52209	1.89226	0.218928
52210	3.78452	0.218739
52211	0.946131	0.190712
52212	0.946131	0.199757
52213	1.18182	0.184262
52214	1.18182	0.177761
52215	0.973819	0.186031
52216	0.94613	0.190418
52217	1.18182	0.18691
52218	0.94613	0.191584
52219	0.973819	0.190881
52220	1.18182	0.184699
52221	0.94613	0.204673
52222	0.94613	0.191886
52223	2.23944	2.1
52224	1.52903	2.1
52225	1.35977	2.1
52226	2.04847	2.1
52227	3.5775	2.1
52228	2.23944	2.1
52229	2.34795	2.1
52230	2.34795	2.1
52231	2.48025	2.1
52232	2.04847	2.1
52233	2.18077	2.1
52234	1.89226	2.1
52235	1.89226	1.92897
52236	1.89226	2.1
52237	1.89226	2.1
52238	3.78452	2.1
52239	0.946127	2.1
52240	0.946127	2.1
52241	0.946131	2.1
52242	0.946131	2.1
52243	0.946128	2.1
52244	0.946128	2.1
52245	0.94613	2.1
52246	0.94613	2.1
52247	0.946129	2.1
52248	0.946129	2.1
52249	0.946129	2.1
52250	0.946129	2.1
52251	1.26956	2.1
52252	2.04847	2.1
52253	1.49576	2.1
52254	1.22377	2.1
52255	1.91172	2.1

52256	1.50198	2.1
52257	1.48568	2.1
52258	2.34795	2.1
52259	2.04847	2.1
52260	2.04847	2.1
52261	2.21155	2.1
52262	2.21155	2.1
52263	1.89226	2.1
52264	1.89226	2.1
52265	1.89226	2.1
52266	1.89226	1.92368
52267	1.89226	2.1
52268	1.89226	1.68128
52269	0.810943	2.1
52270	0.810943	2.1
52271	0.810944	2.1
52272	0.810944	2.1
52273	0.810957	2.1
52274	0.810957	2.1
52275	1.89226	2.1
52276	1.35168	2.1
52277	1.08132	2.1
52278	1.08132	2.1
52279	1.0813	2.1
52280	0.946132	0.301835
52281	1.33855	0.375416
52282	0.946131	0.296803
52283	1.38204	0.410878
52284	0.946131	0.411175
52285	0.946131	0.388502
52286	1.38204	0.308095
52287	1.38204	0.305511
52288	0.946131	0.338898
52289	0.946131	0.361843
52290	1.33855	0.308589
52291	1.38204	0.366896
52292	1.89226	0.43421
52293	2.76407	0.424714
52294	1.89226	0.325069
52295	2.72769	0.398321
52296	1.89226	0.307294
52297	2.72769	0.424794
52298	2.03914	0.287465
52299	1.67727	0.331201
52300	1.94831	0.334559
52301	1.81591	0.277
52302	2.32098	0.336991
52303	2.32098	0.294725
52304	0.801647	0.257271
52305	1.11086	0.297291
52306	0.838634	0.319266
52307	1.16049	0.27849
52308	0.977891	0.317537
52309	0.977891	0.312807
52310	0.984246	0.254488
52311	0.984246	0.276697
52312	1.11086	0.287818
52313	1.16049	0.297387

52314	0.801647	0.295027
52315	0.838634	0.292057
52316	0.946132	0.322689
52317	0.946132	0.410231
52318	0.946131	0.317149
52319	0.946131	0.430542
52320	0.946131	0.43114
52321	0.946131	0.408961
52322	0.946132	0.321467
52323	0.946132	0.316427
52324	0.946131	0.351999
52325	0.946131	0.364492
52326	0.946131	0.349924
52327	0.946131	0.375488
52328	0.946131	0.336578
52329	0.946131	0.456698
52330	0.946131	0.43571
52331	0.946131	0.343982
52332	0.946131	0.437355
52333	0.946131	0.435856
52334	0.946131	0.330372
52335	0.946131	0.345281
52336	0.946131	0.37682
52337	0.946131	0.391899
52338	0.946131	0.402278
52339	0.946131	0.395804
52340	2.0815	2.1
52341	1.20787	2.1
52342	1.92277	2.1
52343	1.3227	2.1
52344	3.29401	2.1
52345	2.16633	2.1
52346	2.13642	2.1
52347	1.40846	2.1
52348	1.40846	2.1
52349	1.3227	2.1
52350	2.08058	2.1
52351	0.938106	2.1
52352	0.790258	2.1
52353	0.742975	2.1
52354	0.742975	2.1
52355	0.874389	2.1
52356	1.17364	2.1
52357	0.525948	2.1
52358	0.52513	2.1
52359	0.553732	2.1
52360	0.876996	2.1
52361	0.530954	2.1
52362	1.095 2.1	
52363	1.00833	2.1
52364	1.70608	2.1
52365	1.33385	2.1
52366	1.33385	2.1
52367	1.07722	2.1
52368	1.07722	2.1
52369	0.953523	1.07984
52370	1.7457	1.05254
52371	1.03218	1.08937

52372	1.03218	1.04438
52373	1.79675	1.10172
52374	1.79675	1.05423
52375	1.13676	1.03741
52376	1.18136	0.969077
52377	0.87285	1.05315
52378	0.898375	1.05242
52379	1.03191	1.04955
52380	1.03191	0.971664
52381	1.0223	1.02645
52382	1.0223	1.06001
52383	1.18136	1.02392
52384	0.898374	1.00985
52385	0.87285	1.02831
52386	1.13676	1.02948
52387	2.36273	0.973472
52388	1.46965	0.964144
52389	1.79675	0.9705
52390	1.79675	0.917431
52391	2.43417	0.988098
52392	2.43417	0.935265
52393	1.4714	1.92297
52394	1.65434	1.72263
52395	1.62322	1.08095
52396	1.86068	1.50922
52397	3.27756	1.54267
52398	1.89226	2.03776
52399	1.89226	1.75026
52400	1.89226	1.1056
52401	1.89226	1.17587
52402	3.78453	1.32114
52403	1.89226	1.40594
52404	1.89226	2.03159
52405	1.89226	1.08396
52406	1.89226	2.04582
52407	3.78453	1.4451
52408	1.65434	1.41085
52409	1.60647	2.1
52410	1.86068	1.22778
52411	1.82649	2.03508
52412	3.48083	1.79997
52413	1.62322	1.86139
52414	1.86068	1.7272
52415	1.84368	1.04289
52416	2.18862	1.28096
52417	3.81184	1.46708
52418	1.89226	1.89725
52419	1.89226	1.62076
52420	1.89226	0.97747
52421	1.89226	1.3196
52422	3.78453	1.42193
52423	1.89226	1.45007
52424	1.89226	2.03397
52425	1.89226	1.18364
52426	1.89226	1.8357
52427	3.78453	1.6488
52428	1.86068	1.33578
52429	1.82649	2.02086

52430	2.18862	1.16313
52431	2.1128	1.9862
52432	4.01511	1.45363
52433	1.74351	2.1
52434	1.13854	2.1
52435	1.40233	2.1
52436	1.922 2.1	
52437	3.14584	2.1
52438	1.922 2.1	
52439	2.00775	2.1
52440	1.7435	2.1
52441	2.09224	2.1
52442	2.00775	2.1
52443	2.09295	2.1
52444	1.69858	2.1
52445	1.922 2.1	
52446	1.40233	2.1
52447	1.40233	2.1
52448	2.18238	2.1
52449	2.18238	2.1
52450	1.00387	2.1
52451	1.10886	2.1
52452	0.961001	2.1
52453	1.09119	2.1
52454	1.02868	2.1
52455	1.02868	2.1
52456	1.04118	2.1
52457	1.04118	2.1
52458	1.10886	2.1
52459	1.09119	2.1
52460	0.960999	2.1
52461	1.00388	2.1
52462	0.885958	0.764442
52463	0.885958	0.73678
52464	0.875826	0.734696
52465	0.875826	0.794495
52466	0.913965	0.790555
52467	0.598609	0.766406
52468	1.16317	0.736313
52469	0.868115	0.737307
52470	0.868115	0.756652
52471	0.598609	0.753694
52472	1.16317	0.759049
52473	0.913965	0.762707
52474	0.946131	0.780867
52475	0.946131	0.760731
52476	0.946131	0.776319
52477	0.946131	0.745158
52478	0.946131	0.775158
52479	0.946131	0.78293
52480	0.946131	0.759674
52481	0.946131	0.743504
52482	0.946131	0.770019
52483	0.946131	0.769664
52484	0.946131	0.76409
52485	0.946131	0.765821
52486	0.946133	0.213325
52487	0.946133	0.216408

52488	1.06246	0.210013
52489	1.06246	0.210303
52490	0.973851	0.217066
52491	0.946133	0.217443
52492	1.06246	0.203228
52493	0.946132	0.214037
52494	0.97385	0.213653
52495	1.06246	0.204024
52496	0.946133	0.210267
52497	0.946133	0.223909
52498	1.89226	0.253713
52499	1.89226	0.234217
52500	1.89226	0.243419
52501	1.89226	0.250508
52502	1.89226	0.230647
52503	1.89226	0.234783
52504	0.946131	0.233343
52505	0.946131	0.235523
52506	0.946127	0.232062
52507	0.946127	0.233529
52508	0.946131	0.251401
52509	0.946131	0.225426
52510	0.946127	0.231166
52511	0.946127	0.234037
52512	0.946128	0.229331
52513	0.946128	0.226839
52514	0.946129	0.23609
52515	0.946129	0.248483
52516	0.946132	0.202823
52517	0.946132	0.21307
52518	1.32446	0.198102
52519	0.794912	0.189606
52520	1.32429	0.202037
52521	0.946131	0.203798
52522	1.32446	0.197798
52523	0.94613	0.201317
52524	1.32429	0.202702
52525	0.794915	0.182523
52526	0.94613	0.201716
52527	0.94613	0.209854
52528	1.89226	0.199913
52529	2.36365	0.192826
52530	2.64857	0.199074
52531	1.89226	0.19569
52532	2.64857	0.182385
52533	1.89226	0.190754
52534	1.18463	2.1
52535	1.27174	2.1
52536	0.946129	2.1
52537	0.946129	2.1
52538	0.946131	2.1
52539	0.946131	2.1
52540	1.27174	2.1
52541	1.27174	2.1
52542	1.18463	2.1
52543	0.94613	2.1
52544	0.946131	2.1
52545	1.27174	2.1

52546	2.57997	2.1
52547	1.89226	2.1
52548	1.89226	2.1
52549	1.89226	2.1
52550	2.41915	2.1
52551	2.41915	2.1
52552	1.89226	2.1
52553	1.89226	2.1
52554	1.89226	2.1
52555	1.89226	2.1
52556	3.78452	2.1
52557	1.08807	2.1
52558	1.18463	2.1
52559	0.946129	2.1
52560	0.946129	2.1
52561	0.946131	2.1
52562	0.946131	2.1
52563	1.18463	2.1
52564	1.18463	2.1
52565	0.946131	2.1
52566	1.08807	2.1
52567	1.18463	2.1
52568	0.94613	2.1
52569	1.07017	2.1
52570	1.07017	2.1
52571	0.946131	2.1
52572	0.946131	2.1
52573	0.946132	2.1
52574	0.946132	2.1
52575	1.20957	2.1
52576	1.07017	2.1
52577	1.07017	2.1
52578	0.946132	2.1
52579	0.946131	2.1
52580	1.20957	2.1
52581	1.89226	2.1
52582	1.89226	2.1
52583	1.89226	2.1
52584	1.89226	2.1
52585	1.89226	2.1
52586	1.89226	2.1
52587	1.16181	0.234
52588	1.0227	0.243737
52589	1.0227	0.2591
52590	0.970959	0.236139
52591	1.24295	0.246656
52592	1.19121	0.227805
52593	1.12076	2.1
52594	2.53614	2.1
52595	1.28413	2.1
52596	1.28413	2.1
52597	2.36423	2.1
52598	2.36423	2.1
52599	1.89226	2.1
52600	1.89226	2.1
52601	1.89226	2.1
52602	1.89226	2.1
52603	3.78452	2.1

52604	1.22557	2.1
52605	2.771	2.1
52606	2.73012	2.1
52607	2.73012	2.1
52608	1.33356	2.1
52609	1.33356	2.1
52610	0.998682	2.1
52611	2.36423	2.1
52612	1.12076	2.1
52613	1.12076	2.1
52614	2.13757	2.1
52615	2.13757	2.1
52616	1.89226	2.1
52617	1.89226	2.1
52618	1.89226	2.1
52619	1.89226	2.1
52620	1.89226	2.1
52621	1.89226	2.1
52622	1.13987	2.1
52623	2.73011	2.1
52624	2.70875	2.1
52625	2.70875	2.1
52626	1.22557	2.1
52627	1.22557	2.1
52628	1.89227	2.1
52629	2.79243	2.1
52630	2.6625	2.1
52631	2.6625	2.1
52632	1.89227	2.1
52633	1.89227	2.1
52634	1.89226	2.1
52635	2.55971	2.1
52636	1.89226	2.1
52637	1.89226	2.1
52638	2.68704	2.1
52639	2.68704	2.1
52640	2.22043	1.50405
52641	2.38975	1.46376
52642	2.28696	1.09405
52643	2.48682	0.988931
52644	4.67671	1.13548
52645	2.38976	1.21058
52646	2.31988	1.4842
52647	2.48683	0.995975
52648	2.4594	1.21997
52649	4.84915	1.33273
52650	2.28696	1.40974
52651	2.48682	1.39024
52652	2.33379	1.45173
52653	2.56149	0.945178
52654	4.84845	1.3122
52655	2.48683	1.40449
52656	2.4594	1.34876
52657	2.5615	0.958031
52658	2.57957	1.18499
52659	5.0209	1.09316
52660	1.06659	1.97947
52661	1.04291	0.961958

52662	1.36595	0.926006
52663	1.36595	1.98912
52664	1.02761	2.1
52665	1.32697	0.923775
52666	0.946132	1.28414
52667	0.946132	1.23633
52668	1.36956	1.94665
52669	1.1229	2.1
52670	1.1229	1.22725
52671	1.1229	2.02224
52672	0.946132	1.08427
52673	0.946132	1.81594
52674	0.946132	1.5429
52675	1.36956	1.59344
52676	1.1229	1.52678
52677	0.946133	1.51918
52678	0.946133	1.03497
52679	0.946133	0.995706
52680	0.946128	1.7277
52681	0.946128	1.55264
52682	0.946131	1.51852
52683	0.946131	1.25712
52684	0.946131	0.865033
52685	0.946131	1.65319
52686	0.94613	1.35841
52687	0.94613	1.34164
52688	0.946131	1.25817
52689	0.946131	1.23758
52690	0.966427	1.16881
52691	0.802265	1.53621
52692	0.803714	1.5623
52693	0.808476	1.36904
52694	1.94371	1.57311
52695	1.36851	1.62834
52696	0.853864	1.77661
52697	1.26635	1.30891
52698	1.09085	1.2439
52699	0.857653	1.58162
52700	0.630243	1.39344
52701	1.20132	0.238451
52702	1.24295	0.254979
52703	1.24295	0.271379
52704	1.16182	0.24477
52705	1.28465	0.261344
52706	1.20351	0.236605
52707	0.600658	0.243502
52708	0.642323	0.269092
52709	0.54424	0.277154
52710	0.576779	0.253502
52711	0.590985	0.277808
52712	0.590985	0.279543
52713	0.586452	0.23867
52714	0.586452	0.255315
52715	0.642323	0.261791
52716	0.576779	0.255469
52717	0.600658	0.26782
52718	0.54424	0.262975
52719	1.28463	0.240179

52720	1.24294	0.255589
52721	1.12328	0.228563
52722	1.15505	0.257155
52723	2.39798	0.244403
52724	1.98431	0.387778
52725	2.18735	0.400252
52726	1.98431	0.432698
52727	1.70303	0.328615
52728	1.70302	0.525373
52729	1.37788	0.390669
52730	1.89226	0.500231
52731	2.37417	0.460035
52732	1.89226	0.507841
52733	2.18735	0.412291
52734	1.89226	0.605325
52735	2.18735	0.485932
52736	1.89226	0.330726
52737	1.89226	0.844142
52738	1.89226	0.542625
52739	1.89226	0.665574
52740	3.78452	0.703005
52741	1.8613	0.693467
52742	2.03576	0.845741
52743	1.61652	0.503413
52744	1.83082	1.03651
52745	3.65228	0.676224
52746	2.03576	0.846843
52747	2.05257	2.1
52748	1.83082	1.02472
52749	1.88487	1.36056
52750	3.92064	1.22642
52751	1.89226	0.844536
52752	1.89226	0.92033
52753	1.89226	0.67717
52754	1.89226	1.39933
52755	3.78453	0.857691
52756	0.946131	0.874867
52757	0.946131	0.465326
52758	0.94613	0.286534
52759	0.94613	0.740292
52760	0.946129	0.688225
52761	0.946129	0.860195
52762	0.946132	0.716834
52763	0.946132	0.293522
52764	0.94613	0.532934
52765	0.94613	0.5816
52766	0.946131	0.42941
52767	0.946131	0.514051
52768	0.804708	1.44919
52769	0.804708	0.706708
52770	0.815274	0.872661
52771	0.815274	0.56857
52772	0.83277	0.869441
52773	0.915407	1.23698
52774	0.80826	0.746722
52775	0.704574	0.548423
52776	0.832771	0.866247
52777	0.704573	0.874768

52778	0.808259	0.8374
52779	0.915409	0.838522
52780	1.83082	1.0454
52781	1.88487	1.47674
52782	1.66555	0.876748
52783	1.75835	2.1
52784	3.55042	1.111
52785	0.810947	0.677722
52786	0.810947	0.919973
52787	0.810947	0.691354
52788	0.810947	0.85052
52789	0.810934	0.873596
52790	0.810934	0.873687
52791	1.89227	1.47132
52792	1.3517	0.957385
52793	1.08132	0.884691
52794	1.08132	0.919709
52795	1.08133	0.889206
52796	1.89226	1.50882
52797	1.70303	1.6157
52798	1.89226	2.02473
52799	2.24712	2.1
52800	2.07363	2.1
52801	1.74617	1.57884
52802	1.89226	1.94681
52803	1.89226	1.26416
52804	1.89226	1.10638
52805	1.89226	1.84932
52806	3.78452	1.29908
52807	1.50108	1.8577
52808	1.43894	1.68753
52809	1.8322	1.04291
52810	1.40967	2.07207
52811	1.50108	1.77422
52812	1.07854	2.1
52813	1.89226	1.08018
52814	1.89226	1.87889
52815	1.89226	1.6617
52816	1.89226	0.933472
52817	3.78452	1.50858
52818	1.89226	2.02723
52819	1.89226	1.25528
52820	1.89226	1.25386
52821	1.89226	1.9306
52822	3.78453	1.35339
52823	1.54457	1.96441
52824	1.35097	1.46141
52825	1.70754	1.14708
52826	1.29629	2.05148
52827	1.54457	1.71788
52828	1.0305	2.1
52829	0.731763	1.92215
52830	1.00043	1.64567
52831	1.13883	1.18695
52832	0.939026	1.54099
52833	1.04003	1.17844
52834	0.853868	2.1
52835	0.927188	1.82268

52836	0.927188	0.923091
52837	0.861565	1.34075
52838	1.13882	1.49729
52839	0.731769	1.55463
52840	0.89319	1.66084
52841	1.46568	2.1
52842	1.20761	2.1
52843	1.51916	2.1
52844	1.36261	2.1
52845	2.72677	2.1
52846	1.51916	2.1
52847	1.60532	2.1
52848	1.36261	2.1
52849	1.36261	2.1
52850	1.64892	2.1
52851	1.64892	2.1
52852	1.3626	2.1
52853	0.98488	2.1
52854	1.60532	2.1
52855	1.32414	2.1
52856	2.68675	2.1
52857	1.78711	2.1
52858	1.14825	2.1
52859	2.16535	2.1
52860	1.6067	2.1
52861	3.3136	2.1
52862	1.89226	2.1
52863	1.89226	2.1
52864	1.89226	2.1
52865	1.89226	2.1
52866	3.78452	2.1
52867	2.34552	2.1
52868	1.53521	2.1
52869	1.53521	2.1
52870	1.60636	2.1
52871	1.92797	2.1
52872	1.99589	2.1
52873	1.34771	2.1
52874	1.34771	2.1
52875	0.946128	2.1
52876	0.946128	2.1
52877	1.3477	2.1
52878	1.31776	2.1
52879	0.946131	2.1
52880	0.946131	2.1
52881	0.94613	2.1
52882	1.31776	2.1
52883	0.946129	2.1
52884	1.3477	2.1
52885	1.89226	2.1
52886	1.89226	2.1
52887	1.89226	2.1
52888	1.89226	2.1
52889	3.78452	2.1
52890	0.92843	2.1
52891	0.672114	2.1
52892	0.89514	2.1
52893	0.89514	2.1

52894	0.720104	2.1
52895	0.755753	2.1
52896	0.789158	2.1
52897	0.967302	2.1
52898	1.06565	2.1
52899	0.7397	2.1
52900	0.758893	2.1
52901	0.94672	2.1
52902	1.89226	2.1
52903	2.588	2.1
52904	2.588	2.1
52905	2.63552	2.1
52906	1.89226	2.1
52907	1.93978	2.1
52908	1.49347	0.380142
52909	2.67757	0.537536
52910	1.80061	0.426987
52911	2.27056	0.454253
52912	4.07117	0.387666
52913	1.1016	0.298757
52914	2.27057	0.407194
52915	1.49348	0.44279
52916	1.81893	0.4587
52917	1.49348	0.314426
52918	1.81893	0.295811
52919	1.81893	0.418009
52920	2.82339	0.578588
52921	2.27057	0.453773
52922	2.25405	0.436113
52923	4.52462	0.38849
52924	1.89226	0.463384
52925	1.89226	0.572524
52926	1.89226	0.610336
52927	1.89226	0.503885
52928	1.89226	0.604864
52929	1.89226	0.532782
52930	1.89226	0.366477
52931	1.89226	0.531346
52932	1.89226	0.388548
52933	1.89226	0.425708
52934	3.78453	0.360292
52935	1.89226	0.561709
52936	1.89226	0.602722
52937	1.89226	0.443525
52938	1.89226	0.655706
52939	3.78452	0.644843
52940	1.89226	0.602792
52941	1.89226	0.802331
52942	1.89226	0.618044
52943	1.89226	0.665204
52944	3.78451	0.607099
52945	1.45633	2.1
52946	1.34678	2.1
52947	1.34678	2.1
52948	1.53735	2.1
52949	1.234	2.1
52950	1.42457	2.1
52951	1.89226	2.1

52952	1.89226	2.1
52953	1.89226	2.1
52954	1.89226	2.1
52955	1.89226	2.1
52956	1.89226	2.1
52957	1.09483	2.1
52958	1.34678	2.1
52959	1.234	2.1
52960	1.234	2.1
52961	1.19	2.1
52962	1.19	2.1
52963	0.946131	2.1
52964	0.946131	2.1
52965	0.946131	2.1
52966	0.946131	2.1
52967	0.946131	2.1
52968	0.946131	2.1
52969	0.946132	2.1
52970	0.946132	2.1
52971	0.946132	2.1
52972	0.946132	2.1
52973	0.946131	2.1
52974	0.946131	2.1
52975	2.82263	2.1
52976	1.89226	2.1
52977	2.71441	2.1
52978	2.71441	2.1
52979	1.89226	2.1
52980	1.89226	2.1
52981	1.1412	1.62438
52982	1.1412	2.1
52983	1.14626	2.1
52984	1.14626	2.1
52985	1.28511	1.80889
52986	1.34086	2.1
52987	0.992242	2.1
52988	0.946593	2.1
52989	0.992242	2.1
52990	1.34086	2.1
52991	0.946594	2.1
52992	1.28511	2.1
52993	1.18954	1.99594
52994	1.18954	2.1
52995	1.21477	2.1
52996	1.21477	2.1
52997	1.41207	2.1
52998	1.34086	2.1
52999	1.01306	2.1
53000	0.992242	2.1
53001	1.01306	2.1
53002	1.41207	2.1
53003	0.992242	2.1
53004	1.34086	2.1
53005	1.09044	1.98065
53006	1.44472	0.915869
53007	1.2955	1.47235
53008	1.2955	1.0665
53009	1.22025	1.44248

53010	1.22025	1.16532
53011	0.810944	0.961588
53012	0.810944	1.8783
53013	0.810946	0.978316
53014	0.810946	0.517397
53015	0.810959	0.960125
53016	0.810959	0.946996
53017	1.89226	0.922528
53018	1.35167	0.965048
53019	1.08132	1.78593
53020	1.08132	0.532345
53021	1.0813	0.964776
53022	1.41894	0.75929
53023	1.28953	0.702288
53024	0.946132	0.765137
53025	0.946132	1.01623
53026	0.946133	0.790732
53027	0.946133	0.706547
53028	1.41894	0.861263
53029	1.41894	0.868257
53030	1.28953	0.750075
53031	0.946132	0.768655
53032	0.946132	0.795043
53033	1.41894	0.758648
53034	1.89226	2.1
53035	2.15444	2.1
53036	1.93239	2.1
53037	1.89226	2.1
53038	2.19457	2.1
53039	2.15444	2.1
53040	1.89226	2.1
53041	1.89226	2.1
53042	1.89226	2.1
53043	1.89226	2.1
53044	1.89226	2.1
53045	1.89226	2.1
53046	0.938507	2.1
53047	0.938507	2.1
53048	1.07871	2.1
53049	1.15801	2.1
53050	1.03236	2.1
53051	1.14766	2.1
53052	0.946133	2.1
53053	0.946133	2.1
53054	0.946131	2.1
53055	1.07009	2.1
53056	0.946131	2.1
53057	1.03237	2.1
53058	1.89226	2.1
53059	2.19457	2.1
53060	1.94492	2.1
53061	1.89226	2.1
53062	2.24723	2.1
53063	2.19457	2.1
53064	0.946132	2.1
53065	0.946132	2.1
53066	0.946131	2.1
53067	0.946131	2.1

53068	0.946132	2.1
53069	0.946132	2.1
53070	0.946132	2.1
53071	0.946132	2.1
53072	0.946132	2.1
53073	0.946132	2.1
53074	0.946132	2.1
53075	0.946132	2.1
53076	2.21345	2.1
53077	1.89226	2.1
53078	1.89226	2.1
53079	2.14017	2.1
53080	2.14017	2.1
53081	1.89226	2.1
53082	1.89226	0.753863
53083	1.89226	0.872787
53084	1.89226	0.780851
53085	1.85439	0.852718
53086	2.23961	0.738987
53087	2.19479	0.774226
53088	1.2916	0.761501
53089	1.2916	0.753067
53090	0.946128	0.733938
53091	0.946128	0.737488
53092	1.2916	0.72621
53093	0.946131	0.736594
53094	0.946126	0.83264
53095	1.1198	0.721071
53096	1.2916	0.725003
53097	1.1198	0.789153
53098	0.946128	0.801411
53099	0.946128	0.768536
53100	1.89226	0.859151
53101	2.71262	0.819435
53102	1.89226	0.828002
53103	2.49959	0.847706
53104	4.39185	0.88562
53105	2.71262	0.815708
53106	1.34592	0.814307
53107	2.49959	0.838186
53108	1.14721	0.78792
53109	3.84551	0.756937
53110	0.946131	0.799765
53111	0.946131	0.811643
53112	1.11247	0.759861
53113	1.11247	0.745583
53114	1.24979	0.751911
53115	1.11247	0.766282
53116	0.946129	0.820791
53117	0.946129	0.7893
53118	0.946131	0.734073
53119	1.11247	0.636669
53120	0.946131	0.672137
53121	1.24979	0.712127
53122	0.946131	2.1
53123	0.946131	2.1
53124	0.94613	2.1
53125	0.94613	2.1

53126	0.946132	2.1
53127	0.946132	2.1
53128	0.946129	2.1
53129	0.946129	2.1
53130	0.946131	2.1
53131	0.946131	2.1
53132	0.946131	2.1
53133	0.946131	2.1
53134	2.77816	2.1
53135	1.89226	2.1
53136	1.89226	2.1
53137	1.89226	2.1
53138	3.78453	2.1
53139	1.89226	2.1
53140	1.89226	2.1
53141	1.89226	2.1
53142	1.89226	2.1
53143	1.89226	2.1
53144	1.89226	2.1
53145	1.89226	2.1
53146	1.89226	2.1
53147	1.89226	2.1
53148	1.89226	2.1
53149	3.78452	2.1
53150	1.55698	2.1
53151	1.89226	2.1
53152	1.89226	2.1
53153	1.89226	2.1
53154	1.8575	2.1
53155	1.8575	2.1
53156	1.89226	2.1
53157	1.89226	2.1
53158	1.89226	2.1
53159	1.89226	2.1
53160	1.89226	2.1
53161	1.89226	2.1
53162	1.89227	2.1
53163	1.89227	2.1
53164	1.89227	2.1
53165	1.89227	2.1
53166	1.89227	2.1
53167	1.89227	2.1
53168	1.35509	2.1
53169	2.06238	2.1
53170	2.12244	2.1
53171	2.12244	2.1
53172	1.34817	2.1
53173	1.34817	2.1
53174	0.946126	1.57287
53175	0.946126	2.1
53176	0.946131	2.1
53177	0.946131	2.1
53178	0.946127	2.1
53179	0.946127	1.67646
53180	0.94613	2.1
53181	0.94613	2.1
53182	0.946128	2.1
53183	0.946128	2.1

53184	0.946128	2.1
53185	0.946128	2.1
53186	1.89226	2.1
53187	1.89226	1.16265
53188	1.89226	1.79299
53189	1.89226	2.08208
53190	1.89226	1.29225
53191	1.89226	2.0024
53192	1.16742	2.1
53193	1.16742	1.85108
53194	1.18122	2.1
53195	1.18122	2.1
53196	1.31745	2.1
53197	1.2597	1.23937
53198	1.03119	2.1
53199	1.06122	2.1
53200	1.06122	2.1
53201	1.31745	2.1
53202	1.03119	2.1
53203	1.2597	2.1
53204	1.36849	2.1
53205	2.12244	2.1
53206	2.20092	2.1
53207	2.20092	2.1
53208	1.35509	2.1
53209	1.35509	2.1
53210	1.89226	2.1
53211	1.89226	2.1
53212	1.89226	2.1
53213	1.89226	2.1
53214	1.89226	2.1
53215	1.89226	2.07111
53216	1.89226	2.1
53217	1.89226	1.162
53218	1.89226	1.90137
53219	1.89226	1.65968
53220	3.78452	1.91258
53221	1.21731	2.1
53222	1.21731	1.56052
53223	1.23412	2.1
53224	1.23412	2.1
53225	1.39021	2.1
53226	1.31745	1.6587
53227	1.06122	2.1
53228	1.10046	2.1
53229	1.10046	2.1
53230	1.39021	2.1
53231	1.06122	2.1
53232	1.31745	2.1
53233	2.16503	0.921482
53234	1.78252	1.05486
53235	1.77293	1.02987
53236	1.56839	1.68161
53237	2.36957	0.792972
53238	2.16503	1.77497
53239	1.89223	0.941231
53240	1.89223	2.05
53241	1.89223	0.934557

53242	1.89223	1.15208
53243	3.78447	1.28114
53244	2.3696	1.15488
53245	1.78254	1.32799
53246	2.63298	0.807331
53247	2.07312	1.36242
53248	4.44272	1.25007
53249	1.89226	1.17094
53250	1.89226	1.26948
53251	1.89226	1.12106
53252	1.89226	1.88491
53253	1.89226	0.832989
53254	1.89226	1.94362
53255	1.89226	0.91027
53256	1.89226	0.749975
53257	1.89226	0.890507
53258	1.89226	0.351314
53259	3.78452	0.750239
53260	2.26369	0.908733
53261	2.2168	0.614883
53262	2.15617	0.817179
53263	2.10864	0.342424
53264	4.37234	0.597151
53265	2.2168	0.654985
53266	1.94499	0.637453
53267	1.94499	0.74785
53268	2.04737	0.6883
53269	2.10864	0.453277
53270	2.21103	0.500094
53271	1.89226	0.75178
53272	1.89226	0.635078
53273	1.89226	0.665805
53274	1.89226	0.726245
53275	1.89226	0.718903
53276	1.89226	0.725475
53277	1.89226	0.875324
53278	1.89226	0.751822
53279	1.89226	0.775056
53280	1.89226	0.892309
53281	1.89226	0.482767
53282	1.89226	0.925769
53283	1.02995	0.365441
53284	1.02995	0.900864
53285	1.03212	0.740354
53286	1.03212	0.865206
53287	0.988256	0.583045
53288	1.05432	0.475904
53289	1.00775	0.866943
53290	1.07809	0.883929
53291	1.00775	0.572595
53292	0.988257	0.647279
53293	1.07808	0.750413
53294	1.05432	0.782805
53295	2.10864	0.734328
53296	1.94499	0.725194
53297	1.97652	0.47541
53298	1.79681	0.576114
53299	3.9215	0.580812

53300	0.810943	0.641046
53301	0.810943	0.745571
53302	0.810943	0.643016
53303	0.810943	0.72861
53304	0.810958	0.480325
53305	0.810958	0.624521
53306	1.89226	0.722476
53307	1.35168	0.674463
53308	1.08132	0.576345
53309	1.08132	0.724504
53310	1.0813	0.709566
53311	0.657403	0.401126
53312	1.35403	0.393346
53313	1.24038	0.372863
53314	0.499144	0.374447
53315	1.24039	0.416775
53316	1.35403	0.389872
53317	0.657402	0.38087
53318	0.499144	0.389274
53319	0.915415	0.410054
53320	0.915415	0.405362
53321	0.937755	0.381999
53322	0.937755	0.379081
53323	1.72526	2.1
53324	1.17989	1.60454
53325	1.96035	1.98819
53326	1.47412	2.1
53327	3.14024	2.1
53328	1.89226	1.27266
53329	1.89226	1.62903
53330	1.89226	1.29619
53331	1.89226	0.949309
53332	3.78452	1.43306
53333	0.946131	1.1899
53334	0.946131	1.1894
53335	1.25764	1.24056
53336	1.25764	1.74227
53337	1.07087	1.06138
53338	1.25765	0.997482
53339	0.946128	1.04434
53340	0.946128	0.83418
53341	0.94613	0.939376
53342	1.25765	0.996308
53343	1.07086	1.18244
53344	0.946129	1.17935
53345	1.96035	1.87233
53346	1.32668	2.1
53347	2.29301	2.1
53348	1.70303	1.40144
53349	3.56428	2.1
53350	1.89226	1.31243
53351	1.89226	1.08571
53352	1.89226	0.861951
53353	1.89226	1.41609
53354	3.78452	0.8486
53355	0.897738	0.708928
53356	0.897738	0.717704
53357	0.946102	0.661949

53358	0.946102	0.575998
53359	1.11247	0.648061
53360	0.89771	0.572182
53361	0.94613	0.679273
53362	0.94613	0.731804
53363	0.897709	0.73087
53364	0.946131	0.752069
53365	0.946131	0.791279
53366	1.11247	0.718618
53367	1.89226	2.1
53368	1.89226	1.79098
53369	1.89226	2.01313
53370	1.89226	1.97012
53371	1.89226	2.0122
53372	1.89226	1.98079
53373	2.15429	2.1
53374	1.89226	2.1
53375	1.89226	2.1
53376	1.89226	2.04593
53377	2.27074	2.1
53378	2.27074	2.0432
53379	0.993311	1.38464
53380	0.993311	1.62013
53381	0.904322	2.1
53382	0.904322	1.77221
53383	0.803324	1.99633
53384	1.36183	1.38079
53385	0.916274	2.1
53386	0.535808	1.53553
53387	0.535808	1.67646
53388	0.803322	1.69834
53389	0.916276	1.60278
53390	1.36183	1.72992
53391	1.89226	2.1
53392	1.89226	1.59819
53393	1.89226	1.99916
53394	1.89226	2.03367
53395	1.89226	2.07264
53396	1.89226	2.08614
53397	2.11955	2.1
53398	2.62097	2.1
53399	1.85542	2.1
53400	2.39016	2.1
53401	4.50971	2.1
53402	2.39016	2.1
53403	2.20242	2.1
53404	2.39016	2.1
53405	1.64008	2.1
53406	1.85542	2.1
53407	1.64008	2.1
53408	1.79667	2.1
53409	1.28267	2.1
53410	1.28267	2.1
53411	1.40117	2.1
53412	1.74514	2.1
53413	1.83994	2.1
53414	0.575914	2.1
53415	0.625292	2.1

53416	0.949144	2.1
53417	0.882782	2.1
53418	0.773697	2.1
53419	0.773697	2.1
53420	0.786497	2.1
53421	0.786494	2.1
53422	0.605477	2.1
53423	0.949148	2.1
53424	0.625291	2.1
53425	0.862374	2.1
53426	1.89226	2.1
53427	2.44918	2.1
53428	2.4616	2.1
53429	2.44918	2.1
53430	1.90468	2.1
53431	1.89226	2.1
53432	1.89226	2.1
53433	1.89226	1.66333
53434	1.89226	2.1
53435	1.89226	1.00515
53436	3.78453	2.1
53437	0.946128	2.1
53438	0.946128	0.974722
53439	1.23631	1.54121
53440	1.23631	2.1
53441	0.946131	1.33092
53442	0.946131	1.47594
53443	1.3147	2.1
53444	1.23631	2.1
53445	1.3147	2.1
53446	0.946131	2.1
53447	1.23631	2.1
53448	0.94613	2.1
53449	1.89226	2.1
53450	2.4616	2.1
53451	2.4717	2.1
53452	2.4616	2.1
53453	1.90237	2.1
53454	1.89226	2.1
53455	0.946132	1.22988
53456	0.946132	2.1
53457	0.946131	2.1
53458	0.946131	1.57738
53459	0.946132	1.28622
53460	0.946132	1.91978
53461	0.946131	2.1
53462	0.946131	2.1
53463	0.946131	2.1
53464	0.946131	2.1
53465	0.946131	2.1
53466	0.946131	2.1
53467	0.946128	2.1
53468	0.946128	1.87625
53469	1.3147	1.13829
53470	1.3147	2.1
53471	0.94613	1.09157
53472	0.94613	1.64471
53473	1.3419	2.1

53474	1.3147	2.1
53475	1.3419	2.1
53476	0.94613	2.1
53477	1.3147	2.05349
53478	0.94613	2.09289
53479	1.89226	2.1
53480	1.89226	2.1
53481	1.89226	2.1
53482	1.89226	2.1
53483	3.78452	2.1
53484	2.3333	2.1
53485	1.3864	2.1
53486	1.3864	2.1
53487	1.4628	2.1
53488	1.95763	2.1
53489	2.03403	2.1
53490	1.294	2.1
53491	1.294	2.1
53492	0.946128	2.1
53493	0.946128	2.1
53494	1.294	2.1
53495	1.27474	2.1
53496	0.946131	2.1
53497	0.946131	2.1
53498	0.946129	2.1
53499	1.27474	2.1
53500	1.294	2.1
53501	0.94613	2.1
53502	1.89226	2.1
53503	1.89226	2.1
53504	1.89226	2.1
53505	1.89226	2.1
53506	1.89226	2.1
53507	1.89226	2.1
53508	0.808088	2.1
53509	0.808088	2.1
53510	0.815623	2.1
53511	0.815623	2.1
53512	0.660053	2.1
53513	0.693199	2.1
53514	0.930512	2.1
53515	0.978817	2.1
53516	0.660053	2.1
53517	0.930513	2.1
53518	0.693198	2.1
53519	0.978817	2.1
53520	1.27474	2.1
53521	1.27474	2.1
53522	0.946128	2.1
53523	0.946128	2.1
53524	1.26221	2.1
53525	1.27474	2.1
53526	0.946131	2.1
53527	0.946131	2.1
53528	0.94613	2.1
53529	1.26221	2.1
53530	0.946129	2.1
53531	1.27474	2.1

53532	0.90207	0.173869
53533	0.90207	0.177789
53534	0.895719	0.176873
53535	0.895719	0.185548
53536	0.927855	0.168422
53537	1.2493	0.175515
53538	0.85723	0.175719
53539	0.548484	0.172713
53540	0.548485	0.17394
53541	0.927855	0.168308
53542	0.85723	0.187934
53543	1.2493	0.179368
53544	1.41319	0.885492
53545	1.22482	0.77064
53546	1.46629	0.79466
53547	1.92622	0.872924
53548	3.15104	0.859983
53549	1.02305	0.731268
53550	1.22482	0.772547
53551	1.80336	0.786784
53552	1.44426	0.720854
53553	2.66908	0.733707
53554	1.3268	2.07942
53555	0.999489	2.1
53556	1.44091	1.87431
53557	1.14657	2.1
53558	2.4404	2.1
53559	1.89226	1.53224
53560	1.89226	2.1
53561	1.89226	1.87602
53562	1.89226	2.1
53563	3.78452	2.1
53564	1.89226	2.1
53565	2.28605	2.1
53566	2.43157	2.1
53567	2.43157	2.1
53568	1.89226	2.1
53569	1.89226	2.1
53570	1.60647	2.02609
53571	1.44091	1.73089
53572	1.31346	2.1
53573	1.31346	2.1
53574	1.14657	2.1
53575	1.44091	2.1
53576	1.89226	1.72169
53577	1.89226	2.1
53578	1.89226	1.52578
53579	1.89226	2.1
53580	3.78452	2.1
53581	0.946132	2.1
53582	0.946132	2.1
53583	1.3048	2.1
53584	1.3048	2.1
53585	1.3048	2.1
53586	1.21578	2.1
53587	0.946132	2.1
53588	0.946132	2.1
53589	1.3048	2.1

53590	0.946132	2.1
53591	0.946132	2.1
53592	1.21578	2.1
53593	1.89227	0.22694
53594	1.89227	0.2236
53595	1.89227	0.195013
53596	1.89227	0.215703
53597	3.78453	0.21813
53598	1.6671	0.188727
53599	1.87338	0.201913
53600	1.6671	0.212748
53601	2.02098	0.227147
53602	1.38328	0.18821
53603	1.73715	0.203617
53604	1.89226	0.191518
53605	1.89226	0.20727
53606	1.89226	0.179036
53607	1.89226	0.187491
53608	3.78453	0.191563
53609	1.6671	0.187843
53610	1.38328	0.183697
53611	1.35562	0.175415
53612	1.01012	0.184826
53613	2.67722	0.185001
53614	0.819377	0.181337
53615	0.647925	0.181688
53616	1.17802	0.174359
53617	1.33174	0.179421
53618	0.98543	0.176448
53619	0.98543	0.185098
53620	0.994237	0.175161
53621	0.994237	0.178149
53622	1.17802	0.176262
53623	0.647926	0.173129
53624	1.33174	0.180967
53625	0.819377	0.179644
53626	1.27806	0.214715
53627	1.68264	0.201221
53628	1.44593	0.19822
53629	1.878	0.217788
53630	3.15606	0.205775
53631	1.878	0.222332
53632	1.9898	0.204765
53633	1.878	0.226045
53634	1.68264	0.211431
53635	2.30533	0.223155
53636	2.10997	0.207912
53637	0.814587	0.202991
53638	0.814596	0.199882
53639	0.82775	0.216971
53640	0.82775	0.224094
53641	1.0107	0.221761
53642	0.91549	0.205713
53643	0.741058	0.216101
53644	0.714748	0.203686
53645	0.714758	0.21215
53646	1.03225	0.209029
53647	0.704864	0.205983

53648	0.915488	0.212031
53649	1.90347	0.226113
53650	2.06584	0.201353
53651	1.878	0.230882
53652	1.50236	0.209411
53653	2.05749	0.209324
53654	2.30533	0.228584
53655	2.24475	2.1
53656	1.85871	2.1
53657	2.13711	2.1
53658	2.13711	2.1
53659	1.9943	2.1
53660	1.9943	2.1
53661	1.85871	2.1
53662	1.7871	2.1
53663	1.41619	2.1
53664	1.41619	2.1
53665	2.13711	2.1
53666	2.13711	2.1
53667	2.24475	2.1
53668	2.13711	2.1
53669	2.54902	2.1
53670	2.46327	2.1
53671	4.70802	2.1
53672	1.06913	2.1
53673	1.06913	2.1
53674	1.0821	2.1
53675	1.0821	2.1
53676	0.893552	2.1
53677	1.08267	2.1
53678	1.06855	2.1
53679	1.23163	2.1
53680	1.23163	2.1
53681	1.08267	2.1
53682	0.893551	2.1
53683	1.06855	2.1
53684	1.67046	0.801932
53685	2.4106	0.710595
53686	2.05835	0.900006
53687	2.4106	0.919904
53688	1.59562	0.753889
53689	1.94788	0.722016
53690	2.34737	0.948775
53691	2.4106	0.73186
53692	2.05834	0.892544
53693	1.99655	0.987316
53694	4.40715	0.839471
53695	1.67046	0.731886
53696	2.05835	0.707497
53697	1.588	0.840619
53698	1.34857	1.15294
53699	3.40692	0.756959
53700	1.73802	0.8163
53701	1.89668	0.871137
53702	1.99655	1.3421
53703	1.99655	0.743082
53704	1.79981	0.868735
53705	2.05834	0.723573

53706	1.89226	1.15851
53707	1.89226	0.972552
53708	1.89226	0.79655
53709	1.89226	1.55058
53710	1.89226	0.819652
53711	1.89226	1.90913
53712	1.89226	1.02785
53713	1.89226	1.84797
53714	1.89226	1.3619
53715	1.89226	0.850094
53716	3.78453	1.33005
53717	0.719473	1.81637
53718	0.916103	1.44812
53719	1.02744	1.11461
53720	0.850328	1.59403
53721	0.888094	1.02362
53722	0.888094	2.1
53723	0.878337	1.77452
53724	0.878337	0.835331
53725	0.916103	1.20551
53726	1.02744	1.2527
53727	0.719473	1.53158
53728	0.850328	1.62152
53729	1.02744	1.22376
53730	0.850329	1.65773
53731	1.0875	1.11051
53732	0.919597	1.27832
53733	0.975841	0.902857
53734	0.975841	2.03323
53735	0.971196	0.894041
53736	0.971196	1.58449
53737	1.02744	1.28113
53738	1.0875	1.05407
53739	0.850328	1.56071
53740	0.919598	1.45328
53741	1.89226	1.51982
53742	1.89226	1.33231
53743	1.89226	0.873049
53744	1.89226	1.30328
53745	3.78453	1.22103
53746	1.89226	1.29566
53747	1.89226	0.975537
53748	1.89226	0.843138
53749	1.89226	1.64932
53750	1.89226	0.828472
53751	1.89226	1.80169
53752	1.11103	1.41697
53753	0.916103	1.80625
53754	1.21177	1.18687
53755	1.02744	1.25329
53756	1.07188	0.945854
53757	1.07188	2.1
53758	1.06659	0.933361
53759	1.06659	1.70456
53760	1.11103	1.50716
53761	1.21177	1.33194
53762	0.916104	1.58627
53763	1.02744	1.63007

53764	2.54158	1.05216
53765	2.42354	1.23644
53766	2.17499	0.868895
53767	2.17499	1.81372
53768	2.42354	1.42852
53769	2.05488	2.00235
53770	1.89226	0.339527
53771	1.89226	0.431296
53772	1.89226	0.362015
53773	1.89226	0.453182
53774	3.78453	0.363361
53775	1.89226	0.315357
53776	1.89226	0.425299
53777	1.89226	0.336405
53778	1.89226	0.400574
53779	1.89226	0.356857
53780	1.89226	0.424487
53781	1.89226	0.499871
53782	1.89226	0.547298
53783	1.89226	0.525428
53784	1.89226	0.44537
53785	1.89226	0.602579
53786	1.89226	0.528663
53787	1.89226	0.507199
53788	1.89226	0.58523
53789	1.89226	0.523112
53790	1.89226	0.60564
53791	3.78452	0.573431
53792	0.802544	0.365759
53793	0.946132	0.291585
53794	0.946132	0.308898
53795	0.787038	0.379852
53796	0.787039	0.348011
53797	0.787039	0.353622
53798	0.961637	0.3148
53799	0.961637	0.318604
53800	0.787038	0.344203
53801	0.802544	0.352093
53802	0.946132	0.326597
53803	0.946132	0.334035
53804	1.89226	0.282135
53805	1.60509	0.357756
53806	1.89226	0.29771
53807	1.89226	0.323644
53808	1.3905	0.320609
53809	1.3905	0.348136
53810	0.946132	0.531404
53811	0.946132	0.37163
53812	0.946131	0.466639
53813	0.946131	0.488027
53814	0.946132	0.497081
53815	0.946132	0.496336
53816	0.946131	0.45215
53817	0.946131	0.451783
53818	0.946131	0.471757
53819	0.946131	0.479972
53820	0.946132	0.466992
53821	0.946132	0.455988

53822	0.946131	0.507956
53823	0.946131	0.528992
53824	0.946131	0.559132
53825	0.946131	0.429223
53826	0.946132	0.517054
53827	0.946132	0.529214
53828	0.946131	0.464047
53829	0.946131	0.460565
53830	0.946131	0.500614
53831	0.946131	0.439088
53832	0.946132	0.473908
53833	0.946132	0.464102
53834	1.42382	1.50113
53835	1.32201	0.959641
53836	1.32096	1.04579
53837	1.42866	1.34348
53838	2.75067	1.14032
53839	1.89226	1.40527
53840	1.89226	0.894244
53841	1.89226	1.01668
53842	1.89226	1.13254
53843	1.89226	0.959939
53844	1.89226	1.06543
53845	1.89226	1.07017
53846	2.79029	0.785769
53847	2.7803	0.897515
53848	2.79029	0.94464
53849	1.88227	0.852574
53850	1.89226	0.90653
53851	0.716065	1.22753
53852	0.714333	1.52824
53853	0.660483	0.980214
53854	0.659126	1.05792
53855	0.687538	0.987022
53856	0.687538	1.18565
53857	0.685921	1.69101
53858	0.685921	1.00935
53859	0.659126	1.10089
53860	0.716065	1.11556
53861	0.660483	1.11021
53862	0.714333	1.11486
53863	1.89226	1.19148
53864	1.89226	0.895372
53865	1.89226	1.00051
53866	1.89226	1.11709
53867	3.78453	1.05892
53868	0.946133	0.917382
53869	0.946133	0.984046
53870	1.39015	0.868916
53871	1.38969	0.711427
53872	0.946132	0.727089
53873	0.946132	0.90845
53874	1.38969	0.99651
53875	1.38969	0.864877
53876	1.38969	0.892078
53877	0.946132	0.893514
53878	0.946133	0.917431
53879	1.39015	0.892026

53880	2.72157	0.700594
53881	1.22176	0.689302
53882	1.59492	0.692349
53883	1.22176	0.713206
53884	2.40743	0.694413
53885	2.03426	0.710686
53886	0.946131	0.737972
53887	0.946131	0.719067
53888	1.36079	0.698516
53889	1.01713	0.71195
53890	0.94613	0.709916
53891	0.94613	0.731891
53892	1.36079	0.710092
53893	1.36079	0.696656
53894	0.946131	0.707863
53895	1.36079	0.708466
53896	1.01713	0.70414
53897	0.946131	0.708321
53898	1.89226	0.732555
53899	1.89226	0.707314
53900	1.89226	0.701441
53901	1.89226	0.751211
53902	3.78453	0.737452
53903	1.89226	0.703233
53904	1.89226	0.67885
53905	1.89226	0.681441
53906	1.89226	0.710747
53907	1.89226	0.675696
53908	1.89226	0.708951
53909	0.946131	0.730797
53910	0.946131	0.712114
53911	0.946131	0.702388
53912	1.36079	0.673001
53913	0.94613	0.702053
53914	0.94613	0.72423
53915	0.946133	0.706206
53916	0.946133	0.628824
53917	0.946131	0.712048
53918	0.946131	0.709887
53919	0.946132	0.686229
53920	1.36079	0.696198
53921	1.89226	0.697128
53922	1.89226	0.744238
53923	1.89226	0.709169
53924	1.89226	0.72317
53925	1.89226	0.707786
53926	1.89226	0.723111
53927	1.89227	0.70114
53928	1.89227	0.662096
53929	1.89227	0.676819
53930	1.89227	0.713814
53931	1.89227	0.666609
53932	1.89227	0.705842
53933	1.60428	2.1
53934	2.2367	2.1
53935	1.50097	2.1
53936	1.80125	1.72195
53937	3.40553	2.1

53938	1.89095	2.1
53939	1.85293	0.89224
53940	1.80125	1.75304
53941	1.76268	0.906285
53942	3.65418	1.09441
53943	0.803177	2.1
53944	0.803177	2.1
53945	0.798644	2.1
53946	0.798644	2.07643
53947	0.84233	2.04306
53948	0.900624	2.0862
53949	0.750483	2.1
53950	0.701197	2.1
53951	0.701197	2.1
53952	0.84233	2.1
53953	0.750483	2.1
53954	0.900625	2.1
53955	0.860925	0.926707
53956	0.860925	2.1
53957	0.862743	2.01512
53958	0.862743	0.927514
53959	0.881338	0.909453
53960	0.826621	0.883751
53961	0.84233	2.06007
53962	0.900625	2.09865
53963	0.84233	1.05511
53964	0.826621	1.0416
53965	0.900624	1.09548
53966	0.881339	1.04491
53967	1.37938	1.01617
53968	1.03319	0.966783
53969	1.00512	1.25933
53970	0.639582	1.25362
53971	1.00463	1.25117
53972	1.00463	1.00772
53973	1.01434	0.956721
53974	1.01434	1.22983
53975	1.37938	1.14415
53976	1.00512	1.23439
53977	1.03319	1.14826
53978	0.639582	1.15571
53979	0.728713	0.412165
53980	0.728713	0.411361
53981	0.73425	0.826332
53982	0.73425	0.914304
53983	0.525122	2.1
53984	0.605622	0.346226
53985	0.948901	0.346226
53986	0.857342	0.346226
53987	0.85734	0.299731
53988	0.525123	0.979475
53989	0.948899	0.299731
53990	0.605624	0.299731
53991	1.75449	0.766237
53992	1.4378	0.794925
53993	1.93916	0.824104
53994	2.22512	0.735945
53995	3.69364	0.796408

53996	1.75448	0.811602
53997	1.89186	0.734001
53998	2.29771	0.739294
53999	2.22512	0.73719
54000	4.05219	0.741619
54001	1.93916	0.814352
54002	2.51749	0.722173
54003	2.62357	0.761115
54004	2.22512	0.740532
54005	2.33761	0.777341
54006	1.93916	0.747929
54007	2.29771	0.750701
54008	2.51749	0.730708
54009	2.22512	0.727178
54010	2.22512	0.720671
54011	2.50905	0.757197
54012	2.50905	0.748072
54013	0.87382	0.2866
54014	1.33662	0.369227
54015	1.20756	0.340916
54016	0.890467	0.448857
54017	1.08859	0.440542
54018	1.08859	0.411809
54019	1.13084	0.286098
54020	1.08793	0.338167
54021	1.34722	0.367597
54022	1.27184	0.389888
54023	0.917678	0.378121
54024	0.97821	0.372373
54025	1.28396	0.327989
54026	1.70302	0.461697
54027	2.30076	0.419033
54028	1.20417	0.339202
54029	2.57506	0.423977
54030	2.2656	0.362905
54031	1.03642	0.936053
54032	1.03642	0.497374
54033	1.07029	0.484565
54034	1.07029	0.517268
54035	1.31531	0.873387
54036	1.23279	0.632894
54037	0.941743	0.550617
54038	0.791399	0.79117
54039	0.791399	0.646537
54040	1.23279	0.627502
54041	0.941744	0.594287
54042	1.31531	0.599479
54043	0.937354	0.952039
54044	0.937354	0.467427
54045	0.954794	0.527855
54046	0.954794	0.534041
54047	1.13563	0.651556
54048	1.23279	0.880364
54049	0.791398	0.570126
54050	0.659359	0.78191
54051	1.13563	0.639248
54052	0.659359	0.647521
54053	0.791399	0.569208

54054	1.23279	0.577266
54055	2.29852	1.03996
54056	1.89227	0.948156
54057	2.14485	0.921994
54058	2.14485	1.05151
54059	1.89227	0.961591
54060	1.89227	1.08973
54061	0.947863	0.965615
54062	1.19854	1.06799
54063	2.16841	1.08412
54064	1.76597	0.955768
54065	2.96451	0.942404
54066	1.89227	0.935099
54067	1.89227	0.885571
54068	1.89227	0.867003
54069	1.89227	0.985232
54070	1.89227	0.879927
54071	1.89227	0.99782
54072	2.46489	2.1
54073	1.98339	2.1
54074	1.81667	2.1
54075	2.36914	2.1
54076	4.35253	2.1
54077	2.46488	2.1
54078	2.70401	2.1
54079	2.64996	2.1
54080	2.36914	2.1
54081	5.07315	2.1
54082	1.81667	2.1
54083	2.29459	2.1
54084	2.36914	2.1
54085	2.36914	2.1
54086	1.68073	2.1
54087	1.68073	2.1
54088	2.64996	2.1
54089	2.29458	2.1
54090	2.29458	2.1
54091	2.36914	2.1
54092	2.61243	2.1
54093	2.68698	2.1
54094	1.89226	1.20458
54095	1.89226	1.15182
54096	1.89226	1.35724
54097	1.89226	1.176
54098	1.89226	1.35532
54099	1.89226	1.14862
54100	1.89224	1.3513
54101	1.89224	1.13006
54102	1.65571	1.27223
54103	1.65571	1.11849
54104	1.89224	1.34726
54105	1.89224	1.19102
54106	1.89224	1.16932
54107	1.89224	1.16177
54108	1.89224	1.28793
54109	1.89224	1.04882
54110	1.89224	1.37495
54111	1.89224	1.08829

54112	1.98252	0.50436
54113	2.20641	0.511796
54114	1.05129	0.477071
54115	1.2951	0.5611
54116	3.27762	0.500007
54117	1.85598	1.67526
54118	1.88635	1.14991
54119	2.12346	0.932774
54120	2.10838	1.62763
54121	3.97943	1.21469
54122	2.10838	1.25279
54123	1.87532	1.89034
54124	1.85597	0.968352
54125	1.85597	2.0996
54126	1.87532	1.29309
54127	1.57914	2.1
54128	2.12348	1.14735
54129	2.35141	1.49807
54130	2.37191	1.26746
54131	2.10841	1.33644
54132	2.38698	1.01916
54133	2.12348	1.22993
54134	1.07811	1.66803
54135	0.937672	2.1
54136	1.17571	1.44592
54137	1.05421	0.958061
54138	1.07087	1.12794
54139	1.07087	2.1
54140	1.06145	1.70401
54141	1.06145	1.09473
54142	1.17571	1.64913
54143	1.07811	1.58459
54144	1.05421	1.5232
54145	0.937672	1.5552
54146	1.67334	2.1
54147	2.76609	2.1
54148	2.74144	2.1
54149	2.74144	2.1
54150	1.71157	2.1
54151	1.71157	2.1
54152	2.76609	2.1
54153	1.89226	2.1
54154	1.89226	2.1
54155	1.89226	2.1
54156	2.74143	2.1
54157	2.74143	2.1
54158	1.10393	2.1
54159	1.10393	2.1
54160	1.10788	2.1
54161	1.10788	2.1
54162	1.38302	2.1
54163	1.37072	2.1
54164	0.836672	2.1
54165	0.841089	2.1
54166	1.38302	2.1
54167	0.84109	2.1
54168	0.836671	2.1
54169	1.37072	2.1

54170	1.38302	2.1
54171	1.38302	2.1
54172	0.946131	2.1
54173	0.946131	2.1
54174	0.946128	2.1
54175	0.946128	2.1
54176	1.38302	2.1
54177	1.37072	2.1
54178	1.38302	2.1
54179	0.946131	2.1
54180	0.94613	2.1
54181	1.37072	2.1
54182	1.28072	0.936325
54183	1.55779	2.1
54184	1.28072	1.6453
54185	1.40765	1.04919
54186	1.47186	1.65395
54187	1.59879	1.45668
54188	1.89227	0.951335
54189	1.89227	0.932293
54190	1.89227	0.824838
54191	1.89227	1.48493
54192	1.89227	0.845036
54193	1.89227	1.42748
54194	2.80057	0.819704
54195	1.89226	0.823169
54196	1.89226	0.793236
54197	1.89226	0.903537
54198	2.63061	0.549241
54199	2.63061	0.945765
54200	1.28072	0.896571
54201	1.38859	2.1
54202	1.47186	1.60524
54203	1.47186	1.08661
54204	1.16777	1.72086
54205	1.16777	1.23827
54206	1.89226	0.900079
54207	1.89226	0.9326
54208	1.89226	0.854473
54209	1.89226	1.57543
54210	1.89226	0.82193
54211	1.89226	1.82411
54212	1.83221	2.1
54213	2.0815	2.1
54214	2.0815	2.1
54215	2.08153	2.1
54216	1.86703	2.1
54217	1.87402	2.1
54218	1.86703	2.1
54219	2.08508	2.1
54220	2.08508	2.1
54221	2.29001	2.1
54222	1.89226	2.1
54223	2.08048	2.1
54224	2.7278	0.404185
54225	1.10909	0.433426
54226	1.50472	0.451641
54227	1.10909	0.469821

54228	2.69881	0.372927
54229	2.30319	0.441595
54230	1.23204	2.1
54231	1.92264	2.1
54232	1.48162	2.1
54233	1.48162	2.1
54234	1.72994	2.1
54235	1.72994	2.1
54236	0.864968	2.1
54237	0.696952	2.1
54238	0.61602	2.1
54239	0.479736	2.1
54240	0.66444	2.1
54241	0.66444	2.1
54242	0.680264	2.1
54243	0.680264	2.1
54244	0.696952	2.1
54245	0.479736	2.1
54246	0.61602	2.1
54247	0.864968	2.1
54248	1.89226	0.821419
54249	2.43219	1.47951
54250	1.89226	1.10953
54251	2.82019	1.08054
54252	1.89226	1.29772
54253	2.82019	1.12174
54254	1.89226	0.912113
54255	2.83701	1.65678
54256	2.2701	1.16974
54257	2.2701	1.01907
54258	1.89226	1.1785
54259	1.89226	1.07388
54260	1.89226	1.20937
54261	1.89226	0.816886
54262	1.89226	0.939372
54263	1.89226	1.00184
54264	1.89226	1.06562
54265	1.89226	0.999883
54266	1.5795	1.27863
54267	1.44091	1.2443
54268	1.05267	1.41582
54269	1.05267	1.09637
54270	1.98408	1.32106
54271	1.98408	1.07911
54272	2.20624	2.1
54273	1.08831	2.1
54274	2.49053	2.1
54275	1.42486	2.1
54276	3.57883	2.1
54277	1.89226	2.1
54278	1.89226	2.1
54279	1.89226	2.1
54280	1.89226	2.1
54281	3.78453	2.1
54282	0.946132	2.1
54283	0.946132	2.1
54284	0.853591	2.1
54285	0.642207	2.1

54286	0.94613	2.1
54287	0.94613	2.1
54288	0.642209	2.1
54289	0.642209	2.1
54290	0.946131	2.1
54291	0.853591	2.1
54292	0.642207	2.1
54293	0.946132	2.1
54294	1.0506	2.1
54295	1.0506	2.1
54296	1.0879	2.1
54297	0.960424	2.1
54298	0.708687	2.1
54299	0.782927	2.1
54300	1.40041	2.1
54301	1.2518	2.1
54302	0.767748	2.1
54303	1.31801	2.1
54304	1.21412	2.1
54305	0.69461	2.1
54306	1.89226	2.1
54307	1.89226	2.1
54308	1.89226	2.1
54309	1.89226	2.1
54310	1.89226	2.1
54311	1.89226	2.1
54312	0.922223	2.1
54313	0.922223	2.1
54314	1.04548	2.1
54315	0.768229	2.1
54316	0.948441	2.1
54317	0.806483	2.1
54318	0.85362	2.1
54319	0.85362	2.1
54320	1.05942	2.1
54321	0.922187	2.1
54322	0.851554	2.1
54323	0.768264	2.1
54324	2.16728	2.1
54325	0.95455	2.1
54326	1.21372	2.1
54327	0.98227	2.1
54328	2.16728	2.1
54329	1.93584	2.1
54330	2.08849	0.335656
54331	1.08732	0.373311
54332	1.3357	0.355636
54333	2.37948	0.374814
54334	3.4668	0.355601
54335	2.57882	0.336986
54336	2.37948	0.35084
54337	2.53055	0.339802
54338	1.48676	0.385439
54339	1.3357	0.382163
54340	1.3357	0.365202
54341	1.89226	2.1
54342	1.89226	2.1
54343	2.17471	2.1

54344	1.89226	2.1
54345	2.60085	2.1
54346	2.26306	2.1
54347	0.946131	1.69252
54348	0.946131	2.1
54349	0.946131	2.01414
54350	0.946131	2.1
54351	0.94613	2.06665
54352	0.94613	2.0131
54353	0.946132	2.1
54354	0.946132	2.1
54355	0.946131	2.1
54356	0.946131	2.1
54357	0.946131	2.1
54358	0.946131	2.1
54359	1.89226	2.1
54360	2.17471	2.1
54361	2.08196	2.1
54362	2.17471	2.1
54363	1.79952	2.1
54364	1.89226	2.1
54365	1.89226	2.1
54366	1.89226	2.1
54367	1.89226	2.1
54368	1.89226	2.1
54369	1.89226	2.1
54370	1.89226	2.1
54371	1.86016	2.1
54372	1.89226	2.1
54373	1.89226	2.1
54374	1.89226	2.1
54375	1.84826	2.1
54376	1.84826	2.1
54377	2.50136	2.1
54378	1.99781	2.1
54379	1.99729	2.1
54380	2.5214	2.1
54381	4.49865	2.1
54382	0.51724	2.1
54383	0.496076	2.1
54384	0.998906	2.1
54385	0.998645	2.1
54386	0.742249	2.1
54387	0.742249	2.1
54388	0.752734	2.1
54389	0.752734	2.1
54390	0.998639	2.1
54391	0.496073	2.1
54392	0.517243	2.1
54393	0.998913	2.1
54394	0.998653	2.1
54395	0.994293	2.1
54396	1.26071	2.1
54397	1.26504	2.1
54398	1.12529	2.1
54399	1.12529	2.1
54400	1.12971	2.1
54401	1.12971	2.1

54402	1.26504	2.1
54403	0.994292	2.1
54404	0.998651	2.1
54405	1.26071	2.1
54406	1.89226	2.1
54407	1.89226	2.1
54408	1.89226	2.1
54409	1.89226	2.1
54410	3.78452	2.1
54411	1.89226	2.1
54412	1.89226	2.1
54413	1.89226	2.1
54414	1.89226	2.1
54415	3.78452	2.1
54416	0.675872	2.1
54417	0.675641	2.1
54418	0.943072	2.1
54419	0.898317	2.1
54420	0.775717	2.1
54421	0.775717	2.1
54422	0.79824	2.1
54423	0.79824	2.1
54424	0.675642	2.1
54425	0.94307	2.1
54426	0.675874	2.1
54427	0.898315	2.1
54428	0.943071	2.1
54429	0.898316	2.1
54430	0.998133	2.1
54431	0.918903	2.1
54432	0.922369	2.1
54433	0.922369	2.1
54434	0.939606	2.1
54435	0.939606	2.1
54436	0.943071	2.1
54437	0.998133	2.1
54438	0.898316	2.1
54439	0.918903	2.1
54440	0.946131	2.1
54441	0.946131	2.1
54442	0.946131	2.1
54443	0.946131	2.1
54444	0.946132	2.1
54445	0.946132	2.1
54446	0.946129	2.1
54447	0.946129	2.1
54448	0.94613	2.1
54449	0.94613	2.1
54450	0.946131	2.1
54451	0.946131	2.1
54452	1.89226	2.1
54453	1.89226	2.1
54454	1.89226	2.1
54455	1.89226	2.1
54456	1.89226	2.1
54457	1.89226	2.1
54458	1.35129	2.1
54459	1.88614	2.1

54460	2.01867	2.1
54461	1.38189	2.1
54462	3.36995	2.1
54463	0.943071	2.1
54464	1.00934	2.1
54465	0.998134	2.1
54466	1.10866	2.1
54467	0.992617	2.1
54468	0.992617	2.1
54469	1.01485	2.1
54470	1.01485	2.1
54471	1.00934	2.1
54472	1.10866	2.1
54473	0.943072	2.1
54474	0.998134	2.1
54475	2.67272	2.1
54476	1.61859	2.1
54477	1.70303	2.1
54478	2.52175	2.1
54479	4.5326	2.1
54480	2.00954	2.1
54481	2.27801	2.1
54482	1.87875	2.1
54483	1.87875	2.1
54484	2.37837	2.1
54485	2.37837	2.1
54486	2.00955	2.1
54487	1.78 2.1	
54488	2.21813	2.1
54489	1.8493	2.1
54490	2.37838	2.1
54491	2.00955	2.1
54492	2.37838	2.1
54493	2.27801	2.1
54494	2.58523	2.1
54495	2.71597	2.1
54496	4.9636	2.1
54497	2.37838	2.1
54498	2.71597	2.1
54499	1.72709	2.1
54500	2.0815	2.1
54501	2.59749	2.1
54502	2.78034	2.1
54503	0.94613	1.00574
54504	0.94613	1.0335
54505	0.946128	0.90484
54506	0.946128	0.885324
54507	0.94613	0.893326
54508	0.94613	0.919145
54509	0.946128	1.02562
54510	0.946128	0.850058
54511	0.946129	0.994334
54512	0.946129	0.976667
54513	0.946129	0.92262
54514	0.946129	0.933163
54515	1.89226	1.11733
54516	1.89226	1.05667
54517	1.89226	1.14682

54518	1.89226	0.967046
54519	3.78452	0.986671
54520	0.946133	0.882776
54521	0.946133	0.889556
54522	0.946132	0.739846
54523	0.946132	0.765635
54524	0.946132	0.796678
54525	0.946132	0.789137
54526	0.946133	0.883375
54527	0.946133	0.863661
54528	0.946132	0.860771
54529	0.946132	0.599476
54530	0.946132	0.774472
54531	0.946132	0.795651
54532	1.89226	0.955684
54533	2.10495	0.755244
54534	1.89226	0.745241
54535	2.30932	0.745083
54536	1.89226	1.03186
54537	2.30932	1.02818
54538	2.30932	0.780185
54539	1.00366	0.534357
54540	2.10496	0.608751
54541	1.24728	0.677583
54542	3.31298	0.679924
54543	1.87557	2.1
54544	1.07167	2.1
54545	2.03045	2.1
54546	1.25185	2.1
54547	3.10212	2.1
54548	1.89226	2.1
54549	1.89226	2.1
54550	1.89226	2.1
54551	1.89226	2.1
54552	3.78453	2.1
54553	0.851541	2.1
54554	0.851541	2.1
54555	1.12236	2.1
54556	1.05783	2.1
54557	0.870663	2.1
54558	0.951856	2.1
54559	0.946132	2.1
54560	0.946132	2.1
54561	1.04073	2.1
54562	1.1296	2.1
54563	0.776062	2.1
54564	0.851527	2.1
54565	0.859395	2.1
54566	0.859395	2.1
54567	0.870165	2.1
54568	0.870165	2.1
54569	0.625924	2.1
54570	0.714333	2.1
54571	1.0821	2.1
54572	1.01523	2.1
54573	1.0821	2.1
54574	0.714333	2.1
54575	1.01523	2.1

54576	0.625924	2.1
54577	1.89226	2.1
54578	1.89226	2.1
54579	1.89226	2.1
54580	1.89226	2.1
54581	3.78453	2.1
54582	1.89226	2.1
54583	1.89226	2.1
54584	2.23164	2.1
54585	2.67802	2.1
54586	2.03663	2.1
54587	1.89226	2.1
54588	2.42221	2.1
54589	1.61933	2.1
54590	2.80896	2.06698
54591	1.87043	2.1
54592	4.29262	2.1
54593	1.89226	1.95698
54594	1.89226	2.1
54595	1.89226	2.1
54596	1.89226	2.1
54597	1.89226	2.06332
54598	1.89226	2.1
54599	2.80892	2.02772
54600	1.87041	2.1
54601	2.67182	1.76221
54602	2.30891	2.1
54603	5.07665	2.1
54604	1.89224	2.00515
54605	1.89224	2.1
54606	1.89224	1.7547
54607	1.89224	2.1
54608	3.78448	2.1
54609	0.946118	2.1
54610	0.946118	2.1
54611	0.946118	2.1
54612	0.946118	2.1
54613	0.946115	2.1
54614	0.946115	2.1
54615	0.94612	2.1
54616	0.94612	2.1
54617	0.94613	2.1
54618	0.94613	2.1
54619	0.946105	2.1
54620	0.946105	2.1
54621	2.3089	2.1
54622	1.02783	2.1
54623	1.98533	2.1
54624	1.76762	2.1
54625	1.24553	2.1
54626	1.02783	2.1
54627	2.2049	0.495122
54628	2.34673	0.476877
54629	1.00763	0.474422
54630	1.23746	0.535526
54631	3.44236	0.490117
54632	1.89226	0.598875
54633	1.89226	0.675099

54634	1.89226	0.622191
54635	1.89226	0.658175
54636	3.78452	0.666239
54637	1.89226	0.678887
54638	1.89226	0.650749
54639	1.89226	0.659749
54640	1.89226	0.705988
54641	3.78453	0.669425
54642	0.891618	0.539233
54643	0.891618	0.523665
54644	0.902158	0.473822
54645	0.902158	0.497702
54646	1.15396	0.530608
54647	0.620408	0.50431
54648	1.17337	0.475166
54649	0.618733	0.537546
54650	0.620408	0.512525
54651	0.618733	0.500896
54652	1.17337	0.516345
54653	1.15396	0.525832
54654	0.69978	0.536416
54655	0.69978	0.530802
54656	0.946102	0.532226
54657	0.946102	0.539819
54658	0.831702	0.555401
54659	0.946129	0.557688
54660	0.699752	0.538145
54661	0.946132	0.509004
54662	0.831703	0.543007
54663	0.699751	0.508376
54664	0.946131	0.518525
54665	0.946131	0.534174
54666	0.831675	0.561657
54667	0.831675	0.563858
54668	0.946163	0.554867
54669	0.864397	0.555278
54670	0.853471	0.593131
54671	0.962623	0.540207
54672	0.748555	0.552821
54673	0.851543	0.55749
54674	0.864714	0.561815
54675	0.760411	0.558154
54676	0.946133	0.560011
54677	0.946133	0.564118
54678	1.89226	0.70029
54679	1.89226	0.743368
54680	1.89226	0.737028
54681	1.89226	0.701251
54682	3.78452	0.703848
54683	1.89226	0.801855
54684	1.89226	0.722772
54685	1.89226	0.732669
54686	1.89226	0.830741
54687	3.78452	0.759742
54688	0.946132	0.802396
54689	0.946132	0.833262
54690	0.946132	0.712464
54691	0.946132	0.731961

54692	0.946132	0.718819
54693	0.946132	0.827941
54694	0.946133	0.721126
54695	0.946133	0.831524
54696	0.946133	0.753793
54697	0.946133	0.752628
54698	0.946132	0.764471
54699	0.946132	0.779572
54700	1.89226	0.771534
54701	2.39715	0.725451
54702	1.89226	0.757484
54703	1.89226	0.838323
54704	1.89226	0.709511
54705	1.89226	0.743749
54706	0.94613	0.831579
54707	0.94613	0.821397
54708	0.94613	0.751379
54709	0.94613	0.732195
54710	0.946131	0.743373
54711	0.946131	0.834003
54712	0.946129	0.822117
54713	0.946129	0.738929
54714	0.94613	0.764425
54715	0.94613	0.770591
54716	0.94613	0.784406
54717	0.94613	0.783282
54718	2.54204	2.1
54719	2.3148	2.1
54720	2.55328	2.1
54721	2.39646	2.1
54722	4.71126	2.1
54723	1.62611	2.1
54724	2.3148	2.1
54725	2.16986	2.1
54726	1.43745	2.1
54727	3.75225	2.1
54728	1.89226	2.1
54729	1.89226	2.1
54730	1.89226	2.1
54731	1.89226	2.1
54732	3.78453	2.1
54733	1.89226	2.1
54734	1.89226	2.1
54735	1.89226	2.1
54736	1.89226	2.1
54737	3.78453	2.1
54738	2.12125	2.1
54739	2.39642	2.1
54740	2.2941	2.1
54741	1.9933	2.1
54742	4.38972	2.1
54743	1.73784	2.1
54744	1.26892	2.1
54745	1.26892	2.1
54746	1.43743	2.1
54747	1.9933	2.1
54748	2.1618	2.1
54749	0.946118	2.1

54750	0.946118	2.1
54751	0.946118	2.1
54752	0.946118	2.1
54753	0.946119	2.1
54754	0.946119	2.1
54755	0.946118	2.1
54756	0.946118	2.1
54757	0.946131	2.1
54758	0.946131	2.1
54759	0.946106	2.1
54760	0.946106	2.1
54761	1.89224	2.1
54762	1.89224	2.1
54763	1.89224	2.1
54764	1.89224	2.1
54765	3.78448	2.1
54766	2.45922	2.1
54767	2.15686	2.1
54768	2.14259	2.1
54769	2.43225	2.1
54770	4.58911	2.1
54771	1.62027	2.1
54772	2.15686	2.1
54773	1.62241	2.1
54774	1.62241	2.1
54775	2.1426	2.1
54776	2.1426	2.1
54777	2.14259	2.1
54778	2.43225	2.1
54779	2.40667	2.1
54780	2.1281	2.1
54781	4.56035	2.1
54782	1.0713	2.1
54783	1.06405	2.1
54784	0.810136	2.1
54785	0.808433	2.1
54786	0.938508	2.1
54787	0.938508	2.1
54788	0.941223	2.1
54789	0.941223	2.1
54790	1.06405	2.1
54791	0.808433	2.1
54792	0.810135	2.1
54793	1.0713	2.1
54794	1.89226	0.809778
54795	1.89226	0.673001
54796	1.89226	0.780115
54797	1.89226	0.714358
54798	3.78453	0.724099
54799	1.89227	0.714476
54800	1.89227	0.668119
54801	1.89227	0.766397
54802	1.89227	0.612859
54803	3.78453	0.694272
54804	1.19	0.640504
54805	1.12469	0.530267
54806	1.01052	0.58606
54807	1.08166	0.561809

54808	2.20051	0.563979
54809	0.594999	0.644259
54810	0.540832	0.587744
54811	0.514182	0.687928
54812	0.567857	0.617754
54813	0.554259	0.604733
54814	0.554259	0.574751
54815	0.55443	0.645859
54816	0.55443	0.673331
54817	0.540832	0.61984
54818	0.514182	0.601691
54819	0.567857	0.609674
54820	0.594999	0.605052
54821	1.38674	0.647858
54822	0.946132	0.707143
54823	1.36361	0.667555
54824	0.946132	0.719212
54825	1.36362	0.659963
54826	1.36362	0.57972
54827	0.946131	0.706522
54828	0.946131	0.748722
54829	1.38674	0.670662
54830	1.36361	0.625724
54831	0.946132	0.707904
54832	0.946132	0.693936
54833	1.89226	0.69512
54834	2.68508	0.567524
54835	2.77349	0.610645
54836	1.89226	0.645335
54837	2.77349	0.619259
54838	1.89226	0.644371
54839	2.15315	0.330062
54840	1.96254	0.389587
54841	1.51377	0.372591
54842	1.89226	0.374533
54843	1.47799	0.353822
54844	1.89226	0.348037
54845	2.49439	0.379917
54846	1.89226	0.45325
54847	2.15315	0.433223
54848	1.89226	0.424003
54849	2.15315	0.41986
54850	1.89226	0.387226
54851	1.89226	0.466028
54852	1.89226	0.509655
54853	1.89226	0.462939
54854	1.89226	0.438883
54855	1.89226	0.549682
54856	1.89226	0.519472
54857	1.89226	0.445881
54858	1.89226	0.441099
54859	1.89226	0.403624
54860	1.89226	0.442435
54861	1.89226	0.39263
54862	1.89226	0.411758
54863	1.375	0.415662
54864	2.01865	0.462627
54865	2.12047	0.414738

54866	1.47354	0.397605
54867	2.12047	0.459199
54868	1.47354	0.436542
54869	0.687499	0.354578
54870	1.06023	0.355973
54871	1.08019	0.35451
54872	0.604229	0.353698
54873	1.08018	0.357881
54874	1.06023	0.370837
54875	0.687502	0.344527
54876	0.60423	0.344042
54877	0.80646	0.342894
54878	0.806461	0.36397
54879	0.858001	0.370774
54880	0.858001	0.345771
54881	2.26414	2.1
54882	1.87342	2.1
54883	1.87342	2.1
54884	1.79667	2.1
54885	2.44045	2.1
54886	2.31924	2.1
54887	2.26414	2.1
54888	2.26616	2.1
54889	2.2355	2.1
54890	2.21858	2.1
54891	4.48474	2.1
54892	2.18516	2.1
54893	1.87344	2.1
54894	2.21859	2.1
54895	2.66233	2.1
54896	1.85196	2.1
54897	2.22236	2.1
54898	2.23551	2.1
54899	2.21859	2.1
54900	2.18516	2.1
54901	2.21189	2.1
54902	4.43049	2.1
54903	1.17171	2.1
54904	2.38924	2.1
54905	2.0815	2.1
54906	2.33348	2.1
54907	1.42851	2.1
54908	1.5959	2.1
54909	1.89226	2.1
54910	1.89226	2.1
54911	1.89226	2.1
54912	1.89226	2.1
54913	3.78452	2.1
54914	2.75425	2.1
54915	1.34366	2.1
54916	1.34366	2.1
54917	1.56512	2.1
54918	2.54186	2.1
54919	2.76332	2.1
54920	1.07751	2.1
54921	2.0815	2.1
54922	1.97259	2.1
54923	1.76455	2.1

54924	1.30191	2.1
54925	1.16461	2.1
54926	0.946131	2.1
54927	0.946131	2.1
54928	0.946129	2.1
54929	0.946129	2.1
54930	0.946131	2.1
54931	0.946131	2.1
54932	0.94613	2.1
54933	0.94613	2.1
54934	0.94613	2.1
54935	0.94613	2.1
54936	0.946131	2.1
54937	0.946131	2.1
54938	1.89226	0.681708
54939	1.89226	0.568707
54940	1.89226	0.530156
54941	1.89226	0.464018
54942	3.78453	0.471446
54943	1.89226	0.700639
54944	1.89226	0.607235
54945	1.89226	0.57266
54946	1.89226	0.465038
54947	3.78453	0.659261
54948	0.946132	0.555803
54949	0.946132	0.598615
54950	1.30327	0.492263
54951	1.33544	0.564013
54952	1.33544	0.494684
54953	1.33544	0.441364
54954	0.946132	0.530457
54955	0.946132	0.462486
54956	0.946132	0.452408
54957	1.30327	0.410898
54958	1.33544	0.5375
54959	0.946132	0.569761
54960	1.30327	0.45125
54961	1.33544	0.484076
54962	0.709426	0.433378
54963	0.790129	0.458659
54964	1.05886	0.368643
54965	1.05886	0.452641
54966	1.03454	0.535509
54967	1.03454	0.451831
54968	1.30327	0.424842
54969	0.709426	0.373889
54970	1.33544	0.540711
54971	0.790128	0.449548
54972	0.946132	0.416494
54973	0.946132	0.461172
54974	0.827866	0.35741
54975	0.946133	0.427047
54976	0.946132	0.345643
54977	0.946132	0.395672
54978	0.946133	0.38197
54979	0.946133	0.494948
54980	0.946132	0.341672
54981	0.827866	0.412743

54982	0.946133	0.511505
54983	0.946133	0.462987
54984	1.89227	0.544897
54985	1.89227	0.374579
54986	1.89227	0.444085
54987	1.89227	0.437043
54988	1.89227	0.419051
54989	1.89227	0.413277
54990	0.946133	0.424423
54991	0.946133	0.446422
54992	1.21602	0.373238
54993	1.30327	0.439267
54994	1.30327	0.375512
54995	1.30327	0.356665
54996	0.946132	0.360005
54997	0.946132	0.409141
54998	0.946132	0.35848
54999	1.21602	0.325862
55000	0.946133	0.440646
55001	1.30327	0.399343
55002	1.04227	0.326747
55003	1.11706	0.386229
55004	0.968413	0.3053
55005	0.945482	0.374112
55006	1.3204	0.349201
55007	1.23215	0.35084
55008	0.810791	0.357715
55009	1.38978	0.307038
55010	1.07728	0.389508
55011	1.11704	0.404499
55012	0.608052	0.361832
55013	1.89226	0.480678
55014	1.89226	0.346158
55015	1.89226	0.387723
55016	1.89226	0.413633
55017	3.78453	0.416617
55018	1.89226	0.308404
55019	1.89226	0.32878
55020	1.89226	0.378003
55021	1.89226	0.271614
55022	3.78451	0.312836
55023	2.58476	0.318285
55024	1.56598	0.252587
55025	1.55045	0.285108
55026	1.5124	0.276554
55027	2.62281	0.274502
55028	2.58476	0.268292
55029	2.58477	0.335367
55030	1.89226	0.358387
55031	1.89226	0.391293
55032	1.89226	0.319284
55033	2.62282	0.38792
55034	2.62282	0.318838
55035	1.89224	0.393203
55036	1.89224	0.425301
55037	1.89224	0.492963
55038	1.89224	0.350029
55039	3.78448	0.389278

55040	1.89223	0.383303
55041	1.89223	0.280267
55042	1.89223	0.327522
55043	1.89223	0.315463
55044	1.89223	0.333209
55045	1.89223	0.323642
55046	2.62278	0.326804
55047	1.45053	0.254946
55048	1.49678	0.271312
55049	1.56596	0.281215
55050	2.5536	0.264829
55051	2.62278	0.275622
55052	1.31139	0.337136
55053	1.2768	0.289488
55054	0.946118	0.426225
55055	0.946118	0.361275
55056	1.2768	0.360134
55057	1.2768	0.31882
55058	0.946116	0.41301
55059	0.946116	0.335173
55060	1.27682	0.335897
55061	0.94613	0.371973
55062	0.946105	0.354687
55063	1.31138	0.342965
55064	1.38506	1.38389
55065	1.53348	1.65015
55066	1.17042	1.38496
55067	1.17042	1.56296
55068	1.73669	1.38567
55069	1.73669	1.54308
55070	1.73669	1.25507
55071	1.79928	0.969803
55072	1.38506	1.19807
55073	1.38506	1.56458
55074	1.79928	1.53705
55075	1.43484	1.61323
55076	1.73669	1.17543
55077	1.93568	1.49491
55078	1.53347	1.0971
55079	1.53347	1.48252
55080	2.14058	1.1231
55081	2.14058	1.52648
55082	0.89964	0.958194
55083	1.10415	1.67163
55084	0.868346	1.21714
55085	1.07029	1.59449
55086	0.98687	1.27211
55087	0.98687	1.965
55088	0.985627	0.81177
55089	0.985627	1.53453
55090	1.07029	1.3776
55091	1.10415	1.26307
55092	0.868344	1.43553
55093	0.899642	1.49021
55094	2.40667	2.1
55095	2.1281	2.1
55096	2.11176	2.1
55097	2.38069	2.1

55098	4.50878	2.1
55099	2.11176	2.1
55100	1.61687	2.1
55101	1.60965	2.1
55102	1.61687	2.1
55103	2.12088	2.1
55104	2.1281	2.1
55105	2.11176	2.1
55106	2.34898	2.1
55107	2.34898	2.1
55108	2.38069	2.1
55109	2.09114	2.1
55110	2.12285	2.1
55111	1.05588	2.1
55112	1.04557	2.1
55113	0.804824	2.1
55114	0.798269	2.1
55115	0.92615	2.1
55116	0.92615	2.1
55117	0.927999	2.1
55118	0.927999	2.1
55119	1.04557	2.1
55120	0.798269	2.1
55121	1.05588	2.1
55122	0.804824	2.1
55123	1.43548	0.478774
55124	1.53873	0.62146
55125	1.25063	0.422291
55126	1.39662	0.447064
55127	2.78937	0.371209
55128	1.25063	0.593903
55129	1.39662	0.520874
55130	1.10476	0.288239
55131	1.28326	0.434764
55132	2.53389	0.437232
55133	1.51147	0.2799
55134	1.86114	0.345906
55135	1.86114	0.329601
55136	2.12926	0.291771
55137	1.3659	0.316884
55138	1.63401	0.27821
55139	1.89226	0.33024
55140	1.89226	0.444172
55141	1.89226	0.394968
55142	1.89226	0.346534
55143	1.89226	0.422058
55144	1.89226	0.399252
55145	1.89226	0.478026
55146	1.89226	0.536936
55147	2.46286	0.439721
55148	1.89226	0.58667
55149	3.78452	0.441216
55150	1.89226	0.54708
55151	1.89226	0.703789
55152	1.89226	0.646246
55153	1.89226	0.644619
55154	1.89226	0.559183
55155	1.89226	0.56331

55156	1.89226	0.504287
55157	1.89226	0.542531
55158	2.28992	0.473736
55159	1.89226	0.59898
55160	3.78452	0.475674
55161	1.13537	0.333295
55162	0.886595	0.518209
55163	0.946131	0.749443
55164	0.946131	0.43718
55165	0.94613	0.544713
55166	0.94613	0.354011
55167	1.25069	0.751826
55168	0.841855	0.679717
55169	1.27436	0.399464
55170	1.06196	0.438288
55171	1.09758	0.360198
55172	0.830306	0.306356
55173	0.826931	0.844605
55174	0.826933	0.286662
55175	0.764631	0.346221
55176	0.764631	0.425238
55177	1.00427	0.800698
55178	0.788034	0.488391
55179	0.726852	0.360771
55180	0.604206	0.581178
55181	0.828475	0.462269
55182	0.604198	0.449728
55183	0.8332	0.401998
55184	1.00428	0.407171
55185	1.89226	0.517627
55186	1.89226	0.361783
55187	1.89226	0.342847
55188	1.89226	0.564687
55189	1.89226	0.377224
55190	1.89226	0.482773
55191	0.768789	1.08967
55192	0.849132	0.147815
55193	0.666423	1.06088
55194	0.688993	0.806327
55195	0.868671	0.147815
55196	0.89117	1.02213
55197	0.705632	2.02425
55198	1.80425	2.1
55199	1.73883	2.1
55200	1.45159	2.1
55201	1.45159	2.1
55202	1.37681	2.1
55203	1.73883	2.1
55204	1.39679	0.216559
55205	2.08149	0.274397
55206	1.89226	0.245661
55207	2.32763	0.220927
55208	1.89226	0.234949
55209	2.31274	0.215102
55210	1.89226	0.249098
55211	1.89226	0.357117
55212	1.89226	0.300117
55213	1.89226	0.260223

55214	1.89226	0.315272
55215	1.89226	0.2725
55216	1.89226	0.251409
55217	1.89226	0.322438
55218	1.89226	0.277397
55219	1.89226	0.267242
55220	1.89226	0.277013
55221	1.89226	0.266832
55222	1.95181	0.219071
55223	1.89226	0.261656
55224	1.89226	0.240962
55225	1.89226	0.223096
55226	3.78451	0.220023
55227	0.946131	0.242633
55228	0.946131	0.230228
55229	0.946125	0.261965
55230	0.946125	0.284885
55231	0.946127	0.291278
55232	0.946127	0.270515
55233	0.946128	0.267786
55234	0.946128	0.236981
55235	0.946128	0.256575
55236	0.946128	0.269719
55237	0.946128	0.238839
55238	0.946128	0.263007
55239	1.89226	0.274394
55240	1.89226	0.384696
55241	1.89226	0.319483
55242	1.89226	0.304814
55243	1.89226	0.325293
55244	1.89226	0.3107
55245	1.89226	0.76246
55246	1.89226	0.761593
55247	1.89226	0.738725
55248	1.89226	0.722698
55249	3.78453	0.747925
55250	1.89226	0.823264
55251	1.89226	0.773222
55252	1.89226	0.767574
55253	1.89226	0.774028
55254	3.78452	0.767788
55255	1.21387	0.77012
55256	0.8164	0.748514
55257	0.946133	0.746264
55258	0.946133	0.758332
55259	0.946102	0.727674
55260	0.946102	0.79707
55261	0.816431	0.798956
55262	0.816431	0.77196
55263	0.816401	0.758942
55264	0.94613	0.735424
55265	0.946131	0.842
55266	1.21388	0.772389
55267	0.946132	0.72098
55268	0.946132	0.72574
55269	0.946132	0.730418
55270	0.946132	0.733173
55271	0.946132	0.71323

55272	0.946132	0.730808
55273	0.946132	0.748745
55274	0.946132	0.718431
55275	0.946131	0.712252
55276	0.946131	0.722325
55277	0.946132	0.747235
55278	0.946132	0.732758
55279	1.89226	0.76329
55280	1.89226	0.709244
55281	1.89226	0.710411
55282	1.89226	0.758871
55283	1.89226	0.721765
55284	1.89226	0.816558
55285	1.89226	0.80495
55286	1.89226	0.81102
55287	2.15871	0.75094
55288	2.15871	0.824593
55289	1.81454	0.738765
55290	1.54809	0.844003
55291	1.89226	0.793073
55292	1.89226	1.00466
55293	1.89226	0.840068
55294	1.89226	0.768614
55295	3.78453	0.893167
55296	1.89226	0.868335
55297	1.89226	0.764153
55298	1.89226	0.746586
55299	1.89226	0.83455
55300	1.89226	0.999923
55301	1.89226	0.914202
55302	2.29205	0.869387
55303	1.42633	0.836582
55304	1.69015	0.959056
55305	1.69015	0.886084
55306	2.10364	0.763818
55307	2.05344	0.760211
55308	2.29205	0.768933
55309	1.58147	0.873092
55310	2.16529	0.777011
55311	1.56339	0.751941
55312	2.29205	0.992173
55313	1.69015	0.907815
55314	1.89226	0.910457
55315	1.89226	0.810335
55316	1.89226	0.765444
55317	1.89226	0.83722
55318	3.78453	0.788273
55319	1.89226	0.819302
55320	1.89226	0.966333
55321	1.89226	0.853075
55322	1.89226	0.781041
55323	3.78452	0.940943
55324	2.29205	0.975925
55325	2.85335	0.731865
55326	2.55409	0.817435
55327	2.04345	0.763681
55328	2.76338	0.835779
55329	2.29205	0.778585

55330	2.29205	0.81586
55331	2.16529	0.915003
55332	2.72418	0.841454
55333	2.76338	0.721764
55334	5.01623	0.839501
55335	1.89227	0.363703
55336	1.89227	0.341006
55337	1.89227	0.276424
55338	1.89227	0.299108
55339	3.78453	0.306091
55340	2.39588	0.272452
55341	2.14918	0.273325
55342	2.14918	0.28859
55343	2.37907	0.337744
55344	2.20531	0.225306
55345	2.4352	0.265363
55346	1.89226	0.309695
55347	2.64327	0.332428
55348	2.36175	0.270816
55349	1.89226	0.274527
55350	4.25402	0.314077
55351	1.89226	0.274898
55352	1.89226	0.240588
55353	1.89226	0.24273
55354	1.89226	0.26377
55355	1.89226	0.250173
55356	1.89226	0.273538
55357	1.05395	0.216334
55358	1.05395	0.223141
55359	1.03114	0.229911
55360	1.03114	0.242403
55361	0.936689	0.217398
55362	1.07459	0.243386
55363	1.10265	0.218351
55364	1.01049	0.221906
55365	0.93669	0.204265
55366	1.01049	0.220493
55367	1.07459	0.25234
55368	1.10265	0.218684
55369	1.18088	0.240948
55370	0.776509	0.245119
55371	0.946132	0.23099
55372	0.946132	0.248319
55373	1.04225	0.237658
55374	0.830392	0.278897
55375	0.931352	0.237123
55376	0.931352	0.240717
55377	1.13006	0.224723
55378	1.04074	0.230648
55379	1.0628	0.24443
55380	0.851529	0.257296
55381	2.40032	2.1
55382	1.89226	2.1
55383	1.89226	2.1
55384	1.89226	2.1
55385	2.38918	2.1
55386	2.38918	2.1
55387	1.89226	2.1

55388	1.89226	1.83023
55389	1.89226	2.1
55390	1.89226	1.63093
55391	1.89226	2.1
55392	1.89226	1.79017
55393	1.6585	2.1
55394	2.37364	1.89758
55395	2.39622	2.1
55396	2.39622	1.88265
55397	1.6488	2.1
55398	1.6488	1.88727
55399	2.44603	2.1
55400	1.89224	2.1
55401	1.89224	2.1
55402	1.89224	2.1
55403	2.40029	2.1
55404	2.40029	2.1
55405	1.89224	2.1
55406	1.89224	1.61486
55407	1.89224	2.1
55408	1.89224	1.43823
55409	1.89224	2.1
55410	1.89224	1.51558
55411	1.01946	1.72733
55412	1.01946	2.1
55413	1.02497	2.1
55414	1.02497	1.99048
55415	1.21519	1.49412
55416	1.19809	1.37268
55417	0.835303	2.1
55418	0.82924	2.1
55419	0.835314	2.1
55420	1.2152	2.1
55421	0.829228	2.02695
55422	1.19808	2.06594
55423	1.91027	2.1
55424	1.05809	2.1
55425	1.00659	2.1
55426	2.06727	2.1
55427	3.11819	2.1
55428	2.37166	2.1
55429	2.06726	2.1
55430	1.66994	2.1
55431	1.67623	2.1
55432	2.40308	2.1
55433	2.40308	2.1
55434	0.960194	2.1
55435	0.939664	2.1
55436	0.531131	2.1
55437	0.503298	2.1
55438	0.729927	2.1
55439	0.729927	2.1
55440	0.733565	2.1
55441	0.733565	2.1
55442	0.960195	2.1
55443	0.531132	2.1
55444	0.503297	2.1
55445	0.939663	2.1

55446	1.20154	2.1
55447	1.21754	2.1
55448	0.960195	2.1
55449	0.939665	2.1
55450	1.07745	2.1
55451	1.07745	2.1
55452	1.07976	2.1
55453	1.07976	2.1
55454	0.960194	2.1
55455	1.21754	2.1
55456	0.939662	2.1
55457	1.20155	2.1
55458	1.89224	2.1
55459	1.89224	2.1
55460	2.08609	2.1
55461	2.08609	2.1
55462	2.09527	2.1
55463	1.90142	2.1
55464	1.65815	2.1
55465	1.71398	2.1
55466	2.2001	2.1
55467	2.23791	2.1
55468	1.4502	2.1
55469	1.4502	2.1
55470	0.793587	2.1
55471	0.793587	2.1
55472	0.792408	2.1
55473	0.792408	2.1
55474	0.952221	2.1
55475	0.858585	2.1
55476	0.725101	2.1
55477	0.633774	2.1
55478	0.633775	2.1
55479	0.858585	2.1
55480	0.952221	2.1
55481	0.725101	2.1
55482	1.5735	2.1
55483	2.56975	2.1
55484	2.63166	2.1
55485	2.63166	2.1
55486	1.54711	2.1
55487	1.54711	2.1
55488	0.946131	2.1
55489	0.946131	2.1
55490	1.28487	2.1
55491	1.28487	2.1
55492	0.946127	1.99085
55493	0.946127	2.1
55494	1.28488	2.1
55495	1.31583	2.1
55496	1.31583	2.1
55497	0.94613	2.1
55498	1.28487	2.1
55499	0.946129	2.1
55500	1.0596	2.1
55501	1.0596	2.1
55502	1.06939	2.1
55503	1.06939	2.1

55504	1.34225	2.1
55505	1.31583	2.1
55506	0.793563	2.1
55507	0.786748	2.1
55508	0.793563	2.1
55509	1.34225	2.1
55510	0.786748	2.1
55511	1.31583	2.1
55512	0.946131	2.1
55513	0.946131	2.1
55514	1.31582	2.1
55515	1.31582	2.1
55516	0.946127	2.1
55517	0.946127	2.1
55518	1.34225	2.1
55519	1.31583	2.1
55520	1.34225	2.1
55521	0.946129	2.1
55522	1.31583	2.1
55523	0.946129	2.1
55524	1.89226	2.1
55525	1.89226	2.1
55526	1.89226	2.1
55527	1.89226	2.1
55528	1.89226	2.1
55529	1.89226	2.1
55530	1.89226	2.1
55531	1.89226	2.1
55532	1.89226	2.1
55533	1.89226	2.1
55534	1.89226	2.1
55535	1.89226	2.1
55536	1.26992	2.1
55537	1.75003	2.1
55538	1.79883	2.1
55539	1.26732	2.1
55540	3.06875	2.1
55541	0.875017	2.1
55542	0.899414	2.1
55543	0.811147	2.1
55544	0.85053	2.1
55545	0.851513	2.1
55546	0.851513	2.1
55547	0.859048	2.1
55548	0.859048	2.1
55549	0.899413	2.1
55550	0.85053	2.1
55551	0.811147	2.1
55552	0.875016	2.1
55553	1.89227	2.1
55554	1.89227	2.1
55555	1.89227	2.1
55556	1.89227	2.1
55557	1.89227	2.1
55558	1.89227	2.1
55559	1.89226	2.1
55560	1.89226	2.1
55561	1.89226	2.1

55562	1.89226	2.1
55563	1.89226	2.1
55564	1.89226	2.1
55565	0.633661	2.1
55566	0.642005	2.1
55567	0.93285	2.1
55568	0.899413	2.1
55569	0.764487	2.1
55570	0.764487	2.1
55571	0.776931	2.1
55572	0.776931	2.1
55573	0.642004	2.1
55574	0.93285	2.1
55575	0.899414	2.1
55576	0.633661	2.1
55577	0.932851	2.1
55578	0.899414	2.1
55579	0.905419	2.1
55580	0.85053	2.1
55581	0.886336	2.1
55582	0.886336	2.1
55583	0.897045	2.1
55584	0.897045	2.1
55585	0.93285	2.1
55586	0.905419	2.1
55587	0.899414	2.1
55588	0.850531	2.1
55589	2.39322	1.99447
55590	2.74149	1.03498
55591	2.70944	2.1
55592	2.6849	1.07343
55593	2.41776	1.86646
55594	2.39322	0.876403
55595	2.41776	1.9751
55596	2.80992	1.62897
55597	2.77256	2.1
55598	2.74149	1.29294
55599	2.44882	2.02875
55600	2.41776	0.950523
55601	2.80992	1.78025
55602	1.89226	1.47262
55603	2.74149	1.60572
55604	2.74149	1.72136
55605	1.89226	1.04614
55606	1.89226	1.2158
55607	1.89226	0.331115
55608	1.89226	0.296237
55609	1.89226	0.386651
55610	1.89226	0.26221
55611	1.89226	0.381062
55612	1.89226	0.268206
55613	2.38796	0.21582
55614	2.0815	0.292933
55615	2.0815	0.261485
55616	2.49452	0.227841
55617	1.55994	0.265259
55618	2.14721	0.223309
55619	1.89227	0.37535

55620	1.89227	0.315486
55621	1.89227	0.414953
55622	1.89227	0.291484
55623	1.89227	0.413355
55624	1.89227	0.303872
55625	1.13387	0.278651
55626	2.27494	0.249398
55627	1.13387	0.329225
55628	2.19489	0.250772
55629	1.60867	0.312851
55630	2.6994	0.239967
55631	1.89226	0.37493
55632	1.89226	0.305645
55633	1.89226	0.290692
55634	1.89226	0.429191
55635	3.78453	0.323321
55636	1.42381	1.29538
55637	1.10747	2.03484
55638	1.10747	1.2435
55639	1.08911	2.04098
55640	1.42866	1.21167
55641	1.4103	2.08874
55642	1.89226	1.93681
55643	1.88181	2.1
55644	1.88181	1.80235
55645	1.76249	2.1
55646	2.0815	1.72274
55647	1.97539	2.1
55648	0.946128	2.00227
55649	0.946128	1.71644
55650	0.946133	1.24424
55651	0.946133	1.07482
55652	0.94613	1.09057
55653	0.94613	1.66872
55654	0.946131	2.0367
55655	0.946131	1.18034
55656	0.946131	1.37229
55657	0.946131	1.39151
55658	0.946131	1.40062
55659	0.946131	1.4039
55660	1.42866	1.254
55661	1.43213	1.22825
55662	1.10748	1.75674
55663	1.10748	2.1
55664	1.13641	2.07252
55665	1.43213	2.07273
55666	2.0815	1.98679
55667	2.10287	2.1
55668	2.10287	1.81146
55669	2.0921	2.1
55670	1.89227	1.78728
55671	1.83938	2.1
55672	0.810944	1.18802
55673	0.810944	1.08739
55674	0.810945	1.10865
55675	0.810945	1.70366
55676	0.810933	1.26011
55677	0.810933	1.27878

55678	1.89226	2.0194
55679	1.3517	1.51833
55680	1.08132	1.25667
55681	1.08132	1.80059
55682	1.08133	1.30634
55683	2.33097	1.79609
55684	1.53822	2.1
55685	1.53822	2.1
55686	1.68437	2.1
55687	2.17996	2.1
55688	2.32612	2.1
55689	0.99189	1.84468
55690	1.18948	0.772134
55691	1.19756	0.803857
55692	1.19756	1.98085
55693	0.978851	1.84145
55694	0.926995	1.54434
55695	1.26924	0.54983
55696	1.34642	0.78748
55697	1.3228	0.856974
55698	0.990899	0.856921
55699	1.0627	0.872857
55700	1.33473	0.864004
55701	1.96196	1.65547
55702	1.39702	2.1
55703	1.39702	2.01476
55704	1.53822	2.1
55705	2.17372	1.9767
55706	2.31011	2.1
55707	1.89226	0.809324
55708	1.89226	0.573442
55709	1.89226	0.785121
55710	1.89226	0.415206
55711	3.78453	0.534218
55712	2.38475	0.866125
55713	1.73957	0.639381
55714	1.88452	0.801943
55715	1.58695	0.321816
55716	3.6241	0.623567
55717	1.73958	0.542324
55718	1.3126	0.812444
55719	1.58695	0.344342
55720	1.14946	0.839624
55721	2.88903	0.559315
55722	1.89226	0.524156
55723	1.89226	0.830339
55724	1.89226	0.342681
55725	1.89226	0.835955
55726	3.78453	0.637398
55727	1.89225	0.75322
55728	1.89225	0.551136
55729	1.89225	0.666249
55730	1.89225	0.41573
55731	3.7845	0.463614
55732	1.88451	0.780598
55733	1.58694	0.322474
55734	1.74649	0.827658
55735	1.44604	0.4032

55736	3.33055	0.380051
55737	1.58694	0.381084
55738	0.995727	0.792832
55739	0.995727	0.78332
55740	1.14945	0.757821
55741	1.44604	0.370356
55742	1.59976	0.789751
55743	1.89225	0.464507
55744	1.89225	0.787161
55745	1.89225	0.829146
55746	1.89225	0.58171
55747	1.89225	0.827481
55748	1.89225	0.539572
55749	2.01197	1.48131
55750	2.0961	1.44924
55751	2.13283	1.05908
55752	2.25399	0.973195
55753	4.22894	1.25983
55754	2.09611	1.18683
55755	2.37014	1.1295
55756	2.29031	1.41636
55757	2.10084	1.55224
55758	2.25399	0.985716
55759	2.09611	1.43467
55760	2.13283	1.38918
55761	2.25399	1.75043
55762	2.22044	1.21661
55763	2.38975	0.999284
55764	4.52258	1.32433
55765	2.38976	0.886095
55766	2.15466	1.23976
55767	2.31988	0.819115
55768	2.31988	1.5578
55769	2.25399	0.904694
55770	2.25399	1.83036
55771	2.58286	0.867012
55772	2.29193	0.462143
55773	2.40107	0.932037
55774	2.13815	0.303228
55775	4.72101	0.543036
55776	2.29193	0.541739
55777	1.90104	0.759302
55778	2.13815	0.34484
55779	1.77249	0.64673
55780	4.03919	0.421139
55781	2.25197	0.911718
55782	2.13815	0.539068
55783	2.00625	0.798083
55784	2.00625	0.517035
55785	2.40107	0.831449
55786	2.40107	0.611615
55787	2.13815	0.584019
55788	1.77249	0.733035
55789	2.00625	0.373997
55790	1.65815	0.749859
55791	3.7963	0.549814
55792	2.43417	0.916843
55793	2.06653	0.882941

55794	1.64587	0.906284
55795	1.65284	0.851869
55796	2.46898	0.929267
55797	2.46898	0.886318
55798	2.46895	0.866358
55799	1.89016	0.785212
55800	1.87861	0.830071
55801	1.87861	0.740081
55802	2.47161	0.872971
55803	2.47161	0.805031
55804	0.946132	1.05449
55805	0.946132	1.03256
55806	0.863311	1.1657
55807	1.34768	1.12941
55808	0.851542	1.15175
55809	0.851542	0.979415
55810	1.30451	1.0777
55811	1.45304	1.11648
55812	0.946132	1.06061
55813	1.20992	1.08997
55814	0.855162	1.13843
55815	0.946132	1.07319
55816	1.89226	0.994671
55817	1.89226	1.17375
55818	1.89226	1.15038
55819	1.89226	0.996062
55820	1.89226	1.1796
55821	1.89226	1.01758
55822	0.946132	0.896973
55823	0.946132	0.813977
55824	0.946133	0.956756
55825	0.946133	0.943651
55826	0.946132	0.958999
55827	0.946132	0.739803
55828	0.946132	0.835174
55829	0.946132	0.938604
55830	0.946132	0.965924
55831	0.946132	0.869564
55832	0.946133	0.876175
55833	0.946133	0.901727
55834	2.35574	0.976896
55835	1.89226	0.801123
55836	1.89226	1.10443
55837	1.89226	0.79268
55838	2.67456	1.01789
55839	2.67456	0.418312
55840	0.583505	0.540157
55841	0.761972	0.597977
55842	1.17787	0.901934
55843	1.33728	0.49707
55844	0.965128	0.788789
55845	0.965128	0.55911
55846	0.974714	0.758337
55847	0.974715	0.897312
55848	1.17787	0.645241
55849	0.583506	0.799592
55850	1.33728	0.468477
55851	0.761974	0.570922

55852	1.89226	0.788739
55853	1.89226	0.610372
55854	1.89226	0.458001
55855	1.89226	0.425557
55856	1.89226	0.578843
55857	1.89226	0.758456
55858	1.89226	0.675181
55859	1.89226	0.378769
55860	1.89226	0.689943
55861	1.89226	0.63375
55862	3.78453	0.593853
55863	0.946132	0.52838
55864	0.946132	0.541843
55865	0.946132	0.710315
55866	0.946132	0.469057
55867	0.946133	0.727849
55868	0.946133	0.556729
55869	0.946132	0.769762
55870	0.946132	0.656216
55871	0.946133	0.618627
55872	0.946133	0.790859
55873	0.946132	0.502221
55874	0.946132	0.698755
55875	1.01779	2.1
55876	1.50685	2.1
55877	1.12064	2.1
55878	1.65151	2.1
55879	2.6275	2.1
55880	1.35733	2.1
55881	1.53507	2.1
55882	1.50683	2.1
55883	1.73626	2.1
55884	3.09359	2.1
55885	1.73626	2.1
55886	1.52226	2.1
55887	1.75555	2.1
55888	1.75555	2.1
55889	1.53507	2.1
55890	1.53507	2.1
55891	1.65151	2.1
55892	1.73628	2.1
55893	1.69528	2.1
55894	1.50685	2.1
55895	1.92471	2.1
55896	1.73628	2.1
55897	1.73629	2.1
55898	1.75558	2.1
55899	1.9943	2.1
55900	1.9943	2.1
55901	1.92471	2.1
55902	1.73629	2.1
55903	2.5601	1.03995
55904	1.97645	1.51456
55905	2.09605	0.948863
55906	2.09605	1.45632
55907	2.45853	1.05043
55908	2.45853	1.63111
55909	2.33824	1.3765

55910	1.97646	1.25601
55911	2.45853	1.00521
55912	1.94984	1.51132
55913	2.33824	1.33226
55914	1.82955	1.80422
55915	1.22926	1.51451
55916	0.946128	1.47454
55917	0.946133	1.54205
55918	1.28005	1.20921
55919	1.22927	1.20084
55920	1.22926	1.33368
55921	0.946131	1.28693
55922	0.946131	1.22608
55923	0.946131	1.21622
55924	0.946131	1.16736
55925	1.28005	1.25824
55926	1.22926	1.22622
55927	2.35349	0.910199
55928	2.61606	0.607217
55929	2.48209	0.891818
55930	2.48209	0.555932
55931	2.50057	0.891406
55932	2.50057	0.57176
55933	2.61607	0.726899
55934	2.57611	0.47278
55935	2.4821	0.470331
55936	2.44814	0.415011
55937	5.06421	0.449459
55938	2.15721	0.747505
55939	2.48209	0.594912
55940	2.31844	0.909446
55941	2.31844	0.491328
55942	2.35349	0.91003
55943	2.35349	0.448262
55944	2.44814	0.49124
55945	2.30908	0.384377
55946	2.44814	0.433135
55947	2.31844	0.363438
55948	2.4821	0.622821
55949	2.31844	0.495906
55950	0.91766	0.487739
55951	0.91766	0.501189
55952	0.912463	0.452401
55953	0.912463	0.529372
55954	1.16525	0.50403
55955	1.26377	0.518757
55956	0.576692	0.50216
55957	0.664871	0.505589
55958	1.16525	0.468386
55959	0.576692	0.479691
55960	0.664871	0.510113
55961	1.26377	0.517809
55962	1.72901	1.7623
55963	1.62414	1.99933
55964	1.88971	1.99622
55965	1.8411	1.27587
55966	3.57011	1.89685
55967	1.89226	1.83821

55968	1.89226	1.29751
55969	1.89226	1.01473
55970	1.89226	1.40867
55971	3.78452	1.04725
55972	1.89226	1.11771
55973	1.89226	2.04002
55974	1.89226	1.37426
55975	1.89226	1.12495
55976	3.78452	1.81102
55977	1.62414	1.8632
55978	1.30896	1.81542
55979	1.8411	1.19074
55980	1.52776	1.65759
55981	3.15006	1.32836
55982	1.51448	1.81467
55983	1.8411	1.59668
55984	2.02311	1.66022
55985	1.99309	0.988582
55986	3.86421	1.32216
55987	1.89226	1.06505
55988	1.89226	1.86193
55989	1.89226	1.53362
55990	1.89226	1.1254
55991	3.78452	1.7429
55992	1.89226	1.85758
55993	1.89226	1.3697
55994	1.89226	1.26961
55995	1.89226	1.87046
55996	3.78452	1.51017
55997	1.8411	1.4765
55998	1.52776	2.08402
55999	1.99309	2.1
56000	1.72526	1.74243
56001	3.56636	2.04546
56002	1.89226	1.99747
56003	1.89226	1.59352
56004	1.89226	1.57148
56005	1.89226	2.09403
56006	3.78453	1.75722
56007	1.0424	2.1
56008	1.12935	2.1
56009	0.929383	2.08907
56010	0.929383	2.1
56011	1.24941	2.1
56012	1.12686	2.1
56013	0.946132	2.1
56014	0.946132	2.05492
56015	1.12944	2.1
56016	0.851554	2.09885
56017	1.05017	2.1
56018	0.929315	2.1
56019	0.898029	2.07598
56020	0.973994	2.1
56021	0.946131	1.62518
56022	0.946131	1.91839
56023	1.0221	1.94427
56024	1.0221	2.1
56025	0.898028	1.57665

56026	0.898028	2.09246
56027	0.973994	2.09111
56028	0.946131	2.1
56029	0.946131	2.08377
56030	0.898029	2.08763
56031	1.89226	1.53879
56032	1.89226	1.90852
56033	1.89226	1.50476
56034	1.89226	1.7658
56035	1.89226	1.52653
56036	1.89226	1.78411
56037	1.13537	1.89174
56038	0.879308	2.1
56039	0.946131	1.6106
56040	0.946131	1.75066
56041	1.13537	1.79353
56042	0.85187	2.1
56043	0.94613	1.89745
56044	0.94613	1.47779
56045	1.05777	1.93331
56046	1.05777	2.03284
56047	0.799318	1.97035
56048	0.776452	1.99337
56049	0.946116	2.1
56050	0.946116	2.1
56051	1.09531	2.1
56052	1.09531	2.1
56053	0.946118	2.1
56054	0.946118	2.1
56055	1.09135	2.1
56056	1.09531	2.1
56057	1.09134	2.1
56058	0.946105	2.1
56059	1.09532	2.1
56060	0.946131	2.1
56061	0.946129	2.1
56062	0.946129	2.1
56063	1.09137	2.1
56064	1.09137	2.1
56065	0.946131	2.1
56066	0.946131	2.1
56067	1.07713	2.1
56068	1.09137	2.1
56069	0.946131	2.1
56070	1.07713	2.1
56071	1.09137	2.1
56072	0.946131	2.1
56073	0.938916	2.1
56074	0.938916	2.1
56075	0.936995	2.1
56076	1.00447	2.1
56077	0.979819	2.1
56078	1.16821	2.1
56079	1.01263	2.1
56080	0.719167	2.1
56081	1.04536	2.1
56082	1.18901	2.1
56083	0.758057	2.1

56084	0.924111	2.1
56085	1.00532	2.1
56086	1.00532	2.1
56087	0.9925	2.1
56088	0.9925	2.1
56089	1.29322	2.1
56090	1.01985	2.1
56091	1.00365	2.1
56092	0.704601	2.1
56093	1.00365	2.1
56094	1.29322	2.1
56095	1.01985	2.1
56096	0.704602	2.1
56097	2.20011	2.1
56098	1.401	2.1
56099	1.401	2.1
56100	1.52008	2.1
56101	2.11084	2.1
56102	2.22991	2.1
56103	0.854068	2.1
56104	0.854068	2.1
56105	0.851357	2.1
56106	0.851357	2.1
56107	0.644681	2.1
56108	0.700493	2.1
56109	1.0554	2.1
56110	1.00493	2.1
56111	1.00495	2.1
56112	0.64469	2.1
56113	0.700483	2.1
56114	1.05539	2.1
56115	2.46665	0.155836
56116	2.22713	0.169625
56117	1.85877	0.159013
56118	1.8967	0.157579
56119	1.56002	0.166181
56120	2.22713	0.165601
56121	1.89225	2.1
56122	1.89225	2.1
56123	1.89225	2.1
56124	1.89225	2.1
56125	1.89225	2.1
56126	1.89225	2.1
56127	1.89225	2.1
56128	1.89225	2.1
56129	1.89225	2.1
56130	1.89225	2.1
56131	1.89225	2.1
56132	1.89225	2.1
56133	1.284	2.1
56134	1.86569	2.1
56135	1.95398	2.1
56136	1.31091	2.1
56137	3.23798	2.1
56138	0.932844	2.1
56139	0.976992	2.1
56140	0.960128	2.1
56141	0.905413	2.1

56142	0.938531	2.1
56143	0.938531	2.1
56144	0.943874	2.1
56145	0.943874	2.1
56146	0.976985	2.1
56147	0.960122	2.1
56148	0.93285	2.1
56149	0.905419	2.1
56150	1.89227	2.1
56151	1.89227	2.1
56152	1.89227	2.1
56153	1.89227	2.1
56154	3.78453	2.1
56155	1.89226	2.1
56156	1.89226	2.1
56157	1.89226	2.1
56158	1.89226	2.1
56159	1.89226	2.1
56160	1.89226	2.1
56161	0.65546	2.1
56162	0.665451	2.1
56163	1.00694	2.1
56164	0.976998	2.1
56165	0.816261	2.1
56166	0.816261	2.1
56167	0.826189	2.1
56168	0.826189	2.1
56169	1.00694	2.1
56170	0.665451	2.1
56171	0.976998	2.1
56172	0.65546	2.1
56173	2.01555	2.1
56174	2.01388	2.1
56175	1.92027	2.1
56176	1.92027	2.1
56177	2.01388	2.1
56178	1.954 2.1	
56179	2.75466	2.1
56180	1.89226	2.1
56181	1.89226	2.1
56182	2.54931	2.1
56183	4.44158	2.1
56184	2.47917	2.1
56185	1.92772	2.1
56186	1.80682	2.1
56187	2.54931	2.1
56188	4.47702	2.1
56189	0.946132	2.1
56190	0.946132	2.1
56191	1.17602	2.1
56192	1.27466	2.1
56193	0.946132	2.1
56194	0.946132	2.1
56195	1.17602	2.1
56196	1.17602	2.1
56197	0.946132	2.1
56198	1.17602	2.1
56199	0.946131	2.1

56200	1.27466	2.1
56201	2.35204	2.1
56202	2.54931	2.1
56203	2.40783	2.1
56204	1.50108	2.1
56205	1.64256	2.1
56206	1.64256	2.1
56207	1.89226	2.09727
56208	1.89226	1.20011
56209	2.31012	1.72093
56210	2.17534	1.84182
56211	4.0676	1.68993
56212	2.17533	1.80101
56213	1.89226	1.09607
56214	2.17533	1.23402
56215	1.89226	1.18068
56216	1.66635	1.72169
56217	1.38328	1.6617
56218	1.92027	2.1
56219	2.17597	1.96031
56220	2.14619	2.1
56221	2.15388	1.8985
56222	1.91087	2.1
56223	1.92027	1.20011
56224	1.08622	1.21181
56225	1.08622	2.1
56226	0.971184	2.1
56227	0.972761	2.1
56228	1.14845	1.57739
56229	1.16321	2.1
56230	0.894769	2.1
56231	0.894967	2.1
56232	0.894775	2.1
56233	1.28563	2.1
56234	1.14844	2.1
56235	1.0167	2.1
56236	1.89226	1.20171
56237	1.89226	1.98549
56238	1.89226	1.87294
56239	1.89226	1.26161
56240	1.89226	1.87197
56241	1.89226	1.29177
56242	0.937933	1.77728
56243	0.937933	1.8727
56244	1.26192	2.1
56245	1.26192	2.1
56246	0.937903	1.66332
56247	1.25372	2.1
56248	0.946132	1.79373
56249	0.946132	2.1
56250	0.946133	2.06606
56251	1.25372	2.1
56252	0.946132	2.08404
56253	0.937902	2.08374
56254	0.946132	1.18344
56255	0.946132	1.0883
56256	0.946128	1.84886
56257	0.946128	1.82692

56258	0.94613	1.71221
56259	0.94613	1.17765
56260	0.94613	1.05628
56261	0.94613	1.67449
56262	0.946131	1.32779
56263	0.946131	1.3594
56264	0.94613	1.37162
56265	0.94613	1.36021
56266	0.946132	1.27034
56267	0.946132	1.16136
56268	1.2778	1.92776
56269	1.00191	2.06903
56270	1.00191	1.25025
56271	1.00191	2.03225
56272	0.94613	1.93884
56273	0.94613	1.24487
56274	0.946131	1.55219
56275	1.2778	1.64952
56276	1.00191	1.47018
56277	0.94613	1.42783
56278	1.50847	0.234715
56279	2.0815	0.288855
56280	1.56806	0.240435
56281	2.0815	0.272508
56282	1.52043	0.240671
56283	1.9224	0.272131
56284	1.06882	0.22918
56285	0.69567	0.27916
56286	0.773112	0.230359
56287	1.09473	0.262355
56288	0.8941	0.270321
56289	0.8941	0.280492
56290	0.937064	0.231199
56291	0.981131	0.232445
56292	0.763762	0.254452
56293	0.722535	0.252055
56294	1.2153	0.252769
56295	1.07279	0.256684
56296	1.89226	0.314804
56297	1.89226	0.362734
56298	1.89226	0.342851
56299	1.89226	0.295195
56300	1.89226	0.38338
56301	1.89226	0.340596
56302	1.89226	0.310692
56303	1.89226	0.385328
56304	1.89226	0.324162
56305	1.89226	0.397399
56306	3.78453	0.368037
56307	2.51518	0.296212
56308	1.89226	0.319144
56309	1.89226	0.309724
56310	1.89226	0.271128
56311	2.37584	0.336731
56312	2.37584	0.306465
56313	1.25759	0.313936
56314	0.946131	0.335891
56315	0.946131	0.284039

56316	1.24489	0.366678
56317	0.946131	0.33468
56318	0.946131	0.344706
56319	1.25759	0.301857
56320	1.25759	0.292504
56321	1.25759	0.316811
56322	1.24489	0.31368
56323	0.946131	0.314933
56324	0.946131	0.319911
56325	1.08028	0.747586
56326	1.09362	0.790564
56327	0.596616	0.832684
56328	0.606432	0.86608
56329	0.844265	0.875513
56330	0.844265	0.489757
56331	0.855786	0.910836
56332	0.855786	0.788503
56333	1.08028	0.862848
56334	0.606433	0.782277
56335	0.596616	0.727973
56336	1.09362	0.717202
56337	2.837	0.819254
56338	2.16055	0.597585
56339	2.12741	0.812769
56340	2.79312	0.79831
56341	4.95367	0.799827
56342	2.12741	0.837238
56343	1.21286	0.642992
56344	1.20859	0.881196
56345	1.21286	0.923927
56346	2.15628	0.745471
56347	2.16056	0.618698
56348	1.89226	2.1
56349	1.89226	2.1
56350	1.89226	2.1
56351	1.89226	2.1
56352	3.78453	2.1
56353	0.946102	2.1
56354	0.946102	2.1
56355	0.850039	2.1
56356	0.850039	2.1
56357	0.914891	2.1
56358	0.85001	2.1
56359	0.946131	2.1
56360	0.946131	2.1
56361	0.94613	2.1
56362	0.91489	2.1
56363	0.850011	2.1
56364	0.946131	2.1
56365	0.71107	2.1
56366	0.784813	2.1
56367	1.28453	2.1
56368	1.20328	2.1
56369	0.992128	2.1
56370	0.992128	2.1
56371	0.995965	2.1
56372	0.995965	2.1
56373	0.784813	2.1

56374	1.28453	2.1
56375	0.711071	2.1
56376	1.20328	2.1
56377	1.89226	2.1
56378	1.89226	2.1
56379	1.89226	2.1
56380	1.89226	2.1
56381	3.78453	2.1
56382	0.946132	2.1
56383	0.946132	2.1
56384	0.914889	2.1
56385	0.914889	2.1
56386	0.985255	2.1
56387	0.914891	2.1
56388	0.946131	2.1
56389	0.946131	2.1
56390	0.946131	2.1
56391	0.985255	2.1
56392	0.94613	2.1
56393	0.91489	2.1
56394	0.784813	2.1
56395	0.871551	2.1
56396	1.37791	2.1
56397	1.28453	2.1
56398	1.07644	2.1
56399	1.07644	2.1
56400	1.07964	2.1
56401	1.07964	2.1
56402	0.871551	2.1
56403	1.37791	2.1
56404	0.784813	2.1
56405	1.28453	2.1
56406	1.73075	1.32751
56407	2.10582	1.05697
56408	1.89319	1.01198
56409	1.38766	1.66267
56410	3.27777	1.0681
56411	1.08715	1.66327
56412	1.54185	1.50919
56413	1.73074	1.3642
56414	1.73074	1.88089
56415	1.18948	1.21207
56416	1.18948	1.64026
56417	1.89319	1.01213
56418	1.41689	1.57118
56419	1.11619	1.12438
56420	1.38766	1.32779
56421	1.6052	1.0656
56422	1.89319	1.24284
56423	0.647894	1.51091
56424	0.594738	1.72314
56425	0.708445	1.49083
56426	0.770926	1.26266
56427	0.738326	1.51549
56428	0.738326	1.7943
56429	0.680494	1.47936
56430	0.680494	1.16197
56431	0.708443	1.258

56432	0.647895	1.28016
56433	0.770925	1.30517
56434	0.59474	1.19695
56435	1.89111	1.07614
56436	1.06994	2.1
56437	2.06689	1.66108
56438	1.37063	2.1
56439	3.13683	2.08542
56440	2.06689	1.26568
56441	1.39295	2.01022
56442	1.56818	1.6412
56443	1.37063	2.1
56444	2.26444	1.27749
56445	2.06689	2.1
56446	1.49808	2.1
56447	1.50201	2.1
56448	2.57784	2.1
56449	2.58615	2.1
56450	4.08424	2.1
56451	0.946133	1.69288
56452	0.946133	1.65092
56453	0.946132	2.05439
56454	0.946132	2.1
56455	0.946132	2.1
56456	0.946132	1.67302
56457	0.946132	1.87664
56458	0.946132	1.658
56459	0.946133	1.6496
56460	0.946133	1.94165
56461	0.946132	1.89263
56462	0.946132	2.07309
56463	1.89226	1.69844
56464	1.89226	2.1
56465	1.89226	2.1
56466	1.89226	1.93764
56467	3.78452	2.07659
56468	0.977201	2.1
56469	0.977201	2.1
56470	0.980489	2.1
56471	0.980489	2.1
56472	0.657973	2.1
56473	1.20669	2.1
56474	0.751002	2.1
56475	1.29307	2.1
56476	1.29308	2.1
56477	1.20669	2.1
56478	0.751003	2.1
56479	0.657972	2.1
56480	0.946131	2.1
56481	0.946131	2.1
56482	1.29308	2.1
56483	1.29308	2.1
56484	0.946132	2.1
56485	1.29308	2.1
56486	0.946132	2.02723
56487	1.28892	2.1
56488	0.946132	1.97501
56489	0.946132	2.1

56490	1.28892	2.09615
56491	1.29308	2.1
56492	0.946132	1.22958
56493	0.946132	1.16172
56494	0.946131	1.43109
56495	0.946131	1.35788
56496	0.946132	1.44532
56497	0.946132	1.26006
56498	0.946132	1.24358
56499	0.946132	1.16286
56500	0.946132	1.20098
56501	0.946132	1.28462
56502	0.946132	1.26773
56503	0.946132	1.50037
56504	1.89226	1.48997
56505	1.89226	1.47493
56506	1.89226	1.73224
56507	1.89226	1.26554
56508	1.89226	1.68812
56509	1.89226	1.2312
56510	0.94613	2.1
56511	0.94613	2.1
56512	1.20669	2.1
56513	1.20669	2.1
56514	1.20669	2.1
56515	0.946132	2.1
56516	0.946129	2.1
56517	1.29307	2.1
56518	0.946131	2.1
56519	0.946131	2.1
56520	1.29308	2.1
56521	1.20669	2.1
56522	1.89226	0.492497
56523	1.89226	0.351436
56524	1.89226	0.386244
56525	1.89226	0.428203
56526	3.78453	0.446948
56527	0.946132	0.28032
56528	0.946132	0.314131
56529	0.946126	0.330974
56530	0.946126	0.38789
56531	0.946129	0.33708
56532	0.946129	0.297708
56533	0.946129	0.329116
56534	0.946129	0.389097
56535	0.946128	0.318237
56536	0.946128	0.32477
56537	0.946129	0.329791
56538	0.946129	0.33329
56539	0.725277	0.26724
56540	0.588155	0.253982
56541	1.19403	0.328949
56542	1.27681	0.275095
56543	0.918903	0.27371
56544	0.918903	0.260628
56545	0.946068	0.31006
56546	0.946068	0.267807
56547	1.19404	0.28184

56548	0.588153	0.277588
56549	0.725275	0.275953
56550	1.27682	0.272627
56551	1.19404	0.339141
56552	1.27682	0.285657
56553	0.946131	0.403665
56554	0.946131	0.353185
56555	1.19404	0.355955
56556	1.19404	0.317143
56557	0.946129	0.337716
56558	0.946129	0.375627
56559	1.19404	0.339892
56560	0.94613	0.340742
56561	1.27682	0.315884
56562	0.946131	0.338191
56563	1.89226	0.367658
56564	1.89226	0.43598
56565	1.89226	0.466324
56566	1.89226	0.37304
56567	1.89226	0.438812
56568	1.89226	0.340702
56569	1.4263	1.25096
56570	1.67651	0.803194
56571	1.5955	0.912249
56572	1.31022	1.45127
56573	2.98674	1.01636
56574	1.18824	2.1
56575	1.28802	2.1
56576	0.946132	1.15579
56577	0.946132	1.1654
56578	0.946132	1.19289
56579	0.946132	2.1
56580	1.28802	1.09957
56581	1.28802	2.1
56582	1.18824	1.61075
56583	0.946132	1.68788
56584	0.946132	1.68157
56585	1.28802	1.59203
56586	1.89226	0.825552
56587	1.89226	1.15014
56588	1.89226	1.43449
56589	1.89226	0.856312
56590	3.78452	0.970584
56591	1.5955	0.912777
56592	1.52909	0.899503
56593	1.31022	0.957106
56594	1.31022	1.4409
56595	1.2087	1.36119
56596	1.52909	1.36703
56597	1.11825	2.1
56598	1.18824	2.1
56599	0.946132	1.22181
56600	0.946132	1.17215
56601	0.946132	1.23559
56602	0.946132	2.1
56603	1.18824	1.13253
56604	1.18824	2.1
56605	0.946132	1.31234

56606	1.11825	1.64695
56607	1.18824	1.6427
56608	0.946133	1.66417
56609	0.810943	1.26353
56610	0.810943	1.17185
56611	0.810945	0.897242
56612	0.810945	1.16483
56613	1.89226	0.910008
56614	1.35168	0.974243
56615	1.08132	0.872478
56616	1.0813	1.04755
56617	1.08132	1.21218
56618	0.810959	1.00436
56619	0.810959	1.00897
56620	1.89226	0.768424
56621	1.89226	0.507434
56622	1.89226	0.594463
56623	1.89226	0.685484
56624	3.78453	0.730649
56625	1.89226	0.423476
56626	1.89226	0.465171
56627	1.89226	0.488016
56628	1.89226	0.354836
56629	1.89226	0.488107
56630	1.89226	0.358116
56631	0.946131	0.338606
56632	0.946131	0.351797
56633	0.946126	0.485033
56634	0.946126	0.404758
56635	0.946128	0.468778
56636	0.946128	0.357029
56637	0.946128	0.480076
56638	0.946128	0.408361
56639	0.946128	0.423031
56640	0.946128	0.422847
56641	0.946128	0.436932
56642	0.946128	0.409079
56643	1.89226	0.703966
56644	1.89226	0.416197
56645	1.89226	0.592076
56646	1.89226	0.5729
56647	1.89226	0.531544
56648	1.89226	0.516672
56649	1.89226	0.515457
56650	1.89226	0.62256
56651	1.89226	0.858185
56652	1.89226	0.48436
56653	3.78453	0.487073
56654	1.07176	0.355292
56655	1.07176	0.395166
56656	0.946126	0.463254
56657	0.946126	0.42697
56658	1.07176	0.413729
56659	0.946129	0.331961
56660	1.27474	0.432274
56661	0.94613	0.465507
56662	1.27474	0.410056
56663	1.07176	0.289932

56664	0.94613	0.370975
56665	0.94613	0.3615
56666	0.723486	0.310446
56667	0.94613	0.32671
56668	0.946126	0.39607
56669	0.946126	0.354393
56670	0.946129	0.373389
56671	0.723484	0.315945
56672	1.07176	0.391268
56673	0.946129	0.305967
56674	1.32398	0.370451
56675	1.19835	0.298842
56676	0.723471	0.325358
56677	0.723471	0.336018
56678	0.810946	0.700899
56679	0.810946	0.511506
56680	0.810946	0.699371
56681	0.810946	0.595863
56682	0.810933	0.390345
56683	0.810933	0.599846
56684	1.89226	0.351942
56685	1.3517	0.569658
56686	1.08132	0.437335
56687	1.08132	0.546101
56688	1.08133	0.553723
56689	2.39923	2.1
56690	1.89226	2.1
56691	1.89226	2.1
56692	1.89226	2.1
56693	2.44606	2.1
56694	2.44606	2.1
56695	1.89226	2.1
56696	1.89226	1.9772
56697	1.89226	2.1
56698	1.89226	1.84549
56699	1.89226	2.1
56700	1.89226	1.89733
56701	1.70302	2.1
56702	2.43041	2.05676
56703	2.25275	2.1
56704	2.4747	1.83777
56705	1.52079	2.1
56706	1.67063	1.99006
56707	2.40644	2.1
56708	1.89226	2.1
56709	1.89226	2.1
56710	1.89226	2.1
56711	2.39923	2.1
56712	2.39923	2.1
56713	0.946131	1.1026
56714	0.946131	2.1
56715	0.946131	2.1
56716	0.946131	1.84376
56717	0.94613	1.52782
56718	0.94613	1.47802
56719	0.946131	2.1
56720	0.946131	2.1
56721	0.946131	2.06274

56722	0.946131	2.07163
56723	0.946131	2.1
56724	0.946131	2.1
56725	1.04938	2.1
56726	1.04938	1.93626
56727	1.111	2.1
56728	1.06978	2.1
56729	1.2748	1.92468
56730	1.25472	1.43564
56731	0.88782	2.1
56732	0.882987	2.1
56733	0.973779	2.1
56734	1.31997	2.1
56735	0.913905	2.1
56736	1.31312	2.1
56737	2.6536	2.1
56738	2.15287	2.1
56739	2.72238	2.1
56740	2.22887	2.1
56741	4.87525	2.1
56742	2.15288	2.1
56743	1.40719	2.1
56744	1.41839	2.1
56745	1.34239	2.1
56746	2.22887	2.1
56747	2.15288	2.1
56748	2.72237	2.1
56749	2.22887	2.1
56750	2.77314	2.1
56751	2.28748	2.1
56752	5.00985	2.1
56753	0.922802	2.1
56754	0.922802	2.1
56755	0.92107	2.1
56756	0.92107	2.1
56757	0.703593	2.1
56758	0.729436	2.1
56759	1.11444	2.1
56760	1.14374	2.1
56761	1.14374	2.1
56762	0.729436	2.1
56763	0.703593	2.1
56764	1.11444	2.1
56765	1.89226	2.00934
56766	1.89226	2.1
56767	1.89226	1.71931
56768	1.89226	2.1
56769	1.89226	1.87331
56770	1.89226	2.1
56771	0.489927	2.1
56772	0.63008	2.1
56773	0.697385	2.1
56774	0.55524	2.04344
56775	0.592141	2.1
56776	0.592141	2.1
56777	0.593179	2.03978
56778	0.593179	2.1
56779	0.63008	2.1

56780	0.697385	2.1
56781	0.55524	2.1
56782	0.489927	2.1
56783	0.946133	2.0433
56784	0.946133	1.73256
56785	0.946132	1.26345
56786	0.946132	1.01155
56787	0.946133	1.13602
56788	0.946133	2.08341
56789	0.946132	1.77353
56790	0.946132	1.07157
56791	0.946132	1.32375
56792	0.946132	1.25645
56793	0.946132	1.17731
56794	0.946132	1.2247
56795	1.89226	2.1
56796	1.89226	1.78978
56797	1.89226	1.48012
56798	1.89226	2.1
56799	1.89226	1.50356
56800	1.89226	2.1
56801	0.805746	2.1
56802	0.63008	2.1
56803	0.881598	1.95569
56804	0.697385	2.1
56805	0.749417	2.1
56806	0.749417	2.1
56807	0.753714	1.83722
56808	0.753714	2.1
56809	0.881598	2.1
56810	0.805746	2.1
56811	0.63008	2.1
56812	0.697385	2.1
56813	0.881599	2.04175
56814	0.697385	2.1
56815	0.805746	1.52748
56816	0.626731	1.16411
56817	0.755479	1.38228
56818	0.755479	2.1
56819	0.752851	1.14671
56820	0.752851	2.06819
56821	0.881598	1.92847
56822	0.805747	1.89413
56823	0.697385	1.99856
56824	0.626731	1.94948
56825	2.65348	2.1
56826	2.12314	2.1
56827	2.63899	2.1
56828	2.13898	2.1
56829	4.76213	2.1
56830	0.763518	2.1
56831	0.765889	2.1
56832	0.84086	2.1
56833	0.84086	2.1
56834	0.632078	2.1
56835	0.597565	2.1
56836	1.08813	2.1
56837	0.985643	2.1

56838	0.982464	2.1
56839	0.66404	2.1
56840	1.0358	2.1
56841	0.598023	2.1
56842	1.29293	2.1
56843	1.29628	2.1
56844	1.19266	2.1
56845	1.19266	2.1
56846	1.07109	2.1
56847	1.13093	2.1
56848	1.34467	2.1
56849	1.35251	2.1
56850	1.34369	2.1
56851	1.12525	2.1
56852	1.28746	2.1
56853	1.13511	2.1
56854	2.19717	2.1
56855	2.16547	2.1
56856	1.37392	2.1
56857	1.37392	2.1
56858	1.2966	2.1
56859	2.11985	2.1
56860	1.89226	2.1
56861	1.89226	2.1
56862	1.89226	2.1
56863	1.89226	2.1
56864	1.89226	2.1
56865	1.89226	2.1
56866	1.65637	2.1
56867	1.41758	2.1
56868	1.65637	2.1
56869	1.74391	2.1
56870	1.35504	2.1
56871	1.44258	2.1
56872	1.40774	2.1
56873	1.40774	2.1
56874	0.946131	2.1
56875	0.946131	2.1
56876	1.40774	2.1
56877	1.38882	2.1
56878	0.946132	2.1
56879	0.946132	2.1
56880	0.946132	2.1
56881	1.38882	2.1
56882	0.946132	2.1
56883	1.40774	2.1
56884	1.89227	2.1
56885	1.89227	2.1
56886	1.89227	2.1
56887	1.89227	2.1
56888	3.78453	2.1
56889	0.603148	2.1
56890	0.600177	2.1
56891	0.558615	2.1
56892	0.580714	2.1
56893	0.558606	2.1
56894	0.67883	2.1
56895	1.65637	2.1

56896	1.07933	2.1
56897	0.842649	2.1
56898	0.905168	2.1
56899	0.774334	2.1
56900	1.38882	2.1
56901	1.38882	2.1
56902	0.946132	2.1
56903	0.946132	2.1
56904	1.38882	2.1
56905	1.37381	2.1
56906	0.946132	2.1
56907	0.946132	2.1
56908	0.946132	2.1
56909	1.37381	2.1
56910	0.946133	2.1
56911	1.38882	2.1
56912	2.3771	2.1
56913	1.9339	2.1
56914	1.70782	2.1
56915	1.9339	2.1
56916	2.42693	2.1
56917	2.83279	2.1
56918	2.34901	0.810189
56919	1.45833	0.843036
56920	1.86695	0.800782
56921	1.03101	0.847872
56922	3.32528	0.819589
56923	1.89226	0.669655
56924	1.89226	0.634402
56925	1.89226	0.527063
56926	1.89226	0.455394
56927	3.78453	0.617554
56928	2.71753	0.455437
56929	1.89226	0.604546
56930	2.76228	0.388117
56931	2.76228	0.443662
56932	1.89226	0.656414
56933	1.89226	0.698897
56934	0.946131	0.499529
56935	0.946131	0.491867
56936	0.946132	0.48979
56937	0.946132	0.519237
56938	0.946132	0.581394
56939	0.946132	0.434248
56940	0.946132	0.591055
56941	0.946132	0.429857
56942	0.946132	0.415727
56943	0.946132	0.454212
56944	0.946132	0.582076
56945	0.946132	0.535441
56946	1.89227	0.560124
56947	1.89227	0.341033
56948	1.89227	0.40024
56949	1.89227	0.508061
56950	1.89227	0.326858
56951	1.89227	0.414545
56952	2.71753	0.412201
56953	2.37907	0.292356

56954	2.37907	0.370101
56955	2.76228	0.480516
56956	2.39588	0.251116
56957	2.77909	0.334006
56958	1.6571	0.916725
56959	1.11682	1.27921
56960	1.47132	0.890787
56961	1.93763	0.713552
56962	3.12842	1.11902
56963	1.93762	0.811055
56964	2.12345	0.969889
56965	1.65709	0.74278
56966	1.65709	1.40413
56967	2.12345	1.00775
56968	1.88635	1.59116
56969	1.47134	1.45514
56970	2.219	0.794507
56971	2.25734	1.67024
56972	1.93765	0.882949
56973	1.79103	1.13881
56974	1.47134	0.748039
56975	1.06174	0.95824
56976	1.19349	1.51416
56977	1.1095	1.36275
56978	0.968827	0.904196
56979	1.07892	0.861957
56980	1.07892	1.97151
56981	1.0834	0.838102
56982	1.0834	1.07816
56983	1.1095	1.13634
56984	1.19349	0.993626
56985	0.968825	1.12097
56986	1.06174	1.08823
56987	2.40627	1.89319
56988	2.08288	1.21851
56989	2.5444	0.930598
56990	2.29285	1.29512
56991	4.62729	1.01138
56992	2.08289	0.965029
56993	1.65703	1.01228
56994	1.71105	1.89862
56995	1.50108	1.3879
56996	2.29286	1.31752
56997	2.08289	1.42579
56998	2.5444	1.07077
56999	2.29285	1.94086
57000	2.71429	1.66846
57001	2.50288	0.911364
57002	5.04728	1.70332
57003	2.50288	0.899199
57004	2.00927	1.52808
57005	2.02116	1.03678
57006	2.02116	1.73181
57007	2.29286	0.949966
57008	2.29286	1.4125
57009	1.89226	1.71514
57010	1.89226	2.01059
57011	1.89226	0.974027

57012	1.89226	2.1
57013	3.78453	1.70427
57014	1.17418	1.70672
57015	1.29741	2.1
57016	1.04921	2.1
57017	1.19352	2.1
57018	2.3677	2.1
57019	1.29741	2.1
57020	1.27575	2.1
57021	1.19352	2.1
57022	1.19473	2.1
57023	2.46927	2.1
57024	1.89226	2.1
57025	1.89226	2.1
57026	1.89226	2.0579
57027	1.89226	2.1
57028	1.89226	2.1
57029	1.89226	2.1
57030	0.561332	2.1
57031	0.561332	2.1
57032	0.567597	2.1
57033	0.567597	2.1
57034	0.597365	2.1
57035	0.544699	2.1
57036	0.596761	2.1
57037	0.531564	2.1
57038	0.5447	2.1
57039	0.531563	2.1
57040	0.596759	2.1
57041	0.597366	2.1
57042	0.946131	2.1
57043	0.946131	2.1
57044	0.946131	1.90053
57045	0.946131	2.1
57046	0.946129	2.1
57047	0.946129	2.1
57048	0.946133	2.1
57049	0.946133	1.93568
57050	0.946131	2.1
57051	0.946131	2.1
57052	0.946131	2.1
57053	0.946131	2.1
57054	1.10938	0.38731
57055	1.17614	0.893662
57056	1.02478	0.556154
57057	1.09056	0.863232
57058	2.19994	0.834173
57059	1.17614	0.898401
57060	1.11868	1.696
57061	1.03408	0.868808
57062	1.03408	1.83765
57063	1.09056	0.86443
57064	1.17516	2.01685
57065	1.89226	0.846915
57066	1.89226	0.799489
57067	1.89226	0.86091
57068	1.89226	0.754362
57069	1.89226	0.883678

57070	1.89226	0.799379
57071	1.89226	0.747882
57072	1.89226	0.798013
57073	1.89226	0.810948
57074	1.89226	0.682228
57075	1.89226	0.857172
57076	1.89226	0.677975
57077	0.579551	0.661682
57078	0.563873	0.602567
57079	0.611341	0.74371
57080	0.598693	0.679454
57081	0.589856	0.667474
57082	0.589856	0.615319
57083	0.588387	0.729598
57084	0.588387	0.672961
57085	0.57955	0.681877
57086	0.611341	0.67789
57087	0.563873	0.663959
57088	0.598693	0.659227
57089	0.611341	0.737019
57090	0.598693	0.672014
57091	0.586048	0.735876
57092	0.572737	0.775496
57093	0.591824	0.734404
57094	0.591824	0.684847
57095	0.592254	0.782367
57096	0.592254	0.747502
57097	0.611341	0.740699
57098	0.586048	0.730069
57099	0.598693	0.73059
57100	0.572737	0.727291
57101	0.946119	0.731703
57102	0.946119	0.828706
57103	0.946119	0.75782
57104	0.946119	0.845038
57105	0.94612	0.753326
57106	0.94612	0.717282
57107	0.946119	0.824916
57108	0.946119	0.86421
57109	0.946132	0.792853
57110	0.946132	0.787675
57111	0.946107	0.807816
57112	0.946107	0.814155
57113	1.89224	0.750598
57114	1.89224	0.702305
57115	1.89224	0.784395
57116	1.89224	0.647866
57117	1.89224	0.821412
57118	1.89224	0.702549
57119	0.562335	0.62838
57120	0.579543	0.570073
57121	0.59499	0.682003
57122	0.611333	0.623725
57123	0.586619	0.614291
57124	0.586619	0.582837
57125	0.587049	0.669618
57126	0.587049	0.634759
57127	0.594999	0.644971

57128	0.562342	0.64413
57129	0.579535	0.628324
57130	0.611325	0.624303
57131	0.59499	0.677613
57132	0.611333	0.619365
57133	0.567849	0.675733
57134	0.58604	0.719
57135	0.590978	0.671772
57136	0.590978	0.630969
57137	0.590052	0.72413
57138	0.590052	0.688419
57139	0.567857	0.70203
57140	0.594998	0.697197
57141	0.611325	0.690167
57142	0.586032	0.681351
57143	1.69661	0.847825
57144	1.9339	0.451154
57145	1.39379	0.863604
57146	1.69107	0.261472
57147	3.32768	0.555403
57148	1.9339	0.551172
57149	1.98414	0.453571
57150	1.69107	0.305852
57151	1.72774	0.395445
57152	3.66164	0.402127
57153	1.39379	0.924275
57154	1.69107	0.334259
57155	1.14397	0.688629
57156	1.43548	0.302863
57157	2.82927	0.561719
57158	1.69107	0.392047
57159	1.72774	0.474935
57160	1.53873	0.408394
57161	1.53873	0.545189
57162	1.43548	0.368284
57163	1.62449	0.461909
57164	2.58431	0.38256
57165	1.34072	0.363121
57166	1.80841	0.398704
57167	1.34072	0.402017
57168	2.51501	0.346745
57169	2.04732	0.357748
57170	1.89226	0.400647
57171	1.89226	0.400354
57172	1.89226	0.429505
57173	1.89226	0.439294
57174	1.89226	0.368474
57175	1.89226	0.384968
57176	0.946131	0.412053
57177	0.946131	0.412949
57178	1.3639	0.430006
57179	1.1516	0.426324
57180	0.946129	0.424868
57181	0.946129	0.439401
57182	1.36391	0.410553
57183	1.36391	0.391212
57184	0.946131	0.437106
57185	1.3639	0.425444

57186	1.1516	0.404961
57187	0.946131	0.407117
57188	0.946131	0.42675
57189	0.946131	0.394
57190	0.94613	0.432636
57191	0.94613	0.372397
57192	0.946128	0.446561
57193	0.946128	0.414173
57194	0.946133	0.382723
57195	0.946133	0.362912
57196	0.946131	0.405501
57197	0.946131	0.469237
57198	0.946131	0.359583
57199	0.946131	0.39066
57200	1.89226	1.15569
57201	2.63634	1.62176
57202	1.89226	1.19884
57203	1.83902	1.27292
57204	3.73128	1.1788
57205	1.47059	1.91742
57206	2.02928	2.1
57207	1.47059	2.1
57208	1.65706	2.1
57209	1.98628	2.1
57210	2.17274	2.1
57211	2.43618	2.1
57212	2.41176	2.1
57213	1.98627	2.1
57214	2.34084	2.1
57215	4.39803	2.1
57216	1.47059	1.88427
57217	1.82453	2.1
57218	1.98627	2.1
57219	1.98627	2.1
57220	1.26929	2.1
57221	1.26929	2.1
57222	1.98627	2.1
57223	2.34084	2.1
57224	1.47264	2.1
57225	2.24937	2.1
57226	4.23564	2.1
57227	1.33026	1.01344
57228	1.51171	1.20388
57229	1.51171	0.987688
57230	1.4617	1.22809
57231	1.35382	0.986711
57232	1.30382	1.44792
57233	1.89226	0.955222
57234	1.89226	1.03914
57235	1.89226	0.924559
57236	1.89226	1.14458
57237	1.89226	0.914001
57238	1.89226	1.19026
57239	1.39263	0.996164
57240	0.962704	0.731743
57241	1.0171	0.869131
57242	0.962704	0.948095
57243	1.40822	0.819466

57244	1.35382	0.905572
57245	0.755853	1.32939
57246	0.789808	1.09989
57247	0.696315	0.995717
57248	0.676912	0.926925
57249	0.729751	0.923412
57250	0.729751	1.07811
57251	0.722417	1.4944
57252	0.722417	0.959216
57253	0.696315	1.05706
57254	0.789808	1.04937
57255	0.755853	1.04738
57256	0.676912	1.04408
57257	0.946132	1.02742
57258	0.946132	1.07779
57259	0.946132	0.933005
57260	0.946132	0.839015
57261	0.946131	0.839759
57262	0.946131	1.02187
57263	0.946132	0.936062
57264	0.946132	1.07708
57265	0.946132	0.957725
57266	0.946132	0.963465
57267	0.946132	0.976971
57268	0.946132	0.975533
57269	0.946133	0.911609
57270	0.946133	0.804001
57271	0.946132	0.650921
57272	0.946132	0.792644
57273	0.946132	0.639618
57274	0.946132	0.791333
57275	0.946133	0.740484
57276	0.946133	0.925592
57277	0.946132	0.826038
57278	0.946132	0.821196
57279	0.946132	0.803076
57280	0.946132	0.850091
57281	1.89227	1.76405
57282	2.53014	1.89426
57283	1.89227	2.01511
57284	1.98241	1.67956
57285	1.89227	2.04387
57286	1.98241	1.74028
57287	1.89226	1.43297
57288	1.89226	1.99886
57289	1.89226	1.61029
57290	1.89226	1.63231
57291	1.89226	1.95388
57292	1.89226	1.95846
57293	1.89226	1.3179
57294	1.89226	1.8576
57295	1.89226	1.44972
57296	1.89226	1.48401
57297	3.78452	1.50647
57298	2.45304	1.34339
57299	1.89226	1.75273
57300	1.89226	1.55448
57301	1.89226	1.54935

57302	1.89226	1.39649
57303	1.89226	1.41118
57304	1.89227	1.67212
57305	1.89227	1.65107
57306	1.89227	1.90747
57307	1.89227	1.42664
57308	1.89227	1.92987
57309	1.89227	1.46007
57310	1.89226	1.41088
57311	1.89226	1.93091
57312	1.89226	1.52472
57313	1.89226	1.60901
57314	1.89226	1.78473
57315	1.89226	1.85371
57316	2.09871	0.785099
57317	2.71891	0.845372
57318	1.31126	0.781239
57319	1.31126	0.838108
57320	2.55341	0.782423
57321	1.76597	0.903228
57322	1.89226	0.884025
57323	1.89226	0.856849
57324	1.89226	0.810185
57325	1.89226	0.961465
57326	1.89226	0.814687
57327	1.89226	0.965353
57328	0.946128	0.857279
57329	0.946128	0.80802
57330	0.946133	0.778532
57331	0.946133	0.764398
57332	0.946131	0.775868
57333	0.946131	0.83338
57334	0.94613	0.774325
57335	0.94613	0.815801
57336	0.946131	0.796524
57337	0.946131	0.788567
57338	0.946131	0.802118
57339	0.946131	0.811828
57340	2.71891	0.774941
57341	1.89226	0.755255
57342	2.09871	0.736278
57343	2.09871	0.827449
57344	1.89226	0.737656
57345	1.89226	0.827511
57346	1.89227	0.840561
57347	1.89227	0.829412
57348	1.89227	0.793485
57349	1.89227	0.915601
57350	1.89227	0.795845
57351	1.89227	0.917171
57352	0.946129	0.830679
57353	0.946129	0.791776
57354	0.946133	0.758667
57355	0.946133	0.744485
57356	0.946131	0.756973
57357	0.946131	0.812517
57358	0.946131	0.753055
57359	0.946131	0.800274

57360	0.946131	0.775403
57361	0.946131	0.769776
57362	0.946131	0.786729
57363	0.946131	0.780883
57364	2.32462	0.277869
57365	1.89226	0.215748
57366	1.89226	0.218101
57367	1.89226	0.232686
57368	2.2291	0.252861
57369	2.2291	0.275152
57370	0.847669	0.214858
57371	0.847669	0.222786
57372	0.796966	0.212359
57373	0.796966	0.234377
57374	0.833552	0.199126
57375	1.01049	0.220534
57376	0.709709	0.205513
57377	0.634147	0.206627
57378	0.634148	0.201395
57379	0.833549	0.218036
57380	0.709711	0.218772
57381	1.01049	0.234497
57382	1.89226	0.20621
57383	1.89226	0.26281
57384	1.89226	0.223351
57385	1.89226	0.260234
57386	1.89226	0.209429
57387	1.89226	0.23406
57388	1.03611	0.207871
57389	0.936655	0.217629
57390	0.946132	0.218745
57391	0.946132	0.242854
57392	0.941918	0.202975
57393	0.941918	0.216789
57394	1.00308	0.230405
57395	1.04117	0.217136
57396	1.1406	0.194428
57397	1.04074	0.207572
57398	0.851529	0.217344
57399	1.00311	0.25492
57400	1.01814	0.189865
57401	1.6671	0.21165
57402	1.35561	0.189046
57403	1.35561	0.210885
57404	1.2683	0.184572
57405	1.2683	0.193899
57406	0.946128	0.211313
57407	0.946128	0.188497
57408	0.946129	0.197991
57409	0.946129	0.222155
57410	0.946132	0.211274
57411	0.946132	0.19726
57412	0.946126	0.200247
57413	0.946126	0.212317
57414	0.946129	0.181321
57415	0.946129	0.196403
57416	0.946129	0.2043
57417	0.946129	0.222849

57418	2.23338	0.253858
57419	1.96	0.213222
57420	2.03438	0.211985
57421	1.87835	0.238884
57422	4.11172	0.233977
57423	1.96	0.211656
57424	1.52661	0.232128
57425	1.87834	0.23803
57426	1.53175	0.20467
57427	3.40495	0.212433
57428	2.03435	0.216787
57429	1.65813	0.242196
57430	1.65813	0.25437
57431	1.87832	0.224858
57432	1.72067	0.244587
57433	1.94086	0.211749
57434	1.65813	0.242633
57435	1.40572	0.203505
57436	1.87832	0.229692
57437	1.87832	0.213798
57438	1.31154	0.21506
57439	1.53173	0.22383
57440	1.86662	2.1
57441	2.0815	2.1
57442	2.0815	2.1
57443	2.0815	2.1
57444	1.87246	2.1
57445	1.87362	2.1
57446	1.0273	2.1
57447	0.996347	2.1
57448	0.946129	2.1
57449	0.946129	2.1
57450	1.04427	2.1
57451	1.04196	2.1
57452	0.910674	2.1
57453	0.910674	2.1
57454	1.04072	2.1
57455	1.10931	2.1
57456	1.03132	2.1
57457	0.933698	2.1
57458	2.63789	0.659084
57459	1.89226	0.781603
57460	2.3033	0.659631
57461	2.3033	0.772345
57462	1.89226	0.633992
57463	1.89226	0.775871
57464	1.89226	0.809866
57465	1.89226	0.854481
57466	1.89226	0.784834
57467	1.89226	0.80524
57468	3.78452	0.832653
57469	1.51898	0.779061
57470	1.36006	0.865648
57471	2.03143	0.858003
57472	2.03143	0.859757
57473	1.04026	0.808524
57474	1.04026	0.812668
57475	0.94613	0.628711

57476	0.94613	0.700034
57477	1.31894	0.562848
57478	1.31894	0.638048
57479	0.94613	0.550596
57480	0.94613	0.727595
57481	1.31894	0.616083
57482	1.41232	0.698861
57483	0.946129	0.628775
57484	1.41232	0.650651
57485	1.31895	0.626645
57486	0.946129	0.617926
57487	1.89226	0.709178
57488	1.89226	0.768313
57489	1.89226	0.680339
57490	1.89226	0.768536
57491	1.89226	0.639007
57492	1.89226	0.764171
57493	1.89804	0.669945
57494	2.03143	0.693004
57495	2.46286	0.730002
57496	2.46287	0.685951
57497	1.51898	0.688671
57498	1.51898	0.653406
57499	1.89226	2.1
57500	1.89226	2.1
57501	1.89226	2.1
57502	1.89226	2.1
57503	3.78452	2.1
57504	1.3864	2.1
57505	1.3864	2.1
57506	0.946132	2.1
57507	0.946132	2.1
57508	1.32905	2.1
57509	1.3864	2.1
57510	0.946132	2.1
57511	0.946132	2.1
57512	0.946132	2.1
57513	1.32905	2.1
57514	0.946133	2.1
57515	1.3864	2.1
57516	2.77279	2.1
57517	1.67883	2.1
57518	1.67883	2.1
57519	1.73415	2.1
57520	2.65811	2.1
57521	2.71343	2.1
57522	0.946131	2.1
57523	0.946131	2.1
57524	0.946128	2.1
57525	0.946128	2.1
57526	0.946127	2.1
57527	0.946127	2.1
57528	0.946131	2.1
57529	0.946131	2.1
57530	0.946129	2.1
57531	0.946129	2.1
57532	0.94613	2.1
57533	0.94613	2.1

57534	1.89226	2.1
57535	1.89226	2.1
57536	1.89226	2.1
57537	1.89226	2.1
57538	3.78452	2.1
57539	1.89226	2.1
57540	2.54394	2.1
57541	2.54394	2.1
57542	2.65811	2.1
57543	1.89226	2.1
57544	2.00643	2.1
57545	2.54394	2.1
57546	2.6581	2.1
57547	1.62743	2.1
57548	1.62743	2.1
57549	1.67883	2.1
57550	2.59533	2.1
57551	1.89226	2.1
57552	1.89226	2.1
57553	1.89226	2.1
57554	1.89226	2.1
57555	1.89226	2.1
57556	1.89226	2.1
57557	1.89226	1.3533
57558	1.89226	1.26051
57559	1.89226	1.7496
57560	1.89226	1.09165
57561	1.89226	1.6547
57562	1.89226	1.07471
57563	1.89226	1.29573
57564	1.89226	1.22727
57565	1.89226	1.67276
57566	1.89226	1.11595
57567	1.89226	1.56381
57568	1.89226	1.07766
57569	1.89226	1.05384
57570	1.89226	0.933504
57571	1.89226	1.27576
57572	1.89226	0.903971
57573	1.89226	1.25966
57574	1.89226	0.900795
57575	2.68537	0.536985
57576	2.22575	0.781745
57577	2.47464	0.311274
57578	1.79093	0.565476
57579	4.70039	0.395546
57580	2.81217	0.893936
57581	2.47464	0.505205
57582	2.58286	0.846569
57583	2.29193	0.318005
57584	5.0575	0.548654
57585	2.47464	0.533995
57586	2.20365	0.678908
57587	2.29193	0.376169
57588	1.90104	0.727619
57589	4.37568	0.458193
57590	1.89226	0.845756
57591	2.16957	1.87363

57592	2.52823	1.0902
57593	2.52823	1.78632
57594	1.89226	0.963464
57595	1.89226	1.39043
57596	1.36226	2.1
57597	1.55369	2.1
57598	1.33309	2.1
57599	1.62593	2.1
57600	2.98819	2.1
57601	0.802281	2.1
57602	0.802281	2.1
57603	0.835143	2.1
57604	0.835143	2.1
57605	0.824458	2.1
57606	0.92641	2.1
57607	0.776847	2.1
57608	0.812967	2.1
57609	0.812967	2.1
57610	0.926411	2.1
57611	0.776847	2.1
57612	0.824458	2.1
57613	1.33309	2.1
57614	1.62593	2.1
57615	1.32813	2.1
57616	1.7237	2.1
57617	2.95406	2.1
57618	0.814544	2.1
57619	0.875312	2.1
57620	0.910502	2.1
57621	0.910502	2.1
57622	0.886633	2.1
57623	0.975739	2.1
57624	0.792623	2.1
57625	0.840283	2.1
57626	0.796372	2.1
57627	1.06159	2.1
57628	0.81948	2.1
57629	0.933833	2.1
57630	2.63997	2.1
57631	1.29694	2.1
57632	1.28141	2.1
57633	2.62623	2.1
57634	3.92317	2.1
57635	2.63998	2.1
57636	1.89226	2.1
57637	1.89226	2.1
57638	2.62623	2.1
57639	4.5185	2.1
57640	1.89226	2.1
57641	1.89226	2.1
57642	1.89226	2.1
57643	1.89226	2
57644	3.78453	2.1
57645	1.89226	2.1
57646	1.89226	2.1
57647	1.89226	2.1
57648	1.89226	2.1
57649	1.89226	2.1

57650	1.89226	2.1
57651	1.25468	2.1
57652	2.62623	2.1
57653	1.28141	2.1
57654	1.28141	2.1
57655	2.60665	2.1
57656	2.60665	2.1
57657	1.89226	2.1
57658	2.62623	2.1
57659	2.60666	2.1
57660	2.62623	2.1
57661	1.87269	2.1
57662	1.89226	2.1
57663	1.89226	2.1
57664	1.89226	2.1
57665	1.89226	2.1
57666	1.89226	2.1
57667	3.78453	2.1
57668	1.89226	2.1
57669	1.89226	1.87998
57670	1.89226	2.1
57671	1.89226	2.1
57672	1.89226	2.1
57673	1.89226	2.06465
57674	2.30147	1.4024
57675	2.27703	1.63897
57676	1.89227	1.29051
57677	1.89227	1.54013
57678	4.1693	1.52543
57679	1.38617	1.4063
57680	1.24613	1.41053
57681	2.59886	1.26449
57682	2.6238	1.40753
57683	3.86993	1.24397
57684	0.946133	1.36356
57685	0.946133	1.121
57686	0.946132	1.07371
57687	0.946132	1.55655
57688	0.946133	1.52435
57689	0.946133	1.43967
57690	0.946132	1.10223
57691	0.946132	1.15603
57692	0.946133	1.24806
57693	0.946133	1.21054
57694	0.946132	1.17126
57695	0.946132	1.18802
57696	2.21773	2.1
57697	2.59216	2.1
57698	1.68541	2.1
57699	1.68541	2.1
57700	1.84814	2.1
57701	2.38046	2.1
57702	1.89966	2.1
57703	1.70303	2.1
57704	1.2028	2.1
57705	1.29897	2.1
57706	2.02873	2.1
57707	2.19093	2.1

57708	2.75218	2.1
57709	1.70303	2.1
57710	3.10254	2.1
57711	2.24783	2.1
57712	2.56839	2.1
57713	1.70303	2.1
57714	2.56839	2.1
57715	1.08276	2.1
57716	2.24061	2.1
57717	1.67491	2.1
57718	1.60948	2.1
57719	1.08276	2.1
57720	1.89226	0.767645
57721	1.89226	0.863828
57722	1.89226	0.741
57723	1.89226	0.772125
57724	3.78453	0.745816
57725	1.89226	0.754906
57726	1.89226	0.858347
57727	1.89226	0.752727
57728	1.89226	0.78119
57729	3.78452	0.854167
57730	1.89226	0.837184
57731	2.7535	0.784288
57732	1.89226	0.963985
57733	1.89226	0.914538
57734	1.89226	0.786603
57735	1.89226	0.780347
57736	1.89226	0.844216
57737	1.89226	0.779523
57738	1.89226	0.743939
57739	1.89226	0.91706
57740	1.89226	0.767379
57741	1.89226	1.02703
57742	1.89227	0.751117
57743	1.89227	0.783013
57744	1.89227	0.735716
57745	1.89227	0.750894
57746	3.78453	0.887592
57747	1.89226	0.806682
57748	2.53412	0.737639
57749	1.89226	1.09848
57750	1.89226	0.750445
57751	1.89226	0.778431
57752	1.89226	0.739098
57753	2.21126	0.472357
57754	1.89226	0.530914
57755	2.57464	0.536716
57756	1.89226	0.525393
57757	2.57464	0.464847
57758	1.89226	0.468735
57759	1.89226	0.615419
57760	1.89226	0.716732
57761	1.89226	0.960565
57762	1.89226	0.621305
57763	1.89226	0.986187
57764	1.89226	0.601933
57765	1.1418	0.40432

57766	0.653021	0.397345
57767	1.37282	0.459194
57768	0.931386	0.460625
57769	1.34568	0.524488
57770	1.17669	0.435248
57771	0.666595	0.450776
57772	0.786895	0.511983
57773	0.933982	0.457129
57774	1.05351	0.483117
57775	1.00792	0.387635
57776	1.00792	0.463797
57777	1.89226	2.1
57778	1.89226	2.1
57779	1.89226	2.1
57780	1.89226	2.1
57781	3.78452	2.1
57782	1.89226	2.1
57783	1.89226	2.1
57784	1.89226	2.1
57785	1.89226	2.1
57786	1.89226	2.1
57787	1.89226	2.1
57788	1.65642	2.1
57789	1.30248	2.1
57790	1.38888	2.1
57791	1.25987	2.1
57792	1.70611	2.1
57793	1.65642	2.1
57794	1.65642	2.1
57795	1.81407	2.1
57796	2.19428	2.1
57797	1.92555	2.1
57798	3.8507	2.1
57799	1.89226	2.1
57800	1.89226	2.1
57801	1.89226	2.1
57802	1.89226	2.1
57803	3.78452	2.1
57804	1.89226	2.1
57805	1.89226	2.1
57806	1.89226	2.1
57807	1.89226	2.1
57808	1.89226	2.1
57809	1.89226	2.1
57810	0.705495	2.1
57811	0.809317	2.1
57812	1.1655	2.1
57813	1.09605	2.1
57814	0.983375	2.1
57815	0.797301	2.1
57816	0.906633	2.1
57817	0.906633	2.1
57818	0.85085	2.1
57819	1.16548	2.1
57820	0.705501	2.1
57821	1.00314	2.1
57822	2.19428	2.1
57823	2.31862	2.1

57824	2.27408	2.1
57825	2.45893	2.1
57826	4.46836	2.1
57827	1.89226	0.835284
57828	1.89226	0.955372
57829	1.89226	0.917846
57830	1.89226	0.834262
57831	1.89226	1.0499
57832	1.89226	0.885063
57833	2.0815	0.838064
57834	2.22136	0.930629
57835	1.5515	0.896579
57836	1.86323	0.822524
57837	2.74986	0.991926
57838	3.30238	0.853244
57839	1.89224	0.857533
57840	1.89224	0.959027
57841	1.89224	0.965944
57842	1.89224	0.849508
57843	1.89224	1.10927
57844	1.89224	0.887228
57845	1.89224	0.82959
57846	1.89224	0.952121
57847	1.89224	0.884817
57848	1.89224	0.819812
57849	1.89224	1.02827
57850	1.89224	0.86967
57851	1.17846	0.799908
57852	0.810935	0.80578
57853	0.93347	0.989769
57854	0.93347	0.826888
57855	0.810932	0.874999
57856	0.810932	0.837959
57857	2.22133	0.865287
57858	1.71919	0.864316
57859	1.32629	0.811802
57860	1.0813	0.905963
57861	1.57137	0.840273
57862	2.21149	2.1
57863	1.89226	1.92011
57864	1.89226	2.1
57865	1.89226	1.65026
57866	2.17159	2.1
57867	2.17159	1.76732
57868	0.946131	0.894365
57869	0.946131	2.07739
57870	0.946131	1.47086
57871	0.946131	1.91473
57872	0.94613	1.22065
57873	0.94613	1.62869
57874	0.946132	1.94865
57875	0.946132	1.76622
57876	0.946131	1.30827
57877	0.946131	1.24201
57878	0.946131	1.66093
57879	0.946131	1.54612
57880	1.12266	1.47637
57881	1.4714	1.79491

57882	1.62322	1.4287
57883	1.62322	1.64469
57884	1.02958	1.64736
57885	1.02958	1.9521
57886	2.2671	2.1
57887	1.89226	1.98827
57888	1.89226	2.1
57889	1.89226	1.82891
57890	2.21149	2.1
57891	2.21149	2.01357
57892	1.89226	1.63516
57893	1.89226	1.64495
57894	1.89226	1.54414
57895	1.89226	1.32439
57896	1.89226	1.80317
57897	1.89226	1.70379
57898	0.736914	1.76789
57899	0.736914	1.70258
57900	0.822839	1.69816
57901	0.822836	2.09896
57902	0.791298	1.15755
57903	1.00003	1.72452
57904	0.640254	1.75213
57905	0.575379	1.89415
57906	0.609001	1.39261
57907	1.00005	1.34533
57908	0.575371	1.77208
57909	0.831908	1.48043
57910	1.89226	2.1
57911	1.89226	2.1
57912	1.89226	2.1
57913	1.89226	2.1
57914	1.89226	2.1
57915	1.89226	2.1
57916	1.89226	2.1
57917	1.89226	2.1
57918	1.89226	2.1
57919	1.89226	2.1
57920	1.89226	2.1
57921	1.89226	2.1
57922	0.730617	2.1
57923	0.71052	2.1
57924	0.7329	2.1
57925	0.712083	2.1
57926	0.701051	2.1
57927	0.701051	2.1
57928	0.721552	2.1
57929	0.721552	2.1
57930	0.710521	2.1
57931	0.732898	2.1
57932	0.730619	2.1
57933	0.712081	2.1
57934	0.7329	2.1
57935	0.712082	2.1
57936	0.559601	2.1
57937	0.495993	2.1
57938	0.603718	2.1
57939	0.603718	2.1

57940	0.625174	2.1
57941	0.625174	2.1
57942	0.732899	2.1
57943	0.559601	2.1
57944	0.712082	2.1
57945	0.495993	2.1
57946	0.94613	2.1
57947	0.94613	2.1
57948	0.94613	2.1
57949	0.94613	2.1
57950	0.946131	2.1
57951	0.946131	2.1
57952	0.946129	2.1
57953	0.946129	2.1
57954	0.94613	2.1
57955	0.94613	2.1
57956	0.94613	2.1
57957	0.94613	2.1
57958	1.89226	2.1
57959	1.89226	2.1
57960	1.89226	2.1
57961	1.89226	2.1
57962	1.89226	2.1
57963	1.89226	2.1
57964	1.42104	2.1
57965	1.4658	2.1
57966	1.51223	2.1
57967	1.42283	2.1
57968	2.93327	2.1
57969	0.732899	2.1
57970	0.756115	2.1
57971	0.629763	2.1
57972	0.559601	2.1
57973	0.646134	2.1
57974	0.646134	2.1
57975	0.669582	2.1
57976	0.669582	2.1
57977	0.756115	2.1
57978	0.629763	2.1
57979	0.732899	2.1
57980	0.559601	2.1
57981	2.566 2.1	
57982	1.20242	1.38623
57983	2.64084	2.1
57984	2.18297	1.91145
57985	1.66029	1.96777
57986	1.20242	1.50664
57987	0.946133	1.0888
57988	0.946133	1.25356
57989	0.946129	1.75447
57990	0.946129	1.75891
57991	0.946131	1.50066
57992	0.946131	1.05195
57993	0.946131	0.818498
57994	0.946131	1.61905
57995	0.946131	1.03069
57996	0.946131	1.2537
57997	0.946131	1.18307

57998	0.946131	1.2276
57999	1.89226	0.22113
58000	1.89226	0.27511
58001	1.89226	0.247962
58002	1.89226	0.23464
58003	3.78453	0.247116
58004	1.03073	0.194263
58005	1.03073	0.195915
58006	1.04066	0.211651
58007	1.04066	0.22868
58008	1.20802	0.223464
58009	1.32689	0.196511
58010	0.883301	0.209455
58011	0.744506	0.194198
58012	1.20802	0.207784
58013	0.744506	0.204571
58014	0.883301	0.20007
58015	1.32689	0.205721
58016	1.20802	0.215331
58017	1.20802	0.204431
58018	0.946131	0.233089
58019	0.946131	0.256658
58020	0.94613	0.254317
58021	0.94613	0.215751
58022	1.20802	0.231827
58023	1.32689	0.211074
58024	1.20802	0.219446
58025	0.946131	0.224127
58026	0.946131	0.212195
58027	1.32689	0.221627
58028	1.89226	0.239515
58029	1.89226	0.313868
58030	1.89226	0.279393
58031	1.89226	0.269966
58032	1.89226	0.276448
58033	1.89226	0.265195
58034	0.946119	0.263672
58035	0.946119	0.240331
58036	0.946118	0.286896
58037	0.946118	0.33184
58038	0.946118	0.328411
58039	0.946118	0.258156
58040	0.946119	0.238093
58041	0.946119	0.281434
58042	0.946131	0.27988
58043	0.946131	0.280237
58044	0.946106	0.278577
58045	0.946106	0.28066
58046	1.3939	2.1
58047	1.49641	2.1
58048	1.15096	2.1
58049	1.10101	2.1
58050	2.59742	2.1
58051	1.22106	2.1
58052	2.60663	2.1
58053	1.25467	2.1
58054	1.25467	2.1
58055	2.58377	2.1

58056	2.58377	2.1
58057	1.89225	2.1
58058	2.60664	2.1
58059	2.58377	2.1
58060	2.60664	2.1
58061	1.86938	2.1
58062	1.89225	2.1
58063	1.89225	2.1
58064	1.89225	2.1
58065	1.89225	2.1
58066	1.89225	2.1
58067	1.89225	2.1
58068	1.89225	2.1
58069	0.946121	2.1
58070	0.946121	2.1
58071	0.946126	2.1
58072	0.946126	2.1
58073	0.946124	1.95636
58074	0.946124	1.93224
58075	0.946123	2.1
58076	0.946123	2.1
58077	0.946117	2.1
58078	0.946117	2.1
58079	0.94613	2.1
58080	0.94613	2.1
58081	1.28017	2.1
58082	1.29189	2.1
58083	0.610534	2.1
58084	0.592373	2.1
58085	0.943764	2.1
58086	0.943764	2.1
58087	0.940501	2.1
58088	0.940501	2.1
58089	1.28017	2.1
58090	0.592371	2.1
58091	0.610533	2.1
58092	1.29189	2.1
58093	0.946133	2.1
58094	0.946133	2.1
58095	1.28017	2.1
58096	1.29189	2.1
58097	0.946132	2.1
58098	0.946132	2.1
58099	1.28017	2.1
58100	1.28017	2.1
58101	1.28017	2.1
58102	0.946132	2.1
58103	0.946133	2.1
58104	1.2919	2.1
58105	1.89226	2.1
58106	1.89226	2.1
58107	1.89226	2.1
58108	1.89226	2.1
58109	1.89226	2.1
58110	1.89226	2.1
58111	1.89226	2.1
58112	1.89226	2.1
58113	1.89226	2.1

58114	1.89226	2.1
58115	3.78452	2.1
58116	1.89226	2.1
58117	2.08196	2.1
58118	1.97854	2.1
58119	2.08196	2.1
58120	1.78884	2.1
58121	1.89226	2.1
58122	1.89226	2.1
58123	1.89226	2.1
58124	1.89226	2.1
58125	1.89226	2.1
58126	1.89226	2.1
58127	1.89226	2.1
58128	1.83688	2.1
58129	1.89226	2.1
58130	1.89226	2.1
58131	1.89226	2.1
58132	1.86015	2.1
58133	1.86015	2.1
58134	1.89226	2.1
58135	1.97854	2.1
58136	1.85883	2.1
58137	1.97854	2.1
58138	1.77255	2.1
58139	1.89226	2.1
58140	0.946131	2.03765
58141	0.946131	2.1
58142	0.946131	2.1
58143	0.946131	2.1
58144	0.94613	2.1
58145	0.94613	2.1
58146	0.946132	2.1
58147	0.946132	2.1
58148	0.946131	2.1
58149	0.946131	2.1
58150	0.946131	2.1
58151	0.946131	2.1
58152	0.918412	2.1
58153	0.918412	2.1
58154	0.94893	2.1
58155	0.750211	2.1
58156	0.851541	2.1
58157	0.851541	2.1
58158	0.970566	2.1
58159	0.826567	2.1
58160	0.970554	2.1
58161	1.04071	2.1
58162	0.826632	2.1
58163	0.851554	2.1
58164	1.89226	0.305243
58165	1.89226	0.334457
58166	1.89226	0.285911
58167	1.89226	0.287
58168	3.78453	0.314426
58169	0.946131	0.285177
58170	0.946131	0.379592
58171	0.811232	0.343442

58172	0.811232	0.303807
58173	0.94613	0.34758
58174	0.94613	0.343963
58175	0.959701	0.339179
58176	0.811233	0.378237
58177	0.94613	0.278136
58178	0.811233	0.30873
58179	0.959702	0.325769
58180	0.946131	0.366118
58181	1.89226	0.278021
58182	1.89226	0.307753
58183	1.89226	0.30938
58184	1.89226	0.29319
58185	1.89226	0.262404
58186	1.89226	0.25657
58187	1.89227	0.265376
58188	1.89227	0.310665
58189	1.89227	0.291616
58190	1.89227	0.291083
58191	1.89227	0.281419
58192	1.89227	0.284397
58193	1.89226	0.283297
58194	1.89226	0.278903
58195	1.89226	0.248657
58196	1.89226	0.237725
58197	3.78453	0.251399
58198	1.89226	0.25584
58199	1.89226	0.233151
58200	1.89226	0.238137
58201	1.89226	0.266493
58202	1.89226	0.230243
58203	1.89226	0.259278
58204	1.89226	2.1
58205	2.36204	2.1
58206	2.39853	2.1
58207	2.36204	2.1
58208	1.92876	2.1
58209	1.89226	2.1
58210	1.89226	2.1
58211	1.89226	1.48961
58212	1.89226	2.1
58213	1.89226	0.928165
58214	3.78453	2.1
58215	0.946128	2.1
58216	0.946128	0.916382
58217	1.13182	1.19952
58218	1.13182	2.1
58219	0.94613	1.1174
58220	0.94613	1.2973
58221	1.1749	2.1
58222	1.13182	2.1
58223	1.1749	2.1
58224	0.94613	1.98356
58225	1.13182	2.01064
58226	0.94613	2.06247
58227	1.89226	2.1
58228	2.39853	2.1
58229	2.44918	2.1

58230	2.39853	2.1
58231	1.94291	2.1
58232	1.89226	2.1
58233	0.946132	0.982682
58234	0.946132	2.1
58235	0.946131	2.1
58236	0.946131	1.58419
58237	0.946132	1.31172
58238	0.946132	1.82919
58239	0.946131	2.1
58240	0.946131	2.1
58241	0.946131	2.1
58242	0.946131	2.1
58243	0.946132	2.1
58244	0.946132	2.1
58245	0.946128	2.1
58246	0.946128	1.94716
58247	1.1749	1.26323
58248	1.1749	2.1
58249	0.946131	1.20635
58250	0.946131	1.39636
58251	1.23631	2.1
58252	1.1749	2.1
58253	1.23631	2.1
58254	0.94613	2.1
58255	1.1749	2.00689
58256	0.946131	1.52222
58257	1.89226	0.946558
58258	1.89226	2.1
58259	1.89226	1.81806
58260	1.89226	1.82169
58261	3.78453	1.9509
58262	1.38859	2.1
58263	1.45333	2.1
58264	1.28909	1.76131
58265	1.38091	2.1
58266	2.74242	2.1
58267	1.45332	2.1
58268	1.38414	2.1
58269	1.38091	2.1
58270	1.33506	2.1
58271	2.78838	2.1
58272	1.89226	2.1
58273	1.89226	2.1
58274	1.89226	1.85018
58275	1.89226	2.1
58276	3.78452	2.1
58277	1.89226	1.94374
58278	1.89226	1.87697
58279	1.89226	1.15101
58280	1.89226	2.1
58281	1.89226	1.18816
58282	1.89226	2.1
58283	1.17418	1.93477
58284	1.38091	2.1
58285	1.17418	2.1
58286	1.28909	2.1
58287	1.29741	2.1

58288	1.41232	2.1
58289	1.38091	2.1
58290	1.33506	2.1
58291	1.27575	2.1
58292	1.27575	2.1
58293	1.29741	2.1
58294	1.35671	2.1
58295	1.89226	1.90015
58296	1.89226	2.1
58297	1.89226	2.1
58298	1.89226	2.1
58299	3.78452	2.1
58300	1.92737	0.53977
58301	2.40223	0.610988
58302	1.56178	0.52405
58303	1.97605	0.636757
58304	3.90343	0.545839
58305	1.89226	0.571841
58306	1.89226	0.673678
58307	1.89226	0.615996
58308	1.89226	0.667702
58309	3.78452	0.667162
58310	1.89226	0.674685
58311	1.89226	0.695902
58312	1.89226	0.690589
58313	1.89226	0.708424
58314	1.89226	0.668856
58315	1.89226	0.692798
58316	1.13641	0.662781
58317	1.13641	0.635767
58318	1.12763	0.606335
58319	1.12763	0.66677
58320	1.29348	0.667011
58321	1.06293	0.674544
58322	1.20111	0.606716
58323	0.988028	0.638974
58324	0.988028	0.647344
58325	1.06293	0.649129
58326	1.29348	0.644073
58327	1.20111	0.634934
58328	1.56176	0.489886
58329	1.46387	0.5969
58330	1.97603	0.5522
58331	1.97603	0.527977
58332	1.08211	0.550555
58333	1.08211	0.519468
58334	1.89224	0.579003
58335	1.89224	0.62781
58336	1.89224	0.602859
58337	1.89224	0.638573
58338	1.89224	0.561824
58339	1.89224	0.60773
58340	1.89224	0.641807
58341	1.89224	0.682429
58342	1.89224	0.629584
58343	1.89224	0.698761
58344	3.78448	0.639611
58345	0.820185	0.551743

58346	0.818947	0.631883
58347	0.911041	0.645624
58348	0.695265	0.633999
58349	1.46388	0.599151
58350	1.23607	0.605664
58351	0.927068	0.603994
58352	1.02691	0.650583
58353	1.12918	0.555365
58354	0.846843	0.609582
58355	0.911039	0.617141
58356	1.29129	2.1
58357	1.45547	2.1
58358	1.67327	2.1
58359	1.66698	2.1
58360	1.20149	2.1
58361	1.20149	2.1
58362	0.946133	1.13817
58363	0.946133	2.07388
58364	0.946129	2.1
58365	0.946129	2.1
58366	0.946131	2.01079
58367	0.946131	1.12283
58368	0.946131	2.1
58369	0.946131	1.90682
58370	0.946131	2.1
58371	0.946131	2.06882
58372	0.946131	2.02048
58373	0.946131	2.0813
58374	1.89226	1.69227
58375	1.89226	1.55865
58376	1.89226	2.01446
58377	1.89226	1.0588
58378	3.78452	1.41321
58379	0.86875	1.49923
58380	0.86875	2.05
58381	0.899002	2.1
58382	0.966245	2.1
58383	0.849364	1.18246
58384	1.01918	1.94828
58385	0.764737	2.1
58386	0.89249	2.03379
58387	0.764624	1.96535
58388	0.967869	2.01424
58389	0.808606	1.86809
58390	0.864506	1.72584
58391	0.773929	2.1
58392	0.773929	2.1
58393	0.781991	2.1
58394	0.836559	2.1
58395	0.983761	2.1
58396	0.834687	2.1
58397	0.680545	2.1
58398	0.640799	2.1
58399	0.70286	2.1
58400	0.935844	2.1
58401	0.629479	2.1
58402	0.823655	2.1
58403	0.946133	1.25888

58404	0.946133	2.09814
58405	0.946128	2.1
58406	0.946128	2.1
58407	0.94613	2.1
58408	0.94613	1.21453
58409	0.946131	2.1
58410	0.946131	2.0308
58411	0.946131	2.1
58412	0.946131	2.1
58413	0.946131	2.1
58414	0.946131	2.1
58415	1.89226	2.1
58416	1.89226	1.11514
58417	1.89226	1.68701
58418	1.89226	1.53802
58419	1.89226	1.28375
58420	1.89226	1.45673
58421	0.931638	2.1
58422	0.931638	1.36688
58423	1.03559	2.1
58424	0.977442	1.7288
58425	0.826487	1.35345
58426	0.993902	1.32678
58427	1.04922	2.1
58428	0.894324	2.1
58429	0.912984	2.06163
58430	1.01156	2.06225
58431	0.944692	1.77085
58432	1.15456	1.80059
58433	1.89226	0.351443
58434	1.89226	0.410188
58435	1.89226	0.482418
58436	1.89226	0.319575
58437	3.78453	0.33574
58438	1.89226	0.345167
58439	1.89226	0.314558
58440	2.64262	0.351953
58441	2.64262	0.284216
58442	1.78052	0.264364
58443	1.03015	0.305959
58444	0.999721	0.297887
58445	0.659011	0.271132
58446	1.15564	0.399255
58447	1.41388	0.315955
58448	1.01583	0.317433
58449	1.01583	0.286191
58450	1.05706	0.396504
58451	1.05706	0.317593
58452	1.15564	0.311054
58453	0.65901	0.300522
58454	1.41388	0.31561
58455	0.999721	0.307583
58456	1.89226	2.1
58457	1.89226	2.1
58458	1.89226	2.1
58459	1.89226	2.1
58460	3.78453	2.1
58461	2.39022	2.1

58462	1.90734	2.1
58463	2.27634	2.1
58464	1.82666	2.1
58465	4.18368	2.1
58466	1.90734	2.1
58467	1.21564	2.1
58468	1.82666	2.1
58469	1.16938	2.1
58470	3.07672	2.1
58471	1.89226	2.1
58472	1.89226	2.1
58473	1.89226	2.1
58474	1.89226	2.1
58475	1.89226	2.1
58476	1.89226	2.1
58477	1.89227	2.1
58478	1.89227	2.1
58479	1.89227	2.1
58480	1.89227	2.1
58481	3.78453	2.1
58482	2.27634	2.1
58483	1.82666	2.1
58484	2.18042	2.1
58485	1.74738	2.1
58486	4.02372	2.1
58487	1.74738	2.1
58488	1.82666	2.1
58489	1.1181	2.1
58490	1.1181	2.1
58491	1.16938	2.1
58492	1.79865	2.1
58493	1.89226	2.1
58494	1.89226	2.1
58495	1.89226	2.1
58496	1.89226	2.1
58497	1.89226	2.1
58498	1.89226	2.1
58499	1.89226	0.997948
58500	1.89226	0.771206
58501	1.89226	0.829451
58502	1.89226	0.815034
58503	1.89226	1.01316
58504	1.89226	0.83833
58505	0.790736	0.823687
58506	1.08265	0.790535
58507	0.938909	1.02604
58508	0.638683	0.882089
58509	0.858588	0.928495
58510	0.858588	0.797457
58511	0.862743	1.02249
58512	0.862743	0.857576
58513	1.08265	0.945562
58514	0.938911	0.807415
58515	0.790735	0.923749
58516	0.638685	0.812817
58517	1.89226	0.808941
58518	1.89226	0.979739
58519	1.89226	0.87254

58520	1.89226	0.817078
58521	1.89226	1.00944
58522	1.89226	0.892008
58523	1.89226	0.786568
58524	1.89226	0.951121
58525	1.89226	0.811819
58526	1.89226	0.809776
58527	1.89226	0.895925
58528	1.89226	0.913141
58529	1.08265	0.792828
58530	1.36209	0.776627
58531	1.25233	0.830873
58532	0.938909	0.863705
58533	1.14195	0.808308
58534	1.14195	0.774865
58535	1.15904	0.960108
58536	1.15904	0.817639
58537	1.36209	0.862997
58538	1.25233	0.762431
58539	1.08265	0.871171
58540	0.938911	0.793503
58541	1.22077	0.800747
58542	0.915244	0.815324
58543	0.967835	0.969144
58544	0.742495	0.938137
58545	0.78648	0.93397
58546	0.985041	0.820759
58547	0.989098	1.07176
58548	0.989098	0.841711
58549	1.03206	0.870098
58550	0.967897	0.965772
58551	0.915404	0.92046
58552	0.706105	0.860047
58553	2.69662	0.219064
58554	1.89226	0.238054
58555	2.35499	0.204685
58556	1.89226	0.185881
58557	4.24725	0.21867
58558	1.89227	0.223245
58559	1.89227	0.227812
58560	1.89227	0.194903
58561	1.89227	0.202787
58562	3.78453	0.216739
58563	2.35499	0.202718
58564	1.70302	0.172265
58565	1.70302	0.17981
58566	1.703	0.183382
58567	1.97374	0.182529
58568	1.97372	0.186801
58569	0.946133	0.194302
58570	0.946133	0.177922
58571	0.946132	0.184714
58572	0.946132	0.201398
58573	0.946133	0.188583
58574	0.946133	0.187732
58575	0.946132	0.188272
58576	0.946132	0.187816
58577	0.946133	0.173688

58578	0.946133	0.18225
58579	0.946132	0.192177
58580	0.946132	0.196929
58581	0.6794	2.1
58582	0.6794	2.1
58583	0.670812	2.1
58584	0.62182	2.1
58585	0.648326	2.1
58586	0.792154	2.1
58587	0.719167	2.1
58588	0.494515	2.1
58589	0.649421	2.1
58590	0.804869	2.1
58591	0.485585	2.1
58592	0.627666	2.1
58593	1.79347	2.1
58594	1.03968	1.77136
58595	1.70303	1.96206
58596	0.933265	1.63549
58597	2.62068	1.49279
58598	1.89226	2.1
58599	1.89226	2.1
58600	1.89226	2.1
58601	1.89226	1.33586
58602	3.78452	2.1
58603	1.89226	1.52337
58604	2.24604	2.1
58605	2.0554	2.08861
58606	2.0554	1.8824
58607	1.89226	2.1
58608	1.89226	2.03222
58609	1.89226	2.1
58610	1.89226	0.927022
58611	1.89226	1.00923
58612	1.89226	1.93701
58613	1.89226	1.021
58614	1.89226	1.98213
58615	2.0815	2.03307
58616	2.64424	0.649541
58617	2.80807	0.897669
58618	3.232	1.46948
58619	1.89417	0.90756
58620	2.18012	1.54916
58621	0.946131	2.03684
58622	0.946131	0.903621
58623	0.946131	2.0965
58624	0.946131	1.05964
58625	0.94613	1.01802
58626	0.94613	0.926632
58627	0.946132	2.06018
58628	0.946132	2.1
58629	0.946131	1.22371
58630	0.946131	1.31791
58631	0.946131	1.18763
58632	0.946131	1.20215
58633	1.89227	0.317615
58634	1.89227	0.393054
58635	1.89227	0.43954

58636	1.89227	0.306121
58637	3.78453	0.330162
58638	2.70881	0.306411
58639	2.68687	0.211239
58640	2.03046	0.234596
58641	1.9682	0.285971
58642	4.67701	0.273338
58643	1.08518	0.204121
58644	1.08518	0.219662
58645	1.09033	0.287542
58646	1.09033	0.25347
58647	0.842273	0.207573
58648	1.19141	0.240014
58649	1.34343	0.219997
58650	0.9841	0.276432
58651	0.9841	0.244078
58652	0.842273	0.25475
58653	1.34344	0.252833
58654	1.19141	0.242173
58655	0.946133	0.301157
58656	0.946133	0.273729
58657	0.946132	0.418952
58658	0.946132	0.354211
58659	0.946132	0.330143
58660	0.946132	0.273068
58661	0.946132	0.417877
58662	0.946132	0.34383
58663	0.946132	0.291515
58664	0.946132	0.336069
58665	0.946133	0.364735
58666	0.946133	0.336254
58667	1.89227	0.416248
58668	1.89227	0.278685
58669	1.89227	0.378464
58670	1.89227	0.316109
58671	1.89227	0.373359
58672	1.89227	0.308172
58673	2.03046	0.237992
58674	1.9682	0.262396
58675	1.23886	0.310266
58676	1.12971	0.224618
58677	3.20706	0.237593
58678	1.89226	0.763707
58679	1.89226	0.946201
58680	1.89226	0.747662
58681	1.89226	0.840465
58682	3.78452	0.802752
58683	2.45569	0.698938
58684	1.89226	0.725415
58685	2.22922	0.692928
58686	2.0815	0.763609
58687	1.85074	0.616235
58688	1.70303	0.741265
58689	2.72856	0.698934
58690	1.89226	0.789882
58691	1.89226	0.677166
58692	1.89226	0.74328
58693	3.78452	0.730366

58694	1.89226	0.763973
58695	1.89226	0.852245
58696	1.89226	0.729916
58697	1.89226	0.850239
58698	3.78452	0.788544
58699	0.946131	0.852408
58700	0.946131	0.727434
58701	0.946131	0.798535
58702	0.946131	0.717617
58703	0.94613	0.781057
58704	0.94613	0.813203
58705	0.946132	0.728632
58706	0.946132	0.736054
58707	0.946131	0.752502
58708	0.946131	0.755094
58709	0.946131	0.768448
58710	0.946131	0.770859
58711	2.0595	0.398751
58712	2.58581	0.453352
58713	1.48694	0.322938
58714	1.48694	0.338161
58715	2.60157	0.374392
58716	2.02901	0.360066
58717	1.89226	0.336838
58718	1.89226	0.40995
58719	1.89226	0.300857
58720	1.89226	0.309503
58721	1.89226	0.388882
58722	1.89226	0.400193
58723	2.04728	0.406421
58724	2.64161	0.414936
58725	1.47606	0.323858
58726	1.47606	0.352041
58727	2.63069	0.35919
58728	2.05947	0.354156
58729	1.89224	0.383675
58730	1.89224	0.363953
58731	1.89224	0.398836
58732	1.89224	0.37961
58733	1.89224	0.338591
58734	1.89224	0.326738
58735	0.946114	0.353633
58736	0.946114	0.350671
58737	0.946118	0.408964
58738	0.946118	0.330432
58739	0.946114	0.418924
58740	0.946114	0.375435
58741	0.946118	0.385855
58742	0.946118	0.362434
58743	0.946128	0.420806
58744	0.946128	0.432857
58745	0.946103	0.344957
58746	0.946103	0.346834
58747	1.1233	1.89554
58748	1.15009	2.1
58749	2.36036	2.04969
58750	2.38225	2.1
58751	3.50554	2.1

58752	2.38224	2.1
58753	0.977775	2.1
58754	2.20473	2.1
58755	2.20473	2.1
58756	1.15009	2.1
58757	1.15009	2.1
58758	0.946119	1.68874
58759	0.946119	1.68753
58760	1.19111	1.86726
58761	1.19111	1.74999
58762	0.946118	1.80889
58763	1.19111	1.89844
58764	1.18016	1.64441
58765	0.946118	1.19072
58766	0.94613	0.900144
58767	0.94613	1.63188
58768	1.18015	1.62144
58769	1.19109	1.66476
58770	0.946118	2.1
58771	0.946118	2.02718
58772	1.10235	2.1
58773	1.10235	2.1
58774	0.946118	2.1
58775	1.10235	2.1
58776	0.946115	1.88297
58777	1.1911	1.97927
58778	0.946129	1.6485
58779	0.946129	2.1
58780	1.19109	2.08377
58781	1.10234	2.1
58782	1.89226	2.1
58783	2.23932	2.1
58784	2.11568	2.1
58785	2.23932	2.1
58786	1.76862	2.1
58787	1.89226	2.1
58788	1.89226	2.1
58789	1.89226	2.1
58790	1.89226	2.1
58791	1.89226	2.1
58792	1.89226	2.1
58793	1.89226	1.5971
58794	2.07838	2.1
58795	1.89226	2.1
58796	1.89226	2.1
58797	1.89226	2.1
58798	2.10264	2.1
58799	2.10264	2.1
58800	1.89226	2.1
58801	2.11568	2.1
58802	1.99753	2.1
58803	2.11568	2.1
58804	1.77411	2.1
58805	1.89226	2.1
58806	0.946128	1.26411
58807	0.946128	2.1
58808	0.946133	2.1
58809	0.946133	2.1

58810	0.94613	2.1
58811	0.94613	1.22025
58812	0.946131	2.1
58813	0.946131	2.1
58814	0.946131	2.1
58815	0.946131	2.1
58816	0.946131	2.1
58817	0.946131	2.1
58818	0.946132	2.1
58819	0.946132	2.1
58820	1.03919	2.1
58821	1.03919	2.1
58822	0.946131	2.1
58823	0.946131	2.09469
58824	1.02744	2.1
58825	1.03919	2.1
58826	1.02744	2.1
58827	0.946132	2.1
58828	1.03919	2.1
58829	0.946132	2.1
58830	1.64834	1.5459
58831	1.82955	1.29452
58832	2.33824	1.11807
58833	2.19301	1.48021
58834	3.98658	1.27307
58835	1.40574	1.60087
58836	2.19301	1.20874
58837	1.64834	1.40932
58838	1.64834	1.34302
58839	1.99961	1.19848
58840	1.99961	1.21167
58841	2.33824	1.15121
58842	2.19301	1.43795
58843	2.68524	1.33783
58844	2.81847	1.17202
58845	5.02349	1.32292
58846	1.99961	1.51301
58847	2.19301	1.13362
58848	2.68524	1.22222
58849	2.50732	1.26959
58850	4.68485	1.21838
58851	1.89226	2.1
58852	1.89226	1.41041
58853	1.89226	1.35127
58854	1.89226	2.1
58855	3.78453	1.77497
58856	1.75142	2.1
58857	2.47026	2.1
58858	1.46811	2.1
58859	2.26652	2.1
58860	4.01794	2.1
58861	1.89226	1.50129
58862	1.89226	2.1
58863	1.89226	1.84693
58864	1.89226	2.1
58865	1.89226	1.75914
58866	1.89226	2.1
58867	1.89226	1.58616

58868	1.89226	2.1
58869	1.89226	1.80764
58870	1.89226	2.1
58871	1.89226	1.61858
58872	1.89226	2.1
58873	0.812082	2.1
58874	0.878759	2.1
58875	0.882782	2.1
58876	0.882782	2.1
58877	1.05572	2.1
58878	1.04037	2.1
58879	0.73849	2.1
58880	0.626857	2.1
58881	0.638677	2.1
58882	1.05581	2.1
58883	0.715695	2.1
58884	1.10492	2.1
58885	1.17058	2.1
58886	1.07863	2.1
58887	1.21113	2.1
58888	1.21113	2.1
58889	1.2811	2.1
58890	1.37319	2.1
58891	1.01788	2.1
58892	0.999492	2.1
58893	1.04352	2.1
58894	1.32112	2.1
58895	1.10492	2.1
58896	1.34699	2.1
58897	1.89226	1.70024
58898	1.89226	2.1
58899	1.89226	2.00637
58900	1.89226	2.1
58901	1.89226	1.88793
58902	1.89226	2.1
58903	1.19813	0.147815
58904	1.19813	0.147815
58905	2.43432	0.147815
58906	1.21716	0.147815
58907	0.946124	0.147815
58908	1.21715	0.147815
58909	0.946118	0.147815
58910	2.56627	2.1
58911	1.89226	2.1
58912	1.89226	2.1
58913	1.89226	2.1
58914	2.43434	2.1
58915	2.43434	2.1
58916	1.89227	0.264204
58917	1.89227	0.292558
58918	1.89227	0.285852
58919	1.89227	0.273184
58920	1.89227	0.278616
58921	1.89227	0.265298
58922	1.89226	0.292074
58923	1.89226	0.355218
58924	1.89226	0.354525
58925	1.89226	0.264957

58926	3.78453	0.310323
58927	1.89226	0.252631
58928	2.0764	0.215644
58929	1.89226	0.240927
58930	2.81674	0.235178
58931	1.89226	0.229774
58932	2.81674	0.218818
58933	0.946132	0.272909
58934	0.946132	0.252907
58935	0.946131	0.34192
58936	0.946131	0.297571
58937	0.946132	0.269107
58938	0.946132	0.285871
58939	0.946132	0.350626
58940	0.946132	0.269234
58941	0.946132	0.28335
58942	0.946132	0.295773
58943	0.946132	0.294836
58944	0.946132	0.28501
58945	0.578684	1.27537
58946	0.578684	1.9354
58947	0.589487	1.36759
58948	0.589487	2.09759
58949	0.625461	1.7311
58950	0.5378	1.75559
58951	0.608714	2.01061
58952	0.54271	1.42151
58953	0.608714	1.35602
58954	0.625461	1.16809
58955	0.54271	1.62404
58956	0.537801	1.6027
58957	0.946128	1.07746
58958	0.946128	1.60984
58959	0.946133	1.094
58960	0.946133	1.95638
58961	0.946131	1.25606
58962	0.946131	1.06566
58963	0.946131	1.06463
58964	0.946131	1.28235
58965	0.94613	1.2317
58966	0.94613	1.05141
58967	0.946131	1.28306
58968	0.946131	1.37898
58969	1.21743	1.58101
58970	1.25092	1.88114
58971	1.38847	1.12364
58972	1.46031	1.15125
58973	2.67774	1.44092
58974	0.946128	1.02785
58975	0.946128	1.64845
58976	0.946132	1.67815
58977	0.946132	1.17793
58978	0.94613	1.36754
58979	0.94613	1.05135
58980	0.94613	1.24233
58981	0.94613	1.11202
58982	0.94613	1.08207
58983	0.94613	1.03252

58984	0.94613	1.38844
58985	0.94613	1.18739
58986	1.89226	1.00406
58987	1.89226	1.21295
58988	1.89226	0.897848
58989	1.89226	1.8271
58990	1.89226	1.80487
58991	1.89226	1.52117
58992	0.539566	1.03015
58993	0.534046	1.99135
58994	0.577914	1.58343
58995	0.471623	1.61241
58996	1.46031	0.955706
58997	0.945865	1.13575
58998	0.770603	0.97835
58999	0.737201	1.74492
59000	0.714829	1.16173
59001	0.536085	1.40945
59002	0.471617	1.36824
59003	1.89226	1.78288
59004	1.89226	1.07148
59005	1.89226	1.06881
59006	1.89226	1.32047
59007	1.89226	1.29623
59008	1.89226	1.79013
59009	1.89225	1.40195
59010	1.89225	0.92566
59011	1.65572	0.945105
59012	1.65572	1.10778
59013	1.89225	1.075
59014	1.89225	1.44933
59015	1.89226	1.36006
59016	1.89226	1.21525
59017	1.89226	1.19267
59018	1.89226	0.824703
59019	3.78453	1.47072
59020	1.89226	1.56356
59021	1.89226	0.922885
59022	1.89226	0.947467
59023	1.89226	1.20216
59024	1.89226	1.10452
59025	1.89226	1.6445
59026	1.89225	1.24843
59027	1.89225	0.807078
59028	1.89225	0.84893
59029	1.89225	0.989424
59030	1.89225	0.912015
59031	1.89225	1.30312
59032	0.763592	0.919275
59033	0.763592	1.26072
59034	0.987417	1.04461
59035	0.987417	1.07591
59036	0.763592	0.860076
59037	0.804881	0.816766
59038	0.946126	1.06404
59039	0.946126	0.888888
59040	0.804879	0.807458
59041	0.94613	0.966497

59042	0.946129	1.14916
59043	0.76359	0.876336
59044	1.46055	2.1
59045	2.04945	2.1
59046	2.04587	2.1
59047	2.04587	2.1
59048	1.50714	2.1
59049	1.50714	2.1
59050	2.04587	2.1
59051	2.38681	2.1
59052	2.42082	2.1
59053	2.42082	2.1
59054	2.04945	2.1
59055	2.04945	1.98194
59056	0.872964	2.1
59057	0.872964	2.1
59058	0.884806	2.1
59059	0.884806	2.1
59060	1.0275	2.1
59061	1.02293	2.1
59062	0.711243	2.1
59063	0.730273	2.1
59064	0.711244	2.1
59065	1.0275	2.1
59066	0.730273	2.1
59067	1.02293	2.1
59068	2.04587	2.1
59069	2.47753	2.1
59070	2.42994	2.1
59071	2.42082	2.07074
59072	2.05499	2.1
59073	2.04587	1.71792
59074	0.946132	2.1
59075	0.946132	2.1
59076	1.13078	2.1
59077	1.07817	2.1
59078	0.946132	2.1
59079	0.946132	2.1
59080	1.07817	2.1
59081	1.07817	2.1
59082	0.946132	2.1
59083	1.07817	2.1
59084	1.13078	2.1
59085	0.946132	2.1
59086	1.89226	2.1
59087	1.89226	2.1
59088	1.89226	2.1
59089	1.89226	2.1
59090	1.89226	2.1
59091	1.89226	2.1
59092	1.89226	2.1
59093	1.89226	2.1
59094	1.89226	2.1
59095	1.89226	2.1
59096	1.89226	2.1
59097	1.89226	2.1
59098	1.89226	2.1
59099	1.89226	2.1

59100	1.8173	2.1
59101	2.66885	2.1
59102	2.7438	2.1
59103	2.7438	2.1
59104	1.89226	0.728159
59105	1.89226	0.7
59106	1.89226	0.701575
59107	1.89226	0.791463
59108	3.78453	0.710647
59109	1.89226	0.7186
59110	1.89226	0.77066
59111	1.89226	0.734375
59112	1.89226	0.855467
59113	1.89226	0.713183
59114	1.89226	0.771004
59115	1.89226	0.720852
59116	1.97385	0.809369
59117	1.7531	0.755319
59118	1.89226	1.00933
59119	1.7531	0.706145
59120	1.89226	0.764052
59121	0.946131	0.853607
59122	0.840904	0.710435
59123	0.684333	0.748545
59124	0.94613	0.74424
59125	0.972337	0.767889
59126	0.946278	1.25976
59127	0.684304	0.700329
59128	0.684304	0.778252
59129	0.999838	0.717318
59130	0.759605	1.22893
59131	0.851528	0.700011
59132	0.851528	0.873668
59133	1.89226	0.700066
59134	2.23818	0.858781
59135	1.79771	0.725466
59136	1.89226	0.919807
59137	1.79771	0.7
59138	1.89226	0.732356
59139	2.79647	2.1
59140	2.01613	2.1
59141	1.46826	2.1
59142	2.54504	2.1
59143	4.56117	2.1
59144	1.89226	2.1
59145	1.89226	2.1
59146	1.89226	2.1
59147	1.89226	2.1
59148	1.89226	2.1
59149	1.89226	2.1
59150	1.89226	2.1
59151	2.66884	2.1
59152	2.56022	2.1
59153	2.66884	2.1
59154	1.78364	2.1
59155	1.89226	2.1
59156	1.14282	2.1
59157	1.24068	2.1

59158	0.880748	2.1
59159	0.834916	2.1
59160	1.05539	2.1
59161	1.05539	2.1
59162	1.04312	2.1
59163	1.04313	2.1
59164	1.2015	2.1
59165	0.83492	2.1
59166	0.789149	2.1
59167	1.24066	2.1
59168	1.89226	2.1
59169	1.89226	2.1
59170	1.89226	2.1
59171	1.89226	2.1
59172	3.78453	2.1
59173	0.946128	2.1
59174	0.946128	2.1
59175	1.28011	2.1
59176	1.22372	2.1
59177	0.946131	2.1
59178	0.946131	2.1
59179	1.22372	2.1
59180	1.22372	2.1
59181	1.22372	2.1
59182	0.94613	2.1
59183	0.946131	2.1
59184	1.28011	2.1
59185	2.28604	2.1
59186	1.34268	2.1
59187	2.43157	2.1
59188	1.50172	2.1
59189	3.77425	2.1
59190	2.6096	2.1
59191	2.43157	2.1
59192	1.66774	2.1
59193	1.66774	2.1
59194	1.50172	2.1
59195	2.43157	2.1
59196	2.16067	2.1
59197	1.08975	2.1
59198	2.19336	2.1
59199	2.06984	2.1
59200	1.21327	2.1
59201	1.08975	2.1
59202	2.28604	2.1
59203	1.21327	2.1
59204	2.29008	2.1
59205	2.16067	2.1
59206	1.34268	2.1
59207	1.21327	2.1
59208	1.89226	0.672784
59209	1.89226	0.624899
59210	1.89226	0.712569
59211	1.89226	0.682611
59212	3.78452	0.631161
59213	1.89804	0.618621
59214	2.21656	0.466112
59215	1.70537	0.667176

59216	2.50023	0.592488
59217	3.8982	0.591999
59218	1.03136	0.558146
59219	1.89486	0.508025
59220	0.949364	0.577321
59221	1.82283	0.497894
59222	2.84422	0.553092
59223	1.89226	0.553588
59224	1.89226	0.563688
59225	1.89226	0.641295
59226	1.89226	0.615822
59227	3.78452	0.622095
59228	0.946132	0.714221
59229	0.946132	0.71098
59230	0.946129	0.70665
59231	0.946129	0.696757
59232	0.94613	0.699771
59233	0.94613	0.737815
59234	0.946131	0.696035
59235	0.946131	0.713194
59236	0.946131	0.71761
59237	0.946131	0.71577
59238	0.946131	0.637175
59239	0.946131	0.738416
59240	1.09466	0.687265
59241	1.09466	0.593708
59242	0.866512	0.696012
59243	1.08352	0.612571
59244	1.19189	0.630105
59245	1.21883	0.66634
59246	0.971139	0.674007
59247	0.873213	0.634786
59248	0.873202	0.640675
59249	1.00855	0.673439
59250	0.923735	0.646621
59251	1.21885	0.623622
59252	1.89225	0.147815
59253	2.40019	0.147815
59254	1.89225	0.263967
59255	1.89225	0.275934
59256	1.89225	0.25691
59257	1.89225	0.359765
59258	1.89225	0.263967
59259	1.89225	0.325335
59260	2.05088	0.258368
59261	1.89225	0.210237
59262	1.89225	0.228089
59263	1.89225	0.294554
59264	2.03956	0.258368
59265	2.03956	0.275934
59266	1.89226	2.1
59267	2.40021	1.25449
59268	2.38473	0.836989
59269	2.40021	1.90263
59270	1.87679	0.892175
59271	1.89226	1.52135
59272	1.89226	1.98401
59273	1.89226	0.664288

59274	1.89226	1.60309
59275	1.89226	0.691087
59276	1.89226	1.02119
59277	1.89226	0.562586
59278	2.0692	1.50975
59279	1.89226	0.845129
59280	1.89226	2.01598
59281	1.89226	0.722921
59282	2.05089	1.9995
59283	2.05089	0.648841
59284	1.89226	1.58901
59285	1.89226	2.1
59286	1.89226	1.71144
59287	1.89226	2.1
59288	3.78452	2.07894
59289	1.89226	0.933772
59290	2.50051	2.1
59291	1.89226	1.65709
59292	2.30076	2.1
59293	4.39277	2.1
59294	2.50052	2.1
59295	1.70377	2.1
59296	2.30076	2.1
59297	1.54231	2.1
59298	4.04283	2.1
59299	1.89226	2.1
59300	1.89226	2.1
59301	1.89226	2.1
59302	1.89226	2.1
59303	1.89226	2.1
59304	1.89226	2.1
59305	1.89226	0.981724
59306	1.89226	2.1
59307	1.89226	1.34536
59308	1.89226	2.1
59309	3.78452	2.1
59310	2.07948	2.1
59311	2.30076	2.1
59312	1.3294	2.1
59313	1.3294	2.1
59314	1.54232	2.1
59315	2.29239	2.1
59316	0.810947	2.1
59317	0.810947	2.1
59318	0.810946	2.1
59319	0.810946	2.1
59320	1.89226	2.1
59321	1.35169	2.1
59322	1.08132	2.1
59323	1.08132	2.1
59324	1.08132	2.1
59325	0.810946	2.1
59326	0.810946	2.1
59327	1.34851	1.21089
59328	0.997471	1.87583
59329	1.62287	0.95124
59330	1.27096	1.38373
59331	2.62034	1.16752

59332	1.89226	1.0926
59333	1.89226	1.87983
59334	1.89226	1.04298
59335	1.89226	1.69825
59336	3.78452	1.27749
59337	0.946128	2.1
59338	0.946128	1.55159
59339	1.29331	0.970264
59340	1.29331	1.75094
59341	1.29331	1.38086
59342	1.10699	2.1
59343	0.946131	0.955304
59344	0.946131	1.27785
59345	0.94613	1.51107
59346	1.29331	1.4895
59347	1.10699	1.3554
59348	0.946131	1.32244
59349	1.62287	1.09935
59350	1.27096	1.60459
59351	1.86067	0.959917
59352	1.58407	1.45887
59353	3.20694	1.43524
59354	1.89226	1.24295
59355	1.89226	1.6516
59356	1.89226	0.965388
59357	1.89226	1.4618
59358	3.78452	1.17329
59359	2.62761	0.727837
59360	2.55849	0.878363
59361	2.12261	1.08832
59362	2.22939	0.830229
59363	4.78788	0.863745
59364	0.772522	1.35021
59365	0.839588	1.21262
59366	1.31126	0.846607
59367	1.08787	0.821514
59368	1.01318	0.905981
59369	1.01318	1.42143
59370	0.900815	1.63179
59371	1.10799	0.755072
59372	0.772511	1.02447
59373	1.03473	1.04399
59374	1.31126	0.99675
59375	0.841464	1.05157
59376	1.89227	0.884708
59377	1.89227	1.38604
59378	1.89227	0.924546
59379	1.89227	1.2097
59380	1.89227	0.92468
59381	1.89227	1.30134
59382	1.89226	0.833982
59383	1.89226	0.924175
59384	1.89226	0.80172
59385	1.89226	0.886649
59386	1.89226	0.804868
59387	1.89226	0.916305
59388	2.12261	0.938598
59389	2.22939	0.762338

59390	1.97004	0.818245
59391	1.8072	1.02553
59392	4.03658	0.846014
59393	1.5847	1.39043
59394	1.8072	0.921182
59395	2.12262	1.0107
59396	2.12262	1.37833
59397	1.23267	0.955467
59398	1.23267	1.38011
59399	1.89227	0.923583
59400	1.89227	1.64119
59401	1.89227	0.951061
59402	1.89227	1.32384
59403	1.89227	0.950801
59404	1.89227	1.43623
59405	1.89226	0.766771
59406	1.89226	0.936292
59407	1.89226	1.03066
59408	1.89226	0.807959
59409	3.78452	0.865519
59410	2.49093	2.1
59411	2.67393	2.1
59412	2.47932	2.1
59413	2.47932	2.1
59414	2.67994	2.1
59415	2.67994	2.1
59416	2.49873	2.1
59417	2.67393	2.1
59418	2.67993	2.1
59419	2.69807	2.1
59420	2.49873	2.1
59421	2.51686	2.1
59422	2.49093	2.1
59423	2.67994	2.1
59424	2.69784	2.1
59425	2.51651	2.1
59426	5.18877	2.1
59427	1.24359	2.1
59428	1.24936	2.1
59429	1.33997	2.1
59430	1.34892	2.1
59431	1.28805	2.1
59432	1.28805	2.1
59433	1.2955	2.1
59434	1.2955	2.1
59435	1.34892	2.1
59436	1.24359	2.1
59437	1.24936	2.1
59438	1.33997	2.1
59439	1.15506	1.6214
59440	1.15506	2.1
59441	0.946128	2.1
59442	0.946128	2.09091
59443	0.946131	2.1
59444	1.15506	1.67267
59445	1.06997	2.04648
59446	0.946128	2.1
59447	1.06997	1.86561

59448	1.15506	1.95356
59449	0.946129	1.98411
59450	0.946129	2.02965
59451	1.70608	2.1
59452	2.13994	2.1
59453	1.33384	2.1
59454	1.43635	2.1
59455	3.14243	2.1
59456	2.13994	2.1
59457	2.31012	2.1
59458	1.43635	2.1
59459	1.23007	2.084
59460	3.74646	2.1
59461	2.46361	2.1
59462	1.89226	1.92657
59463	1.89226	2.1
59464	1.89226	1.89398
59465	2.39045	2.1
59466	2.39045	2.08232
59467	1.89226	2.1
59468	1.89226	1.47238
59469	1.89226	1.82861
59470	1.89226	1.12095
59471	3.78452	1.70822
59472	1.52984	2.09435
59473	2.25682	1.07055
59474	2.20831	1.0511
59475	2.20831	2.06453
59476	1.61178	0.980741
59477	1.61178	1.94509
59478	2.47395	2.1
59479	1.89226	2.1
59480	1.89226	2.1
59481	1.89226	1.99111
59482	2.46362	2.1
59483	2.46362	2.00657
59484	0.946131	0.905813
59485	0.946131	2.1
59486	0.946131	1.94387
59487	0.946131	1.41307
59488	0.94613	1.22028
59489	0.94613	1.46731
59490	0.946132	2.1
59491	0.946132	1.76033
59492	0.946131	1.32361
59493	0.946131	1.45585
59494	0.946131	1.78373
59495	0.946131	1.94499
59496	0.982398	2.07259
59497	0.982398	1.51491
59498	0.931703	1.88765
59499	0.97549	1.70538
59500	1.11509	1.59444
59501	1.10017	1.18051
59502	0.774576	2.09988
59503	0.789859	2.08715
59504	0.716502	1.60514
59505	1.12793	1.71944

59506	1.14398	1.69594
59507	0.817011	1.36619
59508	1.82499	2.1
59509	1.50524	2.1
59510	1.21304	2.1
59511	1.54191	2.1
59512	3.04715	2.1
59513	1.54191	2.1
59514	1.49823	2.1
59515	1.82499	2.1
59516	1.82499	2.1
59517	1.21147	2.1
59518	1.48977	2.1
59519	1.89226	2.1
59520	1.89226	2.1
59521	1.89226	2.1
59522	1.89226	2.1
59523	3.78452	2.1
59524	1.89226	2.1
59525	1.89226	2.1
59526	1.89226	2.1
59527	1.89226	2.1
59528	1.89226	2.1
59529	1.89226	2.1
59530	0.566674	2.1
59531	0.770957	2.1
59532	0.60652	2.1
59533	0.473557	2.1
59534	0.604441	2.1
59535	0.604441	2.1
59536	0.640073	2.1
59537	0.640073	2.1
59538	0.473557	2.1
59539	0.566674	2.1
59540	0.60652	2.1
59541	0.770956	2.1
59542	1.36203	2.1
59543	1.13335	2.1
59544	1.54191	2.1
59545	1.54191	2.1
59546	1.00965	2.1
59547	1.00965	2.1
59548	1.89226	2.1
59549	1.89226	2.1
59550	1.89226	2.1
59551	1.89226	2.1
59552	1.89226	2.1
59553	1.89226	2.1
59554	0.946131	2.1
59555	0.946131	2.1
59556	0.946131	2.1
59557	0.946131	2.1
59558	0.946132	2.1
59559	0.946132	2.1
59560	0.946131	2.1
59561	0.946131	2.1
59562	0.946132	2.1
59563	0.946132	2.1

59564	0.946131	2.1
59565	0.946131	2.1
59566	0.946132	0.231493
59567	0.946132	0.249459
59568	0.946133	0.218076
59569	0.946133	0.241183
59570	0.946132	0.223629
59571	0.946132	0.232241
59572	0.946132	0.226249
59573	0.946132	0.251636
59574	0.946132	0.225917
59575	0.946132	0.225494
59576	0.946133	0.248008
59577	0.946133	0.242539
59578	1.89226	0.277142
59579	1.89226	0.265258
59580	1.89226	0.24343
59581	1.89226	0.230172
59582	3.78453	0.233181
59583	1.89226	0.261728
59584	1.89226	0.221774
59585	1.89226	0.228464
59586	1.89226	0.247652
59587	1.89226	0.235366
59588	1.89226	0.263172
59589	0.946132	0.210083
59590	0.946132	0.222729
59591	0.946131	0.199533
59592	0.946131	0.214444
59593	0.946132	0.207289
59594	0.946132	0.212395
59595	0.946132	0.207459
59596	0.946132	0.226353
59597	0.946132	0.204676
59598	0.946132	0.207916
59599	0.946132	0.235816
59600	0.946132	0.220904
59601	0.946129	0.206846
59602	0.946129	0.219974
59603	0.946128	0.195287
59604	0.946128	0.206905
59605	0.946132	0.200067
59606	0.946132	0.206821
59607	0.946126	0.21198
59608	0.946126	0.21623
59609	0.946129	0.200621
59610	0.946129	0.202177
59611	0.946129	0.214707
59612	0.946129	0.227875
59613	1.20674	0.298843
59614	1.79877	0.351287
59615	1.22586	0.320342
59616	1.22586	0.300184
59617	1.7919	0.334275
59618	1.7919	0.316608
59619	2.27622	0.293309
59620	1.79877	0.354521
59621	1.7919	0.293072

59622	2.27102	0.353665
59623	4.06979	0.308191
59624	1.89226	0.355083
59625	1.89226	0.479589
59626	1.89226	0.35505
59627	1.89226	0.455017
59628	3.78452	0.43912
59629	1.89226	0.351779
59630	1.89226	0.417185
59631	1.89226	0.336067
59632	1.89226	0.454426
59633	3.78453	0.361703
59634	1.13259	0.291914
59635	1.79187	0.337892
59636	1.32741	0.306778
59637	1.18784	0.298874
59638	1.703	0.321083
59639	1.49575	0.311416
59640	1.46201	0.287776
59641	2.27099	0.35874
59642	1.79187	0.315204
59643	1.79187	0.295448
59644	2.12923	0.337184
59645	2.12923	0.318921
59646	1.89224	0.358609
59647	1.89224	0.446678
59648	1.89224	0.34133
59649	1.89224	0.453474
59650	3.78447	0.369921
59651	0.946119	0.46147
59652	0.946119	0.369937
59653	0.946118	0.418632
59654	0.946118	0.337403
59655	0.946118	0.42098
59656	0.946118	0.44901
59657	0.946119	0.33945
59658	0.946119	0.320296
59659	0.946131	0.375978
59660	0.946131	0.352556
59661	0.946106	0.383044
59662	0.946106	0.395888
59663	2.07526	0.741048
59664	1.89226	0.625672
59665	1.89226	0.843553
59666	2.13445	0.83789
59667	4.02671	0.685803
59668	1.89226	0.622175
59669	1.89226	0.698853
59670	1.89226	0.767294
59671	1.89226	0.452062
59672	1.89226	0.861836
59673	1.89226	0.590214
59674	2.13446	0.793911
59675	1.00013	0.834725
59676	1.1475	0.99675
59677	2.20046	0.838303
59678	3.20059	0.895378
59679	0.946133	0.707272

59680	0.946133	0.767733
59681	1.06722	0.749128
59682	1.10023	0.669917
59683	0.946131	0.714074
59684	0.946131	0.520755
59685	1.10023	0.803981
59686	1.10023	0.699482
59687	0.946131	0.785693
59688	1.10023	0.666939
59689	0.94613	0.728625
59690	1.06723	0.652224
59691	1.89226	0.554512
59692	1.89226	0.557605
59693	1.89226	0.746791
59694	1.89226	0.624771
59695	3.78452	0.653852
59696	1.89226	0.838908
59697	1.89226	0.760513
59698	1.89226	0.874606
59699	1.89226	0.776988
59700	1.89226	0.796915
59701	1.89226	0.685726
59702	1.89226	2.1
59703	1.89226	0.552788
59704	1.89226	0.858078
59705	1.89226	0.567667
59706	1.89226	2.1
59707	0.828588	0.147815
59708	0.828588	0.147815
59709	0.841316	0.147815
59710	0.841316	1.81369
59711	0.528387	2.1
59712	0.565893	0.147815
59713	1.10401	0.147815
59714	1.10401	1.81369
59715	0.528387	2.1
59716	1.89226	2.1
59717	1.36047	0.858078
59718	1.33973	2.1
59719	1.33973	2.1
59720	1.89226	0.147815
59721	2.28951	0.597513
59722	1.89226	1.11962
59723	2.80796	1.73625
59724	2.01584	1.546
59725	2.01584	1.2396
59726	1.89226	1.46025
59727	1.89226	1.2167
59728	1.89226	0.933854
59729	1.89226	1.32157
59730	1.89226	1.16466
59731	1.89226	0.993603
59732	1.89226	1.16374
59733	1.89226	0.996947
59734	2.15223	1.01037
59735	1.30664	1.68295
59736	1.96011	1.49092
59737	2.76516	1.08489

59738	4.11234	1.46059
59739	1.89226	0.893287
59740	1.89226	1.23688
59741	1.89226	1.06755
59742	1.89226	0.951115
59743	1.89226	1.0727
59744	1.89226	0.961243
59745	2.81143	2.1
59746	2.58517	2.1
59747	2.55318	2.1
59748	2.7542	2.1
59749	5.33937	2.1
59750	2.7777	2.1
59751	2.7542	2.1
59752	2.71672	2.1
59753	2.70401	2.1
59754	5.45821	2.1
59755	2.55318	2.1
59756	2.7542	2.1
59757	2.71672	2.1
59758	2.54198	2.1
59759	5.29618	2.1
59760	1.95416	2.1
59761	2.162 2.1	
59762	2.11563	2.1
59763	1.91813	2.1
59764	4.08013	2.1
59765	1.95417	2.1
59766	1.47712	2.1
59767	1.45182	2.1
59768	1.91813	2.1
59769	3.39525	2.1
59770	2.11562	2.1
59771	1.91813	2.1
59772	1.50979	2.1
59773	2.03974	2.1
59774	3.95787	2.1
59775	1.85605	2.1
59776	1.91813	2.1
59777	1.88885	2.1
59778	1.42255	2.1
59779	1.45182	2.1
59780	1.45182	2.1
59781	0.94613	0.844369
59782	0.94613	0.711125
59783	0.946128	0.540674
59784	0.946128	0.826262
59785	0.946127	0.809974
59786	0.946127	0.824273
59787	0.946131	0.705051
59788	0.946131	0.599836
59789	0.946129	0.747384
59790	0.946129	0.746765
59791	0.946128	0.765472
59792	0.946128	0.766192
59793	0.543052	0.850985
59794	0.54608	0.59303
59795	0.602293	0.491905

59796	0.535325	0.677456
59797	1.05446	0.835289
59798	0.850496	0.788889
59799	0.699838	0.731391
59800	0.679751	0.826225
59801	0.713797	0.718557
59802	0.509794	0.683361
59803	0.602304	0.656342
59804	1.40017	0.826035
59805	1.40017	1.857
59806	0.94613	0.819782
59807	0.94613	1.84508
59808	1.40017	1.80941
59809	1.33997	1.63682
59810	0.946131	0.810239
59811	0.946131	0.823968
59812	1.33997	0.921329
59813	0.946131	0.92065
59814	0.946131	0.922567
59815	1.40017	0.922839
59816	1.89226	0.292508
59817	1.89226	0.353684
59818	1.89226	0.368824
59819	1.89226	0.266142
59820	3.78452	0.302048
59821	0.946133	0.246141
59822	0.946133	0.267075
59823	0.946132	0.35681
59824	0.946132	0.295746
59825	0.946132	0.26413
59826	0.946132	0.296631
59827	0.946133	0.356074
59828	0.946133	0.27246
59829	0.946132	0.280351
59830	0.946132	0.301386
59831	0.946132	0.292879
59832	0.946132	0.2909
59833	1.89224	0.374212
59834	1.89224	0.276176
59835	1.89224	0.319265
59836	1.89224	0.310944
59837	1.89224	0.335447
59838	1.89224	0.330503
59839	0.94612	0.2501
59840	0.94612	0.274226
59841	0.946119	0.345093
59842	0.946119	0.298822
59843	0.946119	0.269095
59844	0.946119	0.302803
59845	0.94612	0.285122
59846	0.94612	0.341051
59847	0.946132	0.281174
59848	0.946132	0.272676
59849	0.946107	0.268729
59850	0.946107	0.292803
59851	2.63939	2.1
59852	1.2862	2.1
59853	1.29891	2.1

59854	2.64419	2.1
59855	3.93039	2.1
59856	2.6394	2.1
59857	1.89226	2.1
59858	1.89226	2.1
59859	2.64419	2.1
59860	4.53646	2.1
59861	1.89226	2.1
59862	1.89226	2.1
59863	1.89226	2.1
59864	1.89226	1.96445
59865	3.78453	2.1
59866	1.89226	2.1
59867	1.89226	1.97574
59868	1.89226	2.1
59869	1.89226	2.09206
59870	1.89226	2.1
59871	1.89226	2.1
59872	1.31998	2.1
59873	1.32209	2.1
59874	0.649456	2.1
59875	0.648474	2.1
59876	0.984995	2.1
59877	0.984995	2.1
59878	0.985573	2.1
59879	0.985573	2.1
59880	1.31999	2.1
59881	0.648472	2.1
59882	0.649455	2.1
59883	1.32209	2.1
59884	0.946133	2.1
59885	0.946133	2.1
59886	1.31999	2.1
59887	1.3221	2.1
59888	0.946132	2.1
59889	0.946132	2.1
59890	1.31999	2.1
59891	1.31999	2.1
59892	1.31999	2.1
59893	0.946132	2.1
59894	0.946133	2.1
59895	1.3221	2.1
59896	0.946132	2.03056
59897	0.946132	2.1
59898	0.946131	2.1
59899	0.946131	2.1
59900	0.946132	1.89656
59901	0.946132	2.1
59902	0.946131	2.1
59903	0.946131	2.1
59904	0.946131	2.1
59905	0.946131	2.1
59906	0.946132	2.1
59907	0.946132	2.1
59908	0.946128	2.1
59909	0.946128	2.1
59910	0.946132	1.84375
59911	0.946132	2.1

59912	0.946131	1.86102
59913	0.946131	1.88952
59914	0.94613	2.1
59915	0.94613	2.1
59916	0.94613	2.1
59917	0.94613	2.1
59918	0.946131	2.1
59919	0.946131	2.1
59920	1.89226	0.622681
59921	1.89226	0.888772
59922	1.89226	0.846785
59923	1.89226	0.83899
59924	1.89226	0.719566
59925	1.89226	0.705457
59926	1.89226	0.784931
59927	1.89226	1.03981
59928	1.89226	0.903675
59929	1.89226	1.00662
59930	3.78453	1.05374
59931	0.946133	0.800069
59932	0.946133	0.810334
59933	0.946132	0.772143
59934	0.946132	0.747777
59935	0.946133	0.857665
59936	0.946133	0.854888
59937	0.946132	0.795552
59938	0.946132	0.685335
59939	0.946132	0.808197
59940	0.946132	0.873631
59941	0.946132	0.771425
59942	0.946132	0.920588
59943	2.23638	0.654796
59944	1.89226	0.725356
59945	2.66014	0.872802
59946	2.66014	0.898268
59947	1.89226	0.872005
59948	1.89226	0.758326
59949	1.11819	0.589542
59950	1.33007	0.588542
59951	0.660255	0.63118
59952	0.91151	0.611345
59953	0.911511	0.679412
59954	1.33007	0.646571
59955	1.11819	0.526439
59956	0.660255	0.588262
59957	0.985327	0.558998
59958	0.985327	0.654952
59959	1.005	0.584705
59960	1.005	0.650748
59961	1.89226	2.1
59962	1.89226	2.1
59963	1.89226	2.1
59964	1.89226	2.1
59965	3.78453	2.1
59966	1.59533	2.1
59967	2.68571	2.1
59968	1.28291	2.1
59969	2.46974	2.1

59970	4.06506	2.1
59971	2.68571	2.1
59972	1.89226	2.1
59973	1.89226	2.1
59974	1.89226	2.1
59975	2.46973	2.1
59976	2.46973	2.1
59977	1.89226	2.1
59978	1.89226	2.1
59979	1.89226	2.1
59980	1.89226	2.1
59981	1.89226	2.1
59982	1.89226	2.1
59983	1.89226	2.1
59984	1.89226	2.1
59985	1.89226	2.1
59986	1.89226	2.1
59987	1.89226	2.1
59988	1.89226	2.1
59989	0.883594	2.1
59990	0.883594	2.1
59991	0.876484	2.1
59992	0.876484	2.1
59993	1.10442	2.1
59994	1.23487	2.1
59995	0.641457	2.1
59996	0.52521	2.1
59997	1.10442	2.1
59998	0.52521	2.1
59999	0.641457	2.1
60000	1.23487	2.1
60001	0.946131	2.1
60002	0.946131	2.1
60003	0.946131	2.1
60004	0.946131	2.1
60005	0.946131	2.1
60006	0.946131	2.1
60007	0.946131	2.1
60008	0.946131	2.1
60009	0.946131	2.1
60010	0.946131	2.1
60011	0.946131	2.1
60012	0.946131	2.1
60013	2.04016	0.444364
60014	2.13042	0.501995
60015	2.11465	0.51919
60016	2.11465	0.562056
60017	1.95908	0.427287
60018	1.95908	0.448319
60019	1.66953	0.428421
60020	2.13041	0.50591
60021	1.95908	0.398751
60022	2.21952	0.449521
60023	1.66953	0.536221
60024	1.92997	0.572686
60025	2.04016	0.418733
60026	1.95908	0.410021
60027	1.82014	0.3348

60028	2.08757	0.357803
60029	3.86029	0.366647
60030	0.834764	0.411448
60031	0.722102	0.355511
60032	0.979537	0.397988
60033	0.910065	0.349134
60034	0.840053	0.345403
60035	0.840052	0.446171
60036	0.861587	0.354365
60037	0.861587	0.399154
60038	0.910067	0.346965
60039	0.722101	0.323981
60040	0.834763	0.436384
60041	0.979539	0.408112
60042	1.89226	0.380999
60043	1.89226	0.388912
60044	1.89226	0.415332
60045	1.89226	0.350433
60046	1.89226	0.424096
60047	1.89226	0.365551
60048	1.89226	0.393652
60049	1.89226	0.422754
60050	1.89226	0.496062
60051	1.89226	0.334349
60052	3.78453	0.408272
60053	1.89226	0.302798
60054	1.89226	0.317748
60055	1.89226	0.347105
60056	1.89226	0.290152
60057	1.89226	0.347892
60058	1.89226	0.29442
60059	1.20691	0.271922
60060	2.27379	0.270702
60061	2.58476	0.306498
60062	1.5124	0.24349
60063	3.79167	0.272859
60064	1.89226	0.414596
60065	2.2738	0.30015
60066	2.58477	0.322816
60067	1.89226	0.354014
60068	4.47703	0.350132
60069	0.952225	1.65969
60070	0.952225	1.9574
60071	0.909865	1.77092
60072	0.909865	1.99066
60073	0.909895	2.04903
60074	0.946132	1.75211
60075	0.946132	1.8364
60076	0.915958	1.65356
60077	0.946132	1.84437
60078	0.946132	1.80847
60079	0.909894	1.85349
60080	0.915958	1.94747
60081	0.983493	2.1
60082	2.40055	2.1
60083	1.02986	2.1
60084	1.02986	2.1
60085	2.34217	2.1

60086	2.34217	2.1
60087	1.89226	2.1
60088	2.40056	2.1
60089	2.34217	2.1
60090	2.40056	2.1
60091	1.83388	2.1
60092	1.89226	2.1
60093	1.89226	2.1
60094	1.89226	2.1
60095	1.89226	2.1
60096	1.89226	2.1
60097	1.89226	2.1
60098	1.89226	2.1
60099	1.89226	2.1
60100	1.89226	2.1
60101	1.89226	2.1
60102	1.89226	2.1
60103	1.89226	2.1
60104	1.89226	2.1
60105	0.946133	2.1
60106	0.946133	2.1
60107	1.17109	2.1
60108	1.13078	2.1
60109	0.946132	2.1
60110	0.946132	2.1
60111	1.13078	2.1
60112	1.13078	2.1
60113	1.13078	2.1
60114	0.946133	2.1
60115	0.946132	2.1
60116	1.17109	2.1
60117	1.89226	2.1
60118	1.89226	2.1
60119	1.89226	2.1
60120	1.89226	2.1
60121	1.89226	2.1
60122	1.89226	2.1
60123	2.33963	1.10485
60124	1.89227	0.880954
60125	1.74565	0.918674
60126	1.74565	0.985599
60127	1.89227	0.836871
60128	1.89227	0.914776
60129	1.89227	0.960399
60130	1.89227	0.88579
60131	1.89227	1.19296
60132	1.89227	0.912978
60133	3.78453	0.903513
60134	1.31398	0.846793
60135	1.10392	0.865384
60136	0.796878	0.859594
60137	0.524356	0.840548
60138	0.870614	0.835127
60139	0.945739	0.827628
60140	0.89208	0.877278
60141	0.89208	0.929045
60142	1.23425	0.833382
60143	0.774658	0.90424

60144	1.03182	0.840467
60145	0.557289	0.813802
60146	2.33963	1.03347
60147	1.89226	0.83271
60148	1.89226	1.01473
60149	2.77245	0.870596
60150	4.23189	0.955312
60151	1.89226	0.990315
60152	1.89226	0.863504
60153	1.89226	1.05543
60154	1.89226	1.034
60155	1.89226	0.863095
60156	1.89226	0.860719
60157	1.89226	0.70468
60158	1.89226	0.724592
60159	1.89226	0.686216
60160	1.89226	0.762177
60161	1.89226	0.675156
60162	1.89226	0.756927
60163	1.89226	0.681542
60164	1.89226	0.694505
60165	1.89226	0.650512
60166	1.89226	0.728733
60167	1.89226	0.629534
60168	1.89226	0.723657
60169	0.946128	0.647935
60170	0.946128	0.555778
60171	0.963918	0.620138
60172	1.37543	0.502535
60173	0.946129	0.608815
60174	0.946129	0.662523
60175	1.37542	0.536947
60176	1.37542	0.501325
60177	0.94613	0.608859
60178	1.37542	0.604875
60179	0.963916	0.614224
60180	0.94613	0.612018
60181	0.946132	0.700848
60182	0.946132	0.629375
60183	0.946131	0.659926
60184	0.946131	0.57097
60185	0.946131	0.661027
60186	0.946131	0.705262
60187	0.946132	0.616526
60188	0.946132	0.533584
60189	0.946131	0.647781
60190	0.946131	0.652497
60191	0.946131	0.63342
60192	0.946131	0.625334
60193	1.89226	0.711996
60194	1.89226	0.724558
60195	1.89226	0.654139
60196	1.89226	0.745611
60197	3.78452	0.717904
60198	0.946127	0.723561
60199	0.946127	0.655954
60200	0.946131	0.691482
60201	0.946131	0.625401

60202	0.946128	0.692606
60203	0.946128	0.719581
60204	0.94613	0.605147
60205	0.94613	0.66271
60206	0.946129	0.671091
60207	0.946129	0.67947
60208	0.946129	0.669801
60209	0.946129	0.68095
60210	1.89226	0.995984
60211	1.89226	1.08998
60212	1.89226	1.88689
60213	1.89226	0.841499
60214	3.78453	0.852
60215	1.89226	0.928621
60216	1.89226	0.470679
60217	1.89226	0.761691
60218	1.89226	0.808816
60219	3.78453	0.930313
60220	0.946131	0.674607
60221	0.946131	0.679853
60222	0.946132	1.21137
60223	0.946132	0.876751
60224	0.946132	1.07199
60225	0.946132	0.840778
60226	0.946132	1.09707
60227	0.946132	0.938989
60228	0.946132	0.962314
60229	0.946132	1.04035
60230	0.946132	1.06032
60231	0.946132	0.891936
60232	0.548169	0.54173
60233	0.736603	0.528229
60234	1.18322	0.62372
60235	1.01139	0.550737
60236	1.01139	0.52425
60237	0.548168	0.500319
60238	0.736603	0.520711
60239	1.18322	0.512078
60240	0.861589	0.537217
60241	0.861589	0.620857
60242	0.869798	0.521103
60243	0.869798	0.511629
60244	1.16037	0.629721
60245	0.942012	0.530508
60246	0.942012	0.556875
60247	0.62356	0.570573
60248	0.891932	0.568165
60249	0.900255	0.606484
60250	2.02279	0.542919
60251	1.48677	0.555978
60252	1.54724	0.538637
60253	0.831456	0.549616
60254	1.19775	0.582311
60255	1.89226	1.22402
60256	2.76291	1.33979
60257	1.89226	1.44487
60258	1.89226	1.19615
60259	3.78452	1.38223

60260	1.89226	0.991034
60261	1.89226	1.22796
60262	1.89226	1.26903
60263	1.89226	1.0504
60264	1.89226	1.26207
60265	1.89226	1.04446
60266	1.89226	1.32496
60267	1.89226	1.08155
60268	1.89226	1.16856
60269	1.89226	1.20952
60270	3.78452	1.12633
60271	1.89226	0.935564
60272	1.89226	1.21493
60273	1.89226	1.22415
60274	1.89226	0.969714
60275	3.78453	1.12823
60276	0.946128	0.986533
60277	0.946128	1.00651
60278	0.946129	1.2553
60279	0.946129	1.21144
60280	0.946132	0.970353
60281	0.946132	1.18373
60282	0.946126	1.20458
60283	0.946126	0.97899
60284	0.946129	1.11081
60285	0.946129	1.10514
60286	0.946129	1.08907
60287	0.946129	1.06483
60288	2.80495	1.26996
60289	1.89225	1.10117
60290	1.89225	1.30374
60291	1.87794	1.30168
60292	1.89225	1.15472
60293	1.87794	1.1442
60294	2.25195	0.923525
60295	2.00624	0.732655
60296	2.13132	0.869571
60297	1.90239	0.34813
60298	4.13756	0.534332
60299	0.804661	0.640226
60300	0.804663	0.475572
60301	0.889591	0.35344
60302	0.889591	0.769305
60303	0.808324	0.691285
60304	0.761765	0.750212
60305	1.02822	0.626102
60306	0.879624	0.466427
60307	0.879617	0.439262
60308	0.800864	0.494703
60309	0.978023	0.630665
60310	0.808329	0.567623
60311	2.13133	0.874742
60312	2.00558	0.305616
60313	2.09316	0.285905
60314	2.09316	0.909695
60315	2.03253	0.406158
60316	2.03253	0.925279
60317	0.84577	0.603713

60318	0.845767	0.661919
60319	0.843917	0.360951
60320	0.843917	0.776939
60321	0.76177	0.606746
60322	0.719345	0.753955
60323	0.934435	0.486173
60324	0.884197	0.511487
60325	0.934439	0.395479
60326	0.75627	0.435114
60327	0.849424	0.467415
60328	0.76177	0.631386
60329	2.61855	0.372397
60330	1.17793	0.38386
60331	1.42965	0.419269
60332	1.17793	0.399714
60333	2.50057	0.35774
60334	2.24885	0.360293
60335	0.946133	0.403094
60336	0.946133	0.390226
60337	1.30928	0.409875
60338	1.12443	0.386106
60339	0.946132	0.390595
60340	0.946132	0.368945
60341	1.30928	0.363398
60342	1.30928	0.356002
60343	0.946132	0.391038
60344	1.30928	0.377258
60345	1.12443	0.358791
60346	0.946133	0.36787
60347	1.89226	0.362796
60348	1.89226	0.436809
60349	1.89226	0.429958
60350	1.89226	0.4564
60351	3.78452	0.437584
60352	1.89226	0.464756
60353	1.89226	0.407144
60354	1.89226	0.385262
60355	1.89226	0.423561
60356	1.89226	0.394909
60357	1.89226	0.384098
60358	1.89226	0.400156
60359	1.89226	0.424676
60360	1.89226	0.413459
60361	1.89226	0.501315
60362	3.78452	0.415281
60363	0.94613	0.414275
60364	0.94613	0.442033
60365	0.946131	0.453294
60366	0.946131	0.43887
60367	0.946128	0.440842
60368	0.946128	0.431229
60369	0.946133	0.398771
60370	0.946133	0.390349
60371	0.946131	0.411381
60372	0.946131	0.472495
60373	0.946131	0.422015
60374	0.946131	0.375443
60375	1.89226	2.1

60376	1.89226	2.1
60377	1.89226	2.1
60378	1.89226	2.1
60379	3.78453	2.1
60380	1.0465	2.1
60381	2.73926	2.1
60382	2.03721	2.1
60383	2.03721	2.1
60384	1.44709	2.1
60385	1.44709	2.1
60386	0.946131	2.1
60387	0.946131	2.1
60388	0.946131	2.1
60389	0.946131	2.1
60390	0.946132	2.1
60391	0.946132	2.1
60392	0.94613	2.1
60393	0.94613	2.1
60394	0.946131	2.1
60395	0.946131	2.1
60396	0.946131	2.1
60397	0.946131	2.1
60398	0.946132	2.1
60399	0.946132	2.1
60400	0.946131	2.1
60401	0.946131	2.1
60402	0.946132	2.1
60403	0.946132	2.1
60404	0.946132	2.1
60405	0.946132	2.1
60406	0.946132	2.1
60407	0.946132	2.1
60408	0.946132	2.1
60409	0.946132	2.1
60410	1.89226	2.1
60411	1.89226	2.1
60412	1.89226	2.1
60413	1.89226	2.1
60414	3.78452	2.1
60415	1.61969	0.501279
60416	1.79374	0.384968
60417	1.29382	0.396975
60418	1.29382	0.449681
60419	2.05759	0.415945
60420	2.05759	0.466239
60421	1.89226	0.436608
60422	1.89226	0.378723
60423	1.89226	0.368377
60424	1.89226	0.426893
60425	1.89226	0.371403
60426	1.89226	0.413708
60427	1.89226	0.894101
60428	1.89226	0.915163
60429	1.89226	0.905454
60430	1.89226	0.907944
60431	1.89226	0.865884
60432	1.89226	0.869584
60433	1.89226	1.28424

60434	1.89226	0.999939
60435	1.89226	1.12729
60436	1.89226	1.17331
60437	3.78453	1.28287
60438	0.946131	0.979769
60439	0.946131	1.00129
60440	0.946131	0.914015
60441	0.946131	0.960857
60442	0.946131	0.915178
60443	0.946131	0.899278
60444	0.946131	0.986059
60445	0.946131	1.05289
60446	0.946131	0.969042
60447	0.946131	0.997097
60448	0.946131	0.964878
60449	0.946131	0.926086
60450	2.45177	0.523688
60451	2.68507	0.465891
60452	2.3376	0.570435
60453	2.22086	0.434326
60454	4.78937	0.501945
60455	2.45178	0.500928
60456	2.35552	0.402995
60457	1.68062	0.471059
60458	1.81902	0.421633
60459	4.13239	0.448935
60460	2.3376	0.503211
60461	1.55612	0.39651
60462	1.69811	0.416693
60463	2.31527	0.457574
60464	1.79553	0.40996
60465	2.3376	0.455452
60466	1.55613	0.379465
60467	1.06336	0.412593
60468	1.97516	0.421335
60469	1.88966	0.382272
60470	1.06336	0.477343
60471	1.68062	0.376361
60472	1.89226	0.983992
60473	1.91634	1.00245
60474	2.03709	1.19538
60475	1.91634	0.987078
60476	1.83185	1.00619
60477	1.70303	0.772472
60478	1.89226	0.917465
60479	1.89226	0.974613
60480	1.89226	1.13469
60481	1.89226	0.950814
60482	1.89226	0.957127
60483	1.89226	0.704665
60484	1.89226	1.32724
60485	2.03472	1.6859
60486	2.22183	2.05116
60487	2.03472	1.23149
60488	2.07936	1.61452
60489	1.89226	1.02384
60490	1.89226	0.934183
60491	1.8519	0.921308

60492	1.92119	1.05942
60493	1.8519	0.929898
60494	1.96156	0.905642
60495	1.89226	0.711497
60496	1.89226	0.872988
60497	1.89226	0.930755
60498	1.89226	1.05095
60499	1.89226	0.9162
60500	1.89226	0.889264
60501	1.89226	0.622354
60502	1.89226	1.14992
60503	2.22183	1.24526
60504	2.39541	1.69623
60505	2.22183	1.11216
60506	2.06585	1.21037
60507	1.89226	0.976202
60508	1.47001	1.89644
60509	1.57962	1.08172
60510	1.65238	1.23423
60511	1.60502	1.44918
60512	3.18464	1.38581
60513	1.02415	2.1
60514	1.60503	1.60149
60515	1.47001	1.98507
60516	1.47001	2.1
60517	1.28228	2.1
60518	1.28228	2.1
60519	0.946133	2.1
60520	0.946133	1.86595
60521	0.946132	1.46381
60522	0.946132	1.24481
60523	0.946132	1.24639
60524	0.946132	1.82045
60525	0.946133	1.64059
60526	0.946133	2.1
60527	0.946132	1.67802
60528	0.946132	1.5137
60529	0.946133	1.7604
60530	0.946133	1.81689
60531	1.89226	1.07633
60532	1.89226	1.18751
60533	1.89226	1.43932
60534	1.89226	1.00592
60535	3.78452	1.08953
60536	1.76897	1.17307
60537	1.60502	1.28303
60538	1.65238	1.08776
60539	1.65238	1.67016
60540	1.76932	1.06947
60541	1.76932	1.56196
60542	1.55998	2.1
60543	1.60503	1.35629
60544	1.76932	1.81937
60545	1.60503	1.99355
60546	1.44657	1.83956
60547	1.28228	2.00527
60548	0.946133	1.6081
60549	0.946133	1.93172

60550	0.946132	1.51585
60551	0.946132	1.15578
60552	0.946132	1.14232
60553	0.946132	1.49989
60554	0.946133	1.35531
60555	0.946133	1.96312
60556	0.946132	1.62486
60557	0.946132	1.60436
60558	0.946132	1.7467
60559	0.946132	1.62394
60560	0.946128	1.29516
60561	0.946128	1.09414
60562	0.946133	0.953839
60563	0.946133	1.02443
60564	0.94613	0.96228
60565	0.94613	1.0944
60566	0.946131	1.29679
60567	0.946131	1.06766
60568	0.946131	1.06096
60569	0.946131	1.04764
60570	0.946131	1.10207
60571	0.946131	1.09865
60572	1.85779	0.469888
60573	1.00763	0.404266
60574	1.74874	0.432327
60575	1.74873	0.415407
60576	1.14467	0.490459
60577	1.14466	0.455767
60578	1.85779	0.438688
60579	1.61597	0.392295
60580	1.00053	0.447146
60581	1.03018	0.379446
60582	2.65819	0.396258
60583	1.89226	0.781703
60584	1.89226	0.643898
60585	1.89226	0.796534
60586	1.89226	0.74766
60587	3.78451	0.770421
60588	1.89226	0.784459
60589	1.89226	0.506712
60590	1.89226	0.613639
60591	1.89226	0.655861
60592	1.89226	0.590948
60593	1.89226	0.633144
60594	1.11983	0.477762
60595	0.810945	0.509056
60596	0.913906	0.462565
60597	0.913906	0.491447
60598	2.23303	0.405767
60599	1.66057	0.418891
60600	1.28724	0.433587
60601	1.08132	0.443544
60602	1.49316	0.470337
60603	0.810958	0.484844
60604	0.810958	0.473778
60605	0.827861	0.624008
60606	0.946126	0.649223
60607	0.946132	0.750134

60608	0.946132	0.596897
60609	0.827863	0.632045
60610	0.827863	0.632115
60611	0.946129	0.671781
60612	0.946129	0.652836
60613	0.827863	0.634392
60614	0.946128	0.655255
60615	0.946129	0.589419
60616	0.946129	0.638497
60617	0.974718	0.408448
60618	1.53671	0.585591
60619	2.80542	0.420343
60620	2.27259	0.531716
60621	3.8093	0.491421
60622	1.89226	0.605162
60623	1.89226	0.722787
60624	1.89226	0.562795
60625	1.89226	0.709387
60626	3.78453	0.681462
60627	0.946132	0.593632
60628	0.946132	0.697828
60629	0.946131	0.691815
60630	0.946131	0.613185
60631	0.946132	0.693451
60632	0.946132	0.710621
60633	0.946131	0.57361
60634	0.946131	0.576792
60635	0.946132	0.664649
60636	0.946132	0.683304
60637	0.946131	0.645322
60638	0.946131	0.648832
60639	2.80542	0.481979
60640	1.89226	0.558232
60641	2.27259	0.500484
60642	2.27259	0.622281
60643	1.89226	0.393438
60644	1.89226	0.558866
60645	1.89226	0.564975
60646	1.89226	0.717523
60647	1.89226	0.577823
60648	1.89226	0.715668
60649	1.89226	0.581555
60650	1.89226	0.716285
60651	0.946132	0.717119
60652	0.946132	0.584848
60653	0.946131	0.704329
60654	0.946131	0.618365
60655	0.946132	0.706297
60656	0.946132	0.707997
60657	0.946131	0.573754
60658	0.946131	0.592408
60659	0.946131	0.650508
60660	0.946131	0.662121
60661	0.946131	0.633235
60662	0.946131	0.655769
60663	2.68213	2.1
60664	2.58234	2.1
60665	2.58234	2.1

60666	2.61243	2.1
60667	2.67035	2.1
60668	2.70044	2.1
60669	2.68213	2.1
60670	2.53921	2.1
60671	2.53303	2.1
60672	2.67035	2.1
60673	5.20956	2.1
60674	2.67033	2.1
60675	2.58232	2.1
60676	2.55004	2.1
60677	2.64567	2.1
60678	5.228	2.1
60679	2.53301	2.1
60680	2.64567	2.1
60681	2.64567	2.1
60682	2.67033	2.1
60683	2.51603	2.1
60684	2.54069	2.1
60685	0.907497	2.1
60686	0.907497	2.1
60687	0.926641	2.1
60688	0.926641	2.1
60689	1.14989	2.1
60690	1.10046	2.1
60691	0.695334	2.1
60692	0.684247	2.1
60693	0.695333	2.1
60694	1.14989	2.1
60695	0.684246	2.1
60696	1.10046	2.1
60697	0.946126	1.2868
60698	0.946126	2.1
60699	0.946131	2.1
60700	0.946131	2.1
60701	0.946127	2.1
60702	0.946127	1.56223
60703	0.94613	2.1
60704	0.94613	2.1
60705	0.946128	2.1
60706	0.946128	2.1
60707	0.946128	2.1
60708	0.946128	2.1
60709	1.89226	1.96662
60710	1.89226	1.67499
60711	1.89226	2.1
60712	1.89226	1.20682
60713	1.89226	2.1
60714	1.89226	1.05966
60715	1.42174	2.1
60716	2.29978	2.1
60717	2.41233	2.1
60718	2.41233	2.1
60719	1.39067	2.1
60720	1.39067	2.1
60721	1.89226	2.1
60722	1.89226	2.1
60723	1.89226	2.1

60724	1.89226	2.04735
60725	3.78452	2.1
60726	1.89226	2.08031
60727	1.89226	2.0369
60728	1.89226	2.1
60729	1.89226	1.87354
60730	1.89226	2.1
60731	1.89226	1.37256
60732	2.41234	2.1
60733	1.89226	2.05477
60734	2.29979	2.1
60735	1.89226	2.1
60736	1.89226	2.1
60737	2.29979	2.1
60738	1.09506	0.367728
60739	2.82921	0.42817
60740	2.53251	0.379023
60741	2.53251	0.414725
60742	1.42901	0.38295
60743	1.42901	0.419551
60744	0.946132	0.437231
60745	0.946132	0.427949
60746	0.946128	0.39572
60747	0.946128	0.443913
60748	0.94613	0.463944
60749	0.94613	0.474012
60750	0.946131	0.435258
60751	0.946131	0.4113
60752	0.94613	0.430845
60753	0.94613	0.408519
60754	0.94613	0.45391
60755	0.94613	0.477161
60756	1.89226	0.489886
60757	1.89226	0.553854
60758	1.89226	0.499224
60759	1.89226	0.536425
60760	3.78452	0.49468
60761	2.53251	0.408432
60762	1.89226	0.494976
60763	2.82921	0.422162
60764	1.89226	0.437797
60765	1.89226	0.545499
60766	2.82921	0.547476
60767	1.89226	0.467714
60768	1.89226	0.429954
60769	1.89226	0.484966
60770	1.89226	0.485929
60771	1.89226	0.438046
60772	1.89226	0.448454
60773	2.53251	0.382297
60774	1.89226	0.436664
60775	2.06158	0.359971
60776	1.89226	0.380446
60777	4.42477	0.353998
60778	1.23296	2.1
60779	2.55352	2.1
60780	2.47476	2.1
60781	2.47476	2.1

60782	1.32368	2.1
60783	1.32368	2.1
60784	1.89226	2.1
60785	1.89226	2.1
60786	1.89226	2.1
60787	1.89226	2.1
60788	1.89226	2.1
60789	1.89226	2.1
60790	1.89226	2.1
60791	1.89226	2.1
60792	1.89226	2.1
60793	1.89226	2.08958
60794	1.89226	2.1
60795	1.89226	1.52137
60796	2.47476	2.1
60797	1.89226	2.1
60798	1.89226	2.1
60799	1.89226	2.1
60800	2.55353	2.1
60801	2.55353	2.1
60802	1.05983	2.1
60803	2.47476	2.1
60804	2.37664	2.1
60805	2.37664	2.1
60806	1.23296	2.1
60807	1.23296	2.1
60808	0.946127	2.00567
60809	0.946127	2.1
60810	0.946131	2.1
60811	0.946131	2.1
60812	0.946127	2.1
60813	0.946127	1.99283
60814	0.946131	2.1
60815	0.946131	2.1
60816	0.946129	2.1
60817	0.946129	2.1
60818	0.946128	2.1
60819	0.946128	2.1
60820	1.89226	2.1
60821	1.89226	2.1
60822	1.89226	2.1
60823	1.89226	1.24983
60824	3.78452	2.1
60825	2.37665	2.1
60826	1.89226	2.1
60827	1.89226	2.1
60828	1.89226	2.1
60829	2.47476	2.1
60830	2.47476	2.1
60831	1.89226	0.880541
60832	1.92119	0.847978
60833	1.99586	0.974384
60834	1.92119	0.893907
60835	1.96693	0.805959
60836	1.89226	0.667203
60837	1.89226	0.847596
60838	1.89226	0.853806
60839	1.89226	0.97245

60840	1.89226	0.869304
60841	1.89226	0.826996
60842	1.89226	0.568052
60843	1.89226	1.03469
60844	2.39541	1.13646
60845	2.57183	1.3946
60846	2.39541	1.02796
60847	2.06868	1.02176
60848	1.89226	0.899944
60849	0.946133	0.773104
60850	0.946133	0.784565
60851	0.997928	0.829924
60852	1.03763	0.858333
60853	0.946131	0.879121
60854	0.946131	0.524921
60855	1.03763	0.810954
60856	1.03763	0.758369
60857	1.03763	0.833127
60858	0.946131	0.832956
60859	0.94613	0.776041
60860	0.99793	0.69534
60861	1.89226	0.594729
60862	1.89226	0.858181
60863	1.89226	0.88232
60864	1.89226	0.505407
60865	1.89226	0.938733
60866	1.89226	0.611504
60867	0.946131	0.853384
60868	0.946131	0.88412
60869	1.36939	1.0038
60870	1.28592	1.07201
60871	0.946129	1.02888
60872	0.946129	0.598017
60873	1.36939	0.907934
60874	1.36939	0.996215
60875	0.946131	0.90716
60876	1.36939	0.920773
60877	0.946131	0.931224
60878	1.28592	0.889495
60879	1.89226	0.723476
60880	1.89226	0.797174
60881	1.89226	0.764094
60882	1.89226	0.747199
60883	3.78453	0.758394
60884	1.89226	0.796496
60885	1.89226	0.777306
60886	1.89226	0.74971
60887	1.89226	0.818261
60888	3.78452	0.77409
60889	1.91005	0.770834
60890	1.89226	0.809173
60891	2.60215	0.725847
60892	2.60215	0.759265
60893	1.89226	0.76062
60894	1.89226	0.834001
60895	0.946132	0.788999
60896	0.946132	0.763127
60897	0.946132	0.76637

60898	0.946132	0.740162
60899	0.946132	0.767483
60900	0.946132	0.790834
60901	0.946132	0.7653
60902	0.946132	0.73265
60903	0.946132	0.763553
60904	0.946132	0.765496
60905	0.946131	0.756911
60906	0.946131	0.758155
60907	1.89226	0.767634
60908	1.89226	0.821284
60909	1.89226	0.818217
60910	1.89226	0.795531
60911	1.89226	0.793537
60912	1.89226	0.818881
60913	0.780483	0.807206
60914	0.78928	0.916015
60915	0.818566	0.839462
60916	0.460387	0.917493
60917	1.82793	0.76879
60918	1.32039	0.822568
60919	0.613889	0.808322
60920	1.0592	0.838362
60921	1.48698	0.816179
60922	0.818555	0.82854
60923	1.11515	0.82536
60924	1.2802	0.929645
60925	2.20046	0.685313
60926	1.1475	0.892271
60927	1.1475	0.70178
60928	2.27062	0.864073
60929	2.27062	0.690938
60930	1.89226	0.8086
60931	2.20046	0.595829
60932	2.27062	0.759487
60933	2.20046	0.715283
60934	1.96242	0.658821
60935	1.89226	0.629578
60936	1.89226	0.592353
60937	1.89226	0.519748
60938	1.89226	0.727234
60939	1.89226	0.584143
60940	3.78452	0.572316
60941	1.89226	0.683874
60942	1.89226	0.62277
60943	1.89226	0.832362
60944	1.89226	0.651762
60945	3.78452	0.692078
60946	1.17403	0.677477
60947	1.13531	0.779936
60948	0.6401	0.697763
60949	0.714566	0.763542
60950	0.915987	0.743345
60951	0.915987	0.502805
60952	0.933891	0.85027
60953	0.933891	0.735181
60954	1.17403	0.802258
60955	0.714566	0.727642

60956	0.6401	0.763981
60957	1.13531	0.683511
60958	0.946133	0.621411
60959	0.946133	0.709001
60960	1.17403	0.577346
60961	1.13531	0.643269
60962	0.946131	0.60376
60963	0.946131	0.521944
60964	1.17403	0.755292
60965	1.17403	0.595311
60966	1.17403	0.657627
60967	0.94613	0.650259
60968	0.946131	0.701099
60969	1.13531	0.608648
60970	1.89226	0.524095
60971	1.89226	0.512767
60972	1.89226	0.523881
60973	1.89226	0.412174
60974	1.89226	0.657305
60975	1.89226	0.515111
60976	0.946131	0.557133
60977	0.946131	0.572217
60978	0.946131	0.604891
60979	0.946131	0.66641
60980	0.946129	0.612697
60981	0.946129	0.469613
60982	0.946133	0.798283
60983	0.946133	0.534773
60984	0.946131	0.595353
60985	0.946131	0.581749
60986	0.946131	0.537634
60987	0.946131	0.552439
60988	2.29413	2.1
60989	1.99332	2.1
60990	1.84774	2.1
60991	2.21582	2.1
60992	4.20915	2.1
60993	0.517359	2.1
60994	0.634469	2.1
60995	0.996661	2.1
60996	0.92387	2.1
60997	0.768098	2.1
60998	0.768098	2.1
60999	0.790242	2.1
61000	0.790242	2.1
61001	0.923871	2.1
61002	0.517359	2.1
61003	0.634469	2.1
61004	0.996661	2.1
61005	1.89226	2.1
61006	1.89226	2.1
61007	1.89226	2.1
61008	1.89226	2.1
61009	3.78453	2.1
61010	0.946132	2.1
61011	0.946132	2.1
61012	0.946131	2.1
61013	0.946131	2.1

61014	0.946132	2.1
61015	0.946132	2.1
61016	0.946131	2.1
61017	0.946131	2.1
61018	0.946132	2.1
61019	0.946132	2.1
61020	0.946131	2.1
61021	0.946131	2.1
61022	1.62708	2.1
61023	2.21582	2.1
61024	2.13925	2.1
61025	2.21582	2.1
61026	1.77117	2.1
61027	1.84774	2.1
61028	0.946132	2.1
61029	0.946132	2.1
61030	0.946131	2.1
61031	0.946131	2.1
61032	0.946132	2.1
61033	0.946132	2.1
61034	0.946131	2.1
61035	0.946131	2.1
61036	0.946131	2.1
61037	0.946131	2.1
61038	0.946131	2.1
61039	0.946131	2.1
61040	1.89226	1.30516
61041	1.89226	0.969215
61042	1.89226	0.958556
61043	1.89226	1.1061
61044	3.78453	1.14079
61045	1.89226	0.965894
61046	1.89226	1.21096
61047	1.89226	1.13447
61048	1.89226	1.47093
61049	1.89226	0.924586
61050	1.89226	1.09938
61051	1.89226	0.960061
61052	1.89226	1.3959
61053	1.89226	0.919384
61054	1.89226	1.27623
61055	1.89226	0.959959
61056	1.89226	1.47958
61057	0.914369	1.16024
61058	0.914369	1.22513
61059	0.810945	1.85342
61060	0.810945	1.18772
61061	2.26581	2.06854
61062	1.66199	1.8097
61063	1.49503	1.27757
61064	1.28816	2.00015
61065	1.08133	1.40425
61066	0.810933	1.42132
61067	1.1212	1.43779
61068	1.89227	0.748391
61069	1.89227	0.705694
61070	1.89227	0.706973
61071	1.89227	0.77981

61072	3.78453	0.717232
61073	1.89226	0.736701
61074	1.89226	0.819987
61075	1.89226	0.805647
61076	1.89226	0.752219
61077	3.78453	0.784211
61078	1.89226	0.822869
61079	1.89226	0.754023
61080	1.89226	0.755273
61081	1.89226	0.85281
61082	3.78453	0.780944
61083	1.89226	0.707698
61084	1.89226	0.826998
61085	1.89226	0.783336
61086	2.25318	0.731466
61087	3.78453	0.785774
61088	1.89226	0.729341
61089	1.89226	0.832307
61090	1.89226	0.772865
61091	1.9637	0.746967
61092	3.78453	0.792622
61093	1.89226	0.81588
61094	1.89226	0.779439
61095	1.89226	0.753423
61096	1.89226	0.839537
61097	3.78453	0.783802
61098	1.89226	0.783829
61099	1.89226	0.888534
61100	1.89226	0.840428
61101	2.53384	0.805546
61102	3.78453	0.849584
61103	1.55405	0.728986
61104	1.03818	1.07886
61105	1.03818	0.743348
61106	1.34857	0.790953
61107	1.46338	0.743088
61108	1.77377	0.927483
61109	1.86273	0.74148
61110	1.73802	0.786218
61111	1.50969	1.20775
61112	1.46337	0.884076
61113	3.20139	0.811285
61114	1.2377	0.898117
61115	1.46337	0.742367
61116	1.27408	0.817908
61117	1.32039	1.05989
61118	1.50969	0.874504
61119	1.50969	0.744083
61120	1.89226	1.89729
61121	1.89226	2.1
61122	1.89226	2.1
61123	1.89226	2.1
61124	1.89226	2.1
61125	1.89226	1.21465
61126	1.89169	2.1
61127	1.89226	2.1
61128	1.89226	2.1
61129	1.89226	2.1

61130	1.96624	2.1
61131	1.96624	2.1
61132	2.62871	2.1
61133	1.6201	2.1
61134	1.49149	2.1
61135	1.6201	2.1
61136	2.62559	2.1
61137	2.75419	1.5622
61138	0.946129	1.01836
61139	0.946129	1.82698
61140	0.946133	2.1
61141	0.946133	2.06963
61142	0.946131	1.98994
61143	0.946131	0.985743
61144	0.946131	2.1
61145	0.946131	1.88917
61146	0.946131	1.85555
61147	0.946131	1.88212
61148	0.946131	1.92284
61149	0.946131	1.86207
61150	0.851543	2.1
61151	0.851543	2.1
61152	1.04043	2.1
61153	0.997939	2.1
61154	0.946132	2.1
61155	0.946132	1.70551
61156	0.894185	2.1
61157	0.893901	2.1
61158	0.998267	2.1
61159	1.04074	2.1
61160	0.85127	2.1
61161	0.85153	2.1
61162	1.89226	2.1
61163	1.89226	1.96368
61164	1.89226	2.1
61165	1.89226	2.1
61166	1.89226	2.1
61167	1.89226	2.1
61168	0.928691	2.1
61169	0.928691	2.1
61170	0.935332	2.1
61171	0.935332	2.1
61172	1.398	2.1
61173	1.14418	2.1
61174	0.479331	2.1
61175	0.719848	2.1
61176	1.398	2.1
61177	0.719848	2.1
61178	1.14417	2.1
61179	0.47933	2.1
61180	2.28835	2.1
61181	1.89226	2.1
61182	1.89226	2.1
61183	1.89226	2.1
61184	2.796	2.1
61185	2.796	2.1
61186	0.946131	2.1
61187	0.946131	2.1

61188	0.946131	2.06976
61189	0.946131	1.76288
61190	0.946128	1.78152
61191	0.946128	2.1
61192	0.946133	1.96538
61193	0.946133	2.1
61194	0.946131	2.1
61195	0.946131	2.1
61196	0.94613	2.1
61197	0.94613	2.1
61198	0.946131	2.1
61199	0.946131	2.1
61200	0.946131	2.1
61201	0.946131	1.83732
61202	0.946131	1.85296
61203	0.946131	2.1
61204	0.946131	2.1
61205	0.946131	2.04792
61206	0.946131	2.1
61207	0.946131	2.1
61208	0.946131	2.1
61209	0.946131	2.1
61210	1.89226	2.1
61211	1.89226	1.55351
61212	1.89226	1.88035
61213	1.89226	2.1
61214	3.78452	2.1
61215	2.05207	0.766394
61216	1.70302	0.891745
61217	1.33794	1.22054
61218	1.98194	0.850434
61219	3.36873	0.900151
61220	2.62761	0.76203
61221	1.98194	0.844453
61222	2.28009	1.02315
61223	2.28009	0.748273
61224	2.22939	0.899777
61225	2.22939	0.732755
61226	1.89226	0.853351
61227	1.89226	0.754004
61228	1.89226	0.802618
61229	1.89226	0.754698
61230	1.89226	0.828307
61231	1.89226	0.797447
61232	1.89227	0.858523
61233	1.89227	0.870324
61234	1.89227	1.13122
61235	1.89227	0.964722
61236	1.89227	0.8145
61237	1.89227	0.590942
61238	0.888355	0.768931
61239	0.990973	0.884563
61240	0.71133	0.907281
61241	0.634324	0.996138
61242	0.806209	0.954039
61243	0.806209	0.615676
61244	0.819087	1.10474
61245	0.819087	0.958211

61246	0.888355	0.970687
61247	0.634324	0.965424
61248	0.71133	0.876946
61249	0.990972	0.865518
61250	1.11469	0.873923
61251	0.985023	0.844327
61252	0.888354	0.77606
61253	0.990972	0.913738
61254	0.994754	0.858652
61255	0.994754	0.784846
61256	1.0083	0.946656
61257	1.0083	0.755639
61258	0.888354	0.796574
61259	0.985024	0.848673
61260	0.990971	0.769917
61261	1.11469	0.728688
61262	0.946132	0.867158
61263	0.946132	0.788692
61264	0.946132	0.799261
61265	0.946132	0.780733
61266	0.946132	0.797988
61267	0.946132	0.762451
61268	0.946133	1.00031
61269	0.946133	0.807929
61270	0.946133	0.835206
61271	0.946133	0.829246
61272	0.946132	0.758106
61273	0.946132	0.769141
61274	0.810944	0.814051
61275	0.810944	0.768172
61276	0.810947	0.897157
61277	0.810947	0.611133
61278	1.89227	1.07168
61279	1.35168	0.884969
61280	1.08132	0.879332
61281	1.08131	0.890436
61282	1.08132	0.758589
61283	0.810959	0.748168
61284	0.810959	0.776774
61285	1.70303	2.1
61286	2.03975	2.1
61287	1.99324	2.1
61288	2.17615	2.1
61289	1.40549	2.1
61290	1.53446	2.1
61291	0.946133	1.93054
61292	0.946133	2.1
61293	0.946129	2.1
61294	0.946129	2.1
61295	0.946131	2.1
61296	0.946131	1.46349
61297	0.946131	2.1
61298	0.946131	2.1
61299	0.946131	2.1
61300	0.946131	2.1
61301	0.946131	2.1
61302	0.946131	2.1
61303	1.89226	2.1

61304	1.89226	1.62839
61305	1.89226	2.1
61306	1.89226	1.57672
61307	1.89226	2.1
61308	1.89226	1.76232
61309	2.17615	2.1
61310	2.3111	2.1
61311	2.50288	2.1
61312	2.50288	2.1
61313	2.03975	2.1
61314	2.03975	2.1
61315	1.76401	2.1
61316	1.95852	2.1
61317	2.32134	2.1
61318	2.12306	2.1
61319	1.8513	2.1
61320	1.70303	2.1
61321	1.89226	2.08589
61322	1.89226	2.1
61323	1.89226	2.1
61324	1.89226	1.77687
61325	3.78452	2.1
61326	1.89226	2.1
61327	1.89226	1.07802
61328	1.89226	1.75028
61329	1.89226	2.1
61330	3.78452	2.08988
61331	1.2243	2.1
61332	1.2243	2.1
61333	1.24189	2.1
61334	1.24189	2.1
61335	1.37811	2.1
61336	1.25144	2.09912
61337	1.08807	2.1
61338	1.17949	2.1
61339	1.17949	2.1
61340	1.37811	2.1
61341	1.08807	2.1
61342	1.25144	2.1
61343	1.89226	2.1
61344	1.89226	2.1
61345	1.89226	2.1
61346	1.89226	2.1
61347	1.89226	2.1
61348	1.89226	2.1
61349	1.89227	2.1
61350	1.89227	2.1
61351	1.89227	2.1
61352	1.89227	2.1
61353	1.89227	2.1
61354	1.89227	2.1
61355	1.89224	2.1
61356	1.89224	2.1
61357	1.89224	2.1
61358	1.89224	2.1
61359	3.78447	2.1
61360	1.89224	2.1
61361	1.89224	2.1

61362	1.89224	2.1
61363	1.89224	2.1
61364	1.89224	2.1
61365	1.89224	2.1
61366	1.89224	2.1
61367	1.89224	2.1
61368	1.89224	2.1
61369	1.89224	2.1
61370	3.78448	2.1
61371	1.89224	2.1
61372	1.703 2.1	
61373	1.89224	2.1
61374	1.72991	2.1
61375	1.07643	2.1
61376	1.36657	2.1
61377	1.44472	2.1
61378	2.41233	2.1
61379	2.51628	2.1
61380	2.51628	2.1
61381	1.42174	2.1
61382	1.42174	2.1
61383	1.89226	2.1
61384	1.89226	1.99104
61385	1.89226	2.1
61386	1.89226	2.1
61387	1.89226	2.1
61388	1.89226	2.1
61389	1.89226	2.03582
61390	1.89226	2.1
61391	1.89226	2.1
61392	1.89226	1.14827
61393	3.78452	2.07981
61394	2.51628	2.1
61395	1.89226	2.1
61396	1.89226	2.1
61397	1.89226	2.1
61398	2.41234	2.1
61399	2.41234	2.1
61400	0.996254	2.1
61401	0.996254	2.1
61402	1.01153	2.1
61403	1.01153	2.1
61404	1.28542	2.1
61405	1.25814	2.1
61406	0.719184	2.1
61407	0.72236	2.1
61408	0.719183	2.1
61409	1.28542	2.1
61410	0.722359	2.1
61411	1.25814	2.1
61412	0.946126	1.98452
61413	0.946126	2.1
61414	0.946131	2.1
61415	0.946131	2.1
61416	0.946127	2.1
61417	0.946127	1.95387
61418	0.94613	2.1
61419	0.94613	2.1

61420	0.946128	2.1
61421	0.946128	2.1
61422	0.946128	2.1
61423	0.946128	2.1
61424	1.89226	2.1
61425	1.89226	1.30989
61426	1.89226	2.09481
61427	1.89226	2.1
61428	1.89226	1.80642
61429	1.89226	2.1
61430	0.946132	2.1
61431	0.946132	2.1
61432	1.25814	2.1
61433	1.25814	2.1
61434	0.946131	2.1
61435	0.946131	1.77937
61436	1.28543	2.1
61437	1.25814	2.1
61438	1.28543	2.1
61439	0.946132	2.1
61440	1.25814	2.1
61441	0.946132	2.1
61442	1.89226	1.35684
61443	1.89226	0.961071
61444	1.89226	1.30854
61445	1.89226	1.17643
61446	1.89226	1.07745
61447	1.89226	0.982265
61448	1.89226	1.30075
61449	2.27719	0.985581
61450	1.89226	0.960317
61451	1.89226	1.11057
61452	3.78452	1.07067
61453	1.89225	1.37493
61454	1.89225	0.982834
61455	1.89225	1.05505
61456	1.89225	1.20503
61457	3.78451	1.17225
61458	1.89226	0.883539
61459	1.89226	0.991018
61460	1.89226	1.0562
61461	1.89226	0.782432
61462	3.78452	0.907383
61463	0.946132	0.730343
61464	0.946132	0.910834
61465	0.946132	0.986925
61466	0.946132	0.915301
61467	0.946131	0.86646
61468	0.946131	0.775687
61469	0.946132	0.984969
61470	0.946132	0.778048
61471	0.946132	0.829425
61472	0.946132	0.863966
61473	0.946132	0.859299
61474	0.946132	0.770947
61475	0.946126	0.941236
61476	0.946126	0.862942
61477	0.946132	1.35079

61478	0.946132	1.05734
61479	0.946128	0.915046
61480	0.946128	1.01504
61481	0.946129	0.981132
61482	0.946129	1.1937
61483	0.946129	1.06625
61484	0.946129	1.12595
61485	0.946129	0.981536
61486	0.946129	0.988821
61487	1.39858	2.1
61488	1.39858	2.1
61489	0.94613	2.1
61490	0.94613	2.1
61491	0.946132	2.1
61492	0.946132	2.1
61493	1.39858	2.1
61494	1.25002	2.1
61495	1.39858	2.1
61496	0.946131	2.1
61497	1.25002	2.1
61498	0.946131	2.1
61499	2.0815	2.1
61500	1.89226	2.1
61501	1.89226	2.1
61502	2.05298	2.1
61503	2.5593	2.1
61504	2.77667	2.1
61505	0.946133	2.1
61506	0.946133	2.1
61507	0.946132	2.1
61508	0.946132	2.1
61509	0.946132	2.1
61510	0.946132	2.1
61511	0.946133	2.1
61512	0.946133	2.1
61513	0.946132	2.1
61514	0.946132	2.1
61515	0.946132	2.1
61516	0.946132	2.1
61517	1.38908	2.1
61518	1.38908	2.1
61519	0.946132	2.1
61520	0.946132	2.1
61521	0.946132	2.1
61522	0.946132	2.1
61523	1.38908	2.1
61524	1.27965	2.1
61525	1.38908	2.1
61526	0.946132	2.1
61527	0.946133	2.1
61528	1.27965	2.1
61529	0.946133	2.1
61530	0.946133	2.1
61531	0.946132	2.1
61532	0.946132	2.1
61533	0.946132	2.1
61534	0.946132	2.1
61535	0.946133	2.1

61536	0.946133	2.1
61537	0.946132	2.1
61538	0.946132	2.1
61539	0.946133	2.1
61540	0.946133	2.1
61541	1.89227	0.916096
61542	1.89227	1.11606
61543	1.89227	0.874885
61544	1.89227	0.785376
61545	3.78453	0.884782
61546	1.33154	1.00593
61547	1.33154	1.16446
61548	0.946132	1.06319
61549	0.946132	0.931405
61550	1.19095	0.876585
61551	1.33154	1.21943
61552	0.946133	1.02787
61553	0.946133	1.08533
61554	0.946133	1.28286
61555	1.19095	0.913227
61556	0.946132	1.23358
61557	1.33154	1.0054
61558	1.0888	0.878215
61559	1.04745	0.909077
61560	0.946132	0.83248
61561	0.946132	0.857639
61562	1.04745	0.780906
61563	1.04745	1.11535
61564	0.946132	1.12409
61565	0.946132	0.784882
61566	1.04745	1.01673
61567	0.946133	0.791667
61568	0.946132	1.05804
61569	1.0888	0.882939
61570	1.89226	1.25592
61571	1.89226	0.785026
61572	1.89226	0.772207
61573	1.89226	0.870226
61574	1.89226	0.817926
61575	1.89226	1.02902
61576	1.89226	1.28155
61577	1.87592	0.807496
61578	1.87592	0.841136
61579	2.3819	0.96425
61580	1.89226	1.02484
61581	2.39825	1.23983
61582	1.89226	0.792802
61583	2.0949	1.00254
61584	1.88741	0.75684
61585	1.89226	0.762931
61586	1.89226	0.800018
61587	1.88741	1.05633
61588	1.07508	1.17983
61589	1.63419	1.36102
61590	1.63419	1.21695
61591	1.09795	1.52082
61592	1.56137	1.15132
61593	1.02513	1.68608

61594	0.967672	0.980287
61595	1.56137	1.09168
61596	1.56137	0.982592
61597	1.07508	1.1322
61598	1.41319	0.950536
61599	0.926902	1.23151
61600	1.56137	1.14346
61601	1.63419	1.19849
61602	2.29852	1.43459
61603	2.14485	1.02816
61604	3.77904	1.04448
61605	1.41319	0.894203
61606	1.92622	0.881607
61607	1.56137	0.862188
61608	1.56137	1.13494
61609	2.14485	1.02988
61610	1.92622	0.949222
61611	1.89227	2.1
61612	1.89227	2.1
61613	1.89227	2.1
61614	1.89227	2.1
61615	3.78453	2.1
61616	2.56618	2.1
61617	2.08612	2.1
61618	2.48048	2.1
61619	1.99644	2.1
61620	4.5666	2.1
61621	2.08612	2.1
61622	1.30659	2.1
61623	1.99644	2.1
61624	1.25404	2.1
61625	3.34016	2.1
61626	1.89226	2.1
61627	1.89226	2.1
61628	1.89226	2.1
61629	1.89226	2.1
61630	1.89226	2.1
61631	1.89226	2.1
61632	1.89227	2.1
61633	1.89227	2.1
61634	1.89227	2.1
61635	1.89227	2.1
61636	3.78453	2.1
61637	2.48048	2.1
61638	1.99644	2.1
61639	2.39022	2.1
61640	1.90734	2.1
61641	4.38782	2.1
61642	1.90734	2.1
61643	1.99644	2.1
61644	1.21564	2.1
61645	1.21564	2.1
61646	1.25404	2.1
61647	1.94574	2.1
61648	0.810946	2.1
61649	0.810946	2.1
61650	0.810947	2.1
61651	0.810947	2.1

61652	1.89226	2.1
61653	1.3517	2.1
61654	1.08133	2.1
61655	1.08132	2.1
61656	1.08132	2.1
61657	0.810934	2.1
61658	0.810934	2.1
61659	1.89226	2.1
61660	1.89226	0.989714
61661	1.89226	1.37041
61662	1.89226	1.95172
61663	3.78453	1.94056
61664	1.89226	0.756844
61665	1.89226	0.494525
61666	1.89226	1.30339
61667	1.89226	0.688083
61668	1.89226	1.08369
61669	1.89226	0.485731
61670	0.946131	0.803815
61671	0.946131	0.691462
61672	0.94613	0.998511
61673	0.94613	0.906093
61674	0.946128	0.79389
61675	0.946128	0.685649
61676	0.946133	0.507203
61677	0.946133	1.08624
61678	0.946131	0.925962
61679	0.946131	0.873039
61680	0.946131	0.961129
61681	0.946131	0.810153
61682	1.89226	2.09415
61683	1.89226	0.916402
61684	1.89226	1.28044
61685	1.89226	1.16884
61686	1.89226	1.12542
61687	1.89226	1.05149
61688	2.56348	1.03719
61689	1.89226	1.61834
61690	1.89226	2.1
61691	2.53234	1.2848
61692	4.45574	1.10482
61693	2.53234	0.570357
61694	1.65583	0.414952
61695	2.56348	0.5481
61696	2.56348	0.604135
61697	1.66295	0.424221
61698	1.65583	0.394297
61699	0.736602	0.553989
61700	0.827916	0.601897
61701	1.26617	0.641457
61702	1.18322	0.446088
61703	0.7366	0.532887
61704	1.18321	0.52841
61705	1.26617	0.537204
61706	0.827918	0.531612
61707	0.99929	0.567986
61708	0.99929	0.617875
61709	1.00348	0.535407

61710	1.00348	0.529867
61711	1.26617	1.01796
61712	1.18322	0.948404
61713	0.946131	2.05769
61714	0.946131	0.998066
61715	0.946129	0.967058
61716	1.18321	0.829598
61717	0.946133	1.88927
61718	1.26617	1.4184
61719	1.26617	1.43658
61720	1.26617	0.592871
61721	0.946131	1.58113
61722	0.946131	1.10296
61723	2.23267	0.462322
61724	1.89226	0.514273
61725	2.07099	0.49355
61726	1.89226	0.507789
61727	2.07099	0.49729
61728	1.89226	0.502539
61729	0.946132	0.641904
61730	1.35403	0.493441
61731	1.24038	0.495394
61732	0.946131	0.502723
61733	0.946131	0.582892
61734	0.946131	0.592849
61735	1.24039	0.508268
61736	1.35403	0.513421
61737	1.24038	0.579347
61738	1.24038	0.615634
61739	0.946131	0.529211
61740	0.946131	0.677856
61741	1.89226	0.69033
61742	1.89226	0.857317
61743	1.89226	0.648021
61744	1.89226	0.626005
61745	1.89226	0.925242
61746	1.89226	0.909846
61747	1.89226	0.546493
61748	1.89226	0.822628
61749	1.89226	0.706463
61750	1.89226	0.76487
61751	3.78452	0.809272
61752	1.56971	0.519572
61753	2.80086	0.690101
61754	2.59806	0.55752
61755	1.93489	0.539566
61756	2.59806	0.718029
61757	1.93489	0.635158
61758	0.827467	0.522654
61759	1.26652	0.540914
61760	1.13679	0.529039
61761	0.639035	0.529461
61762	1.11547	0.608575
61763	1.30656	0.574243
61764	0.869062	0.504303
61765	0.696608	0.528474
61766	1.07205	0.541997
61767	0.953656	0.609818

61768	0.970744	0.511536
61769	0.970744	0.53759
61770	2.38681	2.1
61771	2.50438	1.23236
61772	2.42082	1.63314
61773	2.59106	2.08068
61774	4.92521	1.81589
61775	2.50438	1.17793
61776	2.40627	2.00309
61777	2.59106	2.1
61778	2.5444	1.04379
61779	5.04879	1.88566
61780	2.42082	2.0505
61781	2.70575	2.1
61782	2.64778	2.1
61783	2.59106	1.49834
61784	2.47753	2.1
61785	2.42082	1.1954
61786	2.70575	2.1
61787	2.5444	1.256
61788	2.76095	2.1
61789	2.59106	1.49579
61790	2.71429	1.90234
61791	2.5444	1.29558
61792	1.89226	2.1
61793	2.38473	2.1
61794	2.36689	2.1
61795	2.38473	2.1
61796	1.87442	2.1
61797	1.89226	2.1
61798	1.89226	2.1
61799	1.89226	0.917788
61800	1.89226	1.3528
61801	1.89226	0.912812
61802	1.89226	1.83868
61803	1.89226	0.938401
61804	2.14952	1.61565
61805	1.89226	0.841991
61806	1.89226	1.48842
61807	1.89226	0.824723
61808	2.0692	2.07436
61809	2.0692	0.967405
61810	1.89226	2.1
61811	2.36689	2.1
61812	2.36204	2.1
61813	2.36689	2.1
61814	1.88742	2.1
61815	1.89226	2.1
61816	0.946132	0.883228
61817	0.946132	2.1
61818	0.946131	2.1
61819	0.946131	1.71989
61820	0.946132	1.19035
61821	0.946132	1.72137
61822	0.946131	2.1
61823	0.946131	2.1
61824	0.946132	1.8796
61825	0.946132	2.02981

61826	0.946131	1.80009
61827	0.946131	2.1
61828	0.946128	2.1
61829	0.946128	1.97414
61830	1.07476	1.22292
61831	1.07476	2.1
61832	0.946131	1.28303
61833	0.946131	1.71796
61834	1.13182	2.1
61835	1.07476	2.1
61836	1.13182	1.74778
61837	0.946131	1.72053
61838	1.07476	1.74511
61839	0.94613	1.0213
61840	1.89227	0.648882
61841	1.89227	0.679337
61842	1.89227	0.673458
61843	1.89227	0.674364
61844	3.78453	0.666392
61845	1.89226	0.672636
61846	1.89226	0.677491
61847	1.89226	0.658582
61848	1.89226	0.682991
61849	3.78452	0.672042
61850	1.89226	0.676271
61851	1.89226	0.697749
61852	1.89226	0.6834
61853	1.89226	0.683817
61854	3.78452	0.681787
61855	1.89226	0.678714
61856	1.89226	0.690996
61857	1.89226	0.691676
61858	1.89226	0.699804
61859	1.89226	0.675024
61860	1.89226	0.680924
61861	2.58344	0.705318
61862	2.00267	0.671964
61863	2.14218	0.689349
61864	2.22674	0.810722
61865	2.14218	0.640363
61866	2.22674	0.665162
61867	2.83531	0.908454
61868	1.56043	0.683528
61869	2.22674	0.89436
61870	1.90912	1.08727
61871	2.22674	0.666182
61872	1.90912	0.68055
61873	0.780217	0.687634
61874	1.11337	0.697219
61875	1.00133	0.697647
61876	0.804472	0.689755
61877	0.804472	0.753942
61878	0.780217	0.694955
61879	1.00133	0.668732
61880	1.11337	0.716159
61881	0.856668	0.701388
61882	0.856668	0.934419
61883	0.924883	0.693809

61884	0.924883	0.675237
61885	1.93107	2.1
61886	1.68657	2.1
61887	1.7237	2.1
61888	1.89226	2.1
61889	1.53436	2.1
61890	1.68657	2.1
61891	1.89226	2.1
61892	1.89226	2.1
61893	1.89226	2.1
61894	1.89226	2.1
61895	3.78453	2.1
61896	0.946131	2.1
61897	0.946131	2.1
61898	0.855092	2.1
61899	0.83953	2.1
61900	0.946131	2.1
61901	0.946131	2.1
61902	0.839531	2.1
61903	0.839531	2.1
61904	0.946131	2.1
61905	0.855093	2.1
61906	0.839531	2.1
61907	0.946131	2.1
61908	1.89226	2.1
61909	1.70302	2.1
61910	2.21419	2.1
61911	1.93107	2.1
61912	1.97872	2.1
61913	1.70302	2.1
61914	0.946133	2.1
61915	0.946133	2.1
61916	0.946132	2.1
61917	0.946132	2.1
61918	0.946132	2.1
61919	0.946132	2.1
61920	0.946132	2.1
61921	0.946132	2.1
61922	0.946133	2.1
61923	0.946133	2.1
61924	0.946132	2.1
61925	0.946132	2.1
61926	1.90081	2.1
61927	1.89226	2.1
61928	1.51379	2.1
61929	1.36813	2.1
61930	1.71019	2.1
61931	1.89226	2.1
61932	1.89227	0.963857
61933	1.89227	0.796456
61934	1.89227	0.780188
61935	1.89227	0.908396
61936	1.89227	0.94552
61937	1.89227	1.2496
61938	1.36428	1.01989
61939	1.37869	0.961181
61940	0.946132	0.865189
61941	0.946132	0.949359

61942	1.37869	0.857281
61943	1.37869	1.41857
61944	0.946133	1.00278
61945	0.946133	1.07713
61946	1.37869	0.858988
61947	0.946132	0.914999
61948	1.36428	1.072
61949	0.946133	0.806357
61950	0.94612	0.925317
61951	0.94612	0.883121
61952	0.946119	0.86658
61953	0.946119	0.902866
61954	0.946119	0.792161
61955	0.946119	1.15153
61956	0.94612	0.922261
61957	0.94612	1.08555
61958	0.946132	0.852742
61959	0.946132	0.797723
61960	0.946107	0.818519
61961	0.946107	1.01425
61962	1.89225	0.147815
61963	1.89225	2.00471
61964	1.63564	0.147815
61965	1.42278	2.1
61966	1.42278	2.1
61967	1.57534	0.147815
61968	1.53735	2.1
61969	1.68991	0.147815
61970	1.57535	0.147815
61971	1.04442	2.1
61972	1.04442	2.1
61973	1.22031	0.147815
61974	1.42278	2.1
61975	1.59868	0.147815
61976	0.946132	2.1
61977	0.946132	2.1
61978	0.946131	2.1
61979	0.946131	2.1
61980	0.946131	2.1
61981	0.946131	2.1
61982	0.946132	2.1
61983	0.946132	2.1
61984	0.946132	2.1
61985	0.946132	2.1
61986	0.946131	2.1
61987	0.946131	2.1
61988	1.89226	2.1
61989	1.89226	2.1
61990	1.89226	2.1
61991	1.89226	2.1
61992	1.89226	2.1
61993	1.89226	2.1
61994	2.03143	0.932315
61995	1.47111	1.32721
61996	2.28685	1.24344
61997	2.28685	1.20649
61998	1.36006	0.990268
61999	1.36006	0.994254

62000	1.89226	0.849826
62001	1.89226	1.1445
62002	1.89226	1.0545
62003	1.89226	1.07852
62004	1.89226	0.865497
62005	1.89226	0.911766
62006	1.89226	1.05145
62007	1.89226	2.07681
62008	1.89226	1.9501
62009	1.89226	2.07567
62010	1.89226	1.09969
62011	1.89226	1.42478
62012	0.902821	1.94847
62013	0.902821	1.58464
62014	0.889218	2.1
62015	0.889218	2.1
62016	0.675759	2.1
62017	1.05649	2.1
62018	0.735554	1.84858
62019	1.14342	1.77533
62020	1.14343	1.2666
62021	1.05649	2.1
62022	0.735554	1.98001
62023	0.675758	2.1
62024	2.46286	0.78331
62025	2.28685	0.911164
62026	2.67843	0.884341
62027	2.67843	0.853314
62028	2.03143	0.816207
62029	2.03143	0.804283
62030	1.89226	0.798268
62031	1.89226	0.845496
62032	1.89226	0.794821
62033	1.89226	0.787788
62034	3.78452	0.847627
62035	0.94613	0.971877
62036	0.94613	0.967977
62037	0.946128	1.22571
62038	0.946128	0.983985
62039	0.94613	1.9014
62040	0.94613	0.976441
62041	0.946128	0.829655
62042	0.946128	0.943668
62043	0.946129	0.7395
62044	0.946129	1.02634
62045	0.946129	0.844934
62046	0.946129	1.68455
62047	2.67843	0.927662
62048	2.11297	1.39498
62049	2.52453	1.24764
62050	2.52453	1.40199
62051	2.28685	1.03018
62052	2.28685	0.986224
62053	1.89226	0.749485
62054	1.89226	0.903767
62055	1.89226	0.600659
62056	1.89226	0.903439
62057	3.78451	0.800812

62058	2.39524	0.643182
62059	2.10188	0.881641
62060	2.29839	0.540751
62061	2.02242	0.866172
62062	4.40028	0.786526
62063	1.71839	2.1
62064	1.65261	2.1
62065	2.10189	0.872612
62066	2.10189	1.82245
62067	2.02243	0.870618
62068	1.63893	0.868181
62069	0.946132	2.1
62070	0.946132	0.903502
62071	0.946129	0.88875
62072	0.946129	2.1
62073	0.94613	2.1
62074	0.94613	2.1
62075	0.946131	0.866291
62076	0.946131	0.881779
62077	0.946131	1.34749
62078	0.946131	1.38264
62079	0.946131	1.53799
62080	0.946131	1.59602
62081	1.89226	0.635342
62082	1.89226	0.97878
62083	1.89226	0.509926
62084	1.89226	0.845277
62085	3.78452	0.781778
62086	2.2984	0.553685
62087	2.02242	0.855955
62088	2.21582	0.343961
62089	1.94412	0.846108
62090	4.24251	0.791714
62091	1.94412	0.852386
62092	1.5854	2.07516
62093	2.02243	2.1
62094	2.02243	0.882761
62095	1.57431	0.866804
62096	1.65261	2.1
62097	0.810943	0.88749
62098	0.810943	2.1
62099	0.810945	2.09228
62100	0.810945	2.1
62101	1.89226	0.853714
62102	1.3517	1.19558
62103	1.08133	1.47473
62104	1.08132	2.1
62105	1.08132	0.870624
62106	0.810933	1.52106
62107	0.810933	1.4611
62108	1.89226	0.360375
62109	1.89226	0.510399
62110	1.89226	0.461516
62111	1.89226	0.42018
62112	1.89226	0.441139
62113	1.89226	0.40135
62114	1.89226	0.461798
62115	1.89226	0.612757

62116	1.89226	0.518378
62117	1.89226	0.576002
62118	3.78452	0.591939
62119	1.89226	0.432305
62120	1.89226	0.564506
62121	1.89226	0.517396
62122	1.89226	0.481358
62123	1.89226	0.498066
62124	1.89226	0.462299
62125	0.946128	0.563576
62126	0.946128	0.475678
62127	0.946132	0.458265
62128	0.946132	0.524249
62129	0.94613	0.569178
62130	0.94613	0.589728
62131	0.946129	0.495529
62132	0.946129	0.452025
62133	0.94613	0.455873
62134	0.94613	0.507936
62135	0.94613	0.478675
62136	0.94613	0.507307
62137	1.89226	0.384265
62138	1.89226	0.533637
62139	1.89226	0.45447
62140	1.89226	0.444043
62141	1.89226	0.509966
62142	1.89226	0.506739
62143	2.81478	2.1
62144	1.89226	2.1
62145	2.65557	2.1
62146	1.89226	2.1
62147	4.54783	2.1
62148	1.89226	2.1
62149	1.89226	2.1
62150	1.89226	2.1
62151	1.89226	2.1
62152	3.78452	2.1
62153	2.65557	2.1
62154	1.89226	2.1
62155	1.89226	2.1
62156	1.89226	2.1
62157	2.57997	2.1
62158	2.57997	2.1
62159	0.946131	2.1
62160	0.946131	2.1
62161	0.946128	2.1
62162	0.946128	2.1
62163	0.94613	2.1
62164	0.94613	2.1
62165	0.946129	2.1
62166	0.946129	2.1
62167	0.94613	2.1
62168	0.94613	2.1
62169	0.94613	2.1
62170	0.94613	2.1
62171	1.89226	1.15548
62172	2.27703	1.47853
62173	2.35615	1.47718

62174	2.35615	1.22335
62175	1.89226	1.46158
62176	1.89226	1.21381
62177	0.946132	1.11792
62178	1.17807	1.45795
62179	1.14926	1.04872
62180	0.946132	1.11631
62181	0.946133	1.11317
62182	0.946133	1.20867
62183	1.14926	1.14054
62184	1.14926	1.14502
62185	0.946132	1.20139
62186	0.946132	1.10241
62187	1.17807	1.30804
62188	1.14926	1.11887
62189	2.16841	1.10711
62190	1.35487	1.20615
62191	1.19854	1.10491
62192	1.19854	1.19944
62193	2.46078	1.11265
62194	2.46078	1.18236
62195	0.69308	1.23551
62196	1.3119	1.30807
62197	0.677433	1.2692
62198	1.23039	1.24189
62199	0.978178	1.27773
62200	0.978178	1.27344
62201	0.945294	1.26779
62202	0.945294	1.25317
62203	1.3119	1.23844
62204	1.23039	1.18091
62205	0.693081	1.20178
62206	0.677432	1.20198
62207	1.89226	1.03292
62208	1.89226	1.4791
62209	1.89226	1.3407
62210	1.89226	1.08034
62211	1.89226	1.2917
62212	1.89226	1.06855
62213	0.946132	1.01341
62214	0.946132	1.35757
62215	0.946132	0.943915
62216	0.946132	1.02646
62217	0.946133	1.02488
62218	0.946133	1.12815
62219	0.946132	1.03089
62220	0.946132	0.977256
62221	0.946133	1.04754
62222	0.946133	0.992994
62223	0.946132	1.09026
62224	0.946132	1.02455
62225	2.60637	1.75727
62226	1.89226	2.0929
62227	1.89226	2.1
62228	1.89226	1.40345
62229	3.78452	2.1
62230	1.89226	1.13177
62231	1.8396	1.51133

62232	1.89226	1.64676
62233	1.89226	1.12602
62234	1.60993	1.95109
62235	1.60993	1.18161
62236	0.946131	1.25296
62237	0.946131	1.47295
62238	0.946128	2.04765
62239	0.946128	1.71892
62240	0.946131	1.24061
62241	0.946131	1.57789
62242	0.946127	2.07886
62243	0.946127	1.4212
62244	0.946129	1.68027
62245	0.946129	1.6628
62246	0.94613	1.56404
62247	0.94613	1.54929
62248	1.89226	2.1
62249	1.89226	1.42493
62250	1.89226	1.97782
62251	1.89226	1.88069
62252	1.89226	1.94521
62253	1.89226	1.8396
62254	1.89226	1.0718
62255	1.89226	1.33436
62256	1.89226	1.55385
62257	1.89226	1.02699
62258	1.89226	1.72082
62259	1.89226	1.0216
62260	0.946131	1.21924
62261	0.946131	1.46912
62262	0.946128	1.96819
62263	0.946128	1.56946
62264	0.946131	1.20465
62265	0.946131	1.46514
62266	0.946127	1.46142
62267	0.946127	1.97483
62268	0.94613	1.58725
62269	0.94613	1.58453
62270	0.946129	1.5047
62271	0.946129	1.51741
62272	0.946133	2.1
62273	0.946133	2.1
62274	1.28153	2.1
62275	1.26781	2.1
62276	0.946132	2.1
62277	0.946132	2.1
62278	1.28153	2.1
62279	1.28153	2.1
62280	0.946132	2.1
62281	1.28153	2.1
62282	0.946133	2.1
62283	1.26781	2.1
62284	1.89226	2.1
62285	1.89226	1.36018
62286	1.89226	2.1
62287	1.89226	2.1
62288	1.89226	2.1
62289	1.89226	1.79071

62290	2.5844	2.1
62291	1.09563	2.1
62292	1.1468	2.1
62293	1.09563	2.1
62294	2.6142	2.1
62295	2.56304	2.1
62296	0.946126	2.1
62297	0.946126	2.1
62298	1.2922	2.1
62299	1.28152	2.1
62300	0.946126	2.1
62301	0.946126	2.1
62302	1.2922	2.1
62303	1.2922	2.1
62304	1.29221	2.1
62305	0.946132	2.1
62306	0.946119	2.1
62307	1.28151	2.1
62308	1.89225	2.1
62309	1.89225	1.72545
62310	1.89225	2.1
62311	1.89225	2.1
62312	1.89225	2.1
62313	1.89225	1.87439
62314	0.946121	1.53717
62315	0.946121	2.1
62316	0.946126	1.45015
62317	0.946126	2.1
62318	0.946124	1.25471
62319	0.946124	2.1
62320	0.946123	2.1
62321	0.946123	2.1
62322	0.94613	2.1
62323	0.94613	2.1
62324	0.946117	2.1
62325	0.946117	2.1
62326	1.89226	1.09886
62327	1.89226	1.07562
62328	1.89226	0.966961
62329	1.89226	1.96146
62330	1.89226	0.847662
62331	1.89226	2.1
62332	1.89226	1.39697
62333	1.89226	1.28682
62334	1.89226	0.919818
62335	1.89226	1.94694
62336	1.89226	0.842609
62337	1.89226	2.1
62338	1.89226	1.5982
62339	2.81281	1.84092
62340	2.50369	1.04926
62341	2.50369	2.04657
62342	1.89226	1.0858
62343	1.89226	2.1
62344	1.89226	2.1
62345	1.89226	2.1
62346	1.89226	1.86418
62347	1.89226	2.1

62348	1.89226	1.87933
62349	1.89226	2.1
62350	1.89226	2.1
62351	1.89226	2.1
62352	1.89226	2.1
62353	1.89226	2.1
62354	1.89226	2.1
62355	1.89226	2.1
62356	0.907036	2.1
62357	1.09714	2.1
62358	0.994609	2.1
62359	0.838808	2.1
62360	0.976536	2.1
62361	0.976536	2.1
62362	0.959412	2.1
62363	0.959412	2.1
62364	1.09714	2.1
62365	0.994609	2.1
62366	0.907036	2.1
62367	0.838808	2.1
62368	0.99461	2.1
62369	0.838808	2.1
62370	0.738212	2.1
62371	0.565634	2.1
62372	0.775921	2.1
62373	0.775921	2.1
62374	0.784322	2.1
62375	0.784322	2.1
62376	0.994609	2.1
62377	0.738212	2.1
62378	0.838808	2.1
62379	0.565635	2.1
62380	1.89226	2.1
62381	1.89226	2.1
62382	1.89226	1.67001
62383	1.89226	2.1
62384	1.89226	1.46494
62385	1.89226	2.1
62386	1.89226	2.1
62387	1.89226	2.1
62388	1.89226	2.1
62389	1.89226	2.1
62390	1.89226	2.1
62391	1.89226	2.1
62392	2.19428	2.1
62393	1.98922	2.1
62394	2.27322	2.1
62395	2.45893	2.1
62396	4.4675	2.1
62397	0.99461	2.1
62398	1.13661	2.1
62399	0.902272	2.1
62400	0.738212	2.1
62401	0.931874	2.1
62402	0.931874	2.1
62403	0.942953	2.1
62404	0.942953	2.1
62405	1.13658	2.1

62406	0.902248	2.1
62407	0.994637	2.1
62408	0.738233	2.1
62409	1.89226	1.45496
62410	1.89226	1.47072
62411	1.89226	1.70391
62412	1.89226	1.60823
62413	1.89226	1.53645
62414	1.89226	1.43734
62415	1.89227	1.63525
62416	2.58246	1.32427
62417	1.89227	1.48362
62418	1.89227	1.55943
62419	3.78453	1.43641
62420	1.89226	1.81145
62421	1.89226	1.57474
62422	1.65573	1.71419
62423	1.65573	1.74272
62424	1.89226	1.70646
62425	1.89226	1.74014
62426	1.89226	1.20047
62427	1.89226	1.30885
62428	1.89226	1.2951
62429	1.89226	1.29185
62430	1.89226	1.26355
62431	1.89226	1.24539
62432	1.89226	1.30505
62433	1.89226	1.3
62434	1.89226	1.29897
62435	1.89226	1.30139
62436	1.89226	1.28441
62437	1.89226	1.29759
62438	0.946132	1.70918
62439	0.946132	1.64982
62440	1.01023	1.3876
62441	1.13651	1.65943
62442	1.01023	1.36523
62443	0.946133	1.57216
62444	1.0644	1.58006
62445	1.0644	1.70117
62446	0.946132	1.72717
62447	0.946132	1.64851
62448	1.13651	1.54176
62449	0.946132	1.54946
62450	1.89226	1.0316
62451	1.89226	1.65052
62452	1.89226	1.25642
62453	1.89226	0.971276
62454	3.78451	1.07127
62455	1.00934	0.875131
62456	0.741561	0.853329
62457	0.946131	1.0479
62458	0.946131	1.13883
62459	0.946131	1.35957
62460	0.741561	0.684711
62461	1.00934	0.742337
62462	0.946131	1.19104
62463	0.741586	0.949855

62464	0.741586	0.965186
62465	0.946106	1.06216
62466	0.946106	0.90892
62467	1.14895	1.07777
62468	0.957449	1.12611
62469	0.934806	1.38469
62470	0.934806	1.34109
62471	1.13536	0.969197
62472	1.02829	1.67865
62473	0.946129	1.70884
62474	0.946129	1.0108
62475	0.946127	1.24448
62476	0.946127	1.31582
62477	0.946127	1.23811
62478	1.0283	1.33649
62479	1.89226	1.50357
62480	1.89226	1.04075
62481	1.89226	0.981823
62482	1.89226	1.37386
62483	3.78452	1.23281
62484	1.89226	0.892709
62485	1.89226	1.15879
62486	1.89226	1.04217
62487	1.89226	0.844195
62488	3.78453	0.912853
62489	1.89226	1.15607
62490	2.69068	0.928853
62491	1.89226	0.846512
62492	1.89226	1.06786
62493	3.78452	1.02645
62494	1.89226	1.03772
62495	1.89226	1.52131
62496	1.89226	1.48064
62497	1.89226	1.15029
62498	1.89226	1.33841
62499	1.89226	1.05593
62500	1.89226	2.1
62501	1.89226	2.1
62502	1.89226	2.1
62503	1.89226	2.1
62504	1.89226	2.1
62505	1.89226	2.1
62506	1.89226	2.1
62507	1.89226	2.1
62508	1.89226	2.1
62509	1.89226	2.1
62510	1.89226	2.1
62511	1.89226	2.1
62512	0.985109	2.1
62513	1.31144	2.1
62514	1.45864	2.1
62515	1.10667	2.1
62516	2.44375	2.1
62517	0.655718	2.1
62518	0.729316	2.1
62519	0.684594	2.1
62520	0.782707	2.1
62521	0.700849	2.1

62522	0.700849	2.1
62523	0.71306	2.1
62524	0.71306	2.1
62525	0.729318	2.1
62526	0.782705	2.1
62527	0.684592	2.1
62528	0.655719	2.1
62529	0.946128	2.1
62530	0.946128	2.1
62531	0.946133	2.1
62532	0.946133	2.1
62533	0.946131	2.1
62534	0.946131	2.1
62535	0.946131	2.1
62536	0.946131	2.1
62537	0.94613	2.1
62538	0.94613	2.1
62539	0.946131	2.1
62540	0.946131	2.1
62541	1.89226	2.1
62542	1.89226	2.1
62543	1.89226	2.1
62544	1.89226	2.1
62545	1.89226	2.1
62546	1.89226	2.1
62547	0.553334	2.1
62548	0.629936	2.1
62549	0.828211	2.1
62550	0.729319	2.1
62551	0.674025	2.1
62552	0.674025	2.1
62553	0.68523	2.1
62554	0.68523	2.1
62555	0.828211	2.1
62556	0.629935	2.1
62557	0.72932	2.1
62558	0.553334	2.1
62559	0.828208	2.1
62560	0.729316	2.1
62561	0.907037	2.1
62562	0.782707	2.1
62563	0.799048	2.1
62564	0.799048	2.1
62565	0.811867	2.1
62566	0.811867	2.1
62567	0.828209	2.1
62568	0.907035	2.1
62569	0.729318	2.1
62570	0.782706	2.1
62571	2.32981	2.1
62572	1.89226	2.01655
62573	1.89226	2.1
62574	1.89226	1.888
62575	2.2671	2.1
62576	2.2671	2.1
62577	1.89226	1.72195
62578	1.89226	1.85296
62579	1.89226	1.73545

62580	1.89226	1.46408
62581	1.89226	1.82583
62582	1.89226	1.72435
62583	1.3991	1.66614
62584	1.81793	1.71918
62585	2.07226	1.46817
62586	2.07226	1.48529
62587	1.24924	1.80864
62588	1.24924	1.92005
62589	0.946131	1.08847
62590	0.946131	2.1
62591	1.16491	2.1
62592	1.16491	2.1
62593	0.946131	1.96601
62594	0.946131	1.36259
62595	1.16491	2.1
62596	1.19523	2.1
62597	0.946132	2.1
62598	1.19523	2.1
62599	1.16491	2.1
62600	0.946131	2.1
62601	1.89226	2.1
62602	1.89226	1.50214
62603	1.89226	1.92319
62604	1.89226	0.948978
62605	3.78452	1.2766
62606	0.901204	1.59172
62607	0.901204	1.96971
62608	0.902504	1.71759
62609	0.902504	1.92598
62610	1.10416	1.59852
62611	1.03613	1.20005
62612	0.764922	2.02387
62613	0.699552	1.99554
62614	0.764921	1.49351
62615	1.10416	1.58092
62616	0.699551	1.70079
62617	1.03613	1.40138
62618	1.89226	0.98628
62619	1.89226	1.1161
62620	1.89226	1.10537
62621	1.89226	1.02314
62622	3.78453	1.09157
62623	1.89226	1.10762
62624	1.89226	1.22177
62625	1.89226	1.11725
62626	1.89226	1.28151
62627	1.89226	1.12558
62628	1.89226	1.28354
62629	0.946131	1.12867
62630	0.946131	1.11133
62631	0.946131	1.03635
62632	0.946131	1.01465
62633	0.946131	1.04114
62634	0.946131	1.12871
62635	0.946131	1.11252
62636	0.946131	0.989244
62637	0.946131	1.03094

62638	0.946131	1.03426
62639	0.946131	1.01757
62640	0.946131	1.03721
62641	1.89226	1.10224
62642	1.89226	1.21623
62643	1.89226	1.13542
62644	1.89226	1.21429
62645	1.89226	1.13241
62646	1.89226	1.21221
62647	0.946127	1.20057
62648	0.946127	1.17853
62649	1.22788	1.13959
62650	1.35469	1.1879
62651	1.35469	1.23184
62652	1.35469	1.20283
62653	0.946128	1.17934
62654	0.946128	1.10416
62655	0.946129	1.16458
62656	1.22788	1.19881
62657	1.35469	1.18278
62658	0.946129	1.14398
62659	1.22788	1.15808
62660	1.35469	1.20844
62661	1.02602	1.05276
62662	0.794861	1.09793
62663	1.15301	1.16137
62664	1.15301	1.21064
62665	1.10089	1.04648
62666	1.10089	1.13557
62667	0.794861	1.0767
62668	1.22788	1.15866
62669	1.02602	1.16255
62670	1.35469	1.15936
62671	1.89226	0.624011
62672	1.89226	0.80324
62673	1.89226	0.842308
62674	1.89226	0.739733
62675	3.78452	0.767643
62676	1.13536	1.28359
62677	0.942524	0.840815
62678	0.946131	0.807618
62679	0.946131	0.732583
62680	0.946127	0.839963
62681	0.946127	1.28268
62682	1.26282	0.726953
62683	0.917471	1.21662
62684	1.21023	0.892796
62685	1.00851	0.890682
62686	1.17955	0.834048
62687	0.883746	0.857862
62688	1.89226	0.823711
62689	1.89226	1.56393
62690	1.89226	0.931489
62691	1.89226	1.01664
62692	1.89226	0.962996
62693	1.89226	1.26621
62694	1.89226	0.740703
62695	1.89226	0.938015

62696	1.89226	0.786562
62697	1.89226	0.801504
62698	1.89226	0.806486
62699	1.89226	0.895067
62700	0.94613	0.862869
62701	0.94613	0.759442
62702	0.94613	0.701456
62703	0.94613	0.739699
62704	0.946128	0.764461
62705	0.946128	0.867707
62706	0.946131	0.864829
62707	0.946131	0.699063
62708	0.94613	0.754987
62709	0.94613	0.786369
62710	0.94613	0.731531
62711	0.94613	0.738008
62712	0.883758	1.22823
62713	1.03041	0.954666
62714	0.946131	0.848228
62715	0.946131	0.76255
62716	0.851537	0.869163
62717	0.851537	1.21723
62718	1.23942	0.746545
62719	1.14906	1.23002
62720	0.851484	0.864135
62721	0.985518	0.828153
62722	1.0156	0.840659
62723	0.851526	0.848523
62724	1.89226	0.847521
62725	1.89226	1.74044
62726	1.89226	0.934041
62727	1.89226	1.05355
62728	1.89226	0.976015
62729	1.89226	1.26626
62730	1.89226	0.763336
62731	1.89226	0.946149
62732	1.89226	0.805305
62733	1.89226	0.835657
62734	1.89226	0.814934
62735	1.89226	0.912625
62736	1.89227	0.566548
62737	1.89227	0.647288
62738	1.89227	0.601811
62739	1.89227	0.658911
62740	3.78453	0.601088
62741	1.89226	0.632785
62742	1.89226	0.66448
62743	1.89226	0.650262
62744	1.89226	0.677869
62745	3.78452	0.669086
62746	0.946132	0.68502
62747	0.946132	0.686193
62748	0.946128	0.687218
62749	0.946128	0.684769
62750	0.94613	0.694702
62751	0.94613	0.684205
62752	0.94613	0.663274
62753	0.94613	0.674716

62754	0.946131	0.67086
62755	0.946131	0.686234
62756	0.94613	0.671604
62757	0.94613	0.691646
62758	1.89226	0.66523
62759	1.89226	0.687676
62760	1.89226	0.671804
62761	1.89226	0.662433
62762	1.89226	0.681518
62763	1.89226	0.675785
62764	1.98874	0.508181
62765	2.33455	0.599591
62766	2.01865	0.618615
62767	2.07024	0.606924
62768	4.32329	0.660137
62769	1.98874	0.594311
62770	2.00266	0.674601
62771	2.14218	0.67955
62772	2.07024	0.68241
62773	3.9914	0.653744
62774	1.00133	0.688761
62775	0.804471	0.679336
62776	1.03512	0.683721
62777	0.995176	0.684889
62778	0.80447	0.697971
62779	0.995178	0.690145
62780	1.00133	0.682734
62781	1.03512	0.691034
62782	0.880587	0.691871
62783	0.880587	0.686178
62784	0.959002	0.671406
62785	0.959002	0.688368
62786	2.33455	0.617344
62787	1.99036	0.677781
62788	2.07024	0.622355
62789	2.58645	0.666746
62790	4.32491	0.542926
62791	1.89226	0.832018
62792	1.89226	0.802962
62793	1.89226	0.859532
62794	1.89226	0.754645
62795	3.78453	0.751642
62796	1.89226	0.926607
62797	1.89226	0.815994
62798	1.89226	0.878203
62799	1.89226	0.85747
62800	3.78453	0.925827
62801	1.89226	0.772399
62802	1.89226	0.70448
62803	1.89226	0.723178
62804	1.89226	0.764778
62805	1.89226	0.720587
62806	1.89226	0.766044
62807	1.89226	0.756763
62808	2.1523	0.532596
62809	2.53257	0.609665
62810	2.53257	0.59449
62811	1.89226	0.744958

62812	1.89226	0.732636
62813	0.946133	0.726719
62814	0.946133	0.78335
62815	0.946132	0.648696
62816	0.946132	0.680586
62817	0.946132	0.643394
62818	0.946132	0.667511
62819	0.946132	0.786964
62820	0.946132	0.682625
62821	0.946133	0.748154
62822	0.946133	0.755276
62823	0.946132	0.80195
62824	0.946132	0.74265
62825	0.946132	0.818267
62826	0.946132	0.856824
62827	0.946132	0.759257
62828	0.946132	0.809762
62829	0.946132	0.758539
62830	0.946132	0.780133
62831	0.946132	0.856978
62832	0.946132	0.815042
62833	0.946132	0.823795
62834	0.946132	0.82546
62835	0.946131	0.81167
62836	0.946131	0.809052
62837	1.62772	0.428237
62838	1.70303	0.494418
62839	1.70303	0.481068
62840	1.70303	0.470486
62841	1.90116	0.44673
62842	1.90116	0.432107
62843	1.89226	0.436203
62844	1.89226	0.554054
62845	1.89226	0.571625
62846	1.89226	0.581327
62847	1.89226	0.451935
62848	1.89226	0.460082
62849	1.89226	0.600015
62850	1.89226	0.667011
62851	1.89226	0.544222
62852	1.89226	0.673777
62853	3.78452	0.654545
62854	1.89226	0.57715
62855	1.89226	0.585771
62856	1.89226	0.56428
62857	1.89226	0.588237
62858	1.89226	0.548126
62859	1.89226	0.579019
62860	0.946132	0.489402
62861	0.946132	0.47638
62862	0.946129	0.497882
62863	0.946129	0.498594
62864	0.94613	0.512861
62865	0.94613	0.522477
62866	0.946131	0.47989
62867	0.946131	0.456381
62868	0.94613	0.461541
62869	0.94613	0.485603

62870	0.946131	0.412718
62871	0.946131	0.533412
62872	1.89226	0.528057
62873	1.89226	0.588262
62874	1.89226	0.647576
62875	1.89226	0.656923
62876	1.89226	0.51301
62877	1.89226	0.52309
62878	1.89226	0.766399
62879	1.89226	0.880083
62880	1.89226	0.873828
62881	1.89226	0.755572
62882	3.78452	0.805509
62883	1.89226	0.859855
62884	1.89226	0.972344
62885	1.89226	0.844849
62886	1.89226	0.988334
62887	1.89226	0.848937
62888	1.89226	0.996898
62889	1.30165	0.842424
62890	0.946127	0.876939
62891	1.09659	0.757714
62892	0.946131	0.770172
62893	1.09659	0.763828
62894	1.09659	0.983442
62895	0.946128	0.940427
62896	0.946128	0.774486
62897	1.30165	0.812875
62898	1.09659	0.872238
62899	0.946129	0.823133
62900	0.946129	0.85367
62901	0.946128	0.882074
62902	0.946128	0.896601
62903	0.946133	0.774434
62904	0.946133	0.796579
62905	0.946131	0.773439
62906	0.946131	0.904303
62907	0.946131	0.894112
62908	0.946131	0.776391
62909	0.94613	0.807755
62910	0.94613	0.807035
62911	0.946131	0.799872
62912	0.946131	0.818309
62913	0.946133	0.986669
62914	0.946133	0.984748
62915	0.946132	0.872558
62916	0.946132	0.886727
62917	0.946132	0.869052
62918	0.946132	0.991137
62919	0.946132	0.984773
62920	0.946132	0.872148
62921	0.946132	0.913516
62922	0.946132	0.911223
62923	0.946133	0.932168
62924	0.946133	0.933045
62925	1.30165	0.946523
62926	0.972228	0.926168
62927	1.09659	0.808088

62928	0.656268	0.825429
62929	1.06217	0.816374
62930	1.06217	1.16094
62931	1.00665	0.803197
62932	1.00665	0.980436
62933	0.97223	0.817859
62934	0.656267	0.801377
62935	1.30165	0.829073
62936	1.09659	0.853115
62937	0.946132	1.22058
62938	0.946132	1.4799
62939	1.19895	1.86604
62940	1.19895	2.1
62941	0.94613	1.90226
62942	0.94613	1.08859
62943	1.39356	1.5937
62944	1.19895	2.1
62945	0.94613	1.51915
62946	1.39356	1.57669
62947	1.19895	1.68929
62948	0.946131	1.53764
62949	1.89226	1.14879
62950	1.89226	1.90422
62951	1.89226	1.97997
62952	1.89226	1.13193
62953	1.89226	2.01517
62954	1.89226	1.16356
62955	0.946132	1.05264
62956	0.946132	1.04638
62957	0.946128	1.28985
62958	0.946128	1.42552
62959	0.94613	1.29329
62960	0.94613	0.966577
62961	0.94613	1.07008
62962	0.94613	1.3034
62963	0.946131	1.09737
62964	0.946131	1.08556
62965	0.94613	1.13471
62966	0.94613	1.15524
62967	1.89226	0.341377
62968	1.89226	0.368157
62969	1.89226	0.43732
62970	1.89226	0.293064
62971	1.89226	0.440807
62972	1.89226	0.289613
62973	1.29881	0.248149
62974	1.09985	0.238523
62975	0.946126	0.351026
62976	0.946126	0.281375
62977	0.851539	0.348912
62978	1.20421	0.286077
62979	0.851483	0.246931
62980	0.999426	0.306744
62981	1.47172	0.282888
62982	0.999413	0.246706
62983	0.851552	0.279252
62984	0.851552	0.279355
62985	1.89226	0.421418

62986	1.89226	0.411763
62987	1.89226	0.524062
62988	1.89226	0.362302
62989	1.89226	0.521042
62990	1.89226	0.358626
62991	1.89226	0.382383
62992	1.89226	0.39536
62993	1.89226	0.472907
62994	1.89226	0.331005
62995	1.89226	0.477103
62996	1.89226	0.322898
62997	1.33915	2.1
62998	1.99297	2.1
62999	2.05216	2.1
63000	2.60163	2.1
63001	4.04513	2.1
63002	1.89226	1.45316
63003	1.89226	2.03253
63004	1.89226	2.1
63005	1.89226	1.58866
63006	3.78452	1.8999
63007	0.946132	1.69238
63008	0.946132	1.57752
63009	0.946133	2.07968
63010	0.946133	2.1
63011	0.946132	2.08793
63012	0.946132	1.30534
63013	0.946133	2.05503
63014	0.946133	1.62687
63015	0.946132	1.94218
63016	0.946132	1.83393
63017	0.946132	1.97163
63018	0.946132	1.97519
63019	1.89226	1.4868
63020	1.89226	1.15368
63021	1.89226	1.22277
63022	1.89226	1.59173
63023	3.78452	1.30514
63024	1.89227	1.19133
63025	1.89227	1.55151
63026	1.89227	1.5915
63027	1.89227	1.16101
63028	1.89227	1.68419
63029	1.89227	1.19905
63030	2.29301	2.1
63031	1.70303	2.02934
63032	2.46749	2.1
63033	2.30728	2.1
63034	4.53937	1.97153
63035	1.89226	0.993378
63036	1.89226	2.05213
63037	1.89226	2.1
63038	1.89226	0.980095
63039	3.78452	2.05204
63040	1.89226	0.85245
63041	1.89226	1.27729
63042	1.89226	1.05359
63043	1.89226	1.39549

63044	1.89226	0.905558
63045	1.89226	1.04587
63046	2.30728	2.1
63047	1.00757	1.09261
63048	2.0833	2.1
63049	1.65318	1.52869
63050	1.417	1.36775
63051	1.00757	1.45843
63052	2.46749	2.1
63053	2.0975	2.1
63054	2.79653	2.1
63055	2.44785	2.05992
63056	4.91534	2.1
63057	1.89226	2.1
63058	1.89226	1.45684
63059	1.89226	1.00522
63060	1.89226	2.06681
63061	3.78452	1.11503
63062	1.89226	1.06257
63063	1.89226	1.37041
63064	1.89226	2.09239
63065	1.89226	1.22366
63066	1.89226	1.97286
63067	1.89226	1.20151
63068	0.970559	1.39851
63069	0.970559	2.08443
63070	0.979305	2.1
63071	0.979305	2.1
63072	0.708502	1.36622
63073	0.901117	2.1
63074	1.04875	2.1
63075	1.22392	1.49047
63076	1.22392	2.0083
63077	0.901117	2.1
63078	1.04875	2.1
63079	0.7085	1.9837
63080	1.89226	1.3312
63081	1.89226	1.00141
63082	1.89226	1.12857
63083	1.89226	1.19608
63084	3.78452	1.41868
63085	1.89226	1.22221
63086	1.89226	1.00107
63087	1.89226	1.2101
63088	1.89226	1.08091
63089	3.78452	1.0684
63090	1.89226	0.802697
63091	1.89226	0.66381
63092	1.89226	0.855184
63093	1.89226	0.786523
63094	3.78453	0.798476
63095	1.89226	0.875295
63096	1.89226	0.936851
63097	1.89226	0.821595
63098	1.89226	0.805562
63099	1.89226	0.792956
63100	1.89226	0.750104
63101	1.89226	0.941485

63102	2.35441	0.936789
63103	1.89226	1.14514
63104	2.30931	0.780962
63105	4.24667	0.874762
63106	1.11612	0.877724
63107	0.946131	0.855928
63108	1.1772	0.853005
63109	0.946131	0.95188
63110	1.17721	0.953761
63111	1.11612	0.870576
63112	0.94613	0.916525
63113	0.94613	0.919669
63114	1.11612	0.919434
63115	1.11612	0.86282
63116	0.946131	0.869841
63117	0.946131	0.848472
63118	1.11612	0.560179
63119	0.582675	0.557303
63120	1.17721	0.597173
63121	0.651969	0.558063
63122	0.651969	0.564337
63123	0.582674	0.531499
63124	1.17721	0.556458
63125	1.11612	0.539192
63126	0.877955	0.575522
63127	0.877955	0.591666
63128	0.881925	0.550535
63129	0.881925	0.565651
63130	1.17721	0.682024
63131	0.651969	0.641792
63132	1.15466	0.631152
63133	0.623642	0.629564
63134	0.623642	0.636896
63135	0.651969	0.578609
63136	1.17721	0.668249
63137	1.15466	0.658432
63138	0.904714	0.694911
63139	0.904714	0.602675
63140	0.901914	0.732728
63141	0.901914	0.63041
63142	1.89226	1.02212
63143	1.89226	0.833456
63144	1.89226	0.796619
63145	1.89226	1.16868
63146	1.89226	0.750463
63147	1.89226	1.46898
63148	1.89226	1.08213
63149	1.89226	0.794539
63150	1.89226	0.772406
63151	1.89226	1.67638
63152	1.89226	0.761103
63153	1.89226	1.75597
63154	2.33986	1.45355
63155	1.97819	0.966949
63156	2.39166	0.740967
63157	2.70141	1.47304
63158	4.73151	0.966789
63159	0.989095	0.941398

63160	1.19583	0.793045
63161	0.851194	0.756559
63162	0.581197	0.833037
63163	0.872704	0.708977
63164	0.872704	1.05019
63165	0.904322	0.754722
63166	0.904322	0.913602
63167	1.19583	0.728936
63168	0.851193	0.728236
63169	0.989095	0.86366
63170	0.581197	0.80981
63171	1.89226	0.895469
63172	1.89226	0.874061
63173	1.89226	0.855991
63174	1.89226	1.24389
63175	1.89226	0.80498
63176	1.89226	1.46517
63177	1.89226	1.08412
63178	1.89226	0.782768
63179	1.89226	0.780863
63180	1.89226	1.58692
63181	1.89226	0.744902
63182	1.89226	1.75104
63183	2.39166	0.876589
63184	1.70239	0.977845
63185	2.13665	0.760339
63186	2.82684	0.821239
63187	4.52831	0.886253
63188	1.89226	0.363209
63189	1.89226	0.562258
63190	1.89226	0.484052
63191	1.89226	0.506083
63192	3.78453	0.540971
63193	1.89226	0.364656
63194	1.89226	0.415658
63195	1.89226	0.481772
63196	1.89226	0.309299
63197	1.89226	0.498614
63198	1.89226	0.313062
63199	1.89226	0.468325
63200	1.89226	0.505616
63201	1.89226	0.581041
63202	1.89226	0.40985
63203	1.89226	0.59449
63204	1.89226	0.409268
63205	1.89226	0.593564
63206	1.89226	0.631454
63207	1.89226	0.789217
63208	1.89226	0.489653
63209	1.89226	0.803047
63210	1.89226	0.493215
63211	1.89226	0.523241
63212	1.89226	0.596117
63213	1.89226	0.689218
63214	1.89226	0.458998
63215	1.89226	0.744186
63216	1.89226	0.483821
63217	1.5169	0.631899

63218	1.61969	0.45406
63219	1.17262	0.48422
63220	1.17262	0.548078
63221	1.88868	0.500591
63222	1.88868	0.573994
63223	1.79981	0.755899
63224	1.88868	0.564024
63225	1.5169	0.619892
63226	1.5169	0.687437
63227	2.12891	0.628834
63228	2.12891	0.705202
63229	1.89226	0.675701
63230	1.89226	0.490035
63231	1.89226	0.546425
63232	1.89226	0.612078
63233	3.78452	0.600155
63234	0.946132	0.45946
63235	0.946132	0.547135
63236	0.946132	0.391688
63237	0.946132	0.424548
63238	0.946132	0.38953
63239	0.946132	0.480049
63240	0.946133	0.437305
63241	0.946133	0.551211
63242	0.946133	0.439781
63243	0.946133	0.456284
63244	0.946132	0.471099
63245	0.946132	0.459048
63246	1.88868	0.624482
63247	2.05759	0.456928
63248	1.61969	0.486503
63249	1.61969	0.552783
63250	2.29747	0.499153
63251	2.29747	0.566283
63252	2.12891	0.74987
63253	2.29747	0.558916
63254	1.88868	0.614296
63255	1.88868	0.667211
63256	2.50894	0.619176
63257	2.50894	0.682646
63258	1.89226	0.54107
63259	1.89226	0.617286
63260	1.89226	0.47729
63261	1.89226	0.65785
63262	1.89226	0.489882
63263	1.89226	0.67177
63264	0.946132	0.542301
63265	0.946132	0.467068
63266	0.946132	0.439819
63267	0.946132	0.402855
63268	0.946131	0.397681
63269	0.946131	0.507348
63270	0.946132	0.44524
63271	0.946132	0.54966
63272	0.946132	0.451506
63273	0.946132	0.472327
63274	0.946132	0.463368
63275	0.946132	0.491578

63276	2.42758	1.29858
63277	2.14895	2.1
63278	2.56975	1.19315
63279	2.28142	2.1
63280	4.71869	1.71364
63281	1.89226	1.16561
63282	1.89226	2.1
63283	1.89226	1.21125
63284	1.89226	2.1
63285	3.78452	1.79569
63286	1.89226	1.86802
63287	1.89226	2.1
63288	1.89226	1.85619
63289	1.89226	2.1
63290	1.89226	1.84168
63291	1.89226	2.1
63292	0.960916	2.1
63293	0.960916	1.86112
63294	0.978118	2.00603
63295	0.875674	2.1
63296	0.775661	2.1
63297	0.773613	2.1
63298	1.07631	1.89941
63299	1.14266	2.06763
63300	1.16927	2.1
63301	0.898612	2.1
63302	1.04757	2.1
63303	0.757536	2.1
63304	2.56975	1.17669
63305	2.28142	2.1
63306	2.64384	1.24982
63307	2.40621	2.06316
63308	4.97596	1.78838
63309	1.89226	1.19941
63310	1.89226	2.1
63311	1.89226	0.923642
63312	1.89226	2.06148
63313	3.78452	1.33397
63314	1.89226	1.68974
63315	1.89226	2.1
63316	1.89226	2.04645
63317	1.89226	2.1
63318	1.89226	1.83148
63319	1.89226	2.1
63320	1.03328	2.1
63321	1.03328	2.05733
63322	1.04321	1.70846
63323	1.15424	2.1
63324	0.936183	2.1
63325	0.944491	2.1
63326	1.14266	1.79506
63327	1.20517	1.8802
63328	1.23323	2.1
63329	0.961268	2.1
63330	1.11215	2.1
63331	0.827247	2.1
63332	1.89226	2.1
63333	1.89226	1.20511

63334	1.89226	2.1
63335	1.89226	1.35611
63336	3.78453	2.1
63337	2.14647	2.1
63338	2.20589	1.95994
63339	1.95567	2.1
63340	2.03224	1.38136
63341	4.17871	2.1
63342	2.06035	1.9784
63343	1.88452	0.764948
63344	1.88452	1.73399
63345	2.26997	0.840608
63346	2.03224	1.32434
63347	2.42272	0.901208
63348	1.89226	1.29234
63349	1.89226	0.647195
63350	1.89226	0.492497
63351	1.89226	1.38562
63352	1.89226	0.611399
63353	1.89226	1.55421
63354	1.89225	2.1
63355	1.89225	1.8867
63356	1.89225	1.82851
63357	1.89225	2.1
63358	1.89225	1.54173
63359	1.89225	2.1
63360	0.952418	2.1
63361	0.952418	2.1
63362	0.95883	1.88302
63363	0.95883	2.1
63364	0.946182	1.77532
63365	1.01611	2.01994
63366	0.895134	2.1
63367	0.977828	2.1
63368	0.89514	2.1
63369	0.946189	2.1
63370	0.977821	2.1
63371	1.01611	2.1
63372	2.03223	2.1
63373	1.88451	0.722792
63374	1.89236	1.80637
63375	1.74649	0.743585
63376	3.77687	0.94593
63377	0.81094	1.66686
63378	0.81094	0.80919
63379	0.810939	1.79721
63380	0.810939	1.52632
63381	0.810945	0.975382
63382	0.810945	0.986068
63383	1.89225	0.604477
63384	1.35167	0.903181
63385	1.08131	1.88732
63386	1.08131	0.574921
63387	1.0813	0.941376
63388	1.89226	0.270803
63389	1.89226	0.301345
63390	1.89226	0.317543
63391	1.89226	0.275007

63392	1.89226	0.308435
63393	1.89226	0.265585
63394	1.01842	0.223448
63395	0.936656	0.220268
63396	0.946131	0.252063
63397	0.946131	0.241651
63398	1.11157	0.218899
63399	1.04281	0.240802
63400	0.925803	0.225116
63401	0.925803	0.254945
63402	0.851553	0.222619
63403	1.11158	0.233644
63404	1.05017	0.246451
63405	0.925798	0.234231
63406	1.89226	0.282624
63407	1.89226	0.337036
63408	1.89226	0.35866
63409	1.89226	0.289688
63410	1.89226	0.343975
63411	1.89226	0.271286
63412	1.23504	0.238875
63413	1.23504	0.213381
63414	0.946131	0.266729
63415	0.946131	0.249239
63416	1.37358	0.247098
63417	1.23504	0.227114
63418	0.946129	0.241867
63419	0.946129	0.276328
63420	1.37358	0.234047
63421	0.946131	0.24091
63422	0.94613	0.238251
63423	1.23504	0.251117
63424	1.00261	0.207889
63425	1.00261	0.209381
63426	1.02952	0.248862
63427	1.02952	0.227205
63428	0.850964	0.225355
63429	0.658553	0.218179
63430	1.37358	0.236183
63431	1.23504	0.212029
63432	1.37358	0.220537
63433	0.850965	0.233236
63434	1.23504	0.221845
63435	0.658553	0.216648
63436	0.946133	0.260982
63437	0.946133	0.239398
63438	0.946132	0.342537
63439	0.946132	0.280637
63440	0.946132	0.251635
63441	0.946132	0.276027
63442	0.946133	0.31999
63443	0.946133	0.270039
63444	0.946133	0.284166
63445	0.946133	0.281404
63446	0.946132	0.274612
63447	0.946132	0.269188
63448	1.89226	0.423125
63449	1.89226	0.448898

63450	1.89226	0.36278
63451	1.89226	0.385176
63452	3.78452	0.445853
63453	1.89226	0.462326
63454	1.89226	0.489503
63455	1.89226	0.386198
63456	1.89226	0.415475
63457	3.78452	0.38959
63458	1.89227	0.410366
63459	1.89227	0.418815
63460	1.89227	0.364817
63461	1.89227	0.385255
63462	1.89227	0.375908
63463	1.89227	0.41092
63464	0.94613	0.311881
63465	0.94613	0.324139
63466	0.946127	0.314221
63467	0.946127	0.324496
63468	0.94613	0.315905
63469	0.94613	0.32998
63470	0.946127	0.307817
63471	0.946127	0.356746
63472	0.946128	0.30394
63473	0.946128	0.285277
63474	0.946128	0.34063
63475	0.946128	0.331076
63476	0.946132	0.339547
63477	0.946132	0.348926
63478	0.946128	0.326617
63479	0.946128	0.340143
63480	0.94613	0.333535
63481	0.94613	0.364851
63482	0.946131	0.31743
63483	0.946131	0.332372
63484	0.94613	0.318199
63485	0.94613	0.301271
63486	0.94613	0.348603
63487	0.94613	0.376248
63488	1.89226	2.1
63489	1.89226	1.78601
63490	1.89226	1.91271
63491	1.89226	2.1
63492	3.78453	2.1
63493	2.72844	2.1
63494	1.89226	2.0934
63495	2.26536	2.1
63496	2.26536	2.1
63497	1.89226	2.1
63498	1.89226	2.1
63499	1.89226	2.09862
63500	1.89226	1.53051
63501	1.89226	1.80754
63502	1.89226	1.89967
63503	1.89226	1.79734
63504	1.89226	1.8795
63505	1.89226	1.90697
63506	1.89226	2.1
63507	1.89226	1.97089

63508	1.89226	2.1
63509	1.89226	1.79804
63510	1.89226	2.1
63511	2.72844	2.1
63512	2.26536	2.1
63513	2.28367	2.1
63514	1.5791	2.1
63515	4.54903	2.1
63516	0.810945	1.91597
63517	0.810945	1.6317
63518	0.810943	1.60809
63519	0.810943	1.9988
63520	1.89226	2.1
63521	1.3517	1.8062
63522	1.08133	1.94699
63523	1.08132	1.94718
63524	1.08132	1.85496
63525	0.810933	2.02353
63526	0.810933	1.89161
63527	1.89227	2.1
63528	1.89227	2.1
63529	1.89227	2.1
63530	1.89227	2.1
63531	3.78453	2.1
63532	2.71897	2.1
63533	2.27495	2.1
63534	2.648	2.1
63535	2.20832	2.1
63536	4.92729	2.1
63537	2.27495	2.1
63538	1.38455	2.1
63539	1.38455	2.1
63540	1.46586	2.1
63541	2.20831	2.1
63542	2.28962	2.1
63543	0.946133	2.1
63544	0.946133	2.1
63545	0.946132	2.1
63546	0.946132	2.1
63547	0.946132	2.1
63548	0.946132	2.1
63549	0.946132	2.1
63550	0.946132	2.1
63551	0.946132	2.1
63552	0.946132	2.1
63553	0.946133	2.1
63554	0.946133	2.1
63555	1.89226	2.1
63556	1.89226	2.1
63557	1.89226	2.1
63558	1.89226	2.1
63559	1.89226	2.1
63560	1.89226	2.1
63561	2.648	2.1
63562	2.08612	2.1
63563	2.08612	2.1
63564	2.20831	2.1
63565	2.56618	2.1

63566	2.68837	2.1
63567	2.08612	2.1
63568	2.20831	2.1
63569	1.30659	2.1
63570	1.30659	2.1
63571	1.38455	2.1
63572	2.16408	2.1
63573	0.810946	2.1
63574	0.810946	2.1
63575	0.810946	2.1
63576	0.810946	2.1
63577	1.89226	2.1
63578	1.3517	2.1
63579	1.08133	2.1
63580	1.08132	2.1
63581	1.08132	2.1
63582	0.810934	2.1
63583	0.810934	2.1
63584	1.89226	1.4529
63585	1.89226	1.27232
63586	1.89226	1.00467
63587	1.89226	1.64701
63588	3.78452	1.20012
63589	1.89226	1.13964
63590	1.89226	1.85085
63591	1.89226	1.82439
63592	1.89226	1.20909
63593	3.78453	1.62518
63594	1.08733	1.7467
63595	0.856615	2.01692
63596	1.25964	1.2865
63597	1.12378	1.26596
63598	1.23551	1.14012
63599	0.969894	2.1
63600	0.985977	1.80781
63601	0.985977	1.18561
63602	1.16169	1.37579
63603	1.21072	1.50479
63604	0.765175	1.50734
63605	0.863419	1.45962
63606	2.39934	1.61911
63607	2.44427	1.34174
63608	2.64003	1.01233
63609	2.39077	1.6984
63610	2.39934	1.49058
63611	2.10194	1.95646
63612	1.89226	1.03017
63613	1.89226	1.77172
63614	1.89226	1.35878
63615	1.89226	0.911646
63616	3.78452	1.38643
63617	0.946132	1.59981
63618	0.946132	1.70934
63619	0.946131	1.11222
63620	0.946131	1.14947
63621	0.946132	1.1131
63622	0.946132	2.08464
63623	0.946132	1.61157

63624	0.946132	1.21372
63625	0.946132	1.36244
63626	0.946132	1.42766
63627	0.946132	1.35431
63628	0.946132	1.50868
63629	1.19967	1.67748
63630	0.946128	1.47226
63631	0.946133	1.09385
63632	1.32002	1.33702
63633	1.19967	1.57783
63634	1.19967	1.55049
63635	0.94613	1.68566
63636	0.94613	0.897685
63637	0.946131	1.20915
63638	0.946131	1.28837
63639	1.19967	1.39462
63640	1.32001	1.42252
63641	1.89226	0.771785
63642	1.89226	0.894818
63643	1.89226	0.666975
63644	1.89226	1.12379
63645	3.78451	0.817737
63646	2.6402	0.708682
63647	2.28552	0.89496
63648	2.50958	0.604788
63649	2.18948	0.854884
63650	4.79509	0.793869
63651	2.28552	0.868948
63652	1.86102	2.1
63653	2.18949	0.882204
63654	1.78376	2.1
63655	4.06928	1.75443
63656	1.89226	0.871352
63657	1.89226	2.1
63658	1.89226	1.00092
63659	1.89226	2.1
63660	3.78452	1.89656
63661	1.89226	0.738515
63662	1.89226	1.26999
63663	1.89226	0.584202
63664	1.89226	0.934881
63665	3.78452	0.797719
63666	2.50958	0.708429
63667	2.18948	0.829779
63668	2.39524	0.445198
63669	2.10188	0.938096
63670	4.61146	0.783403
63671	2.18949	0.837847
63672	1.78376	2.1
63673	1.71839	0.91765
63674	1.71839	2.03088
63675	2.10189	0.963543
63676	2.16726	2.1
63677	0.810943	0.929167
63678	0.810943	2.1
63679	0.810945	0.917058
63680	0.810945	0.89316
63681	0.810959	1.56465

63682	0.810959	1.66823
63683	1.89226	2.1
63684	1.35168	1.80434
63685	1.08132	0.954847
63686	1.08132	2.1
63687	1.0813	1.57355
63688	1.89226	0.805236
63689	1.89226	1.22817
63690	1.89226	0.938859
63691	1.89226	0.910473
63692	1.89226	0.972498
63693	1.89226	1.08034
63694	1.28421	1.12798
63695	0.992041	0.84354
63696	1.07707	1.59984
63697	0.789746	1.26199
63698	1.03815	1.43532
63699	1.03815	1.23328
63700	1.03581	1.28045
63701	1.03581	1.04169
63702	1.28421	1.23088
63703	1.07707	1.38354
63704	0.789748	1.21166
63705	0.992038	1.08236
63706	1.89226	0.865621
63707	1.89226	1.28429
63708	1.89226	1.03388
63709	1.89226	0.915185
63710	1.89226	1.04556
63711	1.89226	0.928494
63712	1.89226	0.799587
63713	1.89226	1.02225
63714	1.89226	0.888076
63715	1.89226	0.872284
63716	1.89226	0.904483
63717	1.89226	0.975578
63718	0.92236	1.62684
63719	0.655971	1.38687
63720	0.916772	1.51048
63721	0.913834	1.15484
63722	1.18074	1.3493
63723	0.922346	1.50339
63724	2.15414	0.926863
63725	1.53528	1.24667
63726	1.22748	1.40081
63727	1.57442	0.996379
63728	0.874683	1.29143
63729	1.25935	2.1
63730	2.61433	2.1
63731	1.22262	2.1
63732	1.22262	2.1
63733	2.62813	2.1
63734	2.62813	2.1
63735	1.89226	2.1
63736	2.61434	2.1
63737	2.62814	2.1
63738	2.61434	2.1
63739	1.90607	2.1

63740	1.89226	2.1
63741	1.89226	2.1
63742	1.89226	2.05472
63743	1.89226	2.1
63744	1.89226	2.1
63745	1.89226	2.1
63746	1.89226	2.1
63747	1.89226	1.34328
63748	1.89226	2.01218
63749	1.89226	2.1
63750	1.89226	2.1
63751	1.89226	2.1
63752	1.89226	2.1
63753	1.31969	2.1
63754	1.31406	2.1
63755	0.629677	2.1
63756	0.643104	2.1
63757	0.976621	2.1
63758	0.976621	2.1
63759	0.98055	2.1
63760	0.98055	2.1
63761	1.3197	2.1
63762	0.643102	2.1
63763	0.629675	2.1
63764	1.31407	2.1
63765	0.946133	2.1
63766	0.946133	2.1
63767	1.3197	2.1
63768	1.31407	2.1
63769	0.946132	2.1
63770	0.946132	2.1
63771	1.3197	2.1
63772	1.3197	2.1
63773	1.3197	2.1
63774	0.946133	2.1
63775	0.946132	2.1
63776	1.31407	2.1
63777	1.89226	2.1
63778	1.89226	1.83642
63779	1.89226	2.1
63780	1.89226	2.1
63781	1.89226	2.1
63782	1.89226	2.08007
63783	1.89226	2.1
63784	1.89226	1.57827
63785	1.89226	2.1
63786	1.89226	2.1
63787	1.89226	2.1
63788	1.89226	1.72133
63789	1.89226	0.505629
63790	1.89226	0.417583
63791	1.89226	0.361119
63792	1.89226	0.373938
63793	3.78452	0.379244
63794	1.89226	0.424815
63795	1.89226	0.345339
63796	1.89226	0.350707
63797	1.89226	0.381399

63798	1.89226	0.387817
63799	1.89226	0.432524
63800	0.946131	0.290154
63801	0.946131	0.298279
63802	0.946128	0.28851
63803	0.946128	0.293282
63804	0.94613	0.282448
63805	0.94613	0.288609
63806	0.94613	0.26564
63807	0.94613	0.332693
63808	0.94613	0.289936
63809	0.94613	0.289348
63810	0.94613	0.322739
63811	0.94613	0.301373
63812	1.89226	0.286477
63813	1.89226	0.306058
63814	1.89226	0.293176
63815	1.89226	0.26567
63816	1.89226	0.274189
63817	1.89226	0.303772
63818	0.924614	0.247326
63819	0.914275	0.241076
63820	0.906003	0.24439
63821	0.62479	0.234097
63822	2.02733	0.250122
63823	1.48332	0.235011
63824	0.833106	0.237015
63825	1.19364	0.238841
63826	1.57482	0.246488
63827	1.18103	0.237875
63828	0.905988	0.242041
63829	1.89226	2.1
63830	1.89226	2.1
63831	1.89226	2.1
63832	1.89226	2.1
63833	3.78452	2.1
63834	1.89226	2.1
63835	1.89226	2.1
63836	1.89226	2.1
63837	1.89226	2.1
63838	1.89226	2.1
63839	1.89226	2.1
63840	0.816805	2.1
63841	0.928516	2.1
63842	1.4045	2.1
63843	1.26268	2.1
63844	1.0881	2.1
63845	1.0881	2.1
63846	1.10309	2.1
63847	1.10309	2.1
63848	0.928518	2.1
63849	1.4045	2.1
63850	0.816807	2.1
63851	1.26267	2.1
63852	1.89226	2.1
63853	2.52535	2.1
63854	1.89226	2.1
63855	1.89226	2.1

63856	2.809	2.1
63857	2.809	2.1
63858	1.89226	2.1
63859	1.89226	2.1
63860	1.89226	2.1
63861	1.89226	2.1
63862	3.78452	2.1
63863	0.946127	2.1
63864	0.946127	2.1
63865	0.946131	2.1
63866	0.946131	2.1
63867	0.94613	2.1
63868	0.94613	2.1
63869	0.946128	2.1
63870	0.946128	2.1
63871	0.946129	2.1
63872	0.946129	2.1
63873	0.946129	2.1
63874	0.946129	2.1
63875	1.89224	0.905724
63876	1.89224	2.1
63877	1.89224	1.29331
63878	1.89224	1.97626
63879	3.78448	1.74535
63880	2.39244	1.93448
63881	2.27036	2.1
63882	2.25945	1.24375
63883	2.27398	2.1
63884	4.52981	2.1
63885	2.27036	2.1
63886	1.97088	2.1
63887	1.86302	2.1
63888	1.84991	2.1
63889	4.12027	2.1
63890	1.89224	2.1
63891	1.89224	2.1
63892	1.89224	2.00456
63893	1.89224	2.1
63894	3.78447	2.1
63895	1.89226	1.32242
63896	1.89226	1.90881
63897	1.89226	0.916358
63898	1.89226	2.1
63899	3.78453	1.57815
63900	2.25948	1.34133
63901	2.33611	2.1
63902	2.05257	2.1
63903	1.94441	2.1
63904	4.20389	2.1
63905	1.92514	2.1
63906	1.84993	2.1
63907	1.94441	2.1
63908	1.74391	2.1
63909	3.79434	2.1
63910	1.89226	1.93538
63911	1.89226	2.1
63912	1.89226	2.1
63913	1.89226	2.1

63914	3.78452	2.1
63915	15.1381	0.735128
63916	15.1381	0.721471
63917	15.1381	0.711229
63918	15.1381	0.826265
63919	30.2762	0.712662
63920	15.1381	0.663994
63921	15.1381	0.76946
63922	15.1381	0.718172
63923	15.1381	0.72258
63924	30.2762	0.727716
63925	15.1381	1.47439
63926	15.1381	1.66818
63927	15.1381	1.25687
63928	15.1381	1.82598
63929	30.2762	1.63616
63930	15.1381	0.557317
63931	15.1381	0.74125
63932	15.1381	0.720009
63933	15.1381	0.707357
63934	30.2762	0.57236
63935	1.89225	1.7414
63936	1.89225	2.1
63937	1.89225	2.02562
63938	1.89225	2.1
63939	3.78449	2.1
63940	1.39909	2.1
63941	1.39909	2.1
63942	0.946123	2.1
63943	0.946123	2.1
63944	1.3291	2.1
63945	1.39908	2.1
63946	0.946126	2.1
63947	0.946126	1.985
63948	0.946118	2.1
63949	1.32909	2.1
63950	0.94613	2.1
63951	1.3991	2.1
63952	3.07801	2.1
63953	1.84322	2.1
63954	1.84322	2.1
63955	1.68788	2.1
63956	2.6582	2.1
63957	2.50287	2.1
63958	1.89225	2.1
63959	1.89225	2.1
63960	1.89225	2.1
63961	1.89225	2.1
63962	3.7845	2.1
63963	1.89226	1.73875
63964	1.89226	2.1
63965	1.89226	1.75792
63966	1.89226	2.1
63967	3.78452	2.1
63968	1.89226	1.68436
63969	2.50051	2.1
63970	2.50051	1.49768
63971	2.65822	2.1

63972	1.89226	1.85045
63973	2.04997	2.1
63974	2.50052	2.1
63975	2.65822	2.1
63976	1.70377	2.1
63977	1.70377	2.1
63978	1.84324	2.1
63979	2.63998	2.1
63980	0.810947	2.1
63981	0.810947	2.1
63982	0.810946	2.1
63983	0.810946	2.1
63984	1.89226	2.1
63985	1.35169	2.1
63986	1.08132	2.1
63987	1.08132	2.1
63988	1.08132	2.1
63989	0.810946	2.1
63990	0.810946	2.1
63991	1.89226	2.1
63992	1.89226	2.1
63993	1.89226	2.1
63994	1.89226	2.1
63995	3.78453	2.1
63996	0.946164	2.1
63997	0.946164	2.1
63998	0.929842	2.1
63999	0.929842	2.1
64000	0.946132	2.1
64001	0.946132	2.1
64002	1.04101	2.1
64003	0.929874	2.1
64004	0.946132	2.1
64005	0.929874	2.1
64006	1.04101	2.1
64007	0.946132	2.1
64008	1.89226	2.1
64009	1.89226	2.1
64010	1.89226	2.1
64011	1.89226	2.1
64012	3.78453	2.1
64013	1.89226	2.1
64014	1.89226	2.1
64015	1.89226	2.1
64016	1.89226	2.1
64017	1.89226	2.1
64018	1.89226	2.1
64019	1.89226	2.1
64020	1.89226	2.1
64021	1.89226	2.1
64022	1.89226	2.1
64023	1.89226	2.1
64024	1.89226	2.1
64025	0.946131	2.1
64026	0.946131	2.1
64027	0.946131	2.1
64028	0.946131	2.1
64029	0.946132	2.1

64030	0.946132	2.1
64031	0.946131	2.1
64032	0.946131	2.1
64033	0.946131	2.1
64034	0.946131	2.1
64035	0.946132	2.1
64036	0.946132	2.1
64037	1.89226	0.788607
64038	1.89226	0.760651
64039	1.89226	0.745368
64040	1.89226	0.777104
64041	3.78453	0.745791
64042	2.73497	0.708539
64043	1.93823	0.905467
64044	1.93823	0.752834
64045	2.20046	0.875519
64046	2.42279	0.771944
64047	2.68501	1.11954
64048	1.89226	0.751623
64049	1.89226	0.903325
64050	1.89226	1.02054
64051	1.89226	0.727888
64052	1.89226	0.970969
64053	1.89226	0.611037
64054	1.89226	0.753505
64055	1.89226	0.649885
64056	1.89226	0.734246
64057	1.89226	0.755435
64058	1.89226	0.704693
64059	1.89226	0.742731
64060	2.82984	0.727978
64061	2.15276	0.656639
64062	2.15276	0.763951
64063	2.42278	0.676692
64064	2.50074	0.611588
64065	2.77077	0.578776
64066	2.42279	0.591988
64067	1.93823	0.85492
64068	1.70423	0.533461
64069	1.70423	0.826197
64070	2.15276	0.571026
64071	2.38676	0.865322
64072	0.810946	0.727536
64073	0.810946	0.9147
64074	0.810944	0.563477
64075	0.810944	0.571973
64076	0.810946	0.72855
64077	0.810946	0.770748
64078	1.89226	0.852649
64079	1.35169	0.750833
64080	1.08132	0.648067
64081	1.08132	0.871221
64082	1.08132	0.785993
64083	1.89226	2.1
64084	1.89226	2.1
64085	1.89226	2.1
64086	1.89226	2.1
64087	3.78452	2.1

64088	1.89226	2.1
64089	1.89226	2.1
64090	1.89226	2.1
64091	1.89226	2.1
64092	3.78453	2.1
64093	1.04144	2.1
64094	1.04144	2.1
64095	0.938972	2.1
64096	0.938972	2.1
64097	0.969661	2.1
64098	1.03285	2.1
64099	0.946133	2.1
64100	0.946133	2.1
64101	0.946131	2.1
64102	0.969663	2.1
64103	0.946131	2.1
64104	1.03284	2.1
64105	0.946131	2.1
64106	0.946131	2.1
64107	0.946128	2.1
64108	0.946128	2.1
64109	0.946127	2.1
64110	0.946127	2.1
64111	0.946131	2.1
64112	0.946131	2.1
64113	0.94613	2.1
64114	0.94613	2.1
64115	0.946129	2.1
64116	0.946129	2.1
64117	1.89226	2.1
64118	1.89226	2.1
64119	1.89226	2.1
64120	1.89226	2.1
64121	3.78452	2.1
64122	1.89226	2.1
64123	1.89226	2.1
64124	1.89226	2.1
64125	1.89226	2.1
64126	3.78453	2.1
64127	1.89226	2.1
64128	1.89226	2.1
64129	1.85882	2.1
64130	1.85882	2.1
64131	1.93932	2.1
64132	1.97276	2.1
64133	0.94613	2.1
64134	0.94613	2.1
64135	0.946128	2.1
64136	0.946128	2.1
64137	0.946127	2.1
64138	0.946127	2.1
64139	0.946131	2.1
64140	0.946131	2.1
64141	0.946129	2.1
64142	0.946129	2.1
64143	0.946129	2.1
64144	0.946129	2.1
64145	1.89225	2.1

64146	1.89225	2.1
64147	1.89225	2.1
64148	1.89225	2.1
64149	3.7845	2.1
64150	1.0086	2.1
64151	1.09028	2.1
64152	0.946125	2.1
64153	0.946125	2.1
64154	0.916906	2.1
64155	0.916906	2.1
64156	1.13051	2.1
64157	1.1505	2.1
64158	1.11557	2.1
64159	1.04071	2.1
64160	0.851542	2.1
64161	0.991196	2.1
64162	1.89225	2.1
64163	1.89225	2.1
64164	1.89225	2.1
64165	1.89225	2.1
64166	3.7845	2.1
64167	1.89225	2.1
64168	1.89225	2.1
64169	1.89225	2.1
64170	1.89225	2.1
64171	1.89225	2.1
64172	1.89225	2.1
64173	1.89226	2.1
64174	1.89226	2.1
64175	1.89226	2.1
64176	1.89226	2.1
64177	3.78452	2.1
64178	0.890962	2.1
64179	0.952547	2.1
64180	0.946132	2.1
64181	0.946132	2.1
64182	0.851543	2.1
64183	0.851543	2.1
64184	1.11558	2.1
64185	1.06304	2.1
64186	0.994315	2.1
64187	1.04072	2.1
64188	0.900943	2.1
64189	0.851542	2.1
64190	1.89226	2.1
64191	1.89226	2.1
64192	1.89226	2.1
64193	1.89226	2.1
64194	1.89226	2.1
64195	1.89226	2.1
64196	1.89226	2.1
64197	1.89226	2.1
64198	1.89226	2.1
64199	1.89226	2.1
64200	3.78452	2.1
64201	1.89226	2.1
64202	1.89226	2.1
64203	1.89226	2.1

64204	1.89226	2.1
64205	3.78452	2.1
64206	0.782015	2.1
64207	0.929718	2.1
64208	0.946132	2.1
64209	0.946132	2.1
64210	0.782016	2.1
64211	0.782016	2.1
64212	1.06706	2.1
64213	1.11785	2.1
64214	0.782015	2.1
64215	0.946133	2.1
64216	0.878931	2.1
64217	0.946132	2.1
64218	1.89226	2.1
64219	1.89226	2.1
64220	1.89226	2.1
64221	1.89226	2.1
64222	1.89226	2.1
64223	1.89226	2.1
64224	0.946131	2.1
64225	0.946131	2.1
64226	0.946129	2.1
64227	0.946129	2.1
64228	0.946131	2.1
64229	0.946131	2.1
64230	0.94613	2.1
64231	0.94613	2.1
64232	0.946131	2.1
64233	0.946131	2.1
64234	0.94613	2.1
64235	0.94613	2.1
64236	0.827866	2.1
64237	0.946131	2.1
64238	0.946129	2.1
64239	0.946129	2.1
64240	0.94613	2.1
64241	0.94613	2.1
64242	0.94613	2.1
64243	0.94613	2.1
64244	0.827865	2.1
64245	0.94613	2.1
64246	0.94613	2.1
64247	0.94613	2.1
64248	1.89226	2.1
64249	1.89226	2.1
64250	1.89226	2.1
64251	1.89226	2.1
64252	3.78452	2.1
64253	1.89226	2.1
64254	1.89226	2.1
64255	1.89226	2.1
64256	1.89226	2.1
64257	1.89226	2.1
64258	1.89226	2.1
64259	1.89226	2.1
64260	1.89226	2.1
64261	1.89226	2.1

64262	1.89226	2.1
64263	3.78453	2.1
64264	1.89226	2.1
64265	1.89226	2.1
64266	1.89226	2.1
64267	1.89226	2.1
64268	3.78452	2.1
64269	0.744377	2.1
64270	0.541919	2.1
64271	0.652022	2.1
64272	0.544198	2.1
64273	0.764633	2.1
64274	0.673254	2.1
64275	1.89226	2.1
64276	1.31149	2.1
64277	1.05423	2.1
64278	1.02189	2.1
64279	0.870602	2.1
64280	1.89226	2.1
64281	1.89226	2.1
64282	1.89226	2.1
64283	1.89226	2.1
64284	1.89226	2.1
64285	1.89226	2.1
64286	1.89226	2.1
64287	1.89226	2.1
64288	1.89226	2.1
64289	1.89226	2.1
64290	1.89226	2.1
64291	1.89226	2.1
64292	1.89226	2.1
64293	1.89226	2.1
64294	1.89226	2.1
64295	1.89226	2.1
64296	1.89226	2.1
64297	1.89226	2.1
64298	0.815314	2.1
64299	0.815313	2.1
64300	0.714029	2.1
64301	0.782951	2.1
64302	0.810944	2.1
64303	0.782954	2.1
64304	1.89226	2.1
64305	1.30999	2.1
64306	1.08132	2.1
64307	1.03962	2.1
64308	0.95209	2.1
64309	1.89226	2.1
64310	1.89226	2.1
64311	1.89226	2.1
64312	1.89226	2.1
64313	1.89226	2.1
64314	1.89226	2.1
64315	1.89226	2.1
64316	1.89226	2.1
64317	1.89226	2.1
64318	1.89226	2.1
64319	1.89226	2.1

64320	1.89226	2.1
64321	1.89226	2.1
64322	1.89226	2.1
64323	1.89226	2.1
64324	1.89226	2.1
64325	1.89226	2.1
64326	1.89226	2.1
64327	0.871558	2.1
64328	0.713078	2.1
64329	0.756858	2.1
64330	0.703674	2.1
64331	0.838025	2.1
64332	0.862196	2.1
64333	1.89226	2.1
64334	1.33194	2.1
64335	1.10834	2.1
64336	1.06163	2.1
64337	1.01785	2.1
64338	1.89226	2.1
64339	1.89226	2.1
64340	1.89226	2.1
64341	1.89226	2.1
64342	3.78452	2.1
64343	1.89226	2.1
64344	1.89226	2.1
64345	1.89226	2.1
64346	1.89226	2.1
64347	1.89226	2.1
64348	1.89226	2.1
64349	1.89226	2.1
64350	1.89226	2.1
64351	1.89226	2.1
64352	1.89226	2.1
64353	1.89226	2.1
64354	1.89226	2.1
64355	0.958406	2.1
64356	1.1383	2.1
64357	0.865636	2.1
64358	1.10093	2.1
64359	0.838024	2.1
64360	0.975154	2.1
64361	1.89226	2.1
64362	1.38553	2.1
64363	1.10834	2.1
64364	1.11522	2.1
64365	1.15271	2.1
64366	1.89227	2.1
64367	1.89227	2.1
64368	1.89227	2.1
64369	1.89227	2.1
64370	3.78453	2.1
64371	0.946132	2.1
64372	0.946132	2.1
64373	0.946132	2.1
64374	0.946132	2.1
64375	0.946132	2.1
64376	0.946132	2.1
64377	0.946133	2.1

64378	0.946133	2.1
64379	0.946132	2.1
64380	0.946132	2.1
64381	0.946133	2.1
64382	0.946133	2.1
64383	0.719271	2.1
64384	0.774968	2.1
64385	1.28401	2.1
64386	1.17415	2.1
64387	0.960999	2.1
64388	0.960999	2.1
64389	0.988115	2.1
64390	0.988115	2.1
64391	0.774968	2.1
64392	1.28401	2.1
64393	0.719271	2.1
64394	1.17415	2.1
64395	2.77811	2.1
64396	2.34829	2.1
64397	2.56802	2.1
64398	2.52333	2.1
64399	2.56802	2.1
64400	2.52333	2.1
64401	1.89227	2.1
64402	1.89227	2.1
64403	1.89227	2.1
64404	1.89227	2.1
64405	3.78453	2.1
64406	1.89226	2.1
64407	1.89226	2.1
64408	1.89226	2.1
64409	1.89226	2.1
64410	1.89226	2.1
64411	1.89226	2.1
64412	1.89226	2.1
64413	2.77811	2.1
64414	1.89226	2.1
64415	2.56802	2.1
64416	1.89226	2.1
64417	2.56802	2.1
64418	1.89226	0.755599
64419	1.89226	1.5629
64420	1.89226	0.724618
64421	1.89226	1.16766
64422	3.78452	0.81175
64423	2.41802	0.86464
64424	1.95764	2.1
64425	1.95764	1.17285
64426	2.27727	2.1
64427	2.39311	0.965229
64428	2.73961	2.1
64429	0.946132	2.1
64430	0.946132	1.04404
64431	0.94613	1.11614
64432	0.94613	2.1
64433	0.946131	2.1
64434	0.946131	2.1
64435	0.946131	1.00348

64436	0.946131	1.00542
64437	0.946131	1.74712
64438	0.946131	1.85183
64439	0.946131	1.65506
64440	0.946131	1.75619
64441	1.89226	0.769538
64442	1.89226	0.895525
64443	1.89226	1.15397
64444	1.89226	0.764502
64445	1.89226	1.13763
64446	1.89226	0.764064
64447	2.78856	0.618863
64448	2.28552	0.890717
64449	2.28552	0.759574
64450	2.3931	0.835726
64451	2.6402	0.603648
64452	2.74778	0.845466
64453	2.39311	0.831506
64454	1.95764	2.1
64455	2.28552	0.884823
64456	1.86103	2.1
64457	4.24316	1.81785
64458	1.89226	0.891783
64459	1.89226	2.1
64460	1.89226	1.1083
64461	1.89226	2.1
64462	1.89226	1.05413
64463	1.89226	2.1
64464	1.47412	1.72568
64465	2.18862	1.55689
64466	2.07226	1.09962
64467	2.33645	1.26325
64468	4.26088	1.34056
64469	1.89226	1.02868
64470	1.89226	1.60082
64471	1.89226	1.10877
64472	1.89226	1.62125
64473	1.89226	1.30353
64474	1.89226	1.90341
64475	1.89226	1.35262
64476	1.89226	1.94785
64477	1.89226	0.836649
64478	1.89226	1.94735
64479	3.78453	1.30189
64480	2.18862	1.33226
64481	2.1128	1.94727
64482	2.33645	1.12363
64483	2.28188	1.90632
64484	4.47051	1.65788
64485	2.07226	1.89168
64486	2.33645	1.58269
64487	2.20831	1.00808
64488	2.4646	1.15948
64489	4.53686	1.26703
64490	0.946131	0.965588
64491	0.946131	2.05955
64492	0.946131	2.01458
64493	0.946131	1.26049

64494	0.94613	1.81449
64495	0.94613	1.19655
64496	0.946133	1.72414
64497	0.946133	1.79311
64498	0.946132	1.04322
64499	0.946132	0.991604
64500	0.946131	1.67982
64501	0.946131	1.44674
64502	1.89226	1.60999
64503	1.89226	1.94042
64504	1.89226	1.08753
64505	1.89226	1.65044
64506	3.78453	1.43846
64507	2.33646	1.33073
64508	2.28189	1.89995
64509	2.4646	1.06534
64510	2.42758	1.84809
64511	4.74648	1.34823
64512	1.89226	2.1
64513	1.89226	2.1
64514	1.89226	2.1
64515	1.89226	2.1
64516	1.89226	2.1
64517	1.89226	2.1
64518	1.89226	2.1
64519	1.89226	2.1
64520	1.89226	2.1
64521	1.89226	2.1
64522	1.89226	2.1
64523	1.89226	2.1
64524	1.89226	2.1
64525	1.89226	2.1
64526	1.89226	2.1
64527	1.89226	2.1
64528	3.78453	2.1
64529	1.89226	2.1
64530	1.89226	2.1
64531	1.89226	2.1
64532	1.89226	2.1
64533	1.89226	2.1
64534	1.89226	2.1
64535	1.89226	2.1
64536	1.89226	2.1
64537	1.89226	2.1
64538	1.89226	2.1
64539	3.78452	2.1
64540	0.877238	2.1
64541	0.877238	2.1
64542	1.24746	2.1
64543	1.04764	2.1
64544	0.964969	2.1
64545	1.1521	2.1
64546	0.946132	2.1
64547	0.946132	2.1
64548	0.946132	2.1
64549	1.12659	2.1
64550	0.964969	2.1
64551	0.946131	2.1

64552	1.89226	2.1
64553	1.89226	2.1
64554	1.89226	2.1
64555	1.89226	2.1
64556	1.89226	2.1
64557	1.89226	2.1
64558	1.89226	2.1
64559	1.89226	2.1
64560	1.89226	2.1
64561	1.89226	2.1
64562	3.78453	2.1
64563	0.868144	2.1
64564	0.992762	2.1
64565	0.946131	2.1
64566	0.946131	2.1
64567	1.07072	2.1
64568	1.07072	2.1
64569	0.868171	2.1
64570	0.868171	2.1
64571	0.992762	2.1
64572	0.946131	2.1
64573	0.946131	2.1
64574	0.868144	2.1
64575	0.946132	2.1
64576	0.946132	2.1
64577	0.946131	2.1
64578	0.946131	2.1
64579	0.946132	2.1
64580	0.946132	2.1
64581	0.946132	2.1
64582	0.946132	2.1
64583	0.946132	2.1
64584	0.946132	2.1
64585	0.946132	2.1
64586	0.946132	2.1
64587	1.89227	2.1
64588	1.89227	2.1
64589	1.89227	2.1
64590	1.89227	2.1
64591	1.89227	2.1
64592	1.89227	2.1
64593	1.89226	2.1
64594	1.89226	2.1
64595	1.89226	2.1
64596	1.89226	2.1
64597	1.89226	2.1
64598	1.89226	2.1
64599	1.98552	2.1
64600	2.22298	2.1
64601	2.22298	2.1
64602	1.89226	2.1
64603	1.89226	2.1
64604	1.54525	2.1
64605	0.810946	2.1
64606	0.810946	2.1
64607	0.810947	2.1
64608	0.810947	2.1
64609	0.81096	2.1

64610	0.81096	2.1
64611	1.89226	2.1
64612	1.35168	2.1
64613	1.08132	2.1
64614	1.08132	2.1
64615	1.0813	2.1
64616	1.89226	2.1
64617	1.89226	2.04223
64618	1.89226	2.1
64619	1.89226	1.84335
64620	3.78452	2.1
64621	1.7753	2.1
64622	2.39703	2.05255
64623	1.90577	2.1
64624	2.03445	2.07656
64625	3.82335	2.1
64626	2.39703	2.00966
64627	2.47949	0.912595
64628	2.26051	2.07808
64629	2.36891	0.863796
64630	4.76594	1.1139
64631	1.89226	2.0184
64632	1.89226	0.881336
64633	1.89226	1.83779
64634	1.89226	0.871552
64635	3.78452	1.02221
64636	1.89226	2.1
64637	1.89226	2.02151
64638	1.89226	2.1
64639	1.89226	1.95446
64640	1.89226	2.1
64641	1.89226	1.99275
64642	0.883745	2.0052
64643	0.973143	2.1
64644	0.988173	2.1
64645	0.988173	1.90153
64646	0.941927	1.7257
64647	1.02971	2.02349
64648	0.854065	2.1
64649	0.82702	2.1
64650	0.847835	2.1
64651	0.981126	2.1
64652	1.02996	2.1
64653	1.10704	2.1
64654	2.28031	2.1
64655	2.26369	0.899391
64656	2.26369	2.0572
64657	2.36891	0.937686
64658	2.30788	1.67202
64659	2.42455	0.93326
64660	1.89226	2.02264
64661	1.89226	0.901151
64662	1.89226	0.88381
64663	1.89226	1.93677
64664	1.89226	0.886693
64665	1.89226	1.90921
64666	2.71406	2.1
64667	1.83244	2.1

64668	1.83244	2.1
64669	2.07567	2.1
64670	2.52044	2.1
64671	2.76367	2.1
64672	1.89226	2.1
64673	1.89226	2.1
64674	1.89226	2.1
64675	1.89226	2.1
64676	3.78453	2.1
64677	1.89226	2.1
64678	1.89226	2.1
64679	1.89226	2.1
64680	1.89226	2.1
64681	1.89226	2.1
64682	1.89226	2.1
64683	2.70714	2.1
64684	2.3148	2.1
64685	2.3148	2.1
64686	2.52044	2.1
64687	2.54204	2.1
64688	2.74768	2.1
64689	2.52044	2.1
64690	2.3148	2.1
64691	1.83244	2.1
64692	1.83244	2.1
64693	1.62611	2.1
64694	2.3148	2.1
64695	0.946131	2.1
64696	0.946131	2.1
64697	0.946131	2.1
64698	0.946131	2.1
64699	0.946131	2.1
64700	0.946131	2.1
64701	0.946131	2.1
64702	0.946131	2.1
64703	0.946131	2.1
64704	0.946131	2.1
64705	0.946131	2.1
64706	0.946131	2.1
64707	1.89226	2.1
64708	1.89226	2.1
64709	1.89226	2.1
64710	1.89226	2.1
64711	3.78453	2.1
64712	1.89226	2.1
64713	1.89226	2.1
64714	1.89226	2.1
64715	1.89226	2.1
64716	1.89226	2.1
64717	1.89226	2.1
64718	1.89227	2.1
64719	1.89227	2.1
64720	1.89227	2.1
64721	1.89227	2.1
64722	3.78453	2.1
64723	1.89226	2.1
64724	1.89226	2.1
64725	1.89226	2.1

64726	1.89226	2.1
64727	3.78453	2.1
64728	0.915781	2.1
64729	0.863524	2.1
64730	0.717056	2.1
64731	0.830182	2.1
64732	0.865094	2.1
64733	0.918003	2.1
64734	1.89226	2.1
64735	1.29933	2.1
64736	1.11215	2.1
64737	1.05227	2.1
64738	0.966269	2.1
64739	1.89226	2.1
64740	1.89226	2.1
64741	1.89226	2.1
64742	1.89226	2.1
64743	1.89226	2.1
64744	1.89226	2.1
64745	1.89226	2.1
64746	1.89226	2.1
64747	1.89226	2.1
64748	1.89226	2.1
64749	1.89226	2.1
64750	1.89226	2.1
64751	1.89226	2.1
64752	1.89226	2.1
64753	1.89226	2.1
64754	1.89226	2.1
64755	1.89226	2.1
64756	1.89226	2.1
64757	1.01725	2.1
64758	0.997048	2.1
64759	0.818657	2.1
64760	0.960724	2.1
64761	0.838013	2.1
64762	1.04859	2.1
64763	1.89226	2.1
64764	1.41754	2.1
64765	1.17625	2.1
64766	1.0793	2.1
64767	1.18822	2.1
64768	2.16402	2.1
64769	1.89452	2.1
64770	1.50084	2.1
64771	2.09288	2.1
64772	3.9874	2.1
64773	2.16402	2.1
64774	2.13925	2.1
64775	2.04483	2.1
64776	2.09288	2.1
64777	4.23212	2.1
64778	1.89226	2.1
64779	1.89226	2.1
64780	1.89226	2.1
64781	1.89226	2.1
64782	3.78452	2.1
64783	1.89226	2.1

64784	1.89226	2.1
64785	1.89226	2.1
64786	1.89226	2.1
64787	1.89226	2.1
64788	1.89226	2.1
64789	1.9824	2.1
64790	2.09288	2.1
64791	1.69889	2.1
64792	1.6817	2.1
64793	2.00695	2.1
64794	2.00695	2.1
64795	1.93592	2.1
64796	2.09288	2.1
64797	2.00695	2.1
64798	2.09288	2.1
64799	1.9589	2.1
64800	2.04483	2.1
64801	0.946128	2.1
64802	0.946128	2.1
64803	0.946132	2.1
64804	0.946132	2.1
64805	0.94613	2.1
64806	0.94613	2.1
64807	0.946129	2.1
64808	0.946129	2.1
64809	0.94613	2.1
64810	0.94613	2.1
64811	0.94613	2.1
64812	0.94613	2.1
64813	0.946131	2.1
64814	0.946131	2.1
64815	0.946131	2.1
64816	0.946131	2.1
64817	0.946131	2.1
64818	0.946131	2.1
64819	0.946131	2.1
64820	0.946131	2.1
64821	0.946131	2.1
64822	0.946131	2.1
64823	0.946131	2.1
64824	0.946131	2.1
64825	1.89226	1.28989
64826	2.48083	2.1
64827	2.6968	1.23301
64828	2.6968	2.1
64829	1.89226	1.27275
64830	1.89226	2.1
64831	1.89226	1.43928
64832	1.89226	2.1
64833	1.89226	1.31439
64834	1.89226	2.1
64835	3.78453	1.9582
64836	1.89226	2.1
64837	1.89226	2.1
64838	1.89226	2.1
64839	1.89226	2.1
64840	1.89226	2.1
64841	1.89226	2.1

64842	1.1017	2.1
64843	1.1017	2.1
64844	1.10605	2.1
64845	1.01647	2.1
64846	0.868255	2.1
64847	0.883424	2.1
64848	1.24624	2.1
64849	1.35473	2.1
64850	0.942231	2.1
64851	1.31464	2.1
64852	0.910064	2.1
64853	1.27147	2.1
64854	1.89226	1.27557
64855	1.89226	2.1
64856	1.89226	0.986946
64857	1.89226	2.1
64858	3.78453	1.89133
64859	1.89226	2.1
64860	1.89226	2.1
64861	1.89226	2.1
64862	1.89226	2.1
64863	1.89226	2.1
64864	1.89226	2.1
64865	1.89226	2.1
64866	1.89226	2.1
64867	1.89226	2.1
64868	1.89226	2.1
64869	3.78453	2.1
64870	1.89226	2.1
64871	2.70015	2.1
64872	1.89226	2.1
64873	2.61064	2.1
64874	4.59241	2.1
64875	2.70015	2.1
64876	2.3372	2.1
64877	2.61064	2.1
64878	1.85184	2.1
64879	4.55198	2.1
64880	1.89226	2.1
64881	1.89226	2.1
64882	1.89226	2.1
64883	1.89226	2.1
64884	1.89226	2.1
64885	1.89226	2.1
64886	1.89227	2.1
64887	1.89227	2.1
64888	1.89227	2.1
64889	1.89227	2.1
64890	3.78453	2.1
64891	1.89226	2.1
64892	2.52194	2.1
64893	2.61064	2.1
64894	2.61064	2.1
64895	1.89226	2.1
64896	1.89226	2.1
64897	1.095	2.1
64898	1.095	2.1
64899	1.09189	2.1

64900	1.09189	2.1
64901	0.875334	2.1
64902	0.925919	2.1
64903	1.26097	2.1
64904	1.30532	2.1
64905	1.26097	2.1
64906	0.875334	2.1
64907	1.30532	2.1
64908	0.92592	2.1
64909	1.89226	2.1
64910	1.89226	2.1
64911	1.89226	2.1
64912	1.89226	2.1
64913	1.89226	2.1
64914	1.89226	2.1
64915	1.89226	2.1
64916	1.89226	2.1
64917	1.89226	2.1
64918	1.89226	2.1
64919	3.78453	2.1
64920	1.26097	2.1
64921	1.26097	2.1
64922	0.946132	2.1
64923	0.946132	2.1
64924	1.22176	2.1
64925	1.26097	2.1
64926	0.946132	2.1
64927	0.946132	2.1
64928	0.946132	2.1
64929	1.22176	2.1
64930	1.26097	2.1
64931	0.946132	2.1
64932	2.52194	2.1
64933	1.67381	2.1
64934	1.67381	2.1
64935	1.75067	2.1
64936	2.44352	2.1
64937	2.52038	2.1
64938	1.89226	2.1
64939	1.89226	2.1
64940	1.89226	2.1
64941	1.89226	2.1
64942	1.89226	2.1
64943	1.89226	2.1
64944	1.89227	2.1
64945	1.89227	2.1
64946	1.89227	2.1
64947	1.89227	2.1
64948	1.89227	2.1
64949	1.89227	2.1
64950	1.22176	2.1
64951	1.22176	2.1
64952	0.946132	2.1
64953	0.946132	2.1
64954	1.19095	2.1
64955	1.22176	2.1
64956	0.946133	2.1
64957	0.946133	2.1

64958	1.19095	2.1
64959	0.946132	2.1
64960	0.946133	2.1
64961	1.22176	2.1
64962	2.44352	2.1
64963	1.6287	2.1
64964	1.6287	2.1
64965	1.67381	2.1
64966	2.3819	2.1
64967	2.427	2.1
64968	1.89226	2.1
64969	1.89226	2.1
64970	1.89226	2.1
64971	1.89226	2.1
64972	1.89226	2.1
64973	1.89226	2.1
64974	1.89226	1.91078
64975	1.89226	2.1
64976	1.89226	1.6754
64977	1.89226	2.1
64978	3.78453	2.1
64979	1.89226	2.0604
64980	1.89226	2.1
64981	1.89226	2.02241
64982	1.89226	2.1
64983	1.89226	1.7702
64984	1.89226	2.1
64985	1.89226	2.1
64986	1.89226	2.1
64987	1.89226	2.1
64988	1.89226	2.1
64989	1.89226	2.1
64990	1.89226	2.1
64991	0.851542	2.1
64992	0.851542	2.1
64993	1.1736	2.1
64994	1.29261	2.1
64995	0.852708	2.1
64996	1.18843	2.1
64997	0.946132	2.1
64998	0.946132	2.1
64999	0.946132	2.1
65000	1.07901	2.1
65001	1.04208	2.1
65002	0.936669	2.1
65003	1.89227	1.2725
65004	1.89227	2.1
65005	1.89227	1.51809
65006	1.89227	2.1
65007	3.78453	2.05096
65008	1.89226	1.96503
65009	1.89226	2.1
65010	1.89226	1.58445
65011	1.89226	2.1
65012	3.78453	2.1
65013	1.89226	2.1
65014	1.89226	2.1
65015	1.89226	2.1

65016	1.89226	2.1
65017	1.89226	2.1
65018	1.89226	2.1
65019	0.946133	2.1
65020	0.946133	2.1
65021	1.28525	2.1
65022	1.28525	2.1
65023	1.07901	2.1
65024	1.28525	2.1
65025	0.946132	2.1
65026	0.946132	2.0776
65027	0.946132	2.1
65028	1.28525	2.1
65029	0.946133	2.1
65030	1.07901	2.1
65031	1.89226	2.1
65032	1.89226	2.1
65033	1.89226	2.1
65034	1.89226	2.1
65035	1.89226	2.1
65036	1.89226	2.1
65037	1.89226	2.1
65038	1.89226	2.1
65039	1.89226	2.1
65040	1.89226	2.1
65041	1.89226	2.1
65042	1.89226	2.1
65043	1.24419	2.1
65044	1.09093	2.1
65045	1.22722	2.1
65046	1.18154	2.1
65047	1.27455	2.1
65048	1.08473	2.1
65049	1.15541	2.1
65050	1.15541	2.1
65051	1.29607	2.1
65052	1.23814	2.1
65053	1.13024	2.1
65054	1.12096	2.1
65055	1.18858	2.1
65056	1.13181	2.1
65057	0.988718	2.1
65058	0.802498	2.1
65059	0.994034	2.1
65060	1.15773	2.1
65061	1.0029	2.1
65062	1.0029	2.1
65063	1.23814	2.1
65064	0.997523	2.1
65065	1.11498	2.1
65066	0.899532	2.1
65067	1.89226	2.1
65068	1.89226	2.1
65069	1.89226	2.1
65070	1.89226	2.1
65071	1.89226	2.1
65072	1.89226	2.1
65073	1.89226	2.1

65074	1.89226	2.1
65075	1.89226	2.1
65076	1.89226	2.1
65077	1.89226	2.1
65078	1.89226	2.1
65079	0.993139	2.1
65080	0.834108	2.1
65081	1.01147	2.1
65082	1.01358	2.1
65083	1.20067	2.1
65084	0.993156	2.1
65085	2.4158	2.1
65086	1.69956	2.1
65087	1.3544	2.1
65088	1.60095	2.1
65089	1.11219	2.1
65090	1.89226	2.1
65091	1.89226	2.1
65092	1.89226	2.0229
65093	1.89226	2.1
65094	3.78452	2.1
65095	1.89226	2.1
65096	1.89226	2.1
65097	1.89226	2.1
65098	1.89226	2.1
65099	1.89226	2.1
65100	1.89226	2.1
65101	1.89226	2.1
65102	2.83014	2.1
65103	2.32404	2.1
65104	2.32404	2.1
65105	1.89226	2.1
65106	1.89226	2.1
65107	1.37966	2.1
65108	1.13294	2.1
65109	0.881723	2.1
65110	0.582304	2.1
65111	0.977944	2.1
65112	0.977948	2.1
65113	1.0264	2.1
65114	1.0264	2.1
65115	1.37967	2.1
65116	1.03896	2.1
65117	1.19108	2.1
65118	0.582298	2.1
65119	1.89226	1.78446
65120	1.89226	2.1
65121	1.89226	2.1
65122	1.89226	2.1
65123	3.78452	2.1
65124	0.946131	2.1
65125	0.946131	2.1
65126	0.946131	2.1
65127	0.946131	2.1
65128	0.946132	2.1
65129	0.946132	2.1
65130	0.946129	2.1
65131	0.946129	2.1

65132	0.946131	2.1
65133	0.946131	2.1
65134	0.94613	2.1
65135	0.94613	2.1
65136	1.89226	2.1
65137	1.89226	1.97813
65138	1.89226	2.1
65139	1.89226	2.1
65140	3.78453	2.1
65141	1.89226	2.1
65142	1.89226	2.1
65143	1.89226	2.1
65144	1.89226	2.1
65145	1.89226	2.1
65146	1.89226	2.1
65147	2.65216	2.1
65148	1.93528	2.1
65149	1.78595	2.1
65150	2.48544	2.1
65151	4.42072	2.1
65152	1.89226	2.1
65153	1.89226	2.1
65154	1.89226	2.1
65155	1.89226	2.1
65156	1.89226	2.1
65157	1.89226	2.1
65158	1.89226	2.1
65159	1.89226	2.1
65160	1.89226	2.1
65161	1.89226	2.1
65162	3.78452	2.1
65163	1.66537	2.1
65164	2.48544	2.1
65165	1.78595	2.1
65166	1.78595	2.1
65167	2.36197	2.1
65168	2.36197	2.1
65169	2.80975	2.1
65170	2.48544	2.1
65171	2.36198	2.1
65172	2.65135	2.1
65173	5.13679	2.1
65174	1.89226	2.1
65175	1.89226	2.1
65176	1.89226	2.1
65177	1.89226	2.1
65178	1.89226	2.1
65179	1.89226	2.1
65180	0.946127	2.1
65181	0.946127	2.1
65182	0.946131	2.1
65183	0.946131	2.1
65184	0.946128	2.1
65185	0.946128	2.1
65186	0.94613	2.1
65187	0.94613	2.1
65188	0.946129	2.1
65189	0.946129	2.1

65190	0.946129	2.1
65191	0.946129	2.1
65192	1.11972	2.1
65193	1.18099	2.1
65194	0.832687	2.1
65195	0.764517	2.1
65196	0.974426	2.1
65197	0.974426	2.1
65198	0.971078	2.1
65199	0.971078	2.1
65200	1.11972	2.1
65201	0.764515	2.1
65202	0.832686	2.1
65203	1.18099	2.1
65204	2.36198	2.1
65205	2.48025	2.1
65206	2.48025	2.1
65207	2.65135	2.1
65208	2.23944	2.1
65209	2.41055	2.1
65210	0.946132	1.6761
65211	0.946132	2.1
65212	0.946131	2.1
65213	0.946131	2.1
65214	0.946131	2.1
65215	0.946131	1.85466
65216	0.946132	2.1
65217	0.946132	2.1
65218	0.946131	2.1
65219	0.946131	2.1
65220	0.946132	2.1
65221	0.946132	2.1
65222	0.810942	2.1
65223	0.810942	2.1
65224	0.810944	2.1
65225	0.810944	2.1
65226	1.89226	2.1
65227	1.3517	2.1
65228	1.08132	2.1
65229	1.08133	2.1
65230	1.08132	2.1
65231	0.810932	2.1
65232	0.810932	2.1
65233	1.89249	1.84298
65234	1.9857	1.50337
65235	2.02433	1.3235
65236	2.15807	1.27341
65237	4.01003	1.3807
65238	1.89226	1.23739
65239	1.89226	1.58909
65240	1.89226	1.4159
65241	1.89226	1.02211
65242	3.78452	1.49076
65243	1.89226	1.45003
65244	1.89226	1.4348
65245	1.89226	0.905663
65246	1.89226	1.28744
65247	3.78452	1.03437

65248	1.98569	1.31986
65249	1.86067	1.54526
65250	2.15807	1.16686
65251	2.07965	1.19652
65252	4.06534	1.55796
65253	2.02433	1.48873
65254	2.15807	1.70647
65255	2.12608	1.96534
65256	2.3025	1.0694
65257	4.32683	1.64366
65258	1.89226	1.577
65259	1.89226	1.33306
65260	1.89226	1.03983
65261	1.89226	1.09879
65262	3.78452	1.1316
65263	1.89226	1.12627
65264	1.89226	1.58353
65265	1.89226	1.06236
65266	1.89226	1.08485
65267	3.78452	1.43799
65268	2.15807	1.53658
65269	2.07965	1.45251
65270	2.3025	1.01195
65271	2.28384	1.20562
65272	4.38215	1.09201
65273	1.89226	0.981538
65274	1.89226	0.469752
65275	1.89226	0.797384
65276	1.89226	0.515288
65277	3.78453	0.701329
65278	2.16905	0.482917
65279	2.56369	0.46932
65280	1.88394	0.742693
65281	2.41938	0.288754
65282	4.44763	0.47487
65283	2.56369	0.466028
65284	2.80663	0.597947
65285	2.41938	0.303055
65286	2.69045	0.372081
65287	5.25414	0.413555
65288	1.89226	0.461259
65289	1.89226	0.546464
65290	1.89226	0.562284
65291	1.89226	0.381251
65292	3.78453	0.41681
65293	1.89226	0.762334
65294	1.89226	0.576964
65295	1.89226	0.945068
65296	1.89226	0.60693
65297	1.89226	0.920302
65298	1.89226	0.41866
65299	1.01988	0.337627
65300	1.01988	0.342065
65301	1.03333	0.666457
65302	1.03333	0.502159
65303	1.13811	0.34205
65304	1.20969	0.346939
65305	0.941974	0.690318

65306	0.843515	0.408326
65307	0.843514	0.35184
65308	1.13811	0.340913
65309	0.941973	0.431004
65310	1.20969	0.30938
65311	2.41938	0.257417
65312	2.57414	0.380903
65313	2.57414	0.683337
65314	2.69045	0.646719
65315	2.27623	0.276624
65316	2.39253	0.66098
65317	1.89227	0.625354
65318	1.89227	0.379457
65319	1.89227	0.351595
65320	1.89227	0.404971
65321	1.89227	0.33327
65322	1.89227	0.414205
65323	2.28385	1.26751
65324	2.09114	1.35386
65325	2.46968	0.913932
65326	2.32438	1.24197
65327	4.56082	0.96155
65328	1.89226	1.0072
65329	1.89226	1.48169
65330	1.89226	0.986593
65331	1.89226	1.25549
65332	3.78452	1.32587
65333	1.89226	1.01849
65334	1.89226	1.62311
65335	1.89226	0.984195
65336	1.89226	1.20522
65337	3.78452	1.06946
65338	2.09114	0.949347
65339	1.5249	1.53989
65340	2.55684	0.905419
65341	1.86899	1.23344
65342	3.96011	1.24669
65343	2.46968	1.10841
65344	2.32439	1.47096
65345	2.62768	0.985953
65346	2.506	1.18699
65347	4.97568	1.52233
65348	1.89226	1.25358
65349	1.89226	1.30311
65350	1.89226	0.812253
65351	1.89226	1.20345
65352	3.78452	0.934962
65353	1.89226	0.99437
65354	1.89226	1.21676
65355	1.89226	1.09978
65356	1.89226	1.8847
65357	1.89226	0.84711
65358	1.89226	1.88386
65359	2.55684	1.09166
65360	2.16506	1.17689
65361	1.86901	1.40906
65362	1.86899	1.13286
65363	2.75664	1.0459

65364	2.75662	0.924166
65365	1.89224	0.38132
65366	1.89224	0.661805
65367	1.89224	0.326614
65368	1.89224	0.861724
65369	3.78447	0.39855
65370	2.24206	0.405102
65371	2.38862	0.959261
65372	2.07107	0.666203
65373	2.23097	0.853051
65374	4.47303	0.837104
65375	2.38863	0.971504
65376	2.25945	1.19847
65377	2.25945	0.855455
65378	2.39244	1.95408
65379	2.23098	0.856236
65380	2.36397	2.05056
65381	1.89224	0.605665
65382	1.89224	1.27206
65383	1.89224	0.907185
65384	1.89224	0.844566
65385	1.89224	0.901393
65386	1.89224	0.856637
65387	1.89226	0.356535
65388	1.89226	0.826076
65389	1.89226	0.744684
65390	1.89226	0.425295
65391	1.89226	0.66766
65392	1.89226	0.668911
65393	0.920975	1.13597
65394	0.920979	0.720791
65395	1.03015	0.829445
65396	1.03015	0.416048
65397	0.992407	0.836629
65398	1.08759	1.0173
65399	0.854346	0.722288
65400	0.87241	0.410132
65401	0.872397	0.821952
65402	1.04334	0.819231
65403	1.00965	0.832764
65404	1.0876	0.828733
65405	2.23101	0.963016
65406	2.25948	1.30693
65407	2.03577	0.850144
65408	2.05257	2.1
65409	4.29524	1.00942
65410	0.810947	0.652249
65411	0.810947	0.901645
65412	0.810947	0.828535
65413	0.810947	0.669634
65414	0.810934	0.851017
65415	0.810934	0.854462
65416	1.89227	1.30983
65417	1.3517	0.932685
65418	1.08132	0.862949
65419	1.08132	0.90913
65420	1.08133	0.867823
65421	1.89226	1.20181

65422	1.89226	1.38473
65423	1.89226	1.04704
65424	1.89226	1.98514
65425	1.89226	0.854216
65426	1.89226	2.06935
65427	1.89226	1.50428
65428	1.89226	1.4121
65429	1.89226	1.08562
65430	1.89226	2.1
65431	1.89226	1.03963
65432	1.89226	2.1
65433	0.879432	1.63966
65434	0.879432	1.1739
65435	0.900223	0.832729
65436	0.900223	1.66461
65437	0.54571	1.6016
65438	0.743092	1.28554
65439	1.03656	1.59761
65440	1.19234	0.956755
65441	1.19234	1.27234
65442	0.743092	1.42406
65443	1.03656	1.4647
65444	0.545711	1.34136
65445	1.89226	0.86549
65446	2.38467	1.47466
65447	2.69293	0.866623
65448	2.69293	1.63171
65449	1.89226	0.811896
65450	1.89226	1.39759
65451	1.89226	1.10503
65452	1.89226	1.41546
65453	1.89226	0.884433
65454	1.89226	1.5129
65455	3.78452	1.20012
65456	0.946129	2.08094
65457	0.946129	1.53436
65458	0.946133	0.908065
65459	0.946133	1.83034
65460	0.946131	1.19618
65461	0.946131	2.07046
65462	0.946131	0.899508
65463	0.946131	1.25179
65464	0.946131	1.33571
65465	0.946131	1.31568
65466	0.946131	1.20822
65467	0.946131	1.20059
65468	1.04846	0.856072
65469	1.04846	1.5165
65470	1.05594	0.847255
65471	1.05594	1.59696
65472	0.743092	1.43346
65473	0.912061	1.14028
65474	1.19234	0.865713
65475	1.34647	0.929696
65476	0.912061	0.896623
65477	1.34646	1.02966
65478	1.19234	1.0131
65479	0.743093	1.00137

65480	1.89226	1.00223
65481	1.89226	1.52552
65482	1.89226	0.831008
65483	1.89226	1.01564
65484	3.78452	0.989554
65485	0.946128	2.1
65486	0.946128	1.20201
65487	0.946133	1.32525
65488	0.946133	0.951088
65489	0.94613	2.09973
65490	0.94613	1.73187
65491	0.946131	1.2733
65492	0.946131	1.01275
65493	0.946131	1.56828
65494	0.946131	1.53057
65495	0.946131	1.19085
65496	0.946131	1.1803
65497	1.89226	1.21016
65498	1.89226	1.82621
65499	1.89226	0.922821
65500	1.89226	1.17313
65501	3.78452	1.37639
65502	1.89226	1.1186
65503	1.89226	1.1514
65504	1.89226	1.05811
65505	1.89226	1.88955
65506	1.89226	0.80641
65507	1.89226	1.90297
65508	1.89226	1.23209
65509	1.89226	1.30234
65510	1.89226	1.01563
65511	1.89226	2.0233
65512	1.89226	0.931671
65513	1.89226	2.09544
65514	0.946132	1.6417
65515	0.946132	1.1847
65516	1.17758	0.91353
65517	1.17759	1.80965
65518	1.04514	1.59083
65519	1.17758	1.09087
65520	0.946133	0.890251
65521	0.946133	0.963933
65522	0.946107	1.12527
65523	1.17755	1.11212
65524	1.04517	1.16412
65525	0.946158	1.11794
65526	1.89226	2.1
65527	2.10171	2.1
65528	1.95064	2.1
65529	2.10171	2.1
65530	1.7412	2.1
65531	1.89226	2.1
65532	1.89226	2.1
65533	1.89226	2.1
65534	1.89226	2.1
65535	1.89226	2.1
65536	1.89226	2.1
65537	1.89226	2.1

65538	1.89226	2.1
65539	1.89226	1.1503
65540	1.89226	2.1
65541	1.89226	1.96543
65542	3.78453	2.1
65543	0.946133	2.1
65544	0.946133	1.84522
65545	0.946132	2.1
65546	0.946132	2.1
65547	0.946133	2.1
65548	0.946133	1.95055
65549	0.946132	2.1
65550	0.946132	2.1
65551	0.946132	2.1
65552	0.946132	2.1
65553	0.946132	2.1
65554	0.946132	2.1
65555	1.70303	2.1
65556	1.95064	2.1
65557	1.74039	2.1
65558	1.95064	2.1
65559	1.69368	2.1
65560	1.89226	2.1
65561	1.89226	2.1
65562	1.89226	2.1
65563	1.89226	2.1
65564	1.89226	2.1
65565	3.78453	2.1
65566	0.946132	1.08361
65567	0.946132	2.1
65568	0.946132	2.1
65569	0.946132	1.99728
65570	0.946133	1.85503
65571	0.946133	1.13548
65572	0.946132	2.1
65573	0.946132	2.1
65574	0.946107	2.08309
65575	0.946107	2.08927
65576	0.946158	2.06239
65577	0.946158	2.1
65578	0.946133	2.1
65579	0.946133	2.1
65580	0.946132	2.1
65581	0.946132	2.1
65582	0.946133	2.1
65583	0.946133	1.792
65584	0.946132	2.1
65585	0.946132	2.1
65586	0.946107	2.1
65587	0.946107	2.1
65588	0.946158	2.1
65589	0.946158	2.1
65590	1.89226	2.1
65591	1.89226	2.1
65592	1.89226	1.64031
65593	1.89226	2.1
65594	1.89226	1.54805
65595	1.89226	2.1

65596	1.89226	2.1
65597	1.89226	2.1
65598	1.89226	2.1
65599	1.89226	2.1
65600	1.89226	2.09613
65601	1.89226	2.1
65602	2.5198	2.1
65603	2.45893	2.1
65604	2.27322	2.1
65605	2.27322	2.1
65606	2.63576	2.1
65607	2.63576	2.1
65608	1.24045	2.1
65609	1.11906	2.1
65610	1.13578	2.1
65611	0.916205	2.1
65612	1.12539	2.1
65613	1.08404	2.1
65614	1.08484	2.1
65615	1.08484	2.1
65616	1.22836	2.1
65617	0.919363	2.1
65618	1.16507	2.1
65619	0.924864	2.1
65620	1.89226	2.1
65621	1.89226	1.85017
65622	1.89226	1.29613
65623	1.89226	2.1
65624	1.89226	1.20239
65625	1.89226	2.1
65626	1.89226	2.1
65627	1.89226	2.1
65628	1.89226	2.1
65629	1.89226	2.1
65630	1.89226	2.07909
65631	1.89226	2.1
65632	2.6946	2.1
65633	2.63576	2.1
65634	2.5198	2.1
65635	2.5198	2.1
65636	2.8362	2.1
65637	2.8362	2.1
65638	1.3265	2.1
65639	1.24045	2.1
65640	0.983223	1.89952
65641	0.954429	2.1
65642	1.06046	2.1
65643	1.10594	2.1
65644	1.1915	1.858
65645	1.1915	2.1
65646	1.31357	2.1
65647	1.09005	2.1
65648	1.29145	2.1
65649	0.995825	2.1
65650	1.89226	2.1
65651	1.89226	2.1
65652	1.89226	2.1
65653	1.89226	2.1

65654	3.78452	2.1
65655	1.89226	2.1
65656	1.89226	2.1
65657	1.89226	2.1
65658	1.89226	2.1
65659	3.78453	2.1
65660	1.91802	2.1
65661	2.27409	2.1
65662	2.48048	2.1
65663	2.22264	2.1
65664	1.91802	2.1
65665	1.66018	2.1
65666	1.13704	2.1
65667	1.24023	2.1
65668	1.22947	2.1
65669	1.31788	2.1
65670	1.23859	2.1
65671	1.23859	2.1
65672	1.23111	2.1
65673	1.23111	2.1
65674	1.24024	2.1
65675	1.31788	2.1
65676	1.13704	2.1
65677	1.22947	2.1
65678	1.89226	2.1
65679	1.89226	2.1
65680	1.89226	2.1
65681	1.89226	2.1
65682	1.89226	2.1
65683	1.89226	2.1
65684	1.89226	2.1
65685	1.89226	2.1
65686	1.89226	2.1
65687	1.89226	2.1
65688	3.78453	2.1
65689	1.05568	2.1
65690	0.959008	2.1
65691	1.32068	2.1
65692	1.24024	2.1
65693	1.15206	2.1
65694	1.15206	2.1
65695	1.14386	2.1
65696	1.14386	2.1
65697	1.05568	2.1
65698	1.32068	2.1
65699	0.959008	2.1
65700	1.24024	2.1
65701	1.32068	2.1
65702	1.24023	2.1
65703	1.31788	2.1
65704	1.4181	2.1
65705	1.31436	2.1
65706	1.31436	2.1
65707	1.3242	2.1
65708	1.3242	2.1
65709	1.32068	2.1
65710	1.4181	2.1
65711	1.24024	2.1

65712	1.31788	2.1
65713	1.89226	1.14423
65714	1.89226	0.586932
65715	1.89226	0.905457
65716	1.89226	0.911011
65717	1.89226	0.905615
65718	1.89226	0.950087
65719	1.02925	0.398751
65720	1.16678	0.559942
65721	0.946132	0.53367
65722	0.946132	0.969331
65723	0.935677	0.695969
65724	0.935677	0.437233
65725	1.18902	0.897975
65726	1.21562	0.817457
65727	1.13433	0.520352
65728	1.04071	0.596331
65729	0.851553	0.667132
65730	1.06075	0.591174
65731	1.89226	0.671563
65732	1.89226	0.496753
65733	1.89226	0.423754
65734	1.89226	0.341447
65735	1.89226	0.541422
65736	1.89226	0.769633
65737	1.89226	0.923898
65738	1.89226	0.323488
65739	1.89226	0.594297
65740	1.89226	0.579124
65741	3.78453	0.512989
65742	0.946128	0.751351
65743	0.946128	0.611594
65744	0.946133	0.546647
65745	0.946133	0.395889
65746	0.946131	0.545344
65747	0.946131	0.569555
65748	0.94613	0.729466
65749	0.94613	0.54069
65750	0.946131	0.677244
65751	0.946131	0.552693
65752	0.946131	0.356573
65753	0.946131	0.451353
65754	0.946132	0.798661
65755	0.946132	0.512609
65756	0.946132	0.887942
65757	0.946132	0.512214
65758	0.946132	0.674454
65759	0.946132	0.38054
65760	0.946131	0.752991
65761	0.946131	0.876227
65762	0.946132	0.656851
65763	0.946132	0.797243
65764	0.946132	0.480349
65765	0.946132	0.625198
65766	0.946131	1.54945
65767	0.946131	1.15845
65768	0.946131	1.8342
65769	0.946131	0.920882

65770	0.946131	0.965907
65771	0.946131	1.45121
65772	0.946131	0.898945
65773	0.946131	0.891104
65774	0.946131	1.05143
65775	0.946131	1.04698
65776	0.946131	1.11627
65777	0.946131	1.13054
65778	1.89226	1.15483
65779	1.89226	1.1736
65780	1.89226	0.944781
65781	1.89226	1.91741
65782	1.89226	0.883264
65783	1.89226	2.0867
65784	1.89226	1.06775
65785	1.89226	1.0011
65786	1.89226	1.00743
65787	1.89226	1.62073
65788	1.89226	0.885932
65789	1.89226	1.83789
65790	1.89226	1.21068
65791	1.89226	1.03893
65792	1.89226	0.902879
65793	1.89226	1.8468
65794	1.89226	0.826062
65795	1.89226	2.06784
65796	1.89226	1.49663
65797	1.89226	1.76197
65798	1.89226	0.923074
65799	1.89226	1.97247
65800	3.78452	1.37826
65801	0.946131	1.61873
65802	0.946131	0.939898
65803	1.17195	0.914717
65804	1.17195	1.83587
65805	0.958489	1.75535
65806	1.17195	1.25619
65807	0.946129	0.922772
65808	0.946129	1.23436
65809	0.946131	1.10014
65810	1.17195	1.11547
65811	0.958487	1.20067
65812	0.94613	1.20087
65813	2.69656	2.00882
65814	2.17516	2.00104
65815	2.41885	2.1
65816	2.41885	1.40098
65817	2.46476	2.1
65818	2.46476	1.13584
65819	2.69657	1.85805
65820	2.30233	0.892255
65821	2.30233	1.25988
65822	2.59476	1.042
65823	2.46477	1.30767
65824	2.7572	1.10449
65825	1.89226	1.39179
65826	1.89226	0.663932
65827	1.89226	0.705384

65828	1.89226	0.824103
65829	1.89226	0.722914
65830	1.89226	0.908665
65831	1.89226	1.41792
65832	1.89226	1.07276
65833	1.89226	1.91412
65834	1.89226	0.69788
65835	3.78452	1.11475
65836	2.46477	1.79506
65837	2.17516	1.96096
65838	1.80252	2.1
65839	2.09715	0.983901
65840	4.27231	1.96971
65841	2.46477	1.74981
65842	2.09715	0.962429
65843	2.30233	1.06136
65844	2.30233	1.00703
65845	1.96416	0.728027
65846	2.09715	0.755229
65847	1.89227	0.949938
65848	1.89227	0.487573
65849	1.89227	0.722691
65850	1.89227	0.718801
65851	1.89227	0.848266
65852	1.89227	0.891936
65853	1.89226	1.84338
65854	1.89226	0.639862
65855	1.89226	0.968053
65856	1.89226	1.00552
65857	3.78452	0.97643
65858	0.946132	2.1
65859	0.946132	2.1
65860	0.946132	2.1
65861	0.946132	2.1
65862	0.946132	2.1
65863	0.946132	2.1
65864	0.946132	2.1
65865	0.946132	2.1
65866	0.946132	2.1
65867	0.946132	2.1
65868	0.946131	2.1
65869	0.946131	2.1
65870	1.89226	2.1
65871	1.89226	2.1
65872	1.89226	2.1
65873	1.89226	2.1
65874	3.78453	2.1
65875	1.89226	2.1
65876	1.89226	2.1
65877	1.89226	2.1
65878	1.89226	2.1
65879	1.89226	2.1
65880	1.89226	2.1
65881	1.89226	2.1
65882	1.63707	2.1
65883	1.63707	2.1
65884	1.38859	2.1
65885	1.89226	2.1

65886	1.64377	2.1
65887	0.94613	2.1
65888	0.94613	2.1
65889	0.94613	2.1
65890	0.94613	2.1
65891	0.946131	2.1
65892	0.946131	2.1
65893	0.946129	2.1
65894	0.946129	2.1
65895	0.94613	2.1
65896	0.94613	2.1
65897	0.94613	2.1
65898	0.94613	2.1
65899	2.15888	2.1
65900	1.99309	1.98077
65901	2.16841	2.1
65902	2.16841	2.0487
65903	2.02311	1.06996
65904	2.02311	0.998698
65905	1.89226	1.13487
65906	1.89226	1.23338
65907	1.89226	2.06626
65908	1.89226	1.18087
65909	1.89226	1.73769
65910	1.89226	0.975382
65911	1.89226	1.45599
65912	1.89226	1.96667
65913	1.89226	2.03754
65914	1.89226	1.35641
65915	1.89226	2.1
65916	1.89226	1.43441
65917	1.99309	2.1
65918	1.96035	2.1
65919	1.72526	2.1
65920	1.72526	1.59135
65921	2.16841	2.1
65922	2.16841	2.1
65923	2.15888	2.1
65924	2.16841	2.1
65925	2.3111	2.1
65926	2.35938	2.1
65927	4.51826	2.1
65928	1.89226	1.89927
65929	1.89226	1.13554
65930	1.89226	1.05222
65931	1.89226	0.739474
65932	3.78452	1.96102
65933	1.89226	1.54577
65934	1.89226	2.00374
65935	1.89226	2.1
65936	1.89226	0.843727
65937	3.78452	1.98678
65938	2.16841	2.1
65939	1.96035	2.1
65940	2.35938	2.1
65941	2.18493	2.1
65942	4.31973	2.1
65943	1.89226	2.1

65944	1.89226	2.1
65945	1.89226	2.1
65946	1.89226	2.1
65947	3.78453	2.1
65948	1.89226	2.1
65949	1.89226	2.1
65950	1.89226	2.1
65951	1.89226	2.1
65952	3.78452	2.1
65953	0.946158	2.1
65954	0.946158	2.1
65955	0.818509	2.1
65956	0.818509	2.1
65957	0.933975	2.1
65958	0.818539	2.1
65959	0.946128	2.1
65960	0.946128	2.1
65961	0.94613	2.1
65962	0.933974	2.1
65963	0.818537	2.1
65964	0.946129	2.1
65965	0.946131	2.1
65966	0.946131	2.1
65967	0.946131	2.1
65968	0.946131	2.1
65969	0.946132	2.1
65970	0.946132	2.1
65971	0.946129	2.1
65972	0.946129	2.1
65973	0.946131	2.1
65974	0.946131	2.1
65975	0.94613	2.1
65976	0.94613	2.1
65977	1.89226	2.1
65978	1.89226	2.1
65979	1.89226	2.1
65980	1.89226	2.1
65981	3.78453	2.1
65982	1.89226	2.1
65983	1.89226	2.1
65984	1.89226	2.1
65985	1.89226	2.1
65986	3.78452	2.1
65987	1.89226	2.1
65988	1.8706	2.1
65989	1.8706	2.1
65990	1.68112	2.1
65991	2.0815	2.1
65992	1.88715	2.1
65993	0.94613	2.1
65994	0.94613	2.1
65995	0.94613	2.1
65996	0.94613	2.1
65997	0.946131	2.1
65998	0.946131	2.1
65999	0.946129	2.1
66000	0.946129	2.1
66001	0.94613	2.1

66002	0.94613	2.1
66003	0.94613	2.1
66004	0.94613	2.1
66005	1.76401	2.1
66006	2.60983	2.1
66007	2.50461	2.1
66008	2.35897	2.1
66009	1.90965	2.1
66010	1.76401	2.1
66011	1.89226	2.1
66012	1.89226	1.63936
66013	1.89226	2.1
66014	1.89226	2.1
66015	3.78452	2.1
66016	1.89226	1.60542
66017	1.89226	2.1
66018	1.89226	2.1
66019	1.89226	1.12458
66020	1.89226	2.1
66021	1.89226	1.30875
66022	1.90965	2.1
66023	2.83834	2.1
66024	2.7538	2.1
66025	2.60983	2.1
66026	2.05361	2.1
66027	1.90965	2.1
66028	1.89226	2.05942
66029	1.89226	2.1
66030	1.89226	2.1
66031	1.89226	1.66764
66032	1.89226	2.1
66033	1.89226	1.69029
66034	1.89226	1.80111
66035	1.89226	2.1
66036	1.89226	2.1
66037	1.89226	1.14579
66038	3.78452	2.1
66039	2.83834	2.1
66040	1.89226	2.1
66041	2.60983	2.1
66042	2.60983	2.1
66043	1.89226	2.1
66044	1.89226	2.1
66045	1.89226	1.8818
66046	1.89226	1.91777
66047	1.89226	2.1
66048	1.89226	1.51999
66049	1.89226	2.1
66050	1.89226	1.27878
66051	1.89226	1.21566
66052	1.89226	1.80689
66053	1.89226	2.1
66054	1.89226	1.14131
66055	1.89226	2.00596
66056	1.89226	1.16514
66057	2.44785	2.1
66058	1.80223	2.1
66059	2.80894	2.1

66060	2.18296	1.63899
66061	4.63081	2.1
66062	1.89226	1.61699
66063	1.89226	2.1
66064	1.89226	2.1
66065	1.89226	1.32356
66066	1.89226	2.1
66067	1.89226	1.32373
66068	0.946131	1.05723
66069	0.946131	1.33852
66070	0.946127	1.6199
66071	0.946127	2.1
66072	0.946131	1.22634
66073	0.946131	2.1
66074	0.946128	2.1
66075	0.946128	1.44161
66076	0.94613	1.54106
66077	0.94613	1.56757
66078	0.946129	1.61873
66079	0.946129	1.52653
66080	1.89226	1.42674
66081	1.89226	2.1
66082	1.89226	2.1
66083	1.89226	1.61633
66084	3.78452	2.1
66085	1.89226	2.1
66086	1.89226	2.1
66087	1.89226	2.1
66088	1.89226	2.1
66089	3.78453	2.1
66090	2.41083	2.1
66091	2.93937	2.1
66092	1.68761	2.1
66093	1.68761	2.1
66094	1.67248	2.1
66095	2.3957	2.1
66096	1.89226	2.1
66097	1.89226	1.39034
66098	1.89226	1.27426
66099	1.89226	2.1
66100	1.89226	1.24381
66101	1.89226	2.1
66102	1.89227	2.1
66103	1.89227	2.1
66104	1.89227	2.1
66105	1.89227	2.1
66106	1.89227	2.1
66107	1.89227	2.1
66108	1.89224	0.814524
66109	1.89224	0.718473
66110	1.89224	0.874687
66111	1.89224	0.820432
66112	3.78448	0.766823
66113	1.89224	1.06399
66114	1.89224	0.829226
66115	1.89224	1.01495
66116	1.89224	0.72666
66117	1.89224	1.02458

66118	1.89224	0.891978
66119	1.11152	0.776302
66120	1.29346	0.901889
66121	0.978596	0.970616
66122	0.918324	1.08748
66123	1.06821	1.03021
66124	1.06821	0.727247
66125	0.985654	1.10427
66126	1.15278	1.03612
66127	1.07693	0.982635
66128	0.902432	1.03315
66129	0.968939	0.966768
66130	1.34644	0.909771
66131	0.946132	0.759474
66132	0.946132	0.797513
66133	0.946132	0.811565
66134	0.946132	0.77099
66135	0.946131	0.784122
66136	0.946131	0.697888
66137	0.946132	0.9002
66138	0.946132	0.752968
66139	0.946132	0.801746
66140	0.946132	0.794565
66141	0.946132	0.728767
66142	0.946132	0.738736
66143	0.946133	0.855858
66144	0.946133	0.779531
66145	0.946132	0.906079
66146	0.946132	0.980892
66147	0.946132	0.923128
66148	0.946132	0.584751
66149	0.946133	1.20011
66150	0.946133	0.810655
66151	0.946132	0.938431
66152	0.946132	0.901158
66153	0.946132	0.787292
66154	0.946132	0.780551
66155	1.89224	0.980476
66156	1.89224	2.1
66157	1.89224	1.15981
66158	1.89224	1.74162
66159	1.89224	1.17455
66160	1.89224	1.97639
66161	1.89224	1.14341
66162	1.89224	2.1
66163	1.89224	1.646
66164	1.89224	2.097
66165	1.89224	1.54946
66166	1.89224	2.1
66167	1.89226	1.07397
66168	1.89226	2.1
66169	1.89226	2.09459
66170	1.89226	1.23271
66171	3.78453	1.89146
66172	1.11056	2.1
66173	1.21595	2.1
66174	1.12763	2.1
66175	1.12763	2.05145

66176	1.23339	2.1
66177	1.35697	2.1
66178	1.03581	2.1
66179	0.95954	2.1
66180	0.89761	2.1
66181	1.26602	2.1
66182	1.12749	2.1
66183	1.45307	2.1
66184	2.71775	2.1
66185	1.89226	2.1
66186	1.89226	2.1
66187	1.89226	2.1
66188	2.47025	2.1
66189	2.47025	2.1
66190	0.94613	2.1
66191	0.94613	2.1
66192	0.94613	1.25705
66193	0.94613	1.77576
66194	0.946128	1.66632
66195	0.946128	2.1
66196	0.946132	2.1
66197	0.946132	1.48668
66198	0.94613	2.02004
66199	0.94613	2.00435
66200	0.94613	2.09443
66201	0.94613	2.1
66202	1.89226	1.01123
66203	1.89226	1.66668
66204	1.89226	1.97239
66205	1.89226	1.10044
66206	1.89226	1.82843
66207	1.89226	1.17095
66208	1.89226	1.0098
66209	1.89226	1.63436
66210	1.89226	1.58477
66211	1.89226	0.998008
66212	1.89226	1.52568
66213	1.89226	1.0897
66214	0.946132	1.31325
66215	0.946132	1.55147
66216	0.946133	2.1
66217	0.946133	2.1
66218	0.946133	2.1
66219	0.946133	1.26515
66220	0.946132	1.22606
66221	0.946132	2.1
66222	0.946133	2.05505
66223	0.946133	2.04815
66224	0.946132	2.02778
66225	0.946132	2.02576
66226	1.89226	1.04202
66227	1.89226	1.665
66228	1.89226	1.78948
66229	1.89226	1.20595
66230	1.89226	1.79679
66231	1.89226	1.22408
66232	0.946131	1.22801
66233	0.946131	1.01501

66234	0.946127	1.54199
66235	0.946127	1.7418
66236	0.946131	1.20001
66237	0.946131	2.04657
66238	0.946127	1.83211
66239	0.946127	1.20498
66240	0.94613	1.38499
66241	0.94613	1.36724
66242	0.946129	1.3588
66243	0.946129	1.34923
66244	1.94291	2.1
66245	2.00118	2.1
66246	2.36337	2.1
66247	2.41217	2.1
66248	4.35508	2.1
66249	1.89226	2.1
66250	1.89226	1.82788
66251	1.89226	2.03872
66252	1.89226	2.1
66253	3.78452	2.1
66254	1.89226	1.83624
66255	1.89226	2.1
66256	1.89226	2.1
66257	1.89226	1.80075
66258	3.78452	2.1
66259	1.06209	2.1
66260	1.06209	2.1
66261	1.05987	2.1
66262	1.05987	2.1
66263	0.920378	2.1
66264	1.12137	2.1
66265	1.00059	2.1
66266	1.20608	2.1
66267	1.20608	2.1
66268	1.12137	2.1
66269	1.00059	2.1
66270	0.920376	2.1
66271	2.77222	2.1
66272	2.41216	2.1
66273	2.77002	2.1
66274	2.36337	2.1
66275	2.81882	2.1
66276	2.41216	2.1
66277	1.89226	1.79972
66278	1.89226	2.1
66279	1.89226	2.1
66280	1.89226	2.05285
66281	1.89226	2.1
66282	1.89226	1.6097
66283	1.89226	2.1
66284	1.89226	1.53763
66285	1.89226	1.73207
66286	1.89226	2.1
66287	3.78451	1.96246
66288	2.41216	2.1
66289	2.24273	2.1
66290	2.81882	2.1
66291	2.68473	2.1

66292	5.09689	2.1
66293	1.89226	2.1
66294	1.89226	2.1
66295	1.89226	2.1
66296	1.89226	2.1
66297	3.78453	2.1
66298	2.17614	2.1
66299	2.65326	2.1
66300	1.93101	2.1
66301	2.58552	2.1
66302	4.58427	2.1
66303	2.65326	2.1
66304	1.89226	2.1
66305	1.89226	2.1
66306	1.89226	2.1
66307	2.58552	2.1
66308	2.58552	2.1
66309	1.89226	2.1
66310	1.89226	2.1
66311	1.89226	2.1
66312	1.89226	2.1
66313	3.78453	2.1
66314	1.89226	2.1
66315	1.89226	2.1
66316	1.89226	2.1
66317	1.89226	2.1
66318	3.78453	2.1
66319	1.93101	2.1
66320	2.58552	2.1
66321	1.46507	2.1
66322	2.52893	2.1
66323	4.45994	2.1
66324	1.26446	2.1
66325	1.26446	2.1
66326	0.946132	2.1
66327	0.946132	2.1
66328	0.94613	2.1
66329	0.94613	2.1
66330	1.29276	2.1
66331	1.26446	2.1
66332	1.26446	2.1
66333	0.94613	2.1
66334	0.94613	2.1
66335	1.29276	2.1
66336	1.89226	2.1
66337	1.89226	2.1
66338	1.89226	2.1
66339	1.89226	2.08074
66340	3.78453	2.1
66341	1.89226	2.1
66342	1.89226	2.1
66343	1.89226	2.1
66344	1.89226	2.09192
66345	3.78453	2.1
66346	1.83378	2.1
66347	2.52893	2.1
66348	1.75592	2.1
66349	2.49844	2.1

66350	4.28485	2.1
66351	1.24922	2.1
66352	1.24922	2.1
66353	0.946133	2.1
66354	0.946133	2.1
66355	0.946131	2.1
66356	0.946131	2.1
66357	1.26446	2.1
66358	1.24922	2.1
66359	1.24922	2.1
66360	0.946131	2.1
66361	1.26446	2.1
66362	0.94613	2.1
66363	0.946132	2.1
66364	0.946132	2.1
66365	0.946132	2.0875
66366	0.946132	2.07443
66367	0.946132	2.05637
66368	0.946132	2.1
66369	0.946132	2.1
66370	0.946132	2.1
66371	0.946132	2.1
66372	0.946132	2.1
66373	0.946131	2.1
66374	0.946131	2.1
66375	1.89226	2.1
66376	1.89226	2.1
66377	1.89226	2.1
66378	1.89226	2.1
66379	1.89226	2.1
66380	1.89226	2.1
66381	1.0465	2.1
66382	1.0465	2.1
66383	1.06047	2.1
66384	1.06047	2.1
66385	1.25687	2.1
66386	1.24922	2.1
66387	0.87796	2.1
66388	0.857749	2.1
66389	0.857749	2.1
66390	1.25687	2.1
66391	0.877959	2.1
66392	1.24922	2.1
66393	2.49843	2.1
66394	1.89226	2.1
66395	2.51374	2.1
66396	1.89226	2.1
66397	4.406	2.1
66398	1.89226	2.1
66399	1.89226	2.06981
66400	1.89226	2.1
66401	1.89226	2.1
66402	1.89226	2.1
66403	1.89226	2.1
66404	1.89226	1.29147
66405	1.89226	2.1
66406	1.89226	2.1
66407	1.89226	1.39193

66408	1.89226	2.1
66409	1.89226	1.46131
66410	1.89226	2.1
66411	1.89226	1.39045
66412	1.89226	2.08556
66413	1.89226	1.40131
66414	1.89226	2.09058
66415	1.89226	1.41809
66416	1.03835	2.1
66417	1.03835	2.09167
66418	1.15388	2.1
66419	0.94267	2.1
66420	0.736433	1.98984
66421	0.849239	2.1
66422	1.15014	2.1
66423	1.39058	2.1
66424	0.838639	2.1
66425	1.39056	2.1
66426	1.09399	2.1
66427	0.736444	2.1
66428	1.89226	1.21867
66429	1.89226	1.96822
66430	1.89226	2.01494
66431	1.89226	1.24367
66432	1.89226	1.97576
66433	1.89226	1.26687
66434	0.94613	1.25604
66435	0.94613	1.24406
66436	0.946127	2.05479
66437	0.946127	1.93121
66438	0.946131	1.96068
66439	0.946131	1.23302
66440	0.946126	2.05315
66441	0.946126	1.29784
66442	0.946128	1.70606
66443	0.946128	1.71284
66444	0.946128	1.52722
66445	0.946128	1.61371
66446	1.89226	1.57572
66447	1.89226	1.11396
66448	1.89226	1.01123
66449	1.89226	1.76444
66450	3.78453	1.16885
66451	0.946133	1.18299
66452	0.946133	0.872813
66453	0.946128	1.3678
66454	0.946128	1.22763
66455	0.946131	1.23238
66456	0.946131	1.20787
66457	0.946131	0.825421
66458	0.946131	1.22685
66459	0.946131	1.06787
66460	0.946131	1.06955
66461	0.94613	1.15151
66462	0.94613	1.13759
66463	1.89226	1.00682
66464	1.89226	1.63923
66465	1.89226	1.39917

66466	1.89226	1.08002
66467	1.89226	1.39278
66468	1.89226	1.11508
66469	1.89226	0.880776
66470	1.89226	1.27693
66471	1.89226	1.08504
66472	1.89226	0.978586
66473	1.89226	1.08852
66474	1.89226	1.09096
66475	1.89226	0.90446
66476	1.89226	1.46701
66477	1.89226	1.12661
66478	1.89226	1.15547
66479	1.89226	1.14691
66480	1.89226	1.23227
66481	1.89226	1.03151
66482	2.67388	1.93063
66483	2.23684	1.54468
66484	2.23684	1.20831
66485	1.89226	1.56685
66486	1.89226	1.25588
66487	1.89226	2.1
66488	1.89226	2.07501
66489	1.89226	2.1
66490	1.89226	2.1
66491	1.89226	2.1
66492	1.89226	2.1
66493	1.72197	2.1
66494	2.51374	2.1
66495	2.58004	2.1
66496	2.58004	2.1
66497	1.7155	2.1
66498	1.7155	2.1
66499	2.51374	2.1
66500	1.89226	2.1
66501	1.89226	2.1
66502	1.89226	2.1
66503	2.58003	2.1
66504	2.58003	2.1
66505	0.946132	2.1
66506	0.946132	2.1
66507	0.946132	2.09843
66508	0.946132	1.99661
66509	0.946132	1.96692
66510	0.946132	2.1
66511	0.946132	2.0336
66512	0.946132	2.1
66513	0.946131	2.1
66514	0.946131	2.1
66515	0.946132	2.1
66516	0.946132	2.1
66517	1.89226	2.1
66518	1.89226	2.09945
66519	1.89226	2.1
66520	1.89226	2.1
66521	3.78453	2.1
66522	1.08262	2.1
66523	1.08262	2.1

66524	1.09674	2.1
66525	1.09674	2.1
66526	1.34652	2.1
66527	1.29002	2.1
66528	0.860984	2.1
66529	0.889338	2.1
66530	1.34652	2.1
66531	0.889338	2.1
66532	0.860983	2.1
66533	1.29002	2.1
66534	1.34652	2.1
66535	1.34652	2.1
66536	0.946133	2.1
66537	0.946133	2.1
66538	0.946131	2.1
66539	0.946131	2.1
66540	1.34652	2.1
66541	1.29002	2.1
66542	1.34652	2.1
66543	0.946131	2.1
66544	0.94613	2.1
66545	1.29002	2.1
66546	1.89226	2.1
66547	1.89226	1.98638
66548	1.89226	2.1
66549	1.89226	2.1
66550	1.89226	2.05614
66551	1.89226	2.1
66552	1.89226	2.1
66553	1.89226	2.1
66554	1.89226	2.1
66555	1.89226	2.1
66556	1.89226	2.09459
66557	1.89226	2.1
66558	0.946131	2.1
66559	0.946131	2.1
66560	0.946131	2.1
66561	0.946131	1.81316
66562	0.946131	1.82889
66563	0.946131	2.1
66564	0.946131	2.1
66565	0.946131	2.05383
66566	0.946131	2.1
66567	0.946131	2.1
66568	0.946131	2.1
66569	0.946131	2.1
66570	1.89226	2.1
66571	1.89226	2.1
66572	1.89226	2.04112
66573	1.89226	2.1
66574	1.89226	2.06535
66575	1.89226	2.1
66576	1.04103	2.1
66577	1.13849	2.1
66578	0.943014	2.1
66579	0.943014	2.1
66580	0.943591	2.1
66581	0.943591	2.1

66582	1.03757	2.1
66583	1.04098	2.1
66584	1.03761	2.1
66585	0.946131	2.1
66586	0.943041	2.1
66587	0.946131	2.1
66588	0.946128	2.1
66589	0.946128	2.1
66590	0.946133	2.1
66591	0.946133	2.1
66592	0.946131	2.1
66593	0.946131	2.1
66594	0.946131	2.1
66595	0.946131	2.1
66596	0.946131	2.1
66597	0.946131	2.1
66598	0.94613	2.1
66599	0.94613	2.1
66600	0.946132	2.1
66601	0.946132	2.1
66602	0.946132	2.092
66603	0.946132	1.83424
66604	0.946132	1.85126
66605	0.946132	2.1
66606	0.946132	2.1
66607	0.946132	2.09129
66608	0.946132	2.1
66609	0.946132	2.1
66610	0.946131	2.1
66611	0.946131	2.1
66612	1.89226	0.89873
66613	1.89226	1.34675
66614	1.89226	0.936281
66615	1.89226	0.886071
66616	3.78452	1.08585
66617	0.946132	1.05624
66618	0.946132	1.07889
66619	0.946131	0.92504
66620	0.946131	0.928694
66621	0.946132	0.928373
66622	0.946132	1.48734
66623	0.946132	1.05357
66624	0.946132	1.03401
66625	0.946132	0.973808
66626	0.946132	0.993522
66627	0.946132	1.03088
66628	0.946132	1.10142
66629	0.935482	1.21707
66630	1.08999	1.20296
66631	1.24315	1.06974
66632	1.12559	1.1613
66633	1.21343	0.998073
66634	0.889397	1.6239
66635	1.01265	0.884782
66636	1.01265	1.28708
66637	1.07332	0.942548
66638	1.34292	1.10506
66639	0.865649	1.19652

66640	0.840024	1.33571
66641	1.15433	1.17569
66642	1.02858	1.27036
66643	1.34082	1.07355
66644	1.08357	1.07607
66645	1.10938	0.882376
66646	1.33349	1.49862
66647	1.12232	1.29665
66648	1.12232	0.846843
66649	1.21357	1.0432
66650	1.3408	1.08706
66651	1.02859	1.22643
66652	1.24211	1.28693
66653	1.89226	1.04186
66654	1.89226	0.803747
66655	1.89226	0.864103
66656	1.89226	1.33083
66657	1.89226	0.802218
66658	1.89226	1.02832
66659	1.89226	0.885646
66660	1.89226	0.991886
66661	1.89226	0.912514
66662	1.89226	1.21914
66663	1.89226	1.00585
66664	1.89226	1.20298
66665	2.0815	1.16383
66666	2.79029	1.02002
66667	2.36793	1.0074
66668	2.58428	1.40895
66669	2.38161	0.872549
66670	2.59922	1.17987
66671	1.89226	1.97621
66672	1.89226	1.4047
66673	1.89226	1.70434
66674	1.89226	1.74792
66675	1.89226	1.69451
66676	1.89226	1.75892
66677	1.89226	1.40933
66678	1.89226	0.866915
66679	1.89226	1.00737
66680	1.89226	1.1016
66681	3.78452	1.05594
66682	1.89226	1.69687
66683	1.89226	1.0519
66684	1.89226	1.40423
66685	1.89226	1.38543
66686	1.89226	1.38419
66687	1.89226	1.38366
66688	0.946132	1.18222
66689	0.946132	1.61447
66690	0.94613	2.01141
66691	0.94613	1.53124
66692	0.946131	1.78362
66693	0.946131	1.18187
66694	0.946131	1.59359
66695	0.946131	2.05035
66696	0.946131	1.5941
66697	0.946131	1.68904

66698	0.946131	1.66921
66699	0.946131	1.61664
66700	1.85178	1.57977
66701	1.89226	1.09608
66702	2.0815	0.976985
66703	2.27054	1.38103
66704	1.70303	0.87
66705	1.5217	0.953633
66706	1.89226	1.74607
66707	1.89226	1.04324
66708	1.89226	1.28298
66709	1.89226	1.17147
66710	1.89226	1.63322
66711	1.89226	1.29549
66712	2.58823	0.622773
66713	2.02924	0.452859
66714	1.79398	0.495633
66715	1.79398	0.394429
66716	2.76708	0.614501
66717	2.76708	0.510081
66718	0.946133	0.484481
66719	0.946133	0.564107
66720	1.29411	0.517011
66721	1.38353	0.42782
66722	0.946131	0.424983
66723	0.946131	0.436121
66724	1.38354	0.590309
66725	1.38354	0.509582
66726	0.946131	0.468199
66727	1.38354	0.479149
66728	0.94613	0.555196
66729	1.29411	0.48821
66730	1.89226	0.41748
66731	1.89226	0.314108
66732	1.89226	0.425462
66733	1.89226	0.387628
66734	3.78452	0.409653
66735	0.946131	0.358809
66736	0.946131	0.431062
66737	0.946131	0.400747
66738	0.946131	0.350744
66739	0.946129	0.354591
66740	0.946129	0.317965
66741	0.946133	0.447868
66742	0.946133	0.428889
66743	0.946131	0.443519
66744	0.946131	0.371218
66745	0.946131	0.319262
66746	0.946131	0.384588
66747	1.89226	0.336481
66748	1.89226	0.326872
66749	1.89226	0.407293
66750	1.89226	0.344144
66751	3.78452	0.319481
66752	0.94613	0.37859
66753	0.94613	0.355172
66754	0.946131	0.330115
66755	0.946131	0.335909

66756	0.946128	0.322713
66757	0.946128	0.30455
66758	0.946133	0.395285
66759	0.946133	0.358275
66760	0.946131	0.376672
66761	0.946131	0.322447
66762	0.946131	0.374415
66763	0.946131	0.315748
66764	0.946129	1.00457
66765	0.946129	0.969718
66766	1.13486	0.993712
66767	0.859753	1.04097
66768	0.851541	1.0292
66769	0.851541	0.913137
66770	1.34346	0.988894
66771	1.26212	1.00414
66772	0.851553	0.934377
66773	1.12403	0.970008
66774	0.851474	0.865159
66775	0.956163	0.976994
66776	0.946132	0.698076
66777	0.946132	0.690126
66778	0.946133	0.787235
66779	0.946133	0.654757
66780	0.946132	0.807486
66781	0.946132	0.568587
66782	0.946133	0.744697
66783	0.946133	0.708796
66784	0.946133	0.748905
66785	0.946133	0.698035
66786	0.946132	0.606061
66787	0.946132	0.716327
66788	1.89226	0.615325
66789	1.89226	0.784349
66790	1.89226	0.847254
66791	1.89226	0.673896
66792	3.78453	0.678768
66793	0.946128	0.988794
66794	0.946128	0.962488
66795	0.946132	1.02911
66796	0.946132	1.01407
66797	0.94613	1.03094
66798	0.94613	0.96038
66799	0.94613	1.05857
66800	0.94613	0.955505
66801	0.946131	1.03158
66802	0.946131	1.00552
66803	0.94613	0.983601
66804	0.94613	0.994871
66805	0.946132	0.547044
66806	0.946132	0.497496
66807	0.946133	0.56587
66808	0.946133	0.49357
66809	0.946132	0.549826
66810	0.946132	0.476542
66811	0.946133	0.503371
66812	0.946133	0.527847
66813	0.946132	0.563159

66814	0.946132	0.516593
66815	0.946132	0.519292
66816	0.946132	0.478937
66817	1.89226	0.577325
66818	1.89226	0.524568
66819	1.89226	0.698577
66820	1.89226	0.48232
66821	1.89226	0.691703
66822	1.89226	0.47516
66823	1.89226	0.917677
66824	1.89226	0.740986
66825	1.89226	0.776431
66826	1.89226	0.73068
66827	1.89226	0.796174
66828	1.89226	0.780721
66829	1.89226	2.1
66830	1.89226	2.1
66831	1.89226	2.1
66832	1.89226	2.1
66833	3.78452	2.1
66834	1.07652	2.1
66835	1.07652	2.1
66836	0.928749	2.1
66837	0.928749	2.1
66838	0.946132	2.1
66839	0.946132	2.1
66840	1.05914	2.1
66841	0.928746	2.1
66842	1.05914	2.1
66843	0.946131	2.1
66844	0.94613	2.1
66845	0.928747	2.1
66846	1.89226	2.1
66847	1.89226	2.1
66848	1.89226	2.1
66849	1.89226	2.1
66850	1.89226	2.1
66851	1.89226	2.1
66852	1.89227	2.1
66853	1.89227	2.1
66854	1.89227	2.04811
66855	1.89227	2.1
66856	1.89227	2.06717
66857	1.89227	2.1
66858	1.89226	2.1
66859	1.89226	2.1
66860	1.89226	2.1
66861	1.89226	2.1
66862	1.89226	2.1
66863	1.89226	2.1
66864	2.11828	2.1
66865	1.89226	2.1
66866	2.35845	2.1
66867	1.89226	2.1
66868	4.25071	2.1
66869	1.89226	2.1
66870	1.89226	2.1
66871	1.89226	2.1

66872	1.89226	2.1
66873	3.78453	2.1
66874	1.89226	2.1
66875	1.89226	2.1
66876	1.89226	2.09995
66877	1.89226	2.1
66878	1.89226	2.06572
66879	1.89226	2.1
66880	1.89226	0.966084
66881	1.89226	1.38811
66882	1.65573	1.21419
66883	1.65573	1.01699
66884	1.89226	1.2117
66885	1.89226	1.02128
66886	1.89226	0.86159
66887	1.89226	1.11585
66888	1.89226	1.00405
66889	1.89226	0.912165
66890	1.89226	1.01479
66891	1.89226	0.919976
66892	1.89226	0.918672
66893	1.89226	1.21296
66894	1.89226	1.13591
66895	1.89226	0.976883
66896	1.89226	1.1359
66897	1.89226	0.979055
66898	1.89226	0.836264
66899	1.89226	1.11216
66900	1.89226	0.952377
66901	1.89226	0.872342
66902	1.89226	0.969877
66903	1.89226	0.883145
66904	0.946128	0.902476
66905	0.946128	0.858768
66906	0.946125	1.14547
66907	0.946125	1.08153
66908	0.94613	0.912742
66909	0.94613	1.34851
66910	0.946124	1.16044
66911	0.946124	0.894858
66912	0.946127	0.966081
66913	0.946127	0.969878
66914	0.946127	0.969108
66915	0.946127	0.973726
66916	1.89226	0.860985
66917	1.89226	0.988363
66918	1.89226	0.87009
66919	1.89226	0.83524
66920	3.78452	0.876073
66921	1.89226	1.04321
66922	1.89226	1.05558
66923	1.89226	0.927186
66924	1.89226	1.0732
66925	3.78453	1.03786
66926	1.05926	1.23438
66927	0.891358	1.83738
66928	0.946129	1.12805
66929	0.946129	1.2958

66930	0.946101	1.14825
66931	0.946101	1.9231
66932	0.891386	1.12814
66933	0.891386	1.1752
66934	0.891357	1.20028
66935	0.946129	1.17867
66936	0.94613	1.19022
66937	1.05926	1.21449
66938	0.946126	0.949448
66939	0.946126	1.02286
66940	0.94613	0.917881
66941	0.94613	0.991708
66942	0.946131	0.987571
66943	0.946131	1.02318
66944	0.946125	1.0061
66945	0.946125	0.837077
66946	0.946127	0.919708
66947	0.946127	0.921394
66948	0.946128	0.997301
66949	0.946128	0.991567
66950	1.89226	0.934591
66951	1.89226	0.867656
66952	1.89226	0.821969
66953	1.89226	1.02171
66954	1.89226	0.849316
66955	1.89226	1.09097
66956	1.89226	1.00317
66957	1.89226	1.19793
66958	1.89226	1.11054
66959	1.89226	1.16951
66960	1.89226	1.00301
66961	1.89226	1.10541
66962	1.16109	1.443
66963	1.23243	1.86063
66964	0.659819	1.22621
66965	0.659819	1.45865
66966	0.697965	1.10993
66967	1.00087	1.95444
66968	0.891386	1.39968
66969	0.891386	1.23118
66970	0.946129	1.3911
66971	0.65979	1.19128
66972	0.891357	1.3452
66973	0.946129	1.32293
66974	1.89225	0.887226
66975	1.89225	1.01998
66976	1.89225	1.10206
66977	1.89225	0.859706
66978	1.89225	1.26184
66979	1.89225	0.962399
66980	1.79981	0.767811
66981	2.02514	0.818968
66982	2.33247	0.876568
66983	2.12891	0.700679
66984	4.15405	0.763916
66985	2.48284	0.996268
66986	2.02514	0.827651
66987	2.33247	0.87552

66988	2.02514	0.940951
66989	2.48567	0.86977
66990	2.17834	0.928783
66991	1.89226	0.816104
66992	1.89226	0.882439
66993	1.89226	0.927873
66994	1.89226	0.771169
66995	3.78453	0.838025
66996	1.89227	0.80748
66997	1.89227	0.606711
66998	1.89227	0.666555
66999	1.89227	0.759829
67000	1.89227	0.688506
67001	1.89227	0.769194
67002	2.50894	0.681023
67003	2.70118	0.83255
67004	2.12891	0.749707
67005	2.12891	0.795555
67006	2.7125	0.748781
67007	2.33247	0.796759
67008	1.89226	0.883812
67009	1.89226	0.762782
67010	1.89226	0.795891
67011	1.89226	0.855993
67012	1.89226	0.783169
67013	1.89226	0.844123
67014	0.946133	0.751462
67015	0.946133	0.790652
67016	0.946132	0.614457
67017	0.946132	0.673792
67018	0.946132	0.604948
67019	0.946132	0.763838
67020	0.946133	0.670205
67021	0.946133	0.796962
67022	0.946132	0.703472
67023	0.946132	0.707217
67024	0.946132	0.721799
67025	0.946132	0.716042
67026	1.90098	0.482599
67027	1.36463	0.513827
67028	1.56656	0.518388
67029	1.36463	0.543759
67030	2.04386	0.448163
67031	1.89227	0.515502
67032	1.89226	0.488607
67033	1.89226	0.504418
67034	1.89226	0.521701
67035	1.89226	0.559373
67036	1.89226	0.456507
67037	1.89226	0.507642
67038	1.89226	0.579357
67039	1.89226	0.606984
67040	1.89226	0.626932
67041	1.89226	0.692751
67042	1.89226	0.528574
67043	1.89226	0.619708
67044	1.89226	0.577484
67045	1.89226	0.601162

67046	1.89226	0.627833
67047	1.89226	0.661974
67048	1.89226	0.545074
67049	1.89226	0.608058
67050	1.89226	0.448015
67051	1.70303	0.477192
67052	2.1292	0.505856
67053	1.70303	0.499966
67054	2.32302	0.429323
67055	1.90098	0.458175
67056	1.89226	0.45404
67057	1.89226	0.487265
67058	1.89226	0.509956
67059	1.89226	0.519973
67060	1.89226	0.435676
67061	1.89226	0.462409
67062	1.89226	0.544273
67063	1.89226	0.580079
67064	1.89226	0.609457
67065	1.89226	0.618131
67066	1.89226	0.512982
67067	1.89226	0.544362
67068	1.89226	0.534922
67069	1.89226	0.567264
67070	1.89226	0.588892
67071	1.89226	0.59585
67072	1.89226	0.509309
67073	1.89226	0.536304
67074	1.89226	0.969179
67075	1.89226	1.16813
67076	1.89226	1.13952
67077	1.89226	0.966443
67078	3.78453	0.963226
67079	1.89226	1.13638
67080	1.89226	1.46739
67081	1.89226	1.0502
67082	1.89226	1.57664
67083	1.89226	1.15549
67084	1.89226	1.82499
67085	1.89226	1.16516
67086	1.89226	0.989868
67087	1.89226	0.96386
67088	1.89226	1.05862
67089	3.78452	1.10211
67090	1.89224	1.15015
67091	1.89224	0.941311
67092	1.89224	0.995054
67093	1.89224	1.17886
67094	1.89224	0.965555
67095	1.89224	1.10382
67096	1.89224	1.14919
67097	1.89224	1.63564
67098	1.89224	1.26442
67099	1.89224	1.944
67100	1.89224	1.05025
67101	1.89224	1.58512
67102	1.31307	1.86846
67103	1.31307	1.88257

67104	0.888948	1.29252
67105	0.888948	1.18426
67106	1.2559	1.35964
67107	0.888979	1.85376
67108	0.946114	1.60074
67109	0.946114	1.17588
67110	0.946129	1.13153
67111	0.888989	1.24713
67112	0.946104	1.07058
67113	1.25588	1.19672
67114	1.89224	0.936675
67115	1.89224	1.21723
67116	1.89224	0.946083
67117	1.89224	1.177
67118	1.89224	1.01405
67119	1.89224	1.35676
67120	1.79375	0.420012
67121	1.47689	0.332175
67122	1.97732	0.363113
67123	2.26687	0.407761
67124	3.74375	0.386704
67125	2.05759	0.511207
67126	2.26686	0.392679
67127	1.79374	0.401869
67128	1.79374	0.459928
67129	2.49889	0.41896
67130	2.49889	0.471165
67131	1.89226	0.446576
67132	1.89226	0.379124
67133	1.89226	0.379445
67134	1.89226	0.437082
67135	3.78452	0.428341
67136	1.89227	0.366723
67137	1.89227	0.394402
67138	1.89227	0.356701
67139	1.89227	0.382886
67140	1.89227	0.315821
67141	1.89227	0.350129
67142	1.97732	0.361692
67143	2.26687	0.399792
67144	2.6607	0.439878
67145	2.41464	0.37288
67146	4.68151	0.366118
67147	1.89226	0.378222
67148	1.89226	0.453116
67149	1.89226	0.479296
67150	1.89226	0.403502
67151	3.78452	0.39242
67152	1.89226	0.390094
67153	1.89226	0.384518
67154	1.89226	0.377168
67155	1.89226	0.417819
67156	1.89226	0.362038
67157	1.89226	0.391942
67158	1.89226	0.873622
67159	1.89226	1.01596
67160	1.89226	0.918525
67161	1.89226	0.882714

67162	3.78452	0.890209
67163	1.89226	0.929968
67164	1.89226	1.15497
67165	1.89226	0.923441
67166	1.89226	1.08093
67167	1.89226	0.966307
67168	1.89226	1.15482
67169	1.89226	1.05332
67170	1.89226	0.916097
67171	1.89226	0.867929
67172	1.89226	1.06205
67173	1.89226	0.889521
67174	1.89226	1.12243
67175	1.89226	0.925537
67176	1.89226	0.888077
67177	1.89226	0.852574
67178	1.89226	1.01962
67179	1.89226	0.846492
67180	1.89226	1.06895
67181	1.89226	0.939441
67182	2.45282	1.10262
67183	1.89226	1.09479
67184	1.89226	1.13091
67185	1.89226	0.918952
67186	1.89226	1.01187
67187	1.89226	0.906537
67188	1.89226	1.05056
67189	1.89226	0.909793
67190	1.89226	0.94242
67191	1.89226	1.15261
67192	1.89226	1.42137
67193	1.87308	0.363025
67194	1.88631	0.37034
67195	1.63908	0.359735
67196	1.70302	0.359122
67197	2.08745	0.362886
67198	2.16916	0.355906
67199	2.41019	0.376696
67200	2.733	0.400485
67201	2.08121	0.365587
67202	2.08121	0.377392
67203	2.74362	0.349563
67204	2.41464	0.363732
67205	1.89226	0.38042
67206	1.89226	0.394861
67207	1.89226	0.37478
67208	1.89226	0.387294
67209	1.89226	0.400974
67210	1.89226	0.407753
67211	0.946133	0.378203
67212	0.946133	0.359344
67213	0.946132	0.400832
67214	0.946132	0.382908
67215	0.946132	0.398204
67216	0.946132	0.39002
67217	0.946133	0.372637
67218	0.946133	0.366934
67219	0.946132	0.383062

67220	0.946132	0.440579
67221	0.946133	0.33067
67222	0.946133	0.386687
67223	2.02912	0.390376
67224	2.57016	0.373618
67225	1.88631	0.383579
67226	1.88631	0.384012
67227	2.55301	0.339297
67228	2.4102	0.358762
67229	1.89226	0.374302
67230	1.89226	0.424368
67231	1.89226	0.439342
67232	1.89226	0.436362
67233	3.78452	0.404861
67234	1.89227	0.379993
67235	1.89227	0.433554
67236	1.89227	0.41088
67237	1.89227	0.417478
67238	1.89227	0.39755
67239	1.89227	0.394148
67240	1.89226	2.08652
67241	1.89226	2.07064
67242	1.89226	1.91857
67243	1.89226	2.1
67244	3.78452	2.06672
67245	1.2515	2.06327
67246	1.34788	2.1
67247	0.946132	2.1
67248	0.946132	2.1
67249	0.973043	2.1
67250	1.16766	2.1
67251	0.919222	1.95615
67252	1.2159	2.1
67253	0.946132	2.1
67254	0.946132	2.1
67255	1.33746	2.1
67256	1.13537	2.1
67257	1.89226	2.06163
67258	1.89226	2.1
67259	1.89226	2.1
67260	1.89226	2.1
67261	1.89226	2.1
67262	1.89226	2.1
67263	1.89226	1.85199
67264	1.89226	2.05766
67265	1.89226	2.01966
67266	1.89226	1.91864
67267	3.78452	1.9375
67268	1.89227	2.08599
67269	1.89227	1.69498
67270	1.89227	2.058
67271	1.89227	1.94297
67272	1.89227	2.09798
67273	1.89227	1.93613
67274	0.946132	2.00155
67275	0.946132	1.91434
67276	0.946129	2.04593
67277	0.946129	2.07762

67278	0.946131	2.03781
67279	0.946131	2.08127
67280	0.946131	2.05096
67281	0.946131	1.91867
67282	0.946131	2.03287
67283	0.946131	2.05897
67284	0.94613	2.02993
67285	0.94613	2.01501
67286	1.89226	0.897694
67287	1.89226	0.928133
67288	1.89226	0.901759
67289	1.89226	0.845154
67290	3.78453	0.882363
67291	0.946132	1.29701
67292	0.946132	0.972168
67293	0.946132	1.02661
67294	0.946132	0.953385
67295	0.946132	0.879751
67296	0.946132	1.2941
67297	0.946132	0.966662
67298	0.946132	0.97802
67299	0.946131	1.25495
67300	0.946131	0.925354
67301	0.946132	1.03188
67302	0.946132	0.967619
67303	1.89226	1.01984
67304	1.89226	0.814255
67305	1.89226	0.7909
67306	1.89226	0.872747
67307	1.89226	0.879399
67308	1.89226	1.138
67309	1.89226	1.03635
67310	1.89226	0.866568
67311	1.89226	0.840557
67312	1.89226	0.883949
67313	3.78453	0.87675
67314	0.96614	1.09989
67315	0.96614	0.976046
67316	0.946131	0.84259
67317	0.946131	0.925572
67318	0.946131	0.957559
67319	0.96614	0.942056
67320	1.37736	1.05223
67321	0.946131	1.08981
67322	1.37736	1.25855
67323	0.96614	0.917508
67324	0.946131	1.14562
67325	0.946131	0.887643
67326	0.946129	1.20277
67327	0.946129	0.913617
67328	0.946128	0.924126
67329	0.946128	1.07091
67330	0.946132	0.825456
67331	0.946132	1.30598
67332	0.946126	0.933496
67333	0.946126	0.895013
67334	0.946129	0.8349
67335	0.946129	0.870456

67336	0.946129	0.833996
67337	0.946129	0.950836
67338	1.89226	0.370506
67339	1.65723	0.401159
67340	1.83117	0.430001
67341	1.65723	0.415562
67342	2.0662	0.366266
67343	1.89226	0.355772
67344	1.89226	0.380382
67345	1.89226	0.416667
67346	1.89226	0.450674
67347	1.89226	0.442053
67348	1.89226	0.363438
67349	1.89226	0.358034
67350	1.89226	0.43108
67351	1.89226	0.483678
67352	1.89226	0.52508
67353	1.89226	0.522453
67354	1.89226	0.406298
67355	1.89226	0.406429
67356	1.89226	0.411243
67357	1.89226	0.443638
67358	1.89226	0.495445
67359	1.89226	0.487302
67360	1.89226	0.397588
67361	1.89226	0.394794
67362	1.89226	0.379016
67363	1.83117	0.413512
67364	1.92807	0.430042
67365	1.83117	0.415556
67366	1.98916	0.368696
67367	1.89226	0.356687
67368	1.89226	0.356454
67369	1.89226	0.432226
67370	1.89226	0.402954
67371	1.89226	0.433611
67372	3.78453	0.397064
67373	1.89226	0.446408
67374	1.89226	0.511738
67375	1.89226	0.542745
67376	1.89226	0.551447
67377	1.89226	0.414617
67378	1.89226	0.421704
67379	1.89226	0.408294
67380	1.89226	0.481827
67381	1.89226	0.508368
67382	1.89226	0.501555
67383	1.89226	0.405491
67384	1.89226	0.402052
67385	1.88037	0.438409
67386	1.89227	0.473068
67387	1.89227	0.497843
67388	1.89227	0.474089
67389	1.98599	0.443639
67390	1.98599	0.42548
67391	1.89226	0.452158
67392	1.89226	0.517106
67393	1.89226	0.557467

67394	1.89226	0.55451
67395	1.89226	0.452638
67396	1.89226	0.455978
67397	1.89226	0.566702
67398	1.89226	0.60658
67399	1.89226	0.562549
67400	1.89226	0.687855
67401	3.78452	0.588452
67402	1.89226	0.540807
67403	1.89226	0.601367
67404	1.89226	0.563757
67405	1.89226	0.56381
67406	1.89226	0.561873
67407	1.89226	0.558664
67408	1.90116	0.43482
67409	1.89227	0.486945
67410	1.89226	0.500916
67411	1.89227	0.478341
67412	1.88036	0.448556
67413	1.88037	0.43224
67414	1.89226	0.453945
67415	1.89226	0.549178
67416	1.89226	0.557241
67417	1.89226	0.544354
67418	1.89226	0.458576
67419	1.89226	0.451755
67420	0.946132	0.629014
67421	0.946132	0.609215
67422	0.946128	0.625788
67423	0.946128	0.627168
67424	0.94613	0.642961
67425	0.94613	0.659411
67426	0.94613	0.590795
67427	0.94613	0.590337
67428	0.946131	0.612754
67429	0.946131	0.632694
67430	0.94613	0.537554
67431	0.94613	0.675518
67432	1.89226	0.579011
67433	1.89226	0.604222
67434	1.89226	0.566271
67435	1.89226	0.588788
67436	1.89226	0.575833
67437	1.89226	0.596899
67438	1.89226	0.937044
67439	1.89226	1.36768
67440	2.35239	1.35577
67441	2.27873	1.0345
67442	1.89226	1.2729
67443	1.83301	1.01708
67444	2.68449	1.11841
67445	1.89226	1.76141
67446	1.89226	1.12233
67447	1.89226	1.21763
67448	3.78453	1.15737
67449	0.946132	1.60745
67450	0.946132	1.10947
67451	0.946129	1.02636

67452	0.946129	1.51494
67453	0.946131	1.50622
67454	0.946131	1.26506
67455	0.94613	1.10728
67456	0.94613	1.18638
67457	0.946131	1.26952
67458	0.946131	1.12444
67459	0.946131	1.20026
67460	0.946131	1.3607
67461	1.89226	0.883862
67462	1.89226	1.137
67463	1.89226	1.27386
67464	1.89226	0.925631
67465	1.89226	1.2
67466	1.89226	0.915179
67467	1.70303	0.926109
67468	1.89226	1.24205
67469	1.89226	0.887972
67470	1.89226	0.996743
67471	3.78453	0.942432
67472	1.89227	1.12453
67473	1.89227	1.22053
67474	1.89227	0.954196
67475	1.89227	1.43292
67476	3.78453	1.18753
67477	1.89226	1.04475
67478	1.89226	1.48132
67479	1.89226	1.06055
67480	1.89226	1.31074
67481	3.78452	1.20551
67482	1.89226	1.88772
67483	1.89226	2.02954
67484	1.89226	2.00447
67485	1.89226	1.92748
67486	3.78452	1.83264
67487	0.946132	1.78184
67488	0.946132	1.94156
67489	0.946129	2.08015
67490	0.946129	2.06169
67491	0.94613	2.06329
67492	0.94613	1.92593
67493	0.946131	2.0112
67494	0.946131	1.914
67495	0.946131	1.93401
67496	0.946131	2.02612
67497	0.946131	1.97772
67498	0.946131	1.82988
67499	1.89226	1.95489
67500	1.89226	1.71916
67501	1.89226	1.7822
67502	1.89226	1.94631
67503	3.78452	1.96774
67504	1.89226	1.51087
67505	1.89226	1.98581
67506	1.89226	2.03906
67507	1.89226	1.63992
67508	1.89226	2.00248
67509	1.89226	1.59283

67510	1.89227	1.97203
67511	2.48308	1.6173
67512	1.89227	1.6444
67513	1.89227	1.93785
67514	3.78453	2.01706
67515	1.89226	1.6964
67516	1.89226	1.89791
67517	1.89226	1.98286
67518	1.89226	1.69103
67519	1.89226	1.91205
67520	1.89226	1.59457
67521	1.89226	1.46761
67522	1.89226	1.66838
67523	1.89226	1.72548
67524	1.89226	1.52551
67525	3.78453	1.80516
67526	0.946131	1.82504
67527	0.946131	1.86973
67528	0.946131	1.86094
67529	0.946131	1.92579
67530	0.946131	1.88123
67531	0.946131	1.92089
67532	0.946131	1.88499
67533	0.946131	1.80014
67534	0.946131	1.84538
67535	0.946131	1.95747
67536	0.946131	1.89134
67537	0.946131	1.77834
67538	1.23719	2.03692
67539	1.28439	1.73719
67540	0.94613	1.5127
67541	0.94613	1.73189
67542	1.28439	1.83349
67543	1.6431	2.03677
67544	0.946129	1.6708
67545	0.946129	1.48529
67546	1.28439	1.46112
67547	0.946131	1.38221
67548	1.21038	1.40715
67549	1.21038	1.48044
67550	1.89226	1.65465
67551	1.89226	1.43036
67552	1.89226	1.45585
67553	1.89226	1.59298
67554	1.89226	1.39171
67555	1.89226	1.53751
67556	1.89226	1.66371
67557	1.89226	1.75775
67558	1.89226	1.84932
67559	1.89226	1.63711
67560	1.89226	1.75006
67561	1.89226	1.53996
67562	1.89226	1.08843
67563	1.89226	0.801594
67564	1.89226	0.833947
67565	1.89226	0.916416
67566	1.89226	0.90324
67567	1.89226	1.122

67568	0.946128	0.796102
67569	0.946128	0.886546
67570	0.946125	0.734244
67571	0.946125	0.822935
67572	0.94613	0.75804
67573	0.94613	0.821639
67574	0.946124	0.848782
67575	0.946124	0.915491
67576	0.946127	0.756785
67577	0.946127	0.740427
67578	0.946127	0.851542
67579	0.946127	0.904077
67580	1.89226	0.727197
67581	2.78671	0.760871
67582	2.59302	0.752626
67583	2.59302	0.742006
67584	1.89226	0.791653
67585	1.89226	0.752215
67586	1.89226	0.753389
67587	1.89226	0.795632
67588	1.89226	0.758497
67589	1.89226	0.749688
67590	1.89226	0.828258
67591	1.89226	0.785141
67592	1.89225	0.736933
67593	1.89225	0.609628
67594	1.89225	0.78992
67595	1.89225	0.72052
67596	1.89225	0.791155
67597	1.89225	0.721815
67598	0.979536	0.713114
67599	0.816597	0.721681
67600	1.15446	0.707327
67601	1.07423	0.618804
67602	1.08452	0.686375
67603	0.603522	0.651267
67604	1.70302	0.649182
67605	1.29344	0.614806
67606	1.21806	0.643022
67607	0.723758	0.609549
67608	1.37562	0.722861
67609	1.89226	0.355994
67610	1.92807	0.418415
67611	1.90843	0.415211
67612	1.92807	0.418614
67613	1.87263	0.363152
67614	1.89226	0.366441
67615	0.946132	0.410682
67616	0.946132	0.395006
67617	0.946131	0.398713
67618	0.946131	0.411803
67619	0.946132	0.43205
67620	0.946132	0.42194
67621	0.946132	0.363379
67622	0.946132	0.374371
67623	0.946132	0.400222
67624	0.946132	0.46608
67625	0.946132	0.392744

67626	0.946132	0.426316
67627	1.89226	0.421768
67628	1.89226	0.529911
67629	1.89226	0.470391
67630	1.89226	0.52945
67631	3.78452	0.473206
67632	1.89226	0.411805
67633	1.89226	0.499152
67634	1.89226	0.502438
67635	1.89226	0.487285
67636	1.89226	0.410326
67637	1.89226	0.399615
67638	1.70303	0.35564
67639	1.90843	0.403006
67640	1.77002	0.395654
67641	1.90843	0.393974
67642	1.75709	0.353838
67643	1.89226	0.349145
67644	1.89226	0.353638
67645	1.89226	0.429344
67646	1.89226	0.389715
67647	1.89226	0.42597
67648	3.78453	0.387175
67649	1.89226	0.445505
67650	1.89226	0.509841
67651	1.89226	0.434094
67652	1.89226	0.554537
67653	3.78452	0.50114
67654	0.946131	0.410635
67655	0.946131	0.431264
67656	0.946131	0.418726
67657	0.946131	0.448406
67658	0.946129	0.467412
67659	0.946129	0.463158
67660	0.946133	0.411216
67661	0.946133	0.400106
67662	0.946131	0.488391
67663	0.946131	0.418701
67664	0.946131	0.389641
67665	0.946131	0.466345
67666	1.89226	0.702252
67667	2.10275	0.845198
67668	1.76377	0.823535
67669	1.76377	0.743332
67670	1.89226	0.77541
67671	1.89226	0.716508
67672	1.89226	0.702522
67673	1.89226	0.865075
67674	2.10275	0.839858
67675	2.55889	0.739642
67676	1.89226	0.795139
67677	2.30274	0.721533
67678	1.89226	0.771672
67679	1.89226	1.01388
67680	1.89226	0.987724
67681	1.89226	0.804301
67682	1.89226	0.921715
67683	1.89226	0.783978

67684	1.89226	0.754313
67685	1.89226	0.998796
67686	1.89226	0.942212
67687	1.89226	0.799781
67688	1.89226	0.887571
67689	1.89226	0.778613
67690	1.89226	0.686019
67691	1.89226	0.807831
67692	1.89226	0.789702
67693	1.89226	0.720811
67694	1.89226	0.756127
67695	1.89226	0.703819
67696	1.89226	0.687332
67697	1.89226	0.82571
67698	1.89226	0.804304
67699	1.89226	0.728575
67700	1.89226	0.771385
67701	1.89226	0.714579
67702	1.89226	0.761511
67703	1.89226	0.957702
67704	1.89226	0.920774
67705	1.89226	0.79416
67706	1.89226	0.881921
67707	1.89226	0.779446
67708	1.89226	0.857224
67709	1.89226	0.926117
67710	1.89226	0.750651
67711	1.89226	0.786177
67712	1.89226	0.75287
67713	1.89226	0.884575
67714	0.946131	0.657174
67715	0.976399	0.616062
67716	1.10124	0.5883
67717	0.946131	0.807952
67718	0.887633	0.780446
67719	0.887633	0.723881
67720	1.16933	0.657693
67721	1.23163	0.608732
67722	0.946131	0.697694
67723	0.946131	0.708491
67724	0.976399	0.705401
67725	1.14992	0.705958
67726	1.89226	0.75806
67727	1.89226	1.00518
67728	1.89226	0.74767
67729	1.89226	0.803446
67730	3.78452	0.756987
67731	1.89226	0.634274
67732	1.89226	0.794687
67733	1.89226	0.618163
67734	1.89226	0.708473
67735	3.78453	0.647438
67736	0.946132	0.533969
67737	0.946132	0.705112
67738	0.946131	0.655655
67739	0.946131	0.732924
67740	0.946131	0.734306
67741	0.946131	0.685236

67742	0.946132	0.520194
67743	0.946132	0.560023
67744	0.946131	0.622478
67745	0.946131	0.656932
67746	0.946132	0.637974
67747	0.946132	0.564312
67748	0.946132	0.787315
67749	0.946132	0.669093
67750	0.946131	0.710149
67751	0.946131	0.830694
67752	0.946131	0.830558
67753	0.946131	0.78576
67754	0.946132	0.714545
67755	0.946132	0.659203
67756	0.946131	0.740246
67757	0.946131	0.765886
67758	0.946132	0.76474
67759	0.946132	0.727824
67760	1.89226	0.789399
67761	1.89226	0.851655
67762	1.89226	0.708286
67763	1.89226	0.875888
67764	3.78452	0.837244
67765	1.89226	0.771005
67766	1.89226	0.756423
67767	1.89226	0.728266
67768	1.89226	0.713847
67769	3.78453	0.733767
67770	1.89226	0.726853
67771	1.89226	0.789371
67772	1.89226	0.737542
67773	1.89226	0.7951
67774	1.89226	0.719813
67775	1.89226	0.751504
67776	1.89225	0.710846
67777	1.89225	0.740849
67778	1.89225	0.720456
67779	1.89225	0.772572
67780	1.89225	0.705972
67781	1.89225	0.7307
67782	1.89226	0.71117
67783	1.89226	0.739082
67784	1.89226	0.717086
67785	1.89226	0.747402
67786	1.89226	0.70708
67787	1.89226	0.722105
67788	1.89226	0.764904
67789	1.89226	0.716986
67790	1.89226	0.702951
67791	1.89226	0.702716
67792	3.78453	0.705722
67793	1.39491	0.740629
67794	1.39491	0.739116
67795	0.924649	0.725409
67796	0.924649	0.719924
67797	0.946131	0.723545
67798	1.37343	0.707532
67799	0.924678	0.694801

67800	0.946131	0.874272
67801	0.924678	0.730182
67802	1.37343	0.706404
67803	0.946131	0.781896
67804	0.946131	0.757479
67805	1.89226	0.714155
67806	1.89226	0.700358
67807	1.89226	0.70543
67808	1.89226	0.715492
67809	1.89226	0.700155
67810	1.89226	0.704947
67811	0.946126	0.313644
67812	0.975461	0.297178
67813	0.946132	0.291455
67814	1.12674	0.401592
67815	0.946129	0.39962
67816	0.946129	0.356539
67817	1.12673	0.314927
67818	1.12673	0.297304
67819	0.946129	0.346143
67820	0.946129	0.379282
67821	0.975464	0.353284
67822	1.12673	0.354762
67823	1.89226	0.369781
67824	1.89226	0.363428
67825	1.89226	0.441816
67826	1.89226	0.351588
67827	1.89226	0.425009
67828	1.89226	0.359021
67829	0.946126	0.374135
67830	0.946126	0.438051
67831	0.946131	0.390393
67832	0.946131	0.551514
67833	0.946129	0.548743
67834	0.946129	0.505153
67835	0.946128	0.414375
67836	0.946128	0.372652
67837	0.946129	0.478024
67838	0.946129	0.520219
67839	0.946128	0.477969
67840	0.946128	0.476101
67841	1.89226	0.33246
67842	1.89226	0.500942
67843	1.89226	0.335193
67844	1.89226	0.385186
67845	3.78451	0.341763
67846	1.89226	0.431235
67847	1.89226	0.442537
67848	1.89226	0.337259
67849	1.89226	0.490978
67850	3.78452	0.460101
67851	0.946125	0.418225
67852	0.946125	0.489971
67853	0.946131	0.457701
67854	0.946131	0.587401
67855	0.946128	0.589331
67856	0.946128	0.555462
67857	0.946128	0.48585

67858	0.946128	0.419183
67859	0.946128	0.498719
67860	0.946128	0.535072
67861	0.946128	0.469648
67862	0.946128	0.514768
67863	1.89226	0.420901
67864	1.89226	0.574301
67865	1.89226	0.608616
67866	1.89226	0.47877
67867	3.78452	0.497097
67868	1.89226	0.418729
67869	1.89226	0.555637
67870	1.89226	0.489715
67871	1.89226	0.551658
67872	1.89226	0.453957
67873	1.89226	0.529584
67874	1.12492	0.549555
67875	1.41201	0.561372
67876	1.12082	0.475311
67877	0.771416	0.46681
67878	1.10725	0.49157
67879	1.10725	0.513416
67880	1.07618	0.671165
67881	1.07618	0.40348
67882	1.12492	0.521876
67883	0.771415	0.522547
67884	1.12082	0.496393
67885	1.41201	0.511972
67886	2.82401	0.468392
67887	1.89226	0.646882
67888	1.89226	0.473248
67889	1.89226	0.549297
67890	2.24983	0.513496
67891	2.24983	0.58844
67892	0.946133	0.610153
67893	0.946133	0.596757
67894	0.946129	0.520221
67895	0.946129	0.515438
67896	0.946131	0.556723
67897	0.946131	0.6323
67898	0.946131	0.638807
67899	0.946131	0.441696
67900	0.946131	0.52318
67901	0.946131	0.571561
67902	0.946131	0.529278
67903	0.946131	0.577174
67904	0.946132	0.551948
67905	0.946132	0.534904
67906	0.946133	0.496362
67907	0.946133	0.493103
67908	0.946132	0.52805
67909	0.946132	0.551481
67910	0.946133	0.50621
67911	0.946133	0.52255
67912	0.946132	0.543809
67913	0.946132	0.491846
67914	0.946133	0.542667
67915	0.946133	0.502272

67916	1.89227	0.781187
67917	1.89227	0.871377
67918	1.89227	0.878777
67919	1.89227	0.765815
67920	1.89227	0.873617
67921	1.89227	0.766617
67922	1.42982	0.51879
67923	1.19944	0.432169
67924	0.936311	0.621947
67925	0.936311	0.542421
67926	0.946132	0.552792
67927	1.02993	0.544082
67928	1.41513	0.56677
67929	0.946133	0.60922
67930	0.946133	0.547549
67931	0.946133	0.582813
67932	1.02993	0.586721
67933	1.18181	0.517735
67934	1.89226	0.796081
67935	1.89226	0.917794
67936	1.89226	0.92732
67937	1.89226	0.698257
67938	3.78453	0.816365
67939	1.89226	0.590665
67940	1.89226	0.67259
67941	1.89226	0.685168
67942	1.89226	0.593485
67943	1.89226	0.677067
67944	1.89226	0.584792
67945	1.89226	0.584679
67946	1.89226	0.63133
67947	1.89226	0.667032
67948	1.89226	0.574935
67949	1.89226	0.661162
67950	1.89226	0.569695
67951	0.946131	0.702942
67952	0.946131	0.782824
67953	0.946132	0.894185
67954	0.946132	0.846597
67955	0.946132	0.741931
67956	0.946132	0.816404
67957	0.946132	0.918322
67958	0.946132	0.703061
67959	0.946132	0.776646
67960	0.946132	0.805662
67961	0.946132	0.797991
67962	0.946132	0.745925
67963	1.89226	1.22138
67964	1.89226	1.2802
67965	1.89226	1.43699
67966	1.89226	1.13286
67967	1.89226	1.42506
67968	1.89226	1.11561
67969	1.89226	1.42149
67970	2.22749	1.03424
67971	1.89226	1.04735
67972	1.89226	1.14162
67973	3.78453	1.14621

67974	1.89226	1.22041
67975	1.89226	1.31328
67976	1.89226	1.41794
67977	1.89226	1.05617
67978	3.78453	1.16011
67979	1.89226	1.0546
67980	1.89226	1.10514
67981	1.89226	1.22076
67982	1.89226	0.97714
67983	1.89226	1.23823
67984	1.89226	0.991373
67985	0.94613	0.969633
67986	0.94613	1.17462
67987	0.94613	1.36344
67988	0.94613	1.14363
67989	0.946131	1.08754
67990	0.946131	0.983974
67991	0.946129	1.31829
67992	0.946129	1.0584
67993	0.94613	1.09726
67994	0.94613	1.12045
67995	0.94613	1.08459
67996	0.94613	1.05321
67997	1.89226	0.426213
67998	1.89226	0.444084
67999	1.89226	0.454061
68000	1.89226	0.408281
68001	1.89226	0.455832
68002	1.89226	0.412828
68003	1.89226	0.453382
68004	1.89226	0.536989
68005	1.89226	0.547391
68006	1.89226	0.385314
68007	3.78452	0.475784
68008	1.89225	0.338518
68009	1.89225	0.356021
68010	1.89225	0.372917
68011	1.89225	0.320726
68012	1.89225	0.38119
68013	1.89225	0.335207
68014	1.89226	0.302942
68015	1.89226	0.310185
68016	1.89226	0.343269
68017	1.89226	0.297453
68018	1.89226	0.339495
68019	1.89226	0.298373
68020	0.946132	0.373229
68021	0.946132	0.428318
68022	0.946132	0.516485
68023	0.946132	0.437431
68024	0.946131	0.409896
68025	0.946131	0.374009
68026	0.946132	0.532743
68027	0.946132	0.383114
68028	0.946132	0.413357
68029	0.946132	0.426618
68030	0.946132	0.428659
68031	0.946132	0.42337

68032	1.89226	0.559678
68033	1.89226	0.652184
68034	1.89226	0.553225
68035	1.89226	0.691742
68036	1.89226	0.540536
68037	1.89226	0.679871
68038	1.89226	0.607395
68039	1.89226	0.543375
68040	1.89226	0.537726
68041	1.89226	0.653953
68042	1.89226	0.460141
68043	1.89226	0.641201
68044	1.89226	0.42979
68045	1.89226	0.5248
68046	1.89226	0.455159
68047	1.89226	0.563991
68048	1.89226	0.396684
68049	1.89226	0.518578
68050	1.89226	0.562038
68051	1.89226	0.654521
68052	1.89226	0.52844
68053	1.89226	0.681985
68054	3.78452	0.584401
68055	1.89226	0.525679
68056	1.89226	0.586792
68057	1.89226	0.501999
68058	1.89226	0.667071
68059	1.89226	0.485645
68060	1.89226	0.646754
68061	1.89226	0.238089
68062	1.89226	0.278912
68063	1.89226	0.264977
68064	1.89226	0.24
68065	3.78453	0.244389
68066	1.89226	0.23745
68067	2.48862	0.220028
68068	1.89226	0.234549
68069	2.22062	0.222173
68070	1.89226	0.235295
68071	2.22062	0.224954
68072	1.89227	0.246434
68073	1.89227	0.309314
68074	1.89227	0.277832
68075	1.89227	0.247657
68076	1.89227	0.285559
68077	1.89227	0.252177
68078	0.946132	0.252053
68079	0.946132	0.24151
68080	0.946131	0.275303
68081	0.946131	0.300221
68082	0.94613	0.300367
68083	0.94613	0.263677
68084	0.946133	0.272098
68085	0.946133	0.24783
68086	0.946131	0.273731
68087	0.946131	0.277807
68088	0.946131	0.272882
68089	0.946131	0.269629

68090	1.89226	0.250361
68091	1.89226	0.231208
68092	1.89226	0.21958
68093	1.89226	0.260285
68094	3.78453	0.242614
68095	1.89226	0.262479
68096	1.89226	0.339429
68097	1.89226	0.306617
68098	1.89226	0.267468
68099	3.78453	0.279847
68100	1.89226	0.675279
68101	1.89226	0.631133
68102	1.89226	0.640519
68103	1.89226	0.677433
68104	1.89226	0.634116
68105	1.89226	0.67749
68106	1.89226	0.675995
68107	1.89226	0.629306
68108	1.89226	0.626665
68109	1.89226	0.701062
68110	3.78452	0.65135
68111	1.89226	0.487655
68112	1.89226	0.668709
68113	1.89226	0.67208
68114	1.89226	0.586688
68115	3.78453	0.619908
68116	2.57454	0.652787
68117	1.89226	0.697274
68118	1.3663	0.702213
68119	1.89226	0.662464
68120	3.25856	0.675097
68121	1.89226	0.687712
68122	1.89226	0.659378
68123	1.89226	0.659188
68124	1.89226	0.698058
68125	3.78451	0.669512
68126	1.89226	0.656306
68127	1.89226	0.705282
68128	1.89226	0.665141
68129	1.89226	0.697327
68130	1.89226	0.665358
68131	1.89226	0.695921
68132	0.810946	0.662773
68133	0.810946	0.651783
68134	0.810946	0.682569
68135	0.810946	0.705645
68136	0.810959	0.684818
68137	0.810959	0.662342
68138	1.89226	0.69552
68139	1.35168	0.68116
68140	1.08132	0.647211
68141	1.08132	0.706477
68142	1.0813	0.674462
68143	1.89226	0.460294
68144	1.89226	0.629547
68145	1.89226	0.681641
68146	1.89226	0.642499
68147	3.78453	0.606355

68148	1.89226	0.665002
68149	1.89226	0.586365
68150	1.89226	0.65797
68151	1.89226	0.677026
68152	1.89226	0.488341
68153	1.89226	0.65872
68154	1.89226	0.582367
68155	1.89226	0.672801
68156	1.89226	0.67874
68157	1.89226	0.657148
68158	3.78452	0.657094
68159	1.89226	0.624953
68160	1.89226	0.646534
68161	1.89226	0.643435
68162	1.89226	0.673191
68163	3.78452	0.655974
68164	2.142	0.719344
68165	1.93949	0.547156
68166	1.39296	0.692724
68167	2.55711	0.707916
68168	1.39296	0.494424
68169	2.55711	0.646568
68170	1.89226	0.674302
68171	2.5571	0.552548
68172	2.142	0.680691
68173	1.89226	0.703309
68174	4.44936	0.684119
68175	0.946131	0.681066
68176	0.946131	0.682917
68177	1.27855	0.671712
68178	1.29171	0.665655
68179	1.29171	0.67848
68180	0.946127	0.679662
68181	1.27855	0.546282
68182	0.946131	0.696183
68183	1.29172	0.685193
68184	1.29172	0.676253
68185	0.946129	0.65598
68186	0.946129	0.670023
68187	1.27855	0.689381
68188	1.29172	0.676756
68189	0.969743	0.671719
68190	1.07109	0.66458
68191	1.29172	0.70485
68192	1.07109	0.660843
68193	0.969745	0.53725
68194	1.27855	0.696416
68195	1.10873	0.704319
68196	1.10873	0.697219
68197	1.15273	0.641957
68198	1.15273	0.662815
68199	1.89227	0.370409
68200	1.89227	0.38136
68201	1.89227	0.408685
68202	1.89227	0.38336
68203	2.1288	0.357064
68204	2.1288	0.339064
68205	1.89227	0.432328

68206	1.89227	0.460728
68207	1.89227	0.398158
68208	1.89227	0.439079
68209	3.78453	0.44111
68210	1.89226	0.486107
68211	1.89226	0.532811
68212	1.89226	0.446579
68213	1.89226	0.470282
68214	3.78453	0.459659
68215	1.89226	0.424422
68216	1.89226	0.465069
68217	1.89226	0.407758
68218	1.89226	0.400337
68219	1.89226	0.431612
68220	1.89226	0.470582
68221	1.94432	0.335814
68222	1.89226	0.390455
68223	2.07911	0.347812
68224	1.89226	0.34612
68225	2.07911	0.365818
68226	1.89226	0.365143
68227	1.89226	0.397877
68228	1.89226	0.366254
68229	1.89226	0.449015
68230	1.89226	0.348331
68231	1.89226	0.381892
68232	1.89226	0.430758
68233	1.89226	0.922314
68234	1.89226	0.939461
68235	1.89226	1.00834
68236	1.89226	0.845093
68237	1.89226	1.02164
68238	1.89226	0.864009
68239	1.89226	0.769502
68240	1.89226	0.76765
68241	1.89226	0.852601
68242	1.89226	0.674355
68243	1.89226	0.875561
68244	1.89226	0.722243
68245	1.86778	0.679259
68246	2.72348	0.67343
68247	1.89226	0.763344
68248	1.89226	0.485937
68249	3.76004	0.684513
68250	1.89226	0.901662
68251	1.89226	0.942326
68252	1.89226	1.04008
68253	1.89226	0.813933
68254	3.78451	0.895632
68255	1.89226	0.77342
68256	1.89226	0.797421
68257	1.89226	0.850172
68258	1.89226	0.690949
68259	1.89226	0.863001
68260	1.89226	0.723515
68261	0.94613	0.580289
68262	0.94613	0.704653
68263	0.94613	0.848228

68264	0.94613	0.691646
68265	0.946131	0.722834
68266	0.946131	0.532987
68267	0.946129	0.792404
68268	0.946129	0.684759
68269	0.94613	0.696053
68270	0.94613	0.72399
68271	0.94613	0.680985
68272	0.94613	0.702022
68273	0.946124	0.814787
68274	0.946124	0.654998
68275	0.94613	0.933736
68276	0.94613	0.821522
68277	0.946128	0.706705
68278	0.946128	0.784021
[truncated: 352,486 more chars]
