# Supplementary material for: On the Use of Bone Remodelling Models to Estimate the Density Distribution of Bones. Uniqueness of the Solution
Source: PLoS One. 2016 Feb 9;11(2):e0148603. doi: 10.1371/journal.pone.0148603 (PMC4747586; doi:10.1371/journal.pone.0148603)
Supplement: S3 File — (PDF) [file pone.0148603.s003.pdf]

N° Elem	Volume (mm3)	Density (g/cm3)
1	1.32415	2.1
2	1.75823	2.1
3	0.984099	2.1
4	1.46442	2.1
5	1.53082	2.1
6	1.32068	2.1
7	1.91404	0.05
8	1.17036	0.05
9	0.929209	0.05
10	1.27254	0.05
11	2.0926	0.05
12	2.20407	0.05
13	1.21488	0.05
14	1.26024	0.05
15	0.794454	0.05
16	1.95087	0.05
17	0.953925	2.1
18	0.98949	2.1
19	1.27875	2.1
20	1.04598	2.1
21	0.594005	2.1
22	0.578861	2.1
23	1.09506	2.1
24	1.45327	2.1
25	2.58396	2.1
26	1.58581	2.1
27	1.5858	2.1
28	0.636946	2.1
29	0.94202	2.1
30	0.946119	2.1
31	0.636953	2.1
32	0.946107	2.1
33	1.09505	2.1
34	1.27389	2.1
35	1.00232	2.1
36	1.00231	2.1
37	1.83583	2.1
38	1.00231	2.1
39	1.28828	0.05
40	1.81696	0.05
41	1.28828	0.05
42	1.28828	0.05
43	1.0044	0.05
44	0.85148	0.05
45	0.851539	0.05
46	1.57767	0.05
47	1.52227	0.05
48	0.730657	0.05
49	0.94613	0.05
50	1.28228	2.1
51	1.64268	2.1
52	1.10101	2.1
53	1.3939	2.1
54	0.959471	2.1
55	1.10101	2.1

56	1.2873	2.1
57	1.59659	2.1
58	1.80841	0.05
59	1.80841	0.0933975
60	0.946129	0.05
61	1.36991	0.05
62	1.32262	0.433534
63	1.32261	0.05
64	1.38464	0.0656971
65	0.962877	0.05
66	0.962878	0.0543863
67	1.82129	0.05
68	1.82129	0.05
69	0.94613	0.0607368
70	1.39751	0.0515494
71	0.946131	0.0816357
72	1.34493	0.05
73	1.39751	0.05
74	0.837577	0.467605
75	0.823478	0.0976257
76	1.10889	2.1
77	1.01686	0.514394
78	1.1942	2.1
79	0.836129	0.398843
80	0.937165	0.0964749
81	0.969438	1.13192
82	0.895386	0.256941
83	1.30554	2.1
84	1.15798	0.306292
85	1.22578	2.1
86	1.15255	1.02026
87	0.9765	0.118165
88	2.02178	2.1
89	1.63755	2.05395
90	0.727713	1.28463
91	0.810945	1.32565
92	1.00559	2.1
93	0.879098	1.13404
94	1.07852	2.1
95	0.657764	0.788034
96	0.829406	0.324049
97	0.720201	0.05
98	0.946131	0.05
99	1.11827	0.05
100	1.50754	0.05
101	1.64835	0.05
102	1.71903	0.05
103	0.851554	0.05
104	1.75626	0.05
105	2.1646	0.05
106	1.5563	0.05
107	1.5563	0.05
108	2.10876	0.05
109	1.05438	0.100318
110	0.946133	0.05
111	1.05438	0.40251
112	1.20256	0.05
113	1.47879	0.112436

114	1.47879	1.61729
115	0.77815	1.47172
116	0.947114	2.1
117	1.31257	2.1
118	1.09235	2.1
119	0.946132	2.1
120	1.54246	2.1
121	1.54246	2.1
122	1.09235	2.1
123	0.946131	2.1
124	1.29507	1.83461
125	1.0353	2.1
126	1.03529	2.1
127	1.28667	2.1
128	1.44777	2.1
129	1.61121	2.1
130	1.22176	0.0570764
131	0.946129	0.05
132	1.08848	0.05
133	0.946131	0.0570445
134	1.61201	0.05
135	1.61201	0.05
136	1.19993	0.05
137	1.35822	0.05
138	1.1539	0.05
139	1.00765	0.05
140	1.28228	2.1
141	2.65339	2.1
142	1.28228	2.1
143	1.55998	2.1
144	1.00509	2.1
145	0.93845	2.1
146	1.27674	2.1
147	0.810958	2.1
148	1.91381	2.1
149	1.1861	2.1
150	1.24886	2.1
151	2.02842	2.1
152	0.991436	0.05
153	1.34588	0.05
154	2.02729	0.05
155	0.72904	0.05
156	1.06139	0.05
157	0.835368	0.05
158	1.63819	0.05
159	1.10745	0.05
160	1.79925	0.05
161	1.00085	0.05
162	0.946133	2.1
163	1.41123	2.1
164	1.8327	2.1
165	1.8327	2.1
166	0.946132	0.613531
167	1.41123	2.1
168	1.3676	2.1
169	1.11048	2.1
170	1.11048	2.1
171	1.65815	2.1

172	1.9004	2.1
173	1.88691	2.1
174	1.82232	2.1
175	1.46591	2.1
176	1.09115	2.1
177	1.02051	0.691381
178	1.03373	1.22266
179	1.10307	2.1
180	1.05556	2.1
181	1.82533	0.745002
182	1.19496	0.259026
183	1.28597	2.1
184	1.20421	2.1
185	2.23644	2.1
186	1.08378	2.1
187	0.983724	2.1
188	0.602075	2.1
189	1.45876	2.1
190	1.45875	2.1
191	0.602103	2.1
192	1.11822	2.1
193	0.946133	2.1
194	0.602103	2.1
195	1.20421	2.1
196	1.26371	0.0656221
197	1.55179	0.0521326
198	1.14042	0.05
199	1.56876	0.05
200	2.27496	0.0536717
201	1.13748	0.05
202	1.02884	0.112192
203	1.25124	0.0501913
204	0.82703	0.0500431
205	0.607775	0.05
206	1.49242	0.05
207	1.35674	0.0804325
208	1.6697	2.1
209	1.81655	2.1
210	0.900108	1.09259
211	0.851537	0.179373
212	0.703076	0.750292
213	0.639143	0.380237
214	1.42358	1.17086
215	1.4976	1.44254
216	0.86284	0.271583
217	1.2965	0.630903
218	1.27835	2.1
219	1.00012	0.586283
220	1.74332	1.4261
221	1.08576	2.1
222	1.19 2.1	
223	1.08576	2.1
224	1.20854	2.1
225	0.946131	2.1
226	0.946131	2.1
227	1.75985	2.1
228	1.75985	2.1
229	1.41232	2.1

230	1.29365	2.1
231	0.973559	2.1
232	1.23698	0.05
233	1.45055	0.05
234	1.26947	0.05
235	0.856218	0.05
236	1.75303	0.05
237	1.14938	0.05
238	0.984711	0.05
239	2.05771	0.05
240	0.625171	0.05
241	0.965675	0.05
242	1.17631	0.05
243	1.4677	0.05
244	1.35403	0.05
245	0.975149	0.05
246	0.946131	0.05
247	1.80893	0.05
248	1.80893	0.05
249	0.975149	0.05
250	0.946132	0.05
251	1.9503	0.05
252	2.80207	0.05
253	1.76411	0.05
254	2.77469	0.05
255	1.9503	0.05
256	1.26784	0.05
257	2.31093	0.05
258	0.851541	0.05
259	1.39521	0.05
260	1.45613	0.05
261	0.851541	0.05
262	0.996013	0.05
263	1.07989	0.05
264	0.946131	0.05
265	1.08213	0.05
266	1.74289	0.05
267	1.75838	0.05
268	2.11163	0.05
269	1.23773	2.1
270	1.25398	2.1
271	1.0304	2.1
272	1.32368	2.1
273	1.11712	0.867658
274	1.07999	0.398733
275	1.1766	2.1
276	1.35735	2.1
277	1.089 0.05	
278	1.81193	0.05
279	1.11081	0.05
280	1.78069	0.05
281	0.717512	0.05
282	0.810946	0.05
283	0.703901	0.05
284	1.09028	0.05
285	0.958031	0.05
286	0.892585	0.05
287	0.873431	0.05

288	0.997755	0.05
289	1.85479	0.05
290	0.983029	0.05
291	1.27922	0.05
292	1.64346	0.05
293	1.27922	0.05
294	1.09795	2.03339
295	1.07508	0.550187
296	1.02975	1.51042
297	1.01121	2.1
298	1.72369	0.05
299	1.12035	0.05
300	2.13511	0.05
301	0.9705	0.05
302	0.946131	0.05
303	0.86182	0.05
304	1.51379	0.05
305	1.51379	0.05
306	0.946132	0.05
307	0.861849	0.05
308	1.34591	0.0854606
309	1.14721	0.05
310	1.15539	2.1
311	1.90938	2.1
312	2.19436	1.95585
313	1.41563	2.1
314	0.785641	2.1
315	0.736384	2.1
316	1.46489	2.1
317	1.46488	2.1
318	0.736372	2.1
319	0.946132	2.1
320	1.90941	1.26392
321	1.18907	2.1
322	1.20409	2.1
323	0.973559	2.1
324	1.29461	2.1
325	0.946129	2.1
326	0.94613	2.1
327	1.78751	2.1
328	1.78751	2.1
329	1.38885	2.1
330	1.34479	2.1
331	1.07283	1.92646
332	1.6869	2.1
333	1.52544	2.1
334	1.07797	2.1
335	1.13819	0.545587
336	0.776179	2.1
337	1.61092	1.49274
338	1.36155	2.1
339	0.77618	2.1
340	1.2933	1.07227
341	1.15946	2.1
342	0.69857	2.1
343	1.87607	2.1
344	1.07612	2.1
345	1.31976	0.881373

346	1.01243	2.1
347	1.41279	1.32199
348	1.35146	0.05
349	1.90664	0.05
350	1.35145	0.05
351	1.22811	0.05
352	1.93147	0.05
353	1.42486	2.1
354	1.56558	2.1
355	1.0476	0.05
356	2.31605	0.05
357	1.89367	0.05
358	0.804706	0.05
359	0.783919	0.05
360	1.3116	0.05
361	1.03891	0.05
362	0.960473	0.05
363	1.08462	0.05
364	0.785994	0.05
365	1.1318	0.05
366	1.29576	0.05
367	1.3946	0.05
368	1.11924	0.05
369	0.834322	2.1
370	1.0256	2.1
371	1.74321	0.400712
372	1.41798	0.343797
373	0.871608	0.344865
374	1.47253	0.290094
375	1.47252	0.331258
376	0.817064	0.276564
377	0.817064	0.341659
378	0.946129	0.236865
379	1.63413	0.262089
380	1.9194	0.05
381	1.93562	0.05
382	2.06307	0.05
383	1.46021	0.05
384	0.955002	0.639018
385	0.833409	1.53834
386	0.838039	0.623547
387	1.48702	0.644851
388	1.1697	0.577422
389	1.23549	0.779206
390	1.18373	0.546448
391	2.02254	0.481526
392	1.68867	0.811356
393	0.875481	0.165511
394	0.821635	1.19795
395	0.858878	0.131625
396	1.30245	0.299378
397	1.07476	0.263245
398	1.11907	0.252534
399	1.10453	0.180704
400	1.70233	0.256803
401	1.70233	0.165686
402	2.6558	1.02112
403	1.7818	2.1

404	1.87059	2.1
405	1.34412	0.05
406	1.98068	0.05
407	2.56369	0.05
408	1.49512	0.05
409	1.35977	0.05
410	1.34254	0.05
411	1.75618	0.05
412	1.75618	0.05
413	0.946129	0.05
414	0.946129	0.05
415	1.17665	0.05
416	1.01976	0.05
417	1.27376	0.05
418	1.35978	0.05
419	1.68741	0.05
420	1.68741	0.05
421	0.946132	0.05
422	0.946133	0.05
423	1.14241	0.05
424	0.996831	0.05
425	0.996828	0.05
426	1.12774	0.05
427	1.13664	2.1
428	1.09627	2.1
429	2.8814	2.1
430	3.49751	2.1
431	1.79109	0.05
432	1.54197	0.05
433	2.75153	0.05
434	1.93019	0.05
435	1.51569	0.18627
436	1.55145	0.625069
437	1.78595	2.1
438	3.74452	2.1
439	1.66537	2.1
440	3.55161	2.1
441	1.35002	0.107636
442	1.19901	0.142496
443	1.61028	2.1
444	1.35002	0.234282
445	1.65712	0.206753
446	1.10585	0.731556
447	0.961347	0.861716
448	0.810944	1.23196
449	1.16148	1.93355
450	1.08066	1.66295
451	0.934577	0.345735
452	1.05164	1.29316
453	0.79216	0.672162
454	2.99194	2.1
455	1.2832	2.1
456	2.97842	2.1
457	1.39517	2.1
458	1.03391	2.1
459	1.74416	2.1
460	1.05071	2.1
461	0.923782	2.1

462	1.55369	2.1
463	1.86444	2.1
464	1.01617	2.1
465	1.27174	2.1
466	1.20573	2.1
467	1.01616	2.1
468	1.13621	2.1
469	1.75366	2.1
470	2.27009	1.8706
471	1.6813	2.03377
472	1.47904	0.05
473	2.62714	0.05
474	1.80085	0.05
475	1.80085	0.05
476	2.74104	0.05
477	1.37052	0.05
478	0.946131	0.05
479	1.37052	0.05
480	1.31357	0.05
481	1.7837	0.05
482	1.7837	0.05
483	0.900397	0.05
484	1.74487	0.05
485	2.88672	2.1
486	2.88249	2.1
487	1.0961	0.0695863
488	1.20143	0.0943661
489	1.66739	0.05
490	1.85795	0.05
491	1.45789	0.05
492	2.11407	0.05
493	0.713524	0.05
494	1.58391	0.05
495	1.58391	0.05
496	1.04765	0.05
497	1.24979	0.05
498	0.713551	0.05
499	0.946127	0.05
500	1.36768	0.05
501	1.14433	0.05
502	1.66923	0.05
503	0.699479	0.05
504	0.70351	0.05
505	0.758416	0.05
506	1.07678	0.05
507	0.909874	0.05
508	0.940218	0.05
509	0.876604	0.05
510	1.27148	0.05
511	2.42348	0.05
512	1.19837	0.05
513	0.764764	0.05
514	0.829259	0.05
515	0.740584	0.05
516	1.2772	0.05
517	0.964465	0.05
518	1.06231	0.05
519	1.06924	0.05

520	1.17566	0.05
521	1.3063	0.05
522	2.51253	0.05
523	2.42267	0.05
524	1.89226	0.05
525	1.46492	0.05
526	0.946132	0.05
527	0.653151	0.05
528	0.653124	0.05
529	1.53995	0.05
530	1.53995	0.05
531	0.936806	0.05
532	1.25626	0.05
533	1.1893	0.05
534	1.62206	0.05
535	1.04921	2.1
536	1.22927	2.1
537	0.801765	2.1
538	0.774917	2.1
539	0.844623	2.1
540	0.737481	2.1
541	3.24379	2.1
542	2.48573	2.1
543	1.40967	0.767349
544	1.47203	1.62276
545	2.07659	0.826713
546	1.40967	1.52896
547	2.06638	0.793292
548	1.17418	2.1
549	1.04921	2.1
550	1.74593	2.1
551	1.74593	2.1
552	0.946132	1.56648
553	1.38651	2.1
554	1.20761	0.05
555	1.20761	1.21312
556	1.30555	0.441153
557	1.46812	0.107288
558	2.41602	0.415952
559	1.46812	0.372241
560	1.46812	0.0514888
561	0.734059	0.0634667
562	0.946132	1.69779
563	1.21261	0.497472
564	1.34225	0.536456
565	0.734084	0.590699
566	1.82077	0.308357
567	1.82077	0.234103
568	1.13751	0.05
569	0.946131	0.354561
570	1.13751	0.103804
571	1.13748	0.0911037
572	0.784392	0.121679
573	1.4906	0.0624027
574	1.4906	0.05
575	1.17879	1.36969
576	1.28291	1.22553
577	0.626495	2.1

578	1.45334	2.1
579	1.45334	2.1
580	0.626494	2.1
581	1.3852	2.1
582	0.946116	2.1
583	0.626499	2.1
584	1.47203	0.482867
585	1.42639	2.1
586	1.42639	2.1
587	0.795469	2.1
588	0.795469	2.1
589	0.946132	2.1
590	0.850877	2.1
591	1.37098	2.1
592	1.87194	2.1
593	1.76988	2.1
594	3.16295	0.05
595	3.26725	0.05
596	1.13322	2.1
597	1.20761	2.1
598	0.686933	1.65829
599	0.493248	0.747483
600	1.2053	2.1
601	0.947149	1.18712
602	1.27591	1.40615
603	1.75732	2.1
604	0.493261	1.44409
605	1.34337	2.1
606	2.73207	0.602802
607	1.19857	1.24011
608	0.671688	0.373727
609	0.946134	2.1
610	1.36603	0.53091
611	2.07817	1.23351
612	1.82573	2.1
613	0.671699	1.83248
614	1.47772	0.636387
615	0.975349	2.1
616	1.22615	2.1
617	1.47868	2.1
618	2.29351	1.99323
619	1.47642	2.1
620	2.94443	1.77523
621	1.80454	2.1
622	0.949135	2.1
623	0.949134	2.1
624	1.10476	0.05
625	0.982857	0.05
626	1.10938	0.05
627	1.19052	0.0536833
628	1.01012	0.05
629	1.35562	0.05
630	0.691742	0.05
631	2.39596	0.05
632	2.35604	0.05
633	0.974081	0.05
634	3.22444	0.05
635	1.3294	0.142151

636	1.37265	1.08032
637	1.3294	0.222549
638	1.51113	2.1
639	1.43643	2.1
640	1.72726	2.1
641	1.634 2.1	
642	0.755566	2.1
643	1.29319	2.1
644	0.755567	2.1
645	0.946131	2.1
646	1.29319	2.1
647	0.973563	2.1
648	0.904398	2.1
649	0.904406	2.1
650	1.1538	2.1
651	1.10437	2.1
652	0.935418	2.1
653	1.16649	2.1
654	0.975667	2.1
655	1.17952	2.1
656	1.95053	2.1
657	0.948676	2.1
658	1.44287	2.1
659	0.746095	2.1
660	1.51224	2.1
661	1.27354	2.1
662	0.746066	2.1
663	1.20573	2.1
664	1.12442	2.1
665	0.671487	2.1
666	1.04355	2.1
667	0.948675	2.1
668	0.967673	0.378954
669	1.12931	0.05
670	1.38212	0.05
671	2.40113	0.05
672	1.08054	0.05
673	1.04072	0.05
674	0.691062	0.05
675	1.58052	0.05
676	1.33502	0.05
677	0.946131	0.05
678	0.691062	0.05
679	1.02663	0.05
680	1.80569	0.05
681	1.26062	0.05
682	1.18203	0.05
683	0.946131	0.05
684	0.795065	0.05
685	1.58753	0.05
686	1.58753	0.05
687	0.946131	0.05
688	0.795093	0.05
689	0.805057	2.1
690	0.812415	2.1
691	1.23868	0.05
692	1.34448	0.05
693	1.67201	2.1

694	1.61202	2.1
695	1.41103	2.1
696	1.41103	2.1
697	0.841088	2.1
698	1.38302	2.1
699	0.841089	2.1
700	0.869097	2.1
701	1.38302	2.1
702	0.949941	2.1
703	0.94994	2.1
704	1.88608	2.1
705	0.854942	2.1
706	1.68893	2.1
707	1.70002	2.1
708	2.02107	0.05
709	2.04982	0.05
710	3.11063	0.05
711	2.03805	0.05
712	1.67638	2.1
713	0.967492	2.1
714	2.00209	2.1
715	0.967491	2.1
716	0.870742	2.1
717	1.30266	0.05
718	0.651334	0.05
719	1.69413	0.05
720	1.69413	0.05
721	0.946132	0.05
722	1.03079	0.05
723	1.31468	0.05
724	0.946132	0.05
725	0.919055	0.05
726	1.16815	0.05
727	2.79004	0.05
728	1.2136	0.05
729	2.62935	0.05
730	0.947692	0.05
731	1.51835	0.05
732	0.947693	0.05
733	0.947692	0.05
734	0.980841	2.1
735	1.43577	2.1
736	0.980841	2.1
737	1.67858	2.1
738	1.34124	0.076113
739	1.39818	0.0510566
740	1.2065	2.1
741	0.946133	2.1
742	1.2079	2.1
743	1.65229	2.1
744	1.65229	2.1
745	0.946132	2.1
746	1.2079	2.1
747	1.39053	2.1
748	1.20651	2.1
749	1.36919	2.1
750	1.12717	2.1
751	1.09483	2.1

752	0.994294	2.1
753	0.97356	2.1
754	1.06827	2.1
755	1.40571	2.1
756	1.29259	2.1
757	0.946131	2.1
758	1.7787	2.1
759	1.7787	2.1
760	1.29259	2.1
761	0.946132	2.1
762	1.09408	0.132069
763	1.03824	0.245612
764	0.851541	0.062542
765	1.23608	0.0602294
766	0.851539	0.0673178
767	1.5021	0.105696
768	1.54839	0.0769788
769	1.12373	0.0521878
770	1.22617	0.05
771	1.42762	0.05
772	2.24746	0.0949009
773	1.05475	0.05
774	1.46845	0.05
775	1.05475	0.05
776	0.929726	0.05
777	1.52072	0.05
778	1.82488	0.05
779	1.02271	0.05
780	1.19225	0.05
781	1.43071	0.05
782	0.94613	0.05
783	1.05475	0.05
784	1.39223	2.1
785	1.71255	2.1
786	0.946132	0.579005
787	1.10531	0.363562
788	1.46355	0.413174
789	1.46355	0.227729
790	0.78086	0.986321
791	1.10531	0.413142
792	1.1391	0.468287
793	1.56166	0.6196
794	2.21062	0.347017
795	1.0361	2.1
796	1.73577	2.1
797	1.73577	2.1
798	0.946116	2.1
799	1.28174	2.1
800	0.946118	2.1
801	1.22883	2.1
802	1.28172	2.1
803	1.03609	2.1
804	0.553567	0.05
805	1.60581	0.05
806	1.60581	0.05
807	0.553594	0.05
808	0.966748	0.05
809	0.966748	0.05

810	0.946133	0.05
811	1.05602	0.05
812	2.38525	0.05
813	1.14503	0.05
814	1.24678	0.05
815	0.56262	0.05
816	1.24678	0.05
817	0.978467	0.05
818	1.66262	0.05
819	1.66262	0.05
820	0.562633	0.05
821	1.09522	0.05
822	2.49356	0.05
823	0.592547	0.05
824	1.04321	0.33483
825	1.25133	1.07951
826	1.70508	1.43347
827	1.81043	0.911843
828	0.494934	1.21465
829	0.601047	0.651033
830	0.993428	1.13289
831	0.946573	1.10096
832	1.35039	0.672928
833	0.816895	1.57175
834	1.5063	0.768535
835	0.816895	0.457777
836	0.946132	0.862061
837	1.9067	1.24831
838	2.14504	1.60794
839	0.816895	0.441913
840	2.29624	0.489644
841	2.76933	0.434982
842	1.254 1.3162	
843	0.946128	2.1
844	1.38813	2.1
845	1.79998	2.1
846	1.79998	2.1
847	0.94613	2.1
848	1.38813	2.1
849	1.35798	2.1
850	1.27809	2.1
851	1.35798	2.1
852	1.68994	2.1
853	1.68994	2.1
854	0.946132	2.1
855	0.946132	2.1
856	1.18067	2.1
857	0.958256	2.1
858	0.958255	2.1
859	0.958257	2.1
860	0.958255	2.1
861	1.06457	2.1
862	1.14195	2.1
863	1.70423	0.0583524
864	1.14195	2.1
865	1.64285	2.1
866	2.02057	2.1
867	0.946132	2.1

868	1.44186	2.1
869	0.946133	2.1
870	1.16366	2.1
871	1.17233	2.1
872	1.31259	0.0946923
873	1.2016	2.1
874	1.17129	2.1
875	1.05724	0.05
876	1.32697	0.969114
877	1.05724	0.401269
878	1.04292	1.24308
879	3.39037	2.1
880	2.57275	2.1
881	0.629212	2.1
882	2.32525	2.1
883	1.76949	0.32055
884	1.65809	1.09623
885	1.27665	2.1
886	1.19861	2.1
887	1.20045	2.1
888	1.1635	2.1
889	0.953633	0.05
890	0.953634	0.05
891	1.21408	2.1
892	1.25716	2.1
893	2.01791	2.1
894	1.89318	2.1
895	0.947921	1.332
896	0.939049	2.1
897	0.947921	2.07118
898	0.740753	2.1
899	0.916104	2.1
900	1.16941	2.1
901	1.72038	2.1
902	2.04441	2.1
903	0.973182	2.1
904	1.11751	2.1
905	0.611089	2.1
906	1.53584	0.05
907	0.767923	0.05
908	1.38925	0.05
909	1.38926	0.05
910	0.946131	0.0571774
911	0.961347	0.05
912	1.19583	0.0822273
913	0.946131	0.0866924
914	1.3663	0.0993338
915	1.21067	0.05
916	1.00166	0.05
917	1.40707	0.05
918	1.50435	0.05
919	1.47954	0.216119
920	1.1233	2.1
921	2.36036	1.72161
922	1.04024	0.218689
923	0.739729	0.503915
924	1.45533	0.11757
925	1.45532	0.0612048

926	0.946118	0.238193
927	1.15163	0.05
928	1.15165	0.340807
929	0.739748	0.111502
930	1.04025	0.953561
931	1.47608	0.05
932	0.946131	0.0853173
933	1.02028	0.05
934	0.946129	0.0554826
935	1.78223	0.05
936	1.78223	0.05
937	1.34153	0.05
938	1.38683	0.05
939	1.3407	0.05
940	1.3407	0.05
941	2.04729	0.05
942	0.851529	0.0560624
943	1.26671	0.05
944	0.946116	0.0516701
945	1.4869	0.05
946	1.29553	0.05
947	1.03645	0.05
948	1.20731	0.05
949	0.73665	2.1
950	0.693596	2.1
951	2.11152	2.1
952	1.73627	2.08942
953	1.06191	2.1
954	1.52226	2.1
955	1.76869	2.1
956	1.1881	2.1
957	1.32349	2.1
958	1.51041	2.1
959	1.61591	2.1
960	0.946132	2.1
961	0.946132	2.1
962	1.13537	2.1
963	3.0048	2.1
964	0.985109	2.1
965	1.68899	2.1
966	3.23261	2.1
967	1.08831	2.1
968	2.20623	2.1
969	0.91749	2.1
970	1.15515	2.1
971	0.550438	2.1
972	1.66294	2.1
973	1.08257	2.1
974	0.87851	2.1
975	2.09046	2.1
976	0.738711	2.1
977	1.31242	0.05
978	1.26311	0.05
979	1.67652	0.05
980	0.968594	2.1
981	0.968594	2.1
982	0.981099	0.05
983	1.32418	0.05

984	2.67175	0.05
985	2.09678	0.05
986	1.2511	0.05
987	0.912698	0.05
988	0.810946	0.05
989	0.810959	0.05
990	1.67479	0.05
991	1.65677	0.05
992	1.28469	0.05
993	1.48817	0.05
994	1.08131	0.05
995	1.04349	0.150844
996	1.42492	0.191136
997	1.05724	0.139355
998	1.65723	0.05
999	1.95313	0.05
1000	1.9293	0.05
1001	2.02729	0.05
1002	0.963393	0.426763
1003	0.963392	0.125669
1004	2.90171	2.1
1005	2.65447	2.1
1006	1.64158	2.1
1007	1.6488	2.1
1008	1.15991	0.0702275
1009	0.946132	0.117653
1010	1.58199	0.0912086
1011	1.58199	0.103383
1012	1.03333	0.100695
1013	1.25436	0.0997118
1014	1.25436	0.125857
1015	0.946132	0.16239
1016	1.3771	0.05
1017	0.687745	0.05
1018	1.37709	0.05
1019	0.812386	0.05
1020	1.50172	0.05
1021	1.50172	0.05
1022	0.687758	0.05
1023	1.17506	0.05
1024	2.00539	0.05
1025	2.2441	0.05
1026	1.09667	0.05
1027	1.43577	2.1
1028	2.24741	2.1
1029	1.26103	2.1
1030	0.851544	2.1
1031	1.5543	2.1
1032	1.59756	2.1
1033	0.851543	2.1
1034	1.44673	2.1
1035	1.31947	2.1
1036	1.1237	2.1
1037	1.26103	2.1
1038	1.089 0.05	
1039	0.946128	0.05
1040	0.965824	0.05
1041	0.94613	0.05

1042	1.51344	0.05
1043	1.51344	0.05
1044	1.13508	0.05
1045	1.32449	0.05
1046	1.089	0.05
1047	3.33706	0.05
1048	2.7542	0.05
1049	1.27256	0.05
1050	0.954172	0.05
1051	1.55527	0.05
1052	1.05765	0.05
1053	1.57326	0.05
1054	1.38251	0.05
1055	0.94613	0.05
1056	2.38829	0.05
1057	2.00539	0.05
1058	1.53798	0.05
1059	1.20969	2.1
1060	1.15864	2.1
1061	1.7986	2.1
1062	1.74533	2.1
1063	0.944827	1.92345
1064	0.946131	1.25907
1065	0.851543	1.27648
1066	1.52816	0.70194
1067	1.5314	1.26958
1068	1.39266	0.201573
1069	0.851528	0.377379
1070	1.39589	0.251433
1071	1.7348	0.60202
1072	1.85439	1.33761
1073	1.86367	1.19598
1074	1.90905	0.57645
1075	1.22176	0.0512526
1076	1.59492	0.05
1077	2.97929	2.1
1078	2.1905	2.1
1079	1.24128	2.1
1080	0.994293	2.1
1081	2.49767	0.05
1082	2.37105	0.05
1083	1.10834	0.879458
1084	1.53214	1.09616
1085	1.417	2.1
1086	2.13599	0.291641
1087	1.61727	2.1
1088	1.75004	2.1
1089	1.96301	2.1
1090	0.966631	2.1
1091	1.37791	2.1
1092	1.47302	2.1
1093	1.47302	2.1
1094	0.871519	2.1
1095	0.94613	2.1
1096	0.87155	2.1
1097	2.38731	2.1
1098	2.12399	0.774656
1099	1.74865	0.615577

1100	1.46724	0.11424
1101	2.38056	0.0931116
1102	1.46724	0.125103
1103	0.818887	0.103594
1104	0.810944	1.28587
1105	0.805227	0.0963706
1106	1.06558	0.298746
1107	0.841487	0.124983
1108	1.08456	0.319931
1109	0.903083	0.284944
1110	1.08929	0.05
1111	1.68454	0.05
1112	1.08929	0.05
1113	1.07658	0.05
1114	0.904439	0.05
1115	1.58501	0.05
1116	1.19729	0.05
1117	1.05427	0.05
1118	1.47764	0.05
1119	0.810959	0.05
1120	1.60946	0.480506
1121	2.28887	0.508644
1122	0.946133	0.890643
1123	1.14444	0.370119
1124	1.67964	0.18287
1125	1.67964	0.243864
1126	0.755334	0.978806
1127	1.14444	0.624082
1128	1.29054	0.490267
1129	1.37762	0.342106
1130	0.946132	0.706505
1131	0.533645	0.280446
1132	1.48361	0.129474
1133	1.48361	0.356271
1134	0.533645	0.722246
1135	0.872823	0.709476
1136	1.14444	0.250887
1137	1.37762	0.323551
1138	2.10101	0.354458
1139	1.02311	0.544749
1140	1.04083	2.1
1141	1.04084	2.1
1142	1.04084	2.1
1143	1.30116	2.1
1144	1.23477	0.05
1145	1.81893	0.05
1146	1.23477	0.05
1147	1.23477	0.05
1148	1.11967	0.05
1149	0.946131	0.05
1150	1.11966	0.05
1151	1.12628	0.05
1152	0.629641	0.05
1153	1.6163	0.05
1154	1.6163	0.05
1155	1.11117	2.1
1156	1.13006	2.1
1157	1.15149	2.1

1158	1.13179	2.1
1159	0.585368	0.05
1160	0.986036	1.29643
1161	1.94788	0.0633529
1162	1.34857	0.0527239
1163	2.31806	2.1
1164	2.06363	2.1
1165	1.00423	2.1
1166	0.829065	2.1
1167	0.551816	2.1
1168	0.99406	2.1
1169	1.98339	2.1
1170	1.68073	2.1
1171	2.558	2.1
1172	0.711814	1.46411
1173	2.97316	2.1
1174	2.41579	2.1
1175	1.15248	0.05
1176	1.14305	0.05
1177	1.15483	0.05
1178	1.90981	0.05
1179	0.998957	0.05
1180	1.83742	0.05
1181	1.27581	2.1
1182	0.94613	2.1
1183	1.6315	2.1
1184	1.6315	2.1
1185	0.946132	2.1
1186	1.28441	2.1
1187	1.28441	2.1
1188	1.00365	2.1
1189	0.968536	2.1
1190	1.57575	2.1
1191	1.27581	2.1
1192	1.99297	2.1
1193	1.32784	2.1
1194	1.4259	2.1
1195	2.27952	2.1
1196	1.5685	2.1
1197	1.54231	2.1
1198	3.54638	2.1
1199	1.3294	2.1
1200	2.98237	2.1
1201	2.01633	0.31492
1202	2.0985	0.532176
1203	2.26047	0.140202
1204	1.7913	0.0875059
1205	1.09504	2.1
1206	1.74126	2.1
1207	1.05983	2.1
1208	2.37664	2.1
1209	2.28084	2.1
1210	2.19948	2.1
1211	1.33593	0.05
1212	1.31508	0.05
1213	1.30647	2.1
1214	1.28846	2.1
1215	1.2955	2.1

1216	1.09044	2.1
1217	1.4099	2.1
1218	1.14984	2.1
1219	0.946132	0.754875
1220	1.70727	2.1
1221	1.70727	2.1
1222	1.14984	2.1
1223	0.946132	2.1
1224	0.911818	2.1
1225	2.12209	2.1
1226	2.4158	2.1
1227	1.20867	2.1
1228	2.84085	2.1
1229	1.42284	2.1
1230	2.45689	2.1
1231	1.41562	2.1
1232	0.918358	2.1
1233	1.11222	2.1
1234	0.999083	2.1
1235	0.696781	2.1
1236	1.30869	2.1
1237	1.66132	2.1
1238	0.696807	2.1
1239	0.745315	2.1
1240	1.23296	2.1
1241	0.780548	2.1
1242	1.93242	0.565774
1243	1.27362	1.15784
1244	2.77828	1.53878
1245	1.02653	0.12086
1246	0.808576	0.147494
1247	0.744012	0.702456
1248	0.605174	0.0509925
1249	1.16501	0.05
1250	0.803432	0.05
1251	0.961016	0.05
1252	1.10425	0.0802846
1253	1.3756	0.273218
1254	2.17765	0.494834
1255	1.84578	0.301052
1256	1.32801	0.134128
1257	1.76377	0.123506
1258	0.946131	0.0711724
1259	1.3022	0.285864
1260	0.694814	0.354242
1261	1.53028	0.0786413
1262	1.53028	0.0953947
1263	1.3022	0.141515
1264	0.922889	0.211442
1265	1.57766	2.1
1266	2.06911	2.01063
1267	1.57766	2.1
1268	1.37721	0.05
1269	1.72174	0.05
1270	1.4468	0.05
1271	1.37721	0.05
1272	1.73972	0.05
1273	1.62324	0.05

1274	0.946125	0.05
1275	1.34259	0.05
1276	1.04187	0.05
1277	1.05019	0.05
1278	1.2361	0.05
1279	1.69055	0.05
1280	2.25056	0.0501808
1281	1.10574	0.0513541
1282	1.21632	0.793622
1283	1.10574	0.803624
1284	1.46181	2.07978
1285	0.946131	0.101296
1286	1.74111	1.64505
1287	1.74111	0.413589
1288	0.94613	0.306949
1289	1.26891	0.187013
1290	1.26891	0.0884963
1291	1.23452	1.14389
1292	1.00255	0.414528
1293	1.72286	0.05
1294	1.12233	0.05
1295	1.30682	0.05
1296	1.97397	0.05
1297	1.67425	2.1
1298	1.77775	2.1
1299	1.73571	2.1
1300	0.426265	2.1
1301	1.31817	1.91037
1302	1.81042	2.1
1303	1.85456	2.1
1304	0.466532	1.90084
1305	1.28025	1.78642
1306	0.964467	1.71079
1307	1.27921	2.1
1308	2.11851	2.00836
1309	2.37121	2.1
1310	1.49357	0.112821
1311	1.42486	2.1
1312	1.00278	2.1
1313	1.00278	2.1
1314	1.12503	2.1
1315	0.568697	2.1
1316	0.555211	2.1
1317	0.982272	2.06449
1318	1.57558	1.92841
1319	0.851539	2.1
1320	1.07307	2.1
1321	1.58292	2.1
1322	1.50731	2.1
1323	0.851541	1.26466
1324	1.00304	2.1
1325	1.36003	2.1
1326	1.13127	2.1
1327	0.672234	2.1
1328	0.763044	2.1
1329	0.966979	2.1
1330	1.31502	2.1
1331	1.67969	2.1

1332	1.62408	2.1
1333	0.661955	2.1
1334	1.35048	2.1
1335	1.40447	2.1
1336	1.40447	2.1
1337	0.607961	2.1
1338	0.946131	2.1
1339	0.607963	2.1
1340	1.01531	2.1
1341	1.01531	2.1
1342	1.91802	2.1
1343	0.777749	2.1
1344	1.22585	0.05
1345	1.20672	0.05
1346	1.03166	0.05
1347	0.553805	2.1
1348	2.00551	2.1
1349	2.07203	2.1
1350	0.589479	2.1
1351	1.22352	2.1
1352	0.984227	2.1
1353	0.802455	2.1
1354	1.40942	2.1
1355	1.6662	2.1
1356	0.802485	2.1
1357	0.936669	2.1
1358	1.31127	0.176317
1359	0.946132	0.167204
1360	1.09729	0.0607527
1361	0.946133	0.206063
1362	1.68703	0.0680616
1363	1.68703	0.0523146
1364	1.3689	0.0920369
1365	1.26426	0.0936511
1366	1.26923	2.1
1367	1.23614	2.1
1368	0.828371	2.1
1369	2.04556	2.1
1370	0.772648	2.1
1371	0.791059	2.1
1372	0.809958	2.1
1373	1.70012	2.1
1374	0.267032	2.1
1375	1.67604	2.1
1376	0.803823	2.1
1377	0.267305	2.1
1378	0.80246	2.1
1379	0.874582	2.1
1380	1.00188	2.1
1381	0.709588	2.1
1382	1.50679	2.1
1383	1.22456	2.1
1384	0.870622	2.1
1385	1.05754	2.1
1386	1.1689	2.1
1387	1.54556	2.1
1388	1.89226	2.1
1389	0.995311	2.1

1390	1.26195	2.1
1391	1.9	2.1
1392	0.845055	2.1
1393	0.737836	2.1
1394	0.823707	2.1
1395	0.626587	2.1
1396	1.16109	2.1
1397	0.85517	2.1
1398	0.932036	2.1
1399	1.08321	2.1
1400	1.43863	2.1
1401	2.72297	2.1
1402	2.90798	2.1
1403	1.47818	1.49866
1404	1.47819	1.32936
1405	0.694357	1.07758
1406	0.694382	1.13955
1407	0.946132	0.553661
1408	0.694382	0.799138
1409	1.22641	0.664538
1410	1.31155	1.57275
1411	2.45282	1.30054
1412	0.787843	1.03047
1413	0.808984	0.536285
1414	1.14232	1.71759
1415	0.795037	1.38511
1416	0.887382	1.57915
1417	1.14493	1.45594
1418	0.78393	0.829123
1419	1.09129	1.24477
1420	0.791482	1.17163
1421	1.1137	1.58239
1422	1.11819	1.13964
1423	0.960173	1.18851
1424	1.09447	1.69984
1425	0.874365	0.935554
1426	1.2474	0.05
1427	1.75038	0.05
1428	1.75038	0.0817151
1429	0.94613	0.326816
1430	1.30076	0.05
1431	1.12817	0.781002
1432	0.94613	0.178664
1433	1.30076	0.109838
1434	1.51841	0.0640807
1435	1.51841	0.106553
1436	0.946132	0.849523
1437	1.39575	0.074559
1438	0.970358	1.41249
1439	0.946133	0.42068
1440	1.39575	0.24501
1441	1.06486	0.05
1442	1.06486	0.05
1443	1.38622	0.05
1444	1.04521	0.05
1445	1.00809	0.05
1446	1.28952	0.05
1447	0.849316	0.05

1448	0.946132	0.05
1449	0.849344	0.05
1450	1.53631	0.05
1451	1.53631	0.05
1452	1.77843	0.05
1453	1.69863	0.05
1454	1.91646	2.1
1455	1.8582	2.1
1456	2.8349	0.05
1457	1.36307	0.05
1458	1.23886	0.05
1459	1.12971	0.05
1460	1.59662	0.05
1461	1.59662	0.05
1462	0.640304	0.05
1463	0.640333	0.05
1464	0.886276	0.05
1465	0.886276	0.05
1466	1.35065	0.05
1467	2.09002	0.05
1468	1.35543	0.05
1469	1.94688	2.1
1470	1.97221	2.1
1471	0.719563	2.1
1472	1.20358	2.1
1473	0.874264	2.1
1474	1.27676	2.1
1475	1.46557	2.1
1476	1.46557	2.1
1477	0.68546	2.1
1478	0.94613	2.1
1479	0.685489	2.1
1480	1.39615	0.05
1481	1.05404	0.05
1482	1.06474	0.05
1483	1.00713	0.05
1484	0.714535	0.05
1485	0.714563	0.05
1486	1.41646	0.05
1487	1.52582	0.05
1488	0.721694	0.05
1489	0.93665	0.05
1490	1.21067	0.05
1491	1.21067	0.05
1492	1.73577	0.0666154
1493	1.17799	0.05
1494	3.21432	0.05
1495	2.41452	0.05
1496	1.36208	0.05
1497	2.53256	0.05
1498	0.834593	0.05
1499	2.5853	0.05
1500	1.77259	0.05
1501	0.615724	0.05
1502	1.11374	0.0675441
1503	0.615726	0.05
1504	0.946129	0.228635
1505	0.615714	0.147774

1506	1.75489	0.0723988
1507	1.75489	0.0514958
1508	1.23146	0.05
1509	2.2772	0.05
1510	0.74463	0.05
1511	1.3546	0.05
1512	1.80252	0.05
1513	2.38304	0.05
1514	1.19151	0.05
1515	1.1231	0.05
1516	1.82345	0.05
1517	1.79805	0.05
1518	0.761337	0.111058
1519	0.946127	0.05
1520	0.557726	0.0568606
1521	1.05353	0.05
1522	1.05353	0.05
1523	1.68599	0.05
1524	1.05353	0.05
1525	1.40588	2.1
1526	1.09373	2.1
1527	1.18382	1.00502
1528	1.46903	0.779977
1529	1.25103	2.1
1530	1.10378	2.1
1531	1.58941	2.1
1532	1.29639	2.1
1533	1.14874	0.05
1534	0.899817	0.05
1535	0.666898	0.05
1536	1.76519	0.05
1537	1.17289	0.05
1538	1.03526	0.05
1539	1.97708	0.05
1540	1.31711	0.05
1541	0.992517	0.05
1542	1.70193	0.05
1543	1.13852	0.05
1544	1.23943	0.05
1545	1.40014	1.70309
1546	1.04598	2.1
1547	1.1483	1.78502
1548	1.11433	0.653026
1549	1.32037	0.0567409
1550	1.00381	0.1059
1551	1.11435	0.05
1552	0.973502	0.195308
1553	0.946128	1.78843
1554	1.83697	0.876824
1555	1.83697	2.1
1556	1.31721	1.72657
1557	1.38614	1.23122
1558	1.39696	0.901614
1559	1.31721	1.58469
1560	1.0756	1.92528
1561	1.08542	0.303241
1562	0.946127	2.1
1563	1.81052	0.151

1564	1.81052	2.1
1565	1.24315	2.1
1566	1.39696	0.114432
1567	1.35969	1.37849
1568	1.24315	1.12606
1569	1.0756	0.05
1570	1.20669	0.05
1571	1.18508	0.05
1572	1.71059	2.1
1573	1.66762	2.1
1574	2.50853	2.1
1575	1.8236	2.1
1576	1.78872	2.1
1577	1.41721	2.1
1578	1.01335	1.76184
1579	1.42439	0.727658
1580	0.946129	0.823641
1581	1.33266	0.947321
1582	1.79776	1.47959
1583	1.79776	2.1
1584	0.946131	0.125347
1585	1.33266	1.5692
1586	1.41123	1.26367
1587	1.25346	2.1
1588	0.946132	0.206885
1589	0.827287	2.0941
1590	1.57878	1.11392
1591	1.57878	1.28055
1592	0.827287	0.883564
1593	1.07341	1.10989
1594	1.33266	0.895616
1595	1.25346	1.09751
1596	1.61149	2.1
1597	1.15644	2.09597
1598	1.27558	0.504985
1599	1.3764	0.12804
1600	1.89526	1.12408
1601	1.37641	0.115859
1602	1.2064	0.066557
1603	1.38426	2.1
1604	1.01595	2.1
1605	1.15067	2.1
1606	1.82276	2.1
1607	1.82276	0.05
1608	0.946131	0.0678994
1609	1.40609	0.05
1610	0.946132	2.1
1611	1.29143	0.890257
1612	1.40609	0.207974
1613	1.30272	2.1
1614	1.19548	2.1
1615	2.95451	2.1
1616	1.46124	2.1
1617	2.93215	2.1
1618	1.42105	2.1
1619	1.75477	0.589368
1620	2.31301	0.798112
1621	2.10275	0.184674

1622	1.80099	0.259324
1623	0.97541	2.1
1624	1.49733	2.1
1625	0.97541	2.1
1626	1.30751	2.1
1627	1.28614	0.05
1628	1.74759	0.05
1629	1.19756	0.0646074
1630	1.71053	0.0514457
1631	1.19757	0.0909517
1632	1.28954	0.05
1633	2.02703	0.05
1634	2.29453	0.05
1635	1.28265	0.05
1636	0.793267	0.05
1637	1.19974	0.05
1638	0.509551	0.05
1639	1.04072	0.05
1640	1.35302	0.05
1641	1.53133	0.05
1642	0.566153	0.05
1643	1.09634	0.05
1644	1.12792	0.05
1645	1.68908	2.1
1646	1.68908	2.1
1647	0.768212	2.1
1648	0.768213	2.1
1649	0.946132	2.1
1650	0.768212	2.1
1651	1.32461	2.1
1652	1.87291	2.1
1653	2.64921	2.1
1654	1.53642	2.1
1655	2.26537	2.1
1656	1.53642	2.1
1657	1.79874	2.1
1658	1.79874	2.1
1659	0.726635	1.96626
1660	0.726636	2.1
1661	0.946128	2.1
1662	0.726634	2.01507
1663	1.23339	1.64046
1664	0.992674	2.1
1665	1.8023	2.1
1666	1.12324	0.05
1667	1.29163	0.05
1668	0.996831	0.05
1669	1.0019	0.05
1670	0.993831	0.05
1671	1.07283	2.1
1672	1.04788	2.1
1673	1.44489	0.992598
1674	1.04788	2.1
1675	1.07652	2.1
1676	0.991406	2.1
1677	1.37	2.1
1678	1.14195	2.1
1679	1.47904	0.05

1680	1.26593	2.00908
1681	1.75366	1.60639
1682	1.57621	0.05
1683	1.24538	0.05
1684	0.969118	2.1
1685	0.946133	0.737349
1686	1.69387	2.1
1687	1.69387	2.1
1688	0.94613	2.1
1689	1.28713	2.1
1690	1.28713	2.1
1691	1.23877	2.1
1692	0.969118	2.1
1693	1.22615	2.1
1694	0.98084	2.1
1695	1.01843	2.1
1696	1.50876	2.1
1697	1.85391	2.1
1698	1.84322	2.1
1699	1.70377	2.1
1700	1.78919	2.1
1701	2.66787	2.1
1702	1.41549	2.1
1703	1.41549	2.1
1704	0.86707	2.1
1705	0.896172	2.1
1706	0.946126	2.1
1707	0.867074	2.1
1708	0.946118	2.1
1709	1.54468	0.05
1710	1.54468	0.05
1711	2.27259	0.05
1712	0.946131	0.05
1713	1.25837	0.05
1714	0.663171	0.05
1715	1.68931	0.05
1716	1.68931	0.05
1717	1.25837	0.05
1718	1.09411	0.05
1719	1.73571	0.05
1720	2.34894	0.05
1721	0.946128	0.05
1722	1.30497	0.05
1723	0.772312	0.05
1724	1.66895	0.05
1725	1.66895	0.05
1726	1.30497	0.05
1727	1.13629	0.05
1728	1.22852	0.634252
1729	1.578	0.451152
1730	2.07781	2.1
1731	1.5478	2.1
1732	1.70048	2.1
1733	1.42329	2.1
1734	1.05481	2.06911
1735	1.1032	0.749576
1736	1.19267	2.1
1737	1.77324	2.1

1738	1.01121	0.05
1739	0.991867	0.05
1740	2.0744	1.11241
1741	1.75142	2.1
1742	1.35418	1.17877
1743	1.40041	2.1
1744	1.50045	2.1
1745	1.37704	2.1
1746	1.50045	2.1
1747	1.87875	2.1
1748	1.35308	0.267104
1749	1.29983	0.330716
1750	1.62784	2.1
1751	1.44807	2.1
1752	1.00965	2.1
1753	2.69264	2.1
1754	1.04075	2.1
1755	0.946131	2.1
1756	0.946132	2.1
1757	1.52704	2.1
1758	1.83246	2.1
1759	1.56757	2.1
1760	1.16687	2.1
1761	2.04857	2.1
1762	1.32707	1.93271
1763	1.25519	0.05
1764	1.32101	0.05
1765	1.25055	0.31245
1766	0.827853	0.05
1767	1.11835	0.05
1768	1.07281	1.85177
1769	1.54302	0.0508561
1770	0.724064	0.226038
1771	1.32709	2.1
1772	3.44123	0.827275
1773	2.46889	0.05
1774	0.730076	0.05
1775	1.25809	0.05
1776	0.730077	0.05
1777	0.946129	0.05
1778	1.72777	0.05
1779	1.72777	0.05
1780	0.73005	0.05
1781	1.7522	0.05
1782	2.51618	0.05
1783	0.628177	0.05
1784	1.1068	0.05
1785	0.747435	0.05
1786	0.946133	0.05
1787	1.84668	0.05
1788	1.8563	0.05
1789	0.628177	0.05
1790	1.45501	0.05
1791	2.19809	0.05
1792	1.45501	0.05
1793	1.48974	0.05
1794	1.7709	2.1
1795	2.63288	2.1

1796	1.5362	2.1
1797	1.56327	2.1
1798	3.03945	0.05
1799	0.899979	0.05
1800	0.869386	0.05
1801	1.85864	0.05
1802	1.78028	0.05
1803	1.28719	0.05
1804	1.09532	0.05
1805	0.881898	0.05
1806	1.28911	0.05
1807	1.39644	0.05
1808	0.688829	0.05
1809	2.14611	0.05
1810	1.80313	0.05
1811	0.68883	0.05
1812	0.946131	0.05
1813	1.25334	0.05
1814	1.88987	0.05
1815	1.94432	0.05
1816	1.37592	0.05
1817	1.11691	1.86128
1818	1.02923	2.02374
1819	0.45824	0.05
1820	0.646512	0.05
1821	2.46292	0.05
1822	0.493451	0.05
1823	2.00826	2.1
1824	2.49784	2.1
1825	1.17054	2.1
1826	1.01485	2.1
1827	1.26045	2.1
1828	1.17054	2.1
1829	1.44247	2.1
1830	2.02687	0.05
1831	1.66365	0.05
1832	1.64656	0.05
1833	1.56597	0.05
1834	1.21419	2.1
1835	1.5008	2.1
1836	1.10711	2.1
1837	1.44287	2.1
1838	1.44287	2.1
1839	0.750368	2.1
1840	1.25876	2.1
1841	0.82542	2.1
1842	1.04074	2.1
1843	1.25874	2.1
1844	1.04159	0.05
1845	1.2489	0.05
1846	0.980547	0.05
1847	1.62235	0.05
1848	1.15991	0.05
1849	0.980547	0.05
1850	0.94613	0.05
1851	1.54662	0.05
1852	1.54661	0.05
1853	1.07912	0.05

1854	1.1032	0.05
1855	1.1032	0.05
1856	0.94613	0.05
1857	1.12468	2.1
1858	1.2724	2.1
1859	1.06521	2.09659
1860	1.0752	2.1
1861	1.94936	0.05
1862	1.62772	0.05
1863	1.94733	0.05
1864	1.42901	0.05
1865	1.33022	2.1
1866	1.70839	2.1
1867	1.75048	2.1
1868	1.41201	2.1
1869	1.52314	2.1
1870	1.44039	2.1
1871	1.51113	2.1
1872	2.58638	2.1
1873	0.987247	0.05
1874	1.38622	0.05
1875	0.858359	0.05
1876	1.19967	0.05
1877	1.06414	0.05
1878	0.946132	0.05
1879	0.94613	0.05
1880	0.687079	0.0876095
1881	0.946132	0.05
1882	0.822611	0.05
1883	1.70242	0.05
1884	1.70242	0.05
1885	0.946132	0.149905
1886	1.11155	0.05
1887	0.946132	0.231851
1888	1.11155	0.0769288
1889	1.37981	0.0642304
1890	1.18348	0.05
1891	2.44179	0.05
1892	2.00403	0.0927097
1893	1.10961	2.1
1894	1.85415	2.1
1895	1.44825	2.1
1896	0.932272	2.1
1897	0.810942	2.1
1898	1.00189	2.1
1899	1.11779	2.1
1900	1.3359	2.1
1901	0.965279	2.1
1902	0.833779	2.1
1903	1.05337	2.1
1904	0.526656	2.1
1905	1.44599	2.1
1906	1.44599	2.1
1907	1.24853	2.1
1908	0.946132	2.1
1909	1.24853	2.1
1910	0.724122	2.1
1911	1.91547	2.1

1912	2.49705	2.1
1913	1.05931	2.1
1914	1.1729	2.1
1915	1.34526	2.1
1916	1.5379	2.1
1917	1.90855	2.1
1918	2.08597	2.1
1919	1.68165	2.1
1920	1.45887	2.1
1921	1.50022	2.1
1922	1.4748	2.1
1923	2.63645	1.90551
1924	1.74431	2.0363
1925	1.25288	1.97434
1926	1.25289	1.49119
1927	0.970025	1.30597
1928	0.747542	2.1
1929	0.810959	1.19108
1930	1.15369	1.36787
1931	0.977472	1.33865
1932	1.12695	1.41783
1933	0.996762	1.28305
1934	1.28249	0.148108
1935	0.946132	0.234585
1936	0.528621	0.331766
1937	1.59186	0.10372
1938	1.44072	0.147696
1939	0.633488	0.115181
1940	0.598506	0.208026
1941	1.51618	0.241898
1942	1.19999	0.131922
1943	1.46511	0.0956181
1944	1.44056	0.109484
1945	0.912754	0.0608889
1946	1.4725	0.05
1947	1.4725	0.05
1948	0.912753	0.05
1949	1.23351	0.0747597
1950	1.23351	0.0918258
1951	0.946131	0.13606
1952	1.17129	0.05
1953	1.82551	0.05
1954	1.27575	1.85873
1955	0.946129	2.1
1956	0.946129	2.1
1957	0.946128	1.92241
1958	0.792754	2.1
1959	0.94613	1.63205
1960	0.792752	1.60477
1961	2.08054	2.1
1962	1.20091	2.1
1963	2.1669	2.06585
1964	1.24383	1.57887
1965	0.904669	2.1
1966	0.83804	1.09576
1967	1.90486	1.3948
1968	1.29162	1.18714
1969	1.37383	1.79843

1970	1.86533	1.23968
1971	1.76472	1.83939
1972	1.60428	1.28521
1973	2.43993	2.07056
1974	1.37833	2.1
1975	1.18632	2.1
1976	0.946132	2.1
1977	1.27544	2.1
1978	1.05085	2.1
1979	0.961178	2.1
1980	0.859844	2.1
1981	1.0499	2.1
1982	1.75592	2.1
1983	2.08668	2.1
1984	1.75592	2.1
1985	1.4402	2.1
1986	1.72285	2.1
1987	0.83312	0.05
1988	1.07295	0.05
1989	1.37607	0.05
1990	1.32097	0.05
1991	1.11332	0.05
1992	1.18568	0.05
1993	0.877482	0.05
1994	1.4458	0.05
1995	2.34112	0.05
1996	2.68021	0.05
1997	0.681677	0.05
1998	0.946131	0.05
1999	0.681649	0.05
2000	1.83115	0.05
2001	1.68029	0.05
2002	1.05482	0.05
2003	1.31485	0.05
2004	2.08247	0.517296
2005	1.33153	0.300551
2006	0.935062	0.517329
2007	0.806958	1.75571
2008	0.827683	0.402736
2009	1.01041	0.629419
2010	0.741832	0.266851
2011	1.15668	0.89789
2012	0.908649	0.464622
2013	0.59561	0.05
2014	1.19028	0.141764
2015	0.59561	0.0781943
2016	0.946132	0.243946
2017	0.595623	0.327381
2018	1.58164	0.160138
2019	1.58164	0.217916
2020	0.946131	2.1
2021	1.55201	2.1
2022	1.55201	2.1
2023	0.946127	2.1
2024	1.13312	2.1
2025	1.13312	2.1
2026	1.08108	2.1
2027	1.04973	2.1

2028	1.20149	2.1
2029	1.86902	0.417186
2030	0.613447	2.1
2031	1.22618	0.946232
2032	1.38663	1.55521
2033	1.38662	2.1
2034	0.613447	0.973267
2035	1.22617	2.1
2036	0.773899	2.09447
2037	1.23515	1.58998
2038	1.87823	1.46461
2039	1.70303	0.160464
2040	1.4714	2.1
2041	1.74067	2.1
2042	0.735699	2.1
2043	1.73461	2.1
2044	1.47224	2.1
2045	1.22827	2.1
2046	1.1634	2.1
2047	1.36471	2.1
2048	0.735701	2.1
2049	2.8386	0.094286
2050	2.39056	2.1
2051	2.44128	0.435444
2052	1.54102	2.1
2053	1.54102	2.1
2054	0.936141	1.59945
2055	1.40594	2.1
2056	1.07121	0.197178
2057	0.936142	2.1
2058	1.07121	1.93058
2059	1.22691	0.05
2060	1.42907	0.05
2061	2.18048	0.05
2062	0.947662	0.05
2063	2.18394	2.1
2064	1.21292	2.1
2065	2.18394	2.1
2066	2.19064	2.1
2067	1.27229	2.1
2068	1.20859	0.397314
2069	1.21073	0.59216
2070	1.73899	2.1
2071	1.3164	2.1
2072	1.61245	2.1
2073	1.41444	2.1
2074	2.55116	2.1
2075	1.26193	2.1
2076	1.89553	2.1
2077	0.916438	2.1
2078	1.26113	2.1
2079	0.810934	2.1
2080	2.00419	2.1
2081	1.27872	2.1
2082	1.35306	2.1
2083	1.96749	2.1
2084	1.07955	2.1
2085	1.31854	2.1

2086	1.29505	0.263365
2087	1.29504	0.234732
2088	0.612469	0.551387
2089	0.990053	0.454066
2090	1.04072	0.541978
2091	0.985266	0.268871
2092	0.990039	0.177973
2093	1.06892	0.322642
2094	1.57158	0.126103
2095	1.895	0.221993
2096	1.05698	0.173201
2097	0.925053	2.1
2098	0.624712	2.1
2099	1.42465	2.1
2100	1.49014	2.1
2101	0.687198	2.1
2102	1.22058	2.1
2103	0.851488	2.1
2104	0.588128	2.1
2105	1.12452	2.1
2106	1.02783	2.1
2107	1.7083	2.1
2108	1.06977	1.69912
2109	0.818569	0.909193
2110	1.14386	1.72932
2111	0.983791	0.942857
2112	1.12214	1.91424
2113	1.0915	1.38974
2114	0.810934	1.57003
2115	0.764114	1.72449
2116	1.87956	0.818374
2117	1.87956	1.69045
2118	0.764141	0.814387
2119	1.37675	0.939822
2120	0.764141	0.628156
2121	0.946132	1.73709
2122	1.9637	2.04351
2123	2.10107	0.348171
2124	1.67814	1.67719
2125	3.02886	1.39862
2126	0.908982	0.05
2127	0.621727	0.05
2128	0.525963	0.05
2129	0.946128	0.05
2130	1.60979	0.05
2131	1.45596	0.05
2132	0.556172	0.05
2133	2.06779	0.05
2134	2.17911	0.05
2135	1.00205	0.05
2136	1.30685	0.05
2137	1.16534	0.05
2138	1.65532	2.1
2139	2.20346	2.1
2140	0.993889	2.1
2141	1.09328	2.1
2142	2.09372	2.1
2143	2.10036	2.1

2144	1.33727	1.97316
2145	2.53383	1.8947
2146	0.893379	1.64715
2147	1.52828	1.38587
2148	1.09599	0.05
2149	1.09599	0.05
2150	1.50524	2.1
2151	1.54711	2.1
2152	1.41469	2.1
2153	1.99459	2.04817
2154	1.79805	2.1
2155	1.79805	2.1
2156	0.707344	2.1
2157	1.30318	2.1
2158	0.707344	2.1
2159	0.946129	2.1
2160	1.30318	2.1
2161	2.40442	2.1
2162	1.89226	2.1
2163	1.40794	2.1
2164	0.898721	2.1
2165	0.791921	1.14644
2166	0.810933	2.1
2167	1.10904	2.04786
2168	0.991102	2.1
2169	0.808502	0.98679
2170	1.1398	2.1
2171	1.31346	2.1
2172	1.11833	2.1
2173	1.669 2.1	
2174	1.25715	2.1
2175	1.3199	2.1
2176	1.63092	2.1
2177	1.63092	2.1
2178	0.946131	2.1
2179	0.946131	2.1
2180	1.13534	2.1
2181	0.979853	2.1
2182	2.19994	2.1
2183	0.725844	2.1
2184	2.19994	2.1
2185	2.35055	2.1
2186	1.52204	2.1
2187	1.40118	2.1
2188	3.42285	2.1
2189	2.05217	2.1
2190	1.50319	2.1
2191	0.947516	0.966896
2192	0.903572	0.785596
2193	0.76996	1.06737
2194	1.90959	2.1
2195	1.18897	2.1
2196	1.24166	1.97914
2197	2.26785	1.6771
2198	2.05217	2.1
2199	2.73306	2.1
2200	1.42729	0.05
2201	1.387 0.05	

2202	1.59458	0.0688512
2203	1.91051	0.077442
2204	1.68108	0.0866406
2205	2.03357	0.21818
2206	0.928982	0.05
2207	1.55687	0.05
2208	1.55687	0.05
2209	0.92898	0.05
2210	1.3495	0.05
2211	1.3495	0.05
2212	0.946133	0.05
2213	1.85795	0.05
2214	2.2727	0.05
2215	1.20324	1.71127
2216	1.07784	0.05
2217	0.957792	0.05
2218	1.2745	0.05
2219	1.4659	0.05
2220	0.946129	0.05
2221	0.95778	0.05
2222	0.851553	0.05
2223	1.74103	0.05
2224	2.28994	0.05
2225	2.08213	0.05
2226	2.1556	0.05
2227	2.20372	0.05
2228	0.838027	0.05
2229	1.11373	0.05
2230	1.2909	0.05
2231	0.998424	0.05
2232	1.40621	0.05
2233	1.19478	0.05
2234	1.11783	0.05
2235	1.6695	0.05
2236	1.7498	0.05
2237	0.477652	0.05
2238	1.77291	0.05
2239	0.590448	0.05
2240	1.07668	0.05
2241	0.810946	0.05
2242	1.1946	0.05
2243	2.03914	0.05
2244	1.25585	0.05
2245	2.02622	0.05
2246	1.39091	0.05
2247	1.31871	0.05
2248	1.15789	2.1
2249	1.15755	2.1
2250	1.82268	2.1
2251	1.87119	2.1
2252	3.10636	2.1
2253	2.96285	2.1
2254	1.73443	0.289082
2255	2.33812	0.351389
2256	1.59457	0.137261
2257	1.73443	1.09114
2258	2.00665	2.1
2259	1.99841	2.1

2260	1.19901	2.1
2261	1.16661	2.1
2262	2.15883	0.05
2263	1.20951	0.05
2264	2.49192	0.05
2265	0.65112	0.05
2266	2.45317	0.05
2267	1.73091	0.05
2268	2.0795	0.05
2269	1.45891	0.05
2270	1.6531	0.05
2271	1.78304	0.05
2272	0.775132	0.05
2273	1.35051	0.05
2274	1.0461	0.05
2275	1.37837	0.05
2276	1.00259	0.05
2277	1.25796	0.105175
2278	1.10719	0.233882
2279	1.51067	2.1
2280	2.65632	2.09886
2281	1.30521	2.1
2282	2.456	2.1
2283	3.19218	2.1
2284	2.67387	2.1
2285	0.988862	2.1
2286	1.917	2.1
2287	0.946132	2.1
2288	1.16202	2.1
2289	1.53871	2.1
2290	1.53871	2.1
2291	0.946132	2.1
2292	1.16202	2.1
2293	1.32282	2.1
2294	2.46356	2.1
2295	2.32404	2.1
2296	1.37562	0.05
2297	0.985546	0.05
2298	1.28983	0.05
2299	1.05566	0.05
2300	0.895312	0.05
2301	1.53325	0.05
2302	1.04342	0.05
2303	1.80881	0.05
2304	0.876865	0.05
2305	0.5689	0.05
2306	1.09506	0.05
2307	1.51789	2.1
2308	1.49872	2.1
2309	0.949364	0.05
2310	2.01233	0.05
2311	1.55063	0.05
2312	1.57783	0.106631
2313	1.57783	0.271881
2314	0.674605	0.209839
2315	0.98537	0.05
2316	0.674633	0.05
2317	0.98537	0.05

2318	1.26706	0.224363
2319	0.980664	0.232389
2320	1.03298	0.05
2321	1.84936	0.236614
2322	1.51696	0.05
2323	1.97074	0.05
2324	1.03298	0.0601126
2325	1.51324	0.05
2326	1.51324	0.05
2327	0.758454	0.05
2328	1.28632	0.05
2329	0.758482	0.05
2330	0.758482	0.05
2331	0.985372	0.05
2332	1.14105	0.05
2333	1.59787	0.05
2334	2.46244	0.05
2335	1.70367	0.05
2336	2.13757	1.41328
2337	1.98142	1.45144
2338	1.35878	0.05
2339	2.39947	0.05
2340	1.24554	0.05
2341	1.7522	0.05
2342	1.49693	1.69737
2343	1.49652	1.82027
2344	1.6211	1.70093
2345	1.45938	1.81543
2346	0.737431	2.1
2347	1.18643	1.86896
2348	1.04072	2.1
2349	0.9459	1.98968
2350	0.66364	2.1
2351	0.985109	1.2901
2352	0.638368	2.1
2353	0.83804	1.16188
2354	0.998024	1.26371
2355	0.914471	0.809894
2356	1.14281	1.47959
2357	0.833972	1.21409
2358	0.991865	0.0947044
2359	1.42208	0.0651524
2360	1.70718	2.1
2361	1.79888	2.1
2362	1.16851	0.165234
2363	1.16851	0.0838955
2364	1.51021	0.0736316
2365	0.946132	0.0675197
2366	1.14897	0.05
2367	0.946132	0.05
2368	1.77177	0.065101
2369	1.77177	0.05
2370	1.33038	0.05
2371	1.38752	0.05
2372	1.1726	0.0610358
2373	1.51688	0.052479
2374	1.06455	2.1
2375	0.98511	2.1

2376	1.31144	2.1
2377	1.79825	2.1
2378	1.79825	2.1
2379	1.73044	2.1
2380	1.59589	0.05
2381	1.59589	0.05
2382	2.78009	2.1
2383	1.68761	2.1
2384	3.05511	2.1
2385	2.25318	2.1
2386	0.578998	2.1
2387	1.03512	2.1
2388	2.26156	2.1
2389	1.44859	1.20299
2390	1.35897	1.62724
2391	1.18301	2.1
2392	1.25173	2.1
2393	1.98851	2.1
2394	2.70685	2.1
2395	0.682342	2.1
2396	1.52491	2.1
2397	1.37119	2.1
2398	0.682313	2.1
2399	1.35342	2.1
2400	0.947555	2.1
2401	0.614107	2.1
2402	1.23007	2.1
2403	1.5735	2.1
2404	1.58713	2.1
2405	1.57979	0.05
2406	1.30723	0.05
2407	0.935658	0.649413
2408	0.8952	0.735867
2409	1.27352	1.57133
2410	1.0985	0.923438
2411	1.07163	0.875144
2412	1.15823	1.21854
2413	0.836451	0.504209
2414	2.08051	0.24856
2415	1.92601	1.5039
2416	2.0644	0.419028
2417	1.37259	2.1
2418	1.37259	2.1
2419	0.752217	2.1
2420	1.28569	2.1
2421	0.752186	2.1
2422	0.839124	2.1
2423	1.28568	2.1
2424	1.04199	2.1
2425	1.04199	2.1
2426	2.59268	2.1
2427	1.04199	2.1
2428	1.5098	2.1
2429	1.5098	2.1
2430	0.520997	2.1
2431	1.29634	2.1
2432	0.520996	2.1
2433	0.73446	2.1

2434	1.29634	2.1
2435	0.983724	2.1
2436	1.23914	2.1
2437	2.65095	2.1
2438	1.52851	2.1
2439	2.399 2.1	
2440	1.68137	2.1
2441	1.11285	2.1
2442	1.04921	2.1
2443	1.30711	2.1
2444	1.04921	2.1
2445	1.07496	0.671584
2446	1.55831	0.0979568
2447	1.07497	0.05
2448	1.07497	0.22827
2449	1.27391	2.1
2450	2.5615	2.1
2451	1.2739	2.1
2452	1.75253	2.1
2453	1.12907	0.05
2454	1.9194	0.05
2455	1.96617	0.05
2456	1.01225	2.1
2457	0.947979	2.1
2458	1.01225	1.22474
2459	1.06561	2.1
2460	1.01225	2.1
2461	3.56876	2.1
2462	3.68767	2.1
2463	1.52301	2.1
2464	1.61013	2.1
2465	1.43726	2.1
2466	2.04275	2.1
2467	0.995734	2.1
2468	1.16199	2.1
2469	1.73704	0.05
2470	1.04019	0.05
2471	0.956294	2.1
2472	1.81488	2.1
2473	1.63719	2.1
2474	1.50103	0.05
2475	1.75289	0.05
2476	1.31491	0.05
2477	1.03567	0.05
2478	0.672257	0.05
2479	1.04072	0.05
2480	0.746903	0.05
2481	1.43317	0.05
2482	1.39986	0.05
2483	1.06385	0.05
2484	1.1624	0.05
2485	1.61667	0.05
2486	1.27258	0.05
2487	2.559 0.05	
2488	1.66093	0.05
2489	1.10719	0.176213
2490	2.03235	0.507149
2491	1.17105	0.852434

2492	1.10719	0.552802
2493	1.3028	2.1
2494	0.946131	2.1
2495	0.946127	2.1
2496	1.52912	2.1
2497	1.52912	2.1
2498	0.946128	1.98811
2499	0.949997	2.1
2500	1.31565	2.1
2501	1.46643	2.1
2502	1.5482	2.1
2503	1.47102	2.1
2504	1.47102	2.1
2505	0.774101	2.1
2506	1.30583	2.1
2507	0.774103	2.1
2508	0.939287	2.1
2509	1.30584	2.1
2510	0.958661	2.1
2511	2.28835	2.1
2512	1.43213	0.05
2513	2.13255	0.05
2514	1.43213	0.05
2515	1.10476	0.05
2516	1.93165	0.05
2517	1.03235	0.491626
2518	1.31	0.633964
2519	1.22082	2.1
2520	1.35827	2.1
2521	0.790936	2.1
2522	0.716644	2.1
2523	1.43257	2.1
2524	1.43257	2.1
2525	0.716615	2.1
2526	0.946131	2.1
2527	0.730157	2.1
2528	1.47212	2.1
2529	1.47212	2.1
2530	0.946131	2.1
2531	0.790792	2.1
2532	1.41149	2.1
2533	0.790791	2.1
2534	1.01006	2.1
2535	1.53045	0.0611417
2536	1.53045	0.132615
2537	0.946132	0.813321
2538	1.40779	0.0861674
2539	1.06879	1.27016
2540	0.990713	0.333643
2541	1.06879	0.39821
2542	1.06486	0.05
2543	1.18347	0.05
2544	1.38622	0.05
2545	1.18347	0.05
2546	1.7304	0.05
2547	1.7304	0.0516217
2548	0.946131	0.294712
2549	1.26874	0.0598324

2550	1.13337	0.969293
2551	1.13337	0.199667
2552	1.40779	0.164759
2553	1.695 2.1	
2554	1.77071	2.1
2555	0.649313	2.1
2556	0.692829	2.1
2557	1.40395	2.1
2558	1.34243	2.1
2559	0.813292	0.05
2560	0.632691	0.05
2561	1.75766	0.0718576
2562	2.25727	0.759769
2563	1.74206	0.05
2564	0.826073	0.05
2565	1.30022	0.05
2566	1.45641	0.05
2567	0.946131	0.05
2568	0.752245	0.05
2569	0.960952	0.05
2570	0.743477	0.05
2571	1.04355	0.05
2572	1.6253	0.05
2573	0.960577	0.05
2574	1.34852	0.80987
2575	1.03235	0.475406
2576	1.78168	0.121737
2577	1.2966	0.05
2578	2.21116	0.05
2579	1.6395	0.05
2580	1.6395	0.05
2581	0.94613	0.05
2582	1.22187	0.05
2583	0.962737	0.05
2584	0.962735	0.05
2585	1.36376	0.05
2586	1.2966	0.05
2587	1.2966	0.05
2588	1.19669	1.09547
2589	2.37636	0.978925
2590	1.08894	0.962297
2591	1.62551	1.45424
2592	2.51472	1.30564
2593	1.89226	1.05228
2594	0.980038	0.800696
2595	1.10187	1.55722
2596	1.01479	1.70094
2597	1.81663	2.1
2598	1.83247	2.1
2599	0.921851	0.0761516
2600	0.983148	2.1
2601	1.82816	2.1
2602	1.26103	2.1
2603	2.34818	2.1
2604	1.23152	2.1
2605	2.15738	2.1
2606	1.23152	2.1
2607	1.51042	2.1

2608	0.842995	2.1
2609	1.22257	2.1
2610	0.810959	2.1
2611	1.68242	2.1
2612	0.95701	2.1
2613	1.12389	2.1
2614	1.95255	2.1
2615	1.57974	2.1
2616	1.94632	2.1
2617	0.81534	2.1
2618	1.13996	2.1
2619	1.18958	0.05
2620	1.25929	0.05
2621	3.02933	0.507045
2622	2.37434	0.05
2623	1.10609	0.292725
2624	1.64132	0.136811
2625	1.64132	0.0763722
2626	0.946132	0.15331
2627	1.40029	0.0717705
2628	1.08946	0.0664161
2629	1.08946	0.18022
2630	1.18717	0.05
2631	2.71421	0.05
2632	1.08189	0.0764381
2633	0.94613	0.05
2634	1.37109	0.05
2635	1.17925	0.05
2636	0.851541	0.05
2637	0.942512	0.05
2638	1.18541	0.05
2639	0.851526	0.05
2640	1.0819	0.05
2641	1.86009	0.05
2642	0.946132	0.05
2643	1.67409	0.05
2644	1.67409	0.05
2645	0.946131	0.05
2646	1.31716	0.05
2647	1.30307	0.05
2648	0.946131	0.05
2649	1.18601	0.05
2650	3.15646	2.1
2651	0.985109	2.1
2652	3.37048	2.1
2653	1.10667	2.1
2654	1.77065	0.973881
2655	1.45837	0.639641
2656	1.77063	0.757286
2657	2.58104	0.627482
2658	1.37652	2.1
2659	1.54168	2.1
2660	1.00224	0.05
2661	1.05851	0.05
2662	1.39093	0.05
2663	1.43358	0.05
2664	0.575741	0.05
2665	0.518161	0.05

2666	1.04071	0.05
2667	1.13092	1.44915
2668	1.17042	2.1
2669	1.53347	1.81059
2670	1.52938	1.45555
2671	1.00613	0.05
2672	1.81618	0.102475
2673	1.81618	0.05
2674	0.946127	0.808088
2675	1.36479	0.118769
2676	0.94613	0.05
2677	1.31724	0.199945
2678	1.3648	0.0790442
2679	1.00613	0.05
2680	0.781375	0.05
2681	1.13431	0.05
2682	1.03547	0.05
2683	1.40668	0.0740281
2684	0.94613	0.0642407
2685	0.713285	0.075129
2686	1.72885	0.0808243
2687	1.72885	0.0742812
2688	0.713298	0.0535442
2689	3.30075	0.05
2690	2.81337	0.0717893
2691	1.39296	0.0964283
2692	0.830164	2.07444
2693	1.50493	2.1
2694	1.50494	2.1
2695	0.830136	1.84089
2696	1.28298	2.1
2697	1.0521	2.1
2698	0.830147	2.1
2699	2.0799	2.1
2700	1.52115	1.62493
2701	2.5142	2.03421
2702	1.31253	0.367495
2703	1.94446	1.0934
2704	1.18817	1.2912
2705	0.598348	1.98927
2706	1.69315	1.13822
2707	1.69315	1.64746
2708	0.598377	0.597717
2709	0.598347	1.05609
2710	0.94613	0.736508
2711	1.28418	0.488185
2712	2.05205	0.694859
2713	1.28418	1.60997
2714	1.0737	2.1
2715	1.81024	2.1
2716	2.73834	2.1
2717	1.71369	2.1
2718	1.71368	2.1
2719	0.946118	2.1
2720	1.29063	2.1
2721	1.36917	2.1
2722	0.94613	2.1
2723	1.29062	2.1

2724	0.947921	2.1
2725	1.58661	0.113624
2726	1.37658	1.8342
2727	1.2364	1.73575
2728	1.66685	0.912327
2729	1.66685	2.03275
2730	0.946133	0.834788
2731	0.946133	1.39042
2732	1.11978	1.66568
2733	1.09194	1.19647
2734	1.21858	2.07797
2735	1.37658	1.62006
2736	1.64903	0.371001
2737	1.64903	2.1
2738	0.946132	0.876015
2739	0.946132	1.48074
2740	1.05496	1.22817
2741	1.22851	0.347225
2742	1.22852	2.1
2743	1.58482	0.414027
2744	1.60578	0.05
2745	1.06336	0.05
2746	1.68062	0.05
2747	1.14721	0.05
2748	1.11924	0.05
2749	1.18421	0.05
2750	2.01276	0.05
2751	1.23502	2.1
2752	1.34926	2.07383
2753	0.546751	1.5894
2754	0.790446	2.03163
2755	1.04072	1.60208
2756	0.555868	1.33708
2757	0.930305	1.40863
2758	1.09344	1.72713
2759	1.18351	1.85407
2760	1.97215	1.54168
2761	1.07416	2.01983
2762	1.33204	1.37172
2763	1.22038	1.10503
2764	0.597449	0.885344
2765	0.821422	1.44533
2766	1.04071	1.19411
2767	0.608026	0.439741
2768	0.929427	1.15916
2769	1.64929	0.05
2770	1.68541	0.05
2771	0.496093	0.05
2772	1.3385	0.05
2773	0.790798	0.05
2774	1.22971	0.05
2775	0.898853	0.05
2776	2.14361	0.05
2777	2.10813	0.05
2778	1.05417	0.05
2779	1.80768	0.05
2780	1.09725	2.1
2781	1.41131	2.1

2782	1.1656	2.1
2783	0.946129	2.1
2784	1.68365	2.1
2785	1.68365	2.1
2786	1.1656	2.1
2787	0.94613	2.1
2788	1.21269	0.05
2789	1.48867	0.05
2790	1.27806	0.05
2791	1.68264	0.05
2792	1.37813	2.01393
2793	1.50743	1.36937
2794	1.04121	0.62271
2795	1.12434	0.134328
2796	1.39553	0.0782602
2797	1.44721	1.53128
2798	0.946132	0.171794
2799	1.23943	0.196374
2800	1.05292	0.312232
2801	1.28607	0.113005
2802	1.16915	0.312649
2803	1.76306	0.603787
2804	1.42266	0.825721
2805	1.36844	2.1
2806	1.36844	2.1
2807	1.67352	2.1
2808	1.11256	0.564615
2809	1.03217	0.367686
2810	1.18422	0.05
2811	1.04159	0.05
2812	1.60139	0.05
2813	1.04159	0.05
2814	0.988805	2.1
2815	1.05481	2.1
2816	1.52938	2.0935
2817	1.94724	1.85818
2818	0.896336	0.05
2819	0.911484	0.05
2820	1.18315	0.05
2821	1.10515	0.05
2822	1.04067	0.05
2823	1.12758	0.05
2824	0.904106	0.05
2825	1.6901	0.05
2826	1.68671	0.05
2827	1.99908	0.05
2828	0.772426	0.574424
2829	0.810945	2.03458
2830	0.667562	1.06461
2831	0.496617	0.063754
2832	0.847689	0.861492
2833	0.914378	1.53444
2834	0.934003	0.457175
2835	1.07459	1.08377
2836	1.40225	2.1
2837	1.07043	2.1
2838	0.94613	2.1
2839	1.63072	2.1

2840	1.63072	2.1
2841	1.07043	2.1
2842	0.946131	2.1
2843	1.53082	2.1
2844	1.69916	2.1
2845	1.75211	0.0890906
2846	1.96831	2.1
2847	1.75211	0.299549
2848	0.946127	0.178988
2849	0.817065	0.215519
2850	1.41798	0.185301
2851	1.41798	0.0899852
2852	0.791656	0.197134
2853	0.791656	0.252796
2854	1.41798	0.158612
2855	1.58331	0.161341
2856	1.41776	0.0899979
2857	0.791647	0.0987861
2858	1.4512	0.0779255
2859	1.45119	0.0833658
2860	0.75821	0.11224
2861	0.758248	0.110659
2862	0.946104	0.0903712
2863	1.36482	0.0865526
2864	1.29389	2.1
2865	1.04164	1.8559
2866	1.40574	1.86442
2867	1.64833	1.6495
2868	1.71183	0.05
2869	1.71183	0.05
2870	0.946131	0.199501
2871	1.35721	0.05
2872	0.946131	0.628439
2873	0.946131	0.229239
2874	1.30076	0.12984
2875	1.2474	0.05
2876	2.85225	0.0574898
2877	2.71441	0.05
2878	1.2474	0.05
2879	1.38622	0.05
2880	0.858359	0.05
2881	1.3682	0.05
2882	1.57364	0.05
2883	0.729695	0.05
2884	0.729723	0.05
2885	0.822611	0.169389
2886	0.946133	0.0513601
2887	0.729723	0.05
2888	0.974704	0.151163
2889	0.974702	0.225118
2890	1.81603	2.1
2891	1.96348	2.1
2892	1.43612	0.581831
2893	1.43612	0.95079
2894	0.634295	1.03602
2895	0.93796	1.25754
2896	0.946133	1.25745
2897	0.634326	0.386824

2898	0.946133	0.803779
2899	1.2654	0.192233
2900	1.26865	0.797515
2901	2.2649	0.494914
2902	1.00864	0.66603
2903	1.09689	0.336325
2904	0.832021	0.33319
2905	1.53229	0.257685
2906	1.1145	0.402678
2907	0.929583	0.260808
2908	1.613	0.16678
2909	0.644729	0.295826
2910	1.03471	1.18097
2911	2.7739	2.1
2912	1.36133	1.35118
2913	2.50215	2.1
2914	2.52195	2.1
2915	1.0465	2.1
2916	1.42673	2.1
2917	1.42673	2.1
2918	0.946132	2.1
2919	1.31904	2.1
2920	0.986499	2.1
2921	0.946132	2.1
2922	1.31904	2.1
2923	0.966978	2.1
2924	1.42237	0.05
2925	2.60925	0.05
2926	0.753659	0.05
2927	1.77241	0.05
2928	1.77241	0.05
2929	0.946129	0.05
2930	0.75366	0.05
2931	1.22144	0.05
2932	0.94613	0.05
2933	1.50732	0.05
2934	1.18514	0.05
2935	0.902187	0.05
2936	1.89858	0.05
2937	1.24294	0.05
2938	1.67211	0.05
2939	1.41786	0.05
2940	0.810959	0.05
2941	1.50732	0.05
2942	2.44288	0.05
2943	1.40969	0.05
2944	2.06654	0.05
2945	1.40969	0.05
2946	1.40882	0.05
2947	1.94063	0.05
2948	0.867921	0.05
2949	1.29289	0.05
2950	0.867918	0.05
2951	0.946128	0.05
2952	0.867944	0.05
2953	1.66589	0.05
2954	1.66589	0.05
2955	0.968595	2.1

2956	1.5534	2.1
2957	1.27793	2.1
2958	1.13433	2.1
2959	1.3439	0.05
2960	2.53709	0.05
2961	0.571998	0.05
2962	0.64837	0.05
2963	1.99755	0.05
2964	1.99854	0.05
2965	0.764229	2.1
2966	1.61842	2.1
2967	1.61842	2.1
2968	1.18315	2.1
2969	1.1995	2.1
2970	1.18315	2.1
2971	0.764256	2.1
2972	1.52364	2.1
2973	2.36631	2.1
2974	0.641912	1.87948
2975	1.7181	2.1
2976	1.459 2.1	
2977	0.634845	1.6284
2978	1.18315	2.1
2979	1.17615	2.1
2980	0.571367	2.1
2981	1.3324	0.05
2982	0.798214	0.05
2983	0.946128	0.05
2984	0.627252	0.05
2985	1.50337	0.05
2986	1.50337	0.05
2987	0.62725	0.05
2988	1.07763	0.05
2989	1.07763	0.05
2990	2.16109	0.05
2991	1.07763	0.05
2992	1.12486	2.1
2993	1.11827	2.1
2994	1.0491	2.1
2995	1.09034	2.1
2996	1.09431	2.1
2997	0.946131	2.1
2998	0.748607	2.1
2999	1.44002	2.1
3000	1.44002	2.1
3001	0.748635	2.1
3002	0.782505	2.1
3003	1.40615	2.1
3004	1.19539	2.1
3005	1.29389	2.1
3006	1.4867	1.22516
3007	1.50103	0.05
3008	1.63025	0.05
3009	2.72109	0.05
3010	0.915755	0.05
3011	0.946132	0.05
3012	0.915755	0.05
3013	1.70236	0.05

3014	1.70236	0.05
3015	1.26155	0.05
3016	1.35657	0.05
3017	1.83151	0.05
3018	1.83151	0.05
3019	2.52309	0.05
3020	0.983607	2.1
3021	1.06838	0.0566062
3022	0.983609	0.811734
3023	0.983596	0.445775
3024	2.74384	0.05
3025	1.6261	0.05
3026	1.15477	2.1
3027	1.02136	2.1
3028	1.83561	2.1
3029	1.83561	2.1
3030	0.946131	2.1
3031	1.39523	2.1
3032	1.29313	1.45694
3033	1.29313	2.1
3034	1.38651	2.1
3035	1.06313	2.1
3036	0.974888	2.1
3037	1.0894	2.1
3038	0.974888	2.1
3039	1.82282	2.1
3040	1.82282	2.1
3041	0.946131	2.1
3042	1.37372	2.1
3043	1.32374	2.1
3044	1.32374	2.1
3045	1.39523	2.1
3046	1.40882	0.05
3047	0.861998	0.05
3048	1.24448	0.05
3049	0.861999	0.05
3050	0.946129	0.05
3051	0.862011	0.05
3052	1.67537	0.05
3053	1.67537	0.05
3054	1.72399	0.05
3055	2.58579	0.05
3056	1.72399	0.05
3057	1.50951	0.05
3058	2.48897	0.05
3059	1.26755	2.1
3060	1.03824	2.1
3061	1.27875	2.1
3062	1.48901	2.1
3063	1.18902	2.1
3064	1.11764	2.1
3065	1.87436	2.1
3066	0.95345	2.1
3067	0.724191	1.46293
3068	0.86668	1.43421
3069	0.811523	1.70901
3070	1.04074	0.562351
3071	0.843097	1.14028

3072	0.833434	1.61535
3073	0.965727	1.16861
3074	0.85346	1.57677
3075	1.00185	0.217285
3076	1.00184	1.28481
3077	0.980651	1.67143
3078	0.980664	1.16301
3079	0.475737	0.465472
3080	0.719696	0.708083
3081	0.946109	0.513525
3082	0.946116	0.956394
3083	0.719693	0.560642
3084	0.946117	0.940461
3085	1.14879	1.56326
3086	0.719683	1.39957
3087	0.900977	0.824987
3088	0.972364	1.04276
3089	1.22961	0.963896
3090	0.405384	0.793256
3091	1.34406	2.1
3092	1.2187	2.1
3093	0.963571	0.05
3094	0.946131	0.05
3095	1.57246	0.132353
3096	1.57246	2.1
3097	0.946131	0.176733
3098	1.1235	2.03501
3099	1.1235	1.64773
3100	1.07439	1.70558
3101	1.19612	1.11781
3102	1.24844	0.98969
3103	1.00007	1.70535
3104	1.02429	2.1
3105	1.10459	2.1
3106	1.06724	0.05
3107	0.67519	0.16563
3108	0.49762	0.05
3109	1.43435	0.05
3110	0.833315	0.05
3111	0.58135	0.05
3112	1.14749	0.371943
3113	1.85091	0.626269
3114	1.2802	0.284137
3115	1.27737	0.0593618
3116	2.3655	0.155996
3117	1.30584	2.1
3118	2.06424	2.1
3119	0.946131	0.0748844
3120	1.04257	0.05
3121	0.94613	0.05
3122	1.67201	0.0736986
3123	1.67201	0.05
3124	1.27696	0.05
3125	1.34118	0.05
3126	1.3413	0.05
3127	2.36238	0.05
3128	0.6036	0.05
3129	1.11745	0.05

3130	1.46126	0.05
3131	1.39612	0.05
3132	0.670678	0.05
3133	1.03402	0.05
3134	1.11271	0.05
3135	0.838028	0.05
3136	0.807019	0.05
3137	1.27787	0.05
3138	0.994705	0.05
3139	1.20014	0.05
3140	0.964793	0.05
3141	0.863868	0.05
3142	1.3413	0.05
3143	1.79549	0.05
3144	1.35877	2.1
3145	1.63648	2.1
3146	1.08171	2.1
3147	1.27839	2.1
3148	1.52601	2.1
3149	1.27841	2.1
3150	1.71255	2.1
3151	2.16307	2.1
3152	1.91592	2.1
3153	1.78508	2.1
3154	0.946128	2.1
3155	1.41199	2.1
3156	0.946128	2.1
3157	0.901088	2.1
3158	1.55244	2.1
3159	1.59475	2.1
3160	1.40921	2.1
3161	0.960106	2.1
3162	1.40921	2.1
3163	2.0073	2.1
3164	2.2973	0.05
3165	1.90705	0.05
3166	1.53671	0.05
3167	1.86035	0.05
3168	1.33747	2.1
3169	1.14007	2.1
3170	1.3212	2.1
3171	1.66867	0.05
3172	1.30203	0.05
3173	1.27696	2.1
3174	1.05025	2.1
3175	1.05025	2.1
3176	1.3704	2.1
3177	1.15677	2.1
3178	0.946129	2.1
3179	1.65879	2.1
3180	1.65879	2.1
3181	1.15676	2.1
3182	0.94613	2.1
3183	0.867217	1.58607
3184	1.28684	1.13417
3185	1.58866	1.18812
3186	1.58866	0.566269
3187	0.867246	1.91438

3188	1.28684	0.783188
3189	1.16906	1.45646
3190	1.13438	1.18105
3191	1.37224	1.40783
3192	1.61519	1.42288
3193	0.883939	2.04575
3194	1.40092	1.15749
3195	1.80383	2.03157
3196	1.80383	1.77088
3197	0.883935	1.89742
3198	1.40092	1.12181
3199	1.28684	1.88595
3200	1.76788	0.798255
3201	2.57368	1.15166
3202	1.37224	2.1
3203	1.65411	0.379953
3204	1.77042	2.1
3205	1.77042	2.1
3206	1.90446	2.1
3207	1.28667	2.1
3208	1.55635	2.1
3209	1.30646	1.03434
3210	2.31836	1.64078
3211	1.30645	0.983719
3212	1.86429	1.18085
3213	1.48676	0.05
3214	0.743379	0.05
3215	1.41848	0.05
3216	1.41848	0.05
3217	0.946131	0.05
3218	0.812649	0.05
3219	1.34921	0.05
3220	0.812649	0.05
3221	0.949366	0.05
3222	1.32974	0.05
3223	0.794204	0.05
3224	0.634352	2.1
3225	0.838025	2.1
3226	1.08802	2.1
3227	0.838038	2.1
3228	1.18314	2.1
3229	1.12495	2.1
3230	1.22774	2.1
3231	1.04343	2.1
3232	0.921111	0.05
3233	2.1609	0.05
3234	1.09987	0.05
3235	2.5043	0.05
3236	1.05492	0.05
3237	0.590344	0.05
3238	1.05492	0.05
3239	1.25215	0.05
3240	1.71674	0.05
3241	1.71674	0.05
3242	0.590331	0.05
3243	1.17078	0.05
3244	0.625542	0.05
3245	0.724673	2.1

3246	1.08011	2.1
3247	1.87598	2.1
3248	1.50738	2.1
3249	1.44951	0.05
3250	1.50102	0.05
3251	1.8042	0.05
3252	0.687769	0.0812902
3253	1.66483	0.05
3254	1.66483	0.0617142
3255	0.946132	0.0971054
3256	0.687798	0.0670087
3257	1.20885	0.149993
3258	0.687799	0.094682
3259	0.886063	0.0612291
3260	0.798751	0.0694772
3261	0.584813	0.05
3262	1.49483	0.05
3263	0.888005	0.05
3264	1.03589	0.05
3265	1.70435	0.05
3266	0.710414	2.1
3267	0.917609	1.25485
3268	0.810947	1.49057
3269	0.81096	1.43125
3270	1.48658	1.32238
3271	1.53653	1.43058
3272	1.19329	1.7608
3273	0.946153	1.40698
3274	1.50798	1.49386
3275	1.85017	2.1
3276	1.19768	2.1
3277	0.864304	2.1
3278	0.792906	1.72091
3279	0.989025	1.29302
3280	1.19768	1.71163
3281	1.54548	1.57409
3282	1.18798	1.94993
3283	1.31875	1.09183
3284	1.05725	1.39006
3285	2.17365	0.05
3286	2.48106	0.05
3287	1.87451	0.05
3288	1.27189	1.0317
3289	2.79399	1.88228
3290	0.859565	1.20811
3291	0.926174	0.914154
3292	0.604744	0.865647
3293	1.41832	0.101855
3294	1.05494	0.521625
3295	0.874749	0.499138
3296	1.50067	0.230447
3297	0.917817	1.30939
3298	0.63591	1.12541
3299	1.72849	0.710698
3300	1.72849	0.194679
3301	0.635943	0.408558
3302	1.39699	1.08153
3303	0.967411	1.8783

3304	0.635943	0.967768
3305	1.39908	0.581889
3306	1.26971	0.965516
3307	1.02969	1.18669
3308	1.15326	2.1
3309	2.30741	1.88035
3310	0.93545	1.33891
3311	0.802747	0.746403
3312	1.15466	1.68185
3313	0.859164	1.77132
3314	1.02616	1.87889
3315	1.23149	1.66167
3316	0.83804	0.897435
3317	1.13266	2.1
3318	1.64142	2.1
3319	1.21223	2.1
3320	1.43051	2.1
3321	1.32418	0.70029
3322	0.621319	0.523009
3323	1.47134	2.1
3324	1.79103	0.0896015
3325	3.25951	0.05
3326	3.16764	0.05
3327	1.65583	0.05
3328	1.4732	0.05
3329	1.76476	2.1
3330	1.80114	1.20537
3331	0.754121	2.1
3332	0.94613	0.198101
3333	0.754119	2.1
3334	1.45636	2.1
3335	1.45636	0.127968
3336	1.30029	0.726731
3337	0.910183	0.8075
3338	1.29099	1.99129
3339	1.08039	2.1
3340	1.05498	2.1
3341	1.24445	2.1
3342	1.2873	2.1
3343	0.987703	2.1
3344	0.964782	2.04591
3345	1.73645	0.05
3346	0.871279	0.05
3347	0.884744	0.05
3348	1.85193	0.05
3349	1.68681	0.05
3350	1.33689	0.05
3351	1.23493	0.05
3352	0.892482	0.05
3353	2.95185	0.05
3354	2.10496	0.05
3355	1.87334	2.1
3356	0.94411	2.1
3357	0.754196	2.1
3358	1.43122	2.1
3359	1.58684	2.1
3360	0.838027	2.1
3361	1.1536	2.1

3362	1.24878	2.1
3363	1.23125	2.1
3364	2.86862	2.1
3365	1.57636	2.1
3366	0.998451	0.189966
3367	1.5176	0.11628
3368	1.13271	0.05
3369	1.60248	0.05
3370	1.22083	2.1
3371	1.79996	2.1
3372	1.19087	2.1
3373	2.00342	2.1
3374	1.3715	2.1
3375	2.79227	2.1
3376	2.7873	2.1
3377	0.987265	0.05
3378	1.62715	2.1
3379	1.70117	2.1
3380	1.27806	0.05
3381	1.45246	2.1
3382	1.45244	2.1
3383	1.87214	2.1
3384	0.995796	2.1
3385	1.68189	0.05
3386	1.49155	2.1
3387	1.58054	2.1
3388	1.59169	2.1
3389	0.736364	0.05
3390	0.70236	0.05
3391	2.24367	0.05
3392	2.57085	0.05
3393	1.70952	0.05
3394	2.60516	0.05
3395	1.45158	2.1
3396	1.00371	2.1
3397	1.65748	2.1
3398	1.23706	0.05
3399	1.55834	0.718757
3400	1.72671	0.644285
3401	0.597656	0.721148
3402	1.39137	0.402858
3403	0.855578	0.05
3404	1.48398	0.188677
3405	0.745117	0.224719
3406	1.23706	1.36787
3407	1.57916	0.0807438
3408	0.893025	0.05
3409	3.22433	0.132908
3410	2.47324	1.51609
3411	1.63852	2.1
3412	1.63852	2.1
3413	0.946132	2.1
3414	1.19095	2.1
3415	0.946133	2.1
3416	1.16684	2.1
3417	1.19095	2.1
3418	1.06698	0.05
3419	1.48692	0.05

3420	1.08334	0.560185
3421	1.07283	2.1
3422	1.28517	0.258075
3423	1.2746	0.05
3424	1.30723	0.05
3425	2.02069	0.05
3426	1.05579	0.05
3427	1.59475	2.1
3428	2.40113	2.1
3429	1.58875	2.1
3430	1.6881	2.1
3431	1.6881	2.1
3432	0.819953	2.1
3433	0.819954	2.1
3434	0.946131	2.1
3435	0.946131	2.1
3436	1.34782	2.1
3437	1.65619	2.1
3438	1.65619	2.1
3439	0.79437	2.1
3440	1.25	2.1
3441	0.94613	2.1
3442	0.946131	2.1
3443	0.794371	2.1
3444	1.58875	2.1
3445	2.5	2.1
3446	1.04147	2.1
3447	0.946131	0.206222
3448	1.79686	2.1
3449	1.79686	2.1
3450	1.25144	0.220471
3451	1.35714	1.869
3452	1.38585	1.8354
3453	1.25144	2.1
3454	1.15564	2.1
3455	1.34389	2.1
3456	0.946133	1.11948
3457	1.74353	2.1
3458	1.74353	2.1
3459	1.14553	0.911024
3460	1.38585	0.66717
3461	1.30382	1.30593
3462	1.14553	2.1
3463	1.15564	2.1
3464	1.90618	2.1
3465	2.11056	2.1
3466	1.032	2.1
3467	0.953636	2.1
3468	1.23131	1.16575
3469	1.39763	1.41824
3470	2.01024	0.761049
3471	1.9186	0.791818
3472	1.83658	0.05
3473	1.81656	0.05
3474	2.2049	0.05
3475	2.16081	0.05
3476	1.12219	0.05
3477	0.599771	0.05

3478	1.61401	0.05
3479	1.05912	0.05
3480	0.951648	0.05
3481	1.89328	0.05
3482	0.913682	0.05
3483	1.00763	0.05
3484	1.29129	2.1
3485	1.37138	2.1
3486	1.41986	0.05
3487	1.41986	0.05
3488	0.944166	0.05
3489	1.41789	0.05
3490	0.944167	0.05
3491	0.944166	0.05
3492	0.946132	0.05
3493	1.98713	0.05
3494	1.23626	0.05
3495	0.932791	0.05
3496	0.810251	0.05
3497	0.986748	0.05
3498	0.898155	0.05
3499	0.92624	0.05
3500	1.1349	0.05
3501	0.934572	0.05
3502	1.23104	0.376084
3503	1.23104	0.481154
3504	0.799994	0.0503708
3505	0.94613	0.268503
3506	0.802822	0.05
3507	1.40944	0.675084
3508	0.800035	0.152071
3509	1.41223	0.163385
3510	1.41223	0.179035
3511	1.6	0.0920693
3512	1.60565	0.0535244
3513	1.23042	2.1
3514	1.0645	2.1
3515	1.00319	2.1
3516	1.29435	2.1
3517	1.2243	2.1
3518	1.2243	2.1
3519	1.05655	1.08696
3520	1.29764	1.60627
3521	2.55252	2.1
3522	1.15676	2.1
3523	0.855295	2.1
3524	0.810959	2.1
3525	1.65857	2.1
3526	1.00849	2.1
3527	1.16021	2.1
3528	1.75869	2.1
3529	1.13775	2.1
3530	1.20091	2.1
3531	1.61582	2.1
3532	1.88031	2.1
3533	0.968536	2.1
3534	0.946131	2.1
3535	1.61481	2.1

3536	1.61481	2.1
3537	1.09547	2.1
3538	1.18486	2.1
3539	1.37609	2.1
3540	1.09546	2.1
3541	1.02461	2.1
3542	1.83223	2.1
3543	1.74002	2.1
3544	1.70939	2.1
3545	1.7094	2.1
3546	0.726287	2.1
3547	1.01712	2.05046
3548	1.30318	2.1
3549	0.946131	2.1
3550	0.87155	2.1
3551	1.77093	1.86595
3552	2.02356	1.91188
3553	1.32449	2.1
3554	1.10003	2.1
3555	1.5161	2.1
3556	1.5161	2.1
3557	0.946132	2.1
3558	1.09206	2.1
3559	0.946132	2.1
3560	1.04404	2.1
3561	1.09206	2.1
3562	0.635628	1.1875
3563	1.14623	0.226442
3564	1.73197	0.321032
3565	1.73197	1.2056
3566	0.529655	0.265214
3567	1.14623	0.673769
3568	0.992085	0.392538
3569	1.16915	1.29016
3570	2.29245	0.0902857
3571	0.584573	0.73816
3572	0.956756	0.244876
3573	1.52471	0.206035
3574	1.51409	1.33809
3575	0.584575	0.652986
3576	0.94613	0.583178
3577	1.14622	0.176336
3578	1.28096	1.1929
3579	1.34695	1.77411
3580	1.04193	2.1
3581	1.05378	2.1
3582	0.798209	2.1
3583	1.32007	2.1
3584	1.64327	0.05
3585	2.64674	2.1
3586	2.38936	0.05
3587	2.88475	0.05
3588	1.07745	2.1
3589	1.08911	2.1
3590	1.49127	2.1
3591	1.49127	2.1
3592	0.709714	2.1
3593	1.37199	2.1

3594	0.82899	2.1
3595	0.709714	2.1
3596	0.946132	2.1
3597	1.00133	2.1
3598	1.40182	2.1
3599	1.53278	2.1
3600	1.40182	2.1
3601	1.31764	0.05
3602	1.00691	0.05
3603	1.24094	0.05
3604	0.64868	0.05
3605	0.946133	0.05
3606	0.64868	0.05
3607	1.71762	0.05
3608	1.71762	0.05
3609	1.41662	0.05
3610	0.975477	0.05
3611	1.73584	0.05
3612	2.48188	0.05
3613	2.2045	0.103789
3614	2.08134	0.0877254
3615	0.993366	0.05
3616	0.993368	0.440467
3617	1.14187	0.129572
3618	0.946132	0.11423
3619	0.946133	1.18189
3620	1.68951	0.456213
3621	1.68951	1.92035
3622	1.34612	0.166539
3623	1.28953	0.174892
3624	1.34611	1.47861
3625	1.31892	0.05
3626	0.946131	0.266705
3627	1.80988	0.05
3628	1.80988	2.1
3629	1.31892	0.712521
3630	0.946131	0.176644
3631	2.13214	2.1
3632	1.94691	2.1
3633	1.52661	0.05
3634	1.53175	0.05
3635	0.956005	0.05
3636	0.955991	0.05
3637	1.40572	0.05
3638	0.955992	0.05
3639	1.51084	1.22485
3640	1.67115	2.08225
3641	1.73143	2.1
3642	1.55234	2.1
3643	1.69377	2.1
3644	2.51483	2.1
3645	1.25987	2.1
3646	1.4472	2.1
3647	1.83151	0.150594
3648	2.01001	0.440219
3649	0.993998	0.05
3650	1.83151	0.10386
3651	1.40915	0.05

3652	1.24653	0.0623942
3653	1.74472	2.1
3654	1.74472	2.1
3655	1.74244	2.1
3656	1.04788	2.1
3657	1.28522	2.1
3658	1.03785	2.1
3659	0.896519	2.1
3660	1.60428	2.1
3661	1.73305	2.1
3662	1.39072	0.05
3663	2.27501	0.050083
3664	1.39073	0.05
3665	1.39073	0.05
3666	1.06838	2.1
3667	0.968462	0.152013
3668	1.06837	1.35683
3669	1.13455	1.25774
3670	1.23527	0.10123
3671	2.71686	0.0624749
3672	1.86881	0.05
3673	1.10586	0.862581
3674	1.03852	1.66165
3675	1.03899	2.1
3676	0.963973	2.1
3677	1.00319	2.1
3678	1.00319	2.1
3679	0.987132	2.1
3680	0.974889	2.1
3681	0.987131	2.1
3682	1.7677	2.1
3683	1.7677	2.1
3684	0.946131	2.1
3685	1.31185	2.1
3686	0.946132	2.1
3687	1.30203	2.1
3688	1.31185	2.1
3689	1.82958	2.1
3690	1.82958	2.1
3691	0.946132	2.1
3692	1.40199	2.1
3693	0.946133	2.1
3694	1.36994	2.1
3695	1.40199	2.1
3696	0.97489	2.1
3697	1.05939	2.1
3698	1.01922	2.1
3699	0.810941	1.89969
3700	1.05372	2.1
3701	0.754065	2.1
3702	0.926131	2.1
3703	1.35905	2.1
3704	0.810931	2.1
3705	1.75647	0.770086
3706	1.68265	2.1
3707	1.41995	2.1
3708	1.36457	1.68688
3709	0.933392	1.27654

3710	1.42255	2.1
3711	1.3868	2.1
3712	1.08074	2.1
3713	1.07196	2.1
3714	1.13537	1.19066
3715	1.01179	0.592411
3716	1.60196	0.194807
3717	1.72798	0.313647
3718	1.3715	0.398689
3719	1.56409	0.72347
3720	1.56409	0.146776
3721	1.97819	1.28991
3722	1.47954	0.162898
3723	0.798655	0.05
3724	1.47952	0.115807
3725	2.30327	0.05
3726	1.49111	2.1
3727	1.96013	2.1
3728	1.54599	2.1
3729	2.06632	2.1
3730	1.54601	2.1
3731	1.10557	0.579355
3732	1.17624	0.142014
3733	1.60497	2.1
3734	1.61587	2.1
3735	1.65977	0.05
3736	1.69044	0.05
3737	1.00659	2.1
3738	1.06226	2.1
3739	1.15171	2.1
3740	1.50853	2.1
3741	1.80252	2.1
3742	1.41432	2.1
3743	1.04687	2.1
3744	0.940878	2.1
3745	1.52031	2.1
3746	1.52031	2.1
3747	0.940877	2.1
3748	0.946132	2.1
3749	1.50853	2.1
3750	0.963973	2.1
3751	1.70562	0.05
3752	2.43203	0.05
3753	1.36004	0.05
3754	1.06648	0.05
3755	1.66009	0.05
3756	1.66009	0.05
3757	0.766427	0.05
3758	0.766452	0.05
3759	0.946132	0.05
3760	1.98012	0.05
3761	1.20357	2.1
3762	1.20356	2.1
3763	1.67324	2.1
3764	1.52008	2.1
3765	1.19964	2.1
3766	1.36861	2.1
3767	1.19964	2.1

3768	2.85415	2.1
3769	1.43687	2.1
3770	0.935852	2.1
3771	1.09972	2.1
3772	0.810933	2.1
3773	2.25974	2.1
3774	1.74149	2.1
3775	1.36984	2.1
3776	1.87602	2.1
3777	0.890748	2.1
3778	1.20587	2.04438
3779	0.810956	1.74693
3780	2.03174	1.98432
3781	1.34403	2.09275
3782	1.58825	2.02327
3783	1.63096	2.02234
3784	2.53735	2.09358
3785	1.98241	2.1
3786	2.53014	2.1
3787	1.1775	1.14272
3788	1.06538	0.524574
3789	0.946132	1.34191
3790	1.4101	2.1
3791	1.75162	2.1
3792	1.75162	1.16599
3793	0.946132	1.13422
3794	1.4101	1.09638
3795	1.28765	1.18651
3796	1.1427	0.788392
3797	1.1427	0.275946
3798	1.3109	2.1
3799	1.02721	0.895512
3800	0.846447	1.3471
3801	0.862076	2.1
3802	0.644098	1.49693
3803	0.581308	1.74677
3804	1.02306	1.40207
3805	0.322641	0.240998
3806	0.589346	0.222414
3807	2.25572	1.63229
3808	1.31372	2.1
3809	1.18873	2.1
3810	1.78225	2.1
3811	1.764 2.1	
3812	1.17503	2.1
3813	1.06821	2.1
3814	1.06821	1.94533
3815	1.7777	2.00386
3816	1.9096	0.777973
3817	1.70239	1.41087
3818	1.16182	0.05
3819	1.20132	0.05
3820	1.23475	2.1
3821	1.56859	1.91544
3822	2.46263	2.1
3823	0.528744	2.1
3824	2.93337	2.1
3825	2.42504	2.1

3826	1.38646	0.05
3827	1.61963	0.05
3828	1.88016	0.192357
3829	0.622283	0.457333
3830	1.03243	0.415806
3831	0.710307	1.117
3832	0.798124	0.331623
3833	0.931073	1.16813
3834	0.892862	1.32165
3835	1.06791	0.252015
3836	0.552787	1.42414
3837	0.946132	0.583334
3838	1.36665	0.67261
3839	1.36665	1.29287
3840	0.506966	0.815965
3841	0.506964	1.4756
3842	1.36665	0.503909
3843	0.653469	0.48365
3844	1.36665	0.179752
3845	1.21339	0.172791
3846	1.21339	1.14441
3847	0.552757	0.343677
3848	0.552786	1.4417
3849	1.21336	0.081407
3850	1.70744	1.14282
3851	1.0775	0.05
3852	1.59527	0.0525156
3853	1.48312	0.069639
3854	2.01868	0.05
3855	1.18947	0.05
3856	1.48312	0.0714524
3857	0.785626	0.05
3858	1.19601	0.05
3859	0.785626	0.05
3860	0.946132	0.05
3861	0.7856	0.05
3862	1.59087	0.05
3863	1.51158	0.05
3864	1.74098	0.05
3865	2.39202	0.05
3866	0.870489	0.05
3867	1.34534	0.05
3868	0.870488	0.05
3869	0.946133	0.0565661
3870	0.870489	0.085839
3871	1.67086	0.05
3872	1.67086	0.05
3873	2.11311	2.1
3874	1.98765	1.62885
3875	0.946133	0.539108
3876	1.30881	2.1
3877	1.72211	2.1
3878	1.72211	1.55819
3879	0.94613	2.07539
3880	1.30881	1.48622
3881	1.35943	0.487467
3882	1.01294	0.05
3883	1.01294	0.05

3884	1.38963	0.147626
3885	1.01294	0.05
3886	1.60056	0.05
3887	1.60056	0.05
3888	0.946132	0.073238
3889	1.204	0.05
3890	0.946132	0.0512879
3891	1.11288	0.05
3892	1.204	0.05
3893	1.14599	0.05
3894	1.00065	0.05
3895	1.74685	0.0681312
3896	1.74685	2.1
3897	0.946128	0.0578321
3898	1.38463	0.05
3899	1.2275	2.1
3900	1.2275	0.52337
3901	1.30835	0.542852
3902	2.04407	2.1
3903	2.50425	2.1
3904	1.19647	0.116411
3905	1.19647	0.326346
3906	1.19645	0.515269
3907	1.75446	0.266931
3908	1.1315	0.05
3909	1.1315	0.791065
3910	1.45569	0.05
3911	1.1315	0.05
3912	1.14357	2.1
3913	1.25793	2.1
3914	1.14357	2.1
3915	2.08181	2.1
3916	1.4714	2.1
3917	0.71295	2.1
3918	1.68285	2.1
3919	1.68285	2.1
3920	1.25603	2.1
3921	1.13976	2.1
3922	1.25603	2.1
3923	0.71295	2.1
3924	1.61095	2.1
3925	2.51207	2.1
3926	1.63762	2.1
3927	2.74451	2.1
3928	0.671255	1.86018
3929	1.87601	2.1
3930	1.87601	2.1
3931	0.671226	1.93294
3932	1.25603	2.1
3933	1.29123	2.1
3934	0.671226	2.1
3935	1.63723	2.1
3936	0.872928	0.05
3937	1.47904	0.05
3938	1.18793	0.05
3939	2.57464	0.05
3940	1.14986	2.1
3941	1.19611	2.1

3942	0.96029	0.0833561
3943	1.7961	2.1
3944	0.928721	2.1
3945	1.34352	2.1
3946	1.37935	2.1
3947	1.37935	2.1
3948	0.892888	2.1
3949	0.946131	2.1
3950	0.892888	2.1
3951	1.75422	2.1
3952	1.05556	2.1
3953	1.27362	0.05
3954	1.27421	0.05
3955	1.3003	0.889294
3956	1.31415	0.256192
3957	1.01431	2.1
3958	0.673951	2.1
3959	1.63384	2.1
3960	1.63661	2.1
3961	1.52245	2.1
3962	1.50714	2.1
3963	0.996778	0.05
3964	1.56091	0.05
3965	1.06543	2.1
3966	2.66689	2.1
3967	1.01474	2.1
3968	2.76422	2.1
3969	1.45321	0.05
3970	0.974716	0.05
3971	0.810943	0.05
3972	0.909982	0.05
3973	0.937793	0.05
3974	1.05138	0.05
3975	1.25044	0.05
3976	0.92095	0.05
3977	0.830414	0.05
3978	2.1402	0.05
3979	1.75938	2.1
3980	1.05556	2.1
3981	1.78558	2.1
3982	1.379 2.1	
3983	0.970214	0.38396
3984	0.9702	0.27249
3985	1.08894	0.583152
3986	2.17251	0.50717
3987	1.51569	0.176824
3988	1.06012	0.121039
3989	0.98966	0.109987
3990	1.55926	0.05
3991	1.92645	0.0978337
3992	1.37427	0.05
3993	2.16338	0.05
3994	1.5456	0.05
3995	1.5456	0.05
3996	1.94002	0.05
3997	1.33135	0.05
3998	1.31948	0.05
3999	2.23255	0.05

4000	2.34061	0.05
4001	1.52903	2.1
4002	3.38327	2.1
4003	1.35977	2.1
4004	2.22414	2.1
4005	1.6812	0.05
4006	2.33397	0.05
4007	1.16699	0.05
4008	0.946131	0.05
4009	1.58115	0.05
4010	1.58115	0.05
4011	0.519726	0.05
4012	0.946131	0.05
4013	0.519697	0.05
4014	1.26868	0.05
4015	1.16699	0.05
4016	1.5951	0.05
4017	1.5951	0.05
4018	0.840568	0.05
4019	0.946131	0.05
4020	0.840598	0.05
4021	1.30047	2.1
4022	1.5586	2.1
4023	1.31825	0.513469
4024	1.31924	0.201275
4025	1.15818	2.1
4026	1.52782	1.14699
4027	1.15818	1.4954
4028	1.15818	1.41886
4029	0.99518	0.108469
4030	0.94613	0.198552
4031	0.922045	0.358556
4032	1.29322	0.39854
4033	1.29321	0.0971667
4034	1.29321	0.402037
4035	0.922056	0.326272
4036	1.06848	0.125984
4037	1.84409	0.113155
4038	1.9186	1.22652
4039	2.72244	0.685009
4040	0.946132	0.932817
4041	1.36122	0.739857
4042	1.79658	1.03582
4043	1.79658	0.478846
4044	0.946133	1.29208
4045	1.36122	0.851897
4046	1.38149	0.77487
4047	1.59106	0.322867
4048	0.946116	0.83591
4049	1.29052	0.505813
4050	1.76641	0.546532
4051	1.76641	0.369899
4052	0.885309	1.19892
4053	1.29054	0.692399
4054	1.36118	0.617147
4055	1.59103	0.888737
4056	2.27816	0.384996
4057	1.56164	0.322897

4058	1.41114	0.05
4059	2.04128	0.05
4060	0.986723	2.1
4061	1.03645	0.05
4062	1.79224	0.05
4063	1.2627	2.1
4064	1.23036	2.1
4065	1.45257	2.1
4066	1.41469	2.1
4067	1.41469	2.1
4068	2.60636	2.1
4069	1.45789	0.05
4070	0.851541	0.05
4071	1.57877	0.05
4072	1.53134	0.05
4073	0.851479	0.05
4074	1.08982	0.05
4075	0.716284	0.05
4076	0.946131	0.05
4077	1.73193	0.05
4078	2.57309	0.05
4079	1.36307	0.05
4080	2.42175	0.05
4081	1.72948	0.05
4082	0.978698	2.1
4083	0.978699	2.1
4084	2.08398	2.1
4085	0.978698	2.1
4086	1.44096	2.1
4087	1.44096	2.1
4088	0.638164	2.1
4089	0.681588	2.1
4090	0.946133	2.1
4091	0.638165	2.1
4092	0.94613	2.1
4093	1.55432	2.1
4094	0.901147	2.1
4095	0.85777	2.1
4096	1.36073	2.1
4097	1.44004	2.1
4098	0.946133	2.1
4099	1.09982	2.1
4100	1.27562	2.1
4101	1.55432	2.1
4102	1.62229	2.1
4103	1.0101	2.1
4104	2.28459	2.1
4105	2.50554	2.06623
4106	0.946131	0.570695
4107	1.25276	2.1
4108	1.73721	2.1
4109	1.73721	1.45294
4110	0.93406	2.1
4111	1.25277	1.8048
4112	1.4185	1.13514
4113	1.53064	0.90366
4114	0.946133	1.0245
4115	0.609462	2.1

4116	1.65123	2.1
4117	1.65123	1.75617
4118	0.609466	2.1
4119	1.00792	1.88443
4120	1.25277	1.50299
4121	1.53064	2.1
4122	2.15223	1.15846
4123	1.30664	1.74987
4124	1.10674	2.1
4125	1.22862	2.1
4126	1.49358	0.0645231
4127	2.0207	0.05
4128	2.09374	2.1
4129	1.88175	2.1
4130	1.10938	0.05
4131	1.02478	0.0508536
4132	1.13272	0.05
4133	1.13272	0.05
4134	1.03935	0.894474
4135	0.810945	1.49585
4136	0.527573	0.161373
4137	0.925011	0.775164
4138	1.03193	0.179401
4139	1.38587	0.414789
4140	0.703469	0.300686
4141	0.912282	0.786969
4142	1.75505	2.1
4143	1.78529	2.1
4144	1.60017	0.151512
4145	1.35608	0.05
4146	1.01479	1.18366
4147	1.47561	0.05
4148	1.01479	0.997678
4149	1.28418	0.05
4150	2.62605	2.1
4151	1.71233	2.1
4152	3.22222	2.1
4153	1.97526	2.1
4154	0.949712	0.05
4155	1.78986	0.05
4156	2.72879	0.05
4157	2.66428	0.05
4158	1.68108	0.05
4159	1.67265	0.05
4160	2.24877	0.0929615
4161	1.15558	0.103911
4162	1.39708	2.1
4163	1.39327	2.1
4164	1.69788	0.05
4165	1.1273	0.05
4166	1.17302	0.05
4167	1.04349	0.05
4168	0.979853	2.1
4169	1.19094	2.1
4170	1.39408	2.1
4171	1.61857	2.1
4172	1.66523	2.1
4173	0.946132	2.1

4174	0.838554	2.1
4175	1.05151	2.1
4176	1.18901	2.1
4177	1.18901	2.1
4178	1.61149	2.1
4179	1.22857	0.277205
4180	1.25848	0.580332
4181	1.09154	2.1
4182	2.20358	2.1
4183	1.23013	2.1
4184	1.55698	2.1
4185	1.55828	2.1
4186	1.52391	2.1
4187	0.60346	1.88451
4188	1.06336	2.1
4189	0.870714	1.09054
4190	0.543109	1.87132
4191	1.10367	1.1964
4192	1.20686	1.97142
4193	1.20684	2.1
4194	2.5118	1.65263
4195	1.20684	2.1
4196	1.52468	2.1
4197	1.52469	2.1
4198	0.532308	1.68473
4199	0.903301	2.1
4200	0.946119	1.51284
4201	0.532315	1.40412
4202	0.946107	1.59921
4203	1.46129	2.1
4204	0.804766	2.1
4205	1.4809	2.1
4206	1.4809	2.1
4207	1.11822	2.1
4208	1.16744	2.1
4209	1.11822	2.1
4210	0.804737	2.1
4211	1.04193	2.1
4212	0.983723	2.1
4213	1.8014	2.1
4214	1.1729	2.1
4215	1.03951	2.1
4216	1.03951	2.1
4217	1.17583	2.1
4218	1.42866	2.1
4219	1.78503	2.1
4220	1.17822	1.52948
4221	1.89121	2.1
4222	1.44472	2.1
4223	1.62143	2.1
4224	1.43837	2.1
4225	1.01121	0.05
4226	0.962703	1.19989
4227	0.946129	0.0992146
4228	1.10941	0.05
4229	0.946131	0.0541754
4230	1.60326	0.323504
4231	1.60326	0.0967044

4232	1.14215	0.106155
4233	1.40724	0.369089
4234	0.962703	0.0742048
4235	1.53336	0.05
4236	1.40272	0.05
4237	1.64984	0.05
4238	1.67345	0.05
4239	1.08212	2.1
4240	1.40905	2.1
4241	0.946132	0.05
4242	0.946132	0.05
4243	0.81268	0.05
4244	0.946131	0.05
4245	0.812681	0.05
4246	0.946131	0.05
4247	0.946132	0.05
4248	0.997755	0.05
4249	1.94585	0.05
4250	1.2925	0.05
4251	2.52454	0.05
4252	1.69182	0.05
4253	1.69182	0.05
4254	0.92361	0.05
4255	1.35316	0.05
4256	1.26227	0.05
4257	0.946131	0.05
4258	1.35316	0.05
4259	1.48676	0.05
4260	1.33917	0.05
4261	0.757292	2.1
4262	1.02415	2.1
4263	1.48495	2.1
4264	0.97587	2.1
4265	0.946132	2.1
4266	0.747365	2.1
4267	1.55432	2.1
4268	1.55432	2.1
4269	0.946132	2.1
4270	0.747394	2.1
4271	1.17464	0.05
4272	1.0372	0.05
4273	2.16553	0.05
4274	2.00616	0.05
4275	1.12184	0.05
4276	1.33177	0.05
4277	0.936674	0.05
4278	0.946129	0.05
4279	0.851552	0.05
4280	1.60205	0.05
4281	1.77391	0.05
4282	1.03327	0.05
4283	1.41512	0.05
4284	1.03327	0.05
4285	0.946133	0.05
4286	0.946131	0.05
4287	1.80077	0.05
4288	1.80077	0.05
4289	1.54848	2.1

4290	1.29931	2.1
4291	1.04482	0.052488
4292	2.08069	0.052317
4293	0.946127	0.05
4294	0.515539	0.0638124
4295	0.515566	0.108022
4296	1.38423	0.05
4297	1.38423	0.05
4298	0.972058	0.05
4299	0.927737	0.0590529
4300	0.94971	0.05
4301	2.19682	0.05
4302	0.946132	0.0606281
4303	1.09841	0.05
4304	0.522382	0.0837772
4305	1.5962	0.05
4306	1.72791	0.05
4307	1.07428	0.05
4308	1.05215	0.05
4309	1.54632	1.58744
4310	1.15693	1.66187
4311	1.41238	2.1
4312	1.41238	2.1
4313	1.77966	2.1
4314	1.77966	2.1
4315	0.946129	2.1
4316	1.40525	2.1
4317	0.946131	2.1
4318	0.94613	2.1
4319	1.32054	2.1
4320	1.41238	2.1
4321	1.94026	2.1
4322	1.0171	0.131589
4323	1.1211	0.060307
4324	0.993022	2.1
4325	0.773813	2.1
4326	1.4073	2.1
4327	1.4073	2.1
4328	0.94613	2.1
4329	0.787615	2.1
4330	0.94613	2.1
4331	0.787615	2.1
4332	1.764	2.1
4333	1.90964	2.1
4334	1.05787	2.1
4335	1.403	0.05
4336	0.744189	0.177748
4337	2.32834	2.1
4338	2.34986	2.1
4339	1.17106	1.80858
4340	1.94826	0.05
4341	2.40606	0.05
4342	1.26387	0.05
4343	0.954013	0.05
4344	0.946131	0.05
4345	1.71	0.05
4346	1.51926	0.05
4347	0.953997	0.05

4348	0.851554	0.05
4349	1.96502	0.05
4350	1.72954	0.05
4351	2.89117	0.05
4352	0.971958	0.05
4353	0.985575	0.05
4354	1.41943	0.05
4355	1.20021	0.05
4356	1.29599	0.05
4357	1.15687	0.05
4358	0.810958	0.05
4359	1.12919	0.05
4360	1.98859	2.1
4361	1.82044	2.1
4362	1.90488	2.1
4363	0.851542	2.1
4364	1.02973	2.1
4365	1.39286	2.1
4366	1.44873	2.1
4367	0.946132	2.1
4368	1.11826	2.1
4369	1.26842	2.1
4370	2.09461	2.1
4371	1.76952	0.05
4372	1.6473	0.05
4373	0.946131	0.05
4374	1.36486	0.05
4375	1.04243	0.05
4376	1.05017	0.05
4377	1.24059	0.05
4378	2.11646	0.05
4379	1.50368	0.05
4380	2.38796	0.05
4381	1.50368	0.05
4382	1.71844	0.05
4383	1.71844	0.05
4384	0.914836	0.05
4385	1.24416	0.05
4386	0.914805	0.05
4387	1.24416	0.05
4388	1.38911	0.05
4389	1.46859	0.05
4390	1.35348	0.05
4391	0.946131	0.05
4392	1.1226	0.05
4393	0.857078	0.05
4394	0.9746	0.05
4395	1.37624	0.05
4396	1.15131	0.05
4397	1.139 0.05	
4398	1.90456	0.05
4399	2.09692	0.05
4400	1.16655	2.1
4401	1.23017	2.1
4402	2.14592	2.1
4403	1.23017	2.1
4404	1.0752	1.62622
4405	1.52482	0.05

4406	1.09252	2.1
4407	1.10725	2.1
4408	1.14305	0.05
4409	0.851541	0.05
4410	1.48073	0.05
4411	1.45607	0.05
4412	0.946131	0.05
4413	0.932823	0.05
4414	1.45828	0.05
4415	0.851528	0.05
4416	1.14305	0.05
4417	1.92737	0.05
4418	1.08211	0.05
4419	1.69831	0.05
4420	1.69831	0.05
4421	0.946131	0.05
4422	1.26868	0.05
4423	1.23377	0.05
4424	1.23377	0.05
4425	0.94723	0.05
4426	1.64322	0.05
4427	1.64322	0.05
4428	0.946131	0.05
4429	1.32068	0.05
4430	0.946129	0.05
4431	1.18789	0.05
4432	1.32068	0.05
4433	1.32813	2.1
4434	1.32461	2.1
4435	1.42729	0.05
4436	2.47846	0.05
4437	1.00024	0.05
4438	1.23923	0.05
4439	0.946131	0.05
4440	0.712304	0.05
4441	1.77501	0.05
4442	1.77501	0.05
4443	0.593593	0.05
4444	1.23923	0.05
4445	1.23175	0.05
4446	0.94613	0.05
4447	0.713646	0.05
4448	1.75735	0.05
4449	1.75735	0.05
4450	0.713633	0.05
4451	1.37157	0.05
4452	1.47169	0.05
4453	2.21808	0.05
4454	1.47169	0.05
4455	1.04759	2.1
4456	0.987132	2.1
4457	1.11181	2.1
4458	2.61682	2.0834
4459	1.11181	2.1
4460	1.06461	1.32547
4461	1.69733	2.1
4462	2.79705	2.1
4463	1.08245	2.1

4464	1.40583	2.1
4465	1.39852	2.1
4466	2.0239	2.1
4467	2.0239	2.1
4468	0.780455	2.1
4469	0.946132	2.1
4470	0.780455	2.1
4471	1.01885	2.1
4472	0.564217	2.1
4473	0.733461	2.1
4474	0.537126	2.1
4475	0.683838	2.1
4476	0.779662	2.1
4477	0.634141	2.1
4478	0.685337	2.1
4479	1.58848	2.1
4480	1.17129	0.05
4481	0.663168	0.05
4482	1.55587	0.05
4483	1.31342	0.05
4484	0.596868	0.05
4485	1.1666	0.05
4486	0.867261	0.05
4487	0.946132	0.05
4488	1.28843	0.05
4489	1.31248	0.05
4490	2.59921	0.05
4491	1.32634	0.05
4492	1.35564	0.05
4493	1.52661	0.05
4494	1.12323	0.05
4495	1.17701	0.536578
4496	1.36405	0.58152
4497	1.19837	0.587043
4498	2.25636	0.438238
4499	1.0823	0.05
4500	1.31774	0.05
4501	1.0823	0.05
4502	1.01121	0.05
4503	1.27408	0.05
4504	0.946131	0.05
4505	0.566074	0.05
4506	0.79576	0.05
4507	1.24596	0.05
4508	1.47567	0.05
4509	1.47567	0.05
4510	0.56605	0.05
4511	1.6567	0.05
4512	0.967792	0.05
4513	1.00366	0.05
4514	1.00366	0.05
4515	1.56409	0.05
4516	1.56409	0.05
4517	0.774911	0.05
4518	1.09483	0.05
4519	0.774912	0.05
4520	1.09483	0.05
4521	1.24417	0.05

4522	1.13387	0.05
4523	1.13387	0.05
4524	1.30572	0.05
4525	2.18966	0.05
4526	0.98575	0.05
4527	1.54982	0.05
4528	2.48833	0.05
4529	1.54982	0.05
4530	1.28954	0.05
4531	1.64002	0.05
4532	1.64002	0.05
4533	1.85594	0.05
4534	1.12968	2.1
4535	1.23536	2.1
4536	1.4882	2.1
4537	1.4882	2.1
4538	0.876836	2.1
4539	0.946131	2.1
4540	0.876835	2.1
4541	1.43166	2.1
4542	2.25936	2.1
4543	1.0305	2.1
4544	0.992095	2.1
4545	1.12968	2.1
4546	1.53813	2.1
4547	1.53812	2.1
4548	0.583653	2.1
4549	0.94613	2.1
4550	0.583651	2.1
4551	0.964033	2.1
4552	0.964034	2.1
4553	1.54457	2.1
4554	3.54109	0.05
4555	0.99974	0.05
4556	0.78814	0.05
4557	1.65729	0.05
4558	1.1283	0.05
4559	1.09407	0.05
4560	1.55091	0.05
4561	0.870773	0.05
4562	1.19967	0.05
4563	1.56521	0.05
4564	1.30896	1.38418
4565	1.17989	2.1
4566	1.72526	2.1
4567	0.851537	1.80463
4568	1.58944	2.1
4569	1.56915	2.1
4570	0.891039	1.66977
4571	1.4172	1.80623
4572	0.963818	1.49845
4573	0.851474	2.05283
4574	1.17989	2.1
4575	1.56261	2.1
4576	1.56261	2.1
4577	0.761574	2.1
4578	1.31323	2.1
4579	0.761573	2.1

4580	0.946133	2.1
4581	1.31323	2.1
4582	1.16759	2.1
4583	1.16759	2.1
4584	2.11829	2.1
4585	1.16759	2.1
4586	1.10748	0.05
4587	1.47688	0.05
4588	0.946133	0.05
4589	0.953319	0.05
4590	0.946132	0.05
4591	1.51168	0.05
4592	1.51168	0.05
4593	1.25797	0.05
4594	1.19984	0.05
4595	1.95729	2.1
4596	2.34853	2.1
4597	1.94343	2.1
4598	2.00551	2.1
4599	1.20067	0.841048
4600	1.87244	0.851043
4601	1.4178	2.1
4602	1.33538	2.1
4603	0.946131	2.1
4604	1.22895	2.1
4605	1.12433	2.1
4606	0.956757	2.1
4607	0.91826	2.1
4608	1.44221	2.1
4609	1.14206	2.1
4610	1.80514	0.303086
4611	0.890304	1.20441
4612	1.1362	0.175715
4613	1.32819	0.184564
4614	1.46311	2.05286
4615	0.8654	0.212466
4616	1.1699	1.03058
4617	1.00267	0.976472
4618	1.02882	0.268857
4619	1.00832	2.1
4620	0.974137	2.1
4621	1.02368	2.1
4622	0.974138	2.1
4623	0.995734	0.05
4624	1.44605	0.05
4625	1.18549	0.05
4626	1.33621	0.142873
4627	1.5176	0.151365
4628	1.91762	0.221207
4629	1.33621	0.110552
4630	1.6638	0.148563
4631	1.66774	2.1
4632	1.11833	2.1
4633	0.926499	2.1
4634	1.51229	2.1
4635	1.51229	2.1
4636	0.926528	2.1
4637	1.40335	2.1

4638	1.03544	2.1
4639	0.926527	2.1
4640	1.85305	2.1
4641	1.5812	2.1
4642	1.15529	2.1
4643	1.31086	2.1
4644	0.999206	2.1
4645	1.46714	2.1
4646	1.73087	2.1
4647	0.772299	2.1
4648	1.24341	2.1
4649	0.997867	2.1
4650	0.772311	2.1
4651	0.936657	2.1
4652	1.35986	2.1
4653	0.959414	2.1
4654	1.36541	2.1
4655	1.49268	2.1
4656	1.49268	2.1
4657	0.832137	2.1
4658	0.946132	2.1
4659	0.832138	2.1
4660	1.64523	2.1
4661	1.11764	2.1
4662	1.05776	2.1
4663	0.972465	2.1
4664	0.972464	2.1
4665	1.98425	2.1
4666	0.969822	2.1
4667	1.30566	2.1
4668	1.52724	2.1
4669	2.32237	0.05
4670	2.30274	0.05
4671	2.27761	0.05
4672	3.33012	0.05
4673	1.24001	0.603521
4674	1.41821	1.34475
4675	0.978584	2.1
4676	1.18076	2.1
4677	1.07645	2.1
4678	0.877351	0.05
4679	0.946132	0.534574
4680	0.877353	0.05
4681	1.58444	2.1
4682	1.49614	2.1
4683	1.28355	1.52248
4684	1.13491	0.627624
4685	1.56875	0.05
4686	0.499592	0.05
4687	0.946131	0.05
4688	1.20538	0.05
4689	1.09913	0.05
4690	1.8049	0.05
4691	1.8049	0.05
4692	0.499606	0.05
4693	2.41076	0.05
4694	0.860202	0.05
4695	0.906798	0.05

4696	1.8626	0.05
4697	1.33986	0.05
4698	1.2992	0.05
4699	1.58702	0.05
4700	1.05939	0.05
4701	1.02074	2.1
4702	1.07866	2.1
4703	1.5082	0.05
4704	1.5522	0.05
4705	1.65903	0.05
4706	1.86766	2.1
4707	2.11684	2.1
4708	0.946132	2.1
4709	1.6585	2.1
4710	1.6585	2.1
4711	0.94613	2.1
4712	1.1872	2.1
4713	1.1872	2.1
4714	1.05842	2.1
4715	1.20455	2.1
4716	1.34141	2.1
4717	1.34141	2.1
4718	1.42416	2.1
4719	1.75818	2.1
4720	2.63715	0.988115
4721	1.80454	2.1
4722	2.26198	2.1
4723	0.696255	2.1
4724	0.624075	2.1
4725	1.50905	0.265405
4726	1.50905	2.1
4727	0.624075	2.1
4728	1.31857	0.161781
4729	0.946131	2.1
4730	0.624075	1.02346
4731	0.914628	2.1
4732	1.14721	2.1
4733	1.16153	2.1
4734	1.47664	2.1
4735	1.54791	2.1
4736	1.19098	2.1
4737	1.13341	2.1
4738	1.00503	0.05
4739	1.00503	1.06538
4740	1.22655	2.1
4741	0.999489	2.1
4742	1.3268	2.1
4743	0.946132	2.1
4744	1.60061	2.1
4745	1.60061	2.1
4746	1.08034	2.1
4747	1.40372	2.1
4748	1.14302	2.1
4749	1.08034	2.1
4750	0.999491	2.1
4751	1.17152	0.05
4752	1.17152	0.05
4753	1.17152	0.05

4754	2.05423	0.05
4755	1.40984	2.05192
4756	1.2489	2.1
4757	0.885089	2.1
4758	0.810931	2.1
4759	1.84128	2.1
4760	1.11206	2.1
4761	1.21926	2.1
4762	1.99084	2.1
4763	0.976334	0.306392
4764	0.599363	0.0975176
4765	1.02621	0.0731227
4766	0.462959	0.209315
4767	1.42612	0.0628325
4768	0.299682	0.0582342
4769	1.51379	2.1
4770	0.719531	2.1
4771	1.3702	2.1
4772	1.33293	2.1
4773	0.851539	2.1
4774	0.62998	2.1
4775	0.851543	2.1
4776	0.629966	2.1
4777	1.43132	2.1
4778	1.13328	1.06312
4779	1.93212	0.568715
4780	1.62345	0.824936
4781	1.38511	1.17674
4782	1.40013	0.258658
4783	1.04132	0.0995594
4784	0.83473	0.884102
4785	0.868732	0.673922
4786	0.810595	1.62794
4787	0.729919	0.491036
4788	0.992428	0.580333
4789	0.577107	0.138669
4790	1.18464	0.832523
4791	0.791199	0.631714
4792	0.810943	0.05
4793	0.860723	0.05
4794	1.33348	0.05
4795	1.57467	0.05
4796	0.981032	0.05
4797	0.731569	0.05
4798	0.770312	0.05
4799	1.46055	2.1
4800	1.42249	2.1
4801	1.75999	0.05
4802	1.6868	0.05
4803	1.17042	0.306488
4804	1.01485	2.1
4805	1.19952	0.05
4806	1.40592	0.0828061
4807	1.19952	0.0573906
4808	2.20145	0.05
4809	1.33649	0.05
4810	1.11612	0.05
4811	0.957588	0.05

4812	0.946126	0.05
4813	1.50648	0.05
4814	1.50648	0.05
4815	0.946128	0.05
4816	2.10671	0.05
4817	1.91518	0.05
4818	2.23223	0.05
4819	1.53556	2.1
4820	1.56253	2.1
4821	1.27806	0.05
4822	1.44593	0.05
4823	1.03818	0.0600901
4824	1.03818	0.05
4825	1.38969	0.287175
4826	1.85548	0.116068
4827	2.6044	0.241936
4828	1.85548	0.0865486
4829	1.49346	1.96161
4830	1.83192	1.74373
4831	0.834043	2.07239
4832	0.810943	0.509272
4833	0.887113	0.807838
4834	0.893827	0.710178
4835	0.991061	0.845142
4836	0.91939	1.01828
4837	0.793307	0.976201
4838	1.75291	1.41561
4839	1.35435	0.99048
4840	1.16989	0.05
4841	1.36717	0.05
4842	2.12276	0.05
4843	2.30493	0.05
4844	2.47857	0.0650313
4845	1.92269	0.05
4846	1.66426	0.05
4847	0.956698	0.371583
4848	0.956698	2.1
4849	0.857957	2.1
4850	1.4706	2.1
4851	1.65706	2.1
4852	1.47059	2.1
4853	1.04713	0.05
4854	0.946131	0.05
4855	1.47374	0.05
4856	1.47374	0.05
4857	0.951675	0.05
4858	1.2582	0.05
4859	1.2582	0.05
4860	0.94613	0.05
4861	1.4468	0.05
4862	1.90335	0.05
4863	1.14886	2.1
4864	2.59689	2.1
4865	1.02747	2.1
4866	0.946131	2.1
4867	0.934061	2.1
4868	1.63979	2.1
4869	1.65206	2.1

4870	1.48	2.1
4871	1.27467	2.1
4872	2.20449	2.1
4873	1.1333	0.05
4874	1.95334	2.1
4875	2.20984	2.1
4876	1.46389	0.05
4877	1.4639	0.05
4878	1.4639	0.05
4879	0.946132	0.05
4880	1.5144	0.05
4881	1.5144	0.05
4882	0.946132	0.05
4883	1.35732	0.05
4884	1.10321	0.05
4885	0.946132	0.05
4886	0.980549	0.05
4887	1.32819	2.1
4888	1.30171	1.16715
4889	1.68377	0.69114
4890	1.68377	2.1
4891	0.946132	0.354345
4892	0.946132	2.08909
4893	1.1952	1.56471
4894	0.990019	1.3765
4895	1.08195	1.92
4896	1.2269	2.1
4897	1.19219	2.07444
4898	1.01485	2.1
4899	1.57125	0.05
4900	2.10696	0.05
4901	1.29666	0.05
4902	1.57125	0.05
4903	1.47712	2.1
4904	1.45182	2.1
4905	1.17816	0.05
4906	1.4516	0.05
4907	1.17816	0.05
4908	1.17816	0.05
4909	0.596954	1.19748
4910	1.65133	0.174674
4911	0.583859	0.195866
4912	1.04072	1.04453
4913	1.28795	0.222703
4914	1.59423	0.188657
4915	0.483559	0.298365
4916	1.74088	0.130172
4917	1.09545	0.699768
4918	1.02519	1.78299
4919	2.47579	0.652544
4920	1.54732	0.919306
4921	2.17663	0.582997
4922	1.75006	0.525787
4923	1.32354	1.03399
4924	1.09962	2.1
4925	1.85057	2.1
4926	1.91046	2.1
4927	2.17037	2.1

4928	1.99528	0.05
4929	1.97969	0.05
4930	3.82765	2.1
4931	1.85767	2.1
4932	2.77042	2.1
4933	1.37236	0.05
4934	2.23254	0.05
4935	1.10089	0.05
4936	0.994294	2.1
4937	1.81921	2.1
4938	1.00073	2.1
4939	0.681043	2.1
4940	0.946131	2.1
4941	0.681073	2.1
4942	1.51804	2.1
4943	1.60653	2.1
4944	1.30188	2.1
4945	0.919622	2.1
4946	1.00965	2.1
4947	1.93821	2.1
4948	2.60377	2.1
4949	1.36416	0.05
4950	1.36416	0.05
4951	1.36416	0.05
4952	2.44132	0.05
4953	2.15871	0.123481
4954	2.22171	0.05
4955	1.70747	0.05
4956	1.49515	0.05
4957	0.773007	0.0587219
4958	0.946125	0.0707068
4959	0.946131	0.117767
4960	0.773034	0.05
4961	1.15844	0.05
4962	1.98104	0.05
4963	2.44283	0.0584705
4964	1.05417	0.05
4965	1.1596	0.05
4966	0.829921	0.05
4967	0.946131	0.05
4968	0.951911	0.05
4969	1.39027	0.05
4970	0.829946	0.05
4971	1.51223	0.05
4972	1.51223	0.05
4973	1.65984	0.05
4974	1.90382	0.05
4975	1.93228	1.01559
4976	2.47924	1.04065
4977	1.73904	1.01581
4978	1.40547	0.278517
4979	2.37589	0.433937
4980	0.662605	1.07888
4981	2.33293	0.548156
4982	1.72249	2.1
4983	1.68478	2.1
4984	1.00514	0.05
4985	1.4537	2.1

4986	1.92698	2.1
4987	1.46041	0.05
4988	2.04817	0.05
4989	1.65954	2.1
4990	1.42342	2.1
4991	1.04405	0.05
4992	1.17056	0.05
4993	1.11339	0.05
4994	0.946133	0.05
4995	0.929724	0.05
4996	1.35424	0.05
4997	1.35424	0.05
4998	0.929713	0.05
4999	1.85945	0.05
5000	1.38905	2.1
5001	1.39056	2.1
5002	1.52852	2.1
5003	1.06472	2.1
5004	0.749096	2.1
5005	1.46076	2.1
5006	1.29263	2.1
5007	0.94613	2.1
5008	0.69097	2.1
5009	0.982284	2.1
5010	0.67419	2.1
5011	1.52852	2.1
5012	1.91121	2.1
5013	1.00775	2.1
5014	1.61201	2.1
5015	0.529987	2.1
5016	0.506125	2.1
5017	1.44696	2.1
5018	2.32981	2.1
5019	1.32784	2.1
5020	1.94158	2.1
5021	1.31595	2.1
5022	0.663922	2.1
5023	1.44663	2.1
5024	1.44663	2.1
5025	1.13976	2.1
5026	0.970788	2.1
5027	1.13976	2.1
5028	0.663921	2.1
5029	0.966114	2.1
5030	1.05498	0.761727
5031	1.38086	2.1
5032	0.946127	2.1
5033	0.922513	2.1
5034	1.49903	2.1
5035	1.49903	2.1
5036	0.92251	2.1
5037	1.06105	2.1
5038	1.36049	2.1
5039	1.38086	2.1
5040	1.52534	2.1
5041	1.04101	2.1
5042	1.30861	0.05
5043	0.949112	0.05

5044	0.894275	0.05
5045	1.10232	0.05
5046	0.830889	0.05
5047	1.26554	0.05
5048	0.948347	0.05
5049	0.81096	0.05
5050	1.18338	0.05
5051	0.883764	0.05
5052	1.54793	0.05
5053	1.02867	0.05
5054	1.8573	0.05
5055	1.06065	0.05
5056	0.64842	0.05
5057	1.99319	0.05
5058	1.9476	0.05
5059	2.51436	0.05
5060	1.68337	0.05
5061	1.92321	0.05
5062	0.810942	0.05
5063	0.921001	0.05
5064	0.99758	0.05
5065	0.960785	0.05
5066	1.01565	0.05
5067	0.908624	0.05
5068	0.912835	0.05
5069	1.18351	0.05
5070	1.40239	2.1
5071	1.26091	2.1
5072	0.947979	2.1
5073	0.946131	2.1
5074	1.81959	2.1
5075	1.81959	0.622789
5076	0.94613	2.1
5077	1.38415	0.581745
5078	1.38414	2.09385
5079	1.33266	2.1
5080	0.946132	2.1
5081	1.76225	2.1
5082	1.76225	2.1
5083	1.27599	2.1
5084	1.38158	2.1
5085	1.3268	2.1
5086	1.27599	2.1
5087	0.947979	2.1
5088	1.0142	0.0563747
5089	1.01422	0.0549378
5090	1.11435	0.524703
5091	1.42186	0.365456
5092	1.24001	1.19117
5093	1.77342	1.38035
5094	1.42186	1.50062
5095	1.99817	0.833866
5096	1.27361	2.1
5097	1.68914	2.1
5098	1.30116	2.1
5099	1.32819	1.68905
5100	1.68322	1.60752
5101	1.68322	2.1

5102	0.94613	0.240873
5103	0.94613	2.1
5104	1.14666	1.97312
5105	1.07612	1.42131
5106	1.31151	2.1
5107	1.22095	2.05099
5108	1.57256	2.1
5109	1.6679	2.1
5110	0.946132	0.863733
5111	0.946133	2.1
5112	1.13537	2.1
5113	1.07855	2.1
5114	1.07855	2.1
5115	1.43895	1.03132
5116	1.38481	0.05
5117	1.72385	0.05
5118	0.550441	0.05
5119	0.79807	0.05
5120	1.11627	0.05
5121	1.30349	0.05
5122	0.94613	0.05
5123	0.686176	0.05
5124	1.11627	0.05
5125	1.73363	0.05
5126	1.73363	0.05
5127	0.686139	0.05
5128	1.24932	0.05
5129	0.899582	0.05
5130	2.00472	0.05
5131	1.17121	0.05
5132	1.2874	0.05
5133	2.0194	0.05
5134	0.88601	0.05
5135	1.77409	0.05
5136	1.73293	0.05
5137	2.74045	0.05
5138	1.83787	0.05
5139	1.45448	0.05
5140	1.27778	0.05
5141	1.1192	2.1
5142	1.25953	2.1
5143	1.93611	2.1
5144	1.92104	2.1
5145	1.15506	0.05
5146	1.15506	0.05
5147	1.23943	0.523971
5148	0.851543	2.1
5149	1.6061	2.1
5150	1.80058	2.1
5151	0.869471	2.1
5152	1.3931	2.1
5153	1.06454	2.1
5154	0.93667	2.1
5155	2.16916	2.1
5156	3.10246	2.1
5157	1.91539	2.1
5158	1.91539	2.1
5159	0.620263	1.74185

5160	0.620265	2.1
5161	0.946132	1.86793
5162	0.620265	1.48375
5163	1.30226	1.28454
5164	1.87292	2.1
5165	1.18769	2.1
5166	2.60452	2.1
5167	1.26574	2.1
5168	2.46679	2.1
5169	1.36459	2.1
5170	1.26865	1.08249
5171	1.1744	0.351917
5172	1.15841	0.0547947
5173	1.02247	0.05
5174	1.93649	2.1
5175	1.64718	2.1
5176	1.59654	2.1
5177	1.57823	2.1
5178	1.00665	0.05
5179	1.28239	0.05
5180	0.887895	2.1
5181	1.22083	2.1
5182	1.42127	2.1
5183	1.07374	2.1
5184	1.00503	1.48061
5185	1.23082	0.46185
5186	2.78663	2.1
5187	2.20883	2.1
5188	2.54588	2.1
5189	0.946128	2.1
5190	1.13716	2.1
5191	1.58724	2.1
5192	1.58724	2.1
5193	0.946131	2.1
5194	1.13716	2.1
5195	1.39621	2.1
5196	1.02438	2.1
5197	1.49543	0.05
5198	2.78336	0.05
5199	1.27148	0.05
5200	1.0906	0.05
5201	1.04072	0.05
5202	0.63574	0.05
5203	1.45506	0.05
5204	1.5863	0.05
5205	0.928352	0.05
5206	0.647687	0.05
5207	2.2375	1.79934
5208	1.50998	1.51441
5209	2.75021	1.3257
5210	1.91912	1.16534
5211	1.99872	0.937075
5212	0.918127	2.02745
5213	1.03031	0.831675
5214	0.951169	0.598251
5215	1.27975	0.552258
5216	1.12735	0.914441
5217	1.15741	0.778221

5218	1.24998	0.690315
5219	1.65953	0.05
5220	0.967555	0.149739
5221	0.967542	0.05
5222	1.03824	0.0537218
5223	1.48693	0.0811725
5224	1.48693	0.0689582
5225	2.02901	0.0620528
5226	0.946127	0.105222
5227	1.07139	0.0733284
5228	0.946126	0.0594363
5229	1.84644	0.05
5230	1.84644	0.05
5231	1.38683	0.05
5232	1.40574	0.05
5233	1.36866	1.19922
5234	0.649325	1.1058
5235	1.93631	2.1
5236	1.50145	2.1
5237	1.6287	2.1
5238	1.31302	2.1
5239	1.18041	2.1
5240	1.18041	2.1
5241	0.974196	2.1
5242	0.946131	2.1
5243	1.74169	2.1
5244	1.74169	2.1
5245	1.23163	2.1
5246	1.27451	2.1
5247	1.41331	2.1
5248	1.23163	2.1
5249	1.28037	2.1
5250	1.28158	2.1
5251	1.28037	2.1
5252	2.28015	0.05
5253	2.06561	0.05
5254	2.61343	0.05
5255	1.11712	0.05
5256	1.63689	1.27919
5257	1.01393	0.652911
5258	0.996775	2.1
5259	1.54277	2.1
5260	1.26397	2.1
5261	1.46129	2.1
5262	1.62391	2.1
5263	1.08848	0.05
5264	1.11833	2.1
5265	1.24041	2.1
5266	2.31884	2.1
5267	1.335 2.1	
5268	1.48589	0.05
5269	1.77131	0.05
5270	1.8422	0.553734
5271	2.47453	1.74825
5272	1.23726	1.25544
5273	1.21996	1.09014
5274	1.65512	1.31031
5275	1.65512	1.16406

5276	0.802107	1.86271
5277	0.946129	0.81485
5278	0.802137	0.738464
5279	1.3279	0.745492
5280	1.23726	0.617097
5281	1.79759	0.647207
5282	1.79759	0.522942
5283	0.767577	1.49425
5284	0.946131	0.473096
5285	0.767577	0.374308
5286	1.03534	0.05
5287	1.09962	0.05
5288	0.929217	1.37206
5289	1.70126	1.51487
5290	0.991407	2.1
5291	1.41077	2.1
5292	0.991406	2.1
5293	1.62966	2.1
5294	1.62966	2.1
5295	0.946128	2.1
5296	1.18364	2.1
5297	0.946131	2.1
5298	1.14186	2.1
5299	1.18364	2.1
5300	1.16858	2.1
5301	1.66137	0.0710889
5302	1.90165	0.05
5303	1.66139	0.05
5304	1.26893	0.0681813
5305	1.15918	0.277161
5306	0.653228	0.609597
5307	0.946133	0.596584
5308	1.77488	2.1
5309	1.77489	1.24392
5310	0.653228	1.38112
5311	1.56043	0.327894
5312	1.60894	1.431
5313	1.56043	0.267519
5314	1.06848	1.48977
5315	1.04307	2.1
5316	1.14188	2.1
5317	1.09195	2.1
5318	0.615121	2.1
5319	1.50867	2.1
5320	1.27152	2.1
5321	0.615094	2.09535
5322	1.07297	2.1
5323	1.1372	2.1
5324	0.553608	2.1
5325	1.23019	2.1
5326	0.992675	2.1
5327	1.40984	1.31891
5328	2.55682	2.1
5329	3.66069	2.1
5330	3.58726	2.1
5331	1.4375	0.05
5332	1.88424	0.05
5333	1.56714	2.1

5334	1.6721	2.1
5335	1.18474	2.1
5336	1.2295	2.1
5337	1.28754	2.1
5338	1.36838	2.1
5339	2.78661	2.1
5340	2.64055	2.1
5341	1.35065	0.947862
5342	2.26675	0.153747
5343	1.35065	0.422756
5344	1.21558	0.214337
5345	1.49709	0.05
5346	1.4971	0.05
5347	0.85472	0.05
5348	0.854718	0.05
5349	0.946129	0.05
5350	0.854717	0.05
5351	1.1604	0.05
5352	1.25265	0.05
5353	2.32081	0.05
5354	1.15371	0.05
5355	1.14945	0.05
5356	0.995725	0.05
5357	1.00307	0.05
5358	1.10907	0.05
5359	2.30315	0.05
5360	1.10908	0.05
5361	1.27558	0.554902
5362	1.71613	1.56995
5363	1.32977	2.1
5364	0.815276	2.1
5365	1.41697	2.1
5366	1.41697	2.1
5367	0.815276	2.1
5368	1.38897	2.1
5369	0.946131	2.1
5370	0.815275	2.1
5371	1.44684	2.1
5372	1.21489	1.90617
5373	1.22476	1.86377
5374	0.870366	0.259339
5375	0.862572	0.157042
5376	0.835435	0.110983
5377	1.6993	0.0668354
5378	1.23618	0.05
5379	1.18624	0.114284
5380	1.53058	0.0710688
5381	2.16108	0.593892
5382	2.21589	0.408325
5383	1.04037	2.09672
5384	1.03933	2.03033
5385	1.57753	0.05
5386	1.87644	0.05
5387	1.24728	0.05
5388	1.30394	0.05
5389	1.3538	2.1
5390	1.69858	2.1
5391	1.1726	0.121793

5392	0.94612	0.0557309
5393	1.09805	0.05
5394	0.946118	0.05
5395	1.72576	0.0557623
5396	1.72575	0.05
5397	1.34152	0.05
5398	1.33034	0.05
5399	0.784151	2.09373
5400	0.946131	2.1
5401	0.784151	2.1
5402	1.47963	2.1
5403	1.47963	2.1
5404	1.3335	2.1
5405	0.930281	2.1
5406	1.29435	1.88472
5407	0.883308	1.16173
5408	1.31589	2.1
5409	1.67846	2.1
5410	0.993598	0.05
5411	2.23193	0.05
5412	0.9936	0.05
5413	0.615629	0.05
5414	1.65925	2.1
5415	2.05992	2.1
5416	1.65925	2.1
5417	4.08735	1.77321
5418	2.72856	1.86371
5419	0.869602	2.1
5420	3.42833	0.785857
5421	1.14397	0.05
5422	1.34563	0.0525522
5423	1.08189	0.0547314
5424	0.782128	2.00113
5425	1.0022	2.1
5426	0.900427	1.81174
5427	1.51499	1.88726
5428	1.05588	1.89062
5429	1.13739	2.1
5430	1.37004	1.93324
5431	1.14603	2.09457
5432	0.851541	1.00881
5433	0.946799	1.79331
5434	1.14854	1.67486
5435	1.33793	1.1946
5436	0.94613	1.17247
5437	1.24336	1.69838
5438	0.851473	1.77395
5439	2.08792	1.71662
5440	1.01869	1.71627
5441	0.986953	0.05
5442	0.896238	0.05
5443	0.906574	0.05
5444	0.852145	0.05
5445	2.14191	0.05
5446	1.15979	0.05
5447	1.18327	0.05
5448	2.32742	0.05
5449	2.35961	0.05

5450	2.38126	0.05
5451	1.13632	0.05
5452	1.17897	0.05
5453	1.76199	0.05
5454	1.00573	2.1
5455	0.946127	0.0713694
5456	1.7077	2.1
5457	1.7077	2.1
5458	0.946127	1.82006
5459	1.24887	2.1
5460	1.24887	2.1
5461	1.22441	2.1
5462	1.08542	2.1
5463	1.21743	2.01525
5464	1.12572	2.1
5465	1.05499	0.290539
5466	1.00521	0.433085
5467	2.86191	0.05
5468	2.27972	0.05
5469	1.01169	2.1
5470	1.03373	2.1
5471	1.39319	2.1
5472	0.636837	2.1
5473	1.63908	2.1
5474	1.45713	2.02049
5475	1.04072	2.1
5476	1.25025	2.06964
5477	1.38914	2.1
5478	0.636807	2.1
5479	1.50211	2.1
5480	1.36555	2.1
5481	2.77545	1.66854
5482	0.682805	2.1
5483	1.50267	2.1
5484	1.50267	2.1
5485	0.797749	2.1
5486	1.38773	2.09288
5487	1.38773	2.1
5488	0.682776	2.1
5489	2.01245	0.05
5490	0.925918	0.05
5491	0.75689	0.05
5492	1.25823	0.05
5493	1.16505	0.05
5494	0.838779	0.05
5495	1.14722	0.05
5496	0.756849	0.05
5497	1.52298	0.05
5498	2.11034	0.05
5499	1.92174	0.05
5500	1.92174	0.05
5501	0.576083	0.05
5502	1.23146	0.05
5503	1.26637	0.05
5504	1.23146	0.05
5505	1.26637	0.05
5506	0.946133	0.156168
5507	1.37034	0.0728191

5508	1.84145	0.0916895
5509	1.84145	0.05
5510	0.946132	0.243198
5511	1.37034	0.11244
5512	1.41724	0.114154
5513	1.0685	0.05
5514	1.14547	0.0528222
5515	1.24976	2.1
5516	1.30682	2.1
5517	2.12095	2.1
5518	1.46392	2.1
5519	1.46392	2.1
5520	0.935738	2.1
5521	1.32949	2.1
5522	1.07017	2.1
5523	0.935766	2.1
5524	1.07017	2.1
5525	1.78095	2.1
5526	1.05619	0.234978
5527	1.05793	0.517802
5528	1.77677	2.1
5529	2.04766	2.1
5530	1.02524	2.1
5531	1.18803	2.1
5532	1.70047	2.1
5533	1.556 2.1	
5534	1.24201	2.09586
5535	1.3592	2.1
5536	2.11824	0.05
5537	2.20248	0.05
5538	3.43878	0.05
5539	3.12466	0.05
5540	2.09351	2.1
5541	0.946128	2.1
5542	1.51269	2.1
5543	1.51269	2.1
5544	0.946131	2.1
5545	1.01306	2.1
5546	1.04676	2.1
5547	1.01306	2.1
5548	1.07444	2.1
5549	2.17465	2.1
5550	1.14808	2.1
5551	1.39067	1.447
5552	1.20958	1.21888
5553	0.662832	0.285829
5554	1.93512	0.957378
5555	1.15471	2.1
5556	1.75119	2.1
5557	1.75118	2.1
5558	0.946131	2.1
5559	1.28606	2.1
5560	1.27087	2.1
5561	0.946131	2.1
5562	1.28606	2.1
5563	1.58916	0.05
5564	0.927822	0.05
5565	2.24367	0.05

5566	1.8734	0.05
5567	3.51668	0.05
5568	2.68986	0.05
5569	2.32395	2.1
5570	1.39564	2.1
5571	2.10862	2.1
5572	1.36763	2.1
5573	0.970963	0.05
5574	0.97096	0.05
5575	1.11897	0.05
5576	1.26885	0.05
5577	1.11605	0.05
5578	2.06612	0.05
5579	1.717 0.05	
5580	0.59502	0.05
5581	0.825755	0.05
5582	0.709624	0.05
5583	1.65741	0.05
5584	0.307486	0.05
5585	1.4785	0.05
5586	0.983143	2.1
5587	1.08247	2.1
5588	1.42353	2.1
5589	1.42353	2.1
5590	0.781667	2.1
5591	0.797634	2.1
5592	0.946131	2.1
5593	0.781638	2.1
5594	0.946131	2.1
5595	0.98315	2.1
5596	1.083 2.1	
5597	1.61975	0.05
5598	1.11946	0.05
5599	1.18977	0.05
5600	0.996484	0.05
5601	2.24343	0.05
5602	1.4787	0.05
5603	1.66127	0.05
5604	1.86028	0.05
5605	1.09128	0.05
5606	1.03115	0.05
5607	0.838025	0.05
5608	1.3736	0.05
5609	1.44009	0.05
5610	0.92917	0.05
5611	1.03889	0.05
5612	0.956974	0.05
5613	3.23468	0.05
5614	0.953922	0.05
5615	0.957104	0.05
5616	1.66231	0.05
5617	1.26778	0.05
5618	1.47615	0.05
5619	1.28621	0.05
5620	1.08043	0.05
5621	2.35026	2.1
5622	2.35025	2.1
5623	2.60659	2.1

5624	1.57627	2.1
5625	1.5493	2.1
5626	1.24682	0.05
5627	2.14277	0.0849958
5628	1.24682	0.161723
5629	1.00307	0.05
5630	2.04056	0.0697215
5631	1.45408	2.1
5632	1.54249	2.1
5633	1.24474	1.68436
5634	1.37813	1.93021
5635	0.924158	0.05
5636	0.916623	0.05
5637	1.35344	0.05
5638	1.00859	0.05
5639	1.34786	0.05
5640	0.952457	0.05
5641	0.85404	0.05
5642	1.06341	2.1
5643	1.09194	2.1
5644	1.93707	2.1
5645	2.14087	2.1
5646	1.93707	2.1
5647	1.07519	1.2766
5648	1.07519	0.157416
5649	1.62518	2.1
5650	1.3592	2.1
5651	1.13183	2.1
5652	1.20357	2.1
5653	1.4858	2.1
5654	1.23694	2.1
5655	1.44011	2.1
5656	1.23989	2.1
5657	2.21604	2.1
5658	1.85574	0.05
5659	1.55649	2.1
5660	1.48861	0.05
5661	1.15968	0.05
5662	0.946132	0.05
5663	0.663056	0.05
5664	1.70837	0.05
5665	1.70837	0.05
5666	0.946131	0.05
5667	0.663026	0.05
5668	2.09604	0.05
5669	0.81094	0.05
5670	1.06559	0.05
5671	0.810956	0.05
5672	1.15474	0.05
5673	0.990051	0.05
5674	0.894971	0.05
5675	1.32536	0.05
5676	0.946133	2.1
5677	1.33125	2.1
5678	1.75823	2.1
5679	1.75823	2.1
5680	0.946133	2.1
5681	1.33125	2.1

5682	1.37312	2.1
5683	1.27985	2.1
5684	1.37312	2.1
5685	1.70684	2.1
5686	1.70684	2.1
5687	0.946132	2.1
5688	0.946132	2.1
5689	1.25081	2.1
5690	0.956293	2.1
5691	0.956294	2.1
5692	1.36035	2.1
5693	1.41944	2.1
5694	0.830638	2.1
5695	0.747568	2.1
5696	0.888498	2.1
5697	1.04071	2.1
5698	1.17128	2.1
5699	1.92339	2.1
5700	2.91208	2.1
5701	1.82208	0.870433
5702	1.89653	2.1
5703	1.82588	1.34449
5704	1.26703	0.111918
5705	1.26703	0.816414
5706	0.6157	0.0807553
5707	1.26706	1.50001
5708	0.61567	0.05
5709	0.615671	0.05
5710	1.26706	0.202158
5711	1.89158	0.752854
5712	1.23134	0.05
5713	2.49497	0.05
5714	1.68103	0.05
5715	2.0554	2.1
5716	1.72647	2.1
5717	1.67375	2.1
5718	1.65037	2.1
5719	1.04349	0.05
5720	1.69055	0.05
5721	1.53463	0.05
5722	1.19022	2.07821
5723	1.89226	0.907872
5724	1.15009	1.12531
5725	1.7054	0.05
5726	1.31612	0.05
5727	1.31611	0.05
5728	1.67345	0.05
5729	1.06577	0.05
5730	1.40193	0.05
5731	1.72411	0.05
5732	1.46123	0.05
5733	0.808808	0.05
5734	0.897714	0.05
5735	0.851554	0.05
5736	1.3499	0.05
5737	1.29001	0.05
5738	1.22083	2.1
5739	1.06191	2.1

5740	1.05758	1.36112
5741	1.348	2.04403
5742	1.43485	1.24726
5743	1.34799	1.9042
5744	1.36307	0.05
5745	2.28188	0.05
5746	1.7926	0.05
5747	1.38957	2.1
5748	1.47446	2.1
5749	0.953638	2.1
5750	1.11868	2.1
5751	0.953636	2.1
5752	1.03408	2.1
5753	1.52443	0.05
5754	1.38539	0.05
5755	0.946132	0.05
5756	1.12747	0.05
5757	1.04393	0.05
5758	1.05017	0.05
5759	1.20529	0.05
5760	1.3663	0.101478
5761	1.89226	0.0838239
5762	1.17885	0.216646
5763	0.867881	0.0539464
5764	1.32917	0.05
5765	1.32917	0.0654747
5766	0.946127	0.0745208
5767	1.19583	0.0718888
5768	1.00122	0.0876316
5769	0.946128	0.103579
5770	1.71405	0.335056
5771	0.871815	2.09302
5772	1.16435	2.1
5773	0.686619	2.1
5774	1.63102	2.1
5775	1.06589	2.1
5776	1.05375	2.1
5777	1.9023	2.1
5778	1.27394	0.483729
5779	1.88345	0.561836
5780	2.05636	0.741424
5781	0.672977	0.18893
5782	1.41274	0.313214
5783	1.65606	0.219556
5784	0.869329	0.220541
5785	1.2207	0.380433
5786	1.35631	0.306569
5787	0.908776	0.223387
5788	0.955081	0.0637636
5789	1.2243	2.1
5790	1.23169	2.1
5791	1.52228	2.1
5792	1.23169	2.1
5793	0.990892	1.69673
5794	1.08376	0.480921
5795	1.79348	2.1
5796	1.50043	1.09444
5797	1.32599	2.1

5798	1.37924	1.38823
5799	0.838014	0.694156
5800	0.87737	1.90519
5801	0.947086	0.860444
5802	1.27199	2.0091
5803	1.04918	1.03208
5804	1.04851	2.1
5805	1.1699	1.65811
5806	0.810934	1.24625
5807	1.83602	2.1
5808	2.21909	2.1
5809	0.619441	1.82741
5810	2.03755	0.893726
5811	1.83472	2.06277
5812	1.20416	1.77596
5813	1.26691	0.7199
5814	0.655795	0.558923
5815	0.94613	1.74814
5816	2.35169	1.73178
5817	3.54705	2.1
5818	2.60741	2.1
5819	1.59019	0.05
5820	1.24526	0.05
5821	1.59019	0.05
5822	2.36406	0.05
5823	1.16776	0.160219
5824	1.67311	0.817441
5825	1.67311	0.185914
5826	0.94613	0.221169
5827	1.38645	0.600979
5828	1.13563	0.05
5829	1.13563	0.221763
5830	1.23279	0.0587292
5831	1.05042	2.1
5832	1.17418	1.71691
5833	1.28909	2.1
5834	0.973448	2.1
5835	1.02958	2.1
5836	3.3005	2.1
5837	2.86325	2.1
5838	1.35879	2.1
5839	1.69332	2.1
5840	1.69332	2.1
5841	0.946128	2.1
5842	1.39659	2.1
5843	1.12035	2.1
5844	1.12035	2.1
5845	1.24286	2.1
5846	1.10407	2.1
5847	1.14599	1.8889
5848	1.32259	0.0689292
5849	0.788592	0.05
5850	2.4588	2.1
5851	0.909581	2.06635
5852	1.35656	1.32639
5853	0.986895	1.75712
5854	2.30602	0.90365
5855	1.46873	1.61274

5856	1.55584	0.951956
5857	2.1547	1.35475
5858	1.87794	1.95085
5859	0.808286	2.05005
5860	0.928464	1.76663
5861	0.810959	1.60987
5862	1.50962	0.673887
5863	1.1053	1.35894
5864	1.0712	1.27879
5865	1.40504	1.20405
5866	1.87794	1.00051
5867	2.80496	1.34885
5868	1.41479	1.77602
5869	1.38634	2.1
5870	1.38634	2.1
5871	1.67762	2.1
5872	0.946131	2.1
5873	1.188	2.1
5874	1.62585	2.1
5875	1.62585	2.1
5876	0.946129	2.1
5877	1.18801	2.1
5878	1.38397	2.1
5879	1.04546	2.1
5880	1.16817	2.1
5881	1.06007	2.1
5882	1.19785	2.1
5883	1.43652	2.1
5884	1.06994	2.1
5885	1.89111	2.1
5886	1.07144	2.1
5887	1.51754	2.1
5888	2.0873	2.1
5889	0.758799	2.1
5890	1.59518	2.1
5891	1.59518	2.1
5892	0.946132	2.1
5893	0.758771	2.1
5894	1.06957	2.1
5895	0.758771	2.1
5896	1.33916	2.1
5897	2.03045	1.30157
5898	2.80184	1.74942
5899	1.76788	2.09287
5900	1.27442	2.1
5901	1.06756	2.1
5902	0.966979	2.1
5903	1.7054	2.1
5904	1.07156	2.1
5905	0.986096	2.1
5906	1.32813	2.1
5907	1.3331	2.1
5908	2.08369	0.667382
5909	2.23818	2.0807
5910	1.10335	0.945511
5911	0.622314	1.87193
5912	1.72451	0.868079
5913	1.72451	1.99077

5914	0.622283	0.421003
5915	0.622314	0.579901
5916	0.946131	0.573783
5917	1.31254	2.1
5918	1.15704	2.1
5919	1.24463	0.725105
5920	2.2067	1.60669
5921	1.30001	0.239679
5922	1.29175	0.732173
5923	1.1233	0.05
5924	1.8689	0.05
5925	1.29494	0.05
5926	1.11895	0.05
5927	0.851539	0.05
5928	0.851474	0.05
5929	1.58352	0.05
5930	1.58228	0.05
5931	0.851527	0.05
5932	2.6389	0.05
5933	1.77548	0.05
5934	1.24555	2.1
5935	2.0512	2.1
5936	1.46101	2.1
5937	2.60635	2.1
5938	1.10476	0.05
5939	1.44103	0.05
5940	0.652391	2.1
5941	0.618322	2.1
5942	2.22915	2.1
5943	1.39067	2.1
5944	1.99737	2.1
5945	1.42174	2.1
5946	0.946132	0.275416
5947	1.29735	1.57744
5948	1.61765	2.1
5949	1.61765	0.887252
5950	0.946132	1.87669
5951	1.29735	0.703022
5952	1.26643	0.335184
5953	1.01335	0.935699
5954	1.01335	0.0570485
5955	0.947669	2.1
5956	2.17167	2.1
5957	2.59733	1.9771
5958	1.50954	2.09238
5959	1.02638	1.53911
5960	0.555785	2.1
5961	1.91101	2.08245
5962	2.09809	1.87572
5963	0.641974	2.04901
5964	1.2022	1.94552
5965	1.36359	1.83344
5966	1.8128	2.08413
5967	2.34348	0.05
5968	1.66343	0.05
5969	0.974717	0.388331
5970	1.53908	0.205363
5971	1.27737	0.36287

5972	1.09408	0.0585009
5973	2.07382	0.141963
5974	1.10466	1.14428
5975	1.05654	1.49011
5976	1.23498	0.561355
5977	1.48104	1.16781
5978	1.23498	0.7343
5979	1.17261	2.1
5980	1.18059	2.1
5981	2.43005	2.1
5982	1.04546	2.1
5983	2.81222	2.1
5984	1.16817	2.1
5985	1.75766	0.05141
5986	1.47021	0.148848
5987	1.07236	0.05
5988	1.81759	0.323353
5989	1.42913	0.180535
5990	1.00636	0.644847
5991	1.00636	0.0972929
5992	1.79397	0.141281
5993	0.946131	0.0553621
5994	1.03691	0.0873347
5995	0.851539	0.123328
5996	1.46001	0.227763
5997	1.37878	0.0846593
5998	0.933258	0.0675055
5999	1.32423	0.0507571
6000	1.32614	0.05
6001	1.11356	0.05
6002	1.32614	0.05
6003	1.28895	0.05
6004	1.66895	0.05
6005	1.66895	0.05
6006	0.946132	0.05
6007	1.29576	0.05
6008	1.1318	0.05
6009	1.37621	1.69215
6010	1.94071	1.55309
6011	1.10517	2.1
6012	1.97098	2.1
6013	0.811 2.1	
6014	0.956127	2.1
6015	1.18647	2.1
6016	0.988455	1.6162
6017	1.12457	0.05
6018	1.7453	0.05
6019	0.92492	2.1
6020	1.24353	2.1
6021	1.84536	0.229033
6022	1.67662	0.12269
6023	1.38247	0.05
6024	1.02663	0.05
6025	0.946132	0.0942932
6026	1.41723	0.0513739
6027	1.72976	0.0697172
6028	1.72976	0.05
6029	0.946131	0.23542

6030	1.41723	0.0894201
6031	1.25866	0.0755867
6032	1.34254	0.05
6033	1.25866	0.05
6034	1.65507	0.05
6035	1.65507	0.05
6036	0.946132	0.102291
6037	0.946132	0.05
6038	1.11929	0.05
6039	0.961779	0.05
6040	0.961779	0.05
6041	0.946132	2.1
6042	1.69361	2.1
6043	1.69361	2.1
6044	0.94613	2.1
6045	1.36523	2.1
6046	1.36523	2.1
6047	1.19802	2.1
6048	0.974196	2.1
6049	1.09518	2.1
6050	0.946131	2.1
6051	1.7161	2.1
6052	1.7161	2.1
6053	0.94613	2.1
6054	1.297	2.1
6055	1.297	2.1
6056	1.14599	2.1
6057	0.974197	2.1
6058	0.974197	2.1
6059	1.80778	2.1
6060	2.20473	2.1
6061	1.80777	2.1
6062	1.3515	2.1
6063	0.583237	2.1
6064	1.39359	2.1
6065	1.39359	2.1
6066	1.07296	2.1
6067	0.946114	2.1
6068	1.07297	2.1
6069	0.903862	2.1
6070	0.956572	2.1
6071	1.03437	2.1
6072	0.975409	2.1
6073	1.31167	0.05
6074	2.70246	0.05
6075	1.12018	0.05
6076	0.655836	0.05
6077	0.655837	0.05
6078	1.81557	0.05
6079	1.81557	0.05
6080	0.655836	0.05
6081	0.946131	0.05
6082	1.01847	0.05
6083	1.61334	0.05
6084	0.810946	0.05
6085	0.8311	0.05
6086	1.2466	0.05
6087	1.20286	0.05

6088	0.97508	0.05
6089	0.870805	0.05
6090	0.760087	0.05
6091	0.853389	2.1
6092	0.820894	2.1
6093	0.928291	2.1
6094	1.06922	2.1
6095	1.32829	2.1
6096	1.12173	2.1
6097	1.16615	2.1
6098	0.957748	2.1
6099	0.612491	1.98748
6100	0.548141	2.1
6101	0.979614	2.1
6102	0.658184	2.1
6103	0.678456	1.64662
6104	0.529544	1.86893
6105	0.739013	2.1
6106	0.694037	2.1
6107	0.475716	1.9714
6108	1.58794	1.67815
6109	1.58794	0.973562
6110	0.475743	1.35925
6111	1.08301	2.1
6112	0.980664	2.1
6113	0.475737	2.04237
6114	1.32709	2.1
6115	1.59396	2.1
6116	1.19719	0.05
6117	1.34921	0.05
6118	1.69061	0.05
6119	1.69061	0.05
6120	0.946132	0.05
6121	1.34921	0.05
6122	1.28753	0.05
6123	0.95895	0.05
6124	1.01051	0.05
6125	1.12468	0.05
6126	1.22588	0.05
6127	1.34254	0.05
6128	1.74561	0.05
6129	1.74561	0.05
6130	0.946128	0.05
6131	1.34253	0.05
6132	1.3492	0.05
6133	1.01051	0.05
6134	1.922 2.1	
6135	1.922 2.1	
6136	2.12556	2.1
6137	0.946131	2.1
6138	1.06278	2.1
6139	1.47631	2.1
6140	1.47631	2.1
6141	0.946129	2.1
6142	1.06278	2.1
6143	1.35966	2.1
6144	0.991985	2.1
6145	0.738507	0.78112

6146	0.536935	1.99117
6147	0.799004	1.02121
6148	1.04281	0.560368
6149	0.879197	0.454372
6150	1.06538	0.434562
6151	0.715942	0.898434
6152	1.00636	0.148332
6153	1.03824	0.0737011
6154	1.70147	0.117649
6155	1.21223	2.1
6156	1.01243	2.1
6157	1.01242	2.1
6158	1.06935	2.1
6159	1.54241	2.1
6160	0.998971	0.05
6161	1.60312	0.05
6162	1.44836	0.05
6163	2.05632	0.05
6164	1.44836	0.05
6165	0.973765	0.05
6166	0.946131	0.05
6167	0.851541	0.05
6168	1.73854	0.05
6169	1.91752	0.05
6170	1.49622	0.05
6171	1.23351	0.05
6172	3.33289	2.1
6173	1.86606	2.1
6174	2.71233	2.1
6175	0.788816	2.1
6176	0.963972	2.1
6177	1.25866	2.1
6178	1.10817	2.1
6179	1.41908	2.1
6180	1.48734	2.1
6181	1.40822	0.0802108
6182	1.40822	0.0548128
6183	2.09871	0.114663
6184	0.946133	0.0745017
6185	1.14926	0.05
6186	0.946133	0.112636
6187	1.78098	0.05
6188	1.78098	0.05
6189	1.35822	0.05
6190	1.3689	0.05
6191	1.22176	0.05
6192	2.03426	0.0545541
6193	0.946131	0.0855476
6194	1.01713	0.05
6195	0.704112	0.104385
6196	1.36237	0.05
6197	1.36237	0.05
6198	1.01713	0.0565485
6199	1.04936	0.0613314
6200	1.87377	2.1
6201	1.60501	2.1
6202	0.981584	0.05
6203	1.69435	0.05

6204	0.85154	2.1
6205	1.47841	2.1
6206	1.4952	2.1
6207	1.31941	2.1
6208	1.04023	2.1
6209	1.1995	2.1
6210	0.914791	2.1
6211	1.1427	1.90377
6212	1.64787	1.34359
6213	1.07526	1.1312
6214	1.07527	0.210695
6215	1.07527	1.60045
6216	1.58551	0.247082
6217	2.77362	0.951419
6218	2.17974	0.24877
6219	2.71723	0.406287
6220	1.29868	0.05
6221	1.3594	0.05
6222	1.04786	0.05
6223	0.527876	0.05
6224	1.4085	0.05
6225	1.48538	0.05
6226	0.76829	0.05
6227	1.18514	0.05
6228	0.617954	0.05
6229	0.864161	0.05
6230	1.18835	0.05
6231	1.16817	2.1
6232	1.04263	2.1
6233	1.50524	2.1
6234	1.85355	2.1
6235	1.23963	2.1
6236	1.06279	2.1
6237	0.94613	2.1
6238	1.35229	2.1
6239	1.15947	2.1
6240	0.965593	2.1
6241	0.851553	2.1
6242	2.21296	2.1
6243	1.11764	2.1
6244	2.46399	2.1
6245	1.39292	2.1
6246	1.34701	2.1
6247	1.34701	2.1
6248	1.47001	2.1
6249	0.963166	0.05
6250	0.893445	0.05
6251	1.21056	0.05
6252	1.67375	2.1
6253	2.0977	2.1
6254	1.09154	2.1
6255	1.09154	2.1
6256	1.57122	0.849792
6257	1.60976	0.0638043
6258	1.69533	0.05
6259	1.6951	0.05
6260	1.5831	0.05
6261	1.20969	2.1

6262	1.12076	2.1
6263	1.49808	2.1
6264	1.50201	2.1
6265	1.00278	2.1
6266	1.22811	0.05
6267	1.11545	2.1
6268	1.09646	2.1
6269	1.03107	0.05
6270	1.44293	0.05
6271	1.66752	1.56594
6272	1.11023	1.52896
6273	2.57831	2.1
6274	2.20191	0.926206
6275	2.18051	2.1
6276	1.48035	0.181476
6277	1.0961	0.0835304
6278	1.63241	0.0714474
6279	1.0961	0.30585
6280	1.20959	2.1
6281	2.86136	2.1
6282	1.64933	2.1
6283	1.47217	2.1
6284	0.915178	2.1
6285	1.19074	2.1
6286	0.897276	2.1
6287	0.915177	2.1
6288	0.987037	2.1
6289	1.04288	2.1
6290	2.39323	2.1
6291	0.976218	0.05
6292	2.23933	0.05
6293	0.976215	0.05
6294	1.07384	0.05
6295	1.07652	2.1
6296	1.55678	2.1
6297	1.54168	2.1
6298	1.69136	2.1
6299	1.64391	1.58627
6300	1.76477	1.48762
6301	0.611486	1.80212
6302	0.683421	0.694993
6303	0.946131	1.46521
6304	1.1019	1.24331
6305	1.12946	1.02989
6306	1.54226	2.0326
6307	1.19496	1.30891
6308	1.96199	1.1122
6309	2.0775	1.74422
6310	1.22297	1.14589
6311	1.3469	0.884739
6312	1.25366	1.24054
6313	1.65443	1.36964
6314	1.65443	1.61499
6315	0.946132	1.59312
6316	0.946132	1.93534
6317	1.12873	1.59129
6318	1.07468	1.11997
6319	1.2854	0.250559

6320	1.3469	0.915241
6321	1.68616	0.847844
6322	1.68616	0.575362
6323	0.946132	1.63356
6324	0.946132	1.55923
6325	1.16109	1.16602
6326	1.17879	0.609269
6327	1.17879	0.895402
6328	1.45304	1.31436
6329	2.21058	2.1
6330	2.81552	2.1
6331	1.45535	0.05
6332	1.49906	0.05
6333	1.04413	2.1
6334	1.74586	2.1
6335	1.34526	2.1
6336	1.17402	0.657507
6337	1.74564	0.300459
6338	1.52724	2.1
6339	1.68142	2.1
6340	1.09651	2.1
6341	1.49149	2.1
6342	1.36814	2.1
6343	1.46783	1.92827
6344	2.18116	2.09567
6345	1.23602	1.30279
6346	1.74538	0.606584
6347	0.851539	2.1
6348	1.45775	2.05378
6349	1.28919	2.1
6350	0.851483	2.1
6351	1.26102	1.95598
6352	0.869889	2.1
6353	0.85151	2.1
6354	1.23451	0.644021
6355	1.23602	1.67646
6356	1.11955	2.1
6357	1.08022	2.1
6358	0.977776	2.1
6359	1.27896	2.1
6360	1.64268	2.1
6361	1.97548	2.1
6362	1.72527	2.1
6363	0.946132	2.1
6364	1.69702	2.1
6365	1.69702	2.1
6366	1.13635	2.1
6367	1.40274	1.90832
6368	1.24041	2.1
6369	1.13635	2.1
6370	1.78427	0.05
6371	1.68338	0.05
6372	1.00681	0.05
6373	1.12988	0.05
6374	0.946132	0.05
6375	0.957543	0.05
6376	1.40515	0.05
6377	1.40515	0.05

6378	0.797932	0.05
6379	1.30187	0.05
6380	1.14846	0.05
6381	0.665137	0.05
6382	1.09575	0.05
6383	0.596894	0.05
6384	1.04072	0.05
6385	0.663171	0.0530868
6386	1.47218	0.05
6387	1.32889	0.05
6388	1.32639	0.05
6389	1.94887	0.05
6390	1.32639	0.05
6391	1.19323	0.05
6392	2.19794	0.05
6393	0.754756	0.05
6394	1.11015	0.05
6395	0.754755	0.05
6396	0.946133	0.05
6397	0.754768	0.05
6398	1.59986	0.05
6399	1.59986	0.05
6400	1.84082	2.1
6401	1.79044	2.1
6402	1.75922	2.1
6403	1.86766	2.1
6404	1.58995	2.1
6405	1.58995	2.1
6406	1.74351	2.1
6407	1.04442	2.1
6408	1.42278	2.1
6409	1.08576	2.1
6410	0.994294	2.1
6411	1.44884	2.05074
6412	1.71036	1.17856
6413	0.714101	0.249471
6414	1.17541	1.81166
6415	1.00872	0.05
6416	0.714072	0.284829
6417	0.936677	0.106291
6418	0.966979	2.1
6419	1.4908	2.1
6420	1.03108	0.0745934
6421	0.949712	0.05
6422	0.810946	0.05
6423	0.88209	0.0559157
6424	0.646702	0.05
6425	0.972663	0.05
6426	0.862301	0.05
6427	0.868143	0.05
6428	1.11094	0.05
6429	1.35809	0.05
6430	1.32455	2.1
6431	1.32328	2.1
6432	1.69586	2.1
6433	1.78301	2.1
6434	1.67906	2.1
6435	1.60635	2.1

6436	1.57234	2.1
6437	1.47012	2.1
6438	1.22811	0.05
6439	1.08732	0.05
6440	2.08849	0.05
6441	1.08732	0.05
6442	1.30635	0.05
6443	0.77315	0.05
6444	1.25732	0.05
6445	2.43845	2.1
6446	2.38577	1.8386
6447	0.946132	0.636864
6448	1.19288	2.1
6449	1.54508	2.1
6450	1.54508	1.87415
6451	0.94515	2.1
6452	1.19288	2.04838
6453	1.29735	1.49847
6454	1.12087	0.336355
6455	1.54699	1.11993
6456	1.43635	2.1
6457	1.33385	2.1
6458	1.43635	2.1
6459	1.02368	2.1
6460	1.035 2.1	
6461	1.00128	2.1
6462	1.04009	2.1
6463	1.14052	2.1
6464	0.443552	2.1
6465	0.984351	2.1
6466	0.591443	2.1
6467	0.929561	2.1
6468	1.52079	2.1
6469	1.035 2.1	
6470	3.5282	2.1
6471	1.88288	2.1
6472	2.73877	0.886899
6473	0.906059	2.1
6474	1.23107	0.05
6475	0.906712	0.05
6476	0.841322	0.05
6477	1.53005	0.05
6478	1.10007	0.05
6479	1.38033	0.05
6480	1.03442	0.05
6481	0.787223	0.05
6482	1.42589	0.05
6483	2.35665	0.05
6484	1.17793	0.05
6485	1.81356	0.05
6486	1.42964	0.05
6487	1.95641	0.05
6488	1.39139	0.05
6489	1.39139	0.0925036
6490	0.564533	0.05
6491	1.20016	0.122504
6492	0.755765	0.05
6493	0.755766	0.05

6494	1.20016	0.05
6495	1.91011	0.38891
6496	1.4695	0.05
6497	1.70048	2.1
6498	1.93765	2.1
6499	0.741307	2.1
6500	0.780371	2.1
6501	0.946133	0.7158
6502	1.28918	1.1355
6503	1.5111	2.04626
6504	1.5111	0.554601
6505	0.946131	1.61857
6506	1.28918	0.757992
6507	1.16805	0.404495
6508	1.01179	0.154594
6509	0.946132	0.804464
6510	1.35328	1.13505
6511	1.69633	1.90303
6512	1.69633	0.676357
6513	0.946133	1.67862
6514	1.35328	0.832806
6515	1.28918	0.360815
6516	1.01179	1.18486
6517	1.58147	1.28841
6518	1.27737	0.250584
6519	2.17223	0.05
6520	0.697168	0.05
6521	1.62495	0.05
6522	1.62495	0.05
6523	1.93948	0.266881
6524	0.601592	2.1
6525	1.93528	2.1
6526	2.13757	2.1
6527	1.67561	2.1
6528	2.70875	2.1
6529	1.93528	2.1
6530	3.98475	2.1
6531	1.12376	2.1
6532	1.19016	2.1
6533	1.67323	0.05
6534	1.74019	0.05
6535	1.84719	0.05
6536	1.00764	0.05
6537	1.23475	2.1
6538	1.06757	2.1
6539	1.57916	2.1
6540	1.06758	2.1
6541	1.28014	2.1
6542	1.33339	2.1
6543	1.2679	2.1
6544	1.23504	2.1
6545	1.50108	2.1
6546	1.3715	2.1
6547	0.58831	0.81102
6548	0.865971	1.28266
6549	0.813704	1.31918
6550	0.658856	1.4551
6551	0.495869	1.1745

6552	1.13845	1.1184
6553	0.780804	1.46048
6554	0.642023	0.851361
6555	0.707748	0.989131
6556	1.64476	0.757419
6557	1.64476	1.17647
6558	0.99611	0.774932
6559	1.35639	1.03491
6560	1.3564	1.75174
6561	0.946129	1.82617
6562	1.99222	0.586404
6563	1.4777	2.1
6564	1.03968	2.1
6565	1.82516	2.1
6566	0.851539	2.1
6567	1.63693	2.1
6568	1.59416	2.1
6569	0.878566	2.1
6570	1.40796	2.1
6571	1.13172	2.1
6572	0.851527	1.29123
6573	1.14366	2.1
6574	0.823822	0.05
6575	0.882001	0.05
6576	1.09756	0.05
6577	1.11857	0.05
6578	1.0934	0.05
6579	0.960104	0.05
6580	0.982001	0.05
6581	1.74319	0.05
6582	1.03043	0.05
6583	0.967194	0.05
6584	1.16938	2.1
6585	2.48377	2.1
6586	1.1181	2.1
6587	2.57349	2.1
6588	1.0465	2.1
6589	1.44709	2.1
6590	0.946132	2.1
6591	1.29302	2.1
6592	1.73502	2.1
6593	1.73502	2.1
6594	0.946132	2.1
6595	1.29302	2.1
6596	1.38813	2.1
6597	1.03933	2.1
6598	1.01976	0.05
6599	1.11764	0.05
6600	0.961779	0.0590159
6601	1.0685	0.05
6602	1.08426	1.06366
6603	0.99695	1.94834
6604	2.25593	1.857
6605	1.47992	1.96753
6606	1.88989	1.36315
6607	1.65813	0.998749
6608	1.20278	1.66777
6609	0.981842	1.22961

6610	0.802655	2.1
6611	0.953489	2.1
6612	0.961719	2.1
6613	0.975614	2.1
6614	0.991617	1.30097
6615	0.940748	1.73686
6616	2.25019	2.1
6617	2.30712	1.75243
6618	1.98823	2.1
6619	0.522844	1.61167
6620	0.946122	2.1
6621	0.946132	1.96103
6622	0.593846	1.30286
6623	1.26501	1.45591
6624	1.21171	0.05
6625	0.851542	0.05
6626	0.940327	0.05
6627	0.940356	0.05
6628	1.52117	0.05
6629	1.58072	0.05
6630	1.35169	0.05
6631	1.13889	0.05
6632	1.35007	0.05
6633	1.35007	0.05
6634	1.78381	0.05
6635	0.946132	0.05
6636	1.06627	0.05
6637	0.946131	0.05
6638	1.73005	0.05
6639	1.73005	0.05
6640	1.32449	0.05
6641	1.35169	0.05
6642	1.42446	0.222961
6643	1.86697	0.230683
6644	1.13413	0.0598753
6645	1.42446	0.0585622
6646	1.33575	0.05
6647	1.20473	0.05
6648	1.87517	0.05
6649	1.87517	0.05
6650	0.488712	0.05
6651	1.13242	0.05
6652	0.488713	0.05
6653	0.488713	0.05
6654	1.23146	0.05
6655	1.57932	0.05
6656	1.01698	0.05
6657	1.42085	0.05
6658	0.246725	0.05
6659	0.996283	0.05
6660	1.36965	0.05
6661	0.86361	0.05
6662	0.810945	0.05
6663	1.72938	0.05
6664	1.21649	0.05
6665	1.1865	0.05
6666	1.62387	0.05
6667	1.05302	0.05

6668	1.10049	0.0547569
6669	0.946129	2.1
6670	1.70184	0.321933
6671	1.70184	2.1
6672	1.12538	2.1
6673	1.35969	0.189704
6674	1.28828	2.1
6675	1.12538	1.57132
6676	0.99747	1.57908
6677	1.34851	0.232702
6678	0.923724	2.1
6679	1.47154	0.523487
6680	1.47154	2.1
6681	1.10698	2.1
6682	1.28828	0.304556
6683	1.10698	2.1
6684	0.923695	2.1
6685	0.99747	0.0950295
6686	1.65677	2.1
6687	1.77677	2.1
6688	1.05689	0.05
6689	2.11031	0.05
6690	1.07367	0.05
6691	0.656239	0.05
6692	1.4563	0.05
6693	1.4563	0.05
6694	0.656238	0.05
6695	0.946133	0.05
6696	0.812937	0.05
6697	0.946131	0.05
6698	1.05689	0.05
6699	1.34009	2.1
6700	1.398 2.1	
6701	3.29047	2.1
6702	2.53897	2.1
6703	1.24509	2.1
6704	3.06162	2.1
6705	1.01595	2.1
6706	1.52661	2.1
6707	1.5266	2.1
6708	0.946131	2.1
6709	1.31049	2.1
6710	1.16225	2.1
6711	1.05978	2.1
6712	1.16225	2.1
6713	1.06561	2.1
6714	0.953927	2.1
6715	1.15114	2.1
6716	1.1807	0.05
6717	0.507259	0.05
6718	1.47709	0.05
6719	1.36192	0.05
6720	0.487293	0.05
6721	1.65569	0.05
6722	0.679725	0.05
6723	0.721928	0.05
6724	1.03931	0.05
6725	1.0775	0.05

6726	1.0775	0.05
6727	2.30207	0.204353
6728	1.01814	0.206456
6729	2.33963	0.129256
6730	2.42589	0.261312
6731	1.33962	2.1
6732	1.27956	2.1
6733	2.14624	0.05
6734	1.6812	0.05
6735	1.25698	0.05
6736	1.35725	0.05
6737	1.86387	0.05
6738	1.86387	0.05
6739	0.750365	0.05
6740	0.946128	0.05
6741	0.750365	0.05
6742	1.72271	0.05
6743	1.72272	0.05
6744	2.53736	0.05
6745	1.07327	2.1
6746	1.05376	2.1
6747	1.00387	0.434284
6748	2.0554	0.127841
6749	1.00387	0.382946
6750	1.00387	0.48814
6751	1.27119	2.1
6752	1.51748	2.1
6753	3.15271	2.1
6754	3.45801	2.1
6755	1.99871	2.1
6756	1.41186	2.1
6757	1.07624	2.1
6758	1.5954	2.1
6759	1.19323	0.315196
6760	1.21286	0.321329
6761	0.946114	2.1
6762	1.38395	2.1
6763	1.73389	2.1
6764	1.7339	2.1
6765	0.946115	2.1
6766	1.38397	2.1
6767	1.29604	2.1
6768	1.04544	2.1
6769	1.60578	0.05
6770	1.93407	0.05
6771	1.0525	0.448814
6772	1.0525	1.15967
6773	1.0525	1.95407
6774	1.38506	1.84705
6775	2.34737	0.05
6776	1.3531	0.05
6777	1.2925	0.05
6778	0.871466	0.05
6779	1.26623	0.05
6780	0.700631	0.05
6781	1.88774	0.05
6782	1.14167	0.05
6783	1.18606	0.05

6784	2.1238	0.05
6785	1.42762	0.312466
6786	1.67791	0.05
6787	1.47118	0.05
6788	0.946128	2.1
6789	1.31641	2.1
6790	1.75933	2.1
6791	1.75933	2.1
6792	0.946131	2.1
6793	1.31641	2.1
6794	1.38905	2.1
6795	1.38437	2.1
6796	1.51829	2.1
6797	0.947114	2.1
6798	1.13335	2.1
6799	1.47521	2.1
6800	1.14911	2.1
6801	1.53822	2.1
6802	1.39702	2.1
6803	0.739166	2.1
6804	0.706056	2.1
6805	0.964033	2.1
6806	1.58993	2.1
6807	2.51892	2.1
6808	0.595905	0.05
6809	1.18155	0.05
6810	1.73364	0.05
6811	0.952735	0.05
6812	2.10381	0.05
6813	1.12115	0.05
6814	1.27826	0.05
6815	2.74021	0.05
6816	1.78416	2.1
6817	1.78416	2.1
6818	0.946132	2.1
6819	1.41126	2.1
6820	1.21275	2.1
6821	0.946133	2.1
6822	1.41126	2.1
6823	1.33125	2.1
6824	1.33125	2.1
6825	1.78918	2.1
6826	1.0465	2.1
6827	1.99715	2.1
6828	1.17683	2.1
6829	1.25889	2.1
6830	0.860834	0.05
6831	0.946131	0.05
6832	1.05008	0.05
6833	0.970444	0.05
6834	1.19401	0.05
6835	1.25989	0.05
6836	0.702942	0.05
6837	1.40591	0.141658
6838	1.72168	0.0559051
6839	0.446352	0.05
6840	1.04072	0.05
6841	0.711038	0.068999

6842	0.980737	0.05
6843	1.05099	0.05
6844	1.26003	0.05
6845	0.495934	0.061879
6846	1.03114	0.05
6847	1.09911	0.05
6848	1.24825	0.05
6849	2.19827	0.05
6850	2.0953	0.05
6851	2.30437	0.05
6852	0.775923	0.05
6853	2.2283	0.05
6854	1.19583	2.1
6855	1.14496	2.1
6856	1.62183	2.1
6857	1.62316	2.1
6858	1.02305	0.08155
6859	1.21136	0.0517008
6860	1.44426	0.0617477
6861	1.21137	0.05
6862	1.20143	0.144175
6863	1.15009	0.143376
6864	1.24965	2.1
6865	1.41077	2.1
6866	1.00052	0.05
6867	1.79553	0.05
6868	1.06336	0.05
6869	1.00053	0.05
6870	1.18041	2.1
6871	1.80818	2.07228
6872	1.73571	1.53867
6873	1.94429	1.76792
6874	1.4375	0.05
6875	1.44421	0.05
6876	1.66953	0.05
6877	1.09529	0.05
6878	0.952395	2.1
6879	0.717337	2.1
6880	1.03609	2.1
6881	1.33949	0.05
6882	1.2774	0.0843387
6883	2.24983	0.0507939
6884	1.28727	0.05
6885	0.583592	0.0541879
6886	0.583591	0.0933447
6887	1.82859	0.0726015
6888	1.82859	0.0571861
6889	0.583593	0.0960906
6890	0.946128	0.053693
6891	0.692233	0.135329
6892	1.12226	0.0825355
6893	2.57454	0.0991639
6894	0.810944	0.0820281
6895	0.810945	0.108812
6896	1.12226	0.0763968
6897	1.45867	0.0744553
6898	1.22403	0.0686255
6899	1.15262	0.132343

6900	1.08132	0.0733317
6901	0.846671	0.107622
6902	0.480954	0.0862612
6903	0.917975	0.0776651
6904	0.917975	0.295821
6905	0.720957	0.0979147
6906	0.795998	0.081484
6907	0.69316	0.17405
6908	0.641306	0.124916
6909	0.690817	0.0547613
6910	0.961899	2.1
6911	2.49579	2.1
6912	2.31106	2.1
6913	1.70482	0.05
6914	1.93156	0.05
6915	1.23868	0.05
6916	1.73878	0.0506016
6917	1.26267	2.1
6918	1.26441	2.1
6919	1.58095	2.1
6920	1.58095	2.1
6921	0.946132	2.1
6922	0.946132	2.1
6923	1.11077	2.1
6924	1.62905	2.1
6925	1.49692	2.1
6926	3.49966	2.1
6927	2.39213	2.1
6928	2.12603	2.1
6929	0.946129	2.1
6930	0.461661	2.1
6931	1.52841	2.1
6932	1.39091	2.1
6933	0.550679	2.1
6934	0.509894	2.1
6935	1.49508	2.1
6936	2.05862	2.1
6937	2.47851	2.1
6938	1.07213	2.1
6939	1.09148	0.214077
6940	1.99776	0.117587
6941	1.38333	0.158322
6942	1.04412	2.1
6943	1.04412	2.1
6944	1.1893	0.05
6945	0.946132	0.05
6946	0.965737	0.05
6947	0.946132	0.05
6948	1.54061	0.05
6949	1.54061	0.05
6950	1.22878	0.05
6951	1.25797	0.05
6952	1.00665	0.05
6953	1.00665	0.05
6954	1.80369	0.05
6955	0.946128	0.05
6956	1.04424	0.05
6957	0.85154	0.05

6958	1.39322	0.05
6959	1.35464	0.05
6960	0.939857	0.05
6961	1.28272	0.05
6962	1.19323	0.05
6963	1.03498	0.05
6964	2.22029	0.05
6965	0.540005	0.05
6966	0.970382	0.05
6967	2.14514	0.05
6968	1.31542	0.05
6969	1.05575	0.05
6970	0.810943	0.05
6971	1.31542	0.05
6972	1.70493	0.05
6973	1.22582	0.05
6974	1.31681	0.05
6975	1.40775	0.05
6976	0.928633	0.05
6977	1.2545	0.05
6978	2.66481	0.05
6979	1.32051	0.05
6980	1.32051	0.05
6981	2.66305	2.1
6982	1.3551	2.1
6983	2.45325	2.1
6984	1.3685	2.1
6985	1.51748	2.1
6986	1.78941	2.1
6987	1.02963	0.05
6988	1.28643	0.05
6989	0.86131	0.05
6990	0.851539	0.05
6991	1.46585	0.05
6992	1.38822	0.05
6993	0.996402	0.05
6994	0.851553	0.05
6995	1.73156	0.05
6996	1.91695	0.05
6997	1.92535	0.05
6998	2.57287	0.05
6999	1.77743	0.05
7000	1.02836	0.05
7001	1.36362	0.05
7002	1.36936	0.05
7003	1.78684	0.05
7004	1.78685	0.05
7005	0.946132	0.05
7006	1.27581	0.05
7007	0.946132	0.05
7008	1.02836	0.05
7009	1.55398	2.1
7010	1.07167	2.1
7011	1.87557	2.1
7012	0.751639	2.1
7013	1.50884	2.1
7014	1.33793	2.1
7015	0.751664	2.1

7016	1.37277	2.1
7017	0.979515	2.1
7018	0.676508	2.1
7019	1.09578	2.1
7020	0.953925	2.1
7021	0.94613	2.1
7022	1.64707	2.1
7023	1.64707	2.1
7024	1.16658	2.1
7025	1.40638	2.1
7026	1.18682	2.1
7027	1.16658	2.1
7028	0.543765	0.05
7029	1.57462	0.05
7030	1.57462	0.05
7031	1.00083	0.05
7032	1.16834	0.05
7033	1.16834	0.05
7034	0.889626	0.05
7035	1.25944	0.05
7036	0.824061	0.05
7037	1.9397	0.05
7038	1.21893	0.05
7039	1.91364	0.05
7040	1.33672	0.05
7041	0.928069	0.05
7042	1.41484	0.05
7043	0.538379	0.05
7044	1.41484	0.05
7045	1.35594	0.05
7046	2.11414	0.05
7047	2.11414	0.05
7048	0.538378	0.05
7049	1.38532	0.05
7050	0.962877	0.0764088
7051	1.07497	0.05
7052	1.00613	0.05
7053	0.962878	0.05
7054	0.711447	2.1
7055	1.40008	0.05
7056	2.51674	0.05
7057	1.40008	0.05
7058	1.7263	0.05
7059	1.40008	0.05
7060	1.9216	0.05
7061	2.7013	0.05
7062	2.58584	0.05
7063	0.979296	0.05
7064	1.46459	0.05
7065	0.899254	0.05
7066	1.15038	2.1
7067	1.24687	2.1
7068	1.16738	2.1
7069	0.942323	2.1
7070	1.65573	1.50559
7071	0.844791	2.1
7072	0.54674	2.05097
7073	0.795811	2.1

7074	1.08131	2.1
7075	1.03553	2.1
7076	0.758334	2.1
7077	0.708665	2.00867
7078	1.22626	1.97086
7079	0.835439	1.96993
7080	0.861131	1.7422
7081	0.68296	2.09228
7082	1.65224	2.05588
7083	2.82624	2.1
7084	1.0151	1.2906
7085	1.15905	1.87578
7086	1.71076	1.56907
7087	1.4529	0.94235
7088	0.494895	2.1
7089	0.946132	1.91036
7090	0.613132	1.71582
7091	0.863631	1.90999
7092	1.78391	2.1
7093	1.1706	0.05
7094	2.02734	0.05
7095	1.1706	0.05
7096	0.681532	0.05
7097	1.50316	0.05
7098	1.50316	0.05
7099	0.681534	0.05
7100	0.973819	0.05
7101	0.97382	0.05
7102	0.94613	0.05
7103	1.1706	0.05
7104	1.67727	0.05
7105	1.60329	0.05
7106	1.3227	2.1
7107	1.40846	2.1
7108	1.07889	2.1
7109	1.07721	2.1
7110	0.953523	0.110461
7111	1.79675	0.155546
7112	2.09859	0.640602
7113	1.21309	2.1
7114	1.54567	0.28973
7115	1.19722	0.751406
7116	1.82793	0.807578
7117	1.1599	0.182261
7118	1.73621	0.180109
7119	0.933474	0.326546
7120	1.51477	0.229114
7121	1.51477	0.255952
7122	0.93347	0.207646
7123	1.27374	0.3409
7124	1.27376	0.501573
7125	0.946105	0.684987
7126	1.1972	0.211522
7127	0.851541	0.05
7128	1.36971	0.05
7129	1.27832	0.05
7130	0.853489	0.05
7131	0.973847	0.05

7132	0.976308	0.05
7133	0.851528	0.05
7134	2.72654	0.05
7135	1.77867	0.05
7136	3.23032	2.1
7137	2.78208	2.1
7138	1.0227	0.05
7139	1.02269	0.05
7140	1.02269	0.05
7141	1.24293	0.05
7142	0.831695	2.1
7143	0.67513	2.1
7144	0.956294	2.1
7145	1.08623	2.1
7146	1.05556	2.1
7147	1.38859	2.1
7148	1.13179	2.1
7149	1.02438	2.1
7150	1.06659	2.1
7151	1.36595	2.1
7152	1.06659	2.1
7153	1.06659	0.222206
7154	0.946131	1.16505
7155	0.875912	2.1
7156	1.65971	2.1
7157	1.65971	2.1
7158	0.875912	2.1
7159	1.1229	2.1
7160	1.41273	2.1
7161	1.54699	1.91448
7162	1.93655	1.76979
7163	1.42307	1.15497
7164	1.97907	1.70117
7165	0.996774	2.1
7166	1.15505	0.05
7167	1.28463	0.05
7168	1.86353	0.05
7169	2.71398	0.05
7170	1.56787	0.05
7171	1.66295	0.05
7172	1.73432	1.23336
7173	1.05019	0.05
7174	1.42117	0.05
7175	0.888299	0.05
7176	1.38127	0.05
7177	1.72993	0.05
7178	0.664129	0.05
7179	0.99302	0.591967
7180	0.993023	0.421475
7181	1.13491	2.1
7182	0.876316	2.1
7183	1.2588	2.1
7184	0.895613	2.1
7185	2.07526	2.1
7186	1.23369	2.1
7187	1.25146	2.1
7188	2.15282	2.1
7189	1.22239	2.1

7190	1.10962	2.1
7191	1.77402	2.1
7192	1.0305	2.1
7193	1.50108	1.68751
7194	1.20761	2.1
7195	1.3626	2.1
7196	1.14825	2.1
7197	1.78711	2.1
7198	1.17933	2.1
7199	1.14824	2.1
7200	1.09651	0.05
7201	1.23851	0.05
7202	1.83284	0.05
7203	1.23851	0.05
7204	1.53521	2.1
7205	1.46279	2.1
7206	1.9233	0.05
7207	1.2966	0.05
7208	2.11443	0.05
7209	1.30265	0.05
7210	1.13528	0.05
7211	0.946131	0.05
7212	1.41169	0.05
7213	1.33879	0.05
7214	0.946128	0.05
7215	1.80435	0.05
7216	1.80435	0.05
7217	1.234 2.1	
7218	1.45633	2.1
7219	1.234 2.1	
7220	1.98448	2.1
7221	2.02612	2.1
7222	1.23527	0.759922
7223	1.44472	0.0975464
7224	0.993366	0.238738
7225	1.22025	0.143382
7226	0.993368	0.35375
7227	2.43669	2.1
7228	2.56707	2.1
7229	1.80348	1.11339
7230	1.74406	0.392471
7231	1.90937	1.07494
7232	1.49357	0.05
7233	2.5832	0.170541
7234	1.82274	0.123393
7235	1.6957	0.088389
7236	2.22495	0.05
7237	2.49959	0.0858773
7238	2.01183	0.05
7239	1.37427	0.05
7240	1.15069	2.01201
7241	1.39379	1.05954
7242	1.69661	0.05
7243	1.39379	0.05
7244	1.39654	2.1
7245	1.93505	2.1
7246	1.5525	2.1
7247	1.5525	2.1

7248	0.615065	2.1
7249	1.38908	2.1
7250	0.615065	2.1
7251	0.77849	2.1
7252	1.38908	2.1
7253	1.56839	1.33439
7254	1.09142	1.9007
7255	2.07313	1.17196
7256	0.998288	0.05
7257	1.31481	0.05
7258	1.88175	0.05
7259	2.23267	0.05
7260	1.06935	2.1
7261	1.06936	2.1
7262	1.17989	2.1
7263	1.00757	1.5231
7264	1.65318	1.64478
7265	1.4185	0.05
7266	2.22495	0.05
7267	1.02011	0.05
7268	0.59676	0.05
7269	1.43606	0.05
7270	1.43606	0.05
7271	0.89771	0.05
7272	1.13511	0.05
7273	0.59676	0.05
7274	0.897709	0.05
7275	1.79542	0.05
7276	2.38566	2.1
7277	1.63838	0.829939
7278	1.63837	1.69403
7279	0.916275	2.1
7280	1.36183	1.20608
7281	1.19282	2.1
7282	0.916275	2.06707
7283	1.19283	2.1
7284	2.01582	2.1
7285	1.00215	0.05
7286	2.60643	2.1
7287	2.15971	2.1
7288	1.28267	2.1
7289	1.29954	2.1
7290	3.40327	2.1
7291	3.49128	2.1
7292	1.3864	2.1
7293	1.3201	2.1
7294	1.45666	0.05
7295	1.55798	0.05
7296	0.857202	0.05
7297	1.24374	0.05
7298	1.04157	0.05
7299	0.85723	0.05
7300	1.02722	0.05
7301	1.01361	0.05
7302	0.974077	0.05
7303	1.01814	0.05
7304	0.974077	0.05
7305	1.43987	0.05

7306	1.43986	0.05
7307	0.927881	0.05
7308	0.946132	0.05
7309	0.927849	0.05
7310	0.927852	0.05
7311	1.2493	0.05
7312	1.22482	0.106561
7313	1.41319	0.261684
7314	1.22482	0.0727982
7315	0.999492	2.1
7316	1.14657	2.1
7317	1.3617	0.05
7318	0.983269	0.05
7319	1.33174	0.05
7320	1.62816	0.05
7321	1.62817	0.05
7322	0.81938	0.05
7323	1.33174	0.05
7324	1.01332	0.05
7325	0.874788	0.05
7326	1.16725	2.1
7327	1.63991	2.1
7328	1.08848	0.05
7329	1.15354	0.05
7330	1.12665	2.1
7331	1.58742	2.1
7332	1.57523	2.1
7333	1.32201	0.494005
7334	1.32097	0.384192
7335	0.851542	0.05
7336	0.877188	0.05
7337	0.797489	0.0574941
7338	1.44721	0.05
7339	1.61761	0.05
7340	0.943799	0.05
7341	1.36079	0.05
7342	1.16361	2.1
7343	1.15852	1.80976
7344	1.07624	2.1
7345	1.07624	2.1
7346	0.946132	0.763802
7347	1.37938	1.03456
7348	1.78653	1.73655
7349	1.78653	0.65507
7350	0.946131	1.66769
7351	1.37938	0.923076
7352	1.35328	0.432933
7353	1.44847	0.252942
7354	0.946133	0.878686
7355	1.38149	0.96074
7356	1.81474	1.31104
7357	1.81474	0.62682
7358	0.946133	1.653
7359	1.38149	0.916905
7360	1.37938	0.557081
7361	1.44847	1.0919
7362	1.93665	0.699662
7363	1.5515	0.270508

7364	1.05025	2.1
7365	1.05025	2.1
7366	1.71468	2.1
7367	1.04759	2.1
7368	1.04646	0.05
7369	1.00739	0.05
7370	1.37086	0.05
7371	1.12547	0.05
7372	1.03853	0.05
7373	1.34824	0.05
7374	0.799786	0.05
7375	2.10105	0.05
7376	2.72007	0.05
7377	0.589918	0.05
7378	0.589918	0.05
7379	1.1509	0.05
7380	0.619832	0.05
7381	1.1509	0.05
7382	0.705903	0.05
7383	1.53703	0.05
7384	1.53703	0.05
7385	0.705904	0.05
7386	0.946133	0.05
7387	0.946132	0.05
7388	0.946132	0.05
7389	0.588467	0.05
7390	1.1078	0.05
7391	0.408061	0.05
7392	1.38703	0.05
7393	1.68328	0.05
7394	1.87031	0.05
7395	1.86268	0.05
7396	1.27472	0.05
7397	0.947862	0.577971
7398	1.19854	0.928718
7399	0.946129	0.449434
7400	0.963111	0.18391
7401	0.946131	0.527792
7402	1.39055	0.14706
7403	1.39055	0.143668
7404	1.26426	0.239506
7405	1.07242	0.25025
7406	1.31126	0.155259
7407	1.76597	0.489742
7408	0.94613	1.24507
7409	1.27214	0.644554
7410	1.90751	0.573083
7411	1.90751	0.395246
7412	0.767114	1.08532
7413	1.27214	1.06631
7414	1.40248	0.926373
7415	1.41479	0.857662
7416	2.22625	1.03817
7417	0.913373	1.28679
7418	1.0158	0.464697
7419	1.61259	0.350298
7420	1.69051	0.163953
7421	0.560949	1.10716

7422	0.898559	1.15295
7423	1.28011	0.890798
7424	1.51026	0.05
7425	1.98252	0.05
7426	1.05129	0.05
7427	1.2951	0.05
7428	1.46041	0.05
7429	1.7155	2.1
7430	1.72196	2.1
7431	0.933885	2.1
7432	0.986723	2.1
7433	1.28072	0.05
7434	1.40765	1.70799
7435	1.28072	0.359593
7436	1.61687	2.1
7437	1.60965	2.1
7438	1.21424	2.1
7439	2.07297	2.1
7440	1.19943	2.1
7441	2.38784	2.1
7442	1.10278	0.05
7443	1.43678	0.05
7444	1.1142	0.05
7445	1.18034	0.05
7446	0.94613	0.05
7447	0.752356	0.05
7448	0.752329	0.05
7449	1.46634	0.05
7450	1.31789	0.05
7451	0.958159	0.05
7452	1.22752	0.05
7453	1.50472	0.05
7454	1.98974	2.1
7455	2.54186	2.1
7456	0.76512	2.1
7457	2.08777	2.1
7458	1.7753	2.1
7459	3.07372	2.1
7460	1.9756	2.1
7461	1.48162	2.1
7462	1.23204	2.1
7463	0.946131	0.814833
7464	1.13505	2.1
7465	1.47432	2.1
7466	1.47432	1.56227
7467	0.876831	2.1
7468	1.13505	1.90588
7469	1.2161	1.27436
7470	1.05267	1.78683
7471	1.05267	0.704652
7472	1.5795	1.15743
7473	0.98186	2.1
7474	1.56735	2.1
7475	0.859099	2.1
7476	1.69858	2.1
7477	1.93586	2.1
7478	1.09233	2.1
7479	1.08732	0.05

7480	1.3357	0.05
7481	1.84826	2.1
7482	0.924132	2.1
7483	1.39188	2.1
7484	1.43685	2.1
7485	0.851541	2.1
7486	1.16251	2.1
7487	0.946131	2.1
7488	1.05685	2.1
7489	2.13781	2.1
7490	1.98859	2.1
7491	1.99731	2.1
7492	1.51379	2.1
7493	1.48796	2.1
7494	0.851542	2.1
7495	0.802558	2.1
7496	1.09244	2.1
7497	1.33891	2.1
7498	0.844958	2.1
7499	0.710864	2.1
7500	1.26087	2.1
7501	1.03679	2.1
7502	1.51026	0.05
7503	1.05248	0.05
7504	0.827258	0.05
7505	1.05248	0.05
7506	1.28435	0.05
7507	1.50953	0.05
7508	1.50953	0.05
7509	0.827298	0.05
7510	1.00366	0.05
7511	2.10495	0.05
7512	1.17885	2.1
7513	1.25185	2.1
7514	1.02784	2.1
7515	1.76764	2.1
7516	1.23746	0.05
7517	1.24082	0.05
7518	1.46506	0.05
7519	1.33061	0.05
7520	0.768407	1.62404
7521	0.972058	0.645848
7522	1.16536	2.1
7523	1.13325	1.42538
7524	0.978906	2.1
7525	1.02291	1.12355
7526	0.838015	0.763336
7527	1.01052	0.05
7528	1.08166	0.05
7529	1.42607	0.05
7530	1.47354	0.05
7531	1.21703	2.1
7532	1.29897	2.1
7533	1.83293	2.1
7534	1.29897	2.1
7535	1.24688	0.05
7536	0.782563	2.1
7537	0.946129	2.1

7538	0.782591	2.1
7539	1.49487	2.1
7540	1.49487	2.1
7541	1.37713	2.1
7542	0.900337	2.1
7543	1.31284	2.1
7544	0.924468	2.1
7545	1.40602	2.1
7546	1.25669	2.1
7547	0.986901	2.1
7548	0.958565	2.1
7549	1.37266	2.1
7550	1.64146	2.1
7551	0.49345	2.1
7552	1.08276	2.1
7553	1.08276	2.1
7554	1.26929	2.1
7555	1.09847	2.1
7556	0.827081	0.05
7557	1.07236	0.0702833
7558	1.25063	0.05
7559	1.43548	0.05
7560	1.25063	0.05
7561	1.3659	0.05
7562	1.72441	0.05
7563	1.3659	0.05
7564	1.4677	0.05
7565	1.86114	0.05
7566	1.40104	0.05
7567	0.962675	0.05
7568	0.946131	0.05
7569	1.74134	0.05
7570	1.74134	0.05
7571	0.962676	0.05
7572	0.946131	0.05
7573	1.05548	0.05
7574	0.902187	0.05
7575	2.14178	0.05
7576	1.74998	0.05
7577	1.38541	0.05
7578	1.64468	0.05
7579	0.949317	0.05
7580	2.06654	0.05
7581	0.839626	0.05
7582	0.810945	0.05
7583	1.29203	0.05
7584	1.18091	0.05
7585	1.06872	0.05
7586	1.11956	0.05
7587	0.926911	0.05
7588	0.901716	0.05
7589	0.909146	0.05
7590	1.33776	0.05
7591	1.02048	0.05
7592	1.08365	0.05
7593	1.21042	0.05
7594	0.865634	0.05
7595	0.590354	2.1

7596	0.61173	2.1
7597	3.46825	2.1
7598	3.28404	2.1
7599	1.56004	0.05
7600	1.69344	0.05
7601	1.7151	0.112698
7602	0.946131	0.05
7603	1.40265	0.05
7604	1.13537	0.05
7605	0.965708	0.05
7606	1.13537	0.0692185
7607	1.29681	0.05
7608	1.40915	0.05
7609	1.61652	0.122925
7610	1.33171	0.141535
7611	1.37681	2.1
7612	0.81094	0.05
7613	0.598606	0.05
7614	0.723111	0.05
7615	1.04562	0.05
7616	0.979052	0.05
7617	0.798185	0.05
7618	0.880117	0.05
7619	0.979341	0.0602635
7620	0.800229	0.05
7621	1.39126	0.0763332
7622	1.13005	0.117065
7623	0.913877	0.0791492
7624	1.26959	0.05
7625	0.699741	0.0975694
7626	1.19496	1.29391
7627	1.13392	0.05
7628	1.11913	0.05
7629	1.06866	0.05
7630	0.615546	0.05
7631	0.831006	0.05
7632	1.53286	0.05
7633	0.690977	0.05
7634	1.01956	0.05
7635	2.33818	0.05
7636	1.6585	2.1
7637	1.67061	2.1
7638	1.41414	2.1
7639	1.16756	2.1
7640	1.00898	0.05
7641	0.966964	2.1
7642	2.85443	2.1
7643	0.966977	2.1
7644	2.08427	2.1
7645	2.94901	2.1
7646	1.10735	0.05
7647	1.65815	2.1
7648	1.4502	2.1
7649	1.45026	2.1
7650	1.53446	2.1
7651	0.946129	0.316496
7652	1.81075	2.1
7653	1.81075	0.210935

7654	0.946131	1.74227
7655	1.37075	2.1
7656	1.37075	0.302927
7657	1.34245	1.97094
7658	0.973502	0.345943
7659	1.08542	2.1
7660	1.5814	0.05
7661	1.43836	0.05
7662	0.946132	0.05
7663	1.13747	0.05
7664	1.04429	0.05
7665	1.05017	0.05
7666	1.24732	0.05
7667	1.13387	0.05
7668	2.19489	0.05
7669	1.10748	2.1
7670	1.25006	2.1
7671	1.58834	2.1
7672	1.58834	0.05
7673	0.94613	2.1
7674	1.16548	2.1
7675	0.946132	0.05
7676	1.08998	0.657508
7677	1.16548	0.0984178
7678	0.995732	0.05
7679	0.657014	2.1
7680	0.957173	2.1
7681	1.01485	2.1
7682	1.12468	2.1
7683	1.43871	2.1
7684	1.23716	2.1
7685	1.89277	0.201553
7686	2.06653	0.0797484
7687	0.967544	0.05
7688	1.89016	0.0507232
7689	2.22165	2.09647
7690	0.83514	2.1
7691	1.76565	2.1
7692	1.76565	2.1
7693	0.94613	2.01761
7694	0.835112	2.1
7695	1.23426	2.07491
7696	1.36653	2.1
7697	0.895892	0.329194
7698	0.859278	0.31147
7699	0.791461	0.623055
7700	1.86249	1.25351
7701	1.14607	0.579025
7702	1.19904	1.14373
7703	1.84898	0.708627
7704	1.03452	1.09429
7705	0.946131	0.05
7706	0.940472	0.05
7707	1.53478	0.411578
7708	1.84175	0.109559
7709	1.55687	0.0729535
7710	1.17787	0.05
7711	1.60589	0.223794

7712	2.35574	0.05
7713	1.3305	0.05
7714	1.15008	0.162677
7715	1.12457	0.05
7716	1.12457	0.05
7717	1.01778	2.1
7718	1.35733	2.1
7719	1.50683	2.1
7720	1.12064	2.1
7721	1.42716	0.209398
7722	1.42717	0.330213
7723	1.45927	0.765821
7724	1.04321	1.7607
7725	1.32634	0.05
7726	2.27022	0.05
7727	1.19352	0.05
7728	1.32634	0.05
7729	1.40924	2.1
7730	1.22927	1.4603
7731	1.69237	1.95928
7732	1.69237	2.1
7733	0.946132	0.741243
7734	0.946133	1.81967
7735	1.16912	1.62703
7736	1.0417	1.46473
7737	1.14605	2.00918
7738	1.1233	2.1
7739	1.96882	1.52695
7740	1.03804	0.05
7741	1.15338	0.05
7742	1.15338	0.05
7743	2.33051	0.05
7744	1.81507	2.06713
7745	2.42949	2.1
7746	0.725879	0.149415
7747	1.88394	0.273044
7748	2.2699	0.05
7749	2.78952	2.1
7750	1.88394	0.05
7751	1.79606	2.1
7752	1.20091	2.1
7753	1.9614	2.1
7754	1.19901	2.1
7755	3.07915	2.1
7756	1.32592	2.1
7757	2.86461	2.1
7758	1.34817	2.1
7759	1.44859	2.1
7760	2.03969	2.1
7761	0.946132	2.1
7762	1.50709	2.1
7763	1.50709	2.1
7764	0.946132	2.1
7765	1.29322	2.1
7766	1.29322	2.1
7767	1.01985	2.1
7768	0.968537	2.1
7769	1.401 2.1	

7770	1.28936	2.1
7771	1.56002	0.05
7772	1.92548	0.05
7773	1.46041	0.05
7774	2.22712	0.05
7775	1.56001	0.05
7776	1.50524	2.1
7777	1.21304	2.1
7778	1.60665	1.95232
7779	1.85178	0.964291
7780	2.17534	2.1
7781	2.31012	2.1
7782	2.17533	1.89503
7783	0.986724	2.1
7784	2.01895	2.1
7785	2.00433	2.1
7786	1.45752	0.05
7787	1.12695	0.05
7788	0.652605	2.1
7789	1.5133	2.1
7790	1.5133	2.1
7791	0.652603	2.1
7792	1.228	2.1
7793	0.946132	2.1
7794	0.652604	2.1
7795	0.946132	1.19165
7796	0.716815	2.1
7797	1.62203	2.1
7798	1.62203	2.1
7799	0.716814	2.1
7800	1.00191	2.1
7801	1.33694	2.1
7802	1.38216	2.1
7803	2.53338	2.1
7804	1.46932	0.05
7805	1.50848	0.05
7806	1.50108	1.44249
7807	1.18382	0.210985
7808	2.02115	0.106372
7809	0.921823	0.05
7810	2.08356	0.05
7811	1.543	0.05
7812	1.00763	0.170007
7813	1.4185	0.231033
7814	1.08028	0.105018
7815	0.946131	0.712459
7816	1.56599	0.191576
7817	1.56599	0.663868
7818	1.08028	0.292194
7819	0.946131	0.0848698
7820	1.37518	2.1
7821	1.63453	2.1
7822	1.04627	0.708231
7823	1.04626	0.475264
7824	1.35111	1.04536
7825	1.22297	0.769793
7826	1.64499	2.1
7827	1.87598	2.07512

7828	1.06994	2.1
7829	1.37063	2.1
7830	1.99943	0.05
7831	2.38807	0.05
7832	0.99972	0.05
7833	1.41388	0.05
7834	1.66179	0.05
7835	1.66179	0.05
7836	0.946129	0.05
7837	1.41388	0.05
7838	1.19404	0.05
7839	1.31023	0.29275
7840	1.20871	0.666927
7841	1.69731	2.1
7842	1.67971	2.1
7843	0.998971	0.05
7844	1.32988	0.05
7845	0.701079	0.05
7846	0.946131	0.05
7847	0.582785	0.05
7848	1.48592	0.05
7849	1.48592	0.05
7850	1.23351	0.05
7851	0.835199	0.05
7852	0.92427	0.05
7853	1.68262	0.05
7854	2.46702	0.05
7855	1.08715	2.06898
7856	1.01606	1.6812
7857	1.29579	1.72572
7858	1.01606	2.04094
7859	1.63823	2.1
7860	1.67687	2.1
7861	1.11048	2.1
7862	1.39477	2.1
7863	0.991114	2.1
7864	2.90978	0.367797
7865	0.906811	0.988451
7866	0.872849	0.0501124
7867	1.17091	0.190991
7868	0.685763	0.30595
7869	1.6386	2.02134
7870	1.06907	0.559193
7871	1.05516	0.785707
7872	1.9189	0.757895
7873	1.04615	0.196688
7874	1.2966	2.1
7875	1.37392	2.1
7876	1.35504	2.1
7877	1.30347	2.1
7878	1.50877	2.1
7879	1.11193	2.1
7880	1.40984	2.1
7881	1.67682	2.1
7882	1.00371	2.1
7883	1.15412	2.1
7884	1.41638	2.1
7885	1.03796	2.1

7886	0.946117	2.1
7887	1.50823	2.1
7888	1.50822	2.1
7889	0.966923	2.1
7890	0.946131	2.1
7891	2.07593	2.1
7892	1.9339	2.1
7893	1.1817	0.338858
7894	0.729178	0.731097
7895	1.5079	0.746454
7896	1.26991	0.840138
7897	0.994049	0.47146
7898	1.17452	0.946057
7899	1.05711	1.20643
7900	1.04071	1.30852
7901	1.80299	0.453195
7902	1.60417	0.35579
7903	0.943405	0.05
7904	1.01129	0.05
7905	1.42972	0.05
7906	1.15904	0.05
7907	0.680409	0.05
7908	1.2816	0.05
7909	0.806176	0.05
7910	1.18954	0.05
7911	1.26055	0.05
7912	1.06313	2.1
7913	1.19352	2.1
7914	1.55766	2.1
7915	1.53729	2.1
7916	0.993021	0.05
7917	1.09056	0.124832
7918	1.03408	2.1
7919	0.993023	0.0957499
7920	0.659901	2.1
7921	0.632905	2.1
7922	1.34072	0.05
7923	1.17793	0.05
7924	2.24885	0.05
7925	0.946132	0.05
7926	1.12443	0.05
7927	0.904207	0.05
7928	1.51237	0.05
7929	1.51237	0.05
7930	1.12443	0.05
7931	1.29215	0.05
7932	1.6943	2.1
7933	2.43858	2.1
7934	2.0589	1.95676
7935	1.67204	2.1
7936	1.67204	2.1
7937	0.555875	2.1
7938	0.91951	2.1
7939	0.946131	1.66576
7940	0.555904	2.1
7941	0.946131	2.07892
7942	1.6943	2.1
7943	1.04744	1.93709

7944	1.50938	2.1
7945	1.60861	2.1
7946	1.92406	0.05
7947	1.18716	0.05
7948	0.962705	0.0562839
7949	1.35382	0.546255
7950	1.43632	0.05
7951	1.09386	0.05
7952	1.92547	0.05
7953	0.994164	0.05
7954	0.903974	2.1
7955	0.928829	1.79608
7956	0.810958	1.24322
7957	1.92853	1.31143
7958	1.598	1.45045
7959	1.28967	1.70527
7960	1.44993	1.53295
7961	0.946132	0.605194
7962	0.882984	0.452073
7963	0.882955	0.777415
7964	1.56071	0.108547
7965	1.56071	0.165539
7966	1.35946	0.334102
7967	1.08421	0.37512
7968	1.41943	0.05
7969	1.26265	0.05
7970	1.03235	0.0502618
7971	1.50778	2.1
7972	1.70387	2.1
7973	1.65608	2.1
7974	1.39076	2.1
7975	1.6104	2.1
7976	1.37456	2.1
7977	1.51898	0.05
7978	1.9726	0.05
7979	2.13757	0.860384
7980	1.74856	0.96414
7981	0.635949	0.173342
7982	2.46448	0.805342
7983	1.23224	0.713713
7984	1.12786	1.13199
7985	1.82721	0.533329
7986	1.82721	0.144397
7987	0.532892	2.03827
7988	0.946131	1.2411
7989	0.53292	1.21058
7990	1.41394	2.1
7991	1.45506	2.1
7992	1.39523	2.1
7993	1.09006	2.1
7994	1.80264	0.05
7995	1.80264	0.0819235
7996	0.946131	0.05
7997	1.34268	0.05
7998	0.946132	0.05
7999	1.23732	0.05
8000	1.34268	0.05
8001	1.2064	0.312243

8002	1.58164	2.1
8003	1.73853	2.1
8004	1.01243	1.02373
8005	0.910672	1.7742
8006	1.47793	2.07598
8007	1.47793	2.1
8008	0.910702	1.69602
8009	1.30382	1.10133
8010	1.08478	1.12163
8011	0.910702	2.1
8012	2.60709	1.17742
8013	1.01243	1.13554
8014	1.85516	1.48876
8015	2.52823	0.160116
8016	1.24001	0.212036
8017	1.89793	0.35357
8018	1.73584	0.05
8019	1.24613	2.1
8020	1.37074	1.67521
8021	1.23763	1.28696
8022	1.04072	2.1
8023	0.75499	1.13661
8024	1.72329	1.44986
8025	1.46195	1.07383
8026	0.946132	1.26107
8027	0.747438	2.02184
8028	1.24613	1.60268
8029	2.59886	0.933522
8030	1.87279	2.1
8031	1.07977	2.1
8032	1.29606	2.1
8033	1.45178	2.1
8034	1.45177	2.1
8035	0.92406	2.1
8036	0.946131	2.1
8037	0.924047	2.1
8038	2.04529	2.1
8039	1.04544	2.1
8040	1.08276	2.1
8041	1.67491	2.1
8042	0.961826	1.12308
8043	0.810942	0.770005
8044	0.826622	1.6756
8045	0.914378	1.78397
8046	0.89022	0.342666
8047	0.922383	1.01328
8048	0.988165	0.299327
8049	1.31357	0.05
8050	0.946131	0.05
8051	1.31357	0.05
8052	1.10564	0.05
8053	1.6797	0.05
8054	1.6797	0.05
8055	0.739506	0.05
8056	1.33957	0.05
8057	1.33957	0.05
8058	1.33957	0.05
8059	2.34223	0.05

8060	1.5515	0.815343
8061	2.22137	0.547615
8062	0.94612	0.600093
8063	1.13908	0.45069
8064	1.45423	0.279876
8065	1.45422	0.823497
8066	0.795519	1.16495
8067	1.1391	0.497497
8068	1.11065	0.661134
8069	1.12266	2.1
8070	1.24925	2.1
8071	1.20242	2.09668
8072	2.18297	2.1
8073	3.00107	1.88657
8074	2.58297	0.0794127
8075	0.884783	2.1
8076	0.851538	0.392661
8077	0.713587	2.1
8078	1.47949	0.518043
8079	1.66673	2.1
8080	0.696199	2.1
8081	0.9383	1.53506
8082	1.29149	0.813159
8083	1.32266	1.866
8084	2.11428	0.05
8085	2.17439	0.05
8086	1.40489	0.05
8087	1.21071	0.05
8088	0.946118	0.05
8089	1.0746	0.05
8090	0.973119	0.05
8091	1.31333	0.05
8092	1.08723	0.05
8093	0.994292	2.1
8094	1.4964	2.1
8095	1.15096	2.1
8096	1.10101	2.1
8097	1.50438	2.1
8098	1.67825	2.1
8099	2.02103	2.1
8100	2.01978	2.1
8101	1.50931	0.05
8102	1.50931	0.05
8103	0.65023	0.05
8104	1.34831	0.05
8105	0.811233	0.05
8106	1.34831	0.05
8107	0.94613	0.05
8108	1.00754	1.03051
8109	0.714132	2.1
8110	1.28877	1.51238
8111	1.39905	1.36446
8112	1.03282	1.61315
8113	0.997769	0.923301
8114	1.11743	1.41837
8115	1.86863	1.07591
8116	3.30723	1.73458
8117	1.66635	2.1

8118	1.35609	0.05
8119	1.38212	0.05
8120	1.95597	2.1
8121	1.64257	2.1
8122	2.93316	2.1
8123	3.13417	2.1
8124	0.733947	2.1
8125	1.15259	2.1
8126	1.07976	2.1
8127	1.30237	2.1
8128	1.99943	0.05
8129	0.907431	0.05
8130	0.878161	0.05
8131	1.07544	0.05
8132	1.03401	0.05
8133	0.991975	0.05
8134	1.03635	0.05
8135	0.788549	0.05
8136	1.44984	0.05
8137	1.84007	0.05
8138	2.31127	0.05
8139	1.27362	2.1
8140	1.27737	1.19863
8141	1.87783	0.796447
8142	2.35499	0.05
8143	2.69662	0.05
8144	2.24065	0.05
8145	0.996082	2.1
8146	0.996083	2.1
8147	1.44859	2.1
8148	1.03968	2.09983
8149	1.60018	0.217384
8150	1.73082	1.23161
8151	0.946132	1.12689
8152	1.49742	1.19004
8153	1.49742	0.05
8154	1.00501	0.321543
8155	1.41585	1.97803
8156	1.41585	0.325411
8157	0.946131	1.44988
8158	1.03697	0.190972
8159	2.95786	2.1
8160	2.32185	2.1
8161	1.65783	0.05
8162	1.772 2.1	
8163	0.946131	0.05
8164	1.40404	0.05
8165	1.13537	2.1
8166	1.04559	0.166043
8167	1.16091	0.137848
8168	2.63751	2.1
8169	1.68455	0.05
8170	1.9682	0.05
8171	2.16834	0.0559528
8172	2.45569	0.0957578
8173	0.946128	0.05
8174	1.36428	0.101725
8175	0.663978	0.112039

8176	1.58218	0.05
8177	1.58219	0.05
8178	1.36428	0.0634456
8179	0.881886	0.0745383
8180	1.73849	2.1
8181	1.35668	2.1
8182	1.1233	2.1
8183	1.15009	2.1
8184	1.16482	0.192251
8185	1.29313	0.277746
8186	1.47608	0.0546496
8187	2.05949	0.05
8188	0.946119	0.05
8189	1.02974	0.05
8190	0.946119	0.05
8191	1.66758	0.05
8192	1.66757	0.05
8193	1.32082	0.05
8194	1.29287	0.05
8195	1.36005	2.1
8196	1.47109	2.1
8197	2.45958	2.1
8198	2.2975	2.1
8199	0.953521	0.273371
8200	0.953521	0.0817147
8201	1.67054	2.1
8202	1.67054	2.1
8203	0.946131	2.1
8204	1.38154	2.1
8205	1.13326	2.1
8206	1.13326	2.1
8207	1.23513	2.1
8208	1.50998	2.08145
8209	2.47982	0.05
8210	1.45091	0.05
8211	0.958953	0.05
8212	0.79239	0.05
8213	2.11929	0.05
8214	1.26511	0.05
8215	1.27733	0.05
8216	2.18127	0.05
8217	1.30169	0.05
8218	1.19259	0.05
8219	0.963395	0.68196
8220	0.999947	2.0083
8221	1.62241	2.1
8222	1.62027	2.1
8223	0.981586	0.05
8224	0.981585	0.05
8225	0.721961	2.1
8226	0.891321	2.1
8227	1.04442	2.1
8228	1.66554	2.1
8229	2.43434	2.1
8230	1.73944	0.05
8231	1.88032	0.05
8232	0.711391	0.05
8233	1.40837	0.05

8234	0.946133	0.05
8235	0.92252	0.05
8236	1.13964	0.05
8237	1.83394	0.05
8238	2.81674	0.05
8239	1.10049	1.56119
8240	1.25092	0.115836
8241	0.760299	0.40582
8242	1.48017	0.626901
8243	1.48017	0.862375
8244	0.760271	0.256299
8245	1.35861	0.517123
8246	0.946129	0.293423
8247	0.760269	0.336692
8248	2.26155	2.1
8249	2.15634	2.1
8250	1.39824	2.1
8251	1.07817	2.1
8252	0.946132	2.1
8253	1.53028	2.1
8254	1.53028	2.1
8255	1.00807	2.1
8256	0.946133	2.1
8257	2.06126	0.222927
8258	1.97385	1.68461
8259	1.75754	0.245449
8260	2.01613	2.1
8261	1.97995	2.1
8262	1.47696	2.1
8263	1.50172	2.1
8264	1.5177	0.05
8265	1.44146	0.05
8266	1.8807	0.05
8267	2.07068	0.05
8268	1.84267	0.05
8269	1.08975	2.1
8270	1.21327	2.1
8271	1.10106	0.05
8272	1.34496	0.05
8273	2.08452	0.05
8274	1.70537	0.05
8275	2.62464	2.1
8276	1.5726	1.29954
8277	1.5726	2.1
8278	0.94613	2.1
8279	1.15038	2.1
8280	0.946132	0.517262
8281	1.03974	2.1
8282	1.15038	2.1
8283	0.99747	0.392844
8284	1.03472	1.89388
8285	1.58407	0.370508
8286	1.21148	0.066138
8287	1.35025	0.842719
8288	1.3428	0.0746366
8289	1.00775	2.1
8290	1.00775	2.1
8291	1.3879	2.1

8292	1.39958	2.1
8293	0.853042	2.1
8294	1.06997	2.1
8295	0.853042	2.1
8296	0.853042	2.1
8297	1.12491	2.1
8298	1.17879	2.1
8299	1.07162	2.1
8300	1.61179	2.1
8301	1.76016	2.1
8302	0.946132	0.126353
8303	1.23449	0.061578
8304	1.70392	0.0663114
8305	1.70392	0.05
8306	0.94613	0.150655
8307	1.23449	0.0763538
8308	1.41556	0.0792207
8309	1.14547	0.0674685
8310	1.1721	0.05
8311	0.96478	0.0506738
8312	1.41843	0.0517265
8313	1.2358	0.05
8314	1.70678	0.05
8315	1.70678	0.05
8316	0.946118	0.0876
8317	1.23582	0.0686084
8318	0.946105	0.0955899
8319	0.964769	0.05
8320	1.1357	0.05
8321	0.964767	0.05
8322	0.619856	0.05
8323	1.72366	0.05
8324	1.72367	0.05
8325	0.619831	0.05
8326	1.19262	0.05
8327	0.619829	0.05
8328	0.946129	0.05
8329	1.57459	2.1
8330	1.86512	2.1
8331	0.93841	2.1
8332	1.57459	2.1
8333	1.10476	0.05
8334	1.00013	0.210566
8335	2.13446	0.393449
8336	0.759781	2.1
8337	1.96011	1.0993
8338	0.946129	0.68623
8339	1.32056	1.88647
8340	1.75702	1.14465
8341	1.75702	2.1
8342	0.946124	1.86579
8343	1.32056	1.33711
8344	1.38258	0.695787
8345	1.96011	1.32438
8346	2.31099	1.17527
8347	0.97356	2.1
8348	1.38885	2.1
8349	1.3771	2.1

8350	0.946131	2.1
8351	1.84843	2.1
8352	1.84843	2.1
8353	1.3771	2.1
8354	0.946131	2.1
8355	1.00278	2.1
8356	1.05447	0.0545483
8357	1.05446	0.177425
8358	1.24913	0.05
8359	1.05447	0.05
8360	1.80882	0.05
8361	1.80882	2.1
8362	0.946129	2.1
8363	1.33997	2.1
8364	0.946131	0.05
8365	1.28831	0.786124
8366	1.33997	0.219551
8367	1.44391	0.05
8368	1.44391	0.05
8369	0.850964	0.05
8370	0.850963	0.05
8371	0.946132	0.05
8372	0.921299	0.05
8373	1.37358	0.05
8374	1.46297	0.05
8375	1.46298	0.05
8376	0.823988	0.05
8377	0.921287	0.05
8378	0.94612	0.05
8379	0.823995	0.05
8380	0.946107	0.05
8381	1.44437	2.1
8382	1.06203	2.1
8383	2.21397	2.1
8384	0.637677	2.1
8385	1.8892	2.1
8386	2.075	2.1
8387	1.32051	0.05
8388	2.23637	0.05
8389	1.10904	0.05
8390	1.11819	0.05
8391	0.94613	0.05
8392	0.735845	0.05
8393	1.4914	0.05
8394	1.4914	0.05
8395	0.735831	0.05
8396	1.61683	2.1
8397	1.61683	2.1
8398	0.946131	2.1
8399	1.3281	2.1
8400	1.10441	2.1
8401	1.10442	2.1
8402	1.23487	2.1
8403	1.09914	2.1
8404	1.20691	0.05
8405	2.27379	0.05
8406	1.1369	0.05
8407	1.26868	0.05

8408	1.54422	0.05
8409	1.54422	0.05
8410	0.861357	0.05
8411	0.94613	0.05
8412	0.861359	0.05
8413	1.2858	0.102394
8414	1.31011	0.557893
8415	1.294 0.11051	
8416	1.03079	0.315033
8417	1.81979	2.07347
8418	1.57829	2.1
8419	1.19023	1.54097
8420	1.19022	1.95285
8421	1.46458	1.7233
8422	1.46458	2.0877
8423	0.789173	2.1
8424	1.34386	2.09102
8425	0.909896	2.1
8426	0.946131	1.99681
8427	0.909893	2.03289
8428	1.27725	2.1
8429	1.68451	2.1
8430	2.26155	2.1
8431	2.34217	2.1
8432	1.55485	0.338893
8433	0.90854	0.279173
8434	0.858256	0.137105
8435	1.47042	0.0874158
8436	1.62771	0.11082
8437	0.777398	0.4416
8438	0.982525	0.163225
8439	1.23865	0.194006
8440	2.09928	0.151183
8441	0.946128	0.05
8442	0.474856	0.05
8443	0.474855	0.0519668
8444	1.58747	0.05
8445	1.58747	0.05
8446	0.963917	0.05
8447	1.09841	0.05
8448	2.23904	2.1
8449	2.60055	0.446326
8450	1.02599	2.1
8451	2.17837	2.1
8452	2.07122	2.1
8453	2.7309	0.316263
8454	2.08482	2.1
8455	1.18322	0.05
8456	1.35381	0.138288
8457	1.01139	0.05
8458	0.946133	0.251668
8459	1.59089	0.122806
8460	1.59089	0.05
8461	0.946133	0.0533027
8462	1.09634	0.05
8463	2.02279	0.05
8464	1.03108	0.0797767
8465	0.808978	1.41189

8466	1.07317	1.36425
8467	0.831695	1.24935
8468	1.22583	1.14644
8469	1.10215	1.09693
8470	1.07508	1.34588
8471	1.22204	1.13188
8472	1.04679	2.1
8473	1.92535	0.05
8474	1.11081	0.05
8475	0.946131	0.05
8476	0.714824	0.05
8477	0.714795	0.05
8478	1.4231	0.05
8479	1.4231	0.05
8480	0.828617	0.05
8481	1.30928	0.05
8482	1.49894	2.1
8483	1.55702	2.1
8484	0.946131	2.1
8485	1.10689	2.1
8486	0.946227	2.1
8487	0.946131	2.1
8488	1.21754	2.1
8489	1.504 2.1	
8490	1.504 2.1	
8491	0.723573	2.1
8492	1.24512	2.1
8493	0.723544	2.1
8494	0.946131	2.1
8495	1.24512	2.1
8496	1.29383	0.0632718
8497	1.29383	0.05
8498	1.61969	0.05
8499	0.946132	0.05
8500	1.12818	0.05
8501	0.946132	0.05
8502	1.80922	0.05
8503	1.80922	0.05
8504	1.41383	0.05
8505	1.34153	0.05
8506	1.47689	0.05
8507	1.79375	0.05
8508	1.24333	0.05
8509	0.768989	0.05
8510	1.24333	0.05
8511	1.0027	0.05
8512	1.47705	0.05
8513	1.47705	0.05
8514	0.768977	0.05
8515	1.28436	0.05
8516	0.928583	0.05
8517	1.28436	0.05
8518	1.24333	0.05
8519	1.5991	0.05
8520	1.5991	0.05
8521	0.928583	0.05
8522	1.85717	0.05
8523	2.48665	0.05

8524	1.18423	0.05
8525	1.9493	1.7041
8526	1.8519	0.82855
8527	1.68802	2.1
8528	1.0302	0.05
8529	0.900473	0.05
8530	1.99663	0.05
8531	2.61301	0.05
8532	0.745772	0.05
8533	0.946131	0.05
8534	1.48881	0.05
8535	1.48881	0.05
8536	0.745745	0.05
8537	0.809786	0.05
8538	1.3065	0.05
8539	0.946132	0.05
8540	0.768357	0.05
8541	0.768387	0.05
8542	1.76897	0.05
8543	1.76896	0.05
8544	1.40271	0.05
8545	1.00777	0.05
8546	1.37427	0.05
8547	1.1967	2.1
8548	0.946132	2.1
8549	1.59397	2.1
8550	1.59397	2.1
8551	0.946131	2.1
8552	1.14989	2.1
8553	1.14989	2.1
8554	1.10046	2.1
8555	1.09506	0.05
8556	2.53251	0.05
8557	1.9212	0.398422
8558	1.99586	0.624307
8559	0.913967	0.385643
8560	1.55028	0.341211
8561	1.55028	0.568217
8562	0.913966	0.142459
8563	1.16318	0.288144
8564	1.16318	0.346698
8565	0.946132	0.245741
8566	1.20478	2.1
8567	1.09662	2.1
8568	1.03472	2.1
8569	1.84774	2.1
8570	1.7984	2.1
8571	2.1221	2.1
8572	1.68223	2.1
8573	1.68224	2.1
8574	0.514872	2.01251
8575	0.888991	2.1
8576	0.946127	0.567992
8577	0.514845	1.98496
8578	0.946129	1.24635
8579	1.10127	1.83029
8580	2.61623	0.852147
8581	0.983581	1.50486

8582	0.869663	0.505179
8583	1.1818	2.1
8584	1.10406	0.782682
8585	1.07133	2.1
8586	1.09405	1.22463
8587	0.838014	1.09373
8588	0.842998	1.30805
8589	1.03818	0.896966
8590	1.46338	0.0733355
8591	1.903 2.1	
8592	1.23942	2.1
8593	0.965933	2.1
8594	0.810075	2.1
8595	1.40571	2.1
8596	1.66219	2.1
8597	0.810047	2.1
8598	0.936678	2.1
8599	1.45808	2.1
8600	1.6384	2.1
8601	1.54699	2.1
8602	0.719877	2.1
8603	1.33256	2.1
8604	0.753547	2.1
8605	1.04073	2.1
8606	1.25822	2.1
8607	0.851542	2.1
8608	0.714102	2.1
8609	1.45886	2.1
8610	1.80171	2.1
8611	0.597337	2.1
8612	0.957713	2.1
8613	1.3033	2.1
8614	0.596613	0.05
8615	1.1386	0.05
8616	0.596612	0.05
8617	0.946133	0.101272
8618	0.596652	0.113136
8619	1.64092	0.0541758
8620	1.64092	0.0501041
8621	1.991 2.1	
8622	1.23013	2.1
8623	2.77816	2.1
8624	2.74107	0.529422
8625	2.07681	0.207915
8626	1.47764	0.05
8627	1.72677	0.05
8628	1.52161	2.1
8629	1.28526	2.1
8630	1.90502	2.1
8631	1.87359	0.05
8632	1.93489	0.05
8633	2.70895	2.1
8634	2.83141	2.1
8635	1.41766	0.24684
8636	1.11337	0.223067
8637	1.41765	1.76859
8638	1.29172	0.354362
8639	1.76324	0.447189

8640	1.76324	1.27907
8641	0.946131	0.221051
8642	1.52428	2.1
8643	1.71459	2.1
8644	1.93852	0.15306
8645	1.07119	1.47841
8646	1.29698	1.21695
8647	1.42203	0.250693
8648	1.42203	1.39954
8649	0.946132	0.585383
8650	0.946132	0.989059
8651	0.96926	1.08287
8652	0.769913	1.99257
8653	0.946119	1.78227
8654	1.37867	1.06636
8655	1.94028	0.860703
8656	0.94612	0.358839
8657	0.946132	1.56109
8658	1.37865	0.641524
8659	2.14234	1.14153
8660	3.06459	1.25542
8661	1.44421	0.05
8662	1.33924	0.05
8663	1.33671	0.05
8664	1.10733	0.05
8665	0.68396	0.05
8666	1.76008	0.05
8667	1.76008	0.05
8668	0.68393	0.05
8669	0.946131	0.05
8670	3.79242	2.1
8671	2.46293	2.1
8672	3.56947	2.1
8673	1.35487	1.53816
8674	1.38616	1.91073
8675	0.954048	0.05
8676	1.36663	0.05
8677	0.909487	0.05
8678	0.887288	2.1
8679	0.810947	0.554282
8680	0.810934	1.74035
8681	1.07046	1.87732
8682	1.13979	2.04575
8683	0.927838	0.573755
8684	0.919995	1.426
8685	1.61447	2.1
8686	2.56305	2.1
8687	0.943414	2.1
8688	1.17016	2.1
8689	1.7238	2.1
8690	1.89334	2.1
8691	0.946128	0.55993
8692	1.25184	2.1
8693	1.55639	2.1
8694	3.12098	1.53074
8695	3.57307	0.448054
8696	2.81281	2.1
8697	0.851542	2.1

8698	1.27689	2.1
8699	1.52568	2.1
8700	0.839689	2.1
8701	1.36471	2.1
8702	0.827865	2.1
8703	0.93667	2.1
8704	0.900861	2.1
8705	0.943152	2.1
8706	1.00667	2.1
8707	1.18141	2.1
8708	1.15298	2.1
8709	1.20335	2.1
8710	1.00713	2.1
8711	0.597537	2.1
8712	1.0577	2.1
8713	0.967342	0.155318
8714	0.838027	0.860473
8715	0.893587	0.0907328
8716	1.06436	0.228385
8717	0.850048	0.0731441
8718	1.22026	0.432329
8719	0.988077	0.139145
8720	1.18769	0.05
8721	1.3991	2.1
8722	1.52984	2.1
8723	1.35469	0.917047
8724	1.25083	0.492262
8725	1.6594	1.32
8726	1.6594	0.628225
8727	0.946129	0.331495
8728	1.02602	0.40501
8729	0.94613	0.248206
8730	1.29724	0.05
8731	1.14501	0.05
8732	1.13078	0.05
8733	1.48381	0.05
8734	1.45921	0.05
8735	0.851539	0.05
8736	0.890836	0.05
8737	0.946131	0.05
8738	1.60428	2.1
8739	1.50097	2.1
8740	0.714507	0.05
8741	1.51396	0.05
8742	1.51396	0.05
8743	0.946132	0.05
8744	0.813862	0.05
8745	1.41461	0.05
8746	0.813862	0.05
8747	0.921311	2.1
8748	1.25084	0.530451
8749	1.30165	0.364159
8750	1.60636	1.39327
8751	1.60636	0.366049
8752	0.94613	0.293936
8753	0.972232	0.344053
8754	0.946131	0.19938
8755	2.35003	2.1

8756	0.92546	2.1
8757	1.00713	2.1
8758	0.810947	2.1
8759	0.810934	2.1
8760	1.056	2.1
8761	0.926811	2.1
8762	0.766408	2.1
8763	1.34292	2.1
8764	1.57932	0.05
8765	2.67595	0.05
8766	1.77737	0.05
8767	1.77738	0.05
8768	0.789633	0.05
8769	1.22903	0.05
8770	0.789663	0.05
8771	0.789662	0.05
8772	1.33797	0.05
8773	0.946128	2.1
8774	1.47351	2.1
8775	1.47351	2.1
8776	0.94613	2.1
8777	1.30082	2.1
8778	1.30082	2.1
8779	0.996486	2.1
8780	0.946127	2.1
8781	1.72122	2.1
8782	1.72122	2.1
8783	0.94613	2.1
8784	1.36653	2.1
8785	1.36653	2.1
8786	1.02608	2.1
8787	0.709123	0.341266
8788	0.946127	0.572566
8789	1.41342	0.921212
8790	1.818	1.34538
8791	1.818	0.896245
8792	0.946127	0.074938
8793	1.41342	0.940744
8794	1.35071	0.443116
8795	1.8993	1.43154
8796	1.4748	2.1
8797	1.18711	0.05
8798	0.946131	0.05
8799	1.18711	0.05
8800	1.20416	0.05
8801	1.91358	0.05
8802	1.91358	0.05
8803	0.477691	0.05
8804	2.16798	0.05
8805	2.22307	0.05
8806	0.758971	0.05
8807	1.80694	0.05
8808	1.80694	0.05
8809	0.946132	0.05
8810	0.75897	0.05
8811	1.25124	0.05
8812	0.946131	0.05
8813	1.76837	2.1

8814	1.76837	2.1
8815	0.946131	2.1
8816	1.36422	2.1
8817	0.946131	2.1
8818	1.14184	2.1
8819	1.36422	2.1
8820	0.793303	0.05
8821	1.79584	0.05
8822	1.35333	2.1
8823	1.19967	1.52396
8824	1.55088	1.91447
8825	1.64246	2.1
8826	0.946132	0.414546
8827	0.886293	2.1
8828	1.25018	2.1
8829	1.5795	1.82475
8830	2.15414	1.44761
8831	0.94613	0.993717
8832	1.37758	1.77913
8833	1.71567	1.04325
8834	1.71567	2.1
8835	0.946125	1.3522
8836	1.37759	0.94291
8837	1.28421	0.574436
8838	0.933959	0.583653
8839	0.946133	0.05
8840	1.7813	0.05
8841	1.7813	0.05
8842	1.05704	0.05
8843	1.37794	0.05
8844	1.37794	0.05
8845	0.946132	0.05
8846	0.946132	0.05
8847	1.68197	0.05
8848	1.68197	0.05
8849	1.01367	0.05
8850	1.25017	0.05
8851	1.25017	0.05
8852	0.946132	0.05
8853	0.838953	2.1
8854	1.4045	2.1
8855	1.56388	2.1
8856	1.3182	2.1
8857	0.928518	2.1
8858	1.04073	2.1
8859	0.835676	2.1
8860	1.47507	2.1
8861	1.47506	2.1
8862	0.797664	2.1
8863	1.34285	2.1
8864	0.929875	2.1
8865	0.797663	2.1
8866	0.946133	2.1
8867	1.83628	0.131223
8868	1.83627	0.539698
8869	0.946131	0.565717
8870	1.36748	0.248684
8871	0.946133	0.0633691

8872	1.21139	0.281749
8873	1.36748	0.05
8874	1.06795	2.1
8875	1.08192	2.1
8876	0.827866	2.1
8877	1.02548	2.1
8878	0.95006	2.1
8879	1.53933	2.1
8880	0.586511	2.1
8881	0.868145	2.1
8882	1.28401	2.1
8883	1.37719	2.1
8884	1.37719	2.1
8885	0.77497	2.1
8886	0.946132	2.1
8887	0.774968	2.1
8888	1.7053	0.05
8889	1.7053	0.537086
8890	0.94613	2.1
8891	1.25715	0.372106
8892	0.946132	0.0640489
8893	1.19655	0.16853
8894	1.25716	0.0532262
8895	0.851542	2.1
8896	0.80043	2.1
8897	1.27239	2.1
8898	1.02719	2.1
8899	0.946131	2.1
8900	0.981318	2.1
8901	1.06628	2.1
8902	1.26022	2.1
8903	0.946132	2.1
8904	0.946132	2.1
8905	1.76447	2.1
8906	1.76447	2.1
8907	1.35357	2.1
8908	1.35703	2.1
8909	0.946131	2.1
8910	1.50538	2.1
8911	1.50538	2.1
8912	1.10311	2.1
8913	1.3484	2.1
8914	1.10312	2.1
8915	0.966428	2.1
8916	0.946133	2.1
8917	1.4008	2.1
8918	1.71908	2.1
8919	1.71908	2.1
8920	0.946133	2.1
8921	1.40081	2.1
8922	1.26441	2.1
8923	0.851543	2.1
8924	0.961732	2.1
8925	1.54576	2.1
8926	1.35364	2.1
8927	0.946133	2.1
8928	1.02913	2.1
8929	1.2735	2.1

8930	1.32608	2.1
8931	0.967643	2.1
8932	0.94613	2.1
8933	1.44873	2.1
8934	1.44873	2.1
8935	0.967641	2.1
8936	0.946131	2.1
8937	1.40488	2.1
8938	1.24272	2.1
8939	0.946131	2.1
8940	1.78483	2.1
8941	1.78483	2.1
8942	1.24272	2.1
8943	0.946131	2.1
8944	0.94613	2.1
8945	1.5627	0.145359
8946	1.5627	2.1
8947	1.03656	2.1
8948	1.31649	0.0651963
8949	1.19233	1.31435
8950	1.03656	0.71153
8951	0.912059	2.1
8952	1.47954	0.225117
8953	1.47954	2.1
8954	0.912061	2.1
8955	1.34647	0.133209
8956	1.04514	1.92209
8957	0.912061	0.974506
8958	0.761972	0.05
8959	0.946132	0.05
8960	0.761974	0.0625858
8961	1.70225	0.116169
8962	1.70225	0.14599
8963	1.33728	0.05
8964	1.01188	0.061962
8965	0.94613	0.806151
8966	0.746361	1.61786
8967	1.6369	2.02692
8968	1.62454	1.69592
8969	0.736738	0.723153
8970	0.958487	1.07369
8971	1.40641	1.26063
8972	1.2284	2.1
8973	0.92609	2.1
8974	0.595102	2.1
8975	1.80871	2.1
8976	1.1516	2.1
8977	0.962474	2.1
8978	2.24024	2.1
8979	0.946131	2.1
8980	1.73689	2.1
8981	1.73689	2.1
8982	0.946132	2.1
8983	1.30492	2.1
8984	1.30492	2.1
8985	1.17949	2.1
8986	0.946131	2.1
8987	1.8566	2.1

8988	1.8566	2.1
8989	1.22392	2.1
8990	1.39826	2.1
8991	1.40447	2.1
8992	1.22392	2.1
8993	0.946131	2.1
8994	1.74134	2.1
8995	1.74134	2.1
8996	1.09148	2.1
8997	1.40447	2.1
8998	1.283	2.1
8999	1.09148	2.1
9000	1.33607	2.1
9001	1.41022	2.1
9002	1.80016	2.1
9003	1.80015	2.1
9004	0.946127	2.1
9005	0.946129	2.1
9006	1.20541	2.1
9007	1.13078	2.1
9008	1.33607	2.1
9009	1.54624	2.1
9010	1.85553	2.1
9011	0.920606	2.1
9012	0.946131	2.1
9013	1.01271	2.1
9014	1.32664	1.85568
9015	0.954692	1.34434
9016	0.946116	0.791329
9017	1.47771	0.549377
9018	1.4777	2.1
9019	0.95468	1.16748
9020	0.94613	0.811641
9021	1.31381	1.23398
9022	1.14005	0.174793
9023	0.946128	1.14426
9024	1.69433	0.0633114
9025	1.69433	1.42309
9026	1.14005	0.719483
9027	0.94613	0.173774
9028	1.521	2.1
9029	1.411	2.1
9030	0.94612	2.1
9031	1.35115	2.1
9032	1.04127	2.1
9033	1.05017	2.1
9034	1.03564	2.1
9035	0.607156	2.1
9036	1.54549	2.1
9037	1.36715	2.1
9038	0.607127	2.0303
9039	1.2571	2.1
9040	0.998701	2.1
9041	0.546436	2.1
9042	0.946133	2.1
9043	1.75286	2.1
9044	1.75286	2.1
9045	1.12206	2.1

9046	1.34237	2.1
9047	1.35663	2.1
9048	1.12206	2.1
9049	0.919455	2.1
9050	1.66517	2.1
9051	1.99822	2.1
9052	1.228 2.1	
9053	1.35662	2.1
9054	1.228 2.1	
9055	1.01143	2.1
9056	0.851543	0.72359
9057	0.986856	2.1
9058	1.42762	1.96658
9059	1.23095	2.1
9060	0.946132	2.1
9061	1.00659	2.00475
9062	1.1779	1.56281
9063	1.40025	2.1
9064	1.40025	2.1
9065	0.889308	2.1
9066	1.34652	2.1
9067	0.943042	2.1
9068	0.943041	2.1
9069	1.34652	2.1
9070	1.39514	1.77592
9071	1.30809	1.46106
9072	1.75711	1.33298
9073	1.7571	2.1
9074	0.946125	0.214175
9075	0.946127	1.48491
9076	1.18396	1.41626
9077	1.4844	0.642106
9078	1.4844	1.31354
9079	0.803322	2.00289
9080	0.946133	0.368238
9081	0.803324	2.1
9082	0.803324	1.58084
9083	1.36183	1.70321
9084	0.946131	0.0789148
9085	1.01462	0.0570492
9086	0.946132	0.0508484
9087	1.59749	0.129736
9088	1.59749	0.0571339
9089	1.16009	0.0664013
9090	1.38354	0.05
9091	0.851539	0.797475
9092	1.36005	1.72496
9093	1.5532	1.07974
9094	1.80402	2.0847
9095	0.851537	1.93917
9096	0.902632	1.57181
9097	1.44437	1.22909
9098	0.633098	2.1
9099	1.00307	0.0569125
9100	0.94613	0.136006
9101	1.16623	0.05
9102	0.946128	0.156855
9103	1.64588	0.0988251

9104	1.64588	0.0684331
9105	1.35059	0.105225
9106	1.24142	0.112972
9107	0.946131	0.05
9108	1.13343	0.05
9109	0.946127	0.05
9110	1.63367	0.05
9111	1.63367	0.05
9112	1.33035	0.05
9113	1.24945	0.05
9114	1.78733	1.66255
9115	1.70856	1.97043
9116	0.637995	1.09649
9117	0.712454	0.974835
9118	0.946132	1.23404
9119	0.637964	0.772085
9120	1.13021	1.13013
9121	0.94613	0.05
9122	1.20509	0.05
9123	0.946127	0.05
9124	1.70545	0.05
9125	1.70545	0.05
9126	1.28508	0.05
9127	1.3665	0.05
9128	1.58612	2.1
9129	1.51845	2.1
9130	0.797374	2.1
9131	1.20057	2.1
9132	0.946132	2.1
9133	0.946132	2.1
9134	0.899819	2.1
9135	1.50471	0.867129
9136	1.50472	1.02489
9137	0.59387	1.20534
9138	0.593844	0.31721
9139	0.946126	1.04393
9140	0.966137	0.517283
9141	1.13245	0.560493
9142	1.20801	0.2268
9143	0.946131	1.01109
9144	1.47644	0.334696
9145	0.733223	0.300099
9146	0.563609	0.291069
9147	1.69218	0.265949
9148	1.6173	0.655238
9149	1.8126	1.97625
9150	1.8126	2.05587
9151	0.678938	2.08155
9152	0.678935	2.0175
9153	0.946133	2.1
9154	0.946132	2.06907
9155	0.678936	2.1
9156	1.21997	1.75605
9157	1.28439	1.63145
9158	1.65282	1.96515
9159	1.86735	1.89719
9160	0.851539	1.99175
9161	0.94613	0.982732

9162	0.87688	0.826512
9163	0.804877	0.0965901
9164	1.40747	0.226258
9165	1.41312	0.05
9166	0.885349	0.0517393
9167	1.25405	0.204828
9168	1.34463	0.0660048
9169	0.804878	0.0514771
9170	0.810299	0.05
9171	1.04072	0.05
9172	1.25601	0.05
9173	1.04943	0.05
9174	0.900294	0.05
9175	1.38777	0.05
9176	1.33131	0.05
9177	1.39168	0.05
9178	0.946132	0.05
9179	0.747712	0.05
9180	1.61944	0.05
9181	1.61944	0.05
9182	0.946132	0.05
9183	0.747712	0.05
9184	1.41201	0.05
9185	1.12082	0.05
9186	0.946128	0.05
9187	1.79415	0.05
9188	1.79415	0.05
9189	1.12082	0.05
9190	0.94613	0.05
9191	0.711721	0.05
9192	1.12316	0.05
9193	0.707038	0.05
9194	1.04072	0.05
9195	0.704409	0.05
9196	1.49036	0.05
9197	1.27689	0.05
9198	0.793044	0.0723545
9199	0.810886	0.373504
9200	0.885957	0.0883423
9201	1.38254	0.0798122
9202	1.35991	0.0589439
9203	1.01375	0.130341
9204	1.03169	0.111969
9205	0.946131	0.0971322
9206	1.3279	0.234793
9207	1.42284	0.11781
9208	1.42284	0.087932
9209	0.851192	0.815783
9210	0.946131	0.503546
9211	0.851165	0.178588
9212	0.748062	0.05
9213	1.27511	0.05
9214	0.74806	0.05
9215	0.94613	0.05
9216	0.748059	0.05
9217	1.86293	0.05
9218	1.86293	0.05
9219	0.851542	0.05

9220	1.38186	0.05
9221	0.851542	0.05
9222	1.67869	0.05
9223	1.66579	0.05
9224	1.25626	0.05
9225	1.23046	0.05
9226	1.46023	0.05
9227	1.27083	0.05
9228	0.946131	0.05
9229	0.999304	0.05
9230	0.946803	0.05
9231	0.851554	0.05
9232	0.999227	0.05
9233	0.750097	0.05
9234	1.91815	0.05
9235	0.946132	0.05
9236	0.768117	0.05
9237	1.78003	0.05
9238	1.99572	0.05
9239	0.650453	0.05
9240	0.946132	0.05
9241	1.23845	0.05
9242	0.946131	0.05
9243	1.75477	0.05
9244	1.75477	0.05
9245	1.33393	0.05
9246	1.36697	0.05
9247	0.749018	1.41667
9248	1.02366	1.7706
9249	1.43924	1.35727
9250	1.00018	1.3831
9251	1.06438	0.916095
9252	0.946127	1.56665
9253	0.74906	1.08013
9254	0.946131	1.59921
9255	1.26488	0.05
9256	0.946131	0.05
9257	0.94613	0.05
9258	1.49051	0.05
9259	1.59837	0.05
9260	0.907797	0.05
9261	1.04455	0.05
9262	1.25214	0.05
9263	0.68452	0.05
9264	1.25215	0.05
9265	1.24677	0.05
9266	1.8144	0.05
9267	1.81441	0.05
9268	0.684518	0.05
9269	0.851542	0.211601
9270	1.52275	0.185242
9271	1.45223	0.148012
9272	1.0269	0.129218
9273	1.29689	0.345315
9274	1.32822	0.469635
9275	0.851553	0.631237
9276	1.21756	0.05
9277	0.946131	0.05

9278	0.946127	0.05
9279	1.78014	0.05
9280	1.78014	0.05
9281	1.32076	0.05
9282	1.40551	0.05
9283	0.711214	0.05
9284	1.82057	0.05
9285	1.82057	0.05
9286	0.946129	0.05
9287	1.22716	0.05
9288	1.30463	0.05
9289	0.94613	0.05
9290	0.568927	0.05
9291	0.946132	0.05
9292	0.568927	0.05
9293	1.99266	0.05
9294	1.99266	0.05
9295	1.26201	0.05
9296	1.29957	0.05
9297	1.21614	0.05
9298	0.851484	0.05
9299	0.851543	0.05
9300	1.85815	0.05
9301	1.81937	0.05
9302	0.697705	0.05
9303	0.946132	0.05
9304	0.593295	0.05
9305	1.53642	0.05
9306	1.53642	0.05
9307	0.908276	0.05
9308	0.593296	0.05
9309	0.593295	0.05
9310	0.94613	0.05
9311	0.867593	0.05
9312	1.42107	0.05
9313	1.42107	0.05
9314	0.867621	0.05
9315	1.05516	0.05
9316	0.867624	0.05
9317	0.946129	0.05
9318	0.766681	0.05
9319	1.18119	0.05
9320	1.57018	0.05
9321	1.57018	0.05
9322	0.766652	0.05
9323	1.18119	0.05
9324	1.15564	0.05
9325	0.946131	0.05
9326	1.239 0.05	
9327	0.851541	0.05
9328	0.851487	0.05
9329	1.40021	0.05
9330	1.38373	0.05
9331	0.851554	0.05
9332	1.12266	0.05
9333	0.946131	0.05
9334	1.08234	0.05
9335	1.2882	0.05

9336	1.49963	0.10836
9337	1.46188	0.05
9338	0.851555	0.0504914
9339	1.33007	0.05
9340	1.08054	0.05
9341	0.946129	0.05
9342	0.91151	0.05
9343	1.49914	0.05
9344	1.49914	0.05
9345	0.911471	0.05
9346	0.946132	0.799584
9347	1.35943	2.09585
9348	1.68117	1.61674
9349	1.68117	2.1
9350	0.946132	1.78023
9351	1.35943	1.15042
9352	1.26787	0.44351
9353	1.81612	0.790509
9354	1.81612	0.05
9355	0.946131	0.05
9356	1.41492	0.05
9357	0.946132	0.678046
9358	1.25037	0.05
9359	1.41492	0.0514008
9360	1.30541	0.05
9361	1.23646	0.05
9362	0.94613	0.05
9363	1.77304	0.05
9364	1.77304	0.05
9365	1.23646	0.05
9366	0.946132	0.05
9367	0.946131	2.1
9368	1.26689	2.1
9369	1.66806	2.1
9370	1.66806	2.1
9371	0.94613	2.1
9372	1.26689	2.1
9373	1.3473	2.1
9374	1.39384	2.1
9375	1.4181	2.1
9376	1.86581	2.1
9377	1.86581	2.1
9378	0.946128	2.1
9379	0.946129	2.1
9380	1.32068	2.1
9381	1.20328	2.1
9382	1.39384	2.1
9383	1.651 2.1	
9384	1.651 2.1	
9385	0.946129	2.1
9386	0.946131	2.1
9387	1.12913	2.1
9388	1.21474	2.1
9389	0.946132	2.1
9390	0.94613	2.1
9391	1.79724	2.1
9392	1.79724	2.1
9393	1.40987	2.1

9394	1.3335	2.1
9395	1.65642	2.1
9396	1.65642	2.1
9397	0.946132	2.1
9398	1.19063	2.1
9399	0.946133	1.19981
9400	1.15203	2.1
9401	1.19063	2.1
9402	0.946131	0.160818
9403	1.77815	2.1
9404	1.77815	2.1
9405	0.946131	1.91956
9406	1.31745	2.1
9407	1.31745	2.1
9408	1.2597	2.1
9409	0.946131	0.0944651
9410	1.85184	1.18556
9411	1.85184	2.1
9412	0.946128	1.5074
9413	1.40684	2.1
9414	1.40684	1.09281
9415	1.34049	1.84804
9416	0.946132	1.78307
9417	1.71333	1.09312
9418	1.71333	2.1
9419	1.1848	1.78954
9420	1.34297	0.897889
9421	1.31649	1.01853
9422	1.1848	1.85261
9423	1.36604	0.414487
9424	0.954559	0.659019
9425	1.36604	0.502868
9426	1.41766	0.423506
9427	1.83756	0.963339
9428	1.83756	2.1
9429	0.946131	0.748963
9430	0.946127	0.0527445
9431	1.14874	0.05
9432	0.946127	0.0692655
9433	1.66393	0.0546299
9434	1.66393	0.05
9435	1.35559	0.0522724
9436	1.25447	0.0661228
9437	0.94613	0.05
9438	1.20732	0.05
9439	0.946129	0.05
9440	1.75073	0.05
9441	1.75073	0.05
9442	1.3665	0.05
9443	1.33035	0.05
9444	1.73256	2.1
9445	1.73256	2.1
9446	0.946131	2.1
9447	1.34921	2.1
9448	1.19462	2.1
9449	1.19462	2.1
9450	1.32949	2.1
9451	1.75754	0.0971201

9452	1.75754	0.0531568
9453	0.946132	0.280178
9454	1.3201	0.0964795
9455	0.946132	0.711863
9456	1.25479	0.05
9457	1.3201	0.0770017
9458	1.8087	0.265682
9459	1.8087	0.05
9460	0.946131	0.05
9461	1.38357	0.05
9462	0.946133	0.206038
9463	1.31034	0.05
9464	1.38357	0.0729675
9465	1.12702	0.05
9466	0.946131	0.05
9467	1.23923	0.05
9468	1.4117	0.05
9469	0.946131	0.05
9470	1.70479	0.05
9471	1.70479	0.05
9472	0.946119	2.1
9473	1.55309	2.1
9474	1.55309	2.1
9475	0.946119	2.1
9476	1.08006	2.1
9477	1.08005	2.1
9478	1.02681	2.1
9479	1.36581	2.1
9480	1.93075	2.1
9481	0.946133	2.1
9482	0.772538	2.1
9483	0.946131	2.1
9484	0.946131	2.1
9485	1.33748	2.1
9486	1.44991	2.1
9487	1.31183	2.1
9488	0.946132	2.1
9489	0.944104	2.1
9490	0.915236	2.1
9491	1.05017	2.1
9492	0.944148	2.1
9493	0.946132	0.05
9494	1.60171	0.05
9495	1.60171	0.05
9496	0.946131	0.05
9497	1.16234	0.05
9498	1.3855	0.05
9499	0.946132	0.05
9500	0.946133	0.05
9501	1.73285	0.05
9502	1.73285	0.05
9503	0.946133	0.05
9504	1.3855	0.05
9505	1.29348	0.0687947
9506	0.946132	0.0759973
9507	1.34828	2.1
9508	1.20943	2.1
9509	0.946129	1.73624

9510	1.71013	2.1
9511	1.71013	2.1
9512	1.20943	2.1
9513	0.94613	2.1
9514	1.19613	0.05
9515	0.946118	0.05
9516	0.946117	0.05
9517	1.70868	0.05
9518	1.70869	0.05
9519	1.40551	0.05
9520	1.2493	0.05
9521	0.946131	2.1
9522	1.73123	2.1
9523	1.73123	2.1
9524	0.946132	2.1
9525	1.37811	2.1
9526	1.37811	2.1
9527	1.25144	2.1
9528	0.946132	2.1
9529	1.75139	2.1
9530	1.75139	2.1
9531	1.23374	2.1
9532	1.29926	2.1
9533	1.39827	2.1
9534	1.23374	2.1
9535	1.41375	0.05
9536	1.25697	0.05
9537	0.946118	0.05
9538	1.80377	0.05
9539	1.80377	0.05
9540	1.25699	0.05
9541	0.946106	0.05
9542	0.946133	1.97144
9543	1.81806	0.619499
9544	1.81806	2.1
9545	0.946133	2.1
9546	1.37089	2.1
9547	1.37089	0.996048
9548	1.34999	2.1
9549	0.946131	2.1
9550	1.80855	0.741153
9551	1.80855	2.1
9552	1.31184	2.1
9553	1.3933	1.1084
9554	1.36139	1.91285
9555	1.31184	2.00749
9556	1.01175	1.21077
9557	0.94613	0.287554
9558	0.919796	1.89086
9559	1.50484	0.124658
9560	1.80584	2.03867
9561	1.53512	0.217065
9562	1.14539	0.509845
9563	0.946133	2.1
9564	1.72797	1.73093
9565	1.72797	2.1
9566	1.2111	2.1
9567	1.39731	1.1704

9568	1.27679	2.1
9569	1.21111	2.1
9570	0.946119	0.05
9571	1.67673	0.733648
9572	1.67673	2.1
9573	0.946118	1.07342
9574	1.21519	2.1
9575	1.2152	2.09833
9576	1.19808	2.1
9577	0.946127	2.1
9578	1.54442	2.1
9579	1.54442	2.1
9580	1.04806	2.1
9581	1.13485	2.1
9582	1.3557	2.1
9583	1.04805	2.1
9584	0.946129	2.1
9585	1.32282	2.1
9586	1.77749	2.1
9587	1.77749	2.1
9588	0.946131	2.1
9589	1.32282	2.1
9590	1.4008	2.1
9591	0.946128	0.05
9592	1.02366	0.05
9593	0.94613	0.05
9594	1.66684	0.05
9595	1.66684	0.05
9596	1.29215	0.05
9597	1.32082	0.05
9598	0.946133	0.0570143
9599	1.01451	0.05
9600	0.946132	0.05
9601	1.62374	0.0545636
9602	1.62374	0.05
9603	1.2929	0.05
9604	1.27697	0.05
9605	1.37869	2.1
9606	1.17215	2.1
9607	0.946132	2.1
9608	1.70508	2.1
9609	1.70508	2.1
9610	1.17215	2.1
9611	0.946132	2.1
9612	1.03784	2.1
9613	0.946132	2.1
9614	0.946132	2.1
9615	1.56718	2.1
9616	1.56717	2.1
9617	1.35703	2.1
9618	1.15628	2.1
9619	1.35356	2.1
9620	1.28976	2.1
9621	0.946131	2.1
9622	1.78612	2.1
9623	1.78612	2.1
9624	1.28976	2.1
9625	0.94613	2.1

9626	0.946132	2.1
9627	1.36373	2.1
9628	1.92382	2.1
9629	0.946131	2.1
9630	1.92382	2.1
9631	1.33471	2.1
9632	0.946132	2.1
9633	0.94613	0.171794
9634	1.29149	0.740615
9635	1.75878	0.50022
9636	1.75878	2.1
9637	0.946128	0.906126
9638	1.29149	0.213598
9639	1.41342	0.0761636
9640	1.41022	2.1
9641	1.37034	1.86778
9642	1.83443	2.1
9643	1.83443	2.1
9644	0.946128	1.2271
9645	0.946131	2.1
9646	1.26928	2.1
9647	0.946128	0.829603
9648	1.38259	1.93709
9649	1.81405	1.06248
9650	1.81405	2.1
9651	0.946126	1.64014
9652	1.38259	1.14877
9653	1.37759	0.469494
9654	1.68131	0.05
9655	1.68131	0.05
9656	0.946131	0.05
9657	1.38896	0.05
9658	1.17438	0.227614
9659	1.17438	0.05
9660	1.23848	0.0609295
9661	1.80088	0.05
9662	1.80088	0.05
9663	0.946132	0.05
9664	1.40332	0.05
9665	1.28185	0.0546436
9666	1.28185	0.05
9667	1.34369	0.05
9668	1.71939	2.1
9669	1.71939	2.1
9670	0.946131	2.1
9671	1.28308	2.1
9672	0.946133	2.1
9673	1.24024	2.1
9674	1.28309	2.1
9675	1.8525	2.1
9676	1.8525	2.1
9677	0.946132	2.1
9678	1.38244	2.1
9679	0.946132	1.23137
9680	1.33469	2.1
9681	1.38244	2.1
9682	1.26654	1.64158
9683	1.39514	1.3794

9684	1.71556	1.26351
9685	1.71556	2.1
9686	0.946126	0.223685
9687	0.946129	1.76506
9688	1.1908	1.36518
9689	1.49995	2.1
9690	1.63875	2.1
9691	0.93608	2.1
9692	0.937208	2.1
9693	0.943708	2.1
9694	0.936134	2.1
9695	1.14341	2.1
9696	1.17802	0.05
9697	1.3542	0.05
9698	1.73981	0.05
9699	1.73981	0.05
9700	0.94613	0.05
9701	1.3542	0.05
9702	1.33174	0.05
9703	1.59452	2.1
9704	1.59452	2.1
9705	0.946132	2.1
9706	1.25 2.1	
9707	0.975266	2.1
9708	0.946132	2.1
9709	1.25 2.1	
9710	0.946131	2.1
9711	1.48685	2.1
9712	1.48685	2.1
9713	0.946132	2.1
9714	1.17273	2.1
9715	1.17273	2.1
9716	0.955602	2.1
9717	1.32827	0.05
9718	1.11166	0.05
9719	0.946127	0.05
9720	1.6136	0.05
9721	1.6136	0.05
9722	1.11166	0.05
9723	0.946129	0.05
9724	0.851541	0.429975
9725	1.22107	0.297986
9726	1.64594	0.196871
9727	1.5271	0.211669
9728	0.94613	0.710554
9729	1.22112	0.390471
9730	1.25002	0.295597
9731	1.65795	0.05
9732	1.65795	1.79269
9733	0.946132	2.01343
9734	1.2366	0.115219
9735	0.946132	0.0536359
9736	1.10023	0.479942
9737	1.2366	0.0590768
9738	1.35856	0.05
9739	0.946132	0.05
9740	1.35856	0.05
9741	1.08019	0.05

9742	1.49261	0.05
9743	1.49261	0.05
9744	0.94613	0.05
9745	1.29322	0.0883325
9746	0.946132	0.0898686
9747	1.29322	0.05
9748	1.35856	0.139617
9749	1.70565	0.05
9750	1.70565	0.109913
9751	0.946132	0.0972376
9752	1.66635	2.1
9753	1.66635	2.1
9754	0.946133	2.1
9755	1.39558	2.1
9756	1.14793	2.1
9757	1.14793	2.1
9758	1.21691	2.1
9759	1.35381	0.05
9760	1.3365	0.0545384
9761	1.09905	0.05
9762	0.946132	0.0701706
9763	1.74418	0.05
9764	1.74418	0.05
9765	0.946132	0.05
9766	1.84759	0.0552187
9767	1.84759	0.05
9768	0.94613	0.05
9769	1.3904	0.05
9770	1.34522	0.05
9771	1.34522	0.05
9772	1.40331	0.05
9773	1.86047	0.102228
9774	1.86047	2.1
9775	0.946132	2.02053
9776	1.41619	2.1
9777	0.946132	0.05
9778	1.3659	0.20253
9779	1.41619	0.379886
9780	1.28464	2.1
9781	1.14496	1.63889
9782	1.57806	1.87999
9783	1.7753	2.1
9784	0.851541	1.73737
9785	0.946131	2.06529
9786	0.872786	2.00917
9787	0.85062	0.636058
9788	1.52635	0.603735
9789	1.63785	0.284472
9790	1.69972	1.15404
9791	0.946132	1.44391
9792	1.37493	0.526604
9793	1.23854	0.464778
9794	1.41134	2.1
9795	1.27079	1.97518
9796	1.736	1.35909
9797	1.736	2.1
9798	0.946132	0.317068
9799	0.946133	1.84038

9800	1.21177	2.05814
9801	0.946131	0.05
9802	0.946129	0.05
9803	0.94613	0.05
9804	0.946131	0.05
9805	0.946131	0.05
9806	1.35403	0.05
9807	0.946131	0.05
9808	1.37052	0.05
9809	0.946132	0.05
9810	0.94613	0.05
9811	0.946133	0.05
9812	0.946131	0.05
9813	0.946131	0.05
9814	0.946131	0.05
9815	1.24249	0.05
9816	0.946132	0.05
9817	0.946133	0.05
9818	0.946132	0.05
9819	0.946132	0.05
9820	0.946132	0.05
9821	0.946132	0.05
9822	0.946132	2.1
9823	0.946132	2.1
9824	0.946131	2.1
9825	0.946132	2.1
9826	0.946132	2.1
9827	0.946132	2.1
9828	1.20608	2.1
9829	1.41232	2.1
9830	0.946128	2.1
9831	0.946129	2.1
9832	0.94613	2.1
9833	0.94613	2.1
9834	0.94613	2.1
9835	0.94613	2.1
9836	0.946132	0.05
9837	0.946131	0.05
9838	0.946131	0.05
9839	0.946132	0.05
9840	0.946132	0.05
9841	0.946131	0.05
9842	0.946132	0.05
9843	0.946116	0.0574318
9844	0.94612	2.02805
9845	0.946118	0.673855
9846	0.946124	2.1
9847	0.946118	1.06435
9848	1.41561	1.96421
9849	0.946131	0.952438
9850	0.945846	2.1
9851	1.03508	2.1
9852	0.77579	2.1
9853	0.658908	2.1
9854	1.17474	2.1
9855	0.94303	2.1
9856	0.849189	2.1
9857	0.94298	2.1

9858	1.03963	2.1
9859	0.946131	1.88726
9860	0.946131	1.5965
9861	0.946131	2.1
9862	0.946131	0.867979
9863	0.946131	0.05
9864	0.946131	1.32225
9865	1.32819	1.57745
9866	0.946133	1.00311
9867	0.946129	2.1
9868	0.946128	2.1
9869	0.94613	2.09886
9870	0.946131	2.1
9871	0.946131	0.992496
9872	0.946131	2.00404
9873	0.954072	0.05
9874	1.28895	0.05
9875	0.946133	0.05
9876	1.15803	0.05
9877	1.29689	0.05
9878	1.29689	0.05
9879	0.946132	0.05
9880	0.946132	0.483564
9881	0.946132	0.615851
9882	0.946132	0.139699
9883	0.946132	0.744455
9884	0.946132	1.51143
9885	0.946131	1.18973
9886	1.0264	1.29116
9887	0.946126	0.05
9888	0.946132	0.05
9889	0.946131	0.05
9890	0.94613	0.05
9891	0.946129	0.05
9892	0.94613	0.05
9893	1.35977	0.05
9894	0.946132	0.336572
9895	0.946133	0.326741
9896	0.876632	1.15213
9897	0.946132	1.10554
9898	1.01566	1.51272
9899	1.01566	0.910186
9900	0.876606	1.22005
9901	0.946131	0.05
9902	0.946128	0.05
9903	0.946128	0.05
9904	0.94613	0.05
9905	0.94613	0.05
9906	1.24979	0.05
9907	0.94613	0.05
9908	0.946131	0.05
9909	0.946131	0.05
9910	0.921762	0.05
9911	0.9705	0.05
9912	0.9705	0.05
9913	0.946131	0.05
9914	0.921791	0.05
9915	0.810944	0.05

9916	0.810945	0.05
9917	0.810959	0.05
9918	2.42267	0.05
9919	1.35168	0.05
9920	1.08132	0.05
9921	1.08132	0.05
9922	1.08131	0.05
9923	0.946133	0.05
9924	0.946132	0.05
9925	0.946132	0.05
9926	0.946133	0.05
9927	0.946133	0.05
9928	0.946132	0.05
9929	0.946133	0.05
9930	0.946131	0.162683
9931	0.946131	0.551238
9932	0.94613	0.0829127
9933	0.851514	0.109338
9934	0.946131	0.249986
9935	0.965268	0.352267
9936	0.851513	0.0890756
9937	0.946122	2.1
9938	0.946123	2.1
9939	0.946126	2.1
9940	0.946124	2.1
9941	0.946124	2.1
9942	0.946118	2.1
9943	0.94613	2.1
9944	0.946131	0.18775
9945	1.30448	0.588767
9946	0.946132	0.858879
9947	0.946129	0.417053
9948	0.946131	1.95629
9949	0.946131	0.199374
9950	0.94613	1.68049
9951	0.946133	0.347192
9952	0.946128	2.1
9953	0.946128	2.1
9954	0.94613	1.75023
9955	1.36503	2.1
9956	0.946131	2.1
9957	0.946131	2.1
9958	0.810947	0.05
9959	0.810947	0.05
9960	2.48233	0.05
9961	1.3517	0.05
9962	1.08132	0.05
9963	1.08132	0.05
9964	1.08133	0.05
9965	0.810934	0.05
9966	1.48874	0.05
9967	2.48233	0.05
9968	2.2369	0.05
9969	0.946133	0.157402
9970	0.946132	0.0904253
9971	0.946132	0.338178
9972	0.946133	0.396155
9973	0.946133	0.563995

9974	1.41851	0.129136
9975	0.946132	0.252088
9976	1.03925	2.1
9977	0.946131	2.1
9978	0.94613	2.1
9979	0.946128	2.1
9980	0.946133	2.1
9981	0.944743	2.1
9982	1.13703	2.1
9983	0.946132	2.1
9984	0.946128	2.1
9985	0.946128	2.1
9986	0.946131	2.1
9987	0.94613	2.1
9988	0.94613	2.1
9989	0.94613	2.1
9990	0.946131	0.05
9991	0.946132	0.05
9992	0.946131	0.05
9993	0.946132	0.05
9994	0.946132	0.05
9995	0.946131	0.05
9996	1.20057	0.05
9997	0.946129	2.1
9998	0.94613	2.1
9999	0.94613	2.1
10000	0.946128	2.1
10001	0.946132	2.1
10002	0.94613	2.1
10003	1.38302	2.1
10004	0.856567	0.05
10005	1.14319	0.05
10006	0.716526	0.05
10007	1.20631	0.05
10008	0.626936	0.05
10009	0.946131	0.05
10010	0.94613	0.05
10011	1.39502	0.05
10012	0.881445	0.05
10013	0.946128	0.05
10014	0.94613	0.05
10015	0.881447	0.05
10016	0.946117	0.05
10017	0.946116	0.05
10018	0.88146	0.05
10019	0.946132	2.1
10020	1.39053	2.1
10021	0.946132	2.1
10022	0.946132	2.1
10023	0.946132	2.1
10024	0.946132	2.1
10025	0.946131	2.1
10026	0.946131	2.1
10027	0.946131	2.1
10028	0.946131	2.1
10029	0.946132	2.1
10030	0.946132	2.1
10031	1.40571	2.1

10032	0.946132	2.1
10033	0.94613	0.05
10034	0.946127	0.065105
10035	0.946128	0.05
10036	0.946131	0.05
10037	0.946127	0.05
10038	1.25819	0.05
10039	0.946129	0.05
10040	0.946129	0.570081
10041	1.29054	0.497803
10042	0.94613	0.385228
10043	0.94613	0.46417
10044	0.946131	1.58964
10045	0.946131	0.797613
10046	0.94613	0.655137
10047	0.810944	0.05
10048	0.810941	0.05
10049	1.61558	0.05
10050	1.3517	0.05
10051	1.08131	0.05
10052	1.08132	0.05
10053	1.08133	0.05
10054	0.810931	0.05
10055	1.02036	1.78033
10056	0.946132	1.33409
10057	1.02036	1.01311
10058	0.946132	0.390293
10059	1.1576	0.875032
10060	0.964656	1.25366
10061	0.927608	0.330017
10062	0.946128	2.1
10063	1.38813	2.1
10064	0.946129	2.1
10065	0.946131	2.1
10066	0.946131	2.1
10067	0.94613	2.1
10068	0.946131	2.1
10069	0.946131	2.1
10070	0.94613	0.074078
10071	0.94613	2.1
10072	1.41665	2.1
10073	0.946132	0.108834
10074	0.946131	2.1
10075	0.94613	1.99168
10076	0.946118	0.056362
10077	0.946118	0.05
10078	0.946116	0.05
10079	0.946126	0.05
10080	0.94612	0.05
10081	0.946131	0.05
10082	0.946105	0.05
10083	0.946132	2.1
10084	0.946132	2.1
10085	0.946132	2.1
10086	0.946131	2.1
10087	0.946132	2.1
10088	0.946132	2.1
10089	0.946132	2.1

10090	0.864997	0.05
10091	0.786573	0.05
10092	0.978561	0.05
10093	1.47385	0.05
10094	1.68652	0.05
10095	1.27803	0.05
10096	1.19771	0.05
10097	1.27595	0.05
10098	0.946127	0.05
10099	1.3771	0.05
10100	0.946127	0.05
10101	0.946131	0.05
10102	0.946129	0.05
10103	0.946129	0.05
10104	0.946129	0.05
10105	0.95697	0.05
10106	1.48133	0.05
10107	1.24499	0.05
10108	1.5295	0.05
10109	0.938161	0.05
10110	0.808717	0.05
10111	1.3542	0.05
10112	0.946133	0.561086
10113	1.14444	0.299518
10114	0.946132	0.217856
10115	0.946132	0.436463
10116	0.946133	1.4609
10117	0.946132	0.804974
10118	0.946132	0.486477
10119	0.946131	0.05
10120	0.946132	0.05
10121	0.946131	0.05
10122	0.946132	0.05
10123	0.946131	0.05
10124	0.946132	0.05
10125	0.946132	0.05
10126	0.946132	2.1
10127	0.946133	2.1
10128	0.946133	2.1
10129	0.946132	2.1
10130	1.29322	2.1
10131	0.946132	2.1
10132	0.946132	2.1
10133	0.946131	2.1
10134	0.946132	2.1
10135	0.946131	2.1
10136	0.946132	2.1
10137	1.28441	2.1
10138	0.946131	2.1
10139	0.946131	2.1
10140	0.946113	2.1
10141	0.946118	2.1
10142	0.946118	2.00558
10143	1.28073	2.1
10144	0.946117	2.1
10145	0.946131	2.1
10146	0.946105	2.1
10147	0.810945	2.1

10148	0.810942	2.1
10149	2.75904	2.1
10150	1.3517	2.1
10151	1.08131	2.1
10152	1.08133	2.1
10153	1.08132	2.1
10154	0.81093	2.1
10155	0.946127	2.1
10156	1.37952	2.1
10157	1.86965	2.1
10158	0.946127	2.1
10159	1.86965	2.1
10160	1.28228	2.1
10161	1.37952	2.1
10162	0.946128	2.1
10163	0.946132	2.1
10164	1.14042	2.1
10165	0.946132	2.1
10166	1.14042	2.1
10167	1.09974	2.1
10168	1.09974	2.1
10169	0.946132	2.1
10170	0.946132	0.05
10171	0.946132	0.05
10172	0.946133	0.05
10173	0.946132	0.05
10174	0.946132	0.05
10175	0.946132	0.05
10176	0.946133	0.05
10177	1.31817	1.87963
10178	0.946131	1.16733
10179	0.946131	1.715
10180	0.946131	1.60119
10181	0.946131	0.584654
10182	0.946131	1.34933
10183	0.946131	1.30465
10184	0.946132	2.1
10185	0.946132	2.1
10186	0.946132	2.1
10187	0.946132	2.1
10188	0.946132	2.1
10189	0.946132	2.1
10190	1.35048	2.1
10191	0.946131	2.1
10192	0.946131	2.1
10193	0.94613	2.1
10194	0.946133	2.1
10195	0.946133	2.1
10196	1.35943	2.1
10197	0.946131	2.1
10198	0.946133	2.1
10199	0.946132	2.1
10200	0.946132	2.1
10201	0.946133	2.1
10202	0.946133	2.1
10203	0.946133	2.1
10204	0.990712	2.1
10205	0.965725	2.1

10206	0.946129	0.72723
10207	0.946133	1.62182
10208	1.21721	1.13832
10209	0.877931	0.518179
10210	0.946131	1.63215
10211	0.946131	0.649436
10212	0.946132	0.0699352
10213	0.946131	0.05
10214	0.946131	0.346606
10215	0.946133	0.0705846
10216	0.946128	0.983856
10217	0.946131	0.205849
10218	1.39575	0.148441
10219	0.810943	0.05
10220	0.810942	0.0552989
10221	2.58794	0.05
10222	1.21652	0.05
10223	0.946154	0.05
10224	0.979952	0.05
10225	1.0813	0.05
10226	0.810959	0.05
10227	0.924071	0.05
10228	1.16453	0.05
10229	1.28586	0.05
10230	0.946131	0.05
10231	1.1227	0.05
10232	1.16462	0.05
10233	0.851553	0.05
10234	0.709578	0.05
10235	0.810943	0.0621674
10236	0.810959	0.05
10237	1.82615	0.05
10238	1.21651	0.05
10239	1.08132	0.05
10240	0.946155	0.05
10241	0.979937	0.05
10242	0.946128	1.38425
10243	0.946132	2.1
10244	0.946132	0.284471
10245	0.94613	2.1
10246	0.94613	2.07077
10247	0.94613	0.226727
10248	0.94613	1.5791
10249	0.946131	2.05728
10250	1.29735	2.1
10251	0.946131	2.1
10252	0.946131	0.857403
10253	0.946131	1.74699
10254	0.946131	0.610762
10255	0.946131	2.1
10256	0.946128	2.1
10257	1.33266	0.914353
10258	0.94613	1.0803
10259	0.946131	2.1
10260	0.94613	0.0905317
10261	0.946131	1.06467
10262	0.946131	0.604325
10263	0.946131	0.05

10264	0.946131	2.1
10265	0.946131	0.05
10266	1.40608	0.05
10267	0.946131	2.1
10268	0.946131	0.106021
10269	0.946131	0.601185
10270	0.946131	0.545552
10271	0.946131	2.1
10272	0.946131	1.18845
10273	1.2334	1.19837
10274	0.946129	1.74945
10275	0.946131	1.22688
10276	0.946131	2.07443
10277	0.946133	2.1
10278	0.946133	2.1
10279	0.946132	1.68089
10280	1.29198	2.1
10281	0.946132	2.1
10282	0.946132	2.1
10283	0.946132	2.1
10284	0.946131	2.1
10285	0.946128	2.1
10286	0.946128	2.1
10287	0.946131	2.1
10288	0.946127	2.1
10289	0.94613	2.1
10290	0.946129	2.1
10291	0.946123	2.1
10292	0.946126	2.1
10293	0.946125	2.1
10294	1.38639	2.1
10295	0.946126	2.1
10296	0.946132	2.1
10297	0.946119	2.1
10298	0.946128	0.05
10299	0.946132	0.05
10300	0.946131	0.05
10301	0.94613	0.05
10302	0.94613	0.05
10303	0.946131	0.05
10304	0.94613	0.05
10305	0.946131	0.05
10306	0.946131	0.05
10307	0.946131	0.05
10308	0.946131	0.05
10309	0.946131	0.05
10310	0.946131	0.05
10311	0.946131	0.05
10312	0.810945	2.1
10313	0.810943	2.1
10314	0.810933	2.1
10315	2.24688	2.1
10316	1.3517	2.1
10317	1.08132	2.1
10318	1.08132	2.1
10319	1.08133	2.1
10320	1.25809	0.05
10321	0.946131	0.0580803

10322	1.25809	0.05
10323	0.946129	0.0582542
10324	0.94613	0.05
10325	0.946131	0.05
10326	0.946131	0.05
10327	0.946131	0.05
10328	0.946131	0.05
10329	0.946129	0.05
10330	1.28911	0.05
10331	0.946131	0.05
10332	0.946131	0.05
10333	0.946131	0.05
10334	0.94613	0.0502425
10335	0.946131	0.0709182
10336	0.94613	0.05
10337	0.946128	0.0568132
10338	0.946132	0.05
10339	1.38954	0.0577314
10340	0.946131	0.05
10341	0.946131	0.05
10342	0.946131	0.066711
10343	0.946131	0.106455
10344	1.37981	0.05
10345	0.946131	0.48811
10346	0.946131	0.0845867
10347	0.946131	0.0768137
10348	0.946131	2.1
10349	0.946127	2.1
10350	0.946127	2.1
10351	0.946131	2.1
10352	0.946127	2.1
10353	0.946129	2.1
10354	0.946128	2.1
10355	0.94613	2.1
10356	0.946126	2.1
10357	0.946127	2.1
10358	0.946131	2.1
10359	0.946126	2.1
10360	0.946128	2.1
10361	0.946128	2.1
10362	0.946133	0.05
10363	0.946132	0.05
10364	0.946132	0.05
10365	0.946132	0.05
10366	0.946132	0.0564893
10367	1.13511	0.05
10368	0.946132	0.05
10369	0.946132	1.93324
10370	0.946129	1.88458
10371	0.94613	0.836733
10372	0.946131	1.98937
10373	0.946131	2.1
10374	0.94613	2.1
10375	1.27575	2.1
10376	0.946129	2.1
10377	0.946131	2.1
10378	0.946131	2.1
10379	0.946128	2.1

10380	0.946133	2.1
10381	0.946131	2.1
10382	0.94613	2.1
10383	1.15581	0.05
10384	0.946132	0.05
10385	0.946131	0.05
10386	0.94613	0.05
10387	1.15581	0.05
10388	0.94613	0.05
10389	0.946131	0.05
10390	0.611407	0.94383
10391	0.946131	0.28182
10392	1.37335	0.451624
10393	1.15086	1.21028
10394	0.617549	0.512572
10395	0.851553	0.776214
10396	1.14592	0.927361
10397	1.22618	1.66495
10398	0.946132	0.381881
10399	0.946132	1.79298
10400	0.946132	0.353328
10401	0.946131	0.376098
10402	0.946132	1.22438
10403	0.946132	0.610653
10404	0.946131	2.1
10405	0.94613	2.1
10406	0.946131	2.1
10407	0.946133	2.1
10408	0.946129	2.1
10409	0.946131	2.1
10410	0.946131	2.1
10411	0.810945	0.789278
10412	0.810943	1.67003
10413	2.50715	1.45443
10414	1.3517	0.496459
10415	1.08133	0.339946
10416	1.08132	1.35506
10417	1.08132	0.0595065
10418	0.810932	0.457713
10419	1.07811	1.24486
10420	0.94613	0.320547
10421	0.946128	1.53355
10422	1.25358	0.0636005
10423	0.946131	0.320035
10424	1.25358	0.244463
10425	0.94613	0.331644
10426	0.946129	2.1
10427	1.09197	2.1
10428	0.946131	2.1
10429	1.09197	2.1
10430	1.09532	2.1
10431	1.09532	2.1
10432	0.946131	2.1
10433	1.58788	2.1
10434	1.34255	2.1
10435	0.594912	2.1
10436	0.58894	2.1
10437	0.946129	2.1

10438	1.16679	2.1
10439	1.04071	2.1
10440	0.535418	2.1
10441	0.946131	2.1
10442	0.946128	2.1
10443	0.946131	2.1
10444	1.20221	2.1
10445	0.946128	2.1
10446	0.94613	2.1
10447	1.20221	2.1
10448	0.946132	2.1
10449	0.946133	2.1
10450	0.946132	2.1
10451	0.946132	2.1
10452	0.946133	2.1
10453	0.946133	2.1
10454	0.946132	2.1
10455	0.946132	0.169563
10456	0.946132	1.46991
10457	0.946133	2.09432
10458	0.946132	0.438863
10459	1.411	1.65455
10460	0.946132	1.02065
10461	0.946133	1.45266
10462	1.05575	0.05
10463	0.563734	0.05
10464	1.05575	0.05
10465	0.773833	0.05
10466	0.751686	0.05
10467	0.75884	0.05
10468	0.815612	0.05
10469	0.793465	0.05
10470	0.768563	0.05
10471	0.946129	0.05
10472	0.946125	0.05
10473	0.946126	0.05
10474	0.94613	0.05
10475	0.946124	0.05
10476	1.31678	0.05
10477	0.946128	0.05
10478	0.946132	0.05
10479	1.29914	0.05
10480	0.946132	0.05
10481	0.946132	0.05
10482	0.946132	0.05
10483	1.29914	0.05
10484	0.946132	0.05
10485	0.810946	0.05
10486	0.810943	0.05
10487	2.21082	0.05
10488	1.35168	0.05
10489	1.08132	0.05
10490	1.08132	0.05
10491	1.0813	0.05
10492	0.81096	0.05
10493	0.625813	0.05
10494	0.946132	0.05
10495	0.946131	0.05

10496	0.946132	0.05
10497	0.946131	0.05
10498	1.39301	0.05
10499	0.625812	0.05
10500	0.810946	1.92684
10501	0.810945	2.1
10502	0.810931	2.1
10503	2.70043	2.1
10504	1.3517	2.1
10505	1.08131	2.02849
10506	1.08131	2.1
10507	1.08133	2.1
10508	0.810943	2.1
10509	0.810944	2.1
10510	0.810958	2.1
10511	2.44802	2.1
10512	1.35168	2.1
10513	1.08132	2.1
10514	1.08132	2.1
10515	1.0813	2.1
10516	0.946131	0.05
10517	0.946129	0.0567712
10518	0.946128	0.05
10519	0.946131	0.05
10520	1.3492	0.05
10521	1.3492	0.05
10522	0.94613	0.05
10523	0.946132	0.158797
10524	0.946132	0.05
10525	0.946133	0.136979
10526	0.946133	0.05
10527	0.946132	0.380238
10528	0.946132	0.05
10529	0.946132	0.275117
10530	0.946129	0.05
10531	0.946133	0.05
10532	0.946131	0.05
10533	0.94613	0.05
10534	0.946131	0.05
10535	0.946131	0.05
10536	0.946131	0.05
10537	0.946119	0.05
10538	0.946119	0.05
10539	0.946119	0.05
10540	0.946125	0.05
10541	0.94612	0.05
10542	0.946132	0.05
10543	0.946107	0.05
10544	0.946133	2.1
10545	0.946132	2.1
10546	0.946132	2.1
10547	0.946132	2.1
10548	0.946132	2.1
10549	0.946133	2.1
10550	1.3868	2.1
10551	0.810947	2.1
10552	0.810946	2.1
10553	0.81096	2.1

10554	2.66094	2.1
10555	1.35168	2.1
10556	1.08132	2.1
10557	1.08132	2.1
10558	1.0813	2.1
10559	0.810946	2.1
10560	0.810946	2.1
10561	0.810933	2.1
10562	2.75737	2.1
10563	1.3517	2.1
10564	1.08132	2.1
10565	1.08132	2.1
10566	1.08133	2.1
10567	0.946133	2.1
10568	0.946132	2.1
10569	0.946132	2.1
10570	0.946132	2.1
10571	0.946133	2.1
10572	0.946132	2.1
10573	0.946133	2.1
10574	0.946133	2.1
10575	0.946133	2.1
10576	0.946132	2.1
10577	0.946132	2.1
10578	0.946133	2.1
10579	0.946132	2.1
10580	1.28569	2.1
10581	0.946132	1.85635
10582	0.946133	2.1
10583	0.946133	2.1
10584	0.946132	2.1
10585	0.946132	2.1
10586	0.946132	2.1
10587	0.946133	2.1
10588	0.946132	0.05
10589	0.946131	0.05
10590	0.94613	0.05
10591	0.946133	0.05
10592	0.946128	0.05
10593	0.946131	0.05
10594	0.946131	0.05
10595	0.946131	2.1
10596	0.94613	2.1
10597	0.946131	2.1
10598	0.94613	2.1
10599	1.41833	1.43671
10600	0.946131	1.75629
10601	0.946131	2.1
10602	1.26155	0.05
10603	0.946131	0.05
10604	0.946131	0.05
10605	0.946132	0.05
10606	0.946132	0.05
10607	0.946131	0.05
10608	0.946132	0.05
10609	0.810946	2.1
10610	0.810946	2.1
10611	0.810959	2.1

10612	2.47938	2.1
10613	1.35168	2.1
10614	1.08132	2.1
10615	1.08132	2.1
10616	1.08131	2.1
10617	0.946131	0.0662412
10618	0.94613	0.0508867
10619	0.94613	0.294363
10620	0.946132	0.05
10621	0.946128	0.725789
10622	0.94613	0.191008
10623	0.94613	0.173286
10624	0.94613	0.05
10625	0.946131	0.05
10626	0.94613	0.05
10627	0.946129	0.05
10628	0.946132	0.05
10629	0.94613	0.05
10630	0.946131	0.05
10631	0.946132	2.1
10632	0.946131	2.1
10633	0.946128	2.1
10634	1.40756	2.1
10635	0.946131	2.1
10636	0.946129	2.1
10637	0.94613	2.1
10638	0.946131	1.72913
10639	0.946131	1.62988
10640	0.946131	2.1
10641	0.946132	2.00876
10642	1.31947	1.97153
10643	0.946131	2.05991
10644	0.946131	2.04647
10645	0.946131	2.1
10646	1.25768	2.1
10647	0.946132	2.1
10648	0.946132	2.1
10649	0.946129	2.1
10650	0.946131	2.1
10651	0.94613	2.1
10652	0.946132	0.0643744
10653	0.946129	0.05
10654	0.946129	0.0660725
10655	0.94613	0.05
10656	1.31716	0.05
10657	1.31716	0.05
10658	0.946131	0.0760802
10659	0.810943	0.954375
10660	0.810942	2.1
10661	0.81093	2.1
10662	2.37052	2.1
10663	1.3517	2.1
10664	1.08132	0.537548
10665	1.08132	2.1
10666	1.08133	1.23242
10667	0.946128	1.00953
10668	1.10335	0.641367
10669	0.94613	0.289428

10670	0.946131	1.41558
10671	0.946131	0.240902
10672	1.10335	0.502028
10673	0.94613	0.998101
10674	0.946132	1.08929
10675	1.18818	0.712627
10676	0.946132	0.439299
10677	0.946132	1.0719
10678	0.946132	0.341095
10679	1.18818	0.445502
10680	0.946132	0.901698
10681	0.946125	2.1
10682	0.94612	2.1
10683	0.946119	2.1
10684	0.946119	2.1
10685	0.946119	2.1
10686	0.946132	2.1
10687	1.31522	2.1
10688	1.28803	2.1
10689	0.946132	0.162597
10690	0.946132	2.1
10691	0.946133	2.1
10692	0.946133	0.0947264
10693	0.946133	2.1
10694	0.946132	1.80051
10695	0.946133	1.27036
10696	0.946132	1.64984
10697	0.946132	1.13563
10698	0.946132	0.701326
10699	0.946133	0.751107
10700	0.946132	0.626845
10701	1.37658	0.912919
10702	1.02239	1.92023
10703	0.946128	0.799088
10704	0.946132	1.70062
10705	1.02239	1.01206
10706	0.94613	0.866745
10707	0.96283	1.74607
10708	0.92943	0.658889
10709	0.946132	0.05
10710	0.946131	0.05
10711	0.946126	0.05
10712	0.946129	0.05
10713	0.946128	0.05
10714	0.946128	0.05
10715	0.946129	0.05
10716	0.810946	0.05
10717	0.810944	0.05
10718	2.22493	0.05
10719	1.21651	0.05
10720	1.0813	0.05
10721	0.946154	0.05
10722	0.979951	0.05
10723	0.70959	0.05
10724	0.946132	0.05
10725	0.946131	0.05
10726	0.946131	0.05
10727	0.946133	0.05

10728	0.946131	0.05
10729	0.946132	0.05
10730	0.946132	0.05
10731	0.946132	2.1
10732	0.946131	2.03154
10733	0.946132	2.1
10734	0.946132	1.32806
10735	0.946132	0.372028
10736	0.946132	1.43732
10737	1.40924	2.02049
10738	0.946131	2.1
10739	0.946128	2.1
10740	0.946127	2.1
10741	0.94613	2.1
10742	1.41743	2.1
10743	0.946128	2.1
10744	0.946129	2.1
10745	1.37675	0.248205
10746	0.946128	0.374475
10747	0.94613	0.273346
10748	0.94613	1.03039
10749	0.94613	0.335656
10750	0.946129	0.358406
10751	0.94613	0.840271
10752	0.946132	0.05
10753	0.946132	0.0555144
10754	0.946132	0.115068
10755	1.35721	0.05
10756	0.946132	0.430905
10757	0.946132	0.114298
10758	1.35721	0.0605437
10759	0.946133	0.841042
10760	0.946133	0.289341
10761	0.946132	0.725471
10762	1.10003	0.112454
10763	0.946133	0.234382
10764	0.946132	0.534949
10765	0.946132	0.308943
10766	0.946132	0.05
10767	0.946131	0.05
10768	0.946131	0.05
10769	0.946131	0.05
10770	1.0616	0.05
10771	1.0616	0.05
10772	0.946132	0.05
10773	0.946132	0.05
10774	0.946131	0.05
10775	0.946131	0.05
10776	0.946131	0.05
10777	1.09402	0.05
10778	1.09402	0.05
10779	0.946131	0.05
10780	0.946132	0.05
10781	0.946126	0.05
10782	1.24093	0.05
10783	0.946129	0.05
10784	0.946129	0.05
10785	0.946128	0.05

10786	0.946129	0.05
10787	0.946131	0.05
10788	0.946131	0.05
10789	1.2929	0.05
10790	0.946131	0.05
10791	0.946131	0.05
10792	0.946131	0.05
10793	0.946131	0.05
10794	0.810946	0.05
10795	0.810945	0.05
10796	0.810959	0.05
10797	2.41233	0.05
10798	1.35168	0.05
10799	1.08132	0.05
10800	1.08132	0.05
10801	1.0813	0.05
10802	0.810946	1.40241
10803	0.810943	2.1
10804	0.750103	2.09598
10805	2.22778	2.1
10806	1.27059	2.1
10807	1.00021	1.83519
10808	1.02049	2.1
10809	1.08133	2.00045
10810	0.946128	2.1
10811	0.946132	2.1
10812	0.946132	2.1
10813	0.94613	2.1
10814	0.94613	2.1
10815	0.94613	2.1
10816	0.946131	2.1
10817	0.946126	0.05
10818	0.946131	0.05
10819	0.946127	0.05
10820	1.3324	0.05
10821	0.946127	0.05
10822	0.946128	0.05
10823	0.946128	0.0507789
10824	0.94613	2.1
10825	0.946131	2.1
10826	0.94613	2.1
10827	0.946128	2.1
10828	0.946133	2.1
10829	0.946131	2.1
10830	0.946131	2.1
10831	0.946133	2.1
10832	0.946132	2.1
10833	0.946133	2.1
10834	0.946133	2.1
10835	0.946132	2.1
10836	0.946132	2.1
10837	1.36541	2.1
10838	0.946131	0.06941
10839	0.94613	2.1
10840	0.946131	0.422617
10841	0.94613	0.23611
10842	1.39509	1.88441
10843	0.946131	0.0571651

10844	0.946131	2.00635
10845	0.946133	2.1
10846	0.946133	2.1
10847	0.946132	2.1
10848	0.946132	2.1
10849	0.946133	2.1
10850	0.946132	2.1
10851	1.29319	2.1
10852	1.01196	0.05
10853	0.946131	0.05
10854	0.946132	0.05
10855	0.946132	0.05
10856	0.946132	0.05
10857	0.946132	0.05
10858	0.946132	0.05
10859	0.946112	2.1
10860	0.946118	2.1
10861	0.946116	2.1
10862	0.946117	2.1
10863	0.946118	2.1
10864	0.94613	2.1
10865	0.946105	2.1
10866	1.28684	1.19717
10867	0.946131	1.19467
10868	0.946132	1.24094
10869	0.946132	1.62944
10870	0.946132	1.70104
10871	0.946132	1.33067
10872	0.946132	1.78469
10873	0.94613	2.1
10874	0.94613	2.1
10875	0.946128	2.05321
10876	0.946131	1.40356
10877	0.946131	2.04351
10878	0.94613	2.06006
10879	1.10185	2.1
10880	0.810947	0.05
10881	0.810945	0.05
10882	1.57348	0.05
10883	1.3517	0.05
10884	1.08131	0.05
10885	1.08132	0.05
10886	1.08133	0.05
10887	0.810932	0.05
10888	0.946119	0.109023
10889	0.946116	0.05
10890	0.946117	0.0986421
10891	0.946121	0.05
10892	0.946118	0.05
10893	0.94613	0.053172
10894	0.946105	0.05
10895	0.94612	0.171155
10896	0.946122	0.05
10897	0.946119	0.05
10898	0.946119	0.221434
10899	1.20884	0.05
10900	0.946132	0.0798911
10901	0.946107	0.05

10902	2.67849	0.72531
10903	1.17709	0.544412
10904	0.605418	1.33981
10905	0.286984	0.609459
10906	0.172654	0.842388
10907	0.810946	0.173159
10908	0.940878	0.123128
10909	0.941235	0.0929996
10910	1.26696	0.0829917
10911	1.56837	0.131733
10912	1.13737	0.0722129
10913	1.34115	0.182943
10914	1.24889	0.0702464
10915	0.946132	1.19039
10916	0.946129	0.725267
10917	0.946128	0.96749
10918	0.94613	0.857572
10919	0.94613	1.26626
10920	0.946131	1.04807
10921	0.94613	1.04337
10922	0.946116	2.1
10923	0.94612	2.1
10924	0.946118	2.1
10925	0.946123	2.1
10926	0.946118	2.1
10927	0.946131	2.1
10928	1.23836	2.1
10929	0.810946	0.05
10930	0.810946	0.05
10931	0.810959	0.05
10932	2.80677	0.05
10933	1.35168	0.05
10934	1.08132	0.05
10935	1.08132	0.05
10936	1.08131	0.05
10937	0.810947	0.05
10938	0.810947	0.810878
10939	2.41574	0.942571
10940	1.21653	0.861657
10941	1.08133	0.204185
10942	0.946153	1.05255
10943	0.979949	0.123455
10944	0.709567	0.218626
10945	0.94613	2.1
10946	0.946131	2.1
10947	0.946128	2.1
10948	1.34782	2.1
10949	0.946128	2.1
10950	0.946129	2.1
10951	1.34782	2.1
10952	0.946132	2.1
10953	0.946132	2.1
10954	0.94613	2.1
10955	1.16023	2.1
10956	0.946131	2.1
10957	0.946131	2.1
10958	1.16023	2.1
10959	0.946131	0.840524

10960	0.946127	2.1
10961	0.946127	2.1
10962	0.946131	1.9669
10963	0.946128	2.1
10964	0.94613	1.14234
10965	0.946129	2.02841
10966	0.946133	0.05
10967	0.946132	0.05
10968	0.946132	0.05
10969	0.946133	0.05
10970	1.20045	0.05
10971	1.20045	0.05
10972	0.946132	0.05
10973	0.918871	0.05
10974	0.971961	0.05
10975	1.37324	0.05
10976	1.78255	0.05
10977	1.25155	0.05
10978	1.42172	0.05
10979	1.40554	0.05
10980	1.08862	0.05
10981	1.03803	0.05
10982	0.946132	0.05
10983	0.946126	0.05
10984	0.943647	0.05
10985	0.948611	0.05
10986	0.946129	0.05
10987	0.946129	0.05
10988	1.1699	0.228742
10989	0.946132	0.162854
10990	0.946133	0.572342
10991	0.946133	0.146223
10992	0.946132	0.159172
10993	0.946133	0.262664
10994	0.946132	0.490794
10995	0.946132	2.1
10996	0.946131	2.1
10997	0.946131	2.1
10998	0.946132	2.1
10999	0.946131	2.1
11000	0.946131	2.1
11001	0.946132	2.1
11002	0.946132	0.544719
11003	1.38623	0.336183
11004	0.946132	0.136384
11005	0.946132	0.169971
11006	0.946133	0.457096
11007	0.946132	0.134998
11008	0.946133	0.851304
11009	0.946132	2.1
11010	1.40098	2.1
11011	0.946131	2.1
11012	0.946131	2.1
11013	0.946132	2.1
11014	0.946132	2.1
11015	0.946132	2.1
11016	1.01777	0.206003
11017	0.946132	0.0783015

11018	0.946133	1.21175
11019	1.68068	0.153713
11020	1.68068	1.8166
11021	1.41894	0.0602094
11022	1.20787	0.0600057
11023	0.946132	0.127349
11024	0.946133	2.1
11025	0.946133	2.1
11026	0.946132	2.1
11027	1.40199	2.1
11028	0.946133	2.1
11029	0.946132	2.1
11030	0.946132	2.1
11031	0.810946	1.39487
11032	0.810947	0.393034
11033	2.37827	1.73044
11034	1.3517	1.14253
11035	1.08132	1.19045
11036	1.08132	1.43418
11037	1.08133	1.22838
11038	0.810934	1.05163
11039	0.946124	2.1
11040	0.94613	1.92824
11041	1.40248	1.19101
11042	0.946131	1.22735
11043	0.946125	0.470341
11044	0.946127	1.82365
11045	0.946128	0.480491
11046	1.25703	1.98184
11047	0.87821	1.0875
11048	1.18912	1.91111
11049	0.946119	0.997444
11050	0.87825	1.15823
11051	1.1891	1.43312
11052	0.946107	2.01755
11053	0.946132	2.1
11054	1.28543	2.1
11055	0.946132	2.00602
11056	0.946132	1.84842
11057	0.946131	0.992882
11058	0.946132	0.488622
11059	0.946132	0.770458
11060	0.946115	2.1
11061	0.946119	2.1
11062	0.946116	2.1
11063	0.946123	2.1
11064	0.946118	2.1
11065	1.41636	2.1
11066	0.946129	2.1
11067	0.946132	0.220286
11068	0.946132	2.1
11069	0.946132	1.25802
11070	0.946131	2.1
11071	0.946131	2.1
11072	1.41432	2.1
11073	0.946132	1.98962
11074	0.810947	0.05
11075	0.810947	0.05

11076	2.44433	0.05
11077	1.21654	0.05
11078	0.946154	0.05
11079	0.97995	0.05
11080	1.08133	0.05
11081	0.810934	0.05
11082	0.851542	0.05
11083	0.835698	0.05
11084	1.22216	0.05
11085	0.946133	0.05
11086	1.01217	0.05
11087	1.09996	0.05
11088	0.851529	0.05
11089	0.946132	1.29682
11090	0.946132	2.1
11091	0.946129	2.1
11092	0.94613	1.87415
11093	0.946131	2.1
11094	0.946131	2.1
11095	1.2254	2.1
11096	0.800897	1.18086
11097	0.838029	2.1
11098	0.838013	1.68473
11099	0.93679	0.356169
11100	1.20217	0.625097
11101	1.16923	1.08394
11102	0.94948	1.31507
11103	0.958118	1.52763
11104	0.36751	0.917212
11105	0.800535	1.77252
11106	0.272087	0.150725
11107	0.428084	0.536906
11108	0.986335	1.69504
11109	0.946132	0.05
11110	0.946133	0.0698117
11111	1.19601	0.05
11112	0.946132	0.05
11113	0.946132	0.05
11114	0.946133	0.05
11115	0.946133	0.05
11116	0.946132	2.01889
11117	1.36956	2.1
11118	0.946132	2.1
11119	0.946132	1.87512
11120	0.946132	2.1
11121	0.946133	2.1
11122	0.946132	2.1
11123	0.946132	0.05
11124	0.946131	0.144013
11125	0.946131	0.05
11126	1.34268	0.05
11127	0.946133	0.05
11128	0.946132	0.05
11129	0.946131	0.05
11130	0.946132	2.1
11131	0.946131	2.1
11132	0.946131	2.1
11133	0.946132	2.1

11134	0.946132	2.1
11135	0.946132	2.1
11136	0.946132	2.1
11137	0.810946	0.85269
11138	0.810943	0.537524
11139	2.51472	0.663488
11140	1.3517	0.374848
11141	1.08132	0.422722
11142	1.08132	0.773006
11143	1.08133	0.555619
11144	0.810933	0.63071
11145	0.902316	0.0896329
11146	1.04517	0.12256
11147	0.974924	0.05
11148	0.530063	0.330835
11149	1.1272	0.159667
11150	0.68207	0.110043
11151	0.477061	0.227791
11152	0.810946	0.05
11153	0.810943	0.05
11154	0.810959	0.05
11155	1.99211	0.05
11156	1.35168	0.05
11157	1.08132	0.05
11158	1.08132	0.05
11159	1.08131	0.05
11160	0.94613	0.05
11161	0.946131	0.05
11162	0.946132	0.05
11163	0.946132	0.05
11164	0.946129	0.05
11165	0.946131	0.05
11166	1.16699	0.05
11167	0.810943	1.31669
11168	0.810942	1.17237
11169	1.6943	2.07598
11170	1.27059	2.1
11171	1.00021	2.0928
11172	1.02049	1.93085
11173	1.08133	1.29096
11174	0.750103	1.36898
11175	0.957813	1.49403
11176	0.946128	1.80788
11177	0.946132	0.649777
11178	1.04832	1.95609
11179	0.870714	0.82202
11180	0.94613	0.724643
11181	0.94613	1.31599
11182	1.86374	2.1
11183	2.0815	1.34319
11184	1.15918	0.928685
11185	0.946129	0.792581
11186	1.15918	0.14425
11187	0.946131	1.23355
11188	0.946131	0.145072
11189	0.946131	0.687057
11190	0.94613	1.13775
11191	0.946119	0.639486

11192	1.3612	0.730933
11193	0.946125	0.685799
11194	0.946119	0.555934
11195	0.94612	1.48461
11196	0.946132	0.866462
11197	0.946107	0.787161
11198	0.810947	0.05
11199	0.810947	0.101483
11200	2.50398	0.05
11201	1.3517	0.05
11202	1.08132	0.05
11203	1.08133	0.066914
11204	1.08132	0.05
11205	0.810934	0.05
11206	0.946132	2.1
11207	0.946132	2.1
11208	0.946132	2.1
11209	1.39754	2.1
11210	0.946132	2.1
11211	0.946132	2.1
11212	0.946131	2.1
11213	0.946129	2.1
11214	1.41732	2.1
11215	0.946129	2.1
11216	0.946131	2.1
11217	0.946131	2.1
11218	0.946131	2.1
11219	0.946131	2.1
11220	0.946132	1.18966
11221	1.25276	2.09015
11222	0.946131	2.1
11223	0.94613	1.19237
11224	0.94613	1.98755
11225	0.94613	1.43839
11226	0.946131	1.64558
11227	1.21261	0.237678
11228	0.94613	0.603175
11229	0.94613	1.84537
11230	0.946128	0.185569
11231	0.946131	1.32223
11232	0.94613	1.10442
11233	0.946129	1.28481
11234	0.946128	2.1
11235	0.946133	2.1
11236	0.946132	2.1
11237	0.946131	2.1
11238	0.94613	2.1
11239	0.946131	2.1
11240	1.39407	2.1
11241	0.946116	1.62463
11242	0.946119	1.67485
11243	0.946119	0.936321
11244	1.15369	1.52719
11245	0.946119	0.647702
11246	0.946131	1.33071
11247	0.946106	1.12083
11248	0.946131	2.1
11249	0.946131	2.1

11250	0.946131	2.1
11251	0.946133	2.1
11252	0.946129	2.1
11253	0.946131	2.1
11254	0.946131	2.1
11255	1.19564	0.05
11256	0.498881	0.05
11257	1.33964	0.05
11258	0.498878	0.05
11259	0.923583	0.05
11260	0.831259	0.05
11261	0.553735	0.05
11262	0.946132	0.05
11263	0.946132	0.05
11264	0.946131	0.05
11265	0.946131	0.05
11266	0.946132	0.05
11267	0.946131	0.05
11268	1.28673	0.05
11269	0.946132	0.05
11270	0.946132	0.05
11271	1.33178	0.05
11272	0.946132	0.05
11273	0.946132	0.05
11274	0.946131	0.05
11275	0.946131	0.05
11276	0.946128	0.0774721
11277	0.946132	0.05
11278	0.946129	0.111825
11279	0.946131	0.05
11280	0.946131	0.05
11281	0.946129	0.0524555
11282	0.94613	0.05
11283	0.946131	2.1
11284	0.946131	2.1
11285	0.946129	2.1
11286	1.40525	2.1
11287	0.94613	2.1
11288	0.94613	2.1
11289	1.40525	2.1
11290	0.946131	2.1
11291	0.946126	2.1
11292	0.946127	2.1
11293	0.946127	2.1
11294	1.4073	2.1
11295	0.946129	2.1
11296	0.946129	2.1
11297	0.946131	0.05
11298	0.946131	0.05
11299	0.946132	0.05
11300	0.946131	0.05
11301	0.946131	0.05
11302	1.05016	0.05
11303	0.946132	0.05
11304	0.946128	0.05
11305	0.946132	0.05
11306	0.946131	0.05
11307	0.946129	0.05

11308	0.94613	0.05
11309	1.13398	0.05
11310	0.94613	0.05
11311	0.946132	0.05
11312	0.946132	0.05
11313	0.946131	0.05
11314	0.946131	0.05
11315	0.946132	0.05
11316	0.946131	0.05
11317	0.946132	0.05
11318	0.946131	0.05
11319	0.94613	0.05
11320	0.946131	0.05
11321	0.94613	0.05
11322	1.38645	0.05
11323	1.38645	0.05
11324	0.946131	0.05
11325	0.946131	0.05
11326	0.946131	0.05
11327	0.94613	0.05
11328	1.23923	0.05
11329	0.94613	0.05
11330	0.946131	0.05
11331	0.946131	0.05
11332	0.946132	0.05
11333	0.946131	0.05
11334	0.946131	0.05
11335	0.946132	0.05
11336	0.946132	0.05
11337	0.946132	0.05
11338	1.40511	0.05
11339	0.946132	2.1
11340	0.946132	2.1
11341	0.946132	2.1
11342	0.946132	2.1
11343	0.946132	2.1
11344	0.946131	2.1
11345	1.12968	2.1
11346	0.946132	1.5929
11347	0.946128	2.1
11348	0.946128	2.1
11349	0.94613	2.02624
11350	0.94613	2.1
11351	0.94613	1.788
11352	0.94613	2.1
11353	0.946133	2.1
11354	0.946133	2.1
11355	0.946132	2.1
11356	0.946132	2.1
11357	0.946132	2.1
11358	0.946133	2.1
11359	1.31323	2.1
11360	0.94613	2.1
11361	0.946131	0.064643
11362	0.946131	0.0668397
11363	0.94613	0.05
11364	0.946131	2.1
11365	0.946131	1.28958

11366	0.946131	0.535725
11367	0.946132	0.05
11368	0.946132	0.05
11369	0.946132	0.0502322
11370	1.36899	0.05
11371	0.946132	0.0918273
11372	0.946132	0.05
11373	0.946133	0.05
11374	1.03543	0.05
11375	0.946131	0.05
11376	0.946131	0.05
11377	0.946132	0.05
11378	0.94613	0.05
11379	0.950987	0.05
11380	0.941275	0.05
11381	0.946131	0.05
11382	0.94613	0.05
11383	1.28952	0.05
11384	0.946132	0.05
11385	0.94613	0.05
11386	0.946131	0.05
11387	0.946131	0.05
11388	0.94613	2.1
11389	1.35966	2.1
11390	0.946131	2.1
11391	0.946131	2.1
11392	0.946129	2.1
11393	0.94613	2.1
11394	0.94613	2.1
11395	0.810946	2.1
11396	0.810942	0.501249
11397	0.810932	2.1
11398	2.6655	2.1
11399	1.3517	2.1
11400	1.08132	2.1
11401	1.08131	0.292609
11402	1.08133	2.1
11403	0.94613	2.1
11404	0.946131	2.1
11405	0.94613	2.1
11406	0.946128	2.1
11407	0.946131	2.1
11408	0.94613	2.1
11409	0.94613	2.1
11410	0.946128	2.1
11411	0.946128	2.1
11412	0.946133	2.1
11413	1.25375	2.1
11414	0.94613	2.1
11415	0.946131	2.1
11416	1.25375	2.1
11417	0.94613	2.1
11418	0.94613	2.1
11419	0.946132	2.1
11420	0.946132	2.1
11421	0.946128	2.1
11422	0.94613	2.1
11423	1.31453	2.1

11424	0.946131	0.05
11425	0.946129	0.05
11426	0.94613	0.05
11427	0.94613	0.05
11428	0.94613	0.05
11429	1.12018	0.05
11430	0.94613	0.05
11431	0.946131	0.05
11432	0.946129	0.05
11433	0.946131	0.05
11434	1.16373	0.05
11435	0.94613	0.05
11436	0.94613	0.05
11437	0.946131	0.05
11438	1.36811	0.05
11439	0.946132	0.05
11440	1.36811	0.05
11441	0.946129	0.05
11442	0.94613	0.0510526
11443	0.946129	0.05
11444	0.946129	0.05
11445	0.946132	0.05
11446	0.946133	0.05
11447	0.946132	0.05
11448	0.946133	0.05
11449	1.35732	0.05
11450	1.35732	0.05
11451	0.946133	0.05
11452	1.36399	0.192669
11453	0.946129	0.692628
11454	0.94613	0.332046
11455	0.946131	0.902291
11456	0.94613	1.84609
11457	0.946131	0.554969
11458	0.946131	1.17166
11459	0.946133	2.1
11460	0.946132	2.1
11461	0.946132	2.1
11462	1.38302	2.1
11463	0.946133	2.1
11464	0.946132	2.1
11465	0.946133	2.1
11466	0.810943	2.1
11467	0.810944	2.1
11468	0.810945	2.1
11469	2.80542	2.1
11470	1.35169	2.1
11471	1.08132	2.1
11472	1.08132	2.1
11473	1.08132	2.1
11474	1.30189	2.1
11475	0.946131	2.1
11476	0.946132	2.1
11477	0.946131	2.1
11478	0.946131	2.1
11479	0.946131	2.1
11480	0.946132	2.1
11481	0.810946	0.997281

11482	0.810947	0.350704
11483	2.33293	0.363762
11484	1.24357	0.44973
11485	1.08132	0.617812
11486	0.973183	0.889528
11487	1.00023	0.593273
11488	0.810934	0.883627
11489	1.06584	0.328438
11490	0.946129	0.567668
11491	0.84731	0.140936
11492	1.16646	0.344263
11493	0.946126	0.913446
11494	1.2831	0.206518
11495	0.851527	0.528296
11496	0.946131	2.1
11497	0.946128	2.1
11498	0.946128	2.1
11499	0.94613	2.1
11500	1.31961	2.1
11501	0.94613	2.1
11502	0.94613	2.1
11503	0.946131	2.1
11504	1.3605	2.1
11505	0.946133	2.1
11506	0.946132	2.1
11507	0.94613	2.1
11508	0.946131	2.1
11509	0.946131	2.1
11510	0.946128	2.1
11511	0.946133	1.88615
11512	0.946132	2.1
11513	0.946131	1.37429
11514	0.946131	0.0908409
11515	0.946131	2.02675
11516	1.30116	2.1
11517	0.946132	0.05
11518	0.946129	0.05
11519	0.94613	0.05
11520	1.3035	0.05
11521	0.946131	0.05
11522	0.94613	0.05
11523	0.946131	0.05
11524	0.946132	2.1
11525	1.2051	2.1
11526	1.2051	2.1
11527	0.946132	2.1
11528	1.37869	2.1
11529	0.946132	2.1
11530	0.946131	2.1
11531	0.810946	2.1
11532	0.810945	2.1
11533	2.69247	2.1
11534	1.35169	2.1
11535	1.08132	2.1
11536	1.08132	2.1
11537	1.08132	2.1
11538	0.810946	2.1
11539	1.22993	2.1

11540	0.946131	2.1
11541	0.946131	2.1
11542	1.34623	2.1
11543	0.946131	2.1
11544	1.34623	2.1
11545	0.946131	2.1
11546	0.946131	0.05
11547	0.946132	0.05
11548	0.946132	0.05
11549	0.946132	0.05
11550	0.946131	0.05
11551	0.946132	0.05
11552	1.32299	0.05
11553	0.94613	0.05
11554	0.946131	0.0581401
11555	0.946128	0.05
11556	0.946132	0.05
11557	0.946133	0.05
11558	0.946131	0.05
11559	0.946131	0.05
11560	0.946132	2.1
11561	0.946129	2.1
11562	0.946128	2.1
11563	0.94613	2.1
11564	0.94613	2.1
11565	0.946131	2.1
11566	0.94613	2.1
11567	0.94613	2.1
11568	0.94613	2.1
11569	0.946131	2.1
11570	0.946132	2.1
11571	0.946129	2.1
11572	0.946131	2.1
11573	0.946131	2.1
11574	0.946132	1.17776
11575	0.946131	1.25207
11576	1.13731	0.507821
11577	0.94775	1.5027
11578	0.944514	2.04621
11579	0.946132	2.03334
11580	1.039	2.02876
11581	0.946131	1.19659
11582	0.946128	0.929423
11583	0.94613	0.326488
11584	0.946131	1.11447
11585	0.946128	1.75428
11586	0.946129	1.89168
11587	1.23726	1.84692
11588	0.946132	2.1
11589	0.946132	2.1
11590	0.946129	2.1
11591	1.39214	2.1
11592	0.946131	2.1
11593	0.946131	2.1
11594	0.94613	2.1
11595	0.946125	0.0675283
11596	0.946119	0.05
11597	0.946118	0.05

11598	0.946118	0.05
11599	0.94612	0.05
11600	0.946106	0.05
11601	0.946131	0.05
11602	1.26893	0.469788
11603	0.946131	0.461489
11604	1.26893	0.365427
11605	0.94613	0.743418
11606	0.946131	0.636351
11607	0.946131	0.393874
11608	0.946131	1.24895
11609	0.946133	0.05
11610	0.946133	0.05
11611	0.946132	0.05
11612	1.28924	0.05
11613	0.946133	0.05
11614	0.946132	0.05
11615	0.946132	0.05
11616	0.946133	0.0859621
11617	0.946132	0.05
11618	0.946132	0.0755009
11619	0.946133	0.05
11620	0.946133	0.0841164
11621	0.946132	0.0802489
11622	0.946133	0.0584314
11623	0.946132	0.304987
11624	0.946133	0.05
11625	0.946133	0.0941183
11626	0.946132	0.262685
11627	1.15388	0.0842668
11628	0.946132	0.249892
11629	0.946133	0.133864
11630	0.946132	0.113056
11631	0.946133	0.05
11632	0.946132	0.104289
11633	0.946133	0.05
11634	0.946133	0.0689193
11635	0.946132	0.0774982
11636	0.946132	0.05
11637	1.0644	0.808794
11638	1.12486	1.77434
11639	1.08674	1.88669
11640	0.946132	1.05836
11641	0.946132	1.86156
11642	0.946133	1.47243
11643	0.946132	1.2636
11644	0.808978	1.1364
11645	0.911537	1.59164
11646	0.932832	1.33418
11647	1.14604	1.84103
11648	1.56953	1.45776
11649	1.25866	1.1179
11650	1.18488	1.72543
11651	1.341	0.999094
11652	0.946131	0.05
11653	0.946131	0.05
11654	0.946131	0.05
11655	0.94613	0.05

11656	1.33922	0.05
11657	0.946131	0.05
11658	0.946131	0.05
11659	0.946132	0.05
11660	0.946133	0.05
11661	0.946132	0.05
11662	0.946132	0.05
11663	0.946132	0.05
11664	0.946133	0.05
11665	0.946132	0.05
11666	0.946129	0.164877
11667	0.946133	2.1
11668	0.946133	0.449659
11669	0.946131	1.13208
11670	1.40496	2.1
11671	0.946131	0.234492
11672	0.946131	2.1
11673	0.94613	1.29808
11674	0.946127	0.0706094
11675	0.946127	0.446469
11676	0.94613	1.43214
11677	1.38913	0.244674
11678	0.946128	1.14429
11679	0.946128	0.463992
11680	1.98863	0.05
11681	2.59915	0.05
11682	1.40338	0.05
11683	0.946131	0.05
11684	1.22266	0.05
11685	1.40338	0.05
11686	0.946132	0.05
11687	0.946132	0.05
11688	1.22266	0.05
11689	0.94613	0.05
11690	0.94613	0.05
11691	0.946124	0.05
11692	1.21428	0.05
11693	0.946126	0.05
11694	0.946127	0.05
11695	0.946127	0.05
11696	1.03411	0.05
11697	0.946131	0.05
11698	1.03411	0.05
11699	0.946129	0.05
11700	1.14258	0.05
11701	0.952143	0.05
11702	0.940118	0.05
11703	0.810943	2.1
11704	0.810945	2.1
11705	0.810958	2.1
11706	2.82603	2.1
11707	1.35168	2.1
11708	1.08132	2.1
11709	1.08132	2.1
11710	1.0813	2.1
11711	0.946133	0.05
11712	0.946132	0.05
11713	0.946132	0.05

11714	0.946132	0.05
11715	0.946132	0.05
11716	1.11539	0.05
11717	0.946133	0.05
11718	0.946133	2.1
11719	0.946132	2.1
11720	0.946133	2.1
11721	0.946133	2.1
11722	0.946132	2.1
11723	1.41195	2.1
11724	0.946133	2.1
11725	0.946131	0.05
11726	0.946131	0.05
11727	0.946131	0.05
11728	0.946131	0.05
11729	0.946131	0.05
11730	0.946131	0.05
11731	1.15968	0.05
11732	0.946131	0.649684
11733	0.94613	1.21898
11734	0.94613	2.1
11735	1.34386	1.38456
11736	0.946132	2.1
11737	0.94613	2.1
11738	1.34386	2.03501
11739	0.946131	0.642684
11740	0.935202	0.441232
11741	0.946131	2.06789
11742	1.25054	0.799971
11743	0.850155	1.94807
11744	0.946131	1.81077
11745	0.935205	1.5051
11746	0.946131	0.05
11747	1.40193	0.05
11748	0.946132	0.05
11749	0.946132	0.05
11750	0.946129	0.05
11751	1.40193	0.05
11752	0.946131	0.05
11753	0.808779	0.05
11754	0.561937	0.05
11755	1.40193	0.05
11756	1.40193	0.05
11757	0.561936	0.05
11758	0.561937	0.05
11759	1.40193	0.05
11760	0.946132	0.05
11761	0.946132	0.05
11762	0.946132	0.05
11763	0.946132	0.05
11764	0.946133	0.05
11765	0.946132	0.05
11766	0.946133	0.05
11767	0.946127	0.0583642
11768	0.946131	0.0620831
11769	0.946131	0.05
11770	0.946127	0.0579808
11771	1.19583	0.05

11772	1.19583	0.05
11773	0.946129	0.0506135
11774	0.589428	0.0716282
11775	1.08737	0.05
11776	1.10961	0.0942666
11777	0.872901	0.071943
11778	0.541327	0.05
11779	0.541312	0.05
11780	0.85153	0.0931945
11781	0.946132	1.87072
11782	0.946131	2.1
11783	0.946131	2.1
11784	0.946132	2.1
11785	0.946132	2.1
11786	0.946132	2.1
11787	0.946132	2.1
11788	0.946132	1.18937
11789	1.2778	2.1
11790	0.946131	2.1
11791	0.946131	1.41199
11792	0.94613	2.1
11793	0.94613	1.77829
11794	0.94613	1.89451
11795	0.946118	0.687786
11796	0.946112	0.762405
11797	0.946118	0.463148
11798	0.946119	0.450333
11799	0.946117	0.710343
11800	1.35631	0.19606
11801	0.946105	0.169434
11802	0.946133	1.19417
11803	0.946133	2.1
11804	0.946132	1.73672
11805	0.946132	2.1
11806	0.946133	0.05
11807	0.946132	0.80064
11808	0.946132	0.69169
11809	0.94613	2.1
11810	0.946131	0.0660967
11811	0.946128	0.0966499
11812	0.946128	0.05
11813	0.94613	2.1
11814	0.946129	1.48742
11815	0.946129	0.515713
11816	0.946132	1.09612
11817	1.15139	0.912403
11818	0.946132	0.559795
11819	0.946132	1.2051
11820	0.946132	1.37178
11821	0.946132	1.4555
11822	0.946132	1.11522
11823	0.946133	2.1
11824	1.36151	2.1
11825	0.946132	2.1
11826	0.946132	2.1
11827	0.946132	2.1
11828	0.946132	2.1
11829	0.946133	2.1

11830	0.946127	2.1
11831	1.38397	2.1
11832	0.94613	2.1
11833	0.946131	2.1
11834	0.946128	2.1
11835	0.94613	2.1
11836	0.946129	2.1
11837	0.946131	0.05
11838	0.946127	0.0758175
11839	0.946127	0.0881672
11840	0.946132	0.0946871
11841	0.946131	0.0743635
11842	0.946128	0.05
11843	0.946129	0.0641973
11844	1.0268	0.05
11845	1.23823	0.05
11846	1.2171	0.05
11847	0.883446	0.05
11848	1.23827	0.05
11849	1.32614	0.05
11850	0.94613	0.05
11851	0.946132	2.1
11852	0.946129	2.1
11853	0.946129	2.1
11854	0.94613	2.1
11855	1.36523	2.1
11856	0.946131	2.1
11857	0.94613	2.1
11858	0.946132	0.05
11859	0.946131	0.05
11860	0.946132	0.05
11861	0.946131	0.05
11862	0.946131	0.05
11863	1.01653	0.05
11864	0.946131	0.05
11865	0.94612	2.1
11866	0.946121	2.07317
11867	0.946119	2.1
11868	0.946119	2.1
11869	0.946119	2.1
11870	0.946132	2.1
11871	0.946107	2.1
11872	0.810942	1.19173
11873	0.810944	0.105088
11874	2.60324	1.81031
11875	1.21651	1.6215
11876	1.0813	1.27209
11877	0.946153	0.103802
11878	0.979948	2.1
11879	0.709588	1.67312
11880	1.3492	0.05
11881	0.946132	0.05
11882	0.946131	0.05
11883	0.94613	0.05
11884	0.946129	0.05
11885	0.946129	0.05
11886	0.94613	0.05
11887	0.946131	0.05

11888	0.94613	0.072182
11889	0.946128	0.05
11890	0.946133	0.0592242
11891	0.946133	0.061658
11892	0.946131	0.0528195
11893	0.946131	0.0596912
11894	1.23351	0.05
11895	0.946131	0.05
11896	0.946131	0.05
11897	0.946132	0.05
11898	0.946132	0.05
11899	0.946131	0.05
11900	0.946132	0.05
11901	1.35657	0.05
11902	0.946132	0.05
11903	0.946131	0.05
11904	0.946132	0.05
11905	0.946132	0.05
11906	0.946131	0.05
11907	0.946131	0.05
11908	0.946132	0.111483
11909	0.946132	0.0578376
11910	0.946132	0.131834
11911	0.946131	0.05
11912	0.946131	0.0679142
11913	0.946132	0.0692881
11914	0.946132	0.0606453
11915	0.946133	0.0585508
11916	0.946132	0.05
11917	0.946133	0.0843448
11918	0.946132	0.05
11919	0.946132	0.05
11920	0.946132	0.05
11921	0.946133	0.05
11922	0.946131	1.3569
11923	1.4101	2.03944
11924	0.946131	2.1
11925	0.946131	0.351676
11926	0.946131	1.33028
11927	0.946131	0.296406
11928	0.946131	2.06119
11929	0.810944	1.18459
11930	0.810939	1.21684
11931	0.709586	0.45591
11932	2.71261	0.548981
11933	1.21651	1.00125
11934	0.946151	0.311503
11935	0.979943	1.12642
11936	1.0813	1.46411
11937	0.946132	2.1
11938	0.946131	2.1
11939	0.946132	2.1
11940	0.946131	2.1
11941	0.946131	2.1
11942	1.37733	2.1
11943	0.946131	2.1
11944	0.946133	2.1
11945	0.946132	1.76085

11946	0.946132	2.1
11947	0.946133	2.1
11948	0.946133	2.1
11949	0.946132	2.1
11950	1.32582	2.1
11951	0.946132	2.1
11952	0.946132	2.1
11953	0.946132	2.1
11954	0.946132	2.1
11955	0.946132	2.1
11956	0.946132	2.1
11957	0.946131	2.1
11958	0.946133	2.0307
11959	0.946132	1.42726
11960	0.946132	1.88394
11961	0.946132	1.24305
11962	0.946132	1.10354
11963	0.946133	1.28704
11964	1.3469	1.34805
11965	0.946126	0.420319
11966	0.94613	2.1
11967	0.946131	2.1
11968	0.946127	1.98144
11969	1.41206	2.1
11970	0.946128	2.1
11971	0.946128	2.1
11972	0.946127	2.1
11973	0.94613	2.1
11974	0.946131	0.588293
11975	0.94613	2.1
11976	0.946128	0.965775
11977	0.946129	2.1
11978	0.946129	2.1
11979	0.946131	0.05
11980	0.94613	0.05
11981	1.24448	0.05
11982	0.946129	0.05
11983	0.946131	0.05
11984	0.94613	0.05
11985	0.946131	0.05
11986	0.946132	0.05
11987	0.946133	0.05
11988	1.11015	0.05
11989	0.946133	0.05
11990	0.946132	0.05
11991	0.946132	0.05
11992	0.946132	0.05
11993	1.38775	2.1
11994	0.946131	2.1
11995	0.946131	2.1
11996	0.946132	2.1
11997	0.946132	2.1
11998	0.946131	2.1
11999	0.946131	2.1
12000	0.946129	2.0147
12001	0.946129	2.1
12002	0.946133	1.10347
12003	0.946131	2.1

12004	0.946131	0.0567734
12005	0.946131	1.03176
12006	0.946131	0.846036
12007	0.946132	2.1
12008	0.946133	2.1
12009	0.946132	2.1
12010	1.40774	2.1
12011	0.946132	2.1
12012	0.946133	2.1
12013	0.946132	2.1
12014	0.610968	0.05
12015	0.688233	0.05
12016	0.715226	0.05
12017	0.792409	0.05
12018	1.13418	0.05
12019	0.956667	0.05
12020	0.757321	0.05
12021	0.968845	0.05
12022	0.595679	0.05
12023	0.946133	1.3953
12024	1.28917	1.54305
12025	0.946131	1.9998
12026	0.94613	1.04907
12027	0.946131	1.46856
12028	0.946131	1.32103
12029	0.946131	1.76444
12030	0.946132	0.0659426
12031	0.946128	0.148713
12032	0.94613	0.337938
12033	1.40668	0.123138
12034	0.946131	0.258846
12035	0.946131	0.102737
12036	0.94613	0.210704
12037	0.810946	2.1
12038	0.810945	2.1
12039	2.78163	2.1
12040	1.3517	2.1
12041	1.08132	2.1
12042	1.08133	2.1
12043	1.08132	2.1
12044	0.810933	2.1
12045	0.946131	2.1
12046	1.39081	2.1
12047	0.94613	2.1
12048	1.30339	2.1
12049	1.30339	2.1
12050	1.39081	2.1
12051	0.946131	2.1
12052	0.946127	1.49939
12053	0.821631	1.89532
12054	0.946131	0.784574
12055	0.946131	1.11818
12056	0.946128	1.10864
12057	0.996109	0.535734
12058	0.827876	0.34302
12059	0.946131	2.1
12060	0.946129	2.1
12061	0.946129	2.1

12062	0.94613	2.1
12063	0.94613	2.1
12064	0.94613	2.1
12065	0.94613	2.1
12066	0.946132	0.05
12067	1.41723	0.0579203
12068	0.946132	0.05
12069	0.946133	0.05
12070	0.946132	0.156784
12071	0.946132	0.0725537
12072	0.946133	0.0675262
12073	0.946133	0.05
12074	0.946132	0.05
12075	0.946133	0.05
12076	0.946133	0.05
12077	0.946132	0.0881174
12078	0.946132	0.0559474
12079	1.34254	0.0502033
12080	1.26501	0.764871
12081	0.946132	1.10469
12082	0.946133	1.09008
12083	0.946133	1.76642
12084	0.946132	1.64131
12085	0.946133	1.13914
12086	0.946132	1.92886
12087	0.946131	0.05
12088	0.946131	0.05
12089	0.946131	0.05
12090	0.946131	0.05
12091	0.946131	0.05
12092	0.946131	0.05
12093	0.946131	0.05
12094	0.878843	0.05
12095	0.82561	0.05
12096	0.586974	0.05
12097	1.05343	0.05
12098	0.80631	0.05
12099	0.782663	0.05
12100	0.789405	0.05
12101	0.902491	0.05
12102	1.05344	0.05
12103	0.94613	0.05
12104	0.946131	0.05
12105	0.946131	0.05
12106	0.94613	0.05
12107	0.946131	0.05
12108	0.946131	0.05
12109	0.946131	0.05
12110	0.946128	2.1
12111	0.946133	2.1
12112	0.946133	0.212241
12113	0.94613	2.1
12114	0.946131	0.805906
12115	0.946131	1.43399
12116	0.946131	1.59061
12117	0.946131	0.0512489
12118	0.946131	0.05
12119	0.94613	0.05

12120	0.946128	0.05
12121	0.946133	0.05
12122	1.2996	0.05
12123	0.946131	0.05
12124	0.946132	2.1
12125	0.946132	2.1
12126	0.946132	2.1
12127	0.946132	2.1
12128	0.946131	2.1
12129	0.946132	2.1
12130	0.946132	2.1
12131	1.99211	0.05
12132	2.55024	0.05
12133	0.946131	0.05
12134	0.946131	0.05
12135	0.946131	0.05
12136	0.946131	0.05
12137	0.946131	0.05
12138	0.946131	0.05
12139	1.35726	0.05
12140	0.946128	2.1
12141	0.946132	2.03482
12142	0.946131	2.1
12143	0.94613	2.09286
12144	0.94613	2.09903
12145	0.94613	2.1
12146	1.39852	2.1
12147	1.31666	0.05
12148	0.946131	0.05
12149	0.946128	0.05
12150	0.94613	0.05
12151	0.946128	0.05
12152	0.946128	0.05
12153	0.946128	0.05
12154	1.32275	0.05
12155	0.946131	0.05
12156	0.946129	0.05
12157	0.946128	0.05
12158	0.946129	0.05
12159	0.946128	0.05
12160	0.946129	0.05
12161	0.946132	2.1
12162	0.946131	2.1
12163	0.946131	2.1
12164	0.946132	2.1
12165	0.94613	2.1
12166	0.946131	2.1
12167	1.41125	2.1
12168	0.946132	2.1
12169	0.946132	2.1
12170	0.946131	2.1
12171	0.946132	2.1
12172	0.946132	2.1
12173	0.946132	2.1
12174	1.31904	2.1
12175	1.02425	0.05
12176	0.946132	0.05
12177	0.94613	0.05

12178	0.946131	0.05
12179	1.1534	0.05
12180	0.961161	0.05
12181	0.9311	0.05
12182	1.40092	1.09122
12183	0.946133	1.14268
12184	0.946132	1.1903
12185	0.946131	1.71662
12186	0.946131	0.930063
12187	0.94613	1.26765
12188	0.946131	1.80112
12189	0.946113	2.1
12190	0.946119	0.05
12191	0.946117	2.1
12192	1.40015	2.1
12193	0.946118	0.0821645
12194	0.946131	1.81724
12195	0.946105	0.21433
12196	0.946132	0.0661895
12197	0.946133	0.080214
12198	0.946132	0.282635
12199	1.03547	0.0813213
12200	0.946133	0.0590648
12201	0.946133	0.154643
12202	0.946132	0.0948558
12203	0.946133	2.1
12204	1.36694	2.1
12205	0.946133	2.1
12206	0.946132	2.1
12207	0.946133	2.1
12208	0.946133	2.1
12209	0.946132	2.1
12210	0.946132	0.05
12211	0.946133	0.05
12212	0.946132	0.05
12213	0.946133	0.05
12214	0.946133	0.05
12215	0.946132	0.05
12216	0.946132	0.05
12217	0.491552	0.05
12218	0.913078	0.05
12219	0.946132	0.05
12220	0.491552	0.05
12221	0.946132	0.05
12222	0.49154	0.05
12223	1.53168	0.05
12224	1.53168	0.05
12225	0.946127	2.1
12226	1.38752	2.1
12227	0.946129	2.1
12228	0.94613	2.1
12229	0.946128	2.1
12230	0.946129	2.1
12231	0.946129	2.1
12232	0.946132	2.1
12233	0.946132	2.1
12234	0.946132	2.1
12235	0.946132	2.1

12236	0.946132	2.1
12237	0.946131	2.1
12238	0.946132	2.1
12239	0.94613	0.05
12240	0.94613	0.05
12241	0.94613	0.05
12242	0.946131	0.05
12243	0.946129	0.05
12244	1.16834	0.05
12245	0.94613	0.05
12246	0.946127	0.05
12247	1.35594	0.05
12248	0.946131	0.05
12249	0.946128	0.05
12250	0.946127	0.05
12251	0.946129	0.05
12252	0.94613	0.05
12253	0.94613	0.140195
12254	0.946131	0.05
12255	0.946128	0.05
12256	0.946128	0.05
12257	0.946131	0.252448
12258	0.94613	0.05
12259	0.94613	0.0986517
12260	0.946132	0.05
12261	0.946132	0.0779297
12262	0.946129	0.0514211
12263	1.39751	0.05
12264	0.94613	0.153173
12265	0.946131	0.05
12266	0.946131	0.058089
12267	0.946132	0.05
12268	0.700041	0.05
12269	0.700013	0.05
12270	1.7697	0.05
12271	1.7697	0.05
12272	1.21134	0.05
12273	0.946131	0.05
12274	1.25837	0.05
12275	0.946133	2.1
12276	0.946132	1.99999
12277	0.946132	1.76154
12278	0.946132	2.09175
12279	0.946132	2.1
12280	0.946133	2.1
12281	1.40583	2.1
12282	0.946131	1.04557
12283	0.946131	2.03331
12284	0.946131	1.09527
12285	0.946131	2.1
12286	0.946131	2.07685
12287	0.827865	1.94534
12288	0.946131	2.00802
12289	0.946132	0.05
12290	0.946129	0.05
12291	0.946129	0.05
12292	0.946131	0.05
12293	0.94613	0.05

12294	1.21087	0.05
12295	0.946131	0.05
12296	0.94613	0.831111
12297	0.946131	1.22203
12298	0.94613	0.315594
12299	0.946128	0.464413
12300	0.946132	0.556281
12301	1.17452	0.343797
12302	0.94613	0.171093
12303	0.946119	0.281751
12304	0.946114	0.418212
12305	0.946119	0.154145
12306	0.946119	0.194198
12307	0.946119	0.214973
12308	1.27376	0.167693
12309	0.946106	0.106292
12310	0.946133	2.1
12311	1.39621	2.1
12312	0.946132	2.1
12313	0.946132	2.1
12314	0.946133	2.1
12315	0.946132	2.1
12316	0.946132	2.1
12317	0.946132	2.1
12318	0.946132	2.1
12319	0.946131	2.1
12320	0.946131	2.1
12321	0.946132	2.1
12322	0.946132	2.1
12323	1.34352	2.1
12324	0.946132	2.1
12325	1.41272	2.1
12326	0.946131	2.1
12327	0.94613	1.93745
12328	0.94613	2.1
12329	0.94613	1.9924
12330	0.94613	2.1
12331	0.946132	2.1
12332	0.946131	2.05632
12333	0.946132	2.1
12334	0.946132	1.92158
12335	0.946132	1.09821
12336	0.946132	2.1
12337	1.23536	2.1
12338	0.946132	2.1
12339	0.946131	2.1
12340	0.946131	2.1
12341	0.946132	2.1
12342	0.946132	2.1
12343	1.41131	2.1
12344	0.946131	2.1
12345	1.41894	1.62457
12346	0.946132	0.0864291
12347	0.946133	0.277187
12348	0.946132	0.288506
12349	0.946132	0.0989295
12350	0.946133	0.308297
12351	0.946132	0.165595

12352	0.946128	0.05
12353	0.946133	0.242423
12354	0.946131	0.145348
12355	0.946129	2.1
12356	0.94613	0.05
12357	1.4099	0.417586
12358	0.946131	0.05
12359	0.810947	2.1
12360	0.810945	1.19139
12361	0.810933	2.1
12362	2.62883	2.1
12363	1.3517	2.1
12364	1.08132	2.1
12365	1.08132	0.780408
12366	1.08133	2.1
12367	0.946131	1.21791
12368	0.946126	2.1
12369	0.946127	1.33715
12370	0.94613	1.28567
12371	0.946128	2.1
12372	0.946129	0.22577
12373	0.946129	0.966759
12374	1.21347	2.1
12375	0.946129	2.1
12376	0.94613	2.1
12377	1.21347	2.1
12378	1.33064	2.1
12379	0.94613	2.1
12380	1.33064	2.1
12381	0.94613	0.05
12382	0.946129	0.05
12383	0.94613	0.05
12384	0.946132	0.05
12385	0.946126	0.05
12386	0.946129	0.05
12387	0.94613	0.05
12388	0.946132	0.981692
12389	1.37938	1.18659
12390	0.946131	1.44649
12391	0.94613	0.714666
12392	0.946131	1.7903
12393	0.94613	1.1561
12394	0.94613	1.22188
12395	0.946133	0.05
12396	0.946133	0.05
12397	1.2968	0.05
12398	0.946132	0.05
12399	0.946133	0.05
12400	1.2968	0.05
12401	0.946133	0.05
12402	1.43643	0.05
12403	0.946129	0.35483
12404	0.946133	0.175976
12405	0.946131	0.397061
12406	0.94613	0.0605617
12407	0.946131	0.160948
12408	0.946131	0.190706
12409	0.946131	0.165378

12410	0.872447	0.950724
12411	0.946132	0.649124
12412	0.872447	0.403289
12413	0.827867	0.881519
12414	0.946132	1.11962
12415	0.827867	1.39756
12416	0.872447	1.3979
12417	0.946117	0.711467
12418	1.27213	0.531754
12419	0.946124	0.265929
12420	0.946119	0.680202
12421	0.946113	1.39173
12422	0.946129	1.05366
12423	0.946104	0.86782
12424	0.94613	0.0529096
12425	0.946131	0.567045
12426	0.94613	0.135526
12427	0.946129	0.559754
12428	0.946132	0.05
12429	0.946131	0.05
12430	0.94613	0.05
12431	0.810942	2.1
12432	0.810943	2.1
12433	2.80212	2.1
12434	1.3517	2.1
12435	1.08133	2.1
12436	1.08132	2.1
12437	1.08132	2.1
12438	0.810932	2.1
12439	1.29521	2.1
12440	0.946129	2.1
12441	0.946131	2.1
12442	1.40106	2.1
12443	0.94613	2.1
12444	1.40106	2.1
12445	0.946131	2.1
12446	0.946132	0.05
12447	1.24596	0.05
12448	0.94613	0.05
12449	0.946131	0.05
12450	0.946131	0.05
12451	0.946131	0.05
12452	0.946131	0.05
12453	0.946131	2.1
12454	0.946131	2.1
12455	0.946131	2.1
12456	0.946128	2.1
12457	0.946133	2.1
12458	0.94613	2.1
12459	0.946131	2.1
12460	0.879548	0.38205
12461	1.21527	0.406353
12462	0.967506	0.4542
12463	0.946132	1.12778
12464	0.946133	0.214255
12465	0.967511	1.38865
12466	0.946132	1.23003
12467	0.946132	1.16536

12468	0.946132	0.52596
12469	0.974253	0.63966
12470	0.974253	1.69538
12471	0.91801	0.494563
12472	0.918009	0.767791
12473	0.946132	1.02434
12474	0.946132	0.05
12475	0.946131	0.05
12476	0.946131	0.05
12477	0.946133	0.05
12478	0.946132	0.05
12479	1.28644	0.05
12480	0.946132	0.05
12481	0.946131	0.05
12482	0.946131	0.05
12483	0.946131	0.05
12484	0.946131	0.05
12485	0.946131	0.05
12486	1.40103	0.05
12487	0.946131	0.05
12488	0.946128	0.05
12489	0.946132	0.05
12490	0.946129	0.05
12491	1.03327	0.05
12492	0.946129	0.05
12493	0.946129	0.05
12494	0.94613	0.05
12495	0.946134	0.100524
12496	0.946133	0.0612103
12497	0.946132	0.05
12498	0.946133	0.05
12499	0.946132	0.05
12500	0.946132	0.05
12501	0.946133	0.05
12502	0.856654	0.05
12503	0.946131	0.05
12504	0.946131	0.05
12505	0.946132	0.05
12506	0.856616	0.05
12507	1.03565	0.05
12508	1.03565	0.05
12509	1.15844	0.164501
12510	0.946132	0.181732
12511	0.946132	0.05
12512	0.946132	0.101607
12513	0.946132	0.0606442
12514	0.946132	0.130388
12515	0.946131	0.0769234
12516	1.4582	2.1
12517	1.35067	2.1
12518	0.946131	2.1
12519	1.04106	2.1
12520	0.938748	2.1
12521	0.943015	2.1
12522	1.05017	2.1
12523	0.938793	2.1
12524	0.946131	0.05
12525	0.946132	0.05

12526	0.946131	0.05
12527	0.94613	0.05
12528	0.946133	0.05
12529	0.946131	0.05
12530	0.946131	0.05
12531	0.946132	0.05
12532	0.946133	0.05
12533	0.946132	0.05
12534	0.946133	0.0604947
12535	0.946133	0.104142
12536	0.946132	0.0540465
12537	0.946133	0.0713026
12538	0.946128	0.518065
12539	0.946132	1.05762
12540	0.946132	0.219355
12541	0.94613	0.45652
12542	1.23426	0.589078
12543	0.94613	0.706752
12544	0.946131	0.756731
12545	0.810943	0.3232
12546	0.810946	0.477501
12547	2.60388	0.949606
12548	1.3517	0.341511
12549	1.08133	0.173906
12550	1.08132	0.469025
12551	1.08132	0.171871
12552	0.810933	0.151778
12553	0.810945	0.0854047
12554	0.810945	0.05
12555	2.72377	1.77651
12556	1.3517	0.46723
12557	1.08133	0.114361
12558	1.08132	0.05
12559	1.08132	2.1
12560	0.810932	0.851168
12561	1.25104	0.05
12562	0.946131	0.234339
12563	0.946131	0.05
12564	1.36188	2.1
12565	0.946131	0.181925
12566	1.36188	0.820534
12567	0.946132	0.565131
12568	0.946132	2.1
12569	0.946132	2.1
12570	0.946132	2.1
12571	0.946132	2.1
12572	0.946132	2.1
12573	0.946132	2.1
12574	1.25488	2.1
12575	1.32132	0.05
12576	0.946126	0.05
12577	0.946127	0.05
12578	0.946128	0.05
12579	0.946128	0.05
12580	0.946128	0.05
12581	0.946128	0.05
12582	0.946133	2.1
12583	1.41123	1.8867

12584	0.946134	2.1
12585	0.946133	2.1
12586	0.946132	0.305373
12587	0.946133	2.1
12588	0.946132	2.1
12589	1.32163	0.05
12590	0.946133	0.05
12591	0.946133	0.05
12592	0.946132	0.05
12593	0.946132	0.05
12594	0.946132	0.05
12595	0.946133	0.05
12596	0.946133	2.1
12597	0.946133	2.1
12598	0.946132	2.1
12599	0.946132	2.1
12600	0.946132	0.932492
12601	0.946133	2.1
12602	0.946132	2.1
12603	0.94613	0.05
12604	0.946131	0.05
12605	0.946128	0.05
12606	0.946133	0.05
12607	0.946133	0.05
12608	0.946131	0.05
12609	0.946131	0.05
12610	0.946133	0.0967015
12611	0.946132	0.05
12612	0.946132	0.125305
12613	0.946134	0.05
12614	0.946133	0.450462
12615	0.946133	0.05
12616	0.946132	0.125393
12617	0.94613	2.00495
12618	0.946128	2.1
12619	0.946127	1.38829
12620	0.94613	0.681855
12621	0.946131	1.87506
12622	0.946128	1.92429
12623	1.21099	2.1
12624	0.946132	0.443281
12625	0.946131	0.330697
12626	0.946131	0.602863
12627	0.946133	0.05
12628	0.946133	0.109394
12629	0.946131	0.25543
12630	0.946132	0.191336
12631	1.34794	0.244534
12632	0.517994	0.35494
12633	0.517965	0.118466
12634	0.946132	0.566521
12635	0.9198	1.83745
12636	0.946132	1.05683
12637	0.9198	0.752324
12638	0.946132	1.20239
12639	0.94613	1.18083
12640	0.946131	0.188052
12641	0.946131	1.11026

12642	0.946131	1.78348
12643	0.946131	1.14479
12644	1.23224	0.55573
12645	0.946132	1.96766
12646	0.946132	2.0929
12647	0.946133	1.7125
12648	0.946132	0.399702
12649	0.946132	1.13531
12650	0.946133	1.64422
12651	1.37511	1.80067
12652	0.946133	0.289299
12653	0.946129	0.418084
12654	0.851104	1.03393
12655	0.946131	0.698186
12656	1.0412	0.706645
12657	1.0412	0.914508
12658	0.851066	0.750788
12659	1.31357	0.05
12660	0.946131	0.05
12661	0.94613	0.05
12662	0.946131	0.05
12663	0.94613	0.05
12664	0.946131	0.05
12665	0.946131	0.05
12666	0.946133	0.730808
12667	1.38149	0.968751
12668	0.946132	1.15988
12669	0.946132	0.579033
12670	0.946133	1.79076
12671	0.946132	1.03369
12672	0.946133	1.04133
12673	0.810944	1.18551
12674	0.810945	2.1
12675	0.810958	2.1
12676	2.65782	2.1
12677	1.35168	2.1
12678	1.08132	1.15991
12679	1.08132	2.1
12680	1.0813	1.38064
12681	0.946113	0.05
12682	0.946119	0.05
12683	0.946118	0.05
12684	0.946118	0.05
12685	0.946119	0.05
12686	0.946131	0.05
12687	0.946106	0.05
12688	0.946132	0.05
12689	0.946132	0.05
12690	0.946128	0.05
12691	0.946131	0.05
12692	0.94613	0.05
12693	0.94613	0.05
12694	0.94613	0.05
12695	1.00099	0.05
12696	0.946132	0.05
12697	1.17876	0.05
12698	0.982282	0.05
12699	0.909981	0.05

12700	0.946132	0.05
12701	0.946131	0.05
12702	0.946132	1.15739
12703	1.35328	1.33575
12704	0.946132	1.87569
12705	0.946132	0.72101
12706	0.946133	1.71848
12707	0.946132	1.16016
12708	0.946133	1.54743
12709	0.946133	0.05
12710	0.946133	0.05
12711	0.946132	0.05
12712	0.946132	0.05
12713	0.946132	0.05
12714	0.946132	0.05
12715	0.946133	0.05
12716	0.900195	0.05
12717	0.946132	0.05
12718	0.900167	0.05
12719	1.41963	0.05
12720	0.900195	0.05
12721	0.946133	0.05
12722	1.35385	0.05
12723	0.946132	0.877259
12724	0.946129	1.36079
12725	0.946129	1.65667
12726	0.946131	0.738908
12727	0.946131	1.78813
12728	1.41585	0.713691
12729	0.94613	2.1
12730	0.810946	2.1
12731	0.810946	2.1
12732	2.68895	2.1
12733	1.3517	2.1
12734	1.08133	2.1
12735	1.08132	2.1
12736	1.08132	2.1
12737	0.810933	2.1
12738	1.19886	2.1
12739	0.946131	2.1
12740	0.946131	2.1
12741	1.34447	2.1
12742	0.946131	2.1
12743	1.34447	2.1
12744	0.946131	2.1
12745	0.946133	0.05
12746	0.946133	0.05
12747	0.946132	0.05
12748	1.35065	0.05
12749	0.946132	0.05
12750	0.946133	0.05
12751	0.946132	0.05
12752	0.946131	0.255332
12753	0.946131	0.248112
12754	0.94613	0.382108
12755	0.946131	0.05
12756	0.946131	0.0618413
12757	0.946131	0.183554

12758	0.946131	0.131974
12759	0.946127	0.05
12760	0.946131	0.0510214
12761	0.946127	0.05
12762	0.94613	0.05
12763	0.946131	0.05
12764	0.946128	0.05
12765	0.946129	0.05
12766	0.810946	0.05
12767	0.810947	0.05
12768	0.810959	0.05
12769	2.4391	0.05
12770	1.35168	0.05
12771	1.08132	0.05
12772	1.08132	0.05
12773	1.08131	0.05
12774	0.946132	0.05
12775	0.946132	0.05
12776	0.946132	0.05
12777	1.40837	0.05
12778	0.946132	0.05
12779	0.946132	0.05
12780	1.40837	0.05
12781	0.946127	2.1
12782	0.946132	2.1
12783	0.946131	0.136549
12784	0.946127	2.1
12785	0.946131	1.61578
12786	0.946129	0.664131
12787	0.94613	1.67808
12788	0.946133	2.08194
12789	0.946132	1.54055
12790	0.946132	1.46083
12791	0.946132	0.853253
12792	0.946132	1.85567
12793	0.946132	0.325865
12794	0.946133	2.1
12795	0.946132	0.412468
12796	1.24344	0.347481
12797	0.946132	0.116043
12798	0.946132	0.918958
12799	0.946132	0.221584
12800	1.24344	0.29098
12801	0.946132	0.932029
12802	0.946131	0.05
12803	0.946128	0.05
12804	0.946128	0.05
12805	0.946131	0.05
12806	1.41232	0.05
12807	0.946129	0.05
12808	0.94613	0.05
12809	0.946128	2.1
12810	0.946133	2.1
12811	0.946133	0.376605
12812	0.946131	2.1
12813	0.946131	0.82919
12814	0.946131	2.01522
12815	0.94613	2.02585

12816	0.946133	0.0541846
12817	1.41556	0.0525167
12818	0.946133	0.05
12819	0.946133	0.05
12820	0.946132	0.13815
12821	0.946132	0.0610208
12822	0.946132	0.0820557
12823	0.94612	0.05
12824	1.41843	0.05
12825	0.946114	0.05
12826	0.94612	0.05
12827	0.946119	0.102398
12828	1.41845	0.05
12829	0.946107	0.0741811
12830	0.946129	0.05
12831	0.946129	0.05
12832	0.94613	0.05
12833	0.946132	0.05
12834	0.946126	0.05
12835	1.1509	0.05
12836	0.94613	0.05
12837	0.946131	0.05
12838	0.946132	0.05
12839	0.946132	0.05
12840	0.946132	0.05
12841	0.946132	0.05
12842	1.19263	0.05
12843	0.946132	0.05
12844	0.94613	0.0884766
12845	0.946131	0.11929
12846	0.946128	0.212196
12847	0.946133	0.149657
12848	0.946133	0.188142
12849	0.946131	0.0927975
12850	0.946131	0.179103
12851	0.946133	1.06791
12852	1.22848	1.60244
12853	1.00336	1.53883
12854	0.827866	1.14165
12855	0.946132	1.98231
12856	0.827865	1.59647
12857	0.946133	1.2507
12858	0.946131	0.206951
12859	0.94613	0.05
12860	0.946127	0.195748
12861	1.41498	0.125091
12862	0.94613	0.0794171
12863	0.946128	0.111821
12864	0.946128	0.0533842
12865	0.94613	0.05
12866	0.94613	0.05
12867	0.94613	0.05
12868	1.37358	0.05
12869	0.946129	0.05
12870	0.94613	0.05
12871	1.37357	0.05
12872	0.946111	0.05
12873	0.946118	0.05

12874	0.946118	0.05
12875	1.36568	0.05
12876	0.946116	0.05
12877	0.94613	0.05
12878	0.946105	0.05
12879	0.946132	0.05
12880	0.946131	0.05
12881	0.94613	0.05
12882	1.23175	0.05
12883	0.946131	0.05
12884	0.946131	0.05
12885	0.946131	0.05
12886	0.946132	0.05
12887	0.946126	0.05
12888	0.946128	0.05
12889	0.946129	0.05
12890	0.946129	0.05
12891	0.946128	0.05
12892	1.25698	0.05
12893	0.810943	2.1
12894	0.810944	2.1
12895	2.81171	2.1
12896	1.3517	2.1
12897	1.08132	2.1
12898	1.08133	2.1
12899	1.08132	2.1
12900	0.810932	2.1
12901	0.946132	0.05
12902	0.946131	0.05
12903	0.946131	0.0610629
12904	0.946132	0.05
12905	0.946132	0.05
12906	0.946132	0.05
12907	0.946131	0.05
12908	0.810936	0.258641
12909	0.810935	2.1
12910	2.80914	1.96147
12911	1.35167	1.24763
12912	1.0813	0.895567
12913	1.0813	2.1
12914	1.0813	0.234962
12915	0.810934	0.950871
12916	1.23851	2.1
12917	0.946132	0.51052
12918	0.946131	2.1
12919	1.40459	0.260497
12920	0.946132	2.02865
12921	1.40459	1.82541
12922	0.946132	1.95965
12923	1.19974	0.05
12924	0.946131	0.05
12925	1.19974	0.05
12926	0.946132	0.05
12927	0.946132	0.05
12928	0.946132	0.05
12929	0.946132	0.05
12930	0.939141	0.742495
12931	1.14375	1.32594

12932	1.03306	1.38684
12933	0.946132	1.05732
12934	0.946126	1.6855
12935	0.946129	1.2738
12936	0.94613	1.09746
12937	0.946132	0.05
12938	0.946131	0.05
12939	0.946132	0.05
12940	0.946132	0.05
12941	0.946132	0.05
12942	0.946132	0.05
12943	0.946132	0.05
12944	0.946131	2.1
12945	0.94613	2.1
12946	0.94613	2.1
12947	0.946131	2.1
12948	0.946129	2.1
12949	0.94613	2.1
12950	1.24512	2.1
12951	0.946133	0.05
12952	0.946129	0.05
12953	0.946131	0.05
12954	0.946132	0.05
12955	0.946131	0.05
12956	0.946131	0.05
12957	0.946131	0.05
12958	0.946132	0.05
12959	0.946133	0.05
12960	0.946132	0.05
12961	0.946132	0.05
12962	0.946133	0.05
12963	0.946132	0.05
12964	0.946132	0.05
12965	0.946132	0.05
12966	1.41392	0.05
12967	0.946132	0.05
12968	0.946132	0.0727705
12969	0.946132	0.05
12970	0.946132	0.05
12971	0.946132	0.0514643
12972	0.946132	0.0908065
12973	1.24333	0.0568242
12974	0.946132	0.05
12975	0.946133	0.240833
12976	0.946132	0.05
12977	0.946132	0.153513
12978	0.946133	0.0760273
12979	1.30649	0.05
12980	0.94613	0.05
12981	0.94613	0.05
12982	0.946128	0.05
12983	0.946126	0.05
12984	0.946127	0.05
12985	0.946127	0.05
12986	0.709578	0.05
12987	0.810943	0.05
12988	2.53303	0.05
12989	1.21651	0.05

12990	0.979951	0.05
12991	1.08132	0.05
12992	0.946141	0.05
12993	0.81096	0.05
12994	0.946131	0.05
12995	0.946131	0.05
12996	0.946131	0.05
12997	0.946131	0.05
12998	0.946131	0.05
12999	0.946131	0.05
13000	0.946131	0.05
13001	0.946132	0.05
13002	0.946129	0.05
13003	0.946129	0.05
13004	0.94613	0.05
13005	1.20634	0.05
13006	1.20634	0.05
13007	0.946131	0.05
13008	0.946133	0.419321
13009	0.946132	0.435032
13010	0.946132	0.174581
13011	0.946132	0.446964
13012	0.946132	0.166518
13013	1.30107	0.237797
13014	0.946133	0.293464
13015	0.810946	2.1
13016	0.810944	2.1
13017	2.73936	2.1
13018	1.35168	2.1
13019	1.0813	2.1
13020	1.08132	2.1
13021	1.08132	2.1
13022	0.810958	2.1
13023	0.946129	2.1
13024	0.946129	2.1
13025	0.946133	0.481669
13026	1.30812	2.1
13027	0.946131	1.11489
13028	0.946131	1.87431
13029	0.946131	1.7082
13030	1.00901	1.13198
13031	0.94613	1.84186
13032	0.94613	0.289005
13033	1.17001	1.46153
13034	0.917259	0.20338
13035	0.94613	0.751957
13036	0.94613	0.684831
13037	0.946126	2.1
13038	0.946127	2.1
13039	0.946131	2.1
13040	0.946127	2.1
13041	0.946131	2.1
13042	0.946129	2.1
13043	1.398	2.1
13044	0.946133	2.1
13045	1.3033	2.1
13046	0.946132	2.1
13047	0.946132	2.1

13048	0.946132	2.1
13049	0.946132	2.1
13050	0.946133	2.1
13051	0.946131	0.0676674
13052	0.946131	0.0935688
13053	1.09897	0.05
13054	0.946129	0.233739
13055	0.946131	0.127783
13056	0.94613	0.171515
13057	0.94613	0.0718869
13058	0.946132	2.1
13059	0.946131	2.1
13060	0.946131	2.1
13061	0.946132	2.1
13062	0.94613	2.1
13063	0.946131	2.1
13064	1.39858	2.1
13065	1.07117	0.400293
13066	1.08878	0.941881
13067	0.946119	1.30327
13068	1.08878	1.25316
13069	0.946119	0.204757
13070	0.946132	1.15959
13071	1.07116	0.4403
13072	0.946132	0.05
13073	0.946132	0.05
13074	0.946132	0.05
13075	0.946132	0.05
13076	0.946132	0.05
13077	1.10733	0.05
13078	0.946132	0.05
13079	0.946128	0.05
13080	0.946132	0.05
13081	0.94613	0.05
13082	0.946129	0.05
13083	0.94613	0.05
13084	1.3367	0.05
13085	0.94613	0.05
13086	0.810943	2.1
13087	0.810944	2.1
13088	2.70234	2.1
13089	1.21653	2.1
13090	1.08132	2.1
13091	0.946155	2.1
13092	0.979951	2.1
13093	0.709578	2.1
13094	1.27174	2.1
13095	0.946129	2.1
13096	0.94613	2.1
13097	1.35117	2.1
13098	0.94613	2.1
13099	1.35117	2.1
13100	0.94613	2.1
13101	0.946133	0.874672
13102	0.946133	0.777109
13103	0.946132	2.02068
13104	0.946133	0.628868
13105	0.946132	2.1

13106	0.946133	1.95082
13107	0.997296	1.96404
13108	0.810943	2.1
13109	0.810944	2.1
13110	0.810945	2.1
13111	2.78833	2.1
13112	1.35169	2.1
13113	1.08132	2.1
13114	1.08132	2.1
13115	1.08132	2.1
13116	0.810946	2.1
13117	0.810943	0.771998
13118	0.810933	1.87037
13119	2.70049	2.1
13120	1.3517	1.42979
13121	1.08132	2.1
13122	1.08132	0.124702
13123	1.08133	0.941005
13124	0.946127	1.01987
13125	1.35469	0.705911
13126	0.94613	0.664706
13127	0.946131	1.02458
13128	0.946128	0.521658
13129	1.35469	1.15923
13130	0.946129	1.28428
13131	0.946131	0.05
13132	1.2722	0.05
13133	0.946132	0.05
13134	0.946132	0.05
13135	0.946129	0.05
13136	1.2722	0.05
13137	0.94613	0.05
13138	0.946133	0.0568874
13139	0.946132	0.05
13140	0.946132	0.0539595
13141	0.946132	0.0602949
13142	1.41461	0.05
13143	1.41461	0.05
13144	0.946133	0.0808625
13145	0.946131	0.495846
13146	1.30165	0.184064
13147	0.946132	0.0681832
13148	0.946132	0.849031
13149	0.946129	0.20355
13150	1.30165	0.525831
13151	0.946131	0.721947
13152	0.946132	0.152742
13153	0.946129	1.85296
13154	0.94613	0.171872
13155	1.39356	2.02966
13156	0.946131	0.655427
13157	0.946131	0.51156
13158	0.94613	1.67815
13159	0.810946	2.1
13160	0.810947	2.1
13161	0.810934	2.1
13162	2.35003	2.1
13163	1.3517	2.1

13164	1.08132	2.1
13165	1.08132	2.1
13166	1.08133	2.1
13167	0.946131	0.05
13168	0.946131	0.05
13169	0.946125	0.05
13170	0.946127	0.05
13171	0.946129	0.05
13172	0.946128	0.05
13173	0.946128	0.05
13174	0.946131	0.05
13175	0.946131	0.05
13176	0.946131	0.05
13177	0.946131	0.05
13178	0.946132	0.05
13179	0.946132	0.05
13180	0.946131	0.05
13181	0.946129	2.1
13182	0.946132	2.1
13183	0.946132	2.1
13184	0.94613	2.1
13185	1.30082	2.1
13186	0.94613	2.1
13187	0.946131	2.1
13188	1.20416	0.05
13189	0.946132	0.05
13190	0.946131	0.05
13191	0.946132	0.05
13192	0.946131	0.05
13193	0.946131	0.05
13194	0.946132	0.05
13195	1.18711	0.05
13196	0.946131	0.05
13197	0.946131	0.05
13198	0.946132	0.05
13199	0.946131	0.05
13200	0.946131	0.05
13201	0.946132	0.05
13202	0.946133	0.05
13203	0.946133	0.05
13204	0.946132	0.05
13205	1.30212	0.05
13206	0.946133	0.05
13207	0.946132	0.05
13208	1.30212	0.05
13209	0.946131	0.05
13210	0.946131	0.05
13211	0.946131	0.05
13212	0.946132	0.05
13213	1.31467	0.05
13214	1.31467	0.05
13215	0.946131	0.05
13216	0.946133	0.05
13217	0.946132	0.05
13218	0.946132	0.05
13219	0.946132	0.05
13220	1.25124	0.05
13221	1.25124	0.05

13222	0.946132	0.05
13223	0.946132	2.1
13224	0.946132	2.1
13225	0.946132	2.1
13226	1.36422	2.1
13227	0.946132	2.1
13228	0.946131	2.1
13229	0.946132	2.1
13230	0.946131	1.57797
13231	1.41851	2.06214
13232	0.946132	2.1
13233	0.946132	1.20846
13234	0.946132	1.88496
13235	0.946132	1.31633
13236	0.946132	1.87052
13237	0.946133	0.05
13238	0.946132	0.05
13239	0.946132	0.05
13240	0.946132	0.05
13241	0.946132	0.05
13242	1.37794	0.05
13243	0.946132	0.05
13244	0.946132	2.1
13245	0.946132	2.1
13246	0.946131	2.1
13247	0.946131	2.1
13248	0.946132	2.1
13249	0.946132	2.1
13250	1.4045	2.1
13251	0.946133	2.1
13252	0.946132	2.1
13253	0.946131	2.1
13254	0.946132	2.1
13255	0.946131	2.1
13256	0.946131	2.1
13257	0.946132	2.1
13258	0.946132	2.1
13259	0.946132	2.1
13260	0.946131	2.1
13261	0.946132	2.1
13262	0.946131	2.1
13263	0.946131	2.1
13264	0.946131	2.1
13265	0.946128	2.1
13266	1.31641	2.1
13267	0.946129	2.1
13268	0.94613	2.1
13269	0.94613	2.1
13270	0.94613	2.1
13271	0.94613	2.1
13272	0.946132	2.1
13273	0.946131	2.1
13274	0.946131	1.35532
13275	0.946132	2.1
13276	0.946132	1.99799
13277	0.946132	2.1
13278	0.946132	2.1
13279	0.946132	2.1

13280	1.41507	2.1
13281	0.946132	2.1
13282	0.946132	2.1
13283	0.946133	2.1
13284	0.946133	2.1
13285	0.946132	2.1
13286	0.946132	2.1
13287	0.946131	2.1
13288	0.946131	2.1
13289	0.946133	2.1
13290	0.946132	2.1
13291	1.32608	2.1
13292	0.946132	2.1
13293	0.94613	2.1
13294	0.94613	2.1
13295	0.94613	0.3645
13296	0.946131	2.1
13297	0.946129	1.78729
13298	0.94613	0.701408
13299	0.94613	1.42405
13300	1.33728	0.930644
13301	0.946131	0.214672
13302	0.94613	0.0731341
13303	0.946132	0.361965
13304	0.946132	1.4605
13305	0.946131	0.131858
13306	0.946131	0.206916
13307	0.946127	2.1
13308	1.40641	2.1
13309	0.946129	2.1
13310	0.94613	2.1
13311	0.946128	1.08442
13312	0.946129	2.1
13313	0.946129	2.1
13314	0.946129	2.1
13315	0.946128	2.1
13316	0.946133	2.1
13317	1.37467	2.1
13318	0.946131	2.1
13319	0.946131	2.1
13320	1.37467	2.1
13321	0.946131	2.1
13322	0.946131	2.1
13323	0.946132	2.1
13324	0.946132	2.1
13325	0.946129	2.1
13326	0.946131	2.1
13327	1.34912	2.1
13328	0.946132	2.1
13329	0.946133	2.1
13330	0.946133	2.1
13331	0.946132	2.1
13332	0.946132	2.1
13333	0.946133	2.1
13334	0.946132	2.1
13335	0.946127	2.1
13336	0.946131	2.1
13337	0.946132	2.1

13338	0.946131	2.1
13339	0.946127	2.1
13340	0.94613	2.1
13341	1.33607	2.1
13342	0.946117	0.05
13343	0.946117	0.283433
13344	0.946115	1.50439
13345	0.946124	2.1
13346	0.946119	0.05
13347	1.32662	0.63741
13348	0.94613	0.102147
13349	0.946119	2.1
13350	0.946114	2.1
13351	0.946118	2.1
13352	0.946114	2.1
13353	0.946118	2.1
13354	0.946104	2.1
13355	0.946129	2.1
13356	0.946132	2.1
13357	0.946133	2.1
13358	0.946133	2.1
13359	0.946133	2.1
13360	0.946132	2.1
13361	0.946132	2.1
13362	0.946133	2.1
13363	0.946133	2.1
13364	0.946132	2.1
13365	0.946132	2.1
13366	0.946133	2.1
13367	0.946132	2.1
13368	0.946132	2.1
13369	0.946132	2.1
13370	0.946133	2.03912
13371	1.30881	2.1
13372	0.946132	2.1
13373	0.946131	1.50491
13374	0.946131	1.70937
13375	0.946131	1.27742
13376	0.946131	2.06601
13377	0.946128	2.1
13378	0.946128	2.1
13379	0.946132	2.1
13380	0.94613	2.1
13381	0.94613	2.1
13382	0.94613	2.1
13383	1.34652	2.1
13384	0.946133	0.923512
13385	0.946133	0.627066
13386	0.946132	2.02312
13387	0.946133	0.5781
13388	0.946132	2.1
13389	0.946132	1.45472
13390	0.946132	1.82031
13391	0.946132	0.05
13392	0.946128	0.0524288
13393	0.94613	0.0650348
13394	0.946131	0.05
13395	0.94613	0.0833273

13396	0.94613	0.05
13397	0.94613	0.05
13398	0.946128	0.0833709
13399	0.946132	0.0509334
13400	0.94613	0.0698064
13401	0.94613	0.05
13402	0.946131	0.0846563
13403	0.94613	0.0540489
13404	0.94613	0.05
13405	0.946129	0.919224
13406	1.32056	1.57041
13407	0.946131	2.05349
13408	0.94613	0.724174
13409	0.946124	1.7717
13410	0.946128	1.03872
13411	0.946127	1.30507
13412	0.946129	0.796561
13413	1.20351	1.44548
13414	0.946132	1.76432
13415	0.946132	0.908143
13416	0.946126	1.82243
13417	0.946129	1.20333
13418	0.946129	1.14206
13419	0.946127	1.66848
13420	0.946127	2.1
13421	0.946131	0.447095
13422	1.25592	1.83129
13423	0.946127	0.745178
13424	0.946129	0.979421
13425	0.94613	1.4467
13426	1.02332	2.1
13427	0.946133	1.30243
13428	0.946132	1.40305
13429	1.02332	1.47924
13430	0.946132	0.657697
13431	0.96196	1.6864
13432	0.930306	0.952426
13433	0.946133	2.07467
13434	0.946133	2.1
13435	0.946132	2.1
13436	1.20057	2.1
13437	0.946132	2.1
13438	0.946133	2.1
13439	1.20057	2.1
13440	0.946128	1.89718
13441	0.946129	0.628603
13442	0.946129	1.51456
13443	1.13245	0.560125
13444	0.946126	0.32611
13445	0.946128	1.23888
13446	0.946129	0.687916
13447	0.946131	0.916505
13448	0.946131	2.02483
13449	0.94613	2.1
13450	1.25	1.80057
13451	0.946129	2.1
13452	0.946131	2.1
13453	1.25	2.1

13454	0.540425	2.03585
13455	0.748493	1.90676
13456	1.46266	2.1
13457	1.20046	2.09718
13458	0.600454	2.09295
13459	1.41283	2.1
13460	0.582567	2.00581
13461	0.946131	1.85291
13462	0.946131	1.46489
13463	0.946132	1.08021
13464	0.946132	1.55847
13465	0.94613	1.50612
13466	0.946131	2.1
13467	1.28439	2.1
13468	0.946132	0.148643
13469	0.946131	0.172047
13470	0.946131	0.287823
13471	0.946132	0.0958071
13472	0.946132	0.413853
13473	1.39335	0.0558104
13474	0.946132	0.109147
13475	0.946131	0.05
13476	0.946131	0.109958
13477	0.94613	0.05
13478	0.946132	0.05
13479	0.946131	0.0587132
13480	0.946131	0.0514748
13481	0.946131	0.05
13482	0.946131	0.0550503
13483	0.94613	0.0683125
13484	0.946126	0.0989968
13485	0.946131	0.05
13486	0.946125	0.333531
13487	0.946127	0.05
13488	0.946128	0.166003
13489	0.946131	0.05
13490	0.946131	0.05
13491	0.946131	0.05
13492	0.946131	0.05
13493	0.946131	0.05
13494	0.946131	0.05
13495	1.39168	0.05
13496	0.946131	0.05
13497	0.946126	0.05
13498	0.946127	0.0580505
13499	0.946131	0.05
13500	0.94613	0.05
13501	1.412 0.05	
13502	0.946128	0.0621519
13503	0.948901	0.05
13504	0.943364	0.05
13505	1.03768	0.05
13506	0.946132	0.0749288
13507	0.946133	0.0678258
13508	1.03767	0.0597126
13509	0.946132	0.05
13510	1.00902	0.098801
13511	0.88324	0.0670866

13512	0.971584	0.05
13513	0.946132	0.0888279
13514	0.946129	0.388496
13515	0.946131	0.212944
13516	0.971572	0.272198
13517	0.946131	0.05
13518	0.946125	0.05
13519	1.33587	0.05
13520	0.946126	0.05
13521	0.946127	0.05
13522	0.946126	0.05
13523	0.946128	0.05
13524	0.946132	0.05
13525	0.946132	0.05
13526	1.27512	0.05
13527	0.946133	0.05
13528	0.946132	0.05
13529	0.946133	0.05
13530	0.946133	0.05
13531	0.946128	0.05
13532	0.946133	0.05
13533	0.946131	0.05
13534	0.94613	0.05
13535	0.946131	0.05
13536	0.94613	0.05
13537	0.946131	0.05
13538	0.946131	0.05
13539	0.946131	0.05
13540	0.946131	0.05
13541	0.946131	0.05
13542	0.946131	0.05
13543	0.946131	0.05
13544	0.946131	0.05
13545	0.94613	0.05
13546	0.946131	0.05
13547	0.946131	0.05
13548	0.94613	0.05
13549	0.946132	0.05
13550	0.946131	0.05
13551	1.35316	0.05
13552	0.94613	0.05
13553	0.94613	0.05
13554	0.946128	0.05
13555	0.946132	0.05
13556	0.946132	0.05
13557	1.28727	0.05
13558	0.94613	0.0509114
13559	0.946131	0.05
13560	0.946132	0.05
13561	0.946132	0.05
13562	1.39502	0.05
13563	0.946132	0.05
13564	0.946132	0.05
13565	0.946132	0.05
13566	0.946125	0.05
13567	0.946131	0.05
13568	0.94613	0.05
13569	0.946129	0.05

13570	0.946126	0.129689
13571	0.946127	0.0854035
13572	1.36173	0.0812099
13573	0.946129	1.52503
13574	1.02363	1.4569
13575	0.94613	0.956513
13576	0.946131	1.42504
13577	0.946131	0.443364
13578	1.02363	0.525778
13579	0.946131	0.78013
13580	0.946128	0.05
13581	1.24678	0.05
13582	0.94613	0.05
13583	0.94613	0.05
13584	0.94613	0.05
13585	0.946131	0.05
13586	0.94613	0.05
13587	0.946129	0.05
13588	1.25215	0.05
13589	0.946132	0.05
13590	0.946126	0.05
13591	0.946129	0.05
13592	0.94613	0.05
13593	0.946129	0.05
13594	0.946129	0.397753
13595	0.946133	0.604977
13596	0.946133	0.242914
13597	0.946131	0.216471
13598	0.946131	0.453776
13599	1.29689	0.113518
13600	0.946131	0.117623
13601	0.946128	0.05
13602	0.946132	0.05
13603	0.946132	0.05
13604	0.94613	0.05
13605	1.22716	0.05
13606	1.22716	0.05
13607	0.946131	0.05
13608	0.946127	0.05
13609	0.946131	0.05
13610	0.946131	0.05
13611	0.946128	0.05
13612	1.30463	0.05
13613	1.30463	0.05
13614	0.946129	0.05
13615	1.29958	0.05
13616	0.946132	0.05
13617	0.946132	0.05
13618	0.946132	0.05
13619	0.946132	0.05
13620	0.946132	0.05
13621	0.946133	0.05
13622	1.26201	0.05
13623	0.946133	0.05
13624	0.946132	0.05
13625	0.946132	0.05
13626	0.946132	0.05
13627	0.946133	0.05

13628	0.946132	0.05
13629	0.946133	0.05
13630	0.946132	0.05
13631	0.946132	0.05
13632	0.946133	0.05
13633	0.946132	0.05
13634	1.40427	0.05
13635	0.946133	0.05
13636	0.946132	0.05
13637	0.946132	0.05
13638	0.946132	0.05
13639	0.946132	0.05
13640	0.946132	0.05
13641	1.35123	0.05
13642	0.946131	0.05
13643	0.946128	0.05
13644	0.946132	0.05
13645	0.946132	0.05
13646	0.946129	0.05
13647	1.22144	0.05
13648	1.22144	0.05
13649	0.946131	0.05
13650	0.946127	0.0816121
13651	0.946132	0.0963817
13652	0.946131	0.0679647
13653	0.946127	0.0665268
13654	0.946131	0.0846941
13655	1.23351	0.05
13656	0.94613	0.0558687
13657	1.18119	0.05
13658	0.946131	0.05
13659	0.946132	0.05
13660	0.946132	0.05
13661	0.94613	0.05
13662	0.946131	0.05
13663	0.946131	0.05
13664	0.946132	0.05
13665	0.946132	0.05
13666	0.946133	0.05
13667	1.30563	0.05
13668	0.946132	0.05
13669	0.946132	0.05
13670	0.946133	0.05
13671	1.20256	0.0767373
13672	0.946132	1.15876
13673	0.946133	1.44468
13674	0.946132	0.05
13675	0.946132	0.282356
13676	0.946132	1.22719
13677	0.946133	0.0766305
13678	0.94613	0.105512
13679	0.94613	0.0661848
13680	0.946128	0.215875
13681	1.33007	0.05
13682	0.946129	0.0990243
13683	0.94613	0.14029
13684	0.946131	0.259702
13685	0.946127	2.1

13686	0.946127	2.1
13687	0.946131	2.1
13688	1.30136	2.1
13689	0.946128	2.1
13690	0.946129	2.1
13691	1.30136	2.1
13692	0.810942	2.1
13693	0.810942	2.1
13694	0.81093	2.1
13695	2.3208	2.1
13696	1.3517	2.1
13697	1.08132	2.1
13698	1.08132	2.1
13699	1.08133	2.1
13700	0.946132	2.1
13701	0.946132	2.1
13702	0.946132	2.1
13703	0.946132	2.1
13704	0.946132	2.1
13705	1.39823	2.1
13706	0.946132	2.1
13707	1.37713	2.1
13708	0.946131	2.1
13709	0.946131	2.1
13710	0.946131	2.1
13711	0.946131	2.1
13712	0.946131	2.1
13713	0.946131	2.1
13714	0.946128	2.1
13715	0.946128	2.1
13716	0.946133	2.1
13717	0.946131	2.1
13718	0.946131	1.44627
13719	0.946131	2.1
13720	0.94613	2.1
13721	0.946129	2.1
13722	0.946129	2.1
13723	0.946133	2.1
13724	0.946131	2.1
13725	0.946131	2.1
13726	0.946131	2.1
13727	0.946131	2.1
13728	0.946132	1.42221
13729	1.33694	2.1
13730	0.946133	2.1
13731	0.946133	1.13686
13732	0.946132	2.1
13733	0.946132	1.41845
13734	0.946133	1.87684
13735	0.946131	2.1
13736	0.94613	2.1
13737	0.946131	2.1
13738	0.946133	2.1
13739	0.946129	2.1
13740	0.946131	2.1
13741	0.946131	2.1
13742	0.946132	2.07795
13743	1.35943	2.1

13744	0.946132	2.1
13745	0.946132	1.2465
13746	0.946131	1.41641
13747	0.946132	1.02742
13748	0.946132	2.1
13749	0.946132	0.05
13750	0.946132	0.05
13751	0.946133	0.05
13752	0.946133	0.05
13753	0.946132	0.05
13754	0.946132	0.05
13755	0.946132	0.05
13756	1.29322	0.0889014
13757	0.946131	0.072513
13758	0.946132	0.0632199
13759	0.946129	0.141474
13760	0.946131	0.0638802
13761	0.946131	0.0678397
13762	0.946131	0.0949013
13763	0.946132	0.05
13764	0.946132	2.1
13765	0.946131	0.05
13766	1.41492	0.05
13767	0.946132	0.253816
13768	0.946132	0.05
13769	0.946131	0.0783826
13770	0.946132	2.1
13771	0.946132	2.1
13772	0.946132	2.1
13773	1.35028	2.1
13774	0.946132	2.1
13775	0.946132	2.1
13776	0.946132	2.1
13777	0.946132	1.66959
13778	0.946132	2.1
13779	0.946132	2.1
13780	1.32461	2.08739
13781	0.946131	2.1
13782	0.946132	2.1
13783	0.946132	2.1
13784	0.946131	2.1
13785	0.946131	2.1
13786	0.946131	2.1
13787	0.946132	2.1
13788	0.946129	2.1
13789	0.94613	2.1
13790	1.40369	2.1
13791	0.946132	0.05
13792	0.946131	0.05
13793	0.946131	0.05
13794	0.979598	0.05
13795	0.946131	0.05
13796	0.946131	0.05
13797	0.946132	0.05
13798	0.946131	2.1
13799	1.41897	2.1
13800	0.946132	2.1
13801	0.946132	2.1

13802	0.94613	2.1
13803	0.946131	2.1
13804	0.946131	2.1
13805	0.946131	2.1
13806	0.94613	2.1
13807	0.946132	2.1
13808	0.946132	2.1
13809	0.946129	2.1
13810	0.946131	2.1
13811	1.37791	2.1
13812	0.946127	2.1
13813	0.946131	2.1
13814	0.946131	2.1
13815	0.94613	2.1
13816	0.946128	2.1
13817	0.946129	2.1
13818	1.39384	2.1
13819	0.810945	2.1
13820	0.810944	1.55693
13821	0.810958	1.96932
13822	2.70852	2.1
13823	1.35168	1.83468
13824	1.08132	2.1
13825	1.08132	0.839816
13826	1.0813	1.67146
13827	1.3335	2.1
13828	0.946132	2.1
13829	0.94613	2.1
13830	0.946132	2.1
13831	0.946131	2.1
13832	0.94613	2.1
13833	0.946131	2.1
13834	0.946132	2.01297
13835	0.946131	0.05
13836	0.94613	1.74607
13837	1.41192	1.52774
13838	0.946132	0.05
13839	0.946131	0.976361
13840	0.94613	0.05
13841	0.946133	0.442068
13842	0.946133	0.684701
13843	0.946132	0.142747
13844	1.39428	0.0793897
13845	0.946132	0.130076
13846	0.946132	0.0603191
13847	0.946133	0.270819
13848	0.94613	0.35808
13849	0.946129	2.1
13850	0.94613	0.821255
13851	0.946128	1.1609
13852	1.40684	2.1
13853	0.94613	0.808068
13854	0.94613	2.1
13855	0.946131	0.292164
13856	0.946131	2.1
13857	0.946131	2.1
13858	0.946131	1.83615
13859	1.39021	2.1

13860	0.946131	2.1
13861	0.946131	2.1
13862	0.946131	2.06548
13863	0.946131	2.1
13864	0.946131	1.1817
13865	0.946131	2.1
13866	0.946131	2.1
13867	0.946131	0.93943
13868	0.946131	1.53732
13869	0.810946	2.1
13870	0.810946	2.1
13871	2.69264	2.1
13872	1.35168	2.1
13873	1.0813	2.1
13874	1.08132	2.1
13875	1.08132	2.1
13876	0.810958	2.1
13877	1.16687	2.1
13878	0.946131	2.1
13879	0.946131	2.1
13880	1.34632	2.1
13881	0.946131	2.1
13882	1.34632	2.1
13883	0.946131	2.1
13884	0.946128	1.47217
13885	0.946133	1.70788
13886	0.946133	0.639726
13887	0.946131	0.889724
13888	0.946131	1.1048
13889	1.3564	0.380116
13890	0.94613	0.384104
13891	0.946128	2.1
13892	0.946132	2.1
13893	0.94613	2.1
13894	0.94613	2.1
13895	0.946131	2.1
13896	1.35827	2.1
13897	0.94613	2.1
13898	0.946133	0.05
13899	0.946132	0.076286
13900	0.946132	0.070091
13901	0.946133	0.0550921
13902	0.946133	0.088182
13903	0.946132	0.0650205
13904	0.946133	0.0849188
13905	0.946132	2.1
13906	0.946132	2.1
13907	0.946132	0.362937
13908	0.946131	2.1
13909	0.946132	1.49753
13910	0.946132	1.40423
13911	0.946132	1.37325
13912	0.94613	0.11996
13913	0.94613	0.05
13914	0.946128	0.136133
13915	0.946132	0.05
13916	0.946132	0.14094
13917	0.94613	0.0757175

13918	0.946131	0.0610959
13919	0.946126	0.05
13920	0.946131	0.05
13921	0.946127	0.068352
13922	0.946129	0.05
13923	0.94613	0.05
13924	0.946128	0.05
13925	0.946128	0.05
13926	0.946131	0.05
13927	0.946127	0.05
13928	0.946127	0.05
13929	0.946131	0.05
13930	0.946131	0.05
13931	0.946128	0.05
13932	0.946129	0.05
13933	0.94613	0.05
13934	0.946131	0.05
13935	0.946129	0.05
13936	0.946132	0.05
13937	0.946132	0.05
13938	0.946131	0.05
13939	0.94613	0.05
13940	0.94613	0.05
13941	0.946131	0.05
13942	0.946129	0.050988
13943	0.946132	0.05
13944	0.946132	0.05
13945	1.32827	0.05
13946	0.946131	0.05
13947	0.946132	2.1
13948	0.946132	2.1
13949	0.946131	2.1
13950	0.946131	2.1
13951	0.946132	2.1
13952	0.946131	2.1
13953	0.946131	2.1
13954	0.946132	0.05
13955	0.946132	0.685388
13956	0.946131	0.05
13957	1.38356	0.05
13958	0.946133	0.173376
13959	0.946131	0.05
13960	0.946131	0.0766134
13961	0.94613	2.1
13962	0.946131	2.1
13963	0.946131	2.1
13964	0.946128	2.1
13965	0.946133	2.1
13966	0.946131	2.1
13967	1.36918	2.1
13968	0.946131	0.0640982
13969	0.94613	2.1
13970	0.946129	1.02478
13971	0.946133	2.1
13972	0.946132	0.963444
13973	1.3771	2.07761
13974	0.946131	1.38414
13975	1.30029	1.27397

13976	0.94613	0.470539
13977	0.946129	1.79357
13978	0.946132	1.793
13979	0.946132	0.0641432
13980	0.946131	1.12948
13981	0.94613	0.966646
13982	0.946133	0.05
13983	0.946132	0.05
13984	0.946132	0.05
13985	0.946132	0.05
13986	0.946132	0.05
13987	0.946132	0.05
13988	1.21174	0.05
13989	0.810946	2.1
13990	0.810945	2.1
13991	2.63045	2.1
13992	1.35169	2.1
13993	1.08132	2.1
13994	1.08132	2.1
13995	1.08132	2.1
13996	0.810946	2.1
13997	1.1796	2.1
13998	0.946131	2.1
13999	0.946132	2.1
14000	1.31522	2.1
14001	0.946132	2.1
14002	1.31522	2.1
14003	0.946132	2.1
14004	0.946131	2.1
14005	1.40615	2.1
14006	0.946129	2.1
14007	0.946128	2.1
14008	0.946133	2.1
14009	0.946131	2.1
14010	0.94613	2.1
14011	0.810945	2.1
14012	0.810944	2.1
14013	2.80479	2.1
14014	1.35169	2.1
14015	1.08132	2.1
14016	1.08132	2.1
14017	1.08132	2.1
14018	0.810945	2.1
14019	1.35562	2.1
14020	0.94613	2.1
14021	1.35561	2.1
14022	0.946133	2.1
14023	0.946131	2.1
14024	1.40239	2.1
14025	0.946131	2.1
14026	0.946125	0.05
14027	0.946131	0.0719943
14028	0.946127	0.05
14029	0.946129	0.05
14030	0.946128	0.0621598
14031	0.946129	0.0784357
14032	0.946128	0.05
14033	0.946119	2.1

14034	0.946113	2.1
14035	0.94612	2.1
14036	0.946119	2.1
14037	1.41915	2.1
14038	0.946107	2.1
14039	0.946132	2.1
14040	0.810946	1.40524
14041	0.810943	2.1
14042	0.810934	2.05263
14043	2.31411	2.1
14044	1.35171	2.1
14045	1.08132	1.78401
14046	1.08132	2.1
14047	1.08133	1.8352
14048	0.946127	2.1
14049	0.94613	2.1
14050	0.94613	2.1
14051	0.946131	2.1
14052	0.946126	2.1
14053	0.946128	2.1
14054	0.946128	2.1
14055	0.810944	2.1
14056	0.810947	2.1
14057	0.81096	2.1
14058	2.78019	2.1
14059	1.35168	2.1
14060	1.08132	2.1
14061	1.08132	2.1
14062	1.08131	2.1
14063	0.946133	0.05
14064	0.946132	0.05
14065	0.946132	0.05
14066	0.946133	0.05
14067	0.946132	0.05
14068	1.3495	0.05
14069	0.946133	0.05
14070	0.946133	2.1
14071	0.946132	2.1
14072	0.946132	2.1
14073	1.39529	2.1
14074	0.946133	2.1
14075	0.946132	2.1
14076	0.946133	2.1
14077	0.946133	2.1
14078	0.946132	2.1
14079	0.946132	2.1
14080	0.946132	2.1
14081	0.946132	2.1
14082	0.946133	2.1
14083	0.946132	2.1
14084	0.810946	2.1
14085	0.810945	2.1
14086	2.67861	2.1
14087	1.35169	2.1
14088	1.08132	2.1
14089	1.08132	2.1
14090	1.08132	2.1
14091	0.810946	2.1

14092	1.24616	2.1
14093	0.946132	2.1
14094	0.946132	2.1
14095	1.33931	2.1
14096	0.946132	2.1
14097	1.33931	2.1
14098	0.946133	2.1
14099	0.946133	0.0549098
14100	0.946133	0.05
14101	0.946132	0.05
14102	0.946133	0.056077
14103	1.3855	0.05
14104	1.3855	0.05
14105	0.946133	0.0575347
14106	0.946128	1.62862
14107	0.946132	2.1
14108	0.946128	2.1
14109	0.94613	2.1
14110	0.946131	2.1
14111	1.40225	2.1
14112	0.94613	2.1
14113	1.40548	0.05
14114	0.946118	0.05
14115	0.946114	0.05
14116	0.946112	0.05
14117	0.946117	0.05
14118	0.946128	0.05
14119	0.946103	0.05
14120	0.94613	2.1
14121	0.946132	2.1
14122	0.946131	2.1
14123	0.946132	2.1
14124	1.37811	2.1
14125	0.946131	2.1
14126	0.946131	2.1
14127	0.946132	2.1
14128	0.946133	2.1
14129	0.946133	2.1
14130	0.946132	2.1
14131	0.946133	2.1
14132	0.946132	2.1
14133	0.946132	2.1
14134	0.946118	2.1
14135	0.946113	2.1
14136	0.946118	2.1
14137	0.946119	2.1
14138	0.946117	2.1
14139	0.946105	2.1
14140	0.946131	2.1
14141	0.946118	0.05
14142	0.946118	0.05
14143	0.946117	0.05
14144	0.946113	0.05
14145	0.94612	0.05
14146	1.41377	0.05
14147	0.946106	0.05
14148	0.946132	0.0853827
14149	1.28436	0.05

14150	0.946131	0.05
14151	0.946131	0.098208
14152	0.946131	0.05
14153	0.94613	0.100213
14154	0.946131	0.0569765
14155	0.810947	0.05
14156	0.810946	0.05
14157	2.68675	0.05
14158	1.35168	0.05
14159	1.08132	0.05
14160	1.08132	0.05
14161	1.0813	0.05
14162	0.81096	0.05
14163	0.946132	0.05
14164	1.34338	0.05
14165	0.946132	0.05
14166	1.34338	0.05
14167	0.946132	0.05
14168	1.34337	0.05
14169	0.946132	0.05
14170	0.946127	2.1
14171	0.946131	2.1
14172	0.946127	2.1
14173	0.946131	1.93915
14174	0.946131	2.1
14175	0.946128	2.1
14176	1.30281	2.1
14177	0.946132	0.0894379
14178	0.946132	0.10608
14179	0.946132	0.0797552
14180	0.946131	0.0622707
14181	0.946132	0.121066
14182	1.37973	0.05
14183	0.946132	0.0548034
14184	0.946131	0.225524
14185	1.41798	0.109911
14186	0.946132	0.0705007
14187	0.946132	0.154318
14188	0.946129	0.317457
14189	0.946131	0.125667
14190	0.946131	0.330336
14191	0.946118	0.147112
14192	1.41776	0.102373
14193	0.946113	0.109962
14194	0.946119	0.0884075
14195	0.946117	0.209317
14196	1.41778	0.0762191
14197	0.946105	0.222097
14198	0.946131	0.293672
14199	1.41798	0.37412
14200	0.946132	0.475643
14201	0.946132	0.222005
14202	0.94613	0.351564
14203	1.41799	0.16253
14204	0.946131	0.185384
14205	0.946133	2.1
14206	0.946132	2.1
14207	0.946132	0.491214

14208	0.946132	2.1
14209	0.946133	1.46626
14210	0.946132	2.1
14211	0.946132	2.1
14212	0.946131	2.1
14213	0.946131	2.1
14214	0.94613	0.10959
14215	0.946129	2.1
14216	0.946132	1.00051
14217	0.94613	1.01999
14218	0.946131	2.08369
14219	0.94612	1.18634
14220	0.946126	2.1
14221	0.946119	0.720213
14222	0.94612	1.74132
14223	1.40766	2.1
14224	0.946132	0.500133
14225	0.946107	2.1
14226	0.946128	2.1
14227	1.31213	2.1
14228	0.946129	2.1
14229	0.94613	2.1
14230	0.94613	2.1
14231	0.94613	2.1
14232	0.94613	2.1
14233	0.946127	2.1
14234	0.946131	2.1
14235	0.94613	2.1
14236	0.94613	2.1
14237	1.36653	2.1
14238	0.946128	2.1
14239	0.946128	2.1
14240	0.946127	2.1
14241	0.94613	2.1
14242	0.946131	2.1
14243	0.94613	2.1
14244	0.946128	2.1
14245	0.946128	2.1
14246	0.946129	2.1
14247	0.946133	2.1
14248	1.40081	2.1
14249	0.946132	2.1
14250	0.946132	2.1
14251	0.946133	2.1
14252	0.946132	2.1
14253	0.946133	2.1
14254	0.946128	2.1
14255	1.32282	2.1
14256	0.946129	2.1
14257	0.94613	2.1
14258	0.946131	2.1
14259	0.946131	2.1
14260	0.946131	2.1
14261	0.810945	2.1
14262	0.810943	2.1
14263	0.810933	2.1
14264	2.44097	2.1
14265	1.3517	2.1

14266	1.08132	2.1
14267	1.08132	2.1
14268	1.08133	2.1
14269	0.946131	2.1
14270	0.94613	2.1
14271	0.946133	2.1
14272	0.946133	2.1
14273	0.946128	2.1
14274	0.946131	2.1
14275	1.27676	2.1
14276	0.946126	0.434825
14277	0.946126	0.593662
14278	0.946131	0.584562
14279	0.946127	0.48607
14280	0.94613	0.0646452
14281	0.946128	0.102002
14282	0.946128	0.0592159
14283	0.946132	0.05
14284	0.946133	0.05
14285	0.946132	0.05
14286	0.946132	0.05
14287	0.946132	0.05
14288	0.946133	0.05
14289	0.946132	0.05
14290	0.946128	0.05
14291	0.946131	0.05
14292	0.94613	0.05
14293	0.946129	0.05
14294	0.94613	0.05
14295	0.94613	0.05
14296	0.94613	0.05
14297	0.946132	0.05
14298	0.946128	0.05
14299	0.94613	0.05
14300	0.946131	0.05
14301	0.94613	0.05
14302	0.94613	0.05
14303	0.946131	0.05
14304	0.94612	0.05
14305	0.946119	0.05
14306	0.946119	0.05
14307	0.946124	0.05
14308	0.946119	0.05
14309	0.946132	0.05
14310	0.946107	0.05
14311	0.946128	2.1
14312	0.946133	2.1
14313	0.946131	2.1
14314	0.94613	2.1
14315	0.946131	2.1
14316	1.37869	2.1
14317	0.946131	2.1
14318	1.35703	2.1
14319	0.946132	2.1
14320	0.946132	2.1
14321	0.946132	2.1
14322	0.946132	2.1
14323	0.946132	2.1

14324	0.946133	2.1
14325	0.946133	2.1
14326	0.946133	2.1
14327	0.946132	2.1
14328	1.37017	2.1
14329	0.946133	2.1
14330	0.946132	2.1
14331	0.946133	2.1
14332	0.946114	0.05
14333	0.946119	0.05
14334	0.946119	0.05
14335	0.94612	0.05
14336	0.946119	0.05
14337	0.946132	0.05
14338	0.946107	0.05
14339	0.946131	0.05
14340	0.946131	0.05
14341	0.946126	0.05
14342	0.946127	0.05
14343	0.94613	0.05
14344	0.946129	0.05
14345	0.946128	0.05
14346	0.946131	0.682958
14347	0.946131	0.510721
14348	0.946132	0.115745
14349	0.946132	0.647439
14350	0.94613	1.43971
14351	0.946131	1.22294
14352	1.3279	1.21391
14353	0.946132	2.1
14354	1.40932	2.1
14355	0.946132	2.1
14356	0.946132	2.1
14357	0.946132	2.1
14358	0.946131	2.1
14359	0.946132	2.1
14360	0.946131	0.05
14361	0.946131	0.05
14362	0.946132	0.05
14363	0.946132	0.05
14364	0.94613	0.05
14365	0.946131	0.05
14366	1.26868	0.05
14367	0.810943	2.1
14368	0.810945	2.1
14369	0.810932	2.1
14370	2.82321	2.1
14371	1.3517	2.1
14372	1.08132	2.1
14373	1.08132	2.1
14374	1.08133	2.1
14375	0.810943	0.05
14376	0.810944	0.05
14377	2.63079	0.0583543
14378	1.35168	0.05
14379	1.0813	0.05
14380	1.08132	0.05
14381	1.08132	0.122448

14382	0.810957	0.0832637
14383	1.17856	0.05
14384	0.946132	0.05
14385	0.946128	0.0516133
14386	1.3154	0.231967
14387	0.94613	0.0574055
14388	1.31539	0.102272
14389	0.94613	0.80326
14390	0.94613	1.60052
14391	0.94613	1.29396
14392	0.946131	0.652787
14393	0.946131	1.49401
14394	0.946129	1.84977
14395	0.94613	2.1
14396	1.21997	2.09745
14397	0.946131	0.935859
14398	1.25083	0.329812
14399	0.946132	0.198292
14400	0.946132	1.1602
14401	0.946129	0.220565
14402	1.25083	0.958664
14403	0.946131	1.0103
14404	0.946127	1.51661
14405	1.41342	0.323012
14406	0.94613	1.23134
14407	0.946131	0.749911
14408	0.946127	0.117419
14409	0.946129	0.330434
14410	0.946129	0.578509
14411	0.946131	2.00853
14412	1.29149	2.00811
14413	0.946133	2.1
14414	0.946133	1.18624
14415	0.946128	1.64442
14416	0.946131	0.377059
14417	0.94613	1.60992
14418	0.94613	2.1
14419	0.94613	1.87625
14420	0.946132	2.1
14421	0.946132	1.39816
14422	0.946128	0.290807
14423	0.94613	2.1
14424	1.41022	2.1
14425	0.946132	0.0503205
14426	0.946132	0.05
14427	0.946128	0.05
14428	0.94613	0.05
14429	0.94613	0.0862661
14430	0.94613	0.05
14431	0.946131	0.0742128
14432	0.946129	1.25528
14433	1.37758	1.85715
14434	0.946131	2.1
14435	0.94613	0.519545
14436	0.946124	1.74445
14437	0.946127	0.720711
14438	0.946127	1.93009
14439	0.946129	1.19987

14440	1.38259	1.72869
14441	0.946132	2.1
14442	0.946132	0.593566
14443	0.946126	1.81644
14444	0.946129	0.847692
14445	0.94613	1.67779
14446	0.946131	0.05
14447	0.946132	0.05
14448	0.946132	0.05
14449	0.946132	0.05
14450	0.946132	0.05
14451	0.946132	0.05
14452	0.946132	0.05
14453	0.946131	0.05
14454	0.94613	0.05
14455	0.94613	0.05
14456	0.946133	0.05
14457	0.946128	0.05
14458	1.25017	0.05
14459	0.946131	0.05
14460	0.946131	2.1
14461	0.946131	2.1
14462	0.946131	2.1
14463	1.3937	2.1
14464	0.946133	2.1
14465	0.946131	2.1
14466	0.94613	2.1
14467	0.946131	0.05
14468	0.946131	0.05
14469	0.946131	0.05
14470	0.94613	0.05
14471	0.946133	0.0903559
14472	0.946132	0.05
14473	0.946131	0.05
14474	0.946132	0.05
14475	0.946132	0.072361
14476	0.946132	0.05
14477	0.946132	0.05
14478	0.946131	0.05
14479	0.946132	0.05
14480	0.946132	0.05
14481	0.946129	0.05
14482	0.94613	0.181295
14483	0.94613	0.0571967
14484	0.946128	0.16386
14485	0.946132	0.05
14486	0.946131	0.0673707
14487	0.94613	0.05
14488	0.946132	2.1
14489	0.946132	0.151432
14490	0.946132	1.06381
14491	1.41619	2.1
14492	0.946133	0.05
14493	0.946133	1.39114
14494	0.946132	0.125675
14495	0.946132	2.1
14496	0.946132	2.1
14497	0.946132	2.1

14498	1.38244	2.1
14499	0.946132	1.70756
14500	0.946131	2.1
14501	0.946132	2.1
14502	0.810946	0.296569
14503	0.810943	0.897916
14504	0.810934	0.300512
14505	2.42741	0.0577846
14506	1.35171	0.11259
14507	1.08132	0.265138
14508	1.08132	0.279814
14509	1.08133	0.242091
14510	0.810947	0.152606
14511	0.810943	1.06481
14512	2.44179	0.0614696
14513	1.35171	0.150148
14514	1.08132	0.135618
14515	1.08132	0.192263
14516	1.08133	0.164702
14517	0.810934	0.140525
14518	0.946132	1.89219
14519	0.946132	1.78502
14520	0.946133	0.810135
14521	0.946133	1.74983
14522	0.946132	1.98584
14523	0.946132	1.7668
14524	1.12786	1.39204
14525	1.27806	0.38006
14526	0.946132	1.09868
14527	0.946133	0.742757
14528	0.946133	1.44127
14529	0.946132	1.94465
14530	0.946132	1.07198
14531	0.946133	1.83971
14532	0.946132	2.1
14533	0.946132	2.1
14534	0.946132	2.1
14535	1.37098	2.1
14536	0.946133	2.1
14537	0.946132	2.1
14538	1.37098	2.1
14539	0.946125	1.36744
14540	0.946129	1.19985
14541	0.946131	2.1
14542	0.94613	0.700908
14543	0.946124	0.457932
14544	0.946127	0.857816
14545	1.39514	1.48998
14546	0.946132	2.1
14547	0.946131	2.1
14548	0.94613	2.1
14549	1.39909	2.1
14550	0.946132	1.46829
14551	0.946131	2.1
14552	0.94613	2.1
14553	0.946133	2.1
14554	0.946133	0.05
14555	0.946132	2.1

14556	1.36835	1.86924
14557	0.946132	0.707121
14558	0.946133	2.03885
14559	0.946132	0.0756515
14560	0.946132	2.1
14561	0.946133	2.1
14562	0.946133	2.1
14563	0.946132	2.1
14564	1.39047	2.1
14565	0.946132	2.1
14566	0.946132	2.1
14567	1.33174	0.05
14568	0.946128	0.05
14569	0.94613	0.05
14570	0.94613	0.05
14571	0.94613	0.05
14572	0.94613	0.05
14573	0.946131	0.05
14574	0.946128	0.05
14575	1.36004	0.05
14576	0.946129	0.05
14577	0.94613	0.05
14578	0.94613	0.05
14579	1.36003	0.05
14580	0.94613	0.05
14581	0.946124	2.1
14582	0.946119	2.1
14583	0.946119	2.1
14584	0.946119	2.1
14585	0.94612	2.1
14586	0.946132	2.1
14587	1.29062	2.1
14588	0.946128	2.1
14589	0.946128	2.1
14590	0.946133	2.1
14591	0.94613	2.1
14592	0.946131	2.1
14593	0.946131	2.1
14594	1.30584	2.1
14595	0.946131	2.1
14596	0.946131	2.1
14597	0.946131	2.1
14598	0.946132	2.1
14599	1.39549	2.1
14600	0.946131	2.1
14601	0.946131	2.1
14602	0.913386	0.653423
14603	1.17466	0.413731
14604	1.00473	0.215485
14605	0.946132	0.515211
14606	0.946126	1.36177
14607	0.94613	0.93187
14608	0.946129	0.744255
14609	0.946132	0.4682
14610	1.38894	0.332338
14611	0.946132	0.185095
14612	0.946132	0.289072
14613	0.946132	1.14034

14614	0.946132	0.417599
14615	0.946133	0.414982
14616	0.946128	0.280135
14617	1.23865	0.269637
14618	0.946129	0.124356
14619	0.94613	0.190376
14620	0.94613	0.404203
14621	0.94613	0.221482
14622	0.94613	0.271801
14623	0.946126	0.675699
14624	1.40248	1.01653
14625	0.946129	0.815221
14626	0.94613	0.710763
14627	0.946124	1.70584
14628	0.946127	1.17155
14629	0.946128	0.926569
14630	0.946133	0.465196
14631	1.1391	0.447105
14632	0.946132	0.535433
14633	0.946132	0.259377
14634	0.946132	1.37935
14635	0.946133	0.443401
14636	0.946132	0.546152
14637	1.17156	1.37682
14638	0.946133	1.38172
14639	1.33893	0.474182
14640	0.827864	0.794026
14641	0.995232	0.344659
14642	0.827864	1.2348
14643	0.94613	0.491488
14644	1.21387	0.567017
14645	0.946132	0.571452
14646	0.946131	0.0908646
14647	0.94613	0.285342
14648	0.94613	0.255202
14649	0.94613	0.425955
14650	0.94613	0.207743
14651	0.810943	0.05
14652	0.810945	0.05
14653	2.42463	0.05
14654	1.3517	0.05
14655	1.08132	0.05
14656	1.08132	0.05
14657	1.08133	0.05
14658	0.810932	0.05
14659	0.946133	1.58321
14660	0.946132	0.190765
14661	0.946131	0.412195
14662	1.36748	1.1575
14663	0.946133	0.865339
14664	0.946131	0.266872
14665	0.946132	0.05
14666	1.35856	0.05
14667	0.946131	0.05
14668	0.94613	0.05
14669	0.94613	0.05
14670	0.946131	0.05
14671	0.94613	0.05

14672	0.94613	0.0511741
14673	0.946132	2.1
14674	0.946131	2.1
14675	0.946131	2.1
14676	0.946131	2.1
14677	1.41149	2.1
14678	1.41149	2.1
14679	0.946131	2.1
14680	0.946128	2.1
14681	0.946128	2.1
14682	0.946133	2.1
14683	0.946131	2.1
14684	0.946131	2.1
14685	0.946131	2.1
14686	0.94613	2.1
14687	1.41388	0.05
14688	0.94613	0.05
14689	0.946132	0.05
14690	0.946132	0.05
14691	0.946129	0.05
14692	0.946131	0.05
14693	0.946131	0.05
14694	1.02798	0.05
14695	0.946132	0.05
14696	1.02798	0.05
14697	0.946133	0.05
14698	1.14931	0.05
14699	0.957746	0.05
14700	0.93452	0.05
14701	0.946129	2.1
14702	0.946134	2.1
14703	0.946133	2.1
14704	0.946131	2.1
14705	0.946131	2.1
14706	0.946131	2.1
14707	0.946131	2.1
14708	0.946133	2.1
14709	0.946132	0.919314
14710	0.946132	1.66579
14711	0.946132	2.1
14712	1.38773	1.08331
14713	0.946132	2.04057
14714	0.946133	1.40974
14715	0.946131	0.05
14716	0.946131	0.05
14717	1.41512	0.05
14718	0.946133	0.05
14719	0.946131	0.05
14720	0.946131	0.05
14721	0.946131	0.05
14722	1.35381	0.140874
14723	0.946132	2.1
14724	1.35381	0.0511452
14725	0.946133	2.1
14726	0.946132	0.820014
14727	0.946132	2.1
14728	0.946133	1.47111
14729	1.3365	0.137634

14730	0.946132	0.757691
14731	1.3365	0.0564414
14732	0.946129	0.592044
14733	0.94613	0.134824
14734	0.946128	0.695502
14735	0.946129	0.183767
14736	0.939895	1.65463
14737	1.14285	2.1
14738	0.952366	2.1
14739	0.946131	1.96843
14740	0.94613	2.1
14741	0.946131	2.05634
14742	0.94613	2.07343
14743	0.946131	2.1
14744	0.946131	2.1
14745	0.946131	2.1
14746	0.946129	2.1
14747	0.946133	2.03595
14748	0.946131	2.1
14749	0.946131	2.1
14750	0.946133	2.1
14751	0.946132	2.1
14752	0.946132	2.1
14753	0.946133	2.1
14754	0.946132	2.1
14755	0.946132	2.1
14756	0.946133	2.1
14757	0.946133	2.1
14758	0.946132	1.43082
14759	0.946132	2.1
14760	0.946133	1.90446
14761	0.946132	2.1
14762	0.946132	2.1
14763	0.946132	1.68956
14764	0.946132	0.555263
14765	0.946131	2.1
14766	0.946131	1.50134
14767	0.946131	1.55933
14768	1.38969	2.1
14769	0.946131	2.1
14770	0.946132	2.1
14771	0.946128	2.1
14772	0.946133	1.90749
14773	0.946132	2.1
14774	0.946131	1.80753
14775	0.94613	1.86283
14776	0.946131	2.08397
14777	1.28464	2.05812
14778	0.946119	0.678122
14779	1.37492	0.825387
14780	0.946125	1.11934
14781	0.946119	0.288676
14782	0.946119	1.50946
14783	0.946132	0.488981
14784	0.946107	1.00291
14785	0.810942	2.1
14786	0.810943	2.1
14787	0.709587	2.1

14788	2.64488	2.1
14789	1.21651	2.1
14790	0.946151	2.1
14791	0.979948	2.1
14792	1.0813	2.1
14793	0.946133	1.78854
14794	0.946132	2.1
14795	0.946132	2.1
14796	0.946133	0.779868
14797	0.946132	0.191199
14798	0.946132	0.554878
14799	1.41134	1.51361
14800	0.946133	2.1
14801	0.946132	1.99154
14802	0.946133	2.1
14803	0.946133	1.26134
14804	0.946132	0.209342
14805	0.946133	1.78544
14806	1.31338	2.01682
14807	2.07612	2.1
14808	0.946102	0.05
14809	0.752448	0.05
14810	1.20617	0.05
14811	0.94613	0.05
14812	0.75242	0.05
14813	0.946131	0.05
14814	0.752419	0.05
14815	0.946132	1.04079
14816	1.25736	0.581593
14817	1.25736	0.433402
14818	0.946132	0.821176
14819	1.08054	0.246993
14820	1.25736	0.49538
14821	0.946132	1.2457
14822	0.946128	2.1
14823	1.1896	2.1
14824	1.18959	2.1
14825	0.946131	0.871939
14826	1.33271	2.1
14827	0.946105	2.1
14828	0.946156	2.1
14829	0.741946	0.05
14830	1.33587	0.05
14831	0.741947	0.05
14832	1.21954	0.05
14833	0.94613	0.05
14834	0.741946	0.05
14835	1.81347	0.05
14836	1.81347	0.05
14837	0.653322	0.05
14838	1.36935	0.05
14839	0.661882	0.05
14840	1.04753	0.05
14841	0.507464	0.05
14842	0.945844	1.27598
14843	1.59507	2.1
14844	1.04043	1.11607
14845	0.946131	2.1

14846	1.32891	2.0457
14847	0.946131	1.35535
14848	0.946131	0.942044
14849	1.28866	1.85706
14850	1.12682	0.05
14851	0.851543	0.0531742
14852	1.04072	0.05
14853	0.946132	0.0813768
14854	1.13437	0.05
14855	1.04167	0.05
14856	0.936652	0.05
14857	1.258	2.1
14858	0.946131	2.1
14859	0.946132	2.1
14860	1.36968	2.1
14861	0.946132	2.1
14862	1.36968	2.1
14863	0.946131	2.1
14864	2.30046	0.05
14865	2.82969	0.05
14866	0.946132	2.1
14867	1.35022	2.1
14868	1.12356	2.02533
14869	0.94613	2.1
14870	1.35022	2.1
14871	0.946131	2.1
14872	0.94613	2.1
14873	0.946131	2.1
14874	1.22401	2.1
14875	0.952456	2.1
14876	0.94613	2.1
14877	1.22401	2.1
14878	0.946131	2.1
14879	0.946131	2.1
14880	1.33047	2.1
14881	0.946132	2.1
14882	1.33047	2.1
14883	0.946131	2.1
14884	1.33047	2.1
14885	0.946132	2.1
14886	1.2107	2.1
14887	0.61957	2.09967
14888	1.80666	2.1
14889	1.53743	2.1
14890	0.619572	1.78643
14891	1.32547	2.1
14892	1.16166	2.1
14893	1.04074	2.1
14894	0.557621	2.1
14895	0.946128	1.94503
14896	1.36607	2.1
14897	0.94613	2.1
14898	1.36607	2.1
14899	1.39416	2.1
14900	0.94613	2.1
14901	0.94613	2.1
14902	1.23969	2.1
14903	0.946131	2.1

14904	0.946131	2.1
14905	1.13537	2.1
14906	1.05719	2.1
14907	0.806848	2.1
14908	1.30251	2.1
14909	0.851526	1.94702
14910	1.14176	2.1
14911	0.93666	1.46338
14912	0.946114	2.1
14913	1.15832	2.03859
14914	0.924624	1.98088
14915	0.967609	1.68059
14916	0.771764	0.05
14917	1.26652	0.05
14918	0.946133	0.05
14919	1.17957	0.05
14920	0.771735	0.05
14921	0.946122	0.05
14922	0.946133	0.05
14923	0.924325	0.333173
14924	0.656739	0.802347
14925	0.637876	0.893531
14926	0.530092	0.548434
14927	0.914415	0.395074
14928	0.821311	1.60908
14929	0.630589	1.34166
14930	0.738823	1.06723
14931	0.839109	0.823605
14932	1.55029	0.05
14933	1.13537	0.05
14934	0.946131	0.465845
14935	0.946128	0.0506223
14936	1.30162	2.1
14937	0.952476	0.762094
14938	1.29289	1.47945
14939	0.939783	1.43092
14940	0.94612	2.1
14941	0.946131	2.1
14942	1.07998	2.1
14943	0.836985	2.1
14944	1.1604	2.1
14945	0.94613	2.1
14946	0.837016	2.1
14947	0.712461	0.198724
14948	1.36399	0.263877
14949	1.8724	0.166661
14950	1.8724	0.0777992
14951	0.623426	1.14817
14952	1.36399	0.106727
14953	1.22089	0.114006
14954	0.946132	0.121691
14955	0.946131	2.1
14956	1.32241	2.1
14957	1.32241	2.1
14958	0.946133	2.1
14959	1.40271	2.1
14960	0.946131	2.1
14961	0.946131	2.1

14962	1.1989	1.07906
14963	0.954106	1.49534
14964	0.820068	0.913875
14965	0.563139	1.39135
14966	1.06448	1.45053
14967	0.892472	1.94494
14968	0.862598	0.994747
14969	0.745851	1.95787
14970	1.13336	1.35357
14971	0.946129	2.1
14972	1.28254	2.1
14973	1.28254	2.1
14974	0.946131	2.1
14975	1.41302	2.1
14976	0.946131	2.1
14977	0.946131	2.1
14978	0.851543	1.93904
14979	1.21961	2.1
14980	0.851484	1.69333
14981	0.851543	2.1
14982	1.15705	2.1
14983	0.946133	2.1
14984	0.909417	2.1
14985	1.13808	0.768774
14986	0.946133	0.05
14987	0.946131	0.927779
14988	1.30194	0.0590677
14989	0.946131	0.239217
14990	1.30194	0.05
14991	0.946131	0.0596931
14992	0.930941	1.07875
14993	1.20185	0.476232
14994	1.02404	0.428295
14995	0.946124	1.28217
14996	1.3563	0.744202
14997	0.946127	1.41462
14998	0.946128	1.06106
14999	0.946116	2.1
15000	1.3144	2.1
15001	1.13088	2.1
15002	0.946118	0.492269
15003	1.31438	2.1
15004	0.946105	2.1
15005	0.946131	2.1
15006	1.76972	0.05
15007	1.42091	0.05
15008	0.946128	0.05
15009	0.946101	0.05
15010	0.863552	0.05
15011	0.94613	0.05
15012	1.21232	0.05
15013	0.94613	0.0527838
15014	1.2137	0.0754944
15015	1.23224	0.325239
15016	1.86512	0.11948
15017	1.86512	0.0548739
15018	0.580825	1.41767
15019	1.2137	0.507308

15020	0.946133	0.521403
15021	0.580822	0.286852
15022	0.946128	2.1
15023	1.40586	2.1
15024	0.94613	2.1
15025	1.3719	2.1
15026	1.3719	2.1
15027	1.40586	2.1
15028	0.94613	2.1
15029	0.946132	2.1
15030	1.35025	2.1
15031	1.1794	2.1
15032	0.946132	0.32881
15033	1.35025	1.25249
15034	0.946131	1.33803
15035	0.946132	1.15819
15036	0.946133	2.1
15037	1.60149	2.1
15038	1.67058	2.1
15039	0.851542	2.1
15040	1.20296	2.1
15041	1.17501	2.1
15042	0.946132	2.1
15043	1.04993	2.1
15044	0.851541	1.92263
15045	1.48219	2.1
15046	0.874565	1.77638
15047	0.946129	2.1
15048	1.22049	2.1
15049	0.946131	2.1
15050	0.946131	2.1
15051	0.94613	2.1
15052	0.848719	2.1
15053	1.17195	2.1
15054	0.946129	1.34753
15055	1.35426	1.55046
15056	0.94613	1.83762
15057	0.94613	2.08113
15058	0.946133	2.1
15059	1.14342	2.1
15060	1.14342	2.1
15061	0.946133	2.1
15062	1.39009	2.1
15063	0.946132	2.1
15064	0.946132	2.1
15065	1.38819	2.1
15066	0.946133	2.1
15067	1.38819	2.1
15068	0.946131	2.1
15069	1.4116	2.1
15070	0.946131	2.1
15071	1.38819	2.1
15072	0.946127	2.1
15073	1.32244	2.1
15074	1.2176	2.1
15075	0.946131	2.1
15076	1.32244	2.1
15077	0.94613	2.1

15078	0.946129	2.1
15079	1.32415	2.1
15080	1.11239	2.1
15081	0.984099	2.1
15082	1.65532	2.1
15083	1.53082	2.1
15084	1.32068	2.1
15085	1.32068	2.1
15086	1.08172	2.1
15087	3.76293	0.05
15088	1.36335	0.05
15089	0.953925	2.1
15090	0.989497	2.1
15091	0.98949	2.1
15092	1.06341	2.1
15093	1.27875	2.1
15094	1.04598	2.1
15095	0.603154	2.1
15096	0.582457	2.1
15097	0.582457	2.1
15098	0.568994	2.1
15099	1.5791	2.1
15100	1.09506	2.1
15101	1.45327	2.1
15102	1.88406	2.1
15103	1.88404	2.1
15104	1.27389	2.1
15105	1.14206	2.1
15106	1.00232	2.1
15107	1.00231	2.1
15108	1.68436	2.1
15109	1.4677	0.05
15110	1.28828	0.05
15111	1.28828	0.05
15112	1.46932	0.05
15113	1.28228	2.1
15114	1.64268	1.14195
15115	0.959473	2.1
15116	1.10101	2.1
15117	1.07976	2.1
15118	1.2873	2.1
15119	1.70303	0.05
15120	1.40066	0.05
15121	1.5563	0.05
15122	1.13727	0.05
15123	1.03529	2.1
15124	1.11285	2.1
15125	1.03529	2.1
15126	1.28667	2.1
15127	1.32119	2.1
15128	1.44777	2.1
15129	1.44778	2.1
15130	1.61121	2.1
15131	1.09372	0.05
15132	1.17664	0.05
15133	1.17664	0.05
15134	0.872931	0.05
15135	1.02415	2.1

15136	1.28228	2.1
15137	2.14692	2.1
15138	3.90469	2.1
15139	1.00509	2.1
15140	1.28228	2.1
15141	3.32936	0.05
15142	1.1333	0.05
15143	1.11048	2.1
15144	0.979854	2.1
15145	1.65815	2.1
15146	1.00185	2.1
15147	1.84887	2.1
15148	1.59597	2.1
15149	1.78468	2.1
15150	1.87508	2.1
15151	1.82716	2.1
15152	1.33429	2.1
15153	1.34626	2.1
15154	0.797142	2.1
15155	1.00007	1.18315
15156	1.02051	2.1
15157	1.02051	1.40892
15158	1.03373	2.1
15159	1.05786	2.1
15160	0.887631	2.1
15161	0.992773	2.1
15162	1.28462	2.1
15163	2.35274	1.94535
15164	1.82533	1.04518
15165	1.82533	1.66917
15166	1.19496	0.383215
15167	1.28597	0.05
15168	1.09725	2.1
15169	1.60948	2.1
15170	1.20421	2.1
15171	1.16566	2.1
15172	1.88522	2.1
15173	1.81948	0.0527674
15174	1.14042	0.05
15175	1.21558	0.051605
15176	1.56876	0.05
15177	1.6697	2.1
15178	1.81655	2.1
15179	1.81655	2.1
15180	1.5956	2.1
15181	0.906059	2.1
15182	1.63045	2.1
15183	0.9533	0.553106
15184	1.1475	0.793572
15185	1.72231	2.1
15186	1.94936	1.52471
15187	1.06826	2.1
15188	1.08576	2.1
15189	1.06826	2.1
15190	0.973559	2.1
15191	1.62737	0.05
15192	0.965676	0.05
15193	1.45055	0.05

15194	1.17631	0.05
15195	1.92535	0.05
15196	1.9503	0.05
15197	1.99828	0.05
15198	1.70302	0.05
15199	1.65139	0.05
15200	1.26784	0.05
15201	1.35923	0.05
15202	1.70302	0.05
15203	1.33033	2.1
15204	1.12481	2.1
15205	1.29452	2.1
15206	1.25399	2.1
15207	1.12481	2.1
15208	0.918358	2.1
15209	1.39332	2.1
15210	1.32368	2.1
15211	1.11712	0.434452
15212	1.07999	0.868725
15213	1.07999	0.129262
15214	1.05619	0.796514
15215	1.1766	2.1
15216	1.35735	2.1
15217	1.42456	0.05
15218	1.089 0.05	
15219	1.11081	0.05
15220	2.88521	0.05
15221	1.09754	0.05
15222	1.85479	0.05
15223	1.27922	0.0769385
15224	1.03556	0.05
15225	1.07508	1.67928
15226	1.09795	2.1
15227	0.967672	0.38027
15228	1.07508	1.43109
15229	1.03933	1.96756
15230	1.02975	2.1
15231	1.02975	2.078
15232	1.01121	2.1
15233	1.24526	0.05
15234	1.72368	0.05
15235	1.89607	0.05
15236	2.13512	0.05
15237	1.34591	0.05
15238	1.14721	0.0930031
15239	1.15539	1.48583
15240	1.90938	0.351646
15241	1.90941	2.1
15242	1.89242	2.1
15243	1.18907	2.1
15244	1.20408	2.1
15245	1.20409	2.1
15246	1.22655	2.1
15247	0.701447	2.1
15248	1.57567	2.1
15249	1.38806	2.1
15250	2.09407	2.1
15251	1.07612	0.996265

15252	0.990019	0.922808
15253	1.15564	2.1
15254	1.01243	1.8592
15255	1.28956	0.05
15256	1.35146	0.05
15257	1.35145	0.05
15258	1.22811	0.05
15259	1.09662	2.1
15260	1.42486	2.1
15261	1.10106	0.05
15262	1.90814	0.05
15263	1.3946	0.05
15264	1.11926	0.05
15265	0.725839	2.1
15266	0.925068	2.1
15267	0.925074	2.1
15268	1.43282	2.1
15269	1.91756	0.587294
15270	1.74321	0.645673
15271	1.74321	0.359375
15272	1.63413	0.435758
15273	1.9194	0.05
15274	1.62246	0.05
15275	1.90456	0.05
15276	1.06578	0.05
15277	1.46019	0.05
15278	1.17036	0.05
15279	1.70233	0.817643
15280	1.53515	0.434583
15281	2.03494	2.1
15282	1.78441	2.1
15283	1.75992	2.1
15284	1.94351	2.1
15285	1.51057	0.05
15286	0.991435	0.05
15287	1.98068	0.05
15288	1.64283	0.05
15289	2.96126	0.05
15290	2.20692	0.05
15291	1.95054	0.05
15292	1.21325	0.05
15293	0.996828	0.05
15294	1.01976	0.05
15295	1.17129	2.1
15296	1.13664	2.1
15297	1.13664	2.1
15298	1.09627	2.1
15299	2.65123	2.1
15300	3.23062	2.1
15301	3.23062	2.1
15302	3.92038	2.1
15303	1.89019	0.05
15304	1.79109	0.05
15305	1.79109	0.05
15306	1.54197	0.05
15307	3.41824	1.25155
15308	1.93553	0.05
15309	2.26215	1.28865

15310	2.0103	0.05
15311	1.51569	0.598647
15312	1.01683	0.398572
15313	1.78595	2.1
15314	1.66537	2.1
15315	3.73426	2.1
15316	3.53905	2.1
15317	1.66537	2.1
15318	1.52903	2.1
15319	3.53905	2.1
15320	3.3837	2.1
15321	1.19901	0.511611
15322	1.35002	1.97593
15323	1.35002	0.0993041
15324	1.11683	0.119079
15325	1.75326	1.94553
15326	0.712404	1.39879
15327	2.97768	2.1
15328	2.98949	2.1
15329	1.2832	2.1
15330	1.39518	2.1
15331	2.98949	2.1
15332	2.99339	2.1
15333	1.39517	2.1
15334	1.46124	2.1
15335	1.28534	2.1
15336	1.03391	2.1
15337	1.75366	0.882428
15338	1.26247	1.85363
15339	1.13621	2.1
15340	2.43994	2.1
15341	1.47904	0.05
15342	1.80085	0.05
15343	1.80085	0.05
15344	1.64656	0.05
15345	2.87331	2.1
15346	2.82863	2.1
15347	2.82859	2.1
15348	2.84673	2.1
15349	1.0961	0.05
15350	1.39448	0.127156
15351	1.21366	0.187339
15352	1.20143	0.05
15353	1.85795	0.05
15354	1.36768	0.05
15355	1.36769	0.05
15356	1.45789	0.05
15357	1.32605	0.05
15358	1.27148	0.05
15359	1.70218	0.05
15360	1.3063	0.05
15361	1.45603	0.05
15362	1.89226	0.05
15363	1.2742	0.05
15364	2.10112	0.05
15365	1.10748	0.05
15366	1.1893	0.05
15367	1.04921	2.1

15368	1.22927	2.1
15369	1.22927	2.1
15370	1.46568	2.1
15371	0.811186	2.1
15372	0.792162	2.1
15373	0.792162	2.1
15374	0.758749	2.1
15375	1.03581	2.1
15376	0.737481	2.1
15377	2.24071	2.1
15378	1.34868	2.1
15379	1.47203	0.538699
15380	1.40967	0.63216
15381	1.40967	0.795935
15382	1.27916	0.71045
15383	1.17418	1.38051
15384	1.04921	2.1
15385	1.28291	0.404146
15386	1.3213	0.05
15387	1.46812	0.201079
15388	2.42522	0.654187
15389	1.17879	0.661174
15390	1.28291	0.576832
15391	1.28291	0.710656
15392	1.13438	0.405903
15393	1.75004	2.1
15394	1.78247	2.1
15395	1.78247	2.1
15396	1.76421	2.1
15397	2.25975	0.05
15398	3.66111	0.05
15399	3.6611	0.05
15400	3.44043	0.05
15401	1.13339	2.1
15402	1.13322	2.1
15403	1.13323	2.1
15404	1.20761	2.1
15405	1.34337	0.562156
15406	1.90912	2.1
15407	1.04399	2.1
15408	2.00578	2.1
15409	1.04973	2.1
15410	1.22615	2.1
15411	1.93384	2.1
15412	2.62816	0.165083
15413	1.13127	2.1
15414	1.47642	2.1
15415	2.62816	2.1
15416	3.31755	0.177554
15417	1.47642	2.1
15418	1.80454	1.96546
15419	0.964782	2.1
15420	0.949135	2.1
15421	0.949134	2.1
15422	0.949134	2.1
15423	1.10476	0.0727216
15424	0.982858	0.05
15425	1.10938	0.082348

15426	0.982858	0.05
15427	1.01012	0.05
15428	0.974081	0.05
15429	1.82643	0.05
15430	1.29585	0.05
15431	1.00763	1.26683
15432	1.3294	0.659009
15433	1.3294	0.29393
15434	1.30001	1.14684
15435	1.59151	2.1
15436	1.99443	2.1
15437	1.07369	2.1
15438	1.30584	2.1
15439	1.05071	2.1
15440	0.948676	2.1
15441	1.13842	2.1
15442	1.42884	2.1
15443	1.59019	0.05
15444	1.38212	0.05
15445	1.34102	0.05
15446	1.02663	0.05
15447	0.797011	2.1
15448	0.81301	2.1
15449	0.81301	2.1
15450	0.811187	2.1
15451	0.991869	0.05
15452	1.23868	0.05
15453	1.67201	2.1
15454	1.61201	2.1
15455	1.61202	2.1
15456	1.57627	2.1
15457	0.94994	2.1
15458	1.77867	2.1
15459	0.854945	2.1
15460	0.973905	2.1
15461	1.64193	2.1
15462	1.68893	2.1
15463	1.68893	2.1
15464	1.70002	2.1
15465	2.08149	0.05
15466	1.69456	0.05
15467	1.67399	0.05
15468	1.83922	2.00854
15469	1.91703	2.1
15470	0.967492	2.1
15471	1.13317	2.1
15472	0.980766	2.1
15473	2.06158	0.05
15474	0.986498	0.05
15475	1.76289	0.05
15476	1.17248	0.05
15477	1.51794	0.05
15478	1.30266	0.05
15479	1.03165	0.05
15480	0.947692	0.05
15481	1.20473	0.05
15482	0.947693	0.05
15483	1.54357	2.1

15484	0.980841	2.1
15485	1.34124	0.0645607
15486	1.39818	0.48012
15487	1.39818	0.05
15488	1.48035	0.531066
15489	1.03932	2.1
15490	1.2065	2.1
15491	1.20651	2.1
15492	1.12717	2.1
15493	1.09483	2.1
15494	0.994293	2.1
15495	0.994293	2.1
15496	0.97356	2.1
15497	0.995252	0.0642234
15498	1.66294	0.257201
15499	1.82775	0.17672
15500	1.42762	0.0668729
15501	1.04713	0.05
15502	1.05475	0.05
15503	1.29002	0.05
15504	1.05475	0.05
15505	1.17585	2.1
15506	1.39223	2.1
15507	1.39223	2.1
15508	1.71255	2.1
15509	1.56166	0.524724
15510	1.37762	0.314824
15511	1.16858	2.1
15512	1.0361	2.1
15513	1.23966	0.05
15514	1.10718	0.05
15515	1.99957	0.05
15516	0.658392	0.05
15517	1.36904	0.05
15518	1.12524	0.05
15519	1.04321	1.3154
15520	1.04627	0.975383
15521	1.91351	0.840697
15522	1.63379	0.935581
15523	1.79718	1.23403
15524	0.59634	1.16576
15525	0.958257	2.1
15526	1.06457	2.1
15527	0.958255	2.1
15528	1.03933	2.1
15529	1.14195	0.0565639
15530	1.49438	0.0508734
15531	1.49438	0.05
15532	1.31259	2.1
15533	1.23036	2.1
15534	1.2016	2.1
15535	1.2016	2.1
15536	1.17129	2.1
15537	1.05724	1.06179
15538	1.04291	1.48039
15539	1.12341	2.1
15540	2.32525	2.1
15541	3.39038	2.1

15542	2.09374	2.1
15543	1.76185	0.307035
15544	1.7934	0.399941
15545	1.65809	0.420174
15546	1.54852	1.44134
15547	1.38766	2.1
15548	1.28765	2.1
15549	1.26599	2.1
15550	1.21397	2.1
15551	1.28765	2.1
15552	1.20045	2.1
15553	1.21398	2.1
15554	1.15234	2.1
15555	0.953634	0.05
15556	1.39132	0.05
15557	1.17285	2.1
15558	1.21408	2.1
15559	1.21408	2.1
15560	0.982848	2.1
15561	2.00101	2.1
15562	2.00973	2.1
15563	2.20314	2.1
15564	1.89318	2.1
15565	1.23267	1.83289
15566	0.947921	2.1
15567	1.18872	2.1
15568	0.740752	2.1
15569	1.92269	0.05
15570	1.53584	0.05
15571	1.53584	0.05
15572	1.21067	0.05
15573	1.40707	0.05
15574	1.50437	0.05
15575	1.50435	0.05
15576	1.45446	0.05
15577	1.47954	0.412229
15578	2.08687	0.814025
15579	2.08687	2.1
15580	1.1233	2.1
15581	1.04025	0.796155
15582	1.36005	1.68715
15583	1.47606	0.05
15584	1.3407	0.05
15585	0.758743	2.1
15586	0.716917	2.1
15587	0.716922	2.1
15588	0.672964	2.1
15589	2.40014	2.1
15590	1.96848	2.1
15591	1.96848	2.1
15592	1.53555	1.66577
15593	1.06191	2.1
15594	1.29579	2.1
15595	1.29579	2.1
15596	1.71289	2.1
15597	1.78584	2.1
15598	2.15211	2.1
15599	2.00427	2.1

15600	3.41615	2.1
15601	1.66408	2.1
15602	1.73954	2.1
15603	2.91663	2.1
15604	3.65818	2.1
15605	1.93286	2.1
15606	1.08831	2.1
15607	1.31242	0.05
15608	1.11712	0.05
15609	1.26311	0.05
15610	1.31242	0.05
15611	1.10378	2.1
15612	0.968594	2.1
15613	1.48389	0.05
15614	1.49613	0.05
15615	1.04349	0.662029
15616	1.26409	0.132054
15617	1.26409	0.0854005
15618	1.05724	0.1965
15619	1.65723	0.05
15620	1.83116	0.05
15621	2.06284	0.05
15622	2.00512	0.05
15623	1.83116	0.05
15624	2.03139	0.05
15625	2.20564	0.05
15626	1.90048	0.05
15627	0.963392	2.1
15628	1.01346	0.084604
15629	2.98092	2.1
15630	2.70295	2.1
15631	2.70295	2.1
15632	2.48828	2.1
15633	1.63661	2.1
15634	1.64158	2.1
15635	1.64158	2.1
15636	1.6488	2.1
15637	1.17506	0.05
15638	1.09667	0.05
15639	2.00539	0.05
15640	1.09667	0.05
15641	1.54946	2.1
15642	1.43577	2.1
15643	1.26103	2.1
15644	1.49808	2.1
15645	1.089 0.05	
15646	1.22586	0.05
15647	2.35027	0.05
15648	1.37549	0.05
15649	3.33706	0.05
15650	1.69894	0.05
15651	2.00539	0.05
15652	1.53798	0.05
15653	1.28938	2.1
15654	1.20969	2.1
15655	1.20969	2.1
15656	1.15864	2.1
15657	1.7986	2.1

15658	1.75869	2.1
15659	1.58281	2.1
15660	1.7392	2.1
15661	1.30733	1.08012
15662	1.5419	1.26359
15663	1.50883	1.21314
15664	1.85439	1.07912
15665	1.73531	0.742526
15666	1.70225	0.786378
15667	1.22176	0.05
15668	1.59492	0.0531616
15669	1.59492	0.05
15670	2.15542	0.05
15671	3.24379	2.1
15672	2.64775	2.1
15673	3.16707	2.1
15674	1.93032	2.1
15675	1.50738	2.1
15676	1.24128	2.1
15677	1.24128	2.1
15678	0.994293	2.1
15679	2.90808	0.05
15680	2.45797	0.05
15681	1.85501	0.05
15682	2.90809	0.05
15683	0.892835	1.91869
15684	1.55871	2.1
15685	1.04615	1.60677
15686	1.66643	0.10882
15687	1.70044	2.1
15688	1.80224	2.1
15689	1.70186	0.74904
15690	3.30123	0.530472
15691	1.74789	2.1
15692	1.77446	2.1
15693	1.77901	2.1
15694	1.57995	2.1
15695	1.81518	2.1
15696	2.17716	2.1
15697	2.17716	2.1
15698	2.65123	2.1
15699	2.01899	0.854994
15700	1.77479	0.559655
15701	1.19122	0.0673973
15702	1.46724	0.0539508
15703	1.70944	0.05
15704	1.08929	0.05
15705	1.68454	0.05
15706	1.08929	0.05
15707	1.51067	0.874208
15708	1.06729	0.441545
15709	1.37762	0.469914
15710	1.13992	0.293648
15711	1.26397	2.1
15712	1.04083	2.1
15713	1.00278	2.1
15714	1.04084	2.1
15715	1.1016	0.05

15716	1.23477	0.05
15717	1.23477	0.05
15718	1.64985	0.05
15719	1.11117	2.1
15720	1.13006	2.1
15721	1.13006	2.1
15722	1.14721	2.1
15723	1.21946	2.1
15724	1.0186	2.1
15725	1.13178	2.1
15726	1.00133	2.1
15727	1.67046	0.05
15728	0.986036	0.0518613
15729	1.67046	0.121905
15730	1.34857	1.96021
15731	2.46016	2.1
15732	2.07161	2.1
15733	2.10732	2.1
15734	2.08371	2.1
15735	1.35602	2.1
15736	0.829065	2.1
15737	1.81667	2.1
15738	0.99406	2.1
15739	1.81667	2.1
15740	1.68073	2.1
15741	2.05736	2.1
15742	2.97311	2.1
15743	2.14011	2.1
15744	1.23411	2.1
15745	1.13632	0.05
15746	1.40862	0.05
15747	1.28055	0.05
15748	1.14305	0.05
15749	1.12035	0.05
15750	1.15483	0.05
15751	1.94099	0.05
15752	1.82126	0.05
15753	1.15481	0.05
15754	0.998957	0.05
15755	2.10078	0.05
15756	1.81617	0.05
15757	1.27581	2.1
15758	0.968536	2.1
15759	1.33916	2.1
15760	1.27581	2.1
15761	1.32784	2.1
15762	1.4259	2.1
15763	1.70377	2.1
15764	1.54231	2.1
15765	3.77569	2.1
15766	3.42809	2.1
15767	1.54231	2.1
15768	1.51298	2.1
15769	3.55866	2.1
15770	2.86136	2.1
15771	1.83301	0.294429
15772	1.26371	0.0980553
15773	1.98301	0.43561

15774	2.0609	0.201813
15775	2.26047	0.138874
15776	2.00537	0.0842666
15777	1.7913	0.0861206
15778	1.48864	0.05
15779	1.09504	2.1
15780	1.39827	2.1
15781	2.28084	2.1
15782	1.05983	2.1
15783	1.33593	0.05
15784	1.31508	0.05
15785	1.31508	0.05
15786	1.09599	0.05
15787	1.30647	2.1
15788	1.28846	2.1
15789	1.28846	2.1
15790	1.26923	2.1
15791	1.2955	2.1
15792	1.09044	2.1
15793	2.12209	2.1
15794	1.26438	2.1
15795	0.911819	2.1
15796	1.38752	2.1
15797	2.57704	2.1
15798	2.71736	2.1
15799	1.42284	2.1
15800	1.41562	2.1
15801	2.69131	2.1
15802	2.36178	2.1
15803	1.41562	2.1
15804	1.39564	2.1
15805	1.32368	2.1
15806	0.908053	2.1
15807	0.908053	2.1
15808	0.629213	2.1
15809	1.8758	1.68764
15810	1.27362	1.47703
15811	1.3756	0.142105
15812	2.28749	0.664425
15813	1.84577	0.584326
15814	2.17765	0.512858
15815	1.38969	0.336507
15816	1.84578	0.497593
15817	1.32801	0.138906
15818	1.76377	0.182414
15819	1.22845	2.05782
15820	1.57766	0.173656
15821	1.57766	2.1
15822	1.39067	2.1
15823	1.15743	0.05
15824	1.37721	0.05
15825	1.69055	0.05
15826	1.14858	0.05
15827	1.38333	1.81758
15828	1.10575	0.05
15829	1.22418	2.1
15830	1.10574	2.01647
15831	1.91509	0.05

15832	1.02001	0.05
15833	1.03074	0.05
15834	1.19122	0.05
15835	1.67887	2.1
15836	1.67597	2.1
15837	1.84984	2.1
15838	1.77775	2.1
15839	1.73571	2.1
15840	1.27921	2.1
15841	2.11851	2.00019
15842	1.27921	2.1
15843	1.42486	2.1
15844	1.12503	2.1
15845	1.12503	2.1
15846	1.30116	2.1
15847	0.568995	2.1
15848	0.557348	2.1
15849	0.557348	2.1
15850	0.53907	2.1
15851	0.633575	2.1
15852	1.33756	0.856573
15853	1.51074	2.1
15854	1.93384	0.316012
15855	0.655202	2.1
15856	0.731934	2.1
15857	0.731934	2.1
15858	1.09061	2.1
15859	0.966979	2.1
15860	1.31502	2.1
15861	1.31502	2.1
15862	1.72116	2.1
15863	1.70002	2.1
15864	1.67969	2.1
15865	1.67969	2.1
15866	1.62408	2.1
15867	1.01531	2.1
15868	1.66018	2.1
15869	1.01531	2.1
15870	0.886968	2.1
15871	1.22585	0.05
15872	1.20674	0.05
15873	1.20672	0.05
15874	1.02962	0.05
15875	0.956467	2.1
15876	2.00551	2.02804
15877	0.553801	2.1
15878	0.617006	2.1
15879	1.26923	2.1
15880	1.23614	2.1
15881	1.23614	2.1
15882	1.16817	2.1
15883	2.14482	2.1
15884	1.72637	2.1
15885	1.09368	2.1
15886	1.26195	2.1
15887	0.760546	2.1
15888	1.84094	2.1
15889	2.62868	2.1

15890	2.79784	2.1
15891	2.79784	2.1
15892	3.00129	2.1
15893	1.27593	0.270801
15894	1.38877	1.96374
15895	1.06486	0.05
15896	1.2474	0.05
15897	1.76234	0.05
15898	1.8685	0.05
15899	1.69863	0.05
15900	1.29666	0.05
15901	1.91646	2.1
15902	1.8582	2.1
15903	1.8582	2.1
15904	1.7986	2.1
15905	3.44043	0.05
15906	1.94764	0.05
15907	1.94764	0.05
15908	1.36307	0.05
15909	1.32075	0.05
15910	1.12971	0.05
15911	2.06319	0.05
15912	1.94982	0.05
15913	1.77255	0.05
15914	0.979297	0.05
15915	1.59997	2.1
15916	1.93506	2.1
15917	1.94409	2.1
15918	1.9597	2.1
15919	0.57899	2.1
15920	0.953036	2.1
15921	0.953049	2.1
15922	1.53503	2.1
15923	1.39615	0.05
15924	1.06474	0.05
15925	0.947564	0.05
15926	1.45506	0.05
15927	1.17799	0.107793
15928	1.21067	0.05
15929	1.35945	0.05
15930	2.05648	0.05
15931	1.36208	0.05
15932	2.15229	0.05
15933	2.89288	0.05
15934	1.95719	0.05
15935	2.25169	0.05
15936	1.52756	0.05
15937	1.19322	0.05
15938	1.23146	0.05
15939	2.45024	0.0621069
15940	0.794122	0.05
15941	1.6343	0.05
15942	1.34161	0.05
15943	1.05353	0.05
15944	1.50795	0.05
15945	1.05353	0.05
15946	1.20669	0.05
15947	1.59289	2.1

15948	0.989543	2.1
15949	1.40588	2.1
15950	0.991427	2.1
15951	0.921822	1.95133
15952	1.46903	0.404236
15953	1.46903	2.1
15954	1.8214	0.0640169
15955	1.39702	2.1
15956	1.25103	2.1
15957	1.25103	2.1
15958	1.10378	2.1
15959	1.71813	2.1
15960	1.39595	2.1
15961	1.386 2.1	
15962	1.13008	2.1
15963	1.31711	0.05
15964	1.70191	0.05
15965	0.893249	0.05
15966	1.42669	0.05
15967	1.23943	2.1
15968	1.1483	1.46045
15969	1.04598	2.1
15970	1.1483	1.59032
15971	1.00381	0.05
15972	1.11433	0.05
15973	0.973501	0.257622
15974	1.0756	0.336381
15975	1.20669	0.05
15976	1.18508	0.05
15977	1.18508	0.05
15978	0.983027	0.05
15979	1.43687	2.1
15980	1.71059	2.1
15981	1.71059	2.1
15982	1.66762	2.1
15983	2.8415	2.1
15984	2.40555	2.1
15985	1.91546	2.1
15986	1.61385	2.1
15987	2.01569	2.1
15988	1.33183	2.1
15989	1.23218	2.1
15990	1.23999	2.1
15991	1.01335	0.144164
15992	1.12088	0.364643
15993	1.25346	2.1
15994	1.15644	2.1
15995	1.3764	0.995211
15996	1.27558	0.428989
15997	1.2064	2.1
15998	1.15067	2.1
15999	1.01595	2.1
16000	1.15067	2.1
16001	1.41902	1.99101
16002	1.30272	2.1
16003	1.30272	1.49668
16004	1.19548	2.1
16005	2.99339	2.1

16006	2.98041	2.1
16007	1.46124	2.1
16008	1.42105	2.1
16009	2.98041	2.1
16010	2.86339	2.1
16011	1.42105	2.1
16012	1.42284	2.1
16013	2.31301	0.451444
16014	1.80273	0.342543
16015	2.17765	0.650975
16016	2.31301	0.33003
16017	1.76378	0.21003
16018	2.10275	0.149283
16019	2.10275	0.198932
16020	2.26047	0.116354
16021	0.97541	2.1
16022	1.40863	2.1
16023	1.40863	2.1
16024	1.30751	2.1
16025	1.20143	0.104814
16026	1.19756	0.0597495
16027	1.43707	0.05
16028	1.28954	0.05
16029	3.34254	0.05
16030	1.92916	0.05
16031	1.80089	0.05
16032	1.1323	0.05
16033	1.47321	0.05
16034	1.23756	0.05
16035	1.87291	2.1
16036	1.53642	2.1
16037	1.53642	2.1
16038	1.5791	2.1
16039	1.0446	2.1
16040	0.992674	2.1
16041	1.12324	0.05
16042	1.29163	0.05
16043	1.29163	0.05
16044	1.15371	0.05
16045	0.996831	0.05
16046	1.00192	0.05
16047	1.0019	0.05
16048	0.958937	0.05
16049	1.07283	2.1
16050	1.25294	1.04932
16051	1.25294	1.28679
16052	1.04788	1.23652
16053	1.14496	2.1
16054	1.07652	2.1
16055	1.07652	2.1
16056	0.991406	2.1
16057	1.60976	2.1
16058	1.37	2.1
16059	1.37	2.1
16060	1.14195	2.1
16061	1.26593	1.9475
16062	1.75366	2.1
16063	1.57621	0.05

16064	1.24538	0.05
16065	0.969118	2.1
16066	1.04147	2.1
16067	0.969118	2.1
16068	1.22615	2.1
16069	0.98084	2.1
16070	1.01843	2.1
16071	1.09435	2.1
16072	1.50876	2.1
16073	1.5976	2.1
16074	1.84322	2.1
16075	1.84324	2.1
16076	1.70377	2.1
16077	1.20709	2.1
16078	2.54729	2.1
16079	0.974716	0.05
16080	1.54468	0.05
16081	1.39021	0.05
16082	1.70303	0.05
16083	1.22852	1.91215
16084	1.578	1.18477
16085	1.578	1.95103
16086	1.93852	0.611189
16087	2.2506	2.1
16088	1.39302	2.1
16089	1.5478	2.1
16090	1.2269	2.1
16091	1.70048	2.1
16092	1.45477	2.06134
16093	1.30929	2.1
16094	1.15069	1.72352
16095	1.00255	2.07614
16096	1.05481	0.05
16097	1.05481	2.1
16098	1.1032	0.05
16099	0.993888	2.1
16100	1.19294	2.1
16101	1.01121	0.05
16102	0.991866	0.05
16103	2.20052	0.178387
16104	1.7409	2.1
16105	1.75142	2.1
16106	1.46811	2.1
16107	1.7409	1.16358
16108	1.23267	2.1
16109	1.46811	2.1
16110	1.18872	2.1
16111	1.12665	2.1
16112	1.50045	2.1
16113	1.50045	2.1
16114	1.37704	2.1
16115	1.35308	1.07479
16116	1.29983	0.119412
16117	1.29983	1.20632
16118	0.970212	0.187148
16119	1.82272	2.1
16120	1.62784	2.1
16121	1.62784	2.1

16122	1.87691	2.1
16123	3.65294	2.1
16124	2.33374	2.1
16125	2.12888	2.1
16126	1.62229	2.1
16127	1.41496	2.1
16128	1.32707	0.256156
16129	2.18871	0.0672083
16130	1.4235	0.05
16131	1.46015	0.05
16132	1.25635	0.05
16133	1.45501	0.05
16134	1.3543	0.05
16135	1.38139	2.1
16136	1.98159	2.1
16137	2.15868	2.1
16138	4.04308	2.1
16139	1.5362	2.1
16140	1.56328	2.1
16141	1.56327	2.1
16142	1.58163	2.1
16143	1.88987	0.05
16144	1.08761	0.05
16145	0.862154	0.05
16146	1.77688	0.05
16147	1.14238	2.1
16148	1.11691	2.1
16149	1.01537	2.1
16150	1.02923	2.1
16151	0.977425	0.05
16152	1.15211	0.05
16153	2.0815	2.1
16154	1.18621	2.1
16155	1.01485	2.1
16156	1.17054	2.1
16157	1.17054	2.1
16158	1.12665	2.1
16159	1.87213	0.05
16160	1.82244	0.05
16161	1.45039	0.05
16162	1.7226	0.05
16163	1.841 0.05	
16164	1.79011	0.05
16165	1.8792	0.05
16166	1.45053	0.05
16167	1.08947	2.1
16168	1.5008	2.1
16169	1.5008	2.1
16170	1.83186	2.1
16171	1.04159	0.05
16172	1.2489	0.05
16173	1.2489	0.05
16174	1.33592	0.05
16175	1.51973	0.05
16176	0.980547	0.05
16177	1.51974	0.05
16178	1.15991	0.0593981
16179	1.34965	2.1

16180	1.2724	2.1
16181	1.2724	2.1
16182	1.38438	2.1
16183	1.02923	2.1
16184	1.06521	2.1
16185	1.06521	2.1
16186	1.0752	2.1
16187	2.03139	0.05
16188	1.92951	0.05
16189	1.74852	0.05
16190	1.62772	0.05
16191	1.73655	0.05
16192	1.76809	0.05
16193	1.62772	0.05
16194	1.1432	0.05
16195	1.09235	2.1
16196	1.48081	2.1
16197	1.46712	2.1
16198	1.89321	2.1
16199	1.94548	2.1
16200	1.5636	2.1
16201	1.5636	2.1
16202	1.21124	2.1
16203	1.52314	2.1
16204	2.0219	2.1
16205	1.51113	2.1
16206	2.0219	2.1
16207	0.987247	0.0630482
16208	1.18347	0.05
16209	1.89226	0.141469
16210	1.61441	0.05
16211	1.42884	2.1
16212	1.10961	2.1
16213	1.56991	2.1
16214	1.05337	2.1
16215	1.05337	2.1
16216	1.91547	2.1
16217	1.05931	2.1
16218	1.1729	2.1
16219	1.34526	2.1
16220	1.0593	2.1
16221	1.0856	2.1
16222	1.90854	2.1
16223	1.91674	2.1
16224	1.11345	2.1
16225	1.75067	2.1
16226	1.50022	2.1
16227	1.50022	2.1
16228	1.53619	2.1
16229	1.4748	2.1
16230	1.74431	1.82558
16231	2.02581	1.51979
16232	1.25288	1.48783
16233	1.01814	0.238969
16234	1.2881	0.05
16235	1.2881	0.105235
16236	1.54433	0.0564895
16237	1.19999	0.134862

16238	1.44056	0.05
16239	1.02011	0.05
16240	1.17129	0.05
16241	1.13775	2.01958
16242	3.34815	2.0793
16243	1.60428	2.02583
16244	1.85928	1.94716
16245	1.75592	2.1
16246	1.25989	2.1
16247	1.75592	2.1
16248	1.71549	2.1
16249	1.37634	2.1
16250	1.64693	2.1
16251	1.64693	2.1
16252	1.64193	2.1
16253	1.18351	0.05
16254	2.19398	0.05
16255	2.16272	0.426983
16256	1.19837	0.271566
16257	1.04973	2.1
16258	1.20149	2.1
16259	1.60574	2.1
16260	1.11164	0.153263
16261	1.87823	0.340997
16262	1.11164	0.968294
16263	0.772882	2.1
16264	1.4714	2.1
16265	2.14242	0.284984
16266	1.12336	2.1
16267	2.8386	0.145718
16268	2.13346	2.1
16269	1.06788	0.05
16270	0.947662	0.05
16271	1.05297	0.05
16272	1.42907	0.05
16273	2.17465	2.1
16274	2.18394	2.1
16275	1.14808	2.1
16276	1.21292	2.1
16277	1.21292	2.1
16278	1.27229	2.1
16279	1.20859	0.147234
16280	1.21073	0.769752
16281	1.21073	0.108025
16282	1.22857	1.59408
16283	1.79158	2.1
16284	1.73899	2.1
16285	1.34012	2.1
16286	1.39991	2.1
16287	1.73898	2.1
16288	1.61245	2.1
16289	1.40136	2.1
16290	1.57234	2.1
16291	1.82162	2.1
16292	3.09875	2.1
16293	1.10264	2.1
16294	1.02339	2.1
16295	1.02339	2.1

16296	1.23042	2.1
16297	2.82266	0.306267
16298	1.05698	0.0604889
16299	1.96384	0.225284
16300	1.10244	0.24746
16301	0.929061	2.1
16302	1.47007	2.1
16303	0.985303	2.1
16304	1.24554	2.1
16305	1.05993	2.1
16306	2.02074	2.1
16307	2.10106	1.11428
16308	1.18171	0.937621
16309	1.68111	0.961839
16310	1.84226	0.54568
16311	1.82131	0.05
16312	1.32371	0.05
16313	1.41902	0.05
16314	1.00205	0.05
16315	1.65532	2.1
16316	1.18977	2.1
16317	1.15471	2.1
16318	0.993889	2.1
16319	2.08521	2.1
16320	2.09885	2.1
16321	2.09497	2.1
16322	2.10604	2.1
16323	0.881028	1.45479
16324	1.52828	0.961864
16325	1.40574	0.05
16326	1.09599	0.05
16327	1.45506	2.1
16328	1.50524	2.1
16329	1.50524	2.1
16330	1.54711	2.1
16331	1.41469	2.1
16332	2.40442	2.1
16333	1.40794	2.1
16334	1.89226	2.1
16335	1.31346	2.1
16336	1.51067	2.1
16337	1.31363	2.1
16338	1.11833	2.1
16339	2.3373	2.1
16340	2.19994	2.1
16341	2.19994	2.1
16342	1.09616	2.1
16343	1.63719	2.1
16344	1.52204	2.1
16345	1.52205	2.1
16346	1.40118	2.1
16347	2.5946	2.1
16348	4.07065	2.1
16349	1.33916	2.1
16350	2.05217	2.1
16351	1.67023	2.1
16352	2.05217	2.1
16353	1.59458	0.163109

16354	1.02247	0.0820698
16355	1.68108	0.251633
16356	1.03079	0.0629952
16357	1.1643	0.05
16358	1.85795	0.05
16359	1.70302	0.05
16360	1.42076	0.05
16361	1.91351	0.05
16362	1.01148	0.05
16363	2.52402	0.05
16364	0.752625	0.05
16365	0.886968	2.1
16366	1.28845	2.1
16367	1.28845	2.1
16368	1.27766	2.1
16369	1.82268	2.1
16370	1.87118	2.1
16371	1.87119	2.1
16372	1.91704	2.1
16373	3.13938	2.1
16374	2.97839	2.1
16375	2.97839	2.1
16376	2.87331	2.1
16377	1.73443	0.980469
16378	1.59457	0.414715
16379	2.01827	2.1
16380	2.04586	2.1
16381	2.04586	2.1
16382	2.00865	2.1
16383	1.19901	2.1
16384	1.16661	2.1
16385	1.13215	0.05
16386	1.96256	0.05
16387	2.34043	0.05
16388	2.04154	0.05
16389	1.58991	0.05
16390	1.45893	0.05
16391	2.56818	0.05
16392	1.70532	0.05
16393	1.17798	0.05
16394	1.25796	0.124476
16395	1.25797	0.117832
16396	1.10719	0.434723
16397	1.51067	2.1
16398	1.60707	2.1
16399	2.06946	2.1
16400	3.19218	2.1
16401	2.19965	2.1
16402	1.30521	2.1
16403	2.23684	1.38583
16404	1.43363	2.1
16405	1.51007	2.1
16406	2.46356	2.1
16407	1.90428	2.1
16408	1.13618	2.1
16409	2.13276	0.05
16410	0.998345	0.05
16411	1.2861	0.05

16412	1.20457	0.05
16413	1.51789	2.1
16414	1.49872	2.1
16415	1.49872	2.1
16416	1.47712	2.1
16417	1.87129	0.05
16418	0.949364	0.05
16419	1.83142	0.05
16420	1.55063	0.05
16421	1.03298	0.05
16422	0.980664	0.093394
16423	1.51696	0.05
16424	1.34927	0.141464
16425	1.70302	0.05
16426	1.65952	0.05
16427	1.76658	0.05
16428	0.986848	0.05
16429	2.13757	1.63111
16430	1.98142	0.131025
16431	1.98142	1.26216
16432	1.65411	0.258944
16433	1.1323	0.05
16434	1.46015	0.05
16435	2.15376	1.71685
16436	1.64622	1.81655
16437	0.991867	0.0717935
16438	1.58074	0.05
16439	1.69368	2.1
16440	1.76403	2.1
16441	1.76403	2.1
16442	1.83318	2.1
16443	1.28418	0.185239
16444	1.16851	0.05
16445	1.1685	0.107001
16446	1.1726	0.0524436
16447	1.06455	2.1
16448	1.18773	2.1
16449	0.98511	2.1
16450	1.18774	2.1
16451	1.76476	2.1
16452	1.79825	2.1
16453	1.79825	2.1
16454	0.961895	2.1
16455	1.59589	0.05
16456	2.25975	0.05
16457	2.76339	2.1
16458	3.0551	2.1
16459	1.68761	2.1
16460	1.59561	2.1
16461	2.17257	2.1
16462	1.05845	2.1
16463	1.03512	2.1
16464	2.19011	2.1
16465	1.44859	2.1
16466	1.35897	0.668852
16467	1.35897	2.1
16468	1.17822	1.40508
16469	1.13641	2.1

16470	1.18301	2.1
16471	1.18301	2.1
16472	1.25173	2.1
16473	2.41297	2.1
16474	1.36463	2.1
16475	1.54711	2.1
16476	1.5735	2.1
16477	1.5735	2.1
16478	1.58713	2.1
16479	1.69044	0.05
16480	1.57979	0.05
16481	1.57979	0.05
16482	1.14387	0.05
16483	1.04199	2.1
16484	1.96491	2.1
16485	1.04199	2.1
16486	1.46892	2.1
16487	0.983724	2.1
16488	1.70579	2.1
16489	1.88522	2.1
16490	1.23914	2.1
16491	1.79424	2.1
16492	1.52851	2.1
16493	1.04921	2.1
16494	1.11285	2.1
16495	1.25334	0.294753
16496	1.07496	0.0935546
16497	1.07497	2.1
16498	1.34852	0.268645
16499	1.27391	2.1
16500	2.20307	2.1
16501	1.81311	2.1
16502	2.10305	2.1
16503	1.21059	0.05
16504	1.44253	0.05
16505	1.9194	0.05
16506	1.21159	0.05
16507	0.947979	2.1
16508	1.01225	0.05
16509	0.947979	2.1
16510	1.01225	2.1
16511	1.00255	2.1
16512	1.01225	2.1
16513	1.01225	2.1
16514	1.06561	2.1
16515	3.50562	2.1
16516	3.61336	2.1
16517	3.61336	2.1
16518	3.73635	2.1
16519	1.61245	2.1
16520	1.52301	2.1
16521	1.57612	2.1
16522	1.83035	2.1
16523	1.52301	2.1
16524	1.24789	2.1
16525	1.85156	2.1
16526	1.89072	2.1
16527	0.995735	2.1

16528	1.57738	2.1
16529	1.87141	0.05
16530	1.73388	0.05
16531	1.61344	0.05
16532	0.922676	0.05
16533	1.56614	2.1
16534	0.956294	2.1
16535	1.82589	2.1
16536	1.63719	2.1
16537	1.12399	0.05
16538	1.50103	0.05
16539	1.56976	0.05
16540	1.03567	0.05
16541	1.21776	0.05
16542	1.89708	0.05
16543	1.16323	0.05
16544	1.63533	0.05
16545	2.29397	0.05
16546	3.01596	0.05
16547	1.80145	0.05
16548	1.7666	0.05
16549	1.91621	0.436228
16550	1.10719	0.0946351
16551	1.57828	1.21232
16552	1.17105	1.46539
16553	1.15806	2.1
16554	1.31565	2.1
16555	1.31565	2.1
16556	1.46643	2.1
16557	1.47507	2.1
16558	1.87858	2.1
16559	0.958661	2.1
16560	1.87858	2.1
16561	1.44293	0.05
16562	1.43213	0.05
16563	1.43213	0.05
16564	1.10476	0.05
16565	1.03235	2.1
16566	1.31 0.05	
16567	1.31 2.1	
16568	1.50108	0.631274
16569	1.32215	2.1
16570	1.10611	2.1
16571	1.10611	2.1
16572	0.904978	2.1
16573	1.06486	0.05
16574	1.18347	0.05
16575	1.70535	2.1
16576	1.94779	2.1
16577	1.77071	2.1
16578	1.82268	2.1
16579	0.61701	2.1
16580	0.672495	2.1
16581	0.672494	2.1
16582	0.713174	2.1
16583	1.40395	2.1
16584	1.34245	2.1
16585	1.34243	2.1

16586	1.30646	2.1
16587	1.06204	0.05
16588	0.632691	0.0536951
16589	1.75766	0.0585799
16590	1.14287	0.495904
16591	1.52101	0.05
16592	1.71414	0.05
16593	1.86411	0.05
16594	1.6253	0.05
16595	1.04355	0.05
16596	1.0443	0.05
16597	1.34852	2.1
16598	1.56016	0.0716151
16599	1.56016	0.0788406
16600	1.03235	0.0855024
16601	1.2966	0.05
16602	1.82354	0.05
16603	1.2966	0.05
16604	1.67687	0.05
16605	1.19669	1.47655
16606	1.82979	1.67111
16607	1.58972	1.2353
16608	1.09219	1.26821
16609	1.42077	0.553632
16610	1.89226	0.48051
16611	1.19547	1.02316
16612	1.10187	2.1
16613	1.10188	0.684883
16614	1.01479	2.1
16615	1.81663	2.1
16616	1.81533	2.1
16617	1.83247	2.1
16618	1.51511	2.1
16619	0.983148	2.1
16620	1.7513	2.1
16621	1.85017	2.1
16622	1.26103	2.1
16623	1.23152	2.1
16624	1.65508	2.1
16625	1.23152	2.1
16626	2.15082	2.1
16627	1.57974	2.1
16628	1.07289	2.1
16629	0.81534	2.1
16630	1.51007	2.1
16631	1.00898	0.05
16632	1.18959	0.05
16633	1.32178	0.05
16634	1.25929	0.05
16635	2.17892	0.05
16636	1.22938	0.05
16637	3.02933	0.277284
16638	2.34619	0.42232
16639	1.47679	0.0503778
16640	1.0819	0.060605
16641	3.10558	2.1
16642	3.31457	2.1
16643	0.985109	2.1

16644	1.10667	2.1
16645	3.31457	2.1
16646	3.54443	2.1
16647	1.10667	2.1
16648	1.25987	2.1
16649	1.77063	1.06264
16650	1.51065	0.615446
16651	1.24688	2.1
16652	1.37652	2.1
16653	1.37652	2.1
16654	1.54168	2.1
16655	1.17042	0.693375
16656	1.13092	1.31643
16657	1.13092	1.89275
16658	1.52938	2.1
16659	1.11787	0.05
16660	1.00613	0.112949
16661	0.781373	0.0622217
16662	1.6885	0.0591425
16663	2.49439	0.05
16664	1.42657	0.05
16665	1.77903	2.1
16666	2.63606	2.1
16667	2.10425	2.1
16668	1.21425	2.03307
16669	1.31253	1.50585
16670	1.28418	1.62387
16671	1.28418	1.09908
16672	1.58972	1.16358
16673	0.858948	2.1
16674	1.90196	2.1
16675	1.81024	2.1
16676	1.90194	2.1
16677	1.4263	0.128823
16678	0.947921	0.0943753
16679	1.22852	0.979936
16680	1.09194	0.563761
16681	1.60578	0.05
16682	1.14721	0.05
16683	1.06336	0.05
16684	1.14721	0.05
16685	1.11924	0.05
16686	1.53814	0.05
16687	1.18421	0.05
16688	1.8458	0.05
16689	1.7565	2.1
16690	1.09344	1.39635
16691	1.09344	0.786279
16692	1.16971	2.1
16693	1.71465	0.05
16694	1.19412	0.05
16695	1.64985	0.05
16696	1.05417	0.05
16697	1.2127	0.05
16698	1.48867	0.05
16699	1.27806	0.05
16700	1.21269	0.05
16701	1.37813	1.16812

16702	1.0417	1.24362
16703	1.88626	0.26831
16704	1.14977	0.768965
16705	1.78803	2.1
16706	1.33793	1.14724
16707	1.42266	2.01197
16708	1.26865	1.78615
16709	1.09518	2.1
16710	1.36844	2.1
16711	1.20455	2.1
16712	1.36844	2.1
16713	1.22297	0.551671
16714	1.11256	0.834338
16715	1.11256	0.395534
16716	1.03217	0.56347
16717	1.18422	0.05
16718	1.4282	0.05
16719	1.04159	0.05
16720	1.4282	0.05
16721	0.988805	2.1
16722	1.05481	2.1
16723	1.52938	0.350551
16724	1.26593	1.61707
16725	1.53082	2.1
16726	1.09847	2.1
16727	1.75211	0.136244
16728	1.22222	2.1
16729	1.75211	0.0593932
16730	1.51592	2.1
16731	1.63413	0.193021
16732	1.26662	0.225906
16733	1.42495	0.078183
16734	1.4356	0.100113
16735	1.29389	1.84441
16736	1.04164	2.1
16737	1.40574	1.57754
16738	1.04164	1.17336
16739	2.22344	0.29539
16740	1.45945	0.05
16741	0.987248	0.0550197
16742	1.2474	0.05
16743	0.974702	0.60888
16744	1.43777	0.184403
16745	1.71289	2.1
16746	1.91529	2.1
16747	1.91529	2.1
16748	2.01827	2.1
16749	1.87111	1.01611
16750	1.00864	0.134154
16751	1.26865	0.250002
16752	1.18769	0.825315
16753	1.24167	2.1
16754	1.36133	0.163599
16755	2.28168	2.1
16756	3.98524	2.1
16757	1.36133	2.1
16758	1.69436	0.482728
16759	2.21926	2.1

16760	1.70302	2.1
16761	1.50732	0.05
16762	1.42237	0.05
16763	1.18659	0.05
16764	1.50732	0.05
16765	1.19785	0.05
16766	1.40969	0.05
16767	1.28339	0.05
16768	1.40882	0.05
16769	0.968595	2.1
16770	1.41903	2.05006
16771	1.12567	2.1
16772	1.15898	2.1
16773	1.27241	2.1
16774	0.995796	2.1
16775	1.89983	0.05
16776	1.05417	0.05
16777	0.571999	0.05
16778	0.743597	0.05
16779	1.89353	0.05
16780	0.924068	0.05
16781	1.52851	2.1
16782	1.26969	2.1
16783	1.07763	0.05
16784	1.82301	0.05
16785	1.07763	0.05
16786	1.17506	0.05
16787	1.20045	2.1
16788	1.12486	2.1
16789	1.15234	2.1
16790	1.11598	2.1
16791	1.12486	2.1
16792	1.0491	2.1
16793	1.11598	2.1
16794	1.1047	2.1
16795	1.05902	2.1
16796	1.14361	2.1
16797	1.14361	2.1
16798	1.26851	2.1
16799	1.29389	1.09315
16800	0.988456	1.17849
16801	1.83688	0.05
16802	1.70302	0.05
16803	1.49386	0.05
16804	1.83151	0.05
16805	1.01346	0.0574168
16806	0.983607	0.0839592
16807	0.983596	2.1
16808	1.08332	0.327384
16809	2.36848	0.05
16810	2.97066	0.05
16811	1.12352	0.05
16812	2.5311	0.05
16813	1.09062	2.1
16814	0.962182	2.1
16815	0.962181	2.1
16816	1.2258	2.1
16817	0.974888	2.1

16818	1.06312	2.1
16819	1.73583	0.05
16820	1.72399	0.05
16821	1.724	0.05
16822	1.50951	0.05
16823	1.26755	2.1
16824	1.03824	2.1
16825	1.27875	2.1
16826	1.03824	2.1
16827	1.18902	2.1
16828	1.534	2.1
16829	1.11764	2.1
16830	1.534	2.1
16831	1.52008	2.1
16832	1.34406	2.1
16833	1.34406	2.1
16834	1.2187	2.1
16835	0.963571	0.373491
16836	1.19612	0.152929
16837	0.963571	2.07552
16838	1.00007	2.1
16839	0.903333	2.1
16840	1.12724	2.1
16841	1.23771	2.1
16842	1.17683	2.1
16843	1.20059	0.404813
16844	0.772023	0.05
16845	1.14749	0.160457
16846	1.2802	0.513105
16847	1.85091	1.29232
16848	2.11193	0.708419
16849	1.2802	0.118408
16850	1.42913	0.830249
16851	2.11193	0.642666
16852	1.27737	0.0912455
16853	1.30584	2.1
16854	1.60936	2.1
16855	1.3413	0.05
16856	1.53336	0.05
16857	2.60679	0.05
16858	1.3413	0.05
16859	1.35877	2.1
16860	1.11024	2.1
16861	1.27839	2.1
16862	1.08171	2.1
16863	1.27841	2.1
16864	1.0577	2.1
16865	1.71255	2.1
16866	1.10611	2.1
16867	0.960106	2.1
16868	1.40921	2.1
16869	1.90705	0.0509044
16870	2.79564	0.05
16871	1.45321	0.05
16872	1.90705	0.05
16873	0.974718	0.05
16874	1.53671	0.05
16875	1.69039	0.05

16876	1.78229	0.05
16877	1.0101	2.1
16878	1.3167	2.1
16879	1.19699	2.1
16880	1.3212	2.1
16881	1.60811	0.05
16882	1.43224	0.05
16883	1.35401	0.05
16884	1.17464	0.05
16885	1.27696	2.1
16886	1.05025	2.1
16887	1.05025	2.1
16888	1.18076	2.1
16889	1.37224	1.72518
16890	1.13438	1.63303
16891	1.76788	1.58569
16892	1.73444	1.38347
16893	1.37704	2.1
16894	1.77042	2.1
16895	1.77042	2.1
16896	1.09778	2.1
16897	1.28667	2.1
16898	1.28228	2.1
16899	1.30646	0.64237
16900	1.68867	0.458551
16901	1.30645	1.39746
16902	0.986494	2.07255
16903	1.6253	0.05
16904	1.48676	0.05
16905	1.48676	0.05
16906	1.32974	0.05
16907	0.634347	0.05
16908	1.15142	0.05
16909	1.11395	0.05
16910	1.18068	0.05
16911	1.36904	0.05
16912	0.764563	0.05
16913	2.09213	0.05
16914	1.56631	2.1
16915	1.08011	2.1
16916	1.80283	2.1
16917	1.50738	2.1
16918	1.44951	0.05
16919	1.50102	0.05
16920	1.50102	0.05
16921	1.39615	0.05
16922	1.02653	0.05
16923	2.98258	0.0542043
16924	2.0815	0.05
16925	1.39525	0.05
16926	1.82176	0.05
16927	0.977607	0.05
16928	1.96133	1.48078
16929	1.27189	0.931564
16930	0.826032	0.438015
16931	2.12831	0.487821
16932	1.77798	2.1
16933	1.02969	1.92186

16934	1.06463	1.28308
16935	1.49219	2.1
16936	1.10003	2.1
16937	1.21223	2.1
16938	1.00513	1.13697
16939	1.43788	2.03225
16940	1.47134	0.0574178
16941	0.968249	0.0846309
16942	3.22585	0.05
16943	3.36933	0.05
16944	3.36932	0.05
16945	3.03865	0.05
16946	1.66295	0.05
16947	1.65583	0.05
16948	1.65583	0.05
16949	1.4732	0.05
16950	1.73704	0.05
16951	1.76476	1.1715
16952	2.13345	0.294347
16953	1.55816	2.1
16954	1.55816	0.522682
16955	1.02601	2.1
16956	1.08039	2.1
16957	1.05498	0.620023
16958	1.08039	2.1
16959	1.2873	2.1
16960	1.01121	2.1
16961	0.987703	2.1
16962	0.98771	2.1
16963	0.964782	2.1
16964	1.35719	2.1
16965	2.61092	2.1
16966	2.6607	2.1
16967	3.41693	2.1
16968	1.23125	2.1
16969	1.73772	2.1
16970	0.99845	0.123791
16971	1.5176	0.171813
16972	1.13271	0.05
16973	1.332 0.05	
16974	1.63159	2.1
16975	1.61342	2.1
16976	1.03679	2.1
16977	1.19087	2.1
16978	1.97172	2.1
16979	2.68634	2.1
16980	1.19086	2.1
16981	1.50091	2.1
16982	2.84677	2.1
16983	2.77894	2.1
16984	2.77894	2.1
16985	2.74206	2.1
16986	1.3284	2.1
16987	1.83524	2.1
16988	1.65853	2.1
16989	1.56741	2.1
16990	1.00509	2.1
16991	1.45246	2.1

16992	1.26193	2.1
16993	1.45244	2.1
16994	1.61963	0.05
16995	1.68189	0.196388
16996	1.68189	1.56317
16997	1.49155	0.0591223
16998	1.58054	2.1
16999	1.59169	2.1
17000	1.59169	2.1
17001	1.60497	2.1
17002	0.743595	0.05
17003	0.702359	0.05
17004	1.15317	0.05
17005	2.24367	0.05
17006	1.15252	0.05
17007	2.60354	0.05
17008	1.45158	2.1
17009	1.56004	2.1
17010	1.64527	2.1
17011	1.00371	2.1
17012	1.38287	0.05
17013	0.893024	0.50023
17014	1.23527	0.525946
17015	1.23706	0.177492
17016	2.20993	0.0763654
17017	1.54858	1.60686
17018	0.798666	0.05
17019	1.22487	0.05
17020	1.33158	0.05
17021	1.85902	0.05
17022	1.08334	2.1
17023	1.17885	0.994448
17024	1.17885	0.120214
17025	1.07283	0.475597
17026	1.30723	0.05
17027	1.62507	0.05
17028	1.62507	0.05
17029	1.08854	0.05
17030	1.59475	2.1
17031	1.58874	2.1
17032	1.58875	2.1
17033	1.35787	2.1
17034	1.04147	2.1
17035	1.15564	2.1
17036	1.78301	2.1
17037	1.90618	2.1
17038	1.71555	2.1
17039	2.13143	2.1
17040	1.10817	2.1
17041	1.032	2.1
17042	1.032	2.1
17043	0.953636	2.1
17044	1.27916	1.08895
17045	1.11938	0.685272
17046	1.11937	0.586853
17047	1.9186	1.12737
17048	1.19967	0.05
17049	1.63489	0.05

17050	1.00763	0.05
17051	1.81656	0.05
17052	1.31814	0.05
17053	2.98183	0.05
17054	1.20149	2.1
17055	1.29129	2.1
17056	1.29129	2.1
17057	1.37138	2.1
17058	1.23104	0.257927
17059	1.75236	0.898565
17060	1.6	0.0717083
17061	1.60565	0.139261
17062	1.60565	0.108604
17063	1.40013	0.170067
17064	1.23042	2.1
17065	1.18278	2.1
17066	1.10853	2.1
17067	1.04724	2.1
17068	1.1766	2.1
17069	1.00319	2.1
17070	1.29435	2.1
17071	1.00319	2.1
17072	1.05655	1.49456
17073	1.01606	1.51473
17074	1.80391	2.1
17075	2.7699	2.1
17076	1.52073	2.1
17077	2.06835	2.1
17078	2.00202	2.1
17079	2.04906	2.1
17080	0.968536	2.1
17081	1.02461	2.1
17082	1.59784	2.1
17083	1.78249	2.1
17084	1.81409	2.1
17085	1.79844	2.1
17086	1.77093	2.00818
17087	2.02356	1.33238
17088	1.32449	2.1
17089	1.10003	2.1
17090	1.16915	0.206268
17091	1.05937	0.05
17092	1.28096	2.1
17093	1.34696	2.1
17094	1.34695	2.1
17095	1.36457	2.1
17096	1.06478	2.1
17097	1.06239	2.1
17098	1.06239	2.1
17099	1.09629	2.1
17100	0.798209	2.1
17101	1.8532	2.1
17102	2.10112	0.05
17103	2.8704	0.05
17104	2.8704	0.05
17105	2.92457	0.05
17106	1.0752	2.1
17107	1.07745	2.1

17108	1.07745	2.1
17109	1.08911	2.1
17110	1.40182	2.1
17111	1.00133	2.1
17112	1.40182	2.1
17113	1.24509	2.1
17114	2.22243	0.05
17115	1.07303	0.05
17116	1.29736	0.05
17117	1.73584	0.05
17118	2.0815	0.111819
17119	1.07999	0.0599877
17120	2.1692	2.1
17121	1.99992	2.1
17122	1.99992	2.1
17123	1.78327	2.1
17124	1.52661	0.05
17125	0.956003	0.05
17126	1.53172	0.05
17127	0.955991	0.05
17128	1.35025	1.15904
17129	1.51084	2.1
17130	1.51084	2.03469
17131	1.67115	2.1
17132	2.00977	2.1
17133	1.63632	2.1
17134	1.63632	2.1
17135	2.66128	2.1
17136	1.13525	2.1
17137	2.37566	2.1
17138	1.25987	2.1
17139	1.4472	2.1
17140	1.4472	2.1
17141	1.66018	2.1
17142	0.994001	0.360161
17143	1.83151	0.25823
17144	1.83151	0.0746263
17145	1.57621	0.0822534
17146	1.40915	0.05
17147	1.24653	0.200462
17148	1.24653	0.05
17149	1.10609	0.42525
17150	1.61121	2.1
17151	1.74472	2.1
17152	1.74472	2.1
17153	1.0368	2.1
17154	1.04788	2.1
17155	1.28522	2.1
17156	1.28522	2.1
17157	1.54196	2.1
17158	1.23387	2.1
17159	0.896519	2.1
17160	1.60428	2.1
17161	1.08467	2.1
17162	1.56875	0.05
17163	1.39072	0.05
17164	1.39073	0.05
17165	1.6	0.120156

17166	1.01346	2.1
17167	1.06838	0.464371
17168	0.968463	0.521127
17169	1.06837	0.507145
17170	3.22433	0.05
17171	2.05993	1.82582
17172	2.01836	0.05
17173	1.66154	1.93868
17174	1.20871	1.9524
17175	1.10586	2.1
17176	1.10586	1.76757
17177	1.03853	2.1
17178	1.12076	2.1
17179	1.03899	2.1
17180	1.03899	2.1
17181	0.963973	2.1
17182	0.987132	2.1
17183	1.0894	2.1
17184	2.37823	2.1
17185	1.75647	1.68075
17186	1.64625	2.1
17187	1.56195	2.1
17188	1.41995	2.1
17189	1.15171	2.1
17190	1.36457	2.1
17191	1.33847	2.08349
17192	1.33847	2.1
17193	0.869601	1.82865
17194	1.42255	2.1
17195	1.3868	2.1
17196	1.3868	2.1
17197	1.33962	2.1
17198	1.05481	2.1
17199	1.08074	2.1
17200	1.08074	2.1
17201	0.857557	2.1
17202	1.13537	1.95258
17203	1.09194	0.813559
17204	1.01179	2.06467
17205	1.13537	0.332326
17206	1.15645	2.1
17207	1.56409	0.05
17208	1.56409	0.470228
17209	1.16239	1.15222
17210	1.10746	0.05
17211	1.47952	0.159173
17212	1.14733	2.04898
17213	3.77068	2.1
17214	1.38497	2.1
17215	1.54599	2.1
17216	1.54601	2.1
17217	1.27697	2.1
17218	1.01393	1.65563
17219	1.10558	1.74802
17220	0.995012	0.609776
17221	1.17624	1.32847
17222	1.60497	2.1
17223	1.61588	2.1

17224	1.61587	2.1
17225	1.62182	2.1
17226	1.48866	0.05
17227	1.65977	0.05
17228	1.65977	0.05
17229	1.69044	0.05
17230	0.961892	2.1
17231	1.00659	2.1
17232	1.0066	2.1
17233	1.06226	2.1
17234	1.15171	2.1
17235	1.50853	2.02927
17236	1.50853	2.1
17237	1.2955	2.1
17238	1.3204	0.05
17239	2.13295	0.05
17240	2.75524	0.05
17241	1.18156	0.05
17242	1.73729	2.1
17243	1.20356	2.1
17244	1.62483	2.1
17245	1.52008	2.1
17246	1.19964	2.1
17247	1.0577	2.1
17248	1.19964	2.1
17249	1.07156	2.1
17250	1.98241	2.1
17251	1.43687	2.1
17252	1.27863	2.1
17253	1.19915	1.43973
17254	0.97269	1.52187
17255	1.0739	0.670237
17256	1.1427	2.1
17257	1.22476	0.718292
17258	1.06584	1.76785
17259	1.22626	1.00668
17260	1.31388	2.1
17261	1.16932	2.1
17262	1.28819	2.1
17263	1.0499	2.1
17264	1.55894	2.1
17265	1.78709	2.1
17266	1.75986	2.1
17267	1.764	2.1
17268	1.44801	2.1
17269	1.28186	2.1
17270	1.20797	2.03979
17271	1.06821	2.1
17272	1.87287	0.448865
17273	2.08014	0.871871
17274	1.63225	1.08526
17275	1.39501	1.67723
17276	1.20132	0.05
17277	1.16182	0.05
17278	1.08848	0.05
17279	1.20132	0.05
17280	1.23475	1.82833
17281	1.16661	2.1

17282	2.31317	2.1
17283	2.50145	2.1
17284	1.04367	2.1
17285	2.23875	2.1
17286	0.953632	0.05
17287	1.38646	0.05
17288	1.38646	0.05
17289	1.61963	0.05
17290	1.62934	1.25346
17291	1.88626	0.904405
17292	1.0775	0.104541
17293	1.119	0.0595581
17294	1.08133	0.05
17295	1.48312	0.05
17296	1.57125	0.05
17297	1.74098	0.05
17298	1.94372	1.12197
17299	1.22237	1.48466
17300	1.18549	0.0686682
17301	1.01294	0.05
17302	1.01294	0.105617
17303	1.2064	0.193389
17304	1.14599	2.1
17305	1.00065	0.05
17306	1.76357	2.1
17307	2.23468	2.1
17308	2.21735	2.1
17309	3.32769	2.1
17310	1.19647	1.00655
17311	1.35307	0.141597
17312	1.19645	0.242236
17313	1.43778	0.0776613
17314	1.34412	0.05
17315	1.1315	2.1
17316	1.1315	0.194897
17317	1.23943	0.0735229
17318	1.57829	2.1
17319	1.22537	2.1
17320	1.14357	2.1
17321	1.32772	2.1
17322	1.4259	2.1
17323	1.34245	2.1
17324	1.47953	2.1
17325	1.70303	2.1
17326	2.21127	0.05
17327	1.47904	0.05
17328	1.18793	0.05
17329	2.21126	0.05
17330	1.1032	2.02009
17331	1.14986	0.0561332
17332	1.14986	2.1
17333	1.19611	0.136679
17334	1.91883	2.1
17335	1.83019	2.1
17336	1.83019	2.1
17337	1.82462	2.1
17338	1.27362	0.05
17339	1.27421	0.05

17340	1.27421	0.05
17341	1.10736	0.05
17342	1.3003	0.073185
17343	1.31415	1.61004
17344	1.31415	0.13666
17345	1.32201	1.91484
17346	1.21124	2.1
17347	0.81568	2.1
17348	0.81568	2.1
17349	0.551816	2.1
17350	1.67119	2.1
17351	1.63384	2.1
17352	1.63384	2.1
17353	1.63661	2.1
17354	1.48734	2.1
17355	1.52245	2.1
17356	1.52245	2.1
17357	1.50714	2.1
17358	0.996778	0.05
17359	1.56091	0.05
17360	1.56091	0.05
17361	1.90457	0.05
17362	1.1181	2.1
17363	1.06543	2.1
17364	2.66828	2.1
17365	2.76932	2.1
17366	1.06543	2.1
17367	1.01474	2.1
17368	2.76932	2.1
17369	2.86704	2.1
17370	1.29724	0.05
17371	1.96938	0.05
17372	1.82462	2.1
17373	1.84553	2.1
17374	1.05556	2.1
17375	1.25363	2.1
17376	1.84552	2.1
17377	1.53208	2.1
17378	1.12826	2.1
17379	1.39075	2.1
17380	0.9702	1.0356
17381	1.16481	0.262195
17382	1.08894	0.519728
17383	1.91756	0.73682
17384	1.50865	0.0846195
17385	1.06012	0.05
17386	1.5456	0.05
17387	1.37427	0.05
17388	1.1211	0.05
17389	1.5456	0.05
17390	1.33135	0.05
17391	1.07956	0.05
17392	2.23255	0.05
17393	1.21031	0.05
17394	1.52903	2.1
17395	1.35977	2.1
17396	3.3837	2.1
17397	3.20581	2.1

17398	1.35977	2.1
17399	1.15414	2.1
17400	3.20582	2.1
17401	2.07596	2.1
17402	1.6812	0.05
17403	1.03939	0.05
17404	1.30047	2.1
17405	1.5586	2.1
17406	1.5586	2.1
17407	1.84214	2.1
17408	1.31825	0.05
17409	1.31923	1.98306
17410	1.31924	0.0875724
17411	1.33026	1.1417
17412	1.15818	0.740219
17413	1.17822	1.27223
17414	1.15818	2.1
17415	1.28096	1.75017
17416	1.06848	0.161089
17417	1.89639	0.082307
17418	1.9186	0.924651
17419	1.77065	0.745853
17420	1.59103	0.593704
17421	1.56164	0.31193
17422	1.09697	0.05
17423	2.38719	0.05
17424	2.22123	0.05
17425	2.46059	0.05
17426	1.45506	0.05
17427	1.03645	0.05
17428	1.30347	2.1
17429	1.2627	2.1
17430	1.2627	2.1
17431	1.23036	2.1
17432	1.41469	2.1
17433	1.45257	2.1
17434	1.46907	0.05
17435	1.70302	0.05
17436	1.49196	0.05
17437	1.36307	0.05
17438	1.05677	2.1
17439	0.978698	2.1
17440	0.978699	2.1
17441	2.01822	2.1
17442	1.47446	2.1
17443	1.55432	2.1
17444	1.55432	2.1
17445	1.06053	2.1
17446	1.86813	1.48644
17447	1.21892	2.07806
17448	1.53064	0.930048
17449	1.30664	0.980171
17450	1.09629	2.1
17451	1.19244	2.1
17452	1.19244	2.1
17453	1.37635	2.1
17454	1.49358	0.0654438
17455	1.57702	0.05

17456	2.09374	2.1
17457	1.88175	2.1
17458	1.88175	2.1
17459	1.51085	2.1
17460	1.10938	0.05
17461	1.02478	0.05
17462	1.33061	0.05
17463	1.13272	0.05
17464	1.75505	2.1
17465	1.78529	2.1
17466	1.6353	2.1
17467	1.59307	2.1
17468	1.60017	0.0750731
17469	1.35608	0.224302
17470	1.35608	0.0592708
17471	1.04713	0.05
17472	1.01479	0.05
17473	1.3868	0.05
17474	1.3868	0.05
17475	1.28418	1.1214
17476	2.15949	2.1
17477	2.90429	2.1
17478	1.51902	2.1
17479	1.54755	2.1
17480	2.90429	2.1
17481	3.49966	2.1
17482	1.70842	2.1
17483	1.97526	2.1
17484	0.949712	0.05
17485	1.92784	0.05
17486	1.34093	0.05
17487	2.53426	0.05
17488	1.92783	0.05
17489	3.89373	0.05
17490	2.53426	0.05
17491	2.35961	0.05
17492	1.68108	0.0784643
17493	1.15558	0.0647005
17494	1.67265	0.0866183
17495	1.27608	0.0534183
17496	1.42249	2.1
17497	1.39708	2.1
17498	1.39708	2.1
17499	1.39327	2.1
17500	1.97046	0.05
17501	1.11688	0.05
17502	1.28418	0.05
17503	1.17302	1.03952
17504	1.17302	0.05
17505	1.04349	1.02933
17506	0.979853	2.1
17507	1.26016	2.1
17508	1.18901	2.1
17509	1.26016	2.1
17510	1.22857	0.118697
17511	1.25848	0.936156
17512	1.25848	0.125203
17513	1.2858	1.07745

17514	1.09154	2.1
17515	2.01915	2.1
17516	1.23013	2.1
17517	1.55698	2.1
17518	1.55698	2.1
17519	1.8575	2.1
17520	2.01854	2.1
17521	1.20686	2.1
17522	1.20684	2.1
17523	1.77796	2.1
17524	1.46129	2.1
17525	1.04193	2.1
17526	1.10051	2.1
17527	0.983723	2.1
17528	1.1729	2.1
17529	0.98505	2.1
17530	0.985052	2.1
17531	1.03951	2.1
17532	1.03949	2.1
17533	1.17583	2.1
17534	1.42866	2.1
17535	1.97261	2.1
17536	1.73274	2.1
17537	1.53629	2.1
17538	1.98182	2.1
17539	1.72511	2.1
17540	1.42174	2.1
17541	1.44472	2.1
17542	1.72511	2.1
17543	1.45411	2.1
17544	1.44472	2.1
17545	1.43836	2.1
17546	0.962703	0.098359
17547	1.0171	0.55497
17548	1.53336	0.05
17549	1.40274	0.05
17550	1.40272	0.05
17551	1.00213	0.05
17552	1.50892	0.05
17553	1.64984	0.05
17554	1.67464	0.05
17555	1.67481	0.05
17556	1.08212	2.1
17557	1.28095	2.1
17558	1.46956	2.1
17559	1.70303	2.1
17560	1.2925	0.05
17561	1.84716	0.05
17562	1.48676	0.05
17563	1.48797	0.05
17564	1.33917	0.05
17565	1.22691	0.05
17566	0.528744	2.1
17567	1.13728	2.1
17568	1.13728	2.1
17569	1.99843	2.1
17570	1.17464	0.05
17571	1.0372	0.05

17572	2.0815	0.05
17573	0.933462	0.05
17574	1.54931	2.1
17575	1.5485	2.1
17576	1.4077	2.1
17577	1.29931	2.1
17578	1.14931	0.0538342
17579	2.02223	0.0537325
17580	1.04482	0.05
17581	0.94971	0.05
17582	1.54632	1.47044
17583	1.15693	1.65114
17584	1.66763	2.1
17585	1.41238	2.1
17586	1.41238	2.1
17587	1.48068	2.1
17588	1.0171	0.05
17589	1.1211	0.247824
17590	1.1211	0.05
17591	1.28418	0.152525
17592	0.993021	2.1
17593	1.764 2.1	
17594	0.993023	2.1
17595	1.05787	2.1
17596	1.90914	0.05
17597	1.12229	1.84544
17598	1.02025	0.0748775
17599	0.585368	2.07175
17600	1.99323	2.1
17601	2.34988	2.1
17602	2.34988	2.1
17603	2.36987	2.1
17604	1.23107	0.05
17605	1.90664	0.05
17606	1.53496	0.05
17607	1.70302	0.05
17608	1.98859	2.1
17609	1.61057	2.1
17610	1.79844	2.1
17611	2.10811	2.1
17612	1.4618	2.1
17613	1.96722	2.1
17614	1.95191	2.1
17615	2.13418	2.1
17616	1.50368	0.05
17617	1.61417	0.05
17618	1.15008	0.05
17619	1.19685	0.05
17620	1.90456	0.05
17621	1.90899	0.05
17622	2.08864	0.05
17623	2.10447	0.05
17624	1.16653	2.1
17625	1.23017	2.1
17626	1.0752	0.05
17627	1.34412	0.05
17628	1.06226	2.1
17629	1.09252	2.1

17630	1.09252	2.1
17631	0.88579	2.1
17632	1.56178	0.05
17633	1.14305	0.05
17634	1.56175	0.05
17635	1.08211	0.05
17636	1.32813	2.1
17637	1.32461	2.1
17638	1.32461	2.1
17639	1.32455	2.1
17640	1.42729	0.05
17641	1.18716	0.05
17642	1.47169	0.05
17643	1.24687	0.05
17644	1.09627	2.1
17645	1.04759	2.1
17646	1.04759	2.1
17647	0.987132	2.1
17648	1.11181	1.87659
17649	1.80662	2.1
17650	1.8066	2.1
17651	1.06461	1.99207
17652	1.56091	2.1
17653	1.9842	2.1
17654	1.73525	0.05
17655	1.40557	0.05
17656	2.26906	0.05
17657	1.31248	0.05
17658	1.66832	0.05
17659	1.32634	0.05
17660	1.03314	0.05
17661	1.24682	0.05
17662	1.38536	0.05
17663	1.52661	0.05
17664	1.177	0.598974
17665	1.36405	0.279924
17666	1.19837	0.57506
17667	1.17701	0.05
17668	0.993831	0.05
17669	1.0823	0.05
17670	1.0823	0.05
17671	1.01121	0.05
17672	1.49653	0.05
17673	1.89583	0.05
17674	0.67242	0.05
17675	1.36047	0.05
17676	1.00366	0.05
17677	1.24728	0.05
17678	1.13387	0.05
17679	1.60867	0.05
17680	1.30572	0.05
17681	1.54982	0.05
17682	0.788592	0.05
17683	1.82961	0.05
17684	1.54982	0.05
17685	1.82961	0.05
17686	1.26062	0.05
17687	1.64002	0.05

17688	1.12035	0.05
17689	1.64002	0.05
17690	1.1673	2.1
17691	1.75367	2.1
17692	0.964034	2.1
17693	1.0305	2.1
17694	1.30896	2.1
17695	1.52776	2.06462
17696	1.52776	2.1
17697	1.17989	2.1
17698	1.16759	2.1
17699	1.8575	2.1
17700	1.16759	2.1
17701	1.60936	2.1
17702	1.10747	0.05
17703	1.47688	0.05
17704	1.40794	2.1
17705	1.95729	1.8204
17706	1.95729	2.1
17707	3.18156	2.06663
17708	1.87598	2.1
17709	1.94343	2.1
17710	1.94343	2.09205
17711	2.00551	2.1
17712	1.20067	0.817282
17713	1.70744	1.88215
17714	1.87244	1.73471
17715	1.32075	1.08906
17716	1.64955	2.1
17717	1.44221	2.1
17718	1.31109	2.1
17719	1.14206	2.1
17720	1.60452	2.05198
17721	1.12111	0.267514
17722	1.00832	2.1
17723	0.974137	2.1
17724	0.974137	2.1
17725	0.98505	2.1
17726	1.02368	2.1
17727	0.985051	2.1
17728	0.995734	0.05
17729	1.31993	0.05
17730	1.31993	0.05
17731	1.18549	0.05
17732	1.5176	0.229905
17733	1.33622	0.123244
17734	1.33621	0.150512
17735	1.02782	0.0822327
17736	1.66774	2.1
17737	1.85305	2.1
17738	1.85305	2.1
17739	2.07088	2.1
17740	1.78873	2.1
17741	1.47533	2.1
17742	1.23152	2.1
17743	0.99922	2.1
17744	1.48493	2.1
17745	1.1394	2.1

17746	1.19179	2.1
17747	1.64289	2.1
17748	1.47858	2.1
17749	1.89687	2.1
17750	0.972464	2.1
17751	1.79473	2.1
17752	1.06971	2.1
17753	1.21089	2.1
17754	1.12572	2.1
17755	1.30566	2.1
17756	1.30566	2.1
17757	1.52724	2.1
17758	2.32237	0.05
17759	2.20248	0.05
17760	2.79564	0.05
17761	2.32237	0.05
17762	1.59354	0.05
17763	3.098 0.05	
17764	3.09801	0.05
17765	3.45132	0.05
17766	1.24001	2.1
17767	1.41822	0.319366
17768	1.41821	2.1
17769	1.44859	0.833625
17770	1.34539	2.1
17771	1.06991	2.1
17772	1.18076	2.1
17773	0.978586	2.1
17774	0.999189	0.05
17775	2.41076	0.05
17776	0.968475	2.1
17777	1.02074	2.1
17778	1.02074	2.1
17779	1.07866	2.1
17780	0.817702	0.05
17781	1.5082	0.05
17782	1.48376	0.05
17783	1.18947	0.05
17784	1.10638	2.1
17785	1.86766	2.1
17786	1.19785	2.1
17787	1.34141	2.1
17788	1.34141	2.1
17789	0.991985	2.1
17790	2.35516	0.115554
17791	1.24815	0.264017
17792	1.80454	2.1
17793	1.22633	2.1
17794	0.696255	2.1
17795	1.19379	0.39843
17796	1.14721	2.1
17797	1.16153	2.1
17798	1.16153	2.1
17799	1.17261	2.1
17800	1.20814	2.1
17801	1.54791	2.1
17802	1.54791	2.1
17803	1.60477	2.1

17804	1.29931	2.1
17805	1.19098	2.1
17806	1.19098	2.1
17807	1.13341	2.1
17808	1.00503	2.1
17809	1.25334	0.0672789
17810	1.22655	2.1
17811	1.26282	2.1
17812	1.26282	2.1
17813	0.999489	2.1
17814	1.54197	0.05
17815	1.17152	0.05
17816	1.17152	0.05
17817	1.49627	0.05
17818	1.36405	0.354763
17819	0.976334	0.0847599
17820	1.12495	2.1
17821	1.54096	2.1
17822	1.75263	2.1
17823	1.49114	2.1
17824	0.842998	2.1
17825	1.60244	0.325022
17826	1.48911	1.94395
17827	2.18685	0.57999
17828	1.75236	0.674437
17829	1.54143	1.39065
17830	1.40013	0.136899
17831	0.946646	0.110629
17832	1.50714	2.1
17833	1.46055	2.1
17834	1.46055	2.1
17835	1.42249	2.1
17836	2.12839	0.05
17837	1.75211	0.05
17838	1.76297	0.05
17839	1.70736	0.05
17840	1.19952	0.05
17841	1.56475	0.05
17842	1.74312	0.05
17843	1.48973	0.05
17844	1.91518	0.05
17845	1.16534	0.05
17846	1.53556	2.1
17847	1.56253	2.1
17848	1.56253	2.1
17849	1.58054	2.1
17850	1.27806	0.05
17851	1.44593	0.05
17852	1.44593	0.05
17853	1.3946	0.05
17854	1.34857	0.05
17855	1.03818	2.0436
17856	1.38969	0.0740987
17857	1.85548	0.162723
17858	1.03108	0.0809824
17859	1.85548	0.11228
17860	1.49346	1.65377
17861	1.83192	2.08488

17862	1.75291	0.929417
17863	1.35435	1.84779
17864	0.921851	0.0550382
17865	1.09646	0.05
17866	1.2183	0.05
17867	1.36717	0.05
17868	2.14179	0.05
17869	1.79805	0.0536036
17870	1.3428	0.06098
17871	2.30493	0.0562381
17872	1.81545	0.05
17873	1.92269	0.05
17874	2.30492	0.0663757
17875	1.3663	0.0832171
17876	1.22418	2.1
17877	0.956698	0.157959
17878	1.4706	2.1
17879	0.953295	2.1
17880	1.47059	1.92138
17881	1.26929	2.1
17882	1.24538	0.05
17883	1.4468	0.05
17884	2.86762	2.1
17885	2.32911	2.1
17886	2.10864	2.1
17887	1.94548	2.1
17888	1.83134	2.1
17889	2.19519	2.1
17890	2.19853	2.1
17891	2.63855	2.1
17892	1.08213	0.05
17893	1.46389	0.05
17894	1.62235	0.05
17895	0.980549	0.05
17896	1.08195	2.04967
17897	0.99002	2.1
17898	1.2269	2.1
17899	1.0838	2.1
17900	0.889491	2.1
17901	1.19587	2.1
17902	1.18801	0.05
17903	1.57125	0.05
17904	1.47712	2.1
17905	1.45182	2.1
17906	1.45182	2.1
17907	1.42255	2.1
17908	1.17816	0.05
17909	1.00982	0.05
17910	1.21056	0.05
17911	1.17816	0.05
17912	3.80902	0.302708
17913	0.976991	0.183681
17914	1.09962	2.1
17915	1.60976	2.1
17916	1.87029	2.1
17917	2.33806	2.1
17918	2.12549	2.1
17919	2.21472	2.1

17920	2.16221	0.05
17921	1.99528	0.05
17922	1.99528	0.05
17923	1.97969	0.05
17924	3.60854	2.1
17925	4.00847	2.1
17926	2.70372	2.1
17927	1.253	2.1
17928	1.65739	2.1
17929	0.994294	2.1
17930	1.36203	2.1
17931	1.00965	2.1
17932	2.33374	2.1
17933	1.36209	2.1
17934	1.64983	0.05
17935	1.36416	0.05
17936	2.19766	0.05
17937	1.36416	0.05
17938	2.15871	0.178778
17939	1.81454	0.05
17940	1.54607	0.05
17941	1.57702	0.0878196
17942	0.948748	0.05
17943	1.04522	0.05
17944	1.60994	0.05
17945	1.90382	0.05
17946	1.90382	0.05
17947	1.57999	0.05
17948	1.73905	0.173542
17949	1.26535	1.2408
17950	2.12552	1.02972
17951	1.06891	0.479113
17952	1.13999	1.31603
17953	2.29812	0.940829
17954	2.83329	0.594753
17955	2.16272	0.330975
17956	1.71277	2.1
17957	1.68401	2.1
17958	1.68401	2.1
17959	1.69368	2.1
17960	1.06472	2.1
17961	1.56017	2.1
17962	1.2765	2.1
17963	1.99541	2.1
17964	1.46041	0.05
17965	2.04817	0.05
17966	2.04817	0.05
17967	2.33181	0.05
17968	2.06353	2.1
17969	1.35449	2.1
17970	1.52045	2.1
17971	1.45437	2.1
17972	1.04405	0.05
17973	1.85945	0.05
17974	1.85945	0.05
17975	2.22679	0.05
17976	1.33339	2.1
17977	1.19376	2.1

17978	1.33266	2.1
17979	1.44244	2.1
17980	1.20455	2.1
17981	1.52852	2.1
17982	1.03882	2.1
17983	1.52852	2.1
17984	1.44801	2.1
17985	1.00775	2.1
17986	0.53907	2.1
17987	0.506124	2.1
17988	2.39045	2.1
17989	0.973794	2.1
17990	1.11193	2.1
17991	1.32784	2.1
17992	1.31595	2.1
17993	0.966115	2.1
17994	1.25953	2.1
17995	1.38086	2.1
17996	1.38086	2.1
17997	1.04101	2.1
17998	1.99319	0.05
17999	1.23107	0.05
18000	3.3606	0.05
18001	1.23112	0.05
18002	1.40239	2.1
18003	1.26091	2.1
18004	1.26091	2.1
18005	1.05776	2.1
18006	1.23768	0.0744366
18007	1.0142	1.55622
18008	1.11435	1.38824
18009	1.01422	0.495471
18010	1.24001	0.23402
18011	1.42186	1.18747
18012	1.42186	0.362952
18013	1.47204	0.596295
18014	1.27361	2.1
18015	1.56642	2.1
18016	1.71676	2.1
18017	2.16815	2.1
18018	1.07855	1.33517
18019	1.07612	1.18673
18020	1.703 0.05	
18021	1.46853	0.05
18022	1.45448	0.05
18023	1.27778	0.05
18024	0.991986	2.1
18025	1.1192	2.1
18026	1.1192	2.1
18027	1.25953	2.1
18028	2.08506	2.1
18029	1.75162	1.20392
18030	1.94523	2.1
18031	1.88405	2.1
18032	1.15506	0.05
18033	1.1233	0.05
18034	2.09736	2.1
18035	2.98303	2.1

18036	2.71184	2.1
18037	3.51614	2.1
18038	1.18769	2.1
18039	1.24053	2.1
18040	1.24053	2.1
18041	1.45327	2.1
18042	1.26865	1.69259
18043	1.1744	1.27506
18044	1.1744	0.354992
18045	1.11712	1.21866
18046	1.15841	0.05
18047	1.02247	0.05
18048	1.93649	2.1
18049	1.81191	2.1
18050	1.64718	2.1
18051	1.47521	2.1
18052	1.59654	2.1
18053	1.57823	2.1
18054	1.57823	2.1
18055	1.55768	2.1
18056	1.00665	0.05
18057	1.28239	0.05
18058	1.28239	0.05
18059	1.44951	0.05
18060	1.22083	2.1
18061	0.957622	2.1
18062	0.887896	2.1
18063	1.27387	2.1
18064	1.00521	0.480661
18065	1.00503	0.873993
18066	1.95844	2.1
18067	1.05042	2.1
18068	2.78662	2.1
18069	2.22399	2.1
18070	2.3356	0.05
18071	1.49543	0.05
18072	1.96854	0.05
18073	1.21681	0.05
18074	2.17803	1.54857
18075	1.50998	2.1
18076	2.35239	1.22131
18077	2.26987	0.507622
18078	1.03824	0.05
18079	1.51396	0.0949179
18080	1.51396	0.0910383
18081	1.48693	0.05
18082	1.08784	2.1
18083	1.36865	2.07742
18084	0.712405	2.1
18085	1.08784	2.1
18086	2.02522	2.1
18087	1.6738	2.1
18088	1.35175	2.1
18089	1.50094	2.1
18090	1.6738	2.1
18091	1.62871	2.1
18092	1.35084	2.1
18093	1.34276	2.1

18094	1.18041	2.1
18095	1.43652	2.1
18096	0.974196	2.1
18097	1.28037	2.1
18098	1.28037	2.1
18099	1.05071	2.1
18100	2.44737	0.05
18101	1.4672	0.05
18102	1.22883	0.05
18103	2.08149	0.05
18104	1.82208	1.91702
18105	1.59826	1.78441
18106	1.59826	0.969493
18107	1.01393	2.08098
18108	1.42757	2.1
18109	0.996775	2.1
18110	1.42757	2.1
18111	1.26397	2.1
18112	1.02461	2.1
18113	1.46129	2.1
18114	1.11833	2.1
18115	1.335	2.1
18116	2.07087	2.1
18117	1.24041	2.1
18118	1.335	2.1
18119	1.55398	2.1
18120	1.21171	0.05
18121	1.48589	0.05
18122	1.32886	0.05
18123	1.8027	0.05
18124	1.53515	1.61194
18125	1.60428	1.25087
18126	1.00035	0.05
18127	1.03534	0.05
18128	1.03535	0.902892
18129	1.09962	2.1
18130	0.62132	1.35783
18131	1.21033	2.1
18132	1.30326	2.1
18133	2.6823	2.1
18134	0.991407	2.1
18135	1.29729	2.1
18136	1.29729	2.1
18137	1.16858	2.1
18138	1.66137	0.05
18139	1.23012	0.05
18140	1.66139	0.0602293
18141	1.41821	0.0580107
18142	1.56043	0.244514
18143	1.06848	0.843902
18144	1.56043	2.09939
18145	1.90911	1.6296
18146	0.982847	2.1
18147	1.05863	2.1
18148	1.05863	2.1
18149	1.22352	2.1
18150	1.23019	1.51311
18151	1.60509	2.1

18152	1.19122	2.1
18153	1.47769	2.1
18154	2.18868	2.1
18155	1.40984	2.1
18156	3.55197	2.1
18157	3.57227	2.1
18158	3.57227	2.1
18159	3.48926	2.1
18160	1.4375	0.05
18161	1.11314	0.05
18162	1.62408	2.1
18163	1.74438	2.1
18164	1.72387	2.1
18165	1.50018	2.1
18166	1.1486	2.1
18167	1.18474	2.1
18168	1.18474	2.1
18169	1.2295	2.1
18170	1.28754	2.1
18171	1.36838	2.1
18172	1.36838	2.1
18173	1.21703	2.1
18174	2.74206	2.1
18175	2.70795	2.1
18176	2.70795	2.1
18177	2.55918	2.1
18178	1.35065	0.571096
18179	1.65411	0.150379
18180	1.48572	0.442594
18181	1.28393	0.440608
18182	1.25265	0.05
18183	1.70944	0.05
18184	1.31259	0.05
18185	1.14945	2.1
18186	1.14945	0.05
18187	0.995725	2.1
18188	1.00307	0.05
18189	2.05786	0.05
18190	1.56357	0.05
18191	1.10907	0.05
18192	1.27558	1.58961
18193	1.05175	0.770091
18194	1.2685	2.1
18195	1.41369	2.1
18196	1.4137	2.1
18197	1.49654	2.1
18198	1.21489	2.1
18199	1.14605	1.7719
18200	1.22476	2.1
18201	1.21489	1.1442
18202	2.51472	1.01882
18203	2.52091	1.05303
18204	2.52091	0.762648
18205	1.79171	0.815986
18206	1.03373	2.1
18207	1.04037	2.1
18208	1.04038	2.1
18209	1.03933	2.1

18210	1.74123	0.05
18211	1.41902	0.05
18212	1.89777	0.05
18213	1.89899	0.05
18214	1.24728	0.05
18215	1.30394	0.05
18216	1.30394	0.05
18217	1.16535	0.05
18218	1.09914	2.1
18219	1.54259	2.1
18220	1.40233	2.1
18221	1.69858	2.1
18222	1.53555	2.1
18223	1.09859	0.937526
18224	1.09859	2.1
18225	0.725879	0.160968
18226	1.07213	2.1
18227	1.31589	2.1
18228	1.36904	2.1
18229	1.69711	2.1
18230	1.51028	0.05
18231	0.993598	0.05
18232	0.61563	0.05
18233	1.26311	0.05
18234	1.19496	2.1
18235	1.65925	2.1
18236	1.65925	2.1
18237	1.48162	2.1
18238	2.75738	1.67688
18239	1.24699	1.35094
18240	4.08729	1.96797
18241	2.35271	1.68082
18242	1.55063	0.101056
18243	1.34563	0.05
18244	1.34563	0.0795555
18245	1.08189	0.05
18246	1.09662	0.05
18247	2.35961	0.05
18248	0.986952	0.05
18249	0.813251	0.05
18250	1.17897	0.05
18251	1.67685	0.05
18252	1.00573	1.62604
18253	1.08542	0.321532
18254	1.00573	2.1
18255	1.12572	2.1
18256	1.05499	2.1
18257	1.00521	0.721786
18258	2.22679	0.05
18259	2.86746	0.05
18260	2.86746	0.05
18261	1.99958	0.05
18262	0.996517	2.1
18263	1.01169	2.1
18264	1.01169	2.1
18265	1.26346	2.1
18266	1.68274	2.1
18267	1.75505	2.1

18268	2.28748	2.0242
18269	1.36555	2.1
18270	3.47888	0.05
18271	0.692788	0.05
18272	1.0685	0.05
18273	1.14547	0.0598727
18274	1.19017	2.1
18275	1.24976	2.1
18276	1.24976	2.1
18277	1.30682	2.1
18278	2.2153	2.1
18279	1.85457	2.1
18280	2.03267	2.1
18281	1.51042	2.1
18282	1.05619	0.0772796
18283	1.05793	0.510984
18284	1.05793	0.0802965
18285	1.00763	0.747066
18286	1.77677	2.1
18287	1.02524	2.1
18288	1.02524	2.1
18289	1.18803	2.1
18290	1.68669	2.1
18291	1.71162	2.1
18292	1.556	2.1
18293	1.39523	2.1
18294	0.960288	0.607108
18295	1.24201	1.40297
18296	1.24202	2.1
18297	1.3592	2.1
18298	2.14045	0.05
18299	1.62054	0.05
18300	2.20248	0.05
18301	2.34699	0.05
18302	3.45132	0.05
18303	3.38241	0.05
18304	3.38241	0.05
18305	2.96127	0.05
18306	2.02612	2.1
18307	2.09351	2.1
18308	2.09351	2.1
18309	2.17465	2.1
18310	1.07445	2.1
18311	1.14808	2.1
18312	1.39067	2.1
18313	1.20958	1.96533
18314	1.20958	2.1
18315	1.00382	1.70411
18316	0.986496	2.1
18317	1.27142	2.1
18318	1.39857	2.1
18319	2.26482	2.1
18320	2.24367	0.05
18321	1.9234	0.05
18322	1.59076	0.05
18323	1.185	0.05
18324	3.86179	0.05
18325	3.0638	0.05

18326	2.7574	0.05
18327	1.83266	0.05
18328	2.59418	2.1
18329	2.23048	2.1
18330	1.39564	2.1
18331	1.36763	2.1
18332	2.23048	2.1
18333	2.02522	2.1
18334	1.36763	2.1
18335	1.35175	2.1
18336	1.16181	0.05
18337	0.97096	0.05
18338	2.05665	0.05
18339	0.483583	0.05
18340	0.601928	0.05
18341	3.88545	0.05
18342	1.04909	2.1
18343	0.983143	2.1
18344	1.10469	2.1
18345	1.10516	2.1
18346	1.10517	2.1
18347	1.09951	2.1
18348	2.33985	2.1
18349	2.35026	2.1
18350	2.35025	2.1
18351	1.19462	2.1
18352	1.57627	2.1
18353	1.5493	2.1
18354	1.5493	2.1
18355	1.53556	2.1
18356	1.42763	0.118212
18357	1.24682	0.0815979
18358	1.24682	0.076178
18359	1.00307	0.0879798
18360	1.37392	2.1
18361	1.45408	2.1
18362	1.45408	2.1
18363	1.54249	2.1
18364	1.24474	1.57447
18365	1.07467	1.68525
18366	1.37813	1.93989
18367	1.24474	1.86017
18368	1.06341	2.1
18369	1.09194	2.1
18370	1.09194	2.1
18371	1.11117	2.1
18372	1.1658	2.1
18373	1.93707	2.1
18374	1.93707	2.1
18375	1.47729	2.1
18376	1.25669	0.178736
18377	1.07519	2.1
18378	1.65417	2.1
18379	1.50956	2.1
18380	1.37231	2.1
18381	1.27285	2.1
18382	1.23612	2.1
18383	1.1899	2.1

18384	1.08172	2.1
18385	1.20357	2.1
18386	1.5664	2.1
18387	1.7472	2.1
18388	1.48434	2.1
18389	1.23989	2.1
18390	1.58836	2.1
18391	1.38766	2.1
18392	1.23989	2.1
18393	1.26599	2.1
18394	2.56248	2.1
18395	2.08673	0.935095
18396	1.87216	1.09026
18397	1.60476	0.05
18398	1.84355	2.1
18399	1.25971	0.763488
18400	1.44141	0.545791
18401	1.25521	0.05
18402	1.19837	0.05
18403	2.31936	0.05
18404	1.38153	2.1
18405	2.41734	2.1
18406	2.70856	2.1
18407	3.48385	2.1
18408	1.89226	1.28973
18409	1.64617	1.39102
18410	1.89112	1.21674
18411	1.23135	0.05
18412	1.23134	0.144714
18413	1.34927	0.123976
18414	3.60096	0.05
18415	1.68103	0.05
18416	2.25644	2.1
18417	1.91955	2.1
18418	1.91955	2.1
18419	1.47688	2.1
18420	1.67375	2.1
18421	1.50033	2.1
18422	1.5098	2.1
18423	1.46908	2.1
18424	1.69055	0.05
18425	1.53463	0.05
18426	1.53463	0.05
18427	1.41943	0.05
18428	1.19022	2.05125
18429	1.95981	0.979279
18430	1.15009	0.879899
18431	1.31612	0.338082
18432	1.31611	0.05
18433	1.10735	0.05
18434	1.18601	0.05
18435	1.3499	0.05
18436	1.3499	0.05
18437	1.29001	0.05
18438	1.22083	2.1
18439	1.06191	2.1
18440	1.348	0.768715
18441	1.05758	1.60946

18442	1.34799	2.09779
18443	1.18647	1.77016
18444	2.92457	0.05
18445	1.99182	0.05
18446	2.25313	0.05
18447	1.30859	0.05
18448	1.30682	2.1
18449	1.38957	2.1
18450	1.38957	2.1
18451	1.47446	2.1
18452	0.953638	2.1
18453	1.03408	2.1
18454	1.17885	0.0703061
18455	1.3663	0.05
18456	1.08265	0.213444
18457	1.71948	0.360711
18458	1.29501	0.786053
18459	0.955093	0.909835
18460	0.955081	0.163563
18461	1.88345	0.429991
18462	1.2243	2.1
18463	1.23169	2.1
18464	2.04726	1.77325
18465	0.842869	1.9416
18466	3.18156	2.1
18467	3.60786	1.82663
18468	3.60786	2.1
18469	2.38566	1.42649
18470	1.24526	0.05
18471	1.59019	0.05
18472	1.16776	0.05
18473	1.05042	2.1
18474	1.17418	2.1
18475	1.05042	2.1
18476	0.949135	2.1
18477	0.973448	2.1
18478	0.973447	2.1
18479	1.02958	2.1
18480	3.57768	2.1
18481	3.04246	2.1
18482	3.04246	2.1
18483	2.47824	2.1
18484	1.35879	2.1
18485	1.10407	2.1
18486	1.14599	0.05
18487	1.10407	0.0865333
18488	1.87794	1.66167
18489	1.53429	1.34211
18490	1.12717	2.1
18491	1.38634	2.1
18492	1.38634	2.1
18493	1.13127	2.1
18494	1.04546	2.1
18495	1.06007	2.1
18496	1.06007	2.1
18497	1.19785	2.1
18498	1.43652	2.1
18499	1.71741	2.1

18500	1.70921	2.01066
18501	1.06994	2.1
18502	0.960106	2.1
18503	1.48077	2.1
18504	1.48077	2.1
18505	2.5946	2.1
18506	2.03045	1.90118
18507	1.76788	2.09932
18508	2.03045	2.1
18509	0.633098	2.1
18510	1.06756	2.1
18511	1.38667	2.1
18512	0.966979	2.1
18513	1.38669	2.1
18514	1.07156	2.1
18515	0.986095	2.1
18516	1.32813	2.1
18517	0.986095	2.1
18518	1.98697	1.57136
18519	1.12016	2.1
18520	1.24463	1.72635
18521	1.1967	2.06171
18522	1.30001	0.05
18523	1.29175	1.53285
18524	1.29175	0.0629994
18525	1.3003	1.80853
18526	1.5606	0.05
18527	2.32098	0.05
18528	2.57888	0.05
18529	2.97463	0.05
18530	1.24555	2.1
18531	1.46101	2.1
18532	1.90723	2.1
18533	2.29126	2.1
18534	1.46101	2.1
18535	1.84089	2.1
18536	2.5519	2.1
18537	2.91663	2.1
18538	1.10476	0.05
18539	1.44103	0.05
18540	1.44103	0.05
18541	1.53336	0.05
18542	0.672964	2.1
18543	0.633884	2.1
18544	0.633884	2.1
18545	0.603154	2.1
18546	2.29663	2.1
18547	2.06892	2.1
18548	1.3685	2.1
18549	1.39067	2.1
18550	2.06892	2.1
18551	1.78208	2.1
18552	1.39067	2.1
18553	1.42174	2.1
18554	1.01335	1.92707
18555	1.08195	0.562859
18556	1.86463	2.00003
18557	1.39827	1.99614

18558	0.861515	2.1
18559	2.10074	2.1
18560	2.09511	0.548879
18561	2.04853	0.352276
18562	2.21488	0.296566
18563	1.38287	0.563475
18564	0.974717	0.215785
18565	0.998451	0.128187
18566	1.42281	0.364796
18567	1.82595	0.495129
18568	1.50458	0.481345
18569	1.09408	0.0991469
18570	1.23498	1.13008
18571	1.05654	0.893074
18572	1.17261	2.1
18573	1.18058	2.1
18574	1.18059	2.1
18575	1.18907	2.1
18576	2.38998	2.1
18577	2.77437	2.1
18578	1.04546	2.1
18579	1.16817	2.1
18580	2.77437	2.1
18581	2.97768	2.1
18582	1.16817	2.1
18583	1.2832	2.1
18584	1.75766	0.05
18585	1.4934	0.682981
18586	1.4934	0.05
18587	1.20324	1.48708
18588	1.07236	0.265499
18589	1.37641	0.121185
18590	1.42913	0.07135
18591	1.93373	0.380263
18592	1.42629	0.566022
18593	1.00636	0.0664163
18594	0.933959	2.1
18595	2.22873	0.429605
18596	2.22873	1.20903
18597	2.13757	0.109252
18598	1.10517	2.1
18599	1.86737	2.1
18600	0.811 2.1	
18601	1.19179	2.1
18602	1.18647	2.1
18603	0.988453	2.1
18604	1.12457	0.05
18605	1.23851	0.05
18606	0.92492	2.1
18607	1.45656	2.1
18608	1.61251	0.380491
18609	1.56696	0.419557
18610	1.90245	0.153638
18611	1.46046	0.116831
18612	1.60329	0.05
18613	1.38247	0.05
18614	1.38247	0.05
18615	1.23198	0.05

18616	1.09518	2.1
18617	1.28159	2.1
18618	0.977777	2.1
18619	1.80778	2.1
18620	1.80777	2.1
18621	1.16655	2.1
18622	1.3515	2.1
18623	0.956573	2.1
18624	1.08022	2.1
18625	1.03437	2.1
18626	1.03437	2.1
18627	0.975409	2.1
18628	1.47369	0.05
18629	1.31167	0.05
18630	1.01847	0.05
18631	2.75562	0.05
18632	1.13443	2.1
18633	1.32709	2.1
18634	1.01051	0.05
18635	0.958947	0.05
18636	1.7211	2.1
18637	1.922 2.1	
18638	1.922 2.1	
18639	0.991113	2.1
18640	1.00636	0.0751324
18641	1.31658	0.255061
18642	1.44627	0.206836
18643	1.03824	0.0591393
18644	1.21223	2.1
18645	1.01242	2.1
18646	1.01243	2.1
18647	0.956698	2.1
18648	1.23167	2.1
18649	1.06935	2.1
18650	1.29215	0.05
18651	0.998971	0.05
18652	1.50103	0.05
18653	1.44836	0.05
18654	3.02513	2.1
18655	3.7335	2.1
18656	2.49988	2.1
18657	1.24948	2.1
18658	0.963972	2.1
18659	1.1822	2.1
18660	1.1822	2.1
18661	1.10817	2.1
18662	1.30237	2.1
18663	1.41908	2.1
18664	1.41908	2.1
18665	1.48734	2.1
18666	1.31127	0.0950865
18667	1.40822	0.089766
18668	1.40822	0.05
18669	1.22176	0.0530488
18670	1.82525	2.1
18671	1.59394	2.1
18672	1.22731	0.05
18673	0.981584	0.05

18674	1.1427	0.162385
18675	1.44091	0.924314
18676	1.49154	0.0989499
18677	1.07526	0.345002
18678	1.07527	0.076767
18679	0.96029	0.11214
18680	2.40568	0.643194
18681	3.46486	0.417309
18682	2.417	0.519057
18683	1.52054	0.185213
18684	1.51638	0.05
18685	1.04786	0.05
18686	1.51404	0.05
18687	1.3461	0.05
18688	1.27696	0.05
18689	0.736457	0.05
18690	1.16817	2.1
18691	1.04263	2.1
18692	1.13449	2.1
18693	1.50524	2.1
18694	2.1182	2.1
18695	2.49001	2.1
18696	1.11764	2.1
18697	1.39292	2.1
18698	2.49001	2.1
18699	2.76339	2.1
18700	1.39292	2.1
18701	1.68761	2.1
18702	1.25173	2.1
18703	1.34701	2.1
18704	1.02415	2.1
18705	1.34701	2.1
18706	1.332	0.05
18707	0.989764	0.05
18708	0.963167	0.05
18709	1.21056	0.05
18710	1.67375	2.1
18711	1.17965	2.1
18712	1.09154	2.1
18713	1.52314	2.1
18714	1.54433	0.0785692
18715	1.81969	0.05
18716	1.3598	0.05
18717	1.71041	0.05
18718	1.70934	0.05
18719	1.33871	0.05
18720	1.79485	0.05
18721	1.51404	0.05
18722	1.30751	2.1
18723	1.20969	2.1
18724	1.20969	2.1
18725	1.12076	2.1
18726	1.49808	2.1
18727	1.50201	2.1
18728	1.50201	2.1
18729	1.31594	2.1
18730	1.2187	2.1
18731	1.11545	2.1

18732	1.227	2.1
18733	1.09645	2.1
18734	1.05902	0.05
18735	1.41595	0.05
18736	1.35696	2.1
18737	1.29446	0.1112
18738	0.857957	2.1
18739	1.21125	0.05
18740	2.43115	2.1
18741	2.1151	0.05
18742	2.0815	0.998155
18743	1.1723	2.1
18744	1.48035	0.0573565
18745	1.59971	0.62454
18746	1.33668	0.234944
18747	1.0961	0.0851456
18748	1.46235	2.1
18749	0.882195	2.1
18750	2.46624	2.1
18751	2.72161	2.1
18752	2.96094	2.1
18753	2.25644	2.1
18754	1.25923	0.05
18755	0.976218	0.05
18756	1.10319	0.05
18757	1.82583	0.05
18758	0.888955	2.1
18759	1.30817	2.1
18760	1.30817	2.1
18761	1.60787	2.1
18762	0.988805	2.1
18763	1.54168	0.454141
18764	1.19496	0.355535
18765	1.12224	1.69491
18766	1.93597	1.45632
18767	1.31113	1.25096
18768	1.17879	1.00769
18769	1.07468	1.08141
18770	2.0523	2.1
18771	2.41385	2.1
18772	2.41385	2.1
18773	3.0355	2.1
18774	1.36717	0.05
18775	1.45535	0.05
18776	1.45535	0.05
18777	1.49906	0.05
18778	1.37785	1.9521
18779	1.04413	2.1
18780	1.06729	0.196844
18781	1.54305	0.156294
18782	1.07444	2.1
18783	1.52724	2.1
18784	1.49151	2.1
18785	1.36814	2.1
18786	1.36814	2.1
18787	1.15541	2.1
18788	1.19379	2.1
18789	1.78657	0.138185

18790	1.6079	2.1
18791	2.59469	0.169021
18792	1.35545	0.0516336
18793	1.23602	0.346225
18794	1.23452	2.1
18795	1.56842	0.687657
18796	1.15864	2.1
18797	1.11955	2.1
18798	1.11955	2.1
18799	1.08022	2.1
18800	1.64268	2.1
18801	1.26247	2.1
18802	1.16912	2.1
18803	2.1095	0.451056
18804	1.75549	0.05
18805	1.7491	0.05
18806	1.59833	0.05
18807	1.68338	0.05
18808	1.14846	0.05
18809	1.06515	0.05
18810	1.2721	0.05
18811	1.32639	0.05
18812	1.32639	0.05
18813	1.19323	0.05
18814	1.81034	2.1
18815	1.9034	2.1
18816	2.08245	2.1
18817	1.84887	2.1
18818	1.41448	2.1
18819	1.77326	2.1
18820	1.76872	2.1
18821	1.86766	2.1
18822	1.46643	2.1
18823	1.58995	2.1
18824	1.58995	2.1
18825	1.13854	2.1
18826	1.23654	2.1
18827	1.04442	2.1
18828	1.23654	2.1
18829	1.08576	2.1
18830	0.966979	2.1
18831	1.41758	2.1
18832	1.03108	0.05
18833	1.94412	0.05
18834	1.32455	2.1
18835	1.32328	2.1
18836	1.32328	2.1
18837	1.34008	2.1
18838	1.60861	2.1
18839	1.69586	2.1
18840	1.69586	2.1
18841	1.78301	2.1
18842	1.7513	2.1
18843	1.67906	2.1
18844	1.67906	2.1
18845	1.60635	2.1
18846	1.68073	2.1
18847	1.57234	2.1

18848	1.57233	2.1
18849	1.47012	2.1
18850	1.22811	0.05
18851	1.79683	0.05
18852	1.53417	0.05
18853	1.08732	0.05
18854	1.8903	1.57342
18855	1.20247	1.79963
18856	1.12087	1.76692
18857	1.54699	2.1
18858	1.43635	2.1
18859	1.02368	2.1
18860	1.43635	2.1
18861	1.23007	2.1
18862	1.035	2.1
18863	1.07889	2.1
18864	3.0355	2.1
18865	3.78805	2.1
18866	2.57183	1.93254
18867	1.27835	1.00906
18868	1.29813	0.05
18869	1.42965	0.05
18870	1.69832	0.05
18871	2.18359	0.05
18872	1.42964	0.05
18873	1.65723	0.05
18874	1.98508	0.05
18875	2.06284	0.05
18876	1.50705	0.452742
18877	1.51153	0.05
18878	1.36037	0.05
18879	1.09767	0.05
18880	1.70048	2.1
18881	1.15177	2.1
18882	0.713174	2.1
18883	0.763575	2.1
18884	0.763575	2.1
18885	0.797011	2.1
18886	1.01179	0.7456
18887	1.27737	0.711555
18888	3.16531	0.05
18889	1.0141	0.05
18890	1.47354	0.141846
18891	1.62495	0.05
18892	1.62495	0.05
18893	1.39296	0.195845
18894	0.998682	2.1
18895	1.93528	2.1
18896	2.71383	2.1
18897	1.13987	2.1
18898	1.93528	2.1
18899	1.78595	2.1
18900	3.98933	2.1
18901	3.73426	2.1
18902	1.02438	2.1
18903	1.12376	2.1
18904	1.12375	2.1
18905	1.19016	2.1

18906	1.37157	0.05
18907	1.60814	0.05
18908	1.70303	0.05
18909	1.01219	0.05
18910	1.06757	2.1
18911	1.23475	2.1
18912	1.2295	2.1
18913	1.28014	2.1
18914	1.28014	2.1
18915	1.33339	2.1
18916	1.3201	2.1
18917	1.2679	2.1
18918	1.2679	2.1
18919	1.23504	2.1
18920	1.50108	2.1
18921	1.3715	2.1
18922	1.3715	2.1
18923	1.25075	2.1
18924	1.99222	1.09821
18925	1.4155	1.05151
18926	1.4777	2.1
18927	1.52613	2.1
18928	1.91031	2.1
18929	1.03968	2.1
18930	2.04289	0.05
18931	0.967192	0.05
18932	1.03043	0.05
18933	1.65452	0.05
18934	1.21564	2.1
18935	1.16938	2.1
18936	2.47056	2.1
18937	2.56853	2.1
18938	1.16938	2.1
18939	1.1181	2.1
18940	2.56853	2.1
18941	2.66828	2.1
18942	1.0465	2.1
18943	1.44709	2.1
18944	1.44709	2.1
18945	1.96491	2.1
18946	1.01976	0.05
18947	0.961779	0.05
18948	1.11764	0.05
18949	0.961779	0.05
18950	1.21171	0.05
18951	1.35007	0.05
18952	1.03102	0.104498
18953	1.42446	0.151597
18954	1.42446	0.0809949
18955	1.03166	0.0731111
18956	1.2951	0.05
18957	1.33575	0.05
18958	1.33575	0.05
18959	1.20473	0.05
18960	1.10049	0.164969
18961	0.99747	0.104525
18962	1.54249	2.1
18963	1.65677	2.1

18964	1.65677	2.1
18965	1.77677	2.1
18966	1.73525	0.05
18967	1.05689	0.05
18968	1.26051	0.05
18969	1.16259	0.05
18970	1.34009	2.1
18971	1.398	2.1
18972	1.398	2.1
18973	1.50437	2.1
18974	2.3245	2.1
18975	1.18237	2.1
18976	1.24509	2.1
18977	1.01595	2.1
18978	3.29047	2.1
18979	2.79168	2.1
18980	1.06561	2.1
18981	1.11077	2.1
18982	1.11077	2.1
18983	0.953927	2.1
18984	1.30509	0.05
18985	1.04685	0.05
18986	1.04685	0.05
18987	0.834593	0.05
18988	1.0775	0.05
18989	1.27362	0.05
18990	1.71762	0.148194
18991	2.5736	0.0849162
18992	2.04717	0.233536
18993	1.45521	0.146977
18994	1.33962	2.1
18995	1.27956	2.1
18996	1.27956	2.1
18997	1.20478	2.1
18998	3.65841	0.05
18999	1.18656	0.05
19000	1.72272	0.05
19001	1.6812	0.05
19002	1.09951	2.1
19003	1.08231	2.1
19004	1.08231	2.1
19005	1.06478	2.1
19006	2.16196	0.943061
19007	1.00387	0.41406
19008	1.00387	0.601133
19009	0.993999	0.0811275
19010	1.04101	2.1
19011	1.27119	2.1
19012	1.27119	2.1
19013	1.51748	2.1
19014	3.00129	2.1
19015	3.27303	2.1
19016	3.27303	2.1
19017	3.60855	2.1
19018	2.22399	2.1
19019	1.71995	2.1
19020	1.54793	2.1
19021	1.227	2.1

19022	1.07624	2.1
19023	1.264 2.1	
19024	1.19323	0.698523
19025	1.21286	0.827902
19026	1.21286	0.172969
19027	1.20859	0.944678
19028	1.60578	0.05
19029	1.27778	0.05
19030	1.0525	2.1
19031	1.05758	1.05125
19032	1.0525	0.593764
19033	1.17042	1.35981
19034	1.62536	0.05
19035	3.71662	0.05
19036	1.78169	0.05
19037	1.91434	0.05
19038	2.12331	0.05
19039	1.33406	0.05
19040	1.38437	2.1
19041	1.51829	2.1
19042	1.51829	2.1
19043	1.6697	2.1
19044	0.947114	2.1
19045	1.13335	2.1
19046	1.13335	2.1
19047	1.00965	2.1
19048	1.47521	2.1
19049	1.27679	2.1
19050	1.14911	2.1
19051	0.93841	2.1
19052	1.68437	2.1
19053	1.53822	2.1
19054	1.53822	2.1
19055	1.39702	2.1
19056	0.726453	2.1
19057	0.754101	2.1
19058	0.754101	2.1
19059	0.69776	2.1
19060	0.964033	2.1
19061	1.62397	2.1
19062	1.70302	2.1
19063	1.78637	1.9833
19064	3.68158	0.05
19065	2.11685	0.05
19066	1.33125	2.1
19067	1.15471	2.1
19068	1.0465	2.1
19069	1.33125	2.1
19070	1.17683	2.1
19071	1.25889	2.1
19072	1.25889	2.1
19073	1.34239	2.1
19074	1.40591	0.05
19075	0.79348	0.05
19076	1.04019	0.05
19077	0.999187	0.05
19078	1.30218	0.05
19079	1.83339	0.05

19080	1.88574	0.05
19081	1.5177	0.05
19082	1.23504	2.1
19083	1.19583	2.1
19084	1.19583	2.1
19085	1.14496	2.1
19086	1.62183	2.1
19087	1.62316	2.1
19088	1.62316	2.1
19089	1.62241	2.1
19090	1.02305	0.0787965
19091	1.21136	0.0605208
19092	0.993832	0.05
19093	1.21137	0.0579883
19094	1.12064	2.1
19095	1.24965	2.1
19096	1.24965	2.1
19097	1.41077	2.1
19098	1.00052	0.05
19099	1.49842	0.05
19100	1.49843	0.05
19101	1.10801	0.05
19102	1.54196	0.599711
19103	1.18041	2.03206
19104	1.73571	1.91101
19105	1.37224	2.1
19106	1.4375	0.05
19107	1.09529	0.05
19108	1.44421	0.05
19109	1.0953	0.05
19110	1.0753	2.1
19111	0.815365	2.1
19112	0.815365	2.1
19113	0.597537	2.1
19114	1.03609	0.0500841
19115	1.18952	0.05
19116	1.54283	0.0597971
19117	1.16719	0.0611976
19118	1.16719	0.126181
19119	1.89226	0.0863509
19120	2.55919	2.1
19121	2.37492	2.1
19122	2.37492	2.1
19123	2.07435	2.1
19124	1.36016	0.05
19125	1.70302	0.05
19126	1.85252	0.05
19127	1.2111	0.0842327
19128	1.62905	2.1
19129	1.49692	2.1
19130	1.49692	2.1
19131	1.40395	2.1
19132	2.19083	2.1
19133	1.30542	2.1
19134	1.58736	2.1
19135	2.16579	2.1
19136	2.0815	2.1
19137	1.17935	2.1

19138	1.53803	2.1
19139	1.09148	0.311949
19140	1.81301	1.82727
19141	1.38333	0.05
19142	1.04412	2.1
19143	1.44437	2.1
19144	1.4675	0.05
19145	1.00665	0.05
19146	1.50951	0.05
19147	0.983105	0.05
19148	1.80252	0.05
19149	0.540005	0.05
19150	1.2545	0.05
19151	2.14514	0.05
19152	1.32051	0.05
19153	1.82302	0.05
19154	2.71057	2.1
19155	2.51107	2.1
19156	1.34817	2.1
19157	1.3551	2.1
19158	2.51107	2.1
19159	2.29663	2.1
19160	1.3551	2.1
19161	1.3685	2.1
19162	1.51748	2.1
19163	1.23389	2.1
19164	0.901928	0.05
19165	1.70302	0.05
19166	1.66787	0.05
19167	1.92535	0.05
19168	1.13571	0.05
19169	1.02836	0.05
19170	1.55398	2.1
19171	1.54179	2.1
19172	1.89608	2.1
19173	1.07167	2.1
19174	3.3488	0.05
19175	0.550044	0.05
19176	0.962877	0.05
19177	1.00065	0.05
19178	1.00613	0.0942466
19179	1.07497	0.120274
19180	1.00486	0.05
19181	1.00486	0.05
19182	0.726453	2.1
19183	1.32634	0.05
19184	1.40008	0.05
19185	1.40008	0.05
19186	2.42267	0.05
19187	1.9216	0.05
19188	1.28066	0.05
19189	1.79102	0.503307
19190	1.18219	0.05
19191	1.18219	0.700184
19192	0.709123	0.05
19193	1.07866	2.1
19194	1.15038	2.1
19195	1.15038	2.1

19196	1.24687	2.1
19197	1.83613	2.1
19198	1.99157	2.1
19199	1.45789	0.05
19200	1.1706	0.05
19201	1.74746	0.05
19202	1.1706	0.05
19203	1.96383	0.05
19204	1.67727	0.05
19205	1.67727	0.05
19206	1.60329	0.05
19207	1.18803	2.1
19208	1.3227	2.1
19209	1.3227	2.1
19210	1.40844	2.1
19211	1.07889	2.1
19212	1.07721	2.1
19213	1.07721	2.1
19214	1.00832	2.1
19215	0.953523	0.153562
19216	1.62875	0.103544
19217	1.07427	0.343144
19218	1.19722	0.214992
19219	1.1972	0.151357
19220	1.1599	0.105189
19221	2.97463	0.05
19222	1.9477	0.05
19223	1.95222	0.05
19224	1.75083	0.05
19225	3.46787	2.1
19226	3.02896	2.1
19227	3.02896	2.1
19228	2.46145	2.1
19229	0.970962	0.05
19230	1.0227	0.05
19231	1.12328	0.05
19232	1.02269	0.05
19233	0.904978	2.1
19234	0.725887	2.1
19235	0.725887	2.1
19236	0.601592	2.1
19237	0.956294	2.1
19238	1.13179	2.1
19239	0.956294	2.1
19240	1.13179	2.1
19241	1.05556	2.1
19242	1.13179	2.1
19243	1.38859	2.1
19244	1.13179	2.1
19245	1.06659	2.1
19246	1.05776	2.1
19247	1.06659	2.1
19248	1.04291	0.239188
19249	1.54699	0.950286
19250	1.42307	1.13395
19251	1.5654	2.01172
19252	1.97906	2.1
19253	1.15354	0.05

19254	1.15505	0.05
19255	1.26455	0.05
19256	2.50042	0.05
19257	2.50042	0.05
19258	3.22585	0.05
19259	1.19258	0.05
19260	1.34562	0.05
19261	1.49515	0.05
19262	1.66295	0.05
19263	1.02395	2.1
19264	1.51067	2.1
19265	1.35821	2.1
19266	2.10671	2.1
19267	1.0305	1.93121
19268	1.07855	1.72385
19269	1.20761	2.1
19270	0.98488	2.1
19271	1.4162	2.1
19272	1.14825	2.1
19273	1.29415	2.1
19274	1.6067	2.1
19275	1.23851	0.05
19276	1.09651	0.05
19277	1.60636	2.1
19278	1.53521	2.1
19279	1.53521	2.1
19280	1.46279	2.1
19281	1.28044	0.05
19282	1.64147	0.05
19283	1.18422	0.05
19284	1.68208	0.05
19285	1.09726	2.1
19286	1.234 2.1	
19287	1.234 2.1	
19288	1.09483	2.1
19289	1.89318	2.1
19290	1.98448	2.1
19291	1.98448	2.1
19292	2.02612	2.1
19293	1.22026	1.10244
19294	1.23527	0.130287
19295	1.09044	0.114391
19296	0.993366	0.05
19297	1.22026	1.54103
19298	0.993368	1.46331
19299	2.61357	2.1
19300	2.48208	2.1
19301	2.48208	2.1
19302	2.62868	2.1
19303	1.85439	0.877953
19304	1.81776	0.232601
19305	1.81776	0.148523
19306	2.1587	0.258145
19307	1.8914	0.720123
19308	2.23961	0.516872
19309	1.49357	0.05
19310	2.23961	0.371284
19311	1.78014	0.231932

19312	1.67635	0.132355
19313	1.66219	0.0831484
19314	1.56036	0.0871443
19315	1.14721	0.05
19316	2.22495	0.0524046
19317	1.88509	0.05
19318	1.95249	0.05
19319	2.11959	0.05
19320	1.37427	0.05
19321	1.39379	0.05
19322	1.17614	0.05
19323	1.39379	2.1
19324	1.14397	0.05
19325	1.09651	2.1
19326	1.42843	2.1
19327	1.7069	2.1
19328	2.31293	2.1
19329	1.56839	2.1
19330	1.78252	0.241241
19331	1.78254	0.074985
19332	1.09142	0.501129
19333	0.99829	0.05
19334	1.09372	0.05
19335	1.88175	0.05
19336	0.898452	0.05
19337	1.06936	2.1
19338	1.4162	2.1
19339	1.17989	2.1
19340	1.47411	2.1
19341	1.25376	2.1
19342	1.00757	0.618441
19343	1.4185	0.05
19344	1.02011	0.05
19345	2.22495	0.05
19346	1.79542	0.05
19347	1.79542	0.05
19348	1.19352	0.05
19349	2.38566	2.1
19350	1.83255	1.69037
19351	1.62667	2.1
19352	1.17879	1.84326
19353	2.79168	2.1
19354	2.28162	2.1
19355	2.28162	2.1
19356	1.90905	2.1
19357	1.40117	2.1
19358	1.28268	2.1
19359	1.41096	2.1
19360	1.22978	2.1
19361	3.20034	2.1
19362	3.42052	2.1
19363	3.42052	2.1
19364	3.50562	2.1
19365	1.46279	2.1
19366	1.3864	2.1
19367	1.3864	2.1
19368	1.3201	2.1
19369	1.35561	0.05

19370	0.974077	0.05
19371	0.967673	0.3609
19372	1.22482	0.186226
19373	1.02305	0.0704383
19374	1.22482	0.136446
19375	0.999492	2.1
19376	1.14657	2.1
19377	1.14657	2.1
19378	1.31346	2.1
19379	1.84417	0.05
19380	1.31777	0.05
19381	1.19796	0.05
19382	0.691742	0.05
19383	1.16725	2.1
19384	1.9679	2.1
19385	1.08848	0.05
19386	1.15356	0.05
19387	1.58713	2.1
19388	1.58742	2.1
19389	1.58742	2.1
19390	1.49662	2.1
19391	1.32201	0.05
19392	1.32097	1.55486
19393	1.32097	0.11086
19394	1.31825	1.83957
19395	1.12028	2.1
19396	1.15852	2.1
19397	1.15852	2.1
19398	1.16239	2.08735
19399	1.07624	2.1
19400	1.30047	2.1
19401	1.44847	0.584526
19402	1.55292	0.515364
19403	1.21125	0.05
19404	1.15262	2.1
19405	1.05025	2.1
19406	1.55351	2.1
19407	1.5329	0.05
19408	2.26758	0.05
19409	1.94968	0.05
19410	1.65747	0.05
19411	1.42549	0.05
19412	1.57656	0.05
19413	2.11157	0.16567
19414	1.56531	0.335375
19415	1.69279	0.0773852
19416	1.06204	0.20916
19417	0.947863	0.707429
19418	1.19854	1.22827
19419	0.947862	0.276058
19420	1.31126	0.249242
19421	1.3425	1.42551
19422	1.29976	1.06562
19423	1.05129	0.05
19424	1.51026	0.05
19425	1.05129	0.05
19426	1.2951	0.05
19427	1.7155	2.1

19428	1.72196	2.1
19429	1.72196	2.1
19430	1.77867	2.1
19431	0.964755	2.1
19432	1.14142	2.1
19433	1.03766	2.1
19434	0.986723	2.1
19435	1.28072	0.394667
19436	1.10609	1.80353
19437	1.28072	0.05
19438	1.16776	2.1
19439	1.61687	2.1
19440	1.60965	2.1
19441	1.60965	2.1
19442	1.59654	2.1
19443	1.26512	2.1
19444	1.23964	2.1
19445	2.07997	2.1
19446	2.60048	2.1
19447	1.23732	2.1
19448	1.3372	2.1
19449	2.62253	2.1
19450	2.47056	2.1
19451	0.92782	0.05
19452	1.40335	0.05
19453	1.26301	0.05
19454	1.52668	0.05
19455	0.99818	0.05
19456	1.51165	0.05
19457	1.66213	0.05
19458	1.55246	0.05
19459	2.31255	2.1
19460	1.34366	2.1
19461	1.90577	2.1
19462	0.969739	2.1
19463	3.4245	2.1
19464	2.8415	2.1
19465	1.97559	2.1
19466	1.82362	2.1
19467	1.48162	2.1
19468	1.23204	2.1
19469	1.23204	2.1
19470	0.95947	2.1
19471	1.05267	2.1
19472	1.44091	1.16458
19473	1.05267	1.61776
19474	1.5795	2.1
19475	0.738711	2.1
19476	1.28549	2.1
19477	1.15694	2.1
19478	1.72061	2.1
19479	1.69858	2.1
19480	0.954563	2.1
19481	0.859087	2.1
19482	0.788886	2.1
19483	1.08732	0.05
19484	1.3357	0.05
19485	1.3357	0.05

19486	1.48676	0.05
19487	2.15079	2.1
19488	2.12414	2.1
19489	2.12414	2.1
19490	2.04664	2.1
19491	1.98859	2.1
19492	0.992159	2.1
19493	1.48064	2.1
19494	1.2163	2.1
19495	1.26337	2.1
19496	1.63159	2.1
19497	1.00366	0.05
19498	1.65452	0.05
19499	1.10408	2.1
19500	1.37704	2.1
19501	1.50224	2.1
19502	1.42866	2.1
19503	1.43939	2.1
19504	1.02784	2.1
19505	1.00763	0.05
19506	1.23746	0.05
19507	1.23747	0.05
19508	1.13642	0.05
19509	1.49906	0.05
19510	1.46506	0.05
19511	1.46506	0.05
19512	1.33061	0.05
19513	1.08166	0.05
19514	1.01052	0.05
19515	1.02836	0.05
19516	1.08166	0.05
19517	1.25334	0.05
19518	1.23749	0.05
19519	1.37501	0.05
19520	1.47354	0.05
19521	1.30167	2.1
19522	1.29897	2.1
19523	1.51042	2.1
19524	1.07684	2.1
19525	1.07684	2.1
19526	0.765121	2.1
19527	1.5535	1.68957
19528	1.40602	2.1
19529	1.40603	2.01004
19530	1.25669	2.1
19531	1.08276	2.1
19532	1.60948	2.1
19533	1.26929	2.1
19534	1.09847	2.1
19535	0.827082	0.05
19536	1.15841	0.05
19537	1.25063	0.05
19538	1.14397	0.05
19539	1.25063	0.0725454
19540	1.10476	0.05
19541	1.14872	0.05
19542	1.3659	0.05
19543	1.36589	0.05

19544	1.4677	0.05
19545	0.590354	2.1
19546	0.655202	2.1
19547	3.48926	2.1
19548	3.32709	2.1
19549	3.32709	2.1
19550	3.13938	2.1
19551	1.24458	0.091893
19552	1.29681	0.0783288
19553	1.40915	0.05
19554	1.17891	0.0893826
19555	1.33171	0.05
19556	1.37681	0.05
19557	1.37681	2.1
19558	1.4516	2.1
19559	2.68241	0.05
19560	1.61031	0.05
19561	1.6488	2.1
19562	1.6585	2.1
19563	1.65848	2.1
19564	1.67061	2.1
19565	1.15152	2.1
19566	1.32548	2.1
19567	1.43677	2.1
19568	1.10264	2.1
19569	1.01472	2.1
19570	0.966964	2.1
19571	2.867 2.1	
19572	2.94897	2.1
19573	1.65815	2.1
19574	1.4502	2.1
19575	1.4502	2.1
19576	1.26755	2.1
19577	1.37138	2.1
19578	1.45026	2.1
19579	1.45026	2.1
19580	1.53446	2.1
19581	0.973502	2.09931
19582	1.08542	2.1
19583	1.13387	0.05
19584	1.71031	0.05
19585	1.08911	2.1
19586	1.10748	2.1
19587	1.21823	2.1
19588	1.25005	2.1
19589	0.657014	2.1
19590	1.38139	2.1
19591	1.01485	2.1
19592	1.26045	2.1
19593	1.12468	2.1
19594	1.26045	2.1
19595	1.89278	0.10862
19596	1.06433	0.0668597
19597	1.87861	0.06623
19598	0.967544	0.05
19599	1.50319	2.1
19600	3.47245	2.07832
19601	2.2354	0.167914

19602	1.16701	0.0992949
19603	1.3592	2.1
19604	1.3305	0.115488
19605	1.3305	1.95136
19606	1.15008	0.161277
19607	1.12457	0.05
19608	1.40707	0.05
19609	1.01778	2.1
19610	1.35733	2.1
19611	1.01779	2.1
19612	1.12064	2.1
19613	1.30694	1.02666
19614	1.42716	1.30112
19615	1.42717	1.77668
19616	1.04321	1.26781
19617	1.19352	0.05
19618	1.32634	0.05
19619	1.14605	2.1
19620	1.0417	2.06448
19621	1.19676	0.05
19622	1.03804	0.05
19623	2.09455	0.05
19624	1.15338	0.05
19625	2.21119	2.1
19626	1.26097	2.1
19627	1.88394	0.05
19628	1.24106	0.05
19629	2.98143	2.1
19630	2.56248	1.62005
19631	1.88394	1.52203
19632	1.68703	0.05
19633	1.5846	2.1
19634	1.94799	2.1
19635	1.20091	2.1
19636	1.40393	2.1
19637	3.09031	2.1
19638	2.90243	2.1
19639	1.27227	2.1
19640	1.32592	2.1
19641	2.90247	2.1
19642	2.71057	2.1
19643	1.32594	2.1
19644	1.34817	2.1
19645	1.4092	2.1
19646	1.44859	2.1
19647	1.52008	2.1
19648	1.401	2.1
19649	1.40099	2.1
19650	1.28936	2.1
19651	1.56002	0.05
19652	1.08811	0.05
19653	1.46041	0.05
19654	1.56002	0.05
19655	1.50524	2.1
19656	1.21304	2.1
19657	1.21304	2.1
19658	0.947115	2.1
19659	1.60665	2.1

19660	1.85179	1.89332
19661	1.85178	1.98192
19662	2.06277	0.882171
19663	2.17534	2.1
19664	1.23007	2.1
19665	2.17533	2.1
19666	1.66635	2.1
19667	2.00869	2.1
19668	0.986724	2.1
19669	2.00475	2.1
19670	1.99394	2.1
19671	1.67244	0.05
19672	1.39604	0.05
19673	1.39604	0.05
19674	0.954046	0.05
19675	0.988863	2.1
19676	1.9864	2.1
19677	1.9864	2.1
19678	3.38645	2.1
19679	1.46932	0.05
19680	1.50847	0.05
19681	1.50848	0.05
19682	1.42705	0.05
19683	1.50108	2.1
19684	1.51621	0.201666
19685	1.94317	0.247092
19686	1.18382	0.05
19687	2.05091	0.05
19688	1.88593	0.05
19689	1.54299	0.05
19690	0.987265	0.05
19691	1.27766	2.1
19692	1.5178	2.1
19693	1.5178	2.1
19694	1.81518	2.1
19695	1.04626	0.906595
19696	1.31057	0.871228
19697	1.31057	0.737703
19698	1.22297	0.871153
19699	1.67844	2.1
19700	1.62862	2.1
19701	1.62112	2.1
19702	1.87598	2.1
19703	1.17844	2.1
19704	1.37063	2.1
19705	1.37063	2.1
19706	1.68723	2.1
19707	1.99943	0.05
19708	1.17631	0.05
19709	1.4263	0.621665
19710	1.31023	2.1
19711	1.31023	0.803126
19712	1.20871	2.1
19713	1.50356	2.1
19714	1.69311	2.1
19715	1.69599	2.1
19716	1.6801	2.1
19717	1.5843	0.05

19718	0.840233	0.05
19719	1.95358	0.05
19720	1.40216	0.05
19721	1.39078	2.1
19722	1.18948	2.02497
19723	1.01606	1.48673
19724	1.18948	1.88614
19725	1.63823	2.1
19726	1.67687	2.1
19727	1.67687	2.1
19728	1.52855	2.1
19729	1.11048	2.1
19730	1.25346	2.1
19731	2.42984	2.08268
19732	3.41731	0.784392
19733	1.2258	2.1
19734	1.2966	2.1
19735	1.2966	2.1
19736	1.37392	2.1
19737	1.41758	2.1
19738	1.35504	2.1
19739	1.35504	2.1
19740	1.30347	2.1
19741	1.50877	2.1
19742	1.11193	2.1
19743	1.40984	2.1
19744	1.54537	1.97837
19745	1.71638	2.1
19746	1.77795	1.33497
19747	2.07593	2.1
19748	1.93387	2.1
19749	1.9339	2.1
19750	1.75669	2.1
19751	1.80299	1.04172
19752	1.31251	0.917248
19753	1.31249	0.590069
19754	1.13411	0.647044
19755	1.06313	2.1
19756	1.04921	2.1
19757	1.55766	2.1
19758	1.53728	2.1
19759	1.53729	2.1
19760	1.51789	2.1
19761	0.993021	0.120122
19762	1.02478	0.100295
19763	1.03408	0.10946
19764	1.09056	0.103481
19765	0.697755	2.1
19766	0.632901	2.1
19767	1.34072	0.05
19768	1.80841	0.05
19769	1.80841	0.05
19770	1.17793	0.05
19771	1.75032	2.1
19772	1.13076	2.1
19773	2.11851	2.1
19774	1.73255	2.1
19775	1.73255	2.1

19776	1.11181	2.1
19777	1.43772	2.1
19778	1.67381	2.1
19779	1.52164	2.1
19780	1.60861	2.1
19781	1.30588	0.05
19782	1.75241	0.05
19783	1.33026	1.44814
19784	0.962705	0.457422
19785	0.99678	0.05
19786	1.30572	0.05
19787	1.41943	0.05
19788	1.2683	0.05
19789	1.14145	0.05
19790	1.01361	0.05
19791	1.29943	2.1
19792	1.3483	2.1
19793	1.34832	2.1
19794	1.83488	2.1
19795	1.71065	2.1
19796	1.66178	2.1
19797	1.39076	2.1
19798	1.37456	2.1
19799	1.66126	2.1
19800	1.562 2.1	
19801	1.37456	2.1
19802	1.36065	2.1
19803	1.04026	0.05
19804	1.51898	0.0552765
19805	1.67088	0.05
19806	1.9726	0.05
19807	1.74852	0.698975
19808	1.95512	0.940819
19809	1.16165	0.666584
19810	1.06584	0.141295
19811	1.39327	2.1
19812	1.41394	2.1
19813	1.41394	2.1
19814	1.45506	2.1
19815	1.39523	2.1
19816	1.21119	2.1
19817	1.09005	2.1
19818	0.921311	2.1
19819	1.58164	2.1
19820	1.77574	2.1
19821	1.73184	2.1
19822	1.59423	2.1
19823	1.8214	2.1
19824	3.10544	0.799823
19825	1.01243	2.1
19826	1.30896	0.533815
19827	2.16956	0.842565
19828	1.29608	0.181322
19829	1.38617	2.02105
19830	1.49536	1.56019
19831	2.35239	0.967681
19832	1.24613	1.90357
19833	1.94351	2.1

19834	2.05361	2.1
19835	2.05358	2.1
19836	2.38994	2.1
19837	1.13533	2.1
19838	1.08276	2.1
19839	1.55611	0.05
19840	1.33957	0.05
19841	1.33957	0.05
19842	1.69011	0.05
19843	1.5515	0.48925
19844	1.59106	0.398217
19845	1.02958	2.1
19846	1.12266	2.1
19847	1.12266	2.1
19848	1.24925	2.1
19849	1.80223	2.1
19850	1.20242	2.00476
19851	2.13665	0.0991789
19852	1.3924	0.0787579
19853	0.904894	1.31758
19854	1.82633	2.1
19855	1.94138	0.05
19856	1.34009	0.05
19857	1.15096	2.1
19858	0.994292	2.1
19859	1.10101	2.1
19860	1.15096	2.1
19861	1.50438	2.1
19862	1.67825	2.1
19863	1.67825	2.1
19864	1.91894	2.1
19865	2.04664	2.1
19866	1.92063	2.1
19867	1.72857	2.1
19868	1.89757	2.1
19869	1.61843	0.05
19870	1.49431	0.05
19871	2.33925	2.1
19872	1.80683	2.1
19873	1.64257	2.1
19874	1.50108	2.1
19875	2.82561	2.1
19876	2.97847	2.1
19877	2.97848	2.1
19878	3.20034	2.1
19879	0.66055	2.1
19880	1.95919	2.1
19881	1.07976	2.1
19882	1.30237	2.1
19883	1.99943	0.05
19884	1.31802	0.05
19885	1.53336	0.05
19886	1.31802	0.05
19887	1.27737	0.533953
19888	1.44848	0.413553
19889	2.35499	0.05
19890	1.30041	0.05
19891	2.35499	0.05

19892	1.65381	0.05
19893	0.996083	2.1
19894	1.28754	2.1
19895	0.996081	2.1
19896	1.44859	2.1
19897	1.24763	1.80178
19898	1.03697	2.09527
19899	0.951273	0.95475
19900	1.60018	0.706257
19901	2.0815	2.1
19902	1.20488	2.1
19903	2.95785	2.1
19904	2.52245	2.1
19905	1.68455	0.05
19906	1.12971	0.05
19907	1.1952	0.05
19908	2.0815	0.0769834
19909	1.3963	2.1
19910	1.4934	2.1
19911	1.47973	2.1
19912	1.25559	2.1
19913	1.1233	2.1
19914	1.15009	2.1
19915	1.15009	2.1
19916	0.977775	2.1
19917	1.16482	0.927069
19918	1.29313	0.18517
19919	1.29313	0.356491
19920	1.294	0.227283
19921	1.48693	0.052651
19922	1.47608	0.05
19923	1.36005	2.1
19924	1.47109	2.1
19925	1.47109	2.1
19926	1.3515	2.1
19927	2.48828	2.1
19928	2.32378	2.1
19929	2.32378	2.1
19930	2.1692	2.1
19931	1.03217	0.226167
19932	0.953521	0.351072
19933	1.4515	0.05
19934	3.72406	0.05
19935	0.963395	1.85563
19936	1.01346	0.42716
19937	1.62241	2.1
19938	1.62027	2.1
19939	1.62027	2.1
19940	1.61687	2.1
19941	1.2593	0.05
19942	0.981586	0.05
19943	0.721961	2.1
19944	0.888955	2.1
19945	1.22031	0.05
19946	1.27566	2.1
19947	2.50066	2.1
19948	1.19814	2.1
19949	1.42278	0.05

19950	2.02657	0.05
19951	1.0756	0.0923725
19952	1.10049	0.0866599
19953	2.26155	2.1
19954	2.15634	2.1
19955	2.15634	2.1
19956	2.01613	2.1
19957	1.94233	1.3491
19958	1.18127	1.71153
19959	1.36865	1.81024
19960	1.75755	1.52264
19961	2.01613	2.1
19962	1.78958	2.1
19963	1.97995	2.1
19964	1.79198	2.1
19965	1.20841	2.1
19966	1.6519	2.1
19967	1.80207	2.1
19968	1.66774	2.1
19969	1.17935	0.05
19970	1.88071	0.05
19971	1.58723	0.05
19972	1.7361	0.05
19973	0.794165	2.1
19974	1.21327	2.1
19975	1.21327	2.1
19976	1.34268	2.1
19977	1.34497	0.05
19978	0.793303	0.05
19979	1.12323	0.05
19980	1.34496	0.05
19981	0.928226	0.05
19982	1.89804	0.05
19983	2.60141	0.05
19984	2.60142	2.1
19985	2.60772	0.05
19986	0.99747	2.1
19987	1.27096	0.291792
19988	1.27096	0.0741933
19989	1.03472	0.256359
19990	1.09962	0.0620406
19991	1.21148	2.1
19992	1.21148	0.996232
19993	1.35025	2.1
19994	1.09795	2.1
19995	1.00775	2.1
19996	1.07162	2.1
19997	1.60665	2.1
19998	1.52984	2.1
19999	1.61179	2.1
20000	1.70057	2.1
20001	1.76063	2.1
20002	1.14547	0.05
20003	0.964782	0.05
20004	1.17208	0.05
20005	0.964769	0.05
20006	1.04268	2.1
20007	1.57459	2.1

20008	1.57459	2.1
20009	1.32449	2.1
20010	2.07527	0.883424
20011	1.00013	0.266615
20012	0.813711	0.05
20013	1.05677	2.1
20014	1.30664	1.20605
20015	1.96011	2.08904
20016	1.7151	1.091
20017	1.61309	1.0279
20018	1.18954	0.05
20019	1.05447	2.1
20020	1.05446	0.05
20021	1.11787	0.05
20022	1.44437	2.1
20023	1.84739	2.1
20024	1.84739	2.1
20025	1.06203	2.1
20026	1.78391	2.1
20027	1.89267	2.1
20028	1.9212	2.1
20029	2.02997	2.1
20030	1.32051	0.05
20031	1.47169	0.05
20032	1.20691	0.05
20033	1.72271	0.05
20034	1.2858	0.0668216
20035	1.31011	1.18117
20036	1.31011	0.05
20037	1.34124	1.21947
20038	1.294 0.519035	
20039	1.03079	0.298655
20040	1.83192	2.08063
20041	1.81979	2.1
20042	1.81979	2.1
20043	1.57829	2.1
20044	1.35435	1.64823
20045	1.19023	2.04266
20046	0.991115	2.1
20047	1.27725	2.1
20048	1.38204	2.1
20049	1.48013	2.1
20050	0.983493	2.1
20051	2.26155	2.1
20052	1.02311	0.211084
20053	1.55485	0.125141
20054	1.71033	0.110749
20055	2.07319	0.0875318
20056	2.29074	0.0976998
20057	1.50822	0.30036
20058	2.08147	2.1
20059	1.1585	2.1
20060	3.74092	0.560258
20061	2.40799	2.1
20062	2.05349	2.1
20063	1.92656	2.1
20064	1.09634	0.05
20065	1.45502	0.05

20066	1.17261	0.0706383
20067	1.29383	0.05
20068	1.29383	0.05
20069	1.47689	0.05
20070	1.85717	0.05
20071	1.53798	0.05
20072	1.9493	2.1
20073	2.0371	1.86007
20074	1.8519	2.1
20075	1.92119	1.06514
20076	1.00764	0.05
20077	1.0302	0.05
20078	1.0302	0.05
20079	0.90047	0.05
20080	1.49155	0.05
20081	2.26403	0.05
20082	2.06158	0.05
20083	1.09506	0.05
20084	1.9212	1.07695
20085	1.99586	0.64632
20086	1.99586	0.613686
20087	2.07527	1.08126
20088	1.20478	2.1
20089	1.09662	2.1
20090	1.55961	2.1
20091	1.03472	2.1
20092	1.54983	2.1
20093	2.02953	2.1
20094	1.84502	2.1
20095	2.1221	2.1
20096	1.02969	2.06581
20097	2.23523	2.1
20098	1.2377	0.05
20099	1.03818	0.0516272
20100	1.978	2.1
20101	1.78159	2.1
20102	1.45762	2.1
20103	1.34234	2.1
20104	2.01624	2.1
20105	2.52072	2.1
20106	1.23013	2.1
20107	2.5593	2.1
20108	3.06464	1.23401
20109	2.63697	0.537524
20110	2.63697	1.01843
20111	1.87111	0.213506
20112	1.04574	0.05
20113	2.13428	0.05
20114	1.61715	2.1
20115	1.44137	2.1
20116	1.44867	2.1
20117	1.18283	2.1
20118	1.50584	0.05
20119	1.45466	0.05
20120	1.4959	0.05
20121	1.93489	0.05
20122	2.60772	2.1
20123	2.68868	2.1

20124	2.68868	2.1
20125	2.82561	2.1
20126	1.49654	2.1
20127	1.38672	2.1
20128	1.36813	2.1
20129	1.98396	2.1
20130	1.93852	1.60736
20131	2.14237	0.880917
20132	1.44421	0.05
20133	1.21749	0.05
20134	1.09572	0.05
20135	1.11081	0.05
20136	2.32363	2.1
20137	1.24375	2.1
20138	3.79242	2.1
20139	3.46787	2.1
20140	1.19854	1.50242
20141	1.35487	1.47293
20142	1.35486	1.77216
20143	1.38616	1.71662
20144	1.67265	0.05
20145	0.909487	0.05
20146	2.53562	2.1
20147	1.09564	2.1
20148	2.9068	2.1
20149	3.57307	0.102043
20150	2.50369	1.57021
20151	1.47348	2.1
20152	1.24925	2.1
20153	1.3991	2.1
20154	1.3991	2.1
20155	1.52984	2.1
20156	1.60428	2.1
20157	1.50097	2.1
20158	1.50097	2.1
20159	1.40239	2.1
20160	2.19621	2.1
20161	2.35003	2.1
20162	1.57932	0.05
20163	1.61194	0.05
20164	2.0815	0.05
20165	1.18539	0.05
20166	1.5795	0.79203
20167	1.53064	1.09599
20168	1.89227	0.05
20169	3.01037	0.05
20170	2.08149	2.1
20171	2.06678	2.1
20172	1.89226	0.05
20173	3.47888	0.05
20174	1.89226	0.05
20175	3.65842	0.05
20176	3.88546	0.05
20177	1.89226	0.05
20178	1.11239	2.1
20179	1.29133	0.05
20180	0.889309	0.05
20181	0.881934	0.05

20182	1.88406	2.1
20183	0.636946	2.1
20184	0.636945	2.09915
20185	1.28073	2.1
20186	1.28075	2.1
20187	0.942007	2.1
20188	1.68436	2.1
20189	1.4677	0.05
20190	0.851542	0.05
20191	1.39063	0.05
20192	0.851539	0.05
20193	1.11597	0.05
20194	1.35403	0.05
20195	0.959473	2.1
20196	1.07976	2.1
20197	0.946129	1.61734
20198	0.946129	0.05
20199	1.38464	0.140874
20200	1.36991	0.05
20201	0.946131	0.05
20202	0.94613	0.952465
20203	1.34493	0.0970882
20204	1.36991	0.0662262
20205	1.36991	0.05
20206	0.946131	0.05
20207	0.78392	0.578924
20208	0.80282	0.460337
20209	1.10351	0.521908
20210	0.80846	0.888376
20211	0.838026	0.621266
20212	1.08558	0.53303
20213	0.718203	0.970174
20214	0.501866	0.2734
20215	0.85426	0.712002
20216	1.24249	0.05
20217	1.37052	0.05
20218	0.851541	0.05
20219	1.0092	0.05
20220	0.851554	0.05
20221	1.49826	0.05
20222	1.13727	0.05
20223	0.778149	0.05
20224	1.20257	0.0553669
20225	0.946133	0.05
20226	0.778149	0.05
20227	0.77815	0.321728
20228	0.946132	2.1
20229	1.17602	2.1
20230	0.946132	2.1
20231	1.31257	2.1
20232	1.17602	2.1
20233	1.35821	0.05
20234	1.19993	0.05
20235	0.946131	0.05
20236	0.946131	0.05
20237	1.08848	0.0503224
20238	1.00509	2.1
20239	1.19629	2.1

20240	0.867593	2.1
20241	0.757921	2.1
20242	0.729116	0.05
20243	0.89162	0.05
20244	1.16811	0.05
20245	1.3676	2.1
20246	1.2638	0.530448
20247	0.946132	2.1
20248	0.946132	2.1
20249	1.2638	2.1
20250	1.00185	2.1
20251	1.60948	2.1
20252	0.602075	2.1
20253	0.942611	2.1
20254	0.946132	2.1
20255	0.942611	2.1
20256	1.11822	2.1
20257	1.21558	0.05
20258	0.607788	0.05
20259	0.827029	0.05
20260	1.00691	0.11697
20261	0.607789	0.05
20262	0.607775	0.0797814
20263	1.23248	0.583491
20264	0.902653	1.08595
20265	0.639143	1.26825
20266	0.851545	0.465853
20267	0.605153	0.658112
20268	1.06826	2.1
20269	1.29365	2.1
20270	1.41232	2.1
20271	0.946131	2.1
20272	1.20854	2.1
20273	0.946131	2.1
20274	0.909005	0.05
20275	1.16672	0.05
20276	0.810959	0.05
20277	0.946131	0.05
20278	1.40104	0.05
20279	0.946131	0.05
20280	1.35403	0.05
20281	1.40104	0.05
20282	1.92535	0.05
20283	2.14534	0.05
20284	1.77532	0.05
20285	0.851541	0.05
20286	1.05585	0.05
20287	1.27098	0.05
20288	0.870925	0.05
20289	1.15547	0.05
20290	1.47583	0.05
20291	1.35007	0.05
20292	0.740095	0.05
20293	0.644912	0.05
20294	0.819489	0.05
20295	0.983028	0.05
20296	0.861849	0.05
20297	1.40511	0.05

20298	0.86182	0.05
20299	0.9705	0.05
20300	1.40511	0.05
20301	1.15539	2.1
20302	0.946119	2.1
20303	0.736382	2.1
20304	0.736384	2.1
20305	1.41561	2.1
20306	0.785651	2.1
20307	1.34479	2.1
20308	1.38885	2.1
20309	0.94613	2.1
20310	1.29461	2.1
20311	0.94613	2.1
20312	0.776179	2.1
20313	0.858611	2.1
20314	0.946128	2.1
20315	1.04073	2.1
20316	1.16399	2.1
20317	0.990019	2.1
20318	1.15564	2.1
20319	1.28956	0.05
20320	1.35145	0.05
20321	1.09662	2.1
20322	1.8155	0.05
20323	0.811459	0.05
20324	0.731982	0.05
20325	0.900554	0.05
20326	0.946127	0.24308
20327	0.817066	0.250243
20328	0.817064	0.274624
20329	1.41798	0.29577
20330	0.871606	0.288731
20331	1.30265	0.05
20332	0.849402	1.85785
20333	0.868582	0.538305
20334	0.909137	0.869147
20335	0.807016	0.99809
20336	0.83393	0.0784066
20337	0.9034	0.212466
20338	1.53515	0.190508
20339	1.17665	0.05
20340	0.946132	0.0605376
20341	0.946129	0.05
20342	1.35977	0.05
20343	1.34254	0.05
20344	1.14241	0.05
20345	0.946132	0.05
20346	0.946132	0.05
20347	1.27376	0.05
20348	1.35978	0.05
20349	1.01976	0.05
20350	1.23498	0.186879
20351	1.35002	0.627572
20352	1.11683	1.27849
20353	0.88359	1.54974
20354	0.751374	1.45347
20355	0.791714	1.42544

20356	1.6067	2.1
20357	0.923782	2.1
20358	1.44689	2.1
20359	0.94613	2.1
20360	0.946131	2.1
20361	1.5261	2.1
20362	1.26247	2.1
20363	1.80085	0.05
20364	1.7693	0.05
20365	0.900422	0.05
20366	1.31357	0.05
20367	0.94613	0.05
20368	0.900424	0.05
20369	0.900397	0.05
20370	1.39448	0.05
20371	1.36769	0.05
20372	0.713524	0.05
20373	0.713554	0.05
20374	0.946124	0.05
20375	1.24979	0.05
20376	1.04765	0.05
20377	0.70054	0.05
20378	0.776991	0.05
20379	0.738905	0.05
20380	1.32605	0.05
20381	0.784957	0.05
20382	0.803955	0.05
20383	0.819486	0.05
20384	1.79306	0.05
20385	1.18275	0.05
20386	1.25626	0.05
20387	0.936806	0.05
20388	0.653124	0.05
20389	0.946132	0.05
20390	0.653151	0.05
20391	1.10748	0.05
20392	1.95244	2.1
20393	1.40967	2.01621
20394	1.27916	1.78141
20395	0.946132	0.05
20396	0.946132	0.950316
20397	1.30555	0.830753
20398	1.38651	1.56642
20399	0.946132	0.380003
20400	1.23571	0.0869656
20401	1.21261	0.646375
20402	1.34225	0.423998
20403	0.946132	1.58519
20404	0.734059	0.05
20405	0.734084	0.598253
20406	0.78438	0.05
20407	1.13748	0.0747621
20408	0.946131	0.325717
20409	0.78438	0.05
20410	0.784392	0.118525
20411	0.626495	2.1
20412	0.694635	2.1
20413	0.946125	2.1

20414	0.694632	2.1
20415	1.38521	2.1
20416	0.795469	2.1
20417	0.850877	2.1
20418	1.37098	2.1
20419	0.946131	2.1
20420	0.79547	2.1
20421	0.946132	0.815478
20422	1.30448	1.85787
20423	0.628361	0.37219
20424	0.686964	0.652947
20425	0.493261	0.653008
20426	1.90912	2.1
20427	0.946127	1.74074
20428	1.36604	0.311353
20429	0.671687	0.851794
20430	0.946128	1.14298
20431	0.671699	0.506809
20432	1.04973	2.1
20433	0.982858	0.05
20434	0.974081	0.05
20435	1.69993	0.05
20436	1.00763	0.463935
20437	0.755566	2.1
20438	0.946129	2.1
20439	1.04184	2.1
20440	1.13509	2.1
20441	0.755566	2.1
20442	1.114 2.1	
20443	0.810946	2.1
20444	0.786259	2.1
20445	1.43455	2.1
20446	1.05071	2.1
20447	0.746095	2.1
20448	0.82737	2.1
20449	0.946126	2.1
20450	1.04071	2.1
20451	1.0852	2.1
20452	1.59019	0.05
20453	0.621972	0.05
20454	1.14475	0.05
20455	0.691062	0.05
20456	1.20057	0.05
20457	0.846868	0.05
20458	1.26062	0.05
20459	0.795093	0.05
20460	1.20056	0.05
20461	0.795065	0.05
20462	1.18203	0.05
20463	1.20057	0.05
20464	0.991869	0.05
20465	0.841088	2.1
20466	0.946131	2.1
20467	0.869097	2.1
20468	0.946131	2.1
20469	0.841089	2.1
20470	1.77867	2.1
20471	1.99296	0.05

20472	1.91703	2.1
20473	0.651334	0.05
20474	1.31467	0.05
20475	0.651332	0.05
20476	0.651332	0.05
20477	1.03079	0.05
20478	0.808519	0.05
20479	1.41417	0.05
20480	1.03165	0.05
20481	1.43577	2.1
20482	1.39053	2.1
20483	1.07006	2.1
20484	0.946132	2.1
20485	0.946133	2.1
20486	1.07006	2.1
20487	1.12717	2.1
20488	0.994293	2.1
20489	0.946132	2.1
20490	1.31912	2.1
20491	0.946131	2.1
20492	1.40571	2.1
20493	1.31912	2.1
20494	1.12292	0.05
20495	1.07755	0.0717826
20496	0.851539	0.0592134
20497	0.946131	0.05
20498	0.929834	0.05
20499	1.83919	0.050641
20500	1.04713	0.05
20501	0.929726	0.05
20502	1.50985	0.05
20503	0.946127	0.05
20504	1.0227	0.05
20505	1.2582	0.05
20506	1.1391	0.376375
20507	0.780831	0.575245
20508	0.78086	0.354693
20509	0.946133	0.573086
20510	0.780831	0.313296
20511	1.37762	0.357671
20512	0.946116	2.1
20513	1.22882	2.1
20514	1.40015	2.1
20515	1.40017	2.1
20516	0.946105	2.1
20517	0.553567	0.05
20518	1.19263	0.05
20519	0.946132	0.05
20520	0.553594	0.05
20521	1.19263	0.05
20522	1.18237	0.05
20523	0.946133	0.05
20524	0.978467	0.05
20525	0.56262	0.05
20526	0.946132	0.05
20527	0.562633	0.05
20528	0.900175	0.05
20529	1.04627	0.435495

20530	0.549928	0.446177
20531	1.17765	0.537028
20532	1.11479	1.06968
20533	0.398261	0.716177
20534	0.927608	1.27528
20535	1.38467	0.529914
20536	1.04824	0.198013
20537	1.33893	0.56308
20538	1.55775	0.806407
20539	0.816895	1.32642
20540	1.96057	1.30969
20541	1.35798	2.1
20542	1.29262	2.1
20543	0.94613	2.1
20544	0.94613	2.1
20545	1.29261	2.1
20546	1.18067	2.1
20547	0.946131	2.1
20548	0.946132	2.1
20549	1.27809	2.1
20550	1.35799	2.1
20551	1.03933	2.1
20552	1.49438	2.1
20553	0.946132	2.1
20554	1.13537	2.1
20555	1.41666	2.1
20556	1.74237	2.1
20557	0.946132	2.1
20558	1.04291	0.05
20559	1.63802	2.1
20560	1.24714	2.02008
20561	0.842013	2.1
20562	0.810959	2.1
20563	0.767923	0.05
20564	1.19583	0.05
20565	0.767921	0.05
20566	0.767921	0.05
20567	0.961348	0.0568981
20568	2.08687	2.07463
20569	0.739729	0.181955
20570	1.04342	0.090873
20571	0.739757	0.180399
20572	0.946129	0.203267
20573	1.04341	0.269629
20574	1.38683	0.05
20575	1.34153	0.0523351
20576	0.946129	0.05
20577	0.946131	0.05
20578	1.02028	0.05
20579	1.47606	0.05
20580	1.20739	0.05
20581	0.957684	0.05
20582	0.946116	0.05
20583	0.851528	0.05
20584	0.957685	0.05
20585	1.04504	2.1
20586	0.902938	2.1
20587	0.946132	2.1

20588	1.07566	2.1
20589	1.4558	2.1
20590	1.93286	2.1
20591	1.27241	2.1
20592	0.828671	2.1
20593	0.810933	2.1
20594	1.31335	0.05
20595	1.40305	0.05
20596	0.912698	0.05
20597	0.810946	0.05
20598	1.11608	0.05
20599	1.26409	0.0533611
20600	0.946132	0.0692514
20601	1.27376	0.109486
20602	0.946132	0.079631
20603	1.03333	0.12576
20604	1.27376	0.099551
20605	0.94613	0.05
20606	0.812384	0.05
20607	0.687744	0.05
20608	0.946132	0.05
20609	0.687758	0.05
20610	1.09667	0.05
20611	1.56977	2.1
20612	0.851544	2.1
20613	1.01136	2.1
20614	0.851481	2.1
20615	0.946131	2.1
20616	0.705409	2.1
20617	1.32449	0.05
20618	1.13508	0.05
20619	0.94613	0.05
20620	0.94613	0.05
20621	0.965822	0.05
20622	1.95301	0.05
20623	0.851538	0.05
20624	1.29262	0.05
20625	0.95417	0.05
20626	0.851541	0.05
20627	0.94613	0.05
20628	1.02727	0.437106
20629	1.37675	1.41671
20630	0.955646	0.266252
20631	0.95114	0.8444
20632	0.851528	0.876552
20633	1.76513	1.40707
20634	0.87155	2.1
20635	0.94613	2.1
20636	0.871519	2.1
20637	0.966631	2.1
20638	1.37791	2.1
20639	1.19122	0.05
20640	0.818971	0.483182
20641	0.628799	0.05
20642	0.861027	0.149335
20643	0.800034	0.05
20644	0.91846	0.05
20645	0.765645	0.05

20646	1.13709	0.201276
20647	1.29054	0.514274
20648	0.755336	0.758181
20649	0.755334	0.450529
20650	0.946133	0.793287
20651	0.755336	0.273625
20652	1.14444	0.456098
20653	0.872823	0.652415
20654	0.533645	0.355629
20655	0.946132	0.414197
20656	0.533645	0.0888892
20657	1.02311	0.183531
20658	1.00278	2.1
20659	1.1016	0.05
20660	0.629615	0.05
20661	1.12628	0.05
20662	0.946131	0.05
20663	0.629615	0.05
20664	0.629641	0.05
20665	1.67046	1.80559
20666	1.81667	2.1
20667	1.7145	2.1
20668	0.94613	2.1
20669	1.29322	2.1
20670	1.00365	2.1
20671	0.946131	2.1
20672	1.29322	2.1
20673	1.27581	2.1
20674	1.33916	2.1
20675	1.32784	2.1
20676	1.74787	2.1
20677	2.28084	2.1
20678	2.98092	2.1
20679	0.946132	0.377007
20680	1.2435	2.1
20681	0.946132	2.1
20682	1.4099	1.41829
20683	1.2435	2.1
20684	1.26438	2.1
20685	0.745287	2.1
20686	0.696827	2.1
20687	0.696781	2.1
20688	1.41195	2.1
20689	0.946131	2.1
20690	1.8758	1.07803
20691	0.832979	0.110839
20692	0.75054	0.05
20693	0.810958	0.05
20694	0.922889	0.642233
20695	0.694844	0.0951716
20696	0.694814	0.299077
20697	0.946131	0.162682
20698	0.694844	0.230723
20699	1.02369	2.1
20700	1.4468	0.05
20701	0.946125	0.05
20702	0.93487	0.05
20703	1.23608	0.0972005

20704	1.34257	0.05
20705	0.851525	0.05
20706	1.03298	0.05
20707	1.22418	1.3094
20708	0.946131	2.1
20709	1.41833	1.09382
20710	1.23452	0.697783
20711	0.946131	0.0839299
20712	1.41833	0.261289
20713	1.13077	0.05
20714	0.942874	2.02217
20715	0.946132	1.67786
20716	0.504366	1.97244
20717	0.593647	2.1
20718	0.898829	2.01487
20719	1.27921	2.1
20720	1.12503	2.1
20721	1.22539	2.1
20722	1.10332	1.44539
20723	0.851541	2.1
20724	0.94613	2.1
20725	0.945109	2.1
20726	0.607962	2.1
20727	0.946133	2.1
20728	0.607961	2.1
20729	0.661956	2.1
20730	1.35048	2.1
20731	1.66018	2.1
20732	0.956467	2.1
20733	0.851542	2.1
20734	0.890734	2.1
20735	0.802455	2.1
20736	1.35943	2.1
20737	1.04798	2.1
20738	1.26426	0.0786486
20739	1.3689	0.0509299
20740	0.946133	0.0663854
20741	0.946133	0.0906579
20742	1.09729	0.0789269
20743	0.765259	2.1
20744	0.810945	2.1
20745	0.810957	2.1
20746	1.10284	2.09333
20747	1.09368	2.1
20748	0.772601	2.1
20749	0.788011	2.1
20750	0.810934	2.1
20751	0.694357	0.785835
20752	1.22641	0.95434
20753	0.946132	2.03716
20754	0.946132	1.25294
20755	0.946133	0.690675
20756	1.42754	1.89499
20757	0.776414	0.329097
20758	0.806525	1.60539
20759	0.575339	0.408236
20760	0.97154	0.263311
20761	0.8217	1.273

20762	0.838039	1.72389
20763	0.94613	0.779356
20764	0.946132	0.291027
20765	1.39575	0.0658101
20766	1.39575	0.166264
20767	1.12818	0.115715
20768	0.946132	1.35521
20769	0.946133	0.660827
20770	1.06879	0.0986434
20771	1.06879	0.426276
20772	0.970358	0.305191
20773	1.2474	0.05
20774	1.01919	0.05
20775	0.94613	0.05
20776	1.28952	0.05
20777	1.00809	0.05
20778	0.849344	0.05
20779	0.640304	0.05
20780	1.35065	0.05
20781	0.946132	0.05
20782	0.640333	0.05
20783	0.946132	0.05
20784	0.685489	2.1
20785	0.94613	2.1
20786	0.68546	2.1
20787	0.874264	2.1
20788	1.27676	2.1
20789	0.946129	0.05
20790	1.09024	0.05
20791	0.714563	0.05
20792	1.12335	0.05
20793	1.07931	0.05
20794	1.17799	0.05
20795	1.8795	0.05
20796	2.15229	0.05
20797	1.13859	0.05
20798	0.946132	0.442868
20799	1.11374	0.0621167
20800	1.1386	0.070582
20801	0.615713	0.100535
20802	1.19322	0.05
20803	1.3377	0.05
20804	0.670801	0.05
20805	0.94613	0.164786
20806	0.66617	0.055282
20807	1.36173	0.0514596
20808	0.980473	0.05
20809	1.39132	0.05
20810	0.864653	0.05
20811	1.23837	0.05
20812	0.810959	0.05
20813	1.1483	0.05
20814	1.00381	1.84432
20815	0.946128	1.902
20816	1.39696	0.920663
20817	0.946131	0.685253
20818	0.946131	1.0495
20819	1.38613	1.19197

20820	0.973501	1.95972
20821	0.946127	0.915457
20822	1.35968	0.84863
20823	0.946131	0.35908
20824	0.946129	1.34196
20825	1.39696	1.26129
20826	1.12088	2.1
20827	1.41122	2.1
20828	1.14464	0.109792
20829	0.946131	2.1
20830	0.946131	1.95372
20831	1.14464	2.1
20832	1.33266	1.85282
20833	1.07341	0.570452
20834	0.827287	1.88699
20835	0.946132	1.48497
20836	0.827287	2.1
20837	1.15644	1.45537
20838	1.27558	0.133076
20839	1.15067	0.0898624
20840	0.946131	2.1
20841	1.29143	0.05
20842	1.36281	2.1
20843	1.36281	0.373526
20844	0.946131	0.253283
20845	1.40863	2.1
20846	1.20143	0.05
20847	1.19755	0.05
20848	0.566153	0.05
20849	0.946131	0.05
20850	1.07979	0.05
20851	1.00463	0.05
20852	0.566153	0.05
20853	0.768212	2.1
20854	1.32461	2.1
20855	1.13268	2.1
20856	1.13268	2.1
20857	0.946133	2.1
20858	1.53642	2.1
20859	1.5791	2.1
20860	0.726635	2.1
20861	1.2334	2.1
20862	1.29198	2.1
20863	1.29198	1.44696
20864	0.946131	0.894782
20865	0.956585	2.1
20866	1.25294	2.1
20867	0.946133	2.1
20868	1.35287	2.1
20869	1.23877	2.1
20870	0.946131	2.1
20871	1.35287	2.1
20872	1.01843	2.1
20873	3.77569	2.1
20874	0.86707	2.1
20875	0.867066	2.1
20876	1.38639	2.1
20877	1.38639	2.1

20878	0.896168	2.1
20879	0.974716	0.05
20880	1.09411	0.05
20881	0.663171	0.05
20882	0.663171	0.05
20883	0.946132	0.05
20884	0.66317	0.05
20885	1.47104	0.05
20886	1.13629	0.05
20887	0.772342	0.05
20888	0.772312	0.05
20889	0.94613	0.05
20890	0.77234	0.05
20891	1.43154	2.1
20892	1.37704	2.1
20893	1.40025	2.1
20894	1.3063	2.1
20895	0.946132	2.1
20896	1.04075	2.1
20897	0.946131	2.1
20898	0.946118	2.08127
20899	0.750011	0.05
20900	1.0971	0.219391
20901	0.889667	0.0779857
20902	0.709598	0.208429
20903	2.39737	0.351066
20904	1.19974	0.05
20905	0.94613	0.05
20906	1.25809	0.05
20907	1.19974	0.05
20908	0.73005	0.05
20909	1.50764	0.05
20910	1.25809	0.05
20911	0.946133	0.05
20912	1.10357	0.05
20913	1.25809	0.05
20914	0.628177	0.05
20915	1.3543	0.05
20916	0.793711	0.05
20917	1.18455	0.05
20918	1.03966	0.05
20919	0.946131	0.05
20920	0.767453	0.05
20921	0.612292	0.05
20922	0.946131	0.05
20923	1.24128	0.05
20924	1.08761	0.05
20925	0.762059	0.05
20926	1.005 2.1	
20927	1.17054	2.1
20928	1.12665	2.1
20929	0.750368	2.1
20930	0.851542	2.1
20931	0.975436	2.1
20932	0.975442	2.1
20933	0.675367	2.1
20934	1.51973	0.05
20935	0.94613	0.05

20936	1.38954	0.05
20937	0.946131	0.05
20938	1.07912	0.05
20939	1.38954	0.05
20940	2.0219	2.1
20941	1.18347	0.05
20942	0.946132	0.164583
20943	0.94613	0.05
20944	1.00202	0.05
20945	1.00201	0.05
20946	0.946132	0.05
20947	0.946132	0.378613
20948	1.37981	0.115959
20949	1.26874	0.0524846
20950	1.26874	0.0517202
20951	0.946132	0.0649921
20952	2.8687	0.05
20953	1.42884	2.1
20954	1.05188	2.1
20955	0.625307	2.1
20956	0.810956	2.1
20957	0.526656	2.1
20958	0.526684	2.1
20959	0.724122	2.1
20960	0.946132	2.1
20961	0.526684	2.1
20962	1.05337	2.1
20963	1.0593	2.1
20964	1.47981	2.1
20965	1.74431	2.04769
20966	0.885612	2.1
20967	0.810946	0.843652
20968	0.845185	1.48375
20969	1.38622	0.203572
20970	0.723194	0.109643
20971	0.554302	0.225944
20972	0.709597	0.100875
20973	0.909507	0.119143
20974	0.912754	0.0628335
20975	1.15174	0.05
20976	0.946131	0.0719722
20977	0.912753	0.0724818
20978	1.15174	0.0659395
20979	1.02011	0.05
20980	0.792754	2.06658
20981	0.946129	2.1
20982	0.792754	2.00541
20983	1.27575	1.80373
20984	0.94613	1.93075
20985	1.60132	2.08151
20986	0.922455	2.1
20987	1.18713	1.07075
20988	0.926957	1.65832
20989	1.66525	1.61203
20990	0.946132	2.1
20991	0.851541	2.1
20992	0.961191	2.1
20993	1.27534	2.1

20994	0.851554	2.1
20995	1.0499	2.1
20996	1.01776	0.05
20997	0.97622	0.05
20998	0.810934	0.05
20999	2.6802	0.05
21000	1.31485	0.05
21001	1.09699	0.05
21002	0.681649	0.05
21003	0.790369	0.05
21004	0.946131	0.05
21005	0.838026	1.18614
21006	0.567833	0.0893868
21007	0.891492	0.598793
21008	0.986984	0.05
21009	0.946132	0.716911
21010	1.19028	0.0954724
21011	0.986984	0.0703181
21012	0.595623	0.13846
21013	0.946131	2.1
21014	1.36502	2.1
21015	1.08109	2.1
21016	0.946129	2.1
21017	1.36503	2.1
21018	0.773897	2.1
21019	0.946133	1.19618
21020	0.613447	2.1
21021	0.613449	2.09909
21022	0.946131	2.1
21023	1.11163	2.1
21024	0.966114	2.1
21025	0.735699	2.1
21026	0.662147	2.1
21027	1.04072	2.1
21028	0.946131	2.1
21029	0.862978	2.1
21030	1.48839	0.474313
21031	0.936141	0.238726
21032	0.946131	1.44023
21033	0.936142	2.1
21034	1.40595	2.1
21035	0.946131	1.91251
21036	1.05297	0.05
21037	3.09035	2.1
21038	1.19952	2.1
21039	0.907198	2.1
21040	0.849144	2.1
21041	0.612469	0.0678293
21042	0.551237	0.182417
21043	0.985256	0.289461
21044	0.551209	0.29339
21045	0.85153	0.78602
21046	0.624712	2.1
21047	0.900018	2.1
21048	0.851542	2.1
21049	0.864787	2.1
21050	1.27669	2.1
21051	0.946711	0.628134

21052	0.810947	0.912374
21053	0.841547	0.688502
21054	0.764114	0.705614
21055	1.26692	1.73522
21056	0.946132	1.02988
21057	1.37675	1.56117
21058	1.26692	0.71074
21059	1.37545	1.27959
21060	1.49637	0.05
21061	0.709598	0.05
21062	0.751252	0.05
21063	1.36811	0.05
21064	0.635625	0.05
21065	1.31713	0.05
21066	1.18977	2.1
21067	0.893377	1.55357
21068	0.707344	2.1
21069	0.946131	2.1
21070	1.20221	2.1
21071	1.20221	2.1
21072	0.707345	2.1
21073	1.26786	2.1
21074	0.988048	2.1
21075	0.751293	2.1
21076	0.632008	2.1
21077	1.51067	2.1
21078	1.13534	2.1
21079	0.946133	2.1
21080	0.946131	2.1
21081	1.25716	2.1
21082	1.3199	2.1
21083	1.09616	2.1
21084	1.36677	1.10085
21085	1.34752	1.86399
21086	0.838041	0.629023
21087	1.67023	2.1
21088	1.02247	0.0695471
21089	1.03079	0.05
21090	0.928982	0.05
21091	1.13635	0.05
21092	0.946132	0.05
21093	0.92898	0.05
21094	1.13635	0.05
21095	1.1643	0.05
21096	0.851541	0.05
21097	1.39939	0.05
21098	0.946129	0.05
21099	1.07775	0.05
21100	1.14503	0.05
21101	1.78573	0.05
21102	1.29827	0.05
21103	0.872373	0.05
21104	1.01162	0.05
21105	0.83804	0.05
21106	1.48851	0.05
21107	1.47385	0.05
21108	0.843858	0.05
21109	0.938626	0.05

21110	0.89567	0.05
21111	1.59457	0.348597
21112	2.09666	0.05
21113	0.775132	0.05
21114	0.927005	0.05
21115	0.854224	0.05
21116	0.775142	0.05
21117	0.946106	0.05
21118	1.80784	2.1
21119	2.04593	2.1
21120	1.32282	2.1
21121	0.952138	2.1
21122	0.946132	2.1
21123	0.946132	2.1
21124	0.952138	2.1
21125	1.46988	2.1
21126	0.80465	0.05
21127	1.14155	0.05
21128	0.810931	0.05
21129	1.66203	0.05
21130	0.674605	0.143405
21131	0.946132	0.521059
21132	1.26706	0.444872
21133	0.674633	0.105038
21134	0.946132	0.653814
21135	0.980664	0.244557
21136	1.34927	0.176082
21137	0.758454	0.05
21138	0.946133	0.05
21139	0.985371	0.05
21140	1.28632	0.05
21141	0.946133	0.0541671
21142	1.46868	0.05
21143	1.7522	0.05
21144	0.737431	2.1
21145	0.663679	2.1
21146	1.16541	1.77793
21147	1.3179	2.08346
21148	1.04081	2.1
21149	0.887866	1.7701
21150	0.806961	0.564772
21151	0.929061	1.43027
21152	1.28418	0.05
21153	1.38752	0.0822397
21154	1.33038	0.110399
21155	0.946132	0.05
21156	0.946132	0.05
21157	1.14897	0.05
21158	1.1685	0.05
21159	1.18774	2.1
21160	0.961899	2.1
21161	1.48841	2.1
21162	1.79105	2.1
21163	3.51614	2.1
21164	0.682342	2.1
21165	0.783184	2.1
21166	0.946132	2.1
21167	0.868396	2.1

21168	1.21813	2.1
21169	0.848305	0.145418
21170	0.94559	0.649547
21171	0.805691	1.03348
21172	0.752217	2.1
21173	0.946132	2.1
21174	0.839123	2.1
21175	0.946131	2.1
21176	0.752187	2.1
21177	1.96491	2.1
21178	0.520997	2.1
21179	0.946132	2.1
21180	0.73446	2.1
21181	0.946133	2.1
21182	0.520995	2.1
21183	1.88522	2.1
21184	1.8361	2.1
21185	1.11285	2.1
21186	1.25334	1.143
21187	2.00756	2.1
21188	1.44252	0.05
21189	1.00255	1.37033
21190	1.64516	2.1
21191	1.12399	0.05
21192	1.13542	0.05
21193	1.09038	0.05
21194	0.746903	0.05
21195	0.672241	0.05
21196	0.871774	0.05
21197	1.73513	1.08685
21198	0.949995	2.1
21199	1.17244	2.1
21200	0.946127	2.1
21201	1.3028	2.1
21202	1.17244	2.1
21203	0.774101	2.1
21204	0.946127	2.1
21205	0.939289	2.1
21206	0.946129	2.1
21207	0.774101	2.1
21208	1.87858	2.1
21209	1.44293	0.05
21210	1.43213	0.05
21211	0.946131	2.1
21212	0.716614	2.1
21213	0.716644	2.1
21214	1.35828	2.1
21215	0.790935	2.1
21216	0.730157	2.1
21217	1.41149	2.1
21218	0.730186	2.1
21219	0.730186	2.1
21220	0.946131	2.1
21221	0.946132	1.37712
21222	0.946132	0.850143
21223	0.990713	0.115792
21224	1.40779	0.342313
21225	0.946132	0.38737

21226	1.06486	0.05
21227	0.946131	1.01393
21228	0.946133	0.288238
21229	1.40779	0.0834604
21230	1.26874	0.218348
21231	0.946131	0.187087
21232	1.02588	0.165207
21233	0.826073	0.05
21234	1.41233	0.05
21235	0.861269	0.05
21236	1.04073	0.05
21237	1.27111	0.05
21238	1.56016	1.79594
21239	1.60866	0.05
21240	0.94613	0.05
21241	0.946129	0.05
21242	1.36376	0.05
21243	1.22187	0.05
21244	0.94613	0.05
21245	1.82581	1.22936
21246	1.19705	0.740765
21247	1.7513	2.1
21248	1.85017	2.1
21249	1.72116	2.1
21250	1.12058	2.1
21251	0.899991	2.1
21252	0.616288	2.1
21253	1.07289	2.1
21254	1.7092	0.165911
21255	0.946132	0.11611
21256	0.946133	0.05
21257	1.18717	0.05
21258	1.40029	0.05
21259	0.946132	0.05
21260	0.94613	0.05
21261	1.18547	0.05
21262	0.960326	0.05
21263	0.960345	0.05
21264	0.942557	0.05
21265	1.18601	0.05
21266	0.946132	0.05
21267	1.30307	0.05
21268	1.08169	0.05
21269	1.08169	0.05
21270	1.31716	0.05
21271	1.51065	0.341901
21272	0.877642	0.05
21273	0.617384	0.05
21274	0.575741	0.05
21275	0.972448	0.05
21276	1.11334	0.05
21277	1.13092	2.1
21278	0.946127	0.476657
21279	1.31724	0.689227
21280	1.39751	0.05
21281	1.39751	0.163643
21282	0.946128	0.24021
21283	0.946132	0.0803349

21284	0.713284	0.05
21285	1.40668	0.11479
21286	1.03547	0.05
21287	0.713298	0.0677167
21288	1.88774	0.05
21289	0.830164	2.1
21290	1.05211	2.1
21291	0.946119	1.93392
21292	0.946107	2.1
21293	1.283	2.1
21294	2.63609	2.1
21295	1.28418	0.546978
21296	0.946128	0.843008
21297	1.10335	0.473419
21298	0.598377	1.61799
21299	1.18818	0.734163
21300	1.10335	0.439341
21301	1.58972	0.652186
21302	1.90194	2.1
21303	0.946118	2.1
21304	0.946115	2.1
21305	0.950969	2.1
21306	1.36918	2.1
21307	0.950955	2.1
21308	1.4263	2.1
21309	1.11978	0.669683
21310	0.946132	1.43795
21311	0.946133	1.64632
21312	1.37658	0.760387
21313	1.2364	0.995436
21314	1.05496	0.46625
21315	0.946132	1.70811
21316	0.946132	1.25284
21317	1.21858	0.42976
21318	1.37658	1.06501
21319	1.09194	1.67695
21320	1.14721	0.05
21321	1.53814	0.05
21322	0.546751	0.250697
21323	0.492063	1.17292
21324	1.16889	2.09807
21325	1.00349	2.04548
21326	0.863559	0.477389
21327	1.38102	1.78983
21328	0.597449	1.07775
21329	0.537748	0.223732
21330	1.16297	1.55568
21331	0.999366	1.19648
21332	0.896554	0.497103
21333	0.566962	0.05
21334	0.946129	0.05
21335	0.452729	0.05
21336	0.696552	0.05
21337	0.852524	0.05
21338	1.57992	0.05
21339	1.64985	0.05
21340	0.946128	2.1
21341	1.21847	2.1

21342	0.946129	2.1
21343	1.41131	2.1
21344	1.21847	2.1
21345	1.21269	0.05
21346	1.0417	2.1
21347	1.15821	0.752034
21348	0.946132	0.60262
21349	0.946132	2.08024
21350	1.37967	0.146849
21351	0.851529	0.2754
21352	1.20455	2.1
21353	1.4282	0.05
21354	1.26593	2.1
21355	0.838026	0.05
21356	0.985096	0.05
21357	0.83804	0.05
21358	0.822395	1.22795
21359	0.420272	0.0779637
21360	0.946131	2.1
21361	1.17461	2.1
21362	0.94613	2.1
21363	1.40225	2.1
21364	1.17461	2.1
21365	1.09847	2.1
21366	1.22222	1.1856
21367	1.41798	0.0859681
21368	0.791658	0.180209
21369	0.791656	0.21046
21370	0.946129	0.206226
21371	0.817064	0.185432
21372	0.946114	0.107437
21373	0.75824	0.108926
21374	0.75821	0.125747
21375	1.41778	0.0923902
21376	0.791635	0.113289
21377	1.04164	2.06216
21378	0.946131	0.676322
21379	1.11172	0.372365
21380	1.30076	0.0686387
21381	1.35721	0.15543
21382	1.11172	0.135657
21383	1.87218	0.05
21384	0.987248	0.05
21385	0.729695	0.237151
21386	0.974389	0.05
21387	1.18002	0.05
21388	0.822612	0.05
21389	1.35721	0.05
21390	0.634295	0.323136
21391	0.634326	0.282904
21392	1.13245	1.20775
21393	1.13245	0.757061
21394	0.937961	0.143564
21395	1.18769	0.327244
21396	0.854949	0.151547
21397	0.967357	0.21818
21398	0.810934	0.150754
21399	3.91082	2.1

21400	0.946132	2.1
21401	0.946132	2.1
21402	1.05383	2.1
21403	1.05383	2.1
21404	0.986498	2.1
21405	1.50732	0.05
21406	0.753659	0.05
21407	1.22144	0.05
21408	1.30463	0.05
21409	0.753659	0.05
21410	1.30463	0.05
21411	0.886189	0.05
21412	1.04321	0.05
21413	0.909928	0.05
21414	1.18659	0.05
21415	1.19785	0.05
21416	1.40969	0.05
21417	1.24094	0.05
21418	0.946126	0.05
21419	1.2929	0.05
21420	1.24093	0.05
21421	0.867944	0.05
21422	1.41903	2.1
21423	2.47881	0.05
21424	1.12943	0.05
21425	0.764229	2.1
21426	0.764256	2.1
21427	0.94613	2.1
21428	0.94613	2.1
21429	1.1995	2.1
21430	1.83423	2.1
21431	0.634816	2.1
21432	0.877837	2.1
21433	0.94613	2.1
21434	1.04073	2.1
21435	1.06485	2.1
21436	0.94613	0.05
21437	0.62725	0.05
21438	0.798212	0.05
21439	1.33241	0.05
21440	0.62725	0.05
21441	1.82301	0.05
21442	1.40615	2.1
21443	0.782504	2.1
21444	0.748635	2.1
21445	0.946131	2.1
21446	0.748607	2.1
21447	0.988456	2.1
21448	1.64833	0.05
21449	1.35657	0.05
21450	1.26155	0.05
21451	0.915755	0.05
21452	0.915755	0.05
21453	0.946132	0.05
21454	1.49386	0.05
21455	1.01346	2.1
21456	0.946131	1.29882
21457	0.946132	2.1

21458	1.38651	2.1
21459	1.39523	2.1
21460	0.946132	2.1
21461	1.06312	2.1
21462	0.946131	2.1
21463	0.946129	2.1
21464	1.39523	2.1
21465	1.37372	2.1
21466	0.946131	2.1
21467	1.2929	0.05
21468	0.94613	0.05
21469	1.24448	0.05
21470	1.2929	0.05
21471	0.862011	0.05
21472	1.73583	0.05
21473	1.724	0.05
21474	1.03824	2.1
21475	1.534	2.1
21476	0.725492	1.85232
21477	0.771967	2.02686
21478	0.61731	0.825783
21479	0.475743	1.61294
21480	0.719694	1.6807
21481	1.14878	0.700126
21482	0.946117	1.78781
21483	0.94613	1.88334
21484	0.946105	1.88296
21485	0.482666	0.791853
21486	0.609172	0.919301
21487	0.946131	2.1
21488	1.39509	0.169386
21489	1.07439	2.1
21490	0.946131	1.52722
21491	1.39509	1.63987
21492	0.963571	1.82494
21493	0.591011	0.133687
21494	1.1357	0.05
21495	2.11193	0.141297
21496	1.60936	2.1
21497	1.34118	0.073318
21498	1.27696	0.107355
21499	0.94613	0.0502087
21500	0.94613	0.05
21501	1.04257	0.05
21502	1.53336	0.05
21503	1.0631	0.05
21504	1.04072	0.05
21505	0.670678	0.05
21506	0.709531	0.05
21507	0.891275	0.05
21508	0.683583	0.05
21509	0.902478	0.05
21510	0.807671	0.05
21511	1.11024	2.1
21512	1.08171	2.1
21513	1.16725	2.1
21514	0.946128	2.1
21515	1.13537	2.1

21516	1.40525	2.1
21517	1.28365	2.1
21518	1.04523	2.1
21519	0.960106	2.1
21520	0.946128	2.1
21521	1.23452	2.1
21522	0.946129	2.1
21523	1.3704	2.1
21524	1.23452	2.1
21525	1.16906	0.874328
21526	0.946131	1.82525
21527	0.867246	1.12756
21528	0.867217	1.97803
21529	0.946131	1.36606
21530	1.13438	1.6333
21531	1.28684	1.72675
21532	0.946132	1.95609
21533	0.883935	1.56205
21534	0.883938	2.1
21535	0.946133	1.57618
21536	1.73444	1.2218
21537	1.21835	2.1
21538	1.28228	2.1
21539	1.8643	1.20192
21540	0.743379	0.05
21541	1.34921	0.05
21542	0.743381	0.05
21543	0.743381	0.05
21544	0.946131	0.05
21545	0.915528	2.1
21546	0.982742	2.1
21547	0.975965	2.1
21548	0.891132	0.05
21549	1.27533	0.05
21550	0.946132	0.05
21551	1.25215	0.05
21552	0.590343	0.05
21553	0.946133	0.05
21554	0.590331	0.05
21555	1.62375	2.1
21556	0.687769	0.133536
21557	1.14374	0.0741736
21558	1.20885	0.0771395
21559	0.946132	0.131802
21560	1.14374	0.163368
21561	1.09308	0.05
21562	1.02055	0.05
21563	0.810934	0.05
21564	0.917609	1.4704
21565	1.13094	1.77106
21566	0.70959	1.04687
21567	1.11223	2.1
21568	0.83051	1.20876
21569	0.810946	1.83651
21570	0.810959	1.17251
21571	1.5773	0.05
21572	1.96133	0.455204
21573	1.0214	0.911136

21574	0.762783	1.62
21575	0.838036	0.570921
21576	0.63591	1.7919
21577	0.967411	0.322324
21578	0.946132	2.1
21579	0.946132	2.1
21580	1.39699	1.67966
21581	1.61642	2.1
21582	0.860954	0.798628
21583	0.957411	1.98328
21584	0.660856	1.09917
21585	1.10003	2.1
21586	0.968249	2.1
21587	0.910184	1.1873
21588	1.30029	0.0998336
21589	0.754119	0.0681428
21590	0.754121	0.451849
21591	0.94613	1.09146
21592	1.08039	2.1
21593	0.810942	0.05
21594	1.15153	0.05
21595	1.06968	0.05
21596	1.38456	2.1
21597	1.04118	2.1
21598	0.838027	2.1
21599	1.04073	2.1
21600	0.754209	2.1
21601	1.332	0.05
21602	1.26193	2.1
21603	1.15317	0.05
21604	3.86179	0.05
21605	1.56005	2.1
21606	0.683035	0.0984628
21607	0.931463	0.0688485
21608	0.556019	0.837776
21609	0.929172	0.257014
21610	0.756874	0.05
21611	1.23527	0.574296
21612	2.05961	0.573439
21613	0.946132	2.1
21614	1.16684	2.1
21615	1.3937	2.1
21616	1.3937	2.1
21617	0.946132	2.1
21618	1.17885	2.1
21619	1.62507	0.05
21620	1.58874	2.1
21621	0.819953	2.1
21622	1.16023	2.1
21623	1.34782	2.1
21624	1.16023	2.1
21625	0.819955	2.1
21626	0.79437	2.1
21627	0.794373	2.1
21628	1.20056	2.1
21629	1.25	2.1
21630	1.20056	2.1
21631	1.35787	2.1

21632	0.946131	2.1
21633	1.38585	2.1
21634	0.946128	0.251614
21635	0.946129	1.69285
21636	1.35714	1.7888
21637	1.04147	2.1
21638	0.946133	1.81509
21639	1.30382	1.3275
21640	0.946131	0.0691266
21641	0.946131	1.20018
21642	1.38585	1.51344
21643	1.22305	1.37951
21644	1.00763	0.05
21645	0.813199	0.05
21646	0.810948	0.05
21647	1.15928	0.05
21648	0.944166	0.05
21649	0.94613	0.05
21650	0.946133	0.05
21651	1.4179	0.05
21652	0.946132	0.05
21653	1.36965	0.05
21654	1.05444	0.05
21655	0.690302	0.05
21656	0.838038	0.05
21657	0.802821	0.0686089
21658	1.40944	0.198832
21659	0.946126	0.560947
21660	0.799998	0.05
21661	0.800035	0.115506
21662	1.1766	2.1
21663	1.00319	2.1
21664	1.01606	1.40065
21665	0.872158	2.1
21666	1.04884	2.1
21667	0.679494	2.1
21668	0.946131	2.1
21669	1.37609	2.1
21670	0.94613	2.1
21671	0.946131	2.1
21672	1.18486	2.1
21673	0.726287	2.1
21674	0.946131	2.1
21675	0.726286	2.1
21676	1.30318	2.1
21677	1.01712	2.1
21678	0.946132	2.1
21679	1.04404	2.1
21680	1.37017	2.1
21681	1.37017	2.1
21682	0.946133	2.1
21683	0.992084	0.986802
21684	0.946131	0.725352
21685	0.529655	2.02789
21686	0.529686	0.895216
21687	0.946131	0.626367
21688	1.05937	1.2231
21689	1.14622	0.298689

21690	0.946131	0.581187
21691	0.584575	1.59249
21692	0.584575	0.459605
21693	0.946129	0.423264
21694	0.709714	2.1
21695	0.946132	2.1
21696	0.709714	2.1
21697	1.37199	2.1
21698	0.82899	2.1
21699	1.00133	2.1
21700	0.778424	0.05
21701	0.946132	0.05
21702	1.24094	0.05
21703	1.00691	0.05
21704	0.64868	0.05
21705	1.29736	0.05
21706	1.24767	0.0790971
21707	1.28953	0.474203
21708	1.34612	0.606551
21709	0.946133	0.10743
21710	1.14187	1.18192
21711	0.946132	0.497742
21712	0.946129	0.207368
21713	1.4099	2.1
21714	0.946131	0.0813828
21715	1.34612	0.066769
21716	1.4099	0.05
21717	0.956003	0.05
21718	1.40572	0.05
21719	3.54443	2.1
21720	0.994001	0.05
21721	1.03679	2.1
21722	1.08467	2.1
21723	1.56875	0.05
21724	0.968463	2.1
21725	0.974889	2.1
21726	0.946131	2.1
21727	1.30203	2.1
21728	1.40199	2.1
21729	1.40199	2.1
21730	0.946132	2.1
21731	0.946132	2.1
21732	1.36994	2.1
21733	1.37372	2.1
21734	1.37372	2.1
21735	0.946132	2.1
21736	0.928979	0.829883
21737	0.565509	1.31854
21738	1.60196	0.221408
21739	1.16239	0.652773
21740	1.10746	0.05
21741	1.15412	2.1
21742	1.15171	2.1
21743	0.946133	2.1
21744	0.940877	2.1
21745	0.940878	2.1
21746	1.41432	2.1
21747	1.04687	2.1

21748	2.75524	0.05
21749	0.946132	0.05
21750	0.766452	0.05
21751	0.766427	0.05
21752	1.36004	0.05
21753	1.06648	0.05
21754	1.62483	2.1
21755	1.0577	2.1
21756	1.39158	2.1
21757	0.94255	2.1
21758	1.05029	2.1
21759	1.03735	2.1
21760	0.931055	2.1
21761	1.03797	2.1
21762	1.43687	2.06478
21763	1.28765	1.80008
21764	1.1811	0.956953
21765	0.946132	1.53433
21766	0.946132	1.53322
21767	1.1811	1.41016
21768	0.67805	0.177129
21769	1.50998	1.96876
21770	1.16661	2.1
21771	1.36749	2.1
21772	0.810945	0.699393
21773	1.14745	1.30254
21774	0.865588	0.34127
21775	1.36665	0.387084
21776	0.506964	1.345
21777	0.506966	0.928629
21778	0.552786	0.441223
21779	0.946133	0.204669
21780	1.21336	0.136685
21781	0.552786	0.550673
21782	0.552757	1.80172
21783	0.653468	0.273374
21784	1.36665	1.49446
21785	1.27922	0.05
21786	1.08133	0.05
21787	1.05348	0.05
21788	0.946132	0.05
21789	1.19601	0.05
21790	1.13276	0.05
21791	0.7856	0.05
21792	1.57125	0.05
21793	1.19601	0.05
21794	0.946131	0.171044
21795	1.34534	0.0824304
21796	1.19601	0.05
21797	0.870489	0.05
21798	1.49404	2.1
21799	1.35943	1.41038
21800	1.10427	1.99891
21801	0.94613	1.13496
21802	0.946131	1.52403
21803	1.10427	1.41836
21804	1.2064	0.05
21805	0.946132	0.05

21806	1.11288	0.104099
21807	1.34268	0.05
21808	1.34268	0.05
21809	0.946132	0.05
21810	0.946128	2.1
21811	0.946128	0.05
21812	1.30835	2.1
21813	1.38463	0.359399
21814	0.946129	0.285153
21815	1.43778	0.588492
21816	1.23943	0.480278
21817	1.4748	2.1
21818	0.71295	2.1
21819	0.712951	2.1
21820	0.946132	2.1
21821	0.946133	2.1
21822	1.13976	2.1
21823	1.71109	2.1
21824	1.32426	2.1
21825	0.671255	2.1
21826	1.29123	2.1
21827	0.946132	2.1
21828	0.946132	2.1
21829	1.25603	2.1
21830	2.21126	0.05
21831	0.892889	2.1
21832	0.946129	2.1
21833	0.892888	2.1
21834	0.92872	2.1
21835	1.34352	2.1
21836	0.622784	0.078046
21837	1.02947	0.05
21838	0.810957	0.05
21839	1.91756	0.711201
21840	1.06012	0.181319
21841	1.09569	0.05
21842	1.5456	0.05
21843	1.1211	0.05
21844	1.21031	0.05
21845	1.03939	0.05
21846	0.519697	0.05
21847	0.933889	0.05
21848	0.519726	0.05
21849	1.16699	0.05
21850	0.933889	0.05
21851	0.840599	0.05
21852	0.94613	0.05
21853	0.840568	0.05
21854	1.26868	0.05
21855	1.16699	0.05
21856	1.17822	2.1
21857	0.922044	0.0810493
21858	1.29322	0.207314
21859	0.946132	0.104864
21860	0.995178	0.194602
21861	0.922056	0.193519
21862	1.76232	0.406171
21863	1.77065	0.959632

21864	1.38149	0.557035
21865	0.959301	1.02229
21866	0.946133	0.595212
21867	0.946132	0.921667
21868	0.959301	0.755256
21869	1.3612	0.534433
21870	0.885314	0.942073
21871	0.885309	0.482197
21872	0.94613	0.830353
21873	0.8853	0.511375
21874	1.56164	0.598799
21875	1.45506	0.05
21876	1.45257	2.1
21877	0.851541	0.05
21878	1.29018	0.05
21879	0.851541	0.05
21880	1.21087	0.05
21881	1.24979	0.05
21882	1.56983	0.05
21883	1.60151	0.05
21884	1.78952	2.1
21885	0.638164	2.1
21886	0.638163	2.1
21887	1.39754	2.1
21888	1.39754	2.1
21889	0.681589	2.1
21890	1.34993	2.1
21891	1.04525	2.1
21892	0.946133	2.1
21893	1.04072	2.1
21894	0.857805	2.1
21895	1.06053	2.1
21896	1.49066	2.1
21897	1.4185	0.780204
21898	0.934059	2.07698
21899	0.93406	0.91399
21900	0.94613	1.67297
21901	0.93406	1.20942
21902	1.25277	1.09069
21903	1.00792	2.1
21904	0.609466	1.20124
21905	0.946132	1.84852
21906	0.609463	2.01635
21907	1.30664	2.1
21908	0.810943	1.67131
21909	1.05741	0.888865
21910	1.3868	1.15378
21911	1.15558	0.05
21912	1.13537	2.1
21913	0.946132	2.1
21914	0.946132	2.1
21915	1.08266	2.1
21916	1.54934	2.1
21917	1.26016	2.1
21918	2.01915	2.1
21919	0.60346	1.45728
21920	0.651529	1.56967
21921	1.15581	2.07477

21922	1.13037	1.18001
21923	1.04071	0.83052
21924	1.77796	2.1
21925	0.532308	1.4692
21926	0.532308	0.810483
21927	1.15369	2.1
21928	1.1537	1.92732
21929	0.903288	1.48026
21930	0.804766	2.1
21931	0.804739	2.1
21932	0.946128	2.1
21933	0.946131	2.1
21934	1.16745	2.1
21935	1.04193	2.1
21936	0.98505	2.1
21937	1.40724	0.245365
21938	1.14216	0.304665
21939	0.946131	0.05
21940	0.946131	0.0776634
21941	1.10941	0.0766587
21942	0.81268	0.05
21943	0.946131	0.05
21944	1.28673	0.05
21945	1.28673	0.05
21946	0.812681	0.05
21947	1.84716	0.05
21948	1.84716	0.05
21949	0.92361	0.05
21950	0.94613	0.05
21951	0.923582	0.05
21952	1.26227	0.05
21953	0.923581	0.05
21954	0.747394	2.1
21955	1.32582	2.1
21956	0.747365	2.1
21957	0.97587	2.1
21958	1.32582	2.1
21959	1.04182	0.05
21960	0.961743	0.05
21961	0.946126	0.05
21962	1.47463	0.05
21963	1.12183	0.05
21964	0.851552	0.05
21965	1.33177	0.05
21966	0.946131	0.05
21967	1.41512	0.05
21968	1.33178	0.05
21969	0.946131	0.05
21970	0.927735	0.12577
21971	0.97206	0.05
21972	0.515566	0.062616
21973	0.946129	0.055553
21974	0.515538	0.0515385
21975	1.04482	0.05
21976	0.975535	0.05
21977	0.522411	0.05
21978	0.522382	0.05
21979	0.925346	0.05

21980	0.575817	0.05
21981	0.946129	2.1
21982	1.14256	2.1
21983	1.32054	2.1
21984	1.40525	2.1
21985	1.14256	2.1
21986	1.59475	2.1
21987	0.773813	2.1
21988	1.4073	2.1
21989	0.773813	2.1
21990	0.773813	2.1
21991	1.4073	2.1
21992	0.993021	2.1
21993	1.50466	0.05
21994	0.851539	0.05
21995	1.20299	0.05
21996	0.946131	0.05
21997	1.54464	0.05
21998	1.20309	0.05
21999	2.00701	0.05
22000	0.810946	0.05
22001	1.07678	0.05
22002	0.900128	0.05
22003	1.26842	2.1
22004	1.11206	2.1
22005	0.946132	2.1
22006	1.04074	2.1
22007	0.948809	2.1
22008	0.946131	0.05
22009	0.929617	0.05
22010	1.24057	0.05
22011	1.36485	0.05
22012	0.851554	0.05
22013	1.96823	0.05
22014	0.914836	0.05
22015	0.94613	0.05
22016	1.38911	0.05
22017	0.914805	0.05
22018	0.946131	0.05
22019	0.946131	0.05
22020	1.04072	0.05
22021	1.26837	0.05
22022	0.970054	0.05
22023	0.923958	0.05
22024	1.16653	2.1
22025	1.34412	2.1
22026	0.851541	0.05
22027	1.38645	0.05
22028	0.891637	0.05
22029	0.851487	0.05
22030	0.890871	0.05
22031	1.56178	0.05
22032	0.946128	0.05
22033	1.26868	0.05
22034	0.946131	0.05
22035	1.18789	0.05
22036	1.26868	0.05
22037	1.26868	0.05

22038	0.946128	0.05
22039	1.18716	0.05
22040	0.946131	0.05
22041	0.59358	0.05
22042	1.23923	0.05
22043	1.00024	0.05
22044	0.593593	0.05
22045	0.946131	0.05
22046	0.713645	0.05
22047	1.23175	0.05
22048	1.23923	0.05
22049	0.713633	0.05
22050	1.24687	0.05
22051	1.80662	2.1
22052	2.15762	2.1
22053	0.780455	2.1
22054	0.946131	2.1
22055	0.780455	2.1
22056	1.40583	2.1
22057	1.39852	2.1
22058	0.565619	2.1
22059	1.1428	2.1
22060	0.663168	0.05
22061	1.02163	0.05
22062	1.04072	0.05
22063	0.663169	0.05
22064	1.13511	0.05
22065	1.85277	0.05
22066	1.17701	0.187637
22067	0.993831	0.05
22068	1.0823	0.05
22069	0.795761	0.05
22070	1.24596	0.05
22071	0.566075	0.05
22072	0.94613	0.05
22073	0.56605	0.05
22074	0.774911	0.05
22075	0.946131	0.05
22076	1.24417	0.05
22077	0.774911	0.05
22078	0.946132	0.05
22079	1.54982	0.05
22080	1.82961	0.05
22081	1.12035	0.05
22082	0.876835	2.1
22083	0.946131	2.1
22084	0.876836	2.1
22085	1.12968	2.1
22086	1.23536	2.1
22087	2.15082	2.1
22088	0.58365	2.1
22089	0.946133	2.1
22090	0.583653	2.1
22091	0.992097	2.1
22092	1.12968	2.1
22093	1.0305	2.1
22094	0.82613	0.05
22095	0.879644	0.05

22096	1.00688	0.05
22097	1.52776	2.1
22098	0.851537	1.81066
22099	1.00851	1.55007
22100	0.946128	0.382963
22101	0.851552	1.33033
22102	1.4895	1.92603
22103	0.761574	2.1
22104	0.946132	2.1
22105	1.01095	2.1
22106	1.01095	2.1
22107	0.761572	2.1
22108	1.8575	2.1
22109	1.19984	0.05
22110	1.25797	0.05
22111	0.946132	0.05
22112	0.946132	0.05
22113	0.95332	0.05
22114	0.946131	2.1
22115	0.851542	2.1
22116	1.03172	2.1
22117	1.22889	2.1
22118	1.0313	2.1
22119	1.15977	1.48674
22120	0.918875	0.669625
22121	0.8654	2.1
22122	0.865374	0.606277
22123	0.946132	0.616384
22124	0.985051	2.1
22125	1.31993	0.05
22126	1.33622	0.131651
22127	0.959992	0.0826697
22128	0.926499	2.1
22129	1.03544	2.1
22130	0.946132	2.1
22131	0.946132	2.1
22132	1.40335	2.1
22133	0.772299	2.1
22134	0.851529	2.1
22135	0.857243	2.1
22136	1.38155	2.1
22137	1.05462	2.1
22138	0.832138	2.1
22139	0.946132	2.1
22140	0.832137	2.1
22141	0.959414	2.1
22142	1.36541	2.1
22143	1.91894	2.1
22144	1.04661	1.46763
22145	1.28355	2.1
22146	0.877353	0.392541
22147	0.877353	1.28689
22148	0.94613	0.565286
22149	1.20537	0.05
22150	1.09914	0.05
22151	0.94613	0.05
22152	0.499593	0.05
22153	0.499606	0.05

22154	1.0959	0.05
22155	0.838027	0.05
22156	0.883644	0.05
22157	0.95019	0.05
22158	1.10638	2.1
22159	0.946132	2.1
22160	1.41744	2.1
22161	1.05842	2.1
22162	0.94613	2.1
22163	1.41744	2.1
22164	0.991985	2.1
22165	3.31755	1.64864
22166	1.05556	2.1
22167	0.624075	1.16337
22168	0.814554	0.710286
22169	0.946131	1.38976
22170	0.814554	1.71678
22171	1.31857	2.1
22172	1.26282	2.1
22173	0.946132	2.1
22174	1.14302	2.1
22175	0.946132	2.1
22176	0.946132	2.1
22177	1.40372	2.1
22178	1.24688	0.05
22179	0.89889	2.1
22180	1.17286	2.1
22181	0.71741	2.1
22182	0.491096	0.280082
22183	1.05432	0.0522002
22184	0.719531	2.1
22185	1.25145	2.1
22186	0.864686	2.1
22187	0.867974	2.1
22188	1.21734	2.1
22189	0.405517	0.0700451
22190	0.818878	0.591072
22191	0.502457	0.05
22192	1.05868	0.05
22193	0.912018	0.05
22194	1.40591	0.05
22195	0.957584	0.05
22196	0.946131	0.05
22197	1.11612	0.05
22198	1.3365	0.05
22199	0.946128	0.05
22200	1.16534	0.05
22201	1.38969	0.102235
22202	0.891597	1.08389
22203	0.640032	2.1
22204	0.956758	1.36174
22205	1.3428	0.05
22206	0.953295	2.1
22207	0.946131	0.05
22208	1.16167	0.05
22209	0.94613	0.05
22210	0.951675	0.05
22211	1.16167	0.05

22212	1.24538	0.05
22213	1.13482	2.1
22214	1.38775	2.1
22215	0.934061	2.1
22216	0.91408	2.1
22217	1.06099	2.1
22218	0.980549	0.05
22219	0.946132	0.05
22220	1.10321	0.05
22221	0.99126	0.05
22222	0.991261	0.05
22223	1.35732	0.05
22224	1.1952	2.1
22225	0.946132	1.3346
22226	0.946132	2.1
22227	1.32819	1.63697
22228	1.30171	1.07853
22229	1.29667	0.05
22230	1.00982	0.05
22231	0.407589	0.0956232
22232	1.18266	1.70281
22233	1.04037	0.137296
22234	0.956694	0.446547
22235	0.552639	0.0998821
22236	0.810943	2.1
22237	1.43144	0.578883
22238	0.918074	0.509125
22239	1.60976	2.1
22240	4.00847	2.1
22241	2.3995	0.05
22242	1.36203	2.1
22243	0.919619	2.1
22244	1.30188	2.1
22245	0.681073	2.1
22246	0.681043	2.1
22247	1.04687	2.1
22248	3.32085	2.1
22249	1.80396	0.05
22250	0.773007	0.05
22251	0.773034	0.05
22252	1.22141	0.0672564
22253	1.22141	0.0793523
22254	0.946131	0.13925
22255	2.02071	0.05
22256	0.951912	0.05
22257	1.39027	0.05
22258	0.94613	0.05
22259	0.829922	0.05
22260	0.829946	0.05
22261	1.24902	1.28372
22262	2.8333	0.993488
22263	0.946131	0.05
22264	0.929725	0.05
22265	1.1134	0.05
22266	1.17056	0.05
22267	0.929713	0.05
22268	0.749096	2.1
22269	1.31961	2.1

22270	0.870191	2.1
22271	1.04071	2.1
22272	1.1877	2.1
22273	1.03882	2.1
22274	1.44801	2.1
22275	3.55198	2.1
22276	1.11193	2.1
22277	0.663922	2.1
22278	0.663922	2.1
22279	0.946129	2.1
22280	0.946131	2.1
22281	0.97079	2.1
22282	1.36049	2.1
22283	1.06105	2.1
22284	0.92251	2.1
22285	0.94613	2.1
22286	0.922511	2.1
22287	1.04101	2.1
22288	0.810944	0.05
22289	0.994109	0.05
22290	0.623143	0.05
22291	0.864244	0.05
22292	1.14627	0.05
22293	0.64586	0.05
22294	0.721422	0.05
22295	0.870885	0.05
22296	0.855175	0.05
22297	0.946131	0.808754
22298	1.38157	2.1
22299	1.33266	0.121073
22300	0.946131	2.1
22301	1.38158	1.99054
22302	0.946132	2.1
22303	1.3268	2.1
22304	0.946132	2.1
22305	0.946132	2.1
22306	1.38158	2.1
22307	1.42186	2.1
22308	1.47204	2.03723
22309	1.14665	2.1
22310	0.946132	1.08669
22311	0.94613	2.1
22312	1.30116	2.1
22313	1.32819	1.97631
22314	1.25388	2.1
22315	0.887808	1.57704
22316	0.946132	2.1
22317	1.23536	2.1
22318	1.34309	2.1
22319	1.07612	2.1
22320	0.728982	0.05
22321	1.3157	0.05
22322	0.946131	0.05
22323	0.686176	0.05
22324	1.3035	0.05
22325	0.686139	0.05
22326	0.906806	0.05
22327	0.794122	0.05

22328	1.17173	0.05
22329	1.8194	0.05
22330	0.851543	2.1
22331	1.19345	2.1
22332	0.946133	2.1
22333	0.946132	2.1
22334	1.56179	2.1
22335	0.620263	2.1
22336	1.30226	2.1
22337	1.2334	1.48917
22338	1.2334	0.558162
22339	0.946133	0.949534
22340	1.24053	2.1
22341	1.4828	2.1
22342	0.957622	2.1
22343	1.00521	1.71336
22344	1.49463	2.1
22345	1.39621	2.1
22346	1.09861	2.1
22347	0.946131	2.1
22348	0.946131	2.1
22349	1.09861	2.1
22350	2.16987	0.05
22351	0.572182	0.05
22352	1.15802	0.05
22353	0.63574	0.05
22354	1.18897	0.05
22355	0.996733	0.05
22356	1.82402	1.41787
22357	0.969602	0.914893
22358	0.794777	1.99317
22359	1.02626	1.34483
22360	1.51396	0.05
22361	1.40574	0.05
22362	1.38683	0.0675091
22363	0.946126	0.0543053
22364	0.946128	0.05
22365	1.07138	0.05
22366	0.946131	2.1
22367	1.41331	2.1
22368	0.94613	2.1
22369	0.94613	2.1
22370	1.27451	2.1
22371	0.974196	2.1
22372	1.2049	0.05
22373	1.42757	2.1
22374	1.02461	2.1
22375	2.07087	2.1
22376	1.92515	0.713949
22377	0.802138	0.773916
22378	0.946128	2.03292
22379	0.802107	1.14831
22380	1.23726	1.04559
22381	1.21997	0.842546
22382	0.767577	0.225699
22383	0.946131	1.81519
22384	0.767577	0.761552
22385	1.3279	0.638889

22386	1.23726	0.46192
22387	1.29729	2.1
22388	0.946128	2.1
22389	1.14186	2.1
22390	1.39214	2.1
22391	1.39214	2.1
22392	0.946129	2.1
22393	1.23012	0.05
22394	0.653228	0.05
22395	0.946133	1.65486
22396	1.15918	1.76519
22397	1.26893	0.262597
22398	0.653228	1.51997
22399	1.06848	1.93772
22400	0.615121	2.1
22401	0.841488	1.58988
22402	0.946131	2.1
22403	1.04071	2.1
22404	0.965717	2.1
22405	1.77893	1.38789
22406	1.11314	0.05
22407	1.65411	0.400119
22408	0.85472	0.05
22409	1.1604	0.05
22410	1.19141	0.05
22411	1.19141	0.05
22412	0.94613	0.05
22413	1.70944	0.05
22414	1.89082	0.05
22415	1.05175	0.551256
22416	0.815276	2.1
22417	0.843283	2.1
22418	0.946131	2.1
22419	0.843284	2.1
22420	1.38897	2.1
22421	1.01019	0.115053
22422	1.05197	0.0517072
22423	0.810959	0.0878083
22424	1.33037	0.0700825
22425	1.3415	0.0891582
22426	0.946118	0.05
22427	0.946131	0.05
22428	1.09803	0.05
22429	0.930281	2.1
22430	1.3335	2.1
22431	0.784151	2.1
22432	0.78415	2.1
22433	0.946132	2.1
22434	1.6299	0.05
22435	0.995795	2.1
22436	1.84846	1.51308
22437	0.929873	1.90239
22438	0.889245	1.92088
22439	0.808072	1.98521
22440	0.851541	1.80918
22441	1.51956	1.32217
22442	0.94613	1.43559
22443	0.851553	1.65099

22444	0.946787	1.51309
22445	1.15544	1.73265
22446	1.30289	0.05
22447	1.28323	0.05
22448	0.85024	0.05
22449	1.09662	0.05
22450	1.67687	0.05
22451	0.946127	2.1
22452	1.40496	2.1
22453	1.22442	2.1
22454	0.946128	2.1
22455	1.40496	2.1
22456	1.00573	2.1
22457	0.636837	2.1
22458	0.988642	2.1
22459	0.573141	2.07982
22460	0.946128	2.1
22461	0.786538	2.1
22462	2.28748	1.69021
22463	0.682805	2.1
22464	0.946132	2.1
22465	0.682776	2.1
22466	0.946132	2.1
22467	0.79775	2.1
22468	1.05403	0.05
22469	0.888273	0.05
22470	0.876373	0.05
22471	0.576083	0.05
22472	0.946132	0.05
22473	0.576057	0.05
22474	0.576058	0.05
22475	0.946131	0.05
22476	1.41724	0.05
22477	1.31678	0.208293
22478	0.946132	0.184045
22479	0.946132	0.1271
22480	1.31678	0.105049
22481	0.935738	2.1
22482	0.946132	2.1
22483	0.935765	2.1
22484	1.32949	2.1
22485	0.946131	2.1
22486	1.02525	2.1
22487	0.946128	2.1
22488	1.41207	2.1
22489	1.04675	2.1
22490	0.946131	2.1
22491	1.41207	2.1
22492	0.946131	2.1
22493	0.946132	2.1
22494	1.41125	2.1
22495	1.41125	2.1
22496	1.27087	2.1
22497	0.924071	0.05
22498	0.608893	0.05
22499	0.528907	0.05
22500	1.78249	0.05
22501	0.720094	0.05

22502	0.781667	2.1
22503	0.781638	2.1
22504	1.40756	2.1
22505	1.40756	2.1
22506	0.797635	2.1
22507	1.11946	0.05
22508	1.32068	0.05
22509	0.88757	0.05
22510	1.27891	0.05
22511	0.810957	0.05
22512	0.914656	0.05
22513	0.640819	0.05
22514	0.745643	0.05
22515	0.865108	0.05
22516	1.06883	0.05
22517	0.806001	0.05
22518	1.19462	2.1
22519	1.42763	0.05
22520	1.24682	0.05
22521	0.78392	0.05
22522	1.02874	0.05
22523	0.884512	0.05
22524	1.1658	2.1
22525	0.663026	0.05
22526	1.21174	0.05
22527	0.663056	0.05
22528	1.15968	0.05
22529	1.21174	0.05
22530	0.993976	0.05
22531	0.945495	0.05
22532	0.671202	0.05
22533	1.37312	2.1
22534	1.28499	2.1
22535	0.946133	2.1
22536	0.946132	2.1
22537	1.28499	2.1
22538	1.25081	2.1
22539	0.946132	2.1
22540	0.946132	2.1
22541	1.27985	2.1
22542	1.37312	2.1
22543	0.830638	2.1
22544	1.05358	2.1
22545	1.22209	2.1
22546	1.09874	2.1
22547	0.74758	2.1
22548	2.50011	1.74914
22549	0.6157	0.05
22550	0.674634	0.05
22551	0.946128	1.20485
22552	0.674633	0.0983429
22553	0.946131	0.568545
22554	1.96192	1.7226
22555	1.31612	0.05
22556	1.10735	0.05
22557	0.936676	0.05
22558	0.808779	0.05
22559	0.808808	0.05

22560	1.00245	0.05
22561	1.26179	0.05
22562	1.05758	2.1
22563	1.03408	2.1
22564	0.946132	0.05
22565	0.914368	0.05
22566	1.20524	0.05
22567	1.12743	0.05
22568	0.851555	0.05
22569	1.17885	0.0988401
22570	0.867881	0.0692387
22571	1.00122	0.05
22572	0.867883	0.05
22573	0.867881	0.0505355
22574	1.19583	0.0641386
22575	1.17743	2.1
22576	0.878684	1.91258
22577	0.687087	2.1
22578	0.955079	0.0598463
22579	0.672977	0.17754
22580	0.735937	0.244537
22581	0.851531	0.323307
22582	0.672957	0.204552
22583	0.923974	0.225808
22584	1.2243	2.1
22585	0.808463	0.949958
22586	0.971078	1.36033
22587	1.06161	1.3884
22588	0.810947	0.949997
22589	0.886074	1.58935
22590	0.786829	1.59529
22591	0.55062	0.813023
22592	0.574458	1.87934
22593	0.946129	1.2362
22594	1.42526	1.40547
22595	1.15962	1.04704
22596	1.24526	0.05
22597	0.94613	0.05
22598	0.946128	0.05
22599	1.23279	0.0808009
22600	1.38645	0.0895483
22601	0.94613	0.0606292
22602	1.05042	2.1
22603	0.946128	2.1
22604	0.946129	2.1
22605	1.24286	2.1
22606	1.39659	2.1
22607	0.94613	2.1
22608	1.10407	2.1
22609	1.32809	1.88757
22610	0.993999	2.1
22611	0.810959	1.68568
22612	0.983643	1.80572
22613	0.869552	2.1
22614	0.747801	1.50144
22615	1.53429	0.73765
22616	1.13127	2.1
22617	1.38397	2.1

22618	1.08366	2.1
22619	0.946129	2.1
22620	0.946131	2.1
22621	1.08366	2.1
22622	1.06007	2.1
22623	1.79641	2.1
22624	0.758799	2.1
22625	1.28442	2.1
22626	1.06957	2.1
22627	0.946133	2.1
22628	1.28442	2.1
22629	1.76788	2.1
22630	1.38669	2.1
22631	0.986095	2.1
22632	1.38478	2.05116
22633	0.946132	0.761259
22634	1.24344	0.289827
22635	0.622283	2.0807
22636	1.10335	0.527821
22637	1.24344	0.506414
22638	1.1967	1.30883
22639	0.946131	0.05
22640	0.901005	0.05
22641	1.05751	0.05
22642	1.37771	0.05
22643	0.851527	0.05
22644	1.26643	1.17834
22645	1.08992	1.67295
22646	0.946132	1.57908
22647	0.946132	0.857102
22648	1.08992	1.71266
22649	1.53534	2.1
22650	1.58825	1.46981
22651	1.0447	2.06245
22652	0.593818	1.66649
22653	0.89883	1.88626
22654	0.734088	1.9678
22655	0.998451	0.346274
22656	1.64156	0.0913518
22657	1.05654	0.605628
22658	1.37641	0.05
22659	1.74553	0.0875756
22660	1.31112	0.0620056
22661	0.74518	0.167325
22662	0.851539	0.0933749
22663	0.851554	0.0534056
22664	0.851475	0.05
22665	0.946132	0.05
22666	1.28895	0.05
22667	1.11356	0.05
22668	0.946132	0.05
22669	0.946132	0.05
22670	1.84214	2.1
22671	1.23851	0.05
22672	1.25866	0.05
22673	1.28655	0.221656
22674	0.946131	0.123053
22675	0.946132	0.0919109

22676	1.28655	0.0749496
22677	1.11929	0.0584612
22678	0.946132	0.143813
22679	0.946132	0.0510554
22680	1.34254	0.05
22681	1.25866	0.05
22682	0.946132	2.1
22683	1.27451	2.1
22684	1.19803	2.1
22685	0.94613	2.1
22686	1.27451	2.1
22687	0.946131	2.1
22688	1.36523	2.1
22689	1.14599	2.1
22690	0.946129	2.1
22691	1.36523	2.1
22692	1.09518	2.1
22693	0.977777	2.1
22694	0.583237	2.1
22695	0.583267	2.1
22696	0.903872	2.1
22697	0.946129	2.1
22698	0.583258	2.1
22699	1.58179	0.05
22700	0.946131	0.05
22701	1.35123	0.05
22702	0.655837	0.05
22703	1.12018	0.05
22704	1.35123	0.05
22705	0.62967	0.05
22706	0.860497	0.05
22707	0.816639	0.05
22708	0.746716	2.1
22709	0.810947	2.1
22710	0.810959	2.1
22711	0.653422	2.1
22712	0.289127	1.20546
22713	0.475716	2.1
22714	0.980651	1.33707
22715	0.946119	2.1
22716	0.946132	2.1
22717	1.08299	2.1
22718	1.34539	2.1
22719	1.28753	0.05
22720	0.946132	0.05
22721	0.946132	0.05
22722	1.19719	0.05
22723	0.946133	0.05
22724	0.958947	0.05
22725	1.3492	0.05
22726	0.946132	0.05
22727	0.946128	0.05
22728	1.22588	0.05
22729	0.946129	0.05
22730	0.991113	2.1
22731	1.35966	2.1
22732	0.961 2.1	
22733	0.946129	2.1

22734	0.946131	2.1
22735	0.961001	2.1
22736	0.727096	1.97066
22737	0.810946	0.381867
22738	0.810959	0.73686
22739	1.31658	0.0614101
22740	1.23167	2.1
22741	1.44836	0.05
22742	1.50103	0.05
22743	1.23351	0.05
22744	1.35657	0.05
22745	0.851541	0.05
22746	0.93668	0.05
22747	0.946131	0.05
22748	3.7335	2.1
22749	1.1822	2.1
22750	1.31127	0.0589411
22751	1.3689	0.05
22752	1.35822	0.05
22753	0.946133	0.05
22754	0.946132	0.05
22755	1.14926	0.0531625
22756	1.40822	0.05
22757	1.04935	0.05
22758	0.704112	0.05
22759	0.704112	0.05
22760	0.946131	0.058768
22761	0.704112	0.0508089
22762	1.37721	0.05
22763	0.85154	2.1
22764	1.06949	2.1
22765	0.851541	2.1
22766	0.946128	2.1
22767	1.1175	2.1
22768	1.44091	2.1
22769	0.96029	0.473261
22770	3.46486	0.66397
22771	0.603286	0.05
22772	0.432283	0.05
22773	0.851541	0.05
22774	1.09355	0.05
22775	0.851534	0.05
22776	1.04263	2.1
22777	0.851541	2.1
22778	0.965604	2.1
22779	0.94613	2.1
22780	1.23953	2.1
22781	0.869615	2.1
22782	1.02415	2.1
22783	0.983032	2.1
22784	1.35385	1.73504
22785	1.59971	0.05
22786	0.915178	2.1
22787	0.962435	2.1
22788	1.3116	2.1
22789	1.38302	2.1
22790	1.13227	2.1
22791	1.25923	0.05

22792	0.988805	2.1
22793	0.611486	0.961587
22794	1.1019	0.828627
22795	1.11124	1.73401
22796	0.716713	0.711276
22797	0.946131	1.53571
22798	1.22297	0.95292
22799	1.12873	1.74127
22800	0.946131	1.67825
22801	0.946132	1.77448
22802	1.3469	1.94271
22803	1.25366	2.09588
22804	1.1611	1.12511
22805	0.946132	1.51145
22806	0.946132	1.20317
22807	1.2854	1.57233
22808	1.3469	1.72278
22809	1.07468	1.28438
22810	1.79317	2.1
22811	1.07444	2.1
22812	1.69436	2.1
22813	0.851539	2.1
22814	0.870925	2.1
22815	0.851541	2.1
22816	0.870951	2.1
22817	1.26097	2.1
22818	1.26247	2.1
22819	0.946132	2.1
22820	1.24041	2.1
22821	0.946132	2.1
22822	0.946132	2.1
22823	1.40274	2.1
22824	0.946131	0.05
22825	0.797945	0.05
22826	1.12988	0.05
22827	1.00681	0.05
22828	0.797932	0.05
22829	0.977483	0.05
22830	0.943362	0.0886228
22831	0.9891	0.05
22832	1.1703	0.05
22833	0.663171	0.05
22834	1.40882	0.05
22835	1.32639	0.05
22836	1.24448	0.05
22837	0.946132	0.05
22838	1.11015	0.05
22839	1.24448	0.05
22840	0.754768	0.05
22841	1.13854	2.1
22842	1.23654	2.1
22843	0.714101	0.0784323
22844	0.851538	0.0590005
22845	0.79261	1.57159
22846	1.30598	0.329158
22847	1.05338	0.0967114
22848	1.41758	2.1
22849	0.83317	0.05

22850	1.00058	0.05
22851	0.810958	0.05
22852	1.63791	0.05
22853	1.55116	2.1
22854	1.29735	1.31128
22855	0.945147	2.1
22856	0.94515	1.48972
22857	0.94613	1.96436
22858	0.945149	1.97635
22859	1.02368	2.1
22860	1.12787	2.1
22861	1.04007	2.1
22862	3.78805	2.1
22863	0.816891	0.05
22864	0.943996	0.05
22865	0.870002	0.05
22866	0.564533	0.05
22867	0.564536	0.05
22868	0.946128	0.37788
22869	0.564535	0.05
22870	0.946128	0.230578
22871	1.15177	2.1
22872	1.16806	0.967259
22873	0.999083	1.16691
22874	0.946131	1.22751
22875	0.946131	1.15881
22876	0.999084	1.39953
22877	1.28918	0.816869
22878	1.0383	1.12931
22879	0.946133	1.13405
22880	0.946132	1.14587
22881	1.0383	1.23487
22882	1.27737	1.61535
22883	1.39296	0.0871512
22884	0.998682	2.1
22885	3.98933	2.1
22886	1.05328	0.05
22887	1.23475	2.1
22888	0.900856	1.70362
22889	0.94001	0.898187
22890	0.707748	0.606229
22891	0.707751	1.33423
22892	0.946128	0.831547
22893	0.70775	1.26433
22894	0.996109	1.15974
22895	1.68551	2.1
22896	0.851539	2.1
22897	0.962688	2.1
22898	0.94613	1.75903
22899	1.04073	2.1
22900	1.44824	2.1
22901	0.794313	0.05
22902	0.838031	0.05
22903	1.11076	0.05
22904	1.38813	2.1
22905	1.17204	2.1
22906	0.946132	2.1
22907	0.946131	2.1

22908	1.17204	2.1
22909	0.961779	0.05
22910	1.0685	0.0550615
22911	0.810947	2.1
22912	1.2899	1.04639
22913	0.890404	0.992794
22914	0.909981	2.1
22915	0.810946	1.45928
22916	0.890402	1.09936
22917	0.522844	2.1
22918	0.593846	2.1
22919	1.30226	1.17531
22920	1.30226	0.991859
22921	0.946132	1.61734
22922	1.03537	0.05
22923	1.50035	0.05
22924	0.940356	0.05
22925	0.946131	0.05
22926	1.03441	0.05
22927	1.21171	0.05
22928	1.35169	0.05
22929	1.32449	0.05
22930	0.946131	0.05
22931	0.946131	0.05
22932	1.06627	0.05
22933	1.03102	0.0552412
22934	0.488712	0.05
22935	0.946131	0.05
22936	1.23146	0.05
22937	1.13242	0.05
22938	0.946131	0.05
22939	0.409199	0.05
22940	0.429959	0.05
22941	1.08357	0.05
22942	0.80926	0.05
22943	0.879791	0.05
22944	0.946129	1.16615
22945	1.28828	2.09983
22946	0.946131	0.845475
22947	0.946131	2.1
22948	1.35969	2.1
22949	1.10049	1.92631
22950	0.923724	1.91636
22951	0.923695	2.1
22952	0.94613	1.72108
22953	0.94613	2.1
22954	1.28828	2.1
22955	1.73525	0.05
22956	0.656239	0.05
22957	1.2996	0.05
22958	0.812939	0.05
22959	0.65624	0.05
22960	1.2996	0.05
22961	1.67372	2.1
22962	0.946131	2.1
22963	0.946132	2.1
22964	1.05978	2.1
22965	1.31049	2.1

22966	0.946132	2.1
22967	1.11077	2.1
22968	0.579725	0.05
22969	0.373532	0.05
22970	1.13537	0.05
22971	1.20726	0.05
22972	0.946131	0.05
22973	1.45694	0.0862126
22974	0.750367	0.05
22975	0.946126	0.05
22976	0.750365	0.05
22977	1.25698	0.05
22978	1.35726	0.05
22979	1.6812	0.05
22980	1.78665	1.42196
22981	1.46568	2.1
22982	1.29606	2.1
22983	1.25298	2.1
22984	0.946115	2.1
22985	0.946129	2.1
22986	1.25296	2.1
22987	1.27778	0.05
22988	1.17042	2.1
22989	1.25858	0.05
22990	0.93106	0.05
22991	0.810959	0.05
22992	1.38905	2.1
22993	1.19216	2.1
22994	0.946131	2.1
22995	0.946131	2.1
22996	1.19216	2.1
22997	1.58339	2.1
22998	0.81094	0.05
22999	0.845129	0.05
23000	0.918535	0.05
23001	0.946132	2.1
23002	0.946132	2.1
23003	1.31904	2.1
23004	1.31904	2.1
23005	1.21275	2.1
23006	1.15471	2.1
23007	1.33125	2.1
23008	0.702954	0.05
23009	0.942973	0.05
23010	0.94613	0.05
23011	0.641772	0.05
23012	0.702942	0.05
23013	0.991865	0.05
23014	0.639952	0.05
23015	1.17333	0.05
23016	0.946132	0.05
23017	0.490975	0.05
23018	0.495934	0.05
23019	1.19904	0.05
23020	1.30488	0.05
23021	1.02305	0.05
23022	1.49842	0.05
23023	1.54196	2.1

23024	1.37224	2.1
23025	1.0953	0.05
23026	1.18952	2.1
23027	1.68851	0.0817018
23028	0.946131	0.05
23029	1.12491	0.05
23030	0.583591	0.05
23031	1.28727	0.05
23032	1.12491	0.0566125
23033	0.884641	0.0874612
23034	0.810944	0.063623
23035	0.917973	0.111474
23036	0.846671	0.100711
23037	0.596968	0.05
23038	0.716347	0.084164
23039	1.36255	0.05
23040	1.11077	2.1
23041	0.946132	2.1
23042	0.946132	2.1
23043	1.26267	2.1
23044	1.26441	2.1
23045	1.85238	2.1
23046	1.36693	2.1
23047	0.616123	2.1
23048	0.481844	2.1
23049	0.709599	2.1
23050	0.815889	2.1
23051	1.1502	2.1
23052	1.63937	0.12141
23053	1.25797	0.05
23054	1.22878	0.05
23055	0.946132	0.05
23056	0.946132	0.05
23057	0.965737	0.05
23058	1.1893	0.05
23059	1.22877	0.05
23060	0.735217	0.05
23061	0.85154	0.05
23062	0.851553	0.05
23063	0.851473	0.05
23064	1.58916	0.05
23065	1.25545	0.05
23066	0.810942	0.05
23067	0.919314	0.05
23068	0.928633	0.05
23069	2.14514	0.05
23070	1.23389	2.1
23071	0.946128	0.05
23072	0.969504	0.05
23073	0.851539	0.05
23074	1.41503	0.05
23075	0.958517	0.05
23076	0.990615	0.05
23077	1.7943	0.05
23078	0.946133	0.05
23079	1.27581	0.05
23080	0.946132	0.05
23081	1.36362	0.05

23082	1.36936	0.05
23083	1.71903	2.1
23084	0.751639	2.1
23085	0.77464	2.1
23086	0.946133	2.1
23087	1.04073	2.1
23088	1.23551	2.1
23089	0.94613	2.1
23090	1.18682	2.1
23091	0.946131	2.1
23092	0.94613	2.1
23093	1.40638	2.1
23094	0.483346	0.05
23095	0.432527	0.05
23096	0.946132	0.05
23097	0.483312	0.05
23098	0.946132	0.05
23099	0.878798	0.05
23100	0.810944	0.05
23101	1.14604	0.05
23102	0.946129	0.05
23103	1.35594	0.05
23104	0.538378	0.05
23105	0.946131	0.05
23106	0.538378	0.05
23107	1.00065	0.0796938
23108	0.962878	0.05
23109	1.32634	0.05
23110	1.28066	0.05
23111	0.785651	2.1
23112	1.20327	2.1
23113	1.47058	2.07868
23114	0.551834	0.939791
23115	1.04072	2.1
23116	0.506719	1.66229
23117	1.12786	1.93602
23118	1.45789	0.05
23119	0.681532	0.05
23120	1.21087	0.05
23121	0.94613	0.05
23122	0.681534	0.05
23123	1.21087	0.05
23124	1.98618	0.109039
23125	1.71742	1.30579
23126	1.07427	0.286828
23127	1.1972	0.202418
23128	0.933474	0.169978
23129	1.1745	0.270523
23130	0.94612	0.213599
23131	0.933485	0.444679
23132	1.17449	0.261624
23133	0.851541	0.05
23134	1.12774	0.05
23135	0.946129	0.05
23136	0.851486	0.05
23137	1.12517	0.05
23138	1.12328	0.05
23139	1.13179	2.1

23140	1.02438	2.1
23141	1.05776	2.1
23142	1.41273	1.55078
23143	1.1229	2.1
23144	0.875912	1.65124
23145	0.946132	2.1
23146	0.875912	2.1
23147	1.42307	2.1
23148	1.15354	0.05
23149	0.848382	0.05
23150	1.2685	2.1
23151	0.938104	2.09661
23152	0.793166	2.1
23153	1.07855	2.1
23154	0.98488	2.1
23155	1.4162	2.1
23156	1.09651	0.05
23157	1.98502	0.05
23158	1.41168	0.05
23159	1.33879	0.05
23160	0.946127	0.05
23161	1.13528	0.05
23162	0.946128	0.05
23163	1.09726	2.1
23164	1.22026	0.902055
23165	1.09044	2.1
23166	2.23961	0.382823
23167	1.14721	0.05
23168	1.17614	1.45287
23169	0.615065	2.1
23170	0.946131	2.1
23171	0.778488	2.1
23172	0.94613	2.1
23173	0.615066	2.1
23174	1.78254	2.1
23175	0.898452	0.05
23176	1.47411	2.1
23177	0.59676	0.05
23178	0.59676	0.05
23179	0.94613	0.05
23180	1.13511	0.05
23181	0.946131	0.05
23182	0.916275	2.1
23183	0.946132	2.1
23184	0.916274	1.42775
23185	1.36182	2.1
23186	0.946131	2.1
23187	0.857202	0.05
23188	0.941918	0.05
23189	0.943349	0.05
23190	1.2493	0.05
23191	0.946133	0.05
23192	1.01814	0.05
23193	0.927881	0.05
23194	1.11845	0.05
23195	1.2493	0.05
23196	1.11845	0.05
23197	0.946129	0.05

23198	0.967673	0.101512
23199	1.01332	0.05
23200	0.946133	0.05
23201	0.81938	0.05
23202	0.819377	0.05
23203	0.946133	0.05
23204	1.22474	0.05
23205	1.15321	0.05
23206	0.797489	0.05
23207	0.946131	0.05
23208	0.797461	0.05
23209	1.35328	0.639158
23210	1.03318	1.16403
23211	0.946131	0.934553
23212	0.94613	1.03158
23213	1.03319	1.16645
23214	1.37938	0.63519
23215	1.00512	1.17182
23216	0.946133	0.770159
23217	0.946132	0.99469
23218	1.00512	1.0144
23219	1.5515	1.20167
23220	1.55351	2.1
23221	0.94261	0.05
23222	0.828156	0.05
23223	0.916495	0.05
23224	3.06309	0.05
23225	0.619832	0.05
23226	0.705903	0.05
23227	1.2968	0.05
23228	0.946133	0.05
23229	0.705904	0.05
23230	1.2968	0.05
23231	1.01971	0.05
23232	0.60302	0.05
23233	1.07242	0.160421
23234	1.26426	0.143806
23235	0.946131	0.190407
23236	0.946131	0.224168
23237	0.963109	0.192805
23238	0.947862	0.150767
23239	1.40248	0.967145
23240	0.767143	1.07251
23241	0.767114	1.0729
23242	0.946129	1.11158
23243	0.767143	0.702457
23244	1.22637	0.600201
23245	1.2281	0.968265
23246	0.590909	1.17613
23247	0.490832	0.892444
23248	0.804174	1.16314
23249	0.747368	0.723218
23250	1.05129	0.05
23251	1.10609	0.177022
23252	1.3639	0.05
23253	0.776117	0.05
23254	0.752329	0.05
23255	1.04073	0.05

23256	0.67713	0.05
23257	3.4245	2.1
23258	1.18526	2.1
23259	1.2161	1.26574
23260	0.876834	2.07327
23261	0.876831	1.15778
23262	0.946132	1.92076
23263	0.876834	1.4692
23264	0.954563	2.1
23265	0.924132	2.1
23266	1.25075	2.1
23267	1.01652	2.1
23268	0.92413	2.1
23269	1.37579	2.1
23270	0.992159	2.1
23271	1.26092	2.1
23272	0.929435	2.1
23273	0.844958	2.1
23274	0.79598	2.1
23275	0.852559	2.1
23276	0.946131	0.05
23277	1.28435	0.05
23278	0.827261	0.05
23279	0.946128	0.05
23280	0.827298	0.05
23281	1.65452	0.05
23282	1.79929	2.1
23283	0.791947	2.06297
23284	0.902072	1.66491
23285	0.882672	1.98672
23286	1.21703	2.1
23287	0.900338	2.1
23288	1.37712	2.1
23289	0.782591	2.1
23290	0.782562	2.1
23291	0.94613	2.1
23292	1.34749	2.1
23293	0.61359	2.1
23294	1.14397	0.05
23295	1.02963	0.05
23296	1.36589	0.05
23297	0.946132	0.05
23298	1.28644	0.05
23299	0.946131	0.05
23300	1.40104	0.05
23301	1.28644	0.05
23302	0.810946	0.05
23303	1.09178	0.05
23304	1.26956	0.05
23305	0.810943	0.05
23306	0.885633	0.05
23307	1.00844	0.05
23308	0.825671	0.05
23309	0.792093	0.05
23310	0.925037	0.05
23311	0.946131	0.101726
23312	0.946131	0.05
23313	1.18052	0.05

23314	1.40265	0.05
23315	0.946132	0.0744183
23316	1.17891	0.05
23317	0.734248	0.05
23318	0.810943	0.05
23319	0.731621	0.05
23320	0.799857	0.05
23321	0.834507	0.203862
23322	0.810932	0.0613888
23323	1.01316	0.05
23324	1.23884	0.05
23325	1.88438	2.1
23326	0.946129	2.1
23327	1.38614	2.1
23328	1.34245	1.25451
23329	0.946131	1.06361
23330	1.38614	1.00989
23331	0.946132	0.05
23332	0.910733	0.05
23333	1.24726	0.05
23334	1.13742	0.05
23335	0.851555	0.05
23336	1.50368	0.05
23337	0.94613	0.05
23338	1.08998	2.1
23339	1.36899	0.05
23340	1.36898	0.187296
23341	0.946131	0.0692286
23342	1.26045	2.1
23343	1.06432	0.208917
23344	1.89016	0.0501561
23345	0.83514	1.83393
23346	1.36653	2.1
23347	1.23426	2.08436
23348	0.94613	1.88637
23349	0.835112	2.1
23350	1.14567	0.716397
23351	1.12096	0.384647
23352	0.881106	1.08587
23353	1.17787	0.05
23354	1.29738	1.01985
23355	0.940472	0.05
23356	1.03452	1.08592
23357	0.946131	0.0624287
23358	2.7489	0.529326
23359	1.01778	2.1
23360	1.04321	0.580376
23361	1.19352	0.05
23362	1.16912	1.76447
23363	0.946132	1.33213
23364	0.946132	2.01419
23365	1.40924	1.97437
23366	1.22927	2.1
23367	2.09455	0.05
23368	3.00575	2.1
23369	1.08928	0.106662
23370	1.94799	2.1
23371	1.4092	2.1

23372	0.946132	2.1
23373	1.16	2.1
23374	1.01985	2.1
23375	0.946133	2.1
23376	1.16	2.1
23377	1.15371	0.05
23378	1.56002	0.05
23379	1.23007	2.1
23380	1.9992	2.1
23381	0.652605	2.1
23382	0.937903	2.1
23383	0.946132	2.1
23384	0.937902	2.1
23385	1.228	2.1
23386	1.33694	1.63405
23387	1.00191	2.1
23388	0.716814	1.72734
23389	0.94613	2.1
23390	0.716816	2.1
23391	1.70388	1.93733
23392	0.946129	0.0836412
23393	1.09362	0.131885
23394	0.946131	0.128758
23395	1.4185	0.13779
23396	1.09362	0.086942
23397	1.31057	0.674586
23398	1.17631	0.05
23399	1.19404	0.05
23400	0.94613	0.05
23401	0.946129	0.05
23402	0.99972	0.05
23403	0.94613	0.05
23404	0.835199	0.05
23405	1.23351	0.05
23406	0.582785	0.05
23407	0.701079	0.05
23408	0.946132	0.05
23409	2.17385	0.05
23410	1.18948	2.06403
23411	1.25346	2.1
23412	1.18586	0.305067
23413	0.880876	0.05
23414	0.68632	0.205536
23415	0.946118	2.1
23416	0.966936	2.1
23417	0.946117	2.1
23418	1.41636	2.1
23419	1.03798	2.1
23420	0.729178	0.34198
23421	0.721888	0.893736
23422	0.946132	0.511969
23423	0.656286	1.07219
23424	1.13898	0.62704
23425	1.02351	0.05
23426	1.48803	0.05
23427	0.946131	0.05
23428	1.04921	2.1
23429	1.02478	0.05

23430	0.993023	2.1
23431	1.80841	0.05
23432	1.29215	0.05
23433	0.904206	0.05
23434	0.904207	0.05
23435	0.946132	0.05
23436	0.904207	0.05
23437	1.07347	2.1
23438	0.555875	1.44831
23439	0.555904	1.79702
23440	1.30841	2.1
23441	1.30841	2.1
23442	0.91951	2.1
23443	1.33026	0.05
23444	1.43289	0.05
23445	1.15703	1.63228
23446	0.886594	2.1
23447	1.05129	1.91118
23448	1.08421	0.130975
23449	1.35946	0.160707
23450	0.882955	0.393092
23451	0.946131	0.307148
23452	0.882985	0.238037
23453	0.583501	0.133591
23454	0.532921	0.154077
23455	0.94613	1.87745
23456	0.532892	1.40206
23457	1.23224	1.27459
23458	1.12786	1.10283
23459	0.946131	0.308059
23460	1.23732	0.05
23461	1.40609	0.102347
23462	1.40609	0.05
23463	0.946131	0.05
23464	0.910672	1.66733
23465	1.08478	1.35184
23466	0.94613	0.102825
23467	0.94613	1.17414
23468	1.30382	1.14498
23469	3.10544	2.0997
23470	0.679509	2.1
23471	1.15966	1.51419
23472	0.75499	2.1
23473	1.37511	1.85438
23474	1.01776	1.52828
23475	2.35239	0.875195
23476	0.92406	2.1
23477	0.946118	2.1
23478	0.92406	2.1
23479	1.07979	2.1
23480	1.29604	2.1
23481	1.29897	2.1
23482	0.902645	0.43017
23483	0.729493	1.48314
23484	0.906844	0.994672
23485	0.739518	0.05
23486	1.10564	0.05
23487	0.946131	0.05

23488	0.739519	0.05
23489	0.739506	0.05
23490	1.79907	0.05
23491	1.59106	0.81144
23492	1.11067	0.415648
23493	0.795516	0.67605
23494	0.795519	0.544425
23495	0.946132	0.695467
23496	0.795506	0.432156
23497	1.80223	2.1
23498	1.95572	2.1
23499	1.16237	0.450006
23500	1.07518	2.1
23501	0.696199	0.458068
23502	0.94613	1.59677
23503	0.696199	1.05081
23504	1.6379	0.05
23505	0.946118	0.05
23506	0.851529	0.05
23507	1.08716	0.05
23508	0.973118	0.05
23509	0.851528	0.05
23510	1.15096	2.1
23511	0.65023	0.05
23512	0.94613	0.05
23513	0.650205	0.05
23514	0.650205	0.05
23515	0.811233	0.05
23516	0.778721	2.1
23517	0.810945	1.02198
23518	0.997518	1.07182
23519	0.838028	0.05
23520	0.952893	0.05
23521	0.838037	0.05
23522	1.58166	0.05
23523	1.44848	1.48502
23524	1.30041	0.05
23525	1.03697	0.05
23526	0.946132	1.56888
23527	1.0277	0.80784
23528	0.946131	1.63947
23529	1.00501	1.04245
23530	1.0277	0.567434
23531	1.64988	2.1
23532	0.946131	2.1
23533	0.94613	0.05
23534	1.27702	2.1
23535	1.55163	0.113014
23536	0.897905	0.0842355
23537	1.12971	0.05
23538	1.35715	0.05
23539	0.881884	0.203979
23540	0.664007	0.05
23541	0.663978	0.0983306
23542	0.94613	0.0679724
23543	0.664006	0.094903
23544	1.48693	0.05
23545	1.29289	0.05

23546	1.32081	0.0522518
23547	0.946119	0.05
23548	0.946132	0.05
23549	1.02972	0.05
23550	0.946131	2.1
23551	0.946127	2.1
23552	1.23513	2.1
23553	1.38154	2.1
23554	0.94613	2.1
23555	1.25933	0.05
23556	0.917954	0.05
23557	0.868589	0.05
23558	3.0957	2.1
23559	0.711391	0.05
23560	0.711391	0.05
23561	0.991303	0.05
23562	1.37322	0.05
23563	0.729145	0.05
23564	2.55648	0.05
23565	1.0756	2.00421
23566	0.760299	1.19199
23567	0.763592	0.0759034
23568	0.946126	0.161223
23569	0.76359	0.0665793
23570	1.35862	0.944278
23571	0.946133	2.1
23572	1.00807	2.1
23573	0.946132	2.1
23574	1.39823	2.1
23575	1.07817	2.1
23576	1.44379	1.10073
23577	1.17934	0.05
23578	1.13451	0.05
23579	0.94613	0.40391
23580	1.03974	2.1
23581	1.36835	0.290785
23582	1.36835	2.1
23583	0.94613	2.1
23584	1.27096	2.1
23585	0.853042	2.1
23586	0.946131	2.1
23587	1.13658	2.1
23588	1.06997	2.1
23589	0.94613	2.1
23590	1.41556	0.05
23591	1.21708	0.130088
23592	0.94613	0.140039
23593	0.946131	0.0861488
23594	1.21708	0.0846118
23595	0.964782	0.0535993
23596	0.946119	0.0970687
23597	1.23447	0.0751343
23598	0.946118	0.0981433
23599	1.41844	0.0709551
23600	1.23446	0.0838463
23601	1.1357	0.05
23602	0.619856	0.05
23603	1.1509	0.05

23604	0.946126	0.05
23605	1.19262	0.05
23606	1.1509	0.05
23607	1.04268	2.1
23608	2.07527	0.276763
23609	1.38259	0.715812
23610	0.980054	1.64982
23611	0.946124	0.876109
23612	0.946127	1.26627
23613	0.980056	1.0394
23614	1.62916	2.05982
23615	0.946131	2.1
23616	1.40571	2.1
23617	0.946131	2.1
23618	1.38885	2.1
23619	1.40571	2.1
23620	1.11787	0.166022
23621	0.946129	0.05
23622	1.28831	2.1
23623	1.41498	0.05
23624	1.41498	0.668945
23625	0.946131	0.134747
23626	0.850964	0.05
23627	0.921299	0.05
23628	1.37358	0.05
23629	0.946132	0.05
23630	0.850964	0.05
23631	0.823988	0.05
23632	0.823984	0.05
23633	1.36568	0.05
23634	1.36569	0.05
23635	0.921275	0.05
23636	1.84739	2.1
23637	1.47169	0.05
23638	0.946131	0.05
23639	0.735844	0.05
23640	1.11819	0.05
23641	1.10904	0.05
23642	0.735831	0.05
23643	0.946131	2.1
23644	0.946132	2.1
23645	1.23487	2.1
23646	1.3281	2.1
23647	0.946131	2.1
23648	1.72271	0.05
23649	0.861359	0.05
23650	0.946131	0.05
23651	0.861357	0.05
23652	1.1369	0.05
23653	1.26868	0.05
23654	0.789173	2.1
23655	0.789143	2.1
23656	0.946133	1.35545
23657	1.34386	2.05317
23658	0.789144	2.1
23659	0.983493	2.1
23660	1.18944	0.259653
23661	1.05121	0.200363

23662	0.777398	0.357817
23663	0.946132	0.550101
23664	0.777423	0.700768
23665	1.09841	0.05
23666	0.963919	0.05
23667	0.474855	0.05
23668	0.946131	0.05
23669	0.474855	0.05
23670	3.40074	1.1274
23671	1.29453	2.1
23672	1.01139	0.05
23673	0.946133	0.171048
23674	1.35381	0.05
23675	1.18322	0.0521562
23676	0.946133	0.124321
23677	1.45502	0.05
23678	0.970088	1.10935
23679	0.960333	1.3914
23680	0.838036	1.42542
23681	1.30927	0.05
23682	0.828617	0.05
23683	0.714795	0.05
23684	0.946132	0.05
23685	0.714824	0.05
23686	0.946131	2.1
23687	0.851542	2.1
23688	1.38204	2.1
23689	1.24512	2.1
23690	1.04086	2.1
23691	0.723573	2.1
23692	0.946131	2.1
23693	0.982454	2.1
23694	0.982454	2.1
23695	0.723544	2.1
23696	1.17261	0.05
23697	1.34153	0.0527792
23698	1.41383	0.0611087
23699	0.946132	0.05
23700	0.946133	0.05
23701	1.12818	0.05
23702	1.29383	0.05
23703	0.946133	0.05
23704	1.0027	0.05
23705	0.768989	0.05
23706	0.946133	0.05
23707	0.768977	0.05
23708	0.946132	0.05
23709	1.24333	0.05
23710	0.928583	0.05
23711	0.946132	0.05
23712	0.928583	0.05
23713	1.53798	0.05
23714	2.95436	0.05
23715	1.30649	0.05
23716	0.809789	0.05
23717	0.745744	0.05
23718	0.745774	0.05
23719	0.946128	0.05

23720	1.00777	0.05
23721	1.40271	0.05
23722	0.768387	0.05
23723	0.946132	0.05
23724	0.922045	0.05
23725	0.946132	2.1
23726	1.39021	2.1
23727	1.10046	2.1
23728	0.946132	2.1
23729	1.39021	2.1
23730	2.06158	0.05
23731	0.913967	0.200047
23732	1.30107	0.633755
23733	0.946132	0.21577
23734	0.913966	0.395791
23735	1.30107	0.171675
23736	1.64117	2.1
23737	0.514872	1.93694
23738	0.514846	0.723819
23739	1.30811	2.1
23740	1.30812	0.702546
23741	0.888989	1.18437
23742	1.99279	1.86163
23743	0.910271	0.744908
23744	0.838027	1.09204
23745	0.928444	0.891685
23746	1.2377	1.51637
23747	0.851537	2.1
23748	0.899142	2.1
23749	0.810075	2.1
23750	1.3771	2.1
23751	1.03281	2.1
23752	0.719877	2.1
23753	0.901838	2.1
23754	1.01748	2.1
23755	1.12315	2.1
23756	0.64787	2.1
23757	1.173 2.1	
23758	0.876903	2.1
23759	0.597337	2.1
23760	1.00291	2.1
23761	0.597309	2.1
23762	1.09897	0.05
23763	0.946132	0.251838
23764	1.1386	0.05
23765	1.09897	0.05
23766	0.596652	0.0606216
23767	2.5593	2.1
23768	0.946133	0.849042
23769	1.29172	0.537867
23770	1.11337	0.0856182
23771	0.946131	0.218869
23772	0.946131	0.0871351
23773	0.96926	0.489177
23774	0.946132	1.15275
23775	0.946132	1.20681
23776	1.07118	0.255879
23777	1.29698	0.319144

23778	1.37867	0.699177
23779	0.946119	1.67907
23780	0.94612	2.02774
23781	1.33154	0.740369
23782	0.946107	1.30713
23783	2.17757	1.3633
23784	0.946131	0.05
23785	0.68393	0.05
23786	0.68396	0.05
23787	1.33671	0.05
23788	1.10733	0.05
23789	2.31367	2.1
23790	0.989326	1.65205
23791	0.689952	2.1
23792	0.854787	1.67141
23793	3.73635	2.1
23794	1.41489	2.1
23795	1.25544	0.479839
23796	0.946128	2.1
23797	0.946129	2.1
23798	1.13536	2.1
23799	2.10284	1.46283
23800	0.851542	2.1
23801	0.828523	2.1
23802	0.946132	2.1
23803	0.946132	2.1
23804	1.50873	2.1
23805	0.989048	2.1
23806	0.925055	2.1
23807	0.838041	2.1
23808	0.807714	0.272696
23809	0.656831	0.05
23810	0.997603	0.15784
23811	0.946131	0.684174
23812	1.02602	0.334985
23813	0.946129	0.916091
23814	1.35469	0.382606
23815	1.25083	0.313012
23816	0.851541	0.05
23817	0.851483	0.05
23818	0.851539	0.05
23819	1.19785	0.05
23820	1.07615	0.05
23821	0.714507	0.05
23822	1.41461	0.05
23823	0.714507	0.05
23824	0.714507	0.05
23825	0.946133	0.05
23826	0.946131	0.382699
23827	0.972232	0.367721
23828	0.94613	1.53176
23829	1.25083	0.231149
23830	1.30165	0.28944
23831	1.05332	2.1
23832	0.574768	2.1
23833	1.94541	0.05
23834	0.789633	0.05
23835	0.946127	0.05

23836	1.33798	0.05
23837	1.22903	0.05
23838	0.946128	0.05
23839	0.946128	2.1
23840	1.11882	2.1
23841	0.996487	2.1
23842	0.94613	2.1
23843	1.11883	2.1
23844	0.946127	2.1
23845	1.30082	2.1
23846	1.02608	2.1
23847	0.946128	2.1
23848	1.30081	2.1
23849	1.3507	1.78227
23850	1.19583	0.0709905
23851	0.946127	2.07529
23852	0.94613	1.31595
23853	1.19583	1.71007
23854	0.477652	0.05
23855	1.20416	0.05
23856	0.946131	0.05
23857	0.477652	0.05
23858	0.477691	0.05
23859	1.34536	0.05
23860	0.758971	0.05
23861	1.25124	0.05
23862	1.31467	0.05
23863	0.758971	0.05
23864	1.31467	0.05
23865	0.946131	2.1
23866	1.14184	2.1
23867	1.35028	2.1
23868	1.35028	2.1
23869	0.946131	2.1
23870	1.13537	2.1
23871	0.946132	1.02476
23872	0.946132	2.1
23873	1.23031	2.1
23874	1.27555	2.1
23875	1.53064	2.1
23876	1.28421	1.26028
23877	1.07707	0.966039
23878	0.946125	1.18495
23879	0.946127	1.23531
23880	1.07707	1.19367
23881	0.946133	0.05
23882	1.3495	0.05
23883	0.946132	0.05
23884	1.05704	0.05
23885	1.3495	0.05
23886	0.946132	0.05
23887	1.37794	0.05
23888	0.946132	0.05
23889	1.01367	0.05
23890	1.37794	0.05
23891	0.928516	2.1
23892	0.946133	2.1
23893	0.928518	2.1

23894	1.14105	2.1
23895	1.26406	2.1
23896	0.797664	2.1
23897	0.946132	2.1
23898	0.797663	2.1
23899	1.34285	2.1
23900	0.929875	2.1
23901	0.946131	1.51635
23902	1.21139	0.460203
23903	1.41492	0.0722035
23904	1.41492	0.143102
23905	0.946131	0.0625817
23906	0.946131	2.1
23907	0.689388	2.1
23908	0.94365	2.1
23909	0.777345	2.1
23910	0.709599	2.1
23911	0.774968	2.1
23912	0.946132	2.1
23913	0.77497	2.1
23914	0.868145	2.1
23915	1.28401	2.1
23916	0.94613	0.123021
23917	1.19655	1.93276
23918	1.39428	0.05
23919	1.39428	0.210367
23920	0.946131	0.05
23921	1.1848	2.1
23922	1.07591	2.1
23923	0.946131	2.1
23924	0.946131	2.1
23925	0.874788	2.1
23926	1.35703	2.1
23927	1.35357	2.1
23928	0.946132	2.1
23929	1.26022	2.1
23930	0.946132	2.1
23931	0.946131	2.1
23932	0.966426	2.1
23933	0.946133	2.1
23934	0.946131	2.1
23935	1.3484	2.1
23936	1.26441	2.1
23937	1.2079	2.1
23938	0.946133	2.1
23939	0.946132	2.1
23940	1.2079	2.1
23941	1.2736	2.1
23942	1.25774	2.1
23943	0.946133	2.1
23944	0.851555	2.1
23945	0.96172	2.1
23946	0.946128	2.1
23947	1.06879	2.1
23948	0.94613	2.1
23949	1.32608	2.1
23950	1.06879	2.1
23951	0.946132	2.1

23952	1.32608	2.1
23953	0.946131	2.1
23954	1.40488	2.1
23955	1.32608	2.1
23956	0.94613	0.809249
23957	1.19234	0.0911053
23958	0.946129	0.2888
23959	0.94613	1.14092
23960	1.31649	1.4733
23961	0.912059	0.614541
23962	1.04514	0.655371
23963	0.946132	0.395092
23964	0.946132	1.90753
23965	1.34646	2.04574
23966	1.01187	0.34257
23967	1.33728	0.342853
23968	0.761974	0.05
23969	0.761974	0.515338
23970	0.94613	0.106834
23971	1.40641	2.05198
23972	0.955752	0.492584
23973	0.736738	2.07088
23974	0.94613	0.921183
23975	0.736739	1.16864
23976	0.861471	2.1
23977	1.34556	2.1
23978	0.810957	2.1
23979	0.946131	2.1
23980	1.37811	2.1
23981	1.17949	2.1
23982	0.946132	2.1
23983	1.37811	2.1
23984	0.946131	2.1
23985	1.40447	2.1
23986	0.946132	2.1
23987	0.946132	2.1
23988	1.39826	2.1
23989	0.946131	2.1
23990	1.283 2.1	
23991	0.94613	2.1
23992	0.946131	2.1
23993	1.40447	2.1
23994	1.20541	2.1
23995	0.946131	2.1
23996	0.946127	2.1
23997	1.33607	2.1
23998	1.41021	2.1
23999	1.01271	2.1
24000	0.946131	2.1
24001	0.920606	2.1
24002	1.13078	2.1
24003	1.60331	2.1
24004	0.946118	0.462594
24005	1.09718	2.1
24006	0.946116	1.34478
24007	1.32662	0.299786
24008	1.0972	0.994258
24009	0.94613	0.470971

24010	1.32666	1.35727
24011	0.946128	0.136949
24012	1.31381	0.116744
24013	1.32666	0.076109
24014	0.94612	2.1
24015	0.940748	2.1
24016	1.03561	2.1
24017	1.35111	2.1
24018	0.851527	2.1
24019	0.607156	2.1
24020	0.789158	2.1
24021	0.946129	1.37849
24022	1.04071	2.1
24023	1.13144	2.1
24024	0.946133	2.1
24025	1.35663	2.1
24026	0.946132	2.1
24027	0.946133	2.1
24028	1.34237	2.1
24029	0.919455	2.1
24030	1.01143	2.1
24031	0.946128	2.1
24032	0.946131	2.1
24033	1.62796	2.1
24034	1.17796	1.64884
24035	1.23024	2.1
24036	0.946132	1.21439
24037	0.85153	1.9627
24038	0.986867	1.98671
24039	0.889308	2.1
24040	0.94613	2.1
24041	0.889338	2.1
24042	0.946131	2.1
24043	0.889337	2.1
24044	1.18396	1.18336
24045	0.94613	0.832953
24046	0.946125	1.95891
24047	1.39514	1.26022
24048	1.30809	1.56608
24049	0.803322	2.1
24050	0.925893	2.0674
24051	1.36183	1.48569
24052	0.925892	1.64094
24053	0.946133	1.6614
24054	1.38354	0.0801543
24055	1.16009	0.156511
24056	0.946132	0.0600162
24057	0.946132	0.05
24058	1.01462	0.05
24059	1.18854	0.668907
24060	0.888265	1.91356
24061	0.745095	1.11175
24062	0.709597	1.65755
24063	0.936541	1.46653
24064	1.24142	0.107173
24065	1.35059	0.077789
24066	0.946128	0.05
24067	0.94613	0.105097

24068	1.16623	0.0834312
24069	1.24945	0.05
24070	1.33035	0.05
24071	0.946127	0.05
24072	0.946128	0.05
24073	1.13343	0.05
24074	0.637995	0.706271
24075	1.10455	0.762956
24076	1.22641	1.64953
24077	1.22641	2.03083
24078	0.946132	1.44961
24079	1.3665	0.05
24080	1.28507	0.05
24081	0.946127	0.05
24082	0.946129	0.05
24083	1.2051	0.05
24084	0.797374	2.1
24085	0.797376	2.1
24086	1.13113	2.1
24087	1.20057	2.1
24088	1.12049	2.1
24089	0.59387	0.550979
24090	1.13245	0.402866
24091	0.966134	0.896704
24092	0.593842	0.210727
24093	0.946128	1.04177
24094	0.641455	0.0859032
24095	0.916507	0.0862655
24096	1.04072	1.04742
24097	0.415585	0.0501763
24098	0.507244	0.776456
24099	0.678938	2.1
24100	1.24154	2.1
24101	1.25	1.75031
24102	1.24154	2.08553
24103	1.25	2.07761
24104	0.851009	1.30393
24105	0.946131	2.09968
24106	0.851539	1.61166
24107	1.21997	1.20651
24108	1.45109	1.14036
24109	0.804877	0.419234
24110	1.00062	0.05
24111	0.851536	0.283899
24112	0.909673	0.05
24113	0.946129	0.338772
24114	1.20494	0.05
24115	1.09088	0.05
24116	0.887634	0.05
24117	0.81027	0.05
24118	0.900294	0.05
24119	0.747713	0.05
24120	0.975467	0.05
24121	0.747712	0.05
24122	1.39168	0.05
24123	0.975467	0.05
24124	0.94613	0.05
24125	1.32827	0.05

24126	0.946128	0.05
24127	1.41201	0.05
24128	1.32827	0.05
24129	0.981121	0.05
24130	0.936311	0.05
24131	1.00715	0.05
24132	1.15914	0.05
24133	0.704409	0.05
24134	0.713444	0.344072
24135	0.954538	0.0823159
24136	0.944508	0.11022
24137	0.851165	0.510796
24138	0.946131	1.43059
24139	0.851192	0.378711
24140	0.946131	0.302092
24141	1.3279	0.229163
24142	1.33587	0.05
24143	0.946126	0.05
24144	1.27512	0.05
24145	1.33587	0.05
24146	0.748059	0.05
24147	1.17451	0.05
24148	1.04299	0.05
24149	0.851542	0.05
24150	0.946131	0.05
24151	0.892109	0.05
24152	0.946131	0.05
24153	0.851541	0.05
24154	1.2178	0.05
24155	1.2179	0.05
24156	0.946793	0.05
24157	0.709599	0.05
24158	0.987426	0.05
24159	1.39502	0.05
24160	1.13624	0.05
24161	0.73176	0.05
24162	1.36697	0.05
24163	1.33393	0.05
24164	0.946131	0.05
24165	0.946132	0.05
24166	1.23845	0.05
24167	0.50702	0.599628
24168	1.06442	0.481501
24169	0.946131	0.988499
24170	1.02363	0.965807
24171	1.13537	0.05
24172	1.25049	0.05
24173	0.94613	0.05
24174	1.14993	0.05
24175	1.38056	0.05
24176	0.946126	0.05
24177	1.24678	0.05
24178	0.684521	0.05
24179	0.946124	0.05
24180	0.684518	0.05
24181	0.851542	0.189814
24182	0.925459	0.138191
24183	0.946131	0.227854

24184	0.851474	0.370202
24185	0.923305	0.294869
24186	1.4055	0.05
24187	1.32077	0.05
24188	0.946127	0.05
24189	1.21757	0.05
24190	0.946129	0.05
24191	0.711214	0.05
24192	1.30463	0.05
24193	0.711185	0.05
24194	0.711185	0.05
24195	1.22716	0.05
24196	1.29958	0.05
24197	1.26201	0.05
24198	0.568927	0.05
24199	0.568927	0.05
24200	0.946132	0.05
24201	0.851542	0.05
24202	1.44968	0.05
24203	0.851543	0.05
24204	1.35123	0.05
24205	1.40427	0.05
24206	0.593295	0.05
24207	0.946129	0.05
24208	1.22144	0.05
24209	0.908278	0.05
24210	1.22144	0.05
24211	0.867593	0.05
24212	1.23351	0.05
24213	0.946131	0.05
24214	1.05516	0.05
24215	1.23351	0.05
24216	1.15564	0.05
24217	0.946131	0.05
24218	0.766652	0.05
24219	0.766681	0.05
24220	0.946131	0.05
24221	0.946131	0.05
24222	0.897851	0.05
24223	1.1751	0.05
24224	1.00273	0.05
24225	0.851554	0.05
24226	0.883272	0.05
24227	1.17111	0.110116
24228	0.851541	0.05
24229	1.01665	0.05
24230	0.851555	0.05
24231	0.946131	0.05
24232	0.911508	0.05
24233	1.08054	0.05
24234	1.33007	0.05
24235	0.911471	0.05
24236	1.26787	2.06741
24237	1.16641	1.76205
24238	0.946132	1.72096
24239	0.946133	1.32606
24240	1.16641	1.91309
24241	0.946131	2.1

24242	1.25037	0.05
24243	1.34733	1.12164
24244	1.34733	0.05
24245	0.946131	0.354443
24246	0.946131	0.05
24247	1.41377	0.05
24248	0.94613	0.05
24249	1.30541	0.05
24250	1.41377	0.05
24251	1.3473	2.1
24252	1.11804	2.1
24253	0.94613	2.1
24254	0.946131	2.1
24255	1.11805	2.1
24256	1.32068	2.1
24257	0.946131	2.1
24258	0.946128	2.1
24259	1.39384	2.1
24260	1.4181	2.1
24261	1.12913	2.1
24262	0.94613	2.1
24263	0.946129	2.1
24264	1.20328	2.1
24265	1.39385	2.1
24266	1.3335	2.1
24267	1.40987	2.1
24268	0.94613	2.1
24269	1.21474	2.1
24270	0.94613	2.1
24271	0.946132	2.1
24272	1.15203	2.1
24273	1.41192	1.26074
24274	1.41192	2.1
24275	0.946132	2.1
24276	0.946131	2.1
24277	1.40684	2.1
24278	1.2597	2.1
24279	0.946131	2.1
24280	1.40684	2.04613
24281	0.946131	2.1
24282	1.39113	2.1
24283	1.34049	1.42742
24284	0.946131	1.67375
24285	1.39113	1.38566
24286	0.946132	2.03784
24287	1.31649	0.474626
24288	0.946132	0.42336
24289	0.946132	1.03833
24290	1.34297	0.964729
24291	0.946131	1.09907
24292	1.41766	1.07175
24293	0.954559	2.1
24294	0.946131	0.615805
24295	0.946131	0.243785
24296	1.25447	0.0574605
24297	1.35559	0.0599313
24298	0.946127	0.05
24299	0.946128	0.0547133

24300	1.14874	0.0581929
24301	1.33035	0.05
24302	1.3665	0.05
24303	0.946129	0.05
24304	0.94613	0.05
24305	1.20732	0.05
24306	0.946131	2.1
24307	0.946131	2.1
24308	1.32949	2.1
24309	1.34921	2.1
24310	0.946131	2.1
24311	0.946132	0.678506
24312	1.25479	0.80666
24313	1.38357	0.519624
24314	1.38357	0.05
24315	0.946132	0.071276
24316	0.946131	1.70565
24317	1.31034	0.05
24318	1.37127	0.305363
24319	1.37127	0.05
24320	0.946131	0.123737
24321	1.23923	0.05
24322	1.41169	0.05
24323	0.94613	0.05
24324	1.12703	0.05
24325	0.946131	0.05
24326	0.946119	2.1
24327	1.41915	2.1
24328	1.02679	2.1
24329	0.946107	2.1
24330	1.41917	2.1
24331	0.946133	2.1
24332	1.36581	2.1
24333	0.946131	2.1
24334	1.36581	2.1
24335	0.946131	2.1
24336	0.946132	2.1
24337	1.04384	2.1
24338	1.34973	2.1
24339	1.34967	2.1
24340	0.851555	2.1
24341	0.946132	0.05
24342	1.3855	0.05
24343	1.08603	0.05
24344	1.08602	0.05
24345	1.16234	0.05
24346	0.946133	0.05
24347	1.29348	0.0534771
24348	1.20111	0.05
24349	1.20112	0.05
24350	1.3855	0.05
24351	0.946128	1.88806
24352	1.30798	2.1
24353	0.946129	2.1
24354	1.34828	2.1
24355	1.30797	2.1
24356	1.24932	0.05
24357	1.40549	0.05

24358	0.946117	0.05
24359	1.19615	0.05
24360	0.946106	0.05
24361	0.946131	2.1
24362	1.29926	2.1
24363	1.25144	2.1
24364	0.946131	2.1
24365	1.29925	2.1
24366	0.946132	2.1
24367	1.39826	2.1
24368	0.946133	2.1
24369	0.946133	2.1
24370	1.29926	2.1
24371	0.946119	0.05
24372	1.33614	0.05
24373	0.946118	0.05
24374	1.41377	0.05
24375	1.33612	0.05
24376	0.946133	1.55825
24377	1.3933	2.1
24378	1.34999	0.414653
24379	0.946133	1.76543
24380	1.3933	0.894286
24381	0.946131	1.95665
24382	1.36139	2.1
24383	0.946132	1.06045
24384	0.946131	2.1
24385	1.39329	1.43556
24386	1.37449	1.53336
24387	1.27925	0.118835
24388	0.919796	0.148905
24389	1.01176	0.396651
24390	0.94613	0.993593
24391	0.946133	2.1
24392	1.27679	2.1
24393	0.946133	1.85319
24394	0.946132	2.1
24395	1.39731	2.1
24396	0.946119	2.1
24397	1.40765	1.91334
24398	1.1981	2.1
24399	0.946131	1.79178
24400	1.40764	2.1
24401	0.946127	2.1
24402	1.3557	2.1
24403	0.946127	2.1
24404	0.946128	2.1
24405	1.13485	2.1
24406	1.4008	2.1
24407	1.1587	2.1
24408	0.946131	2.1
24409	0.946131	2.1
24410	1.1587	2.1
24411	1.32082	0.05
24412	1.29215	0.05
24413	0.94613	0.05
24414	0.946131	0.05
24415	1.02366	0.05

24416	1.27697	0.0569785
24417	1.2929	0.0762741
24418	0.946132	0.05
24419	0.946132	0.05
24420	1.01451	0.05
24421	0.946132	2.1
24422	1.27252	2.1
24423	0.946132	2.1
24424	1.37869	2.1
24425	1.27252	2.1
24426	1.15627	2.1
24427	1.35703	2.1
24428	0.946132	2.1
24429	1.03783	2.1
24430	0.946132	2.1
24431	0.946128	2.1
24432	1.37869	2.1
24433	0.946131	2.1
24434	1.35357	2.1
24435	1.37869	2.1
24436	0.946132	2.1
24437	0.946131	2.1
24438	1.33471	2.1
24439	0.946131	2.1
24440	1.36373	2.1
24441	1.41342	0.551953
24442	1.06832	0.854347
24443	0.946128	1.7022
24444	0.94613	0.148429
24445	1.06832	1.3602
24446	1.26929	2.1
24447	0.946131	1.26193
24448	0.946128	2.1
24449	1.41022	2.1
24450	1.37034	2.1
24451	1.37759	1.01693
24452	1.07612	1.19895
24453	0.946126	0.931979
24454	0.946129	1.24157
24455	1.07612	1.07076
24456	0.946131	0.43884
24457	0.94613	0.05
24458	1.23848	0.05
24459	1.38897	0.05
24460	0.946131	0.05
24461	0.946132	0.122441
24462	0.946133	0.05
24463	1.34369	0.05
24464	1.40332	0.05
24465	0.946132	0.05
24466	0.946131	2.1
24467	1.24023	2.1
24468	1.38244	2.1
24469	1.38244	2.1
24470	0.946131	2.1
24471	0.946132	2.1
24472	1.33468	2.1
24473	1.41619	2.1

24474	1.41619	2.1
24475	0.946131	2.1
24476	1.1908	1.23652
24477	0.946128	0.972519
24478	0.946126	1.76351
24479	1.26654	1.80503
24480	1.39514	2.1
24481	0.93608	2.1
24482	1.04105	2.1
24483	1.39551	2.1
24484	1.52471	2.1
24485	1.04072	2.1
24486	1.33174	0.05
24487	0.946128	0.05
24488	0.94613	0.05
24489	1.17801	0.05
24490	0.94613	0.05
24491	0.946132	2.1
24492	0.946132	2.1
24493	1.29065	2.1
24494	1.29065	2.1
24495	0.975266	2.1
24496	0.946131	2.1
24497	1.26025	2.1
24498	0.955601	2.1
24499	0.946131	2.1
24500	1.26025	2.1
24501	0.94613	0.05
24502	1.23146	0.05
24503	0.946127	0.05
24504	1.32827	0.05
24505	1.23146	0.05
24506	1.25008	0.351309
24507	1.05018	0.346127
24508	0.94613	0.211796
24509	0.932744	0.673911
24510	1.04207	0.386806
24511	0.946132	0.05495
24512	1.10023	0.156563
24513	1.36748	0.0523782
24514	1.36748	0.339342
24515	0.946133	0.1016
24516	1.06023	0.05
24517	1.08019	0.05
24518	0.94613	0.05
24519	1.06023	0.05
24520	0.94613	0.05
24521	1.16727	0.0561092
24522	1.35856	0.073391
24523	0.946132	0.142593
24524	1.16727	0.0679336
24525	0.946132	0.063647
24526	0.946133	2.1
24527	0.946131	2.1
24528	1.21691	2.1
24529	1.39558	2.1
24530	0.946131	2.1
24531	1.09905	0.05

24532	0.946132	0.0558784
24533	1.3365	0.05
24534	1.35381	0.05
24535	0.946132	0.0627957
24536	0.94613	0.05
24537	0.946128	0.05
24538	1.40332	0.05
24539	1.39041	0.05
24540	0.94613	0.05
24541	0.946132	0.05
24542	1.3659	1.90791
24543	1.39041	0.0537147
24544	1.39041	0.247415
24545	0.946132	0.270468
24546	0.840292	2.1
24547	0.946133	1.8897
24548	0.851541	2.1
24549	1.28464	2.06871
24550	1.28806	2.03888
24551	1.12591	0.57159
24552	1.05033	0.747764
24553	0.946132	0.631966
24554	0.946132	0.998189
24555	1.13537	0.883712
24556	1.21177	0.965794
24557	0.946132	1.31166
24558	0.946132	2.1
24559	1.41134	1.22399
24560	1.27079	1.50602
24561	0.946131	0.05
24562	1.35403	0.05
24563	0.94613	0.05
24564	0.94613	0.05
24565	0.946131	0.05
24566	0.946131	0.05
24567	0.946132	0.05
24568	0.94613	0.05
24569	1.37052	0.05
24570	0.946131	0.05
24571	0.946133	0.05
24572	0.946132	0.05
24573	0.946133	0.05
24574	1.24249	0.05
24575	0.946132	0.05
24576	1.20608	2.1
24577	0.946132	2.1
24578	0.946131	2.1
24579	0.946132	2.1
24580	0.946132	2.1
24581	0.946131	2.1
24582	0.946128	2.1
24583	0.946129	2.1
24584	1.41232	2.1
24585	0.94613	2.1
24586	0.946132	0.05
24587	1.32449	0.05
24588	0.946131	0.05
24589	0.946131	0.05

24590	1.32449	0.05
24591	0.946116	1.33491
24592	1.41563	2.1
24593	0.946118	2.09926
24594	0.946105	2.01672
24595	0.946131	1.14729
24596	1.26563	2.1
24597	1.32819	2.1
24598	0.946131	0.0780737
24599	0.946131	2.1
24600	0.946131	0.928322
24601	0.946131	2.08851
24602	0.946133	1.61018
24603	1.30382	2.1
24604	0.946131	1.43826
24605	0.946131	1.38743
24606	1.30382	2.07434
24607	0.946133	0.05
24608	1.15803	0.05
24609	1.28895	0.05
24610	0.954071	0.05
24611	0.946132	0.05
24612	1.0264	0.0709428
24613	0.946132	1.54412
24614	0.946132	0.60253
24615	0.946131	0.992502
24616	0.946132	0.636822
24617	1.35977	0.05
24618	0.946132	0.05
24619	0.946129	0.05
24620	0.94613	0.05
24621	0.946129	0.05
24622	0.876631	0.114978
24623	0.946133	0.448767
24624	0.946132	1.08031
24625	0.946132	1.56571
24626	0.876606	1.18284
24627	0.946131	0.05
24628	1.24979	0.05
24629	0.94613	0.05
24630	0.94613	0.05
24631	0.94613	0.05
24632	0.921791	0.05
24633	0.946131	0.05
24634	0.921762	0.05
24635	0.946131	0.05
24636	0.946131	0.05
24637	0.810944	0.05
24638	0.810945	0.05
24639	0.810959	0.05
24640	0.946133	0.05
24641	1.25797	0.05
24642	0.946132	0.05
24643	0.946132	0.05
24644	1.25797	0.05
24645	1.13748	0.05
24646	0.851512	0.144244
24647	0.94613	0.635083

24648	1.02373	0.05
24649	0.946131	0.298882
24650	0.946122	2.1
24651	1.3852	2.1
24652	0.946124	2.1
24653	0.946118	2.1
24654	1.38521	2.1
24655	0.946131	0.476762
24656	0.946131	1.43098
24657	1.30448	1.12971
24658	0.946129	0.578704
24659	0.94613	0.667348
24660	0.946133	2.1
24661	0.94613	2.1
24662	0.946131	2.1
24663	0.946131	2.1
24664	1.36503	2.1
24665	0.810947	0.05
24666	0.810947	0.05
24667	0.810934	0.05
24668	3.34066	0.05
24669	0.946133	0.417632
24670	1.4185	0.234491
24671	0.946132	0.124743
24672	0.946133	0.14429
24673	0.946132	0.279707
24674	0.94613	2.1
24675	0.946128	2.1
24676	1.03925	2.1
24677	0.944743	2.1
24678	0.947518	2.1
24679	0.946132	2.1
24680	1.20573	2.1
24681	0.94613	2.1
24682	0.94613	2.1
24683	1.20573	2.1
24684	1.20056	0.05
24685	0.946132	0.05
24686	0.946131	0.05
24687	0.946131	0.05
24688	0.946131	0.05
24689	0.94613	2.1
24690	0.946128	2.1
24691	1.38302	2.1
24692	0.94613	2.1
24693	0.94613	2.1
24694	0.827866	0.05
24695	0.913552	0.05
24696	0.716526	0.05
24697	0.82784	0.05
24698	1.20634	0.05
24699	0.946131	0.05
24700	0.94613	0.05
24701	1.39502	0.05
24702	0.88146	0.05
24703	0.946132	2.1
24704	0.946132	2.1
24705	0.946132	2.1

24706	0.946132	2.1
24707	1.39053	2.1
24708	0.946131	2.1
24709	1.40571	2.1
24710	0.946131	2.1
24711	0.946131	2.1
24712	0.946132	2.1
24713	0.94613	0.05
24714	1.2582	0.0501159
24715	0.946127	0.05
24716	0.946129	0.05
24717	0.946129	0.05
24718	0.946129	1.10044
24719	0.946132	0.972483
24720	0.946131	0.42816
24721	0.946131	1.16191
24722	1.29054	0.531234
24723	0.810944	0.05
24724	0.810941	0.05
24725	0.810931	0.05
24726	0.946132	0.383117
24727	0.946132	0.232652
24728	0.946132	1.48911
24729	0.946132	1.01823
24730	0.927608	0.396575
24731	0.946128	2.1
24732	0.946133	2.1
24733	0.946131	2.1
24734	0.94613	2.1
24735	1.38813	2.1
24736	0.94613	0.0883249
24737	0.946128	2.1
24738	0.946132	0.12925
24739	1.41666	2.1
24740	0.94613	1.64871
24741	0.946118	0.05
24742	1.34151	0.05
24743	0.946116	0.05
24744	0.946131	0.05
24745	1.34149	0.05
24746	0.946132	2.1
24747	1.31889	2.1
24748	0.946132	2.1
24749	0.946132	2.1
24750	1.31889	2.1
24751	0.864997	0.05
24752	0.928981	0.05
24753	0.872219	0.05
24754	0.946127	0.0531442
24755	0.946131	0.05
24756	1.37709	0.05
24757	0.946131	0.0547175
24758	0.946129	0.05
24759	1.21882	0.05
24760	0.938168	0.05
24761	0.938161	0.05
24762	1.06327	0.05
24763	1.33457	0.05

24764	0.946133	0.97954
24765	0.946132	0.689743
24766	0.946133	0.361316
24767	0.946132	1.26339
24768	1.14444	0.337079
24769	1.12628	0.05
24770	0.946132	0.05
24771	0.946131	0.05
24772	1.12628	0.05
24773	0.946131	0.05
24774	0.946132	2.1
24775	0.946132	2.1
24776	0.946133	2.1
24777	0.946132	2.1
24778	1.29322	2.1
24779	0.946131	2.1
24780	0.946132	2.1
24781	0.94613	2.1
24782	0.946131	2.1
24783	1.28441	2.1
24784	0.946118	2.1
24785	0.946119	2.1
24786	0.946117	2.1
24787	1.28075	2.1
24788	0.946105	2.1
24789	0.810945	2.1
24790	0.810942	2.1
24791	0.81093	2.1
24792	0.946127	2.1
24793	0.946127	2.1
24794	1.28228	2.1
24795	0.946129	2.1
24796	0.946132	2.1
24797	1.14042	2.1
24798	0.946132	2.1
24799	0.946133	2.1
24800	1.14042	2.1
24801	0.946133	0.05
24802	0.946132	0.05
24803	1.28632	0.05
24804	1.28632	0.05
24805	0.946133	0.05
24806	0.946131	1.5088
24807	0.946131	1.09675
24808	0.946131	1.39493
24809	1.31817	1.52121
24810	0.946131	1.0704
24811	1.35048	2.1
24812	0.946132	2.1
24813	0.946132	2.1
24814	0.946132	2.1
24815	0.946131	2.1
24816	0.946131	2.1
24817	1.35943	2.1
24818	0.94613	2.1
24819	0.946132	2.1
24820	0.946131	2.1
24821	0.990713	2.1

24822	0.946132	2.1
24823	0.946132	2.1
24824	0.946133	2.1
24825	0.946132	2.1
24826	0.946133	1.36514
24827	1.01433	1.26509
24828	0.877931	1.56085
24829	0.96573	0.567346
24830	0.946131	0.612307
24831	0.946131	1.0232
24832	0.946133	0.571192
24833	1.39575	0.110007
24834	0.946131	0.182398
24835	0.94613	0.163138
24836	0.810943	0.0577556
24837	0.810942	0.0674268
24838	0.709589	0.05
24839	0.924071	0.05
24840	1.04293	0.05
24841	1.15747	0.05
24842	0.946131	0.05
24843	1.04071	0.05
24844	0.810946	0.0645318
24845	0.810943	0.05
24846	0.810959	0.05
24847	0.946128	1.25896
24848	1.39696	1.93467
24849	0.94613	0.330747
24850	0.94613	0.345373
24851	1.39696	1.47751
24852	0.946131	0.282113
24853	0.946131	2.00013
24854	0.946131	1.65824
24855	0.946131	1.02526
24856	1.29735	1.96103
24857	0.946128	1.37796
24858	0.946133	0.0810824
24859	0.94613	2.1
24860	0.946131	1.38852
24861	1.33266	0.336143
24862	0.946131	2.1
24863	0.94613	0.05
24864	0.946131	2.1
24865	1.40609	0.0714384
24866	0.946131	0.668595
24867	0.946131	1.82439
24868	0.946133	1.93205
24869	0.946129	2.00937
24870	1.23339	2.1
24871	0.946131	1.65165
24872	0.946132	2.1
24873	0.946133	2.1
24874	0.946132	2.1
24875	1.29198	2.1
24876	0.946132	2.09009
24877	0.946131	2.1
24878	1.07297	2.1
24879	0.946127	2.1

24880	0.94613	2.1
24881	1.07297	2.1
24882	0.946125	2.1
24883	0.946126	2.1
24884	0.946126	2.1
24885	1.3864	2.1
24886	0.946119	2.1
24887	0.946128	0.05
24888	1.09411	0.05
24889	0.946131	0.05
24890	0.946131	0.05
24891	1.09411	0.05
24892	0.946131	0.05
24893	1.25837	0.05
24894	0.946131	0.05
24895	0.946131	0.05
24896	1.25837	0.05
24897	0.810945	2.1
24898	0.810943	2.1
24899	0.810933	2.1
24900	0.94613	0.05
24901	0.946131	0.092737
24902	0.946132	0.05
24903	0.946129	0.05
24904	0.946131	0.05
24905	0.946131	0.05
24906	1.28911	0.05
24907	0.946129	0.05
24908	0.946133	0.05
24909	0.946131	0.05
24910	0.94613	0.05
24911	1.38954	0.0664685
24912	0.946132	0.05
24913	0.94613	0.0545441
24914	0.946131	0.05
24915	0.946131	0.499858
24916	0.946131	0.111986
24917	0.946131	0.0744106
24918	1.37981	0.0823504
24919	0.946131	0.0807753
24920	0.946131	2.1
24921	1.26958	2.1
24922	0.946127	2.1
24923	0.946129	2.1
24924	1.26957	2.1
24925	0.94613	2.1
24926	1.16889	2.1
24927	0.946126	2.1
24928	0.946128	2.1
24929	1.16889	2.1
24930	0.946133	0.05
24931	1.13511	0.05
24932	0.946132	0.0599422
24933	0.946133	0.05
24934	0.946132	0.05
24935	1.27575	0.75156
24936	0.946129	2.1
24937	0.946131	1.85211

24938	0.94613	2.1
24939	0.946131	2.05951
24940	0.946131	2.1
24941	0.946128	2.1
24942	1.41712	2.1
24943	1.41712	2.1
24944	0.94613	2.1
24945	0.946129	0.05
24946	0.946132	0.05
24947	0.946131	0.05
24948	0.946131	0.05
24949	0.946131	0.05
24950	0.979045	2.00863
24951	0.946131	0.899866
24952	0.617549	2.1
24953	0.555844	2.1
24954	1.04071	0.846868
24955	0.946132	1.75297
24956	0.946132	1.31342
24957	0.946131	2.1
24958	1.22618	0.954426
24959	0.946132	0.338727
24960	0.946131	2.1
24961	1.13976	2.1
24962	0.946129	2.1
24963	0.946131	2.1
24964	1.13976	2.1
24965	0.810945	0.255112
24966	0.810943	0.067004
24967	0.810932	0.639397
24968	1.25358	0.912225
24969	0.94613	0.119511
24970	0.946128	0.087127
24971	1.25358	2.08238
24972	1.07811	0.313811
24973	0.946129	2.1
24974	1.09197	2.1
24975	0.946131	2.1
24976	0.946131	2.1
24977	1.09197	2.1
24978	0.594912	2.1
24979	0.993703	2.1
24980	1.10173	2.1
24981	0.9916	2.1
24982	0.946131	2.1
24983	0.946128	2.1
24984	0.946131	2.1
24985	0.94613	2.1
24986	0.946129	2.1
24987	0.946132	2.1
24988	1.40335	2.1
24989	0.946133	2.1
24990	0.946133	2.1
24991	1.40335	2.1
24992	0.946132	0.513635
24993	0.946132	2.00887
24994	0.946132	0.781551
24995	0.946132	1.01881

24996	1.411	1.7542
24997	0.611675	0.05
24998	0.946129	0.05
24999	1.31678	0.05
25000	0.946124	0.05
25001	0.946127	0.05
25002	0.946128	0.05
25003	0.946132	0.05
25004	0.946131	0.05
25005	0.946132	0.05
25006	0.946132	0.05
25007	0.946132	0.05
25008	0.810946	0.05
25009	0.810943	0.05
25010	0.81096	0.05
25011	1.39301	0.05
25012	0.946132	0.05
25013	0.625812	0.05
25014	0.946132	0.05
25015	0.625812	0.05
25016	0.810946	1.1318
25017	0.810945	1.95271
25018	0.810931	2.1
25019	0.810943	2.1
25020	0.810944	2.1
25021	0.810958	2.1
25022	0.946131	0.05
25023	0.946131	0.05
25024	0.946128	0.05
25025	0.946129	0.05
25026	0.94613	0.05
25027	0.946133	0.0939669
25028	0.946133	0.224791
25029	0.98537	0.05
25030	0.985371	0.05
25031	0.946132	0.31662
25032	0.946129	0.05
25033	1.38752	0.05
25034	0.946131	0.05
25035	0.946131	0.05
25036	1.38752	0.05
25037	0.946119	0.05
25038	1.33036	0.05
25039	0.946119	0.05
25040	0.946132	0.05
25041	1.33035	0.05
25042	1.3868	2.1
25043	0.946132	2.1
25044	0.946132	2.1
25045	0.946133	2.1
25046	0.946132	2.1
25047	0.810947	2.1
25048	0.810946	2.1
25049	0.81096	2.1
25050	0.810946	2.1
25051	0.810946	2.1
25052	0.810933	2.1
25053	0.946133	2.1

25054	1.35342	2.1
25055	0.946133	2.1
25056	0.946132	2.1
25057	1.35342	2.1
25058	0.946132	2.1
25059	0.946132	2.1
25060	1.28569	2.1
25061	0.946132	2.1
25062	0.946133	2.1
25063	0.946132	2.07515
25064	1.32547	2.1
25065	0.946132	2.1
25066	0.946132	2.1
25067	1.32547	2.1
25068	0.94613	0.05
25069	0.946133	0.05
25070	1.39664	0.05
25071	1.39664	0.05
25072	0.946131	0.05
25073	0.946131	1.34565
25074	0.94613	2.1
25075	0.946132	0.474261
25076	0.946131	2.09102
25077	1.41833	1.83941
25078	0.946131	0.05
25079	0.946131	0.05
25080	0.946131	0.05
25081	1.26155	0.05
25082	0.946132	0.05
25083	0.810946	2.1
25084	0.810946	2.1
25085	0.810959	2.1
25086	0.94613	1.06817
25087	0.946132	0.274932
25088	1.40779	0.0756245
25089	1.40779	0.168345
25090	0.94613	0.205433
25091	0.94613	0.05
25092	0.946129	0.05
25093	1.36376	0.05
25094	1.36376	0.05
25095	0.946131	0.05
25096	0.946128	2.1
25097	0.946127	2.1
25098	0.946131	2.1
25099	1.40756	2.1
25100	0.94613	2.1
25101	0.946131	1.79013
25102	0.946132	2.1
25103	0.94613	1.80255
25104	0.946131	1.92053
25105	1.31947	2.1
25106	0.946131	2.1
25107	0.946131	2.1
25108	0.946129	2.1
25109	0.946131	2.1
25110	1.25768	2.1
25111	0.946132	0.0787958

25112	0.94613	0.0831644
25113	0.946131	0.052556
25114	0.946131	0.0594141
25115	0.946131	0.0637162
25116	0.810943	0.415492
25117	0.810942	1.19524
25118	0.81093	2.09227
25119	0.946128	0.93845
25120	0.946133	0.344853
25121	0.946131	1.23708
25122	0.946131	0.779288
25123	0.94613	0.870486
25124	0.946132	0.984799
25125	0.946131	0.33587
25126	0.946132	1.15958
25127	0.946132	0.803155
25128	0.946132	0.979218
25129	0.946119	2.1
25130	0.946119	2.1
25131	1.31523	2.1
25132	0.946132	2.1
25133	0.946107	2.1
25134	0.946133	2.1
25135	0.946132	0.211699
25136	0.946132	0.147911
25137	1.28803	1.87106
25138	0.946132	1.89922
25139	1.37658	1.22655
25140	0.946132	1.05953
25141	0.946133	1.68872
25142	0.946132	1.36483
25143	0.946132	1.51826
25144	0.946132	1.06123
25145	0.94613	1.14295
25146	0.94613	1.71935
25147	1.15541	0.743808
25148	0.92943	1.41183
25149	0.946126	0.05
25150	0.946129	0.05
25151	1.33849	0.05
25152	1.33849	0.05
25153	0.946129	0.05
25154	0.810946	0.05
25155	0.810944	0.05
25156	0.810959	0.05
25157	1.39027	0.05
25158	0.946131	0.05
25159	0.946131	0.05
25160	1.39027	0.05
25161	0.946131	0.05
25162	1.40924	2.1
25163	0.946131	1.39834
25164	0.946132	2.1
25165	0.946132	1.11714
25166	0.946132	1.85943
25167	0.946131	2.1
25168	0.94613	2.1
25169	0.946128	2.1

25170	0.946128	2.1
25171	1.41744	2.1
25172	0.946132	0.203579
25173	0.946128	0.169609
25174	0.94613	0.935846
25175	1.37675	0.241776
25176	0.94613	0.947796
25177	0.946132	0.698748
25178	0.946132	0.101704
25179	0.946132	0.0549813
25180	0.946132	0.104071
25181	0.946131	0.0809691
25182	0.946132	0.798206
25183	0.946132	0.225728
25184	0.946133	1.37183
25185	1.10003	0.0928832
25186	0.946132	0.998627
25187	0.946132	0.05
25188	0.946131	0.05
25189	0.946132	0.05
25190	0.946131	0.05
25191	0.946132	0.05
25192	0.946132	0.05
25193	0.946131	0.05
25194	0.946132	0.05
25195	0.946132	0.05
25196	0.946131	0.05
25197	1.24094	0.05
25198	0.946126	0.05
25199	0.946128	0.05
25200	0.946129	0.05
25201	0.946129	0.05
25202	1.2929	0.05
25203	0.946131	0.05
25204	0.946131	0.05
25205	0.946131	0.05
25206	0.946131	0.05
25207	0.810946	0.05
25208	0.810945	0.05
25209	0.810959	0.05
25210	0.810946	1.90244
25211	0.810943	1.9066
25212	0.810933	2.07481
25213	0.946128	2.1
25214	1.18315	2.1
25215	0.94613	2.1
25216	0.94613	2.1
25217	1.18316	2.1
25218	0.946126	0.05
25219	1.3324	0.05
25220	0.946127	0.05
25221	0.94613	0.05
25222	0.946128	0.05
25223	0.94613	2.1
25224	0.946128	2.1
25225	1.39523	2.1
25226	1.39523	2.1
25227	0.946131	2.1

25228	1.36541	2.1
25229	0.946132	2.1
25230	0.946132	2.1
25231	0.946132	2.1
25232	0.946132	2.1
25233	0.946131	2.1
25234	0.94613	0.6931
25235	0.946132	1.78838
25236	0.946131	0.05
25237	1.39509	1.51505
25238	0.946132	2.1
25239	0.946132	2.1
25240	1.29319	2.1
25241	0.946132	2.1
25242	0.946133	2.1
25243	0.946132	0.05
25244	0.946131	0.05
25245	0.946132	0.05
25246	1.01196	0.05
25247	0.946132	0.05
25248	0.946116	2.1
25249	0.946117	2.1
25250	1.38153	2.1
25251	1.38155	2.1
25252	0.946105	2.1
25253	0.946132	1.96283
25254	0.946131	1.99435
25255	0.946132	1.82422
25256	1.28684	1.80642
25257	0.946132	1.46353
25258	1.10185	1.44384
25259	0.94613	1.93348
25260	0.946128	2.1
25261	0.94613	2.1
25262	0.94613	1.89724
25263	0.810947	0.05
25264	0.810945	0.05
25265	0.810932	0.05
25266	0.946119	0.05
25267	1.16717	0.05
25268	0.946117	0.05
25269	0.94613	0.05
25270	1.16715	0.05
25271	0.94612	0.0917155
25272	0.946119	0.0519838
25273	0.946119	0.061339
25274	0.946132	0.05
25275	1.20882	0.05
25276	1.07043	0.0941467
25277	0.823268	0.0681023
25278	0.810959	0.0832128
25279	0.946132	1.71952
25280	1.14879	0.243462
25281	0.94613	1.31577
25282	0.946131	1.62635
25283	1.14879	0.552623
25284	1.23837	2.1
25285	0.94612	2.1

25286	0.946118	2.1
25287	0.946131	2.1
25288	0.946105	2.1
25289	0.810946	0.05
25290	0.810946	0.05
25291	0.810959	0.05
25292	0.810947	1.53043
25293	0.810947	0.05
25294	0.810934	0.05
25295	0.946128	2.1
25296	0.946131	2.1
25297	0.946128	2.1
25298	0.946129	2.1
25299	0.94613	2.1
25300	0.94613	2.1
25301	0.946131	2.1
25302	0.946131	2.1
25303	0.946131	2.1
25304	0.946131	2.1
25305	0.946131	1.95679
25306	1.38585	2.1
25307	0.946128	1.34481
25308	0.94613	1.91111
25309	1.38585	2.06398
25310	0.946133	0.05
25311	0.946133	0.05
25312	0.946132	0.05
25313	0.946132	0.05
25314	0.946132	0.05
25315	0.918871	0.05
25316	0.810248	0.05
25317	0.838037	0.05
25318	0.946126	0.05
25319	0.943647	0.05
25320	1.13836	0.05
25321	1.03802	0.05
25322	0.946129	0.05
25323	0.946133	0.889166
25324	0.946132	0.598041
25325	0.946132	2.09336
25326	1.1699	0.297165
25327	0.946132	0.29984
25328	0.946131	2.1
25329	0.946132	2.1
25330	1.37199	2.1
25331	1.37199	2.1
25332	0.946132	2.1
25333	0.946132	0.748977
25334	0.946133	0.234439
25335	0.946133	1.2108
25336	0.946132	0.367559
25337	1.38623	0.233157
25338	0.946132	2.1
25339	0.946132	2.1
25340	0.946132	2.1
25341	0.946132	2.1
25342	1.40098	2.1
25343	1.20787	0.154848

25344	1.41894	0.254206
25345	0.946133	0.05
25346	1.01777	0.301575
25347	0.946132	2.1
25348	0.946132	2.1
25349	0.946133	2.1
25350	1.40199	2.1
25351	0.946132	2.1
25352	0.810946	0.955107
25353	0.810947	1.93224
25354	0.810934	1.24702
25355	0.946126	0.518911
25356	0.94613	1.55279
25357	0.946125	1.50534
25358	1.40247	0.808176
25359	0.946128	0.91133
25360	1.25703	1.48752
25361	0.87821	0.8057
25362	0.878238	0.438476
25363	0.946119	1.99294
25364	0.946132	2.1
25365	0.946132	0.165421
25366	0.946132	0.351802
25367	0.946131	1.69846
25368	0.946132	0.464405
25369	1.28543	0.450066
25370	0.946115	2.1
25371	1.41638	2.1
25372	0.946116	2.1
25373	0.946104	2.1
25374	0.946129	2.1
25375	0.946132	2.1
25376	1.41432	2.1
25377	0.946132	2.1
25378	0.946132	2.1
25379	0.946132	2.1
25380	0.810947	0.05
25381	0.810947	0.05
25382	0.709567	0.05
25383	0.851542	0.05
25384	1.01217	0.05
25385	0.697993	0.05
25386	0.946133	0.05
25387	0.851488	0.05
25388	0.946129	2.1
25389	0.94613	2.1
25390	1.2254	1.76457
25391	0.946131	2.1
25392	0.946131	2.08604
25393	0.700785	2.1
25394	0.915893	1.95051
25395	0.838013	1.56155
25396	0.760188	0.779355
25397	1.19601	0.05
25398	0.946133	0.05
25399	0.946132	0.0617713
25400	0.946132	0.05
25401	0.946132	0.05

25402	0.946132	0.525885
25403	0.946133	2.1
25404	0.946132	1.38762
25405	0.946133	2.1
25406	1.36956	2.1
25407	0.946131	0.197163
25408	0.94613	0.05
25409	0.946133	0.0850288
25410	1.34268	0.05
25411	0.946131	0.05
25412	0.946132	2.1
25413	1.25603	2.1
25414	0.946132	2.1
25415	0.946132	2.1
25416	1.25603	2.1
25417	0.810946	0.786199
25418	0.810943	0.906119
25419	0.810933	0.734628
25420	0.496545	0.05
25421	0.805767	0.330861
25422	0.936671	0.156971
25423	0.530063	0.113214
25424	1.05954	0.144786
25425	0.810946	0.05
25426	0.810943	0.05
25427	0.810959	0.05
25428	1.16699	0.05
25429	0.946131	0.05
25430	0.946129	0.05
25431	0.946131	0.05
25432	0.946131	0.05
25433	0.810943	1.24549
25434	0.810942	1.61574
25435	0.810933	1.41464
25436	0.946132	1.23055
25437	1.04832	1.09146
25438	0.870714	1.28625
25439	0.957819	1.8031
25440	0.94613	0.698853
25441	1.82931	2.1
25442	0.946132	1.14259
25443	0.946129	0.931607
25444	0.94613	1.19262
25445	0.946131	1.06384
25446	0.94613	0.714572
25447	0.946119	1.01175
25448	0.94612	1.09724
25449	0.94612	0.48018
25450	0.946132	0.975321
25451	1.36118	0.674438
25452	0.810947	0.05
25453	0.810947	0.05
25454	0.810934	0.05
25455	0.946132	2.1
25456	0.946132	2.1
25457	0.946132	2.1
25458	1.39754	2.1
25459	0.946131	2.1

25460	0.946129	2.1
25461	0.946133	2.1
25462	0.946131	2.1
25463	0.946131	2.1
25464	1.41732	2.1
25465	0.946132	0.634348
25466	0.946128	1.99728
25467	0.94613	1.03844
25468	0.94613	1.25565
25469	1.25277	1.63996
25470	0.94613	1.37001
25471	0.946128	2.0371
25472	1.21261	0.310679
25473	0.94613	1.4308
25474	0.94613	0.555986
25475	1.39407	2.1
25476	0.946133	2.1
25477	0.94613	2.1
25478	0.946131	2.1
25479	0.946131	2.1
25480	0.946119	1.04716
25481	0.946119	1.53641
25482	0.946119	1.765
25483	1.1537	1.45004
25484	0.946106	0.616954
25485	0.946131	2.1
25486	1.11822	2.1
25487	0.946129	2.1
25488	0.946131	2.1
25489	1.11822	2.1
25490	1.46806	0.05
25491	0.498881	0.05
25492	0.996658	0.05
25493	1.2179	0.05
25494	0.991519	0.05
25495	0.946131	0.05
25496	0.946131	0.05
25497	1.28673	0.05
25498	0.946131	0.05
25499	0.946131	0.05
25500	1.33178	0.05
25501	0.946132	0.05
25502	0.946132	0.05
25503	0.946132	0.05
25504	0.946132	0.05
25505	0.946128	0.05
25506	0.972061	0.05
25507	0.946129	0.0521485
25508	0.946129	0.05
25509	0.972059	0.05
25510	0.946129	2.1
25511	0.94613	2.1
25512	0.94613	2.1
25513	0.94613	2.1
25514	0.94613	2.1
25515	0.946131	2.1
25516	0.946127	2.1
25517	0.946131	2.1

25518	0.946129	2.1
25519	1.4073	2.1
25520	0.946131	0.05
25521	1.05016	0.05
25522	0.946132	0.05
25523	0.946131	0.05
25524	0.946132	0.05
25525	0.946128	0.05
25526	1.13398	0.05
25527	0.946131	0.05
25528	0.946131	0.05
25529	0.94613	0.05
25530	0.946131	0.05
25531	0.946131	0.05
25532	1.38911	0.05
25533	1.38911	0.05
25534	0.946132	0.05
25535	0.946131	0.05
25536	0.94613	0.05
25537	0.946131	0.05
25538	0.946131	0.05
25539	0.946131	0.05
25540	0.946131	0.05
25541	1.23923	0.05
25542	0.94613	0.05
25543	0.946131	0.05
25544	0.946131	0.05
25545	1.40511	0.05
25546	0.946131	0.05
25547	0.946131	0.05
25548	0.946132	0.05
25549	0.946131	0.05
25550	1.12968	2.1
25551	0.946132	2.1
25552	0.946132	2.1
25553	0.946131	2.1
25554	0.946132	2.1
25555	0.946132	1.67397
25556	1.4172	2.1
25557	0.94613	1.1694
25558	0.94613	1.93357
25559	1.4172	2.1
25560	0.946132	2.1
25561	0.946132	2.1
25562	1.31323	2.1
25563	0.946133	2.1
25564	0.946132	2.1
25565	0.946131	2.1
25566	0.94613	0.05
25567	1.36546	2.1
25568	1.36546	1.48298
25569	0.946131	0.642338
25570	0.946132	0.281653
25571	0.946133	0.145345
25572	0.946132	0.05
25573	1.36899	0.05
25574	0.946133	0.0531975
25575	0.946131	0.05

25576	0.946132	0.05
25577	1.03543	0.05
25578	1.1412	0.05
25579	0.941275	0.05
25580	1.28952	0.05
25581	0.94613	0.05
25582	0.946129	0.05
25583	0.946132	0.05
25584	0.94613	0.05
25585	0.94613	2.1
25586	0.94613	2.1
25587	0.946129	2.1
25588	0.94613	2.1
25589	1.35966	2.1
25590	0.810946	2.1
25591	0.810942	2.1
25592	0.810932	2.1
25593	0.94613	2.1
25594	1.40372	2.1
25595	0.946131	2.1
25596	0.94613	2.1
25597	1.40372	2.1
25598	0.946133	2.1
25599	0.946131	2.1
25600	0.94613	2.1
25601	0.946131	2.1
25602	0.946131	2.1
25603	1.31453	2.1
25604	0.94613	2.1
25605	0.946128	2.1
25606	0.94613	2.1
25607	0.94613	2.1
25608	0.946131	0.05
25609	1.12018	0.05
25610	0.94613	0.05
25611	0.94613	0.05
25612	0.94613	0.05
25613	0.946131	0.05
25614	1.16373	0.05
25615	0.946131	0.05
25616	0.94613	0.05
25617	0.946131	0.05
25618	0.946126	0.05
25619	0.946132	0.05
25620	0.946128	0.05
25621	0.946129	0.0900183
25622	0.946129	0.0688452
25623	0.946132	0.05
25624	0.946133	0.05
25625	0.946132	0.05
25626	0.946132	0.05
25627	0.946133	0.05
25628	0.946132	1.06176
25629	0.946129	1.79643
25630	0.94613	0.824259
25631	1.36399	1.2001
25632	0.946131	0.856901
25633	0.946132	2.1

25634	0.946132	2.1
25635	0.946133	2.1
25636	1.38302	2.1
25637	0.946133	2.1
25638	0.810943	2.1
25639	0.810944	2.1
25640	0.810945	2.1
25641	0.946132	2.1
25642	0.946131	2.1
25643	0.946132	2.1
25644	1.30188	2.1
25645	0.946132	2.1
25646	0.810946	0.757733
25647	0.72985	1.83295
25648	0.810934	1.25797
25649	0.856243	0.30648
25650	0.946129	0.388306
25651	0.946132	0.454829
25652	1.19686	0.148294
25653	0.851527	0.816987
25654	0.946131	2.1
25655	0.94613	2.1
25656	0.94613	2.1
25657	0.94613	2.1
25658	1.31961	2.1
25659	0.946131	2.1
25660	0.946131	2.1
25661	0.94613	2.1
25662	0.946131	2.1
25663	1.3605	2.1
25664	1.30116	2.1
25665	0.946133	0.183745
25666	0.946131	2.1
25667	0.946131	1.81686
25668	0.94613	2.1
25669	0.946132	0.05
25670	1.3035	0.05
25671	0.94613	0.05
25672	0.946131	0.05
25673	0.946131	0.05
25674	0.946132	2.1
25675	1.37869	2.1
25676	1.2051	2.1
25677	0.946132	2.1
25678	1.2051	2.1
25679	0.810946	2.1
25680	0.810945	2.1
25681	0.810946	2.1
25682	1.34623	2.1
25683	0.946131	2.1
25684	0.946131	2.1
25685	1.34623	2.1
25686	1.22993	2.1
25687	1.32299	0.05
25688	0.946132	0.05
25689	0.946132	0.05
25690	0.946132	0.05
25691	0.946131	0.05

25692	0.94613	0.05
25693	1.40574	0.05
25694	0.946128	0.0550155
25695	0.946131	0.100302
25696	1.40574	0.0687433
25697	0.946132	2.1
25698	1.41331	2.1
25699	0.94613	2.1
25700	0.946131	2.1
25701	1.41331	2.1
25702	0.94613	2.1
25703	1.37609	2.1
25704	0.946129	2.1
25705	0.946131	2.1
25706	1.37609	2.1
25707	1.039	0.222295
25708	0.946131	2.07581
25709	0.944514	1.18892
25710	0.946132	1.86484
25711	0.946132	1.48989
25712	1.23726	0.288511
25713	0.946128	1.79702
25714	0.946128	1.14787
25715	0.946129	1.68933
25716	0.94613	1.33374
25717	0.946129	2.1
25718	0.946131	2.1
25719	0.946131	2.1
25720	1.39214	2.1
25721	0.94613	2.1
25722	0.946118	0.0971356
25723	0.946118	0.05
25724	1.41078	0.05
25725	1.41076	0.05
25726	0.946131	0.05
25727	0.946131	1.15327
25728	0.946131	0.661857
25729	0.946132	0.99233
25730	0.94613	0.629639
25731	0.946131	0.466725
25732	0.946132	0.05
25733	0.946132	0.05
25734	0.946133	0.05
25735	1.28924	0.05
25736	0.946132	0.05
25737	0.946133	0.0801066
25738	1.09812	0.05
25739	0.946132	0.05
25740	0.946132	0.107186
25741	1.09812	0.0904711
25742	0.946132	0.317285
25743	0.946132	0.091182
25744	0.946132	0.2763
25745	0.946132	0.262812
25746	1.15388	0.212024
25747	0.946132	0.0761819
25748	0.974688	0.05
25749	0.946132	0.05

25750	0.946132	0.0719253
25751	0.974687	0.0606331
25752	1.0644	1.26895
25753	0.946133	1.87485
25754	0.946132	1.11331
25755	0.946133	1.20041
25756	1.26547	1.34847
25757	1.02905	0.866751
25758	0.911537	1.00041
25759	0.838038	1.24536
25760	0.946131	0.05
25761	0.94613	0.05
25762	0.946132	0.05
25763	0.946131	0.05
25764	1.33922	0.05
25765	0.946132	0.05
25766	0.946132	0.05
25767	1.40926	0.05
25768	1.40927	0.05
25769	0.946132	0.05
25770	0.946129	2.1
25771	0.946131	2.1
25772	0.946131	1.00755
25773	0.946131	0.55184
25774	1.40496	1.92049
25775	0.94613	1.40395
25776	0.94613	0.477277
25777	0.946127	1.16198
25778	0.946128	0.504794
25779	1.38914	0.526643
25780	2.675 0.05	
25781	1.22266	0.05
25782	0.946131	0.05
25783	0.946132	0.05
25784	0.946132	0.05
25785	1.22266	0.05
25786	0.946124	0.05
25787	0.946128	0.05
25788	0.946126	0.05
25789	1.21428	0.05
25790	0.946127	0.05
25791	0.946131	0.05
25792	0.946131	0.05
25793	0.946132	0.05
25794	0.946129	0.05
25795	0.940118	0.05
25796	0.810943	2.1
25797	0.810945	2.1
25798	0.810958	2.1
25799	0.946133	0.05
25800	1.11539	0.05
25801	0.946132	0.05
25802	0.946132	0.05
25803	0.946133	0.05
25804	0.946133	2.1
25805	1.41195	2.1
25806	0.946133	2.1
25807	0.946132	2.1

25808	0.946133	2.1
25809	1.15968	0.05
25810	0.946131	0.05
25811	0.946131	0.05
25812	0.946131	0.05
25813	0.946131	0.05
25814	0.94613	2.1
25815	0.946128	2.1
25816	0.946132	1.12956
25817	0.94613	2.1
25818	0.94613	1.83287
25819	0.946131	2.07748
25820	1.04211	2.03975
25821	0.850155	0.695553
25822	0.946131	1.67304
25823	0.946131	1.17546
25824	0.946131	0.05
25825	0.94613	0.05
25826	0.946129	0.05
25827	0.946131	0.05
25828	0.946131	0.05
25829	1.40193	0.05
25830	1.19756	0.05
25831	0.561936	0.05
25832	0.808778	0.05
25833	1.19756	0.05
25834	0.946132	0.05
25835	0.946132	0.05
25836	1.33916	0.05
25837	1.33916	0.05
25838	0.946133	0.05
25839	0.946127	0.0555435
25840	0.946127	0.05
25841	0.946131	0.05
25842	0.946128	0.05
25843	0.946129	0.05
25844	0.589428	0.0981887
25845	0.851501	0.05
25846	0.868924	0.0708203
25847	0.877703	0.0591857
25848	0.85153	0.0694849
25849	0.946132	1.78388
25850	1.25372	2.1
25851	0.946132	2.09085
25852	0.946132	2.1
25853	1.25372	2.1
25854	0.946132	0.317319
25855	0.946128	2.1
25856	0.94613	0.821572
25857	0.94613	1.52787
25858	1.2778	1.98507
25859	0.946118	0.455586
25860	1.35629	0.510583
25861	0.946117	0.586033
25862	0.946131	0.427143
25863	0.946105	0.229346
25864	0.946132	0.0633988
25865	0.946132	1.09874

25866	1.38646	0.524215
25867	1.38646	0.776395
25868	0.946132	0.829339
25869	0.946128	2.1
25870	0.946128	0.0769222
25871	1.39659	2.1
25872	1.39659	1.60529
25873	0.946129	0.612356
25874	0.946132	1.77065
25875	0.946131	1.30663
25876	0.946132	1.49974
25877	0.946132	1.50311
25878	1.15139	1.15545
25879	0.946133	2.1
25880	0.946132	2.1
25881	0.946132	2.1
25882	0.946132	2.1
25883	1.36151	2.1
25884	0.946127	2.1
25885	0.946131	2.1
25886	0.946128	2.1
25887	0.94613	2.1
25888	1.38397	2.1
25889	0.946131	0.0514248
25890	1.31112	0.0506614
25891	0.946127	0.0828952
25892	0.946128	0.143705
25893	1.31112	0.0691357
25894	1.29778	0.05
25895	0.883417	0.05
25896	1.32613	0.05
25897	0.94613	0.05
25898	0.94613	0.05
25899	0.946132	2.1
25900	0.94613	2.1
25901	0.946131	2.1
25902	0.946131	2.1
25903	1.36523	2.1
25904	0.946132	0.05
25905	1.01653	0.05
25906	0.946132	0.05
25907	0.946131	0.05
25908	0.946131	0.05
25909	0.94612	2.1
25910	1.08301	1.72233
25911	0.946119	2.1
25912	0.946132	2.1
25913	1.08299	2.1
25914	0.810942	2.1
25915	0.810944	1.0168
25916	0.810957	1.82252
25917	0.946126	0.05
25918	0.946132	0.05
25919	0.946129	0.05
25920	1.3492	0.05
25921	0.94613	0.05
25922	0.946131	0.05
25923	1.24766	0.05

25924	0.946128	0.0723591
25925	0.946131	0.141897
25926	1.24766	0.0842609
25927	0.946132	0.05
25928	0.946131	0.05
25929	0.946131	0.05
25930	1.23351	0.05
25931	0.946132	0.05
25932	0.946131	0.05
25933	0.946132	0.05
25934	0.946131	0.05
25935	1.35657	0.05
25936	0.946131	0.05
25937	0.946132	0.0710858
25938	1.3689	0.05
25939	0.946132	0.0606551
25940	0.946132	0.0715773
25941	1.3689	0.0589481
25942	0.946133	0.05
25943	1.35822	0.05
25944	0.946133	0.05
25945	0.946132	0.05
25946	1.35822	0.05
25947	0.946131	0.255917
25948	0.946131	1.39292
25949	0.946131	1.39273
25950	0.946131	0.743223
25951	1.4101	1.63548
25952	0.810944	1.70393
25953	0.810939	1.72335
25954	0.810955	1.11336
25955	0.946132	2.1
25956	1.37733	2.1
25957	0.946132	2.1
25958	0.946131	2.1
25959	0.946131	2.1
25960	1.32582	2.1
25961	0.946132	2.1
25962	0.946132	1.87189
25963	0.946132	2.1
25964	0.946132	2.1
25965	0.946132	2.1
25966	0.946132	2.1
25967	1.31401	2.1
25968	1.31401	2.1
25969	0.946131	2.1
25970	1.3469	1.59501
25971	0.946132	1.85768
25972	0.946132	1.977
25973	0.946133	0.87928
25974	0.946132	1.27128
25975	0.946126	2.1
25976	0.946127	2.1
25977	0.94613	2.1
25978	0.946128	2.1
25979	1.41206	2.1
25980	0.946127	2.00232
25981	1.40109	2.1

25982	0.946128	2.00846
25983	0.946129	2.1
25984	1.40109	2.1
25985	1.24448	0.05
25986	0.94613	0.05
25987	0.946132	0.05
25988	0.946129	0.05
25989	0.946131	0.05
25990	1.11015	0.05
25991	0.946133	0.05
25992	0.946132	0.05
25993	0.946133	0.05
25994	0.946132	0.05
25995	0.946132	2.1
25996	0.946131	2.1
25997	0.946131	2.1
25998	1.38775	2.1
25999	0.946131	2.1
26000	0.946133	0.127638
26001	0.946131	0.293367
26002	1.30598	1.69959
26003	1.30598	1.0169
26004	0.946131	0.953006
26005	0.946132	2.1
26006	0.946132	2.1
26007	0.946132	2.1
26008	1.40774	2.1
26009	0.946132	2.1
26010	0.798396	0.05
26011	0.946133	0.656611
26012	0.946128	1.54282
26013	0.946131	1.24108
26014	0.946131	1.2397
26015	1.28918	1.27749
26016	0.946132	0.069673
26017	1.40668	0.0548818
26018	0.94613	0.0971438
26019	0.94613	0.0652853
26020	0.94613	0.0987544
26021	0.810946	2.1
26022	0.810945	2.1
26023	0.810933	2.1
26024	0.946131	2.1
26025	1.30338	2.1
26026	0.94613	2.1
26027	1.30339	2.1
26028	0.946131	2.1
26029	0.946127	0.768834
26030	0.87161	1.5456
26031	0.946128	1.07795
26032	0.946129	1.31288
26033	0.827876	0.624081
26034	0.946131	2.1
26035	1.40796	2.1
26036	0.94613	2.1
26037	0.94613	2.1
26038	1.40796	2.1
26039	0.946132	0.119237

26040	0.946132	0.197766
26041	0.946132	0.107501
26042	0.946132	0.0753728
26043	1.41724	0.0655147
26044	1.34254	0.05
26045	0.946132	0.0834711
26046	0.946132	0.0548721
26047	0.946132	0.051301
26048	0.946133	0.05
26049	0.946133	2.03593
26050	0.946132	1.73308
26051	0.946132	1.71475
26052	1.26501	2.03763
26053	0.946132	1.4565
26054	0.946131	0.05
26055	1.35169	0.05
26056	0.946131	0.05
26057	0.946131	0.05
26058	1.35169	0.05
26059	0.676844	0.05
26060	0.946131	0.05
26061	0.94613	0.05
26062	1.13242	0.05
26063	1.13242	0.05
26064	0.946131	0.05
26065	0.946128	0.989685
26066	1.28828	2.1
26067	0.946131	1.21544
26068	0.946131	1.42558
26069	1.28828	1.75158
26070	0.946131	0.0564319
26071	1.2996	0.05
26072	0.946133	0.0562095
26073	0.946131	0.05
26074	0.946131	0.05
26075	0.946132	2.1
26076	1.40638	2.1
26077	0.946131	1.44392
26078	0.946132	2.1
26079	1.40638	2.1
26080	2.84909	0.05
26081	1.35726	0.05
26082	0.946131	0.05
26083	0.946131	0.05
26084	0.946131	0.05
26085	0.946131	0.05
26086	1.39852	2.1
26087	0.946132	2.1
26088	0.94613	2.1
26089	0.94613	2.1
26090	0.94613	2.1
26091	0.946126	0.05
26092	0.946131	0.05
26093	0.946128	0.05
26094	1.31666	0.05
26095	0.946128	0.05
26096	0.946126	0.05
26097	0.946131	0.05

26098	0.946129	0.05
26099	1.32275	0.05
26100	0.946129	0.05
26101	0.946131	2.1
26102	0.946132	2.1
26103	1.41125	2.1
26104	0.946131	2.1
26105	0.946131	2.1
26106	0.946131	2.1
26107	0.946132	2.1
26108	1.31904	2.1
26109	0.946132	2.1
26110	0.946132	2.1
26111	0.946129	0.05
26112	0.946132	0.05
26113	0.94613	0.05
26114	1.02426	0.05
26115	0.9311	0.05
26116	0.946128	1.94534
26117	0.946133	1.24084
26118	0.946131	1.76666
26119	1.40092	1.86504
26120	0.946131	1.48189
26121	0.946117	0.126089
26122	0.946118	2.1
26123	0.946118	0.05
26124	1.40017	1.84047
26125	0.946105	0.201336
26126	0.946132	0.0593724
26127	1.03547	0.05
26128	0.946132	0.05
26129	0.946133	0.0737111
26130	0.946132	0.0734099
26131	0.946133	2.1
26132	0.946132	2.1
26133	0.946133	2.1
26134	0.946133	2.1
26135	1.36694	2.1
26136	0.946132	0.05
26137	1.22878	0.05
26138	0.946132	0.05
26139	0.946132	0.05
26140	1.22878	0.05
26141	1.11014	0.05
26142	0.913078	0.05
26143	1.11015	0.05
26144	0.49154	0.05
26145	0.946127	2.1
26146	0.946131	2.1
26147	0.946128	2.1
26148	0.946129	2.1
26149	1.38752	2.1
26150	0.946132	2.1
26151	1.37277	2.1
26152	0.946132	2.1
26153	0.946131	2.1
26154	1.37277	2.1
26155	0.94613	0.05

26156	1.16834	0.05
26157	0.946129	0.05
26158	0.94613	0.05
26159	0.94613	0.05
26160	0.946127	0.05
26161	0.946131	0.05
26162	1.35594	0.05
26163	0.946128	0.05
26164	0.94613	0.05
26165	0.946128	1.28463
26166	0.946128	0.05
26167	1.38464	0.0918488
26168	1.38463	0.05
26169	0.94613	0.0705476
26170	0.946129	1.37535
26171	0.946131	0.0548433
26172	0.94613	0.0680313
26173	1.39751	0.05
26174	0.946131	0.138952
26175	1.25837	0.05
26176	1.21134	0.05
26177	0.700013	0.05
26178	0.700041	0.05
26179	1.40583	2.1
26180	0.946132	2.1
26181	0.946132	2.1
26182	0.946133	2.1
26183	0.946132	2.1
26184	0.827865	2.1
26185	0.946131	2.1
26186	0.946131	2.04673
26187	0.946131	2.1
26188	0.946131	1.99073
26189	0.946132	0.05
26190	1.21087	0.05
26191	0.94613	0.05
26192	0.94613	0.05
26193	0.946131	0.05
26194	0.94613	0.406409
26195	1.17452	0.883682
26196	0.946132	0.444329
26197	0.94613	0.944409
26198	0.94613	0.230144
26199	0.946119	0.186747
26200	1.27374	0.305131
26201	0.946119	0.187008
26202	0.946131	0.309878
26203	0.946106	0.121943
26204	0.946133	2.1
26205	0.946132	2.1
26206	0.946133	2.1
26207	0.946132	2.1
26208	1.39621	2.1
26209	1.34352	2.1
26210	0.946132	2.1
26211	0.946132	2.1
26212	0.946132	2.1
26213	0.946132	2.1

26214	0.946132	0.201369
26215	0.946128	2.1
26216	0.94613	1.27391
26217	0.94613	1.992
26218	1.41273	2.1
26219	1.23536	2.1
26220	0.946131	1.66918
26221	0.946132	2.1
26222	0.946132	2.1
26223	0.946132	2.1
26224	0.946132	2.1
26225	1.41131	2.1
26226	0.946131	2.1
26227	0.946131	2.1
26228	0.946131	2.1
26229	0.946133	0.365277
26230	0.946132	2.1
26231	0.946133	0.160584
26232	1.41894	0.102733
26233	0.946132	0.05
26234	0.946128	0.0670154
26235	1.4099	1.49733
26236	0.946131	0.209006
26237	0.946131	2.1
26238	0.946131	0.05
26239	0.810947	2.05431
26240	0.810945	2.0723
26241	0.810933	2.1
26242	0.946131	0.136637
26243	1.25765	2.1
26244	0.946128	0.991436
26245	0.946129	0.162532
26246	1.25765	1.2745
26247	1.21347	2.1
26248	0.946129	2.1
26249	0.94613	2.1
26250	1.21347	2.1
26251	0.94613	2.1
26252	0.94613	0.05
26253	0.946132	0.05
26254	1.2493	0.05
26255	1.2493	0.05
26256	0.94613	0.05
26257	0.946132	0.869995
26258	0.946128	1.38698
26259	0.946131	0.663524
26260	0.94613	1.19971
26261	1.37938	1.03102
26262	0.946133	0.05
26263	0.946132	0.05
26264	0.946133	0.05
26265	0.946132	0.05
26266	0.946133	0.05
26267	0.946129	0.171461
26268	1.26426	0.081511
26269	0.946131	0.186434
26270	0.946131	0.205775
26271	1.26426	0.17157

26272	0.872447	1.9455
26273	0.946132	1.17192
26274	0.946132	1.6713
26275	0.946132	1.86563
26276	0.946133	1.47358
26277	0.946117	1.3514
26278	0.946116	1.12559
26279	0.946113	0.962556
26280	0.946129	1.18132
26281	1.27211	0.706968
26282	0.94613	0.05
26283	0.946129	0.05
26284	1.40029	0.05
26285	1.40028	0.05
26286	0.94613	0.0995784
26287	0.810942	2.1
26288	0.810943	2.1
26289	0.810932	2.1
26290	1.29521	2.1
26291	0.946129	2.1
26292	0.946131	2.1
26293	1.29521	2.1
26294	1.29521	2.1
26295	0.946132	0.05
26296	0.946129	0.05
26297	1.24596	0.05
26298	0.946131	0.05
26299	0.946131	0.05
26300	0.946131	2.1
26301	1.35616	2.1
26302	0.946133	2.1
26303	0.94613	2.1
26304	1.35616	2.1
26305	0.879548	0.445504
26306	1.01272	0.615013
26307	0.946133	0.917848
26308	0.946133	0.281116
26309	0.946132	0.788493
26310	0.946132	0.840366
26311	0.918009	0.272879
26312	0.91801	1.68745
26313	0.946132	0.613651
26314	0.946132	1.21452
26315	0.946132	0.05
26316	1.28644	0.05
26317	0.946131	0.05
26318	0.946131	0.05
26319	0.946132	0.05
26320	0.946131	0.05
26321	1.40103	0.05
26322	0.946131	0.05
26323	0.946131	0.05
26324	0.946131	0.05
26325	0.946128	0.05
26326	1.03327	0.05
26327	0.946129	0.05
26328	0.946131	0.05
26329	0.94613	0.05

26330	0.946132	0.131076
26331	0.946133	0.05
26332	1.40265	0.05
26333	1.40265	0.05
26334	0.946133	0.05
26335	0.946131	0.05
26336	0.946131	0.05
26337	0.946131	0.05
26338	0.856655	0.05
26339	0.856616	0.05
26340	0.946132	0.0710814
26341	0.946132	0.0924823
26342	0.946132	0.275088
26343	1.15844	0.05
26344	0.946131	0.24216
26345	0.946131	2.1
26346	1.4031	2.1
26347	1.40304	2.1
26348	0.851554	2.1
26349	0.946131	0.05
26350	0.94613	0.05
26351	1.24417	0.05
26352	1.24416	0.05
26353	0.946131	0.05
26354	0.946132	0.0706636
26355	1.20992	0.0557428
26356	0.946132	0.05
26357	0.946132	0.190868
26358	1.20992	0.0879894
26359	0.946128	0.425061
26360	0.94613	0.896396
26361	0.94613	0.492991
26362	0.94613	1.32083
26363	1.23426	1.24558
26364	0.810943	0.645037
26365	0.810946	0.0736565
26366	0.810933	0.0806568
26367	0.810945	2.1
26368	0.810945	0.052274
26369	0.810932	0.962913
26370	1.25104	1.35236
26371	0.946131	2.1
26372	0.946131	0.196461
26373	1.25104	1.53864
26374	1.25104	0.113064
26375	1.25488	2.1
26376	0.946132	2.1
26377	0.946132	2.1
26378	0.946132	2.1
26379	0.946131	2.1
26380	0.946131	0.05
26381	0.946126	0.05
26382	0.946128	0.05
26383	1.32131	0.05
26384	0.946128	0.05
26385	0.946133	2.1
26386	0.946132	0.250061
26387	0.946132	2.1

26388	0.946133	2.1
26389	1.41123	1.90348
26390	0.946132	0.05
26391	0.946133	0.05
26392	0.946132	0.05
26393	1.32163	0.05
26394	0.946133	0.05
26395	0.946132	1.43584
26396	0.946132	2.1
26397	1.38651	2.1
26398	1.38651	2.1
26399	0.946132	2.1
26400	0.94613	0.05
26401	1.3639	0.05
26402	0.946128	0.05
26403	0.946131	0.05
26404	1.3639	0.05
26405	0.946133	0.383276
26406	1.40724	0.0565928
26407	0.946132	0.05
26408	0.946133	0.0590515
26409	1.40724	0.076153
26410	1.21099	0.706138
26411	0.946128	1.74853
26412	0.946127	2.1
26413	0.946128	2.1
26414	0.946129	1.37312
26415	0.946132	0.117067
26416	1.35946	0.0803331
26417	0.946131	0.339485
26418	0.946131	0.239236
26419	1.35946	0.20696
26420	1.34794	0.0990324
26421	0.517994	1.47364
26422	0.946132	0.16891
26423	0.946132	0.483529
26424	0.517965	0.665746
26425	1.23224	0.313938
26426	0.94613	2.03272
26427	0.946131	1.43684
26428	0.946131	0.773783
26429	0.946131	0.633927
26430	1.37511	0.580242
26431	0.946132	1.0377
26432	0.946133	2.08912
26433	0.946133	1.94961
26434	0.946132	1.05138
26435	0.851106	0.154926
26436	0.946129	0.230003
26437	0.946131	0.462677
26438	0.946131	1.15613
26439	0.851066	0.784384
26440	0.946131	0.05
26441	0.946131	0.05
26442	0.94613	0.05
26443	1.31357	0.05
26444	0.946131	0.05
26445	0.946133	1.00826

26446	0.946132	1.30701
26447	0.946133	0.576014
26448	0.946132	1.12316
26449	1.38149	0.846497
26450	0.810944	0.323691
26451	0.810945	1.47702
26452	0.810958	2.1
26453	0.946118	0.05
26454	0.946118	0.05
26455	1.37835	0.05
26456	1.37837	0.05
26457	0.946106	0.05
26458	0.946128	0.05
26459	0.946131	0.05
26460	1.34831	0.05
26461	1.34831	0.05
26462	0.94613	0.05
26463	0.946132	0.05
26464	0.946132	0.05
26465	0.909981	0.05
26466	1.00099	0.05
26467	0.946131	0.05
26468	0.946132	0.577771
26469	0.946133	1.49338
26470	0.946133	0.886096
26471	0.946132	1.20009
26472	1.35328	1.17514
26473	0.946132	0.05
26474	0.946132	0.05
26475	1.35386	0.05
26476	1.35385	0.05
26477	0.946133	0.05
26478	0.946121	0.05
26479	0.946132	0.05
26480	0.900167	0.05
26481	1.01189	0.05
26482	0.946132	0.05
26483	0.946132	2.1
26484	1.41585	0.949824
26485	0.946131	1.88859
26486	0.946131	0.982894
26487	0.94613	1.62846
26488	0.810946	2.1
26489	0.810946	2.1
26490	0.810933	2.1
26491	1.34447	2.1
26492	0.946131	2.1
26493	0.946131	2.1
26494	1.34447	2.1
26495	1.19886	2.1
26496	0.946132	0.05
26497	0.946132	0.05
26498	0.946132	0.05
26499	1.35065	0.05
26500	0.946132	0.05
26501	0.946131	0.068544
26502	1.3022	0.0577961
26503	0.94613	0.269204

26504	0.946131	0.15859
26505	1.3022	0.134728
26506	0.946127	0.05
26507	1.38683	0.05
26508	0.946127	0.05
26509	0.946128	0.0544845
26510	1.38683	0.05
26511	0.810946	0.05
26512	0.810947	0.05
26513	0.810959	0.05
26514	0.946132	0.05
26515	0.946131	0.05
26516	0.946132	0.05
26517	0.946132	0.05
26518	0.946132	0.05
26519	0.946127	1.00965
26520	1.35968	2.02638
26521	0.946131	0.892099
26522	0.946129	0.57905
26523	1.35969	1.78324
26524	0.946133	2.1
26525	1.35862	1.68318
26526	0.946132	2.1
26527	0.946132	0.94026
26528	1.35862	1.6591
26529	0.946132	0.882718
26530	0.946132	0.610157
26531	0.946132	1.56469
26532	0.946131	0.300364
26533	0.946132	0.483943
26534	0.946131	0.05
26535	0.946131	0.05
26536	0.946128	0.05
26537	0.946129	0.05
26538	1.41232	0.05
26539	0.946128	1.15496
26540	1.29331	2.1
26541	0.946131	1.27392
26542	0.946131	2.00715
26543	1.29331	2.00938
26544	0.946133	0.112188
26545	0.946132	0.138882
26546	0.946132	0.11032
26547	0.946132	0.0761156
26548	1.41556	0.0664668
26549	0.94612	0.0959245
26550	0.946119	0.0739705
26551	0.946119	0.0951372
26552	0.946132	0.0538774
26553	0.946107	0.058799
26554	0.946129	0.05
26555	1.1509	0.05
26556	0.946126	0.05
26557	0.946129	0.05
26558	0.94613	0.05
26559	0.946131	0.05
26560	1.19263	0.05
26561	0.946132	0.05

26562	0.946132	0.05
26563	0.946132	0.05
26564	0.94613	0.145803
26565	1.36939	0.194711
26566	0.946128	0.170624
26567	0.946131	0.478971
26568	1.36939	0.166615
26569	0.946133	0.845614
26570	0.827865	1.93375
26571	0.946132	0.858487
26572	0.946132	1.46751
26573	1.40398	1.3787
26574	0.946127	0.108592
26575	0.946127	0.142175
26576	0.94613	0.05
26577	1.41498	0.101126
26578	0.946128	0.0599728
26579	0.94613	0.05
26580	0.946131	0.05
26581	0.946129	0.05
26582	0.94613	0.05
26583	0.94613	0.05
26584	0.946118	0.05
26585	0.946119	0.05
26586	0.946116	0.05
26587	1.36569	0.05
26588	0.946105	0.05
26589	0.946132	0.05
26590	1.23175	0.05
26591	0.94613	0.05
26592	0.946133	0.05
26593	0.946131	0.05
26594	1.25698	0.05
26595	0.946126	0.05
26596	0.946129	0.05
26597	0.946128	0.05
26598	0.946129	0.05
26599	0.810943	2.1
26600	0.810944	2.1
26601	0.810932	2.1
26602	0.946132	0.05
26603	1.09841	0.05
26604	0.946131	0.05
26605	0.946132	0.05
26606	1.09841	0.05
26607	0.810936	0.388041
26608	0.810935	0.165069
26609	0.810934	1.07409
26610	1.23851	2.1
26611	0.946132	0.296829
26612	0.946131	0.586417
26613	1.23851	2.1
26614	1.23851	2.04286
26615	0.946132	0.05
26616	0.946131	0.05
26617	0.946132	0.05
26618	0.946132	0.05
26619	0.946132	0.05

26620	0.939141	1.19494
26621	0.953118	1.67574
26622	0.946126	0.889523
26623	0.946129	1.13451
26624	1.03306	1.19665
26625	0.946132	0.05
26626	1.30927	0.05
26627	0.946132	0.05
26628	0.946132	0.05
26629	1.30928	0.05
26630	0.94613	2.1
26631	0.946131	2.1
26632	1.24511	2.1
26633	0.94613	2.1
26634	0.94613	2.1
26635	0.946133	0.05
26636	1.34152	0.05
26637	0.946131	0.05
26638	0.946131	0.05
26639	1.34152	0.05
26640	0.946132	0.05
26641	1.41383	0.05
26642	0.946132	0.05
26643	0.946132	0.05
26644	1.41383	0.05
26645	0.946132	0.0896196
26646	0.946131	0.05
26647	1.41392	0.05
26648	0.946132	0.0968678
26649	0.946132	0.0602755
26650	0.946132	0.160008
26651	0.946132	0.111742
26652	1.24333	0.05
26653	0.946133	0.252842
26654	0.946133	0.151592
26655	0.946124	0.05
26656	0.94613	0.05
26657	0.946126	0.05
26658	1.3065	0.05
26659	0.946127	0.05
26660	0.810946	0.05
26661	0.810943	0.05
26662	0.81096	0.05
26663	0.946131	0.05
26664	1.40271	0.05
26665	0.946131	0.05
26666	0.946131	0.05
26667	1.40271	0.05
26668	0.946132	0.05
26669	0.94613	0.05
26670	0.946131	0.05
26671	0.94613	0.05
26672	0.946131	0.05
26673	0.946133	0.232268
26674	1.30107	0.297653
26675	0.946132	0.284107
26676	0.946132	0.372597
26677	0.946133	0.339648

26678	0.810946	2.1
26679	0.810944	2.1
26680	0.810958	2.1
26681	0.946133	1.38735
26682	0.946131	1.19834
26683	0.946131	2.1
26684	1.30812	2.00929
26685	0.946131	1.22143
26686	0.94613	0.346893
26687	0.975002	1.19656
26688	0.917259	2.1
26689	1.00902	1.58031
26690	0.94613	0.272114
26691	0.946131	2.1
26692	0.946127	2.1
26693	1.398 2.1	
26694	0.946129	2.1
26695	0.946129	2.1
26696	0.946133	2.1
26697	0.946132	2.1
26698	0.946132	2.1
26699	0.946132	2.1
26700	1.3033	2.1
26701	1.09897	0.05
26702	0.946131	0.213404
26703	0.946132	0.0990066
26704	0.946129	0.0889473
26705	0.946131	0.123699
26706	0.946131	2.1
26707	0.946132	2.1
26708	1.39858	2.1
26709	0.946131	2.1
26710	0.946131	2.1
26711	0.946119	0.294041
26712	1.08878	0.408028
26713	0.946119	1.39175
26714	1.0888	0.415807
26715	0.946107	1.3609
26716	0.946132	0.05
26717	1.10733	0.05
26718	0.946132	0.05
26719	0.946131	0.05
26720	0.946132	0.05
26721	0.946128	0.05
26722	1.33671	0.05
26723	0.94613	0.05
26724	0.94613	0.05
26725	0.94613	0.05
26726	0.810943	2.1
26727	0.810944	2.1
26728	0.810945	2.1
26729	1.35117	2.1
26730	0.946129	2.1
26731	0.94613	2.1
26732	1.35117	2.1
26733	1.27174	2.1
26734	0.946132	2.1
26735	0.946133	2.1

26736	0.997296	1.3507
26737	0.946133	2.02625
26738	0.946132	1.72016
26739	0.810943	2.1
26740	0.810944	2.1
26741	0.810945	2.1
26742	0.810946	0.577617
26743	0.810943	2.1
26744	0.810933	1.55185
26745	0.946127	1.0999
26746	0.946131	0.536979
26747	0.946128	1.0417
26748	0.94613	1.07937
26749	0.946129	1.22126
26750	0.946131	0.05
26751	0.946131	0.05
26752	0.946129	0.05
26753	0.946131	0.05
26754	0.94613	0.05
26755	0.946133	0.0606995
26756	0.946132	0.0782708
26757	0.946132	0.05
26758	0.946132	0.0573603
26759	0.946133	0.0597982
26760	0.946131	0.701943
26761	0.94613	0.392494
26762	0.946129	0.876521
26763	0.946131	0.377199
26764	0.946131	0.470244
26765	0.946132	0.37319
26766	0.946129	2.1
26767	0.94613	1.76095
26768	0.946131	1.12239
26769	1.39356	1.89222
26770	0.810946	2.1
26771	0.810947	2.1
26772	0.810934	2.1
26773	0.946125	0.05
26774	0.946127	0.05
26775	1.33798	0.05
26776	1.33797	0.05
26777	0.946128	0.05
26778	0.946131	0.05
26779	0.946131	0.05
26780	1.23146	0.05
26781	1.23146	0.05
26782	0.946131	0.05
26783	0.946129	2.1
26784	0.94613	2.1
26785	0.946131	2.1
26786	0.94613	2.1
26787	1.30082	2.1
26788	0.946131	0.05
26789	0.946132	0.05
26790	0.946131	0.05
26791	1.20416	0.05
26792	0.946132	0.05
26793	0.946132	0.05

26794	0.946131	0.05
26795	0.946131	0.05
26796	1.18711	0.05
26797	0.946132	0.05
26798	0.946132	0.05
26799	0.946132	0.05
26800	0.946133	0.05
26801	0.946132	0.05
26802	0.946133	0.05
26803	0.946131	0.05
26804	0.946132	0.05
26805	0.94613	0.05
26806	0.946131	0.05
26807	0.946131	0.05
26808	0.946133	0.05
26809	0.946132	0.05
26810	0.946133	0.05
26811	0.946132	0.05
26812	0.946132	0.05
26813	0.946132	2.1
26814	0.946132	2.1
26815	0.946132	2.1
26816	1.36422	2.1
26817	0.946132	2.1
26818	0.946131	0.71732
26819	0.946132	1.90818
26820	0.946132	1.19839
26821	0.946132	1.23482
26822	1.4185	1.72454
26823	0.946133	0.05
26824	1.37794	0.05
26825	0.946132	0.05
26826	0.946133	0.05
26827	0.946132	0.05
26828	1.4045	2.1
26829	0.946132	2.1
26830	0.946132	2.1
26831	0.946132	2.1
26832	0.946132	2.1
26833	0.946131	2.1
26834	0.946132	2.1
26835	1.34285	2.1
26836	1.34285	2.1
26837	0.946132	2.1
26838	0.946131	2.1
26839	0.946132	2.1
26840	1.40739	2.1
26841	1.40739	2.1
26842	0.946131	2.1
26843	0.946128	2.1
26844	0.946132	2.1
26845	0.94613	2.1
26846	0.94613	2.1
26847	1.3164	2.1
26848	0.946132	2.088
26849	1.3484	2.1
26850	0.946132	1.75632
26851	0.946132	2.1

26852	1.3484	2.1
26853	0.946132	2.1
26854	0.946132	2.1
26855	0.946133	2.1
26856	0.946133	2.1
26857	1.41507	2.1
26858	0.946132	2.1
26859	1.32608	2.1
26860	0.946131	2.1
26861	0.946131	2.1
26862	0.946132	2.1
26863	0.94613	0.572162
26864	1.34646	2.1
26865	0.946129	0.964323
26866	0.94613	0.587535
26867	1.34646	1.61667
26868	0.946131	0.05
26869	0.946131	2.1
26870	0.94613	0.4183
26871	1.33728	0.05
26872	0.946131	0.0830314
26873	0.946127	2.1
26874	0.946131	0.843405
26875	0.946128	2.1
26876	0.946129	2.1
26877	1.4064	2.0138
26878	0.946133	2.1
26879	0.946131	2.1
26880	0.946131	2.1
26881	0.946131	2.1
26882	0.94613	2.1
26883	1.34912	2.1
26884	0.946131	2.1
26885	0.946129	2.1
26886	0.946131	2.1
26887	0.94613	2.1
26888	0.946132	2.1
26889	1.40447	2.1
26890	0.946132	2.1
26891	0.946133	2.1
26892	1.40447	2.1
26893	1.33607	2.1
26894	0.946131	2.1
26895	0.946127	2.1
26896	0.94613	2.1
26897	0.946129	2.1
26898	0.946117	0.103692
26899	1.32664	1.23202
26900	0.946115	0.257951
26901	0.946105	1.70727
26902	0.94613	0.05
26903	0.946118	2.1
26904	0.946114	2.1
26905	1.35886	2.1
26906	1.35884	2.1
26907	0.946129	2.1
26908	0.946132	2.1
26909	1.2571	2.1

26910	0.946132	2.1
26911	0.946132	2.1
26912	1.2571	2.1
26913	0.946133	2.1
26914	1.35663	2.1
26915	0.946132	2.1
26916	0.946132	2.1
26917	1.35663	2.1
26918	0.946133	0.473397
26919	0.946129	1.94286
26920	0.946131	1.3474
26921	0.946131	1.22471
26922	1.30881	2.02457
26923	0.946132	2.1
26924	0.94613	2.1
26925	1.34652	2.1
26926	0.94613	2.1
26927	0.94613	2.1
26928	0.946132	2.1
26929	0.946133	1.86945
26930	1.36183	1.12489
26931	1.36183	1.52034
26932	0.946132	1.80396
26933	0.946132	0.055643
26934	1.38353	0.05
26935	0.94613	0.0576015
26936	0.94613	0.0673059
26937	1.38354	0.05
26938	0.946128	0.0602217
26939	1.24887	0.05
26940	0.94613	0.05
26941	0.94613	0.119923
26942	1.24887	0.0593601
26943	0.946129	0.807106
26944	0.946126	1.63954
26945	0.946124	0.809736
26946	0.946128	0.996204
26947	1.32056	1.24813
26948	0.946129	0.955433
26949	0.946128	1.70841
26950	0.946126	0.746826
26951	0.946129	1.16027
26952	1.20351	1.18352
26953	0.946131	1.42968
26954	0.946131	1.58363
26955	0.946127	1.98482
26956	1.25591	1.95708
26957	0.94613	0.418187
26958	0.946132	1.51293
26959	0.946133	1.25668
26960	0.946132	1.92618
26961	1.15436	1.07471
26962	0.930306	0.914421
26963	0.946132	2.1
26964	0.946132	2.1
26965	0.946132	2.1
26966	0.946133	2.1
26967	0.946132	2.1

26968	0.946129	1.44206
26969	0.946132	0.999289
26970	0.946126	1.64446
26971	1.13245	0.447207
26972	0.946129	0.956202
26973	0.94613	2.1
26974	0.946132	2.1
26975	0.946129	1.97004
26976	0.946131	2.1
26977	0.946131	2.1
26978	1.41279	2.1
26979	1.76254	2.1
26980	0.600454	2.03739
26981	0.858991	2.01268
26982	0.940722	2.07222
26983	1.28439	1.16035
26984	0.946131	1.65564
26985	0.94613	1.75133
26986	0.946131	2.1
26987	0.946131	2.09752
26988	0.946132	0.684059
26989	1.39335	0.0588515
26990	0.946132	0.623713
26991	0.946132	0.105107
26992	0.946132	0.463213
26993	1.33879	0.05
26994	0.946131	0.05
26995	0.94613	0.129412
26996	1.33879	0.05
26997	0.946131	0.0551945
26998	0.946126	0.0828747
26999	0.946131	0.117843
27000	1.37342	0.0663775
27001	1.37343	0.0537752
27002	0.946128	0.417539
27003	1.39168	0.05
27004	0.946131	0.05
27005	0.946131	0.05
27006	0.946131	0.05
27007	0.946131	0.05
27008	0.946131	0.0503756
27009	1.412	0.05
27010	0.946127	0.05
27011	0.946128	0.05
27012	0.946128	0.05
27013	1.1387	0.05
27014	0.943364	0.0871317
27015	0.946132	0.05
27016	0.946132	0.05
27017	0.946133	0.05
27018	1.21085	0.05
27019	0.88324	0.350724
27020	0.946129	0.132209
27021	0.946131	0.173925
27022	0.946131	0.111166
27023	1.33587	0.05
27024	0.946125	0.05
27025	0.946129	0.05

27026	0.946126	0.05
27027	0.946127	0.05
27028	1.27512	0.05
27029	0.946132	0.05
27030	0.946132	0.05
27031	0.946133	0.05
27032	0.946132	0.05
27033	0.946128	0.05
27034	1.30498	0.05
27035	0.946131	0.05
27036	0.94613	0.05
27037	1.30497	0.05
27038	0.946131	0.05
27039	1.25626	0.05
27040	0.946131	0.05
27041	0.946131	0.05
27042	1.25626	0.05
27043	0.946131	0.05
27044	0.94613	0.05
27045	1.35316	0.05
27046	0.946131	0.05
27047	0.946131	0.05
27048	0.94613	0.0501024
27049	1.28727	0.05
27050	0.946128	0.05
27051	0.946131	0.05
27052	0.94613	0.05
27053	0.946131	0.05
27054	1.39502	0.05
27055	0.946132	0.05
27056	0.946132	0.05
27057	0.946132	0.05
27058	1.36173	0.05
27059	0.946131	0.128997
27060	0.946126	0.0555387
27061	0.946127	0.0703106
27062	0.946128	0.0517343
27063	0.946129	0.855802
27064	0.946133	0.405419
27065	0.946131	0.998026
27066	0.946131	1.18239
27067	0.946131	0.943828
27068	0.946128	0.05
27069	0.946132	0.05
27070	1.24678	0.05
27071	0.94613	0.05
27072	0.94613	0.05
27073	0.946129	0.05
27074	0.94613	0.05
27075	1.25215	0.05
27076	0.946126	0.05
27077	0.946129	0.05
27078	0.946129	0.409863
27079	1.29689	0.331531
27080	0.946131	0.460726
27081	0.946131	0.308467
27082	0.946131	0.152185
27083	0.946128	0.05

27084	0.94613	0.05
27085	0.946131	0.05
27086	0.94613	0.05
27087	0.946131	0.05
27088	0.946127	0.05
27089	0.946128	0.05
27090	0.94613	0.05
27091	0.946129	0.05
27092	0.946129	0.05
27093	0.946133	0.05
27094	0.946132	0.05
27095	0.946132	0.05
27096	1.29957	0.05
27097	0.946133	0.05
27098	0.946132	0.05
27099	0.946133	0.05
27100	0.946132	0.05
27101	1.26201	0.05
27102	0.946132	0.05
27103	0.946133	0.05
27104	1.40427	0.05
27105	0.946132	0.05
27106	0.946132	0.05
27107	0.946133	0.05
27108	0.946132	0.05
27109	1.35123	0.05
27110	0.946132	0.05
27111	0.946132	0.05
27112	0.946131	0.05
27113	0.946128	0.05
27114	0.946129	0.05
27115	0.946131	0.05
27116	0.94613	0.05
27117	0.946131	0.05
27118	0.946127	0.0898307
27119	1.23351	0.0690148
27120	0.946131	0.0951827
27121	0.946129	0.0674351
27122	0.94613	0.0635762
27123	0.946131	0.05
27124	0.946131	0.05
27125	0.94613	0.05
27126	1.18119	0.05
27127	0.946131	0.05
27128	0.946132	0.05
27129	1.30563	0.05
27130	0.946133	0.05
27131	0.946132	0.05
27132	0.946133	0.05
27133	0.946132	0.05
27134	0.946132	0.05
27135	0.946133	0.398565
27136	1.20256	0.05
27137	0.946133	0.0523404
27138	0.94613	0.0619551
27139	1.33007	0.05
27140	0.946128	0.123122
27141	0.946132	0.05

27142	0.946131	0.0570818
27143	0.946131	2.1
27144	0.946131	2.1
27145	0.946128	2.1
27146	0.946129	2.1
27147	0.94613	2.1
27148	0.810942	2.1
27149	0.810942	2.1
27150	0.81093	2.1
27151	0.946132	2.1
27152	1.39823	2.1
27153	0.946132	2.1
27154	0.946131	2.1
27155	0.946132	2.1
27156	0.946131	2.1
27157	0.946131	2.1
27158	0.946131	2.1
27159	1.37713	2.1
27160	0.946131	2.1
27161	0.946133	1.6322
27162	0.946131	2.1
27163	1.40594	2.1
27164	1.40594	2.1
27165	0.94613	2.1
27166	0.946133	2.1
27167	0.946131	2.1
27168	1.38155	2.1
27169	1.38155	2.1
27170	0.946131	2.1
27171	0.946132	0.499626
27172	0.946133	2.1
27173	0.946132	0.994582
27174	0.946132	1.15099
27175	1.33694	1.87696
27176	0.946131	2.1
27177	1.228 2.1	
27178	0.946129	2.1
27179	0.946131	2.1
27180	1.228 2.1	
27181	0.946132	1.12065
27182	0.946132	1.69669
27183	0.946131	2.071
27184	0.946132	1.33095
27185	1.35943	2.00942
27186	0.946133	0.05
27187	0.946133	0.05
27188	1.02377	0.05
27189	1.02377	0.05
27190	0.946132	0.05
27191	0.94613	0.0796802
27192	0.946131	0.0584513
27193	0.946132	0.0959312
27194	1.29322	0.05
27195	0.946131	0.0978838
27196	0.946131	1.46981
27197	0.946131	0.05
27198	0.946132	1.43714
27199	1.41492	0.05

27200	0.946131	0.219493
27201	0.946132	2.1
27202	0.946131	2.1
27203	0.946132	2.1
27204	1.35028	2.1
27205	0.946132	2.1
27206	0.946132	2.1
27207	0.946132	2.1
27208	0.946131	2.1
27209	1.32461	2.1
27210	0.946132	2.1
27211	0.946131	2.1
27212	0.946132	2.1
27213	1.40369	2.1
27214	0.94613	2.1
27215	0.946131	2.1
27216	0.946132	0.05
27217	0.979597	0.05
27218	0.946131	0.05
27219	0.946132	0.05
27220	0.946132	0.05
27221	0.946131	2.1
27222	0.946131	2.1
27223	0.94613	2.1
27224	0.946131	2.1
27225	1.41897	2.1
27226	1.37791	2.1
27227	0.94613	2.1
27228	0.946129	2.1
27229	0.946131	2.1
27230	0.946131	2.1
27231	1.39384	2.1
27232	0.946131	2.1
27233	0.946128	2.1
27234	0.946129	2.1
27235	0.946129	2.1
27236	0.810945	1.59375
27237	0.810944	2.1
27238	0.810958	1.85797
27239	0.946128	2.1
27240	0.946132	2.1
27241	0.94613	2.1
27242	1.3335	2.1
27243	0.946131	2.1
27244	0.94613	0.05
27245	0.946129	1.90529
27246	0.946132	0.05
27247	1.41192	0.97112
27248	0.94613	0.05
27249	0.946132	0.723781
27250	0.946133	0.241756
27251	0.946132	0.131746
27252	1.39428	0.134389
27253	0.946133	0.0819011
27254	0.94613	2.03946
27255	0.946128	2.1
27256	0.946132	0.964282
27257	0.94613	1.98189

27258	1.40684	2.1
27259	0.946131	2.1
27260	0.946131	2.1
27261	0.946131	2.1
27262	0.946131	2.1
27263	1.39021	2.1
27264	0.946131	1.1152
27265	1.31649	2.1
27266	0.946131	0.653369
27267	0.946131	0.609772
27268	1.31649	1.53423
27269	0.810946	2.1
27270	0.810946	2.1
27271	0.810958	2.1
27272	1.16687	2.1
27273	0.946131	2.1
27274	0.946131	2.1
27275	1.16687	2.1
27276	1.16687	2.1
27277	0.946128	1.08554
27278	1.3564	1.11042
27279	0.946131	1.26863
27280	0.946131	1.19697
27281	0.94613	0.563941
27282	0.946128	2.1
27283	1.35828	2.1
27284	0.94613	2.1
27285	0.946131	2.1
27286	0.94613	2.1
27287	0.946133	0.072807
27288	1.36477	0.063932
27289	0.946132	0.0973912
27290	0.946132	0.209278
27291	1.36477	0.081843
27292	0.946132	0.570208
27293	1.31857	2.1
27294	0.946132	1.31873
27295	0.946132	1.67326
27296	1.31857	1.51927
27297	0.94613	0.142536
27298	1.35059	0.05
27299	0.946128	0.0512357
27300	0.94613	0.0772054
27301	1.35059	0.0602808
27302	0.946126	0.05
27303	1.35559	0.05
27304	0.946127	0.05
27305	0.946128	0.05
27306	1.35559	0.05
27307	0.946131	0.05
27308	1.33035	0.05
27309	0.946127	0.05
27310	0.946128	0.05
27311	1.33035	0.05
27312	0.94613	0.05
27313	1.3665	0.05
27314	0.946129	0.05
27315	0.946131	0.05

27316	1.3665	0.05
27317	0.94613	0.05
27318	1.32828	0.05
27319	0.946129	0.05
27320	0.94613	0.05
27321	0.946131	0.05
27322	0.946131	2.1
27323	0.946131	2.1
27324	1.32949	2.1
27325	1.32949	2.1
27326	0.946131	2.1
27327	0.946131	1.51128
27328	0.94613	0.05
27329	0.946133	0.2885
27330	1.38357	0.05
27331	0.946131	0.130477
27332	0.946131	2.1
27333	0.946128	2.1
27334	1.36919	2.1
27335	0.946131	2.1
27336	0.94613	2.1
27337	0.946131	1.1099
27338	1.3771	2.1
27339	0.946129	2.1
27340	0.94613	2.09867
27341	0.946131	1.4524
27342	0.946131	1.35482
27343	0.94613	0.809344
27344	0.946129	0.13636
27345	1.30029	0.674467
27346	0.94613	0.39717
27347	1.21174	0.05
27348	0.946132	0.05
27349	0.946132	0.05
27350	0.946132	0.05
27351	0.946133	0.05
27352	0.810946	2.1
27353	0.810945	2.1
27354	0.810946	2.1
27355	1.1796	2.1
27356	0.946131	2.1
27357	0.946132	2.1
27358	1.1796	2.1
27359	1.1796	2.1
27360	0.946131	2.1
27361	0.946131	2.1
27362	0.946133	2.1
27363	0.946131	2.1
27364	1.40615	2.1
27365	0.810945	2.1
27366	0.810944	2.1
27367	0.810945	2.1
27368	1.40239	2.1
27369	0.94613	2.1
27370	1.35561	2.1
27371	0.946133	2.1
27372	1.35562	2.1
27373	1.41169	0.05

27374	0.946131	0.05
27375	0.946127	0.10467
27376	1.41169	0.05
27377	0.946128	0.0568693
27378	0.946119	2.1
27379	0.946119	2.1
27380	0.946119	2.1
27381	0.946107	2.1
27382	1.41917	2.1
27383	0.810946	1.21231
27384	0.810943	1.45815
27385	0.810934	2.03655
27386	0.946127	2.1
27387	1.1995	2.1
27388	0.946126	2.1
27389	0.946128	2.1
27390	1.1995	2.1
27391	0.810944	2.1
27392	0.810947	2.1
27393	0.81096	2.1
27394	0.946133	0.05
27395	1.3495	0.05
27396	0.946132	0.05
27397	0.946132	0.05
27398	0.946133	0.05
27399	0.946132	2.1
27400	0.946132	2.1
27401	0.946133	2.1
27402	1.39529	2.1
27403	0.946133	2.1
27404	0.946133	2.1
27405	1.28525	2.1
27406	0.946132	2.1
27407	0.946133	2.1
27408	1.28525	2.1
27409	0.810946	2.1
27410	0.810945	2.1
27411	0.810946	2.1
27412	1.33931	2.1
27413	0.946132	2.1
27414	0.946132	2.1
27415	1.33931	2.1
27416	1.24616	2.1
27417	0.946133	0.05
27418	0.946133	0.0543623
27419	0.946132	0.05
27420	0.946132	0.05
27421	0.946133	0.05
27422	0.946128	2.1
27423	1.40225	2.1
27424	0.946128	2.1
27425	0.94613	2.1
27426	0.94613	2.1
27427	0.946113	0.05
27428	0.946118	0.05
27429	0.946114	0.05
27430	1.40551	0.05
27431	0.946103	0.05

27432	0.94613	2.1
27433	0.946132	2.1
27434	0.946129	2.1
27435	0.946131	2.1
27436	1.37811	2.1
27437	0.946132	2.1
27438	1.39826	2.1
27439	0.946133	2.1
27440	0.946132	2.1
27441	1.39826	2.1
27442	0.946118	2.1
27443	1.28298	2.1
27444	0.946117	2.1
27445	0.946105	2.1
27446	1.283 2.1	
27447	0.946118	0.05
27448	1.41375	0.05
27449	0.946117	0.05
27450	0.946131	0.05
27451	0.946106	0.05
27452	0.946132	0.102567
27453	0.946129	0.0519982
27454	1.28436	0.05
27455	0.946131	0.111237
27456	0.946131	0.0673688
27457	0.810947	0.05
27458	0.810946	0.05
27459	0.81096	0.05
27460	0.946132	0.05
27461	1.34338	0.05
27462	0.961435	0.05
27463	0.961435	0.05
27464	0.946132	0.05
27465	1.3028	2.04031
27466	0.946131	2.02889
27467	0.946127	2.1
27468	0.946128	2.1
27469	0.946129	2.07559
27470	0.946132	0.14087
27471	1.37973	0.0550794
27472	0.946132	0.173652
27473	0.946132	0.05
27474	0.946132	0.0654981
27475	0.946131	0.410042
27476	0.94613	0.240305
27477	0.946129	0.413676
27478	0.946131	0.200828
27479	1.41798	0.199612
27480	0.946118	0.229696
27481	0.946118	0.124532
27482	0.946117	0.231667
27483	0.946131	0.13711
27484	0.946105	0.133209
27485	0.946131	0.477616
27486	0.946131	0.266677
27487	0.94613	0.470644
27488	0.946131	0.25696
27489	0.946131	0.197733

27490	0.946133	0.897169
27491	1.40274	2.1
27492	0.946133	0.228007
27493	0.946132	2.1
27494	1.40274	2.1
27495	0.946131	1.21749
27496	1.39731	2.1
27497	0.946132	0.597801
27498	0.94613	1.18935
27499	1.39731	1.95759
27500	0.94612	2.1
27501	0.94612	1.7242
27502	0.946119	1.62483
27503	0.946132	1.72253
27504	1.40764	2.1
27505	0.946128	2.1
27506	0.946132	2.1
27507	0.94613	2.1
27508	0.94613	2.1
27509	1.31213	2.1
27510	0.946127	1.99362
27511	0.94613	2.1
27512	0.946127	2.01669
27513	0.946128	2.1
27514	1.36653	2.1
27515	0.946127	2.1
27516	1.3557	2.1
27517	0.946128	2.1
27518	0.946128	2.1
27519	1.3557	2.1
27520	0.946133	2.1
27521	0.946132	2.1
27522	0.946133	2.1
27523	0.946132	2.1
27524	1.40081	2.1
27525	0.946128	2.1
27526	0.946133	2.1
27527	0.946131	2.1
27528	0.946131	2.1
27529	1.32282	2.1
27530	0.810945	2.1
27531	0.810943	2.1
27532	0.810933	2.1
27533	1.27676	2.1
27534	0.94613	2.1
27535	0.946128	2.1
27536	0.946131	2.1
27537	0.946131	2.1
27538	0.946131	0.337391
27539	0.946127	0.05
27540	1.41328	0.303738
27541	1.41327	0.110405
27542	0.946128	0.141227
27543	0.946132	0.05
27544	1.32083	0.05
27545	0.946132	0.05
27546	0.946133	0.05
27547	1.32082	0.05

27548	0.946128	0.05
27549	1.29215	0.05
27550	0.94613	0.05
27551	0.94613	0.05
27552	1.29215	0.05
27553	0.946132	0.05
27554	1.27696	0.05
27555	0.94613	0.05
27556	0.94613	0.05
27557	1.27696	0.05
27558	0.94612	0.05
27559	1.29289	0.05
27560	0.946119	0.05
27561	0.946132	0.05
27562	1.29287	0.05
27563	0.946128	2.1
27564	1.37869	2.1
27565	0.946131	2.1
27566	0.94613	2.1
27567	0.946131	2.1
27568	0.946133	2.1
27569	0.946132	2.1
27570	0.946132	2.1
27571	1.35703	2.1
27572	0.946133	2.1
27573	0.946132	2.1
27574	0.946132	2.1
27575	0.946133	2.1
27576	1.37017	2.1
27577	0.946133	2.1
27578	0.946119	0.05
27579	0.94612	0.05
27580	1.38583	0.05
27581	1.38585	0.05
27582	0.946107	0.05
27583	0.946126	0.05
27584	0.946127	0.05
27585	1.22904	0.05
27586	1.22904	0.05
27587	0.946128	0.05
27588	1.3279	0.0939179
27589	0.946131	1.43072
27590	0.94613	0.75135
27591	0.946131	1.08409
27592	0.946131	0.707971
27593	0.946132	2.1
27594	0.946132	2.1
27595	0.946132	2.1
27596	0.946131	2.1
27597	1.40932	2.1
27598	1.26868	0.05
27599	0.946131	0.05
27600	0.94613	0.05
27601	0.946131	0.05
27602	0.946131	0.05
27603	0.810943	2.1
27604	0.810945	2.1
27605	0.810932	2.1

27606	0.810943	0.272359
27607	0.810944	0.0758989
27608	0.810957	0.05
27609	1.31539	0.158244
27610	0.946132	1.85169
27611	0.946128	0.05
27612	1.3154	0.0988748
27613	1.17856	0.0545748
27614	1.21997	0.604976
27615	0.94613	1.92893
27616	0.946129	1.47725
27617	0.94613	2.05347
27618	0.94613	1.89469
27619	0.946131	0.919147
27620	0.946131	0.249408
27621	0.946129	1.16538
27622	0.94613	0.785483
27623	0.946131	1.14589
27624	0.946127	0.213749
27625	0.946131	0.0944979
27626	0.946127	2.05824
27627	0.946129	0.723273
27628	1.41342	0.218993
27629	0.946131	0.120006
27630	0.946131	1.59828
27631	0.946128	1.90091
27632	0.946131	0.417593
27633	1.29149	1.52483
27634	1.41022	2.1
27635	0.94613	0.386864
27636	0.946128	2.1
27637	0.94613	2.1
27638	0.94613	2.1
27639	0.946128	0.05
27640	0.94613	0.0591451
27641	1.27864	0.05
27642	1.27864	0.05
27643	0.946131	0.0623644
27644	0.946129	0.608511
27645	0.946125	1.72199
27646	0.946124	1.30813
27647	0.946127	0.98101
27648	1.37759	1.47077
27649	0.946129	0.739437
27650	0.94613	1.70467
27651	0.946126	1.08242
27652	0.946129	1.00644
27653	1.38259	1.31225
27654	0.946131	0.05
27655	1.34118	0.05
27656	0.946132	0.05
27657	0.946132	0.05
27658	1.34118	0.05
27659	0.946131	0.05
27660	1.25017	0.05
27661	0.946128	0.05
27662	0.946131	0.05
27663	0.946131	0.05

27664	0.946131	2.1
27665	0.946128	2.1
27666	0.946133	2.1
27667	1.3937	2.1
27668	0.94613	2.1
27669	0.946131	0.511723
27670	0.94613	0.05
27671	1.40404	0.05
27672	1.40404	0.05
27673	0.946131	0.05
27674	0.946132	0.314432
27675	0.946132	0.05
27676	1.38897	0.05
27677	1.38897	0.05
27678	0.946132	0.05
27679	0.94613	0.05
27680	0.946128	0.05
27681	1.40332	0.05
27682	1.40331	0.05
27683	0.94613	0.127453
27684	0.946132	0.05
27685	0.946132	1.24044
27686	0.946133	0.212771
27687	1.41619	1.59731
27688	0.946132	0.100792
27689	0.946132	1.83812
27690	0.946132	2.1
27691	0.946132	2.1
27692	1.38244	2.1
27693	0.946132	2.1
27694	0.810946	1.28389
27695	0.810943	0.482711
27696	0.810934	0.17598
27697	0.810947	1.20554
27698	0.810943	0.201976
27699	0.810934	0.213952
27700	1.12786	1.23566
27701	0.946132	2.1
27702	0.946132	1.95137
27703	0.946132	1.39397
27704	0.946133	1.39127
27705	0.946132	1.90437
27706	0.946132	1.93912
27707	0.946132	1.54168
27708	1.27806	1.91119
27709	0.946133	1.41472
27710	0.946132	2.1
27711	0.946132	2.1
27712	0.946133	2.1
27713	0.946132	2.1
27714	0.946133	2.1
27715	1.39514	1.81212
27716	0.946129	1.20502
27717	0.946124	1.63859
27718	0.946127	0.797908
27719	0.946127	1.70703
27720	0.94613	2.1
27721	0.946129	2.1

27722	0.946132	2.1
27723	1.3991	2.1
27724	0.94613	2.1
27725	0.946132	0.0657359
27726	0.946133	2.1
27727	0.946132	2.00324
27728	1.36835	0.470156
27729	0.946132	0.313616
27730	0.946132	2.1
27731	0.946132	2.1
27732	0.946133	2.1
27733	0.946132	2.1
27734	1.39047	2.1
27735	0.946132	0.05
27736	0.946128	0.05
27737	0.94613	0.05
27738	1.33174	0.05
27739	0.946131	0.05
27740	0.946128	0.05
27741	0.946131	0.05
27742	0.94613	0.05
27743	0.94613	0.05
27744	0.94613	0.05
27745	0.946119	2.1
27746	0.946119	2.1
27747	1.29064	2.1
27748	0.946132	2.1
27749	0.946107	2.1
27750	0.946133	2.1
27751	0.94613	2.1
27752	1.30584	2.1
27753	0.946131	2.1
27754	0.946131	2.1
27755	0.946131	2.1
27756	0.946132	2.1
27757	0.94613	2.1
27758	0.946131	2.1
27759	1.39549	2.1
27760	0.913386	1.29486
27761	0.978873	0.950776
27762	0.946126	0.789374
27763	0.94613	1.19777
27764	1.00473	0.470584
27765	0.946132	0.591671
27766	0.946133	0.405013
27767	0.946132	0.274353
27768	0.946132	0.776546
27769	1.38894	0.209591
27770	0.946128	0.346831
27771	0.946131	0.257251
27772	0.94613	0.246626
27773	0.94613	0.342336
27774	1.23865	0.157607
27775	0.946126	1.38211
27776	0.946129	1.30362
27777	0.946124	0.805297
27778	0.946127	1.19626
27779	1.40248	0.858265

27780	0.946133	0.727529
27781	0.946132	0.679895
27782	0.946132	0.332691
27783	0.946133	0.757179
27784	1.1391	0.374543
27785	0.827862	0.378361
27786	0.946133	0.706368
27787	0.946131	1.25566
27788	0.946131	0.860937
27789	0.94613	0.654652
27790	0.946128	0.187736
27791	0.946132	0.46926
27792	0.94613	0.612219
27793	1.21388	0.095768
27794	0.94613	0.566901
27795	0.810943	0.05
27796	0.810945	0.0832374
27797	0.810932	0.0759305
27798	0.946131	0.254941
27799	0.946131	0.820101
27800	0.946133	1.31865
27801	1.36748	0.156411
27802	0.946132	0.05
27803	0.946128	0.0545839
27804	0.946131	0.05
27805	0.94613	0.0534726
27806	1.35856	0.05
27807	0.94613	0.05
27808	0.946132	2.1
27809	0.946131	2.1
27810	0.946133	2.1
27811	0.946132	2.1
27812	0.946131	2.1
27813	0.946133	2.1
27814	0.946131	2.1
27815	1.39558	2.1
27816	1.39558	2.1
27817	0.94613	2.1
27818	0.946131	0.05
27819	0.94613	0.05
27820	0.946129	0.05
27821	1.41388	0.05
27822	0.946131	0.05
27823	0.946133	0.05
27824	0.946132	0.05
27825	0.946132	0.05
27826	0.946133	0.05
27827	0.93452	0.05
27828	0.946129	2.1
27829	1.38897	2.1
27830	0.946131	2.1
27831	0.946131	2.1
27832	1.38897	2.1
27833	0.946133	2.08803
27834	0.946132	1.67408
27835	0.946133	2.00126
27836	0.946132	0.637017
27837	1.38773	1.26645

27838	1.41512	0.05
27839	0.946131	0.05
27840	0.946129	0.05
27841	0.946133	0.05
27842	0.946131	0.05
27843	0.946132	0.191525
27844	0.946132	2.1
27845	0.946132	0.274135
27846	0.946133	1.31295
27847	0.946133	2.1
27848	0.946126	0.135063
27849	0.946132	0.603372
27850	0.946129	0.130332
27851	0.946129	0.360539
27852	0.946129	1.06384
27853	0.939895	2.06046
27854	1.03386	2.1
27855	0.94613	2.05034
27856	0.946131	1.91327
27857	1.03386	1.99937
27858	0.946131	2.1
27859	1.39309	2.1
27860	0.946133	1.95362
27861	0.946131	2.1
27862	1.39309	2.1
27863	0.946133	2.1
27864	1.24853	2.1
27865	0.946132	2.1
27866	0.946132	2.1
27867	1.24853	2.1
27868	0.946133	2.1
27869	1.39699	0.303337
27870	0.946132	1.84951
27871	0.946132	2.1
27872	1.39699	0.967451
27873	0.946132	2.1
27874	0.946131	1.9863
27875	0.946133	2.1
27876	0.946131	2.1
27877	1.38969	2.1
27878	1.28464	2.1
27879	0.946133	2.1
27880	0.94613	2.1
27881	0.946131	2.01864
27882	0.946131	2.04054
27883	0.946119	0.782538
27884	0.94612	0.948137
27885	0.946119	0.624132
27886	0.946132	0.830983
27887	1.3749	0.48332
27888	0.810942	2.1
27889	0.810943	2.1
27890	0.810957	2.1
27891	1.41134	2.1
27892	0.946132	0.44259
27893	0.946132	2.06942
27894	0.946132	0.88119
27895	0.946132	2.00605

27896	1.31338	2.1
27897	0.946132	0.979796
27898	0.946132	2.1
27899	0.946133	1.62637
27900	0.946132	2.05411
27901	0.946103	0.05
27902	0.752448	0.05
27903	0.946132	0.05
27904	0.946131	0.05
27905	1.20617	0.05
27906	0.946132	0.948625
27907	1.08054	0.301716
27908	1.25736	0.533087
27909	0.946132	0.975195
27910	0.946131	0.748754
27911	0.946128	2.1
27912	1.33275	0.69648
27913	1.1896	2.1
27914	0.946131	2.1
27915	1.18962	2.1
27916	1.21955	0.05
27917	0.946131	0.05
27918	1.33587	0.05
27919	0.741946	0.05
27920	1.07387	0.05
27921	0.945844	0.297418
27922	1.3292	2.1
27923	1.59472	2.05728
27924	0.946131	0.712208
27925	1.04043	1.06364
27926	1.13529	0.05
27927	0.946132	0.05
27928	1.24893	0.05
27929	1.12675	0.05
27930	0.936652	0.05
27931	1.36968	2.1
27932	0.946131	2.1
27933	0.946132	2.1
27934	1.36968	2.1
27935	1.258 2.1	
27936	3.17458	0.05
27937	0.946132	1.86776
27938	1.35022	2.1
27939	1.35022	2.1
27940	0.94613	2.0788
27941	1.12356	2.1
27942	0.946131	2.1
27943	1.22401	2.1
27944	1.22401	2.1
27945	0.94613	2.1
27946	0.952455	2.1
27947	1.2107	2.1
27948	0.946132	2.1
27949	1.33047	2.1
27950	0.946131	2.1
27951	0.946132	2.1
27952	0.61957	2.1
27953	0.8636	2.1

27954	0.946132	2.1
27955	1.19294	2.1
27956	0.946128	2.1
27957	1.36607	2.1
27958	0.94613	2.1
27959	1.39416	2.1
27960	1.36607	2.1
27961	1.21474	2.1
27962	0.946131	2.1
27963	0.946131	2.1
27964	1.16288	2.1
27965	1.08542	2.1
27966	0.851526	0.688169
27967	1.30374	2.1
27968	1.01708	2.1
27969	0.946114	0.845326
27970	0.96976	1.82661
27971	0.946132	0.05
27972	1.49997	0.05
27973	0.771735	0.05
27974	0.771764	0.05
27975	0.946133	0.05
27976	0.700863	0.906842
27977	1.42222	1.98533
27978	0.946131	2.1
27979	0.946128	0.674557
27980	1.42509	2.1
27981	1.14298	0.813391
27982	0.837014	2.1
27983	0.946131	2.1
27984	1.29426	2.1
27985	0.836985	2.1
27986	0.946129	2.1
27987	1.22089	0.0593653
27988	0.946132	1.17144
27989	0.712486	0.120998
27990	0.6234	0.0589365
27991	0.946131	2.1
27992	1.40271	2.1
27993	1.32241	2.1
27994	0.946133	2.1
27995	1.32241	2.1
27996	0.769373	1.21115
27997	0.946129	2.1
27998	1.41302	2.1
27999	1.28254	2.1
28000	0.946131	2.1
28001	1.28254	2.1
28002	0.851543	1.88471
28003	1.23056	2.1
28004	1.24356	2.1
28005	0.851543	2.01867
28006	0.909417	2.1
28007	1.13808	0.12302
28008	0.946133	0.17039
28009	0.946131	0.05
28010	1.13808	1.30308
28011	1.13808	0.208838

28012	0.930941	1.19252
28013	1.37808	0.800284
28014	1.17573	0.321307
28015	0.946124	1.22522
28016	1.02404	0.418025
28017	0.946116	2.1
28018	1.3144	0.39151
28019	1.3144	2.1
28020	0.946118	2.1
28021	1.13089	2.1
28022	0.946128	0.05
28023	0.946101	0.05
28024	1.2606	0.05
28025	1.26055	0.05
28026	0.580823	0.313893
28027	0.946132	1.21888
28028	0.580825	0.348845
28029	1.23224	0.372043
28030	0.946128	2.1
28031	1.3719	2.1
28032	0.94613	2.1
28033	1.3719	2.1
28034	0.94613	2.1
28035	0.946132	1.3331
28036	1.35025	0.228755
28037	1.35025	0.700115
28038	0.946132	1.93868
28039	1.1794	1.19596
28040	0.946133	2.1
28041	1.25598	2.1
28042	1.29249	2.1
28043	1.38154	2.1
28044	0.851541	2.1
28045	1.31508	2.1
28046	1.37558	2.1
28047	0.946129	2.1
28048	0.867663	2.1
28049	0.94613	1.48654
28050	1.35426	1.10756
28051	0.84872	0.913929
28052	0.946129	0.969092
28053	1.17195	2.09559
28054	0.946133	2.1
28055	1.39009	2.1
28056	1.14342	2.1
28057	0.946133	2.1
28058	1.14343	2.1
28059	1.38819	2.1
28060	0.946133	2.1
28061	1.4116	2.1
28062	0.946131	2.1
28063	0.946131	2.1
28064	0.946127	2.1
28065	1.32244	2.1
28066	1.32244	2.1
28067	0.946131	2.1
28068	1.2176	2.1
28069	1.75823	2.1

28070	1.11239	2.1
28071	2.17979	2.1
28072	1.65533	2.1
28073	3.41356	2.1
28074	1.94841	2.1
28075	1.91767	2.1
28076	1.76726	2.1
28077	1.70302	2.1
28078	3.76137	2.1
28079	1.94841	2.1
28080	1.33962	2.1
28081	1.31365	2.1
28082	1.70302	2.1
28083	3.2123	2.1
28084	1.76726	2.1
28085	1.70302	2.1
28086	1.47581	2.1
28087	1.7457	2.1
28088	3.22764	2.1
28089	1.20478	2.1
28090	1.70302	2.1
28091	1.27956	2.1
28092	1.18569	2.1
28093	1.63979	2.1
28094	1.51949	2.1
28095	1.53082	2.1
28096	1.92911	2.1
28097	1.32068	2.1
28098	1.74454	2.1
28099	3.27536	2.1
28100	1.08172	2.1
28101	1.74454	2.1
28102	1.52603	2.1
28103	1.52603	2.1
28104	1.32068	2.1
28105	1.32068	2.1
28106	1.89226	2.1
28107	2.37364	2.1
28108	1.89226	2.1
28109	2.42769	2.1
28110	4.31995	2.1
28111	2.37365	2.1
28112	1.5362	2.1
28113	2.4277	2.1
28114	1.56328	2.1
28115	3.9639	2.1
28116	1.89225	2.1
28117	2.42768	2.1
28118	1.89213	2.1
28119	1.89225	2.1
28120	2.42756	2.1
28121	2.42768	2.1
28122	2.42768	2.1
28123	1.58163	2.1
28124	1.56315	2.1
28125	1.56327	2.1
28126	2.42757	2.1
28127	2.42768	2.1

28128	1.01641	2.1
28129	2.15005	2.1
28130	1.00099	2.1
28131	2.15114	2.1
28132	3.15104	2.1
28133	0.994813	2.1
28134	2.15114	2.1
28135	2.17159	2.1
28136	2.17159	2.1
28137	1.00099	2.1
28138	1.00099	2.1
28139	1.29608	2.1
28140	1.29608	2.1
28141	0.942035	2.1
28142	0.942035	1.89688
28143	1.29198	2.1
28144	0.942033	2.1
28145	0.946132	2.1
28146	0.946132	2.1
28147	0.946132	2.09294
28148	0.942033	2.1
28149	0.946133	1.60892
28150	1.29198	2.1
28151	1.99985	2.1
28152	1.14207	2.1
28153	1.83584	2.1
28154	1.00232	2.1
28155	3.00217	2.1
28156	1.99867	0.05
28157	2.0815	0.05
28158	1.56806	0.05
28159	1.28828	0.05
28160	3.58519	0.05
28161	1.89226	0.05
28162	1.89226	0.05
28163	1.89226	0.05
28164	1.89226	0.05
28165	1.89226	0.05
28166	1.89226	0.05
28167	1.89226	0.05
28168	1.89226	0.05
28169	2.23193	0.05
28170	2.48978	0.05
28171	4.38204	0.05
28172	1.85293	0.698342
28173	1.64279	0.05
28174	1.76268	0.0694352
28175	1.54214	0.05
28176	3.39507	0.05
28177	1.6428	0.0551225
28178	1.17302	0.05
28179	1.17302	0.707639
28180	1.28419	0.0980398
28181	1.54214	0.05
28182	1.65331	0.0871515
28183	1.76268	0.775923
28184	1.54214	0.109632
28185	1.65324	0.0668297

28186	1.42491	0.05
28187	3.19538	0.05
28188	0.651043	0.159895
28189	0.651043	0.59293
28190	0.647925	0.0804794
28191	0.647925	0.260241
28192	0.521747	0.0776395
28193	0.58651	0.118096
28194	0.77107	0.529204
28195	0.712457	0.101909
28196	0.712457	0.05
28197	0.521747	0.0604301
28198	0.58651	0.05
28199	0.77107	0.458963
28200	1.2873	2.1
28201	1.48751	2.1
28202	1.59659	2.1
28203	1.77596	2.00931
28204	3.06326	2.1
28205	1.07976	2.1
28206	1.59659	2.1
28207	1.30237	2.1
28208	1.77723	2.1
28209	2.89897	2.1
28210	1.77723	2.1
28211	1.77596	2.1
28212	1.82614	2.1
28213	1.59659	2.1
28214	2.0055	2.1
28215	1.77596	2.1
28216	2.64523	0.769879
28217	2.73982	0.05
28218	2.50057	0.145479
28219	2.61606	0.05
28220	5.26129	0.05
28221	2.73982	0.0736683
28222	2.68987	0.0778256
28223	2.61607	0.0943137
28224	2.57611	0.05
28225	5.30593	0.05
28226	1.89226	0.0547097
28227	1.89226	0.356768
28228	1.89226	0.153226
28229	2.02178	0.163702
28230	3.78452	0.238055
28231	1.89226	0.05
28232	1.89226	0.05
28233	1.89226	1.3193
28234	1.89226	0.05
28235	1.89226	0.948056
28236	1.89226	0.05
28237	2.35204	2.1
28238	1.50108	2.1
28239	1.3715	2.1
28240	2.18469	2.1
28241	3.68577	2.1
28242	2.62514	2.1
28243	2.1847	2.1

28244	2.0487	2.1
28245	2.45213	2.1
28246	4.63682	2.1
28247	1.25075	2.1
28248	2.18469	2.1
28249	1.3715	2.1
28250	1.3715	2.1
28251	2.0487	2.1
28252	2.0487	2.1
28253	1.28667	2.1
28254	1.11285	2.1
28255	1.03529	2.1
28256	1.03529	2.1
28257	1.30711	2.1
28258	1.30711	2.1
28259	2.35053	2.1
28260	1.89225	2.1
28261	1.89225	2.1
28262	2.51216	2.1
28263	4.24278	2.1
28264	1.89225	2.1
28265	1.89225	2.1
28266	1.89225	2.1
28267	1.89225	2.1
28268	3.7845	2.1
28269	1.92025	2.1
28270	1.32119	2.1
28271	1.44777	2.1
28272	1.33834	2.1
28273	1.92025	2.1
28274	1.81082	2.1
28275	1.89226	2.1
28276	2.67272	2.1
28277	2.51218	2.1
28278	2.51218	2.1
28279	1.89226	2.1
28280	1.89226	2.1
28281	1.89226	2.1
28282	1.89226	2.1
28283	1.89226	2.1
28284	1.89226	2.1
28285	3.78452	2.1
28286	1.92027	2.1
28287	1.44778	2.1
28288	1.61121	2.1
28289	2.01555	2.1
28290	3.53147	2.1
28291	1.90727	0.05
28292	2.29851	0.05
28293	2.71643	0.05
28294	2.17695	0.05
28295	4.47547	0.05
28296	1.90728	0.05
28297	1.56339	0.05
28298	1.58143	0.05
28299	2.17696	0.05
28300	3.74035	0.05
28301	1.91368	0.05

28302	2.17696	0.05
28303	2.39986	0.05
28304	1.75904	0.05
28305	3.936	0.05
28306	1.18793	0.05
28307	2.74744	0.05
28308	1.38716	0.05
28309	2.57465	0.05
28310	3.93537	0.05
28311	1.3148	0.05
28312	2.74744	0.05
28313	2.70805	0.05
28314	1.38716	0.05
28315	2.70805	0.05
28316	1.38716	0.05
28317	1.20608	2.1
28318	0.975871	2.1
28319	0.946132	2.1
28320	0.946132	2.1
28321	0.946132	2.1
28322	0.946132	2.1
28323	0.975871	2.1
28324	0.975871	2.1
28325	1.20608	2.1
28326	0.946132	2.1
28327	0.946133	2.1
28328	0.975871	2.1
28329	2.8198	0.870807
28330	2.29967	2.1
28331	2.09542	2.1
28332	2.63784	0.167549
28333	4.93751	2.1
28334	2.09542	2.1
28335	1.39523	2.1
28336	1.21119	2.1
28337	1.39523	2.1
28338	2.11563	2.1
28339	2.29967	2.1
28340	1.88584	2.1
28341	2.63784	0.114442
28342	2.11262	1.70759
28343	1.91132	1.22171
28344	4.02153	2.02936
28345	1.88584	2.1
28346	1.21119	2.1
28347	1.12606	2.1
28348	1.91132	1.5181
28349	3.1225	2.1
28350	2.18077	2.1
28351	1.78167	2.1
28352	2.2947	0.375715
28353	1.93139	1.62576
28354	4.07637	0.594877
28355	1.5709	1.25592
28356	1.03235	2.1
28357	2.05933	2.04423
28358	1.78168	0.884468
28359	1.31	0.103931

28360	1.03235	0.164326
28361	2.2947	1.06895
28362	2.31785	2.1
28363	2.40627	2.1
28364	2.08288	0.767198
28365	4.37758	2.1
28366	1.95737	1.92995
28367	1.31	2.1
28368	2.08289	0.16913
28369	1.50108	0.665511
28370	3.39289	0.435082
28371	2.49676	2.1
28372	2.24781	2.1
28373	2.5276	1.19896
28374	2.7352	2.1
28375	5.02436	2.1
28376	2.49676	2.1
28377	2.43549	2.1
28378	2.6398	2.1
28379	2.7352	2.1
28380	5.13656	2.1
28381	1.89226	2.1
28382	2.7352	2.1
28383	1.89226	2.1
28384	2.6398	2.1
28385	1.89226	2.1
28386	2.6398	2.1
28387	2.16843	0.05
28388	1.00185	0.05
28389	1.90039	2.1
28390	3.1547	0.05
28391	2.54064	0.05
28392	2.46752	2.1
28393	1.90039	2.1
28394	2.26044	2.1
28395	4.36791	0.05
28396	1.65815	2.1
28397	2.48652	2.1
28398	1.65815	2.1
28399	1.9004	2.1
28400	2.21711	2.1
28401	2.5034	2.1
28402	2.37167	2.1
28403	2.59965	2.1
28404	2.62812	2.1
28405	2.40308	2.1
28406	4.99979	2.1
28407	2.50137	2.1
28408	2.59965	2.1
28409	2.62812	2.1
28410	2.5214	2.1
28411	5.12948	2.1
28412	2.62814	2.1
28413	2.4031	2.1
28414	2.43509	2.1
28415	2.65066	2.1
28416	5.06323	2.1
28417	2.62814	2.1

28418	2.52142	2.1
28419	2.53008	2.1
28420	2.65066	2.1
28421	5.15822	2.1
28422	2.0815	2.1
28423	1.92067	2.1
28424	1.59611	2.1
28425	1.89226	2.1
28426	1.62453	2.1
28427	1.92067	2.1
28428	1.89226	2.1
28429	1.89226	2.1
28430	1.89226	2.1
28431	1.89226	2.1
28432	3.78453	2.1
28433	0.946131	2.1
28434	0.946131	2.1
28435	0.929501	2.1
28436	0.910761	2.1
28437	0.9461	2.1
28438	0.9461	2.1
28439	0.910792	2.1
28440	0.910792	2.1
28441	0.946131	2.1
28442	0.929501	2.1
28443	0.910761	2.1
28444	0.946131	2.1
28445	2.0815	2.1
28446	1.81661	2.1
28447	2.1341	2.1
28448	2.0815	2.1
28449	1.86921	2.1
28450	1.81661	2.1
28451	1.89226	2.1
28452	1.89226	2.1
28453	1.89226	2.1
28454	1.89226	2.1
28455	1.89226	2.1
28456	1.89226	2.1
28457	0.946131	2.1
28458	0.946131	2.1
28459	0.949104	2.1
28460	0.9295	2.1
28461	0.946132	2.1
28462	0.946132	2.1
28463	0.929499	2.1
28464	0.929499	2.1
28465	0.946131	2.1
28466	0.949105	2.1
28467	0.946131	2.1
28468	0.929501	2.1
28469	1.66104	2.1
28470	2.34044	2.1
28471	2.08889	2.1
28472	1.33264	2.1
28473	3.67308	2.1
28474	2.0889	2.1
28475	1.08011	2.1

28476	1.08011	2.1
28477	1.33264	2.1
28478	1.87598	2.1
28479	2.12851	2.1
28480	2.27472	2.1
28481	1.89226	1.49718
28482	2.27703	2.1
28483	1.89226	0.42714
28484	4.16699	0.774194
28485	1.89226	2.1
28486	1.89226	1.43378
28487	1.89226	1.71197
28488	1.89226	0.851156
28489	3.78452	2.02281
28490	1.00007	2.04077
28491	1.32316	0.756691
28492	1.29417	0.244081
28493	1.29417	1.85645
28494	1.02051	0.198974
28495	1.02051	1.45714
28496	2.2846	2.1
28497	1.89226	1.05645
28498	2.27703	1.17916
28499	2.27703	2.1
28500	1.89226	0.0966377
28501	1.89226	2.1
28502	1.89226	2.1
28503	1.89226	2.03276
28504	1.89226	0.549518
28505	1.89226	0.113234
28506	3.78452	0.448348
28507	1.02051	2.1
28508	1.32316	2.00739
28509	1.03373	0.405186
28510	1.34066	0.744821
28511	2.35689	2.1
28512	1.703	2.1
28513	1.89224	2.1
28514	1.52266	2.1
28515	1.89224	2.1
28516	1.23344	2.1
28517	1.53282	2.1
28518	1.89223	2.1
28519	1.89223	2.1
28520	1.89223	2.1
28521	1.89223	2.1
28522	3.78447	2.1
28523	0.796845	2.1
28524	0.796845	2.1
28525	0.812146	2.1
28526	0.812146	2.1
28527	1.08006	2.1
28528	1.02679	2.1
28529	0.551538	2.1
28530	0.528929	2.1
28531	0.551529	2.1
28532	1.08005	2.1
28533	0.528935	2.1

28534	1.0268	2.1
28535	1.5001	2.1
28536	1.70302	2.1
28537	2.0726	2.1
28538	1.70302	2.1
28539	1.99601	2.1
28540	1.70303	2.1
28541	1.89226	2.1
28542	1.89226	2.1
28543	1.89226	2.1
28544	1.89226	2.1
28545	1.89226	2.1
28546	1.89226	2.1
28547	1.17285	2.1
28548	2.16015	2.1
28549	2.23563	2.1
28550	2.23563	2.1
28551	1.10309	2.1
28552	1.10309	2.1
28553	0.946128	2.1
28554	0.946128	2.1
28555	1.21847	0.0998569
28556	1.2884	2.1
28557	0.94613	2.1
28558	0.94613	2.1
28559	1.21847	2.1
28560	1.21847	0.0615372
28561	0.946131	2.1
28562	1.21847	2.1
28563	0.94613	2.1
28564	1.2884	2.1
28565	1.83922	0.80343
28566	2.5768	2.1
28567	1.83488	2.1
28568	1.82846	0.05
28569	2.43693	2.1
28570	2.43693	2.1
28571	2.36134	2.1
28572	1.81656	2.1
28573	1.86454	2.1
28574	1.6697	2.1
28575	2.55618	2.1
28576	2.36134	2.1
28577	2.55618	2.1
28578	1.9323	2.1
28579	1.9302	2.1
28580	1.81656	2.1
28581	2.66983	2.1
28582	2.55618	2.1
28583	1.89226	0.698746
28584	1.70302	0.115275
28585	1.94935	0.632294
28586	1.70302	0.413955
28587	1.95569	0.157538
28588	1.70302	0.05599
28589	2.82465	2.1
28590	2.41707	2.1
28591	2.24481	2.1

28592	2.68958	2.1
28593	5.10665	2.1
28594	1.41232	2.1
28595	1.34479	2.1
28596	0.946129	2.1
28597	0.946129	2.1
28598	0.94613	2.1
28599	0.94613	2.1
28600	1.41232	2.1
28601	1.41232	2.1
28602	1.34479	2.1
28603	0.94613	2.1
28604	0.946131	2.1
28605	1.41232	2.1
28606	1.86859	0.05
28607	1.89226	0.05
28608	1.89226	0.05
28609	1.89226	0.05
28610	1.45875	0.05
28611	1.45875	0.05
28612	2.1769	0.196459
28613	2.50259	0.179554
28614	1.94955	0.0525402
28615	2.25959	0.367845
28616	4.45214	0.571211
28617	1.89226	0.360038
28618	1.89226	0.266014
28619	1.89226	0.169568
28620	1.89226	0.550413
28621	3.78452	0.142081
28622	1.89226	0.338856
28623	1.89226	0.989081
28624	1.89226	0.458637
28625	1.89226	0.471796
28626	3.78452	1.56156
28627	2.50259	0.237874
28628	2.49445	0.198949
28629	2.25959	0.203219
28630	2.20843	0.153845
28631	4.71102	0.0954494
28632	1.94955	0.0667117
28633	2.25959	0.0660643
28634	1.24689	0.05
28635	1.79906	0.0547805
28636	3.74861	0.05
28637	1.89226	0.242758
28638	1.89226	0.269989
28639	1.89226	0.0621468
28640	1.89226	0.101348
28641	3.78452	0.482259
28642	1.89226	0.23424
28643	1.89226	0.113711
28644	1.89226	0.0871392
28645	1.89226	0.0680073
28646	3.78452	0.0622489
28647	2.25959	0.05
28648	2.20843	0.05
28649	1.79906	0.05

28650	1.98391	0.0786265
28651	4.00748	0.0668697
28652	1.89226	2.1
28653	1.89226	2.1
28654	1.89226	2.1
28655	1.89226	2.1
28656	3.78452	2.1
28657	1.89226	2.1
28658	1.89226	2.1
28659	1.89226	2.1
28660	1.89226	2.1
28661	3.78452	2.1
28662	1.64718	2.1
28663	1.23085	2.1
28664	1.46028	2.1
28665	1.39564	2.1
28666	1.68372	2.1
28667	1.64718	2.1
28668	1.64718	2.1
28669	1.64981	2.1
28670	1.70303	2.1
28671	1.6991	2.1
28672	3.3199	2.1
28673	1.89226	2.1
28674	1.89226	2.1
28675	1.89226	2.1
28676	1.89226	2.1
28677	3.78452	2.1
28678	1.89226	2.1
28679	1.89226	2.1
28680	1.89226	2.1
28681	1.89226	2.1
28682	3.78452	2.1
28683	0.666701	2.1
28684	0.65896	2.1
28685	0.920797	2.1
28686	0.895441	2.1
28687	0.775871	2.1
28688	0.687493	2.1
28689	0.777393	2.1
28690	0.777393	2.1
28691	0.677856	2.1
28692	0.90095	2.1
28693	0.621435	2.1
28694	0.858124	2.1
28695	1.70303	2.1
28696	1.70731	2.1
28697	1.79663	2.1
28698	1.68783	2.1
28699	3.17337	2.1
28700	1.22724	2.1
28701	2.0021	2.1
28702	1.90427	2.1
28703	1.08698	2.1
28704	3.08907	2.1
28705	2.41689	2.1
28706	2.00209	2.1
28707	1.90427	2.1

28708	2.35245	2.1
28709	4.35454	2.1
28710	1.90427	2.1
28711	0.956294	2.1
28712	0.956294	2.1
28713	1.08698	2.1
28714	1.81488	2.1
28715	1.94556	2.1
28716	1.90427	2.1
28717	2.35245	2.1
28718	2.28979	2.1
28719	1.81488	2.1
28720	4.16733	2.1
28721	1.6272	2.1
28722	1.70302	2.1
28723	1.70303	2.1
28724	1.70302	2.1
28725	1.91167	2.1
28726	1.91167	2.1
28727	1.49063	2.1
28728	1.89226	2.1
28729	1.89226	2.1
28730	1.89226	2.1
28731	1.6272	2.1
28732	1.6272	2.1
28733	2.25988	1.17688
28734	1.89226	0.05
28735	1.89226	0.869786
28736	2.23152	0.280258
28737	4.12379	0.129232
28738	2.25988	0.280537
28739	1.16361	0.98685
28740	1.15853	2.1
28741	2.23153	0.0574197
28742	3.39513	1.03376
28743	1.89226	0.752218
28744	2.23153	0.134967
28745	2.21022	1.12062
28746	2.23153	0.410028
28747	1.87095	0.0507535
28748	1.89226	0.05
28749	1.16239	1.73316
28750	2.23153	0.05
28751	1.15853	0.849953
28752	1.15853	0.0588765
28753	2.21022	0.995933
28754	2.21022	0.063781
28755	1.78381	0.05
28756	1.70302	0.05
28757	1.15453	0.05
28758	1.35007	0.05
28759	1.82021	0.05
28760	2.12851	0.05
28761	1.89226	0.05
28762	1.89226	0.05
28763	1.89226	0.05
28764	1.89226	0.05
28765	1.89226	0.05

28766	1.89226	0.05
28767	1.89258	0.05
28768	1.089 0.05	
28769	1.22586	0.05
28770	1.79877	0.05
28771	2.88777	0.05
28772	1.91564	0.05
28773	1.79877	0.05
28774	1.96356	0.05
28775	1.70302	0.05
28776	2.37828	0.05
28777	2.04859	0.05
28778	1.89226	0.05
28779	1.89226	0.05
28780	1.89226	0.05
28781	1.89226	0.05
28782	3.78453	0.05
28783	1.89226	0.05
28784	1.89226	0.05
28785	1.89226	0.05
28786	1.89226	0.05
28787	1.89226	0.05
28788	1.89226	0.05
28789	2.28593	2.1
28790	1.89226	2.04395
28791	2.2731	2.1
28792	1.89226	0.367206
28793	4.17819	1.51049
28794	1.89226	2.1
28795	1.89226	2.1
28796	1.89226	1.43675
28797	1.89226	0.918872
28798	3.78452	1.88366
28799	1.03933	2.1
28800	1.34355	0.6848
28801	1.35048	0.28552
28802	1.35048	2.1
28803	1.02975	0.139275
28804	1.02975	1.86235
28805	2.25105	2.1
28806	1.89226	1.87891
28807	2.27311	2.1
28808	2.27311	2.1
28809	1.89226	0.69695
28810	1.89226	2.1
28811	1.89226	2.1
28812	1.89226	2.08971
28813	1.89226	1.64806
28814	1.89226	0.0586478
28815	3.78452	0.739595
28816	1.02975	2.1
28817	1.34355	2.05641
28818	1.01121	1.0978
28819	1.33079	0.551786
28820	2.35476	2.1
28821	1.24526	0.05
28822	2.36406	0.05
28823	2.81021	0.05

28824	1.7237	0.05
28825	4.08776	0.05
28826	1.89226	2.1
28827	1.89226	2.1
28828	1.89226	2.1
28829	1.89226	2.1
28830	3.78453	2.1
28831	1.79028	2.1
28832	1.89238	2.1
28833	1.65533	2.1
28834	1.77821	2.1
28835	3.5477	2.1
28836	1.89238	2.1
28837	1.7465	0.05
28838	1.77821	2.1
28839	1.63557	0.05
28840	3.52795	0.312716
28841	1.89226	2.1
28842	1.89226	0.05
28843	1.89226	2.1
28844	1.89226	0.05
28845	3.78452	0.0764402
28846	1.89226	2.1
28847	1.89226	2.1
28848	1.89226	2.1
28849	1.89226	2.1
28850	3.78452	2.1
28851	1.65532	2.1
28852	1.77821	2.1
28853	1.53278	2.1
28854	1.67571	2.1
28855	3.33103	2.1
28856	1.77821	2.1
28857	1.63557	0.05
28858	1.67571	2.1
28859	1.52615	0.05
28860	3.31128	0.34118
28861	1.89226	2.1
28862	1.89226	0.05
28863	1.89226	2.1
28864	1.89226	0.05
28865	3.78452	0.368931
28866	2.83129	2.1
28867	1.89226	0.05
28868	1.89226	0.392399
28869	2.6491	1.63392
28870	4.54136	0.142375
28871	2.83129	2.1
28872	1.47278	2.1
28873	1.3909	2.1
28874	2.6491	2.09592
28875	4.12188	2.1
28876	1.89225	2.1
28877	2.65393	2.1
28878	1.89225	2.1
28879	2.69591	2.1
28880	4.58816	2.1
28881	1.91703	2.1

28882	2.00209	2.1
28883	2.65393	2.1
28884	2.65393	2.1
28885	2.69591	2.1
28886	2.00209	2.1
28887	1.89226	2.1
28888	2.69593	2.1
28889	1.93967	2.1
28890	1.89226	2.1
28891	2.74334	2.1
28892	2.69593	2.1
28893	2.74335	2.1
28894	2.0021	2.1
28895	2.76367	2.1
28896	2.69593	2.1
28897	2.06984	2.1
28898	2.0021	2.1
28899	2.24481	2.1
28900	2.58921	2.1
28901	2.68958	2.1
28902	2.68958	2.1
28903	2.11984	2.1
28904	2.11984	2.1
28905	2.11984	2.1
28906	2.58921	2.1
28907	2.46488	2.1
28908	1.98339	2.1
28909	4.5726	2.1
28910	2.7777	2.1
28911	2.58921	2.1
28912	2.46488	2.1
28913	2.70401	2.1
28914	5.29322	2.1
28915	2.17741	2.1
28916	2.54197	2.1
28917	2.54018	2.1
28918	2.18608	2.1
28919	4.72805	2.1
28920	2.17742	2.1
28921	1.58054	2.1
28922	1.59169	2.1
28923	2.18608	2.1
28924	3.76662	2.1
28925	2.54018	2.1
28926	2.18608	2.1
28927	2.19104	2.1
28928	2.5392	2.1
28929	4.72528	2.1
28930	1.60497	2.1
28931	2.18608	2.1
28932	1.59169	2.1
28933	1.59169	2.1
28934	2.19105	2.1
28935	2.19105	2.1
28936	2.58661	1.90486
28937	1.06203	2.1
28938	2.7043	2.1
28939	2.21397	2.1

28940	1.55236	2.1
28941	1.06203	2.1
28942	1.89226	1.43074
28943	1.89226	2.02478
28944	1.89226	0.292995
28945	1.89226	2.07923
28946	3.78453	1.53092
28947	1.08195	1.72142
28948	0.990019	2.1
28949	1.31976	2.02934
28950	1.4539	0.336936
28951	2.40171	2.1
28952	1.89226	1.11908
28953	1.89226	2.1
28954	1.89226	1.05257
28955	1.89226	0.104266
28956	3.78453	1.35166
28957	1.89226	1.86374
28958	1.89226	1.49415
28959	1.89226	0.595801
28960	1.89226	2.1
28961	3.78453	1.2341
28962	1.43895	2.1
28963	1.31976	2.1
28964	1.70066	0.200431
28965	1.33783	2.1
28966	1.43895	2.1
28967	1.07612	2.1
28968	0.65988	2.0281
28969	0.850329	1.39703
28970	0.919598	0.625961
28971	0.726952	1.78938
28972	0.788106	0.215775
28973	0.788106	2.1
28974	0.789175	2.1
28975	0.789175	0.0805595
28976	0.850329	0.794783
28977	0.919598	0.588561
28978	0.659879	1.7889
28979	0.726952	1.36591
28980	1.34389	2.1
28981	1.41279	2.05733
28982	1.15564	2.1
28983	1.15564	2.1
28984	1.55998	2.03655
28985	1.55998	2.1
28986	1.89226	1.80931
28987	1.89226	2.1
28988	1.89226	2.1
28989	1.89226	1.22473
28990	1.89226	2.1
28991	1.89226	1.34747
28992	1.55998	2.1
28993	1.41279	2.1
28994	1.72901	2.00177
28995	1.62414	2.1
28996	3.1418	2.1
28997	1.89226	1.84796

28998	1.89226	2.1
28999	1.89226	2.03618
29000	1.89226	0.977871
29001	3.78452	2.1
29002	1.89226	0.295211
29003	1.89226	2.1
29004	1.89226	1.66699
29005	1.89226	2.1
29006	1.89226	0.74137
29007	1.89226	1.2922
29008	1.62414	2.1
29009	1.01243	2.1
29010	1.70932	2.1
29011	1.41279	1.82225
29012	1.30896	2.07236
29013	1.01243	1.40696
29014	1.9719	2.1
29015	1.10725	2.1
29016	1.12411	2.1
29017	1.98691	2.1
29018	3.09601	2.1
29019	1.97189	2.1
29020	2.47932	2.1
29021	2.49092	2.1
29022	1.98691	2.1
29023	4.46282	2.1
29024	1.1486	2.1
29025	1.98691	2.1
29026	1.12411	2.1
29027	1.12411	2.1
29028	2.01602	2.1
29029	2.01602	2.1
29030	2.49093	2.1
29031	1.98691	2.1
29032	2.01601	2.1
29033	2.51651	2.1
29034	4.50694	2.1
29035	1.93147	0.05
29036	2.51593	0.05
29037	1.35146	0.05
29038	1.35146	0.05
29039	2.48665	0.05
29040	1.90664	0.05
29041	1.63979	2.1
29042	1.7457	2.1
29043	1.66046	2.1
29044	1.56558	2.1
29045	3.31128	2.1
29046	0.819895	2.1
29047	0.782792	2.1
29048	0.60239	2.1
29049	0.54831	2.1
29050	0.688347	2.1
29051	0.688347	2.1
29052	0.679857	2.1
29053	0.679857	2.1
29054	0.782792	2.1
29055	0.54831	2.1

29056	0.60239	2.1
29057	0.819895	2.1
29058	1.66046	2.1
29059	1.42485	2.1
29060	1.56558	2.1
29061	1.56558	2.1
29062	1.52995	2.1
29063	1.52995	2.1
29064	2.58101	0.05
29065	2.09803	2.1
29066	2.408 0.05	
29067	1.86893	2.1
29068	4.44994	0.154961
29069	2.36062	2.1
29070	1.25104	2.1
29071	1.25104	2.1
29072	1.39703	2.1
29073	1.86893	2.1
29074	2.01492	2.1
29075	2.408 0.05	
29076	1.86893	2.1
29077	2.22575	0.05
29078	1.70677	2.1
29079	4.09468	0.128408
29080	0.737605	2.1
29081	0.737605	2.1
29082	0.741302	2.1
29083	0.741302	2.1
29084	0.55189	2.1
29085	0.625517	2.1
29086	0.934468	2.1
29087	0.853389	2.1
29088	0.853389	2.1
29089	0.55189	2.1
29090	0.625517	2.1
29091	0.934467	2.1
29092	2.35053	2.1
29093	1.09615	2.1
29094	1.25998	2.1
29095	2.51216	2.1
29096	3.61051	2.1
29097	1.33636	2.1
29098	1.25609	2.1
29099	0.698455	2.1
29100	0.629995	2.1
29101	0.986092	2.1
29102	0.986092	2.1
29103	0.980263	2.1
29104	0.980263	2.1
29105	1.33636	2.1
29106	0.698455	2.1
29107	0.629995	2.1
29108	1.25609	2.1
29109	1.89226	0.481719
29110	1.89226	0.805333
29111	1.89226	0.912161
29112	1.89226	0.629026
29113	3.78452	0.594018

29114	1.74321	0.353406
29115	1.89226	0.374755
29116	1.91755	0.488605
29117	1.91755	0.455941
29118	1.89226	0.419509
29119	1.89226	0.426758
29120	0.946132	0.780928
29121	0.946132	0.600116
29122	0.949798	0.225448
29123	1.08054	0.427486
29124	1.08054	0.301951
29125	1.08054	0.421615
29126	0.946132	0.628411
29127	0.946132	0.694815
29128	0.946132	0.610117
29129	0.949798	0.204462
29130	1.08054	0.325494
29131	0.946131	1.01606
29132	1.89226	0.904739
29133	1.89226	0.469391
29134	1.89226	0.391761
29135	1.89226	0.490244
29136	1.89226	0.556445
29137	1.89226	0.695911
29138	2.39378	0.05
29139	2.29234	0.05
29140	2.29234	0.05
29141	2.7933	0.05
29142	1.18422	0.05
29143	1.68518	0.05
29144	2.79329	0.05
29145	1.89226	0.05
29146	2.29233	0.05
29147	1.89226	0.05
29148	4.18459	0.05
29149	0.857046	0.05
29150	0.857037	0.05
29151	0.94613	0.05
29152	0.94613	0.05
29153	0.851539	0.05
29154	0.851539	0.05
29155	1.0315	0.05
29156	0.952867	0.05
29157	0.851486	0.05
29158	0.952869	0.05
29159	1.03156	0.05
29160	0.851528	0.05
29161	1.61981	2.1
29162	1.93096	2.1
29163	2.00776	2.1
29164	1.62218	2.1
29165	3.62757	2.1
29166	2.00776	2.1
29167	1.68541	2.1
29168	1.83215	2.1
29169	1.62218	2.1
29170	2.21773	2.1
29171	2.00776	2.1

29172	1.89188	0.05
29173	1.70303	0.05
29174	1.89226	0.05
29175	1.72465	0.05
29176	1.89226	0.05
29177	1.70552	0.05
29178	2.48659	0.05
29179	2.0815	0.05
29180	1.89226	0.05
29181	2.08111	0.05
29182	1.89226	0.05
29183	2.08111	0.05
29184	2.35332	0.05
29185	1.83284	0.05
29186	2.28483	0.05
29187	2.71955	0.05
29188	4.63815	0.05
29189	1.89227	2.1
29190	1.89227	2.1
29191	1.89227	2.1
29192	1.89227	2.1
29193	3.78453	2.1
29194	1.33506	2.1
29195	1.17129	2.1
29196	1.33506	2.1
29197	1.38415	2.1
29198	1.13664	2.1
29199	1.18573	2.1
29200	1.89226	2.1
29201	2.68785	2.1
29202	1.89226	2.1
29203	2.6692	2.1
29204	4.58011	2.1
29205	1.89226	2.1
29206	1.89226	2.1
29207	1.89226	2.1
29208	1.89226	2.1
29209	3.78453	2.1
29210	1.33506	2.1
29211	1.13664	2.1
29212	1.27575	2.1
29213	1.09627	2.1
29214	2.43133	2.1
29215	1.89226	2.1
29216	2.65349	2.1
29217	2.66919	2.1
29218	2.66919	2.1
29219	1.89226	2.1
29220	1.89226	2.1
29221	1.89226	2.1
29222	1.89226	2.1
29223	1.89226	2.1
29224	1.89226	2.1
29225	3.78453	2.1
29226	1.16834	2.1
29227	1.16834	2.1
29228	0.946132	2.1
29229	0.946132	2.1

29230	1.26978	2.1
29231	1.16834	2.1
29232	0.94613	2.1
29233	0.94613	2.1
29234	0.94613	2.1
29235	1.26978	2.1
29236	0.94613	2.1
29237	1.16834	2.1
29238	2.14698	2.1
29239	1.89226	2.1
29240	1.89226	2.1
29241	1.89226	2.1
29242	2.40719	2.1
29243	2.40719	2.1
29244	1.89226	2.1
29245	1.89226	2.1
29246	1.89226	2.1
29247	1.89226	2.1
29248	3.78453	2.1
29249	1.26977	2.1
29250	1.26977	2.1
29251	0.946133	2.1
29252	0.946133	2.1
29253	1.37467	2.1
29254	1.26978	2.1
29255	0.946131	2.1
29256	0.946131	2.1
29257	1.37467	2.1
29258	0.94613	2.1
29259	0.946131	2.1
29260	1.26978	2.1
29261	1.2036	2.1
29262	1.34912	2.1
29263	0.946131	2.1
29264	0.946131	2.1
29265	1.34912	2.1
29266	1.34912	2.1
29267	0.946129	2.1
29268	0.946129	2.1
29269	1.34912	2.1
29270	0.94613	2.1
29271	0.946131	2.1
29272	1.2036	2.1
29273	2.47164	0.0552209
29274	1.89019	0.05
29275	1.79109	0.05
29276	2.41632	0.05
29277	4.26273	0.0524298
29278	1.54197	0.05
29279	2.41632	0.05
29280	1.79109	0.05
29281	1.79109	0.05
29282	2.20929	0.05
29283	2.20929	0.05
29284	1.70302	0.439146
29285	1.48104	0.730293
29286	1.99365	0.929156
29287	1.32897	0.371661

29288	1.70302	0.943557
29289	1.14034	0.373102
29290	1.70302	0.51074
29291	1.99365	0.487073
29292	1.99297	0.745821
29293	2.36337	0.184129
29294	4.04804	0.343555
29295	1.51569	0.298765
29296	2.49093	0.229347
29297	1.6026	0.420211
29298	1.43229	0.120223
29299	2.0815	0.508534
29300	1.83402	0.151577
29301	2.54665	2.1
29302	2.34044	2.1
29303	2.08889	2.1
29304	2.37653	2.1
29305	4.71697	2.1
29306	2.54665	2.1
29307	2.45212	2.1
29308	2.31012	2.1
29309	2.37653	2.1
29310	4.82865	2.1
29311	2.08889	2.1
29312	2.37653	2.1
29313	2.23366	2.1
29314	1.87598	2.1
29315	4.25251	2.1
29316	2.31012	2.1
29317	2.37653	2.1
29318	2.23367	2.1
29319	2.17384	2.1
29320	4.55037	2.1
29321	0.946131	2.1
29322	0.946131	2.1
29323	1.30338	2.1
29324	1.23186	2.1
29325	0.94613	2.1
29326	0.94613	2.1
29327	1.23187	2.1
29328	1.23187	2.1
29329	0.946131	2.1
29330	1.23186	2.1
29331	1.30338	2.1
29332	0.946131	2.1
29333	1.89226	2.1
29334	2.46373	2.1
29335	2.34725	2.1
29336	2.46373	2.1
29337	1.77579	2.1
29338	1.89226	2.1
29339	2.61901	2.1
29340	2.45899	2.1
29341	2.64067	2.1
29342	2.52708	2.1
29343	5.14609	2.1
29344	2.41795	2.1
29345	2.0815	2.1

29346	2.52708	2.1
29347	1.92277	2.1
29348	4.31368	2.1
29349	2.64067	2.1
29350	2.52708	2.1
29351	2.58638	2.1
29352	2.51039	2.1
29353	5.11346	2.1
29354	2.52708	2.1
29355	2.13642	2.1
29356	2.51039	2.1
29357	2.16633	2.1
29358	4.69341	2.1
29359	1.61028	1.4934
29360	1.65712	0.156159
29361	1.35002	0.05
29362	1.35002	1.62417
29363	1.88637	0.121965
29364	1.88637	2.1
29365	2.57663	2.1
29366	2.24227	2.1
29367	2.48354	2.1
29368	2.14317	2.1
29369	4.7198	2.1
29370	2.24227	2.1
29371	1.77353	2.1
29372	2.14317	2.1
29373	1.69956	2.1
29374	3.9167	2.1
29375	2.48353	2.1
29376	2.14317	2.1
29377	2.39282	2.1
29378	1.79824	2.1
29379	4.53599	2.1
29380	2.14317	2.1
29381	1.69956	2.1
29382	1.91427	2.1
29383	1.6347	2.1
29384	3.77787	2.1
29385	1.89226	0.947228
29386	1.89226	1.25296
29387	1.89226	1.25885
29388	1.89226	1.04841
29389	3.78453	1.84509
29390	1.89226	0.968039
29391	1.89226	0.681107
29392	1.89226	0.992281
29393	1.89226	0.817976
29394	3.78452	0.290909
29395	1.89226	0.679926
29396	1.89652	1.6193
29397	1.89226	0.176737
29398	1.89226	0.95806
29399	1.19352	0.931542
29400	1.19352	2.1
29401	1.89226	1.08003
29402	1.89653	0.362387
29403	1.89226	1.67937

29404	1.89226	1.27793
29405	1.89226	0.731714
29406	1.89226	0.811295
29407	1.89226	1.58115
29408	1.75326	0.688137
29409	1.89226	2.1
29410	1.12948	1.6363
29411	1.89226	0.743454
29412	1.12948	0.254718
29413	2.61064	2.1
29414	1.89226	2.1
29415	1.89226	2.1
29416	1.89226	2.1
29417	2.6085	2.1
29418	2.6085	2.1
29419	2.6085	2.1
29420	1.89226	2.1
29421	1.89226	2.1
29422	1.89226	2.1
29423	2.62161	2.1
29424	2.62161	2.1
29425	2.16535	2.1
29426	1.44603	2.1
29427	2.79784	2.1
29428	1.84756	2.1
29429	4.01292	2.1
29430	2.65262	2.1
29431	1.03391	2.1
29432	2.20239	2.1
29433	1.74416	2.1
29434	1.49213	2.1
29435	1.03391	2.1
29436	2.29066	2.1
29437	2.49174	2.1
29438	2.56502	2.1
29439	2.33802	2.1
29440	4.85568	2.1
29441	2.39663	2.1
29442	2.49174	2.1
29443	2.56502	2.1
29444	2.45929	2.1
29445	4.96165	2.1
29446	2.56502	2.1
29447	2.33802	2.1
29448	2.37168	2.1
29449	2.59967	2.1
29450	4.9367	2.1
29451	2.56502	2.1
29452	2.45929	2.1
29453	2.50138	2.1
29454	2.59967	2.1
29455	5.0664	2.1
29456	2.27009	0.943369
29457	1.26247	2.1
29458	1.86812	2.1
29459	2.837	1.53749
29460	4.13822	2.1
29461	1.89226	0.30917

29462	1.89226	2.1
29463	1.89226	2.1
29464	1.89226	2.1
29465	3.78453	2.1
29466	1.75835	2.1
29467	1.72866	2.1
29468	1.65313	2.1
29469	1.64822	2.1
29470	3.3818	2.1
29471	1.72866	2.1
29472	1.58401	2.1
29473	1.64822	2.1
29474	1.52441	2.1
29475	3.25307	2.1
29476	1.89226	2.1
29477	1.89226	2.1
29478	1.89226	2.1
29479	1.89226	2.1
29480	3.78452	2.1
29481	1.89226	2.1
29482	1.89226	2.1
29483	1.89226	0.289122
29484	1.89226	2.1
29485	3.78453	2.1
29486	1.65313	2.1
29487	1.64822	2.1
29488	1.55779	2.1
29489	1.57968	2.1
29490	3.23281	2.1
29491	1.64822	2.1
29492	1.52441	2.1
29493	1.57967	2.1
29494	1.474 2.1	
29495	3.10408	2.1
29496	1.89226	2.1
29497	1.89226	2.1
29498	1.89226	2.1
29499	1.89226	2.1
29500	3.78452	2.1
29501	1.86067	1.20521
29502	1.58407	2.1
29503	2.07965	0.394584
29504	1.83123	1.7935
29505	3.66372	0.896416
29506	1.89226	0.533494
29507	1.89226	2.1
29508	1.89226	0.953241
29509	1.89226	1.99746
29510	3.78452	1.41664
29511	1.89226	0.607564
29512	1.89226	2.1
29513	1.89226	0.248332
29514	1.89226	2.1
29515	1.89226	0.131448
29516	1.89226	2.1
29517	1.83123	0.602247
29518	1.03472	2.1
29519	1.91068	2.03805

29520	1.58406	0.996611
29521	1.36133	1.09465
29522	1.03472	0.42638
29523	2.07965	0.872015
29524	1.83123	1.90497
29525	2.28385	0.846899
29526	2.09114	1.46336
29527	4.1708	1.81505
29528	1.89226	1.43033
29529	1.89226	2.1
29530	1.89226	0.459088
29531	1.89226	1.90246
29532	3.78452	0.874183
29533	1.89226	0.605197
29534	1.89226	2.1
29535	1.89226	0.857974
29536	1.89226	2.1
29537	3.78452	1.20114
29538	1.83123	0.501401
29539	1.36133	2.1
29540	2.09114	0.435015
29541	1.69436	1.98726
29542	3.45248	1.09572
29543	1.89226	2.1
29544	1.89226	2.1
29545	1.89226	2.1
29546	1.89226	2.1
29547	3.78453	2.1
29548	1.53278	2.1
29549	1.67571	2.1
29550	1.40182	2.1
29551	1.55686	2.1
29552	3.07752	2.1
29553	1.67571	2.1
29554	1.52615	0.05
29555	1.55686	2.1
29556	1.38963	0.05
29557	3.06534	0.350744
29558	1.89226	2.1
29559	1.89226	0.05
29560	1.89226	2.1
29561	1.89226	0.05
29562	3.78452	0.368561
29563	1.89226	2.1
29564	1.89226	2.1
29565	1.89226	2.1
29566	1.89226	2.1
29567	3.78453	2.1
29568	1.40182	2.1
29569	1.55686	2.1
29570	1.24509	2.1
29571	1.38426	2.1
29572	2.78608	2.1
29573	1.55686	2.1
29574	1.38963	0.05
29575	1.2064	2.1
29576	1.2064	0.05
29577	1.38426	2.1

29578	1.56749	0.125367
29579	0.810946	2.1
29580	0.810946	0.05
29581	0.810945	2.1
29582	0.810945	2.1
29583	0.810945	1.5421
29584	0.810945	0.834522
29585	1.89226	0.05
29586	1.35169	0.0594226
29587	1.08132	2.1
29588	1.08132	0.05
29589	1.08132	0.200492
29590	1.28418	0.121415
29591	1.59382	0.0613362
29592	1.78289	0.137606
29593	1.51021	0.05
29594	3.10403	0.053756
29595	1.76897	0.766206
29596	1.59382	0.05
29597	1.78289	0.126667
29598	1.94302	0.101702
29599	3.53684	0.138694
29600	1.89226	0.05
29601	1.89226	0.0551339
29602	1.89226	0.0775179
29603	1.89226	0.05
29604	3.78453	0.05
29605	1.89227	0.0685202
29606	1.89227	0.05
29607	1.89227	0.05
29608	1.89227	0.05
29609	1.89227	0.05
29610	1.89227	0.0620873
29611	1.78286	0.149411
29612	1.51019	0.05
29613	1.79979	0.0845207
29614	2.02511	0.0616287
29615	3.5353	0.0952315
29616	1.78286	0.129139
29617	1.94299	0.0853377
29618	2.17831	0.400045
29619	2.02511	0.05
29620	3.9681	0.05
29621	1.89224	0.0678007
29622	1.89224	0.05
29623	1.89224	0.05
29624	1.89224	0.105281
29625	3.78448	0.0748137
29626	1.89224	0.0506728
29627	1.89224	0.05
29628	1.89224	0.05
29629	1.89224	0.05
29630	1.89224	0.05
29631	1.89224	0.0654671
29632	1.83198	0.139948
29633	1.0961	0.05
29634	1.39448	0.144508
29635	1.99568	0.0576109

29636	3.09178	0.110356
29637	1.25503	0.0766179
29638	1.99568	0.05
29639	2.3661	0.148564
29640	1.70302	0.05
29641	3.55833	0.05
29642	2.69899	0.05
29643	1.85796	0.05
29644	2.11407	0.05
29645	1.36769	0.05
29646	3.97203	0.05
29647	1.89226	0.05
29648	1.89226	0.05
29649	1.89226	0.05
29650	1.89226	0.05
29651	3.78452	0.05
29652	1.89225	0.05
29653	2.63355	0.05
29654	2.09529	0.05
29655	2.09529	0.05
29656	1.89225	0.05
29657	1.89225	0.05
29658	1.89226	0.05
29659	1.89226	0.05
29660	1.89226	0.05
29661	1.89226	0.05
29662	1.89226	0.05
29663	1.89226	0.05
29664	2.12204	0.05
29665	1.89226	0.05
29666	2.05798	0.05
29667	2.01954	0.05
29668	1.70302	0.05
29669	1.3865	0.05
29670	1.28326	0.05
29671	1.33326	0.143965
29672	1.19052	0.05
29673	1.25369	0.104117
29674	2.53694	0.0583918
29675	1.33327	0.0896935
29676	1.27339	2.1
29677	1.25369	0.05
29678	1.19733	2.1
29679	2.52708	0.395313
29680	1.19052	0.05
29681	1.25369	0.072949
29682	1.10938	0.05
29683	1.17614	0.108267
29684	2.36307	0.05
29685	1.25369	0.05
29686	1.19733	2.1
29687	1.17614	0.120494
29688	1.11868	2.1
29689	2.37237	1.6574
29690	1.10747	0.05
29691	1.47688	0.05
29692	1.97732	0.05
29693	1.62206	0.05

29694	3.09894	0.05
29695	1.89226	0.05
29696	1.89226	0.05
29697	1.89226	0.05
29698	1.89226	0.05
29699	1.89226	0.05
29700	1.89226	0.05
29701	1.62206	0.05
29702	1.70302	0.05
29703	1.22962	0.05
29704	1.1893	0.05
29705	2.15177	0.05
29706	2.08121	0.05
29707	1.97732	0.05
29708	2.08121	0.05
29709	1.62206	0.05
29710	1.62206	0.05
29711	2.41464	0.05
29712	2.41464	0.05
29713	1.89226	0.05
29714	1.89226	0.05
29715	1.89226	0.05
29716	1.89226	0.05
29717	3.78452	0.05
29718	0.946133	0.05
29719	0.946133	0.05
29720	0.946132	0.05
29721	0.946132	0.05
29722	0.946132	0.05
29723	0.946132	0.05
29724	0.946133	0.05
29725	0.946133	0.05
29726	0.946132	0.05
29727	0.946132	0.05
29728	0.946132	0.05
29729	0.946132	0.05
29730	1.83157	0.0800866
29731	2.19989	0.0766622
29732	2.20664	0.05
29733	1.81487	0.05
29734	4.03146	0.0683637
29735	2.41055	2.1
29736	2.19989	2.1
29737	2.20665	1.91468
29738	2.38023	1.57906
29739	4.61044	1.73593
29740	2.19989	2.1
29741	2.22558	2.1
29742	2.38023	1.94008
29743	2.07065	0.127635
29744	4.42547	1.53631
29745	2.19988	0.124634
29746	1.70303	0.05
29747	1.89939	0.0572467
29748	1.86357	0.0590442
29749	3.78536	0.05
29750	1.04921	2.1
29751	1.30711	2.1

29752	1.53076	2.1
29753	1.22927	2.1
29754	2.57997	2.1
29755	1.53076	2.1
29756	1.46568	2.1
29757	1.56056	2.1
29758	1.22927	2.1
29759	1.86206	2.1
29760	1.53076	2.1
29761	1.18353	2.1
29762	2.28593	2.1
29763	1.16979	2.1
29764	2.2731	2.1
29765	3.45571	2.1
29766	0.854776	2.1
29767	0.854776	2.1
29768	0.855641	2.1
29769	0.855641	2.1
29770	1.12552	2.1
29771	1.13655	2.1
29772	0.57216	2.1
29773	0.584893	2.1
29774	0.57216	2.1
29775	1.12552	2.1
29776	0.584893	2.1
29777	1.13655	2.1
29778	1.54462	2.1
29779	2.76309	2.1
29780	1.34869	2.1
29781	2.48573	2.1
29782	4.03035	2.1
29783	1.35328	1.14515
29784	1.0383	0.937839
29785	1.03318	1.73091
29786	0.704834	1.61145
29787	1.0257	1.55025
29788	1.0257	0.88014
29789	1.03241	0.463194
29790	1.03241	1.87529
29791	1.35328	1.17715
29792	1.03319	1.84173
29793	1.0383	1.57117
29794	0.704835	1.39418
29795	2.27126	0.05
29796	2.77291	2.1
29797	1.81771	0.372584
29798	2.6111	0.0708155
29799	4.88236	0.23889
29800	2.77291	1.835
29801	1.89227	0.54239
29802	1.89227	0.142504
29803	1.89227	2.1
29804	2.6111	0.0536282
29805	2.6111	2.1
29806	2.22061	0.0990956
29807	2.41522	0.0863215
29808	2.41522	0.05
29809	2.6111	1.548

29810	1.75765	0.05
29811	1.95353	2.1
29812	2.27502	0.148975
29813	1.89227	1.51991
29814	1.89227	0.200559
29815	2.8189	0.0795372
29816	4.71116	0.121397
29817	1.89226	0.0900787
29818	1.89226	1.68749
29819	1.89226	0.35209
29820	1.89226	0.137581
29821	1.89226	1.01246
29822	1.89226	0.370658
29823	1.89226	1.93985
29824	1.89226	1.84695
29825	1.89226	1.47889
29826	1.89226	1.18263
29827	3.78453	1.8736
29828	1.89226	1.81665
29829	2.57079	0.575884
29830	2.5932	1.35538
29831	1.89226	1.09319
29832	4.48546	1.11568
29833	0.589396	1.29717
29834	0.641454	0.729683
29835	0.814062	1.548
29836	0.726518	1.19961
29837	0.675126	1.41598
29838	0.675125	1.40981
29839	0.692847	1.59003
29840	0.692847	1.31316
29841	0.641454	0.989134
29842	0.814062	1.46957
29843	0.726518	1.60791
29844	0.589396	1.23971
29845	1.89226	1.64338
29846	1.89226	1.19425
29847	1.89226	1.60492
29848	1.89226	1.97732
29849	1.89226	1.23841
29850	1.89226	1.94194
29851	1.16906	0.177063
29852	1.2966	1.26531
29853	0.946132	0.992816
29854	0.946132	1.87036
29855	1.16906	1.03363
29856	1.16906	0.371323
29857	0.946132	1.32815
29858	0.946132	1.44517
29859	1.16906	1.61113
29860	0.946131	1.45203
29861	1.2966	1.1939
29862	0.946132	1.86305
29863	0.641455	1.33107
29864	0.567191	0.581826
29865	0.807594	1.61769
29866	0.814062	1.22421
29867	0.673735	1.62983

29868	0.673734	1.08184
29869	0.707519	1.3418
29870	0.707519	1.73161
29871	0.807594	1.5306
29872	0.56719	1.88453
29873	0.641455	1.54971
29874	0.814063	1.94095
29875	1.89225	2.1
29876	1.89225	2.1
29877	1.89225	2.1
29878	1.89225	2.1
29879	3.7845	2.1
29880	1.89225	2.1
29881	1.89225	2.1
29882	1.89225	2.1
29883	1.89225	2.1
29884	1.89225	2.1
29885	1.89225	2.1
29886	1.89226	2.1
29887	1.89226	2.1
29888	1.89226	2.1
29889	1.89226	2.1
29890	3.78452	2.1
29891	1.89226	2.1
29892	1.89226	2.1
29893	1.89226	2.1
29894	1.89226	2.1
29895	1.89226	2.1
29896	1.89226	2.1
29897	1.89226	2.1
29898	1.89226	2.1
29899	1.89226	2.1
29900	1.89226	2.1
29901	3.78452	2.1
29902	1.89226	2.1
29903	1.38928	2.1
29904	1.89226	2.1
29905	1.51378	2.1
29906	1.89061	2.1
29907	1.89226	2.1
29908	1.89226	2.1
29909	1.89226	2.1
29910	1.89226	2.1
29911	1.89226	2.1
29912	3.78453	2.1
29913	1.04572	2.1
29914	0.994213	2.1
29915	0.912466	2.1
29916	0.912466	2.1
29917	0.947743	2.1
29918	1.00388	2.1
29919	0.900913	2.1
29920	0.900913	2.1
29921	0.900915	2.1
29922	0.946131	2.1
29923	0.946131	2.1
29924	0.999196	2.1
29925	1.89226	2.09813

29926	1.89226	2.1
29927	1.89226	2.1
29928	1.89226	2.1
29929	3.78453	2.1
29930	0.946161	2.1
29931	1.1354	2.1
29932	0.850847	2.1
29933	0.850847	2.1
29934	1.01445	2.1
29935	1.02106	2.1
29936	0.946131	2.1
29937	0.946131	2.1
29938	1.13537	2.1
29939	1.01444	2.1
29940	0.850877	2.1
29941	0.946131	2.1
29942	1.80183	2.1
29943	1.89226	2.1
29944	1.89226	2.1
29945	1.89226	2.1
29946	1.79606	2.1
29947	1.79606	2.1
29948	1.24001	2.1
29949	1.41822	1.74606
29950	1.95665	0.813312
29951	1.77342	1.58393
29952	3.19666	1.31772
29953	1.95665	1.31526
29954	1.44859	2.05126
29955	1.41821	1.94876
29956	1.41821	1.97104
29957	1.95543	1.21948
29958	1.95543	1.65914
29959	1.95665	1.19987
29960	1.77342	1.70806
29961	2.33611	1.46626
29962	2.49694	0.695573
29963	4.29276	1.78753
29964	1.95665	1.7374
29965	1.95544	1.68156
29966	2.4728	0.350546
29967	2.49693	1.41324
29968	4.42945	1.27561
29969	2.24937	2.1
29970	2.49053	2.1
29971	2.16323	2.1
29972	2.36377	2.1
29973	4.61314	2.1
29974	2.49053	2.1
29975	2.58639	2.1
29976	2.36377	2.1
29977	2.47395	2.1
29978	4.95016	2.1
29979	2.45054	2.1
29980	2.36377	2.1
29981	1.92911	2.1
29982	2.181	2.1
29983	4.29288	2.1

29984	2.36377	2.1
29985	2.47395	2.1
29986	2.181	2.1
29987	2.29881	2.1
29988	4.66258	2.1
29989	1.57885	2.1
29990	1.13339	2.1
29991	1.48594	2.1
29992	1.13322	2.1
29993	2.71207	2.1
29994	1.48596	2.1
29995	1.20761	2.1
29996	1.20761	2.1
29997	1.13323	2.1
29998	1.46568	2.1
29999	1.3913	2.1
30000	1.89226	0.3126
30001	1.89226	1.67995
30002	1.89226	0.364687
30003	1.89226	1.49075
30004	1.89226	0.100692
30005	1.89226	0.475181
30006	1.11712	0.917658
30007	0.946129	1.34479
30008	0.686934	0.522972
30009	0.946133	0.445639
30010	0.9461	0.882146
30011	1.10729	2.1
30012	0.686963	1.52811
30013	0.686963	0.161007
30014	1.10945	1.09887
30015	0.80397	2.1
30016	0.946131	0.444794
30017	0.946131	1.50586
30018	1.89226	2.1
30019	1.89226	1.83553
30020	1.89226	1.19868
30021	1.89226	2.02499
30022	3.78452	1.86515
30023	1.22615	2.1
30024	1.3439	2.1
30025	1.47868	2.1
30026	1.55998	1.96593
30027	2.78613	2.1
30028	1.04973	2.1
30029	1.47868	2.1
30030	1.20149	2.1
30031	1.61721	2.1
30032	2.68017	2.1
30033	1.89226	2.1
30034	1.89226	2.1
30035	1.89226	2.1
30036	1.89226	0.16918
30037	3.78452	2.1
30038	0.946133	0.182328
30039	0.946133	2.1
30040	0.946128	2.1
30041	0.946128	2.1

30042	0.946131	0.119567
30043	0.946131	1.935
30044	0.946131	2.1
30045	0.946131	0.394111
30046	0.94613	2.1
30047	0.94613	1.70789
30048	0.946131	2.1
30049	0.946131	1.62498
30050	0.798126	2.1
30051	0.798126	2.1
30052	0.805719	2.1
30053	0.805719	2.1
30054	0.779992	2.1
30055	0.864507	2.1
30056	0.739338	2.1
30057	0.808607	2.1
30058	0.808606	2.1
30059	0.864506	2.1
30060	0.739339	2.1
30061	0.779993	2.1
30062	0.946128	2.1
30063	0.946128	2.1
30064	1.18959	1.25437
30065	1.00304	2.1
30066	1.00304	2.1
30067	1.00304	2.1
30068	0.94613	2.1
30069	0.94613	1.39798
30070	0.94613	2.1
30071	1.18959	2.1
30072	1.00304	2.1
30073	0.94613	2.1
30074	2.19163	2.1
30075	1.89226	2.1
30076	2.16541	2.1
30077	1.89226	1.53835
30078	4.08389	2.1
30079	1.89226	2.1
30080	1.89226	1.78813
30081	1.89226	2.1
30082	1.89226	0.881335
30083	3.78452	2.1
30084	0.964782	2.1
30085	1.30151	0.713664
30086	1.30318	0.400648
30087	1.30318	2.1
30088	0.949134	0.177797
30089	0.949134	2.1
30090	2.15005	2.1
30091	1.89226	2.01457
30092	2.16541	2.1
30093	2.16541	2.1
30094	1.89226	1.36594
30095	1.89226	2.1
30096	1.89226	2.1
30097	1.89226	2.1
30098	1.89226	2.1
30099	1.89226	0.112229

30100	3.78452	1.25277
30101	0.949134	2.1
30102	1.30151	2.1
30103	0.949134	2.1
30104	1.32143	0.663862
30105	2.25064	2.1
30106	0.982859	0.05
30107	1.28326	0.05
30108	1.19052	0.05
30109	1.19052	0.0598293
30110	1.10476	0.05
30111	1.10476	0.0542083
30112	1.37265	0.333766
30113	1.38247	0.464443
30114	1.36636	1.10089
30115	1.3294	0.0815836
30116	2.71187	0.172511
30117	1.89226	0.774662
30118	1.89226	0.0517288
30119	1.89226	0.143243
30120	1.89226	0.128206
30121	1.89226	0.179229
30122	1.89226	0.517865
30123	1.36636	1.49314
30124	1.3294	0.05
30125	1.30001	0.284055
30126	1.36416	0.955046
30127	2.69356	0.556068
30128	1.89226	1.19519
30129	1.89226	0.0669765
30130	1.89226	0.106531
30131	1.89226	0.594092
30132	1.89226	0.0628894
30133	1.89226	0.251846
30134	2.83701	0.435658
30135	1.89226	0.264766
30136	1.89226	1.04228
30137	2.79312	0.0523188
30138	4.68539	0.0862097
30139	1.44288	2.1
30140	0.948677	2.1
30141	1.84076	2.1
30142	1.42884	2.1
30143	2.78943	2.1
30144	1.89226	2.1
30145	1.89226	2.1
30146	1.89226	2.1
30147	1.89226	2.1
30148	3.78452	2.1
30149	0.909402	2.1
30150	0.909402	2.1
30151	1.3063	2.1
30152	1.56757	2.1
30153	1.00038	2.1
30154	1.5235	2.1
30155	0.946127	2.1
30156	0.946127	2.1
30157	0.946128	2.1

30158	1.26957	2.1
30159	1.00038	2.1
30160	0.946129	2.1
30161	1.17256	2.1
30162	2.2846	2.1
30163	1.1844	2.1
30164	2.28904	2.1
30165	3.469	2.1
30166	0.867886	2.1
30167	0.867886	2.1
30168	0.867277	2.1
30169	0.867277	2.1
30170	1.14296	2.1
30171	1.14452	2.1
30172	0.591766	2.1
30173	0.592199	2.1
30174	0.591766	2.1
30175	1.14296	2.1
30176	0.5922	2.1
30177	1.14452	2.1
30178	1.27408	0.05
30179	1.56339	0.05
30180	1.52522	0.05
30181	1.34447	0.05
30182	2.90786	0.05
30183	1.27408	0.05
30184	1.01121	0.05
30185	0.991868	0.05
30186	1.34447	0.05
30187	2.35569	0.05
30188	0.944818	0.05
30189	0.883008	0.05
30190	0.66957	0.05
30191	0.616883	0.05
30192	0.755679	0.05
30193	0.755679	0.0505962
30194	0.791912	0.0677079
30195	0.701943	0.05
30196	0.901541	0.05
30197	0.634847	0.05
30198	0.82781	0.05
30199	0.655405	0.05
30200	1.73819	2.1
30201	1.70303	2.1
30202	1.70303	2.1
30203	1.70303	2.1
30204	1.94516	2.1
30205	1.94517	2.1
30206	0.94994	2.1
30207	2.07469	2.1
30208	1.08506	2.1
30209	2.0815	2.1
30210	3.12902	2.1
30211	0.946131	2.1
30212	0.946131	2.1
30213	0.946131	2.1
30214	0.946131	2.1
30215	0.946128	2.1

30216	0.946128	2.1
30217	0.946133	2.1
30218	0.946133	2.1
30219	0.946131	2.1
30220	0.946131	2.1
30221	0.94613	2.1
30222	0.94613	2.1
30223	2.51796	2.1
30224	1.64193	2.1
30225	2.51172	2.1
30226	1.68893	2.1
30227	4.20689	2.1
30228	2.51172	2.1
30229	1.70002	2.1
30230	1.70002	2.1
30231	1.68893	2.1
30232	2.48689	2.1
30233	2.4758	2.1
30234	2.27963	0.05
30235	2.21582	0.05
30236	2.60486	0.05
30237	2.5105	0.05
30238	4.79013	0.05
30239	2.21582	0.05
30240	2.02069	0.05
30241	2.5105	0.05
30242	2.28615	0.05
30243	4.53119	0.05
30244	2.60486	0.05
30245	2.5105	0.05
30246	2.55791	0.05
30247	2.47141	0.05
30248	5.06842	0.05
30249	2.5105	0.05
30250	2.28615	0.05
30251	2.47141	0.05
30252	2.23337	0.05
30253	4.74387	0.05
30254	2.06984	2.1
30255	0.967499	2.1
30256	2.12435	2.1
30257	2.0021	2.1
30258	1.08975	2.1
30259	0.967499	2.1
30260	1.89226	0.05
30261	1.89226	0.05
30262	1.89226	0.05
30263	1.89226	0.05
30264	1.89226	0.05
30265	1.89226	0.05
30266	1.89226	2.1
30267	1.89226	2.1
30268	1.89226	2.1
30269	1.89226	2.1
30270	3.78452	2.1
30271	1.89227	2.1
30272	1.89227	2.1
30273	1.89227	2.1

30274	1.89227	2.1
30275	3.78453	2.1
30276	0.55882	2.1
30277	0.696462	2.1
30278	1.07003	2.1
30279	0.937179	2.1
30280	0.818077	2.1
30281	0.818077	2.1
30282	0.815565	2.1
30283	0.815565	2.1
30284	0.696462	2.1
30285	1.07003	2.1
30286	0.93718	2.1
30287	0.55882	2.1
30288	2.53857	2.1
30289	1.87436	2.1
30290	2.27541	2.1
30291	2.27541	2.1
30292	2.14005	2.1
30293	2.14005	2.1
30294	1.89226	2.1
30295	1.89226	2.1
30296	1.89226	2.1
30297	1.89226	2.1
30298	3.78452	2.1
30299	1.89227	2.1
30300	1.89227	2.1
30301	1.89227	2.1
30302	1.89227	2.1
30303	3.78453	2.1
30304	1.39292	2.1
30305	2.14006	2.1
30306	2.41083	2.1
30307	1.68761	2.1
30308	3.80376	2.1
30309	2.53857	2.1
30310	2.14005	2.1
30311	2.41083	2.1
30312	2.82043	2.1
30313	4.9494	2.1
30314	1.90439	0.05
30315	1.51835	0.05
30316	1.20473	0.05
30317	0.947693	0.05
30318	2.85208	0.05
30319	2.586	2.1
30320	1.46262	2.1
30321	2.14009	2.1
30322	2.78101	2.1
30323	4.72609	2.1
30324	1.89224	2.1
30325	2.78101	2.1
30326	2.586	2.1
30327	2.586	2.1
30328	1.89224	2.1
30329	1.89224	2.1
30330	0.994293	2.1
30331	1.19	2.1

30332	1.09483	2.1
30333	1.09483	2.1
30334	1.06827	2.1
30335	1.06827	2.1
30336	1.89226	2.1
30337	1.89226	2.1
30338	1.89226	2.1
30339	1.89226	2.1
30340	3.78452	2.1
30341	0.946132	2.1
30342	0.946132	2.1
30343	1.31912	2.1
30344	1.3572	2.1
30345	0.946131	2.1
30346	0.946131	2.1
30347	1.31912	2.1
30348	1.31912	2.1
30349	0.946131	2.1
30350	1.31912	2.1
30351	1.35721	2.1
30352	0.946131	2.1
30353	2.24577	0.19679
30354	1.09408	0.05
30355	1.60239	0.0674143
30356	1.09408	0.121849
30357	2.39404	0.0624782
30358	2.07382	0.05
30359	1.70303	0.05
30360	1.3216	0.05
30361	1.23104	0.05
30362	1.05475	0.05
30363	2.60408	0.05
30364	1.89226	0.0691528
30365	1.89226	0.06057
30366	1.89226	0.05
30367	1.89226	0.0577717
30368	3.78452	0.0763671
30369	1.89226	0.05
30370	2.3845	0.05
30371	2.6875	0.05
30372	1.89226	0.05
30373	2.6875	0.05
30374	1.89226	0.05
30375	1.46759	2.1
30376	1.17585	2.1
30377	1.85189	2.1
30378	1.39223	2.1
30379	2.85981	2.1
30380	1.85189	2.1
30381	1.71255	2.1
30382	1.71255	2.1
30383	1.39223	2.1
30384	2.28511	2.1
30385	1.96479	2.1
30386	1.89227	0.492317
30387	1.89227	0.913394
30388	1.89227	1.24323
30389	1.89227	0.375555

30390	1.89227	1.70773
30391	1.89227	0.728132
30392	1.89226	0.566269
30393	1.89226	0.813683
30394	1.89226	1.23292
30395	1.89226	0.270352
30396	1.89226	1.74524
30397	1.89226	0.516757
30398	1.89226	0.529554
30399	1.89226	1.17153
30400	1.89226	1.17395
30401	1.89226	0.267277
30402	1.89226	1.83514
30403	1.89226	0.672582
30404	1.89226	0.277088
30405	2.77788	0.44084
30406	2.21062	0.704873
30407	2.21062	0.17062
30408	1.89226	1.20778
30409	1.89226	0.29926
30410	0.868548	0.20579
30411	0.610104	0.388736
30412	0.796678	0.331449
30413	0.865021	0.160133
30414	1.0883	0.259932
30415	0.904938	0.37541
30416	2.21062	0.291454
30417	1.49548	0.330529
30418	1.2068	0.746943
30419	1.54764	0.344314
30420	0.790005	0.20086
30421	1.89226	2.1
30422	2.67457	2.1
30423	1.89226	2.1
30424	2.56352	2.1
30425	4.56683	2.1
30426	2.67456	2.1
30427	2.20011	2.1
30428	2.56351	2.1
30429	2.11083	2.1
30430	4.78539	2.1
30431	2.56348	2.1
30432	2.1108	2.1
30433	2.45763	2.1
30434	2.00986	2.1
30435	4.56843	2.1
30436	0.946133	0.05
30437	0.946133	0.05
30438	0.946132	0.05
30439	0.946132	0.05
30440	0.946132	0.05
30441	0.946132	0.05
30442	0.946132	0.05
30443	0.946132	0.05
30444	0.946132	0.05
30445	0.946132	0.05
30446	0.946133	0.05
30447	0.946133	0.05

30448	1.45927	0.290335
30449	1.25133	1.08608
30450	1.00082	0.629382
30451	1.41689	1.09604
30452	1.0432	0.383593
30453	1.45927	0.952747
30454	1.41689	0.583505
30455	1.29579	1.39655
30456	1.34874	0.983823
30457	1.25133	0.936725
30458	2.76563	1.63209
30459	0.675555	1.21739
30460	0.674371	1.07796
30461	0.625664	0.877009
30462	0.523133	0.768
30463	0.676509	0.990774
30464	0.676509	1.48521
30465	0.62471	1.04649
30466	0.62471	0.63754
30467	0.675556	0.574015
30468	0.523132	0.364724
30469	0.674373	0.466996
30470	0.625663	0.656001
30471	1.89226	0.798053
30472	1.89226	0.742517
30473	1.89226	0.66506
30474	1.89226	0.707473
30475	3.78453	1.25334
30476	0.851517	1.39158
30477	0.946133	0.736167
30478	0.946132	1.15713
30479	0.946132	1.41916
30480	0.946133	0.336279
30481	0.851517	1.94912
30482	0.946132	1.06614
30483	0.946132	0.54444
30484	0.851516	0.804535
30485	0.851516	0.518833
30486	0.946133	1.46281
30487	0.946133	0.948099
30488	0.569995	0.540468
30489	0.816895	1.33913
30490	1.33893	1.35631
30491	1.18795	0.784565
30492	1.18795	0.711562
30493	0.569995	1.65105
30494	0.816895	1.43567
30495	1.33893	1.27542
30496	0.859572	1.56873
30497	0.859572	1.16171
30498	1.04936	1.04476
30499	1.04936	0.715983
30500	1.89226	2.1
30501	1.89226	2.1
30502	2.55618	2.1
30503	2.55618	2.1
30504	2.66983	2.1
30505	2.00591	2.1

30506	1.89226	0.0737374
30507	2.64586	2.1
30508	2.83331	2.1
30509	2.83331	0.0520899
30510	1.89226	2.1
30511	1.89226	0.05
30512	2.64586	2.1
30513	2.0815	2.1
30514	1.99985	2.1
30515	1.94005	2.1
30516	2.83332	2.1
30517	2.74859	2.1
30518	1.98061	2.1
30519	1.68356	2.1
30520	1.83879	2.1
30521	1.55987	2.1
30522	3.54048	2.1
30523	1.68356	2.1
30524	1.20969	2.1
30525	1.20969	2.1
30526	1.30751	2.1
30527	1.55987	2.1
30528	1.65769	2.1
30529	1.83879	2.1
30530	1.55987	2.1
30531	1.68443	2.1
30532	1.4449	2.1
30533	3.2443	2.1
30534	0.660368	2.1
30535	0.660368	2.1
30536	0.666922	2.1
30537	0.666922	2.1
30538	0.560377	2.1
30539	0.604842	2.1
30540	0.779935	2.1
30541	0.722448	2.1
30542	0.722446	2.1
30543	0.560377	2.1
30544	0.604844	2.1
30545	0.779934	2.1
30546	1.89227	2.1
30547	1.89227	2.1
30548	1.89227	2.1
30549	1.89227	2.1
30550	3.78453	2.1
30551	1.42896	2.1
30552	1.23037	2.1
30553	1.42896	2.1
30554	1.474 2.1	
30555	1.2016	2.1
30556	1.24665	2.1
30557	1.89226	2.1
30558	2.7188	2.1
30559	1.89226	2.1
30560	2.70702	2.1
30561	4.61106	2.1
30562	1.89226	2.1
30563	1.89226	2.1

30564	1.89226	2.1
30565	1.89226	2.1
30566	3.78453	2.1
30567	0.644603	2.1
30568	0.644603	2.1
30569	0.648272	2.1
30570	0.648272	2.1
30571	0.585644	2.1
30572	0.600802	2.1
30573	0.692073	2.1
30574	0.714478	2.1
30575	0.585644	2.1
30576	0.692073	2.1
30577	0.714478	2.1
30578	0.600802	2.1
30579	1.35351	2.1
30580	1.35351	2.1
30581	0.946131	2.1
30582	0.946131	2.1
30583	1.34392	2.1
30584	1.35351	2.1
30585	0.946132	2.1
30586	0.946132	2.1
30587	0.946132	2.1
30588	1.34392	2.1
30589	0.946132	2.1
30590	1.35351	2.1
30591	1.87609	2.1
30592	1.79155	2.1
30593	1.79155	2.1
30594	1.89226	2.1
30595	1.89226	2.1
30596	1.99211	2.1
30597	1.89226	2.1
30598	1.71706	2.1
30599	1.71706	2.1
30600	1.79155	2.1
30601	1.89226	2.1
30602	1.96675	2.1
30603	1.46869	2.1
30604	1.89226	2.1
30605	1.89226	2.1
30606	1.89226	2.1
30607	1.5001	2.1
30608	1.5001	2.1
30609	1.89226	2.1
30610	1.89226	2.1
30611	1.89226	2.1
30612	1.89226	2.1
30613	1.89226	2.1
30614	1.89226	2.1
30615	1.21408	2.1
30616	2.23563	2.1
30617	2.34021	2.1
30618	2.34021	2.1
30619	1.17285	2.1
30620	1.17285	2.1
30621	1.89226	2.1

30622	1.89226	2.1
30623	1.47937	2.1
30624	1.89226	2.1
30625	1.14822	2.1
30626	1.46869	2.1
30627	1.89226	2.1
30628	1.89226	2.1
30629	1.89226	2.1
30630	1.89226	2.1
30631	1.89226	2.1
30632	1.89226	2.09123
30633	1.25716	2.1
30634	2.34021	2.1
30635	2.4542	2.1
30636	2.4542	2.1
30637	1.21408	2.1
30638	1.21408	2.1
30639	1.66824	2.1
30640	2.44883	2.1
30641	1.93003	2.1
30642	2.24795	2.1
30643	3.88907	2.1
30644	1.89319	2.1
30645	2.24795	2.1
30646	2.82724	2.1
30647	2.31318	2.1
30648	2.30868	2.1
30649	1.93003	2.1
30650	1.89226	1.24971
30651	1.42589	2.1
30652	1.42589	2.1
30653	1.80501	2.1
30654	1.89226	2.1
30655	2.27137	2.1
30656	1.21067	0.05
30657	2.39166	0.0799654
30658	1.73577	0.0528972
30659	1.73577	0.05
30660	1.53584	0.05
30661	1.53584	0.05
30662	1.35025	1.91437
30663	2.0599	0.805435
30664	1.51084	0.742654
30665	2.27218	0.05
30666	3.57074	0.812441
30667	2.0599	0.273261
30668	2.51818	1.48891
30669	2.27218	0.05
30670	2.66532	0.999719
30671	4.72522	0.151405
30672	1.67115	1.65505
30673	2.27218	0.432965
30674	2.39605	1.42191
30675	2.39605	0.368943
30676	1.51084	1.24956
30677	1.51084	1.03097
30678	2.27218	0.194854
30679	2.66532	1.80194

30680	2.39605	0.05
30681	2.76829	0.196933
30682	5.06137	0.205287
30683	2.08684	0.876854
30684	2.36033	1.76207
30685	1.89224	0.461065
30686	1.89224	1.15518
30687	4.25257	0.763607
30688	2.41473	0.05
30689	1.00307	0.05
30690	2.01365	0.05
30691	1.00307	0.0674661
30692	3.05113	0.05
30693	2.04056	0.05
30694	1.11099	2.1
30695	2.25103	2.1
30696	2.22227	2.1
30697	2.22227	2.1
30698	1.14431	2.1
30699	1.14431	2.1
30700	1.07485	2.1
30701	2.22229	2.1
30702	2.19163	2.1
30703	2.19163	2.1
30704	1.111	2.1
30705	1.111	2.1
30706	1.0547	2.1
30707	1.19886	2.1
30708	0.946131	2.1
30709	0.946131	2.1
30710	0.946131	2.1
30711	0.946131	2.1
30712	1.19886	2.1
30713	1.19886	2.1
30714	1.0547	2.1
30715	0.946131	2.1
30716	0.946132	2.1
30717	1.19886	2.1
30718	1.89417	2.1
30719	2.64424	0.05
30720	1.76585	2.08598
30721	2.47695	0.126713
30722	4.37112	0.552662
30723	1.89226	2.1
30724	1.89226	0.26233
30725	1.89226	2.1
30726	1.89226	0.13713
30727	3.78453	1.70301
30728	0.930281	2.1
30729	1.0547	2.1
30730	0.946131	2.1
30731	0.946131	2.1
30732	0.930309	2.1
30733	0.930309	2.1
30734	1.07052	2.1
30735	1.07052	2.1
30736	0.93028	2.1
30737	0.946131	2.1

30738	1.0547	2.1
30739	0.946131	2.1
30740	1.00015	0.05
30741	1.00015	1.45377
30742	1.01918	2.08975
30743	1.01918	0.0666179
30744	1.17437	0.0737956
30745	1.23847	0.05
30746	0.882926	2.08121
30747	0.780859	1.45198
30748	0.780859	0.1113
30749	1.17438	0.180396
30750	0.882925	0.256551
30751	1.23848	0.26917
30752	1.89226	2.1
30753	1.89226	0.0770619
30754	1.89226	0.388691
30755	1.89226	2.1
30756	1.89226	0.298212
30757	1.89226	2.1
30758	1.22083	2.1
30759	1.29579	2.1
30760	1.26236	2.1
30761	1.06191	2.1
30762	1.42128	2.1
30763	1.22083	2.1
30764	1.42128	2.1
30765	1.45806	2.1
30766	1.43502	2.1
30767	1.29579	2.1
30768	1.5605	2.1
30769	1.42128	2.1
30770	1.02813	2.1
30771	1.12121	2.1
30772	1.40557	2.1
30773	1.35298	2.1
30774	1.1314	2.1
30775	1.28679	2.1
30776	1.1715	2.1
30777	1.1715	2.1
30778	0.975656	2.1
30779	1.24908	2.1
30780	0.98956	2.1
30781	1.2681	2.1
30782	0.946132	2.1
30783	0.946132	2.1
30784	1.31889	2.1
30785	1.31889	2.1
30786	1.16546	2.1
30787	1.31889	2.1
30788	0.946133	2.1
30789	0.946133	2.1
30790	0.946133	2.1
30791	1.31889	2.1
30792	0.946132	2.1
30793	1.16546	2.1
30794	2.51518	0.05
30795	2.37584	0.05

30796	1.11712	0.05
30797	1.31242	0.05
30798	3.68826	0.05
30799	2.51518	0.05
30800	1.26311	0.05
30801	2.48977	0.05
30802	2.48977	0.05
30803	1.31242	0.05
30804	1.31242	0.05
30805	1.89226	0.17881
30806	1.89226	0.773968
30807	1.89226	0.05
30808	1.89226	0.967962
30809	3.78452	0.127817
30810	2.04737	0.05
30811	1.77122	0.707009
30812	1.94498	0.05
30813	1.65174	0.737151
30814	3.71621	0.0533916
30815	1.41077	2.1
30816	1.29729	2.1
30817	1.77123	0.0605715
30818	1.77123	2.1
30819	1.65175	0.34748
30820	1.29129	0.55955
30821	1.89226	0.148665
30822	1.89226	2.1
30823	1.89226	2.1
30824	1.89226	0.0730572
30825	1.89226	2.1
30826	1.89226	0.304404
30827	1.89226	0.05
30828	1.89226	0.772088
30829	1.89226	0.05
30830	1.89226	0.358139
30831	3.78451	0.05
30832	1.94498	0.05
30833	1.65174	0.790942
30834	1.7968	0.05
30835	1.49831	0.267505
30836	3.44329	0.05
30837	1.49831	0.095457
30838	1.29729	2.1
30839	1.49831	2.1
30840	1.65175	0.0797915
30841	1.16858	2.1
30842	1.32201	0.20722
30843	1.89226	0.05
30844	1.89226	2.1
30845	1.89226	0.108112
30846	1.89226	2.1
30847	3.78452	0.727197
30848	1.89226	0.05
30849	1.89226	0.05
30850	1.89226	0.05
30851	1.89226	0.05
30852	1.89226	0.05
30853	1.89226	0.05

30854	1.65324	0.37722
30855	1.26409	0.05
30856	1.42491	0.05
30857	1.42491	0.583749
30858	1.52268	0.05
30859	1.52268	0.603391
30860	1.52268	1.39373
30861	1.26409	0.0798563
30862	1.32697	0.0562253
30863	1.05724	0.05
30864	2.57991	0.0503839
30865	1.89226	0.05
30866	1.96548	0.05
30867	2.0178	0.05
30868	1.96548	0.05
30869	1.94458	0.05
30870	1.89226	0.05
30871	1.99366	0.05
30872	2.0178	0.05
30873	1.89226	0.05
30874	2.0178	0.05
30875	1.75999	0.05
30876	1.89226	0.05
30877	0.946126	2.1
30878	0.946126	2.1
30879	1.28228	2.1
30880	1.19444	2.1
30881	0.946127	2.1
30882	0.946127	2.1
30883	1.19444	2.1
30884	1.19444	2.1
30885	0.946128	2.1
30886	1.19444	2.1
30887	1.28228	2.1
30888	0.946128	2.1
30889	1.89226	2.1
30890	1.89226	2.1
30891	1.89226	2.1
30892	1.89226	2.1
30893	3.78452	2.1
30894	2.14104	2.1
30895	1.89226	2.1
30896	2.19948	2.1
30897	1.89226	2.1
30898	1.89226	2.1
30899	2.19948	2.1
30900	1.89226	2.1
30901	2.38888	2.1
30902	2.23932	2.1
30903	2.38888	2.1
30904	1.74269	2.1
30905	1.89226	2.1
30906	1.89226	2.1
30907	1.89226	2.1
30908	1.89226	2.1
30909	1.89226	2.1
30910	3.78452	2.1
30911	0.946132	2.1

30912	0.946132	2.1
30913	1.07052	2.1
30914	1.07052	2.1
30915	0.946132	2.1
30916	0.946132	2.1
30917	1.05132	2.1
30918	1.07052	2.1
30919	1.05132	2.1
30920	0.946132	2.1
30921	1.07052	2.1
30922	0.946133	2.1
30923	2.40171	2.1
30924	1.89226	2.1
30925	1.89226	2.1
30926	1.89226	2.1
30927	2.42723	2.1
30928	2.42723	2.1
30929	1.89226	2.1
30930	1.89226	1.92091
30931	1.89226	2.1
30932	1.89226	2.1
30933	3.78452	2.1
30934	1.63661	2.1
30935	2.3532	1.62694
30936	2.33287	1.57758
30937	2.33287	2.1
30938	1.64158	1.23008
30939	1.64158	2.1
30940	2.38918	2.1
30941	1.89226	2.1
30942	1.89226	2.1
30943	1.89226	2.1
30944	2.40171	2.1
30945	2.40171	2.1
30946	1.89226	2.1
30947	1.89226	2.1
30948	1.89226	2.1
30949	1.89226	2.1
30950	1.89226	2.1
30951	1.89226	2.1
30952	1.64158	2.1
30953	2.3532	2.1
30954	1.6488	2.1
30955	2.37364	2.1
30956	4.002 2.1	
30957	2.54752	0.124315
30958	1.42445	0.141457
30959	1.86697	0.205897
30960	1.86697	0.109301
30961	2.06666	0.202682
30962	2.06666	0.116051
30963	2.50872	0.05
30964	2.06666	0.0611505
30965	1.90439	0.05
30966	1.51835	0.0579237
30967	3.97105	0.05
30968	0.755161	0.0624472
30969	0.755161	0.0573916

30970	0.793994	0.0572787
30971	0.793994	0.0764748
30972	0.712225	0.0582346
30973	0.515826	0.0683861
30974	1.03333	0.0635575
30975	0.759175	0.0656302
30976	0.759173	0.0528317
30977	0.515827	0.05
30978	0.712226	0.05
30979	1.03333	0.0555949
30980	1.69633	0.05
30981	1.89226	0.05
30982	1.62477	0.05
30983	1.89226	0.05
30984	1.62477	0.05
30985	1.89226	0.05
30986	1.89226	0.05
30987	1.89226	0.05
30988	1.89226	0.05
30989	1.89226	0.05
30990	3.78452	0.05
30991	2.28622	0.05
30992	1.09667	0.05
30993	1.17506	0.05
30994	2.24411	0.05
30995	1.17506	0.05
30996	2.24411	0.05
30997	2.24741	2.1
30998	1.70303	2.1
30999	2.49168	2.1
31000	1.57936	2.1
31001	2.53729	2.1
31002	1.70303	2.1
31003	2.24741	2.1
31004	2.47557	2.1
31005	1.89227	2.1
31006	1.89227	2.1
31007	4.36784	2.1
31008	2.27016	0.05
31009	1.10476	0.05
31010	1.44103	0.05
31011	1.10476	0.05
31012	2.26791	0.05
31013	1.93165	0.05
31014	2.27016	0.05
31015	1.89226	0.05
31016	1.89226	0.05
31017	2.4065	0.05
31018	4.29876	0.05
31019	2.27016	0.05
31020	1.44103	0.05
31021	1.53336	0.05
31022	2.4065	0.05
31023	3.84753	0.05
31024	2.7542	0.05
31025	1.89226	0.05
31026	2.35027	0.05
31027	1.89226	0.05

31028	2.35027	0.05
31029	1.89226	0.05
31030	2.3301	2.1
31031	2.36071	2.1
31032	2.07629	2.1
31033	2.01105	2.1
31034	4.37176	2.1
31035	1.86512	2.1
31036	2.01105	2.1
31037	2.01105	2.1
31038	2.3301	2.1
31039	1.57459	2.1
31040	1.89364	2.1
31041	2.52671	2.1
31042	2.01105	2.1
31043	1.78251	2.1
31044	1.70302	2.1
31045	3.98362	2.1
31046	1.227 2.1	
31047	2.01105	2.1
31048	1.45869	2.1
31049	1.41713	2.1
31050	1.70302	2.1
31051	1.64994	2.1
31052	2.00989	2.1
31053	1.28938	2.1
31054	1.8769	2.1
31055	1.20969	2.1
31056	3.21958	2.1
31057	1.8769	2.1
31058	1.15864	2.1
31059	1.15864	2.1
31060	1.20969	2.1
31061	1.77353	2.1
31062	1.82458	2.1
31063	1.7986	2.1
31064	2.48717	2.1
31065	2.50161	2.1
31066	1.75869	2.1
31067	4.30021	2.1
31068	2.50161	2.1
31069	1.75251	2.1
31070	1.81678	2.1
31071	1.75869	2.1
31072	2.5597	2.1
31073	2.50161	2.1
31074	1.91334	0.182388
31075	1.85439	0.94449
31076	1.91334	0.298978
31077	1.70303	0.847454
31078	1.70303	0.184418
31079	1.49692	0.443104
31080	2.39986	0.05
31081	1.89226	0.0560422
31082	1.89226	0.05
31083	2.50756	0.05
31084	4.39982	0.05
31085	2.39986	0.05

31086	1.70303	0.05
31087	1.75564	0.05
31088	1.75904	0.05
31089	2.50272	0.05
31090	2.50756	0.05
31091	1.89226	0.05
31092	2.50756	0.05
31093	2.47857	0.05
31094	2.50756	0.0615657
31095	1.86327	0.05
31096	1.89226	0.05
31097	1.22241	0.05
31098	1.20827	0.05
31099	0.772416	0.05
31100	0.555208	0.05
31101	1.004	0.05
31102	1.004	0.05
31103	0.93504	0.0773946
31104	0.744364	0.05
31105	1.2089	0.05
31106	0.654935	0.05
31107	0.773927	0.05
31108	1.03618	0.05
31109	1.89227	0.05
31110	1.89227	0.05
31111	1.89227	0.05
31112	1.89227	0.05
31113	3.78453	0.05
31114	1.89226	0.05
31115	1.89226	0.05
31116	1.89226	0.05
31117	1.89226	0.05
31118	3.78452	0.05
31119	1.82643	0.05
31120	2.7084	0.05
31121	2.12654	0.05
31122	2.65614	0.05
31123	1.82643	0.05
31124	2.35603	0.05
31125	1.29385	0.05
31126	0.946133	0.05
31127	1.05492	0.05
31128	0.946132	0.05
31129	1.05492	0.05
31130	1.29385	0.05
31131	0.946133	0.05
31132	0.946133	0.05
31133	1.29385	0.05
31134	1.29385	0.05
31135	0.946132	0.05
31136	0.946132	0.05
31137	0.946132	0.05
31138	1.27329	0.05
31139	1.29385	0.05
31140	0.946129	0.05
31141	1.29385	0.05
31142	1.27328	0.05
31143	0.946131	0.05

31144	0.946131	0.05
31145	1.27328	0.05
31146	1.27328	0.05
31147	0.94613	0.05
31148	0.94613	0.05
31149	1.89226	2.02465
31150	1.89226	2.08301
31151	1.89226	0.70656
31152	1.89226	1.37682
31153	3.78453	1.64861
31154	1.89226	1.74194
31155	1.89226	2.07354
31156	1.89226	0.532142
31157	1.89226	1.78554
31158	3.78453	1.16672
31159	1.21488	2.03578
31160	1.14605	2.1
31161	1.62974	1.59881
31162	1.52399	1.18831
31163	2.84463	2.07647
31164	0.577078	1.39995
31165	0.81251	2.1
31166	0.821003	1.78089
31167	0.543413	2.1
31168	0.683929	1.48584
31169	0.722433	2.1
31170	0.730674	2.1
31171	0.730674	1.20741
31172	0.857766	1.80124
31173	0.862119	1.44099
31174	0.600938	2.09305
31175	0.596094	1.86705
31176	1.89226	1.18385
31177	1.89226	1.92815
31178	1.89226	0.583027
31179	1.89226	1.5895
31180	3.78453	1.31666
31181	1.89226	0.998234
31182	1.89226	2.1
31183	1.89226	0.8468
31184	1.89226	1.37747
31185	3.78453	1.70452
31186	1.62974	2.1
31187	1.56958	2.1
31188	2.2362	1.16408
31189	2.11424	1.70479
31190	3.86595	1.71727
31191	2.2362	1.25005
31192	1.85414	2.1
31193	2.28211	0.499153
31194	1.78367	0.816213
31195	2.2362	2.02308
31196	1.73256	2.1
31197	0.851539	1.74961
31198	0.851539	0.19502
31199	1.14454	1.37716
31200	0.938988	2.1
31201	0.851485	1.70697

31202	0.920593	0.199973
31203	0.851541	0.05
31204	0.851541	0.494378
31205	1.04073	0.138756
31206	1.16342	0.227768
31207	0.825315	0.642646
31208	0.825316	0.23209
31209	1.89226	2.1
31210	1.89226	2.1
31211	1.89226	2.1
31212	1.89226	2.1
31213	3.78453	2.1
31214	2.57016	2.1
31215	1.74789	2.1
31216	2.57016	2.1
31217	2.522	2.1
31218	1.61727	2.1
31219	1.56911	2.1
31220	1.28075	2.1
31221	1.406	2.1
31222	0.946131	2.1
31223	0.946131	2.1
31224	1.28075	2.1
31225	1.00333	2.1
31226	0.94613	2.1
31227	0.94613	2.1
31228	1.041	2.1
31229	1.041	2.1
31230	0.943484	2.1
31231	1.27717	2.1
31232	1.89226	2.1
31233	1.89226	2.1
31234	1.89226	2.1
31235	1.89226	2.1
31236	1.89226	2.1
31237	1.89226	2.1
31238	2.57016	2.1
31239	1.61727	2.1
31240	2.74196	2.1
31241	1.59094	2.1
31242	4.16109	2.1
31243	1.89226	2.1
31244	2.0815	2.1
31245	2.2034	2.1
31246	1.82493	2.1
31247	1.87847	2.1
31248	2.16142	2.1
31249	1.89226	2.1
31250	1.89226	2.1
31251	1.89226	2.1
31252	1.89226	2.1
31253	3.78453	2.1
31254	0.985252	2.1
31255	0.985252	2.1
31256	0.946132	2.1
31257	0.946132	2.1
31258	1.0655	2.1
31259	0.985255	2.1

31260	0.94613	2.1
31261	0.94613	2.1
31262	0.94613	2.1
31263	1.0655	2.1
31264	0.94613	2.1
31265	0.985255	2.1
31266	1.89226	2.1
31267	1.89226	2.1
31268	1.89226	2.1
31269	1.89226	2.1
31270	3.78453	2.1
31271	1.06549	2.1
31272	1.06549	2.1
31273	0.946133	2.1
31274	0.946133	2.1
31275	1.16834	2.1
31276	1.0655	2.1
31277	0.946131	2.1
31278	0.946131	2.1
31279	0.946131	2.1
31280	1.16834	2.1
31281	0.94613	2.1
31282	1.0655	2.1
31283	0.966631	2.1
31284	1.07349	2.1
31285	0.946131	2.1
31286	0.946131	2.1
31287	1.07349	2.1
31288	1.07349	2.1
31289	0.946129	2.1
31290	0.946129	2.1
31291	1.07349	2.1
31292	0.946131	2.1
31293	0.94613	2.1
31294	0.966631	2.1
31295	2.38055	0.164346
31296	1.89226	1.22445
31297	1.89226	1.00387
31298	1.46724	0.05
31299	3.35949	0.234295
31300	1.42249	2.1
31301	2.05499	2.1
31302	1.39708	2.1
31303	2.09033	2.1
31304	3.45207	2.1
31305	2.05499	2.1
31306	2.57425	2.1
31307	2.51287	2.1
31308	2.47753	2.1
31309	2.09033	2.1
31310	2.05499	2.1
31311	1.39327	2.1
31312	2.09033	2.1
31313	2.16217	2.1
31314	2.16217	2.1
31315	1.39708	2.1
31316	1.39708	2.1
31317	2.16217	2.1

31318	2.57426	2.1
31319	2.73006	2.1
31320	2.73006	2.1
31321	2.09033	2.1
31322	2.09033	2.1
31323	1.89226	0.05
31324	1.89226	0.05
31325	1.89226	0.05
31326	1.89226	0.05
31327	3.78452	0.05
31328	0.838749	0.05
31329	0.838749	0.05
31330	0.85824	0.05
31331	0.85824	0.05
31332	0.544644	0.05
31333	0.854718	0.05
31334	0.842271	0.05
31335	1.19141	0.05
31336	0.842272	0.05
31337	0.544643	0.05
31338	0.854718	0.05
31339	1.19141	0.05
31340	1.34214	0.05
31341	1.34214	0.05
31342	0.893236	0.05
31343	0.893236	0.05
31344	0.893265	0.05
31345	1.28924	0.05
31346	0.946133	0.05
31347	0.946133	0.05
31348	0.946132	0.05
31349	0.893265	0.05
31350	0.946133	0.05
31351	1.28924	0.05
31352	2.7013	0.05
31353	1.89226	0.05
31354	1.9216	0.05
31355	1.9216	0.05
31356	1.89226	0.05
31357	1.89226	0.05
31358	1.26397	2.1
31359	1.52995	2.1
31360	1.30116	2.1
31361	1.04084	2.1
31362	2.57079	2.1
31363	1.47013	2.1
31364	2.17459	2.1
31365	2.10668	2.1
31366	1.38328	2.1
31367	3.55787	2.1
31368	2.55006	2.1
31369	2.17459	2.1
31370	2.10668	2.1
31371	2.49168	2.1
31372	4.66626	2.1
31373	0.613622	2.1
31374	0.691639	2.1
31375	1.00105	2.1

31376	1.05334	2.1
31377	0.839874	2.1
31378	0.839874	2.1
31379	0.852814	2.1
31380	0.852814	2.1
31381	0.613622	2.1
31382	1.00105	2.1
31383	1.05334	2.1
31384	0.691639	2.1
31385	1.00105	2.1
31386	1.05334	2.1
31387	1.20844	2.1
31388	1.24584	2.1
31389	1.12717	2.1
31390	1.12717	2.1
31391	1.13462	2.1
31392	1.13462	2.1
31393	1.20845	2.1
31394	1.00105	2.1
31395	1.05334	2.1
31396	1.24584	2.1
31397	2.22558	2.1
31398	1.85981	2.01741
31399	2.18943	1.50413
31400	1.89142	0.0705402
31401	4.0854	0.791085
31402	2.02641	2.1
31403	1.85981	1.60024
31404	2.18943	1.65937
31405	1.67346	0.715451
31406	3.88622	1.19999
31407	1.85981	0.05
31408	1.10736	0.05
31409	1.27421	0.05
31410	1.67346	0.05
31411	1.27421	0.05
31412	1.67346	0.628936
31413	1.85981	0.05
31414	1.27362	0.05
31415	1.89142	0.18037
31416	1.27421	0.05
31417	3.13343	0.05
31418	1.81893	0.05
31419	1.64985	0.05
31420	1.23477	0.05
31421	2.25405	0.05
31422	3.48882	0.05
31423	2.23933	0.05
31424	1.89226	0.05
31425	1.89226	0.05
31426	2.78053	0.05
31427	1.89226	0.05
31428	2.78053	0.05
31429	0.946131	0.05
31430	0.946131	0.05
31431	0.946132	0.05
31432	0.946132	0.05
31433	0.946131	0.05

31434	0.946131	0.05
31435	0.946132	0.05
31436	0.946132	0.05
31437	0.946131	0.05
31438	0.946131	0.05
31439	0.946132	0.05
31440	0.946132	0.05
31441	1.89226	2.1
31442	2.48596	2.1
31443	1.89226	2.1
31444	2.51506	2.1
31445	4.40732	2.1
31446	2.48596	2.1
31447	1.63823	2.1
31448	2.51507	2.1
31449	1.67687	2.1
31450	4.1533	2.1
31451	1.89226	2.1
31452	2.51506	2.1
31453	1.92044	2.1
31454	1.89226	2.1
31455	2.54325	2.1
31456	2.51506	2.1
31457	2.51507	2.1
31458	1.5704	2.1
31459	1.70505	2.1
31460	1.67687	2.1
31461	2.54325	2.1
31462	2.51507	2.1
31463	2.0815	2.1
31464	1.70303	2.1
31465	1.70303	2.1
31466	1.80354	2.1
31467	1.98172	2.1
31468	2.09868	2.1
31469	1.27944	2.1
31470	1.79028	2.1
31471	1.13179	2.1
31472	1.65533	2.1
31473	2.93477	2.1
31474	1.89226	2.1
31475	1.89226	2.1
31476	1.89226	2.1
31477	1.89226	2.1
31478	1.89226	2.1
31479	1.89226	2.1
31480	1.98171	2.1
31481	1.89226	2.1
31482	1.89226	2.1
31483	1.89226	2.1
31484	1.65798	2.1
31485	1.65797	2.1
31486	1.00133	2.1
31487	1.65532	2.1
31488	1.53278	2.1
31489	1.53278	2.1
31490	1.13179	2.1
31491	1.13179	2.1

31492	0.810946	2.1
31493	0.810946	2.1
31494	0.810944	2.1
31495	0.810944	2.1
31496	0.810946	2.1
31497	0.810946	2.1
31498	1.89226	2.1
31499	1.35169	2.1
31500	1.08132	2.1
31501	1.08132	2.1
31502	1.08132	2.1
31503	1.99528	0.05
31504	1.70303	0.05
31505	2.08152	0.05
31506	1.70303	0.05
31507	2.33877	0.05
31508	1.93967	0.05
31509	1.89226	0.05
31510	1.89226	0.05
31511	1.89226	0.05
31512	1.89226	0.05
31513	3.78452	0.05
31514	1.26259	0.05
31515	2.17205	0.05
31516	2.32468	0.05
31517	2.32468	0.05
31518	1.28055	0.05
31519	1.28055	0.05
31520	0.946131	0.05
31521	0.946131	0.05
31522	0.997642	0.05
31523	0.997642	0.05
31524	0.946131	0.05
31525	0.946131	0.05
31526	0.989846	0.05
31527	0.997642	0.05
31528	0.946132	0.05
31529	0.989846	0.05
31530	0.997641	0.05
31531	0.946131	0.05
31532	1.89226	0.05
31533	1.89226	0.05
31534	1.89226	0.05
31535	1.89226	0.05
31536	3.78452	0.05
31537	1.28055	0.05
31538	2.17205	0.05
31539	1.14305	0.05
31540	1.92737	0.05
31541	3.3151	0.05
31542	0.954908	0.05
31543	0.9705	0.05
31544	0.946131	0.05
31545	0.946131	0.05
31546	0.946131	0.05
31547	0.946131	0.05
31548	0.9705	0.05
31549	0.9705	0.05

31550	0.954908	0.05
31551	0.946131	0.05
31552	0.946131	0.05
31553	0.9705	0.05
31554	0.835187	0.05
31555	0.954896	0.05
31556	0.946118	0.05
31557	0.946118	0.05
31558	0.835186	0.05
31559	0.835186	0.05
31560	1.06583	0.05
31561	1.06583	0.05
31562	0.835198	0.05
31563	0.946131	0.05
31564	0.954883	0.05
31565	0.946106	0.05
31566	1.89227	2.1
31567	1.89227	2.1
31568	1.89227	2.1
31569	1.89227	2.1
31570	1.89227	2.1
31571	1.89227	2.1
31572	0.885265	2.1
31573	0.885265	2.1
31574	0.871467	2.1
31575	0.871467	2.1
31576	1.11883	2.1
31577	0.787875	2.1
31578	0.996487	2.1
31579	0.637907	2.1
31580	0.996487	2.1
31581	1.11883	2.1
31582	0.787875	2.1
31583	0.637906	2.1
31584	1.89226	2.1
31585	2.39258	2.1
31586	2.39258	2.1
31587	2.54729	2.1
31588	1.89226	2.1
31589	2.04697	2.1
31590	1.89226	2.1
31591	2.0815	2.1
31592	2.0815	2.1
31593	2.48371	2.1
31594	2.0815	2.1
31595	2.51469	2.1
31596	1.00035	0.05
31597	1.55755	0.05
31598	1.03534	0.05
31599	1.61333	0.05
31600	2.59289	0.05
31601	1.55755	0.05
31602	1.85217	1.9572
31603	1.61333	0.05
31604	1.92875	0.293633
31605	3.48629	0.05
31606	1.09962	0.127052
31607	1.61334	0.05

31608	1.70868	0.962556
31609	1.70868	0.05
31610	1.03535	1.04926
31611	1.03535	0.05
31612	1.61334	0.05
31613	1.92876	0.425378
31614	1.70868	0.05
31615	2.05274	1.64161
31616	3.63744	0.05
31617	1.82233	0.05
31618	2.05768	0.259024
31619	1.89226	0.415508
31620	1.65406	0.0507656
31621	1.89226	0.34188
31622	1.65406	0.0576538
31623	2.26047	0.0751949
31624	2.0815	0.627651
31625	1.89226	0.538711
31626	2.01156	0.0844347
31627	1.89226	0.39369
31628	2.01156	0.0821186
31629	1.89226	2.1
31630	1.89226	2.1
31631	1.89226	2.1
31632	1.89226	2.1
31633	3.78453	2.1
31634	2.70055	2.1
31635	2.28367	2.1
31636	2.56456	2.1
31637	2.33565	2.1
31638	4.84823	2.1
31639	2.28367	2.1
31640	1.5791	2.1
31641	1.96966	2.1
31642	1.09506	2.1
31643	3.37873	2.1
31644	1.89226	2.1
31645	1.89226	2.1
31646	1.89226	2.1
31647	1.89226	2.1
31648	3.78452	2.1
31649	0.94612	2.1
31650	0.94612	2.1
31651	0.946119	2.1
31652	0.946119	2.1
31653	0.946119	2.1
31654	0.946119	2.1
31655	0.946119	2.1
31656	0.946119	2.1
31657	0.946132	2.1
31658	0.946132	2.1
31659	0.946107	2.1
31660	0.946107	2.1
31661	2.56452	2.1
31662	1.57307	2.1
31663	2.10299	2.1
31664	2.1115	2.1
31665	2.52196	2.1

31666	2.52196	2.1
31667	1.89226	2.1
31668	1.89226	1.85954
31669	1.89226	2.1
31670	1.89226	2.1
31671	3.78452	2.1
31672	2.37665	2.1
31673	1.89226	2.1
31674	2.28084	2.1
31675	1.89226	2.1
31676	4.26891	2.1
31677	0.946129	1.74641
31678	0.946129	2.1
31679	0.946133	2.1
31680	0.946133	2.1
31681	0.946131	2.1
31682	0.946131	1.72944
31683	0.946131	2.1
31684	0.946131	2.1
31685	0.946131	2.1
31686	0.946131	2.1
31687	0.946131	2.1
31688	0.946131	2.1
31689	1.7207	0.05
31690	1.65815	0.05
31691	1.33593	0.05
31692	1.31508	0.05
31693	2.99408	0.05
31694	0.676047	0.05
31695	0.676047	0.05
31696	0.684362	0.05
31697	0.684362	0.05
31698	0.547994	0.05
31699	0.702871	0.05
31700	0.829076	0.05
31701	0.657538	0.05
31702	0.657538	0.05
31703	0.547994	0.05
31704	0.829077	0.05
31705	0.702871	0.05
31706	1.9518	2.1
31707	1.30648	2.1
31708	1.28846	2.1
31709	1.92033	2.1
31710	3.22681	2.1
31711	1.89226	2.1
31712	1.89226	2.1
31713	1.89226	2.1
31714	1.89226	2.1
31715	3.78453	2.1
31716	0.946128	2.1
31717	0.946128	2.1
31718	1.10113	2.1
31719	1.09587	2.1
31720	0.946131	2.1
31721	0.946131	2.1
31722	1.09587	2.1
31723	1.09587	2.1

31724	0.946131	2.1
31725	1.09587	2.1
31726	1.10112	2.1
31727	0.94613	2.1
31728	1.26923	2.1
31729	1.92033	2.1
31730	1.28846	2.1
31731	1.28846	2.1
31732	1.89452	2.1
31733	1.89452	2.1
31734	1.89226	2.1
31735	1.89226	2.1
31736	1.89226	2.1
31737	1.89226	2.1
31738	3.78453	2.1
31739	1.89226	2.1
31740	2.19174	2.1
31741	2.15616	2.1
31742	2.19174	2.1
31743	1.85669	2.1
31744	1.89226	2.1
31745	1.89226	2.1
31746	1.89226	2.1
31747	1.89226	2.1
31748	1.89226	2.1
31749	3.78452	2.1
31750	1.89226	2.1
31751	1.89226	2.1
31752	1.89226	2.1
31753	1.89226	2.1
31754	3.78452	2.1
31755	1.51223	2.1
31756	1.41562	2.1
31757	1.5139	2.1
31758	1.42284	2.1
31759	1.6033	2.1
31760	1.51223	2.1
31761	1.51223	2.1
31762	1.25953	2.1
31763	1.38085	2.1
31764	1.6033	2.1
31765	2.89309	2.1
31766	0.946131	2.1
31767	0.946131	2.1
31768	0.946131	2.1
31769	0.946131	2.1
31770	0.946132	2.1
31771	0.946132	2.1
31772	0.946129	2.1
31773	0.946129	2.1
31774	0.946131	2.1
31775	0.946131	2.1
31776	0.94613	2.1
31777	0.94613	2.1
31778	1.89226	2.1
31779	1.89226	2.1
31780	1.89226	2.1
31781	1.89226	2.1

31782	3.78452	2.1
31783	0.707806	2.1
31784	0.697816	2.1
31785	0.823593	2.1
31786	0.801649	2.1
31787	0.74182	2.1
31788	0.74182	2.1
31789	0.757645	2.1
31790	0.757645	2.1
31791	0.823592	2.1
31792	0.697818	2.1
31793	0.707807	2.1
31794	0.801647	2.1
31795	1.52534	2.1
31796	1.6033	2.1
31797	1.64719	2.1
31798	1.38085	2.1
31799	1.64719	2.1
31800	1.38085	2.1
31801	2.67041	2.1
31802	1.89226	0.213838
31803	1.89226	2.1
31804	1.89226	2.08855
31805	2.487	2.1
31806	2.487	2.1
31807	2.70097	2.1
31808	1.70303	2.1
31809	1.71163	2.1
31810	2.487	2.1
31811	4.46723	2.1
31812	1.14984	2.1
31813	1.2435	2.1
31814	0.778	2.1
31815	0.697616	2.1
31816	0.967211	2.1
31817	0.967211	2.1
31818	0.973907	2.1
31819	0.973907	2.1
31820	0.697616	2.1
31821	1.14984	2.1
31822	0.778	2.1
31823	1.2435	2.1
31824	2.4158	2.1
31825	1.26438	2.1
31826	1.676	2.1
31827	2.77505	2.1
31828	4.0918	2.1
31829	2.58258	2.1
31830	1.89226	2.1
31831	1.89226	2.1
31832	1.89226	2.1
31833	2.47032	2.1
31834	2.47032	2.1
31835	1.23516	2.1
31836	1.18734	2.1
31837	0.946131	2.1
31838	0.946131	2.1
31839	1.18734	2.1

31840	1.18734	2.1
31841	0.94613	2.1
31842	0.94613	2.1
31843	1.18734	2.1
31844	0.946131	2.1
31845	0.946131	2.1
31846	1.23516	2.1
31847	2.8239	2.1
31848	1.39361	2.1
31849	1.12342	2.1
31850	2.57275	2.1
31851	3.96637	2.1
31852	0.937929	0.667317
31853	0.937929	0.211021
31854	1.39734	0.0762141
31855	1.39734	0.0724831
31856	1.38914	0.0963812
31857	0.94613	0.391866
31858	0.937902	0.48308
31859	0.94613	0.05
31860	0.94613	0.224396
31861	0.94613	0.255669
31862	1.38914	0.0919299
31863	0.937902	0.0775747
31864	1.89226	0.0693442
31865	2.17765	0.660935
31866	1.84577	0.512459
31867	1.84577	0.150823
31868	1.89226	0.326291
31869	1.89226	0.0932498
31870	2.11262	1.35978
31871	1.57766	1.48597
31872	2.22689	2.1
31873	1.91132	0.05
31874	2.19624	2.1
31875	1.79689	0.05
31876	1.99656	0.05
31877	1.57766	0.758967
31878	1.39067	2.1
31879	1.73802	0.199865
31880	3.31568	0.898454
31881	1.89226	0.188563
31882	1.89226	0.05
31883	1.89226	0.0556598
31884	1.89226	0.246107
31885	1.89226	0.05
31886	1.89226	0.0643393
31887	1.84936	0.254401
31888	2.0815	0.05
31889	2.0815	0.0556141
31890	2.47217	0.159293
31891	1.04528	0.05
31892	1.62774	0.0819586
31893	1.89226	0.0605904
31894	1.89226	0.05
31895	1.89226	0.05
31896	1.89226	0.0993715
31897	1.89226	0.05

31898	1.89226	0.05
31899	1.69055	0.05
31900	2.68518	0.05
31901	1.69055	0.05
31902	2.25055	0.05
31903	2.39587	0.05
31904	3.01801	0.05
31905	1.89226	0.05
31906	1.89226	0.05
31907	1.89226	0.05
31908	1.89226	0.05
31909	3.78452	0.05
31910	1.24499	1.82814
31911	1.70302	2.09531
31912	1.41207	2.1
31913	1.10574	0.0812864
31914	2.66056	1.76454
31915	1.70303	2.1
31916	2.39605	1.33342
31917	2.72674	1.50814
31918	2.46904	0.313548
31919	1.84557	1.90479
31920	1.67115	1.12156
31921	2.46904	0.0527999
31922	2.76829	1.94866
31923	2.83666	0.05
31924	2.83666	0.299187
31925	2.39605	0.153587
31926	2.39605	1.95075
31927	1.07323	0.05
31928	1.07323	2.1
31929	1.06715	2.1
31930	1.18052	0.627727
31931	1.30067	0.130069
31932	1.26542	0.327257
31933	0.914516	2.09675
31934	0.839633	2.1
31935	0.936958	1.17794
31936	1.26751	1.05863
31937	0.847764	0.648703
31938	1.35395	1.01321
31939	2.69069	0.0810378
31940	1.03074	0.05
31941	1.74098	0.05
31942	1.97397	0.05
31943	3.71495	0.05
31944	2.44554	2.1
31945	1.89226	2.1
31946	1.89226	2.1
31947	1.89226	2.1
31948	2.40645	2.1
31949	2.40645	2.1
31950	1.89226	2.1
31951	1.89226	2.1
31952	1.89226	2.1
31953	1.89226	2.08905
31954	3.78452	2.1
31955	1.82117	2.1

31956	2.55786	2.03868
31957	2.51431	2.1
31958	2.51431	2.1
31959	1.85054	2.1
31960	1.84375	2.1
31961	2.38467	2.1
31962	1.89226	2.1
31963	1.89226	2.1
31964	1.89226	2.1
31965	2.44554	2.1
31966	2.44554	2.1
31967	1.89226	2.1
31968	1.89226	2.1
31969	1.89226	2.1
31970	1.89226	2.1
31971	1.89226	2.1
31972	1.89226	2.1
31973	2.02397	2.1
31974	2.55786	2.1
31975	1.77775	2.1
31976	2.61404	2.1
31977	4.33561	2.1
31978	1.89226	1.61518
31979	1.89226	1.57612
31980	1.89226	0.708324
31981	1.89226	1.14363
31982	3.78453	1.41492
31983	1.89226	1.72278
31984	2.80184	1.94373
31985	1.89226	1.13756
31986	1.89226	1.82472
31987	2.03045	1.97141
31988	2.03045	2.1
31989	2.52263	1.67252
31990	1.27921	2.1
31991	1.58429	2.1
31992	1.73571	2.1
31993	2.37121	1.96446
31994	2.52263	1.78042
31995	0.764974	2.1
31996	0.650577	2.1
31997	0.712428	2.1
31998	0.562516	2.1
31999	0.67264	2.1
32000	0.67264	2.1
32001	0.654851	2.1
32002	0.654851	2.1
32003	0.562515	2.1
32004	0.650579	2.1
32005	0.712427	2.1
32006	0.764976	2.1
32007	1.89227	2.1
32008	1.89227	2.1
32009	1.89227	2.1
32010	1.89227	2.1
32011	3.78453	2.1
32012	1.89226	2.1
32013	1.89226	2.1

32014	1.89226	2.1
32015	1.89226	2.1
32016	3.78453	2.1
32017	1.49248	2.1
32018	1.24959	2.1
32019	1.28211	2.1
32020	1.26432	2.1
32021	1.51027	2.1
32022	1.49248	2.1
32023	1.49248	2.1
32024	1.19017	2.1
32025	1.24977	2.1
32026	1.51027	2.1
32027	2.74225	2.1
32028	1.89226	2.1
32029	1.89226	2.1
32030	1.89226	2.1
32031	1.89226	2.1
32032	3.78453	2.1
32033	1.89226	2.1
32034	1.89226	2.1
32035	1.89226	2.1
32036	1.89226	2.1
32037	3.78453	2.1
32038	0.624796	2.1
32039	0.618472	2.1
32040	0.770147	2.1
32041	0.755132	2.1
32042	0.681445	2.1
32043	0.681445	2.1
32044	0.69216	2.1
32045	0.69216	2.1
32046	0.618472	2.1
32047	0.770147	2.1
32048	0.624796	2.1
32049	0.755132	2.1
32050	1.54029	2.1
32051	1.51027	2.1
32052	1.54029	2.1
32053	1.30682	2.1
32054	1.24977	2.1
32055	1.24977	2.1
32056	0.994293	2.1
32057	2.17159	2.1
32058	2.21149	2.1
32059	2.21149	2.1
32060	0.994814	2.1
32061	0.994814	2.1
32062	0.990481	2.1
32063	2.21149	2.1
32064	2.2671	2.1
32065	2.2671	2.1
32066	0.994293	2.1
32067	0.994293	2.1
32068	2.41579	2.1
32069	1.09847	2.1
32070	1.70302	1.70157
32071	2.45069	2.1

32072	3.61169	2.1
32073	2.48689	2.1
32074	1.67969	2.1
32075	1.67969	2.1
32076	1.70002	2.1
32077	2.43826	2.1
32078	2.45859	2.1
32079	2.43827	2.1
32080	1.62408	2.1
32081	1.62408	2.1
32082	1.67969	2.1
32083	2.36729	2.1
32084	2.4229	2.1
32085	2.47199	2.1
32086	1.0823	2.1
32087	1.0607	2.1
32088	2.44276	2.1
32089	3.52506	2.1
32090	2.47199	2.1
32091	1.89226	2.1
32092	1.89226	2.1
32093	2.44277	2.1
32094	4.33503	2.1
32095	1.89226	2.1
32096	1.89226	2.1
32097	1.89226	2.1
32098	1.89226	2.1
32099	3.78453	2.1
32100	1.89226	2.1
32101	1.89226	2.1
32102	1.89226	2.1
32103	1.89226	2.1
32104	1.89226	2.1
32105	1.89226	2.1
32106	1.02986	2.1
32107	2.44276	2.1
32108	1.0607	2.1
32109	1.0607	2.1
32110	2.40055	2.1
32111	2.40055	2.1
32112	1.89226	2.1
32113	2.44277	2.1
32114	2.40056	2.1
32115	2.44277	2.1
32116	1.85005	2.1
32117	1.89226	2.1
32118	1.89226	2.1
32119	1.89226	2.1
32120	1.89226	2.1
32121	1.89226	2.1
32122	3.78453	2.1
32123	1.89226	2.1
32124	1.89226	2.1
32125	1.89226	2.1
32126	1.89226	2.1
32127	3.78452	2.1
32128	2.79502	0.05
32129	2.63448	0.500555

32130	2.68986	0.05
32131	2.53868	0.115107
32132	5.32434	0.05
32133	2.63449	0.05
32134	2.39282	2.1
32135	2.53869	0.0519969
32136	2.3085	2.1
32137	4.94299	0.31975
32138	2.68986	0.05
32139	2.53868	0.100466
32140	2.5761	0.05
32141	2.43404	0.142924
32142	5.1239	0.05
32143	2.53869	0.05
32144	2.3085	2.1
32145	2.43405	0.05
32146	2.22575	2.1
32147	4.74255	0.258135
32148	1.89226	2.1
32149	1.89226	2.1
32150	1.89226	2.1
32151	1.89226	2.1
32152	3.78453	2.1
32153	1.32391	2.1
32154	1.44951	2.1
32155	1.44951	2.1
32156	1.89226	2.1
32157	1.89226	2.1
32158	2.20203	2.1
32159	0.507656	2.1
32160	0.578945	2.1
32161	1.05568	2.1
32162	0.959007	2.1
32163	0.76264	2.1
32164	0.76264	2.1
32165	0.775313	2.1
32166	0.775313	2.1
32167	0.578945	2.1
32168	1.05568	2.1
32169	0.959007	2.1
32170	0.507656	2.1
32171	1.89226	0.05
32172	2.4065	0.05
32173	2.36561	0.05
32174	2.4065	0.05
32175	1.85138	0.05
32176	1.89226	0.05
32177	1.40274	0.05
32178	2.4065	0.05
32179	1.53336	0.05
32180	1.53336	0.05
32181	2.36562	0.05
32182	2.36562	0.05
32183	0.946115	0.05
32184	0.946115	0.05
32185	1.06496	0.05
32186	1.18279	0.05
32187	0.946117	0.05

32188	0.946117	0.05
32189	1.06496	0.05
32190	1.06496	0.05
32191	0.94613	0.05
32192	1.06497	0.05
32193	0.946105	0.05
32194	1.18278	0.05
32195	1.00213	0.05
32196	2.36559	0.05
32197	1.40272	0.05
32198	1.40272	0.05
32199	2.12992	0.05
32200	2.12992	0.05
32201	0.95646	2.1
32202	2.07201	2.1
32203	1.01808	2.1
32204	2.14876	1.85374
32205	3.09009	2.1
32206	1.89226	2.1
32207	1.89226	1.29341
32208	1.89226	2.1
32209	1.89226	2.1
32210	3.78453	2.1
32211	1.89227	2.1
32212	1.89227	2.1
32213	1.89227	2.1
32214	1.89227	2.1
32215	1.89227	2.1
32216	1.89227	2.1
32217	2.71886	2.1
32218	1.60497	2.1
32219	1.43935	2.1
32220	2.55151	2.1
32221	4.15648	2.1
32222	2.71886	2.1
32223	1.89226	2.1
32224	1.89226	2.1
32225	2.55151	2.1
32226	4.44377	2.1
32227	1.89226	2.1
32228	1.89226	2.1
32229	1.89226	2.1
32230	1.89226	1.74012
32231	3.78453	2.1
32232	1.89226	2.1
32233	1.89226	2.1
32234	1.89226	2.1
32235	1.89226	2.1
32236	3.78453	2.1
32237	1.58942	0.0712891
32238	1.92622	0.17177
32239	2.52852	0.156766
32240	2.19457	0.0678706
32241	4.12079	0.0683311
32242	1.92649	0.077106
32243	1.44426	0.05
32244	1.87529	0.05
32245	2.19457	0.0791274

32246	3.63883	0.0646168
32247	1.87529	0.05
32248	2.19457	0.0732355
32249	2.7378	0.0721407
32250	2.29852	0.05
32251	4.49309	0.05
32252	1.35976	2.1
32253	1.89452	2.1
32254	1.39616	2.1
32255	1.1423	2.1
32256	1.8996	2.1
32257	1.52068	2.1
32258	1.89226	2.1
32259	1.89226	2.1
32260	1.89226	2.1
32261	1.89226	2.1
32262	1.89226	2.1
32263	1.89226	2.1
32264	1.89226	2.1
32265	2.15617	2.1
32266	2.0953	2.1
32267	2.15617	2.1
32268	1.83139	2.1
32269	1.89226	2.1
32270	1.16817	2.1
32271	1.8996	2.1
32272	1.23614	2.1
32273	1.48338	2.1
32274	1.80218	2.1
32275	2.16263	2.1
32276	1.89226	2.1
32277	1.89226	2.1
32278	1.89226	2.1
32279	1.89226	2.1
32280	1.89226	2.1
32281	1.89226	2.1
32282	1.89226	2.1
32283	2.0953	2.1
32284	2.03074	2.1
32285	2.0953	2.1
32286	1.8277	2.1
32287	1.89226	2.1
32288	1.74455	2.1
32289	2.181	2.1
32290	1.74455	2.1
32291	1.92911	2.1
32292	1.98195	2.1
32293	2.16651	2.1
32294	2.181	2.1
32295	2.29881	2.1
32296	1.98195	2.1
32297	1.80729	2.1
32298	4.28075	2.1
32299	1.74455	2.1
32300	1.98195	2.1
32301	1.52603	2.1
32302	1.76603	2.1
32303	3.51058	2.1

32304	1.76603	2.1
32305	1.94506	2.1
32306	1.76603	2.1
32307	1.98195	2.1
32308	1.86795	2.1
32309	2.08387	2.1
32310	0.984305	2.1
32311	1.9	2.1
32312	2.86619	2.1
32313	1.86189	2.1
32314	3.76189	2.1
32315	1.4621	2.1
32316	1.89226	2.1
32317	2.81374	2.1
32318	1.89226	2.1
32319	3.35437	2.1
32320	0.946133	2.1
32321	0.946133	2.1
32322	0.946132	2.1
32323	0.946132	2.1
32324	0.946132	2.1
32325	0.946132	2.1
32326	0.946133	2.1
32327	0.946133	2.1
32328	0.946132	2.1
32329	0.946132	2.1
32330	0.946132	2.1
32331	0.946132	2.1
32332	1.64268	2.1
32333	1.93673	2.1
32334	2.25284	2.1
32335	1.97548	2.1
32336	3.89552	2.1
32337	1.86206	2.1
32338	1.93673	2.1
32339	2.25284	2.1
32340	2.20075	2.1
32341	4.1149	2.1
32342	2.25284	2.1
32343	2.24781	2.1
32344	1.97548	2.1
32345	1.97548	2.1
32346	2.49676	2.1
32347	2.49676	2.1
32348	2.25284	2.1
32349	2.43549	2.1
32350	2.44467	2.1
32351	2.20075	2.1
32352	2.49676	2.1
32353	2.25284	2.1
32354	2.57928	2.1
32355	2.32606	2.1
32356	2.59522	2.1
32357	2.39022	2.1
32358	4.9695	2.1
32359	2.32606	2.1
32360	1.77677	2.1
32361	2.39022	2.1

32362	2.28669	2.1
32363	4.16699	2.1
32364	2.59522	2.1
32365	2.39022	2.1
32366	2.61901	2.1
32367	2.45899	2.1
32368	5.00923	2.1
32369	2.39022	2.1
32370	2.11575	2.1
32371	2.41795	2.1
32372	2.0815	2.1
32373	4.67085	2.1
32374	1.89226	2.1
32375	2.24723	2.1
32376	1.95405	2.1
32377	1.89226	2.1
32378	2.30902	2.1
32379	2.24723	2.1
32380	1.89226	2.1
32381	1.89226	2.1
32382	1.89226	2.1
32383	1.89226	2.1
32384	3.78453	2.1
32385	0.946131	2.1
32386	0.946131	2.1
32387	1.14643	2.1
32388	1.10673	2.1
32389	1.10673	2.1
32390	1.10673	2.1
32391	0.946133	2.1
32392	0.946133	2.1
32393	0.946131	2.1
32394	1.14643	2.1
32395	1.10673	2.1
32396	0.946131	2.1
32397	1.89226	2.1
32398	2.30902	2.1
32399	1.96635	2.1
32400	1.89226	2.1
32401	2.38311	2.1
32402	2.30902	2.1
32403	1.89226	2.1
32404	1.89226	2.1
32405	1.89226	2.1
32406	1.89226	2.1
32407	1.89226	2.1
32408	1.89226	2.1
32409	0.946131	2.1
32410	0.946131	2.1
32411	1.19156	2.1
32412	1.14643	2.1
32413	1.14643	2.1
32414	1.14643	2.1
32415	0.946133	2.1
32416	0.946133	2.1
32417	0.946131	2.1
32418	1.19156	2.1
32419	0.946131	2.1

32420	1.14643	2.1
32421	1.89226	1.52603
32422	1.89226	0.453666
32423	1.89226	0.30445
32424	1.89226	1.37882
32425	1.89226	0.610792
32426	1.89226	1.85885
32427	1.89226	1.05451
32428	1.89226	0.589236
32429	1.89226	0.558907
32430	1.9645	0.997235
32431	3.78452	1.20825
32432	2.39489	2.1
32433	2.15715	2.1
32434	2.07821	2.1
32435	2.33541	2.1
32436	4.49256	2.1
32437	2.39489	2.1
32438	2.34898	2.1
32439	2.30729	2.1
32440	2.33541	2.1
32441	4.6844	2.1
32442	2.07821	2.1
32443	2.33541	2.1
32444	2.26415	2.1
32445	1.99632	2.1
32446	4.33174	2.1
32447	2.30729	2.1
32448	2.33542	2.1
32449	2.26416	2.1
32450	2.26618	2.1
32451	4.60159	2.1
32452	1.19837	1.76233
32453	2.79151	0.0655003
32454	2.25636	0.907071
32455	2.25636	0.32438
32456	1.94072	0.611386
32457	1.94072	0.425843
32458	2.53725	0.05
32459	1.16699	0.05
32460	1.69864	0.05
32461	2.0815	0.05
32462	3.58273	0.05
32463	1.91646	2.1
32464	2.51686	2.1
32465	2.49872	2.1
32466	1.85819	2.1
32467	4.41518	2.1
32468	0.929098	2.1
32469	0.8993	2.1
32470	1.24359	2.1
32471	1.24936	2.1
32472	1.06832	2.1
32473	1.06832	2.1
32474	1.08034	2.1
32475	1.08034	2.1
32476	0.899298	2.1
32477	1.24359	2.1

32478	1.24936	2.1
32479	0.929097	2.1
32480	2.0815	0.05
32481	1.89226	0.05
32482	2.0815	0.05
32483	1.82811	0.05
32484	1.94982	0.05
32485	1.71246	0.05
32486	2.3256	2.1
32487	1.89226	2.1
32488	1.89226	2.1
32489	1.89226	2.1
32490	2.38467	2.1
32491	2.38467	2.1
32492	1.89226	2.1
32493	1.89226	2.1
32494	1.89226	2.1
32495	1.89226	2.1
32496	1.89226	2.1
32497	1.89226	2.1
32498	1.89763	2.1
32499	2.61405	2.1
32500	2.15035	2.1
32501	2.68796	2.1
32502	1.42219	2.1
32503	1.77775	2.1
32504	2.26306	2.1
32505	1.89226	2.1
32506	1.89226	2.1
32507	1.89226	2.1
32508	2.3256	2.1
32509	2.3256	2.1
32510	1.89226	2.1
32511	1.89226	2.1
32512	1.89226	2.1
32513	1.89226	2.1
32514	1.89226	2.1
32515	1.89226	2.1
32516	1.92887	2.1
32517	2.41915	2.1
32518	2.50142	2.1
32519	2.50142	2.1
32520	1.90388	2.1
32521	1.89763	2.1
32522	1.89224	2.1
32523	1.89224	2.1
32524	1.89224	2.1
32525	1.89224	2.1
32526	3.78448	2.1
32527	2.25315	2.1
32528	1.05843	2.1
32529	2.38995	2.1
32530	1.31541	2.1
32531	3.56856	2.1
32532	0.517552	2.1
32533	0.68548	2.1
32534	1.27675	2.1
32535	1.13076	2.1

32536	0.913611	2.1
32537	0.913612	2.1
32538	0.902633	2.1
32539	0.902633	2.1
32540	0.68547	2.1
32541	1.27673	2.1
32542	1.13078	2.1
32543	0.517559	2.1
32544	1.89226	2.1
32545	1.89226	2.1
32546	1.89226	2.1
32547	1.89226	2.1
32548	3.78453	2.1
32549	0.962936	2.1
32550	0.962936	2.1
32551	0.996141	2.1
32552	0.996141	2.1
32553	0.830609	2.1
32554	0.657714	2.1
32555	1.30136	2.1
32556	1.19499	2.1
32557	1.30136	2.1
32558	0.830609	2.1
32559	1.19499	2.1
32560	0.657713	2.1
32561	1.60057	0.05
32562	1.39615	0.05
32563	1.06474	0.05
32564	1.39615	0.05
32565	1.64655	0.05
32566	2.04424	0.05
32567	1.89226	0.05
32568	1.89226	0.0506514
32569	1.89226	0.05
32570	1.89226	0.05
32571	1.89226	0.05
32572	1.89226	0.05
32573	2.56322	0.05
32574	1.89226	0.05
32575	1.89226	0.05
32576	1.89226	0.05
32577	2.18047	0.05
32578	2.18047	0.05
32579	1.89226	0.0813984
32580	1.89226	0.0983085
32581	1.89226	0.169668
32582	1.65573	0.05
32583	1.89226	0.171197
32584	1.65573	0.0519389
32585	1.05353	0.05
32586	1.83158	0.05
32587	1.50282	0.05
32588	1.20669	0.05
32589	2.88511	0.05
32590	1.44807	2.1
32591	1.93592	2.1
32592	1.80703	2.1
32593	1.27806	2.1

32594	3.21398	2.1
32595	2.0815	2.1
32596	1.87465	2.1
32597	1.87465	2.1
32598	1.50566	2.1
32599	2.0815	2.1
32600	1.69263	2.1
32601	1.89226	2.1
32602	1.89226	2.1
32603	1.89226	2.1
32604	1.89226	2.1
32605	1.89226	2.1
32606	1.89226	2.1
32607	1.62632	2.1
32608	0.994294	2.1
32609	0.994294	2.1
32610	1.27806	2.1
32611	1.81921	2.1
32612	2.10776	2.1
32613	2.0815	2.1
32614	1.70303	2.1
32615	1.70303	2.1
32616	1.85749	2.1
32617	1.90451	2.1
32618	2.07724	2.1
32619	1.89226	2.1
32620	1.89226	2.1
32621	1.89226	2.1
32622	1.89226	2.1
32623	1.89226	2.1
32624	1.89226	2.1
32625	2.69773	2.1
32626	1.88129	2.1
32627	2.55196	2.1
32628	2.26469	2.1
32629	4.94367	2.1
32630	1.88129	2.1
32631	1.25889	2.1
32632	1.26614	2.1
32633	1.05914	2.1
32634	2.26469	2.1
32635	1.88129	2.1
32636	2.55196	2.1
32637	2.0588	2.1
32638	2.6536	2.1
32639	2.15287	2.1
32640	4.70484	2.1
32641	0.8516	2.1
32642	0.8516	2.1
32643	0.849002	2.1
32644	0.849002	2.1
32645	0.629445	2.1
32646	0.671197	2.1
32647	1.0294	2.1
32648	1.07644	2.1
32649	1.07644	2.1
32650	0.671197	2.1
32651	0.629446	2.1

32652	1.0294	2.1
32653	2.02115	2.1
32654	1.18382	2.1
32655	2.29107	0.101754
32656	1.46903	1.62826
32657	3.47488	0.689834
32658	2.60763	1.25023
32659	2.29107	1.85879
32660	1.82141	1.48305
32661	1.82141	2.1
32662	1.46903	2.08749
32663	2.29107	2.09347
32664	1.64007	2.1
32665	2.2024	2.1
32666	1.44044	2.1
32667	2.02634	2.1
32668	3.6664	2.1
32669	1.25565	2.1
32670	2.02635	2.1
32671	1.87228	2.1
32672	1.87228	2.1
32673	1.44045	2.1
32674	1.44045	2.1
32675	1.89226	0.05
32676	1.78503	0.05
32677	1.78503	0.05
32678	1.40048	0.05
32679	2.0815	0.05
32680	1.62972	0.05
32681	1.27875	2.1
32682	1.1483	0.508911
32683	1.40014	0.808241
32684	1.40014	2.1
32685	1.04598	1.50656
32686	1.04598	2.1
32687	2.63443	1.21083
32688	2.26444	2.1
32689	2.79392	0.0921682
32690	2.4863	2.1
32691	5.12073	1.9294
32692	1.83158	0.05
32693	1.18508	0.05
32694	1.20669	0.05
32695	1.81488	0.05
32696	1.20669	0.05
32697	1.81488	0.05
32698	1.08134	0.05
32699	1.70303	0.05
32700	1.24027	0.05
32701	1.30359	0.05
32702	1.89939	0.05
32703	1.70303	0.05
32704	1.89226	2.1
32705	2.69027	2.1
32706	1.89226	2.1
32707	2.2078	2.1
32708	1.89226	2.1
32709	2.2078	2.1

32710	1.89226	2.1
32711	2.78977	2.1
32712	1.89226	2.1
32713	2.69027	2.1
32714	1.89226	2.1
32715	2.69027	2.1
32716	1.89226	2.1
32717	1.89226	2.1
32718	1.89226	1.89539
32719	1.89226	2.1
32720	3.78452	2.1
32721	1.43687	2.1
32722	1.71059	2.1
32723	2.53014	2.1
32724	2.6521	2.1
32725	4.24073	2.1
32726	2.6521	2.1
32727	1.66762	2.1
32728	2.50299	2.1
32729	2.50299	2.1
32730	1.71059	2.1
32731	1.71059	2.1
32732	1.89226	2.1
32733	1.89226	2.1
32734	1.89226	2.1
32735	1.89226	2.1
32736	1.89226	2.1
32737	1.89226	2.1
32738	2.10662	0.272321
32739	2.77417	0.14293
32740	2.78873	1.38499
32741	2.11661	0.178062
32742	4.89078	0.147146
32743	2.10662	1.03047
32744	1.22857	0.05
32745	1.25849	1.29582
32746	2.11661	0.284546
32747	3.34518	0.89205
32748	2.78873	0.954781
32749	2.11661	0.1938
32750	2.12475	0.363533
32751	2.79029	0.140633
32752	4.9069	0.339383
32753	1.2858	0.908792
32754	2.11661	0.276055
32755	1.25849	0.644754
32756	1.25849	0.680499
32757	2.12475	0.27449
32758	2.12475	0.463722
32759	1.13537	2.1
32760	1.20714	2.1
32761	0.946129	2.1
32762	0.946129	2.1
32763	0.946131	2.1
32764	0.946131	2.1
32765	1.29521	2.1
32766	1.20713	2.1
32767	1.13537	2.1

32768	0.946131	2.1
32769	0.94613	2.1
32770	1.29521	2.1
32771	1.00533	2.1
32772	0.876055	2.1
32773	0.946128	2.1
32774	0.946128	2.1
32775	0.913908	2.1
32776	0.913908	2.1
32777	1.17081	2.1
32778	0.896413	2.1
32779	0.955905	2.1
32780	0.893715	2.1
32781	1.20327	2.1
32782	0.998544	2.1
32783	1.89226	1.95997
32784	1.89226	2.09379
32785	1.89226	0.226069
32786	1.89226	1.33911
32787	3.78452	0.67453
32788	0.540975	1.23672
32789	0.726949	2.1
32790	0.712198	2.1
32791	0.506675	0.996645
32792	0.611916	1.10847
32793	0.611916	2.1
32794	0.621708	2.00838
32795	0.621708	0.919735
32796	0.726951	1.5193
32797	0.712196	1.08239
32798	0.506674	1.79244
32799	0.540976	1.54004
32800	1.89226	1.12862
32801	1.89226	2.1
32802	1.89226	1.12692
32803	1.89226	1.29899
32804	3.78453	1.95354
32805	1.89226	0.923339
32806	1.89226	1.25161
32807	1.89226	0.178552
32808	1.89226	2.1
32809	1.89226	0.249759
32810	1.89226	2.1
32811	0.919594	0.979258
32812	0.726949	1.97127
32813	0.898318	1.16979
32814	0.712198	1.92291
32815	0.817559	0.434837
32816	0.817559	2.1
32817	0.814234	0.478905
32818	0.814234	2.05581
32819	0.919597	0.748693
32820	0.898316	0.308966
32821	0.726951	2.00483
32822	0.712196	1.4018
32823	1.79663	0.530155
32824	1.12088	2.1
32825	1.54699	1.14809

32826	1.17475	1.31064
32827	1.79663	2.1
32828	1.42439	2.1
32829	2.28928	0.886543
32830	1.23077	1.73461
32831	1.65458	0.544937
32832	2.66533	2.0019
32833	3.94385	1.37855
32834	1.89226	2.1
32835	1.89226	2.1
32836	1.89226	2.1
32837	1.89226	2.1
32838	3.78452	2.1
32839	1.38426	2.1
32840	1.15067	2.1
32841	1.38426	2.1
32842	1.01595	2.1
32843	1.24509	2.1
32844	1.01595	2.1
32845	2.62097	2.1
32846	1.89226	2.1
32847	2.72561	2.1
32848	2.72561	2.1
32849	1.89226	2.1
32850	1.89226	2.1
32851	1.90103	0.05
32852	1.41902	2.02449
32853	1.77249	0.05
32854	1.30272	2.1
32855	3.20375	0.100589
32856	1.77249	0.05
32857	1.19548	2.03621
32858	1.19548	0.101908
32859	1.30272	1.70992
32860	1.65815	0.05
32861	1.76539	1.05148
32862	2.62161	2.1
32863	1.89226	2.1
32864	1.89226	2.1
32865	1.89226	2.1
32866	2.6446	2.1
32867	2.6446	2.1
32868	2.64459	2.1
32869	1.89226	2.1
32870	1.89226	2.1
32871	1.89226	2.1
32872	2.58257	2.1
32873	2.58257	2.1
32874	1.89226	0.080468
32875	1.89226	0.777453
32876	2.31301	0.692038
32877	2.76309	0.1159
32878	1.89226	0.506817
32879	2.26047	0.101716
32880	1.89226	0.0827596
32881	2.31301	0.784058
32882	2.17765	0.625379
32883	2.17765	0.139615

32884	1.89226	0.465038
32885	1.89226	0.101532
32886	2.18516	2.1
32887	1.85196	2.1
32888	1.78595	2.1
32889	2.14606	2.1
32890	3.99802	2.1
32891	2.18516	2.1
32892	2.21189	2.1
32893	2.18937	2.1
32894	2.14606	2.1
32895	4.35796	2.1
32896	1.60735	2.1
32897	2.14606	2.1
32898	2.15714	2.1
32899	1.70302	2.1
32900	3.70055	2.1
32901	2.18937	2.1
32902	2.14606	2.1
32903	2.0927	2.1
32904	2.162 2.1	
32905	4.30806	2.1
32906	2.17326	2.1
32907	2.58517	2.1
32908	2.55318	2.1
32909	2.1665	2.1
32910	4.75167	2.1
32911	2.17326	2.1
32912	1.53556	2.1
32913	1.56253	2.1
32914	2.1665	2.1
32915	3.70206	2.1
32916	2.55317	2.1
32917	2.1665	2.1
32918	2.17741	2.1
32919	2.54197	2.1
32920	4.70847	2.1
32921	2.17742	2.1
32922	2.1665	2.1
32923	2.18452	2.1
32924	1.58054	2.1
32925	1.56253	2.1
32926	1.56253	2.1
32927	1.71051	0.0500878
32928	1.89807	0.05
32929	1.19755	0.05
32930	1.19755	0.05
32931	2.25632	0.0565461
32932	2.25632	0.05
32933	0.876046	0.05
32934	1.27293	0.05
32935	0.946132	0.05
32936	0.946132	0.05
32937	0.946131	0.05
32938	0.869312	0.05
32939	1.12767	0.05
32940	0.946132	0.05
32941	1.22225	0.05

32942	1.37968	0.05
32943	0.851555	0.05
32944	0.851555	0.05
32945	0.946133	1.85693
32946	0.946133	2.1
32947	0.946132	2.08478
32948	0.946132	1.02985
32949	0.946133	1.87877
32950	0.946133	1.98115
32951	0.946132	2.1
32952	0.946132	2.03154
32953	0.946132	2.1
32954	0.946132	2.09262
32955	0.946133	2.04722
32956	0.946133	2.1
32957	1.35152	2.1
32958	0.948936	2.1
32959	1.90166	2.1
32960	1.70303	2.1
32961	2.90858	2.1
32962	1.89226	2.1
32963	1.89226	2.1
32964	1.89226	2.1
32965	1.89226	2.1
32966	3.78452	2.1
32967	1.90166	2.1
32968	1.70303	2.1
32969	2.52454	2.1
32970	2.13919	2.1
32971	3.99529	2.1
32972	1.89226	1.41766
32973	1.89226	2.1
32974	1.89226	2.1
32975	1.89226	2.1
32976	3.78452	2.1
32977	1.89226	2.1
32978	1.89226	2.1
32979	1.89226	2.1
32980	1.89226	2.1
32981	1.89226	0.615457
32982	1.89226	2.1
32983	2.13918	2.1
32984	0.992674	2.1
32985	2.3397	2.1
32986	1.8023	2.1
32987	1.47769	2.1
32988	0.992674	2.1
32989	1.89226	0.05
32990	1.89226	0.05
32991	1.89226	0.05
32992	1.89226	0.05
32993	3.78452	0.05
32994	1.89226	0.05
32995	2.54752	0.05
32996	2.63974	0.05
32997	1.89226	0.05
32998	4.53201	0.05
32999	0.498412	0.05

33000	0.500955	0.05
33001	0.57955	0.05
33002	0.563873	0.05
33003	0.529107	0.05
33004	0.529107	0.05
33005	0.53572	0.05
33006	0.53572	0.05
33007	0.500956	0.05
33008	0.579548	0.05
33009	0.563871	0.05
33010	0.498414	0.05
33011	1.89223	0.05
33012	1.89223	0.05
33013	1.89223	0.05
33014	1.89223	0.05
33015	1.89223	0.05
33016	1.89223	0.05
33017	1.28752	0.05
33018	1.31985	0.05
33019	0.946119	0.05
33020	0.946119	0.05
33021	1.28752	0.05
33022	1.28752	0.05
33023	0.946119	0.05
33024	0.946119	0.05
33025	1.28753	0.05
33026	0.946132	0.05
33027	1.31984	0.05
33028	0.946107	0.05
33029	0.500948	0.05
33030	0.479465	0.05
33031	0.562335	0.05
33032	0.579542	0.05
33033	0.528436	0.05
33034	0.528436	0.05
33035	0.530571	0.05
33036	0.530571	0.05
33037	0.56234	0.05
33038	0.479473	0.05
33039	0.500943	0.05
33040	0.579533	0.05
33041	1.89226	2.1
33042	1.89226	2.1
33043	1.89226	2.1
33044	1.89226	2.1
33045	3.78452	2.1
33046	1.50767	2.1
33047	1.14496	2.1
33048	1.50767	2.1
33049	1.58539	2.1
33050	1.07653	2.1
33051	1.15425	2.1
33052	1.89226	2.1
33053	2.43826	2.1
33054	2.43826	2.1
33055	2.48688	2.1
33056	1.89226	2.1
33057	1.94088	2.1

33058	1.89226	2.1
33059	1.89226	2.1
33060	1.89226	2.1
33061	1.89226	2.1
33062	3.78452	2.1
33063	1.50767	2.1
33064	1.07653	2.1
33065	1.41077	2.1
33066	0.991407	2.1
33067	2.49907	2.1
33068	1.21913	2.1
33069	1.21913	2.1
33070	0.946128	2.1
33071	0.946128	2.1
33072	1.21913	2.1
33073	1.18364	2.1
33074	0.946131	2.1
33075	0.946131	2.1
33076	1.18364	2.1
33077	0.946129	2.1
33078	1.21913	2.1
33079	0.94613	2.1
33080	1.89226	0.11955
33081	1.89226	2.1
33082	1.89226	0.310421
33083	1.89226	2.1
33084	3.78452	2.05647
33085	1.93823	0.310453
33086	1.60976	2.1
33087	1.93823	2.1
33088	2.20045	0.134962
33089	1.36999	2.1
33090	1.63222	1.17738
33091	1.38302	2.1
33092	1.38302	2.1
33093	0.946132	2.1
33094	0.946132	2.1
33095	1.27258	2.1
33096	1.38302	2.1
33097	0.946133	2.1
33098	0.946133	2.1
33099	0.946133	2.1
33100	1.27258	2.1
33101	0.946132	2.1
33102	1.38302	2.1
33103	1.89226	0.0789255
33104	1.89226	2.1
33105	1.89226	0.141569
33106	1.89226	2.1
33107	3.78452	2.1
33108	1.93823	0.0808992
33109	1.36999	2.1
33110	1.70423	0.05
33111	1.14195	2.1
33112	3.08018	1.57859
33113	1.27258	2.1
33114	1.27258	2.1
33115	0.946132	2.1

33116	0.946132	2.1
33117	1.17233	2.1
33118	1.27258	2.1
33119	0.946132	2.1
33120	0.946132	2.1
33121	1.17233	2.1
33122	0.946132	2.1
33123	0.946132	2.1
33124	1.27258	2.1
33125	2.70575	2.1
33126	1.89226	0.698963
33127	2.71429	2.1
33128	2.71429	2.1
33129	1.89226	2.1
33130	1.89226	0.700047
33131	0.946133	0.583719
33132	0.946133	2.1
33133	1.28713	2.1
33134	1.28713	2.1
33135	0.946131	2.1
33136	0.946131	0.48865
33137	1.28713	2.1
33138	1.36503	2.1
33139	0.946131	2.1
33140	1.36503	2.1
33141	1.28713	2.1
33142	0.946131	2.1
33143	1.89226	2.1
33144	1.89226	1.99154
33145	1.89226	2.1
33146	1.89226	1.98073
33147	1.89226	2.1
33148	1.89226	1.83684
33149	1.22615	2.1
33150	1.04147	2.1
33151	1.3439	2.1
33152	1.3439	2.1
33153	0.969118	2.1
33154	0.969118	2.1
33155	0.980841	2.1
33156	0.992505	2.1
33157	1.34289	2.1
33158	1.70303	2.1
33159	2.56081	2.1
33160	1.89225	2.1
33161	2.66785	2.1
33162	2.79506	2.1
33163	2.79506	2.1
33164	1.89225	2.1
33165	1.89225	2.1
33166	0.978778	2.1
33167	0.978778	2.1
33168	0.993303	2.1
33169	0.993303	2.1
33170	0.603538	2.1
33171	0.638158	2.1
33172	1.33393	2.1
33173	1.39753	2.1

33174	1.33391	2.1
33175	0.603535	2.1
33176	0.638164	2.1
33177	1.39754	2.1
33178	1.33393	2.1
33179	1.33393	2.1
33180	0.946132	2.1
33181	0.946132	2.1
33182	1.33393	2.1
33183	1.27365	2.1
33184	0.946132	2.1
33185	0.946132	2.1
33186	0.946132	2.1
33187	1.27365	2.1
33188	1.33393	2.1
33189	0.946131	2.1
33190	1.89226	2.1
33191	1.89226	2.1
33192	1.89226	2.1
33193	1.89226	2.1
33194	3.78452	2.1
33195	1.89226	2.1
33196	1.89226	2.1
33197	1.89226	2.1
33198	1.89226	2.1
33199	3.78453	2.1
33200	0.979364	2.1
33201	0.979364	2.1
33202	0.89618	2.1
33203	0.89618	2.1
33204	0.896178	2.1
33205	0.929411	2.1
33206	0.946133	2.1
33207	0.946133	2.1
33208	0.946131	2.1
33209	0.89618	2.1
33210	0.946131	2.1
33211	0.929413	2.1
33212	1.89226	2.1
33213	1.89226	2.1
33214	1.89226	2.1
33215	1.89226	2.1
33216	1.89226	2.1
33217	1.89226	2.1
33218	1.89225	2.1
33219	1.89225	2.1
33220	1.89225	2.1
33221	1.89225	2.1
33222	3.7845	2.1
33223	1.89225	2.1
33224	1.89225	2.1
33225	1.89225	2.1
33226	1.89225	2.1
33227	1.89225	2.1
33228	1.89225	2.1
33229	0.946128	0.05
33230	0.946128	0.05
33231	0.946132	0.05

33232	0.946132	0.05
33233	0.94613	0.05
33234	0.94613	0.05
33235	0.946129	0.05
33236	0.946129	0.05
33237	0.94613	0.05
33238	0.94613	0.05
33239	0.94613	0.05
33240	0.94613	0.05
33241	1.89227	1.46467
33242	1.89227	1.45487
33243	1.89227	1.15194
33244	1.89227	0.807069
33245	3.78453	1.19743
33246	1.89226	1.48343
33247	2.43717	1.96657
33248	2.63259	1.17387
33249	1.89226	0.484422
33250	4.52485	1.52013
33251	0.616808	1.18601
33252	0.792277	1.42527
33253	1.04986	1.03742
33254	0.72303	1.35941
33255	0.702675	0.11539
33256	0.76862	2.1
33257	0.809154	1.82393
33258	0.809154	1.0023
33259	0.808757	1.40316
33260	1.07789	0.532188
33261	0.772568	0.954432
33262	0.598878	1.5806
33263	1.89226	1.49663
33264	1.89226	1.28714
33265	1.89226	1.07822
33266	1.89226	1.0068
33267	3.78453	1.40156
33268	1.36428	2.03238
33269	1.31629	1.19129
33270	0.946132	1.38321
33271	0.946132	1.25083
33272	1.36428	0.638575
33273	1.36428	2.07075
33274	0.946132	1.35662
33275	0.946132	1.93177
33276	1.36428	1.99135
33277	0.946132	0.849528
33278	1.31629	1.76538
33279	0.946132	0.475497
33280	1.578	1.48856
33281	2.85336	0.719375
33282	2.11368	0.426116
33283	2.14375	1.1696
33284	1.93852	1.24816
33285	1.93852	1.60528
33286	2.34806	0.193102
33287	1.42913	0.05
33288	1.61539	0.535069
33289	2.45322	0.0562708

33290	3.88235	0.15759
33291	2.34806	0.0845788
33292	1.89226	0.05
33293	1.89226	0.351872
33294	2.45322	0.0868402
33295	4.34548	0.05
33296	1.89226	0.0711498
33297	1.89226	0.05
33298	1.89226	0.127182
33299	1.89226	0.0966205
33300	3.78452	0.172742
33301	1.89226	0.0513315
33302	1.89226	0.05
33303	1.89226	0.0795489
33304	1.89226	0.100997
33305	3.78452	0.05
33306	1.25972	0.123605
33307	2.45322	0.05
33308	2.58823	0.364886
33309	1.79398	0.05
33310	4.2472	0.05
33311	1.89226	0.252344
33312	2.45322	0.05
33313	2.58823	0.0637864
33314	2.45322	0.05
33315	2.02727	0.05
33316	1.89226	0.05
33317	1.89226	0.0582648
33318	1.89226	0.0507732
33319	1.89226	0.217772
33320	1.89226	0.05
33321	3.78452	0.05
33322	1.89226	0.05
33323	1.89226	0.05
33324	1.89226	0.05
33325	1.89226	0.05
33326	1.89226	0.208087
33327	1.89226	0.0558473
33328	1.70048	2.1
33329	2.1572	0.05
33330	1.45477	1.87458
33331	1.93973	0.05
33332	3.64021	0.261996
33333	1.17614	1.77437
33334	1.93973	0.05
33335	1.69661	2.1
33336	1.69661	0.05
33337	1.45477	2.1
33338	1.45477	0.0574319
33339	1.94343	2.1
33340	2.53782	1.60768
33341	2.59903	2.1
33342	2.59903	0.269795
33343	1.87598	2.1
33344	1.87598	0.649697
33345	0.946131	0.11188
33346	0.946131	1.35748
33347	1.26891	2.1

33348	1.26891	0.05
33349	0.94613	1.68698
33350	0.94613	0.970769
33351	1.26891	1.67341
33352	1.29952	0.389663
33353	1.29952	0.0743395
33354	0.946131	0.05
33355	1.26891	0.573285
33356	0.946131	0.724661
33357	2.00551	2.1
33358	2.59903	1.9817
33359	2.65811	2.1
33360	2.65811	0.976752
33361	1.94343	2.1
33362	1.94343	1.40644
33363	2.65811	0.215431
33364	1.89226	2.08083
33365	1.89226	0.120699
33366	1.89226	1.36061
33367	2.59903	0.567157
33368	2.59903	2.1
33369	1.89227	2.1
33370	1.89227	2.1
33371	1.89227	2.1
33372	1.89227	2.1
33373	3.78453	2.1
33374	1.15471	2.1
33375	0.993889	2.1
33376	1.78919	2.1
33377	1.77325	2.1
33378	2.78308	2.1
33379	1.89226	2.1
33380	1.89226	2.1
33381	1.89226	2.1
33382	1.89226	2.1
33383	3.78453	2.1
33384	1.78919	2.1
33385	1.77325	2.1
33386	2.45969	2.1
33387	2.70835	2.1
33388	4.49753	2.1
33389	0.939314	2.1
33390	0.939315	2.1
33391	1.01133	2.1
33392	1.01133	2.1
33393	0.596471	2.1
33394	1.20813	2.1
33395	0.886619	2.1
33396	1.35417	2.1
33397	1.35417	2.1
33398	1.20813	2.1
33399	0.886621	2.1
33400	0.59647	2.1
33401	0.946131	2.1
33402	0.946131	2.1
33403	0.94613	2.1
33404	0.94613	2.1
33405	0.946133	2.1

33406	0.946133	2.1
33407	0.946128	2.1
33408	0.946128	2.1
33409	0.946131	2.1
33410	0.946131	2.1
33411	0.946131	2.1
33412	0.946131	2.1
33413	1.03939	2.1
33414	1.03939	2.1
33415	0.946132	0.737538
33416	0.946132	1.4906
33417	0.946132	1.76119
33418	0.946132	2.1
33419	1.23851	0.846529
33420	1.03939	2.1
33421	1.03939	2.1
33422	0.946132	2.1
33423	0.946133	2.1
33424	1.23851	2.1
33425	0.902503	2.1
33426	0.946129	2.1
33427	0.946132	0.832719
33428	0.946132	2.1
33429	0.902472	1.99789
33430	0.902472	2.1
33431	1.08736	1.02372
33432	0.994093	2.1
33433	0.946133	2.1
33434	0.902504	2.1
33435	1.03939	2.1
33436	0.946132	2.1
33437	1.35306	1.26513
33438	1.29982	0.263085
33439	1.7216	0.298697
33440	1.89183	0.281419
33441	3.07466	0.3623
33442	0.485098	0.386716
33443	0.582401	0.403211
33444	0.649908	0.759171
33445	0.8608	0.519035
33446	0.587739	0.125894
33447	0.58774	0.589649
33448	0.644567	0.433312
33449	0.644567	1.50204
33450	0.58241	0.881937
33451	0.86081	0.218187
33452	0.649898	1.09895
33453	0.485093	0.40751
33454	2.25936	2.1
33455	2.71441	2.1
33456	2.63824	2.1
33457	2.20652	2.1
33458	4.92092	2.1
33459	2.25936	2.1
33460	1.57627	2.1
33461	1.5493	2.1
33462	2.20652	2.1
33463	3.78279	2.1

33464	2.63824	2.1
33465	2.20652	2.1
33466	2.17326	2.1
33467	2.58517	2.1
33468	4.79169	2.1
33469	2.17326	2.1
33470	2.20652	2.1
33471	2.19278	2.1
33472	1.53556	2.1
33473	1.5493	2.1
33474	1.5493	2.1
33475	1.97465	2.1
33476	2.13925	2.1
33477	2.04483	2.1
33478	1.62784	2.1
33479	3.76709	2.1
33480	1.89226	2.1
33481	1.89226	2.1
33482	1.89226	2.1
33483	1.89226	2.1
33484	1.89226	2.1
33485	1.89226	2.1
33486	0.724037	2.1
33487	0.81392	2.1
33488	0.96796	2.1
33489	1.02242	2.1
33490	0.882091	2.1
33491	0.882091	2.1
33492	0.899789	2.1
33493	0.899789	2.1
33494	0.724036	2.1
33495	0.96796	2.1
33496	1.02242	2.1
33497	0.81392	2.1
33498	1.13537	2.1
33499	1.63054	2.1
33500	0.946131	2.1
33501	0.946131	2.1
33502	0.946131	2.1
33503	0.946131	2.1
33504	1.258	2.1
33505	1.63054	2.1
33506	1.22632	2.1
33507	1.22632	2.1
33508	0.946131	2.1
33509	1.258	2.1
33510	1.89226	2.1
33511	1.89226	2.1
33512	1.89226	2.1
33513	1.89226	2.1
33514	1.89226	2.1
33515	1.89226	2.1
33516	0.809739	2.1
33517	0.862674	2.1
33518	1.1995	2.1
33519	1.33899	2.1
33520	1.06324	2.1
33521	1.06324	2.1

33522	0.981932	2.1
33523	1.19563	2.1
33524	0.809727	2.1
33525	1.14092	2.1
33526	1.33902	2.1
33527	0.863494	2.1
33528	2.23742	2.1
33529	1.84902	2.1
33530	1.88088	2.1
33531	2.20392	2.1
33532	4.08644	2.1
33533	1.3592	2.1
33534	1.84902	2.1
33535	1.88088	2.1
33536	1.3305	2.1
33537	1.88088	2.1
33538	1.3305	2.1
33539	1.84902	2.1
33540	1.15008	0.05
33541	1.3305	2.1
33542	1.70539	2.1
33543	2.9991	2.1
33544	1.84901	2.1
33545	2.0264	2.1
33546	2.20391	2.1
33547	1.70539	2.1
33548	3.87541	2.1
33549	1.03852	2.1
33550	1.38247	0.147171
33551	1.36635	0.602266
33552	1.01537	1.91094
33553	2.39784	1.50466
33554	2.0815	2.06794
33555	1.91744	1.83845
33556	1.91744	1.36012
33557	2.06496	2.1
33558	1.89227	1.45225
33559	2.03474	2.1
33560	1.89226	0.273389
33561	1.89226	1.41143
33562	1.89226	0.196811
33563	1.89226	2.07671
33564	1.89226	0.112896
33565	1.89226	1.96479
33566	1.36635	0.797667
33567	1.02923	2.1
33568	1.02923	0.85922
33569	1.01537	2.1
33570	1.36416	0.346916
33571	1.3503	2.1
33572	1.89226	2.07881
33573	1.89359	2.1
33574	1.89359	1.85834
33575	1.91744	2.1
33576	1.89226	1.72207
33577	1.91611	2.1
33578	0.946128	2.1
33579	0.946128	1.7892

33580	0.946133	0.466955
33581	0.946133	0.162997
33582	0.94613	0.160368
33583	0.94613	1.50979
33584	0.946131	2.1
33585	0.946131	0.244582
33586	0.946131	1.21329
33587	0.946131	1.22031
33588	0.946131	1.30091
33589	0.946131	1.32212
33590	2.689 2.1	
33591	2.0815	2.1
33592	2.72786	2.1
33593	2.48295	2.1
33594	5.02397	2.1
33595	2.72786	2.1
33596	1.86424	2.1
33597	2.7188	2.1
33598	2.08981	2.1
33599	4.81768	2.1
33600	2.08982	2.1
33601	0.986987	2.1
33602	2.08982	2.1
33603	1.87249	2.1
33604	1.01964	2.1
33605	0.906115	2.1
33606	1.44247	2.1
33607	1.43872	2.1
33608	1.17054	2.1
33609	1.17054	2.1
33610	1.43872	2.1
33611	1.26045	2.1
33612	2.0815	0.05
33613	2.0815	0.05
33614	2.0815	0.05
33615	1.81468	0.05
33616	1.89065	0.05
33617	1.55625	0.05
33618	1.89062	0.05
33619	1.89224	0.05
33620	1.89224	0.05
33621	2.27073	0.05
33622	1.86856	0.05
33623	2.24232	0.05
33624	1.21419	2.1
33625	2.79716	2.1
33626	2.50005	2.1
33627	2.50005	2.1
33628	1.5008	2.1
33629	1.5008	2.1
33630	1.60139	0.05
33631	1.7103	0.05
33632	1.04159	0.05
33633	1.2489	0.05
33634	2.75189	0.05
33635	0.732812	0.05
33636	0.732812	0.05
33637	0.751986	0.05

33638	0.751986	0.05
33639	0.860346	0.05
33640	0.667961	0.05
33641	0.85515	0.05
33642	0.624452	0.05
33643	0.62445	0.05
33644	0.667962	0.05
33645	0.855148	0.05
33646	0.860348	0.05
33647	1.89226	0.0624411
33648	2.50872	0.0814474
33649	2.77909	0.0672586
33650	1.89226	0.069516
33651	2.77909	0.070504
33652	1.89226	0.0732544
33653	2.15825	0.05
33654	2.50872	0.05
33655	1.90439	0.05
33656	1.90439	0.05
33657	2.77909	0.05
33658	2.77909	0.05
33659	1.12468	2.1
33660	1.43872	2.1
33661	1.64926	2.1
33662	1.2724	2.1
33663	2.77395	2.1
33664	1.64926	2.1
33665	1.38438	2.1
33666	1.47244	2.1
33667	1.2724	2.1
33668	1.8493	2.1
33669	1.64926	2.1
33670	1.36416	0.630615
33671	1.0652	2.1
33672	1.0652	1.19838
33673	1.02923	2.1
33674	1.37496	0.723917
33675	1.33898	2.1
33676	1.89227	2.1
33677	1.90433	2.1
33678	1.90433	2.1
33679	1.89359	2.1
33680	1.89227	2.06363
33681	1.88153	2.1
33682	0.946129	2.1
33683	0.946129	2.08189
33684	0.946133	0.937938
33685	0.946133	0.292105
33686	0.946131	0.306291
33687	0.946131	2.01301
33688	0.946131	2.1
33689	0.946131	0.466942
33690	0.946131	1.44943
33691	0.946131	1.53715
33692	0.946131	1.443
33693	0.946131	1.48829
33694	1.37496	0.826816
33695	1.07519	2.1

33696	1.07519	1.29635
33697	1.0652	2.1
33698	1.39725	0.836202
33699	1.38726	2.1
33700	1.89227	2.1
33701	1.9052	2.1
33702	1.9052	2.1
33703	1.90433	2.1
33704	1.89227	2.1
33705	1.8914	2.1
33706	0.946129	2.1
33707	0.946129	2.1
33708	0.946133	1.16761
33709	0.946133	0.332894
33710	0.946131	0.354173
33711	0.946131	2.1
33712	0.946131	2.1
33713	0.946131	0.541816
33714	0.946131	1.62901
33715	0.946131	1.7891
33716	0.946131	1.61032
33717	0.946131	1.70252
33718	1.91316	0.05
33719	1.89226	0.05
33720	1.89226	0.05
33721	1.89226	0.05
33722	1.99366	0.05
33723	1.99366	0.05
33724	1.93083	0.05
33725	1.89226	0.05
33726	1.51379	0.05
33727	1.89226	0.05
33728	1.53051	0.05
33729	1.91316	0.05
33730	2.16731	2.1
33731	1.21373	2.1
33732	1.47804	2.1
33733	2.37601	2.1
33734	3.64535	2.1
33735	1.7211	2.1
33736	2.37601	2.1
33737	1.47804	2.1
33738	1.47804	2.1
33739	2.56843	2.1
33740	2.56843	2.1
33741	2.58731	2.1
33742	1.59094	2.1
33743	1.59094	2.1
33744	1.76816	2.1
33745	2.41707	2.1
33746	2.5943	2.1
33747	0.718346	2.1
33748	0.795468	2.1
33749	1.20854	2.1
33750	1.1224	2.1
33751	0.961203	2.1
33752	0.961203	2.1
33753	0.95667	2.1

33754	0.95667	2.1
33755	0.718346	2.1
33756	1.1224	2.1
33757	0.795469	2.1
33758	1.20854	2.1
33759	1.84076	2.1
33760	1.42884	2.1
33761	2.24274	2.1
33762	1.85415	2.1
33763	3.69491	2.1
33764	1.89226	2.1
33765	1.89226	2.1
33766	1.89226	2.1
33767	1.89226	2.1
33768	3.78452	2.1
33769	2.24273	2.1
33770	2.24412	2.1
33771	1.85415	2.1
33772	1.85415	2.1
33773	2.68473	2.1
33774	2.68473	2.1
33775	1.89226	2.04377
33776	1.89226	2.1
33777	1.89226	2.1
33778	1.89226	1.81723
33779	1.89226	2.1
33780	1.89226	1.88391
33781	1.89226	1.81276
33782	1.89226	2.1
33783	1.89226	2.1
33784	1.89226	2.1
33785	3.78451	2.1
33786	1.85415	2.1
33787	1.10961	2.1
33788	2.24411	2.1
33789	1.51067	2.1
33790	3.35373	2.1
33791	1.70302	2.1
33792	1.89226	2.1
33793	1.76399	2.1
33794	1.43577	2.1
33795	2.08149	2.1
33796	1.70302	2.1
33797	1.89226	2.1
33798	1.89226	2.1
33799	1.89226	2.1
33800	1.89226	2.1
33801	1.89226	2.1
33802	1.89226	2.1
33803	1.0593	2.1
33804	1.17289	2.1
33805	1.5379	2.1
33806	1.46757	2.1
33807	2.71079	2.1
33808	0.931235	2.1
33809	1.1151	2.1
33810	0.975236	2.1
33811	0.975236	2.1

33812	1.31147	2.1
33813	0.762882	2.1
33814	1.13152	2.1
33815	0.505799	2.1
33816	1.31149	2.1
33817	1.07629	2.1
33818	0.884817	2.1
33819	0.505793	2.1
33820	2.69027	2.1
33821	1.60441	2.1
33822	2.69027	2.1
33823	1.64771	2.1
33824	2.78977	2.1
33825	1.72731	2.1
33826	0.77878	0.05
33827	0.843226	0.05
33828	0.845997	0.05
33829	0.926091	0.05
33830	0.77878	0.05
33831	0.926093	0.05
33832	1.3922	1.35439
33833	1.3922	1.00368
33834	0.872156	2.1
33835	0.872156	2.1
33836	0.872157	2.1
33837	0.946132	0.752004
33838	0.946129	0.607075
33839	1.31822	2.1
33840	0.94613	2.06243
33841	0.94613	2.08605
33842	1.31823	1.8714
33843	0.872157	2.06674
33844	1.81534	0.179474
33845	1.89226	0.151057
33846	1.59602	0.208734
33847	1.59602	0.465333
33848	1.89226	0.05
33849	1.89226	0.0509624
33850	1.89227	0.164039
33851	1.89227	0.212877
33852	1.89227	0.235557
33853	1.89227	0.110766
33854	3.78453	0.103552
33855	1.89226	0.21174
33856	1.89226	0.0604175
33857	1.89226	0.101682
33858	1.89226	0.0688142
33859	3.78453	0.245518
33860	2.30348	0.05
33861	1.4185	0.05
33862	1.82551	0.05
33863	1.02011	0.05
33864	3.24401	0.05
33865	2.46702	0.05
33866	1.17129	0.05
33867	1.82551	0.05
33868	1.82551	0.05
33869	1.73525	0.05

33870	1.73525	0.05
33871	1.89227	0.126609
33872	1.89227	0.0515363
33873	1.89227	0.0515957
33874	1.89227	0.0581004
33875	1.89227	0.10738
33876	1.89227	0.121056
33877	2.80034	2.1
33878	2.45767	2.1
33879	2.67994	2.1
33880	2.35395	2.1
33881	5.1376	2.1
33882	2.45766	2.1
33883	1.8769	2.1
33884	2.00989	2.1
33885	2.00989	2.1
33886	2.35395	2.1
33887	2.35395	2.1
33888	2.57662	2.1
33889	2.35395	2.1
33890	2.57662	2.1
33891	2.67993	2.1
33892	2.24227	2.1
33893	2.34558	2.1
33894	1.02874	2.1
33895	1.02874	2.1
33896	1.03084	2.1
33897	1.03084	2.1
33898	0.938448	2.1
33899	0.886762	2.1
33900	1.17698	2.1
33901	1.12114	2.1
33902	1.12113	2.1
33903	0.886764	2.1
33904	0.93845	2.1
33905	1.17697	2.1
33906	1.91969	2.1
33907	2.5508	2.1
33908	1.88273	2.1
33909	2.76609	2.1
33910	4.68578	2.1
33911	2.83423	2.1
33912	1.89226	2.1
33913	2.76609	2.1
33914	1.89226	2.1
33915	4.65835	2.1
33916	1.56685	0.189341
33917	1.11712	0.0769661
33918	1.07999	1.51063
33919	1.51448	0.389797
33920	2.6316	0.549224
33921	1.56685	0.0542094
33922	1.67652	0.662104
33923	1.5955	0.943246
33924	1.51448	0.370013
33925	3.19099	0.483449
33926	1.89226	1.14957
33927	1.89226	0.05

33928	1.89226	0.378104
33929	1.89226	0.40362
33930	3.78453	0.76494
33931	1.89226	0.368737
33932	1.89226	0.106426
33933	1.89226	0.458134
33934	1.89226	0.688948
33935	1.89226	0.053053
33936	1.89226	0.0540569
33937	1.0562	0.71876
33938	1.51448	0.21262
33939	1.07999	0.377601
33940	1.07999	0.13164
33941	1.46441	0.530868
33942	1.46441	0.258549
33943	1.5955	1.4235
33944	1.51448	0.305148
33945	1.46441	0.345484
33946	1.52909	0.410257
33947	3.04357	0.58816
33948	1.89226	0.297456
33949	1.89226	0.454394
33950	1.89226	0.471462
33951	1.89226	0.0925575
33952	3.78453	0.112634
33953	1.89227	0.2013
33954	1.89227	0.05
33955	1.89227	0.886082
33956	1.89227	0.224704
33957	3.78453	0.440852
33958	1.89994	0.05
33959	1.70303	0.05
33960	1.68576	0.05
33961	1.89608	0.05
33962	3.8237	0.05
33963	1.89994	0.05
33964	2.31116	0.770577
33965	1.9386	0.0923955
33966	1.88653	0.327524
33967	3.74885	0.273898
33968	2.31117	1.56671
33969	2.20665	1.21651
33970	2.09617	0.951841
33971	2.41055	2.1
33972	2.09617	1.0263
33973	2.41055	2.1
33974	1.10777	0.05
33975	1.0523	0.05
33976	0.912098	0.05
33977	0.757533	0.05
33978	0.89287	0.0534642
33979	1.07571	0.05
33980	0.77485	0.05
33981	0.98645	0.05
33982	0.989171	0.05
33983	0.875089	0.05
33984	0.979686	0.05
33985	0.979686	0.0798554

33986	2.52442	2.1
33987	1.49998	2.1
33988	2.51692	2.1
33989	1.56957	2.1
33990	4.09399	2.1
33991	1.01298	2.1
33992	1.01298	2.1
33993	1.03079	2.1
33994	1.03079	2.1
33995	0.820964	2.1
33996	0.784786	2.1
33997	1.25898	2.1
33998	1.25846	2.1
33999	1.25898	2.1
34000	0.820964	2.1
34001	1.25846	2.1
34002	0.784786	2.1
34003	1.89226	0.05
34004	1.89226	0.05
34005	2.6297	0.05
34006	1.89226	0.05
34007	3.78452	0.05
34008	1.89226	0.05
34009	1.89226	0.05
34010	1.89226	0.05
34011	1.89226	0.05
34012	1.89226	0.05
34013	1.89226	0.05
34014	2.19397	0.05
34015	1.89226	0.05
34016	2.34112	0.05
34017	2.34112	0.05
34018	1.89226	0.05
34019	1.89226	0.05
34020	2.23366	2.1
34021	1.87598	2.1
34022	1.64716	2.1
34023	2.06048	2.1
34024	3.93645	2.1
34025	2.23367	2.1
34026	2.17384	2.1
34027	2.04818	2.1
34028	2.06048	2.1
34029	4.23432	2.1
34030	1.9855	2.1
34031	2.06048	2.1
34032	1.93163	2.1
34033	1.50737	2.1
34034	3.56785	2.1
34035	2.04818	2.1
34036	2.06048	2.1
34037	2.27465	2.1
34038	1.92264	2.1
34039	3.98312	2.1
34040	2.08149	0.0784109
34041	1.89226	1.62476
34042	1.89226	0.918135
34043	1.33153	0.0653076

34044	1.68038	1.97451
34045	1.18244	0.132515
34046	1.89226	0.478875
34047	1.89226	1.87716
34048	1.89226	1.64891
34049	1.89226	0.183519
34050	3.78452	0.60552
34051	2.69069	0.0712939
34052	1.89226	0.253477
34053	1.89226	0.330459
34054	1.97397	0.0674425
34055	1.89226	0.293523
34056	1.97397	0.0614953
34057	1.89226	1.90185
34058	2.38056	0.162239
34059	1.89226	0.193568
34060	1.89226	0.993487
34061	3.78452	0.672316
34062	0.904435	2.1
34063	0.904435	2.1
34064	0.925315	2.1
34065	0.925315	2.1
34066	1.13312	2.1
34067	1.08109	2.1
34068	0.70697	2.1
34069	0.696633	2.1
34070	0.70697	2.1
34071	1.13312	2.1
34072	0.696633	2.1
34073	1.08109	2.1
34074	1.45506	2.1
34075	2.26623	2.1
34076	2.38363	2.1
34077	2.38363	2.1
34078	1.41394	2.1
34079	1.41394	2.1
34080	0.946131	2.1
34081	0.946131	2.1
34082	1.13311	2.1
34083	1.13311	2.1
34084	0.946127	2.1
34085	0.946127	2.1
34086	1.13312	2.1
34087	1.19182	2.1
34088	1.19181	2.1
34089	0.946129	2.1
34090	1.13312	2.1
34091	0.94613	2.1
34092	1.89226	0.172532
34093	1.89226	2.07836
34094	1.89226	0.562818
34095	1.89226	2.03069
34096	1.89226	0.494701
34097	1.89226	2.00529
34098	1.05168	2.1
34099	0.696527	2.1
34100	0.907442	0.619535
34101	0.907442	1.03573

34102	0.953145	0.346509
34103	0.782285	2.1
34104	0.773928	2.1
34105	0.773928	0.636475
34106	0.696538	0.960764
34107	0.851554	1.20202
34108	1.04071	1.30154
34109	1.12335	1.30466
34110	1.89226	0.520467
34111	1.89226	0.106957
34112	1.89226	0.140399
34113	1.89226	0.05
34114	3.78453	0.0609749
34115	1.89226	0.459764
34116	1.89226	0.102428
34117	1.89226	0.192954
34118	1.89226	0.0739424
34119	3.78453	0.316301
34120	2.50912	0.0873032
34121	1.89226	0.0527312
34122	1.89226	0.0645767
34123	2.63195	0.05
34124	4.40139	0.05
34125	2.50912	0.0901919
34126	1.3598	0.05
34127	1.70303	0.05
34128	2.36874	0.05
34129	3.82055	0.0523645
34130	1.89227	0.134839
34131	1.89227	0.05
34132	1.89227	0.05
34133	1.89227	0.05
34134	3.78453	0.0899868
34135	1.89227	0.18477
34136	1.89227	0.0641431
34137	1.89227	0.0563945
34138	1.89227	0.05
34139	3.78453	0.05
34140	1.89226	0.0553531
34141	2.67088	0.05
34142	2.63195	0.05
34143	2.63195	0.05
34144	1.89226	0.05
34145	1.89226	0.0569117
34146	1.30202	0.05
34147	1.28304	0.05
34148	0.809902	0.05
34149	0.888801	0.05
34150	1.13262	0.05
34151	0.968276	0.05
34152	1.0733	0.05
34153	1.0733	0.05
34154	1.35419	0.05
34155	0.801222	0.05
34156	0.899732	0.05
34157	1.23967	0.05
34158	1.31595	2.1
34159	0.869501	2.1

34160	2.65473	2.1
34161	1.88782	2.1
34162	3.20377	2.1
34163	1.89227	2.1
34164	1.89227	2.1
34165	1.89227	2.1
34166	1.89227	2.1
34167	3.78453	2.1
34168	2.65473	2.1
34169	1.89226	2.1
34170	1.88781	2.1
34171	1.88782	2.1
34172	2.0815	2.1
34173	2.0815	2.1
34174	1.89226	2.1
34175	1.89226	2.1
34176	1.89226	2.1
34177	1.89226	2.1
34178	1.89226	2.1
34179	1.89226	2.1
34180	1.89226	2.1
34181	1.89226	2.1
34182	1.89226	2.1
34183	1.89226	2.1
34184	1.89226	2.1
34185	1.89226	2.1
34186	2.39057	2.1
34187	1.89226	1.02195
34188	1.89226	0.288892
34189	1.89226	2.1
34190	2.14243	0.199715
34191	2.14243	2.1
34192	1.89226	0.0648464
34193	1.89226	2.00874
34194	1.89226	0.264866
34195	1.89226	0.443659
34196	1.89226	0.767228
34197	1.89226	2.0015
34198	1.89226	0.0756263
34199	1.89226	2.1
34200	1.89226	0.0955674
34201	1.89226	0.331375
34202	1.89226	0.190479
34203	1.89226	1.90046
34204	2.56323	0.05
34205	1.36324	0.05
34206	1.05296	0.05
34207	2.18048	0.05
34208	3.54372	0.05
34209	0.946128	2.1
34210	0.946128	2.1
34211	1.08733	2.1
34212	1.08733	2.1
34213	0.946131	2.1
34214	0.946131	2.1
34215	1.09197	2.1
34216	1.08732	2.1
34217	0.946131	2.1

34218	1.09197	2.1
34219	1.08732	2.1
34220	0.946131	2.1
34221	1.35111	0.958712
34222	1.51327	0.621587
34223	1.64493	0.636866
34224	1.31058	0.917569
34225	2.99605	1.22772
34226	1.8124	0.547053
34227	1.51327	0.885082
34228	1.64493	1.12254
34229	1.31358	0.539616
34230	1.8124	0.979901
34231	1.48105	0.417171
34232	1.64493	0.687238
34233	1.31058	0.708252
34234	1.22297	0.605026
34235	1.70101	0.581857
34236	2.8679	0.411743
34237	1.70101	0.764808
34238	1.99297	0.535894
34239	1.64493	0.935936
34240	1.64493	0.398298
34241	1.99297	0.965243
34242	1.8124	0.266581
34243	2.0815	2.1
34244	1.80258	2.1
34245	1.80258	2.1
34246	1.83506	2.1
34247	2.0815	2.1
34248	2.1212	2.1
34249	2.0815	2.1
34250	1.82129	2.1
34251	1.82129	2.1
34252	1.80445	2.1
34253	2.0815	2.1
34254	2.06092	2.1
34255	1.89224	2.1
34256	1.703 2.1	
34257	1.97839	2.1
34258	1.703 2.1	
34259	1.97402	2.1
34260	1.703 2.1	
34261	1.89226	0.724212
34262	1.89226	1.22787
34263	1.89226	0.279999
34264	2.75351	0.697786
34265	3.78453	0.844161
34266	1.89226	0.567764
34267	3.02887	1.21278
34268	2.03149	0.233259
34269	2.03149	0.454084
34270	1.89226	0.932581
34271	1.89226	1.61459
34272	2.17979	2.1
34273	1.65533	2.1
34274	2.57212	2.1
34275	2.20346	2.1

34276	4.22745	2.1
34277	1.89227	2.1
34278	2.20347	2.1
34279	1.89227	2.1
34280	2.57212	2.1
34281	1.89227	2.1
34282	2.57212	2.1
34283	2.05274	0.888551
34284	2.16783	2.1
34285	2.24429	0.48043
34286	2.38404	2.1
34287	4.41212	2.1
34288	2.16783	2.1
34289	2.05967	2.1
34290	2.38404	2.1
34291	2.24695	2.1
34292	4.41478	2.1
34293	2.24429	0.301368
34294	2.38404	2.1
34295	2.51819	0.696472
34296	2.60723	2.1
34297	4.85152	2.1
34298	2.38404	2.1
34299	2.24695	2.1
34300	2.60723	2.1
34301	2.45247	2.1
34302	4.85418	2.1
34303	1.89227	2.1
34304	1.89227	2.1
34305	1.89227	2.1
34306	1.89227	2.1
34307	3.78453	2.1
34308	1.89226	2.1
34309	1.89226	2.1
34310	1.89226	2.1
34311	1.89226	2.1
34312	3.78453	2.1
34313	0.618473	2.1
34314	0.619945	2.1
34315	0.797692	2.1
34316	0.770147	2.1
34317	0.68852	2.1
34318	0.68852	2.1
34319	0.701573	2.1
34320	0.701573	2.1
34321	0.619945	2.1
34322	0.797691	2.1
34323	0.618473	2.1
34324	0.770147	2.1
34325	0.797692	2.1
34326	0.770148	2.1
34327	0.694787	2.1
34328	0.653411	2.1
34329	0.722102	2.1
34330	0.722102	2.1
34331	0.729001	2.1
34332	0.729001	2.1
34333	0.797692	2.1

34334	0.694787	2.1
34335	0.770148	2.1
34336	0.653412	2.1
34337	1.89227	2.1
34338	1.89227	2.1
34339	1.89227	2.1
34340	1.89227	2.1
34341	3.78453	2.1
34342	1.89226	2.1
34343	1.89226	2.1
34344	1.89226	2.1
34345	1.89226	2.1
34346	3.78453	2.1
34347	1.23989	2.1
34348	1.59538	2.1
34349	1.66647	2.1
34350	1.26599	2.1
34351	2.90636	2.1
34352	1.66647	2.1
34353	1.38957	2.1
34354	1.47446	2.1
34355	1.40337	2.1
34356	1.66647	2.1
34357	1.59538	2.1
34358	2.80619	2.1
34359	2.0815	2.1
34360	2.0815	2.1
34361	2.0815	2.1
34362	2.73721	2.1
34363	2.74192	2.1
34364	2.73721	2.1
34365	2.0815	2.1
34366	2.0815	2.1
34367	2.0815	2.1
34368	2.689	2.1
34369	2.69166	2.1
34370	2.62369	2.1
34371	2.19717	2.1
34372	2.60405	2.1
34373	2.20566	2.1
34374	4.80122	2.1
34375	2.19717	2.1
34376	1.37392	2.1
34377	2.20566	2.1
34378	1.45408	2.1
34379	3.65125	2.1
34380	2.60405	2.1
34381	2.20566	2.1
34382	2.57466	2.1
34383	2.21715	2.1
34384	4.8212	2.1
34385	2.20566	2.1
34386	1.54249	2.1
34387	1.54249	2.1
34388	1.45408	2.1
34389	2.21715	2.1
34390	2.12874	2.1
34391	1.60647	2.1

34392	1.31346	2.1
34393	1.82649	2.1
34394	1.51067	2.1
34395	3.13995	2.1
34396	1.89226	2.1
34397	1.89226	2.1
34398	1.89226	2.1
34399	1.89226	2.1
34400	3.78452	2.1
34401	1.89226	2.1
34402	2.6096	2.1
34403	2.8067	2.1
34404	2.8067	2.1
34405	1.89226	2.1
34406	1.89226	2.1
34407	1.82649	2.09985
34408	1.3596	2.1
34409	2.17708	2.1
34410	1.70302	2.1
34411	3.40243	2.1
34412	1.89226	2.09732
34413	1.89226	2.1
34414	1.89226	1.35813
34415	1.89226	2.1
34416	3.78452	2.1
34417	2.27067	2.1
34418	2.43549	2.1
34419	2.63979	2.1
34420	2.41828	2.1
34421	2.27067	2.1
34422	2.04916	2.1
34423	1.30047	2.1
34424	2.04916	2.1
34425	2.27068	2.1
34426	1.5586	2.1
34427	3.57115	2.1
34428	2.27068	2.1
34429	1.84214	2.1
34430	1.80224	2.1
34431	1.5586	2.1
34432	2.51432	2.1
34433	2.27068	2.1
34434	1.63719	2.1
34435	2.15714	2.1
34436	2.07821	2.1
34437	1.52204	2.1
34438	3.67919	2.1
34439	2.07821	2.1
34440	1.40118	2.1
34441	1.40118	2.1
34442	1.52204	2.1
34443	1.99632	2.1
34444	2.11718	2.1
34445	0.946131	2.1
34446	0.946131	2.1
34447	1.06957	2.1
34448	1.06957	2.1
34449	0.946132	2.1

34450	0.946132	1.44044
34451	1.411	2.1
34452	1.06957	2.1
34453	0.946132	2.1
34454	1.411	2.1
34455	1.06957	2.1
34456	0.946132	2.1
34457	1.99875	0.575369
34458	2.57079	0.31895
34459	2.59319	1.31203
34460	1.9105	0.107595
34461	4.59194	0.530377
34462	1.99875	0.482346
34463	1.02247	0.05
34464	1.07017	0.0788143
34465	1.15841	0.0837848
34466	1.91051	0.0622214
34467	1.99875	0.0910451
34468	2.59319	1.21332
34469	1.9105	0.138931
34470	2.33812	0.38831
34471	2.59319	0.392121
34472	1.59457	0.603764
34473	1.59457	0.624676
34474	2.50906	0.413198
34475	2.0467	0.125753
34476	2.03357	0.2134
34477	2.53176	0.109257
34478	4.54263	0.165666
34479	1.294	0.385238
34480	1.66636	0.231509
34481	2.03357	0.16133
34482	1.03079	0.0852269
34483	3.32757	0.216144
34484	1.68108	0.157759
34485	2.53176	0.0934968
34486	2.36417	0.0727718
34487	2.71666	0.0650292
34488	1.68108	0.473857
34489	2.03357	0.286564
34490	1.89226	0.05
34491	2.59302	0.05
34492	2.2727	0.05
34493	2.2727	0.05
34494	1.89226	0.0514522
34495	1.89226	0.05
34496	1.89226	0.0629914
34497	1.89226	0.05
34498	1.89226	0.05
34499	1.89226	0.05
34500	3.78452	0.0597531
34501	1.89225	0.114707
34502	1.89225	0.05
34503	1.89225	0.05
34504	1.89225	0.05
34505	1.89225	0.05
34506	1.89225	0.05
34507	0.934114	0.05

34508	0.934114	0.05
34509	0.944544	0.05
34510	0.944544	0.05
34511	0.721493	0.05
34512	0.58215	0.05
34513	1.2965	0.05
34514	1.13634	0.05
34515	1.13635	0.05
34516	0.582148	0.05
34517	1.29651	0.05
34518	0.721491	0.05
34519	1.89226	0.05
34520	1.89226	0.05
34521	1.89226	0.05
34522	1.89226	0.05
34523	3.78452	0.05
34524	0.946131	0.05
34525	0.946131	0.05
34526	0.946131	0.05
34527	0.946131	0.05
34528	0.946131	0.05
34529	0.946131	0.05
34530	0.946131	0.05
34531	0.946131	0.05
34532	0.946131	0.05
34533	0.946131	0.05
34534	0.946131	0.05
34535	0.946131	0.05
34536	1.89226	0.05
34537	1.89226	0.05
34538	2.78602	0.05
34539	1.89226	0.05
34540	3.78453	0.05
34541	1.52601	2.1
34542	1.76601	2.1
34543	1.27839	2.1
34544	1.56095	2.1
34545	3.0444	2.1
34546	1.76601	2.1
34547	1.86793	2.1
34548	1.56095	2.1
34549	1.68152	2.1
34550	3.44753	2.1
34551	1.27841	2.1
34552	1.56097	2.1
34553	1.0577	2.1
34554	1.36861	2.1
34555	2.64702	2.1
34556	1.56097	2.1
34557	1.68154	2.1
34558	1.36861	2.1
34559	1.53296	2.1
34560	3.05015	2.1
34561	1.89226	2.1
34562	1.89226	2.1
34563	1.89226	2.1
34564	1.89226	2.1
34565	3.78453	2.1

34566	0.983075	2.1
34567	0.788361	2.1
34568	0.724726	2.1
34569	0.724726	2.1
34570	0.983027	2.1
34571	0.724734	2.1
34572	0.788364	2.1
34573	0.788364	2.1
34574	0.788335	2.1
34575	0.94613	2.1
34576	0.724757	2.1
34577	0.946131	2.1
34578	1.15789	2.1
34579	2.11135	2.1
34580	2.25826	2.1
34581	1.28845	2.1
34582	3.41615	2.1
34583	1.89226	2.1
34584	1.89226	2.1
34585	1.89226	2.1
34586	1.89226	2.1
34587	3.78453	2.1
34588	1.57667	2.1
34589	1.70002	2.1
34590	1.70002	2.1
34591	1.89226	2.1
34592	1.89226	2.1
34593	2.05244	2.1
34594	2.25826	2.1
34595	1.42214	2.1
34596	1.43675	2.1
34597	1.28845	2.1
34598	2.40656	2.1
34599	2.25826	2.1
34600	1.58066	2.1
34601	1.74247	2.1
34602	1.85097	2.1
34603	1.61155	2.1
34604	3.43163	2.1
34605	0.785612	2.1
34606	0.789638	2.1
34607	0.882561	2.1
34608	0.763529	2.1
34609	0.686921	2.1
34610	0.86048	2.1
34611	0.871812	2.1
34612	0.871812	2.1
34613	0.830183	2.1
34614	0.882572	2.1
34615	0.902315	2.1
34616	0.785602	2.1
34617	1.01972	0.05
34618	0.936657	0.05
34619	0.946131	0.05
34620	0.946131	0.05
34621	0.927018	0.05
34622	0.927018	0.05
34623	1.1376	0.05

34624	0.953663	0.05
34625	1.1257	0.05
34626	1.04073	0.05
34627	1.00358	0.05
34628	0.851528	0.05
34629	1.73576	0.0534857
34630	2.00244	0.227662
34631	1.17798	0.0838305
34632	1.25796	0.05
34633	2.99373	0.05
34634	2.00244	0.203899
34635	1.10719	0.0778113
34636	2.03236	0.348987
34637	2.03236	0.140231
34638	1.25797	0.240433
34639	1.25797	0.0700301
34640	1.89226	2.1
34641	1.89226	2.1
34642	1.89226	2.1
34643	1.89226	2.1
34644	3.78452	2.1
34645	2.3174	2.1
34646	1.50005	2.1
34647	1.90428	2.1
34648	2.64564	2.1
34649	4.22168	2.1
34650	1.89226	2.1
34651	1.89226	2.1
34652	1.89226	2.1
34653	1.89226	2.1
34654	1.89226	2.1
34655	1.89226	2.1
34656	1.32749	0.05
34657	1.70302	0.05
34658	2.08149	0.05
34659	1.70302	0.05
34660	2.19634	0.05
34661	1.93083	0.05
34662	1.89226	1.55525
34663	1.89226	2.1
34664	1.89226	0.279918
34665	1.89226	2.1
34666	3.78453	0.931652
34667	1.89226	1.41029
34668	1.89226	2.09909
34669	1.89226	0.727061
34670	1.89226	1.61137
34671	3.78452	1.96594
34672	2.17499	0.852969
34673	1.79663	2.01787
34674	2.1538	0.141234
34675	1.818	0.730519
34676	2.17499	1.77346
34677	1.83919	2.1
34678	0.898316	2.1
34679	1.0769	1.11062
34680	0.968276	2.1
34681	0.773495	1.65126

34682	0.921126	2.02741
34683	0.921126	2.1
34684	0.92927	1.89826
34685	0.92927	1.92771
34686	1.0769	1.83797
34687	0.968276	1.21332
34688	0.898316	2.1
34689	0.773495	1.56797
34690	1.89226	0.792325
34691	1.89226	2.1
34692	1.89226	1.30969
34693	1.89226	1.16042
34694	3.78453	2.1
34695	1.89226	1.47283
34696	1.89226	2.1
34697	1.89226	0.435973
34698	1.89226	1.82544
34699	3.78452	1.12424
34700	2.17499	1.96842
34701	2.1538	2.1
34702	2.53574	0.992555
34703	2.54157	0.567862
34704	4.71073	1.97417
34705	1.0769	1.73214
34706	1.26787	1.10826
34707	1.16641	1.42303
34708	0.968276	2.1
34709	1.11625	1.4578
34710	1.11625	1.89494
34711	1.11989	2.08848
34712	1.11989	0.420647
34713	1.26787	1.25655
34714	1.16641	2.1
34715	1.0769	2.1
34716	0.968277	1.95895
34717	1.89226	0.0702461
34718	1.89226	0.060482
34719	1.89226	0.0724375
34720	1.89226	0.0641671
34721	3.78452	0.0741317
34722	1.91739	0.05
34723	2.20045	0.05
34724	1.79005	0.0647739
34725	2.33802	0.0525093
34726	4.25539	0.05
34727	2.52754	0.05
34728	1.89226	0.0512255
34729	2.69841	0.05
34730	2.69841	0.05
34731	1.89226	0.05
34732	1.89226	0.05
34733	1.89226	0.093261
34734	1.89226	0.0929331
34735	1.89226	0.0832262
34736	1.89226	0.278053
34737	3.78452	0.0907509
34738	1.93544	0.0953071
34739	2.33802	0.0740214

34740	1.55063	0.05
34741	2.1769	0.0564898
34742	3.88865	0.105911
34743	2.20929	0.05
34744	2.55162	0.05
34745	2.27807	0.05
34746	1.48831	0.05
34747	4.48736	0.05
34748	2.27807	0.05
34749	2.72723	0.05
34750	2.55162	0.05
34751	2.55162	0.05
34752	2.43157	0.05
34753	2.43157	0.05
34754	2.27807	0.05
34755	1.51612	0.05
34756	1.96048	0.05
34757	1.73022	0.05
34758	1.70302	0.05
34759	1.61016	0.05
34760	2.27807	0.05
34761	2.08048	0.05
34762	1.98522	0.05
34763	2.43157	0.05
34764	1.83798	0.05
34765	2.27807	0.05
34766	1.70302	0.05
34767	2.68507	0.05
34768	2.23856	0.05
34769	2.52174	0.05
34770	2.35331	0.05
34771	2.65099	0.05
34772	1.70303	0.05
34773	0.986848	0.05
34774	2.41566	0.05
34775	1.70303	0.05
34776	1.88139	0.05
34777	1.64955	0.05
34778	1.89226	2.1
34779	2.34978	1.7775
34780	1.89226	2.1
34781	2.68773	1.31534
34782	1.89226	2.1
34783	2.68773	1.7762
34784	1.89226	2.1
34785	1.89226	1.35133
34786	1.89226	1.63552
34787	1.89226	2.1
34788	3.78453	2.1
34789	1.00825	0.451108
34790	1.09847	0.720893
34791	0.939987	0.651421
34792	0.783516	1.96173
34793	0.838041	1.17561
34794	0.838041	1.93443
34795	1.49693	2.06713
34796	1.26602	1.20385
34797	1.10875	0.498666

34798	1.05187	2.1
34799	1.28743	1.38937
34800	0.946132	0.725496
34801	0.946132	1.45651
34802	0.946132	2.1
34803	0.946132	2.1
34804	0.946132	0.508487
34805	1.31823	2.1
34806	0.946132	1.34774
34807	0.946132	2.1
34808	0.946131	2.1
34809	0.946131	2.1
34810	0.946132	1.95774
34811	1.31823	2.1
34812	1.51688	0.05
34813	1.79978	0.0714206
34814	1.1685	0.05
34815	1.1685	0.05
34816	1.85857	0.05
34817	1.51018	0.0622291
34818	0.946116	0.05
34819	0.946116	0.05
34820	0.94612	0.05
34821	0.94612	0.05
34822	0.946118	0.05
34823	0.946118	0.05
34824	0.946118	0.05
34825	0.946118	0.0603011
34826	0.946131	0.05
34827	0.946131	0.0553559
34828	0.946105	0.05
34829	0.946105	0.05
34830	1.89224	2.1
34831	1.89224	2.1
34832	1.89224	2.1
34833	1.89224	2.1
34834	1.89224	2.1
34835	1.89224	2.1
34836	1.38678	2.1
34837	1.3349	2.1
34838	0.946119	2.1
34839	0.946119	2.1
34840	1.38678	2.1
34841	1.38678	2.1
34842	0.946119	2.1
34843	0.946119	2.1
34844	1.38676	2.1
34845	0.946107	2.1
34846	0.946132	2.1
34847	1.33491	2.1
34848	1.06455	2.1
34849	1.03932	2.1
34850	1.20649	2.1
34851	1.18773	2.1
34852	2.27105	2.1
34853	1.89226	2.1
34854	1.89226	2.1
34855	1.89226	2.1

34856	1.89226	2.1
34857	1.89226	2.1
34858	1.89226	2.1
34859	1.36918	2.1
34860	1.18774	2.1
34861	1.31144	2.1
34862	1.20651	2.1
34863	1.31144	2.1
34864	1.20651	2.1
34865	2.29066	2.1
34866	1.76476	2.1
34867	1.79825	2.1
34868	2.33801	2.1
34869	4.08891	2.1
34870	1.79825	2.1
34871	2.33801	2.1
34872	2.37168	2.1
34873	1.49325	2.1
34874	4.16993	2.1
34875	1.13641	2.1
34876	1.43213	0.63878
34877	1.44085	1.95525
34878	1.18301	2.1
34879	2.61513	2.1
34880	1.89226	2.1
34881	2.23875	2.1
34882	2.23875	2.1
34883	2.10287	2.1
34884	1.89226	2.1
34885	1.75639	2.1
34886	1.89226	0.978618
34887	1.89226	2.1
34888	1.89226	1.25884
34889	1.89226	2.1
34890	1.89226	0.822027
34891	1.89226	2.1
34892	1.44085	2.1
34893	1.25173	2.1
34894	1.25173	1.7681
34895	1.18301	2.1
34896	1.4617	1.34275
34897	1.39298	2.1
34898	1.89227	2.1
34899	2.23875	2.1
34900	2.42504	2.1
34901	1.89227	2.1
34902	4.13101	2.1
34903	1.89226	0.853194
34904	1.89226	2.0594
34905	1.89226	0.686298
34906	1.89226	2.1
34907	1.89226	0.470906
34908	1.89226	2.1
34909	1.89226	2.1
34910	2.41297	2.1
34911	2.70685	2.1
34912	2.70685	2.1
34913	1.89226	2.1

34914	1.89226	2.1
34915	1.89226	2.1
34916	1.89226	2.1
34917	1.89226	2.1
34918	1.89226	2.1
34919	1.89226	2.1
34920	1.89226	2.1
34921	1.89226	2.1
34922	1.89226	2.1
34923	1.89226	2.1
34924	1.89226	2.1
34925	3.78453	2.1
34926	1.89226	2.1
34927	1.89226	2.1
34928	1.89226	2.1
34929	1.89226	2.1
34930	1.89226	2.1
34931	1.89226	2.1
34932	1.69044	0.05
34933	1.57979	0.05
34934	2.27963	0.05
34935	2.21582	0.05
34936	3.85942	0.05
34937	2.21582	0.05
34938	1.30722	0.05
34939	2.02069	0.05
34940	2.02069	0.05
34941	1.57979	0.05
34942	1.57979	0.05
34943	1.67825	2.1
34944	1.89226	2.1
34945	1.89226	2.1
34946	1.89226	2.1
34947	1.91894	2.1
34948	1.91894	2.1
34949	0.946133	2.1
34950	0.946133	2.1
34951	0.946132	2.1
34952	0.946132	2.1
34953	0.946132	2.1
34954	0.946132	2.1
34955	0.946133	2.1
34956	0.946133	2.1
34957	0.946133	2.1
34958	0.946133	2.1
34959	0.946132	2.1
34960	0.946132	2.1
34961	0.942609	2.1
34962	0.942609	2.1
34963	1.329 2.1	
34964	1.329 2.1	
34965	1.32547	2.1
34966	0.94261	2.1
34967	0.946132	2.1
34968	0.946132	2.1
34969	0.946132	2.1
34970	1.32547	2.1
34971	0.946132	2.1

34972	0.94261	2.1
34973	1.55831	2.1
34974	1.07496	2.1
34975	1.80327	0.212126
34976	1.34851	0.537044
34977	2.87824	0.957591
34978	2.14346	0.735057
34979	2.77937	0.143238
34980	2.79173	1.01627
34981	2.17129	0.0909784
34982	4.95066	0.0852391
34983	2.14346	0.949433
34984	1.34124	0.05
34985	1.39818	0.685871
34986	2.1713	0.146522
34987	3.51253	0.526768
34988	2.79173	0.606554
34989	2.21882	0.0583109
34990	2.17129	0.118579
34991	2.17129	0.126743
34992	2.81448	0.228868
34993	2.81448	0.244586
34994	1.10941	0.0960873
34995	1.08565	0.121173
34996	0.699089	0.0711773
34997	0.740176	0.379266
34998	0.908594	0.0529504
34999	0.908594	0.0643237
35000	0.91723	0.8108
35001	0.91723	0.109196
35002	1.10941	0.113683
35003	0.740176	0.0826655
35004	0.699089	0.0936416
35005	1.08565	0.0566398
35006	1.89227	0.148434
35007	1.89227	0.05
35008	1.89227	0.05
35009	1.89227	0.05
35010	3.78453	0.05
35011	2.40032	0.0606774
35012	2.19526	0.05
35013	1.12907	0.05
35014	1.44253	0.05
35015	3.32433	0.05
35016	2.19526	0.05
35017	2.71093	0.05
35018	1.29826	0.05
35019	2.0815	0.05
35020	4.18911	0.05
35021	1.89226	0.05
35022	1.89226	0.05
35023	1.89226	0.05
35024	1.89226	0.05
35025	3.78452	0.05
35026	2.30822	0.05
35027	1.89226	0.05
35028	2.79329	0.05
35029	2.79329	0.05

35030	1.89226	0.05
35031	1.89226	0.05
35032	0.946131	2.1
35033	0.946131	0.533102
35034	1.38414	2.1
35035	1.38414	0.0504258
35036	0.94613	2.1
35037	0.94613	2.1
35038	1.38415	0.648611
35039	1.41833	0.0868777
35040	0.946132	1.75269
35041	1.41833	1.50744
35042	1.38414	1.2047
35043	0.946131	1.88248
35044	1.89226	2.1
35045	2.6536	2.1
35046	1.89226	2.1
35047	2.72238	2.1
35048	4.61465	2.1
35049	0.946132	2.1
35050	0.946132	2.1
35051	1.38657	2.1
35052	1.38657	2.1
35053	1.38657	2.1
35054	1.36119	2.1
35055	0.946131	1.99716
35056	0.946131	2.1
35057	0.946132	2.1
35058	1.38657	2.1
35059	0.946132	2.1
35060	1.36119	2.1
35061	1.89226	2.1
35062	2.4717	2.1
35063	2.5026	2.1
35064	2.4717	2.1
35065	1.92316	2.1
35066	1.89226	2.1
35067	1.89226	2.1
35068	1.89226	2.1
35069	1.89226	2.1
35070	1.89226	2.01816
35071	3.78453	2.1
35072	0.946128	2.1
35073	0.946128	1.94393
35074	1.3419	2.1
35075	1.3419	2.1
35076	0.946131	2.1
35077	0.946131	2.1
35078	1.36607	2.1
35079	1.3419	2.1
35080	0.946131	2.1
35081	1.36607	2.1
35082	1.3419	2.1
35083	0.94613	2.1
35084	1.89226	2.1
35085	2.50259	2.1
35086	2.53563	2.1
35087	2.50259	2.1

35088	1.92529	2.1
35089	1.89226	2.1
35090	0.946132	2.1
35091	0.946132	2.1
35092	0.946131	2.1
35093	0.946131	2.1
35094	0.946132	2.1
35095	0.946132	2.1
35096	0.946131	2.1
35097	0.946131	2.1
35098	0.946131	2.1
35099	0.946131	2.1
35100	0.946131	2.1
35101	0.946131	2.1
35102	2.0815	2.1
35103	1.86506	2.1
35104	1.86506	2.1
35105	1.82567	2.1
35106	2.0815	2.1
35107	2.03335	2.1
35108	2.0815	2.1
35109	2.0815	2.1
35110	2.0815	2.1
35111	1.88668	2.1
35112	1.83221	2.1
35113	1.57275	2.1
35114	2.34061	0.05
35115	1.89226	0.05
35116	2.23254	0.05
35117	1.89226	0.05
35118	2.23254	0.05
35119	1.89226	0.05
35120	1.8619	0.05
35121	1.89226	0.05
35122	2.34061	0.05
35123	1.89226	0.05
35124	2.34061	0.05
35125	1.89226	0.05
35126	1.89227	0.05
35127	1.89227	0.05
35128	1.89227	0.05
35129	1.89227	0.05
35130	1.89227	0.05
35131	1.89227	0.05
35132	1.89227	0.05
35133	1.89227	0.05
35134	1.89227	0.05
35135	1.89227	0.05
35136	1.89227	0.05
35137	1.89227	0.05
35138	1.06365	0.05
35139	0.503305	0.05
35140	0.794912	0.05
35141	1.42752	0.05
35142	1.34826	0.05
35143	1.07163	0.05
35144	0.533113	0.05
35145	0.761277	0.05

35146	0.848403	0.05
35147	0.876191	0.05
35148	0.963372	0.05
35149	0.963372	0.05
35150	1.55571	0.05
35151	2.76574	0.05
35152	2.92453	0.05
35153	1.70301	0.05
35154	2.33089	0.05
35155	1.06905	0.05
35156	2.28979	2.1
35157	1.81488	2.1
35158	1.63722	2.1
35159	2.22627	2.1
35160	4.04115	2.1
35161	1.98246	2.1
35162	2.15715	2.1
35163	2.22627	2.1
35164	2.22627	2.1
35165	1.63719	2.1
35166	1.63719	2.1
35167	2.1644	0.05
35168	1.903 0.05	
35169	1.23639	0.05
35170	1.42704	0.05
35171	3.61546	0.05
35172	1.89226	0.05
35173	1.89226	0.05
35174	1.89226	0.05
35175	1.89226	0.05
35176	3.78451	0.05
35177	2.61343	0.05
35178	1.89226	0.05
35179	2.08149	0.05
35180	1.74357	0.05
35181	2.08149	0.05
35182	1.74357	0.05
35183	1.63483	0.05
35184	1.8631	0.05
35185	1.8631	0.05
35186	1.70301	0.05
35187	2.0815	0.05
35188	1.94319	0.05
35189	2.0815	0.05
35190	1.89226	0.05
35191	1.89226	0.05
35192	2.05236	0.05
35193	2.23014	0.05
35194	2.41882	0.05
35195	2.14993	2.1
35196	1.87217	2.1
35197	2.34488	2.1
35198	2.5254	2.1
35199	4.39757	2.1
35200	0.949995	2.1
35201	0.630972	2.1
35202	0.936084	2.1
35203	1.17244	2.1

35204	0.922394	2.1
35205	0.922394	2.1
35206	0.88102	2.1
35207	0.88102	2.1
35208	0.949998	2.1
35209	1.17244	2.1
35210	0.936081	2.1
35211	0.630973	2.1
35212	1.89226	2.1
35213	1.89226	2.1
35214	1.89226	2.1
35215	1.89226	2.1
35216	3.78452	2.1
35217	1.89226	1.96571
35218	1.89226	2.1
35219	1.89226	1.94332
35220	1.89226	2.1
35221	1.89226	1.87046
35222	1.89226	2.1
35223	2.34488	2.1
35224	2.5254	1.97979
35225	1.89226	1.97031
35226	1.89226	2.1
35227	4.41766	2.1
35228	1.89226	2.1
35229	1.89226	2.1
35230	1.89226	2.1
35231	1.89226	2.1
35232	3.78452	2.1
35233	1.89226	1.71673
35234	1.89226	2.1
35235	1.89226	1.72988
35236	1.89226	2.0307
35237	1.89226	1.77859
35238	1.89226	2.05493
35239	1.5605	2.1
35240	1.15806	2.1
35241	1.31565	2.1
35242	1.65642	2.1
35243	2.87615	2.1
35244	1.46643	2.1
35245	1.65642	2.1
35246	1.31565	2.1
35247	1.31565	2.1
35248	1.74246	2.1
35249	1.74246	2.1
35250	1.89226	2.1
35251	1.89226	2.1
35252	1.89226	2.1
35253	1.89226	2.1
35254	1.89226	2.1
35255	1.89226	2.1
35256	1.15099	2.1
35257	1.15099	2.1
35258	0.939316	2.1
35259	0.939316	2.1
35260	0.946127	2.1
35261	0.946127	2.1

35262	1.14418	2.1
35263	0.939289	2.1
35264	1.14417	2.1
35265	0.946129	2.1
35266	0.939287	2.1
35267	0.94613	2.1
35268	1.89226	2.1
35269	1.89226	2.1
35270	1.89226	2.1
35271	1.89226	2.1
35272	3.78452	2.1
35273	1.93164	0.05
35274	2.64898	0.05
35275	1.43213	0.05
35276	1.43213	0.05
35277	2.63206	0.05
35278	2.13254	0.05
35279	1.89226	2.1
35280	1.89226	2.1
35281	1.89226	2.1
35282	1.89226	2.1
35283	3.78452	2.1
35284	2.71655	2.1
35285	1.43323	2.1
35286	1.28413	2.1
35287	2.53614	2.1
35288	3.96937	2.1
35289	1.89226	2.1
35290	1.89226	2.1
35291	1.89226	2.1
35292	1.89226	2.1
35293	1.89226	2.1
35294	1.89226	2.1
35295	1.33356	2.1
35296	2.82297	2.1
35297	2.771 2.1	
35298	2.771 2.1	
35299	1.46037	2.1
35300	1.46037	2.1
35301	2.56034	2.1
35302	1.18474	2.1
35303	1.15113	2.1
35304	2.53724	2.1
35305	3.72198	2.1
35306	2.56034	2.1
35307	1.89226	2.1
35308	1.89226	2.1
35309	2.53725	2.1
35310	4.42951	2.1
35311	1.89226	2.1
35312	1.89226	2.1
35313	1.89226	2.1
35314	1.89226	2.1
35315	3.78453	2.1
35316	1.89226	2.1
35317	1.89226	2.1
35318	1.89226	2.1
35319	1.89226	2.1

35320	3.78452	2.1
35321	1.1229	2.1
35322	2.53724	2.1
35323	1.15113	2.1
35324	1.15113	2.1
35325	2.51559	2.1
35326	2.51559	2.1
35327	1.89226	2.1
35328	2.53724	2.1
35329	2.51559	2.1
35330	2.53724	2.1
35331	1.87061	2.1
35332	1.89226	2.1
35333	1.89226	2.1
35334	1.89226	2.1
35335	1.89226	2.1
35336	1.89226	2.1
35337	3.78453	2.1
35338	1.89226	2.1
35339	1.89226	2.1
35340	1.89226	2.1
35341	1.89226	2.1
35342	3.78452	2.1
35343	1.98143	1.34231
35344	2.81559	0.0638696
35345	1.65411	0.212456
35346	2.26675	0.863457
35347	4.24818	0.341552
35348	0.810656	1.25292
35349	0.810656	2.1
35350	0.821888	2.1
35351	0.821888	2.1
35352	1.1235	1.50263
35353	1.07439	2.1
35354	0.535693	2.1
35355	0.509042	2.1
35356	0.535693	2.1
35357	1.1235	2.1
35358	0.509043	2.1
35359	1.07439	2.1
35360	1.10378	2.1
35361	2.247	2.1
35362	2.27472	2.1
35363	2.27472	2.1
35364	1.07139	2.1
35365	1.07139	2.1
35366	2.08081	2.1
35367	1.40395	2.1
35368	1.34245	2.1
35369	2.00239	2.1
35370	3.40634	2.1
35371	1.89226	2.1
35372	1.89226	2.1
35373	1.89226	2.1
35374	1.89226	2.1
35375	3.78453	2.1
35376	0.946128	2.1
35377	0.946128	2.1

35378	1.13785	2.1
35379	1.11496	2.1
35380	0.946131	2.1
35381	0.946131	2.1
35382	1.11495	2.1
35383	1.11495	2.1
35384	0.94613	2.1
35385	1.11495	2.1
35386	1.13785	2.1
35387	0.946131	2.1
35388	0.975888	2.1
35389	1.00118	2.1
35390	0.671218	2.1
35391	0.653229	2.1
35392	0.825372	2.1
35393	0.825372	2.1
35394	0.829038	2.1
35395	0.829038	2.1
35396	0.975901	2.1
35397	0.653238	2.1
35398	0.671209	2.1
35399	1.00117	2.1
35400	1.89224	2.1
35401	1.89224	2.1
35402	1.89224	2.1
35403	1.89224	2.1
35404	3.78448	2.1
35405	0.946115	2.1
35406	0.946115	2.1
35407	1.11494	2.1
35408	1.10111	2.1
35409	0.946118	2.1
35410	0.946118	2.1
35411	1.10111	2.1
35412	1.10111	2.1
35413	1.10112	2.1
35414	0.94613	2.1
35415	0.946105	2.1
35416	1.11492	2.1
35417	1.04028	0.0575075
35418	2.27126	0.05
35419	2.0815	0.05
35420	1.82249	0.286705
35421	1.33723	0.05
35422	1.18684	0.172671
35423	0.812616	0.05
35424	0.812616	0.05
35425	0.997777	0.05
35426	1.08122	0.05
35427	0.94613	0.05
35428	0.94613	0.05
35429	0.812648	0.05
35430	0.932779	0.05
35431	0.812648	0.05
35432	0.94613	0.05
35433	0.849335	0.05
35434	0.94613	0.05
35435	1.80327	0.67191

35436	1.56016	0.539369
35437	1.57731	2.05592
35438	1.34851	2.01713
35439	2.03207	0.672799
35440	1.80327	2.1
35441	2.03207	1.92804
35442	1.56016	1.78128
35443	2.18076	0.889554
35444	1.78167	1.02596
35445	3.81374	1.8936
35446	1.37597	2.034
35447	1.60058	2.1
35448	1.70302	0.544338
35449	1.85448	1.14709
35450	3.15028	1.06058
35451	1.60058	0.596977
35452	1.4054	2.1
35453	1.66202	0.709107
35454	1.70302	2.1
35455	3.09979	1.30838
35456	1.70302	1.65688
35457	1.85448	1.65264
35458	2.21317	1.11647
35459	2.0961	1.06799
35460	4.01757	1.76932
35461	1.66202	1.14177
35462	1.70302	2.1
35463	2.09611	0.4846
35464	2.00892	1.89692
35465	3.6468	1.00159
35466	1.2966	0.05
35467	2.25495	0.05
35468	1.2966	0.05
35469	2.21117	0.05
35470	1.67687	0.05
35471	2.6167	0.05
35472	1.89227	0.05
35473	1.89227	0.05
35474	1.89227	0.05
35475	1.89227	0.05
35476	3.78453	0.05
35477	2.44375	0.05
35478	1.89227	0.05
35479	2.81853	0.05
35480	2.81853	0.05
35481	1.89227	0.05
35482	1.89227	0.05
35483	1.89226	0.998045
35484	1.75853	1.1016
35485	2.37636	0.590459
35486	2.37636	1.13483
35487	1.89226	0.547809
35488	1.89226	1.18727
35489	1.65814	0.05
35490	1.10187	1.45994
35491	1.10187	0.141908
35492	1.19547	1.59871
35493	1.56263	0.05

35494	1.65624	1.23018
35495	1.56264	0.05
35496	1.01479	1.54541
35497	1.01479	0.575708
35498	1.10187	1.73042
35499	1.47561	0.05
35500	1.5627	1.42706
35501	1.89225	2.1
35502	1.89225	2.1
35503	1.89225	2.1
35504	1.89225	2.1
35505	3.7845	2.1
35506	2.54386	2.1
35507	1.91546	2.1
35508	2.46008	2.1
35509	1.82815	2.1
35510	4.37554	2.1
35511	1.91546	2.1
35512	1.04909	2.1
35513	1.82815	2.1
35514	0.983141	2.1
35515	2.8986	2.1
35516	1.89225	2.1
35517	1.89225	2.1
35518	1.89225	2.1
35519	1.89225	2.1
35520	3.78449	2.1
35521	1.89226	2.1
35522	1.89226	2.1
35523	1.89226	2.1
35524	1.89226	2.1
35525	1.89226	2.1
35526	1.89226	2.1
35527	2.46009	2.1
35528	1.7513	2.1
35529	1.82817	2.1
35530	1.82817	2.1
35531	2.38127	2.1
35532	2.38127	2.1
35533	2.55006	2.1
35534	2.64569	2.1
35535	2.60128	2.1
35536	2.49168	2.1
35537	5.13737	2.1
35538	2.51605	2.1
35539	2.64569	2.1
35540	2.60128	2.1
35541	2.48891	2.1
35542	5.1346	2.1
35543	2.60128	2.1
35544	2.49168	2.1
35545	2.41689	2.1
35546	2.55248	2.1
35547	5.04416	2.1
35548	2.60128	2.1
35549	2.48891	2.1
35550	2.45922	2.1
35551	2.55249	2.1

35552	5.04139	2.1
35553	1.89227	2.1
35554	1.89227	2.1
35555	1.89227	2.1
35556	1.89227	2.1
35557	1.89227	2.1
35558	1.89227	2.1
35559	0.96063	2.1
35560	0.96063	2.1
35561	0.958806	2.1
35562	0.958806	2.1
35563	1.28892	2.1
35564	0.749042	2.1
35565	1.17409	2.1
35566	0.630516	2.1
35567	1.17409	2.1
35568	1.28892	2.1
35569	0.630515	2.1
35570	0.749042	2.1
35571	0.92511	2.1
35572	0.92511	2.1
35573	1.19511	2.1
35574	1.19511	2.1
35575	0.946132	2.1
35576	1.17409	2.1
35577	0.925081	2.09864
35578	0.946129	2.1
35579	0.946131	1.98038
35580	0.946131	2.1
35581	1.17409	2.1
35582	0.925083	2.1
35583	1.89226	0.576414
35584	1.89226	0.714273
35585	1.89226	0.296225
35586	1.89226	0.954854
35587	1.89226	0.22882
35588	1.89226	0.750391
35589	1.89226	1.62819
35590	1.89226	1.75119
35591	1.89226	1.12586
35592	1.89226	1.91722
35593	3.78453	1.30955
35594	0.946132	2.1
35595	0.946132	2.1
35596	1.28892	2.1
35597	1.28892	2.1
35598	0.946132	2.1
35599	1.28892	2.1
35600	0.946133	2.1
35601	1.17409	2.1
35602	0.946132	2.1
35603	0.946132	2.1
35604	1.17409	2.1
35605	1.28892	2.1
35606	0.625229	2.1
35607	0.890321	2.1
35608	1.21636	2.1
35609	1.13022	2.1

35610	1.07094	2.1
35611	1.03541	2.1
35612	0.942207	2.1
35613	0.942207	2.1
35614	0.898766	2.1
35615	1.26626	2.1
35616	1.0853	2.1
35617	0.600358	2.1
35618	0.719012	2.1
35619	0.719012	2.1
35620	1.17957	2.1
35621	1.17957	2.1
35622	0.719012	2.1
35623	0.952456	2.1
35624	0.946129	2.1
35625	0.946129	2.1
35626	0.946131	2.1
35627	0.952454	2.1
35628	0.946131	2.1
35629	0.719011	2.1
35630	0.94613	2.1
35631	0.94613	2.1
35632	0.94613	2.1
35633	0.94613	2.1
35634	0.946131	2.1
35635	0.946131	2.1
35636	0.946129	2.1
35637	0.946129	2.1
35638	0.94613	2.1
35639	0.94613	2.1
35640	0.94613	2.1
35641	0.94613	2.1
35642	1.1587	2.1
35643	0.973158	2.1
35644	0.750023	2.1
35645	0.536443	2.1
35646	0.84054	2.1
35647	0.84054	2.1
35648	0.854602	2.1
35649	0.854602	2.1
35650	1.1587	2.1
35651	0.750023	2.1
35652	0.973158	2.1
35653	0.536443	2.1
35654	1.89227	0.126194
35655	1.89227	2.1
35656	1.89227	1.98401
35657	1.89227	0.386779
35658	3.78453	1.50233
35659	1.89226	2.1
35660	1.89226	2.1
35661	1.89226	1.57595
35662	1.89226	2.1
35663	3.78453	2.1
35664	1.7632	2.1
35665	1.61149	2.1
35666	1.98749	2.1
35667	2.11615	2.1

35668	3.75068	2.1
35669	1.7632	2.1
35670	1.61149	2.1
35671	1.97733	0.279646
35672	2.11615	2.1
35673	3.74052	2.1
35674	1.89226	1.88406
35675	1.89226	0.714628
35676	1.89226	0.227784
35677	1.89226	2.1
35678	3.78453	0.888688
35679	1.89226	1.03917
35680	1.89226	2.1
35681	1.89226	2.1
35682	1.89226	2.08499
35683	3.78453	2.1
35684	1.98749	2.1
35685	2.11615	2.1
35686	2.43151	2.1
35687	2.27541	2.1
35688	4.41899	2.1
35689	1.97733	0.413543
35690	2.11615	2.1
35691	2.43151	2.1
35692	2.33986	0.302887
35693	4.40883	2.1
35694	2.17892	0.05
35695	2.80057	0.348389
35696	1.88349	0.311661
35697	2.63062	0.0546037
35698	4.80954	0.14268
35699	1.89226	1.1524
35700	1.89226	0.05
35701	1.89226	0.167584
35702	1.89226	0.05
35703	3.78452	0.056677
35704	2.0155	0.751502
35705	1.97652	0.05
35706	1.8247	0.162847
35707	1.80645	0.05
35708	3.80122	0.05
35709	1.97652	0.12578
35710	1.79681	0.136155
35711	1.80645	0.05
35712	1.59244	0.0668373
35713	3.56896	0.05
35714	1.89226	0.117222
35715	1.89226	0.784931
35716	1.89226	0.05
35717	1.89226	0.0845182
35718	3.78452	0.05
35719	1.89223	0.140017
35720	1.89223	0.074655
35721	1.89223	0.981207
35722	1.89223	0.05
35723	3.78447	0.0656245
35724	1.82467	0.145875
35725	1.80643	0.05

35726	1.55159	0.0981971
35727	1.53969	0.05
35728	3.36437	0.05
35729	1.80643	0.05
35730	1.59242	0.120535
35731	1.5397	0.05
35732	1.40538	0.05
35733	3.13211	0.05
35734	1.89224	0.12811
35735	1.89224	0.155689
35736	1.89224	0.05
35737	1.89224	0.125519
35738	3.78447	0.0806182
35739	1.37427	0.05
35740	2.37086	0.05
35741	2.37972	0.05
35742	1.94703	0.05
35743	2.19299	0.05
35744	1.91813	0.05
35745	1.07566	2.1
35746	1.12954	2.1
35747	0.946131	2.1
35748	0.946131	2.1
35749	1.12954	2.1
35750	1.12954	2.1
35751	0.946131	2.1
35752	0.946131	2.1
35753	1.12954	2.1
35754	0.946131	2.1
35755	0.946131	2.1
35756	1.07566	2.1
35757	2.25908	2.1
35758	1.89226	2.1
35759	1.89226	2.1
35760	1.89226	2.1
35761	2.37567	2.1
35762	2.37567	2.1
35763	1.37597	1.93864
35764	1.70302	0.301847
35765	1.24162	2.1
35766	1.12219	2.1
35767	1.37072	0.944937
35768	1.70302	2.1
35769	1.37072	2.1
35770	1.70302	1.16697
35771	1.54168	1.89127
35772	2.21318	1.12722
35773	3.39238	1.89387
35774	1.89226	0.05
35775	1.89226	0.05
35776	1.89226	0.05
35777	1.89226	0.05
35778	3.78452	0.05
35779	1.89227	0.05
35780	1.89227	0.05
35781	1.89227	0.05
35782	1.89227	0.05
35783	3.78453	0.05

35784	1.89226	0.05
35785	1.89226	0.05
35786	1.89226	0.05
35787	1.89226	0.05
35788	3.78453	0.05
35789	1.89226	0.05
35790	2.21654	0.05
35791	2.72007	0.05
35792	2.72007	0.05
35793	1.89226	0.05
35794	1.89226	0.05
35795	0.946128	0.05
35796	0.946128	0.05
35797	0.946132	0.05
35798	0.946132	0.05
35799	0.94613	0.05
35800	0.94613	0.05
35801	0.946131	0.05
35802	0.946131	0.05
35803	0.94613	0.05
35804	0.94613	0.05
35805	0.94613	0.05
35806	0.94613	0.05
35807	1.89226	0.05
35808	1.89226	0.05
35809	1.89226	0.05
35810	1.89226	0.05
35811	3.78453	0.05
35812	1.33457	0.05
35813	0.946131	0.05
35814	1.08045	0.05
35815	0.946132	0.05
35816	1.08045	0.05
35817	1.08045	0.05
35818	0.946132	0.05
35819	0.946132	0.05
35820	1.33457	0.05
35821	1.08045	0.05
35822	0.946132	0.05
35823	0.946132	0.05
35824	1.53347	1.113
35825	1.13092	2.1
35826	1.52938	2.04736
35827	1.93568	1.7763
35828	3.06285	2.1
35829	1.89226	0.0962489
35830	2.72959	0.122692
35831	2.82996	0.160979
35832	2.82996	0.05
35833	1.89226	0.373494
35834	1.89226	0.0809927
35835	2.7296	0.051795
35836	2.57663	2.1
35837	2.48354	0.05
35838	2.48354	2.1
35839	2.82996	0.05
35840	2.82996	2.1
35841	2.63449	0.0783698

35842	2.7296	0.0517007
35843	2.39282	0.096817
35844	2.39282	2.1
35845	2.48354	2.1
35846	2.72521	2.1
35847	1.89224	2.1
35848	1.89224	2.06484
35849	1.89224	0.399543
35850	1.89224	2.1
35851	3.78447	1.46706
35852	1.89226	2.1
35853	2.10425	2.1
35854	2.5142	2.1
35855	2.5142	2.1
35856	1.89226	2.1
35857	1.89226	2.1
35858	0.946133	0.640171
35859	0.946133	1.57244
35860	0.946128	2.1
35861	0.946128	2.1
35862	0.946131	2.1
35863	0.946131	0.615612
35864	0.94613	0.0558972
35865	0.94613	2.1
35866	0.946131	1.94762
35867	0.946131	2.0896
35868	0.946131	1.96258
35869	0.946131	2.00911
35870	1.89226	0.299122
35871	1.89226	0.902962
35872	1.89226	0.439974
35873	1.89226	1.23124
35874	1.89226	0.284386
35875	1.89226	0.872261
35876	1.94446	0.365695
35877	2.50167	1.27973
35878	1.28418	0.606922
35879	1.94446	0.750986
35880	2.05205	0.538688
35881	2.05205	0.87762
35882	1.27835	2.1
35883	1.08341	2.1
35884	0.946132	2.1
35885	0.946132	2.1
35886	0.851542	2.1
35887	0.851542	2.1
35888	0.977356	2.1
35889	1.18368	2.1
35890	1.18378	2.1
35891	0.851555	2.1
35892	0.977346	2.1
35893	0.851476	2.1
35894	1.07369	2.1
35895	2.5813	2.1
35896	1.95053	2.1
35897	1.95053	2.1
35898	1.90196	2.1
35899	1.90196	2.1

35900	1.89226	2.1
35901	1.89226	2.1
35902	1.89226	2.1
35903	1.89226	2.1
35904	3.78452	2.1
35905	1.89224	2.1
35906	1.89224	2.1
35907	1.89224	2.1
35908	1.89224	2.1
35909	1.89224	2.1
35910	1.89224	2.1
35911	1.97004	0.060932
35912	1.8072	1.63112
35913	1.5866	1.11512
35914	1.79478	0.108422
35915	3.60198	0.506025
35916	1.23267	2.1
35917	1.8072	0.343005
35918	1.58661	0.0960854
35919	0.947921	2.08324
35920	2.75512	1.3627
35921	1.89226	0.383156
35922	1.89226	2.1
35923	1.89226	0.199021
35924	1.89226	1.72811
35925	1.89226	0.136171
35926	1.89226	2.1
35927	1.89226	1.63956
35928	1.89226	0.126238
35929	1.89226	0.130798
35930	1.89226	1.00213
35931	3.78452	0.491981
35932	1.4263	1.56099
35933	1.79478	0.22798
35934	1.67651	0.199569
35935	1.79478	1.80062
35936	1.46834	0.05
35937	1.5866	0.391887
35938	1.89226	0.274678
35939	1.89226	0.813576
35940	1.89226	0.0868344
35941	1.89226	0.474777
35942	1.89226	0.110248
35943	1.89226	1.6647
35944	2.23956	1.81899
35945	1.81847	1.93049
35946	2.10991	1.02889
35947	2.75317	1.19359
35948	4.34947	1.56744
35949	1.60578	0.05
35950	1.94974	0.05
35951	1.68062	0.05
35952	1.14721	0.05
35953	3.2864	0.05
35954	1.3946	0.05
35955	1.692 0.05	
35956	2.0154	0.05
35957	2.0154	0.05

35958	1.11926	0.05
35959	1.11926	0.05
35960	1.90347	0.05
35961	1.83626	0.05
35962	1.84583	0.05
35963	1.89434	0.05
35964	3.79781	0.05
35965	1.89226	1.52385
35966	1.89226	1.15045
35967	1.89226	0.431377
35968	1.89226	1.3908
35969	3.78452	1.03142
35970	1.20986	1.61351
35971	1.20986	1.27282
35972	0.912635	1.29985
35973	0.912635	1.8186
35974	0.912667	0.681616
35975	1.17637	1.76724
35976	0.946125	1.85221
35977	0.946125	1.9357
35978	0.946128	1.96312
35979	0.912664	1.1847
35980	0.946127	2.1
35981	1.17637	1.37552
35982	1.89226	0.05
35983	1.65573	0.05
35984	1.65573	0.05
35985	1.89226	0.05
35986	1.89226	0.05
35987	2.12879	0.05
35988	1.89226	0.05
35989	1.89226	0.05
35990	1.89226	0.05
35991	1.89226	0.05
35992	1.89226	0.05
35993	1.89226	0.05
35994	2.40928	0.05
35995	1.89226	0.05
35996	1.95959	0.05
35997	1.70302	0.05
35998	3.47338	0.05
35999	1.89226	0.05
36000	1.89226	0.05
36001	1.89226	0.05
36002	1.89226	0.05
36003	3.78452	0.05
36004	1.64985	0.05
36005	2.47846	0.05
36006	2.25405	0.05
36007	1.80768	0.05
36008	4.06173	0.05
36009	1.98846	0.05
36010	0.912729	0.05
36011	1.05417	0.05
36012	1.70303	0.05
36013	2.82107	0.05
36014	2.47846	0.05
36015	1.70303	0.05

36016	2.86918	0.05
36017	2.05973	0.05
36018	2.51808	0.05
36019	1.80768	0.05
36020	0.946131	0.05
36021	0.946131	0.05
36022	0.946132	0.05
36023	0.946132	0.05
36024	0.946131	0.05
36025	0.946131	0.05
36026	0.946133	0.05
36027	0.946133	0.05
36028	0.946131	0.05
36029	0.946131	0.05
36030	0.946132	0.05
36031	0.946132	0.05
36032	0.946133	0.05
36033	0.946133	0.05
36034	0.946132	0.05
36035	0.946132	0.05
36036	0.946133	0.05
36037	0.946133	0.05
36038	0.946132	0.05
36039	0.946132	0.05
36040	0.946132	0.05
36041	0.946132	0.05
36042	0.946133	0.05
36043	0.946133	0.05
36044	1.89227	0.05
36045	1.89227	0.05
36046	1.89227	0.05
36047	1.89227	0.05
36048	1.89227	0.05
36049	1.89227	0.05
36050	1.1656	2.1
36051	1.21847	2.1
36052	0.836006	2.1
36053	0.806007	2.1
36054	1.00653	2.1
36055	1.00653	2.1
36056	1.01794	2.1
36057	1.01794	2.1
36058	1.1656	2.1
36059	0.806007	2.1
36060	0.836006	2.1
36061	1.21847	2.1
36062	2.82263	2.1
36063	2.3312	2.1
36064	2.25936	2.1
36065	2.71441	2.1
36066	5.0456	2.1
36067	2.25936	2.1
36068	1.61201	2.1
36069	1.57627	2.1
36070	1.61201	2.1
36071	2.29545	2.1
36072	2.3312	2.1
36073	1.21269	0.05

36074	1.48867	0.05
36075	1.68264	0.05
36076	1.9898	0.05
36077	3.17131	0.05
36078	2.0487	2.1
36079	2.45212	2.1
36080	2.31012	2.1
36081	1.94095	2.1
36082	4.39307	2.1
36083	1.02435	2.1
36084	0.970469	2.1
36085	0.625375	2.1
36086	0.567166	2.1
36087	0.796854	2.1
36088	0.796854	2.1
36089	0.79466	2.1
36090	0.79466	2.1
36091	0.970471	2.1
36092	0.567165	2.1
36093	0.625374	2.1
36094	1.02435	2.1
36095	2.31012	2.1
36096	1.94095	2.1
36097	1.4825	2.1
36098	2.17384	2.1
36099	4.11479	2.1
36100	0.946187	2.1
36101	0.803763	2.1
36102	0.581357	2.1
36103	0.510356	2.1
36104	0.745632	2.1
36105	0.745632	2.1
36106	0.734566	2.1
36107	0.734563	2.1
36108	0.896142	2.1
36109	0.510366	2.1
36110	0.946173	2.1
36111	0.552965	2.1
36112	1.89226	0.851362
36113	1.89226	1.74247
36114	1.89226	1.95416
36115	1.89226	2.0438
36116	1.89226	1.06305
36117	1.89226	1.78883
36118	1.50743	1.65647
36119	1.0417	2.1
36120	1.50743	2.1
36121	1.62974	1.16235
36122	1.14605	1.15538
36123	1.14605	2.1
36124	1.89226	0.705672
36125	1.89226	2.05348
36126	1.89226	0.312903
36127	1.89226	1.74213
36128	1.89226	0.416151
36129	1.89226	1.95732
36130	1.89226	1.9174
36131	1.89226	1.63441

36132	1.89226	0.213004
36133	1.89226	2.03017
36134	3.78453	1.15044
36135	1.77186	1.83776
36136	1.35668	2.1
36137	1.70254	0.832506
36138	1.38756	2.1
36139	1.77186	2.03311
36140	1.37814	2.1
36141	0.734857	2.1
36142	1.00549	1.93773
36143	0.835261	1.13012
36144	1.08357	2.1
36145	0.97665	1.68069
36146	0.795559	2.1
36147	0.891385	1.89641
36148	0.891385	0.287213
36149	0.874231	1.21126
36150	1.08356	1.57066
36151	0.734863	1.55793
36152	0.79449	1.96751
36153	1.89226	1.5249
36154	1.89226	0.149241
36155	2.42671	0.484452
36156	1.98783	1.12106
36157	4.31897	0.490868
36158	0.579854	0.117062
36159	0.965582	0.391822
36160	0.634403	0.95764
36161	1.17796	0.265659
36162	1.00592	0.20231
36163	0.797259	1.91907
36164	0.905246	1.11544
36165	0.905246	0.20813
36166	0.695715	0.213245
36167	0.637106	0.721759
36168	1.24374	0.82063
36169	0.95617	0.500068
36170	2.0815	0.190064
36171	1.60521	1.01485
36172	1.29556	0.41212
36173	2.32205	1.718
36174	1.28445	0.0677853
36175	2.0815	0.866382
36176	2.3085	2.1
36177	2.21147	2.1
36178	2.3085	2.1
36179	2.39282	2.1
36180	1.98899	2.1
36181	2.0733	2.1
36182	2.3275	2.1
36183	1.63471	2.1
36184	1.98899	2.1
36185	1.56801	2.1
36186	3.62369	2.1
36187	2.3085	2.1
36188	1.98899	2.1
36189	2.22575	2.1

36190	1.90502	2.1
36191	4.21353	2.1
36192	1.90502	2.1
36193	1.56801	2.1
36194	1.90502	2.1
36195	1.98899	2.1
36196	1.49733	2.1
36197	1.58129	2.1
36198	1.89226	0.980448
36199	2.6491	0.83491
36200	2.49266	2.1
36201	2.6491	0.779361
36202	1.73582	0.6528
36203	1.89226	0.05
36204	1.31854	2.1
36205	2.6491	1.32762
36206	1.3909	2.1
36207	1.3909	2.1
36208	2.49266	2.1
36209	2.49266	2.1
36210	1.89226	1.0942
36211	2.49266	0.747485
36212	2.38634	2.1
36213	2.49266	0.542881
36214	1.78595	0.521168
36215	1.89226	0.05
36216	1.23042	2.1
36217	2.49266	0.988926
36218	1.31854	2.1
36219	1.31854	1.90098
36220	2.38635	2.1
36221	2.38635	2.00264
36222	2.83487	2.1
36223	1.89226	2.1
36224	2.594	2.1
36225	2.594	2.1
36226	1.89226	2.1
36227	1.89226	2.1
36228	1.89226	2.1
36229	1.89226	2.1
36230	1.89226	2.1
36231	1.89226	2.1
36232	3.78452	2.1
36233	1.09518	2.1
36234	1.28158	2.1
36235	1.36844	2.1
36236	1.59972	2.1
36237	2.65003	2.1
36238	1.89226	2.1
36239	1.89226	2.1
36240	1.89226	2.1
36241	1.89226	2.1
36242	3.78452	2.1
36243	1.36844	2.1
36244	1.59972	2.1
36245	1.67352	2.1
36246	1.94291	2.1
36247	3.31135	2.1

36248	1.52938	2.1
36249	1.93568	2.09891
36250	2.36221	0.466513
36251	1.94723	2.1
36252	3.89158	1.89955
36253	1.94724	1.19123
36254	1.26593	2.1
36255	1.75367	2.1
36256	2.4322	1.59601
36257	3.7009	2.1
36258	2.36221	0.961893
36259	2.43219	2.1
36260	1.94723	1.45234
36261	1.94723	1.73671
36262	2.8202	1.77251
36263	2.8202	2.02337
36264	2.51604	2.1
36265	2.18533	2.1
36266	2.17228	2.1
36267	2.4889	2.1
36268	4.67423	2.1
36269	2.17228	2.1
36270	1.62183	2.1
36271	1.62316	2.1
36272	1.62183	2.1
36273	2.18666	2.1
36274	2.18533	2.1
36275	2.17227	2.1
36276	2.4889	2.1
36277	2.45922	2.1
36278	2.15686	2.1
36279	4.64576	2.1
36280	2.17228	2.1
36281	1.62316	2.1
36282	1.62241	2.1
36283	2.15686	2.1
36284	3.78002	2.1
36285	1.89226	0.822759
36286	1.89226	0.902389
36287	1.89226	0.680444
36288	1.89226	0.662277
36289	3.78453	1.38591
36290	1.89226	1.04091
36291	1.89226	0.925767
36292	1.89226	0.644921
36293	1.89226	0.134815
36294	3.78452	0.293817
36295	1.89226	0.937214
36296	1.89226	0.472329
36297	1.89226	0.969827
36298	1.89226	0.743042
36299	1.89226	0.595149
36300	1.89226	0.659698
36301	1.89226	0.897016
36302	1.89226	1.11711
36303	2.53413	0.149543
36304	1.89226	0.214613
36305	3.78453	0.171929

36306	1.38048	0.225628
36307	1.38048	0.385331
36308	0.490332	0.348499
36309	0.490332	0.248961
36310	0.924679	0.05
36311	0.946131	1.74824
36312	0.946131	1.33921
36313	0.490332	0.05
36314	0.924678	0.05
36315	0.490332	0.05
36316	0.946131	0.679824
36317	0.946131	0.208205
36318	1.89227	0.147138
36319	1.89227	0.783126
36320	1.89227	0.552767
36321	1.89227	1.11615
36322	1.89227	0.145319
36323	1.89227	0.310533
36324	1.30803	2.1
36325	2.55151	2.1
36326	1.43935	2.1
36327	1.43935	2.1
36328	2.34922	2.1
36329	2.34922	2.1
36330	0.946131	2.1
36331	0.946131	2.1
36332	1.27575	2.1
36333	1.17461	2.1
36334	0.94613	2.1
36335	0.94613	2.1
36336	1.17461	2.1
36337	1.17461	2.1
36338	0.946131	2.1
36339	1.17461	2.1
36340	0.946132	2.1
36341	1.27575	2.1
36342	1.89226	2.1
36343	1.89226	1.46903
36344	1.89226	2.1
36345	1.89226	2.1
36346	1.89226	2.1
36347	1.89226	2.1
36348	0.946133	2.1
36349	0.946133	2.1
36350	0.946132	2.1
36351	0.946132	2.1
36352	0.946133	2.1
36353	0.946133	2.1
36354	0.946132	2.1
36355	0.946132	2.1
36356	0.946132	2.1
36357	0.946132	2.1
36358	0.946133	2.1
36359	0.946133	2.1
36360	1.07043	2.1
36361	1.17461	2.1
36362	0.654016	2.1
36363	0.5829	2.1

36364	0.870481	2.1
36365	0.870481	2.1
36366	0.887027	2.1
36367	0.887027	2.1
36368	1.07043	2.1
36369	0.5829	2.1
36370	0.654016	2.1
36371	1.17461	2.1
36372	1.89226	2.1
36373	1.89226	1.20334
36374	1.89226	2.1
36375	1.89226	2.1
36376	3.78453	2.1
36377	1.89226	2.1
36378	1.89226	2.1
36379	1.89226	2.1
36380	1.89226	2.1
36381	3.78453	2.1
36382	1.26929	2.1
36383	2.19313	2.1
36384	1.09847	2.1
36385	1.69916	2.1
36386	2.96844	2.1
36387	1.84124	2.1
36388	2.24937	2.1
36389	1.69915	2.1
36390	1.75691	2.1
36391	3.94852	2.1
36392	1.53082	2.1
36393	2.04422	2.1
36394	1.53082	2.1
36395	1.69915	2.1
36396	1.92911	2.1
36397	2.09744	2.1
36398	1.39263	0.255916
36399	1.0171	0.05
36400	1.1211	0.0682657
36401	1.46482	0.0761235
36402	2.48192	0.0915474
36403	1.39263	0.108654
36404	1.57962	0.256818
36405	1.65238	1.27168
36406	1.46482	0.0511331
36407	3.04443	0.0890117
36408	1.89226	0.235588
36409	1.89226	0.05
36410	1.89226	0.05
36411	1.89226	0.0720112
36412	3.78453	0.0820293
36413	1.89227	0.05
36414	1.89227	0.05
36415	1.89227	0.0615042
36416	1.89227	0.05
36417	3.78453	0.05
36418	1.28418	0.136965
36419	1.46482	0.080701
36420	1.1211	0.0700571
36421	1.1211	0.332757

36422	1.59382	0.05
36423	1.59382	0.166657
36424	1.76897	0.821595
36425	1.46482	0.0551379
36426	1.59382	0.0899213
36427	1.46482	0.26199
36428	1.78138	0.0711794
36429	1.65238	0.192534
36430	1.89226	0.05
36431	1.89226	0.0651897
36432	1.89226	0.05
36433	1.89226	0.209935
36434	1.89226	0.05
36435	1.89226	0.215596
36436	1.89226	0.0631012
36437	1.89226	0.05
36438	1.89226	0.05
36439	1.89226	0.05
36440	1.89226	0.05
36441	1.89226	0.0657936
36442	1.96832	2.04761
36443	2.40858	1.90309
36444	1.75211	0.119042
36445	2.20797	2.1
36446	4.1607	1.59815
36447	1.75211	0.155002
36448	2.20797	2.1
36449	1.51592	2.1
36450	2.02064	2.04252
36451	3.77275	2.1
36452	2.71793	0.173284
36453	2.67099	0.057095
36454	2.55105	0.115693
36455	2.71297	0.05
36456	5.26898	0.0772881
36457	2.36417	0.24282
36458	2.67098	0.0617077
36459	2.55104	0.121932
36460	1.83418	0.05
36461	4.91522	0.0790387
36462	2.55105	0.0985321
36463	2.71297	0.05
36464	2.58032	0.0731768
36465	2.28922	0.05
36466	5.13137	0.055564
36467	2.55104	0.101746
36468	2.23787	0.05
36469	1.67265	0.05
36470	2.28922	0.05
36471	4.22369	0.05
36472	0.851542	0.500756
36473	0.851542	0.45007
36474	0.981419	0.156568
36475	0.854774	0.288263
36476	0.949431	0.195638
36477	0.753763	0.296612
36478	0.946131	0.479978
36479	0.946131	0.453965

36480	1.04073	0.420876
36481	0.981433	0.107256
36482	0.851528	0.696709
36483	0.854828	0.289022
36484	1.89226	0.798727
36485	1.89226	0.32451
36486	1.89226	0.305601
36487	1.89226	0.352977
36488	1.89226	0.613839
36489	1.89226	0.707978
36490	0.814227	0.243769
36491	0.814227	0.244378
36492	0.946117	0.0976418
36493	1.07285	0.122135
36494	0.879132	0.0983862
36495	0.923293	0.112354
36496	0.946119	0.253613
36497	0.946119	0.299204
36498	0.946131	0.212749
36499	0.814236	0.0535121
36500	0.946106	0.384501
36501	0.870405	0.160082
36502	1.89224	0.483102
36503	1.89224	0.176096
36504	1.89224	0.179627
36505	1.89224	0.201179
36506	1.89224	0.396563
36507	1.89224	0.445211
36508	1.29388	1.25499
36509	1.04164	2.1
36510	1.64834	2.07101
36511	1.82955	1.17916
36512	2.94222	2.1
36513	1.4934	0.137063
36514	2.41522	0.05
36515	2.20387	0.05
36516	2.20387	1.9248
36517	1.75765	0.0523528
36518	1.75765	1.50795
36519	2.41522	0.05
36520	2.77303	2.1
36521	2.20387	2.08495
36522	2.58627	1.5611
36523	5.00149	2.02694
36524	1.22222	0.203271
36525	2.20387	0.233861
36526	1.96831	0.0522588
36527	1.96831	2.01942
36528	1.4934	0.0842753
36529	1.4934	1.99366
36530	1.96832	2.1
36531	2.58627	1.46342
36532	2.40858	0.326447
36533	2.40858	2.1
36534	2.20387	0.876012
36535	2.20387	2.1
36536	1.5605	2.1
36537	1.53775	2.1

36538	1.55398	2.1
36539	1.45806	2.1
36540	1.65642	2.1
36541	1.5605	2.1
36542	0.768876	2.1
36543	0.79033	2.1
36544	0.828211	2.1
36545	0.871233	2.1
36546	0.803843	2.1
36547	0.803843	2.1
36548	0.814698	2.1
36549	0.814698	2.1
36550	0.79033	2.1
36551	0.871233	2.1
36552	0.768876	2.1
36553	0.828212	2.1
36554	1.80252	2.1
36555	2.09715	0.247007
36556	1.9394	2.1
36557	1.50853	2.1
36558	3.60567	2.1
36559	1.96416	0.142409
36560	2.09715	0.59577
36561	1.88484	2.1
36562	1.57916	0.747691
36563	3.67631	1.12964
36564	1.89226	0.488486
36565	1.89226	0.05
36566	1.89226	0.402428
36567	1.89226	0.89404
36568	3.78453	0.0866565
36569	1.89226	0.186908
36570	1.89226	0.406359
36571	1.89226	2.1
36572	1.89226	0.05
36573	1.89226	2.1
36574	1.89226	0.05
36575	1.5852	2.1
36576	1.50853	2.1
36577	1.2955	2.1
36578	1.44472	0.251907
36579	2.95325	2.09492
36580	1.53065	1.73317
36581	1.57916	0.0799547
36582	1.44472	0.407479
36583	1.78861	0.622887
36584	1.23527	1.23076
36585	1.23527	0.391901
36586	1.89227	0.0629269
36587	1.89227	0.187405
36588	1.89227	1.26367
36589	1.89227	0.554239
36590	3.78453	0.209762
36591	1.89226	0.235525
36592	1.89226	0.171974
36593	1.89226	2.1
36594	1.89226	0.231166
36595	1.89226	1.08555

36596	1.89226	0.05
36597	1.89227	1.27901
36598	1.89227	0.660418
36599	1.89227	0.731203
36600	1.89227	0.476693
36601	3.78453	0.545366
36602	0.943708	0.483996
36603	0.752219	0.288109
36604	0.946132	0.522085
36605	0.946132	0.570572
36606	0.946133	0.156129
36607	0.946133	1.16961
36608	0.752218	1.32138
36609	0.752218	0.680561
36610	0.752218	0.575855
36611	0.946132	0.268418
36612	0.946133	0.937686
36613	0.943708	0.560032
36614	0.943992	2.1
36615	0.943992	2.1
36616	1.23507	0.652552
36617	1.48209	2.1
36618	1.0384	2.1
36619	1.47953	2.1
36620	0.946128	0.623768
36621	0.946128	2.1
36622	0.946129	2.1
36623	1.23293	2.1
36624	1.0384	2.1
36625	0.946129	2.1
36626	1.89226	2.1
36627	2.46586	2.1
36628	2.80218	0.579865
36629	2.80218	2.1
36630	1.89226	0.653741
36631	1.89226	2.1
36632	1.32455	2.1
36633	1.89128	2.1
36634	1.32328	2.1
36635	1.97299	2.1
36636	3.29754	2.1
36637	1.89128	2.1
36638	2.4255	2.1
36639	1.973	2.1
36640	2.63807	2.1
36641	4.3985	2.1
36642	1.34008	2.1
36643	1.97299	2.1
36644	2.10766	2.1
36645	2.10766	2.1
36646	1.32328	2.1
36647	1.32328	2.1
36648	2.41055	2.1
36649	2.37491	2.1
36650	2.40754	2.1
36651	2.38023	2.1
36652	4.78546	2.1
36653	2.23743	2.1

36654	2.37491	2.1
36655	2.40754	2.1
36656	2.20392	2.1
36657	4.61234	2.1
36658	2.37491	2.1
36659	2.02641	2.1
36660	2.20392	2.1
36661	2.18943	2.1
36662	4.40132	2.1
36663	2.37491	2.1
36664	2.22558	2.1
36665	2.38023	2.1
36666	2.18943	2.1
36667	4.60049	2.1
36668	1.89226	0.05
36669	1.89226	0.05
36670	1.89226	0.05
36671	1.89226	0.05
36672	1.89226	0.05
36673	1.89226	0.05
36674	1.03599	0.05
36675	0.706698	0.05
36676	1.41141	0.05
36677	1.17573	0.05
36678	1.11957	0.05
36679	0.765034	0.05
36680	1.05913	0.05
36681	1.45054	0.05
36682	1.04847	0.05
36683	1.04847	0.05
36684	1.15323	0.05
36685	1.05825	0.05
36686	1.89226	0.05
36687	1.89226	0.05
36688	1.89226	0.05
36689	1.89226	0.05
36690	1.89226	0.05
36691	1.89226	0.05
36692	2.22575	0.05
36693	1.70677	2.1
36694	1.90157	0.05
36695	1.5534	2.1
36696	3.77914	0.115269
36697	1.70678	2.1
36698	0.968594	2.1
36699	0.968594	2.1
36700	1.10378	2.1
36701	1.5534	2.1
36702	1.68859	2.1
36703	2.31429	0.05
36704	1.5534	2.1
36705	1.90103	0.05
36706	1.41902	2.03006
36707	3.45443	0.0991176
36708	2.53709	0.05
36709	1.209	0.05
36710	1.05417	0.05
36711	2.6513	0.05

36712	3.70547	0.05
36713	1.02675	0.05
36714	1.16384	0.05
36715	2.22062	0.05
36716	2.48862	0.05
36717	3.38446	0.05
36718	1.89226	0.05
36719	1.89226	0.05
36720	1.89226	0.05
36721	1.89226	0.05
36722	1.89226	0.05
36723	1.89226	0.05
36724	1.89226	1.12846
36725	1.89226	2.1
36726	1.89226	2.1
36727	1.89226	1.59182
36728	1.89226	2.1
36729	1.89226	1.32452
36730	1.89226	0.786515
36731	1.89226	1.7211
36732	1.89226	1.94969
36733	1.89226	1.17235
36734	1.89226	1.78568
36735	1.89226	0.917359
36736	1.89227	1.19775
36737	1.89227	1.84124
36738	1.75032	1.99632
36739	1.75032	1.30257
36740	1.89227	1.82329
36741	1.89227	1.10591
36742	1.89226	0.05
36743	1.89226	0.05
36744	1.89226	0.05
36745	1.89226	0.05
36746	1.89226	0.05
36747	1.89226	0.05
36748	0.946132	0.05
36749	0.946132	0.05
36750	0.798215	0.05
36751	0.848163	0.05
36752	0.848163	0.05
36753	0.946131	0.05
36754	0.946132	0.05
36755	0.798216	0.05
36756	0.996105	0.05
36757	0.996105	0.05
36758	0.79819	0.05
36759	0.79819	0.05
36760	1.07763	0.05
36761	2.28622	0.05
36762	2.16109	0.05
36763	1.17506	0.05
36764	3.36385	0.05
36765	1.89226	2.1
36766	1.6663	2.1
36767	1.6663	2.1
36768	1.71706	2.1
36769	1.89226	2.1

36770	1.94302	2.1
36771	1.89226	2.1
36772	1.62801	2.1
36773	1.62801	2.1
36774	1.6663	2.1
36775	1.89226	2.1
36776	1.93055	2.1
36777	1.48734	2.1
36778	1.99609	2.1
36779	1.52245	2.1
36780	2.04137	2.1
36781	3.51854	2.1
36782	1.99609	2.1
36783	2.28563	2.1
36784	2.04136	2.1
36785	2.35019	2.1
36786	4.34628	2.1
36787	1.50714	2.1
36788	2.04137	2.1
36789	2.04945	2.1
36790	2.04945	2.1
36791	1.52245	2.1
36792	1.52245	2.1
36793	2.04136	2.1
36794	2.35019	2.1
36795	2.04945	2.1
36796	2.38681	2.1
36797	4.39964	2.1
36798	2.50924	0.05
36799	1.38305	2.1
36800	2.56918	2.1
36801	1.44535	2.1
36802	3.95222	2.1
36803	1.89226	2.1
36804	1.89226	2.1
36805	1.89226	2.1
36806	1.89226	2.1
36807	3.78453	2.1
36808	1.40615	2.1
36809	1.38851	2.1
36810	0.748607	2.1
36811	0.724468	2.1
36812	1.06362	2.1
36813	1.06362	2.1
36814	1.067 2.1	
36815	1.067 2.1	
36816	1.40615	2.1
36817	0.748607	2.1
36818	0.724468	2.1
36819	1.38851	2.1
36820	2.56918	2.1
36821	1.53377	2.1
36822	1.53706	2.1
36823	1.44535	2.1
36824	2.66088	2.1
36825	2.56918	2.1
36826	1.89226	2.1
36827	1.89226	2.1

36828	1.89226	2.1
36829	1.89226	2.1
36830	3.78453	2.1
36831	1.40239	2.1
36832	1.68466	2.1
36833	1.26091	2.1
36834	1.54641	2.1
36835	2.94881	2.1
36836	1.68466	2.1
36837	1.65324	0.0519688
36838	1.54641	2.1
36839	1.52268	0.05
36840	3.19965	0.970652
36841	1.05776	2.1
36842	1.54641	2.1
36843	1.36595	2.1
36844	1.36595	2.1
36845	1.26091	2.1
36846	1.26091	2.1
36847	1.54641	2.1
36848	1.32697	0.05
36849	1.52268	0.0718464
36850	1.52268	2.1
36851	1.36595	0.0706555
36852	1.36595	2.1
36853	1.18647	1.75049
36854	0.988454	2.1
36855	1.4867	2.1
36856	1.63892	1.78168
36857	2.67317	2.1
36858	1.4867	2.1
36859	1.63892	1.99814
36860	2.09605	0.310059
36861	1.97645	2.1
36862	3.58275	1.52452
36863	1.29389	2.1
36864	1.97645	1.86923
36865	1.4867	1.8348
36866	1.4867	2.1
36867	1.82955	1.16041
36868	1.82955	2.1
36869	1.13454	0.954601
36870	0.983594	2.1
36871	1.16809	2.1
36872	1.06836	2.05423
36873	1.08332	2.1
36874	0.983594	1.25615
36875	1.48751	2.1
36876	1.48047	1.92721
36877	1.77596	1.9213
36878	1.75506	1.98641
36879	3.25643	2.07396
36880	1.48047	1.84919
36881	1.25334	2.1
36882	1.75505	1.53098
36883	1.55831	0.187699
36884	3.03878	2.01383
36885	1.77596	2.04882

36886	1.75506	2.1
36887	2.00551	2.1
36888	2.00083	0.60325
36889	3.77679	2.1
36890	1.75506	2.1
36891	1.80328	0.37878
36892	1.55831	2.0616
36893	1.55831	2.1
36894	2.00083	0.35956
36895	2.00083	1.31268
36896	2.58627	1.52677
36897	2.79047	2.1
36898	2.40858	2.1
36899	2.64748	2.1
36900	5.23375	2.1
36901	1.26755	2.1
36902	1.71717	2.1
36903	1.03824	2.1
36904	1.48901	2.1
36905	2.75656	2.1
36906	1.89226	2.1
36907	1.89226	2.1
36908	1.89226	2.1
36909	1.89226	2.1
36910	1.89226	2.1
36911	1.89226	2.1
36912	1.36541	2.1
36913	1.22156	2.1
36914	0.946132	2.1
36915	0.946132	2.1
36916	1.36541	2.1
36917	1.36541	2.1
36918	0.946132	2.1
36919	0.946132	2.1
36920	1.36541	2.1
36921	0.946133	2.1
36922	0.946132	2.1
36923	1.22156	2.1
36924	1.18901	2.1
36925	1.61149	2.1
36926	1.98748	2.1
36927	1.534	2.1
36928	3.1765	2.1
36929	1.89226	2.1
36930	1.89226	2.1
36931	1.89226	2.1
36932	1.89226	2.1
36933	3.78452	2.1
36934	1.98748	2.1
36935	1.534	2.1
36936	2.27541	2.1
36937	1.98748	2.1
36938	1.87436	2.1
36939	1.87436	2.1
36940	2.2988	2.1
36941	2.2604	2.1
36942	2.20831	2.1
36943	2.02802	2.1

36944	4.32683	2.1
36945	2.26041	2.1
36946	1.98957	2.1
36947	2.02803	2.1
36948	1.75021	2.1
36949	4.0176	2.1
36950	2.34607	2.1
36951	2.02803	2.1
36952	1.86795	2.1
36953	1.81448	2.1
36954	3.89597	2.1
36955	2.02803	2.1
36956	1.75021	2.1
36957	1.81448	2.1
36958	1.57887	2.1
36959	3.6069	2.1
36960	0.946131	0.05
36961	0.946131	2.1
36962	1.1235	2.1
36963	1.1235	2.1
36964	0.946131	1.73372
36965	0.946131	0.438688
36966	1.1235	2.1
36967	1.13736	2.1
36968	1.13736	0.338552
36969	0.946131	1.66811
36970	1.1235	1.96146
36971	0.946131	1.74707
36972	1.89226	2.1
36973	1.89226	2.1
36974	1.89226	0.487281
36975	1.89226	0.316508
36976	3.78452	0.201201
36977	0.563266	2.1
36978	0.563266	0.934493
36979	0.565606	0.0863177
36980	0.565606	2.1
36981	0.647086	2.0543
36982	0.624219	2.01908
36983	0.500035	0.986796
36984	0.481785	0.943418
36985	0.500034	0.0911306
36986	0.647087	0.120926
36987	0.624219	1.81195
36988	0.481785	0.786395
36989	0.915788	0.05
36990	0.915788	0.0569864
36991	1.02732	0.392585
36992	1.02732	0.136043
36993	0.660831	0.199876
36994	1.11155	0.05
36995	0.713295	0.05
36996	1.26874	0.270182
36997	0.713295	0.127322
36998	0.660831	0.0941081
36999	1.11155	0.106043
37000	1.26874	0.0768069
37001	1.89226	0.544751

37002	1.85091	0.05
37003	2.11193	0.219742
37004	1.85091	0.439795
37005	2.15328	0.0607272
37006	1.89226	0.0626518
37007	0.946131	0.0504587
37008	0.946131	0.0861092
37009	1.18275	0.0711025
37010	1.05596	0.178643
37011	0.946127	0.0884102
37012	0.946127	0.05
37013	1.18275	0.15073
37014	1.18275	0.0718064
37015	0.946128	0.357746
37016	1.18275	0.05
37017	0.946129	0.114779
37018	1.05596	0.05
37019	1.60936	2.1
37020	2.35846	2.1
37021	2.06424	2.1
37022	2.57651	2.1
37023	4.42269	2.1
37024	1.89226	2.1
37025	1.89226	2.1
37026	1.89226	2.1
37027	1.89226	2.1
37028	3.78453	2.1
37029	1.89226	2.1
37030	1.89226	2.1
37031	1.89226	2.1
37032	1.89226	2.1
37033	1.89226	2.1
37034	1.89226	2.1
37035	1.95053	2.1
37036	2.06424	2.1
37037	1.95053	2.1
37038	1.30584	2.1
37039	2.5	2.1
37040	1.8553	2.1
37041	2.49999	2.1
37042	2.57651	2.1
37043	2.49999	2.1
37044	2.06423	2.1
37045	2.80738	2.1
37046	2.37162	2.1
37047	1.89226	2.1
37048	1.89226	2.1
37049	1.89226	2.1
37050	1.89226	2.1
37051	1.89226	2.1
37052	1.89226	2.1
37053	1.49295	0.0634651
37054	1.03824	0.05
37055	1.51396	0.132281
37056	2.08514	0.0547431
37057	3.12338	0.0573359
37058	1.49798	0.0551805
37059	2.32017	0.05

37060	2.68236	0.0991743
37061	2.08514	0.0686198
37062	4.4053	0.05
37063	1.89226	0.05
37064	1.89226	0.05
37065	1.89226	0.0997106
37066	1.89226	0.05
37067	3.78452	0.060903
37068	1.89226	0.0810282
37069	1.89226	0.05
37070	1.89226	0.0696329
37071	1.89226	0.05
37072	1.89226	0.0503078
37073	1.89226	0.05
37074	1.51396	0.0512731
37075	2.08514	0.05
37076	2.55393	0.0874895
37077	2.02901	0.05
37078	4.11415	0.05
37079	1.89226	0.05
37080	1.89226	0.05
37081	1.89226	0.0642772
37082	1.89226	0.05
37083	3.78451	0.05
37084	1.89226	0.05
37085	1.89226	0.0531499
37086	1.89226	0.05
37087	1.89226	0.05
37088	1.89226	0.074153
37089	1.89226	0.0657368
37090	0.946131	0.05
37091	0.946131	0.05
37092	0.946132	0.05
37093	0.946132	0.05
37094	0.946132	0.05
37095	0.946132	0.05
37096	0.946132	0.05
37097	0.946132	0.05
37098	0.946132	0.05
37099	0.946132	0.05
37100	0.946132	0.05
37101	0.946132	0.05
37102	1.89226	2.1
37103	1.89226	2.1
37104	1.89226	2.1
37105	1.89226	2.1
37106	3.78452	2.1
37107	1.28367	2.1
37108	1.82707	2.1
37109	1.11025	2.1
37110	1.6365	2.09337
37111	2.92016	2.1
37112	1.82707	2.1
37113	1.8247	0.0544725
37114	1.8247	2.1
37115	2.01549	0.0816855
37116	1.6365	2.03377
37117	1.82729	0.0758649

37118	1.89226	2.1
37119	1.89226	0.0518317
37120	1.89226	0.05
37121	1.89226	2.1
37122	1.89226	0.05
37123	1.89226	2.1
37124	1.63648	2.1
37125	1.82467	0.05
37126	1.35877	2.1
37127	1.55159	0.05
37128	3.18345	0.378375
37129	1.89223	2.1
37130	1.89223	0.05
37131	1.89223	2.1
37132	1.89223	0.0900377
37133	3.78447	1.01242
37134	1.59972	2.1
37135	1.28037	2.1
37136	1.63702	2.1
37137	1.28158	2.1
37138	1.63581	2.1
37139	1.28037	2.1
37140	1.89226	2.1
37141	1.89226	2.1
37142	1.89226	2.1
37143	1.89226	2.1
37144	1.89226	2.1
37145	1.89226	2.1
37146	1.89226	2.1
37147	1.89226	2.1
37148	1.89226	2.1
37149	1.89226	2.1
37150	3.78451	2.1
37151	1.28037	2.1
37152	1.05071	2.1
37153	1.63581	2.1
37154	1.44287	2.1
37155	2.72324	2.1
37156	1.59972	2.1
37157	1.63581	2.1
37158	1.94291	2.1
37159	2.00118	2.1
37160	3.57872	2.1
37161	1.89226	2.1
37162	1.89226	2.1
37163	1.89226	2.1
37164	1.89226	2.1
37165	3.78452	2.1
37166	0.946131	2.1
37167	0.946131	2.1
37168	0.946127	2.1
37169	0.946127	2.1
37170	0.94613	2.1
37171	0.94613	2.1
37172	0.946128	2.1
37173	0.946128	2.1
37174	0.946128	2.1
37175	0.946128	2.1

37176	0.946129	2.1
37177	0.946129	2.1
37178	0.865056	2.1
37179	0.865056	2.1
37180	0.873224	2.1
37181	0.873224	2.1
37182	0.721437	2.1
37183	0.920378	2.1
37184	0.817903	2.1
37185	1.00059	2.1
37186	1.00059	2.1
37187	0.920376	2.1
37188	0.817904	2.1
37189	0.721437	2.1
37190	3.09157	2.1
37191	1.71255	2.1
37192	2.0815	2.1
37193	1.9918	2.1
37194	2.51364	2.1
37195	2.39396	2.1
37196	2.0815	2.1
37197	1.05051	2.1
37198	2.56724	2.1
37199	1.63991	2.1
37200	3.47929	2.1
37201	1.89226	0.05
37202	2.2973	0.0708388
37203	1.90705	0.0520625
37204	1.90705	0.05
37205	1.89226	0.05
37206	1.89226	0.05
37207	1.89226	0.05
37208	1.90705	0.0576416
37209	1.45321	0.0591039
37210	1.45321	0.05
37211	1.89226	0.05
37212	1.89226	0.05
37213	0.901146	2.1
37214	0.901146	2.1
37215	1.15227	2.1
37216	1.21279	2.1
37217	0.991265	2.1
37218	1.15003	2.1
37219	0.946132	2.1
37220	0.946132	2.1
37221	0.946132	2.1
37222	1.09997	2.1
37223	0.991266	2.1
37224	0.946132	2.1
37225	1.89226	2.1
37226	1.89226	2.1
37227	1.89226	2.1
37228	1.89226	2.1
37229	1.89226	2.1
37230	1.89226	2.1
37231	1.62229	2.1
37232	1.06053	2.1
37233	1.19698	2.1

37234	1.70106	2.1
37235	2.81928	2.1
37236	0.946132	2.1
37237	0.946132	2.1
37238	1.17527	2.1
37239	1.09997	2.1
37240	1.09997	2.1
37241	1.09997	2.1
37242	0.946133	2.1
37243	0.946133	2.1
37244	1.17527	2.1
37245	0.946132	2.1
37246	0.946133	2.1
37247	1.09997	2.1
37248	1.89226	2.1
37249	1.89226	2.1
37250	1.89226	2.1
37251	1.89226	2.1
37252	1.89226	2.1
37253	1.89226	2.1
37254	0.905416	2.1
37255	0.850527	2.1
37256	0.660601	2.1
37257	0.598494	2.1
37258	0.750137	2.1
37259	0.750137	2.1
37260	0.753774	2.1
37261	0.753774	2.1
37262	0.660599	2.1
37263	0.905418	2.1
37264	0.598493	2.1
37265	0.850529	2.1
37266	2.43509	2.1
37267	2.65066	2.1
37268	2.66602	2.1
37269	2.46339	2.1
37270	5.10111	2.1
37271	2.53008	2.1
37272	2.65066	2.1
37273	2.66602	2.1
37274	2.52852	2.1
37275	5.1961	2.1
37276	2.66602	2.1
37277	2.46339	2.1
37278	2.47932	2.1
37279	2.67393	2.1
37280	5.14535	2.1
37281	2.66602	2.1
37282	2.52853	2.1
37283	2.51686	2.1
37284	2.67393	2.1
37285	5.18288	2.1
37286	1.89226	1.08827
37287	1.89226	0.05
37288	1.89226	0.190545
37289	1.89226	0.05
37290	3.78452	0.0848134
37291	1.55161	0.12111

37292	1.53972	0.05
37293	1.32259	0.194482
37294	1.35082	0.05
37295	2.8623	0.05
37296	1.53972	0.0754093
37297	1.4054	0.11183
37298	1.35083	0.05
37299	1.24913	0.0627426
37300	2.78885	0.05
37301	1.89226	0.0772334
37302	1.89226	0.88495
37303	1.89226	0.0914847
37304	1.89226	0.0679258
37305	3.78452	0.0584621
37306	1.89226	0.0586723
37307	1.89226	0.06644
37308	1.89226	0.850355
37309	1.89226	0.08553
37310	1.89226	0.106381
37311	1.89226	0.05
37312	1.14599	0.0541996
37313	1.35082	0.05
37314	1.14599	0.0513284
37315	1.32259	0.733193
37316	1.19993	0.05
37317	1.37652	0.452219
37318	1.35083	0.05
37319	1.24913	0.281519
37320	1.19993	0.0503899
37321	1.11787	0.05
37322	2.44906	0.05
37323	1.89226	0.374829
37324	1.89226	0.319898
37325	1.89226	0.0771919
37326	1.89226	0.05
37327	3.78452	0.0889009
37328	1.51697	0.05
37329	2.57264	0.05
37330	1.30203	0.05
37331	2.41701	0.05
37332	3.93398	0.05
37333	1.17464	0.05
37334	2.41701	0.05
37335	2.32462	0.05
37336	2.32462	0.05
37337	1.30203	0.05
37338	1.30203	0.05
37339	2.08149	2.1
37340	1.9839	2.1
37341	1.71398	2.1
37342	1.99182	0.0624954
37343	3.71121	2.1
37344	2.16061	2.1
37345	2.01399	0.05
37346	1.99182	0.470652
37347	1.83895	0.195511
37348	4.00581	0.100448
37349	1.90444	2.1

37350	1.99182	0.141304
37351	1.71717	2.1
37352	1.805	1.3656
37353	3.70899	2.1
37354	1.99182	0.839212
37355	1.83896	0.254799
37356	1.805	2.1
37357	1.65243	0.125776
37358	3.64425	0.1061
37359	1.18076	2.1
37360	1.27697	2.1
37361	1.05025	2.1
37362	1.27697	2.1
37363	1.25	2.1
37364	1.47671	2.1
37365	1.89226	2.1
37366	1.89226	2.1
37367	1.89226	2.1
37368	1.89226	2.1
37369	3.78452	2.1
37370	2.64124	2.1
37371	1.89226	2.1
37372	1.89226	2.1
37373	1.89226	2.1
37374	2.46904	2.1
37375	2.46904	2.1
37376	1.89226	2.1
37377	1.89226	2.1
37378	2.7408	2.1
37379	2.82858	2.1
37380	4.72084	2.1
37381	1.87875	2.1
37382	1.37704	2.1
37383	1.77042	2.1
37384	2.27801	2.1
37385	3.64916	2.1
37386	1.77042	2.1
37387	2.27801	2.1
37388	2.58523	2.1
37389	1.90178	2.1
37390	4.35565	2.1
37391	1.28667	2.1
37392	1.30711	2.1
37393	1.53077	2.1
37394	1.55635	2.1
37395	2.81743	2.1
37396	1.55635	2.1
37397	1.28228	2.1
37398	1.64268	2.1
37399	1.93673	2.1
37400	3.19903	2.1
37401	1.53077	2.1
37402	1.93673	2.1
37403	1.55635	2.1
37404	1.55635	2.1
37405	1.86206	2.1
37406	1.86206	2.1
37407	1.64892	2.1

37408	1.60532	2.1
37409	1.85282	2.1
37410	1.96716	2.1
37411	3.61608	2.1
37412	1.60531	2.1
37413	1.32414	2.1
37414	1.96716	2.1
37415	1.75823	2.1
37416	3.2913	2.1
37417	1.91219	2.1
37418	1.96716	2.1
37419	2.0815	2.1
37420	2.51396	2.1
37421	4.30843	2.1
37422	1.96716	2.1
37423	2.17979	2.1
37424	1.75823	2.1
37425	1.75823	2.1
37426	2.28541	2.1
37427	2.28541	2.1
37428	1.89226	1.27676
37429	1.89226	2.05925
37430	1.89226	1.2804
37431	1.89226	1.21053
37432	3.78453	1.48873
37433	1.89226	1.62306
37434	1.89226	1.67226
37435	1.89226	1.21556
37436	1.89226	1.76157
37437	1.89226	1.39259
37438	1.89226	1.89597
37439	1.61016	1.3483
37440	1.45303	1.99258
37441	1.62812	1.53935
37442	1.86379	1.51618
37443	3.23828	1.60124
37444	1.61016	1.87812
37445	1.78716	2.08189
37446	2.0815	1.01851
37447	1.6774	1.95288
37448	3.54454	1.89321
37449	1.89226	1.75356
37450	1.89226	1.63802
37451	1.89226	0.838504
37452	1.89226	1.79923
37453	3.78453	1.39591
37454	1.89226	1.79164
37455	1.89226	1.79027
37456	1.89226	1.46599
37457	1.89226	2.1
37458	1.89226	0.634641
37459	1.89226	1.3549
37460	0.807591	1.90702
37461	0.814059	1.55912
37462	1.00217	1.93998
37463	0.931896	1.61241
37464	0.850586	1.65847
37465	0.850586	2.0859

37466	0.8889	1.24676
37467	0.8889	1.86019
37468	0.807592	1.09933
37469	1.00217	1.90803
37470	0.814061	1.66749
37471	0.931894	2.09728
37472	2.0815	1.3419
37473	1.6774	2.1
37474	2.00435	1.49115
37475	2.36104	1.41528
37476	3.86809	1.61515
37477	1.00532	0.05
37478	1.00532	0.05
37479	1.00183	0.05
37480	1.00183	0.05
37481	1.26377	0.05
37482	1.34921	0.05
37483	0.664872	0.05
37484	0.743381	0.05
37485	0.664872	0.05
37486	1.26377	0.05
37487	1.34921	0.05
37488	0.743381	0.05
37489	1.26826	0.05
37490	0.946131	2.1
37491	1.02404	2.1
37492	0.94613	2.1
37493	1.30281	2.1
37494	0.930941	2.1
37495	0.930941	2.1
37496	1.318 2.1	
37497	1.58161	2.1
37498	0.94613	2.1
37499	0.94613	2.0765
37500	1.56338	2.1
37501	1.02404	2.1
37502	2.81374	2.1
37503	1.89226	2.1
37504	1.89226	2.1
37505	1.89226	2.1
37506	3.78453	2.1
37507	0.946133	2.1
37508	0.946133	2.1
37509	0.946132	2.1
37510	0.946132	2.1
37511	0.946132	2.1
37512	0.946132	2.1
37513	0.946133	2.1
37514	0.946133	2.1
37515	0.946132	2.1
37516	0.946132	2.1
37517	0.946133	2.1
37518	0.946133	2.1
37519	1.89226	1.68068
37520	1.89226	2.1
37521	1.89226	1.99101
37522	1.89226	2.1
37523	3.78452	2.1

37524	1.89226	2.1
37525	1.89226	2.1
37526	1.89226	2.1
37527	1.89226	2.05291
37528	1.89226	2.1
37529	1.89226	2.06093
37530	0.946133	2.08389
37531	0.946133	2.1
37532	0.946132	2.05092
37533	0.946132	2.1
37534	0.946132	2.1
37535	0.946132	2.1
37536	0.946133	2.1
37537	0.946133	2.09319
37538	0.946132	2.1
37539	0.946132	2.1
37540	0.946132	2.1
37541	0.946132	2.1
37542	2.56953	2.1
37543	2.64148	2.1
37544	2.48631	2.1
37545	2.62213	2.1
37546	5.19166	2.1
37547	2.64148	2.1
37548	2.57929	2.1
37549	2.62213	2.1
37550	2.59522	2.1
37551	5.20142	2.1
37552	2.48631	2.1
37553	2.62213	2.1
37554	2.41176	2.1
37555	2.59262	2.1
37556	5.03389	2.1
37557	2.62213	2.1
37558	2.59522	2.1
37559	2.59262	2.1
37560	2.61901	2.1
37561	5.24114	2.1
37562	0.946132	0.05
37563	0.946132	0.05
37564	0.946133	0.05
37565	0.946133	0.05
37566	0.946132	0.05
37567	0.946132	0.05
37568	0.946133	0.05
37569	0.946133	0.05
37570	0.946132	0.05
37571	0.946132	0.05
37572	0.946133	0.05
37573	0.946133	0.05
37574	1.5681	0.05
37575	1.01248	0.05
37576	1.10749	0.05
37577	0.570913	0.05
37578	1.15589	0.05
37579	1.15588	0.05
37580	0.977549	0.05
37581	0.977549	0.05

37582	1.01249	0.05
37583	0.654445	0.05
37584	1.36795	0.05
37585	1.10748	0.05
37586	1.73883	0.05
37587	1.87638	0.05
37588	1.85219	0.05
37589	1.93113	2.1
37590	3.7407	0.05
37591	1.87638	0.05
37592	1.73883	2.1
37593	1.93113	2.1
37594	1.80426	2.1
37595	3.66996	2.1
37596	2.02912	0.05
37597	1.44951	0.05
37598	1.50103	0.05
37599	2.02248	0.05
37600	3.47199	0.05
37601	1.89226	0.05
37602	1.89226	0.05
37603	1.89226	0.05
37604	1.89226	0.05
37605	3.78453	0.05
37606	2.57881	0.05
37607	1.89226	0.05
37608	1.89226	0.05
37609	1.89226	0.05
37610	2.6428	0.05
37611	2.6428	0.05
37612	1.01379	0.05
37613	0.985937	0.05
37614	0.769295	0.05
37615	0.715547	0.05
37616	0.853042	0.05
37617	0.853042	0.05
37618	0.864117	0.05
37619	0.864114	0.05
37620	0.715556	0.05
37621	1.03047	0.05
37622	0.731721	0.05
37623	0.985921	0.05
37624	1.89227	0.05
37625	1.89227	0.05
37626	1.89227	0.05
37627	1.89227	0.0593424
37628	3.78453	0.05
37629	0.946129	0.05
37630	0.946129	0.05
37631	1.28161	0.05
37632	1.3214	0.05
37633	0.94613	0.05
37634	0.94613	0.05
37635	1.28161	0.05
37636	1.28161	0.05
37637	1.28161	0.05
37638	0.94613	0.05
37639	0.946131	0.05

37640	1.3214	0.05
37641	0.946132	0.103983
37642	0.946132	0.0782235
37643	0.706045	0.05
37644	1.16718	0.05
37645	0.706017	0.05
37646	0.706017	0.239164
37647	1.4073	0.05
37648	1.4073	0.05
37649	0.94613	0.05
37650	1.16719	0.05
37651	0.706046	0.05
37652	0.946131	0.05
37653	1.89226	0.212838
37654	1.89226	0.05
37655	1.89226	0.05
37656	1.89226	0.187734
37657	1.89226	0.05
37658	1.89226	0.222717
37659	0.946119	0.138482
37660	0.946119	0.10964
37661	0.946115	0.0610067
37662	0.946115	0.05
37663	0.946117	0.060466
37664	0.946117	0.184106
37665	0.946117	0.0828045
37666	0.946117	0.05
37667	0.94613	0.05
37668	0.94613	0.0781048
37669	0.946105	0.0701261
37670	0.946105	0.0515168
37671	0.946132	0.323809
37672	0.946132	0.199648
37673	0.946131	0.199949
37674	0.946131	0.0741279
37675	0.946132	0.489597
37676	0.946132	0.208321
37677	0.946132	0.192088
37678	0.827865	0.10272
37679	0.946132	0.0957074
37680	0.946132	0.153779
37681	0.946132	0.209574
37682	0.827865	0.182934
37683	1.89226	1.3408
37684	1.89226	0.864695
37685	1.89226	1.53565
37686	1.89226	0.614515
37687	1.89226	2.00689
37688	1.89226	1.1054
37689	0.93094	0.589926
37690	0.93094	1.058
37691	1.04649	0.609191
37692	0.614468	0.206994
37693	0.950358	0.305739
37694	0.799012	0.264312
37695	0.682742	0.685966
37696	0.682742	0.60227
37697	1.04073	1.5885

37698	1.15596	1.16785
37699	0.614478	1.19862
37700	0.851524	0.930859
37701	2.22575	2.1
37702	1.90502	2.1
37703	2.10893	2.1
37704	1.80258	2.1
37705	4.01395	2.1
37706	1.90502	2.1
37707	1.40863	2.1
37708	1.49733	2.1
37709	1.49733	2.1
37710	1.80258	2.1
37711	1.80258	2.1
37712	2.10893	2.1
37713	1.68356	2.1
37714	1.80258	2.1
37715	1.80258	2.1
37716	1.98061	2.1
37717	1.98061	2.1
37718	1.80258	2.1
37719	1.40863	2.1
37720	1.68356	2.1
37721	1.30751	2.1
37722	3.11009	2.1
37723	1.35374	1.92346
37724	1.35374	2.05791
37725	0.746096	1.27601
37726	0.746096	1.29654
37727	1.1537	1.2354
37728	0.746095	1.83617
37729	0.946132	1.62957
37730	0.946132	0.91152
37731	0.946132	1.11346
37732	0.746095	1.18391
37733	0.946131	0.688305
37734	1.1537	1.05816
37735	2.15247	2.1
37736	1.70303	2.1
37737	1.41207	2.1
37738	1.43052	2.1
37739	3.22793	2.1
37740	0.940246	2.1
37741	0.728442	2.1
37742	0.650037	2.1
37743	0.539874	2.1
37744	0.694756	2.1
37745	0.694756	2.1
37746	0.776346	2.1
37747	0.656772	2.1
37748	0.733146	2.1
37749	0.563769	2.1
37750	0.66316	2.1
37751	0.899692	2.1
37752	1.21223	2.1
37753	1.22418	2.1
37754	1.43052	2.1
37755	1.43052	2.1

37756	1.24353	2.1
37757	1.46181	2.1
37758	1.89226	0.05
37759	2.39894	0.05
37760	1.89226	0.05
37761	2.37417	0.05
37762	1.89226	0.05
37763	2.37417	0.05
37764	1.12767	0.05
37765	1.19947	0.05
37766	0.94613	0.05
37767	0.94613	0.05
37768	1.12766	0.05
37769	0.946128	0.05
37770	1.19947	0.05
37771	0.946132	0.05
37772	1.19947	0.05
37773	1.19947	0.05
37774	0.94613	0.05
37775	0.94613	0.05
37776	2.22621	0.05
37777	1.14533	2.1
37778	1.1131	2.1
37779	0.88238	2.1
37780	0.868521	2.1
37781	1.01153	2.1
37782	1.01153	2.1
37783	1.00232	2.1
37784	1.00232	2.1
37785	1.14533	2.1
37786	0.882378	2.1
37787	0.868519	2.1
37788	1.11311	2.1
37789	0.910183	1.90604
37790	1.07811	0.917438
37791	0.94613	0.0693605
37792	0.94613	0.146827
37793	0.910212	0.178223
37794	0.910212	2.03119
37795	1.11403	0.0701236
37796	1.11403	2.1
37797	0.910183	0.437595
37798	0.94613	0.921591
37799	0.94613	1.0375
37800	1.07811	1.04374
37801	1.87228	0.450654
37802	2.81189	2.1
37803	1.6197	2.1
37804	2.56716	2.1
37805	4.43944	2.1
37806	0.946132	0.92073
37807	0.946132	2.1
37808	0.946132	0.0882347
37809	0.946132	0.19375
37810	0.946131	0.414394
37811	0.946131	2.1
37812	0.946132	0.113761
37813	0.946132	1.24138

37814	0.946132	0.424016
37815	0.946132	0.513457
37816	0.946132	0.75131
37817	0.946132	1.0378
37818	1.28724	2.1
37819	2.56716	2.1
37820	2.3014	1.58965
37821	2.3014	2.1
37822	1.6197	2.1
37823	1.6197	2.1
37824	0.946132	2.1
37825	0.946132	2.01701
37826	0.946132	0.0876476
37827	0.946132	0.449665
37828	0.946132	0.624982
37829	0.946132	2.1
37830	0.946133	2.1
37831	0.946133	0.0869313
37832	0.946133	1.13194
37833	0.946133	1.41146
37834	0.946132	1.26907
37835	0.946132	1.39526
37836	1.08039	2.1
37837	1.24445	2.1
37838	1.2873	2.1
37839	1.48751	2.1
37840	2.53175	2.1
37841	0.946125	0.21129
37842	0.946125	2.1
37843	1.12552	2.1
37844	1.12552	2.1
37845	0.946124	2.01435
37846	0.946124	2.1
37847	1.12552	2.1
37848	1.11114	2.1
37849	1.11113	2.1
37850	0.946118	2.1
37851	1.12552	2.1
37852	0.946131	2.1
37853	1.89225	0.605622
37854	1.89225	0.933547
37855	1.89225	2.1
37856	1.89225	2.1
37857	1.89225	0.120244
37858	1.89225	2.1
37859	0.987703	0.423541
37860	1.33078	1.90695
37861	1.31565	0.998769
37862	1.31565	2.1
37863	1.01121	0.993708
37864	1.01121	2.1
37865	2.19163	2.1
37866	1.89226	2.1
37867	1.89226	2.1
37868	1.89226	1.99829
37869	2.22229	2.1
37870	2.22229	1.93012
37871	1.89226	2.1

37872	1.89226	0.103111
37873	1.89226	0.0720431
37874	1.89226	2.1
37875	1.89226	0.091033
37876	1.89226	2.1
37877	0.57141	2.1
37878	0.57141	0.162468
37879	0.574036	0.848326
37880	0.574036	2.1
37881	0.651591	2.1
37882	0.657827	0.82341
37883	0.482391	1.29786
37884	0.493855	2.04004
37885	0.482391	0.151245
37886	0.651591	0.289987
37887	0.493855	1.74883
37888	0.657828	1.7234
37889	1.88428	2.1
37890	2.03975	2.1
37891	1.97316	2.1
37892	1.8266	2.1
37893	3.86635	2.1
37894	2.23053	2.1
37895	1.42255	2.1
37896	1.3868	2.1
37897	1.8266	2.1
37898	3.24915	2.1
37899	1.97316	2.1
37900	1.8266	2.1
37901	1.77128	2.1
37902	1.91767	2.1
37903	3.74427	2.1
37904	1.77128	2.1
37905	1.8266	2.1
37906	1.77942	2.1
37907	1.33962	2.1
37908	1.3868	2.1
37909	1.3868	2.1
37910	0.944111	2.1
37911	0.944111	2.1
37912	1.26036	2.1
37913	1.16968	2.1
37914	1.03853	2.1
37915	1.16646	2.1
37916	0.946129	2.1
37917	0.946129	2.1
37918	0.94613	2.1
37919	1.25768	2.1
37920	1.03853	2.1
37921	0.946131	2.1
37922	1.82441	0.05
37923	1.33061	0.05
37924	1.13272	0.05
37925	1.60249	0.05
37926	2.95712	0.05
37927	1.82441	0.05
37928	2.08757	0.05
37929	1.81292	0.05

37930	1.60249	0.05
37931	3.63733	0.05
37932	1.81292	0.05
37933	1.332 0.05	
37934	1.60249	0.05
37935	1.60249	0.05
37936	1.5344	0.05
37937	1.5344	0.05
37938	1.10107	2.1
37939	2.51559	2.1
37940	1.1229	2.1
37941	1.1229	2.1
37942	2.49456	2.1
37943	2.49456	2.1
37944	1.89226	2.1
37945	2.51559	2.1
37946	2.49457	2.1
37947	2.51559	2.1
37948	1.87124	2.1
37949	1.89226	2.1
37950	1.89226	2.1
37951	1.89226	2.1
37952	1.89226	2.1
37953	1.89226	2.1
37954	1.89226	2.1
37955	1.89226	2.1
37956	1.89226	2.1
37957	1.89226	2.1
37958	1.89226	2.1
37959	1.89226	2.1
37960	1.89226	2.1
37961	1.89226	2.1
37962	1.23599	2.1
37963	1.24728	2.1
37964	0.550534	2.1
37965	0.541151	2.1
37966	0.893754	2.1
37967	0.893754	2.1
37968	0.894678	2.1
37969	0.894678	2.1
37970	1.23599	2.1
37971	0.541149	2.1
37972	0.550533	2.1
37973	1.24728	2.1
37974	0.946132	2.1
37975	0.946132	2.1
37976	1.23599	2.1
37977	1.24728	2.1
37978	0.946132	2.1
37979	0.946132	2.1
37980	1.23599	2.1
37981	1.23599	2.1
37982	1.23599	2.1
37983	0.946132	2.1
37984	0.946132	2.1
37985	1.24728	2.1
37986	1.89226	2.1
37987	1.89226	2.1

37988	1.89226	2.1
37989	1.89226	2.1
37990	1.89226	2.1
37991	1.89226	2.1
37992	0.946128	2.1
37993	0.946128	2.1
37994	0.946132	2.1
37995	0.946132	2.1
37996	0.94613	2.1
37997	0.94613	2.1
37998	0.946129	2.1
37999	0.946129	2.1
38000	0.94613	2.1
38001	0.94613	2.1
38002	0.94613	2.1
38003	0.94613	2.1
38004	2.58696	0.05
38005	2.57073	0.0700243
38006	2.12585	0.05
38007	2.07607	0.116858
38008	4.69658	0.0835559
38009	1.89226	0.0696235
38010	1.89226	0.115394
38011	1.89226	0.062362
38012	1.89226	0.112472
38013	3.78452	0.0640025
38014	1.89226	0.120176
38015	1.89226	0.244498
38016	1.89226	0.119634
38017	1.89226	0.224993
38018	3.78453	0.211665
38019	2.57074	0.0724746
38020	2.32635	0.177453
38021	2.07608	0.123163
38022	1.73623	0.109581
38023	4.30697	0.0904028
38024	2.12582	0.05
38025	1.51971	0.0502555
38026	1.57255	0.05
38027	2.07604	0.05
38028	1.62233	0.05
38029	2.12582	0.05
38030	1.89223	0.05
38031	1.89223	0.0886281
38032	1.89223	0.10068
38033	1.89223	0.0629272
38034	1.89223	0.068252
38035	1.89223	0.05
38036	0.946119	0.146358
38037	0.946119	0.10622
38038	0.946119	0.0686041
38039	0.946119	0.151376
38040	0.946119	0.166304
38041	0.946119	0.146471
38042	0.94612	0.0995141
38043	0.94612	0.118109
38044	0.946132	0.125434
38045	0.946132	0.0906407

38046	0.946107	0.129483
38047	0.946107	0.114801
38048	0.811456	0.0619437
38049	0.811456	0.132733
38050	0.806521	0.0530301
38051	0.806521	0.12413
38052	0.868104	0.144588
38053	0.579949	0.085938
38054	0.759859	0.0507712
38055	1.03802	0.05
38056	0.579957	0.0757791
38057	0.759868	0.065035
38058	1.03801	0.0771545
38059	0.868093	0.0928241
38060	2.7408	2.1
38061	2.82858	2.1
38062	2.57368	2.1
38063	2.52633	2.1
38064	5.3549	2.1
38065	2.7408	2.1
38066	2.31353	2.1
38067	2.14063	2.1
38068	2.52632	2.1
38069	4.83985	2.1
38070	2.57368	2.1
38071	2.52633	2.1
38072	2.3301	2.1
38073	2.36071	2.1
38074	4.88704	2.1
38075	1.86512	2.1
38076	2.52632	2.1
38077	2.14063	2.1
38078	2.14063	2.1
38079	2.3301	2.1
38080	2.3301	2.1
38081	1.86431	2.1
38082	1.89227	2.1
38083	1.68992	2.1
38084	1.68992	2.1
38085	1.89227	2.1
38086	1.89227	2.1
38087	2.0815	2.1
38088	1.89226	2.1
38089	2.04022	2.1
38090	1.86431	2.1
38091	2.07081	2.1
38092	1.89226	2.1
38093	1.91686	1.47727
38094	1.65683	1.93927
38095	1.86871	2.1
38096	1.65683	1.47135
38097	2.13075	2.02096
38098	1.89226	0.105312
38099	1.89226	2.01589
38100	1.89226	0.0603252
38101	1.89226	0.336241
38102	1.89226	1.71522
38103	3.78452	0.626147

38104	0.851541	2.1
38105	0.851541	2.1
38106	0.952803	2.1
38107	0.851573	2.1
38108	0.946129	2.1
38109	0.946129	1.50243
38110	0.929184	2.1
38111	0.757482	2.1
38112	0.836274	2.1
38113	0.851528	2.1
38114	0.952816	2.1
38115	1.04073	2.1
38116	1.70303	1.09818
38117	1.70302	0.481332
38118	1.5544	2.07151
38119	1.70302	0.865175
38120	1.74957	0.728424
38121	1.91686	0.0917958
38122	1.89226	0.235792
38123	1.89226	0.770246
38124	1.89226	1.72391
38125	1.89226	0.461911
38126	1.89226	0.249675
38127	1.89226	0.05
38128	1.89226	2.1
38129	1.89226	2.1
38130	2.03472	2.1
38131	1.89226	2.1
38132	1.99827	2.1
38133	1.85836	1.63264
38134	1.00509	2.1
38135	1.55998	2.1
38136	1.83347	2.1
38137	1.45246	2.1
38138	3.01244	2.1
38139	1.23839	2.1
38140	0.884251	2.1
38141	0.946133	2.1
38142	0.946133	2.1
38143	0.884226	2.1
38144	0.884226	2.1
38145	0.946158	2.1
38146	0.946159	2.1
38147	1.23839	2.1
38148	0.946131	2.1
38149	0.946131	2.1
38150	0.884254	2.1
38151	1.89226	2.1
38152	1.89226	2.1
38153	1.89226	2.1
38154	1.89226	2.1
38155	1.89226	2.1
38156	1.89226	2.1
38157	2.1499	2.1
38158	1.45244	2.1
38159	1.83345	2.1
38160	1.83345	2.1
38161	1.87214	2.1

38162	1.87214	2.1
38163	1.89224	2.1
38164	1.89224	2.01224
38165	1.89224	2.1
38166	1.89224	2.1
38167	3.78448	2.1
38168	1.49155	0.174558
38169	2.0815	1.59988
38170	1.77515	1.43863
38171	1.72377	2.1
38172	3.50659	0.882714
38173	1.69876	0.05
38174	2.0815	2.08418
38175	1.9133	0.0807348
38176	1.68189	0.05
38177	2.17628	2.1
38178	1.94335	2.1
38179	2.65129	0.05
38180	1.15317	0.05
38181	2.57085	0.05
38182	1.209 0.05	
38183	2.57085	0.05
38184	1.209 0.05	
38185	1.89226	0.05
38186	1.89226	0.05
38187	1.89226	0.05
38188	1.89226	0.05
38189	3.78452	0.05
38190	2.48861	0.05
38191	1.15252	0.05
38192	2.60515	0.05
38193	2.60515	0.05
38194	1.16384	0.05
38195	1.16384	0.05
38196	1.59903	0.499514
38197	1.26865	0.106863
38198	1.30486	1.44407
38199	1.70302	1.03549
38200	2.98573	0.543745
38201	1.59903	0.31741
38202	1.79478	0.477567
38203	1.79478	0.230576
38204	1.97005	0.812479
38205	1.50365	0.127492
38206	1.64192	1.36482
38207	1.89226	0.108314
38208	1.89226	0.551049
38209	1.89226	0.51882
38210	1.89226	0.130731
38211	3.78453	0.140356
38212	1.89226	0.27326
38213	1.89226	0.472495
38214	1.89226	0.430511
38215	1.89226	0.112418
38216	1.89226	0.566956
38217	1.89226	0.956499
38218	0.761672	0.137743
38219	0.896406	0.224777

38220	0.603509	0.174658
38221	0.574071	0.752037
38222	0.695159	0.700683
38223	0.695159	0.0544931
38224	0.762564	1.68445
38225	0.770159	0.107871
38226	0.803021	0.456062
38227	0.572529	1.20542
38228	0.696466	0.0871886
38229	0.899733	0.124295
38230	1.66034	0.05
38231	1.79478	0.785624
38232	1.56685	0.132092
38233	1.68512	1.76534
38234	1.67652	0.310964
38235	1.67652	0.762723
38236	1.89226	0.824044
38237	1.89226	0.282635
38238	1.89226	0.732436
38239	1.89226	0.433652
38240	1.89226	0.726362
38241	1.89226	0.619122
38242	0.946133	0.0984397
38243	0.946133	0.255377
38244	0.946132	1.25066
38245	0.946132	0.465992
38246	0.946132	0.710795
38247	0.946132	0.0651029
38248	0.946133	1.81234
38249	0.946133	0.132176
38250	0.946133	0.715101
38251	0.946133	0.298169
38252	0.946132	0.164009
38253	0.946132	0.115754
38254	1.80424	2.1
38255	1.45158	2.1
38256	1.9098	2.1
38257	1.56003	2.1
38258	3.36138	2.1
38259	1.90982	2.1
38260	1.71606	2.1
38261	2.15314	2.1
38262	1.70302	2.1
38263	3.89051	2.1
38264	1.96416	0.879258
38265	1.23706	1.24703
38266	1.00053	0.146364
38267	1.38287	0.132031
38268	1.57916	0.527315
38269	1.9615	1.09203
38270	0.827865	0.186852
38271	0.946132	0.0980777
38272	0.946132	0.0637151
38273	0.946132	0.345005
38274	0.946132	0.608833
38275	0.946132	0.226221
38276	0.946131	1.07407
38277	0.946131	0.177374

38278	0.827865	0.801445
38279	0.946132	0.05
38280	0.946132	0.0593925
38281	0.946132	0.154062
38282	1.89227	2.1
38283	1.89227	2.1
38284	1.89227	2.1
38285	1.89227	2.1
38286	3.78453	2.1
38287	2.3819	2.1
38288	1.58089	2.1
38289	1.58089	2.1
38290	1.6287	2.1
38291	2.33368	2.1
38292	2.3815	2.1
38293	0.946133	2.1
38294	0.946133	2.1
38295	0.946132	2.1
38296	0.946132	2.1
38297	0.946132	2.1
38298	0.946132	2.1
38299	0.946132	2.1
38300	0.946132	2.1
38301	0.946133	2.1
38302	0.946133	2.1
38303	0.946132	2.1
38304	0.946132	2.1
38305	1.89226	2.1
38306	1.89226	2.1
38307	1.89226	2.1
38308	1.89226	2.1
38309	3.78453	2.1
38310	2.7874	2.1
38311	2.33368	2.1
38312	2.71897	2.1
38313	2.27495	2.1
38314	5.05266	2.1
38315	0.970991	2.1
38316	0.970991	2.1
38317	0.956929	2.1
38318	0.956929	2.1
38319	0.732928	2.1
38320	0.790444	2.1
38321	1.16684	2.1
38322	1.13748	2.1
38323	1.13748	2.1
38324	0.732928	2.1
38325	0.790444	2.1
38326	1.16684	2.1
38327	0.946132	2.1
38328	0.946132	2.1
38329	0.946131	2.1
38330	0.946131	2.1
38331	0.946132	2.1
38332	0.946132	2.1
38333	0.946132	2.1
38334	0.946132	2.1
38335	0.946132	2.1

38336	0.946132	2.1
38337	0.946132	2.1
38338	0.946132	2.1
38339	1.10747	0.05
38340	2.30331	0.05
38341	1.47954	0.05
38342	2.63789	0.05
38343	3.78285	0.05
38344	1.65215	0.05
38345	2.63789	0.05
38346	2.82465	0.05
38347	2.82465	0.05
38348	1.47954	0.05
38349	1.47954	0.05
38350	1.13456	2.1
38351	1.08334	2.1
38352	1.20674	0.323849
38353	1.17885	2.1
38354	2.29008	2.00934
38355	1.20674	1.66037
38356	1.17885	2.1
38357	1.28649	0.667668
38358	1.28516	1.92213
38359	2.4919	1.80482
38360	1.89226	2.1
38361	1.89226	2.1
38362	1.89226	2.1
38363	1.89226	2.1
38364	1.89226	2.1
38365	1.89226	2.1
38366	2.50288	1.81507
38367	2.02115	2.1
38368	2.7717	1.72119
38369	2.29107	0.350168
38370	4.79395	2.1
38371	1.27339	2.1
38372	1.10817	2.1
38373	1.19733	2.1
38374	1.032	2.1
38375	2.30539	2.1
38376	1.19733	2.1
38377	0.953637	2.1
38378	0.953637	2.1
38379	1.032	2.1
38380	1.11868	2.1
38381	1.19704	2.1
38382	2.01024	0.751542
38383	1.11938	0.992008
38384	1.9186	1.43988
38385	2.76298	0.699481
38386	3.92884	1.18838
38387	0.851541	0.05
38388	0.851541	0.05
38389	0.957577	0.05
38390	0.848952	0.05
38391	1.08043	0.05
38392	0.851541	0.05
38393	0.851484	0.05

38394	0.957578	0.05
38395	1.20045	0.05
38396	0.726289	0.05
38397	0.946131	0.05
38398	0.946131	0.05
38399	1.50524	2.1
38400	2.38363	2.1
38401	2.49018	2.1
38402	2.49018	2.1
38403	1.45506	2.1
38404	1.45506	2.1
38405	2.49017	2.1
38406	1.89226	2.1
38407	1.89226	2.1
38408	1.89226	2.1
38409	2.38363	2.1
38410	2.38363	2.1
38411	1.54711	2.1
38412	2.49018	2.1
38413	2.56975	2.1
38414	2.56975	2.1
38415	1.50524	2.1
38416	1.50524	2.1
38417	2.56975	2.1
38418	1.89226	2.1
38419	1.89226	2.1
38420	1.89226	2.1
38421	2.49017	2.1
38422	2.49017	2.1
38423	1.89226	0.05
38424	1.89226	0.05
38425	1.89226	0.05
38426	1.89226	0.05
38427	1.89226	0.05
38428	1.89226	0.05
38429	1.89226	0.05
38430	1.89226	0.05
38431	1.89226	0.05
38432	1.89226	0.05
38433	1.89226	0.05
38434	1.89226	0.05
38435	1.89226	0.05
38436	1.89226	0.05
38437	2.8358	0.05
38438	1.89226	0.05
38439	3.78453	0.05
38440	1.60565	0.05
38441	1.89226	1.09073
38442	1.89226	0.476237
38443	1.40013	0.105095
38444	1.89226	0.736905
38445	1.40013	0.189756
38446	1.1766	2.1
38447	1.29435	2.1
38448	1.35735	2.1
38449	1.53509	2.1
38450	2.65169	2.1
38451	0.69701	2.1

38452	0.69701	2.1
38453	0.711303	2.1
38454	0.711303	2.1
38455	0.761138	2.1
38456	0.612149	2.1
38457	0.647174	2.1
38458	0.767548	2.1
38459	0.767548	2.1
38460	0.761139	2.1
38461	0.612149	2.1
38462	0.647174	2.1
38463	1.29764	1.04186
38464	1.29579	1.59746
38465	1.34875	1.41505
38466	1.06717	1.53336
38467	1.29764	1.16763
38468	1.01606	1.00453
38469	0.756635	0.910371
38470	0.648821	1.06665
38471	0.675557	1.4716
38472	0.674374	1.28864
38473	0.74214	1.53804
38474	0.74214	1.11383
38475	0.688868	0.891886
38476	0.688868	1.21626
38477	0.675557	0.71548
38478	0.756634	0.900147
38479	0.648821	0.504367
38480	0.674374	0.723184
38481	1.51327	1.23923
38482	1.48105	0.715138
38483	1.29764	1.36087
38484	1.29764	0.940251
38485	1.48105	1.37391
38486	1.05655	0.647144
38487	1.13537	2.1
38488	1.07326	2.1
38489	0.94613	2.1
38490	0.94613	2.1
38491	0.946132	2.1
38492	0.946132	2.1
38493	1.13537	2.1
38494	1.08927	2.1
38495	1.13537	2.1
38496	0.946131	2.1
38497	0.946131	2.1
38498	0.95947	2.1
38499	1.46892	2.1
38500	1.89226	2.1
38501	1.89226	2.1
38502	1.89226	2.1
38503	1.88031	2.1
38504	1.88031	2.1
38505	1.89226	2.1
38506	1.89226	2.1
38507	1.89226	2.1
38508	1.89226	2.1
38509	3.78453	2.1

38510	2.18254	2.1
38511	1.89226	2.1
38512	2.50005	2.1
38513	1.89226	2.1
38514	4.39231	2.1
38515	1.88031	2.1
38516	2.0815	2.1
38517	2.0815	2.1
38518	1.92078	2.1
38519	2.0815	2.1
38520	1.9218	2.1
38521	1.89227	2.1
38522	1.89227	2.1
38523	1.89227	2.1
38524	1.89227	2.1
38525	1.89227	2.1
38526	1.89227	2.1
38527	1.98693	2.1
38528	1.82857	2.1
38529	2.32	2.1
38530	2.36972	2.1
38531	4.14857	2.1
38532	1.82857	2.1
38533	1.58688	2.1
38534	2.36971	2.1
38535	2.19093	2.1
38536	4.0195	2.1
38537	0.946132	2.1
38538	0.946132	2.1
38539	1.18486	2.1
38540	1.18486	2.1
38541	1.18486	2.1
38542	0.946132	2.1
38543	0.946132	2.1
38544	1.16	2.1
38545	0.946133	2.1
38546	0.946133	2.1
38547	1.16	2.1
38548	1.18486	2.1
38549	1.07798	1.81865
38550	1.08101	1.10391
38551	0.910786	2.1
38552	0.910786	2.1
38553	0.851537	2.1
38554	0.901377	1.10754
38555	1.14652	2.1
38556	1.04072	2.1
38557	0.946129	2.1
38558	0.946129	2.1
38559	1.0019	2.092
38560	1.04263	2.1
38561	1.89226	2.1
38562	1.89226	2.1
38563	1.89226	2.1
38564	1.89226	2.1
38565	3.78452	2.1
38566	1.89226	2.1
38567	2.30146	2.1

38568	1.89226	2.1
38569	2.18412	2.1
38570	4.19372	2.1
38571	2.30146	2.1
38572	1.39962	2.1
38573	2.18411	2.1
38574	1.28765	2.1
38575	3.5891	2.1
38576	1.89226	2.1
38577	1.89226	2.1
38578	1.89226	2.1
38579	1.89226	2.1
38580	1.89226	2.1
38581	1.89226	2.1
38582	1.89226	2.1
38583	1.89226	2.1
38584	1.89226	2.1
38585	1.89226	2.1
38586	3.78452	2.1
38587	2.18412	2.1
38588	1.20045	2.1
38589	1.20045	2.1
38590	1.28765	2.1
38591	2.08808	2.1
38592	2.17528	2.1
38593	0.946131	2.1
38594	0.946131	2.1
38595	0.946128	2.1
38596	0.946128	2.1
38597	0.946127	2.1
38598	0.946127	2.1
38599	0.946131	2.1
38600	0.946131	2.1
38601	0.946129	2.1
38602	0.946129	2.1
38603	0.94613	2.1
38604	0.94613	2.1
38605	1.89226	0.383829
38606	1.89226	1.49798
38607	1.89226	1.4349
38608	1.89226	0.426143
38609	3.78452	0.186584
38610	1.89227	0.226378
38611	1.89227	1.88153
38612	1.89227	0.145987
38613	1.89227	1.33256
38614	1.89227	0.161574
38615	1.89227	1.59356
38616	1.89226	1.70714
38617	1.89226	0.445393
38618	1.89226	0.562368
38619	2.29245	0.480639
38620	3.78452	1.24287
38621	1.89226	1.57926
38622	1.89226	0.447047
38623	1.89226	0.348968
38624	1.89226	1.05
38625	1.89226	0.123249

38626	1.89226	0.260566
38627	1.28095	2.02087
38628	1.81846	2.1
38629	2.10991	0.98755
38630	1.34695	1.83414
38631	3.39086	1.97598
38632	2.10991	1.40278
38633	1.36457	1.90361
38634	1.67421	2.1
38635	1.34695	2.00085
38636	2.43717	1.56749
38637	2.10991	1.73892
38638	2.69541	2.1
38639	1.48572	2.1
38640	2.63553	2.1
38641	1.45385	2.1
38642	4.14926	2.1
38643	2.63553	2.1
38644	1.44207	2.1
38645	1.44207	2.1
38646	1.45385	2.1
38647	2.588	2.1
38648	2.59978	2.1
38649	1.89227	2.1
38650	1.89227	2.1
38651	1.89227	2.1
38652	1.89227	2.1
38653	3.78453	2.1
38654	1.89226	2.1
38655	1.89226	2.1
38656	1.89226	2.1
38657	1.89226	2.1
38658	3.78453	2.1
38659	2.15929	2.1
38660	1.38276	2.1
38661	1.43617	2.1
38662	1.35614	2.1
38663	2.23932	2.1
38664	2.15929	2.1
38665	2.15929	2.1
38666	2.25168	2.1
38667	2.3558	2.1
38668	2.23932	2.1
38669	4.51509	2.1
38670	1.89226	2.1
38671	1.89226	2.1
38672	1.89226	2.1
38673	1.89226	2.1
38674	3.78453	2.1
38675	1.89226	2.1
38676	1.89226	2.1
38677	1.89226	2.1
38678	1.89226	2.1
38679	1.89226	2.1
38680	1.89226	2.1
38681	2.23932	2.1
38682	1.43854	2.1
38683	1.49173	2.1

38684	1.38276	2.1
38685	2.34829	2.1
38686	2.23932	2.1
38687	2.3558	2.1
38688	2.34829	2.1
38689	2.40685	2.1
38690	2.23932	2.1
38691	2.52332	2.1
38692	2.3558	2.1
38693	1.89226	0.05
38694	1.87361	0.05
38695	2.25082	0.05
38696	1.87361	0.05
38697	2.26947	0.05
38698	1.89226	0.05
38699	1.89226	0.05
38700	1.89226	0.05
38701	1.89226	0.05
38702	1.89226	0.05
38703	1.89226	0.05
38704	1.89226	0.05
38705	2.41314	0.05
38706	1.89226	0.05
38707	1.89226	0.05
38708	1.89226	0.05
38709	2.12204	0.05
38710	2.12204	0.05
38711	1.89226	0.05
38712	2.25082	0.05
38713	2.28189	0.05
38714	2.25082	0.05
38715	1.92333	0.05
38716	1.89226	0.05
38717	1.89226	0.05
38718	1.89226	0.05
38719	1.89226	0.05
38720	1.89226	0.05
38721	1.89226	0.05
38722	1.89226	0.05
38723	2.42521	0.05
38724	1.89226	0.05
38725	1.89226	0.05
38726	1.89226	0.05
38727	2.41314	0.05
38728	2.41314	0.05
38729	1.39725	1.25002
38730	1.07745	2.1
38731	1.07745	1.29772
38732	1.07519	2.1
38733	1.41336	0.707053
38734	1.41111	2.1
38735	1.89226	2.1
38736	1.92039	2.1
38737	1.92039	2.1
38738	1.9052	2.1
38739	1.89226	2.1
38740	1.87708	2.1
38741	0.946128	2.1

38742	0.946128	2.1
38743	0.946133	1.0023
38744	0.946133	0.472342
38745	0.94613	0.480124
38746	0.94613	2.1
38747	0.946131	2.1
38748	0.946131	0.508939
38749	0.946131	1.64088
38750	0.946131	1.6804
38751	0.946131	1.5842
38752	0.946131	1.7142
38753	1.41336	1.15433
38754	1.08911	2.1
38755	1.08911	1.69999
38756	1.07745	2.1
38757	1.42381	1.16363
38758	1.41215	2.1
38759	1.89227	2.1
38760	1.95833	2.1
38761	1.95833	2.1
38762	1.92039	2.1
38763	1.89227	2.1
38764	1.85433	2.1
38765	0.946129	2.1
38766	0.946129	2.1
38767	0.946133	1.59092
38768	0.946133	0.558175
38769	0.946131	0.5481
38770	0.946131	2.1
38771	0.946131	2.1
38772	0.946131	0.902529
38773	0.946131	1.9525
38774	0.946131	1.98043
38775	0.946131	1.93944
38776	0.946131	1.97836
38777	1.41943	2.1
38778	2.74398	2.1
38779	1.18238	2.1
38780	2.53897	2.1
38781	3.9584	2.1
38782	2.74398	2.1
38783	1.89226	2.1
38784	1.89226	2.1
38785	1.89226	2.1
38786	2.53897	2.1
38787	2.53897	2.1
38788	1.89227	1.0086
38789	1.89227	0.377581
38790	1.89227	1.07947
38791	1.89227	0.248072
38792	3.78453	0.387558
38793	1.89226	0.328343
38794	2.08134	0.10757
38795	2.00632	0.0981831
38796	2.00632	0.0972374
38797	1.89226	0.226209
38798	1.89226	0.22082
38799	1.89227	0.562587

38800	1.89227	0.130278
38801	1.89227	0.307731
38802	1.89227	0.115233
38803	3.78453	0.318414
38804	1.89227	0.931013
38805	1.89227	0.112745
38806	1.89227	0.12008
38807	1.89227	0.229476
38808	1.89227	0.378485
38809	1.89227	0.813368
38810	2.50912	0.0982196
38811	1.89226	0.188293
38812	1.89226	0.05
38813	2.31261	0.125982
38814	2.31261	0.0976065
38815	1.89226	0.247461
38816	2.50912	0.0995456
38817	1.19999	0.05
38818	1.44056	0.05
38819	1.24405	0.0560686
38820	2.50912	0.0615764
38821	2.31261	0.0860509
38822	2.69224	0.44657
38823	2.28373	0.205428
38824	1.94788	1.59014
38825	2.4106	0.20512
38826	4.69434	1.19134
38827	2.69223	0.05
38828	2.63783	0.775349
38829	2.34737	1.59522
38830	2.4106	0.509817
38831	5.04843	0.742634
38832	1.89226	2.1
38833	1.99753	2.1
38834	1.87366	2.1
38835	1.99753	2.1
38836	1.76839	2.1
38837	1.89226	2.1
38838	1.89226	2.1
38839	1.89226	1.62109
38840	1.89226	2.1
38841	1.89226	2.1
38842	3.78452	2.1
38843	0.946132	2.1
38844	0.946132	2.1
38845	1.02744	2.1
38846	1.02744	2.1
38847	0.946132	2.1
38848	0.946132	2.1
38849	1.00989	2.1
38850	1.02744	2.1
38851	1.00989	2.1
38852	0.946133	2.1
38853	1.02744	2.1
38854	0.946132	2.1
38855	1.70302	2.1
38856	1.87366	2.1
38857	1.71618	2.1

38858	1.87366	2.1
38859	1.73105	2.1
38860	1.89226	2.1
38861	1.89226	2.1
38862	1.89226	2.1
38863	1.89226	2.1
38864	1.89226	2.1
38865	1.89226	2.1
38866	1.89226	1.35292
38867	1.96624	2.1
38868	1.89226	2.1
38869	1.89226	2.1
38870	1.89226	2.1
38871	2.01977	2.1
38872	2.01977	2.1
38873	1.1032	1.77015
38874	1.15114	2.1
38875	1.14986	0.05
38876	1.19242	2.1
38877	2.301	2.1
38878	1.89226	1.47764
38879	1.89226	2.1
38880	1.89226	0.05
38881	1.89226	2.1
38882	3.78453	1.26428
38883	0.946132	2.1
38884	0.946132	2.1
38885	0.946132	2.1
38886	0.946132	2.1
38887	0.946133	2.1
38888	0.946133	2.1
38889	0.946132	2.1
38890	0.946132	2.1
38891	0.946132	2.1
38892	0.946132	2.1
38893	0.946133	2.1
38894	0.946133	2.1
38895	1.19243	2.1
38896	0.953928	2.1
38897	1.18671	2.1
38898	1.15114	2.1
38899	0.9895	2.1
38900	0.953928	2.1
38901	1.19611	0.0555237
38902	1.19242	2.1
38903	1.24693	0.0677582
38904	1.24693	2.1
38905	1.14986	0.139643
38906	1.14986	2.1
38907	1.89225	0.0634001
38908	1.89225	2.1
38909	1.89225	2.1
38910	1.89225	0.616738
38911	1.89225	2.1
38912	1.89225	0.790904
38913	1.89225	2.1
38914	1.89225	2.1
38915	1.89225	2.1

38916	1.89225	2.1
38917	1.89225	1.33165
38918	1.89225	2.1
38919	0.561532	2.1
38920	0.561532	2.1
38921	0.566382	2.1
38922	0.566382	2.1
38923	0.494747	2.1
38924	0.531705	2.1
38925	0.596208	2.1
38926	0.623464	1.93991
38927	0.623469	2.1
38928	0.531708	2.1
38929	0.596205	2.1
38930	0.494743	2.1
38931	1.25742	2.1
38932	1.18783	2.1
38933	0.946131	2.1
38934	0.946131	2.1
38935	1.25742	2.1
38936	1.25742	2.1
38937	0.946131	2.1
38938	0.946131	2.1
38939	1.25742	2.1
38940	0.946131	2.1
38941	0.946131	2.1
38942	1.18783	2.1
38943	1.35048	2.1
38944	1.25742	2.1
38945	0.946132	2.1
38946	0.946132	2.1
38947	1.35048	2.1
38948	1.35048	2.1
38949	0.946132	2.1
38950	0.946132	2.1
38951	1.35044	2.1
38952	0.946106	2.1
38953	1.25745	2.1
38954	0.946158	2.1
38955	1.13525	2.1
38956	2.70095	2.1
38957	2.51483	2.1
38958	2.51483	2.1
38959	1.21593	2.1
38960	1.21593	2.1
38961	1.93142	0.05
38962	2.8053	0.05
38963	1.559	0.202156
38964	2.60359	0.05
38965	4.53501	0.0779899
38966	2.80531	0.0606282
38967	1.89226	0.05
38968	2.60359	0.05
38969	1.89226	0.0866143
38970	4.49586	0.05
38971	1.22938	0.0752267
38972	2.60359	0.05
38973	2.37434	0.177738

38974	2.37434	0.05
38975	1.55901	0.379652
38976	1.55901	0.05
38977	1.18717	0.14722
38978	1.18717	0.05
38979	0.946132	0.05
38980	0.946132	0.05
38981	0.946133	0.0559236
38982	0.946133	0.13779
38983	1.3018	0.05
38984	1.18717	0.178042
38985	1.18714	0.05
38986	0.946107	0.0501799
38987	1.30183	0.05
38988	0.946158	0.05
38989	1.89226	2.1
38990	2.80196	2.1
38991	2.67272	2.1
38992	2.67272	2.1
38993	1.89226	2.1
38994	1.89226	2.1
38995	1.89226	2.1
38996	1.89226	2.1
38997	1.89226	2.1
38998	1.89226	2.1
38999	1.89226	2.1
39000	1.89226	2.1
39001	1.74471	2.1
39002	2.01555	2.1
39003	1.61121	2.1
39004	1.61121	2.1
39005	2.13878	2.1
39006	2.13878	2.1
39007	1.89226	2.1
39008	1.89226	2.1
39009	1.89226	2.1
39010	1.89226	2.1
39011	1.89226	2.1
39012	1.89226	2.1
39013	1.09766	2.1
39014	1.04263	2.1
39015	0.881794	2.1
39016	0.894188	2.1
39017	0.795402	2.1
39018	0.99524	2.1
39019	1.00647	2.1
39020	1.00647	2.1
39021	1.15401	2.1
39022	0.881789	2.1
39023	0.850526	2.1
39024	1.04263	2.1
39025	1.4054	0.782354
39026	1.04788	2.1
39027	1.70302	1.0698
39028	1.41375	2.1
39029	2.82706	2.1
39030	1.70302	0.670193
39031	1.38776	2.1

39032	1.59457	2.02543
39033	1.41375	2.1
39034	1.77261	0.557813
39035	1.70302	2.1
39036	1.89226	2.1
39037	1.89226	2.1
39038	1.89226	2.1
39039	1.89226	2.1
39040	3.78453	2.1
39041	2.18042	2.1
39042	1.74738	2.1
39043	2.08433	2.1
39044	1.66347	2.1
39045	3.83171	2.1
39046	1.74738	2.1
39047	1.1181	2.1
39048	1.66347	2.1
39049	1.06543	2.1
39050	2.81281	2.1
39051	1.89226	2.1
39052	1.89226	2.1
39053	1.89226	2.1
39054	1.89226	2.1
39055	3.78453	2.1
39056	1.89227	2.1
39057	1.89227	2.1
39058	1.89227	2.1
39059	1.89227	2.1
39060	3.78453	2.1
39061	2.08433	2.1
39062	1.66347	2.1
39063	1.97092	2.1
39064	1.57431	2.1
39065	3.65864	2.1
39066	1.66347	2.1
39067	1.01474	2.1
39068	1.01474	2.1
39069	1.06544	2.1
39070	1.57431	2.1
39071	1.625 2.1	
39072	1.89226	2.1
39073	1.89226	2.1
39074	1.89226	2.1
39075	1.89226	2.1
39076	1.89226	2.1
39077	1.89226	2.1
39078	1.08467	2.1
39079	1.87228	2.1
39080	1.73305	2.1
39081	1.73305	2.1
39082	1.25565	2.1
39083	1.25565	2.1
39084	0.695361	0.05
39085	0.799996	0.104203
39086	1.13751	0.108403
39087	1.40945	0.179119
39088	1.40944	0.31976
39089	0.799999	0.05

39090	1.13751	0.0857353
39091	0.695363	0.05
39092	1.01059	0.0741026
39093	1.01059	0.0965171
39094	0.926908	0.179302
39095	0.926909	0.05
39096	1.05481	2.1
39097	1.78069	2.1
39098	1.08074	2.1
39099	1.83284	2.1
39100	2.86143	2.1
39101	1.78069	2.1
39102	2.22044	2.06633
39103	1.83284	2.1
39104	2.28696	1.03335
39105	4.06766	2.1
39106	1.07196	2.1
39107	1.64955	2.1
39108	2.02665	2.1
39109	2.0284	2.1
39110	1.08074	2.1
39111	1.08074	2.1
39112	1.64955	2.1
39113	2.28696	1.7054
39114	1.92033	2.1
39115	2.1004	2.1
39116	3.74988	2.1
39117	2.83787	0.0960855
39118	1.70303	1.62203
39119	1.70303	0.135483
39120	1.83195	0.193329
39121	2.64075	0.288855
39122	2.70231	1.46111
39123	1.2087	2.091
39124	1.52909	0.176266
39125	1.43063	0.131448
39126	1.10586	1.96519
39127	2.63494	0.786959
39128	1.89227	1.07057
39129	2.10732	2.1
39130	2.10732	1.78373
39131	2.2365	2.1
39132	1.89227	1.21831
39133	2.02145	2.1
39134	1.89226	0.259615
39135	1.89226	1.78431
39136	1.89226	0.28316
39137	1.89226	1.58221
39138	1.89226	0.115395
39139	1.89226	2.03898
39140	0.51926	2.00018
39141	0.552927	2.07643
39142	0.691235	0.425492
39143	0.715314	0.175376
39144	0.619684	0.107237
39145	0.619684	2.1
39146	0.624477	2.1
39147	0.624477	0.108401

39148	0.519262	0.758808
39149	0.691234	0.777232
39150	0.715313	1.13584
39151	0.552928	0.835861
39152	1.89226	1.87222
39153	1.87321	2.1
39154	1.87321	2.1
39155	1.89658	2.1
39156	2.0815	1.75791
39157	2.14789	2.1
39158	0.946128	2.1
39159	0.946128	1.90864
39160	0.946133	0.171781
39161	0.946133	0.159701
39162	0.94613	0.0928849
39163	0.94613	1.79171
39164	0.946131	0.301352
39165	0.946131	2.08247
39166	0.946131	0.402494
39167	0.946131	0.415919
39168	0.946131	0.925779
39169	0.946131	0.811772
39170	1.99297	0.770376
39171	2.19514	0.118397
39172	2.46153	0.383311
39173	2.187	0.288253
39174	4.45451	0.34354
39175	2.0815	0.225833
39176	2.33402	0.136684
39177	2.46154	0.424719
39178	2.83046	0.0693405
39179	4.94496	0.155774
39180	2.46153	0.335565
39181	2.187	0.196335
39182	2.27351	0.481337
39183	2.63356	0.0954181
39184	4.73504	0.243138
39185	2.46154	0.374924
39186	2.83447	0.0580652
39187	2.74513	0.125833
39188	2.5731	0.304802
39189	2.63357	0.105235
39190	2.46154	0.22663
39191	2.80398	2.1
39192	2.60406	2.1
39193	2.73988	2.1
39194	2.57466	2.1
39195	5.34394	2.1
39196	1.89226	1.33571
39197	1.89226	0.483198
39198	1.89226	0.0976591
39199	1.89226	1.49459
39200	1.89226	0.197419
39201	1.89226	2.09278
39202	1.89224	1.59278
39203	1.89224	0.540591
39204	1.89224	0.342463
39205	1.89224	1.85555

39206	1.89224	0.59189
39207	1.89224	2.02537
39208	1.40245	1.06272
39209	1.40246	1.91209
39210	0.946117	1.41495
39211	0.946117	2.02878
39212	1.17635	0.683247
39213	1.40246	1.94747
39214	0.946112	0.724983
39215	0.946112	1.96705
39216	0.946127	1.46385
39217	1.17637	0.664319
39218	0.946102	2.067
39219	1.40244	1.10612
39220	2.53269	2.1
39221	1.70302	2.1
39222	1.56194	2.1
39223	2.17516	2.1
39224	4.19807	2.1
39225	1.89226	2.1
39226	1.89226	1.29624
39227	1.89226	2.1
39228	1.89226	2.1
39229	3.78453	2.1
39230	2.57276	2.1
39231	1.89226	2.1
39232	1.89226	2.1
39233	1.89226	2.1
39234	2.32525	2.1
39235	2.32525	2.1
39236	1.15171	2.1
39237	2.17517	2.1
39238	1.41995	2.1
39239	1.41995	2.1
39240	1.80252	2.1
39241	1.80252	2.1
39242	1.89226	2.1
39243	1.89226	2.1
39244	1.89226	2.1
39245	1.89226	0.20136
39246	3.78453	2.1
39247	0.946133	2.1
39248	0.946133	2.1
39249	1.16263	2.1
39250	1.04687	2.1
39251	0.946133	2.1
39252	0.946133	2.1
39253	1.04687	2.1
39254	1.04687	2.1
39255	0.946133	2.1
39256	1.04687	2.1
39257	0.946132	2.1
39258	1.16263	2.1
39259	0.682281	1.87806
39260	0.669233	2.1
39261	1.21859	2.1
39262	1.31629	1.56761
39263	0.916158	1.12412

39264	0.916158	2.1
39265	0.971658	1.85797
39266	0.971658	2.02381
39267	0.669234	2.03578
39268	1.31629	1.58921
39269	0.682283	1.91469
39270	1.21858	2.07607
39271	0.669232	1.63162
39272	0.623495	1.9903
39273	1.36428	1.83143
39274	1.31629	1.1501
39275	0.946475	0.610581
39276	0.946476	2.1
39277	0.99331	1.77524
39278	0.99331	1.84371
39279	1.36428	1.68351
39280	0.623497	1.12912
39281	0.669234	1.80604
39282	1.31629	1.31004
39283	2.02069	0.05
39284	1.46253	0.05
39285	2.51479	0.05
39286	1.87136	0.05
39287	3.89203	0.05
39288	1.46253	0.05
39289	1.38536	0.05
39290	1.38536	0.05
39291	1.05579	0.05
39292	1.73281	0.05
39293	1.46916	0.05
39294	2.51479	0.05
39295	1.87136	0.05
39296	2.23338	0.05
39297	2.15603	0.05
39298	4.55146	0.05
39299	0.822553	0.05
39300	0.822563	0.05
39301	0.842935	0.05
39302	0.842935	0.05
39303	0.744203	0.05
39304	0.675346	0.05
39305	0.874795	0.05
39306	1.00453	0.05
39307	1.00454	0.05
39308	0.782417	0.05
39309	0.675337	0.05
39310	0.858007	0.05
39311	0.946132	1.50053
39312	1.24847	0.940087
39313	0.946132	1.29542
39314	1.16806	1.51034
39315	1.24847	0.467111
39316	1.24847	1.97465
39317	0.946133	0.353221
39318	0.946133	1.84687
39319	0.946133	1.20377
39320	0.946133	0.645886
39321	1.16805	1.5631

39322	1.24847	0.875062
39323	1.2364	1.49862
39324	0.946129	1.20121
39325	0.946133	1.46036
39326	1.24847	1.17752
39327	1.2364	0.879652
39328	1.2364	1.7755
39329	0.946131	0.552504
39330	0.946131	2.09353
39331	0.946131	1.42407
39332	0.946131	0.972333
39333	1.2364	1.86406
39334	1.24847	1.00683
39335	1.89226	1.55454
39336	1.89226	1.3399
39337	1.89226	0.870662
39338	1.89226	0.973683
39339	1.89226	1.4903
39340	1.89226	1.67501
39341	1.89226	1.40194
39342	1.89226	1.02396
39343	1.89226	0.824134
39344	1.89226	1.40427
39345	1.89226	1.03373
39346	1.89226	1.59736
39347	1.69014	1.19992
39348	1.09194	1.47123
39349	1.13537	1.6197
39350	1.13537	1.53134
39351	1.58481	0.766037
39352	1.58481	1.12987
39353	1.58147	0.950231
39354	1.13537	1.19286
39355	1.69015	1.26325
39356	1.01179	0.660442
39357	1.69015	1.21732
39358	1.01179	1.40891
39359	0.946132	1.93116
39360	0.946132	1.06303
39361	1.28543	0.226234
39362	1.07341	2.03218
39363	1.07341	1.86643
39364	1.07341	0.051071
39365	0.946132	0.557632
39366	0.946132	0.331014
39367	0.946133	0.511952
39368	1.28543	0.488381
39369	1.07341	0.413717
39370	0.946132	0.923046
39371	1.89226	0.606331
39372	1.89226	0.124657
39373	1.89226	0.0819994
39374	1.89226	2.08313
39375	1.89226	0.0828037
39376	1.89226	2.1
39377	0.805744	2.1
39378	0.988659	1.00909
39379	0.782044	0.0884976

39380	0.578223	0.651314
39381	0.77823	0.104176
39382	0.778231	2.1
39383	0.788653	0.0635865
39384	0.788653	1.79783
39385	0.988662	0.925758
39386	0.782042	0.855685
39387	0.805745	1.05129
39388	0.578222	0.699952
39389	1.89226	0.616633
39390	1.89226	0.618121
39391	1.89226	0.108231
39392	1.89226	2.09892
39393	1.89226	0.0545282
39394	1.89226	2.1
39395	1.97732	1.62157
39396	1.97819	0.340793
39397	1.56408	0.12985
39398	1.56408	2.09402
39399	2.33986	0.294208
39400	2.33986	2.1
39401	1.84516	2.1
39402	2.21152	2.1
39403	1.90702	2.1
39404	1.54599	2.1
39405	3.75751	2.1
39406	1.89224	2.1
39407	1.89224	2.1
39408	1.89224	2.1
39409	1.89224	2.1
39410	1.89224	2.1
39411	1.89224	2.1
39412	1.47672	2.1
39413	1.54601	2.1
39414	1.27697	2.1
39415	1.54601	2.1
39416	1.638	2.1
39417	1.90705	2.1
39418	0.946132	2.1
39419	0.946132	2.1
39420	0.946131	2.1
39421	0.946131	2.1
39422	0.946131	2.1
39423	0.946131	2.1
39424	0.946132	2.1
39425	0.946132	2.1
39426	0.946132	2.1
39427	0.946132	2.1
39428	0.946131	2.1
39429	0.946131	2.1
39430	0.946128	2.1
39431	0.946128	2.1
39432	1.32062	2.1
39433	1.4164	2.1
39434	0.946129	2.1
39435	0.946129	2.1
39436	1.32062	2.1
39437	1.32062	2.1

39438	1.32062	2.1
39439	0.94613	2.1
39440	1.41639	2.1
39441	0.946129	2.1
39442	1.98979	0.05
39443	1.65977	0.05
39444	2.21772	0.05
39445	2.21772	0.05
39446	1.48866	0.05
39447	1.48866	0.05
39448	0.973129	0.05
39449	0.973129	0.05
39450	0.980954	0.05
39451	0.980954	0.05
39452	0.845218	0.05
39453	1.13981	0.05
39454	0.829886	0.05
39455	1.10886	0.05
39456	1.10886	0.05
39457	1.13982	0.05
39458	0.845219	0.05
39459	0.829886	0.05
39460	0.946132	1.13288
39461	0.946132	2.1
39462	1.3352	2.1
39463	1.41432	2.1
39464	0.946133	2.1
39465	0.946133	0.104175
39466	1.3352	2.1
39467	1.3352	2.1
39468	1.3352	2.1
39469	0.946132	2.1
39470	1.41432	2.1
39471	0.946133	2.1
39472	2.70097	2.1
39473	1.88175	2.1
39474	1.51482	2.1
39475	1.88175	2.1
39476	2.37784	2.1
39477	2.82864	2.1
39478	1.89227	0.05
39479	1.89227	0.05
39480	1.89227	0.05
39481	1.89227	0.05
39482	3.78453	0.05
39483	1.89226	0.05
39484	2.13295	0.05
39485	2.43203	0.05
39486	2.43203	0.05
39487	1.89226	0.05
39488	1.89226	0.05
39489	0.946133	0.05
39490	0.946133	0.05
39491	0.946132	0.05
39492	0.946132	0.05
39493	0.946132	0.05
39494	0.946132	0.05
39495	0.946133	0.05

39496	0.946133	0.05
39497	0.946132	0.05
39498	0.946132	0.05
39499	0.946132	0.05
39500	0.946132	0.05
39501	1.54987	2.1
39502	1.24128	2.1
39503	1.24128	2.1
39504	1.50738	2.1
39505	1.75702	2.1
39506	2.02312	2.1
39507	1.89289	2.1
39508	1.92264	2.1
39509	1.72994	2.1
39510	1.75702	2.1
39511	3.67966	2.1
39512	1.75702	2.1
39513	0.994291	2.1
39514	0.994291	2.1
39515	1.24128	2.1
39516	1.4964	2.1
39517	1.74339	2.1
39518	1.72993	2.1
39519	1.49641	2.1
39520	1.49641	2.1
39521	1.75702	2.1
39522	1.3939	2.1
39523	1.65452	2.1
39524	1.19964	2.1
39525	1.53296	2.1
39526	1.19964	2.1
39527	1.36861	2.1
39528	1.42624	2.1
39529	1.59521	2.1
39530	1.19964	2.1
39531	1.42624	2.1
39532	1.07156	2.1
39533	1.36226	2.1
39534	2.5619	2.1
39535	2.14058	1.16799
39536	1.93569	2.1
39537	2.36221	1.60152
39538	2.5753	1.55279
39539	4.50279	2.1
39540	2.14057	1.97831
39541	2.20831	2.1
39542	2.64725	1.09638
39543	2.57529	1.91729
39544	4.78782	1.86177
39545	2.64725	1.3992
39546	2.57529	2.07617
39547	1.89226	1.13165
39548	1.89226	0.538432
39549	4.53951	1.77805
39550	2.0815	2.1
39551	1.86137	2.1
39552	1.86137	2.1
39553	1.86159	2.1

39554	2.0815	2.1
39555	2.10235	2.1
39556	1.33552	2.1
39557	2.32417	2.1
39558	1.18872	2.1
39559	1.93101	2.1
39560	3.26653	2.1
39561	1.89226	2.1
39562	1.89226	2.1
39563	1.89226	2.1
39564	1.89226	2.1
39565	1.89226	2.1
39566	1.89226	2.1
39567	0.955191	2.1
39568	1.03372	2.1
39569	0.946131	2.1
39570	0.946131	2.1
39571	0.930654	2.1
39572	0.930654	2.1
39573	1.04233	2.1
39574	1.05247	2.1
39575	0.955341	2.1
39576	0.946131	2.1
39577	1.02375	2.1
39578	1.13537	2.1
39579	1.1549	2.1
39580	1.93101	2.1
39581	1.91108	2.1
39582	1.52487	2.1
39583	1.3076	2.1
39584	1.06985	2.1
39585	1.89226	2.1
39586	1.89226	2.1
39587	1.89226	2.1
39588	1.89226	2.1
39589	1.89226	2.1
39590	1.89226	2.1
39591	1.58713	2.1
39592	2.6845	2.1
39593	1.58742	2.1
39594	2.74201	2.1
39595	4.27191	2.1
39596	2.68449	2.1
39597	1.89226	2.1
39598	2.74201	2.1
39599	1.89226	2.1
39600	4.57675	2.1
39601	1.57523	2.1
39602	2.74201	2.1
39603	2.8146	2.1
39604	2.8146	2.1
39605	1.58742	2.1
39606	1.58742	2.1
39607	0.946131	2.1
39608	0.946131	2.1
39609	1.371	2.1
39610	1.371	2.1
39611	0.946127	2.1

39612	0.946127	2.1
39613	1.37101	2.1
39614	1.4073	2.1
39615	0.94613	2.1
39616	1.4073	2.1
39617	1.371 2.1	
39618	0.946129	2.1
39619	1.44801	2.05603
39620	1.06821	2.06524
39621	2.00542	1.92441
39622	2.27703	2.1
39623	3.34524	1.83049
39624	1.70301	0.05
39625	1.89226	0.12957
39626	1.65809	0.05
39627	1.89226	0.108427
39628	1.62758	0.05
39629	1.85744	0.0886295
39630	1.89226	0.05
39631	1.89226	0.134605
39632	1.89226	0.0744011
39633	1.89226	0.0689936
39634	3.78452	0.0819231
39635	1.89226	0.136598
39636	1.89226	0.05
39637	1.89226	0.0665195
39638	1.89226	0.17475
39639	3.78452	0.0953085
39640	0.851541	0.184662
39641	0.851541	0.138336
39642	0.91193	0.0747342
39643	0.824837	0.0968709
39644	0.854559	0.0820641
39645	0.851534	0.351834
39646	0.81851	0.13241
39647	0.717219	0.05
39648	0.871743	0.122441
39649	0.837985	0.0815175
39650	0.82902	0.0861322
39651	0.82902	0.146325
39652	1.89226	0.12875
39653	1.92659	0.101953
39654	2.03235	0.120115
39655	1.89226	0.290392
39656	2.03235	0.0625241
39657	1.89226	0.11994
39658	0.585557	0.408665
39659	0.585557	0.135486
39660	1.22612	0.181176
39661	1.09436	0.391564
39662	0.851537	0.70062
39663	0.526987	0.14435
39664	0.983879	0.121985
39665	1.04072	0.320554
39666	0.585526	0.179915
39667	0.792325	0.221285
39668	0.946129	0.242725
39669	0.946129	0.203773

39670	1.68152	2.1
39671	1.81445	2.1
39672	1.68152	2.1
39673	1.86792	2.1
39674	1.65888	2.1
39675	1.84529	2.1
39676	1.81445	2.1
39677	1.57885	2.1
39678	1.65889	2.1
39679	1.48594	2.1
39680	3.23773	2.1
39681	1.68154	2.1
39682	1.65891	2.1
39683	1.53296	2.1
39684	1.56299	2.1
39685	3.24453	2.1
39686	1.56299	2.1
39687	1.48596	2.1
39688	1.56299	2.1
39689	1.65891	2.1
39690	1.46568	2.1
39691	1.5616	2.1
39692	2.68785	2.1
39693	2.08831	2.1
39694	2.66919	2.1
39695	2.1024	2.1
39696	4.79025	2.1
39697	2.08832	2.1
39698	1.15478	2.1
39699	1.15478	2.1
39700	1.09062	2.1
39701	2.10241	2.1
39702	2.03825	2.1
39703	2.65348	2.1
39704	2.1024	2.1
39705	2.12313	2.1
39706	2.12313	2.1
39707	2.66919	2.1
39708	2.66919	2.1
39709	0.813169	2.1
39710	0.813169	2.1
39711	0.825788	2.1
39712	0.825788	2.1
39713	0.612902	2.1
39714	0.577387	2.1
39715	1.0512	2.1
39716	1.06157	2.1
39717	1.06157	2.1
39718	0.612902	2.1
39719	0.577387	2.1
39720	1.0512	2.1
39721	1.27227	2.1
39722	1.91724	2.1
39723	1.32591	2.1
39724	1.99837	2.1
39725	3.24315	2.1
39726	1.89223	2.1
39727	1.89223	2.1

39728	1.89223	2.1
39729	1.89223	2.1
39730	3.78446	2.1
39731	1.89224	2.1
39732	1.89224	1.20012
39733	1.89224	2.1
39734	1.89224	2.1
39735	3.78447	2.1
39736	1.91724	2.1
39737	2.321	2.1
39738	1.99838	2.1
39739	2.4149	2.1
39740	4.33214	2.1
39741	1.34817	2.1
39742	1.9984	2.1
39743	2.06238	2.1
39744	2.06238	2.1
39745	1.32593	2.1
39746	1.32593	2.1
39747	1.89226	2.1
39748	1.89226	2.1
39749	1.89226	2.1
39750	1.89226	2.1
39751	1.89226	2.1
39752	1.89226	2.1
39753	1.89226	2.1
39754	1.89226	2.1
39755	1.89226	2.1
39756	1.89226	1.33095
39757	3.78452	2.1
39758	1.9984	2.1
39759	2.41494	2.1
39760	2.06239	2.1
39761	2.5194	2.1
39762	4.47732	2.1
39763	1.19901	1.03018
39764	1.16661	2.1
39765	1.56859	2.1
39766	1.61029	1.33662
39767	2.7676	2.1
39768	1.56859	2.1
39769	1.88637	0.213693
39770	1.61029	0.0927781
39771	1.61029	2.1
39772	1.856	0.0993539
39773	1.856	2.1
39774	1.856	0.406247
39775	1.57916	2.1
39776	1.56859	1.00086
39777	1.56859	2.1
39778	1.57916	2.1
39779	1.23475	2.1
39780	0.890924	0.05
39781	1.07608	0.05
39782	0.72877	0.059427
39783	0.910106	0.05
39784	1.00196	0.05
39785	1.09519	0.167547

39786	0.839967	0.05
39787	0.719043	0.05
39788	0.893859	0.05
39789	0.942408	0.11144
39790	0.873497	0.05
39791	0.873497	0.0803898
39792	1.53046	0.05
39793	1.89994	0.05
39794	1.049	0.05
39795	1.049	0.05
39796	1.52512	0.05
39797	1.89994	0.05
39798	1.65573	1.08951
39799	1.89226	0.304001
39800	1.89226	0.316878
39801	2.73329	1.21885
39802	3.548	1.26287
39803	1.65573	0.46702
39804	2.42671	0.781291
39805	2.73329	0.271989
39806	1.89226	0.122598
39807	2.73329	1.0636
39808	1.89226	0.647257
39809	0.941078	0.05
39810	1.01599	0.05
39811	0.818936	0.05
39812	0.651554	0.05
39813	0.904729	0.0568456
39814	0.665756	0.0557036
39815	0.843423	0.0514846
39816	0.918649	0.075506
39817	0.855806	0.107979
39818	0.814384	0.05
39819	0.844437	0.0760256
39820	0.844437	0.063675
39821	2.22558	1.09933
39822	1.95785	0.0860279
39823	2.07065	0.209079
39824	1.89142	0.175195
39825	2.07065	1.69114
39826	1.89142	1.51821
39827	0.640813	0.0891381
39828	0.951654	0.25404
39829	0.797442	0.0565189
39830	0.535364	0.0984795
39831	0.525269	0.13627
39832	0.620874	0.123171
39833	0.916953	0.162253
39834	0.969374	0.153668
39835	0.788925	0.496894
39836	0.69596	0.05
39837	0.743154	0.197457
39838	0.743154	0.282294
39839	1.89226	2.1
39840	1.89226	1.84473
39841	1.89226	0.489242
39842	1.89226	2.1
39843	3.78452	1.15212

39844	1.32541	1.73657
39845	1.13722	1.12412
39846	0.91103	2.1
39847	0.881123	2.1
39848	1.03363	2.1
39849	1.03364	2.00805
39850	1.1505	1.95301
39851	1.1505	2.1
39852	1.32539	2.1
39853	1.07662	2.1
39854	0.881121	2.1
39855	0.962303	1.95086
39856	1.89226	1.0534
39857	1.89226	2.1
39858	1.89226	2.1
39859	1.89226	1.42241
39860	3.78453	2.1
39861	1.89226	0.406541
39862	1.89226	2.1
39863	1.89226	1.72357
39864	1.89226	1.45737
39865	1.89226	1.8097
39866	1.89226	1.83232
39867	1.27606	1.95359
39868	1.07664	1.48912
39869	0.905895	2.1
39870	0.696099	2.1
39871	0.984902	2.1
39872	0.984905	1.97289
39873	1.01878	2.1
39874	1.01878	0.491002
39875	1.27604	2.1
39876	1.06044	2.1
39877	1.13192	2.1
39878	0.696111	2.1
39879	1.89226	0.0709233
39880	1.89226	0.05
39881	1.89226	0.05
39882	1.89226	0.0812926
39883	1.89226	0.05
39884	1.89226	0.0772322
39885	1.52615	0.05
39886	1.01294	0.05
39887	1.01294	0.225634
39888	1.18549	0.05
39889	1.38963	0.05
39890	1.56218	0.05
39891	1.89226	0.05
39892	2.40801	0.05
39893	2.58101	0.0857166
39894	2.58101	0.0946907
39895	1.89226	0.05
39896	1.89226	0.05
39897	2.24071	2.1
39898	2.79317	2.1
39899	1.99136	2.1
39900	2.6167	2.1
39901	4.85741	2.1

39902	2.79317	2.1
39903	1.89226	0.05
39904	1.89226	2.1
39905	1.89226	0.095925
39906	2.6167	2.1
39907	2.6167	0.0552248
39908	1.08354	2.1
39909	1.08354	2.1
39910	1.10222	2.1
39911	1.10222	2.1
39912	1.2275	2.1
39913	1.30835	2.1
39914	0.995678	2.1
39915	0.877412	2.1
39916	0.877411	2.1
39917	1.2275	2.1
39918	0.995678	2.1
39919	1.30835	2.1
39920	1.05793	0.749317
39921	1.46441	0.126739
39922	1.0562	0.44019
39923	1.0562	0.289219
39924	1.41787	0.448403
39925	1.41787	0.330689
39926	1.41787	0.241838
39927	1.52909	0.843714
39928	1.46441	0.179221
39929	1.46441	0.756785
39930	1.43063	0.100448
39931	1.43063	0.452784
39932	1.89226	0.890104
39933	1.89226	0.150709
39934	1.89226	0.159676
39935	1.89226	0.611544
39936	3.78453	0.494433
39937	1.89227	0.678085
39938	1.89227	0.298608
39939	1.89227	0.239902
39940	1.89227	0.214387
39941	1.89227	0.182187
39942	1.89227	0.261167
39943	1.00763	0.287604
39944	1.41787	0.27448
39945	1.05793	0.27294
39946	1.05793	0.621849
39947	1.37265	0.234782
39948	1.37265	0.523924
39949	1.37265	0.381476
39950	1.43063	0.362762
39951	1.41787	0.246485
39952	1.41787	1.09023
39953	1.38247	0.0807537
39954	1.38247	0.720098
39955	0.946132	0.343331
39956	0.946132	0.170999
39957	0.946132	0.184902
39958	0.946132	0.05
39959	0.946131	0.05

39960	0.946131	0.160209
39961	0.946132	0.586602
39962	0.946132	0.148421
39963	0.946132	0.0929291
39964	0.946132	0.072426
39965	0.946132	0.171856
39966	0.946132	0.114314
39967	1.89226	0.447678
39968	1.89226	0.0961709
39969	1.89226	0.191324
39970	1.89226	0.433377
39971	1.89226	0.142045
39972	1.89226	0.461857
39973	1.19645	1.04521
39974	1.89183	0.212096
39975	1.91106	0.608716
39976	1.35306	0.205952
39977	1.75446	0.583462
39978	1.19645	0.189865
39979	2.37576	0.05
39980	1.90139	1.51092
39981	2.1845	2.1
39982	1.86568	1.24317
39983	4.24145	1.93237
39984	2.29435	0.534397
39985	1.40602	2.1
39986	1.40602	0.144471
39987	1.55351	1.30625
39988	1.86568	2.1
39989	2.01317	2.1
39990	2.18452	2.1
39991	1.8657	0.05
39992	2.01399	0.182492
39993	1.70141	2.1
39994	3.87969	0.155627
39995	0.774999	2.1
39996	0.774999	0.0761308
39997	0.778721	0.869479
39998	0.778721	2.1
39999	0.628348	2.1
40000	0.703016	2.03966
40001	0.932848	0.223931
40002	0.850704	1.32243
40003	0.850704	1.8288
40004	0.628348	1.29293
40005	0.703015	0.168009
40006	0.932848	1.00086
40007	1.65243	0.143277
40008	1.34412	0.05
40009	1.45569	0.603705
40010	1.1315	1.18763
40011	2.78393	0.142155
40012	1.48901	2.1
40013	1.805	1.51562
40014	1.48901	1.72119
40015	1.71717	2.1
40016	1.60635	0.0949719
40017	1.83451	2.1

40018	1.805	1.99695
40019	1.65243	0.117071
40020	1.60635	1.17953
40021	1.45569	0.05
40022	3.25879	0.0827809
40023	1.27875	2.1
40024	1.60635	0.449269
40025	1.27875	2.04291
40026	1.48901	2.1
40027	1.40014	0.373425
40028	1.6104	2.1
40029	0.715199	0.0620288
40030	0.715199	1.97847
40031	0.712714	1.5853
40032	0.712714	0.05
40033	0.727843	0.05
40034	0.619713	0.05
40035	0.803177	1.44218
40036	0.70007	0.535823
40037	0.700069	0.0533051
40038	0.619713	0.05
40039	0.803177	0.070142
40040	0.727844	0.0507134
40041	1.14357	2.1
40042	2.3506	2.1
40043	1.14357	2.1
40044	2.68773	2.09327
40045	1.57829	2.1
40046	2.68773	2.09541
40047	2.00551	2.1
40048	2.65811	1.83059
40049	2.07203	2.1
40050	2.72117	0.05
40051	4.72667	2.04933
40052	0.946131	0.0944398
40053	0.946131	2.1
40054	1.32905	2.1
40055	1.32905	0.0542615
40056	0.94613	1.07143
40057	0.94613	1.79416
40058	1.32905	1.66923
40059	1.36058	1.24981
40060	1.36058	0.0730413
40061	0.946131	0.05
40062	1.32905	1.15064
40063	0.946131	1.78633
40064	1.21649	0.05
40065	1.21649	2.1
40066	1.21459	2.1
40067	1.21459	2.1
40068	1.39508	1.36541
40069	1.36057	0.378966
40070	1.03601	2.1
40071	1.07438	2.1
40072	1.07439	1.73337
40073	1.39509	1.80406
40074	1.036	1.8263
40075	1.36056	1.2122

40076	2.79015	0.198709
40077	2.72114	2.1
40078	1.89225	0.333017
40079	1.89225	2.1
40080	1.89225	1.9196
40081	2.72114	0.101438
40082	0.876258	2.1
40083	0.892889	2.1
40084	1.34352	2.1
40085	1.27985	2.1
40086	1.07462	2.1
40087	1.07462	2.1
40088	1.09812	2.1
40089	1.09812	2.1
40090	0.892888	2.1
40091	1.34352	2.1
40092	0.876256	2.1
40093	1.27985	2.1
40094	1.39725	2.03998
40095	1.30029	0.100295
40096	1.31415	0.398884
40097	1.41336	1.53055
40098	2.71366	1.03214
40099	1.89226	0.157123
40100	1.89226	0.658646
40101	1.89226	1.84054
40102	1.89226	0.119108
40103	3.78453	0.24284
40104	0.946133	0.788922
40105	0.946133	0.326907
40106	1.38709	0.0681918
40107	1.39437	0.6112
40108	0.946132	0.0697785
40109	0.946132	0.254503
40110	1.39437	1.00783
40111	1.39437	0.0783674
40112	0.946132	0.115781
40113	1.39437	0.0543073
40114	1.38709	0.239118
40115	0.946132	0.3016
40116	0.706681	1.96363
40117	0.711908	1.65236
40118	0.657077	0.170964
40119	0.661003	0.795001
40120	0.684188	0.135976
40121	0.684188	1.51326
40122	0.683496	2.1
40123	0.683496	0.247611
40124	0.711908	0.704092
40125	0.661003	0.689928
40126	0.657076	0.978995
40127	0.706681	0.878435
40128	0.946132	1.83696
40129	0.946132	0.688799
40130	0.946132	0.247716
40131	0.946132	0.102445
40132	0.946132	0.105931
40133	0.946132	0.73496

40134	0.946133	0.344678
40135	0.946133	1.87063
40136	0.946133	0.275813
40137	0.946133	0.211413
40138	0.946132	0.78863
40139	0.946132	0.735897
40140	2.79029	0.0582253
40141	1.89227	1.26109
40142	2.78873	0.181738
40143	2.78873	0.115973
40144	1.89383	1.23746
40145	1.89227	0.414384
40146	1.43669	2.1
40147	2.11984	2.1
40148	2.24481	2.1
40149	2.24481	2.1
40150	1.20311	2.1
40151	1.20311	2.1
40152	0.49703	2.1
40153	0.601553	2.1
40154	1.05992	2.1
40155	0.991694	2.1
40156	0.787558	2.1
40157	0.787558	2.1
40158	0.805689	2.1
40159	0.805689	2.1
40160	0.991694	2.1
40161	0.49703	2.1
40162	1.05992	2.1
40163	0.601553	2.1
40164	2.45905	2.1
40165	1.89226	2.1
40166	1.89226	2.1
40167	1.89226	2.1
40168	2.47395	2.1
40169	2.47395	2.1
40170	1.89226	2.1
40171	1.89226	2.02337
40172	1.89226	2.1
40173	1.89226	1.60139
40174	3.78452	2.1
40175	1.38427	2.1
40176	2.31203	1.08338
40177	2.28743	0.811754
40178	2.28743	2.1
40179	1.63384	0.463157
40180	1.63384	2.1
40181	2.42723	2.1
40182	1.89226	2.1
40183	1.89226	2.1
40184	1.89226	2.1
40185	2.45905	2.1
40186	2.45905	2.1
40187	0.946131	0.126877
40188	0.946131	2.1
40189	0.946131	2.1
40190	0.946131	2.1
40191	0.94613	1.48199

40192	0.94613	2.1
40193	0.946132	2.1
40194	0.946132	2.1
40195	0.946131	2.1
40196	0.946131	2.1
40197	0.946131	2.1
40198	0.946131	2.1
40199	0.989441	2.1
40200	0.989441	2.1
40201	0.993918	2.1
40202	0.993918	2.1
40203	1.16644	2.1
40204	1.15601	1.01069
40205	0.818308	2.1
40206	0.816922	2.1
40207	0.818307	2.1
40208	1.16644	2.1
40209	1.15601	2.1
40210	0.816922	2.1
40211	0.996778	0.05
40212	2.44375	0.05
40213	0.996778	0.05
40214	1.92547	0.05
40215	1.56091	0.05
40216	2.4896	0.05
40217	1.56091	0.05
40218	2.44375	0.05
40219	1.90456	0.05
40220	1.90456	0.05
40221	2.81853	0.05
40222	2.47487	0.05
40223	1.32143	2.06886
40224	1.43294	2.1
40225	1.37444	0.201122
40226	1.51691	2.1
40227	2.80738	1.94241
40228	1.89226	1.53418
40229	1.89226	2.1
40230	1.89226	0.319656
40231	1.89226	1.63979
40232	3.78453	1.16961
40233	1.89226	1.74553
40234	1.89226	2.1
40235	1.89226	0.997588
40236	1.89226	2.1
40237	3.78453	2.1
40238	1.43294	1.6732
40239	1.3268	2.1
40240	1.51691	1.4844
40241	1.44091	2.1
40242	2.87385	2.1
40243	1.37444	2.1
40244	1.51691	2.1
40245	1.4714	0.197704
40246	1.65434	1.76144
40247	3.02878	1.76286
40248	1.89226	2.1
40249	1.89226	2.05707

40250	1.89226	0.960626
40251	1.89226	1.81657
40252	3.78452	1.75144
40253	1.89226	1.36518
40254	1.89226	2.1
40255	1.89226	1.0264
40256	1.89226	2.1
40257	3.78453	2.1
40258	1.51691	1.9859
40259	1.44091	2.1
40260	1.65434	1.08709
40261	1.60647	2.1
40262	3.09526	2.1
40263	1.05013	2.1
40264	1.05013	2.1
40265	0.94613	2.1
40266	0.94613	2.1
40267	1.04959	2.1
40268	1.05014	2.1
40269	0.946129	2.1
40270	0.946129	2.1
40271	0.946131	2.1
40272	1.04958	2.1
40273	0.946131	2.1
40274	1.05013	2.1
40275	1.04959	2.1
40276	1.04959	2.1
40277	0.946131	2.1
40278	0.946131	2.1
40279	1.04765	2.1
40280	1.04959	2.1
40281	0.94613	2.1
40282	0.94613	2.1
40283	0.946131	2.1
40284	1.04765	2.1
40285	0.946131	2.1
40286	1.04959	2.1
40287	1.85744	2.1
40288	1.89226	2.1
40289	1.89226	2.1
40290	1.89226	2.1
40291	1.94839	2.1
40292	1.94839	2.1
40293	0.97419	2.1
40294	0.822054	2.1
40295	0.946123	2.1
40296	0.946123	2.1
40297	1.04202	2.1
40298	0.84898	2.1
40299	0.933307	2.1
40300	0.933307	2.1
40301	0.933285	2.1
40302	1.05018	2.1
40303	0.851528	2.1
40304	0.876787	2.1
40305	1.87528	0.05
40306	1.21137	0.05
40307	1.57893	0.05

40308	1.21137	0.076232
40309	1.81182	0.05
40310	1.44426	0.0500774
40311	0.993833	0.05
40312	1.21137	0.05
40313	1.57893	0.057807
40314	1.31774	0.05
40315	2.5291	0.05
40316	1.87528	0.05
40317	1.57893	0.05
40318	1.90728	0.05
40319	2.29851	0.05
40320	3.87744	0.0500672
40321	1.90728	0.05
40322	1.31774	0.05
40323	1.56339	0.05
40324	1.31774	0.05
40325	1.82458	0.05
40326	1.57893	0.05
40327	1.89226	0.319604
40328	1.89226	0.891882
40329	1.89226	0.711275
40330	1.89226	0.972663
40331	1.89226	0.823077
40332	1.89226	1.10258
40333	1.89226	0.586801
40334	1.89226	1.11296
40335	1.89226	1.28293
40336	1.89226	0.70645
40337	3.78452	0.982469
40338	1.89226	0.772176
40339	2.17251	0.633982
40340	2.45575	0.432205
40341	2.45575	0.597617
40342	1.89226	0.481973
40343	1.89226	0.672062
40344	1.08625	0.834048
40345	1.22788	0.535949
40346	0.54447	0.443817
40347	0.794861	0.550502
40348	0.967729	0.64387
40349	0.967729	0.784516
40350	0.913386	0.565468
40351	0.913386	0.431622
40352	1.08625	0.665308
40353	0.544469	0.533831
40354	0.794862	0.659632
40355	1.22788	0.546639
40356	0.946128	0.836112
40357	0.946128	1.20293
40358	0.946132	0.731889
40359	0.946132	0.679161
40360	0.94613	0.724351
40361	0.94613	0.866537
40362	0.94613	1.25324
40363	0.94613	0.440242
40364	0.94613	0.67694
40365	0.94613	0.674968

40366	0.94613	0.750932
40367	0.94613	0.924595
40368	0.946127	0.508282
40369	0.946127	0.514895
40370	1.08625	0.390994
40371	0.958779	0.593525
40372	1.08625	0.443831
40373	1.08625	0.499203
40374	0.946128	0.437244
40375	0.946128	0.52728
40376	0.946129	0.601318
40377	0.958777	0.647444
40378	1.08625	0.545088
40379	0.946129	0.681243
40380	2.16338	0.05
40381	1.94002	0.05
40382	1.98836	0.05
40383	2.60614	0.05
40384	1.5456	0.05
40385	2.16338	0.05
40386	1.19952	0.05
40387	2.34061	0.05
40388	1.21031	0.05
40389	2.1711	0.05
40390	3.54013	0.05
40391	1.13624	2.1
40392	1.70302	2.1
40393	1.8267	2.1
40394	1.11545	2.1
40395	2.85455	2.1
40396	1.82671	2.1
40397	1.91767	2.1
40398	1.81563	2.1
40399	1.91767	2.1
40400	1.71362	2.1
40401	1.83277	2.1
40402	1.66064	2.1
40403	0.996776	2.1
40404	0.996776	2.1
40405	1.11545	2.1
40406	1.54278	2.1
40407	1.66145	2.1
40408	1.66064	2.1
40409	1.7457	2.1
40410	1.81563	2.1
40411	1.81563	2.1
40412	1.54278	2.1
40413	1.54278	2.1
40414	2.71672	2.1
40415	2.70402	2.1
40416	2.64996	2.1
40417	2.69443	2.1
40418	5.39845	2.1
40419	2.71672	2.1
40420	2.54198	2.1
40421	2.54019	2.1
40422	2.69443	2.1
40423	5.23641	2.1

40424	2.64996	2.1
40425	2.69443	2.1
40426	2.68213	2.1
40427	2.61243	2.1
40428	5.30686	2.1
40429	2.54019	2.1
40430	2.69443	2.1
40431	2.68213	2.1
40432	2.5392	2.1
40433	5.23363	2.1
40434	1.89226	2.1
40435	2.34725	2.1
40436	2.22414	2.1
40437	2.34725	2.1
40438	1.76914	2.1
40439	1.89226	2.1
40440	0.946131	2.1
40441	0.946131	2.1
40442	1.11207	2.1
40443	1.03798	2.1
40444	0.94613	2.1
40445	0.94613	2.1
40446	1.03798	2.1
40447	1.03798	2.1
40448	0.946131	2.1
40449	1.03798	2.1
40450	0.946131	2.1
40451	1.11207	2.1
40452	1.89226	0.05
40453	1.89226	0.05
40454	1.89226	0.05
40455	1.89226	0.05
40456	1.89226	0.05
40457	1.89226	0.05
40458	1.89226	0.05
40459	1.89226	0.05
40460	1.89226	0.05
40461	1.89226	0.05
40462	1.89226	0.05
40463	1.89226	0.05
40464	1.89226	0.05
40465	1.89226	0.05
40466	1.89226	0.05
40467	1.89226	0.05
40468	1.89226	0.05
40469	1.89226	0.05
40470	1.44085	2.08101
40471	1.31825	0.0953544
40472	1.31923	0.463655
40473	1.31825	1.71196
40474	1.43311	0.2635
40475	1.43213	1.20235
40476	1.89226	0.123713
40477	1.89226	0.963611
40478	1.89226	0.221319
40479	1.89226	1.22189
40480	1.89226	0.0983291
40481	1.89226	1.47933

40482	0.946133	0.614921
40483	0.946133	0.254614
40484	1.38969	0.0863156
40485	1.39587	0.235022
40486	0.946132	0.0918024
40487	0.946132	0.289918
40488	1.39587	0.683982
40489	1.39587	0.0561821
40490	0.946132	0.106735
40491	1.39587	0.0660116
40492	1.38969	0.241055
40493	0.946132	0.379492
40494	1.44085	2.1
40495	1.31923	0.0642583
40496	1.33026	0.534254
40497	1.4617	1.57403
40498	2.78093	1.29145
40499	1.89226	0.100074
40500	1.89226	0.597248
40501	1.89226	0.101919
40502	1.89226	1.01571
40503	1.89226	0.0591053
40504	1.89226	1.36116
40505	0.946133	0.435337
40506	0.946133	0.138161
40507	1.39587	0.1155
40508	1.40724	0.05
40509	0.946132	0.05
40510	0.946132	0.11922
40511	1.40724	0.374635
40512	1.40724	0.1471
40513	0.946132	0.140685
40514	1.40724	0.0773484
40515	0.946133	0.502817
40516	1.39587	0.139439
40517	1.15818	2.09861
40518	1.81846	1.94928
40519	1.52782	1.19981
40520	1.52782	2.1
40521	1.28095	0.810798
40522	1.28095	2.1
40523	2.56709	2.1
40524	1.4349	0.05
40525	1.4349	0.273553
40526	1.75471	0.510004
40527	2.29909	0.340354
40528	2.61889	1.81476
40529	2.5671	2.1
40530	2.82858	2.1
40531	2.57368	2.1
40532	2.29909	0.486604
40533	5.12767	2.1
40534	2.29909	2.1
40535	1.09148	0.05
40536	1.09148	0.233182
40537	1.4349	0.21594
40538	1.99776	0.124954
40539	2.34119	0.806648

40540	2.57368	2.1
40541	1.99777	0.217404
40542	1.99777	2.1
40543	2.29909	2.1
40544	2.36071	2.1
40545	2.66203	2.1
40546	1.89226	0.258736
40547	1.89226	0.837558
40548	1.89226	0.49082
40549	1.89226	0.239839
40550	3.78453	0.318599
40551	0.946128	0.618972
40552	0.946128	1.74253
40553	0.946133	0.378199
40554	0.946133	0.568718
40555	0.946131	0.798419
40556	0.946131	1.16965
40557	0.946131	1.44529
40558	0.946131	0.647359
40559	0.94613	1.61208
40560	0.94613	0.863219
40561	0.946131	0.624334
40562	0.946131	1.15512
40563	1.89226	0.926999
40564	1.89226	1.20093
40565	1.89226	0.258495
40566	1.89226	1.14947
40567	3.78453	1.12651
40568	1.89226	1.26954
40569	1.89226	0.608212
40570	1.89226	0.345934
40571	1.89226	0.681416
40572	3.78453	0.760702
40573	1.89226	0.706228
40574	2.61682	1.78905
40575	1.89226	1.51206
40576	1.89226	1.68786
40577	1.89226	0.549932
40578	1.89226	1.63323
40579	1.89226	0.411804
40580	1.89226	0.461339
40581	1.89226	0.732497
40582	1.89226	0.872442
40583	3.78452	1.11419
40584	1.89226	0.342163
40585	1.89226	1.3028
40586	1.89226	0.369522
40587	1.89226	0.786687
40588	1.89226	0.8698
40589	1.89226	1.42957
40590	1.89227	0.427923
40591	1.89227	0.107921
40592	1.89227	0.959495
40593	1.89227	0.693231
40594	1.89227	0.458527
40595	1.89227	0.447116
40596	0.80447	1.26745
40597	0.534241	0.789698

40598	0.995175	1.08614
40599	0.92204	0.886623
40600	0.922042	0.133426
40601	0.534241	0.227557
40602	0.804468	0.815073
40603	0.995176	0.125038
40604	0.71543	0.301793
40605	0.71543	0.0515264
40606	0.813988	0.310326
40607	0.813988	0.100297
40608	0.922045	0.135579
40609	0.942899	0.475289
40610	0.946132	0.309346
40611	0.946132	0.197165
40612	1.05169	0.05
40613	0.936661	0.294005
40614	0.922045	0.530048
40615	0.946133	0.156564
40616	1.01665	0.179693
40617	1.13008	0.05
40618	0.851529	0.104994
40619	0.851529	0.252385
40620	1.41908	2.1
40621	1.77723	2.1
40622	1.90485	2.1
40623	1.90485	2.1
40624	1.30237	2.1
40625	1.30237	2.1
40626	1.77723	2.1
40627	2.0055	2.1
40628	1.90485	2.1
40629	2.17158	2.1
40630	3.91035	2.1
40631	0.850906	2.1
40632	0.850906	2.1
40633	0.85668	2.1
40634	0.85668	2.1
40635	0.998045	2.1
40636	0.952425	2.1
40637	0.74367	2.1
40638	0.709541	2.1
40639	0.74367	2.1
40640	0.998045	2.1
40641	0.709541	2.1
40642	0.952425	2.1
40643	1.90485	2.1
40644	2.28563	2.1
40645	2.26282	2.1
40646	2.17158	2.1
40647	1.99609	2.1
40648	1.90485	1.88455
40649	2.00098	0.05
40650	1.90462	0.05
40651	1.60057	0.05
40652	1.79224	0.05
40653	3.69686	0.05
40654	2.65654	0.05
40655	1.79224	0.05

40656	2.22332	0.05
40657	2.22332	0.05
40658	2.24163	0.05
40659	2.24163	0.05
40660	1.89226	0.0501043
40661	1.89226	0.05
40662	1.89226	0.05
40663	1.89226	0.0842976
40664	3.78452	0.05
40665	1.89226	0.05
40666	1.89226	0.0641364
40667	1.89226	0.05
40668	1.89226	0.0555515
40669	1.89226	0.0575352
40670	1.89226	0.0592543
40671	2.24163	0.0514451
40672	1.03645	0.05
40673	1.79224	0.0581218
40674	1.79224	0.05
40675	1.54283	0.0576061
40676	1.54283	0.05
40677	0.946132	0.0533739
40678	0.946132	0.05
40679	0.946129	0.0700307
40680	0.946129	0.0564194
40681	0.94613	0.0569796
40682	0.94613	0.0559763
40683	0.946131	0.0601449
40684	0.946131	0.0541054
40685	0.94613	0.0606284
40686	0.94613	0.0727478
40687	0.946131	0.0505342
40688	0.946131	0.05
40689	1.89226	2.1
40690	1.89226	2.1
40691	1.89226	2.1
40692	1.89226	2.1
40693	3.78453	2.1
40694	1.52441	2.1
40695	1.30347	2.1
40696	1.52441	2.1
40697	1.58401	2.1
40698	1.2627	2.1
40699	1.3223	2.1
40700	1.89226	2.1
40701	2.72787	2.1
40702	2.72787	2.1
40703	2.74762	2.1
40704	1.89226	2.1
40705	1.91202	2.1
40706	1.89227	2.1
40707	1.89227	2.1
40708	1.89227	2.1
40709	1.89227	2.1
40710	3.78453	2.1
40711	0.56414	2.1
40712	0.561557	2.1
40713	0.527284	2.1

40714	0.541143	2.1
40715	0.527276	2.1
40716	0.631685	2.1
40717	1.52441	2.1
40718	1.00232	2.1
40719	0.784495	2.1
40720	0.842304	2.1
40721	0.721569	2.1
40722	1.36393	2.1
40723	1.36393	2.1
40724	0.946132	2.1
40725	0.946132	2.1
40726	1.36393	2.1
40727	1.3594	2.1
40728	0.946132	2.1
40729	0.946132	2.1
40730	0.946107	2.1
40731	1.35936	2.1
40732	1.36397	2.1
40733	0.946158	2.1
40734	1.89226	2.1
40735	1.89226	2.1
40736	1.89226	2.1
40737	1.89226	2.1
40738	3.78453	2.1
40739	2.20802	2.1
40740	1.05677	2.1
40741	2.30479	2.1
40742	0.978699	2.1
40743	3.18672	2.1
40744	0.94613	2.1
40745	0.94613	2.1
40746	0.669866	2.1
40747	0.669866	2.1
40748	0.681588	2.1
40749	0.669865	2.1
40750	0.946133	2.1
40751	0.946133	2.1
40752	0.94613	2.1
40753	0.681589	2.1
40754	0.946131	2.1
40755	0.669867	2.1
40756	2.73514	2.1
40757	1.78301	2.1
40758	1.90618	2.1
40759	2.83464	2.1
40760	4.64132	2.1
40761	2.73514	2.1
40762	1.89226	2.1
40763	1.89226	2.1
40764	2.83464	2.1
40765	4.6274	2.1
40766	2.03915	2.1
40767	2.26616	2.1
40768	2.23549	2.1
40769	2.01704	2.1
40770	4.2832	2.1
40771	2.03916	2.1

40772	1.55766	2.1
40773	1.53728	2.1
40774	2.01704	2.1
40775	3.5747	2.1
40776	2.23551	2.1
40777	2.01705	2.1
40778	1.99823	2.1
40779	2.21189	2.1
40780	4.22894	2.1
40781	1.51788	2.1
40782	2.01705	2.1
40783	1.53729	2.1
40784	1.53729	2.1
40785	1.99823	2.1
40786	1.99823	2.1
40787	2.588	2.1
40788	1.45581	2.1
40789	1.45581	2.1
40790	1.44207	2.1
40791	2.54949	2.1
40792	2.53574	2.1
40793	0.98641	2.1
40794	0.98641	2.1
40795	1.00371	2.1
40796	1.00371	2.1
40797	0.749992	2.1
40798	0.727904	2.1
40799	1.26221	2.1
40800	1.27474	2.1
40801	1.26221	2.1
40802	0.749993	2.1
40803	0.727904	2.1
40804	1.27474	2.1
40805	1.23105	0.151743
40806	1.35435	1.96797
40807	1.89226	0.937436
40808	1.89226	0.458362
40809	1.89226	1.67254
40810	1.7043	0.805097
40811	1.89226	2.1
40812	1.89226	0.803855
40813	1.89226	0.181275
40814	1.89226	2.08106
40815	3.78453	1.60422
40816	1.89226	0.121564
40817	1.89226	2.05776
40818	1.89226	0.791013
40819	1.89226	0.893823
40820	1.89226	1.53623
40821	1.89226	1.64909
40822	0.946131	0.221278
40823	1.40945	0.150146
40824	0.94613	0.217476
40825	0.94613	1.64134
40826	0.946129	1.73143
40827	0.946129	0.509914
40828	0.946132	1.788
40829	1.40945	0.116508

40830	0.94613	0.586804
40831	0.94613	0.47834
40832	0.946131	0.316206
40833	0.94613	0.22638
40834	0.946133	0.386746
40835	0.946133	0.295622
40836	0.946132	1.36672
40837	0.946132	1.99938
40838	0.946132	1.9756
40839	0.946132	0.510513
40840	0.946133	1.37634
40841	0.946133	0.332334
40842	0.946132	1.12542
40843	0.946132	1.02046
40844	0.946133	0.57967
40845	0.946133	0.638247
40846	1.89226	0.771742
40847	1.89226	2.1
40848	1.89226	1.65317
40849	1.89226	1.30823
40850	1.89226	1.98506
40851	1.89226	1.74447
40852	1.89226	2.1
40853	1.89226	2.1
40854	1.89226	2.1
40855	1.89226	2.1
40856	1.89226	2.1
40857	1.89226	2.1
40858	1.89226	2.1
40859	1.89226	2.1
40860	1.89226	2.1
40861	1.89226	2.1
40862	3.78453	2.1
40863	1.66647	2.1
40864	1.15399	2.1
40865	1.25443	2.1
40866	1.26599	2.1
40867	1.58697	2.1
40868	1.66647	2.1
40869	1.66647	2.1
40870	1.47446	2.1
40871	1.55433	2.1
40872	1.74241	2.1
40873	3.2208	2.1
40874	1.89227	2.1
40875	1.89227	2.1
40876	1.89227	2.1
40877	1.89227	2.1
40878	3.78453	2.1
40879	0.946132	2.1
40880	0.946132	2.1
40881	0.946132	2.1
40882	0.946132	2.1
40883	0.946132	2.1
40884	0.946132	2.1
40885	0.946133	2.1
40886	0.946133	2.1
40887	0.946132	2.1

40888	0.946132	2.1
40889	0.946133	2.1
40890	0.946133	2.1
40891	0.625067	2.1
40892	0.61907	2.1
40893	0.896913	2.1
40894	0.946199	2.1
40895	0.806287	2.1
40896	0.677685	2.1
40897	0.750657	2.1
40898	0.750657	2.1
40899	0.646004	2.1
40900	0.890236	2.1
40901	0.61049	2.1
40902	0.888979	2.1
40903	1.6223	2.1
40904	1.98034	2.1
40905	1.75003	2.1
40906	1.55433	2.1
40907	1.75003	2.1
40908	1.55433	2.1
40909	1.88602	2.1
40910	2.08149	2.1
40911	2.07991	2.1
40912	1.88473	2.1
40913	2.08149	2.1
40914	1.88602	2.1
40915	1.88458	2.1
40916	2.08149	2.1
40917	1.7069	2.1
40918	1.56284	2.1
40919	2.08149	2.1
40920	1.88458	2.1
40921	1.5955	2.1
40922	1.89226	2.1
40923	1.91975	2.1
40924	1.62299	2.1
40925	1.89226	2.1
40926	1.5955	2.1
40927	1.89226	2.1
40928	1.89226	2.1
40929	1.89226	2.1
40930	1.89226	2.1
40931	1.89226	1.473
40932	1.89226	2.1
40933	1.89226	2.1
40934	1.89226	2.1
40935	1.89226	2.1
40936	1.89226	2.1
40937	1.89226	2.1
40938	1.89226	2.1
40939	1.62299	2.1
40940	1.89226	2.1
40941	1.71752	2.1
40942	1.44824	2.1
40943	1.89226	2.1
40944	1.62299	2.1
40945	1.89226	0.292856

40946	2.8317	0.129838
40947	1.89226	0.276435
40948	2.64055	0.0559663
40949	4.53281	0.262067
40950	2.8317	0.280248
40951	1.83151	0.05
40952	2.01001	0.0958472
40953	2.01001	0.537595
40954	2.64055	0.05
40955	2.64055	0.101192
40956	0.946132	0.05
40957	0.946132	0.0910434
40958	1.16167	0.05
40959	1.16167	0.0923051
40960	1.16167	0.0703372
40961	1.32027	0.05
40962	0.946131	0.05
40963	0.946131	0.108632
40964	0.94613	0.05
40965	1.16167	0.05
40966	0.946131	0.05
40967	1.32027	0.05
40968	2.32335	0.104325
40969	1.83151	0.05
40970	2.38525	0.0775173
40971	2.64055	0.056968
40972	1.57621	0.05
40973	1.83151	0.05
40974	2.25677	2.1
40975	1.88158	2.1
40976	1.86856	2.1
40977	2.0815	2.1
40978	1.70303	2.1
40979	1.88158	2.1
40980	2.50381	2.1
40981	1.89227	2.1
40982	2.25677	2.1
40983	2.25677	2.1
40984	1.89227	2.1
40985	1.89227	2.1
40986	1.89226	0.05
40987	1.50899	0.05
40988	2.13659	0.05
40989	1.50899	0.0547721
40990	2.51986	0.05
40991	1.89226	0.05
40992	0.946131	0.05
40993	0.946131	0.05
40994	1.06829	0.05
40995	0.895375	0.05
40996	0.94613	0.05
40997	0.94613	0.05
40998	1.33922	0.05
40999	0.895376	0.05
41000	0.946131	0.05
41001	1.33922	0.05
41002	0.946131	0.05
41003	1.06829	0.05

41004	0.841467	0.0754137
41005	0.578428	0.135644
41006	1.18079	0.0808614
41007	0.920237	0.131089
41008	0.772317	0.05
41009	0.856555	0.173557
41010	0.910271	0.0824889
41011	0.910271	0.204874
41012	0.975752	0.095073
41013	0.536929	0.0516037
41014	0.81952	0.162775
41015	1.15252	0.0720719
41016	1.89226	2.1
41017	2.51431	2.1
41018	1.89226	2.1
41019	1.89226	2.1
41020	2.78815	2.1
41021	2.78815	2.1
41022	2.0815	2.1
41023	2.0516	2.1
41024	2.0815	2.1
41025	3.06699	2.1
41026	2.76576	2.1
41027	2.8002	2.1
41028	1.94112	2.1
41029	1.89226	2.1
41030	2.20358	2.1
41031	1.89226	2.1
41032	4.09584	2.1
41033	1.09154	2.1
41034	2.62647	2.1
41035	2.20358	2.1
41036	2.20358	2.1
41037	1.52314	2.1
41038	1.52314	2.1
41039	1.31323	2.1
41040	1.31323	2.1
41041	0.946132	2.1
41042	0.946132	2.1
41043	0.946132	2.1
41044	0.946132	2.1
41045	1.31323	2.1
41046	1.10179	2.1
41047	1.31323	2.1
41048	0.946132	2.1
41049	1.10179	2.1
41050	0.946133	2.1
41051	1.89226	0.234586
41052	1.89226	1.19656
41053	1.89226	1.21176
41054	1.89226	1.75923
41055	1.89226	0.173963
41056	1.89226	0.988347
41057	1.35126	1.98588
41058	1.35126	2.1
41059	0.903285	1.22394
41060	0.903285	1.87228
41061	1.30841	1.76959

41062	0.903313	2.1
41063	0.946132	1.67306
41064	0.946132	1.05731
41065	0.946132	1.69328
41066	0.903312	1.63006
41067	1.30841	1.49532
41068	0.946133	1.49711
41069	2.62097	2.1
41070	2.72561	2.1
41071	2.39016	2.1
41072	2.51062	2.1
41073	5.11577	2.1
41074	2.51062	2.1
41075	2.58286	0.05
41076	2.40107	2.1
41077	2.40107	0.196319
41078	2.72561	2.1
41079	2.72561	0.295278
41080	2.39016	2.1
41081	2.51062	2.1
41082	2.20242	2.1
41083	2.34107	2.1
41084	4.73124	2.1
41085	2.51062	2.1
41086	2.40107	0.05
41087	2.34107	2.1
41088	2.25197	0.0643257
41089	4.74215	0.579087
41090	2.75218	2.1
41091	1.89226	2.1
41092	2.3349	2.1
41093	2.3349	2.1
41094	1.89226	2.1
41095	1.89226	2.1
41096	1.46129	2.1
41097	0.996308	2.1
41098	1.88649	2.1
41099	1.70302	2.1
41100	2.95419	2.1
41101	1.89227	2.1
41102	1.89227	2.1
41103	1.89227	2.1
41104	1.89227	2.1
41105	3.78453	2.1
41106	0.646558	2.1
41107	0.646557	2.1
41108	0.60697	2.1
41109	0.60697	2.1
41110	0.587917	2.1
41111	0.519747	2.1
41112	0.586443	2.1
41113	0.733782	2.1
41114	0.733793	2.1
41115	0.587924	2.1
41116	0.519739	2.1
41117	0.586436	2.1
41118	2.1642	2.1
41119	1.42867	2.1

41120	2.28847	2.1
41121	1.68299	2.1
41122	3.71714	2.1
41123	1.89226	2.1
41124	1.89226	2.1
41125	1.89226	2.1
41126	1.89226	2.1
41127	3.78453	2.1
41128	0.946131	2.1
41129	0.946131	2.1
41130	1.24994	2.1
41131	1.24994	2.1
41132	1.11582	2.1
41133	1.24994	2.1
41134	0.946133	2.1
41135	0.946133	2.1
41136	0.946131	2.1
41137	1.24994	2.1
41138	0.94613	2.1
41139	1.11582	2.1
41140	2.28847	2.1
41141	1.53629	2.1
41142	1.57498	2.1
41143	1.47417	2.1
41144	2.42221	2.1
41145	2.28847	2.1
41146	1.89226	2.1
41147	1.89226	2.1
41148	1.89226	2.1
41149	1.89226	2.1
41150	3.78453	2.1
41151	1.89226	2.1
41152	2.49988	2.1
41153	2.71233	2.1
41154	2.71233	2.1
41155	1.89226	2.1
41156	1.89226	2.1
41157	0.924534	2.1
41158	0.924534	2.1
41159	0.990395	2.1
41160	0.990395	2.1
41161	0.946131	2.1
41162	0.946131	2.1
41163	0.924505	2.1
41164	0.968797	2.1
41165	0.924505	2.1
41166	0.946131	2.1
41167	0.968797	2.1
41168	0.946131	2.1
41169	0.924533	2.1
41170	0.924533	2.1
41171	0.948264	2.1
41172	0.945345	2.1
41173	0.851541	2.1
41174	0.851541	2.1
41175	0.958934	2.1
41176	1.02617	2.1
41177	0.936668	2.1

41178	1.04829	2.1
41179	0.924505	2.1
41180	0.946131	2.1
41181	2.28431	0.586076
41182	1.48035	0.05
41183	1.59971	0.0657689
41184	1.48035	0.05
41185	2.33818	0.0931042
41186	2.21882	0.0655902
41187	2.28431	0.0836267
41188	1.89226	0.05
41189	1.89226	0.195602
41190	2.3968	0.05
41191	4.28906	0.05
41192	2.28431	0.21583
41193	1.70303	0.05
41194	1.5428	0.05
41195	1.59971	0.05
41196	2.31152	0.202372
41197	2.3968	0.193779
41198	1.99823	2.1
41199	2.21189	2.1
41200	2.18937	2.1
41201	1.979 2.1	
41202	4.19089	2.1
41203	0.999114	2.1
41204	0.9895	2.1
41205	0.758942	2.1
41206	0.749358	2.1
41207	0.874236	2.1
41208	0.874236	2.1
41209	0.874236	2.1
41210	0.874236	2.1
41211	0.9895	2.1
41212	0.749358	2.1
41213	0.758942	2.1
41214	0.999114	2.1
41215	2.18937	2.1
41216	1.979 2.1	
41217	1.95416	2.1
41218	2.162 2.1	
41219	4.14099	2.1
41220	1.95416	2.1
41221	1.49871	2.1
41222	1.47712	2.1
41223	1.49871	2.1
41224	1.9574	2.1
41225	1.979 2.1	
41226	1.68508	0.05
41227	1.64984	0.05
41228	2.67711	0.05
41229	2.76407	0.05
41230	4.41391	0.05
41231	1.84227	0.05
41232	2.76407	0.05
41233	2.76407	0.05
41234	1.85007	0.05
41235	2.72769	0.05

41236	1.80812	0.05
41237	1.94516	2.1
41238	1.89226	2.1
41239	1.89226	2.1
41240	1.89226	2.1
41241	1.98853	2.1
41242	1.98852	2.1
41243	1.28095	2.1
41244	2.0815	2.1
41245	2.29123	2.1
41246	2.06833	2.1
41247	1.08212	2.1
41248	0.976848	2.1
41249	1.89226	2.1
41250	1.89226	2.1
41251	1.89226	2.1
41252	1.89226	2.1
41253	1.89226	2.1
41254	1.89226	2.1
41255	1.13537	2.1
41256	1.18233	2.1
41257	0.946132	2.1
41258	0.946132	2.1
41259	0.946132	2.1
41260	0.946132	2.1
41261	0.994264	2.1
41262	1.18233	2.1
41263	1.13537	2.1
41264	0.946131	2.1
41265	0.946132	2.1
41266	0.994264	2.1
41267	0.953204	2.1
41268	0.953204	2.1
41269	0.966515	2.1
41270	0.966515	2.1
41271	1.30583	2.1
41272	1.14561	2.1
41273	0.640475	2.1
41274	0.774103	2.1
41275	1.30584	2.1
41276	0.774101	2.1
41277	0.640474	2.1
41278	1.14562	2.1
41279	0.946131	2.1
41280	0.946131	2.1
41281	0.946131	2.1
41282	0.946131	2.1
41283	0.946128	2.1
41284	0.946128	2.1
41285	0.946133	2.1
41286	0.946133	2.1
41287	0.94613	2.1
41288	0.94613	2.1
41289	0.946131	2.1
41290	0.946131	2.1
41291	0.946132	0.05
41292	0.946132	0.05
41293	0.946131	0.05

41294	0.946131	0.05
41295	0.946131	0.05
41296	0.946131	0.05
41297	0.946132	0.05
41298	0.946132	0.05
41299	0.946131	0.05
41300	0.946131	0.05
41301	0.946132	0.05
41302	0.946132	0.05
41303	0.946131	0.05
41304	0.946131	0.05
41305	0.946131	0.05
41306	0.946131	0.05
41307	0.94613	0.05
41308	0.94613	0.05
41309	0.946132	0.05
41310	0.946132	0.05
41311	0.946131	0.05
41312	0.946131	0.05
41313	0.946131	0.05
41314	0.946131	0.05
41315	2.57882	0.05
41316	1.48676	0.05
41317	1.48797	0.05
41318	2.64281	0.05
41319	4.12957	0.05
41320	2.56323	0.05
41321	2.64281	0.05
41322	2.51807	0.05
41323	1.36324	0.05
41324	1.48797	0.05
41325	1.48797	0.05
41326	1.04367	2.1
41327	2.42504	2.1
41328	2.65164	2.1
41329	1.49479	2.1
41330	3.91983	2.1
41331	2.15622	1.85367
41332	1.70539	2.1
41333	2.02369	2.1
41334	2.39039	2.1
41335	4.17992	2.1
41336	0.966979	2.1
41337	1.7054	2.1
41338	2.0237	2.1
41339	1.31502	2.1
41340	2.99067	2.1
41341	2.02369	2.1
41342	2.65637	2.1
41343	2.39039	1.66424
41344	2.39039	2.1
41345	2.29332	1.62409
41346	2.29332	2.1
41347	2.0237	2.1
41348	1.72116	2.1
41349	1.58465	2.1
41350	1.31502	2.1
41351	2.29332	2.1

41352	2.0237	2.1
41353	1.0372	0.05
41354	2.32462	0.05
41355	2.2291	0.05
41356	2.2291	0.05
41357	1.17464	0.05
41358	1.17464	0.05
41359	2.68289	0.05
41360	1.07805	0.05
41361	1.70302	0.05
41362	2.06653	0.05
41363	3.5779	0.05
41364	1.89226	0.05
41365	1.89226	0.05
41366	1.89226	0.05
41367	1.89226	0.05
41368	3.78453	0.05
41369	1.89226	0.05
41370	1.89226	0.05
41371	1.89226	0.05
41372	1.89226	0.05
41373	1.89226	0.05
41374	1.89226	0.05
41375	1.89225	0.05
41376	1.89225	0.05
41377	1.89225	0.05
41378	1.89225	0.05
41379	1.89225	0.05
41380	1.89225	0.05
41381	0.94613	0.05
41382	0.94613	0.05
41383	0.94613	0.05
41384	0.94613	0.05
41385	0.946128	0.05
41386	0.946128	0.05
41387	0.946132	0.05
41388	0.946132	0.05
41389	0.94613	0.05
41390	0.94613	0.05
41391	0.94613	0.05
41392	0.94613	0.05
41393	1.29417	2.1
41394	1.32732	2.1
41395	1.32316	0.683051
41396	1.34973	2.1
41397	2.65048	2.1
41398	1.89226	1.3658
41399	1.89226	2.1
41400	1.89226	0.646834
41401	1.89226	2.1
41402	3.78453	1.70788
41403	1.89226	2.1
41404	1.89226	2.1
41405	1.89226	1.36969
41406	1.89226	2.1
41407	3.78453	2.1
41408	1.32732	2.1
41409	1.13006	2.1

41410	1.13358	2.1
41411	1.11117	2.1
41412	1.34973	2.1
41413	1.32732	2.1
41414	1.32316	1.96864
41415	1.34973	2.1
41416	1.34066	0.909578
41417	1.36601	2.1
41418	2.68918	2.1
41419	1.89226	1.91065
41420	1.89226	2.1
41421	1.89226	0.13903
41422	1.89226	2.1
41423	3.78453	1.99935
41424	1.89226	1.99392
41425	1.89226	2.1
41426	1.89226	2.1
41427	1.89226	2.1
41428	1.89226	1.85187
41429	1.89226	2.1
41430	0.624134	2.1
41431	0.624134	2.1
41432	0.624334	2.1
41433	0.624334	2.1
41434	0.56503	2.1
41435	0.573605	2.1
41436	0.674864	2.1
41437	0.683007	2.1
41438	0.683007	2.1
41439	0.573605	2.1
41440	0.565029	2.1
41441	0.674864	2.1
41442	2.16633	2.1
41443	1.40846	2.1
41444	2.13977	2.1
41445	1.40771	2.1
41446	3.57404	2.1
41447	2.13977	2.1
41448	1.29932	2.1
41449	1.29932	2.1
41450	1.40771	2.1
41451	1.98957	2.1
41452	2.09796	2.1
41453	2.6044	0.0502476
41454	1.85548	0.121354
41455	1.89226	0.05
41456	1.89226	0.198335
41457	3.74774	0.0799321
41458	1.89226	0.05
41459	1.89226	0.220365
41460	1.89226	0.05
41461	1.89226	0.0872476
41462	3.78453	0.0639467
41463	1.89226	2.1
41464	2.64107	2.1
41465	1.89226	2.1
41466	2.78977	2.1
41467	1.89226	2.1

41468	2.78977	2.1
41469	1.89226	2.1
41470	1.89226	2.1
41471	1.89226	2.1
41472	1.89226	2.1
41473	1.89226	2.1
41474	1.89226	2.1
41475	2.43173	2.1
41476	1.41238	2.1
41477	2.0815	2.1
41478	1.98048	2.1
41479	1.62014	2.1
41480	1.50086	2.1
41481	0.946129	0.0758004
41482	0.946129	0.0562152
41483	1.27636	0.0951293
41484	1.1984	0.05
41485	0.946131	0.05
41486	0.946131	0.05
41487	1.27636	0.0922488
41488	1.27636	0.05
41489	0.946131	0.0758354
41490	1.27636	0.05
41491	0.946131	0.05
41492	1.1984	0.05
41493	1.99569	0.0580729
41494	2.55272	0.191964
41495	1.77403	0.05
41496	1.70303	0.05
41497	3.05772	0.141766
41498	2.55121	0.0807883
41499	0.946128	0.05
41500	0.946128	0.0757958
41501	1.38752	0.113807
41502	1.27636	0.05
41503	0.94613	0.05
41504	0.94613	0.05
41505	1.38752	0.110506
41506	1.38752	0.05
41507	0.946131	0.05
41508	1.38752	0.0556778
41509	0.946131	0.122943
41510	1.27636	0.05
41511	2.77504	0.111267
41512	1.99569	0.05
41513	2.29794	0.05
41514	1.99569	0.05
41515	2.85497	0.05
41516	2.55272	0.05
41517	1.53284	2.1
41518	1.89226	2.1
41519	1.89226	2.1
41520	1.89226	2.1
41521	1.54763	2.1
41522	1.54763	2.1
41523	0.946131	2.1
41524	0.946131	2.1
41525	0.946127	2.1

41526	0.946127	2.1
41527	0.946127	2.1
41528	0.946127	2.1
41529	0.946131	2.1
41530	0.946131	2.1
41531	0.94613	2.1
41532	0.94613	2.1
41533	0.946129	2.1
41534	0.946129	2.1
41535	0.751754	2.1
41536	0.751754	2.1
41537	0.771562	2.1
41538	0.771561	2.1
41539	1.0268	2.1
41540	0.95482	2.1
41541	0.528937	2.1
41542	0.496512	2.1
41543	0.528936	2.1
41544	1.0268	2.1
41545	0.954821	2.1
41546	0.49651	2.1
41547	2.64075	0.0675891
41548	1.35181	1.37417
41549	1.35181	0.05
41550	1.83823	0.157219
41551	2.28373	0.183721
41552	2.88566	1.69595
41553	0.493018	1.11579
41554	0.675903	0.81817
41555	1.14187	0.236604
41556	0.973937	0.123488
41557	0.821166	0.305738
41558	0.821166	1.56274
41559	0.828674	2.1
41560	0.828674	0.0533859
41561	0.973937	0.694101
41562	0.493018	0.865916
41563	0.675903	0.362806
41564	1.14187	0.680561
41565	1.89226	2.1
41566	2.11667	2.1
41567	1.91184	2.1
41568	1.89226	2.1
41569	2.13625	2.1
41570	2.11667	2.1
41571	1.89226	2.1
41572	1.89226	2.1
41573	1.89226	2.1
41574	1.89226	2.1
41575	3.78453	2.1
41576	0.907729	2.1
41577	0.907729	2.1
41578	1.04459	2.1
41579	0.890955	2.1
41580	0.988181	2.1
41581	0.841856	2.1
41582	0.946133	2.1
41583	0.946133	2.1

41584	1.04072	2.1
41585	1.1064	2.1
41586	0.89099	2.1
41587	0.851541	2.1
41588	1.89225	2.1
41589	2.13623	2.1
41590	1.91044	2.1
41591	1.89225	2.1
41592	2.15442	2.1
41593	2.13623	2.1
41594	1.89225	2.1
41595	1.89225	2.1
41596	1.89225	2.1
41597	1.89225	2.1
41598	1.89225	2.1
41599	1.89225	2.1
41600	0.907723	2.1
41601	0.907723	2.1
41602	1.07536	2.1
41603	1.07814	2.1
41604	0.998499	2.1
41605	1.03575	2.1
41606	0.946126	2.1
41607	0.946126	2.1
41608	1.04072	2.1
41609	1.14025	2.1
41610	0.851528	2.1
41611	0.898284	2.1
41612	1.89226	0.05
41613	1.89226	0.05
41614	1.89226	0.05
41615	1.89226	0.05
41616	1.89226	0.05
41617	1.89226	0.05
41618	2.53008	2.1
41619	1.94534	2.1
41620	1.98702	2.1
41621	1.98858	2.1
41622	2.52852	2.1
41623	2.53008	2.1
41624	1.03267	2.1
41625	1.12591	2.1
41626	1.2959	2.1
41627	1.28992	2.1
41628	1.10851	2.1
41629	1.33078	2.1
41630	1.10661	2.1
41631	1.10661	2.1
41632	0.982208	2.1
41633	1.28992	2.1
41634	1.03267	2.1
41635	1.23262	2.1
41636	2.2712	2.1
41637	1.89226	2.1
41638	1.95954	2.1
41639	1.95954	2.1
41640	1.89226	2.1
41641	1.89226	2.1

41642	1.89226	2.1
41643	1.89226	2.1
41644	1.89226	2.1
41645	1.89226	2.1
41646	3.78453	2.1
41647	1.33887	2.1
41648	1.25395	2.1
41649	0.88327	2.1
41650	0.676665	2.1
41651	0.84881	2.1
41652	0.987503	2.1
41653	1.13243	2.1
41654	1.13243	2.1
41655	1.15338	2.1
41656	0.849966	2.1
41657	0.793074	2.1
41658	1.11957	2.1
41659	0.946129	2.1
41660	0.946129	2.1
41661	1.28254	2.1
41662	1.1356	2.1
41663	1.1356	2.1
41664	1.1356	2.1
41665	0.946131	2.1
41666	0.946131	2.1
41667	0.946131	2.1
41668	1.28254	2.1
41669	1.1356	2.1
41670	0.946131	2.1
41671	1.89227	2.1
41672	1.89227	2.1
41673	1.89227	2.1
41674	1.89227	2.1
41675	3.78453	2.1
41676	0.936287	0.05
41677	1.01399	0.05
41678	0.959415	0.05
41679	0.959415	0.05
41680	0.910786	0.05
41681	1.38301	0.05
41682	1.0286	0.05
41683	0.535742	0.05
41684	0.505202	0.05
41685	0.937685	0.05
41686	1.11066	0.05
41687	1.46907	0.05
41688	1.00546	0.05
41689	1.01524	0.05
41690	0.94613	0.05
41691	0.94613	0.05
41692	1.05018	0.05
41693	0.914025	0.05
41694	0.913512	0.05
41695	0.851542	0.05
41696	1.05385	0.05
41697	1.05494	0.05
41698	0.91404	0.05
41699	0.91404	0.05

41700	2.03253	0.5478
41701	1.82325	0.05
41702	1.93938	0.221633
41703	1.73756	0.05
41704	3.77009	0.05
41705	1.82325	0.05
41706	1.47562	0.408484
41707	1.73756	0.05
41708	1.3868	0.05
41709	3.21317	0.05
41710	1.93938	0.253156
41711	1.73756	0.05
41712	1.85293	0.581457
41713	1.64279	0.05
41714	3.59049	0.05
41715	1.73756	0.0589169
41716	1.3868	0.280001
41717	1.64279	0.05
41718	1.28419	0.121585
41719	3.02174	0.0601703
41720	1.35048	2.1
41721	1.37999	2.1
41722	1.34355	0.785084
41723	1.37854	2.1
41724	2.72354	2.03446
41725	1.89226	1.08952
41726	1.89226	2.1
41727	1.89226	0.540068
41728	1.89226	1.95346
41729	3.78452	0.941384
41730	1.89226	2.1
41731	1.89226	2.1
41732	1.89226	0.484506
41733	1.89226	2.1
41734	3.78453	2.1
41735	1.37999	2.1
41736	1.18059	2.1
41737	1.17117	2.1
41738	1.17262	2.1
41739	1.37855	2.1
41740	1.37999	2.1
41741	1.34355	2.04697
41742	1.37854	2.1
41743	1.33079	0.862533
41744	1.3741	2.1
41745	2.71765	2.03816
41746	1.89226	1.77319
41747	1.89226	2.1
41748	1.89226	0.216689
41749	1.89226	2.1
41750	3.78453	1.94841
41751	1.89226	1.24019
41752	1.89226	2.1
41753	1.89226	1.5263
41754	1.89226	2.1
41755	3.78453	2.1
41756	0.640305	2.1
41757	0.640305	2.1

41758	0.643507	0.398637
41759	0.643507	2.1
41760	0.590294	2.1
41761	0.594539	2.1
41762	0.689273	1.60695
41763	0.687048	2.1
41764	0.687048	2.1
41765	0.594538	2.1
41766	0.590294	2.1
41767	0.689273	2.1
41768	2.01399	0.05
41769	1.70141	0.114877
41770	1.83896	1.81136
41771	1.52482	1.78159
41772	3.53881	0.575594
41773	1.70141	0.172413
41774	1.0752	2.1
41775	1.0752	0.0852285
41776	1.25669	0.290664
41777	1.52482	0.455685
41778	1.70632	2.1
41779	1.83896	1.29587
41780	1.52482	0.050482
41781	1.65244	0.277149
41782	1.34412	1.67054
41783	3.17725	0.310015
41784	1.26782	0.05
41785	2.90353	0.05
41786	2.54203	0.05
41787	2.41979	0.05
41788	1.77007	0.05
41789	1.703 0.05	
41790	0.946118	0.05
41791	0.946118	0.05
41792	1.38643	0.05
41793	1.38644	0.05
41794	0.946117	0.05
41795	0.946117	0.05
41796	1.38644	0.05
41797	1.15545	0.05
41798	0.946131	0.05
41799	1.15546	0.05
41800	1.38642	0.05
41801	0.946105	0.05
41802	2.42256	0.072729
41803	2.63056	0.05
41804	2.43174	0.05
41805	2.25035	0.05
41806	4.8543	0.05
41807	2.58031	0.0632552
41808	2.63056	0.05
41809	2.43174	0.05
41810	2.32127	0.05
41811	5.01205	0.05
41812	2.43174	0.05
41813	2.25035	0.05
41814	2.11465	0.05
41815	2.13042	0.05

41816	4.54639	0.05
41817	2.43174	0.05
41818	2.32127	0.05
41819	1.92997	0.05
41820	2.13041	0.05
41821	4.36171	0.05
41822	1.89227	2.1
41823	1.89227	2.1
41824	1.89227	2.1
41825	1.89227	2.1
41826	3.78453	2.1
41827	0.568724	2.1
41828	0.568724	2.1
41829	0.57678	2.1
41830	0.57678	2.1
41831	0.523796	2.1
41832	0.548137	2.1
41833	0.597368	2.1
41834	0.637877	2.1
41835	0.597367	2.1
41836	0.523796	2.1
41837	0.637877	2.1
41838	0.548137	2.1
41839	1.32674	2.1
41840	1.32674	2.1
41841	0.946132	2.1
41842	0.946132	2.1
41843	1.3195	2.1
41844	1.32674	2.1
41845	0.946132	2.1
41846	0.946132	2.1
41847	0.946132	2.1
41848	1.3195	2.1
41849	1.32674	2.1
41850	0.946133	2.1
41851	1.89227	2.1
41852	1.89227	2.1
41853	1.89227	2.1
41854	1.89227	2.1
41855	3.78453	2.1
41856	1.19473	2.1
41857	0.987132	2.1
41858	0.987132	2.1
41859	1.04759	2.1
41860	1.0894	2.1
41861	1.14986	2.1
41862	1.3195	2.1
41863	1.3195	2.1
41864	0.946132	2.1
41865	0.946132	2.1
41866	1.31185	2.1
41867	1.3195	2.1
41868	0.946132	2.1
41869	0.946132	2.1
41870	1.31185	2.1
41871	0.946133	2.1
41872	0.946132	2.1
41873	1.3195	2.1

41874	1.11256	0.46249
41875	1.70101	0.381995
41876	1.22297	0.45543
41877	1.22297	0.495469
41878	1.7252	0.517967
41879	1.7252	0.552517
41880	1.99298	0.542369
41881	1.70101	0.514501
41882	1.7252	0.614358
41883	2.18701	0.239258
41884	3.71818	0.347165
41885	1.03218	0.357662
41886	1.7252	0.272013
41887	1.11256	0.382292
41888	1.11256	0.391027
41889	1.7457	0.399582
41890	1.7457	0.413226
41891	1.7457	0.468203
41892	2.187	0.163426
41893	1.7252	0.321149
41894	1.7252	0.403965
41895	2.27351	0.301195
41896	2.27351	0.381423
41897	1.95544	1.08728
41898	1.35897	2.1
41899	1.44859	1.64633
41900	1.44859	1.93649
41901	1.80709	1.18184
41902	1.80709	1.86041
41903	1.80709	0.85996
41904	1.17822	2.07204
41905	1.35897	1.64573
41906	1.35897	2.09049
41907	1.52782	0.712311
41908	1.52782	1.82644
41909	1.11978	1.84107
41910	0.903546	1.19939
41911	0.977719	1.95773
41912	1.2364	1.20965
41913	1.03812	0.890059
41914	1.03812	2.1
41915	1.05938	0.607344
41916	1.05938	2.04524
41917	1.11978	1.46835
41918	1.2364	0.747061
41919	0.97772	1.70123
41920	0.903546	1.35493
41921	1.80709	2.04216
41922	1.81847	1.52169
41923	1.96029	2.00158
41924	1.52782	1.7738
41925	2.23956	1.23849
41926	1.80709	1.5423
41927	1.89226	0.0584178
41928	2.32962	0.05
41929	2.5992	0.05
41930	1.89226	0.05
41931	2.5992	0.0543582

41932	1.89226	0.0568067
41933	2.55791	0.05
41934	2.47141	0.05
41935	2.13515	0.05
41936	2.10939	0.05
41937	4.66731	0.05
41938	2.47141	0.05
41939	2.23337	0.05
41940	2.10939	0.05
41941	2.03438	0.05
41942	4.34276	0.05
41943	2.13512	0.05
41944	1.71027	0.05
41945	1.71027	0.05
41946	2.10936	0.05
41947	1.60137	0.05
41948	2.00046	0.05
41949	1.71027	0.05
41950	2.03435	0.05
41951	1.71027	0.05
41952	2.10936	0.05
41953	1.72067	0.05
41954	2.11976	0.05
41955	1.177 0.05	
41956	1.36405	0.584928
41957	2.25635	0.638287
41958	2.60151	0.05
41959	3.6204	0.228666
41960	0.658869	0.05
41961	0.781697	0.05
41962	0.637037	0.05
41963	0.541148	0.05
41964	0.654682	0.05
41965	0.654682	0.05
41966	0.641224	0.0565532
41967	0.641224	0.05
41968	0.637039	0.05
41969	0.781696	0.05
41970	0.658867	0.05
41971	0.541149	0.05
41972	1.89226	0.05
41973	1.59152	0.05
41974	1.89226	0.05
41975	1.82125	0.05
41976	1.89226	0.05
41977	1.82125	0.05
41978	2.18967	0.05
41979	1.89227	0.05
41980	2.72752	0.05
41981	2.72752	0.05
41982	1.89227	0.05
41983	1.89227	0.05
41984	2.18967	0.05
41985	1.92547	0.05
41986	1.92547	0.05
41987	2.72752	0.05
41988	1.30572	0.05
41989	2.10777	0.05

41990	2.39663	2.1
41991	1.92293	2.1
41992	1.96889	2.1
41993	2.45928	2.1
41994	4.36552	2.1
41995	1.02605	2.1
41996	1.92293	2.1
41997	1.9689	2.1
41998	1.04679	2.1
41999	2.99495	2.1
42000	1.96889	2.1
42001	2.45928	2.1
42002	2.50138	2.1
42003	1.99782	2.1
42004	4.47027	2.1
42005	1.9689	2.1
42006	1.03449	2.1
42007	1.07572	2.1
42008	1.04679	2.1
42009	1.99783	2.1
42010	1.9689	2.1
42011	1.72162	0.769109
42012	1.89186	0.280588
42013	2.29771	0.502304
42014	1.80149	0.116572
42015	4.01933	0.341535
42016	1.16482	0.828511
42017	1.97003	0.200049
42018	1.72162	0.123788
42019	1.72162	0.260273
42020	1.29313	0.308441
42021	1.29313	0.595655
42022	2.29771	0.587229
42023	2.84159	0.0746951
42024	2.19622	0.172522
42025	1.97366	0.153206
42026	2.50906	0.262992
42027	2.29771	0.304518
42028	1.29313	0.675386
42029	2.4483	0.182397
42030	2.14223	0.163507
42031	2.14228	0.224952
42032	1.294	0.342292
42033	1.294	0.459059
42034	1.21495	0.05
42035	1.70303	0.05
42036	1.7924	0.05
42037	1.64002	0.05
42038	3.11963	0.05
42039	2.81021	0.05
42040	1.89226	0.05
42041	1.89226	0.05
42042	1.89226	0.05
42043	2.36406	0.05
42044	2.36406	0.05
42045	1.89226	0.05
42046	1.89226	0.05
42047	1.89226	0.05

42048	1.89226	0.05
42049	1.89226	0.05
42050	1.89226	0.05
42051	2.29976	0.05
42052	1.64002	0.05
42053	1.8959	0.05
42054	1.90873	0.05
42055	1.85594	0.05
42056	1.85594	0.05
42057	1.89226	0.05
42058	1.89226	0.05
42059	1.89226	0.05
42060	1.89226	0.05
42061	3.78453	0.05
42062	1.89226	0.449959
42063	2.52823	2.1
42064	2.83441	1.88641
42065	2.83441	2.1
42066	1.89226	1.27446
42067	1.89226	2.08032
42068	1.04522	1.9165
42069	1.04522	1.57039
42070	1.12831	2.01173
42071	1.18364	2.1
42072	0.640525	1.86198
42073	0.924882	1.86447
42074	1.27877	2.08092
42075	1.43363	0.357452
42076	0.93474	1.88473
42077	1.58926	1.65713
42078	1.29573	2.0778
42079	0.664247	2.05939
42080	2.02191	2.1
42081	1.89226	2.1
42082	1.89226	2.1
42083	1.89226	2.1
42084	2.58639	2.1
42085	2.58639	2.1
42086	1.16759	2.1
42087	2.11828	2.1
42088	1.60936	2.1
42089	2.35846	2.1
42090	3.52605	2.1
42091	1.89226	2.1
42092	1.89226	2.1
42093	1.89226	2.1
42094	1.89226	2.1
42095	3.78453	2.1
42096	2.82765	0.05
42097	1.89809	0.05
42098	2.15971	0.05
42099	1.89809	0.05
42100	2.29233	0.05
42101	2.03071	0.05
42102	1.90664	0.05
42103	2.15971	0.05
42104	1.28956	0.05
42105	1.28956	0.05

42106	2.64414	0.05
42107	2.02706	0.05
42108	2.82765	0.05
42109	1.89226	0.05
42110	2.39968	0.05
42111	2.39968	0.05
42112	1.89226	0.05
42113	1.89226	0.05
42114	0.978643	2.1
42115	0.703968	2.1
42116	0.946129	2.1
42117	0.946129	2.1
42118	0.703939	2.1
42119	0.703939	2.1
42120	0.946159	2.1
42121	0.946159	2.1
42122	0.978642	2.1
42123	0.94613	2.1
42124	0.94613	2.1
42125	0.703968	2.1
42126	1.89226	2.1
42127	2.17309	1.1635
42128	2.17309	2.1
42129	1.47475	2.0971
42130	1.89226	2.1
42131	1.19393	1.5431
42132	1.89226	2.1
42133	1.89226	2.1
42134	1.89226	2.1
42135	1.89226	2.1
42136	3.78452	2.1
42137	1.17426	2.1
42138	0.978644	2.1
42139	0.946129	2.1
42140	0.946129	2.1
42141	0.946131	2.1
42142	0.946131	2.1
42143	0.978643	2.1
42144	0.978642	2.1
42145	1.17426	2.1
42146	0.946131	2.1
42147	0.978642	2.1
42148	0.94613	2.1
42149	1.89226	2.1
42150	1.89226	1.18971
42151	1.89226	2.1
42152	2.17309	1.41386
42153	2.56346	2.1
42154	2.94389	1.91312
42155	0.946131	2.1
42156	0.946131	2.1
42157	0.946128	2.1
42158	0.946128	2.1
42159	0.946131	2.1
42160	0.946131	2.1
42161	0.946128	2.1
42162	0.946128	2.1
42163	0.94613	2.1

42164	0.94613	2.1
42165	0.946129	2.1
42166	0.946129	2.1
42167	1.89226	0.680009
42168	1.89226	0.508737
42169	1.89226	0.998863
42170	1.89226	0.567316
42171	3.78453	0.202879
42172	1.89226	0.942155
42173	1.89226	0.437943
42174	1.89226	1.07587
42175	1.89226	1.04016
42176	1.89226	0.656884
42177	1.89226	0.528827
42178	1.89226	0.503729
42179	1.30733	0.934293
42180	1.89226	1.21514
42181	1.92403	0.660717
42182	3.38635	0.319016
42183	1.89226	0.577547
42184	3.02887	1.04119
42185	2.0815	0.151795
42186	1.88422	0.674022
42187	2.0815	0.697212
42188	1.88422	1.79056
42189	1.89226	1.26676
42190	1.70221	0.594474
42191	1.89226	1.73306
42192	1.20068	1.12162
42193	1.89226	1.02846
42194	1.20068	0.511161
42195	1.01393	1.01435
42196	1.20067	0.609833
42197	1.89226	1.35057
42198	2.44958	1.65255
42199	1.01393	0.457455
42200	1.89226	0.303055
42201	1.89226	2.1
42202	1.89226	2.1
42203	1.89226	2.1
42204	1.89226	2.1
42205	1.89226	2.1
42206	1.89226	2.1
42207	1.89226	2.1
42208	1.89226	0.0581654
42209	1.89226	0.0955012
42210	1.89226	2.1
42211	1.89226	0.153227
42212	1.89226	2.1
42213	1.89226	2.1
42214	1.89226	2.1
42215	1.89226	2.1
42216	1.89226	2.1
42217	1.89226	2.1
42218	1.89226	2.1
42219	1.91161	2.1
42220	2.45782	2.1
42221	1.39653	2.1

42222	2.44652	2.1
42223	4.35812	2.1
42224	2.73092	1.98133
42225	1.89226	0.0594912
42226	1.89226	2.1
42227	1.89226	0.06517
42228	2.44652	2.1
42229	2.44652	0.0501939
42230	1.89226	2.1
42231	1.89226	0.113003
42232	1.89226	0.333269
42233	1.89226	2.1
42234	1.89226	0.43434
42235	1.89226	2.1
42236	2.64924	2.1
42237	1.31109	2.1
42238	1.31109	2.1
42239	1.49958	2.1
42240	1.95039	2.1
42241	2.14451	2.1
42242	0.825157	2.1
42243	0.634856	2.1
42244	0.816836	2.1
42245	0.816836	2.1
42246	0.556743	2.1
42247	0.639142	2.1
42248	0.985106	2.1
42249	0.795045	2.1
42250	0.967461	2.1
42251	0.552494	2.1
42252	0.676829	2.1
42253	0.98803	2.1
42254	2.28564	2.1
42255	2.34084	1.62245
42256	2.3502	2.04631
42257	2.42908	2.1
42258	4.69104	2.1
42259	2.34084	2.1
42260	2.18077	2.1
42261	2.42908	2.1
42262	2.2947	0.973579
42263	4.63554	2.1
42264	2.3502	2.1
42265	2.42908	2.1
42266	2.38681	2.1
42267	2.50438	1.54242
42268	4.85458	2.1
42269	2.42908	2.1
42270	2.2947	1.81593
42271	2.50438	1.27131
42272	2.40627	2.1
42273	4.79908	1.71052
42274	1.89226	0.16886
42275	2.20711	1.4003
42276	1.89226	0.174571
42277	1.89226	1.86867
42278	2.02693	0.156879
42279	2.02693	1.86434

42280	1.2269	2.1
42281	2.20711	0.884095
42282	2.0815	0.640941
42283	1.11506	2.1
42284	3.22109	2.1
42285	0.545802	1.85226
42286	0.547489	2.1
42287	0.85914	1.05453
42288	1.11641	1.15521
42289	0.899974	1.29969
42290	0.849893	2.1
42291	0.747885	1.89814
42292	0.747885	0.767866
42293	0.557184	2.1
42294	0.887039	2.07944
42295	1.08948	2.02496
42296	0.59815	2.06736
42297	0.974139	2.1
42298	1.33385	2.1
42299	1.02368	2.1
42300	1.02368	2.1
42301	1.00833	2.1
42302	1.00833	2.1
42303	1.89226	0.2225
42304	1.89226	0.051729
42305	1.89226	0.0510964
42306	1.89226	0.05
42307	3.78453	0.05
42308	1.74651	0.0564978
42309	1.44605	0.05
42310	1.63557	0.05
42311	1.31993	0.05
42312	3.08162	0.05
42313	1.89226	0.05
42314	1.89226	0.0795712
42315	1.89226	0.0668784
42316	1.89226	0.05
42317	3.78452	0.05
42318	1.63557	0.05
42319	1.31992	0.05
42320	1.52615	0.05
42321	1.18549	0.05
42322	2.82106	0.05
42323	1.89226	0.05
42324	2.58101	0.05
42325	2.73797	0.0900301
42326	2.73797	0.05
42327	1.89226	0.05
42328	1.89226	0.05
42329	1.91762	0.134245
42330	1.44354	0.0950704
42331	1.33622	0.097611
42332	1.33622	0.0781375
42333	2.20248	0.227628
42334	2.20248	0.149487
42335	1.11712	2.1
42336	1.11712	2.1
42337	1.11421	2.1

42338	1.11421	2.1
42339	0.833871	2.1
42340	0.926528	2.1
42341	1.3048	2.1
42342	1.40335	2.1
42343	0.926527	2.1
42344	1.40335	2.1
42345	1.3048	2.1
42346	0.833871	2.1
42347	0.879657	2.1
42348	0.937695	2.1
42349	0.946129	2.1
42350	0.946129	2.1
42351	0.851541	2.1
42352	0.851541	2.1
42353	1.1126	2.1
42354	1.04519	2.1
42355	0.851486	2.1
42356	0.980501	2.1
42357	0.898251	2.1
42358	0.851528	2.1
42359	1.37756	2.1
42360	2.73081	2.1
42361	2.44311	2.1
42362	2.44311	2.1
42363	1.66428	2.1
42364	1.66428	2.1
42365	1.89226	2.1
42366	1.89226	2.1
42367	1.89226	2.1
42368	1.89226	2.1
42369	3.78452	2.1
42370	1.65781	2.1
42371	1.89837	2.1
42372	1.46713	2.1
42373	1.98027	2.1
42374	3.63808	2.1
42375	2.29054	2.1
42376	2.26369	0.05
42377	1.98027	2.1
42378	2.15617	0.05
42379	4.24396	0.491579
42380	1.89226	2.1
42381	1.89226	0.05
42382	1.89226	2.1
42383	1.89226	0.0564503
42384	3.78452	1.37481
42385	0.946131	2.1
42386	0.946131	2.1
42387	0.946128	2.1
42388	0.946128	2.1
42389	0.946129	2.1
42390	0.946129	2.1
42391	0.94613	2.1
42392	0.94613	2.1
42393	0.94613	2.1
42394	0.94613	2.1
42395	0.94613	2.1

42396	0.94613	2.1
42397	0.812186	2.1
42398	0.812186	2.1
42399	0.819781	2.1
42400	0.819781	2.1
42401	0.913532	2.1
42402	0.990134	2.1
42403	0.733564	2.1
42404	0.641833	2.1
42405	0.641832	2.1
42406	0.913533	2.1
42407	0.733564	2.1
42408	0.990134	2.1
42409	0.994262	0.05
42410	0.994262	2.1
42411	0.997354	2.1
42412	0.997354	0.05
42413	1.00775	0.05
42414	1.07808	0.05
42415	0.913533	2.1
42416	0.990135	2.1
42417	0.913532	0.432252
42418	1.00775	0.35567
42419	0.990134	0.24486
42420	1.07808	0.188354
42421	0.946131	2.1
42422	0.946131	0.0609625
42423	0.946128	2.1
42424	0.946128	0.0547373
42425	0.946128	0.05
42426	0.946128	0.05
42427	0.94613	2.1
42428	0.94613	2.1
42429	0.946129	1.53608
42430	0.946129	1.63255
42431	0.946129	0.529775
42432	0.946129	0.914732
42433	0.816232	2.1
42434	0.760038	2.1
42435	0.845999	2.1
42436	0.845999	2.1
42437	1.0631	2.1
42438	1.28131	2.1
42439	0.474065	2.1
42440	0.591905	2.1
42441	0.601756	2.1
42442	1.23903	2.1
42443	0.988592	2.1
42444	0.49051	2.1
42445	2.57021	2.1
42446	1.89226	2.1
42447	2.68172	2.1
42448	1.89226	1.66195
42449	4.46247	2.1
42450	1.89226	2.1
42451	1.89226	1.89842
42452	1.89226	1.83734
42453	1.89226	1.76931

42454	3.78452	1.92107
42455	1.12573	2.1
42456	1.65504	1.62814
42457	1.38848	2.1
42458	1.38848	2.1
42459	1.30567	1.2769
42460	1.30567	2.1
42461	2.82413	2.1
42462	1.89226	1.96401
42463	2.68172	2.1
42464	2.68172	2.1
42465	1.89226	2.1
42466	1.89226	2.1
42467	1.89226	2.04739
42468	1.89226	2.1
42469	1.89226	2.1
42470	1.89226	0.930958
42471	3.78452	2.00605
42472	1.30567	2.1
42473	1.65504	2.04283
42474	1.52724	2.1
42475	1.89249	2.1
42476	3.18228	2.1
42477	2.57386	0.05
42478	1.89226	0.05
42479	1.89226	0.05
42480	2.66753	0.05
42481	1.89226	0.05
42482	2.66753	0.05
42483	1.89226	0.05
42484	1.89226	0.0575037
42485	2.2973	0.0551589
42486	3.12479	0.05
42487	1.89226	0.05
42488	2.57385	0.05
42489	0.489293	2.1
42490	0.672698	2.1
42491	0.59038	2.1
42492	0.738357	2.1
42493	0.62269	2.1
42494	0.62269	2.1
42495	0.640388	2.1
42496	0.640388	2.1
42497	0.59038	2.1
42498	0.489293	2.1
42499	0.738357	2.1
42500	0.672698	2.1
42501	1.89226	2.1
42502	1.89226	2.1
42503	1.89226	2.1
42504	1.89226	0.485524
42505	3.78452	2.1
42506	2.82858	2.1
42507	1.89226	0.514554
42508	2.5671	2.1
42509	1.89226	2.1
42510	2.5671	2.1
42511	1.89226	2.1

42512	2.06581	0.05
42513	2.47308	0.05
42514	1.89433	0.05
42515	1.67571	0.05
42516	3.87274	0.05
42517	2.55791	0.05
42518	2.10276	0.05
42519	2.47308	0.05
42520	2.47308	0.05
42521	1.83415	0.05
42522	1.90922	0.05
42523	1.18421	0.05
42524	2.03277	0.05
42525	1.18421	0.05
42526	2.01275	0.05
42527	1.42818	0.05
42528	2.25673	0.05
42529	0.882904	0.05
42530	0.958492	0.05
42531	0.905756	0.05
42532	0.905756	0.05
42533	1.06352	0.05
42534	0.797654	0.05
42535	1.11319	0.05
42536	0.716795	0.05
42537	0.820721	0.05
42538	0.731963	0.05
42539	1.13143	0.05
42540	1.10528	0.05
42541	1.89226	0.05
42542	1.89226	0.05
42543	1.89226	0.05
42544	1.89226	0.05
42545	1.89226	0.05
42546	1.89226	0.05
42547	1.01241	0.05
42548	0.703929	0.05
42549	0.946133	0.05
42550	0.946133	0.05
42551	1.04072	0.05
42552	1.163 0.05	
42553	0.639952	0.05
42554	0.851543	0.05
42555	0.946132	0.05
42556	0.920761	0.05
42557	0.711038	0.05
42558	0.711038	0.05
42559	1.89226	0.05
42560	1.89226	0.05
42561	1.89226	0.05
42562	1.89226	0.05
42563	1.89226	0.05
42564	1.89226	0.05
42565	1.89226	0.05
42566	1.89226	0.05
42567	2.41075	0.05
42568	1.89226	0.05
42569	3.78451	0.05

42570	0.968476	1.98735
42571	1.20674	0.497932
42572	1.13456	0.318162
42573	1.13456	2.1
42574	1.02074	0.159669
42575	1.02074	2.04093
42576	1.02074	2.1
42577	1.20674	1.97897
42578	1.07866	0.925564
42579	1.28649	0.738135
42580	2.2854	2.1
42581	1.94221	0.05
42582	1.36717	0.05
42583	1.45535	0.05
42584	1.93771	0.05
42585	3.39756	0.05
42586	1.94222	0.0607001
42587	2.25035	0.05
42588	2.41806	0.05
42589	2.42256	0.05
42590	1.93772	0.05
42591	1.94222	0.05
42592	1.49906	0.05
42593	1.93771	0.05
42594	1.45535	0.05
42595	1.45535	0.05
42596	1.91484	0.05
42597	1.91484	0.05
42598	1.91484	0.05
42599	2.25035	0.05
42600	1.93772	0.05
42601	1.93772	0.05
42602	2.11465	0.05
42603	2.11465	0.05
42604	2.61735	0.05
42605	1.5082	0.05
42606	2.36481	0.05
42607	1.58597	0.05
42608	2.36481	0.05
42609	1.46653	0.05
42610	2.36482	0.05
42611	1.08133	0.05
42612	1.5082	0.05
42613	2.01868	0.05
42614	3.52688	0.05
42615	0.94613	0.05
42616	0.94613	0.05
42617	0.946131	0.05
42618	0.946131	0.05
42619	0.946129	0.05
42620	0.946129	0.05
42621	0.946132	0.05
42622	0.946132	0.05
42623	0.94613	0.05
42624	0.94613	0.05
42625	0.946131	0.05
42626	0.946131	0.05
42627	2.53725	0.05

42628	1.89227	0.05
42629	1.89227	0.05
42630	2.27073	0.05
42631	1.89227	0.05
42632	2.27073	0.05
42633	1.89227	0.05
42634	1.89227	0.05
42635	1.89227	0.05
42636	1.89227	0.05
42637	1.89227	0.05
42638	1.89227	0.05
42639	1.86766	2.1
42640	2.83487	2.1
42641	2.84318	2.1
42642	2.59401	2.1
42643	2.11684	2.1
42644	1.86766	2.1
42645	1.10638	2.1
42646	2.11684	2.1
42647	1.29111	2.1
42648	2.37439	2.1
42649	3.40796	2.1
42650	2.56843	2.1
42651	1.89226	2.1
42652	1.89226	2.1
42653	2.71932	2.1
42654	4.46069	2.1
42655	1.89226	2.1
42656	1.89226	2.1
42657	1.89226	2.1
42658	1.89226	2.1
42659	3.78452	2.1
42660	0.6416	2.1
42661	0.697585	2.1
42662	0.670706	2.1
42663	0.598927	2.1
42664	0.644313	2.1
42665	0.644313	2.1
42666	0.652199	2.1
42667	0.652199	2.1
42668	0.697587	2.1
42669	0.670705	2.1
42670	0.598926	2.1
42671	0.641601	2.1
42672	1.89226	2.1
42673	1.89226	2.1
42674	1.89226	2.1
42675	1.89226	2.1
42676	1.89226	2.1
42677	1.89226	2.1
42678	0.697585	2.1
42679	0.730617	2.1
42680	0.712082	2.1
42681	0.670706	2.1
42682	0.69854	2.1
42683	0.69854	2.1
42684	0.702783	2.1
42685	0.702783	2.1

42686	0.730618	2.1
42687	0.712081	2.1
42688	0.697586	2.1
42689	0.670705	2.1
42690	1.89226	2.1
42691	1.89226	2.1
42692	1.89226	0.6948
42693	1.89226	2.1
42694	1.89226	0.455124
42695	1.89226	2.1
42696	2.0815	2.1
42697	1.12076	2.1
42698	1.38662	1.89768
42699	2.01244	2.1
42700	3.41508	2.1
42701	1.89226	2.1
42702	2.54324	2.1
42703	1.91981	2.1
42704	1.89226	2.1
42705	2.57079	2.1
42706	2.54324	2.1
42707	2.54325	2.1
42708	1.94779	2.1
42709	1.76879	2.1
42710	1.73811	2.1
42711	2.5708	2.1
42712	2.54325	2.1
42713	1.89226	2.1
42714	2.57079	2.1
42715	1.91894	2.1
42716	1.89226	2.1
42717	2.59747	2.1
42718	2.57079	2.1
42719	2.5708	2.1
42720	1.82268	2.1
42721	1.79738	2.1
42722	1.77071	2.1
42723	2.59747	2.1
42724	2.5708	2.1
42725	1.89226	0.05
42726	1.89226	0.583224
42727	1.89226	0.05
42728	1.89226	0.75655
42729	3.78451	0.05
42730	2.21582	0.05
42731	1.94412	0.490255
42732	2.13428	0.05
42733	1.86292	0.0799233
42734	4.07839	0.05
42735	1.94412	0.05
42736	1.5854	2.1
42737	1.86293	0.05
42738	1.50767	2.1
42739	3.45179	1.8987
42740	1.89226	0.05
42741	1.89226	2.1
42742	1.89226	0.230768
42743	1.89226	2.1

42744	3.78452	2.1
42745	1.89226	0.05
42746	1.89226	1.22243
42747	1.89226	0.05
42748	1.89226	0.492347
42749	3.78452	0.0595274
42750	2.13428	0.05
42751	1.86293	0.0751188
42752	2.04737	0.05
42753	1.77122	0.463751
42754	3.9055	0.05
42755	1.86293	0.05
42756	1.50767	2.1
42757	1.41077	0.159799
42758	1.41077	2.1
42759	1.77123	0.202012
42760	1.86813	2.1
42761	0.810943	0.0847904
42762	0.810943	2.1
42763	0.810945	0.0658099
42764	0.810945	0.164008
42765	0.810958	2.07843
42766	0.810958	1.80742
42767	1.89226	2.1
42768	1.35168	2.1
42769	1.08132	0.24436
42770	1.08132	2.1
42771	1.0813	1.88667
42772	0.745979	2.1
42773	0.745979	2.1
42774	0.778781	2.1
42775	0.778781	2.1
42776	0.59549	2.1
42777	0.649658	2.1
42778	0.994784	2.1
42779	0.875102	2.1
42780	0.875102	2.1
42781	0.59549	2.1
42782	0.649658	2.1
42783	0.994784	2.1
42784	1.57887	2.1
42785	1.75021	2.1
42786	1.13341	2.1
42787	1.13341	2.1
42788	1.19098	2.1
42789	1.63644	2.1
42790	2.5761	0.05
42791	2.43404	0.127019
42792	2.44813	0.05
42793	2.31751	0.0840483
42794	4.88218	0.05
42795	2.43405	0.05
42796	2.22575	2.1
42797	2.31751	0.05
42798	2.10893	2.1
42799	4.54298	0.267994
42800	2.44813	0.05
42801	2.31751	0.0749371

42802	2.30908	0.05
42803	2.19815	0.0718249
42804	4.64628	0.05
42805	2.31751	0.05
42806	2.10893	2.1
42807	2.19815	0.05
42808	1.98061	2.1
42809	4.30708	0.210703
42810	0.94613	2.1
42811	0.94613	2.1
42812	1.40372	2.1
42813	1.40372	2.1
42814	1.40372	2.1
42815	1.37167	2.1
42816	0.946132	2.1
42817	0.946132	2.1
42818	0.946131	2.1
42819	1.40372	2.1
42820	0.94613	2.1
42821	1.37167	2.1
42822	2.80744	2.1
42823	2.06984	2.1
42824	2.83418	2.1
42825	2.74335	2.1
42826	2.16067	2.1
42827	2.06984	2.1
42828	1.077 0.05	
42829	0.809116	0.05
42830	0.600419	0.05
42831	0.790277	0.05
42832	0.927647	0.05
42833	0.758598	0.05
42834	0.85749	0.05
42835	0.85749	0.05
42836	1.02417	0.05
42837	0.600411	0.05
42838	0.751701	0.05
42839	1.07702	0.05
42840	1.89226	2.1
42841	1.89226	2.1
42842	1.89226	2.1
42843	1.89226	2.1
42844	1.89226	2.1
42845	1.89226	2.1
42846	1.89226	2.1
42847	1.89226	2.1
42848	1.89226	2.1
42849	1.89226	2.1
42850	3.78453	2.1
42851	1.89226	2.1
42852	1.89226	2.1
42853	1.89226	2.1
42854	1.89226	2.1
42855	1.89226	2.1
42856	1.89226	2.1
42857	0.946131	2.1
42858	0.946131	2.1
42859	0.946131	2.1

42860	0.946131	2.1
42861	0.946133	2.1
42862	0.946133	2.1
42863	0.946128	2.1
42864	0.946128	2.1
42865	0.946131	2.1
42866	0.946131	2.1
42867	0.94613	2.1
42868	0.94613	2.1
42869	2.17159	2.1
42870	2.00083	2.1
42871	2.21305	2.1
42872	2.00551	2.06758
42873	2.20837	2.1
42874	2.00083	1.97184
42875	2.20837	2.0947
42876	1.80328	1.04168
42877	2.22962	2.1
42878	2.00083	1.71116
42879	2.03207	0.755415
42880	1.80328	0.463279
42881	2.28564	2.1
42882	2.20837	1.85915
42883	2.30406	2.1
42884	2.17159	2.1
42885	2.34084	2.1
42886	2.20837	2.1
42887	1.09523	0.35589
42888	1.09523	2.1
42889	1.09934	2.1
42890	1.09934	2.1
42891	1.01603	0.281623
42892	1.09038	2.1
42893	1.10419	0.942215
42894	1.17042	2.1
42895	1.17042	2.1
42896	1.09038	1.89148
42897	1.10419	1.35069
42898	1.01603	2.1
42899	2.60152	0.05
42900	0.976334	0.2156
42901	2.22344	0.296934
42902	2.22344	0.0654832
42903	1.36405	0.329092
42904	1.36405	0.05
42905	1.89226	2.1
42906	1.70302	2.1
42907	1.69019	2.1
42908	1.70302	2.1
42909	1.73933	2.1
42910	1.70303	1.19707
42911	1.89226	2.1
42912	1.89226	0.198308
42913	1.89226	1.95898
42914	1.89226	2.1
42915	3.78452	2.1
42916	1.89226	1.9263
42917	1.59036	2.1

42918	1.65683	2.1
42919	1.59036	2.09978
42920	1.95873	2.1
42921	1.89226	0.339898
42922	0.946131	0.222669
42923	0.946131	0.503474
42924	0.946127	2.1
42925	0.946127	1.94967
42926	0.946127	1.96634
42927	0.946127	0.0621855
42928	0.946131	2.1
42929	0.946131	0.557017
42930	0.946129	0.675197
42931	0.946129	0.768892
42932	0.94613	1.13226
42933	0.94613	0.862859
42934	0.851541	2.1
42935	0.851541	2.1
42936	0.851483	2.1
42937	1.15713	2.1
42938	0.851539	2.1
42939	0.851539	2.01498
42940	0.942127	2.1
42941	1.14455	2.1
42942	0.838683	2.1
42943	0.946131	2.1
42944	0.957503	2.1
42945	0.957504	2.1
42946	1.89226	0.229041
42947	1.89226	0.05
42948	1.89226	0.0865681
42949	1.89226	1.85824
42950	3.78452	0.05
42951	1.89226	0.05
42952	1.89226	0.05
42953	1.89226	0.0608121
42954	1.89226	0.05
42955	1.89226	0.05
42956	1.89226	0.05
42957	1.89226	0.05
42958	1.89226	0.05
42959	1.89226	0.05
42960	1.89226	0.05
42961	3.78453	0.05
42962	1.89226	0.05
42963	1.89226	2.1
42964	1.89226	2.1
42965	1.89226	0.338572
42966	3.78453	2.06163
42967	1.22575	0.145236
42968	1.05438	0.150304
42969	0.946131	0.278883
42970	0.946131	0.0635245
42971	0.946129	1.9414
42972	1.22575	0.05
42973	1.05438	0.546306
42974	0.946132	0.05
42975	1.05438	0.311446

42976	1.05438	0.566955
42977	0.946131	0.189218
42978	0.946131	0.633231
42979	2.4515	0.13734
42980	1.13726	0.05
42981	2.10875	0.05
42982	1.49515	0.05
42983	2.10875	0.05
42984	1.49515	0.05
42985	0.831477	0.05
42986	0.747575	0.05
42987	1.22575	0.0644874
42988	1.28174	0.05
42989	1.28174	0.05
42990	0.831476	0.05
42991	1.22575	0.05
42992	0.747575	0.05
42993	1.03561	0.05
42994	1.03561	0.05
42995	1.02162	0.05
42996	1.02162	0.05
42997	1.89227	2.1
42998	2.56348	0.109579
42999	1.89227	2.1
43000	2.45151	0.380102
43001	1.89227	2.1
43002	2.45151	0.164294
43003	1.40013	0.106176
43004	1.89226	1.57895
43005	1.89226	0.418505
43006	0.946643	0.0529571
43007	1.89226	1.0365
43008	0.946643	0.132032
43009	1.89226	0.05
43010	1.89226	0.05
43011	1.89226	0.05
43012	1.89226	0.05
43013	3.78453	0.05
43014	1.89226	0.05
43015	1.89226	0.05
43016	1.89226	0.05
43017	1.89226	0.05
43018	3.78453	0.05
43019	0.946131	0.05
43020	0.946131	0.05
43021	0.946128	0.05
43022	0.946128	0.05
43023	0.94613	0.05
43024	0.94613	0.05
43025	0.946129	0.05
43026	0.946129	0.05
43027	0.94613	0.05
43028	0.94613	0.05
43029	0.94613	0.05
43030	0.94613	0.05
43031	1.89226	0.05
43032	1.89226	0.05
43033	1.17244	0.05

43034	1.89226	0.05
43035	0.975291	0.05
43036	1.57408	0.05
43037	0.946132	0.05
43038	0.946132	0.05
43039	0.946131	0.05
43040	0.946131	0.05
43041	0.946132	0.05
43042	0.946132	0.05
43043	0.946131	0.05
43044	0.946131	0.05
43045	0.946131	0.05
43046	0.946131	0.05
43047	0.946132	0.05
43048	0.946132	0.05
43049	2.74744	0.05
43050	1.89226	0.05
43051	2.70805	0.05
43052	1.89226	0.05
43053	2.70805	0.05
43054	1.89226	0.05
43055	2.74744	0.05
43056	1.89226	0.05
43057	2.57465	0.05
43058	1.89226	0.05
43059	4.6397	0.05
43060	1.89226	0.05
43061	1.89226	0.05
43062	1.89226	0.05
43063	1.89226	0.19462
43064	3.78452	0.05
43065	1.89226	0.05
43066	1.89226	0.0863098
43067	1.89226	0.05
43068	1.89226	0.05
43069	1.89226	0.170927
43070	1.89226	0.102483
43071	2.16206	0.05
43072	2.67861	0.05
43073	3.11996	0.05
43074	1.70303	0.05
43075	2.89015	0.05
43076	1.486 0.05	
43077	2.12839	0.05
43078	2.83631	0.05
43079	3.08096	0.05
43080	1.84157	0.05
43081	3.08096	0.05
43082	1.84157	0.05
43083	1.89225	0.513466
43084	1.89225	0.134957
43085	1.89225	0.174075
43086	1.89225	1.03115
43087	1.89225	0.11255
43088	1.89225	0.566367
43089	1.89226	0.0852019
43090	1.89226	0.121632
43091	1.89226	0.592971

43092	1.89226	0.052629
43093	3.78452	0.0687539
43094	1.89226	0.05
43095	2.49041	0.05
43096	1.89226	0.05
43097	1.89226	0.05
43098	2.27073	0.05
43099	2.27073	0.05
43100	1.09904	0.05
43101	0.677147	0.05
43102	1.3365	0.05
43103	0.95759	0.05
43104	0.957591	0.05
43105	0.677148	0.05
43106	1.09904	0.05
43107	1.3365	0.05
43108	0.996015	0.05
43109	0.996015	0.05
43110	1.01764	0.05
43111	1.01764	0.05
43112	2.77349	0.05
43113	2.72723	0.05
43114	2.43156	0.05
43115	2.4564	0.05
43116	5.20505	0.05
43117	2.77349	0.05
43118	2.68508	0.05
43119	2.33761	0.05
43120	2.4564	0.05
43121	5.1111	0.05
43122	2.43156	0.05
43123	2.70208	0.05
43124	1.85779	0.05
43125	2.10695	0.05
43126	4.77572	0.05
43127	2.33761	0.05
43128	2.70208	0.05
43129	1.85779	0.05
43130	1.61597	0.05
43131	4.19542	0.05
43132	0.946132	0.621293
43133	0.946132	1.34484
43134	0.746729	1.61803
43135	0.746729	2.09442
43136	0.946132	2.1
43137	0.915958	1.44091
43138	0.946133	0.589423
43139	0.74673	2.1
43140	0.946133	1.60647
43141	0.946133	1.59919
43142	0.915958	1.53259
43143	0.746729	1.41281
43144	1.50743	2.1
43145	2.45501	2.1
43146	2.29118	2.1
43147	2.29118	2.1
43148	1.75482	2.1
43149	1.75482	2.1

43150	2.45501	2.1
43151	2.76927	0.05
43152	2.29118	2.1
43153	2.64523	0.0630758
43154	5.10024	0.750446
43155	1.25559	2.1
43156	2.29118	2.1
43157	2.0815	2.1
43158	2.26387	2.1
43159	1.35668	2.1
43160	1.47667	2.1
43161	2.28481	2.1
43162	2.64523	0.0975
43163	2.50057	2.1
43164	2.50057	0.0695458
43165	2.29118	2.1
43166	2.29118	0.096626
43167	0.946129	0.05
43168	0.946129	0.05
43169	1.23929	0.05
43170	1.15246	0.05
43171	0.946131	0.05
43172	0.946131	0.0527428
43173	1.15246	0.05
43174	1.15246	0.05
43175	0.946131	0.05
43176	1.15246	0.05
43177	0.946131	0.05
43178	1.23928	0.05
43179	0.946128	0.05
43180	0.946128	0.05
43181	1.15246	0.05
43182	0.942814	0.0513056
43183	0.946131	0.05
43184	0.946131	0.0618111
43185	0.961347	0.05
43186	0.942811	0.05
43187	0.946131	0.05
43188	0.961347	0.05
43189	0.946131	0.05
43190	1.15246	0.05
43191	2.32334	0.05
43192	1.57621	0.05
43193	1.90335	0.05
43194	1.24538	0.05
43195	3.47956	0.05
43196	0.965762	0.05
43197	0.965762	0.05
43198	1.00245	0.05
43199	1.00245	0.05
43200	0.92786	0.05
43201	0.705297	0.05
43202	0.856414	0.05
43203	1.28968	0.05
43204	1.00306	0.05
43205	0.705303	0.05
43206	1.28967	0.05
43207	0.975478	0.05

43208	3.05308	2.1
43209	1.76816	2.1
43210	1.76816	2.1
43211	1.69129	2.1
43212	2.58731	2.1
43213	2.51043	2.1
43214	1.89226	0.05
43215	2.71464	0.05
43216	2.34627	0.05
43217	1.89226	0.05
43218	2.34627	0.05
43219	1.89226	0.05
43220	2.34627	0.05
43221	2.71464	0.05
43222	1.98252	0.05
43223	1.98252	0.05
43224	1.51026	0.05
43225	2.34627	0.05
43226	2.38698	1.16415
43227	2.35142	2.1
43228	2.60343	1.22338
43229	2.6636	1.04614
43230	4.99041	1.49507
43231	1.07811	2.1
43232	1.1952	2.1
43233	1.17571	2.09549
43234	1.30171	0.757475
43235	1.18322	0.604236
43236	1.18322	2.1
43237	1.18769	2.1
43238	1.18769	1.14421
43239	1.1952	2.1
43240	1.30171	2.1
43241	1.17571	2.1
43242	1.07811	2.1
43243	1.89226	1.57618
43244	2.6636	1.97009
43245	1.89226	0.135232
43246	2.60343	1.36801
43247	1.89226	1.49819
43248	2.60343	2.1
43249	1.5344	0.05
43250	1.4516	0.05
43251	1.17816	0.05
43252	1.21056	0.05
43253	2.71257	0.05
43254	1.89226	2.1
43255	1.89226	0.594391
43256	1.89226	1.3211
43257	1.89226	1.38252
43258	3.78452	1.08923
43259	1.89226	1.4508
43260	1.89226	0.128241
43261	1.89226	0.569573
43262	1.89226	0.400779
43263	1.65573	0.942485
43264	1.65573	0.59132
43265	1.89226	1.18256

43266	1.89226	1.47562
43267	1.89226	2.1
43268	2.64212	0.352481
43269	3.78452	1.7468
43270	1.89226	1.46007
43271	1.89226	2.1
43272	1.89226	1.62094
43273	1.89226	2.1
43274	3.78452	2.1
43275	2.7367	0.0764918
43276	2.07947	2.1
43277	2.47319	1.50064
43278	1.85057	2.1
43279	4.55267	2.1
43280	2.07948	2.1
43281	1.3294	2.1
43282	1.85057	2.1
43283	1.09962	2.1
43284	3.1791	2.1
43285	1.89226	2.1
43286	1.89226	2.1
43287	1.89226	2.1
43288	1.89226	2.1
43289	3.78453	2.1
43290	0.94613	2.1
43291	0.94613	0.184849
43292	0.94613	0.920087
43293	0.94613	2.1
43294	0.946128	2.1
43295	0.946128	2.1
43296	0.946132	0.134879
43297	0.946132	0.191357
43298	0.94613	2.1
43299	0.94613	2.1
43300	0.946131	2.1
43301	0.946131	2.1
43302	1.00875	2.1
43303	1.00875	0.0752676
43304	1.01676	2.1
43305	1.01676	0.110505
43306	0.925282	2.1
43307	0.804877	2.1
43308	1.2366	0.454007
43309	1.10023	0.433267
43310	0.804878	2.1
43311	1.10023	2.1
43312	1.2366	2.1
43313	0.925284	2.1
43314	1.89226	2.1
43315	1.89226	2.1
43316	1.89226	2.1
43317	1.89226	2.1
43318	3.78453	2.1
43319	1.89226	2.1
43320	1.89226	2.1
43321	1.89226	2.1
43322	1.89226	2.1
43323	1.89226	2.1

43324	1.89226	2.1
43325	1.09766	2.1
43326	1.14842	2.1
43327	1.02909	2.1
43328	1.07659	2.1
43329	1.16718	2.1
43330	0.954189	2.1
43331	1.06653	2.1
43332	1.06653	2.1
43333	1.20738	2.1
43334	1.02909	2.1
43335	1.09767	2.1
43336	0.815951	2.1
43337	2.00793	2.1
43338	1.14048	2.1
43339	1.19086	2.1
43340	1.13723	2.1
43341	2.00793	2.1
43342	2.11772	2.1
43343	1.89226	2.1
43344	1.89226	2.1
43345	1.89226	2.1
43346	1.89226	2.1
43347	1.89226	2.1
43348	1.89226	2.1
43349	1.89226	2.1
43350	1.89226	2.1
43351	1.89226	2.1
43352	1.89226	2.1
43353	1.89226	2.1
43354	1.89226	2.1
43355	1.1779	2.1
43356	1.26166	2.1
43357	1.0875	2.1
43358	1.00397	2.1
43359	1.13286	2.1
43360	1.13286	2.1
43361	1.13277	2.1
43362	1.13277	2.1
43363	1.26166	2.1
43364	1.08749	2.1
43365	1.1779	2.1
43366	1.00396	2.1
43367	1.0875	2.1
43368	1.00397	2.1
43369	0.685749	2.1
43370	0.595432	2.1
43371	0.83976	2.1
43372	0.83976	2.1
43373	0.843169	2.1
43374	0.843169	2.1
43375	1.0875	2.1
43376	0.685749	2.1
43377	1.00397	2.1
43378	0.595432	2.1
43379	1.89226	2.1
43380	1.86443	2.1
43381	2.4988	2.1

43382	2.0815	2.1
43383	2.28173	2.1
43384	1.86443	2.1
43385	1.89226	2.1
43386	1.89226	2.1
43387	1.89226	2.1
43388	1.89226	2.1
43389	1.89226	2.1
43390	1.89226	2.1
43391	0.946131	2.1
43392	0.946131	2.1
43393	0.969491	2.1
43394	0.949105	2.1
43395	0.949103	2.1
43396	0.949103	2.1
43397	0.946133	2.1
43398	0.946133	2.1
43399	0.946131	2.1
43400	0.969492	2.1
43401	0.946131	2.1
43402	0.949105	2.1
43403	1.89226	2.1
43404	2.07428	2.1
43405	1.93464	2.1
43406	1.89226	2.1
43407	2.11667	2.1
43408	2.07428	2.1
43409	0.946132	2.1
43410	0.946132	2.1
43411	0.946131	2.1
43412	0.946131	2.1
43413	0.946132	2.1
43414	0.946132	2.1
43415	0.946132	2.1
43416	0.946132	2.1
43417	0.946132	2.1
43418	0.946132	2.1
43419	0.946132	2.1
43420	0.946132	2.1
43421	1.97992	2.1
43422	1.89226	2.1
43423	1.89226	2.1
43424	1.93898	2.1
43425	1.93898	2.1
43426	1.89226	2.1
43427	2.12475	0.119972
43428	2.79029	0.319216
43429	2.7803	1.19138
43430	2.13052	0.0790117
43431	4.92081	0.108174
43432	1.06238	0.518483
43433	1.06526	0.128947
43434	0.642901	0.138077
43435	0.655055	1.18319
43436	0.856391	0.136671
43437	0.856391	0.120577
43438	0.86104	2.07472
43439	0.86104	0.129657

43440	1.06526	0.164066
43441	0.655055	0.0733768
43442	0.642901	0.178489
43443	1.06238	0.156156
43444	2.7803	1.21288
43445	2.13053	0.05
43446	2.14346	0.382981
43447	2.77937	0.144018
43448	4.9099	0.428332
43449	2.14346	0.569165
43450	1.31011	0.05
43451	1.34124	0.0912818
43452	1.31011	0.144856
43453	2.16166	0.111838
43454	2.13053	0.114941
43455	1.89226	2.1
43456	2.58176	2.1
43457	2.01422	2.1
43458	1.89226	2.1
43459	2.70373	2.1
43460	2.58176	2.1
43461	1.89226	2.1
43462	1.89226	2.1
43463	1.89226	2.1
43464	1.89226	2.1
43465	1.89226	2.1
43466	1.89226	2.1
43467	0.946128	2.1
43468	0.946128	2.1
43469	1.38521	2.1
43470	1.38521	2.1
43471	1.38521	2.1
43472	1.35186	2.1
43473	0.946131	2.1
43474	0.946131	2.1
43475	0.94613	2.1
43476	1.38521	2.1
43477	0.946131	2.1
43478	1.35186	2.1
43479	0.946133	2.1
43480	0.946133	2.1
43481	0.946132	2.1
43482	0.946132	2.1
43483	0.946132	2.1
43484	0.946132	2.1
43485	0.946132	2.1
43486	0.946132	2.1
43487	0.946132	2.1
43488	0.946132	2.1
43489	0.946133	2.1
43490	0.946133	2.1
43491	2.33374	2.1
43492	1.89226	2.1
43493	1.89226	2.1
43494	1.89226	2.1
43495	2.60377	2.1
43496	2.60377	2.1
43497	2.6771	0.05

43498	1.36416	0.05
43499	1.64983	0.05
43500	1.64983	0.05
43501	2.44131	0.05
43502	2.44131	0.05
43503	2.58321	0.0538004
43504	1.89226	0.113093
43505	2.44283	0.05
43506	2.44283	0.05
43507	1.89226	0.309889
43508	1.89226	0.791789
43509	2.58321	0.143043
43510	2.44283	0.0564293
43511	1.49358	0.05
43512	1.57702	0.05
43513	3.93641	0.05
43514	0.951913	0.05
43515	0.81556	0.05
43516	0.946132	0.05
43517	0.946132	0.05
43518	1.04072	0.05
43519	1.12182	0.05
43520	0.856745	0.05
43521	0.851542	0.05
43522	0.923163	0.05
43523	0.923163	0.05
43524	1.04304	0.05
43525	0.85098	0.05
43526	2.70125	0.751187
43527	2.37734	1.06671
43528	2.63495	0.980955
43529	2.30539	2.06041
43530	5.01229	1.91376
43531	1.89226	2.00239
43532	1.89226	2.1
43533	1.89226	1.57879
43534	1.89226	2.1
43535	3.78452	0.982933
43536	1.89226	2.1
43537	1.89226	2.1
43538	1.89226	2.1
43539	1.89226	2.1
43540	3.78452	2.1
43541	2.37734	1.97307
43542	1.79346	2.1
43543	2.53594	1.75366
43544	1.70302	2.1
43545	4.27682	2.1
43546	2.63495	0.433399
43547	2.30539	0.787758
43548	2.49445	0.690604
43549	2.12089	0.434075
43550	4.75584	0.370774
43551	1.89226	0.990207
43552	1.89226	2.07031
43553	1.89226	1.16079
43554	1.89226	2.0911
43555	3.78452	2.1

43556	1.89226	1.98312
43557	1.89226	2.1
43558	1.89226	2.1
43559	1.89226	2.1
43560	1.89226	1.19715
43561	1.89226	1.43572
43562	2.53594	0.48218
43563	1.60017	1.07327
43564	1.57079	0.885726
43565	1.70302	0.443703
43566	2.15772	0.609352
43567	2.33299	0.268114
43568	2.09114	2.1
43569	2.34898	2.1
43570	2.30729	2.1
43571	2.06556	2.1
43572	4.41454	2.1
43573	2.09114	2.1
43574	1.59654	2.1
43575	1.57823	2.1
43576	2.06556	2.1
43577	3.6621	2.1
43578	2.30729	2.1
43579	2.06556	2.1
43580	2.03917	2.1
43581	2.26617	2.1
43582	4.33173	2.1
43583	2.03917	2.1
43584	2.06556	2.1
43585	2.045 2.1	
43586	1.55767	2.1
43587	1.57823	2.1
43588	1.57823	2.1
43589	1.89225	2.1
43590	1.09726	2.1
43591	1.61196	2.1
43592	1.28597	2.1
43593	1.28597	0.05
43594	1.45633	2.1
43595	1.45633	2.1
43596	1.9544	0.05
43597	1.61196	2.1
43598	1.45633	2.1
43599	1.53735	2.1
43600	3.14931	2.1
43601	1.89226	2.1
43602	1.89226	2.1
43603	1.89226	2.1
43604	1.89226	2.1
43605	3.78453	2.1
43606	0.810946	2.1
43607	0.810946	2.1
43608	0.810947	2.1
43609	0.810947	2.1
43610	0.810933	2.1
43611	0.810933	2.1
43612	1.89226	2.1
43613	1.3517	2.1

43614	1.08132	2.1
43615	1.08132	2.1
43616	1.08133	2.1
43617	0.851539	2.1
43618	0.851539	2.1
43619	0.972053	2.1
43620	1.05632	2.1
43621	0.94613	2.1
43622	0.94613	2.1
43623	1.0279	2.1
43624	0.94991	2.1
43625	1.0279	2.1
43626	0.946131	2.1
43627	0.865643	2.1
43628	0.94613	2.1
43629	1.89226	2.1
43630	1.89226	2.1
43631	1.89226	2.1
43632	1.89226	2.1
43633	3.78452	2.1
43634	1.03882	2.1
43635	1.91121	2.1
43636	1.41833	2.1
43637	2.34547	2.1
43638	3.32954	2.1
43639	2.3979	2.1
43640	1.89226	2.1
43641	1.89226	2.1
43642	1.89226	2.1
43643	2.0558	2.1
43644	2.0558	2.04628
43645	1.89226	2.1
43646	1.89226	1.31404
43647	1.89226	2.1
43648	1.89226	2.1
43649	3.78452	2.1
43650	1.05816	2.1
43651	1.05816	2.1
43652	0.842045	2.1
43653	1.05252	2.1
43654	1.36057	2.1
43655	1.14339	2.1
43656	0.873397	2.1
43657	0.585073	2.1
43658	0.873388	2.1
43659	1.15129	2.1
43660	0.69143	2.1
43661	1.1434	2.1
43662	1.93338	2.1
43663	1.85883	2.1
43664	1.51714	2.1
43665	1.85883	2.1
43666	1.54795	2.1
43667	1.89226	2.1
43668	1.89226	2.1
43669	1.89226	2.1
43670	1.89226	2.1
43671	1.89226	2.1

43672	3.78452	2.1
43673	0.844726	2.1
43674	0.844726	2.1
43675	0.960524	2.1
43676	1.16288	2.1
43677	0.851541	2.1
43678	0.851541	2.1
43679	0.929147	2.1
43680	1.15533	2.1
43681	0.844697	2.1
43682	0.946131	2.1
43683	0.851632	2.1
43684	0.946131	2.1
43685	1.89226	2.1
43686	1.70303	2.1
43687	1.74006	2.1
43688	1.70303	2.1
43689	1.99471	2.1
43690	1.93338	2.1
43691	0.946131	2.1
43692	0.946131	2.1
43693	0.946131	2.1
43694	0.946131	2.1
43695	0.94613	2.1
43696	0.94613	2.1
43697	0.946132	2.1
43698	0.946132	2.1
43699	0.946131	2.1
43700	0.946131	2.1
43701	0.946131	2.1
43702	0.946131	2.1
43703	1.58158	2.1
43704	1.89226	2.1
43705	1.89226	2.1
43706	1.89226	2.1
43707	1.68939	2.1
43708	1.68939	2.1
43709	0.946132	0.05
43710	0.946132	0.05
43711	1.12988	0.05
43712	1.30783	0.05
43713	1.30783	0.05
43714	0.946133	0.05
43715	0.946132	0.05
43716	1.12988	0.05
43717	1.30783	0.05
43718	1.30783	0.05
43719	0.946133	0.05
43720	0.946133	0.05
43721	1.89227	0.05
43722	1.89227	0.05
43723	1.89227	0.05
43724	1.89227	0.05
43725	1.89227	0.05
43726	1.89227	0.05
43727	1.89226	0.05
43728	1.89226	0.05
43729	1.89226	0.05

43730	1.89226	0.05
43731	1.89226	0.05
43732	1.89226	0.05
43733	0.946131	0.05
43734	0.946131	0.05
43735	1.30783	0.05
43736	1.37715	0.05
43737	1.37715	0.05
43738	0.946133	0.05
43739	1.30783	0.05
43740	0.946129	0.05
43741	1.37715	0.05
43742	1.37715	0.05
43743	0.946131	0.05
43744	0.946131	0.05
43745	1.17056	0.05
43746	0.591757	0.05
43747	0.522025	0.05
43748	0.929727	0.05
43749	0.929728	0.05
43750	1.17056	0.05
43751	0.591756	0.05
43752	0.522025	0.05
43753	0.889054	0.05
43754	0.889054	0.05
43755	0.803535	0.05
43756	0.803535	0.05
43757	1.49814	2.1
43758	2.37439	2.1
43759	2.63923	2.1
43760	2.63923	2.1
43761	1.29111	2.1
43762	1.29111	2.1
43763	0.946132	2.1
43764	0.946132	2.1
43765	1.18719	2.1
43766	1.18719	2.1
43767	0.94613	2.1
43768	0.94613	2.1
43769	1.1872	2.1
43770	1.31961	2.1
43771	0.946131	2.1
43772	1.31961	2.1
43773	1.1872	2.1
43774	0.94613	2.1
43775	2.49445	0.113852
43776	2.12089	0.114748
43777	2.20843	0.126767
43778	1.82805	0.207441
43779	4.32932	0.135812
43780	1.89226	0.717179
43781	1.89226	1.03298
43782	1.89226	0.412754
43783	1.89226	0.49467
43784	3.78452	0.192513
43785	0.94613	0.390893
43786	0.94613	0.343822
43787	0.946128	0.105457

43788	0.946128	0.444517
43789	0.946131	0.510421
43790	0.946131	0.472918
43791	0.946127	0.378014
43792	0.946127	0.244619
43793	0.946129	0.401875
43794	0.946129	0.16461
43795	0.946129	0.317556
43796	0.946129	0.287498
43797	1.82804	0.132135
43798	1.60017	0.212245
43799	1.87679	0.252032
43800	2.12088	0.0561389
43801	1.35608	0.281317
43802	1.60017	0.121459
43803	2.20843	0.05
43804	1.3216	0.05
43805	1.44426	0.05
43806	1.82805	0.05
43807	1.80399	0.0624638
43808	2.20843	0.0555942
43809	1.89226	0.0989129
43810	1.89226	0.143041
43811	1.89226	0.493286
43812	1.89226	0.201139
43813	1.89226	0.140158
43814	1.89226	0.0806957
43815	1.89226	0.163283
43816	1.89226	0.0621516
43817	1.89226	0.112332
43818	1.89226	0.164681
43819	1.89226	0.288675
43820	1.89226	0.0820065
43821	0.627705	0.05
43822	0.634941	0.0555339
43823	0.629317	0.05
43824	0.783422	0.05
43825	0.629328	0.05
43826	0.448764	0.05
43827	1.35608	0.05
43828	1.00805	0.05
43829	0.814295	0.05
43830	0.598375	0.0530641
43831	1.04461	0.05
43832	1.09795	2.02186
43833	1.00775	2.1
43834	1.61202	2.1
43835	1.63419	1.93182
43836	2.64194	1.74472
43837	1.44801	1.6781
43838	1.61201	2.1
43839	2.35614	1.9316
43840	2.27703	2.07781
43841	3.88904	2.05468
43842	2.35615	1.71624
43843	1.63419	1.61854
43844	2.29852	1.55547
43845	1.63419	1.68149

43846	2.27634	1.63044
43847	1.61202	2.05836
43848	0.973793	2.1
43849	2.2671	2.1
43850	2.32981	2.1
43851	2.32981	2.1
43852	0.990481	2.1
43853	0.990481	2.1
43854	1.50877	2.1
43855	1.11193	2.1
43856	2.29667	2.1
43857	1.94158	2.1
43858	3.45035	2.1
43859	2.29667	2.1
43860	1.89227	2.1
43861	1.94158	2.1
43862	1.94158	2.1
43863	1.89227	2.1
43864	1.89227	2.1
43865	2.51379	2.1
43866	1.30026	2.1
43867	1.84502	2.1
43868	2.72099	2.1
43869	4.35882	2.1
43870	1.89226	2.1
43871	2.721	2.1
43872	2.5138	2.1
43873	2.5138	2.1
43874	1.89226	2.1
43875	1.89226	2.1
43876	1.89226	0.05
43877	1.89226	0.05
43878	2.59101	0.05
43879	1.89226	0.05
43880	3.78453	0.05
43881	0.851541	0.05
43882	0.851541	0.05
43883	1.42502	0.05
43884	0.948679	0.05
43885	0.946132	0.05
43886	0.946132	0.05
43887	0.853617	0.05
43888	0.853618	0.05
43889	0.851475	0.05
43890	0.948666	0.05
43891	1.166	0.05
43892	0.851554	0.05
43893	2.51818	0.424347
43894	2.60723	2.1
43895	2.66532	0.336682
43896	2.76315	2.1
43897	5.27255	1.64145
43898	2.60723	2.1
43899	2.45247	2.1
43900	2.76315	2.1
43901	2.55197	2.1
43902	5.1592	2.1
43903	1.23768	0.254038

43904	1.11433	0.441592
43905	1.32037	1.74218
43906	1.32037	0.249405
43907	1.0142	0.873367
43908	1.0142	0.138404
43909	0.886711	1.02037
43910	1.16806	1.00337
43911	0.999083	2.03079
43912	0.710926	1.42331
43913	0.937757	1.66325
43914	0.937757	1.19972
43915	0.941224	0.417241
43916	0.941224	2.09276
43917	1.16805	1.26645
43918	0.999084	2.1
43919	0.886709	1.83375
43920	0.710928	1.48842
43921	2.29332	2.1
43922	1.54904	2.1
43923	2.0815	2.1
43924	2.44199	2.1
43925	4.00418	2.1
43926	1.53873	0.05
43927	1.53422	0.0566433
43928	1.39661	0.05
43929	1.42387	0.0684876
43930	2.9626	0.05
43931	1.53423	0.05
43932	1.44091	2.1
43933	1.42387	0.05
43934	1.35215	2.1
43935	2.86479	0.307122
43936	1.39661	0.05
43937	1.42387	0.0623051
43938	1.28325	0.05
43939	1.33326	0.120938
43940	2.70713	0.05
43941	1.42387	0.05
43942	1.35215	2.1
43943	1.33327	0.0776382
43944	1.27339	2.1
43945	2.69726	0.920367
43946	2.58442	2.1
43947	1.1468	2.1
43948	1.18775	2.1
43949	2.60047	2.1
43950	3.74728	2.1
43951	2.58442	2.1
43952	1.89226	2.1
43953	1.89226	2.1
43954	2.60048	2.1
43955	4.49274	2.1
43956	1.89226	2.1
43957	1.89226	2.1
43958	1.89226	2.1
43959	1.89226	2.1
43960	3.78453	2.1
43961	1.89226	2.1

43962	1.89226	2.1
43963	1.89226	2.1
43964	1.89226	1.49791
43965	3.78452	2.1
43966	1.22262	2.1
43967	2.60047	2.1
43968	1.18775	2.1
43969	1.18775	2.1
43970	2.61433	2.1
43971	2.61433	2.1
43972	1.89226	2.1
43973	2.60048	2.1
43974	2.61434	2.1
43975	2.60048	2.1
43976	1.90612	2.1
43977	1.89226	2.1
43978	1.89226	2.1
43979	1.89226	2.1
43980	1.89226	2.1
43981	1.89226	2.1
43982	3.78453	2.1
43983	1.89226	2.1
43984	1.89226	2.1
43985	1.89226	2.1
43986	1.89226	2.1
43987	3.78452	2.1
43988	2.12556	2.1
43989	0.991113	2.1
43990	1.27725	2.1
43991	2.31081	2.1
43992	3.40281	2.1
43993	1.89226	2.1
43994	2.31082	2.1
43995	2.12556	2.1
43996	2.12556	2.1
43997	1.89226	2.1
43998	1.89226	2.1
43999	1.22544	2.1
44000	1.12649	2.1
44001	0.775254	2.1
44002	0.643133	2.1
44003	0.917924	2.1
44004	0.934955	2.1
44005	0.975579	2.1
44006	0.975579	2.1
44007	1.23113	2.1
44008	0.685272	2.1
44009	0.651719	2.1
44010	1.1791	2.1
44011	1.89226	2.1
44012	2.5138	2.1
44013	2.31082	2.1
44014	2.31082	2.1
44015	1.89226	2.1
44016	1.89226	2.1
44017	1.89226	2.1
44018	1.89226	2.1
44019	1.89226	2.1

44020	1.89226	2.1
44021	3.78453	2.1
44022	0.922224	2.1
44023	0.922224	2.1
44024	1.23555	2.1
44025	1.19715	2.1
44026	1.01445	2.1
44027	1.16061	2.1
44028	0.946132	2.1
44029	0.946132	2.1
44030	0.946131	2.1
44031	1.2051	2.1
44032	1.13537	2.1
44033	1.01445	2.1
44034	0.946133	2.1
44035	0.946133	2.1
44036	1.20648	2.1
44037	1.20648	2.1
44038	1.06454	2.1
44039	1.20648	2.1
44040	0.946133	2.1
44041	0.946133	2.1
44042	1.20649	2.1
44043	0.946133	2.1
44044	1.06454	2.1
44045	0.946132	2.1
44046	0.946132	2.1
44047	0.946132	2.1
44048	0.946132	2.1
44049	0.946132	2.1
44050	0.946132	2.1
44051	0.946132	2.1
44052	0.946133	2.1
44053	0.946133	2.1
44054	0.946133	2.1
44055	0.946133	2.1
44056	0.946132	2.1
44057	0.946132	2.1
44058	1.33078	2.05027
44059	1.37409	2.1
44060	1.31565	0.0870598
44061	1.37114	2.1
44062	2.68973	2.1
44063	1.89225	2.00363
44064	1.89225	2.1
44065	1.89225	0.0920509
44066	1.89225	2.05301
44067	3.7845	0.966128
44068	1.89225	1.57365
44069	1.89225	2.1
44070	1.89225	0.933343
44071	1.89225	2.1
44072	3.7845	2.1
44073	1.37409	1.88387
44074	1.20408	2.1
44075	1.18612	2.1
44076	1.18907	2.1
44077	1.37114	1.57078

44078	1.37409	2.1
44079	1.31566	2.1
44080	1.37115	2.1
44081	1.30318	0.0766044
44082	1.37433	2.1
44083	2.68998	1.82494
44084	1.89226	2.1
44085	1.89226	2.1
44086	1.89226	0.149246
44087	1.89226	2.1
44088	3.78453	1.29929
44089	1.89226	1.30914
44090	1.89226	2.1
44091	1.89226	2.1
44092	1.89226	2.1
44093	1.89226	1.01065
44094	1.89226	2.1
44095	0.647001	2.1
44096	0.647001	2.1
44097	0.651851	0.742249
44098	0.651851	2.1
44099	0.602046	2.1
44100	0.613277	2.1
44101	0.685576	1.41326
44102	0.687164	1.88461
44103	0.687164	2.1
44104	0.613276	2.1
44105	0.602046	2.1
44106	0.685576	2.1
44107	0.946128	0.781695
44108	0.946128	0.0567638
44109	1.15307	2.00377
44110	1.19317	0.903797
44111	0.94613	1.41987
44112	0.94613	0.05
44113	1.15307	1.39912
44114	1.15307	1.9465
44115	0.946131	1.155
44116	1.15307	0.565312
44117	0.946131	0.412561
44118	1.19317	0.174064
44119	1.18278	2.1
44120	2.38635	0.279207
44121	1.23042	2.1
44122	1.23042	1.71292
44123	2.30614	2.1
44124	2.30614	1.84593
44125	0.946129	0.353224
44126	0.946129	0.141217
44127	1.12994	0.761691
44128	1.15307	0.339326
44129	0.946131	0.760923
44130	0.946131	0.05
44131	1.12994	0.43198
44132	1.12994	0.424312
44133	1.12994	0.397957
44134	0.946131	0.437494
44135	0.946131	0.21252

44136	1.15307	0.126298
44137	2.25988	1.30852
44138	2.30614	0.0550868
44139	2.28696	2.1
44140	1.16361	2.1
44141	1.18278	2.1
44142	1.18278	1.44106
44143	2.3771	2.1
44144	1.90121	2.1
44145	1.81191	2.1
44146	2.46904	2.1
44147	4.37025	2.1
44148	1.15676	2.1
44149	1.23452	2.1
44150	0.823592	2.1
44151	0.737606	2.1
44152	0.988113	2.1
44153	0.988113	2.1
44154	0.984014	2.1
44155	0.984014	2.1
44156	1.15676	2.1
44157	0.737605	2.1
44158	0.823592	2.1
44159	1.23452	2.1
44160	1.30318	2.1
44161	1.37433	2.1
44162	1.30151	0.664391
44163	1.39067	2.1
44164	2.67584	1.87526
44165	1.89226	1.93897
44166	1.89226	2.1
44167	1.89226	0.49464
44168	1.89226	1.71691
44169	3.78453	1.02417
44170	1.89226	0.462188
44171	1.89226	2.1
44172	1.89226	0.29119
44173	1.89226	2.1
44174	1.89226	1.06173
44175	1.89226	2.1
44176	0.656819	2.1
44177	0.656819	1.01377
44178	0.661755	1.07524
44179	0.661755	2.1
44180	0.613277	2.1
44181	0.63141	2.1
44182	0.687164	1.1566
44183	0.695336	2.03629
44184	0.695337	2.1
44185	0.63141	2.1
44186	0.687164	2.1
44187	0.613276	2.1
44188	1.30151	2.0716
44189	1.39067	2.1
44190	1.32143	0.564819
44191	1.43294	2.1
44192	2.73445	2.1
44193	1.89226	1.96125

44194	1.89226	2.1
44195	1.89226	0.254487
44196	1.89226	2.1
44197	3.78452	1.45649
44198	1.89226	1.55318
44199	1.89226	1.21634
44200	1.89226	0.960658
44201	1.89226	2.1
44202	1.89226	2.1
44203	1.89226	2.1
44204	1.43294	1.43548
44205	1.26282	2.1
44206	1.3268	1.15138
44207	1.3268	2.1
44208	1.39067	1.86394
44209	1.39067	2.1
44210	1.5511	0.05
44211	1.00665	0.05
44212	1.28239	0.05
44213	1.88631	0.05
44214	2.89296	0.05
44215	1.89227	0.05
44216	1.89227	0.05
44217	1.89227	0.05
44218	1.89227	0.05
44219	1.89227	0.05
44220	1.89227	0.05
44221	2.08848	0.05
44222	1.89226	0.05
44223	1.89226	0.05
44224	2.37947	0.05
44225	4.27173	0.05
44226	1.01456	0.05
44227	0.943157	0.05
44228	0.641198	0.05
44229	0.724757	0.05
44230	0.830923	0.05
44231	0.830923	0.05
44232	0.836991	0.05
44233	0.836991	0.05
44234	1.01456	0.05
44235	0.724757	0.05
44236	0.641197	0.05
44237	0.943157	0.05
44238	1.89227	0.05
44239	1.89227	0.05
44240	1.89227	0.05
44241	1.89227	0.05
44242	3.78453	0.05
44243	0.946129	0.05
44244	0.946129	0.05
44245	1.28941	0.05
44246	1.18974	0.05
44247	0.94613	0.05
44248	0.94613	0.05
44249	1.28941	0.05
44250	1.28941	0.05
44251	1.28941	0.05

44252	0.946131	0.05
44253	0.94613	0.05
44254	1.18974	0.05
44255	0.710636	2.1
44256	0.780249	2.1
44257	0.579033	2.1
44258	0.478812	2.1
44259	0.621822	2.1
44260	0.621822	2.1
44261	0.63724	2.1
44262	0.63724	2.1
44263	0.780251	2.1
44264	0.579032	2.1
44265	0.710637	2.1
44266	0.478811	2.1
44267	1.05499	2.1
44268	1.00521	2.1
44269	1.24445	1.97555
44270	1.23082	0.637769
44271	2.28581	2.1
44272	1.48751	2.1
44273	1.23083	1.75297
44274	1.4941	2.1
44275	1.24445	1.53884
44276	1.48047	1.24815
44277	1.23083	1.27773
44278	0.621185	1.95535
44279	0.621185	2.1
44280	0.620899	1.08578
44281	0.620899	2.1
44282	0.502515	1.84194
44283	0.626672	1.21874
44284	0.615412	0.739868
44285	0.740234	2.1
44286	0.740233	0.796356
44287	0.626672	0.446064
44288	0.615413	1.08979
44289	0.502516	1.51997
44290	1.95844	2.1
44291	2.6562	2.1
44292	1.95844	2.1
44293	2.20883	2.1
44294	2.38225	2.1
44295	2.63264	2.1
44296	1.89226	2.1
44297	1.89226	2.1
44298	1.89226	2.1
44299	1.89226	2.1
44300	3.78453	2.1
44301	1.95844	2.1
44302	2.38225	2.1
44303	1.71082	2.1
44304	2.14647	2.1
44305	4.1049	2.1
44306	0.946131	2.1
44307	0.946131	2.1
44308	0.946131	2.1
44309	0.946131	2.1

44310	0.946131	2.1
44311	0.946131	2.1
44312	0.946131	2.1
44313	0.946131	2.1
44314	0.946131	2.1
44315	0.946131	2.1
44316	0.946131	2.1
44317	0.946131	2.1
44318	2.12597	2.1
44319	1.2295	2.1
44320	1.28014	2.1
44321	2.19723	2.1
44322	3.40611	2.1
44323	2.12597	2.1
44324	2.79242	2.1
44325	2.73375	2.1
44326	2.66249	2.1
44327	2.19723	2.1
44328	2.12597	2.1
44329	1.13716	2.1
44330	1.09861	2.1
44331	0.666692	2.1
44332	0.640071	2.1
44333	0.891617	2.1
44334	0.891617	2.1
44335	0.885614	2.1
44336	0.885614	2.1
44337	1.13716	2.1
44338	0.666692	2.1
44339	0.640071	2.1
44340	1.09861	2.1
44341	1.89226	0.05
44342	1.70303	0.05
44343	1.89226	0.05
44344	2.64598	0.05
44345	1.81091	0.05
44346	2.53222	0.05
44347	2.32422	0.05
44348	1.89226	0.05
44349	2.78336	0.05
44350	2.78336	0.05
44351	1.89226	0.05
44352	1.89226	0.05
44353	0.946132	0.05
44354	0.946132	0.05
44355	0.946131	0.05
44356	0.946131	0.05
44357	0.946132	0.05
44358	0.946132	0.05
44359	0.946131	0.05
44360	0.946131	0.05
44361	0.946131	0.05
44362	0.946131	0.05
44363	0.946131	0.05
44364	0.946131	0.05
44365	0.946131	0.05
44366	0.946131	0.05
44367	0.946132	0.05

44368	0.946132	0.05
44369	0.946132	0.05
44370	0.946132	0.05
44371	0.946131	0.05
44372	0.946131	0.05
44373	0.946131	0.05
44374	0.946131	0.05
44375	0.946131	0.05
44376	0.946131	0.05
44377	2.24323	0.599429
44378	1.89226	2.06792
44379	2.75021	1.36901
44380	2.75021	1.76314
44381	1.89226	0.376455
44382	1.89226	0.862715
44383	2.30908	0.05
44384	2.19815	0.0509508
44385	2.1609	0.05
44386	1.83796	0.05
44387	4.35904	0.05
44388	2.19815	0.05
44389	2.02268	2.1
44390	2.17869	2.1
44391	1.9559	0.05
44392	2.0815	2.1
44393	1.83424	0.05
44394	2.1609	0.05
44395	1.88877	0.16795
44396	1.98413	0.05
44397	1.87355	0.0867724
44398	4.03445	0.05
44399	1.87355	0.05
44400	2.02268	2.1
44401	1.87355	2.1
44402	2.10488	0.05
44403	1.68443	2.1
44404	1.89448	0.113048
44405	0.946131	0.05
44406	0.946131	0.05
44407	1.40574	0.0731581
44408	1.12373	0.0604098
44409	0.946129	0.05
44410	0.946129	0.05
44411	1.40574	0.084333
44412	1.40574	0.0521597
44413	0.946131	0.05
44414	1.40574	0.0729836
44415	1.12373	0.058733
44416	0.946131	0.05
44417	2.14277	0.0607262
44418	2.81148	0.0921072
44419	1.42762	0.05
44420	1.42762	0.05
44421	2.96261	0.05
44422	2.24746	0.05
44423	1.89226	0.864192
44424	1.89226	0.86111
44425	1.89226	0.700642

44426	1.89226	1.0278
44427	3.78453	1.58431
44428	1.89226	0.491724
44429	1.89226	0.817043
44430	1.89226	0.775818
44431	1.89226	0.799295
44432	3.78452	0.386361
44433	0.68433	0.458273
44434	0.946127	1.21645
44435	0.543921	1.57848
44436	0.946131	0.623348
44437	0.946131	0.715137
44438	0.946131	2.07337
44439	0.543918	1.16585
44440	0.543918	1.0888
44441	0.684332	0.810469
44442	0.54392	2.1
44443	0.946129	0.281278
44444	0.946129	0.832768
44445	1.89226	0.865504
44446	1.08784	1.30877
44447	1.19352	0.108451
44448	1.89226	0.936438
44449	1.19352	1.99591
44450	1.89226	1.64908
44451	0.635711	0.713291
44452	0.946132	1.52777
44453	0.946132	1.82873
44454	0.56474	0.954068
44455	0.750045	1.15962
44456	1.25657	2.1
44457	0.635682	0.963639
44458	0.635682	0.483855
44459	0.891954	0.822485
44460	1.32751	2.1
44461	0.564766	0.52424
44462	0.564766	1.46923
44463	0.858345	0.858968
44464	0.946128	1.44768
44465	0.635712	0.781542
44466	0.946133	0.80674
44467	0.977198	1.18733
44468	0.944521	2.1
44469	0.635682	1.32256
44470	0.635682	0.156088
44471	1.02924	0.996257
44472	0.705625	2.1
44473	0.851528	0.188191
44474	0.851528	2.08678
44475	2.18903	2.1
44476	2.10182	2.1
44477	2.10182	2.1
44478	1.89226	2.1
44479	1.89226	2.1
44480	1.64983	2.1
44481	1.89226	2.1
44482	1.83867	2.1
44483	1.83867	2.1

44484	1.89164	2.1
44485	2.0815	2.1
44486	2.17638	2.1
44487	2.57957	0.671364
44488	2.3506	2.07092
44489	2.69056	0.202075
44490	2.49439	1.77801
44491	5.04116	0.409646
44492	2.3506	0.655137
44493	1.8911	2.1
44494	2.49439	0.873992
44495	2.06689	2.1
44496	4.41748	2.1
44497	2.69056	0.529803
44498	2.49439	1.86835
44499	2.77227	0.111105
44500	2.63443	1.5724
44501	5.32499	1.78841
44502	2.49439	0.735344
44503	2.06689	2.1
44504	2.63443	0.166587
44505	2.26444	2.1
44506	4.70131	1.81774
44507	2.46327	2.1
44508	2.54347	2.1
44509	2.52869	2.1
44510	2.16534	2.1
44511	2.82661	2.1
44512	2.46327	2.1
44513	0.946131	2.1
44514	0.946131	2.1
44515	1.27174	2.1
44516	1.27174	2.1
44517	1.27174	2.1
44518	0.94613	2.1
44519	0.946128	2.1
44520	1.4133	2.1
44521	0.946129	2.1
44522	0.946129	2.1
44523	1.27174	2.1
44524	1.4133	2.1
44525	1.42757	2.1
44526	1.7457	2.1
44527	1.66047	2.1
44528	1.7457	2.1
44529	1.45754	2.1
44530	1.54278	2.1
44531	1.42757	2.1
44532	1.52996	2.1
44533	1.66047	2.1
44534	1.66047	2.1
44535	1.26397	2.1
44536	1.26397	2.1
44537	0.968536	2.1
44538	1.02461	2.1
44539	1.57575	2.1
44540	1.62391	2.1
44541	2.59245	2.1

44542	0.946131	2.1
44543	0.946131	2.1
44544	0.946132	2.1
44545	0.946132	2.1
44546	0.946132	2.1
44547	0.946132	2.1
44548	0.946132	2.1
44549	0.946132	2.1
44550	0.946132	2.1
44551	0.946132	2.1
44552	0.946132	2.1
44553	0.946132	2.1
44554	2.23765	2.1
44555	1.62391	2.1
44556	2.22154	2.1
44557	1.57575	2.1
44558	2.2697	2.1
44559	1.62391	2.1
44560	1.89226	2.1
44561	1.89226	2.1
44562	1.89226	2.1
44563	1.89226	2.1
44564	1.89226	2.1
44565	1.89226	2.1
44566	1.89226	2.1
44567	1.89226	2.1
44568	1.89226	2.1
44569	1.89226	2.1
44570	1.89226	2.1
44571	1.89226	2.1
44572	2.2697	2.1
44573	1.46129	2.1
44574	2.25873	2.1
44575	1.62391	2.1
44576	2.09611	2.1
44577	1.46129	2.1
44578	1.89226	2.1
44579	2.07088	2.1
44580	1.89226	2.1
44581	2.31884	2.1
44582	4.21111	2.1
44583	1.89226	2.1
44584	2.31884	2.1
44585	2.11713	2.1
44586	1.89226	2.1
44587	2.54371	2.1
44588	2.31884	2.1
44589	2.54371	2.1
44590	1.24041	2.1
44591	2.55208	2.1
44592	2.31884	2.1
44593	1.23712	2.1
44594	1.00388	2.1
44595	2.72769	0.05
44596	2.57507	0.05
44597	1.89226	0.05
44598	1.89226	0.05
44599	4.46733	0.05

44600	1.89226	0.05
44601	2.57506	0.05
44602	1.89226	0.05
44603	2.30076	0.05
44604	1.89226	0.05
44605	2.30076	0.05
44606	1.21171	0.05
44607	1.48589	0.05
44608	2.09166	0.05
44609	1.78381	0.05
44610	3.2697	0.05
44611	2.09166	0.05
44612	1.70302	0.05
44613	1.48589	0.05
44614	1.18027	0.05
44615	2.26571	0.05
44616	1.79969	0.05
44617	1.89226	0.05
44618	1.89226	0.05
44619	1.89226	0.05
44620	1.89226	0.05
44621	1.89226	0.05
44622	1.89226	0.05
44623	0.946131	0.05
44624	0.946131	0.05
44625	0.946128	0.05
44626	0.946128	0.05
44627	0.946129	0.05
44628	0.946129	0.05
44629	0.94613	0.05
44630	0.94613	0.05
44631	0.94613	0.05
44632	0.94613	0.05
44633	0.94613	0.05
44634	0.94613	0.05
44635	1.89226	1.818
44636	1.89226	1.92757
44637	1.89226	2.1
44638	1.89226	1.1511
44639	1.89226	2.1
44640	1.89226	1.04728
44641	1.89226	1.87565
44642	1.89226	1.89606
44643	1.89226	2.1
44644	1.89226	1.23577
44645	1.89226	2.09648
44646	1.89226	1.16973
44647	1.89226	1.34298
44648	1.89226	1.48054
44649	1.89226	2.02961
44650	1.89226	0.72317
44651	1.89226	2.03916
44652	1.89226	0.710073
44653	1.79103	0.365077
44654	0.968249	2.1
44655	1.44807	1.98039
44656	2.17984	0.0787553
44657	3.2391	2.1

44658	1.29735	1.55773
44659	1.08992	1.43688
44660	0.945147	2.1
44661	0.724033	2.1
44662	1.00723	2.1
44663	1.00723	1.86577
44664	1.01416	0.175586
44665	1.01416	2.1
44666	1.29735	1.93655
44667	0.94515	2.1
44668	0.724034	2.07216
44669	1.08992	2.1
44670	1.39214	2.1
44671	1.39214	2.1
44672	0.946129	2.1
44673	0.946129	2.1
44674	1.33728	2.1
44675	1.39214	2.1
44676	0.94613	2.1
44677	0.94613	2.1
44678	0.94613	2.1
44679	1.33728	2.1
44680	0.94613	2.1
44681	1.39214	2.1
44682	2.78428	2.1
44683	2.28373	2.1
44684	2.67456	2.1
44685	2.20011	2.1
44686	4.95829	2.1
44687	1.90165	0.05
44688	2.24206	0.05
44689	1.66137	0.05
44690	2.07108	0.05
44691	3.90343	0.05
44692	1.89224	0.05
44693	1.89224	0.05
44694	1.89224	0.05
44695	1.89224	0.05
44696	3.78448	0.05
44697	2.82159	0.05
44698	1.89226	0.05
44699	2.50138	0.0514342
44700	1.89226	0.05
44701	4.39365	0.05
44702	1.66139	0.05
44703	1.86399	0.063298
44704	1.56005	0.05
44705	1.90324	0.05
44706	3.56463	0.05
44707	0.946133	0.05
44708	0.946133	0.05
44709	0.946132	0.05
44710	0.946132	0.05
44711	0.946133	0.05
44712	0.946133	0.0501182
44713	0.946132	0.05
44714	0.946132	0.0554521
44715	0.946133	0.05

44716	0.946133	0.05
44717	0.946132	0.05
44718	0.946132	0.05
44719	1.5176	0.34596
44720	1.97964	0.128322
44721	2.32889	0.253835
44722	1.91762	0.187094
44723	3.84649	0.172847
44724	2.3376	0.209796
44725	1.97963	0.125791
44726	2.32889	0.342471
44727	2.58303	0.12889
44728	4.66649	0.252997
44729	2.32889	0.218125
44730	2.20248	0.109076
44731	1.91762	0.0957205
44732	1.91762	0.0781359
44733	2.5615	0.252596
44734	2.5615	0.180349
44735	2.32889	0.306223
44736	2.58303	0.101358
44737	2.71793	0.260897
44738	2.56149	0.0991963
44739	5.04681	0.149557
44740	1.89226	0.628762
44741	2.53785	2.1
44742	1.89226	0.288324
44743	2.60895	0.243792
44744	1.89226	1.44747
44745	2.60895	1.18198
44746	0.493248	0.180653
44747	1.30448	1.65356
44748	1.26893	2.1
44749	0.653226	2.0187
44750	0.653227	1.03142
44751	0.493247	2.1
44752	1.26893	2.1
44753	1.30448	0.509778
44754	0.832232	2.1
44755	0.832233	2.1
44756	0.929944	0.179712
44757	0.929944	0.857405
44758	1.89226	2.1
44759	1.47937	2.1
44760	1.50143	2.1
44761	1.47937	2.1
44762	1.91432	2.1
44763	1.89226	2.1
44764	1.89226	2.1
44765	1.89226	2.1
44766	1.89226	2.1
44767	1.89226	2.1
44768	1.89226	2.1
44769	1.89226	1.35711
44770	1.33419	2.1
44771	2.4542	2.1
44772	2.59834	2.1
44773	2.59834	2.1

44774	1.25716	2.1
44775	1.25716	2.1
44776	1.70303	2.1
44777	1.50143	2.1
44778	1.60878	2.1
44779	1.50143	2.1
44780	1.93599	2.1
44781	1.89226	1.84307
44782	0.946131	1.45798
44783	0.946131	2.1
44784	0.946127	2.1
44785	0.946127	2.1
44786	0.946127	2.1
44787	0.946127	0.440513
44788	0.946131	2.1
44789	0.946131	2.1
44790	0.94613	2.1
44791	0.94613	2.1
44792	0.946129	2.1
44793	0.946129	2.1
44794	1.43912	2.1
44795	2.59834	2.1
44796	2.78093	2.1
44797	2.78093	2.1
44798	1.33419	2.1
44799	1.33419	2.1
44800	1.89226	2.1
44801	2.26322	2.1
44802	2.55682	2.1
44803	2.55682	2.1
44804	1.89226	2.1
44805	1.89226	2.1
44806	2.58032	0.0746577
44807	2.28922	0.05
44808	1.88424	0.05
44809	2.32127	0.05
44810	4.46456	0.05
44811	1.67265	0.0549194
44812	2.28922	0.05
44813	1.88424	0.05
44814	1.11314	0.05
44815	3.55689	0.05
44816	1.43751	0.05
44817	2.32127	0.05
44818	1.92998	0.05
44819	2.37671	0.05
44820	1.43751	0.05
44821	1.88424	0.0516672
44822	2.36729	2.1
44823	1.56714	2.1
44824	1.56714	2.1
44825	1.62408	2.1
44826	2.28373	2.1
44827	2.34067	2.1
44828	0.937268	2.1
44829	0.937268	2.1
44830	0.94636	2.1
44831	0.94636	2.1

44832	0.76004	2.1
44833	0.783572	2.1
44834	1.14187	2.1
44835	1.10006	2.1
44836	1.10006	2.1
44837	0.76004	2.1
44838	0.783572	2.1
44839	1.14187	2.1
44840	1.28753	2.1
44841	1.36838	2.1
44842	1.62617	2.1
44843	1.82857	2.1
44844	3.1161	2.1
44845	1.82856	2.1
44846	1.17996	2.1
44847	1.53417	2.1
44848	1.56618	2.1
44849	1.36838	2.1
44850	1.36838	2.1
44851	1.35065	0.818658
44852	1.42659	0.05
44853	2.26675	0.244552
44854	2.26675	0.250986
44855	1.62137	0.140096
44856	2.53747	0.240381
44857	1.89226	0.05
44858	1.89226	0.05
44859	1.89226	0.05
44860	1.89226	0.05
44861	3.78452	0.05
44862	1.36569	0.05
44863	1.36569	0.05
44864	0.946131	0.05
44865	0.946131	0.05
44866	1.16041	0.05
44867	1.36569	0.05
44868	0.946129	0.05
44869	0.946129	0.05
44870	1.1604	0.05
44871	0.94613	0.05
44872	0.946131	0.05
44873	1.36569	0.05
44874	2.73139	0.05
44875	1.25265	0.05
44876	1.25265	0.05
44877	1.64799	0.05
44878	2.32081	0.05
44879	2.71615	0.05
44880	0.946133	0.05
44881	0.946133	0.05
44882	0.946132	0.05
44883	0.946132	0.05
44884	0.946132	0.05
44885	0.946132	0.05
44886	0.946133	0.05
44887	0.946133	0.05
44888	0.946132	0.05
44889	0.946132	0.05

44890	0.946133	0.05
44891	0.946133	0.05
44892	0.946131	0.05
44893	0.946131	0.05
44894	0.94613	0.05
44895	0.94613	0.05
44896	0.946132	0.05
44897	0.946132	0.05
44898	0.946129	0.05
44899	0.946129	0.05
44900	0.946131	0.05
44901	0.946131	0.05
44902	0.946131	0.05
44903	0.946131	0.05
44904	1.89226	0.05
44905	2.48503	2.1
44906	2.64586	2.1
44907	2.64586	0.05
44908	1.89226	2.1
44909	1.89226	0.05
44910	2.48504	2.1
44911	1.99985	2.1
44912	1.83584	2.1
44913	1.83584	2.1
44914	2.64586	2.1
44915	2.64586	2.1
44916	1.89225	0.067249
44917	2.33095	2.1
44918	2.48501	2.1
44919	2.48501	0.05
44920	1.89225	2.1
44921	1.89225	0.05
44922	2.33096	2.1
44923	1.83583	2.1
44924	1.68436	2.1
44925	1.68436	2.1
44926	2.48502	2.1
44927	2.48502	2.1
44928	1.14807	2.1
44929	1.76545	2.1
44930	1.21292	2.1
44931	1.83936	2.1
44932	2.97837	2.1
44933	1.89226	2.1
44934	1.89226	2.1
44935	1.89226	2.1
44936	1.89226	2.1
44937	3.78451	2.1
44938	1.89226	2.1
44939	1.89226	1.11548
44940	1.89226	2.1
44941	1.89226	2.06294
44942	3.78452	2.1
44943	1.76545	2.1
44944	2.12608	2.1
44945	1.83937	2.1
44946	2.22529	2.038
44947	3.99074	2.1

44948	0.780222	2.1
44949	0.780222	2.1
44950	0.784871	2.1
44951	0.784871	2.1
44952	0.95863	2.1
44953	0.919681	2.1
44954	0.636145	2.1
44955	0.606461	2.1
44956	0.636144	2.1
44957	0.958631	2.1
44958	0.60646	2.1
44959	0.919683	2.1
44960	1.89226	2.1
44961	1.89226	2.1
44962	1.89226	2.1
44963	1.89226	2.1
44964	3.78452	2.1
44965	1.89226	2.1
44966	1.89226	2.1
44967	1.89226	2.1
44968	1.89226	1.09258
44969	3.78452	2.1
44970	1.03789	2.1
44971	1.03789	2.1
44972	1.04231	2.1
44973	1.04231	2.1
44974	1.16052	2.1
44975	1.11265	0.936375
44976	0.958634	2.1
44977	0.919685	2.1
44978	0.958634	2.1
44979	1.16052	2.1
44980	0.919685	2.1
44981	1.11265	2.1
44982	1.43837	2.1
44983	2.57085	2.1
44984	1.39332	2.1
44985	2.58228	2.1
44986	3.96417	2.1
44987	1.89226	2.1
44988	1.89226	2.1
44989	1.89226	2.1
44990	1.89226	2.1
44991	1.89226	2.1
44992	1.89226	2.1
44993	1.89226	2.1
44994	1.89226	1.58209
44995	1.89226	2.1
44996	1.89226	2.1
44997	3.78452	2.1
44998	2.57085	2.1
44999	1.89226	2.1
45000	2.58229	2.1
45001	1.89226	2.1
45002	4.46312	2.1
45003	1.32368	2.1
45004	2.58228	2.1
45005	2.55352	2.1

45006	2.55352	2.1
45007	1.39332	2.1
45008	1.39332	2.1
45009	1.89226	2.1
45010	1.89226	2.1
45011	1.89226	2.1
45012	1.89226	2.1
45013	1.89226	2.1
45014	1.89226	2.1
45015	1.89226	2.1
45016	1.89226	2.1
45017	1.89226	2.1
45018	1.89226	2.1
45019	1.89226	2.1
45020	1.89226	2.1
45021	2.55353	2.1
45022	1.89226	2.1
45023	2.58228	2.1
45024	1.89226	2.1
45025	1.89226	2.1
45026	2.58228	2.1
45027	1.40574	1.71553
45028	1.05175	1.32323
45029	1.71613	1.83565
45030	1.99961	1.16416
45031	3.12187	1.60154
45032	1.71613	1.82296
45033	2.50733	1.24585
45034	1.99961	2.00322
45035	1.99961	1.86473
45036	2.25746	1.08195
45037	2.25746	1.24722
45038	2.25746	1.11279
45039	1.89526	1.26334
45040	1.71613	1.70801
45041	1.71613	0.634658
45042	1.89526	1.23867
45043	1.27558	0.66705
45044	1.07536	2.1
45045	1.07536	2.1
45046	1.07034	2.1
45047	1.07034	2.1
45048	0.766879	2.1
45049	0.81527	2.1
45050	1.38896	2.1
45051	1.33043	2.1
45052	0.815264	2.1
45053	1.38895	2.1
45054	0.766884	2.1
45055	1.33044	2.1
45056	1.89225	2.1
45057	1.89225	2.1
45058	1.89225	2.1
45059	1.89225	2.1
45060	1.89225	2.1
45061	1.89225	2.1
45062	1.64059	2.1
45063	1.89225	2.1

45064	1.565	2.1
45065	1.565	2.1
45066	1.89225	2.1
45067	1.89225	2.1
45068	1.89226	2.1
45069	1.89226	2.1
45070	1.89226	2.1
45071	1.89226	2.1
45072	1.89226	2.1
45073	1.89226	2.1
45074	1.67906	2.1
45075	1.89226	2.1
45076	1.6406	2.1
45077	1.6406	2.1
45078	1.89226	2.1
45079	1.89226	2.1
45080	2.18106	1.77448
45081	2.20831	2.1
45082	2.64725	1.08428
45083	2.63102	1.56851
45084	4.82831	1.78839
45085	2.18106	1.47585
45086	2.09605	2.1
45087	2.5601	1.6718
45088	2.63102	1.56975
45089	4.74116	2.1
45090	2.64725	1.38044
45091	1.89226	1.62005
45092	1.87603	2.1
45093	2.63102	1.39972
45094	1.89226	1.95945
45095	2.64725	0.959502
45096	0.946132	1.73865
45097	1.28005	1.32153
45098	0.946132	2.1
45099	1.31551	1.06216
45100	1.28005	1.79692
45101	1.28005	2.1
45102	0.946132	1.09947
45103	0.946132	1.6329
45104	0.946132	1.18299
45105	0.946132	1.43164
45106	1.28005	1.75142
45107	1.31551	1.86147
45108	2.0815	2.1
45109	1.59318	2.1
45110	2.0815	2.1
45111	1.40161	2.1
45112	2.49782	2.1
45113	1.68553	2.1
45114	0.819885	2.1
45115	0.935082	2.1
45116	0.867363	2.1
45117	0.867363	2.1
45118	0.715431	2.1
45119	0.902777	2.1
45120	0.738933	2.1
45121	0.980053	2.1

45122	0.936716	2.1
45123	0.917651	2.1
45124	0.775853	2.1
45125	0.74037	2.1
45126	2.78977	2.1
45127	2.38239	2.1
45128	2.78977	2.1
45129	1.96468	2.1
45130	2.64107	2.1
45131	1.80733	2.1
45132	1.80141	2.1
45133	2.28512	2.1
45134	2.28512	2.1
45135	1.8519	2.1
45136	2.64107	2.1
45137	2.20785	2.1
45138	0.946133	0.16852
45139	0.946133	0.0849606
45140	0.946132	0.0741073
45141	0.946132	0.0539953
45142	0.946132	0.0736763
45143	0.946132	0.217169
45144	0.946132	0.0921425
45145	0.946132	0.051232
45146	0.946132	0.0779801
45147	0.946132	0.0797307
45148	0.946133	0.0824928
45149	0.946133	0.124841
45150	1.89226	0.0607831
45151	1.89226	0.0548735
45152	1.89226	0.05
45153	1.89226	0.05
45154	1.89226	0.05
45155	1.89226	0.115951
45156	1.89226	0.0549617
45157	1.89226	0.051976
45158	1.89226	0.0553051
45159	1.89226	0.137037
45160	3.78453	0.0834312
45161	1.89226	0.0784258
45162	1.89226	0.119473
45163	1.89226	0.05
45164	1.89226	0.159624
45165	1.89226	0.0518574
45166	1.89226	0.174343
45167	2.12925	0.05
45168	2.51397	0.05
45169	2.22488	0.05
45170	1.86113	0.05
45171	4.3751	0.05
45172	2.82754	0.05
45173	2.51397	0.05
45174	2.22489	0.05
45175	2.47291	0.05
45176	4.98688	0.05
45177	1.89226	0.05
45178	1.89226	0.05
45179	1.89226	0.05

45180	1.89226	0.05
45181	3.78453	0.05
45182	1.89226	0.05
45183	1.89226	0.05
45184	1.89226	0.05
45185	1.89226	0.05
45186	3.78452	0.05
45187	0.90848	0.05
45188	1.11244	0.05
45189	0.930566	0.05
45190	0.73385	0.05
45191	0.921298	0.05
45192	0.921298	0.05
45193	0.924994	0.05
45194	0.924994	0.05
45195	0.908478	0.05
45196	0.733851	0.05
45197	1.11244	0.05
45198	0.930567	0.05
45199	2.22489	0.05
45200	2.0815	0.05
45201	2.0815	0.05
45202	2.30674	0.05
45203	1.99867	0.05
45204	2.33358	0.05
45205	1.89226	0.05
45206	1.89226	0.05
45207	1.89226	0.05
45208	1.89226	0.05
45209	1.89226	0.05
45210	1.89226	0.05
45211	0.946131	0.05
45212	0.946131	0.05
45213	0.946128	0.05
45214	0.946128	0.05
45215	0.946129	0.05
45216	0.946129	0.05
45217	0.94613	0.05
45218	0.94613	0.05
45219	0.94613	0.05
45220	0.94613	0.05
45221	0.94613	0.05
45222	0.94613	0.05
45223	2.28904	2.1
45224	1.89226	1.70808
45225	2.2846	1.96814
45226	2.2846	2.1
45227	1.89226	0.208016
45228	1.89226	2.1
45229	1.89226	1.09248
45230	1.89226	1.15816
45231	1.89226	2.1
45232	1.89226	2.1
45233	1.89226	0.167258
45234	1.89226	2.1
45235	1.04037	0.440635
45236	1.34066	1.90374
45237	1.34955	0.438692

45238	1.34955	2.1
45239	1.03373	0.900979
45240	1.03373	2.1
45241	0.946131	0.0588658
45242	0.946131	2.1
45243	1.14452	2.1
45244	1.14452	2.1
45245	0.946131	1.52946
45246	0.946131	1.3023
45247	1.14296	2.1
45248	1.14452	2.1
45249	1.14296	0.965777
45250	0.946131	2.08938
45251	1.14452	2.1
45252	0.946132	1.92981
45253	1.89226	0.776779
45254	1.89226	2.1
45255	1.89226	1.86147
45256	1.89226	2.1
45257	1.89226	0.0966646
45258	1.89226	2.1
45259	0.597454	0.68137
45260	0.597454	2.0655
45261	0.597972	0.439061
45262	0.597972	2.1
45263	0.675238	2.1
45264	0.674776	0.956608
45265	0.519667	1.04215
45266	0.520188	1.45737
45267	0.519667	0.200745
45268	0.675238	0.357979
45269	0.520188	1.42102
45270	0.674777	1.47182
45271	1.89226	0.05
45272	1.82125	0.05
45273	1.89226	0.05
45274	1.84314	0.05
45275	1.89226	0.05
45276	1.84314	0.05
45277	1.89226	0.05
45278	1.84314	0.05
45279	1.89226	0.05
45280	1.65794	0.05
45281	1.89226	0.05
45282	1.65794	0.05
45283	1.348	2.1
45284	1.43485	2.1
45285	1.79929	0.817579
45286	1.75309	1.94316
45287	3.14728	1.96609
45288	1.348	1.43769
45289	1.63892	2.06613
45290	1.59157	2.1
45291	1.18647	2.09906
45292	1.75309	1.56558
45293	1.348	1.96873
45294	1.79929	1.77488
45295	1.75309	2.1

45296	2.18106	1.39312
45297	2.20831	1.37426
45298	3.98034	2.05357
45299	2.18106	1.13648
45300	1.63892	2.1
45301	1.75309	1.27767
45302	1.75309	2.1
45303	2.09605	1.16944
45304	2.09605	2.1
45305	2.66076	0.0955643
45306	1.70302	0.05
45307	2.41575	0.05
45308	1.70302	0.05
45309	3.08598	0.05
45310	2.3661	0.05
45311	1.71053	0.05
45312	2.41575	0.102348
45313	1.20143	0.05
45314	1.20143	0.05
45315	2.2123	0.05
45316	1.66739	0.05
45317	2.25633	0.05
45318	2.68302	0.0683611
45319	1.71051	0.05
45320	1.71051	0.05
45321	2.74192	0.05
45322	2.1961	0.05
45323	1.32553	0.681541
45324	2.34875	0.05
45325	2.19139	1.79796
45326	2.19139	0.05
45327	1.56172	1.84246
45328	1.56172	0.05
45329	1.89226	2.1
45330	1.89226	0.05
45331	1.89226	2.1
45332	1.89226	0.0930701
45333	3.78453	0.926438
45334	2.667 2.1	
45335	1.26097	2.03888
45336	1.26097	2.1
45337	1.5683	2.1
45338	2.42949	2.1
45339	2.73682	2.1
45340	1.13213	0.190129
45341	2.19139	0.05
45342	2.0815	1.23763
45343	1.77615	0.05
45344	1.37767	1.20557
45345	1.19297	0.05
45346	0.946132	0.0560839
45347	0.946132	2.1
45348	0.946131	2.1
45349	0.946131	0.136487
45350	0.946131	0.0897097
45351	0.946131	0.05
45352	0.946132	2.09873
45353	0.946132	2.1

45354	0.946132	0.870801
45355	0.946132	1.13612
45356	0.946131	0.604733
45357	0.946131	0.671012
45358	2.02064	2.1
45359	2.3606	2.1
45360	1.47478	2.1
45361	2.22997	2.1
45362	4.25061	2.1
45363	2.36059	2.1
45364	2.56952	2.1
45365	2.22997	2.1
45366	2.48631	2.1
45367	4.79949	2.1
45368	1.84261	2.1
45369	2.22997	2.1
45370	1.65706	2.1
45371	1.78837	2.1
45372	3.88703	2.1
45373	2.22997	2.1
45374	2.48631	2.1
45375	2.19528	2.1
45376	2.41175	2.1
45377	4.64172	2.1
45378	1.6466	2.1
45379	1.89226	2.1
45380	1.35972	2.1
45381	1.35972	2.1
45382	1.89226	2.1
45383	1.89226	2.1
45384	1.89226	2.1
45385	1.89226	2.1
45386	1.89226	2.1
45387	1.89226	2.1
45388	3.78453	2.1
45389	2.44797	2.1
45390	1.07214	2.1
45391	1.31589	2.1
45392	1.1211	2.1
45393	2.44797	2.1
45394	2.25318	2.1
45395	1.95954	2.1
45396	1.89226	2.1
45397	1.6466	2.1
45398	1.6466	2.1
45399	1.89226	2.1
45400	1.89226	2.1
45401	0.946133	2.1
45402	0.946133	2.1
45403	0.946132	2.1
45404	0.946132	2.1
45405	0.946132	2.1
45406	0.946132	2.1
45407	0.946133	2.1
45408	0.946133	2.1
45409	0.946132	2.1
45410	0.946132	2.1
45411	0.946133	2.1

45412	0.946133	2.1
45413	2.44797	2.1
45414	1.36904	2.1
45415	1.70303	2.1
45416	2.37586	2.1
45417	4.00507	2.1
45418	0.631553	0.05
45419	1.24489	0.05
45420	1.11597	0.05
45421	0.496799	0.05
45422	0.872272	0.05
45423	0.872272	0.05
45424	0.869415	0.05
45425	0.869415	0.05
45426	0.4968	0.05
45427	0.631553	0.05
45428	1.11597	0.05
45429	1.24489	0.05
45430	1.84253	2.1
45431	2.17384	2.1
45432	2.04818	2.1
45433	1.65925	2.1
45434	3.83309	2.1
45435	2.04818	2.1
45436	1.65925	2.1
45437	1.48162	2.1
45438	1.92264	2.1
45439	3.58189	2.1
45440	2.3305	0.05
45441	1.89226	0.0565291
45442	2.52753	0.05
45443	2.52753	0.0558029
45444	1.89226	0.05
45445	1.89226	0.0606293
45446	1.89226	0.056969
45447	1.89226	0.351355
45448	1.89226	0.139025
45449	1.89226	0.176304
45450	1.89226	0.11717
45451	1.89226	0.230464
45452	1.34563	0.05
45453	2.1769	0.0551873
45454	1.94955	0.0641777
45455	1.94955	0.269751
45456	1.55063	0.05
45457	1.55063	0.146552
45458	2.09455	0.05
45459	1.89226	0.0752541
45460	2.3305	0.05
45461	2.3305	0.0502024
45462	1.89226	0.05
45463	1.89226	0.0564591
45464	1.89226	0.0618057
45465	1.89226	0.0862911
45466	1.89226	0.146672
45467	1.89226	0.062528
45468	1.89226	0.0841758
45469	1.89226	0.158951

45470	0.773392	0.05
45471	0.612765	0.36145
45472	0.79504	0.0749134
45473	0.575323	0.225347
45474	1.0819	0.05
45475	1.06106	0.171606
45476	1.04675	0.05
45477	0.86858	0.117391
45478	0.796873	0.0686796
45479	0.557469	0.05
45480	0.807663	0.0748773
45481	2.2024	2.1
45482	2.34106	2.1
45483	2.02634	2.1
45484	2.20125	2.1
45485	4.36739	2.1
45486	2.34106	2.1
45487	2.25195	0.05
45488	2.20125	2.1
45489	2.13132	0.0536904
45490	4.47237	1.50233
45491	2.02635	2.1
45492	2.22465	2.1
45493	1.68504	2.1
45494	2.0815	2.1
45495	3.92673	2.1
45496	2.22465	2.1
45497	2.13133	0.05
45498	2.0815	2.1
45499	2.23579	0.0645127
45500	4.44862	1.53366
45501	1.89226	0.901391
45502	1.89226	1.4024
45503	1.89226	1.89227
45504	1.89226	1.03921
45505	1.89226	1.75132
45506	1.89226	0.856552
45507	1.93967	0.05
45508	1.70302	0.05
45509	1.21883	0.05
45510	1.70302	0.05
45511	1.19889	0.05
45512	1.70302	0.05
45513	0.946131	0.05
45514	0.946131	0.05
45515	0.946131	0.05
45516	0.946131	0.05
45517	0.94613	0.05
45518	0.94613	0.05
45519	0.946131	0.05
45520	0.946131	0.05
45521	0.946131	0.05
45522	0.946131	0.05
45523	0.946131	0.05
45524	0.946131	0.05
45525	0.883128	0.05
45526	0.883128	0.05
45527	0.827519	0.05

45528	0.82752	0.05
45529	1.16234	0.05
45530	1.19063	0.05
45531	0.631294	0.05
45532	0.548311	0.05
45533	0.631293	0.05
45534	1.16234	0.05
45535	1.19063	0.05
45536	0.54831	0.05
45537	1.89226	0.05
45538	1.89226	0.05
45539	1.89226	0.05
45540	1.89226	0.05
45541	3.78453	0.05
45542	2.33328	0.05
45543	1.87991	0.05
45544	1.87991	0.05
45545	2.82938	0.05
45546	1.84457	0.05
45547	2.79403	0.05
45548	2.82937	0.05
45549	2.70881	0.05
45550	1.8799	0.05
45551	1.82739	0.05
45552	4.58874	0.05
45553	1.89226	0.05
45554	1.89226	0.05
45555	1.89226	0.05
45556	1.89226	0.05
45557	3.78452	0.05
45558	1.17897	0.05
45559	1.82739	0.05
45560	1.17897	0.05
45561	1.76199	0.05
45562	1.23885	0.05
45563	1.80989	0.05
45564	0.946131	0.05
45565	0.946131	0.05
45566	0.946131	0.05
45567	0.946131	0.05
45568	0.946129	0.05
45569	0.946129	0.05
45570	0.946133	0.05
45571	0.946133	0.05
45572	0.946131	0.05
45573	0.946131	0.05
45574	0.946131	0.05
45575	0.946131	0.05
45576	0.946126	0.0714125
45577	0.946126	2.1
45578	1.24887	2.1
45579	1.24887	2.1
45580	0.946127	2.1
45581	0.946127	0.190691
45582	1.24887	2.1
45583	1.28511	2.1
45584	1.2851	2.1
45585	0.946128	2.1

45586	1.24887	2.1
45587	0.946128	2.1
45588	1.89226	2.1
45589	1.89226	2.04154
45590	1.89226	2.1
45591	1.89226	0.21603
45592	3.78452	1.61048
45593	0.592142	2.1
45594	0.592142	2.1
45595	0.60496	2.1
45596	0.60496	2.1
45597	0.694237	2.1
45598	0.608714	0.758329
45599	0.562863	2.1
45600	0.502864	2.1
45601	0.562863	2.1
45602	0.694237	2.1
45603	0.608714	2.1
45604	0.502864	2.1
45605	1.89226	0.39627
45606	1.89226	2.1
45607	1.89226	2.1
45608	1.89226	2.1
45609	3.78453	2.1
45610	1.55779	2.1
45611	1.57968	2.1
45612	1.47186	2.1
45613	1.51725	2.1
45614	3.05154	2.1
45615	1.57967	2.1
45616	1.474	2.1
45617	1.51725	2.1
45618	1.42895	2.1
45619	3.00863	2.1
45620	1.89226	2.1
45621	1.89226	2.1
45622	1.89226	2.1
45623	1.89226	2.1
45624	3.78452	2.1
45625	1.89226	2.1
45626	1.89226	2.1
45627	1.89226	0.342851
45628	1.89226	2.1
45629	3.78453	2.1
45630	1.47186	2.1
45631	1.51725	2.1
45632	1.38859	2.1
45633	1.45332	2.1
45634	2.92519	2.1
45635	1.51725	2.1
45636	1.42895	2.1
45637	1.45332	2.1
45638	1.38414	2.1
45639	2.88228	2.1
45640	1.89226	2.1
45641	1.89226	2.1
45642	1.89226	2.1
45643	1.89226	2.1

45644	3.78452	2.1
45645	1.34497	0.05
45646	1.36747	0.05
45647	1.18919	0.05
45648	2.08149	0.05
45649	1.23556	0.05
45650	2.13702	0.05
45651	1.10106	0.05
45652	2.08001	0.05
45653	1.34497	0.05
45654	2.31605	0.05
45655	1.34497	0.05
45656	2.31605	0.05
45657	2.31605	0.05
45658	2.0815	0.05
45659	1.9419	0.05
45660	2.46665	0.05
45661	4.52922	0.05
45662	1.20349	0.05
45663	2.08149	0.05
45664	1.36948	0.05
45665	1.34737	0.05
45666	2.08149	0.05
45667	2.08149	0.05
45668	0.946132	0.05
45669	0.946132	0.05
45670	1.37715	0.05
45671	0.823199	0.05
45672	1.21598	0.05
45673	0.946132	0.05
45674	1.37715	0.05
45675	0.946132	0.05
45676	1.21598	0.05
45677	0.823198	0.05
45678	0.946133	0.05
45679	0.946133	0.05
45680	1.89226	0.05
45681	1.89226	0.05
45682	1.89226	0.05
45683	1.89226	0.05
45684	3.78453	0.05
45685	1.89227	0.05
45686	1.89227	0.05
45687	1.89227	0.05
45688	1.89227	0.05
45689	3.78453	0.05
45690	0.946132	0.05
45691	0.946132	0.05
45692	1.21599	0.05
45693	1.21599	0.05
45694	0.966749	0.05
45695	0.946133	0.05
45696	1.21598	0.05
45697	0.946132	0.05
45698	0.966748	0.05
45699	1.21598	0.05
45700	0.946133	0.05
45701	0.946133	0.05

45702	0.946133	0.05
45703	0.946133	0.05
45704	1.1134	0.05
45705	1.11555	0.05
45706	1.11555	0.05
45707	0.946132	0.05
45708	0.946132	0.05
45709	1.1134	0.05
45710	1.11555	0.05
45711	1.11555	0.05
45712	0.946132	0.05
45713	0.946132	0.05
45714	0.946133	0.05
45715	0.946133	0.05
45716	1.11555	0.05
45717	0.978467	0.05
45718	0.978468	0.05
45719	0.946133	0.05
45720	1.11555	0.05
45721	0.946132	0.05
45722	0.978468	0.05
45723	0.978468	0.05
45724	0.946132	0.05
45725	0.946132	0.05
45726	0.851542	2.1
45727	0.851542	2.1
45728	1.14168	2.1
45729	1.27897	2.1
45730	1.02817	2.1
45731	1.13536	2.1
45732	0.946131	2.1
45733	0.861207	2.1
45734	0.94613	2.1
45735	0.94613	2.1
45736	0.868751	2.1
45737	1.15181	2.1
45738	0.946132	2.1
45739	0.946132	2.06904
45740	0.946128	2.07436
45741	0.946128	0.829946
45742	0.94613	2.1
45743	0.94613	2.08371
45744	0.946131	1.96079
45745	0.946131	0.928441
45746	0.94613	0.751526
45747	0.94613	2.1
45748	0.94613	1.70328
45749	0.94613	2.1
45750	2.53273	0.05
45751	1.89226	0.05
45752	1.89226	0.05
45753	1.89226	0.05
45754	1.89226	0.05
45755	1.89226	0.05
45756	1.89226	2.1
45757	1.89226	2.1
45758	1.89226	2.1
45759	1.89226	2.1

45760	3.78452	2.1
45761	1.89226	2.1
45762	2.54394	2.1
45763	1.89226	2.1
45764	2.42221	2.1
45765	4.43621	2.1
45766	2.54394	2.1
45767	1.62743	2.1
45768	2.42221	2.1
45769	1.5437	2.1
45770	4.08764	2.1
45771	1.89226	2.1
45772	1.89226	2.1
45773	1.89226	2.1
45774	1.89226	2.1
45775	3.78452	2.1
45776	1.89226	2.1
45777	1.89226	2.1
45778	1.89226	2.1
45779	1.89226	2.1
45780	3.78452	2.1
45781	1.89226	2.1
45782	2.30146	2.1
45783	2.42221	2.1
45784	1.89226	2.1
45785	1.89226	2.1
45786	2.42221	2.1
45787	2.30146	2.1
45788	2.42221	2.1
45789	1.39962	2.1
45790	1.39962	2.1
45791	1.5437	2.1
45792	2.44553	2.1
45793	1.89226	2.1
45794	1.89226	2.1
45795	1.89226	2.1
45796	1.89226	2.1
45797	3.78452	2.1
45798	1.62196	2.1
45799	2.00695	2.1
45800	1.95288	2.1
45801	1.70303	2.1
45802	3.53316	2.1
45803	1.80703	2.1
45804	2.00695	2.1
45805	1.92991	2.1
45806	2.00695	2.1
45807	1.85888	2.1
45808	1.93592	2.1
45809	1.89226	2.1
45810	1.89226	2.1
45811	1.89226	2.1
45812	1.89226	2.1
45813	1.89226	2.1
45814	1.89226	2.1
45815	1.89226	2.1
45816	1.89226	2.1
45817	1.89226	2.1

45818	1.89226	2.1
45819	3.78453	2.1
45820	1.95288	2.1
45821	1.50524	2.1
45822	1.83564	2.1
45823	1.70303	2.1
45824	2.00749	2.1
45825	1.84671	2.1
45826	1.62632	2.1
45827	1.82498	2.1
45828	1.92991	2.1
45829	1.92991	2.1
45830	1.47928	2.1
45831	1.47751	2.1
45832	0.946128	2.1
45833	0.946128	2.1
45834	0.946132	2.1
45835	0.946132	2.1
45836	0.94613	2.1
45837	0.94613	2.1
45838	0.946129	2.1
45839	0.946129	2.1
45840	0.94613	2.1
45841	0.94613	2.1
45842	0.94613	2.1
45843	0.94613	2.1
45844	1.89226	2.1
45845	1.89226	2.1
45846	1.89226	2.1
45847	1.89226	2.1
45848	1.89226	2.1
45849	1.89226	2.1
45850	2.27351	0.319518
45851	2.74068	0.0806492
45852	2.72278	0.128068
45853	2.63356	0.24197
45854	2.36273	0.120024
45855	2.27351	0.223816
45856	2.36273	0.207197
45857	2.83112	0.0658887
45858	2.81212	0.101594
45859	2.74068	0.177837
45860	2.43416	0.0977819
45861	2.36273	0.170273
45862	1.89227	0.142157
45863	2.83112	0.0602068
45864	2.74069	0.123798
45865	2.74069	0.0530742
45866	1.89227	0.198361
45867	1.89227	0.0809054
45868	2.40887	2.1
45869	1.4494	2.1
45870	1.52164	2.1
45871	2.47534	2.1
45872	3.93051	2.1
45873	1.89226	2.1
45874	2.47534	2.1
45875	2.40887	2.1

45876	2.40887	2.1
45877	1.89226	2.1
45878	1.89226	2.1
45879	1.60861	2.1
45880	2.47534	2.1
45881	1.52164	2.1
45882	1.52164	2.1
45883	2.55301	2.1
45884	2.55301	2.1
45885	0.946128	2.1
45886	0.946128	2.1
45887	1.23767	2.1
45888	1.2765	2.1
45889	1.23767	2.1
45890	1.23767	2.1
45891	0.946131	2.1
45892	0.946131	2.1
45893	0.946131	2.1
45894	1.2765	2.1
45895	1.23767	2.1
45896	0.946131	2.1
45897	1.89226	2.1
45898	1.87511	2.1
45899	1.87511	2.1
45900	1.95852	2.1
45901	2.0815	2.1
45902	2.22169	2.1
45903	1.89226	2.1
45904	1.89226	2.1
45905	1.89226	2.1
45906	1.89226	2.1
45907	1.89226	2.1
45908	1.89226	2.1
45909	2.0815	2.1
45910	1.80067	2.1
45911	1.80067	2.1
45912	2.05518	2.1
45913	1.89226	2.1
45914	2.16509	2.1
45915	1.87153	2.1
45916	2.65897	2.1
45917	1.58724	2.1
45918	2.38924	2.1
45919	4.26077	2.1
45920	1.89226	2.1
45921	1.89226	2.1
45922	1.89226	2.1
45923	1.89226	2.1
45924	1.89226	2.1
45925	1.89226	2.1
45926	1.89226	0.703778
45927	2.21022	0.05
45928	2.19769	0.812447
45929	2.21022	0.197694
45930	1.87973	0.0589916
45931	1.89226	0.05
45932	1.18376	1.01712
45933	2.21022	0.05

45934	1.16239	0.624373
45935	1.16239	0.05
45936	2.19769	0.631505
45937	2.19769	0.05
45938	0.946128	0.340803
45939	0.946128	0.0951176
45940	1.09362	0.991441
45941	1.09884	0.0815592
45942	0.94613	0.141344
45943	0.94613	0.05
45944	1.09362	0.780333
45945	1.09362	0.0912336
45946	0.946131	0.504613
45947	1.09362	0.460794
45948	0.946131	0.0765687
45949	1.09884	0.05
45950	1.19323	0.892464
45951	2.19769	0.05
45952	1.18376	0.50457
45953	1.18376	0.05
45954	2.18724	0.531547
45955	2.18724	0.0569589
45956	2.0815	2.1
45957	0.922714	2.1
45958	2.0815	2.1
45959	1.84882	2.1
45960	1.20787	2.1
45961	1.08321	2.1
45962	2.6238	0.05
45963	1.89226	0.05
45964	1.89226	0.05
45965	2.4866	0.05
45966	1.89226	0.05
45967	2.4866	0.05
45968	2.66753	0.05
45969	1.89226	0.05
45970	1.89226	0.05
45971	2.6238	0.05
45972	1.89226	0.05
45973	2.6238	0.05
45974	0.946129	2.1
45975	0.946129	2.1
45976	1.04676	2.1
45977	1.04676	2.1
45978	0.946131	2.1
45979	0.946131	2.1
45980	1.08732	2.1
45981	1.04676	2.1
45982	1.08732	2.1
45983	0.946131	2.1
45984	1.04676	2.1
45985	0.946131	2.1
45986	1.20958	2.1
45987	1.73802	0.375476
45988	1.39067	0.509865
45989	1.39067	0.0855397
45990	1.50969	0.638308
45991	1.50969	0.749612

45992	1.00382	1.29466
45993	1.50969	0.05
45994	1.20958	0.741459
45995	1.20958	0.05
45996	1.32039	1.46313
45997	1.32039	0.207079
45998	2.0815	2.1
45999	2.51396	2.1
46000	2.19826	2.1
46001	2.54175	2.1
46002	4.79599	2.1
46003	2.54174	2.1
46004	2.17978	2.1
46005	2.54174	2.1
46006	2.2854	2.1
46007	2.57212	2.1
46008	2.31578	2.1
46009	2.27103	2.1
46010	2.54175	2.1
46011	2.4255	2.1
46012	2.82251	2.1
46013	4.96725	2.1
46014	2.60614	0.05
46015	2.75374	0.05
46016	1.94002	0.05
46017	2.08392	0.05
46018	4.69006	0.05
46019	2.75373	0.05
46020	2.6875	0.05
46021	2.08392	0.05
46022	1.99944	0.05
46023	4.77142	0.05
46024	1.1211	0.05
46025	2.08392	0.05
46026	1.17787	0.05
46027	1.94002	0.05
46028	1.32178	0.05
46029	2.08392	0.05
46030	2.08392	0.05
46031	1.99944	0.05
46032	1.32178	0.05
46033	1.25929	0.05
46034	3.34321	0.05
46035	1.22266	0.05
46036	0.779473	0.05
46037	0.94613	0.05
46038	0.94613	0.05
46039	1.04072	0.05
46040	1.1407	0.05
46041	1.10042	0.05
46042	0.851542	0.05
46043	0.942046	0.05
46044	0.942046	0.05
46045	1.04114	0.05
46046	0.826602	0.05
46047	2.37468	2.1
46048	1.89226	2.1
46049	1.89226	2.1

46050	1.89226	2.1
46051	2.27917	2.1
46052	2.27917	2.1
46053	1.13959	2.1
46054	1.09451	2.1
46055	0.946131	2.1
46056	0.946131	2.1
46057	1.09451	2.1
46058	1.09451	2.1
46059	0.94613	2.1
46060	0.94613	2.1
46061	1.09451	2.1
46062	0.946131	2.1
46063	0.946131	2.1
46064	1.13959	2.1
46065	2.52454	0.382859
46066	2.12221	2.1
46067	2.67624	0.647087
46068	2.33865	2.1
46069	4.86319	2.1
46070	1.89226	2.06181
46071	1.89226	2.1
46072	1.89226	1.98021
46073	1.89226	2.1
46074	3.78452	2.1
46075	1.89226	2.1
46076	1.89226	2.1
46077	1.89226	2.1
46078	1.89226	2.1
46079	3.78452	2.1
46080	2.12221	2.1
46081	1.47769	2.1
46082	2.33865	2.1
46083	1.62898	2.1
46084	3.81634	2.1
46085	2.67624	2.1
46086	2.37734	2.00566
46087	2.33865	2.1
46088	2.33865	2.1
46089	2.70125	2.1
46090	2.70125	2.1
46091	1.89226	2.1
46092	1.89226	2.1
46093	1.89226	2.1
46094	1.89226	2.1
46095	1.89226	0.755987
46096	1.89226	1.57269
46097	1.89226	2.1
46098	1.89226	2.1
46099	1.89226	2.1
46100	1.89226	2.1
46101	1.89226	2.1
46102	1.89226	2.1
46103	2.37733	2.1
46104	1.95927	2.1
46105	1.82516	1.73033
46106	1.82516	2.1
46107	2.33865	2.1

46108	2.33865	2.1
46109	2.57466	2.1
46110	2.21715	2.1
46111	2.55653	2.1
46112	2.24775	2.1
46113	4.77367	2.1
46114	0.937095	2.1
46115	0.937095	2.1
46116	0.958028	2.1
46117	0.958028	2.1
46118	0.828386	2.1
46119	0.771244	2.1
46120	1.10858	2.1
46121	1.12388	2.1
46122	1.12388	2.1
46123	0.828386	2.1
46124	0.771244	2.1
46125	1.10858	2.1
46126	2.55653	2.1
46127	2.32606	2.1
46128	2.24775	2.1
46129	2.24775	2.1
46130	2.57928	2.1
46131	2.57928	2.1
46132	0.990481	2.1
46133	0.990481	2.1
46134	1.00094	2.1
46135	1.00094	2.1
46136	0.888385	2.1
46137	0.828386	2.1
46138	1.16303	2.1
46139	1.12388	2.1
46140	1.16303	2.1
46141	0.888385	2.1
46142	1.12388	2.1
46143	0.828387	2.1
46144	0.962524	2.1
46145	0.962524	2.1
46146	0.7976	2.1
46147	0.7976	2.1
46148	0.797628	2.1
46149	0.814 2.1	
46150	0.946125	2.1
46151	0.946125	2.1
46152	0.946118	2.1
46153	0.797623	2.1
46154	0.814006	2.1
46155	0.946131	2.1
46156	1.8978	0.05
46157	2.23892	0.05
46158	1.71469	0.05
46159	4.13672	0.05
46160	2.64136	0.05
46161	2.23892	0.05
46162	2.37578	0.05
46163	1.77489	2.1
46164	4.6147	0.05
46165	1.02284	2.1

46166	0.898796	0.0554282
46167	0.948554	0.931559
46168	0.948554	2.1
46169	0.878797	2.1
46170	0.796197	2.1
46171	1.09145	0.223213
46172	0.963109	0.564128
46173	1.12114	2.1
46174	0.782739	2.1
46175	1.11084	2.1
46176	0.850742	2.1
46177	2.48024	2.1
46178	2.405 2.1	
46179	2.24585	2.1
46180	2.34795	2.1
46181	4.75295	2.1
46182	1.93794	2.1
46183	2.405 2.1	
46184	2.24585	2.1
46185	1.67594	2.1
46186	4.08094	2.1
46187	1.89226	2.1
46188	1.89226	0.0672011
46189	1.89226	2.1
46190	1.89226	1.2943
46191	3.78452	2.02022
46192	1.89226	2.1
46193	1.89226	2.1
46194	1.89226	2.1
46195	1.89226	1.57274
46196	3.78452	2.1
46197	2.24585	2.1
46198	2.34795	2.1
46199	2.21155	2.1
46200	2.06626	2.1
46201	4.4142	2.1
46202	2.24585	2.1
46203	1.41498	2.1
46204	1.41498	2.1
46205	1.67594	2.1
46206	2.06625	2.1
46207	2.32721	2.1
46208	1.89226	2.1
46209	1.89226	1.26
46210	1.89226	2.1
46211	1.89226	0.611816
46212	1.89226	2.1
46213	1.89226	0.242014
46214	1.89226	2.1
46215	1.89226	1.52251
46216	1.89226	2.1
46217	1.89226	2.1
46218	3.78452	2.1
46219	1.89226	0.05
46220	1.89226	0.05
46221	1.89226	0.05
46222	1.89226	0.05
46223	3.78452	0.05

46224	1.89226	0.05
46225	2.53655	0.05
46226	1.89226	0.05
46227	1.89226	0.05
46228	1.89226	0.05
46229	1.89226	0.05
46230	1.89227	2.1
46231	1.89227	2.1
46232	1.89227	2.1
46233	1.89227	2.1
46234	1.89227	2.1
46235	1.89227	2.1
46236	0.91339	2.1
46237	0.91339	2.1
46238	1.2158	2.1
46239	1.20629	2.1
46240	1.00473	2.1
46241	1.15749	2.1
46242	0.946132	2.1
46243	0.946132	2.1
46244	0.946133	2.1
46245	1.17513	2.1
46246	1.00473	2.1
46247	0.946132	2.1
46248	1.89226	2.1
46249	1.89226	2.1
46250	1.89226	2.1
46251	1.89226	2.1
46252	1.89226	2.1
46253	1.89226	2.1
46254	1.89226	2.1
46255	2.6066	2.1
46256	2.35026	2.1
46257	2.35026	2.1
46258	1.89226	2.1
46259	1.89226	2.1
46260	2.04056	0.05
46261	2.77366	0.0658272
46262	1.24682	0.05
46263	1.24682	0.0507426
46264	2.93651	0.05
46265	2.14277	0.052184
46266	2.68524	1.91171
46267	2.81847	1.88962
46268	1.89226	0.851054
46269	1.89226	1.84672
46270	4.57751	1.26436
46271	2.68524	1.48648
46272	1.89226	2.00337
46273	1.89226	2.05026
46274	2.50732	1.70698
46275	1.89226	1.87293
46276	2.50732	1.98737
46277	1.89226	1.11867
46278	1.89226	2.02473
46279	1.89226	1.7753
46280	1.89226	2.06511
46281	1.89226	1.23124

46282	1.89226	1.97865
46283	1.89226	2.01752
46284	1.89226	1.63263
46285	1.89226	1.73588
46286	1.89226	2.1
46287	1.89226	0.680617
46288	1.89226	1.32934
46289	1.61016	1.30019
46290	1.07467	1.78966
46291	1.24474	1.18354
46292	1.24474	2.06902
46293	1.45303	1.31501
46294	1.45303	2.08483
46295	1.61016	1.80818
46296	1.24474	2.1
46297	1.61016	1.92781
46298	1.77186	1.4847
46299	1.37813	1.43781
46300	1.37813	1.94515
46301	1.73305	2.1
46302	2.0815	2.1
46303	1.97658	2.1
46304	1.75791	2.1
46305	1.87228	2.1
46306	1.66515	2.1
46307	2.0815	2.1
46308	1.93938	0.129686
46309	2.03253	0.05
46310	2.2142	2.1
46311	1.97658	0.05
46312	2.15324	2.1
46313	1.73305	2.1
46314	1.97658	2.1
46315	1.60428	2.1
46316	1.8589	2.1
46317	3.58085	2.1
46318	1.97657	2.1
46319	1.93938	0.0583592
46320	1.51316	2.1
46321	1.85293	0.153189
46322	3.82951	1.62299
46323	1.70303	0.05
46324	1.96087	0.05
46325	1.42308	0.05
46326	1.96087	0.05
46327	1.25023	0.05
46328	1.70303	0.05
46329	0.946132	0.05
46330	0.946132	0.05
46331	0.946131	0.05
46332	0.946131	0.05
46333	0.946132	0.05
46334	0.946132	0.05
46335	0.946132	0.05
46336	0.946132	0.05
46337	0.946132	0.05
46338	0.946132	0.05
46339	0.946132	0.05

46340	0.946132	0.05
46341	1.89226	0.05
46342	1.89226	0.0806778
46343	1.89226	0.0505896
46344	1.89226	0.092377
46345	3.78452	0.0533573
46346	1.89226	0.05
46347	1.89226	0.05
46348	1.89226	0.05
46349	1.89226	0.0551164
46350	1.89226	0.05
46351	1.89226	0.05
46352	1.89226	0.05
46353	1.89226	0.052311
46354	1.89226	0.0563477
46355	1.89226	0.05
46356	1.89226	0.05
46357	1.89226	0.05
46358	1.89226	0.05
46359	1.89226	0.05
46360	2.48255	0.05
46361	1.89226	0.05
46362	3.78452	0.05
46363	1.89226	2.1
46364	2.42758	2.1
46365	1.9209	2.1
46366	1.89226	2.1
46367	2.45622	2.1
46368	2.42758	2.1
46369	2.42758	2.1
46370	1.49987	2.1
46371	1.61028	2.1
46372	1.58164	2.1
46373	2.45622	2.1
46374	2.42758	2.1
46375	0.94613	2.1
46376	0.94613	2.1
46377	1.24298	2.1
46378	1.24298	2.1
46379	1.24298	2.1
46380	1.22811	2.1
46381	0.946131	2.1
46382	0.946131	2.1
46383	0.94613	2.1
46384	1.24298	2.1
46385	0.94613	2.1
46386	1.22811	2.1
46387	2.48596	2.1
46388	2.45622	2.1
46389	1.63823	2.1
46390	1.4304	2.1
46391	1.45739	2.1
46392	2.45622	2.1
46393	2.4169	2.1
46394	2.55249	2.1
46395	2.51125	2.1
46396	2.35245	2.1
46397	4.90494	2.1

46398	2.45922	2.1
46399	2.55249	2.1
46400	2.51126	2.1
46401	2.43226	2.1
46402	4.98474	2.1
46403	2.51125	2.1
46404	2.35245	2.1
46405	2.28979	2.1
46406	2.47228	2.1
46407	4.82473	2.1
46408	2.51126	2.1
46409	2.43226	2.1
46410	2.40668	2.1
46411	2.47228	2.1
46412	4.90453	2.1
46413	2.8045	2.1
46414	1.93707	2.1
46415	2.14087	2.1
46416	2.14087	2.1
46417	2.61595	2.1
46418	2.61595	2.1
46419	1.89226	2.1
46420	1.89226	2.1
46421	1.89226	2.1
46422	1.89226	1.68552
46423	1.89226	2.1
46424	1.89226	0.864987
46425	1.22368	2.1
46426	1.32339	2.1
46427	0.957125	2.1
46428	0.750074	2.1
46429	1.08438	2.1
46430	1.08438	2.1
46431	1.13094	2.1
46432	0.970797	2.1
46433	1.17568	2.1
46434	0.764392	2.1
46435	0.944286	2.1
46436	1.27523	2.1
46437	1.89226	2.1
46438	1.89226	2.1
46439	1.89226	2.1
46440	1.89226	1.47465
46441	3.78453	2.1
46442	2.8239	2.1
46443	1.89227	2.1
46444	2.57276	2.1
46445	2.57276	2.1
46446	1.89227	2.1
46447	1.89227	2.1
46448	1.18139	2.1
46449	1.85195	2.1
46450	1.78595	2.1
46451	1.08172	2.1
46452	2.93367	2.1
46453	1.60735	2.1
46454	1.20356	2.1
46455	0.967034	2.1

46456	1.08172	2.1
46457	1.67324	2.1
46458	1.77293	2.1
46459	1.89226	2.1
46460	1.89226	2.1
46461	1.8213	2.1
46462	1.82129	2.1
46463	2.14171	2.1
46464	2.40192	2.1
46465	0.810943	2.1
46466	0.810943	2.1
46467	0.798469	2.1
46468	0.788336	2.1
46469	0.804015	2.1
46470	0.810945	2.1
46471	1.947 2.1	
46472	1.36506	2.1
46473	1.08686	2.1
46474	1.07208	2.1
46475	1.08132	2.1
46476	1.13537	0.05
46477	1.14754	1.34007
46478	0.946131	0.0660295
46479	0.946131	2.06734
46480	0.94613	0.05
46481	0.94613	0.05
46482	1.25104	2.1
46483	1.14754	0.805382
46484	1.13537	0.179124
46485	0.946131	0.175913
46486	0.946131	0.572978
46487	1.25104	0.27832
46488	0.96542	0.05
46489	0.922284	0.489942
46490	0.946131	2.0481
46491	0.946131	0.144309
46492	0.946131	0.144113
46493	0.946131	0.05
46494	1.13537	2.08471
46495	0.924824	0.759759
46496	0.859476	0.197039
46497	0.842305	0.216988
46498	1.25996	0.178573
46499	1.04996	0.186632
46500	1.71007	0.05
46501	2.73283	0.05
46502	2.73283	0.05
46503	1.89226	0.05
46504	1.89226	0.05
46505	1.13262	0.05
46506	0.946131	0.05
46507	0.946131	0.05
46508	0.946131	0.05
46509	0.946131	0.05
46510	0.946132	0.05
46511	0.946132	0.05
46512	0.946131	0.05
46513	0.946131	0.05

46514	0.946131	0.05
46515	0.946131	0.05
46516	0.946132	0.05
46517	0.946132	0.05
46518	0.946131	0.05
46519	1.36642	0.05
46520	0.946125	0.05
46521	0.946125	0.05
46522	0.946128	0.05
46523	0.946128	0.05
46524	0.946126	0.05
46525	0.946128	0.05
46526	0.946128	0.05
46527	0.946128	0.05
46528	1.36641	0.05
46529	0.946128	0.05
46530	1.89226	0.05
46531	1.89226	0.05
46532	1.89226	0.05
46533	1.89226	0.05
46534	1.89226	0.05
46535	1.89226	0.05
46536	2.69784	2.1
46537	2.51651	2.1
46538	2.56999	2.1
46539	2.74623	2.1
46540	5.26783	2.1
46541	2.69784	2.1
46542	2.48718	2.1
46543	2.50161	2.1
46544	2.74623	2.1
46545	5.19945	2.1
46546	1.89226	2.1
46547	1.89226	2.1
46548	1.89226	2.1
46549	1.89226	2.1
46550	3.78453	2.1
46551	0.874264	2.1
46552	1.09879	2.1
46553	0.946131	2.1
46554	0.946131	2.1
46555	1.17062	2.1
46556	1.17062	2.1
46557	0.874293	2.1
46558	0.874293	2.1
46559	1.09879	2.1
46560	0.94613	2.1
46561	0.946131	2.1
46562	0.874265	2.1
46563	1.89226	2.1
46564	1.89226	2.1
46565	1.89226	2.1
46566	1.89226	2.1
46567	3.78453	2.1
46568	0.977382	2.1
46569	1.16424	2.1
46570	0.946133	2.1
46571	0.946133	2.1

46572	1.33012	2.1
46573	1.25589	2.1
46574	0.888498	2.1
46575	0.888498	2.1
46576	1.25375	2.1
46577	0.946131	2.1
46578	0.946131	2.1
46579	0.977384	2.1
46580	1.89226	2.1
46581	2.19757	2.1
46582	2.62906	2.1
46583	1.89226	2.1
46584	1.89226	2.1
46585	2.62906	2.1
46586	1.27944	1.27292
46587	1.27731	2.1
46588	0.615701	1.9726
46589	0.615701	1.33136
46590	0.615673	0.05
46591	0.946133	1.85767
46592	0.948264	0.355823
46593	0.946132	1.3107
46594	0.94752	0.430971
46595	0.61706	0.05
46596	0.946132	0.84908
46597	0.946132	0.746393
46598	2.76604	2.1
46599	1.66728	2.1
46600	1.66728	2.1
46601	1.83036	2.1
46602	2.54515	2.1
46603	2.70823	2.1
46604	2.54515	2.1
46605	1.49958	2.1
46606	1.49958	2.1
46607	1.66728	2.1
46608	2.34465	2.1
46609	2.51235	2.1
46610	1.21128	2.1
46611	1.21128	2.1
46612	0.946131	2.1
46613	0.946131	2.1
46614	0.946131	2.1
46615	0.946131	2.1
46616	1.31401	2.1
46617	1.21128	2.1
46618	1.21128	2.1
46619	0.946131	2.1
46620	0.946131	2.1
46621	1.31401	2.1
46622	1.67374	2.1
46623	2.29586	2.1
46624	1.50033	2.1
46625	2.16598	2.1
46626	3.83972	2.1
46627	1.89226	2.1
46628	1.89226	2.1
46629	1.89226	2.1

46630	1.89226	2.1
46631	3.78452	2.1
46632	1.13537	2.1
46633	1.02188	2.1
46634	0.946132	2.1
46635	0.946132	2.1
46636	0.946132	2.1
46637	0.946132	2.1
46638	1.21128	2.1
46639	1.02188	2.1
46640	1.04672	2.1
46641	1.04672	2.1
46642	1.21128	2.1
46643	0.946132	2.1
46644	0.815411	2.1
46645	0.870464	2.1
46646	0.885437	2.1
46647	0.885437	2.1
46648	1.06395	2.1
46649	1.00483	2.1
46650	0.757125	2.1
46651	0.673956	2.1
46652	0.684461	2.1
46653	1.06979	2.1
46654	0.731403	2.1
46655	1.0559	2.1
46656	1.89226	2.1
46657	1.89226	2.1
46658	1.89226	2.1
46659	1.89226	2.1
46660	1.89226	2.1
46661	1.89226	2.1
46662	1.76897	0.952319
46663	1.76932	1.24821
46664	1.97455	2.1
46665	1.94302	0.112404
46666	3.71233	0.562834
46667	1.83347	2.1
46668	1.76932	1.44954
46669	1.97455	2.1
46670	1.76932	2.1
46671	1.76522	2.1
46672	1.55999	2.1
46673	1.89227	1.42643
46674	1.89227	2.0274
46675	1.89227	2.1
46676	1.89227	0.581467
46677	3.78453	1.23352
46678	1.89226	1.22582
46679	1.89226	0.0509992
46680	1.89226	0.0886618
46681	1.89226	0.452384
46682	3.78452	0.376138
46683	1.97452	2.1
46684	1.94299	0.0849249
46685	2.17831	0.394432
46686	2.23195	0.441004
46687	4.17495	0.6463

46688	2.23196	0.605761
46689	2.1499	2.1
46690	1.97452	2.1
46691	1.97452	2.1
46692	2.09088	2.1
46693	1.83345	2.1
46694	0.94612	1.87598
46695	0.94612	2.1
46696	0.946119	0.373039
46697	0.946119	0.396517
46698	0.946119	0.420858
46699	0.946119	1.76839
46700	0.94612	0.563429
46701	0.94612	2.1
46702	0.946132	0.916962
46703	0.946132	0.652395
46704	0.946107	1.29725
46705	0.946107	1.19989
46706	1.89224	0.0701626
46707	1.89224	0.331355
46708	1.89224	0.351531
46709	1.89224	0.0760351
46710	3.78447	0.0754905
46711	1.70302	0.0906506
46712	1.056	0.0546824
46713	1.21829	0.148065
46714	1.89906	0.0696358
46715	3.07892	0.0712716
46716	2.20248	0.0935615
46717	1.56498	0.0721371
46718	1.46539	0.0684336
46719	1.48787	0.0509197
46720	2.37625	0.169799
46721	2.37625	0.105445
46722	1.14104	0.0564616
46723	1.00675	0.0615512
46724	0.700689	0.0502074
46725	0.624389	0.0604721
46726	0.782149	0.05
46727	0.940197	0.0534719
46728	0.803437	0.05
46729	0.803437	0.066311
46730	0.995423	0.05
46731	0.700699	0.05
46732	0.593895	0.11519
46733	1.00673	0.0535953
46734	1.18097	0.0698597
46735	1.15839	0.0616129
46736	0.995407	0.0608116
46737	0.865932	0.0544538
46738	1.17299	0.05
46739	0.957054	0.0534006
46740	1.08016	0.0727373
46741	1.08016	0.110872
46742	0.995425	0.0816282
46743	1.24161	0.05
46744	0.796771	0.05
46745	1.15837	0.100564

46746	2.57264	0.05
46747	1.89226	0.05
46748	2.41701	0.05
46749	1.89226	0.05
46750	4.30928	0.05
46751	1.69055	0.05
46752	2.39587	0.05
46753	1.53463	0.05
46754	2.2053	0.05
46755	3.89585	0.05
46756	0.946129	0.05
46757	0.946129	0.05
46758	0.94613	0.05
46759	0.94613	0.05
46760	0.946132	0.05
46761	0.946132	0.05
46762	0.946126	0.05
46763	0.946126	0.05
46764	0.94613	0.05
46765	0.94613	0.05
46766	0.946129	0.05
46767	0.946129	0.05
46768	2.41701	0.05
46769	1.89226	0.05
46770	1.89226	0.05
46771	1.89226	0.05
46772	2.32462	0.05
46773	2.32462	0.05
46774	1.41942	0.05
46775	2.2053	0.05
46776	2.02097	0.05
46777	2.02097	0.05
46778	1.53463	0.05
46779	1.53463	0.05
46780	1.89226	0.05
46781	1.89226	0.05
46782	1.89226	0.05
46783	1.89226	0.05
46784	1.89226	0.05
46785	1.89226	0.05
46786	1.89226	0.702997
46787	1.89226	2.1
46788	1.89226	2.1
46789	1.89226	1.2753
46790	1.89226	2.0348
46791	1.89226	0.888956
46792	1.02816	0.579326
46793	1.02816	1.24763
46794	0.595141	1.53694
46795	0.595141	1.94511
46796	0.946132	0.926591
46797	0.595113	2.05672
46798	0.677174	1.94405
46799	0.946132	0.52844
46800	0.946132	1.44563
46801	0.946132	1.43197
46802	0.595113	1.33146
46803	0.677174	1.47745

46804	1.89226	1.27405
46805	1.89226	2.1
46806	1.89226	2.05896
46807	1.89226	1.41912
46808	1.89226	2.1
46809	1.89226	1.66295
46810	0.946133	0.713488
46811	0.946133	0.306864
46812	0.946132	2.0366
46813	0.946132	2.06038
46814	0.946132	2.03695
46815	0.946132	0.818011
46816	0.946132	1.73837
46817	0.946132	0.342738
46818	0.946132	1.39254
46819	0.946132	1.71346
46820	0.946133	1.06616
46821	0.946133	1.07126
46822	0.946131	1.63243
46823	0.946131	1.61734
46824	0.946131	2.1
46825	0.946131	2.1
46826	0.946128	2.1
46827	0.946128	1.6291
46828	0.946133	2.1
46829	0.946133	1.49685
46830	0.94613	2.04592
46831	0.94613	2.1
46832	0.946131	2.0704
46833	0.946131	1.93039
46834	2.0264	2.05711
46835	1.67345	0.778803
46836	2.0264	1.40434
46837	1.70539	0.0831614
46838	1.31611	0.300651
46839	1.31611	0.415314
46840	1.89226	0.05
46841	1.89226	0.05
46842	1.89226	0.0780196
46843	1.89226	0.05
46844	3.78452	0.05
46845	1.89226	0.0769929
46846	1.89226	0.0590297
46847	1.89226	0.0550624
46848	1.89226	0.105825
46849	1.89226	0.0614745
46850	1.89226	0.131416
46851	1.02645	0.05
46852	0.810942	0.05
46853	0.882781	0.05
46854	0.882781	0.05
46855	2.80386	0.05
46856	1.56719	0.05
46857	1.22499	0.05
46858	1.08132	0.05
46859	1.36865	0.05
46860	0.810957	0.05
46861	0.810957	0.05

46862	0.946132	0.05
46863	0.946132	0.05
46864	0.946129	0.05
46865	0.946129	0.05
46866	0.946131	0.05
46867	0.946131	0.05
46868	0.946131	0.05
46869	0.946131	0.05
46870	0.94613	0.05
46871	0.94613	0.05
46872	0.946131	0.05
46873	0.946131	0.05
46874	1.89226	0.05
46875	1.89226	0.05
46876	1.89226	0.0545758
46877	1.89226	0.05
46878	3.78453	0.05
46879	1.30462	0.05
46880	1.79906	0.05
46881	1.01299	0.05
46882	1.44185	0.05
46883	1.3499	0.05
46884	1.79906	0.05
46885	1.89226	0.05
46886	1.89226	0.05
46887	1.89226	0.05
46888	1.89226	0.05
46889	1.89226	0.101268
46890	1.89226	0.218749
46891	1.89226	0.05
46892	1.89226	0.05
46893	1.89226	0.0644276
46894	1.89226	0.05
46895	1.89226	0.05
46896	1.89226	0.05
46897	1.3499	0.05
46898	1.79905	0.05
46899	1.29001	0.05
46900	1.36005	0.05
46901	1.88294	0.05
46902	1.79905	0.05
46903	1.89226	0.05
46904	2.75374	0.05
46905	2.60614	0.05
46906	1.89226	0.05
46907	2.60614	0.05
46908	1.89226	0.05
46909	0.946131	0.05
46910	0.946131	0.05
46911	1.37687	0.05
46912	1.37687	0.05
46913	0.946131	0.05
46914	1.34375	0.05
46915	1.37686	0.05
46916	0.946128	0.05
46917	1.37687	0.05
46918	1.34375	0.05
46919	0.94613	0.05

46920	0.94613	0.05
46921	1.44247	2.1
46922	1.12665	2.1
46923	1.50044	2.1
46924	1.73651	2.1
46925	2.94291	2.1
46926	1.73652	2.1
46927	1.64926	2.1
46928	1.44247	2.1
46929	1.44247	2.1
46930	1.64926	2.1
46931	1.43872	2.1
46932	1.50044	2.1
46933	1.73651	2.1
46934	2.00955	2.1
46935	1.87875	2.1
46936	3.50999	2.1
46937	1.64926	2.1
46938	1.73652	2.1
46939	2.00955	2.1
46940	1.8493	2.1
46941	3.65881	2.1
46942	1.89226	0.05
46943	2.28188	0.05
46944	1.99182	0.05
46945	2.28188	0.05
46946	1.6022	0.05
46947	1.89226	0.05
46948	1.89226	0.05
46949	1.89226	0.05
46950	1.89226	0.05
46951	1.89226	0.05
46952	1.89226	0.05
46953	1.89226	0.05
46954	1.89226	0.05
46955	1.89226	0.05
46956	2.19578	0.05
46957	1.89226	0.05
46958	2.81422	0.05
46959	2.42521	0.05
46960	1.89224	0.05
46961	1.99179	0.05
46962	1.454	0.05
46963	1.99179	0.05
46964	1.35444	0.05
46965	1.89224	0.05
46966	1.89224	0.05
46967	1.89224	0.05
46968	1.89224	0.05
46969	1.89224	0.05
46970	1.89224	0.05
46971	1.89224	0.05
46972	1.703	0.05
46973	2.19575	0.05
46974	1.9911	0.05
46975	2.19575	0.05
46976	1.67811	0.05
46977	1.89223	0.05

46978	1.69586	2.1
46979	2.55301	2.1
46980	1.60861	2.1
46981	1.60861	2.1
46982	2.65233	2.1
46983	2.65233	2.1
46984	0.946129	2.1
46985	0.946129	2.1
46986	1.2765	2.1
46987	1.32617	2.1
46988	1.2765	2.1
46989	1.2765	2.1
46990	0.946131	2.1
46991	0.946131	2.1
46992	0.946131	2.1
46993	1.32616	2.1
46994	0.946131	2.1
46995	1.2765	2.1
46996	1.36757	2.1
46997	1.32617	2.1
46998	0.891503	2.1
46999	0.847932	2.1
47000	1.10722	2.1
47001	1.10722	2.1
47002	1.10829	2.1
47003	1.10829	2.1
47004	1.36757	2.1
47005	0.891504	2.1
47006	0.847932	2.1
47007	1.32617	2.1
47008	0.946129	2.1
47009	0.946129	2.1
47010	1.36757	2.1
47011	1.32617	2.1
47012	1.32616	2.1
47013	1.32616	2.1
47014	0.946131	2.1
47015	0.946131	2.1
47016	1.36757	2.1
47017	0.946131	2.1
47018	0.946131	2.1
47019	1.32616	2.1
47020	1.89226	0.05
47021	1.89226	0.05
47022	1.89226	0.05
47023	1.89226	0.05
47024	3.78452	0.05
47025	2.36336	0.05
47026	1.89226	0.05
47027	2.67831	0.05
47028	2.67831	0.05
47029	1.89226	0.05
47030	1.89226	0.05
47031	1.89227	0.05
47032	1.89227	0.05
47033	1.89227	0.05
47034	1.89227	0.05
47035	3.78453	0.05

47036	1.89226	0.05
47037	1.89226	0.05
47038	1.89226	0.05
47039	1.89226	0.05
47040	3.78452	0.05
47041	2.36336	0.05
47042	2.0815	0.05
47043	2.0815	0.05
47044	2.41048	0.05
47045	1.66267	0.05
47046	2.13452	0.05
47047	1.89226	0.05
47048	1.89226	0.05
47049	1.89226	0.05
47050	1.89226	0.05
47051	1.89226	0.05
47052	1.89226	0.05
47053	1.89226	0.05
47054	1.89226	0.0732447
47055	1.89226	0.05
47056	1.89226	0.0696753
47057	1.89226	0.05
47058	1.89226	0.0627771
47059	0.946128	0.0927714
47060	0.946128	0.0861218
47061	1.00122	0.05
47062	1.00122	0.05
47063	0.94613	0.134478
47064	1.01618	0.0679609
47065	0.946131	0.0680382
47066	1.00122	0.05
47067	1.00122	0.05
47068	1.01618	0.0570424
47069	0.946131	0.111986
47070	0.946131	0.0692819
47071	1.89226	1.87042
47072	1.89226	0.464531
47073	1.89226	0.357648
47074	1.89226	2.1
47075	3.78452	0.671438
47076	1.89226	1.21885
47077	1.89226	2.1
47078	1.89226	2.1
47079	1.89226	1.59502
47080	1.89226	2.1
47081	1.89226	1.54388
47082	1.89226	0.149428
47083	1.89226	2.06787
47084	1.89226	1.86394
47085	1.89226	0.57547
47086	1.89226	1.8497
47087	1.89226	0.558435
47088	1.89226	0.483834
47089	1.89226	2.1
47090	1.89226	2.1
47091	1.89226	1.11662
47092	1.89226	2.05586
47093	1.89226	1.22694

47094	2.71279	0.91166
47095	1.4155	0.712145
47096	2.28488	0.528825
47097	1.06122	0.699093
47098	3.70038	0.416739
47099	1.89226	1.31321
47100	1.89226	1.00648
47101	1.89226	0.831242
47102	1.89226	0.789354
47103	3.78452	1.68497
47104	0.851542	0.38159
47105	0.851542	0.299177
47106	0.851484	0.579196
47107	0.865567	0.907994
47108	0.981616	0.344214
47109	0.864767	0.498447
47110	0.851542	0.800181
47111	0.851542	0.452965
47112	0.851477	0.507709
47113	0.851477	0.837544
47114	0.865635	0.504126
47115	0.816472	0.261989
47116	1.68443	2.1
47117	1.44489	2.1
47118	1.54924	2.1
47119	1.34389	2.1
47120	3.02832	2.1
47121	1.44489	2.1
47122	1.03899	2.1
47123	1.03899	2.1
47124	1.12075	2.1
47125	1.34389	2.1
47126	1.42566	2.1
47127	1.54924	2.1
47128	1.34389	2.1
47129	1.44091	2.1
47130	1.25866	2.1
47131	2.7848	2.1
47132	0.573141	2.1
47133	0.573141	2.1
47134	0.575681	2.1
47135	0.575681	2.1
47136	0.481985	2.1
47137	0.519492	2.1
47138	0.671948	2.1
47139	0.629331	2.1
47140	0.629329	2.1
47141	0.481986	2.1
47142	0.519494	2.1
47143	0.671947	2.1
47144	1.89226	0.348412
47145	1.89226	1.52111
47146	1.89226	0.76139
47147	2.31925	0.646107
47148	3.78452	1.25007
47149	1.30916	2.1
47150	1.17426	2.1
47151	0.946129	2.1

47152	0.946129	2.1
47153	0.94613	2.1
47154	0.94613	2.1
47155	1.17426	2.1
47156	1.17426	2.1
47157	1.30916	2.1
47158	0.94613	2.1
47159	1.17426	2.1
47160	0.94613	2.1
47161	2.56346	2.1
47162	1.89226	1.29045
47163	1.89226	2.1
47164	1.89226	1.58682
47165	2.6074	2.1
47166	2.6074	1.58594
47167	1.89226	2.1
47168	1.89226	2.1
47169	1.89226	2.1
47170	1.89226	2.1
47171	3.78452	2.1
47172	1.33064	2.1
47173	1.84121	2.1
47174	0.946129	2.1
47175	0.946129	2.1
47176	0.946131	2.1
47177	0.946131	2.1
47178	1.30916	2.1
47179	1.8412	2.1
47180	1.33064	2.1
47181	0.94613	2.1
47182	1.30916	2.1
47183	0.946131	2.1
47184	1.19283	1.34524
47185	1.19283	1.15423
47186	0.946131	2.1
47187	0.946131	2.1
47188	0.946132	2.1
47189	0.946132	1.23996
47190	1.3037	2.1
47191	1.19283	1.19276
47192	1.19283	2.0877
47193	0.946131	2.08652
47194	0.946131	2.05272
47195	1.3037	2.1
47196	1.89226	2.1
47197	1.89226	2.1
47198	1.89226	2.1
47199	1.89226	2.1
47200	1.89226	2.1
47201	1.89226	2.1
47202	2.17708	2.05451
47203	1.70302	2.1
47204	2.28188	1.96351
47205	2.13621	2.1
47206	4.28253	2.1
47207	1.89226	1.35091
47208	1.89226	2.1
47209	1.89226	2.01132

47210	1.89226	2.1
47211	3.78452	2.1
47212	0.946132	2.1
47213	0.946132	2.1
47214	0.946132	2.1
47215	0.946132	2.1
47216	0.946131	2.1
47217	0.946131	2.1
47218	0.946132	2.1
47219	0.946132	2.1
47220	0.946132	2.1
47221	0.946132	2.1
47222	0.946132	2.1
47223	0.946132	2.1
47224	1.74933	2.1
47225	1.11833	2.1
47226	1.90074	2.1
47227	1.669	2.1
47228	1.335	2.1
47229	1.11833	2.1
47230	2.28188	1.41423
47231	2.31804	2.1
47232	2.42758	1.93497
47233	2.14895	2.1
47234	4.43083	2.1
47235	1.89226	1.49109
47236	1.89226	2.1
47237	1.89226	1.2556
47238	1.89226	2.1
47239	3.78452	2.1
47240	1.89226	2.1
47241	1.89226	2.1
47242	1.89226	2.1
47243	1.89226	2.1
47244	1.89226	2.1
47245	1.89226	2.1
47246	0.864133	2.1
47247	0.864133	2.1
47248	0.967939	2.1
47249	0.793739	2.1
47250	0.650797	2.1
47251	0.757546	2.1
47252	1.08941	2.1
47253	1.10136	2.1
47254	1.10138	2.1
47255	0.796445	2.1
47256	0.866907	2.1
47257	0.650788	2.1
47258	2.63062	0.0514198
47259	1.89226	0.0611013
47260	1.89226	0.05
47261	1.89226	0.292217
47262	2.46557	0.05
47263	2.46557	0.384805
47264	1.16777	0.0832427
47265	1.38859	2.1
47266	1.05042	2.09966
47267	1.28909	2.1

47268	2.45686	2.1
47269	1.89227	0.140609
47270	1.89227	0.441968
47271	1.89227	0.05
47272	1.89227	2.1
47273	3.78453	0.15086
47274	2.15114	2.1
47275	1.89226	2.1
47276	2.15005	2.1
47277	2.15005	2.1
47278	1.89226	1.73677
47279	1.89226	2.1
47280	1.89226	2.1
47281	1.89226	0.850898
47282	1.89226	2.1
47283	1.89226	2.1
47284	1.89226	0.237862
47285	1.89226	2.1
47286	0.973447	2.1
47287	1.32143	2.1
47288	1.37444	2.1
47289	1.37444	2.1
47290	0.949134	2.1
47291	0.949134	2.1
47292	0.946131	0.894596
47293	0.946131	2.1
47294	1.07557	2.1
47295	1.07557	2.1
47296	0.946131	2.1
47297	0.946131	2.1
47298	1.07557	2.1
47299	1.08579	2.1
47300	1.08579	2.1
47301	0.946131	2.1
47302	1.07557	2.1
47303	0.946131	2.1
47304	0.946131	0.0766037
47305	0.946131	2.1
47306	0.946131	2.01225
47307	0.946131	2.03152
47308	0.94613	0.739913
47309	0.94613	1.37925
47310	0.946131	2.1
47311	0.946131	2.1
47312	0.946131	1.82323
47313	0.946131	1.70548
47314	0.946131	1.79074
47315	0.946131	2.08213
47316	0.606115	2.1
47317	0.606115	2.01993
47318	0.616308	2.1
47319	0.616308	2.1
47320	0.68722	0.650636
47321	0.735699	2.1
47322	0.514789	2.1
47323	0.486724	2.1
47324	0.514788	1.50334
47325	0.735699	1.8411

47326	0.486723	2.1
47327	0.687221	2.02366
47328	2.33761	0.587716
47329	2.51749	0.124106
47330	2.68092	0.289622
47331	2.58304	0.139125
47332	5.01853	0.199327
47333	2.50906	0.326489
47334	2.51749	0.141431
47335	2.68091	0.309918
47336	2.53176	0.086414
47337	5.18997	0.227773
47338	2.68092	0.287086
47339	2.58304	0.108228
47340	2.71793	0.250392
47341	2.67098	0.0698919
47342	5.39885	0.161835
47343	2.68091	0.31021
47344	2.53175	0.0758973
47345	2.36417	0.202323
47346	2.67098	0.0772803
47347	5.04508	0.130729
47348	2.76309	2.1
47349	1.89226	2.1
47350	1.89226	2.1
47351	1.89226	2.1
47352	2.48573	2.1
47353	2.48573	2.1
47354	1.35879	2.1
47355	1.55161	0.05
47356	1.10407	2.1
47357	1.32259	0.0510092
47358	2.68138	1.40598
47359	1.89226	2.1
47360	1.89226	0.051694
47361	1.89226	2.1
47362	1.89226	0.0507782
47363	3.78452	0.532208
47364	1.19612	2.06384
47365	1.24694	2.1
47366	1.24844	0.0687469
47367	1.29747	2.1
47368	2.49358	2.1
47369	1.89226	2.1
47370	1.89226	2.1
47371	1.89226	0.05
47372	1.89226	2.1
47373	3.78453	1.91197
47374	1.89226	2.1
47375	1.89226	2.1
47376	1.89226	2.1
47377	1.89226	2.1
47378	1.89226	2.1
47379	1.89226	2.1
47380	0.587463	2.1
47381	0.587463	2.1
47382	0.581977	2.1
47383	0.581977	2.1

47384	0.531709	2.1
47385	0.545972	2.1
47386	0.623469	2.1
47387	0.648733	2.1
47388	0.648733	2.1
47389	0.545971	2.1
47390	0.623469	2.1
47391	0.531709	2.1
47392	1.29417	0.085655
47393	1.29747	2.1
47394	1.27829	2.1
47395	1.24844	1.12246
47396	1.32732	2.1
47397	1.29747	0.878667
47398	1.89226	0.321951
47399	1.89226	2.1
47400	1.89226	0.0548541
47401	1.89226	1.26909
47402	1.89226	1.13209
47403	1.89226	2.1
47404	1.89226	2.1
47405	1.89226	2.1
47406	1.89226	1.91479
47407	1.89226	2.1
47408	1.89226	2.1
47409	1.89226	1.37371
47410	0.60346	2.1
47411	0.60346	2.1
47412	0.60086	1.02827
47413	0.60086	2.1
47414	0.545971	2.1
47415	0.555587	2.1
47416	0.648733	2.1
47417	0.663661	2.1
47418	0.663661	2.1
47419	0.555587	2.1
47420	0.648733	2.1
47421	0.545971	2.1
47422	1.34066	1.99548
47423	1.36601	2.1
47424	1.34955	0.0575086
47425	1.37623	2.1
47426	2.71556	2.1
47427	1.89226	1.2584
47428	1.89226	2.1
47429	1.89226	0.0589967
47430	1.89226	2.09022
47431	3.78452	0.946182
47432	0.946132	2.1
47433	0.946132	2.1
47434	0.946132	0.271688
47435	0.946132	2.1
47436	0.946132	2.1
47437	0.946132	2.1
47438	0.946131	1.59351
47439	0.946131	1.41393
47440	0.946132	2.1
47441	0.946132	2.1

47442	0.946132	2.1
47443	0.946132	2.1
47444	0.631351	1.06325
47445	0.631351	2.1
47446	0.632421	2.1
47447	0.632421	2.1
47448	0.573604	2.1
47449	0.580765	2.1
47450	0.683006	0.679658
47451	0.688118	2.1
47452	0.688118	2.1
47453	0.580765	2.1
47454	0.683007	2.1
47455	0.573604	2.1
47456	1.34955	1.92485
47457	1.37624	2.1
47458	1.35048	0.0676404
47459	1.37999	2.1
47460	2.72954	1.80593
47461	0.946131	1.97799
47462	0.946131	0.985133
47463	0.946131	2.1
47464	0.946131	2.1
47465	0.94613	2.1
47466	0.94613	1.89694
47467	0.946133	0.494796
47468	0.946133	1.30522
47469	0.946131	1.78157
47470	0.946131	0.940138
47471	0.946132	1.01728
47472	0.946132	1.11857
47473	1.89226	2.08399
47474	1.89226	2.1
47475	1.89226	2.1
47476	1.89226	2.1
47477	1.89226	2.1
47478	1.89226	2.1
47479	0.532189	1.51358
47480	0.531141	2.1
47481	0.497784	2.1
47482	0.502536	2.1
47483	1.37999	2.1
47484	0.92433	2.1
47485	0.670081	2.1
47486	0.727155	2.1
47487	0.786438	1.45876
47488	0.589798	2.1
47489	0.497784	2.1
47490	1.89226	2.1
47491	2.72301	2.1
47492	2.4158	2.1
47493	2.4158	2.1
47494	1.89226	2.1
47495	1.89226	2.1
47496	1.89226	2.1
47497	1.89226	2.1
47498	1.89226	2.1
47499	1.89226	2.1

47500	1.89226	2.1
47501	1.89226	2.1
47502	1.36919	2.1
47503	1.12717	2.1
47504	1.38634	2.1
47505	1.56541	2.1
47506	2.75552	2.1
47507	1.89226	2.1
47508	1.89226	2.1
47509	1.89226	2.1
47510	1.89226	2.1
47511	1.89226	2.1
47512	1.89226	2.1
47513	0.907036	2.1
47514	0.782706	2.1
47515	0.838808	2.1
47516	0.693168	2.1
47517	0.794747	2.1
47518	0.794747	2.1
47519	0.805458	2.1
47520	0.805458	2.1
47521	0.907036	2.1
47522	0.838808	2.1
47523	0.693169	2.1
47524	0.782707	2.1
47525	0.94613	2.1
47526	0.94613	2.1
47527	1.188	2.1
47528	1.28421	2.1
47529	1.18801	2.1
47530	1.18801	2.1
47531	0.946129	2.1
47532	0.946129	2.1
47533	1.28421	2.1
47534	0.94613	2.1
47535	1.188	2.1
47536	0.94613	2.1
47537	1.89226	2.1
47538	1.89226	2.1
47539	1.89226	2.1
47540	1.89226	2.1
47541	1.89226	2.1
47542	1.89226	2.1
47543	1.16817	2.1
47544	1.06007	2.1
47545	1.19785	2.1
47546	1.2832	2.1
47547	2.36602	2.1
47548	0.960106	2.1
47549	2.0073	2.1
47550	1.51754	2.1
47551	2.56883	2.1
47552	3.52484	2.1
47553	1.06756	2.1
47554	1.57913	2.1
47555	1.87532	2.1
47556	1.38666	2.1
47557	2.94287	2.1

47558	2.15622	1.73
47559	1.38668	2.1
47560	1.87534	2.1
47561	1.87534	2.1
47562	1.70539	2.1
47563	1.70539	2.1
47564	0.986095	2.1
47565	1.36226	2.1
47566	1.3331	2.1
47567	1.3331	2.1
47568	1.07156	2.1
47569	1.07156	2.1
47570	0.682081	1.48041
47571	0.68748	1.14249
47572	0.650003	0.10125
47573	0.645873	0.347888
47574	0.666344	0.0885193
47575	0.666344	0.904607
47576	0.66161	2.05632
47577	0.66161	0.132666
47578	0.68748	0.368131
47579	0.645873	0.335981
47580	0.650004	0.552027
47581	0.682082	0.476869
47582	0.946132	1.24464
47583	0.946132	0.382209
47584	0.946132	0.126056
47585	0.946132	0.0526871
47586	0.946132	0.0543065
47587	0.946132	0.35186
47588	0.946133	0.183727
47589	0.946133	1.26624
47590	0.946132	0.145571
47591	0.946132	0.109578
47592	0.946133	0.447028
47593	0.946133	0.341558
47594	1.89227	1.1111
47595	2.79312	0.0546879
47596	2.77591	0.168956
47597	2.79312	0.264043
47598	1.87505	0.0748347
47599	1.89227	0.120512
47600	0.68748	1.68116
47601	0.698626	1.22945
47602	0.645873	0.165431
47603	0.650148	0.315878
47604	0.670561	0.148513
47605	0.670561	1.19671
47606	0.667067	2.05208
47607	0.667067	0.116031
47608	0.698627	0.494821
47609	0.650148	0.483418
47610	0.68748	0.669838
47611	0.645873	0.644724
47612	0.946132	1.48471
47613	0.946132	0.433949
47614	0.946132	0.343707
47615	0.946132	0.0673992

47616	0.946132	0.0647045
47617	0.946132	0.401531
47618	0.946133	0.356585
47619	0.946133	1.57907
47620	0.946133	0.258619
47621	0.946133	0.276222
47622	0.946132	0.787575
47623	0.946132	0.533031
47624	0.946133	0.684669
47625	0.946133	0.0950036
47626	1.38709	0.427864
47627	1.38795	0.0617781
47628	0.946132	0.0646949
47629	0.946132	0.162253
47630	1.38709	0.83327
47631	1.38709	0.0698309
47632	1.38709	0.162362
47633	0.946133	0.208263
47634	0.946132	0.447114
47635	1.38795	0.228563
47636	1.70301	0.05
47637	2.07659	0.05
47638	1.89226	0.05
47639	1.89226	0.05
47640	1.76332	0.05
47641	1.76331	0.05
47642	1.89226	0.05
47643	1.89226	0.05
47644	1.89226	0.05
47645	1.89226	0.05
47646	1.89226	0.05
47647	1.89226	0.05
47648	1.89226	0.05
47649	1.89226	0.05
47650	1.89226	0.05
47651	1.89226	0.05
47652	3.78452	0.05
47653	1.89226	0.05
47654	2.12492	0.05
47655	1.89226	0.05
47656	1.89226	0.05
47657	2.07659	0.05
47658	2.07659	0.05
47659	0.94613	0.05
47660	0.94613	0.05
47661	1.17498	0.05
47662	1.32446	0.05
47663	1.32446	0.05
47664	0.94613	0.05
47665	0.946127	0.05
47666	1.17498	0.05
47667	1.32446	0.05
47668	1.32446	0.05
47669	0.946128	0.05
47670	0.946128	0.05
47671	1.89226	2.1
47672	1.86472	2.1
47673	2.11049	2.1

47674	1.89226	2.1
47675	2.08295	2.1
47676	1.86472	2.1
47677	1.89226	2.1
47678	2.08295	2.1
47679	2.14023	2.1
47680	1.89226	2.1
47681	2.33091	2.1
47682	2.08295	2.1
47683	0.809215	2.1
47684	0.809215	2.1
47685	0.810915	2.1
47686	0.810915	2.1
47687	1.09581	2.1
47688	1.08271	2.1
47689	0.520996	2.1
47690	0.537426	2.1
47691	0.520997	2.1
47692	1.08271	2.1
47693	0.537426	2.1
47694	1.09581	2.1
47695	0.79674	2.1
47696	0.79674	2.1
47697	0.799279	2.1
47698	0.799279	2.1
47699	1.07502	2.1
47700	1.0827	2.1
47701	0.508206	2.1
47702	0.520996	2.1
47703	0.508206	2.1
47704	1.07502	2.1
47705	0.520996	2.1
47706	1.0827	2.1
47707	2.15714	2.1
47708	1.70302	2.1
47709	1.78732	2.1
47710	2.02219	2.1
47711	3.98971	2.1
47712	2.0927	2.1
47713	2.162	2.1
47714	2.11563	2.1
47715	2.02219	2.1
47716	4.18419	2.1
47717	1.62483	2.1
47718	2.02219	2.1
47719	1.93617	2.1
47720	1.52008	2.1
47721	3.54227	2.1
47722	2.11563	2.1
47723	2.02219	2.1
47724	2.29218	2.1
47725	2.03975	2.1
47726	4.06194	2.1
47727	2.007	2.1
47728	1.89226	2.1
47729	1.89226	2.1
47730	1.89226	2.1
47731	2.06776	2.1

47732	2.06776	2.1
47733	0.946129	2.1
47734	0.946129	2.1
47735	1.0035	2.1
47736	1.0035	2.1
47737	0.946131	2.1
47738	0.946131	2.1
47739	0.968797	2.1
47740	1.0035	2.1
47741	0.968771	2.1
47742	0.946105	2.1
47743	1.00353	2.1
47744	0.946157	2.1
47745	1.79103	2.1
47746	2.53286	0.0941823
47747	2.60781	2.1
47748	2.219	0.566719
47749	2.17985	1.59727
47750	1.79103	0.0936994
47751	2.219	1.78561
47752	2.66359	1.68433
47753	2.70084	2.1
47754	2.38698	1.2683
47755	2.53285	0.598475
47756	2.219	0.196825
47757	1.33179	1.51712
47758	0.946128	0.512446
47759	0.946133	1.06993
47760	1.26643	2.08163
47761	1.3318	0.722977
47762	1.3318	1.9487
47763	0.946131	0.24618
47764	0.946131	1.5201
47765	0.946131	0.314803
47766	0.946131	0.147396
47767	1.33179	0.758132
47768	1.26643	2.1
47769	2.28979	2.1
47770	2.47227	2.1
47771	2.43451	2.1
47772	2.22627	2.1
47773	4.69855	2.1
47774	2.40667	2.1
47775	2.47228	2.1
47776	2.43451	2.1
47777	2.38069	2.1
47778	4.85296	2.1
47779	2.43451	2.1
47780	2.15715	2.1
47781	2.15715	2.1
47782	2.22627	2.1
47783	2.39489	2.1
47784	2.46402	2.1
47785	2.43451	2.1
47786	2.34899	2.1
47787	2.38069	2.1
47788	2.38069	2.1
47789	2.3949	2.1

47790	2.3949	2.1
47791	2.85425	1.61162
47792	1.70302	0.325066
47793	1.70302	0.0685224
47794	1.52254	0.120581
47795	2.34181	0.104895
47796	2.08499	0.463323
47797	1.89226	0.0893349
47798	1.89226	0.373146
47799	1.89226	0.05
47800	1.89226	0.107306
47801	1.89226	0.0638791
47802	1.89226	1.01551
47803	2.34181	0.313365
47804	1.38287	0.994613
47805	1.38287	0.140847
47806	1.84132	0.352909
47807	1.96416	0.124314
47808	2.4907	0.637241
47809	0.931432	1.05679
47810	0.946135	0.0529139
47811	0.946133	0.0546101
47812	0.946133	0.05
47813	0.931464	0.309071
47814	0.931464	1.02387
47815	1.15573	0.05
47816	0.96378	1.53431
47817	0.931434	0.433563
47818	0.946131	0.05
47819	0.946131	0.0536898
47820	1.13808	0.05
47821	0.946132	0.658497
47822	0.946132	0.11705
47823	0.946132	0.371884
47824	0.946132	0.05
47825	0.946133	0.485893
47826	0.946133	0.0558382
47827	0.946132	1.13677
47828	0.946132	0.174233
47829	0.946133	0.130978
47830	0.946133	0.748412
47831	0.946132	0.0516063
47832	0.946132	0.05
47833	1.68074	2.1
47834	2.29459	2.1
47835	2.23361	2.1
47836	1.57234	2.1
47837	3.86693	2.1
47838	2.61243	2.1
47839	2.29458	2.1
47840	2.2336	2.1
47841	2.58234	2.1
47842	4.87692	2.1
47843	0.73506	2.1
47844	0.786166	2.1
47845	1.08729	2.1
47846	1.11679	2.1
47847	0.93134	2.1

47848	0.93134	2.1
47849	0.942113	2.1
47850	0.942113	2.1
47851	0.735065	2.1
47852	1.08729	2.1
47853	1.11679	2.1
47854	0.78616	2.1
47855	1.08729	2.1
47856	1.11679	2.1
47857	1.27502	2.1
47858	1.29116	2.1
47859	1.19259	2.1
47860	1.19259	2.1
47861	1.19923	2.1
47862	1.19923	2.1
47863	1.27503	2.1
47864	1.08729	2.1
47865	1.11679	2.1
47866	1.29115	2.1
47867	0.974715	0.753777
47868	1.43779	0.243187
47869	1.93915	0.298431
47870	1.53908	0.283683
47871	2.91387	0.26232
47872	1.53908	0.173972
47873	0.99845	0.198897
47874	1.5176	0.71896
47875	1.97963	0.166603
47876	3.05668	0.250784
47877	1.93915	0.274496
47878	1.97963	0.157073
47879	1.53908	0.20117
47880	1.53908	0.079939
47881	2.3376	0.760604
47882	2.3376	0.208955
47883	1.89226	2.1
47884	2.59747	2.1
47885	1.91802	2.1
47886	1.89226	2.1
47887	2.62322	2.1
47888	2.59747	2.1
47889	2.59747	2.1
47890	1.87119	2.1
47891	1.84844	2.1
47892	1.82268	2.1
47893	2.62323	2.1
47894	2.59747	2.1
47895	0.94613	2.1
47896	0.94613	2.1
47897	1.32697	2.1
47898	1.32697	2.1
47899	1.32697	2.1
47900	1.31161	2.1
47901	0.946132	2.1
47902	0.946132	2.1
47903	0.94613	2.1
47904	1.32697	2.1
47905	0.946131	2.1

47906	1.31161	2.1
47907	2.62323	2.1
47908	1.91704	2.1
47909	1.90191	2.1
47910	1.87119	2.1
47911	2.65395	2.1
47912	2.62323	2.1
47913	1.89226	2.1
47914	1.89226	2.1
47915	2.15958	2.1
47916	2.15958	2.1
47917	2.46558	2.1
47918	2.19826	2.1
47919	2.46558	2.1
47920	1.89226	2.1
47921	1.89226	2.1
47922	1.89226	2.1
47923	2.61064	2.1
47924	2.61064	2.1
47925	1.81759	0.402594
47926	1.89526	0.470815
47927	2.32219	1.14018
47928	1.881	0.220326
47929	1.81759	0.671325
47930	1.3764	0.152841
47931	1.81759	0.477213
47932	2.57079	0.283254
47933	2.32219	1.42051
47934	2.32219	0.367557
47935	1.99875	0.727645
47936	1.99875	0.159891
47937	0.536182	0.0946861
47938	0.579206	0.05
47939	0.908796	0.389038
47940	0.999375	0.166421
47941	0.732091	0.243964
47942	0.732091	0.05
47943	0.755911	0.0884634
47944	0.755911	0.266767
47945	0.999376	0.141706
47946	0.579206	0.216095
47947	0.908796	0.157177
47948	0.536182	0.136921
47949	0.94613	0.0867475
47950	0.94613	0.104937
47951	1.31112	0.162046
47952	1.18275	0.0607743
47953	0.946126	0.0792249
47954	0.946126	0.05
47955	1.31112	0.143821
47956	1.31112	0.0512297
47957	0.946128	0.0978625
47958	1.31112	0.130004
47959	0.946128	0.0878716
47960	1.18275	0.05
47961	2.64617	0.32306
47962	1.27737	0.05
47963	1.83076	0.142089

47964	1.27737	0.186218
47965	3.03764	0.0887839
47966	2.3655	0.05
47967	1.12991	0.05
47968	1.22375	0.05
47969	1.35931	0.05
47970	1.04971	0.05
47971	1.0419	0.05
47972	1.15237	0.05
47973	1.19593	0.05
47974	1.30306	0.05
47975	1.12937	0.05
47976	1.31934	0.05
47977	1.15203	0.05
47978	1.15203	0.05
47979	2.65227	0.05
47980	1.46041	0.05
47981	2.22712	0.05
47982	2.04816	0.05
47983	2.22712	0.05
47984	2.04816	0.05
47985	1.89226	0.05
47986	1.89226	0.112417
47987	1.89226	0.05
47988	1.89226	0.05
47989	3.78452	0.052399
47990	1.24653	0.0693408
47991	1.66554	0.0811247
47992	1.53538	0.05
47993	1.53538	1.22494
47994	1.40915	0.05
47995	1.40915	0.986811
47996	1.66555	0.0754269
47997	1.75835	2.1
47998	1.53538	1.80909
47999	1.65313	2.1
48000	3.31868	2.1
48001	1.89226	0.100715
48002	1.89226	0.270636
48003	1.89226	0.05
48004	1.89226	2.1
48005	3.78453	0.11804
48006	1.89226	0.05
48007	1.89226	0.05
48008	1.89226	0.05
48009	1.89226	0.31519
48010	3.78452	0.05
48011	0.658778	2.1
48012	0.658778	0.05
48013	0.661956	0.05
48014	0.661956	0.184902
48015	0.703822	0.125106
48016	0.767689	2.03116
48017	0.623266	0.05
48018	0.553046	0.0548223
48019	0.703804	0.0765801
48020	0.55303	0.0837363
48021	0.623282	0.0525289

48022	0.767711	0.0526287
48023	1.53538	1.24871
48024	1.65313	2.1
48025	1.40765	0.113695
48026	1.55779	2.1
48027	3.06078	2.06761
48028	1.89227	0.05
48029	1.89227	2.1
48030	1.89227	0.224773
48031	1.89227	0.254104
48032	3.78453	0.476251
48033	1.10517	2.1
48034	2.16532	2.1
48035	1.94374	2.1
48036	1.94001	2.1
48037	1.37756	2.1
48038	1.37756	2.1
48039	1.7453	0.05
48040	1.83284	0.05
48041	2.28483	0.05
48042	1.77804	0.05
48043	1.7453	0.05
48044	1.23851	0.05
48045	1.7453	0.05
48046	2.54752	0.05
48047	2.28483	0.05
48048	2.28483	0.05
48049	2.0189	0.05
48050	2.0189	0.05
48051	1.12457	0.05
48052	2.0189	0.05
48053	1.7453	0.05
48054	1.7453	0.05
48055	1.40707	0.05
48056	1.40707	0.05
48057	2.41176	2.1
48058	2.59262	2.1
48059	2.34085	2.1
48060	2.55827	2.1
48061	4.97003	2.1
48062	2.59262	2.1
48063	2.61901	2.1
48064	2.55827	2.1
48065	2.64067	2.1
48066	5.17728	2.1
48067	2.34084	2.1
48068	2.55827	2.1
48069	2.24937	2.1
48070	2.49053	2.1
48071	4.80764	2.1
48072	2.55826	2.1
48073	2.64067	2.1
48074	2.49053	2.1
48075	2.58639	2.1
48076	5.14465	2.1
48077	2.27918	0.05
48078	2.44132	0.05
48079	1.89226	0.05

48080	1.89226	0.05
48081	4.33359	0.05
48082	1.89226	0.05
48083	2.44132	0.05
48084	2.6771	0.05
48085	2.6771	0.05
48086	1.89226	0.05
48087	1.89226	0.05
48088	1.60329	0.05
48089	1.38246	0.05
48090	1.98182	0.05
48091	2.2217	0.05
48092	3.60417	0.05
48093	2.05362	0.05
48094	1.02663	0.05
48095	1.43255	0.05
48096	1.52072	0.05
48097	1.70302	0.05
48098	1.7685	0.05
48099	0.946131	0.05
48100	0.946131	0.05
48101	0.946131	0.05
48102	0.946131	0.05
48103	0.946131	0.05
48104	0.946131	0.05
48105	0.946131	0.05
48106	0.946131	0.05
48107	0.946131	0.05
48108	0.946131	0.05
48109	0.946131	0.05
48110	0.946131	0.05
48111	1.89226	0.05
48112	1.89226	0.05
48113	1.89226	0.05
48114	1.89226	0.05
48115	1.89226	0.05
48116	1.89226	0.05
48117	1.92645	0.112641
48118	2.83045	0.0797905
48119	1.92225	0.151651
48120	2.01873	0.0516648
48121	2.51732	0.204252
48122	2.51732	0.0668612
48123	2.51732	0.0519235
48124	2.23857	0.05
48125	1.92645	0.0847729
48126	1.92645	0.05
48127	2.23857	0.130513
48128	1.50865	0.05
48129	2.02687	2.1
48130	2.39604	2.1
48131	2.29198	2.1
48132	2.73046	2.1
48133	4.68803	2.1
48134	1.21148	0.05
48135	1.87957	0.180345
48136	2.03555	0.05
48137	2.03555	0.116005

48138	1.09962	0.05
48139	1.09962	0.849416
48140	1.92558	0.05
48141	2.05274	1.69695
48142	2.2443	0.05
48143	2.24429	0.67505
48144	1.70869	0.05
48145	1.70868	1.40906
48146	0.818397	0.05
48147	0.818397	0.219279
48148	0.827911	0.05
48149	0.827908	0.260129
48150	1.00418	0.151348
48151	1.02054	0.102512
48152	0.692018	0.0757784
48153	0.620899	0.0662445
48154	0.65823	0.05
48155	1.00418	0.05
48156	0.620899	0.0702521
48157	1.0262	0.05
48158	1.92558	0.509252
48159	2.51818	1.40664
48160	1.94816	0.0576548
48161	2.01985	0.695633
48162	1.85389	0.05
48163	1.92558	0.727495
48164	0.903844	2.1
48165	0.903844	2.1
48166	0.946148	2.1
48167	0.946151	2.1
48168	0.946118	2.1
48169	0.903876	2.1
48170	0.946118	2.1
48171	1.10235	2.1
48172	0.946131	2.1
48173	0.946131	2.1
48174	1.10234	2.1
48175	0.903863	2.1
48176	1.63471	2.1
48177	1.08022	2.1
48178	1.56801	2.1
48179	1.03437	2.1
48180	2.66908	2.1
48181	1.56801	2.1
48182	0.97541	2.1
48183	0.97541	2.1
48184	1.03437	2.1
48185	1.49733	2.1
48186	1.55629	2.1
48187	1.89226	0.05
48188	1.89226	0.05
48189	1.89226	0.05
48190	1.89226	0.05
48191	3.78452	0.05
48192	0.946131	0.05
48193	1.16373	0.05
48194	0.946131	0.05
48195	0.734637	0.05

48196	0.946132	0.05
48197	0.946132	0.05
48198	1.16373	0.05
48199	0.734636	0.05
48200	0.946093	0.05
48201	0.946093	0.05
48202	0.734675	0.05
48203	0.734675	0.05
48204	1.98413	0.05
48205	1.87355	0.0604305
48206	1.72774	0.05
48207	1.67865	0.0566233
48208	3.60128	0.05
48209	1.87355	0.05
48210	1.54924	2.1
48211	1.68443	2.1
48212	1.68443	0.05
48213	1.67866	2.1
48214	1.67866	0.05
48215	1.72774	0.05
48216	1.53422	0.0518609
48217	1.67865	0.05
48218	1.67865	0.05
48219	1.53873	0.05
48220	1.53873	0.05
48221	1.67866	0.05
48222	1.54924	2.1
48223	1.53423	0.05
48224	1.44091	2.1
48225	3.11957	0.385843
48226	1.89226	2.1
48227	1.89226	1.79128
48228	1.65573	2.08286
48229	1.65573	2.1
48230	1.89226	2.1
48231	1.89226	2.1
48232	1.89226	2.1
48233	1.89614	1.1185
48234	1.7508	1.8026
48235	1.72666	1.86112
48236	1.89226	1.92159
48237	1.89226	2.04737
48238	1.89223	2.1
48239	1.89223	1.45137
48240	1.89223	1.60733
48241	1.89223	1.96399
48242	1.89223	1.74811
48243	1.89223	2.1
48244	1.89224	2.05892
48245	1.89224	0.709961
48246	1.89224	1.38592
48247	1.89224	1.38141
48248	1.89224	1.72629
48249	1.89224	1.86551
48250	2.09773	2.1
48251	2.06623	2.1
48252	2.43268	2.1
48253	2.33713	2.1

48254	1.703	2.1
48255	1.61372	2.1
48256	0.646929	2.1
48257	0.689776	2.1
48258	1.05897	2.1
48259	0.956364	2.1
48260	0.905594	2.1
48261	0.904315	2.1
48262	0.727014	2.1
48263	0.850757	2.1
48264	0.798445	2.1
48265	0.654101	2.1
48266	0.717539	2.1
48267	1.0478	2.1
48268	1.89223	2.09896
48269	1.89223	0.706429
48270	1.89223	2.1
48271	1.6557	0.05
48272	3.54794	1.13993
48273	0.946118	1.94425
48274	0.946118	2.1
48275	0.946118	2.1
48276	0.946118	2.1
48277	0.946117	2.1
48278	0.946117	0.592535
48279	0.946119	2.1
48280	0.946119	2.1
48281	0.946105	2.1
48282	0.946105	2.1
48283	0.946131	2.1
48284	0.946131	2.1
48285	1.47671	2.1
48286	1.70303	2.1
48287	1.90705	2.1
48288	1.98945	2.1
48289	1.3454	2.1
48290	1.40353	2.1
48291	1.89226	2.1
48292	1.89226	2.1
48293	1.89226	2.1
48294	1.89226	1.84036
48295	1.89226	2.1
48296	1.89226	0.903576
48297	2.39438	0.05
48298	2.69842	0.05
48299	2.45177	0.05
48300	2.28563	0.05
48301	4.84615	0.05
48302	1.28421	2.1
48303	1.35966	2.1
48304	0.961001	2.1
48305	0.860549	2.1
48306	1.1039	2.1
48307	1.1039	2.1
48308	1.11631	2.1
48309	1.11631	2.1
48310	1.35966	2.1
48311	0.961001	2.1

48312	0.860549	2.1
48313	1.28421	2.1
48314	1.89226	1.44737
48315	1.89226	2.1
48316	1.89226	2.1
48317	1.89226	0.728946
48318	3.78452	1.60563
48319	1.48702	0.299984
48320	1.40656	0.388306
48321	0.626442	1.75448
48322	0.626442	1.34595
48323	0.626442	1.9812
48324	0.946132	0.371557
48325	1.05489	0.14179
48326	1.05012	2.05015
48327	0.946131	1.14778
48328	0.946131	1.23618
48329	0.626442	1.33208
48330	1.05012	0.94698
48331	1.89226	2.1
48332	1.97212	0.488076
48333	1.89226	0.714092
48334	1.89226	1.48883
48335	3.78453	1.6479
48336	1.89226	2.1
48337	1.89226	0.871465
48338	1.89226	1.21976
48339	1.89226	2.03048
48340	3.78452	1.95571
48341	1.89226	0.817572
48342	1.89226	1.51824
48343	1.89226	1.94139
48344	1.89226	0.251601
48345	1.89226	1.98331
48346	1.89226	0.285487
48347	0.946161	0.130837
48348	0.946161	0.654656
48349	0.932156	1.58424
48350	0.932156	0.719472
48351	0.932184	1.2286
48352	0.946132	0.0790376
48353	0.946133	1.5608
48354	0.946133	0.255781
48355	0.946132	0.416487
48356	0.946132	0.537936
48357	0.932185	0.438954
48358	0.946133	0.247644
48359	0.946132	0.70727
48360	0.946132	0.238661
48361	0.946132	2.1
48362	0.946132	1.70699
48363	0.946132	1.55559
48364	0.946132	0.613523
48365	0.946133	0.852959
48366	0.946133	1.98325
48367	0.946132	1.24099
48368	0.946132	1.62397
48369	0.946133	0.833314

48370	0.946133	1.19882
48371	1.79397	0.0789291
48372	1.00636	0.05
48373	1.31658	0.441772
48374	2.02924	0.0620312
48375	3.0356	0.0934026
48376	1.89226	0.0984873
48377	1.89226	0.05
48378	1.89226	0.0963262
48379	1.89226	0.05
48380	1.89226	0.0721035
48381	1.89226	0.05
48382	0.946131	0.05
48383	0.946131	0.0603132
48384	1.24766	0.12231
48385	1.03691	0.060013
48386	0.946129	0.0559895
48387	0.946129	0.05
48388	1.24766	0.131194
48389	1.24766	0.0564618
48390	0.946131	0.0699481
48391	1.24766	0.123484
48392	1.03691	0.05
48393	0.946131	0.05
48394	1.44824	0.0617936
48395	2.02924	0.05
48396	1.7065	0.07755
48397	1.38604	0.33362
48398	2.32017	0.0540479
48399	2.32017	0.05
48400	1.89226	0.05
48401	1.89226	0.05
48402	1.89226	0.05
48403	1.89226	0.05
48404	1.89226	0.130596
48405	1.89226	0.0671722
48406	0.478349	0.396817
48407	0.612092	2.1
48408	0.606115	2.1
48409	0.506213	2.1
48410	0.550677	2.1
48411	0.550677	0.390758
48412	0.567628	2.1
48413	0.567628	2.1
48414	0.506213	2.1
48415	0.478349	2.1
48416	0.606116	2.1
48417	0.612092	2.1
48418	1.52226	2.1
48419	1.54241	2.1
48420	1.23167	2.1
48421	1.23167	2.1
48422	1.75555	2.1
48423	1.75555	2.1
48424	1.75557	2.1
48425	1.54243	2.1
48426	1.9943	2.1
48427	1.85871	2.1

48428	3.53673	2.1
48429	1.85871	2.1
48430	1.06936	2.1
48431	1.88926	2.1
48432	1.54243	2.1
48433	1.41619	2.1
48434	1.06936	2.1
48435	1.28649	1.91216
48436	1.28516	2.1
48437	1.38986	0.0809533
48438	1.42399	1.8039
48439	2.67502	1.5384
48440	1.42399	0.45023
48441	1.07283	2.1
48442	1.46528	2.1
48443	1.28517	0.987408
48444	1.25294	2.1
48445	1.07283	0.60493
48446	1.38986	2.1
48447	1.42399	2.1
48448	1.52885	0.419568
48449	1.60058	1.02104
48450	2.99043	1.45601
48451	0.715316	2.1
48452	0.715316	1.73076
48453	0.719126	0.069684
48454	0.719126	2.1
48455	0.722447	2.1
48456	0.626469	2.1
48457	0.711995	0.315661
48458	0.800291	0.721241
48459	0.80029	1.83911
48460	0.722447	1.27039
48461	0.711995	1.94215
48462	0.62647	1.94205
48463	2.05632	0.05
48464	1.70303	0.05
48465	2.3923	0.05
48466	2.61277	0.05
48467	1.44836	0.05
48468	1.58184	0.05
48469	1.89226	0.05
48470	1.89226	0.05
48471	1.89226	0.05
48472	1.89226	0.05
48473	3.78453	0.05
48474	1.44091	2.1
48475	1.1822	2.1
48476	1.25866	2.1
48477	1.25866	2.1
48478	1.35215	2.1
48479	1.35215	2.1
48480	1.27338	2.1
48481	1.1822	2.1
48482	1.27338	2.1
48483	1.35215	2.1
48484	1.10817	2.1
48485	1.18694	2.1

48486	0.946132	0.100866
48487	0.946132	0.0658187
48488	0.946132	0.05
48489	0.946132	0.05
48490	0.946133	0.05
48491	0.946133	0.101638
48492	0.946132	0.0649806
48493	0.946132	0.05
48494	0.946132	0.0555103
48495	0.946132	0.060642
48496	0.946133	0.0579446
48497	0.946133	0.0578582
48498	2.6875	0.05
48499	2.40558	0.05
48500	2.1994	0.05
48501	1.70303	0.05
48502	4.68305	0.05
48503	0.915523	0.05
48504	0.915523	0.05
48505	0.861057	0.05
48506	0.946796	0.05
48507	0.838652	0.05
48508	0.654182	0.05
48509	1.14044	0.05
48510	0.80252	0.05
48511	0.836435	0.05
48512	0.608659	0.05
48513	1.22198	0.05
48514	0.863472	0.05
48515	2.1994	0.05
48516	0.981586	0.05
48517	1.59619	0.05
48518	1.70303	0.05
48519	1.24816	0.05
48520	1.38523	0.05
48521	2.71141	2.1
48522	1.70302	2.1
48523	2.56527	2.1
48524	1.88649	2.1
48525	2.41279	2.1
48526	1.70302	2.1
48527	1.89226	2.1
48528	1.89226	2.1
48529	1.89226	2.1
48530	1.89226	2.1
48531	3.78453	2.1
48532	1.89226	2.1
48533	1.89226	2.1
48534	1.89226	2.1
48535	1.89226	2.1
48536	1.89226	2.1
48537	1.89226	2.1
48538	2.18867	2.1
48539	0.983723	2.1
48540	2.42263	2.1
48541	1.8014	2.1
48542	1.62992	2.1
48543	0.983723	2.1

48544	2.71141	2.1
48545	1.89226	2.1
48546	2.31157	2.1
48547	2.31157	2.1
48548	1.89226	2.1
48549	1.89226	2.1
48550	1.89227	2.1
48551	1.89227	1.67634
48552	1.89227	1.6165
48553	1.89227	2.1
48554	3.78453	2.1
48555	1.89226	2.00937
48556	1.89226	2.1
48557	1.89226	2.1
48558	1.89226	1.86284
48559	1.89226	2.1
48560	1.89226	1.81051
48561	1.89226	1.76066
48562	1.89226	1.42177
48563	1.89226	2.1
48564	1.89226	0.626692
48565	1.89226	1.55912
48566	1.89226	0.178721
48567	1.1427	2.0982
48568	1.14956	2.1
48569	1.70302	1.34824
48570	1.81267	2.0828
48571	2.88983	2.06579
48572	1.89226	0.933671
48573	1.89226	2.1
48574	1.89226	0.814419
48575	1.89226	1.46326
48576	3.78453	1.56002
48577	1.89226	1.74981
48578	1.89226	1.95323
48579	1.89226	0.465746
48580	1.89226	1.4969
48581	3.78452	1.1824
48582	0.884197	2.1
48583	1.1125	1.55684
48584	1.13273	1.51664
48585	0.844557	1.88716
48586	0.981137	1.04256
48587	1.13971	2.01702
48588	0.980432	2.02901
48589	0.980432	1.20036
48590	1.15859	1.3778
48591	1.12206	0.730738
48592	0.898619	2.1
48593	0.94426	1.59996
48594	2.21016	1.31838
48595	1.44091	2.1
48596	1.98408	1.28946
48597	1.4218	1.12059
48598	2.21016	2.1
48599	1.64788	2.1
48600	1.07526	0.105951
48601	1.91531	2.1

48602	1.49155	2.1
48603	1.58551	0.136618
48604	1.49155	0.178769
48605	1.58551	2.02591
48606	1.98979	0.05
48607	2.21773	0.05
48608	2.30533	0.05
48609	2.55895	0.05
48610	4.52305	0.05
48611	2.55896	0.05
48612	2.27963	0.05
48613	2.60486	0.05
48614	2.60486	0.05
48615	2.21773	0.05
48616	2.21773	0.05
48617	2.30533	0.05
48618	2.55895	0.05
48619	2.29539	0.05
48620	2.47308	0.05
48621	4.7784	0.05
48622	2.55896	0.05
48623	2.55792	0.05
48624	2.55792	0.05
48625	2.60486	0.05
48626	2.47308	0.05
48627	2.52003	0.05
48628	0.946133	1.87204
48629	0.946133	2.01707
48630	1.20851	1.19495
48631	1.20851	1.10183
48632	0.967411	1.01352
48633	1.20851	0.595273
48634	0.946132	2.1
48635	0.946132	1.81852
48636	0.946132	1.98025
48637	1.20851	1.13328
48638	0.967412	1.00608
48639	0.946133	1.80038
48640	1.89226	1.90631
48641	1.89226	0.968809
48642	1.89226	1.043
48643	1.89226	1.01632
48644	1.89226	1.97093
48645	1.89226	1.97442
48646	1.89226	1.96498
48647	2.41701	1.70905
48648	2.71724	0.775875
48649	2.71724	1.5818
48650	1.89226	2.1
48651	1.89226	2.1
48652	0.946131	2.07397
48653	0.946131	2.1
48654	0.946128	1.76745
48655	0.946128	1.90623
48656	0.94613	0.63366
48657	0.94613	1.07905
48658	0.94613	2.07737
48659	0.94613	2.1

48660	0.94613	1.87424
48661	0.94613	1.0323
48662	0.94613	1.39125
48663	0.94613	2.1
48664	0.851541	0.05
48665	0.851541	0.05
48666	0.851481	0.05
48667	0.997219	0.05
48668	0.997112	0.05
48669	0.992853	0.05
48670	0.85154	0.05
48671	0.85154	0.05
48672	0.894824	0.05
48673	0.894824	0.05
48674	0.94613	0.05
48675	0.906485	0.05
48676	0.802947	2.1
48677	0.845556	2.1
48678	0.582339	2.1
48679	0.519756	2.1
48680	0.714508	2.1
48681	0.714508	2.1
48682	0.628932	2.1
48683	0.708518	2.1
48684	0.801358	2.1
48685	0.476016	2.1
48686	0.569477	2.1
48687	0.878553	2.1
48688	1.89226	2.1
48689	1.89226	2.1
48690	1.89226	2.1
48691	1.89226	2.1
48692	1.89226	2.1
48693	1.89226	2.1
48694	0.946128	2.1
48695	0.946128	2.1
48696	1.01537	2.1
48697	0.9662	2.1
48698	0.946131	2.1
48699	0.946131	2.1
48700	0.966198	2.1
48701	0.966198	2.1
48702	0.94613	2.1
48703	0.966198	2.1
48704	0.946131	2.1
48705	1.01537	2.1
48706	1.89226	2.1
48707	1.89226	2.1
48708	1.91883	2.1
48709	1.91883	2.1
48710	2.15905	2.1
48711	2.13249	2.1
48712	1.07953	2.1
48713	1.2107	2.1
48714	0.946132	2.1
48715	0.946132	2.1
48716	1.2107	2.1
48717	1.2107	2.1

48718	0.946132	2.1
48719	0.946132	2.1
48720	1.2107	2.1
48721	0.946133	2.1
48722	0.946132	2.1
48723	1.07953	2.1
48724	2.46752	2.1
48725	2.53733	2.1
48726	2.26044	2.1
48727	2.33222	1.90521
48728	4.79974	2.1
48729	2.53733	2.1
48730	2.37576	0.05
48731	2.33222	2.1
48732	2.1845	2.1
48733	4.70798	2.1
48734	2.48652	2.1
48735	2.33224	1.78377
48736	2.08149	2.1
48737	1.9839	2.1
48738	4.45623	2.1
48739	2.33223	2.01692
48740	2.18452	1.55231
48741	2.16061	2.1
48742	2.01399	0.0917789
48743	4.34623	1.21888
48744	1.4617	1.38049
48745	1.34701	2.1
48746	1.34701	1.76469
48747	1.25173	2.1
48748	1.5117	1.2813
48749	1.41642	2.1
48750	2.65164	2.1
48751	1.89227	2.1
48752	1.89227	2.1
48753	1.89227	2.1
48754	2.42504	2.1
48755	2.42504	2.1
48756	1.89226	0.742833
48757	1.89226	2.00752
48758	1.89226	2.1
48759	1.89226	0.153905
48760	3.78452	1.09269
48761	1.5117	1.62615
48762	1.34701	2.1
48763	1.47001	2.1
48764	1.57961	0.283206
48765	2.92663	2.01667
48766	1.89226	2.1
48767	1.89226	0.149526
48768	1.89226	0.286153
48769	1.89226	1.74123
48770	3.78452	1.27369
48771	0.412773	0.05
48772	0.570839	0.05
48773	0.50721	0.05
48774	0.513638	0.05
48775	0.518805	0.05

48776	0.41278	0.05
48777	1.5344	0.05
48778	0.939282	0.05
48779	0.720051	0.05
48780	0.691767	0.05
48781	0.761152	0.05
48782	1.89226	2.1
48783	1.89226	2.1
48784	1.89226	2.1
48785	1.89226	2.1
48786	1.89226	2.1
48787	1.89226	2.1
48788	1.24618	2.1
48789	2.10585	2.1
48790	1.08134	2.1
48791	1.93032	2.1
48792	3.1765	2.1
48793	1.98474	2.1
48794	2.57777	2.1
48795	1.93031	2.1
48796	2.19041	2.1
48797	3.99251	2.1
48798	1.89226	2.1
48799	1.89226	2.1
48800	1.89226	2.1
48801	1.89226	2.1
48802	3.78452	2.1
48803	1.67374	2.1
48804	2.19042	2.1
48805	1.67374	2.1
48806	2.09769	2.1
48807	2.29586	2.1
48808	2.7198	2.1
48809	1.89226	2.1
48810	1.89226	2.1
48811	1.89226	2.1
48812	1.89226	2.1
48813	1.89226	2.1
48814	1.89226	2.1
48815	0.946132	0.0968589
48816	0.946132	0.0743301
48817	0.947763	0.0522193
48818	0.909304	0.0632269
48819	0.946127	0.05
48820	0.947831	0.05
48821	0.907673	0.05
48822	0.907673	0.0920233
48823	0.94613	0.0509939
48824	0.947834	0.05
48825	0.907671	0.183004
48826	0.94613	0.05
48827	0.906519	0.05
48828	0.906519	0.05
48829	0.946097	0.05
48830	0.946097	0.05
48831	0.947834	0.05
48832	0.906485	0.05
48833	0.946131	0.05

48834	0.946131	0.05
48835	0.94613	0.05
48836	0.906485	0.05
48837	0.946131	0.05
48838	0.947835	0.05
48839	2.58638	2.1
48840	2.51039	2.1
48841	2.47395	2.1
48842	2.41539	2.1
48843	5.00178	2.1
48844	2.51039	2.1
48845	2.16633	2.1
48846	2.4154	2.1
48847	2.13977	2.1
48848	4.58173	2.1
48849	2.47395	2.1
48850	2.41539	2.1
48851	2.2988	2.1
48852	2.2604	2.1
48853	4.7142	2.1
48854	2.4154	2.1
48855	2.13977	2.1
48856	2.26041	2.1
48857	1.98957	2.1
48858	4.40497	2.1
48859	2.15721	0.05
48860	2.31843	0.05
48861	1.93973	0.0562361
48862	2.13243	0.05
48863	4.25816	0.05
48864	2.31844	0.0687628
48865	2.30908	0.05
48866	2.13243	0.05
48867	2.1609	0.05
48868	4.47934	0.05
48869	1.93973	0.146053
48870	2.13243	0.05
48871	1.69661	0.0612783
48872	1.9339	0.05
48873	3.87363	0.05
48874	2.13243	0.05
48875	2.1609	0.05
48876	1.9339	0.05
48877	1.98414	0.05
48878	4.0948	0.05
48879	1.88857	0.05
48880	2.34107	0.05
48881	0.502431	0.05
48882	0.71416	0.05
48883	0.50243	0.05
48884	0.944284	0.05
48885	1.88856	2.1
48886	2.34107	2.1
48887	2.39662	2.1
48888	1.92293	2.1
48889	4.28519	2.1
48890	1.00486	2.1
48891	1.92293	2.1

48892	1.88857	2.1
48893	1.88857	2.1
48894	1.02605	2.1
48895	1.02605	2.1
48896	2.53728	2.1
48897	1.70303	2.1
48898	2.03734	2.1
48899	1.3624	2.1
48900	2.54706	2.1
48901	1.70303	2.1
48902	2.54706	2.1
48903	1.50876	2.1
48904	2.74067	2.1
48905	1.85391	2.1
48906	2.29667	2.1
48907	1.50876	2.1
48908	0.946133	2.1
48909	0.946133	2.1
48910	1.24191	2.1
48911	1.24191	2.1
48912	0.946132	2.1
48913	1.24191	2.1
48914	1.23779	2.1
48915	0.946132	2.1
48916	0.946132	2.1
48917	0.946132	2.1
48918	1.23779	2.1
48919	1.24191	2.1
48920	0.946131	2.1
48921	0.946131	2.1
48922	1.14833	2.1
48923	1.14833	2.1
48924	1.14834	2.1
48925	0.946132	2.1
48926	0.94613	2.1
48927	1.24191	2.1
48928	0.946131	2.1
48929	0.946131	2.1
48930	1.24191	2.1
48931	1.14833	2.1
48932	2.08149	2.1
48933	2.31116	2.1
48934	1.9701	1.13707
48935	2.18328	2.1
48936	4.49563	2.1
48937	2.08149	2.1
48938	2.14059	2.1
48939	1.72377	2.1
48940	2.18328	2.1
48941	3.88931	2.1
48942	2.14059	2.1
48943	2.40755	2.1
48944	2.31145	2.1
48945	2.23743	2.1
48946	2.31145	2.1
48947	2.23743	2.1
48948	2.31117	2.1
48949	2.40754	2.1

48950	2.41055	2.1
48951	2.31145	2.1
48952	2.41055	2.1
48953	2.31145	2.1
48954	2.5615	0.0881833
48955	2.37625	0.0669342
48956	2.20248	0.0598531
48957	2.20248	0.0501428
48958	2.65967	0.166196
48959	2.65967	0.126511
48960	2.71793	0.185368
48961	2.65966	0.0562078
48962	2.56149	0.05
48963	2.56149	0.05
48964	2.71296	0.0919101
48965	2.71296	0.111649
48966	1.31528	0.0804585
48967	1.32983	0.0620199
48968	1.21128	0.0707943
48969	1.18812	0.0600445
48970	1.24232	0.05
48971	1.24232	0.0556004
48972	1.26109	0.0797819
48973	1.26109	0.122105
48974	1.21128	0.0983057
48975	1.31528	0.05
48976	1.18812	0.05
48977	1.32983	0.107546
48978	2.63056	0.05
48979	2.58032	0.0629564
48980	2.65966	0.05
48981	2.65966	0.050785
48982	2.58032	0.0800467
48983	2.71296	0.103308
48984	1.92039	2.1
48985	1.06226	2.1
48986	1.09252	2.1
48987	1.96052	2.1
48988	3.01291	2.1
48989	1.92039	2.1
48990	2.46338	2.1
48991	2.47522	2.1
48992	2.43508	2.1
48993	1.96052	2.1
48994	1.92039	2.1
48995	0.985949	2.1
48996	0.980261	2.1
48997	0.553625	2.1
48998	0.546262	2.1
48999	0.765671	2.1
49000	0.765671	2.1
49001	0.766539	2.1
49002	0.766539	2.1
49003	0.985949	2.1
49004	0.553625	2.1
49005	0.546261	2.1
49006	0.980261	2.1
49007	1.96052	2.1

49008	2.46338	2.1
49009	1.97189	2.1
49010	1.96052	2.1
49011	2.47932	2.1
49012	2.47932	2.1
49013	0.964608	0.05
49014	0.691524	0.05
49015	1.25462	0.05
49016	0.691524	0.05
49017	1.89226	0.05
49018	1.89226	0.05
49019	1.44894	0.05
49020	2.77655	0.05
49021	1.4237	0.05
49022	2.77701	0.05
49023	2.77701	0.05
49024	1.38847	2.1
49025	1.46031	1.98053
49026	1.65504	1.31965
49027	1.75396	1.05803
49028	3.11535	1.68554
49029	1.89226	1.85208
49030	1.89226	2.06786
49031	1.89226	1.49985
49032	1.89226	0.783206
49033	3.78452	1.9161
49034	1.89226	1.55066
49035	1.89226	2.1
49036	1.89226	0.299441
49037	1.89226	1.89727
49038	3.78452	0.601411
49039	1.46031	1.14463
49040	1.34851	2.1
49041	1.75395	0.859547
49042	1.62287	1.34715
49043	3.08318	1.96048
49044	1.65504	1.85625
49045	1.75396	1.99177
49046	1.89249	2.01258
49047	1.9857	0.982716
49048	3.64073	1.93465
49049	1.89226	1.99233
49050	1.89226	1.80073
49051	1.89226	0.93536
49052	1.89226	1.14585
49053	3.78452	1.03214
49054	1.89226	0.969484
49055	1.89226	2.1
49056	1.89226	1.00506
49057	1.89226	1.78714
49058	3.78452	2.06757
49059	1.75395	1.72148
49060	1.62287	1.96583
49061	1.98569	0.643214
49062	1.86067	1.15095
49063	3.60856	1.02422
49064	1.35697	2.1
49065	2.02064	2.1

49066	1.89989	0.743828
49067	1.49571	2.1
49068	1.66753	1.32371
49069	1.36431	2.1
49070	0.744004	2.1
49071	0.743997	2.1
49072	0.717972	0.506838
49073	0.717972	2.1
49074	0.997982	2.1
49075	0.807796	2.1
49076	0.632235	0.785833
49077	0.488575	2.1
49078	0.849275	2.1
49079	0.488581	2.1
49080	0.724729	2.1
49081	0.997967	2.1
49082	2.5276	2.08326
49083	1.70791	2.02954
49084	1.82872	0.269165
49085	1.56362	2.1
49086	2.5276	1.05425
49087	2.24781	2.1
49088	1.37591	2.1
49089	1.23217	1.86804
49090	1.00442	2.02862
49091	0.945282	0.462915
49092	1.15446	0.817593
49093	1.09123	2.1
49094	1.21506	2.02704
49095	1.21506	1.0307
49096	1.39452	1.07662
49097	1.13108	2.0333
49098	1.27876	1.40568
49099	0.929006	0.743605
49100	1.46505	0.05
49101	1.91484	0.05
49102	1.49906	0.05
49103	1.49906	0.05
49104	1.90196	0.05
49105	1.90196	0.05
49106	1.90196	0.05
49107	2.11465	0.05
49108	1.91484	0.05
49109	1.91484	0.05
49110	2.04016	0.05
49111	2.04016	0.05
49112	0.912201	0.05
49113	0.950978	0.05
49114	0.665307	0.05
49115	0.732528	0.05
49116	0.829512	0.05
49117	0.829512	0.05
49118	0.815217	0.05
49119	0.815217	0.05
49120	0.912202	0.05
49121	0.665305	0.05
49122	0.732527	0.05
49123	0.95098	0.05

49124	1.90196	0.05
49125	2.08757	0.05
49126	1.9626	0.05
49127	2.04016	0.05
49128	1.82441	0.05
49129	1.90196	0.05
49130	1.13048	2.1
49131	0.821162	2.1
49132	0.946131	2.1
49133	0.946131	2.1
49134	1.04567	2.1
49135	0.882347	2.1
49136	0.897276	2.1
49137	0.897276	2.1
49138	0.863109	2.1
49139	1.01055	2.1
49140	0.940305	2.1
49141	0.942166	2.1
49142	2.23933	0.05
49143	0.976217	0.05
49144	2.78053	0.05
49145	2.78053	0.05
49146	0.976217	0.05
49147	1.65984	0.05
49148	1.54168	2.1
49149	2.01197	1.87945
49150	1.69136	2.1
49151	2.13284	0.617164
49152	3.67452	2.1
49153	0.988805	2.1
49154	1.78069	2.1
49155	1.69136	2.1
49156	1.69136	2.1
49157	1.05481	2.1
49158	1.05481	2.1
49159	1.69136	2.1
49160	2.13284	1.24887
49161	1.78069	2.1
49162	2.22044	1.65749
49163	3.91353	2.1
49164	2.69379	0.79541
49165	2.32219	1.20559
49166	2.25746	1.97124
49167	2.25746	0.944803
49168	2.32219	1.19017
49169	1.89526	0.71321
49170	1.4556	2.1
49171	1.85028	2.1
49172	2.4909	0.215359
49173	2.03799	2.1
49174	2.73495	1.6821
49175	1.85028	2.1
49176	2.71938	0.264108
49177	1.85028	2.1
49178	3.36051	2.1
49179	2.73495	1.99846
49180	2.47584	2.1
49181	1.85028	1.2

49182	2.47584	2.1
49183	1.04413	2.1
49184	2.1461	2.1
49185	1.74586	2.1
49186	1.44437	2.1
49187	1.04413	2.1
49188	1.89226	2.1
49189	1.89226	1.42622
49190	1.89226	2.07613
49191	1.89226	1.92447
49192	3.78452	2.1
49193	1.52724	2.1
49194	1.89249	2.1
49195	1.68143	2.1
49196	2.02433	1.73503
49197	3.55157	2.1
49198	1.07444	2.1
49199	1.76545	2.1
49200	1.68142	2.1
49201	1.68142	2.1
49202	1.14807	2.1
49203	1.14807	2.1
49204	1.89226	2.1
49205	1.89226	2.1
49206	1.89226	2.1
49207	1.89226	2.1
49208	1.89226	2.1
49209	1.89226	1.16425
49210	1.89226	2.1
49211	1.89226	2.1
49212	1.89226	2.1
49213	1.89226	0.656522
49214	3.78452	2.05075
49215	1.68143	2.1
49216	2.02433	2.01826
49217	1.76545	2.1
49218	2.12608	2.1
49219	3.78978	2.1
49220	2.62871	2.1
49221	1.49149	2.1
49222	1.36814	2.1
49223	2.44761	2.02532
49224	3.9391	2.1
49225	1.89226	2.1
49226	1.89226	0.0788133
49227	1.89226	2.1
49228	1.89226	2.1
49229	3.78452	2.1
49230	0.851542	2.1
49231	0.851542	2.1
49232	1.00897	2.1
49233	0.931224	2.1
49234	0.838952	2.1
49235	0.838952	2.09291
49236	0.956848	2.1
49237	0.942474	2.1
49238	0.946132	2.1
49239	0.838952	2.1

49240	0.878743	2.1
49241	0.946132	2.1
49242	1.15541	2.1
49243	2.44761	0.355749
49244	1.36814	2.1
49245	1.36814	2.1
49246	2.19439	2.1
49247	2.19439	2.1
49248	1.89226	0.485426
49249	1.89226	2.1
49250	1.89226	2.1
49251	1.89226	0.166735
49252	3.78452	2.1
49253	1.89226	2.1
49254	1.67791	2.1
49255	1.5713	2.1
49256	1.67791	2.1
49257	1.78566	2.1
49258	1.89226	2.1
49259	1.89226	0.102042
49260	1.62911	2.1
49261	2.24602	2.06312
49262	1.89226	2.1
49263	1.98287	0.823639
49264	1.62911	1.04008
49265	1.89226	2.1
49266	1.89226	2.1
49267	1.89226	0.764324
49268	1.89226	2.1
49269	3.78452	1.64286
49270	1.26688	2.1
49271	1.11804	2.1
49272	0.852467	1.55106
49273	0.693314	2.1
49274	0.977459	2.1
49275	0.977459	2.1
49276	0.982741	1.37651
49277	0.982741	2.1
49278	1.26689	2.1
49279	0.852464	2.1
49280	0.693312	2.1
49281	1.11804	2.1
49282	0.946131	2.1
49283	0.946131	0.364911
49284	1.1794	0.381019
49285	1.1794	2.1
49286	0.991434	2.1
49287	1.1794	0.839732
49288	0.946131	0.191225
49289	0.946131	0.325016
49290	0.946131	2.04159
49291	1.1794	1.7776
49292	0.946131	2.05027
49293	0.991434	1.34038
49294	1.89226	0.362417
49295	1.89226	2.1
49296	1.89226	0.812705
49297	1.89226	1.56487

49298	3.78452	2.02735
49299	2.53377	2.1
49300	1.53443	2.1
49301	2.0815	0.830114
49302	2.82979	2.1
49303	4.35462	2.1
49304	1.96192	0.309055
49305	1.23602	2.1
49306	2.16506	0.599101
49307	1.56841	1.9415
49308	3.40108	1.6271
49309	1.89226	1.77529
49310	1.89226	2.1
49311	1.89226	0.709403
49312	1.89226	2.1
49313	3.78452	1.76681
49314	0.946129	2.1
49315	0.946129	2.1
49316	1.13067	1.34251
49317	1.12952	2.1
49318	0.946129	2.1
49319	1.13089	2.1
49320	0.944978	1.33361
49321	0.944978	2.1
49322	0.946131	2.1
49323	1.13089	2.1
49324	0.944977	2.1
49325	0.946131	2.1
49326	0.710208	2.1
49327	0.710208	2.1
49328	0.718894	2.1
49329	0.718894	2.1
49330	0.559773	2.1
49331	0.579321	2.1
49332	0.886767	2.1
49333	0.849781	2.1
49334	0.849781	2.1
49335	0.559774	2.1
49336	0.579321	2.1
49337	0.886766	2.1
49338	1.63471	2.1
49339	1.69956	2.1
49340	1.08022	2.1
49341	1.08022	2.1
49342	1.11955	2.1
49343	1.67403	2.1
49344	1.03766	2.1
49345	2.02825	2.1
49346	2.02794	2.1
49347	2.0348	2.1
49348	1.07196	2.1
49349	1.07196	2.1
49350	1.9276	2.1
49351	2.1004	2.1
49352	2.1306	2.1
49353	2.1306	2.1
49354	1.92178	2.1
49355	1.92032	2.1

49356	0.986724	2.1
49357	2.03064	2.1
49358	2.01385	2.1
49359	2.02404	2.1
49360	1.03766	2.1
49361	1.03766	2.1
49362	1.92027	2.1
49363	2.1306	2.1
49364	2.15388	2.1
49365	2.15388	2.1
49366	1.92614	2.1
49367	1.9276	2.1
49368	1.09578	2.1
49369	0.963018	2.1
49370	0.782956	1.20022
49371	0.529693	2.1
49372	0.6994	1.65763
49373	0.862532	2.1
49374	0.898605	2.1
49375	0.898605	1.68843
49376	0.776756	2.1
49377	1.14805	2.1
49378	0.966521	2.1
49379	0.617676	2.1
49380	2.67185	2.1
49381	2.099 2.1	
49382	2.80548	2.1
49383	2.2727	2.1
49384	4.94455	2.1
49385	1.89226	2.1
49386	1.89226	2.1
49387	1.89226	2.1
49388	1.89226	2.1
49389	3.78453	2.1
49390	1.89226	2.1
49391	1.89226	2.1
49392	1.89226	2.1
49393	1.89226	2.1
49394	1.89226	2.1
49395	1.89226	2.1
49396	0.884801	2.1
49397	0.884801	2.1
49398	0.895199	2.1
49399	0.895199	2.1
49400	0.622774	2.1
49401	0.730501	2.1
49402	1.0495	2.1
49403	1.13635	2.1
49404	1.13635	2.1
49405	0.730503	2.1
49406	1.0495	2.1
49407	0.622775	2.1
49408	1.89226	2.1
49409	1.89226	2.1
49410	1.89226	2.1
49411	1.89226	2.1
49412	1.89226	2.1
49413	1.89226	2.1

49414	1.89226	2.1
49415	1.89226	2.1
49416	1.89226	2.1
49417	1.89226	2.1
49418	1.89226	2.1
49419	1.89226	2.1
49420	2.2727	2.1
49421	1.89586	2.1
49422	1.66913	2.1
49423	1.46101	2.1
49424	2.48082	2.1
49425	2.2727	2.1
49426	1.89226	2.1
49427	1.89226	2.1
49428	1.84641	2.1
49429	1.89226	2.1
49430	1.30868	2.1
49431	1.34118	1.19784
49432	1.89226	0.40831
49433	1.89226	0.529761
49434	1.89226	0.903787
49435	1.89226	0.312756
49436	1.89226	0.371317
49437	1.89226	0.111417
49438	1.89226	0.578782
49439	1.89226	0.05
49440	1.89226	0.248357
49441	1.89226	0.478073
49442	3.78452	0.356895
49443	0.946132	2.02631
49444	0.946132	1.55257
49445	0.946131	2.1
49446	0.946131	2.1
49447	0.946132	2.1
49448	0.946132	1.04333
49449	0.946132	2.1
49450	0.946132	2.1
49451	0.946132	2.1
49452	0.946132	2.1
49453	0.946132	2.1
49454	0.946132	2.1
49455	1.89227	1.5347
49456	1.84641	1.71585
49457	2.29517	2.1
49458	1.84641	1.14946
49459	2.34102	1.6265
49460	1.89227	0.372586
49461	1.89226	0.239178
49462	1.89226	0.094856
49463	1.89226	0.362933
49464	1.89226	0.182122
49465	1.89226	0.10609
49466	1.89226	0.05
49467	1.89226	0.085405
49468	1.89226	0.179932
49469	1.89226	0.263979
49470	1.89226	0.151466
49471	1.89226	0.086838

49472	1.89226	0.05
49473	1.89226	1.74834
49474	1.89226	0.881534
49475	1.89226	2.08183
49476	1.89226	0.914661
49477	1.89226	1.53725
49478	1.89226	0.750955
49479	0.851544	0.05
49480	0.757542	0.05
49481	1.13421	0.05
49482	1.04072	0.05
49483	0.946132	0.05
49484	0.946132	0.05
49485	0.841686	0.05
49486	0.834428	0.05
49487	1.13649	0.05
49488	0.937687	0.05
49489	0.841713	0.05
49490	0.841713	0.05
49491	1.89226	0.05
49492	1.89226	0.05
49493	1.89226	0.0543881
49494	1.89226	0.05
49495	1.89226	0.0548333
49496	1.89226	0.05
49497	2.31988	1.17205
49498	2.00082	2.1
49499	2.4594	0.439715
49500	2.17638	2.1
49501	4.46022	1.17793
49502	2.00082	1.1373
49503	1.41829	2.1
49504	1.61207	1.85133
49505	1.43652	2.1
49506	2.17637	0.845155
49507	2.00082	2.1
49508	2.4594	0.335432
49509	2.17638	2.1
49510	2.57957	0.517739
49511	2.3506	2.1
49512	4.81	1.35668
49513	2.3506	1.19269
49514	1.57368	2.1
49515	1.89111	0.176167
49516	1.89111	2.1
49517	2.17638	0.201518
49518	2.17638	2.1
49519	1.32813	2.1
49520	1.7237	2.1
49521	1.32461	2.1
49522	1.81656	2.1
49523	3.14468	2.1
49524	1.55132	2.1
49525	2.0815	2.1
49526	1.81656	2.1
49527	2.19826	2.1
49528	3.65818	2.1
49529	0.775951	2.1

49530	0.775951	2.1
49531	0.794601	2.1
49532	0.794601	2.1
49533	0.945641	2.1
49534	0.90828	2.1
49535	0.662302	2.1
49536	0.662273	2.1
49537	0.662274	2.1
49538	0.945639	2.1
49539	0.662304	2.1
49540	0.908279	2.1
49541	1.03062	2.1
49542	1.03062	2.1
49543	1.05054	2.1
49544	1.05054	2.1
49545	1.13551	2.1
49546	1.21275	2.1
49547	0.945641	2.1
49548	0.90828	2.1
49549	0.945641	2.1
49550	1.21275	2.1
49551	0.90828	2.1
49552	1.13552	2.1
49553	1.89226	2.1
49554	1.79883	2.1
49555	1.95341	2.1
49556	1.89226	2.1
49557	1.85998	2.1
49558	1.79883	2.1
49559	1.89226	2.1
49560	1.89226	2.1
49561	1.89226	2.1
49562	1.89226	2.1
49563	3.78453	2.1
49564	0.851541	2.1
49565	0.851541	2.1
49566	0.970398	2.1
49567	0.954365	2.1
49568	0.855356	2.1
49569	0.855357	2.1
49570	0.946133	2.1
49571	0.946133	2.1
49572	1.04072	2.1
49573	0.970397	2.1
49574	0.950003	2.1
49575	0.851538	2.1
49576	1.89226	2.1
49577	1.85998	2.1
49578	1.95296	2.1
49579	1.89226	2.1
49580	1.92067	2.1
49581	1.85998	2.1
49582	0.946133	2.1
49583	0.946133	2.1
49584	0.946132	2.1
49585	0.946132	2.1
49586	0.946132	2.1
49587	0.946132	2.1

49588	0.946132	2.1
49589	0.946132	2.1
49590	0.946132	2.1
49591	0.946132	2.1
49592	0.946133	2.1
49593	0.946133	2.1
49594	1.82152	2.1
49595	1.89226	2.1
49596	1.89226	2.1
49597	2.11152	2.1
49598	1.7472	2.1
49599	1.89226	2.1
49600	2.02687	2.1
49601	2.29199	2.1
49602	1.2734	2.1
49603	1.41077	2.1
49604	1.47381	2.1
49605	2.29199	2.1
49606	1.46889	2.1
49607	2.29199	2.1
49608	1.86766	2.1
49609	2.59401	2.1
49610	4.15965	2.1
49611	1.58995	2.1
49612	1.74246	2.1
49613	1.46643	2.1
49614	1.46643	2.1
49615	1.85097	2.1
49616	1.85097	2.1
49617	1.58995	2.1
49618	1.66584	2.1
49619	1.89214	2.1
49620	1.91787	2.1
49621	3.48208	2.1
49622	1.18474	2.1
49623	1.8144	2.1
49624	1.1486	2.1
49625	1.1486	2.1
49626	2.13071	2.1
49627	2.1257	2.1
49628	2.0815	2.1
49629	2.51651	2.1
49630	1.73681	2.1
49631	2.01601	2.1
49632	2.21406	2.1
49633	2.56998	2.1
49634	1.19995	2.1
49635	1.06256	2.1
49636	0.620677	2.1
49637	0.598084	2.1
49638	0.831426	2.1
49639	0.946056	2.1
49640	0.827692	2.1
49641	0.827692	2.1
49642	1.08958	2.1
49643	0.630132	2.1
49644	0.577547	2.1
49645	1.06104	2.1

49646	2.0815	2.1
49647	2.66249	2.1
49648	2.78966	2.1
49649	2.47108	2.1
49650	2.33858	2.1
49651	2.0815	2.1
49652	1.53735	2.1
49653	1.42278	2.1
49654	1.23654	2.1
49655	1.34678	2.1
49656	2.76955	2.1
49657	1.89226	2.1
49658	1.89226	2.1
49659	1.89226	2.1
49660	1.89226	2.1
49661	3.78452	2.1
49662	1.23654	2.1
49663	1.34678	2.1
49664	1.19 2.1	
49665	1.08576	2.1
49666	2.43254	2.1
49667	1.38776	2.1
49668	1.29366	2.1
49669	0.946131	2.1
49670	0.946131	2.1
49671	0.946131	2.1
49672	0.946131	2.1
49673	1.38775	2.1
49674	1.38775	2.1
49675	1.29366	2.1
49676	0.946132	2.1
49677	0.946131	2.1
49678	1.38775	2.1
49679	0.946131	2.1
49680	0.946131	2.1
49681	0.946129	2.1
49682	0.946129	2.1
49683	0.94613	2.1
49684	0.94613	2.1
49685	0.946131	2.1
49686	0.946131	2.1
49687	0.946131	2.1
49688	0.946131	2.1
49689	0.94613	2.1
49690	0.94613	2.1
49691	1.89226	0.05
49692	1.89226	1.11033
49693	1.89226	0.150446
49694	1.89226	0.247242
49695	1.89226	0.344208
49696	1.89226	1.24996
49697	1.89226	0.094357
49698	1.89226	1.79278
49699	1.89226	0.0699304
49700	1.89226	0.146163
49701	1.89226	0.0676332
49702	1.89226	1.01301
49703	1.89226	0.195448

49704	1.89226	1.43404
49705	1.89226	0.121245
49706	1.89226	0.771891
49707	1.89226	0.110882
49708	1.89226	1.45999
49709	0.92338	2.1
49710	0.92338	0.409898
49711	0.94428	0.387438
49712	0.94428	0.10705
49713	1.19528	0.923293
49714	1.30598	2.1
49715	0.714073	0.151683
49716	0.561679	1.83206
49717	1.19528	0.987907
49718	0.561678	1.06537
49719	0.714072	1.33668
49720	1.30598	1.28691
49721	2.61197	2.1
49722	1.89226	1.3009
49723	1.89226	1.02223
49724	1.89226	2.1
49725	2.39056	0.428281
49726	2.39056	2.1
49727	0.946132	1.76672
49728	0.946132	0.478414
49729	0.946132	0.05
49730	0.946132	0.0912472
49731	0.946131	0.125296
49732	0.946131	1.79099
49733	0.946132	0.925595
49734	0.946132	0.0923161
49735	0.946132	0.368551
49736	0.946132	0.222964
49737	0.946132	0.307033
49738	0.946132	0.515355
49739	1.89224	2.1
49740	1.89224	2.1
49741	1.89224	2.1
49742	1.89224	2.1
49743	3.78448	2.1
49744	1.97089	2.1
49745	1.57428	2.1
49746	1.84991	2.1
49747	1.49078	2.1
49748	3.42419	2.1
49749	0.621927	2.1
49750	0.621927	2.1
49751	0.630824	2.1
49752	0.630824	2.1
49753	0.483483	2.1
49754	0.507361	2.1
49755	0.787142	2.1
49756	0.745391	2.1
49757	0.745381	2.1
49758	0.483476	2.1
49759	0.507368	2.1
49760	0.787152	2.1
49761	1.89224	2.1

49762	1.89224	2.1
49763	1.89224	2.1
49764	1.89224	2.1
49765	1.89224	2.1
49766	1.89224	2.1
49767	1.89227	2.1
49768	1.89227	2.1
49769	1.89227	2.1
49770	1.89227	2.1
49771	1.89227	2.1
49772	1.89227	2.1
49773	1.84994	2.1
49774	1.41758	2.1
49775	1.4908	2.1
49776	1.4908	2.1
49777	1.74391	2.1
49778	1.74391	2.1
49779	1.89226	2.1
49780	1.89226	2.1
49781	1.89226	2.1
49782	1.89226	2.1
49783	3.78452	2.1
49784	1.01323	2.1
49785	1.01323	2.1
49786	1.01445	2.1
49787	1.01445	2.1
49788	0.839531	2.1
49789	0.875652	2.1
49790	1.19063	2.1
49791	1.15203	2.1
49792	1.15203	2.1
49793	0.839531	2.1
49794	0.875652	2.1
49795	1.19063	2.1
49796	1.89226	2.1
49797	2.75587	2.1
49798	2.81512	2.1
49799	2.81512	2.1
49800	1.89226	2.1
49801	1.89226	2.1
49802	1.89226	2.1
49803	1.89226	2.1
49804	1.89226	2.1
49805	1.89226	2.1
49806	3.78452	2.1
49807	0.974831	2.1
49808	0.974831	2.1
49809	0.976681	2.1
49810	0.976681	2.1
49811	0.803179	2.1
49812	0.83953	2.1
49813	1.11198	2.1
49814	1.15203	2.1
49815	1.11198	2.1
49816	0.803179	2.1
49817	1.15203	2.1
49818	0.83953	2.1
49819	1.37794	2.1

49820	1.37794	2.1
49821	0.946128	2.1
49822	0.946128	2.1
49823	1.3477	2.1
49824	1.37793	2.1
49825	0.946131	2.1
49826	0.946131	2.1
49827	0.946129	2.1
49828	1.3477	2.1
49829	1.37793	2.1
49830	0.946129	2.1
49831	2.38577	1.70281
49832	1.20247	2.1
49833	1.75182	2.1
49834	2.82546	1.77536
49835	4.1376	2.1
49836	2.82546	2.1
49837	1.89226	0.325057
49838	2.38577	2.07141
49839	2.38577	1.86566
49840	1.89226	2.1
49841	1.89226	1.73212
49842	1.035	2.1
49843	2.15429	2.1
49844	0.970998	2.1
49845	2.0815	2.1
49846	2.89672	2.1
49847	0.946131	0.05
49848	0.946131	0.05
49849	0.853249	0.05
49850	1.02715	0.05
49851	0.851537	0.05
49852	0.851537	0.05
49853	1.02663	0.05
49854	1.13139	0.05
49855	0.946128	0.05
49856	0.932039	0.05
49857	0.843985	0.05
49858	0.946129	0.05
49859	1.89226	0.05
49860	1.86408	0.05
49861	1.96548	0.05
49862	1.86408	0.05
49863	1.99366	0.05
49864	1.89226	0.05
49865	2.70702	2.1
49866	2.08981	2.1
49867	2.08693	2.1
49868	2.08693	2.1
49869	2.7188	2.1
49870	2.7188	2.1
49871	2.08982	2.1
49872	1.01964	2.1
49873	2.08693	2.1
49874	1.04615	2.1
49875	3.13597	2.1
49876	2.70702	2.1
49877	2.08693	2.1

49878	2.68784	2.1
49879	2.08831	2.1
49880	4.77477	2.1
49881	0.778233	2.1
49882	0.778233	2.1
49883	0.789001	2.1
49884	0.789001	2.1
49885	0.545307	2.1
49886	0.523075	2.1
49887	1.04347	2.1
49888	1.04416	2.1
49889	1.04416	2.1
49890	0.545308	2.1
49891	1.04347	2.1
49892	0.523075	2.1
49893	1.89226	0.437382
49894	1.89226	0.491687
49895	1.89226	0.136416
49896	1.89226	0.0938434
49897	3.78453	0.124209
49898	1.39237	0.221584
49899	1.17933	1.70878
49900	0.755796	0.89686
49901	0.755796	0.933932
49902	0.755767	0.05
49903	0.94613	1.4477
49904	1.15918	0.113112
49905	0.94613	0.741115
49906	1.11632	0.155015
49907	0.92595	0.05
49908	0.94613	0.672264
49909	0.94613	0.396001
49910	1.89226	0.49362
49911	1.89226	0.180476
49912	1.89226	0.0813879
49913	1.89226	0.05
49914	3.78452	0.267204
49915	0.950067	0.05
49916	0.75071	0.05
49917	0.906516	0.0539745
49918	0.906516	0.05
49919	0.81586	0.05
49920	0.851541	0.147105
49921	0.962101	0.05
49922	0.851476	0.0744893
49923	0.962105	0.05
49924	0.815808	0.05
49925	0.851529	0.0659059
49926	0.851529	0.0539439
49927	1.05032	0.05
49928	0.972326	0.05
49929	0.906515	0.05
49930	0.906515	0.05
49931	1.12245	0.05
49932	1.04072	0.05
49933	0.815857	0.05
49934	0.851539	0.108627
49935	1.04451	0.05

49936	0.815853	0.05
49937	0.919795	0.0637743
49938	0.919795	0.05
49939	1.5855	2.1
49940	2.14058	2.1
49941	1.9153	2.1
49942	1.79922	2.1
49943	3.72608	2.1
49944	1.58551	1.9845
49945	1.24201	2.1
49946	0.960287	2.04707
49947	1.79922	2.1
49948	2.82752	2.1
49949	0.679602	2.1
49950	0.621009	2.1
49951	0.899614	2.1
49952	0.940441	2.1
49953	0.679602	2.1
49954	0.94044	2.1
49955	0.621009	2.1
49956	0.899615	2.1
49957	0.794027	2.1
49958	0.794027	2.1
49959	0.785189	2.1
49960	0.785189	0.588012
49961	2.14058	2.1
49962	1.79922	2.1
49963	2.23742	2.1
49964	2.23742	2.1
49965	1.78404	2.1
49966	1.88088	2.1
49967	1.15177	2.1
49968	2.0815	2.1
49969	1.93765	2.1
49970	1.78342	2.1
49971	1.39511	2.1
49972	1.28406	2.1
49973	1.97719	2.1
49974	2.50057	0.05
49975	2.35349	2.1
49976	2.35349	0.0501796
49977	2.31686	2.1
49978	2.32857	0.0727101
49979	1.93765	2.1
49980	2.35349	0.079564
49981	1.70048	2.1
49982	2.15721	0.05
49983	4.05397	0.263518
49984	0.850388	2.1
49985	0.850388	2.1
49986	0.840019	2.1
49987	0.840019	2.1
49988	1.13736	2.1
49989	1.13852	2.1
49990	0.573661	2.1
49991	0.551891	2.1
49992	0.573661	2.1
49993	1.13852	2.1

49994	0.551892	2.1
49995	1.13736	2.1
49996	0.860203	2.1
49997	0.860203	2.1
49998	0.855758	2.1
49999	0.855758	2.1
50000	1.1423	2.1
50001	1.13852	2.1
50002	0.58628	2.1
50003	0.573661	2.1
50004	0.586281	2.1
50005	1.1423	2.1
50006	0.573661	2.1
50007	1.13852	2.1
50008	1.28918	1.1976
50009	0.999086	1.04353
50010	1.0383	1.93738
50011	0.736019	1.5609
50012	1.00954	1.55717
50013	1.00954	1.01122
50014	1.01566	0.418906
50015	1.01566	1.98622
50016	1.28918	1.17238
50017	1.0383	2.02188
50018	0.999086	1.70138
50019	0.736019	1.44058
50020	2.20797	2.1
50021	2.64748	2.1
50022	2.50375	2.01256
50023	2.50375	2.1
50024	2.40858	2.1
50025	2.40858	2.1
50026	2.64748	2.1
50027	2.74744	2.1
50028	2.50375	2.1
50029	2.6521	2.1
50030	5.29958	2.1
50031	2.20797	2.1
50032	2.50375	2.1
50033	2.02064	1.94849
50034	2.3606	2.1
50035	4.52439	2.1
50036	2.36059	2.1
50037	2.6521	2.1
50038	2.56952	2.1
50039	2.56952	2.1
50040	2.50375	2.1
50041	2.50375	2.1
50042	2.49439	0.05
50043	1.89226	0.0652281
50044	2.81337	0.0713328
50045	1.89226	0.0748296
50046	4.38665	0.0714909
50047	1.89226	0.0805545
50048	1.89226	0.219373
50049	1.89226	0.270025
50050	1.89226	0.408644
50051	3.78452	0.194946

50052	1.89226	0.069356
50053	1.89226	0.110727
50054	1.89226	0.1025
50055	1.89226	0.158067
50056	3.78452	0.103867
50057	1.93948	0.297143
50058	1.98874	0.0933393
50059	1.62494	0.279644
50060	2.14218	0.485033
50061	1.62494	0.05
50062	2.14218	0.0561351
50063	1.47354	0.137891
50064	1.98874	0.0502579
50065	2.01865	0.137963
50066	1.62495	0.0504245
50067	2.01865	0.251043
50068	1.62495	0.05
50069	1.89226	0.341525
50070	1.89226	2.1
50071	1.89226	2.1
50072	1.89226	2.1
50073	3.78453	2.1
50074	2.05257	2.1
50075	1.94441	2.1
50076	1.88487	2.1
50077	1.82441	2.1
50078	3.82928	2.1
50079	1.94441	2.1
50080	1.74391	2.1
50081	1.82441	2.1
50082	1.65636	2.1
50083	3.60077	2.1
50084	1.89226	2.1
50085	1.89226	2.1
50086	1.89226	2.1
50087	1.89226	2.1
50088	3.78452	2.1
50089	1.89226	2.1
50090	1.89226	2.1
50091	1.89226	0.26665
50092	1.89226	2.1
50093	3.78453	2.1
50094	1.88487	2.1
50095	1.82441	2.1
50096	1.75835	2.1
50097	1.72866	2.1
50098	3.61354	2.1
50099	1.82441	2.1
50100	1.65636	2.1
50101	1.72866	2.1
50102	1.58401	2.1
50103	3.38503	2.1
50104	1.89226	2.1
50105	1.89226	2.1
50106	1.89226	2.1
50107	1.89226	2.1
50108	3.78452	2.1
50109	2.27432	2.1

50110	1.33339	2.1
50111	1.38905	2.1
50112	2.34471	2.1
50113	3.66337	2.1
50114	1.89226	2.1
50115	2.34471	2.1
50116	2.27432	2.1
50117	2.27432	2.1
50118	1.89226	2.1
50119	1.89226	2.1
50120	1.20443	2.1
50121	1.17235	2.1
50122	0.724695	2.1
50123	0.694522	2.1
50124	0.949994	2.1
50125	0.949993	2.1
50126	0.948955	2.1
50127	0.948955	2.1
50128	1.20443	2.1
50129	0.7247	2.1
50130	0.694517	2.1
50131	1.17234	2.1
50132	1.89225	2.1
50133	2.40885	2.1
50134	2.3447	2.1
50135	2.3447	2.1
50136	1.89225	2.1
50137	1.89225	2.1
50138	1.37138	2.1
50139	1.82597	2.1
50140	1.45027	2.1
50141	1.92772	2.1
50142	3.27624	2.1
50143	1.89226	2.1
50144	1.89226	2.1
50145	1.89226	2.1
50146	1.89226	2.1
50147	3.78452	2.1
50148	1.89226	2.1
50149	1.89226	1.21053
50150	1.89226	2.1
50151	1.89226	1.97692
50152	3.78452	2.1
50153	1.82597	2.1
50154	2.02312	0.106041
50155	1.92772	2.1
50156	2.15888	2.1
50157	3.98485	2.1
50158	1.53446	2.1
50159	1.92772	2.1
50160	2.03975	2.1
50161	2.03975	2.1
50162	1.45027	2.1
50163	1.45027	2.1
50164	1.89226	2.1
50165	1.89226	2.1
50166	1.89226	2.1
50167	1.89226	2.1

50168	1.89226	2.1
50169	1.89226	2.1
50170	1.89226	2.1
50171	1.89226	2.1
50172	1.89226	2.1
50173	1.89226	1.96462
50174	3.78452	2.1
50175	1.92772	2.1
50176	2.15888	2.1
50177	2.03975	2.1
50178	2.3111	2.1
50179	4.19863	2.1
50180	1.89226	2.1
50181	1.89226	2.1
50182	1.89226	2.1
50183	1.89226	2.1
50184	3.78452	2.1
50185	1.78376	2.1
50186	1.3201	2.1
50187	1.78376	2.1
50188	1.86102	2.1
50189	1.2679	2.1
50190	1.34517	2.1
50191	1.89226	2.1
50192	2.52442	2.1
50193	1.89226	2.1
50194	2.51691	2.1
50195	4.41667	2.1
50196	1.89226	2.1
50197	1.89226	2.1
50198	1.89226	2.1
50199	1.89226	2.1
50200	3.78452	2.1
50201	1.78376	2.1
50202	1.2679	2.1
50203	1.71839	2.1
50204	1.23504	2.1
50205	3.0188	2.1
50206	1.89226	2.1
50207	2.51795	2.1
50208	2.51691	2.1
50209	2.51691	2.1
50210	1.89226	2.1
50211	1.89226	2.1
50212	1.89226	0.656502
50213	1.89226	1.65156
50214	1.89226	1.07812
50215	1.89226	1.01092
50216	3.78452	0.925826
50217	1.89227	0.722487
50218	1.89227	0.637685
50219	3.26088	1.31838
50220	2.79543	0.9919
50221	2.20733	0.546413
50222	1.89227	0.521719
50223	1.89226	1.38813
50224	1.89226	0.951294
50225	1.89226	0.687741

50226	1.89226	1.02174
50227	1.89226	1.04047
50228	1.89226	1.48966
50229	1.89227	0.706492
50230	2.20733	0.641782
50231	1.46973	1.31606
50232	1.89227	0.734201
50233	1.7848	1.15561
50234	2.20733	0.508198
50235	1.89226	2.1
50236	2.55682	2.1
50237	2.81593	2.1
50238	2.81593	2.1
50239	1.89226	2.1
50240	1.89226	2.1
50241	1.0607	2.1
50242	1.0607	2.1
50243	1.12617	2.1
50244	0.986957	2.1
50245	0.699328	2.1
50246	0.785567	2.1
50247	1.28855	2.1
50248	1.41913	2.1
50249	0.766737	1.35803
50250	1.34711	2.1
50251	1.24643	2.1
50252	0.687289	2.1
50253	3.11063	0.05
50254	1.89226	0.05
50255	1.96536	0.05
50256	1.89226	0.05
50257	1.96536	0.05
50258	1.89226	0.05
50259	1.89226	0.141445
50260	1.89226	0.05
50261	1.89226	0.0554266
50262	1.89226	0.05
50263	3.78452	0.142221
50264	1.89226	0.05
50265	1.96256	0.05
50266	1.89226	0.05
50267	1.89226	0.05
50268	2.49192	0.05
50269	2.49192	0.05
50270	1.85716	0.05
50271	2.56871	0.05
50272	1.03043	0.05
50273	1.03043	0.05
50274	2.48125	0.05
50275	1.65452	0.05
50276	1.89226	2.1
50277	2.09622	2.1
50278	2.09622	2.1
50279	2.08508	2.1
50280	1.89226	2.1
50281	1.88112	2.1
50282	1.04811	2.1
50283	1.04811	2.1

50284	0.946131	2.1
50285	0.946131	2.1
50286	1.05014	2.1
50287	1.04811	2.1
50288	0.94613	2.1
50289	0.94613	2.1
50290	0.946131	2.1
50291	1.05013	2.1
50292	1.04811	2.1
50293	0.946131	2.1
50294	1.398	2.1
50295	2.10766	2.1
50296	2.3018	2.1
50297	2.3018	2.1
50298	1.34008	2.1
50299	1.34008	2.1
50300	1.1509	2.1
50301	1.1509	2.1
50302	0.946132	2.1
50303	0.946132	2.1
50304	0.946132	2.1
50305	0.946132	2.1
50306	1.05383	2.1
50307	1.1509	2.1
50308	1.1509	2.1
50309	0.946133	2.1
50310	0.946132	2.1
50311	1.05383	2.1
50312	0.931141	2.1
50313	0.931141	2.1
50314	0.971946	2.1
50315	0.971946	2.1
50316	1.28569	2.1
50317	1.1509	2.1
50318	0.699	2.1
50319	0.752185	2.1
50320	1.28568	2.1
50321	0.752186	2.1
50322	0.699001	2.1
50323	1.1509	2.1
50324	2.30181	2.1
50325	1.89226	2.1
50326	1.89226	2.1
50327	1.89226	2.1
50328	2.57137	2.1
50329	2.57137	2.1
50330	2.0815	2.1
50331	1.21962	2.1
50332	1.55173	2.1
50333	2.57849	2.1
50334	3.79001	2.1
50335	1.38779	2.1
50336	1.2923	2.1
50337	1.03974	2.1
50338	1.28832	2.1
50339	1.33566	2.1
50340	1.21116	2.1
50341	1.22343	2.1

50342	1.22343	2.1
50343	1.42288	2.1
50344	1.20138	2.1
50345	1.26025	2.1
50346	1.00644	2.1
50347	1.26066	2.1
50348	1.14271	2.1
50349	0.886635	2.1
50350	0.795277	2.1
50351	1.02966	2.1
50352	1.02967	2.1
50353	1.05216	2.1
50354	1.05216	2.1
50355	1.26068	2.1
50356	0.790042	2.1
50357	0.795269	2.1
50358	1.20135	2.1
50359	1.89226	0.081967
50360	1.89226	0.0544883
50361	1.89226	0.0952465
50362	1.89226	0.05
50363	1.89226	0.128757
50364	1.89226	0.0529501
50365	1.89227	0.05
50366	1.89227	0.05
50367	1.89227	0.0607347
50368	1.89227	0.05
50369	1.89227	0.071516
50370	1.89227	0.05
50371	0.563873	0.05
50372	0.50988	0.05
50373	0.598693	0.05
50374	0.558819	0.05
50375	0.564855	0.05
50376	0.564855	0.05
50377	0.557838	0.05
50378	0.557838	0.05
50379	0.563873	0.05
50380	0.598694	0.05
50381	0.558819	0.05
50382	0.50988	0.05
50383	0.598693	0.05
50384	0.558819	0.05
50385	0.572737	0.0553074
50386	0.534248	0.05
50387	0.566788	0.05
50388	0.566788	0.05
50389	0.566153	0.05
50390	0.566153	0.05
50391	0.598694	0.05
50392	0.572737	0.05
50393	0.534248	0.05
50394	0.558819	0.05
50395	1.85217	1.9572
50396	1.93111	2.1
50397	1.92875	0.898755
50398	2.02391	2.1
50399	3.85986	2.1

50400	1.93112	2.1
50401	1.80425	2.1
50402	2.02392	2.1
50403	1.90981	2.1
50404	3.84092	2.1
50405	1.92876	0.291998
50406	2.02393	2.1
50407	2.05274	1.43029
50408	2.16783	2.1
50409	4.09659	2.1
50410	2.02393	2.1
50411	1.90982	2.1
50412	2.16784	2.1
50413	2.05967	2.1
50414	4.07766	2.1
50415	1.89226	1.53957
50416	1.89226	1.15943
50417	1.89226	1.54038
50418	1.89226	1.33616
50419	3.78453	0.79482
50420	1.89226	1.07059
50421	1.89226	1.68688
50422	1.89226	2.1
50423	2.44829	0.565164
50424	3.78453	2.04503
50425	1.89226	1.55895
50426	1.89226	1.7897
50427	1.89226	1.24246
50428	2.60452	0.695798
50429	3.78453	2.1
50430	1.58723	0.05
50431	1.88071	0.05
50432	2.97376	0.05
50433	2.13255	0.05
50434	4.43982	0.05
50435	2.20641	0.05
50436	1.33575	0.05
50437	2.15824	0.05
50438	2.15824	0.05
50439	1.2951	0.05
50440	1.2951	0.05
50441	0.825383	0.05
50442	0.825383	0.05
50443	0.856104	0.05
50444	0.856104	0.05
50445	0.952196	0.05
50446	0.602363	0.05
50447	0.667877	0.05
50448	1.07913	0.05
50449	0.602363	0.05
50450	0.667876	0.05
50451	1.07913	0.05
50452	0.952197	0.05
50453	2.57657	1.18269
50454	1.44437	2.1
50455	2.65378	2.1
50456	2.25076	2.1
50457	1.84739	2.1

50458	1.44437	2.1
50459	2.0189	0.05
50460	2.54752	0.05
50461	2.63975	0.05
50462	2.31049	0.05
50463	4.65865	0.05
50464	1.40707	0.05
50465	1.89605	0.05
50466	2.0189	0.05
50467	2.0189	0.05
50468	1.50438	0.05
50469	1.50438	0.05
50470	2.63971	0.05
50471	2.38246	0.05
50472	2.13402	0.05
50473	2.57503	0.05
50474	4.77373	0.05
50475	1.50436	0.05
50476	1.96803	0.05
50477	2.13402	0.05
50478	1.45446	0.05
50479	3.63838	0.05
50480	2.30793	0.05
50481	2.11032	0.05
50482	1.24082	0.05
50483	1.05689	0.05
50484	3.36482	0.05
50485	1.89226	0.05
50486	1.89226	0.0511172
50487	1.89226	0.05
50488	1.89226	0.05
50489	3.78453	0.05
50490	0.812967	0.05
50491	0.812967	0.05
50492	1.00181	0.05
50493	1.08141	0.05
50494	0.946132	0.05
50495	0.812938	0.05
50496	0.932815	0.05
50497	1.05577	0.05
50498	0.812938	0.05
50499	0.932812	0.05
50500	0.946133	0.05
50501	0.946133	0.05
50502	2.53897	2.1
50503	1.89226	2.1
50504	1.89226	2.1
50505	1.89226	2.1
50506	2.3245	2.1
50507	2.3245	2.1
50508	1.00255	1.28618
50509	1.06561	2.1
50510	1.05481	0.05
50511	1.11077	2.1
50512	2.12042	2.1
50513	1.89226	1.73287
50514	1.89226	2.1
50515	1.89226	0.0605883

50516	1.89226	2.1
50517	3.78453	1.49415
50518	1.89226	2.1
50519	2.77314	2.1
50520	2.81276	2.1
50521	2.81276	2.1
50522	1.89226	2.1
50523	1.89226	2.1
50524	1.11077	2.1
50525	1.15114	2.1
50526	1.10274	0.53492
50527	1.1032	2.1
50528	1.05481	2.04775
50529	1.1032	0.719577
50530	1.89226	1.14165
50531	1.89226	2.1
50532	1.89226	0.05
50533	1.89226	2.1
50534	3.78452	1.23444
50535	0.721957	0.0781218
50536	0.721957	0.079197
50537	0.946103	0.0843009
50538	0.946103	0.05
50539	0.814237	0.05
50540	0.721929	0.05
50541	0.946132	0.0842349
50542	0.946132	0.136402
50543	0.721928	0.122388
50544	0.946131	0.161713
50545	0.946132	0.202028
50546	0.814237	0.112017
50547	1.89226	0.286419
50548	1.89226	0.0815111
50549	1.89226	0.0833455
50550	1.89226	0.0960437
50551	1.89226	0.203665
50552	1.89226	0.240416
50553	1.37196	0.0580857
50554	2.83556	0.05
50555	1.5165	0.0502938
50556	1.5165	0.0667399
50557	2.74923	0.05
50558	2.74923	0.0569115
50559	0.946128	0.0650386
50560	0.946128	0.094424
50561	0.827866	0.05
50562	0.946132	0.0515995
50563	0.94613	0.05
50564	0.94613	0.05
50565	0.94613	0.101207
50566	0.94613	0.0738928
50567	0.94613	0.100826
50568	0.827864	0.14429
50569	0.94613	0.0794192
50570	0.94613	0.180669
50571	1.36208	0.05
50572	2.74923	0.05
50573	1.37196	0.05

50574	1.37196	0.05
50575	2.53256	0.05
50576	2.53256	0.05
50577	1.07705	2.1
50578	1.07705	2.1
50579	1.08253	2.1
50580	1.08253	2.1
50581	0.762985	2.1
50582	0.781638	2.1
50583	1.37794	2.1
50584	1.40756	2.1
50585	1.37794	2.1
50586	0.762986	2.1
50587	0.781638	2.1
50588	1.40756	2.1
50589	2.69541	2.1
50590	2.75587	2.1
50591	1.48572	2.1
50592	1.48572	2.1
50593	1.52597	2.1
50594	2.73566	2.1
50595	2.0554	0.876339
50596	1.00387	0.436622
50597	2.01001	0.124366
50598	0.994002	0.125272
50599	3.01389	0.066114
50600	1.89226	2.1
50601	2.41579	2.1
50602	2.12209	2.1
50603	2.12209	2.1
50604	1.89226	2.1
50605	1.89226	2.1
50606	1.89226	2.1
50607	1.89226	2.1
50608	1.89226	2.1
50609	1.89226	2.1
50610	1.89226	2.1
50611	1.89226	2.1
50612	0.88209	2.1
50613	0.899471	2.1
50614	0.651501	2.1
50615	0.533531	2.1
50616	0.724105	2.1
50617	0.711243	2.1
50618	0.692622	2.1
50619	0.692622	2.1
50620	0.890668	2.1
50621	0.738066	2.1
50622	0.778223	2.1
50623	0.531121	2.1
50624	1.89226	2.1
50625	2.77504	2.1
50626	2.4158	2.1
50627	2.4158	2.1
50628	1.89226	2.1
50629	1.89226	2.1
50630	1.89226	2.1
50631	1.89226	2.1

50632	1.89226	2.1
50633	1.89226	2.1
50634	1.89226	2.1
50635	1.89226	2.1
50636	1.70303	2.1
50637	1.39832	2.1
50638	1.51749	2.1
50639	1.68783	2.1
50640	3.24844	2.1
50641	1.89226	2.1
50642	2.38311	2.1
50643	1.98321	2.1
50644	1.89226	2.1
50645	2.47406	2.1
50646	2.38311	2.1
50647	1.89226	2.1
50648	1.89226	2.1
50649	1.89226	2.1
50650	1.89226	2.1
50651	1.89226	2.1
50652	1.89226	2.1
50653	0.946131	2.1
50654	0.946131	2.1
50655	1.25167	2.1
50656	1.19156	2.1
50657	1.19156	2.1
50658	1.19156	2.1
50659	0.946133	2.1
50660	0.946133	2.1
50661	0.946131	2.1
50662	1.25167	2.1
50663	0.946131	2.1
50664	1.19156	2.1
50665	1.89226	2.1
50666	2.47406	2.1
50667	1.99996	2.1
50668	1.89226	2.1
50669	2.58176	2.1
50670	2.47406	2.1
50671	1.89226	2.1
50672	1.89226	2.1
50673	1.89226	2.1
50674	1.89226	2.1
50675	1.89226	2.1
50676	1.89226	2.1
50677	0.946131	2.1
50678	0.946131	2.1
50679	1.32241	2.1
50680	1.25167	2.1
50681	1.25167	2.1
50682	1.25167	2.1
50683	0.946133	2.1
50684	0.946133	2.1
50685	0.946131	2.1
50686	1.32241	2.1
50687	0.946131	2.1
50688	1.25167	2.1
50689	1.65151	2.1

50690	1.92471	2.1
50691	1.81834	2.1
50692	2.13272	2.1
50693	3.74306	2.1
50694	1.92472	2.1
50695	1.9943	2.1
50696	2.13273	2.1
50697	2.24475	2.1
50698	4.16947	2.1
50699	1.81834	2.1
50700	2.13272	2.1
50701	2.02687	2.1
50702	2.39605	2.1
50703	4.21439	2.1
50704	2.13273	2.1
50705	2.24475	2.1
50706	2.39605	2.1
50707	2.54903	2.1
50708	4.6408	2.1
50709	1.10534	2.1
50710	1.22993	2.1
50711	0.946132	2.1
50712	0.946132	2.1
50713	0.946132	2.1
50714	0.946132	2.1
50715	1.22993	2.1
50716	1.22993	2.1
50717	1.10534	2.1
50718	0.946131	2.1
50719	0.946132	2.1
50720	1.22993	2.1
50721	1.47221	2.1
50722	2.14647	2.1
50723	1.95567	2.1
50724	1.95567	2.1
50725	1.71082	2.1
50726	1.71082	2.1
50727	0.946131	2.1
50728	0.946131	2.1
50729	0.946131	2.1
50730	0.946131	2.1
50731	0.946132	2.1
50732	0.946132	2.1
50733	0.946131	2.1
50734	0.946131	2.1
50735	0.946131	2.1
50736	0.946131	2.1
50737	0.946132	2.1
50738	0.946132	2.1
50739	0.998086	2.1
50740	1.09429	2.1
50741	0.946125	2.1
50742	0.946125	2.1
50743	0.90732	2.1
50744	0.90732	2.1
50745	1.14414	2.1
50746	1.16553	2.1
50747	1.05018	2.1

50748	0.907332	2.1
50749	0.99482	2.1
50750	0.851529	2.1
50751	0.80534	2.1
50752	0.80534	2.1
50753	0.812206	2.1
50754	0.812206	2.1
50755	0.895134	2.1
50756	0.977828	2.1
50757	0.736099	2.1
50758	0.639718	2.1
50759	0.639722	2.1
50760	0.89514	2.1
50761	0.736095	2.1
50762	0.977821	2.1
50763	0.946125	2.1
50764	0.946125	2.1
50765	0.946125	2.1
50766	0.946125	2.1
50767	0.946125	2.1
50768	0.946125	2.1
50769	0.946124	2.1
50770	0.946124	2.1
50771	0.946131	2.1
50772	0.946131	2.1
50773	0.946118	2.1
50774	0.946118	2.1
50775	2.22622	0.05
50776	2.43347	0.05
50777	2.34108	0.05
50778	1.24587	2.1
50779	1.21674	2.1
50780	1.14533	2.1
50781	1.11311	2.1
50782	1.17874	2.1
50783	1.17874	2.1
50784	1.18024	2.1
50785	1.18024	2.1
50786	1.14533	2.1
50787	1.24587	2.1
50788	1.21674	2.1
50789	1.11311	2.1
50790	2.43347	2.1
50791	2.34107	2.1
50792	2.39663	2.1
50793	2.49174	2.1
50794	4.8301	2.1
50795	1.86206	2.1
50796	1.41058	2.1
50797	1.70302	2.1
50798	2.42083	2.1
50799	3.68129	2.1
50800	1.70302	2.1
50801	2.43549	2.1
50802	2.20075	2.1
50803	2.50358	2.1
50804	2.04916	2.1
50805	2.33113	2.1

50806	1.07624	2.1
50807	2.12621	2.1
50808	1.57297	2.1
50809	1.60383	2.1
50810	1.30047	2.1
50811	1.30047	2.1
50812	2.33379	2.1
50813	2.56149	1.21286
50814	2.36734	1.51362
50815	2.62796	1.45291
50816	4.92883	1.73675
50817	2.5615	1.35027
50818	2.57957	1.47537
50819	2.62796	1.08384
50820	2.69056	0.968671
50821	5.25206	1.8769
50822	2.39322	2.06296
50823	2.62796	2.09571
50824	2.6849	2.1
50825	2.6849	2.06212
50826	2.36734	2.1
50827	2.36734	2.07113
50828	2.62796	1.85347
50829	2.69056	1.8954
50830	2.6849	0.25027
50831	2.77227	0.169064
50832	5.37546	0.600484
50833	1.89226	0.05
50834	1.89226	0.05
50835	1.89226	0.05
50836	1.89226	0.05
50837	1.89226	0.05
50838	1.89226	0.05
50839	1.06974	0.05
50840	1.06974	0.05
50841	1.02299	0.05
50842	1.02299	0.05
50843	1.35299	0.05
50844	1.26227	0.05
50845	0.646252	0.05
50846	0.830464	0.05
50847	0.830463	0.05
50848	1.35299	0.05
50849	1.26227	0.05
50850	0.646252	0.05
50851	2.52454	0.05
50852	1.89226	0.05
50853	2.70598	0.05
50854	1.89226	0.05
50855	4.59824	0.05
50856	1.89226	0.05
50857	1.89226	0.05
50858	1.89226	0.05
50859	1.89226	0.05
50860	1.89226	0.05
50861	1.89226	0.05
50862	1.89226	0.05
50863	1.89226	0.05

50864	1.89226	0.05
50865	1.89226	0.05
50866	1.89226	0.05
50867	1.89226	0.05
50868	1.7666	0.05
50869	2.70598	0.05
50870	2.65378	0.05
50871	2.65378	0.05
50872	1.66093	0.05
50873	1.66093	0.05
50874	2.70598	0.05
50875	1.89226	0.05
50876	1.89226	0.05
50877	1.89226	0.05
50878	2.65377	0.05
50879	2.65377	0.05
50880	0.946132	0.05
50881	0.946132	0.05
50882	0.946131	0.05
50883	0.946131	0.05
50884	0.946131	0.05
50885	0.946131	0.05
50886	0.946132	0.05
50887	0.946132	0.05
50888	0.946132	0.05
50889	0.946132	0.05
50890	0.946131	0.05
50891	0.946131	0.05
50892	1.69858	2.1
50893	2.18238	2.1
50894	2.506	2.1
50895	1.93586	2.1
50896	4.20458	2.1
50897	2.21772	2.1
50898	2.18238	2.1
50899	2.506	2.1
50900	2.59215	2.1
50901	4.72372	2.1
50902	2.50596	2.1
50903	1.93584	2.1
50904	2.16728	2.1
50905	2.7679	2.1
50906	4.67325	2.1
50907	1.53296	2.1
50908	1.56299	2.1
50909	1.42624	2.1
50910	1.52961	2.1
50911	2.98923	2.1
50912	1.56299	2.1
50913	1.46568	2.1
50914	1.52961	2.1
50915	1.51916	2.1
50916	3.08215	2.1
50917	1.36226	2.1
50918	1.52961	2.1
50919	1.36226	2.1
50920	1.42624	2.1
50921	1.55369	2.1

50922	1.61768	2.1
50923	1.52961	2.1
50924	1.51916	2.1
50925	1.55369	2.1
50926	1.64892	2.1
50927	3.07285	2.1
50928	2.13405	0.05
50929	2.57507	0.05
50930	2.39438	0.05
50931	1.93408	0.05
50932	4.52843	0.05
50933	2.13405	0.05
50934	1.27778	0.05
50935	1.2545	0.05
50936	1.45448	0.05
50937	1.93408	0.05
50938	2.13405	0.05
50939	2.39438	0.05
50940	1.93407	0.05
50941	1.60578	0.05
50942	1.87985	0.05
50943	4.00016	0.05
50944	1.0525	2.1
50945	1.05758	2.1
50946	1.43484	0.628309
50947	1.38506	1.99143
50948	2.48734	2.08895
50949	1.20455	2.1
50950	1.67352	2.1
50951	1.52852	2.1
50952	2.05164	2.1
50953	3.20203	2.1
50954	1.89226	2.1
50955	1.89226	2.1
50956	1.89226	2.1
50957	1.89226	2.1
50958	3.78452	2.1
50959	1.89226	2.1
50960	1.89226	2.1
50961	1.89226	2.1
50962	1.89226	2.1
50963	1.89226	2.1
50964	1.89226	2.1
50965	2.05165	2.1
50966	1.94291	2.1
50967	2.09398	2.1
50968	1.67352	2.1
50969	2.36337	2.1
50970	1.94291	2.1
50971	1.52851	2.1
50972	2.52049	2.1
50973	2.43433	2.1
50974	2.05164	2.1
50975	1.9112	2.1
50976	1.52851	2.1
50977	1.89226	2.1
50978	1.89226	2.1
50979	1.89226	2.1

50980	1.89226	2.1
50981	3.78451	2.1
50982	1.89226	2.1
50983	1.89226	2.1
50984	1.89226	2.1
50985	1.89226	2.1
50986	3.78452	2.1
50987	2.5205	2.1
50988	2.36337	2.1
50989	2.4605	2.1
50990	2.05165	2.1
50991	2.77222	2.1
50992	2.36337	2.1
50993	1.89226	2.1
50994	1.89226	2.1
50995	1.89226	2.1
50996	1.89226	2.1
50997	1.89226	2.1
50998	1.89226	2.1
50999	1.89226	2.1
51000	1.89226	2.1
51001	1.89226	2.1
51002	1.89226	2.1
51003	3.78452	2.1
51004	1.38905	2.1
51005	1.26166	2.1
51006	1.19216	2.1
51007	1.0875	2.1
51008	1.24393	2.1
51009	1.24393	2.1
51010	1.23261	2.1
51011	1.23261	2.1
51012	1.38905	2.1
51013	1.19216	2.1
51014	1.26166	2.1
51015	1.0875	2.1
51016	1.16233	2.1
51017	1.06028	2.1
51018	0.777395	2.1
51019	0.705508	2.1
51020	0.940018	2.1
51021	0.936465	2.1
51022	0.954216	2.1
51023	0.954216	2.1
51024	1.16125	2.1
51025	0.685505	2.1
51026	1.1157	2.1
51027	0.703534	2.1
51028	1.89226	2.1
51029	1.89226	2.1
51030	1.89226	2.1
51031	1.89226	2.1
51032	1.89226	2.1
51033	1.89226	2.1
51034	1.89226	2.1
51035	1.89226	2.1
51036	1.89226	2.1
51037	1.89226	2.1

51038	1.89226	2.1
51039	1.89226	2.1
51040	2.38433	2.1
51041	1.43456	2.1
51042	1.93881	2.1
51043	2.36952	2.1
51044	4.32313	2.1
51045	1.8493	2.1
51046	1.5927	2.1
51047	1.45535	2.1
51048	1.38438	2.1
51049	1.89657	2.1
51050	1.8493	2.1
51051	2.0815	2.1
51052	1.6697	2.1
51053	2.0657	2.1
51054	1.5927	2.1
51055	2.59749	2.1
51056	2.0815	2.1
51057	2.76597	2.1
51058	1.89226	2.1
51059	1.89226	2.1
51060	1.89226	2.1
51061	2.6126	2.1
51062	2.6126	2.1
51063	2.62513	2.1
51064	1.89226	2.1
51065	1.89226	2.1
51066	1.89226	2.1
51067	2.76598	2.1
51068	2.76598	2.1
51069	2.54665	2.1
51070	2.6126	2.1
51071	2.76598	2.1
51072	2.76598	2.1
51073	2.34044	2.1
51074	2.34044	2.1
51075	2.62514	2.1
51076	2.76598	2.1
51077	2.54666	2.1
51078	2.45212	2.1
51079	5.2181	2.1
51080	2.14063	2.1
51081	1.47521	2.1
51082	1.27679	2.1
51083	1.47521	2.1
51084	2.11511	2.1
51085	2.31353	2.1
51086	0.93256	2.1
51087	1.07032	2.1
51088	0.638396	2.1
51089	0.521342	2.1
51090	0.790674	2.1
51091	0.790674	2.1
51092	0.80098	2.1
51093	0.800981	2.1
51094	0.932535	2.1
51095	0.521328	2.1

51096	1.07034	2.1
51097	0.638414	2.1
51098	1.89226	2.1
51099	1.89226	1.66189
51100	1.89226	1.85741
51101	1.89226	2.1
51102	3.78453	2.1
51103	1.89226	2.1
51104	1.9842	2.1
51105	1.89226	2.1
51106	1.89226	2.1
51107	2.79705	2.1
51108	2.79705	2.1
51109	0.469955	2.1
51110	0.791665	2.1
51111	0.989115	1.95969
51112	0.763235	2.1
51113	0.776356	2.1
51114	0.805461	2.1
51115	0.776759	2.1
51116	0.776759	2.1
51117	0.799151	2.1
51118	0.940869	2.1
51119	0.78449	2.1
51120	0.489635	2.1
51121	1.89226	1.48364
51122	1.89226	2.1
51123	1.89226	1.46491
51124	1.89226	2.1
51125	1.89226	1.24112
51126	1.89226	2.1
51127	2.46058	2.05466
51128	2.28991	2.1
51129	1.8525	2.1
51130	1.87697	2.1
51131	2.28991	2.07803
51132	1.62397	2.1
51133	0.946131	0.05
51134	0.974033	0.05
51135	1.31667	0.05
51136	0.946131	0.05
51137	0.885497	0.05
51138	0.885497	0.05
51139	1.0468	0.05
51140	1.04678	0.05
51141	1.31667	0.05
51142	0.974019	0.05
51143	0.946131	0.05
51144	0.946131	0.05
51145	1.89226	0.05
51146	1.89226	0.05
51147	1.89226	0.05
51148	1.89226	0.05
51149	3.78452	0.05
51150	0.946125	0.05
51151	0.946125	0.05
51152	0.946131	0.05
51153	0.946131	0.05

51154	0.946128	0.05
51155	0.946128	0.05
51156	0.946128	0.05
51157	0.946128	0.05
51158	0.946128	0.05
51159	0.946128	0.05
51160	0.946128	0.05
51161	0.946128	0.05
51162	0.946131	0.05
51163	0.98453	0.05
51164	1.32276	0.05
51165	0.946131	0.05
51166	0.946132	0.05
51167	0.946132	0.05
51168	0.98453	0.05
51169	1.32276	0.05
51170	1.32276	0.05
51171	1.32276	0.05
51172	0.946132	0.05
51173	0.946132	0.05
51174	1.89227	2.1
51175	1.89227	2.1
51176	1.89227	2.1
51177	1.89227	2.1
51178	1.89227	2.1
51179	1.89227	2.1
51180	1.89226	2.1
51181	1.89226	2.1
51182	1.89226	2.1
51183	1.89226	2.1
51184	1.89226	2.1
51185	1.89226	2.1
51186	1.05556	2.1
51187	1.38859	2.1
51188	1.46996	2.1
51189	1.25364	2.1
51190	2.52552	2.1
51191	0.694296	2.1
51192	0.73498	2.1
51193	0.561881	2.1
51194	0.512189	2.1
51195	0.621304	2.1
51196	0.621304	2.1
51197	0.625865	2.1
51198	0.625865	2.1
51199	0.734979	2.1
51200	0.561881	2.1
51201	0.51219	2.1
51202	0.694296	2.1
51203	0.946126	2.1
51204	0.946126	2.1
51205	0.946126	2.1
51206	0.946126	2.1
51207	0.946126	2.1
51208	0.946126	2.1
51209	0.946126	2.1
51210	0.946126	2.1
51211	0.946132	2.1

51212	0.946132	2.1
51213	0.94612	2.1
51214	0.94612	2.1
51215	1.89225	2.1
51216	1.89225	2.1
51217	1.89225	2.1
51218	1.89225	2.1
51219	1.89225	2.1
51220	1.89225	2.1
51221	0.626814	2.1
51222	0.632155	2.1
51223	0.746234	2.1
51224	0.734974	2.1
51225	0.68202	2.1
51226	0.68202	2.1
51227	0.685108	2.1
51228	0.685108	2.1
51229	0.746239	2.1
51230	0.632159	2.1
51231	0.734969	2.1
51232	0.62681	2.1
51233	0.746235	2.1
51234	0.734975	2.1
51235	0.595081	2.1
51236	0.561877	2.1
51237	0.648584	2.1
51238	0.648584	2.1
51239	0.659528	2.1
51240	0.659528	2.1
51241	0.746239	2.1
51242	0.595085	2.1
51243	0.734969	2.1
51244	0.561874	2.1
51245	1.89249	2.1
51246	2.2137	2.1
51247	1.33125	2.1
51248	1.06499	2.1
51249	2.12571	2.1
51250	1.78918	2.1
51251	0.946131	2.1
51252	0.946131	2.1
51253	0.94613	2.1
51254	0.94613	2.1
51255	0.946132	2.1
51256	0.946132	2.1
51257	0.946129	2.1
51258	0.946129	2.1
51259	0.946131	2.1
51260	0.946131	2.1
51261	0.946131	2.1
51262	0.946131	2.1
51263	1.89227	2.1
51264	1.89227	2.1
51265	1.89227	2.1
51266	1.89227	2.1
51267	3.78453	2.1
51268	1.89226	2.1
51269	1.89226	2.1

51270	1.89226	2.1
51271	1.89226	2.1
51272	1.89226	2.1
51273	1.89226	2.1
51274	2.01388	2.1
51275	1.38757	2.1
51276	1.24229	2.1
51277	1.3309	2.1
51278	1.88011	2.1
51279	2.01388	2.1
51280	2.01388	2.1
51281	2.01555	2.1
51282	2.13879	2.1
51283	1.80766	2.1
51284	4.15267	2.1
51285	0.946133	2.1
51286	0.946133	2.1
51287	0.946132	2.1
51288	0.946132	2.1
51289	0.946132	2.1
51290	0.946132	2.1
51291	0.946133	2.1
51292	0.946133	2.1
51293	0.946132	2.1
51294	0.946132	2.1
51295	0.946132	2.1
51296	0.946132	2.1
51297	1.89226	2.1
51298	1.89226	2.1
51299	1.89226	2.1
51300	1.89226	2.1
51301	1.89226	2.1
51302	1.89226	2.1
51303	0.676745	2.1
51304	0.68382	2.1
51305	1.07048	2.1
51306	1.1327	2.1
51307	0.929324	2.1
51308	0.871438	2.1
51309	0.867362	2.1
51310	0.867362	2.1
51311	1.10666	2.1
51312	0.695036	2.1
51313	0.744098	2.1
51314	1.10144	2.1
51315	2.25168	2.1
51316	2.22499	2.1
51317	2.15929	2.1
51318	2.13879	2.1
51319	2.15929	2.1
51320	2.13879	2.1
51321	2.15314	2.1
51322	1.70302	2.1
51323	2.24694	2.1
51324	1.65245	2.1
51325	3.82722	2.1
51326	1.65245	2.1
51327	1.00371	2.1

51328	1.7437	2.1
51329	1.65748	2.1
51330	1.10459	2.1
51331	1.00371	2.1
51332	2.24694	2.1
51333	1.65246	2.1
51334	2.69773	2.1
51335	1.88129	2.1
51336	4.12824	2.1
51337	1.65246	2.1
51338	1.10459	2.1
51339	1.88129	2.1
51340	1.05914	2.1
51341	2.76496	2.1
51342	1.89226	2.1
51343	1.89226	2.1
51344	1.89226	2.1
51345	1.89226	2.1
51346	3.78452	2.1
51347	0.718577	2.1
51348	0.718577	2.1
51349	0.725249	2.1
51350	0.725249	2.1
51351	0.597916	2.1
51352	0.61752	2.1
51353	0.826306	2.1
51354	0.859194	2.1
51355	0.826306	2.1
51356	0.597916	2.1
51357	0.859194	2.1
51358	0.61752	2.1
51359	1.25898	2.1
51360	1.25898	2.1
51361	0.946128	2.1
51362	0.946128	2.1
51363	1.25897	2.1
51364	1.25585	2.1
51365	0.946131	2.1
51366	0.946131	2.1
51367	0.946129	2.1
51368	1.25586	2.1
51369	1.25898	2.1
51370	0.946129	2.1
51371	1.89226	2.1
51372	1.89226	2.1
51373	1.89226	2.1
51374	1.89226	2.1
51375	3.78452	2.1
51376	0.693258	2.1
51377	0.693258	2.1
51378	0.697356	2.1
51379	0.697356	2.1
51380	0.572479	2.1
51381	0.597916	2.1
51382	0.792698	2.1
51383	0.826306	2.1
51384	0.572478	2.1
51385	0.792697	2.1

51386	0.826307	2.1
51387	0.597917	2.1
51388	1.25586	2.1
51389	1.25586	2.1
51390	0.946128	2.1
51391	0.946128	2.1
51392	1.25585	2.1
51393	1.24344	2.1
51394	0.946131	2.1
51395	0.946131	2.1
51396	0.946129	2.1
51397	1.24344	2.1
51398	0.94613	2.1
51399	1.25586	2.1
51400	1.65151	2.1
51401	1.81835	2.1
51402	1.12064	2.1
51403	1.12064	2.1
51404	1.24965	2.1
51405	1.81835	2.1
51406	1.24965	2.1
51407	1.81835	2.1
51408	1.41077	2.1
51409	2.02687	2.1
51410	3.22911	2.1
51411	1.89122	1.0993
51412	1.38776	2.1
51413	2.37014	0.693139
51414	1.80818	2.1
51415	3.6994	2.09684
51416	2.15466	0.626414
51417	2.00083	2.1
51418	1.80818	1.93836
51419	1.80818	0.632862
51420	2.31988	2.08034
51421	2.31988	0.64465
51422	1.80818	0.382707
51423	1.18041	2.1
51424	2.00082	1.92385
51425	1.43652	2.1
51426	3.18123	2.1
51427	1.89226	0.891397
51428	1.89226	1.86257
51429	1.89226	1.15197
51430	1.89226	0.854096
51431	3.78453	1.20087
51432	1.89226	1.82052
51433	1.89226	1.83496
51434	1.89226	1.07712
51435	1.89226	1.17963
51436	3.78452	1.89972
51437	0.80759	1.50629
51438	0.686119	1.86309
51439	0.972147	1.58978
51440	1.00217	1.84344
51441	0.821334	2.0797
51442	0.821334	1.88454
51443	0.866957	1.53305

51444	0.866957	1.4741
51445	0.972145	1.93021
51446	0.686121	2.1
51447	0.807592	2.00519
51448	1.00217	2.1
51449	0.972146	1.64715
51450	1.00217	1.83279
51451	1.26654	2.00525
51452	1.1908	1.61812
51453	1.05501	1.80274
51454	1.05501	2.1
51455	1.10794	1.5575
51456	1.10794	0.582691
51457	0.972146	0.842357
51458	1.26654	1.85568
51459	1.00217	1.58341
51460	1.1908	2.1
51461	1.89226	1.50676
51462	1.89226	0.525501
51463	1.89226	0.855264
51464	1.89226	2.03498
51465	1.89226	0.301193
51466	1.89226	1.19707
51467	0.972147	1.69898
51468	0.867854	1.90707
51469	1.26132	1.22089
51470	1.26654	1.8121
51471	1.0424	1.95169
51472	1.0424	2.1
51473	1.092	0.442636
51474	1.092	1.78455
51475	1.26132	1.86152
51476	0.867854	2.1
51477	0.972147	2.05875
51478	1.26654	2.1
51479	1.4375	0.05
51480	1.92998	0.05
51481	1.66953	0.05
51482	1.0953	0.05
51483	3.10703	0.05
51484	1.89226	2.1
51485	1.89226	2.1
51486	1.89226	2.1
51487	1.89226	2.1
51488	3.78452	2.1
51489	2.74034	2.1
51490	2.08808	2.1
51491	2.63859	2.1
51492	2.00343	2.1
51493	4.72667	2.1
51494	0.799825	2.1
51495	0.799825	2.1
51496	0.80211	2.1
51497	0.80211	2.1
51498	0.562428	2.1
51499	0.600223	2.1
51500	1.04404	2.1
51501	1.00171	2.1

51502	1.00171	2.1
51503	0.56243	2.1
51504	0.600225	2.1
51505	1.04404	2.1
51506	0.94613	2.1
51507	0.94613	2.1
51508	0.946128	2.1
51509	0.946128	2.1
51510	0.946127	2.1
51511	0.946127	2.1
51512	0.946131	2.1
51513	0.946131	2.1
51514	0.946129	2.1
51515	0.946129	2.1
51516	0.946129	2.1
51517	0.946129	2.1
51518	1.89226	2.1
51519	1.89226	2.1
51520	1.89226	2.1
51521	1.89226	2.1
51522	3.78452	2.1
51523	2.63859	2.1
51524	2.00343	2.1
51525	2.54388	2.1
51526	1.91548	2.1
51527	4.55407	2.1
51528	1.91548	2.1
51529	2.00342	2.1
51530	1.04909	2.1
51531	1.04909	2.1
51532	1.12486	2.1
51533	1.99124	2.1
51534	0.810942	2.1
51535	0.810942	2.1
51536	0.810942	2.1
51537	0.810942	2.1
51538	1.89226	2.1
51539	1.35169	2.1
51540	1.08131	2.1
51541	1.08132	2.1
51542	1.08132	2.1
51543	0.810944	2.1
51544	0.810944	2.1
51545	1.89226	0.05
51546	1.89226	0.326932
51547	1.89226	0.05
51548	1.89226	0.637287
51549	3.78452	0.0692688
51550	1.7968	0.05
51551	1.49831	0.279633
51552	1.59244	0.05
51553	1.3395	0.0786368
51554	3.09074	0.05
51555	1.49831	0.05
51556	1.16858	2.1
51557	1.33951	0.05
51558	1.0361	2.1
51559	2.53442	0.916751

51560	1.89226	0.151378
51561	1.89226	0.05
51562	1.89226	0.283581
51563	1.89226	2.1
51564	1.89226	2.1
51565	1.89226	2.1
51566	1.89223	0.05
51567	1.89223	0.217589
51568	1.89223	0.211845
51569	1.89223	0.530336
51570	1.89223	0.05
51571	1.89223	0.116502
51572	1.59241	0.05
51573	1.18952	0.0923299
51574	1.33949	0.0828811
51575	1.33949	0.05
51576	1.40538	0.0919099
51577	1.40538	0.05
51578	1.89226	0.05
51579	1.89226	0.0790837
51580	1.89226	0.05
51581	1.89226	0.0627593
51582	1.89226	0.05
51583	1.89226	0.0785771
51584	1.89226	2.1
51585	1.89226	2.1
51586	1.89226	2.1
51587	1.89226	2.1
51588	3.78452	2.1
51589	1.89226	2.1
51590	1.89226	2.1
51591	1.89226	2.1
51592	1.89226	2.1
51593	3.78452	2.1
51594	2.01867	2.1
51595	1.46955	2.1
51596	1.58476	2.1
51597	1.38189	2.1
51598	2.22153	2.1
51599	2.01867	2.1
51600	2.01867	2.1
51601	2.52881	2.1
51602	2.21732	2.1
51603	2.21732	2.1
51604	2.22154	2.1
51605	2.22154	2.1
51606	1.89226	2.1
51607	1.89226	2.1
51608	1.89226	2.1
51609	1.89226	2.1
51610	1.89226	2.1
51611	1.89226	2.1
51612	1.89226	2.1
51613	1.89226	2.1
51614	1.89226	2.1
51615	1.89226	2.1
51616	1.89226	2.1
51617	1.89226	2.1

51618	2.22153	2.1
51619	1.63361	2.1
51620	1.77336	2.1
51621	1.46955	2.1
51622	2.52534	2.1
51623	2.22153	2.1
51624	2.34431	2.1
51625	1.62905	2.1
51626	1.49693	2.1
51627	2.19197	2.1
51628	3.82103	2.1
51629	1.89226	2.1
51630	1.89226	2.1
51631	1.89226	2.1
51632	1.89226	2.1
51633	3.78453	2.1
51634	0.946128	2.1
51635	0.946128	2.1
51636	1.22372	2.1
51637	1.17331	2.1
51638	0.946131	2.1
51639	0.946131	2.1
51640	1.17331	2.1
51641	1.17331	2.1
51642	0.946131	2.1
51643	1.17331	2.1
51644	1.22372	2.1
51645	0.94613	2.1
51646	1.04041	2.1
51647	1.09599	2.1
51648	0.748462	2.1
51649	0.701976	2.1
51650	0.896701	2.1
51651	0.896701	2.1
51652	0.901262	2.1
51653	0.901262	2.1
51654	1.04041	2.1
51655	0.701977	2.1
51656	0.748463	2.1
51657	1.09599	2.1
51658	1.89226	2.1
51659	1.89226	2.1
51660	1.89226	2.1
51661	1.89226	2.1
51662	3.78453	2.1
51663	0.946128	2.1
51664	0.946128	2.1
51665	1.17331	2.1
51666	1.13785	2.1
51667	0.94613	2.1
51668	0.94613	2.1
51669	1.13785	2.1
51670	1.13785	2.1
51671	1.13785	2.1
51672	0.94613	2.1
51673	0.94613	2.1
51674	1.17331	2.1
51675	2.50381	2.1

51676	1.89226	2.1
51677	1.89226	2.1
51678	2.73387	2.1
51679	4.39607	2.1
51680	1.80045	2.1
51681	1.99777	0.171913
51682	2.45134	2.1
51683	2.12463	2.1
51684	1.70302	2.1
51685	1.42655	2.1
51686	2.20231	2.1
51687	1.70302	2.02295
51688	1.93128	2.1
51689	1.66768	2.1
51690	1.24499	0.386231
51691	1.70302	0.377008
51692	2.55572	0.05
51693	1.22811	0.05
51694	1.82584	0.05
51695	1.22811	0.05
51696	2.62764	0.05
51697	1.93147	0.05
51698	2.14513	0.05
51699	1.89226	0.05
51700	2.66481	0.05
51701	1.89226	0.05
51702	2.66481	0.05
51703	1.89226	0.05
51704	2.11649	2.1
51705	1.89226	2.1
51706	1.89226	2.1
51707	1.89226	2.1
51708	2.15426	2.1
51709	2.15426	2.1
51710	2.06776	2.1
51711	1.89226	2.1
51712	1.89226	2.1
51713	1.89226	2.1
51714	2.11649	2.1
51715	2.11649	2.1
51716	1.89226	2.1
51717	1.89226	2.1
51718	1.89226	2.1
51719	1.89226	2.1
51720	1.89226	2.1
51721	1.89226	2.1
51722	0.998134	2.1
51723	0.918904	2.1
51724	0.894708	2.1
51725	0.758742	2.1
51726	0.864276	2.1
51727	0.864276	2.1
51728	0.8926	2.1
51729	0.8926	2.1
51730	0.998134	2.1
51731	0.894709	2.1
51732	0.758742	2.1
51733	0.918904	2.1

51734	1.89226	2.1
51735	1.89226	2.1
51736	1.89226	2.1
51737	1.89226	2.1
51738	1.89226	2.1
51739	1.89226	2.1
51740	1.89226	2.1
51741	1.89226	2.1
51742	1.89226	2.1
51743	1.89226	2.1
51744	1.89226	2.1
51745	1.89226	2.1
51746	1.99627	2.1
51747	1.61047	2.1
51748	2.0815	2.1
51749	2.07569	2.1
51750	3.75063	2.1
51751	0.848077	2.1
51752	1.07748	2.1
51753	0.802498	2.1
51754	0.626808	2.1
51755	0.875552	2.1
51756	0.737527	2.1
51757	0.835602	2.1
51758	0.835602	2.1
51759	0.983245	2.1
51760	0.781108	2.1
51761	0.872306	2.1
51762	0.601497	2.1
51763	2.74745	2.1
51764	2.73988	2.1
51765	2.6521	2.1
51766	2.67792	2.1
51767	5.39198	2.1
51768	2.73988	2.1
51769	2.57466	2.1
51770	2.67791	2.1
51771	2.55653	2.1
51772	5.29641	2.1
51773	2.6521	2.1
51774	2.64148	2.1
51775	2.67792	2.1
51776	2.67792	2.1
51777	2.56953	2.1
51778	2.56953	2.1
51779	2.64148	2.1
51780	2.55653	2.1
51781	2.57929	2.1
51782	2.57929	2.1
51783	2.67792	2.1
51784	2.67792	2.1
51785	1.89226	0.145813
51786	1.89226	0.05
51787	1.89226	0.0642275
51788	1.89226	0.05
51789	3.78453	0.05
51790	1.68703	0.14066
51791	2.50385	0.05

51792	1.45958	0.05
51793	2.0815	0.05
51794	3.93806	0.05
51795	2.50385	0.05
51796	2.57414	0.05
51797	2.0815	0.05
51798	2.39921	0.05
51799	4.91181	0.05
51800	1.89226	0.115409
51801	1.89226	0.05
51802	1.89226	0.05
51803	1.89226	0.05
51804	3.78453	0.05
51805	1.89226	0.16745
51806	1.89226	0.05
51807	1.89226	0.05
51808	1.89226	0.05
51809	1.89226	0.05
51810	1.89226	0.10179
51811	1.23013	0.05
51812	2.0815	0.05
51813	1.90167	0.05
51814	1.71243	0.05
51815	1.45887	0.0555096
51816	1.31369	0.05
51817	2.0815	0.05
51818	2.39921	0.05
51819	1.7115	0.05
51820	2.2421	0.05
51821	4.09829	0.05
51822	1.89227	0.05
51823	1.89227	0.05
51824	1.89227	0.05
51825	1.89227	0.05
51826	3.78453	0.05
51827	2.39697	0.05
51828	1.85594	0.05
51829	1.91161	0.05
51830	2.49867	0.05
51831	4.35461	0.05
51832	1.12035	0.05
51833	1.85594	0.05
51834	1.91161	0.05
51835	1.15483	0.05
51836	3.01077	0.05
51837	1.89226	0.05
51838	1.89226	0.05
51839	1.89226	0.05
51840	1.89226	0.05
51841	1.89226	0.05
51842	1.89226	0.05
51843	1.89226	0.05
51844	1.89226	0.05
51845	1.89226	0.05
51846	1.89226	0.05
51847	1.89226	0.05
51848	1.89226	0.05
51849	0.936516	0.05

51850	1.03496	0.05
51851	1.22607	0.05
51852	1.28058	0.05
51853	1.06317	0.05
51854	1.06317	0.05
51855	1.19735	0.05
51856	1.0813	0.05
51857	0.858168	0.05
51858	1.32278	0.05
51859	0.957674	0.05
51860	1.25178	0.05
51861	1.92659	0.05
51862	0.998957	0.05
51863	0.998957	0.05
51864	1.15481	0.05
51865	1.6031	0.05
51866	1.74459	0.05
51867	0.946119	0.05
51868	0.946119	0.05
51869	0.946118	0.05
51870	0.946118	0.05
51871	0.946118	0.05
51872	0.946118	0.05
51873	0.946119	0.05
51874	0.946119	0.05
51875	0.946131	0.05
51876	0.946131	0.05
51877	0.946106	0.05
51878	0.946106	0.05
51879	0.946118	0.05
51880	0.946118	0.05
51881	0.946119	0.05
51882	0.946119	0.05
51883	0.946118	0.05
51884	0.946118	0.05
51885	0.946119	0.05
51886	0.946119	0.05
51887	0.946131	0.05
51888	0.946131	0.05
51889	0.946106	0.05
51890	0.946106	0.05
51891	2.47164	0.0545914
51892	2.83689	0.05
51893	2.73872	0.0677649
51894	2.41632	0.05
51895	5.21036	0.05
51896	0.946133	0.05
51897	0.946133	0.0667135
51898	1.41845	0.05
51899	1.36936	0.0545921
51900	1.41845	0.05
51901	1.41845	0.05
51902	0.946132	0.0657059
51903	0.946132	0.0652103
51904	0.946132	0.05
51905	1.36936	0.05
51906	1.41845	0.05
51907	0.946133	0.0538351

51908	1.36936	0.05
51909	1.27581	0.05
51910	1.20816	0.05
51911	1.10464	0.05
51912	1.24454	0.05
51913	1.24454	0.05
51914	1.23943	0.05
51915	1.23943	0.05
51916	1.10465	0.05
51917	1.27581	0.05
51918	1.36936	0.05
51919	1.20816	0.05
51920	0.946131	2.1
51921	0.946131	2.1
51922	1.37277	2.1
51923	1.37277	2.1
51924	1.37277	2.1
51925	1.27186	2.1
51926	0.946131	2.1
51927	0.946131	2.1
51928	0.946131	2.1
51929	1.37277	2.1
51930	0.946131	2.1
51931	1.27186	2.1
51932	2.74554	2.1
51933	1.23712	2.1
51934	2.80992	2.1
51935	2.54371	2.1
51936	1.50333	2.1
51937	1.23712	2.1
51938	2.77314	2.1
51939	2.33316	2.1
51940	2.28748	2.1
51941	2.28748	2.1
51942	2.81275	2.1
51943	2.81275	2.1
51944	2.28749	2.1
51945	1.45887	2.1
51946	2.33317	2.1
51947	1.50022	2.1
51948	3.79204	2.1
51949	2.37364	2.1
51950	2.33317	2.1
51951	1.5362	2.1
51952	1.5362	2.1
51953	1.50022	2.1
51954	2.33317	2.1
51955	1.89226	0.0598528
51956	1.89226	0.0632937
51957	1.89226	0.05
51958	1.89226	0.0645495
51959	1.89226	0.0508455
51960	1.89226	0.0834945
51961	0.946128	0.05
51962	0.946128	0.05
51963	1.0644	0.05
51964	0.946133	0.05
51965	1.0644	0.05

51966	0.94613	0.05
51967	0.946131	0.05
51968	0.946131	0.05
51969	1.0644	0.05
51970	1.0644	0.05
51971	0.946131	0.05
51972	0.946131	0.05
51973	1.89226	1.00378
51974	1.89226	0.0596295
51975	1.89226	0.175831
51976	1.89226	0.05
51977	3.78452	0.05
51978	1.14599	0.886292
51979	1.19993	0.05
51980	1.00065	0.0788771
51981	1.07497	0.05
51982	2.22096	0.05
51983	0.546551	0.05
51984	0.546551	0.139169
51985	0.54987	0.0585381
51986	0.54987	0.509875
51987	0.503067	0.05
51988	0.558937	0.05
51989	0.599966	0.05
51990	0.537484	0.110017
51991	0.503067	0.05
51992	0.537485	0.05
51993	0.599966	0.0957008
51994	0.558936	0.05
51995	0.946132	0.05456
51996	0.946132	0.24175
51997	0.94613	0.05
51998	0.94613	0.877991
51999	0.946131	0.122172
52000	0.946131	0.0513041
52001	0.946131	0.206528
52002	0.946131	0.204092
52003	0.946131	0.05
52004	0.946131	0.05
52005	0.946131	0.170227
52006	0.946131	0.120357
52007	2.19104	2.1
52008	2.5392	2.1
52009	2.53302	2.1
52010	2.19226	2.1
52011	4.73146	2.1
52012	1.09552	2.1
52013	1.09613	2.1
52014	0.802484	2.1
52015	0.807941	2.1
52016	0.95052	2.1
52017	0.95052	2.1
52018	0.952944	2.1
52019	0.952944	2.1
52020	1.09613	2.1
52021	0.807941	2.1
52022	0.802484	2.1
52023	1.09552	2.1

52024	2.533	2.1
52025	2.18531	2.1
52026	2.19224	2.1
52027	2.19224	2.1
52028	2.51603	2.1
52029	2.51603	2.1
52030	1.09266	2.1
52031	1.09612	2.1
52032	0.807935	2.1
52033	0.81091	2.1
52034	0.951897	2.1
52035	0.951897	2.1
52036	0.955134	2.1
52037	0.955134	2.1
52038	0.810915	2.1
52039	1.09266	2.1
52040	1.09611	2.1
52041	0.80793	2.1
52042	2.12741	0.593809
52043	2.79312	0.212299
52044	2.77591	1.29631
52045	2.10714	0.168136
52046	4.90026	0.110015
52047	2.12741	0.307778
52048	1.20859	0.05
52049	1.21073	1.6126
52050	2.10714	0.338503
52051	3.31573	0.687195
52052	1.38708	0.478326
52053	1.38795	0.0809348
52054	1.05331	0.301013
52055	1.05357	0.2553
52056	1.22049	0.294885
52057	1.22049	0.429338
52058	1.22078	1.92383
52059	1.22078	0.05
52060	1.05331	0.195354
52061	1.38709	0.276194
52062	1.38795	0.202611
52063	1.05357	0.585347
52064	2.10662	0.981127
52065	2.10714	0.137421
52066	2.12499	0.200661
52067	1.22857	0.362527
52068	1.21073	0.130745
52069	1.21073	0.05
52070	1.82014	0.05
52071	2.08757	0.05
52072	1.81292	0.05
52073	1.67485	0.05
52074	3.63306	0.05
52075	1.82013	0.05
52076	1.21748	0.05
52077	1.29891	0.05
52078	1.4442	0.05
52079	1.67484	0.05
52080	1.82013	0.05
52081	1.81292	0.05

52082	1.4516	0.05
52083	1.39633	0.05
52084	1.67485	0.05
52085	1.5344	0.05
52086	1.81292	0.05
52087	1.67484	0.05
52088	1.00982	0.05
52089	0.994235	0.05
52090	1.21748	0.05
52091	1.45159	0.05
52092	1.67484	0.05
52093	1.55216	2.1
52094	1.34407	2.1
52095	1.34407	2.1
52096	1.52008	2.1
52097	1.81783	2.1
52098	1.99384	2.1
52099	1.90817	2.1
52100	2.03975	2.1
52101	1.97317	2.1
52102	1.81783	2.1
52103	3.85758	2.1
52104	0.599049	2.1
52105	0.660673	2.1
52106	0.785063	2.1
52107	0.924278	2.1
52108	0.760416	2.1
52109	0.760416	2.1
52110	0.719088	2.1
52111	0.694532	2.1
52112	0.522441	2.1
52113	0.796789	2.1
52114	0.655207	2.1
52115	0.886155	2.1
52116	1.97317	2.1
52117	1.81783	2.1
52118	1.65098	2.1
52119	1.91767	2.1
52120	3.7355	2.1
52121	1.15038	1.07581
52122	1.28649	2.01204
52123	1.38986	1.54231
52124	1.38986	2.1
52125	1.07866	1.7643
52126	1.07866	2.1
52127	0.664498	0.0814141
52128	0.664498	2.1
52129	0.675122	2.1
52130	0.675122	2.1
52131	0.69493	0.452601
52132	0.764429	1.90454
52133	0.623438	1.55036
52134	0.575191	2.1
52135	0.764429	1.03492
52136	0.623438	0.718839
52137	0.575191	2.1
52138	0.69493	1.98384
52139	1.89226	0.64692

52140	1.89226	0.0950211
52141	1.89226	0.654473
52142	1.89226	0.0806746
52143	3.78453	0.05
52144	2.50075	0.16152
52145	2.15276	0.0928372
52146	2.24902	0.787665
52147	2.30422	0.0632672
52148	4.40178	0.05
52149	1.70424	0.231261
52150	1.34494	0.300321
52151	2.15276	0.05
52152	2.15276	0.245511
52153	2.0793	0.05
52154	1.63078	0.100521
52155	1.89226	0.05
52156	1.89226	0.416028
52157	1.89226	1.06359
52158	1.89226	0.05
52159	1.89226	0.732736
52160	1.89226	0.201571
52161	1.89226	0.174745
52162	1.89226	0.163534
52163	1.89226	0.05
52164	1.89226	0.05
52165	3.78452	0.05
52166	2.24902	0.203305
52167	1.91497	0.05
52168	1.97135	0.174167
52169	1.73957	0.05
52170	3.98859	0.05
52171	1.73957	0.05
52172	1.34494	0.107588
52173	1.73957	0.900574
52174	1.69006	0.05
52175	1.3126	0.176192
52176	1.34848	0.0790478
52177	0.946132	0.05
52178	0.946132	0.446844
52179	0.946132	0.05
52180	0.946132	1.24433
52181	0.946132	0.268367
52182	0.946132	0.0740176
52183	0.946131	0.05
52184	0.946131	0.05
52185	0.946132	0.05
52186	0.946132	0.05
52187	0.946132	0.0811562
52188	0.946132	0.211621
52189	1.89226	2.1
52190	1.89226	1.89774
52191	1.89226	2.1
52192	1.89226	2.03922
52193	3.78453	2.1
52194	1.89226	2.1
52195	2.82624	1.78469
52196	1.89226	2.09308
52197	2.02536	1.92895

52198	1.89226	2.05725
52199	2.02536	1.70674
52200	2.25027	0.05
52201	1.1706	0.05
52202	2.02733	0.05
52203	2.02733	0.05
52204	1.61462	0.05
52205	1.69832	0.05
52206	1.89226	0.05
52207	1.89226	0.05
52208	1.89226	0.05
52209	1.89226	0.05
52210	3.78452	0.05
52211	0.946131	0.05
52212	0.946131	0.05
52213	1.18182	0.05
52214	1.18182	0.05
52215	0.973819	0.05
52216	0.94613	0.05
52217	1.18182	0.05
52218	0.94613	0.05
52219	0.973819	0.05
52220	1.18182	0.05
52221	0.94613	0.05
52222	0.94613	0.05
52223	2.23944	2.1
52224	1.52903	2.1
52225	1.35977	2.1
52226	2.04847	2.1
52227	3.5775	2.1
52228	2.23944	2.1
52229	2.34795	2.1
52230	2.34795	2.1
52231	2.48025	2.1
52232	2.04847	2.1
52233	2.18077	2.1
52234	1.89226	2.1
52235	1.89226	2.1
52236	1.89226	2.1
52237	1.89226	2.1
52238	3.78452	2.1
52239	0.946127	2.1
52240	0.946127	2.1
52241	0.946131	2.1
52242	0.946131	2.1
52243	0.946128	2.1
52244	0.946128	2.1
52245	0.94613	2.1
52246	0.94613	2.1
52247	0.946129	2.1
52248	0.946129	2.1
52249	0.946129	2.1
52250	0.946129	2.1
52251	1.26956	2.1
52252	2.04847	2.1
52253	1.49576	2.1
52254	1.22377	2.1
52255	1.91172	2.1

52256	1.50198	2.1
52257	1.48568	2.1
52258	2.34795	2.1
52259	2.04847	2.1
52260	2.04847	2.1
52261	2.21155	2.1
52262	2.21155	2.1
52263	1.89226	2.1
52264	1.89226	2.1
52265	1.89226	2.1
52266	1.89226	2.1
52267	1.89226	2.1
52268	1.89226	2.1
52269	0.810943	2.1
52270	0.810943	2.1
52271	0.810944	2.1
52272	0.810944	2.1
52273	0.810957	2.1
52274	0.810957	2.1
52275	1.89226	2.1
52276	1.35168	2.1
52277	1.08132	2.1
52278	1.08132	2.1
52279	1.0813	2.1
52280	0.946132	0.05
52281	1.33855	0.05
52282	0.946131	0.05
52283	1.38204	0.05
52284	0.946131	0.05
52285	0.946131	0.05
52286	1.38204	0.05
52287	1.38204	0.05
52288	0.946131	0.05
52289	0.946131	0.05
52290	1.33855	0.05
52291	1.38204	0.05
52292	1.89226	0.05
52293	2.76407	0.05
52294	1.89226	0.05
52295	2.72769	0.05
52296	1.89226	0.05
52297	2.72769	0.05
52298	2.03914	0.05
52299	1.67727	0.05
52300	1.94831	0.05
52301	1.81591	0.05
52302	2.32098	0.05
52303	2.32098	0.05
52304	0.801647	0.05
52305	1.11086	0.05
52306	0.838634	0.05
52307	1.16049	0.05
52308	0.977891	0.05
52309	0.977891	0.05
52310	0.984246	0.05
52311	0.984246	0.05
52312	1.11086	0.05
52313	1.16049	0.05

52314	0.801647	0.05
52315	0.838634	0.05
52316	0.946132	0.05
52317	0.946132	0.05
52318	0.946131	0.05
52319	0.946131	0.05
52320	0.946131	0.05
52321	0.946131	0.05
52322	0.946132	0.05
52323	0.946132	0.05
52324	0.946131	0.05
52325	0.946131	0.05
52326	0.946131	0.05
52327	0.946131	0.05
52328	0.946131	0.05
52329	0.946131	0.05
52330	0.946131	0.05
52331	0.946131	0.05
52332	0.946131	0.05
52333	0.946131	0.05
52334	0.946131	0.05
52335	0.946131	0.05
52336	0.946131	0.05
52337	0.946131	0.05
52338	0.946131	0.05
52339	0.946131	0.05
52340	2.0815	2.1
52341	1.20787	2.1
52342	1.92277	2.1
52343	1.3227	2.1
52344	3.29401	2.1
52345	2.16633	2.1
52346	2.13642	2.1
52347	1.40846	2.1
52348	1.40846	2.1
52349	1.3227	2.1
52350	2.08058	2.1
52351	0.938106	2.1
52352	0.790258	2.1
52353	0.742975	2.1
52354	0.742975	2.1
52355	0.874389	2.1
52356	1.17364	2.1
52357	0.525948	2.1
52358	0.52513	2.1
52359	0.553732	2.1
52360	0.876996	2.1
52361	0.530954	2.1
52362	1.095 2.1	
52363	1.00833	2.1
52364	1.70608	2.1
52365	1.33385	2.1
52366	1.33385	2.1
52367	1.07722	2.1
52368	1.07722	2.1
52369	0.953523	0.299814
52370	1.7457	0.198853
52371	1.03218	0.260009

52372	1.03218	0.262384
52373	1.79675	0.259203
52374	1.79675	0.266009
52375	1.13676	0.299582
52376	1.18136	0.122376
52377	0.87285	0.210175
52378	0.898375	0.295251
52379	1.03191	0.197635
52380	1.03191	0.112168
52381	1.0223	0.270832
52382	1.0223	0.291595
52383	1.18136	0.195301
52384	0.898374	0.160267
52385	0.87285	0.194756
52386	1.13676	0.191954
52387	2.36273	0.185046
52388	1.46965	0.102819
52389	1.79675	0.12872
52390	1.79675	0.0757298
52391	2.43417	0.18327
52392	2.43417	0.108773
52393	1.4714	2.1
52394	1.65434	2.1
52395	1.62322	0.320836
52396	1.86068	1.98986
52397	3.27756	1.76782
52398	1.89226	2.1
52399	1.89226	2.1
52400	1.89226	0.398032
52401	1.89226	1.19285
52402	3.78453	1.24071
52403	1.89226	1.35872
52404	1.89226	2.1
52405	1.89226	0.785433
52406	1.89226	2.1
52407	3.78453	1.87952
52408	1.65434	1.46367
52409	1.60647	2.1
52410	1.86068	1.08945
52411	1.82649	2.1
52412	3.48083	2.1
52413	1.62322	2.1
52414	1.86068	2.09712
52415	1.84368	0.350769
52416	2.18862	1.38491
52417	3.81184	1.67086
52418	1.89226	2.1
52419	1.89226	1.98367
52420	1.89226	0.183526
52421	1.89226	1.52129
52422	3.78453	1.64262
52423	1.89226	1.53439
52424	1.89226	2.1
52425	1.89226	0.915756
52426	1.89226	2.1
52427	3.78453	2.1
52428	1.86068	1.08795
52429	1.82649	2.1

52430	2.18862	0.869601
52431	2.1128	2.1
52432	4.01511	1.82785
52433	1.74351	2.1
52434	1.13854	2.1
52435	1.40233	2.1
52436	1.922 2.1	
52437	3.14584	2.1
52438	1.922 2.1	
52439	2.00775	2.1
52440	1.7435	2.1
52441	2.09224	2.1
52442	2.00775	2.1
52443	2.09295	2.1
52444	1.69858	2.1
52445	1.922 2.1	
52446	1.40233	2.1
52447	1.40233	2.1
52448	2.18238	2.1
52449	2.18238	2.1
52450	1.00387	2.1
52451	1.10886	2.1
52452	0.961001	2.1
52453	1.09119	2.1
52454	1.02868	2.1
52455	1.02868	2.1
52456	1.04118	2.1
52457	1.04118	2.1
52458	1.10886	2.1
52459	1.09119	2.1
52460	0.960999	2.1
52461	1.00388	2.1
52462	0.885958	0.352171
52463	0.885958	0.193838
52464	0.875826	0.136188
52465	0.875826	0.541872
52466	0.913965	0.610429
52467	0.598609	0.158882
52468	1.16317	0.227283
52469	0.868115	0.146941
52470	0.868115	0.405618
52471	0.598609	0.281695
52472	1.16317	0.190445
52473	0.913965	0.210665
52474	0.946131	0.347487
52475	0.946131	0.263771
52476	0.946131	0.573222
52477	0.946131	0.304245
52478	0.946131	0.44777
52479	0.946131	0.360687
52480	0.946131	0.293823
52481	0.946131	0.293803
52482	0.946131	0.404466
52483	0.946131	0.505633
52484	0.946131	0.328684
52485	0.946131	0.301787
52486	0.946133	0.05
52487	0.946133	0.05

52488	1.06246	0.05
52489	1.06246	0.05
52490	0.973851	0.05
52491	0.946133	0.05
52492	1.06246	0.05
52493	0.946132	0.05
52494	0.97385	0.05
52495	1.06246	0.05
52496	0.946133	0.05
52497	0.946133	0.05
52498	1.89226	0.05
52499	1.89226	0.05
52500	1.89226	0.05
52501	1.89226	0.05
52502	1.89226	0.05
52503	1.89226	0.05
52504	0.946131	0.05
52505	0.946131	0.05
52506	0.946127	0.05
52507	0.946127	0.05
52508	0.946131	0.05
52509	0.946131	0.05
52510	0.946127	0.05
52511	0.946127	0.05
52512	0.946128	0.05
52513	0.946128	0.05
52514	0.946129	0.05
52515	0.946129	0.05
52516	0.946132	0.05
52517	0.946132	0.05
52518	1.32446	0.05
52519	0.794912	0.05
52520	1.32429	0.05
52521	0.946131	0.05
52522	1.32446	0.05
52523	0.94613	0.05
52524	1.32429	0.05
52525	0.794915	0.05
52526	0.94613	0.05
52527	0.94613	0.05
52528	1.89226	0.05
52529	2.36365	0.05
52530	2.64857	0.05
52531	1.89226	0.05
52532	2.64857	0.05
52533	1.89226	0.05
52534	1.18463	2.1
52535	1.27174	2.1
52536	0.946129	2.1
52537	0.946129	2.1
52538	0.946131	2.1
52539	0.946131	2.1
52540	1.27174	2.1
52541	1.27174	2.1
52542	1.18463	2.1
52543	0.94613	2.1
52544	0.946131	2.1
52545	1.27174	2.1

52546	2.57997	2.1
52547	1.89226	2.1
52548	1.89226	2.1
52549	1.89226	2.1
52550	2.41915	2.1
52551	2.41915	2.1
52552	1.89226	2.1
52553	1.89226	2.1
52554	1.89226	2.1
52555	1.89226	2.1
52556	3.78452	2.1
52557	1.08807	2.1
52558	1.18463	2.1
52559	0.946129	2.1
52560	0.946129	2.1
52561	0.946131	2.1
52562	0.946131	2.1
52563	1.18463	2.1
52564	1.18463	2.1
52565	0.946131	2.1
52566	1.08807	2.1
52567	1.18463	2.1
52568	0.94613	2.1
52569	1.07017	2.1
52570	1.07017	2.1
52571	0.946131	2.1
52572	0.946131	2.1
52573	0.946132	2.1
52574	0.946132	2.1
52575	1.20957	2.1
52576	1.07017	2.1
52577	1.07017	2.1
52578	0.946132	2.1
52579	0.946131	2.1
52580	1.20957	2.1
52581	1.89226	2.1
52582	1.89226	2.1
52583	1.89226	2.1
52584	1.89226	2.1
52585	1.89226	2.1
52586	1.89226	2.1
52587	1.16181	0.05
52588	1.0227	0.05
52589	1.0227	0.05
52590	0.970959	0.05
52591	1.24295	0.05
52592	1.19121	0.05
52593	1.12076	2.1
52594	2.53614	2.1
52595	1.28413	2.1
52596	1.28413	2.1
52597	2.36423	2.1
52598	2.36423	2.1
52599	1.89226	2.1
52600	1.89226	2.1
52601	1.89226	2.1
52602	1.89226	2.1
52603	3.78452	2.1

52604	1.22557	2.1
52605	2.771	2.1
52606	2.73012	2.1
52607	2.73012	2.1
52608	1.33356	2.1
52609	1.33356	2.1
52610	0.998682	2.1
52611	2.36423	2.1
52612	1.12076	2.1
52613	1.12076	2.1
52614	2.13757	2.1
52615	2.13757	2.1
52616	1.89226	2.1
52617	1.89226	2.1
52618	1.89226	2.1
52619	1.89226	2.1
52620	1.89226	2.1
52621	1.89226	2.1
52622	1.13987	2.1
52623	2.73011	2.1
52624	2.70875	2.1
52625	2.70875	2.1
52626	1.22557	2.1
52627	1.22557	2.1
52628	1.89227	2.1
52629	2.79243	2.1
52630	2.6625	2.1
52631	2.6625	2.1
52632	1.89227	2.1
52633	1.89227	2.1
52634	1.89226	2.1
52635	2.55971	2.1
52636	1.89226	2.1
52637	1.89226	2.1
52638	2.68704	2.1
52639	2.68704	2.1
52640	2.22043	1.9662
52641	2.38975	1.86135
52642	2.28696	1.20707
52643	2.48682	0.690306
52644	4.67671	1.05075
52645	2.38976	0.964358
52646	2.31988	1.819
52647	2.48683	0.739556
52648	2.4594	1.56269
52649	4.84915	1.50949
52650	2.28696	1.90624
52651	2.48682	1.67398
52652	2.33379	2.09119
52653	2.56149	0.394288
52654	4.84845	1.54799
52655	2.48683	1.74303
52656	2.4594	1.65281
52657	2.5615	0.453081
52658	2.57957	1.1932
52659	5.0209	0.869922
52660	1.06659	2.1
52661	1.04291	0.180127

52662	1.36595	0.146031
52663	1.36595	2.1
52664	1.02761	2.1
52665	1.32697	0.094557
52666	0.946132	1.93759
52667	0.946132	1.81289
52668	1.36956	2.1
52669	1.1229	2.1
52670	1.1229	1.68774
52671	1.1229	2.1
52672	0.946132	0.574237
52673	0.946132	2.1
52674	0.946132	2.1
52675	1.36956	2.1
52676	1.1229	2.1
52677	0.946133	2.1
52678	0.946133	0.939716
52679	0.946133	0.685567
52680	0.946128	2.1
52681	0.946128	2.1
52682	0.946131	2.1
52683	0.946131	1.64204
52684	0.946131	0.119679
52685	0.946131	2.1
52686	0.94613	2.08433
52687	0.94613	1.97408
52688	0.946131	1.876
52689	0.946131	1.84206
52690	0.966427	1.22565
52691	0.802265	2.1
52692	0.803714	2.1
52693	0.808476	1.79395
52694	1.94371	2.1
52695	1.36851	2.1
52696	0.853864	2.1
52697	1.26635	1.80275
52698	1.09085	1.5338
52699	0.857653	2.1
52700	0.630243	1.94029
52701	1.20132	0.05
52702	1.24295	0.05
52703	1.24295	0.05
52704	1.16182	0.05
52705	1.28465	0.05
52706	1.20351	0.05
52707	0.600658	0.05
52708	0.642323	0.05
52709	0.54424	0.05
52710	0.576779	0.05
52711	0.590985	0.05
52712	0.590985	0.05
52713	0.586452	0.05
52714	0.586452	0.05
52715	0.642323	0.05
52716	0.576779	0.05
52717	0.600658	0.05
52718	0.54424	0.05
52719	1.28463	0.05

52720	1.24294	0.05
52721	1.12328	0.05
52722	1.15505	0.05
52723	2.39798	0.05
52724	1.98431	0.05
52725	2.18735	0.05
52726	1.98431	0.05
52727	1.70303	0.05
52728	1.70302	0.05
52729	1.37788	0.05
52730	1.89226	0.05
52731	2.37417	0.05
52732	1.89226	0.05
52733	2.18735	0.05
52734	1.89226	0.05
52735	2.18735	0.05
52736	1.89226	0.05
52737	1.89226	0.0782402
52738	1.89226	0.05
52739	1.89226	0.05
52740	3.78452	0.0519848
52741	1.8613	0.05
52742	2.03576	0.0684565
52743	1.61652	0.05
52744	1.83082	1.61143
52745	3.65228	0.05
52746	2.03576	0.05
52747	2.05257	2.1
52748	1.83082	1.2513
52749	1.88487	2.1
52750	3.92064	1.93045
52751	1.89226	0.0561753
52752	1.89226	0.335145
52753	1.89226	0.05
52754	1.89226	2.1
52755	3.78453	0.0948115
52756	0.946131	0.092807
52757	0.946131	0.05
52758	0.94613	0.05
52759	0.94613	0.124084
52760	0.946129	0.05
52761	0.946129	0.099443
52762	0.946132	0.0584406
52763	0.946132	0.05
52764	0.94613	0.05
52765	0.94613	0.05
52766	0.946131	0.05
52767	0.946131	0.05
52768	0.804708	2.1
52769	0.804708	0.05
52770	0.815274	0.0954984
52771	0.815274	0.05
52772	0.83277	0.0696349
52773	0.915407	1.84114
52774	0.80826	0.05
52775	0.704574	0.05
52776	0.832771	0.05
52777	0.704573	0.05

52778	0.808259	0.11627
52779	0.915409	0.0747983
52780	1.83082	1.1236
52781	1.88487	2.1
52782	1.66555	0.0648351
52783	1.75835	2.1
52784	3.55042	1.95136
52785	0.810947	0.05
52786	0.810947	0.296194
52787	0.810947	0.05
52788	0.810947	0.0581419
52789	0.810934	0.124813
52790	0.810934	0.13207
52791	1.89227	2.1
52792	1.3517	0.227478
52793	1.08132	0.11777
52794	1.08132	0.274641
52795	1.08133	0.0794543
52796	1.89226	2.04754
52797	1.70303	2.1
52798	1.89226	2.1
52799	2.24712	2.1
52800	2.07363	2.1
52801	1.74617	2.1
52802	1.89226	2.1
52803	1.89226	1.61206
52804	1.89226	0.901252
52805	1.89226	2.1
52806	3.78452	1.7292
52807	1.50108	2.1
52808	1.43894	2.1
52809	1.8322	0.478356
52810	1.40967	2.1
52811	1.50108	2.1
52812	1.07854	2.1
52813	1.89226	0.582676
52814	1.89226	2.1
52815	1.89226	2.1
52816	1.89226	0.19594
52817	3.78452	2.05495
52818	1.89226	2.1
52819	1.89226	1.43238
52820	1.89226	1.30551
52821	1.89226	2.1
52822	3.78453	1.81565
52823	1.54457	2.1
52824	1.35097	2.05737
52825	1.70754	0.758187
52826	1.29629	2.1
52827	1.54457	2.1
52828	1.0305	2.1
52829	0.731763	2.1
52830	1.00043	2.1
52831	1.13883	1.0583
52832	0.939026	2.0528
52833	1.04003	1.07468
52834	0.853868	2.1
52835	0.927188	2.1

52836	0.927188	0.154202
52837	0.861565	1.78538
52838	1.13882	2.06669
52839	0.731769	2.09425
52840	0.89319	2.1
52841	1.46568	2.1
52842	1.20761	2.1
52843	1.51916	2.1
52844	1.36261	2.1
52845	2.72677	2.1
52846	1.51916	2.1
52847	1.60532	2.1
52848	1.36261	2.1
52849	1.36261	2.1
52850	1.64892	2.1
52851	1.64892	2.1
52852	1.3626	2.1
52853	0.98488	2.1
52854	1.60532	2.1
52855	1.32414	2.1
52856	2.68675	2.1
52857	1.78711	2.1
52858	1.14825	2.1
52859	2.16535	2.1
52860	1.6067	2.1
52861	3.3136	2.1
52862	1.89226	2.1
52863	1.89226	2.1
52864	1.89226	2.1
52865	1.89226	2.1
52866	3.78452	2.1
52867	2.34552	2.1
52868	1.53521	2.1
52869	1.53521	2.1
52870	1.60636	2.1
52871	1.92797	2.1
52872	1.99589	2.1
52873	1.34771	2.1
52874	1.34771	2.1
52875	0.946128	2.1
52876	0.946128	2.1
52877	1.3477	2.1
52878	1.31776	2.1
52879	0.946131	2.1
52880	0.946131	2.1
52881	0.94613	2.1
52882	1.31776	2.1
52883	0.946129	2.1
52884	1.3477	2.1
52885	1.89226	2.1
52886	1.89226	2.1
52887	1.89226	2.1
52888	1.89226	2.1
52889	3.78452	2.1
52890	0.92843	2.1
52891	0.672114	2.1
52892	0.89514	2.1
52893	0.89514	2.1

52894	0.720104	2.1
52895	0.755753	2.1
52896	0.789158	2.1
52897	0.967302	2.1
52898	1.06565	2.1
52899	0.7397	2.1
52900	0.758893	2.1
52901	0.94672	2.1
52902	1.89226	2.1
52903	2.588 2.1	
52904	2.588 2.1	
52905	2.63552	2.1
52906	1.89226	2.1
52907	1.93978	2.1
52908	1.49347	0.05
52909	2.67757	0.05
52910	1.80061	0.05
52911	2.27056	0.05
52912	4.07117	0.05
52913	1.1016	0.05
52914	2.27057	0.05
52915	1.49348	0.05
52916	1.81893	0.05
52917	1.49348	0.05
52918	1.81893	0.05
52919	1.81893	0.05
52920	2.82339	0.05
52921	2.27057	0.05
52922	2.25405	0.05
52923	4.52462	0.05
52924	1.89226	0.05
52925	1.89226	0.05
52926	1.89226	0.05
52927	1.89226	0.05
52928	1.89226	0.05
52929	1.89226	0.05
52930	1.89226	0.05
52931	1.89226	0.05
52932	1.89226	0.05
52933	1.89226	0.05
52934	3.78453	0.05
52935	1.89226	0.05
52936	1.89226	0.05
52937	1.89226	0.05
52938	1.89226	0.05
52939	3.78452	0.05
52940	1.89226	0.05
52941	1.89226	0.073175
52942	1.89226	0.05
52943	1.89226	0.05
52944	3.78451	0.05
52945	1.45633	2.1
52946	1.34678	2.1
52947	1.34678	2.1
52948	1.53735	2.1
52949	1.234 2.1	
52950	1.42457	2.1
52951	1.89226	2.1

52952	1.89226	2.1
52953	1.89226	2.1
52954	1.89226	2.1
52955	1.89226	2.1
52956	1.89226	2.1
52957	1.09483	2.1
52958	1.34678	2.1
52959	1.234	2.1
52960	1.234	2.1
52961	1.19	2.1
52962	1.19	2.1
52963	0.946131	2.1
52964	0.946131	2.1
52965	0.946131	2.1
52966	0.946131	2.1
52967	0.946131	2.1
52968	0.946131	2.1
52969	0.946132	2.1
52970	0.946132	2.1
52971	0.946132	2.1
52972	0.946132	2.1
52973	0.946131	2.1
52974	0.946131	2.1
52975	2.82263	2.1
52976	1.89226	2.1
52977	2.71441	2.1
52978	2.71441	2.1
52979	1.89226	2.1
52980	1.89226	2.1
52981	1.1412	2.1
52982	1.1412	2.1
52983	1.14626	2.1
52984	1.14626	2.1
52985	1.28511	2.1
52986	1.34086	2.1
52987	0.992242	2.1
52988	0.946593	2.1
52989	0.992242	2.1
52990	1.34086	2.1
52991	0.946594	2.1
52992	1.28511	2.1
52993	1.18954	2.1
52994	1.18954	2.1
52995	1.21477	2.1
52996	1.21477	2.1
52997	1.41207	2.1
52998	1.34086	2.1
52999	1.01306	2.1
53000	0.992242	2.1
53001	1.01306	2.1
53002	1.41207	2.1
53003	0.992242	2.1
53004	1.34086	2.1
53005	1.09044	2.1
53006	1.44472	0.078072
53007	1.2955	2.1
53008	1.2955	1.07749
53009	1.22025	2.1

53010	1.22025	2.04226
53011	0.810944	0.0912558
53012	0.810944	2.1
53013	0.810946	0.609728
53014	0.810946	0.05
53015	0.810959	0.320937
53016	0.810959	0.0791874
53017	1.89226	0.0727577
53018	1.35167	0.252812
53019	1.08132	2.1
53020	1.08132	0.05
53021	1.0813	0.190579
53022	1.41894	0.479669
53023	1.28953	0.223543
53024	0.946132	0.05
53025	0.946132	1.72919
53026	0.946133	1.42561
53027	0.946133	0.196235
53028	1.41894	0.370494
53029	1.41894	1.5197
53030	1.28953	0.394539
53031	0.946132	1.22153
53032	0.946132	0.852676
53033	1.41894	0.174425
53034	1.89226	2.1
53035	2.15444	2.1
53036	1.93239	2.1
53037	1.89226	2.1
53038	2.19457	2.1
53039	2.15444	2.1
53040	1.89226	2.1
53041	1.89226	2.1
53042	1.89226	2.1
53043	1.89226	2.1
53044	1.89226	2.1
53045	1.89226	2.1
53046	0.938507	2.1
53047	0.938507	2.1
53048	1.07871	2.1
53049	1.15801	2.1
53050	1.03236	2.1
53051	1.14766	2.1
53052	0.946133	2.1
53053	0.946133	2.1
53054	0.946131	2.1
53055	1.07009	2.1
53056	0.946131	2.1
53057	1.03237	2.1
53058	1.89226	2.1
53059	2.19457	2.1
53060	1.94492	2.1
53061	1.89226	2.1
53062	2.24723	2.1
53063	2.19457	2.1
53064	0.946132	2.1
53065	0.946132	2.1
53066	0.946131	2.1
53067	0.946131	2.1

53068	0.946132	2.1
53069	0.946132	2.1
53070	0.946132	2.1
53071	0.946132	2.1
53072	0.946132	2.1
53073	0.946132	2.1
53074	0.946132	2.1
53075	0.946132	2.1
53076	2.21345	2.1
53077	1.89226	2.1
53078	1.89226	2.1
53079	2.14017	2.1
53080	2.14017	2.1
53081	1.89226	2.1
53082	1.89226	0.0897057
53083	1.89226	0.987398
53084	1.89226	0.159143
53085	1.85439	0.851598
53086	2.23961	0.0688834
53087	2.19479	0.23525
53088	1.2916	0.11521
53089	1.2916	0.0799825
53090	0.946128	0.148502
53091	0.946128	0.0993521
53092	1.2916	0.0543917
53093	0.946131	0.13747
53094	0.946126	0.718807
53095	1.1198	0.05
53096	1.2916	0.0534087
53097	1.1198	0.205854
53098	0.946128	0.511765
53099	0.946128	0.261749
53100	1.89226	0.381654
53101	2.71262	0.216651
53102	1.89226	0.178303
53103	2.49959	0.173534
53104	4.39185	0.621926
53105	2.71262	0.188204
53106	1.34592	0.10947
53107	2.49959	0.145296
53108	1.14721	0.0752318
53109	3.84551	0.0618167
53110	0.946131	0.0824011
53111	0.946131	0.107398
53112	1.11247	0.107345
53113	1.11247	0.0891247
53114	1.24979	0.0906565
53115	1.11247	0.0554185
53116	0.946129	0.14291
53117	0.946129	0.154062
53118	0.946131	0.05
53119	1.11247	0.05
53120	0.946131	0.0514059
53121	1.24979	0.0665354
53122	0.946131	2.1
53123	0.946131	2.1
53124	0.94613	2.1
53125	0.94613	2.1

53126	0.946132	2.1
53127	0.946132	2.1
53128	0.946129	2.1
53129	0.946129	2.1
53130	0.946131	2.1
53131	0.946131	2.1
53132	0.946131	2.1
53133	0.946131	2.1
53134	2.77816	2.1
53135	1.89226	2.1
53136	1.89226	2.1
53137	1.89226	2.1
53138	3.78453	2.1
53139	1.89226	2.1
53140	1.89226	2.1
53141	1.89226	2.1
53142	1.89226	2.1
53143	1.89226	2.1
53144	1.89226	2.1
53145	1.89226	2.1
53146	1.89226	2.1
53147	1.89226	2.1
53148	1.89226	2.1
53149	3.78452	2.1
53150	1.55698	2.1
53151	1.89226	2.1
53152	1.89226	2.1
53153	1.89226	2.1
53154	1.8575	2.1
53155	1.8575	2.1
53156	1.89226	2.1
53157	1.89226	2.1
53158	1.89226	2.1
53159	1.89226	2.1
53160	1.89226	2.1
53161	1.89226	2.1
53162	1.89227	2.1
53163	1.89227	2.1
53164	1.89227	2.1
53165	1.89227	2.1
53166	1.89227	2.1
53167	1.89227	2.1
53168	1.35509	2.1
53169	2.06238	2.1
53170	2.12244	2.1
53171	2.12244	2.1
53172	1.34817	2.1
53173	1.34817	2.1
53174	0.946126	2.1
53175	0.946126	2.1
53176	0.946131	2.1
53177	0.946131	2.1
53178	0.946127	2.1
53179	0.946127	2.1
53180	0.94613	2.1
53181	0.94613	2.1
53182	0.946128	2.1
53183	0.946128	2.1

53184	0.946128	2.1
53185	0.946128	2.1
53186	1.89226	2.1
53187	1.89226	1.55956
53188	1.89226	2.1
53189	1.89226	2.1
53190	1.89226	2.09352
53191	1.89226	2.1
53192	1.16742	2.1
53193	1.16742	2.1
53194	1.18122	2.1
53195	1.18122	2.1
53196	1.31745	2.1
53197	1.2597	1.82852
53198	1.03119	2.1
53199	1.06122	2.1
53200	1.06122	2.1
53201	1.31745	2.1
53202	1.03119	2.1
53203	1.2597	2.1
53204	1.36849	2.1
53205	2.12244	2.1
53206	2.20092	2.1
53207	2.20092	2.1
53208	1.35509	2.1
53209	1.35509	2.1
53210	1.89226	2.1
53211	1.89226	2.1
53212	1.89226	2.1
53213	1.89226	2.1
53214	1.89226	2.1
53215	1.89226	2.1
53216	1.89226	2.1
53217	1.89226	1.30069
53218	1.89226	2.1
53219	1.89226	2.1
53220	3.78452	2.1
53221	1.21731	2.1
53222	1.21731	2.1
53223	1.23412	2.1
53224	1.23412	2.1
53225	1.39021	2.1
53226	1.31745	2.1
53227	1.06122	2.1
53228	1.10046	2.1
53229	1.10046	2.1
53230	1.39021	2.1
53231	1.06122	2.1
53232	1.31745	2.1
53233	2.16503	0.344003
53234	1.78252	1.30237
53235	1.77293	0.89572
53236	1.56839	2.1
53237	2.36957	0.0530785
53238	2.16503	2.1
53239	1.89223	0.420931
53240	1.89223	2.1
53241	1.89223	0.246968

53242	1.89223	1.39602
53243	3.78447	2.01909
53244	2.3696	1.17109
53245	1.78254	1.69539
53246	2.63298	0.0794547
53247	2.07312	2.1
53248	4.44272	1.57187
53249	1.89226	1.5396
53250	1.89226	2.00436
53251	1.89226	1.28067
53252	1.89226	2.1
53253	1.89226	0.0707279
53254	1.89226	2.1
53255	1.89226	0.286783
53256	1.89226	0.0810988
53257	1.89226	0.321724
53258	1.89226	0.05
53259	3.78452	0.05
53260	2.26369	0.329833
53261	2.2168	0.05
53262	2.15617	0.05
53263	2.10864	0.05
53264	4.37234	0.05
53265	2.2168	0.0796797
53266	1.94499	0.0628181
53267	1.94499	0.354493
53268	2.04737	0.116789
53269	2.10864	0.05
53270	2.21103	0.05
53271	1.89226	0.816415
53272	1.89226	0.0638853
53273	1.89226	0.277712
53274	1.89226	0.146633
53275	1.89226	0.477126
53276	1.89226	0.214453
53277	1.89226	0.112127
53278	1.89226	0.0677677
53279	1.89226	0.0844704
53280	1.89226	0.675655
53281	1.89226	0.05
53282	1.89226	0.51302
53283	1.02995	0.05
53284	1.02995	0.605256
53285	1.03212	0.06875
53286	1.03212	0.0984328
53287	0.988256	0.05
53288	1.05432	0.05
53289	1.00775	0.060938
53290	1.07809	0.0995925
53291	1.00775	0.05
53292	0.988257	0.05
53293	1.07808	0.0503425
53294	1.05432	0.0962635
53295	2.10864	0.449872
53296	1.94499	0.0843896
53297	1.97652	0.05
53298	1.79681	0.135613
53299	3.9215	0.0886127

53300	0.810943	0.05
53301	0.810943	0.523662
53302	0.810943	0.05
53303	0.810943	0.0537543
53304	0.810958	0.05
53305	0.810958	0.05
53306	1.89226	0.088934
53307	1.35168	0.05
53308	1.08132	0.05
53309	1.08132	0.0812324
53310	1.0813	0.059831
53311	0.657403	0.05
53312	1.35403	0.05
53313	1.24038	0.05
53314	0.499144	0.05
53315	1.24039	0.05
53316	1.35403	0.05
53317	0.657402	0.05
53318	0.499144	0.05
53319	0.915415	0.05
53320	0.915415	0.05
53321	0.937755	0.05
53322	0.937755	0.05
53323	1.72526	2.1
53324	1.17989	2.1
53325	1.96035	2.1
53326	1.47412	2.1
53327	3.14024	2.1
53328	1.89226	1.56774
53329	1.89226	2.1
53330	1.89226	2.1
53331	1.89226	0.862034
53332	3.78452	2.1
53333	0.946131	1.7468
53334	0.946131	1.69703
53335	1.25764	2.04877
53336	1.25764	2.1
53337	1.07087	1.14137
53338	1.25765	1.28266
53339	0.946128	1.02618
53340	0.946128	0.0847298
53341	0.94613	0.605497
53342	1.25765	1.16471
53343	1.07086	1.45851
53344	0.946129	1.38529
53345	1.96035	2.1
53346	1.32668	2.1
53347	2.29301	2.1
53348	1.70303	2.1
53349	3.56428	2.1
53350	1.89226	2.1
53351	1.89226	1.82053
53352	1.89226	0.251893
53353	1.89226	2.1
53354	3.78452	0.115831
53355	0.897738	0.0594099
53356	0.897738	0.058805
53357	0.946102	0.05

53358	0.946102	0.05
53359	1.11247	0.05
53360	0.89771	0.05
53361	0.94613	0.0750215
53362	0.94613	0.0618407
53363	0.897709	0.0778906
53364	0.946131	0.0971686
53365	0.946131	0.146863
53366	1.11247	0.0508037
53367	1.89226	2.1
53368	1.89226	2.1
53369	1.89226	2.1
53370	1.89226	2.1
53371	1.89226	2.1
53372	1.89226	2.1
53373	2.15429	2.1
53374	1.89226	2.1
53375	1.89226	2.1
53376	1.89226	2.1
53377	2.27074	2.1
53378	2.27074	2.1
53379	0.993311	1.41366
53380	0.993311	2.0523
53381	0.904322	2.1
53382	0.904322	2.1
53383	0.803324	2.1
53384	1.36183	1.40427
53385	0.916274	2.1
53386	0.535808	1.60593
53387	0.535808	2.03693
53388	0.803322	2.1
53389	0.916276	2.06299
53390	1.36183	2.1
53391	1.89226	2.1
53392	1.89226	2.0965
53393	1.89226	2.1
53394	1.89226	2.1
53395	1.89226	2.1
53396	1.89226	2.1
53397	2.11955	2.1
53398	2.62097	2.1
53399	1.85542	2.1
53400	2.39016	2.1
53401	4.50971	2.1
53402	2.39016	2.1
53403	2.20242	2.1
53404	2.39016	2.1
53405	1.64008	2.1
53406	1.85542	2.1
53407	1.64008	2.1
53408	1.79667	2.1
53409	1.28267	2.1
53410	1.28267	2.1
53411	1.40117	2.1
53412	1.74514	2.1
53413	1.83994	2.1
53414	0.575914	2.1
53415	0.625292	2.1

53416	0.949144	2.1
53417	0.882782	2.1
53418	0.773697	2.1
53419	0.773697	2.1
53420	0.786497	2.1
53421	0.786494	2.1
53422	0.605477	2.1
53423	0.949148	2.1
53424	0.625291	2.1
53425	0.862374	2.1
53426	1.89226	2.1
53427	2.44918	2.1
53428	2.4616	2.1
53429	2.44918	2.1
53430	1.90468	2.1
53431	1.89226	2.1
53432	1.89226	2.1
53433	1.89226	2.1
53434	1.89226	2.1
53435	1.89226	1.20679
53436	3.78453	2.1
53437	0.946128	2.1
53438	0.946128	0.841926
53439	1.23631	2.1
53440	1.23631	2.1
53441	0.946131	2.1
53442	0.946131	2.1
53443	1.3147	2.1
53444	1.23631	2.1
53445	1.3147	2.1
53446	0.946131	2.1
53447	1.23631	2.1
53448	0.94613	2.1
53449	1.89226	2.1
53450	2.4616	2.1
53451	2.4717	2.1
53452	2.4616	2.1
53453	1.90237	2.1
53454	1.89226	2.1
53455	0.946132	2.1
53456	0.946132	2.1
53457	0.946131	2.1
53458	0.946131	2.1
53459	0.946132	2.1
53460	0.946132	2.1
53461	0.946131	2.1
53462	0.946131	2.1
53463	0.946131	2.1
53464	0.946131	2.1
53465	0.946131	2.1
53466	0.946131	2.1
53467	0.946128	2.1
53468	0.946128	2.1
53469	1.3147	2.04646
53470	1.3147	2.1
53471	0.94613	1.71547
53472	0.94613	2.1
53473	1.3419	2.1

53474	1.3147	2.1
53475	1.3419	2.1
53476	0.94613	2.1
53477	1.3147	2.1
53478	0.94613	2.1
53479	1.89226	2.1
53480	1.89226	2.1
53481	1.89226	2.1
53482	1.89226	2.1
53483	3.78452	2.1
53484	2.3333	2.1
53485	1.3864	2.1
53486	1.3864	2.1
53487	1.4628	2.1
53488	1.95763	2.1
53489	2.03403	2.1
53490	1.294	2.1
53491	1.294	2.1
53492	0.946128	2.1
53493	0.946128	2.1
53494	1.294	2.1
53495	1.27474	2.1
53496	0.946131	2.1
53497	0.946131	2.1
53498	0.946129	2.1
53499	1.27474	2.1
53500	1.294	2.1
53501	0.94613	2.1
53502	1.89226	2.1
53503	1.89226	2.1
53504	1.89226	2.1
53505	1.89226	2.1
53506	1.89226	2.1
53507	1.89226	2.1
53508	0.808088	2.1
53509	0.808088	2.1
53510	0.815623	2.1
53511	0.815623	2.1
53512	0.660053	2.1
53513	0.693199	2.1
53514	0.930512	2.1
53515	0.978817	2.1
53516	0.660053	2.1
53517	0.930513	2.1
53518	0.693198	2.1
53519	0.978817	2.1
53520	1.27474	2.1
53521	1.27474	2.1
53522	0.946128	2.1
53523	0.946128	2.1
53524	1.26221	2.1
53525	1.27474	2.1
53526	0.946131	2.1
53527	0.946131	2.1
53528	0.94613	2.1
53529	1.26221	2.1
53530	0.946129	2.1
53531	1.27474	2.1

53532	0.90207	0.05
53533	0.90207	0.05
53534	0.895719	0.05
53535	0.895719	0.05
53536	0.927855	0.05
53537	1.2493	0.05
53538	0.85723	0.05
53539	0.548484	0.05
53540	0.548485	0.05
53541	0.927855	0.05
53542	0.85723	0.05
53543	1.2493	0.05
53544	1.41319	0.183022
53545	1.22482	0.05
53546	1.46629	0.081248
53547	1.92622	0.197912
53548	3.15104	0.142994
53549	1.02305	0.0534785
53550	1.22482	0.0748234
53551	1.80336	0.0867476
53552	1.44426	0.05
53553	2.66908	0.05
53554	1.3268	2.1
53555	0.999489	2.1
53556	1.44091	2.1
53557	1.14657	2.1
53558	2.4404	2.1
53559	1.89226	2.1
53560	1.89226	2.1
53561	1.89226	2.1
53562	1.89226	2.1
53563	3.78452	2.1
53564	1.89226	2.1
53565	2.28605	2.1
53566	2.43157	2.1
53567	2.43157	2.1
53568	1.89226	2.1
53569	1.89226	2.1
53570	1.60647	2.1
53571	1.44091	2.1
53572	1.31346	2.1
53573	1.31346	2.1
53574	1.14657	2.1
53575	1.44091	2.1
53576	1.89226	2.1
53577	1.89226	2.1
53578	1.89226	2.1
53579	1.89226	2.1
53580	3.78452	2.1
53581	0.946132	2.1
53582	0.946132	2.1
53583	1.3048	2.1
53584	1.3048	2.1
53585	1.3048	2.1
53586	1.21578	2.1
53587	0.946132	2.1
53588	0.946132	2.1
53589	1.3048	2.1

53590	0.946132	2.1
53591	0.946132	2.1
53592	1.21578	2.1
53593	1.89227	0.05
53594	1.89227	0.05
53595	1.89227	0.05
53596	1.89227	0.05
53597	3.78453	0.05
53598	1.6671	0.05
53599	1.87338	0.05
53600	1.6671	0.05
53601	2.02098	0.05
53602	1.38328	0.05
53603	1.73715	0.05
53604	1.89226	0.05
53605	1.89226	0.05
53606	1.89226	0.05
53607	1.89226	0.05
53608	3.78453	0.05
53609	1.6671	0.05
53610	1.38328	0.05
53611	1.35562	0.05
53612	1.01012	0.05
53613	2.67722	0.05
53614	0.819377	0.05
53615	0.647925	0.05
53616	1.17802	0.05
53617	1.33174	0.05
53618	0.98543	0.05
53619	0.98543	0.05
53620	0.994237	0.05
53621	0.994237	0.05
53622	1.17802	0.05
53623	0.647926	0.05
53624	1.33174	0.05
53625	0.819377	0.05
53626	1.27806	0.05
53627	1.68264	0.05
53628	1.44593	0.05
53629	1.878 0.05	
53630	3.15606	0.05
53631	1.878 0.05	
53632	1.9898	0.05
53633	1.878 0.05	
53634	1.68264	0.05
53635	2.30533	0.05
53636	2.10997	0.05
53637	0.814587	0.05
53638	0.814596	0.05
53639	0.82775	0.05
53640	0.82775	0.05
53641	1.0107	0.05
53642	0.91549	0.05
53643	0.741058	0.05
53644	0.714748	0.05
53645	0.714758	0.05
53646	1.03225	0.05
53647	0.704864	0.05

53648	0.915488	0.05
53649	1.90347	0.05
53650	2.06584	0.05
53651	1.878	0.05
53652	1.50236	0.05
53653	2.05749	0.05
53654	2.30533	0.05
53655	2.24475	2.1
53656	1.85871	2.1
53657	2.13711	2.1
53658	2.13711	2.1
53659	1.9943	2.1
53660	1.9943	2.1
53661	1.85871	2.1
53662	1.7871	2.1
53663	1.41619	2.1
53664	1.41619	2.1
53665	2.13711	2.1
53666	2.13711	2.1
53667	2.24475	2.1
53668	2.13711	2.1
53669	2.54902	2.1
53670	2.46327	2.1
53671	4.70802	2.1
53672	1.06913	2.1
53673	1.06913	2.1
53674	1.0821	2.1
53675	1.0821	2.1
53676	0.893552	2.1
53677	1.08267	2.1
53678	1.06855	2.1
53679	1.23163	2.1
53680	1.23163	2.1
53681	1.08267	2.1
53682	0.893551	2.1
53683	1.06855	2.1
53684	1.67046	1.14209
53685	2.4106	0.0663528
53686	2.05835	1.02783
53687	2.4106	1.45419
53688	1.59562	0.0724762
53689	1.94788	0.0912614
53690	2.34737	1.20597
53691	2.4106	0.111084
53692	2.05834	1.45929
53693	1.99655	1.15632
53694	4.40715	0.668285
53695	1.67046	0.508094
53696	2.05835	0.05
53697	1.588	1.21926
53698	1.34857	1.77329
53699	3.40692	0.518412
53700	1.73802	0.166797
53701	1.89668	0.942017
53702	1.99655	2.1
53703	1.99655	0.096938
53704	1.79981	0.274981
53705	2.05834	0.0543657

53706	1.89226	1.16805
53707	1.89226	0.677239
53708	1.89226	0.0683584
53709	1.89226	2.06352
53710	1.89226	0.0937729
53711	1.89226	2.1
53712	1.89226	0.748526
53713	1.89226	2.1
53714	1.89226	1.89368
53715	1.89226	0.0901439
53716	3.78453	1.86759
53717	0.719473	2.1
53718	0.916103	1.90831
53719	1.02744	0.846592
53720	0.850328	2.0927
53721	0.888094	0.559714
53722	0.888094	2.1
53723	0.878337	2.1
53724	0.878337	0.0719956
53725	0.916103	1.26253
53726	1.02744	1.50496
53727	0.719473	1.9324
53728	0.850328	2.0488
53729	1.02744	1.19805
53730	0.850329	2.1
53731	1.0875	1.03696
53732	0.919597	1.36723
53733	0.975841	0.225689
53734	0.975841	2.1
53735	0.971196	0.265048
53736	0.971196	2.08871
53737	1.02744	1.79032
53738	1.0875	0.995272
53739	0.850328	2.04089
53740	0.919598	1.80949
53741	1.89226	2.0842
53742	1.89226	1.69223
53743	1.89226	0.357224
53744	1.89226	1.88736
53745	3.78453	1.44368
53746	1.89226	1.59005
53747	1.89226	0.598902
53748	1.89226	0.0901941
53749	1.89226	2.1
53750	1.89226	0.0741015
53751	1.89226	2.1
53752	1.11103	1.77752
53753	0.916103	2.1
53754	1.21177	1.08024
53755	1.02744	1.22909
53756	1.07188	0.350685
53757	1.07188	2.1
53758	1.06659	0.343621
53759	1.06659	2.1
53760	1.11103	2.09305
53761	1.21177	1.67925
53762	0.916104	2.07381
53763	1.02744	2.1

53764	2.54158	0.860907
53765	2.42354	1.47847
53766	2.17499	0.195224
53767	2.17499	2.1
53768	2.42354	1.94003
53769	2.05488	2.1
53770	1.89226	0.05
53771	1.89226	0.05
53772	1.89226	0.05
53773	1.89226	0.05
53774	3.78453	0.05
53775	1.89226	0.05
53776	1.89226	0.05
53777	1.89226	0.05
53778	1.89226	0.05
53779	1.89226	0.05
53780	1.89226	0.05
53781	1.89226	0.05
53782	1.89226	0.05
53783	1.89226	0.05
53784	1.89226	0.05
53785	1.89226	0.05
53786	1.89226	0.05
53787	1.89226	0.05
53788	1.89226	0.05
53789	1.89226	0.05
53790	1.89226	0.05
53791	3.78452	0.05
53792	0.802544	0.05
53793	0.946132	0.05
53794	0.946132	0.05
53795	0.787038	0.05
53796	0.787039	0.05
53797	0.787039	0.05
53798	0.961637	0.05
53799	0.961637	0.05
53800	0.787038	0.05
53801	0.802544	0.05
53802	0.946132	0.05
53803	0.946132	0.05
53804	1.89226	0.05
53805	1.60509	0.05
53806	1.89226	0.05
53807	1.89226	0.05
53808	1.3905	0.05
53809	1.3905	0.05
53810	0.946132	0.05
53811	0.946132	0.05
53812	0.946131	0.05
53813	0.946131	0.05
53814	0.946132	0.05
53815	0.946132	0.05
53816	0.946131	0.05
53817	0.946131	0.05
53818	0.946131	0.05
53819	0.946131	0.05
53820	0.946132	0.05
53821	0.946132	0.05

53822	0.946131	0.05
53823	0.946131	0.05
53824	0.946131	0.05
53825	0.946131	0.05
53826	0.946132	0.05
53827	0.946132	0.05
53828	0.946131	0.05
53829	0.946131	0.05
53830	0.946131	0.05
53831	0.946131	0.05
53832	0.946132	0.05
53833	0.946132	0.05
53834	1.42382	2.1
53835	1.32201	0.0884587
53836	1.32096	0.505932
53837	1.42866	1.86948
53838	2.75067	1.14656
53839	1.89226	2.06205
53840	1.89226	0.0538899
53841	1.89226	0.192231
53842	1.89226	0.695408
53843	1.89226	0.115677
53844	1.89226	0.378929
53845	1.89226	1.55744
53846	2.79029	0.123564
53847	2.7803	0.102668
53848	2.79029	0.206531
53849	1.88227	0.0622561
53850	1.89226	0.129935
53851	0.716065	1.59736
53852	0.714333	2.1
53853	0.660483	0.26897
53854	0.659126	0.884389
53855	0.687538	0.193987
53856	0.687538	1.46941
53857	0.685921	2.1
53858	0.685921	0.316932
53859	0.659126	0.421113
53860	0.716065	0.661633
53861	0.660483	1.19884
53862	0.714333	1.136
53863	1.89226	1.49006
53864	1.89226	0.05
53865	1.89226	0.331851
53866	1.89226	0.704445
53867	3.78453	0.778821
53868	0.946133	0.0859156
53869	0.946133	0.65713
53870	1.39015	0.130641
53871	1.38969	0.05
53872	0.946132	0.05
53873	0.946132	0.116357
53874	1.38969	0.67988
53875	1.38969	0.201887
53876	1.38969	0.0873659
53877	0.946132	0.136063
53878	0.946133	0.581309
53879	1.39015	0.144897

53880	2.72157	0.05
53881	1.22176	0.05
53882	1.59492	0.05
53883	1.22176	0.0572474
53884	2.40743	0.05
53885	2.03426	0.05
53886	0.946131	0.0722792
53887	0.946131	0.05
53888	1.36079	0.05
53889	1.01713	0.05
53890	0.94613	0.05
53891	0.94613	0.0771685
53892	1.36079	0.05
53893	1.36079	0.05
53894	0.946131	0.0521576
53895	1.36079	0.0509554
53896	1.01713	0.051997
53897	0.946131	0.0551069
53898	1.89226	0.0646284
53899	1.89226	0.05
53900	1.89226	0.05
53901	1.89226	0.111578
53902	3.78453	0.0519874
53903	1.89226	0.05
53904	1.89226	0.05
53905	1.89226	0.05
53906	1.89226	0.0813276
53907	1.89226	0.05
53908	1.89226	0.0534003
53909	0.946131	0.0642383
53910	0.946131	0.05
53911	0.946131	0.05
53912	1.36079	0.05
53913	0.94613	0.05
53914	0.94613	0.0749358
53915	0.946133	0.05
53916	0.946133	0.05
53917	0.946131	0.05
53918	0.946131	0.05
53919	0.946132	0.05
53920	1.36079	0.05
53921	1.89226	0.05
53922	1.89226	0.101552
53923	1.89226	0.05
53924	1.89226	0.0632657
53925	1.89226	0.05
53926	1.89226	0.0664978
53927	1.89227	0.05
53928	1.89227	0.05
53929	1.89227	0.05
53930	1.89227	0.0709491
53931	1.89227	0.05
53932	1.89227	0.05
53933	1.60428	2.1
53934	2.2367	2.1
53935	1.50097	2.1
53936	1.80125	2.1
53937	3.40553	2.1

53938	1.89095	2.1
53939	1.85293	0.0737049
53940	1.80125	2.1
53941	1.76268	0.05
53942	3.65418	1.50545
53943	0.803177	2.1
53944	0.803177	2.1
53945	0.798644	2.1
53946	0.798644	2.1
53947	0.84233	2.1
53948	0.900624	2.1
53949	0.750483	2.1
53950	0.701197	2.1
53951	0.701197	2.1
53952	0.84233	2.1
53953	0.750483	2.1
53954	0.900625	2.1
53955	0.860925	0.0538935
53956	0.860925	2.1
53957	0.862743	2.1
53958	0.862743	0.124881
53959	0.881338	0.0537574
53960	0.826621	0.05
53961	0.84233	2.1
53962	0.900625	2.1
53963	0.84233	0.630695
53964	0.826621	0.515314
53965	0.900624	0.916717
53966	0.881339	0.32192
53967	1.37938	1.0071
53968	1.03319	1.08295
53969	1.00512	1.33538
53970	0.639582	1.50892
53971	1.00463	1.38043
53972	1.00463	0.732078
53973	1.01434	0.703286
53974	1.01434	1.56512
53975	1.37938	1.14053
53976	1.00512	1.48605
53977	1.03319	1.30708
53978	0.639582	1.28775
53979	0.728713	0.05
53980	0.728713	0.05
53981	0.73425	0.05
53982	0.73425	0.850205
53983	0.525122	2.1
53984	0.605622	0.05
53985	0.948901	0.05
53986	0.857342	0.05
53987	0.85734	0.05
53988	0.525123	0.529562
53989	0.948899	0.05
53990	0.605624	0.05
53991	1.75449	0.381368
53992	1.4378	0.377627
53993	1.93916	0.689505
53994	2.22512	0.13109
53995	3.69364	0.493015

53996	1.75448	0.884334
53997	1.89186	0.206849
53998	2.29771	0.393376
53999	2.22512	0.2253
54000	4.05219	0.338105
54001	1.93916	0.674502
54002	2.51749	0.121326
54003	2.62357	0.2124
54004	2.22512	0.129589
54005	2.33761	0.634623
54006	1.93916	0.332649
54007	2.29771	0.459096
54008	2.51749	0.135793
54009	2.22512	0.146213
54010	2.22512	0.0813811
54011	2.50905	0.660151
54012	2.50905	0.288492
54013	0.87382	0.05
54014	1.33662	0.05
54015	1.20756	0.05
54016	0.890467	0.05
54017	1.08859	0.05
54018	1.08859	0.05
54019	1.13084	0.05
54020	1.08793	0.05
54021	1.34722	0.05
54022	1.27184	0.05
54023	0.917678	0.05
54024	0.97821	0.05
54025	1.28396	0.05
54026	1.70302	0.05
54027	2.30076	0.05
54028	1.20417	0.05
54029	2.57506	0.05
54030	2.2656	0.05
54031	1.03642	0.928724
54032	1.03642	0.05
54033	1.07029	0.05
54034	1.07029	0.0903717
54035	1.31531	0.403123
54036	1.23279	0.05
54037	0.941743	0.0560731
54038	0.791399	0.747629
54039	0.791399	0.05
54040	1.23279	0.05
54041	0.941744	0.0906395
54042	1.31531	0.151839
54043	0.937354	1.20308
54044	0.937354	0.05
54045	0.954794	0.05
54046	0.954794	0.05
54047	1.13563	0.05
54048	1.23279	0.309905
54049	0.791398	0.05
54050	0.659359	0.702738
54051	1.13563	0.05
54052	0.659359	0.05
54053	0.791399	0.05

54054	1.23279	0.05
54055	2.29852	0.419324
54056	1.89227	0.514617
54057	2.14485	0.197587
54058	2.14485	1.09494
54059	1.89227	0.210978
54060	1.89227	1.15859
54061	0.947863	0.318019
54062	1.19854	0.871499
54063	2.16841	0.499316
54064	1.76597	0.51441
54065	2.96451	0.372478
54066	1.89227	0.297485
54067	1.89227	0.409842
54068	1.89227	0.211699
54069	1.89227	0.898699
54070	1.89227	0.215684
54071	1.89227	0.905428
54072	2.46489	2.1
54073	1.98339	2.1
54074	1.81667	2.1
54075	2.36914	2.1
54076	4.35253	2.1
54077	2.46488	2.1
54078	2.70401	2.1
54079	2.64996	2.1
54080	2.36914	2.1
54081	5.07315	2.1
54082	1.81667	2.1
54083	2.29459	2.1
54084	2.36914	2.1
54085	2.36914	2.1
54086	1.68073	2.1
54087	1.68073	2.1
54088	2.64996	2.1
54089	2.29458	2.1
54090	2.29458	2.1
54091	2.36914	2.1
54092	2.61243	2.1
54093	2.68698	2.1
54094	1.89226	1.33841
54095	1.89226	0.831851
54096	1.89226	1.56418
54097	1.89226	1.05551
54098	1.89226	1.66631
54099	1.89226	1.08666
54100	1.89224	1.60497
54101	1.89224	0.73354
54102	1.65571	1.20148
54103	1.65571	0.744112
54104	1.89224	1.48512
54105	1.89224	1.10686
54106	1.89224	1.3417
54107	1.89224	1.15454
54108	1.89224	1.56663
54109	1.89224	0.758756
54110	1.89224	1.8119
54111	1.89224	0.985164

54112	1.98252	0.05
54113	2.20641	0.05
54114	1.05129	0.05
54115	1.2951	0.05
54116	3.27762	0.05
54117	1.85598	2.1
54118	1.88635	0.941656
54119	2.12346	0.753797
54120	2.10838	2.1
54121	3.97943	1.38001
54122	2.10838	1.86627
54123	1.87532	2.1
54124	1.85597	0.353253
54125	1.85597	2.1
54126	1.87532	1.98587
54127	1.57914	2.1
54128	2.12348	1.22296
54129	2.35141	2.1
54130	2.37191	1.39739
54131	2.10841	1.75115
54132	2.38698	0.801049
54133	2.12348	1.78894
54134	1.07811	2.1
54135	0.937672	2.1
54136	1.17571	2.1
54137	1.05421	0.307294
54138	1.07087	1.35742
54139	1.07087	2.1
54140	1.06145	2.1
54141	1.06145	1.08034
54142	1.17571	2.1
54143	1.07811	2.1
54144	1.05421	2.1
54145	0.937672	2.1
54146	1.67334	2.1
54147	2.76609	2.1
54148	2.74144	2.1
54149	2.74144	2.1
54150	1.71157	2.1
54151	1.71157	2.1
54152	2.76609	2.1
54153	1.89226	2.1
54154	1.89226	2.1
54155	1.89226	2.1
54156	2.74143	2.1
54157	2.74143	2.1
54158	1.10393	2.1
54159	1.10393	2.1
54160	1.10788	2.1
54161	1.10788	2.1
54162	1.38302	2.1
54163	1.37072	2.1
54164	0.836672	2.1
54165	0.841089	2.1
54166	1.38302	2.1
54167	0.84109	2.1
54168	0.836671	2.1
54169	1.37072	2.1

54170	1.38302	2.1
54171	1.38302	2.1
54172	0.946131	2.1
54173	0.946131	2.1
54174	0.946128	2.1
54175	0.946128	2.1
54176	1.38302	2.1
54177	1.37072	2.1
54178	1.38302	2.1
54179	0.946131	2.1
54180	0.94613	2.1
54181	1.37072	2.1
54182	1.28072	0.318155
54183	1.55779	2.1
54184	1.28072	2.1
54185	1.40765	1.3381
54186	1.47186	2.1
54187	1.59879	2.1
54188	1.89227	0.628476
54189	1.89227	0.317862
54190	1.89227	0.05
54191	1.89227	2.1
54192	1.89227	0.05
54193	1.89227	2.1
54194	2.80057	0.0955858
54195	1.89226	0.0605903
54196	1.89226	0.0737484
54197	1.89226	0.608484
54198	2.63061	0.05
54199	2.63061	0.615681
54200	1.28072	0.132494
54201	1.38859	2.1
54202	1.47186	2.1
54203	1.47186	1.62595
54204	1.16777	2.1
54205	1.16777	2.02726
54206	1.89226	0.209712
54207	1.89226	0.322873
54208	1.89226	0.0953881
54209	1.89226	2.1
54210	1.89226	0.05
54211	1.89226	2.1
54212	1.83221	2.1
54213	2.0815	2.1
54214	2.0815	2.1
54215	2.08153	2.1
54216	1.86703	2.1
54217	1.87402	2.1
54218	1.86703	2.1
54219	2.08508	2.1
54220	2.08508	2.1
54221	2.29001	2.1
54222	1.89226	2.1
54223	2.08048	2.1
54224	2.7278	0.05
54225	1.10909	0.05
54226	1.50472	0.05
54227	1.10909	0.05

54228	2.69881	0.05
54229	2.30319	0.05
54230	1.23204	2.1
54231	1.92264	2.1
54232	1.48162	2.1
54233	1.48162	2.1
54234	1.72994	2.1
54235	1.72994	2.1
54236	0.864968	2.1
54237	0.696952	2.1
54238	0.61602	2.1
54239	0.479736	2.1
54240	0.66444	2.1
54241	0.66444	2.1
54242	0.680264	2.1
54243	0.680264	2.1
54244	0.696952	2.1
54245	0.479736	2.1
54246	0.61602	2.1
54247	0.864968	2.1
54248	1.89226	0.16865
54249	2.43219	2.1
54250	1.89226	1.38133
54251	2.82019	1.51319
54252	1.89226	2.00395
54253	2.82019	1.72178
54254	1.89226	0.350233
54255	2.83701	2.1
54256	2.2701	1.6325
54257	2.2701	1.17276
54258	1.89226	1.77455
54259	1.89226	1.53838
54260	1.89226	1.79222
54261	1.89226	0.559159
54262	1.89226	0.604539
54263	1.89226	1.41068
54264	1.89226	1.5604
54265	1.89226	1.45995
54266	1.5795	1.86965
54267	1.44091	1.80206
54268	1.05267	2.09873
54269	1.05267	1.497
54270	1.98408	1.89026
54271	1.98408	1.30683
54272	2.20624	2.1
54273	1.08831	2.1
54274	2.49053	2.1
54275	1.42486	2.1
54276	3.57883	2.1
54277	1.89226	2.1
54278	1.89226	2.1
54279	1.89226	2.1
54280	1.89226	2.1
54281	3.78453	2.1
54282	0.946132	2.1
54283	0.946132	2.1
54284	0.853591	2.1
54285	0.642207	2.1

54286	0.94613	2.1
54287	0.94613	2.1
54288	0.642209	2.1
54289	0.642209	2.1
54290	0.946131	2.1
54291	0.853591	2.1
54292	0.642207	2.1
54293	0.946132	2.1
54294	1.0506	2.1
54295	1.0506	2.1
54296	1.0879	2.1
54297	0.960424	2.1
54298	0.708687	2.1
54299	0.782927	2.1
54300	1.40041	2.1
54301	1.2518	2.1
54302	0.767748	2.1
54303	1.31801	2.1
54304	1.21412	2.1
54305	0.69461	2.1
54306	1.89226	2.1
54307	1.89226	2.1
54308	1.89226	2.1
54309	1.89226	2.1
54310	1.89226	2.1
54311	1.89226	2.1
54312	0.922223	2.1
54313	0.922223	2.1
54314	1.04548	2.1
54315	0.768229	2.1
54316	0.948441	2.1
54317	0.806483	2.1
54318	0.85362	2.1
54319	0.85362	2.1
54320	1.05942	2.1
54321	0.922187	2.1
54322	0.851554	2.1
54323	0.768264	2.1
54324	2.16728	2.1
54325	0.95455	2.1
54326	1.21372	2.1
54327	0.98227	2.1
54328	2.16728	2.1
54329	1.93584	2.1
54330	2.08849	0.05
54331	1.08732	0.05
54332	1.3357	0.05
54333	2.37948	0.05
54334	3.4668	0.05
54335	2.57882	0.05
54336	2.37948	0.05
54337	2.53055	0.05
54338	1.48676	0.05
54339	1.3357	0.05
54340	1.3357	0.05
54341	1.89226	2.1
54342	1.89226	2.1
54343	2.17471	2.1

54344	1.89226	2.1
54345	2.60085	2.1
54346	2.26306	2.1
54347	0.946131	2.1
54348	0.946131	2.1
54349	0.946131	2.1
54350	0.946131	2.1
54351	0.94613	2.1
54352	0.94613	2.1
54353	0.946132	2.1
54354	0.946132	2.1
54355	0.946131	2.1
54356	0.946131	2.1
54357	0.946131	2.1
54358	0.946131	2.1
54359	1.89226	2.1
54360	2.17471	2.1
54361	2.08196	2.1
54362	2.17471	2.1
54363	1.79952	2.1
54364	1.89226	2.1
54365	1.89226	2.1
54366	1.89226	2.1
54367	1.89226	2.1
54368	1.89226	2.1
54369	1.89226	2.1
54370	1.89226	2.1
54371	1.86016	2.1
54372	1.89226	2.1
54373	1.89226	2.1
54374	1.89226	2.1
54375	1.84826	2.1
54376	1.84826	2.1
54377	2.50136	2.1
54378	1.99781	2.1
54379	1.99729	2.1
54380	2.5214	2.1
54381	4.49865	2.1
54382	0.51724	2.1
54383	0.496076	2.1
54384	0.998906	2.1
54385	0.998645	2.1
54386	0.742249	2.1
54387	0.742249	2.1
54388	0.752734	2.1
54389	0.752734	2.1
54390	0.998639	2.1
54391	0.496073	2.1
54392	0.517243	2.1
54393	0.998913	2.1
54394	0.998653	2.1
54395	0.994293	2.1
54396	1.26071	2.1
54397	1.26504	2.1
54398	1.12529	2.1
54399	1.12529	2.1
54400	1.12971	2.1
54401	1.12971	2.1

54402	1.26504	2.1
54403	0.994292	2.1
54404	0.998651	2.1
54405	1.26071	2.1
54406	1.89226	2.1
54407	1.89226	2.1
54408	1.89226	2.1
54409	1.89226	2.1
54410	3.78452	2.1
54411	1.89226	2.1
54412	1.89226	2.1
54413	1.89226	2.1
54414	1.89226	2.1
54415	3.78452	2.1
54416	0.675872	2.1
54417	0.675641	2.1
54418	0.943072	2.1
54419	0.898317	2.1
54420	0.775717	2.1
54421	0.775717	2.1
54422	0.79824	2.1
54423	0.79824	2.1
54424	0.675642	2.1
54425	0.94307	2.1
54426	0.675874	2.1
54427	0.898315	2.1
54428	0.943071	2.1
54429	0.898316	2.1
54430	0.998133	2.1
54431	0.918903	2.1
54432	0.922369	2.1
54433	0.922369	2.1
54434	0.939606	2.1
54435	0.939606	2.1
54436	0.943071	2.1
54437	0.998133	2.1
54438	0.898316	2.1
54439	0.918903	2.1
54440	0.946131	2.1
54441	0.946131	2.1
54442	0.946131	2.1
54443	0.946131	2.1
54444	0.946132	2.1
54445	0.946132	2.1
54446	0.946129	2.1
54447	0.946129	2.1
54448	0.94613	2.1
54449	0.94613	2.1
54450	0.946131	2.1
54451	0.946131	2.1
54452	1.89226	2.1
54453	1.89226	2.1
54454	1.89226	2.1
54455	1.89226	2.1
54456	1.89226	2.1
54457	1.89226	2.1
54458	1.35129	2.1
54459	1.88614	2.1

54460	2.01867	2.1
54461	1.38189	2.1
54462	3.36995	2.1
54463	0.943071	2.1
54464	1.00934	2.1
54465	0.998134	2.1
54466	1.10866	2.1
54467	0.992617	2.1
54468	0.992617	2.1
54469	1.01485	2.1
54470	1.01485	2.1
54471	1.00934	2.1
54472	1.10866	2.1
54473	0.943072	2.1
54474	0.998134	2.1
54475	2.67272	2.1
54476	1.61859	2.1
54477	1.70303	2.1
54478	2.52175	2.1
54479	4.5326	2.1
54480	2.00954	2.1
54481	2.27801	2.1
54482	1.87875	2.1
54483	1.87875	2.1
54484	2.37837	2.1
54485	2.37837	2.1
54486	2.00955	2.1
54487	1.78	2.1
54488	2.21813	2.1
54489	1.8493	2.1
54490	2.37838	2.1
54491	2.00955	2.1
54492	2.37838	2.1
54493	2.27801	2.1
54494	2.58523	2.1
54495	2.71597	2.1
54496	4.9636	2.1
54497	2.37838	2.1
54498	2.71597	2.1
54499	1.72709	2.1
54500	2.0815	2.1
54501	2.59749	2.1
54502	2.78034	2.1
54503	0.94613	0.141171
54504	0.94613	0.229084
54505	0.946128	0.102768
54506	0.946128	0.0593753
54507	0.94613	0.0554574
54508	0.94613	0.0950168
54509	0.946128	0.230382
54510	0.946128	0.0515958
54511	0.946129	0.0896309
54512	0.946129	0.088177
54513	0.946129	0.136
54514	0.946129	0.148439
54515	1.89226	0.725675
54516	1.89226	0.342898
54517	1.89226	0.610706

54518	1.89226	0.18
54519	3.78452	0.206936
54520	0.946133	0.0523227
54521	0.946133	0.0726331
54522	0.946132	0.05
54523	0.946132	0.05
54524	0.946132	0.05
54525	0.946132	0.05
54526	0.946133	0.0677881
54527	0.946133	0.0520524
54528	0.946132	0.05
54529	0.946132	0.05
54530	0.946132	0.05
54531	0.946132	0.0522103
54532	1.89226	0.091171
54533	2.10495	0.05
54534	1.89226	0.05
54535	2.30932	0.05
54536	1.89226	0.109686
54537	2.30932	0.142968
54538	2.30932	0.05
54539	1.00366	0.05
54540	2.10496	0.05
54541	1.24728	0.05
54542	3.31298	0.05
54543	1.87557	2.1
54544	1.07167	2.1
54545	2.03045	2.1
54546	1.25185	2.1
54547	3.10212	2.1
54548	1.89226	2.1
54549	1.89226	2.1
54550	1.89226	2.1
54551	1.89226	2.1
54552	3.78453	2.1
54553	0.851541	2.1
54554	0.851541	2.1
54555	1.12236	2.1
54556	1.05783	2.1
54557	0.870663	2.1
54558	0.951856	2.1
54559	0.946132	2.1
54560	0.946132	2.1
54561	1.04073	2.1
54562	1.1296	2.1
54563	0.776062	2.1
54564	0.851527	2.1
54565	0.859395	2.1
54566	0.859395	2.1
54567	0.870165	2.1
54568	0.870165	2.1
54569	0.625924	2.1
54570	0.714333	2.1
54571	1.0821	2.1
54572	1.01523	2.1
54573	1.0821	2.1
54574	0.714333	2.1
54575	1.01523	2.1

54576	0.625924	2.1
54577	1.89226	2.1
54578	1.89226	2.1
54579	1.89226	2.1
54580	1.89226	2.1
54581	3.78453	2.1
54582	1.89226	2.1
54583	1.89226	2.1
54584	2.23164	2.1
54585	2.67802	2.1
54586	2.03663	2.1
54587	1.89226	2.1
54588	2.42221	2.1
54589	1.61933	2.1
54590	2.80896	2.1
54591	1.87043	2.1
54592	4.29262	2.1
54593	1.89226	2.1
54594	1.89226	2.1
54595	1.89226	2.1
54596	1.89226	2.1
54597	1.89226	2.1
54598	1.89226	2.1
54599	2.80892	2.1
54600	1.87041	2.1
54601	2.67182	2.1
54602	2.30891	2.1
54603	5.07665	2.1
54604	1.89224	2.1
54605	1.89224	2.1
54606	1.89224	2.1
54607	1.89224	2.1
54608	3.78448	2.1
54609	0.946118	2.1
54610	0.946118	2.1
54611	0.946118	2.1
54612	0.946118	2.1
54613	0.946115	2.1
54614	0.946115	2.1
54615	0.94612	2.1
54616	0.94612	2.1
54617	0.94613	2.1
54618	0.94613	2.1
54619	0.946105	2.1
54620	0.946105	2.1
54621	2.3089	2.1
54622	1.02783	2.1
54623	1.98533	2.1
54624	1.76762	2.1
54625	1.24553	2.1
54626	1.02783	2.1
54627	2.2049	0.05
54628	2.34673	0.05
54629	1.00763	0.05
54630	1.23746	0.05
54631	3.44236	0.05
54632	1.89226	0.05
54633	1.89226	0.05

54634	1.89226	0.05
54635	1.89226	0.05
54636	3.78452	0.05
54637	1.89226	0.05
54638	1.89226	0.05
54639	1.89226	0.05
54640	1.89226	0.05
54641	3.78453	0.05
54642	0.891618	0.05
54643	0.891618	0.05
54644	0.902158	0.05
54645	0.902158	0.05
54646	1.15396	0.05
54647	0.620408	0.05
54648	1.17337	0.05
54649	0.618733	0.05
54650	0.620408	0.05
54651	0.618733	0.05
54652	1.17337	0.05
54653	1.15396	0.05
54654	0.69978	0.05
54655	0.69978	0.05
54656	0.946102	0.05
54657	0.946102	0.05
54658	0.831702	0.05
54659	0.946129	0.05
54660	0.699752	0.05
54661	0.946132	0.05
54662	0.831703	0.05
54663	0.699751	0.05
54664	0.946131	0.05
54665	0.946131	0.05
54666	0.831675	0.05
54667	0.831675	0.05
54668	0.946163	0.05
54669	0.864397	0.05
54670	0.853471	0.05
54671	0.962623	0.05
54672	0.748555	0.05
54673	0.851543	0.05
54674	0.864714	0.05
54675	0.760411	0.05
54676	0.946133	0.05
54677	0.946133	0.05
54678	1.89226	0.141287
54679	1.89226	0.90708
54680	1.89226	0.540021
54681	1.89226	0.209129
54682	3.78452	0.460014
54683	1.89226	0.826727
54684	1.89226	0.475551
54685	1.89226	0.394973
54686	1.89226	1.06546
54687	3.78452	0.637691
54688	0.946132	0.608247
54689	0.946132	0.991871
54690	0.946132	0.352409
54691	0.946132	0.569975

54692	0.946132	0.218318
54693	0.946132	1.40206
54694	0.946133	0.39995
54695	0.946133	0.951128
54696	0.946133	0.354656
54697	0.946133	0.397976
54698	0.946132	0.726555
54699	0.946132	0.798238
54700	1.89226	0.847454
54701	2.39715	0.523191
54702	1.89226	1.00036
54703	1.89226	1.41761
54704	1.89226	0.273989
54705	1.89226	0.450756
54706	0.94613	0.959796
54707	0.94613	0.747224
54708	0.94613	0.535851
54709	0.94613	0.425843
54710	0.946131	0.451002
54711	0.946131	1.36717
54712	0.946129	0.812267
54713	0.946129	0.591425
54714	0.94613	0.430142
54715	0.94613	0.554451
54716	0.94613	0.888849
54717	0.94613	0.699918
54718	2.54204	2.1
54719	2.3148	2.1
54720	2.55328	2.1
54721	2.39646	2.1
54722	4.71126	2.1
54723	1.62611	2.1
54724	2.3148	2.1
54725	2.16986	2.1
54726	1.43745	2.1
54727	3.75225	2.1
54728	1.89226	2.1
54729	1.89226	2.1
54730	1.89226	2.1
54731	1.89226	2.1
54732	3.78453	2.1
54733	1.89226	2.1
54734	1.89226	2.1
54735	1.89226	2.1
54736	1.89226	2.1
54737	3.78453	2.1
54738	2.12125	2.1
54739	2.39642	2.1
54740	2.2941	2.1
54741	1.9933	2.1
54742	4.38972	2.1
54743	1.73784	2.1
54744	1.26892	2.1
54745	1.26892	2.1
54746	1.43743	2.1
54747	1.9933	2.1
54748	2.1618	2.1
54749	0.946118	2.1

54750	0.946118	2.1
54751	0.946118	2.1
54752	0.946118	2.1
54753	0.946119	2.1
54754	0.946119	2.1
54755	0.946118	2.1
54756	0.946118	2.1
54757	0.946131	2.1
54758	0.946131	2.1
54759	0.946106	2.1
54760	0.946106	2.1
54761	1.89224	2.1
54762	1.89224	2.1
54763	1.89224	2.1
54764	1.89224	2.1
54765	3.78448	2.1
54766	2.45922	2.1
54767	2.15686	2.1
54768	2.14259	2.1
54769	2.43225	2.1
54770	4.58911	2.1
54771	1.62027	2.1
54772	2.15686	2.1
54773	1.62241	2.1
54774	1.62241	2.1
54775	2.1426	2.1
54776	2.1426	2.1
54777	2.14259	2.1
54778	2.43225	2.1
54779	2.40667	2.1
54780	2.1281	2.1
54781	4.56035	2.1
54782	1.0713	2.1
54783	1.06405	2.1
54784	0.810136	2.1
54785	0.808433	2.1
54786	0.938508	2.1
54787	0.938508	2.1
54788	0.941223	2.1
54789	0.941223	2.1
54790	1.06405	2.1
54791	0.808433	2.1
54792	0.810135	2.1
54793	1.0713	2.1
54794	1.89226	0.0572773
54795	1.89226	0.05
54796	1.89226	0.0504397
54797	1.89226	0.05
54798	3.78453	0.05
54799	1.89227	0.05
54800	1.89227	0.05
54801	1.89227	0.05
54802	1.89227	0.05
54803	3.78453	0.05
54804	1.19 0.05	
54805	1.12469	0.05
54806	1.01052	0.05
54807	1.08166	0.05

54808	2.20051	0.05
54809	0.594999	0.05
54810	0.540832	0.05
54811	0.514182	0.05
54812	0.567857	0.05
54813	0.554259	0.05
54814	0.554259	0.05
54815	0.55443	0.05
54816	0.55443	0.05
54817	0.540832	0.05
54818	0.514182	0.05
54819	0.567857	0.05
54820	0.594999	0.05
54821	1.38674	0.05
54822	0.946132	0.05
54823	1.36361	0.05
54824	0.946132	0.05
54825	1.36362	0.05
54826	1.36362	0.05
54827	0.946131	0.05
54828	0.946131	0.05
54829	1.38674	0.05
54830	1.36361	0.05
54831	0.946132	0.05
54832	0.946132	0.05
54833	1.89226	0.05
54834	2.68508	0.05
54835	2.77349	0.05
54836	1.89226	0.05
54837	2.77349	0.05
54838	1.89226	0.05
54839	2.15315	0.05
54840	1.96254	0.05
54841	1.51377	0.05
54842	1.89226	0.05
54843	1.47799	0.05
54844	1.89226	0.05
54845	2.49439	0.05
54846	1.89226	0.0661934
54847	2.15315	0.0693581
54848	1.89226	0.05
54849	2.15315	0.05
54850	1.89226	0.05
54851	1.89226	0.0785247
54852	1.89226	0.0955629
54853	1.89226	0.0716066
54854	1.89226	0.054306
54855	1.89226	0.175521
54856	1.89226	0.118134
54857	1.89226	0.0566809
54858	1.89226	0.05
54859	1.89226	0.05
54860	1.89226	0.074557
54861	1.89226	0.05
54862	1.89226	0.05
54863	1.375	0.05
54864	2.01865	0.05
54865	2.12047	0.05

54866	1.47354	0.05
54867	2.12047	0.057324
54868	1.47354	0.05
54869	0.687499	0.05
54870	1.06023	0.05
54871	1.08019	0.05
54872	0.604229	0.05
54873	1.08018	0.05
54874	1.06023	0.05
54875	0.687502	0.05
54876	0.60423	0.05
54877	0.80646	0.05
54878	0.806461	0.05
54879	0.858001	0.05
54880	0.858001	0.05
54881	2.26414	2.1
54882	1.87342	2.1
54883	1.87342	2.1
54884	1.79667	2.1
54885	2.44045	2.1
54886	2.31924	2.1
54887	2.26414	2.1
54888	2.26616	2.1
54889	2.2355	2.1
54890	2.21858	2.1
54891	4.48474	2.1
54892	2.18516	2.1
54893	1.87344	2.1
54894	2.21859	2.1
54895	2.66233	2.1
54896	1.85196	2.1
54897	2.22236	2.1
54898	2.23551	2.1
54899	2.21859	2.1
54900	2.18516	2.1
54901	2.21189	2.1
54902	4.43049	2.1
54903	1.17171	2.1
54904	2.38924	2.1
54905	2.0815	2.1
54906	2.33348	2.1
54907	1.42851	2.1
54908	1.5959	2.1
54909	1.89226	2.1
54910	1.89226	2.1
54911	1.89226	2.1
54912	1.89226	2.1
54913	3.78452	2.1
54914	2.75425	2.1
54915	1.34366	2.1
54916	1.34366	2.1
54917	1.56512	2.1
54918	2.54186	2.1
54919	2.76332	2.1
54920	1.07751	2.1
54921	2.0815	2.1
54922	1.97259	2.1
54923	1.76455	2.1

54924	1.30191	2.1
54925	1.16461	2.1
54926	0.946131	2.1
54927	0.946131	2.1
54928	0.946129	2.1
54929	0.946129	2.1
54930	0.946131	2.1
54931	0.946131	2.1
54932	0.94613	2.1
54933	0.94613	2.1
54934	0.94613	2.1
54935	0.94613	2.1
54936	0.946131	2.1
54937	0.946131	2.1
54938	1.89226	0.0504541
54939	1.89226	0.05
54940	1.89226	0.05
54941	1.89226	0.05
54942	3.78453	0.05
54943	1.89226	0.051324
54944	1.89226	0.05
54945	1.89226	0.05
54946	1.89226	0.05
54947	3.78453	0.05
54948	0.946132	0.05
54949	0.946132	0.05
54950	1.30327	0.05
54951	1.33544	0.05
54952	1.33544	0.05
54953	1.33544	0.05
54954	0.946132	0.05
54955	0.946132	0.05
54956	0.946132	0.05
54957	1.30327	0.05
54958	1.33544	0.05
54959	0.946132	0.05
54960	1.30327	0.05
54961	1.33544	0.05
54962	0.709426	0.05
54963	0.790129	0.05
54964	1.05886	0.05
54965	1.05886	0.05
54966	1.03454	0.05
54967	1.03454	0.05
54968	1.30327	0.05
54969	0.709426	0.05
54970	1.33544	0.05
54971	0.790128	0.05
54972	0.946132	0.05
54973	0.946132	0.05
54974	0.827866	0.05
54975	0.946133	0.05
54976	0.946132	0.05
54977	0.946132	0.05
54978	0.946133	0.05
54979	0.946133	0.05
54980	0.946132	0.05
54981	0.827866	0.05

54982	0.946133	0.05
54983	0.946133	0.05
54984	1.89227	0.05
54985	1.89227	0.05
54986	1.89227	0.05
54987	1.89227	0.05
54988	1.89227	0.05
54989	1.89227	0.05
54990	0.946133	0.05
54991	0.946133	0.05
54992	1.21602	0.05
54993	1.30327	0.05
54994	1.30327	0.05
54995	1.30327	0.05
54996	0.946132	0.05
54997	0.946132	0.05
54998	0.946132	0.05
54999	1.21602	0.05
55000	0.946133	0.05
55001	1.30327	0.05
55002	1.04227	0.05
55003	1.11706	0.05
55004	0.968413	0.05
55005	0.945482	0.05
55006	1.3204	0.05
55007	1.23215	0.05
55008	0.810791	0.05
55009	1.38978	0.05
55010	1.07728	0.05
55011	1.11704	0.05
55012	0.608052	0.05
55013	1.89226	0.05
55014	1.89226	0.05
55015	1.89226	0.05
55016	1.89226	0.05
55017	3.78453	0.05
55018	1.89226	0.05
55019	1.89226	0.05
55020	1.89226	0.05
55021	1.89226	0.05
55022	3.78451	0.05
55023	2.58476	0.05
55024	1.56598	0.05
55025	1.55045	0.05
55026	1.5124	0.05
55027	2.62281	0.05
55028	2.58476	0.05
55029	2.58477	0.05
55030	1.89226	0.05
55031	1.89226	0.05
55032	1.89226	0.05
55033	2.62282	0.05
55034	2.62282	0.05
55035	1.89224	0.05
55036	1.89224	0.05
55037	1.89224	0.05
55038	1.89224	0.05
55039	3.78448	0.05

55040	1.89223	0.05
55041	1.89223	0.05
55042	1.89223	0.05
55043	1.89223	0.05
55044	1.89223	0.05
55045	1.89223	0.05
55046	2.62278	0.05
55047	1.45053	0.05
55048	1.49678	0.05
55049	1.56596	0.05
55050	2.5536	0.05
55051	2.62278	0.05
55052	1.31139	0.05
55053	1.2768	0.05
55054	0.946118	0.05
55055	0.946118	0.05
55056	1.2768	0.05
55057	1.2768	0.05
55058	0.946116	0.05
55059	0.946116	0.05
55060	1.27682	0.05
55061	0.94613	0.05
55062	0.946105	0.05
55063	1.31138	0.05
55064	1.38506	1.81537
55065	1.53348	2.1
55066	1.17042	1.80805
55067	1.17042	2.08935
55068	1.73669	1.86188
55069	1.73669	2.09468
55070	1.73669	1.54155
55071	1.79928	0.648783
55072	1.38506	1.26074
55073	1.38506	2.0979
55074	1.79928	2.06926
55075	1.43484	2.1
55076	1.73669	1.31358
55077	1.93568	2.06429
55078	1.53347	0.955663
55079	1.53347	2.0368
55080	2.14058	1.14159
55081	2.14058	2.1
55082	0.89964	0.617507
55083	1.10415	2.1
55084	0.868346	1.46959
55085	1.07029	2.1
55086	0.98687	1.69191
55087	0.98687	2.1
55088	0.985627	0.155374
55089	0.985627	2.1
55090	1.07029	2.00183
55091	1.10415	1.6761
55092	0.868344	2.06201
55093	0.899642	2.1
55094	2.40667	2.1
55095	2.1281	2.1
55096	2.11176	2.1
55097	2.38069	2.1

55098	4.50878	2.1
55099	2.11176	2.1
55100	1.61687	2.1
55101	1.60965	2.1
55102	1.61687	2.1
55103	2.12088	2.1
55104	2.1281	2.1
55105	2.11176	2.1
55106	2.34898	2.1
55107	2.34898	2.1
55108	2.38069	2.1
55109	2.09114	2.1
55110	2.12285	2.1
55111	1.05588	2.1
55112	1.04557	2.1
55113	0.804824	2.1
55114	0.798269	2.1
55115	0.92615	2.1
55116	0.92615	2.1
55117	0.927999	2.1
55118	0.927999	2.1
55119	1.04557	2.1
55120	0.798269	2.1
55121	1.05588	2.1
55122	0.804824	2.1
55123	1.43548	0.05
55124	1.53873	0.05
55125	1.25063	0.05
55126	1.39662	0.05
55127	2.78937	0.05
55128	1.25063	0.0595399
55129	1.39662	0.05
55130	1.10476	0.05
55131	1.28326	0.05
55132	2.53389	0.05
55133	1.51147	0.05
55134	1.86114	0.05
55135	1.86114	0.05
55136	2.12926	0.05
55137	1.3659	0.05
55138	1.63401	0.05
55139	1.89226	0.05
55140	1.89226	0.05
55141	1.89226	0.05
55142	1.89226	0.05
55143	1.89226	0.05
55144	1.89226	0.05
55145	1.89226	0.05
55146	1.89226	0.05
55147	2.46286	0.05
55148	1.89226	0.05
55149	3.78452	0.05
55150	1.89226	0.05
55151	1.89226	0.05
55152	1.89226	0.05
55153	1.89226	0.05
55154	1.89226	0.05
55155	1.89226	0.05

55156	1.89226	0.05
55157	1.89226	0.05
55158	2.28992	0.05
55159	1.89226	0.05
55160	3.78452	0.05
55161	1.13537	0.05
55162	0.886595	0.0762473
55163	0.946131	0.05
55164	0.946131	0.05
55165	0.94613	0.0981553
55166	0.94613	0.05
55167	1.25069	0.05
55168	0.841855	0.104189
55169	1.27436	0.05
55170	1.06196	0.05
55171	1.09758	0.05
55172	0.830306	0.05
55173	0.826931	0.239897
55174	0.826933	0.05
55175	0.764631	0.05
55176	0.764631	0.0644737
55177	1.00427	0.0737236
55178	0.788034	0.05
55179	0.726852	0.05
55180	0.604206	0.16131
55181	0.828475	0.05
55182	0.604198	0.05
55183	0.8332	0.05
55184	1.00428	0.05
55185	1.89226	0.121216
55186	1.89226	0.05
55187	1.89226	0.05
55188	1.89226	0.05
55189	1.89226	0.05
55190	1.89226	0.05
55191	0.768789	1.10181
55192	0.849132	0.05
55193	0.666423	0.976964
55194	0.688993	0.0653859
55195	0.868671	0.05
55196	0.89117	0.828262
55197	0.705632	2.1
55198	1.80425	2.1
55199	1.73883	2.1
55200	1.45159	2.1
55201	1.45159	2.1
55202	1.37681	2.1
55203	1.73883	2.1
55204	1.39679	0.05
55205	2.08149	0.05
55206	1.89226	0.05
55207	2.32763	0.05
55208	1.89226	0.05
55209	2.31274	0.05
55210	1.89226	0.05
55211	1.89226	0.05
55212	1.89226	0.05
55213	1.89226	0.05

55214	1.89226	0.05
55215	1.89226	0.05
55216	1.89226	0.05
55217	1.89226	0.05
55218	1.89226	0.05
55219	1.89226	0.05
55220	1.89226	0.05
55221	1.89226	0.05
55222	1.95181	0.05
55223	1.89226	0.05
55224	1.89226	0.05
55225	1.89226	0.05
55226	3.78451	0.05
55227	0.946131	0.05
55228	0.946131	0.05
55229	0.946125	0.05
55230	0.946125	0.05
55231	0.946127	0.05
55232	0.946127	0.05
55233	0.946128	0.05
55234	0.946128	0.05
55235	0.946128	0.05
55236	0.946128	0.05
55237	0.946128	0.05
55238	0.946128	0.05
55239	1.89226	0.05
55240	1.89226	0.05
55241	1.89226	0.05
55242	1.89226	0.05
55243	1.89226	0.05
55244	1.89226	0.05
55245	1.89226	0.716845
55246	1.89226	0.577469
55247	1.89226	0.412903
55248	1.89226	0.194427
55249	3.78453	0.472105
55250	1.89226	0.633064
55251	1.89226	0.369751
55252	1.89226	0.505741
55253	1.89226	0.196646
55254	3.78452	0.435415
55255	1.21387	0.159248
55256	0.8164	0.0614612
55257	0.946133	0.211092
55258	0.946133	0.278243
55259	0.946102	0.0545934
55260	0.946102	0.341307
55261	0.816431	0.865527
55262	0.816431	0.250536
55263	0.816401	0.162543
55264	0.94613	0.0923484
55265	0.946131	0.904831
55266	1.21388	0.203796
55267	0.946132	0.115762
55268	0.946132	0.141926
55269	0.946132	0.230801
55270	0.946132	0.245972
55271	0.946132	0.0636826

55272	0.946132	0.161007
55273	0.946132	0.588563
55274	0.946132	0.177181
55275	0.946131	0.0648981
55276	0.946131	0.189686
55277	0.946132	0.583656
55278	0.946132	0.178445
55279	1.89226	0.532769
55280	1.89226	0.0745441
55281	1.89226	0.0609317
55282	1.89226	0.347553
55283	1.89226	0.0970934
55284	1.89226	0.60739
55285	1.89226	0.615059
55286	1.89226	0.296877
55287	2.15871	0.05
55288	2.15871	0.322238
55289	1.81454	0.05
55290	1.54809	0.395771
55291	1.89226	1.05168
55292	1.89226	1.58201
55293	1.89226	1.10843
55294	1.89226	0.915102
55295	3.78453	1.30428
55296	1.89226	1.27869
55297	1.89226	0.715535
55298	1.89226	0.585246
55299	1.89226	1.21308
55300	1.89226	1.75722
55301	1.89226	1.31362
55302	2.29205	1.24007
55303	1.42633	1.07764
55304	1.69015	1.42889
55305	1.69015	1.37101
55306	2.10364	0.445526
55307	2.05344	0.573535
55308	2.29205	0.885622
55309	1.58147	1.0196
55310	2.16529	0.462693
55311	1.56339	0.250627
55312	2.29205	1.52172
55313	1.69015	1.31781
55314	1.89226	1.33882
55315	1.89226	1.16005
55316	1.89226	0.855263
55317	1.89226	1.1067
55318	3.78453	0.904836
55319	1.89226	1.13546
55320	1.89226	1.50236
55321	1.89226	1.27113
55322	1.89226	0.623525
55323	3.78452	1.54336
55324	2.29205	1.49682
55325	2.85335	0.259837
55326	2.55409	1.18639
55327	2.04345	0.545001
55328	2.76338	1.05198
55329	2.29205	0.662528

55330	2.29205	1.16747
55331	2.16529	1.27239
55332	2.72418	1.08781
55333	2.76338	0.126012
55334	5.01623	1.07924
55335	1.89227	0.05
55336	1.89227	0.05
55337	1.89227	0.05
55338	1.89227	0.05
55339	3.78453	0.05
55340	2.39588	0.05
55341	2.14918	0.05
55342	2.14918	0.05
55343	2.37907	0.05
55344	2.20531	0.05
55345	2.4352	0.05
55346	1.89226	0.05
55347	2.64327	0.05
55348	2.36175	0.05
55349	1.89226	0.05
55350	4.25402	0.05
55351	1.89226	0.05
55352	1.89226	0.05
55353	1.89226	0.05
55354	1.89226	0.05
55355	1.89226	0.05
55356	1.89226	0.05
55357	1.05395	0.05
55358	1.05395	0.05
55359	1.03114	0.05
55360	1.03114	0.05
55361	0.936689	0.05
55362	1.07459	0.05
55363	1.10265	0.05
55364	1.01049	0.05
55365	0.93669	0.05
55366	1.01049	0.05
55367	1.07459	0.05
55368	1.10265	0.05
55369	1.18088	0.05
55370	0.776509	0.05
55371	0.946132	0.05
55372	0.946132	0.05
55373	1.04225	0.05
55374	0.830392	0.05
55375	0.931352	0.05
55376	0.931352	0.05
55377	1.13006	0.05
55378	1.04074	0.05
55379	1.0628	0.05
55380	0.851529	0.05
55381	2.40032	2.1
55382	1.89226	2.1
55383	1.89226	2.1
55384	1.89226	2.1
55385	2.38918	2.1
55386	2.38918	2.1
55387	1.89226	2.1

55388	1.89226	2.1
55389	1.89226	2.1
55390	1.89226	2.1
55391	1.89226	2.1
55392	1.89226	2.1
55393	1.6585	2.1
55394	2.37364	2.1
55395	2.39622	2.1
55396	2.39622	2.1
55397	1.6488	2.1
55398	1.6488	2.1
55399	2.44603	2.1
55400	1.89224	2.1
55401	1.89224	2.1
55402	1.89224	2.1
55403	2.40029	2.1
55404	2.40029	2.1
55405	1.89224	2.1
55406	1.89224	2.1
55407	1.89224	2.1
55408	1.89224	2.1
55409	1.89224	2.1
55410	1.89224	2.1
55411	1.01946	2.1
55412	1.01946	2.1
55413	1.02497	2.1
55414	1.02497	2.1
55415	1.21519	2.1
55416	1.19809	2.07611
55417	0.835303	2.1
55418	0.82924	2.1
55419	0.835314	2.1
55420	1.2152	2.1
55421	0.829228	2.1
55422	1.19808	2.1
55423	1.91027	2.1
55424	1.05809	2.1
55425	1.00659	2.1
55426	2.06727	2.1
55427	3.11819	2.1
55428	2.37166	2.1
55429	2.06726	2.1
55430	1.66994	2.1
55431	1.67623	2.1
55432	2.40308	2.1
55433	2.40308	2.1
55434	0.960194	2.1
55435	0.939664	2.1
55436	0.531131	2.1
55437	0.503298	2.1
55438	0.729927	2.1
55439	0.729927	2.1
55440	0.733565	2.1
55441	0.733565	2.1
55442	0.960195	2.1
55443	0.531132	2.1
55444	0.503297	2.1
55445	0.939663	2.1

55446	1.20154	2.1
55447	1.21754	2.1
55448	0.960195	2.1
55449	0.939665	2.1
55450	1.07745	2.1
55451	1.07745	2.1
55452	1.07976	2.1
55453	1.07976	2.1
55454	0.960194	2.1
55455	1.21754	2.1
55456	0.939662	2.1
55457	1.20155	2.1
55458	1.89224	2.1
55459	1.89224	2.1
55460	2.08609	2.1
55461	2.08609	2.1
55462	2.09527	2.1
55463	1.90142	2.1
55464	1.65815	2.1
55465	1.71398	2.1
55466	2.2001	2.1
55467	2.23791	2.1
55468	1.4502	2.1
55469	1.4502	2.1
55470	0.793587	2.1
55471	0.793587	2.1
55472	0.792408	2.1
55473	0.792408	2.1
55474	0.952221	2.1
55475	0.858585	2.1
55476	0.725101	2.1
55477	0.633774	2.1
55478	0.633775	2.1
55479	0.858585	2.1
55480	0.952221	2.1
55481	0.725101	2.1
55482	1.5735	2.1
55483	2.56975	2.1
55484	2.63166	2.1
55485	2.63166	2.1
55486	1.54711	2.1
55487	1.54711	2.1
55488	0.946131	2.1
55489	0.946131	2.1
55490	1.28487	2.1
55491	1.28487	2.1
55492	0.946127	2.1
55493	0.946127	2.1
55494	1.28488	2.1
55495	1.31583	2.1
55496	1.31583	2.1
55497	0.94613	2.1
55498	1.28487	2.1
55499	0.946129	2.1
55500	1.0596	2.1
55501	1.0596	2.1
55502	1.06939	2.1
55503	1.06939	2.1

55504	1.34225	2.1
55505	1.31583	2.1
55506	0.793563	2.1
55507	0.786748	2.1
55508	0.793563	2.1
55509	1.34225	2.1
55510	0.786748	2.1
55511	1.31583	2.1
55512	0.946131	2.1
55513	0.946131	2.1
55514	1.31582	2.1
55515	1.31582	2.1
55516	0.946127	2.1
55517	0.946127	2.1
55518	1.34225	2.1
55519	1.31583	2.1
55520	1.34225	2.1
55521	0.946129	2.1
55522	1.31583	2.1
55523	0.946129	2.1
55524	1.89226	2.1
55525	1.89226	2.1
55526	1.89226	2.1
55527	1.89226	2.1
55528	1.89226	2.1
55529	1.89226	2.1
55530	1.89226	2.1
55531	1.89226	2.1
55532	1.89226	2.1
55533	1.89226	2.1
55534	1.89226	2.1
55535	1.89226	2.1
55536	1.26992	2.1
55537	1.75003	2.1
55538	1.79883	2.1
55539	1.26732	2.1
55540	3.06875	2.1
55541	0.875017	2.1
55542	0.899414	2.1
55543	0.811147	2.1
55544	0.85053	2.1
55545	0.851513	2.1
55546	0.851513	2.1
55547	0.859048	2.1
55548	0.859048	2.1
55549	0.899413	2.1
55550	0.85053	2.1
55551	0.811147	2.1
55552	0.875016	2.1
55553	1.89227	2.1
55554	1.89227	2.1
55555	1.89227	2.1
55556	1.89227	2.1
55557	1.89227	2.1
55558	1.89227	2.1
55559	1.89226	2.1
55560	1.89226	2.1
55561	1.89226	2.1

55562	1.89226	2.1
55563	1.89226	2.1
55564	1.89226	2.1
55565	0.633661	2.1
55566	0.642005	2.1
55567	0.93285	2.1
55568	0.899413	2.1
55569	0.764487	2.1
55570	0.764487	2.1
55571	0.776931	2.1
55572	0.776931	2.1
55573	0.642004	2.1
55574	0.93285	2.1
55575	0.899414	2.1
55576	0.633661	2.1
55577	0.932851	2.1
55578	0.899414	2.1
55579	0.905419	2.1
55580	0.85053	2.1
55581	0.886336	2.1
55582	0.886336	2.1
55583	0.897045	2.1
55584	0.897045	2.1
55585	0.93285	2.1
55586	0.905419	2.1
55587	0.899414	2.1
55588	0.850531	2.1
55589	2.39322	2.1
55590	2.74149	0.765731
55591	2.70944	2.1
55592	2.6849	0.9197
55593	2.41776	2.1
55594	2.39322	0.0898645
55595	2.41776	2.1
55596	2.80992	2.1
55597	2.77256	2.1
55598	2.74149	2.1
55599	2.44882	2.1
55600	2.41776	0.391779
55601	2.80992	2.1
55602	1.89226	1.86856
55603	2.74149	1.95836
55604	2.74149	2.1
55605	1.89226	0.467558
55606	1.89226	1.5635
55607	1.89226	0.05
55608	1.89226	0.05
55609	1.89226	0.05
55610	1.89226	0.05
55611	1.89226	0.05
55612	1.89226	0.05
55613	2.38796	0.05
55614	2.0815	0.05
55615	2.0815	0.05
55616	2.49452	0.05
55617	1.55994	0.05
55618	2.14721	0.05
55619	1.89227	0.05

55620	1.89227	0.05
55621	1.89227	0.05
55622	1.89227	0.05
55623	1.89227	0.05
55624	1.89227	0.05
55625	1.13387	0.05
55626	2.27494	0.05
55627	1.13387	0.05
55628	2.19489	0.05
55629	1.60867	0.05
55630	2.6994	0.05
55631	1.89226	0.05
55632	1.89226	0.05
55633	1.89226	0.05
55634	1.89226	0.05
55635	3.78453	0.05
55636	1.42381	1.63768
55637	1.10747	2.1
55638	1.10747	1.35742
55639	1.08911	2.1
55640	1.42866	0.899867
55641	1.4103	2.1
55642	1.89226	2.1
55643	1.88181	2.1
55644	1.88181	2.1
55645	1.76249	2.1
55646	2.0815	2.1
55647	1.97539	2.1
55648	0.946128	2.1
55649	0.946128	2.1
55650	0.946133	1.2554
55651	0.946133	0.281128
55652	0.94613	0.297411
55653	0.94613	2.1
55654	0.946131	2.1
55655	0.946131	0.64842
55656	0.946131	1.89412
55657	0.946131	1.94942
55658	0.946131	1.93877
55659	0.946131	1.95962
55660	1.42866	1.45692
55661	1.43213	1.23083
55662	1.10748	2.1
55663	1.10748	2.1
55664	1.13641	2.1
55665	1.43213	2.1
55666	2.0815	2.1
55667	2.10287	2.1
55668	2.10287	2.1
55669	2.0921	2.1
55670	1.89227	2.1
55671	1.83938	2.1
55672	0.810944	1.09337
55673	0.810944	0.389013
55674	0.810945	0.404775
55675	0.810945	2.1
55676	0.810933	1.68559
55677	0.810933	1.72526

55678	1.89226	2.1
55679	1.3517	2.1
55680	1.08132	1.69383
55681	1.08132	2.1
55682	1.08133	1.58433
55683	2.33097	2.1
55684	1.53822	2.1
55685	1.53822	2.1
55686	1.68437	2.1
55687	2.17996	2.1
55688	2.32612	2.1
55689	0.99189	2.1
55690	1.18948	0.05
55691	1.19756	0.05
55692	1.19756	2.1
55693	0.978851	2.1
55694	0.926995	2.1
55695	1.26924	0.05
55696	1.34642	0.05
55697	1.3228	0.129178
55698	0.990899	0.153919
55699	1.0627	0.714632
55700	1.33473	0.602957
55701	1.96196	2.1
55702	1.39702	2.1
55703	1.39702	2.1
55704	1.53822	2.1
55705	2.17372	2.1
55706	2.31011	2.1
55707	1.89226	0.532135
55708	1.89226	0.05
55709	1.89226	0.116795
55710	1.89226	0.05
55711	3.78453	0.0571252
55712	2.38475	0.262801
55713	1.73957	0.0697283
55714	1.88452	0.0997615
55715	1.58695	0.05
55716	3.6241	0.0822121
55717	1.73958	0.05
55718	1.3126	0.597498
55719	1.58695	0.05
55720	1.14946	0.0923901
55721	2.88903	0.05
55722	1.89226	0.05
55723	1.89226	0.505038
55724	1.89226	0.05
55725	1.89226	0.120359
55726	3.78453	0.05
55727	1.89225	0.0633376
55728	1.89225	0.0917543
55729	1.89225	0.13457
55730	1.89225	0.05
55731	3.7845	0.05
55732	1.88451	0.0596352
55733	1.58694	0.05
55734	1.74649	0.0641546
55735	1.44604	0.05

55736	3.33055	0.05
55737	1.58694	0.05
55738	0.995727	0.0555579
55739	0.995727	0.136983
55740	1.14945	0.078828
55741	1.44604	0.05
55742	1.59976	0.121927
55743	1.89225	0.05
55744	1.89225	0.0514655
55745	1.89225	0.213682
55746	1.89225	0.05
55747	1.89225	0.291285
55748	1.89225	0.05
55749	2.01197	1.87764
55750	2.0961	1.72092
55751	2.13283	0.993232
55752	2.25399	0.310569
55753	4.22894	1.35239
55754	2.09611	0.980024
55755	2.37014	1.08493
55756	2.29031	1.73648
55757	2.10084	2.0966
55758	2.25399	0.28352
55759	2.09611	2.0612
55760	2.13283	1.666
55761	2.25399	2.1
55762	2.22044	1.47162
55763	2.38975	0.53047
55764	4.52258	1.42471
55765	2.38976	0.152323
55766	2.15466	1.15144
55767	2.31988	0.0503124
55768	2.31988	2.02919
55769	2.25399	0.123534
55770	2.25399	2.1
55771	2.58286	0.284511
55772	2.29193	0.05
55773	2.40107	0.319054
55774	2.13815	0.05
55775	4.72101	0.05
55776	2.29193	0.05
55777	1.90104	0.0541325
55778	2.13815	0.05
55779	1.77249	0.058658
55780	4.03919	0.05
55781	2.25197	0.0890322
55782	2.13815	0.05
55783	2.00625	0.0598863
55784	2.00625	0.05
55785	2.40107	0.05
55786	2.40107	0.05
55787	2.13815	0.05
55788	1.77249	0.0644378
55789	2.00625	0.05
55790	1.65815	0.05
55791	3.7963	0.05
55792	2.43417	0.144162
55793	2.06653	0.0733722

55794	1.64587	0.092638
55795	1.65284	0.0599132
55796	2.46898	0.134544
55797	2.46898	0.0846888
55798	2.46895	0.084036
55799	1.89016	0.05
55800	1.87861	0.0539145
55801	1.87861	0.05
55802	2.47161	0.0879113
55803	2.47161	0.0511234
55804	0.946132	0.170615
55805	0.946132	0.103627
55806	0.863311	0.195296
55807	1.34768	0.148795
55808	0.851542	0.179002
55809	0.851542	0.0993778
55810	1.30451	0.153981
55811	1.45304	0.15506
55812	0.946132	0.12591
55813	1.20992	0.144846
55814	0.855162	0.209116
55815	0.946132	0.11528
55816	1.89226	0.0916378
55817	1.89226	0.257295
55818	1.89226	0.151753
55819	1.89226	0.122199
55820	1.89226	0.187704
55821	1.89226	0.155778
55822	0.946132	0.113242
55823	0.946132	0.05
55824	0.946133	0.0587865
55825	0.946133	0.05
55826	0.946132	0.0573949
55827	0.946132	0.05
55828	0.946132	0.05
55829	0.946132	0.05
55830	0.946132	0.0854608
55831	0.946132	0.05
55832	0.946133	0.05
55833	0.946133	0.0532165
55834	2.35574	0.529799
55835	1.89226	0.0624086
55836	1.89226	2.1
55837	1.89226	0.053979
55838	2.67456	0.850787
55839	2.67456	0.05
55840	0.583505	0.05
55841	0.761972	0.149152
55842	1.17787	1.27772
55843	1.33728	0.05
55844	0.965128	1.21332
55845	0.965128	0.05
55846	0.974714	0.525681
55847	0.974715	0.468656
55848	1.17787	0.0902356
55849	0.583506	1.02252
55850	1.33728	0.05
55851	0.761974	0.0904078

55852	1.89226	0.831838
55853	1.89226	0.0801453
55854	1.89226	0.0675506
55855	1.89226	0.05
55856	1.89226	0.0645399
55857	1.89226	0.706937
55858	1.89226	0.05
55859	1.89226	0.05
55860	1.89226	0.05
55861	1.89226	0.0561305
55862	3.78453	0.05
55863	0.946132	0.05
55864	0.946132	0.05
55865	0.946132	0.064276
55866	0.946132	0.05
55867	0.946133	0.173113
55868	0.946133	0.0537973
55869	0.946132	0.653576
55870	0.946132	0.05
55871	0.946133	0.05
55872	0.946133	0.498766
55873	0.946132	0.05
55874	0.946132	0.253861
55875	1.01779	2.1
55876	1.50685	2.1
55877	1.12064	2.1
55878	1.65151	2.1
55879	2.6275	2.1
55880	1.35733	2.1
55881	1.53507	2.1
55882	1.50683	2.1
55883	1.73626	2.1
55884	3.09359	2.1
55885	1.73626	2.1
55886	1.52226	2.1
55887	1.75555	2.1
55888	1.75555	2.1
55889	1.53507	2.1
55890	1.53507	2.1
55891	1.65151	2.1
55892	1.73628	2.1
55893	1.69528	2.1
55894	1.50685	2.1
55895	1.92471	2.1
55896	1.73628	2.1
55897	1.73629	2.1
55898	1.75558	2.1
55899	1.9943	2.1
55900	1.9943	2.1
55901	1.92471	2.1
55902	1.73629	2.1
55903	2.5601	1.13918
55904	1.97645	2.1
55905	2.09605	1.07193
55906	2.09605	2.05286
55907	2.45853	1.23758
55908	2.45853	2.1
55909	2.33824	1.98743

55910	1.97646	1.88754
55911	2.45853	0.774494
55912	1.94984	2.1
55913	2.33824	2.01551
55914	1.82955	2.1
55915	1.22926	2.1
55916	0.946128	2.1
55917	0.946133	2.1
55918	1.28005	1.64735
55919	1.22927	1.66026
55920	1.22926	2.04366
55921	0.946131	1.88597
55922	0.946131	1.67998
55923	0.946131	1.71437
55924	0.946131	1.49114
55925	1.28005	1.82157
55926	1.22926	1.7463
55927	2.35349	0.09134
55928	2.61606	0.05
55929	2.48209	0.14908
55930	2.48209	0.05
55931	2.50057	0.0991145
55932	2.50057	0.05
55933	2.61607	0.111795
55934	2.57611	0.05
55935	2.4821	0.05
55936	2.44814	0.05
55937	5.06421	0.05
55938	2.15721	0.05
55939	2.48209	0.05
55940	2.31844	0.122053
55941	2.31844	0.05
55942	2.35349	0.101421
55943	2.35349	0.05
55944	2.44814	0.05
55945	2.30908	0.05
55946	2.44814	0.05
55947	2.31844	0.05
55948	2.4821	0.0751394
55949	2.31844	0.05
55950	0.91766	0.05
55951	0.91766	0.05
55952	0.912463	0.05
55953	0.912463	0.05
55954	1.16525	0.05
55955	1.26377	0.05
55956	0.576692	0.05
55957	0.664871	0.05
55958	1.16525	0.05
55959	0.576692	0.05
55960	0.664871	0.05
55961	1.26377	0.05
55962	1.72901	2.1
55963	1.62414	2.1
55964	1.88971	2.1
55965	1.8411	1.82585
55966	3.57011	2.1
55967	1.89226	2.1

55968	1.89226	1.94113
55969	1.89226	0.556857
55970	1.89226	2.08383
55971	3.78452	0.871766
55972	1.89226	1.09794
55973	1.89226	2.1
55974	1.89226	2.06108
55975	1.89226	1.0946
55976	3.78452	2.1
55977	1.62414	2.1
55978	1.30896	2.1
55979	1.8411	1.3905
55980	1.52776	2.1
55981	3.15006	1.97327
55982	1.51448	2.1
55983	1.8411	2.1
55984	2.02311	2.1
55985	1.99309	0.544362
55986	3.86421	1.87486
55987	1.89226	0.69781
55988	1.89226	2.1
55989	1.89226	2.05556
55990	1.89226	1.25214
55991	3.78452	2.1
55992	1.89226	2.1
55993	1.89226	1.84173
55994	1.89226	1.47151
55995	1.89226	2.1
55996	3.78452	2.05966
55997	1.8411	2.1
55998	1.52776	2.1
55999	1.99309	2.1
56000	1.72526	2.1
56001	3.56636	2.1
56002	1.89226	2.1
56003	1.89226	1.99231
56004	1.89226	2.07942
56005	1.89226	2.1
56006	3.78453	2.1
56007	1.0424	2.1
56008	1.12935	2.1
56009	0.929383	2.1
56010	0.929383	2.1
56011	1.24941	2.1
56012	1.12686	2.1
56013	0.946132	2.1
56014	0.946132	2.1
56015	1.12944	2.1
56016	0.851554	2.1
56017	1.05017	2.1
56018	0.929315	2.1
56019	0.898029	2.1
56020	0.973994	2.1
56021	0.946131	2.03061
56022	0.946131	2.1
56023	1.0221	2.1
56024	1.0221	2.1
56025	0.898028	1.94086

56026	0.898028	2.1
56027	0.973994	2.1
56028	0.946131	2.1
56029	0.946131	2.1
56030	0.898029	2.1
56031	1.89226	2.05656
56032	1.89226	2.1
56033	1.89226	1.80683
56034	1.89226	2.1
56035	1.89226	1.88733
56036	1.89226	2.1
56037	1.13537	2.1
56038	0.879308	2.1
56039	0.946131	2.00033
56040	0.946131	1.95935
56041	1.13537	2.00388
56042	0.85187	2.1
56043	0.94613	2.1
56044	0.94613	1.70397
56045	1.05777	2.1
56046	1.05777	2.1
56047	0.799318	2.1
56048	0.776452	2.1
56049	0.946116	2.1
56050	0.946116	2.1
56051	1.09531	2.1
56052	1.09531	2.1
56053	0.946118	2.1
56054	0.946118	2.1
56055	1.09135	2.1
56056	1.09531	2.1
56057	1.09134	2.1
56058	0.946105	2.1
56059	1.09532	2.1
56060	0.946131	2.1
56061	0.946129	2.1
56062	0.946129	2.1
56063	1.09137	2.1
56064	1.09137	2.1
56065	0.946131	2.1
56066	0.946131	2.1
56067	1.07713	2.1
56068	1.09137	2.1
56069	0.946131	2.1
56070	1.07713	2.1
56071	1.09137	2.1
56072	0.946131	2.1
56073	0.938916	2.1
56074	0.938916	2.1
56075	0.936995	2.1
56076	1.00447	2.1
56077	0.979819	2.1
56078	1.16821	2.1
56079	1.01263	2.1
56080	0.719167	2.1
56081	1.04536	2.1
56082	1.18901	2.1
56083	0.758057	2.1

56084	0.924111	2.1
56085	1.00532	2.1
56086	1.00532	2.1
56087	0.9925	2.1
56088	0.9925	2.1
56089	1.29322	2.1
56090	1.01985	2.1
56091	1.00365	2.1
56092	0.704601	2.1
56093	1.00365	2.1
56094	1.29322	2.1
56095	1.01985	2.1
56096	0.704602	2.1
56097	2.20011	2.1
56098	1.401	2.1
56099	1.401	2.1
56100	1.52008	2.1
56101	2.11084	2.1
56102	2.22991	2.1
56103	0.854068	2.1
56104	0.854068	2.1
56105	0.851357	2.1
56106	0.851357	2.1
56107	0.644681	2.1
56108	0.700493	2.1
56109	1.0554	2.1
56110	1.00493	2.1
56111	1.00495	2.1
56112	0.64469	2.1
56113	0.700483	2.1
56114	1.05539	2.1
56115	2.46665	0.05
56116	2.22713	0.05
56117	1.85877	0.05
56118	1.8967	0.05
56119	1.56002	0.05
56120	2.22713	0.05
56121	1.89225	2.1
56122	1.89225	2.1
56123	1.89225	2.1
56124	1.89225	2.1
56125	1.89225	2.1
56126	1.89225	2.1
56127	1.89225	2.1
56128	1.89225	2.1
56129	1.89225	2.1
56130	1.89225	2.1
56131	1.89225	2.1
56132	1.89225	2.1
56133	1.284	2.1
56134	1.86569	2.1
56135	1.95398	2.1
56136	1.31091	2.1
56137	3.23798	2.1
56138	0.932844	2.1
56139	0.976992	2.1
56140	0.960128	2.1
56141	0.905413	2.1

56142	0.938531	2.1
56143	0.938531	2.1
56144	0.943874	2.1
56145	0.943874	2.1
56146	0.976985	2.1
56147	0.960122	2.1
56148	0.93285	2.1
56149	0.905419	2.1
56150	1.89227	2.1
56151	1.89227	2.1
56152	1.89227	2.1
56153	1.89227	2.1
56154	3.78453	2.1
56155	1.89226	2.1
56156	1.89226	2.1
56157	1.89226	2.1
56158	1.89226	2.1
56159	1.89226	2.1
56160	1.89226	2.1
56161	0.65546	2.1
56162	0.665451	2.1
56163	1.00694	2.1
56164	0.976998	2.1
56165	0.816261	2.1
56166	0.816261	2.1
56167	0.826189	2.1
56168	0.826189	2.1
56169	1.00694	2.1
56170	0.665451	2.1
56171	0.976998	2.1
56172	0.65546	2.1
56173	2.01555	2.1
56174	2.01388	2.1
56175	1.92027	2.1
56176	1.92027	2.1
56177	2.01388	2.1
56178	1.954 2.1	
56179	2.75466	2.1
56180	1.89226	2.1
56181	1.89226	2.1
56182	2.54931	2.1
56183	4.44158	2.1
56184	2.47917	2.1
56185	1.92772	2.1
56186	1.80682	2.1
56187	2.54931	2.1
56188	4.47702	2.1
56189	0.946132	2.1
56190	0.946132	2.1
56191	1.17602	2.1
56192	1.27466	2.1
56193	0.946132	2.1
56194	0.946132	2.1
56195	1.17602	2.1
56196	1.17602	2.1
56197	0.946132	2.1
56198	1.17602	2.1
56199	0.946131	2.1

56200	1.27466	2.1
56201	2.35204	2.1
56202	2.54931	2.1
56203	2.40783	2.1
56204	1.50108	2.1
56205	1.64256	2.1
56206	1.64256	2.1
56207	1.89226	2.1
56208	1.89226	0.883463
56209	2.31012	2.1
56210	2.17534	2.1
56211	4.0676	2.1
56212	2.17533	2.1
56213	1.89226	0.599188
56214	2.17533	1.51704
56215	1.89226	0.937535
56216	1.66635	2.1
56217	1.38328	2.05353
56218	1.92027	2.1
56219	2.17597	2.1
56220	2.14619	2.1
56221	2.15388	2.1
56222	1.91087	2.1
56223	1.92027	1.68262
56224	1.08622	1.60062
56225	1.08622	2.1
56226	0.971184	2.1
56227	0.972761	2.1
56228	1.14845	2.1
56229	1.16321	2.1
56230	0.894769	2.1
56231	0.894967	2.1
56232	0.894775	2.1
56233	1.28563	2.1
56234	1.14844	2.1
56235	1.0167	2.1
56236	1.89226	0.972458
56237	1.89226	2.1
56238	1.89226	2.1
56239	1.89226	1.28706
56240	1.89226	2.1
56241	1.89226	1.59942
56242	0.937933	2.1
56243	0.937933	2.1
56244	1.26192	2.1
56245	1.26192	2.1
56246	0.937903	2.1
56247	1.25372	2.1
56248	0.946132	2.1
56249	0.946132	2.1
56250	0.946133	2.1
56251	1.25372	2.1
56252	0.946132	2.1
56253	0.937902	2.1
56254	0.946132	0.966681
56255	0.946132	0.380454
56256	0.946128	2.1
56257	0.946128	2.1

56258	0.94613	2.1
56259	0.94613	1.00107
56260	0.94613	0.177566
56261	0.94613	2.1
56262	0.946131	1.76327
56263	0.946131	1.89387
56264	0.94613	1.81759
56265	0.94613	1.78154
56266	0.946132	1.37561
56267	0.946132	0.885113
56268	1.2778	2.1
56269	1.00191	2.1
56270	1.00191	1.20991
56271	1.00191	2.1
56272	0.94613	2.1
56273	0.94613	1.3125
56274	0.946131	2.06765
56275	1.2778	2.1
56276	1.00191	2.03609
56277	0.94613	1.98791
56278	1.50847	0.05
56279	2.0815	0.05
56280	1.56806	0.05
56281	2.0815	0.05
56282	1.52043	0.05
56283	1.9224	0.05
56284	1.06882	0.05
56285	0.69567	0.05
56286	0.773112	0.05
56287	1.09473	0.05
56288	0.8941	0.05
56289	0.8941	0.05
56290	0.937064	0.05
56291	0.981131	0.05
56292	0.763762	0.05
56293	0.722535	0.05
56294	1.2153	0.05
56295	1.07279	0.05
56296	1.89226	0.05
56297	1.89226	0.05
56298	1.89226	0.05
56299	1.89226	0.05
56300	1.89226	0.05
56301	1.89226	0.05
56302	1.89226	0.05
56303	1.89226	0.05
56304	1.89226	0.05
56305	1.89226	0.05
56306	3.78453	0.05
56307	2.51518	0.05
56308	1.89226	0.05
56309	1.89226	0.05
56310	1.89226	0.05
56311	2.37584	0.05
56312	2.37584	0.05
56313	1.25759	0.05
56314	0.946131	0.05
56315	0.946131	0.05

56316	1.24489	0.05
56317	0.946131	0.05
56318	0.946131	0.05
56319	1.25759	0.05
56320	1.25759	0.05
56321	1.25759	0.05
56322	1.24489	0.05
56323	0.946131	0.05
56324	0.946131	0.05
56325	1.08028	0.110441
56326	1.09362	0.201331
56327	0.596616	0.162371
56328	0.606432	0.505025
56329	0.844265	0.461944
56330	0.844265	0.05
56331	0.855786	1.55674
56332	0.855786	0.085648
56333	1.08028	0.30322
56334	0.606433	0.0828657
56335	0.596616	0.080223
56336	1.09362	0.0820242
56337	2.837	0.944101
56338	2.16055	0.05
56339	2.12741	0.611222
56340	2.79312	0.259807
56341	4.95367	0.642151
56342	2.12741	0.405261
56343	1.21286	0.05
56344	1.20859	0.794138
56345	1.21286	0.916383
56346	2.15628	0.157375
56347	2.16056	0.05
56348	1.89226	2.1
56349	1.89226	2.1
56350	1.89226	2.1
56351	1.89226	2.1
56352	3.78453	2.1
56353	0.946102	2.1
56354	0.946102	2.1
56355	0.850039	2.1
56356	0.850039	2.1
56357	0.914891	2.1
56358	0.85001	2.1
56359	0.946131	2.1
56360	0.946131	2.1
56361	0.94613	2.1
56362	0.91489	2.1
56363	0.850011	2.1
56364	0.946131	2.1
56365	0.71107	2.1
56366	0.784813	2.1
56367	1.28453	2.1
56368	1.20328	2.1
56369	0.992128	2.1
56370	0.992128	2.1
56371	0.995965	2.1
56372	0.995965	2.1
56373	0.784813	2.1

56374	1.28453	2.1
56375	0.711071	2.1
56376	1.20328	2.1
56377	1.89226	2.1
56378	1.89226	2.1
56379	1.89226	2.1
56380	1.89226	2.1
56381	3.78453	2.1
56382	0.946132	2.1
56383	0.946132	2.1
56384	0.914889	2.1
56385	0.914889	2.1
56386	0.985255	2.1
56387	0.914891	2.1
56388	0.946131	2.1
56389	0.946131	2.1
56390	0.946131	2.1
56391	0.985255	2.1
56392	0.94613	2.1
56393	0.91489	2.1
56394	0.784813	2.1
56395	0.871551	2.1
56396	1.37791	2.1
56397	1.28453	2.1
56398	1.07644	2.1
56399	1.07644	2.1
56400	1.07964	2.1
56401	1.07964	2.1
56402	0.871551	2.1
56403	1.37791	2.1
56404	0.784813	2.1
56405	1.28453	2.1
56406	1.73075	1.57601
56407	2.10582	0.354794
56408	1.89319	0.307656
56409	1.38766	1.97288
56410	3.27777	0.385948
56411	1.08715	1.7662
56412	1.54185	1.42001
56413	1.73074	1.13398
56414	1.73074	2.02348
56415	1.18948	0.602865
56416	1.18948	1.6111
56417	1.89319	0.230791
56418	1.41689	1.71244
56419	1.11619	0.38877
56420	1.38766	1.33137
56421	1.6052	0.274272
56422	1.89319	1.26227
56423	0.647894	1.46283
56424	0.594738	1.7078
56425	0.708445	1.35047
56426	0.770926	0.868676
56427	0.738326	1.3434
56428	0.738326	1.95476
56429	0.680494	1.34452
56430	0.680494	0.439028
56431	0.708443	0.764522

56432	0.647895	0.769039
56433	0.770925	0.814685
56434	0.59474	0.342122
56435	1.89111	1.03675
56436	1.06994	2.1
56437	2.06689	2.1
56438	1.37063	2.1
56439	3.13683	2.1
56440	2.06689	2.07141
56441	1.39295	2.1
56442	1.56818	2.1
56443	1.37063	2.1
56444	2.26444	1.84929
56445	2.06689	2.1
56446	1.49808	2.1
56447	1.50201	2.1
56448	2.57784	2.1
56449	2.58615	2.1
56450	4.08424	2.1
56451	0.946133	2.1
56452	0.946133	2.1
56453	0.946132	2.1
56454	0.946132	2.1
56455	0.946132	2.1
56456	0.946132	2.1
56457	0.946132	2.1
56458	0.946132	2.1
56459	0.946133	2.1
56460	0.946133	2.1
56461	0.946132	2.1
56462	0.946132	2.1
56463	1.89226	2.1
56464	1.89226	2.1
56465	1.89226	2.1
56466	1.89226	2.1
56467	3.78452	2.1
56468	0.977201	2.1
56469	0.977201	2.1
56470	0.980489	2.1
56471	0.980489	2.1
56472	0.657973	2.1
56473	1.20669	2.1
56474	0.751002	2.1
56475	1.29307	2.1
56476	1.29308	2.1
56477	1.20669	2.1
56478	0.751003	2.1
56479	0.657972	2.1
56480	0.946131	2.1
56481	0.946131	2.1
56482	1.29308	2.1
56483	1.29308	2.1
56484	0.946132	2.1
56485	1.29308	2.1
56486	0.946132	2.1
56487	1.28892	2.1
56488	0.946132	2.1
56489	0.946132	2.1

56490	1.28892	2.1
56491	1.29308	2.1
56492	0.946132	2.1
56493	0.946132	1.65759
56494	0.946131	2.1
56495	0.946131	2.07583
56496	0.946132	2.1
56497	0.946132	2.1
56498	0.946132	1.91097
56499	0.946132	1.63955
56500	0.946132	1.80541
56501	0.946132	2.1
56502	0.946132	2.0627
56503	0.946132	2.1
56504	1.89226	2.1
56505	1.89226	2.1
56506	1.89226	2.1
56507	1.89226	2.07858
56508	1.89226	2.1
56509	1.89226	2.00733
56510	0.94613	2.1
56511	0.94613	2.1
56512	1.20669	2.1
56513	1.20669	2.1
56514	1.20669	2.1
56515	0.946132	2.1
56516	0.946129	2.1
56517	1.29307	2.1
56518	0.946131	2.1
56519	0.946131	2.1
56520	1.29308	2.1
56521	1.20669	2.1
56522	1.89226	0.05
56523	1.89226	0.05
56524	1.89226	0.05
56525	1.89226	0.05
56526	3.78453	0.05
56527	0.946132	0.05
56528	0.946132	0.05
56529	0.946126	0.05
56530	0.946126	0.05
56531	0.946129	0.05
56532	0.946129	0.05
56533	0.946129	0.05
56534	0.946129	0.05
56535	0.946128	0.05
56536	0.946128	0.05
56537	0.946129	0.05
56538	0.946129	0.05
56539	0.725277	0.05
56540	0.588155	0.05
56541	1.19403	0.05
56542	1.27681	0.05
56543	0.918903	0.05
56544	0.918903	0.05
56545	0.946068	0.05
56546	0.946068	0.05
56547	1.19404	0.05

56548	0.588153	0.05
56549	0.725275	0.05
56550	1.27682	0.05
56551	1.19404	0.05
56552	1.27682	0.05
56553	0.946131	0.05
56554	0.946131	0.05
56555	1.19404	0.05
56556	1.19404	0.05
56557	0.946129	0.05
56558	0.946129	0.05
56559	1.19404	0.05
56560	0.94613	0.05
56561	1.27682	0.05
56562	0.946131	0.05
56563	1.89226	0.05
56564	1.89226	0.05
56565	1.89226	0.05
56566	1.89226	0.05
56567	1.89226	0.05
56568	1.89226	0.05
56569	1.4263	1.95995
56570	1.67651	0.0626486
56571	1.5955	0.269318
56572	1.31022	1.95677
56573	2.98674	0.929736
56574	1.18824	2.1
56575	1.28802	2.1
56576	0.946132	0.628622
56577	0.946132	1.36069
56578	0.946132	1.09447
56579	0.946132	2.1
56580	1.28802	0.247267
56581	1.28802	2.1
56582	1.18824	2.1
56583	0.946132	2.1
56584	0.946132	2.1
56585	1.28802	2.0919
56586	1.89226	0.0960899
56587	1.89226	1.34165
56588	1.89226	1.99814
56589	1.89226	0.0628523
56590	3.78452	0.179949
56591	1.5955	0.651419
56592	1.52909	0.0832088
56593	1.31022	0.188604
56594	1.31022	2.1
56595	1.2087	1.94207
56596	1.52909	1.93276
56597	1.11825	2.1
56598	1.18824	2.1
56599	0.946132	1.50323
56600	0.946132	0.948421
56601	0.946132	0.963428
56602	0.946132	2.1
56603	1.18824	0.559513
56604	1.18824	2.1
56605	0.946132	1.9449

56606	1.11825	2.1
56607	1.18824	2.1
56608	0.946133	2.1
56609	0.810943	1.78696
56610	0.810943	0.973393
56611	0.810945	0.147137
56612	0.810945	1.30884
56613	1.89226	0.121933
56614	1.35168	0.320186
56615	1.08132	0.138219
56616	1.0813	0.551967
56617	1.08132	1.65464
56618	0.810959	0.401483
56619	0.810959	0.635151
56620	1.89226	0.05
56621	1.89226	0.05
56622	1.89226	0.05
56623	1.89226	0.05
56624	3.78453	0.05
56625	1.89226	0.05
56626	1.89226	0.05
56627	1.89226	0.05
56628	1.89226	0.05
56629	1.89226	0.05
56630	1.89226	0.05
56631	0.946131	0.05
56632	0.946131	0.05
56633	0.946126	0.05
56634	0.946126	0.05
56635	0.946128	0.05
56636	0.946128	0.05
56637	0.946128	0.05
56638	0.946128	0.05
56639	0.946128	0.05
56640	0.946128	0.05
56641	0.946128	0.05
56642	0.946128	0.05
56643	1.89226	0.05
56644	1.89226	0.05
56645	1.89226	0.05
56646	1.89226	0.05
56647	1.89226	0.05
56648	1.89226	0.05
56649	1.89226	0.05
56650	1.89226	0.05
56651	1.89226	0.0619625
56652	1.89226	0.05
56653	3.78453	0.05
56654	1.07176	0.05
56655	1.07176	0.05
56656	0.946126	0.05
56657	0.946126	0.05
56658	1.07176	0.05
56659	0.946129	0.05
56660	1.27474	0.05
56661	0.94613	0.05
56662	1.27474	0.05
56663	1.07176	0.05

56664	0.94613	0.05
56665	0.94613	0.05
56666	0.723486	0.05
56667	0.94613	0.05
56668	0.946126	0.05
56669	0.946126	0.05
56670	0.946129	0.05
56671	0.723484	0.05
56672	1.07176	0.05
56673	0.946129	0.05
56674	1.32398	0.05
56675	1.19835	0.05
56676	0.723471	0.05
56677	0.723471	0.05
56678	0.810946	0.05
56679	0.810946	0.05
56680	0.810946	0.05
56681	0.810946	0.05
56682	0.810933	0.05
56683	0.810933	0.05
56684	1.89226	0.05
56685	1.3517	0.05
56686	1.08132	0.05
56687	1.08132	0.05
56688	1.08133	0.05
56689	2.39923	2.1
56690	1.89226	2.1
56691	1.89226	2.1
56692	1.89226	2.1
56693	2.44606	2.1
56694	2.44606	2.1
56695	1.89226	2.1
56696	1.89226	2.1
56697	1.89226	2.1
56698	1.89226	2.1
56699	1.89226	2.1
56700	1.89226	2.1
56701	1.70302	2.1
56702	2.43041	2.1
56703	2.25275	2.1
56704	2.4747	2.1
56705	1.52079	2.1
56706	1.67063	2.1
56707	2.40644	2.1
56708	1.89226	2.1
56709	1.89226	2.1
56710	1.89226	2.1
56711	2.39923	2.1
56712	2.39923	2.1
56713	0.946131	1.11606
56714	0.946131	2.1
56715	0.946131	2.1
56716	0.946131	2.1
56717	0.94613	2.1
56718	0.94613	2.1
56719	0.946131	2.1
56720	0.946131	2.1
56721	0.946131	2.1

56722	0.946131	2.1
56723	0.946131	2.1
56724	0.946131	2.1
56725	1.04938	2.1
56726	1.04938	2.1
56727	1.111 2.1	
56728	1.06978	2.1
56729	1.2748	2.1
56730	1.25472	2.1
56731	0.88782	2.1
56732	0.882987	2.1
56733	0.973779	2.1
56734	1.31997	2.1
56735	0.913905	2.1
56736	1.31312	2.1
56737	2.6536	2.1
56738	2.15287	2.1
56739	2.72238	2.1
56740	2.22887	2.1
56741	4.87525	2.1
56742	2.15288	2.1
56743	1.40719	2.1
56744	1.41839	2.1
56745	1.34239	2.1
56746	2.22887	2.1
56747	2.15288	2.1
56748	2.72237	2.1
56749	2.22887	2.1
56750	2.77314	2.1
56751	2.28748	2.1
56752	5.00985	2.1
56753	0.922802	2.1
56754	0.922802	2.1
56755	0.92107	2.1
56756	0.92107	2.1
56757	0.703593	2.1
56758	0.729436	2.1
56759	1.11444	2.1
56760	1.14374	2.1
56761	1.14374	2.1
56762	0.729436	2.1
56763	0.703593	2.1
56764	1.11444	2.1
56765	1.89226	2.1
56766	1.89226	2.1
56767	1.89226	2.1
56768	1.89226	2.1
56769	1.89226	2.1
56770	1.89226	2.1
56771	0.489927	2.1
56772	0.63008	2.1
56773	0.697385	2.1
56774	0.55524	2.1
56775	0.592141	2.1
56776	0.592141	2.1
56777	0.593179	2.1
56778	0.593179	2.1
56779	0.63008	2.1

56780	0.697385	2.1
56781	0.55524	2.1
56782	0.489927	2.1
56783	0.946133	2.1
56784	0.946133	2.1
56785	0.946132	1.81989
56786	0.946132	0.347309
56787	0.946133	1.19931
56788	0.946133	2.1
56789	0.946132	2.1
56790	0.946132	0.61725
56791	0.946132	1.77367
56792	0.946132	1.43613
56793	0.946132	1.14297
56794	0.946132	1.51371
56795	1.89226	2.1
56796	1.89226	2.1
56797	1.89226	2.1
56798	1.89226	2.1
56799	1.89226	2.1
56800	1.89226	2.1
56801	0.805746	2.1
56802	0.63008	2.1
56803	0.881598	2.1
56804	0.697385	2.1
56805	0.749417	2.1
56806	0.749417	2.1
56807	0.753714	2.1
56808	0.753714	2.1
56809	0.881598	2.1
56810	0.805746	2.1
56811	0.63008	2.1
56812	0.697385	2.1
56813	0.881599	2.1
56814	0.697385	2.1
56815	0.805746	2.1
56816	0.626731	1.23494
56817	0.755479	2.01311
56818	0.755479	2.1
56819	0.752851	1.20037
56820	0.752851	2.1
56821	0.881598	2.1
56822	0.805747	2.1
56823	0.697385	2.1
56824	0.626731	2.1
56825	2.65348	2.1
56826	2.12314	2.1
56827	2.63899	2.1
56828	2.13898	2.1
56829	4.76213	2.1
56830	0.763518	2.1
56831	0.765889	2.1
56832	0.84086	2.1
56833	0.84086	2.1
56834	0.632078	2.1
56835	0.597565	2.1
56836	1.08813	2.1
56837	0.985643	2.1

56838	0.982464	2.1
56839	0.66404	2.1
56840	1.0358	2.1
56841	0.598023	2.1
56842	1.29293	2.1
56843	1.29628	2.1
56844	1.19266	2.1
56845	1.19266	2.1
56846	1.07109	2.1
56847	1.13093	2.1
56848	1.34467	2.1
56849	1.35251	2.1
56850	1.34369	2.1
56851	1.12525	2.1
56852	1.28746	2.1
56853	1.13511	2.1
56854	2.19717	2.1
56855	2.16547	2.1
56856	1.37392	2.1
56857	1.37392	2.1
56858	1.2966	2.1
56859	2.11985	2.1
56860	1.89226	2.1
56861	1.89226	2.1
56862	1.89226	2.1
56863	1.89226	2.1
56864	1.89226	2.1
56865	1.89226	2.1
56866	1.65637	2.1
56867	1.41758	2.1
56868	1.65637	2.1
56869	1.74391	2.1
56870	1.35504	2.1
56871	1.44258	2.1
56872	1.40774	2.1
56873	1.40774	2.1
56874	0.946131	2.1
56875	0.946131	2.1
56876	1.40774	2.1
56877	1.38882	2.1
56878	0.946132	2.1
56879	0.946132	2.1
56880	0.946132	2.1
56881	1.38882	2.1
56882	0.946132	2.1
56883	1.40774	2.1
56884	1.89227	2.1
56885	1.89227	2.1
56886	1.89227	2.1
56887	1.89227	2.1
56888	3.78453	2.1
56889	0.603148	2.1
56890	0.600177	2.1
56891	0.558615	2.1
56892	0.580714	2.1
56893	0.558606	2.1
56894	0.67883	2.1
56895	1.65637	2.1

56896	1.07933	2.1
56897	0.842649	2.1
56898	0.905168	2.1
56899	0.774334	2.1
56900	1.38882	2.1
56901	1.38882	2.1
56902	0.946132	2.1
56903	0.946132	2.1
56904	1.38882	2.1
56905	1.37381	2.1
56906	0.946132	2.1
56907	0.946132	2.1
56908	0.946132	2.1
56909	1.37381	2.1
56910	0.946133	2.1
56911	1.38882	2.1
56912	2.3771	2.1
56913	1.9339	2.1
56914	1.70782	2.1
56915	1.9339	2.1
56916	2.42693	2.1
56917	2.83279	2.1
56918	2.34901	0.303445
56919	1.45833	0.415455
56920	1.86695	0.23261
56921	1.03101	0.456801
56922	3.32528	0.28025
56923	1.89226	0.05
56924	1.89226	0.05
56925	1.89226	0.05
56926	1.89226	0.05
56927	3.78453	0.05
56928	2.71753	0.05
56929	1.89226	0.05
56930	2.76228	0.05
56931	2.76228	0.05
56932	1.89226	0.05
56933	1.89226	0.111511
56934	0.946131	0.05
56935	0.946131	0.05
56936	0.946132	0.05
56937	0.946132	0.05
56938	0.946132	0.05
56939	0.946132	0.05
56940	0.946132	0.05
56941	0.946132	0.05
56942	0.946132	0.05
56943	0.946132	0.05
56944	0.946132	0.05
56945	0.946132	0.05
56946	1.89227	0.05
56947	1.89227	0.05
56948	1.89227	0.05
56949	1.89227	0.05
56950	1.89227	0.05
56951	1.89227	0.05
56952	2.71753	0.05
56953	2.37907	0.05

56954	2.37907	0.05
56955	2.76228	0.05
56956	2.39588	0.05
56957	2.77909	0.05
56958	1.6571	0.469676
56959	1.11682	1.73993
56960	1.47132	0.671432
56961	1.93763	0.05
56962	3.12842	1.4295
56963	1.93762	0.11586
56964	2.12345	0.895875
56965	1.65709	0.0594705
56966	1.65709	1.91616
56967	2.12345	0.707904
56968	1.88635	2.1
56969	1.47134	2.087
56970	2.219	0.118652
56971	2.25734	2.1
56972	1.93765	0.273213
56973	1.79103	1.41638
56974	1.47134	0.0621578
56975	1.06174	0.52325
56976	1.19349	2.1
56977	1.1095	1.99841
56978	0.968827	0.388884
56979	1.07892	0.22756
56980	1.07892	2.1
56981	1.0834	0.149822
56982	1.0834	1.00564
56983	1.1095	1.14454
56984	1.19349	0.830135
56985	0.968825	1.19065
56986	1.06174	1.01268
56987	2.40627	2.1
56988	2.08288	1.56884
56989	2.5444	0.414519
56990	2.29285	1.88297
56991	4.62729	0.897099
56992	2.08289	0.468428
56993	1.65703	1.04878
56994	1.71105	2.1
56995	1.50108	2.02911
56996	2.29286	2.06044
56997	2.08289	2.08659
56998	2.5444	1.00304
56999	2.29285	2.1
57000	2.71429	2.1
57001	2.50288	0.274829
57002	5.04728	2.1
57003	2.50288	0.354183
57004	2.00927	2.1
57005	2.02116	1.01177
57006	2.02116	2.1
57007	2.29286	0.587053
57008	2.29286	2.01119
57009	1.89226	2.1
57010	1.89226	2.1
57011	1.89226	0.4735

57012	1.89226	2.1
57013	3.78453	2.1
57014	1.17418	2.1
57015	1.29741	2.1
57016	1.04921	2.1
57017	1.19352	2.1
57018	2.3677	2.1
57019	1.29741	2.1
57020	1.27575	2.1
57021	1.19352	2.1
57022	1.19473	2.1
57023	2.46927	2.1
57024	1.89226	2.1
57025	1.89226	2.1
57026	1.89226	2.1
57027	1.89226	2.1
57028	1.89226	2.1
57029	1.89226	2.1
57030	0.561332	2.1
57031	0.561332	2.1
57032	0.567597	2.1
57033	0.567597	2.1
57034	0.597365	2.1
57035	0.544699	2.1
57036	0.596761	2.1
57037	0.531564	2.1
57038	0.5447	2.1
57039	0.531563	2.1
57040	0.596759	2.1
57041	0.597366	2.1
57042	0.946131	2.1
57043	0.946131	2.1
57044	0.946131	2.1
57045	0.946131	2.1
57046	0.946129	2.1
57047	0.946129	2.1
57048	0.946133	2.1
57049	0.946133	2.1
57050	0.946131	2.1
57051	0.946131	2.1
57052	0.946131	2.1
57053	0.946131	2.1
57054	1.10938	0.05
57055	1.17614	0.165786
57056	1.02478	0.05
57057	1.09056	0.126595
57058	2.19994	0.0599655
57059	1.17614	0.151817
57060	1.11868	2.1
57061	1.03408	0.108416
57062	1.03408	2.1
57063	1.09056	0.115472
57064	1.17516	2.1
57065	1.89226	0.0702939
57066	1.89226	0.05
57067	1.89226	0.0730158
57068	1.89226	0.05
57069	1.89226	0.0970131

57070	1.89226	0.05
57071	1.89226	0.05
57072	1.89226	0.0576609
57073	1.89226	0.0530574
57074	1.89226	0.05
57075	1.89226	0.0756528
57076	1.89226	0.05
57077	0.579551	0.05
57078	0.563873	0.05
57079	0.611341	0.05
57080	0.598693	0.05
57081	0.589856	0.05
57082	0.589856	0.05
57083	0.588387	0.05
57084	0.588387	0.05
57085	0.57955	0.05
57086	0.611341	0.05
57087	0.563873	0.05
57088	0.598693	0.05
57089	0.611341	0.05
57090	0.598693	0.05
57091	0.586048	0.05
57092	0.572737	0.05
57093	0.591824	0.05
57094	0.591824	0.05
57095	0.592254	0.05
57096	0.592254	0.05
57097	0.611341	0.05
57098	0.586048	0.05
57099	0.598693	0.05
57100	0.572737	0.05
57101	0.946119	0.05
57102	0.946119	0.0707928
57103	0.946119	0.05
57104	0.946119	0.0741246
57105	0.94612	0.05
57106	0.94612	0.05
57107	0.946119	0.0683421
57108	0.946119	0.087565
57109	0.946132	0.05
57110	0.946132	0.05
57111	0.946107	0.0533574
57112	0.946107	0.0526156
57113	1.89224	0.05
57114	1.89224	0.05
57115	1.89224	0.05
57116	1.89224	0.05
57117	1.89224	0.0666404
57118	1.89224	0.05
57119	0.562335	0.05
57120	0.579543	0.05
57121	0.59499	0.05
57122	0.611333	0.05
57123	0.586619	0.05
57124	0.586619	0.05
57125	0.587049	0.05
57126	0.587049	0.05
57127	0.594999	0.05

57128	0.562342	0.05
57129	0.579535	0.05
57130	0.611325	0.05
57131	0.59499	0.05
57132	0.611333	0.05
57133	0.567849	0.05
57134	0.58604	0.05
57135	0.590978	0.05
57136	0.590978	0.05
57137	0.590052	0.05
57138	0.590052	0.05
57139	0.567857	0.05
57140	0.594998	0.05
57141	0.611325	0.05
57142	0.586032	0.05
57143	1.69661	0.0578577
57144	1.9339	0.05
57145	1.39379	0.05
57146	1.69107	0.05
57147	3.32768	0.05
57148	1.9339	0.05
57149	1.98414	0.05
57150	1.69107	0.05
57151	1.72774	0.05
57152	3.66164	0.05
57153	1.39379	0.169145
57154	1.69107	0.05
57155	1.14397	0.05
57156	1.43548	0.05
57157	2.82927	0.05
57158	1.69107	0.05
57159	1.72774	0.05
57160	1.53873	0.05
57161	1.53873	0.05
57162	1.43548	0.05
57163	1.62449	0.05
57164	2.58431	0.05
57165	1.34072	0.05
57166	1.80841	0.05
57167	1.34072	0.05
57168	2.51501	0.05
57169	2.04732	0.05
57170	1.89226	0.05
57171	1.89226	0.05
57172	1.89226	0.05
57173	1.89226	0.05
57174	1.89226	0.05
57175	1.89226	0.05
57176	0.946131	0.05
57177	0.946131	0.05
57178	1.3639	0.05
57179	1.1516	0.05
57180	0.946129	0.05
57181	0.946129	0.05
57182	1.36391	0.05
57183	1.36391	0.05
57184	0.946131	0.05
57185	1.3639	0.05

57186	1.1516	0.05
57187	0.946131	0.05
57188	0.946131	0.05
57189	0.946131	0.05
57190	0.94613	0.05
57191	0.94613	0.05
57192	0.946128	0.05
57193	0.946128	0.05
57194	0.946133	0.05
57195	0.946133	0.05
57196	0.946131	0.05
57197	0.946131	0.05
57198	0.946131	0.05
57199	0.946131	0.05
57200	1.89226	1.26656
57201	2.63634	2.1
57202	1.89226	1.49852
57203	1.83902	1.7051
57204	3.73128	1.35897
57205	1.47059	2.1
57206	2.02928	2.1
57207	1.47059	2.1
57208	1.65706	2.1
57209	1.98628	2.1
57210	2.17274	2.1
57211	2.43618	2.1
57212	2.41176	2.1
57213	1.98627	2.1
57214	2.34084	2.1
57215	4.39803	2.1
57216	1.47059	2.1
57217	1.82453	2.1
57218	1.98627	2.1
57219	1.98627	2.1
57220	1.26929	2.1
57221	1.26929	2.1
57222	1.98627	2.1
57223	2.34084	2.1
57224	1.47264	2.1
57225	2.24937	2.1
57226	4.23564	2.1
57227	1.33026	0.186811
57228	1.51171	1.34989
57229	1.51171	0.206234
57230	1.4617	1.64607
57231	1.35382	0.155392
57232	1.30382	2.1
57233	1.89226	0.086902
57234	1.89226	0.156118
57235	1.89226	0.0678546
57236	1.89226	0.928581
57237	1.89226	0.05
57238	1.89226	1.16839
57239	1.39263	0.78401
57240	0.962704	0.05
57241	1.0171	0.0757733
57242	0.962704	0.165241
57243	1.40822	0.05

57244	1.35382	0.0718532
57245	0.755853	2.01494
57246	0.789808	0.268502
57247	0.696315	0.370909
57248	0.676912	0.0742436
57249	0.729751	0.0541244
57250	0.729751	0.271185
57251	0.722417	2.1
57252	0.722417	0.101296
57253	0.696315	0.208295
57254	0.789808	0.203992
57255	0.755853	0.336406
57256	0.676912	0.387995
57257	0.946132	0.154727
57258	0.946132	0.202185
57259	0.946132	0.0948274
57260	0.946132	0.05
57261	0.946131	0.05
57262	0.946131	0.141588
57263	0.946132	0.0635292
57264	0.946132	0.220901
57265	0.946132	0.0589536
57266	0.946132	0.0790453
57267	0.946132	0.0884889
57268	0.946132	0.083479
57269	0.946133	0.0926935
57270	0.946133	0.05
57271	0.946132	0.05
57272	0.946132	0.05
57273	0.946132	0.05
57274	0.946132	0.05061
57275	0.946133	0.05
57276	0.946133	0.114456
57277	0.946132	0.0544831
57278	0.946132	0.0556318
57279	0.946132	0.0558534
57280	0.946132	0.107021
57281	1.89227	2.05198
57282	2.53014	2.01369
57283	1.89227	2.1
57284	1.98241	1.42677
57285	1.89227	2.1
57286	1.98241	1.63214
57287	1.89226	1.385
57288	1.89226	2.1
57289	1.89226	2.0864
57290	1.89226	2.02878
57291	1.89226	2.1
57292	1.89226	2.1
57293	1.89226	1.21224
57294	1.89226	2.1
57295	1.89226	1.90343
57296	1.89226	1.87331
57297	3.78452	1.93665
57298	2.45304	0.631262
57299	1.89226	2.1
57300	1.89226	1.80375
57301	1.89226	1.69022

57302	1.89226	1.26525
57303	1.89226	1.15349
57304	1.89227	2.08856
57305	1.89227	1.9515
57306	1.89227	2.1
57307	1.89227	1.39272
57308	1.89227	2.1
57309	1.89227	1.52486
57310	1.89226	1.78552
57311	1.89226	2.1
57312	1.89226	2.07186
57313	1.89226	2.07434
57314	1.89226	2.1
57315	1.89226	2.1
57316	2.09871	0.105491
57317	2.71891	0.17065
57318	1.31126	0.0937917
57319	1.31126	0.229354
57320	2.55341	0.0962721
57321	1.76597	0.448092
57322	1.89226	0.335212
57323	1.89226	0.40394
57324	1.89226	0.143273
57325	1.89226	0.858242
57326	1.89226	0.153608
57327	1.89226	0.910264
57328	0.946128	0.388855
57329	0.946128	0.134977
57330	0.946133	0.115215
57331	0.946133	0.0729434
57332	0.946131	0.111609
57333	0.946131	0.301035
57334	0.94613	0.075903
57335	0.94613	0.146522
57336	0.946131	0.137315
57337	0.946131	0.135354
57338	0.946131	0.141077
57339	0.946131	0.149158
57340	2.71891	0.0737144
57341	1.89226	0.0868678
57342	2.09871	0.0524914
57343	2.09871	0.300174
57344	1.89226	0.05
57345	1.89226	0.245244
57346	1.89227	0.347245
57347	1.89227	0.295482
57348	1.89227	0.175329
57349	1.89227	0.761035
57350	1.89227	0.179619
57351	1.89227	0.778603
57352	0.946129	0.290916
57353	0.946129	0.163264
57354	0.946133	0.107339
57355	0.946133	0.0686614
57356	0.946131	0.104677
57357	0.946131	0.248286
57358	0.946131	0.0715112
57359	0.946131	0.176107

57360	0.946131	0.11935
57361	0.946131	0.1173
57362	0.946131	0.129428
57363	0.946131	0.129686
57364	2.32462	0.05
57365	1.89226	0.05
57366	1.89226	0.05
57367	1.89226	0.05
57368	2.2291	0.05
57369	2.2291	0.05
57370	0.847669	0.05
57371	0.847669	0.05
57372	0.796966	0.05
57373	0.796966	0.05
57374	0.833552	0.05
57375	1.01049	0.05
57376	0.709709	0.05
57377	0.634147	0.05
57378	0.634148	0.05
57379	0.833549	0.05
57380	0.709711	0.05
57381	1.01049	0.05
57382	1.89226	0.05
57383	1.89226	0.05
57384	1.89226	0.05
57385	1.89226	0.05
57386	1.89226	0.05
57387	1.89226	0.05
57388	1.03611	0.05
57389	0.936655	0.05
57390	0.946132	0.05
57391	0.946132	0.05
57392	0.941918	0.05
57393	0.941918	0.05
57394	1.00308	0.05
57395	1.04117	0.05
57396	1.1406	0.05
57397	1.04074	0.05
57398	0.851529	0.05
57399	1.00311	0.05
57400	1.01814	0.05
57401	1.6671	0.05
57402	1.35561	0.05
57403	1.35561	0.05
57404	1.2683	0.05
57405	1.2683	0.05
57406	0.946128	0.05
57407	0.946128	0.05
57408	0.946129	0.05
57409	0.946129	0.05
57410	0.946132	0.05
57411	0.946132	0.05
57412	0.946126	0.05
57413	0.946126	0.05
57414	0.946129	0.05
57415	0.946129	0.05
57416	0.946129	0.05
57417	0.946129	0.05

57418	2.23338	0.05
57419	1.96	0.05
57420	2.03438	0.05
57421	1.87835	0.05
57422	4.11172	0.05
57423	1.96	0.05
57424	1.52661	0.05
57425	1.87834	0.05
57426	1.53175	0.05
57427	3.40495	0.05
57428	2.03435	0.05
57429	1.65813	0.05
57430	1.65813	0.05
57431	1.87832	0.05
57432	1.72067	0.05
57433	1.94086	0.05
57434	1.65813	0.05
57435	1.40572	0.05
57436	1.87832	0.05
57437	1.87832	0.05
57438	1.31154	0.05
57439	1.53173	0.05
57440	1.86662	2.1
57441	2.0815	2.1
57442	2.0815	2.1
57443	2.0815	2.1
57444	1.87246	2.1
57445	1.87362	2.1
57446	1.0273	2.1
57447	0.996347	2.1
57448	0.946129	2.1
57449	0.946129	2.1
57450	1.04427	2.1
57451	1.04196	2.1
57452	0.910674	2.1
57453	0.910674	2.1
57454	1.04072	2.1
57455	1.10931	2.1
57456	1.03132	2.1
57457	0.933698	2.1
57458	2.63789	0.05
57459	1.89226	0.142942
57460	2.3033	0.05
57461	2.3033	0.157097
57462	1.89226	0.05
57463	1.89226	0.160949
57464	1.89226	0.1898
57465	1.89226	0.132318
57466	1.89226	0.067791
57467	1.89226	0.173443
57468	3.78452	0.150435
57469	1.51898	0.0574744
57470	1.36006	0.0921621
57471	2.03143	0.111703
57472	2.03143	0.0816392
57473	1.04026	0.131367
57474	1.04026	0.0982758
57475	0.94613	0.05

57476	0.94613	0.0870337
57477	1.31894	0.05
57478	1.31894	0.0526759
57479	0.94613	0.05
57480	0.94613	0.110203
57481	1.31894	0.05
57482	1.41232	0.0838719
57483	0.946129	0.05
57484	1.41232	0.0502984
57485	1.31895	0.05
57486	0.946129	0.05
57487	1.89226	0.05
57488	1.89226	0.186157
57489	1.89226	0.05
57490	1.89226	0.136804
57491	1.89226	0.05
57492	1.89226	0.145655
57493	1.89804	0.05
57494	2.03143	0.05
57495	2.46286	0.0650572
57496	2.46287	0.05
57497	1.51898	0.0543743
57498	1.51898	0.05
57499	1.89226	2.1
57500	1.89226	2.1
57501	1.89226	2.1
57502	1.89226	2.1
57503	3.78452	2.1
57504	1.3864	2.1
57505	1.3864	2.1
57506	0.946132	2.1
57507	0.946132	2.1
57508	1.32905	2.1
57509	1.3864	2.1
57510	0.946132	2.1
57511	0.946132	2.1
57512	0.946132	2.1
57513	1.32905	2.1
57514	0.946133	2.1
57515	1.3864	2.1
57516	2.77279	2.1
57517	1.67883	2.1
57518	1.67883	2.1
57519	1.73415	2.1
57520	2.65811	2.1
57521	2.71343	2.1
57522	0.946131	2.1
57523	0.946131	2.1
57524	0.946128	2.1
57525	0.946128	2.1
57526	0.946127	2.1
57527	0.946127	2.1
57528	0.946131	2.1
57529	0.946131	2.1
57530	0.946129	2.1
57531	0.946129	2.1
57532	0.94613	2.1
57533	0.94613	2.1

57534	1.89226	2.1
57535	1.89226	2.1
57536	1.89226	2.1
57537	1.89226	2.1
57538	3.78452	2.1
57539	1.89226	2.1
57540	2.54394	2.1
57541	2.54394	2.1
57542	2.65811	2.1
57543	1.89226	2.1
57544	2.00643	2.1
57545	2.54394	2.1
57546	2.6581	2.1
57547	1.62743	2.1
57548	1.62743	2.1
57549	1.67883	2.1
57550	2.59533	2.1
57551	1.89226	2.1
57552	1.89226	2.1
57553	1.89226	2.1
57554	1.89226	2.1
57555	1.89226	2.1
57556	1.89226	2.1
57557	1.89226	1.64462
57558	1.89226	1.27662
57559	1.89226	2.1
57560	1.89226	0.817293
57561	1.89226	2.09218
57562	1.89226	0.578798
57563	1.89226	1.79076
57564	1.89226	1.30097
57565	1.89226	2.1
57566	1.89226	1.22571
57567	1.89226	2.0646
57568	1.89226	0.929723
57569	1.89226	0.817401
57570	1.89226	0.272981
57571	1.89226	1.72244
57572	1.89226	0.209488
57573	1.89226	1.40993
57574	1.89226	0.164897
57575	2.68537	0.0629056
57576	2.22575	0.0864928
57577	2.47464	0.05
57578	1.79093	0.05
57579	4.70039	0.05
57580	2.81217	0.0624496
57581	2.47464	0.05
57582	2.58286	0.0682891
57583	2.29193	0.05
57584	5.0575	0.05
57585	2.47464	0.05
57586	2.20365	0.05
57587	2.29193	0.05
57588	1.90104	0.05
57589	4.37568	0.05
57590	1.89226	0.100964
57591	2.16957	2.1

57592	2.52823	1.15709
57593	2.52823	2.1
57594	1.89226	0.530361
57595	1.89226	1.93016
57596	1.36226	2.1
57597	1.55369	2.1
57598	1.33309	2.1
57599	1.62593	2.1
57600	2.98819	2.1
57601	0.802281	2.1
57602	0.802281	2.1
57603	0.835143	2.1
57604	0.835143	2.1
57605	0.824458	2.1
57606	0.92641	2.1
57607	0.776847	2.1
57608	0.812967	2.1
57609	0.812967	2.1
57610	0.926411	2.1
57611	0.776847	2.1
57612	0.824458	2.1
57613	1.33309	2.1
57614	1.62593	2.1
57615	1.32813	2.1
57616	1.7237	2.1
57617	2.95406	2.1
57618	0.814544	2.1
57619	0.875312	2.1
57620	0.910502	2.1
57621	0.910502	2.1
57622	0.886633	2.1
57623	0.975739	2.1
57624	0.792623	2.1
57625	0.840283	2.1
57626	0.796372	2.1
57627	1.06159	2.1
57628	0.81948	2.1
57629	0.933833	2.1
57630	2.63997	2.1
57631	1.29694	2.1
57632	1.28141	2.1
57633	2.62623	2.1
57634	3.92317	2.1
57635	2.63998	2.1
57636	1.89226	2.1
57637	1.89226	2.1
57638	2.62623	2.1
57639	4.5185	2.1
57640	1.89226	2.1
57641	1.89226	2.1
57642	1.89226	2.1
57643	1.89226	2.1
57644	3.78453	2.1
57645	1.89226	2.1
57646	1.89226	2.1
57647	1.89226	2.1
57648	1.89226	2.1
57649	1.89226	2.1

57650	1.89226	2.1
57651	1.25468	2.1
57652	2.62623	2.1
57653	1.28141	2.1
57654	1.28141	2.1
57655	2.60665	2.1
57656	2.60665	2.1
57657	1.89226	2.1
57658	2.62623	2.1
57659	2.60666	2.1
57660	2.62623	2.1
57661	1.87269	2.1
57662	1.89226	2.1
57663	1.89226	2.1
57664	1.89226	2.1
57665	1.89226	2.1
57666	1.89226	2.1
57667	3.78453	2.1
57668	1.89226	2.1
57669	1.89226	2.1
57670	1.89226	2.1
57671	1.89226	2.1
57672	1.89226	2.1
57673	1.89226	2.1
57674	2.30147	1.10644
57675	2.27703	2.1
57676	1.89227	0.9341
57677	1.89227	1.98499
57678	4.1693	1.76562
57679	1.38617	0.967217
57680	1.24613	1.62629
57681	2.59886	0.727828
57682	2.6238	1.71121
57683	3.86993	0.924316
57684	0.946133	1.77992
57685	0.946133	0.622504
57686	0.946132	0.852336
57687	0.946132	2.1
57688	0.946133	2.1
57689	0.946133	1.97181
57690	0.946132	0.594167
57691	0.946132	1.10516
57692	0.946133	1.53479
57693	0.946133	1.43796
57694	0.946132	1.19126
57695	0.946132	1.24051
57696	2.21773	2.1
57697	2.59216	2.1
57698	1.68541	2.1
57699	1.68541	2.1
57700	1.84814	2.1
57701	2.38046	2.1
57702	1.89966	2.1
57703	1.70303	2.1
57704	1.2028	2.1
57705	1.29897	2.1
57706	2.02873	2.1
57707	2.19093	2.1

57708	2.75218	2.1
57709	1.70303	2.1
57710	3.10254	2.1
57711	2.24783	2.1
57712	2.56839	2.1
57713	1.70303	2.1
57714	2.56839	2.1
57715	1.08276	2.1
57716	2.24061	2.1
57717	1.67491	2.1
57718	1.60948	2.1
57719	1.08276	2.1
57720	1.89226	0.880936
57721	1.89226	1.20446
57722	1.89226	0.848558
57723	1.89226	0.786635
57724	3.78453	0.440039
57725	1.89226	0.816187
57726	1.89226	1.1227
57727	1.89226	0.652809
57728	1.89226	0.721091
57729	3.78452	1.68928
57730	1.89226	1.09097
57731	2.7535	0.293359
57732	1.89226	1.38758
57733	1.89226	1.13947
57734	1.89226	0.525883
57735	1.89226	0.552363
57736	1.89226	1.18195
57737	1.89226	0.8126
57738	1.89226	0.269313
57739	1.89226	1.09304
57740	1.89226	0.607475
57741	1.89226	1.60887
57742	1.89227	1.08245
57743	1.89227	0.875014
57744	1.89227	0.756218
57745	1.89227	0.579522
57746	3.78453	1.76799
57747	1.89226	0.993783
57748	2.53412	0.141665
57749	1.89226	1.83497
57750	1.89226	0.336348
57751	1.89226	0.703058
57752	1.89226	0.250303
57753	2.21126	0.05
57754	1.89226	0.05
57755	2.57464	0.05
57756	1.89226	0.05
57757	2.57464	0.05
57758	1.89226	0.05
57759	1.89226	0.05
57760	1.89226	0.05
57761	1.89226	0.269091
57762	1.89226	0.05
57763	1.89226	0.133049
57764	1.89226	0.05
57765	1.1418	0.05

57766	0.653021	0.05
57767	1.37282	0.05
57768	0.931386	0.05
57769	1.34568	0.05
57770	1.17669	0.05
57771	0.666595	0.05
57772	0.786895	0.05
57773	0.933982	0.05
57774	1.05351	0.05
57775	1.00792	0.05
57776	1.00792	0.05
57777	1.89226	2.1
57778	1.89226	2.1
57779	1.89226	2.1
57780	1.89226	2.1
57781	3.78452	2.1
57782	1.89226	2.1
57783	1.89226	2.1
57784	1.89226	2.1
57785	1.89226	2.1
57786	1.89226	2.1
57787	1.89226	2.1
57788	1.65642	2.1
57789	1.30248	2.1
57790	1.38888	2.1
57791	1.25987	2.1
57792	1.70611	2.1
57793	1.65642	2.1
57794	1.65642	2.1
57795	1.81407	2.1
57796	2.19428	2.1
57797	1.92555	2.1
57798	3.8507	2.1
57799	1.89226	2.1
57800	1.89226	2.1
57801	1.89226	2.1
57802	1.89226	2.1
57803	3.78452	2.1
57804	1.89226	2.1
57805	1.89226	2.1
57806	1.89226	2.1
57807	1.89226	2.1
57808	1.89226	2.1
57809	1.89226	2.1
57810	0.705495	2.1
57811	0.809317	2.1
57812	1.1655	2.1
57813	1.09605	2.1
57814	0.983375	2.1
57815	0.797301	2.1
57816	0.906633	2.1
57817	0.906633	2.1
57818	0.85085	2.1
57819	1.16548	2.1
57820	0.705501	2.1
57821	1.00314	2.1
57822	2.19428	2.1
57823	2.31862	2.1

57824	2.27408	2.1
57825	2.45893	2.1
57826	4.46836	2.1
57827	1.89226	0.644401
57828	1.89226	1.11342
57829	1.89226	1.22995
57830	1.89226	0.63605
57831	1.89226	1.58833
57832	1.89226	1.14655
57833	2.0815	0.702193
57834	2.22136	0.711213
57835	1.5515	0.563413
57836	1.86323	0.324416
57837	2.74986	1.17593
57838	3.30238	0.678973
57839	1.89224	0.591436
57840	1.89224	1.03428
57841	1.89224	1.24132
57842	1.89224	0.51233
57843	1.89224	1.68715
57844	1.89224	0.997366
57845	1.89224	0.595012
57846	1.89224	1.37467
57847	1.89224	0.925344
57848	1.89224	0.390079
57849	1.89224	1.61422
57850	1.89224	1.05689
57851	1.17846	0.467404
57852	0.810935	0.593349
57853	0.93347	1.38726
57854	0.93347	0.391632
57855	0.810932	0.815902
57856	0.810932	0.486087
57857	2.22133	0.545243
57858	1.71919	0.465025
57859	1.32629	0.267881
57860	1.0813	0.785844
57861	1.57137	0.566203
57862	2.21149	2.1
57863	1.89226	2.1
57864	1.89226	2.1
57865	1.89226	2.1
57866	2.17159	2.1
57867	2.17159	2.1
57868	0.946131	0.0606436
57869	0.946131	2.1
57870	0.946131	2.1
57871	0.946131	2.1
57872	0.94613	0.785411
57873	0.94613	2.00806
57874	0.946132	2.1
57875	0.946132	2.1
57876	0.946131	1.71638
57877	0.946131	1.51511
57878	0.946131	2.0778
57879	0.946131	1.9082
57880	1.12266	2.1
57881	1.4714	2.05927

57882	1.62322	2.06271
57883	1.62322	1.98735
57884	1.02958	2.1
57885	1.02958	2.1
57886	2.2671	2.1
57887	1.89226	2.1
57888	1.89226	2.1
57889	1.89226	2.1
57890	2.21149	2.1
57891	2.21149	2.1
57892	1.89226	2.1
57893	1.89226	1.87155
57894	1.89226	2.1
57895	1.89226	1.06219
57896	1.89226	2.1
57897	1.89226	2.1
57898	0.736914	2.08518
57899	0.736914	2.1
57900	0.822839	2.1
57901	0.822836	2.1
57902	0.791298	0.441599
57903	1.00003	2.1
57904	0.640254	2.1
57905	0.575379	2.1
57906	0.609001	1.78198
57907	1.00005	1.88284
57908	0.575371	2.1
57909	0.831908	1.74737
57910	1.89226	2.1
57911	1.89226	2.1
57912	1.89226	2.1
57913	1.89226	2.1
57914	1.89226	2.1
57915	1.89226	2.1
57916	1.89226	2.1
57917	1.89226	2.1
57918	1.89226	2.1
57919	1.89226	2.1
57920	1.89226	2.1
57921	1.89226	2.1
57922	0.730617	2.1
57923	0.71052	2.1
57924	0.7329	2.1
57925	0.712083	2.1
57926	0.701051	2.1
57927	0.701051	2.1
57928	0.721552	2.1
57929	0.721552	2.1
57930	0.710521	2.1
57931	0.732898	2.1
57932	0.730619	2.1
57933	0.712081	2.1
57934	0.7329	2.1
57935	0.712082	2.1
57936	0.559601	2.1
57937	0.495993	2.1
57938	0.603718	2.1
57939	0.603718	2.1

57940	0.625174	2.1
57941	0.625174	2.1
57942	0.732899	2.1
57943	0.559601	2.1
57944	0.712082	2.1
57945	0.495993	2.1
57946	0.94613	2.1
57947	0.94613	2.1
57948	0.94613	2.1
57949	0.94613	2.1
57950	0.946131	2.1
57951	0.946131	2.1
57952	0.946129	2.1
57953	0.946129	2.1
57954	0.94613	2.1
57955	0.94613	2.1
57956	0.94613	2.1
57957	0.94613	2.1
57958	1.89226	2.1
57959	1.89226	2.1
57960	1.89226	2.1
57961	1.89226	2.1
57962	1.89226	2.1
57963	1.89226	2.1
57964	1.42104	2.1
57965	1.4658	2.1
57966	1.51223	2.1
57967	1.42283	2.1
57968	2.93327	2.1
57969	0.732899	2.1
57970	0.756115	2.1
57971	0.629763	2.1
57972	0.559601	2.1
57973	0.646134	2.1
57974	0.646134	2.1
57975	0.669582	2.1
57976	0.669582	2.1
57977	0.756115	2.1
57978	0.629763	2.1
57979	0.732899	2.1
57980	0.559601	2.1
57981	2.566 2.1	
57982	1.20242	2.1
57983	2.64084	2.1
57984	2.18297	2.1
57985	1.66029	2.1
57986	1.20242	2.1
57987	0.946133	1.22395
57988	0.946133	1.73086
57989	0.946129	2.1
57990	0.946129	2.1
57991	0.946131	2.1
57992	0.946131	1.15861
57993	0.946131	0.05
57994	0.946131	2.1
57995	0.946131	0.895864
57996	0.946131	2.06276
57997	0.946131	1.71801

57998	0.946131	1.94716
57999	1.89226	0.05
58000	1.89226	0.05
58001	1.89226	0.05
58002	1.89226	0.05
58003	3.78453	0.05
58004	1.03073	0.05
58005	1.03073	0.05
58006	1.04066	0.05
58007	1.04066	0.05
58008	1.20802	0.05
58009	1.32689	0.05
58010	0.883301	0.05
58011	0.744506	0.05
58012	1.20802	0.05
58013	0.744506	0.05
58014	0.883301	0.05
58015	1.32689	0.05
58016	1.20802	0.05
58017	1.20802	0.05
58018	0.946131	0.05
58019	0.946131	0.05
58020	0.94613	0.05
58021	0.94613	0.05
58022	1.20802	0.05
58023	1.32689	0.05
58024	1.20802	0.05
58025	0.946131	0.05
58026	0.946131	0.05
58027	1.32689	0.05
58028	1.89226	0.05
58029	1.89226	0.05
58030	1.89226	0.05
58031	1.89226	0.05
58032	1.89226	0.05
58033	1.89226	0.05
58034	0.946119	0.05
58035	0.946119	0.05
58036	0.946118	0.05
58037	0.946118	0.05
58038	0.946118	0.05
58039	0.946118	0.05
58040	0.946119	0.05
58041	0.946119	0.05
58042	0.946131	0.05
58043	0.946131	0.05
58044	0.946106	0.05
58045	0.946106	0.05
58046	1.3939	2.1
58047	1.49641	2.1
58048	1.15096	2.1
58049	1.10101	2.1
58050	2.59742	2.1
58051	1.22106	2.1
58052	2.60663	2.1
58053	1.25467	2.1
58054	1.25467	2.1
58055	2.58377	2.1

58056	2.58377	2.1
58057	1.89225	2.1
58058	2.60664	2.1
58059	2.58377	2.1
58060	2.60664	2.1
58061	1.86938	2.1
58062	1.89225	2.1
58063	1.89225	2.1
58064	1.89225	2.1
58065	1.89225	2.1
58066	1.89225	2.1
58067	1.89225	2.1
58068	1.89225	2.1
58069	0.946121	2.1
58070	0.946121	2.1
58071	0.946126	2.1
58072	0.946126	2.1
58073	0.946124	2.1
58074	0.946124	2.1
58075	0.946123	2.1
58076	0.946123	2.1
58077	0.946117	2.1
58078	0.946117	2.1
58079	0.94613	2.1
58080	0.94613	2.1
58081	1.28017	2.1
58082	1.29189	2.1
58083	0.610534	2.1
58084	0.592373	2.1
58085	0.943764	2.1
58086	0.943764	2.1
58087	0.940501	2.1
58088	0.940501	2.1
58089	1.28017	2.1
58090	0.592371	2.1
58091	0.610533	2.1
58092	1.29189	2.1
58093	0.946133	2.1
58094	0.946133	2.1
58095	1.28017	2.1
58096	1.29189	2.1
58097	0.946132	2.1
58098	0.946132	2.1
58099	1.28017	2.1
58100	1.28017	2.1
58101	1.28017	2.1
58102	0.946132	2.1
58103	0.946133	2.1
58104	1.2919	2.1
58105	1.89226	2.1
58106	1.89226	2.1
58107	1.89226	2.1
58108	1.89226	2.1
58109	1.89226	2.1
58110	1.89226	2.1
58111	1.89226	2.1
58112	1.89226	2.1
58113	1.89226	2.1

58114	1.89226	2.1
58115	3.78452	2.1
58116	1.89226	2.1
58117	2.08196	2.1
58118	1.97854	2.1
58119	2.08196	2.1
58120	1.78884	2.1
58121	1.89226	2.1
58122	1.89226	2.1
58123	1.89226	2.1
58124	1.89226	2.1
58125	1.89226	2.1
58126	1.89226	2.1
58127	1.89226	2.1
58128	1.83688	2.1
58129	1.89226	2.1
58130	1.89226	2.1
58131	1.89226	2.1
58132	1.86015	2.1
58133	1.86015	2.1
58134	1.89226	2.1
58135	1.97854	2.1
58136	1.85883	2.1
58137	1.97854	2.1
58138	1.77255	2.1
58139	1.89226	2.1
58140	0.946131	2.1
58141	0.946131	2.1
58142	0.946131	2.1
58143	0.946131	2.1
58144	0.94613	2.1
58145	0.94613	2.1
58146	0.946132	2.1
58147	0.946132	2.1
58148	0.946131	2.1
58149	0.946131	2.1
58150	0.946131	2.1
58151	0.946131	2.1
58152	0.918412	2.1
58153	0.918412	2.1
58154	0.94893	2.1
58155	0.750211	2.1
58156	0.851541	2.1
58157	0.851541	2.1
58158	0.970566	2.1
58159	0.826567	2.1
58160	0.970554	2.1
58161	1.04071	2.1
58162	0.826632	2.1
58163	0.851554	2.1
58164	1.89226	0.05
58165	1.89226	0.05
58166	1.89226	0.05
58167	1.89226	0.05
58168	3.78453	0.05
58169	0.946131	0.05
58170	0.946131	0.05
58171	0.811232	0.05

58172	0.811232	0.05
58173	0.94613	0.05
58174	0.94613	0.05
58175	0.959701	0.05
58176	0.811233	0.05
58177	0.94613	0.05
58178	0.811233	0.05
58179	0.959702	0.05
58180	0.946131	0.05
58181	1.89226	0.05
58182	1.89226	0.05
58183	1.89226	0.05
58184	1.89226	0.05
58185	1.89226	0.05
58186	1.89226	0.05
58187	1.89227	0.05
58188	1.89227	0.05
58189	1.89227	0.05
58190	1.89227	0.05
58191	1.89227	0.05
58192	1.89227	0.05
58193	1.89226	0.05
58194	1.89226	0.05
58195	1.89226	0.05
58196	1.89226	0.05
58197	3.78453	0.05
58198	1.89226	0.05
58199	1.89226	0.05
58200	1.89226	0.05
58201	1.89226	0.05
58202	1.89226	0.05
58203	1.89226	0.05
58204	1.89226	2.1
58205	2.36204	2.1
58206	2.39853	2.1
58207	2.36204	2.1
58208	1.92876	2.1
58209	1.89226	2.1
58210	1.89226	2.1
58211	1.89226	2.1
58212	1.89226	2.1
58213	1.89226	0.108498
58214	3.78453	2.1
58215	0.946128	2.1
58216	0.946128	0.130323
58217	1.13182	1.96667
58218	1.13182	2.1
58219	0.94613	1.30334
58220	0.94613	2.1
58221	1.1749	2.1
58222	1.13182	2.1
58223	1.1749	2.1
58224	0.94613	2.1
58225	1.13182	2.1
58226	0.94613	2.1
58227	1.89226	2.1
58228	2.39853	2.1
58229	2.44918	2.1

58230	2.39853	2.1
58231	1.94291	2.1
58232	1.89226	2.1
58233	0.946132	0.940053
58234	0.946132	2.1
58235	0.946131	2.1
58236	0.946131	2.1
58237	0.946132	2.1
58238	0.946132	2.1
58239	0.946131	2.1
58240	0.946131	2.1
58241	0.946131	2.1
58242	0.946131	2.1
58243	0.946132	2.1
58244	0.946132	2.1
58245	0.946128	2.1
58246	0.946128	2.1
58247	1.1749	2.1
58248	1.1749	2.1
58249	0.946131	2.1
58250	0.946131	2.1
58251	1.23631	2.1
58252	1.1749	2.1
58253	1.23631	2.1
58254	0.94613	2.1
58255	1.1749	2.1
58256	0.946131	2.1
58257	1.89226	0.460681
58258	1.89226	2.1
58259	1.89226	2.1
58260	1.89226	2.1
58261	3.78453	2.1
58262	1.38859	2.1
58263	1.45333	2.1
58264	1.28909	2.1
58265	1.38091	2.1
58266	2.74242	2.1
58267	1.45332	2.1
58268	1.38414	2.1
58269	1.38091	2.1
58270	1.33506	2.1
58271	2.78838	2.1
58272	1.89226	2.1
58273	1.89226	2.1
58274	1.89226	2.1
58275	1.89226	2.1
58276	3.78452	2.1
58277	1.89226	2.1
58278	1.89226	2.1
58279	1.89226	1.55134
58280	1.89226	2.1
58281	1.89226	1.64737
58282	1.89226	2.1
58283	1.17418	2.1
58284	1.38091	2.1
58285	1.17418	2.1
58286	1.28909	2.1
58287	1.29741	2.1

58288	1.41232	2.1
58289	1.38091	2.1
58290	1.33506	2.1
58291	1.27575	2.1
58292	1.27575	2.1
58293	1.29741	2.1
58294	1.35671	2.1
58295	1.89226	2.1
58296	1.89226	2.1
58297	1.89226	2.1
58298	1.89226	2.1
58299	3.78452	2.1
58300	1.92737	0.05
58301	2.40223	0.05
58302	1.56178	0.05
58303	1.97605	0.05
58304	3.90343	0.05
58305	1.89226	0.05
58306	1.89226	0.05
58307	1.89226	0.05
58308	1.89226	0.05
58309	3.78452	0.05
58310	1.89226	0.05
58311	1.89226	0.05947
58312	1.89226	0.0511811
58313	1.89226	0.0700066
58314	1.89226	0.05
58315	1.89226	0.05
58316	1.13641	0.05
58317	1.13641	0.05
58318	1.12763	0.05
58319	1.12763	0.05
58320	1.29348	0.05
58321	1.06293	0.05
58322	1.20111	0.05
58323	0.988028	0.05
58324	0.988028	0.05
58325	1.06293	0.05
58326	1.29348	0.05
58327	1.20111	0.05
58328	1.56176	0.05
58329	1.46387	0.05
58330	1.97603	0.05
58331	1.97603	0.05
58332	1.08211	0.05
58333	1.08211	0.05
58334	1.89224	0.05
58335	1.89224	0.05
58336	1.89224	0.05
58337	1.89224	0.05
58338	1.89224	0.05
58339	1.89224	0.05
58340	1.89224	0.05
58341	1.89224	0.05
58342	1.89224	0.05
58343	1.89224	0.05
58344	3.78448	0.05
58345	0.820185	0.05

58346	0.818947	0.05
58347	0.911041	0.05
58348	0.695265	0.05
58349	1.46388	0.05
58350	1.23607	0.05
58351	0.927068	0.05
58352	1.02691	0.05
58353	1.12918	0.05
58354	0.846843	0.05
58355	0.911039	0.05
58356	1.29129	2.1
58357	1.45547	2.1
58358	1.67327	2.1
58359	1.66698	2.1
58360	1.20149	2.1
58361	1.20149	2.1
58362	0.946133	0.514757
58363	0.946133	2.1
58364	0.946129	2.1
58365	0.946129	2.1
58366	0.946131	2.1
58367	0.946131	0.442687
58368	0.946131	2.1
58369	0.946131	2.1
58370	0.946131	2.1
58371	0.946131	2.1
58372	0.946131	2.1
58373	0.946131	2.1
58374	1.89226	2.1
58375	1.89226	2.1
58376	1.89226	2.1
58377	1.89226	0.727357
58378	3.78452	2.06007
58379	0.86875	2.1
58380	0.86875	2.1
58381	0.899002	2.1
58382	0.966245	2.1
58383	0.849364	1.06111
58384	1.01918	2.1
58385	0.764737	2.1
58386	0.89249	2.1
58387	0.764624	2.1
58388	0.967869	2.1
58389	0.808606	2.1
58390	0.864506	2.1
58391	0.773929	2.1
58392	0.773929	2.1
58393	0.781991	2.1
58394	0.836559	2.1
58395	0.983761	2.1
58396	0.834687	2.1
58397	0.680545	2.1
58398	0.640799	2.1
58399	0.70286	2.1
58400	0.935844	2.1
58401	0.629479	2.1
58402	0.823655	2.1
58403	0.946133	1.81575

58404	0.946133	2.1
58405	0.946128	2.1
58406	0.946128	2.1
58407	0.94613	2.1
58408	0.94613	1.5602
58409	0.946131	2.1
58410	0.946131	2.1
58411	0.946131	2.1
58412	0.946131	2.1
58413	0.946131	2.1
58414	0.946131	2.1
58415	1.89226	2.1
58416	1.89226	0.898294
58417	1.89226	2.1
58418	1.89226	2.1
58419	1.89226	1.36808
58420	1.89226	2.1
58421	0.931638	2.1
58422	0.931638	1.85886
58423	1.03559	2.1
58424	0.977442	2.1
58425	0.826487	1.23832
58426	0.993902	1.84502
58427	1.04922	2.1
58428	0.894324	2.1
58429	0.912984	2.1
58430	1.01156	2.1
58431	0.944692	2.1
58432	1.15456	2.1
58433	1.89226	0.05
58434	1.89226	0.05
58435	1.89226	0.05
58436	1.89226	0.05
58437	3.78453	0.05
58438	1.89226	0.05
58439	1.89226	0.05
58440	2.64262	0.05
58441	2.64262	0.05
58442	1.78052	0.05
58443	1.03015	0.05
58444	0.999721	0.05
58445	0.659011	0.05
58446	1.15564	0.05
58447	1.41388	0.05
58448	1.01583	0.05
58449	1.01583	0.05
58450	1.05706	0.05
58451	1.05706	0.05
58452	1.15564	0.05
58453	0.65901	0.05
58454	1.41388	0.05
58455	0.999721	0.05
58456	1.89226	2.1
58457	1.89226	2.1
58458	1.89226	2.1
58459	1.89226	2.1
58460	3.78453	2.1
58461	2.39022	2.1

58462	1.90734	2.1
58463	2.27634	2.1
58464	1.82666	2.1
58465	4.18368	2.1
58466	1.90734	2.1
58467	1.21564	2.1
58468	1.82666	2.1
58469	1.16938	2.1
58470	3.07672	2.1
58471	1.89226	2.1
58472	1.89226	2.1
58473	1.89226	2.1
58474	1.89226	2.1
58475	1.89226	2.1
58476	1.89226	2.1
58477	1.89227	2.1
58478	1.89227	2.1
58479	1.89227	2.1
58480	1.89227	2.1
58481	3.78453	2.1
58482	2.27634	2.1
58483	1.82666	2.1
58484	2.18042	2.1
58485	1.74738	2.1
58486	4.02372	2.1
58487	1.74738	2.1
58488	1.82666	2.1
58489	1.1181	2.1
58490	1.1181	2.1
58491	1.16938	2.1
58492	1.79865	2.1
58493	1.89226	2.1
58494	1.89226	2.1
58495	1.89226	2.1
58496	1.89226	2.1
58497	1.89226	2.1
58498	1.89226	2.1
58499	1.89226	1.43151
58500	1.89226	0.750923
58501	1.89226	1.0546
58502	1.89226	1.02503
58503	1.89226	1.64105
58504	1.89226	0.966327
58505	0.790736	1.05076
58506	1.08265	0.868345
58507	0.938909	1.31333
58508	0.638683	0.985963
58509	0.858588	1.15358
58510	0.858588	0.734847
58511	0.862743	1.46123
58512	0.862743	1.22475
58513	1.08265	1.3374
58514	0.938911	0.625199
58515	0.790735	1.32464
58516	0.638685	0.727269
58517	1.89226	0.698283
58518	1.89226	1.30819
58519	1.89226	1.18374

58520	1.89226	0.707871
58521	1.89226	1.61345
58522	1.89226	1.29953
58523	1.89226	0.982282
58524	1.89226	1.43279
58525	1.89226	0.843751
58526	1.89226	0.823393
58527	1.89226	1.34116
58528	1.89226	1.38804
58529	1.08265	0.590294
58530	1.36209	0.636112
58531	1.25233	0.659941
58532	0.938909	0.923082
58533	1.14195	0.5787
58534	1.14195	0.279454
58535	1.15904	1.43395
58536	1.15904	0.88943
58537	1.36209	1.14647
58538	1.25233	0.235198
58539	1.08265	1.11385
58540	0.938911	0.394283
58541	1.22077	0.546027
58542	0.915244	0.688185
58543	0.967835	1.01269
58544	0.742495	0.978425
58545	0.78648	0.852563
58546	0.985041	0.560299
58547	0.989098	1.4851
58548	0.989098	0.920928
58549	1.03206	0.561007
58550	0.967897	1.23208
58551	0.915404	1.23127
58552	0.706105	0.693083
58553	2.69662	0.05
58554	1.89226	0.05
58555	2.35499	0.05
58556	1.89226	0.05
58557	4.24725	0.05
58558	1.89227	0.05
58559	1.89227	0.05
58560	1.89227	0.05
58561	1.89227	0.05
58562	3.78453	0.05
58563	2.35499	0.05
58564	1.70302	0.05
58565	1.70302	0.05
58566	1.703	0.05
58567	1.97374	0.05
58568	1.97372	0.05
58569	0.946133	0.05
58570	0.946133	0.05
58571	0.946132	0.05
58572	0.946132	0.05
58573	0.946133	0.05
58574	0.946133	0.05
58575	0.946132	0.05
58576	0.946132	0.05
58577	0.946133	0.05

58578	0.946133	0.05
58579	0.946132	0.05
58580	0.946132	0.05
58581	0.6794	2.1
58582	0.6794	2.1
58583	0.670812	2.1
58584	0.62182	2.1
58585	0.648326	2.1
58586	0.792154	2.1
58587	0.719167	2.1
58588	0.494515	2.1
58589	0.649421	2.1
58590	0.804869	2.1
58591	0.485585	2.1
58592	0.627666	2.1
58593	1.79347	2.1
58594	1.03968	2.06177
58595	1.70303	2.1
58596	0.933265	1.23387
58597	2.62068	1.18753
58598	1.89226	2.1
58599	1.89226	2.1
58600	1.89226	2.1
58601	1.89226	1.35976
58602	3.78452	2.1
58603	1.89226	2.0748
58604	2.24604	2.1
58605	2.0554	2.1
58606	2.0554	2.1
58607	1.89226	2.1
58608	1.89226	2.1
58609	1.89226	2.1
58610	1.89226	0.05
58611	1.89226	0.160845
58612	1.89226	2.1
58613	1.89226	0.221389
58614	1.89226	2.1
58615	2.0815	2.1
58616	2.64424	0.05
58617	2.80807	0.05
58618	3.232	1.92013
58619	1.89417	0.05
58620	2.18012	2.08908
58621	0.946131	2.1
58622	0.946131	0.05
58623	0.946131	2.1
58624	0.946131	0.614514
58625	0.94613	0.479153
58626	0.94613	0.0665734
58627	0.946132	2.1
58628	0.946132	2.1
58629	0.946131	1.21082
58630	0.946131	1.81052
58631	0.946131	1.2102
58632	0.946131	1.37574
58633	1.89227	0.05
58634	1.89227	0.05
58635	1.89227	0.05

58636	1.89227	0.05
58637	3.78453	0.05
58638	2.70881	0.05
58639	2.68687	0.05
58640	2.03046	0.05
58641	1.9682	0.05
58642	4.67701	0.05
58643	1.08518	0.05
58644	1.08518	0.05
58645	1.09033	0.05
58646	1.09033	0.05
58647	0.842273	0.05
58648	1.19141	0.05
58649	1.34343	0.05
58650	0.9841	0.05
58651	0.9841	0.05
58652	0.842273	0.05
58653	1.34344	0.05
58654	1.19141	0.05
58655	0.946133	0.05
58656	0.946133	0.05
58657	0.946132	0.05
58658	0.946132	0.05
58659	0.946132	0.05
58660	0.946132	0.05
58661	0.946132	0.05
58662	0.946132	0.05
58663	0.946132	0.05
58664	0.946132	0.05
58665	0.946133	0.05
58666	0.946133	0.05
58667	1.89227	0.05
58668	1.89227	0.05
58669	1.89227	0.05
58670	1.89227	0.05
58671	1.89227	0.05
58672	1.89227	0.05
58673	2.03046	0.05
58674	1.9682	0.05
58675	1.23886	0.05
58676	1.12971	0.05
58677	3.20706	0.05
58678	1.89226	0.0803558
58679	1.89226	0.650078
58680	1.89226	0.0546426
58681	1.89226	0.336144
58682	3.78452	0.177841
58683	2.45569	0.05
58684	1.89226	0.0656319
58685	2.22922	0.05
58686	2.0815	0.151278
58687	1.85074	0.05
58688	1.70303	0.0736426
58689	2.72856	0.05
58690	1.89226	0.233777
58691	1.89226	0.05
58692	1.89226	0.100214
58693	3.78452	0.074905

58694	1.89226	0.120751
58695	1.89226	0.409386
58696	1.89226	0.0642805
58697	1.89226	0.436006
58698	3.78452	0.170214
58699	0.946131	0.386533
58700	0.946131	0.0595863
58701	0.946131	0.240035
58702	0.946131	0.05
58703	0.94613	0.198031
58704	0.94613	0.281721
58705	0.946132	0.0531988
58706	0.946132	0.0663461
58707	0.946131	0.102388
58708	0.946131	0.117738
58709	0.946131	0.119474
58710	0.946131	0.105314
58711	2.0595	0.05
58712	2.58581	0.0576663
58713	1.48694	0.05
58714	1.48694	0.05
58715	2.60157	0.05
58716	2.02901	0.05
58717	1.89226	0.05
58718	1.89226	0.0533039
58719	1.89226	0.05
58720	1.89226	0.05
58721	1.89226	0.0522501
58722	1.89226	0.065977
58723	2.04728	0.05
58724	2.64161	0.05
58725	1.47606	0.05
58726	1.47606	0.05
58727	2.63069	0.05
58728	2.05947	0.05
58729	1.89224	0.05
58730	1.89224	0.05
58731	1.89224	0.0560376
58732	1.89224	0.05
58733	1.89224	0.05
58734	1.89224	0.05
58735	0.946114	0.05
58736	0.946114	0.05
58737	0.946118	0.05
58738	0.946118	0.05
58739	0.946114	0.05
58740	0.946114	0.05
58741	0.946118	0.05
58742	0.946118	0.05
58743	0.946128	0.05
58744	0.946128	0.05
58745	0.946103	0.05
58746	0.946103	0.05
58747	1.1233	2.1
58748	1.15009	2.1
58749	2.36036	2.1
58750	2.38225	2.1
58751	3.50554	2.1

58752	2.38224	2.1
58753	0.977775	2.1
58754	2.20473	2.1
58755	2.20473	2.1
58756	1.15009	2.1
58757	1.15009	2.1
58758	0.946119	2.1
58759	0.946119	2.1
58760	1.19111	2.1
58761	1.19111	2.1
58762	0.946118	2.1
58763	1.19111	2.1
58764	1.18016	2.1
58765	0.946118	1.36763
58766	0.94613	0.192645
58767	0.94613	2.1
58768	1.18015	2.1
58769	1.19109	2.1
58770	0.946118	2.1
58771	0.946118	2.1
58772	1.10235	2.1
58773	1.10235	2.1
58774	0.946118	2.1
58775	1.10235	2.1
58776	0.946115	2.1
58777	1.1911	2.1
58778	0.946129	2.1
58779	0.946129	2.1
58780	1.19109	2.1
58781	1.10234	2.1
58782	1.89226	2.1
58783	2.23932	2.1
58784	2.11568	2.1
58785	2.23932	2.1
58786	1.76862	2.1
58787	1.89226	2.1
58788	1.89226	2.1
58789	1.89226	2.1
58790	1.89226	2.1
58791	1.89226	2.1
58792	1.89226	2.1
58793	1.89226	2.1
58794	2.07838	2.1
58795	1.89226	2.1
58796	1.89226	2.1
58797	1.89226	2.1
58798	2.10264	2.1
58799	2.10264	2.1
58800	1.89226	2.1
58801	2.11568	2.1
58802	1.99753	2.1
58803	2.11568	2.1
58804	1.77411	2.1
58805	1.89226	2.1
58806	0.946128	1.87959
58807	0.946128	2.1
58808	0.946133	2.1
58809	0.946133	2.1

58810	0.94613	2.1
58811	0.94613	1.71614
58812	0.946131	2.1
58813	0.946131	2.1
58814	0.946131	2.1
58815	0.946131	2.1
58816	0.946131	2.1
58817	0.946131	2.1
58818	0.946132	2.1
58819	0.946132	2.1
58820	1.03919	2.1
58821	1.03919	2.1
58822	0.946131	2.1
58823	0.946131	2.1
58824	1.02744	2.1
58825	1.03919	2.1
58826	1.02744	2.1
58827	0.946132	2.1
58828	1.03919	2.1
58829	0.946132	2.1
58830	1.64834	2.1
58831	1.82955	1.9493
58832	2.33824	1.19267
58833	2.19301	2.1
58834	3.98658	1.81488
58835	1.40574	2.1
58836	2.19301	1.72265
58837	1.64834	1.9659
58838	1.64834	2.03015
58839	1.99961	1.11515
58840	1.99961	1.66207
58841	2.33824	1.34122
58842	2.19301	2.1
58843	2.68524	1.85624
58844	2.81847	1.68956
58845	5.02349	1.97956
58846	1.99961	2.1
58847	2.19301	1.56443
58848	2.68524	1.4717
58849	2.50732	1.91259
58850	4.68485	1.69632
58851	1.89226	2.1
58852	1.89226	2.01238
58853	1.89226	1.77971
58854	1.89226	2.1
58855	3.78453	2.1
58856	1.75142	2.1
58857	2.47026	2.1
58858	1.46811	2.1
58859	2.26652	2.1
58860	4.01794	2.1
58861	1.89226	2.1
58862	1.89226	2.1
58863	1.89226	2.1
58864	1.89226	2.1
58865	1.89226	2.1
58866	1.89226	2.1
58867	1.89226	2.09294

58868	1.89226	2.1
58869	1.89226	2.1
58870	1.89226	2.1
58871	1.89226	2.1
58872	1.89226	2.1
58873	0.812082	2.1
58874	0.878759	2.1
58875	0.882782	2.1
58876	0.882782	2.1
58877	1.05572	2.1
58878	1.04037	2.1
58879	0.73849	2.1
58880	0.626857	2.1
58881	0.638677	2.1
58882	1.05581	2.1
58883	0.715695	2.1
58884	1.10492	2.1
58885	1.17058	2.1
58886	1.07863	2.1
58887	1.21113	2.1
58888	1.21113	2.1
58889	1.2811	2.1
58890	1.37319	2.1
58891	1.01788	2.1
58892	0.999492	2.1
58893	1.04352	2.1
58894	1.32112	2.1
58895	1.10492	2.1
58896	1.34699	2.1
58897	1.89226	2.1
58898	1.89226	2.1
58899	1.89226	2.1
58900	1.89226	2.1
58901	1.89226	2.1
58902	1.89226	2.1
58903	1.19813	0.05
58904	1.19813	0.05
58905	2.43432	0.05
58906	1.21716	0.05
58907	0.946124	0.05
58908	1.21715	0.05
58909	0.946118	0.05
58910	2.56627	2.1
58911	1.89226	2.1
58912	1.89226	2.1
58913	1.89226	2.1
58914	2.43434	2.1
58915	2.43434	2.1
58916	1.89227	0.05
58917	1.89227	0.05
58918	1.89227	0.05
58919	1.89227	0.05
58920	1.89227	0.05
58921	1.89227	0.05
58922	1.89226	0.05
58923	1.89226	0.05
58924	1.89226	0.05
58925	1.89226	0.05

58926	3.78453	0.05
58927	1.89226	0.05
58928	2.0764	0.05
58929	1.89226	0.05
58930	2.81674	0.05
58931	1.89226	0.05
58932	2.81674	0.05
58933	0.946132	0.05
58934	0.946132	0.05
58935	0.946131	0.05
58936	0.946131	0.05
58937	0.946132	0.05
58938	0.946132	0.05
58939	0.946132	0.05
58940	0.946132	0.05
58941	0.946132	0.05
58942	0.946132	0.05
58943	0.946132	0.05
58944	0.946132	0.05
58945	0.578684	1.30801
58946	0.578684	2.1
58947	0.589487	1.85338
58948	0.589487	2.1
58949	0.625461	2.1
58950	0.5378	2.1
58951	0.608714	2.1
58952	0.54271	1.72897
58953	0.608714	1.58396
58954	0.625461	0.816068
58955	0.54271	2.03429
58956	0.537801	1.95057
58957	0.946128	0.80233
58958	0.946128	2.1
58959	0.946133	0.614267
58960	0.946133	2.1
58961	0.946131	1.13495
58962	0.946131	0.678739
58963	0.946131	0.667966
58964	0.946131	1.35625
58965	0.94613	1.14973
58966	0.94613	0.492254
58967	0.946131	1.15534
58968	0.946131	1.44844
58969	1.21743	2.08235
58970	1.25092	2.1
58971	1.38847	0.803764
58972	1.46031	0.954336
58973	2.67774	1.81179
58974	0.946128	0.768434
58975	0.946128	2.1
58976	0.946132	2.1
58977	0.946132	0.962026
58978	0.94613	1.67403
58979	0.94613	0.775408
58980	0.94613	1.40587
58981	0.94613	1.00901
58982	0.94613	0.82189
58983	0.94613	0.522333

58984	0.94613	1.65486
58985	0.94613	0.866158
58986	1.89226	0.590879
58987	1.89226	1.11847
58988	1.89226	0.105599
58989	1.89226	2.1
58990	1.89226	2.1
58991	1.89226	2.1
58992	0.539566	0.813203
58993	0.534046	2.1
58994	0.577914	1.97262
58995	0.471623	1.95711
58996	1.46031	0.327236
58997	0.945865	0.903342
58998	0.770603	0.287422
58999	0.737201	2.1
59000	0.714829	0.942191
59001	0.536085	1.68978
59002	0.471617	1.23924
59003	1.89226	2.1
59004	1.89226	0.501856
59005	1.89226	0.46493
59006	1.89226	1.36128
59007	1.89226	1.46938
59008	1.89226	2.1
59009	1.89225	1.76349
59010	1.89225	0.168872
59011	1.65572	0.163755
59012	1.65572	0.61734
59013	1.89225	0.765147
59014	1.89225	1.91659
59015	1.89226	1.5564
59016	1.89226	0.665338
59017	1.89226	1.00065
59018	1.89226	0.0542443
59019	3.78453	1.66938
59020	1.89226	2.09693
59021	1.89226	0.245783
59022	1.89226	0.288137
59023	1.89226	1.18578
59024	1.89226	1.1387
59025	1.89226	2.1
59026	1.89225	1.52349
59027	1.89225	0.0845318
59028	1.89225	0.122019
59029	1.89225	0.475426
59030	1.89225	0.355617
59031	1.89225	1.61521
59032	0.763592	0.177037
59033	0.763592	0.916533
59034	0.987417	0.470464
59035	0.987417	0.550727
59036	0.763592	0.0978973
59037	0.804881	0.0599592
59038	0.946126	0.520814
59039	0.946126	0.18722
59040	0.804879	0.0539555
59041	0.94613	0.314165

59042	0.946129	0.745393
59043	0.76359	0.112954
59044	1.46055	2.1
59045	2.04945	2.1
59046	2.04587	2.1
59047	2.04587	2.1
59048	1.50714	2.1
59049	1.50714	2.1
59050	2.04587	2.1
59051	2.38681	2.1
59052	2.42082	2.1
59053	2.42082	2.1
59054	2.04945	2.1
59055	2.04945	2.1
59056	0.872964	2.1
59057	0.872964	2.1
59058	0.884806	2.1
59059	0.884806	2.1
59060	1.0275	2.1
59061	1.02293	2.1
59062	0.711243	2.1
59063	0.730273	2.1
59064	0.711244	2.1
59065	1.0275	2.1
59066	0.730273	2.1
59067	1.02293	2.1
59068	2.04587	2.1
59069	2.47753	2.1
59070	2.42994	2.1
59071	2.42082	2.1
59072	2.05499	2.1
59073	2.04587	2.1
59074	0.946132	2.1
59075	0.946132	2.1
59076	1.13078	2.1
59077	1.07817	2.1
59078	0.946132	2.1
59079	0.946132	2.1
59080	1.07817	2.1
59081	1.07817	2.1
59082	0.946132	2.1
59083	1.07817	2.1
59084	1.13078	2.1
59085	0.946132	2.1
59086	1.89226	2.1
59087	1.89226	2.1
59088	1.89226	2.1
59089	1.89226	2.1
59090	1.89226	2.1
59091	1.89226	2.1
59092	1.89226	2.1
59093	1.89226	2.1
59094	1.89226	2.1
59095	1.89226	2.1
59096	1.89226	2.1
59097	1.89226	2.1
59098	1.89226	2.1
59099	1.89226	2.1

59100	1.8173	2.1
59101	2.66885	2.1
59102	2.7438	2.1
59103	2.7438	2.1
59104	1.89226	0.968666
59105	1.89226	0.516379
59106	1.89226	0.583836
59107	1.89226	0.941502
59108	3.78453	0.876707
59109	1.89226	0.312874
59110	1.89226	0.323201
59111	1.89226	0.206187
59112	1.89226	1.6252
59113	1.89226	0.0610364
59114	1.89226	0.444221
59115	1.89226	0.157357
59116	1.97385	0.556072
59117	1.7531	0.269388
59118	1.89226	1.77629
59119	1.7531	0.0543189
59120	1.89226	0.299152
59121	0.946131	1.19817
59122	0.840904	0.202465
59123	0.684333	0.409292
59124	0.94613	0.728577
59125	0.972337	0.578763
59126	0.946278	1.73149
59127	0.684304	0.438034
59128	0.684304	0.724682
59129	0.999838	0.237858
59130	0.759605	1.69688
59131	0.851528	0.483798
59132	0.851528	1.32948
59133	1.89226	0.443484
59134	2.23818	1.23553
59135	1.79771	0.47064
59136	1.89226	1.58103
59137	1.79771	0.128294
59138	1.89226	0.498397
59139	2.79647	2.1
59140	2.01613	2.1
59141	1.46826	2.1
59142	2.54504	2.1
59143	4.56117	2.1
59144	1.89226	2.1
59145	1.89226	2.1
59146	1.89226	2.1
59147	1.89226	2.1
59148	1.89226	2.1
59149	1.89226	2.1
59150	1.89226	2.1
59151	2.66884	2.1
59152	2.56022	2.1
59153	2.66884	2.1
59154	1.78364	2.1
59155	1.89226	2.1
59156	1.14282	2.1
59157	1.24068	2.1

59158	0.880748	2.1
59159	0.834916	2.1
59160	1.05539	2.1
59161	1.05539	2.1
59162	1.04312	2.1
59163	1.04313	2.1
59164	1.2015	2.1
59165	0.83492	2.1
59166	0.789149	2.1
59167	1.24066	2.1
59168	1.89226	2.1
59169	1.89226	2.1
59170	1.89226	2.1
59171	1.89226	2.1
59172	3.78453	2.1
59173	0.946128	2.1
59174	0.946128	2.1
59175	1.28011	2.1
59176	1.22372	2.1
59177	0.946131	2.1
59178	0.946131	2.1
59179	1.22372	2.1
59180	1.22372	2.1
59181	1.22372	2.1
59182	0.94613	2.1
59183	0.946131	2.1
59184	1.28011	2.1
59185	2.28604	2.1
59186	1.34268	2.1
59187	2.43157	2.1
59188	1.50172	2.1
59189	3.77425	2.1
59190	2.6096	2.1
59191	2.43157	2.1
59192	1.66774	2.1
59193	1.66774	2.1
59194	1.50172	2.1
59195	2.43157	2.1
59196	2.16067	2.1
59197	1.08975	2.1
59198	2.19336	2.1
59199	2.06984	2.1
59200	1.21327	2.1
59201	1.08975	2.1
59202	2.28604	2.1
59203	1.21327	2.1
59204	2.29008	2.1
59205	2.16067	2.1
59206	1.34268	2.1
59207	1.21327	2.1
59208	1.89226	0.05
59209	1.89226	0.05
59210	1.89226	0.05
59211	1.89226	0.05
59212	3.78452	0.05
59213	1.89804	0.05
59214	2.21656	0.05
59215	1.70537	0.0612109

59216	2.50023	0.05
59217	3.8982	0.05
59218	1.03136	0.05
59219	1.89486	0.05
59220	0.949364	0.05
59221	1.82283	0.05
59222	2.84422	0.05
59223	1.89226	0.05
59224	1.89226	0.05
59225	1.89226	0.0506315
59226	1.89226	0.05
59227	3.78452	0.05
59228	0.946132	0.0633211
59229	0.946132	0.0672891
59230	0.946129	0.0916891
59231	0.946129	0.05
59232	0.94613	0.05
59233	0.94613	0.0834209
59234	0.946131	0.05
59235	0.946131	0.0785608
59236	0.946131	0.0858148
59237	0.946131	0.0719317
59238	0.946131	0.05
59239	0.946131	0.0998871
59240	1.09466	0.05
59241	1.09466	0.05
59242	0.866512	0.0604955
59243	1.08352	0.05
59244	1.19189	0.05
59245	1.21883	0.05
59246	0.971139	0.05
59247	0.873213	0.05
59248	0.873202	0.05
59249	1.00855	0.05
59250	0.923735	0.05
59251	1.21885	0.05
59252	1.89225	0.05
59253	2.40019	0.05
59254	1.89225	0.05
59255	1.89225	0.05
59256	1.89225	0.05
59257	1.89225	0.05
59258	1.89225	0.05
59259	1.89225	0.05
59260	2.05088	0.05
59261	1.89225	0.05
59262	1.89225	0.05
59263	1.89225	0.05
59264	2.03956	0.05
59265	2.03956	0.05
59266	1.89226	2.1
59267	2.40021	2.1
59268	2.38473	0.05
59269	2.40021	2.1
59270	1.87679	0.0646645
59271	1.89226	2.1
59272	1.89226	2.1
59273	1.89226	0.05

59274	1.89226	2.1
59275	1.89226	0.05
59276	1.89226	1.74608
59277	1.89226	0.05
59278	2.0692	2.1
59279	1.89226	0.1959
59280	1.89226	2.1
59281	1.89226	0.05
59282	2.05089	2.1
59283	2.05089	0.05
59284	1.89226	2.1
59285	1.89226	2.1
59286	1.89226	2.1
59287	1.89226	2.1
59288	3.78452	2.1
59289	1.89226	0.274156
59290	2.50051	2.1
59291	1.89226	2.1
59292	2.30076	2.1
59293	4.39277	2.1
59294	2.50052	2.1
59295	1.70377	2.1
59296	2.30076	2.1
59297	1.54231	2.1
59298	4.04283	2.1
59299	1.89226	2.1
59300	1.89226	2.1
59301	1.89226	2.1
59302	1.89226	2.1
59303	1.89226	2.1
59304	1.89226	2.1
59305	1.89226	0.369281
59306	1.89226	2.1
59307	1.89226	2.09748
59308	1.89226	2.1
59309	3.78452	2.1
59310	2.07948	2.1
59311	2.30076	2.1
59312	1.3294	2.1
59313	1.3294	2.1
59314	1.54232	2.1
59315	2.29239	2.1
59316	0.810947	2.1
59317	0.810947	2.1
59318	0.810946	2.1
59319	0.810946	2.1
59320	1.89226	2.1
59321	1.35169	2.1
59322	1.08132	2.1
59323	1.08132	2.1
59324	1.08132	2.1
59325	0.810946	2.1
59326	0.810946	2.1
59327	1.34851	0.995773
59328	0.997471	2.1
59329	1.62287	0.428743
59330	1.27096	1.96329
59331	2.62034	1.03225

59332	1.89226	0.642236
59333	1.89226	2.1
59334	1.89226	1.01051
59335	1.89226	2.1
59336	3.78452	1.38485
59337	0.946128	2.1
59338	0.946128	2.1
59339	1.29331	0.290823
59340	1.29331	2.1
59341	1.29331	2.03855
59342	1.10699	2.1
59343	0.946131	0.240093
59344	0.946131	1.92178
59345	0.94613	2.1
59346	1.29331	2.1
59347	1.10699	2.1
59348	0.946131	2.09375
59349	1.62287	0.744196
59350	1.27096	2.05676
59351	1.86067	0.676211
59352	1.58407	2.03503
59353	3.20694	1.74682
59354	1.89226	1.31096
59355	1.89226	2.1
59356	1.89226	0.594206
59357	1.89226	2.04999
59358	3.78452	1.25656
59359	2.62761	0.44593
59360	2.55849	0.788869
59361	2.12261	1.50579
59362	2.22939	0.736555
59363	4.78788	0.679104
59364	0.772522	1.94236
59365	0.839588	1.36728
59366	1.31126	0.083113
59367	1.08787	0.0859128
59368	1.01318	0.140429
59369	1.01318	2.06263
59370	0.900815	2.1
59371	1.10799	0.05
59372	0.772511	0.461139
59373	1.03473	0.543022
59374	1.31126	0.745936
59375	0.841464	1.18876
59376	1.89227	0.121494
59377	1.89227	1.94064
59378	1.89227	0.176216
59379	1.89227	1.63292
59380	1.89227	0.133428
59381	1.89227	1.78743
59382	1.89226	0.68362
59383	1.89226	0.501515
59384	1.89226	0.254361
59385	1.89226	0.960429
59386	1.89226	0.124369
59387	1.89226	0.64897
59388	2.12261	1.06665
59389	2.22939	0.142388

59390	1.97004	0.166571
59391	1.8072	1.15073
59392	4.03658	0.295496
59393	1.5847	2.00906
59394	1.8072	0.12252
59395	2.12262	0.674378
59396	2.12262	1.89678
59397	1.23267	0.359599
59398	1.23267	1.91708
59399	1.89227	0.138637
59400	1.89227	2.1
59401	1.89227	0.204854
59402	1.89227	1.75895
59403	1.89227	0.150432
59404	1.89227	1.97172
59405	1.89226	0.148379
59406	1.89226	0.58419
59407	1.89226	1.17279
59408	1.89226	0.125819
59409	3.78452	0.290765
59410	2.49093	2.1
59411	2.67393	2.1
59412	2.47932	2.1
59413	2.47932	2.1
59414	2.67994	2.1
59415	2.67994	2.1
59416	2.49873	2.1
59417	2.67393	2.1
59418	2.67993	2.1
59419	2.69807	2.1
59420	2.49873	2.1
59421	2.51686	2.1
59422	2.49093	2.1
59423	2.67994	2.1
59424	2.69784	2.1
59425	2.51651	2.1
59426	5.18877	2.1
59427	1.24359	2.1
59428	1.24936	2.1
59429	1.33997	2.1
59430	1.34892	2.1
59431	1.28805	2.1
59432	1.28805	2.1
59433	1.2955	2.1
59434	1.2955	2.1
59435	1.34892	2.1
59436	1.24359	2.1
59437	1.24936	2.1
59438	1.33997	2.1
59439	1.15506	2.0921
59440	1.15506	2.1
59441	0.946128	2.1
59442	0.946128	2.1
59443	0.946131	2.1
59444	1.15506	2.1
59445	1.06997	2.1
59446	0.946128	2.1
59447	1.06997	2.1

59448	1.15506	2.1
59449	0.946129	2.1
59450	0.946129	2.1
59451	1.70608	2.1
59452	2.13994	2.1
59453	1.33384	2.1
59454	1.43635	2.1
59455	3.14243	2.1
59456	2.13994	2.1
59457	2.31012	2.1
59458	1.43635	2.1
59459	1.23007	2.1
59460	3.74646	2.1
59461	2.46361	2.1
59462	1.89226	2.1
59463	1.89226	2.1
59464	1.89226	2.1
59465	2.39045	2.1
59466	2.39045	2.1
59467	1.89226	2.1
59468	1.89226	1.88532
59469	1.89226	2.1
59470	1.89226	1.12222
59471	3.78452	2.1
59472	1.52984	2.1
59473	2.25682	0.709363
59474	2.20831	0.290616
59475	2.20831	2.1
59476	1.61178	0.157466
59477	1.61178	2.1
59478	2.47395	2.1
59479	1.89226	2.1
59480	1.89226	2.1
59481	1.89226	2.1
59482	2.46362	2.1
59483	2.46362	2.1
59484	0.946131	0.131982
59485	0.946131	2.1
59486	0.946131	2.1
59487	0.946131	1.93699
59488	0.94613	1.2417
59489	0.94613	2.1
59490	0.946132	2.1
59491	0.946132	2.1
59492	0.946131	1.9998
59493	0.946131	2.1
59494	0.946131	2.1
59495	0.946131	2.1
59496	0.982398	2.1
59497	0.982398	2.1
59498	0.931703	2.1
59499	0.97549	2.1
59500	1.11509	2.05892
59501	1.10017	0.658527
59502	0.774576	2.1
59503	0.789859	2.1
59504	0.716502	2.1
59505	1.12793	2.1

59506	1.14398	2.1
59507	0.817011	1.90313
59508	1.82499	2.1
59509	1.50524	2.1
59510	1.21304	2.1
59511	1.54191	2.1
59512	3.04715	2.1
59513	1.54191	2.1
59514	1.49823	2.1
59515	1.82499	2.1
59516	1.82499	2.1
59517	1.21147	2.1
59518	1.48977	2.1
59519	1.89226	2.1
59520	1.89226	2.1
59521	1.89226	2.1
59522	1.89226	2.1
59523	3.78452	2.1
59524	1.89226	2.1
59525	1.89226	2.1
59526	1.89226	2.1
59527	1.89226	2.1
59528	1.89226	2.1
59529	1.89226	2.1
59530	0.566674	2.1
59531	0.770957	2.1
59532	0.60652	2.1
59533	0.473557	2.1
59534	0.604441	2.1
59535	0.604441	2.1
59536	0.640073	2.1
59537	0.640073	2.1
59538	0.473557	2.1
59539	0.566674	2.1
59540	0.60652	2.1
59541	0.770956	2.1
59542	1.36203	2.1
59543	1.13335	2.1
59544	1.54191	2.1
59545	1.54191	2.1
59546	1.00965	2.1
59547	1.00965	2.1
59548	1.89226	2.1
59549	1.89226	2.1
59550	1.89226	2.1
59551	1.89226	2.1
59552	1.89226	2.1
59553	1.89226	2.1
59554	0.946131	2.1
59555	0.946131	2.1
59556	0.946131	2.1
59557	0.946131	2.1
59558	0.946132	2.1
59559	0.946132	2.1
59560	0.946131	2.1
59561	0.946131	2.1
59562	0.946132	2.1
59563	0.946132	2.1

59564	0.946131	2.1
59565	0.946131	2.1
59566	0.946132	0.05
59567	0.946132	0.05
59568	0.946133	0.05
59569	0.946133	0.05
59570	0.946132	0.05
59571	0.946132	0.05
59572	0.946132	0.05
59573	0.946132	0.05
59574	0.946132	0.05
59575	0.946132	0.05
59576	0.946133	0.05
59577	0.946133	0.05
59578	1.89226	0.05
59579	1.89226	0.05
59580	1.89226	0.05
59581	1.89226	0.05
59582	3.78453	0.05
59583	1.89226	0.05
59584	1.89226	0.05
59585	1.89226	0.05
59586	1.89226	0.05
59587	1.89226	0.05
59588	1.89226	0.05
59589	0.946132	0.05
59590	0.946132	0.05
59591	0.946131	0.05
59592	0.946131	0.05
59593	0.946132	0.05
59594	0.946132	0.05
59595	0.946132	0.05
59596	0.946132	0.05
59597	0.946132	0.05
59598	0.946132	0.05
59599	0.946132	0.05
59600	0.946132	0.05
59601	0.946129	0.05
59602	0.946129	0.05
59603	0.946128	0.05
59604	0.946128	0.05
59605	0.946132	0.05
59606	0.946132	0.05
59607	0.946126	0.05
59608	0.946126	0.05
59609	0.946129	0.05
59610	0.946129	0.05
59611	0.946129	0.05
59612	0.946129	0.05
59613	1.20674	0.05
59614	1.79877	0.05
59615	1.22586	0.05
59616	1.22586	0.05
59617	1.7919	0.05
59618	1.7919	0.05
59619	2.27622	0.05
59620	1.79877	0.05
59621	1.7919	0.05

59622	2.27102	0.05
59623	4.06979	0.05
59624	1.89226	0.05
59625	1.89226	0.05
59626	1.89226	0.05
59627	1.89226	0.05
59628	3.78452	0.05
59629	1.89226	0.05
59630	1.89226	0.05
59631	1.89226	0.05
59632	1.89226	0.05
59633	3.78453	0.05
59634	1.13259	0.05
59635	1.79187	0.05
59636	1.32741	0.05
59637	1.18784	0.05
59638	1.703	0.05
59639	1.49575	0.05
59640	1.46201	0.05
59641	2.27099	0.05
59642	1.79187	0.05
59643	1.79187	0.05
59644	2.12923	0.05
59645	2.12923	0.05
59646	1.89224	0.05
59647	1.89224	0.05
59648	1.89224	0.05
59649	1.89224	0.05
59650	3.78447	0.05
59651	0.946119	0.05
59652	0.946119	0.05
59653	0.946118	0.05
59654	0.946118	0.05
59655	0.946118	0.05
59656	0.946118	0.05
59657	0.946119	0.05
59658	0.946119	0.05
59659	0.946131	0.05
59660	0.946131	0.05
59661	0.946106	0.05
59662	0.946106	0.05
59663	2.07526	0.0949345
59664	1.89226	0.126469
59665	1.89226	0.920965
59666	2.13445	0.153097
59667	4.02671	0.05798
59668	1.89226	0.0644524
59669	1.89226	0.15926
59670	1.89226	0.368664
59671	1.89226	0.05
59672	1.89226	0.82436
59673	1.89226	0.0556763
59674	2.13446	0.155469
59675	1.00013	0.0758588
59676	1.1475	1.19678
59677	2.20046	0.172032
59678	3.20059	0.384714
59679	0.946133	0.105023

59680	0.946133	0.194485
59681	1.06722	0.200007
59682	1.10023	0.117668
59683	0.946131	0.171029
59684	0.946131	0.05
59685	1.10023	0.354382
59686	1.10023	0.0762219
59687	0.946131	0.252742
59688	1.10023	0.113516
59689	0.94613	0.331438
59690	1.06723	0.0831819
59691	1.89226	0.05
59692	1.89226	0.05
59693	1.89226	0.246789
59694	1.89226	0.128631
59695	3.78452	0.136583
59696	1.89226	0.163692
59697	1.89226	0.0684277
59698	1.89226	0.259916
59699	1.89226	0.17619
59700	1.89226	0.0943815
59701	1.89226	0.0628306
59702	1.89226	2.1
59703	1.89226	0.05
59704	1.89226	0.0608291
59705	1.89226	0.05
59706	1.89226	2.1
59707	0.828588	0.05
59708	0.828588	0.05
59709	0.841316	0.05
59710	0.841316	2.1
59711	0.528387	2.1
59712	0.565893	0.05
59713	1.10401	0.05
59714	1.10401	2.1
59715	0.528387	2.1
59716	1.89226	2.1
59717	1.36047	0.0608291
59718	1.33973	2.1
59719	1.33973	2.1
59720	1.89226	0.05
59721	2.28951	0.05
59722	1.89226	0.840999
59723	2.80796	2.04495
59724	2.01584	1.98765
59725	2.01584	1.17532
59726	1.89226	1.84244
59727	1.89226	1.04714
59728	1.89226	0.583815
59729	1.89226	1.91965
59730	1.89226	1.63382
59731	1.89226	0.946038
59732	1.89226	1.69921
59733	1.89226	1.08654
59734	2.15223	0.869797
59735	1.30664	2.1
59736	1.96011	2.06378
59737	2.76516	0.867464

59738	4.11234	2.06889
59739	1.89226	0.463021
59740	1.89226	1.85266
59741	1.89226	1.50191
59742	1.89226	0.804878
59743	1.89226	1.59802
59744	1.89226	0.94301
59745	2.81143	2.1
59746	2.58517	2.1
59747	2.55318	2.1
59748	2.7542	2.1
59749	5.33937	2.1
59750	2.7777	2.1
59751	2.7542	2.1
59752	2.71672	2.1
59753	2.70401	2.1
59754	5.45821	2.1
59755	2.55318	2.1
59756	2.7542	2.1
59757	2.71672	2.1
59758	2.54198	2.1
59759	5.29618	2.1
59760	1.95416	2.1
59761	2.162 2.1	
59762	2.11563	2.1
59763	1.91813	2.1
59764	4.08013	2.1
59765	1.95417	2.1
59766	1.47712	2.1
59767	1.45182	2.1
59768	1.91813	2.1
59769	3.39525	2.1
59770	2.11562	2.1
59771	1.91813	2.1
59772	1.50979	2.1
59773	2.03974	2.1
59774	3.95787	2.1
59775	1.85605	2.1
59776	1.91813	2.1
59777	1.88885	2.1
59778	1.42255	2.1
59779	1.45182	2.1
59780	1.45182	2.1
59781	0.94613	0.308319
59782	0.94613	0.0766373
59783	0.946128	0.05
59784	0.946128	0.968151
59785	0.946127	0.138345
59786	0.946127	0.171161
59787	0.946131	0.05
59788	0.946131	0.05
59789	0.946129	0.152962
59790	0.946129	0.16956
59791	0.946128	0.07754
59792	0.946128	0.153532
59793	0.543052	0.17362
59794	0.54608	0.05
59795	0.602293	0.05

59796	0.535325	0.065417
59797	1.05446	0.26077
59798	0.850496	0.120005
59799	0.699838	0.0784096
59800	0.679751	0.219584
59801	0.713797	0.0530758
59802	0.509794	0.05
59803	0.602304	0.05
59804	1.40017	0.0542575
59805	1.40017	2.1
59806	0.94613	0.05
59807	0.94613	2.1
59808	1.40017	2.1
59809	1.33997	2.1
59810	0.946131	0.05
59811	0.946131	0.05
59812	1.33997	0.587415
59813	0.946131	0.52071
59814	0.946131	0.429976
59815	1.40017	0.484443
59816	1.89226	0.05
59817	1.89226	0.05
59818	1.89226	0.05
59819	1.89226	0.05
59820	3.78452	0.05
59821	0.946133	0.05
59822	0.946133	0.05
59823	0.946132	0.05
59824	0.946132	0.05
59825	0.946132	0.05
59826	0.946132	0.05
59827	0.946133	0.05
59828	0.946133	0.05
59829	0.946132	0.05
59830	0.946132	0.05
59831	0.946132	0.05
59832	0.946132	0.05
59833	1.89224	0.05
59834	1.89224	0.05
59835	1.89224	0.05
59836	1.89224	0.05
59837	1.89224	0.05
59838	1.89224	0.05
59839	0.94612	0.05
59840	0.94612	0.05
59841	0.946119	0.05
59842	0.946119	0.05
59843	0.946119	0.05
59844	0.946119	0.05
59845	0.94612	0.05
59846	0.94612	0.05
59847	0.946132	0.05
59848	0.946132	0.05
59849	0.946107	0.05
59850	0.946107	0.05
59851	2.63939	2.1
59852	1.2862	2.1
59853	1.29891	2.1

59854	2.64419	2.1
59855	3.93039	2.1
59856	2.6394	2.1
59857	1.89226	2.1
59858	1.89226	2.1
59859	2.64419	2.1
59860	4.53646	2.1
59861	1.89226	2.1
59862	1.89226	2.1
59863	1.89226	2.1
59864	1.89226	2.1
59865	3.78453	2.1
59866	1.89226	2.1
59867	1.89226	2.1
59868	1.89226	2.1
59869	1.89226	2.1
59870	1.89226	2.1
59871	1.89226	2.1
59872	1.31998	2.1
59873	1.32209	2.1
59874	0.649456	2.1
59875	0.648474	2.1
59876	0.984995	2.1
59877	0.984995	2.1
59878	0.985573	2.1
59879	0.985573	2.1
59880	1.31999	2.1
59881	0.648472	2.1
59882	0.649455	2.1
59883	1.32209	2.1
59884	0.946133	2.1
59885	0.946133	2.1
59886	1.31999	2.1
59887	1.3221	2.1
59888	0.946132	2.1
59889	0.946132	2.1
59890	1.31999	2.1
59891	1.31999	2.1
59892	1.31999	2.1
59893	0.946132	2.1
59894	0.946133	2.1
59895	1.3221	2.1
59896	0.946132	2.1
59897	0.946132	2.1
59898	0.946131	2.1
59899	0.946131	2.1
59900	0.946132	2.1
59901	0.946132	2.1
59902	0.946131	2.1
59903	0.946131	2.1
59904	0.946131	2.1
59905	0.946131	2.1
59906	0.946132	2.1
59907	0.946132	2.1
59908	0.946128	2.1
59909	0.946128	2.1
59910	0.946132	2.1
59911	0.946132	2.1

59912	0.946131	2.1
59913	0.946131	2.1
59914	0.94613	2.1
59915	0.94613	2.1
59916	0.94613	2.1
59917	0.94613	2.1
59918	0.946131	2.1
59919	0.946131	2.1
59920	1.89226	0.05
59921	1.89226	0.0572721
59922	1.89226	0.0573124
59923	1.89226	0.05
59924	1.89226	0.05
59925	1.89226	0.05
59926	1.89226	0.05
59927	1.89226	0.254789
59928	1.89226	0.0713221
59929	1.89226	0.14878
59930	3.78453	0.248396
59931	0.946133	0.0542143
59932	0.946133	0.05
59933	0.946132	0.05
59934	0.946132	0.05
59935	0.946133	0.0581048
59936	0.946133	0.05889
59937	0.946132	0.0609852
59938	0.946132	0.05
59939	0.946132	0.05
59940	0.946132	0.0734624
59941	0.946132	0.05
59942	0.946132	0.112533
59943	2.23638	0.05
59944	1.89226	0.05
59945	2.66014	0.05
59946	2.66014	0.0520469
59947	1.89226	0.05
59948	1.89226	0.05
59949	1.11819	0.05
59950	1.33007	0.05
59951	0.660255	0.05
59952	0.91151	0.05
59953	0.911511	0.05
59954	1.33007	0.05
59955	1.11819	0.05
59956	0.660255	0.05
59957	0.985327	0.05
59958	0.985327	0.05
59959	1.005 0.05	
59960	1.005 0.05	
59961	1.89226	2.1
59962	1.89226	2.1
59963	1.89226	2.1
59964	1.89226	2.1
59965	3.78453	2.1
59966	1.59533	2.1
59967	2.68571	2.1
59968	1.28291	2.1
59969	2.46974	2.1

59970	4.06506	2.1
59971	2.68571	2.1
59972	1.89226	2.1
59973	1.89226	2.1
59974	1.89226	2.1
59975	2.46973	2.1
59976	2.46973	2.1
59977	1.89226	2.1
59978	1.89226	2.1
59979	1.89226	2.1
59980	1.89226	2.1
59981	1.89226	2.1
59982	1.89226	2.1
59983	1.89226	2.1
59984	1.89226	2.1
59985	1.89226	2.1
59986	1.89226	2.1
59987	1.89226	2.1
59988	1.89226	2.1
59989	0.883594	2.1
59990	0.883594	2.1
59991	0.876484	2.1
59992	0.876484	2.1
59993	1.10442	2.1
59994	1.23487	2.1
59995	0.641457	2.1
59996	0.52521	2.1
59997	1.10442	2.1
59998	0.52521	2.1
59999	0.641457	2.1
60000	1.23487	2.1
60001	0.946131	2.1
60002	0.946131	2.1
60003	0.946131	2.1
60004	0.946131	2.1
60005	0.946131	2.1
60006	0.946131	2.1
60007	0.946131	2.1
60008	0.946131	2.1
60009	0.946131	2.1
60010	0.946131	2.1
60011	0.946131	2.1
60012	0.946131	2.1
60013	2.04016	0.05
60014	2.13042	0.05
60015	2.11465	0.05
60016	2.11465	0.05
60017	1.95908	0.05
60018	1.95908	0.05
60019	1.66953	0.05
60020	2.13041	0.05
60021	1.95908	0.05
60022	2.21952	0.05
60023	1.66953	0.05
60024	1.92997	0.05
60025	2.04016	0.05
60026	1.95908	0.05
60027	1.82014	0.05

60028	2.08757	0.05
60029	3.86029	0.05
60030	0.834764	0.05
60031	0.722102	0.05
60032	0.979537	0.05
60033	0.910065	0.05
60034	0.840053	0.05
60035	0.840052	0.05
60036	0.861587	0.05
60037	0.861587	0.05
60038	0.910067	0.05
60039	0.722101	0.05
60040	0.834763	0.05
60041	0.979539	0.05
60042	1.89226	0.05
60043	1.89226	0.05
60044	1.89226	0.05
60045	1.89226	0.05
60046	1.89226	0.05
60047	1.89226	0.05
60048	1.89226	0.05
60049	1.89226	0.05
60050	1.89226	0.05
60051	1.89226	0.05
60052	3.78453	0.05
60053	1.89226	0.05
60054	1.89226	0.05
60055	1.89226	0.05
60056	1.89226	0.05
60057	1.89226	0.05
60058	1.89226	0.05
60059	1.20691	0.05
60060	2.27379	0.05
60061	2.58476	0.05
60062	1.5124	0.05
60063	3.79167	0.05
60064	1.89226	0.05
60065	2.2738	0.05
60066	2.58477	0.05
60067	1.89226	0.05
60068	4.47703	0.05
60069	0.952225	1.09584
60070	0.952225	1.8688
60071	0.909865	2.06759
60072	0.909865	2.1
60073	0.909895	2.1
60074	0.946132	1.22931
60075	0.946132	2.04822
60076	0.915958	1.15457
60077	0.946132	1.99175
60078	0.946132	2.00958
60079	0.909894	1.89079
60080	0.915958	1.98683
60081	0.983493	2.1
60082	2.40055	2.1
60083	1.02986	2.1
60084	1.02986	2.1
60085	2.34217	2.1

60086	2.34217	2.1
60087	1.89226	2.1
60088	2.40056	2.1
60089	2.34217	2.1
60090	2.40056	2.1
60091	1.83388	2.1
60092	1.89226	2.1
60093	1.89226	2.1
60094	1.89226	2.1
60095	1.89226	2.1
60096	1.89226	2.1
60097	1.89226	2.1
60098	1.89226	2.1
60099	1.89226	2.1
60100	1.89226	2.1
60101	1.89226	2.1
60102	1.89226	2.1
60103	1.89226	2.1
60104	1.89226	2.1
60105	0.946133	2.1
60106	0.946133	2.1
60107	1.17109	2.1
60108	1.13078	2.1
60109	0.946132	2.1
60110	0.946132	2.1
60111	1.13078	2.1
60112	1.13078	2.1
60113	1.13078	2.1
60114	0.946133	2.1
60115	0.946132	2.1
60116	1.17109	2.1
60117	1.89226	2.1
60118	1.89226	2.1
60119	1.89226	2.1
60120	1.89226	2.1
60121	1.89226	2.1
60122	1.89226	2.1
60123	2.33963	1.1673
60124	1.89227	0.332255
60125	1.74565	0.517896
60126	1.74565	0.592989
60127	1.89227	0.100136
60128	1.89227	0.186243
60129	1.89227	0.720449
60130	1.89227	0.377448
60131	1.89227	1.51132
60132	1.89227	0.69432
60133	3.78453	0.628018
60134	1.31398	0.295604
60135	1.10392	0.42873
60136	0.796878	0.232455
60137	0.524356	0.145146
60138	0.870614	0.291652
60139	0.945739	0.122499
60140	0.89208	0.328621
60141	0.89208	0.914196
60142	1.23425	0.232717
60143	0.774658	0.724316

60144	1.03182	0.180155
60145	0.557289	0.121267
60146	2.33963	0.874631
60147	1.89226	0.0826085
60148	1.89226	1.09615
60149	2.77245	0.223718
60150	4.23189	0.664346
60151	1.89226	1.27226
60152	1.89226	0.378579
60153	1.89226	1.14423
60154	1.89226	0.995642
60155	1.89226	0.467135
60156	1.89226	0.375311
60157	1.89226	0.05
60158	1.89226	0.064918
60159	1.89226	0.05
60160	1.89226	0.170154
60161	1.89226	0.05
60162	1.89226	0.135581
60163	1.89226	0.05
60164	1.89226	0.05
60165	1.89226	0.05
60166	1.89226	0.0921619
60167	1.89226	0.05
60168	1.89226	0.060103
60169	0.946128	0.05
60170	0.946128	0.05
60171	0.963918	0.05
60172	1.37543	0.05
60173	0.946129	0.05
60174	0.946129	0.05
60175	1.37542	0.05
60176	1.37542	0.05
60177	0.94613	0.05
60178	1.37542	0.05
60179	0.963916	0.05
60180	0.94613	0.05
60181	0.946132	0.05
60182	0.946132	0.05
60183	0.946131	0.05
60184	0.946131	0.05
60185	0.946131	0.05
60186	0.946131	0.05
60187	0.946132	0.05
60188	0.946132	0.05
60189	0.946131	0.05
60190	0.946131	0.05
60191	0.946131	0.05
60192	0.946131	0.05
60193	1.89226	0.05
60194	1.89226	0.0666249
60195	1.89226	0.05
60196	1.89226	0.127319
60197	3.78452	0.05
60198	0.946127	0.0667377
60199	0.946127	0.05
60200	0.946131	0.05
60201	0.946131	0.05

60202	0.946128	0.05
60203	0.946128	0.0639513
60204	0.94613	0.05
60205	0.94613	0.05
60206	0.946129	0.05
60207	0.946129	0.05
60208	0.946129	0.05
60209	0.946129	0.05
60210	1.89226	0.144339
60211	1.89226	0.474573
60212	1.89226	2.1
60213	1.89226	0.05
60214	3.78453	0.05
60215	1.89226	0.0921032
60216	1.89226	0.05
60217	1.89226	0.05
60218	1.89226	0.05
60219	3.78453	0.0634414
60220	0.946131	0.05
60221	0.946131	0.05
60222	0.946132	0.692288
60223	0.946132	0.0732524
60224	0.946132	0.499968
60225	0.946132	0.05
60226	0.946132	0.519828
60227	0.946132	0.05
60228	0.946132	0.0759693
60229	0.946132	0.149558
60230	0.946132	0.202995
60231	0.946132	0.0610554
60232	0.548169	0.05
60233	0.736603	0.05
60234	1.18322	0.05
60235	1.01139	0.05
60236	1.01139	0.05
60237	0.548168	0.05
60238	0.736603	0.05
60239	1.18322	0.05
60240	0.861589	0.05
60241	0.861589	0.05
60242	0.869798	0.05
60243	0.869798	0.05
60244	1.16037	0.05
60245	0.942012	0.05
60246	0.942012	0.05
60247	0.62356	0.05
60248	0.891932	0.05
60249	0.900255	0.05
60250	2.02279	0.05
60251	1.48677	0.05
60252	1.54724	0.05
60253	0.831456	0.05
60254	1.19775	0.05
60255	1.89226	1.34783
60256	2.76291	1.23814
60257	1.89226	1.65399
60258	1.89226	1.14418
60259	3.78452	1.39038

60260	1.89226	0.729677
60261	1.89226	1.43329
60262	1.89226	1.84539
60263	1.89226	0.82034
60264	1.89226	1.8146
60265	1.89226	0.761289
60266	1.89226	1.612
60267	1.89226	0.886665
60268	1.89226	1.34002
60269	1.89226	1.12758
60270	3.78452	1.18332
60271	1.89226	0.601147
60272	1.89226	1.53703
60273	1.89226	1.67424
60274	1.89226	0.996336
60275	3.78453	1.34079
60276	0.946128	0.891809
60277	0.946128	1.30201
60278	0.946129	1.60615
60279	0.946129	1.23417
60280	0.946132	0.55403
60281	0.946132	1.37881
60282	0.946126	1.42485
60283	0.946126	0.988707
60284	0.946129	1.32371
60285	0.946129	1.24241
60286	0.946129	1.19998
60287	0.946129	1.0289
60288	2.80495	1.12254
60289	1.89225	0.766763
60290	1.89225	1.46878
60291	1.87794	1.49717
60292	1.89225	1.1466
60293	1.87794	1.08893
60294	2.25195	0.102882
60295	2.00624	0.0788912
60296	2.13132	0.0985529
60297	1.90239	0.05
60298	4.13756	0.05
60299	0.804661	0.0684068
60300	0.804663	0.05
60301	0.889591	0.05
60302	0.889591	0.19069
60303	0.808324	0.05
60304	0.761765	0.0672631
60305	1.02822	0.0742831
60306	0.879624	0.05
60307	0.879617	0.05
60308	0.800864	0.05
60309	0.978023	0.0568552
60310	0.808329	0.05
60311	2.13133	0.11334
60312	2.00558	0.05
60313	2.09316	0.05
60314	2.09316	0.284589
60315	2.03253	0.05
60316	2.03253	1.0764
60317	0.84577	0.05

60318	0.845767	0.0558785
60319	0.843917	0.05
60320	0.843917	0.507186
60321	0.76177	0.05
60322	0.719345	0.05
60323	0.934435	0.05
60324	0.884197	0.05
60325	0.934439	0.05
60326	0.75627	0.05
60327	0.849424	0.05
60328	0.76177	0.05
60329	2.61855	0.05
60330	1.17793	0.05
60331	1.42965	0.05
60332	1.17793	0.05
60333	2.50057	0.05
60334	2.24885	0.05
60335	0.946133	0.05
60336	0.946133	0.05
60337	1.30928	0.05
60338	1.12443	0.05
60339	0.946132	0.05
60340	0.946132	0.05
60341	1.30928	0.05
60342	1.30928	0.05
60343	0.946132	0.05
60344	1.30928	0.05
60345	1.12443	0.05
60346	0.946133	0.05
60347	1.89226	0.05
60348	1.89226	0.05
60349	1.89226	0.05
60350	1.89226	0.05
60351	3.78452	0.05
60352	1.89226	0.05
60353	1.89226	0.05
60354	1.89226	0.05
60355	1.89226	0.05
60356	1.89226	0.05
60357	1.89226	0.05
60358	1.89226	0.05
60359	1.89226	0.05
60360	1.89226	0.05
60361	1.89226	0.05
60362	3.78452	0.05
60363	0.94613	0.05
60364	0.94613	0.05
60365	0.946131	0.05
60366	0.946131	0.05
60367	0.946128	0.05
60368	0.946128	0.05
60369	0.946133	0.05
60370	0.946133	0.05
60371	0.946131	0.05
60372	0.946131	0.05
60373	0.946131	0.05
60374	0.946131	0.05
60375	1.89226	2.1

60376	1.89226	2.1
60377	1.89226	2.1
60378	1.89226	2.1
60379	3.78453	2.1
60380	1.0465	2.1
60381	2.73926	2.1
60382	2.03721	2.1
60383	2.03721	2.1
60384	1.44709	2.1
60385	1.44709	2.1
60386	0.946131	2.1
60387	0.946131	2.1
60388	0.946131	2.1
60389	0.946131	2.1
60390	0.946132	2.1
60391	0.946132	2.1
60392	0.94613	2.1
60393	0.94613	2.1
60394	0.946131	2.1
60395	0.946131	2.1
60396	0.946131	2.1
60397	0.946131	2.1
60398	0.946132	2.1
60399	0.946132	2.1
60400	0.946131	2.1
60401	0.946131	2.1
60402	0.946132	2.1
60403	0.946132	2.1
60404	0.946132	2.1
60405	0.946132	2.1
60406	0.946132	2.1
60407	0.946132	2.1
60408	0.946132	2.1
60409	0.946132	2.1
60410	1.89226	2.1
60411	1.89226	2.1
60412	1.89226	2.1
60413	1.89226	2.1
60414	3.78452	2.1
60415	1.61969	0.0523026
60416	1.79374	0.05
60417	1.29382	0.05
60418	1.29382	0.05
60419	2.05759	0.05
60420	2.05759	0.05
60421	1.89226	0.05
60422	1.89226	0.05
60423	1.89226	0.05
60424	1.89226	0.05
60425	1.89226	0.05
60426	1.89226	0.05
60427	1.89226	0.0755795
60428	1.89226	0.174462
60429	1.89226	0.0808104
60430	1.89226	0.0859861
60431	1.89226	0.0664954
60432	1.89226	0.0708589
60433	1.89226	1.31893

60434	1.89226	0.330993
60435	1.89226	0.360739
60436	1.89226	0.840676
60437	3.78453	1.26154
60438	0.946131	0.190832
60439	0.946131	0.204345
60440	0.946131	0.0750916
60441	0.946131	0.209288
60442	0.946131	0.136465
60443	0.946131	0.0842466
60444	0.946131	0.221352
60445	0.946131	0.307162
60446	0.946131	0.117201
60447	0.946131	0.10679
60448	0.946131	0.219924
60449	0.946131	0.149234
60450	2.45177	0.05
60451	2.68507	0.05
60452	2.3376	0.05
60453	2.22086	0.05
60454	4.78937	0.05
60455	2.45178	0.05
60456	2.35552	0.05
60457	1.68062	0.05
60458	1.81902	0.05
60459	4.13239	0.05
60460	2.3376	0.05
60461	1.55612	0.05
60462	1.69811	0.05
60463	2.31527	0.05
60464	1.79553	0.05
60465	2.3376	0.05
60466	1.55613	0.05
60467	1.06336	0.05
60468	1.97516	0.05
60469	1.88966	0.05
60470	1.06336	0.05
60471	1.68062	0.05
60472	1.89226	0.705857
60473	1.91634	0.187607
60474	2.03709	1.56656
60475	1.91634	0.565869
60476	1.83185	0.351907
60477	1.70303	0.05
60478	1.89226	0.2198
60479	1.89226	0.143547
60480	1.89226	1.32438
60481	1.89226	0.286113
60482	1.89226	0.148166
60483	1.89226	0.05
60484	1.89226	1.91049
60485	2.03472	2.1
60486	2.22183	2.1
60487	2.03472	1.49868
60488	2.07936	2.1
60489	1.89226	0.275524
60490	1.89226	1.17068
60491	1.8519	0.0800594

60492	1.92119	0.931581
60493	1.8519	0.420287
60494	1.96156	0.109988
60495	1.89226	0.05
60496	1.89226	0.260829
60497	1.89226	0.137811
60498	1.89226	1.25576
60499	1.89226	0.346473
60500	1.89226	0.0877428
60501	1.89226	0.05
60502	1.89226	1.39662
60503	2.22183	1.21164
60504	2.39541	2.1
60505	2.22183	0.803151
60506	2.06585	1.34022
60507	1.89226	0.190938
60508	1.47001	2.1
60509	1.57962	0.186831
60510	1.65238	1.39701
60511	1.60502	2.0888
60512	3.18464	2.1
60513	1.02415	2.1
60514	1.60503	2.1
60515	1.47001	2.1
60516	1.47001	2.1
60517	1.28228	2.1
60518	1.28228	2.1
60519	0.946133	2.1
60520	0.946133	2.1
60521	0.946132	2.1
60522	0.946132	1.34691
60523	0.946132	1.38548
60524	0.946132	2.1
60525	0.946133	2.1
60526	0.946133	2.1
60527	0.946132	2.1
60528	0.946132	2.1
60529	0.946133	2.1
60530	0.946133	2.1
60531	1.89226	0.184541
60532	1.89226	0.922673
60533	1.89226	2.05312
60534	1.89226	0.0692409
60535	3.78452	0.214564
60536	1.76897	1.09975
60537	1.60502	1.59875
60538	1.65238	0.395114
60539	1.65238	2.1
60540	1.76932	0.311296
60541	1.76932	2.1
60542	1.55998	2.1
60543	1.60503	1.95169
60544	1.76932	2.1
60545	1.60503	2.1
60546	1.44657	2.1
60547	1.28228	2.1
60548	0.946133	2.1
60549	0.946133	2.1

60550	0.946132	2.1
60551	0.946132	0.419377
60552	0.946132	0.36182
60553	0.946132	2.1
60554	0.946133	1.94309
60555	0.946133	2.1
60556	0.946132	2.1
60557	0.946132	2.1
60558	0.946132	2.1
60559	0.946132	2.1
60560	0.946128	1.67157
60561	0.946128	0.216163
60562	0.946133	0.0567983
60563	0.946133	0.178789
60564	0.94613	0.05726
60565	0.94613	0.207367
60566	0.946131	1.66484
60567	0.946131	0.290944
60568	0.946131	0.193696
60569	0.946131	0.170248
60570	0.946131	0.339869
60571	0.946131	0.316752
60572	1.85779	0.05
60573	1.00763	0.05
60574	1.74874	0.05
60575	1.74873	0.05
60576	1.14467	0.05
60577	1.14466	0.05
60578	1.85779	0.05
60579	1.61597	0.05
60580	1.00053	0.05
60581	1.03018	0.05
60582	2.65819	0.05
60583	1.89226	0.05
60584	1.89226	0.05
60585	1.89226	0.0593298
60586	1.89226	0.05
60587	3.78451	0.05
60588	1.89226	0.0508151
60589	1.89226	0.05
60590	1.89226	0.05
60591	1.89226	0.05
60592	1.89226	0.05
60593	1.89226	0.05
60594	1.11983	0.05
60595	0.810945	0.05
60596	0.913906	0.05
60597	0.913906	0.05
60598	2.23303	0.05
60599	1.66057	0.05
60600	1.28724	0.05
60601	1.08132	0.05
60602	1.49316	0.05
60603	0.810958	0.05
60604	0.810958	0.05
60605	0.827861	0.05
60606	0.946126	0.05
60607	0.946132	0.05

60608	0.946132	0.05
60609	0.827863	0.05
60610	0.827863	0.05
60611	0.946129	0.05
60612	0.946129	0.05
60613	0.827863	0.05
60614	0.946128	0.05
60615	0.946129	0.05
60616	0.946129	0.05
60617	0.974718	0.05
60618	1.53671	0.05
60619	2.80542	0.05
60620	2.27259	0.05
60621	3.8093	0.05
60622	1.89226	0.05
60623	1.89226	0.0639252
60624	1.89226	0.05
60625	1.89226	0.0541397
60626	3.78453	0.05
60627	0.946132	0.05
60628	0.946132	0.05
60629	0.946131	0.05
60630	0.946131	0.05
60631	0.946132	0.05
60632	0.946132	0.0559359
60633	0.946131	0.05
60634	0.946131	0.05
60635	0.946132	0.05
60636	0.946132	0.05
60637	0.946131	0.05
60638	0.946131	0.05
60639	2.80542	0.05
60640	1.89226	0.05
60641	2.27259	0.05
60642	2.27259	0.05
60643	1.89226	0.05
60644	1.89226	0.05
60645	1.89226	0.05
60646	1.89226	0.0585189
60647	1.89226	0.05
60648	1.89226	0.0542349
60649	1.89226	0.05
60650	1.89226	0.0557273
60651	0.946132	0.0573499
60652	0.946132	0.05
60653	0.946131	0.0500512
60654	0.946131	0.05
60655	0.946132	0.0510535
60656	0.946132	0.0504702
60657	0.946131	0.05
60658	0.946131	0.05
60659	0.946131	0.05
60660	0.946131	0.05
60661	0.946131	0.05
60662	0.946131	0.05
60663	2.68213	2.1
60664	2.58234	2.1
60665	2.58234	2.1

60666	2.61243	2.1
60667	2.67035	2.1
60668	2.70044	2.1
60669	2.68213	2.1
60670	2.53921	2.1
60671	2.53303	2.1
60672	2.67035	2.1
60673	5.20956	2.1
60674	2.67033	2.1
60675	2.58232	2.1
60676	2.55004	2.1
60677	2.64567	2.1
60678	5.228 2.1	
60679	2.53301	2.1
60680	2.64567	2.1
60681	2.64567	2.1
60682	2.67033	2.1
60683	2.51603	2.1
60684	2.54069	2.1
60685	0.907497	2.1
60686	0.907497	2.1
60687	0.926641	2.1
60688	0.926641	2.1
60689	1.14989	2.1
60690	1.10046	2.1
60691	0.695334	2.1
60692	0.684247	2.1
60693	0.695333	2.1
60694	1.14989	2.1
60695	0.684246	2.1
60696	1.10046	2.1
60697	0.946126	1.69076
60698	0.946126	2.1
60699	0.946131	2.1
60700	0.946131	2.1
60701	0.946127	2.1
60702	0.946127	2.1
60703	0.94613	2.1
60704	0.94613	2.1
60705	0.946128	2.1
60706	0.946128	2.1
60707	0.946128	2.1
60708	0.946128	2.1
60709	1.89226	2.1
60710	1.89226	2.1
60711	1.89226	2.1
60712	1.89226	1.59551
60713	1.89226	2.1
60714	1.89226	0.463599
60715	1.42174	2.1
60716	2.29978	2.1
60717	2.41233	2.1
60718	2.41233	2.1
60719	1.39067	2.1
60720	1.39067	2.1
60721	1.89226	2.1
60722	1.89226	2.1
60723	1.89226	2.1

60724	1.89226	2.1
60725	3.78452	2.1
60726	1.89226	2.1
60727	1.89226	2.1
60728	1.89226	2.1
60729	1.89226	2.1
60730	1.89226	2.1
60731	1.89226	2.1
60732	2.41234	2.1
60733	1.89226	2.1
60734	2.29979	2.1
60735	1.89226	2.1
60736	1.89226	2.1
60737	2.29979	2.1
60738	1.09506	0.05
60739	2.82921	0.05
60740	2.53251	0.05
60741	2.53251	0.05
60742	1.42901	0.05
60743	1.42901	0.05
60744	0.946132	0.05
60745	0.946132	0.05
60746	0.946128	0.05
60747	0.946128	0.05
60748	0.94613	0.05
60749	0.94613	0.05
60750	0.946131	0.05
60751	0.946131	0.05
60752	0.94613	0.05
60753	0.94613	0.05
60754	0.94613	0.05
60755	0.94613	0.05
60756	1.89226	0.05
60757	1.89226	0.0601901
60758	1.89226	0.0601996
60759	1.89226	0.0922698
60760	3.78452	0.0577528
60761	2.53251	0.05
60762	1.89226	0.0502539
60763	2.82921	0.05
60764	1.89226	0.05
60765	1.89226	0.0937463
60766	2.82921	0.0903723
60767	1.89226	0.05
60768	1.89226	0.05
60769	1.89226	0.0535857
60770	1.89226	0.050531
60771	1.89226	0.05
60772	1.89226	0.05
60773	2.53251	0.05
60774	1.89226	0.05
60775	2.06158	0.05
60776	1.89226	0.05
60777	4.42477	0.05
60778	1.23296	2.1
60779	2.55352	2.1
60780	2.47476	2.1
60781	2.47476	2.1

60782	1.32368	2.1
60783	1.32368	2.1
60784	1.89226	2.1
60785	1.89226	2.1
60786	1.89226	2.1
60787	1.89226	2.1
60788	1.89226	2.1
60789	1.89226	2.1
60790	1.89226	2.1
60791	1.89226	2.1
60792	1.89226	2.1
60793	1.89226	2.1
60794	1.89226	2.1
60795	1.89226	2.1
60796	2.47476	2.1
60797	1.89226	2.1
60798	1.89226	2.1
60799	1.89226	2.1
60800	2.55353	2.1
60801	2.55353	2.1
60802	1.05983	2.1
60803	2.47476	2.1
60804	2.37664	2.1
60805	2.37664	2.1
60806	1.23296	2.1
60807	1.23296	2.1
60808	0.946127	2.1
60809	0.946127	2.1
60810	0.946131	2.1
60811	0.946131	2.1
60812	0.946127	2.1
60813	0.946127	2.1
60814	0.946131	2.1
60815	0.946131	2.1
60816	0.946129	2.1
60817	0.946129	2.1
60818	0.946128	2.1
60819	0.946128	2.1
60820	1.89226	2.1
60821	1.89226	2.1
60822	1.89226	2.1
60823	1.89226	1.69792
60824	3.78452	2.1
60825	2.37665	2.1
60826	1.89226	2.1
60827	1.89226	2.1
60828	1.89226	2.1
60829	2.47476	2.1
60830	2.47476	2.1
60831	1.89226	1.00911
60832	1.92119	0.0582552
60833	1.99586	0.822176
60834	1.92119	0.417006
60835	1.96693	0.0788226
60836	1.89226	0.05
60837	1.89226	0.735284
60838	1.89226	0.0705655
60839	1.89226	1.0453

60840	1.89226	0.173251
60841	1.89226	0.108772
60842	1.89226	0.05
60843	1.89226	1.03108
60844	2.39541	0.400079
60845	2.57183	1.71793
60846	2.39541	0.2919
60847	2.06868	0.292142
60848	1.89226	0.0712479
60849	0.946133	0.262297
60850	0.946133	0.238586
60851	0.997928	0.131036
60852	1.03763	0.242253
60853	0.946131	0.540009
60854	0.946131	0.05
60855	1.03763	0.345585
60856	1.03763	0.071079
60857	1.03763	0.310279
60858	0.946131	0.508707
60859	0.94613	0.271816
60860	0.99793	0.0648034
60861	1.89226	0.05
60862	1.89226	0.361863
60863	1.89226	0.479507
60864	1.89226	0.05
60865	1.89226	0.915614
60866	1.89226	0.05
60867	0.946131	0.125868
60868	0.946131	0.151884
60869	1.36939	0.118877
60870	1.28592	0.380026
60871	0.946129	0.330737
60872	0.946129	0.05
60873	1.36939	0.240177
60874	1.36939	0.238976
60875	0.946131	0.139912
60876	1.36939	0.124217
60877	0.946131	0.12789
60878	1.28592	0.05
60879	1.89226	0.0940407
60880	1.89226	0.541117
60881	1.89226	0.214399
60882	1.89226	0.210822
60883	3.78453	0.288654
60884	1.89226	0.558644
60885	1.89226	0.605228
60886	1.89226	0.228699
60887	1.89226	0.280015
60888	3.78452	0.341768
60889	1.91005	0.569493
60890	1.89226	0.865584
60891	2.60215	0.339446
60892	2.60215	0.503344
60893	1.89226	0.48802
60894	1.89226	0.990819
60895	0.946132	0.518754
60896	0.946132	0.362433
60897	0.946132	0.542849

60898	0.946132	0.279491
60899	0.946132	0.464291
60900	0.946132	0.573356
60901	0.946132	0.376102
60902	0.946132	0.228739
60903	0.946132	0.436888
60904	0.946132	0.397056
60905	0.946131	0.335557
60906	0.946131	0.361361
60907	1.89226	0.46369
60908	1.89226	0.606617
60909	1.89226	1.36919
60910	1.89226	0.586821
60911	1.89226	0.305366
60912	1.89226	1.0418
60913	0.780483	0.389604
60914	0.78928	1.06873
60915	0.818566	0.382076
60916	0.460387	0.984434
60917	1.82793	0.176238
60918	1.32039	0.761076
60919	0.613889	0.183466
60920	1.0592	0.793543
60921	1.48698	0.934376
60922	0.818555	0.446754
60923	1.11515	0.98324
60924	1.2802	1.44369
60925	2.20046	0.115584
60926	1.1475	0.640074
60927	1.1475	0.109712
60928	2.27062	0.36381
60929	2.27062	0.114795
60930	1.89226	0.763075
60931	2.20046	0.138763
60932	2.27062	0.195184
60933	2.20046	0.0890289
60934	1.96242	0.189101
60935	1.89226	0.0807179
60936	1.89226	0.134357
60937	1.89226	0.0567001
60938	1.89226	0.588439
60939	1.89226	0.0893385
60940	3.78452	0.0746117
60941	1.89226	0.109288
60942	1.89226	0.05
60943	1.89226	0.464634
60944	1.89226	0.157352
60945	3.78452	0.188787
60946	1.17403	0.0722808
60947	1.13531	0.210685
60948	0.6401	0.139031
60949	0.714566	0.445129
60950	0.915987	0.191716
60951	0.915987	0.05
60952	0.933891	1.2255
60953	0.933891	0.144349
60954	1.17403	0.262265
60955	0.714566	0.0678856

60956	0.6401	0.259969
60957	1.13531	0.116085
60958	0.946133	0.076083
60959	0.946133	0.128005
60960	1.17403	0.0648075
60961	1.13531	0.0970415
60962	0.946131	0.0858771
60963	0.946131	0.05
60964	1.17403	0.234454
60965	1.17403	0.05
60966	1.17403	0.0984447
60967	0.94613	0.102019
60968	0.946131	0.277111
60969	1.13531	0.0618659
60970	1.89226	0.055729
60971	1.89226	0.076567
60972	1.89226	0.05
60973	1.89226	0.05
60974	1.89226	0.24234
60975	1.89226	0.0647986
60976	0.946131	0.0699282
60977	0.946131	0.0554251
60978	0.946131	0.100964
60979	0.946131	0.112934
60980	0.946129	0.151602
60981	0.946129	0.05
60982	0.946133	0.211487
60983	0.946133	0.05
60984	0.946131	0.0726956
60985	0.946131	0.0858179
60986	0.946131	0.05
60987	0.946131	0.0628506
60988	2.29413	2.1
60989	1.99332	2.1
60990	1.84774	2.1
60991	2.21582	2.1
60992	4.20915	2.1
60993	0.517359	2.1
60994	0.634469	2.1
60995	0.996661	2.1
60996	0.92387	2.1
60997	0.768098	2.1
60998	0.768098	2.1
60999	0.790242	2.1
61000	0.790242	2.1
61001	0.923871	2.1
61002	0.517359	2.1
61003	0.634469	2.1
61004	0.996661	2.1
61005	1.89226	2.1
61006	1.89226	2.1
61007	1.89226	2.1
61008	1.89226	2.1
61009	3.78453	2.1
61010	0.946132	2.1
61011	0.946132	2.1
61012	0.946131	2.1
61013	0.946131	2.1

61014	0.946132	2.1
61015	0.946132	2.1
61016	0.946131	2.1
61017	0.946131	2.1
61018	0.946132	2.1
61019	0.946132	2.1
61020	0.946131	2.1
61021	0.946131	2.1
61022	1.62708	2.1
61023	2.21582	2.1
61024	2.13925	2.1
61025	2.21582	2.1
61026	1.77117	2.1
61027	1.84774	2.1
61028	0.946132	2.1
61029	0.946132	2.1
61030	0.946131	2.1
61031	0.946131	2.1
61032	0.946132	2.1
61033	0.946132	2.1
61034	0.946131	2.1
61035	0.946131	2.1
61036	0.946131	2.1
61037	0.946131	2.1
61038	0.946131	2.1
61039	0.946131	2.1
61040	1.89226	1.91142
61041	1.89226	0.291949
61042	1.89226	0.186123
61043	1.89226	0.979496
61044	3.78453	1.33728
61045	1.89226	0.233474
61046	1.89226	1.42632
61047	1.89226	1.51926
61048	1.89226	2.1
61049	1.89226	0.116455
61050	1.89226	0.814485
61051	1.89226	0.245886
61052	1.89226	2.1
61053	1.89226	0.0881121
61054	1.89226	1.74929
61055	1.89226	0.177757
61056	1.89226	2.1
61057	0.914369	0.688303
61058	0.914369	1.08601
61059	0.810945	2.1
61060	0.810945	0.944325
61061	2.26581	2.1
61062	1.66199	2.1
61063	1.49503	1.01647
61064	1.28816	2.1
61065	1.08133	1.65814
61066	0.810933	1.77969
61067	1.1212	1.68032
61068	1.89227	0.38476
61069	1.89227	0.257506
61070	1.89227	0.131316
61071	1.89227	0.507922

61072	3.78453	0.201107
61073	1.89226	0.112337
61074	1.89226	0.737216
61075	1.89226	0.362051
61076	1.89226	0.257697
61077	3.78453	0.413255
61078	1.89226	0.713823
61079	1.89226	0.34421
61080	1.89226	0.251145
61081	1.89226	0.94733
61082	3.78453	0.39312
61083	1.89226	0.265848
61084	1.89226	1.1755
61085	1.89226	0.488119
61086	2.25318	0.328579
61087	3.78453	0.873999
61088	1.89226	0.598109
61089	1.89226	1.10424
61090	1.89226	0.35772
61091	1.9637	0.305729
61092	3.78453	0.714136
61093	1.89226	0.661992
61094	1.89226	0.583988
61095	1.89226	0.245011
61096	1.89226	0.812564
61097	3.78453	0.429072
61098	1.89226	0.57964
61099	1.89226	1.58876
61100	1.89226	0.766135
61101	2.53384	0.59588
61102	3.78453	1.09319
61103	1.55405	0.586123
61104	1.03818	1.57331
61105	1.03818	0.0671544
61106	1.34857	0.167583
61107	1.46338	0.142198
61108	1.77377	1.47271
61109	1.86273	0.185318
61110	1.73802	0.291891
61111	1.50969	1.96006
61112	1.46337	1.29452
61113	3.20139	0.348426
61114	1.2377	1.28451
61115	1.46337	0.298791
61116	1.27408	0.322141
61117	1.32039	1.94357
61118	1.50969	0.772524
61119	1.50969	0.15904
61120	1.89226	2.1
61121	1.89226	2.1
61122	1.89226	2.1
61123	1.89226	2.1
61124	1.89226	2.1
61125	1.89226	1.52994
61126	1.89169	2.1
61127	1.89226	2.1
61128	1.89226	2.1
61129	1.89226	2.1

61130	1.96624	2.1
61131	1.96624	2.1
61132	2.62871	2.1
61133	1.6201	2.1
61134	1.49149	2.1
61135	1.6201	2.1
61136	2.62559	2.1
61137	2.75419	2.07937
61138	0.946129	0.289443
61139	0.946129	2.1
61140	0.946133	2.1
61141	0.946133	2.1
61142	0.946131	2.1
61143	0.946131	0.11028
61144	0.946131	2.1
61145	0.946131	2.1
61146	0.946131	2.1
61147	0.946131	2.1
61148	0.946131	2.1
61149	0.946131	2.1
61150	0.851543	2.1
61151	0.851543	2.1
61152	1.04043	2.1
61153	0.997939	2.1
61154	0.946132	2.1
61155	0.946132	2.1
61156	0.894185	2.1
61157	0.893901	2.1
61158	0.998267	2.1
61159	1.04074	2.1
61160	0.85127	2.1
61161	0.85153	2.1
61162	1.89226	2.1
61163	1.89226	2.1
61164	1.89226	2.1
61165	1.89226	2.1
61166	1.89226	2.1
61167	1.89226	2.1
61168	0.928691	2.1
61169	0.928691	2.1
61170	0.935332	2.1
61171	0.935332	2.1
61172	1.398	2.1
61173	1.14418	2.1
61174	0.479331	2.1
61175	0.719848	2.1
61176	1.398	2.1
61177	0.719848	2.1
61178	1.14417	2.1
61179	0.47933	2.1
61180	2.28835	2.1
61181	1.89226	2.1
61182	1.89226	2.1
61183	1.89226	2.1
61184	2.796	2.1
61185	2.796	2.1
61186	0.946131	2.1
61187	0.946131	2.1

61188	0.946131	2.1
61189	0.946131	2.1
61190	0.946128	2.1
61191	0.946128	2.1
61192	0.946133	2.1
61193	0.946133	2.1
61194	0.946131	2.1
61195	0.946131	2.1
61196	0.94613	2.1
61197	0.94613	2.1
61198	0.946131	2.1
61199	0.946131	2.1
61200	0.946131	2.1
61201	0.946131	2.1
61202	0.946131	2.1
61203	0.946131	2.1
61204	0.946131	2.1
61205	0.946131	2.1
61206	0.946131	2.1
61207	0.946131	2.1
61208	0.946131	2.1
61209	0.946131	2.1
61210	1.89226	2.1
61211	1.89226	2.1
61212	1.89226	2.1
61213	1.89226	2.1
61214	3.78452	2.1
61215	2.05207	0.0563705
61216	1.70302	0.14322
61217	1.33794	2.06092
61218	1.98194	0.502688
61219	3.36873	0.353176
61220	2.62761	0.250743
61221	1.98194	0.4396
61222	2.28009	1.41994
61223	2.28009	0.0971881
61224	2.22939	1.3556
61225	2.22939	0.13254
61226	1.89226	0.587824
61227	1.89226	0.0783973
61228	1.89226	0.254565
61229	1.89226	0.108699
61230	1.89226	0.642898
61231	1.89226	0.439935
61232	1.89227	0.678491
61233	1.89227	0.178539
61234	1.89227	1.94969
61235	1.89227	0.853295
61236	1.89227	0.06814
61237	1.89227	0.05
61238	0.888355	0.06633
61239	0.990973	0.687966
61240	0.71133	0.205794
61241	0.634324	1.01028
61242	0.806209	0.693578
61243	0.806209	0.05
61244	0.819087	1.77535
61245	0.819087	0.774765

61246	0.888355	0.888617
61247	0.634324	1.11519
61248	0.71133	0.383236
61249	0.990972	0.441071
61250	1.11469	1.30953
61251	0.985023	0.453172
61252	0.888354	0.113935
61253	0.990972	1.26678
61254	0.994754	0.612213
61255	0.994754	0.173242
61256	1.0083	1.26135
61257	1.0083	0.0870592
61258	0.888354	0.153039
61259	0.985024	0.699749
61260	0.990971	0.178128
61261	1.11469	0.118657
61262	0.946132	0.842542
61263	0.946132	0.131281
61264	0.946132	0.25879
61265	0.946132	0.410365
61266	0.946132	0.39245
61267	0.946132	0.192854
61268	0.946133	1.64048
61269	0.946133	0.249315
61270	0.946133	0.616779
61271	0.946133	0.405546
61272	0.946132	0.334113
61273	0.946132	0.966019
61274	0.810944	0.276573
61275	0.810944	0.157539
61276	0.810947	0.623439
61277	0.810947	0.05
61278	1.89227	1.77254
61279	1.35168	0.228881
61280	1.08132	0.216623
61281	1.08131	0.347596
61282	1.08132	0.0565221
61283	0.810959	0.101341
61284	0.810959	0.100081
61285	1.70303	2.1
61286	2.03975	2.1
61287	1.99324	2.1
61288	2.17615	2.1
61289	1.40549	2.1
61290	1.53446	2.1
61291	0.946133	2.1
61292	0.946133	2.1
61293	0.946129	2.1
61294	0.946129	2.1
61295	0.946131	2.1
61296	0.946131	2.08343
61297	0.946131	2.1
61298	0.946131	2.1
61299	0.946131	2.1
61300	0.946131	2.1
61301	0.946131	2.1
61302	0.946131	2.1
61303	1.89226	2.1

61304	1.89226	2.1
61305	1.89226	2.1
61306	1.89226	2.1
61307	1.89226	2.1
61308	1.89226	2.1
61309	2.17615	2.1
61310	2.3111	2.1
61311	2.50288	2.1
61312	2.50288	2.1
61313	2.03975	2.1
61314	2.03975	2.1
61315	1.76401	2.1
61316	1.95852	2.1
61317	2.32134	2.1
61318	2.12306	2.1
61319	1.8513	2.1
61320	1.70303	2.1
61321	1.89226	2.1
61322	1.89226	2.1
61323	1.89226	2.1
61324	1.89226	2.1
61325	3.78452	2.1
61326	1.89226	2.1
61327	1.89226	0.623666
61328	1.89226	2.1
61329	1.89226	2.1
61330	3.78452	2.1
61331	1.2243	2.1
61332	1.2243	2.1
61333	1.24189	2.1
61334	1.24189	2.1
61335	1.37811	2.1
61336	1.25144	2.1
61337	1.08807	2.1
61338	1.17949	2.1
61339	1.17949	2.1
61340	1.37811	2.1
61341	1.08807	2.1
61342	1.25144	2.1
61343	1.89226	2.1
61344	1.89226	2.1
61345	1.89226	2.1
61346	1.89226	2.1
61347	1.89226	2.1
61348	1.89226	2.1
61349	1.89227	2.1
61350	1.89227	2.1
61351	1.89227	2.1
61352	1.89227	2.1
61353	1.89227	2.1
61354	1.89227	2.1
61355	1.89224	2.1
61356	1.89224	2.1
61357	1.89224	2.1
61358	1.89224	2.1
61359	3.78447	2.1
61360	1.89224	2.1
61361	1.89224	2.1

61362	1.89224	2.1
61363	1.89224	2.1
61364	1.89224	2.1
61365	1.89224	2.1
61366	1.89224	2.1
61367	1.89224	2.1
61368	1.89224	2.1
61369	1.89224	2.1
61370	3.78448	2.1
61371	1.89224	2.1
61372	1.703	2.1
61373	1.89224	2.1
61374	1.72991	2.1
61375	1.07643	2.1
61376	1.36657	2.1
61377	1.44472	2.1
61378	2.41233	2.1
61379	2.51628	2.1
61380	2.51628	2.1
61381	1.42174	2.1
61382	1.42174	2.1
61383	1.89226	2.1
61384	1.89226	2.1
61385	1.89226	2.1
61386	1.89226	2.1
61387	1.89226	2.1
61388	1.89226	2.1
61389	1.89226	2.1
61390	1.89226	2.1
61391	1.89226	2.1
61392	1.89226	1.00357
61393	3.78452	2.1
61394	2.51628	2.1
61395	1.89226	2.1
61396	1.89226	2.1
61397	1.89226	2.1
61398	2.41234	2.1
61399	2.41234	2.1
61400	0.996254	2.1
61401	0.996254	2.1
61402	1.01153	2.1
61403	1.01153	2.1
61404	1.28542	2.1
61405	1.25814	2.1
61406	0.719184	2.1
61407	0.72236	2.1
61408	0.719183	2.1
61409	1.28542	2.1
61410	0.722359	2.1
61411	1.25814	2.1
61412	0.946126	2.1
61413	0.946126	2.1
61414	0.946131	2.1
61415	0.946131	2.1
61416	0.946127	2.1
61417	0.946127	2.1
61418	0.94613	2.1
61419	0.94613	2.1

61420	0.946128	2.1
61421	0.946128	2.1
61422	0.946128	2.1
61423	0.946128	2.1
61424	1.89226	2.1
61425	1.89226	1.98851
61426	1.89226	2.1
61427	1.89226	2.1
61428	1.89226	2.1
61429	1.89226	2.1
61430	0.946132	2.1
61431	0.946132	2.1
61432	1.25814	2.1
61433	1.25814	2.1
61434	0.946131	2.1
61435	0.946131	2.1
61436	1.28543	2.1
61437	1.25814	2.1
61438	1.28543	2.1
61439	0.946132	2.1
61440	1.25814	2.1
61441	0.946132	2.1
61442	1.89226	1.1899
61443	1.89226	0.104164
61444	1.89226	0.862526
61445	1.89226	0.510447
61446	1.89226	0.296756
61447	1.89226	0.162317
61448	1.89226	0.896365
61449	2.27719	0.0653432
61450	1.89226	0.107476
61451	1.89226	0.242432
61452	3.78452	0.211257
61453	1.89225	1.36694
61454	1.89225	0.1404
61455	1.89225	0.528997
61456	1.89225	0.706413
61457	3.78451	0.668725
61458	1.89226	0.0793336
61459	1.89226	0.135536
61460	1.89226	0.46142
61461	1.89226	0.05
61462	3.78452	0.0894304
61463	0.946132	0.05
61464	0.946132	0.0895669
61465	0.946132	0.162817
61466	0.946132	0.085501
61467	0.946131	0.0648618
61468	0.946131	0.05
61469	0.946132	0.129489
61470	0.946132	0.05
61471	0.946132	0.0519352
61472	0.946132	0.067855
61473	0.946132	0.0560508
61474	0.946132	0.05
61475	0.946126	0.105874
61476	0.946126	0.0596937
61477	0.946132	1.24339

61478	0.946132	0.278892
61479	0.946128	0.0889283
61480	0.946128	0.207379
61481	0.946129	0.140712
61482	0.946129	0.631438
61483	0.946129	0.260495
61484	0.946129	0.407918
61485	0.946129	0.158817
61486	0.946129	0.175555
61487	1.39858	2.1
61488	1.39858	2.1
61489	0.94613	2.1
61490	0.94613	2.1
61491	0.946132	2.1
61492	0.946132	2.1
61493	1.39858	2.1
61494	1.25002	2.1
61495	1.39858	2.1
61496	0.946131	2.1
61497	1.25002	2.1
61498	0.946131	2.1
61499	2.0815	2.1
61500	1.89226	2.1
61501	1.89226	2.1
61502	2.05298	2.1
61503	2.5593	2.1
61504	2.77667	2.1
61505	0.946133	2.1
61506	0.946133	2.1
61507	0.946132	2.1
61508	0.946132	2.1
61509	0.946132	2.1
61510	0.946132	2.1
61511	0.946133	2.1
61512	0.946133	2.1
61513	0.946132	2.1
61514	0.946132	2.1
61515	0.946132	2.1
61516	0.946132	2.1
61517	1.38908	2.1
61518	1.38908	2.1
61519	0.946132	2.1
61520	0.946132	2.1
61521	0.946132	2.1
61522	0.946132	2.1
61523	1.38908	2.1
61524	1.27965	2.1
61525	1.38908	2.1
61526	0.946132	2.1
61527	0.946133	2.1
61528	1.27965	2.1
61529	0.946133	2.1
61530	0.946133	2.1
61531	0.946132	2.1
61532	0.946132	2.1
61533	0.946132	2.1
61534	0.946132	2.1
61535	0.946133	2.1

61536	0.946133	2.1
61537	0.946132	2.1
61538	0.946132	2.1
61539	0.946133	2.1
61540	0.946133	2.1
61541	1.89227	1.54729
61542	1.89227	1.631
61543	1.89227	1.05862
61544	1.89227	0.35561
61545	3.78453	1.20954
61546	1.33154	1.01685
61547	1.33154	1.18453
61548	0.946132	1.27531
61549	0.946132	0.932935
61550	1.19095	0.507106
61551	1.33154	1.22128
61552	0.946133	1.30323
61553	0.946133	1.5335
61554	0.946133	2.02634
61555	1.19095	0.691832
61556	0.946132	1.8558
61557	1.33154	1.0447
61558	1.0888	0.71993
61559	1.04745	0.678557
61560	0.946132	0.800888
61561	0.946132	0.802695
61562	1.04745	0.282663
61563	1.04745	1.1899
61564	0.946132	1.64563
61565	0.946132	0.386778
61566	1.04745	1.53544
61567	0.946133	0.42576
61568	0.946132	1.46073
61569	1.0888	0.726699
61570	1.89226	1.97633
61571	1.89226	0.410206
61572	1.89226	0.335868
61573	1.89226	0.655321
61574	1.89226	0.705762
61575	1.89226	1.13665
61576	1.89226	1.74976
61577	1.87592	0.123764
61578	1.87592	0.346838
61579	2.3819	0.678208
61580	1.89226	1.24418
61581	2.39825	1.23624
61582	1.89226	0.561354
61583	2.0949	1.21179
61584	1.88741	0.176441
61585	1.89226	0.255807
61586	1.89226	0.284876
61587	1.88741	1.15313
61588	1.07508	1.08448
61589	1.63419	1.71426
61590	1.63419	1.31774
61591	1.09795	1.77274
61592	1.56137	1.09592
61593	1.02513	2.01614

61594	0.967672	0.345131
61595	1.56137	0.81797
61596	1.56137	0.372327
61597	1.07508	0.919235
61598	1.41319	0.272955
61599	0.926902	1.23606
61600	1.56137	0.841997
61601	1.63419	1.3603
61602	2.29852	1.53136
61603	2.14485	0.812359
61604	3.77904	0.617432
61605	1.41319	0.186399
61606	1.92622	0.207783
61607	1.56137	0.135165
61608	1.56137	0.779801
61609	2.14485	0.805952
61610	1.92622	0.462372
61611	1.89227	2.1
61612	1.89227	2.1
61613	1.89227	2.1
61614	1.89227	2.1
61615	3.78453	2.1
61616	2.56618	2.1
61617	2.08612	2.1
61618	2.48048	2.1
61619	1.99644	2.1
61620	4.5666	2.1
61621	2.08612	2.1
61622	1.30659	2.1
61623	1.99644	2.1
61624	1.25404	2.1
61625	3.34016	2.1
61626	1.89226	2.1
61627	1.89226	2.1
61628	1.89226	2.1
61629	1.89226	2.1
61630	1.89226	2.1
61631	1.89226	2.1
61632	1.89227	2.1
61633	1.89227	2.1
61634	1.89227	2.1
61635	1.89227	2.1
61636	3.78453	2.1
61637	2.48048	2.1
61638	1.99644	2.1
61639	2.39022	2.1
61640	1.90734	2.1
61641	4.38782	2.1
61642	1.90734	2.1
61643	1.99644	2.1
61644	1.21564	2.1
61645	1.21564	2.1
61646	1.25404	2.1
61647	1.94574	2.1
61648	0.810946	2.1
61649	0.810946	2.1
61650	0.810947	2.1
61651	0.810947	2.1

61652	1.89226	2.1
61653	1.3517	2.1
61654	1.08133	2.1
61655	1.08132	2.1
61656	1.08132	2.1
61657	0.810934	2.1
61658	0.810934	2.1
61659	1.89226	2.1
61660	1.89226	0.144867
61661	1.89226	0.382756
61662	1.89226	2.1
61663	3.78453	2.1
61664	1.89226	0.05
61665	1.89226	0.05
61666	1.89226	0.854293
61667	1.89226	0.05
61668	1.89226	0.156856
61669	1.89226	0.05
61670	0.946131	0.05
61671	0.946131	0.05
61672	0.94613	0.108882
61673	0.94613	0.0767619
61674	0.946128	0.05
61675	0.946128	0.05
61676	0.946133	0.05
61677	0.946133	0.134256
61678	0.946131	0.05
61679	0.946131	0.05
61680	0.946131	0.0633084
61681	0.946131	0.05
61682	1.89226	2.1
61683	1.89226	0.085553
61684	1.89226	1.76177
61685	1.89226	0.856579
61686	1.89226	0.305612
61687	1.89226	0.167614
61688	2.56348	0.1089
61689	1.89226	2.1
61690	1.89226	2.1
61691	2.53234	0.522075
61692	4.45574	0.200711
61693	2.53234	0.05
61694	1.65583	0.05
61695	2.56348	0.05
61696	2.56348	0.05
61697	1.66295	0.05
61698	1.65583	0.05
61699	0.736602	0.05
61700	0.827916	0.05
61701	1.26617	0.05
61702	1.18322	0.05
61703	0.7366	0.05
61704	1.18321	0.05
61705	1.26617	0.05
61706	0.827918	0.05
61707	0.99929	0.05
61708	0.99929	0.05
61709	1.00348	0.05

61710	1.00348	0.05
61711	1.26617	0.05
61712	1.18322	0.05
61713	0.946131	2.1
61714	0.946131	0.10822
61715	0.946129	0.0774616
61716	1.18321	0.05
61717	0.946133	2.1
61718	1.26617	0.701011
61719	1.26617	0.930157
61720	1.26617	0.05
61721	0.946131	1.96907
61722	0.946131	0.278257
61723	2.23267	0.05
61724	1.89226	0.05
61725	2.07099	0.05
61726	1.89226	0.05
61727	2.07099	0.05
61728	1.89226	0.05
61729	0.946132	0.05
61730	1.35403	0.05
61731	1.24038	0.05
61732	0.946131	0.05
61733	0.946131	0.05
61734	0.946131	0.05
61735	1.24039	0.05
61736	1.35403	0.05
61737	1.24038	0.05
61738	1.24038	0.05
61739	0.946131	0.05
61740	0.946131	0.05
61741	1.89226	0.05
61742	1.89226	0.0521378
61743	1.89226	0.05
61744	1.89226	0.05
61745	1.89226	0.0945091
61746	1.89226	0.0741332
61747	1.89226	0.05
61748	1.89226	0.0534899
61749	1.89226	0.05
61750	1.89226	0.05
61751	3.78452	0.05
61752	1.56971	0.05
61753	2.80086	0.05
61754	2.59806	0.05
61755	1.93489	0.05
61756	2.59806	0.05
61757	1.93489	0.05
61758	0.827467	0.05
61759	1.26652	0.05
61760	1.13679	0.05
61761	0.639035	0.05
61762	1.11547	0.05
61763	1.30656	0.05
61764	0.869062	0.05
61765	0.696608	0.05
61766	1.07205	0.05
61767	0.953656	0.05

61768	0.970744	0.05
61769	0.970744	0.05
61770	2.38681	2.1
61771	2.50438	1.87591
61772	2.42082	2.1
61773	2.59106	2.1
61774	4.92521	2.1
61775	2.50438	1.55561
61776	2.40627	2.1
61777	2.59106	2.1
61778	2.5444	1.19311
61779	5.04879	2.1
61780	2.42082	2.1
61781	2.70575	2.1
61782	2.64778	2.1
61783	2.59106	2.1
61784	2.47753	2.1
61785	2.42082	1.69998
61786	2.70575	2.1
61787	2.5444	1.81408
61788	2.76095	2.1
61789	2.59106	2.1
61790	2.71429	2.1
61791	2.5444	2.05137
61792	1.89226	2.1
61793	2.38473	2.1
61794	2.36689	2.1
61795	2.38473	2.1
61796	1.87442	2.1
61797	1.89226	2.1
61798	1.89226	2.1
61799	1.89226	0.270716
61800	1.89226	2.1
61801	1.89226	0.430527
61802	1.89226	2.1
61803	1.89226	1.70812
61804	2.14952	2.1
61805	1.89226	0.0600118
61806	1.89226	2.1
61807	1.89226	0.0555382
61808	2.0692	2.1
61809	2.0692	0.944551
61810	1.89226	2.1
61811	2.36689	2.1
61812	2.36204	2.1
61813	2.36689	2.1
61814	1.88742	2.1
61815	1.89226	2.1
61816	0.946132	0.122783
61817	0.946132	2.1
61818	0.946131	2.1
61819	0.946131	2.1
61820	0.946132	2.01843
61821	0.946132	2.1
61822	0.946131	2.1
61823	0.946131	2.1
61824	0.946132	2.1
61825	0.946132	2.1

61826	0.946131	2.1
61827	0.946131	2.1
61828	0.946128	2.1
61829	0.946128	2.1
61830	1.07476	2.02785
61831	1.07476	2.1
61832	0.946131	1.74987
61833	0.946131	2.1
61834	1.13182	2.1
61835	1.07476	2.1
61836	1.13182	2.1
61837	0.946131	2.1
61838	1.07476	2.1
61839	0.94613	0.854334
61840	1.89227	0.165445
61841	1.89227	0.455704
61842	1.89227	0.631149
61843	1.89227	0.812556
61844	3.78453	0.371952
61845	1.89226	0.18837
61846	1.89226	0.431553
61847	1.89226	0.158184
61848	1.89226	0.938886
61849	3.78452	0.208512
61850	1.89226	0.395991
61851	1.89226	0.630024
61852	1.89226	1.24599
61853	1.89226	1.3783
61854	3.78452	0.393824
61855	1.89226	0.422978
61856	1.89226	0.7532
61857	1.89226	0.427169
61858	1.89226	0.602275
61859	1.89226	1.02038
61860	1.89226	1.10761
61861	2.58344	0.957767
61862	2.00267	0.0780861
61863	2.14218	1.02459
61864	2.22674	1.46856
61865	2.14218	0.05
61866	2.22674	0.061362
61867	2.83531	1.62025
61868	1.56043	0.334551
61869	2.22674	1.57227
61870	1.90912	1.90297
61871	2.22674	0.0544919
61872	1.90912	0.10779
61873	0.780217	0.310134
61874	1.11337	0.392804
61875	1.00133	1.53383
61876	0.804472	0.976568
61877	0.804472	1.34298
61878	0.780217	0.18802
61879	1.00133	0.0727682
61880	1.11337	0.985484
61881	0.856668	0.965539
61882	0.856668	1.84104
61883	0.924883	0.198242

61884	0.924883	0.148481
61885	1.93107	2.1
61886	1.68657	2.1
61887	1.7237	2.1
61888	1.89226	2.1
61889	1.53436	2.1
61890	1.68657	2.1
61891	1.89226	2.1
61892	1.89226	2.1
61893	1.89226	2.1
61894	1.89226	2.1
61895	3.78453	2.1
61896	0.946131	2.1
61897	0.946131	2.1
61898	0.855092	2.1
61899	0.83953	2.1
61900	0.946131	2.1
61901	0.946131	2.1
61902	0.839531	2.1
61903	0.839531	2.1
61904	0.946131	2.1
61905	0.855093	2.1
61906	0.839531	2.1
61907	0.946131	2.1
61908	1.89226	2.1
61909	1.70302	2.1
61910	2.21419	2.1
61911	1.93107	2.1
61912	1.97872	2.1
61913	1.70302	2.1
61914	0.946133	2.1
61915	0.946133	2.1
61916	0.946132	2.1
61917	0.946132	2.1
61918	0.946132	2.1
61919	0.946132	2.1
61920	0.946132	2.1
61921	0.946132	2.1
61922	0.946133	2.1
61923	0.946133	2.1
61924	0.946132	2.1
61925	0.946132	2.1
61926	1.90081	2.1
61927	1.89226	2.1
61928	1.51379	2.1
61929	1.36813	2.1
61930	1.71019	2.1
61931	1.89226	2.1
61932	1.89227	1.53589
61933	1.89227	0.569852
61934	1.89227	0.66454
61935	1.89227	1.20368
61936	1.89227	1.61401
61937	1.89227	1.93878
61938	1.36428	1.28648
61939	1.37869	1.19263
61940	0.946132	0.770399
61941	0.946132	1.39942

61942	1.37869	0.431233
61943	1.37869	2.1
61944	0.946133	1.40578
61945	0.946133	1.72856
61946	1.37869	0.45148
61947	0.946132	1.14288
61948	1.36428	1.47047
61949	0.946133	0.181695
61950	0.94612	1.12065
61951	0.94612	0.982497
61952	0.946119	1.12988
61953	0.946119	1.25972
61954	0.946119	0.301454
61955	0.946119	1.77663
61956	0.94612	1.4463
61957	0.94612	1.88602
61958	0.946132	1.07153
61959	0.946132	0.343546
61960	0.946107	0.402136
61961	0.946107	1.46275
61962	1.89225	0.05
61963	1.89225	2.1
61964	1.63564	0.05
61965	1.42278	2.1
61966	1.42278	2.1
61967	1.57534	0.05
61968	1.53735	2.1
61969	1.68991	0.05
61970	1.57535	0.05
61971	1.04442	2.1
61972	1.04442	2.1
61973	1.22031	0.05
61974	1.42278	2.1
61975	1.59868	0.05
61976	0.946132	2.1
61977	0.946132	2.1
61978	0.946131	2.1
61979	0.946131	2.1
61980	0.946131	2.1
61981	0.946131	2.1
61982	0.946132	2.1
61983	0.946132	2.1
61984	0.946132	2.1
61985	0.946132	2.1
61986	0.946131	2.1
61987	0.946131	2.1
61988	1.89226	2.1
61989	1.89226	2.1
61990	1.89226	2.1
61991	1.89226	2.1
61992	1.89226	2.1
61993	1.89226	2.1
61994	2.03143	0.301702
61995	1.47111	2.00955
61996	2.28685	1.91367
61997	2.28685	1.85046
61998	1.36006	0.827689
61999	1.36006	0.618739

62000	1.89226	0.304678
62001	1.89226	1.58591
62002	1.89226	0.917707
62003	1.89226	1.45142
62004	1.89226	0.29896
62005	1.89226	0.750714
62006	1.89226	1.29888
62007	1.89226	2.1
62008	1.89226	2.1
62009	1.89226	2.1
62010	1.89226	1.50697
62011	1.89226	2.1
62012	0.902821	2.1
62013	0.902821	2.1
62014	0.889218	2.1
62015	0.889218	2.1
62016	0.675759	2.1
62017	1.05649	2.1
62018	0.735554	2.1
62019	1.14342	2.1
62020	1.14343	2.04519
62021	1.05649	2.1
62022	0.735554	2.1
62023	0.675758	2.1
62024	2.46286	0.101439
62025	2.28685	0.268451
62026	2.67843	0.317681
62027	2.67843	0.153226
62028	2.03143	0.195499
62029	2.03143	0.118543
62030	1.89226	0.319058
62031	1.89226	0.2317
62032	1.89226	0.142702
62033	1.89226	0.154977
62034	3.78452	0.482069
62035	0.94613	0.919834
62036	0.94613	1.21236
62037	0.946128	2.08037
62038	0.946128	0.445572
62039	0.94613	2.1
62040	0.94613	0.70745
62041	0.946128	0.119135
62042	0.946128	0.804701
62043	0.946129	0.0625425
62044	0.946129	1.50141
62045	0.946129	0.0893859
62046	0.946129	2.1
62047	2.67843	0.281117
62048	2.11297	1.998
62049	2.52453	1.72861
62050	2.52453	2.1
62051	2.28685	0.915078
62052	2.28685	0.573946
62053	1.89226	0.0600975
62054	1.89226	1.16443
62055	1.89226	0.05
62056	1.89226	1.81329
62057	3.78451	0.12445

62058	2.39524	0.05
62059	2.10188	0.921348
62060	2.29839	0.05
62061	2.02242	1.52038
62062	4.40028	0.0689932
62063	1.71839	2.1
62064	1.65261	2.1
62065	2.10189	0.0853969
62066	2.10189	2.1
62067	2.02243	0.838423
62068	1.63893	0.859231
62069	0.946132	2.1
62070	0.946132	0.217858
62071	0.946129	0.583969
62072	0.946129	2.1
62073	0.94613	2.1
62074	0.94613	2.1
62075	0.946131	0.104366
62076	0.946131	0.445753
62077	0.946131	2.1
62078	0.946131	2.1
62079	0.946131	2.1
62080	0.946131	2.1
62081	1.89226	0.05
62082	1.89226	1.8177
62083	1.89226	0.05
62084	1.89226	0.737031
62085	3.78452	0.0840488
62086	2.2984	0.05
62087	2.02242	1.47292
62088	2.21582	0.05
62089	1.94412	0.634429
62090	4.24251	0.0904437
62091	1.94412	0.147991
62092	1.5854	2.1
62093	2.02243	2.1
62094	2.02243	0.313369
62095	1.57431	0.185515
62096	1.65261	2.1
62097	0.810943	0.164987
62098	0.810943	2.1
62099	0.810945	2.1
62100	0.810945	2.1
62101	1.89226	0.177096
62102	1.3517	1.91719
62103	1.08133	2.1
62104	1.08132	2.1
62105	1.08132	0.0905576
62106	0.810933	2.1
62107	0.810933	2.1
62108	1.89226	0.05
62109	1.89226	0.05
62110	1.89226	0.05
62111	1.89226	0.05
62112	1.89226	0.05
62113	1.89226	0.05
62114	1.89226	0.05
62115	1.89226	0.05

62116	1.89226	0.05
62117	1.89226	0.05
62118	3.78452	0.05
62119	1.89226	0.05
62120	1.89226	0.05
62121	1.89226	0.05
62122	1.89226	0.05
62123	1.89226	0.05
62124	1.89226	0.05
62125	0.946128	0.05
62126	0.946128	0.05
62127	0.946132	0.05
62128	0.946132	0.05
62129	0.94613	0.05
62130	0.94613	0.05
62131	0.946129	0.05
62132	0.946129	0.05
62133	0.94613	0.05
62134	0.94613	0.05
62135	0.94613	0.05
62136	0.94613	0.05
62137	1.89226	0.05
62138	1.89226	0.05
62139	1.89226	0.05
62140	1.89226	0.05
62141	1.89226	0.05
62142	1.89226	0.05
62143	2.81478	2.1
62144	1.89226	2.1
62145	2.65557	2.1
62146	1.89226	2.1
62147	4.54783	2.1
62148	1.89226	2.1
62149	1.89226	2.1
62150	1.89226	2.1
62151	1.89226	2.1
62152	3.78452	2.1
62153	2.65557	2.1
62154	1.89226	2.1
62155	1.89226	2.1
62156	1.89226	2.1
62157	2.57997	2.1
62158	2.57997	2.1
62159	0.946131	2.1
62160	0.946131	2.1
62161	0.946128	2.1
62162	0.946128	2.1
62163	0.94613	2.1
62164	0.94613	2.1
62165	0.946129	2.1
62166	0.946129	2.1
62167	0.94613	2.1
62168	0.94613	2.1
62169	0.94613	2.1
62170	0.94613	2.1
62171	1.89226	0.630229
62172	2.27703	1.99185
62173	2.35615	2.05555

62174	2.35615	0.915689
62175	1.89226	2.04158
62176	1.89226	0.844516
62177	0.946132	0.541636
62178	1.17807	2.09455
62179	1.14926	0.491917
62180	0.946132	1.45882
62181	0.946133	1.40196
62182	0.946133	1.53076
62183	1.14926	0.730842
62184	1.14926	0.664497
62185	0.946132	1.28164
62186	0.946132	0.928212
62187	1.17807	1.48411
62188	1.14926	0.840844
62189	2.16841	0.615097
62190	1.35487	1.32259
62191	1.19854	1.01667
62192	1.19854	0.607544
62193	2.46078	0.98518
62194	2.46078	0.79924
62195	0.69308	0.576711
62196	1.3119	1.43673
62197	0.677433	1.52996
62198	1.23039	0.919601
62199	0.978178	1.53106
62200	0.978178	1.52725
62201	0.945294	0.75689
62202	0.945294	0.913626
62203	1.3119	1.06701
62204	1.23039	0.95754
62205	0.693081	1.06491
62206	0.677432	1.05653
62207	1.89226	0.680482
62208	1.89226	2.08738
62209	1.89226	2.00649
62210	1.89226	0.915081
62211	1.89226	1.95474
62212	1.89226	0.921574
62213	0.946132	0.569478
62214	0.946132	2.0462
62215	0.946132	0.350469
62216	0.946132	1.18154
62217	0.946133	1.17529
62218	0.946133	1.49127
62219	0.946132	0.598126
62220	0.946132	0.453112
62221	0.946133	0.964907
62222	0.946133	0.664722
62223	0.946132	1.19665
62224	0.946132	0.816326
62225	2.60637	2.1
62226	1.89226	2.1
62227	1.89226	2.1
62228	1.89226	1.7199
62229	3.78452	2.1
62230	1.89226	0.492225
62231	1.8396	1.7716

62232	1.89226	2.07012
62233	1.89226	0.506147
62234	1.60993	2.1
62235	1.60993	0.496194
62236	0.946131	1.13753
62237	0.946131	2.00263
62238	0.946128	2.1
62239	0.946128	2.1
62240	0.946131	1.06865
62241	0.946131	2.01044
62242	0.946127	2.1
62243	0.946127	1.8708
62244	0.946129	2.1
62245	0.946129	2.1
62246	0.94613	2.1
62247	0.94613	2.1
62248	1.89226	2.1
62249	1.89226	1.87535
62250	1.89226	2.1
62251	1.89226	2.1
62252	1.89226	2.1
62253	1.89226	2.1
62254	1.89226	0.519367
62255	1.89226	1.52992
62256	1.89226	2.04149
62257	1.89226	0.56326
62258	1.89226	2.1
62259	1.89226	0.397239
62260	0.946131	1.22997
62261	0.946131	2.00606
62262	0.946128	2.1
62263	0.946128	2.01022
62264	0.946131	1.16669
62265	0.946131	1.86649
62266	0.946127	1.98439
62267	0.946127	2.1
62268	0.94613	2.09812
62269	0.94613	2.0961
62270	0.946129	2.06843
62271	0.946129	2.08681
62272	0.946133	2.1
62273	0.946133	2.1
62274	1.28153	2.1
62275	1.26781	2.1
62276	0.946132	2.1
62277	0.946132	2.1
62278	1.28153	2.1
62279	1.28153	2.1
62280	0.946132	2.1
62281	1.28153	2.1
62282	0.946133	2.1
62283	1.26781	2.1
62284	1.89226	2.1
62285	1.89226	2.1
62286	1.89226	2.1
62287	1.89226	2.1
62288	1.89226	2.1
62289	1.89226	2.1

62290	2.5844	2.1
62291	1.09563	2.1
62292	1.1468	2.1
62293	1.09563	2.1
62294	2.6142	2.1
62295	2.56304	2.1
62296	0.946126	2.1
62297	0.946126	2.1
62298	1.2922	2.1
62299	1.28152	2.1
62300	0.946126	2.1
62301	0.946126	2.1
62302	1.2922	2.1
62303	1.2922	2.1
62304	1.29221	2.1
62305	0.946132	2.1
62306	0.946119	2.1
62307	1.28151	2.1
62308	1.89225	2.1
62309	1.89225	2.1
62310	1.89225	2.1
62311	1.89225	2.1
62312	1.89225	2.1
62313	1.89225	2.1
62314	0.946121	2.1
62315	0.946121	2.1
62316	0.946126	2.1
62317	0.946126	2.1
62318	0.946124	2.0899
62319	0.946124	2.1
62320	0.946123	2.1
62321	0.946123	2.1
62322	0.94613	2.1
62323	0.94613	2.1
62324	0.946117	2.1
62325	0.946117	2.1
62326	1.89226	1.05086
62327	1.89226	1.18966
62328	1.89226	0.528326
62329	1.89226	2.1
62330	1.89226	0.0833845
62331	1.89226	2.1
62332	1.89226	2.1
62333	1.89226	2.0264
62334	1.89226	0.21698
62335	1.89226	2.1
62336	1.89226	0.0667643
62337	1.89226	2.1
62338	1.89226	2.1
62339	2.81281	2.1
62340	2.50369	0.950787
62341	2.50369	2.1
62342	1.89226	1.21048
62343	1.89226	2.1
62344	1.89226	2.1
62345	1.89226	2.1
62346	1.89226	2.1
62347	1.89226	2.1

62348	1.89226	2.1
62349	1.89226	2.1
62350	1.89226	2.1
62351	1.89226	2.1
62352	1.89226	2.1
62353	1.89226	2.1
62354	1.89226	2.1
62355	1.89226	2.1
62356	0.907036	2.1
62357	1.09714	2.1
62358	0.994609	2.1
62359	0.838808	2.1
62360	0.976536	2.1
62361	0.976536	2.1
62362	0.959412	2.1
62363	0.959412	2.1
62364	1.09714	2.1
62365	0.994609	2.1
62366	0.907036	2.1
62367	0.838808	2.1
62368	0.99461	2.1
62369	0.838808	2.1
62370	0.738212	2.1
62371	0.565634	2.1
62372	0.775921	2.1
62373	0.775921	2.1
62374	0.784322	2.1
62375	0.784322	2.1
62376	0.994609	2.1
62377	0.738212	2.1
62378	0.838808	2.1
62379	0.565635	2.1
62380	1.89226	2.1
62381	1.89226	2.1
62382	1.89226	2.1
62383	1.89226	2.1
62384	1.89226	2.1
62385	1.89226	2.1
62386	1.89226	2.1
62387	1.89226	2.1
62388	1.89226	2.1
62389	1.89226	2.1
62390	1.89226	2.1
62391	1.89226	2.1
62392	2.19428	2.1
62393	1.98922	2.1
62394	2.27322	2.1
62395	2.45893	2.1
62396	4.4675	2.1
62397	0.99461	2.1
62398	1.13661	2.1
62399	0.902272	2.1
62400	0.738212	2.1
62401	0.931874	2.1
62402	0.931874	2.1
62403	0.942953	2.1
62404	0.942953	2.1
62405	1.13658	2.1

62406	0.902248	2.1
62407	0.994637	2.1
62408	0.738233	2.1
62409	1.89226	2.05898
62410	1.89226	1.83184
62411	1.89226	2.1
62412	1.89226	2.05132
62413	1.89226	2.07197
62414	1.89226	1.88451
62415	1.89227	2.08459
62416	2.58246	0.953996
62417	1.89227	1.80859
62418	1.89227	1.89675
62419	3.78453	1.38222
62420	1.89226	2.1
62421	1.89226	1.85356
62422	1.65573	2.1
62423	1.65573	2.1
62424	1.89226	2.1
62425	1.89226	2.1
62426	1.89226	1.65725
62427	1.89226	1.31308
62428	1.89226	1.60434
62429	1.89226	1.60032
62430	1.89226	1.49586
62431	1.89226	1.5319
62432	1.89226	1.26055
62433	1.89226	1.41423
62434	1.89226	1.19147
62435	1.89226	1.57403
62436	1.89226	1.17836
62437	1.89226	1.56044
62438	0.946132	2.1
62439	0.946132	2.0108
62440	1.01023	1.02865
62441	1.13651	1.9779
62442	1.01023	0.932954
62443	0.946133	1.70313
62444	1.0644	1.88601
62445	1.0644	2.1
62446	0.946132	2.09241
62447	0.946132	1.9008
62448	1.13651	1.69995
62449	0.946132	1.81106
62450	1.89226	0.174154
62451	1.89226	1.81492
62452	1.89226	0.991267
62453	1.89226	0.0989775
62454	3.78451	0.320981
62455	1.00934	0.0710671
62456	0.741561	0.05
62457	0.946131	0.138114
62458	0.946131	0.220836
62459	0.946131	0.603184
62460	0.741561	0.05
62461	1.00934	0.05
62462	0.946131	0.342684
62463	0.741586	0.108909

62464	0.741586	0.105966
62465	0.946106	0.10091
62466	0.946106	0.05
62467	1.14895	0.135051
62468	0.957449	0.250579
62469	0.934806	1.22542
62470	0.934806	0.855496
62471	1.13536	0.077388
62472	1.02829	1.71518
62473	0.946129	1.93812
62474	0.946129	0.130692
62475	0.946127	0.590441
62476	0.946127	0.745262
62477	0.946127	0.450014
62478	1.0283	0.622584
62479	1.89226	1.82897
62480	1.89226	0.26294
62481	1.89226	0.238635
62482	1.89226	1.36767
62483	3.78452	0.880933
62484	1.89226	0.0766152
62485	1.89226	0.477035
62486	1.89226	0.329461
62487	1.89226	0.0660309
62488	3.78453	0.109053
62489	1.89226	0.477712
62490	2.69068	0.0719565
62491	1.89226	0.0680502
62492	1.89226	0.328553
62493	3.78452	0.19962
62494	1.89226	0.263522
62495	1.89226	1.66124
62496	1.89226	1.62908
62497	1.89226	0.595489
62498	1.89226	1.2802
62499	1.89226	0.345639
62500	1.89226	2.1
62501	1.89226	2.1
62502	1.89226	2.1
62503	1.89226	2.1
62504	1.89226	2.1
62505	1.89226	2.1
62506	1.89226	2.1
62507	1.89226	2.1
62508	1.89226	2.1
62509	1.89226	2.1
62510	1.89226	2.1
62511	1.89226	2.1
62512	0.985109	2.1
62513	1.31144	2.1
62514	1.45864	2.1
62515	1.10667	2.1
62516	2.44375	2.1
62517	0.655718	2.1
62518	0.729316	2.1
62519	0.684594	2.1
62520	0.782707	2.1
62521	0.700849	2.1

62522	0.700849	2.1
62523	0.71306	2.1
62524	0.71306	2.1
62525	0.729318	2.1
62526	0.782705	2.1
62527	0.684592	2.1
62528	0.655719	2.1
62529	0.946128	2.1
62530	0.946128	2.1
62531	0.946133	2.1
62532	0.946133	2.1
62533	0.946131	2.1
62534	0.946131	2.1
62535	0.946131	2.1
62536	0.946131	2.1
62537	0.94613	2.1
62538	0.94613	2.1
62539	0.946131	2.1
62540	0.946131	2.1
62541	1.89226	2.1
62542	1.89226	2.1
62543	1.89226	2.1
62544	1.89226	2.1
62545	1.89226	2.1
62546	1.89226	2.1
62547	0.553334	2.1
62548	0.629936	2.1
62549	0.828211	2.1
62550	0.729319	2.1
62551	0.674025	2.1
62552	0.674025	2.1
62553	0.68523	2.1
62554	0.68523	2.1
62555	0.828211	2.1
62556	0.629935	2.1
62557	0.72932	2.1
62558	0.553334	2.1
62559	0.828208	2.1
62560	0.729316	2.1
62561	0.907037	2.1
62562	0.782707	2.1
62563	0.799048	2.1
62564	0.799048	2.1
62565	0.811867	2.1
62566	0.811867	2.1
62567	0.828209	2.1
62568	0.907035	2.1
62569	0.729318	2.1
62570	0.782706	2.1
62571	2.32981	2.1
62572	1.89226	2.1
62573	1.89226	2.1
62574	1.89226	2.1
62575	2.2671	2.1
62576	2.2671	2.1
62577	1.89226	2.1
62578	1.89226	2.1
62579	1.89226	2.1

62580	1.89226	1.67517
62581	1.89226	2.1
62582	1.89226	2.1
62583	1.3991	2.1
62584	1.81793	2.01343
62585	2.07226	2.08641
62586	2.07226	1.64042
62587	1.24924	2.1
62588	1.24924	2.1
62589	0.946131	1.3414
62590	0.946131	2.1
62591	1.16491	2.1
62592	1.16491	2.1
62593	0.946131	2.1
62594	0.946131	2.1
62595	1.16491	2.1
62596	1.19523	2.1
62597	0.946132	2.1
62598	1.19523	2.1
62599	1.16491	2.1
62600	0.946131	2.1
62601	1.89226	2.1
62602	1.89226	1.89185
62603	1.89226	2.1
62604	1.89226	0.163388
62605	3.78452	1.54144
62606	0.901204	2.06956
62607	0.901204	2.1
62608	0.902504	2.1
62609	0.902504	2.1
62610	1.10416	2.02996
62611	1.03613	0.672532
62612	0.764922	2.1
62613	0.699552	2.1
62614	0.764921	2.0609
62615	1.10416	2.1
62616	0.699551	2.1
62617	1.03613	1.68122
62618	1.89226	0.321719
62619	1.89226	0.994503
62620	1.89226	1.08523
62621	1.89226	0.423271
62622	3.78453	0.773015
62623	1.89226	0.776803
62624	1.89226	1.19512
62625	1.89226	0.655226
62626	1.89226	1.62403
62627	1.89226	0.685894
62628	1.89226	1.64232
62629	0.946131	1.00134
62630	0.946131	1.12738
62631	0.946131	0.647436
62632	0.946131	0.584273
62633	0.946131	0.612445
62634	0.946131	1.11992
62635	0.946131	1.1864
62636	0.946131	0.341739
62637	0.946131	0.562637

62638	0.946131	0.561165
62639	0.946131	0.663972
62640	0.946131	0.819564
62641	1.89226	0.756984
62642	1.89226	1.21198
62643	1.89226	0.75282
62644	1.89226	1.42267
62645	1.89226	0.744871
62646	1.89226	1.41065
62647	0.946127	0.718357
62648	0.946127	0.78647
62649	1.22788	0.575363
62650	1.35469	0.572907
62651	1.35469	0.739364
62652	1.35469	0.583198
62653	0.946128	0.805492
62654	0.946128	0.460382
62655	0.946129	0.586937
62656	1.22788	0.635934
62657	1.35469	0.67215
62658	0.946129	0.609142
62659	1.22788	0.831503
62660	1.35469	0.796328
62661	1.02602	0.451421
62662	0.794861	0.79474
62663	1.15301	0.810601
62664	1.15301	0.882233
62665	1.10089	0.445433
62666	1.10089	0.859924
62667	0.794861	0.819725
62668	1.22788	0.863044
62669	1.02602	0.862383
62670	1.35469	0.800093
62671	1.89226	0.05
62672	1.89226	0.21123
62673	1.89226	1.67719
62674	1.89226	0.0802384
62675	3.78452	0.165864
62676	1.13536	1.99885
62677	0.942524	0.05
62678	0.946131	0.095278
62679	0.946131	0.05
62680	0.946127	0.134352
62681	0.946127	1.98567
62682	1.26282	0.05
62683	0.917471	1.60737
62684	1.21023	0.198395
62685	1.00851	0.201676
62686	1.17955	0.208631
62687	0.883746	0.0905559
62688	1.89226	0.0738499
62689	1.89226	2.1
62690	1.89226	0.285263
62691	1.89226	0.864542
62692	1.89226	0.342059
62693	1.89226	1.67279
62694	1.89226	0.0588958
62695	1.89226	0.507885

62696	1.89226	0.0604027
62697	1.89226	0.133136
62698	1.89226	0.0663636
62699	1.89226	0.464804
62700	0.94613	0.932851
62701	0.94613	0.114037
62702	0.94613	0.111094
62703	0.94613	0.119963
62704	0.946128	0.209921
62705	0.946128	1.04327
62706	0.946131	1.43772
62707	0.946131	0.0579228
62708	0.94613	0.193
62709	0.94613	0.649842
62710	0.94613	0.342121
62711	0.94613	0.223766
62712	0.883758	1.87579
62713	1.03041	0.438571
62714	0.946131	0.404369
62715	0.946131	0.0677969
62716	0.851537	0.175339
62717	0.851537	1.82473
62718	1.23942	0.05
62719	1.14906	1.92575
62720	0.851484	0.195272
62721	0.985518	0.155997
62722	1.0156	0.406916
62723	0.851526	0.437926
62724	1.89226	0.0982716
62725	1.89226	2.1
62726	1.89226	0.1418
62727	1.89226	0.812576
62728	1.89226	0.199944
62729	1.89226	1.59078
62730	1.89226	0.194841
62731	1.89226	0.283375
62732	1.89226	0.136643
62733	1.89226	0.395272
62734	1.89226	0.101767
62735	1.89226	0.534222
62736	1.89227	0.113085
62737	1.89227	0.223134
62738	1.89227	0.1533
62739	1.89227	0.623407
62740	3.78453	0.178656
62741	1.89226	0.204392
62742	1.89226	0.154385
62743	1.89226	0.39969
62744	1.89226	0.960972
62745	3.78452	0.45849
62746	0.946132	0.533474
62747	0.946132	0.71861
62748	0.946128	1.23612
62749	0.946128	0.630379
62750	0.94613	0.398264
62751	0.94613	0.639397
62752	0.94613	0.142009
62753	0.94613	0.797859

62754	0.946131	0.520941
62755	0.946131	1.24722
62756	0.94613	0.250697
62757	0.94613	0.405516
62758	1.89226	0.639306
62759	1.89226	0.382784
62760	1.89226	0.672549
62761	1.89226	0.290953
62762	1.89226	0.702283
62763	1.89226	0.287022
62764	1.98874	0.05
62765	2.33455	0.0977409
62766	2.01865	0.266866
62767	2.07024	0.191246
62768	4.32329	0.29135
62769	1.98874	0.0936708
62770	2.00266	0.0905438
62771	2.14218	0.499505
62772	2.07024	0.986533
62773	3.9914	0.0870701
62774	1.00133	0.162955
62775	0.804471	0.0677363
62776	1.03512	0.606386
62777	0.995176	0.668256
62778	0.80447	1.15727
62779	0.995178	0.185053
62780	1.00133	0.149339
62781	1.03512	1.19146
62782	0.880587	0.920584
62783	0.880587	0.384967
62784	0.959002	0.0739641
62785	0.959002	0.166881
62786	2.33455	0.103314
62787	1.99036	0.12188
62788	2.07024	0.183846
62789	2.58645	0.329828
62790	4.32491	0.0775575
62791	1.89226	0.083611
62792	1.89226	0.070023
62793	1.89226	0.0925276
62794	1.89226	0.05
62795	3.78453	0.05
62796	1.89226	0.244482
62797	1.89226	0.0757515
62798	1.89226	0.117578
62799	1.89226	0.0936828
62800	3.78453	0.228019
62801	1.89226	0.0514808
62802	1.89226	0.05
62803	1.89226	0.05
62804	1.89226	0.05
62805	1.89226	0.05
62806	1.89226	0.05
62807	1.89226	0.05
62808	2.1523	0.05
62809	2.53257	0.05
62810	2.53257	0.05
62811	1.89226	0.05

62812	1.89226	0.05
62813	0.946133	0.05
62814	0.946133	0.0592653
62815	0.946132	0.05
62816	0.946132	0.05
62817	0.946132	0.05
62818	0.946132	0.05
62819	0.946132	0.0619852
62820	0.946132	0.05
62821	0.946133	0.05
62822	0.946133	0.05
62823	0.946132	0.0638231
62824	0.946132	0.05
62825	0.946132	0.0545473
62826	0.946132	0.101703
62827	0.946132	0.05
62828	0.946132	0.0748084
62829	0.946132	0.05
62830	0.946132	0.05
62831	0.946132	0.106091
62832	0.946132	0.0740064
62833	0.946132	0.0563366
62834	0.946132	0.0568749
62835	0.946131	0.0785066
62836	0.946131	0.0752044
62837	1.62772	0.05
62838	1.70303	0.05
62839	1.70303	0.05
62840	1.70303	0.05
62841	1.90116	0.05
62842	1.90116	0.05
62843	1.89226	0.05
62844	1.89226	0.05
62845	1.89226	0.0753855
62846	1.89226	0.103466
62847	1.89226	0.05
62848	1.89226	0.05
62849	1.89226	0.05
62850	1.89226	0.0948872
62851	1.89226	0.0533768
62852	1.89226	0.108474
62853	3.78452	0.0822497
62854	1.89226	0.0538513
62855	1.89226	0.0753091
62856	1.89226	0.0560447
62857	1.89226	0.0638724
62858	1.89226	0.05
62859	1.89226	0.0562946
62860	0.946132	0.05
62861	0.946132	0.05
62862	0.946129	0.05
62863	0.946129	0.05
62864	0.94613	0.0502743
62865	0.94613	0.0529714
62866	0.946131	0.05
62867	0.946131	0.05
62868	0.94613	0.05
62869	0.94613	0.05

62870	0.946131	0.05
62871	0.946131	0.0572558
62872	1.89226	0.05
62873	1.89226	0.069843
62874	1.89226	0.121044
62875	1.89226	0.116474
62876	1.89226	0.05
62877	1.89226	0.05
62878	1.89226	0.135871
62879	1.89226	0.921166
62880	1.89226	0.891637
62881	1.89226	0.0789557
62882	3.78452	0.22652
62883	1.89226	0.370224
62884	1.89226	0.811776
62885	1.89226	0.161495
62886	1.89226	1.03859
62887	1.89226	0.205409
62888	1.89226	1.29818
62889	1.30165	0.291571
62890	0.946127	0.590573
62891	1.09659	0.178742
62892	0.946131	0.268029
62893	1.09659	0.17205
62894	1.09659	1.24258
62895	0.946128	1.58704
62896	0.946128	0.0934565
62897	1.30165	0.26625
62898	1.09659	0.734948
62899	0.946129	0.273697
62900	0.946129	0.626044
62901	0.946128	0.512272
62902	0.946128	0.831745
62903	0.946133	0.224317
62904	0.946133	0.557688
62905	0.946131	0.151597
62906	0.946131	1.14879
62907	0.946131	0.926072
62908	0.946131	0.208981
62909	0.94613	0.240155
62910	0.94613	0.194988
62911	0.946131	0.24197
62912	0.946131	0.521998
62913	0.946133	0.609371
62914	0.946133	0.922766
62915	0.946132	0.37645
62916	0.946132	0.684734
62917	0.946132	0.231425
62918	0.946132	1.07356
62919	0.946132	1.04437
62920	0.946132	0.43868
62921	0.946132	0.467136
62922	0.946132	0.501938
62923	0.946133	0.677409
62924	0.946133	0.723063
62925	1.30165	0.971186
62926	0.972228	0.941943
62927	1.09659	0.494864

62928	0.656268	1.15495
62929	1.06217	0.356009
62930	1.06217	1.72955
62931	1.00665	0.172374
62932	1.00665	1.61035
62933	0.97223	0.201205
62934	0.656267	0.222158
62935	1.30165	0.40674
62936	1.09659	0.668626
62937	0.946132	1.41736
62938	0.946132	1.80829
62939	1.19895	2.1
62940	1.19895	2.1
62941	0.94613	2.1
62942	0.94613	0.534679
62943	1.39356	2.0419
62944	1.19895	2.1
62945	0.94613	2.00547
62946	1.39356	2.1
62947	1.19895	2.1
62948	0.946131	2.1
62949	1.89226	0.77833
62950	1.89226	2.1
62951	1.89226	2.1
62952	1.89226	0.729918
62953	1.89226	2.1
62954	1.89226	0.862242
62955	0.946132	0.410484
62956	0.946132	0.107759
62957	0.946128	0.955657
62958	0.946128	1.69
62959	0.94613	0.968036
62960	0.94613	0.0727098
62961	0.94613	0.146503
62962	0.94613	1.31138
62963	0.946131	0.118922
62964	0.946131	0.141739
62965	0.94613	0.440437
62966	0.94613	0.519114
62967	1.89226	0.05
62968	1.89226	0.05
62969	1.89226	0.05
62970	1.89226	0.05
62971	1.89226	0.05
62972	1.89226	0.05
62973	1.29881	0.05
62974	1.09985	0.05
62975	0.946126	0.05
62976	0.946126	0.05
62977	0.851539	0.05
62978	1.20421	0.05
62979	0.851483	0.05
62980	0.999426	0.05
62981	1.47172	0.05
62982	0.999413	0.05
62983	0.851552	0.05
62984	0.851552	0.05
62985	1.89226	0.05

62986	1.89226	0.05
62987	1.89226	0.05
62988	1.89226	0.05
62989	1.89226	0.05
62990	1.89226	0.05
62991	1.89226	0.05
62992	1.89226	0.05
62993	1.89226	0.05
62994	1.89226	0.05
62995	1.89226	0.05
62996	1.89226	0.05
62997	1.33915	2.1
62998	1.99297	2.1
62999	2.05216	2.1
63000	2.60163	2.1
63001	4.04513	2.1
63002	1.89226	1.86305
63003	1.89226	2.1
63004	1.89226	2.1
63005	1.89226	2.1
63006	3.78452	2.1
63007	0.946132	2.1
63008	0.946132	2.07525
63009	0.946133	2.1
63010	0.946133	2.1
63011	0.946132	2.1
63012	0.946132	1.35556
63013	0.946133	2.1
63014	0.946133	2.1
63015	0.946132	2.1
63016	0.946132	2.1
63017	0.946132	2.1
63018	0.946132	2.1
63019	1.89226	1.87743
63020	1.89226	0.790717
63021	1.89226	1.00544
63022	1.89226	2.0299
63023	3.78452	1.46369
63024	1.89227	1.06805
63025	1.89227	2.07714
63026	1.89227	2.08087
63027	1.89227	0.933789
63028	1.89227	2.09338
63029	1.89227	1.05997
63030	2.29301	2.1
63031	1.70303	2.1
63032	2.46749	2.1
63033	2.30728	2.1
63034	4.53937	2.1
63035	1.89226	0.633344
63036	1.89226	2.1
63037	1.89226	2.1
63038	1.89226	1.36074
63039	3.78452	2.1
63040	1.89226	0.217398
63041	1.89226	2.1
63042	1.89226	1.95928
63043	1.89226	2.1

63044	1.89226	0.487771
63045	1.89226	1.46141
63046	2.30728	2.1
63047	1.00757	1.98744
63048	2.0833	2.1
63049	1.65318	2.1
63050	1.417	2.1
63051	1.00757	2.1
63052	2.46749	2.1
63053	2.0975	2.1
63054	2.79653	2.1
63055	2.44785	2.1
63056	4.91534	2.1
63057	1.89226	2.1
63058	1.89226	2.1
63059	1.89226	0.399948
63060	1.89226	2.1
63061	3.78452	1.41383
63062	1.89226	1.23641
63063	1.89226	2.1
63064	1.89226	2.1
63065	1.89226	2.00111
63066	1.89226	2.1
63067	1.89226	1.95408
63068	0.970559	2.1
63069	0.970559	2.1
63070	0.979305	2.1
63071	0.979305	2.1
63072	0.708502	2.1
63073	0.901117	2.1
63074	1.04875	2.1
63075	1.22392	2.1
63076	1.22392	2.1
63077	0.901117	2.1
63078	1.04875	2.1
63079	0.7085	2.1
63080	1.89226	1.48044
63081	1.89226	0.26121
63082	1.89226	0.72254
63083	1.89226	0.392352
63084	3.78452	1.489
63085	1.89226	1.11765
63086	1.89226	0.162387
63087	1.89226	0.530591
63088	1.89226	0.234994
63089	3.78452	0.242234
63090	1.89226	0.05
63091	1.89226	0.05
63092	1.89226	0.05
63093	1.89226	0.05
63094	3.78453	0.05
63095	1.89226	0.0776296
63096	1.89226	0.0561465
63097	1.89226	0.05
63098	1.89226	0.05
63099	1.89226	0.05
63100	1.89226	0.05
63101	1.89226	0.103794

63102	2.35441	0.185902
63103	1.89226	0.686547
63104	2.30931	0.05
63105	4.24667	0.0577956
63106	1.11612	0.05
63107	0.946131	0.05
63108	1.1772	0.0694625
63109	0.946131	0.0700048
63110	1.17721	0.150603
63111	1.11612	0.05
63112	0.94613	0.05
63113	0.94613	0.106338
63114	1.11612	0.0564184
63115	1.11612	0.05
63116	0.946131	0.05
63117	0.946131	0.05
63118	1.11612	0.05
63119	0.582675	0.05
63120	1.17721	0.05
63121	0.651969	0.05
63122	0.651969	0.05
63123	0.582674	0.05
63124	1.17721	0.05
63125	1.11612	0.05
63126	0.877955	0.05
63127	0.877955	0.05
63128	0.881925	0.05
63129	0.881925	0.05
63130	1.17721	0.05
63131	0.651969	0.05
63132	1.15466	0.05
63133	0.623642	0.05
63134	0.623642	0.05
63135	0.651969	0.05
63136	1.17721	0.05
63137	1.15466	0.05
63138	0.904714	0.05
63139	0.904714	0.05
63140	0.901914	0.05
63141	0.901914	0.05
63142	1.89226	1.14278
63143	1.89226	0.551967
63144	1.89226	0.18948
63145	1.89226	1.56354
63146	1.89226	0.0722591
63147	1.89226	2.1
63148	1.89226	1.19575
63149	1.89226	0.112643
63150	1.89226	0.0970416
63151	1.89226	2.1
63152	1.89226	0.0819872
63153	1.89226	2.1
63154	2.33986	2.1
63155	1.97819	0.555839
63156	2.39166	0.0547661
63157	2.70141	2.1
63158	4.73151	0.455439
63159	0.989095	0.902506

63160	1.19583	0.126879
63161	0.851194	0.203543
63162	0.581197	0.355565
63163	0.872704	0.05
63164	0.872704	1.37542
63165	0.904322	0.22605
63166	0.904322	0.75427
63167	1.19583	0.0893657
63168	0.851193	0.119817
63169	0.989095	0.429566
63170	0.581197	0.244289
63171	1.89226	0.434434
63172	1.89226	0.579205
63173	1.89226	0.290175
63174	1.89226	1.73923
63175	1.89226	0.141315
63176	1.89226	2.1
63177	1.89226	1.2954
63178	1.89226	0.110071
63179	1.89226	0.0947305
63180	1.89226	2.1
63181	1.89226	0.0560967
63182	1.89226	2.1
63183	2.39166	0.484211
63184	1.70239	1.21425
63185	2.13665	0.250985
63186	2.82684	0.248223
63187	4.52831	0.675162
63188	1.89226	0.05
63189	1.89226	0.05
63190	1.89226	0.05
63191	1.89226	0.05
63192	3.78453	0.05
63193	1.89226	0.05
63194	1.89226	0.05
63195	1.89226	0.05
63196	1.89226	0.05
63197	1.89226	0.05
63198	1.89226	0.05
63199	1.89226	0.05
63200	1.89226	0.05
63201	1.89226	0.05
63202	1.89226	0.05
63203	1.89226	0.05
63204	1.89226	0.05
63205	1.89226	0.05
63206	1.89226	0.05
63207	1.89226	0.0743587
63208	1.89226	0.05
63209	1.89226	0.0844649
63210	1.89226	0.05
63211	1.89226	0.05
63212	1.89226	0.05
63213	1.89226	0.05
63214	1.89226	0.05
63215	1.89226	0.05
63216	1.89226	0.05
63217	1.5169	0.0863521

63218	1.61969	0.05
63219	1.17262	0.05
63220	1.17262	0.05
63221	1.88868	0.05
63222	1.88868	0.0726528
63223	1.79981	0.0721429
63224	1.88868	0.05
63225	1.5169	0.05
63226	1.5169	0.0589435
63227	2.12891	0.05
63228	2.12891	0.105717
63229	1.89226	0.0803216
63230	1.89226	0.05
63231	1.89226	0.05
63232	1.89226	0.0502927
63233	3.78452	0.0526995
63234	0.946132	0.05
63235	0.946132	0.05
63236	0.946132	0.05
63237	0.946132	0.05
63238	0.946132	0.05
63239	0.946132	0.05
63240	0.946133	0.05
63241	0.946133	0.0576972
63242	0.946133	0.05
63243	0.946133	0.05
63244	0.946132	0.05
63245	0.946132	0.05
63246	1.88868	0.0796221
63247	2.05759	0.05
63248	1.61969	0.05
63249	1.61969	0.0547797
63250	2.29747	0.05
63251	2.29747	0.056376
63252	2.12891	0.107166
63253	2.29747	0.05
63254	1.88868	0.05
63255	1.88868	0.050627
63256	2.50894	0.05
63257	2.50894	0.0681353
63258	1.89226	0.05
63259	1.89226	0.0507322
63260	1.89226	0.05
63261	1.89226	0.0705588
63262	1.89226	0.05
63263	1.89226	0.0718652
63264	0.946132	0.05
63265	0.946132	0.05
63266	0.946132	0.05
63267	0.946132	0.05
63268	0.946131	0.05
63269	0.946131	0.0646936
63270	0.946132	0.05
63271	0.946132	0.0639186
63272	0.946132	0.05
63273	0.946132	0.05
63274	0.946132	0.05
63275	0.946132	0.05

63276	2.42758	1.41293
63277	2.14895	2.1
63278	2.56975	1.33289
63279	2.28142	2.1
63280	4.71869	2.1
63281	1.89226	1.04357
63282	1.89226	2.1
63283	1.89226	1.4105
63284	1.89226	2.1
63285	3.78452	2.1
63286	1.89226	2.1
63287	1.89226	2.1
63288	1.89226	2.1
63289	1.89226	2.1
63290	1.89226	2.1
63291	1.89226	2.1
63292	0.960916	2.1
63293	0.960916	2.1
63294	0.978118	2.1
63295	0.875674	2.1
63296	0.775661	2.1
63297	0.773613	2.1
63298	1.07631	2.1
63299	1.14266	2.1
63300	1.16927	2.1
63301	0.898612	2.1
63302	1.04757	2.1
63303	0.757536	2.1
63304	2.56975	0.979088
63305	2.28142	2.1
63306	2.64384	1.44445
63307	2.40621	2.1
63308	4.97596	2.1
63309	1.89226	1.01156
63310	1.89226	2.1
63311	1.89226	0.19959
63312	1.89226	2.1
63313	3.78452	1.96391
63314	1.89226	2.1
63315	1.89226	2.1
63316	1.89226	2.1
63317	1.89226	2.1
63318	1.89226	2.1
63319	1.89226	2.1
63320	1.03328	2.1
63321	1.03328	2.1
63322	1.04321	2.1
63323	1.15424	2.1
63324	0.936183	2.1
63325	0.944491	2.1
63326	1.14266	2.1
63327	1.20517	2.1
63328	1.23323	2.1
63329	0.961268	2.1
63330	1.11215	2.1
63331	0.827247	2.1
63332	1.89226	2.1
63333	1.89226	1.80761

63334	1.89226	2.1
63335	1.89226	1.97511
63336	3.78453	2.1
63337	2.14647	2.1
63338	2.20589	2.1
63339	1.95567	2.1
63340	2.03224	2.01718
63341	4.17871	2.1
63342	2.06035	2.1
63343	1.88452	0.05
63344	1.88452	2.1
63345	2.26997	0.05
63346	2.03224	1.92911
63347	2.42272	0.0656441
63348	1.89226	2.1
63349	1.89226	0.05
63350	1.89226	0.05
63351	1.89226	2.04146
63352	1.89226	0.05
63353	1.89226	2.1
63354	1.89225	2.1
63355	1.89225	2.1
63356	1.89225	2.1
63357	1.89225	2.1
63358	1.89225	2.1
63359	1.89225	2.1
63360	0.952418	2.1
63361	0.952418	2.1
63362	0.95883	2.1
63363	0.95883	2.1
63364	0.946182	2.1
63365	1.01611	2.1
63366	0.895134	2.1
63367	0.977828	2.1
63368	0.89514	2.1
63369	0.946189	2.1
63370	0.977821	2.1
63371	1.01611	2.1
63372	2.03223	2.1
63373	1.88451	0.05
63374	1.89236	2.1
63375	1.74649	0.05
63376	3.77687	0.0937768
63377	0.81094	2.1
63378	0.81094	0.0600768
63379	0.810939	2.1
63380	0.810939	2.1
63381	0.810945	0.185026
63382	0.810945	0.290646
63383	1.89225	0.05
63384	1.35167	0.0692672
63385	1.08131	2.1
63386	1.08131	0.05
63387	1.0813	0.281537
63388	1.89226	0.05
63389	1.89226	0.05
63390	1.89226	0.05
63391	1.89226	0.05

63392	1.89226	0.05
63393	1.89226	0.05
63394	1.01842	0.05
63395	0.936656	0.05
63396	0.946131	0.05
63397	0.946131	0.05
63398	1.11157	0.05
63399	1.04281	0.05
63400	0.925803	0.05
63401	0.925803	0.05
63402	0.851553	0.05
63403	1.11158	0.05
63404	1.05017	0.05
63405	0.925798	0.05
63406	1.89226	0.05
63407	1.89226	0.05
63408	1.89226	0.05
63409	1.89226	0.05
63410	1.89226	0.05
63411	1.89226	0.05
63412	1.23504	0.05
63413	1.23504	0.05
63414	0.946131	0.05
63415	0.946131	0.05
63416	1.37358	0.05
63417	1.23504	0.05
63418	0.946129	0.05
63419	0.946129	0.05
63420	1.37358	0.05
63421	0.946131	0.05
63422	0.94613	0.05
63423	1.23504	0.05
63424	1.00261	0.05
63425	1.00261	0.05
63426	1.02952	0.05
63427	1.02952	0.05
63428	0.850964	0.05
63429	0.658553	0.05
63430	1.37358	0.05
63431	1.23504	0.05
63432	1.37358	0.05
63433	0.850965	0.05
63434	1.23504	0.05
63435	0.658553	0.05
63436	0.946133	0.05
63437	0.946133	0.05
63438	0.946132	0.05
63439	0.946132	0.05
63440	0.946132	0.05
63441	0.946132	0.05
63442	0.946133	0.05
63443	0.946133	0.05
63444	0.946133	0.05
63445	0.946133	0.05
63446	0.946132	0.05
63447	0.946132	0.05
63448	1.89226	0.05
63449	1.89226	0.05

63450	1.89226	0.05
63451	1.89226	0.05
63452	3.78452	0.05
63453	1.89226	0.05
63454	1.89226	0.05
63455	1.89226	0.05
63456	1.89226	0.05
63457	3.78452	0.05
63458	1.89227	0.05
63459	1.89227	0.05
63460	1.89227	0.05
63461	1.89227	0.05
63462	1.89227	0.05
63463	1.89227	0.05
63464	0.94613	0.05
63465	0.94613	0.05
63466	0.946127	0.05
63467	0.946127	0.05
63468	0.94613	0.05
63469	0.94613	0.05
63470	0.946127	0.05
63471	0.946127	0.05
63472	0.946128	0.05
63473	0.946128	0.05
63474	0.946128	0.05
63475	0.946128	0.05
63476	0.946132	0.05
63477	0.946132	0.05
63478	0.946128	0.05
63479	0.946128	0.05
63480	0.94613	0.05
63481	0.94613	0.05
63482	0.946131	0.05
63483	0.946131	0.05
63484	0.94613	0.05
63485	0.94613	0.05
63486	0.94613	0.05
63487	0.94613	0.05
63488	1.89226	2.1
63489	1.89226	2.1
63490	1.89226	2.1
63491	1.89226	2.1
63492	3.78453	2.1
63493	2.72844	2.1
63494	1.89226	2.1
63495	2.26536	2.1
63496	2.26536	2.1
63497	1.89226	2.1
63498	1.89226	2.1
63499	1.89226	2.1
63500	1.89226	2.07348
63501	1.89226	2.1
63502	1.89226	2.1
63503	1.89226	2.1
63504	1.89226	2.1
63505	1.89226	2.1
63506	1.89226	2.1
63507	1.89226	2.1

63508	1.89226	2.1
63509	1.89226	2.1
63510	1.89226	2.1
63511	2.72844	2.1
63512	2.26536	2.1
63513	2.28367	2.1
63514	1.5791	2.1
63515	4.54903	2.1
63516	0.810945	2.1
63517	0.810945	2.1
63518	0.810943	2.1
63519	0.810943	2.1
63520	1.89226	2.1
63521	1.3517	2.1
63522	1.08133	2.1
63523	1.08132	2.1
63524	1.08132	2.1
63525	0.810933	2.1
63526	0.810933	2.1
63527	1.89227	2.1
63528	1.89227	2.1
63529	1.89227	2.1
63530	1.89227	2.1
63531	3.78453	2.1
63532	2.71897	2.1
63533	2.27495	2.1
63534	2.648	2.1
63535	2.20832	2.1
63536	4.92729	2.1
63537	2.27495	2.1
63538	1.38455	2.1
63539	1.38455	2.1
63540	1.46586	2.1
63541	2.20831	2.1
63542	2.28962	2.1
63543	0.946133	2.1
63544	0.946133	2.1
63545	0.946132	2.1
63546	0.946132	2.1
63547	0.946132	2.1
63548	0.946132	2.1
63549	0.946132	2.1
63550	0.946132	2.1
63551	0.946132	2.1
63552	0.946132	2.1
63553	0.946133	2.1
63554	0.946133	2.1
63555	1.89226	2.1
63556	1.89226	2.1
63557	1.89226	2.1
63558	1.89226	2.1
63559	1.89226	2.1
63560	1.89226	2.1
63561	2.648	2.1
63562	2.08612	2.1
63563	2.08612	2.1
63564	2.20831	2.1
63565	2.56618	2.1

63566	2.68837	2.1
63567	2.08612	2.1
63568	2.20831	2.1
63569	1.30659	2.1
63570	1.30659	2.1
63571	1.38455	2.1
63572	2.16408	2.1
63573	0.810946	2.1
63574	0.810946	2.1
63575	0.810946	2.1
63576	0.810946	2.1
63577	1.89226	2.1
63578	1.3517	2.1
63579	1.08133	2.1
63580	1.08132	2.1
63581	1.08132	2.1
63582	0.810934	2.1
63583	0.810934	2.1
63584	1.89226	1.92598
63585	1.89226	1.58268
63586	1.89226	0.671064
63587	1.89226	2.1
63588	3.78452	1.22771
63589	1.89226	0.557863
63590	1.89226	2.1
63591	1.89226	2.1
63592	1.89226	1.29607
63593	3.78453	2.1
63594	1.08733	2.1
63595	0.856615	2.1
63596	1.25964	1.57766
63597	1.12378	1.53471
63598	1.23551	1.06541
63599	0.969894	2.1
63600	0.985977	2.1
63601	0.985977	1.02931
63602	1.16169	1.88682
63603	1.21072	2.00964
63604	0.765175	2.05
63605	0.863419	2.01383
63606	2.39934	2.1
63607	2.44427	1.70475
63608	2.64003	0.588825
63609	2.39077	2.1
63610	2.39934	2.05175
63611	2.10194	2.1
63612	1.89226	0.719537
63613	1.89226	2.1
63614	1.89226	1.85338
63615	1.89226	0.199953
63616	3.78452	1.97527
63617	0.946132	2.0962
63618	0.946132	2.1
63619	0.946131	0.774363
63620	0.946131	0.871322
63621	0.946132	0.869281
63622	0.946132	2.1
63623	0.946132	2.1

63624	0.946132	1.00095
63625	0.946132	1.81665
63626	0.946132	2.01522
63627	0.946132	1.53318
63628	0.946132	2.01105
63629	1.19967	2.1
63630	0.946128	1.95857
63631	0.946133	0.93906
63632	1.32002	1.75612
63633	1.19967	2.1
63634	1.19967	2.08888
63635	0.94613	2.1
63636	0.94613	0.160919
63637	0.946131	1.32308
63638	0.946131	1.60347
63639	1.19967	1.82532
63640	1.32001	1.78402
63641	1.89226	0.0900053
63642	1.89226	0.926358
63643	1.89226	0.05
63644	1.89226	2.1
63645	3.78451	0.18282
63646	2.6402	0.05
63647	2.28552	0.855401
63648	2.50958	0.05
63649	2.18948	0.580448
63650	4.79509	0.151018
63651	2.28552	0.0702849
63652	1.86102	2.1
63653	2.18949	0.100873
63654	1.78376	2.1
63655	4.06928	2.1
63656	1.89226	0.0926477
63657	1.89226	2.1
63658	1.89226	1.41116
63659	1.89226	2.1
63660	3.78452	2.1
63661	1.89226	0.05
63662	1.89226	2.1
63663	1.89226	0.05
63664	1.89226	1.65061
63665	3.78452	0.0750114
63666	2.50958	0.05
63667	2.18948	0.3348
63668	2.39524	0.05
63669	2.10188	1.74814
63670	4.61146	0.05
63671	2.18949	0.05
63672	1.78376	2.1
63673	1.71839	0.821223
63674	1.71839	2.1
63675	2.10189	1.08057
63676	2.16726	2.1
63677	0.810943	0.407135
63678	0.810943	2.1
63679	0.810945	0.206072
63680	0.810945	0.723874
63681	0.810959	2.1

63682	0.810959	2.1
63683	1.89226	2.1
63684	1.35168	2.1
63685	1.08132	0.992939
63686	1.08132	2.1
63687	1.0813	2.1
63688	1.89226	0.698329
63689	1.89226	1.93519
63690	1.89226	1.12781
63691	1.89226	1.24981
63692	1.89226	1.56639
63693	1.89226	1.98052
63694	1.28421	1.67289
63695	0.992041	0.592156
63696	1.07707	2.1
63697	0.789746	1.89371
63698	1.03815	2.07048
63699	1.03815	1.84432
63700	1.03581	1.97506
63701	1.03581	1.47427
63702	1.28421	1.71768
63703	1.07707	2.1
63704	0.789748	1.86837
63705	0.992038	1.31543
63706	1.89226	0.6846
63707	1.89226	1.99508
63708	1.89226	1.35319
63709	1.89226	1.12423
63710	1.89226	1.56918
63711	1.89226	1.37239
63712	1.89226	0.715284
63713	1.89226	1.47985
63714	1.89226	0.858979
63715	1.89226	1.2005
63716	1.89226	1.31472
63717	1.89226	1.79238
63718	0.92236	2.1
63719	0.655971	1.99665
63720	0.916772	2.08805
63721	0.913834	1.43211
63722	1.18074	1.96082
63723	0.922346	2.1
63724	2.15414	0.996265
63725	1.53528	1.9226
63726	1.22748	2.06516
63727	1.57442	1.08328
63728	0.874683	1.93565
63729	1.25935	2.1
63730	2.61433	2.1
63731	1.22262	2.1
63732	1.22262	2.1
63733	2.62813	2.1
63734	2.62813	2.1
63735	1.89226	2.1
63736	2.61434	2.1
63737	2.62814	2.1
63738	2.61434	2.1
63739	1.90607	2.1

63740	1.89226	2.1
63741	1.89226	2.1
63742	1.89226	2.1
63743	1.89226	2.1
63744	1.89226	2.1
63745	1.89226	2.1
63746	1.89226	2.1
63747	1.89226	2.1
63748	1.89226	2.1
63749	1.89226	2.1
63750	1.89226	2.1
63751	1.89226	2.1
63752	1.89226	2.1
63753	1.31969	2.1
63754	1.31406	2.1
63755	0.629677	2.1
63756	0.643104	2.1
63757	0.976621	2.1
63758	0.976621	2.1
63759	0.98055	2.1
63760	0.98055	2.1
63761	1.3197	2.1
63762	0.643102	2.1
63763	0.629675	2.1
63764	1.31407	2.1
63765	0.946133	2.1
63766	0.946133	2.1
63767	1.3197	2.1
63768	1.31407	2.1
63769	0.946132	2.1
63770	0.946132	2.1
63771	1.3197	2.1
63772	1.3197	2.1
63773	1.3197	2.1
63774	0.946133	2.1
63775	0.946132	2.1
63776	1.31407	2.1
63777	1.89226	2.1
63778	1.89226	2.1
63779	1.89226	2.1
63780	1.89226	2.1
63781	1.89226	2.1
63782	1.89226	2.1
63783	1.89226	2.1
63784	1.89226	2.1
63785	1.89226	2.1
63786	1.89226	2.1
63787	1.89226	2.1
63788	1.89226	2.1
63789	1.89226	0.05
63790	1.89226	0.05
63791	1.89226	0.05
63792	1.89226	0.05
63793	3.78452	0.05
63794	1.89226	0.05
63795	1.89226	0.05
63796	1.89226	0.05
63797	1.89226	0.05

63798	1.89226	0.05
63799	1.89226	0.05
63800	0.946131	0.05
63801	0.946131	0.05
63802	0.946128	0.05
63803	0.946128	0.05
63804	0.94613	0.05
63805	0.94613	0.05
63806	0.94613	0.05
63807	0.94613	0.05
63808	0.94613	0.05
63809	0.94613	0.05
63810	0.94613	0.05
63811	0.94613	0.05
63812	1.89226	0.05
63813	1.89226	0.05
63814	1.89226	0.05
63815	1.89226	0.05
63816	1.89226	0.05
63817	1.89226	0.05
63818	0.924614	0.05
63819	0.914275	0.05
63820	0.906003	0.05
63821	0.62479	0.05
63822	2.02733	0.05
63823	1.48332	0.05
63824	0.833106	0.05
63825	1.19364	0.05
63826	1.57482	0.05
63827	1.18103	0.05
63828	0.905988	0.05
63829	1.89226	2.1
63830	1.89226	2.1
63831	1.89226	2.1
63832	1.89226	2.1
63833	3.78452	2.1
63834	1.89226	2.1
63835	1.89226	2.1
63836	1.89226	2.1
63837	1.89226	2.1
63838	1.89226	2.1
63839	1.89226	2.1
63840	0.816805	2.1
63841	0.928516	2.1
63842	1.4045	2.1
63843	1.26268	2.1
63844	1.0881	2.1
63845	1.0881	2.1
63846	1.10309	2.1
63847	1.10309	2.1
63848	0.928518	2.1
63849	1.4045	2.1
63850	0.816807	2.1
63851	1.26267	2.1
63852	1.89226	2.1
63853	2.52535	2.1
63854	1.89226	2.1
63855	1.89226	2.1

63856	2.809	2.1
63857	2.809	2.1
63858	1.89226	2.1
63859	1.89226	2.1
63860	1.89226	2.1
63861	1.89226	2.1
63862	3.78452	2.1
63863	0.946127	2.1
63864	0.946127	2.1
63865	0.946131	2.1
63866	0.946131	2.1
63867	0.94613	2.1
63868	0.94613	2.1
63869	0.946128	2.1
63870	0.946128	2.1
63871	0.946129	2.1
63872	0.946129	2.1
63873	0.946129	2.1
63874	0.946129	2.1
63875	1.89224	0.266173
63876	1.89224	2.1
63877	1.89224	2.1
63878	1.89224	2.1
63879	3.78448	2.1
63880	2.39244	2.1
63881	2.27036	2.1
63882	2.25945	2.1
63883	2.27398	2.1
63884	4.52981	2.1
63885	2.27036	2.1
63886	1.97088	2.1
63887	1.86302	2.1
63888	1.84991	2.1
63889	4.12027	2.1
63890	1.89224	2.1
63891	1.89224	2.1
63892	1.89224	2.1
63893	1.89224	2.1
63894	3.78447	2.1
63895	1.89226	2.1
63896	1.89226	2.1
63897	1.89226	0.325393
63898	1.89226	2.1
63899	3.78453	2.1
63900	2.25948	2.1
63901	2.33611	2.1
63902	2.05257	2.1
63903	1.94441	2.1
63904	4.20389	2.1
63905	1.92514	2.1
63906	1.84993	2.1
63907	1.94441	2.1
63908	1.74391	2.1
63909	3.79434	2.1
63910	1.89226	2.1
63911	1.89226	2.1
63912	1.89226	2.1
63913	1.89226	2.1

63914	3.78452	2.1
63915	15.1381	0.0911339
63916	15.1381	0.0744127
63917	15.1381	0.1061
63918	15.1381	1.32751
63919	30.2762	0.05
63920	15.1381	0.139525
63921	15.1381	0.685213
63922	15.1381	0.161918
63923	15.1381	0.648749
63924	30.2762	0.754717
63925	15.1381	2.08951
63926	15.1381	2.1
63927	15.1381	1.83081
63928	15.1381	2.1
63929	30.2762	2.1
63930	15.1381	0.056819
63931	15.1381	0.550458
63932	15.1381	0.395369
63933	15.1381	0.239833
63934	30.2762	0.0543265
63935	1.89225	2.1
63936	1.89225	2.1
63937	1.89225	2.1
63938	1.89225	2.1
63939	3.78449	2.1
63940	1.39909	2.1
63941	1.39909	2.1
63942	0.946123	2.1
63943	0.946123	2.1
63944	1.3291	2.1
63945	1.39908	2.1
63946	0.946126	2.1
63947	0.946126	2.1
63948	0.946118	2.1
63949	1.32909	2.1
63950	0.94613	2.1
63951	1.3991	2.1
63952	3.07801	2.1
63953	1.84322	2.1
63954	1.84322	2.1
63955	1.68788	2.1
63956	2.6582	2.1
63957	2.50287	2.1
63958	1.89225	2.1
63959	1.89225	2.1
63960	1.89225	2.1
63961	1.89225	2.1
63962	3.7845	2.1
63963	1.89226	2.1
63964	1.89226	2.1
63965	1.89226	2.1
63966	1.89226	2.1
63967	3.78452	2.1
63968	1.89226	2.1
63969	2.50051	2.1
63970	2.50051	2.1
63971	2.65822	2.1

63972	1.89226	2.1
63973	2.04997	2.1
63974	2.50052	2.1
63975	2.65822	2.1
63976	1.70377	2.1
63977	1.70377	2.1
63978	1.84324	2.1
63979	2.63998	2.1
63980	0.810947	2.1
63981	0.810947	2.1
63982	0.810946	2.1
63983	0.810946	2.1
63984	1.89226	2.1
63985	1.35169	2.1
63986	1.08132	2.1
63987	1.08132	2.1
63988	1.08132	2.1
63989	0.810946	2.1
63990	0.810946	2.1
63991	1.89226	2.1
63992	1.89226	2.1
63993	1.89226	2.1
63994	1.89226	2.1
63995	3.78453	2.1
63996	0.946164	2.1
63997	0.946164	2.1
63998	0.929842	2.1
63999	0.929842	2.1
64000	0.946132	2.1
64001	0.946132	2.1
64002	1.04101	2.1
64003	0.929874	2.1
64004	0.946132	2.1
64005	0.929874	2.1
64006	1.04101	2.1
64007	0.946132	2.1
64008	1.89226	2.1
64009	1.89226	2.1
64010	1.89226	2.1
64011	1.89226	2.1
64012	3.78453	2.1
64013	1.89226	2.1
64014	1.89226	2.1
64015	1.89226	2.1
64016	1.89226	2.1
64017	1.89226	2.1
64018	1.89226	2.1
64019	1.89226	2.1
64020	1.89226	2.1
64021	1.89226	2.1
64022	1.89226	2.1
64023	1.89226	2.1
64024	1.89226	2.1
64025	0.946131	2.1
64026	0.946131	2.1
64027	0.946131	2.1
64028	0.946131	2.1
64029	0.946132	2.1

64030	0.946132	2.1
64031	0.946131	2.1
64032	0.946131	2.1
64033	0.946131	2.1
64034	0.946131	2.1
64035	0.946132	2.1
64036	0.946132	2.1
64037	1.89226	1.28668
64038	1.89226	0.443442
64039	1.89226	0.106318
64040	1.89226	0.641015
64041	3.78453	0.86669
64042	2.73497	0.05
64043	1.93823	0.360463
64044	1.93823	0.05
64045	2.20046	0.940143
64046	2.42279	0.05
64047	2.68501	1.839
64048	1.89226	0.0718543
64049	1.89226	0.331388
64050	1.89226	1.99502
64051	1.89226	0.05
64052	1.89226	1.33828
64053	1.89226	0.05
64054	1.89226	0.169373
64055	1.89226	0.118174
64056	1.89226	0.105386
64057	1.89226	1.06248
64058	1.89226	0.0641754
64059	1.89226	0.222589
64060	2.82984	0.484061
64061	2.15276	0.0962619
64062	2.15276	0.763248
64063	2.42278	0.102046
64064	2.50074	0.05
64065	2.77077	0.0699323
64066	2.42279	0.05
64067	1.93823	0.362275
64068	1.70423	0.05
64069	1.70423	0.37355
64070	2.15276	0.05
64071	2.38676	0.290479
64072	0.810946	0.05
64073	0.810946	0.653251
64074	0.810944	0.05
64075	0.810944	0.05
64076	0.810946	0.0500351
64077	0.810946	0.0789767
64078	1.89226	0.317535
64079	1.35169	0.05
64080	1.08132	0.05
64081	1.08132	0.183033
64082	1.08132	0.05
64083	1.89226	2.1
64084	1.89226	2.1
64085	1.89226	2.1
64086	1.89226	2.1
64087	3.78452	2.1

64088	1.89226	2.1
64089	1.89226	2.1
64090	1.89226	2.1
64091	1.89226	2.1
64092	3.78453	2.1
64093	1.04144	2.1
64094	1.04144	2.1
64095	0.938972	2.1
64096	0.938972	2.1
64097	0.969661	2.1
64098	1.03285	2.1
64099	0.946133	2.1
64100	0.946133	2.1
64101	0.946131	2.1
64102	0.969663	2.1
64103	0.946131	2.1
64104	1.03284	2.1
64105	0.946131	2.1
64106	0.946131	2.1
64107	0.946128	2.1
64108	0.946128	2.1
64109	0.946127	2.1
64110	0.946127	2.1
64111	0.946131	2.1
64112	0.946131	2.1
64113	0.94613	2.1
64114	0.94613	2.1
64115	0.946129	2.1
64116	0.946129	2.1
64117	1.89226	2.1
64118	1.89226	2.1
64119	1.89226	2.1
64120	1.89226	2.1
64121	3.78452	2.1
64122	1.89226	2.1
64123	1.89226	2.1
64124	1.89226	2.1
64125	1.89226	2.1
64126	3.78453	2.1
64127	1.89226	2.1
64128	1.89226	2.1
64129	1.85882	2.1
64130	1.85882	2.1
64131	1.93932	2.1
64132	1.97276	2.1
64133	0.94613	2.1
64134	0.94613	2.1
64135	0.946128	2.1
64136	0.946128	2.1
64137	0.946127	2.1
64138	0.946127	2.1
64139	0.946131	2.1
64140	0.946131	2.1
64141	0.946129	2.1
64142	0.946129	2.1
64143	0.946129	2.1
64144	0.946129	2.1
64145	1.89225	2.1

64146	1.89225	2.1
64147	1.89225	2.1
64148	1.89225	2.1
64149	3.7845	2.1
64150	1.0086	2.1
64151	1.09028	2.1
64152	0.946125	2.1
64153	0.946125	2.1
64154	0.916906	2.1
64155	0.916906	2.1
64156	1.13051	2.1
64157	1.1505	2.1
64158	1.11557	2.1
64159	1.04071	2.1
64160	0.851542	2.1
64161	0.991196	2.1
64162	1.89225	2.1
64163	1.89225	2.1
64164	1.89225	2.1
64165	1.89225	2.1
64166	3.7845	2.1
64167	1.89225	2.1
64168	1.89225	2.1
64169	1.89225	2.1
64170	1.89225	2.1
64171	1.89225	2.1
64172	1.89225	2.1
64173	1.89226	2.1
64174	1.89226	2.1
64175	1.89226	2.1
64176	1.89226	2.1
64177	3.78452	2.1
64178	0.890962	2.1
64179	0.952547	2.1
64180	0.946132	2.1
64181	0.946132	2.1
64182	0.851543	2.1
64183	0.851543	2.1
64184	1.11558	2.1
64185	1.06304	2.1
64186	0.994315	2.1
64187	1.04072	2.1
64188	0.900943	2.1
64189	0.851542	2.1
64190	1.89226	2.1
64191	1.89226	2.1
64192	1.89226	2.1
64193	1.89226	2.1
64194	1.89226	2.1
64195	1.89226	2.1
64196	1.89226	2.1
64197	1.89226	2.1
64198	1.89226	2.1
64199	1.89226	2.1
64200	3.78452	2.1
64201	1.89226	2.1
64202	1.89226	2.1
64203	1.89226	2.1

64204	1.89226	2.1
64205	3.78452	2.1
64206	0.782015	2.1
64207	0.929718	2.1
64208	0.946132	2.1
64209	0.946132	2.1
64210	0.782016	2.1
64211	0.782016	2.1
64212	1.06706	2.1
64213	1.11785	2.1
64214	0.782015	2.1
64215	0.946133	2.1
64216	0.878931	2.1
64217	0.946132	2.1
64218	1.89226	2.1
64219	1.89226	2.1
64220	1.89226	2.1
64221	1.89226	2.1
64222	1.89226	2.1
64223	1.89226	2.1
64224	0.946131	2.1
64225	0.946131	2.1
64226	0.946129	2.1
64227	0.946129	2.1
64228	0.946131	2.1
64229	0.946131	2.1
64230	0.94613	2.1
64231	0.94613	2.1
64232	0.946131	2.1
64233	0.946131	2.1
64234	0.94613	2.1
64235	0.94613	2.1
64236	0.827866	2.1
64237	0.946131	2.1
64238	0.946129	2.1
64239	0.946129	2.1
64240	0.94613	2.1
64241	0.94613	2.1
64242	0.94613	2.1
64243	0.94613	2.1
64244	0.827865	2.1
64245	0.94613	2.1
64246	0.94613	2.1
64247	0.94613	2.1
64248	1.89226	2.1
64249	1.89226	2.1
64250	1.89226	2.1
64251	1.89226	2.1
64252	3.78452	2.1
64253	1.89226	2.1
64254	1.89226	2.1
64255	1.89226	2.1
64256	1.89226	2.1
64257	1.89226	2.1
64258	1.89226	2.1
64259	1.89226	2.1
64260	1.89226	2.1
64261	1.89226	2.1

64262	1.89226	2.1
64263	3.78453	2.1
64264	1.89226	2.1
64265	1.89226	2.1
64266	1.89226	2.1
64267	1.89226	2.1
64268	3.78452	2.1
64269	0.744377	2.1
64270	0.541919	2.1
64271	0.652022	2.1
64272	0.544198	2.1
64273	0.764633	2.1
64274	0.673254	2.1
64275	1.89226	2.1
64276	1.31149	2.1
64277	1.05423	2.1
64278	1.02189	2.1
64279	0.870602	2.1
64280	1.89226	2.1
64281	1.89226	2.1
64282	1.89226	2.1
64283	1.89226	2.1
64284	1.89226	2.1
64285	1.89226	2.1
64286	1.89226	2.1
64287	1.89226	2.1
64288	1.89226	2.1
64289	1.89226	2.1
64290	1.89226	2.1
64291	1.89226	2.1
64292	1.89226	2.1
64293	1.89226	2.1
64294	1.89226	2.1
64295	1.89226	2.1
64296	1.89226	2.1
64297	1.89226	2.1
64298	0.815314	2.1
64299	0.815313	2.1
64300	0.714029	2.1
64301	0.782951	2.1
64302	0.810944	2.1
64303	0.782954	2.1
64304	1.89226	2.1
64305	1.30999	2.1
64306	1.08132	2.1
64307	1.03962	2.1
64308	0.95209	2.1
64309	1.89226	2.1
64310	1.89226	2.1
64311	1.89226	2.1
64312	1.89226	2.1
64313	1.89226	2.1
64314	1.89226	2.1
64315	1.89226	2.1
64316	1.89226	2.1
64317	1.89226	2.1
64318	1.89226	2.1
64319	1.89226	2.1

64320	1.89226	2.1
64321	1.89226	2.1
64322	1.89226	2.1
64323	1.89226	2.1
64324	1.89226	2.1
64325	1.89226	2.1
64326	1.89226	2.1
64327	0.871558	2.1
64328	0.713078	2.1
64329	0.756858	2.1
64330	0.703674	2.1
64331	0.838025	2.1
64332	0.862196	2.1
64333	1.89226	2.1
64334	1.33194	2.1
64335	1.10834	2.1
64336	1.06163	2.1
64337	1.01785	2.1
64338	1.89226	2.1
64339	1.89226	2.1
64340	1.89226	2.1
64341	1.89226	2.1
64342	3.78452	2.1
64343	1.89226	2.1
64344	1.89226	2.1
64345	1.89226	2.1
64346	1.89226	2.1
64347	1.89226	2.1
64348	1.89226	2.1
64349	1.89226	2.1
64350	1.89226	2.1
64351	1.89226	2.1
64352	1.89226	2.1
64353	1.89226	2.1
64354	1.89226	2.1
64355	0.958406	2.1
64356	1.1383	2.1
64357	0.865636	2.1
64358	1.10093	2.1
64359	0.838024	2.1
64360	0.975154	2.1
64361	1.89226	2.1
64362	1.38553	2.1
64363	1.10834	2.1
64364	1.11522	2.1
64365	1.15271	2.1
64366	1.89227	2.1
64367	1.89227	2.1
64368	1.89227	2.1
64369	1.89227	2.1
64370	3.78453	2.1
64371	0.946132	2.1
64372	0.946132	2.1
64373	0.946132	2.1
64374	0.946132	2.1
64375	0.946132	2.1
64376	0.946132	2.1
64377	0.946133	2.1

64378	0.946133	2.1
64379	0.946132	2.1
64380	0.946132	2.1
64381	0.946133	2.1
64382	0.946133	2.1
64383	0.719271	2.1
64384	0.774968	2.1
64385	1.28401	2.1
64386	1.17415	2.1
64387	0.960999	2.1
64388	0.960999	2.1
64389	0.988115	2.1
64390	0.988115	2.1
64391	0.774968	2.1
64392	1.28401	2.1
64393	0.719271	2.1
64394	1.17415	2.1
64395	2.77811	2.1
64396	2.34829	2.1
64397	2.56802	2.1
64398	2.52333	2.1
64399	2.56802	2.1
64400	2.52333	2.1
64401	1.89227	2.1
64402	1.89227	2.1
64403	1.89227	2.1
64404	1.89227	2.1
64405	3.78453	2.1
64406	1.89226	2.1
64407	1.89226	2.1
64408	1.89226	2.1
64409	1.89226	2.1
64410	1.89226	2.1
64411	1.89226	2.1
64412	1.89226	2.1
64413	2.77811	2.1
64414	1.89226	2.1
64415	2.56802	2.1
64416	1.89226	2.1
64417	2.56802	2.1
64418	1.89226	0.0557358
64419	1.89226	2.1
64420	1.89226	0.05
64421	1.89226	2.1
64422	3.78452	0.142179
64423	2.41802	0.254767
64424	1.95764	2.1
64425	1.95764	1.85298
64426	2.27727	2.1
64427	2.39311	0.888643
64428	2.73961	2.1
64429	0.946132	2.1
64430	0.946132	1.30961
64431	0.94613	1.49327
64432	0.94613	2.1
64433	0.946131	2.1
64434	0.946131	2.1
64435	0.946131	0.731226

64436	0.946131	1.1831
64437	0.946131	2.1
64438	0.946131	2.1
64439	0.946131	2.1
64440	0.946131	2.1
64441	1.89226	0.0836719
64442	1.89226	1.01596
64443	1.89226	2.05711
64444	1.89226	0.0510113
64445	1.89226	2.1
64446	1.89226	0.0833947
64447	2.78856	0.05
64448	2.28552	0.919497
64449	2.28552	0.05
64450	2.3931	0.313815
64451	2.6402	0.05
64452	2.74778	0.193355
64453	2.39311	0.0549555
64454	1.95764	2.1
64455	2.28552	0.209219
64456	1.86103	2.1
64457	4.24316	2.1
64458	1.89226	0.306819
64459	1.89226	2.1
64460	1.89226	1.52729
64461	1.89226	2.1
64462	1.89226	1.34521
64463	1.89226	2.1
64464	1.47412	2.1
64465	2.18862	1.82958
64466	2.07226	0.616878
64467	2.33645	1.46114
64468	4.26088	1.38923
64469	1.89226	0.388316
64470	1.89226	1.89743
64471	1.89226	0.671362
64472	1.89226	2.08505
64473	1.89226	1.57457
64474	1.89226	2.1
64475	1.89226	1.08938
64476	1.89226	2.1
64477	1.89226	0.05
64478	1.89226	2.1
64479	3.78453	1.18683
64480	2.18862	1.06295
64481	2.1128	2.1
64482	2.33645	0.925654
64483	2.28188	2.1
64484	4.47051	2.1
64485	2.07226	2.1
64486	2.33645	1.95638
64487	2.20831	0.422833
64488	2.4646	0.791778
64489	4.53686	1.21396
64490	0.946131	0.227711
64491	0.946131	2.1
64492	0.946131	2.1
64493	0.946131	1.26246

64494	0.94613	2.1
64495	0.94613	1.06607
64496	0.946133	2.1
64497	0.946133	2.1
64498	0.946132	0.323475
64499	0.946132	0.186079
64500	0.946131	2.1
64501	0.946131	1.70078
64502	1.89226	1.96189
64503	1.89226	2.1
64504	1.89226	0.445554
64505	1.89226	2.1
64506	3.78453	1.71495
64507	2.33646	1.19946
64508	2.28189	2.1
64509	2.4646	0.398026
64510	2.42758	2.1
64511	4.74648	1.51149
64512	1.89226	2.1
64513	1.89226	2.1
64514	1.89226	2.1
64515	1.89226	2.1
64516	1.89226	2.1
64517	1.89226	2.1
64518	1.89226	2.1
64519	1.89226	2.1
64520	1.89226	2.1
64521	1.89226	2.1
64522	1.89226	2.1
64523	1.89226	2.1
64524	1.89226	2.1
64525	1.89226	2.1
64526	1.89226	2.1
64527	1.89226	2.1
64528	3.78453	2.1
64529	1.89226	2.1
64530	1.89226	2.1
64531	1.89226	2.1
64532	1.89226	2.1
64533	1.89226	2.1
64534	1.89226	2.1
64535	1.89226	2.1
64536	1.89226	2.1
64537	1.89226	2.1
64538	1.89226	2.1
64539	3.78452	2.1
64540	0.877238	2.1
64541	0.877238	2.1
64542	1.24746	2.1
64543	1.04764	2.1
64544	0.964969	2.1
64545	1.1521	2.1
64546	0.946132	2.1
64547	0.946132	2.1
64548	0.946132	2.1
64549	1.12659	2.1
64550	0.964969	2.1
64551	0.946131	2.1

64552	1.89226	2.1
64553	1.89226	2.1
64554	1.89226	2.1
64555	1.89226	2.1
64556	1.89226	2.1
64557	1.89226	2.1
64558	1.89226	2.1
64559	1.89226	2.1
64560	1.89226	2.1
64561	1.89226	2.1
64562	3.78453	2.1
64563	0.868144	2.1
64564	0.992762	2.1
64565	0.946131	2.1
64566	0.946131	2.1
64567	1.07072	2.1
64568	1.07072	2.1
64569	0.868171	2.1
64570	0.868171	2.1
64571	0.992762	2.1
64572	0.946131	2.1
64573	0.946131	2.1
64574	0.868144	2.1
64575	0.946132	2.1
64576	0.946132	2.1
64577	0.946131	2.1
64578	0.946131	2.1
64579	0.946132	2.1
64580	0.946132	2.1
64581	0.946132	2.1
64582	0.946132	2.1
64583	0.946132	2.1
64584	0.946132	2.1
64585	0.946132	2.1
64586	0.946132	2.1
64587	1.89227	2.1
64588	1.89227	2.1
64589	1.89227	2.1
64590	1.89227	2.1
64591	1.89227	2.1
64592	1.89227	2.1
64593	1.89226	2.1
64594	1.89226	2.1
64595	1.89226	2.1
64596	1.89226	2.1
64597	1.89226	2.1
64598	1.89226	2.1
64599	1.98552	2.1
64600	2.22298	2.1
64601	2.22298	2.1
64602	1.89226	2.1
64603	1.89226	2.1
64604	1.54525	2.1
64605	0.810946	2.1
64606	0.810946	2.1
64607	0.810947	2.1
64608	0.810947	2.1
64609	0.81096	2.1

64610	0.81096	2.1
64611	1.89226	2.1
64612	1.35168	2.1
64613	1.08132	2.1
64614	1.08132	2.1
64615	1.0813	2.1
64616	1.89226	2.1
64617	1.89226	2.1
64618	1.89226	2.1
64619	1.89226	2.1
64620	3.78452	2.1
64621	1.7753	2.1
64622	2.39703	2.1
64623	1.90577	2.1
64624	2.03445	2.1
64625	3.82335	2.1
64626	2.39703	2.1
64627	2.47949	0.05
64628	2.26051	2.1
64629	2.36891	0.05
64630	4.76594	1.30238
64631	1.89226	2.1
64632	1.89226	0.058685
64633	1.89226	2.1
64634	1.89226	0.05
64635	3.78452	0.388025
64636	1.89226	2.1
64637	1.89226	2.1
64638	1.89226	2.1
64639	1.89226	2.1
64640	1.89226	2.1
64641	1.89226	2.1
64642	0.883745	2.1
64643	0.973143	2.1
64644	0.988173	2.1
64645	0.988173	2.1
64646	0.941927	2.1
64647	1.02971	2.1
64648	0.854065	2.1
64649	0.82702	2.1
64650	0.847835	2.1
64651	0.981126	2.1
64652	1.02996	2.1
64653	1.10704	2.1
64654	2.28031	2.1
64655	2.26369	0.0901053
64656	2.26369	2.1
64657	2.36891	0.113113
64658	2.30788	2.1
64659	2.42455	0.0907767
64660	1.89226	2.1
64661	1.89226	0.0809874
64662	1.89226	0.05
64663	1.89226	2.1
64664	1.89226	0.0544084
64665	1.89226	2.1
64666	2.71406	2.1
64667	1.83244	2.1

64668	1.83244	2.1
64669	2.07567	2.1
64670	2.52044	2.1
64671	2.76367	2.1
64672	1.89226	2.1
64673	1.89226	2.1
64674	1.89226	2.1
64675	1.89226	2.1
64676	3.78453	2.1
64677	1.89226	2.1
64678	1.89226	2.1
64679	1.89226	2.1
64680	1.89226	2.1
64681	1.89226	2.1
64682	1.89226	2.1
64683	2.70714	2.1
64684	2.3148	2.1
64685	2.3148	2.1
64686	2.52044	2.1
64687	2.54204	2.1
64688	2.74768	2.1
64689	2.52044	2.1
64690	2.3148	2.1
64691	1.83244	2.1
64692	1.83244	2.1
64693	1.62611	2.1
64694	2.3148	2.1
64695	0.946131	2.1
64696	0.946131	2.1
64697	0.946131	2.1
64698	0.946131	2.1
64699	0.946131	2.1
64700	0.946131	2.1
64701	0.946131	2.1
64702	0.946131	2.1
64703	0.946131	2.1
64704	0.946131	2.1
64705	0.946131	2.1
64706	0.946131	2.1
64707	1.89226	2.1
64708	1.89226	2.1
64709	1.89226	2.1
64710	1.89226	2.1
64711	3.78453	2.1
64712	1.89226	2.1
64713	1.89226	2.1
64714	1.89226	2.1
64715	1.89226	2.1
64716	1.89226	2.1
64717	1.89226	2.1
64718	1.89227	2.1
64719	1.89227	2.1
64720	1.89227	2.1
64721	1.89227	2.1
64722	3.78453	2.1
64723	1.89226	2.1
64724	1.89226	2.1
64725	1.89226	2.1

64726	1.89226	2.1
64727	3.78453	2.1
64728	0.915781	2.1
64729	0.863524	2.1
64730	0.717056	2.1
64731	0.830182	2.1
64732	0.865094	2.1
64733	0.918003	2.1
64734	1.89226	2.1
64735	1.29933	2.1
64736	1.11215	2.1
64737	1.05227	2.1
64738	0.966269	2.1
64739	1.89226	2.1
64740	1.89226	2.1
64741	1.89226	2.1
64742	1.89226	2.1
64743	1.89226	2.1
64744	1.89226	2.1
64745	1.89226	2.1
64746	1.89226	2.1
64747	1.89226	2.1
64748	1.89226	2.1
64749	1.89226	2.1
64750	1.89226	2.1
64751	1.89226	2.1
64752	1.89226	2.1
64753	1.89226	2.1
64754	1.89226	2.1
64755	1.89226	2.1
64756	1.89226	2.1
64757	1.01725	2.1
64758	0.997048	2.1
64759	0.818657	2.1
64760	0.960724	2.1
64761	0.838013	2.1
64762	1.04859	2.1
64763	1.89226	2.1
64764	1.41754	2.1
64765	1.17625	2.1
64766	1.0793	2.1
64767	1.18822	2.1
64768	2.16402	2.1
64769	1.89452	2.1
64770	1.50084	2.1
64771	2.09288	2.1
64772	3.9874	2.1
64773	2.16402	2.1
64774	2.13925	2.1
64775	2.04483	2.1
64776	2.09288	2.1
64777	4.23212	2.1
64778	1.89226	2.1
64779	1.89226	2.1
64780	1.89226	2.1
64781	1.89226	2.1
64782	3.78452	2.1
64783	1.89226	2.1

64784	1.89226	2.1
64785	1.89226	2.1
64786	1.89226	2.1
64787	1.89226	2.1
64788	1.89226	2.1
64789	1.9824	2.1
64790	2.09288	2.1
64791	1.69889	2.1
64792	1.6817	2.1
64793	2.00695	2.1
64794	2.00695	2.1
64795	1.93592	2.1
64796	2.09288	2.1
64797	2.00695	2.1
64798	2.09288	2.1
64799	1.9589	2.1
64800	2.04483	2.1
64801	0.946128	2.1
64802	0.946128	2.1
64803	0.946132	2.1
64804	0.946132	2.1
64805	0.94613	2.1
64806	0.94613	2.1
64807	0.946129	2.1
64808	0.946129	2.1
64809	0.94613	2.1
64810	0.94613	2.1
64811	0.94613	2.1
64812	0.94613	2.1
64813	0.946131	2.1
64814	0.946131	2.1
64815	0.946131	2.1
64816	0.946131	2.1
64817	0.946131	2.1
64818	0.946131	2.1
64819	0.946131	2.1
64820	0.946131	2.1
64821	0.946131	2.1
64822	0.946131	2.1
64823	0.946131	2.1
64824	0.946131	2.1
64825	1.89226	2.05872
64826	2.48083	2.1
64827	2.6968	2.08081
64828	2.6968	2.1
64829	1.89226	2.09814
64830	1.89226	2.1
64831	1.89226	2.1
64832	1.89226	2.1
64833	1.89226	2.08061
64834	1.89226	2.1
64835	3.78453	2.1
64836	1.89226	2.1
64837	1.89226	2.1
64838	1.89226	2.1
64839	1.89226	2.1
64840	1.89226	2.1
64841	1.89226	2.1

64842	1.1017	2.1
64843	1.1017	2.1
64844	1.10605	2.1
64845	1.01647	2.1
64846	0.868255	2.1
64847	0.883424	2.1
64848	1.24624	2.1
64849	1.35473	2.1
64850	0.942231	2.1
64851	1.31464	2.1
64852	0.910064	2.1
64853	1.27147	2.1
64854	1.89226	2.02527
64855	1.89226	2.1
64856	1.89226	0.425251
64857	1.89226	2.1
64858	3.78453	2.1
64859	1.89226	2.1
64860	1.89226	2.1
64861	1.89226	2.1
64862	1.89226	2.1
64863	1.89226	2.1
64864	1.89226	2.1
64865	1.89226	2.1
64866	1.89226	2.1
64867	1.89226	2.1
64868	1.89226	2.1
64869	3.78453	2.1
64870	1.89226	2.1
64871	2.70015	2.1
64872	1.89226	2.1
64873	2.61064	2.1
64874	4.59241	2.1
64875	2.70015	2.1
64876	2.3372	2.1
64877	2.61064	2.1
64878	1.85184	2.1
64879	4.55198	2.1
64880	1.89226	2.1
64881	1.89226	2.1
64882	1.89226	2.1
64883	1.89226	2.1
64884	1.89226	2.1
64885	1.89226	2.1
64886	1.89227	2.1
64887	1.89227	2.1
64888	1.89227	2.1
64889	1.89227	2.1
64890	3.78453	2.1
64891	1.89226	2.1
64892	2.52194	2.1
64893	2.61064	2.1
64894	2.61064	2.1
64895	1.89226	2.1
64896	1.89226	2.1
64897	1.095	2.1
64898	1.095	2.1
64899	1.09189	2.1

64900	1.09189	2.1
64901	0.875334	2.1
64902	0.925919	2.1
64903	1.26097	2.1
64904	1.30532	2.1
64905	1.26097	2.1
64906	0.875334	2.1
64907	1.30532	2.1
64908	0.92592	2.1
64909	1.89226	2.1
64910	1.89226	2.1
64911	1.89226	2.1
64912	1.89226	2.1
64913	1.89226	2.1
64914	1.89226	2.1
64915	1.89226	2.1
64916	1.89226	2.1
64917	1.89226	2.1
64918	1.89226	2.1
64919	3.78453	2.1
64920	1.26097	2.1
64921	1.26097	2.1
64922	0.946132	2.1
64923	0.946132	2.1
64924	1.22176	2.1
64925	1.26097	2.1
64926	0.946132	2.1
64927	0.946132	2.1
64928	0.946132	2.1
64929	1.22176	2.1
64930	1.26097	2.1
64931	0.946132	2.1
64932	2.52194	2.1
64933	1.67381	2.1
64934	1.67381	2.1
64935	1.75067	2.1
64936	2.44352	2.1
64937	2.52038	2.1
64938	1.89226	2.1
64939	1.89226	2.1
64940	1.89226	2.1
64941	1.89226	2.1
64942	1.89226	2.1
64943	1.89226	2.1
64944	1.89227	2.1
64945	1.89227	2.1
64946	1.89227	2.1
64947	1.89227	2.1
64948	1.89227	2.1
64949	1.89227	2.1
64950	1.22176	2.1
64951	1.22176	2.1
64952	0.946132	2.1
64953	0.946132	2.1
64954	1.19095	2.1
64955	1.22176	2.1
64956	0.946133	2.1
64957	0.946133	2.1

64958	1.19095	2.1
64959	0.946132	2.1
64960	0.946133	2.1
64961	1.22176	2.1
64962	2.44352	2.1
64963	1.6287	2.1
64964	1.6287	2.1
64965	1.67381	2.1
64966	2.3819	2.1
64967	2.427	2.1
64968	1.89226	2.1
64969	1.89226	2.1
64970	1.89226	2.1
64971	1.89226	2.1
64972	1.89226	2.1
64973	1.89226	2.1
64974	1.89226	2.1
64975	1.89226	2.1
64976	1.89226	2.1
64977	1.89226	2.1
64978	3.78453	2.1
64979	1.89226	2.1
64980	1.89226	2.1
64981	1.89226	2.1
64982	1.89226	2.1
64983	1.89226	2.1
64984	1.89226	2.1
64985	1.89226	2.1
64986	1.89226	2.1
64987	1.89226	2.1
64988	1.89226	2.1
64989	1.89226	2.1
64990	1.89226	2.1
64991	0.851542	2.1
64992	0.851542	2.1
64993	1.1736	2.1
64994	1.29261	2.1
64995	0.852708	2.1
64996	1.18843	2.1
64997	0.946132	2.1
64998	0.946132	2.1
64999	0.946132	2.1
65000	1.07901	2.1
65001	1.04208	2.1
65002	0.936669	2.1
65003	1.89227	2.07612
65004	1.89227	2.1
65005	1.89227	2.1
65006	1.89227	2.1
65007	3.78453	2.1
65008	1.89226	2.1
65009	1.89226	2.1
65010	1.89226	2.1
65011	1.89226	2.1
65012	3.78453	2.1
65013	1.89226	2.1
65014	1.89226	2.1
65015	1.89226	2.1

65016	1.89226	2.1
65017	1.89226	2.1
65018	1.89226	2.1
65019	0.946133	2.1
65020	0.946133	2.1
65021	1.28525	2.1
65022	1.28525	2.1
65023	1.07901	2.1
65024	1.28525	2.1
65025	0.946132	2.1
65026	0.946132	2.1
65027	0.946132	2.1
65028	1.28525	2.1
65029	0.946133	2.1
65030	1.07901	2.1
65031	1.89226	2.1
65032	1.89226	2.1
65033	1.89226	2.1
65034	1.89226	2.1
65035	1.89226	2.1
65036	1.89226	2.1
65037	1.89226	2.1
65038	1.89226	2.1
65039	1.89226	2.1
65040	1.89226	2.1
65041	1.89226	2.1
65042	1.89226	2.1
65043	1.24419	2.1
65044	1.09093	2.1
65045	1.22722	2.1
65046	1.18154	2.1
65047	1.27455	2.1
65048	1.08473	2.1
65049	1.15541	2.1
65050	1.15541	2.1
65051	1.29607	2.1
65052	1.23814	2.1
65053	1.13024	2.1
65054	1.12096	2.1
65055	1.18858	2.1
65056	1.13181	2.1
65057	0.988718	2.1
65058	0.802498	2.1
65059	0.994034	2.1
65060	1.15773	2.1
65061	1.0029	2.1
65062	1.0029	2.1
65063	1.23814	2.1
65064	0.997523	2.1
65065	1.11498	2.1
65066	0.899532	2.1
65067	1.89226	2.1
65068	1.89226	2.1
65069	1.89226	2.1
65070	1.89226	2.1
65071	1.89226	2.1
65072	1.89226	2.1
65073	1.89226	2.1

65074	1.89226	2.1
65075	1.89226	2.1
65076	1.89226	2.1
65077	1.89226	2.1
65078	1.89226	2.1
65079	0.993139	2.1
65080	0.834108	2.1
65081	1.01147	2.1
65082	1.01358	2.1
65083	1.20067	2.1
65084	0.993156	2.1
65085	2.4158	2.1
65086	1.69956	2.1
65087	1.3544	2.1
65088	1.60095	2.1
65089	1.11219	2.1
65090	1.89226	2.1
65091	1.89226	2.1
65092	1.89226	2.1
65093	1.89226	2.1
65094	3.78452	2.1
65095	1.89226	2.1
65096	1.89226	2.1
65097	1.89226	2.1
65098	1.89226	2.1
65099	1.89226	2.1
65100	1.89226	2.1
65101	1.89226	2.1
65102	2.83014	2.1
65103	2.32404	2.1
65104	2.32404	2.1
65105	1.89226	2.1
65106	1.89226	2.1
65107	1.37966	2.1
65108	1.13294	2.1
65109	0.881723	2.1
65110	0.582304	2.1
65111	0.977944	2.1
65112	0.977948	2.1
65113	1.0264	2.1
65114	1.0264	2.1
65115	1.37967	2.1
65116	1.03896	2.1
65117	1.19108	2.1
65118	0.582298	2.1
65119	1.89226	2.1
65120	1.89226	2.1
65121	1.89226	2.1
65122	1.89226	2.1
65123	3.78452	2.1
65124	0.946131	2.1
65125	0.946131	2.1
65126	0.946131	2.1
65127	0.946131	2.1
65128	0.946132	2.1
65129	0.946132	2.1
65130	0.946129	2.1
65131	0.946129	2.1

65132	0.946131	2.1
65133	0.946131	2.1
65134	0.94613	2.1
65135	0.94613	2.1
65136	1.89226	2.1
65137	1.89226	2.1
65138	1.89226	2.1
65139	1.89226	2.1
65140	3.78453	2.1
65141	1.89226	2.1
65142	1.89226	2.1
65143	1.89226	2.1
65144	1.89226	2.1
65145	1.89226	2.1
65146	1.89226	2.1
65147	2.65216	2.1
65148	1.93528	2.1
65149	1.78595	2.1
65150	2.48544	2.1
65151	4.42072	2.1
65152	1.89226	2.1
65153	1.89226	2.1
65154	1.89226	2.1
65155	1.89226	2.1
65156	1.89226	2.1
65157	1.89226	2.1
65158	1.89226	2.1
65159	1.89226	2.1
65160	1.89226	2.1
65161	1.89226	2.1
65162	3.78452	2.1
65163	1.66537	2.1
65164	2.48544	2.1
65165	1.78595	2.1
65166	1.78595	2.1
65167	2.36197	2.1
65168	2.36197	2.1
65169	2.80975	2.1
65170	2.48544	2.1
65171	2.36198	2.1
65172	2.65135	2.1
65173	5.13679	2.1
65174	1.89226	2.1
65175	1.89226	2.1
65176	1.89226	2.1
65177	1.89226	2.1
65178	1.89226	2.1
65179	1.89226	2.1
65180	0.946127	2.1
65181	0.946127	2.1
65182	0.946131	2.1
65183	0.946131	2.1
65184	0.946128	2.1
65185	0.946128	2.1
65186	0.94613	2.1
65187	0.94613	2.1
65188	0.946129	2.1
65189	0.946129	2.1

65190	0.946129	2.1
65191	0.946129	2.1
65192	1.11972	2.1
65193	1.18099	2.1
65194	0.832687	2.1
65195	0.764517	2.1
65196	0.974426	2.1
65197	0.974426	2.1
65198	0.971078	2.1
65199	0.971078	2.1
65200	1.11972	2.1
65201	0.764515	2.1
65202	0.832686	2.1
65203	1.18099	2.1
65204	2.36198	2.1
65205	2.48025	2.1
65206	2.48025	2.1
65207	2.65135	2.1
65208	2.23944	2.1
65209	2.41055	2.1
65210	0.946132	2.1
65211	0.946132	2.1
65212	0.946131	2.1
65213	0.946131	2.1
65214	0.946131	2.1
65215	0.946131	2.1
65216	0.946132	2.1
65217	0.946132	2.1
65218	0.946131	2.1
65219	0.946131	2.1
65220	0.946132	2.1
65221	0.946132	2.1
65222	0.810942	2.1
65223	0.810942	2.1
65224	0.810944	2.1
65225	0.810944	2.1
65226	1.89226	2.1
65227	1.3517	2.1
65228	1.08132	2.1
65229	1.08133	2.1
65230	1.08132	2.1
65231	0.810932	2.1
65232	0.810932	2.1
65233	1.89249	2.1
65234	1.9857	1.84102
65235	2.02433	1.79032
65236	2.15807	1.64819
65237	4.01003	1.81372
65238	1.89226	1.40556
65239	1.89226	2.02174
65240	1.89226	2.00473
65241	1.89226	0.877722
65242	3.78452	2.00926
65243	1.89226	1.87286
65244	1.89226	1.95084
65245	1.89226	0.420434
65246	1.89226	1.63976
65247	3.78452	0.748601

65248	1.98569	1.3887
65249	1.86067	1.86554
65250	2.15807	1.1261
65251	2.07965	1.132
65252	4.06534	2.05405
65253	2.02433	2.0469
65254	2.15807	2.1
65255	2.12608	2.1
65256	2.3025	1.00003
65257	4.32683	2.1
65258	1.89226	2.1
65259	1.89226	1.66316
65260	1.89226	1.01547
65261	1.89226	1.07864
65262	3.78452	1.1738
65263	1.89226	1.06948
65264	1.89226	2.1
65265	1.89226	1.05221
65266	1.89226	1.2039
65267	3.78452	2.06116
65268	2.15807	2.02534
65269	2.07965	1.87708
65270	2.3025	0.758169
65271	2.28384	1.14046
65272	4.38215	0.902515
65273	1.89226	0.200342
65274	1.89226	0.05
65275	1.89226	0.0905124
65276	1.89226	0.05
65277	3.78453	0.05
65278	2.16905	0.0583326
65279	2.56369	0.05
65280	1.88394	0.073559
65281	2.41938	0.05
65282	4.44763	0.05
65283	2.56369	0.05
65284	2.80663	0.0644122
65285	2.41938	0.05
65286	2.69045	0.05
65287	5.25414	0.05
65288	1.89226	0.05
65289	1.89226	0.05
65290	1.89226	0.05
65291	1.89226	0.05
65292	3.78453	0.05
65293	1.89226	0.0536428
65294	1.89226	0.05
65295	1.89226	0.293739
65296	1.89226	0.05
65297	1.89226	0.307794
65298	1.89226	0.05
65299	1.01988	0.05
65300	1.01988	0.05
65301	1.03333	0.05
65302	1.03333	0.05
65303	1.13811	0.0501626
65304	1.20969	0.05
65305	0.941974	0.0503658

65306	0.843515	0.05
65307	0.843514	0.05
65308	1.13811	0.05
65309	0.941973	0.05
65310	1.20969	0.05
65311	2.41938	0.05
65312	2.57414	0.05
65313	2.57414	0.25615
65314	2.69045	0.112428
65315	2.27623	0.05
65316	2.39253	0.0540633
65317	1.89227	0.077329
65318	1.89227	0.05
65319	1.89227	0.05
65320	1.89227	0.05
65321	1.89227	0.05
65322	1.89227	0.05
65323	2.28385	1.50973
65324	2.09114	1.75813
65325	2.46968	0.36522
65326	2.32438	1.55235
65327	4.56082	0.525345
65328	1.89226	0.684085
65329	1.89226	2.06981
65330	1.89226	0.912669
65331	1.89226	1.74975
65332	3.78452	1.75121
65333	1.89226	0.860284
65334	1.89226	2.1
65335	1.89226	0.776851
65336	1.89226	1.67092
65337	3.78452	1.21849
65338	2.09114	0.414561
65339	1.5249	2.08481
65340	2.55684	0.505648
65341	1.86899	1.78643
65342	3.96011	1.35519
65343	2.46968	0.94572
65344	2.32439	1.94759
65345	2.62768	0.646453
65346	2.506	1.4228
65347	4.97568	2.09345
65348	1.89226	1.51757
65349	1.89226	1.77494
65350	1.89226	0.230795
65351	1.89226	1.56353
65352	3.78452	0.466094
65353	1.89226	0.575261
65354	1.89226	1.74017
65355	1.89226	1.14824
65356	1.89226	2.1
65357	1.89226	0.14633
65358	1.89226	2.1
65359	2.55684	1.08505
65360	2.16506	1.61746
65361	1.86901	1.90737
65362	1.86899	1.13926
65363	2.75664	1.12998

65364	2.75662	0.49509
65365	1.89224	0.05
65366	1.89224	0.05
65367	1.89224	0.05
65368	1.89224	0.274828
65369	3.78447	0.05
65370	2.24206	0.05
65371	2.38862	0.598436
65372	2.07107	0.05
65373	2.23097	0.119459
65374	4.47303	0.0643015
65375	2.38863	0.677494
65376	2.25945	2.1
65377	2.25945	0.188928
65378	2.39244	2.1
65379	2.23098	0.123104
65380	2.36397	2.1
65381	1.89224	0.05
65382	1.89224	2.1
65383	1.89224	0.278423
65384	1.89224	0.135757
65385	1.89224	0.21013
65386	1.89224	0.26269
65387	1.89226	0.05
65388	1.89226	0.0516013
65389	1.89226	0.0633331
65390	1.89226	0.05
65391	1.89226	0.05
65392	1.89226	0.05
65393	0.920975	1.54896
65394	0.920979	0.05
65395	1.03015	0.05
65396	1.03015	0.05
65397	0.992407	0.05
65398	1.08759	1.10587
65399	0.854346	0.05
65400	0.87241	0.05
65401	0.872397	0.05
65402	1.04334	0.05
65403	1.00965	0.0835415
65404	1.0876	0.0505241
65405	2.23101	0.950576
65406	2.25948	2.1
65407	2.03577	0.05
65408	2.05257	2.1
65409	4.29524	1.70437
65410	0.810947	0.05
65411	0.810947	0.266096
65412	0.810947	0.05
65413	0.810947	0.05
65414	0.810934	0.0931478
65415	0.810934	0.0938047
65416	1.89227	2.1
65417	1.3517	0.184712
65418	1.08132	0.0906453
65419	1.08132	0.333389
65420	1.08133	0.0610683
65421	1.89226	1.64399

65422	1.89226	2.07527
65423	1.89226	0.722353
65424	1.89226	2.1
65425	1.89226	0.102081
65426	1.89226	2.1
65427	1.89226	2.1
65428	1.89226	2.1
65429	1.89226	1.1406
65430	1.89226	2.1
65431	1.89226	0.82415
65432	1.89226	2.1
65433	0.879432	2.1
65434	0.879432	1.33836
65435	0.900223	0.0773386
65436	0.900223	2.1
65437	0.54571	2.1
65438	0.743092	1.9655
65439	1.03656	2.1
65440	1.19234	0.333429
65441	1.19234	1.84023
65442	0.743092	2.06715
65443	1.03656	2.08637
65444	0.545711	1.9163
65445	1.89226	0.306147
65446	2.38467	2.03116
65447	2.69293	0.252363
65448	2.69293	2.1
65449	1.89226	0.127784
65450	1.89226	1.95187
65451	1.89226	0.8849
65452	1.89226	1.98601
65453	1.89226	0.467437
65454	1.89226	2.1
65455	3.78452	1.27386
65456	0.946129	2.1
65457	0.946129	2.1
65458	0.946133	0.143348
65459	0.946133	2.1
65460	0.946131	1.83851
65461	0.946131	2.1
65462	0.946131	0.134352
65463	0.946131	1.84617
65464	0.946131	1.94796
65465	0.946131	1.92698
65466	0.946131	1.8308
65467	0.946131	1.7407
65468	1.04846	0.0837852
65469	1.04846	2.1
65470	1.05594	0.154192
65471	1.05594	2.1
65472	0.743092	2.1
65473	0.912061	1.59943
65474	1.19234	0.188668
65475	1.34647	0.353517
65476	0.912061	0.265449
65477	1.34646	1.13227
65478	1.19234	0.599851
65479	0.743093	0.66728

65480	1.89226	0.795442
65481	1.89226	2.1
65482	1.89226	0.0664022
65483	1.89226	1.11044
65484	3.78452	1.05398
65485	0.946128	2.1
65486	0.946128	1.62816
65487	0.946133	2.02235
65488	0.946133	0.318804
65489	0.94613	2.1
65490	0.94613	2.1
65491	0.946131	1.89445
65492	0.946131	0.544996
65493	0.946131	2.1
65494	0.946131	2.1
65495	0.946131	1.85438
65496	0.946131	1.87346
65497	1.89226	1.40301
65498	1.89226	2.1
65499	1.89226	0.505719
65500	1.89226	1.7004
65501	3.78452	1.96322
65502	1.89226	1.43564
65503	1.89226	1.66282
65504	1.89226	0.86017
65505	1.89226	2.1
65506	1.89226	0.053604
65507	1.89226	2.1
65508	1.89226	1.49839
65509	1.89226	2.02386
65510	1.89226	0.700873
65511	1.89226	2.1
65512	1.89226	0.306467
65513	1.89226	2.1
65514	0.946132	2.1
65515	0.946132	1.43366
65516	1.17758	0.278531
65517	1.17759	2.1
65518	1.04514	2.1
65519	1.17758	1.08341
65520	0.946133	0.217037
65521	0.946133	0.614405
65522	0.946107	1.23865
65523	1.17755	1.27419
65524	1.04517	1.41109
65525	0.946158	1.1715
65526	1.89226	2.1
65527	2.10171	2.1
65528	1.95064	2.1
65529	2.10171	2.1
65530	1.7412	2.1
65531	1.89226	2.1
65532	1.89226	2.1
65533	1.89226	2.1
65534	1.89226	2.1
65535	1.89226	2.1
65536	1.89226	2.1
65537	1.89226	2.1

65538	1.89226	2.1
65539	1.89226	1.39022
65540	1.89226	2.1
65541	1.89226	2.1
65542	3.78453	2.1
65543	0.946133	2.1
65544	0.946133	2.1
65545	0.946132	2.1
65546	0.946132	2.1
65547	0.946133	2.1
65548	0.946133	2.1
65549	0.946132	2.1
65550	0.946132	2.1
65551	0.946132	2.1
65552	0.946132	2.1
65553	0.946132	2.1
65554	0.946132	2.1
65555	1.70303	2.1
65556	1.95064	2.1
65557	1.74039	2.1
65558	1.95064	2.1
65559	1.69368	2.1
65560	1.89226	2.1
65561	1.89226	2.1
65562	1.89226	2.1
65563	1.89226	2.1
65564	1.89226	2.1
65565	3.78453	2.1
65566	0.946132	0.552934
65567	0.946132	2.1
65568	0.946132	2.1
65569	0.946132	2.1
65570	0.946133	2.1
65571	0.946133	0.965728
65572	0.946132	2.1
65573	0.946132	2.1
65574	0.946107	2.1
65575	0.946107	2.1
65576	0.946158	2.1
65577	0.946158	2.1
65578	0.946133	2.1
65579	0.946133	2.1
65580	0.946132	2.1
65581	0.946132	2.1
65582	0.946133	2.1
65583	0.946133	2.1
65584	0.946132	2.1
65585	0.946132	2.1
65586	0.946107	2.1
65587	0.946107	2.1
65588	0.946158	2.1
65589	0.946158	2.1
65590	1.89226	2.1
65591	1.89226	2.1
65592	1.89226	2.1
65593	1.89226	2.1
65594	1.89226	2.1
65595	1.89226	2.1

65596	1.89226	2.1
65597	1.89226	2.1
65598	1.89226	2.1
65599	1.89226	2.1
65600	1.89226	2.1
65601	1.89226	2.1
65602	2.5198	2.1
65603	2.45893	2.1
65604	2.27322	2.1
65605	2.27322	2.1
65606	2.63576	2.1
65607	2.63576	2.1
65608	1.24045	2.1
65609	1.11906	2.1
65610	1.13578	2.1
65611	0.916205	2.1
65612	1.12539	2.1
65613	1.08404	2.1
65614	1.08484	2.1
65615	1.08484	2.1
65616	1.22836	2.1
65617	0.919363	2.1
65618	1.16507	2.1
65619	0.924864	2.1
65620	1.89226	2.1
65621	1.89226	2.1
65622	1.89226	1.89866
65623	1.89226	2.1
65624	1.89226	1.50926
65625	1.89226	2.1
65626	1.89226	2.1
65627	1.89226	2.1
65628	1.89226	2.1
65629	1.89226	2.1
65630	1.89226	2.1
65631	1.89226	2.1
65632	2.6946	2.1
65633	2.63576	2.1
65634	2.5198	2.1
65635	2.5198	2.1
65636	2.8362	2.1
65637	2.8362	2.1
65638	1.3265	2.1
65639	1.24045	2.1
65640	0.983223	2.1
65641	0.954429	2.1
65642	1.06046	2.1
65643	1.10594	2.1
65644	1.1915	2.1
65645	1.1915	2.1
65646	1.31357	2.1
65647	1.09005	2.1
65648	1.29145	2.1
65649	0.995825	2.1
65650	1.89226	2.1
65651	1.89226	2.1
65652	1.89226	2.1
65653	1.89226	2.1

65654	3.78452	2.1
65655	1.89226	2.1
65656	1.89226	2.1
65657	1.89226	2.1
65658	1.89226	2.1
65659	3.78453	2.1
65660	1.91802	2.1
65661	2.27409	2.1
65662	2.48048	2.1
65663	2.22264	2.1
65664	1.91802	2.1
65665	1.66018	2.1
65666	1.13704	2.1
65667	1.24023	2.1
65668	1.22947	2.1
65669	1.31788	2.1
65670	1.23859	2.1
65671	1.23859	2.1
65672	1.23111	2.1
65673	1.23111	2.1
65674	1.24024	2.1
65675	1.31788	2.1
65676	1.13704	2.1
65677	1.22947	2.1
65678	1.89226	2.1
65679	1.89226	2.1
65680	1.89226	2.1
65681	1.89226	2.1
65682	1.89226	2.1
65683	1.89226	2.1
65684	1.89226	2.1
65685	1.89226	2.1
65686	1.89226	2.1
65687	1.89226	2.1
65688	3.78453	2.1
65689	1.05568	2.1
65690	0.959008	2.1
65691	1.32068	2.1
65692	1.24024	2.1
65693	1.15206	2.1
65694	1.15206	2.1
65695	1.14386	2.1
65696	1.14386	2.1
65697	1.05568	2.1
65698	1.32068	2.1
65699	0.959008	2.1
65700	1.24024	2.1
65701	1.32068	2.1
65702	1.24023	2.1
65703	1.31788	2.1
65704	1.4181	2.1
65705	1.31436	2.1
65706	1.31436	2.1
65707	1.3242	2.1
65708	1.3242	2.1
65709	1.32068	2.1
65710	1.4181	2.1
65711	1.24024	2.1

65712	1.31788	2.1
65713	1.89226	1.5489
65714	1.89226	0.05
65715	1.89226	0.277041
65716	1.89226	0.085457
65717	1.89226	0.251204
65718	1.89226	0.219656
65719	1.02925	0.05
65720	1.16678	0.0967517
65721	0.946132	0.05
65722	0.946132	0.563531
65723	0.935677	0.649094
65724	0.935677	0.05
65725	1.18902	0.170502
65726	1.21562	0.257093
65727	1.13433	0.0655245
65728	1.04071	0.0999072
65729	0.851553	0.0723654
65730	1.06075	0.05
65731	1.89226	0.181474
65732	1.89226	0.05
65733	1.89226	0.0607177
65734	1.89226	0.05
65735	1.89226	0.05
65736	1.89226	0.117711
65737	1.89226	0.650352
65738	1.89226	0.05
65739	1.89226	0.05
65740	1.89226	0.05
65741	3.78453	0.05
65742	0.946128	0.214389
65743	0.946128	0.0702566
65744	0.946133	0.05
65745	0.946133	0.05
65746	0.946131	0.05
65747	0.946131	0.0604529
65748	0.94613	0.351877
65749	0.94613	0.05
65750	0.946131	0.121203
65751	0.946131	0.12973
65752	0.946131	0.05
65753	0.946131	0.05
65754	0.946132	0.553454
65755	0.946132	0.05
65756	0.946132	0.566757
65757	0.946132	0.05
65758	0.946132	0.12641
65759	0.946132	0.05
65760	0.946131	0.118101
65761	0.946131	0.28344
65762	0.946132	0.05
65763	0.946132	0.557466
65764	0.946132	0.05
65765	0.946132	0.0762427
65766	0.946131	2.1
65767	0.946131	1.25094
65768	0.946131	2.1
65769	0.946131	0.3934

65770	0.946131	0.597719
65771	0.946131	2.1
65772	0.946131	0.585027
65773	0.946131	0.299017
65774	0.946131	1.19155
65775	0.946131	1.17932
65776	0.946131	1.23759
65777	0.946131	1.24854
65778	1.89226	1.1744
65779	1.89226	1.41272
65780	1.89226	0.426918
65781	1.89226	2.1
65782	1.89226	0.247263
65783	1.89226	2.1
65784	1.89226	0.896662
65785	1.89226	0.624175
65786	1.89226	0.886192
65787	1.89226	2.1
65788	1.89226	0.261207
65789	1.89226	2.1
65790	1.89226	1.30366
65791	1.89226	0.697392
65792	1.89226	0.176931
65793	1.89226	2.1
65794	1.89226	0.0701671
65795	1.89226	2.1
65796	1.89226	2.06597
65797	1.89226	2.1
65798	1.89226	0.253768
65799	1.89226	2.1
65800	3.78452	1.85441
65801	0.946131	2.1
65802	0.946131	0.292092
65803	1.17195	0.305683
65804	1.17195	2.1
65805	0.958489	2.1
65806	1.17195	1.55743
65807	0.946129	0.327641
65808	0.946129	1.64391
65809	0.946131	1.22502
65810	1.17195	1.27007
65811	0.958487	1.30516
65812	0.94613	1.24871
65813	2.69656	2.1
65814	2.17516	2.1
65815	2.41885	2.1
65816	2.41885	1.98922
65817	2.46476	2.1
65818	2.46476	1.2204
65819	2.69657	2.1
65820	2.30233	0.156383
65821	2.30233	1.72423
65822	2.59476	0.678197
65823	2.46477	1.86687
65824	2.7572	1.31675
65825	1.89226	1.99852
65826	1.89226	0.0560457
65827	1.89226	0.05

65828	1.89226	0.0880566
65829	1.89226	0.05
65830	1.89226	0.316337
65831	1.89226	1.96826
65832	1.89226	0.776462
65833	1.89226	2.1
65834	1.89226	0.05
65835	3.78452	1.13092
65836	2.46477	2.1
65837	2.17516	2.1
65838	1.80252	2.1
65839	2.09715	0.173698
65840	4.27231	2.1
65841	2.46477	2.1
65842	2.09715	0.494212
65843	2.30233	0.964835
65844	2.30233	0.912409
65845	1.96416	0.05
65846	2.09715	0.05
65847	1.89227	0.449024
65848	1.89227	0.05
65849	1.89227	0.0678724
65850	1.89227	0.05
65851	1.89227	0.411326
65852	1.89227	0.73968
65853	1.89226	2.1
65854	1.89226	0.05
65855	1.89226	0.147689
65856	1.89226	0.203116
65857	3.78452	0.154159
65858	0.946132	2.1
65859	0.946132	2.1
65860	0.946132	2.1
65861	0.946132	2.1
65862	0.946132	2.1
65863	0.946132	2.1
65864	0.946132	2.1
65865	0.946132	2.1
65866	0.946132	2.1
65867	0.946132	2.1
65868	0.946131	2.1
65869	0.946131	2.1
65870	1.89226	2.1
65871	1.89226	2.1
65872	1.89226	2.1
65873	1.89226	2.1
65874	3.78453	2.1
65875	1.89226	2.1
65876	1.89226	2.1
65877	1.89226	2.1
65878	1.89226	2.1
65879	1.89226	2.1
65880	1.89226	2.1
65881	1.89226	2.1
65882	1.63707	2.1
65883	1.63707	2.1
65884	1.38859	2.1
65885	1.89226	2.1

65886	1.64377	2.1
65887	0.94613	2.1
65888	0.94613	2.1
65889	0.94613	2.1
65890	0.94613	2.1
65891	0.946131	2.1
65892	0.946131	2.1
65893	0.946129	2.1
65894	0.946129	2.1
65895	0.94613	2.1
65896	0.94613	2.1
65897	0.94613	2.1
65898	0.94613	2.1
65899	2.15888	2.1
65900	1.99309	2.1
65901	2.16841	2.1
65902	2.16841	2.1
65903	2.02311	0.717932
65904	2.02311	0.611891
65905	1.89226	0.670412
65906	1.89226	1.9015
65907	1.89226	2.1
65908	1.89226	1.46542
65909	1.89226	2.1
65910	1.89226	0.295657
65911	1.89226	2.0483
65912	1.89226	2.1
65913	1.89226	2.1
65914	1.89226	2.04908
65915	1.89226	2.1
65916	1.89226	1.97166
65917	1.99309	2.1
65918	1.96035	2.1
65919	1.72526	2.1
65920	1.72526	2.1
65921	2.16841	2.1
65922	2.16841	2.1
65923	2.15888	2.1
65924	2.16841	2.1
65925	2.3111	2.1
65926	2.35938	2.1
65927	4.51826	2.1
65928	1.89226	2.1
65929	1.89226	0.612866
65930	1.89226	0.876478
65931	1.89226	0.05
65932	3.78452	2.1
65933	1.89226	2.1
65934	1.89226	2.1
65935	1.89226	2.1
65936	1.89226	0.428492
65937	3.78452	2.1
65938	2.16841	2.1
65939	1.96035	2.1
65940	2.35938	2.1
65941	2.18493	2.1
65942	4.31973	2.1
65943	1.89226	2.1

65944	1.89226	2.1
65945	1.89226	2.1
65946	1.89226	2.1
65947	3.78453	2.1
65948	1.89226	2.1
65949	1.89226	2.1
65950	1.89226	2.1
65951	1.89226	2.1
65952	3.78452	2.1
65953	0.946158	2.1
65954	0.946158	2.1
65955	0.818509	2.1
65956	0.818509	2.1
65957	0.933975	2.1
65958	0.818539	2.1
65959	0.946128	2.1
65960	0.946128	2.1
65961	0.94613	2.1
65962	0.933974	2.1
65963	0.818537	2.1
65964	0.946129	2.1
65965	0.946131	2.1
65966	0.946131	2.1
65967	0.946131	2.1
65968	0.946131	2.1
65969	0.946132	2.1
65970	0.946132	2.1
65971	0.946129	2.1
65972	0.946129	2.1
65973	0.946131	2.1
65974	0.946131	2.1
65975	0.94613	2.1
65976	0.94613	2.1
65977	1.89226	2.1
65978	1.89226	2.1
65979	1.89226	2.1
65980	1.89226	2.1
65981	3.78453	2.1
65982	1.89226	2.1
65983	1.89226	2.1
65984	1.89226	2.1
65985	1.89226	2.1
65986	3.78452	2.1
65987	1.89226	2.1
65988	1.8706	2.1
65989	1.8706	2.1
65990	1.68112	2.1
65991	2.0815	2.1
65992	1.88715	2.1
65993	0.94613	2.1
65994	0.94613	2.1
65995	0.94613	2.1
65996	0.94613	2.1
65997	0.946131	2.1
65998	0.946131	2.1
65999	0.946129	2.1
66000	0.946129	2.1
66001	0.94613	2.1

66002	0.94613	2.1
66003	0.94613	2.1
66004	0.94613	2.1
66005	1.76401	2.1
66006	2.60983	2.1
66007	2.50461	2.1
66008	2.35897	2.1
66009	1.90965	2.1
66010	1.76401	2.1
66011	1.89226	2.1
66012	1.89226	2.1
66013	1.89226	2.1
66014	1.89226	2.1
66015	3.78452	2.1
66016	1.89226	2.1
66017	1.89226	2.1
66018	1.89226	2.1
66019	1.89226	1.01039
66020	1.89226	2.1
66021	1.89226	1.82293
66022	1.90965	2.1
66023	2.83834	2.1
66024	2.7538	2.1
66025	2.60983	2.1
66026	2.05361	2.1
66027	1.90965	2.1
66028	1.89226	2.1
66029	1.89226	2.1
66030	1.89226	2.1
66031	1.89226	2.1
66032	1.89226	2.1
66033	1.89226	2.1
66034	1.89226	2.1
66035	1.89226	2.1
66036	1.89226	2.1
66037	1.89226	0.771723
66038	3.78452	2.1
66039	2.83834	2.1
66040	1.89226	2.1
66041	2.60983	2.1
66042	2.60983	2.1
66043	1.89226	2.1
66044	1.89226	2.1
66045	1.89226	2.1
66046	1.89226	2.1
66047	1.89226	2.1
66048	1.89226	2.1
66049	1.89226	2.1
66050	1.89226	2.1
66051	1.89226	1.91971
66052	1.89226	2.1
66053	1.89226	2.1
66054	1.89226	1.79797
66055	1.89226	2.1
66056	1.89226	1.99676
66057	2.44785	2.1
66058	1.80223	2.1
66059	2.80894	2.1

66060	2.18296	2.1
66061	4.63081	2.1
66062	1.89226	2.1
66063	1.89226	2.1
66064	1.89226	2.1
66065	1.89226	2.04288
66066	1.89226	2.1
66067	1.89226	2.0567
66068	0.946131	1.24004
66069	0.946131	2.09408
66070	0.946127	2.1
66071	0.946127	2.1
66072	0.946131	2.0293
66073	0.946131	2.1
66074	0.946128	2.1
66075	0.946128	2.1
66076	0.94613	2.1
66077	0.94613	2.1
66078	0.946129	2.1
66079	0.946129	2.1
66080	1.89226	2.07327
66081	1.89226	2.1
66082	1.89226	2.1
66083	1.89226	2.1
66084	3.78452	2.1
66085	1.89226	2.1
66086	1.89226	2.1
66087	1.89226	2.1
66088	1.89226	2.1
66089	3.78453	2.1
66090	2.41083	2.1
66091	2.93937	2.1
66092	1.68761	2.1
66093	1.68761	2.1
66094	1.67248	2.1
66095	2.3957	2.1
66096	1.89226	2.1
66097	1.89226	2.1
66098	1.89226	1.8682
66099	1.89226	2.1
66100	1.89226	1.73383
66101	1.89226	2.1
66102	1.89227	2.1
66103	1.89227	2.1
66104	1.89227	2.1
66105	1.89227	2.1
66106	1.89227	2.1
66107	1.89227	2.1
66108	1.89224	0.131349
66109	1.89224	0.05
66110	1.89224	0.757888
66111	1.89224	0.400745
66112	3.78448	0.106208
66113	1.89224	1.1356
66114	1.89224	0.187082
66115	1.89224	1.07362
66116	1.89224	0.05
66117	1.89224	1.74282

66118	1.89224	0.38784
66119	1.11152	0.0797198
66120	1.29346	0.524997
66121	0.978596	0.264315
66122	0.918324	1.19856
66123	1.06821	1.16665
66124	1.06821	0.05
66125	0.985654	1.87978
66126	1.15278	1.03704
66127	1.07693	1.09117
66128	0.902432	1.32436
66129	0.968939	1.60495
66130	1.34644	0.399379
66131	0.946132	0.108266
66132	0.946132	0.293769
66133	0.946132	0.415503
66134	0.946132	0.0798539
66135	0.946131	0.222277
66136	0.946131	0.05
66137	0.946132	0.935693
66138	0.946132	0.0687734
66139	0.946132	0.368299
66140	0.946132	0.148956
66141	0.946132	0.05
66142	0.946132	0.0840733
66143	0.946133	0.263752
66144	0.946133	0.05
66145	0.946132	0.21768
66146	0.946132	1.1031
66147	0.946132	0.369532
66148	0.946132	0.05
66149	0.946133	2.00055
66150	0.946133	0.0774533
66151	0.946132	0.826435
66152	0.946132	0.265986
66153	0.946132	0.0711797
66154	0.946132	0.0545708
66155	1.89224	0.183948
66156	1.89224	2.1
66157	1.89224	1.26367
66158	1.89224	2.1
66159	1.89224	1.20575
66160	1.89224	2.1
66161	1.89224	1.23168
66162	1.89224	2.1
66163	1.89224	2.1
66164	1.89224	2.1
66165	1.89224	2.1
66166	1.89224	2.1
66167	1.89226	0.65762
66168	1.89226	2.1
66169	1.89226	2.1
66170	1.89226	1.59545
66171	3.78453	2.1
66172	1.11056	2.1
66173	1.21595	2.1
66174	1.12763	2.1
66175	1.12763	2.1

66176	1.23339	2.1
66177	1.35697	2.1
66178	1.03581	2.1
66179	0.95954	2.1
66180	0.89761	2.1
66181	1.26602	2.1
66182	1.12749	2.1
66183	1.45307	2.1
66184	2.71775	2.1
66185	1.89226	2.1
66186	1.89226	2.1
66187	1.89226	2.1
66188	2.47025	2.1
66189	2.47025	2.1
66190	0.94613	2.1
66191	0.94613	2.1
66192	0.94613	1.82536
66193	0.94613	2.1
66194	0.946128	2.1
66195	0.946128	2.1
66196	0.946132	2.1
66197	0.946132	2.1
66198	0.94613	2.1
66199	0.94613	2.1
66200	0.94613	2.1
66201	0.94613	2.1
66202	1.89226	0.41906
66203	1.89226	2.1
66204	1.89226	2.1
66205	1.89226	1.28119
66206	1.89226	2.1
66207	1.89226	1.65213
66208	1.89226	0.800206
66209	1.89226	2.1
66210	1.89226	2.1
66211	1.89226	0.824434
66212	1.89226	2.1
66213	1.89226	1.28544
66214	0.946132	2.1
66215	0.946132	2.1
66216	0.946133	2.1
66217	0.946133	2.1
66218	0.946133	2.1
66219	0.946133	1.85809
66220	0.946132	1.52689
66221	0.946132	2.1
66222	0.946133	2.1
66223	0.946133	2.1
66224	0.946132	2.1
66225	0.946132	2.1
66226	1.89226	1.01516
66227	1.89226	2.1
66228	1.89226	2.1
66229	1.89226	1.77226
66230	1.89226	2.1
66231	1.89226	1.79343
66232	0.946131	1.64913
66233	0.946131	0.872508

66234	0.946127	2.1
66235	0.946127	2.1
66236	0.946131	1.5997
66237	0.946131	2.1
66238	0.946127	2.1
66239	0.946127	1.53592
66240	0.94613	2.08454
66241	0.94613	2.08848
66242	0.946129	2.06537
66243	0.946129	2.06054
66244	1.94291	2.1
66245	2.00118	2.1
66246	2.36337	2.1
66247	2.41217	2.1
66248	4.35508	2.1
66249	1.89226	2.1
66250	1.89226	2.1
66251	1.89226	2.1
66252	1.89226	2.1
66253	3.78452	2.1
66254	1.89226	2.1
66255	1.89226	2.1
66256	1.89226	2.1
66257	1.89226	2.1
66258	3.78452	2.1
66259	1.06209	2.1
66260	1.06209	2.1
66261	1.05987	2.1
66262	1.05987	2.1
66263	0.920378	2.1
66264	1.12137	2.1
66265	1.00059	2.1
66266	1.20608	2.1
66267	1.20608	2.1
66268	1.12137	2.1
66269	1.00059	2.1
66270	0.920376	2.1
66271	2.77222	2.1
66272	2.41216	2.1
66273	2.77002	2.1
66274	2.36337	2.1
66275	2.81882	2.1
66276	2.41216	2.1
66277	1.89226	2.1
66278	1.89226	2.1
66279	1.89226	2.1
66280	1.89226	2.1
66281	1.89226	2.1
66282	1.89226	2.0167
66283	1.89226	2.1
66284	1.89226	1.96559
66285	1.89226	2.1
66286	1.89226	2.1
66287	3.78451	2.1
66288	2.41216	2.1
66289	2.24273	2.1
66290	2.81882	2.1
66291	2.68473	2.1

66292	5.09689	2.1
66293	1.89226	2.1
66294	1.89226	2.1
66295	1.89226	2.1
66296	1.89226	2.1
66297	3.78453	2.1
66298	2.17614	2.1
66299	2.65326	2.1
66300	1.93101	2.1
66301	2.58552	2.1
66302	4.58427	2.1
66303	2.65326	2.1
66304	1.89226	2.1
66305	1.89226	2.1
66306	1.89226	2.1
66307	2.58552	2.1
66308	2.58552	2.1
66309	1.89226	2.1
66310	1.89226	2.1
66311	1.89226	2.1
66312	1.89226	2.1
66313	3.78453	2.1
66314	1.89226	2.1
66315	1.89226	2.1
66316	1.89226	2.1
66317	1.89226	2.1
66318	3.78453	2.1
66319	1.93101	2.1
66320	2.58552	2.1
66321	1.46507	2.1
66322	2.52893	2.1
66323	4.45994	2.1
66324	1.26446	2.1
66325	1.26446	2.1
66326	0.946132	2.1
66327	0.946132	2.1
66328	0.94613	2.1
66329	0.94613	2.1
66330	1.29276	2.1
66331	1.26446	2.1
66332	1.26446	2.1
66333	0.94613	2.1
66334	0.94613	2.1
66335	1.29276	2.1
66336	1.89226	2.1
66337	1.89226	2.1
66338	1.89226	2.1
66339	1.89226	2.1
66340	3.78453	2.1
66341	1.89226	2.1
66342	1.89226	2.1
66343	1.89226	2.1
66344	1.89226	2.1
66345	3.78453	2.1
66346	1.83378	2.1
66347	2.52893	2.1
66348	1.75592	2.1
66349	2.49844	2.1

66350	4.28485	2.1
66351	1.24922	2.1
66352	1.24922	2.1
66353	0.946133	2.1
66354	0.946133	2.1
66355	0.946131	2.1
66356	0.946131	2.1
66357	1.26446	2.1
66358	1.24922	2.1
66359	1.24922	2.1
66360	0.946131	2.1
66361	1.26446	2.1
66362	0.94613	2.1
66363	0.946132	2.1
66364	0.946132	2.1
66365	0.946132	2.1
66366	0.946132	2.1
66367	0.946132	2.1
66368	0.946132	2.1
66369	0.946132	2.1
66370	0.946132	2.1
66371	0.946132	2.1
66372	0.946132	2.1
66373	0.946131	2.1
66374	0.946131	2.1
66375	1.89226	2.1
66376	1.89226	2.1
66377	1.89226	2.1
66378	1.89226	2.1
66379	1.89226	2.1
66380	1.89226	2.1
66381	1.0465	2.1
66382	1.0465	2.1
66383	1.06047	2.1
66384	1.06047	2.1
66385	1.25687	2.1
66386	1.24922	2.1
66387	0.87796	2.1
66388	0.857749	2.1
66389	0.857749	2.1
66390	1.25687	2.1
66391	0.877959	2.1
66392	1.24922	2.1
66393	2.49843	2.1
66394	1.89226	2.1
66395	2.51374	2.1
66396	1.89226	2.1
66397	4.406 2.1	
66398	1.89226	2.1
66399	1.89226	2.1
66400	1.89226	2.1
66401	1.89226	2.1
66402	1.89226	2.1
66403	1.89226	2.1
66404	1.89226	1.33458
66405	1.89226	2.1
66406	1.89226	2.1
66407	1.89226	1.703

66408	1.89226	2.1
66409	1.89226	1.99912
66410	1.89226	2.1
66411	1.89226	1.92884
66412	1.89226	2.1
66413	1.89226	1.73428
66414	1.89226	2.1
66415	1.89226	1.9441
66416	1.03835	2.1
66417	1.03835	2.1
66418	1.15388	2.1
66419	0.94267	2.1
66420	0.736433	2.1
66421	0.849239	2.1
66422	1.15014	2.1
66423	1.39058	2.1
66424	0.838639	2.1
66425	1.39056	2.1
66426	1.09399	2.1
66427	0.736444	2.1
66428	1.89226	1.02618
66429	1.89226	2.1
66430	1.89226	2.1
66431	1.89226	1.06245
66432	1.89226	2.1
66433	1.89226	1.27686
66434	0.94613	1.17084
66435	0.94613	1.20967
66436	0.946127	2.1
66437	0.946127	2.1
66438	0.946131	2.1
66439	0.946131	0.987173
66440	0.946126	2.1
66441	0.946126	1.36813
66442	0.946128	2.1
66443	0.946128	2.1
66444	0.946128	2.1
66445	0.946128	2.1
66446	1.89226	2.1
66447	1.89226	1.29316
66448	1.89226	0.220112
66449	1.89226	2.1
66450	3.78453	1.32605
66451	0.946133	1.91622
66452	0.946133	0.244217
66453	0.946128	2.1
66454	0.946128	1.77189
66455	0.946131	1.91712
66456	0.946131	1.76902
66457	0.946131	0.0766102
66458	0.946131	1.74464
66459	0.946131	1.27992
66460	0.946131	1.24036
66461	0.94613	1.48614
66462	0.94613	1.57798
66463	1.89226	0.654126
66464	1.89226	2.1
66465	1.89226	2.07356

66466	1.89226	1.17745
66467	1.89226	2.07128
66468	1.89226	1.51775
66469	1.89226	0.690232
66470	1.89226	2.03743
66471	1.89226	1.54566
66472	1.89226	1.19382
66473	1.89226	1.69756
66474	1.89226	1.82134
66475	1.89226	0.493768
66476	1.89226	2.1
66477	1.89226	1.24084
66478	1.89226	1.50693
66479	1.89226	1.39676
66480	1.89226	1.86963
66481	1.89226	0.687889
66482	2.67388	2.1
66483	2.23684	2.1
66484	2.23684	1.69764
66485	1.89226	2.1
66486	1.89226	1.91366
66487	1.89226	2.1
66488	1.89226	2.1
66489	1.89226	2.1
66490	1.89226	2.1
66491	1.89226	2.1
66492	1.89226	2.1
66493	1.72197	2.1
66494	2.51374	2.1
66495	2.58004	2.1
66496	2.58004	2.1
66497	1.7155	2.1
66498	1.7155	2.1
66499	2.51374	2.1
66500	1.89226	2.1
66501	1.89226	2.1
66502	1.89226	2.1
66503	2.58003	2.1
66504	2.58003	2.1
66505	0.946132	2.1
66506	0.946132	2.1
66507	0.946132	2.1
66508	0.946132	2.1
66509	0.946132	2.1
66510	0.946132	2.1
66511	0.946132	2.1
66512	0.946132	2.1
66513	0.946131	2.1
66514	0.946131	2.1
66515	0.946132	2.1
66516	0.946132	2.1
66517	1.89226	2.1
66518	1.89226	2.1
66519	1.89226	2.1
66520	1.89226	2.1
66521	3.78453	2.1
66522	1.08262	2.1
66523	1.08262	2.1

66524	1.09674	2.1
66525	1.09674	2.1
66526	1.34652	2.1
66527	1.29002	2.1
66528	0.860984	2.1
66529	0.889338	2.1
66530	1.34652	2.1
66531	0.889338	2.1
66532	0.860983	2.1
66533	1.29002	2.1
66534	1.34652	2.1
66535	1.34652	2.1
66536	0.946133	2.1
66537	0.946133	2.1
66538	0.946131	2.1
66539	0.946131	2.1
66540	1.34652	2.1
66541	1.29002	2.1
66542	1.34652	2.1
66543	0.946131	2.1
66544	0.94613	2.1
66545	1.29002	2.1
66546	1.89226	2.1
66547	1.89226	2.1
66548	1.89226	2.1
66549	1.89226	2.1
66550	1.89226	2.1
66551	1.89226	2.1
66552	1.89226	2.1
66553	1.89226	2.1
66554	1.89226	2.1
66555	1.89226	2.1
66556	1.89226	2.1
66557	1.89226	2.1
66558	0.946131	2.1
66559	0.946131	2.1
66560	0.946131	2.1
66561	0.946131	2.1
66562	0.946131	2.1
66563	0.946131	2.1
66564	0.946131	2.1
66565	0.946131	2.1
66566	0.946131	2.1
66567	0.946131	2.1
66568	0.946131	2.1
66569	0.946131	2.1
66570	1.89226	2.1
66571	1.89226	2.1
66572	1.89226	2.1
66573	1.89226	2.1
66574	1.89226	2.1
66575	1.89226	2.1
66576	1.04103	2.1
66577	1.13849	2.1
66578	0.943014	2.1
66579	0.943014	2.1
66580	0.943591	2.1
66581	0.943591	2.1

66582	1.03757	2.1
66583	1.04098	2.1
66584	1.03761	2.1
66585	0.946131	2.1
66586	0.943041	2.1
66587	0.946131	2.1
66588	0.946128	2.1
66589	0.946128	2.1
66590	0.946133	2.1
66591	0.946133	2.1
66592	0.946131	2.1
66593	0.946131	2.1
66594	0.946131	2.1
66595	0.946131	2.1
66596	0.946131	2.1
66597	0.946131	2.1
66598	0.94613	2.1
66599	0.94613	2.1
66600	0.946132	2.1
66601	0.946132	2.1
66602	0.946132	2.1
66603	0.946132	2.1
66604	0.946132	2.1
66605	0.946132	2.1
66606	0.946132	2.1
66607	0.946132	2.1
66608	0.946132	2.1
66609	0.946132	2.1
66610	0.946131	2.1
66611	0.946131	2.1
66612	1.89226	0.860341
66613	1.89226	2.1
66614	1.89226	1.01802
66615	1.89226	1.10636
66616	3.78452	1.6168
66617	0.946132	1.27733
66618	0.946132	1.7032
66619	0.946131	1.01424
66620	0.946131	0.804292
66621	0.946132	1.02769
66622	0.946132	2.1
66623	0.946132	1.30388
66624	0.946132	1.33267
66625	0.946132	1.34655
66626	0.946132	1.35142
66627	0.946132	1.41184
66628	0.946132	1.61146
66629	0.935482	1.99982
66630	1.08999	1.88416
66631	1.24315	1.5404
66632	1.12559	1.91797
66633	1.21343	1.39457
66634	0.889397	2.1
66635	1.01265	0.473108
66636	1.01265	2.07052
66637	1.07332	1.24223
66638	1.34292	1.80422
66639	0.865649	1.81679

66640	0.840024	2.1
66641	1.15433	1.77597
66642	1.02858	2.07068
66643	1.34082	1.64957
66644	1.08357	1.71985
66645	1.10938	1.02028
66646	1.33349	2.1
66647	1.12232	2.05686
66648	1.12232	0.669247
66649	1.21357	1.69502
66650	1.3408	1.80779
66651	1.02859	1.92382
66652	1.24211	2.06829
66653	1.89226	1.42532
66654	1.89226	0.303255
66655	1.89226	0.919671
66656	1.89226	2.1
66657	1.89226	0.284943
66658	1.89226	1.68568
66659	1.89226	0.608561
66660	1.89226	1.18272
66661	1.89226	0.644591
66662	1.89226	1.97184
66663	1.89226	1.34076
66664	1.89226	1.9693
66665	2.0815	1.77851
66666	2.79029	1.6594
66667	2.36793	1.32029
66668	2.58428	2.1
66669	2.38161	0.473361
66670	2.59922	1.97985
66671	1.89226	2.1
66672	1.89226	1.81882
66673	1.89226	2.1
66674	1.89226	2.1
66675	1.89226	2.1
66676	1.89226	2.1
66677	1.89226	1.90749
66678	1.89226	0.241259
66679	1.89226	0.850113
66680	1.89226	1.16416
66681	3.78452	1.07485
66682	1.89226	2.1
66683	1.89226	0.998136
66684	1.89226	1.98982
66685	1.89226	1.88462
66686	1.89226	1.88171
66687	1.89226	1.8786
66688	0.946132	1.16633
66689	0.946132	2.1
66690	0.94613	2.1
66691	0.94613	2.07109
66692	0.946131	2.1
66693	0.946131	1.14161
66694	0.946131	2.1
66695	0.946131	2.1
66696	0.946131	2.1
66697	0.946131	2.1

66698	0.946131	2.1
66699	0.946131	2.1
66700	1.85178	2.08842
66701	1.89226	0.908281
66702	2.0815	0.452868
66703	2.27054	1.32131
66704	1.70303	0.235645
66705	1.5217	0.563171
66706	1.89226	2.1
66707	1.89226	0.772591
66708	1.89226	1.76785
66709	1.89226	1.24774
66710	1.89226	2.1
66711	1.89226	1.77813
66712	2.58823	0.196392
66713	2.02924	0.0652823
66714	1.79398	0.05
66715	1.79398	0.05
66716	2.76708	0.430816
66717	2.76708	0.142886
66718	0.946133	0.0743513
66719	0.946133	0.089761
66720	1.29411	0.0994738
66721	1.38353	0.0618195
66722	0.946131	0.0524712
66723	0.946131	0.0682297
66724	1.38354	0.136885
66725	1.38354	0.05
66726	0.946131	0.0605496
66727	1.38354	0.0761633
66728	0.94613	0.132455
66729	1.29411	0.0561071
66730	1.89226	0.05
66731	1.89226	0.05
66732	1.89226	0.0876685
66733	1.89226	0.0559056
66734	3.78452	0.0703079
66735	0.946131	0.05
66736	0.946131	0.05
66737	0.946131	0.098516
66738	0.946131	0.05
66739	0.946129	0.05
66740	0.946129	0.05
66741	0.946133	0.104691
66742	0.946133	0.051221
66743	0.946131	0.168432
66744	0.946131	0.05
66745	0.946131	0.05
66746	0.946131	0.05
66747	1.89226	0.05
66748	1.89226	0.05
66749	1.89226	0.0598345
66750	1.89226	0.0522823
66751	3.78452	0.05
66752	0.94613	0.05
66753	0.94613	0.05
66754	0.946131	0.05
66755	0.946131	0.05

66756	0.946128	0.05
66757	0.946128	0.05
66758	0.946133	0.05
66759	0.946133	0.05
66760	0.946131	0.0630423
66761	0.946131	0.05
66762	0.946131	0.082454
66763	0.946131	0.05
66764	0.946129	0.17866
66765	0.946129	0.0800182
66766	1.13486	0.0504161
66767	0.859753	0.104417
66768	0.851541	0.100527
66769	0.851541	0.0957004
66770	1.34346	0.0946315
66771	1.26212	0.0671446
66772	0.851553	0.0644643
66773	1.12403	0.0803411
66774	0.851474	0.05
66775	0.956163	0.0709649
66776	0.946132	0.0713263
66777	0.946132	0.05
66778	0.946133	0.0603581
66779	0.946133	0.05
66780	0.946132	0.0639823
66781	0.946132	0.05
66782	0.946133	0.05
66783	0.946133	0.0613415
66784	0.946133	0.0981003
66785	0.946133	0.05
66786	0.946132	0.05
66787	0.946132	0.05
66788	1.89226	0.05
66789	1.89226	0.05
66790	1.89226	0.0802181
66791	1.89226	0.0580366
66792	3.78453	0.05
66793	0.946128	0.196018
66794	0.946128	0.147353
66795	0.946132	0.0951361
66796	0.946132	0.0963584
66797	0.94613	0.0984619
66798	0.94613	0.120081
66799	0.94613	0.13695
66800	0.94613	0.130782
66801	0.946131	0.174302
66802	0.946131	0.104102
66803	0.94613	0.0948929
66804	0.94613	0.152395
66805	0.946132	0.0670168
66806	0.946132	0.05
66807	0.946133	0.0637326
66808	0.946133	0.05
66809	0.946132	0.05
66810	0.946132	0.05
66811	0.946133	0.05
66812	0.946133	0.05
66813	0.946132	0.0882345

66814	0.946132	0.05
66815	0.946132	0.05
66816	0.946132	0.05
66817	1.89226	0.0503851
66818	1.89226	0.05
66819	1.89226	0.112867
66820	1.89226	0.05
66821	1.89226	0.113611
66822	1.89226	0.05
66823	1.89226	0.0770905
66824	1.89226	0.0835776
66825	1.89226	0.05
66826	1.89226	0.0592419
66827	1.89226	0.0561561
66828	1.89226	0.0871608
66829	1.89226	2.1
66830	1.89226	2.1
66831	1.89226	2.1
66832	1.89226	2.1
66833	3.78452	2.1
66834	1.07652	2.1
66835	1.07652	2.1
66836	0.928749	2.1
66837	0.928749	2.1
66838	0.946132	2.1
66839	0.946132	2.1
66840	1.05914	2.1
66841	0.928746	2.1
66842	1.05914	2.1
66843	0.946131	2.1
66844	0.94613	2.1
66845	0.928747	2.1
66846	1.89226	2.1
66847	1.89226	2.1
66848	1.89226	2.1
66849	1.89226	2.1
66850	1.89226	2.1
66851	1.89226	2.1
66852	1.89227	2.1
66853	1.89227	2.1
66854	1.89227	2.1
66855	1.89227	2.1
66856	1.89227	2.1
66857	1.89227	2.1
66858	1.89226	2.1
66859	1.89226	2.1
66860	1.89226	2.1
66861	1.89226	2.1
66862	1.89226	2.1
66863	1.89226	2.1
66864	2.11828	2.1
66865	1.89226	2.1
66866	2.35845	2.1
66867	1.89226	2.1
66868	4.25071	2.1
66869	1.89226	2.1
66870	1.89226	2.1
66871	1.89226	2.1

66872	1.89226	2.1
66873	3.78453	2.1
66874	1.89226	2.1
66875	1.89226	2.1
66876	1.89226	2.1
66877	1.89226	2.1
66878	1.89226	2.1
66879	1.89226	2.1
66880	1.89226	0.609077
66881	1.89226	1.91566
66882	1.65573	1.78848
66883	1.65573	0.813817
66884	1.89226	1.7599
66885	1.89226	0.774291
66886	1.89226	0.446995
66887	1.89226	1.71241
66888	1.89226	1.47753
66889	1.89226	0.759131
66890	1.89226	1.56092
66891	1.89226	0.861968
66892	1.89226	0.526096
66893	1.89226	1.72712
66894	1.89226	1.68081
66895	1.89226	0.722573
66896	1.89226	1.70651
66897	1.89226	0.752369
66898	1.89226	0.430901
66899	1.89226	1.75318
66900	1.89226	1.47386
66901	1.89226	0.703534
66902	1.89226	1.51369
66903	1.89226	0.763473
66904	0.946128	0.917956
66905	0.946128	0.660977
66906	0.946125	1.33039
66907	0.946125	1.48965
66908	0.94613	0.735992
66909	0.94613	2.09683
66910	0.946124	1.79683
66911	0.946124	0.756233
66912	0.946127	1.18711
66913	0.946127	1.19983
66914	0.946127	1.24308
66915	0.946127	1.30367
66916	1.89226	0.627151
66917	1.89226	1.45622
66918	1.89226	0.386283
66919	1.89226	0.199628
66920	3.78452	0.641782
66921	1.89226	1.08116
66922	1.89226	1.27606
66923	1.89226	0.429908
66924	1.89226	1.16056
66925	3.78453	0.961169
66926	1.05926	1.90727
66927	0.891358	2.1
66928	0.946129	1.45858
66929	0.946129	2.02703

66930	0.946101	1.2714
66931	0.946101	2.1
66932	0.891386	1.68902
66933	0.891386	1.86964
66934	0.891357	1.92648
66935	0.946129	1.69431
66936	0.94613	1.89725
66937	1.05926	1.83973
66938	0.946126	0.690015
66939	0.946126	1.19782
66940	0.94613	0.789928
66941	0.94613	1.43573
66942	0.946131	0.863876
66943	0.946131	1.36136
66944	0.946125	1.44287
66945	0.946125	0.203013
66946	0.946127	0.98473
66947	0.946127	0.648655
66948	0.946128	1.36157
66949	0.946128	1.03233
66950	1.89226	0.631699
66951	1.89226	0.227418
66952	1.89226	0.137373
66953	1.89226	1.188
66954	1.89226	0.222021
66955	1.89226	1.59906
66956	1.89226	0.690295
66957	1.89226	1.45745
66958	1.89226	1.19069
66959	1.89226	1.54431
66960	1.89226	0.594066
66961	1.89226	1.53511
66962	1.16109	2.09054
66963	1.23243	2.1
66964	0.659819	1.61239
66965	0.659819	2.1
66966	0.697965	1.01394
66967	1.00087	2.1
66968	0.891386	2.06652
66969	0.891386	1.68039
66970	0.946129	1.99821
66971	0.65979	1.33582
66972	0.891357	1.93932
66973	0.946129	1.74323
66974	1.89225	0.299852
66975	1.89225	1.16921
66976	1.89225	1.65191
66977	1.89225	0.140151
66978	1.89225	2.1
66979	1.89225	0.510342
66980	1.79981	0.0837127
66981	2.02514	0.0572786
66982	2.33247	0.17076
66983	2.12891	0.05
66984	4.15405	0.05
66985	2.48284	0.316689
66986	2.02514	0.05
66987	2.33247	0.0724756

66988	2.02514	0.157485
66989	2.48567	0.0681989
66990	2.17834	0.145674
66991	1.89226	0.05
66992	1.89226	0.102113
66993	1.89226	0.141677
66994	1.89226	0.0514471
66995	3.78453	0.05
66996	1.89227	0.05
66997	1.89227	0.05
66998	1.89227	0.05
66999	1.89227	0.0614364
67000	1.89227	0.05
67001	1.89227	0.105801
67002	2.50894	0.05
67003	2.70118	0.0935533
67004	2.12891	0.05
67005	2.12891	0.0625018
67006	2.7125	0.05
67007	2.33247	0.0617619
67008	1.89226	0.130366
67009	1.89226	0.0508517
67010	1.89226	0.05
67011	1.89226	0.117991
67012	1.89226	0.05
67013	1.89226	0.0769815
67014	0.946133	0.0528615
67015	0.946133	0.0639529
67016	0.946132	0.05
67017	0.946132	0.05
67018	0.946132	0.05
67019	0.946132	0.0831645
67020	0.946133	0.05
67021	0.946133	0.0719572
67022	0.946132	0.05
67023	0.946132	0.0585495
67024	0.946132	0.0515231
67025	0.946132	0.05
67026	1.90098	0.05
67027	1.36463	0.05
67028	1.56656	0.05
67029	1.36463	0.05
67030	2.04386	0.05
67031	1.89227	0.05
67032	1.89226	0.05
67033	1.89226	0.05
67034	1.89226	0.05
67035	1.89226	0.052813
67036	1.89226	0.05
67037	1.89226	0.05
67038	1.89226	0.060934
67039	1.89226	0.05
67040	1.89226	0.0647863
67041	1.89226	0.0809634
67042	1.89226	0.05
67043	1.89226	0.0582491
67044	1.89226	0.05
67045	1.89226	0.05

67046	1.89226	0.0628125
67047	1.89226	0.0628324
67048	1.89226	0.05
67049	1.89226	0.05
67050	1.89226	0.05
67051	1.70303	0.05
67052	2.1292	0.05
67053	1.70303	0.05
67054	2.32302	0.05
67055	1.90098	0.05
67056	1.89226	0.05
67057	1.89226	0.05
67058	1.89226	0.05
67059	1.89226	0.05
67060	1.89226	0.05
67061	1.89226	0.05
67062	1.89226	0.05
67063	1.89226	0.05
67064	1.89226	0.0568861
67065	1.89226	0.0560445
67066	1.89226	0.05
67067	1.89226	0.05
67068	1.89226	0.05
67069	1.89226	0.05
67070	1.89226	0.0524634
67071	1.89226	0.05
67072	1.89226	0.05
67073	1.89226	0.05
67074	1.89226	0.221679
67075	1.89226	1.45702
67076	1.89226	1.22982
67077	1.89226	0.384059
67078	3.78453	0.190728
67079	1.89226	0.814004
67080	1.89226	1.97247
67081	1.89226	0.241768
67082	1.89226	2.1
67083	1.89226	1.00609
67084	1.89226	2.1
67085	1.89226	1.40959
67086	1.89226	0.343874
67087	1.89226	0.300296
67088	1.89226	0.56636
67089	3.78452	0.863282
67090	1.89224	1.17008
67091	1.89224	0.250481
67092	1.89224	0.572393
67093	1.89224	1.46394
67094	1.89224	0.258542
67095	1.89224	0.495034
67096	1.89224	0.76619
67097	1.89224	2.1
67098	1.89224	1.52754
67099	1.89224	2.1
67100	1.89224	0.190986
67101	1.89224	2.1
67102	1.31307	2.1
67103	1.31307	2.1

67104	0.888948	1.40631
67105	0.888948	1.00459
67106	1.2559	1.62832
67107	0.888979	2.1
67108	0.946114	2.1
67109	0.946114	0.757939
67110	0.946129	0.510502
67111	0.888989	1.36097
67112	0.946104	0.199062
67113	1.25588	0.725827
67114	1.89224	0.185536
67115	1.89224	1.58581
67116	1.89224	0.356043
67117	1.89224	1.23332
67118	1.89224	0.800821
67119	1.89224	1.90621
67120	1.79375	0.05
67121	1.47689	0.05
67122	1.97732	0.05
67123	2.26687	0.05
67124	3.74375	0.05
67125	2.05759	0.05
67126	2.26686	0.05
67127	1.79374	0.05
67128	1.79374	0.05
67129	2.49889	0.05
67130	2.49889	0.05078
67131	1.89226	0.05
67132	1.89226	0.05
67133	1.89226	0.05
67134	1.89226	0.05
67135	3.78452	0.05
67136	1.89227	0.05
67137	1.89227	0.05
67138	1.89227	0.05
67139	1.89227	0.05
67140	1.89227	0.05
67141	1.89227	0.05
67142	1.97732	0.05
67143	2.26687	0.05
67144	2.6607	0.05
67145	2.41464	0.05
67146	4.68151	0.05
67147	1.89226	0.05
67148	1.89226	0.05
67149	1.89226	0.05
67150	1.89226	0.05
67151	3.78452	0.05
67152	1.89226	0.05
67153	1.89226	0.05
67154	1.89226	0.05
67155	1.89226	0.05
67156	1.89226	0.05
67157	1.89226	0.05
67158	1.89226	0.466404
67159	1.89226	1.48715
67160	1.89226	0.799053
67161	1.89226	0.487345

67162	3.78452	0.581065
67163	1.89226	0.575597
67164	1.89226	1.80856
67165	1.89226	0.502449
67166	1.89226	1.24381
67167	1.89226	0.653299
67168	1.89226	1.64516
67169	1.89226	1.50298
67170	1.89226	0.3644
67171	1.89226	0.400974
67172	1.89226	1.32105
67173	1.89226	0.448613
67174	1.89226	1.2764
67175	1.89226	0.871959
67176	1.89226	0.402598
67177	1.89226	0.318423
67178	1.89226	1.36326
67179	1.89226	0.434911
67180	1.89226	1.49382
67181	1.89226	0.663467
67182	2.45282	1.21756
67183	1.89226	1.4487
67184	1.89226	1.81843
67185	1.89226	0.395341
67186	1.89226	1.13968
67187	1.89226	0.434864
67188	1.89226	1.46304
67189	1.89226	0.449485
67190	1.89226	0.621005
67191	1.89226	1.54741
67192	1.89226	2.03323
67193	1.87308	0.05
67194	1.88631	0.05
67195	1.63908	0.05
67196	1.70302	0.05
67197	2.08745	0.05
67198	2.16916	0.05
67199	2.41019	0.05
67200	2.733	0.05
67201	2.08121	0.05
67202	2.08121	0.05
67203	2.74362	0.05
67204	2.41464	0.05
67205	1.89226	0.05
67206	1.89226	0.05
67207	1.89226	0.05
67208	1.89226	0.05
67209	1.89226	0.05
67210	1.89226	0.05
67211	0.946133	0.05
67212	0.946133	0.05
67213	0.946132	0.05
67214	0.946132	0.05
67215	0.946132	0.05
67216	0.946132	0.05
67217	0.946133	0.05
67218	0.946133	0.05
67219	0.946132	0.05

67220	0.946132	0.05
67221	0.946133	0.05
67222	0.946133	0.05
67223	2.02912	0.05
67224	2.57016	0.05
67225	1.88631	0.05
67226	1.88631	0.05
67227	2.55301	0.05
67228	2.4102	0.05
67229	1.89226	0.05
67230	1.89226	0.05
67231	1.89226	0.05
67232	1.89226	0.05
67233	3.78452	0.05
67234	1.89227	0.05
67235	1.89227	0.05
67236	1.89227	0.05
67237	1.89227	0.05
67238	1.89227	0.05
67239	1.89227	0.05
67240	1.89226	2.1
67241	1.89226	2.1
67242	1.89226	2.1
67243	1.89226	2.1
67244	3.78452	2.1
67245	1.2515	2.1
67246	1.34788	2.1
67247	0.946132	2.1
67248	0.946132	2.1
67249	0.973043	2.1
67250	1.16766	2.1
67251	0.919222	2.1
67252	1.2159	2.1
67253	0.946132	2.1
67254	0.946132	2.1
67255	1.33746	2.1
67256	1.13537	2.1
67257	1.89226	2.1
67258	1.89226	2.1
67259	1.89226	2.1
67260	1.89226	2.1
67261	1.89226	2.1
67262	1.89226	2.1
67263	1.89226	2.1
67264	1.89226	2.1
67265	1.89226	2.1
67266	1.89226	2.1
67267	3.78452	2.1
67268	1.89227	2.1
67269	1.89227	1.86383
67270	1.89227	2.1
67271	1.89227	2.1
67272	1.89227	2.1
67273	1.89227	2.1
67274	0.946132	2.1
67275	0.946132	2.1
67276	0.946129	2.1
67277	0.946129	2.1

67278	0.946131	2.1
67279	0.946131	2.1
67280	0.946131	2.1
67281	0.946131	2.1
67282	0.946131	2.1
67283	0.946131	2.1
67284	0.94613	2.1
67285	0.94613	2.1
67286	1.89226	1.26091
67287	1.89226	1.08385
67288	1.89226	1.24662
67289	1.89226	0.914384
67290	3.78453	1.06255
67291	0.946132	1.81307
67292	0.946132	0.975995
67293	0.946132	1.27161
67294	0.946132	1.175
67295	0.946132	0.404259
67296	0.946132	1.87795
67297	0.946132	1.15759
67298	0.946132	1.26171
67299	0.946131	1.95654
67300	0.946131	0.82083
67301	0.946132	1.34218
67302	0.946132	1.01646
67303	1.89226	1.40656
67304	1.89226	0.593505
67305	1.89226	0.530303
67306	1.89226	0.864011
67307	1.89226	1.22806
67308	1.89226	1.44264
67309	1.89226	1.62847
67310	1.89226	0.811678
67311	1.89226	0.554548
67312	1.89226	0.818008
67313	3.78453	0.888944
67314	0.96614	1.02651
67315	0.96614	1.17198
67316	0.946131	0.281434
67317	0.946131	0.732206
67318	0.946131	0.865442
67319	0.96614	0.497973
67320	1.37736	1.17337
67321	0.946131	1.48128
67322	1.37736	1.72265
67323	0.96614	0.424423
67324	0.946131	1.50709
67325	0.946131	0.451163
67326	0.946129	1.45659
67327	0.946129	0.874714
67328	0.946128	1.15747
67329	0.946128	1.37872
67330	0.946132	0.420351
67331	0.946132	1.87058
67332	0.946126	1.30597
67333	0.946126	1.012
67334	0.946129	0.546839
67335	0.946129	0.828272

67336	0.946129	0.451378
67337	0.946129	1.10127
67338	1.89226	0.05
67339	1.65723	0.05
67340	1.83117	0.05
67341	1.65723	0.05
67342	2.0662	0.05
67343	1.89226	0.05
67344	1.89226	0.05
67345	1.89226	0.05
67346	1.89226	0.05
67347	1.89226	0.05
67348	1.89226	0.05
67349	1.89226	0.05
67350	1.89226	0.05
67351	1.89226	0.05
67352	1.89226	0.0519324
67353	1.89226	0.05
67354	1.89226	0.05
67355	1.89226	0.05
67356	1.89226	0.05
67357	1.89226	0.05
67358	1.89226	0.05
67359	1.89226	0.05
67360	1.89226	0.05
67361	1.89226	0.05
67362	1.89226	0.05
67363	1.83117	0.05
67364	1.92807	0.05
67365	1.83117	0.05
67366	1.98916	0.05
67367	1.89226	0.05
67368	1.89226	0.05
67369	1.89226	0.05
67370	1.89226	0.05
67371	1.89226	0.05
67372	3.78453	0.05
67373	1.89226	0.05
67374	1.89226	0.05
67375	1.89226	0.0708806
67376	1.89226	0.0793835
67377	1.89226	0.05
67378	1.89226	0.05
67379	1.89226	0.05
67380	1.89226	0.05
67381	1.89226	0.05
67382	1.89226	0.05
67383	1.89226	0.05
67384	1.89226	0.05
67385	1.88037	0.05
67386	1.89227	0.05
67387	1.89227	0.05
67388	1.89227	0.05
67389	1.98599	0.05
67390	1.98599	0.05
67391	1.89226	0.05
67392	1.89226	0.05
67393	1.89226	0.05

67394	1.89226	0.05
67395	1.89226	0.05
67396	1.89226	0.05
67397	1.89226	0.05
67398	1.89226	0.05
67399	1.89226	0.05
67400	1.89226	0.0658149
67401	3.78452	0.05
67402	1.89226	0.05
67403	1.89226	0.05
67404	1.89226	0.05
67405	1.89226	0.05
67406	1.89226	0.05
67407	1.89226	0.05
67408	1.90116	0.05
67409	1.89227	0.05
67410	1.89226	0.05
67411	1.89227	0.05
67412	1.88036	0.05
67413	1.88037	0.05
67414	1.89226	0.05
67415	1.89226	0.05
67416	1.89226	0.05
67417	1.89226	0.05
67418	1.89226	0.05
67419	1.89226	0.05
67420	0.946132	0.0510367
67421	0.946132	0.05
67422	0.946128	0.0501307
67423	0.946128	0.05
67424	0.94613	0.0517889
67425	0.94613	0.0520085
67426	0.94613	0.05
67427	0.94613	0.05
67428	0.946131	0.0502661
67429	0.946131	0.0572962
67430	0.94613	0.05
67431	0.94613	0.0597397
67432	1.89226	0.05
67433	1.89226	0.05
67434	1.89226	0.05
67435	1.89226	0.05
67436	1.89226	0.05
67437	1.89226	0.05
67438	1.89226	0.137869
67439	1.89226	1.46844
67440	2.35239	1.37436
67441	2.27873	0.194633
67442	1.89226	1.27449
67443	1.83301	0.222001
67444	2.68449	0.333784
67445	1.89226	2.1
67446	1.89226	0.651486
67447	1.89226	0.981935
67448	3.78453	0.783995
67449	0.946132	2.07982
67450	0.946132	0.738164
67451	0.946129	0.475844

67452	0.946129	2.02833
67453	0.946131	2.03442
67454	0.946131	1.43765
67455	0.94613	0.687297
67456	0.94613	1.09539
67457	0.946131	1.52633
67458	0.946131	1.03982
67459	0.946131	1.04378
67460	0.946131	1.58226
67461	1.89226	0.196837
67462	1.89226	1.06888
67463	1.89226	1.53534
67464	1.89226	0.255601
67465	1.89226	1.4498
67466	1.89226	0.286666
67467	1.70303	0.179089
67468	1.89226	1.39599
67469	1.89226	0.188817
67470	1.89226	0.361843
67471	3.78453	0.289385
67472	1.89227	1.06345
67473	1.89227	1.28449
67474	1.89227	0.290912
67475	1.89227	1.94965
67476	3.78453	1.20474
67477	1.89226	0.764978
67478	1.89226	2.06758
67479	1.89226	1.08842
67480	1.89226	1.7358
67481	3.78452	1.55628
67482	1.89226	2.1
67483	1.89226	2.1
67484	1.89226	2.1
67485	1.89226	2.1
67486	3.78452	2.1
67487	0.946132	1.79337
67488	0.946132	2.1
67489	0.946129	2.1
67490	0.946129	2.1
67491	0.94613	2.1
67492	0.94613	2.08006
67493	0.946131	2.1
67494	0.946131	2.1
67495	0.946131	2.1
67496	0.946131	2.1
67497	0.946131	2.1
67498	0.946131	2.06818
67499	1.89226	2.1
67500	1.89226	2.0627
67501	1.89226	2.1
67502	1.89226	2.1
67503	3.78452	2.1
67504	1.89226	1.64408
67505	1.89226	2.1
67506	1.89226	2.1
67507	1.89226	1.69202
67508	1.89226	2.1
67509	1.89226	1.61844

67510	1.89227	2.1
67511	2.48308	1.04456
67512	1.89227	1.71767
67513	1.89227	2.1
67514	3.78453	2.1
67515	1.89226	2.03571
67516	1.89226	2.1
67517	1.89226	2.1
67518	1.89226	1.92567
67519	1.89226	2.1
67520	1.89226	1.74619
67521	1.89226	1.96525
67522	1.89226	1.90449
67523	1.89226	2.1
67524	1.89226	1.879
67525	3.78453	2.1
67526	0.946131	1.91996
67527	0.946131	2.1
67528	0.946131	2.1
67529	0.946131	2.1
67530	0.946131	2.1
67531	0.946131	2.08193
67532	0.946131	2.1
67533	0.946131	2.1
67534	0.946131	2.09296
67535	0.946131	2.1
67536	0.946131	2.1
67537	0.946131	2.0093
67538	1.23719	2.1
67539	1.28439	1.97309
67540	0.94613	1.79734
67541	0.94613	1.92234
67542	1.28439	2.03831
67543	1.6431	2.1
67544	0.946129	1.88655
67545	0.946129	1.67549
67546	1.28439	1.25657
67547	0.946131	1.09665
67548	1.21038	0.854168
67549	1.21038	1.15245
67550	1.89226	2.07617
67551	1.89226	1.75234
67552	1.89226	1.92409
67553	1.89226	1.90243
67554	1.89226	1.80857
67555	1.89226	1.80701
67556	1.89226	2.02141
67557	1.89226	2.08071
67558	1.89226	2.1
67559	1.89226	1.7281
67560	1.89226	2.1
67561	1.89226	1.51273
67562	1.89226	1.35459
67563	1.89226	0.102276
67564	1.89226	0.131613
67565	1.89226	0.430552
67566	1.89226	0.345491
67567	1.89226	1.29943

67568	0.946128	0.134706
67569	0.946128	0.561688
67570	0.946125	0.05
67571	0.946125	0.155335
67572	0.94613	0.0549844
67573	0.94613	0.145287
67574	0.946124	0.29448
67575	0.946124	0.648537
67576	0.946127	0.0719582
67577	0.946127	0.05
67578	0.946127	0.239925
67579	0.946127	0.543294
67580	1.89226	0.05
67581	2.78671	0.05
67582	2.59302	0.05
67583	2.59302	0.05
67584	1.89226	0.100124
67585	1.89226	0.0588894
67586	1.89226	0.0943745
67587	1.89226	0.154129
67588	1.89226	0.0697057
67589	1.89226	0.0704004
67590	1.89226	0.234036
67591	1.89226	0.145309
67592	1.89225	0.0585863
67593	1.89225	0.05
67594	1.89225	0.106357
67595	1.89225	0.0709005
67596	1.89225	0.185163
67597	1.89225	0.05
67598	0.979536	0.05
67599	0.816597	0.05
67600	1.15446	0.05
67601	1.07423	0.05
67602	1.08452	0.05
67603	0.603522	0.05
67604	1.70302	0.05
67605	1.29344	0.05
67606	1.21806	0.05
67607	0.723758	0.05
67608	1.37562	0.05
67609	1.89226	0.05
67610	1.92807	0.05
67611	1.90843	0.05
67612	1.92807	0.05
67613	1.87263	0.05
67614	1.89226	0.05
67615	0.946132	0.05
67616	0.946132	0.05
67617	0.946131	0.05
67618	0.946131	0.05
67619	0.946132	0.05
67620	0.946132	0.05
67621	0.946132	0.05
67622	0.946132	0.05
67623	0.946132	0.05
67624	0.946132	0.05
67625	0.946132	0.05

67626	0.946132	0.05
67627	1.89226	0.05
67628	1.89226	0.0746428
67629	1.89226	0.05
67630	1.89226	0.0791627
67631	3.78452	0.05
67632	1.89226	0.05
67633	1.89226	0.0514225
67634	1.89226	0.0524401
67635	1.89226	0.0527805
67636	1.89226	0.05
67637	1.89226	0.05
67638	1.70303	0.05
67639	1.90843	0.05
67640	1.77002	0.05
67641	1.90843	0.05
67642	1.75709	0.05
67643	1.89226	0.05
67644	1.89226	0.05
67645	1.89226	0.05
67646	1.89226	0.05
67647	1.89226	0.05
67648	3.78453	0.05
67649	1.89226	0.05
67650	1.89226	0.0805686
67651	1.89226	0.05
67652	1.89226	0.0986094
67653	3.78452	0.0748175
67654	0.946131	0.05
67655	0.946131	0.05
67656	0.946131	0.05
67657	0.946131	0.05
67658	0.946129	0.05
67659	0.946129	0.05
67660	0.946133	0.05
67661	0.946133	0.05
67662	0.946131	0.0522529
67663	0.946131	0.05
67664	0.946131	0.05
67665	0.946131	0.05
67666	1.89226	0.05
67667	2.10275	0.247513
67668	1.76377	0.156509
67669	1.76377	0.0584694
67670	1.89226	0.0979067
67671	1.89226	0.05
67672	1.89226	0.05
67673	1.89226	0.228759
67674	2.10275	0.202149
67675	2.55889	0.05
67676	1.89226	0.137443
67677	2.30274	0.05
67678	1.89226	0.0909251
67679	1.89226	0.798241
67680	1.89226	0.718477
67681	1.89226	0.109498
67682	1.89226	0.535856
67683	1.89226	0.0993144

67684	1.89226	0.0653114
67685	1.89226	0.863055
67686	1.89226	0.620852
67687	1.89226	0.134412
67688	1.89226	0.445483
67689	1.89226	0.113479
67690	1.89226	0.05
67691	1.89226	0.206731
67692	1.89226	0.189987
67693	1.89226	0.05
67694	1.89226	0.130423
67695	1.89226	0.05
67696	1.89226	0.05
67697	1.89226	0.220856
67698	1.89226	0.168139
67699	1.89226	0.0513638
67700	1.89226	0.122748
67701	1.89226	0.05
67702	1.89226	0.0942867
67703	1.89226	0.737512
67704	1.89226	0.643414
67705	1.89226	0.138312
67706	1.89226	0.547264
67707	1.89226	0.132576
67708	1.89226	0.488196
67709	1.89226	0.768143
67710	1.89226	0.106451
67711	1.89226	0.195486
67712	1.89226	0.0907205
67713	1.89226	0.414515
67714	0.946131	0.05
67715	0.976399	0.05
67716	1.10124	0.05
67717	0.946131	0.0822151
67718	0.887633	0.0709181
67719	0.887633	0.05
67720	1.16933	0.05
67721	1.23163	0.05
67722	0.946131	0.05
67723	0.946131	0.05
67724	0.976399	0.05
67725	1.14992	0.05
67726	1.89226	0.05
67727	1.89226	0.349697
67728	1.89226	0.05
67729	1.89226	0.0708916
67730	3.78452	0.0543951
67731	1.89226	0.05
67732	1.89226	0.1182
67733	1.89226	0.05
67734	1.89226	0.05
67735	3.78453	0.05
67736	0.946132	0.05
67737	0.946132	0.05
67738	0.946131	0.05
67739	0.946131	0.05
67740	0.946131	0.05
67741	0.946131	0.05

67742	0.946132	0.05
67743	0.946132	0.05
67744	0.946131	0.05
67745	0.946131	0.05
67746	0.946132	0.05
67747	0.946132	0.05
67748	0.946132	0.0749454
67749	0.946132	0.05
67750	0.946131	0.05
67751	0.946131	0.119992
67752	0.946131	0.123209
67753	0.946131	0.0573151
67754	0.946132	0.05
67755	0.946132	0.05
67756	0.946131	0.05
67757	0.946131	0.0503281
67758	0.946132	0.05
67759	0.946132	0.05
67760	1.89226	0.0982842
67761	1.89226	0.179846
67762	1.89226	0.05
67763	1.89226	0.28058
67764	3.78452	0.132736
67765	1.89226	0.808559
67766	1.89226	0.618648
67767	1.89226	0.608459
67768	1.89226	0.274887
67769	3.78453	0.285833
67770	1.89226	0.158107
67771	1.89226	0.780644
67772	1.89226	0.587343
67773	1.89226	0.954247
67774	1.89226	0.189302
67775	1.89226	0.328599
67776	1.89225	0.167339
67777	1.89225	0.297591
67778	1.89225	0.320466
67779	1.89225	0.878453
67780	1.89225	0.0911807
67781	1.89225	0.219015
67782	1.89226	0.315551
67783	1.89226	0.466061
67784	1.89226	0.36514
67785	1.89226	0.725172
67786	1.89226	0.133558
67787	1.89226	0.241088
67788	1.89226	0.990995
67789	1.89226	0.262262
67790	1.89226	0.115064
67791	1.89226	0.0594283
67792	3.78453	0.171254
67793	1.39491	0.0981186
67794	1.39491	0.0995772
67795	0.924649	0.0810003
67796	0.924649	0.095631
67797	0.946131	0.233778
67798	1.37343	0.0546811
67799	0.924678	0.05

67800	0.946131	0.829046
67801	0.924678	0.0612873
67802	1.37343	0.0525738
67803	0.946131	0.379191
67804	0.946131	0.401131
67805	1.89226	0.30863
67806	1.89226	0.0519582
67807	1.89226	0.131436
67808	1.89226	0.203216
67809	1.89226	0.05
67810	1.89226	0.0689074
67811	0.946126	0.05
67812	0.975461	0.05
67813	0.946132	0.05
67814	1.12674	0.05
67815	0.946129	0.05
67816	0.946129	0.05
67817	1.12673	0.05
67818	1.12673	0.05
67819	0.946129	0.05
67820	0.946129	0.05
67821	0.975464	0.05
67822	1.12673	0.05
67823	1.89226	0.05
67824	1.89226	0.05
67825	1.89226	0.05
67826	1.89226	0.05
67827	1.89226	0.05
67828	1.89226	0.05
67829	0.946126	0.05
67830	0.946126	0.05
67831	0.946131	0.05
67832	0.946131	0.05
67833	0.946129	0.05
67834	0.946129	0.05
67835	0.946128	0.05
67836	0.946128	0.05
67837	0.946129	0.05
67838	0.946129	0.05
67839	0.946128	0.05
67840	0.946128	0.05
67841	1.89226	0.05
67842	1.89226	0.05
67843	1.89226	0.05
67844	1.89226	0.05
67845	3.78451	0.05
67846	1.89226	0.05
67847	1.89226	0.05
67848	1.89226	0.05
67849	1.89226	0.05
67850	3.78452	0.05
67851	0.946125	0.05
67852	0.946125	0.05
67853	0.946131	0.05
67854	0.946131	0.05
67855	0.946128	0.05
67856	0.946128	0.05
67857	0.946128	0.05

67858	0.946128	0.05
67859	0.946128	0.05
67860	0.946128	0.05
67861	0.946128	0.05
67862	0.946128	0.05
67863	1.89226	0.05
67864	1.89226	0.0857496
67865	1.89226	0.0580085
67866	1.89226	0.05
67867	3.78452	0.0518377
67868	1.89226	0.05
67869	1.89226	0.0683524
67870	1.89226	0.0574148
67871	1.89226	0.0737661
67872	1.89226	0.05
67873	1.89226	0.0533388
67874	1.12492	0.05
67875	1.41201	0.05
67876	1.12082	0.05
67877	0.771416	0.0516672
67878	1.10725	0.05
67879	1.10725	0.05
67880	1.07618	0.110574
67881	1.07618	0.05
67882	1.12492	0.05
67883	0.771415	0.0596375
67884	1.12082	0.05
67885	1.41201	0.063531
67886	2.82401	0.05
67887	1.89226	0.0576062
67888	1.89226	0.05
67889	1.89226	0.05
67890	2.24983	0.05
67891	2.24983	0.0516961
67892	0.946133	0.0615904
67893	0.946133	0.0518224
67894	0.946129	0.05
67895	0.946129	0.05
67896	0.946131	0.0581881
67897	0.946131	0.0891456
67898	0.946131	0.0606273
67899	0.946131	0.05
67900	0.946131	0.05
67901	0.946131	0.0547064
67902	0.946131	0.0570995
67903	0.946131	0.0672115
67904	0.946132	0.0667023
67905	0.946132	0.0781867
67906	0.946133	0.0613708
67907	0.946133	0.0533044
67908	0.946132	0.0660426
67909	0.946132	0.0854571
67910	0.946133	0.05
67911	0.946133	0.0533657
67912	0.946132	0.0601615
67913	0.946132	0.05
67914	0.946133	0.0746232
67915	0.946133	0.0563092

67916	1.89227	0.05
67917	1.89227	0.0776726
67918	1.89227	0.0863189
67919	1.89227	0.05
67920	1.89227	0.0877602
67921	1.89227	0.05
67922	1.42982	0.05
67923	1.19944	0.05
67924	0.936311	0.05
67925	0.936311	0.05
67926	0.946132	0.05
67927	1.02993	0.05
67928	1.41513	0.05
67929	0.946133	0.05
67930	0.946133	0.05
67931	0.946133	0.05
67932	1.02993	0.05
67933	1.18181	0.05
67934	1.89226	0.05
67935	1.89226	0.102773
67936	1.89226	0.208254
67937	1.89226	0.05
67938	3.78453	0.0541599
67939	1.89226	0.05
67940	1.89226	0.05
67941	1.89226	0.05
67942	1.89226	0.05
67943	1.89226	0.05
67944	1.89226	0.05
67945	1.89226	0.05
67946	1.89226	0.05
67947	1.89226	0.05
67948	1.89226	0.05
67949	1.89226	0.05
67950	1.89226	0.05
67951	0.946131	0.05
67952	0.946131	0.05
67953	0.946132	0.0979674
67954	0.946132	0.0718363
67955	0.946132	0.05
67956	0.946132	0.060421
67957	0.946132	0.106414
67958	0.946132	0.05
67959	0.946132	0.05
67960	0.946132	0.0536431
67961	0.946132	0.05
67962	0.946132	0.05
67963	1.89226	0.706261
67964	1.89226	0.776305
67965	1.89226	1.38814
67966	1.89226	0.331753
67967	1.89226	1.48053
67968	1.89226	0.341617
67969	1.89226	1.21464
67970	2.22749	0.0759463
67971	1.89226	0.162503
67972	1.89226	0.32815
67973	3.78453	0.362847

67974	1.89226	0.957864
67975	1.89226	1.19643
67976	1.89226	1.64087
67977	1.89226	0.311323
67978	3.78453	0.710817
67979	1.89226	0.249459
67980	1.89226	0.384954
67981	1.89226	0.807488
67982	1.89226	0.126237
67983	1.89226	0.86878
67984	1.89226	0.145559
67985	0.94613	0.0991786
67986	0.94613	0.625954
67987	0.94613	1.43529
67988	0.94613	0.503516
67989	0.946131	0.331964
67990	0.946131	0.115338
67991	0.946129	1.19885
67992	0.946129	0.297966
67993	0.94613	0.296229
67994	0.94613	0.341057
67995	0.94613	0.322755
67996	0.94613	0.212877
67997	1.89226	0.05
67998	1.89226	0.05
67999	1.89226	0.05
68000	1.89226	0.05
68001	1.89226	0.05
68002	1.89226	0.05
68003	1.89226	0.05
68004	1.89226	0.05
68005	1.89226	0.05
68006	1.89226	0.05
68007	3.78452	0.05
68008	1.89225	0.05
68009	1.89225	0.05
68010	1.89225	0.05
68011	1.89225	0.05
68012	1.89225	0.05
68013	1.89225	0.05
68014	1.89226	0.05
68015	1.89226	0.05
68016	1.89226	0.05
68017	1.89226	0.05
68018	1.89226	0.05
68019	1.89226	0.05
68020	0.946132	0.05
68021	0.946132	0.05
68022	0.946132	0.05
68023	0.946132	0.05
68024	0.946131	0.05
68025	0.946131	0.05
68026	0.946132	0.05
68027	0.946132	0.05
68028	0.946132	0.05
68029	0.946132	0.05
68030	0.946132	0.05
68031	0.946132	0.05

68032	1.89226	0.05
68033	1.89226	0.05
68034	1.89226	0.05
68035	1.89226	0.05
68036	1.89226	0.05
68037	1.89226	0.05
68038	1.89226	0.05
68039	1.89226	0.05
68040	1.89226	0.05
68041	1.89226	0.05
68042	1.89226	0.05
68043	1.89226	0.05
68044	1.89226	0.05
68045	1.89226	0.05
68046	1.89226	0.05
68047	1.89226	0.05
68048	1.89226	0.05
68049	1.89226	0.05
68050	1.89226	0.05
68051	1.89226	0.05
68052	1.89226	0.05
68053	1.89226	0.05
68054	3.78452	0.05
68055	1.89226	0.05
68056	1.89226	0.05
68057	1.89226	0.05
68058	1.89226	0.05
68059	1.89226	0.05
68060	1.89226	0.05
68061	1.89226	0.05
68062	1.89226	0.05
68063	1.89226	0.05
68064	1.89226	0.05
68065	3.78453	0.05
68066	1.89226	0.05
68067	2.48862	0.05
68068	1.89226	0.05
68069	2.22062	0.05
68070	1.89226	0.05
68071	2.22062	0.05
68072	1.89227	0.05
68073	1.89227	0.05
68074	1.89227	0.05
68075	1.89227	0.05
68076	1.89227	0.05
68077	1.89227	0.05
68078	0.946132	0.05
68079	0.946132	0.05
68080	0.946131	0.05
68081	0.946131	0.05
68082	0.94613	0.05
68083	0.94613	0.05
68084	0.946133	0.05
68085	0.946133	0.05
68086	0.946131	0.05
68087	0.946131	0.05
68088	0.946131	0.05
68089	0.946131	0.05

68090	1.89226	0.05
68091	1.89226	0.05
68092	1.89226	0.05
68093	1.89226	0.05
68094	3.78453	0.05
68095	1.89226	0.05
68096	1.89226	0.05
68097	1.89226	0.05
68098	1.89226	0.05
68099	3.78453	0.05
68100	1.89226	0.05
68101	1.89226	0.05
68102	1.89226	0.05
68103	1.89226	0.0724209
68104	1.89226	0.05
68105	1.89226	0.05
68106	1.89226	0.0792784
68107	1.89226	0.0647122
68108	1.89226	0.05
68109	1.89226	0.108674
68110	3.78452	0.0540178
68111	1.89226	0.05
68112	1.89226	0.0919117
68113	1.89226	0.05
68114	1.89226	0.0506864
68115	3.78453	0.058755
68116	2.57454	0.05
68117	1.89226	0.0643701
68118	1.3663	0.05
68119	1.89226	0.05
68120	3.25856	0.0510597
68121	1.89226	0.0528686
68122	1.89226	0.05
68123	1.89226	0.05
68124	1.89226	0.0714535
68125	3.78451	0.05
68126	1.89226	0.05
68127	1.89226	0.132836
68128	1.89226	0.05
68129	1.89226	0.0648381
68130	1.89226	0.05
68131	1.89226	0.0716826
68132	0.810946	0.0614388
68133	0.810946	0.0501402
68134	0.810946	0.0745243
68135	0.810946	0.117606
68136	0.810959	0.0595374
68137	0.810959	0.05
68138	1.89226	0.060985
68139	1.35168	0.0501471
68140	1.08132	0.05
68141	1.08132	0.0892778
68142	1.0813	0.06811
68143	1.89226	0.05
68144	1.89226	0.155377
68145	1.89226	0.22504
68146	1.89226	0.172667
68147	3.78453	0.119635

68148	1.89226	0.0746245
68149	1.89226	0.117963
68150	1.89226	0.0714995
68151	1.89226	0.336066
68152	1.89226	0.05
68153	1.89226	0.0870292
68154	1.89226	0.123546
68155	1.89226	0.220088
68156	1.89226	0.421255
68157	1.89226	0.161078
68158	3.78452	0.145855
68159	1.89226	0.165123
68160	1.89226	0.175545
68161	1.89226	0.183741
68162	1.89226	0.714729
68163	3.78452	0.232332
68164	2.142	0.282644
68165	1.93949	0.0517797
68166	1.39296	0.253698
68167	2.55711	0.493055
68168	1.39296	0.05
68169	2.55711	0.0533299
68170	1.89226	0.0995281
68171	2.5571	0.05
68172	2.142	0.117812
68173	1.89226	0.671675
68174	4.44936	0.129383
68175	0.946131	0.10448
68176	0.946131	0.0860844
68177	1.27855	0.143477
68178	1.29171	0.142904
68179	1.29171	0.292313
68180	0.946127	0.170977
68181	1.27855	0.05
68182	0.946131	0.516493
68183	1.29172	0.16247
68184	1.29172	0.0716466
68185	0.946129	0.0748011
68186	0.946129	0.110223
68187	1.27855	0.166936
68188	1.29172	0.143551
68189	0.969743	0.315266
68190	1.07109	0.20467
68191	1.29172	0.991649
68192	1.07109	0.0561071
68193	0.969745	0.0509604
68194	1.27855	0.448899
68195	1.10873	0.411122
68196	1.10873	0.520054
68197	1.15273	0.0768614
68198	1.15273	0.0746401
68199	1.89227	0.05
68200	1.89227	0.05
68201	1.89227	0.05
68202	1.89227	0.05
68203	2.1288	0.05
68204	2.1288	0.05
68205	1.89227	0.05

68206	1.89227	0.0500273
68207	1.89227	0.05
68208	1.89227	0.05
68209	3.78453	0.05
68210	1.89226	0.0630529
68211	1.89226	0.0784817
68212	1.89226	0.0536972
68213	1.89226	0.0630742
68214	3.78453	0.056336
68215	1.89226	0.05
68216	1.89226	0.05
68217	1.89226	0.05
68218	1.89226	0.05
68219	1.89226	0.05
68220	1.89226	0.05
68221	1.94432	0.05
68222	1.89226	0.05
68223	2.07911	0.05
68224	1.89226	0.05
68225	2.07911	0.05
68226	1.89226	0.05
68227	1.89226	0.05
68228	1.89226	0.05
68229	1.89226	0.05
68230	1.89226	0.05
68231	1.89226	0.05
68232	1.89226	0.05
68233	1.89226	0.112864
68234	1.89226	0.136259
68235	1.89226	0.269064
68236	1.89226	0.0561881
68237	1.89226	0.299637
68238	1.89226	0.062897
68239	1.89226	0.05
68240	1.89226	0.05
68241	1.89226	0.0687532
68242	1.89226	0.05
68243	1.89226	0.0791942
68244	1.89226	0.05
68245	1.86778	0.05
68246	2.72348	0.05
68247	1.89226	0.05
68248	1.89226	0.05
68249	3.76004	0.05
68250	1.89226	0.118224
68251	1.89226	0.142146
68252	1.89226	0.465991
68253	1.89226	0.0517772
68254	3.78451	0.0974704
68255	1.89226	0.05
68256	1.89226	0.05
68257	1.89226	0.0753833
68258	1.89226	0.05
68259	1.89226	0.0844734
68260	1.89226	0.05
68261	0.94613	0.05
68262	0.94613	0.05
68263	0.94613	0.0680368

68264	0.94613	0.05
68265	0.946131	0.05
68266	0.946131	0.05
68267	0.946129	0.05
68268	0.946129	0.05
68269	0.94613	0.05
68270	0.94613	0.05
68271	0.94613	0.05
68272	0.94613	0.05
68273	0.946124	0.0507168
68274	0.946124	0.05
68275	0.94613	0.157435
68276	0.94613	0.0568017
68277	0.946128	0.05
68278	0.946128	0.05
68279	0.946126	0.137059
68280	0.946126	0.05
68281	0.946127	0.0517237
68282	0.946127	0.0577877
68283	0.946127	0.05
68284	0.946127	0.05
68285	2.09166	0.05
68286	2.1285	0.05
68287	1.78381	0.05
68288	1.78381	0.05
68289	2.47689	0.05
68290	2.47689	0.05
68291	2.09167	0.05
68292	2.26572	0.05
68293	2.73393	0.05
68294	2.4769	0.05
68295	4.74261	0.05
68296	1.89226	0.05
68297	1.89226	0.05
68298	1.89226	0.05
68299	1.89226	0.05
68300	1.89226	0.05
68301	1.89226	0.05
68302	0.946131	0.05
68303	0.946131	0.05
68304	0.946129	0.05
68305	0.946129	0.05
68306	0.94613	0.05
68307	0.94613	0.05
68308	0.946131	0.05
68309	0.946131	0.05
68310	0.94613	0.05
68311	0.94613	0.05
68312	0.946131	0.05
68313	0.946131	0.05
68314	2.27622	0.05
68315	2.66786	0.05
68316	2.1285	0.05
68317	2.1285	0.05
68318	2.62461	0.05
68319	2.47689	0.05
68320	1.89226	0.05
68321	1.89226	0.05

68322	1.89226	0.05
68323	1.89226	0.05
68324	3.78453	0.05
68325	1.89226	0.05
68326	1.89226	0.05
68327	1.89226	0.05
68328	1.89226	0.05
68329	1.89226	0.05
68330	1.89226	0.05
68331	1.89226	0.403773
68332	1.89226	1.16848
68333	1.89226	0.473996
68334	1.89226	1.00136
68335	1.89226	0.462079
68336	1.89226	0.981049
68337	1.89226	1.25803
68338	1.89226	0.580918
68339	1.89226	0.40962
68340	1.89226	1.40098
68341	3.78452	0.797435
68342	1.89227	0.457539
68343	1.89227	1.20729
68344	1.89227	1.11465
68345	1.89227	0.664754
68346	3.78453	0.879481
68347	0.946127	1.21913
68348	0.946127	0.933233
68349	1.06438	1.81806
68350	0.946131	0.770626
68351	1.06438	1.20893
68352	0.94613	1.31702
68353	0.946128	1.07937
68354	0.946128	0.720384
68355	1.06438	1.2107
68356	1.06438	1.78606
68357	0.946129	1.21714
68358	0.946129	0.964691
68359	1.89226	0.465224
68360	1.89226	1.14269
68361	1.89226	0.742363
68362	1.89226	1.07973
68363	1.89226	0.847986
68364	1.89226	1.18862
68365	0.946133	0.843827
68366	0.946133	0.620435
68367	1.39771	1.24464
68368	0.946132	0.492281
68369	1.39771	1.20736
68370	1.39772	0.495951
68371	0.946133	0.764122
68372	0.946133	0.42688
68373	0.946132	1.22806
68374	1.39772	1.30252
68375	0.946132	1.21612
68376	0.946132	0.765778
68377	1.98182	0.05
68378	2.2217	0.05
68379	2.79889	0.05

68380	2.50097	0.05
68381	4.72267	0.05
68382	2.05362	0.05
68383	1.70302	0.05
68384	2.75109	0.05
68385	2.138 0.05	
68386	2.0815	0.05
68387	1.84751	0.05
68388	0.946131	0.05
68389	0.946131	0.05
68390	0.946131	0.05
68391	0.946131	0.05
68392	0.946131	0.05
68393	0.946131	0.05
68394	0.946132	0.05
68395	0.946132	0.05
68396	0.946131	0.05
68397	0.946131	0.05
68398	0.946132	0.05
68399	0.946132	0.05
68400	1.89226	0.05
68401	1.89226	0.05
68402	1.89226	0.05
68403	1.89226	0.05
68404	3.78451	0.05
68405	0.946126	0.05
68406	1.25048	0.05
68407	0.946131	0.05
68408	1.39945	0.05
68409	0.946127	0.05
68410	0.946127	0.05
68411	1.39944	0.05
68412	1.39944	0.05
68413	0.946129	0.05
68414	0.946129	0.05
68415	1.39944	0.05
68416	1.25048	0.05
68417	1.89226	0.05
68418	1.89226	0.05
68419	1.89226	0.05
68420	1.89226	0.05
68421	1.89226	0.05
68422	1.89226	0.05
68423	0.946126	0.05
68424	0.946126	0.05
68425	0.946131	0.05
68426	0.946131	0.05
68427	0.946128	0.05
68428	0.946128	0.05
68429	0.946129	0.05
68430	0.946129	0.05
68431	0.946129	0.05
68432	0.946129	0.05
68433	0.946128	0.05
68434	0.946128	0.05
68435	1.89226	0.05
68436	1.89226	0.05
68437	1.89226	0.05

68438	1.89226	0.05
68439	1.89226	0.05
68440	1.89226	0.05
68441	1.01393	0.05
68442	0.775245	0.05
68443	0.946126	0.05
68444	0.946126	0.05
68445	0.78305	0.05
68446	0.78305	0.05
68447	0.946159	0.05
68448	0.929267	0.05
68449	1.14956	0.05
68450	1.04073	0.05
68451	0.851526	0.05
68452	0.704781	0.05
68453	1.89226	0.05
68454	1.89226	0.05
68455	1.89226	0.05
68456	1.89226	0.05
68457	3.78451	0.05
68458	1.89226	0.05
68459	1.89226	0.05
68460	1.89226	0.05
68461	1.89226	0.05
68462	1.89226	0.05
68463	1.89226	0.05
68464	1.89226	0.05
68465	1.89226	0.05
68466	1.89226	0.05
68467	1.89226	0.05
68468	1.89226	0.05
68469	1.89226	0.05
68470	1.16317	0.05
68471	1.0292	0.05
68472	0.946126	0.05
68473	0.946126	0.05
68474	0.93562	0.05
68475	0.93562	0.05
68476	1.0418	0.05
68477	1.04181	0.05
68478	1.16317	0.05
68479	0.946129	0.05
68480	1.02919	0.05
68481	0.946128	0.05
68482	0.946131	0.05
68483	0.946131	0.05
68484	0.946126	0.05
68485	0.946126	0.05
68486	0.946129	0.05
68487	0.946129	0.05
68488	0.946128	0.05
68489	0.946128	0.05
68490	0.946129	0.05
68491	0.946129	0.05
68492	0.946128	0.05
68493	0.946128	0.05
68494	1.89226	0.05
68495	1.89226	0.05

68496	1.89226	0.05
68497	1.89226	0.05
68498	1.89226	0.05
68499	1.89226	0.05
68500	1.89226	0.05
68501	1.89226	0.05
68502	1.89226	0.05
68503	1.89226	0.05
68504	1.89226	0.05
68505	1.89226	0.05
68506	1.89226	0.05
68507	1.89226	0.05
68508	1.89226	0.05
68509	1.89226	0.05
68510	3.78453	0.05
68511	1.89226	0.05
68512	1.89226	0.05
68513	1.89226	0.05
68514	1.89226	0.05
68515	3.78452	0.05
68516	1.89225	0.05
68517	1.89225	0.05
68518	1.89225	0.05
68519	1.89225	0.05
68520	1.89225	0.05
68521	1.89225	0.05
68522	0.946132	0.05
68523	0.946132	0.05
68524	0.946133	0.05
68525	0.946133	0.05
68526	0.946132	0.05
68527	0.946132	0.05
68528	0.946132	0.05
68529	0.946132	0.05
68530	0.946133	0.05
68531	0.946133	0.05
68532	0.946132	0.05
68533	0.946132	0.05
68534	1.89226	0.05
68535	1.89226	0.05
68536	1.89226	0.05
68537	1.89226	0.05
68538	3.78453	0.05
68539	1.13537	0.05
68540	1.09222	0.05
68541	0.946126	0.05
68542	0.946126	0.05
68543	0.946128	0.05
68544	0.946128	0.05
68545	1.16317	0.05
68546	1.13034	0.05
68547	1.30155	0.05
68548	1.08462	0.05
68549	0.992915	0.05
68550	0.807642	0.05
68551	0.946131	0.05
68552	0.946131	0.05
68553	0.946125	0.05

68554	0.946125	0.05
68555	0.946128	0.05
68556	0.946128	0.05
68557	0.946128	0.05
68558	0.946128	0.05
68559	0.946128	0.05
68560	0.946128	0.05
68561	0.946128	0.05
68562	0.946128	0.05
68563	1.89226	0.05
68564	1.89226	0.05
68565	1.89226	0.05
68566	1.89226	0.05
68567	1.89226	0.05
68568	1.89226	0.05
68569	1.89224	0.05
68570	1.89224	0.05
68571	1.89224	0.05
68572	1.89224	0.05
68573	1.89224	0.05
68574	1.89224	0.05
68575	0.946086	0.05
68576	1.17919	0.05
68577	0.89738	0.05
68578	0.89738	0.05
68579	0.946116	0.05
68580	0.946116	0.05
68581	1.13048	0.05
68582	1.11844	0.05
68583	0.946128	0.05
68584	0.897362	0.05
68585	1.1282	0.05
68586	0.946103	0.05
68587	1.89223	0.05
68588	1.89223	0.05
68589	1.89223	0.05
68590	1.89223	0.05
68591	3.78446	0.05
68592	0.946118	0.05
68593	0.946118	0.05
68594	0.946118	0.05
68595	0.946118	0.05
68596	0.946119	0.05
68597	0.946119	0.05
68598	0.946118	0.05
68599	0.946118	0.05
68600	0.946131	0.05
68601	0.946131	0.05
68602	0.946106	0.05
68603	0.946106	0.05
68604	1.89227	0.735444
68605	1.89227	1.07732
68606	1.89227	0.724048
68607	1.89227	0.499697
68608	3.78453	0.470339
68609	1.89226	1.03694
68610	1.89226	0.244175
68611	1.89226	0.222067

68612	1.89226	0.309868
68613	1.89226	0.733827
68614	1.89226	0.956068
68615	1.89226	0.139681
68616	2.59378	0.0867296
68617	2.30348	0.0630388
68618	2.30348	0.0889923
68619	1.89226	0.142094
68620	1.89226	0.22735
68621	1.89226	0.678711
68622	1.89226	0.283213
68623	1.89226	0.338614
68624	1.89226	0.272942
68625	3.78453	0.681337
68626	1.89226	0.272169
68627	1.89226	0.171967
68628	1.89226	0.116642
68629	1.89226	0.156377
68630	1.89226	0.390193
68631	1.89226	0.54956
68632	0.904406	0.0800546
68633	1.01667	0.115045
68634	1.11974	0.0886632
68635	0.994417	0.0907792
68636	1.02013	0.0902311
68637	0.628203	0.143022
68638	1.70302	0.105589
68639	1.49898	0.0810462
68640	1.14559	0.0842709
68641	0.900571	0.0919302
68642	1.58886	0.116208
68643	2.81102	0.05
68644	2.3923	0.05
68645	2.05632	0.05
68646	2.43514	0.05
68647	4.82744	0.05
68648	1.89226	0.05
68649	1.89226	0.05
68650	1.89226	0.05
68651	1.89226	0.05
68652	1.89226	0.05
68653	1.89226	0.05
68654	1.89226	0.05
68655	1.89226	0.05
68656	1.89226	0.05
68657	1.89226	0.05
68658	3.78451	0.05
68659	2.50803	0.05
68660	1.903 0.05	
68661	1.903 0.05	
68662	2.19162	0.05
68663	2.09934	0.05
68664	2.39063	0.05
68665	1.00243	0.05
68666	0.731731	0.05
68667	0.876616	0.05
68668	1.00451	0.05
68669	0.986901	0.05

68670	0.986901	0.05
68671	0.887729	0.05
68672	0.887732	0.05
68673	0.876605	0.05
68674	0.769283	0.05
68675	1.00245	0.05
68676	1.18712	0.05
68677	0.946131	0.05
68678	0.946131	0.05
68679	0.946131	0.05
68680	0.946131	0.05
68681	0.946131	0.05
68682	0.946131	0.05
68683	0.946131	0.05
68684	0.946131	0.05
68685	0.946131	0.05
68686	0.946131	0.05
68687	0.946131	0.05
68688	0.946131	0.05
68689	1.89226	0.05
68690	1.89226	0.05
68691	1.89226	0.05
68692	1.89226	0.05
68693	1.89226	0.05
68694	1.89226	0.05
68695	1.89226	0.05
68696	1.89226	0.05
68697	1.89226	0.05
68698	1.89226	0.05
68699	3.78453	0.05
68700	1.89226	0.05
68701	1.89226	0.05
68702	1.89226	0.05
68703	1.89226	0.05
68704	3.78453	0.05
68705	1.89226	0.05
68706	1.89226	0.05
68707	1.89226	0.05
68708	1.89226	0.05
68709	1.89226	0.05
68710	1.89226	0.05
68711	0.946132	0.05
68712	0.946132	0.05
68713	0.946131	0.05
68714	0.946131	0.05
68715	0.946131	0.05
68716	0.946131	0.05
68717	0.946132	0.05
68718	0.946132	0.05
68719	0.946131	0.05
68720	0.946131	0.05
68721	0.946131	0.05
68722	0.946131	0.05
68723	0.946131	0.05
68724	0.946131	0.05
68725	0.946131	0.05
68726	0.946131	0.05
68727	0.94613	0.05

68728	0.94613	0.05
68729	0.946132	0.05
68730	0.946132	0.05
68731	0.946131	0.05
68732	0.946131	0.05
68733	0.946131	0.05
68734	0.946131	0.05
68735	1.89226	0.05
68736	1.89226	0.05
68737	1.89226	0.05
68738	1.89226	0.05
68739	1.89226	0.05
68740	1.89226	0.05
68741	1.89226	0.05
68742	1.89226	0.05
68743	1.89226	0.05
68744	1.89226	0.05
68745	3.78453	0.05
68746	1.89226	0.05
68747	1.89226	0.05
68748	1.89226	0.05
68749	1.89226	0.05
68750	1.89226	0.05
68751	1.89226	0.05
68752	0.946132	0.05
68753	0.946132	0.05
68754	0.946131	0.05
68755	0.946131	0.05
68756	0.946132	0.05
68757	0.946132	0.05
68758	0.946131	0.05
68759	0.946131	0.05
68760	0.946132	0.05
68761	0.946132	0.05
68762	0.946131	0.05
68763	0.946131	0.05
68764	0.946131	0.05
68765	0.946131	0.05
68766	0.946132	0.05
68767	0.946132	0.05
68768	0.946132	0.05
68769	0.946132	0.05
68770	0.946131	0.05
68771	0.946131	0.05
68772	0.946132	0.05
68773	0.946132	0.05
68774	0.946131	0.05
68775	0.946131	0.05
68776	1.89226	0.102326
68777	1.89226	0.104145
68778	1.89226	0.137116
68779	1.89226	0.07252
68780	3.78452	0.0654153
68781	1.89226	0.512193
68782	1.89226	0.0872937
68783	1.89226	0.194326
68784	1.89226	0.204584
68785	3.78452	0.519533

68786	1.89226	0.0988329
68787	1.89226	0.120639
68788	1.89226	0.170432
68789	1.89226	0.0775402
68790	3.78452	0.0763668
68791	1.89226	0.118633
68792	2.75945	0.05
68793	1.89226	0.056154
68794	2.35026	0.05
68795	4.24252	0.159445
68796	0.946131	0.0582025
68797	0.946131	0.0923575
68798	0.946131	0.05
68799	0.946131	0.05
68800	0.946132	0.05
68801	0.946132	0.05
68802	0.946129	0.102432
68803	0.946129	0.0552957
68804	0.946131	0.0663361
68805	0.946131	0.0763317
68806	0.94613	0.124448
68807	0.94613	0.0681993
68808	1.89226	0.143591
68809	1.89226	0.0959324
68810	1.89226	0.0809438
68811	1.89226	0.144515
68812	1.89226	0.0982029
68813	1.89226	0.209042
68814	0.946129	0.05
68815	0.946129	0.0552679
68816	0.946133	0.05
68817	0.946133	0.05
68818	0.946131	0.05
68819	0.946131	0.05
68820	0.946131	0.056341
68821	0.946131	0.05
68822	0.946131	0.0552669
68823	0.946131	0.0538508
68824	0.946131	0.0747246
68825	0.946131	0.0545596
68826	1.89226	0.05
68827	2.35026	0.05
68828	1.32126	0.05
68829	1.89226	0.05
68830	1.77925	0.05
68831	2.35026	0.05
68832	1.89226	0.05
68833	1.89226	0.05
68834	1.89226	0.05
68835	1.89226	0.05
68836	1.89226	0.05
68837	1.89226	0.05
68838	1.89226	0.05
68839	1.89226	0.05
68840	1.89226	0.05
68841	1.89226	0.05
68842	3.78453	0.05
68843	1.89226	0.05

68844	1.89226	0.05
68845	1.89226	0.05
68846	1.89226	0.05
68847	1.89226	0.05
68848	1.89226	0.05
68849	0.946131	0.05
68850	0.946131	0.05
68851	0.946131	0.05
68852	0.946131	0.05
68853	0.946132	0.05
68854	0.946132	0.05
68855	0.946131	0.05
68856	0.946131	0.05
68857	0.946132	0.05
68858	0.946132	0.05
68859	0.946131	0.05
68860	0.946131	0.05
68861	1.89226	0.05
68862	1.89226	0.05
68863	1.89226	0.05
68864	1.89226	0.05
68865	1.89226	0.05
68866	1.89226	0.05
68867	1.89226	0.05
68868	1.89226	0.05
68869	1.89226	0.05
68870	1.89226	0.05
68871	3.78453	0.05
68872	1.89227	0.05
68873	1.89227	0.05
68874	1.89227	0.05
68875	1.89227	0.0523435
68876	3.78453	0.05
68877	1.89226	0.05
68878	1.89226	0.05
68879	1.89226	0.05
68880	1.89226	0.05
68881	3.78452	0.05
68882	0.946132	0.05
68883	0.946132	0.05
68884	0.946131	0.05
68885	0.946131	0.05
68886	0.946131	0.05
68887	0.946131	0.05
68888	0.946133	0.05
68889	0.946133	0.05
68890	0.946132	0.05
68891	0.946132	0.05
68892	0.946131	0.05
68893	0.946131	0.05
68894	1.89227	0.05
68895	1.89227	0.05
68896	1.89227	0.05
68897	1.89227	0.05
68898	1.89227	0.05
68899	1.89227	0.05
68900	2.20491	0.05
68901	1.81656	0.05

68902	1.81656	0.05
68903	1.89226	0.05
68904	1.89226	0.05
68905	1.89226	0.05
68906	1.89226	0.0627656
68907	1.89226	0.0916925
68908	1.89226	0.0598483
68909	1.89226	0.0653406
68910	3.78452	0.0583819
68911	1.89226	0.260643
68912	1.89226	0.195365
68913	1.89226	0.146042
68914	1.89226	0.278345
68915	3.78453	0.266488
68916	1.89226	0.21571
68917	1.89226	0.19301
68918	1.89226	0.171578
68919	1.89226	0.227348
68920	1.89226	0.212846
68921	1.89226	0.291363
68922	1.89226	0.05
68923	2.11032	0.05
68924	2.30793	0.05
68925	1.89226	0.05
68926	2.30793	0.05
68927	1.89226	0.05
68928	1.89226	0.0909539
68929	1.89226	0.102697
68930	1.89226	0.0654582
68931	1.89226	0.0749211
68932	1.89226	0.10355
68933	1.89226	0.121262
68934	0.810943	0.0951014
68935	0.810943	0.0748989
68936	0.810943	0.0959336
68937	0.810943	0.0780703
68938	0.810933	0.0788401
68939	0.810933	0.0977597
68940	1.89226	0.068643
68941	1.3517	0.093331
68942	1.08132	0.085118
68943	1.08132	0.110953
68944	1.08133	0.085832
68945	1.89226	0.05
68946	1.89226	0.05
68947	1.89226	0.05
68948	1.89226	0.05
68949	3.78453	0.05
68950	1.89226	0.05
68951	1.89226	0.05
68952	1.89226	0.05
68953	1.89226	0.05
68954	1.89226	0.05
68955	1.89226	0.05
68956	0.810945	0.05
68957	0.810945	0.05
68958	0.870829	0.05
68959	0.870829	0.05

68960	0.990502	0.05
68961	0.810933	0.05
68962	2.82777	0.05
68963	1.53126	0.05
68964	1.32076	0.05
68965	1.201 0.05	
68966	1.08133	0.05
68967	1.89226	0.05
68968	1.89226	0.05
68969	1.89226	0.05
68970	1.89226	0.05
68971	1.89226	0.05
68972	1.89226	0.05
68973	0.946132	0.05
68974	0.946132	0.05
68975	0.946133	0.05
68976	0.946133	0.05
68977	0.946132	0.05
68978	0.946132	0.05
68979	0.946132	0.05
68980	0.946132	0.05
68981	0.946133	0.05
68982	0.946133	0.05
68983	0.946132	0.05
68984	0.946132	0.05
68985	0.946133	0.05
68986	0.946133	0.05
68987	0.946132	0.05
68988	0.946132	0.05
68989	0.946132	0.05
68990	0.946132	0.05
68991	0.946132	0.05
68992	0.946132	0.05
68993	0.946133	0.05
68994	0.946133	0.05
68995	0.946132	0.05
68996	0.946132	0.05
68997	1.89226	0.05
68998	1.89226	0.05
68999	1.89226	0.05
69000	1.89226	0.05
69001	1.89226	0.05
69002	1.89226	0.05
69003	1.89226	0.05
69004	1.89226	0.0591271
69005	1.89226	0.05
69006	1.89226	0.05
69007	3.78452	0.0545251
69008	1.89226	0.0553717
69009	1.89226	0.0664616
69010	1.89226	0.05
69011	1.89226	0.170265
69012	3.78452	0.05
69013	1.89226	0.05
69014	1.89226	0.0611937
69015	1.89226	0.05
69016	1.89226	0.05
69017	1.89226	0.0532112

69018	1.89226	0.0547949
69019	0.946128	0.05
69020	0.946128	0.05
69021	0.946132	0.05
69022	0.946132	0.05
69023	0.946129	0.05
69024	0.946129	0.05
69025	0.946131	0.05
69026	0.946131	0.05
69027	0.94613	0.05
69028	0.94613	0.05
69029	0.946131	0.05
69030	0.946131	0.05
69031	0.946129	0.05
69032	0.946129	0.05
69033	0.946133	0.05
69034	0.946133	0.05
69035	0.946131	0.05
69036	0.946131	0.05
69037	0.946131	0.05
69038	0.946131	0.05
69039	0.946131	0.05
69040	0.946131	0.05
69041	0.946131	0.05
69042	0.946131	0.05
69043	1.89226	0.05
69044	1.89226	0.05
69045	1.89226	0.05
69046	1.89226	0.05
69047	3.78452	0.05
69048	1.89225	0.05
69049	1.89225	0.05
69050	1.89225	0.05
69051	1.89225	0.05
69052	1.89225	0.05
69053	1.89225	0.05
69054	1.89226	0.05
69055	1.89226	0.05
69056	1.89226	0.05
69057	1.89226	0.05
69058	3.78452	0.05
69059	1.89226	0.05
69060	1.89226	0.0690865
69061	1.89226	0.05
69062	1.89226	0.05
69063	3.78453	0.05
69064	1.89226	0.05
69065	1.89226	0.05
69066	1.89226	0.05
69067	1.89226	0.05
69068	3.78452	0.05
69069	1.22164	0.05
69070	1.22164	0.05
69071	0.806032	0.05
69072	0.806032	0.05
69073	1.08154	0.05
69074	0.946126	0.05
69075	0.946129	0.05

69076	0.806065	0.05
69077	0.806063	0.05
69078	1.08155	0.05
69079	0.946128	0.05
69080	0.946128	0.05
69081	1.28554	0.05
69082	1.28554	0.05
69083	0.946126	0.05
69084	0.946126	0.05
69085	1.28554	0.05
69086	0.946129	0.05
69087	1.08155	0.05
69088	0.94613	0.05
69089	1.08155	0.05
69090	1.28554	0.05
69091	0.94613	0.05
69092	0.94613	0.05
69093	0.946132	0.05
69094	0.946132	0.05
69095	0.946132	0.05
69096	0.946132	0.05
69097	0.946132	0.079934
69098	0.946132	0.05
69099	0.946133	0.05
69100	0.946133	0.05
69101	0.946132	0.05
69102	0.946132	0.05
69103	0.946133	0.05
69104	0.946133	0.05
69105	1.89226	0.157448
69106	1.89226	0.05
69107	1.89226	0.15303
69108	1.89226	0.0519224
69109	3.78453	0.124356
69110	1.89226	0.117531
69111	1.89226	0.05
69112	1.89226	0.05
69113	1.89226	0.05
69114	1.89226	0.05
69115	1.89226	0.05
69116	1.89226	0.05
69117	1.89226	0.05
69118	1.89226	0.05
69119	1.89226	0.05
69120	1.89226	0.05
69121	1.89226	0.05
69122	1.89226	0.05
69123	1.89226	0.05
69124	1.89226	0.05
69125	1.89226	0.05
69126	1.89226	0.05
69127	1.89226	0.05
69128	0.946132	0.05
69129	0.946132	0.0575948
69130	0.946132	0.05
69131	0.946132	0.05
69132	0.946132	0.05
69133	0.946132	0.05

69134	0.946132	0.05
69135	0.946132	0.05
69136	0.946131	0.05
69137	0.946131	0.05
69138	0.946132	0.05
69139	0.946132	0.05
69140	1.89226	0.05
69141	1.89226	0.05
69142	1.89226	0.05
69143	1.89226	0.05
69144	3.78453	0.05
69145	0.851541	0.05
69146	0.72883	0.05
69147	0.991276	0.05
69148	0.851485	0.05
69149	1.00003	0.05
69150	1.00003	0.05
69151	0.809788	0.05
69152	0.809788	0.05
69153	0.890738	0.05
69154	0.991264	0.05
69155	0.851554	0.05
69156	0.851554	0.05
69157	0.8628	0.05
69158	0.78387	0.05
69159	0.830233	0.05
69160	0.830233	0.05
69161	0.808312	0.05
69162	0.817238	0.05
69163	1.70303	0.05
69164	1.24116	0.05
69165	0.995299	0.05
69166	1.0989	0.05
69167	1.02714	0.05
69168	1.89226	0.112657
69169	1.89226	0.142569
69170	1.89226	0.0611119
69171	1.89226	0.968101
69172	3.78451	0.074362
69173	0.946131	0.129026
69174	0.946131	0.05
69175	0.946131	0.133655
69176	0.946131	0.0694423
69177	0.94613	0.138372
69178	0.94613	0.05
69179	0.946132	0.05
69180	0.946132	0.05
69181	0.946131	0.05
69182	0.946131	0.081216
69183	0.946131	0.05
69184	0.946131	0.05
69185	1.89226	0.05
69186	1.89226	1.77683
69187	1.89226	0.979928
69188	1.89226	1.36214
69189	3.78453	0.848926
69190	1.89226	2.1
69191	1.89226	1.97491

69192	1.89226	2.1
69193	1.89226	0.471153
69194	1.89226	2.1
69195	1.89226	0.587892
69196	0.946126	0.05
69197	1.17111	0.0703143
69198	1.3393	0.0564129
69199	0.946131	0.0550688
69200	0.946127	0.05
69201	0.946127	0.05
69202	1.3393	0.05
69203	1.17111	0.0617034
69204	1.3393	0.05
69205	1.3393	0.05
69206	0.946129	0.05
69207	0.946129	0.05
69208	0.946126	2.1
69209	0.946126	0.05
69210	0.946132	2.1
69211	0.946132	2.1
69212	0.94613	2.1
69213	0.94613	0.151932
69214	0.946129	1.80491
69215	0.946129	0.504291
69216	0.946129	0.05
69217	0.946129	2.1
69218	0.94613	0.736693
69219	0.94613	0.310778
69220	0.946131	0.0578039
69221	0.946131	0.05
69222	0.94613	0.05
69223	0.94613	0.05
69224	0.946128	0.0559341
69225	0.946128	0.0625728
69226	0.946132	0.05
69227	0.946132	0.05
69228	0.94613	0.0576325
69229	0.94613	0.05
69230	0.94613	0.05
69231	0.94613	0.05
69232	0.94613	0.977151
69233	0.94613	0.261759
69234	0.94613	0.244411
69235	0.94613	0.14549
69236	0.946128	0.79505
69237	0.946128	1.16428
69238	0.946131	0.147387
69239	0.946131	0.106878
69240	0.94613	0.606338
69241	0.94613	0.0984969
69242	0.94613	0.22713
69243	0.94613	0.824788
69244	1.89226	1.20021
69245	1.89226	0.346842
69246	1.89226	0.581167
69247	1.89226	1.80388
69248	3.78453	0.805315
69249	1.89226	0.214731

69250	1.89226	0.33533
69251	1.89226	0.0560506
69252	1.89226	0.236594
69253	1.89226	0.0620458
69254	1.89226	0.386112
69255	1.89226	0.0986588
69256	1.89226	0.558933
69257	1.89226	0.0591662
69258	1.89226	0.118642
69259	1.89226	0.0929671
69260	1.89226	0.204042
69261	1.89227	0.05
69262	2.28622	0.0520161
69263	2.16109	0.05
69264	1.89227	0.0739581
69265	4.17849	0.05
69266	15.1381	0.0518772
69267	15.1381	0.58499
69268	15.1381	0.122835
69269	15.1381	0.159366
69270	30.2762	0.05
69271	15.1381	0.05
69272	15.1381	0.547123
69273	15.1381	0.05
69274	15.1381	0.14287
69275	30.2762	0.05
69276	15.1381	0.151244
69277	15.1381	0.548879
69278	15.1381	0.05
69279	15.1381	0.48599
69280	30.2762	0.256852
69281	15.1381	0.05
69282	15.1381	0.644854
69283	15.1381	0.05
69284	15.1381	0.132257
69285	30.2762	0.19939
69286	15.138	0.651854
69287	15.138	1.9828
69288	15.138	0.577569
69289	15.138	2.1
69290	30.276	1.60814
69291	15.138	2.01773
69292	15.138	2.1
69293	15.138	2.1
69294	15.138	2.1
69295	30.276	2.1
69296	15.138	1.73533
69297	15.138	1.2911
69298	15.138	0.598469
69299	15.138	2.1
69300	30.2759	1.82896
69301	15.138	1.54042
69302	15.138	2.1
69303	15.138	2.1
69304	15.138	1.36509
69305	30.2759	1.92888
69306	15.138	2.1
69307	15.138	2.1

69308	15.138	2.1
69309	15.138	2.1
69310	30.276	2.1
69311	15.1381	2.1
69312	15.1381	2.1
69313	15.1381	2.1
69314	15.1381	2.1
69315	30.2762	2.1
69316	15.1381	2.1
69317	15.1381	2.1
69318	15.1381	2.1
69319	15.1381	2.1
69320	30.2762	2.1
69321	15.138	1.94183
69322	15.138	2.1
69323	15.138	2.1
69324	15.138	2.1
69325	30.276	2.1
69326	15.138	2.1
69327	15.138	2.1
69328	15.138	2.1
69329	15.138	2.1
69330	30.276	2.1
69331	15.1381	1.34665
69332	15.1381	2.1
69333	15.1381	2.06901
69334	15.1381	1.73842
69335	30.2762	1.98419
69336	15.1381	2.1
69337	15.1381	2.1
69338	15.1381	2.00937
69339	15.1381	2.1
69340	30.2762	2.1
69341	15.1381	2.1
69342	15.1381	2.1
69343	15.1381	2.1
69344	15.1381	2.1
69345	30.2762	2.1
69346	15.138	0.0742371
69347	15.138	0.149073
69348	15.138	0.0731931
69349	15.138	0.655645
69350	30.276	0.05
69351	1.89226	2.1
69352	1.89226	2.1
69353	1.89226	2.1
69354	1.89226	2.1
69355	3.78453	2.1
69356	1.89226	2.1
69357	1.89226	2.1
69358	1.89226	2.1
69359	1.89226	2.1
69360	1.89226	2.1
69361	1.89226	2.1
69362	1.89226	2.1
69363	1.89226	2.1
69364	1.89226	2.1
69365	1.89226	2.1

69366	1.89226	2.1
69367	1.89226	2.1
69368	1.89226	2.1
69369	1.89226	2.1
69370	1.89226	2.1
69371	1.89226	2.1
69372	3.78453	2.1
69373	1.89226	2.1
69374	1.89226	2.1
69375	1.89226	2.1
69376	1.89226	2.1
69377	1.89226	2.1
69378	1.89226	2.1
69379	0.946127	2.1
69380	0.946127	2.1
69381	0.946131	2.1
69382	0.946131	2.1
69383	0.946131	2.1
69384	0.946131	2.1
69385	0.946128	2.1
69386	0.946128	2.1
69387	0.94613	2.1
69388	0.94613	2.1
69389	0.946129	2.1
69390	0.946129	2.1
69391	1.89226	2.1
69392	1.89226	2.1
69393	1.89226	2.1
69394	1.89226	2.1
69395	1.89226	2.1
69396	1.89226	2.1
69397	1.89226	2.1
69398	1.89226	2.1
69399	1.89226	2.1
69400	1.89226	2.1
69401	3.78452	2.1
69402	1.89226	2.1
69403	1.89226	2.1
69404	1.89226	2.1
69405	1.89226	2.1
69406	1.89226	2.1
69407	1.89226	2.1
69408	0.946129	2.1
69409	0.946129	2.1
69410	0.946133	2.1
69411	0.946133	2.1
69412	0.946131	2.1
69413	0.946131	2.1
69414	0.946131	2.1
69415	0.946131	2.1
69416	0.946131	2.1
69417	0.946131	2.1
69418	0.946131	2.1
69419	0.946131	2.1
69420	0.946132	2.1
69421	0.946132	2.1
69422	0.946132	2.1
69423	0.946132	2.1

69424	0.946132	2.1
69425	0.946132	2.1
69426	0.946133	2.1
69427	0.946133	2.1
69428	0.946132	2.1
69429	0.946132	2.1
69430	0.946133	2.1
69431	0.946133	2.1
69432	1.89226	2.1
69433	1.89226	2.1
69434	1.89226	2.1
69435	1.89226	2.1
69436	3.78452	2.1
69437	1.89226	2.1
69438	1.89226	2.1
69439	1.89226	2.1
69440	1.89226	2.1
69441	1.89226	2.1
69442	1.89226	2.1
69443	0.946129	2.1
69444	0.946129	2.1
69445	0.946133	2.1
69446	0.946133	2.1
69447	0.946131	2.1
69448	0.946131	2.1
69449	0.946131	2.1
69450	0.946131	2.1
69451	0.946131	2.1
69452	0.946131	2.1
69453	0.946131	2.1
69454	0.946131	2.1
69455	0.946132	2.1
69456	0.946132	2.1
69457	0.946132	2.1
69458	0.946132	2.1
69459	0.946132	2.1
69460	0.946132	2.1
69461	0.946133	2.1
69462	0.946133	2.1
69463	0.946133	2.1
69464	0.946133	2.1
69465	0.946132	2.1
69466	0.946132	2.1
69467	1.89226	2.1
69468	1.89226	2.1
69469	1.89226	2.1
69470	1.89226	2.1
69471	3.78453	2.1
69472	0.946128	2.1
69473	0.946128	2.1
69474	0.946132	2.1
69475	0.946132	2.1
69476	0.94613	2.1
69477	0.94613	2.1
69478	0.94613	2.1
69479	0.94613	2.1
69480	0.94613	2.1
69481	0.94613	2.1

69482	0.94613	2.1
69483	0.94613	2.1
69484	0.946127	2.1
69485	0.946127	2.1
69486	0.946131	2.1
69487	0.946131	2.1
69488	0.94613	2.1
69489	0.94613	2.1
69490	0.946128	2.1
69491	0.946128	2.1
69492	0.946129	2.1
69493	0.946129	2.1
69494	0.946129	2.1
69495	0.946129	2.1
69496	0.94613	2.1
69497	0.94613	2.1
69498	0.94613	2.1
69499	0.94613	2.1
69500	0.946131	2.1
69501	0.946131	2.1
69502	0.946129	2.1
69503	0.946129	2.1
69504	0.94613	2.1
69505	0.94613	2.1
69506	0.94613	2.1
69507	0.94613	2.1
69508	1.89226	2.1
69509	1.89226	2.1
69510	1.89226	2.1
69511	1.89226	2.1
69512	3.78453	2.1
69513	1.89226	2.1
69514	1.89226	2.1
69515	1.89226	2.1
69516	1.89226	2.1
69517	1.89226	2.1
69518	1.89226	2.1
69519	1.89226	2.1
69520	1.89226	2.1
69521	1.89226	2.1
69522	1.89226	2.1
69523	1.89226	2.1
69524	1.89226	2.1
69525	0.946131	2.1
69526	0.946131	2.1
69527	0.946131	2.1
69528	0.946131	2.1
69529	0.946132	2.1
69530	0.946132	2.1
69531	0.946129	2.1
69532	0.946129	2.1
69533	0.946131	2.1
69534	0.946131	2.1
69535	0.94613	2.1
69536	0.94613	2.1
69537	1.89224	2.1
69538	1.89224	2.1
69539	1.89224	2.1

69540	1.89224	2.1
69541	3.78448	2.1
69542	1.89224	2.1
69543	1.89224	2.1
69544	1.89224	2.1
69545	1.89224	2.1
69546	3.78447	2.1
69547	1.89223	2.1
69548	2.38995	2.1
69549	2.25315	2.1
69550	2.25315	2.1
69551	1.89223	2.1
69552	1.89223	2.1
69553	1.89224	2.1
69554	1.89224	2.1
69555	1.89224	2.1
69556	1.89224	2.1
69557	1.89224	2.1
69558	1.89224	2.1
69559	1.89226	2.1
69560	1.89226	2.1
69561	1.89226	2.1
69562	1.89226	2.1
69563	3.78453	2.1
69564	1.89226	2.1
69565	1.89226	2.1
69566	1.89226	2.1
69567	1.89226	2.1
69568	1.89226	2.1
69569	1.89226	2.1
69570	1.89226	2.1
69571	2.60272	2.1
69572	2.38998	2.1
69573	2.38998	2.1
69574	1.89226	2.1
69575	1.89226	2.1
69576	0.94613	2.1
69577	0.94613	2.1
69578	0.94613	2.1
69579	0.94613	2.1
69580	0.946131	2.1
69581	0.946131	2.1
69582	0.946129	2.1
69583	0.946129	2.1
69584	0.94613	2.1
69585	0.94613	2.1
69586	0.94613	2.1
69587	0.94613	2.1
69588	1.89226	2.1
69589	1.89226	2.1
69590	1.89226	2.1
69591	1.89226	2.1
69592	3.78452	2.1
69593	1.89226	2.1
69594	1.89226	2.1
69595	1.89226	2.1
69596	1.89226	2.1
69597	3.78452	2.1

69598	1.89226	2.1
69599	1.89226	2.1
69600	1.89226	2.1
69601	1.89226	2.1
69602	1.89226	2.1
69603	1.89226	2.1
69604	0.946132	2.1
69605	0.946132	2.1
69606	0.946132	2.1
69607	0.946132	2.1
69608	0.946131	2.1
69609	0.946131	2.1
69610	0.946132	2.1
69611	0.946132	2.1
69612	0.946132	2.1
69613	0.946132	2.1
69614	0.946132	2.1
69615	0.946132	2.1
69616	1.89226	2.1
69617	1.89226	2.1
69618	1.89226	2.1
69619	1.89226	2.1
69620	3.78452	2.1
69621	1.89226	2.1
69622	1.89226	2.1
69623	1.89226	2.1
69624	1.89226	2.1
69625	3.78452	2.1
69626	0.946129	2.1
69627	0.946129	2.1
69628	0.946133	2.1
69629	0.946133	2.1
69630	0.946131	2.1
69631	0.946131	2.1
69632	0.946131	2.1
69633	0.946131	2.1
69634	0.946131	2.1
69635	0.946131	2.1
69636	0.946131	2.1
69637	0.946131	2.1
69638	0.946132	2.1
69639	0.946132	2.1
69640	0.946132	2.1
69641	0.946132	2.1
69642	0.946132	2.1
69643	0.946132	2.1
69644	0.946133	2.1
69645	0.946133	2.1
69646	0.946133	2.1
69647	0.946133	2.1
69648	0.946132	2.1
69649	0.946132	2.1
69650	1.89224	2.1
69651	1.89224	2.1
69652	1.89224	2.1
69653	1.89224	2.1
69654	3.78447	2.1
69655	1.89223	2.1

69656	1.89223	2.1
69657	1.89223	2.1
69658	1.89223	2.1
69659	1.89223	2.1
69660	1.89223	2.1
69661	1.89224	2.1
69662	1.89224	2.1
69663	1.89224	2.1
69664	1.89224	2.1
69665	3.78447	2.1
69666	1.89224	2.1
69667	1.89224	2.1
69668	1.89224	2.1
69669	1.89224	2.1
69670	3.78448	2.1
69671	1.89226	2.1
69672	1.89226	2.1
69673	1.89226	2.1
69674	1.89226	2.1
69675	3.78452	2.1
69676	1.89226	2.1
69677	1.89226	2.1
69678	1.89226	2.1
69679	1.89226	2.1
69680	1.89226	2.1
69681	1.89226	2.1
69682	1.89226	2.1
69683	1.89226	2.1
69684	1.89226	2.1
69685	1.89226	2.1
69686	1.89226	2.1
69687	1.89226	2.1
69688	0.810946	2.1
69689	0.810946	2.1
69690	0.810946	2.1
69691	0.810946	2.1
69692	0.810933	2.1
69693	0.810933	2.1
69694	1.89226	2.1
69695	1.3517	2.1
69696	1.08132	2.1
69697	1.08132	2.1
69698	1.08133	2.1
69699	1.89226	2.1
69700	1.89226	2.1
69701	1.89226	2.1
69702	1.89226	2.1
69703	3.78453	2.1
69704	1.89226	2.1
69705	1.89226	2.1
69706	1.89226	2.1
69707	1.89226	2.1
69708	3.78452	2.1
69709	1.89226	2.1
69710	1.89226	2.1
69711	1.89226	2.1
69712	1.89226	2.1
69713	1.89226	2.1

69714	1.89226	2.1
69715	0.94613	2.1
69716	0.94613	2.1
69717	0.94613	2.1
69718	0.94613	2.1
69719	0.946131	2.1
69720	0.946131	2.1
69721	0.946129	2.1
69722	0.946129	2.1
69723	0.94613	2.1
69724	0.94613	2.1
69725	0.94613	2.1
69726	0.94613	2.1
69727	1.89226	2.1
69728	1.89226	2.1
69729	1.89226	2.1
69730	1.89226	2.1
69731	3.78453	2.1
69732	1.89226	2.1
69733	1.89226	2.1
69734	1.89226	2.1
69735	1.89226	2.1
69736	3.78452	2.1
69737	1.89226	2.1
69738	1.89226	2.1
69739	1.89226	2.1
69740	1.89226	2.1
69741	3.78452	2.1
69742	1.89226	2.1
69743	1.89226	2.1
69744	1.89226	2.1
69745	1.89226	2.1
69746	1.89226	2.1
69747	1.89226	2.1
69748	1.89226	2.1
69749	1.89226	2.1
69750	1.89226	2.1
69751	1.89226	2.1
69752	3.78452	2.1
69753	1.89226	2.1
69754	1.89226	2.1
69755	1.89226	2.1
69756	1.89226	2.1
69757	3.78452	2.1
69758	0.946128	2.1
69759	0.946128	2.1
69760	0.946133	2.1
69761	0.946133	2.1
69762	0.94613	2.1
69763	0.94613	2.1
69764	0.946131	2.1
69765	0.946131	2.1
69766	0.946131	2.1
69767	0.946131	2.1
69768	0.946131	2.1
69769	0.946131	2.1
69770	0.946132	2.1
69771	0.946132	2.1

69772	0.946132	2.1
69773	0.946132	2.1
69774	0.946131	2.1
69775	0.946131	2.1
69776	0.946132	2.1
69777	0.946132	2.1
69778	0.946132	2.1
69779	0.946132	2.1
69780	0.946132	2.1
69781	0.946132	2.1
69782	1.89226	2.1
69783	1.89226	2.1
69784	1.89226	2.1
69785	1.89226	2.1
69786	3.78452	2.1
69787	1.89226	2.1
69788	1.89226	2.1
69789	1.89226	2.1
69790	1.89226	2.1
69791	3.78452	2.1
69792	0.946129	2.1
69793	0.946129	2.1
69794	0.946133	2.1
69795	0.946133	2.1
69796	0.946131	2.1
69797	0.946131	2.1
69798	0.946131	2.1
69799	0.946131	2.1
69800	0.946131	2.1
69801	0.946131	2.1
69802	0.946131	2.1
69803	0.946131	2.1
69804	0.946132	2.1
69805	0.946132	2.1
69806	0.946132	2.1
69807	0.946132	2.1
69808	0.946132	2.1
69809	0.946132	2.1
69810	0.946133	2.1
69811	0.946133	2.1
69812	0.946132	2.1
69813	0.946132	2.1
69814	0.946133	2.1
69815	0.946133	2.1
69816	1.89226	2.1
69817	1.89226	2.1
69818	1.89226	2.1
69819	1.89226	2.1
69820	1.89226	2.1
69821	1.89226	2.1
69822	0.946128	2.1
69823	0.946128	2.1
69824	0.946133	2.1
69825	0.946133	2.1
69826	0.946131	2.1
69827	0.946131	2.1
69828	0.94613	2.1
69829	0.94613	2.1

69830	0.946131	2.1
69831	0.946131	2.1
69832	0.946131	2.1
69833	0.946131	2.1
69834	1.89226	2.1
69835	1.89226	2.1
69836	1.89226	2.1
69837	1.89226	2.1
69838	1.89226	2.1
69839	1.89226	2.1
69840	1.89226	2.1
69841	1.89226	2.1
69842	1.89226	2.1
69843	1.89226	2.05836
69844	3.78453	2.1
69845	1.89226	2.1
69846	1.89226	2.1
69847	1.89226	2.1
69848	1.89226	2.1
69849	1.89226	2.1
69850	1.89226	2.1
69851	0.946127	2.1
69852	0.946127	2.1
69853	0.946131	2.1
69854	0.946131	2.1
69855	0.946131	2.1
69856	0.946131	2.1
69857	0.946127	2.1
69858	0.946127	2.1
69859	0.946129	2.1
69860	0.946129	2.1
69861	0.94613	2.1
69862	0.94613	2.1
69863	0.946131	2.1
69864	0.946131	2.1
69865	0.946131	2.1
69866	0.946131	2.08303
69867	0.946133	2.09649
69868	0.946133	2.1
69869	0.946128	2.1
69870	0.946128	2.05095
69871	0.94613	2.1
69872	0.94613	2.1
69873	0.946131	2.1
69874	0.946131	2.1
69875	1.89226	2.1
69876	1.89226	2.1
69877	1.89226	2.1
69878	1.89226	2.1
69879	1.89226	2.1
69880	1.89226	2.1
69881	1.89226	2.1
69882	1.89226	2.1
69883	1.89226	2.1
69884	1.89226	2.1
69885	3.78452	2.1
69886	1.89226	2.1
69887	1.89226	2.1

69888	1.89226	2.1
69889	1.89226	2.1
69890	3.78452	2.1
69891	1.89226	2.1
69892	1.89226	2.1
69893	1.89226	2.1
69894	1.89226	2.1
69895	1.89226	2.1
69896	1.89226	2.1
69897	1.89226	2.1
69898	1.89226	2.1
69899	1.89226	2.1
69900	1.89226	2.1
69901	3.78453	2.1
69902	1.89226	2.1
69903	1.89226	2.1
69904	1.89226	2.1
69905	1.89226	2.1
69906	3.78452	2.1
69907	1.89226	2.1
69908	1.89226	2.1
69909	1.89226	2.1
69910	1.89226	2.1
69911	3.78452	2.1
69912	1.89226	2.1
69913	1.89226	2.1
69914	1.89226	2.1
69915	1.89226	2.1
69916	1.89226	2.1
69917	1.89226	2.1
69918	0.946131	2.1
69919	0.946131	2.1
69920	0.946131	2.1
69921	0.946131	2.1
69922	0.946131	2.1
69923	0.946131	2.1
69924	0.946131	2.1
69925	0.946131	2.1
69926	0.946131	2.1
69927	0.946131	2.1
69928	0.946131	2.1
69929	0.946131	2.1
69930	1.89226	2.1
69931	1.89226	2.1
69932	1.89226	2.1
69933	1.89226	2.1
69934	1.89226	2.1
69935	1.89226	2.1
69936	1.89226	2.1
69937	1.89226	2.1
69938	1.89226	2.1
69939	1.89226	2.1
69940	1.89226	2.1
69941	1.89226	2.1
69942	0.946131	2.1
69943	0.946131	2.1
69944	0.946131	2.1
69945	0.946131	2.1

69946	0.946131	2.1
69947	0.946131	2.1
69948	0.946131	2.1
69949	0.946131	2.1
69950	0.946131	2.1
69951	0.946131	2.1
69952	0.946131	2.1
69953	0.946131	2.1
69954	1.89226	2.1
69955	1.89226	2.1
69956	1.89226	2.1
69957	1.89226	2.1
69958	1.89226	2.1
69959	1.89226	2.1
69960	0.946128	2.1
69961	0.946128	2.1
69962	0.946133	2.1
69963	0.946133	2.1
69964	0.946131	2.1
69965	0.946131	2.1
69966	0.946131	2.1
69967	0.946131	2.1
69968	0.946131	2.1
69969	0.946131	2.1
69970	0.94613	2.1
69971	0.94613	2.1
69972	1.89226	2.1
69973	1.89226	2.1
69974	1.89226	2.1
69975	1.89226	2.1
69976	1.89226	2.1
69977	1.89226	2.1
69978	0.946131	2.1
69979	0.946131	2.1
69980	0.946131	2.1
69981	0.946131	2.1
69982	0.946132	2.1
69983	0.946132	2.1
69984	0.946131	2.1
69985	0.946131	2.1
69986	0.946132	2.1
69987	0.946132	2.1
69988	0.946131	2.1
69989	0.946131	2.1
69990	1.89226	2.1
69991	1.89226	2.1
69992	1.89226	2.1
69993	1.89226	2.1
69994	1.89226	2.1
69995	1.89226	2.1
69996	1.89226	2.1
69997	1.89226	2.1
69998	1.89226	2.1
69999	1.89226	2.1
70000	3.78452	2.1
70001	1.89226	2.1
70002	1.89226	2.1
70003	1.89226	2.1

70004	1.89226	2.1
70005	3.78452	2.1
70006	0.946131	2.1
70007	0.946131	2.1
70008	0.946131	2.1
70009	0.946131	2.1
70010	0.946132	2.1
70011	0.946132	2.1
70012	0.946131	2.1
70013	0.946131	2.1
70014	0.946132	2.1
70015	0.946132	2.1
70016	0.946131	2.1
70017	0.946131	2.1
70018	1.89226	2.1
70019	1.89226	2.1
70020	1.89226	2.1
70021	1.89226	2.1
70022	1.89226	2.1
70023	1.89226	2.1
70024	1.89226	2.1
70025	1.89226	2.1
70026	1.89226	2.1
70027	1.89226	2.1
70028	3.78452	2.1
70029	1.89226	2.1
70030	1.89226	2.1
70031	1.89226	2.1
70032	1.89226	2.1
70033	3.78452	2.1
70034	0.946131	2.1
70035	0.946131	2.1
70036	0.946131	2.1
70037	0.946131	2.1
70038	0.946131	2.1
70039	0.946131	2.1
70040	0.946131	2.1
70041	0.946131	2.1
70042	0.946131	2.1
70043	0.946131	2.1
70044	0.946131	2.1
70045	0.946131	2.1
70046	1.89226	2.1
70047	1.89226	2.1
70048	1.89226	2.1
70049	1.89226	2.1
70050	3.78453	2.1
70051	1.89226	2.1
70052	1.89226	2.1
70053	1.89226	2.1
70054	1.89226	2.1
70055	1.89226	2.1
70056	1.89226	2.1
70057	1.89226	2.1
70058	1.89226	2.1
70059	1.89226	2.1
70060	1.89226	2.1
70061	3.78453	2.1

70062	1.89226	2.1
70063	1.89226	2.1
70064	1.89226	2.1
70065	1.89226	2.1
70066	3.78453	2.1
70067	1.89226	2.1
70068	1.89226	2.1
70069	1.89226	2.1
70070	1.89226	2.1
70071	3.78453	2.1
70072	0.946132	2.1
70073	0.946132	2.1
70074	0.946132	2.1
70075	0.946132	2.1
70076	0.946132	2.1
70077	0.946132	2.1
70078	0.946132	2.1
70079	0.946132	2.1
70080	0.946132	2.1
70081	0.946132	2.1
70082	0.946131	2.1
70083	0.946131	2.1
70084	0.946131	2.1
70085	0.946131	2.1
70086	0.946131	2.1
70087	0.946131	2.1
70088	0.946132	2.1
70089	0.946132	2.1
70090	0.946131	2.1
70091	0.946131	2.1
70092	0.946132	2.1
70093	0.946132	2.1
70094	0.946131	2.1
70095	0.946131	2.1
70096	1.89226	2.1
70097	1.89226	2.1
70098	1.89226	2.1
70099	1.89226	2.1
70100	3.78453	2.1
70101	1.89226	2.1
70102	1.89226	2.1
70103	1.89226	2.1
70104	1.89226	2.1
70105	3.78453	2.1
70106	0.946131	2.1
70107	0.946131	2.1
70108	0.946131	2.1
70109	0.946131	2.1
70110	0.946131	2.1
70111	0.946131	2.1
70112	0.946131	2.1
70113	0.946131	2.1
70114	0.946131	2.1
70115	0.946131	2.1
70116	0.946131	2.1
70117	0.946131	2.1
70118	1.89226	2.1
70119	1.89226	2.1

70120	1.89226	2.1
70121	1.89226	2.1
70122	3.78452	2.1
70123	0.946132	2.1
70124	0.946132	2.1
70125	0.946131	2.1
70126	0.946131	2.1
70127	0.946132	2.1
70128	0.946132	2.1
70129	0.946131	2.1
70130	0.946131	2.1
70131	0.946131	2.1
70132	0.946131	2.1
70133	0.946131	2.1
70134	0.946131	2.1
70135	1.89226	2.1
70136	1.89226	2.1
70137	1.89226	2.1
70138	1.89226	2.1
70139	1.89226	2.1
70140	1.89226	2.1
70141	0.946132	2.1
70142	0.946132	2.1
70143	0.946132	2.1
70144	0.946132	2.1
70145	0.946132	2.1
70146	0.946132	2.1
70147	0.946132	2.1
70148	0.946132	2.1
70149	0.946132	2.1
70150	0.946132	2.1
70151	0.946131	2.1
70152	0.946131	2.1
70153	0.946131	2.1
70154	0.946131	2.1
70155	0.946131	2.1
70156	0.946131	2.1
70157	0.946132	2.1
70158	0.946132	2.1
70159	0.946131	2.1
70160	0.946131	2.1
70161	0.946132	2.1
70162	0.946132	2.1
70163	0.946131	2.1
70164	0.946131	2.1
70165	0.946132	2.1
70166	0.946132	2.1
70167	0.946131	2.1
70168	0.946131	2.1
70169	0.946132	2.1
70170	0.946132	2.1
70171	0.946131	2.1
70172	0.946131	2.1
70173	0.946132	2.1
70174	0.946132	2.1
70175	0.946131	2.1
70176	0.946131	2.1
70177	1.89226	2.1

70178	1.89226	2.1
70179	1.89226	2.1
70180	1.89226	2.1
70181	3.78453	2.1
70182	1.89226	2.1
70183	1.89226	2.1
70184	1.89226	2.1
70185	1.89226	2.1
70186	3.78453	2.1
70187	1.89226	2.1
70188	1.89226	2.1
70189	1.89226	2.1
70190	1.89226	2.1
70191	1.89226	2.1
70192	1.89226	2.1
70193	1.89226	2.1
70194	1.89226	2.1
70195	1.89226	2.1
70196	1.89226	2.1
70197	3.78453	2.1
70198	1.89226	2.1
70199	1.89226	2.1
70200	1.89226	2.1
70201	1.89226	2.1
70202	3.78453	2.1
70203	1.89226	2.1
70204	1.89226	2.1
70205	1.89226	2.1
70206	1.89226	2.1
70207	1.89226	2.1
70208	1.89226	2.1
70209	1.89226	2.1
70210	1.89226	2.1
70211	1.89226	2.1
70212	1.89226	2.1
70213	1.89226	2.1
70214	1.89226	2.1
70215	1.89226	2.1
70216	1.89226	2.1
70217	1.89226	2.1
70218	1.89226	2.1
70219	3.78453	2.1
70220	1.89226	2.1
70221	1.89226	2.1
70222	1.89226	2.1
70223	1.89226	2.1
70224	1.89226	2.1
70225	1.89226	2.1
70226	1.89226	2.1
70227	1.89226	2.1
70228	1.89226	2.1
70229	1.89226	2.1
70230	1.89226	2.1
70231	1.89226	2.1
70232	1.89226	2.1
70233	1.89226	1.27225
70234	1.89226	2.1
70235	1.89226	0.63599

70236	3.78453	1.96122
70237	1.89226	2.1
70238	1.89226	0.298791
70239	1.89226	0.0722101
70240	1.89226	2.1
70241	1.89226	0.0737333
70242	1.89226	2.1
70243	1.89226	2.1
70244	1.89226	2.1
70245	1.89226	2.1
70246	1.89226	2.1
70247	1.89226	2.1
70248	1.89226	2.1
70249	1.89226	2.1
70250	1.89226	2.1
70251	1.89226	2.1
70252	1.89226	2.1
70253	3.78453	2.1
70254	1.89226	2.1
70255	1.89226	1.73356
70256	1.89226	2.1
70257	1.89226	1.13915
70258	1.89226	2.1
70259	1.89226	1.58069
70260	1.89226	2.1
70261	1.89226	1.15018
70262	1.89226	2.1
70263	1.89226	2.1
70264	1.89226	1.99634
70265	1.89226	1.15274
70266	1.89226	2.1
70267	1.89226	2.1
70268	1.89226	2.1
70269	1.89226	2.1
70270	1.89226	2.1
70271	1.89226	2.1
70272	1.89226	2.1
70273	1.89226	2.06767
70274	1.89226	2.1
70275	1.89226	1.94594
70276	3.78452	2.1
70277	1.89226	2.1
70278	1.89226	1.30139
70279	1.89226	2.1
70280	1.89226	0.115513
70281	3.78452	2.06934
70282	0.946131	2.1
70283	0.946131	0.0891183
70284	0.946131	1.71585
70285	0.946131	2.1
70286	0.94613	0.482912
70287	0.94613	1.98484
70288	0.946132	2.1
70289	0.946132	2.1
70290	0.946131	1.84117
70291	0.946131	1.08543
70292	0.946131	1.9467
70293	0.946131	1.41255

70294	1.89226	2.1
70295	1.89226	2.1
70296	1.89226	2.1
70297	1.89226	2.1
70298	1.89226	2.1
70299	1.89226	2.1
70300	1.89226	2.1
70301	1.89226	2.1
70302	1.89226	2.1
70303	1.89226	2.07703
70304	3.78452	2.1
70305	1.89226	2.1
70306	1.89226	0.674028
70307	1.89226	2.1
70308	1.89226	0.05
70309	3.78452	0.667586
70310	0.946131	1.19761
70311	0.946131	2.1
70312	0.946131	2.1
70313	0.946131	1.09549
70314	0.94613	0.410203
70315	0.94613	0.110298
70316	0.946132	2.1
70317	0.946132	2.1
70318	0.946131	2.08377
70319	0.946131	1.89897
70320	0.946131	1.88208
70321	0.946131	0.880259
70322	1.89226	2.1
70323	1.89226	2.1
70324	1.89226	2.1
70325	1.89226	2.1
70326	3.78452	2.1
70327	1.89226	2.1
70328	1.89226	2.1
70329	1.89226	2.1
70330	1.89226	2.1
70331	3.78453	2.1
70332	1.89226	2.1
70333	1.89226	2.1
70334	1.89226	2.1
70335	1.89226	0.308487
70336	3.78452	1.76935
70337	1.89226	2.1
70338	1.89226	2.1
70339	1.89226	2.1
70340	1.89226	1.28214
70341	1.89226	2.1
70342	1.89226	2.1
70343	1.89226	2.1
70344	1.89226	2.1
70345	1.89226	2.1
70346	1.89226	1.4099
70347	3.78452	2.1
70348	1.89226	2.1
70349	1.89226	2.1
70350	1.89226	2.1
70351	1.89226	2.1

70352	3.78453	2.1
70353	1.89226	2.1
70354	1.89226	0.649007
70355	1.89226	2.1
70356	1.89226	0.0954162
70357	3.78453	2.05589
70358	1.89226	2.1
70359	1.89226	0.250095
70360	1.89226	2.1
70361	1.89226	1.18228
70362	1.89226	2.1
70363	1.89226	0.0699841
70364	1.89226	2.1
70365	1.89226	2.1
70366	1.89226	2.1
70367	1.89226	0.881777
70368	3.78452	2.1
70369	1.89226	2.1
70370	1.89226	2.08204
70371	1.89226	2.1
70372	1.89226	1.31651
70373	3.78453	2.1
70374	1.89226	2.1
70375	1.89226	2.09592
70376	1.89226	2.1
70377	1.89226	0.219488
70378	3.78453	1.27351
70379	1.89226	2.1
70380	1.89226	1.15771
70381	1.89226	1.79767
70382	1.89226	0.887259
70383	1.89226	2.1
70384	1.89226	2.1
70385	0.946128	1.46367
70386	0.946128	2.1
70387	0.946132	1.90222
70388	0.946132	2.1
70389	0.946129	2.1
70390	0.946129	2.1
70391	0.94613	2.1
70392	0.94613	2.1
70393	0.94613	2.1
70394	0.94613	2.1
70395	0.94613	2.1
70396	0.94613	2.1
70397	1.89226	2.1
70398	1.89226	2.07483
70399	1.89226	2.1
70400	1.89226	2.1
70401	1.89226	2.1
70402	1.89226	1.29162
70403	1.89226	2.1
70404	1.89226	1.02882
70405	1.89226	1.45088
70406	1.89226	2.1
70407	1.89226	0.05
70408	1.89226	2.1
70409	0.946127	2.1

70410	0.946127	1.39462
70411	0.946131	0.420435
70412	0.946131	2.1
70413	0.946128	2.02516
70414	0.946128	0.287462
70415	0.94613	2.1
70416	0.94613	2.1
70417	0.946129	1.95205
70418	0.946129	1.94396
70419	0.946129	2.07634
70420	0.946129	2.1
70421	1.89226	2.1
70422	1.89226	2.1
70423	1.89226	2.1
70424	1.89226	1.70073
70425	3.78452	2.1
70426	1.89226	2.1
70427	1.89226	1.79303
70428	1.89226	2.1
70429	1.89226	2.1
70430	1.89226	2.1
70431	1.89226	2.1
70432	1.89226	2.1
70433	1.89226	0.796708
70434	1.89226	1.66823
70435	1.89226	2.1
70436	1.89226	0.0661972
70437	1.89226	2.1
70438	1.89226	0.45847
70439	1.89226	2.1
70440	1.89226	2.1
70441	1.89226	1.20499
70442	1.89226	2.1
70443	1.89226	1.98671
70444	0.946131	1.95
70445	0.946131	2.1
70446	0.946131	2.1
70447	0.946131	2.1
70448	0.946131	2.1
70449	0.946131	2.1
70450	0.946132	2.1
70451	0.946132	2.1
70452	0.946132	2.1
70453	0.946132	2.1
70454	0.946131	2.1
70455	0.946131	2.1
70456	1.89226	2.1
70457	1.89226	2.07725
70458	1.89226	2.1
70459	1.89226	2.1
70460	1.89226	2.1
70461	1.89226	2.1
70462	1.89226	2.1
70463	1.89226	0.242702
70464	1.89226	1.10888
70465	1.89226	2.1
70466	1.89226	0.05
70467	1.89226	2.1

70468	0.946131	2.1
70469	0.946131	0.790912
70470	0.946131	0.748445
70471	0.946131	2.1
70472	0.94613	0.185938
70473	0.94613	0.217256
70474	0.946132	2.1
70475	0.946132	2.1
70476	0.946131	2.1
70477	0.946131	2.1
70478	0.946131	2.07907
70479	0.946131	1.57659
70480	1.89226	2.1
70481	1.89226	2.1
70482	1.89226	2.1
70483	1.89226	1.50869
70484	3.78453	2.1
70485	1.89226	2.1
70486	1.89226	1.69669
70487	1.89226	2.1
70488	1.89226	2.1
70489	1.89226	2.1
70490	1.89226	1.38458
70491	1.89226	2.1
70492	1.89226	1.65814
70493	1.89226	2.1
70494	1.89226	0.106812
70495	3.78452	1.20145
70496	1.89226	2.1
70497	1.89226	1.55777
70498	1.89226	2.1
70499	1.89226	0.457137
70500	3.78452	2.07067
70501	0.946131	0.554102
70502	0.946131	2.1
70503	0.946131	2.1
70504	0.946131	1.83618
70505	0.946131	1.793
70506	0.946131	1.90896
70507	0.946131	2.1
70508	0.946131	2.1
70509	0.946131	2.1
70510	0.946131	2.1
70511	0.946131	2.1
70512	0.946131	2.1
70513	0.946128	0.748878
70514	0.946128	2.1
70515	0.946132	1.93361
70516	0.946132	2.1
70517	0.946129	1.91178
70518	0.946129	1.74789
70519	0.94613	2.1
70520	0.94613	2.1
70521	0.94613	2.1
70522	0.94613	2.1
70523	0.94613	2.1
70524	0.94613	2.1
70525	1.89226	2.1

70526	1.89226	0.394404
70527	1.89226	0.406312
70528	1.89226	2.1
70529	1.89226	0.05
70530	1.89226	2.1
70531	0.810945	2.1
70532	0.810945	0.750389
70533	0.810944	0.848304
70534	0.810944	0.910834
70535	1.89226	2.1
70536	1.35169	2.1
70537	1.08132	1.19232
70538	1.08132	1.73371
70539	1.08132	2.1
70540	0.810945	1.40246
70541	0.810945	2.1
70542	0.946131	2.1
70543	0.946131	2.1
70544	0.946131	2.1
70545	0.946131	2.1
70546	0.946131	2.1
70547	0.946131	2.1
70548	0.946131	2.1
70549	0.946131	2.1
70550	0.946131	2.1
70551	0.946131	2.1
70552	0.946131	2.1
70553	0.946131	2.1
70554	1.89226	2.1
70555	1.89226	2.1
70556	1.89226	2.1
70557	1.89226	2.1
70558	1.89226	2.1
70559	1.89226	2.1
70560	1.89226	2.1
70561	1.89226	2.1
70562	1.89226	2.1
70563	1.89226	2.1
70564	1.89226	2.1
70565	1.89226	2.1
70566	0.946131	2.1
70567	0.946131	2.1
70568	0.946131	2.1
70569	0.946131	2.1
70570	0.946131	2.1
70571	0.946131	2.1
70572	0.946131	2.1
70573	0.946131	2.1
70574	0.946131	2.1
70575	0.946131	2.1
70576	0.946131	2.1
70577	0.946131	2.1
70578	1.89226	2.1
70579	1.89226	2.1
70580	1.89226	2.1
70581	1.89226	2.1
70582	1.89226	2.1
70583	1.89226	2.1

70584	1.89226	2.1
70585	1.89226	2.1
70586	1.89226	2.1
70587	1.89226	2.1
70588	1.89226	2.1
70589	1.89226	2.1
70590	1.89226	2.1
70591	1.89226	2.1
70592	1.89226	2.1
70593	1.89226	2.1
70594	1.89226	2.1
70595	1.89226	2.1
70596	0.946132	2.1
70597	0.946132	2.1
70598	0.946131	2.1
70599	0.946131	2.1
70600	0.946132	2.1
70601	0.946132	2.1
70602	0.946132	2.1
70603	0.946132	2.1
70604	0.946132	2.1
70605	0.946132	2.1
70606	0.946132	2.1
70607	0.946132	2.1
70608	0.946131	2.1
70609	0.946131	2.1
70610	0.946131	2.1
70611	0.946131	2.1
70612	0.946131	2.1
70613	0.946131	2.1
70614	0.946131	2.1
70615	0.946131	2.1
70616	0.946131	2.1
70617	0.946131	2.1
70618	0.946131	2.1
70619	0.946131	2.1
70620	0.946132	2.1
70621	0.946132	2.1
70622	0.946131	2.1
70623	0.946131	2.1
70624	0.946132	2.1
70625	0.946132	2.1
70626	0.946131	2.1
70627	0.946131	2.1
70628	0.946131	2.1
70629	0.946131	2.1
70630	0.946131	2.1
70631	0.946131	2.1
70632	1.89226	2.1
70633	1.89226	2.1
70634	1.89226	2.1
70635	1.89226	2.1
70636	1.89226	2.1
70637	1.89226	2.1
70638	1.37713	2.1
70639	1.27093	2.1
70640	0.946132	2.1
70641	0.946132	2.1

70642	0.946132	2.1
70643	0.946132	2.1
70644	1.37713	2.1
70645	1.37713	2.1
70646	1.27093	2.1
70647	0.946131	2.1
70648	0.946132	2.1
70649	1.37713	2.1
70650	0.946131	2.1
70651	0.946131	2.1
70652	0.946131	2.1
70653	0.946131	2.1
70654	0.946132	2.1
70655	0.946132	2.1
70656	0.946131	2.1
70657	0.946131	2.1
70658	0.946131	2.1
70659	0.946131	2.1
70660	0.946132	2.1
70661	0.946132	2.1
70662	0.946132	2.1
70663	0.946132	2.1
70664	0.946131	2.1
70665	0.946131	2.1
70666	0.946132	2.1
70667	0.946132	2.1
70668	0.946131	2.1
70669	0.946131	2.1
70670	0.946131	2.1
70671	0.946131	2.1
70672	0.946132	2.1
70673	0.946132	2.1
70674	1.89226	2.1
70675	1.89226	2.1
70676	1.89226	2.1
70677	1.89226	2.1
70678	1.89226	2.1
70679	1.89226	2.1
70680	1.89226	2.1
70681	1.89226	2.1
70682	1.89226	2.1
70683	1.89226	2.1
70684	1.89226	2.1
70685	1.89226	2.1
70686	1.89226	2.1
70687	1.89226	2.1
70688	1.89226	2.1
70689	1.89226	0.633124
70690	3.78452	2.1
70691	1.89226	2.1
70692	1.89226	2.09123
70693	1.89226	2.1
70694	1.89226	1.48729
70695	1.89226	2.1
70696	1.89226	2.1
70697	1.89226	2.1
70698	1.89226	2.1
70699	1.89226	2.1

70700	1.89226	2.1
70701	3.78452	2.1
70702	1.89226	2.1
70703	1.89226	2.1
70704	1.89226	2.1
70705	1.89226	2.1
70706	3.78453	2.1
70707	1.89226	2.1
70708	1.89226	0.994503
70709	1.89226	1.72784
70710	1.89226	2.1
70711	1.89226	0.133554
70712	1.89226	2.1
70713	1.89226	0.164555
70714	1.89226	2.1
70715	1.89226	2.1
70716	1.89226	1.54132
70717	1.89226	2.1
70718	1.89226	2.02679
70719	1.89226	2.1
70720	1.89226	2.1
70721	1.89226	2.1
70722	1.89226	2.1
70723	3.78452	2.1
70724	1.89226	2.1
70725	1.89226	2.09051
70726	1.89226	2.1
70727	1.89226	2.1
70728	1.89226	2.1
70729	1.89226	2.1
70730	1.89226	2.1
70731	1.89226	1.76158
70732	1.89226	1.74541
70733	1.89226	2.1
70734	1.89226	0.167715
70735	1.89226	2.1
70736	1.89226	1.21079
70737	1.89226	2.1
70738	1.89226	2.1
70739	1.89226	1.82974
70740	1.89226	2.1
70741	1.89226	2.0658
70742	0.946131	2.09706
70743	0.946131	2.1
70744	0.946131	2.1
70745	0.946131	2.1
70746	0.946131	2.1
70747	0.946131	2.1
70748	0.946132	2.1
70749	0.946132	2.1
70750	0.946131	2.1
70751	0.946131	2.1
70752	0.946132	2.1
70753	0.946132	2.1
70754	1.89226	2.1
70755	1.89226	2.1
70756	1.89226	2.1
70757	1.89226	2.1

70758	1.89226	2.1
70759	1.89226	2.1
70760	1.89226	2.1
70761	1.89226	1.00897
70762	1.89226	1.00642
70763	1.89226	2.1
70764	1.89226	0.05
70765	1.89226	2.1
70766	0.946131	2.1
70767	0.946131	1.24392
70768	0.946131	1.31037
70769	0.946131	2.1
70770	0.94613	0.927266
70771	0.94613	0.470517
70772	0.946132	2.1
70773	0.946132	2.1
70774	0.946131	1.53411
70775	0.946131	2.1
70776	0.946131	2.05344
70777	0.946131	1.81757
70778	1.89226	2.1
70779	1.89226	0.834393
70780	1.89226	0.313045
70781	1.89226	2.1
70782	3.78453	1.70011
70783	2.81189	2.1
70784	1.89226	2.1
70785	1.89226	2.1
70786	1.89226	2.1
70787	2.56715	2.1
70788	2.56715	2.1
70789	1.89226	2.1
70790	1.89226	2.1
70791	1.89226	2.1
70792	1.89226	2.1
70793	3.78452	2.1
70794	1.89226	1.02592
70795	1.89226	2.1
70796	1.89226	2.1
70797	1.89226	2.1
70798	3.78452	2.1
70799	1.89226	0.545151
70800	1.89226	2.1
70801	1.89226	1.26146
70802	1.89226	2.1
70803	1.89226	1.11295
70804	1.89226	2.1
70805	1.1507	2.1
70806	1.1507	2.1
70807	0.946133	2.1
70808	0.946133	2.09583
70809	0.946131	2.1
70810	0.946131	2.1
70811	1.28358	2.1
70812	1.1507	2.1
70813	1.1507	2.1
70814	0.946131	2.1
70815	0.94613	2.1

70816	1.28358	2.1
70817	1.89226	2.1
70818	1.89226	2.1
70819	1.89226	2.1
70820	1.89226	2.1
70821	1.89226	2.1
70822	1.89226	2.1
70823	0.946131	2.1
70824	0.946131	2.1
70825	0.946131	1.14852
70826	0.946131	2.1
70827	0.946128	2.1
70828	0.946128	2.1
70829	0.946133	2.1
70830	0.946133	1.70167
70831	0.946131	2.1
70832	0.946131	2.1
70833	0.94613	2.1
70834	0.94613	2.1
70835	1.89226	0.160603
70836	1.89226	2.1
70837	1.89226	1.87704
70838	1.89226	0.475003
70839	3.78453	1.14288
70840	1.89226	2.1
70841	1.89226	2.1
70842	1.89226	0.839804
70843	1.89226	2.1
70844	3.78452	2.1
70845	1.89226	2.1
70846	1.89226	2.1
70847	1.89226	2.1
70848	1.89226	2.1
70849	3.78452	2.1
70850	1.89226	2.1
70851	1.89226	1.65114
70852	1.89226	0.59891
70853	1.89226	2.1
70854	3.78452	2.1
70855	0.946132	2.1
70856	0.946132	1.5965
70857	0.946132	0.0768286
70858	0.946132	0.588879
70859	0.946132	0.697361
70860	0.946132	2.1
70861	0.946132	2.1
70862	0.946132	0.0670018
70863	0.946132	1.2065
70864	0.946132	1.20682
70865	0.946131	1.20024
70866	0.946131	1.22619
70867	1.89226	2.1
70868	1.89226	2.1
70869	1.89226	2.1
70870	1.89226	2.1
70871	1.89226	2.1
70872	1.89226	2.1
70873	1.89226	0.838144

70874	1.89226	2.1
70875	1.89226	2.1
70876	1.89226	2.1
70877	1.89226	2.1
70878	1.89226	2.1
70879	1.89226	2.1
70880	1.89226	2.1
70881	1.89226	2.1
70882	1.89226	2.1
70883	3.78453	2.1
70884	1.89226	2.1
70885	1.89226	2.1
70886	1.89226	2.1
70887	1.89226	2.1
70888	3.78452	2.1
70889	1.89226	2.1
70890	1.89226	2.1
70891	1.89226	2.1
70892	1.89226	2.1
70893	1.89226	2.1
70894	1.89226	2.1
70895	1.89226	2.1
70896	1.89226	2.1
70897	1.89226	2.1
70898	1.89226	2.1
70899	3.78453	2.1
70900	1.89226	2.1
70901	1.89226	2.1
70902	1.89226	2.1
70903	1.89226	2.1
70904	1.89226	2.1
70905	1.89226	2.1
70906	1.89226	2.1
70907	1.89226	2.1
70908	1.89226	2.1
70909	1.89226	2.1
70910	3.78452	2.1
70911	1.89226	2.1
70912	1.89226	2.1
70913	1.89226	2.1
70914	1.89226	2.1
70915	3.78452	2.1
70916	1.89226	2.1
70917	1.89226	2.1
70918	1.89226	2.1
70919	1.89226	2.1
70920	3.78453	2.1
70921	1.89224	2.1
70922	1.89224	2.1
70923	1.89224	2.1
70924	1.89224	2.1
70925	3.78448	2.1
70926	1.89224	2.1
70927	1.89224	2.1
70928	1.89224	2.1
70929	1.89224	2.1
70930	1.89224	2.1
70931	1.89224	2.1

70932	1.89224	2.1
70933	1.89224	2.1
70934	1.89224	2.1
70935	1.89224	2.1
70936	3.78447	2.1
70937	0.946119	2.1
70938	0.946119	2.1
70939	0.946119	2.1
70940	0.946119	2.1
70941	0.946119	2.1
70942	0.946119	2.1
70943	0.946119	2.1
70944	0.946119	2.1
70945	0.946106	2.1
70946	0.946106	2.1
70947	0.946131	2.1
70948	0.946131	2.1
70949	1.89226	2.1
70950	1.89226	2.1
70951	1.89226	2.1
70952	1.89226	2.1
70953	3.78453	2.1
70954	0.810943	2.1
70955	0.810943	2.1
70956	0.810945	2.1
70957	0.810945	2.1
70958	0.810932	2.1
70959	0.810932	2.1
70960	1.89226	2.1
70961	1.3517	2.1
70962	1.08132	2.1
70963	1.08132	2.1
70964	1.08133	2.1
70965	1.89226	2.1
70966	1.89226	2.1
70967	1.89226	2.1
70968	1.89226	2.1
70969	1.89226	2.1
70970	1.89226	2.1
70971	0.946131	2.1
70972	0.946131	2.1
70973	0.946131	2.1
70974	0.946131	2.1
70975	0.946131	2.1
70976	0.946131	2.1
70977	0.946131	2.1
70978	0.946131	2.1
70979	0.946131	2.1
70980	0.946131	2.1
70981	0.946131	2.1
70982	0.946131	2.1
70983	1.89226	1.82839
70984	1.89226	2.1
70985	1.89226	2.1
70986	1.89226	2.1
70987	3.78453	2.1
70988	1.89226	2.1
70989	1.89226	2.1

70990	1.89226	2.1
70991	1.89226	2.1
70992	3.78452	2.1
70993	1.89226	2.1
70994	1.89226	2.1
70995	1.89226	2.1
70996	1.89226	2.1
70997	3.78452	2.1
70998	1.89226	2.1
70999	1.89226	2.1
71000	1.89226	2.1
71001	1.89226	2.1
71002	3.78453	2.1
71003	1.89226	2.1
71004	1.89226	2.1
71005	1.89226	2.1
71006	1.89226	2.1
71007	1.89226	2.1
71008	1.89226	2.1
71009	1.89226	2.1
71010	1.89226	2.1
71011	1.89226	2.1
71012	1.89226	2.1
71013	1.89226	2.1
71014	1.89226	2.1
71015	1.89226	2.1
71016	1.89226	2.1
71017	1.89226	2.1
71018	1.89226	2.1
71019	3.78452	2.1
71020	1.89226	2.1
71021	1.89226	2.1
71022	1.89226	2.1
71023	1.89226	2.1
71024	1.89226	2.1
71025	1.89226	2.1
71026	1.89226	2.1
71027	1.89226	2.1
71028	1.89226	2.1
71029	1.89226	2.1
71030	1.89226	2.1
71031	1.89226	2.1
71032	0.946128	2.1
71033	0.946128	2.1
71034	0.946133	2.1
71035	0.946133	2.1
71036	0.946131	2.1
71037	0.946131	2.1
71038	0.946131	2.1
71039	0.946131	2.1
71040	0.94613	2.1
71041	0.94613	2.1
71042	0.946131	2.1
71043	0.946131	2.1
71044	1.89226	2.1
71045	1.89226	2.1
71046	1.89226	2.1
71047	1.89226	2.1

71048	1.89226	2.1
71049	1.89226	2.1
71050	1.89226	2.1
71051	1.89226	2.1
71052	1.89226	2.1
71053	1.89226	2.1
71054	1.89226	2.1
71055	1.89226	2.1
71056	2.76309	2.1
71057	1.89226	2.1
71058	2.57778	2.1
71059	1.89226	2.1
71060	4.47004	2.1
71061	0.946128	2.1
71062	0.946128	2.1
71063	0.946133	2.1
71064	0.946133	2.1
71065	0.946131	2.1
71066	0.946131	2.1
71067	0.946131	2.1
71068	0.946131	2.1
71069	0.946131	2.1
71070	0.946131	2.1
71071	0.94613	2.1
71072	0.94613	2.1
71073	0.946132	2.1
71074	0.946132	2.1
71075	0.946132	2.1
71076	0.946132	2.1
71077	0.946132	2.1
71078	0.946132	2.1
71079	0.946132	2.1
71080	0.946132	2.1
71081	0.946132	2.1
71082	0.946132	2.1
71083	0.946131	2.1
71084	0.946131	2.1
71085	1.89227	1.95304
71086	1.89227	0.335891
71087	1.89227	0.137572
71088	1.89227	2.1
71089	3.78453	0.65545
71090	1.89226	0.0745936
71091	1.89226	1.91323
71092	1.89226	1.03919
71093	1.89226	0.692083
71094	3.78452	1.00512
71095	1.89226	2.1
71096	1.89226	0.156232
71097	1.89226	1.66625
71098	1.89226	1.84787
71099	1.89226	0.386836
71100	1.89226	0.410418
71101	0.946131	0.278334
71102	0.946131	0.177903
71103	0.946131	2.1
71104	0.946131	2.1
71105	0.946133	2.1

71106	0.946133	0.516212
71107	0.946129	0.483443
71108	0.946129	2.1
71109	0.946131	0.990114
71110	0.946131	1.19909
71111	0.946131	0.869602
71112	0.946131	0.428076
71113	1.89226	0.0981064
71114	1.89226	1.98416
71115	1.89226	1.9022
71116	1.89226	0.255435
71117	1.89226	1.68462
71118	1.89226	0.400294
71119	1.89226	0.899747
71120	1.89226	0.355513
71121	1.89226	0.0922902
71122	1.89226	1.64111
71123	3.78452	0.253568
71124	1.89226	1.49006
71125	1.89226	0.722992
71126	1.89226	1.7083
71127	1.89226	0.217312
71128	1.89226	1.99944
71129	1.89226	0.493185
71130	0.94613	0.257181
71131	0.94613	0.477119
71132	0.946131	2.1
71133	0.946131	1.89155
71134	0.946133	0.687994
71135	0.946133	2.01978
71136	0.946128	1.99458
71137	0.946128	0.328247
71138	0.946131	1.38705
71139	0.946131	1.5218
71140	0.946131	0.69763
71141	0.946131	0.808881
71142	1.89226	0.384856
71143	1.89226	2.1
71144	1.89226	1.98886
71145	1.89226	0.989375
71146	1.89226	1.96896
71147	1.89226	1.18472
71148	1.89226	0.455582
71149	1.89226	1.98589
71150	1.89226	1.2522
71151	1.89226	1.25788
71152	1.89226	1.31745
71153	1.89226	1.62746
71154	0.946133	1.69945
71155	0.946133	0.283977
71156	0.946129	2.1
71157	0.946129	1.38001
71158	0.946131	2.1
71159	0.946131	1.11709
71160	0.946131	1.57006
71161	0.946131	1.02081
71162	0.946131	1.46994
71163	0.946131	1.35248

71164	0.946131	1.3368
71165	0.946131	1.50467
71166	1.89226	0.260006
71167	1.89226	2.1
71168	1.89226	1.60327
71169	1.89226	0.908283
71170	1.89226	1.60529
71171	1.89226	1.21053
71172	1.89226	0.388361
71173	1.89226	1.93045
71174	1.89226	0.872849
71175	1.89226	0.972508
71176	1.89226	1.0229
71177	1.89226	1.483
71178	1.89226	0.51114
71179	1.89226	2.05511
71180	1.89226	1.14378
71181	1.89226	1.27454
71182	1.89226	1.33923
71183	1.89226	1.77161
71184	1.89227	0.485122
71185	1.89227	2.1
71186	1.89227	1.8365
71187	1.89227	1.41542
71188	1.89227	1.89058
71189	1.89227	1.61008
71190	1.89227	2.1
71191	1.89227	2.1
71192	1.89227	2.1
71193	1.89227	2.1
71194	1.89227	2.1
71195	1.89227	2.1
71196	1.89226	0.406333
71197	1.89226	2.1
71198	1.89226	2.1
71199	1.89226	0.628204
71200	1.89226	2.1
71201	1.89226	0.72076
71202	1.89226	0.536411
71203	1.89226	2.1
71204	1.89226	2.1
71205	1.89226	0.871428
71206	1.89226	2.1
71207	1.89226	1.18649
71208	1.89227	2.03175
71209	1.89227	2.1
71210	1.89227	2.1
71211	1.89227	2.07091
71212	3.78453	2.1
71213	1.89226	0.15707
71214	1.89226	1.91768
71215	1.89226	1.99925
71216	1.89226	0.259876
71217	1.89226	1.77828
71218	1.89226	0.32736
71219	1.89226	0.223836
71220	1.89226	2.1
71221	1.89226	1.98898

71222	1.89226	0.284443
71223	3.78452	1.62749
71224	1.89226	2.1
71225	1.89226	2.1
71226	1.89226	2.1
71227	1.89226	2.1
71228	1.89226	2.1
71229	1.89226	2.1
71230	1.89227	0.120347
71231	1.89227	1.77641
71232	1.89227	1.36738
71233	1.89227	0.23013
71234	1.89227	1.23892
71235	1.89227	0.248312
71236	1.89226	0.0759708
71237	1.89226	1.23468
71238	1.89226	0.808292
71239	1.89226	0.306217
71240	1.89226	0.889699
71241	1.89226	0.458646
71242	1.89226	1.37202
71243	1.89226	0.689357
71244	1.89226	1.49331
71245	1.89226	0.269754
71246	1.89226	1.81987
71247	1.89226	0.481481
71248	0.946131	0.236708
71249	0.946131	0.367882
71250	0.946131	1.58896
71251	0.946131	2.1
71252	0.946133	0.534521
71253	0.946133	1.98476
71254	0.946129	1.78194
71255	0.946129	0.298388
71256	0.946131	1.19177
71257	0.946131	1.30127
71258	0.946131	0.554343
71259	0.946131	0.656474
71260	1.89227	0.0746701
71261	1.89227	1.47513
71262	1.89227	0.917041
71263	1.89227	0.200796
71264	1.89227	0.947814
71265	1.89227	0.227479
71266	1.89226	0.341703
71267	1.89226	1.06435
71268	1.89226	0.368445
71269	1.89226	0.202766
71270	1.89226	0.544193
71271	1.89226	0.450871
71272	1.89226	0.890962
71273	1.89226	0.643977
71274	1.89226	1.16998
71275	1.89226	0.218158
71276	1.89226	1.63642
71277	1.89226	0.482491
71278	0.946131	0.225461
71279	0.946131	0.311452

71280	0.946131	1.0146
71281	0.946131	2.06646
71282	0.946133	0.401718
71283	0.946133	1.67224
71284	0.946129	1.13718
71285	0.946129	0.219482
71286	0.946131	0.938639
71287	0.946131	1.08781
71288	0.946131	0.307361
71289	0.946131	0.353291
71290	1.89227	0.378697
71291	1.89227	2.1
71292	1.89227	2.1
71293	1.89227	0.454512
71294	3.78453	2.0863
71295	1.89226	2.01047
71296	1.89226	1.01187
71297	1.89226	0.282995
71298	1.89226	2.1
71299	3.78452	0.564929
71300	1.89226	0.478771
71301	1.89226	2.1
71302	1.89226	2.00066
71303	1.89226	1.34829
71304	3.78452	2.0763
71305	1.89226	2.1
71306	1.89226	1.16379
71307	1.89226	0.337799
71308	1.89226	2.1
71309	3.78452	1.41442
71310	1.89226	2.1
71311	1.89226	0.712286
71312	1.89226	2.1
71313	1.89226	0.630975
71314	1.89226	2.03549
71315	1.89226	0.100734
71316	1.89226	0.313321
71317	1.89226	2.1
71318	1.89226	1.65551
71319	1.89226	0.416429
71320	1.89226	1.59336
71321	1.89226	0.712203
71322	0.946131	1.18473
71323	0.946131	0.25224
71324	0.946127	1.98133
71325	0.946127	2.1
71326	0.946131	0.972
71327	0.946131	1.97905
71328	0.946127	1.96353
71329	0.946127	0.435523
71330	0.946129	1.648
71331	0.946129	1.6015
71332	0.946129	0.967077
71333	0.946129	1.30532
71334	0.94613	0.454996
71335	0.94613	0.551405
71336	0.946131	2.1
71337	0.946131	2.1

71338	0.946133	0.949242
71339	0.946133	2.1
71340	0.946128	0.273384
71341	0.946128	2.0798
71342	0.946131	2.1
71343	0.946131	1.81146
71344	0.946131	1.82195
71345	0.946131	1.91029
71346	1.89226	2.01696
71347	1.89226	1.94756
71348	1.89226	0.2861
71349	1.89226	2.06515
71350	3.78453	0.857487
71351	1.89226	1.72426
71352	1.89226	1.81959
71353	1.89226	0.430732
71354	1.89226	1.48527
[truncated: 354,862 more chars]
