# Supplementary material for: On the Use of Bone Remodelling Models to Estimate the Density Distribution of Bones. Uniqueness of the Solution
Source: PLoS One. 2016 Feb 9;11(2):e0148603. doi: 10.1371/journal.pone.0148603 (PMC4747586; doi:10.1371/journal.pone.0148603)
Supplement: S4 File — (PDF) [file pone.0148603.s004.pdf]

N° Elem	Volume (mm3)	Density (g/cm3)
1	1.32415	2.1
2	1.75823	2.1
3	0.984099	2.1
4	1.46442	2.1
5	1.53082	2.1
6	1.32068	2.1
7	1.91404	0.05
8	1.17036	0.05
9	0.929209	0.05
10	1.27254	0.05
11	2.0926	0.05
12	2.20407	0.05
13	1.21488	0.05
14	1.26024	0.05
15	0.794454	0.05
16	1.95087	0.05
17	0.953925	2.1
18	0.98949	2.1
19	1.27875	2.1
20	1.04598	2.1
21	0.594005	2.1
22	0.578861	2.1
23	1.09506	2.1
24	1.45327	2.1
25	2.58396	2.1
26	1.58581	2.1
27	1.5858	2.1
28	0.636946	2.1
29	0.94202	2.1
30	0.946119	2.1
31	0.636953	2.1
32	0.946107	2.1
33	1.09505	2.1
34	1.27389	2.1
35	1.00232	2.1
36	1.00231	2.1
37	1.83583	2.1
38	1.00231	2.1
39	1.28828	0.05
40	1.81696	0.05
41	1.28828	0.05
42	1.28828	0.05
43	1.0044	0.05
44	0.85148	0.05
45	0.851539	0.05
46	1.57767	0.05
47	1.52227	0.05
48	0.730657	0.05
49	0.94613	0.05
50	1.28228	2.1
51	1.64268	2.1
52	1.10101	2.1
53	1.3939	2.1
54	0.959471	2.1
55	1.10101	2.1

56	1.2873	2.1
57	1.59659	2.1
58	1.80841	0.05
59	1.80841	0.106418
60	0.946129	0.05
61	1.36991	0.05
62	1.32262	0.72968
63	1.32261	0.05
64	1.38464	0.0845139
65	0.962877	0.05
66	0.962878	0.0798341
67	1.82129	0.0695851
68	1.82129	0.05
69	0.94613	0.0510752
70	1.39751	0.0528484
71	0.946131	0.095451
72	1.34493	0.05
73	1.39751	0.05
74	0.837577	0.384498
75	0.823478	0.115607
76	1.10889	1.70151
77	1.01686	0.436947
78	1.1942	1.75764
79	0.836129	0.341071
80	0.937165	0.123352
81	0.969438	0.855669
82	0.895386	0.248585
83	1.30554	1.88962
84	1.15798	0.262488
85	1.22578	2.1
86	1.15255	0.722459
87	0.9765	0.126161
88	2.02178	1.9074
89	1.63755	1.67168
90	0.727713	0.865395
91	0.810945	0.845694
92	1.00559	1.85595
93	0.879098	0.777151
94	1.07852	1.94974
95	0.657764	0.608082
96	0.829406	0.315622
97	0.720201	0.05
98	0.946131	0.05
99	1.11827	0.05
100	1.50754	0.05
101	1.64835	0.05
102	1.71903	0.05
103	0.851554	0.05
104	1.75626	0.05
105	2.1646	0.05
106	1.5563	0.05
107	1.5563	0.05
108	2.10876	0.05
109	1.05438	0.0915982
110	0.946133	0.0521459
111	1.05438	0.556644
112	1.20256	0.05
113	1.47879	0.0814596

114	1.47879	1.26091
115	0.77815	1.17303
116	0.947114	2.1
117	1.31257	2.1
118	1.09235	2.1
119	0.946132	2.1
120	1.54246	2.1
121	1.54246	2.1
122	1.09235	2.1
123	0.946131	2.1
124	1.29507	1.93333
125	1.0353	2.1
126	1.03529	2.1
127	1.28667	2.1
128	1.44777	2.1
129	1.61121	2.1
130	1.22176	0.0685057
131	0.946129	0.0608465
132	1.08848	0.05
133	0.946131	0.0750125
134	1.61201	0.05
135	1.61201	0.05
136	1.19993	0.05
137	1.35822	0.05
138	1.1539	0.05
139	1.00765	0.05
140	1.28228	2.1
141	2.65339	2.1
142	1.28228	2.1
143	1.55998	2.1
144	1.00509	2.1
145	0.93845	2.1
146	1.27674	2.1
147	0.810958	2.1
148	1.91381	2.1
149	1.1861	2.1
150	1.24886	2.1
151	2.02842	2.1
152	0.991436	0.05
153	1.34588	0.05
154	2.02729	0.05
155	0.72904	0.05
156	1.06139	0.05
157	0.835368	0.05
158	1.63819	0.05
159	1.10745	0.05
160	1.79925	0.05
161	1.00085	0.05
162	0.946133	2.1
163	1.41123	2.1
164	1.8327	2.1
165	1.8327	2.1
166	0.946132	1.08115
167	1.41123	2.1
168	1.3676	2.1
169	1.11048	2.1
170	1.11048	2.1
171	1.65815	2.1

172	1.9004	2.1
173	1.88691	2.1
174	1.82232	2.1
175	1.46591	2.1
176	1.09115	2.1
177	1.02051	0.932135
178	1.03373	1.48239
179	1.10307	2.1
180	1.05556	2.1
181	1.82533	0.867898
182	1.19496	0.384447
183	1.28597	2.1
184	1.20421	2.1
185	2.23644	2.1
186	1.08378	2.1
187	0.983724	2.1
188	0.602075	2.1
189	1.45876	2.1
190	1.45875	2.1
191	0.602103	2.1
192	1.11822	2.1
193	0.946133	2.1
194	0.602103	2.1
195	1.20421	2.1
196	1.26371	0.111956
197	1.55179	0.0798729
198	1.14042	0.05
199	1.56876	0.05
200	2.27496	0.0885379
201	1.13748	0.0615793
202	1.02884	0.175002
203	1.25124	0.085505
204	0.82703	0.0798209
205	0.607775	0.0733881
206	1.49242	0.0810078
207	1.35674	0.122138
208	1.6697	2.1
209	1.81655	2.1
210	0.900108	0.865533
211	0.851537	0.238793
212	0.703076	1.05592
213	0.639143	0.448973
214	1.42358	0.95485
215	1.4976	1.52494
216	0.86284	0.428864
217	1.2965	0.802486
218	1.27835	2.1
219	1.00012	0.428887
220	1.74332	1.51727
221	1.08576	2.1
222	1.19 2.1	
223	1.08576	2.1
224	1.20854	2.1
225	0.946131	2.1
226	0.946131	2.1
227	1.75985	2.1
228	1.75985	2.1
229	1.41232	2.1

230	1.29365	2.1
231	0.973559	2.1
232	1.23698	0.05
233	1.45055	0.05
234	1.26947	0.05
235	0.856218	0.05
236	1.75303	0.05
237	1.14938	0.05
238	0.984711	0.05
239	2.05771	0.05
240	0.625171	0.05
241	0.965675	0.05
242	1.17631	0.05
243	1.4677	0.05
244	1.35403	0.05
245	0.975149	0.05
246	0.946131	0.05
247	1.80893	0.05
248	1.80893	0.05
249	0.975149	0.05
250	0.946132	0.05
251	1.9503	0.05
252	2.80207	0.05
253	1.76411	0.05
254	2.77469	0.05
255	1.9503	0.05
256	1.26784	0.05
257	2.31093	0.05
258	0.851541	0.05
259	1.39521	0.05
260	1.45613	0.05
261	0.851541	0.05
262	0.996013	0.05
263	1.07989	0.05
264	0.946131	0.05
265	1.08213	0.05
266	1.74289	0.05
267	1.75838	0.05
268	2.11163	0.05
269	1.23773	2.1
270	1.25398	2.1
271	1.0304	2.1
272	1.32368	2.1
273	1.11712	0.756603
274	1.07999	0.326036
275	1.1766	2.1
276	1.35735	2.1
277	1.089 0.05	
278	1.81193	0.05
279	1.11081	0.05
280	1.78069	0.05
281	0.717512	0.05
282	0.810946	0.05
283	0.703901	0.05
284	1.09028	0.05
285	0.958031	0.05
286	0.892585	0.05
287	0.873431	0.05

288	0.997755	0.05
289	1.85479	0.05
290	0.983029	0.05
291	1.27922	0.05
292	1.64346	0.066355
293	1.27922	0.05
294	1.09795	2.1
295	1.07508	0.860788
296	1.02975	1.65259
297	1.01121	2.1
298	1.72369	0.05
299	1.12035	0.05
300	2.13511	0.05
301	0.9705	0.05
302	0.946131	0.05
303	0.86182	0.05
304	1.51379	0.05
305	1.51379	0.05
306	0.946132	0.05
307	0.861849	0.05
308	1.34591	0.123999
309	1.14721	0.05
310	1.15539	2.1
311	1.90938	2.1
312	2.19436	2.1
313	1.41563	2.1
314	0.785641	2.1
315	0.736384	2.1
316	1.46489	2.1
317	1.46488	2.1
318	0.736372	2.1
319	0.946132	2.1
320	1.90941	1.74606
321	1.18907	2.1
322	1.20409	2.1
323	0.973559	2.1
324	1.29461	2.1
325	0.946129	2.1
326	0.94613	2.1
327	1.78751	2.1
328	1.78751	2.1
329	1.38885	2.1
330	1.34479	2.1
331	1.07283	2.1
332	1.6869	2.1
333	1.52544	2.1
334	1.07797	2.1
335	1.13819	1.14244
336	0.776179	2.1
337	1.61092	2.04511
338	1.36155	2.1
339	0.77618	2.1
340	1.2933	1.70949
341	1.15946	2.1
342	0.69857	2.1
343	1.87607	2.1
344	1.07612	2.1
345	1.31976	0.882151

346	1.01243	2.1
347	1.41279	1.30111
348	1.35146	0.05
349	1.90664	0.05
350	1.35145	0.05
351	1.22811	0.05
352	1.93147	0.05
353	1.42486	2.1
354	1.56558	2.1
355	1.0476	0.05
356	2.31605	0.05
357	1.89367	0.05
358	0.804706	0.05
359	0.783919	0.05
360	1.3116	0.05
361	1.03891	0.05
362	0.960473	0.05
363	1.08462	0.05
364	0.785994	0.05
365	1.1318	0.05
366	1.29576	0.05
367	1.3946	0.05
368	1.11924	0.05
369	0.834322	2.1
370	1.0256	2.1
371	1.74321	0.535206
372	1.41798	0.501201
373	0.871608	0.468649
374	1.47253	0.425306
375	1.47252	0.483812
376	0.817064	0.403042
377	0.817064	0.473255
378	0.946129	0.3762
379	1.63413	0.373202
380	1.9194	0.05
381	1.93562	0.05
382	2.06307	0.05
383	1.46021	0.05
384	0.955002	0.819225
385	0.833409	1.57474
386	0.838039	0.774272
387	1.48702	0.788374
388	1.1697	0.71404
389	1.23549	0.928501
390	1.18373	0.720371
391	2.02254	0.618629
392	1.68867	0.953043
393	0.875481	0.268814
394	0.821635	1.30183
395	0.858878	0.224847
396	1.30245	0.400015
397	1.07476	0.363445
398	1.11907	0.374359
399	1.10453	0.279651
400	1.70233	0.38417
401	1.70233	0.269913
402	2.6558	1.09492
403	1.7818	2.1

404	1.87059	2.1
405	1.34412	0.05
406	1.98068	0.05
407	2.56369	0.05
408	1.49512	0.05
409	1.35977	0.05
410	1.34254	0.05
411	1.75618	0.05
412	1.75618	0.05
413	0.946129	0.0762249
414	0.946129	0.05
415	1.17665	0.05
416	1.01976	0.05
417	1.27376	0.05
418	1.35978	0.05
419	1.68741	0.05
420	1.68741	0.05
421	0.946132	0.05
422	0.946133	0.05
423	1.14241	0.05
424	0.996831	0.05
425	0.996828	0.05
426	1.12774	0.05
427	1.13664	2.1
428	1.09627	2.1
429	2.8814	2.1
430	3.49751	2.1
431	1.79109	0.0682978
432	1.54197	0.05
433	2.75153	0.0542447
434	1.93019	0.05
435	1.51569	0.292894
436	1.55145	0.774108
437	1.78595	2.1
438	3.74452	2.1
439	1.66537	2.1
440	3.55161	2.1
441	1.35002	0.0913406
442	1.19901	0.201885
443	1.61028	2.09346
444	1.35002	0.246655
445	1.65712	0.238393
446	1.10585	0.726318
447	0.961347	0.939351
448	0.810944	1.17535
449	1.16148	1.81501
450	1.08066	1.61822
451	0.934577	0.371536
452	1.05164	1.28446
453	0.79216	0.621853
454	2.99194	2.1
455	1.2832	2.1
456	2.97842	2.1
457	1.39517	2.1
458	1.03391	2.1
459	1.74416	2.1
460	1.05071	2.1
461	0.923782	2.1

462	1.55369	2.1
463	1.86444	2.1
464	1.01617	2.1
465	1.27174	2.1
466	1.20573	2.1
467	1.01616	2.1
468	1.13621	2.1
469	1.75366	2.1
470	2.27009	1.92983
471	1.6813	2.09875
472	1.47904	0.05
473	2.62714	0.05
474	1.80085	0.05
475	1.80085	0.05
476	2.74104	0.05
477	1.37052	0.05
478	0.946131	0.05
479	1.37052	0.05
480	1.31357	0.05
481	1.7837	0.05
482	1.7837	0.05
483	0.900397	0.05
484	1.74487	0.05
485	2.88672	2.1
486	2.88249	2.1
487	1.0961	0.0824844
488	1.20143	0.0976166
489	1.66739	0.0636195
490	1.85795	0.05
491	1.45789	0.05
492	2.11407	0.05
493	0.713524	0.05
494	1.58391	0.05
495	1.58391	0.05
496	1.04765	0.05
497	1.24979	0.05
498	0.713551	0.05
499	0.946127	0.05
500	1.36768	0.05
501	1.14433	0.05
502	1.66923	0.05
503	0.699479	0.05
504	0.70351	0.05
505	0.758416	0.05
506	1.07678	0.05
507	0.909874	0.05
508	0.940218	0.05
509	0.876604	0.05
510	1.27148	0.05
511	2.42348	0.05
512	1.19837	0.05
513	0.764764	0.05
514	0.829259	0.05
515	0.740584	0.05
516	1.2772	0.05
517	0.964465	0.05
518	1.06231	0.05
519	1.06924	0.05

520	1.17566	0.05
521	1.3063	0.05
522	2.51253	0.05
523	2.42267	0.05
524	1.89226	0.05
525	1.46492	0.05
526	0.946132	0.05
527	0.653151	0.05
528	0.653124	0.05
529	1.53995	0.05
530	1.53995	0.05
531	0.936806	0.05
532	1.25626	0.05
533	1.1893	0.05
534	1.62206	0.05
535	1.04921	2.1
536	1.22927	2.1
537	0.801765	2.1
538	0.774917	2.1
539	0.844623	2.1
540	0.737481	2.1
541	3.24379	2.1
542	2.48573	2.1
543	1.40967	0.89784
544	1.47203	1.58708
545	2.07659	0.922043
546	1.40967	1.51825
547	2.06638	0.917152
548	1.17418	2.1
549	1.04921	2.1
550	1.74593	2.1
551	1.74593	2.1
552	0.946132	2.1
553	1.38651	2.1
554	1.20761	0.0787541
555	1.20761	1.78467
556	1.30555	1.56429
557	1.46812	0.173934
558	2.41602	0.550349
559	1.46812	0.525852
560	1.46812	0.0938352
561	0.734059	0.117391
562	0.946132	1.7075
563	1.21261	0.631256
564	1.34225	0.661749
565	0.734084	0.712821
566	1.82077	0.439315
567	1.82077	0.328133
568	1.13751	0.0740399
569	0.946131	0.480978
570	1.13751	0.173839
571	1.13748	0.148464
572	0.784392	0.194492
573	1.4906	0.110326
574	1.4906	0.0541787
575	1.17879	1.29606
576	1.28291	1.19754
577	0.626495	2.1

578	1.45334	2.1
579	1.45334	2.1
580	0.626494	2.1
581	1.3852	2.1
582	0.946116	2.1
583	0.626499	2.1
584	1.47203	0.679585
585	1.42639	2.1
586	1.42639	2.1
587	0.795469	2.1
588	0.795469	2.1
589	0.946132	2.1
590	0.850877	2.1
591	1.37098	2.1
592	1.87194	2.1
593	1.76988	2.1
594	3.16295	0.05
595	3.26725	0.05
596	1.13322	2.1
597	1.20761	2.1
598	0.686933	1.3677
599	0.493248	0.683733
600	1.2053	2.1
601	0.947149	0.879139
602	1.27591	1.07539
603	1.75732	2.1
604	0.493261	0.979166
605	1.34337	2.1
606	2.73207	0.524677
607	1.19857	1.0103
608	0.671688	0.293821
609	0.946134	2.1
610	1.36603	0.44941
611	2.07817	0.9313
612	1.82573	2.03723
613	0.671699	1.38796
614	1.47772	0.466427
615	0.975349	2.1
616	1.22615	2.1
617	1.47868	2.1
618	2.29351	2.1
619	1.47642	2.1
620	2.94443	1.95229
621	1.80454	2.1
622	0.949135	2.1
623	0.949134	2.1
624	1.10476	0.05
625	0.982857	0.05
626	1.10938	0.05
627	1.19052	0.113961
628	1.01012	0.05
629	1.35562	0.05
630	0.691742	0.05
631	2.39596	0.05
632	2.35604	0.05
633	0.974081	0.05
634	3.22444	0.05
635	1.3294	0.15924

636	1.37265	0.780377
637	1.3294	0.218754
638	1.51113	2.1
639	1.43643	2.1
640	1.72726	2.1
641	1.634	2.1
642	0.755566	2.1
643	1.29319	2.1
644	0.755567	2.1
645	0.946131	2.1
646	1.29319	2.1
647	0.973563	2.1
648	0.904398	2.1
649	0.904406	2.1
650	1.1538	2.1
651	1.10437	2.1
652	0.935418	2.1
653	1.16649	2.1
654	0.975667	2.1
655	1.17952	2.1
656	1.95053	2.1
657	0.948676	2.1
658	1.44287	2.1
659	0.746095	2.1
660	1.51224	2.1
661	1.27354	2.1
662	0.746066	2.1
663	1.20573	2.1
664	1.12442	2.1
665	0.671487	2.1
666	1.04355	2.1
667	0.948675	2.1
668	0.967673	0.587542
669	1.12931	0.05
670	1.38212	0.05
671	2.40113	0.05
672	1.08054	0.05
673	1.04072	0.05
674	0.691062	0.05
675	1.58052	0.05
676	1.33502	0.05
677	0.946131	0.05
678	0.691062	0.05
679	1.02663	0.05
680	1.80569	0.05
681	1.26062	0.05
682	1.18203	0.05
683	0.946131	0.05
684	0.795065	0.05
685	1.58753	0.05
686	1.58753	0.05
687	0.946131	0.05
688	0.795093	0.05
689	0.805057	2.1
690	0.812415	2.1
691	1.23868	0.05
692	1.34448	0.05
693	1.67201	2.1

694	1.61202	2.1
695	1.41103	2.1
696	1.41103	2.1
697	0.841088	2.1
698	1.38302	2.1
699	0.841089	2.1
700	0.869097	2.1
701	1.38302	2.1
702	0.949941	2.1
703	0.94994	2.1
704	1.88608	2.1
705	0.854942	2.1
706	1.68893	2.1
707	1.70002	2.1
708	2.02107	0.05
709	2.04982	0.05
710	3.11063	0.05
711	2.03805	0.05
712	1.67638	2.1
713	0.967492	2.1
714	2.00209	2.1
715	0.967491	2.1
716	0.870742	2.1
717	1.30266	0.05
718	0.651334	0.05
719	1.69413	0.05
720	1.69413	0.05
721	0.946132	0.05
722	1.03079	0.05
723	1.31468	0.05
724	0.946132	0.05
725	0.919055	0.05
726	1.16815	0.05
727	2.79004	0.05
728	1.2136	0.05
729	2.62935	0.05
730	0.947692	0.05
731	1.51835	0.0522002
732	0.947693	0.05
733	0.947692	0.05
734	0.980841	2.1
735	1.43577	2.1
736	0.980841	2.1
737	1.67858	2.1
738	1.34124	0.0874479
739	1.39818	0.0612417
740	1.2065	2.1
741	0.946133	2.1
742	1.2079	2.1
743	1.65229	2.1
744	1.65229	2.1
745	0.946132	2.1
746	1.2079	2.1
747	1.39053	2.1
748	1.20651	2.1
749	1.36919	2.1
750	1.12717	2.1
751	1.09483	2.1

752	0.994294	2.1
753	0.97356	2.1
754	1.06827	2.1
755	1.40571	2.1
756	1.29259	2.1
757	0.946131	2.1
758	1.7787	2.1
759	1.7787	2.1
760	1.29259	2.1
761	0.946132	2.1
762	1.09408	0.14171
763	1.03824	0.186053
764	0.851541	0.0771025
765	1.23608	0.0687588
766	0.851539	0.077053
767	1.5021	0.105313
768	1.54839	0.0881274
769	1.12373	0.068742
770	1.22617	0.05
771	1.42762	0.0588581
772	2.24746	0.101591
773	1.05475	0.05
774	1.46845	0.05
775	1.05475	0.05
776	0.929726	0.05
777	1.52072	0.05
778	1.82488	0.05
779	1.02271	0.05
780	1.19225	0.05
781	1.43071	0.05
782	0.94613	0.05
783	1.05475	0.05
784	1.39223	2.1
785	1.71255	2.1
786	0.946132	0.607719
787	1.10531	0.429586
788	1.46355	0.46138
789	1.46355	0.282148
790	0.78086	0.968085
791	1.10531	0.477898
792	1.1391	0.505439
793	1.56166	0.670011
794	2.21062	0.407459
795	1.0361	2.1
796	1.73577	2.1
797	1.73577	2.1
798	0.946116	2.1
799	1.28174	2.1
800	0.946118	2.1
801	1.22883	2.1
802	1.28172	2.1
803	1.03609	2.1
804	0.553567	0.05
805	1.60581	0.05
806	1.60581	0.05
807	0.553594	0.05
808	0.966748	0.05
809	0.966748	0.05

810	0.946133	0.05
811	1.05602	0.05
812	2.38525	0.05
813	1.14503	0.05
814	1.24678	0.05
815	0.56262	0.05
816	1.24678	0.05
817	0.978467	0.05
818	1.66262	0.05
819	1.66262	0.05
820	0.562633	0.05
821	1.09522	0.05
822	2.49356	0.05
823	0.592547	0.05
824	1.04321	0.528528
825	1.25133	1.18575
826	1.70508	1.3626
827	1.81043	1.0704
828	0.494934	1.2129
829	0.601047	0.844999
830	0.993428	1.23665
831	0.946573	1.17564
832	1.35039	0.864333
833	0.816895	1.48598
834	1.5063	0.900796
835	0.816895	0.592088
836	0.946132	0.954277
837	1.9067	1.20599
838	2.14504	1.51975
839	0.816895	0.615296
840	2.29624	0.62442
841	2.76933	0.554741
842	1.254 1.40157	
843	0.946128	2.1
844	1.38813	2.1
845	1.79998	2.1
846	1.79998	2.1
847	0.94613	2.1
848	1.38813	2.1
849	1.35798	2.1
850	1.27809	2.1
851	1.35798	2.1
852	1.68994	2.1
853	1.68994	2.1
854	0.946132	2.1
855	0.946132	2.1
856	1.18067	2.1
857	0.958256	2.1
858	0.958255	2.1
859	0.958257	2.1
860	0.958255	2.1
861	1.06457	2.1
862	1.14195	2.1
863	1.70423	0.208714
864	1.14195	2.1
865	1.64285	2.1
866	2.02057	2.1
867	0.946132	2.1

868	1.44186	2.1
869	0.946133	2.1
870	1.16366	2.1
871	1.17233	2.1
872	1.31259	0.706196
873	1.2016	2.1
874	1.17129	2.1
875	1.05724	0.05
876	1.32697	1.09984
877	1.05724	0.819346
878	1.04292	1.8139
879	3.39037	2.1
880	2.57275	2.1
881	0.629212	2.1
882	2.32525	2.1
883	1.76949	0.304039
884	1.65809	0.890977
885	1.27665	2.1
886	1.19861	2.1
887	1.20045	2.1
888	1.1635	2.1
889	0.953633	0.05
890	0.953634	0.05
891	1.21408	2.1
892	1.25716	2.1
893	2.01791	2.1
894	1.89318	2.1
895	0.947921	2.01302
896	0.939049	2.1
897	0.947921	2.1
898	0.740753	2.1
899	0.916104	2.1
900	1.16941	2.1
901	1.72038	2.1
902	2.04441	2.1
903	0.973182	2.1
904	1.11751	2.1
905	0.611089	2.1
906	1.53584	0.05
907	0.767923	0.0541777
908	1.38925	0.0549534
909	1.38926	0.05
910	0.946131	0.0750092
911	0.961347	0.050193
912	1.19583	0.0986845
913	0.946131	0.0993086
914	1.3663	0.114674
915	1.21067	0.05
916	1.00166	0.05
917	1.40707	0.05
918	1.50435	0.05
919	1.47954	0.344054
920	1.1233	2.1
921	2.36036	1.81072
922	1.04024	0.252207
923	0.739729	0.501858
924	1.45533	0.150211
925	1.45532	0.0858171

926	0.946118	0.300218
927	1.15163	0.05
928	1.15165	0.36971
929	0.739748	0.157093
930	1.04025	1.05805
931	1.47608	0.05
932	0.946131	0.092184
933	1.02028	0.0568689
934	0.946129	0.0691041
935	1.78223	0.05
936	1.78223	0.05
937	1.34153	0.05
938	1.38683	0.05
939	1.3407	0.05
940	1.3407	0.05
941	2.04729	0.05
942	0.851529	0.0647518
943	1.26671	0.05
944	0.946116	0.0612107
945	1.4869	0.05
946	1.29553	0.05
947	1.03645	0.05
948	1.20731	0.05
949	0.73665	2.1
950	0.693596	2.1
951	2.11152	2.1
952	1.73627	2.1
953	1.06191	2.1
954	1.52226	2.1
955	1.76869	2.1
956	1.1881	2.1
957	1.32349	2.1
958	1.51041	2.1
959	1.61591	2.1
960	0.946132	2.1
961	0.946132	2.1
962	1.13537	2.1
963	3.0048	2.1
964	0.985109	2.1
965	1.68899	2.1
966	3.23261	2.1
967	1.08831	2.1
968	2.20623	2.1
969	0.91749	2.1
970	1.15515	2.1
971	0.550438	2.1
972	1.66294	2.1
973	1.08257	2.1
974	0.87851	2.1
975	2.09046	2.1
976	0.738711	2.1
977	1.31242	0.05
978	1.26311	0.05
979	1.67652	0.05
980	0.968594	2.1
981	0.968594	2.1
982	0.981099	0.05
983	1.32418	0.05

984	2.67175	0.05
985	2.09678	0.05
986	1.2511	0.05
987	0.912698	0.05
988	0.810946	0.05
989	0.810959	0.05
990	1.67479	0.05
991	1.65677	0.05
992	1.28469	0.05
993	1.48817	0.05
994	1.08131	0.05
995	1.04349	0.159589
996	1.42492	0.321774
997	1.05724	0.452736
998	1.65723	0.05
999	1.95313	0.05
1000	1.9293	0.05
1001	2.02729	0.05
1002	0.963393	0.678974
1003	0.963392	0.224442
1004	2.90171	2.1
1005	2.65447	2.1
1006	1.64158	2.1
1007	1.6488	2.1
1008	1.15991	0.079801
1009	0.946132	0.1347
1010	1.58199	0.105976
1011	1.58199	0.12087
1012	1.03333	0.121052
1013	1.25436	0.115348
1014	1.25436	0.144993
1015	0.946132	0.179194
1016	1.3771	0.05
1017	0.687745	0.05
1018	1.37709	0.05
1019	0.812386	0.05
1020	1.50172	0.05
1021	1.50172	0.05
1022	0.687758	0.05
1023	1.17506	0.05
1024	2.00539	0.05
1025	2.2441	0.05
1026	1.09667	0.05
1027	1.43577	2.1
1028	2.24741	2.1
1029	1.26103	2.1
1030	0.851544	2.1
1031	1.5543	2.1
1032	1.59756	2.1
1033	0.851543	2.1
1034	1.44673	2.1
1035	1.31947	2.1
1036	1.1237	2.1
1037	1.26103	2.1
1038	1.089 0.05	
1039	0.946128	0.05
1040	0.965824	0.05
1041	0.94613	0.05

1042	1.51344	0.05
1043	1.51344	0.05
1044	1.13508	0.05
1045	1.32449	0.05
1046	1.089	0.05
1047	3.33706	0.05
1048	2.7542	0.05
1049	1.27256	0.05
1050	0.954172	0.05
1051	1.55527	0.05
1052	1.05765	0.05
1053	1.57326	0.05
1054	1.38251	0.05
1055	0.94613	0.05
1056	2.38829	0.05
1057	2.00539	0.05
1058	1.53798	0.05
1059	1.20969	2.1
1060	1.15864	2.1
1061	1.7986	2.1
1062	1.74533	2.1
1063	0.944827	1.83837
1064	0.946131	1.22719
1065	0.851543	1.27458
1066	1.52816	0.917202
1067	1.5314	1.35113
1068	1.39266	0.319751
1069	0.851528	0.529946
1070	1.39589	0.394673
1071	1.7348	0.7385
1072	1.85439	1.3923
1073	1.86367	1.1752
1074	1.90905	0.76248
1075	1.22176	0.0672047
1076	1.59492	0.0638614
1077	2.97929	2.1
1078	2.1905	2.1
1079	1.24128	2.1
1080	0.994293	2.1
1081	2.49767	0.05
1082	2.37105	0.05
1083	1.10834	0.773222
1084	1.53214	0.846722
1085	1.417	2.1
1086	2.13599	0.252085
1087	1.61727	2.1
1088	1.75004	2.1
1089	1.96301	2.1
1090	0.966631	2.1
1091	1.37791	2.1
1092	1.47302	2.1
1093	1.47302	2.1
1094	0.871519	2.1
1095	0.94613	2.1
1096	0.87155	2.1
1097	2.38731	2.1
1098	2.12399	0.853141
1099	1.74865	0.724488

1100	1.46724	0.179166
1101	2.38056	0.144171
1102	1.46724	0.19005
1103	0.818887	0.173389
1104	0.810944	1.34496
1105	0.805227	0.164573
1106	1.06558	0.374528
1107	0.841487	0.195537
1108	1.08456	0.425447
1109	0.903083	0.372043
1110	1.08929	0.05
1111	1.68454	0.05
1112	1.08929	0.05
1113	1.07658	0.05
1114	0.904439	0.05
1115	1.58501	0.05
1116	1.19729	0.05
1117	1.05427	0.05
1118	1.47764	0.05
1119	0.810959	0.05
1120	1.60946	0.585081
1121	2.28887	0.603588
1122	0.946133	0.920212
1123	1.14444	0.456237
1124	1.67964	0.242034
1125	1.67964	0.312564
1126	0.755334	0.992165
1127	1.14444	0.697341
1128	1.29054	0.552566
1129	1.37762	0.422967
1130	0.946132	0.75679
1131	0.533645	0.327868
1132	1.48361	0.175969
1133	1.48361	0.405463
1134	0.533645	0.740844
1135	0.872823	0.742119
1136	1.14444	0.322637
1137	1.37762	0.404289
1138	2.10101	0.416715
1139	1.02311	0.583208
1140	1.04083	2.1
1141	1.04084	2.1
1142	1.04084	2.1
1143	1.30116	2.1
1144	1.23477	0.05
1145	1.81893	0.05
1146	1.23477	0.05
1147	1.23477	0.05
1148	1.11967	0.05
1149	0.946131	0.05
1150	1.11966	0.05
1151	1.12628	0.05
1152	0.629641	0.05
1153	1.6163	0.05
1154	1.6163	0.05
1155	1.11117	2.1
1156	1.13006	2.1
1157	1.15149	2.1

1158	1.13179	2.1
1159	0.585368	0.0562394
1160	0.986036	1.095
1161	1.94788	0.067514
1162	1.34857	0.0694285
1163	2.31806	2.1
1164	2.06363	2.1
1165	1.00423	2.1
1166	0.829065	2.1
1167	0.551816	2.1
1168	0.99406	2.1
1169	1.98339	2.1
1170	1.68073	2.1
1171	2.558	2.1
1172	0.711814	2.09477
1173	2.97316	2.1
1174	2.41579	2.1
1175	1.15248	0.05
1176	1.14305	0.05
1177	1.15483	0.05
1178	1.90981	0.05
1179	0.998957	0.05
1180	1.83742	0.05
1181	1.27581	2.1
1182	0.94613	2.1
1183	1.6315	2.1
1184	1.6315	2.1
1185	0.946132	2.1
1186	1.28441	2.1
1187	1.28441	2.1
1188	1.00365	2.1
1189	0.968536	2.1
1190	1.57575	2.1
1191	1.27581	2.1
1192	1.99297	2.1
1193	1.32784	2.1
1194	1.4259	2.1
1195	2.27952	2.1
1196	1.5685	2.1
1197	1.54231	2.1
1198	3.54638	2.1
1199	1.3294	2.1
1200	2.98237	2.1
1201	2.01633	0.423701
1202	2.0985	0.627588
1203	2.26047	0.207158
1204	1.7913	0.13312
1205	1.09504	2.1
1206	1.74126	2.1
1207	1.05983	2.1
1208	2.37664	2.1
1209	2.28084	2.1
1210	2.19948	2.1
1211	1.33593	0.05
1212	1.31508	0.05
1213	1.30647	2.1
1214	1.28846	2.1
1215	1.2955	2.1

1216	1.09044	2.1
1217	1.4099	2.1
1218	1.14984	2.1
1219	0.946132	1.24537
1220	1.70727	2.1
1221	1.70727	2.1
1222	1.14984	2.1
1223	0.946132	2.1
1224	0.911818	2.1
1225	2.12209	2.1
1226	2.4158	2.1
1227	1.20867	2.1
1228	2.84085	2.1
1229	1.42284	2.1
1230	2.45689	2.1
1231	1.41562	2.1
1232	0.918358	2.1
1233	1.11222	2.1
1234	0.999083	2.1
1235	0.696781	2.1
1236	1.30869	2.1
1237	1.66132	2.1
1238	0.696807	2.1
1239	0.745315	2.1
1240	1.23296	2.1
1241	0.780548	2.1
1242	1.93242	0.675836
1243	1.27362	1.39215
1244	2.77828	1.60819
1245	1.02653	0.160761
1246	0.808576	0.214581
1247	0.744012	0.788182
1248	0.605174	0.0733812
1249	1.16501	0.05
1250	0.803432	0.0515733
1251	0.961016	0.066404
1252	1.10425	0.113591
1253	1.3756	0.366382
1254	2.17765	0.610567
1255	1.84578	0.421329
1256	1.32801	0.207372
1257	1.76377	0.19465
1258	0.946131	0.115219
1259	1.3022	0.405984
1260	0.694814	0.480835
1261	1.53028	0.128592
1262	1.53028	0.151821
1263	1.3022	0.228749
1264	0.922889	0.311106
1265	1.57766	2.1
1266	2.06911	2.1
1267	1.57766	2.1
1268	1.37721	0.05
1269	1.72174	0.05
1270	1.4468	0.05
1271	1.37721	0.05
1272	1.73972	0.0653637
1273	1.62324	0.064371

1274	0.946125	0.05
1275	1.34259	0.05
1276	1.04187	0.05
1277	1.05019	0.05
1278	1.2361	0.0583461
1279	1.69055	0.05
1280	2.25056	0.0717318
1281	1.10574	0.0821336
1282	1.21632	0.879097
1283	1.10574	1.20753
1284	1.46181	2.1
1285	0.946131	0.0941259
1286	1.74111	1.73675
1287	1.74111	0.45372
1288	0.94613	0.46143
1289	1.26891	0.236304
1290	1.26891	0.163647
1291	1.23452	1.25518
1292	1.00255	0.445975
1293	1.72286	0.05
1294	1.12233	0.05
1295	1.30682	0.05
1296	1.97397	0.0615264
1297	1.67425	2.1
1298	1.77775	2.1
1299	1.73571	2.1
1300	0.426265	2.1
1301	1.31817	1.91185
1302	1.81042	2.1
1303	1.85456	2.1
1304	0.466532	1.90424
1305	1.28025	1.7967
1306	0.964467	1.7021
1307	1.27921	2.1
1308	2.11851	1.94928
1309	2.37121	2.1
1310	1.49357	0.159513
1311	1.42486	2.1
1312	1.00278	2.1
1313	1.00278	2.1
1314	1.12503	2.1
1315	0.568697	2.1
1316	0.555211	2.1
1317	0.982272	2.1
1318	1.57558	2.1
1319	0.851539	2.1
1320	1.07307	2.1
1321	1.58292	2.1
1322	1.50731	2.1
1323	0.851541	1.87892
1324	1.00304	2.1
1325	1.36003	2.1
1326	1.13127	2.1
1327	0.672234	2.1
1328	0.763044	2.1
1329	0.966979	2.1
1330	1.31502	2.1
1331	1.67969	2.1

1332	1.62408	2.1
1333	0.661955	2.1
1334	1.35048	2.1
1335	1.40447	2.1
1336	1.40447	2.1
1337	0.607961	2.1
1338	0.946131	2.1
1339	0.607963	2.1
1340	1.01531	2.1
1341	1.01531	2.1
1342	1.91802	2.1
1343	0.777749	2.1
1344	1.22585	0.05
1345	1.20672	0.05
1346	1.03166	0.05
1347	0.553805	2.1
1348	2.00551	2.1
1349	2.07203	2.1
1350	0.589479	2.1
1351	1.22352	2.1
1352	0.984227	2.1
1353	0.802455	2.1
1354	1.40942	2.1
1355	1.6662	2.1
1356	0.802485	2.1
1357	0.936669	2.1
1358	1.31127	0.258235
1359	0.946132	0.260356
1360	1.09729	0.093306
1361	0.946133	0.305289
1362	1.68703	0.110867
1363	1.68703	0.0820879
1364	1.3689	0.148543
1365	1.26426	0.151315
1366	1.26923	2.1
1367	1.23614	2.1
1368	0.828371	2.1
1369	2.04556	2.1
1370	0.772648	2.1
1371	0.791059	2.1
1372	0.809958	2.1
1373	1.70012	2.1
1374	0.267032	2.1
1375	1.67604	2.1
1376	0.803823	2.1
1377	0.267305	2.1
1378	0.80246	2.1
1379	0.874582	2.1
1380	1.00188	2.1
1381	0.709588	2.1
1382	1.50679	2.1
1383	1.22456	2.1
1384	0.870622	2.1
1385	1.05754	2.1
1386	1.1689	2.1
1387	1.54556	2.1
1388	1.89226	2.1
1389	0.995311	2.1

1390	1.26195	2.1
1391	1.9	2.1
1392	0.845055	2.1
1393	0.737836	2.1
1394	0.823707	2.1
1395	0.626587	2.1
1396	1.16109	2.1
1397	0.85517	2.1
1398	0.932036	2.1
1399	1.08321	2.1
1400	1.43863	2.1
1401	2.72297	2.1
1402	2.90798	2.1
1403	1.47818	1.42792
1404	1.47819	1.43912
1405	0.694357	1.14679
1406	0.694382	1.17044
1407	0.946132	0.642193
1408	0.694382	1.06606
1409	1.22641	0.858698
1410	1.31155	1.63408
1411	2.45282	1.39934
1412	0.787843	1.08343
1413	0.808984	0.593038
1414	1.14232	1.54893
1415	0.795037	1.3864
1416	0.887382	1.47286
1417	1.14493	1.28925
1418	0.78393	0.871771
1419	1.09129	1.30933
1420	0.791482	1.19878
1421	1.1137	1.55772
1422	1.11819	1.1766
1423	0.960173	1.20316
1424	1.09447	1.69286
1425	0.874365	1.10879
1426	1.2474	0.05
1427	1.75038	0.060561
1428	1.75038	0.12235
1429	0.94613	0.451894
1430	1.30076	0.0661135
1431	1.12817	0.883167
1432	0.94613	0.263386
1433	1.30076	0.165737
1434	1.51841	0.0998234
1435	1.51841	0.161494
1436	0.946132	1.00547
1437	1.39575	0.119037
1438	0.970358	1.46914
1439	0.946133	0.571584
1440	1.39575	0.362473
1441	1.06486	0.0563463
1442	1.06486	0.0564554
1443	1.38622	0.0637168
1444	1.04521	0.05
1445	1.00809	0.05
1446	1.28952	0.05
1447	0.849316	0.05

1448	0.946132	0.05
1449	0.849344	0.05
1450	1.53631	0.05
1451	1.53631	0.05
1452	1.77843	0.05
1453	1.69863	0.05
1454	1.91646	2.1
1455	1.8582	2.1
1456	2.8349	0.05
1457	1.36307	0.05
1458	1.23886	0.05
1459	1.12971	0.05
1460	1.59662	0.05
1461	1.59662	0.05
1462	0.640304	0.05
1463	0.640333	0.05
1464	0.886276	0.05
1465	0.886276	0.05
1466	1.35065	0.05
1467	2.09002	0.05
1468	1.35543	0.05
1469	1.94688	2.1
1470	1.97221	2.1
1471	0.719563	2.1
1472	1.20358	2.1
1473	0.874264	2.1
1474	1.27676	2.1
1475	1.46557	2.1
1476	1.46557	2.1
1477	0.68546	2.1
1478	0.94613	2.1
1479	0.685489	2.1
1480	1.39615	0.05
1481	1.05404	0.05
1482	1.06474	0.05
1483	1.00713	0.05
1484	0.714535	0.05
1485	0.714563	0.05
1486	1.41646	0.05
1487	1.52582	0.05
1488	0.721694	0.05
1489	0.93665	0.05
1490	1.21067	0.0523556
1491	1.21067	0.05
1492	1.73577	0.0803135
1493	1.17799	0.0522436
1494	3.21432	0.0526266
1495	2.41452	0.0522642
1496	1.36208	0.05
1497	2.53256	0.05
1498	0.834593	0.05
1499	2.5853	0.05
1500	1.77259	0.05
1501	0.615724	0.05
1502	1.11374	0.121823
1503	0.615726	0.05
1504	0.946129	0.343226
1505	0.615714	0.229393

1506	1.75489	0.128738
1507	1.75489	0.0895828
1508	1.23146	0.05
1509	2.2772	0.0530371
1510	0.74463	0.05
1511	1.3546	0.05
1512	1.80252	0.05
1513	2.38304	0.053409
1514	1.19151	0.0780928
1515	1.1231	0.0627359
1516	1.82345	0.0511103
1517	1.79805	0.05
1518	0.761337	0.171127
1519	0.946127	0.070459
1520	0.557726	0.0909132
1521	1.05353	0.05
1522	1.05353	0.05
1523	1.68599	0.05
1524	1.05353	0.05
1525	1.40588	2.1
1526	1.09373	2.1
1527	1.18382	1.02157
1528	1.46903	0.846532
1529	1.25103	2.1
1530	1.10378	2.1
1531	1.58941	2.1
1532	1.29639	2.1
1533	1.14874	0.05
1534	0.899817	0.05
1535	0.666898	0.05
1536	1.76519	0.05
1537	1.17289	0.05
1538	1.03526	0.05
1539	1.97708	0.05
1540	1.31711	0.05
1541	0.992517	0.05
1542	1.70193	0.05
1543	1.13852	0.05
1544	1.23943	0.05
1545	1.40014	2.1
1546	1.04598	2.1
1547	1.1483	2.1
1548	1.11433	0.539617
1549	1.32037	0.0532171
1550	1.00381	0.117586
1551	1.11435	0.05
1552	0.973502	0.271333
1553	0.946128	1.66399
1554	1.83697	0.966211
1555	1.83697	2.1
1556	1.31721	1.6711
1557	1.38614	1.21401
1558	1.39696	1.02713
1559	1.31721	1.6952
1560	1.0756	1.9149
1561	1.08542	0.375643
1562	0.946127	2.1
1563	1.81052	0.231217

1564	1.81052	2.1
1565	1.24315	2.1
1566	1.39696	0.16791
1567	1.35969	1.35346
1568	1.24315	1.15258
1569	1.0756	0.0528565
1570	1.20669	0.05
1571	1.18508	0.05
1572	1.71059	2.1
1573	1.66762	2.1
1574	2.50853	2.1
1575	1.8236	2.1
1576	1.78872	2.1
1577	1.41721	2.1
1578	1.01335	1.67264
1579	1.42439	0.827222
1580	0.946129	0.869843
1581	1.33266	0.71554
1582	1.79776	1.25252
1583	1.79776	2.1
1584	0.946131	0.152585
1585	1.33266	1.57926
1586	1.41123	1.40244
1587	1.25346	2.1
1588	0.946132	0.229231
1589	0.827287	1.93925
1590	1.57878	1.08495
1591	1.57878	1.01525
1592	0.827287	0.815992
1593	1.07341	1.02872
1594	1.33266	0.882933
1595	1.25346	1.09541
1596	1.61149	2.1
1597	1.15644	1.90027
1598	1.27558	0.623703
1599	1.3764	0.167568
1600	1.89526	1.16577
1601	1.37641	0.158326
1602	1.2064	0.434921
1603	1.38426	2.1
1604	1.01595	2.1
1605	1.15067	2.1
1606	1.82276	2.1
1607	1.82276	0.0806099
1608	0.946131	0.288186
1609	1.40609	0.0612134
1610	0.946132	2.1
1611	1.29143	2.1
1612	1.40609	2.03953
1613	1.30272	2.1
1614	1.19548	2.1
1615	2.95451	2.1
1616	1.46124	2.1
1617	2.93215	2.1
1618	1.42105	2.1
1619	1.75477	0.676438
1620	2.31301	0.887625
1621	2.10275	0.264662

1622	1.80099	0.345723
1623	0.97541	2.1
1624	1.49733	2.1
1625	0.97541	2.1
1626	1.30751	2.1
1627	1.28614	0.05
1628	1.74759	0.05
1629	1.19756	0.0667327
1630	1.71053	0.0605764
1631	1.19757	0.088518
1632	1.28954	0.05
1633	2.02703	0.05
1634	2.29453	0.05
1635	1.28265	0.05
1636	0.793267	0.05
1637	1.19974	0.05
1638	0.509551	0.05
1639	1.04072	0.05
1640	1.35302	0.05
1641	1.53133	0.05
1642	0.566153	0.05
1643	1.09634	0.05
1644	1.12792	0.05
1645	1.68908	2.1
1646	1.68908	2.1
1647	0.768212	2.1
1648	0.768213	2.1
1649	0.946132	2.1
1650	0.768212	2.1
1651	1.32461	2.1
1652	1.87291	2.1
1653	2.64921	2.1
1654	1.53642	2.1
1655	2.26537	2.1
1656	1.53642	2.1
1657	1.79874	2.1
1658	1.79874	2.1
1659	0.726635	2.0869
1660	0.726636	2.1
1661	0.946128	2.1
1662	0.726634	2.1
1663	1.23339	1.98869
1664	0.992674	2.1
1665	1.8023	2.1
1666	1.12324	0.05
1667	1.29163	0.05
1668	0.996831	0.05
1669	1.0019	0.05
1670	0.993831	0.05
1671	1.07283	2.1
1672	1.04788	2.1
1673	1.44489	1.34931
1674	1.04788	2.1
1675	1.07652	2.1
1676	0.991406	2.1
1677	1.37	2.1
1678	1.14195	2.1
1679	1.47904	0.05

1680	1.26593	2.0636
1681	1.75366	1.75268
1682	1.57621	0.05
1683	1.24538	0.05
1684	0.969118	2.1
1685	0.946133	1.23713
1686	1.69387	2.1
1687	1.69387	2.1
1688	0.94613	2.1
1689	1.28713	2.1
1690	1.28713	2.1
1691	1.23877	2.1
1692	0.969118	2.1
1693	1.22615	2.1
1694	0.98084	2.1
1695	1.01843	2.1
1696	1.50876	2.1
1697	1.85391	2.1
1698	1.84322	2.1
1699	1.70377	2.1
1700	1.78919	2.1
1701	2.66787	2.1
1702	1.41549	2.1
1703	1.41549	2.1
1704	0.86707	2.1
1705	0.896172	2.1
1706	0.946126	2.1
1707	0.867074	2.1
1708	0.946118	2.1
1709	1.54468	0.05
1710	1.54468	0.05
1711	2.27259	0.05
1712	0.946131	0.05
1713	1.25837	0.05
1714	0.663171	0.060659
1715	1.68931	0.05
1716	1.68931	0.05
1717	1.25837	0.05
1718	1.09411	0.05
1719	1.73571	0.05
1720	2.34894	0.05
1721	0.946128	0.05
1722	1.30497	0.05
1723	0.772312	0.05
1724	1.66895	0.05
1725	1.66895	0.05
1726	1.30497	0.05
1727	1.13629	0.05
1728	1.22852	0.642842
1729	1.578	0.509186
1730	2.07781	2.1
1731	1.5478	2.1
1732	1.70048	2.1
1733	1.42329	2.1
1734	1.05481	2.08998
1735	1.1032	0.9282
1736	1.19267	2.1
1737	1.77324	2.1

1738	1.01121	0.05
1739	0.991867	0.05
1740	2.0744	1.5149
1741	1.75142	2.1
1742	1.35418	1.76278
1743	1.40041	2.1
1744	1.50045	2.1
1745	1.37704	2.1
1746	1.50045	2.1
1747	1.87875	2.1
1748	1.35308	0.322085
1749	1.29983	0.385879
1750	1.62784	2.1
1751	1.44807	2.1
1752	1.00965	2.1
1753	2.69264	2.1
1754	1.04075	2.1
1755	0.946131	2.1
1756	0.946132	2.1
1757	1.52704	2.1
1758	1.83246	2.1
1759	1.56757	2.1
1760	1.16687	2.1
1761	2.04857	2.1
1762	1.32707	2.1
1763	1.25519	0.05
1764	1.32101	0.05
1765	1.25055	0.85629
1766	0.827853	0.0781106
1767	1.11835	0.05
1768	1.07281	2.1
1769	1.54302	0.123397
1770	0.724064	0.578363
1771	1.32709	2.1
1772	3.44123	1.6477
1773	2.46889	0.05
1774	0.730076	0.05
1775	1.25809	0.05
1776	0.730077	0.05
1777	0.946129	0.05
1778	1.72777	0.05
1779	1.72777	0.05
1780	0.73005	0.05
1781	1.7522	0.05
1782	2.51618	0.05
1783	0.628177	0.05
1784	1.1068	0.05
1785	0.747435	0.05
1786	0.946133	0.05
1787	1.84668	0.05
1788	1.8563	0.05
1789	0.628177	0.05
1790	1.45501	0.05
1791	2.19809	0.05
1792	1.45501	0.05
1793	1.48974	0.05
1794	1.7709	2.1
1795	2.63288	2.1

1796	1.5362	2.1
1797	1.56327	2.1
1798	3.03945	0.05
1799	0.899979	0.05
1800	0.869386	0.05
1801	1.85864	0.05
1802	1.78028	0.05
1803	1.28719	0.05
1804	1.09532	0.05
1805	0.881898	0.05
1806	1.28911	0.05
1807	1.39644	0.05
1808	0.688829	0.05
1809	2.14611	0.05
1810	1.80313	0.05
1811	0.68883	0.05
1812	0.946131	0.05
1813	1.25334	0.05
1814	1.88987	0.05
1815	1.94432	0.05
1816	1.37592	0.05
1817	1.11691	2.1
1818	1.02923	2.1
1819	0.45824	0.05
1820	0.646512	0.05
1821	2.46292	0.05
1822	0.493451	0.05
1823	2.00826	2.1
1824	2.49784	2.1
1825	1.17054	2.1
1826	1.01485	2.1
1827	1.26045	2.1
1828	1.17054	2.1
1829	1.44247	2.1
1830	2.02687	0.05
1831	1.66365	0.05
1832	1.64656	0.05
1833	1.56597	0.05
1834	1.21419	2.1
1835	1.5008	2.1
1836	1.10711	2.1
1837	1.44287	2.1
1838	1.44287	2.1
1839	0.750368	2.1
1840	1.25876	2.1
1841	0.82542	2.1
1842	1.04074	2.1
1843	1.25874	2.1
1844	1.04159	0.05
1845	1.2489	0.05
1846	0.980547	0.05
1847	1.62235	0.05
1848	1.15991	0.05
1849	0.980547	0.05
1850	0.94613	0.05
1851	1.54662	0.05
1852	1.54661	0.05
1853	1.07912	0.05

1854	1.1032	0.05
1855	1.1032	0.05
1856	0.94613	0.05
1857	1.12468	2.1
1858	1.2724	2.1
1859	1.06521	2.1
1860	1.0752	2.1
1861	1.94936	0.05
1862	1.62772	0.05
1863	1.94733	0.05
1864	1.42901	0.05
1865	1.33022	2.1
1866	1.70839	2.1
1867	1.75048	2.1
1868	1.41201	2.1
1869	1.52314	2.1
1870	1.44039	2.1
1871	1.51113	2.1
1872	2.58638	2.1
1873	0.987247	0.05
1874	1.38622	0.073465
1875	0.858359	0.05
1876	1.19967	0.05
1877	1.06414	0.05
1878	0.946132	0.0632629
1879	0.94613	0.05
1880	0.687079	0.135149
1881	0.946132	0.0621979
1882	0.822611	0.0582753
1883	1.70242	0.05
1884	1.70242	0.0617424
1885	0.946132	0.210187
1886	1.11155	0.0500923
1887	0.946132	0.315698
1888	1.11155	0.115672
1889	1.37981	0.101856
1890	1.18348	0.05
1891	2.44179	0.0574554
1892	2.00403	0.136072
1893	1.10961	2.1
1894	1.85415	2.1
1895	1.44825	2.1
1896	0.932272	2.1
1897	0.810942	2.1
1898	1.00189	2.1
1899	1.11779	2.1
1900	1.3359	2.1
1901	0.965279	2.1
1902	0.833779	2.1
1903	1.05337	2.1
1904	0.526656	2.1
1905	1.44599	2.1
1906	1.44599	2.1
1907	1.24853	2.1
1908	0.946132	2.1
1909	1.24853	2.1
1910	0.724122	2.1
1911	1.91547	2.1

1912	2.49705	2.1
1913	1.05931	2.1
1914	1.1729	2.1
1915	1.34526	2.1
1916	1.5379	2.1
1917	1.90855	2.1
1918	2.08597	2.1
1919	1.68165	2.1
1920	1.45887	2.1
1921	1.50022	2.1
1922	1.4748	2.09386
1923	2.63645	1.9567
1924	1.74431	2.0027
1925	1.25288	1.9747
1926	1.25289	1.55195
1927	0.970025	1.45871
1928	0.747542	2.1
1929	0.810959	1.28868
1930	1.15369	1.48616
1931	0.977472	1.44072
1932	1.12695	1.52475
1933	0.996762	1.44072
1934	1.28249	0.207263
1935	0.946132	0.305668
1936	0.528621	0.395985
1937	1.59186	0.140485
1938	1.44072	0.191366
1939	0.633488	0.170242
1940	0.598506	0.283793
1941	1.51618	0.31841
1942	1.19999	0.177998
1943	1.46511	0.145169
1944	1.44056	0.149018
1945	0.912754	0.0809567
1946	1.4725	0.0575803
1947	1.4725	0.05
1948	0.912753	0.0642209
1949	1.23351	0.0926566
1950	1.23351	0.112688
1951	0.946131	0.163947
1952	1.17129	0.05
1953	1.82551	0.05
1954	1.27575	1.99679
1955	0.946129	2.1
1956	0.946129	2.1
1957	0.946128	2.02253
1958	0.792754	2.1
1959	0.94613	1.78665
1960	0.792752	1.8395
1961	2.08054	2.1
1962	1.20091	2.1
1963	2.1669	2.06881
1964	1.24383	1.69277
1965	0.904669	2.09009
1966	0.83804	1.24787
1967	1.90486	1.52055
1968	1.29162	1.33316
1969	1.37383	1.8437

1970	1.86533	1.42128
1971	1.76472	1.87847
1972	1.60428	1.47036
1973	2.43993	2.05962
1974	1.37833	2.1
1975	1.18632	2.1
1976	0.946132	2.1
1977	1.27544	2.1
1978	1.05085	2.1
1979	0.961178	2.1
1980	0.859844	2.1
1981	1.0499	2.1
1982	1.75592	2.1
1983	2.08668	2.1
1984	1.75592	2.1
1985	1.4402	2.1
1986	1.72285	2.1
1987	0.83312	0.05
1988	1.07295	0.05
1989	1.37607	0.05
1990	1.32097	0.05
1991	1.11332	0.05
1992	1.18568	0.05
1993	0.877482	0.05
1994	1.4458	0.05
1995	2.34112	0.05
1996	2.68021	0.05
1997	0.681677	0.05
1998	0.946131	0.05
1999	0.681649	0.05
2000	1.83115	0.05
2001	1.68029	0.05
2002	1.05482	0.05
2003	1.31485	0.05
2004	2.08247	0.621159
2005	1.33153	0.406774
2006	0.935062	0.619672
2007	0.806958	1.74342
2008	0.827683	0.497795
2009	1.01041	0.750396
2010	0.741832	0.369174
2011	1.15668	0.985835
2012	0.908649	0.57255
2013	0.59561	0.05
2014	1.19028	0.214912
2015	0.59561	0.134769
2016	0.946132	0.351618
2017	0.595623	0.462816
2018	1.58164	0.238545
2019	1.58164	0.308586
2020	0.946131	2.1
2021	1.55201	2.1
2022	1.55201	2.1
2023	0.946127	2.1
2024	1.13312	2.1
2025	1.13312	2.1
2026	1.08108	2.1
2027	1.04973	2.1

2028	1.20149	2.1
2029	1.86902	0.739481
2030	0.613447	2.1
2031	1.22618	1.24631
2032	1.38663	1.86646
2033	1.38662	2.1
2034	0.613447	1.47127
2035	1.22617	2.1
2036	0.773899	2.1
2037	1.23515	1.88021
2038	1.87823	1.45224
2039	1.70303	0.192927
2040	1.4714	2.1
2041	1.74067	2.1
2042	0.735699	2.1
2043	1.73461	2.1
2044	1.47224	2.1
2045	1.22827	2.1
2046	1.1634	2.1
2047	1.36471	2.1
2048	0.735701	2.1
2049	2.8386	0.122291
2050	2.39056	2.1
2051	2.44128	0.752301
2052	1.54102	2.1
2053	1.54102	2.1
2054	0.936141	2.09866
2055	1.40594	2.1
2056	1.07121	0.474446
2057	0.936142	2.1
2058	1.07121	2.1
2059	1.22691	0.05
2060	1.42907	0.05
2061	2.18048	0.05
2062	0.947662	0.05
2063	2.18394	2.1
2064	1.21292	2.1
2065	2.18394	2.1
2066	2.19064	2.1
2067	1.27229	2.1
2068	1.20859	0.313251
2069	1.21073	0.403151
2070	1.73899	2.1
2071	1.3164	2.1
2072	1.61245	2.1
2073	1.41444	2.1
2074	2.55116	2.1
2075	1.26193	2.1
2076	1.89553	2.1
2077	0.916438	2.1
2078	1.26113	2.1
2079	0.810934	2.1
2080	2.00419	2.1
2081	1.27872	2.1
2082	1.35306	2.1
2083	1.96749	2.1
2084	1.07955	2.1
2085	1.31854	2.1

2086	1.29505	0.342449
2087	1.29504	0.318182
2088	0.612469	0.633942
2089	0.990053	0.528517
2090	1.04072	0.605008
2091	0.985266	0.356407
2092	0.990039	0.255244
2093	1.06892	0.456256
2094	1.57158	0.180679
2095	1.895	0.304902
2096	1.05698	0.233032
2097	0.925053	2.1
2098	0.624712	2.1
2099	1.42465	2.1
2100	1.49014	2.1
2101	0.687198	2.1
2102	1.22058	2.1
2103	0.851488	2.1
2104	0.588128	2.1
2105	1.12452	2.1
2106	1.02783	2.1
2107	1.7083	2.1
2108	1.06977	1.27335
2109	0.818569	0.692301
2110	1.14386	1.51351
2111	0.983791	0.778533
2112	1.12214	1.65787
2113	1.0915	1.08035
2114	0.810934	0.990477
2115	0.764114	1.39626
2116	1.87956	0.792166
2117	1.87956	1.55977
2118	0.764141	0.82484
2119	1.37675	0.775043
2120	0.764141	0.652244
2121	0.946132	1.24274
2122	1.9637	1.83421
2123	2.10107	0.386099
2124	1.67814	1.43117
2125	3.02886	1.21556
2126	0.908982	0.05
2127	0.621727	0.05
2128	0.525963	0.05
2129	0.946128	0.05
2130	1.60979	0.05
2131	1.45596	0.05
2132	0.556172	0.05
2133	2.06779	0.05
2134	2.17911	0.05
2135	1.00205	0.05
2136	1.30685	0.05
2137	1.16534	0.05
2138	1.65532	2.1
2139	2.20346	2.1
2140	0.993889	2.1
2141	1.09328	2.1
2142	2.09372	2.1
2143	2.10036	2.1

2144	1.33727	1.79545
2145	2.53383	1.72344
2146	0.893379	1.64441
2147	1.52828	1.31472
2148	1.09599	0.05
2149	1.09599	0.05
2150	1.50524	2.1
2151	1.54711	2.1
2152	1.41469	2.1
2153	1.99459	2.05505
2154	1.79805	2.1
2155	1.79805	2.1
2156	0.707344	2.1
2157	1.30318	2.1
2158	0.707344	2.1
2159	0.946129	2.1
2160	1.30318	2.1
2161	2.40442	2.1
2162	1.89226	2.1
2163	1.40794	2.1
2164	0.898721	2.1
2165	0.791921	1.2792
2166	0.810933	2.1
2167	1.10904	2.04214
2168	0.991102	2.1
2169	0.808502	1.04479
2170	1.1398	2.1
2171	1.31346	2.1
2172	1.11833	2.1
2173	1.669	2.1
2174	1.25715	2.1
2175	1.3199	2.1
2176	1.63092	2.1
2177	1.63092	2.1
2178	0.946131	2.1
2179	0.946131	2.1
2180	1.13534	2.1
2181	0.979853	2.1
2182	2.19994	2.1
2183	0.725844	2.1
2184	2.19994	2.1
2185	2.35055	2.1
2186	1.52204	2.1
2187	1.40118	2.1
2188	3.42285	2.1
2189	2.05217	2.1
2190	1.50319	2.1
2191	0.947516	1.29331
2192	0.903572	1.13398
2193	0.76996	1.44018
2194	1.90959	2.1
2195	1.18897	2.1
2196	1.24166	2.02745
2197	2.26785	1.82535
2198	2.05217	2.1
2199	2.73306	2.1
2200	1.42729	0.05
2201	1.387	0.05

2202	1.59458	0.0998637
2203	1.91051	0.114914
2204	1.68108	0.122893
2205	2.03357	0.28121
2206	0.928982	0.05
2207	1.55687	0.05
2208	1.55687	0.05
2209	0.92898	0.05
2210	1.3495	0.05
2211	1.3495	0.05
2212	0.946133	0.05
2213	1.85795	0.05
2214	2.2727	0.05
2215	1.20324	1.50935
2216	1.07784	0.05
2217	0.957792	0.05
2218	1.2745	0.05
2219	1.4659	0.05
2220	0.946129	0.05
2221	0.95778	0.05
2222	0.851553	0.05
2223	1.74103	0.05
2224	2.28994	0.05
2225	2.08213	0.05
2226	2.1556	0.05
2227	2.20372	0.05
2228	0.838027	0.05
2229	1.11373	0.05
2230	1.2909	0.05
2231	0.998424	0.05
2232	1.40621	0.05
2233	1.19478	0.05
2234	1.11783	0.05
2235	1.6695	0.05
2236	1.7498	0.05
2237	0.477652	0.05
2238	1.77291	0.05
2239	0.590448	0.05
2240	1.07668	0.05
2241	0.810946	0.05
2242	1.1946	0.05
2243	2.03914	0.05
2244	1.25585	0.05
2245	2.02622	0.05
2246	1.39091	0.05
2247	1.31871	0.05
2248	1.15789	2.1
2249	1.15755	2.1
2250	1.82268	2.1
2251	1.87119	2.1
2252	3.10636	2.1
2253	2.96285	2.1
2254	1.73443	0.361149
2255	2.33812	0.417634
2256	1.59457	0.182119
2257	1.73443	1.05128
2258	2.00665	2.1
2259	1.99841	2.1

2260	1.19901	2.1
2261	1.16661	2.1
2262	2.15883	0.05
2263	1.20951	0.05
2264	2.49192	0.05
2265	0.65112	0.05
2266	2.45317	0.05
2267	1.73091	0.05
2268	2.0795	0.05
2269	1.45891	0.05
2270	1.6531	0.05
2271	1.78304	0.05
2272	0.775132	0.05
2273	1.35051	0.05
2274	1.0461	0.05
2275	1.37837	0.05
2276	1.00259	0.05
2277	1.25796	0.114112
2278	1.10719	0.240868
2279	1.51067	2.1
2280	2.65632	2.1
2281	1.30521	2.1
2282	2.456	2.1
2283	3.19218	2.1
2284	2.67387	2.1
2285	0.988862	2.1
2286	1.917	2.1
2287	0.946132	2.1
2288	1.16202	2.1
2289	1.53871	2.1
2290	1.53871	2.1
2291	0.946132	2.1
2292	1.16202	2.1
2293	1.32282	2.1
2294	2.46356	2.1
2295	2.32404	2.1
2296	1.37562	0.05
2297	0.985546	0.05
2298	1.28983	0.05
2299	1.05566	0.05
2300	0.895312	0.05
2301	1.53325	0.05
2302	1.04342	0.05
2303	1.80881	0.05
2304	0.876865	0.05
2305	0.5689	0.05
2306	1.09506	0.05
2307	1.51789	2.1
2308	1.49872	2.1
2309	0.949364	0.05
2310	2.01233	0.05
2311	1.55063	0.0622843
2312	1.57783	0.175716
2313	1.57783	0.320739
2314	0.674605	0.274634
2315	0.98537	0.05
2316	0.674633	0.05
2317	0.98537	0.05

2318	1.26706	0.315295
2319	0.980664	0.359431
2320	1.03298	0.0727074
2321	1.84936	0.34186
2322	1.51696	0.05
2323	1.97074	0.05
2324	1.03298	0.0888032
2325	1.51324	0.05
2326	1.51324	0.05
2327	0.758454	0.05
2328	1.28632	0.05
2329	0.758482	0.05
2330	0.758482	0.05
2331	0.985372	0.05
2332	1.14105	0.05
2333	1.59787	0.05
2334	2.46244	0.076422
2335	1.70367	0.05
2336	2.13757	1.45494
2337	1.98142	1.47776
2338	1.35878	0.05
2339	2.39947	0.05
2340	1.24554	0.05
2341	1.7522	0.05
2342	1.49693	1.74733
2343	1.49652	1.87383
2344	1.6211	1.78303
2345	1.45938	1.89949
2346	0.737431	2.1
2347	1.18643	1.93326
2348	1.04072	2.1
2349	0.9459	1.98874
2350	0.66364	2.1
2351	0.985109	1.38969
2352	0.638368	2.08425
2353	0.83804	1.24693
2354	0.998024	1.39164
2355	0.914471	0.928589
2356	1.14281	1.56982
2357	0.833972	1.339
2358	0.991865	0.129812
2359	1.42208	0.0922778
2360	1.70718	2.1
2361	1.79888	2.1
2362	1.16851	0.177381
2363	1.16851	0.089002
2364	1.51021	0.0976431
2365	0.946132	0.0826732
2366	1.14897	0.05
2367	0.946132	0.0615079
2368	1.77177	0.0784569
2369	1.77177	0.05
2370	1.33038	0.0588834
2371	1.38752	0.05
2372	1.1726	0.0668224
2373	1.51688	0.06342
2374	1.06455	2.1
2375	0.98511	2.1

2376	1.31144	2.1
2377	1.79825	2.1
2378	1.79825	2.1
2379	1.73044	2.1
2380	1.59589	0.05
2381	1.59589	0.05
2382	2.78009	2.1
2383	1.68761	2.1
2384	3.05511	2.1
2385	2.25318	2.1
2386	0.578998	2.1
2387	1.03512	2.1
2388	2.26156	2.1
2389	1.44859	1.19991
2390	1.35897	1.49307
2391	1.18301	2.1
2392	1.25173	2.1
2393	1.98851	2.1
2394	2.70685	2.1
2395	0.682342	2.1
2396	1.52491	2.1
2397	1.37119	2.1
2398	0.682313	2.1
2399	1.35342	2.1
2400	0.947555	2.1
2401	0.614107	2.1
2402	1.23007	2.1
2403	1.5735	2.1
2404	1.58713	2.1
2405	1.57979	0.05
2406	1.30723	0.05
2407	0.935658	0.839478
2408	0.8952	0.829678
2409	1.27352	1.36894
2410	1.0985	1.06321
2411	1.07163	0.938534
2412	1.15823	1.1924
2413	0.836451	0.682905
2414	2.08051	0.285422
2415	1.92601	1.50999
2416	2.0644	0.513318
2417	1.37259	2.1
2418	1.37259	2.1
2419	0.752217	2.1
2420	1.28569	2.1
2421	0.752186	2.1
2422	0.839124	2.1
2423	1.28568	2.1
2424	1.04199	2.1
2425	1.04199	2.1
2426	2.59268	2.1
2427	1.04199	2.1
2428	1.5098	2.1
2429	1.5098	2.1
2430	0.520997	2.1
2431	1.29634	2.1
2432	0.520996	2.1
2433	0.73446	2.1

2434	1.29634	2.1
2435	0.983724	2.1
2436	1.23914	2.1
2437	2.65095	2.1
2438	1.52851	2.1
2439	2.399	2.1
2440	1.68137	2.1
2441	1.11285	2.1
2442	1.04921	2.1
2443	1.30711	2.1
2444	1.04921	2.1
2445	1.07496	0.73376
2446	1.55831	0.125077
2447	1.07497	0.05
2448	1.07497	0.2996
2449	1.27391	2.1
2450	2.5615	2.1
2451	1.2739	2.1
2452	1.75253	2.1
2453	1.12907	0.05
2454	1.9194	0.05
2455	1.96617	0.05
2456	1.01225	2.1
2457	0.947979	2.1
2458	1.01225	1.81182
2459	1.06561	2.1
2460	1.01225	2.1
2461	3.56876	2.1
2462	3.68767	2.1
2463	1.52301	2.1
2464	1.61013	2.1
2465	1.43726	2.1
2466	2.04275	2.1
2467	0.995734	2.1
2468	1.16199	2.1
2469	1.73704	0.05
2470	1.04019	0.05
2471	0.956294	2.1
2472	1.81488	2.1
2473	1.63719	2.1
2474	1.50103	0.05
2475	1.75289	0.05
2476	1.31491	0.05
2477	1.03567	0.05
2478	0.672257	0.05
2479	1.04072	0.05
2480	0.746903	0.05
2481	1.43317	0.05
2482	1.39986	0.05
2483	1.06385	0.05
2484	1.1624	0.05
2485	1.61667	0.05
2486	1.27258	0.05
2487	2.559	0.05
2488	1.66093	0.05
2489	1.10719	0.210786
2490	2.03235	0.43019
2491	1.17105	0.731004

2492	1.10719	0.452301
2493	1.3028	2.1
2494	0.946131	2.1
2495	0.946127	2.1
2496	1.52912	2.1
2497	1.52912	2.1
2498	0.946128	2.08889
2499	0.949997	2.1
2500	1.31565	2.1
2501	1.46643	2.1
2502	1.5482	2.1
2503	1.47102	2.1
2504	1.47102	2.1
2505	0.774101	2.1
2506	1.30583	2.1
2507	0.774103	2.1
2508	0.939287	2.1
2509	1.30584	2.1
2510	0.958661	2.1
2511	2.28835	2.1
2512	1.43213	0.05
2513	2.13255	0.05
2514	1.43213	0.05
2515	1.10476	0.05
2516	1.93165	0.05
2517	1.03235	0.539056
2518	1.31	0.645332
2519	1.22082	2.1
2520	1.35827	2.1
2521	0.790936	2.1
2522	0.716644	2.1
2523	1.43257	2.1
2524	1.43257	2.1
2525	0.716615	2.1
2526	0.946131	2.1
2527	0.730157	2.1
2528	1.47212	2.1
2529	1.47212	2.1
2530	0.946131	2.1
2531	0.790792	2.1
2532	1.41149	2.1
2533	0.790791	2.1
2534	1.01006	2.1
2535	1.53045	0.0945972
2536	1.53045	0.191206
2537	0.946132	0.990351
2538	1.40779	0.131004
2539	1.06879	1.32467
2540	0.990713	0.484447
2541	1.06879	0.531622
2542	1.06486	0.0539561
2543	1.18347	0.0529161
2544	1.38622	0.0684779
2545	1.18347	0.05
2546	1.7304	0.051287
2547	1.7304	0.0808405
2548	0.946131	0.385816
2549	1.26874	0.0903882

2550	1.13337	1.02737
2551	1.13337	0.260919
2552	1.40779	0.229042
2553	1.695	2.1
2554	1.77071	2.1
2555	0.649313	2.1
2556	0.692829	2.1
2557	1.40395	2.1
2558	1.34243	2.1
2559	0.813292	0.05
2560	0.632691	0.05
2561	1.75766	0.0938019
2562	2.25727	0.661018
2563	1.74206	0.05
2564	0.826073	0.05
2565	1.30022	0.05
2566	1.45641	0.05
2567	0.946131	0.05
2568	0.752245	0.05
2569	0.960952	0.05
2570	0.743477	0.05
2571	1.04355	0.05
2572	1.6253	0.05
2573	0.960577	0.05
2574	1.34852	0.738701
2575	1.03235	0.487797
2576	1.78168	0.154329
2577	1.2966	0.05
2578	2.21116	0.05
2579	1.6395	0.05
2580	1.6395	0.05
2581	0.94613	0.05
2582	1.22187	0.05
2583	0.962737	0.05
2584	0.962735	0.05
2585	1.36376	0.05
2586	1.2966	0.05
2587	1.2966	0.05
2588	1.19669	1.04327
2589	2.37636	0.986324
2590	1.08894	0.971674
2591	1.62551	1.47151
2592	2.51472	1.27938
2593	1.89226	0.995205
2594	0.980038	0.906412
2595	1.10187	2.1
2596	1.01479	2.1
2597	1.81663	2.1
2598	1.83247	2.1
2599	0.921851	0.110643
2600	0.983148	2.1
2601	1.82816	2.1
2602	1.26103	2.1
2603	2.34818	2.1
2604	1.23152	2.1
2605	2.15738	2.1
2606	1.23152	2.1
2607	1.51042	2.1

2608	0.842995	2.1
2609	1.22257	2.1
2610	0.810959	2.1
2611	1.68242	2.1
2612	0.95701	2.1
2613	1.12389	2.1
2614	1.95255	2.1
2615	1.57974	2.1
2616	1.94632	2.1
2617	0.81534	2.1
2618	1.13996	2.1
2619	1.18958	0.05
2620	1.25929	0.05
2621	3.02933	0.644415
2622	2.37434	0.05
2623	1.10609	0.419675
2624	1.64132	0.121587
2625	1.64132	0.0709249
2626	0.946132	0.199422
2627	1.40029	0.0862902
2628	1.08946	0.0715112
2629	1.08946	0.186493
2630	1.18717	0.05
2631	2.71421	0.05
2632	1.08189	0.0937541
2633	0.94613	0.0557056
2634	1.37109	0.0605286
2635	1.17925	0.05
2636	0.851541	0.0593102
2637	0.942512	0.055248
2638	1.18541	0.05
2639	0.851526	0.0635027
2640	1.0819	0.0523057
2641	1.86009	0.0610632
2642	0.946132	0.05
2643	1.67409	0.05
2644	1.67409	0.05
2645	0.946131	0.0500916
2646	1.31716	0.05
2647	1.30307	0.05
2648	0.946131	0.0545181
2649	1.18601	0.05
2650	3.15646	2.1
2651	0.985109	2.1
2652	3.37048	2.1
2653	1.10667	2.1
2654	1.77065	0.993365
2655	1.45837	0.757676
2656	1.77063	0.858948
2657	2.58104	0.703615
2658	1.37652	2.1
2659	1.54168	2.1
2660	1.00224	0.05
2661	1.05851	0.05
2662	1.39093	0.05
2663	1.43358	0.05
2664	0.575741	0.05
2665	0.518161	0.05

2666	1.04071	0.05
2667	1.13092	1.40524
2668	1.17042	2.0963
2669	1.53347	1.71211
2670	1.52938	1.42497
2671	1.00613	0.05
2672	1.81618	0.257488
2673	1.81618	0.05
2674	0.946127	0.801007
2675	1.36479	0.248826
2676	0.94613	0.05
2677	1.31724	0.193686
2678	1.3648	0.0631338
2679	1.00613	0.05
2680	0.781375	0.05
2681	1.13431	0.05
2682	1.03547	0.0525138
2683	1.40668	0.0817231
2684	0.94613	0.0730167
2685	0.713285	0.0828081
2686	1.72885	0.0863623
2687	1.72885	0.077607
2688	0.713298	0.0614799
2689	3.30075	0.05
2690	2.81337	0.0748572
2691	1.39296	0.105258
2692	0.830164	2.1
2693	1.50493	2.1
2694	1.50494	2.1
2695	0.830136	2.1
2696	1.28298	2.1
2697	1.0521	2.1
2698	0.830147	2.1
2699	2.0799	2.1
2700	1.52115	1.81617
2701	2.5142	2.1
2702	1.31253	0.453874
2703	1.94446	1.15994
2704	1.18817	1.19504
2705	0.598348	1.80257
2706	1.69315	1.16893
2707	1.69315	1.63935
2708	0.598377	0.64133
2709	0.598347	0.973081
2710	0.94613	0.695304
2711	1.28418	0.596496
2712	2.05205	0.794133
2713	1.28418	1.59799
2714	1.0737	2.1
2715	1.81024	2.1
2716	2.73834	2.1
2717	1.71369	2.1
2718	1.71368	2.1
2719	0.946118	2.1
2720	1.29063	2.1
2721	1.36917	2.1
2722	0.94613	2.1
2723	1.29062	2.1

2724	0.947921	2.1
2725	1.58661	0.164972
2726	1.37658	1.69601
2727	1.2364	1.59617
2728	1.66685	0.920628
2729	1.66685	1.87971
2730	0.946133	0.862539
2731	0.946133	1.33938
2732	1.11978	1.58388
2733	1.09194	1.15085
2734	1.21858	1.96659
2735	1.37658	1.48945
2736	1.64903	0.452775
2737	1.64903	2.1
2738	0.946132	0.873906
2739	0.946132	1.37692
2740	1.05496	1.23939
2741	1.22851	0.432215
2742	1.22852	2.03228
2743	1.58482	0.472245
2744	1.60578	0.05
2745	1.06336	0.05
2746	1.68062	0.05
2747	1.14721	0.05
2748	1.11924	0.05
2749	1.18421	0.05
2750	2.01276	0.05
2751	1.23502	2.08554
2752	1.34926	2.06369
2753	0.546751	1.59722
2754	0.790446	2.03345
2755	1.04072	1.67218
2756	0.555868	1.4133
2757	0.930305	1.5051
2758	1.09344	1.7129
2759	1.18351	1.86415
2760	1.97215	1.63168
2761	1.07416	1.99763
2762	1.33204	1.44388
2763	1.22038	1.18627
2764	0.597449	0.957354
2765	0.821422	1.46805
2766	1.04071	1.2446
2767	0.608026	0.575701
2768	0.929427	1.13487
2769	1.64929	0.05
2770	1.68541	0.05
2771	0.496093	0.05
2772	1.3385	0.05
2773	0.790798	0.05
2774	1.22971	0.05
2775	0.898853	0.05
2776	2.14361	0.05
2777	2.10813	0.05
2778	1.05417	0.05
2779	1.80768	0.05
2780	1.09725	2.1
2781	1.41131	2.1

2782	1.1656	2.1
2783	0.946129	2.1
2784	1.68365	2.1
2785	1.68365	2.1
2786	1.1656	2.1
2787	0.94613	2.1
2788	1.21269	0.05
2789	1.48867	0.05
2790	1.27806	0.05
2791	1.68264	0.05
2792	1.37813	1.89382
2793	1.50743	1.2926
2794	1.04121	0.663161
2795	1.12434	0.183518
2796	1.39553	0.104785
2797	1.44721	1.54331
2798	0.946132	0.202216
2799	1.23943	0.24123
2800	1.05292	0.355774
2801	1.28607	0.122536
2802	1.16915	0.323475
2803	1.76306	0.778835
2804	1.42266	0.718315
2805	1.36844	2.1
2806	1.36844	2.1
2807	1.67352	2.1
2808	1.11256	0.773757
2809	1.03217	0.56526
2810	1.18422	0.05
2811	1.04159	0.05
2812	1.60139	0.05
2813	1.04159	0.05
2814	0.988805	2.1
2815	1.05481	2.1
2816	1.52938	2.08186
2817	1.94724	1.76029
2818	0.896336	0.05
2819	0.911484	0.05
2820	1.18315	0.05
2821	1.10515	0.05
2822	1.04067	0.05
2823	1.12758	0.05
2824	0.904106	0.05
2825	1.6901	0.05
2826	1.68671	0.05
2827	1.99908	0.05
2828	0.772426	0.695227
2829	0.810945	1.95166
2830	0.667562	1.09359
2831	0.496617	0.105769
2832	0.847689	0.970089
2833	0.914378	1.48632
2834	0.934003	0.583053
2835	1.07459	1.06811
2836	1.40225	2.1
2837	1.07043	2.1
2838	0.94613	2.1
2839	1.63072	2.1

2840	1.63072	2.1
2841	1.07043	2.1
2842	0.946131	2.1
2843	1.53082	2.1
2844	1.69916	2.1
2845	1.75211	0.122951
2846	1.96831	2.1
2847	1.75211	0.580397
2848	0.946127	0.287191
2849	0.817065	0.314728
2850	1.41798	0.291628
2851	1.41798	0.1526
2852	0.791656	0.294432
2853	0.791656	0.363343
2854	1.41798	0.254538
2855	1.58331	0.241253
2856	1.41776	0.151903
2857	0.791647	0.154422
2858	1.4512	0.129808
2859	1.45119	0.140148
2860	0.75821	0.176492
2861	0.758248	0.173228
2862	0.946104	0.152768
2863	1.36482	0.137985
2864	1.29389	2.1
2865	1.04164	1.80612
2866	1.40574	1.74544
2867	1.64833	1.63264
2868	1.71183	0.05
2869	1.71183	0.05
2870	0.946131	0.264028
2871	1.35721	0.0595363
2872	0.946131	0.700522
2873	0.946131	0.287828
2874	1.30076	0.181328
2875	1.2474	0.05
2876	2.85225	0.0839254
2877	2.71441	0.05
2878	1.2474	0.05
2879	1.38622	0.0680447
2880	0.858359	0.05
2881	1.3682	0.05
2882	1.57364	0.05
2883	0.729695	0.0705667
2884	0.729723	0.05
2885	0.822611	0.234451
2886	0.946133	0.0782969
2887	0.729723	0.05
2888	0.974704	0.203451
2889	0.974702	0.275514
2890	1.81603	2.1
2891	1.96348	2.1
2892	1.43612	0.695672
2893	1.43612	1.0195
2894	0.634295	1.05754
2895	0.93796	1.22639
2896	0.946133	1.22046
2897	0.634326	0.494794

2898	0.946133	0.904458
2899	1.2654	0.266759
2900	1.26865	0.852359
2901	2.2649	0.596612
2902	1.00864	0.738281
2903	1.09689	0.407687
2904	0.832021	0.409283
2905	1.53229	0.3284
2906	1.1145	0.464136
2907	0.929583	0.33993
2908	1.613	0.229583
2909	0.644729	0.374666
2910	1.03471	1.5658
2911	2.7739	2.1
2912	1.36133	1.60558
2913	2.50215	2.1
2914	2.52195	2.1
2915	1.0465	2.1
2916	1.42673	2.1
2917	1.42673	2.1
2918	0.946132	2.1
2919	1.31904	2.1
2920	0.986499	2.1
2921	0.946132	2.1
2922	1.31904	2.1
2923	0.966978	2.1
2924	1.42237	0.05
2925	2.60925	0.05
2926	0.753659	0.05
2927	1.77241	0.05
2928	1.77241	0.05
2929	0.946129	0.05
2930	0.75366	0.05
2931	1.22144	0.05
2932	0.94613	0.05
2933	1.50732	0.05
2934	1.18514	0.05
2935	0.902187	0.05
2936	1.89858	0.05
2937	1.24294	0.05
2938	1.67211	0.05
2939	1.41786	0.05
2940	0.810959	0.05
2941	1.50732	0.05
2942	2.44288	0.05
2943	1.40969	0.05
2944	2.06654	0.05
2945	1.40969	0.05
2946	1.40882	0.05
2947	1.94063	0.05
2948	0.867921	0.05
2949	1.29289	0.05
2950	0.867918	0.05
2951	0.946128	0.05
2952	0.867944	0.05
2953	1.66589	0.05
2954	1.66589	0.05
2955	0.968595	2.1

2956	1.5534	2.1
2957	1.27793	2.1
2958	1.13433	2.1
2959	1.3439	0.05
2960	2.53709	0.05
2961	0.571998	0.05
2962	0.64837	0.05
2963	1.99755	0.05
2964	1.99854	0.05
2965	0.764229	2.1
2966	1.61842	2.1
2967	1.61842	2.1
2968	1.18315	2.1
2969	1.1995	2.1
2970	1.18315	2.1
2971	0.764256	2.1
2972	1.52364	2.1
2973	2.36631	2.1
2974	0.641912	2.06514
2975	1.7181	2.1
2976	1.459 2.1	
2977	0.634845	1.89385
2978	1.18315	2.1
2979	1.17615	2.1
2980	0.571367	2.1
2981	1.3324	0.05
2982	0.798214	0.05
2983	0.946128	0.05
2984	0.627252	0.05
2985	1.50337	0.05
2986	1.50337	0.05
2987	0.62725	0.05
2988	1.07763	0.05
2989	1.07763	0.05
2990	2.16109	0.05
2991	1.07763	0.05
2992	1.12486	2.1
2993	1.11827	2.1
2994	1.0491	2.1
2995	1.09034	2.1
2996	1.09431	2.1
2997	0.946131	2.1
2998	0.748607	2.1
2999	1.44002	2.1
3000	1.44002	2.1
3001	0.748635	2.1
3002	0.782505	2.1
3003	1.40615	2.1
3004	1.19539	2.1
3005	1.29389	2.1
3006	1.4867	1.16996
3007	1.50103	0.05
3008	1.63025	0.05
3009	2.72109	0.05
3010	0.915755	0.05
3011	0.946132	0.05
3012	0.915755	0.05
3013	1.70236	0.05

3014	1.70236	0.05
3015	1.26155	0.05
3016	1.35657	0.05
3017	1.83151	0.05
3018	1.83151	0.05
3019	2.52309	0.05
3020	0.983607	2.1
3021	1.06838	0.100901
3022	0.983609	1.42556
3023	0.983596	0.929005
3024	2.74384	0.05
3025	1.6261	0.05
3026	1.15477	2.1
3027	1.02136	2.1
3028	1.83561	2.1
3029	1.83561	2.1
3030	0.946131	2.1
3031	1.39523	2.1
3032	1.29313	2.1
3033	1.29313	2.1
3034	1.38651	2.1
3035	1.06313	2.1
3036	0.974888	2.1
3037	1.0894	2.1
3038	0.974888	2.1
3039	1.82282	2.1
3040	1.82282	2.1
3041	0.946131	2.1
3042	1.37372	2.1
3043	1.32374	2.1
3044	1.32374	2.1
3045	1.39523	2.1
3046	1.40882	0.05
3047	0.861998	0.05
3048	1.24448	0.05
3049	0.861999	0.05
3050	0.946129	0.05
3051	0.862011	0.05
3052	1.67537	0.05
3053	1.67537	0.05
3054	1.72399	0.05
3055	2.58579	0.05
3056	1.72399	0.05
3057	1.50951	0.05
3058	2.48897	0.05
3059	1.26755	2.1
3060	1.03824	2.1
3061	1.27875	2.1
3062	1.48901	2.1
3063	1.18902	2.1
3064	1.11764	2.1
3065	1.87436	2.1
3066	0.95345	2.0899
3067	0.724191	1.58124
3068	0.86668	1.54417
3069	0.811523	1.77549
3070	1.04074	0.761818
3071	0.843097	1.28937

3072	0.833434	1.69821
3073	0.965727	1.35001
3074	0.85346	1.64301
3075	1.00185	0.388721
3076	1.00184	1.38365
3077	0.980651	1.75349
3078	0.980664	1.26484
3079	0.475737	0.67338
3080	0.719696	0.847491
3081	0.946109	0.647924
3082	0.946116	1.02441
3083	0.719693	0.756476
3084	0.946117	1.00591
3085	1.14879	1.61794
3086	0.719683	1.4797
3087	0.900977	0.92989
3088	0.972364	1.14373
3089	1.22961	1.0743
3090	0.405384	0.920737
3091	1.34406	2.1
3092	1.2187	2.1
3093	0.963571	0.05
3094	0.946131	0.05
3095	1.57246	0.180536
3096	1.57246	2.1
3097	0.946131	0.210314
3098	1.1235	2.1
3099	1.1235	1.72401
3100	1.07439	1.83516
3101	1.19612	1.1329
3102	1.24844	1.05536
3103	1.00007	1.84717
3104	1.02429	2.1
3105	1.10459	2.1
3106	1.06724	0.0589984
3107	0.67519	0.228962
3108	0.49762	0.0661148
3109	1.43435	0.05
3110	0.833315	0.0650964
3111	0.58135	0.0674322
3112	1.14749	0.28877
3113	1.85091	0.630706
3114	1.2802	0.232611
3115	1.27737	0.110273
3116	2.3655	0.183326
3117	1.30584	2.1
3118	2.06424	2.1
3119	0.946131	0.0854017
3120	1.04257	0.0609492
3121	0.94613	0.0550567
3122	1.67201	0.0777506
3123	1.67201	0.05
3124	1.27696	0.0626287
3125	1.34118	0.05
3126	1.3413	0.05
3127	2.36238	0.05
3128	0.6036	0.05
3129	1.11745	0.05

3130	1.46126	0.05
3131	1.39612	0.05
3132	0.670678	0.05
3133	1.03402	0.05
3134	1.11271	0.05
3135	0.838028	0.05
3136	0.807019	0.05
3137	1.27787	0.05
3138	0.994705	0.05
3139	1.20014	0.05
3140	0.964793	0.05
3141	0.863868	0.05
3142	1.3413	0.05
3143	1.79549	0.05
3144	1.35877	2.1
3145	1.63648	2.1
3146	1.08171	2.1
3147	1.27839	2.1
3148	1.52601	2.1
3149	1.27841	2.1
3150	1.71255	2.1
3151	2.16307	2.1
3152	1.91592	2.1
3153	1.78508	2.1
3154	0.946128	2.1
3155	1.41199	2.1
3156	0.946128	2.1
3157	0.901088	2.1
3158	1.55244	2.1
3159	1.59475	2.1
3160	1.40921	2.1
3161	0.960106	2.1
3162	1.40921	2.1
3163	2.0073	2.1
3164	2.2973	0.0744988
3165	1.90705	0.066291
3166	1.53671	0.05
3167	1.86035	0.05
3168	1.33747	2.1
3169	1.14007	2.1
3170	1.3212	2.1
3171	1.66867	0.05
3172	1.30203	0.05
3173	1.27696	2.1
3174	1.05025	2.1
3175	1.05025	2.1
3176	1.3704	2.1
3177	1.15677	2.1
3178	0.946129	2.1
3179	1.65879	2.1
3180	1.65879	2.1
3181	1.15676	2.1
3182	0.94613	2.1
3183	0.867217	1.44495
3184	1.28684	1.176
3185	1.58866	1.18018
3186	1.58866	0.639701
3187	0.867246	1.85426

3188	1.28684	0.896583
3189	1.16906	1.37844
3190	1.13438	1.17156
3191	1.37224	1.34786
3192	1.61519	1.39238
3193	0.883939	1.96327
3194	1.40092	1.18252
3195	1.80383	1.98268
3196	1.80383	1.69202
3197	0.883935	1.87799
3198	1.40092	1.11305
3199	1.28684	1.82303
3200	1.76788	0.845313
3201	2.57368	1.15505
3202	1.37224	2.05325
3203	1.65411	0.510764
3204	1.77042	2.1
3205	1.77042	2.1
3206	1.90446	2.1
3207	1.28667	2.1
3208	1.55635	2.1
3209	1.30646	0.91992
3210	2.31836	1.5037
3211	1.30645	1.02766
3212	1.86429	1.29497
3213	1.48676	0.05
3214	0.743379	0.05
3215	1.41848	0.05
3216	1.41848	0.05
3217	0.946131	0.05
3218	0.812649	0.05
3219	1.34921	0.05
3220	0.812649	0.05
3221	0.949366	0.05
3222	1.32974	0.05
3223	0.794204	0.05
3224	0.634352	2.1
3225	0.838025	2.1
3226	1.08802	2.1
3227	0.838038	2.1
3228	1.18314	2.1
3229	1.12495	2.1
3230	1.22774	2.1
3231	1.04343	2.1
3232	0.921111	0.05
3233	2.1609	0.05
3234	1.09987	0.05
3235	2.5043	0.05
3236	1.05492	0.05
3237	0.590344	0.05
3238	1.05492	0.05
3239	1.25215	0.05
3240	1.71674	0.05
3241	1.71674	0.05
3242	0.590331	0.05
3243	1.17078	0.05
3244	0.625542	0.05
3245	0.724673	2.1

3246	1.08011	2.1
3247	1.87598	2.1
3248	1.50738	2.1
3249	1.44951	0.05
3250	1.50102	0.05
3251	1.8042	0.0658699
3252	0.687769	0.126604
3253	1.66483	0.0708004
3254	1.66483	0.0893551
3255	0.946132	0.148755
3256	0.687798	0.077282
3257	1.20885	0.200195
3258	0.687799	0.131427
3259	0.886063	0.0684708
3260	0.798751	0.0782849
3261	0.584813	0.05
3262	1.49483	0.05
3263	0.888005	0.05
3264	1.03589	0.05
3265	1.70435	0.05
3266	0.710414	2.1
3267	0.917609	1.67465
3268	0.810947	1.81975
3269	0.81096	1.66839
3270	1.48658	1.46755
3271	1.53653	1.56791
3272	1.19329	1.88884
3273	0.946153	1.59476
3274	1.50798	1.70323
3275	1.85017	2.1
3276	1.19768	2.1
3277	0.864304	2.1
3278	0.792906	1.9615
3279	0.989025	1.51396
3280	1.19768	1.69254
3281	1.54548	1.68282
3282	1.18798	2.00179
3283	1.31875	1.23918
3284	1.05725	1.58691
3285	2.17365	0.05
3286	2.48106	0.05
3287	1.87451	0.05
3288	1.27189	1.05292
3289	2.79399	1.86694
3290	0.859565	1.18931
3291	0.926174	0.947895
3292	0.604744	0.892003
3293	1.41832	0.174675
3294	1.05494	0.657555
3295	0.874749	0.704496
3296	1.50067	0.381191
3297	0.917817	1.39197
3298	0.63591	1.21844
3299	1.72849	0.815606
3300	1.72849	0.307153
3301	0.635943	0.580466
3302	1.39699	1.20406
3303	0.967411	1.89196

3304	0.635943	1.0377
3305	1.39908	0.713393
3306	1.26971	1.13236
3307	1.02969	1.19561
3308	1.15326	2.1
3309	2.30741	1.89304
3310	0.93545	1.40199
3311	0.802747	0.838725
3312	1.15466	1.60062
3313	0.859164	1.79667
3314	1.02616	1.83147
3315	1.23149	1.55875
3316	0.83804	0.95611
3317	1.13266	2.1
3318	1.64142	2.08908
3319	1.21223	2.1
3320	1.43051	2.1
3321	1.32418	0.690544
3322	0.621319	0.859259
3323	1.47134	2.08883
3324	1.79103	0.101315
3325	3.25951	0.05
3326	3.16764	0.05
3327	1.65583	0.05
3328	1.4732	0.05
3329	1.76476	2.1
3330	1.80114	1.55272
3331	0.754121	2.1
3332	0.94613	0.204252
3333	0.754119	2.1
3334	1.45636	2.08446
3335	1.45636	0.137644
3336	1.30029	0.711656
3337	0.910183	0.743896
3338	1.29099	2.1
3339	1.08039	2.1
3340	1.05498	2.1
3341	1.24445	2.1
3342	1.2873	2.1
3343	0.987703	2.1
3344	0.964782	2.1
3345	1.73645	0.05
3346	0.871279	0.05
3347	0.884744	0.05
3348	1.85193	0.05
3349	1.68681	0.05
3350	1.33689	0.05
3351	1.23493	0.05
3352	0.892482	0.05
3353	2.95185	0.05
3354	2.10496	0.05
3355	1.87334	2.1
3356	0.94411	2.1
3357	0.754196	2.1
3358	1.43122	2.1
3359	1.58684	2.1
3360	0.838027	2.1
3361	1.1536	2.1

3362	1.24878	2.1
3363	1.23125	2.1
3364	2.86862	2.1
3365	1.57636	2.1
3366	0.998451	0.243757
3367	1.5176	0.165242
3368	1.13271	0.05
3369	1.60248	0.05
3370	1.22083	2.1
3371	1.79996	2.1
3372	1.19087	2.1
3373	2.00342	2.1
3374	1.3715	2.1
3375	2.79227	2.1
3376	2.7873	2.1
3377	0.987265	0.05
3378	1.62715	2.1
3379	1.70117	2.1
3380	1.27806	0.05
3381	1.45246	2.1
3382	1.45244	2.1
3383	1.87214	2.1
3384	0.995796	2.1
3385	1.68189	0.05
3386	1.49155	2.1
3387	1.58054	2.1
3388	1.59169	2.1
3389	0.736364	0.05
3390	0.70236	0.05
3391	2.24367	0.05
3392	2.57085	0.05
3393	1.70952	0.05
3394	2.60516	0.05
3395	1.45158	2.1
3396	1.00371	2.1
3397	1.65748	2.1
3398	1.23706	0.05
3399	1.55834	0.380562
3400	1.72671	0.482141
3401	0.597656	0.542224
3402	1.39137	0.241705
3403	0.855578	0.05
3404	1.48398	0.15532
3405	0.745117	0.177259
3406	1.23706	1.02626
3407	1.57916	0.067479
3408	0.893025	0.05
3409	3.22433	0.155357
3410	2.47324	1.33948
3411	1.63852	2.1
3412	1.63852	2.1
3413	0.946132	2.1
3414	1.19095	2.1
3415	0.946133	2.1
3416	1.16684	2.1
3417	1.19095	2.1
3418	1.06698	0.05
3419	1.48692	0.05

3420	1.08334	1.10675
3421	1.07283	2.1
3422	1.28517	0.345691
3423	1.2746	0.05
3424	1.30723	0.05
3425	2.02069	0.05
3426	1.05579	0.05
3427	1.59475	2.1
3428	2.40113	2.1
3429	1.58875	2.1
3430	1.6881	2.1
3431	1.6881	2.1
3432	0.819953	2.1
3433	0.819954	2.1
3434	0.946131	2.1
3435	0.946131	2.1
3436	1.34782	2.1
3437	1.65619	2.1
3438	1.65619	2.1
3439	0.79437	2.1
3440	1.25	2.1
3441	0.94613	2.1
3442	0.946131	2.1
3443	0.794371	2.1
3444	1.58875	2.1
3445	2.5	2.1
3446	1.04147	2.1
3447	0.946131	0.302699
3448	1.79686	2.1
3449	1.79686	2.1
3450	1.25144	0.310511
3451	1.35714	1.78727
3452	1.38585	1.80912
3453	1.25144	2.1
3454	1.15564	2.1
3455	1.34389	2.1
3456	0.946133	1.11739
3457	1.74353	2.09841
3458	1.74353	2.1
3459	1.14553	1.01209
3460	1.38585	0.711227
3461	1.30382	1.23844
3462	1.14553	2.09093
3463	1.15564	2.1
3464	1.90618	2.1
3465	2.11056	2.1
3466	1.032	2.1
3467	0.953636	2.1
3468	1.23131	1.22489
3469	1.39763	1.38694
3470	2.01024	0.885394
3471	1.9186	0.898898
3472	1.83658	0.05
3473	1.81656	0.05
3474	2.2049	0.05
3475	2.16081	0.05
3476	1.12219	0.05
3477	0.599771	0.05

3478	1.61401	0.05
3479	1.05912	0.05
3480	0.951648	0.05
3481	1.89328	0.05
3482	0.913682	0.05
3483	1.00763	0.05
3484	1.29129	2.1
3485	1.37138	2.1
3486	1.41986	0.05
3487	1.41986	0.05
3488	0.944166	0.05
3489	1.41789	0.05
3490	0.944167	0.05
3491	0.944166	0.05
3492	0.946132	0.05
3493	1.98713	0.05
3494	1.23626	0.05
3495	0.932791	0.05
3496	0.810251	0.05
3497	0.986748	0.05
3498	0.898155	0.05
3499	0.92624	0.05
3500	1.1349	0.05
3501	0.934572	0.05
3502	1.23104	0.511258
3503	1.23104	0.628307
3504	0.799994	0.0853935
3505	0.94613	0.382967
3506	0.802822	0.05
3507	1.40944	0.790141
3508	0.800035	0.251192
3509	1.41223	0.260749
3510	1.41223	0.268291
3511	1.6	0.146984
3512	1.60565	0.0940258
3513	1.23042	2.1
3514	1.0645	2.1
3515	1.00319	2.1
3516	1.29435	2.1
3517	1.2243	2.1
3518	1.2243	2.1
3519	1.05655	1.25745
3520	1.29764	1.72713
3521	2.55252	2.1
3522	1.15676	2.1
3523	0.855295	2.1
3524	0.810959	2.1
3525	1.65857	2.1
3526	1.00849	2.1
3527	1.16021	2.1
3528	1.75869	2.1
3529	1.13775	2.1
3530	1.20091	2.1
3531	1.61582	2.1
3532	1.88031	2.1
3533	0.968536	2.1
3534	0.946131	2.1
3535	1.61481	2.1

3536	1.61481	2.1
3537	1.09547	2.1
3538	1.18486	2.1
3539	1.37609	2.1
3540	1.09546	2.1
3541	1.02461	2.1
3542	1.83223	2.1
3543	1.74002	2.1
3544	1.70939	2.1
3545	1.7094	2.1
3546	0.726287	2.1
3547	1.01712	2.1
3548	1.30318	2.1
3549	0.946131	2.1
3550	0.87155	2.1
3551	1.77093	1.92279
3552	2.02356	1.92991
3553	1.32449	2.1
3554	1.10003	2.1
3555	1.5161	2.1
3556	1.5161	2.1
3557	0.946132	2.1
3558	1.09206	2.1
3559	0.946132	2.1
3560	1.04404	2.1
3561	1.09206	2.1
3562	0.635628	1.10675
3563	1.14623	0.27938
3564	1.73197	0.381807
3565	1.73197	1.08523
3566	0.529655	0.286849
3567	1.14623	0.56993
3568	0.992085	0.416034
3569	1.16915	1.24337
3570	2.29245	0.102416
3571	0.584573	0.786046
3572	0.956756	0.337152
3573	1.52471	0.292581
3574	1.51409	1.19952
3575	0.584575	0.612502
3576	0.94613	0.588467
3577	1.14622	0.218815
3578	1.28096	1.13074
3579	1.34695	1.64608
3580	1.04193	2.1
3581	1.05378	2.1
3582	0.798209	2.1
3583	1.32007	2.1
3584	1.64327	0.05
3585	2.64674	2.1
3586	2.38936	0.05
3587	2.88475	0.05
3588	1.07745	2.1
3589	1.08911	2.1
3590	1.49127	2.1
3591	1.49127	2.1
3592	0.709714	2.1
3593	1.37199	2.1

3594	0.82899	2.1
3595	0.709714	2.1
3596	0.946132	2.1
3597	1.00133	2.1
3598	1.40182	2.1
3599	1.53278	2.1
3600	1.40182	2.1
3601	1.31764	0.05
3602	1.00691	0.05
3603	1.24094	0.05
3604	0.64868	0.05
3605	0.946133	0.05
3606	0.64868	0.05
3607	1.71762	0.05
3608	1.71762	0.05
3609	1.41662	0.05
3610	0.975477	0.05
3611	1.73584	0.05
3612	2.48188	0.05
3613	2.2045	0.147931
3614	2.08134	0.127786
3615	0.993366	0.05
3616	0.993368	0.497961
3617	1.14187	0.137262
3618	0.946132	0.120952
3619	0.946133	0.971478
3620	1.68951	0.398287
3621	1.68951	1.78054
3622	1.34612	0.267193
3623	1.28953	0.169839
3624	1.34611	1.27798
3625	1.31892	0.0687205
3626	0.946131	0.241294
3627	1.80988	0.0709707
3628	1.80988	2.1
3629	1.31892	0.585474
3630	0.946131	0.189636
3631	2.13214	2.1
3632	1.94691	2.1
3633	1.52661	0.05
3634	1.53175	0.05
3635	0.956005	0.05
3636	0.955991	0.05
3637	1.40572	0.05
3638	0.955992	0.05
3639	1.51084	2.02606
3640	1.67115	2.1
3641	1.73143	2.1
3642	1.55234	2.1
3643	1.69377	2.1
3644	2.51483	2.1
3645	1.25987	2.1
3646	1.4472	2.1
3647	1.83151	0.201112
3648	2.01001	0.540503
3649	0.993998	0.0822919
3650	1.83151	0.142985
3651	1.40915	0.05

3652	1.24653	0.0598931
3653	1.74472	2.1
3654	1.74472	2.1
3655	1.74244	2.1
3656	1.04788	2.1
3657	1.28522	2.1
3658	1.03785	2.1
3659	0.896519	2.1
3660	1.60428	2.1
3661	1.73305	2.1
3662	1.39072	0.05
3663	2.27501	0.0842751
3664	1.39073	0.0777032
3665	1.39073	0.05
3666	1.06838	2.1
3667	0.968462	0.235312
3668	1.06837	1.47178
3669	1.13455	1.28311
3670	1.23527	0.101298
3671	2.71686	0.064323
3672	1.86881	0.0557862
3673	1.10586	1.44851
3674	1.03852	2.03738
3675	1.03899	2.1
3676	0.963973	2.1
3677	1.00319	2.1
3678	1.00319	2.1
3679	0.987132	2.1
3680	0.974889	2.1
3681	0.987131	2.1
3682	1.7677	2.1
3683	1.7677	2.1
3684	0.946131	2.1
3685	1.31185	2.1
3686	0.946132	2.1
3687	1.30203	2.1
3688	1.31185	2.1
3689	1.82958	2.1
3690	1.82958	2.1
3691	0.946132	2.1
3692	1.40199	2.1
3693	0.946133	2.1
3694	1.36994	2.1
3695	1.40199	2.1
3696	0.97489	2.1
3697	1.05939	2.1
3698	1.01922	2.1
3699	0.810941	1.89202
3700	1.05372	2.1
3701	0.754065	2.1
3702	0.926131	2.1
3703	1.35905	2.1
3704	0.810931	2.08865
3705	1.75647	0.87498
3706	1.68265	2.1
3707	1.41995	2.1
3708	1.36457	1.62502
3709	0.933392	1.29853

3710	1.42255	2.1
3711	1.3868	2.1
3712	1.08074	2.1
3713	1.07196	2.1
3714	1.13537	1.11181
3715	1.01179	0.610664
3716	1.60196	0.198885
3717	1.72798	0.301228
3718	1.3715	0.36583
3719	1.56409	0.6805
3720	1.56409	0.175641
3721	1.97819	1.1269
3722	1.47954	0.212148
3723	0.798655	0.05
3724	1.47952	0.131034
3725	2.30327	0.05
3726	1.49111	2.1
3727	1.96013	2.1
3728	1.54599	2.1
3729	2.06632	2.1
3730	1.54601	2.1
3731	1.10557	0.609158
3732	1.17624	0.16542
3733	1.60497	2.1
3734	1.61587	2.1
3735	1.65977	0.05
3736	1.69044	0.05
3737	1.00659	2.1
3738	1.06226	2.1
3739	1.15171	2.1
3740	1.50853	2.1
3741	1.80252	2.1
3742	1.41432	2.1
3743	1.04687	2.1
3744	0.940878	2.1
3745	1.52031	2.1
3746	1.52031	2.1
3747	0.940877	2.1
3748	0.946132	2.1
3749	1.50853	2.1
3750	0.963973	2.1
3751	1.70562	0.05
3752	2.43203	0.05
3753	1.36004	0.05
3754	1.06648	0.05
3755	1.66009	0.05
3756	1.66009	0.05
3757	0.766427	0.05
3758	0.766452	0.05
3759	0.946132	0.05
3760	1.98012	0.05
3761	1.20357	2.1
3762	1.20356	2.1
3763	1.67324	2.1
3764	1.52008	2.1
3765	1.19964	2.1
3766	1.36861	2.1
3767	1.19964	2.1

3768	2.85415	2.1
3769	1.43687	2.1
3770	0.935852	2.1
3771	1.09972	2.1
3772	0.810933	2.1
3773	2.25974	2.1
3774	1.74149	2.1
3775	1.36984	2.1
3776	1.87602	2.1
3777	0.890748	2.1
3778	1.20587	2.08068
3779	0.810956	1.84613
3780	2.03174	2.03415
3781	1.34403	2.08347
3782	1.58825	2.05993
3783	1.63096	2.06734
3784	2.53735	2.08583
3785	1.98241	2.09439
3786	2.53014	2.1
3787	1.1775	1.17291
3788	1.06538	0.425394
3789	0.946132	1.32
3790	1.4101	2.1
3791	1.75162	2.1
3792	1.75162	1.17579
3793	0.946132	1.11435
3794	1.4101	1.1565
3795	1.28765	1.1935
3796	1.1427	0.850465
3797	1.1427	0.344957
3798	1.3109	2.1
3799	1.02721	1.12996
3800	0.846447	1.36011
3801	0.862076	2.1
3802	0.644098	1.48319
3803	0.581308	1.80519
3804	1.02306	1.60307
3805	0.322641	0.321888
3806	0.589346	0.295023
3807	2.25572	1.69276
3808	1.31372	2.1
3809	1.18873	2.1
3810	1.78225	2.1
3811	1.764 2.1	
3812	1.17503	2.1
3813	1.06821	2.1
3814	1.06821	1.99324
3815	1.7777	2.03244
3816	1.9096	0.661193
3817	1.70239	1.0479
3818	1.16182	0.05
3819	1.20132	0.05
3820	1.23475	2.1
3821	1.56859	2.06409
3822	2.46263	2.1
3823	0.528744	2.1
3824	2.93337	2.1
3825	2.42504	2.1

3826	1.38646	0.05
3827	1.61963	0.05
3828	1.88016	0.235239
3829	0.622283	0.604786
3830	1.03243	0.535926
3831	0.710307	1.08891
3832	0.798124	0.49148
3833	0.931073	1.05598
3834	0.892862	1.261
3835	1.06791	0.383134
3836	0.552787	1.34014
3837	0.946132	0.697967
3838	1.36665	0.783506
3839	1.36665	1.28182
3840	0.506966	0.820003
3841	0.506964	1.43965
3842	1.36665	0.526317
3843	0.653469	0.545679
3844	1.36665	0.227539
3845	1.21339	0.214392
3846	1.21339	1.00958
3847	0.552757	0.419783
3848	0.552786	1.54202
3849	1.21336	0.0969832
3850	1.70744	1.15304
3851	1.0775	0.05
3852	1.59527	0.0835227
3853	1.48312	0.105066
3854	2.01868	0.0709699
3855	1.18947	0.05
3856	1.48312	0.107598
3857	0.785626	0.05
3858	1.19601	0.05
3859	0.785626	0.05
3860	0.946132	0.05
3861	0.7856	0.05
3862	1.59087	0.05
3863	1.51158	0.05
3864	1.74098	0.05
3865	2.39202	0.05
3866	0.870489	0.05
3867	1.34534	0.065806
3868	0.870488	0.05
3869	0.946133	0.0892926
3870	0.870489	0.136157
3871	1.67086	0.0700273
3872	1.67086	0.069883
3873	2.11311	2.1
3874	1.98765	1.59364
3875	0.946133	0.623611
3876	1.30881	2.1
3877	1.72211	2.1
3878	1.72211	1.45766
3879	0.94613	2.05626
3880	1.30881	1.52967
3881	1.35943	0.681611
3882	1.01294	0.0606536
3883	1.01294	0.05

3884	1.38963	0.339302
3885	1.01294	0.05
3886	1.60056	0.0602009
3887	1.60056	0.0632206
3888	0.946132	0.140564
3889	1.204	0.05
3890	0.946132	0.0935581
3891	1.11288	0.05
3892	1.204	0.0586734
3893	1.14599	0.223519
3894	1.00065	0.12351
3895	1.74685	0.346927
3896	1.74685	2.1
3897	0.946128	0.280869
3898	1.38463	0.0738117
3899	1.2275	2.1
3900	1.2275	1.90999
3901	1.30835	1.93842
3902	2.04407	2.1
3903	2.50425	2.1
3904	1.19647	0.161506
3905	1.19647	0.37921
3906	1.19645	0.566405
3907	1.75446	0.307013
3908	1.1315	0.05
3909	1.1315	0.792164
3910	1.45569	0.05
3911	1.1315	0.05
3912	1.14357	2.1
3913	1.25793	2.1
3914	1.14357	2.1
3915	2.08181	2.1
3916	1.4714	2.1
3917	0.71295	2.1
3918	1.68285	2.1
3919	1.68285	2.1
3920	1.25603	2.1
3921	1.13976	2.1
3922	1.25603	2.1
3923	0.71295	2.1
3924	1.61095	2.1
3925	2.51207	2.1
3926	1.63762	2.1
3927	2.74451	2.1
3928	0.671255	2.0126
3929	1.87601	2.1
3930	1.87601	2.1
3931	0.671226	2.02888
3932	1.25603	2.1
3933	1.29123	2.1
3934	0.671226	2.1
3935	1.63723	2.1
3936	0.872928	0.05
3937	1.47904	0.05
3938	1.18793	0.05
3939	2.57464	0.05
3940	1.14986	2.1
3941	1.19611	2.1

3942	0.96029	0.0912632
3943	1.7961	2.1
3944	0.928721	2.1
3945	1.34352	2.1
3946	1.37935	2.1
3947	1.37935	2.1
3948	0.892888	2.1
3949	0.946131	2.1
3950	0.892888	2.1
3951	1.75422	2.1
3952	1.05556	2.1
3953	1.27362	0.05
3954	1.27421	0.05
3955	1.3003	0.783541
3956	1.31415	0.278865
3957	1.01431	2.1
3958	0.673951	2.1
3959	1.63384	2.1
3960	1.63661	2.1
3961	1.52245	2.1
3962	1.50714	2.1
3963	0.996778	0.05
3964	1.56091	0.05
3965	1.06543	2.1
3966	2.66689	2.1
3967	1.01474	2.1
3968	2.76422	2.1
3969	1.45321	0.066997
3970	0.974716	0.05
3971	0.810943	0.05
3972	0.909982	0.0750695
3973	0.937793	0.05
3974	1.05138	0.05
3975	1.25044	0.05
3976	0.92095	0.05
3977	0.830414	0.05
3978	2.1402	0.05
3979	1.75938	2.1
3980	1.05556	2.1
3981	1.78558	2.1
3982	1.379 2.1	
3983	0.970214	0.419626
3984	0.9702	0.32066
3985	1.08894	0.684615
3986	2.17251	0.6749
3987	1.51569	0.275515
3988	1.06012	0.199277
3989	0.98966	0.178505
3990	1.55926	0.0611968
3991	1.92645	0.154533
3992	1.37427	0.05
3993	2.16338	0.05
3994	1.5456	0.05
3995	1.5456	0.05
3996	1.94002	0.05
3997	1.33135	0.05
3998	1.31948	0.05
3999	2.23255	0.05

4000	2.34061	0.05
4001	1.52903	2.1
4002	3.38327	2.1
4003	1.35977	2.1
4004	2.22414	2.1
4005	1.6812	0.05
4006	2.33397	0.05
4007	1.16699	0.05
4008	0.946131	0.05
4009	1.58115	0.05
4010	1.58115	0.05
4011	0.519726	0.05
4012	0.946131	0.05
4013	0.519697	0.05
4014	1.26868	0.05
4015	1.16699	0.05
4016	1.5951	0.05
4017	1.5951	0.05
4018	0.840568	0.05
4019	0.946131	0.05
4020	0.840598	0.05
4021	1.30047	2.1
4022	1.5586	2.1
4023	1.31825	0.566565
4024	1.31924	0.222278
4025	1.15818	2.1
4026	1.52782	1.08028
4027	1.15818	1.33878
4028	1.15818	1.30981
4029	0.99518	0.150284
4030	0.94613	0.165213
4031	0.922045	0.372565
4032	1.29322	0.322984
4033	1.29321	0.108988
4034	1.29321	0.334896
4035	0.922056	0.322434
4036	1.06848	0.167384
4037	1.84409	0.141797
4038	1.9186	1.20442
4039	2.72244	0.757481
4040	0.946132	0.913547
4041	1.36122	0.795431
4042	1.79658	0.982304
4043	1.79658	0.544766
4044	0.946133	1.19722
4045	1.36122	0.873905
4046	1.38149	0.776576
4047	1.59106	0.415285
4048	0.946116	0.859914
4049	1.29052	0.582851
4050	1.76641	0.571492
4051	1.76641	0.436512
4052	0.885309	1.17947
4053	1.29054	0.751782
4054	1.36118	0.664196
4055	1.59103	0.897459
4056	2.27816	0.449525
4057	1.56164	0.407773

4058	1.41114	0.05
4059	2.04128	0.05
4060	0.986723	2.1
4061	1.03645	0.05
4062	1.79224	0.05
4063	1.2627	2.1
4064	1.23036	2.1
4065	1.45257	2.1
4066	1.41469	2.1
4067	1.41469	2.1
4068	2.60636	2.1
4069	1.45789	0.05
4070	0.851541	0.05
4071	1.57877	0.05
4072	1.53134	0.05
4073	0.851479	0.05
4074	1.08982	0.05
4075	0.716284	0.05
4076	0.946131	0.05
4077	1.73193	0.05
4078	2.57309	0.05
4079	1.36307	0.05
4080	2.42175	0.05
4081	1.72948	0.05
4082	0.978698	2.1
4083	0.978699	2.1
4084	2.08398	2.1
4085	0.978698	2.1
4086	1.44096	2.1
4087	1.44096	2.1
4088	0.638164	2.1
4089	0.681588	2.1
4090	0.946133	2.1
4091	0.638165	2.1
4092	0.94613	2.1
4093	1.55432	2.1
4094	0.901147	2.1
4095	0.85777	2.1
4096	1.36073	2.1
4097	1.44004	2.1
4098	0.946133	2.1
4099	1.09982	2.1
4100	1.27562	2.1
4101	1.55432	2.1
4102	1.62229	2.1
4103	1.0101	2.1
4104	2.28459	2.1
4105	2.50554	2.1
4106	0.946131	0.688618
4107	1.25276	2.1
4108	1.73721	2.1
4109	1.73721	1.42762
4110	0.93406	2.09013
4111	1.25277	1.76461
4112	1.4185	1.12547
4113	1.53064	1.05698
4114	0.946133	1.21216
4115	0.609462	2.1

4116	1.65123	2.1
4117	1.65123	1.84019
4118	0.609466	2.1
4119	1.00792	1.93547
4120	1.25277	1.69675
4121	1.53064	2.07821
4122	2.15223	1.18838
4123	1.30664	1.885
4124	1.10674	2.1
4125	1.22862	2.1
4126	1.49358	0.0907221
4127	2.0207	0.05
4128	2.09374	2.1
4129	1.88175	2.1
4130	1.10938	0.05
4131	1.02478	0.12968
4132	1.13272	0.05
4133	1.13272	0.05
4134	1.03935	1.03235
4135	0.810945	1.54523
4136	0.527573	0.264522
4137	0.925011	0.927171
4138	1.03193	0.264576
4139	1.38587	0.532658
4140	0.703469	0.40779
4141	0.912282	0.941668
4142	1.75505	2.1
4143	1.78529	2.1
4144	1.60017	0.196665
4145	1.35608	0.05
4146	1.01479	2.1
4147	1.47561	0.0738424
4148	1.01479	2.1
4149	1.28418	0.0516661
4150	2.62605	2.1
4151	1.71233	2.1
4152	3.22222	2.1
4153	1.97526	2.1
4154	0.949712	0.070518
4155	1.78986	0.05
4156	2.72879	0.05
4157	2.66428	0.05
4158	1.68108	0.0669664
4159	1.67265	0.0596695
4160	2.24877	0.132424
4161	1.15558	0.150405
4162	1.39708	2.1
4163	1.39327	2.1
4164	1.69788	0.05
4165	1.1273	0.05
4166	1.17302	0.05
4167	1.04349	0.05
4168	0.979853	2.1
4169	1.19094	2.1
4170	1.39408	2.1
4171	1.61857	2.1
4172	1.66523	2.1
4173	0.946132	2.1

4174	0.838554	2.1
4175	1.05151	2.1
4176	1.18901	2.1
4177	1.18901	2.1
4178	1.61149	2.1
4179	1.22857	0.263911
4180	1.25848	0.478172
4181	1.09154	2.1
4182	2.20358	2.1
4183	1.23013	2.1
4184	1.55698	2.1
4185	1.55828	2.1
4186	1.52391	2.1
4187	0.60346	1.93492
4188	1.06336	2.1
4189	0.870714	1.27681
4190	0.543109	1.93058
4191	1.10367	1.33996
4192	1.20686	1.982
4193	1.20684	2.1
4194	2.5118	1.77584
4195	1.20684	2.1
4196	1.52468	2.09714
4197	1.52469	2.1
4198	0.532308	1.77154
4199	0.903301	2.1
4200	0.946119	1.60783
4201	0.532315	1.56145
4202	0.946107	1.7143
4203	1.46129	2.1
4204	0.804766	2.1
4205	1.4809	2.1
4206	1.4809	2.1
4207	1.11822	2.1
4208	1.16744	2.1
4209	1.11822	2.1
4210	0.804737	2.1
4211	1.04193	2.1
4212	0.983723	2.1
4213	1.8014	2.1
4214	1.1729	2.1
4215	1.03951	2.1
4216	1.03951	2.1
4217	1.17583	2.1
4218	1.42866	2.1
4219	1.78503	2.1
4220	1.17822	1.35311
4221	1.89121	2.1
4222	1.44472	2.1
4223	1.62143	2.1
4224	1.43837	2.1
4225	1.01121	0.05
4226	0.962703	1.0138
4227	0.946129	0.118139
4228	1.10941	0.0696729
4229	0.946131	0.0881566
4230	1.60326	0.316746
4231	1.60326	0.0926482

4232	1.14215	0.121823
4233	1.40724	0.313705
4234	0.962703	0.0914625
4235	1.53336	0.05
4236	1.40272	0.05
4237	1.64984	0.05
4238	1.67345	0.05
4239	1.08212	2.1
4240	1.40905	2.1
4241	0.946132	0.05
4242	0.946132	0.05
4243	0.81268	0.05
4244	0.946131	0.05
4245	0.812681	0.05
4246	0.946131	0.05
4247	0.946132	0.05
4248	0.997755	0.05
4249	1.94585	0.05
4250	1.2925	0.05
4251	2.52454	0.05
4252	1.69182	0.05
4253	1.69182	0.05
4254	0.92361	0.05
4255	1.35316	0.05
4256	1.26227	0.05
4257	0.946131	0.05
4258	1.35316	0.05
4259	1.48676	0.05
4260	1.33917	0.05
4261	0.757292	2.1
4262	1.02415	2.1
4263	1.48495	2.1
4264	0.97587	2.1
4265	0.946132	2.1
4266	0.747365	2.1
4267	1.55432	2.1
4268	1.55432	2.1
4269	0.946132	2.1
4270	0.747394	2.1
4271	1.17464	0.05
4272	1.0372	0.05
4273	2.16553	0.05
4274	2.00616	0.05
4275	1.12184	0.05
4276	1.33177	0.05
4277	0.936674	0.05
4278	0.946129	0.05
4279	0.851552	0.05
4280	1.60205	0.05
4281	1.77391	0.05
4282	1.03327	0.05
4283	1.41512	0.05
4284	1.03327	0.05
4285	0.946133	0.05
4286	0.946131	0.05
4287	1.80077	0.05
4288	1.80077	0.05
4289	1.54848	2.1

4290	1.29931	2.1
4291	1.04482	0.083425
4292	2.08069	0.0856629
4293	0.946127	0.0549672
4294	0.515539	0.104855
4295	0.515566	0.167426
4296	1.38423	0.0578485
4297	1.38423	0.0652306
4298	0.972058	0.0812792
4299	0.927737	0.0977656
4300	0.94971	0.05
4301	2.19682	0.0632719
4302	0.946132	0.0987309
4303	1.09841	0.065272
4304	0.522382	0.133275
4305	1.5962	0.05
4306	1.72791	0.05
4307	1.07428	0.0586684
4308	1.05215	0.0625823
4309	1.54632	1.508
4310	1.15693	1.65955
4311	1.41238	2.1
4312	1.41238	2.1
4313	1.77966	2.1
4314	1.77966	2.1
4315	0.946129	2.1
4316	1.40525	2.1
4317	0.946131	2.1
4318	0.94613	2.1
4319	1.32054	2.1
4320	1.41238	2.1
4321	1.94026	2.1
4322	1.0171	0.168417
4323	1.1211	0.0830759
4324	0.993022	2.1
4325	0.773813	2.1
4326	1.4073	2.1
4327	1.4073	2.1
4328	0.94613	2.1
4329	0.787615	2.1
4330	0.94613	2.1
4331	0.787615	2.1
4332	1.764	2.1
4333	1.90964	2.1
4334	1.05787	2.1
4335	1.403	0.0524976
4336	0.744189	0.21676
4337	2.32834	2.1
4338	2.34986	2.1
4339	1.17106	1.44077
4340	1.94826	0.05
4341	2.40606	0.05
4342	1.26387	0.05
4343	0.954013	0.05
4344	0.946131	0.05
4345	1.71	0.05
4346	1.51926	0.05
4347	0.953997	0.05

4348	0.851554	0.05
4349	1.96502	0.05
4350	1.72954	0.05
4351	2.89117	0.05
4352	0.971958	0.05
4353	0.985575	0.05
4354	1.41943	0.05
4355	1.20021	0.05
4356	1.29599	0.05
4357	1.15687	0.05
4358	0.810958	0.05
4359	1.12919	0.05
4360	1.98859	2.1
4361	1.82044	2.1
4362	1.90488	2.1
4363	0.851542	2.1
4364	1.02973	2.1
4365	1.39286	2.1
4366	1.44873	2.1
4367	0.946132	2.1
4368	1.11826	2.1
4369	1.26842	2.1
4370	2.09461	2.1
4371	1.76952	0.05
4372	1.6473	0.05
4373	0.946131	0.05
4374	1.36486	0.05
4375	1.04243	0.05
4376	1.05017	0.05
4377	1.24059	0.05
4378	2.11646	0.05
4379	1.50368	0.05
4380	2.38796	0.05
4381	1.50368	0.05
4382	1.71844	0.05
4383	1.71844	0.05
4384	0.914836	0.05
4385	1.24416	0.05
4386	0.914805	0.05
4387	1.24416	0.05
4388	1.38911	0.05
4389	1.46859	0.05
4390	1.35348	0.05
4391	0.946131	0.05
4392	1.1226	0.05
4393	0.857078	0.05
4394	0.9746	0.05
4395	1.37624	0.05
4396	1.15131	0.05
4397	1.139	0.05
4398	1.90456	0.05
4399	2.09692	0.05
4400	1.16655	2.1
4401	1.23017	2.1
4402	2.14592	2.1
4403	1.23017	2.1
4404	1.0752	2.1
4405	1.52482	0.0503068

4406	1.09252	2.1
4407	1.10725	2.1
4408	1.14305	0.05
4409	0.851541	0.05
4410	1.48073	0.05
4411	1.45607	0.05
4412	0.946131	0.05
4413	0.932823	0.05
4414	1.45828	0.05
4415	0.851528	0.05
4416	1.14305	0.05
4417	1.92737	0.05
4418	1.08211	0.05
4419	1.69831	0.05
4420	1.69831	0.05
4421	0.946131	0.05
4422	1.26868	0.05
4423	1.23377	0.05
4424	1.23377	0.05
4425	0.94723	0.05
4426	1.64322	0.05
4427	1.64322	0.05
4428	0.946131	0.05
4429	1.32068	0.05
4430	0.946129	0.05
4431	1.18789	0.05
4432	1.32068	0.05
4433	1.32813	2.1
4434	1.32461	2.1
4435	1.42729	0.05
4436	2.47846	0.05
4437	1.00024	0.05
4438	1.23923	0.05
4439	0.946131	0.05
4440	0.712304	0.05
4441	1.77501	0.05
4442	1.77501	0.05
4443	0.593593	0.05
4444	1.23923	0.05
4445	1.23175	0.05
4446	0.94613	0.05
4447	0.713646	0.05
4448	1.75735	0.05
4449	1.75735	0.05
4450	0.713633	0.05
4451	1.37157	0.05
4452	1.47169	0.05
4453	2.21808	0.05
4454	1.47169	0.05
4455	1.04759	2.1
4456	0.987132	2.1
4457	1.11181	2.1
4458	2.61682	2.07895
4459	1.11181	2.1
4460	1.06461	1.43454
4461	1.69733	2.1
4462	2.79705	2.1
4463	1.08245	2.1

4464	1.40583	2.1
4465	1.39852	2.1
4466	2.0239	2.1
4467	2.0239	2.1
4468	0.780455	2.1
4469	0.946132	2.1
4470	0.780455	2.1
4471	1.01885	2.1
4472	0.564217	2.1
4473	0.733461	2.1
4474	0.537126	2.1
4475	0.683838	2.1
4476	0.779662	2.1
4477	0.634141	2.1
4478	0.685337	2.1
4479	1.58848	2.1
4480	1.17129	0.05
4481	0.663168	0.05
4482	1.55587	0.05
4483	1.31342	0.05
4484	0.596868	0.05
4485	1.1666	0.05
4486	0.867261	0.05
4487	0.946132	0.05
4488	1.28843	0.05
4489	1.31248	0.05
4490	2.59921	0.05
4491	1.32634	0.05
4492	1.35564	0.05
4493	1.52661	0.05
4494	1.12323	0.05
4495	1.17701	0.689954
4496	1.36405	0.682051
4497	1.19837	0.681023
4498	2.25636	0.583635
4499	1.0823	0.05
4500	1.31774	0.05
4501	1.0823	0.05
4502	1.01121	0.05
4503	1.27408	0.05
4504	0.946131	0.05
4505	0.566074	0.05
4506	0.79576	0.05
4507	1.24596	0.05
4508	1.47567	0.05
4509	1.47567	0.05
4510	0.56605	0.05
4511	1.6567	0.05
4512	0.967792	0.05
4513	1.00366	0.05
4514	1.00366	0.05
4515	1.56409	0.05
4516	1.56409	0.05
4517	0.774911	0.05
4518	1.09483	0.05
4519	0.774912	0.05
4520	1.09483	0.05
4521	1.24417	0.05

4522	1.13387	0.05
4523	1.13387	0.05
4524	1.30572	0.05
4525	2.18966	0.05
4526	0.98575	0.05
4527	1.54982	0.05
4528	2.48833	0.05
4529	1.54982	0.05
4530	1.28954	0.05
4531	1.64002	0.05
4532	1.64002	0.05
4533	1.85594	0.05
4534	1.12968	2.1
4535	1.23536	2.1
4536	1.4882	2.1
4537	1.4882	2.1
4538	0.876836	2.1
4539	0.946131	2.1
4540	0.876835	2.1
4541	1.43166	2.1
4542	2.25936	2.1
4543	1.0305	2.1
4544	0.992095	2.1
4545	1.12968	2.1
4546	1.53813	2.1
4547	1.53812	2.1
4548	0.583653	2.1
4549	0.94613	2.1
4550	0.583651	2.1
4551	0.964033	2.1
4552	0.964034	2.1
4553	1.54457	2.1
4554	3.54109	0.05
4555	0.99974	0.05
4556	0.78814	0.05
4557	1.65729	0.05
4558	1.1283	0.05
4559	1.09407	0.05
4560	1.55091	0.05
4561	0.870773	0.05
4562	1.19967	0.05
4563	1.56521	0.05
4564	1.30896	1.44778
4565	1.17989	2.1
4566	1.72526	2.1
4567	0.851537	1.73332
4568	1.58944	2.1
4569	1.56915	2.1
4570	0.891039	1.60924
4571	1.4172	1.62642
4572	0.963818	1.42351
4573	0.851474	2.01053
4574	1.17989	2.1
4575	1.56261	2.1
4576	1.56261	2.1
4577	0.761574	2.1
4578	1.31323	2.1
4579	0.761573	2.1

4580	0.946133	2.1
4581	1.31323	2.1
4582	1.16759	2.1
4583	1.16759	2.1
4584	2.11829	2.1
4585	1.16759	2.1
4586	1.10748	0.05
4587	1.47688	0.05
4588	0.946133	0.05
4589	0.953319	0.05
4590	0.946132	0.05
4591	1.51168	0.05
4592	1.51168	0.05
4593	1.25797	0.05
4594	1.19984	0.05
4595	1.95729	2.1
4596	2.34853	2.1
4597	1.94343	2.1
4598	2.00551	2.1
4599	1.20067	0.957824
4600	1.87244	0.949474
4601	1.4178	2.1
4602	1.33538	2.1
4603	0.946131	2.1
4604	1.22895	2.1
4605	1.12433	2.1
4606	0.956757	2.1
4607	0.91826	2.1
4608	1.44221	2.1
4609	1.14206	2.1
4610	1.80514	0.332994
4611	0.890304	1.47035
4612	1.1362	0.265673
4613	1.32819	0.29768
4614	1.46311	2.07871
4615	0.8654	0.321394
4616	1.1699	1.14494
4617	1.00267	1.1883
4618	1.02882	0.322926
4619	1.00832	2.1
4620	0.974137	2.1
4621	1.02368	2.1
4622	0.974138	2.1
4623	0.995734	0.0667639
4624	1.44605	0.05
4625	1.18549	0.05
4626	1.33621	0.18173
4627	1.5176	0.203368
4628	1.91762	0.264203
4629	1.33621	0.152662
4630	1.6638	0.191341
4631	1.66774	2.1
4632	1.11833	2.1
4633	0.926499	2.1
4634	1.51229	2.1
4635	1.51229	2.1
4636	0.926528	2.1
4637	1.40335	2.1

4638	1.03544	2.1
4639	0.926527	2.1
4640	1.85305	2.1
4641	1.5812	2.1
4642	1.15529	2.1
4643	1.31086	2.1
4644	0.999206	2.1
4645	1.46714	2.1
4646	1.73087	2.1
4647	0.772299	2.1
4648	1.24341	2.1
4649	0.997867	2.1
4650	0.772311	2.1
4651	0.936657	2.1
4652	1.35986	2.1
4653	0.959414	2.1
4654	1.36541	2.1
4655	1.49268	2.1
4656	1.49268	2.1
4657	0.832137	2.1
4658	0.946132	2.1
4659	0.832138	2.1
4660	1.64523	2.1
4661	1.11764	2.1
4662	1.05776	2.1
4663	0.972465	2.1
4664	0.972464	2.1
4665	1.98425	2.1
4666	0.969822	2.1
4667	1.30566	2.1
4668	1.52724	2.1
4669	2.32237	0.0508409
4670	2.30274	0.0709454
4671	2.27761	0.05
4672	3.33012	0.05
4673	1.24001	0.628122
4674	1.41821	1.24902
4675	0.978584	2.1
4676	1.18076	2.1
4677	1.07645	2.1
4678	0.877351	0.0521233
4679	0.946132	1.36816
4680	0.877353	0.106776
4681	1.58444	2.1
4682	1.49614	2.1
4683	1.28355	2.02456
4684	1.13491	1.67297
4685	1.56875	0.0515841
4686	0.499592	0.05
4687	0.946131	0.05
4688	1.20538	0.05
4689	1.09913	0.05
4690	1.8049	0.05
4691	1.8049	0.05
4692	0.499606	0.05
4693	2.41076	0.05
4694	0.860202	0.05
4695	0.906798	0.05

4696	1.8626	0.05
4697	1.33986	0.05
4698	1.2992	0.05
4699	1.58702	0.05
4700	1.05939	0.05
4701	1.02074	2.1
4702	1.07866	2.1
4703	1.5082	0.05
4704	1.5522	0.05
4705	1.65903	0.05
4706	1.86766	2.1
4707	2.11684	2.1
4708	0.946132	2.1
4709	1.6585	2.1
4710	1.6585	2.1
4711	0.94613	2.1
4712	1.1872	2.1
4713	1.1872	2.1
4714	1.05842	2.1
4715	1.20455	2.1
4716	1.34141	2.1
4717	1.34141	2.1
4718	1.42416	2.1
4719	1.75818	2.1
4720	2.63715	1.03152
4721	1.80454	2.1
4722	2.26198	2.1
4723	0.696255	2.1
4724	0.624075	2.1
4725	1.50905	0.300259
4726	1.50905	2.1
4727	0.624075	2.1
4728	1.31857	0.186161
4729	0.946131	2.04363
4730	0.624075	0.98494
4731	0.914628	2.1
4732	1.14721	2.1
4733	1.16153	2.1
4734	1.47664	2.1
4735	1.54791	2.1
4736	1.19098	2.1
4737	1.13341	2.1
4738	1.00503	0.0733764
4739	1.00503	1.04621
4740	1.22655	2.1
4741	0.999489	2.1
4742	1.3268	2.1
4743	0.946132	2.1
4744	1.60061	2.1
4745	1.60061	2.1
4746	1.08034	2.1
4747	1.40372	2.1
4748	1.14302	2.1
4749	1.08034	2.1
4750	0.999491	2.1
4751	1.17152	0.05
4752	1.17152	0.05
4753	1.17152	0.05

4754	2.05423	0.05
4755	1.40984	2.09513
4756	1.2489	2.1
4757	0.885089	2.1
4758	0.810931	2.1
4759	1.84128	2.1
4760	1.11206	2.1
4761	1.21926	2.1
4762	1.99084	2.1
4763	0.976334	0.385619
4764	0.599363	0.154788
4765	1.02621	0.101487
4766	0.462959	0.260255
4767	1.42612	0.0878512
4768	0.299682	0.0837304
4769	1.51379	2.1
4770	0.719531	2.1
4771	1.3702	2.1
4772	1.33293	2.1
4773	0.851539	2.1
4774	0.62998	2.1
4775	0.851543	2.1
4776	0.629966	2.1
4777	1.43132	2.1
4778	1.13328	1.05018
4779	1.93212	0.660619
4780	1.62345	0.935615
4781	1.38511	1.23536
4782	1.40013	0.3589
4783	1.04132	0.172248
4784	0.83473	0.983829
4785	0.868732	0.751898
4786	0.810595	1.59691
4787	0.729919	0.629133
4788	0.992428	0.715441
4789	0.577107	0.23735
4790	1.18464	0.903441
4791	0.791199	0.74936
4792	0.810943	0.05
4793	0.860723	0.05
4794	1.33348	0.05
4795	1.57467	0.05
4796	0.981032	0.05
4797	0.731569	0.05
4798	0.770312	0.05
4799	1.46055	2.1
4800	1.42249	2.1
4801	1.75999	0.05
4802	1.6868	0.05
4803	1.17042	0.419444
4804	1.01485	2.1
4805	1.19952	0.05
4806	1.40592	0.0967071
4807	1.19952	0.0644905
4808	2.20145	0.05
4809	1.33649	0.05
4810	1.11612	0.05
4811	0.957588	0.05

4812	0.946126	0.0523662
4813	1.50648	0.05
4814	1.50648	0.05
4815	0.946128	0.05
4816	2.10671	0.05
4817	1.91518	0.05
4818	2.23223	0.05
4819	1.53556	2.1
4820	1.56253	2.1
4821	1.27806	0.05
4822	1.44593	0.05
4823	1.03818	0.0733849
4824	1.03818	0.0563328
4825	1.38969	0.398717
4826	1.85548	0.186436
4827	2.6044	0.352113
4828	1.85548	0.140674
4829	1.49346	1.97732
4830	1.83192	1.85068
4831	0.834043	2.04504
4832	0.810943	0.673188
4833	0.887113	0.965812
4834	0.893827	0.862699
4835	0.991061	0.989558
4836	0.91939	1.14829
4837	0.793307	1.16217
4838	1.75291	1.48092
4839	1.35435	1.15232
4840	1.16989	0.0749437
4841	1.36717	0.0546985
4842	2.12276	0.05
4843	2.30493	0.05
4844	2.47857	0.0744039
4845	1.92269	0.0577694
4846	1.66426	0.0575921
4847	0.956698	1.20554
4848	0.956698	2.1
4849	0.857957	2.1
4850	1.4706	2.1
4851	1.65706	2.1
4852	1.47059	2.1
4853	1.04713	0.05
4854	0.946131	0.05
4855	1.47374	0.0637014
4856	1.47374	0.05
4857	0.951675	0.05
4858	1.2582	0.0613043
4859	1.2582	0.05
4860	0.94613	0.0550756
4861	1.4468	0.05
4862	1.90335	0.05
4863	1.14886	2.1
4864	2.59689	2.1
4865	1.02747	2.1
4866	0.946131	2.1
4867	0.934061	2.1
4868	1.63979	2.1
4869	1.65206	2.1

4870	1.48	2.1
4871	1.27467	2.1
4872	2.20449	2.1
4873	1.1333	0.05
4874	1.95334	2.1
4875	2.20984	2.1
4876	1.46389	0.05
4877	1.4639	0.05
4878	1.4639	0.05
4879	0.946132	0.05
4880	1.5144	0.05
4881	1.5144	0.05
4882	0.946132	0.05
4883	1.35732	0.05
4884	1.10321	0.05
4885	0.946132	0.05
4886	0.980549	0.05
4887	1.32819	2.1
4888	1.30171	1.11848
4889	1.68377	0.775862
4890	1.68377	2.1
4891	0.946132	0.478283
4892	0.946132	2.04501
4893	1.1952	1.51105
4894	0.990019	1.36284
4895	1.08195	1.80142
4896	1.2269	2.1
4897	1.19219	2.1
4898	1.01485	2.1
4899	1.57125	0.05
4900	2.10696	0.05
4901	1.29666	0.05
4902	1.57125	0.05
4903	1.47712	2.1
4904	1.45182	2.1
4905	1.17816	0.05
4906	1.4516	0.05
4907	1.17816	0.05
4908	1.17816	0.05
4909	0.596954	1.17891
4910	1.65133	0.286239
4911	0.583859	0.243254
4912	1.04072	1.08942
4913	1.28795	0.323096
4914	1.59423	0.282577
4915	0.483559	0.418682
4916	1.74088	0.197816
4917	1.09545	0.872531
4918	1.02519	1.70434
4919	2.47579	0.676289
4920	1.54732	0.942657
4921	2.17663	0.643815
4922	1.75006	0.647869
4923	1.32354	1.08247
4924	1.09962	2.1
4925	1.85057	2.1
4926	1.91046	2.1
4927	2.17037	2.1

4928	1.99528	0.05
4929	1.97969	0.05
4930	3.82765	2.1
4931	1.85767	2.1
4932	2.77042	2.1
4933	1.37236	0.05
4934	2.23254	0.05
4935	1.10089	0.05
4936	0.994294	2.1
4937	1.81921	2.1
4938	1.00073	2.1
4939	0.681043	2.1
4940	0.946131	2.1
4941	0.681073	2.1
4942	1.51804	2.1
4943	1.60653	2.1
4944	1.30188	2.1
4945	0.919622	2.1
4946	1.00965	2.1
4947	1.93821	2.1
4948	2.60377	2.1
4949	1.36416	0.05
4950	1.36416	0.05
4951	1.36416	0.05
4952	2.44132	0.05
4953	2.15871	0.195246
4954	2.22171	0.0574773
4955	1.70747	0.0713326
4956	1.49515	0.0653127
4957	0.773007	0.0899307
4958	0.946125	0.10483
4959	0.946131	0.170751
4960	0.773034	0.05
4961	1.15844	0.0720606
4962	1.98104	0.0600396
4963	2.44283	0.0864381
4964	1.05417	0.05
4965	1.1596	0.05
4966	0.829921	0.05
4967	0.946131	0.05
4968	0.951911	0.05
4969	1.39027	0.05
4970	0.829946	0.05
4971	1.51223	0.05
4972	1.51223	0.05
4973	1.65984	0.05
4974	1.90382	0.05
4975	1.93228	1.06445
4976	2.47924	1.1199
4977	1.73904	1.04687
4978	1.40547	0.392861
4979	2.37589	0.611665
4980	0.662605	1.1218
4981	2.33293	0.660513
4982	1.72249	2.1
4983	1.68478	2.1
4984	1.00514	0.05
4985	1.4537	2.1

4986	1.92698	2.1
4987	1.46041	0.05
4988	2.04817	0.05
4989	1.65954	2.1
4990	1.42342	2.1
4991	1.04405	0.05
4992	1.17056	0.05
4993	1.11339	0.05
4994	0.946133	0.05
4995	0.929724	0.05
4996	1.35424	0.05
4997	1.35424	0.05
4998	0.929713	0.05
4999	1.85945	0.05
5000	1.38905	2.1
5001	1.39056	2.1
5002	1.52852	2.1
5003	1.06472	2.1
5004	0.749096	2.1
5005	1.46076	2.1
5006	1.29263	2.1
5007	0.94613	2.1
5008	0.69097	2.1
5009	0.982284	2.1
5010	0.67419	2.1
5011	1.52852	2.1
5012	1.91121	2.1
5013	1.00775	2.1
5014	1.61201	2.1
5015	0.529987	2.1
5016	0.506125	2.1
5017	1.44696	2.1
5018	2.32981	2.1
5019	1.32784	2.1
5020	1.94158	2.1
5021	1.31595	2.1
5022	0.663922	2.1
5023	1.44663	2.1
5024	1.44663	2.1
5025	1.13976	2.1
5026	0.970788	2.1
5027	1.13976	2.1
5028	0.663921	2.1
5029	0.966114	2.1
5030	1.05498	0.864715
5031	1.38086	2.1
5032	0.946127	2.1
5033	0.922513	2.1
5034	1.49903	2.1
5035	1.49903	2.1
5036	0.92251	2.1
5037	1.06105	2.1
5038	1.36049	2.1
5039	1.38086	2.1
5040	1.52534	2.1
5041	1.04101	2.1
5042	1.30861	0.05
5043	0.949112	0.05

5044	0.894275	0.05
5045	1.10232	0.05
5046	0.830889	0.05
5047	1.26554	0.05
5048	0.948347	0.05
5049	0.81096	0.05
5050	1.18338	0.05
5051	0.883764	0.05
5052	1.54793	0.05
5053	1.02867	0.05
5054	1.8573	0.05
5055	1.06065	0.05
5056	0.64842	0.05
5057	1.99319	0.05
5058	1.9476	0.05
5059	2.51436	0.05
5060	1.68337	0.05
5061	1.92321	0.05
5062	0.810942	0.05
5063	0.921001	0.05
5064	0.99758	0.05
5065	0.960785	0.05
5066	1.01565	0.05
5067	0.908624	0.05
5068	0.912835	0.05
5069	1.18351	0.05
5070	1.40239	2.1
5071	1.26091	2.1
5072	0.947979	2.1
5073	0.946131	2.1
5074	1.81959	2.1
5075	1.81959	1.55193
5076	0.94613	2.1
5077	1.38415	1.02794
5078	1.38414	2.1
5079	1.33266	2.1
5080	0.946132	2.1
5081	1.76225	2.1
5082	1.76225	2.1
5083	1.27599	2.1
5084	1.38158	2.1
5085	1.3268	2.1
5086	1.27599	2.1
5087	0.947979	2.1
5088	1.0142	0.0642017
5089	1.01422	0.0600578
5090	1.11435	0.492772
5091	1.42186	0.496347
5092	1.24001	1.18863
5093	1.77342	1.31859
5094	1.42186	1.48262
5095	1.99817	0.897029
5096	1.27361	2.1
5097	1.68914	2.1
5098	1.30116	2.1
5099	1.32819	1.63317
5100	1.68322	1.62938
5101	1.68322	2.1

5102	0.94613	0.441524
5103	0.94613	2.1
5104	1.14666	2.04467
5105	1.07612	1.50866
5106	1.31151	2.1
5107	1.22095	2.08887
5108	1.57256	2.1
5109	1.6679	2.1
5110	0.946132	1.47602
5111	0.946133	2.1
5112	1.13537	2.1
5113	1.07855	2.1
5114	1.07855	2.1
5115	1.43895	1.05702
5116	1.38481	0.05
5117	1.72385	0.05
5118	0.550441	0.05
5119	0.79807	0.05
5120	1.11627	0.05
5121	1.30349	0.05
5122	0.94613	0.05
5123	0.686176	0.05
5124	1.11627	0.05
5125	1.73363	0.05
5126	1.73363	0.05
5127	0.686139	0.05
5128	1.24932	0.05
5129	0.899582	0.05
5130	2.00472	0.05
5131	1.17121	0.05
5132	1.2874	0.05
5133	2.0194	0.05
5134	0.88601	0.05
5135	1.77409	0.05
5136	1.73293	0.05
5137	2.74045	0.05
5138	1.83787	0.05
5139	1.45448	0.05
5140	1.27778	0.05
5141	1.1192	2.1
5142	1.25953	2.1
5143	1.93611	2.1
5144	1.92104	2.1
5145	1.15506	0.05
5146	1.15506	0.05
5147	1.23943	0.307184
5148	0.851543	2.1
5149	1.6061	2.1
5150	1.80058	2.1
5151	0.869471	2.1
5152	1.3931	2.1
5153	1.06454	2.1
5154	0.93667	2.1
5155	2.16916	2.1
5156	3.10246	2.1
5157	1.91539	2.1
5158	1.91539	2.1
5159	0.620263	1.93955

5160	0.620265	2.1
5161	0.946132	1.98608
5162	0.620265	1.78662
5163	1.30226	1.50517
5164	1.87292	2.1
5165	1.18769	2.1
5166	2.60452	2.1
5167	1.26574	2.1
5168	2.46679	2.1
5169	1.36459	2.1
5170	1.26865	0.815422
5171	1.1744	0.27602
5172	1.15841	0.0825591
5173	1.02247	0.05
5174	1.93649	2.1
5175	1.64718	2.1
5176	1.59654	2.1
5177	1.57823	2.1
5178	1.00665	0.05
5179	1.28239	0.05
5180	0.887895	2.1
5181	1.22083	2.1
5182	1.42127	2.1
5183	1.07374	2.1
5184	1.00503	1.35901
5185	1.23082	0.502606
5186	2.78663	2.1
5187	2.20883	2.1
5188	2.54588	2.1
5189	0.946128	2.1
5190	1.13716	2.1
5191	1.58724	2.1
5192	1.58724	2.1
5193	0.946131	2.1
5194	1.13716	2.1
5195	1.39621	2.1
5196	1.02438	2.1
5197	1.49543	0.05
5198	2.78336	0.05
5199	1.27148	0.05
5200	1.0906	0.05
5201	1.04072	0.05
5202	0.63574	0.05
5203	1.45506	0.05
5204	1.5863	0.05
5205	0.928352	0.05
5206	0.647687	0.05
5207	2.2375	1.85044
5208	1.50998	1.62606
5209	2.75021	1.43445
5210	1.91912	1.25585
5211	1.99872	1.07479
5212	0.918127	2.00249
5213	1.03031	0.989234
5214	0.951169	0.705622
5215	1.27975	0.688382
5216	1.12735	1.05071
5217	1.15741	0.9083

5218	1.24998	0.857301
5219	1.65953	0.05
5220	0.967555	0.242876
5221	0.967542	0.05
5222	1.03824	0.0638481
5223	1.48693	0.075514
5224	1.48693	0.0625668
5225	2.02901	0.0674693
5226	0.946127	0.108928
5227	1.07139	0.083107
5228	0.946126	0.0716612
5229	1.84644	0.05
5230	1.84644	0.05
5231	1.38683	0.0531926
5232	1.40574	0.05
5233	1.36866	1.08064
5234	0.649325	1.15362
5235	1.93631	2.1
5236	1.50145	2.1
5237	1.6287	2.1
5238	1.31302	2.1
5239	1.18041	2.1
5240	1.18041	2.1
5241	0.974196	2.1
5242	0.946131	2.1
5243	1.74169	2.1
5244	1.74169	2.1
5245	1.23163	2.1
5246	1.27451	2.1
5247	1.41331	2.1
5248	1.23163	2.1
5249	1.28037	2.1
5250	1.28158	2.1
5251	1.28037	2.1
5252	2.28015	0.05
5253	2.06561	0.05
5254	2.61343	0.05
5255	1.11712	0.05
5256	1.63689	1.25908
5257	1.01393	0.716342
5258	0.996775	2.1
5259	1.54277	2.1
5260	1.26397	2.1
5261	1.46129	2.1
5262	1.62391	2.1
5263	1.08848	0.05
5264	1.11833	2.1
5265	1.24041	2.1
5266	2.31884	2.1
5267	1.335 2.1	
5268	1.48589	0.05
5269	1.77131	0.05
5270	1.8422	0.730753
5271	2.47453	1.74025
5272	1.23726	1.34148
5273	1.21996	1.26604
5274	1.65512	1.44134
5275	1.65512	1.28422

5276	0.802107	1.86881
5277	0.946129	0.953771
5278	0.802137	0.898015
5279	1.3279	0.844855
5280	1.23726	0.776932
5281	1.79759	0.79872
5282	1.79759	0.629983
5283	0.767577	1.5478
5284	0.946131	0.605156
5285	0.767577	0.513942
5286	1.03534	0.05
5287	1.09962	0.0658294
5288	0.929217	1.67449
5289	1.70126	1.73566
5290	0.991407	2.1
5291	1.41077	2.1
5292	0.991406	2.1
5293	1.62966	2.1
5294	1.62966	2.1
5295	0.946128	2.1
5296	1.18364	2.1
5297	0.946131	2.1
5298	1.14186	2.1
5299	1.18364	2.1
5300	1.16858	2.1
5301	1.66137	0.111498
5302	1.90165	0.05
5303	1.66139	0.05
5304	1.26893	0.0966248
5305	1.15918	0.337734
5306	0.653228	0.650013
5307	0.946133	0.659785
5308	1.77488	1.99399
5309	1.77489	0.975796
5310	0.653228	1.06524
5311	1.56043	0.319219
5312	1.60894	1.08027
5313	1.56043	0.30595
5314	1.06848	1.35073
5315	1.04307	2.1
5316	1.14188	2.1
5317	1.09195	2.1
5318	0.615121	2.1
5319	1.50867	2.1
5320	1.27152	2.1
5321	0.615094	2.1
5322	1.07297	2.1
5323	1.1372	2.1
5324	0.553608	2.1
5325	1.23019	2.1
5326	0.992675	2.1
5327	1.40984	1.68221
5328	2.55682	2.1
5329	3.66069	2.1
5330	3.58726	2.1
5331	1.4375	0.05
5332	1.88424	0.05
5333	1.56714	2.1

5334	1.6721	2.1
5335	1.18474	2.1
5336	1.2295	2.1
5337	1.28754	2.1
5338	1.36838	2.1
5339	2.78661	2.1
5340	2.64055	2.1
5341	1.35065	0.945194
5342	2.26675	0.223265
5343	1.35065	0.553755
5344	1.21558	0.281653
5345	1.49709	0.05
5346	1.4971	0.05
5347	0.85472	0.05
5348	0.854718	0.05
5349	0.946129	0.05
5350	0.854717	0.05
5351	1.1604	0.05
5352	1.25265	0.05
5353	2.32081	0.05
5354	1.15371	0.05
5355	1.14945	0.440005
5356	0.995725	0.340931
5357	1.00307	0.05
5358	1.10907	0.05
5359	2.30315	0.05
5360	1.10908	0.05
5361	1.27558	0.641177
5362	1.71613	1.55305
5363	1.32977	2.1
5364	0.815276	2.1
5365	1.41697	2.1
5366	1.41697	2.1
5367	0.815276	2.1
5368	1.38897	2.1
5369	0.946131	2.1
5370	0.815275	2.1
5371	1.44684	2.1
5372	1.21489	1.81488
5373	1.22476	1.757
5374	0.870366	0.384066
5375	0.862572	0.234561
5376	0.835435	0.164488
5377	1.6993	0.0970209
5378	1.23618	0.0757197
5379	1.18624	0.152779
5380	1.53058	0.113367
5381	2.16108	0.663831
5382	2.21589	0.489293
5383	1.04037	2.1
5384	1.03933	2.1
5385	1.57753	0.05
5386	1.87644	0.05
5387	1.24728	0.05
5388	1.30394	0.05
5389	1.3538	2.1
5390	1.69858	2.1
5391	1.1726	0.126721

5392	0.94612	0.0686914
5393	1.09805	0.05
5394	0.946118	0.0647172
5395	1.72576	0.0666539
5396	1.72575	0.05
5397	1.34152	0.0544054
5398	1.33034	0.05
5399	0.784151	2.1
5400	0.946131	2.1
5401	0.784151	2.1
5402	1.47963	2.1
5403	1.47963	2.1
5404	1.3335	2.1
5405	0.930281	2.1
5406	1.29435	2.1
5407	0.883308	2.00304
5408	1.31589	2.1
5409	1.67846	2.1
5410	0.993598	0.05
5411	2.23193	0.05
5412	0.9936	0.05
5413	0.615629	0.05
5414	1.65925	2.1
5415	2.05992	2.1
5416	1.65925	2.1
5417	4.08735	1.7277
5418	2.72856	1.792
5419	0.869602	2.1
5420	3.42833	0.907132
5421	1.14397	0.05
5422	1.34563	0.0724845
5423	1.08189	0.0724487
5424	0.782128	2.03595
5425	1.0022	2.1
5426	0.900427	1.94737
5427	1.51499	2.00818
5428	1.05588	1.98887
5429	1.13739	2.1
5430	1.37004	2.02785
5431	1.14603	2.1
5432	0.851541	1.15522
5433	0.946799	1.87789
5434	1.14854	1.73729
5435	1.33793	1.3474
5436	0.94613	1.27093
5437	1.24336	1.7711
5438	0.851473	1.82375
5439	2.08792	1.72584
5440	1.01869	1.73722
5441	0.986953	0.05
5442	0.896238	0.05
5443	0.906574	0.05
5444	0.852145	0.05
5445	2.14191	0.05
5446	1.15979	0.05
5447	1.18327	0.05
5448	2.32742	0.05
5449	2.35961	0.05

5450	2.38126	0.05
5451	1.13632	0.05
5452	1.17897	0.05
5453	1.76199	0.05
5454	1.00573	2.1
5455	0.946127	0.126153
5456	1.7077	2.1
5457	1.7077	2.1
5458	0.946127	1.83815
5459	1.24887	2.1
5460	1.24887	2.1
5461	1.22441	2.1
5462	1.08542	2.1
5463	1.21743	1.96413
5464	1.12572	2.1
5465	1.05499	0.307738
5466	1.00521	0.365724
5467	2.86191	0.05
5468	2.27972	0.05
5469	1.01169	2.1
5470	1.03373	2.1
5471	1.39319	2.1
5472	0.636837	2.1
5473	1.63908	2.1
5474	1.45713	1.98295
5475	1.04072	2.1
5476	1.25025	2.02538
5477	1.38914	2.1
5478	0.636807	2.1
5479	1.50211	2.1
5480	1.36555	2.1
5481	2.77545	1.70943
5482	0.682805	2.1
5483	1.50267	2.1
5484	1.50267	2.1
5485	0.797749	2.1
5486	1.38773	2.07213
5487	1.38773	2.1
5488	0.682776	2.1
5489	2.01245	0.05
5490	0.925918	0.05
5491	0.75689	0.05
5492	1.25823	0.05
5493	1.16505	0.05
5494	0.838779	0.05
5495	1.14722	0.05
5496	0.756849	0.05
5497	1.52298	0.05
5498	2.11034	0.05
5499	1.92174	0.05
5500	1.92174	0.05
5501	0.576083	0.05
5502	1.23146	0.05
5503	1.26637	0.05
5504	1.23146	0.05
5505	1.26637	0.05
5506	0.946133	0.258814
5507	1.37034	0.126485

5508	1.84145	0.158246
5509	1.84145	0.081477
5510	0.946132	0.391524
5511	1.37034	0.189507
5512	1.41724	0.188626
5513	1.0685	0.0846358
5514	1.14547	0.093178
5515	1.24976	2.1
5516	1.30682	2.1
5517	2.12095	2.1
5518	1.46392	2.1
5519	1.46392	2.1
5520	0.935738	2.1
5521	1.32949	2.1
5522	1.07017	2.1
5523	0.935766	2.1
5524	1.07017	2.1
5525	1.78095	2.1
5526	1.05619	0.209761
5527	1.05793	0.410374
5528	1.77677	2.1
5529	2.04766	2.1
5530	1.02524	2.1
5531	1.18803	2.1
5532	1.70047	2.1
5533	1.556 2.1	
5534	1.24201	1.94393
5535	1.3592	2.1
5536	2.11824	0.05
5537	2.20248	0.05
5538	3.43878	0.05
5539	3.12466	0.05
5540	2.09351	2.1
5541	0.946128	2.1
5542	1.51269	2.1
5543	1.51269	2.1
5544	0.946131	2.1
5545	1.01306	2.1
5546	1.04676	2.1
5547	1.01306	2.1
5548	1.07444	2.1
5549	2.17465	2.1
5550	1.14808	2.1
5551	1.39067	1.51496
5552	1.20958	1.18237
5553	0.662832	0.329429
5554	1.93512	0.819392
5555	1.15471	2.1
5556	1.75119	2.1
5557	1.75118	2.1
5558	0.946131	2.1
5559	1.28606	2.1
5560	1.27087	2.1
5561	0.946131	2.1
5562	1.28606	2.1
5563	1.58916	0.05
5564	0.927822	0.05
5565	2.24367	0.05

5566	1.8734	0.05
5567	3.51668	0.05
5568	2.68986	0.05
5569	2.32395	2.1
5570	1.39564	2.1
5571	2.10862	2.1
5572	1.36763	2.1
5573	0.970963	0.05
5574	0.97096	0.05
5575	1.11897	0.05
5576	1.26885	0.05
5577	1.11605	0.05
5578	2.06612	0.05
5579	1.717 0.05	
5580	0.59502	0.05
5581	0.825755	0.05
5582	0.709624	0.05
5583	1.65741	0.05
5584	0.307486	0.05
5585	1.4785	0.05
5586	0.983143	2.1
5587	1.08247	2.1
5588	1.42353	2.1
5589	1.42353	2.1
5590	0.781667	2.1
5591	0.797634	2.1
5592	0.946131	2.1
5593	0.781638	2.1
5594	0.946131	2.1
5595	0.98315	2.1
5596	1.083 2.1	
5597	1.61975	0.05
5598	1.11946	0.05
5599	1.18977	0.05
5600	0.996484	0.05
5601	2.24343	0.05
5602	1.4787	0.05
5603	1.66127	0.05
5604	1.86028	0.05
5605	1.09128	0.05
5606	1.03115	0.05
5607	0.838025	0.05
5608	1.3736	0.05
5609	1.44009	0.05
5610	0.92917	0.05
5611	1.03889	0.05
5612	0.956974	0.05
5613	3.23468	0.05
5614	0.953922	0.05
5615	0.957104	0.05
5616	1.66231	0.05
5617	1.26778	0.05
5618	1.47615	0.05
5619	1.28621	0.05
5620	1.08043	0.05
5621	2.35026	2.1
5622	2.35025	2.1
5623	2.60659	2.1

5624	1.57627	2.1
5625	1.5493	2.1
5626	1.24682	0.05
5627	2.14277	0.0993962
5628	1.24682	0.1817
5629	1.00307	0.05
5630	2.04056	0.0817304
5631	1.45408	2.1
5632	1.54249	2.1
5633	1.24474	1.59703
5634	1.37813	1.82841
5635	0.924158	0.05
5636	0.916623	0.05
5637	1.35344	0.05
5638	1.00859	0.05
5639	1.34786	0.05
5640	0.952457	0.05
5641	0.85404	0.05
5642	1.06341	2.1
5643	1.09194	2.1
5644	1.93707	2.1
5645	2.14087	2.1
5646	1.93707	2.1
5647	1.07519	2.1
5648	1.07519	1.38348
5649	1.62518	2.1
5650	1.3592	2.1
5651	1.13183	2.1
5652	1.20357	2.1
5653	1.4858	2.1
5654	1.23694	2.1
5655	1.44011	2.1
5656	1.23989	2.1
5657	2.21604	2.1
5658	1.85574	0.05
5659	1.55649	2.1
5660	1.48861	0.05
5661	1.15968	0.05
5662	0.946132	0.05
5663	0.663056	0.05
5664	1.70837	0.05
5665	1.70837	0.05
5666	0.946131	0.05
5667	0.663026	0.05
5668	2.09604	0.05
5669	0.81094	0.05
5670	1.06559	0.05
5671	0.810956	0.05
5672	1.15474	0.05
5673	0.990051	0.05
5674	0.894971	0.05
5675	1.32536	0.05
5676	0.946133	2.1
5677	1.33125	2.1
5678	1.75823	2.1
5679	1.75823	2.1
5680	0.946133	2.1
5681	1.33125	2.1

5682	1.37312	2.1
5683	1.27985	2.1
5684	1.37312	2.1
5685	1.70684	2.1
5686	1.70684	2.1
5687	0.946132	2.1
5688	0.946132	2.1
5689	1.25081	2.1
5690	0.956293	2.1
5691	0.956294	2.1
5692	1.36035	2.1
5693	1.41944	2.1
5694	0.830638	2.1
5695	0.747568	2.1
5696	0.888498	2.1
5697	1.04071	2.1
5698	1.17128	2.1
5699	1.92339	2.1
5700	2.91208	2.1
5701	1.82208	0.827691
5702	1.89653	2.0738
5703	1.82588	1.36274
5704	1.26703	0.165869
5705	1.26703	0.860735
5706	0.6157	0.142161
5707	1.26706	1.46302
5708	0.61567	0.05
5709	0.615671	0.05
5710	1.26706	0.283033
5711	1.89158	0.934147
5712	1.23134	0.065352
5713	2.49497	0.05
5714	1.68103	0.05
5715	2.0554	2.1
5716	1.72647	2.1
5717	1.67375	2.1
5718	1.65037	2.1
5719	1.04349	0.05
5720	1.69055	0.05
5721	1.53463	0.05
5722	1.19022	2.06453
5723	1.89226	1.02004
5724	1.15009	0.416808
5725	1.7054	0.0702548
5726	1.31612	0.05
5727	1.31611	0.0535185
5728	1.67345	0.0582324
5729	1.06577	0.05
5730	1.40193	0.05
5731	1.72411	0.05
5732	1.46123	0.05
5733	0.808808	0.05
5734	0.897714	0.05
5735	0.851554	0.05
5736	1.3499	0.05
5737	1.29001	0.05
5738	1.22083	2.1
5739	1.06191	2.1

5740	1.05758	1.33328
5741	1.348	1.97612
5742	1.43485	1.22463
5743	1.34799	1.80771
5744	1.36307	0.05
5745	2.28188	0.05
5746	1.7926	0.05
5747	1.38957	2.1
5748	1.47446	2.1
5749	0.953638	2.1
5750	1.11868	2.1
5751	0.953636	2.1
5752	1.03408	2.1
5753	1.52443	0.05
5754	1.38539	0.05
5755	0.946132	0.05
5756	1.12747	0.05
5757	1.04393	0.05
5758	1.05017	0.05
5759	1.20529	0.05
5760	1.3663	0.117975
5761	1.89226	0.106896
5762	1.17885	0.221521
5763	0.867881	0.0708138
5764	1.32917	0.0554147
5765	1.32917	0.0810334
5766	0.946127	0.0939455
5767	1.19583	0.0835872
5768	1.00122	0.11485
5769	0.946128	0.11367
5770	1.71405	0.314097
5771	0.871815	2.1
5772	1.16435	2.1
5773	0.686619	2.1
5774	1.63102	2.1
5775	1.06589	2.1
5776	1.05375	2.1
5777	1.9023	2.1
5778	1.27394	0.515217
5779	1.88345	0.557729
5780	2.05636	0.729684
5781	0.672977	0.264615
5782	1.41274	0.344812
5783	1.65606	0.269372
5784	0.869329	0.29176
5785	1.2207	0.414759
5786	1.35631	0.358
5787	0.908776	0.297384
5788	0.955081	0.107582
5789	1.2243	2.1
5790	1.23169	2.1
5791	1.52228	2.1
5792	1.23169	2.1
5793	0.990892	1.34307
5794	1.08376	0.449845
5795	1.79348	2.08142
5796	1.50043	0.843508
5797	1.32599	2.1

5798	1.37924	1.08182
5799	0.838014	0.519415
5800	0.87737	1.70028
5801	0.947086	0.770619
5802	1.27199	1.83402
5803	1.04918	0.88342
5804	1.04851	2.06181
5805	1.1699	1.47198
5806	0.810934	1.12518
5807	1.83602	2.1
5808	2.21909	2.1
5809	0.619441	1.54007
5810	2.03755	0.816214
5811	1.83472	1.94813
5812	1.20416	1.6608
5813	1.26691	0.613964
5814	0.655795	0.694131
5815	0.94613	1.34392
5816	2.35169	1.62344
5817	3.54705	2.1
5818	2.60741	2.1
5819	1.59019	0.05
5820	1.24526	0.05
5821	1.59019	0.05
5822	2.36406	0.05
5823	1.16776	1.08365
5824	1.67311	1.34053
5825	1.67311	0.404574
5826	0.94613	0.16949
5827	1.38645	1.18379
5828	1.13563	0.05
5829	1.13563	0.234802
5830	1.23279	0.0638167
5831	1.05042	2.1
5832	1.17418	2.1
5833	1.28909	2.1
5834	0.973448	2.1
5835	1.02958	2.1
5836	3.3005	2.1
5837	2.86325	2.1
5838	1.35879	2.1
5839	1.69332	2.1
5840	1.69332	2.1
5841	0.946128	2.1
5842	1.39659	2.1
5843	1.12035	2.1
5844	1.12035	2.1
5845	1.24286	2.1
5846	1.10407	2.1
5847	1.14599	2.1
5848	1.32259	0.365725
5849	0.788592	0.05
5850	2.4588	2.1
5851	0.909581	2.06949
5852	1.35656	1.5158
5853	0.986895	1.84679
5854	2.30602	1.15626
5855	1.46873	1.73717

5856	1.55584	1.18913
5857	2.1547	1.55095
5858	1.87794	1.99542
5859	0.808286	2.04689
5860	0.928464	1.85295
5861	0.810959	1.72788
5862	1.50962	0.854751
5863	1.1053	1.5295
5864	1.0712	1.49167
5865	1.40504	1.45118
5866	1.87794	1.09388
5867	2.80496	1.41731
5868	1.41479	1.85362
5869	1.38634	2.1
5870	1.38634	2.1
5871	1.67762	2.1
5872	0.946131	2.1
5873	1.188 2.1	
5874	1.62585	2.1
5875	1.62585	2.1
5876	0.946129	2.1
5877	1.18801	2.1
5878	1.38397	2.1
5879	1.04546	2.1
5880	1.16817	2.1
5881	1.06007	2.1
5882	1.19785	2.1
5883	1.43652	2.1
5884	1.06994	2.1
5885	1.89111	2.1
5886	1.07144	2.1
5887	1.51754	2.1
5888	2.0873	2.1
5889	0.758799	2.1
5890	1.59518	2.1
5891	1.59518	2.1
5892	0.946132	2.1
5893	0.758771	2.1
5894	1.06957	2.1
5895	0.758771	2.1
5896	1.33916	2.1
5897	2.03045	1.27855
5898	2.80184	1.72097
5899	1.76788	2.0397
5900	1.27442	2.1
5901	1.06756	2.1
5902	0.966979	2.1
5903	1.7054	2.1
5904	1.07156	2.1
5905	0.986096	2.1
5906	1.32813	2.1
5907	1.3331	2.1
5908	2.08369	0.561248
5909	2.23818	2.03474
5910	1.10335	0.866514
5911	0.622314	1.70255
5912	1.72451	0.773395
5913	1.72451	1.89131

5914	0.622283	0.428486
5915	0.622314	0.542554
5916	0.946131	0.57826
5917	1.31254	2.1
5918	1.15704	2.1
5919	1.24463	0.715763
5920	2.2067	1.50131
5921	1.30001	0.245314
5922	1.29175	0.626327
5923	1.1233	0.05
5924	1.8689	0.05
5925	1.29494	0.05
5926	1.11895	0.05
5927	0.851539	0.05
5928	0.851474	0.05
5929	1.58352	0.05
5930	1.58228	0.05
5931	0.851527	0.05
5932	2.6389	0.05
5933	1.77548	0.05
5934	1.24555	2.1
5935	2.0512	2.1
5936	1.46101	2.1
5937	2.60635	2.1
5938	1.10476	0.05
5939	1.44103	0.05
5940	0.652391	2.1
5941	0.618322	2.1
5942	2.22915	2.1
5943	1.39067	2.1
5944	1.99737	2.1
5945	1.42174	2.1
5946	0.946132	0.338978
5947	1.29735	1.50039
5948	1.61765	2.1
5949	1.61765	0.827076
5950	0.946132	1.78619
5951	1.29735	0.703817
5952	1.26643	0.425466
5953	1.01335	0.877113
5954	1.01335	0.0828007
5955	0.947669	2.1
5956	2.17167	2.1
5957	2.59733	2.05678
5958	1.50954	2.1
5959	1.02638	1.70646
5960	0.555785	2.09996
5961	1.91101	2.08034
5962	2.09809	1.92779
5963	0.641974	2.06569
5964	1.2022	1.98057
5965	1.36359	1.91245
5966	1.8128	2.1
5967	2.34348	0.05
5968	1.66343	0.05
5969	0.974717	0.446641
5970	1.53908	0.252926
5971	1.27737	0.441275

5972	1.09408	0.0992983
5973	2.07382	0.14977
5974	1.10466	1.20773
5975	1.05654	1.56854
5976	1.23498	0.710855
5977	1.48104	1.29612
5978	1.23498	0.871298
5979	1.17261	2.1
5980	1.18059	2.1
5981	2.43005	2.1
5982	1.04546	2.1
5983	2.81222	2.1
5984	1.16817	2.1
5985	1.75766	0.05
5986	1.47021	0.147828
5987	1.07236	0.0550747
5988	1.81759	0.391371
5989	1.42913	0.180136
5990	1.00636	0.468363
5991	1.00636	0.111139
5992	1.79397	0.168819
5993	0.946131	0.0777596
5994	1.03691	0.0989794
5995	0.851539	0.129667
5996	1.46001	0.210629
5997	1.37878	0.101161
5998	0.933258	0.0937404
5999	1.32423	0.0595546
6000	1.32614	0.05
6001	1.11356	0.05
6002	1.32614	0.05
6003	1.28895	0.05
6004	1.66895	0.05
6005	1.66895	0.05
6006	0.946132	0.05
6007	1.29576	0.05
6008	1.1318	0.05
6009	1.37621	1.7108
6010	1.94071	1.56071
6011	1.10517	2.1
6012	1.97098	2.1
6013	0.811 2.1	
6014	0.956127	2.1
6015	1.18647	2.1
6016	0.988455	1.50993
6017	1.12457	0.05
6018	1.7453	0.05
6019	0.92492	2.1
6020	1.24353	2.1
6021	1.84536	0.30981
6022	1.67662	0.181123
6023	1.38247	0.05
6024	1.02663	0.05
6025	0.946132	0.155821
6026	1.41723	0.0875689
6027	1.72976	0.117333
6028	1.72976	0.0562407
6029	0.946131	0.354954

6030	1.41723	0.145428
6031	1.25866	0.128722
6032	1.34254	0.05
6033	1.25866	0.0752098
6034	1.65507	0.0550255
6035	1.65507	0.05
6036	0.946132	0.16781
6037	0.946132	0.0707403
6038	1.11929	0.058872
6039	0.961779	0.0566781
6040	0.961779	0.060045
6041	0.946132	2.1
6042	1.69361	2.1
6043	1.69361	2.1
6044	0.94613	2.1
6045	1.36523	2.1
6046	1.36523	2.1
6047	1.19802	2.1
6048	0.974196	2.1
6049	1.09518	2.1
6050	0.946131	2.1
6051	1.7161	2.1
6052	1.7161	2.1
6053	0.94613	2.1
6054	1.297	2.1
6055	1.297	2.1
6056	1.14599	2.1
6057	0.974197	2.1
6058	0.974197	2.1
6059	1.80778	2.1
6060	2.20473	2.1
6061	1.80777	2.1
6062	1.3515	2.1
6063	0.583237	2.1
6064	1.39359	2.1
6065	1.39359	2.1
6066	1.07296	2.1
6067	0.946114	2.1
6068	1.07297	2.1
6069	0.903862	2.1
6070	0.956572	2.1
6071	1.03437	2.1
6072	0.975409	2.1
6073	1.31167	0.05
6074	2.70246	0.05
6075	1.12018	0.05
6076	0.655836	0.05
6077	0.655837	0.05
6078	1.81557	0.05
6079	1.81557	0.05
6080	0.655836	0.05
6081	0.946131	0.05
6082	1.01847	0.05
6083	1.61334	0.05
6084	0.810946	0.05
6085	0.8311	0.05
6086	1.2466	0.05
6087	1.20286	0.05

6088	0.97508	0.05
6089	0.870805	0.05
6090	0.760087	0.05
6091	0.853389	2.1
6092	0.820894	2.1
6093	0.928291	2.1
6094	1.06922	2.1
6095	1.32829	2.1
6096	1.12173	2.1
6097	1.16615	2.1
6098	0.957748	2.1
6099	0.612491	2.02128
6100	0.548141	2.1
6101	0.979614	2.1
6102	0.658184	2.1
6103	0.678456	1.85838
6104	0.529544	1.95165
6105	0.739013	2.1
6106	0.694037	2.1
6107	0.475716	1.99509
6108	1.58794	1.82169
6109	1.58794	1.26141
6110	0.475743	1.59989
6111	1.08301	2.1
6112	0.980664	2.1
6113	0.475737	2.07648
6114	1.32709	2.1
6115	1.59396	2.1
6116	1.19719	0.05
6117	1.34921	0.05
6118	1.69061	0.05
6119	1.69061	0.05
6120	0.946132	0.05
6121	1.34921	0.05
6122	1.28753	0.05
6123	0.95895	0.05
6124	1.01051	0.05
6125	1.12468	0.05
6126	1.22588	0.05
6127	1.34254	0.05
6128	1.74561	0.05
6129	1.74561	0.05
6130	0.946128	0.05
6131	1.34253	0.05
6132	1.3492	0.05
6133	1.01051	0.05
6134	1.922	2.1
6135	1.922	2.1
6136	2.12556	2.1
6137	0.946131	2.1
6138	1.06278	2.1
6139	1.47631	2.1
6140	1.47631	2.1
6141	0.946129	2.1
6142	1.06278	2.1
6143	1.35966	2.1
6144	0.991985	2.1
6145	0.738507	0.920081

6146	0.536935	1.97899
6147	0.799004	1.14777
6148	1.04281	0.664114
6149	0.879197	0.559042
6150	1.06538	0.562593
6151	0.715942	0.999492
6152	1.00636	0.140661
6153	1.03824	0.0776767
6154	1.70147	0.121042
6155	1.21223	2.1
6156	1.01243	2.1
6157	1.01242	2.1
6158	1.06935	2.1
6159	1.54241	2.1
6160	0.998971	0.05
6161	1.60312	0.05
6162	1.44836	0.05
6163	2.05632	0.05
6164	1.44836	0.05
6165	0.973765	0.05
6166	0.946131	0.05
6167	0.851541	0.05
6168	1.73854	0.05
6169	1.91752	0.05
6170	1.49622	0.05
6171	1.23351	0.05
6172	3.33289	2.1
6173	1.86606	2.1
6174	2.71233	2.1
6175	0.788816	2.1
6176	0.963972	2.1
6177	1.25866	2.1
6178	1.10817	2.1
6179	1.41908	2.1
6180	1.48734	2.1
6181	1.40822	0.108957
6182	1.40822	0.0887295
6183	2.09871	0.175819
6184	0.946133	0.107014
6185	1.14926	0.05
6186	0.946133	0.147427
6187	1.78098	0.0554876
6188	1.78098	0.05
6189	1.35822	0.0656934
6190	1.3689	0.0630509
6191	1.22176	0.05
6192	2.03426	0.0793376
6193	0.946131	0.136247
6194	1.01713	0.0663407
6195	0.704112	0.157869
6196	1.36237	0.0692274
6197	1.36237	0.0604845
6198	1.01713	0.0938003
6199	1.04936	0.101513
6200	1.87377	2.1
6201	1.60501	2.1
6202	0.981584	0.05
6203	1.69435	0.05

6204	0.85154	2.1
6205	1.47841	2.1
6206	1.4952	2.1
6207	1.31941	2.1
6208	1.04023	2.1
6209	1.1995	2.1
6210	0.914791	2.1
6211	1.1427	1.81455
6212	1.64787	1.29671
6213	1.07526	1.30299
6214	1.07527	0.0928487
6215	1.07527	1.00749
6216	1.58551	0.189022
6217	2.77362	0.979381
6218	2.17974	0.35574
6219	2.71723	0.557975
6220	1.29868	0.05
6221	1.3594	0.05
6222	1.04786	0.05
6223	0.527876	0.05
6224	1.4085	0.05
6225	1.48538	0.05
6226	0.76829	0.05
6227	1.18514	0.05
6228	0.617954	0.05
6229	0.864161	0.05
6230	1.18835	0.05
6231	1.16817	2.1
6232	1.04263	2.1
6233	1.50524	2.1
6234	1.85355	2.1
6235	1.23963	2.1
6236	1.06279	2.1
6237	0.94613	2.1
6238	1.35229	2.1
6239	1.15947	2.1
6240	0.965593	2.1
6241	0.851553	2.1
6242	2.21296	2.1
6243	1.11764	2.1
6244	2.46399	2.1
6245	1.39292	2.1
6246	1.34701	2.1
6247	1.34701	2.1
6248	1.47001	2.1
6249	0.963166	0.05
6250	0.893445	0.05
6251	1.21056	0.05
6252	1.67375	2.1
6253	2.0977	2.1
6254	1.09154	2.1
6255	1.09154	2.1
6256	1.57122	0.970241
6257	1.60976	0.0930478
6258	1.69533	0.05
6259	1.6951	0.05
6260	1.5831	0.05
6261	1.20969	2.1

6262	1.12076	2.1
6263	1.49808	2.1
6264	1.50201	2.1
6265	1.00278	2.1
6266	1.22811	0.05
6267	1.11545	2.1
6268	1.09646	2.1
6269	1.03107	0.05
6270	1.44293	0.05
6271	1.66752	1.92452
6272	1.11023	2.01937
6273	2.57831	2.08623
6274	2.20191	0.989621
6275	2.18051	2.1
6276	1.48035	0.172101
6277	1.0961	0.0916258
6278	1.63241	0.0870878
6279	1.0961	0.257777
6280	1.20959	2.1
6281	2.86136	2.1
6282	1.64933	2.1
6283	1.47217	2.1
6284	0.915178	2.1
6285	1.19074	2.1
6286	0.897276	2.1
6287	0.915177	2.1
6288	0.987037	2.1
6289	1.04288	2.1
6290	2.39323	2.1
6291	0.976218	0.05
6292	2.23933	0.05
6293	0.976215	0.05
6294	1.07384	0.05
6295	1.07652	2.1
6296	1.55678	2.1
6297	1.54168	2.1
6298	1.69136	2.1
6299	1.64391	1.57751
6300	1.76477	1.52083
6301	0.611486	1.71717
6302	0.683421	0.865983
6303	0.946131	1.47136
6304	1.1019	1.329
6305	1.12946	1.07446
6306	1.54226	2.01636
6307	1.19496	1.30962
6308	1.96199	1.15377
6309	2.0775	1.7505
6310	1.22297	1.22694
6311	1.3469	0.913696
6312	1.25366	1.20475
6313	1.65443	1.31307
6314	1.65443	1.46846
6315	0.946132	1.60963
6316	0.946132	1.85801
6317	1.12873	1.5253
6318	1.07468	1.13704
6319	1.2854	0.321059

6320	1.3469	0.990433
6321	1.68616	0.922546
6322	1.68616	0.63702
6323	0.946132	1.60512
6324	0.946132	1.49113
6325	1.16109	1.16332
6326	1.17879	0.717564
6327	1.17879	0.944246
6328	1.45304	1.30659
6329	2.21058	2.1
6330	2.81552	2.1
6331	1.45535	0.05
6332	1.49906	0.05
6333	1.04413	2.1
6334	1.74586	2.1
6335	1.34526	2.1
6336	1.17402	0.769786
6337	1.74564	0.404478
6338	1.52724	2.1
6339	1.68142	2.1
6340	1.09651	2.1
6341	1.49149	2.1
6342	1.36814	2.1
6343	1.46783	2.1
6344	2.18116	2.1
6345	1.23602	1.35696
6346	1.74538	0.63473
6347	0.851539	2.1
6348	1.45775	2.1
6349	1.28919	2.1
6350	0.851483	2.1
6351	1.26102	2.1
6352	0.869889	2.1
6353	0.85151	2.1
6354	1.23451	1.19671
6355	1.23602	2.08321
6356	1.11955	2.1
6357	1.08022	2.1
6358	0.977776	2.1
6359	1.27896	2.1
6360	1.64268	2.1
6361	1.97548	2.1
6362	1.72527	2.1
6363	0.946132	2.1
6364	1.69702	2.1
6365	1.69702	2.1
6366	1.13635	2.1
6367	1.40274	2.1
6368	1.24041	2.1
6369	1.13635	2.1
6370	1.78427	0.05
6371	1.68338	0.05
6372	1.00681	0.05
6373	1.12988	0.05
6374	0.946132	0.05
6375	0.957543	0.05
6376	1.40515	0.05
6377	1.40515	0.05

6378	0.797932	0.05
6379	1.30187	0.05
6380	1.14846	0.05
6381	0.665137	0.05
6382	1.09575	0.05
6383	0.596894	0.05
6384	1.04072	0.0652736
6385	0.663171	0.0803452
6386	1.47218	0.05
6387	1.32889	0.05
6388	1.32639	0.05
6389	1.94887	0.05
6390	1.32639	0.05
6391	1.19323	0.05
6392	2.19794	0.05
6393	0.754756	0.05
6394	1.11015	0.05
6395	0.754755	0.05
6396	0.946133	0.05
6397	0.754768	0.05
6398	1.59986	0.05
6399	1.59986	0.05
6400	1.84082	2.1
6401	1.79044	2.1
6402	1.75922	2.1
6403	1.86766	2.1
6404	1.58995	2.1
6405	1.58995	2.1
6406	1.74351	2.1
6407	1.04442	2.1
6408	1.42278	2.1
6409	1.08576	2.1
6410	0.994294	2.1
6411	1.44884	2.1
6412	1.71036	1.07397
6413	0.714101	0.223475
6414	1.17541	2.04668
6415	1.00872	0.05
6416	0.714072	0.399093
6417	0.936677	0.119481
6418	0.966979	2.1
6419	1.4908	2.1
6420	1.03108	0.11595
6421	0.949712	0.05
6422	0.810946	0.0777649
6423	0.88209	0.0855763
6424	0.646702	0.0564755
6425	0.972663	0.05
6426	0.862301	0.05
6427	0.868143	0.05
6428	1.11094	0.0511253
6429	1.35809	0.0508285
6430	1.32455	2.1
6431	1.32328	2.1
6432	1.69586	2.1
6433	1.78301	2.1
6434	1.67906	2.1
6435	1.60635	2.1

6436	1.57234	2.1
6437	1.47012	2.1
6438	1.22811	0.05
6439	1.08732	0.05
6440	2.08849	0.05
6441	1.08732	0.05
6442	1.30635	0.05
6443	0.77315	0.05
6444	1.25732	0.05
6445	2.43845	2.1
6446	2.38577	1.94556
6447	0.946132	0.613604
6448	1.19288	2.1
6449	1.54508	2.1
6450	1.54508	1.69661
6451	0.94515	2.1
6452	1.19288	2.01952
6453	1.29735	1.55257
6454	1.12087	0.410891
6455	1.54699	1.10627
6456	1.43635	2.1
6457	1.33385	2.1
6458	1.43635	2.1
6459	1.02368	2.1
6460	1.035 2.1	
6461	1.00128	2.1
6462	1.04009	2.1
6463	1.14052	2.1
6464	0.443552	2.1
6465	0.984351	2.1
6466	0.591443	2.1
6467	0.929561	2.1
6468	1.52079	2.1
6469	1.035 2.1	
6470	3.5282	2.1
6471	1.88288	2.1
6472	2.73877	1.11303
6473	0.906059	2.1
6474	1.23107	0.05
6475	0.906712	0.05
6476	0.841322	0.05
6477	1.53005	0.05
6478	1.10007	0.05
6479	1.38033	0.05
6480	1.03442	0.05
6481	0.787223	0.05
6482	1.42589	0.05
6483	2.35665	0.05
6484	1.17793	0.05
6485	1.81356	0.05
6486	1.42964	0.05
6487	1.95641	0.05
6488	1.39139	0.0604451
6489	1.39139	0.146984
6490	0.564533	0.0522193
6491	1.20016	0.197943
6492	0.755765	0.05
6493	0.755766	0.05

6494	1.20016	0.0802251
6495	1.91011	0.435728
6496	1.4695	0.05
6497	1.70048	2.1
6498	1.93765	2.1
6499	0.741307	2.1
6500	0.780371	2.1
6501	0.946133	0.735736
6502	1.28918	1.1578
6503	1.5111	1.95431
6504	1.5111	0.611545
6505	0.946131	1.54408
6506	1.28918	0.813514
6507	1.16805	0.472875
6508	1.01179	0.208905
6509	0.946132	0.791081
6510	1.35328	1.18193
6511	1.69633	1.81008
6512	1.69633	0.731722
6513	0.946133	1.58578
6514	1.35328	0.902574
6515	1.28918	0.44586
6516	1.01179	1.19651
6517	1.58147	1.23296
6518	1.27737	0.33156
6519	2.17223	0.05
6520	0.697168	0.05
6521	1.62495	0.0532591
6522	1.62495	0.0522219
6523	1.93948	0.245016
6524	0.601592	2.1
6525	1.93528	2.1
6526	2.13757	2.1
6527	1.67561	2.1
6528	2.70875	2.1
6529	1.93528	2.1
6530	3.98475	2.1
6531	1.12376	2.1
6532	1.19016	2.1
6533	1.67323	0.05
6534	1.74019	0.05
6535	1.84719	0.05
6536	1.00764	0.05
6537	1.23475	2.1
6538	1.06757	2.1
6539	1.57916	2.1
6540	1.06758	2.1
6541	1.28014	2.1
6542	1.33339	2.1
6543	1.2679	2.1
6544	1.23504	2.1
6545	1.50108	2.1
6546	1.3715	2.1
6547	0.58831	0.820789
6548	0.865971	1.12772
6549	0.813704	1.28485
6550	0.658856	1.33634
6551	0.495869	1.1918

6552	1.13845	1.05929
6553	0.780804	1.34004
6554	0.642023	0.925326
6555	0.707748	0.938503
6556	1.64476	0.736144
6557	1.64476	1.11017
6558	0.99611	0.788329
6559	1.35639	0.926866
6560	1.3564	1.49332
6561	0.946129	1.55592
6562	1.99222	0.621859
6563	1.4777	2.1
6564	1.03968	2.1
6565	1.82516	2.1
6566	0.851539	2.1
6567	1.63693	2.1
6568	1.59416	2.1
6569	0.878566	2.1
6570	1.40796	2.1
6571	1.13172	2.1
6572	0.851527	1.99987
6573	1.14366	2.1
6574	0.823822	0.05
6575	0.882001	0.05
6576	1.09756	0.05
6577	1.11857	0.05
6578	1.0934	0.05
6579	0.960104	0.05
6580	0.982001	0.05
6581	1.74319	0.05
6582	1.03043	0.05
6583	0.967194	0.05
6584	1.16938	2.1
6585	2.48377	2.1
6586	1.1181	2.1
6587	2.57349	2.1
6588	1.0465	2.1
6589	1.44709	2.1
6590	0.946132	2.1
6591	1.29302	2.1
6592	1.73502	2.1
6593	1.73502	2.1
6594	0.946132	2.1
6595	1.29302	2.1
6596	1.38813	2.1
6597	1.03933	2.1
6598	1.01976	0.05
6599	1.11764	0.0587921
6600	0.961779	0.100708
6601	1.0685	0.0718845
6602	1.08426	1.14781
6603	0.99695	1.91898
6604	2.25593	1.80384
6605	1.47992	1.94473
6606	1.88989	1.26933
6607	1.65813	1.02555
6608	1.20278	1.60625
6609	0.981842	1.49155

6610	0.802655	2.1
6611	0.953489	2.1
6612	0.961719	2.1
6613	0.975614	2.08777
6614	0.991617	1.55744
6615	0.940748	1.83604
6616	2.25019	2.1
6617	2.30712	1.6872
6618	1.98823	2.1
6619	0.522844	1.69309
6620	0.946122	2.1
6621	0.946132	1.97393
6622	0.593846	1.45659
6623	1.26501	1.51861
6624	1.21171	0.05
6625	0.851542	0.05
6626	0.940327	0.05
6627	0.940356	0.05
6628	1.52117	0.05
6629	1.58072	0.05
6630	1.35169	0.05
6631	1.13889	0.05
6632	1.35007	0.05
6633	1.35007	0.05
6634	1.78381	0.05
6635	0.946132	0.05
6636	1.06627	0.05
6637	0.946131	0.05
6638	1.73005	0.05
6639	1.73005	0.05
6640	1.32449	0.05
6641	1.35169	0.05
6642	1.42446	0.238357
6643	1.86697	0.239683
6644	1.13413	0.0905697
6645	1.42446	0.0781548
6646	1.33575	0.05
6647	1.20473	0.05
6648	1.87517	0.05
6649	1.87517	0.05
6650	0.488712	0.05
6651	1.13242	0.05
6652	0.488713	0.05
6653	0.488713	0.05
6654	1.23146	0.05
6655	1.57932	0.05
6656	1.01698	0.05
6657	1.42085	0.05
6658	0.246725	0.05
6659	0.996283	0.05
6660	1.36965	0.05
6661	0.86361	0.05
6662	0.810945	0.05
6663	1.72938	0.05
6664	1.21649	0.05
6665	1.1865	0.05
6666	1.62387	0.05
6667	1.05302	0.05

6668	1.10049	0.0705131
6669	0.946129	2.1
6670	1.70184	0.351967
6671	1.70184	2.1
6672	1.12538	2.1
6673	1.35969	0.2106
6674	1.28828	2.1
6675	1.12538	1.74516
6676	0.99747	1.59752
6677	1.34851	0.215235
6678	0.923724	2.1
6679	1.47154	0.940194
6680	1.47154	2.1
6681	1.10698	2.1
6682	1.28828	0.528946
6683	1.10698	2.1
6684	0.923695	2.1
6685	0.99747	0.170248
6686	1.65677	2.1
6687	1.77677	2.1
6688	1.05689	0.05
6689	2.11031	0.05
6690	1.07367	0.05
6691	0.656239	0.05
6692	1.4563	0.05
6693	1.4563	0.05
6694	0.656238	0.05
6695	0.946133	0.05
6696	0.812937	0.05
6697	0.946131	0.05
6698	1.05689	0.05
6699	1.34009	2.1
6700	1.398 2.1	
6701	3.29047	2.1
6702	2.53897	2.1
6703	1.24509	2.1
6704	3.06162	2.1
6705	1.01595	2.1
6706	1.52661	2.1
6707	1.5266	2.1
6708	0.946131	2.1
6709	1.31049	2.1
6710	1.16225	2.1
6711	1.05978	2.1
6712	1.16225	2.1
6713	1.06561	2.1
6714	0.953927	2.1
6715	1.15114	2.1
6716	1.1807	0.0696853
6717	0.507259	0.05
6718	1.47709	0.05
6719	1.36192	0.05
6720	0.487293	0.05
6721	1.65569	0.0665339
6722	0.679725	0.05
6723	0.721928	0.05
6724	1.03931	0.05
6725	1.0775	0.05

6726	1.0775	0.05
6727	2.30207	0.328141
6728	1.01814	0.281735
6729	2.33963	0.192914
6730	2.42589	0.34929
6731	1.33962	2.1
6732	1.27956	2.1
6733	2.14624	0.05
6734	1.6812	0.05
6735	1.25698	0.05
6736	1.35725	0.05
6737	1.86387	0.05
6738	1.86387	0.05
6739	0.750365	0.05
6740	0.946128	0.05
6741	0.750365	0.05
6742	1.72271	0.05
6743	1.72272	0.05
6744	2.53736	0.05
6745	1.07327	2.1
6746	1.05376	2.1
6747	1.00387	0.561904
6748	2.0554	0.247443
6749	1.00387	0.433215
6750	1.00387	0.533086
6751	1.27119	2.1
6752	1.51748	2.1
6753	3.15271	2.1
6754	3.45801	2.1
6755	1.99871	2.1
6756	1.41186	2.1
6757	1.07624	2.1
6758	1.5954	2.1
6759	1.19323	0.218719
6760	1.21286	0.218784
6761	0.946114	2.1
6762	1.38395	2.1
6763	1.73389	2.1
6764	1.7339	2.1
6765	0.946115	2.1
6766	1.38397	2.1
6767	1.29604	2.1
6768	1.04544	2.1
6769	1.60578	0.05
6770	1.93407	0.05
6771	1.0525	0.600311
6772	1.0525	1.185
6773	1.0525	1.8943
6774	1.38506	1.77031
6775	2.34737	0.05
6776	1.3531	0.05
6777	1.2925	0.05
6778	0.871466	0.05
6779	1.26623	0.05
6780	0.700631	0.05
6781	1.88774	0.05
6782	1.14167	0.05
6783	1.18606	0.05

6784	2.1238	0.05
6785	1.42762	0.325075
6786	1.67791	0.05
6787	1.47118	0.05
6788	0.946128	2.1
6789	1.31641	2.1
6790	1.75933	2.1
6791	1.75933	2.1
6792	0.946131	2.1
6793	1.31641	2.1
6794	1.38905	2.1
6795	1.38437	2.1
6796	1.51829	2.1
6797	0.947114	2.1
6798	1.13335	2.1
6799	1.47521	2.1
6800	1.14911	2.1
6801	1.53822	2.1
6802	1.39702	2.1
6803	0.739166	2.1
6804	0.706056	2.1
6805	0.964033	2.1
6806	1.58993	2.1
6807	2.51892	2.1
6808	0.595905	0.05
6809	1.18155	0.05
6810	1.73364	0.05
6811	0.952735	0.05
6812	2.10381	0.05
6813	1.12115	0.05
6814	1.27826	0.05
6815	2.74021	0.05
6816	1.78416	2.1
6817	1.78416	2.1
6818	0.946132	2.1
6819	1.41126	2.1
6820	1.21275	2.1
6821	0.946133	2.1
6822	1.41126	2.1
6823	1.33125	2.1
6824	1.33125	2.1
6825	1.78918	2.1
6826	1.0465	2.1
6827	1.99715	2.1
6828	1.17683	2.1
6829	1.25889	2.1
6830	0.860834	0.05
6831	0.946131	0.05
6832	1.05008	0.05
6833	0.970444	0.05
6834	1.19401	0.05
6835	1.25989	0.05
6836	0.702942	0.05
6837	1.40591	0.147263
6838	1.72168	0.0745498
6839	0.446352	0.05
6840	1.04072	0.05
6841	0.711038	0.094521

6842	0.980737	0.0589117
6843	1.05099	0.05
6844	1.26003	0.05
6845	0.495934	0.0852777
6846	1.03114	0.05
6847	1.09911	0.05
6848	1.24825	0.05
6849	2.19827	0.05
6850	2.0953	0.05
6851	2.30437	0.05
6852	0.775923	0.05
6853	2.2283	0.05
6854	1.19583	2.1
6855	1.14496	2.1
6856	1.62183	2.1
6857	1.62316	2.1
6858	1.02305	0.0956986
6859	1.21136	0.0575728
6860	1.44426	0.0745345
6861	1.21137	0.05
6862	1.20143	0.135163
6863	1.15009	0.0686482
6864	1.24965	2.1
6865	1.41077	2.1
6866	1.00052	0.05
6867	1.79553	0.05
6868	1.06336	0.05
6869	1.00053	0.05
6870	1.18041	2.1
6871	1.80818	2.1
6872	1.73571	1.47591
6873	1.94429	1.7175
6874	1.4375	0.05
6875	1.44421	0.05
6876	1.66953	0.05
6877	1.09529	0.05
6878	0.952395	2.1
6879	0.717337	2.1
6880	1.03609	2.1
6881	1.33949	0.172211
6882	1.2774	0.0979275
6883	2.24983	0.0583607
6884	1.28727	0.0581201
6885	0.583592	0.0651565
6886	0.583591	0.112643
6887	1.82859	0.0797905
6888	1.82859	0.0629063
6889	0.583593	0.107138
6890	0.946128	0.0629744
6891	0.692233	0.137492
6892	1.12226	0.0917375
6893	2.57454	0.113918
6894	0.810944	0.0980916
6895	0.810945	0.122755
6896	1.12226	0.083468
6897	1.45867	0.0835971
6898	1.22403	0.0783529
6899	1.15262	0.135186

6900	1.08132	0.0891647
6901	0.846671	0.114683
6902	0.480954	0.104519
6903	0.917975	0.0931212
6904	0.917975	0.272734
6905	0.720957	0.103155
6906	0.795998	0.0937153
6907	0.69316	0.172588
6908	0.641306	0.124241
6909	0.690817	0.0676351
6910	0.961899	2.1
6911	2.49579	2.1
6912	2.31106	2.1
6913	1.70482	0.05
6914	1.93156	0.054471
6915	1.23868	0.05
6916	1.73878	0.0600998
6917	1.26267	2.1
6918	1.26441	2.1
6919	1.58095	2.1
6920	1.58095	2.1
6921	0.946132	2.1
6922	0.946132	2.1
6923	1.11077	2.1
6924	1.62905	2.1
6925	1.49692	2.1
6926	3.49966	2.1
6927	2.39213	2.1
6928	2.12603	2.1
6929	0.946129	2.1
6930	0.461661	2.1
6931	1.52841	2.1
6932	1.39091	2.1
6933	0.550679	2.1
6934	0.509894	2.1
6935	1.49508	2.1
6936	2.05862	2.1
6937	2.47851	2.1
6938	1.07213	2.1
6939	1.09148	0.410488
6940	1.99776	0.458424
6941	1.38333	0.365151
6942	1.04412	2.1
6943	1.04412	2.1
6944	1.1893	0.05
6945	0.946132	0.05
6946	0.965737	0.05
6947	0.946132	0.05
6948	1.54061	0.05
6949	1.54061	0.05
6950	1.22878	0.05
6951	1.25797	0.05
6952	1.00665	0.05
6953	1.00665	0.05
6954	1.80369	0.05
6955	0.946128	0.05
6956	1.04424	0.05
6957	0.85154	0.05

6958	1.39322	0.05
6959	1.35464	0.05
6960	0.939857	0.05
6961	1.28272	0.05
6962	1.19323	0.05
6963	1.03498	0.05
6964	2.22029	0.05
6965	0.540005	0.05
6966	0.970382	0.05
6967	2.14514	0.05
6968	1.31542	0.05
6969	1.05575	0.05
6970	0.810943	0.05
6971	1.31542	0.05
6972	1.70493	0.05
6973	1.22582	0.05
6974	1.31681	0.05
6975	1.40775	0.05
6976	0.928633	0.05
6977	1.2545	0.05
6978	2.66481	0.05
6979	1.32051	0.05
6980	1.32051	0.05
6981	2.66305	2.1
6982	1.3551	2.1
6983	2.45325	2.1
6984	1.3685	2.1
6985	1.51748	2.1
6986	1.78941	2.1
6987	1.02963	0.05
6988	1.28643	0.05
6989	0.86131	0.05
6990	0.851539	0.05
6991	1.46585	0.05
6992	1.38822	0.05
6993	0.996402	0.05
6994	0.851553	0.05
6995	1.73156	0.05
6996	1.91695	0.05
6997	1.92535	0.05
6998	2.57287	0.05
6999	1.77743	0.05
7000	1.02836	0.05
7001	1.36362	0.05
7002	1.36936	0.05
7003	1.78684	0.05
7004	1.78685	0.05
7005	0.946132	0.0616696
7006	1.27581	0.0561784
7007	0.946132	0.0831925
7008	1.02836	0.05
7009	1.55398	2.1
7010	1.07167	2.1
7011	1.87557	2.1
7012	0.751639	2.1
7013	1.50884	2.1
7014	1.33793	2.1
7015	0.751664	2.1

7016	1.37277	2.1
7017	0.979515	2.1
7018	0.676508	2.1
7019	1.09578	2.1
7020	0.953925	2.1
7021	0.94613	2.1
7022	1.64707	2.1
7023	1.64707	2.1
7024	1.16658	2.1
7025	1.40638	2.1
7026	1.18682	2.1
7027	1.16658	2.1
7028	0.543765	0.05
7029	1.57462	0.05
7030	1.57462	0.05
7031	1.00083	0.05
7032	1.16834	0.05
7033	1.16834	0.05
7034	0.889626	0.05
7035	1.25944	0.05
7036	0.824061	0.05
7037	1.9397	0.05
7038	1.21893	0.05
7039	1.91364	0.05
7040	1.33672	0.05
7041	0.928069	0.05
7042	1.41484	0.05
7043	0.538379	0.05
7044	1.41484	0.05
7045	1.35594	0.05
7046	2.11414	0.05
7047	2.11414	0.05
7048	0.538378	0.05
7049	1.38532	0.05
7050	0.962877	0.0988035
7051	1.07497	0.05
7052	1.00613	0.05
7053	0.962878	0.0677218
7054	0.711447	2.1
7055	1.40008	0.05
7056	2.51674	0.05
7057	1.40008	0.05
7058	1.7263	0.05
7059	1.40008	0.05
7060	1.9216	0.05
7061	2.7013	0.05
7062	2.58584	0.05
7063	0.979296	0.05
7064	1.46459	0.05
7065	0.899254	0.05
7066	1.15038	2.1
7067	1.24687	2.1
7068	1.16738	2.1
7069	0.942323	2.1
7070	1.65573	1.66038
7071	0.844791	2.1
7072	0.54674	2.1
7073	0.795811	2.1

7074	1.08131	2.1
7075	1.03553	2.1
7076	0.758334	2.1
7077	0.708665	2.03907
7078	1.22626	1.95876
7079	0.835439	1.95669
7080	0.861131	1.7633
7081	0.68296	2.09666
7082	1.65224	2.00164
7083	2.82624	2.1
7084	1.0151	1.30911
7085	1.15905	1.87325
7086	1.71076	1.58744
7087	1.4529	0.929139
7088	0.494895	2.1
7089	0.946132	1.89356
7090	0.613132	1.69666
7091	0.863631	1.88476
7092	1.78391	2.1
7093	1.1706	0.05
7094	2.02734	0.05
7095	1.1706	0.05
7096	0.681532	0.05
7097	1.50316	0.05
7098	1.50316	0.05
7099	0.681534	0.05
7100	0.973819	0.05
7101	0.97382	0.05
7102	0.94613	0.05
7103	1.1706	0.05
7104	1.67727	0.05
7105	1.60329	0.05
7106	1.3227	2.1
7107	1.40846	2.1
7108	1.07889	2.1
7109	1.07721	2.1
7110	0.953523	0.206423
7111	1.79675	0.267345
7112	2.09859	0.721638
7113	1.21309	2.1
7114	1.54567	0.416257
7115	1.19722	0.602734
7116	1.82793	0.627736
7117	1.1599	0.18295
7118	1.73621	0.174462
7119	0.933474	0.313878
7120	1.51477	0.231473
7121	1.51477	0.272784
7122	0.93347	0.23008
7123	1.27374	0.302955
7124	1.27376	0.432807
7125	0.946105	0.565451
7126	1.1972	0.205523
7127	0.851541	0.05
7128	1.36971	0.05
7129	1.27832	0.05
7130	0.853489	0.05
7131	0.973847	0.05

7132	0.976308	0.05
7133	0.851528	0.05
7134	2.72654	0.05
7135	1.77867	0.05
7136	3.23032	2.1
7137	2.78208	2.1
7138	1.0227	0.05
7139	1.02269	0.05
7140	1.02269	0.05
7141	1.24293	0.05
7142	0.831695	2.1
7143	0.67513	2.1
7144	0.956294	2.1
7145	1.08623	2.1
7146	1.05556	2.1
7147	1.38859	2.1
7148	1.13179	2.1
7149	1.02438	2.1
7150	1.06659	2.1
7151	1.36595	2.1
7152	1.06659	2.1
7153	1.06659	1.90517
7154	0.946131	1.25574
7155	0.875912	2.1
7156	1.65971	2.1
7157	1.65971	2.1
7158	0.875912	2.1
7159	1.1229	2.1
7160	1.41273	2.1
7161	1.54699	1.94415
7162	1.93655	1.67024
7163	1.42307	1.23388
7164	1.97907	1.7637
7165	0.996774	2.1
7166	1.15505	0.05
7167	1.28463	0.05
7168	1.86353	0.05
7169	2.71398	0.05
7170	1.56787	0.05
7171	1.66295	0.05
7172	1.73432	1.35803
7173	1.05019	0.05
7174	1.42117	0.05
7175	0.888299	0.05
7176	1.38127	0.05
7177	1.72993	0.05
7178	0.664129	0.05
7179	0.99302	2.00837
7180	0.993023	2.02706
7181	1.13491	2.1
7182	0.876316	2.1
7183	1.2588	2.1
7184	0.895613	2.1
7185	2.07526	2.1
7186	1.23369	2.1
7187	1.25146	2.1
7188	2.15282	2.1
7189	1.22239	2.1

7190	1.10962	2.1
7191	1.77402	2.1
7192	1.0305	2.1
7193	1.50108	1.76732
7194	1.20761	2.1
7195	1.3626	2.1
7196	1.14825	2.1
7197	1.78711	2.1
7198	1.17933	2.1
7199	1.14824	2.1
7200	1.09651	0.05
7201	1.23851	0.05
7202	1.83284	0.05
7203	1.23851	0.05
7204	1.53521	2.1
7205	1.46279	2.1
7206	1.9233	0.05
7207	1.2966	0.05
7208	2.11443	0.05
7209	1.30265	0.05
7210	1.13528	0.05
7211	0.946131	0.0764435
7212	1.41169	0.05
7213	1.33879	0.05
7214	0.946128	0.05
7215	1.80435	0.05
7216	1.80435	0.05
7217	1.234	2.1
7218	1.45633	2.1
7219	1.234	2.1
7220	1.98448	2.1
7221	2.02612	2.1
7222	1.23527	0.518675
7223	1.44472	0.159523
7224	0.993366	0.948118
7225	1.22025	0.631722
7226	0.993368	0.26419
7227	2.43669	2.1
7228	2.56707	2.1
7229	1.80348	1.18486
7230	1.74406	0.512271
7231	1.90937	1.13737
7232	1.49357	0.05
7233	2.5832	0.23414
7234	1.82274	0.163135
7235	1.6957	0.119794
7236	2.22495	0.0785186
7237	2.49959	0.121771
7238	2.01183	0.05
7239	1.37427	0.05
7240	1.15069	2.1
7241	1.39379	1.89944
7242	1.69661	0.0661647
7243	1.39379	0.19919
7244	1.39654	2.1
7245	1.93505	2.1
7246	1.5525	2.1
7247	1.5525	2.1

7248	0.615065	2.1
7249	1.38908	2.1
7250	0.615065	2.1
7251	0.77849	2.1
7252	1.38908	2.1
7253	1.56839	1.59092
7254	1.09142	2.05917
7255	2.07313	1.31015
7256	0.998288	0.05
7257	1.31481	0.05
7258	1.88175	0.05
7259	2.23267	0.05
7260	1.06935	2.1
7261	1.06936	2.1
7262	1.17989	2.02759
7263	1.00757	1.32357
7264	1.65318	2.01117
7265	1.4185	0.0576248
7266	2.22495	0.0558004
7267	1.02011	0.05
7268	0.59676	0.05
7269	1.43606	0.05
7270	1.43606	0.05
7271	0.89771	0.05
7272	1.13511	0.05
7273	0.59676	0.05
7274	0.897709	0.05
7275	1.79542	0.05
7276	2.38566	2.1
7277	1.63838	0.989194
7278	1.63837	1.65634
7279	0.916275	2.1
7280	1.36183	1.17516
7281	1.19282	2.1
7282	0.916275	2.0405
7283	1.19283	2.1
7284	2.01582	2.1
7285	1.00215	0.05
7286	2.60643	2.1
7287	2.15971	2.1
7288	1.28267	2.1
7289	1.29954	2.1
7290	3.40327	2.1
7291	3.49128	2.1
7292	1.3864	2.1
7293	1.3201	2.1
7294	1.45666	0.05
7295	1.55798	0.05
7296	0.857202	0.05
7297	1.24374	0.05
7298	1.04157	0.05
7299	0.85723	0.05
7300	1.02722	0.05
7301	1.01361	0.05
7302	0.974077	0.05
7303	1.01814	0.05
7304	0.974077	0.05
7305	1.43987	0.05

7306	1.43986	0.05
7307	0.927881	0.05
7308	0.946132	0.05
7309	0.927849	0.05
7310	0.927852	0.05
7311	1.2493	0.05
7312	1.22482	0.159953
7313	1.41319	0.398445
7314	1.22482	0.0900875
7315	0.999492	2.1
7316	1.14657	2.1
7317	1.3617	0.05
7318	0.983269	0.05
7319	1.33174	0.05
7320	1.62816	0.05
7321	1.62817	0.05
7322	0.81938	0.05
7323	1.33174	0.05
7324	1.01332	0.05
7325	0.874788	0.05
7326	1.16725	2.1
7327	1.63991	2.1
7328	1.08848	0.05
7329	1.15354	0.05
7330	1.12665	2.1
7331	1.58742	2.1
7332	1.57523	2.1
7333	1.32201	0.540739
7334	1.32097	0.398593
7335	0.851542	0.0666273
7336	0.877188	0.05
7337	0.797489	0.0797741
7338	1.44721	0.05
7339	1.61761	0.05
7340	0.943799	0.05
7341	1.36079	0.05
7342	1.16361	2.1
7343	1.15852	2.00215
7344	1.07624	2.1
7345	1.07624	2.1
7346	0.946132	0.743755
7347	1.37938	1.11089
7348	1.78653	1.64175
7349	1.78653	0.720005
7350	0.946131	1.57208
7351	1.37938	0.966326
7352	1.35328	0.540665
7353	1.44847	0.346482
7354	0.946133	0.828955
7355	1.38149	1.01542
7356	1.81474	1.24183
7357	1.81474	0.697085
7358	0.946133	1.49928
7359	1.38149	0.96042
7360	1.37938	0.663683
7361	1.44847	1.1041
7362	1.93665	0.714972
7363	1.5515	0.372029

7364	1.05025	2.1
7365	1.05025	2.1
7366	1.71468	2.1
7367	1.04759	2.1
7368	1.04646	0.05
7369	1.00739	0.05
7370	1.37086	0.05
7371	1.12547	0.05
7372	1.03853	0.05
7373	1.34824	0.05
7374	0.799786	0.05
7375	2.10105	0.05
7376	2.72007	0.05
7377	0.589918	0.05
7378	0.589918	0.05
7379	1.1509	0.05
7380	0.619832	0.05
7381	1.1509	0.05
7382	0.705903	0.05
7383	1.53703	0.05
7384	1.53703	0.05
7385	0.705904	0.05
7386	0.946133	0.05
7387	0.946132	0.05
7388	0.946132	0.05
7389	0.588467	0.05
7390	1.1078	0.05
7391	0.408061	0.05
7392	1.38703	0.05
7393	1.68328	0.05
7394	1.87031	0.05
7395	1.86268	0.05
7396	1.27472	0.05
7397	0.947862	0.783306
7398	1.19854	1.16116
7399	0.946129	0.630267
7400	0.963111	0.27699
7401	0.946131	0.715587
7402	1.39055	0.246356
7403	1.39055	0.225396
7404	1.26426	0.370334
7405	1.07242	0.395731
7406	1.31126	0.240909
7407	1.76597	0.645573
7408	0.94613	1.25041
7409	1.27214	0.772594
7410	1.90751	0.75671
7411	1.90751	0.518825
7412	0.767114	1.00669
7413	1.27214	1.01888
7414	1.40248	0.926044
7415	1.41479	0.924434
7416	2.22625	1.1289
7417	0.913373	1.2718
7418	1.0158	0.606052
7419	1.61259	0.493792
7420	1.69051	0.252168
7421	0.560949	1.04021

7422	0.898559	1.13379
7423	1.28011	0.935576
7424	1.51026	0.05
7425	1.98252	0.05
7426	1.05129	0.05
7427	1.2951	0.05
7428	1.46041	0.05
7429	1.7155	2.1
7430	1.72196	2.1
7431	0.933885	2.1
7432	0.986723	2.1
7433	1.28072	0.0753992
7434	1.40765	2.1
7435	1.28072	1.13705
7436	1.61687	2.1
7437	1.60965	2.1
7438	1.21424	2.1
7439	2.07297	2.1
7440	1.19943	2.1
7441	2.38784	2.1
7442	1.10278	0.05
7443	1.43678	0.05
7444	1.1142	0.05
7445	1.18034	0.05
7446	0.94613	0.05
7447	0.752356	0.05
7448	0.752329	0.05
7449	1.46634	0.05
7450	1.31789	0.05
7451	0.958159	0.05
7452	1.22752	0.05
7453	1.50472	0.05
7454	1.98974	2.1
7455	2.54186	2.1
7456	0.76512	2.1
7457	2.08777	2.1
7458	1.7753	2.1
7459	3.07372	2.1
7460	1.9756	2.1
7461	1.48162	2.1
7462	1.23204	2.1
7463	0.946131	0.839874
7464	1.13505	2.1
7465	1.47432	2.1
7466	1.47432	1.39323
7467	0.876831	2.1
7468	1.13505	1.86128
7469	1.2161	1.19919
7470	1.05267	1.6462
7471	1.05267	0.796542
7472	1.5795	1.1458
7473	0.98186	2.1
7474	1.56735	2.1
7475	0.859099	2.1
7476	1.69858	2.1
7477	1.93586	2.1
7478	1.09233	2.1
7479	1.08732	0.05

7480	1.3357	0.05
7481	1.84826	2.1
7482	0.924132	2.1
7483	1.39188	2.1
7484	1.43685	2.1
7485	0.851541	2.1
7486	1.16251	2.1
7487	0.946131	2.1
7488	1.05685	2.1
7489	2.13781	2.1
7490	1.98859	2.1
7491	1.99731	2.1
7492	1.51379	2.1
7493	1.48796	2.1
7494	0.851542	2.1
7495	0.802558	2.1
7496	1.09244	2.1
7497	1.33891	2.1
7498	0.844958	2.1
7499	0.710864	2.1
7500	1.26087	2.1
7501	1.03679	2.1
7502	1.51026	0.05
7503	1.05248	0.0562941
7504	0.827258	0.0504895
7505	1.05248	0.0553757
7506	1.28435	0.0548404
7507	1.50953	0.0597942
7508	1.50953	0.05
7509	0.827298	0.0591255
7510	1.00366	0.05
7511	2.10495	0.05
7512	1.17885	2.1
7513	1.25185	2.1
7514	1.02784	2.1
7515	1.76764	2.1
7516	1.23746	0.05
7517	1.24082	0.05
7518	1.46506	0.05
7519	1.33061	0.05
7520	0.768407	1.31038
7521	0.972058	0.555238
7522	1.16536	1.95613
7523	1.13325	1.08733
7524	0.978906	2.1
7525	1.02291	0.876718
7526	0.838015	0.652128
7527	1.01052	0.05
7528	1.08166	0.05
7529	1.42607	0.05
7530	1.47354	0.05
7531	1.21703	2.1
7532	1.29897	2.1
7533	1.83293	2.1
7534	1.29897	2.1
7535	1.24688	0.05
7536	0.782563	2.1
7537	0.946129	2.1

7538	0.782591	2.1
7539	1.49487	2.1
7540	1.49487	2.1
7541	1.37713	2.1
7542	0.900337	2.1
7543	1.31284	2.1
7544	0.924468	2.1
7545	1.40602	2.1
7546	1.25669	2.1
7547	0.986901	2.1
7548	0.958565	2.1
7549	1.37266	2.1
7550	1.64146	2.1
7551	0.49345	2.1
7552	1.08276	2.1
7553	1.08276	2.1
7554	1.26929	2.1
7555	1.09847	2.1
7556	0.827081	0.053166
7557	1.07236	0.101802
7558	1.25063	0.05
7559	1.43548	0.05
7560	1.25063	0.05
7561	1.3659	0.05
7562	1.72441	0.05
7563	1.3659	0.05
7564	1.4677	0.05
7565	1.86114	0.05
7566	1.40104	0.05
7567	0.962675	0.05
7568	0.946131	0.05
7569	1.74134	0.05
7570	1.74134	0.05
7571	0.962676	0.05
7572	0.946131	0.05
7573	1.05548	0.05
7574	0.902187	0.05
7575	2.14178	0.05
7576	1.74998	0.05
7577	1.38541	0.05
7578	1.64468	0.05
7579	0.949317	0.05
7580	2.06654	0.05
7581	0.839626	0.05
7582	0.810945	0.05
7583	1.29203	0.05
7584	1.18091	0.05
7585	1.06872	0.05
7586	1.11956	0.05
7587	0.926911	0.05
7588	0.901716	0.05
7589	0.909146	0.05
7590	1.33776	0.05
7591	1.02048	0.05
7592	1.08365	0.05
7593	1.21042	0.05
7594	0.865634	0.05
7595	0.590354	2.1

7596	0.61173	2.1
7597	3.46825	2.1
7598	3.28404	2.1
7599	1.56004	0.05
7600	1.69344	0.05
7601	1.7151	0.122289
7602	0.946131	0.05
7603	1.40265	0.05
7604	1.13537	0.0525363
7605	0.965708	0.05
7606	1.13537	0.0933467
7607	1.29681	0.05
7608	1.40915	0.05
7609	1.61652	0.172622
7610	1.33171	0.621465
7611	1.37681	2.1
7612	0.81094	0.05
7613	0.598606	0.05
7614	0.723111	0.05
7615	1.04562	0.05
7616	0.979052	0.05
7617	0.798185	0.05
7618	0.880117	0.05
7619	0.979341	0.0961934
7620	0.800229	0.05
7621	1.39126	0.112708
7622	1.13005	0.160503
7623	0.913877	0.120664
7624	1.26959	0.0642759
7625	0.699741	0.139736
7626	1.19496	1.3435
7627	1.13392	0.05
7628	1.11913	0.05
7629	1.06866	0.05
7630	0.615546	0.05
7631	0.831006	0.05
7632	1.53286	0.05
7633	0.690977	0.05
7634	1.01956	0.05
7635	2.33818	0.05
7636	1.6585	2.1
7637	1.67061	2.1
7638	1.41414	2.1
7639	1.16756	2.1
7640	1.00898	0.05
7641	0.966964	2.1
7642	2.85443	2.1
7643	0.966977	2.1
7644	2.08427	2.1
7645	2.94901	2.1
7646	1.10735	0.05
7647	1.65815	2.1
7648	1.4502	2.1
7649	1.45026	2.1
7650	1.53446	2.1
7651	0.946129	0.420139
7652	1.81075	2.1
7653	1.81075	0.333156

7654	0.946131	1.67822
7655	1.37075	2.1
7656	1.37075	0.400198
7657	1.34245	1.92583
7658	0.973502	0.469901
7659	1.08542	2.1
7660	1.5814	0.05
7661	1.43836	0.05
7662	0.946132	0.05
7663	1.13747	0.05
7664	1.04429	0.05
7665	1.05017	0.05
7666	1.24732	0.05
7667	1.13387	0.05
7668	2.19489	0.05
7669	1.10748	2.1
7670	1.25006	2.1
7671	1.58834	2.1
7672	1.58834	0.104648
7673	0.94613	2.1
7674	1.16548	2.1
7675	0.946132	0.0737935
7676	1.08998	2.07552
7677	1.16548	1.71894
7678	0.995732	0.0889424
7679	0.657014	2.1
7680	0.957173	2.1
7681	1.01485	2.1
7682	1.12468	2.1
7683	1.43871	2.1
7684	1.23716	2.1
7685	1.89277	0.31507
7686	2.06653	0.135503
7687	0.967544	0.0564898
7688	1.89016	0.0879201
7689	2.22165	2.1
7690	0.83514	2.1
7691	1.76565	2.1
7692	1.76565	2.1
7693	0.94613	2.1
7694	0.835112	2.1
7695	1.23426	2.1
7696	1.36653	2.1
7697	0.895892	0.643859
7698	0.859278	0.638157
7699	0.791461	0.888337
7700	1.86249	1.30809
7701	1.14607	0.969923
7702	1.19904	1.20605
7703	1.84898	0.9802
7704	1.03452	1.02424
7705	0.946131	0.0628247
7706	0.940472	0.05
7707	1.53478	0.764236
7708	1.84175	0.136323
7709	1.55687	0.124956
7710	1.17787	0.05
7711	1.60589	0.15859

7712	2.35574	0.05
7713	1.3305	0.05
7714	1.15008	0.145996
7715	1.12457	0.05
7716	1.12457	0.05
7717	1.01778	2.1
7718	1.35733	2.1
7719	1.50683	2.1
7720	1.12064	2.1
7721	1.42716	0.265293
7722	1.42717	0.428451
7723	1.45927	0.91489
7724	1.04321	1.77003
7725	1.32634	0.05
7726	2.27022	0.05
7727	1.19352	0.05
7728	1.32634	0.05
7729	1.40924	2.01945
7730	1.22927	1.35643
7731	1.69237	1.84918
7732	1.69237	2.1
7733	0.946132	0.926804
7734	0.946133	1.75505
7735	1.16912	1.57397
7736	1.0417	1.37269
7737	1.14605	1.90757
7738	1.1233	2.1
7739	1.96882	1.60055
7740	1.03804	0.05
7741	1.15338	0.05
7742	1.15338	0.05
7743	2.33051	0.052481
7744	1.81507	2.1
7745	2.42949	2.1
7746	0.725879	0.777515
7747	1.88394	1.09042
7748	2.2699	0.05
7749	2.78952	2.1
7750	1.88394	0.05
7751	1.79606	2.1
7752	1.20091	2.1
7753	1.9614	2.1
7754	1.19901	1.97445
7755	3.07915	2.1
7756	1.32592	2.1
7757	2.86461	2.1
7758	1.34817	2.1
7759	1.44859	2.1
7760	2.03969	2.1
7761	0.946132	2.1
7762	1.50709	2.1
7763	1.50709	2.1
7764	0.946132	2.1
7765	1.29322	2.1
7766	1.29322	2.1
7767	1.01985	2.1
7768	0.968537	2.1
7769	1.401	2.1

7770	1.28936	2.1
7771	1.56002	0.05
7772	1.92548	0.05
7773	1.46041	0.05
7774	2.22712	0.05
7775	1.56001	0.05
7776	1.50524	2.1
7777	1.21304	2.1
7778	1.60665	1.97215
7779	1.85178	1.00751
7780	2.17534	2.1
7781	2.31012	2.1
7782	2.17533	1.9816
7783	0.986724	2.1
7784	2.01895	2.1
7785	2.00433	2.1
7786	1.45752	0.05
7787	1.12695	0.05
7788	0.652605	2.1
7789	1.5133	2.1
7790	1.5133	2.1
7791	0.652603	2.1
7792	1.228 2.1	
7793	0.946132	2.1
7794	0.652604	2.1
7795	0.946132	1.4986
7796	0.716815	2.1
7797	1.62203	2.1
7798	1.62203	2.1
7799	0.716814	2.1
7800	1.00191	2.1
7801	1.33694	2.1
7802	1.38216	2.1
7803	2.53338	2.1
7804	1.46932	0.05
7805	1.50848	0.05
7806	1.50108	1.36141
7807	1.18382	0.225575
7808	2.02115	0.131795
7809	0.921823	0.05
7810	2.08356	0.05
7811	1.543 0.05	
7812	1.00763	0.132837
7813	1.4185	0.184979
7814	1.08028	0.118382
7815	0.946131	0.531772
7816	1.56599	0.163605
7817	1.56599	0.495242
7818	1.08028	0.280739
7819	0.946131	0.104395
7820	1.37518	2.1
7821	1.63453	2.1
7822	1.04627	0.911985
7823	1.04626	0.747316
7824	1.35111	1.17354
7825	1.22297	0.996128
7826	1.64499	2.1
7827	1.87598	2.1

7828	1.06994	2.1
7829	1.37063	2.1
7830	1.99943	0.05
7831	2.38807	0.05
7832	0.99972	0.05
7833	1.41388	0.05
7834	1.66179	0.05
7835	1.66179	0.05
7836	0.946129	0.05
7837	1.41388	0.05
7838	1.19404	0.05
7839	1.31023	0.71274
7840	1.20871	1.11346
7841	1.69731	2.1
7842	1.67971	2.1
7843	0.998971	0.05
7844	1.32988	0.05
7845	0.701079	0.05
7846	0.946131	0.05
7847	0.582785	0.05
7848	1.48592	0.05
7849	1.48592	0.05
7850	1.23351	0.05
7851	0.835199	0.05
7852	0.92427	0.05
7853	1.68262	0.05
7854	2.46702	0.05
7855	1.08715	2.1
7856	1.01606	1.82811
7857	1.29579	1.91864
7858	1.01606	2.06951
7859	1.63823	2.1
7860	1.67687	2.1
7861	1.11048	2.1
7862	1.39477	2.1
7863	0.991114	2.1
7864	2.90978	0.431634
7865	0.906811	1.26185
7866	0.872849	0.05
7867	1.17091	0.157909
7868	0.685763	0.201462
7869	1.6386	1.54621
7870	1.06907	0.395967
7871	1.05516	0.576082
7872	1.9189	0.51344
7873	1.04615	0.189308
7874	1.2966	2.1
7875	1.37392	2.1
7876	1.35504	2.1
7877	1.30347	2.1
7878	1.50877	2.1
7879	1.11193	2.1
7880	1.40984	2.1
7881	1.67682	2.1
7882	1.00371	2.1
7883	1.15412	2.1
7884	1.41638	2.1
7885	1.03796	2.1

7886	0.946117	2.1
7887	1.50823	2.1
7888	1.50822	2.1
7889	0.966923	2.1
7890	0.946131	2.1
7891	2.07593	2.1
7892	1.9339	2.1
7893	1.1817	0.393661
7894	0.729178	0.635511
7895	1.5079	0.632295
7896	1.26991	0.804929
7897	0.994049	0.49022
7898	1.17452	0.732103
7899	1.05711	0.966127
7900	1.04071	1.01378
7901	1.80299	0.46104
7902	1.60417	0.368385
7903	0.943405	0.05
7904	1.01129	0.05
7905	1.42972	0.05
7906	1.15904	0.05
7907	0.680409	0.05
7908	1.2816	0.05
7909	0.806176	0.05
7910	1.18954	0.05
7911	1.26055	0.05
7912	1.06313	2.1
7913	1.19352	2.1
7914	1.55766	2.1
7915	1.53729	2.1
7916	0.993021	0.05
7917	1.09056	1.07491
7918	1.03408	2.1
7919	0.993023	0.842137
7920	0.659901	2.1
7921	0.632905	2.1
7922	1.34072	0.05
7923	1.17793	0.05
7924	2.24885	0.05
7925	0.946132	0.05
7926	1.12443	0.05
7927	0.904207	0.05
7928	1.51237	0.05
7929	1.51237	0.05
7930	1.12443	0.05
7931	1.29215	0.05
7932	1.6943	2.1
7933	2.43858	2.1
7934	2.0589	1.92455
7935	1.67204	2.1
7936	1.67204	2.1
7937	0.555875	2.1
7938	0.91951	2.1
7939	0.946131	1.7361
7940	0.555904	2.1
7941	0.946131	2.08562
7942	1.6943	2.1
7943	1.04744	1.94556

7944	1.50938	2.1
7945	1.60861	2.1
7946	1.92406	0.05
7947	1.18716	0.05
7948	0.962705	0.0644908
7949	1.35382	0.462001
7950	1.43632	0.05
7951	1.09386	0.05
7952	1.92547	0.05
7953	0.994164	0.05
7954	0.903974	2.1
7955	0.928829	1.86404
7956	0.810958	1.41761
7957	1.92853	1.46464
7958	1.598	1.56545
7959	1.28967	1.77891
7960	1.44993	1.66001
7961	0.946132	0.778035
7962	0.882984	0.609921
7963	0.882955	0.931779
7964	1.56071	0.173452
7965	1.56071	0.246658
7966	1.35946	0.487046
7967	1.08421	0.532632
7968	1.41943	0.05
7969	1.26265	0.05
7970	1.03235	0.0678493
7971	1.50778	2.1
7972	1.70387	2.1
7973	1.65608	2.1
7974	1.39076	2.1
7975	1.6104	2.1
7976	1.37456	2.1
7977	1.51898	0.05
7978	1.9726	0.05
7979	2.13757	0.929491
7980	1.74856	1.04489
7981	0.635949	0.21679
7982	2.46448	0.848536
7983	1.23224	0.711153
7984	1.12786	1.12174
7985	1.82721	0.667746
7986	1.82721	0.213453
7987	0.532892	2.02806
7988	0.946131	1.23173
7989	0.53292	1.16468
7990	1.41394	2.1
7991	1.45506	2.1
7992	1.39523	2.1
7993	1.09006	2.1
7994	1.80264	0.05
7995	1.80264	0.178921
7996	0.946131	0.05
7997	1.34268	0.05
7998	0.946132	0.0616696
7999	1.23732	0.05
8000	1.34268	0.05
8001	1.2064	0.870918

8002	1.58164	2.1
8003	1.73853	2.1
8004	1.01243	1.00297
8005	0.910672	1.7138
8006	1.47793	1.89411
8007	1.47793	2.1
8008	0.910702	1.63527
8009	1.30382	1.03954
8010	1.08478	1.14787
8011	0.910702	2.04694
8012	2.60709	1.1812
8013	1.01243	1.08032
8014	1.85516	1.28866
8015	2.52823	0.233772
8016	1.24001	0.270328
8017	1.89793	0.476077
8018	1.73584	0.05
8019	1.24613	2.1
8020	1.37074	1.71143
8021	1.23763	1.38906
8022	1.04072	2.1
8023	0.75499	1.2071
8024	1.72329	1.53431
8025	1.46195	1.2042
8026	0.946132	1.35582
8027	0.747438	2.00322
8028	1.24613	1.64079
8029	2.59886	1.04838
8030	1.87279	2.1
8031	1.07977	2.1
8032	1.29606	2.1
8033	1.45178	2.1
8034	1.45177	2.1
8035	0.92406	2.1
8036	0.946131	2.1
8037	0.924047	2.1
8038	2.04529	2.1
8039	1.04544	2.1
8040	1.08276	2.1
8041	1.67491	2.1
8042	0.961826	1.08592
8043	0.810942	0.835513
8044	0.826622	1.6675
8045	0.914378	1.73337
8046	0.89022	0.494337
8047	0.922383	1.07387
8048	0.988165	0.425434
8049	1.31357	0.05
8050	0.946131	0.05
8051	1.31357	0.05
8052	1.10564	0.05
8053	1.6797	0.05
8054	1.6797	0.05
8055	0.739506	0.05
8056	1.33957	0.05
8057	1.33957	0.05
8058	1.33957	0.05
8059	2.34223	0.05

8060	1.5515	0.859154
8061	2.22137	0.592919
8062	0.94612	0.62015
8063	1.13908	0.52451
8064	1.45423	0.339156
8065	1.45422	0.822076
8066	0.795519	1.11753
8067	1.1391	0.556163
8068	1.11065	0.672744
8069	1.12266	2.1
8070	1.24925	2.1
8071	1.20242	2.1
8072	2.18297	2.1
8073	3.00107	1.85032
8074	2.58297	0.0750721
8075	0.884783	2.1
8076	0.851538	0.306587
8077	0.713587	2.042
8078	1.47949	0.415413
8079	1.66673	2.1
8080	0.696199	2.1
8081	0.9383	1.51562
8082	1.29149	0.611141
8083	1.32266	2.04721
8084	2.11428	0.05
8085	2.17439	0.05
8086	1.40489	0.05
8087	1.21071	0.05
8088	0.946118	0.05
8089	1.0746	0.05
8090	0.973119	0.05
8091	1.31333	0.05
8092	1.08723	0.05
8093	0.994292	2.1
8094	1.4964	2.1
8095	1.15096	2.1
8096	1.10101	2.1
8097	1.50438	2.1
8098	1.67825	2.1
8099	2.02103	2.1
8100	2.01978	2.1
8101	1.50931	0.05
8102	1.50931	0.05
8103	0.65023	0.05
8104	1.34831	0.05
8105	0.811233	0.05
8106	1.34831	0.05
8107	0.94613	0.05
8108	1.00754	1.05965
8109	0.714132	2.1
8110	1.28877	1.52859
8111	1.39905	1.43689
8112	1.03282	1.54189
8113	0.997769	0.991201
8114	1.11743	1.41876
8115	1.86863	1.06214
8116	3.30723	1.67735
8117	1.66635	2.1

8118	1.35609	0.05
8119	1.38212	0.05
8120	1.95597	2.1
8121	1.64257	2.1
8122	2.93316	2.1
8123	3.13417	2.1
8124	0.733947	2.1
8125	1.15259	2.1
8126	1.07976	2.1
8127	1.30237	2.1
8128	1.99943	0.05
8129	0.907431	0.05
8130	0.878161	0.05
8131	1.07544	0.05
8132	1.03401	0.05
8133	0.991975	0.05
8134	1.03635	0.05
8135	0.788549	0.05
8136	1.44984	0.05
8137	1.84007	0.05
8138	2.31127	0.05
8139	1.27362	2.1
8140	1.27737	1.20209
8141	1.87783	0.811897
8142	2.35499	0.05
8143	2.69662	0.05
8144	2.24065	0.05
8145	0.996082	2.1
8146	0.996083	2.1
8147	1.44859	2.1
8148	1.03968	2.1
8149	1.60018	0.219261
8150	1.73082	1.2453
8151	0.946132	1.19126
8152	1.49742	1.52085
8153	1.49742	0.05
8154	1.00501	0.53541
8155	1.41585	2.04755
8156	1.41585	0.510311
8157	0.946131	1.58432
8158	1.03697	0.357101
8159	2.95786	2.1
8160	2.32185	2.1
8161	1.65783	0.080609
8162	1.772 2.1	
8163	0.946131	0.127558
8164	1.40404	0.05
8165	1.13537	2.1
8166	1.04559	1.2048
8167	1.16091	1.11456
8168	2.63751	2.1
8169	1.68455	0.05
8170	1.9682	0.05
8171	2.16834	0.090932
8172	2.45569	0.150494
8173	0.946128	0.0566782
8174	1.36428	0.161168
8175	0.663978	0.180747

8176	1.58218	0.0645609
8177	1.58219	0.0644137
8178	1.36428	0.105081
8179	0.881886	0.121976
8180	1.73849	2.1
8181	1.35668	2.1
8182	1.1233	2.1
8183	1.15009	2.1
8184	1.16482	0.241302
8185	1.29313	0.327336
8186	1.47608	0.0511748
8187	2.05949	0.05
8188	0.946119	0.0550626
8189	1.02974	0.05
8190	0.946119	0.05
8191	1.66758	0.05
8192	1.66757	0.05
8193	1.32082	0.05
8194	1.29287	0.05
8195	1.36005	2.1
8196	1.47109	2.1
8197	2.45958	2.1
8198	2.2975	2.1
8199	0.953521	0.441895
8200	0.953521	0.163611
8201	1.67054	2.1
8202	1.67054	2.1
8203	0.946131	2.1
8204	1.38154	2.1
8205	1.13326	2.1
8206	1.13326	2.1
8207	1.23513	2.1
8208	1.50998	2.07398
8209	2.47982	0.05
8210	1.45091	0.05
8211	0.958953	0.05
8212	0.79239	0.05
8213	2.11929	0.05
8214	1.26511	0.05
8215	1.27733	0.05
8216	2.18127	0.05
8217	1.30169	0.05
8218	1.19259	0.05
8219	0.963395	0.71364
8220	0.999947	2.02459
8221	1.62241	2.1
8222	1.62027	2.1
8223	0.981586	0.05
8224	0.981585	0.05
8225	0.721961	2.1
8226	0.891321	2.1
8227	1.04442	2.1
8228	1.66554	2.1
8229	2.43434	2.1
8230	1.73944	0.05
8231	1.88032	0.05
8232	0.711391	0.05
8233	1.40837	0.05

8234	0.946133	0.05
8235	0.92252	0.05
8236	1.13964	0.05
8237	1.83394	0.05
8238	2.81674	0.05
8239	1.10049	1.58911
8240	1.25092	0.165017
8241	0.760299	0.552417
8242	1.48017	0.696894
8243	1.48017	0.907908
8244	0.760271	0.365611
8245	1.35861	0.604195
8246	0.946129	0.425586
8247	0.760269	0.449683
8248	2.26155	2.1
8249	2.15634	2.1
8250	1.39824	2.1
8251	1.07817	2.1
8252	0.946132	2.1
8253	1.53028	2.1
8254	1.53028	2.1
8255	1.00807	2.1
8256	0.946133	2.1
8257	2.06126	0.264075
8258	1.97385	1.73274
8259	1.75754	0.264577
8260	2.01613	2.1
8261	1.97995	2.1
8262	1.47696	2.1
8263	1.50172	2.1
8264	1.5177	0.05
8265	1.44146	0.05
8266	1.8807	0.05
8267	2.07068	0.05
8268	1.84267	0.05
8269	1.08975	2.1
8270	1.21327	2.1
8271	1.10106	0.05
8272	1.34496	0.05
8273	2.08452	0.0507449
8274	1.70537	0.05
8275	2.62464	2.1
8276	1.5726	2.1
8277	1.5726	2.1
8278	0.94613	2.1
8279	1.15038	2.1
8280	0.946132	2.1
8281	1.03974	2.1
8282	1.15038	2.1
8283	0.99747	0.620166
8284	1.03472	1.88137
8285	1.58407	0.422406
8286	1.21148	0.461544
8287	1.35025	1.43146
8288	1.3428	0.0812243
8289	1.00775	2.1
8290	1.00775	2.1
8291	1.3879	2.1

8292	1.39958	2.1
8293	0.853042	2.1
8294	1.06997	2.1
8295	0.853042	2.1
8296	0.853042	2.1
8297	1.12491	2.1
8298	1.17879	2.1
8299	1.07162	2.1
8300	1.61179	2.1
8301	1.76016	2.1
8302	0.946132	0.216223
8303	1.23449	0.112007
8304	1.70392	0.120984
8305	1.70392	0.0855494
8306	0.94613	0.25613
8307	1.23449	0.137474
8308	1.41556	0.14073
8309	1.14547	0.117721
8310	1.1721	0.053481
8311	0.96478	0.0877702
8312	1.41843	0.0929445
8313	1.2358	0.066154
8314	1.70678	0.063833
8315	1.70678	0.0663081
8316	0.946118	0.15726
8317	1.23582	0.122813
8318	0.946105	0.170137
8319	0.964769	0.05
8320	1.1357	0.05
8321	0.964767	0.05
8322	0.619856	0.05
8323	1.72366	0.05
8324	1.72367	0.05
8325	0.619831	0.05
8326	1.19262	0.05
8327	0.619829	0.05
8328	0.946129	0.05
8329	1.57459	2.1
8330	1.86512	2.1
8331	0.93841	2.1
8332	1.57459	2.1
8333	1.10476	0.05
8334	1.00013	0.204243
8335	2.13446	0.403871
8336	0.759781	2.1
8337	1.96011	1.26175
8338	0.946129	0.747871
8339	1.32056	1.88178
8340	1.75702	1.11815
8341	1.75702	2.1
8342	0.946124	1.82053
8343	1.32056	1.3581
8344	1.38258	0.798037
8345	1.96011	1.3248
8346	2.31099	1.2287
8347	0.97356	2.1
8348	1.38885	2.1
8349	1.3771	2.1

8350	0.946131	2.1
8351	1.84843	2.1
8352	1.84843	2.1
8353	1.3771	2.1
8354	0.946131	2.1
8355	1.00278	2.1
8356	1.05447	0.323312
8357	1.05446	0.538354
8358	1.24913	0.05
8359	1.05447	0.349604
8360	1.80882	0.39679
8361	1.80882	2.1
8362	0.946129	2.1
8363	1.33997	2.1
8364	0.946131	0.0619422
8365	1.28831	2.06799
8366	1.33997	2.06816
8367	1.44391	0.05
8368	1.44391	0.05
8369	0.850964	0.05
8370	0.850963	0.05
8371	0.946132	0.05
8372	0.921299	0.05
8373	1.37358	0.05
8374	1.46297	0.05
8375	1.46298	0.05
8376	0.823988	0.05
8377	0.921287	0.05
8378	0.94612	0.05
8379	0.823995	0.05
8380	0.946107	0.05
8381	1.44437	2.1
8382	1.06203	2.1
8383	2.21397	2.1
8384	0.637677	2.1
8385	1.8892	2.1
8386	2.075	2.1
8387	1.32051	0.05
8388	2.23637	0.05
8389	1.10904	0.05
8390	1.11819	0.05
8391	0.94613	0.05
8392	0.735845	0.05
8393	1.4914	0.05
8394	1.4914	0.05
8395	0.735831	0.05
8396	1.61683	2.1
8397	1.61683	2.1
8398	0.946131	2.1
8399	1.3281	2.1
8400	1.10441	2.1
8401	1.10442	2.1
8402	1.23487	2.1
8403	1.09914	2.1
8404	1.20691	0.05
8405	2.27379	0.05
8406	1.1369	0.05
8407	1.26868	0.05

8408	1.54422	0.05
8409	1.54422	0.05
8410	0.861357	0.05
8411	0.94613	0.05
8412	0.861359	0.05
8413	1.2858	0.111496
8414	1.31011	0.494901
8415	1.294 0.1542	
8416	1.03079	0.389928
8417	1.81979	2.08345
8418	1.57829	2.1
8419	1.19023	1.6691
8420	1.19022	1.99036
8421	1.46458	1.8346
8422	1.46458	2.07575
8423	0.789173	2.1
8424	1.34386	2.08013
8425	0.909896	2.1
8426	0.946131	2.01624
8427	0.909893	2.07685
8428	1.27725	2.1
8429	1.68451	2.1
8430	2.26155	2.1
8431	2.34217	2.1
8432	1.55485	0.393519
8433	0.90854	0.338118
8434	0.858256	0.190939
8435	1.47042	0.125727
8436	1.62771	0.14907
8437	0.777398	0.483286
8438	0.982525	0.221903
8439	1.23865	0.238081
8440	2.09928	0.207825
8441	0.946128	0.067022
8442	0.474856	0.0647073
8443	0.474855	0.0834246
8444	1.58747	0.05
8445	1.58747	0.05
8446	0.963917	0.05
8447	1.09841	0.05
8448	2.23904	2.1
8449	2.60055	0.540107
8450	1.02599	2.1
8451	2.17837	2.1
8452	2.07122	2.1
8453	2.7309	0.612806
8454	2.08482	2.1
8455	1.18322	0.05
8456	1.35381	0.179757
8457	1.01139	0.061408
8458	0.946133	0.405707
8459	1.59089	0.118694
8460	1.59089	0.05
8461	0.946133	0.0893096
8462	1.09634	0.05
8463	2.02279	0.05
8464	1.03108	0.123554
8465	0.808978	1.45722

8466	1.07317	1.39417
8467	0.831695	1.28307
8468	1.22583	1.1303
8469	1.10215	1.14525
8470	1.07508	1.33313
8471	1.22204	1.16039
8472	1.04679	2.1
8473	1.92535	0.05
8474	1.11081	0.05
8475	0.946131	0.05
8476	0.714824	0.05
8477	0.714795	0.05
8478	1.4231	0.05
8479	1.4231	0.05
8480	0.828617	0.05
8481	1.30928	0.05
8482	1.49894	2.1
8483	1.55702	2.1
8484	0.946131	2.1
8485	1.10689	2.1
8486	0.946227	2.1
8487	0.946131	2.1
8488	1.21754	2.1
8489	1.504	2.1
8490	1.504	2.1
8491	0.723573	2.1
8492	1.24512	2.1
8493	0.723544	2.1
8494	0.946131	2.1
8495	1.24512	2.1
8496	1.29383	0.0658317
8497	1.29383	0.0517339
8498	1.61969	0.05
8499	0.946132	0.0533248
8500	1.12818	0.05
8501	0.946132	0.0542437
8502	1.80922	0.05
8503	1.80922	0.05
8504	1.41383	0.05
8505	1.34153	0.05
8506	1.47689	0.05
8507	1.79375	0.05
8508	1.24333	0.05
8509	0.768989	0.05
8510	1.24333	0.05
8511	1.0027	0.0627974
8512	1.47705	0.0518249
8513	1.47705	0.05
8514	0.768977	0.05
8515	1.28436	0.0504866
8516	0.928583	0.0639586
8517	1.28436	0.05
8518	1.24333	0.0600924
8519	1.5991	0.0584621
8520	1.5991	0.05
8521	0.928583	0.0695122
8522	1.85717	0.05
8523	2.48665	0.05

8524	1.18423	0.05
8525	1.9493	1.87601
8526	1.8519	0.87318
8527	1.68802	2.1
8528	1.0302	0.05
8529	0.900473	0.05
8530	1.99663	0.05
8531	2.61301	0.05
8532	0.745772	0.05
8533	0.946131	0.05
8534	1.48881	0.05
8535	1.48881	0.05
8536	0.745745	0.05
8537	0.809786	0.05
8538	1.3065	0.05
8539	0.946132	0.05
8540	0.768357	0.05
8541	0.768387	0.05
8542	1.76897	0.05
8543	1.76896	0.05
8544	1.40271	0.05
8545	1.00777	0.05
8546	1.37427	0.05
8547	1.1967	2.1
8548	0.946132	2.1
8549	1.59397	2.1
8550	1.59397	2.1
8551	0.946131	2.1
8552	1.14989	2.1
8553	1.14989	2.1
8554	1.10046	2.1
8555	1.09506	0.05
8556	2.53251	0.05
8557	1.9212	0.359692
8558	1.99586	0.414652
8559	0.913967	0.341664
8560	1.55028	0.315871
8561	1.55028	0.498666
8562	0.913966	0.191906
8563	1.16318	0.262668
8564	1.16318	0.310428
8565	0.946132	0.24544
8566	1.20478	2.1
8567	1.09662	2.1
8568	1.03472	2.1
8569	1.84774	2.1
8570	1.7984	2.1
8571	2.1221	2.1
8572	1.68223	2.1
8573	1.68224	2.1
8574	0.514872	2.04229
8575	0.888991	2.1
8576	0.946127	0.863259
8577	0.514845	2.04735
8578	0.946129	1.59533
8579	1.10127	1.87361
8580	2.61623	1.14506
8581	0.983581	1.141

8582	0.869663	0.446952
8583	1.1818	1.94842
8584	1.10406	0.640813
8585	1.07133	2.06809
8586	1.09405	0.938032
8587	0.838014	0.68163
8588	0.842998	1.10664
8589	1.03818	0.804476
8590	1.46338	0.0717072
8591	1.903 2.1	
8592	1.23942	2.1
8593	0.965933	2.1
8594	0.810075	2.1
8595	1.40571	2.1
8596	1.66219	2.1
8597	0.810047	2.1
8598	0.936678	2.1
8599	1.45808	2.1
8600	1.6384	2.1
8601	1.54699	2.1
8602	0.719877	2.1
8603	1.33256	2.1
8604	0.753547	2.1
8605	1.04073	2.1
8606	1.25822	2.1
8607	0.851542	2.1
8608	0.714102	2.1
8609	1.45886	2.1
8610	1.80171	2.1
8611	0.597337	2.1
8612	0.957713	2.1
8613	1.3033	2.1
8614	0.596613	0.05
8615	1.1386	0.0829555
8616	0.596612	0.05
8617	0.946133	0.16454
8618	0.596652	0.172755
8619	1.64092	0.0922237
8620	1.64092	0.0825533
8621	1.991 2.1	
8622	1.23013	2.1
8623	2.77816	2.1
8624	2.74107	0.597687
8625	2.07681	0.277722
8626	1.47764	0.05
8627	1.72677	0.05
8628	1.52161	2.1
8629	1.28526	2.1
8630	1.90502	2.1
8631	1.87359	0.05
8632	1.93489	0.05
8633	2.70895	2.1
8634	2.83141	2.1
8635	1.41766	0.234759
8636	1.11337	0.220599
8637	1.41765	1.41291
8638	1.29172	0.293612
8639	1.76324	0.354537

8640	1.76324	0.977874
8641	0.946131	0.235245
8642	1.52428	2.1
8643	1.71459	2.1
8644	1.93852	0.210429
8645	1.07119	1.38501
8646	1.29698	1.15802
8647	1.42203	0.309561
8648	1.42203	1.29152
8649	0.946132	0.625872
8650	0.946132	0.978024
8651	0.96926	1.09157
8652	0.769913	1.90828
8653	0.946119	1.64965
8654	1.37867	1.10373
8655	1.94028	0.926259
8656	0.94612	0.448017
8657	0.946132	1.44562
8658	1.37865	0.740464
8659	2.14234	1.13651
8660	3.06459	1.19954
8661	1.44421	0.05
8662	1.33924	0.05
8663	1.33671	0.05
8664	1.10733	0.05
8665	0.68396	0.05
8666	1.76008	0.05
8667	1.76008	0.05
8668	0.68393	0.05
8669	0.946131	0.05
8670	3.79242	2.1
8671	2.46293	2.1
8672	3.56947	2.1
8673	1.35487	1.67677
8674	1.38616	1.94478
8675	0.954048	0.05
8676	1.36663	0.05
8677	0.909487	0.0613758
8678	0.887288	2.1
8679	0.810947	0.707307
8680	0.810934	1.76374
8681	1.07046	1.90322
8682	1.13979	2.04402
8683	0.927838	0.778118
8684	0.919995	1.55677
8685	1.61447	2.1
8686	2.56305	2.1
8687	0.943414	2.1
8688	1.17016	2.1
8689	1.7238	2.1
8690	1.89334	2.1
8691	0.946128	0.919243
8692	1.25184	2.1
8693	1.55639	2.1
8694	3.12098	1.637
8695	3.57307	0.555311
8696	2.81281	2.1
8697	0.851542	2.1

8698	1.27689	2.1
8699	1.52568	2.1
8700	0.839689	2.1
8701	1.36471	2.1
8702	0.827865	2.1
8703	0.93667	2.1
8704	0.900861	2.1
8705	0.943152	2.1
8706	1.00667	2.1
8707	1.18141	2.1
8708	1.15298	2.1
8709	1.20335	2.1
8710	1.00713	2.1
8711	0.597537	2.1
8712	1.0577	2.1
8713	0.967342	0.236496
8714	0.838027	0.966552
8715	0.893587	0.152784
8716	1.06436	0.322773
8717	0.850048	0.122809
8718	1.22026	0.545361
8719	0.988077	0.21744
8720	1.18769	0.05
8721	1.3991	2.1
8722	1.52984	2.1
8723	1.35469	1.08905
8724	1.25083	0.650116
8725	1.6594	1.38453
8726	1.6594	0.832341
8727	0.946129	0.482234
8728	1.02602	0.500467
8729	0.94613	0.359482
8730	1.29724	0.05
8731	1.14501	0.05
8732	1.13078	0.05
8733	1.48381	0.05
8734	1.45921	0.05
8735	0.851539	0.05
8736	0.890836	0.05
8737	0.946131	0.05
8738	1.60428	2.1
8739	1.50097	2.1
8740	0.714507	0.05
8741	1.51396	0.05
8742	1.51396	0.05
8743	0.946132	0.05
8744	0.813862	0.05
8745	1.41461	0.05
8746	0.813862	0.05
8747	0.921311	2.1
8748	1.25084	0.655495
8749	1.30165	0.45764
8750	1.60636	1.45663
8751	1.60636	0.474948
8752	0.94613	0.38972
8753	0.972232	0.413267
8754	0.946131	0.281319
8755	2.35003	2.1

8756	0.92546	2.1
8757	1.00713	2.1
8758	0.810947	2.1
8759	0.810934	2.1
8760	1.056	2.1
8761	0.926811	2.1
8762	0.766408	2.1
8763	1.34292	2.1
8764	1.57932	0.05
8765	2.67595	0.05
8766	1.77737	0.05
8767	1.77738	0.05
8768	0.789633	0.05
8769	1.22903	0.05
8770	0.789663	0.05
8771	0.789662	0.05
8772	1.33797	0.05
8773	0.946128	2.1
8774	1.47351	2.1
8775	1.47351	2.1
8776	0.94613	2.1
8777	1.30082	2.1
8778	1.30082	2.1
8779	0.996486	2.1
8780	0.946127	2.1
8781	1.72122	2.1
8782	1.72122	2.1
8783	0.94613	2.1
8784	1.36653	2.1
8785	1.36653	2.1
8786	1.02608	2.1
8787	0.709123	0.549957
8788	0.946127	0.562479
8789	1.41342	0.813476
8790	1.818	1.1456
8791	1.818	0.984554
8792	0.946127	0.0900822
8793	1.41342	0.881633
8794	1.35071	0.552078
8795	1.8993	1.07667
8796	1.4748	2.1
8797	1.18711	0.05
8798	0.946131	0.05
8799	1.18711	0.05
8800	1.20416	0.05
8801	1.91358	0.05
8802	1.91358	0.05
8803	0.477691	0.05
8804	2.16798	0.05
8805	2.22307	0.05
8806	0.758971	0.05
8807	1.80694	0.05
8808	1.80694	0.05
8809	0.946132	0.05
8810	0.75897	0.05
8811	1.25124	0.05
8812	0.946131	0.05
8813	1.76837	2.1

8814	1.76837	2.1
8815	0.946131	2.1
8816	1.36422	2.1
8817	0.946131	2.1
8818	1.14184	2.1
8819	1.36422	2.1
8820	0.793303	0.05
8821	1.79584	0.05
8822	1.35333	2.1
8823	1.19967	1.37012
8824	1.55088	1.813
8825	1.64246	2.1
8826	0.946132	0.6254
8827	0.886293	2.08936
8828	1.25018	2.1
8829	1.5795	1.81323
8830	2.15414	1.36879
8831	0.94613	0.982283
8832	1.37758	1.73396
8833	1.71567	1.00779
8834	1.71567	2.1
8835	0.946125	1.28383
8836	1.37759	1.00157
8837	1.28421	0.626126
8838	0.933959	0.704895
8839	0.946133	0.05
8840	1.7813	0.05
8841	1.7813	0.05
8842	1.05704	0.05
8843	1.37794	0.05
8844	1.37794	0.05
8845	0.946132	0.05
8846	0.946132	0.05
8847	1.68197	0.05
8848	1.68197	0.05
8849	1.01367	0.05
8850	1.25017	0.05
8851	1.25017	0.05
8852	0.946132	0.05
8853	0.838953	2.1
8854	1.4045	2.1
8855	1.56388	2.1
8856	1.3182	2.1
8857	0.928518	2.1
8858	1.04073	2.1
8859	0.835676	2.1
8860	1.47507	2.1
8861	1.47506	2.1
8862	0.797664	2.1
8863	1.34285	2.1
8864	0.929875	2.1
8865	0.797663	2.1
8866	0.946133	2.1
8867	1.83628	0.103249
8868	1.83627	0.914977
8869	0.946131	0.37663
8870	1.36748	0.168509
8871	0.946133	0.0643627

8872	1.21139	0.363228
8873	1.36748	0.05
8874	1.06795	2.1
8875	1.08192	2.1
8876	0.827866	2.1
8877	1.02548	2.1
8878	0.95006	2.1
8879	1.53933	2.1
8880	0.586511	2.1
8881	0.868145	2.1
8882	1.28401	2.1
8883	1.37719	2.1
8884	1.37719	2.1
8885	0.77497	2.1
8886	0.946132	2.1
8887	0.774968	2.1
8888	1.7053	0.05
8889	1.7053	1.87972
8890	0.94613	2.1
8891	1.25715	1.68617
8892	0.946132	0.05
8893	1.19655	0.222667
8894	1.25716	0.0898183
8895	0.851542	2.1
8896	0.80043	2.1
8897	1.27239	2.1
8898	1.02719	2.1
8899	0.946131	2.1
8900	0.981318	2.1
8901	1.06628	2.1
8902	1.26022	2.1
8903	0.946132	2.1
8904	0.946132	2.1
8905	1.76447	2.1
8906	1.76447	2.1
8907	1.35357	2.1
8908	1.35703	2.1
8909	0.946131	2.1
8910	1.50538	2.1
8911	1.50538	2.1
8912	1.10311	2.1
8913	1.3484	2.1
8914	1.10312	2.1
8915	0.966428	2.1
8916	0.946133	2.1
8917	1.4008	2.1
8918	1.71908	2.1
8919	1.71908	2.1
8920	0.946133	2.1
8921	1.40081	2.1
8922	1.26441	2.1
8923	0.851543	2.1
8924	0.961732	2.1
8925	1.54576	2.1
8926	1.35364	2.1
8927	0.946133	2.1
8928	1.02913	2.1
8929	1.2735	2.1

8930	1.32608	2.1
8931	0.967643	2.1
8932	0.94613	2.1
8933	1.44873	2.1
8934	1.44873	2.1
8935	0.967641	2.1
8936	0.946131	2.1
8937	1.40488	2.1
8938	1.24272	2.1
8939	0.946131	2.1
8940	1.78483	2.1
8941	1.78483	2.1
8942	1.24272	2.1
8943	0.946131	2.1
8944	0.94613	2.1
8945	1.5627	0.209222
8946	1.5627	2.1
8947	1.03656	2.1
8948	1.31649	0.0929368
8949	1.19233	1.19069
8950	1.03656	0.799287
8951	0.912059	2.1
8952	1.47954	0.272722
8953	1.47954	2.1
8954	0.912061	2.1
8955	1.34647	0.154579
8956	1.04514	1.88554
8957	0.912061	0.969946
8958	0.761972	0.05
8959	0.946132	0.05
8960	0.761974	0.063921
8961	1.70225	0.0977199
8962	1.70225	0.112828
8963	1.33728	0.0500619
8964	1.01188	0.0754743
8965	0.94613	0.947037
8966	0.746361	1.42604
8967	1.6369	1.96108
8968	1.62454	1.60072
8969	0.736738	0.81587
8970	0.958487	1.08814
8971	1.40641	1.28136
8972	1.2284	2.1
8973	0.92609	2.1
8974	0.595102	2.1
8975	1.80871	2.1
8976	1.1516	2.1
8977	0.962474	2.1
8978	2.24024	2.1
8979	0.946131	2.1
8980	1.73689	2.1
8981	1.73689	2.1
8982	0.946132	2.1
8983	1.30492	2.1
8984	1.30492	2.1
8985	1.17949	2.1
8986	0.946131	2.1
8987	1.8566	2.1

8988	1.8566	2.1
8989	1.22392	2.1
8990	1.39826	2.1
8991	1.40447	2.1
8992	1.22392	2.1
8993	0.946131	2.1
8994	1.74134	2.1
8995	1.74134	2.1
8996	1.09148	2.1
8997	1.40447	2.1
8998	1.283 2.1	
8999	1.09148	2.1
9000	1.33607	2.1
9001	1.41022	2.1
9002	1.80016	2.1
9003	1.80015	2.1
9004	0.946127	2.1
9005	0.946129	2.1
9006	1.20541	2.1
9007	1.13078	2.1
9008	1.33607	2.1
9009	1.54624	2.1
9010	1.85553	2.1
9011	0.920606	2.1
9012	0.946131	2.1
9013	1.01271	2.1
9014	1.32664	1.48735
9015	0.954692	1.24997
9016	0.946116	0.506562
9017	1.47771	0.59687
9018	1.4777	2.1
9019	0.95468	0.836672
9020	0.94613	1.01772
9021	1.31381	1.03165
9022	1.14005	0.167801
9023	0.946128	1.04862
9024	1.69433	0.0663507
9025	1.69433	1.22888
9026	1.14005	0.616928
9027	0.94613	0.143838
9028	1.521 2.1	
9029	1.411 2.1	
9030	0.94612	2.1
9031	1.35115	2.1
9032	1.04127	2.1
9033	1.05017	2.1
9034	1.03564	2.1
9035	0.607156	2.1
9036	1.54549	2.1
9037	1.36715	2.1
9038	0.607127	2.08897
9039	1.2571	2.1
9040	0.998701	2.1
9041	0.546436	2.1
9042	0.946133	2.1
9043	1.75286	2.1
9044	1.75286	2.1
9045	1.12206	2.1

9046	1.34237	2.1
9047	1.35663	2.1
9048	1.12206	2.1
9049	0.919455	2.1
9050	1.66517	2.1
9051	1.99822	2.1
9052	1.228 2.1	
9053	1.35662	2.1
9054	1.228 2.1	
9055	1.01143	2.1
9056	0.851543	0.769104
9057	0.986856	2.1
9058	1.42762	1.83016
9059	1.23095	2.1
9060	0.946132	2.1
9061	1.00659	2.00721
9062	1.1779	1.67851
9063	1.40025	2.1
9064	1.40025	2.1
9065	0.889308	2.1
9066	1.34652	2.1
9067	0.943042	2.1
9068	0.943041	2.1
9069	1.34652	2.1
9070	1.39514	1.62766
9071	1.30809	1.34677
9072	1.75711	1.2559
9073	1.7571	2.1
9074	0.946125	0.306215
9075	0.946127	1.40494
9076	1.18396	1.39187
9077	1.4844	0.813875
9078	1.4844	1.30099
9079	0.803322	1.98946
9080	0.946133	0.509887
9081	0.803324	2.1
9082	0.803324	1.61098
9083	1.36183	1.68655
9084	0.946131	0.0941673
9085	1.01462	0.0726617
9086	0.946132	0.0696537
9087	1.59749	0.133562
9088	1.59749	0.0648749
9089	1.16009	0.0885088
9090	1.38354	0.0595613
9091	0.851539	0.878185
9092	1.36005	1.76082
9093	1.5532	1.10856
9094	1.80402	2.02358
9095	0.851537	1.89198
9096	0.902632	1.55238
9097	1.44437	1.17936
9098	0.633098	2.1
9099	1.00307	0.0706038
9100	0.94613	0.229705
9101	1.16623	0.0609728
9102	0.946128	0.261423
9103	1.64588	0.172417

9104	1.64588	0.106661
9105	1.35059	0.164531
9106	1.24142	0.177122
9107	0.946131	0.05
9108	1.13343	0.05
9109	0.946127	0.0558024
9110	1.63367	0.05
9111	1.63367	0.05
9112	1.33035	0.05
9113	1.24945	0.05
9114	1.78733	1.6743
9115	1.70856	1.93984
9116	0.637995	1.16246
9117	0.712454	1.09113
9118	0.946132	1.31279
9119	0.637964	0.937972
9120	1.13021	1.22777
9121	0.94613	0.05
9122	1.20509	0.05
9123	0.946127	0.05
9124	1.70545	0.05
9125	1.70545	0.05
9126	1.28508	0.05
9127	1.3665	0.05
9128	1.58612	2.1
9129	1.51845	2.1
9130	0.797374	2.1
9131	1.20057	2.1
9132	0.946132	2.1
9133	0.946132	2.1
9134	0.899819	2.1
9135	1.50471	0.951616
9136	1.50472	1.05017
9137	0.59387	1.20012
9138	0.593844	0.458079
9139	0.946126	1.09575
9140	0.966137	0.674248
9141	1.13245	0.683383
9142	1.20801	0.340086
9143	0.946131	1.10781
9144	1.47644	0.472657
9145	0.733223	0.425321
9146	0.563609	0.426649
9147	1.69218	0.399177
9148	1.6173	0.748288
9149	1.8126	2.0472
9150	1.8126	2.04896
9151	0.678938	2.1
9152	0.678935	2.03222
9153	0.946133	2.1
9154	0.946132	2.06147
9155	0.678936	2.1
9156	1.21997	1.81148
9157	1.28439	1.7955
9158	1.65282	2.01871
9159	1.86735	1.96455
9160	0.851539	2.01082
9161	0.94613	1.16127

9162	0.87688	1.06839
9163	0.804877	0.152069
9164	1.40747	0.306505
9165	1.41312	0.05
9166	0.885349	0.0822693
9167	1.25405	0.286374
9168	1.34463	0.0986597
9169	0.804878	0.0787504
9170	0.810299	0.05
9171	1.04072	0.05
9172	1.25601	0.05
9173	1.04943	0.05
9174	0.900294	0.05
9175	1.38777	0.05
9176	1.33131	0.05
9177	1.39168	0.05
9178	0.946132	0.05
9179	0.747712	0.05
9180	1.61944	0.05
9181	1.61944	0.05
9182	0.946132	0.05
9183	0.747712	0.05
9184	1.41201	0.05
9185	1.12082	0.05
9186	0.946128	0.0516062
9187	1.79415	0.05
9188	1.79415	0.05
9189	1.12082	0.05
9190	0.94613	0.05
9191	0.711721	0.05
9192	1.12316	0.05
9193	0.707038	0.05
9194	1.04072	0.05
9195	0.704409	0.05
9196	1.49036	0.05
9197	1.27689	0.05
9198	0.793044	0.122008
9199	0.810886	0.492919
9200	0.885957	0.149354
9201	1.38254	0.131199
9202	1.35991	0.10343
9203	1.01375	0.205951
9204	1.03169	0.191093
9205	0.946131	0.161999
9206	1.3279	0.35648
9207	1.42284	0.204188
9208	1.42284	0.147722
9209	0.851192	0.939256
9210	0.946131	0.619437
9211	0.851165	0.286264
9212	0.748062	0.05
9213	1.27511	0.05
9214	0.74806	0.05
9215	0.94613	0.05
9216	0.748059	0.05
9217	1.86293	0.05
9218	1.86293	0.05
9219	0.851542	0.05

9220	1.38186	0.05
9221	0.851542	0.05
9222	1.67869	0.05
9223	1.66579	0.05
9224	1.25626	0.05
9225	1.23046	0.05
9226	1.46023	0.05
9227	1.27083	0.05
9228	0.946131	0.05
9229	0.999304	0.05
9230	0.946803	0.05
9231	0.851554	0.05
9232	0.999227	0.05
9233	0.750097	0.05
9234	1.91815	0.05
9235	0.946132	0.05
9236	0.768117	0.05
9237	1.78003	0.05
9238	1.99572	0.05
9239	0.650453	0.05
9240	0.946132	0.05
9241	1.23845	0.05
9242	0.946131	0.05
9243	1.75477	0.05
9244	1.75477	0.05
9245	1.33393	0.05
9246	1.36697	0.05
9247	0.749018	1.29798
9248	1.02366	1.70473
9249	1.43924	1.342
9250	1.00018	1.36645
9251	1.06438	0.863706
9252	0.946127	1.37491
9253	0.74906	1.05602
9254	0.946131	1.36881
9255	1.26488	0.05
9256	0.946131	0.05
9257	0.94613	0.05
9258	1.49051	0.05
9259	1.59837	0.05
9260	0.907797	0.05
9261	1.04455	0.05
9262	1.25214	0.05
9263	0.68452	0.05
9264	1.25215	0.05
9265	1.24677	0.05
9266	1.8144	0.05
9267	1.81441	0.05
9268	0.684518	0.05
9269	0.851542	0.244713
9270	1.52275	0.203926
9271	1.45223	0.181811
9272	1.0269	0.166257
9273	1.29689	0.332452
9274	1.32822	0.44647
9275	0.851553	0.598305
9276	1.21756	0.05
9277	0.946131	0.05

9278	0.946127	0.05
9279	1.78014	0.05
9280	1.78014	0.05
9281	1.32076	0.05
9282	1.40551	0.05
9283	0.711214	0.05
9284	1.82057	0.05
9285	1.82057	0.05
9286	0.946129	0.05
9287	1.22716	0.05
9288	1.30463	0.05
9289	0.94613	0.05
9290	0.568927	0.05
9291	0.946132	0.05
9292	0.568927	0.05
9293	1.99266	0.05
9294	1.99266	0.05
9295	1.26201	0.05
9296	1.29957	0.05
9297	1.21614	0.05
9298	0.851484	0.05
9299	0.851543	0.05
9300	1.85815	0.05
9301	1.81937	0.05
9302	0.697705	0.05
9303	0.946132	0.05
9304	0.593295	0.05
9305	1.53642	0.05
9306	1.53642	0.05
9307	0.908276	0.05
9308	0.593296	0.05
9309	0.593295	0.05
9310	0.94613	0.05
9311	0.867593	0.0538048
9312	1.42107	0.05
9313	1.42107	0.05
9314	0.867621	0.05
9315	1.05516	0.05
9316	0.867624	0.05
9317	0.946129	0.0621653
9318	0.766681	0.05
9319	1.18119	0.05
9320	1.57018	0.05
9321	1.57018	0.05
9322	0.766652	0.05
9323	1.18119	0.05
9324	1.15564	0.05
9325	0.946131	0.05
9326	1.239 0.05	
9327	0.851541	0.05
9328	0.851487	0.05
9329	1.40021	0.05
9330	1.38373	0.05
9331	0.851554	0.05
9332	1.12266	0.05
9333	0.946131	0.05
9334	1.08234	0.05
9335	1.2882	0.05

9336	1.49963	0.117044
9337	1.46188	0.05
9338	0.851555	0.05
9339	1.33007	0.05
9340	1.08054	0.05
9341	0.946129	0.0599003
9342	0.91151	0.05
9343	1.49914	0.050203
9344	1.49914	0.05
9345	0.911471	0.0604442
9346	0.946132	0.899822
9347	1.35943	2.0545
9348	1.68117	1.53444
9349	1.68117	2.1
9350	0.946132	1.70621
9351	1.35943	1.16314
9352	1.26787	0.618703
9353	1.81612	1.87236
9354	1.81612	0.05
9355	0.946131	0.05
9356	1.41492	0.05
9357	0.946132	2.1
9358	1.25037	0.123801
9359	1.41492	0.108587
9360	1.30541	0.05
9361	1.23646	0.05
9362	0.94613	0.05
9363	1.77304	0.05
9364	1.77304	0.05
9365	1.23646	0.05
9366	0.946132	0.05
9367	0.946131	2.1
9368	1.26689	2.1
9369	1.66806	2.1
9370	1.66806	2.1
9371	0.94613	2.1
9372	1.26689	2.1
9373	1.3473	2.1
9374	1.39384	2.1
9375	1.4181	2.1
9376	1.86581	2.1
9377	1.86581	2.1
9378	0.946128	2.1
9379	0.946129	2.1
9380	1.32068	2.1
9381	1.20328	2.1
9382	1.39384	2.1
9383	1.651 2.1	
9384	1.651 2.1	
9385	0.946129	2.1
9386	0.946131	2.1
9387	1.12913	2.1
9388	1.21474	2.1
9389	0.946132	2.1
9390	0.94613	2.1
9391	1.79724	2.1
9392	1.79724	2.1
9393	1.40987	2.1

9394	1.3335	2.1
9395	1.65642	2.1
9396	1.65642	2.1
9397	0.946132	2.1
9398	1.19063	2.1
9399	0.946133	2.1
9400	1.15203	2.1
9401	1.19063	2.1
9402	0.946131	0.258874
9403	1.77815	2.1
9404	1.77815	2.1
9405	0.946131	1.94722
9406	1.31745	2.1
9407	1.31745	2.1
9408	1.2597	2.1
9409	0.946131	0.12895
9410	1.85184	1.19023
9411	1.85184	2.1
9412	0.946128	1.44554
9413	1.40684	2.1
9414	1.40684	1.21504
9415	1.34049	1.64974
9416	0.946132	1.60528
9417	1.71333	1.03201
9418	1.71333	2.1
9419	1.1848	1.58627
9420	1.34297	0.759294
9421	1.31649	1.03527
9422	1.1848	1.83305
9423	1.36604	0.347966
9424	0.954559	0.536782
9425	1.36604	0.437954
9426	1.41766	0.372193
9427	1.83756	0.64962
9428	1.83756	2.0193
9429	0.946131	0.646409
9430	0.946127	0.066194
9431	1.14874	0.05
9432	0.946127	0.0924263
9433	1.66393	0.0685648
9434	1.66393	0.05
9435	1.35559	0.0655082
9436	1.25447	0.0762888
9437	0.94613	0.05
9438	1.20732	0.05
9439	0.946129	0.05
9440	1.75073	0.05
9441	1.75073	0.05
9442	1.3665	0.05
9443	1.33035	0.05
9444	1.73256	2.1
9445	1.73256	2.1
9446	0.946131	2.1
9447	1.34921	2.1
9448	1.19462	2.1
9449	1.19462	2.1
9450	1.32949	2.1
9451	1.75754	0.195458

9452	1.75754	0.05
9453	0.946132	0.224832
9454	1.3201	0.152971
9455	0.946132	1.00382
9456	1.25479	0.05
9457	1.3201	0.171693
9458	1.8087	0.502837
9459	1.8087	0.05
9460	0.946131	0.05
9461	1.38357	0.05
9462	0.946133	0.300992
9463	1.31034	0.05
9464	1.38357	0.0808302
9465	1.12702	0.05
9466	0.946131	0.0632969
9467	1.23923	0.05
9468	1.4117	0.05
9469	0.946131	0.05
9470	1.70479	0.05
9471	1.70479	0.05
9472	0.946119	2.1
9473	1.55309	2.1
9474	1.55309	2.1
9475	0.946119	2.1
9476	1.08006	2.1
9477	1.08005	2.1
9478	1.02681	2.1
9479	1.36581	2.1
9480	1.93075	2.1
9481	0.946133	2.1
9482	0.772538	2.1
9483	0.946131	2.1
9484	0.946131	2.1
9485	1.33748	2.1
9486	1.44991	2.1
9487	1.31183	2.1
9488	0.946132	2.1
9489	0.944104	2.1
9490	0.915236	2.1
9491	1.05017	2.1
9492	0.944148	2.1
9493	0.946132	0.05
9494	1.60171	0.05
9495	1.60171	0.05
9496	0.946131	0.05
9497	1.16234	0.05
9498	1.3855	0.0513057
9499	0.946132	0.0508661
9500	0.946133	0.05
9501	1.73285	0.05
9502	1.73285	0.05
9503	0.946133	0.0609411
9504	1.3855	0.05
9505	1.29348	0.0811595
9506	0.946132	0.0864573
9507	1.34828	2.1
9508	1.20943	2.1
9509	0.946129	2.1

9510	1.71013	2.1
9511	1.71013	2.1
9512	1.20943	2.1
9513	0.94613	2.1
9514	1.19613	0.05
9515	0.946118	0.05
9516	0.946117	0.05
9517	1.70868	0.05
9518	1.70869	0.05
9519	1.40551	0.05
9520	1.2493	0.05
9521	0.946131	2.1
9522	1.73123	2.1
9523	1.73123	2.1
9524	0.946132	2.1
9525	1.37811	2.1
9526	1.37811	2.1
9527	1.25144	2.1
9528	0.946132	2.1
9529	1.75139	2.1
9530	1.75139	2.1
9531	1.23374	2.1
9532	1.29926	2.1
9533	1.39827	2.1
9534	1.23374	2.1
9535	1.41375	0.05
9536	1.25697	0.05
9537	0.946118	0.05
9538	1.80377	0.05
9539	1.80377	0.05
9540	1.25699	0.05
9541	0.946106	0.05
9542	0.946133	1.75496
9543	1.81806	0.712511
9544	1.81806	2.1
9545	0.946133	2.1
9546	1.37089	2.1
9547	1.37089	1.10715
9548	1.34999	2.1
9549	0.946131	2.1
9550	1.80855	0.900931
9551	1.80855	2.1
9552	1.31184	2.1
9553	1.3933	1.2162
9554	1.36139	1.99782
9555	1.31184	2.06818
9556	1.01175	1.16118
9557	0.94613	0.293699
9558	0.919796	1.77876
9559	1.50484	0.133445
9560	1.80584	1.91262
9561	1.53512	0.236009
9562	1.14539	0.515505
9563	0.946133	2.1
9564	1.72797	2.06006
9565	1.72797	2.1
9566	1.2111	2.1
9567	1.39731	1.41081

9568	1.27679	2.1
9569	1.21111	2.1
9570	0.946119	0.0684773
9571	1.67673	0.84507
9572	1.67673	2.1
9573	0.946118	1.07452
9574	1.21519	2.1
9575	1.2152	2.1
9576	1.19808	2.1
9577	0.946127	2.1
9578	1.54442	2.1
9579	1.54442	2.1
9580	1.04806	2.1
9581	1.13485	2.1
9582	1.3557	2.1
9583	1.04805	2.1
9584	0.946129	2.1
9585	1.32282	2.1
9586	1.77749	2.1
9587	1.77749	2.1
9588	0.946131	2.1
9589	1.32282	2.1
9590	1.4008	2.1
9591	0.946128	0.05
9592	1.02366	0.05
9593	0.94613	0.05
9594	1.66684	0.05
9595	1.66684	0.05
9596	1.29215	0.05
9597	1.32082	0.05
9598	0.946133	0.0652684
9599	1.01451	0.0586576
9600	0.946132	0.0514408
9601	1.62374	0.0590645
9602	1.62374	0.05
9603	1.2929	0.0510439
9604	1.27697	0.05
9605	1.37869	2.1
9606	1.17215	2.1
9607	0.946132	2.1
9608	1.70508	2.1
9609	1.70508	2.1
9610	1.17215	2.1
9611	0.946132	2.1
9612	1.03784	2.1
9613	0.946132	2.1
9614	0.946132	2.1
9615	1.56718	2.1
9616	1.56717	2.1
9617	1.35703	2.1
9618	1.15628	2.1
9619	1.35356	2.1
9620	1.28976	2.1
9621	0.946131	2.1
9622	1.78612	2.1
9623	1.78612	2.1
9624	1.28976	2.1
9625	0.94613	2.1

9626	0.946132	2.1
9627	1.36373	2.1
9628	1.92382	2.1
9629	0.946131	2.1
9630	1.92382	2.1
9631	1.33471	2.1
9632	0.946132	2.1
9633	0.94613	0.202634
9634	1.29149	0.66057
9635	1.75878	0.452962
9636	1.75878	2.02277
9637	0.946128	0.794081
9638	1.29149	0.229327
9639	1.41342	0.102444
9640	1.41022	2.1
9641	1.37034	2.07737
9642	1.83443	2.1
9643	1.83443	2.1
9644	0.946128	1.76485
9645	0.946131	2.1
9646	1.26928	2.1
9647	0.946128	0.807124
9648	1.38259	1.91612
9649	1.81405	1.09887
9650	1.81405	2.1
9651	0.946126	1.56302
9652	1.38259	1.19543
9653	1.37759	0.553972
9654	1.68131	0.05
9655	1.68131	0.05
9656	0.946131	0.05
9657	1.38896	0.05
9658	1.17438	0.555395
9659	1.17438	0.05
9660	1.23848	0.0855142
9661	1.80088	0.0575388
9662	1.80088	0.05
9663	0.946132	0.05
9664	1.40332	0.0517013
9665	1.28185	0.0753345
9666	1.28185	0.05
9667	1.34369	0.05
9668	1.71939	2.1
9669	1.71939	2.1
9670	0.946131	2.1
9671	1.28308	2.1
9672	0.946133	2.1
9673	1.24024	2.1
9674	1.28309	2.1
9675	1.8525	2.1
9676	1.8525	2.1
9677	0.946132	2.1
9678	1.38244	2.1
9679	0.946132	2.1
9680	1.33469	2.1
9681	1.38244	2.1
9682	1.26654	1.50211
9683	1.39514	1.28489

9684	1.71556	1.20601
9685	1.71556	2.1
9686	0.946126	0.320866
9687	0.946129	1.63909
9688	1.1908	1.32737
9689	1.49995	2.1
9690	1.63875	2.1
9691	0.93608	2.1
9692	0.937208	2.1
9693	0.943708	2.1
9694	0.936134	2.1
9695	1.14341	2.1
9696	1.17802	0.05
9697	1.3542	0.05
9698	1.73981	0.05
9699	1.73981	0.05
9700	0.94613	0.05
9701	1.3542	0.05
9702	1.33174	0.05
9703	1.59452	2.1
9704	1.59452	2.1
9705	0.946132	2.1
9706	1.25	2.1
9707	0.975266	2.1
9708	0.946132	2.1
9709	1.25	2.1
9710	0.946131	2.1
9711	1.48685	2.1
9712	1.48685	2.1
9713	0.946132	2.1
9714	1.17273	2.1
9715	1.17273	2.1
9716	0.955602	2.1
9717	1.32827	0.05
9718	1.11166	0.05
9719	0.946127	0.05
9720	1.6136	0.05
9721	1.6136	0.05
9722	1.11166	0.05
9723	0.946129	0.05
9724	0.851541	0.477589
9725	1.22107	0.357799
9726	1.64594	0.237304
9727	1.5271	0.257779
9728	0.94613	0.705167
9729	1.22112	0.438497
9730	1.25002	0.337628
9731	1.65795	0.05
9732	1.65795	2.08766
9733	0.946132	2.1
9734	1.2366	0.522024
9735	0.946132	0.0575382
9736	1.10023	0.519831
9737	1.2366	0.11283
9738	1.35856	0.05
9739	0.946132	0.05
9740	1.35856	0.05
9741	1.08019	0.05

9742	1.49261	0.05
9743	1.49261	0.05
9744	0.94613	0.05
9745	1.29322	0.10558
9746	0.946132	0.0961727
9747	1.29322	0.0586821
9748	1.35856	0.135077
9749	1.70565	0.05
9750	1.70565	0.114733
9751	0.946132	0.114088
9752	1.66635	2.1
9753	1.66635	2.1
9754	0.946133	2.1
9755	1.39558	2.1
9756	1.14793	2.1
9757	1.14793	2.1
9758	1.21691	2.1
9759	1.35381	0.05
9760	1.3365	0.13135
9761	1.09905	0.05
9762	0.946132	0.162642
9763	1.74418	0.0640452
9764	1.74418	0.05
9765	0.946132	0.05
9766	1.84759	0.263828
9767	1.84759	0.05
9768	0.94613	0.0540399
9769	1.3904	0.277349
9770	1.34522	0.05
9771	1.34522	0.05
9772	1.40331	0.05
9773	1.86047	1.26171
9774	1.86047	2.1
9775	0.946132	2.1
9776	1.41619	2.1
9777	0.946132	0.05
9778	1.3659	1.62627
9779	1.41619	1.74232
9780	1.28464	2.1
9781	1.14496	1.61523
9782	1.57806	1.82461
9783	1.7753	2.06434
9784	0.851541	1.89155
9785	0.946131	2.04516
9786	0.872786	1.98006
9787	0.85062	0.642108
9788	1.52635	0.654543
9789	1.63785	0.349881
9790	1.69972	1.12923
9791	0.946132	1.3365
9792	1.37493	0.581102
9793	1.23854	0.514527
9794	1.41134	2.1
9795	1.27079	1.88436
9796	1.736	1.39487
9797	1.736	2.1
9798	0.946132	0.442649
9799	0.946133	1.74188

9800	1.21177	1.99638
9801	0.946131	0.05
9802	0.946129	0.05
9803	0.94613	0.05
9804	0.946131	0.05
9805	0.946131	0.05
9806	1.35403	0.05
9807	0.946131	0.05
9808	1.37052	0.05
9809	0.946132	0.0625903
9810	0.94613	0.05
9811	0.946133	0.05
9812	0.946131	0.05
9813	0.946131	0.05
9814	0.946131	0.05
9815	1.24249	0.05
9816	0.946132	0.05
9817	0.946133	0.05
9818	0.946132	0.05
9819	0.946132	0.05
9820	0.946132	0.05
9821	0.946132	0.05
9822	0.946132	2.1
9823	0.946132	2.1
9824	0.946131	2.1
9825	0.946132	2.1
9826	0.946132	2.1
9827	0.946132	2.1
9828	1.20608	2.1
9829	1.41232	2.1
9830	0.946128	2.1
9831	0.946129	2.1
9832	0.94613	2.1
9833	0.94613	2.1
9834	0.94613	2.1
9835	0.94613	2.1
9836	0.946132	0.05
9837	0.946131	0.05
9838	0.946131	0.05
9839	0.946132	0.05
9840	0.946132	0.05
9841	0.946131	0.05
9842	0.946132	0.05
9843	0.946116	0.162263
9844	0.94612	2.1
9845	0.946118	1.16069
9846	0.946124	2.1
9847	0.946118	1.55125
9848	1.41561	2.1
9849	0.946131	1.88011
9850	0.945846	2.1
9851	1.03508	2.1
9852	0.77579	2.1
9853	0.658908	2.1
9854	1.17474	2.1
9855	0.94303	2.1
9856	0.849189	2.1
9857	0.94298	2.1

9858	1.03963	2.1
9859	0.946131	1.96531
9860	0.946131	1.53494
9861	0.946131	2.1
9862	0.946131	0.921228
9863	0.946131	0.0695157
9864	0.946131	1.39558
9865	1.32819	1.61232
9866	0.946133	1.04455
9867	0.946129	2.1
9868	0.946128	2.1
9869	0.94613	1.98414
9870	0.946131	2.1
9871	0.946131	1.01131
9872	0.946131	1.91302
9873	0.954072	0.05
9874	1.28895	0.05
9875	0.946133	0.05
9876	1.15803	0.05
9877	1.29689	0.05
9878	1.29689	0.05
9879	0.946132	0.05
9880	0.946132	0.596007
9881	0.946132	0.679143
9882	0.946132	0.236575
9883	0.946132	0.843735
9884	0.946132	1.53666
9885	0.946131	1.28604
9886	1.0264	1.34584
9887	0.946126	0.05
9888	0.946132	0.05
9889	0.946131	0.05
9890	0.94613	0.05
9891	0.946129	0.0667512
9892	0.94613	0.0507951
9893	1.35977	0.05
9894	0.946132	0.372812
9895	0.946133	0.335925
9896	0.876632	1.086
9897	0.946132	0.87734
9898	1.01566	1.25865
9899	1.01566	0.803808
9900	0.876606	1.02367
9901	0.946131	0.05
9902	0.946128	0.05
9903	0.946128	0.05
9904	0.94613	0.05
9905	0.94613	0.05
9906	1.24979	0.05
9907	0.94613	0.05
9908	0.946131	0.05
9909	0.946131	0.05
9910	0.921762	0.05
9911	0.9705	0.05
9912	0.9705	0.05
9913	0.946131	0.05
9914	0.921791	0.05
9915	0.810944	0.05

9916	0.810945	0.05
9917	0.810959	0.05
9918	2.42267	0.05
9919	1.35168	0.05
9920	1.08132	0.05
9921	1.08132	0.05
9922	1.08131	0.05
9923	0.946133	0.05
9924	0.946132	0.05
9925	0.946132	0.05
9926	0.946133	0.05
9927	0.946133	0.05
9928	0.946132	0.05
9929	0.946133	0.05
9930	0.946131	0.248088
9931	0.946131	0.685367
9932	0.94613	0.142058
9933	0.851514	0.171306
9934	0.946131	0.369782
9935	0.965268	0.460925
9936	0.851513	0.149704
9937	0.946122	2.1
9938	0.946123	2.1
9939	0.946126	2.1
9940	0.946124	2.1
9941	0.946124	2.1
9942	0.946118	2.1
9943	0.94613	2.1
9944	0.946131	0.221055
9945	1.30448	0.507995
9946	0.946132	0.797556
9947	0.946129	0.363979
9948	0.946131	1.58601
9949	0.946131	0.207219
9950	0.94613	1.38625
9951	0.946133	1.17294
9952	0.946128	2.1
9953	0.946128	2.1
9954	0.94613	2.1
9955	1.36503	2.1
9956	0.946131	2.1
9957	0.946131	2.1
9958	0.810947	0.05
9959	0.810947	0.05
9960	2.48233	0.05
9961	1.3517	0.05
9962	1.08132	0.05
9963	1.08132	0.05
9964	1.08133	0.05
9965	0.810934	0.05
9966	1.48874	0.05
9967	2.48233	0.05
9968	2.2369	0.05
9969	0.946133	0.170823
9970	0.946132	0.103146
9971	0.946132	0.292722
9972	0.946133	0.371863
9973	0.946133	0.379792

9974	1.41851	0.127624
9975	0.946132	0.205124
9976	1.03925	2.1
9977	0.946131	2.1
9978	0.94613	2.1
9979	0.946128	2.1
9980	0.946133	2.1
9981	0.944743	2.1
9982	1.13703	2.1
9983	0.946132	2.1
9984	0.946128	2.1
9985	0.946128	2.1
9986	0.946131	2.1
9987	0.94613	2.1
9988	0.94613	2.1
9989	0.94613	2.1
9990	0.946131	0.05
9991	0.946132	0.05
9992	0.946131	0.05
9993	0.946132	0.05
9994	0.946132	0.05
9995	0.946131	0.05
9996	1.20057	0.05
9997	0.946129	2.1
9998	0.94613	2.1
9999	0.94613	2.1
10000	0.946128	2.1
10001	0.946132	2.1
10002	0.94613	2.1
10003	1.38302	2.1
10004	0.856567	0.05
10005	1.14319	0.05
10006	0.716526	0.05
10007	1.20631	0.05
10008	0.626936	0.05
10009	0.946131	0.05
10010	0.94613	0.05
10011	1.39502	0.05
10012	0.881445	0.05
10013	0.946128	0.05
10014	0.94613	0.05
10015	0.881447	0.05
10016	0.946117	0.05
10017	0.946116	0.05
10018	0.88146	0.05
10019	0.946132	2.1
10020	1.39053	2.1
10021	0.946132	2.1
10022	0.946132	2.1
10023	0.946132	2.1
10024	0.946132	2.1
10025	0.946131	2.1
10026	0.946131	2.1
10027	0.946131	2.1
10028	0.946131	2.1
10029	0.946132	2.1
10030	0.946132	2.1
10031	1.40571	2.1

10032	0.946132	2.1
10033	0.94613	0.051923
10034	0.946127	0.09088
10035	0.946128	0.0629693
10036	0.946131	0.0507626
10037	0.946127	0.0607107
10038	1.25819	0.056187
10039	0.946129	0.05
10040	0.946129	0.590751
10041	1.29054	0.572335
10042	0.94613	0.44396
10043	0.94613	0.514934
10044	0.946131	1.3865
10045	0.946131	0.819501
10046	0.94613	0.694362
10047	0.810944	0.05
10048	0.810941	0.05
10049	1.61558	0.05
10050	1.3517	0.05
10051	1.08131	0.05
10052	1.08132	0.05
10053	1.08133	0.05
10054	0.810931	0.05
10055	1.02036	1.65168
10056	0.946132	1.26127
10057	1.02036	1.07082
10058	0.946132	0.52262
10059	1.1576	0.896418
10060	0.964656	1.20596
10061	0.927608	0.491407
10062	0.946128	2.1
10063	1.38813	2.1
10064	0.946129	2.1
10065	0.946131	2.1
10066	0.946131	2.1
10067	0.94613	2.1
10068	0.946131	2.1
10069	0.946131	2.1
10070	0.94613	0.684894
10071	0.94613	2.1
10072	1.41665	2.1
10073	0.946132	0.667067
10074	0.946131	2.1
10075	0.94613	2.1
10076	0.946118	0.0662341
10077	0.946118	0.05
10078	0.946116	0.0578436
10079	0.946126	0.05
10080	0.94612	0.05
10081	0.946131	0.05
10082	0.946105	0.05
10083	0.946132	2.1
10084	0.946132	2.1
10085	0.946132	2.1
10086	0.946131	2.1
10087	0.946132	2.1
10088	0.946132	2.1
10089	0.946132	2.1

10090	0.864997	0.05
10091	0.786573	0.05
10092	0.978561	0.05
10093	1.47385	0.05
10094	1.68652	0.05
10095	1.27803	0.05
10096	1.19771	0.05
10097	1.27595	0.05
10098	0.946127	0.0510893
10099	1.3771	0.05
10100	0.946127	0.05
10101	0.946131	0.0804613
10102	0.946129	0.05
10103	0.946129	0.0544145
10104	0.946129	0.05417
10105	0.95697	0.05
10106	1.48133	0.05
10107	1.24499	0.05
10108	1.5295	0.05
10109	0.938161	0.05
10110	0.808717	0.05
10111	1.3542	0.05
10112	0.946133	0.579768
10113	1.14444	0.377591
10114	0.946132	0.282254
10115	0.946132	0.484748
10116	0.946133	1.28268
10117	0.946132	0.807307
10118	0.946132	0.571104
10119	0.946131	0.05
10120	0.946132	0.05
10121	0.946131	0.05
10122	0.946132	0.05
10123	0.946131	0.05
10124	0.946132	0.05
10125	0.946132	0.05
10126	0.946132	2.1
10127	0.946133	2.1
10128	0.946133	2.1
10129	0.946132	2.1
10130	1.29322	2.1
10131	0.946132	2.1
10132	0.946132	2.1
10133	0.946131	2.1
10134	0.946132	2.1
10135	0.946131	2.1
10136	0.946132	2.1
10137	1.28441	2.1
10138	0.946131	2.1
10139	0.946131	2.1
10140	0.946113	2.1
10141	0.946118	2.1
10142	0.946118	2.1
10143	1.28073	2.1
10144	0.946117	2.1
10145	0.946131	2.1
10146	0.946105	2.1
10147	0.810945	2.1

10148	0.810942	2.1
10149	2.75904	2.1
10150	1.3517	2.1
10151	1.08131	2.1
10152	1.08133	2.1
10153	1.08132	2.1
10154	0.81093	2.1
10155	0.946127	2.1
10156	1.37952	2.1
10157	1.86965	2.1
10158	0.946127	2.1
10159	1.86965	2.1
10160	1.28228	2.1
10161	1.37952	2.1
10162	0.946128	2.1
10163	0.946132	2.1
10164	1.14042	2.1
10165	0.946132	2.1
10166	1.14042	2.1
10167	1.09974	2.1
10168	1.09974	2.1
10169	0.946132	2.1
10170	0.946132	0.05
10171	0.946132	0.05
10172	0.946133	0.05
10173	0.946132	0.05
10174	0.946132	0.05
10175	0.946132	0.05
10176	0.946133	0.05
10177	1.31817	1.83413
10178	0.946131	1.16824
10179	0.946131	1.71504
10180	0.946131	1.61951
10181	0.946131	0.742411
10182	0.946131	1.34694
10183	0.946131	1.35919
10184	0.946132	2.1
10185	0.946132	2.1
10186	0.946132	2.1
10187	0.946132	2.1
10188	0.946132	2.1
10189	0.946132	2.1
10190	1.35048	2.1
10191	0.946131	2.1
10192	0.946131	2.1
10193	0.94613	2.1
10194	0.946133	2.1
10195	0.946133	2.1
10196	1.35943	2.1
10197	0.946131	2.1
10198	0.946133	2.1
10199	0.946132	2.1
10200	0.946132	2.1
10201	0.946133	2.1
10202	0.946133	2.1
10203	0.946133	2.1
10204	0.990712	2.1
10205	0.965725	2.1

10206	0.946129	0.943719
10207	0.946133	1.554
10208	1.21721	1.16021
10209	0.877931	0.692488
10210	0.946131	1.59655
10211	0.946131	0.890792
10212	0.946132	0.107058
10213	0.946131	0.0733812
10214	0.946131	0.47854
10215	0.946133	0.108072
10216	0.946128	1.07173
10217	0.946131	0.303924
10218	1.39575	0.225905
10219	0.810943	0.0807764
10220	0.810942	0.0904188
10221	2.58794	0.05
10222	1.21652	0.05
10223	0.946154	0.0744696
10224	0.979952	0.0724217
10225	1.0813	0.0579246
10226	0.810959	0.0816389
10227	0.924071	0.05
10228	1.16453	0.05
10229	1.28586	0.05
10230	0.946131	0.0568783
10231	1.1227	0.05
10232	1.16462	0.05
10233	0.851553	0.0724123
10234	0.709578	0.05
10235	0.810943	0.10153
10236	0.810959	0.0744936
10237	1.82615	0.05
10238	1.21651	0.05
10239	1.08132	0.05
10240	0.946155	0.0505663
10241	0.979937	0.077783
10242	0.946128	1.2751
10243	0.946132	2.1
10244	0.946132	0.299856
10245	0.94613	2.1
10246	0.94613	2.00293
10247	0.94613	0.328588
10248	0.94613	1.64241
10249	0.946131	1.99316
10250	1.29735	2.1
10251	0.946131	2.1
10252	0.946131	0.903711
10253	0.946131	1.60535
10254	0.946131	0.648793
10255	0.946131	2.06891
10256	0.946128	2.1
10257	1.33266	0.715569
10258	0.94613	0.965712
10259	0.946131	2.1
10260	0.94613	0.106588
10261	0.946131	1.01883
10262	0.946131	0.536445
10263	0.946131	0.193024

10264	0.946131	2.1
10265	0.946131	0.0692709
10266	1.40608	0.121874
10267	0.946131	2.1
10268	0.946131	1.65566
10269	0.946131	1.92609
10270	0.946131	0.880738
10271	0.946131	2.1
10272	0.946131	1.48921
10273	1.2334	1.54017
10274	0.946129	1.94112
10275	0.946131	1.59355
10276	0.946131	2.1
10277	0.946133	2.1
10278	0.946133	2.1
10279	0.946132	1.99915
10280	1.29198	2.1
10281	0.946132	2.1
10282	0.946132	2.1
10283	0.946132	2.1
10284	0.946131	2.1
10285	0.946128	2.1
10286	0.946128	2.1
10287	0.946131	2.1
10288	0.946127	2.1
10289	0.94613	2.1
10290	0.946129	2.1
10291	0.946123	2.1
10292	0.946126	2.1
10293	0.946125	2.1
10294	1.38639	2.1
10295	0.946126	2.1
10296	0.946132	2.1
10297	0.946119	2.1
10298	0.946128	0.0548182
10299	0.946132	0.0547186
10300	0.946131	0.0667971
10301	0.94613	0.05
10302	0.94613	0.05
10303	0.946131	0.05
10304	0.94613	0.05
10305	0.946131	0.05
10306	0.946131	0.05
10307	0.946131	0.0598381
10308	0.946131	0.05
10309	0.946131	0.05
10310	0.946131	0.05
10311	0.946131	0.05
10312	0.810945	2.1
10313	0.810943	2.1
10314	0.810933	2.1
10315	2.24688	2.1
10316	1.3517	2.1
10317	1.08132	2.1
10318	1.08132	2.1
10319	1.08133	2.1
10320	1.25809	0.05
10321	0.946131	0.101328

10322	1.25809	0.05
10323	0.946129	0.0907358
10324	0.94613	0.05
10325	0.946131	0.0610525
10326	0.946131	0.05
10327	0.946131	0.05
10328	0.946131	0.05
10329	0.946129	0.05
10330	1.28911	0.05
10331	0.946131	0.05
10332	0.946131	0.05
10333	0.946131	0.05
10334	0.94613	0.0663032
10335	0.946131	0.0833423
10336	0.94613	0.05
10337	0.946128	0.0723066
10338	0.946132	0.05
10339	1.38954	0.0688133
10340	0.946131	0.0641233
10341	0.946131	0.05
10342	0.946131	0.0980072
10343	0.946131	0.165444
10344	1.37981	0.062304
10345	0.946131	0.595688
10346	0.946131	0.125642
10347	0.946131	0.119157
10348	0.946131	2.1
10349	0.946127	2.1
10350	0.946127	2.1
10351	0.946131	2.1
10352	0.946127	2.1
10353	0.946129	2.1
10354	0.946128	2.1
10355	0.94613	2.1
10356	0.946126	2.1
10357	0.946127	2.1
10358	0.946131	2.1
10359	0.946126	2.1
10360	0.946128	2.1
10361	0.946128	2.1
10362	0.946133	0.0665218
10363	0.946132	0.0518933
10364	0.946132	0.0598141
10365	0.946132	0.0526266
10366	0.946132	0.078041
10367	1.13511	0.05
10368	0.946132	0.05
10369	0.946132	1.92213
10370	0.946129	1.90079
10371	0.94613	1.07282
10372	0.946131	1.99159
10373	0.946131	2.1
10374	0.94613	2.1
10375	1.27575	2.1
10376	0.946129	2.1
10377	0.946131	2.1
10378	0.946131	2.1
10379	0.946128	2.1

10380	0.946133	2.1
10381	0.946131	2.1
10382	0.94613	2.1
10383	1.15581	0.05
10384	0.946132	0.05
10385	0.946131	0.05
10386	0.94613	0.05
10387	1.15581	0.05
10388	0.94613	0.05
10389	0.946131	0.05
10390	0.611407	0.987848
10391	0.946131	0.336839
10392	1.37335	0.596912
10393	1.15086	1.28195
10394	0.617549	0.570849
10395	0.851553	0.797351
10396	1.14592	1.03104
10397	1.22618	1.76596
10398	0.946132	0.450191
10399	0.946132	1.88679
10400	0.946132	0.461711
10401	0.946131	0.455635
10402	0.946132	1.23963
10403	0.946132	0.809596
10404	0.946131	2.1
10405	0.94613	2.1
10406	0.946131	2.1
10407	0.946133	2.1
10408	0.946129	2.1
10409	0.946131	2.1
10410	0.946131	2.1
10411	0.810945	0.725336
10412	0.810943	1.71768
10413	2.50715	1.25765
10414	1.3517	0.422001
10415	1.08133	0.30482
10416	1.08132	1.42009
10417	1.08132	0.0619498
10418	0.810932	0.434984
10419	1.07811	1.27947
10420	0.94613	0.351857
10421	0.946128	1.54163
10422	1.25358	0.072672
10423	0.946131	0.315351
10424	1.25358	0.299221
10425	0.94613	0.410463
10426	0.946129	2.1
10427	1.09197	2.1
10428	0.946131	2.1
10429	1.09197	2.1
10430	1.09532	2.1
10431	1.09532	2.1
10432	0.946131	2.1
10433	1.58788	2.1
10434	1.34255	2.1
10435	0.594912	2.1
10436	0.58894	2.1
10437	0.946129	2.1

10438	1.16679	2.1
10439	1.04071	2.1
10440	0.535418	2.1
10441	0.946131	2.1
10442	0.946128	2.1
10443	0.946131	2.1
10444	1.20221	2.1
10445	0.946128	2.1
10446	0.94613	2.1
10447	1.20221	2.1
10448	0.946132	2.1
10449	0.946133	2.1
10450	0.946132	2.1
10451	0.946132	2.1
10452	0.946133	2.1
10453	0.946133	2.1
10454	0.946132	2.1
10455	0.946132	0.518715
10456	0.946132	1.80598
10457	0.946133	2.1
10458	0.946132	0.999034
10459	1.411	1.8929
10460	0.946132	1.4772
10461	0.946133	1.81489
10462	1.05575	0.05
10463	0.563734	0.05
10464	1.05575	0.05
10465	0.773833	0.05
10466	0.751686	0.05
10467	0.75884	0.05
10468	0.815612	0.05
10469	0.793465	0.05
10470	0.768563	0.05
10471	0.946129	0.05
10472	0.946125	0.05
10473	0.946126	0.05
10474	0.94613	0.05
10475	0.946124	0.05
10476	1.31678	0.05
10477	0.946128	0.05
10478	0.946132	0.05
10479	1.29914	0.05
10480	0.946132	0.05
10481	0.946132	0.05
10482	0.946132	0.05
10483	1.29914	0.05
10484	0.946132	0.05
10485	0.810946	0.05
10486	0.810943	0.05
10487	2.21082	0.05
10488	1.35168	0.05
10489	1.08132	0.05
10490	1.08132	0.05
10491	1.0813	0.05
10492	0.81096	0.05
10493	0.625813	0.05
10494	0.946132	0.05
10495	0.946131	0.05

10496	0.946132	0.05
10497	0.946131	0.05
10498	1.39301	0.05
10499	0.625812	0.05
10500	0.810946	2.01906
10501	0.810945	2.1
10502	0.810931	2.1
10503	2.70043	2.1
10504	1.3517	2.1
10505	1.08131	2.1
10506	1.08131	2.1
10507	1.08133	2.1
10508	0.810943	2.1
10509	0.810944	2.1
10510	0.810958	2.1
10511	2.44802	2.1
10512	1.35168	2.1
10513	1.08132	2.1
10514	1.08132	2.1
10515	1.0813	2.1
10516	0.946131	0.05
10517	0.946129	0.0630002
10518	0.946128	0.05
10519	0.946131	0.0646711
10520	1.3492	0.05
10521	1.3492	0.0548733
10522	0.94613	0.05
10523	0.946132	0.225329
10524	0.946132	0.0696976
10525	0.946133	0.204329
10526	0.946133	0.0638017
10527	0.946132	0.467626
10528	0.946132	0.0649256
10529	0.946132	0.374247
10530	0.946129	0.05
10531	0.946133	0.05
10532	0.946131	0.0674719
10533	0.94613	0.0543651
10534	0.946131	0.0637993
10535	0.946131	0.05
10536	0.946131	0.05
10537	0.946119	0.05
10538	0.946119	0.05
10539	0.946119	0.0630483
10540	0.946125	0.05
10541	0.94612	0.0631953
10542	0.946132	0.05
10543	0.946107	0.05
10544	0.946133	2.1
10545	0.946132	2.1
10546	0.946132	2.1
10547	0.946132	2.1
10548	0.946132	2.1
10549	0.946133	2.1
10550	1.3868	2.1
10551	0.810947	2.1
10552	0.810946	2.1
10553	0.81096	2.1

10554	2.66094	2.1
10555	1.35168	2.1
10556	1.08132	2.1
10557	1.08132	2.1
10558	1.0813	2.1
10559	0.810946	2.1
10560	0.810946	2.1
10561	0.810933	2.1
10562	2.75737	2.1
10563	1.3517	2.1
10564	1.08132	2.1
10565	1.08132	2.1
10566	1.08133	2.1
10567	0.946133	2.1
10568	0.946132	2.1
10569	0.946132	2.1
10570	0.946132	2.1
10571	0.946133	2.1
10572	0.946132	2.1
10573	0.946133	2.1
10574	0.946133	2.1
10575	0.946133	2.1
10576	0.946132	2.1
10577	0.946132	2.1
10578	0.946133	2.1
10579	0.946132	2.1
10580	1.28569	2.1
10581	0.946132	2.08382
10582	0.946133	2.1
10583	0.946133	2.1
10584	0.946132	2.1
10585	0.946132	2.1
10586	0.946132	2.1
10587	0.946133	2.1
10588	0.946132	0.05
10589	0.946131	0.05
10590	0.94613	0.05
10591	0.946133	0.05
10592	0.946128	0.05
10593	0.946131	0.05
10594	0.946131	0.05
10595	0.946131	2.1
10596	0.94613	2.1
10597	0.946131	2.1
10598	0.94613	2.1
10599	1.41833	1.4615
10600	0.946131	2.09778
10601	0.946131	2.1
10602	1.26155	0.05
10603	0.946131	0.05
10604	0.946131	0.05
10605	0.946132	0.05
10606	0.946132	0.05
10607	0.946131	0.05
10608	0.946132	0.05
10609	0.810946	2.1
10610	0.810946	2.1
10611	0.810959	2.1

10612	2.47938	2.1
10613	1.35168	2.1
10614	1.08132	2.1
10615	1.08132	2.1
10616	1.08131	2.1
10617	0.946131	0.0981301
10618	0.94613	0.0787734
10619	0.94613	0.417626
10620	0.946132	0.0757182
10621	0.946128	0.866499
10622	0.94613	0.275316
10623	0.94613	0.259672
10624	0.94613	0.05
10625	0.946131	0.05
10626	0.94613	0.05
10627	0.946129	0.05
10628	0.946132	0.05
10629	0.94613	0.05
10630	0.946131	0.05
10631	0.946132	2.1
10632	0.946131	2.1
10633	0.946128	2.1
10634	1.40756	2.1
10635	0.946131	2.1
10636	0.946129	2.1
10637	0.94613	2.1
10638	0.946131	2.05123
10639	0.946131	1.79838
10640	0.946131	2.1
10641	0.946132	2.1
10642	1.31947	2.05557
10643	0.946131	2.1
10644	0.946131	2.1
10645	0.946131	2.1
10646	1.25768	2.1
10647	0.946132	2.1
10648	0.946132	2.1
10649	0.946129	2.1
10650	0.946131	2.1
10651	0.94613	2.1
10652	0.946132	0.0815589
10653	0.946129	0.0591057
10654	0.946129	0.0910267
10655	0.94613	0.0646218
10656	1.31716	0.0633984
10657	1.31716	0.05936
10658	0.946131	0.10287
10659	0.810943	0.744862
10660	0.810942	2.1
10661	0.81093	2.07233
10662	2.37052	2.1
10663	1.3517	2.0277
10664	1.08132	0.463472
10665	1.08132	2.1
10666	1.08133	1.08883
10667	0.946128	1.00139
10668	1.10335	0.617528
10669	0.94613	0.337676

10670	0.946131	1.2975
10671	0.946131	0.292661
10672	1.10335	0.671351
10673	0.94613	1.10772
10674	0.946132	1.03275
10675	1.18818	0.66587
10676	0.946132	0.509523
10677	0.946132	1.00853
10678	0.946132	0.418112
10679	1.18818	0.606409
10680	0.946132	0.984597
10681	0.946125	2.1
10682	0.94612	2.1
10683	0.946119	2.1
10684	0.946119	2.1
10685	0.946119	2.1
10686	0.946132	2.1
10687	1.31522	2.1
10688	1.28803	2.1
10689	0.946132	0.414862
10690	0.946132	2.1
10691	0.946133	2.1
10692	0.946133	0.185399
10693	0.946133	2.1
10694	0.946132	2.1
10695	0.946133	1.20896
10696	0.946132	1.53164
10697	0.946132	1.15501
10698	0.946132	0.734446
10699	0.946133	0.77707
10700	0.946132	0.629088
10701	1.37658	0.917122
10702	1.02239	1.86569
10703	0.946128	0.968674
10704	0.946132	1.64253
10705	1.02239	1.06229
10706	0.94613	0.991927
10707	0.96283	1.6798
10708	0.92943	0.880795
10709	0.946132	0.05
10710	0.946131	0.05
10711	0.946126	0.05
10712	0.946129	0.05
10713	0.946128	0.05
10714	0.946128	0.05
10715	0.946129	0.05
10716	0.810946	0.05
10717	0.810944	0.05
10718	2.22493	0.05
10719	1.21651	0.05
10720	1.0813	0.05
10721	0.946154	0.05
10722	0.979951	0.05
10723	0.70959	0.05
10724	0.946132	0.05
10725	0.946131	0.05
10726	0.946131	0.05
10727	0.946133	0.05

10728	0.946131	0.05
10729	0.946132	0.05
10730	0.946132	0.05
10731	0.946132	2.1
10732	0.946131	1.92627
10733	0.946132	2.1
10734	0.946132	1.26622
10735	0.946132	0.537687
10736	0.946132	1.33624
10737	1.40924	1.93579
10738	0.946131	2.1
10739	0.946128	2.1
10740	0.946127	2.1
10741	0.94613	2.1
10742	1.41743	2.1
10743	0.946128	2.1
10744	0.946129	2.1
10745	1.37675	0.369383
10746	0.946128	0.54235
10747	0.94613	0.378323
10748	0.94613	1.08358
10749	0.94613	0.427054
10750	0.946129	0.463998
10751	0.94613	0.941651
10752	0.946132	0.05
10753	0.946132	0.084816
10754	0.946132	0.174281
10755	1.35721	0.05
10756	0.946132	0.53101
10757	0.946132	0.167351
10758	1.35721	0.0959935
10759	0.946133	0.841689
10760	0.946133	0.37048
10761	0.946132	0.760104
10762	1.10003	0.165879
10763	0.946133	0.302851
10764	0.946132	0.600941
10765	0.946132	0.386391
10766	0.946132	0.05
10767	0.946131	0.05
10768	0.946131	0.05
10769	0.946131	0.05
10770	1.0616	0.05
10771	1.0616	0.05
10772	0.946132	0.05
10773	0.946132	0.05
10774	0.946131	0.05
10775	0.946131	0.05
10776	0.946131	0.05
10777	1.09402	0.05
10778	1.09402	0.05
10779	0.946131	0.05
10780	0.946132	0.05
10781	0.946126	0.05
10782	1.24093	0.05
10783	0.946129	0.05
10784	0.946129	0.05
10785	0.946128	0.05

10786	0.946129	0.05
10787	0.946131	0.05
10788	0.946131	0.05
10789	1.2929	0.05
10790	0.946131	0.05
10791	0.946131	0.05
10792	0.946131	0.05
10793	0.946131	0.05
10794	0.810946	0.05
10795	0.810945	0.05
10796	0.810959	0.05
10797	2.41233	0.05
10798	1.35168	0.05
10799	1.08132	0.05
10800	1.08132	0.05
10801	1.0813	0.05
10802	0.810946	1.71683
10803	0.810943	2.1
10804	0.750103	2.1
10805	2.22778	2.1
10806	1.27059	2.1
10807	1.00021	2.01188
10808	1.02049	2.1
10809	1.08133	2.1
10810	0.946128	2.1
10811	0.946132	2.1
10812	0.946132	2.1
10813	0.94613	2.1
10814	0.94613	2.1
10815	0.94613	2.1
10816	0.946131	2.1
10817	0.946126	0.05
10818	0.946131	0.05
10819	0.946127	0.0602876
10820	1.3324	0.05
10821	0.946127	0.05
10822	0.946128	0.05
10823	0.946128	0.0801516
10824	0.94613	2.1
10825	0.946131	2.1
10826	0.94613	2.1
10827	0.946128	2.1
10828	0.946133	2.1
10829	0.946131	2.1
10830	0.946131	2.1
10831	0.946133	2.1
10832	0.946132	2.1
10833	0.946133	2.1
10834	0.946133	2.1
10835	0.946132	2.1
10836	0.946132	2.1
10837	1.36541	2.1
10838	0.946131	0.0817815
10839	0.94613	2.1
10840	0.946131	0.473911
10841	0.94613	0.370488
10842	1.39509	1.93191
10843	0.946131	0.0922661

10844	0.946131	2.02445
10845	0.946133	2.1
10846	0.946133	2.1
10847	0.946132	2.1
10848	0.946132	2.1
10849	0.946133	2.1
10850	0.946132	2.1
10851	1.29319	2.1
10852	1.01196	0.05
10853	0.946131	0.05
10854	0.946132	0.05
10855	0.946132	0.05
10856	0.946132	0.05
10857	0.946132	0.05
10858	0.946132	0.05
10859	0.946112	2.1
10860	0.946118	2.1
10861	0.946116	2.1
10862	0.946117	2.1
10863	0.946118	2.1
10864	0.94613	2.1
10865	0.946105	2.1
10866	1.28684	1.19264
10867	0.946131	1.19763
10868	0.946132	1.20973
10869	0.946132	1.56795
10870	0.946132	1.71717
10871	0.946132	1.31728
10872	0.946132	1.7053
10873	0.94613	2.1
10874	0.94613	2.1
10875	0.946128	2.03875
10876	0.946131	1.59316
10877	0.946131	2.04039
10878	0.94613	2.04617
10879	1.10185	2.1
10880	0.810947	0.05
10881	0.810945	0.05
10882	1.57348	0.05
10883	1.3517	0.05
10884	1.08131	0.05
10885	1.08132	0.05
10886	1.08133	0.05
10887	0.810932	0.05
10888	0.946119	0.112756
10889	0.946116	0.0501044
10890	0.946117	0.109526
10891	0.946121	0.05
10892	0.946118	0.05
10893	0.94613	0.0581765
10894	0.946105	0.05
10895	0.94612	0.189683
10896	0.946122	0.05
10897	0.946119	0.0527207
10898	0.946119	0.241725
10899	1.20884	0.05
10900	0.946132	0.0943755
10901	0.946107	0.0548874

10902	2.67849	0.941237
10903	1.17709	0.752409
10904	0.605418	1.39373
10905	0.286984	0.851088
10906	0.172654	0.997624
10907	0.810946	0.26383
10908	0.940878	0.188012
10909	0.941235	0.143559
10910	1.26696	0.135228
10911	1.56837	0.20212
10912	1.13737	0.120869
10913	1.34115	0.250351
10914	1.24889	0.118732
10915	0.946132	1.28975
10916	0.946129	0.800719
10917	0.946128	0.992827
10918	0.94613	0.928278
10919	0.94613	1.2483
10920	0.946131	1.11549
10921	0.94613	1.07783
10922	0.946116	2.1
10923	0.94612	2.1
10924	0.946118	2.1
10925	0.946123	2.1
10926	0.946118	2.1
10927	0.946131	2.1
10928	1.23836	2.1
10929	0.810946	0.05
10930	0.810946	0.05
10931	0.810959	0.05
10932	2.80677	0.05
10933	1.35168	0.05
10934	1.08132	0.05
10935	1.08132	0.05
10936	1.08131	0.05
10937	0.810947	0.0585753
10938	0.810947	0.641376
10939	2.41574	0.891774
10940	1.21653	0.767877
10941	1.08133	0.240944
10942	0.946153	0.81661
10943	0.979949	0.0955285
10944	0.709567	0.185939
10945	0.94613	2.1
10946	0.946131	2.1
10947	0.946128	2.1
10948	1.34782	2.1
10949	0.946128	2.1
10950	0.946129	2.1
10951	1.34782	2.1
10952	0.946132	2.1
10953	0.946132	2.1
10954	0.94613	2.1
10955	1.16023	2.1
10956	0.946131	2.1
10957	0.946131	2.1
10958	1.16023	2.1
10959	0.946131	0.979415

10960	0.946127	2.1
10961	0.946127	2.1
10962	0.946131	1.80724
10963	0.946128	2.1
10964	0.94613	1.12372
10965	0.946129	1.9303
10966	0.946133	0.05
10967	0.946132	0.05
10968	0.946132	0.05
10969	0.946133	0.05
10970	1.20045	0.05
10971	1.20045	0.05
10972	0.946132	0.05
10973	0.918871	0.05
10974	0.971961	0.05
10975	1.37324	0.05
10976	1.78255	0.05
10977	1.25155	0.05
10978	1.42172	0.05
10979	1.40554	0.05
10980	1.08862	0.05
10981	1.03803	0.05
10982	0.946132	0.05
10983	0.946126	0.05
10984	0.943647	0.05
10985	0.948611	0.05
10986	0.946129	0.05
10987	0.946129	0.05
10988	1.1699	0.320681
10989	0.946132	0.201505
10990	0.946133	0.64589
10991	0.946133	0.181426
10992	0.946132	0.191603
10993	0.946133	0.299472
10994	0.946132	0.548348
10995	0.946132	2.1
10996	0.946131	2.1
10997	0.946131	2.1
10998	0.946132	2.1
10999	0.946131	2.1
11000	0.946131	2.1
11001	0.946132	2.1
11002	0.946132	0.590856
11003	1.38623	0.394436
11004	0.946132	0.194253
11005	0.946132	0.235634
11006	0.946133	0.513173
11007	0.946132	0.198802
11008	0.946133	0.818458
11009	0.946132	2.1
11010	1.40098	2.1
11011	0.946131	2.1
11012	0.946131	2.1
11013	0.946132	2.1
11014	0.946132	2.1
11015	0.946132	2.1
11016	1.01777	0.187872
11017	0.946132	0.0803758

11018	0.946133	1.04666
11019	1.68068	0.139863
11020	1.68068	1.59304
11021	1.41894	0.0799276
11022	1.20787	0.0839889
11023	0.946132	0.158278
11024	0.946133	2.1
11025	0.946133	2.1
11026	0.946132	2.1
11027	1.40199	2.1
11028	0.946133	2.1
11029	0.946132	2.1
11030	0.946132	2.1
11031	0.810946	1.37757
11032	0.810947	0.539341
11033	2.37827	1.77098
11034	1.3517	1.22463
11035	1.08132	1.29379
11036	1.08132	1.49357
11037	1.08133	1.25212
11038	0.810934	1.09989
11039	0.946124	2.1
11040	0.94613	1.81677
11041	1.40248	1.22266
11042	0.946131	1.21416
11043	0.946125	0.560223
11044	0.946127	1.72387
11045	0.946128	0.638228
11046	1.25703	1.98372
11047	0.87821	1.15212
11048	1.18912	1.93955
11049	0.946119	1.05347
11050	0.87825	1.27233
11051	1.1891	1.53859
11052	0.946107	1.98713
11053	0.946132	2.1
11054	1.28543	2.07642
11055	0.946132	1.85395
11056	0.946132	1.58403
11057	0.946131	0.629164
11058	0.946132	0.526563
11059	0.946132	0.794395
11060	0.946115	2.1
11061	0.946119	2.1
11062	0.946116	2.1
11063	0.946123	2.1
11064	0.946118	2.1
11065	1.41636	2.1
11066	0.946129	2.1
11067	0.946132	0.792274
11068	0.946132	2.1
11069	0.946132	2.07664
11070	0.946131	2.1
11071	0.946131	2.1
11072	1.41432	2.1
11073	0.946132	2.1
11074	0.810947	0.05
11075	0.810947	0.05

11076	2.44433	0.05
11077	1.21654	0.05
11078	0.946154	0.05
11079	0.97995	0.05
11080	1.08133	0.05
11081	0.810934	0.05
11082	0.851542	0.05
11083	0.835698	0.05
11084	1.22216	0.05
11085	0.946133	0.05
11086	1.01217	0.05
11087	1.09996	0.05
11088	0.851529	0.05
11089	0.946132	1.56472
11090	0.946132	2.1
11091	0.946129	2.1
11092	0.94613	1.98805
11093	0.946131	2.1
11094	0.946131	2.1
11095	1.2254	2.1
11096	0.800897	1.29923
11097	0.838029	2.1
11098	0.838013	1.72736
11099	0.93679	0.432479
11100	1.20217	0.700081
11101	1.16923	1.0928
11102	0.94948	1.43794
11103	0.958118	1.66427
11104	0.36751	0.988961
11105	0.800535	1.72152
11106	0.272087	0.240199
11107	0.428084	0.592986
11108	0.986335	1.68008
11109	0.946132	0.05
11110	0.946133	0.108931
11111	1.19601	0.05
11112	0.946132	0.0701946
11113	0.946132	0.078036
11114	0.946133	0.0852733
11115	0.946133	0.05
11116	0.946132	1.95008
11117	1.36956	2.1
11118	0.946132	2.1
11119	0.946132	1.83551
11120	0.946132	2.1
11121	0.946133	2.1
11122	0.946132	2.1
11123	0.946132	0.05
11124	0.946131	0.335657
11125	0.946131	0.05
11126	1.34268	0.05
11127	0.946133	0.05
11128	0.946132	0.05
11129	0.946131	0.05
11130	0.946132	2.1
11131	0.946131	2.1
11132	0.946131	2.1
11133	0.946132	2.1

11134	0.946132	2.1
11135	0.946132	2.1
11136	0.946132	2.1
11137	0.810946	0.955208
11138	0.810943	0.602879
11139	2.51472	0.750654
11140	1.3517	0.509509
11141	1.08132	0.492434
11142	1.08132	0.868042
11143	1.08133	0.685113
11144	0.810933	0.793735
11145	0.902316	0.149335
11146	1.04517	0.196087
11147	0.974924	0.0505719
11148	0.530063	0.45163
11149	1.1272	0.242597
11150	0.68207	0.182632
11151	0.477061	0.324976
11152	0.810946	0.05
11153	0.810943	0.05
11154	0.810959	0.05
11155	1.99211	0.05
11156	1.35168	0.05
11157	1.08132	0.05
11158	1.08132	0.05
11159	1.08131	0.05
11160	0.94613	0.05
11161	0.946131	0.05
11162	0.946132	0.05
11163	0.946132	0.05
11164	0.946129	0.05
11165	0.946131	0.05
11166	1.16699	0.05
11167	0.810943	1.4637
11168	0.810942	1.30396
11169	1.6943	2.08014
11170	1.27059	2.1
11171	1.00021	2.09778
11172	1.02049	1.94956
11173	1.08133	1.4457
11174	0.750103	1.46077
11175	0.957813	1.65554
11176	0.946128	1.93436
11177	0.946132	0.864794
11178	1.04832	2.04083
11179	0.870714	1.01374
11180	0.94613	0.987688
11181	0.94613	1.50899
11182	1.86374	2.1
11183	2.0815	1.46019
11184	1.15918	0.902285
11185	0.946129	0.749656
11186	1.15918	0.164663
11187	0.946131	0.952402
11188	0.946131	0.148289
11189	0.946131	0.629214
11190	0.94613	0.972548
11191	0.946119	0.657765

11192	1.3612	0.778322
11193	0.946125	0.709681
11194	0.946119	0.601698
11195	0.94612	1.34117
11196	0.946132	0.891076
11197	0.946107	0.804634
11198	0.810947	0.0540994
11199	0.810947	0.100441
11200	2.50398	0.05
11201	1.3517	0.05
11202	1.08132	0.05
11203	1.08133	0.0721394
11204	1.08132	0.05
11205	0.810934	0.0546435
11206	0.946132	2.1
11207	0.946132	2.1
11208	0.946132	2.1
11209	1.39754	2.1
11210	0.946132	2.1
11211	0.946132	2.1
11212	0.946131	2.1
11213	0.946129	2.1
11214	1.41732	2.1
11215	0.946129	2.1
11216	0.946131	2.1
11217	0.946131	2.1
11218	0.946131	2.1
11219	0.946131	2.1
11220	0.946132	1.14469
11221	1.25276	2.03103
11222	0.946131	2.1
11223	0.94613	1.16059
11224	0.94613	1.95438
11225	0.94613	1.38717
11226	0.946131	1.53386
11227	1.21261	0.352091
11228	0.94613	0.700916
11229	0.94613	1.82813
11230	0.946128	0.293869
11231	0.946131	1.36496
11232	0.94613	1.18301
11233	0.946129	1.34947
11234	0.946128	2.1
11235	0.946133	2.1
11236	0.946132	2.1
11237	0.946131	2.1
11238	0.94613	2.1
11239	0.946131	2.1
11240	1.39407	2.1
11241	0.946116	1.68039
11242	0.946119	1.84025
11243	0.946119	1.01221
11244	1.15369	1.63693
11245	0.946119	0.854434
11246	0.946131	1.37985
11247	0.946106	1.28884
11248	0.946131	2.1
11249	0.946131	2.1

11250	0.946131	2.1
11251	0.946133	2.1
11252	0.946129	2.1
11253	0.946131	2.1
11254	0.946131	2.1
11255	1.19564	0.05
11256	0.498881	0.05
11257	1.33964	0.05
11258	0.498878	0.05
11259	0.923583	0.05
11260	0.831259	0.05
11261	0.553735	0.05
11262	0.946132	0.05
11263	0.946132	0.05
11264	0.946131	0.05
11265	0.946131	0.05
11266	0.946132	0.05
11267	0.946131	0.05
11268	1.28673	0.05
11269	0.946132	0.05
11270	0.946132	0.05
11271	1.33178	0.05
11272	0.946132	0.05
11273	0.946132	0.05
11274	0.946131	0.05
11275	0.946131	0.05
11276	0.946128	0.127631
11277	0.946132	0.0794664
11278	0.946129	0.174235
11279	0.946131	0.05
11280	0.946131	0.05
11281	0.946129	0.0830628
11282	0.94613	0.0691195
11283	0.946131	2.1
11284	0.946131	2.1
11285	0.946129	2.1
11286	1.40525	2.1
11287	0.94613	2.1
11288	0.94613	2.1
11289	1.40525	2.1
11290	0.946131	2.1
11291	0.946126	2.1
11292	0.946127	2.1
11293	0.946127	2.1
11294	1.4073	2.1
11295	0.946129	2.1
11296	0.946129	2.1
11297	0.946131	0.05
11298	0.946131	0.05
11299	0.946132	0.05
11300	0.946131	0.05
11301	0.946131	0.05
11302	1.05016	0.05
11303	0.946132	0.05
11304	0.946128	0.05
11305	0.946132	0.05
11306	0.946131	0.05
11307	0.946129	0.05

11308	0.94613	0.05
11309	1.13398	0.05
11310	0.94613	0.05
11311	0.946132	0.05
11312	0.946132	0.05
11313	0.946131	0.05
11314	0.946131	0.05
11315	0.946132	0.05
11316	0.946131	0.05
11317	0.946132	0.05
11318	0.946131	0.05
11319	0.94613	0.05
11320	0.946131	0.05
11321	0.94613	0.05
11322	1.38645	0.05
11323	1.38645	0.05
11324	0.946131	0.05
11325	0.946131	0.05
11326	0.946131	0.05
11327	0.94613	0.05
11328	1.23923	0.05
11329	0.94613	0.05
11330	0.946131	0.05
11331	0.946131	0.05
11332	0.946132	0.05
11333	0.946131	0.05
11334	0.946131	0.05
11335	0.946132	0.05
11336	0.946132	0.05
11337	0.946132	0.05
11338	1.40511	0.05
11339	0.946132	2.1
11340	0.946132	2.1
11341	0.946132	2.1
11342	0.946132	2.1
11343	0.946132	2.1
11344	0.946131	2.1
11345	1.12968	2.1
11346	0.946132	1.54441
11347	0.946128	2.1
11348	0.946128	2.1
11349	0.94613	1.93017
11350	0.94613	2.1
11351	0.94613	1.71275
11352	0.94613	2.1
11353	0.946133	2.1
11354	0.946133	2.1
11355	0.946132	2.1
11356	0.946132	2.1
11357	0.946132	2.1
11358	0.946133	2.1
11359	1.31323	2.1
11360	0.94613	2.1
11361	0.946131	0.254506
11362	0.946131	0.444672
11363	0.94613	0.0944292
11364	0.946131	2.1
11365	0.946131	2.07982

11366	0.946131	1.80708
11367	0.946132	0.0641055
11368	0.946132	0.05
11369	0.946132	0.12547
11370	1.36899	0.05
11371	0.946132	0.12785
11372	0.946132	0.05
11373	0.946133	0.05
11374	1.03543	0.05
11375	0.946131	0.05
11376	0.946131	0.05
11377	0.946132	0.05
11378	0.94613	0.05
11379	0.950987	0.05
11380	0.941275	0.05
11381	0.946131	0.05
11382	0.94613	0.05
11383	1.28952	0.05
11384	0.946132	0.05
11385	0.94613	0.05
11386	0.946131	0.05
11387	0.946131	0.05
11388	0.94613	2.1
11389	1.35966	2.1
11390	0.946131	2.1
11391	0.946131	2.1
11392	0.946129	2.1
11393	0.94613	2.1
11394	0.94613	2.1
11395	0.810946	2.1
11396	0.810942	0.848162
11397	0.810932	2.1
11398	2.6655	2.1
11399	1.3517	2.1
11400	1.08132	2.1
11401	1.08131	0.472563
11402	1.08133	2.1
11403	0.94613	2.1
11404	0.946131	2.1
11405	0.94613	2.1
11406	0.946128	2.1
11407	0.946131	2.1
11408	0.94613	2.1
11409	0.94613	2.1
11410	0.946128	2.1
11411	0.946128	2.1
11412	0.946133	2.1
11413	1.25375	2.1
11414	0.94613	2.1
11415	0.946131	2.1
11416	1.25375	2.1
11417	0.94613	2.1
11418	0.94613	2.1
11419	0.946132	2.1
11420	0.946132	2.1
11421	0.946128	2.1
11422	0.94613	2.1
11423	1.31453	2.1

11424	0.946131	0.05
11425	0.946129	0.05
11426	0.94613	0.05
11427	0.94613	0.05
11428	0.94613	0.05
11429	1.12018	0.05
11430	0.94613	0.05
11431	0.946131	0.05
11432	0.946129	0.05
11433	0.946131	0.05
11434	1.16373	0.05
11435	0.94613	0.05
11436	0.94613	0.05
11437	0.946131	0.05
11438	1.36811	0.0559927
11439	0.946132	0.0595782
11440	1.36811	0.05
11441	0.946129	0.0701126
11442	0.94613	0.0787549
11443	0.946129	0.0643075
11444	0.946129	0.0610853
11445	0.946132	0.05
11446	0.946133	0.05
11447	0.946132	0.05
11448	0.946133	0.05
11449	1.35732	0.05
11450	1.35732	0.05
11451	0.946133	0.05
11452	1.36399	0.262951
11453	0.946129	0.822722
11454	0.94613	0.377843
11455	0.946131	0.948473
11456	0.94613	1.74554
11457	0.946131	0.647726
11458	0.946131	1.17657
11459	0.946133	2.1
11460	0.946132	2.1
11461	0.946132	2.1
11462	1.38302	2.1
11463	0.946133	2.1
11464	0.946132	2.1
11465	0.946133	2.1
11466	0.810943	2.1
11467	0.810944	2.1
11468	0.810945	2.1
11469	2.80542	2.1
11470	1.35169	2.1
11471	1.08132	2.1
11472	1.08132	2.1
11473	1.08132	2.1
11474	1.30189	2.1
11475	0.946131	2.1
11476	0.946132	2.1
11477	0.946131	2.1
11478	0.946131	2.1
11479	0.946131	2.1
11480	0.946132	2.1
11481	0.810946	1.05181

11482	0.810947	0.488714
11483	2.33293	0.492869
11484	1.24357	0.558495
11485	1.08132	0.709326
11486	0.973183	0.956649
11487	1.00023	0.729609
11488	0.810934	0.950059
11489	1.06584	0.419635
11490	0.946129	0.671716
11491	0.84731	0.226315
11492	1.16646	0.459179
11493	0.946126	0.978031
11494	1.2831	0.299633
11495	0.851527	0.627609
11496	0.946131	2.1
11497	0.946128	2.1
11498	0.946128	2.1
11499	0.94613	2.1
11500	1.31961	2.1
11501	0.94613	2.1
11502	0.94613	2.1
11503	0.946131	2.1
11504	1.3605	2.1
11505	0.946133	2.1
11506	0.946132	2.1
11507	0.94613	2.1
11508	0.946131	2.1
11509	0.946131	2.1
11510	0.946128	2.1
11511	0.946133	1.81273
11512	0.946132	2.1
11513	0.946131	1.35939
11514	0.946131	0.153365
11515	0.946131	2.01677
11516	1.30116	2.1
11517	0.946132	0.05
11518	0.946129	0.05
11519	0.94613	0.05
11520	1.3035	0.05
11521	0.946131	0.05
11522	0.94613	0.05
11523	0.946131	0.05
11524	0.946132	2.1
11525	1.2051	2.1
11526	1.2051	2.1
11527	0.946132	2.1
11528	1.37869	2.1
11529	0.946132	2.1
11530	0.946131	2.1
11531	0.810946	2.1
11532	0.810945	2.1
11533	2.69247	2.1
11534	1.35169	2.1
11535	1.08132	2.1
11536	1.08132	2.1
11537	1.08132	2.1
11538	0.810946	2.1
11539	1.22993	2.1

11540	0.946131	2.1
11541	0.946131	2.1
11542	1.34623	2.1
11543	0.946131	2.1
11544	1.34623	2.1
11545	0.946131	2.1
11546	0.946131	0.05
11547	0.946132	0.05
11548	0.946132	0.05
11549	0.946132	0.05
11550	0.946131	0.05
11551	0.946132	0.05
11552	1.32299	0.05
11553	0.94613	0.053721
11554	0.946131	0.0730588
11555	0.946128	0.05
11556	0.946132	0.05
11557	0.946133	0.05
11558	0.946131	0.0615785
11559	0.946131	0.0557347
11560	0.946132	2.1
11561	0.946129	2.1
11562	0.946128	2.1
11563	0.94613	2.1
11564	0.94613	2.1
11565	0.946131	2.1
11566	0.94613	2.1
11567	0.94613	2.1
11568	0.94613	2.1
11569	0.946131	2.1
11570	0.946132	2.1
11571	0.946129	2.1
11572	0.946131	2.1
11573	0.946131	2.1
11574	0.946132	1.26619
11575	0.946131	1.3292
11576	1.13731	0.669762
11577	0.94775	1.54756
11578	0.944514	2.00732
11579	0.946132	2.02643
11580	1.039	1.99943
11581	0.946131	1.28376
11582	0.946128	1.0605
11583	0.94613	0.469792
11584	0.946131	1.24431
11585	0.946128	1.7661
11586	0.946129	1.90117
11587	1.23726	1.83291
11588	0.946132	2.1
11589	0.946132	2.1
11590	0.946129	2.1
11591	1.39214	2.1
11592	0.946131	2.1
11593	0.946131	2.1
11594	0.94613	2.1
11595	0.946125	0.118147
11596	0.946119	0.05
11597	0.946118	0.05

11598	0.946118	0.05
11599	0.94612	0.0594204
11600	0.946106	0.05
11601	0.946131	0.05
11602	1.26893	0.471718
11603	0.946131	0.477944
11604	1.26893	0.348576
11605	0.94613	0.614491
11606	0.946131	0.562943
11607	0.946131	0.402902
11608	0.946131	1.01467
11609	0.946133	0.05
11610	0.946133	0.05
11611	0.946132	0.05
11612	1.28924	0.05
11613	0.946133	0.05
11614	0.946132	0.05
11615	0.946132	0.05
11616	0.946133	0.116816
11617	0.946132	0.0734174
11618	0.946132	0.106421
11619	0.946133	0.05
11620	0.946133	0.113129
11621	0.946132	0.113743
11622	0.946133	0.0854208
11623	0.946132	0.494151
11624	0.946133	0.0569486
11625	0.946133	0.176294
11626	0.946132	0.457443
11627	1.15388	0.145748
11628	0.946132	0.42323
11629	0.946133	0.232881
11630	0.946132	0.145299
11631	0.946133	0.0507384
11632	0.946132	0.137648
11633	0.946133	0.05
11634	0.946133	0.0926467
11635	0.946132	0.103156
11636	0.946132	0.060691
11637	1.0644	0.873403
11638	1.12486	1.71703
11639	1.08674	1.82255
11640	0.946132	1.14305
11641	0.946132	1.72598
11642	0.946133	1.4722
11643	0.946132	1.29658
11644	0.808978	1.11969
11645	0.911537	1.46176
11646	0.932832	1.31855
11647	1.14604	1.74088
11648	1.56953	1.44458
11649	1.25866	1.17831
11650	1.18488	1.61547
11651	1.341	0.996217
11652	0.946131	0.05
11653	0.946131	0.05
11654	0.946131	0.05
11655	0.94613	0.0540063

11656	1.33922	0.05
11657	0.946131	0.05
11658	0.946131	0.05
11659	0.946132	0.05
11660	0.946133	0.05
11661	0.946132	0.05
11662	0.946132	0.05
11663	0.946132	0.05
11664	0.946133	0.05
11665	0.946132	0.05
11666	0.946129	0.230403
11667	0.946133	2.1
11668	0.946133	0.604962
11669	0.946131	1.15603
11670	1.40496	2.1
11671	0.946131	0.333784
11672	0.946131	2.1
11673	0.94613	1.437
11674	0.946127	0.15524
11675	0.946127	0.688004
11676	0.94613	1.54633
11677	1.38913	0.420939
11678	0.946128	1.31133
11679	0.946128	0.70786
11680	1.98863	0.05
11681	2.59915	0.05
11682	1.40338	0.05
11683	0.946131	0.05
11684	1.22266	0.05
11685	1.40338	0.05
11686	0.946132	0.05
11687	0.946132	0.05
11688	1.22266	0.05
11689	0.94613	0.05
11690	0.94613	0.05
11691	0.946124	0.05
11692	1.21428	0.05
11693	0.946126	0.05
11694	0.946127	0.05
11695	0.946127	0.05
11696	1.03411	0.05
11697	0.946131	0.05
11698	1.03411	0.05
11699	0.946129	0.05
11700	1.14258	0.05
11701	0.952143	0.05
11702	0.940118	0.05
11703	0.810943	2.1
11704	0.810945	2.1
11705	0.810958	2.1
11706	2.82603	2.1
11707	1.35168	2.1
11708	1.08132	2.1
11709	1.08132	2.1
11710	1.0813	2.1
11711	0.946133	0.05
11712	0.946132	0.05
11713	0.946132	0.05

11714	0.946132	0.05
11715	0.946132	0.05
11716	1.11539	0.05
11717	0.946133	0.05
11718	0.946133	2.1
11719	0.946132	2.1
11720	0.946133	2.1
11721	0.946133	2.1
11722	0.946132	2.1
11723	1.41195	2.1
11724	0.946133	2.1
11725	0.946131	0.05
11726	0.946131	0.05
11727	0.946131	0.05
11728	0.946131	0.05
11729	0.946131	0.05
11730	0.946131	0.05
11731	1.15968	0.05
11732	0.946131	0.823673
11733	0.94613	1.29089
11734	0.94613	2.1
11735	1.34386	1.48217
11736	0.946132	2.1
11737	0.94613	2.1
11738	1.34386	2.00467
11739	0.946131	0.775079
11740	0.935202	0.523428
11741	0.946131	2.02997
11742	1.25054	0.907798
11743	0.850155	1.91301
11744	0.946131	1.8401
11745	0.935205	1.56707
11746	0.946131	0.05
11747	1.40193	0.05
11748	0.946132	0.05
11749	0.946132	0.05
11750	0.946129	0.05
11751	1.40193	0.05
11752	0.946131	0.05
11753	0.808779	0.05
11754	0.561937	0.05
11755	1.40193	0.05
11756	1.40193	0.05
11757	0.561936	0.05
11758	0.561937	0.05
11759	1.40193	0.05
11760	0.946132	0.05
11761	0.946132	0.05
11762	0.946132	0.05
11763	0.946132	0.05
11764	0.946133	0.05
11765	0.946132	0.05
11766	0.946133	0.05
11767	0.946127	0.0749876
11768	0.946131	0.0682298
11769	0.946131	0.05
11770	0.946127	0.0831357
11771	1.19583	0.05

11772	1.19583	0.05
11773	0.946129	0.0680185
11774	0.589428	0.094446
11775	1.08737	0.0510949
11776	1.10961	0.0974845
11777	0.872901	0.11035
11778	0.541327	0.0616519
11779	0.541312	0.0573616
11780	0.85153	0.118205
11781	0.946132	2.1
11782	0.946131	2.1
11783	0.946131	2.1
11784	0.946132	2.1
11785	0.946132	2.1
11786	0.946132	2.1
11787	0.946132	2.1
11788	0.946132	1.19775
11789	1.2778	2.1
11790	0.946131	2.1
11791	0.946131	1.46674
11792	0.94613	2.1
11793	0.94613	1.80151
11794	0.94613	1.8957
11795	0.946118	0.696527
11796	0.946112	0.746536
11797	0.946118	0.490081
11798	0.946119	0.50139
11799	0.946117	0.690347
11800	1.35631	0.238865
11801	0.946105	0.238263
11802	0.946133	1.90781
11803	0.946133	2.1
11804	0.946132	2.1
11805	0.946132	2.1
11806	0.946133	0.0545804
11807	0.946132	1.55993
11808	0.946132	1.45092
11809	0.94613	2.1
11810	0.946131	0.285534
11811	0.946128	0.556193
11812	0.946128	0.109947
11813	0.94613	2.1
11814	0.946129	2.1
11815	0.946129	1.91917
11816	0.946132	1.19849
11817	1.15139	0.953632
11818	0.946132	0.664126
11819	0.946132	1.31873
11820	0.946132	1.28848
11821	0.946132	1.48288
11822	0.946132	1.15958
11823	0.946133	2.1
11824	1.36151	2.1
11825	0.946132	2.1
11826	0.946132	2.1
11827	0.946132	2.1
11828	0.946132	2.1
11829	0.946133	2.1

11830	0.946127	2.1
11831	1.38397	2.1
11832	0.94613	2.1
11833	0.946131	2.1
11834	0.946128	2.1
11835	0.94613	2.1
11836	0.946129	2.1
11837	0.946131	0.0581512
11838	0.946127	0.0957408
11839	0.946127	0.0943603
11840	0.946132	0.103968
11841	0.946131	0.0851712
11842	0.946128	0.0678324
11843	0.946129	0.0746764
11844	1.0268	0.05
11845	1.23823	0.05
11846	1.2171	0.05
11847	0.883446	0.05
11848	1.23827	0.05
11849	1.32614	0.05
11850	0.94613	0.05
11851	0.946132	2.1
11852	0.946129	2.1
11853	0.946129	2.1
11854	0.94613	2.1
11855	1.36523	2.1
11856	0.946131	2.1
11857	0.94613	2.1
11858	0.946132	0.05
11859	0.946131	0.05
11860	0.946132	0.05
11861	0.946131	0.05
11862	0.946131	0.05
11863	1.01653	0.05
11864	0.946131	0.05
11865	0.94612	2.1
11866	0.946121	2.1
11867	0.946119	2.1
11868	0.946119	2.1
11869	0.946119	2.1
11870	0.946132	2.1
11871	0.946107	2.1
11872	0.810942	1.71264
11873	0.810944	0.22203
11874	2.60324	2.1
11875	1.21651	2.04511
11876	1.0813	1.83719
11877	0.946153	0.227896
11878	0.979948	2.1
11879	0.709588	2.08311
11880	1.3492	0.05
11881	0.946132	0.05
11882	0.946131	0.05
11883	0.94613	0.05
11884	0.946129	0.05
11885	0.946129	0.05
11886	0.94613	0.05
11887	0.946131	0.05

11888	0.94613	0.086656
11889	0.946128	0.0528887
11890	0.946133	0.0683875
11891	0.946133	0.070643
11892	0.946131	0.0639794
11893	0.946131	0.068122
11894	1.23351	0.05
11895	0.946131	0.05
11896	0.946131	0.05
11897	0.946132	0.05
11898	0.946132	0.05
11899	0.946131	0.05
11900	0.946132	0.05
11901	1.35657	0.05
11902	0.946132	0.05
11903	0.946131	0.05
11904	0.946132	0.05
11905	0.946132	0.05
11906	0.946131	0.05
11907	0.946131	0.05
11908	0.946132	0.17688
11909	0.946132	0.0942335
11910	0.946132	0.197009
11911	0.946131	0.05
11912	0.946131	0.10904
11913	0.946132	0.109348
11914	0.946132	0.0966804
11915	0.946133	0.084761
11916	0.946132	0.05
11917	0.946133	0.112654
11918	0.946132	0.05
11919	0.946132	0.0609958
11920	0.946132	0.0642119
11921	0.946133	0.0567166
11922	0.946131	1.27394
11923	1.4101	1.95634
11924	0.946131	2.1
11925	0.946131	0.443579
11926	0.946131	1.2778
11927	0.946131	0.384366
11928	0.946131	1.98767
11929	0.810944	1.21142
11930	0.810939	1.21316
11931	0.709586	0.619767
11932	2.71261	0.705166
11933	1.21651	0.997398
11934	0.946151	0.462062
11935	0.979943	1.07451
11936	1.0813	1.4617
11937	0.946132	2.1
11938	0.946131	2.1
11939	0.946132	2.1
11940	0.946131	2.1
11941	0.946131	2.1
11942	1.37733	2.1
11943	0.946131	2.1
11944	0.946133	2.1
11945	0.946132	2.09723

11946	0.946132	2.1
11947	0.946133	2.1
11948	0.946133	2.1
11949	0.946132	2.1
11950	1.32582	2.1
11951	0.946132	2.1
11952	0.946132	2.1
11953	0.946132	2.1
11954	0.946132	2.1
11955	0.946132	2.1
11956	0.946132	2.1
11957	0.946131	2.1
11958	0.946133	1.95467
11959	0.946132	1.38356
11960	0.946132	1.70141
11961	0.946132	1.22461
11962	0.946132	1.18083
11963	0.946133	1.27136
11964	1.3469	1.31006
11965	0.946126	1.25801
11966	0.94613	2.1
11967	0.946131	2.1
11968	0.946127	2.1
11969	1.41206	2.1
11970	0.946128	2.1
11971	0.946128	2.1
11972	0.946127	2.1
11973	0.94613	2.1
11974	0.946131	0.707644
11975	0.94613	2.1
11976	0.946128	1.08308
11977	0.946129	2.1
11978	0.946129	2.1
11979	0.946131	0.05
11980	0.94613	0.05
11981	1.24448	0.05
11982	0.946129	0.05
11983	0.946131	0.05
11984	0.94613	0.05
11985	0.946131	0.05
11986	0.946132	0.05
11987	0.946133	0.05
11988	1.11015	0.05
11989	0.946133	0.0764897
11990	0.946132	0.0588311
11991	0.946132	0.0584278
11992	0.946132	0.05
11993	1.38775	2.1
11994	0.946131	2.1
11995	0.946131	2.1
11996	0.946132	2.1
11997	0.946132	2.1
11998	0.946131	2.1
11999	0.946131	2.1
12000	0.946129	2.1
12001	0.946129	2.1
12002	0.946133	1.54074
12003	0.946131	2.1

12004	0.946131	0.0907599
12005	0.946131	1.82468
12006	0.946131	1.60112
12007	0.946132	2.1
12008	0.946133	2.1
12009	0.946132	2.1
12010	1.40774	2.1
12011	0.946132	2.1
12012	0.946133	2.1
12013	0.946132	2.1
12014	0.610968	0.05
12015	0.688233	0.05
12016	0.715226	0.05
12017	0.792409	0.05
12018	1.13418	0.05
12019	0.956667	0.05
12020	0.757321	0.05
12021	0.968845	0.05
12022	0.595679	0.05
12023	0.946133	1.3262
12024	1.28917	1.46419
12025	0.946131	1.87896
12026	0.94613	0.997504
12027	0.946131	1.38728
12028	0.946131	1.26177
12029	0.946131	1.63101
12030	0.946132	0.0781955
12031	0.946128	0.135495
12032	0.94613	0.29832
12033	1.40668	0.126456
12034	0.946131	0.214581
12035	0.946131	0.117652
12036	0.94613	0.180972
12037	0.810946	2.1
12038	0.810945	2.1
12039	2.78163	2.1
12040	1.3517	2.1
12041	1.08132	2.1
12042	1.08133	2.1
12043	1.08132	2.1
12044	0.810933	2.1
12045	0.946131	2.1
12046	1.39081	2.1
12047	0.94613	2.1
12048	1.30339	2.1
12049	1.30339	2.1
12050	1.39081	2.1
12051	0.946131	2.1
12052	0.946127	1.31033
12053	0.821631	1.63246
12054	0.946131	0.750555
12055	0.946131	1.05095
12056	0.946128	0.971451
12057	0.996109	0.591587
12058	0.827876	0.49174
12059	0.946131	2.1
12060	0.946129	2.1
12061	0.946129	2.1

12062	0.94613	2.1
12063	0.94613	2.1
12064	0.94613	2.1
12065	0.94613	2.1
12066	0.946132	0.0756262
12067	1.41723	0.100007
12068	0.946132	0.0838757
12069	0.946133	0.0584775
12070	0.946132	0.261006
12071	0.946132	0.12555
12072	0.946133	0.118168
12073	0.946133	0.0813161
12074	0.946132	0.0578337
12075	0.946133	0.05
12076	0.946133	0.0621925
12077	0.946132	0.148205
12078	0.946132	0.0931941
12079	1.34254	0.083717
12080	1.26501	0.881912
12081	0.946132	1.1827
12082	0.946133	1.12125
12083	0.946133	1.78472
12084	0.946132	1.6874
12085	0.946133	1.1929
12086	0.946132	1.91469
12087	0.946131	0.05
12088	0.946131	0.05
12089	0.946131	0.05
12090	0.946131	0.05
12091	0.946131	0.05
12092	0.946131	0.05
12093	0.946131	0.05
12094	0.878843	0.05
12095	0.82561	0.05
12096	0.586974	0.05
12097	1.05343	0.05
12098	0.80631	0.05
12099	0.782663	0.05
12100	0.789405	0.05
12101	0.902491	0.05
12102	1.05344	0.05
12103	0.94613	0.05
12104	0.946131	0.05
12105	0.946131	0.05
12106	0.94613	0.05
12107	0.946131	0.05
12108	0.946131	0.05
12109	0.946131	0.05
12110	0.946128	2.1
12111	0.946133	2.1
12112	0.946133	0.234618
12113	0.94613	2.1
12114	0.946131	0.789065
12115	0.946131	1.33377
12116	0.946131	1.52533
12117	0.946131	0.0718314
12118	0.946131	0.0611437
12119	0.94613	0.0534066

12120	0.946128	0.0607455
12121	0.946133	0.0632061
12122	1.2996	0.05
12123	0.946131	0.0570422
12124	0.946132	2.1
12125	0.946132	2.1
12126	0.946132	2.1
12127	0.946132	2.1
12128	0.946131	2.1
12129	0.946132	2.1
12130	0.946132	2.1
12131	1.99211	0.05
12132	2.55024	0.05
12133	0.946131	0.05
12134	0.946131	0.05
12135	0.946131	0.05
12136	0.946131	0.05
12137	0.946131	0.05
12138	0.946131	0.05
12139	1.35726	0.05
12140	0.946128	2.1
12141	0.946132	2.05402
12142	0.946131	2.1
12143	0.94613	2.1
12144	0.94613	2.1
12145	0.94613	2.1
12146	1.39852	2.1
12147	1.31666	0.05
12148	0.946131	0.05
12149	0.946128	0.05
12150	0.94613	0.05
12151	0.946128	0.05
12152	0.946128	0.05
12153	0.946128	0.05
12154	1.32275	0.05
12155	0.946131	0.05
12156	0.946129	0.05
12157	0.946128	0.05
12158	0.946129	0.05
12159	0.946128	0.05
12160	0.946129	0.05
12161	0.946132	2.1
12162	0.946131	2.1
12163	0.946131	2.1
12164	0.946132	2.1
12165	0.94613	2.1
12166	0.946131	2.1
12167	1.41125	2.1
12168	0.946132	2.1
12169	0.946132	2.1
12170	0.946131	2.1
12171	0.946132	2.1
12172	0.946132	2.1
12173	0.946132	2.1
12174	1.31904	2.1
12175	1.02425	0.05
12176	0.946132	0.05
12177	0.94613	0.05

12178	0.946131	0.05
12179	1.1534	0.05
12180	0.961161	0.05
12181	0.9311	0.05
12182	1.40092	1.10932
12183	0.946133	1.19461
12184	0.946132	1.20694
12185	0.946131	1.71396
12186	0.946131	1.04334
12187	0.94613	1.29721
12188	0.946131	1.77786
12189	0.946113	2.1
12190	0.946119	0.31478
12191	0.946117	2.1
12192	1.40015	2.1
12193	0.946118	0.352478
12194	0.946131	2.1
12195	0.946105	2.1
12196	0.946132	0.0779286
12197	0.946133	0.0889409
12198	0.946132	0.268829
12199	1.03547	0.0932706
12200	0.946133	0.0686274
12201	0.946133	0.154734
12202	0.946132	0.10141
12203	0.946133	2.1
12204	1.36694	2.1
12205	0.946133	2.1
12206	0.946132	2.1
12207	0.946133	2.1
12208	0.946133	2.1
12209	0.946132	2.1
12210	0.946132	0.05
12211	0.946133	0.05
12212	0.946132	0.05
12213	0.946133	0.05
12214	0.946133	0.05
12215	0.946132	0.05
12216	0.946132	0.05
12217	0.491552	0.05
12218	0.913078	0.05
12219	0.946132	0.0515369
12220	0.491552	0.05
12221	0.946132	0.05
12222	0.49154	0.05
12223	1.53168	0.05
12224	1.53168	0.05
12225	0.946127	2.1
12226	1.38752	2.1
12227	0.946129	2.1
12228	0.94613	2.1
12229	0.946128	2.1
12230	0.946129	2.1
12231	0.946129	2.1
12232	0.946132	2.1
12233	0.946132	2.1
12234	0.946132	2.1
12235	0.946132	2.1

12236	0.946132	2.1
12237	0.946131	2.1
12238	0.946132	2.1
12239	0.94613	0.05
12240	0.94613	0.05
12241	0.94613	0.05
12242	0.946131	0.05
12243	0.946129	0.05
12244	1.16834	0.05
12245	0.94613	0.05
12246	0.946127	0.05
12247	1.35594	0.05
12248	0.946131	0.05
12249	0.946128	0.050831
12250	0.946127	0.05
12251	0.946129	0.05
12252	0.94613	0.05
12253	0.94613	0.250721
12254	0.946131	0.05
12255	0.946128	0.05
12256	0.946128	0.05
12257	0.946131	0.409367
12258	0.94613	0.05
12259	0.94613	0.118978
12260	0.946132	0.05
12261	0.946132	0.129543
12262	0.946129	0.05
12263	1.39751	0.05
12264	0.94613	0.234094
12265	0.946131	0.05
12266	0.946131	0.0976932
12267	0.946132	0.05
12268	0.700041	0.05
12269	0.700013	0.05
12270	1.7697	0.05
12271	1.7697	0.05
12272	1.21134	0.05
12273	0.946131	0.05
12274	1.25837	0.05
12275	0.946133	2.1
12276	0.946132	2.03271
12277	0.946132	1.81275
12278	0.946132	2.1
12279	0.946132	2.1
12280	0.946133	2.1
12281	1.40583	2.1
12282	0.946131	1.10549
12283	0.946131	2.0436
12284	0.946131	1.1317
12285	0.946131	2.1
12286	0.946131	2.08855
12287	0.827865	2.00467
12288	0.946131	2.04601
12289	0.946132	0.05
12290	0.946129	0.05
12291	0.946129	0.05
12292	0.946131	0.05
12293	0.94613	0.05

12294	1.21087	0.05
12295	0.946131	0.05
12296	0.94613	0.661582
12297	0.946131	0.93743
12298	0.94613	0.308767
12299	0.946128	0.443942
12300	0.946132	0.448769
12301	1.17452	0.314817
12302	0.94613	0.208286
12303	0.946119	0.260724
12304	0.946114	0.363879
12305	0.946119	0.166772
12306	0.946119	0.205467
12307	0.946119	0.206813
12308	1.27376	0.170379
12309	0.946106	0.131264
12310	0.946133	2.1
12311	1.39621	2.1
12312	0.946132	2.1
12313	0.946132	2.1
12314	0.946133	2.1
12315	0.946132	2.1
12316	0.946132	2.1
12317	0.946132	2.1
12318	0.946132	2.1
12319	0.946131	2.1
12320	0.946131	2.1
12321	0.946132	2.1
12322	0.946132	2.1
12323	1.34352	2.1
12324	0.946132	2.1
12325	1.41272	2.1
12326	0.946131	2.1
12327	0.94613	1.76958
12328	0.94613	2.1
12329	0.94613	1.88457
12330	0.94613	2.1
12331	0.946132	2.1
12332	0.946131	2.03078
12333	0.946132	2.1
12334	0.946132	1.9447
12335	0.946132	1.47233
12336	0.946132	2.1
12337	1.23536	2.1
12338	0.946132	2.1
12339	0.946131	2.1
12340	0.946131	2.1
12341	0.946132	2.1
12342	0.946132	2.1
12343	1.41131	2.1
12344	0.946131	2.1
12345	1.41894	1.42864
12346	0.946132	0.0605944
12347	0.946133	0.265328
12348	0.946132	0.240757
12349	0.946132	0.132987
12350	0.946133	0.285191
12351	0.946132	0.10865

12352	0.946128	0.05
12353	0.946133	0.385853
12354	0.946131	0.129335
12355	0.946129	2.1
12356	0.94613	0.068824
12357	1.4099	0.821127
12358	0.946131	0.05
12359	0.810947	2.1
12360	0.810945	1.43214
12361	0.810933	2.1
12362	2.62883	2.1
12363	1.3517	2.1
12364	1.08132	2.1
12365	1.08132	1.17636
12366	1.08133	2.1
12367	0.946131	1.17467
12368	0.946126	2.1
12369	0.946127	1.2079
12370	0.94613	1.14662
12371	0.946128	2.1
12372	0.946129	0.193981
12373	0.946129	0.661762
12374	1.21347	2.1
12375	0.946129	2.1
12376	0.94613	2.1
12377	1.21347	2.1
12378	1.33064	2.1
12379	0.94613	2.1
12380	1.33064	2.1
12381	0.94613	0.05
12382	0.946129	0.05
12383	0.94613	0.05
12384	0.946132	0.05
12385	0.946126	0.05
12386	0.946129	0.05
12387	0.94613	0.05
12388	0.946132	0.949317
12389	1.37938	1.18927
12390	0.946131	1.3269
12391	0.94613	0.733266
12392	0.946131	1.6298
12393	0.94613	1.11802
12394	0.94613	1.20014
12395	0.946133	0.05
12396	0.946133	0.05
12397	1.2968	0.05
12398	0.946132	0.05
12399	0.946133	0.05
12400	1.2968	0.05
12401	0.946133	0.05
12402	1.43643	0.05
12403	0.946129	0.509114
12404	0.946133	0.272292
12405	0.946131	0.552582
12406	0.94613	0.104282
12407	0.946131	0.256253
12408	0.946131	0.291132
12409	0.946131	0.25491

12410	0.872447	1.09598
12411	0.946132	0.737943
12412	0.872447	0.557678
12413	0.827867	0.977835
12414	0.946132	1.1231
12415	0.827867	1.49202
12416	0.872447	1.45287
12417	0.946117	0.781886
12418	1.27213	0.644188
12419	0.946124	0.380332
12420	0.946119	0.763515
12421	0.946113	1.24017
12422	0.946129	1.03552
12423	0.946104	0.893323
12424	0.94613	0.0792846
12425	0.946131	1.08308
12426	0.94613	0.172581
12427	0.946129	1.10353
12428	0.946132	0.05
12429	0.946131	0.0636204
12430	0.94613	0.0548842
12431	0.810942	2.1
12432	0.810943	2.1
12433	2.80212	2.1
12434	1.3517	2.1
12435	1.08133	2.1
12436	1.08132	2.1
12437	1.08132	2.1
12438	0.810932	2.1
12439	1.29521	2.1
12440	0.946129	2.1
12441	0.946131	2.1
12442	1.40106	2.1
12443	0.94613	2.1
12444	1.40106	2.1
12445	0.946131	2.1
12446	0.946132	0.0517216
12447	1.24596	0.05
12448	0.94613	0.05
12449	0.946131	0.05
12450	0.946131	0.05
12451	0.946131	0.05
12452	0.946131	0.0517053
12453	0.946131	2.1
12454	0.946131	2.1
12455	0.946131	2.1
12456	0.946128	2.1
12457	0.946133	2.1
12458	0.94613	2.1
12459	0.946131	2.1
12460	0.879548	0.412166
12461	1.21527	0.372957
12462	0.967506	0.394579
12463	0.946132	1.0171
12464	0.946133	0.242667
12465	0.967511	1.1989
12466	0.946132	1.13221
12467	0.946132	1.08147

12468	0.946132	0.512557
12469	0.974253	0.604307
12470	0.974253	1.5024
12471	0.91801	0.4862
12472	0.918009	0.680111
12473	0.946132	1.01736
12474	0.946132	0.05
12475	0.946131	0.05
12476	0.946131	0.05
12477	0.946133	0.05
12478	0.946132	0.05
12479	1.28644	0.05
12480	0.946132	0.05
12481	0.946131	0.05
12482	0.946131	0.05
12483	0.946131	0.05
12484	0.946131	0.05
12485	0.946131	0.05
12486	1.40103	0.05
12487	0.946131	0.05
12488	0.946128	0.05
12489	0.946132	0.05
12490	0.946129	0.05
12491	1.03327	0.05
12492	0.946129	0.05
12493	0.946129	0.05
12494	0.94613	0.05
12495	0.946134	0.125195
12496	0.946133	0.0803102
12497	0.946132	0.05
12498	0.946133	0.0666601
12499	0.946132	0.05
12500	0.946132	0.05
12501	0.946133	0.05
12502	0.856654	0.05
12503	0.946131	0.05
12504	0.946131	0.05
12505	0.946132	0.05
12506	0.856616	0.05
12507	1.03565	0.05
12508	1.03565	0.05
12509	1.15844	0.211301
12510	0.946132	0.242243
12511	0.946132	0.0650763
12512	0.946132	0.142071
12513	0.946132	0.0905783
12514	0.946132	0.179636
12515	0.946131	0.113404
12516	1.4582	2.1
12517	1.35067	2.1
12518	0.946131	2.1
12519	1.04106	2.1
12520	0.938748	2.1
12521	0.943015	2.1
12522	1.05017	2.1
12523	0.938793	2.1
12524	0.946131	0.05
12525	0.946132	0.05

12526	0.946131	0.05
12527	0.94613	0.05
12528	0.946133	0.05
12529	0.946131	0.05
12530	0.946131	0.05
12531	0.946132	0.0661933
12532	0.946133	0.0819894
12533	0.946132	0.0618624
12534	0.946133	0.104573
12535	0.946133	0.136055
12536	0.946132	0.095295
12537	0.946133	0.114833
12538	0.946128	0.80839
12539	0.946132	1.6042
12540	0.946132	0.542314
12541	0.94613	0.85722
12542	1.23426	1.03342
12543	0.94613	1.09315
12544	0.946131	1.16591
12545	0.810943	0.397157
12546	0.810946	0.317227
12547	2.60388	0.731923
12548	1.3517	0.284786
12549	1.08133	0.178611
12550	1.08132	0.299699
12551	1.08132	0.125984
12552	0.810933	0.107961
12553	0.810945	0.431252
12554	0.810945	0.0570596
12555	2.72377	2.1
12556	1.3517	1.66104
12557	1.08133	0.658027
12558	1.08132	0.0983646
12559	1.08132	2.1
12560	0.810932	1.45827
12561	1.25104	0.05
12562	0.946131	0.715064
12563	0.946131	0.0501263
12564	1.36188	2.1
12565	0.946131	0.694035
12566	1.36188	1.45024
12567	0.946132	1.34937
12568	0.946132	2.1
12569	0.946132	2.1
12570	0.946132	2.1
12571	0.946132	2.1
12572	0.946132	2.1
12573	0.946132	2.1
12574	1.25488	2.1
12575	1.32132	0.05
12576	0.946126	0.05
12577	0.946127	0.05
12578	0.946128	0.05
12579	0.946128	0.05
12580	0.946128	0.05
12581	0.946128	0.05
12582	0.946133	2.1
12583	1.41123	2.0319

12584	0.946134	2.1
12585	0.946133	2.1
12586	0.946132	0.5897
12587	0.946133	2.1
12588	0.946132	2.1
12589	1.32163	0.05
12590	0.946133	0.05
12591	0.946133	0.05
12592	0.946132	0.05
12593	0.946132	0.05
12594	0.946132	0.05
12595	0.946133	0.05
12596	0.946133	2.1
12597	0.946133	2.1
12598	0.946132	2.1
12599	0.946132	2.1
12600	0.946132	2.03543
12601	0.946133	2.1
12602	0.946132	2.1
12603	0.94613	0.05
12604	0.946131	0.05
12605	0.946128	0.05
12606	0.946133	0.05
12607	0.946133	0.05
12608	0.946131	0.05
12609	0.946131	0.05
12610	0.946133	0.140287
12611	0.946132	0.05
12612	0.946132	0.183366
12613	0.946134	0.05
12614	0.946133	0.450935
12615	0.946133	0.0633542
12616	0.946132	0.13859
12617	0.94613	1.97053
12618	0.946128	2.1
12619	0.946127	1.43583
12620	0.94613	0.861478
12621	0.946131	1.85232
12622	0.946128	1.89699
12623	1.21099	2.08186
12624	0.946132	0.596892
12625	0.946131	0.462027
12626	0.946131	0.750053
12627	0.946133	0.078604
12628	0.946133	0.175549
12629	0.946131	0.373952
12630	0.946132	0.284482
12631	1.34794	0.343828
12632	0.517994	0.477802
12633	0.517965	0.155057
12634	0.946132	0.666804
12635	0.9198	1.83335
12636	0.946132	1.039
12637	0.9198	0.810973
12638	0.946132	1.19755
12639	0.94613	1.14216
12640	0.946131	0.248414
12641	0.946131	1.1138

12642	0.946131	1.78102
12643	0.946131	1.10101
12644	1.23224	0.642568
12645	0.946132	1.9217
12646	0.946132	2.05788
12647	0.946133	1.68008
12648	0.946132	0.547404
12649	0.946132	1.2094
12650	0.946133	1.65817
12651	1.37511	1.7642
12652	0.946133	0.370214
12653	0.946129	0.504779
12654	0.851104	1.05606
12655	0.946131	0.716658
12656	1.0412	0.720382
12657	1.0412	0.924942
12658	0.851066	0.788058
12659	1.31357	0.05
12660	0.946131	0.05
12661	0.94613	0.05
12662	0.946131	0.05
12663	0.94613	0.05
12664	0.946131	0.05
12665	0.946131	0.05
12666	0.946133	0.723772
12667	1.38149	1.00025
12668	0.946132	1.10104
12669	0.946132	0.618647
12670	0.946133	1.58404
12671	0.946132	1.01318
12672	0.946133	1.00054
12673	0.810944	1.05834
12674	0.810945	2.1
12675	0.810958	2.1
12676	2.65782	2.1
12677	1.35168	2.1
12678	1.08132	0.987233
12679	1.08132	2.1
12680	1.0813	1.19276
12681	0.946113	0.05
12682	0.946119	0.05
12683	0.946118	0.05
12684	0.946118	0.05
12685	0.946119	0.05
12686	0.946131	0.05
12687	0.946106	0.05
12688	0.946132	0.05
12689	0.946132	0.05
12690	0.946128	0.05
12691	0.946131	0.05
12692	0.94613	0.05
12693	0.94613	0.05
12694	0.94613	0.05
12695	1.00099	0.05
12696	0.946132	0.05
12697	1.17876	0.05
12698	0.982282	0.05
12699	0.909981	0.05

12700	0.946132	0.05
12701	0.946131	0.05
12702	0.946132	1.11405
12703	1.35328	1.29901
12704	0.946132	1.73613
12705	0.946132	0.740933
12706	0.946133	1.57698
12707	0.946132	1.10959
12708	0.946133	1.43707
12709	0.946133	0.05
12710	0.946133	0.05
12711	0.946132	0.05
12712	0.946132	0.05
12713	0.946132	0.05
12714	0.946132	0.05
12715	0.946133	0.05
12716	0.900195	0.05
12717	0.946132	0.05
12718	0.900167	0.05
12719	1.41963	0.05
12720	0.900195	0.05
12721	0.946133	0.05
12722	1.35385	0.05
12723	0.946132	1.00594
12724	0.946129	1.58387
12725	0.946129	1.91595
12726	0.946131	0.904438
12727	0.946131	2.00141
12728	1.41585	0.932881
12729	0.94613	2.07583
12730	0.810946	2.1
12731	0.810946	2.1
12732	2.68895	2.1
12733	1.3517	2.1
12734	1.08133	2.1
12735	1.08132	2.1
12736	1.08132	2.1
12737	0.810933	2.1
12738	1.19886	2.1
12739	0.946131	2.1
12740	0.946131	2.1
12741	1.34447	2.1
12742	0.946131	2.1
12743	1.34447	2.1
12744	0.946131	2.1
12745	0.946133	0.05
12746	0.946133	0.05
12747	0.946132	0.05
12748	1.35065	0.05
12749	0.946132	0.05
12750	0.946133	0.05
12751	0.946132	0.05
12752	0.946131	0.375409
12753	0.946131	0.365479
12754	0.94613	0.514712
12755	0.946131	0.0552612
12756	0.946131	0.101686
12757	0.946131	0.275652

12758	0.946131	0.20205
12759	0.946127	0.058097
12760	0.946131	0.0625875
12761	0.946127	0.0513306
12762	0.94613	0.05
12763	0.946131	0.05
12764	0.946128	0.0523656
12765	0.946129	0.05
12766	0.810946	0.05
12767	0.810947	0.05
12768	0.810959	0.05
12769	2.4391	0.05
12770	1.35168	0.05
12771	1.08132	0.05
12772	1.08132	0.05
12773	1.08131	0.05
12774	0.946132	0.05
12775	0.946132	0.05
12776	0.946132	0.05
12777	1.40837	0.05
12778	0.946132	0.05
12779	0.946132	0.05
12780	1.40837	0.05
12781	0.946127	2.0398
12782	0.946132	2.1
12783	0.946131	0.213848
12784	0.946127	2.1
12785	0.946131	1.63036
12786	0.946129	0.666354
12787	0.94613	1.66116
12788	0.946133	2.0723
12789	0.946132	1.6078
12790	0.946132	1.53331
12791	0.946132	0.957817
12792	0.946132	1.84523
12793	0.946132	0.533608
12794	0.946133	2.1
12795	0.946132	0.477169
12796	1.24344	0.372325
12797	0.946132	0.138555
12798	0.946132	0.977459
12799	0.946132	0.245647
12800	1.24344	0.371126
12801	0.946132	1.00784
12802	0.946131	0.05
12803	0.946128	0.05
12804	0.946128	0.05
12805	0.946131	0.0564235
12806	1.41232	0.05
12807	0.946129	0.0500275
12808	0.94613	0.05
12809	0.946128	2.1
12810	0.946133	2.1
12811	0.946133	0.401647
12812	0.946131	2.1
12813	0.946131	0.919741
12814	0.946131	2.06872
12815	0.94613	2.09269

12816	0.946133	0.0969894
12817	1.41556	0.0940128
12818	0.946133	0.0750722
12819	0.946133	0.0678213
12820	0.946132	0.238635
12821	0.946132	0.109217
12822	0.946132	0.147043
12823	0.94612	0.0803918
12824	1.41843	0.0761807
12825	0.946114	0.0753329
12826	0.94612	0.0613283
12827	0.946119	0.181126
12828	1.41845	0.0741559
12829	0.946107	0.131527
12830	0.946129	0.05
12831	0.946129	0.05
12832	0.94613	0.05
12833	0.946132	0.05
12834	0.946126	0.05
12835	1.1509	0.05
12836	0.94613	0.05
12837	0.946131	0.05
12838	0.946132	0.05
12839	0.946132	0.05
12840	0.946132	0.05
12841	0.946132	0.05
12842	1.19263	0.05
12843	0.946132	0.05
12844	0.94613	0.103696
12845	0.946131	0.154041
12846	0.946128	0.209534
12847	0.946133	0.168396
12848	0.946133	0.184834
12849	0.946131	0.129919
12850	0.946131	0.181305
12851	0.946133	1.01109
12852	1.22848	1.47824
12853	1.00336	1.38595
12854	0.827866	1.12935
12855	0.946132	1.84597
12856	0.827865	1.50587
12857	0.946133	1.17856
12858	0.946131	0.303408
12859	0.94613	0.05
12860	0.946127	0.248768
12861	1.41498	0.198961
12862	0.94613	0.0826835
12863	0.946128	0.133983
12864	0.946128	0.0563756
12865	0.94613	0.05
12866	0.94613	0.05
12867	0.94613	0.05
12868	1.37358	0.05
12869	0.946129	0.05
12870	0.94613	0.05
12871	1.37357	0.05
12872	0.946111	0.05
12873	0.946118	0.05

12874	0.946118	0.05
12875	1.36568	0.05
12876	0.946116	0.05
12877	0.94613	0.05
12878	0.946105	0.05
12879	0.946132	0.05
12880	0.946131	0.05
12881	0.94613	0.07551
12882	1.23175	0.05
12883	0.946131	0.05
12884	0.946131	0.05
12885	0.946131	0.05
12886	0.946132	0.05
12887	0.946126	0.05
12888	0.946128	0.05
12889	0.946129	0.05
12890	0.946129	0.05
12891	0.946128	0.05
12892	1.25698	0.05
12893	0.810943	2.1
12894	0.810944	2.1
12895	2.81171	2.1
12896	1.3517	2.1
12897	1.08132	2.1
12898	1.08133	2.1
12899	1.08132	2.1
12900	0.810932	2.1
12901	0.946132	0.0804084
12902	0.946131	0.0735793
12903	0.946131	0.0995245
12904	0.946132	0.05
12905	0.946132	0.05
12906	0.946132	0.0659975
12907	0.946131	0.0510179
12908	0.810936	0.555157
12909	0.810935	2.1
12910	2.80914	2.1
12911	1.35167	1.91519
12912	1.0813	1.50955
12913	1.0813	2.1
12914	1.0813	0.365414
12915	0.810934	1.53194
12916	1.23851	2.1
12917	0.946132	1.05382
12918	0.946131	2.1
12919	1.40459	0.573781
12920	0.946132	2.1
12921	1.40459	2.1
12922	0.946132	2.1
12923	1.19974	0.05
12924	0.946131	0.05
12925	1.19974	0.05
12926	0.946132	0.05
12927	0.946132	0.05
12928	0.946132	0.05
12929	0.946132	0.05
12930	0.939141	0.891666
12931	1.14375	1.32511

12932	1.03306	1.3275
12933	0.946132	1.04868
12934	0.946126	1.59526
12935	0.946129	1.24536
12936	0.94613	1.05839
12937	0.946132	0.05
12938	0.946131	0.05
12939	0.946132	0.05
12940	0.946132	0.05
12941	0.946132	0.05
12942	0.946132	0.05
12943	0.946132	0.05
12944	0.946131	2.1
12945	0.94613	2.1
12946	0.94613	2.1
12947	0.946131	2.1
12948	0.946129	2.1
12949	0.94613	2.1
12950	1.24512	2.1
12951	0.946133	0.05
12952	0.946129	0.05
12953	0.946131	0.062001
12954	0.946132	0.05
12955	0.946131	0.05
12956	0.946131	0.05
12957	0.946131	0.05
12958	0.946132	0.05
12959	0.946133	0.05
12960	0.946132	0.05
12961	0.946132	0.05
12962	0.946133	0.05
12963	0.946132	0.05
12964	0.946132	0.05
12965	0.946132	0.0726705
12966	1.41392	0.05
12967	0.946132	0.05
12968	0.946132	0.119625
12969	0.946132	0.05
12970	0.946132	0.0726646
12971	0.946132	0.0865934
12972	0.946132	0.142665
12973	1.24333	0.0878363
12974	0.946132	0.07323
12975	0.946133	0.335193
12976	0.946132	0.05
12977	0.946132	0.236097
12978	0.946133	0.116485
12979	1.30649	0.05
12980	0.94613	0.05
12981	0.94613	0.05
12982	0.946128	0.05
12983	0.946126	0.05
12984	0.946127	0.05
12985	0.946127	0.05
12986	0.709578	0.05
12987	0.810943	0.05
12988	2.53303	0.05
12989	1.21651	0.05

12990	0.979951	0.05
12991	1.08132	0.05
12992	0.946141	0.05
12993	0.81096	0.05
12994	0.946131	0.05
12995	0.946131	0.05
12996	0.946131	0.05
12997	0.946131	0.05
12998	0.946131	0.05
12999	0.946131	0.05
13000	0.946131	0.05
13001	0.946132	0.05
13002	0.946129	0.05
13003	0.946129	0.05
13004	0.94613	0.05
13005	1.20634	0.05
13006	1.20634	0.05
13007	0.946131	0.05
13008	0.946133	0.323687
13009	0.946132	0.319899
13010	0.946132	0.17639
13011	0.946132	0.350987
13012	0.946132	0.164551
13013	1.30107	0.236323
13014	0.946133	0.302593
13015	0.810946	2.1
13016	0.810944	2.1
13017	2.73936	2.1
13018	1.35168	2.1
13019	1.0813	2.1
13020	1.08132	2.1
13021	1.08132	2.1
13022	0.810958	2.1
13023	0.946129	2.1
13024	0.946129	2.1
13025	0.946133	0.721956
13026	1.30812	2.1
13027	0.946131	1.29813
13028	0.946131	2.04575
13029	0.946131	2.0293
13030	1.00901	1.20384
13031	0.94613	1.9491
13032	0.94613	0.346927
13033	1.17001	1.58991
13034	0.917259	0.258644
13035	0.94613	0.796296
13036	0.94613	0.882118
13037	0.946126	2.1
13038	0.946127	2.1
13039	0.946131	2.1
13040	0.946127	2.1
13041	0.946131	2.1
13042	0.946129	2.1
13043	1.398	2.1
13044	0.946133	2.1
13045	1.3033	2.1
13046	0.946132	2.1
13047	0.946132	2.1

13048	0.946132	2.1
13049	0.946132	2.1
13050	0.946133	2.1
13051	0.946131	0.111031
13052	0.946131	0.151329
13053	1.09897	0.0609696
13054	0.946129	0.336259
13055	0.946131	0.199878
13056	0.94613	0.253969
13057	0.94613	0.116597
13058	0.946132	2.1
13059	0.946131	2.1
13060	0.946131	2.1
13061	0.946132	2.1
13062	0.94613	2.1
13063	0.946131	2.1
13064	1.39858	2.1
13065	1.07117	0.470745
13066	1.08878	0.950841
13067	0.946119	1.2404
13068	1.08878	1.20875
13069	0.946119	0.262913
13070	0.946132	1.08992
13071	1.07116	0.511705
13072	0.946132	0.05
13073	0.946132	0.05
13074	0.946132	0.05
13075	0.946132	0.05
13076	0.946132	0.05
13077	1.10733	0.05
13078	0.946132	0.05
13079	0.946128	0.05
13080	0.946132	0.05
13081	0.94613	0.05
13082	0.946129	0.05
13083	0.94613	0.05
13084	1.3367	0.05
13085	0.94613	0.05
13086	0.810943	2.1
13087	0.810944	2.1
13088	2.70234	2.1
13089	1.21653	2.1
13090	1.08132	2.1
13091	0.946155	2.1
13092	0.979951	2.1
13093	0.709578	2.1
13094	1.27174	2.1
13095	0.946129	2.1
13096	0.94613	2.1
13097	1.35117	2.1
13098	0.94613	2.1
13099	1.35117	2.1
13100	0.94613	2.1
13101	0.946133	0.9517
13102	0.946133	0.903369
13103	0.946132	2.02192
13104	0.946133	0.773524
13105	0.946132	2.1

13106	0.946133	1.97938
13107	0.997296	2.00039
13108	0.810943	2.1
13109	0.810944	2.1
13110	0.810945	2.1
13111	2.78833	2.1
13112	1.35169	2.1
13113	1.08132	2.1
13114	1.08132	2.1
13115	1.08132	2.1
13116	0.810946	2.1
13117	0.810943	0.816369
13118	0.810933	1.71029
13119	2.70049	2.1
13120	1.3517	1.22178
13121	1.08132	2.1
13122	1.08132	0.161374
13123	1.08133	0.901862
13124	0.946127	1.15831
13125	1.35469	1.00668
13126	0.94613	0.958615
13127	0.946131	1.17076
13128	0.946128	0.78904
13129	1.35469	1.29305
13130	0.946129	1.43388
13131	0.946131	0.05
13132	1.2722	0.05
13133	0.946132	0.05
13134	0.946132	0.05
13135	0.946129	0.05
13136	1.2722	0.05
13137	0.94613	0.05
13138	0.946133	0.0696966
13139	0.946132	0.05
13140	0.946132	0.0712205
13141	0.946132	0.0759928
13142	1.41461	0.0592838
13143	1.41461	0.0564096
13144	0.946133	0.0999488
13145	0.946131	0.633033
13146	1.30165	0.228998
13147	0.946132	0.102075
13148	0.946132	0.875266
13149	0.946129	0.279991
13150	1.30165	0.605966
13151	0.946131	0.793418
13152	0.946132	0.467851
13153	0.946129	2.08777
13154	0.94613	0.524105
13155	1.39356	2.1
13156	0.946131	0.985031
13157	0.946131	1.02077
13158	0.94613	1.98229
13159	0.810946	2.1
13160	0.810947	2.1
13161	0.810934	2.1
13162	2.35003	2.1
13163	1.3517	2.1

13164	1.08132	2.1
13165	1.08132	2.1
13166	1.08133	2.1
13167	0.946131	0.05
13168	0.946131	0.05
13169	0.946125	0.05
13170	0.946127	0.05
13171	0.946129	0.05
13172	0.946128	0.05
13173	0.946128	0.05
13174	0.946131	0.05
13175	0.946131	0.05
13176	0.946131	0.05
13177	0.946131	0.05
13178	0.946132	0.05
13179	0.946132	0.05
13180	0.946131	0.05
13181	0.946129	2.1
13182	0.946132	2.1
13183	0.946132	2.1
13184	0.94613	2.1
13185	1.30082	2.1
13186	0.94613	2.1
13187	0.946131	2.1
13188	1.20416	0.05
13189	0.946132	0.05
13190	0.946131	0.05
13191	0.946132	0.05
13192	0.946131	0.05
13193	0.946131	0.05
13194	0.946132	0.05
13195	1.18711	0.05
13196	0.946131	0.05
13197	0.946131	0.05
13198	0.946132	0.05
13199	0.946131	0.05
13200	0.946131	0.05
13201	0.946132	0.05
13202	0.946133	0.05
13203	0.946133	0.05
13204	0.946132	0.05
13205	1.30212	0.05
13206	0.946133	0.05
13207	0.946132	0.05
13208	1.30212	0.05
13209	0.946131	0.05
13210	0.946131	0.05
13211	0.946131	0.05
13212	0.946132	0.05
13213	1.31467	0.05
13214	1.31467	0.05
13215	0.946131	0.05
13216	0.946133	0.05
13217	0.946132	0.05
13218	0.946132	0.05
13219	0.946132	0.05
13220	1.25124	0.05
13221	1.25124	0.05

13222	0.946132	0.05
13223	0.946132	2.1
13224	0.946132	2.1
13225	0.946132	2.1
13226	1.36422	2.1
13227	0.946132	2.1
13228	0.946131	2.1
13229	0.946132	2.1
13230	0.946131	1.44189
13231	1.41851	1.99423
13232	0.946132	2.1
13233	0.946132	1.19965
13234	0.946132	1.83007
13235	0.946132	1.25253
13236	0.946132	1.7581
13237	0.946133	0.05
13238	0.946132	0.05
13239	0.946132	0.05
13240	0.946132	0.05
13241	0.946132	0.05
13242	1.37794	0.05
13243	0.946132	0.05
13244	0.946132	2.1
13245	0.946132	2.1
13246	0.946131	2.1
13247	0.946131	2.1
13248	0.946132	2.1
13249	0.946132	2.1
13250	1.4045	2.1
13251	0.946133	2.1
13252	0.946132	2.1
13253	0.946131	2.1
13254	0.946132	2.1
13255	0.946131	2.1
13256	0.946131	2.1
13257	0.946132	2.1
13258	0.946132	2.1
13259	0.946132	2.1
13260	0.946131	2.1
13261	0.946132	2.1
13262	0.946131	2.1
13263	0.946131	2.1
13264	0.946131	2.1
13265	0.946128	2.1
13266	1.31641	2.1
13267	0.946129	2.1
13268	0.94613	2.1
13269	0.94613	2.1
13270	0.94613	2.1
13271	0.94613	2.1
13272	0.946132	2.1
13273	0.946131	2.1
13274	0.946131	1.82258
13275	0.946132	2.1
13276	0.946132	2.1
13277	0.946132	2.1
13278	0.946132	2.1
13279	0.946132	2.1

13280	1.41507	2.1
13281	0.946132	2.1
13282	0.946132	2.1
13283	0.946133	2.1
13284	0.946133	2.1
13285	0.946132	2.1
13286	0.946132	2.1
13287	0.946131	2.1
13288	0.946131	2.1
13289	0.946133	2.1
13290	0.946132	2.1
13291	1.32608	2.1
13292	0.946132	2.1
13293	0.94613	2.00058
13294	0.94613	2.1
13295	0.94613	0.426807
13296	0.946131	2.1
13297	0.946129	1.61618
13298	0.94613	0.625551
13299	0.94613	1.32531
13300	1.33728	0.689321
13301	0.946131	0.575183
13302	0.94613	0.0792804
13303	0.946132	0.644218
13304	0.946132	2.00346
13305	0.946131	0.135551
13306	0.946131	0.221166
13307	0.946127	2.1
13308	1.40641	2.1
13309	0.946129	2.1
13310	0.94613	2.1
13311	0.946128	1.11136
13312	0.946129	2.1
13313	0.946129	2.1
13314	0.946129	2.1
13315	0.946128	2.1
13316	0.946133	2.1
13317	1.37467	2.1
13318	0.946131	2.1
13319	0.946131	2.1
13320	1.37467	2.1
13321	0.946131	2.1
13322	0.946131	2.1
13323	0.946132	2.1
13324	0.946132	2.1
13325	0.946129	2.1
13326	0.946131	2.1
13327	1.34912	2.1
13328	0.946132	2.1
13329	0.946133	2.1
13330	0.946133	2.1
13331	0.946132	2.1
13332	0.946132	2.1
13333	0.946133	2.1
13334	0.946132	2.1
13335	0.946127	2.1
13336	0.946131	2.1
13337	0.946132	2.1

13338	0.946131	2.1
13339	0.946127	2.1
13340	0.94613	2.1
13341	1.33607	2.1
13342	0.946117	0.05
13343	0.946117	0.288038
13344	0.946115	1.35849
13345	0.946124	2.1
13346	0.946119	0.05
13347	1.32662	0.677925
13348	0.94613	0.0840719
13349	0.946119	2.1
13350	0.946114	2.1
13351	0.946118	2.1
13352	0.946114	2.1
13353	0.946118	2.1
13354	0.946104	2.1
13355	0.946129	2.1
13356	0.946132	2.1
13357	0.946133	2.1
13358	0.946133	2.1
13359	0.946133	2.1
13360	0.946132	2.1
13361	0.946132	2.1
13362	0.946133	2.1
13363	0.946133	2.1
13364	0.946132	2.1
13365	0.946132	2.1
13366	0.946133	2.1
13367	0.946132	2.1
13368	0.946132	2.1
13369	0.946132	2.1
13370	0.946133	1.9278
13371	1.30881	2.1
13372	0.946132	2.1
13373	0.946131	1.39625
13374	0.946131	1.70009
13375	0.946131	1.23983
13376	0.946131	2.00181
13377	0.946128	2.1
13378	0.946128	2.1
13379	0.946132	2.1
13380	0.94613	2.1
13381	0.94613	2.1
13382	0.94613	2.1
13383	1.34652	2.1
13384	0.946133	1.0065
13385	0.946133	0.793689
13386	0.946132	2.01989
13387	0.946133	0.738254
13388	0.946132	2.1
13389	0.946132	1.47319
13390	0.946132	1.81288
13391	0.946132	0.0531344
13392	0.946128	0.0668298
13393	0.94613	0.0859513
13394	0.946131	0.053918
13395	0.94613	0.0976811

13396	0.94613	0.05
13397	0.94613	0.0538602
13398	0.946128	0.116149
13399	0.946132	0.0803661
13400	0.94613	0.10153
13401	0.94613	0.0502507
13402	0.946131	0.105701
13403	0.94613	0.0798507
13404	0.94613	0.0614019
13405	0.946129	0.947629
13406	1.32056	1.52732
13407	0.946131	1.95658
13408	0.94613	0.817678
13409	0.946124	1.66611
13410	0.946128	1.09844
13411	0.946127	1.26104
13412	0.946129	0.855012
13413	1.20351	1.42039
13414	0.946132	1.66239
13415	0.946132	1.00315
13416	0.946126	1.73378
13417	0.946129	1.18992
13418	0.946129	1.1099
13419	0.946127	1.92695
13420	0.946127	2.1
13421	0.946131	0.701897
13422	1.25592	1.99469
13423	0.946127	1.03138
13424	0.946129	1.36016
13425	0.94613	1.72374
13426	1.02332	2.09851
13427	0.946133	1.43561
13428	0.946132	1.45149
13429	1.02332	1.57202
13430	0.946132	0.830464
13431	0.96196	1.68723
13432	0.930306	1.08889
13433	0.946133	2.1
13434	0.946133	2.1
13435	0.946132	2.1
13436	1.20057	2.1
13437	0.946132	2.1
13438	0.946133	2.1
13439	1.20057	2.1
13440	0.946128	1.77549
13441	0.946129	0.743808
13442	0.946129	1.39068
13443	1.13245	0.653055
13444	0.946126	0.426238
13445	0.946128	1.21768
13446	0.946129	0.783421
13447	0.946131	1.26293
13448	0.946131	2.03451
13449	0.94613	2.1
13450	1.25	1.93007
13451	0.946129	2.1
13452	0.946131	2.1
13453	1.25	2.1

13454	0.540425	2.07617
13455	0.748493	2.05362
13456	1.46266	2.1
13457	1.20046	2.09392
13458	0.600454	2.1
13459	1.41283	2.1
13460	0.582567	2.08331
13461	0.946131	1.89436
13462	0.946131	1.72697
13463	0.946132	1.34853
13464	0.946132	1.80199
13465	0.94613	1.68571
13466	0.946131	2.1
13467	1.28439	2.1
13468	0.946132	0.236106
13469	0.946131	0.234847
13470	0.946131	0.387764
13471	0.946132	0.156634
13472	0.946132	0.523542
13473	1.39335	0.0930035
13474	0.946132	0.168689
13475	0.946131	0.0599342
13476	0.946131	0.174159
13477	0.94613	0.05
13478	0.946132	0.05
13479	0.946131	0.0982291
13480	0.946131	0.080913
13481	0.946131	0.05
13482	0.946131	0.0914593
13483	0.94613	0.104044
13484	0.946126	0.152034
13485	0.946131	0.0583501
13486	0.946125	0.419548
13487	0.946127	0.0577686
13488	0.946128	0.223582
13489	0.946131	0.05
13490	0.946131	0.05
13491	0.946131	0.05
13492	0.946131	0.05
13493	0.946131	0.05
13494	0.946131	0.05
13495	1.39168	0.05
13496	0.946131	0.0524842
13497	0.946126	0.05
13498	0.946127	0.072981
13499	0.946131	0.05
13500	0.94613	0.0571479
13501	1.412	0.05
13502	0.946128	0.0725048
13503	0.948901	0.05
13504	0.943364	0.0578231
13505	1.03768	0.05
13506	0.946132	0.119637
13507	0.946133	0.106667
13508	1.03767	0.095566
13509	0.946132	0.0532353
13510	1.00902	0.156564
13511	0.88324	0.110424

13512	0.971584	0.0594351
13513	0.946132	0.143836
13514	0.946129	0.521249
13515	0.946131	0.302363
13516	0.971572	0.363013
13517	0.946131	0.05
13518	0.946125	0.05
13519	1.33587	0.05
13520	0.946126	0.05
13521	0.946127	0.05
13522	0.946126	0.05
13523	0.946128	0.05
13524	0.946132	0.05
13525	0.946132	0.05
13526	1.27512	0.05
13527	0.946133	0.05
13528	0.946132	0.05
13529	0.946133	0.05
13530	0.946133	0.05
13531	0.946128	0.05
13532	0.946133	0.05
13533	0.946131	0.05
13534	0.94613	0.05
13535	0.946131	0.05
13536	0.94613	0.05
13537	0.946131	0.05
13538	0.946131	0.05
13539	0.946131	0.05
13540	0.946131	0.05
13541	0.946131	0.05
13542	0.946131	0.05
13543	0.946131	0.05
13544	0.946131	0.05
13545	0.94613	0.05
13546	0.946131	0.05
13547	0.946131	0.05
13548	0.94613	0.05
13549	0.946132	0.05
13550	0.946131	0.05
13551	1.35316	0.05
13552	0.94613	0.0579935
13553	0.94613	0.05
13554	0.946128	0.0669916
13555	0.946132	0.05
13556	0.946132	0.0606335
13557	1.28727	0.05
13558	0.94613	0.064515
13559	0.946131	0.05
13560	0.946132	0.05
13561	0.946132	0.05
13562	1.39502	0.05
13563	0.946132	0.05
13564	0.946132	0.05
13565	0.946132	0.05
13566	0.946125	0.0758661
13567	0.946131	0.0683536
13568	0.94613	0.05
13569	0.946129	0.0805908

13570	0.946126	0.202374
13571	0.946127	0.131943
13572	1.36173	0.123818
13573	0.946129	1.2629
13574	1.02363	1.18221
13575	0.94613	0.894169
13576	0.946131	1.22097
13577	0.946131	0.471358
13578	1.02363	0.61458
13579	0.946131	0.899137
13580	0.946128	0.05
13581	1.24678	0.05
13582	0.94613	0.05
13583	0.94613	0.05
13584	0.94613	0.05
13585	0.946131	0.05
13586	0.94613	0.05
13587	0.946129	0.05
13588	1.25215	0.05
13589	0.946132	0.05
13590	0.946126	0.05
13591	0.946129	0.05
13592	0.94613	0.05
13593	0.946129	0.05
13594	0.946129	0.385383
13595	0.946133	0.548539
13596	0.946133	0.258719
13597	0.946131	0.245864
13598	0.946131	0.412008
13599	1.29689	0.12908
13600	0.946131	0.155157
13601	0.946128	0.05
13602	0.946132	0.05
13603	0.946132	0.05
13604	0.94613	0.05
13605	1.22716	0.05
13606	1.22716	0.05
13607	0.946131	0.05
13608	0.946127	0.05
13609	0.946131	0.05
13610	0.946131	0.05
13611	0.946128	0.05
13612	1.30463	0.05
13613	1.30463	0.05
13614	0.946129	0.05
13615	1.29958	0.05
13616	0.946132	0.05
13617	0.946132	0.05
13618	0.946132	0.05
13619	0.946132	0.05
13620	0.946132	0.05
13621	0.946133	0.05
13622	1.26201	0.05
13623	0.946133	0.05
13624	0.946132	0.05
13625	0.946132	0.05
13626	0.946132	0.05
13627	0.946133	0.05

13628	0.946132	0.05
13629	0.946133	0.05
13630	0.946132	0.05
13631	0.946132	0.05
13632	0.946133	0.05
13633	0.946132	0.05
13634	1.40427	0.05
13635	0.946133	0.05
13636	0.946132	0.05
13637	0.946132	0.05
13638	0.946132	0.05
13639	0.946132	0.05
13640	0.946132	0.05
13641	1.35123	0.05
13642	0.946131	0.05
13643	0.946128	0.05
13644	0.946132	0.05
13645	0.946132	0.05
13646	0.946129	0.05
13647	1.22144	0.05
13648	1.22144	0.05
13649	0.946131	0.05
13650	0.946127	0.103435
13651	0.946132	0.111896
13652	0.946131	0.0845303
13653	0.946127	0.0865934
13654	0.946131	0.102226
13655	1.23351	0.0546352
13656	0.94613	0.0748609
13657	1.18119	0.05
13658	0.946131	0.05
13659	0.946132	0.05
13660	0.946132	0.05
13661	0.94613	0.05
13662	0.946131	0.05
13663	0.946131	0.05
13664	0.946132	0.05
13665	0.946132	0.05
13666	0.946133	0.05
13667	1.30563	0.05
13668	0.946132	0.05
13669	0.946132	0.05
13670	0.946133	0.0522345
13671	1.20256	0.0677988
13672	0.946132	1.4866
13673	0.946133	1.6404
13674	0.946132	0.05
13675	0.946132	0.200549
13676	0.946132	1.49832
13677	0.946133	0.0952594
13678	0.94613	0.178852
13679	0.94613	0.102718
13680	0.946128	0.328005
13681	1.33007	0.080638
13682	0.946129	0.134303
13683	0.94613	0.245021
13684	0.946131	0.440197
13685	0.946127	2.1

13686	0.946127	2.1
13687	0.946131	2.1
13688	1.30136	2.1
13689	0.946128	2.1
13690	0.946129	2.1
13691	1.30136	2.1
13692	0.810942	2.1
13693	0.810942	2.1
13694	0.81093	2.1
13695	2.3208	2.1
13696	1.3517	2.1
13697	1.08132	2.1
13698	1.08132	2.1
13699	1.08133	2.1
13700	0.946132	2.1
13701	0.946132	2.1
13702	0.946132	2.1
13703	0.946132	2.1
13704	0.946132	2.1
13705	1.39823	2.1
13706	0.946132	2.1
13707	1.37713	2.1
13708	0.946131	2.1
13709	0.946131	2.1
13710	0.946131	2.1
13711	0.946131	2.1
13712	0.946131	2.1
13713	0.946131	2.1
13714	0.946128	2.1
13715	0.946128	2.1
13716	0.946133	2.1
13717	0.946131	2.1
13718	0.946131	2.1
13719	0.946131	2.1
13720	0.94613	2.1
13721	0.946129	2.1
13722	0.946129	2.1
13723	0.946133	2.1
13724	0.946131	2.1
13725	0.946131	2.1
13726	0.946131	2.1
13727	0.946131	2.1
13728	0.946132	1.21354
13729	1.33694	2.1
13730	0.946133	2.1
13731	0.946133	1.09634
13732	0.946132	2.1
13733	0.946132	1.32524
13734	0.946133	1.75039
13735	0.946131	2.1
13736	0.94613	2.1
13737	0.946131	2.1
13738	0.946133	2.1
13739	0.946129	2.1
13740	0.946131	2.1
13741	0.946131	2.1
13742	0.946132	2.00852
13743	1.35943	2.1

13744	0.946132	2.1
13745	0.946132	1.21278
13746	0.946131	1.37183
13747	0.946132	1.09218
13748	0.946132	2.06953
13749	0.946132	0.05
13750	0.946132	0.05
13751	0.946133	0.05
13752	0.946133	0.05
13753	0.946132	0.05
13754	0.946132	0.05
13755	0.946132	0.05
13756	1.29322	0.110333
13757	0.946131	0.0928297
13758	0.946132	0.0672996
13759	0.946129	0.157208
13760	0.946131	0.0725901
13761	0.946131	0.073696
13762	0.946131	0.126506
13763	0.946132	0.062682
13764	0.946132	2.1
13765	0.946131	0.05
13766	1.41492	0.05
13767	0.946132	1.86765
13768	0.946132	0.0683374
13769	0.946131	0.238531
13770	0.946132	2.1
13771	0.946132	2.1
13772	0.946132	2.1
13773	1.35028	2.1
13774	0.946132	2.1
13775	0.946132	2.1
13776	0.946132	2.1
13777	0.946132	1.7848
13778	0.946132	2.1
13779	0.946132	2.1
13780	1.32461	2.1
13781	0.946131	2.1
13782	0.946132	2.1
13783	0.946132	2.1
13784	0.946131	2.1
13785	0.946131	2.1
13786	0.946131	2.1
13787	0.946132	2.1
13788	0.946129	2.1
13789	0.94613	2.1
13790	1.40369	2.1
13791	0.946132	0.05
13792	0.946131	0.05
13793	0.946131	0.05
13794	0.979598	0.05
13795	0.946131	0.05
13796	0.946131	0.05
13797	0.946132	0.05
13798	0.946131	2.1
13799	1.41897	2.1
13800	0.946132	2.1
13801	0.946132	2.1

13802	0.94613	2.1
13803	0.946131	2.1
13804	0.946131	2.1
13805	0.946131	2.1
13806	0.94613	2.1
13807	0.946132	2.1
13808	0.946132	2.1
13809	0.946129	2.1
13810	0.946131	2.1
13811	1.37791	2.1
13812	0.946127	2.1
13813	0.946131	2.1
13814	0.946131	2.1
13815	0.94613	2.1
13816	0.946128	2.1
13817	0.946129	2.1
13818	1.39384	2.1
13819	0.810945	2.1
13820	0.810944	1.47403
13821	0.810958	1.91851
13822	2.70852	2.1
13823	1.35168	1.78406
13824	1.08132	2.0482
13825	1.08132	0.928869
13826	1.0813	1.66106
13827	1.3335	2.1
13828	0.946132	2.1
13829	0.94613	2.1
13830	0.946132	2.1
13831	0.946131	2.1
13832	0.94613	2.1
13833	0.946131	2.1
13834	0.946132	2.1
13835	0.946131	0.05
13836	0.94613	2.1
13837	1.41192	2.1
13838	0.946132	0.0590333
13839	0.946131	1.64813
13840	0.94613	0.114864
13841	0.946133	0.386405
13842	0.946133	0.971922
13843	0.946132	0.0805765
13844	1.39428	0.0690642
13845	0.946132	0.216895
13846	0.946132	0.05
13847	0.946133	0.506955
13848	0.94613	0.393819
13849	0.946129	2.1
13850	0.94613	0.907116
13851	0.946128	1.09638
13852	1.40684	2.1
13853	0.94613	0.903886
13854	0.94613	2.1
13855	0.946131	0.820239
13856	0.946131	2.1
13857	0.946131	2.1
13858	0.946131	2.06049
13859	1.39021	2.1

13860	0.946131	2.1
13861	0.946131	2.1
13862	0.946131	1.95995
13863	0.946131	2.1
13864	0.946131	1.09452
13865	0.946131	2.1
13866	0.946131	2.1
13867	0.946131	0.980716
13868	0.946131	1.50975
13869	0.810946	2.1
13870	0.810946	2.1
13871	2.69264	2.1
13872	1.35168	2.1
13873	1.0813	2.1
13874	1.08132	2.1
13875	1.08132	2.1
13876	0.810958	2.1
13877	1.16687	2.1
13878	0.946131	2.1
13879	0.946131	2.1
13880	1.34632	2.1
13881	0.946131	2.1
13882	1.34632	2.1
13883	0.946131	2.1
13884	0.946128	1.22335
13885	0.946133	1.4138
13886	0.946133	0.60513
13887	0.946131	0.846653
13888	0.946131	0.913646
13889	1.3564	0.421645
13890	0.94613	0.4924
13891	0.946128	2.1
13892	0.946132	2.1
13893	0.94613	2.1
13894	0.94613	2.1
13895	0.946131	2.1
13896	1.35827	2.1
13897	0.94613	2.1
13898	0.946133	0.0681823
13899	0.946132	0.131088
13900	0.946132	0.0819259
13901	0.946133	0.109308
13902	0.946133	0.123738
13903	0.946132	0.11047
13904	0.946133	0.140509
13905	0.946132	2.1
13906	0.946132	2.1
13907	0.946132	0.423651
13908	0.946131	2.1
13909	0.946132	1.40937
13910	0.946132	1.2522
13911	0.946132	1.22451
13912	0.94613	0.195065
13913	0.94613	0.0761849
13914	0.946128	0.212344
13915	0.946132	0.0615804
13916	0.946132	0.231156
13917	0.94613	0.119799

13918	0.946131	0.100117
13919	0.946126	0.0606257
13920	0.946131	0.05
13921	0.946127	0.0889831
13922	0.946129	0.05
13923	0.94613	0.0631028
13924	0.946128	0.0504992
13925	0.946128	0.05
13926	0.946131	0.05
13927	0.946127	0.05
13928	0.946127	0.05
13929	0.946131	0.05
13930	0.946131	0.05
13931	0.946128	0.05
13932	0.946129	0.05
13933	0.94613	0.05
13934	0.946131	0.05
13935	0.946129	0.05
13936	0.946132	0.05
13937	0.946132	0.05
13938	0.946131	0.05
13939	0.94613	0.05
13940	0.94613	0.05
13941	0.946131	0.05
13942	0.946129	0.0603808
13943	0.946132	0.05
13944	0.946132	0.05
13945	1.32827	0.05
13946	0.946131	0.0536278
13947	0.946132	2.1
13948	0.946132	2.1
13949	0.946131	2.1
13950	0.946131	2.1
13951	0.946132	2.1
13952	0.946131	2.1
13953	0.946131	2.1
13954	0.946132	0.05
13955	0.946132	0.996834
13956	0.946131	0.05
13957	1.38356	0.05
13958	0.946133	0.313994
13959	0.946131	0.05
13960	0.946131	0.109548
13961	0.94613	2.1
13962	0.946131	2.1
13963	0.946131	2.1
13964	0.946128	2.1
13965	0.946133	2.1
13966	0.946131	2.1
13967	1.36918	2.1
13968	0.946131	0.161036
13969	0.94613	2.1
13970	0.946129	1.37628
13971	0.946133	2.1
13972	0.946132	1.72342
13973	1.3771	2.1
13974	0.946131	2.0583
13975	1.30029	1.20615

13976	0.94613	0.516946
13977	0.946129	1.61167
13978	0.946132	1.6173
13979	0.946132	0.0809431
13980	0.946131	0.967042
13981	0.94613	0.857059
13982	0.946133	0.05
13983	0.946132	0.05
13984	0.946132	0.05
13985	0.946132	0.05
13986	0.946132	0.05
13987	0.946132	0.05
13988	1.21174	0.05
13989	0.810946	2.1
13990	0.810945	2.1
13991	2.63045	2.1
13992	1.35169	2.1
13993	1.08132	2.1
13994	1.08132	2.1
13995	1.08132	2.1
13996	0.810946	2.1
13997	1.1796	2.1
13998	0.946131	2.1
13999	0.946132	2.1
14000	1.31522	2.1
14001	0.946132	2.1
14002	1.31522	2.1
14003	0.946132	2.1
14004	0.946131	2.1
14005	1.40615	2.1
14006	0.946129	2.1
14007	0.946128	2.1
14008	0.946133	2.1
14009	0.946131	2.1
14010	0.94613	2.1
14011	0.810945	2.1
14012	0.810944	2.1
14013	2.80479	2.1
14014	1.35169	2.1
14015	1.08132	2.1
14016	1.08132	2.1
14017	1.08132	2.1
14018	0.810945	2.1
14019	1.35562	2.1
14020	0.94613	2.1
14021	1.35561	2.1
14022	0.946133	2.1
14023	0.946131	2.1
14024	1.40239	2.1
14025	0.946131	2.1
14026	0.946125	0.0504391
14027	0.946131	0.119069
14028	0.946127	0.05
14029	0.946129	0.0570512
14030	0.946128	0.103573
14031	0.946129	0.120078
14032	0.946128	0.05
14033	0.946119	2.1

14034	0.946113	2.1
14035	0.94612	2.1
14036	0.946119	2.1
14037	1.41915	2.1
14038	0.946107	2.1
14039	0.946132	2.1
14040	0.810946	1.59532
14041	0.810943	2.1
14042	0.810934	2.1
14043	2.31411	2.1
14044	1.35171	2.1
14045	1.08132	1.93985
14046	1.08132	2.1
14047	1.08133	2.01995
14048	0.946127	2.1
14049	0.94613	2.1
14050	0.94613	2.1
14051	0.946131	2.1
14052	0.946126	2.1
14053	0.946128	2.1
14054	0.946128	2.1
14055	0.810944	2.1
14056	0.810947	2.1
14057	0.81096	2.1
14058	2.78019	2.1
14059	1.35168	2.1
14060	1.08132	2.1
14061	1.08132	2.1
14062	1.08131	2.1
14063	0.946133	0.05
14064	0.946132	0.05
14065	0.946132	0.05
14066	0.946133	0.05
14067	0.946132	0.05
14068	1.3495	0.05
14069	0.946133	0.05
14070	0.946133	2.1
14071	0.946132	2.1
14072	0.946132	2.1
14073	1.39529	2.1
14074	0.946133	2.1
14075	0.946132	2.1
14076	0.946133	2.1
14077	0.946133	2.1
14078	0.946132	2.1
14079	0.946132	2.1
14080	0.946132	2.1
14081	0.946132	2.1
14082	0.946133	2.1
14083	0.946132	2.1
14084	0.810946	2.1
14085	0.810945	2.1
14086	2.67861	2.1
14087	1.35169	2.1
14088	1.08132	2.1
14089	1.08132	2.1
14090	1.08132	2.1
14091	0.810946	2.1

14092	1.24616	2.1
14093	0.946132	2.1
14094	0.946132	2.1
14095	1.33931	2.1
14096	0.946132	2.1
14097	1.33931	2.1
14098	0.946133	2.1
14099	0.946133	0.069691
14100	0.946133	0.0502057
14101	0.946132	0.05
14102	0.946133	0.0736972
14103	1.3855	0.05
14104	1.3855	0.05
14105	0.946133	0.0731712
14106	0.946128	2.1
14107	0.946132	2.1
14108	0.946128	2.1
14109	0.94613	2.1
14110	0.946131	2.1
14111	1.40225	2.1
14112	0.94613	2.1
14113	1.40548	0.05
14114	0.946118	0.05
14115	0.946114	0.05
14116	0.946112	0.05
14117	0.946117	0.05
14118	0.946128	0.05
14119	0.946103	0.05
14120	0.94613	2.1
14121	0.946132	2.1
14122	0.946131	2.1
14123	0.946132	2.1
14124	1.37811	2.1
14125	0.946131	2.1
14126	0.946131	2.1
14127	0.946132	2.1
14128	0.946133	2.1
14129	0.946133	2.1
14130	0.946132	2.1
14131	0.946133	2.1
14132	0.946132	2.1
14133	0.946132	2.1
14134	0.946118	2.1
14135	0.946113	2.1
14136	0.946118	2.1
14137	0.946119	2.1
14138	0.946117	2.1
14139	0.946105	2.1
14140	0.946131	2.1
14141	0.946118	0.05
14142	0.946118	0.05
14143	0.946117	0.05
14144	0.946113	0.05
14145	0.94612	0.05
14146	1.41377	0.05
14147	0.946106	0.05
14148	0.946132	0.157118
14149	1.28436	0.0820137

14150	0.946131	0.0848543
14151	0.946131	0.179082
14152	0.946131	0.05
14153	0.94613	0.204451
14154	0.946131	0.1094
14155	0.810947	0.05
14156	0.810946	0.05
14157	2.68675	0.05
14158	1.35168	0.05
14159	1.08132	0.05
14160	1.08132	0.05
14161	1.0813	0.05
14162	0.81096	0.05
14163	0.946132	0.05
14164	1.34338	0.05
14165	0.946132	0.05
14166	1.34338	0.05
14167	0.946132	0.05
14168	1.34337	0.05
14169	0.946132	0.05
14170	0.946127	2.1
14171	0.946131	2.1
14172	0.946127	2.1
14173	0.946131	2.02077
14174	0.946131	2.1
14175	0.946128	2.1
14176	1.30281	2.1
14177	0.946132	0.145451
14178	0.946132	0.149211
14179	0.946132	0.122646
14180	0.946131	0.105314
14181	0.946132	0.175795
14182	1.37973	0.05
14183	0.946132	0.0921911
14184	0.946131	0.354227
14185	1.41798	0.185183
14186	0.946132	0.125349
14187	0.946132	0.24885
14188	0.946129	0.475167
14189	0.946131	0.213275
14190	0.946131	0.484005
14191	0.946118	0.241059
14192	1.41776	0.170536
14193	0.946113	0.180755
14194	0.946119	0.150157
14195	0.946117	0.330168
14196	1.41778	0.134821
14197	0.946105	0.343858
14198	0.946131	0.447746
14199	1.41798	0.54318
14200	0.946132	0.651305
14201	0.946132	0.350609
14202	0.94613	0.529341
14203	1.41799	0.271791
14204	0.946131	0.303234
14205	0.946133	2.1
14206	0.946132	2.1
14207	0.946132	0.587646

14208	0.946132	2.1
14209	0.946133	1.53386
14210	0.946132	2.1
14211	0.946132	2.1
14212	0.946131	2.1
14213	0.946131	2.1
14214	0.94613	0.210225
14215	0.946129	2.1
14216	0.946132	1.12671
14217	0.94613	1.18948
14218	0.946131	2.09887
14219	0.94612	1.24679
14220	0.946126	2.1
14221	0.946119	0.797242
14222	0.94612	1.78166
14223	1.40766	2.1
14224	0.946132	0.678529
14225	0.946107	2.1
14226	0.946128	2.1
14227	1.31213	2.1
14228	0.946129	2.1
14229	0.94613	2.1
14230	0.94613	2.1
14231	0.94613	2.1
14232	0.94613	2.1
14233	0.946127	2.1
14234	0.946131	2.1
14235	0.94613	2.1
14236	0.94613	2.1
14237	1.36653	2.1
14238	0.946128	2.1
14239	0.946128	2.1
14240	0.946127	2.1
14241	0.94613	2.1
14242	0.946131	2.1
14243	0.94613	2.1
14244	0.946128	2.1
14245	0.946128	2.1
14246	0.946129	2.1
14247	0.946133	2.1
14248	1.40081	2.1
14249	0.946132	2.1
14250	0.946132	2.1
14251	0.946133	2.1
14252	0.946132	2.1
14253	0.946133	2.1
14254	0.946128	2.1
14255	1.32282	2.1
14256	0.946129	2.1
14257	0.94613	2.1
14258	0.946131	2.1
14259	0.946131	2.1
14260	0.946131	2.1
14261	0.810945	2.1
14262	0.810943	2.1
14263	0.810933	2.1
14264	2.44097	2.1
14265	1.3517	2.1

14266	1.08132	2.1
14267	1.08132	2.1
14268	1.08133	2.1
14269	0.946131	2.1
14270	0.94613	2.1
14271	0.946133	2.1
14272	0.946133	2.1
14273	0.946128	2.1
14274	0.946131	2.1
14275	1.27676	2.1
14276	0.946126	0.381366
14277	0.946126	0.819405
14278	0.946131	0.596094
14279	0.946127	0.667974
14280	0.94613	0.0657959
14281	0.946128	0.084818
14282	0.946128	0.0734834
14283	0.946132	0.05
14284	0.946133	0.05
14285	0.946132	0.05
14286	0.946132	0.05
14287	0.946132	0.05
14288	0.946133	0.05
14289	0.946132	0.05
14290	0.946128	0.05
14291	0.946131	0.05
14292	0.94613	0.05
14293	0.946129	0.05
14294	0.94613	0.05
14295	0.94613	0.05
14296	0.94613	0.05
14297	0.946132	0.05
14298	0.946128	0.05
14299	0.94613	0.0589045
14300	0.946131	0.05
14301	0.94613	0.0584562
14302	0.94613	0.0539842
14303	0.946131	0.05
14304	0.94612	0.05
14305	0.946119	0.05
14306	0.946119	0.05
14307	0.946124	0.05
14308	0.946119	0.05
14309	0.946132	0.05
14310	0.946107	0.05
14311	0.946128	2.1
14312	0.946133	2.1
14313	0.946131	2.1
14314	0.94613	2.1
14315	0.946131	2.1
14316	1.37869	2.1
14317	0.946131	2.1
14318	1.35703	2.1
14319	0.946132	2.1
14320	0.946132	2.1
14321	0.946132	2.1
14322	0.946132	2.1
14323	0.946132	2.1

14324	0.946133	2.1
14325	0.946133	2.1
14326	0.946133	2.1
14327	0.946132	2.1
14328	1.37017	2.1
14329	0.946133	2.1
14330	0.946132	2.1
14331	0.946133	2.1
14332	0.946114	0.05
14333	0.946119	0.05
14334	0.946119	0.05
14335	0.94612	0.05
14336	0.946119	0.05
14337	0.946132	0.05
14338	0.946107	0.05
14339	0.946131	0.05
14340	0.946131	0.05
14341	0.946126	0.05
14342	0.946127	0.05
14343	0.94613	0.05
14344	0.946129	0.05
14345	0.946128	0.05
14346	0.946131	0.79976
14347	0.946131	0.638809
14348	0.946132	0.20335
14349	0.946132	0.776482
14350	0.94613	1.48894
14351	0.946131	1.32742
14352	1.3279	1.28574
14353	0.946132	2.1
14354	1.40932	2.1
14355	0.946132	2.1
14356	0.946132	2.1
14357	0.946132	2.1
14358	0.946131	2.1
14359	0.946132	2.1
14360	0.946131	0.05
14361	0.946131	0.05
14362	0.946132	0.05
14363	0.946132	0.05
14364	0.94613	0.05
14365	0.946131	0.05
14366	1.26868	0.05
14367	0.810943	2.1
14368	0.810945	2.1
14369	0.810932	2.1
14370	2.82321	2.1
14371	1.3517	2.1
14372	1.08132	2.1
14373	1.08132	2.1
14374	1.08133	2.1
14375	0.810943	0.05
14376	0.810944	0.05
14377	2.63079	0.0958876
14378	1.35168	0.05
14379	1.0813	0.05
14380	1.08132	0.05
14381	1.08132	0.383727

14382	0.810957	0.131365
14383	1.17856	0.05
14384	0.946132	0.05
14385	0.946128	0.05
14386	1.3154	0.377224
14387	0.94613	0.05
14388	1.31539	0.146264
14389	0.94613	0.935643
14390	0.94613	1.66601
14391	0.94613	1.47142
14392	0.946131	0.849536
14393	0.946131	1.63742
14394	0.946129	1.88303
14395	0.94613	2.1
14396	1.21997	2.08212
14397	0.946131	1.089
14398	1.25083	0.444402
14399	0.946132	0.33984
14400	0.946132	1.25721
14401	0.946129	0.334968
14402	1.25083	1.09767
14403	0.946131	1.16161
14404	0.946127	1.259
14405	1.41342	0.367261
14406	0.94613	1.19715
14407	0.946131	0.61161
14408	0.946127	0.124282
14409	0.946129	0.306594
14410	0.946129	0.607237
14411	0.946131	1.89769
14412	1.29149	1.85144
14413	0.946133	2.1
14414	0.946133	1.13399
14415	0.946128	1.55134
14416	0.946131	0.385212
14417	0.94613	1.49943
14418	0.94613	2.1
14419	0.94613	1.96905
14420	0.946132	2.1
14421	0.946132	1.7576
14422	0.946128	0.622978
14423	0.94613	2.1
14424	1.41022	2.1
14425	0.946132	0.0756077
14426	0.946132	0.05
14427	0.946128	0.0572253
14428	0.94613	0.05
14429	0.94613	0.12635
14430	0.94613	0.05
14431	0.946131	0.115651
14432	0.946129	1.21177
14433	1.37758	1.75757
14434	0.946131	2.1
14435	0.94613	0.570795
14436	0.946124	1.63235
14437	0.946127	0.758979
14438	0.946127	1.82115
14439	0.946129	1.14136

14440	1.38259	1.64834
14441	0.946132	2.1
14442	0.946132	0.668727
14443	0.946126	1.70932
14444	0.946129	0.912983
14445	0.94613	1.57189
14446	0.946131	0.05
14447	0.946132	0.0511812
14448	0.946132	0.0633636
14449	0.946132	0.0565927
14450	0.946132	0.0533257
14451	0.946132	0.0505738
14452	0.946132	0.05
14453	0.946131	0.05
14454	0.94613	0.05
14455	0.94613	0.05
14456	0.946133	0.05
14457	0.946128	0.05
14458	1.25017	0.05
14459	0.946131	0.05
14460	0.946131	2.1
14461	0.946131	2.1
14462	0.946131	2.1
14463	1.3937	2.1
14464	0.946133	2.1
14465	0.946131	2.1
14466	0.94613	2.1
14467	0.946131	0.0559674
14468	0.946131	0.05
14469	0.946131	0.05
14470	0.94613	0.05
14471	0.946133	0.192358
14472	0.946132	0.05
14473	0.946131	0.05
14474	0.946132	0.0696135
14475	0.946132	0.121521
14476	0.946132	0.05
14477	0.946132	0.05
14478	0.946131	0.05
14479	0.946132	0.05
14480	0.946132	0.05
14481	0.946129	0.05
14482	0.94613	0.813603
14483	0.94613	0.131957
14484	0.946128	0.851587
14485	0.946132	0.05
14486	0.946131	0.092469
14487	0.94613	0.0695778
14488	0.946132	2.1
14489	0.946132	0.898812
14490	0.946132	2.1
14491	1.41619	2.1
14492	0.946133	0.0695883
14493	0.946133	2.09887
14494	0.946132	1.59337
14495	0.946132	2.1
14496	0.946132	2.1
14497	0.946132	2.1

14498	1.38244	2.1
14499	0.946132	2.1
14500	0.946131	2.1
14501	0.946132	2.1
14502	0.810946	0.37543
14503	0.810943	0.952643
14504	0.810934	0.364753
14505	2.42741	0.0836864
14506	1.35171	0.157083
14507	1.08132	0.307935
14508	1.08132	0.348496
14509	1.08133	0.312177
14510	0.810947	0.227484
14511	0.810943	1.10449
14512	2.44179	0.0935235
14513	1.35171	0.213792
14514	1.08132	0.200594
14515	1.08132	0.250487
14516	1.08133	0.239437
14517	0.810934	0.202606
14518	0.946132	1.83543
14519	0.946132	1.71963
14520	0.946133	0.874975
14521	0.946133	1.69403
14522	0.946132	2.00168
14523	0.946132	1.72799
14524	1.12786	1.41524
14525	1.27806	0.522889
14526	0.946132	1.12263
14527	0.946133	0.782275
14528	0.946133	1.40086
14529	0.946132	1.90331
14530	0.946132	1.12166
14531	0.946133	1.78639
14532	0.946132	2.1
14533	0.946132	2.1
14534	0.946132	2.1
14535	1.37098	2.1
14536	0.946133	2.1
14537	0.946132	2.1
14538	1.37098	2.1
14539	0.946125	1.33363
14540	0.946129	1.19754
14541	0.946131	2.09735
14542	0.94613	0.772876
14543	0.946124	0.537998
14544	0.946127	0.8789
14545	1.39514	1.40602
14546	0.946132	2.1
14547	0.946131	2.1
14548	0.94613	2.1
14549	1.39909	2.1
14550	0.946132	2.1
14551	0.946131	2.1
14552	0.94613	2.1
14553	0.946133	2.1
14554	0.946133	0.0668913
14555	0.946132	2.1

14556	1.36835	2.1
14557	0.946132	0.93085
14558	0.946133	2.1
14559	0.946132	0.248285
14560	0.946132	2.1
14561	0.946133	2.1
14562	0.946133	2.1
14563	0.946132	2.1
14564	1.39047	2.1
14565	0.946132	2.1
14566	0.946132	2.1
14567	1.33174	0.05
14568	0.946128	0.05
14569	0.94613	0.05
14570	0.94613	0.05
14571	0.94613	0.05
14572	0.94613	0.05
14573	0.946131	0.05
14574	0.946128	0.05
14575	1.36004	0.05
14576	0.946129	0.05
14577	0.94613	0.05
14578	0.94613	0.05
14579	1.36003	0.05
14580	0.94613	0.05
14581	0.946124	2.1
14582	0.946119	2.1
14583	0.946119	2.1
14584	0.946119	2.1
14585	0.94612	2.1
14586	0.946132	2.1
14587	1.29062	2.1
14588	0.946128	2.1
14589	0.946128	2.1
14590	0.946133	2.1
14591	0.94613	2.1
14592	0.946131	2.1
14593	0.946131	2.1
14594	1.30584	2.1
14595	0.946131	2.1
14596	0.946131	2.1
14597	0.946131	2.1
14598	0.946132	2.1
14599	1.39549	2.1
14600	0.946131	2.1
14601	0.946131	2.1
14602	0.913386	0.760447
14603	1.17466	0.52287
14604	1.00473	0.318283
14605	0.946132	0.624559
14606	0.946126	1.22689
14607	0.94613	0.954775
14608	0.946129	0.82783
14609	0.946132	0.481975
14610	1.38894	0.372974
14611	0.946132	0.242511
14612	0.946132	0.329767
14613	0.946132	1.05082

14614	0.946132	0.463671
14615	0.946133	0.460167
14616	0.946128	0.327834
14617	1.23865	0.308937
14618	0.946129	0.170613
14619	0.94613	0.235173
14620	0.94613	0.460329
14621	0.94613	0.273729
14622	0.94613	0.323271
14623	0.946126	0.708811
14624	1.40248	0.999319
14625	0.946129	0.917016
14626	0.94613	0.778031
14627	0.946124	1.53169
14628	0.946127	1.13272
14629	0.946128	0.966674
14630	0.946133	0.484532
14631	1.1391	0.501907
14632	0.946132	0.569613
14633	0.946132	0.309323
14634	0.946132	1.26942
14635	0.946133	0.497645
14636	0.946132	0.573451
14637	1.17156	1.36768
14638	0.946133	1.35007
14639	1.33893	0.631861
14640	0.827864	0.86829
14641	0.995232	0.492943
14642	0.827864	1.22763
14643	0.94613	0.634554
14644	1.21387	0.595407
14645	0.946132	0.60121
14646	0.946131	0.129679
14647	0.94613	0.343917
14648	0.94613	0.31603
14649	0.94613	0.472694
14650	0.94613	0.273067
14651	0.810943	0.0613469
14652	0.810945	0.05
14653	2.42463	0.05
14654	1.3517	0.05
14655	1.08132	0.05
14656	1.08132	0.0530228
14657	1.08133	0.05
14658	0.810932	0.0528456
14659	0.946133	1.51291
14660	0.946132	0.167051
14661	0.946131	0.47933
14662	1.36748	1.14182
14663	0.946133	0.96563
14664	0.946131	0.46768
14665	0.946132	0.0514514
14666	1.35856	0.0627805
14667	0.946131	0.0620736
14668	0.94613	0.05
14669	0.94613	0.0541086
14670	0.946131	0.05
14671	0.94613	0.0504367

14672	0.94613	0.0715015
14673	0.946132	2.1
14674	0.946131	2.1
14675	0.946131	2.1
14676	0.946131	2.1
14677	1.41149	2.1
14678	1.41149	2.1
14679	0.946131	2.1
14680	0.946128	2.1
14681	0.946128	2.1
14682	0.946133	2.1
14683	0.946131	2.1
14684	0.946131	2.1
14685	0.946131	2.1
14686	0.94613	2.1
14687	1.41388	0.05
14688	0.94613	0.05
14689	0.946132	0.05
14690	0.946132	0.05
14691	0.946129	0.05
14692	0.946131	0.05
14693	0.946131	0.05
14694	1.02798	0.05
14695	0.946132	0.05
14696	1.02798	0.05
14697	0.946133	0.05
14698	1.14931	0.05
14699	0.957746	0.05
14700	0.93452	0.05
14701	0.946129	2.1
14702	0.946134	2.1
14703	0.946133	2.1
14704	0.946131	2.1
14705	0.946131	2.1
14706	0.946131	2.1
14707	0.946131	2.1
14708	0.946133	2.1
14709	0.946132	1.19993
14710	0.946132	1.72757
14711	0.946132	2.1
14712	1.38773	1.23737
14713	0.946132	1.98742
14714	0.946133	1.51416
14715	0.946131	0.05
14716	0.946131	0.05
14717	1.41512	0.05
14718	0.946133	0.05
14719	0.946131	0.05
14720	0.946131	0.05
14721	0.946131	0.05
14722	1.35381	0.183792
14723	0.946132	2.1
14724	1.35381	0.115526
14725	0.946133	2.1
14726	0.946132	0.378543
14727	0.946132	2.1
14728	0.946133	1.89551
14729	1.3365	0.223618

14730	0.946132	1.19194
14731	1.3365	0.11919
14732	0.946129	0.793883
14733	0.94613	0.2421
14734	0.946128	1.00276
14735	0.946129	0.318771
14736	0.939895	1.85073
14737	1.14285	2.1
14738	0.952366	2.1
14739	0.946131	2.06115
14740	0.94613	2.1
14741	0.946131	2.1
14742	0.94613	2.1
14743	0.946131	2.1
14744	0.946131	2.1
14745	0.946131	2.1
14746	0.946129	2.1
14747	0.946133	2.1
14748	0.946131	2.1
14749	0.946131	2.1
14750	0.946133	2.1
14751	0.946132	2.1
14752	0.946132	2.1
14753	0.946133	2.1
14754	0.946132	2.1
14755	0.946132	2.1
14756	0.946133	2.1
14757	0.946133	2.1
14758	0.946132	1.43706
14759	0.946132	2.1
14760	0.946133	1.95089
14761	0.946132	2.1
14762	0.946132	2.1
14763	0.946132	1.67025
14764	0.946132	1.24376
14765	0.946131	2.1
14766	0.946131	2.0436
14767	0.946131	1.96194
14768	1.38969	2.1
14769	0.946131	2.1
14770	0.946132	2.1
14771	0.946128	2.1
14772	0.946133	1.85142
14773	0.946132	2.0585
14774	0.946131	1.78272
14775	0.94613	1.89359
14776	0.946131	2.0551
14777	1.28464	2.00066
14778	0.946119	0.68033
14779	1.37492	0.818166
14780	0.946125	1.04552
14781	0.946119	0.335743
14782	0.946119	1.36841
14783	0.946132	0.527176
14784	0.946107	0.943103
14785	0.810942	2.1
14786	0.810943	2.1
14787	0.709587	2.1

14788	2.64488	2.1
14789	1.21651	2.1
14790	0.946151	2.1
14791	0.979948	2.1
14792	1.0813	2.1
14793	0.946133	1.7326
14794	0.946132	2.1
14795	0.946132	2.1
14796	0.946133	0.943574
14797	0.946132	0.296352
14798	0.946132	0.713803
14799	1.41134	1.52328
14800	0.946133	2.1
14801	0.946132	1.9135
14802	0.946133	2.1
14803	0.946133	1.23282
14804	0.946132	0.34635
14805	0.946133	1.72866
14806	1.31338	1.95709
14807	2.07612	2.08825
14808	0.946102	0.05
14809	0.752448	0.05
14810	1.20617	0.05
14811	0.94613	0.05
14812	0.75242	0.05
14813	0.946131	0.05
14814	0.752419	0.05
14815	0.946132	1.04008
14816	1.25736	0.695116
14817	1.25736	0.583082
14818	0.946132	0.835696
14819	1.08054	0.359334
14820	1.25736	0.641496
14821	0.946132	1.16914
14822	0.946128	2.1
14823	1.1896	2.1
14824	1.18959	2.1
14825	0.946131	1.17317
14826	1.33271	2.1
14827	0.946105	2.1
14828	0.946156	2.1
14829	0.741946	0.05
14830	1.33587	0.05
14831	0.741947	0.05
14832	1.21954	0.05
14833	0.94613	0.05
14834	0.741946	0.05
14835	1.81347	0.05
14836	1.81347	0.05
14837	0.653322	0.05
14838	1.36935	0.05
14839	0.661882	0.05
14840	1.04753	0.05
14841	0.507464	0.05
14842	0.945844	1.16416
14843	1.59507	2.1
14844	1.04043	0.945121
14845	0.946131	2.1

14846	1.32891	1.98671
14847	0.946131	1.35442
14848	0.946131	0.564
14849	1.28866	1.84019
14850	1.12682	0.05
14851	0.851543	0.0569615
14852	1.04072	0.05
14853	0.946132	0.0798608
14854	1.13437	0.05
14855	1.04167	0.05
14856	0.936652	0.05
14857	1.258	2.1
14858	0.946131	2.1
14859	0.946132	2.1
14860	1.36968	2.1
14861	0.946132	2.1
14862	1.36968	2.1
14863	0.946131	2.1
14864	2.30046	0.05
14865	2.82969	0.05
14866	0.946132	2.1
14867	1.35022	2.1
14868	1.12356	2.1
14869	0.94613	2.1
14870	1.35022	2.1
14871	0.946131	2.1
14872	0.94613	2.1
14873	0.946131	2.1
14874	1.22401	2.1
14875	0.952456	2.1
14876	0.94613	2.1
14877	1.22401	2.1
14878	0.946131	2.1
14879	0.946131	2.1
14880	1.33047	2.1
14881	0.946132	2.1
14882	1.33047	2.1
14883	0.946131	2.1
14884	1.33047	2.1
14885	0.946132	2.1
14886	1.2107	2.1
14887	0.61957	2.1
14888	1.80666	2.1
14889	1.53743	2.1
14890	0.619572	1.98536
14891	1.32547	2.1
14892	1.16166	2.1
14893	1.04074	2.1
14894	0.557621	2.1
14895	0.946128	2.1
14896	1.36607	2.1
14897	0.94613	2.1
14898	1.36607	2.1
14899	1.39416	2.1
14900	0.94613	2.1
14901	0.94613	2.1
14902	1.23969	2.1
14903	0.946131	2.1

14904	0.946131	2.1
14905	1.13537	2.1
14906	1.05719	2.1
14907	0.806848	2.1
14908	1.30251	2.1
14909	0.851526	1.67948
14910	1.14176	2.1
14911	0.93666	1.25824
14912	0.946114	2.1
14913	1.15832	1.99601
14914	0.924624	1.93213
14915	0.967609	1.37766
14916	0.771764	0.05
14917	1.26652	0.05
14918	0.946133	0.05
14919	1.17957	0.05
14920	0.771735	0.05
14921	0.946122	0.05
14922	0.946133	0.05
14923	0.924325	0.516904
14924	0.656739	1.0218
14925	0.637876	1.1555
14926	0.530092	0.760345
14927	0.914415	0.59089
14928	0.821311	1.65992
14929	0.630589	1.46016
14930	0.738823	1.20574
14931	0.839109	1.04497
14932	1.55029	0.05
14933	1.13537	0.0651159
14934	0.946131	1.40174
14935	0.946128	0.150089
14936	1.30162	2.1
14937	0.952476	1.88673
14938	1.29289	2.07957
14939	0.939783	2.1
14940	0.94612	2.1
14941	0.946131	2.1
14942	1.07998	2.1
14943	0.836985	2.1
14944	1.1604	2.1
14945	0.94613	2.1
14946	0.837016	2.1
14947	0.712461	0.269381
14948	1.36399	0.33814
14949	1.8724	0.220923
14950	1.8724	0.109718
14951	0.623426	1.15326
14952	1.36399	0.158215
14953	1.22089	0.176053
14954	0.946132	0.183239
14955	0.946131	2.1
14956	1.32241	2.1
14957	1.32241	2.1
14958	0.946133	2.1
14959	1.40271	2.1
14960	0.946131	2.1
14961	0.946131	2.1

14962	1.1989	1.16728
14963	0.954106	1.57414
14964	0.820068	0.999325
14965	0.563139	1.48668
14966	1.06448	1.51543
14967	0.892472	1.89305
14968	0.862598	1.13589
14969	0.745851	1.92293
14970	1.13336	1.43764
14971	0.946129	2.1
14972	1.28254	2.1
14973	1.28254	2.1
14974	0.946131	2.1
14975	1.41302	2.1
14976	0.946131	2.1
14977	0.946131	2.1
14978	0.851543	2.0748
14979	1.21961	2.1
14980	0.851484	1.90281
14981	0.851543	2.1
14982	1.15705	2.1
14983	0.946133	2.1
14984	0.909417	2.1
14985	1.13808	0.44638
14986	0.946133	0.05
14987	0.946131	0.569126
14988	1.30194	0.0519543
14989	0.946131	0.239684
14990	1.30194	0.05
14991	0.946131	0.0544421
14992	0.930941	1.05244
14993	1.20185	0.580995
14994	1.02404	0.620037
14995	0.946124	1.2605
14996	1.3563	0.812908
14997	0.946127	1.39952
14998	0.946128	1.02072
14999	0.946116	2.1
15000	1.3144	2.1
15001	1.13088	2.1
15002	0.946118	1.05761
15003	1.31438	2.1
15004	0.946105	2.1
15005	0.946131	2.1
15006	1.76972	0.05
15007	1.42091	0.05
15008	0.946128	0.05
15009	0.946101	0.05
15010	0.863552	0.0627579
15011	0.94613	0.05
15012	1.21232	0.05
15013	0.94613	0.075995
15014	1.2137	0.104427
15015	1.23224	0.404659
15016	1.86512	0.167931
15017	1.86512	0.0774898
15018	0.580825	1.37733
15019	1.2137	0.566559

15020	0.946133	0.581747
15021	0.580822	0.354603
15022	0.946128	2.1
15023	1.40586	2.1
15024	0.94613	2.1
15025	1.3719	2.1
15026	1.3719	2.1
15027	1.40586	2.1
15028	0.94613	2.1
15029	0.946132	2.1
15030	1.35025	2.1
15031	1.1794	2.1
15032	0.946132	0.420087
15033	1.35025	1.15805
15034	0.946131	1.2432
15035	0.946132	1.03423
15036	0.946133	2.1
15037	1.60149	2.1
15038	1.67058	2.1
15039	0.851542	2.1
15040	1.20296	2.1
15041	1.17501	2.1
15042	0.946132	2.1
15043	1.04993	2.1
15044	0.851541	2.02787
15045	1.48219	2.1
15046	0.874565	1.95616
15047	0.946129	2.1
15048	1.22049	2.1
15049	0.946131	2.1
15050	0.946131	2.1
15051	0.94613	2.1
15052	0.848719	2.1
15053	1.17195	2.1
15054	0.946129	1.33807
15055	1.35426	1.45528
15056	0.94613	1.72903
15057	0.94613	2.00171
15058	0.946133	2.1
15059	1.14342	2.1
15060	1.14342	2.1
15061	0.946133	2.1
15062	1.39009	2.1
15063	0.946132	2.1
15064	0.946132	2.1
15065	1.38819	2.1
15066	0.946133	2.1
15067	1.38819	2.1
15068	0.946131	2.1
15069	1.4116	2.1
15070	0.946131	2.1
15071	1.38819	2.1
15072	0.946127	2.1
15073	1.32244	2.1
15074	1.2176	2.1
15075	0.946131	2.1
15076	1.32244	2.1
15077	0.94613	2.1

15078	0.946129	2.1
15079	1.32415	2.1
15080	1.11239	2.1
15081	0.984099	2.1
15082	1.65532	2.1
15083	1.53082	2.1
15084	1.32068	2.1
15085	1.32068	2.1
15086	1.08172	2.1
15087	3.76293	0.05
15088	1.36335	0.05
15089	0.953925	2.1
15090	0.989497	2.1
15091	0.98949	2.1
15092	1.06341	2.1
15093	1.27875	2.1
15094	1.04598	2.1
15095	0.603154	2.1
15096	0.582457	2.1
15097	0.582457	2.1
15098	0.568994	2.1
15099	1.5791	2.1
15100	1.09506	2.1
15101	1.45327	2.1
15102	1.88406	2.1
15103	1.88404	2.1
15104	1.27389	2.1
15105	1.14206	2.1
15106	1.00232	2.1
15107	1.00231	2.1
15108	1.68436	2.1
15109	1.4677	0.05
15110	1.28828	0.05
15111	1.28828	0.05
15112	1.46932	0.05
15113	1.28228	2.1
15114	1.64268	1.59166
15115	0.959473	2.1
15116	1.10101	2.1
15117	1.07976	2.1
15118	1.2873	2.1
15119	1.70303	0.05
15120	1.40066	0.05
15121	1.5563	0.05
15122	1.13727	0.05
15123	1.03529	2.1
15124	1.11285	2.1
15125	1.03529	2.1
15126	1.28667	2.1
15127	1.32119	2.1
15128	1.44777	2.1
15129	1.44778	2.1
15130	1.61121	2.1
15131	1.09372	0.05
15132	1.17664	0.05
15133	1.17664	0.05
15134	0.872931	0.05
15135	1.02415	2.1

15136	1.28228	2.1
15137	2.14692	2.1
15138	3.90469	2.1
15139	1.00509	2.1
15140	1.28228	2.1
15141	3.32936	0.05
15142	1.1333	0.05
15143	1.11048	2.1
15144	0.979854	2.1
15145	1.65815	2.1
15146	1.00185	2.1
15147	1.84887	2.1
15148	1.59597	2.1
15149	1.78468	2.1
15150	1.87508	2.1
15151	1.82716	2.1
15152	1.33429	2.1
15153	1.34626	2.1
15154	0.797142	2.1
15155	1.00007	1.37991
15156	1.02051	2.1
15157	1.02051	1.76584
15158	1.03373	2.1
15159	1.05786	2.1
15160	0.887631	2.1
15161	0.992773	2.1
15162	1.28462	2.1
15163	2.35274	1.92433
15164	1.82533	1.01727
15165	1.82533	1.65615
15166	1.19496	0.518098
15167	1.28597	0.05
15168	1.09725	2.1
15169	1.60948	2.1
15170	1.20421	2.1
15171	1.16566	2.1
15172	1.88522	2.1
15173	1.81948	0.0798217
15174	1.14042	0.05
15175	1.21558	0.0884936
15176	1.56876	0.05
15177	1.6697	2.1
15178	1.81655	2.1
15179	1.81655	2.1
15180	1.5956	2.1
15181	0.906059	2.1
15182	1.63045	2.1
15183	0.9533	0.562109
15184	1.1475	0.534712
15185	1.72231	2.1
15186	1.94936	1.58255
15187	1.06826	2.1
15188	1.08576	2.1
15189	1.06826	2.1
15190	0.973559	2.1
15191	1.62737	0.05
15192	0.965676	0.05
15193	1.45055	0.05

15194	1.17631	0.05
15195	1.92535	0.05
15196	1.9503	0.05
15197	1.99828	0.05
15198	1.70302	0.05
15199	1.65139	0.05
15200	1.26784	0.05
15201	1.35923	0.05
15202	1.70302	0.05
15203	1.33033	2.1
15204	1.12481	2.1
15205	1.29452	2.1
15206	1.25399	2.1
15207	1.12481	2.1
15208	0.918358	2.1
15209	1.39332	2.1
15210	1.32368	2.1
15211	1.11712	0.422876
15212	1.07999	0.646051
15213	1.07999	0.126936
15214	1.05619	0.594947
15215	1.1766	2.1
15216	1.35735	2.1
15217	1.42456	0.05
15218	1.089	0.05
15219	1.11081	0.05
15220	2.88521	0.05
15221	1.09754	0.05
15222	1.85479	0.05
15223	1.27922	0.114788
15224	1.03556	0.0645174
15225	1.07508	1.89431
15226	1.09795	2.1
15227	0.967672	0.603918
15228	1.07508	1.70778
15229	1.03933	2.04785
15230	1.02975	2.1
15231	1.02975	2.1
15232	1.01121	2.1
15233	1.24526	0.05
15234	1.72368	0.05
15235	1.89607	0.05
15236	2.13512	0.05
15237	1.34591	0.0658386
15238	1.14721	0.131453
15239	1.15539	2.0638
15240	1.90938	0.826109
15241	1.90941	2.1
15242	1.89242	2.1
15243	1.18907	2.1
15244	1.20408	2.1
15245	1.20409	2.1
15246	1.22655	2.1
15247	0.701447	2.1
15248	1.57567	2.1
15249	1.38806	2.1
15250	2.09407	2.1
15251	1.07612	0.96666

15252	0.990019	0.891327
15253	1.15564	2.09413
15254	1.01243	1.81421
15255	1.28956	0.05
15256	1.35146	0.05
15257	1.35145	0.05
15258	1.22811	0.05
15259	1.09662	2.1
15260	1.42486	2.1
15261	1.10106	0.05
15262	1.90814	0.05
15263	1.3946	0.05
15264	1.11926	0.05
15265	0.725839	2.1
15266	0.925068	2.1
15267	0.925074	2.1
15268	1.43282	2.1
15269	1.91756	0.690819
15270	1.74321	0.749361
15271	1.74321	0.462119
15272	1.63413	0.549245
15273	1.9194	0.05
15274	1.62246	0.05
15275	1.90456	0.05
15276	1.06578	0.05
15277	1.46019	0.05
15278	1.17036	0.05
15279	1.70233	0.924077
15280	1.53515	0.543553
15281	2.03494	2.1
15282	1.78441	2.1
15283	1.75992	2.1
15284	1.94351	2.1
15285	1.51057	0.05
15286	0.991435	0.05
15287	1.98068	0.05
15288	1.64283	0.05
15289	2.96126	0.05
15290	2.20692	0.05
15291	1.95054	0.05
15292	1.21325	0.05
15293	0.996828	0.05
15294	1.01976	0.05
15295	1.17129	2.1
15296	1.13664	2.1
15297	1.13664	2.1
15298	1.09627	2.1
15299	2.65123	2.1
15300	3.23062	2.1
15301	3.23062	2.1
15302	3.92038	2.1
15303	1.89019	0.05
15304	1.79109	0.0652935
15305	1.79109	0.05
15306	1.54197	0.05
15307	3.41824	1.85712
15308	1.93553	0.05
15309	2.26215	1.85898

15310	2.0103	0.05
15311	1.51569	0.763887
15312	1.01683	0.547896
15313	1.78595	2.1
15314	1.66537	2.1
15315	3.73426	2.1
15316	3.53905	2.1
15317	1.66537	2.1
15318	1.52903	2.1
15319	3.53905	2.1
15320	3.3837	2.1
15321	1.19901	0.420433
15322	1.35002	1.88257
15323	1.35002	0.136713
15324	1.11683	0.145283
15325	1.75326	1.80385
15326	0.712404	1.27941
15327	2.97768	2.1
15328	2.98949	2.1
15329	1.2832	2.1
15330	1.39518	2.1
15331	2.98949	2.1
15332	2.99339	2.1
15333	1.39517	2.1
15334	1.46124	2.1
15335	1.28534	2.1
15336	1.03391	2.1
15337	1.75366	0.993658
15338	1.26247	1.94542
15339	1.13621	2.1
15340	2.43994	2.1
15341	1.47904	0.05
15342	1.80085	0.05
15343	1.80085	0.05
15344	1.64656	0.05
15345	2.87331	2.1
15346	2.82863	2.1
15347	2.82859	2.1
15348	2.84673	2.1
15349	1.0961	0.05
15350	1.39448	0.126884
15351	1.21366	0.180397
15352	1.20143	0.0554662
15353	1.85795	0.05
15354	1.36768	0.05
15355	1.36769	0.05
15356	1.45789	0.05
15357	1.32605	0.05
15358	1.27148	0.05
15359	1.70218	0.05
15360	1.3063	0.05
15361	1.45603	0.05
15362	1.89226	0.05
15363	1.2742	0.05
15364	2.10112	0.05
15365	1.10748	0.05
15366	1.1893	0.05
15367	1.04921	2.1

15368	1.22927	2.1
15369	1.22927	2.1
15370	1.46568	2.1
15371	0.811186	2.1
15372	0.792162	2.1
15373	0.792162	2.1
15374	0.758749	2.1
15375	1.03581	2.1
15376	0.737481	2.1
15377	2.24071	2.1
15378	1.34868	2.1
15379	1.47203	0.659343
15380	1.40967	0.75736
15381	1.40967	0.925323
15382	1.27916	0.842314
15383	1.17418	2.08328
15384	1.04921	2.1
15385	1.28291	0.561932
15386	1.3213	0.0721111
15387	1.46812	0.288027
15388	2.42522	0.803639
15389	1.17879	0.694384
15390	1.28291	0.67243
15391	1.28291	0.785648
15392	1.13438	0.534899
15393	1.75004	2.1
15394	1.78247	2.1
15395	1.78247	2.1
15396	1.76421	2.1
15397	2.25975	0.05
15398	3.66111	0.05
15399	3.6611	0.05
15400	3.44043	0.05
15401	1.13339	2.1
15402	1.13322	2.1
15403	1.13323	2.1
15404	1.20761	2.1
15405	1.34337	0.432732
15406	1.90912	2.1
15407	1.04399	1.86029
15408	2.00578	2.09728
15409	1.04973	2.1
15410	1.22615	2.1
15411	1.93384	2.1
15412	2.62816	0.280098
15413	1.13127	2.1
15414	1.47642	2.1
15415	2.62816	2.1
15416	3.31755	0.269629
15417	1.47642	2.1
15418	1.80454	2.1
15419	0.964782	2.1
15420	0.949135	2.1
15421	0.949134	2.1
15422	0.949134	2.1
15423	1.10476	0.0982697
15424	0.982858	0.05
15425	1.10938	0.129879

15426	0.982858	0.0731499
15427	1.01012	0.05
15428	0.974081	0.05
15429	1.82643	0.05
15430	1.29585	0.05
15431	1.00763	0.937885
15432	1.3294	0.488583
15433	1.3294	0.297944
15434	1.30001	0.83781
15435	1.59151	2.1
15436	1.99443	2.1
15437	1.07369	2.1
15438	1.30584	2.1
15439	1.05071	2.1
15440	0.948676	2.1
15441	1.13842	2.1
15442	1.42884	2.1
15443	1.59019	0.05
15444	1.38212	0.05
15445	1.34102	0.05
15446	1.02663	0.05
15447	0.797011	2.1
15448	0.81301	2.1
15449	0.81301	2.1
15450	0.811187	2.1
15451	0.991869	0.05
15452	1.23868	0.05
15453	1.67201	2.1
15454	1.61201	2.1
15455	1.61202	2.1
15456	1.57627	2.1
15457	0.94994	2.1
15458	1.77867	2.1
15459	0.854945	2.1
15460	0.973905	2.1
15461	1.64193	2.1
15462	1.68893	2.1
15463	1.68893	2.1
15464	1.70002	2.1
15465	2.08149	0.05
15466	1.69456	0.05
15467	1.67399	0.05
15468	1.83922	2.1
15469	1.91703	2.1
15470	0.967492	2.1
15471	1.13317	2.1
15472	0.980766	2.1
15473	2.06158	0.05
15474	0.986498	0.05
15475	1.76289	0.05
15476	1.17248	0.05
15477	1.51794	0.05
15478	1.30266	0.05
15479	1.03165	0.05
15480	0.947692	0.05
15481	1.20473	0.05
15482	0.947693	0.05
15483	1.54357	2.1

15484	0.980841	2.1
15485	1.34124	0.0762643
15486	1.39818	0.40861
15487	1.39818	0.05
15488	1.48035	0.417353
15489	1.03932	2.1
15490	1.2065	2.1
15491	1.20651	2.1
15492	1.12717	2.1
15493	1.09483	2.1
15494	0.994293	2.1
15495	0.994293	2.1
15496	0.97356	2.1
15497	0.995252	0.0904662
15498	1.66294	0.230324
15499	1.82775	0.146353
15500	1.42762	0.0840682
15501	1.04713	0.05
15502	1.05475	0.05
15503	1.29002	0.05
15504	1.05475	0.05
15505	1.17585	2.1
15506	1.39223	2.1
15507	1.39223	2.1
15508	1.71255	2.1
15509	1.56166	0.580295
15510	1.37762	0.366885
15511	1.16858	2.1
15512	1.0361	2.1
15513	1.23966	0.05
15514	1.10718	0.05
15515	1.99957	0.05
15516	0.658392	0.05
15517	1.36904	0.05
15518	1.12524	0.05
15519	1.04321	1.33986
15520	1.04627	1.12935
15521	1.91351	0.944104
15522	1.63379	0.994128
15523	1.79718	1.24623
15524	0.59634	1.17903
15525	0.958257	2.1
15526	1.06457	2.1
15527	0.958255	2.1
15528	1.03933	2.1
15529	1.14195	0.347888
15530	1.49438	0.166776
15531	1.49438	0.164962
15532	1.31259	2.1
15533	1.23036	2.1
15534	1.2016	2.1
15535	1.2016	2.1
15536	1.17129	2.1
15537	1.05724	1.32771
15538	1.04291	1.91406
15539	1.12341	2.1
15540	2.32525	2.1
15541	3.39038	2.1

15542	2.09374	2.1
15543	1.76185	0.291222
15544	1.7934	0.374038
15545	1.65809	0.377887
15546	1.54852	1.11325
15547	1.38766	2.1
15548	1.28765	2.1
15549	1.26599	2.1
15550	1.21397	2.1
15551	1.28765	2.1
15552	1.20045	2.1
15553	1.21398	2.1
15554	1.15234	2.1
15555	0.953634	0.05
15556	1.39132	0.05
15557	1.17285	2.1
15558	1.21408	2.1
15559	1.21408	2.1
15560	0.982848	2.1
15561	2.00101	2.1
15562	2.00973	2.1
15563	2.20314	2.1
15564	1.89318	2.1
15565	1.23267	2.05714
15566	0.947921	2.1
15567	1.18872	2.1
15568	0.740752	2.1
15569	1.92269	0.0507264
15570	1.53584	0.05
15571	1.53584	0.0571553
15572	1.21067	0.05
15573	1.40707	0.05
15574	1.50437	0.05
15575	1.50435	0.05
15576	1.45446	0.05
15577	1.47954	0.526765
15578	2.08687	0.99397
15579	2.08687	2.1
15580	1.1233	2.1
15581	1.04025	0.875359
15582	1.36005	1.79904
15583	1.47606	0.05
15584	1.3407	0.05
15585	0.758743	2.1
15586	0.716917	2.1
15587	0.716922	2.1
15588	0.672964	2.1
15589	2.40014	2.1
15590	1.96848	2.1
15591	1.96848	2.1
15592	1.53555	2.1
15593	1.06191	2.1
15594	1.29579	2.1
15595	1.29579	2.1
15596	1.71289	2.1
15597	1.78584	2.1
15598	2.15211	2.1
15599	2.00427	2.1

15600	3.41615	2.1
15601	1.66408	2.1
15602	1.73954	2.1
15603	2.91663	2.1
15604	3.65818	2.1
15605	1.93286	2.1
15606	1.08831	2.1
15607	1.31242	0.05
15608	1.11712	0.05
15609	1.26311	0.05
15610	1.31242	0.05
15611	1.10378	2.1
15612	0.968594	2.1
15613	1.48389	0.05
15614	1.49613	0.05
15615	1.04349	1.14836
15616	1.26409	0.225501
15617	1.26409	0.259776
15618	1.05724	0.150421
15619	1.65723	0.05
15620	1.83116	0.05
15621	2.06284	0.05
15622	2.00512	0.05
15623	1.83116	0.05
15624	2.03139	0.05
15625	2.20564	0.05
15626	1.90048	0.05
15627	0.963392	2.1
15628	1.01346	0.153844
15629	2.98092	2.1
15630	2.70295	2.1
15631	2.70295	2.1
15632	2.48828	2.1
15633	1.63661	2.1
15634	1.64158	2.1
15635	1.64158	2.1
15636	1.6488	2.1
15637	1.17506	0.05
15638	1.09667	0.05
15639	2.00539	0.05
15640	1.09667	0.05
15641	1.54946	2.1
15642	1.43577	2.1
15643	1.26103	2.1
15644	1.49808	2.1
15645	1.089 0.05	
15646	1.22586	0.05
15647	2.35027	0.05
15648	1.37549	0.05
15649	3.33706	0.05
15650	1.69894	0.05
15651	2.00539	0.05
15652	1.53798	0.05
15653	1.28938	2.1
15654	1.20969	2.1
15655	1.20969	2.1
15656	1.15864	2.1
15657	1.7986	2.1

15658	1.75869	2.1
15659	1.58281	2.1
15660	1.7392	2.1
15661	1.30733	1.10946
15662	1.5419	1.27605
15663	1.50883	1.16988
15664	1.85439	1.1596
15665	1.73531	0.877576
15666	1.70225	0.879234
15667	1.22176	0.05
15668	1.59492	0.0652974
15669	1.59492	0.05
15670	2.15542	0.0547
15671	3.24379	2.1
15672	2.64775	2.1
15673	3.16707	2.1
15674	1.93032	2.1
15675	1.50738	2.1
15676	1.24128	2.1
15677	1.24128	2.1
15678	0.994293	2.1
15679	2.90808	0.05
15680	2.45797	0.05
15681	1.85501	0.05
15682	2.90809	0.05
15683	0.892835	1.37757
15684	1.55871	2.1
15685	1.04615	1.40171
15686	1.66643	0.0657318
15687	1.70044	2.1
15688	1.80224	2.1
15689	1.70186	0.629774
15690	3.30123	0.422009
15691	1.74789	2.1
15692	1.77446	2.1
15693	1.77901	2.1
15694	1.57995	2.1
15695	1.81518	2.1
15696	2.17716	2.1
15697	2.17716	2.1
15698	2.65123	2.1
15699	2.01899	0.959162
15700	1.77479	0.664732
15701	1.19122	0.120268
15702	1.46724	0.0913895
15703	1.70944	0.05
15704	1.08929	0.05
15705	1.68454	0.05
15706	1.08929	0.05
15707	1.51067	0.915536
15708	1.06729	0.531575
15709	1.37762	0.524318
15710	1.13992	0.347229
15711	1.26397	2.1
15712	1.04083	2.1
15713	1.00278	2.1
15714	1.04084	2.1
15715	1.1016	0.05

15716	1.23477	0.05
15717	1.23477	0.05
15718	1.64985	0.05
15719	1.11117	2.1
15720	1.13006	2.1
15721	1.13006	2.1
15722	1.14721	2.1
15723	1.21946	2.1
15724	1.0186	2.1
15725	1.13178	2.1
15726	1.00133	2.1
15727	1.67046	0.05
15728	0.986036	0.051024
15729	1.67046	0.14255
15730	1.34857	1.82964
15731	2.46016	2.1
15732	2.07161	2.1
15733	2.10732	2.1
15734	2.08371	2.1
15735	1.35602	2.1
15736	0.829065	2.1
15737	1.81667	2.1
15738	0.99406	2.1
15739	1.81667	2.1
15740	1.68073	2.1
15741	2.05736	2.1
15742	2.97311	2.1
15743	2.14011	2.1
15744	1.23411	2.1
15745	1.13632	0.05
15746	1.40862	0.05
15747	1.28055	0.05
15748	1.14305	0.05
15749	1.12035	0.05
15750	1.15483	0.05
15751	1.94099	0.05
15752	1.82126	0.05
15753	1.15481	0.05
15754	0.998957	0.05
15755	2.10078	0.05
15756	1.81617	0.05
15757	1.27581	2.1
15758	0.968536	2.1
15759	1.33916	2.1
15760	1.27581	2.1
15761	1.32784	2.1
15762	1.4259	2.1
15763	1.70377	2.1
15764	1.54231	2.1
15765	3.77569	2.1
15766	3.42809	2.1
15767	1.54231	2.1
15768	1.51298	2.1
15769	3.55866	2.1
15770	2.86136	2.1
15771	1.83301	0.400168
15772	1.26371	0.164529
15773	1.98301	0.54735

15774	2.0609	0.293268
15775	2.26047	0.203037
15776	2.00537	0.129059
15777	1.7913	0.129871
15778	1.48864	0.0625761
15779	1.09504	2.1
15780	1.39827	2.1
15781	2.28084	2.1
15782	1.05983	2.1
15783	1.33593	0.05
15784	1.31508	0.05
15785	1.31508	0.05
15786	1.09599	0.05
15787	1.30647	2.1
15788	1.28846	2.1
15789	1.28846	2.1
15790	1.26923	2.1
15791	1.2955	2.1
15792	1.09044	2.1
15793	2.12209	2.1
15794	1.26438	2.1
15795	0.911819	2.1
15796	1.38752	2.1
15797	2.57704	2.1
15798	2.71736	2.1
15799	1.42284	2.1
15800	1.41562	2.1
15801	2.69131	2.1
15802	2.36178	2.1
15803	1.41562	2.1
15804	1.39564	2.1
15805	1.32368	2.1
15806	0.908053	2.1
15807	0.908053	2.1
15808	0.629213	2.1
15809	1.8758	1.77317
15810	1.27362	1.76501
15811	1.3756	0.222437
15812	2.28749	0.834459
15813	1.84577	0.684812
15814	2.17765	0.637786
15815	1.38969	0.453949
15816	1.84578	0.623837
15817	1.32801	0.213426
15818	1.76377	0.263812
15819	1.22845	2.1
15820	1.57766	0.657313
15821	1.57766	2.1
15822	1.39067	2.1
15823	1.15743	0.05
15824	1.37721	0.05
15825	1.69055	0.05
15826	1.14858	0.05
15827	1.38333	1.9475
15828	1.10575	0.05
15829	1.22418	2.1
15830	1.10574	2.1
15831	1.91509	0.05

15832	1.02001	0.05
15833	1.03074	0.0630142
15834	1.19122	0.05
15835	1.67887	2.1
15836	1.67597	2.1
15837	1.84984	2.1
15838	1.77775	2.1
15839	1.73571	2.1
15840	1.27921	2.1
15841	2.11851	1.96486
15842	1.27921	2.1
15843	1.42486	2.1
15844	1.12503	2.1
15845	1.12503	2.1
15846	1.30116	2.1
15847	0.568995	2.1
15848	0.557348	2.1
15849	0.557348	2.1
15850	0.53907	2.1
15851	0.633575	2.1
15852	1.33756	1.33084
15853	1.51074	2.1
15854	1.93384	0.66398
15855	0.655202	2.1
15856	0.731934	2.1
15857	0.731934	2.1
15858	1.09061	2.1
15859	0.966979	2.1
15860	1.31502	2.1
15861	1.31502	2.1
15862	1.72116	2.1
15863	1.70002	2.1
15864	1.67969	2.1
15865	1.67969	2.1
15866	1.62408	2.1
15867	1.01531	2.1
15868	1.66018	2.1
15869	1.01531	2.1
15870	0.886968	2.1
15871	1.22585	0.05
15872	1.20674	0.05
15873	1.20672	0.05
15874	1.02962	0.05
15875	0.956467	2.1
15876	2.00551	2.1
15877	0.553801	2.1
15878	0.617006	2.1
15879	1.26923	2.1
15880	1.23614	2.1
15881	1.23614	2.1
15882	1.16817	2.1
15883	2.14482	2.1
15884	1.72637	2.1
15885	1.09368	2.1
15886	1.26195	2.1
15887	0.760546	2.1
15888	1.84094	2.1
15889	2.62868	2.1

15890	2.79784	2.1
15891	2.79784	2.1
15892	3.00129	2.1
15893	1.27593	0.518125
15894	1.38877	1.95878
15895	1.06486	0.0659579
15896	1.2474	0.05
15897	1.76234	0.05
15898	1.8685	0.05
15899	1.69863	0.05
15900	1.29666	0.05
15901	1.91646	2.1
15902	1.8582	2.1
15903	1.8582	2.1
15904	1.7986	2.1
15905	3.44043	0.05
15906	1.94764	0.05
15907	1.94764	0.05
15908	1.36307	0.05
15909	1.32075	0.05
15910	1.12971	0.05
15911	2.06319	0.05
15912	1.94982	0.05
15913	1.77255	0.05
15914	0.979297	0.05
15915	1.59997	2.1
15916	1.93506	2.1
15917	1.94409	2.1
15918	1.9597	2.1
15919	0.57899	2.1
15920	0.953036	2.1
15921	0.953049	2.1
15922	1.53503	2.1
15923	1.39615	0.05
15924	1.06474	0.05
15925	0.947564	0.05
15926	1.45506	0.05
15927	1.17799	0.111048
15928	1.21067	0.05
15929	1.35945	0.05
15930	2.05648	0.053442
15931	1.36208	0.05
15932	2.15229	0.05
15933	2.89288	0.05
15934	1.95719	0.05
15935	2.25169	0.05
15936	1.52756	0.05
15937	1.19322	0.0518283
15938	1.23146	0.05
15939	2.45024	0.107251
15940	0.794122	0.05
15941	1.6343	0.0502198
15942	1.34161	0.05
15943	1.05353	0.05
15944	1.50795	0.05
15945	1.05353	0.05
15946	1.20669	0.05
15947	1.59289	2.1

15948	0.989543	2.1
15949	1.40588	2.1
15950	0.991427	2.1
15951	0.921822	1.77241
15952	1.46903	0.446156
15953	1.46903	2.1
15954	1.8214	0.0993
15955	1.39702	2.1
15956	1.25103	2.1
15957	1.25103	2.1
15958	1.10378	2.1
15959	1.71813	2.1
15960	1.39595	2.1
15961	1.386 2.1	
15962	1.13008	2.1
15963	1.31711	0.05
15964	1.70191	0.05
15965	0.893249	0.05
15966	1.42669	0.05
15967	1.23943	2.1
15968	1.1483	2.1
15969	1.04598	2.1
15970	1.1483	2.1
15971	1.00381	0.05
15972	1.11433	0.05
15973	0.973501	0.32229
15974	1.0756	0.325514
15975	1.20669	0.05
15976	1.18508	0.05
15977	1.18508	0.05
15978	0.983027	0.05
15979	1.43687	2.1
15980	1.71059	2.1
15981	1.71059	2.1
15982	1.66762	2.1
15983	2.8415	2.1
15984	2.40555	2.1
15985	1.91546	2.1
15986	1.61385	2.1
15987	2.01569	2.1
15988	1.33183	2.1
15989	1.23218	2.1
15990	1.23999	2.1
15991	1.01335	0.207018
15992	1.12088	0.461542
15993	1.25346	2.08264
15994	1.15644	2.1
15995	1.3764	1.05659
15996	1.27558	0.533036
15997	1.2064	2.1
15998	1.15067	2.1
15999	1.01595	2.1
16000	1.15067	2.1
16001	1.41902	2.1
16002	1.30272	2.1
16003	1.30272	2.1
16004	1.19548	2.1
16005	2.99339	2.1

16006	2.98041	2.1
16007	1.46124	2.1
16008	1.42105	2.1
16009	2.98041	2.1
16010	2.86339	2.1
16011	1.42105	2.1
16012	1.42284	2.1
16013	2.31301	0.539108
16014	1.80273	0.458054
16015	2.17765	0.750609
16016	2.31301	0.426818
16017	1.76378	0.292313
16018	2.10275	0.217707
16019	2.10275	0.273478
16020	2.26047	0.177471
16021	0.97541	2.1
16022	1.40863	2.1
16023	1.40863	2.1
16024	1.30751	2.1
16025	1.20143	0.0991681
16026	1.19756	0.0696411
16027	1.43707	0.0512862
16028	1.28954	0.05
16029	3.34254	0.05
16030	1.92916	0.05
16031	1.80089	0.05
16032	1.1323	0.05
16033	1.47321	0.05
16034	1.23756	0.05
16035	1.87291	2.1
16036	1.53642	2.1
16037	1.53642	2.1
16038	1.5791	2.1
16039	1.0446	2.1
16040	0.992674	2.1
16041	1.12324	0.05
16042	1.29163	0.05
16043	1.29163	0.05
16044	1.15371	0.05
16045	0.996831	0.05
16046	1.00192	0.05
16047	1.0019	0.05
16048	0.958937	0.05
16049	1.07283	2.1
16050	1.25294	1.57908
16051	1.25294	1.60474
16052	1.04788	1.66097
16053	1.14496	2.1
16054	1.07652	2.1
16055	1.07652	2.1
16056	0.991406	2.1
16057	1.60976	2.1
16058	1.37 2.1	
16059	1.37 2.1	
16060	1.14195	2.1
16061	1.26593	1.97515
16062	1.75366	2.1
16063	1.57621	0.05

16064	1.24538	0.05
16065	0.969118	2.1
16066	1.04147	2.1
16067	0.969118	2.1
16068	1.22615	2.1
16069	0.98084	2.1
16070	1.01843	2.1
16071	1.09435	2.1
16072	1.50876	2.1
16073	1.5976	2.1
16074	1.84322	2.1
16075	1.84324	2.1
16076	1.70377	2.1
16077	1.20709	2.1
16078	2.54729	2.1
16079	0.974716	0.05
16080	1.54468	0.05
16081	1.39021	0.05
16082	1.70303	0.05
16083	1.22852	1.78084
16084	1.578	1.17991
16085	1.578	1.83596
16086	1.93852	0.659843
16087	2.2506	2.1
16088	1.39302	2.1
16089	1.5478	2.1
16090	1.2269	2.1
16091	1.70048	2.1
16092	1.45477	2.1
16093	1.30929	2.1
16094	1.15069	2.1
16095	1.00255	2.1
16096	1.05481	0.05
16097	1.05481	2.1
16098	1.1032	0.05
16099	0.993888	2.1
16100	1.19294	2.1
16101	1.01121	0.05
16102	0.991866	0.05
16103	2.20052	0.375901
16104	1.7409	2.1
16105	1.75142	2.1
16106	1.46811	2.1
16107	1.7409	1.5005
16108	1.23267	2.1
16109	1.46811	2.1
16110	1.18872	2.1
16111	1.12665	2.1
16112	1.50045	2.1
16113	1.50045	2.1
16114	1.37704	2.1
16115	1.35308	1.017
16116	1.29983	0.159604
16117	1.29983	1.10345
16118	0.970212	0.23183
16119	1.82272	2.1
16120	1.62784	2.1
16121	1.62784	2.1

16122	1.87691	2.1
16123	3.65294	2.1
16124	2.33374	2.1
16125	2.12888	2.1
16126	1.62229	2.1
16127	1.41496	2.1
16128	1.32707	1.06112
16129	2.18871	0.133906
16130	1.4235	0.05
16131	1.46015	0.05
16132	1.25635	0.05
16133	1.45501	0.05
16134	1.3543	0.05
16135	1.38139	2.1
16136	1.98159	2.1
16137	2.15868	2.1
16138	4.04308	2.1
16139	1.5362	2.1
16140	1.56328	2.1
16141	1.56327	2.1
16142	1.58163	2.1
16143	1.88987	0.05
16144	1.08761	0.05
16145	0.862154	0.05
16146	1.77688	0.05
16147	1.14238	2.1
16148	1.11691	2.1
16149	1.01537	2.1
16150	1.02923	2.1
16151	0.977425	0.05
16152	1.15211	0.05
16153	2.0815	2.1
16154	1.18621	2.1
16155	1.01485	2.1
16156	1.17054	2.1
16157	1.17054	2.1
16158	1.12665	2.1
16159	1.87213	0.05
16160	1.82244	0.05
16161	1.45039	0.05
16162	1.7226	0.05
16163	1.841	0.05
16164	1.79011	0.05
16165	1.8792	0.05
16166	1.45053	0.05
16167	1.08947	2.1
16168	1.5008	2.1
16169	1.5008	2.1
16170	1.83186	2.1
16171	1.04159	0.05
16172	1.2489	0.05
16173	1.2489	0.05
16174	1.33592	0.05
16175	1.51973	0.05
16176	0.980547	0.05
16177	1.51974	0.05
16178	1.15991	0.0697871
16179	1.34965	2.1

16180	1.2724	2.1
16181	1.2724	2.1
16182	1.38438	2.1
16183	1.02923	2.1
16184	1.06521	2.1
16185	1.06521	2.1
16186	1.0752	2.1
16187	2.03139	0.05
16188	1.92951	0.05
16189	1.74852	0.05
16190	1.62772	0.05
16191	1.73655	0.05
16192	1.76809	0.05
16193	1.62772	0.05
16194	1.1432	0.05
16195	1.09235	2.1
16196	1.48081	2.1
16197	1.46712	2.1
16198	1.89321	2.1
16199	1.94548	2.1
16200	1.5636	2.1
16201	1.5636	2.1
16202	1.21124	2.1
16203	1.52314	2.1
16204	2.0219	2.1
16205	1.51113	2.1
16206	2.0219	2.1
16207	0.987247	0.0919942
16208	1.18347	0.05
16209	1.89226	0.192702
16210	1.61441	0.05
16211	1.42884	2.1
16212	1.10961	2.1
16213	1.56991	2.1
16214	1.05337	2.1
16215	1.05337	2.1
16216	1.91547	2.1
16217	1.05931	2.1
16218	1.1729	2.1
16219	1.34526	2.1
16220	1.0593	2.1
16221	1.0856	2.1
16222	1.90854	2.1
16223	1.91674	2.1
16224	1.11345	2.1
16225	1.75067	2.1
16226	1.50022	2.1
16227	1.50022	2.1
16228	1.53619	2.1
16229	1.4748	2.1
16230	1.74431	1.89424
16231	2.02581	1.60335
16232	1.25288	1.54608
16233	1.01814	0.298743
16234	1.2881	0.0741266
16235	1.2881	0.160866
16236	1.54433	0.0812529
16237	1.19999	0.178222

16238	1.44056	0.05
16239	1.02011	0.05
16240	1.17129	0.05
16241	1.13775	2.02843
16242	3.34815	2.05922
16243	1.60428	2.01836
16244	1.85928	1.9928
16245	1.75592	2.1
16246	1.25989	2.1
16247	1.75592	2.1
16248	1.71549	2.1
16249	1.37634	2.1
16250	1.64693	2.1
16251	1.64693	2.1
16252	1.64193	2.1
16253	1.18351	0.05
16254	2.19398	0.05
16255	2.16272	0.539588
16256	1.19837	0.37813
16257	1.04973	2.1
16258	1.20149	2.1
16259	1.60574	2.1
16260	1.11164	0.295734
16261	1.87823	0.400037
16262	1.11164	1.10659
16263	0.772882	2.1
16264	1.4714	2.1
16265	2.14242	0.624185
16266	1.12336	2.1
16267	2.8386	0.21044
16268	2.13346	2.1
16269	1.06788	0.05
16270	0.947662	0.05
16271	1.05297	0.05
16272	1.42907	0.05
16273	2.17465	2.1
16274	2.18394	2.1
16275	1.14808	2.1
16276	1.21292	2.1
16277	1.21292	2.1
16278	1.27229	2.1
16279	1.20859	0.140466
16280	1.21073	0.494046
16281	1.21073	0.107269
16282	1.22857	1.186
16283	1.79158	2.1
16284	1.73899	2.1
16285	1.34012	2.1
16286	1.39991	2.1
16287	1.73898	2.1
16288	1.61245	2.1
16289	1.40136	2.1
16290	1.57234	2.1
16291	1.82162	2.1
16292	3.09875	2.1
16293	1.10264	2.1
16294	1.02339	2.1
16295	1.02339	2.1

16296	1.23042	2.1
16297	2.82266	0.383755
16298	1.05698	0.0937057
16299	1.96384	0.313228
16300	1.10244	0.318124
16301	0.929061	2.1
16302	1.47007	2.1
16303	0.985303	2.1
16304	1.24554	2.1
16305	1.05993	2.1
16306	2.02074	2.1
16307	2.10106	0.992191
16308	1.18171	0.80707
16309	1.68111	0.928016
16310	1.84226	0.547771
16311	1.82131	0.05
16312	1.32371	0.05
16313	1.41902	0.05
16314	1.00205	0.05
16315	1.65532	2.1
16316	1.18977	2.1
16317	1.15471	2.1
16318	0.993889	2.1
16319	2.08521	2.1
16320	2.09885	2.1
16321	2.09497	2.1
16322	2.10604	2.1
16323	0.881028	1.38535
16324	1.52828	0.930335
16325	1.40574	0.05
16326	1.09599	0.05
16327	1.45506	2.1
16328	1.50524	2.1
16329	1.50524	2.1
16330	1.54711	2.1
16331	1.41469	2.1
16332	2.40442	2.1
16333	1.40794	2.1
16334	1.89226	2.1
16335	1.31346	2.1
16336	1.51067	2.1
16337	1.31363	2.1
16338	1.11833	2.1
16339	2.3373	2.1
16340	2.19994	2.1
16341	2.19994	2.1
16342	1.09616	2.1
16343	1.63719	2.1
16344	1.52204	2.1
16345	1.52205	2.1
16346	1.40118	2.1
16347	2.5946	2.1
16348	4.07065	2.1
16349	1.33916	2.1
16350	2.05217	2.1
16351	1.67023	2.1
16352	2.05217	2.1
16353	1.59458	0.227688

16354	1.02247	0.121373
16355	1.68108	0.307002
16356	1.03079	0.0958368
16357	1.1643	0.05
16358	1.85795	0.05
16359	1.70302	0.05
16360	1.42076	0.05
16361	1.91351	0.05
16362	1.01148	0.05
16363	2.52402	0.05
16364	0.752625	0.05
16365	0.886968	2.1
16366	1.28845	2.1
16367	1.28845	2.1
16368	1.27766	2.1
16369	1.82268	2.1
16370	1.87118	2.1
16371	1.87119	2.1
16372	1.91704	2.1
16373	3.13938	2.1
16374	2.97839	2.1
16375	2.97839	2.1
16376	2.87331	2.1
16377	1.73443	1.01376
16378	1.59457	0.485023
16379	2.01827	2.1
16380	2.04586	2.1
16381	2.04586	2.1
16382	2.00865	2.1
16383	1.19901	2.1
16384	1.16661	2.1
16385	1.13215	0.05
16386	1.96256	0.05
16387	2.34043	0.05
16388	2.04154	0.05
16389	1.58991	0.05
16390	1.45893	0.05
16391	2.56818	0.05
16392	1.70532	0.05
16393	1.17798	0.0551266
16394	1.25796	0.133008
16395	1.25797	0.130496
16396	1.10719	0.379664
16397	1.51067	2.1
16398	1.60707	2.1
16399	2.06946	2.1
16400	3.19218	2.1
16401	2.19965	2.1
16402	1.30521	2.1
16403	2.23684	1.69513
16404	1.43363	2.1
16405	1.51007	2.1
16406	2.46356	2.1
16407	1.90428	2.1
16408	1.13618	2.1
16409	2.13276	0.05
16410	0.998345	0.05
16411	1.2861	0.05

16412	1.20457	0.05
16413	1.51789	2.1
16414	1.49872	2.1
16415	1.49872	2.1
16416	1.47712	2.1
16417	1.87129	0.05
16418	0.949364	0.05
16419	1.83142	0.05
16420	1.55063	0.0504635
16421	1.03298	0.05
16422	0.980664	0.149748
16423	1.51696	0.05
16424	1.34927	0.179067
16425	1.70302	0.0746859
16426	1.65952	0.05
16427	1.76658	0.05
16428	0.986848	0.05
16429	2.13757	1.60933
16430	1.98142	0.230597
16431	1.98142	1.26541
16432	1.65411	0.360783
16433	1.1323	0.05
16434	1.46015	0.05
16435	2.15376	1.79402
16436	1.64622	1.87058
16437	0.991867	0.104363
16438	1.58074	0.05
16439	1.69368	2.1
16440	1.76403	2.1
16441	1.76403	2.1
16442	1.83318	2.1
16443	1.28418	0.194129
16444	1.16851	0.0624427
16445	1.1685	0.111552
16446	1.1726	0.067256
16447	1.06455	2.1
16448	1.18773	2.1
16449	0.98511	2.1
16450	1.18774	2.1
16451	1.76476	2.1
16452	1.79825	2.1
16453	1.79825	2.1
16454	0.961895	2.1
16455	1.59589	0.05
16456	2.25975	0.05
16457	2.76339	2.1
16458	3.0551	2.1
16459	1.68761	2.1
16460	1.59561	2.1
16461	2.17257	2.1
16462	1.05845	2.1
16463	1.03512	2.1
16464	2.19011	2.1
16465	1.44859	2.1
16466	1.35897	0.685058
16467	1.35897	2.1
16468	1.17822	1.29042
16469	1.13641	2.1

16470	1.18301	2.1
16471	1.18301	2.1
16472	1.25173	2.1
16473	2.41297	2.1
16474	1.36463	2.1
16475	1.54711	2.1
16476	1.5735	2.1
16477	1.5735	2.1
16478	1.58713	2.1
16479	1.69044	0.05
16480	1.57979	0.05
16481	1.57979	0.05
16482	1.14387	0.05
16483	1.04199	2.1
16484	1.96491	2.1
16485	1.04199	2.1
16486	1.46892	2.1
16487	0.983724	2.1
16488	1.70579	2.1
16489	1.88522	2.1
16490	1.23914	2.1
16491	1.79424	2.1
16492	1.52851	2.1
16493	1.04921	2.1
16494	1.11285	2.1
16495	1.25334	0.292223
16496	1.07496	0.13412
16497	1.07497	2.1
16498	1.34852	0.321899
16499	1.27391	2.1
16500	2.20307	2.1
16501	1.81311	2.1
16502	2.10305	2.1
16503	1.21059	0.0651007
16504	1.44253	0.05
16505	1.9194	0.05
16506	1.21159	0.05
16507	0.947979	2.1
16508	1.01225	0.109246
16509	0.947979	2.1
16510	1.01225	2.1
16511	1.00255	2.1
16512	1.01225	2.1
16513	1.01225	2.1
16514	1.06561	2.1
16515	3.50562	2.1
16516	3.61336	2.1
16517	3.61336	2.1
16518	3.73635	2.1
16519	1.61245	2.1
16520	1.52301	2.1
16521	1.57612	2.1
16522	1.83035	2.1
16523	1.52301	2.1
16524	1.24789	2.1
16525	1.85156	2.1
16526	1.89072	2.1
16527	0.995735	2.1

16528	1.57738	2.1
16529	1.87141	0.05
16530	1.73388	0.05
16531	1.61344	0.05
16532	0.922676	0.05
16533	1.56614	2.1
16534	0.956294	2.1
16535	1.82589	2.1
16536	1.63719	2.1
16537	1.12399	0.05
16538	1.50103	0.05
16539	1.56976	0.05
16540	1.03567	0.05
16541	1.21776	0.05
16542	1.89708	0.05
16543	1.16323	0.05
16544	1.63533	0.05
16545	2.29397	0.05
16546	3.01596	0.05
16547	1.80145	0.05
16548	1.7666	0.05
16549	1.91621	0.377093
16550	1.10719	0.12768
16551	1.57828	0.936246
16552	1.17105	1.14286
16553	1.15806	2.1
16554	1.31565	2.1
16555	1.31565	2.1
16556	1.46643	2.1
16557	1.47507	2.1
16558	1.87858	2.1
16559	0.958661	2.1
16560	1.87858	2.1
16561	1.44293	0.05
16562	1.43213	0.05
16563	1.43213	0.05
16564	1.10476	0.05
16565	1.03235	2.1
16566	1.31	0.0540636
16567	1.31	2.1
16568	1.50108	0.647396
16569	1.32215	2.1
16570	1.10611	2.1
16571	1.10611	2.1
16572	0.904978	2.1
16573	1.06486	0.0711034
16574	1.18347	0.05
16575	1.70535	2.1
16576	1.94779	2.1
16577	1.77071	2.1
16578	1.82268	2.1
16579	0.61701	2.1
16580	0.672495	2.1
16581	0.672494	2.1
16582	0.713174	2.1
16583	1.40395	2.1
16584	1.34245	2.1
16585	1.34243	2.1

16586	1.30646	2.1
16587	1.06204	0.05
16588	0.632691	0.05
16589	1.75766	0.0640706
16590	1.14287	0.398243
16591	1.52101	0.05
16592	1.71414	0.05
16593	1.86411	0.05
16594	1.6253	0.05
16595	1.04355	0.05
16596	1.0443	0.05
16597	1.34852	2.1
16598	1.56016	0.0874567
16599	1.56016	0.09076
16600	1.03235	0.119984
16601	1.2966	0.05
16602	1.82354	0.05
16603	1.2966	0.05
16604	1.67687	0.05
16605	1.19669	1.37583
16606	1.82979	1.58086
16607	1.58972	1.224
16608	1.09219	1.21265
16609	1.42077	0.70068
16610	1.89226	0.572707
16611	1.19547	2.1
16612	1.10187	2.1
16613	1.10188	2.1
16614	1.01479	2.1
16615	1.81663	2.1
16616	1.81533	2.1
16617	1.83247	2.1
16618	1.51511	2.1
16619	0.983148	2.1
16620	1.7513	2.1
16621	1.85017	2.1
16622	1.26103	2.1
16623	1.23152	2.1
16624	1.65508	2.1
16625	1.23152	2.1
16626	2.15082	2.1
16627	1.57974	2.1
16628	1.07289	2.1
16629	0.81534	2.1
16630	1.51007	2.1
16631	1.00898	0.05
16632	1.18959	0.05
16633	1.32178	0.05
16634	1.25929	0.05
16635	2.17892	0.05
16636	1.22938	0.05
16637	3.02933	0.268179
16638	2.34619	0.42266
16639	1.47679	0.0735836
16640	1.0819	0.0847239
16641	3.10558	2.1
16642	3.31457	2.1
16643	0.985109	2.1

16644	1.10667	2.1
16645	3.31457	2.1
16646	3.54443	2.1
16647	1.10667	2.1
16648	1.25987	2.1
16649	1.77063	1.05728
16650	1.51065	0.675253
16651	1.24688	2.1
16652	1.37652	2.1
16653	1.37652	2.1
16654	1.54168	2.1
16655	1.17042	0.781933
16656	1.13092	1.33741
16657	1.13092	1.74664
16658	1.52938	2.1
16659	1.11787	0.05
16660	1.00613	0.253966
16661	0.781373	0.0685462
16662	1.6885	0.0641372
16663	2.49439	0.05
16664	1.42657	0.0533766
16665	1.77903	2.1
16666	2.63606	2.1
16667	2.10425	2.1
16668	1.21425	2.1
16669	1.31253	1.54865
16670	1.28418	1.60865
16671	1.28418	1.25307
16672	1.58972	1.17957
16673	0.858948	2.1
16674	1.90196	2.1
16675	1.81024	2.1
16676	1.90194	2.1
16677	1.4263	0.148926
16678	0.947921	0.190676
16679	1.22852	0.962242
16680	1.09194	0.602918
16681	1.60578	0.05
16682	1.14721	0.05
16683	1.06336	0.05
16684	1.14721	0.05
16685	1.11924	0.05
16686	1.53814	0.05
16687	1.18421	0.05
16688	1.8458	0.05
16689	1.7565	2.1
16690	1.09344	1.45389
16691	1.09344	0.920567
16692	1.16971	2.1
16693	1.71465	0.05
16694	1.19412	0.05
16695	1.64985	0.05
16696	1.05417	0.05
16697	1.2127	0.05
16698	1.48867	0.05
16699	1.27806	0.05
16700	1.21269	0.05
16701	1.37813	1.16269

16702	1.0417	1.18943
16703	1.88626	0.289904
16704	1.14977	0.738253
16705	1.78803	2.1
16706	1.33793	1.08102
16707	1.42266	2.05284
16708	1.26865	1.35914
16709	1.09518	2.1
16710	1.36844	2.1
16711	1.20455	2.1
16712	1.36844	2.1
16713	1.22297	0.734429
16714	1.11256	1.02691
16715	1.11256	0.57605
16716	1.03217	0.776111
16717	1.18422	0.05
16718	1.4282	0.05
16719	1.04159	0.05
16720	1.4282	0.05
16721	0.988805	2.1
16722	1.05481	2.1
16723	1.52938	0.458705
16724	1.26593	1.63994
16725	1.53082	2.1
16726	1.09847	2.1
16727	1.75211	0.271923
16728	1.22222	2.1
16729	1.75211	0.0928037
16730	1.51592	2.1
16731	1.63413	0.276761
16732	1.26662	0.310479
16733	1.42495	0.122521
16734	1.4356	0.154299
16735	1.29389	1.64434
16736	1.04164	2.1
16737	1.40574	1.60279
16738	1.04164	1.17263
16739	2.22344	0.346921
16740	1.45945	0.05
16741	0.987248	0.0838367
16742	1.2474	0.05
16743	0.974702	0.631076
16744	1.43777	0.227927
16745	1.71289	2.1
16746	1.91529	2.1
16747	1.91529	2.1
16748	2.01827	2.1
16749	1.87111	1.0355
16750	1.00864	0.197112
16751	1.26865	0.346027
16752	1.18769	0.920902
16753	1.24167	2.1
16754	1.36133	0.32517
16755	2.28168	2.1
16756	3.98524	2.1
16757	1.36133	2.1
16758	1.69436	0.598071
16759	2.21926	2.1

16760	1.70302	2.1
16761	1.50732	0.05
16762	1.42237	0.05
16763	1.18659	0.05
16764	1.50732	0.05
16765	1.19785	0.05
16766	1.40969	0.05
16767	1.28339	0.05
16768	1.40882	0.05
16769	0.968595	2.1
16770	1.41903	2.1
16771	1.12567	2.1
16772	1.15898	2.1
16773	1.27241	2.1
16774	0.995796	2.1
16775	1.89983	0.05
16776	1.05417	0.05
16777	0.571999	0.05
16778	0.743597	0.05
16779	1.89353	0.05
16780	0.924068	0.05
16781	1.52851	2.1
16782	1.26969	2.1
16783	1.07763	0.05
16784	1.82301	0.05
16785	1.07763	0.05
16786	1.17506	0.05
16787	1.20045	2.1
16788	1.12486	2.1
16789	1.15234	2.1
16790	1.11598	2.1
16791	1.12486	2.1
16792	1.0491	2.1
16793	1.11598	2.1
16794	1.1047	2.1
16795	1.05902	2.1
16796	1.14361	2.1
16797	1.14361	2.1
16798	1.26851	2.1
16799	1.29389	1.08135
16800	0.988456	1.12403
16801	1.83688	0.05
16802	1.70302	0.05
16803	1.49386	0.05
16804	1.83151	0.05
16805	1.01346	0.107373
16806	0.983607	0.160348
16807	0.983596	2.1
16808	1.08332	0.673184
16809	2.36848	0.05
16810	2.97066	0.05
16811	1.12352	0.05
16812	2.5311	0.05
16813	1.09062	2.1
16814	0.962182	2.1
16815	0.962181	2.1
16816	1.2258	2.1
16817	0.974888	2.1

16818	1.06312	2.1
16819	1.73583	0.05
16820	1.72399	0.05
16821	1.724	0.05
16822	1.50951	0.05
16823	1.26755	2.1
16824	1.03824	2.1
16825	1.27875	2.1
16826	1.03824	2.1
16827	1.18902	2.1
16828	1.534	2.1
16829	1.11764	2.1
16830	1.534	2.1
16831	1.52008	2.1
16832	1.34406	2.1
16833	1.34406	2.1
16834	1.2187	2.1
16835	0.963571	0.493776
16836	1.19612	0.176316
16837	0.963571	2.1
16838	1.00007	2.1
16839	0.903333	2.1
16840	1.12724	2.1
16841	1.23771	2.1
16842	1.17683	2.1
16843	1.20059	0.490158
16844	0.772023	0.0644472
16845	1.14749	0.163768
16846	1.2802	0.380539
16847	1.85091	1.42724
16848	2.11193	0.628012
16849	1.2802	0.133696
16850	1.42913	0.605487
16851	2.11193	0.499175
16852	1.27737	0.121966
16853	1.30584	2.1
16854	1.60936	2.1
16855	1.3413	0.05
16856	1.53336	0.05
16857	2.60679	0.05
16858	1.3413	0.05
16859	1.35877	2.1
16860	1.11024	2.1
16861	1.27839	2.1
16862	1.08171	2.1
16863	1.27841	2.1
16864	1.0577	2.1
16865	1.71255	2.1
16866	1.10611	2.1
16867	0.960106	2.1
16868	1.40921	2.1
16869	1.90705	0.0781531
16870	2.79564	0.059732
16871	1.45321	0.0690947
16872	1.90705	0.0727216
16873	0.974718	0.05
16874	1.53671	0.05
16875	1.69039	0.05

16876	1.78229	0.05
16877	1.0101	2.1
16878	1.3167	2.1
16879	1.19699	2.1
16880	1.3212	2.1
16881	1.60811	0.05
16882	1.43224	0.05
16883	1.35401	0.05
16884	1.17464	0.05
16885	1.27696	2.1
16886	1.05025	2.1
16887	1.05025	2.1
16888	1.18076	2.1
16889	1.37224	1.66216
16890	1.13438	1.57334
16891	1.76788	1.53163
16892	1.73444	1.35821
16893	1.37704	2.1
16894	1.77042	2.1
16895	1.77042	2.1
16896	1.09778	2.1
16897	1.28667	2.1
16898	1.28228	2.1
16899	1.30646	0.67176
16900	1.68867	0.550263
16901	1.30645	1.33248
16902	0.986494	1.95917
16903	1.6253	0.05
16904	1.48676	0.05
16905	1.48676	0.05
16906	1.32974	0.05
16907	0.634347	0.05
16908	1.15142	0.05
16909	1.11395	0.05
16910	1.18068	0.05
16911	1.36904	0.05
16912	0.764563	0.05
16913	2.09213	0.05
16914	1.56631	2.1
16915	1.08011	2.1
16916	1.80283	2.1
16917	1.50738	2.1
16918	1.44951	0.05
16919	1.50102	0.05
16920	1.50102	0.05
16921	1.39615	0.05
16922	1.02653	0.05
16923	2.98258	0.0652499
16924	2.0815	0.05
16925	1.39525	0.05
16926	1.82176	0.05
16927	0.977607	0.05
16928	1.96133	1.62019
16929	1.27189	1.02448
16930	0.826032	0.631941
16931	2.12831	0.642555
16932	1.77798	2.1
16933	1.02969	1.88727

16934	1.06463	1.48397
16935	1.49219	2.1
16936	1.10003	2.1
16937	1.21223	2.1
16938	1.00513	1.08718
16939	1.43788	1.99701
16940	1.47134	0.0638013
16941	0.968249	0.106418
16942	3.22585	0.05
16943	3.36933	0.05
16944	3.36932	0.05
16945	3.03865	0.05
16946	1.66295	0.05
16947	1.65583	0.05
16948	1.65583	0.05
16949	1.4732	0.05
16950	1.73704	0.05
16951	1.76476	2.00848
16952	2.13345	0.53293
16953	1.55816	2.1
16954	1.55816	0.897285
16955	1.02601	2.1
16956	1.08039	2.1
16957	1.05498	0.672879
16958	1.08039	2.1
16959	1.2873	2.1
16960	1.01121	2.1
16961	0.987703	2.1
16962	0.98771	2.1
16963	0.964782	2.1
16964	1.35719	2.1
16965	2.61092	2.1
16966	2.6607	2.1
16967	3.41693	2.1
16968	1.23125	2.1
16969	1.73772	2.1
16970	0.99845	0.165072
16971	1.5176	0.232578
16972	1.13271	0.05
16973	1.332 0.05	
16974	1.63159	2.1
16975	1.61342	2.1
16976	1.03679	2.1
16977	1.19087	2.1
16978	1.97172	2.1
16979	2.68634	2.1
16980	1.19086	2.1
16981	1.50091	2.1
16982	2.84677	2.1
16983	2.77894	2.1
16984	2.77894	2.1
16985	2.74206	2.1
16986	1.3284	2.1
16987	1.83524	2.1
16988	1.65853	2.1
16989	1.56741	2.1
16990	1.00509	2.1
16991	1.45246	2.1

16992	1.26193	2.1
16993	1.45244	2.1
16994	1.61963	0.05
16995	1.68189	0.0883985
16996	1.68189	0.693726
16997	1.49155	0.0508388
16998	1.58054	2.1
16999	1.59169	2.1
17000	1.59169	2.1
17001	1.60497	2.1
17002	0.743595	0.05
17003	0.702359	0.05
17004	1.15317	0.05
17005	2.24367	0.05
17006	1.15252	0.05
17007	2.60354	0.05
17008	1.45158	2.1
17009	1.56004	2.1
17010	1.64527	2.1
17011	1.00371	2.1
17012	1.38287	0.05
17013	0.893024	0.420143
17014	1.23527	0.234453
17015	1.23706	0.108986
17016	2.20993	0.0832828
17017	1.54858	1.39704
17018	0.798666	0.05
17019	1.22487	0.05
17020	1.33158	0.05
17021	1.85902	0.05
17022	1.08334	2.1
17023	1.17885	1.09205
17024	1.17885	0.182479
17025	1.07283	0.599896
17026	1.30723	0.05
17027	1.62507	0.05
17028	1.62507	0.05
17029	1.08854	0.05
17030	1.59475	2.1
17031	1.58874	2.1
17032	1.58875	2.1
17033	1.35787	2.1
17034	1.04147	2.1
17035	1.15564	2.1
17036	1.78301	2.1
17037	1.90618	2.1
17038	1.71555	2.1
17039	2.13143	2.1
17040	1.10817	2.1
17041	1.032	2.1
17042	1.032	2.1
17043	0.953636	2.1
17044	1.27916	1.07984
17045	1.11938	0.846979
17046	1.11937	0.75035
17047	1.9186	1.12037
17048	1.19967	0.05
17049	1.63489	0.05

17050	1.00763	0.05
17051	1.81656	0.05
17052	1.31814	0.05
17053	2.98183	0.05
17054	1.20149	2.1
17055	1.29129	2.1
17056	1.29129	2.1
17057	1.37138	2.1
17058	1.23104	0.360749
17059	1.75236	0.996646
17060	1.6	0.109552
17061	1.60565	0.218796
17062	1.60565	0.168518
17063	1.40013	0.25931
17064	1.23042	2.1
17065	1.18278	2.1
17066	1.10853	2.1
17067	1.04724	2.1
17068	1.1766	2.1
17069	1.00319	2.1
17070	1.29435	2.1
17071	1.00319	2.1
17072	1.05655	1.64903
17073	1.01606	1.68281
17074	1.80391	2.1
17075	2.7699	2.1
17076	1.52073	2.1
17077	2.06835	2.1
17078	2.00202	2.1
17079	2.04906	2.1
17080	0.968536	2.1
17081	1.02461	2.1
17082	1.59784	2.1
17083	1.78249	2.1
17084	1.81409	2.1
17085	1.79844	2.1
17086	1.77093	2.02563
17087	2.02356	1.42162
17088	1.32449	2.1
17089	1.10003	2.1
17090	1.16915	0.224368
17091	1.05937	0.0565829
17092	1.28096	2.1
17093	1.34696	2.1
17094	1.34695	2.1
17095	1.36457	2.1
17096	1.06478	2.1
17097	1.06239	2.1
17098	1.06239	2.1
17099	1.09629	2.1
17100	0.798209	2.1
17101	1.8532	2.1
17102	2.10112	0.05
17103	2.8704	0.05
17104	2.8704	0.05
17105	2.92457	0.05
17106	1.0752	2.1
17107	1.07745	2.1

17108	1.07745	2.1
17109	1.08911	2.1
17110	1.40182	2.1
17111	1.00133	2.1
17112	1.40182	2.1
17113	1.24509	2.1
17114	2.22243	0.05
17115	1.07303	0.05
17116	1.29736	0.05
17117	1.73584	0.05
17118	2.0815	0.159187
17119	1.07999	0.0868171
17120	2.1692	2.1
17121	1.99992	2.1
17122	1.99992	2.1
17123	1.78327	2.1
17124	1.52661	0.05
17125	0.956003	0.05
17126	1.53172	0.05
17127	0.955991	0.05
17128	1.35025	1.6838
17129	1.51084	2.1
17130	1.51084	2.1
17131	1.67115	2.1
17132	2.00977	2.1
17133	1.63632	2.1
17134	1.63632	2.1
17135	2.66128	2.1
17136	1.13525	2.1
17137	2.37566	2.1
17138	1.25987	2.1
17139	1.4472	2.1
17140	1.4472	2.1
17141	1.66018	2.1
17142	0.994001	0.503951
17143	1.83151	0.346419
17144	1.83151	0.104423
17145	1.57621	0.122163
17146	1.40915	0.05
17147	1.24653	0.323909
17148	1.24653	0.05
17149	1.10609	0.684204
17150	1.61121	2.1
17151	1.74472	2.1
17152	1.74472	2.1
17153	1.0368	2.1
17154	1.04788	2.1
17155	1.28522	2.1
17156	1.28522	2.1
17157	1.54196	2.1
17158	1.23387	2.1
17159	0.896519	2.1
17160	1.60428	2.1
17161	1.08467	2.1
17162	1.56875	0.0766241
17163	1.39072	0.05
17164	1.39073	0.0500569
17165	1.6	0.178783

17166	1.01346	2.1
17167	1.06838	0.55587
17168	0.968463	0.733501
17169	1.06837	0.599159
17170	3.22433	0.05
17171	2.05993	1.63686
17172	2.01836	0.05
17173	1.66154	1.77616
17174	1.20871	2.06262
17175	1.10586	2.1
17176	1.10586	2.00601
17177	1.03853	2.1
17178	1.12076	2.1
17179	1.03899	2.1
17180	1.03899	2.1
17181	0.963973	2.1
17182	0.987132	2.1
17183	1.0894	2.1
17184	2.37823	2.1
17185	1.75647	1.70617
17186	1.64625	2.1
17187	1.56195	2.1
17188	1.41995	2.1
17189	1.15171	2.1
17190	1.36457	2.1
17191	1.33847	2.03588
17192	1.33847	2.1
17193	0.869601	1.7936
17194	1.42255	2.1
17195	1.3868	2.1
17196	1.3868	2.1
17197	1.33962	2.1
17198	1.05481	2.1
17199	1.08074	2.1
17200	1.08074	2.1
17201	0.857557	2.1
17202	1.13537	1.81972
17203	1.09194	0.774484
17204	1.01179	1.96227
17205	1.13537	0.378321
17206	1.15645	2.06582
17207	1.56409	0.05
17208	1.56409	0.332871
17209	1.16239	0.892224
17210	1.10746	0.05
17211	1.47952	0.162545
17212	1.14733	2.1
17213	3.77068	2.1
17214	1.38497	2.1
17215	1.54599	2.1
17216	1.54601	2.1
17217	1.27697	2.1
17218	1.01393	1.56879
17219	1.10558	1.73992
17220	0.995012	0.636615
17221	1.17624	1.24819
17222	1.60497	2.1
17223	1.61588	2.1

17224	1.61587	2.1
17225	1.62182	2.1
17226	1.48866	0.05
17227	1.65977	0.05
17228	1.65977	0.05
17229	1.69044	0.05
17230	0.961892	2.1
17231	1.00659	2.1
17232	1.0066	2.1
17233	1.06226	2.1
17234	1.15171	2.1
17235	1.50853	2.1
17236	1.50853	2.1
17237	1.2955	2.1
17238	1.3204	0.05
17239	2.13295	0.05
17240	2.75524	0.05
17241	1.18156	0.05
17242	1.73729	2.1
17243	1.20356	2.1
17244	1.62483	2.1
17245	1.52008	2.1
17246	1.19964	2.1
17247	1.0577	2.1
17248	1.19964	2.1
17249	1.07156	2.1
17250	1.98241	2.1
17251	1.43687	2.1
17252	1.27863	2.1
17253	1.19915	1.41452
17254	0.97269	1.56019
17255	1.0739	0.496135
17256	1.1427	2.1
17257	1.22476	0.82298
17258	1.06584	1.73568
17259	1.22626	1.14716
17260	1.31388	2.1
17261	1.16932	2.1
17262	1.28819	2.1
17263	1.0499	2.1
17264	1.55894	2.1
17265	1.78709	2.1
17266	1.75986	2.1
17267	1.764 2.1	
17268	1.44801	2.1
17269	1.28186	2.1
17270	1.20797	2.06355
17271	1.06821	2.1
17272	1.87287	0.420106
17273	2.08014	0.703145
17274	1.63225	0.847281
17275	1.39501	1.2387
17276	1.20132	0.05
17277	1.16182	0.05
17278	1.08848	0.05
17279	1.20132	0.05
17280	1.23475	2.03914
17281	1.16661	2.1

17282	2.31317	2.1
17283	2.50145	2.1
17284	1.04367	2.1
17285	2.23875	2.1
17286	0.953632	0.05
17287	1.38646	0.05
17288	1.38646	0.05
17289	1.61963	0.05
17290	1.62934	1.12579
17291	1.88626	0.857945
17292	1.0775	0.172102
17293	1.119	0.087336
17294	1.08133	0.0605237
17295	1.48312	0.0519881
17296	1.57125	0.05
17297	1.74098	0.05
17298	1.94372	1.18292
17299	1.22237	1.5269
17300	1.18549	0.12452
17301	1.01294	0.05
17302	1.01294	0.214427
17303	1.2064	1.08141
17304	1.14599	2.1
17305	1.00065	0.0916674
17306	1.76357	2.1
17307	2.23468	2.1
17308	2.21735	2.1
17309	3.32769	2.1
17310	1.19647	0.945998
17311	1.35307	0.184867
17312	1.19645	0.290493
17313	1.43778	0.109114
17314	1.34412	0.05
17315	1.1315	2.1
17316	1.1315	0.156209
17317	1.23943	0.0671117
17318	1.57829	2.1
17319	1.22537	2.1
17320	1.14357	2.1
17321	1.32772	2.1
17322	1.4259	2.1
17323	1.34245	2.1
17324	1.47953	2.1
17325	1.70303	2.1
17326	2.21127	0.05
17327	1.47904	0.05
17328	1.18793	0.05
17329	2.21126	0.05
17330	1.1032	2.02426
17331	1.14986	0.0708693
17332	1.14986	2.1
17333	1.19611	0.130633
17334	1.91883	2.1
17335	1.83019	2.1
17336	1.83019	2.1
17337	1.82462	2.1
17338	1.27362	0.05
17339	1.27421	0.05

17340	1.27421	0.05
17341	1.10736	0.05
17342	1.3003	0.0797074
17343	1.31415	1.26027
17344	1.31415	0.142279
17345	1.32201	1.58374
17346	1.21124	2.1
17347	0.81568	2.1
17348	0.81568	2.1
17349	0.551816	2.1
17350	1.67119	2.1
17351	1.63384	2.1
17352	1.63384	2.1
17353	1.63661	2.1
17354	1.48734	2.1
17355	1.52245	2.1
17356	1.52245	2.1
17357	1.50714	2.1
17358	0.996778	0.05
17359	1.56091	0.05
17360	1.56091	0.05
17361	1.90457	0.05
17362	1.1181	2.1
17363	1.06543	2.1
17364	2.66828	2.1
17365	2.76932	2.1
17366	1.06543	2.1
17367	1.01474	2.1
17368	2.76932	2.1
17369	2.86704	2.1
17370	1.29724	0.05
17371	1.96938	0.05
17372	1.82462	2.1
17373	1.84553	2.1
17374	1.05556	2.1
17375	1.25363	2.1
17376	1.84552	2.1
17377	1.53208	2.1
17378	1.12826	2.1
17379	1.39075	2.1
17380	0.9702	1.00041
17381	1.16481	0.324866
17382	1.08894	0.671201
17383	1.91756	0.840597
17384	1.50865	0.140424
17385	1.06012	0.0602479
17386	1.5456	0.05
17387	1.37427	0.05
17388	1.1211	0.05
17389	1.5456	0.05
17390	1.33135	0.05
17391	1.07956	0.05
17392	2.23255	0.05
17393	1.21031	0.05
17394	1.52903	2.1
17395	1.35977	2.1
17396	3.3837	2.1
17397	3.20581	2.1

17398	1.35977	2.1
17399	1.15414	2.1
17400	3.20582	2.1
17401	2.07596	2.1
17402	1.6812	0.05
17403	1.03939	0.05
17404	1.30047	2.1
17405	1.5586	2.1
17406	1.5586	2.1
17407	1.84214	2.1
17408	1.31825	0.0603254
17409	1.31923	1.77943
17410	1.31924	0.0882313
17411	1.33026	0.962101
17412	1.15818	0.799619
17413	1.17822	1.19239
17414	1.15818	2.1
17415	1.28096	1.57401
17416	1.06848	0.242441
17417	1.89639	0.111684
17418	1.9186	0.985602
17419	1.77065	0.77195
17420	1.59103	0.646087
17421	1.56164	0.375053
17422	1.09697	0.05
17423	2.38719	0.05
17424	2.22123	0.05
17425	2.46059	0.05
17426	1.45506	0.05
17427	1.03645	0.05
17428	1.30347	2.1
17429	1.2627	2.1
17430	1.2627	2.1
17431	1.23036	2.1
17432	1.41469	2.1
17433	1.45257	2.1
17434	1.46907	0.05
17435	1.70302	0.05
17436	1.49196	0.05
17437	1.36307	0.05
17438	1.05677	2.1
17439	0.978698	2.1
17440	0.978699	2.1
17441	2.01822	2.1
17442	1.47446	2.1
17443	1.55432	2.1
17444	1.55432	2.1
17445	1.06053	2.1
17446	1.86813	1.72878
17447	1.21892	2.1
17448	1.53064	0.984572
17449	1.30664	1.0871
17450	1.09629	2.1
17451	1.19244	2.1
17452	1.19244	2.1
17453	1.37635	2.1
17454	1.49358	0.102039
17455	1.57702	0.05

17456	2.09374	2.1
17457	1.88175	2.1
17458	1.88175	2.1
17459	1.51085	2.1
17460	1.10938	0.05
17461	1.02478	0.05
17462	1.33061	0.05
17463	1.13272	0.05
17464	1.75505	2.1
17465	1.78529	2.1
17466	1.6353	2.1
17467	1.59307	2.1
17468	1.60017	0.0994183
17469	1.35608	0.269517
17470	1.35608	0.0914577
17471	1.04713	0.0764479
17472	1.01479	0.05
17473	1.3868	0.05
17474	1.3868	0.05
17475	1.28418	2.1
17476	2.15949	2.1
17477	2.90429	2.1
17478	1.51902	2.1
17479	1.54755	2.1
17480	2.90429	2.1
17481	3.49966	2.1
17482	1.70842	2.1
17483	1.97526	2.1
17484	0.949712	0.05
17485	1.92784	0.05
17486	1.34093	0.05
17487	2.53426	0.05
17488	1.92783	0.05
17489	3.89373	0.05
17490	2.53426	0.05
17491	2.35961	0.05
17492	1.68108	0.116064
17493	1.15558	0.0969715
17494	1.67265	0.125438
17495	1.27608	0.0796194
17496	1.42249	2.1
17497	1.39708	2.1
17498	1.39708	2.1
17499	1.39327	2.1
17500	1.97046	0.05
17501	1.11688	0.05
17502	1.28418	0.05
17503	1.17302	2.00799
17504	1.17302	0.05
17505	1.04349	1.95851
17506	0.979853	2.1
17507	1.26016	2.1
17508	1.18901	2.1
17509	1.26016	2.1
17510	1.22857	0.117155
17511	1.25848	0.683835
17512	1.25848	0.115306
17513	1.2858	0.880296

17514	1.09154	2.1
17515	2.01915	2.1
17516	1.23013	2.1
17517	1.55698	2.1
17518	1.55698	2.1
17519	1.8575	2.1
17520	2.01854	2.1
17521	1.20686	2.1
17522	1.20684	2.1
17523	1.77796	2.1
17524	1.46129	2.1
17525	1.04193	2.1
17526	1.10051	2.1
17527	0.983723	2.1
17528	1.1729	2.1
17529	0.98505	2.1
17530	0.985052	2.1
17531	1.03951	2.1
17532	1.03949	2.1
17533	1.17583	2.1
17534	1.42866	2.1
17535	1.97261	2.1
17536	1.73274	2.1
17537	1.53629	2.1
17538	1.98182	2.1
17539	1.72511	2.1
17540	1.42174	2.1
17541	1.44472	2.1
17542	1.72511	2.1
17543	1.45411	2.1
17544	1.44472	2.1
17545	1.43836	2.1
17546	0.962703	0.0991685
17547	1.0171	0.477645
17548	1.53336	0.05
17549	1.40274	0.05
17550	1.40272	0.05
17551	1.00213	0.05
17552	1.50892	0.05
17553	1.64984	0.05
17554	1.67464	0.05
17555	1.67481	0.05
17556	1.08212	2.1
17557	1.28095	2.1
17558	1.46956	2.1
17559	1.70303	2.1
17560	1.2925	0.05
17561	1.84716	0.05
17562	1.48676	0.05
17563	1.48797	0.05
17564	1.33917	0.05
17565	1.22691	0.05
17566	0.528744	2.1
17567	1.13728	2.1
17568	1.13728	2.1
17569	1.99843	2.1
17570	1.17464	0.05
17571	1.0372	0.05

17572	2.0815	0.05
17573	0.933462	0.05
17574	1.54931	2.1
17575	1.5485	2.1
17576	1.4077	2.1
17577	1.29931	2.1
17578	1.14931	0.0857918
17579	2.02223	0.0866657
17580	1.04482	0.05
17581	0.94971	0.0641069
17582	1.54632	1.44867
17583	1.15693	1.5736
17584	1.66763	2.1
17585	1.41238	2.1
17586	1.41238	2.1
17587	1.48068	2.1
17588	1.0171	0.05
17589	1.1211	0.277733
17590	1.1211	0.05
17591	1.28418	0.171671
17592	0.993021	2.1
17593	1.764 2.1	
17594	0.993023	2.1
17595	1.05787	2.1
17596	1.90914	0.05
17597	1.12229	1.74045
17598	1.02025	0.0876414
17599	0.585368	1.98976
17600	1.99323	2.1
17601	2.34988	2.1
17602	2.34988	2.1
17603	2.36987	2.1
17604	1.23107	0.05
17605	1.90664	0.05
17606	1.53496	0.05
17607	1.70302	0.05
17608	1.98859	2.1
17609	1.61057	2.1
17610	1.79844	2.1
17611	2.10811	2.1
17612	1.4618	2.1
17613	1.96722	2.1
17614	1.95191	2.1
17615	2.13418	2.1
17616	1.50368	0.05
17617	1.61417	0.05
17618	1.15008	0.05
17619	1.19685	0.05
17620	1.90456	0.05
17621	1.90899	0.05
17622	2.08864	0.05
17623	2.10447	0.05
17624	1.16653	2.1
17625	1.23017	2.1
17626	1.0752	0.0604097
17627	1.34412	0.05
17628	1.06226	2.1
17629	1.09252	2.1

17630	1.09252	2.1
17631	0.88579	2.1
17632	1.56178	0.05
17633	1.14305	0.05
17634	1.56175	0.05
17635	1.08211	0.05
17636	1.32813	2.1
17637	1.32461	2.1
17638	1.32461	2.1
17639	1.32455	2.1
17640	1.42729	0.05
17641	1.18716	0.05
17642	1.47169	0.05
17643	1.24687	0.05
17644	1.09627	2.1
17645	1.04759	2.1
17646	1.04759	2.1
17647	0.987132	2.1
17648	1.11181	1.93287
17649	1.80662	2.1
17650	1.8066	2.1
17651	1.06461	1.98438
17652	1.56091	2.1
17653	1.9842	2.1
17654	1.73525	0.05
17655	1.40557	0.05
17656	2.26906	0.05
17657	1.31248	0.05
17658	1.66832	0.05
17659	1.32634	0.05
17660	1.03314	0.05
17661	1.24682	0.05
17662	1.38536	0.05
17663	1.52661	0.05
17664	1.177	0.741571
17665	1.36405	0.390906
17666	1.19837	0.726231
17667	1.17701	0.05
17668	0.993831	0.05
17669	1.0823	0.05
17670	1.0823	0.05
17671	1.01121	0.05
17672	1.49653	0.05
17673	1.89583	0.05
17674	0.67242	0.05
17675	1.36047	0.05
17676	1.00366	0.05
17677	1.24728	0.05
17678	1.13387	0.05
17679	1.60867	0.05
17680	1.30572	0.05
17681	1.54982	0.05
17682	0.788592	0.05
17683	1.82961	0.05
17684	1.54982	0.05
17685	1.82961	0.05
17686	1.26062	0.05
17687	1.64002	0.05

17688	1.12035	0.05
17689	1.64002	0.05
17690	1.1673	2.1
17691	1.75367	2.1
17692	0.964034	2.1
17693	1.0305	2.1
17694	1.30896	2.07373
17695	1.52776	1.9767
17696	1.52776	2.1
17697	1.17989	2.1
17698	1.16759	2.1
17699	1.8575	2.1
17700	1.16759	2.1
17701	1.60936	2.1
17702	1.10747	0.05
17703	1.47688	0.05
17704	1.40794	2.1
17705	1.95729	1.88048
17706	1.95729	2.1
17707	3.18156	2.03071
17708	1.87598	2.1
17709	1.94343	2.1
17710	1.94343	2.1
17711	2.00551	2.1
17712	1.20067	0.887028
17713	1.70744	1.81005
17714	1.87244	1.63561
17715	1.32075	1.14028
17716	1.64955	2.1
17717	1.44221	2.1
17718	1.31109	2.1
17719	1.14206	2.1
17720	1.60452	2.0513
17721	1.12111	0.316651
17722	1.00832	2.1
17723	0.974137	2.1
17724	0.974137	2.1
17725	0.98505	2.1
17726	1.02368	2.1
17727	0.985051	2.1
17728	0.995734	0.05
17729	1.31993	0.05
17730	1.31993	0.05
17731	1.18549	0.05
17732	1.5176	0.276547
17733	1.33622	0.159399
17734	1.33621	0.19366
17735	1.02782	0.116039
17736	1.66774	2.1
17737	1.85305	2.1
17738	1.85305	2.1
17739	2.07088	2.1
17740	1.78873	2.1
17741	1.47533	2.1
17742	1.23152	2.1
17743	0.99922	2.1
17744	1.48493	2.1
17745	1.1394	2.1

17746	1.19179	2.1
17747	1.64289	2.1
17748	1.47858	2.1
17749	1.89687	2.1
17750	0.972464	2.1
17751	1.79473	2.1
17752	1.06971	2.1
17753	1.21089	2.1
17754	1.12572	2.1
17755	1.30566	2.1
17756	1.30566	2.1
17757	1.52724	2.1
17758	2.32237	0.0503824
17759	2.20248	0.05
17760	2.79564	0.0671607
17761	2.32237	0.05
17762	1.59354	0.05
17763	3.098 0.05	
17764	3.09801	0.05
17765	3.45132	0.05
17766	1.24001	2.06728
17767	1.41822	0.363581
17768	1.41821	2.1
17769	1.44859	0.799593
17770	1.34539	2.1
17771	1.06991	2.1
17772	1.18076	2.1
17773	0.978586	2.1
17774	0.999189	0.05
17775	2.41076	0.05
17776	0.968475	2.1
17777	1.02074	2.1
17778	1.02074	2.1
17779	1.07866	2.1
17780	0.817702	0.05
17781	1.5082	0.05
17782	1.48376	0.05
17783	1.18947	0.05
17784	1.10638	2.1
17785	1.86766	2.1
17786	1.19785	2.1
17787	1.34141	2.1
17788	1.34141	2.1
17789	0.991985	2.1
17790	2.35516	0.127327
17791	1.24815	0.423482
17792	1.80454	2.1
17793	1.22633	2.1
17794	0.696255	2.1
17795	1.19379	0.712899
17796	1.14721	2.1
17797	1.16153	2.1
17798	1.16153	2.1
17799	1.17261	2.1
17800	1.20814	2.1
17801	1.54791	2.1
17802	1.54791	2.1
17803	1.60477	2.1

17804	1.29931	2.1
17805	1.19098	2.1
17806	1.19098	2.1
17807	1.13341	2.1
17808	1.00503	2.1
17809	1.25334	0.0777102
17810	1.22655	2.1
17811	1.26282	2.1
17812	1.26282	2.1
17813	0.999489	2.1
17814	1.54197	0.05
17815	1.17152	0.05
17816	1.17152	0.05
17817	1.49627	0.05
17818	1.36405	0.433303
17819	0.976334	0.136986
17820	1.12495	2.1
17821	1.54096	2.1
17822	1.75263	2.1
17823	1.49114	2.1
17824	0.842998	2.02038
17825	1.60244	0.34695
17826	1.48911	1.76491
17827	2.18685	0.465638
17828	1.75236	0.7885
17829	1.54143	1.42541
17830	1.40013	0.211793
17831	0.946646	0.190426
17832	1.50714	2.1
17833	1.46055	2.1
17834	1.46055	2.1
17835	1.42249	2.1
17836	2.12839	0.05
17837	1.75211	0.05
17838	1.76297	0.05
17839	1.70736	0.05
17840	1.19952	0.05
17841	1.56475	0.05
17842	1.74312	0.05
17843	1.48973	0.05
17844	1.91518	0.05
17845	1.16534	0.05
17846	1.53556	2.1
17847	1.56253	2.1
17848	1.56253	2.1
17849	1.58054	2.1
17850	1.27806	0.05
17851	1.44593	0.05
17852	1.44593	0.05
17853	1.3946	0.05
17854	1.34857	0.05
17855	1.03818	1.90692
17856	1.38969	0.120598
17857	1.85548	0.250361
17858	1.03108	0.128446
17859	1.85548	0.177698
17860	1.49346	1.7582
17861	1.83192	2.07423

17862	1.75291	1.05516
17863	1.35435	1.8717
17864	0.921851	0.0820661
17865	1.09646	0.0736606
17866	1.2183	0.0706078
17867	1.36717	0.0517726
17868	2.14179	0.05
17869	1.79805	0.0657924
17870	1.3428	0.0706027
17871	2.30493	0.070602
17872	1.81545	0.05
17873	1.92269	0.0618367
17874	2.30492	0.074129
17875	1.3663	0.0975515
17876	1.22418	2.1
17877	0.956698	0.534028
17878	1.4706	2.1
17879	0.953295	2.1
17880	1.47059	2.1
17881	1.26929	2.1
17882	1.24538	0.05
17883	1.4468	0.05
17884	2.86762	2.1
17885	2.32911	2.1
17886	2.10864	2.1
17887	1.94548	2.1
17888	1.83134	2.1
17889	2.19519	2.1
17890	2.19853	2.1
17891	2.63855	2.1
17892	1.08213	0.05
17893	1.46389	0.05
17894	1.62235	0.05
17895	0.980549	0.05
17896	1.08195	2.03612
17897	0.99002	2.1
17898	1.2269	2.1
17899	1.0838	2.1
17900	0.889491	2.1
17901	1.19587	2.1
17902	1.18801	0.05
17903	1.57125	0.05
17904	1.47712	2.1
17905	1.45182	2.1
17906	1.45182	2.1
17907	1.42255	2.1
17908	1.17816	0.05
17909	1.00982	0.05
17910	1.21056	0.05
17911	1.17816	0.05
17912	3.80902	0.37913
17913	0.976991	0.227209
17914	1.09962	2.1
17915	1.60976	2.1
17916	1.87029	2.1
17917	2.33806	2.1
17918	2.12549	2.1
17919	2.21472	2.1

17920	2.16221	0.05
17921	1.99528	0.05
17922	1.99528	0.05
17923	1.97969	0.05
17924	3.60854	2.1
17925	4.00847	2.1
17926	2.70372	2.1
17927	1.253	2.1
17928	1.65739	2.1
17929	0.994294	2.1
17930	1.36203	2.1
17931	1.00965	2.1
17932	2.33374	2.1
17933	1.36209	2.1
17934	1.64983	0.05
17935	1.36416	0.05
17936	2.19766	0.05
17937	1.36416	0.05
17938	2.15871	0.257784
17939	1.81454	0.0620321
17940	1.54607	0.05
17941	1.57702	0.127883
17942	0.948748	0.05
17943	1.04522	0.05
17944	1.60994	0.05
17945	1.90382	0.05
17946	1.90382	0.05
17947	1.57999	0.05
17948	1.73905	0.279705
17949	1.26535	1.30004
17950	2.12552	1.08447
17951	1.06891	0.644518
17952	1.13999	1.30494
17953	2.29812	1.03779
17954	2.83329	0.707644
17955	2.16272	0.444579
17956	1.71277	2.1
17957	1.68401	2.1
17958	1.68401	2.1
17959	1.69368	2.1
17960	1.06472	2.1
17961	1.56017	2.1
17962	1.2765	2.1
17963	1.99541	2.1
17964	1.46041	0.05
17965	2.04817	0.05
17966	2.04817	0.05
17967	2.33181	0.05
17968	2.06353	2.1
17969	1.35449	2.1
17970	1.52045	2.1
17971	1.45437	2.1
17972	1.04405	0.05
17973	1.85945	0.05
17974	1.85945	0.05
17975	2.22679	0.05
17976	1.33339	2.1
17977	1.19376	2.1

17978	1.33266	2.1
17979	1.44244	2.1
17980	1.20455	2.1
17981	1.52852	2.1
17982	1.03882	2.1
17983	1.52852	2.1
17984	1.44801	2.1
17985	1.00775	2.1
17986	0.53907	2.1
17987	0.506124	2.1
17988	2.39045	2.1
17989	0.973794	2.1
17990	1.11193	2.1
17991	1.32784	2.1
17992	1.31595	2.1
17993	0.966115	2.1
17994	1.25953	2.1
17995	1.38086	2.1
17996	1.38086	2.1
17997	1.04101	2.1
17998	1.99319	0.05
17999	1.23107	0.05
18000	3.3606	0.05
18001	1.23112	0.05
18002	1.40239	2.1
18003	1.26091	2.1
18004	1.26091	2.1
18005	1.05776	2.1
18006	1.23768	0.080684
18007	1.0142	1.41263
18008	1.11435	1.19941
18009	1.01422	0.452627
18010	1.24001	0.295574
18011	1.42186	1.153
18012	1.42186	0.454474
18013	1.47204	0.689385
18014	1.27361	2.1
18015	1.56642	2.1
18016	1.71676	2.1
18017	2.16815	2.1
18018	1.07855	1.34512
18019	1.07612	1.13317
18020	1.703 0.05	
18021	1.46853	0.05
18022	1.45448	0.05
18023	1.27778	0.05
18024	0.991986	2.1
18025	1.1192	2.1
18026	1.1192	2.1
18027	1.25953	2.1
18028	2.08506	2.1
18029	1.75162	2.1
18030	1.94523	2.1
18031	1.88405	1.98795
18032	1.15506	0.05
18033	1.1233	0.05
18034	2.09736	2.1
18035	2.98303	2.1

18036	2.71184	2.1
18037	3.51614	2.1
18038	1.18769	2.1
18039	1.24053	2.1
18040	1.24053	2.1
18041	1.45327	2.1
18042	1.26865	1.508
18043	1.1744	0.968689
18044	1.1744	0.330013
18045	1.11712	0.926833
18046	1.15841	0.05
18047	1.02247	0.05
18048	1.93649	2.1
18049	1.81191	2.1
18050	1.64718	2.1
18051	1.47521	2.1
18052	1.59654	2.1
18053	1.57823	2.1
18054	1.57823	2.1
18055	1.55768	2.1
18056	1.00665	0.05
18057	1.28239	0.05
18058	1.28239	0.05
18059	1.44951	0.05
18060	1.22083	2.1
18061	0.957622	2.1
18062	0.887896	2.1
18063	1.27387	2.1
18064	1.00521	0.434499
18065	1.00503	0.759124
18066	1.95844	2.1
18067	1.05042	2.1
18068	2.78662	2.1
18069	2.22399	2.1
18070	2.3356	0.05
18071	1.49543	0.05
18072	1.96854	0.05
18073	1.21681	0.05
18074	2.17803	1.63443
18075	1.50998	2.1
18076	2.35239	1.31554
18077	2.26987	0.647559
18078	1.03824	0.050829
18079	1.51396	0.0884643
18080	1.51396	0.0748985
18081	1.48693	0.0502315
18082	1.08784	2.1
18083	1.36865	1.93087
18084	0.712405	2.08778
18085	1.08784	2.1
18086	2.02522	2.1
18087	1.6738	2.1
18088	1.35175	2.1
18089	1.50094	2.1
18090	1.6738	2.1
18091	1.62871	2.1
18092	1.35084	2.1
18093	1.34276	2.1

18094	1.18041	2.1
18095	1.43652	2.1
18096	0.974196	2.1
18097	1.28037	2.1
18098	1.28037	2.1
18099	1.05071	2.1
18100	2.44737	0.05
18101	1.4672	0.05
18102	1.22883	0.05
18103	2.08149	0.05
18104	1.82208	1.84068
18105	1.59826	1.7439
18106	1.59826	0.975624
18107	1.01393	2.01064
18108	1.42757	2.1
18109	0.996775	2.1
18110	1.42757	2.1
18111	1.26397	2.1
18112	1.02461	2.1
18113	1.46129	2.1
18114	1.11833	2.1
18115	1.335 2.1	
18116	2.07087	2.1
18117	1.24041	2.1
18118	1.335 2.1	
18119	1.55398	2.1
18120	1.21171	0.05
18121	1.48589	0.05
18122	1.32886	0.05
18123	1.8027	0.05
18124	1.53515	1.63654
18125	1.60428	1.34019
18126	1.00035	0.05
18127	1.03534	0.05
18128	1.03535	1.25086
18129	1.09962	2.1
18130	0.62132	1.39721
18131	1.21033	2.1
18132	1.30326	2.1
18133	2.6823	2.1
18134	0.991407	2.1
18135	1.29729	2.1
18136	1.29729	2.1
18137	1.16858	2.1
18138	1.66137	0.05
18139	1.23012	0.05
18140	1.66139	0.0841704
18141	1.41821	0.0936345
18142	1.56043	0.271349
18143	1.06848	0.736532
18144	1.56043	1.91089
18145	1.90911	1.40055
18146	0.982847	2.1
18147	1.05863	2.1
18148	1.05863	2.1
18149	1.22352	2.1
18150	1.23019	1.73097
18151	1.60509	2.1

18152	1.19122	2.1
18153	1.47769	2.1
18154	2.18868	2.1
18155	1.40984	2.1
18156	3.55197	2.1
18157	3.57227	2.1
18158	3.57227	2.1
18159	3.48926	2.1
18160	1.4375	0.05
18161	1.11314	0.05
18162	1.62408	2.1
18163	1.74438	2.1
18164	1.72387	2.1
18165	1.50018	2.1
18166	1.1486	2.1
18167	1.18474	2.1
18168	1.18474	2.1
18169	1.2295	2.1
18170	1.28754	2.1
18171	1.36838	2.1
18172	1.36838	2.1
18173	1.21703	2.1
18174	2.74206	2.1
18175	2.70795	2.1
18176	2.70795	2.1
18177	2.55918	2.1
18178	1.35065	0.714059
18179	1.65411	0.204822
18180	1.48572	0.559601
18181	1.28393	0.503979
18182	1.25265	0.05
18183	1.70944	0.05
18184	1.31259	0.17761
18185	1.14945	2.1
18186	1.14945	0.101836
18187	0.995725	2.1
18188	1.00307	0.05
18189	2.05786	0.05
18190	1.56357	0.05
18191	1.10907	0.0533053
18192	1.27558	1.58589
18193	1.05175	0.929115
18194	1.2685	2.1
18195	1.41369	2.1
18196	1.4137	2.1
18197	1.49654	2.1
18198	1.21489	2.1
18199	1.14605	1.6887
18200	1.22476	2.1
18201	1.21489	1.15199
18202	2.51472	1.05925
18203	2.52091	1.02373
18204	2.52091	0.807047
18205	1.79171	0.81552
18206	1.03373	2.1
18207	1.04037	2.1
18208	1.04038	2.1
18209	1.03933	2.1

18210	1.74123	0.05
18211	1.41902	0.05
18212	1.89777	0.05
18213	1.89899	0.05
18214	1.24728	0.05
18215	1.30394	0.05
18216	1.30394	0.05
18217	1.16535	0.05
18218	1.09914	2.1
18219	1.54259	2.1
18220	1.40233	2.1
18221	1.69858	2.1
18222	1.53555	2.1
18223	1.09859	1.83359
18224	1.09859	2.1
18225	0.725879	0.777202
18226	1.07213	2.1
18227	1.31589	2.1
18228	1.36904	2.1
18229	1.69711	2.1
18230	1.51028	0.05
18231	0.993598	0.05
18232	0.61563	0.05
18233	1.26311	0.05
18234	1.19496	2.1
18235	1.65925	2.1
18236	1.65925	2.1
18237	1.48162	2.1
18238	2.75738	1.59911
18239	1.24699	1.35786
18240	4.08729	1.94023
18241	2.35271	1.62798
18242	1.55063	0.119157
18243	1.34563	0.05
18244	1.34563	0.102379
18245	1.08189	0.05
18246	1.09662	0.05
18247	2.35961	0.05
18248	0.986952	0.05
18249	0.813251	0.05
18250	1.17897	0.05
18251	1.67685	0.05
18252	1.00573	1.68332
18253	1.08542	0.440604
18254	1.00573	2.1
18255	1.12572	2.1
18256	1.05499	2.1
18257	1.00521	0.662058
18258	2.22679	0.05
18259	2.86746	0.05
18260	2.86746	0.05
18261	1.99958	0.05
18262	0.996517	2.1
18263	1.01169	2.1
18264	1.01169	2.1
18265	1.26346	2.1
18266	1.68274	2.1
18267	1.75505	2.1

18268	2.28748	2.02521
18269	1.36555	2.1
18270	3.47888	0.05
18271	0.692788	0.05
18272	1.0685	0.0699852
18273	1.14547	0.106389
18274	1.19017	2.1
18275	1.24976	2.1
18276	1.24976	2.1
18277	1.30682	2.1
18278	2.2153	2.1
18279	1.85457	2.1
18280	2.03267	2.1
18281	1.51042	2.1
18282	1.05619	0.0698913
18283	1.05793	0.38464
18284	1.05793	0.0656366
18285	1.00763	0.538842
18286	1.77677	2.1
18287	1.02524	2.1
18288	1.02524	2.1
18289	1.18803	2.1
18290	1.68669	2.1
18291	1.71162	2.1
18292	1.556	2.1
18293	1.39523	2.1
18294	0.960288	0.609621
18295	1.24201	1.94258
18296	1.24202	2.1
18297	1.3592	2.1
18298	2.14045	0.05
18299	1.62054	0.05
18300	2.20248	0.05
18301	2.34699	0.05
18302	3.45132	0.05
18303	3.38241	0.05
18304	3.38241	0.05
18305	2.96127	0.05
18306	2.02612	2.1
18307	2.09351	2.1
18308	2.09351	2.1
18309	2.17465	2.1
18310	1.07445	2.1
18311	1.14808	2.1
18312	1.39067	2.1
18313	1.20958	2.04281
18314	1.20958	2.1
18315	1.00382	1.45347
18316	0.986496	2.1
18317	1.27142	2.1
18318	1.39857	2.1
18319	2.26482	1.96353
18320	2.24367	0.05
18321	1.9234	0.05
18322	1.59076	0.05
18323	1.185	0.05
18324	3.86179	0.05
18325	3.0638	0.05

18326	2.7574	0.05
18327	1.83266	0.05
18328	2.59418	2.1
18329	2.23048	2.1
18330	1.39564	2.1
18331	1.36763	2.1
18332	2.23048	2.1
18333	2.02522	2.1
18334	1.36763	2.1
18335	1.35175	2.1
18336	1.16181	0.05
18337	0.97096	0.05
18338	2.05665	0.05
18339	0.483583	0.05
18340	0.601928	0.05
18341	3.88545	0.05
18342	1.04909	2.1
18343	0.983143	2.1
18344	1.10469	2.1
18345	1.10516	2.1
18346	1.10517	2.1
18347	1.09951	2.1
18348	2.33985	2.1
18349	2.35026	2.1
18350	2.35025	2.1
18351	1.19462	2.1
18352	1.57627	2.1
18353	1.5493	2.1
18354	1.5493	2.1
18355	1.53556	2.1
18356	1.42763	0.118228
18357	1.24682	0.102101
18358	1.24682	0.0809723
18359	1.00307	0.0992663
18360	1.37392	2.1
18361	1.45408	2.1
18362	1.45408	2.1
18363	1.54249	2.1
18364	1.24474	1.48048
18365	1.07467	1.59044
18366	1.37813	1.80014
18367	1.24474	1.76291
18368	1.06341	2.1
18369	1.09194	2.1
18370	1.09194	2.1
18371	1.11117	2.1
18372	1.1658	2.1
18373	1.93707	2.1
18374	1.93707	2.1
18375	1.47729	2.1
18376	1.25669	1.98351
18377	1.07519	2.1
18378	1.65417	2.1
18379	1.50956	2.1
18380	1.37231	2.1
18381	1.27285	2.1
18382	1.23612	2.1
18383	1.1899	2.1

18384	1.08172	2.1
18385	1.20357	2.1
18386	1.5664	2.1
18387	1.7472	2.1
18388	1.48434	2.1
18389	1.23989	2.1
18390	1.58836	2.1
18391	1.38766	2.1
18392	1.23989	2.1
18393	1.26599	2.1
18394	2.56248	2.1
18395	2.08673	1.49431
18396	1.87216	1.74367
18397	1.60476	0.05
18398	1.84355	2.1
18399	1.25971	1.5403
18400	1.44141	1.0536
18401	1.25521	0.05
18402	1.19837	0.05
18403	2.31936	0.05
18404	1.38153	2.1
18405	2.41734	2.1
18406	2.70856	2.1
18407	3.48385	2.1
18408	1.89226	1.22709
18409	1.64617	1.51933
18410	1.89112	1.25865
18411	1.23135	0.0843378
18412	1.23134	0.194169
18413	1.34927	0.189309
18414	3.60096	0.05
18415	1.68103	0.05
18416	2.25644	2.1
18417	1.91955	2.1
18418	1.91955	2.1
18419	1.47688	2.1
18420	1.67375	2.1
18421	1.50033	2.1
18422	1.5098	2.1
18423	1.46908	2.1
18424	1.69055	0.05
18425	1.53463	0.05
18426	1.53463	0.05
18427	1.41943	0.05
18428	1.19022	2.02721
18429	1.95981	1.0787
18430	1.15009	1.47437
18431	1.31612	0.506434
18432	1.31611	0.0777774
18433	1.10735	0.05
18434	1.18601	0.05
18435	1.3499	0.05
18436	1.3499	0.05
18437	1.29001	0.05
18438	1.22083	2.1
18439	1.06191	2.1
18440	1.348 0.908579	
18441	1.05758	1.54627

18442	1.34799	2.05259
18443	1.18647	1.667
18444	2.92457	0.05
18445	1.99182	0.05
18446	2.25313	0.05
18447	1.30859	0.05
18448	1.30682	2.1
18449	1.38957	2.1
18450	1.38957	2.1
18451	1.47446	2.1
18452	0.953638	2.1
18453	1.03408	2.1
18454	1.17885	0.0875005
18455	1.3663	0.0595366
18456	1.08265	0.222857
18457	1.71948	0.345745
18458	1.29501	0.776292
18459	0.955093	0.854123
18460	0.955081	0.238271
18461	1.88345	0.451958
18462	1.2243	2.1
18463	1.23169	2.1
18464	2.04726	1.59685
18465	0.842869	1.84497
18466	3.18156	2.1
18467	3.60786	1.80354
18468	3.60786	2.1
18469	2.38566	1.43565
18470	1.24526	0.05
18471	1.59019	0.05
18472	1.16776	0.0511112
18473	1.05042	2.1
18474	1.17418	2.1
18475	1.05042	2.1
18476	0.949135	2.1
18477	0.973448	2.1
18478	0.973447	2.1
18479	1.02958	2.1
18480	3.57768	2.1
18481	3.04246	2.1
18482	3.04246	2.1
18483	2.47824	2.1
18484	1.35879	2.1
18485	1.10407	2.1
18486	1.14599	0.133537
18487	1.10407	0.721149
18488	1.87794	1.67673
18489	1.53429	1.36537
18490	1.12717	2.1
18491	1.38634	2.1
18492	1.38634	2.1
18493	1.13127	2.1
18494	1.04546	2.1
18495	1.06007	2.1
18496	1.06007	2.1
18497	1.19785	2.1
18498	1.43652	2.1
18499	1.71741	2.1

18500	1.70921	2.1
18501	1.06994	2.1
18502	0.960106	2.1
18503	1.48077	2.1
18504	1.48077	2.1
18505	2.5946	2.1
18506	2.03045	1.88285
18507	1.76788	2.0639
18508	2.03045	2.1
18509	0.633098	2.1
18510	1.06756	2.1
18511	1.38667	2.1
18512	0.966979	2.1
18513	1.38669	2.1
18514	1.07156	2.1
18515	0.986095	2.1
18516	1.32813	2.1
18517	0.986095	2.1
18518	1.98697	1.36536
18519	1.12016	2.07762
18520	1.24463	1.63915
18521	1.1967	1.98201
18522	1.30001	0.05
18523	1.29175	1.19992
18524	1.29175	0.0720682
18525	1.3003	1.4541
18526	1.5606	0.05
18527	2.32098	0.05
18528	2.57888	0.05
18529	2.97463	0.05
18530	1.24555	2.1
18531	1.46101	2.1
18532	1.90723	2.1
18533	2.29126	2.1
18534	1.46101	2.1
18535	1.84089	2.1
18536	2.5519	2.1
18537	2.91663	2.1
18538	1.10476	0.05
18539	1.44103	0.05
18540	1.44103	0.05
18541	1.53336	0.05
18542	0.672964	2.1
18543	0.633884	2.1
18544	0.633884	2.1
18545	0.603154	2.1
18546	2.29663	2.1
18547	2.06892	2.1
18548	1.3685	2.1
18549	1.39067	2.1
18550	2.06892	2.1
18551	1.78208	2.1
18552	1.39067	2.1
18553	1.42174	2.1
18554	1.01335	1.91778
18555	1.08195	0.598418
18556	1.86463	2.06527
18557	1.39827	2.06

18558	0.861515	2.1
18559	2.10074	2.1
18560	2.09511	0.302666
18561	2.04853	0.412291
18562	2.21488	0.178414
18563	1.38287	0.564379
18564	0.974717	0.268102
18565	0.998451	0.171317
18566	1.42281	0.569083
18567	1.82595	0.516661
18568	1.50458	0.362151
18569	1.09408	0.127316
18570	1.23498	1.27097
18571	1.05654	1.05379
18572	1.17261	2.1
18573	1.18058	2.1
18574	1.18059	2.1
18575	1.18907	2.1
18576	2.38998	2.1
18577	2.77437	2.1
18578	1.04546	2.1
18579	1.16817	2.1
18580	2.77437	2.1
18581	2.97768	2.1
18582	1.16817	2.1
18583	1.2832	2.1
18584	1.75766	0.05
18585	1.4934	0.479753
18586	1.4934	0.05
18587	1.20324	1.33361
18588	1.07236	0.328921
18589	1.37641	0.162447
18590	1.42913	0.0865407
18591	1.93373	0.307056
18592	1.42629	0.433703
18593	1.00636	0.0827612
18594	0.933959	2.1
18595	2.22873	0.575883
18596	2.22873	1.29007
18597	2.13757	0.196204
18598	1.10517	2.1
18599	1.86737	2.1
18600	0.811 2.1	
18601	1.19179	2.1
18602	1.18647	2.1
18603	0.988453	2.1
18604	1.12457	0.05
18605	1.23851	0.05
18606	0.92492	2.1
18607	1.45656	2.1
18608	1.61251	0.453279
18609	1.56696	0.494597
18610	1.90245	0.210927
18611	1.46046	0.170194
18612	1.60329	0.05
18613	1.38247	0.05
18614	1.38247	0.05
18615	1.23198	0.05

18616	1.09518	2.1
18617	1.28159	2.1
18618	0.977777	2.1
18619	1.80778	2.1
18620	1.80777	2.1
18621	1.16655	2.1
18622	1.3515	2.1
18623	0.956573	2.1
18624	1.08022	2.1
18625	1.03437	2.1
18626	1.03437	2.1
18627	0.975409	2.1
18628	1.47369	0.05
18629	1.31167	0.05
18630	1.01847	0.05
18631	2.75562	0.05
18632	1.13443	2.1
18633	1.32709	2.1
18634	1.01051	0.05
18635	0.958947	0.05
18636	1.7211	2.1
18637	1.922 2.1	
18638	1.922 2.1	
18639	0.991113	2.1
18640	1.00636	0.0890975
18641	1.31658	0.193842
18642	1.44627	0.156606
18643	1.03824	0.069358
18644	1.21223	2.1
18645	1.01242	2.1
18646	1.01243	2.1
18647	0.956698	2.1
18648	1.23167	2.1
18649	1.06935	2.1
18650	1.29215	0.05
18651	0.998971	0.05
18652	1.50103	0.05
18653	1.44836	0.05
18654	3.02513	2.1
18655	3.7335	2.1
18656	2.49988	2.1
18657	1.24948	2.1
18658	0.963972	2.1
18659	1.1822	2.1
18660	1.1822	2.1
18661	1.10817	2.1
18662	1.30237	2.1
18663	1.41908	2.1
18664	1.41908	2.1
18665	1.48734	2.1
18666	1.31127	0.150734
18667	1.40822	0.13897
18668	1.40822	0.0702812
18669	1.22176	0.0734235
18670	1.82525	2.1
18671	1.59394	2.1
18672	1.22731	0.05
18673	0.981584	0.05

18674	1.1427	0.230684
18675	1.44091	0.990455
18676	1.49154	0.0910664
18677	1.07526	0.353197
18678	1.07527	0.352709
18679	0.96029	0.0793993
18680	2.40568	0.760799
18681	3.46486	0.597408
18682	2.417	0.64111
18683	1.52054	0.287509
18684	1.51638	0.05
18685	1.04786	0.05
18686	1.51404	0.05
18687	1.3461	0.05
18688	1.27696	0.05
18689	0.736457	0.05
18690	1.16817	2.1
18691	1.04263	2.1
18692	1.13449	2.1
18693	1.50524	2.1
18694	2.1182	2.1
18695	2.49001	2.1
18696	1.11764	2.1
18697	1.39292	2.1
18698	2.49001	2.1
18699	2.76339	2.1
18700	1.39292	2.1
18701	1.68761	2.1
18702	1.25173	2.1
18703	1.34701	2.1
18704	1.02415	2.1
18705	1.34701	2.1
18706	1.332	0.05
18707	0.989764	0.05
18708	0.963167	0.05
18709	1.21056	0.05
18710	1.67375	2.1
18711	1.17965	2.1
18712	1.09154	2.1
18713	1.52314	2.1
18714	1.54433	0.113053
18715	1.81969	0.0533109
18716	1.3598	0.0585909
18717	1.71041	0.05
18718	1.70934	0.05
18719	1.33871	0.05
18720	1.79485	0.05
18721	1.51404	0.05
18722	1.30751	2.1
18723	1.20969	2.1
18724	1.20969	2.1
18725	1.12076	2.1
18726	1.49808	2.1
18727	1.50201	2.1
18728	1.50201	2.1
18729	1.31594	2.1
18730	1.2187	2.1
18731	1.11545	2.1

18732	1.227	2.1
18733	1.09645	2.1
18734	1.05902	0.05
18735	1.41595	0.05
18736	1.35696	2.1
18737	1.29446	0.230133
18738	0.857957	2.1
18739	1.21125	0.05
18740	2.43115	2.1
18741	2.1151	0.05
18742	2.0815	0.984621
18743	1.1723	2.09461
18744	1.48035	0.0661322
18745	1.59971	0.521884
18746	1.33668	0.209396
18747	1.0961	0.0959595
18748	1.46235	2.1
18749	0.882195	2.1
18750	2.46624	2.1
18751	2.72161	2.1
18752	2.96094	2.1
18753	2.25644	2.1
18754	1.25923	0.05
18755	0.976218	0.05
18756	1.10319	0.05
18757	1.82583	0.05
18758	0.888955	2.1
18759	1.30817	2.1
18760	1.30817	2.1
18761	1.60787	2.1
18762	0.988805	2.1
18763	1.54168	1.23426
18764	1.19496	0.514925
18765	1.12224	1.7502
18766	1.93597	1.51202
18767	1.31113	1.3028
18768	1.17879	1.10453
18769	1.07468	1.14017
18770	2.0523	2.1
18771	2.41385	2.1
18772	2.41385	2.1
18773	3.0355	2.1
18774	1.36717	0.0559299
18775	1.45535	0.05
18776	1.45535	0.05
18777	1.49906	0.05
18778	1.37785	2.1
18779	1.04413	2.1
18780	1.06729	0.28075
18781	1.54305	0.225927
18782	1.07444	2.1
18783	1.52724	2.1
18784	1.49151	2.1
18785	1.36814	2.1
18786	1.36814	2.1
18787	1.15541	2.1
18788	1.19379	2.1
18789	1.78657	0.258434

18790	1.6079	2.1
18791	2.59469	0.261923
18792	1.35545	0.0580987
18793	1.23602	0.474083
18794	1.23452	2.1
18795	1.56842	1.14905
18796	1.15864	2.1
18797	1.11955	2.1
18798	1.11955	2.1
18799	1.08022	2.1
18800	1.64268	2.1
18801	1.26247	2.1
18802	1.16912	2.1
18803	2.1095	0.536928
18804	1.75549	0.05
18805	1.7491	0.05
18806	1.59833	0.05
18807	1.68338	0.05
18808	1.14846	0.05
18809	1.06515	0.05
18810	1.2721	0.05
18811	1.32639	0.05
18812	1.32639	0.05
18813	1.19323	0.05
18814	1.81034	2.1
18815	1.9034	2.1
18816	2.08245	2.1
18817	1.84887	2.1
18818	1.41448	2.1
18819	1.77326	2.1
18820	1.76872	2.1
18821	1.86766	2.1
18822	1.46643	2.1
18823	1.58995	2.1
18824	1.58995	2.1
18825	1.13854	2.1
18826	1.23654	2.1
18827	1.04442	2.1
18828	1.23654	2.1
18829	1.08576	2.1
18830	0.966979	2.1
18831	1.41758	2.1
18832	1.03108	0.05
18833	1.94412	0.0714633
18834	1.32455	2.1
18835	1.32328	2.1
18836	1.32328	2.1
18837	1.34008	2.1
18838	1.60861	2.1
18839	1.69586	2.1
18840	1.69586	2.1
18841	1.78301	2.1
18842	1.7513	2.1
18843	1.67906	2.1
18844	1.67906	2.1
18845	1.60635	2.1
18846	1.68073	2.1
18847	1.57234	2.1

18848	1.57233	2.1
18849	1.47012	2.1
18850	1.22811	0.05
18851	1.79683	0.05
18852	1.53417	0.05
18853	1.08732	0.05
18854	1.8903	1.79904
18855	1.20247	1.94476
18856	1.12087	1.64431
18857	1.54699	2.1
18858	1.43635	2.1
18859	1.02368	2.1
18860	1.43635	2.1
18861	1.23007	2.1
18862	1.035	2.1
18863	1.07889	2.1
18864	3.0355	2.1
18865	3.78805	2.1
18866	2.57183	1.98969
18867	1.27835	1.26131
18868	1.29813	0.05
18869	1.42965	0.05
18870	1.69832	0.05
18871	2.18359	0.05
18872	1.42964	0.05
18873	1.65723	0.05
18874	1.98508	0.05
18875	2.06284	0.05
18876	1.50705	0.484902
18877	1.51153	0.05
18878	1.36037	0.0683579
18879	1.09767	0.05
18880	1.70048	2.1
18881	1.15177	2.1
18882	0.713174	2.1
18883	0.763575	2.1
18884	0.763575	2.1
18885	0.797011	2.1
18886	1.01179	0.748088
18887	1.27737	0.732122
18888	3.16531	0.05
18889	1.0141	0.05
18890	1.47354	0.139085
18891	1.62495	0.0582716
18892	1.62495	0.057734
18893	1.39296	0.192065
18894	0.998682	2.1
18895	1.93528	2.1
18896	2.71383	2.1
18897	1.13987	2.1
18898	1.93528	2.1
18899	1.78595	2.1
18900	3.98933	2.1
18901	3.73426	2.1
18902	1.02438	2.1
18903	1.12376	2.1
18904	1.12375	2.1
18905	1.19016	2.1

18906	1.37157	0.05
18907	1.60814	0.05
18908	1.70303	0.05
18909	1.01219	0.05
18910	1.06757	2.1
18911	1.23475	2.1
18912	1.2295	2.1
18913	1.28014	2.1
18914	1.28014	2.1
18915	1.33339	2.1
18916	1.3201	2.1
18917	1.2679	2.1
18918	1.2679	2.1
18919	1.23504	2.1
18920	1.50108	2.1
18921	1.3715	2.1
18922	1.3715	2.1
18923	1.25075	2.1
18924	1.99222	1.04735
18925	1.4155	1.02374
18926	1.4777	2.1
18927	1.52613	2.1
18928	1.91031	2.1
18929	1.03968	2.1
18930	2.04289	0.05
18931	0.967192	0.05
18932	1.03043	0.05
18933	1.65452	0.05
18934	1.21564	2.1
18935	1.16938	2.1
18936	2.47056	2.1
18937	2.56853	2.1
18938	1.16938	2.1
18939	1.1181	2.1
18940	2.56853	2.1
18941	2.66828	2.1
18942	1.0465	2.1
18943	1.44709	2.1
18944	1.44709	2.1
18945	1.96491	2.1
18946	1.01976	0.0594217
18947	0.961779	0.05
18948	1.11764	0.0563581
18949	0.961779	0.05
18950	1.21171	0.05
18951	1.35007	0.05
18952	1.03102	0.137679
18953	1.42446	0.173061
18954	1.42446	0.103173
18955	1.03166	0.0905382
18956	1.2951	0.05
18957	1.33575	0.05
18958	1.33575	0.05
18959	1.20473	0.05
18960	1.10049	0.198107
18961	0.99747	0.130756
18962	1.54249	2.1
18963	1.65677	2.1

18964	1.65677	2.1
18965	1.77677	2.1
18966	1.73525	0.05
18967	1.05689	0.05
18968	1.26051	0.05
18969	1.16259	0.05
18970	1.34009	2.1
18971	1.398	2.1
18972	1.398	2.1
18973	1.50437	2.1
18974	2.3245	2.1
18975	1.18237	2.1
18976	1.24509	2.1
18977	1.01595	2.1
18978	3.29047	2.1
18979	2.79168	2.1
18980	1.06561	2.1
18981	1.11077	2.1
18982	1.11077	2.1
18983	0.953927	2.1
18984	1.30509	0.059378
18985	1.04685	0.0558164
18986	1.04685	0.05
18987	0.834593	0.05
18988	1.0775	0.05
18989	1.27362	0.05
18990	1.71762	0.233552
18991	2.5736	0.12771
18992	2.04717	0.327068
18993	1.45521	0.194742
18994	1.33962	2.1
18995	1.27956	2.1
18996	1.27956	2.1
18997	1.20478	2.1
18998	3.65841	0.05
18999	1.18656	0.05
19000	1.72272	0.05
19001	1.6812	0.05
19002	1.09951	2.1
19003	1.08231	2.1
19004	1.08231	2.1
19005	1.06478	2.1
19006	2.16196	1.14412
19007	1.00387	0.561462
19008	1.00387	0.625067
19009	0.993999	0.130576
19010	1.04101	2.1
19011	1.27119	2.1
19012	1.27119	2.1
19013	1.51748	2.1
19014	3.00129	2.1
19015	3.27303	2.1
19016	3.27303	2.1
19017	3.60855	2.1
19018	2.22399	2.1
19019	1.71995	2.1
19020	1.54793	2.1
19021	1.227	2.1

19022	1.07624	2.1
19023	1.264	2.1
19024	1.19323	0.659126
19025	1.21286	0.457004
19026	1.21286	0.16715
19027	1.20859	0.60629
19028	1.60578	0.05
19029	1.27778	0.05
19030	1.0525	2.1
19031	1.05758	1.10619
19032	1.0525	0.723157
19033	1.17042	1.36906
19034	1.62536	0.05
19035	3.71662	0.05
19036	1.78169	0.05
19037	1.91434	0.05
19038	2.12331	0.05
19039	1.33406	0.05
19040	1.38437	2.1
19041	1.51829	2.1
19042	1.51829	2.1
19043	1.6697	2.1
19044	0.947114	2.1
19045	1.13335	2.1
19046	1.13335	2.1
19047	1.00965	2.1
19048	1.47521	2.1
19049	1.27679	2.1
19050	1.14911	2.1
19051	0.93841	2.1
19052	1.68437	2.1
19053	1.53822	2.1
19054	1.53822	2.1
19055	1.39702	2.1
19056	0.726453	2.1
19057	0.754101	2.1
19058	0.754101	2.1
19059	0.69776	2.1
19060	0.964033	2.1
19061	1.62397	2.1
19062	1.70302	2.1
19063	1.78637	2.00027
19064	3.68158	0.05
19065	2.11685	0.05
19066	1.33125	2.1
19067	1.15471	2.1
19068	1.0465	2.1
19069	1.33125	2.1
19070	1.17683	2.1
19071	1.25889	2.1
19072	1.25889	2.1
19073	1.34239	2.1
19074	1.40591	0.05
19075	0.79348	0.05
19076	1.04019	0.05
19077	0.999187	0.05
19078	1.30218	0.05
19079	1.83339	0.05

19080	1.88574	0.05
19081	1.5177	0.05
19082	1.23504	2.1
19083	1.19583	2.1
19084	1.19583	2.1
19085	1.14496	2.1
19086	1.62183	2.1
19087	1.62316	2.1
19088	1.62316	2.1
19089	1.62241	2.1
19090	1.02305	0.091105
19091	1.21136	0.0746816
19092	0.993832	0.05
19093	1.21137	0.0651416
19094	1.12064	2.1
19095	1.24965	2.1
19096	1.24965	2.1
19097	1.41077	2.1
19098	1.00052	0.05
19099	1.49842	0.05
19100	1.49843	0.05
19101	1.10801	0.05
19102	1.54196	1.21591
19103	1.18041	2.1
19104	1.73571	1.87609
19105	1.37224	2.06639
19106	1.4375	0.05
19107	1.09529	0.05
19108	1.44421	0.05
19109	1.0953	0.05
19110	1.0753	2.1
19111	0.815365	2.1
19112	0.815365	2.1
19113	0.597537	2.1
19114	1.03609	0.359612
19115	1.18952	0.149771
19116	1.54283	0.0658783
19117	1.16719	0.0715958
19118	1.16719	0.137708
19119	1.89226	0.102526
19120	2.55919	2.1
19121	2.37492	2.1
19122	2.37492	2.1
19123	2.07435	2.1
19124	1.36016	0.0542236
19125	1.70302	0.0568557
19126	1.85252	0.05
19127	1.2111	0.092628
19128	1.62905	2.1
19129	1.49692	2.1
19130	1.49692	2.1
19131	1.40395	2.1
19132	2.19083	2.1
19133	1.30542	2.1
19134	1.58736	2.1
19135	2.16579	2.1
19136	2.0815	2.1
19137	1.17935	2.1

19138	1.53803	2.1
19139	1.09148	0.64882
19140	1.81301	2.1
19141	1.38333	0.102094
19142	1.04412	2.1
19143	1.44437	2.1
19144	1.4675	0.05
19145	1.00665	0.05
19146	1.50951	0.05
19147	0.983105	0.05
19148	1.80252	0.05
19149	0.540005	0.05
19150	1.2545	0.05
19151	2.14514	0.05
19152	1.32051	0.05
19153	1.82302	0.05
19154	2.71057	2.1
19155	2.51107	2.1
19156	1.34817	2.1
19157	1.3551	2.1
19158	2.51107	2.1
19159	2.29663	2.1
19160	1.3551	2.1
19161	1.3685	2.1
19162	1.51748	2.1
19163	1.23389	2.1
19164	0.901928	0.05
19165	1.70302	0.05
19166	1.66787	0.05
19167	1.92535	0.05
19168	1.13571	0.05
19169	1.02836	0.05
19170	1.55398	2.1
19171	1.54179	2.1
19172	1.89608	2.1
19173	1.07167	2.1
19174	3.3488	0.05
19175	0.550044	0.05
19176	0.962877	0.05
19177	1.00065	0.05
19178	1.00613	0.145577
19179	1.07497	0.187644
19180	1.00486	0.05
19181	1.00486	0.05
19182	0.726453	2.1
19183	1.32634	0.05
19184	1.40008	0.05
19185	1.40008	0.05
19186	2.42267	0.05
19187	1.9216	0.05
19188	1.28066	0.05
19189	1.79102	0.937015
19190	1.18219	0.05
19191	1.18219	0.988341
19192	0.709123	0.05
19193	1.07866	2.1
19194	1.15038	2.1
19195	1.15038	2.1

19196	1.24687	2.1
19197	1.83613	2.1
19198	1.99157	2.09124
19199	1.45789	0.05
19200	1.1706	0.05
19201	1.74746	0.05
19202	1.1706	0.05
19203	1.96383	0.05
19204	1.67727	0.05
19205	1.67727	0.05
19206	1.60329	0.05
19207	1.18803	2.1
19208	1.3227	2.1
19209	1.3227	2.1
19210	1.40844	2.1
19211	1.07889	2.1
19212	1.07721	2.1
19213	1.07721	2.1
19214	1.00832	2.1
19215	0.953523	0.265013
19216	1.62875	0.189846
19217	1.07427	0.34429
19218	1.19722	0.229678
19219	1.1972	0.15506
19220	1.1599	0.119578
19221	2.97463	0.05
19222	1.9477	0.05
19223	1.95222	0.05
19224	1.75083	0.05
19225	3.46787	2.1
19226	3.02896	2.1
19227	3.02896	2.1
19228	2.46145	2.1
19229	0.970962	0.05
19230	1.0227	0.05
19231	1.12328	0.05
19232	1.02269	0.05
19233	0.904978	2.1
19234	0.725887	2.1
19235	0.725887	2.1
19236	0.601592	2.1
19237	0.956294	2.1
19238	1.13179	2.1
19239	0.956294	2.1
19240	1.13179	2.1
19241	1.05556	2.1
19242	1.13179	2.1
19243	1.38859	2.1
19244	1.13179	2.1
19245	1.06659	2.1
19246	1.05776	2.1
19247	1.06659	2.1
19248	1.04291	1.26182
19249	1.54699	0.941784
19250	1.42307	1.15821
19251	1.5654	1.96979
19252	1.97906	2.1
19253	1.15354	0.05

19254	1.15505	0.05
19255	1.26455	0.05
19256	2.50042	0.05
19257	2.50042	0.05
19258	3.22585	0.05
19259	1.19258	0.05
19260	1.34562	0.05
19261	1.49515	0.05
19262	1.66295	0.05
19263	1.02395	2.1
19264	1.51067	2.1
19265	1.35821	2.1
19266	2.10671	2.1
19267	1.0305	1.94992
19268	1.07855	1.69789
19269	1.20761	2.1
19270	0.98488	2.1
19271	1.4162	2.1
19272	1.14825	2.1
19273	1.29415	2.1
19274	1.6067	2.1
19275	1.23851	0.05
19276	1.09651	0.05
19277	1.60636	2.1
19278	1.53521	2.1
19279	1.53521	2.1
19280	1.46279	2.1
19281	1.28044	0.05
19282	1.64147	0.05
19283	1.18422	0.05
19284	1.68208	0.0514471
19285	1.09726	2.1
19286	1.234 2.1	
19287	1.234 2.1	
19288	1.09483	2.1
19289	1.89318	2.1
19290	1.98448	2.1
19291	1.98448	2.1
19292	2.02612	2.1
19293	1.22026	1.21479
19294	1.23527	0.183718
19295	1.09044	0.700933
19296	0.993366	0.05
19297	1.22026	1.77846
19298	0.993368	1.15893
19299	2.61357	2.1
19300	2.48208	2.1
19301	2.48208	2.1
19302	2.62868	2.1
19303	1.85439	0.925634
19304	1.81776	0.353974
19305	1.81776	0.239707
19306	2.1587	0.365218
19307	1.8914	0.79881
19308	2.23961	0.600712
19309	1.49357	0.05
19310	2.23961	0.464013
19311	1.78014	0.255084

19312	1.67635	0.166579
19313	1.66219	0.113667
19314	1.56036	0.113504
19315	1.14721	0.0650387
19316	2.22495	0.0886413
19317	1.88509	0.05
19318	1.95249	0.05
19319	2.11959	0.05
19320	1.37427	0.05
19321	1.39379	0.112478
19322	1.17614	0.0839596
19323	1.39379	2.1
19324	1.14397	0.05
19325	1.09651	2.1
19326	1.42843	2.1
19327	1.7069	2.1
19328	2.31293	2.1
19329	1.56839	2.1
19330	1.78252	0.347091
19331	1.78254	0.0865554
19332	1.09142	0.86083
19333	0.99829	0.05
19334	1.09372	0.05
19335	1.88175	0.05
19336	0.898452	0.05
19337	1.06936	2.1
19338	1.4162	2.1
19339	1.17989	2.1
19340	1.47411	2.1
19341	1.25376	2.1
19342	1.00757	1.05645
19343	1.4185	0.05
19344	1.02011	0.0554479
19345	2.22495	0.0517692
19346	1.79542	0.05
19347	1.79542	0.05
19348	1.19352	0.05
19349	2.38566	2.1
19350	1.83255	1.68746
19351	1.62667	2.1
19352	1.17879	1.84703
19353	2.79168	2.1
19354	2.28162	2.1
19355	2.28162	2.1
19356	1.90905	2.1
19357	1.40117	2.1
19358	1.28268	2.1
19359	1.41096	2.1
19360	1.22978	2.1
19361	3.20034	2.1
19362	3.42052	2.1
19363	3.42052	2.1
19364	3.50562	2.1
19365	1.46279	2.1
19366	1.3864	2.1
19367	1.3864	2.1
19368	1.3201	2.1
19369	1.35561	0.05

19370	0.974077	0.05
19371	0.967673	0.533385
19372	1.22482	0.298168
19373	1.02305	0.0820522
19374	1.22482	0.180116
19375	0.999492	2.1
19376	1.14657	2.1
19377	1.14657	2.1
19378	1.31346	2.1
19379	1.84417	0.05
19380	1.31777	0.05
19381	1.19796	0.05
19382	0.691742	0.05
19383	1.16725	2.1
19384	1.9679	2.1
19385	1.08848	0.05
19386	1.15356	0.05
19387	1.58713	2.1
19388	1.58742	2.1
19389	1.58742	2.1
19390	1.49662	2.1
19391	1.32201	0.0597643
19392	1.32097	1.25104
19393	1.32097	0.118736
19394	1.31825	1.53014
19395	1.12028	2.1
19396	1.15852	2.1
19397	1.15852	2.1
19398	1.16239	2.1
19399	1.07624	2.1
19400	1.30047	2.1
19401	1.44847	0.628576
19402	1.55292	0.568187
19403	1.21125	0.05
19404	1.15262	2.1
19405	1.05025	2.1
19406	1.55351	2.1
19407	1.5329	0.05
19408	2.26758	0.05
19409	1.94968	0.05
19410	1.65747	0.05
19411	1.42549	0.05
19412	1.57656	0.05
19413	2.11157	0.11077
19414	1.56531	0.391014
19415	1.69279	0.0636909
19416	1.06204	0.220079
19417	0.947863	0.920048
19418	1.19854	1.41387
19419	0.947862	0.432334
19420	1.31126	0.371832
19421	1.3425	1.48065
19422	1.29976	1.08133
19423	1.05129	0.05
19424	1.51026	0.05
19425	1.05129	0.05
19426	1.2951	0.05
19427	1.7155	2.1

19428	1.72196	2.1
19429	1.72196	2.1
19430	1.77867	2.1
19431	0.964755	2.1
19432	1.14142	2.1
19433	1.03766	2.1
19434	0.986723	2.1
19435	1.28072	0.942684
19436	1.10609	2.1
19437	1.28072	0.0922718
19438	1.16776	2.1
19439	1.61687	2.1
19440	1.60965	2.1
19441	1.60965	2.1
19442	1.59654	2.1
19443	1.26512	2.1
19444	1.23964	2.1
19445	2.07997	2.1
19446	2.60048	2.1
19447	1.23732	2.1
19448	1.3372	2.1
19449	2.62253	2.1
19450	2.47056	2.1
19451	0.92782	0.05
19452	1.40335	0.05
19453	1.26301	0.05
19454	1.52668	0.05
19455	0.99818	0.05
19456	1.51165	0.05
19457	1.66213	0.05
19458	1.55246	0.05
19459	2.31255	2.1
19460	1.34366	2.1
19461	1.90577	2.1
19462	0.969739	2.1
19463	3.4245	2.1
19464	2.8415	2.1
19465	1.97559	2.1
19466	1.82362	2.1
19467	1.48162	2.1
19468	1.23204	2.1
19469	1.23204	2.1
19470	0.95947	2.1
19471	1.05267	2.1
19472	1.44091	1.17457
19473	1.05267	1.51444
19474	1.5795	2.1
19475	0.738711	2.1
19476	1.28549	2.1
19477	1.15694	2.1
19478	1.72061	2.1
19479	1.69858	2.1
19480	0.954563	2.1
19481	0.859087	2.1
19482	0.788886	2.1
19483	1.08732	0.05
19484	1.3357	0.05
19485	1.3357	0.05

19486	1.48676	0.05
19487	2.15079	2.1
19488	2.12414	2.1
19489	2.12414	2.1
19490	2.04664	2.1
19491	1.98859	2.1
19492	0.992159	2.1
19493	1.48064	2.1
19494	1.2163	2.1
19495	1.26337	2.1
19496	1.63159	2.1
19497	1.00366	0.05
19498	1.65452	0.05
19499	1.10408	2.1
19500	1.37704	2.1
19501	1.50224	2.1
19502	1.42866	2.1
19503	1.43939	2.1
19504	1.02784	2.1
19505	1.00763	0.05
19506	1.23746	0.05
19507	1.23747	0.05
19508	1.13642	0.05
19509	1.49906	0.05
19510	1.46506	0.05
19511	1.46506	0.05
19512	1.33061	0.05
19513	1.08166	0.05
19514	1.01052	0.05
19515	1.02836	0.05
19516	1.08166	0.05
19517	1.25334	0.05
19518	1.23749	0.05
19519	1.37501	0.05
19520	1.47354	0.05
19521	1.30167	2.1
19522	1.29897	2.1
19523	1.51042	2.1
19524	1.07684	2.1
19525	1.07684	2.1
19526	0.765121	2.1
19527	1.5535	2.1
19528	1.40602	2.1
19529	1.40603	2.1
19530	1.25669	2.1
19531	1.08276	2.1
19532	1.60948	2.1
19533	1.26929	2.1
19534	1.09847	2.1
19535	0.827082	0.05
19536	1.15841	0.05
19537	1.25063	0.05
19538	1.14397	0.05
19539	1.25063	0.0984915
19540	1.10476	0.05
19541	1.14872	0.05
19542	1.3659	0.05
19543	1.36589	0.05

19544	1.4677	0.05
19545	0.590354	2.1
19546	0.655202	2.1
19547	3.48926	2.1
19548	3.32709	2.1
19549	3.32709	2.1
19550	3.13938	2.1
19551	1.24458	0.0962828
19552	1.29681	0.0901084
19553	1.40915	0.0509456
19554	1.17891	0.114248
19555	1.33171	0.05
19556	1.37681	0.0796976
19557	1.37681	2.1
19558	1.4516	2.1
19559	2.68241	0.05
19560	1.61031	0.05
19561	1.6488	2.1
19562	1.6585	2.1
19563	1.65848	2.1
19564	1.67061	2.1
19565	1.15152	2.1
19566	1.32548	2.1
19567	1.43677	2.1
19568	1.10264	2.1
19569	1.01472	2.1
19570	0.966964	2.1
19571	2.867	2.1
19572	2.94897	2.1
19573	1.65815	2.1
19574	1.4502	2.1
19575	1.4502	2.1
19576	1.26755	2.1
19577	1.37138	2.1
19578	1.45026	2.1
19579	1.45026	2.1
19580	1.53446	2.1
19581	0.973502	2.1
19582	1.08542	2.1
19583	1.13387	0.05
19584	1.71031	0.05
19585	1.08911	2.1
19586	1.10748	2.1
19587	1.21823	2.1
19588	1.25005	2.1
19589	0.657014	2.1
19590	1.38139	2.1
19591	1.01485	2.1
19592	1.26045	2.1
19593	1.12468	2.1
19594	1.26045	2.1
19595	1.89278	0.182201
19596	1.06433	0.115865
19597	1.87861	0.115615
19598	0.967544	0.0717984
19599	1.50319	2.1
19600	3.47245	2.1
19601	2.2354	0.125845

19602	1.16701	0.0558281
19603	1.3592	2.1
19604	1.3305	0.106163
19605	1.3305	2.0905
19606	1.15008	0.066022
19607	1.12457	0.05
19608	1.40707	0.05
19609	1.01778	2.1
19610	1.35733	2.1
19611	1.01779	2.1
19612	1.12064	2.1
19613	1.30694	0.993003
19614	1.42716	1.30643
19615	1.42717	1.80463
19616	1.04321	1.26478
19617	1.19352	0.05
19618	1.32634	0.05
19619	1.14605	2.1
19620	1.0417	1.96559
19621	1.19676	0.05
19622	1.03804	0.05
19623	2.09455	0.0521138
19624	1.15338	0.0594227
19625	2.21119	2.1
19626	1.26097	2.1
19627	1.88394	0.0614452
19628	1.24106	0.05
19629	2.98143	2.1
19630	2.56248	2.03381
19631	1.88394	2.02339
19632	1.68703	0.05
19633	1.5846	2.1
19634	1.94799	2.1
19635	1.20091	2.1
19636	1.40393	2.1
19637	3.09031	2.1
19638	2.90243	2.1
19639	1.27227	2.1
19640	1.32592	2.1
19641	2.90247	2.1
19642	2.71057	2.1
19643	1.32594	2.1
19644	1.34817	2.1
19645	1.4092	2.1
19646	1.44859	2.1
19647	1.52008	2.1
19648	1.401	2.1
19649	1.40099	2.1
19650	1.28936	2.1
19651	1.56002	0.05
19652	1.08811	0.05
19653	1.46041	0.05
19654	1.56002	0.05
19655	1.50524	2.1
19656	1.21304	2.1
19657	1.21304	2.1
19658	0.947115	2.1
19659	1.60665	2.1

19660	1.85179	1.91609
19661	1.85178	1.97876
19662	2.06277	0.948366
19663	2.17534	2.1
19664	1.23007	2.1
19665	2.17533	2.1
19666	1.66635	2.1
19667	2.00869	2.1
19668	0.986724	2.1
19669	2.00475	2.1
19670	1.99394	2.1
19671	1.67244	0.05
19672	1.39604	0.05
19673	1.39604	0.05
19674	0.954046	0.05
19675	0.988863	2.1
19676	1.9864	2.1
19677	1.9864	2.1
19678	3.38645	2.1
19679	1.46932	0.05
19680	1.50847	0.05
19681	1.50848	0.05
19682	1.42705	0.05
19683	1.50108	2.1
19684	1.51621	0.212857
19685	1.94317	0.258525
19686	1.18382	0.05
19687	2.05091	0.05
19688	1.88593	0.05
19689	1.54299	0.05
19690	0.987265	0.05
19691	1.27766	2.1
19692	1.5178	2.1
19693	1.5178	2.1
19694	1.81518	2.1
19695	1.04626	1.07273
19696	1.31057	1.05645
19697	1.31057	0.928884
19698	1.22297	1.08677
19699	1.67844	2.1
19700	1.62862	2.1
19701	1.62112	2.1
19702	1.87598	2.1
19703	1.17844	2.1
19704	1.37063	2.1
19705	1.37063	2.1
19706	1.68723	2.1
19707	1.99943	0.05
19708	1.17631	0.05
19709	1.4263	0.775211
19710	1.31023	2.1
19711	1.31023	1.20481
19712	1.20871	2.1
19713	1.50356	2.1
19714	1.69311	2.1
19715	1.69599	2.1
19716	1.6801	2.1
19717	1.5843	0.05

19718	0.840233	0.05
19719	1.95358	0.05
19720	1.40216	0.05
19721	1.39078	2.1
19722	1.18948	2.1
19723	1.01606	1.76721
19724	1.18948	2.06726
19725	1.63823	2.1
19726	1.67687	2.1
19727	1.67687	2.1
19728	1.52855	2.1
19729	1.11048	2.1
19730	1.25346	2.1
19731	2.42984	1.9655
19732	3.41731	0.536623
19733	1.2258	2.1
19734	1.2966	2.1
19735	1.2966	2.1
19736	1.37392	2.1
19737	1.41758	2.1
19738	1.35504	2.1
19739	1.35504	2.1
19740	1.30347	2.1
19741	1.50877	2.1
19742	1.11193	2.1
19743	1.40984	2.1
19744	1.54537	2.1
19745	1.71638	2.1
19746	1.77795	1.60451
19747	2.07593	2.1
19748	1.93387	2.1
19749	1.9339	2.1
19750	1.75669	2.1
19751	1.80299	0.924573
19752	1.31251	0.824883
19753	1.31249	0.579911
19754	1.13411	0.574782
19755	1.06313	2.1
19756	1.04921	2.1
19757	1.55766	2.1
19758	1.53728	2.1
19759	1.53729	2.1
19760	1.51789	2.1
19761	0.993021	0.71371
19762	1.02478	0.95022
19763	1.03408	0.840079
19764	1.09056	1.25985
19765	0.697755	2.1
19766	0.632901	2.1
19767	1.34072	0.05
19768	1.80841	0.05
19769	1.80841	0.05
19770	1.17793	0.05
19771	1.75032	2.1
19772	1.13076	2.1
19773	2.11851	2.1
19774	1.73255	2.1
19775	1.73255	2.1

19776	1.11181	2.1
19777	1.43772	2.1
19778	1.67381	2.1
19779	1.52164	2.1
19780	1.60861	2.1
19781	1.30588	0.05
19782	1.75241	0.05
19783	1.33026	1.27052
19784	0.962705	0.465237
19785	0.99678	0.05
19786	1.30572	0.05
19787	1.41943	0.05
19788	1.2683	0.05
19789	1.14145	0.05
19790	1.01361	0.05
19791	1.29943	2.1
19792	1.3483	2.1
19793	1.34832	2.1
19794	1.83488	2.1
19795	1.71065	2.1
19796	1.66178	2.1
19797	1.39076	2.1
19798	1.37456	2.1
19799	1.66126	2.1
19800	1.562 2.1	
19801	1.37456	2.1
19802	1.36065	2.1
19803	1.04026	0.05
19804	1.51898	0.0586848
19805	1.67088	0.05
19806	1.9726	0.05
19807	1.74852	0.801077
19808	1.95512	1.03378
19809	1.16165	0.724952
19810	1.06584	0.22077
19811	1.39327	2.1
19812	1.41394	2.1
19813	1.41394	2.1
19814	1.45506	2.1
19815	1.39523	2.1
19816	1.21119	2.1
19817	1.09005	2.1
19818	0.921311	2.1
19819	1.58164	2.1
19820	1.77574	2.1
19821	1.73184	2.1
19822	1.59423	2.1
19823	1.8214	2.1
19824	3.10544	0.696399
19825	1.01243	2.06953
19826	1.30896	0.705681
19827	2.16956	0.752749
19828	1.29608	0.257724
19829	1.38617	2.02035
19830	1.49536	1.64242
19831	2.35239	1.08123
19832	1.24613	1.87919
19833	1.94351	2.1

19834	2.05361	2.1
19835	2.05358	2.1
19836	2.38994	2.1
19837	1.13533	2.1
19838	1.08276	2.1
19839	1.55611	0.05
19840	1.33957	0.05
19841	1.33957	0.05
19842	1.69011	0.05
19843	1.5515	0.551899
19844	1.59106	0.46049
19845	1.02958	2.1
19846	1.12266	2.1
19847	1.12266	2.1
19848	1.24925	2.1
19849	1.80223	2.1
19850	1.20242	2.1
19851	2.13665	0.100125
19852	1.3924	0.0785982
19853	0.904894	1.29047
19854	1.82633	2.1
19855	1.94138	0.05
19856	1.34009	0.05
19857	1.15096	2.1
19858	0.994292	2.1
19859	1.10101	2.1
19860	1.15096	2.1
19861	1.50438	2.1
19862	1.67825	2.1
19863	1.67825	2.1
19864	1.91894	2.1
19865	2.04664	2.1
19866	1.92063	2.1
19867	1.72857	2.1
19868	1.89757	2.1
19869	1.61843	0.05
19870	1.49431	0.05
19871	2.33925	2.1
19872	1.80683	2.1
19873	1.64257	2.1
19874	1.50108	2.1
19875	2.82561	2.1
19876	2.97847	2.1
19877	2.97848	2.1
19878	3.20034	2.1
19879	0.66055	2.1
19880	1.95919	2.1
19881	1.07976	2.1
19882	1.30237	2.1
19883	1.99943	0.05
19884	1.31802	0.05
19885	1.53336	0.05
19886	1.31802	0.05
19887	1.27737	0.582953
19888	1.44848	0.485897
19889	2.35499	0.05
19890	1.30041	0.05
19891	2.35499	0.05

19892	1.65381	0.05
19893	0.996083	2.1
19894	1.28754	2.1
19895	0.996081	2.1
19896	1.44859	2.1
19897	1.24763	2.03292
19898	1.03697	2.1
19899	0.951273	1.05944
19900	1.60018	0.892999
19901	2.0815	2.1
19902	1.20488	2.1
19903	2.95785	2.1
19904	2.52245	2.1
19905	1.68455	0.05
19906	1.12971	0.05
19907	1.1952	0.0544833
19908	2.0815	0.123127
19909	1.3963	2.1
19910	1.4934	2.1
19911	1.47973	2.1
19912	1.25559	2.1
19913	1.1233	2.1
19914	1.15009	2.1
19915	1.15009	2.1
19916	0.977775	2.1
19917	1.16482	0.931371
19918	1.29313	0.236153
19919	1.29313	0.42882
19920	1.294	0.278105
19921	1.48693	0.05
19922	1.47608	0.05
19923	1.36005	2.1
19924	1.47109	2.1
19925	1.47109	2.1
19926	1.3515	2.1
19927	2.48828	2.1
19928	2.32378	2.1
19929	2.32378	2.1
19930	2.1692	2.1
19931	1.03217	0.378146
19932	0.953521	0.54346
19933	1.4515	0.05
19934	3.72406	0.05
19935	0.963395	1.93623
19936	1.01346	0.515294
19937	1.62241	2.1
19938	1.62027	2.1
19939	1.62027	2.1
19940	1.61687	2.1
19941	1.2593	0.05
19942	0.981586	0.05
19943	0.721961	2.1
19944	0.888955	2.1
19945	1.22031	0.05
19946	1.27566	2.1
19947	2.50066	2.1
19948	1.19814	2.1
19949	1.42278	0.05

19950	2.02657	0.05
19951	1.0756	0.144277
19952	1.10049	0.142436
19953	2.26155	2.1
19954	2.15634	2.1
19955	2.15634	2.1
19956	2.01613	2.1
19957	1.94233	1.36965
19958	1.18127	1.73559
19959	1.36865	1.69285
19960	1.75755	1.43925
19961	2.01613	2.1
19962	1.78958	2.1
19963	1.97995	2.1
19964	1.79198	2.1
19965	1.20841	2.1
19966	1.6519	2.1
19967	1.80207	2.1
19968	1.66774	2.1
19969	1.17935	0.05
19970	1.88071	0.05
19971	1.58723	0.05
19972	1.7361	0.05
19973	0.794165	2.1
19974	1.21327	2.1
19975	1.21327	2.1
19976	1.34268	2.1
19977	1.34497	0.05
19978	0.793303	0.05
19979	1.12323	0.05
19980	1.34496	0.05
19981	0.928226	0.05
19982	1.89804	0.05
19983	2.60141	0.05
19984	2.60142	2.1
19985	2.60772	0.0924378
19986	0.99747	2.1
19987	1.27096	0.390278
19988	1.27096	0.0913909
19989	1.03472	0.296944
19990	1.09962	0.296459
19991	1.21148	2.1
19992	1.21148	1.76054
19993	1.35025	2.1
19994	1.09795	2.1
19995	1.00775	2.1
19996	1.07162	2.1
19997	1.60665	2.1
19998	1.52984	2.1
19999	1.61179	2.1
20000	1.70057	2.1
20001	1.76063	2.1
20002	1.14547	0.06391
20003	0.964782	0.0565256
20004	1.17208	0.0526135
20005	0.964769	0.05
20006	1.04268	2.1
20007	1.57459	2.1

20008	1.57459	2.1
20009	1.32449	2.1
20010	2.07527	0.675363
20011	1.00013	0.239831
20012	0.813711	0.05
20013	1.05677	2.1
20014	1.30664	1.35034
20015	1.96011	2.04303
20016	1.7151	1.10308
20017	1.61309	1.12971
20018	1.18954	0.260878
20019	1.05447	2.1
20020	1.05446	0.05
20021	1.11787	0.05
20022	1.44437	2.1
20023	1.84739	2.1
20024	1.84739	2.1
20025	1.06203	2.1
20026	1.78391	2.1
20027	1.89267	2.1
20028	1.9212	2.1
20029	2.02997	2.1
20030	1.32051	0.05
20031	1.47169	0.05
20032	1.20691	0.05
20033	1.72271	0.05
20034	1.2858	0.0773894
20035	1.31011	0.947493
20036	1.31011	0.0673219
20037	1.34124	0.949036
20038	1.294	0.595707
20039	1.03079	0.354111
20040	1.83192	2.0663
20041	1.81979	2.1
20042	1.81979	2.09984
20043	1.57829	2.1
20044	1.35435	1.70925
20045	1.19023	2.0059
20046	0.991115	2.1
20047	1.27725	2.1
20048	1.38204	2.1
20049	1.48013	2.1
20050	0.983493	2.1
20051	2.26155	2.1
20052	1.02311	0.267444
20053	1.55485	0.177568
20054	1.71033	0.161425
20055	2.07319	0.121421
20056	2.29074	0.109419
20057	1.50822	0.420539
20058	2.08147	2.1
20059	1.1585	2.1
20060	3.74092	0.777831
20061	2.40799	2.1
20062	2.05349	2.1
20063	1.92656	2.1
20064	1.09634	0.05
20065	1.45502	0.05

20066	1.17261	0.0737844
20067	1.29383	0.0515125
20068	1.29383	0.051976
20069	1.47689	0.0507237
20070	1.85717	0.0564312
20071	1.53798	0.05
20072	1.9493	2.1
20073	2.0371	1.95258
20074	1.8519	2.1
20075	1.92119	1.0258
20076	1.00764	0.05
20077	1.0302	0.05
20078	1.0302	0.05
20079	0.90047	0.05
20080	1.49155	0.05
20081	2.26403	0.05
20082	2.06158	0.05
20083	1.09506	0.05
20084	1.9212	1.09635
20085	1.99586	0.454831
20086	1.99586	0.554508
20087	2.07527	0.72423
20088	1.20478	2.1
20089	1.09662	2.1
20090	1.55961	2.1
20091	1.03472	2.1
20092	1.54983	2.1
20093	2.02953	2.1
20094	1.84502	2.1
20095	2.1221	2.1
20096	1.02969	2.1
20097	2.23523	2.1
20098	1.2377	0.05
20099	1.03818	0.0687438
20100	1.978	2.1
20101	1.78159	2.1
20102	1.45762	2.1
20103	1.34234	2.1
20104	2.01624	2.1
20105	2.52072	2.1
20106	1.23013	2.1
20107	2.5593	2.1
20108	3.06464	1.21292
20109	2.63697	0.597586
20110	2.63697	1.03967
20111	1.87111	0.284992
20112	1.04574	0.05
20113	2.13428	0.05
20114	1.61715	2.1
20115	1.44137	2.1
20116	1.44867	2.1
20117	1.18283	2.1
20118	1.50584	0.05
20119	1.45466	0.05
20120	1.4959	0.05
20121	1.93489	0.05
20122	2.60772	2.1
20123	2.68868	2.1

20124	2.68868	2.1
20125	2.82561	2.1
20126	1.49654	2.1
20127	1.38672	2.1
20128	1.36813	2.1
20129	1.98396	2.1
20130	1.93852	1.50218
20131	2.14237	0.89846
20132	1.44421	0.05
20133	1.21749	0.05
20134	1.09572	0.05
20135	1.11081	0.05
20136	2.32363	2.1
20137	1.24375	2.1
20138	3.79242	2.1
20139	3.46787	2.1
20140	1.19854	1.6171
20141	1.35487	1.5918
20142	1.35486	1.83267
20143	1.38616	1.77379
20144	1.67265	0.0532466
20145	0.909487	0.05
20146	2.53562	2.1
20147	1.09564	2.1
20148	2.9068	2.1
20149	3.57307	0.136615
20150	2.50369	1.69953
20151	1.47348	2.1
20152	1.24925	2.1
20153	1.3991	2.1
20154	1.3991	2.1
20155	1.52984	2.1
20156	1.60428	2.1
20157	1.50097	2.1
20158	1.50097	2.1
20159	1.40239	2.1
20160	2.19621	2.1
20161	2.35003	2.1
20162	1.57932	0.05
20163	1.61194	0.05
20164	2.0815	0.05
20165	1.18539	0.05
20166	1.5795	0.851541
20167	1.53064	1.11554
20168	1.89227	0.05
20169	3.01037	0.05
20170	2.08149	2.1
20171	2.06678	2.1
20172	1.89226	0.05
20173	3.47888	0.05
20174	1.89226	0.05
20175	3.65842	0.05
20176	3.88546	0.05
20177	1.89226	0.05
20178	1.11239	2.1
20179	1.29133	0.05
20180	0.889309	0.05
20181	0.881934	0.05

20182	1.88406	2.1
20183	0.636946	2.1
20184	0.636945	2.1
20185	1.28073	2.1
20186	1.28075	2.1
20187	0.942007	2.1
20188	1.68436	2.1
20189	1.4677	0.05
20190	0.851542	0.05
20191	1.39063	0.05
20192	0.851539	0.05
20193	1.11597	0.05
20194	1.35403	0.05
20195	0.959473	2.1
20196	1.07976	2.1
20197	0.946129	1.73565
20198	0.946129	0.05
20199	1.38464	0.151561
20200	1.36991	0.05
20201	0.946131	0.0506741
20202	0.94613	0.999207
20203	1.34493	0.108216
20204	1.36991	0.076953
20205	1.36991	0.05
20206	0.946131	0.0794471
20207	0.78392	0.49372
20208	0.80282	0.445518
20209	1.10351	0.475985
20210	0.80846	0.821228
20211	0.838026	0.603089
20212	1.08558	0.418256
20213	0.718203	0.747421
20214	0.501866	0.359483
20215	0.85426	0.541724
20216	1.24249	0.05
20217	1.37052	0.05
20218	0.851541	0.05
20219	1.0092	0.05
20220	0.851554	0.05
20221	1.49826	0.05
20222	1.13727	0.05
20223	0.778149	0.05
20224	1.20257	0.079188
20225	0.946133	0.05
20226	0.778149	0.0514857
20227	0.77815	0.251885
20228	0.946132	2.1
20229	1.17602	2.1
20230	0.946132	2.1
20231	1.31257	2.1
20232	1.17602	2.1
20233	1.35821	0.05
20234	1.19993	0.05
20235	0.946131	0.05
20236	0.946131	0.05
20237	1.08848	0.0621801
20238	1.00509	2.1
20239	1.19629	2.1

20240	0.867593	2.1
20241	0.757921	2.1
20242	0.729116	0.05
20243	0.89162	0.05
20244	1.16811	0.05
20245	1.3676	2.1
20246	1.2638	1.02415
20247	0.946132	2.1
20248	0.946132	2.1
20249	1.2638	2.1
20250	1.00185	2.1
20251	1.60948	2.1
20252	0.602075	2.1
20253	0.942611	2.1
20254	0.946132	2.1
20255	0.942611	2.1
20256	1.11822	2.1
20257	1.21558	0.05
20258	0.607788	0.05
20259	0.827029	0.05
20260	1.00691	0.180588
20261	0.607789	0.05
20262	0.607775	0.126217
20263	1.23248	0.612074
20264	0.902653	1.32979
20265	0.639143	1.48113
20266	0.851545	0.526396
20267	0.605153	0.927557
20268	1.06826	2.1
20269	1.29365	2.1
20270	1.41232	2.1
20271	0.946131	2.1
20272	1.20854	2.1
20273	0.946131	2.1
20274	0.909005	0.05
20275	1.16672	0.05
20276	0.810959	0.05
20277	0.946131	0.05
20278	1.40104	0.05
20279	0.946131	0.05
20280	1.35403	0.05
20281	1.40104	0.05
20282	1.92535	0.05
20283	2.14534	0.05
20284	1.77532	0.05
20285	0.851541	0.05
20286	1.05585	0.05
20287	1.27098	0.05
20288	0.870925	0.05
20289	1.15547	0.05
20290	1.47583	0.05
20291	1.35007	0.05
20292	0.740095	0.05
20293	0.644912	0.05
20294	0.819489	0.05
20295	0.983028	0.05
20296	0.861849	0.05
20297	1.40511	0.05

20298	0.86182	0.05
20299	0.9705	0.05
20300	1.40511	0.05
20301	1.15539	2.1
20302	0.946119	2.1
20303	0.736382	2.1
20304	0.736384	2.1
20305	1.41561	2.1
20306	0.785651	2.1
20307	1.34479	2.1
20308	1.38885	2.1
20309	0.94613	2.1
20310	1.29461	2.1
20311	0.94613	2.1
20312	0.776179	2.1
20313	0.858611	2.1
20314	0.946128	2.1
20315	1.04073	2.1
20316	1.16399	2.1
20317	0.990019	2.1
20318	1.15564	2.1
20319	1.28956	0.05
20320	1.35145	0.05
20321	1.09662	2.1
20322	1.8155	0.05
20323	0.811459	0.05
20324	0.731982	0.05
20325	0.900554	0.05
20326	0.946127	0.381872
20327	0.817066	0.377505
20328	0.817064	0.407138
20329	1.41798	0.439268
20330	0.871606	0.409406
20331	1.30265	0.05
20332	0.849402	1.84284
20333	0.868582	0.689022
20334	0.909137	1.02474
20335	0.807016	1.09021
20336	0.83393	0.132935
20337	0.9034	0.331528
20338	1.53515	0.288426
20339	1.17665	0.05
20340	0.946132	0.101135
20341	0.946129	0.05
20342	1.35977	0.05
20343	1.34254	0.05
20344	1.14241	0.05
20345	0.946132	0.05
20346	0.946132	0.05
20347	1.27376	0.05
20348	1.35978	0.05
20349	1.01976	0.05
20350	1.23498	0.287624
20351	1.35002	0.680921
20352	1.11683	1.19999
20353	0.88359	1.32654
20354	0.751374	1.44584
20355	0.791714	1.3466

20356	1.6067	2.1
20357	0.923782	2.1
20358	1.44689	2.1
20359	0.94613	2.1
20360	0.946131	2.1
20361	1.5261	2.1
20362	1.26247	2.1
20363	1.80085	0.05
20364	1.7693	0.05
20365	0.900422	0.05
20366	1.31357	0.05
20367	0.94613	0.05
20368	0.900424	0.05
20369	0.900397	0.05
20370	1.39448	0.05
20371	1.36769	0.05
20372	0.713524	0.05
20373	0.713554	0.05
20374	0.946124	0.05
20375	1.24979	0.05
20376	1.04765	0.05
20377	0.70054	0.05
20378	0.776991	0.05
20379	0.738905	0.05
20380	1.32605	0.05
20381	0.784957	0.05
20382	0.803955	0.05
20383	0.819486	0.05
20384	1.79306	0.05
20385	1.18275	0.05
20386	1.25626	0.05
20387	0.936806	0.05
20388	0.653124	0.05
20389	0.946132	0.05
20390	0.653151	0.05
20391	1.10748	0.05
20392	1.95244	2.1
20393	1.40967	1.9741
20394	1.27916	1.74637
20395	0.946132	0.099868
20396	0.946132	2.04518
20397	1.30555	1.79942
20398	1.38651	2.00409
20399	0.946132	1.40359
20400	1.23571	0.145942
20401	1.21261	0.772478
20402	1.34225	0.557305
20403	0.946132	1.61026
20404	0.734059	0.0859549
20405	0.734084	0.700332
20406	0.78438	0.05
20407	1.13748	0.130227
20408	0.946131	0.45149
20409	0.78438	0.05
20410	0.784392	0.184647
20411	0.626495	2.1
20412	0.694635	2.1
20413	0.946125	2.1

20414	0.694632	2.1
20415	1.38521	2.1
20416	0.795469	2.1
20417	0.850877	2.1
20418	1.37098	2.1
20419	0.946131	2.1
20420	0.79547	2.1
20421	0.946132	0.587959
20422	1.30448	1.61591
20423	0.628361	0.384296
20424	0.686964	0.623014
20425	0.493261	0.547469
20426	1.90912	2.00422
20427	0.946127	1.53043
20428	1.36604	0.276917
20429	0.671687	0.577696
20430	0.946128	1.07211
20431	0.671699	0.428807
20432	1.04973	2.1
20433	0.982858	0.05
20434	0.974081	0.05
20435	1.69993	0.05
20436	1.00763	0.442901
20437	0.755566	2.1
20438	0.946129	2.1
20439	1.04184	2.1
20440	1.13509	2.1
20441	0.755566	2.1
20442	1.114 2.1	
20443	0.810946	2.1
20444	0.786259	2.1
20445	1.43455	2.1
20446	1.05071	2.1
20447	0.746095	2.1
20448	0.82737	2.1
20449	0.946126	2.1
20450	1.04071	2.1
20451	1.0852	2.1
20452	1.59019	0.05
20453	0.621972	0.05
20454	1.14475	0.05
20455	0.691062	0.05
20456	1.20057	0.05
20457	0.846868	0.05
20458	1.26062	0.05
20459	0.795093	0.05
20460	1.20056	0.05
20461	0.795065	0.05
20462	1.18203	0.05
20463	1.20057	0.05
20464	0.991869	0.05
20465	0.841088	2.1
20466	0.946131	2.1
20467	0.869097	2.1
20468	0.946131	2.1
20469	0.841089	2.1
20470	1.77867	2.1
20471	1.99296	0.05

20472	1.91703	2.1
20473	0.651334	0.05
20474	1.31467	0.05
20475	0.651332	0.05
20476	0.651332	0.05
20477	1.03079	0.05
20478	0.808519	0.05
20479	1.41417	0.05
20480	1.03165	0.05
20481	1.43577	2.1
20482	1.39053	2.1
20483	1.07006	2.1
20484	0.946132	2.1
20485	0.946133	2.1
20486	1.07006	2.1
20487	1.12717	2.1
20488	0.994293	2.1
20489	0.946132	2.1
20490	1.31912	2.1
20491	0.946131	2.1
20492	1.40571	2.1
20493	1.31912	2.1
20494	1.12292	0.05
20495	1.07755	0.0777578
20496	0.851539	0.0649108
20497	0.946131	0.0541831
20498	0.929834	0.05
20499	1.83919	0.0657776
20500	1.04713	0.05
20501	0.929726	0.05
20502	1.50985	0.05
20503	0.946127	0.05
20504	1.0227	0.05
20505	1.2582	0.05
20506	1.1391	0.415286
20507	0.780831	0.633636
20508	0.78086	0.417973
20509	0.946133	0.616923
20510	0.780831	0.360845
20511	1.37762	0.422858
20512	0.946116	2.1
20513	1.22882	2.1
20514	1.40015	2.1
20515	1.40017	2.1
20516	0.946105	2.1
20517	0.553567	0.05
20518	1.19263	0.05
20519	0.946132	0.05
20520	0.553594	0.05
20521	1.19263	0.05
20522	1.18237	0.05
20523	0.946133	0.05
20524	0.978467	0.05
20525	0.56262	0.05
20526	0.946132	0.05
20527	0.562633	0.05
20528	0.900175	0.05
20529	1.04627	0.690283

20530	0.549928	0.622296
20531	1.17765	0.71253
20532	1.11479	1.20826
20533	0.398261	0.936296
20534	0.927608	1.29879
20535	1.38467	0.670953
20536	1.04824	0.309719
20537	1.33893	0.721492
20538	1.55775	0.906264
20539	0.816895	1.32608
20540	1.96057	1.23114
20541	1.35798	2.1
20542	1.29262	2.1
20543	0.94613	2.1
20544	0.94613	2.1
20545	1.29261	2.1
20546	1.18067	2.1
20547	0.946131	2.1
20548	0.946132	2.1
20549	1.27809	2.1
20550	1.35799	2.1
20551	1.03933	2.1
20552	1.49438	2.1
20553	0.946132	2.1
20554	1.13537	2.1
20555	1.41666	2.1
20556	1.74237	2.1
20557	0.946132	2.1
20558	1.04291	0.05
20559	1.63802	2.1
20560	1.24714	2.1
20561	0.842013	2.1
20562	0.810959	2.1
20563	0.767923	0.0553299
20564	1.19583	0.0524517
20565	0.767921	0.05
20566	0.767921	0.05
20567	0.961348	0.075216
20568	2.08687	2.05463
20569	0.739729	0.227234
20570	1.04342	0.13911
20571	0.739757	0.212064
20572	0.946129	0.262479
20573	1.04341	0.367656
20574	1.38683	0.05
20575	1.34153	0.05
20576	0.946129	0.05
20577	0.946131	0.05
20578	1.02028	0.05
20579	1.47606	0.05
20580	1.20739	0.05
20581	0.957684	0.05
20582	0.946116	0.05
20583	0.851528	0.05
20584	0.957685	0.05
20585	1.04504	2.1
20586	0.902938	2.1
20587	0.946132	2.1

20588	1.07566	2.1
20589	1.4558	2.1
20590	1.93286	2.1
20591	1.27241	2.1
20592	0.828671	2.1
20593	0.810933	2.1
20594	1.31335	0.05
20595	1.40305	0.05
20596	0.912698	0.05
20597	0.810946	0.05
20598	1.11608	0.05
20599	1.26409	0.0514022
20600	0.946132	0.0895696
20601	1.27376	0.125588
20602	0.946132	0.099475
20603	1.03333	0.14054
20604	1.27376	0.121795
20605	0.94613	0.05
20606	0.812384	0.05
20607	0.687744	0.05
20608	0.946132	0.05
20609	0.687758	0.05
20610	1.09667	0.05
20611	1.56977	2.1
20612	0.851544	2.1
20613	1.01136	2.1
20614	0.851481	2.1
20615	0.946131	2.1
20616	0.705409	2.1
20617	1.32449	0.05
20618	1.13508	0.05
20619	0.94613	0.05
20620	0.94613	0.05
20621	0.965822	0.05
20622	1.95301	0.05
20623	0.851538	0.05
20624	1.29262	0.05
20625	0.95417	0.05
20626	0.851541	0.05
20627	0.94613	0.05
20628	1.02727	0.589242
20629	1.37675	1.34954
20630	0.955646	0.381714
20631	0.95114	0.990214
20632	0.851528	0.976058
20633	1.76513	1.37293
20634	0.87155	2.1
20635	0.94613	2.1
20636	0.871519	2.1
20637	0.966631	2.1
20638	1.37791	2.1
20639	1.19122	0.0621701
20640	0.818971	0.57925
20641	0.628799	0.0688701
20642	0.861027	0.235428
20643	0.800034	0.05
20644	0.91846	0.05
20645	0.765645	0.05

20646	1.13709	0.279475
20647	1.29054	0.584067
20648	0.755336	0.802885
20649	0.755334	0.539722
20650	0.946133	0.851099
20651	0.755336	0.321852
20652	1.14444	0.530075
20653	0.872823	0.687474
20654	0.533645	0.456493
20655	0.946132	0.446264
20656	0.533645	0.124143
20657	1.02311	0.245752
20658	1.00278	2.1
20659	1.1016	0.05
20660	0.629615	0.05
20661	1.12628	0.05
20662	0.946131	0.05
20663	0.629615	0.05
20664	0.629641	0.05
20665	1.67046	1.64464
20666	1.81667	2.1
20667	1.7145	2.1
20668	0.94613	2.1
20669	1.29322	2.1
20670	1.00365	2.1
20671	0.946131	2.1
20672	1.29322	2.1
20673	1.27581	2.1
20674	1.33916	2.1
20675	1.32784	2.1
20676	1.74787	2.1
20677	2.28084	2.1
20678	2.98092	2.1
20679	0.946132	0.852023
20680	1.2435	2.1
20681	0.946132	2.1
20682	1.4099	2.1
20683	1.2435	2.1
20684	1.26438	2.1
20685	0.745287	2.1
20686	0.696827	2.1
20687	0.696781	2.1
20688	1.41195	2.1
20689	0.946131	2.1
20690	1.8758	1.09212
20691	0.832979	0.136431
20692	0.75054	0.05
20693	0.810958	0.0577414
20694	0.922889	0.768488
20695	0.694844	0.149263
20696	0.694814	0.427459
20697	0.946131	0.254341
20698	0.694844	0.326663
20699	1.02369	2.1
20700	1.4468	0.05
20701	0.946125	0.0581731
20702	0.93487	0.05
20703	1.23608	0.153821

20704	1.34257	0.05
20705	0.851525	0.05
20706	1.03298	0.0607631
20707	1.22418	1.86543
20708	0.946131	2.1
20709	1.41833	1.11203
20710	1.23452	0.810159
20711	0.946131	0.165517
20712	1.41833	0.493219
20713	1.13077	0.05
20714	0.942874	1.98457
20715	0.946132	1.72759
20716	0.504366	1.9313
20717	0.593647	2.1
20718	0.898829	1.98264
20719	1.27921	2.1
20720	1.12503	2.1
20721	1.22539	2.1
20722	1.10332	2.05246
20723	0.851541	2.1
20724	0.94613	2.1
20725	0.945109	2.1
20726	0.607962	2.1
20727	0.946133	2.1
20728	0.607961	2.1
20729	0.661956	2.1
20730	1.35048	2.1
20731	1.66018	2.1
20732	0.956467	2.1
20733	0.851542	2.1
20734	0.890734	2.1
20735	0.802455	2.1
20736	1.35943	2.1
20737	1.04798	2.1
20738	1.26426	0.1317
20739	1.3689	0.0810116
20740	0.946133	0.101626
20741	0.946133	0.147501
20742	1.09729	0.124806
20743	0.765259	2.1
20744	0.810945	2.1
20745	0.810957	2.1
20746	1.10284	2.1
20747	1.09368	2.1
20748	0.772601	2.1
20749	0.788011	2.1
20750	0.810934	2.1
20751	0.694357	1.03632
20752	1.22641	1.06372
20753	0.946132	1.99495
20754	0.946132	1.12626
20755	0.946133	0.796303
20756	1.42754	1.85921
20757	0.776414	0.498145
20758	0.806525	1.49508
20759	0.575339	0.722638
20760	0.97154	0.445089
20761	0.8217	1.37427

20762	0.838039	1.715
20763	0.94613	0.86355
20764	0.946132	0.383606
20765	1.39575	0.104574
20766	1.39575	0.238695
20767	1.12818	0.187399
20768	0.946132	1.36529
20769	0.946133	0.824634
20770	1.06879	0.16535
20771	1.06879	0.549759
20772	0.970358	0.458198
20773	1.2474	0.0585095
20774	1.01919	0.05
20775	0.94613	0.05
20776	1.28952	0.05
20777	1.00809	0.05
20778	0.849344	0.05
20779	0.640304	0.05
20780	1.35065	0.05
20781	0.946132	0.05
20782	0.640333	0.05
20783	0.946132	0.05
20784	0.685489	2.1
20785	0.94613	2.1
20786	0.68546	2.1
20787	0.874264	2.1
20788	1.27676	2.1
20789	0.946129	0.05
20790	1.09024	0.05
20791	0.714563	0.05
20792	1.12335	0.05
20793	1.07931	0.05
20794	1.17799	0.05
20795	1.8795	0.05
20796	2.15229	0.05
20797	1.13859	0.0785797
20798	0.946132	0.576589
20799	1.11374	0.110834
20800	1.1386	0.125122
20801	0.615713	0.165698
20802	1.19322	0.05
20803	1.3377	0.05
20804	0.670801	0.05
20805	0.94613	0.238246
20806	0.66617	0.0908847
20807	1.36173	0.0858514
20808	0.980473	0.0596186
20809	1.39132	0.05
20810	0.864653	0.05
20811	1.23837	0.05
20812	0.810959	0.05
20813	1.1483	0.05
20814	1.00381	1.60054
20815	0.946128	1.93001
20816	1.39696	1.0771
20817	0.946131	0.685896
20818	0.946131	1.15695
20819	1.38613	1.2818

20820	0.973501	1.98727
20821	0.946127	0.972952
20822	1.35968	0.906316
20823	0.946131	0.318463
20824	0.946129	1.42629
20825	1.39696	1.30841
20826	1.12088	2.1
20827	1.41122	2.1
20828	1.14464	0.133061
20829	0.946131	2.1
20830	0.946131	1.95075
20831	1.14464	2.07191
20832	1.33266	1.58783
20833	1.07341	0.656473
20834	0.827287	1.80714
20835	0.946132	1.35304
20836	0.827287	2.08945
20837	1.15644	1.28782
20838	1.27558	0.177386
20839	1.15067	0.768312
20840	0.946131	2.1
20841	1.29143	0.0964641
20842	1.36281	2.1
20843	1.36281	2.07025
20844	0.946131	2.08994
20845	1.40863	2.1
20846	1.20143	0.05
20847	1.19755	0.05
20848	0.566153	0.05
20849	0.946131	0.05
20850	1.07979	0.05
20851	1.00463	0.05
20852	0.566153	0.05
20853	0.768212	2.1
20854	1.32461	2.1
20855	1.13268	2.1
20856	1.13268	2.1
20857	0.946133	2.1
20858	1.53642	2.1
20859	1.5791	2.1
20860	0.726635	2.1
20861	1.2334	2.1
20862	1.29198	2.1
20863	1.29198	1.83608
20864	0.946131	1.36199
20865	0.956585	2.1
20866	1.25294	2.1
20867	0.946133	2.1
20868	1.35287	2.1
20869	1.23877	2.1
20870	0.946131	2.1
20871	1.35287	2.1
20872	1.01843	2.1
20873	3.77569	2.1
20874	0.86707	2.1
20875	0.867066	2.1
20876	1.38639	2.1
20877	1.38639	2.1

20878	0.896168	2.1
20879	0.974716	0.05
20880	1.09411	0.05
20881	0.663171	0.05
20882	0.663171	0.05
20883	0.946132	0.05
20884	0.66317	0.05
20885	1.47104	0.05
20886	1.13629	0.05
20887	0.772342	0.05
20888	0.772312	0.05
20889	0.94613	0.05
20890	0.77234	0.05
20891	1.43154	2.1
20892	1.37704	2.1
20893	1.40025	2.1
20894	1.3063	2.1
20895	0.946132	2.1
20896	1.04075	2.1
20897	0.946131	2.1
20898	0.946118	2.1
20899	0.750011	0.0626157
20900	1.0971	0.764283
20901	0.889667	0.221172
20902	0.709598	0.552473
20903	2.39737	1.01563
20904	1.19974	0.05
20905	0.94613	0.05
20906	1.25809	0.05
20907	1.19974	0.05
20908	0.73005	0.05
20909	1.50764	0.05
20910	1.25809	0.05
20911	0.946133	0.05
20912	1.10357	0.05
20913	1.25809	0.05
20914	0.628177	0.05
20915	1.3543	0.05
20916	0.793711	0.05
20917	1.18455	0.05
20918	1.03966	0.05
20919	0.946131	0.05
20920	0.767453	0.05
20921	0.612292	0.05
20922	0.946131	0.05
20923	1.24128	0.05
20924	1.08761	0.05
20925	0.762059	0.05
20926	1.005 2.1	
20927	1.17054	2.1
20928	1.12665	2.1
20929	0.750368	2.1
20930	0.851542	2.1
20931	0.975436	2.1
20932	0.975442	2.1
20933	0.675367	2.1
20934	1.51973	0.05
20935	0.94613	0.05

20936	1.38954	0.05
20937	0.946131	0.05
20938	1.07912	0.05
20939	1.38954	0.05
20940	2.0219	2.1
20941	1.18347	0.05
20942	0.946132	0.229511
20943	0.94613	0.0615873
20944	1.00202	0.0504241
20945	1.00201	0.05
20946	0.946132	0.0759622
20947	0.946132	0.463482
20948	1.37981	0.167005
20949	1.26874	0.0816765
20950	1.26874	0.0824487
20951	0.946132	0.103377
20952	2.8687	0.05
20953	1.42884	2.1
20954	1.05188	2.1
20955	0.625307	2.1
20956	0.810956	2.1
20957	0.526656	2.1
20958	0.526684	2.1
20959	0.724122	2.1
20960	0.946132	2.1
20961	0.526684	2.1
20962	1.05337	2.1
20963	1.0593	2.1
20964	1.47981	2.1
20965	1.74431	2.01757
20966	0.885612	2.1
20967	0.810946	0.987598
20968	0.845185	1.54856
20969	1.38622	0.267107
20970	0.723194	0.163747
20971	0.554302	0.3365
20972	0.709597	0.137459
20973	0.909507	0.158633
20974	0.912754	0.0838842
20975	1.15174	0.05
20976	0.946131	0.0935509
20977	0.912753	0.0926077
20978	1.15174	0.088338
20979	1.02011	0.05
20980	0.792754	2.09097
20981	0.946129	2.1
20982	0.792754	2.0519
20983	1.27575	1.93077
20984	0.94613	2.03357
20985	1.60132	2.07373
20986	0.922455	2.1
20987	1.18713	1.24661
20988	0.926957	1.72312
20989	1.66525	1.74838
20990	0.946132	2.1
20991	0.851541	2.1
20992	0.961191	2.1
20993	1.27534	2.1

20994	0.851554	2.1
20995	1.0499	2.1
20996	1.01776	0.05
20997	0.97622	0.05
20998	0.810934	0.05
20999	2.6802	0.05
21000	1.31485	0.05
21001	1.09699	0.05
21002	0.681649	0.05
21003	0.790369	0.05
21004	0.946131	0.05
21005	0.838026	1.25962
21006	0.567833	0.156289
21007	0.891492	0.722679
21008	0.986984	0.0851686
21009	0.946132	0.829207
21010	1.19028	0.152549
21011	0.986984	0.119044
21012	0.595623	0.226973
21013	0.946131	2.1
21014	1.36502	2.1
21015	1.08109	2.1
21016	0.946129	2.1
21017	1.36503	2.1
21018	0.773897	2.1
21019	0.946133	1.53326
21020	0.613447	2.1
21021	0.613449	2.1
21022	0.946131	2.1
21023	1.11163	2.1
21024	0.966114	2.1
21025	0.735699	2.1
21026	0.662147	2.1
21027	1.04072	2.1
21028	0.946131	2.1
21029	0.862978	2.1
21030	1.48839	0.544251
21031	0.936141	0.583037
21032	0.946131	2.1
21033	0.936142	2.1
21034	1.40595	2.1
21035	0.946131	2.06967
21036	1.05297	0.05
21037	3.09035	2.1
21038	1.19952	2.1
21039	0.907198	2.1
21040	0.849144	2.1
21041	0.612469	0.111433
21042	0.551237	0.250038
21043	0.985256	0.387679
21044	0.551209	0.3597
21045	0.85153	0.828431
21046	0.624712	2.1
21047	0.900018	2.1
21048	0.851542	2.1
21049	0.864787	2.1
21050	1.27669	2.1
21051	0.946711	0.563654

21052	0.810947	0.689169
21053	0.841547	0.575651
21054	0.764114	0.70536
21055	1.26692	1.571
21056	0.946132	0.871464
21057	1.37675	1.25165
21058	1.26692	0.708265
21059	1.37545	1.25122
21060	1.49637	0.05
21061	0.709598	0.05
21062	0.751252	0.05
21063	1.36811	0.05
21064	0.635625	0.05
21065	1.31713	0.05
21066	1.18977	2.1
21067	0.893377	1.55618
21068	0.707344	2.1
21069	0.946131	2.1
21070	1.20221	2.1
21071	1.20221	2.1
21072	0.707345	2.1
21073	1.26786	2.1
21074	0.988048	2.1
21075	0.751293	2.1
21076	0.632008	2.1
21077	1.51067	2.1
21078	1.13534	2.1
21079	0.946133	2.1
21080	0.946131	2.1
21081	1.25716	2.1
21082	1.3199	2.1
21083	1.09616	2.1
21084	1.36677	1.33431
21085	1.34752	1.98662
21086	0.838041	1.00942
21087	1.67023	2.1
21088	1.02247	0.0989878
21089	1.03079	0.0640272
21090	0.928982	0.05
21091	1.13635	0.05
21092	0.946132	0.05
21093	0.92898	0.05
21094	1.13635	0.05
21095	1.1643	0.05
21096	0.851541	0.05
21097	1.39939	0.05
21098	0.946129	0.05
21099	1.07775	0.05
21100	1.14503	0.05
21101	1.78573	0.05
21102	1.29827	0.05
21103	0.872373	0.05
21104	1.01162	0.05
21105	0.83804	0.05
21106	1.48851	0.05
21107	1.47385	0.05
21108	0.843858	0.05
21109	0.938626	0.05

21110	0.89567	0.05
21111	1.59457	0.391716
21112	2.09666	0.05
21113	0.775132	0.05
21114	0.927005	0.05
21115	0.854224	0.05
21116	0.775142	0.05
21117	0.946106	0.05
21118	1.80784	2.1
21119	2.04593	2.1
21120	1.32282	2.1
21121	0.952138	2.1
21122	0.946132	2.1
21123	0.946132	2.1
21124	0.952138	2.1
21125	1.46988	2.1
21126	0.80465	0.05
21127	1.14155	0.05
21128	0.810931	0.05
21129	1.66203	0.05
21130	0.674605	0.211214
21131	0.946132	0.57345
21132	1.26706	0.52025
21133	0.674633	0.142265
21134	0.946132	0.701613
21135	0.980664	0.299191
21136	1.34927	0.22597
21137	0.758454	0.05
21138	0.946133	0.05
21139	0.985371	0.05
21140	1.28632	0.05
21141	0.946133	0.0795446
21142	1.46868	0.05
21143	1.7522	0.05
21144	0.737431	2.1
21145	0.663679	2.1
21146	1.16541	1.84906
21147	1.3179	2.07571
21148	1.04081	2.1
21149	0.887866	1.75955
21150	0.806961	0.682284
21151	0.929061	1.46758
21152	1.28418	0.05
21153	1.38752	0.0906266
21154	1.33038	0.122506
21155	0.946132	0.05
21156	0.946132	0.0507393
21157	1.14897	0.0535898
21158	1.1685	0.05
21159	1.18774	2.1
21160	0.961899	2.1
21161	1.48841	2.1
21162	1.79105	2.1
21163	3.51614	2.1
21164	0.682342	2.1
21165	0.783184	2.1
21166	0.946132	2.1
21167	0.868396	2.1

21168	1.21813	2.1
21169	0.848305	0.267197
21170	0.94559	0.76903
21171	0.805691	1.11766
21172	0.752217	2.1
21173	0.946132	2.1
21174	0.839123	2.1
21175	0.946131	2.1
21176	0.752187	2.1
21177	1.96491	2.1
21178	0.520997	2.1
21179	0.946132	2.1
21180	0.73446	2.1
21181	0.946133	2.1
21182	0.520995	2.1
21183	1.88522	2.1
21184	1.8361	2.1
21185	1.11285	2.1
21186	1.25334	1.15249
21187	2.00756	2.1
21188	1.44252	0.05
21189	1.00255	1.77492
21190	1.64516	2.1
21191	1.12399	0.05
21192	1.13542	0.05
21193	1.09038	0.05
21194	0.746903	0.05
21195	0.672241	0.05
21196	0.871774	0.05
21197	1.73513	0.876642
21198	0.949995	2.1
21199	1.17244	2.1
21200	0.946127	2.1
21201	1.3028	2.1
21202	1.17244	2.1
21203	0.774101	2.1
21204	0.946127	2.1
21205	0.939289	2.1
21206	0.946129	2.1
21207	0.774101	2.1
21208	1.87858	2.1
21209	1.44293	0.05
21210	1.43213	0.05
21211	0.946131	2.1
21212	0.716614	2.1
21213	0.716644	2.1
21214	1.35828	2.1
21215	0.790935	2.1
21216	0.730157	2.1
21217	1.41149	2.1
21218	0.730186	2.1
21219	0.730186	2.1
21220	0.946131	2.1
21221	0.946132	1.37287
21222	0.946132	0.957683
21223	0.990713	0.183533
21224	1.40779	0.452392
21225	0.946132	0.54884

21226	1.06486	0.0710236
21227	0.946131	1.04262
21228	0.946133	0.362476
21229	1.40779	0.129764
21230	1.26874	0.278337
21231	0.946131	0.267792
21232	1.02588	0.134748
21233	0.826073	0.05
21234	1.41233	0.05
21235	0.861269	0.05
21236	1.04073	0.05
21237	1.27111	0.05
21238	1.56016	1.70009
21239	1.60866	0.05
21240	0.94613	0.05
21241	0.946129	0.05
21242	1.36376	0.05
21243	1.22187	0.05
21244	0.94613	0.05
21245	1.82581	1.22112
21246	1.19705	0.865556
21247	1.7513	2.1
21248	1.85017	2.1
21249	1.72116	2.1
21250	1.12058	2.1
21251	0.899991	2.1
21252	0.616288	2.1
21253	1.07289	2.1
21254	1.7092	0.191054
21255	0.946132	0.142606
21256	0.946133	0.05
21257	1.18717	0.05
21258	1.40029	0.05
21259	0.946132	0.05
21260	0.94613	0.0688581
21261	1.18547	0.0568125
21262	0.960326	0.0562555
21263	0.960345	0.05
21264	0.942557	0.0631743
21265	1.18601	0.05
21266	0.946132	0.05
21267	1.30307	0.05
21268	1.08169	0.05
21269	1.08169	0.05
21270	1.31716	0.05
21271	1.51065	0.442799
21272	0.877642	0.05
21273	0.617384	0.05
21274	0.575741	0.05
21275	0.972448	0.05
21276	1.11334	0.05
21277	1.13092	2.1
21278	0.946127	0.346539
21279	1.31724	0.724327
21280	1.39751	0.05
21281	1.39751	0.198958
21282	0.946128	0.113341
21283	0.946132	0.0885208

21284	0.713284	0.05
21285	1.40668	0.115303
21286	1.03547	0.05
21287	0.713298	0.0719069
21288	1.88774	0.05
21289	0.830164	2.1
21290	1.05211	2.1
21291	0.946119	2.1
21292	0.946107	2.1
21293	1.283 2.1	
21294	2.63609	2.1
21295	1.28418	0.603914
21296	0.946128	0.924941
21297	1.10335	0.577017
21298	0.598377	1.46189
21299	1.18818	0.739895
21300	1.10335	0.516067
21301	1.58972	0.772379
21302	1.90194	2.1
21303	0.946118	2.1
21304	0.946115	2.1
21305	0.950969	2.1
21306	1.36918	2.1
21307	0.950955	2.1
21308	1.4263	2.1
21309	1.11978	0.72968
21310	0.946132	1.34609
21311	0.946133	1.5293
21312	1.37658	0.805399
21313	1.2364	1.02163
21314	1.05496	0.567841
21315	0.946132	1.55743
21316	0.946132	1.23023
21317	1.21858	0.520143
21318	1.37658	1.06523
21319	1.09194	1.51077
21320	1.14721	0.05
21321	1.53814	0.05
21322	0.546751	0.433698
21323	0.492063	1.2573
21324	1.16889	2.08809
21325	1.00349	2.0189
21326	0.863559	0.685723
21327	1.38102	1.81565
21328	0.597449	1.11634
21329	0.537748	0.340035
21330	1.16297	1.59685
21331	0.999366	1.24669
21332	0.896554	0.68494
21333	0.566962	0.05
21334	0.946129	0.05
21335	0.452729	0.05
21336	0.696552	0.05
21337	0.852524	0.05
21338	1.57992	0.05
21339	1.64985	0.05
21340	0.946128	2.1
21341	1.21847	2.1

21342	0.946129	2.1
21343	1.41131	2.1
21344	1.21847	2.1
21345	1.21269	0.05
21346	1.0417	2.1
21347	1.15821	0.909597
21348	0.946132	0.610126
21349	0.946132	2.07955
21350	1.37967	0.182822
21351	0.851529	0.328607
21352	1.20455	2.1
21353	1.4282	0.05
21354	1.26593	2.1
21355	0.838026	0.05
21356	0.985096	0.05
21357	0.83804	0.05
21358	0.822395	1.20178
21359	0.420272	0.131313
21360	0.946131	2.1
21361	1.17461	2.1
21362	0.94613	2.1
21363	1.40225	2.1
21364	1.17461	2.1
21365	1.09847	2.1
21366	1.22222	1.23435
21367	1.41798	0.146978
21368	0.791658	0.275345
21369	0.791656	0.315293
21370	0.946129	0.32313
21371	0.817064	0.278366
21372	0.946114	0.180876
21373	0.75824	0.174812
21374	0.75821	0.200332
21375	1.41778	0.153679
21376	0.791635	0.179493
21377	1.04164	1.95162
21378	0.946131	0.717694
21379	1.11172	0.438295
21380	1.30076	0.109225
21381	1.35721	0.210017
21382	1.11172	0.189388
21383	1.87218	0.05
21384	0.987248	0.05
21385	0.729695	0.304715
21386	0.974389	0.0663557
21387	1.18002	0.0669857
21388	0.822612	0.0585851
21389	1.35721	0.0589423
21390	0.634295	0.421147
21391	0.634326	0.366894
21392	1.13245	1.19029
21393	1.13245	0.833788
21394	0.937961	0.216755
21395	1.18769	0.453482
21396	0.854949	0.210793
21397	0.967357	0.294107
21398	0.810934	0.208942
21399	3.91082	2.1

21400	0.946132	2.1
21401	0.946132	2.1
21402	1.05383	2.1
21403	1.05383	2.1
21404	0.986498	2.1
21405	1.50732	0.05
21406	0.753659	0.05
21407	1.22144	0.05
21408	1.30463	0.05
21409	0.753659	0.05
21410	1.30463	0.05
21411	0.886189	0.05
21412	1.04321	0.05
21413	0.909928	0.05
21414	1.18659	0.05
21415	1.19785	0.05
21416	1.40969	0.05
21417	1.24094	0.05
21418	0.946126	0.05
21419	1.2929	0.05
21420	1.24093	0.05
21421	0.867944	0.05
21422	1.41903	2.1
21423	2.47881	0.05
21424	1.12943	0.05
21425	0.764229	2.1
21426	0.764256	2.1
21427	0.94613	2.1
21428	0.94613	2.1
21429	1.1995	2.1
21430	1.83423	2.1
21431	0.634816	2.1
21432	0.877837	2.1
21433	0.94613	2.1
21434	1.04073	2.1
21435	1.06485	2.1
21436	0.94613	0.05
21437	0.62725	0.05
21438	0.798212	0.05
21439	1.33241	0.05
21440	0.62725	0.05
21441	1.82301	0.05
21442	1.40615	2.1
21443	0.782504	2.1
21444	0.748635	2.1
21445	0.946131	2.1
21446	0.748607	2.1
21447	0.988456	2.1
21448	1.64833	0.05
21449	1.35657	0.05
21450	1.26155	0.05
21451	0.915755	0.05
21452	0.915755	0.05
21453	0.946132	0.05
21454	1.49386	0.05
21455	1.01346	2.1
21456	0.946131	2.1
21457	0.946132	2.1

21458	1.38651	2.1
21459	1.39523	2.1
21460	0.946132	2.1
21461	1.06312	2.1
21462	0.946131	2.1
21463	0.946129	2.1
21464	1.39523	2.1
21465	1.37372	2.1
21466	0.946131	2.1
21467	1.2929	0.05
21468	0.94613	0.05
21469	1.24448	0.05
21470	1.2929	0.05
21471	0.862011	0.05
21472	1.73583	0.05
21473	1.724 0.05	
21474	1.03824	2.1
21475	1.534 2.1	
21476	0.725492	1.84918
21477	0.771967	2.04856
21478	0.61731	0.941937
21479	0.475743	1.76689
21480	0.719694	1.63766
21481	1.14878	0.821584
21482	0.946117	1.81152
21483	0.94613	1.87157
21484	0.946105	1.89113
21485	0.482666	0.932919
21486	0.609172	1.00412
21487	0.946131	2.1
21488	1.39509	0.268822
21489	1.07439	2.1
21490	0.946131	1.81539
21491	1.39509	1.8728
21492	0.963571	1.85022
21493	0.591011	0.176292
21494	1.1357	0.05
21495	2.11193	0.20573
21496	1.60936	2.1
21497	1.34118	0.0776502
21498	1.27696	0.109091
21499	0.94613	0.0602327
21500	0.94613	0.0536494
21501	1.04257	0.05
21502	1.53336	0.05
21503	1.0631	0.05
21504	1.04072	0.05
21505	0.670678	0.05
21506	0.709531	0.05
21507	0.891275	0.05
21508	0.683583	0.05
21509	0.902478	0.05
21510	0.807671	0.05
21511	1.11024	2.1
21512	1.08171	2.1
21513	1.16725	2.1
21514	0.946128	2.1
21515	1.13537	2.1

21516	1.40525	2.1
21517	1.28365	2.1
21518	1.04523	2.1
21519	0.960106	2.1
21520	0.946128	2.1
21521	1.23452	2.1
21522	0.946129	2.1
21523	1.3704	2.1
21524	1.23452	2.1
21525	1.16906	0.880991
21526	0.946131	1.76023
21527	0.867246	1.14463
21528	0.867217	1.8547
21529	0.946131	1.31318
21530	1.13438	1.52927
21531	1.28684	1.6421
21532	0.946132	1.91313
21533	0.883935	1.47652
21534	0.883938	2.1
21535	0.946133	1.53508
21536	1.73444	1.18545
21537	1.21835	2.1
21538	1.28228	2.1
21539	1.8643	1.24214
21540	0.743379	0.05
21541	1.34921	0.05
21542	0.743381	0.05
21543	0.743381	0.05
21544	0.946131	0.05
21545	0.915528	2.1
21546	0.982742	2.1
21547	0.975965	2.1
21548	0.891132	0.05
21549	1.27533	0.05
21550	0.946132	0.05
21551	1.25215	0.05
21552	0.590343	0.05
21553	0.946133	0.05
21554	0.590331	0.05
21555	1.62375	2.1
21556	0.687769	0.164812
21557	1.14374	0.117023
21558	1.20885	0.101582
21559	0.946132	0.183398
21560	1.14374	0.229825
21561	1.09308	0.05
21562	1.02055	0.05
21563	0.810934	0.05
21564	0.917609	1.7049
21565	1.13094	1.93318
21566	0.70959	1.18765
21567	1.11223	2.1
21568	0.83051	1.42207
21569	0.810946	1.93295
21570	0.810959	1.3952
21571	1.5773	0.05
21572	1.96133	0.634928
21573	1.0214	0.943452

21574	0.762783	1.54349
21575	0.838036	0.67686
21576	0.63591	1.76089
21577	0.967411	0.495519
21578	0.946132	2.1
21579	0.946132	2.09196
21580	1.39699	1.72987
21581	1.61642	2.1
21582	0.860954	1.01135
21583	0.957411	1.94461
21584	0.660856	1.35449
21585	1.10003	2.1
21586	0.968249	2.1
21587	0.910184	1.11258
21588	1.30029	0.096396
21589	0.754119	0.106443
21590	0.754121	0.487067
21591	0.94613	0.970645
21592	1.08039	2.1
21593	0.810942	0.05
21594	1.15153	0.05
21595	1.06968	0.05
21596	1.38456	2.1
21597	1.04118	2.1
21598	0.838027	2.1
21599	1.04073	2.1
21600	0.754209	2.1
21601	1.332	0.05
21602	1.26193	2.1
21603	1.15317	0.05
21604	3.86179	0.05
21605	1.56005	2.1
21606	0.683035	0.104562
21607	0.931463	0.0687767
21608	0.556019	0.520752
21609	0.929172	0.182337
21610	0.756874	0.05
21611	1.23527	0.587876
21612	2.05961	0.4036
21613	0.946132	2.1
21614	1.16684	2.1
21615	1.3937	2.1
21616	1.3937	2.1
21617	0.946132	2.1
21618	1.17885	2.1
21619	1.62507	0.05
21620	1.58874	2.1
21621	0.819953	2.1
21622	1.16023	2.1
21623	1.34782	2.1
21624	1.16023	2.1
21625	0.819955	2.1
21626	0.79437	2.1
21627	0.794373	2.1
21628	1.20056	2.1
21629	1.25	2.1
21630	1.20056	2.1
21631	1.35787	2.1

21632	0.946131	2.1
21633	1.38585	2.01668
21634	0.946128	0.352301
21635	0.946129	1.75248
21636	1.35714	1.86772
21637	1.04147	2.1
21638	0.946133	1.73055
21639	1.30382	1.11956
21640	0.946131	0.101748
21641	0.946131	1.20013
21642	1.38585	1.53499
21643	1.22305	1.34999
21644	1.00763	0.05
21645	0.813199	0.05
21646	0.810948	0.05
21647	1.15928	0.05
21648	0.944166	0.05
21649	0.94613	0.05
21650	0.946133	0.05
21651	1.4179	0.05
21652	0.946132	0.05
21653	1.36965	0.05
21654	1.05444	0.05
21655	0.690302	0.05
21656	0.838038	0.05
21657	0.802821	0.116995
21658	1.40944	0.309059
21659	0.946126	0.691255
21660	0.799998	0.0568594
21661	0.800035	0.180231
21662	1.1766	2.1
21663	1.00319	2.1
21664	1.01606	1.51769
21665	0.872158	2.1
21666	1.04884	2.1
21667	0.679494	2.1
21668	0.946131	2.1
21669	1.37609	2.1
21670	0.94613	2.1
21671	0.946131	2.1
21672	1.18486	2.1
21673	0.726287	2.1
21674	0.946131	2.1
21675	0.726286	2.1
21676	1.30318	2.1
21677	1.01712	2.1
21678	0.946132	2.1
21679	1.04404	2.1
21680	1.37017	2.1
21681	1.37017	2.1
21682	0.946133	2.1
21683	0.992084	1.03023
21684	0.946131	0.730672
21685	0.529655	2.00687
21686	0.529686	0.870691
21687	0.946131	0.601634
21688	1.05937	1.21234
21689	1.14622	0.345283

21690	0.946131	0.655258
21691	0.584575	1.47356
21692	0.584575	0.445
21693	0.946129	0.434531
21694	0.709714	2.1
21695	0.946132	2.1
21696	0.709714	2.1
21697	1.37199	2.1
21698	0.82899	2.1
21699	1.00133	2.1
21700	0.778424	0.05
21701	0.946132	0.05
21702	1.24094	0.05
21703	1.00691	0.05
21704	0.64868	0.05
21705	1.29736	0.05
21706	1.24767	0.113984
21707	1.28953	0.30396
21708	1.34612	0.550572
21709	0.946133	0.0960102
21710	1.14187	0.99762
21711	0.946132	0.453008
21712	0.946129	0.175684
21713	1.4099	2.1
21714	0.946131	0.10373
21715	1.34612	0.0590309
21716	1.4099	0.0809731
21717	0.956003	0.05
21718	1.40572	0.05
21719	3.54443	2.1
21720	0.994001	0.05
21721	1.03679	2.1
21722	1.08467	2.1
21723	1.56875	0.05
21724	0.968463	2.1
21725	0.974889	2.1
21726	0.946131	2.1
21727	1.30203	2.1
21728	1.40199	2.1
21729	1.40199	2.1
21730	0.946132	2.1
21731	0.946132	2.1
21732	1.36994	2.1
21733	1.37372	2.1
21734	1.37372	2.1
21735	0.946132	2.1
21736	0.928979	0.939618
21737	0.565509	1.45156
21738	1.60196	0.214844
21739	1.16239	0.716669
21740	1.10746	0.05
21741	1.15412	2.1
21742	1.15171	2.1
21743	0.946133	2.1
21744	0.940877	2.1
21745	0.940878	2.1
21746	1.41432	2.1
21747	1.04687	2.1

21748	2.75524	0.05
21749	0.946132	0.05
21750	0.766452	0.05
21751	0.766427	0.05
21752	1.36004	0.05
21753	1.06648	0.05
21754	1.62483	2.1
21755	1.0577	2.1
21756	1.39158	2.1
21757	0.94255	2.1
21758	1.05029	2.1
21759	1.03735	2.1
21760	0.931055	2.1
21761	1.03797	2.1
21762	1.43687	2.08163
21763	1.28765	1.68769
21764	1.1811	1.05755
21765	0.946132	1.49678
21766	0.946132	1.4536
21767	1.1811	1.34895
21768	0.67805	0.2282
21769	1.50998	2.00515
21770	1.16661	2.1
21771	1.36749	2.1
21772	0.810945	0.720621
21773	1.14745	1.25876
21774	0.865588	0.482231
21775	1.36665	0.430556
21776	0.506964	1.2231
21777	0.506966	0.858144
21778	0.552786	0.532745
21779	0.946133	0.253159
21780	1.21336	0.163038
21781	0.552786	0.631495
21782	0.552757	1.82886
21783	0.653468	0.357795
21784	1.36665	1.56063
21785	1.27922	0.05
21786	1.08133	0.05
21787	1.05348	0.05
21788	0.946132	0.05
21789	1.19601	0.05
21790	1.13276	0.05
21791	0.7856	0.05
21792	1.57125	0.05
21793	1.19601	0.05
21794	0.946131	0.25085
21795	1.34534	0.124492
21796	1.19601	0.05
21797	0.870489	0.0645836
21798	1.49404	2.1
21799	1.35943	1.29097
21800	1.10427	2.01076
21801	0.94613	1.11647
21802	0.946131	1.57125
21803	1.10427	1.3168
21804	1.2064	0.05
21805	0.946132	0.05

21806	1.11288	0.188184
21807	1.34268	0.05
21808	1.34268	0.05
21809	0.946132	0.05
21810	0.946128	2.1
21811	0.946128	0.138638
21812	1.30835	2.1
21813	1.38463	1.48486
21814	0.946129	1.64343
21815	1.43778	0.637885
21816	1.23943	0.572835
21817	1.4748	2.1
21818	0.71295	2.1
21819	0.712951	2.1
21820	0.946132	2.1
21821	0.946133	2.1
21822	1.13976	2.1
21823	1.71109	2.1
21824	1.32426	2.1
21825	0.671255	2.1
21826	1.29123	2.1
21827	0.946132	2.1
21828	0.946132	2.1
21829	1.25603	2.1
21830	2.21126	0.05
21831	0.892889	2.1
21832	0.946129	2.1
21833	0.892888	2.1
21834	0.92872	2.1
21835	1.34352	2.1
21836	0.622784	0.121446
21837	1.02947	0.05
21838	0.810957	0.05
21839	1.91756	0.81913
21840	1.06012	0.290892
21841	1.09569	0.05
21842	1.5456	0.05
21843	1.1211	0.05
21844	1.21031	0.05
21845	1.03939	0.05
21846	0.519697	0.05
21847	0.933889	0.05
21848	0.519726	0.05
21849	1.16699	0.05
21850	0.933889	0.05
21851	0.840599	0.05
21852	0.94613	0.05
21853	0.840568	0.05
21854	1.26868	0.05
21855	1.16699	0.05
21856	1.17822	2.1
21857	0.922044	0.114451
21858	1.29322	0.17675
21859	0.946132	0.105787
21860	0.995178	0.232221
21861	0.922056	0.198384
21862	1.76232	0.432661
21863	1.77065	0.964369

21864	1.38149	0.592825
21865	0.959301	1.04374
21866	0.946133	0.694355
21867	0.946132	0.920757
21868	0.959301	0.825836
21869	1.3612	0.582901
21870	0.885314	0.969919
21871	0.885309	0.578907
21872	0.94613	0.866841
21873	0.8853	0.531526
21874	1.56164	0.647953
21875	1.45506	0.05
21876	1.45257	2.1
21877	0.851541	0.05
21878	1.29018	0.05
21879	0.851541	0.05
21880	1.21087	0.05
21881	1.24979	0.05
21882	1.56983	0.05
21883	1.60151	0.05
21884	1.78952	2.1
21885	0.638164	2.1
21886	0.638163	2.1
21887	1.39754	2.1
21888	1.39754	2.1
21889	0.681589	2.1
21890	1.34993	2.1
21891	1.04525	2.1
21892	0.946133	2.1
21893	1.04072	2.1
21894	0.857805	2.1
21895	1.06053	2.1
21896	1.49066	2.1
21897	1.4185	0.822344
21898	0.934059	2.02954
21899	0.93406	0.923853
21900	0.94613	1.61082
21901	0.93406	1.19265
21902	1.25277	1.27484
21903	1.00792	2.1
21904	0.609466	1.46404
21905	0.946132	1.8893
21906	0.609463	2.01295
21907	1.30664	2.1
21908	0.810943	1.69741
21909	1.05741	1.01152
21910	1.3868	2.1
21911	1.15558	0.0510787
21912	1.13537	2.1
21913	0.946132	2.1
21914	0.946132	2.1
21915	1.08266	2.1
21916	1.54934	2.1
21917	1.26016	2.1
21918	2.01915	2.1
21919	0.60346	1.59365
21920	0.651529	1.71476
21921	1.15581	2.1

21922	1.13037	1.25785
21923	1.04071	1.03648
21924	1.77796	2.1
21925	0.532308	1.62102
21926	0.532308	0.989729
21927	1.15369	2.1
21928	1.1537	1.91386
21929	0.903288	1.68658
21930	0.804766	2.1
21931	0.804739	2.1
21932	0.946128	2.1
21933	0.946131	2.1
21934	1.16745	2.1
21935	1.04193	2.1
21936	0.98505	2.1
21937	1.40724	0.245725
21938	1.14216	0.318751
21939	0.946131	0.0526376
21940	0.946131	0.0946804
21941	1.10941	0.100462
21942	0.81268	0.05
21943	0.946131	0.05
21944	1.28673	0.05
21945	1.28673	0.05
21946	0.812681	0.05
21947	1.84716	0.05
21948	1.84716	0.05
21949	0.92361	0.05
21950	0.94613	0.05
21951	0.923582	0.05
21952	1.26227	0.05
21953	0.923581	0.05
21954	0.747394	2.1
21955	1.32582	2.1
21956	0.747365	2.1
21957	0.97587	2.1
21958	1.32582	2.1
21959	1.04182	0.05
21960	0.961743	0.05
21961	0.946126	0.05
21962	1.47463	0.05
21963	1.12183	0.05
21964	0.851552	0.05
21965	1.33177	0.05
21966	0.946131	0.05
21967	1.41512	0.05
21968	1.33178	0.05
21969	0.946131	0.05
21970	0.927735	0.192814
21971	0.97206	0.0670581
21972	0.515566	0.104177
21973	0.946129	0.090785
21974	0.515538	0.0828519
21975	1.04482	0.05
21976	0.975535	0.05
21977	0.522411	0.05
21978	0.522382	0.0697311
21979	0.925346	0.0699574

21980	0.575817	0.0664042
21981	0.946129	2.1
21982	1.14256	2.1
21983	1.32054	2.1
21984	1.40525	2.1
21985	1.14256	2.1
21986	1.59475	2.1
21987	0.773813	2.1
21988	1.4073	2.1
21989	0.773813	2.1
21990	0.773813	2.1
21991	1.4073	2.1
21992	0.993021	2.1
21993	1.50466	0.05
21994	0.851539	0.05
21995	1.20299	0.05
21996	0.946131	0.05
21997	1.54464	0.05
21998	1.20309	0.05
21999	2.00701	0.05
22000	0.810946	0.05
22001	1.07678	0.05
22002	0.900128	0.05
22003	1.26842	2.1
22004	1.11206	2.1
22005	0.946132	2.1
22006	1.04074	2.1
22007	0.948809	2.1
22008	0.946131	0.05
22009	0.929617	0.05
22010	1.24057	0.05
22011	1.36485	0.05
22012	0.851554	0.05
22013	1.96823	0.05
22014	0.914836	0.05
22015	0.94613	0.05
22016	1.38911	0.05
22017	0.914805	0.05
22018	0.946131	0.05
22019	0.946131	0.05
22020	1.04072	0.05
22021	1.26837	0.05
22022	0.970054	0.05
22023	0.923958	0.05
22024	1.16653	2.1
22025	1.34412	2.1
22026	0.851541	0.05
22027	1.38645	0.05
22028	0.891637	0.05
22029	0.851487	0.05
22030	0.890871	0.05
22031	1.56178	0.05
22032	0.946128	0.05
22033	1.26868	0.05
22034	0.946131	0.05
22035	1.18789	0.05
22036	1.26868	0.05
22037	1.26868	0.05

22038	0.946128	0.05
22039	1.18716	0.05
22040	0.946131	0.05
22041	0.59358	0.05
22042	1.23923	0.05
22043	1.00024	0.05
22044	0.593593	0.05
22045	0.946131	0.05
22046	0.713645	0.05
22047	1.23175	0.05
22048	1.23923	0.05
22049	0.713633	0.05
22050	1.24687	0.05
22051	1.80662	2.1
22052	2.15762	2.1
22053	0.780455	2.1
22054	0.946131	2.1
22055	0.780455	2.1
22056	1.40583	2.1
22057	1.39852	2.1
22058	0.565619	2.1
22059	1.1428	2.1
22060	0.663168	0.05
22061	1.02163	0.05
22062	1.04072	0.05
22063	0.663169	0.05
22064	1.13511	0.05
22065	1.85277	0.05
22066	1.17701	0.295437
22067	0.993831	0.05
22068	1.0823	0.05
22069	0.795761	0.05
22070	1.24596	0.05
22071	0.566075	0.05
22072	0.94613	0.05
22073	0.56605	0.05
22074	0.774911	0.05
22075	0.946131	0.05
22076	1.24417	0.05
22077	0.774911	0.05
22078	0.946132	0.05
22079	1.54982	0.05
22080	1.82961	0.05
22081	1.12035	0.05
22082	0.876835	2.1
22083	0.946131	2.1
22084	0.876836	2.1
22085	1.12968	2.1
22086	1.23536	2.1
22087	2.15082	2.1
22088	0.58365	2.1
22089	0.946133	2.1
22090	0.583653	2.1
22091	0.992097	2.1
22092	1.12968	2.1
22093	1.0305	2.1
22094	0.82613	0.05
22095	0.879644	0.05

22096	1.00688	0.05
22097	1.52776	2.1
22098	0.851537	1.74982
22099	1.00851	1.26231
22100	0.946128	0.497616
22101	0.851552	1.40424
22102	1.4895	1.85821
22103	0.761574	2.1
22104	0.946132	2.1
22105	1.01095	2.1
22106	1.01095	2.1
22107	0.761572	2.1
22108	1.8575	2.1
22109	1.19984	0.05
22110	1.25797	0.05
22111	0.946132	0.05
22112	0.946132	0.05
22113	0.95332	0.05
22114	0.946131	2.1
22115	0.851542	2.1
22116	1.03172	2.1
22117	1.22889	2.1
22118	1.0313	2.1
22119	1.15977	1.75968
22120	0.918875	0.733955
22121	0.8654	2.1
22122	0.865374	0.788906
22123	0.946132	0.768206
22124	0.985051	2.1
22125	1.31993	0.0687487
22126	1.33622	0.179578
22127	0.959992	0.118531
22128	0.926499	2.1
22129	1.03544	2.1
22130	0.946132	2.1
22131	0.946132	2.1
22132	1.40335	2.1
22133	0.772299	2.1
22134	0.851529	2.1
22135	0.857243	2.1
22136	1.38155	2.1
22137	1.05462	2.1
22138	0.832138	2.1
22139	0.946132	2.1
22140	0.832137	2.1
22141	0.959414	2.1
22142	1.36541	2.1
22143	1.91894	2.1
22144	1.04661	1.95686
22145	1.28355	2.1
22146	0.877353	1.18098
22147	0.877353	1.83221
22148	0.94613	1.63195
22149	1.20537	0.05
22150	1.09914	0.05
22151	0.94613	0.05
22152	0.499593	0.05
22153	0.499606	0.05

22154	1.0959	0.05
22155	0.838027	0.05
22156	0.883644	0.05
22157	0.95019	0.05
22158	1.10638	2.1
22159	0.946132	2.1
22160	1.41744	2.1
22161	1.05842	2.1
22162	0.94613	2.1
22163	1.41744	2.1
22164	0.991985	2.1
22165	3.31755	1.61826
22166	1.05556	2.1
22167	0.624075	1.2252
22168	0.814554	0.778651
22169	0.946131	1.38222
22170	0.814554	1.70253
22171	1.31857	2.08616
22172	1.26282	2.1
22173	0.946132	2.1
22174	1.14302	2.1
22175	0.946132	2.1
22176	0.946132	2.1
22177	1.40372	2.1
22178	1.24688	0.05
22179	0.89889	2.1
22180	1.17286	2.1
22181	0.71741	2.1
22182	0.491096	0.321197
22183	1.05432	0.07597
22184	0.719531	2.1
22185	1.25145	2.1
22186	0.864686	2.1
22187	0.867974	2.1
22188	1.21734	2.1
22189	0.405517	0.136802
22190	0.818878	0.738374
22191	0.502457	0.05
22192	1.05868	0.05
22193	0.912018	0.05
22194	1.40591	0.0540975
22195	0.957584	0.05
22196	0.946131	0.05
22197	1.11612	0.05
22198	1.3365	0.05
22199	0.946128	0.0513076
22200	1.16534	0.05
22201	1.38969	0.158764
22202	0.891597	1.25402
22203	0.640032	2.1
22204	0.956758	1.44389
22205	1.3428	0.0526915
22206	0.953295	2.1
22207	0.946131	0.05
22208	1.16167	0.0529934
22209	0.94613	0.05
22210	0.951675	0.05
22211	1.16167	0.05

22212	1.24538	0.05
22213	1.13482	2.1
22214	1.38775	2.1
22215	0.934061	2.1
22216	0.91408	2.1
22217	1.06099	2.1
22218	0.980549	0.05
22219	0.946132	0.05
22220	1.10321	0.05
22221	0.99126	0.05
22222	0.991261	0.05
22223	1.35732	0.05
22224	1.1952	2.1
22225	0.946132	1.3313
22226	0.946132	2.1
22227	1.32819	1.62827
22228	1.30171	1.10313
22229	1.29667	0.05
22230	1.00982	0.05
22231	0.407589	0.150918
22232	1.18266	1.57153
22233	1.04037	0.208917
22234	0.956694	0.556361
22235	0.552639	0.184167
22236	0.810943	2.06302
22237	1.43144	0.661321
22238	0.918074	0.638637
22239	1.60976	2.1
22240	4.00847	2.1
22241	2.3995	0.05
22242	1.36203	2.1
22243	0.919619	2.1
22244	1.30188	2.1
22245	0.681073	2.1
22246	0.681043	2.1
22247	1.04687	2.1
22248	3.32085	2.1
22249	1.80396	0.05
22250	0.773007	0.0665807
22251	0.773034	0.05
22252	1.22141	0.101641
22253	1.22141	0.115212
22254	0.946131	0.197595
22255	2.02071	0.05
22256	0.951912	0.05
22257	1.39027	0.05
22258	0.94613	0.05
22259	0.829922	0.05
22260	0.829946	0.05
22261	1.24902	1.36031
22262	2.8333	1.06279
22263	0.946131	0.05
22264	0.929725	0.05
22265	1.1134	0.05
22266	1.17056	0.05
22267	0.929713	0.05
22268	0.749096	2.1
22269	1.31961	2.1

22270	0.870191	2.1
22271	1.04071	2.1
22272	1.1877	2.1
22273	1.03882	2.1
22274	1.44801	2.1
22275	3.55198	2.1
22276	1.11193	2.1
22277	0.663922	2.1
22278	0.663922	2.1
22279	0.946129	2.1
22280	0.946131	2.1
22281	0.97079	2.1
22282	1.36049	2.1
22283	1.06105	2.1
22284	0.92251	2.1
22285	0.94613	2.1
22286	0.922511	2.1
22287	1.04101	2.1
22288	0.810944	0.05
22289	0.994109	0.05
22290	0.623143	0.05
22291	0.864244	0.05
22292	1.14627	0.05
22293	0.64586	0.05
22294	0.721422	0.05
22295	0.870885	0.05
22296	0.855175	0.05
22297	0.946131	1.00322
22298	1.38157	2.1
22299	1.33266	0.160143
22300	0.946131	2.1
22301	1.38158	2.1
22302	0.946132	2.1
22303	1.3268	2.1
22304	0.946132	2.1
22305	0.946132	2.1
22306	1.38158	2.1
22307	1.42186	2.06947
22308	1.47204	1.99199
22309	1.14665	2.1
22310	0.946132	1.32768
22311	0.94613	2.1
22312	1.30116	2.1
22313	1.32819	2.04002
22314	1.25388	2.1
22315	0.887808	1.94476
22316	0.946132	2.1
22317	1.23536	2.1
22318	1.34309	2.1
22319	1.07612	2.1
22320	0.728982	0.05
22321	1.3157	0.05
22322	0.946131	0.05
22323	0.686176	0.05
22324	1.3035	0.05
22325	0.686139	0.05
22326	0.906806	0.05
22327	0.794122	0.05

22328	1.17173	0.05
22329	1.8194	0.05
22330	0.851543	2.1
22331	1.19345	2.1
22332	0.946133	2.1
22333	0.946132	2.1
22334	1.56179	2.1
22335	0.620263	2.1
22336	1.30226	2.1
22337	1.2334	1.72183
22338	1.2334	0.881986
22339	0.946133	1.1186
22340	1.24053	2.1
22341	1.4828	2.1
22342	0.957622	2.1
22343	1.00521	1.67247
22344	1.49463	2.1
22345	1.39621	2.1
22346	1.09861	2.1
22347	0.946131	2.1
22348	0.946131	2.1
22349	1.09861	2.1
22350	2.16987	0.05
22351	0.572182	0.05
22352	1.15802	0.05
22353	0.63574	0.05
22354	1.18897	0.05
22355	0.996733	0.05
22356	1.82402	1.5285
22357	0.969602	1.071
22358	0.794777	1.96532
22359	1.02626	1.43998
22360	1.51396	0.05
22361	1.40574	0.05
22362	1.38683	0.0607537
22363	0.946126	0.0620664
22364	0.946128	0.05
22365	1.07138	0.05
22366	0.946131	2.1
22367	1.41331	2.1
22368	0.94613	2.1
22369	0.94613	2.1
22370	1.27451	2.1
22371	0.974196	2.1
22372	1.2049	0.05
22373	1.42757	2.1
22374	1.02461	2.1
22375	2.07087	2.1
22376	1.92515	0.86717
22377	0.802138	0.926699
22378	0.946128	2.02043
22379	0.802107	1.25273
22380	1.23726	1.19398
22381	1.21997	1.00261
22382	0.767577	0.32321
22383	0.946131	1.801
22384	0.767577	0.850069
22385	1.3279	0.788461

22386	1.23726	0.593093
22387	1.29729	2.1
22388	0.946128	2.1
22389	1.14186	2.1
22390	1.39214	2.1
22391	1.39214	2.1
22392	0.946129	2.1
22393	1.23012	0.0518528
22394	0.653228	0.0691283
22395	0.946133	1.34166
22396	1.15918	1.65204
22397	1.26893	0.294329
22398	0.653228	1.29963
22399	1.06848	1.70244
22400	0.615121	2.1
22401	0.841488	1.85091
22402	0.946131	2.1
22403	1.04071	2.1
22404	0.965717	2.1
22405	1.77893	1.48257
22406	1.11314	0.05
22407	1.65411	0.519635
22408	0.85472	0.05
22409	1.1604	0.05
22410	1.19141	0.05
22411	1.19141	0.05
22412	0.94613	0.05
22413	1.70944	0.05
22414	1.89082	0.05
22415	1.05175	0.655963
22416	0.815276	2.1
22417	0.843283	2.1
22418	0.946131	2.1
22419	0.843284	2.1
22420	1.38897	2.1
22421	1.01019	0.170161
22422	1.05197	0.084505
22423	0.810959	0.13469
22424	1.33037	0.07836
22425	1.3415	0.0982692
22426	0.946118	0.05
22427	0.946131	0.0526564
22428	1.09803	0.05
22429	0.930281	2.1
22430	1.3335	2.1
22431	0.784151	2.1
22432	0.78415	2.1
22433	0.946132	2.1
22434	1.6299	0.05
22435	0.995795	2.1
22436	1.84846	1.52412
22437	0.929873	1.99528
22438	0.889245	2.00428
22439	0.808072	2.02449
22440	0.851541	1.81857
22441	1.51956	1.42795
22442	0.94613	1.62596
22443	0.851553	1.73977

22444	0.946787	1.67426
22445	1.15544	1.80389
22446	1.30289	0.05
22447	1.28323	0.05
22448	0.85024	0.05
22449	1.09662	0.05
22450	1.67687	0.05
22451	0.946127	2.1
22452	1.40496	2.1
22453	1.22442	2.1
22454	0.946128	2.1
22455	1.40496	2.1
22456	1.00573	2.1
22457	0.636837	2.1
22458	0.988642	2.1
22459	0.573141	2.1
22460	0.946128	2.1
22461	0.786538	2.1
22462	2.28748	1.69654
22463	0.682805	2.1
22464	0.946132	2.1
22465	0.682776	2.1
22466	0.946132	2.1
22467	0.79775	2.1
22468	1.05403	0.05
22469	0.888273	0.05
22470	0.876373	0.05
22471	0.576083	0.05
22472	0.946132	0.05
22473	0.576057	0.05
22474	0.576058	0.05
22475	0.946131	0.05
22476	1.41724	0.0847072
22477	1.31678	0.335533
22478	0.946132	0.302237
22479	0.946132	0.217825
22480	1.31678	0.178663
22481	0.935738	2.1
22482	0.946132	2.1
22483	0.935765	2.1
22484	1.32949	2.1
22485	0.946131	2.1
22486	1.02525	2.1
22487	0.946128	2.1
22488	1.41207	2.1
22489	1.04675	2.1
22490	0.946131	2.1
22491	1.41207	2.1
22492	0.946131	2.1
22493	0.946132	2.1
22494	1.41125	2.1
22495	1.41125	2.1
22496	1.27087	2.1
22497	0.924071	0.05
22498	0.608893	0.05
22499	0.528907	0.05
22500	1.78249	0.05
22501	0.720094	0.05

22502	0.781667	2.1
22503	0.781638	2.1
22504	1.40756	2.1
22505	1.40756	2.1
22506	0.797635	2.1
22507	1.11946	0.05
22508	1.32068	0.05
22509	0.88757	0.05
22510	1.27891	0.05
22511	0.810957	0.05
22512	0.914656	0.05
22513	0.640819	0.05
22514	0.745643	0.05
22515	0.865108	0.05
22516	1.06883	0.05
22517	0.806001	0.05
22518	1.19462	2.1
22519	1.42763	0.05
22520	1.24682	0.05
22521	0.78392	0.05
22522	1.02874	0.05
22523	0.884512	0.05
22524	1.1658	2.1
22525	0.663026	0.05
22526	1.21174	0.05
22527	0.663056	0.05
22528	1.15968	0.05
22529	1.21174	0.05
22530	0.993976	0.05
22531	0.945495	0.05
22532	0.671202	0.05
22533	1.37312	2.1
22534	1.28499	2.1
22535	0.946133	2.1
22536	0.946132	2.1
22537	1.28499	2.1
22538	1.25081	2.1
22539	0.946132	2.1
22540	0.946132	2.1
22541	1.27985	2.1
22542	1.37312	2.1
22543	0.830638	2.1
22544	1.05358	2.1
22545	1.22209	2.1
22546	1.09874	2.1
22547	0.74758	2.1
22548	2.50011	1.70979
22549	0.6157	0.0590304
22550	0.674634	0.074161
22551	0.946128	1.19964
22552	0.674633	0.144525
22553	0.946131	0.670535
22554	1.96192	1.797
22555	1.31612	0.05
22556	1.10735	0.05
22557	0.936676	0.05
22558	0.808779	0.05
22559	0.808808	0.05

22560	1.00245	0.05
22561	1.26179	0.05
22562	1.05758	2.1
22563	1.03408	2.1
22564	0.946132	0.05
22565	0.914368	0.05
22566	1.20524	0.05
22567	1.12743	0.05
22568	0.851555	0.05
22569	1.17885	0.109859
22570	0.867881	0.0742273
22571	1.00122	0.0619598
22572	0.867883	0.0598175
22573	0.867881	0.0678442
22574	1.19583	0.0854056
22575	1.17743	2.1
22576	0.878684	2.1
22577	0.687087	2.1
22578	0.955079	0.103206
22579	0.672977	0.2537
22580	0.735937	0.31131
22581	0.851531	0.379306
22582	0.672957	0.277894
22583	0.923974	0.276051
22584	1.2243	2.1
22585	0.808463	0.88541
22586	0.971078	1.11609
22587	1.06161	1.03928
22588	0.810947	0.920797
22589	0.886074	1.31295
22590	0.786829	1.44919
22591	0.55062	0.808048
22592	0.574458	1.78806
22593	0.946129	1.03635
22594	1.42526	1.17756
22595	1.15962	0.953277
22596	1.24526	0.05
22597	0.94613	0.05
22598	0.946128	0.05
22599	1.23279	0.0878525
22600	1.38645	0.207137
22601	0.94613	0.0623409
22602	1.05042	2.1
22603	0.946128	2.1
22604	0.946129	2.1
22605	1.24286	2.1
22606	1.39659	2.1
22607	0.94613	2.1
22608	1.10407	2.1
22609	1.32809	1.92449
22610	0.993999	2.1
22611	0.810959	1.78243
22612	0.983643	1.87565
22613	0.869552	2.09751
22614	0.747801	1.60531
22615	1.53429	0.845153
22616	1.13127	2.1
22617	1.38397	2.1

22618	1.08366	2.1
22619	0.946129	2.1
22620	0.946131	2.1
22621	1.08366	2.1
22622	1.06007	2.1
22623	1.79641	2.1
22624	0.758799	2.1
22625	1.28442	2.1
22626	1.06957	2.1
22627	0.946133	2.1
22628	1.28442	2.1
22629	1.76788	2.05135
22630	1.38669	2.1
22631	0.986095	2.1
22632	1.38478	1.86412
22633	0.946132	0.741379
22634	1.24344	0.353055
22635	0.622283	2.01846
22636	1.10335	0.488634
22637	1.24344	0.569192
22638	1.1967	1.19136
22639	0.946131	0.05
22640	0.901005	0.05
22641	1.05751	0.05
22642	1.37771	0.05
22643	0.851527	0.05
22644	1.26643	1.15536
22645	1.08992	1.69251
22646	0.946132	1.56498
22647	0.946132	0.852379
22648	1.08992	1.64978
22649	1.53534	2.1
22650	1.58825	1.68512
22651	1.0447	2.05879
22652	0.593818	1.79925
22653	0.89883	1.94977
22654	0.734088	1.95589
22655	0.998451	0.40881
22656	1.64156	0.121061
22657	1.05654	0.741675
22658	1.37641	0.0647773
22659	1.74553	0.0952708
22660	1.31112	0.0772699
22661	0.74518	0.146444
22662	0.851539	0.100415
22663	0.851554	0.0724033
22664	0.851475	0.05
22665	0.946132	0.05
22666	1.28895	0.05
22667	1.11356	0.05
22668	0.946132	0.05
22669	0.946132	0.05
22670	1.84214	2.1
22671	1.23851	0.05
22672	1.25866	0.0578671
22673	1.28655	0.328485
22674	0.946131	0.200055
22675	0.946132	0.153437

22676	1.28655	0.124964
22677	1.11929	0.0970732
22678	0.946132	0.225117
22679	0.946132	0.0856105
22680	1.34254	0.07741
22681	1.25866	0.0605647
22682	0.946132	2.1
22683	1.27451	2.1
22684	1.19803	2.1
22685	0.94613	2.1
22686	1.27451	2.1
22687	0.946131	2.1
22688	1.36523	2.1
22689	1.14599	2.1
22690	0.946129	2.1
22691	1.36523	2.1
22692	1.09518	2.1
22693	0.977777	2.1
22694	0.583237	2.1
22695	0.583267	2.1
22696	0.903872	2.1
22697	0.946129	2.1
22698	0.583258	2.1
22699	1.58179	0.05
22700	0.946131	0.05
22701	1.35123	0.05
22702	0.655837	0.05
22703	1.12018	0.05
22704	1.35123	0.05
22705	0.62967	0.05
22706	0.860497	0.05
22707	0.816639	0.05
22708	0.746716	2.1
22709	0.810947	2.1
22710	0.810959	2.1
22711	0.653422	2.1
22712	0.289127	1.49583
22713	0.475716	2.1
22714	0.980651	1.52603
22715	0.946119	2.1
22716	0.946132	2.1
22717	1.08299	2.1
22718	1.34539	2.1
22719	1.28753	0.05
22720	0.946132	0.05
22721	0.946132	0.05
22722	1.19719	0.05
22723	0.946133	0.05
22724	0.958947	0.05
22725	1.3492	0.05
22726	0.946132	0.05
22727	0.946128	0.05
22728	1.22588	0.05
22729	0.946129	0.05
22730	0.991113	2.1
22731	1.35966	2.1
22732	0.961 2.1	
22733	0.946129	2.1

22734	0.946131	2.1
22735	0.961001	2.1
22736	0.727096	1.93797
22737	0.810946	0.466871
22738	0.810959	0.885183
22739	1.31658	0.0664941
22740	1.23167	2.1
22741	1.44836	0.05
22742	1.50103	0.05
22743	1.23351	0.05
22744	1.35657	0.05
22745	0.851541	0.05
22746	0.93668	0.05
22747	0.946131	0.05
22748	3.7335	2.1
22749	1.1822	2.1
22750	1.31127	0.0931863
22751	1.3689	0.0669441
22752	1.35822	0.05
22753	0.946133	0.0544617
22754	0.946132	0.0698524
22755	1.14926	0.0715729
22756	1.40822	0.05
22757	1.04935	0.0786211
22758	0.704112	0.0602607
22759	0.704112	0.0731983
22760	0.946131	0.0965713
22761	0.704112	0.0840868
22762	1.37721	0.05
22763	0.85154	2.1
22764	1.06949	2.1
22765	0.851541	2.1
22766	0.946128	2.1
22767	1.1175	2.1
22768	1.44091	2.1
22769	0.96029	0.731131
22770	3.46486	0.738006
22771	0.603286	0.05
22772	0.432283	0.05
22773	0.851541	0.05
22774	1.09355	0.05
22775	0.851534	0.05
22776	1.04263	2.1
22777	0.851541	2.1
22778	0.965604	2.1
22779	0.94613	2.1
22780	1.23953	2.1
22781	0.869615	2.1
22782	1.02415	2.1
22783	0.983032	2.1
22784	1.35385	1.65042
22785	1.59971	0.05
22786	0.915178	2.1
22787	0.962435	2.1
22788	1.3116	2.1
22789	1.38302	2.1
22790	1.13227	2.1
22791	1.25923	0.05

22792	0.988805	2.1
22793	0.611486	1.10708
22794	1.1019	0.926714
22795	1.11124	1.74273
22796	0.716713	0.95737
22797	0.946131	1.5533
22798	1.22297	1.08678
22799	1.12873	1.64612
22800	0.946131	1.64092
22801	0.946132	1.67775
22802	1.3469	1.86087
22803	1.25366	2.03156
22804	1.1611	1.13619
22805	0.946132	1.47034
22806	0.946132	1.19802
22807	1.2854	1.49634
22808	1.3469	1.64452
22809	1.07468	1.22651
22810	1.79317	2.1
22811	1.07444	2.1
22812	1.69436	2.03684
22813	0.851539	2.1
22814	0.870925	2.1
22815	0.851541	2.1
22816	0.870951	2.1
22817	1.26097	2.1
22818	1.26247	2.1
22819	0.946132	2.1
22820	1.24041	2.1
22821	0.946132	2.1
22822	0.946132	2.1
22823	1.40274	2.1
22824	0.946131	0.05
22825	0.797945	0.05
22826	1.12988	0.05
22827	1.00681	0.05
22828	0.797932	0.05
22829	0.977483	0.05
22830	0.943362	0.130955
22831	0.9891	0.05
22832	1.1703	0.05
22833	0.663171	0.0543292
22834	1.40882	0.05
22835	1.32639	0.05
22836	1.24448	0.05
22837	0.946132	0.05
22838	1.11015	0.05
22839	1.24448	0.05
22840	0.754768	0.05
22841	1.13854	2.1
22842	1.23654	2.1
22843	0.714101	0.0738821
22844	0.851538	0.0863577
22845	0.79261	1.43047
22846	1.30598	0.507523
22847	1.05338	0.111428
22848	1.41758	2.1
22849	0.83317	0.05

22850	1.00058	0.0574102
22851	0.810958	0.0558542
22852	1.63791	0.05
22853	1.55116	2.1
22854	1.29735	1.15307
22855	0.945147	2.1
22856	0.94515	1.3097
22857	0.94613	1.97669
22858	0.945149	1.80682
22859	1.02368	2.1
22860	1.12787	2.1
22861	1.04007	2.1
22862	3.78805	2.1
22863	0.816891	0.05
22864	0.943996	0.05
22865	0.870002	0.05
22866	0.564533	0.05
22867	0.564536	0.05
22868	0.946128	0.511943
22869	0.564535	0.05
22870	0.946128	0.318266
22871	1.15177	2.1
22872	1.16806	0.962189
22873	0.999083	1.18011
22874	0.946131	1.20757
22875	0.946131	1.16767
22876	0.999084	1.31447
22877	1.28918	0.806069
22878	1.0383	1.15867
22879	0.946133	1.10876
22880	0.946132	1.17302
22881	1.0383	1.20018
22882	1.27737	1.53628
22883	1.39296	0.0949157
22884	0.998682	2.1
22885	3.98933	2.1
22886	1.05328	0.05
22887	1.23475	2.1
22888	0.900856	1.59628
22889	0.94001	0.852033
22890	0.707748	0.638707
22891	0.707751	1.27697
22892	0.946128	0.787785
22893	0.70775	1.15164
22894	0.996109	1.0654
22895	1.68551	2.1
22896	0.851539	2.1
22897	0.962688	2.1
22898	0.94613	2.1
22899	1.04073	2.1
22900	1.44824	2.1
22901	0.794313	0.05
22902	0.838031	0.05
22903	1.11076	0.05
22904	1.38813	2.1
22905	1.17204	2.1
22906	0.946132	2.1
22907	0.946131	2.1

22908	1.17204	2.1
22909	0.961779	0.05
22910	1.0685	0.0939926
22911	0.810947	2.1
22912	1.2899	1.0898
22913	0.890404	1.07835
22914	0.909981	2.1
22915	0.810946	1.50134
22916	0.890402	1.15849
22917	0.522844	2.1
22918	0.593846	2.1
22919	1.30226	1.20008
22920	1.30226	1.07261
22921	0.946132	1.63817
22922	1.03537	0.05
22923	1.50035	0.05
22924	0.940356	0.05
22925	0.946131	0.05
22926	1.03441	0.05
22927	1.21171	0.05
22928	1.35169	0.05
22929	1.32449	0.05
22930	0.946131	0.05
22931	0.946131	0.05
22932	1.06627	0.05
22933	1.03102	0.0849145
22934	0.488712	0.05
22935	0.946131	0.05
22936	1.23146	0.05
22937	1.13242	0.05
22938	0.946131	0.05
22939	0.409199	0.05
22940	0.429959	0.05
22941	1.08357	0.05
22942	0.80926	0.05
22943	0.879791	0.05
22944	0.946129	1.31329
22945	1.28828	2.09275
22946	0.946131	0.826898
22947	0.946131	2.1
22948	1.35969	2.1
22949	1.10049	1.93853
22950	0.923724	2.1
22951	0.923695	2.1
22952	0.94613	2.09303
22953	0.94613	2.1
22954	1.28828	2.1
22955	1.73525	0.05
22956	0.656239	0.05
22957	1.2996	0.05
22958	0.812939	0.05
22959	0.65624	0.05
22960	1.2996	0.05
22961	1.67372	2.1
22962	0.946131	2.1
22963	0.946132	2.1
22964	1.05978	2.1
22965	1.31049	2.1

22966	0.946132	2.1
22967	1.11077	2.1
22968	0.579725	0.0641112
22969	0.373532	0.05
22970	1.13537	0.0747438
22971	1.20726	0.05
22972	0.946131	0.05
22973	1.45694	0.130076
22974	0.750367	0.05
22975	0.946126	0.05
22976	0.750365	0.05
22977	1.25698	0.05
22978	1.35726	0.05
22979	1.6812	0.05
22980	1.78665	1.57534
22981	1.46568	2.1
22982	1.29606	2.1
22983	1.25298	2.1
22984	0.946115	2.1
22985	0.946129	2.1
22986	1.25296	2.1
22987	1.27778	0.05
22988	1.17042	2.1
22989	1.25858	0.05
22990	0.93106	0.05
22991	0.810959	0.05
22992	1.38905	2.1
22993	1.19216	2.1
22994	0.946131	2.1
22995	0.946131	2.1
22996	1.19216	2.1
22997	1.58339	2.1
22998	0.81094	0.05
22999	0.845129	0.05
23000	0.918535	0.05
23001	0.946132	2.1
23002	0.946132	2.1
23003	1.31904	2.1
23004	1.31904	2.1
23005	1.21275	2.1
23006	1.15471	2.1
23007	1.33125	2.1
23008	0.702954	0.05
23009	0.942973	0.05
23010	0.94613	0.05
23011	0.641772	0.05
23012	0.702942	0.05
23013	0.991865	0.06257
23014	0.639952	0.05
23015	1.17333	0.05
23016	0.946132	0.05
23017	0.490975	0.05
23018	0.495934	0.0606473
23019	1.19904	0.05
23020	1.30488	0.05
23021	1.02305	0.05
23022	1.49842	0.05
23023	1.54196	2.1

23024	1.37224	2.08175
23025	1.0953	0.05
23026	1.18952	2.1
23027	1.68851	0.0875691
23028	0.946131	0.052659
23029	1.12491	0.0519557
23030	0.583591	0.05
23031	1.28727	0.05
23032	1.12491	0.0656436
23033	0.884641	0.0930846
23034	0.810944	0.0788747
23035	0.917973	0.117524
23036	0.846671	0.108589
23037	0.596968	0.05
23038	0.716347	0.0949824
23039	1.36255	0.05
23040	1.11077	2.1
23041	0.946132	2.1
23042	0.946132	2.1
23043	1.26267	2.1
23044	1.26441	2.1
23045	1.85238	2.1
23046	1.36693	2.1
23047	0.616123	2.1
23048	0.481844	2.1
23049	0.709599	2.1
23050	0.815889	2.1
23051	1.1502	2.1
23052	1.63937	0.682216
23053	1.25797	0.05
23054	1.22878	0.05
23055	0.946132	0.05
23056	0.946132	0.05
23057	0.965737	0.05
23058	1.1893	0.05
23059	1.22877	0.05
23060	0.735217	0.05
23061	0.85154	0.05
23062	0.851553	0.05
23063	0.851473	0.05
23064	1.58916	0.05
23065	1.25545	0.05
23066	0.810942	0.05
23067	0.919314	0.05
23068	0.928633	0.05
23069	2.14514	0.05
23070	1.23389	2.1
23071	0.946128	0.05
23072	0.969504	0.05
23073	0.851539	0.05
23074	1.41503	0.05
23075	0.958517	0.05
23076	0.990615	0.05
23077	1.7943	0.05
23078	0.946133	0.0677744
23079	1.27581	0.0646002
23080	0.946132	0.0697211
23081	1.36362	0.0605017

23082	1.36936	0.0566871
23083	1.71903	2.1
23084	0.751639	2.1
23085	0.77464	2.1
23086	0.946133	2.1
23087	1.04073	2.1
23088	1.23551	2.1
23089	0.94613	2.1
23090	1.18682	2.1
23091	0.946131	2.1
23092	0.94613	2.1
23093	1.40638	2.1
23094	0.483346	0.05
23095	0.432527	0.05
23096	0.946132	0.05
23097	0.483312	0.05
23098	0.946132	0.05
23099	0.878798	0.05
23100	0.810944	0.05
23101	1.14604	0.05
23102	0.946129	0.05
23103	1.35594	0.05
23104	0.538378	0.05
23105	0.946131	0.05
23106	0.538378	0.05
23107	1.00065	0.1207
23108	0.962878	0.05
23109	1.32634	0.05
23110	1.28066	0.05
23111	0.785651	2.1
23112	1.20327	2.1
23113	1.47058	2.00692
23114	0.551834	0.925942
23115	1.04072	2.1
23116	0.506719	1.64089
23117	1.12786	1.91557
23118	1.45789	0.05
23119	0.681532	0.05
23120	1.21087	0.05
23121	0.94613	0.05
23122	0.681534	0.05
23123	1.21087	0.05
23124	1.98618	0.204215
23125	1.71742	1.34632
23126	1.07427	0.344567
23127	1.1972	0.197955
23128	0.933474	0.187049
23129	1.1745	0.283402
23130	0.94612	0.22049
23131	0.933485	0.390742
23132	1.17449	0.27546
23133	0.851541	0.05
23134	1.12774	0.05
23135	0.946129	0.05
23136	0.851486	0.05
23137	1.12517	0.05
23138	1.12328	0.05
23139	1.13179	2.1

23140	1.02438	2.1
23141	1.05776	2.1
23142	1.41273	1.54667
23143	1.1229	2.1
23144	0.875912	1.83028
23145	0.946132	2.1
23146	0.875912	2.1
23147	1.42307	2.1
23148	1.15354	0.05
23149	0.848382	0.05
23150	1.2685	2.1
23151	0.938104	2.1
23152	0.793166	2.1
23153	1.07855	2.1
23154	0.98488	2.1
23155	1.4162	2.1
23156	1.09651	0.05
23157	1.98502	0.0585033
23158	1.41168	0.05
23159	1.33879	0.05
23160	0.946127	0.0706697
23161	1.13528	0.05
23162	0.946128	0.05
23163	1.09726	2.1
23164	1.22026	0.624007
23165	1.09044	2.1
23166	2.23961	0.486692
23167	1.14721	0.05
23168	1.17614	2.1
23169	0.615065	2.1
23170	0.946131	2.1
23171	0.778488	2.1
23172	0.94613	2.1
23173	0.615066	2.1
23174	1.78254	2.1
23175	0.898452	0.05
23176	1.47411	2.1
23177	0.59676	0.05
23178	0.59676	0.05
23179	0.94613	0.0527428
23180	1.13511	0.05
23181	0.946131	0.05
23182	0.916275	2.1
23183	0.946132	2.1
23184	0.916274	1.44352
23185	1.36182	2.1
23186	0.946131	2.1
23187	0.857202	0.05
23188	0.941918	0.05
23189	0.943349	0.05
23190	1.2493	0.05
23191	0.946133	0.05
23192	1.01814	0.05
23193	0.927881	0.05
23194	1.11845	0.05
23195	1.2493	0.05
23196	1.11845	0.05
23197	0.946129	0.05

23198	0.967673	0.154946
23199	1.01332	0.05
23200	0.946133	0.05
23201	0.81938	0.05
23202	0.819377	0.05
23203	0.946133	0.05
23204	1.22474	0.05
23205	1.15321	0.05
23206	0.797489	0.05
23207	0.946131	0.05
23208	0.797461	0.05
23209	1.35328	0.651538
23210	1.03318	1.21178
23211	0.946131	0.963015
23212	0.94613	1.06651
23213	1.03319	1.08893
23214	1.37938	0.631971
23215	1.00512	1.17592
23216	0.946133	0.848325
23217	0.946132	1.0084
23218	1.00512	1.00237
23219	1.5515	1.17824
23220	1.55351	2.1
23221	0.94261	0.05
23222	0.828156	0.05
23223	0.916495	0.05
23224	3.06309	0.05
23225	0.619832	0.05
23226	0.705903	0.05
23227	1.2968	0.05
23228	0.946133	0.05
23229	0.705904	0.05
23230	1.2968	0.05
23231	1.01971	0.05
23232	0.60302	0.05
23233	1.07242	0.277792
23234	1.26426	0.227913
23235	0.946131	0.291055
23236	0.946131	0.355467
23237	0.963109	0.316236
23238	0.947862	0.228957
23239	1.40248	1.00788
23240	0.767143	1.03951
23241	0.767114	1.08907
23242	0.946129	1.09495
23243	0.767143	0.730095
23244	1.22637	0.692313
23245	1.2281	1.00351
23246	0.590909	1.14027
23247	0.490832	0.986344
23248	0.804174	1.15644
23249	0.747368	0.842942
23250	1.05129	0.05
23251	1.10609	0.30772
23252	1.3639	0.05
23253	0.776117	0.05
23254	0.752329	0.05
23255	1.04073	0.05

23256	0.67713	0.05
23257	3.4245	2.1
23258	1.18526	2.1
23259	1.2161	1.1357
23260	0.876834	2.05092
23261	0.876831	1.08988
23262	0.946132	1.86635
23263	0.876834	1.30957
23264	0.954563	2.1
23265	0.924132	2.1
23266	1.25075	2.1
23267	1.01652	2.1
23268	0.92413	2.1
23269	1.37579	2.1
23270	0.992159	2.1
23271	1.26092	2.1
23272	0.929435	2.1
23273	0.844958	2.1
23274	0.79598	2.1
23275	0.852559	2.1
23276	0.946131	0.056396
23277	1.28435	0.05
23278	0.827261	0.0516831
23279	0.946128	0.0564745
23280	0.827298	0.0618384
23281	1.65452	0.05
23282	1.79929	2.1
23283	0.791947	1.78909
23284	0.902072	1.48476
23285	0.882672	1.69391
23286	1.21703	2.1
23287	0.900338	2.1
23288	1.37712	2.1
23289	0.782591	2.1
23290	0.782562	2.1
23291	0.94613	2.1
23292	1.34749	2.1
23293	0.61359	2.1
23294	1.14397	0.05
23295	1.02963	0.05
23296	1.36589	0.05
23297	0.946132	0.05
23298	1.28644	0.05
23299	0.946131	0.05
23300	1.40104	0.05
23301	1.28644	0.05
23302	0.810946	0.05
23303	1.09178	0.05
23304	1.26956	0.05
23305	0.810943	0.05
23306	0.885633	0.05
23307	1.00844	0.05
23308	0.825671	0.05
23309	0.792093	0.05
23310	0.925037	0.05
23311	0.946131	0.193148
23312	0.946131	0.05
23313	1.18052	0.05

23314	1.40265	0.0512593
23315	0.946132	0.140649
23316	1.17891	0.0554015
23317	0.734248	0.05
23318	0.810943	0.05
23319	0.731621	0.05
23320	0.799857	0.0617111
23321	0.834507	0.274181
23322	0.810932	0.0910125
23323	1.01316	0.05
23324	1.23884	0.05
23325	1.88438	2.1
23326	0.946129	2.1
23327	1.38614	2.1
23328	1.34245	1.35027
23329	0.946131	0.911772
23330	1.38614	1.03636
23331	0.946132	0.05
23332	0.910733	0.05
23333	1.24726	0.05
23334	1.13742	0.05
23335	0.851555	0.05
23336	1.50368	0.05
23337	0.94613	0.471
23338	1.08998	2.1
23339	1.36899	0.05
23340	1.36898	1.21433
23341	0.946131	1.15227
23342	1.26045	2.1
23343	1.06432	0.327038
23344	1.89016	0.0884297
23345	0.83514	2.1
23346	1.36653	2.1
23347	1.23426	2.1
23348	0.94613	2.1
23349	0.835112	2.1
23350	1.14567	0.956495
23351	1.12096	0.795903
23352	0.881106	1.15042
23353	1.17787	0.05
23354	1.29738	1.12872
23355	0.940472	0.05
23356	1.03452	0.815699
23357	0.946131	0.0572978
23358	2.7489	0.438357
23359	1.01778	2.1
23360	1.04321	0.613135
23361	1.19352	0.05
23362	1.16912	1.71529
23363	0.946132	1.2678
23364	0.946132	1.95591
23365	1.40924	1.89594
23366	1.22927	2.09988
23367	2.09455	0.05
23368	3.00575	2.1
23369	1.08928	0.52765
23370	1.94799	2.1
23371	1.4092	2.1

23372	0.946132	2.1
23373	1.16	2.1
23374	1.01985	2.1
23375	0.946133	2.1
23376	1.16	2.1
23377	1.15371	0.05
23378	1.56002	0.05
23379	1.23007	2.1
23380	1.9992	2.1
23381	0.652605	2.1
23382	0.937903	2.1
23383	0.946132	2.1
23384	0.937902	2.1
23385	1.228	2.1
23386	1.33694	1.78099
23387	1.00191	2.1
23388	0.716814	1.94759
23389	0.94613	2.1
23390	0.716816	2.1
23391	1.70388	1.83581
23392	0.946129	0.0855856
23393	1.09362	0.12533
23394	0.946131	0.132781
23395	1.4185	0.153666
23396	1.09362	0.102118
23397	1.31057	0.972274
23398	1.17631	0.05
23399	1.19404	0.05
23400	0.94613	0.05
23401	0.946129	0.05
23402	0.99972	0.05
23403	0.94613	0.05
23404	0.835199	0.05
23405	1.23351	0.05
23406	0.582785	0.05
23407	0.701079	0.05
23408	0.946132	0.05
23409	2.17385	0.05
23410	1.18948	2.09957
23411	1.25346	2.1
23412	1.18586	0.276254
23413	0.880876	0.05
23414	0.68632	0.099617
23415	0.946118	2.1
23416	0.966936	2.1
23417	0.946117	2.1
23418	1.41636	2.1
23419	1.03798	2.1
23420	0.729178	0.367396
23421	0.721888	0.824015
23422	0.946132	0.472635
23423	0.656286	0.866146
23424	1.13898	0.602557
23425	1.02351	0.05
23426	1.48803	0.05
23427	0.946131	0.05
23428	1.04921	2.1
23429	1.02478	0.05

23430	0.993023	2.1
23431	1.80841	0.05
23432	1.29215	0.05
23433	0.904206	0.05
23434	0.904207	0.05
23435	0.946132	0.05
23436	0.904207	0.05
23437	1.07347	2.1
23438	0.555875	1.59007
23439	0.555904	1.84972
23440	1.30841	2.1
23441	1.30841	2.1
23442	0.91951	2.1
23443	1.33026	0.05
23444	1.43289	0.05
23445	1.15703	1.7239
23446	0.886594	2.1
23447	1.05129	1.93103
23448	1.08421	0.212861
23449	1.35946	0.254034
23450	0.882955	0.546518
23451	0.946131	0.454815
23452	0.882985	0.365587
23453	0.583501	0.177254
23454	0.532921	0.196994
23455	0.94613	1.8833
23456	0.532892	1.28613
23457	1.23224	1.27969
23458	1.12786	1.1372
23459	0.946131	0.552295
23460	1.23732	0.05
23461	1.40609	0.222161
23462	1.40609	0.05
23463	0.946131	0.05
23464	0.910672	1.60508
23465	1.08478	1.13225
23466	0.94613	0.148601
23467	0.94613	1.15825
23468	1.30382	1.16507
23469	3.10544	2.00509
23470	0.679509	2.1
23471	1.15966	1.62144
23472	0.75499	2.1
23473	1.37511	1.84401
23474	1.01776	1.61548
23475	2.35239	0.982906
23476	0.92406	2.1
23477	0.946118	2.1
23478	0.92406	2.1
23479	1.07979	2.1
23480	1.29604	2.1
23481	1.29897	2.1
23482	0.902645	0.475496
23483	0.729493	1.44983
23484	0.906844	1.01068
23485	0.739518	0.05
23486	1.10564	0.05
23487	0.946131	0.05

23488	0.739519	0.05
23489	0.739506	0.05
23490	1.79907	0.05
23491	1.59106	0.85435
23492	1.11067	0.449778
23493	0.795516	0.733059
23494	0.795519	0.582587
23495	0.946132	0.719164
23496	0.795506	0.48303
23497	1.80223	2.1
23498	1.95572	2.05138
23499	1.16237	0.326077
23500	1.07518	2.1
23501	0.696199	0.514493
23502	0.94613	1.52091
23503	0.696199	0.834269
23504	1.6379	0.05
23505	0.946118	0.05
23506	0.851529	0.05
23507	1.08716	0.05
23508	0.973118	0.05
23509	0.851528	0.05
23510	1.15096	2.1
23511	0.65023	0.05
23512	0.94613	0.05
23513	0.650205	0.05
23514	0.650205	0.05
23515	0.811233	0.05
23516	0.778721	2.1
23517	0.810945	1.0693
23518	0.997518	1.10166
23519	0.838028	0.05
23520	0.952893	0.05
23521	0.838037	0.05
23522	1.58166	0.05
23523	1.44848	1.42574
23524	1.30041	0.05
23525	1.03697	0.0728666
23526	0.946132	1.66191
23527	1.0277	1.0362
23528	0.946131	1.78924
23529	1.00501	1.22386
23530	1.0277	0.76017
23531	1.64988	2.1
23532	0.946131	2.1
23533	0.94613	0.101799
23534	1.27702	2.1
23535	1.55163	0.86074
23536	0.897905	0.753827
23537	1.12971	0.05
23538	1.35715	0.0674935
23539	0.881884	0.309431
23540	0.664007	0.0645912
23541	0.663978	0.157385
23542	0.94613	0.111186
23543	0.664006	0.149458
23544	1.48693	0.05
23545	1.29289	0.05

23546	1.32081	0.0519178
23547	0.946119	0.05
23548	0.946132	0.05
23549	1.02972	0.05
23550	0.946131	2.1
23551	0.946127	2.1
23552	1.23513	2.1
23553	1.38154	2.1
23554	0.94613	2.1
23555	1.25933	0.05
23556	0.917954	0.05
23557	0.868589	0.05
23558	3.0957	2.1
23559	0.711391	0.05
23560	0.711391	0.05
23561	0.991303	0.05
23562	1.37322	0.05
23563	0.729145	0.05
23564	2.55648	0.05
23565	1.0756	2.0027
23566	0.760299	1.19884
23567	0.763592	0.126503
23568	0.946126	0.253285
23569	0.76359	0.114718
23570	1.35862	0.953192
23571	0.946133	2.1
23572	1.00807	2.1
23573	0.946132	2.1
23574	1.39823	2.1
23575	1.07817	2.1
23576	1.44379	1.01632
23577	1.17934	0.05
23578	1.13451	0.05
23579	0.94613	2.0098
23580	1.03974	2.1
23581	1.36835	1.74967
23582	1.36835	2.1
23583	0.94613	2.1
23584	1.27096	2.1
23585	0.853042	2.1
23586	0.946131	2.1
23587	1.13658	2.1
23588	1.06997	2.1
23589	0.94613	2.1
23590	1.41556	0.0863617
23591	1.21708	0.223034
23592	0.94613	0.236882
23593	0.946131	0.155128
23594	1.21708	0.148662
23595	0.964782	0.0946438
23596	0.946119	0.171235
23597	1.23447	0.135492
23598	0.946118	0.174204
23599	1.41844	0.125985
23600	1.23446	0.149274
23601	1.1357	0.05
23602	0.619856	0.05
23603	1.1509	0.05

23604	0.946126	0.05
23605	1.19262	0.05
23606	1.1509	0.05
23607	1.04268	2.1
23608	2.07527	0.261271
23609	1.38259	0.757625
23610	0.980054	1.68387
23611	0.946124	0.985709
23612	0.946127	1.29329
23613	0.980056	1.05301
23614	1.62916	1.99174
23615	0.946131	2.1
23616	1.40571	2.1
23617	0.946131	2.1
23618	1.38885	2.1
23619	1.40571	2.1
23620	1.11787	0.414765
23621	0.946129	0.31416
23622	1.28831	2.1
23623	1.41498	0.0750602
23624	1.41498	1.9291
23625	0.946131	1.8263
23626	0.850964	0.05
23627	0.921299	0.05
23628	1.37358	0.05
23629	0.946132	0.05
23630	0.850964	0.05
23631	0.823988	0.05
23632	0.823984	0.05
23633	1.36568	0.05
23634	1.36569	0.05
23635	0.921275	0.05
23636	1.84739	2.1
23637	1.47169	0.05
23638	0.946131	0.05
23639	0.735844	0.05
23640	1.11819	0.05
23641	1.10904	0.05
23642	0.735831	0.05
23643	0.946131	2.1
23644	0.946132	2.1
23645	1.23487	2.1
23646	1.3281	2.1
23647	0.946131	2.1
23648	1.72271	0.05
23649	0.861359	0.05
23650	0.946131	0.05
23651	0.861357	0.05
23652	1.1369	0.05
23653	1.26868	0.05
23654	0.789173	2.1
23655	0.789143	2.1
23656	0.946133	1.53833
23657	1.34386	2.06619
23658	0.789144	2.1
23659	0.983493	2.1
23660	1.18944	0.309183
23661	1.05121	0.262067

23662	0.777398	0.407862
23663	0.946132	0.571652
23664	0.777423	0.68653
23665	1.09841	0.05
23666	0.963919	0.05
23667	0.474855	0.0521097
23668	0.946131	0.05
23669	0.474855	0.05
23670	3.40074	1.16043
23671	1.29453	2.1
23672	1.01139	0.05
23673	0.946133	0.324393
23674	1.35381	0.05
23675	1.18322	0.0717118
23676	0.946133	0.221808
23677	1.45502	0.05
23678	0.970088	1.16262
23679	0.960333	1.44926
23680	0.838036	1.43393
23681	1.30927	0.05
23682	0.828617	0.05
23683	0.714795	0.05
23684	0.946132	0.05
23685	0.714824	0.05
23686	0.946131	2.1
23687	0.851542	2.1
23688	1.38204	2.1
23689	1.24512	2.1
23690	1.04086	2.1
23691	0.723573	2.1
23692	0.946131	2.1
23693	0.982454	2.1
23694	0.982454	2.1
23695	0.723544	2.1
23696	1.17261	0.05
23697	1.34153	0.0603371
23698	1.41383	0.0678126
23699	0.946132	0.05
23700	0.946133	0.05
23701	1.12818	0.0510284
23702	1.29383	0.05
23703	0.946133	0.0511679
23704	1.0027	0.05
23705	0.768989	0.05
23706	0.946133	0.0570269
23707	0.768977	0.0526096
23708	0.946132	0.056786
23709	1.24333	0.05
23710	0.928583	0.0588591
23711	0.946132	0.055568
23712	0.928583	0.0667677
23713	1.53798	0.05
23714	2.95436	0.05
23715	1.30649	0.05
23716	0.809789	0.05
23717	0.745744	0.05
23718	0.745774	0.05
23719	0.946128	0.05

23720	1.00777	0.05
23721	1.40271	0.05
23722	0.768387	0.05
23723	0.946132	0.05
23724	0.922045	0.05
23725	0.946132	2.1
23726	1.39021	2.1
23727	1.10046	2.1
23728	0.946132	2.1
23729	1.39021	2.1
23730	2.06158	0.05
23731	0.913967	0.210035
23732	1.30107	0.511104
23733	0.946132	0.219628
23734	0.913966	0.344129
23735	1.30107	0.201372
23736	1.64117	2.1
23737	0.514872	2.00304
23738	0.514846	0.907866
23739	1.30811	2.1
23740	1.30812	0.982187
23741	0.888989	1.43134
23742	1.99279	1.99004
23743	0.910271	0.705819
23744	0.838027	0.871222
23745	0.928444	0.689827
23746	1.2377	1.34675
23747	0.851537	2.1
23748	0.899142	2.1
23749	0.810075	2.1
23750	1.3771	2.1
23751	1.03281	2.1
23752	0.719877	2.1
23753	0.901838	2.1
23754	1.01748	2.1
23755	1.12315	2.1
23756	0.64787	2.1
23757	1.173 2.1	
23758	0.876903	2.1
23759	0.597337	2.1
23760	1.00291	2.1
23761	0.597309	2.1
23762	1.09897	0.05
23763	0.946132	0.359105
23764	1.1386	0.0523365
23765	1.09897	0.0679849
23766	0.596652	0.0998131
23767	2.5593	2.1
23768	0.946133	0.617695
23769	1.29172	0.422588
23770	1.11337	0.109592
23771	0.946131	0.236128
23772	0.946131	0.104184
23773	0.96926	0.574077
23774	0.946132	1.07594
23775	0.946132	1.19992
23776	1.07118	0.327132
23777	1.29698	0.390579

23778	1.37867	0.78219
23779	0.946119	1.58122
23780	0.94612	1.92475
23781	1.33154	0.822786
23782	0.946107	1.24883
23783	2.17757	1.29453
23784	0.946131	0.05
23785	0.68393	0.05
23786	0.68396	0.05
23787	1.33671	0.05
23788	1.10733	0.05
23789	2.31367	2.1
23790	0.989326	1.72287
23791	0.689952	2.1
23792	0.854787	1.73142
23793	3.73635	2.1
23794	1.41489	2.1
23795	1.25544	0.711814
23796	0.946128	2.1
23797	0.946129	2.1
23798	1.13536	2.1
23799	2.10284	1.26027
23800	0.851542	2.1
23801	0.828523	2.1
23802	0.946132	2.1
23803	0.946132	2.1
23804	1.50873	2.1
23805	0.989048	2.1
23806	0.925055	2.1
23807	0.838041	2.1
23808	0.807714	0.377372
23809	0.656831	0.055181
23810	0.997603	0.242532
23811	0.946131	0.862505
23812	1.02602	0.494152
23813	0.946129	1.02331
23814	1.35469	0.500012
23815	1.25083	0.48276
23816	0.851541	0.05
23817	0.851483	0.05
23818	0.851539	0.05
23819	1.19785	0.05
23820	1.07615	0.05
23821	0.714507	0.05
23822	1.41461	0.05
23823	0.714507	0.05
23824	0.714507	0.05
23825	0.946133	0.05
23826	0.946131	0.533322
23827	0.972232	0.448221
23828	0.94613	1.54917
23829	1.25083	0.294784
23830	1.30165	0.375582
23831	1.05332	2.1
23832	0.574768	2.1
23833	1.94541	0.05
23834	0.789633	0.05
23835	0.946127	0.05

23836	1.33798	0.05
23837	1.22903	0.05
23838	0.946128	0.05
23839	0.946128	2.1
23840	1.11882	2.1
23841	0.996487	2.1
23842	0.94613	2.1
23843	1.11883	2.1
23844	0.946127	2.1
23845	1.30082	2.1
23846	1.02608	2.1
23847	0.946128	2.1
23848	1.30081	2.1
23849	1.3507	1.61507
23850	1.19583	0.0952875
23851	0.946127	1.98849
23852	0.94613	1.23555
23853	1.19583	1.52151
23854	0.477652	0.05
23855	1.20416	0.05
23856	0.946131	0.05
23857	0.477652	0.05
23858	0.477691	0.05
23859	1.34536	0.05
23860	0.758971	0.05
23861	1.25124	0.05
23862	1.31467	0.05
23863	0.758971	0.05
23864	1.31467	0.05
23865	0.946131	2.1
23866	1.14184	2.1
23867	1.35028	2.1
23868	1.35028	2.1
23869	0.946131	2.1
23870	1.13537	2.1
23871	0.946132	1.00271
23872	0.946132	2.1
23873	1.23031	2.07489
23874	1.27555	2.05718
23875	1.53064	2.1
23876	1.28421	1.22004
23877	1.07707	1.07474
23878	0.946125	1.18666
23879	0.946127	1.23855
23880	1.07707	1.19773
23881	0.946133	0.05
23882	1.3495	0.05
23883	0.946132	0.05
23884	1.05704	0.05
23885	1.3495	0.05
23886	0.946132	0.05
23887	1.37794	0.05
23888	0.946132	0.05
23889	1.01367	0.05
23890	1.37794	0.05
23891	0.928516	2.1
23892	0.946133	2.1
23893	0.928518	2.1

23894	1.14105	2.1
23895	1.26406	2.1
23896	0.797664	2.1
23897	0.946132	2.1
23898	0.797663	2.1
23899	1.34285	2.1
23900	0.929875	2.1
23901	0.946131	1.40811
23902	1.21139	0.17628
23903	1.41492	0.0631511
23904	1.41492	0.4329
23905	0.946131	0.103383
23906	0.946131	2.1
23907	0.689388	2.1
23908	0.94365	2.1
23909	0.777345	2.1
23910	0.709599	2.1
23911	0.774968	2.1
23912	0.946132	2.1
23913	0.77497	2.1
23914	0.868145	2.1
23915	1.28401	2.1
23916	0.94613	0.168857
23917	1.19655	2.1
23918	1.39428	0.05
23919	1.39428	0.186859
23920	0.946131	0.0585501
23921	1.1848	2.1
23922	1.07591	2.1
23923	0.946131	2.1
23924	0.946131	2.1
23925	0.874788	2.1
23926	1.35703	2.1
23927	1.35357	2.1
23928	0.946132	2.1
23929	1.26022	2.1
23930	0.946132	2.1
23931	0.946131	2.1
23932	0.966426	2.1
23933	0.946133	2.1
23934	0.946131	2.1
23935	1.3484	2.1
23936	1.26441	2.1
23937	1.2079	2.1
23938	0.946133	2.1
23939	0.946132	2.1
23940	1.2079	2.1
23941	1.2736	2.1
23942	1.25774	2.1
23943	0.946133	2.1
23944	0.851555	2.1
23945	0.96172	2.1
23946	0.946128	2.1
23947	1.06879	2.1
23948	0.94613	2.1
23949	1.32608	2.1
23950	1.06879	2.1
23951	0.946132	2.1

23952	1.32608	2.1
23953	0.946131	2.1
23954	1.40488	2.1
23955	1.32608	2.1
23956	0.94613	0.901179
23957	1.19234	0.103673
23958	0.946129	0.249893
23959	0.94613	1.1352
23960	1.31649	1.33697
23961	0.912059	0.710943
23962	1.04514	0.677718
23963	0.946132	0.351545
23964	0.946132	1.9217
23965	1.34646	2.03557
23966	1.01187	0.209205
23967	1.33728	0.224855
23968	0.761974	0.05
23969	0.761974	0.388998
23970	0.94613	0.135928
23971	1.40641	2.06543
23972	0.955752	0.568078
23973	0.736738	2.03987
23974	0.94613	0.957249
23975	0.736739	1.09442
23976	0.861471	2.1
23977	1.34556	2.1
23978	0.810957	2.1
23979	0.946131	2.1
23980	1.37811	2.1
23981	1.17949	2.1
23982	0.946132	2.1
23983	1.37811	2.1
23984	0.946131	2.1
23985	1.40447	2.1
23986	0.946132	2.1
23987	0.946132	2.1
23988	1.39826	2.1
23989	0.946131	2.1
23990	1.283 2.1	
23991	0.94613	2.1
23992	0.946131	2.1
23993	1.40447	2.1
23994	1.20541	2.1
23995	0.946131	2.1
23996	0.946127	2.1
23997	1.33607	2.1
23998	1.41021	2.1
23999	1.01271	2.1
24000	0.946131	2.1
24001	0.920606	2.1
24002	1.13078	2.1
24003	1.60331	2.1
24004	0.946118	0.367067
24005	1.09718	2.1
24006	0.946116	1.31457
24007	1.32662	0.255914
24008	1.0972	1.20758
24009	0.94613	0.414758

24010	1.32666	1.11806
24011	0.946128	0.107658
24012	1.31381	0.126188
24013	1.32666	0.0763253
24014	0.94612	2.1
24015	0.940748	2.1
24016	1.03561	2.1
24017	1.35111	2.1
24018	0.851527	2.1
24019	0.607156	2.1
24020	0.789158	2.1
24021	0.946129	1.90226
24022	1.04071	2.1
24023	1.13144	2.1
24024	0.946133	2.1
24025	1.35663	2.1
24026	0.946132	2.1
24027	0.946133	2.1
24028	1.34237	2.1
24029	0.919455	2.1
24030	1.01143	2.1
24031	0.946128	2.1
24032	0.946131	2.1
24033	1.62796	2.1
24034	1.17796	1.3935
24035	1.23024	2.1
24036	0.946132	1.14629
24037	0.85153	1.96966
24038	0.986867	1.85383
24039	0.889308	2.1
24040	0.94613	2.1
24041	0.889338	2.1
24042	0.946131	2.1
24043	0.889337	2.1
24044	1.18396	1.18593
24045	0.94613	0.847763
24046	0.946125	1.83778
24047	1.39514	1.20776
24048	1.30809	1.49337
24049	0.803322	2.1
24050	0.925893	2.05096
24051	1.36183	1.50982
24052	0.925892	1.64458
24053	0.946133	1.65603
24054	1.38354	0.09028
24055	1.16009	0.161834
24056	0.946132	0.076288
24057	0.946132	0.0683743
24058	1.01462	0.0500469
24059	1.18854	0.779961
24060	0.888265	1.89538
24061	0.745095	1.07229
24062	0.709597	1.5944
24063	0.936541	1.44701
24064	1.24142	0.188834
24065	1.35059	0.116996
24066	0.946128	0.067264
24067	0.94613	0.163343

24068	1.16623	0.136445
24069	1.24945	0.05
24070	1.33035	0.05
24071	0.946127	0.05
24072	0.946128	0.05
24073	1.13343	0.0522215
24074	0.637995	0.963301
24075	1.10455	0.911139
24076	1.22641	1.67971
24077	1.22641	1.98218
24078	0.946132	1.51754
24079	1.3665	0.05
24080	1.28507	0.05
24081	0.946127	0.05
24082	0.946129	0.05
24083	1.2051	0.05
24084	0.797374	2.1
24085	0.797376	2.1
24086	1.13113	2.1
24087	1.20057	2.1
24088	1.12049	2.1
24089	0.59387	0.657661
24090	1.13245	0.523195
24091	0.966134	1.00495
24092	0.593842	0.324032
24093	0.946128	1.08015
24094	0.641455	0.153444
24095	0.916507	0.148315
24096	1.04072	1.13522
24097	0.415585	0.0942551
24098	0.507244	0.866659
24099	0.678938	2.1
24100	1.24154	2.1
24101	1.25	1.90299
24102	1.24154	2.09005
24103	1.25	2.1
24104	0.851009	1.54834
24105	0.946131	2.1
24106	0.851539	1.69543
24107	1.21997	1.418
24108	1.45109	1.37251
24109	0.804877	0.534989
24110	1.00062	0.0514029
24111	0.851536	0.370781
24112	0.909673	0.0570762
24113	0.946129	0.409602
24114	1.20494	0.05
24115	1.09088	0.05
24116	0.887634	0.05
24117	0.81027	0.05
24118	0.900294	0.05
24119	0.747713	0.05
24120	0.975467	0.05
24121	0.747712	0.05
24122	1.39168	0.05
24123	0.975467	0.05
24124	0.94613	0.0547063
24125	1.32827	0.05

24126	0.946128	0.05
24127	1.41201	0.05
24128	1.32827	0.05
24129	0.981121	0.05
24130	0.936311	0.05
24131	1.00715	0.05
24132	1.15914	0.05
24133	0.704409	0.05
24134	0.713444	0.454133
24135	0.954538	0.151731
24136	0.944508	0.189857
24137	0.851165	0.618462
24138	0.946131	1.45792
24139	0.851192	0.488532
24140	0.946131	0.431386
24141	1.3279	0.340115
24142	1.33587	0.05
24143	0.946126	0.05
24144	1.27512	0.05
24145	1.33587	0.05
24146	0.748059	0.05
24147	1.17451	0.05
24148	1.04299	0.05
24149	0.851542	0.05
24150	0.946131	0.05
24151	0.892109	0.05
24152	0.946131	0.05
24153	0.851541	0.05
24154	1.2178	0.05
24155	1.2179	0.05
24156	0.946793	0.05
24157	0.709599	0.05
24158	0.987426	0.05
24159	1.39502	0.05
24160	1.13624	0.05
24161	0.73176	0.05
24162	1.36697	0.05
24163	1.33393	0.05
24164	0.946131	0.05
24165	0.946132	0.05
24166	1.23845	0.05
24167	0.50702	0.674987
24168	1.06442	0.534936
24169	0.946131	0.955389
24170	1.02363	0.953132
24171	1.13537	0.05
24172	1.25049	0.05
24173	0.94613	0.05
24174	1.14993	0.05
24175	1.38056	0.05
24176	0.946126	0.05
24177	1.24678	0.05
24178	0.684521	0.05
24179	0.946124	0.05
24180	0.684518	0.05
24181	0.851542	0.220853
24182	0.925459	0.169554
24183	0.946131	0.251299

24184	0.851474	0.374146
24185	0.923305	0.320499
24186	1.4055	0.05
24187	1.32077	0.05
24188	0.946127	0.05
24189	1.21757	0.05
24190	0.946129	0.05
24191	0.711214	0.05
24192	1.30463	0.05
24193	0.711185	0.05
24194	0.711185	0.05
24195	1.22716	0.05
24196	1.29958	0.05
24197	1.26201	0.05
24198	0.568927	0.05
24199	0.568927	0.05
24200	0.946132	0.05
24201	0.851542	0.05
24202	1.44968	0.05
24203	0.851543	0.05
24204	1.35123	0.05
24205	1.40427	0.05
24206	0.593295	0.05
24207	0.946129	0.05
24208	1.22144	0.05
24209	0.908278	0.05
24210	1.22144	0.05
24211	0.867593	0.0520824
24212	1.23351	0.05
24213	0.946131	0.0539136
24214	1.05516	0.05
24215	1.23351	0.05
24216	1.15564	0.05
24217	0.946131	0.05
24218	0.766652	0.05
24219	0.766681	0.05
24220	0.946131	0.05
24221	0.946131	0.05
24222	0.897851	0.05
24223	1.1751	0.05
24224	1.00273	0.05
24225	0.851554	0.05
24226	0.883272	0.05
24227	1.17111	0.122649
24228	0.851541	0.05
24229	1.01665	0.05
24230	0.851555	0.05
24231	0.946131	0.062392
24232	0.911508	0.05
24233	1.08054	0.0660244
24234	1.33007	0.05
24235	0.911471	0.05
24236	1.26787	1.98595
24237	1.16641	1.73452
24238	0.946132	1.64177
24239	0.946133	1.34435
24240	1.16641	1.82673
24241	0.946131	2.1

24242	1.25037	0.05
24243	1.34733	2.1
24244	1.34733	0.154009
24245	0.946131	0.45371
24246	0.946131	0.05
24247	1.41377	0.05
24248	0.94613	0.05
24249	1.30541	0.05
24250	1.41377	0.05
24251	1.3473	2.1
24252	1.11804	2.1
24253	0.94613	2.1
24254	0.946131	2.1
24255	1.11805	2.1
24256	1.32068	2.1
24257	0.946131	2.1
24258	0.946128	2.1
24259	1.39384	2.1
24260	1.4181	2.1
24261	1.12913	2.1
24262	0.94613	2.1
24263	0.946129	2.1
24264	1.20328	2.1
24265	1.39385	2.1
24266	1.3335	2.1
24267	1.40987	2.1
24268	0.94613	2.1
24269	1.21474	2.1
24270	0.94613	2.1
24271	0.946132	2.1
24272	1.15203	2.1
24273	1.41192	2.1
24274	1.41192	2.1
24275	0.946132	2.1
24276	0.946131	2.1
24277	1.40684	2.1
24278	1.2597	2.1
24279	0.946131	2.1
24280	1.40684	2.1
24281	0.946131	2.1
24282	1.39113	2.1
24283	1.34049	1.64316
24284	0.946131	1.4881
24285	1.39113	1.37128
24286	0.946132	2.02259
24287	1.31649	0.538477
24288	0.946132	0.379795
24289	0.946132	1.07514
24290	1.34297	0.963838
24291	0.946131	0.924736
24292	1.41766	0.836612
24293	0.954559	1.78271
24294	0.946131	0.655767
24295	0.946131	0.257679
24296	1.25447	0.073402
24297	1.35559	0.0697756
24298	0.946127	0.05
24299	0.946128	0.0711528

24300	1.14874	0.0757359
24301	1.33035	0.05
24302	1.3665	0.05
24303	0.946129	0.05
24304	0.94613	0.05
24305	1.20732	0.05
24306	0.946131	2.1
24307	0.946131	2.1
24308	1.32949	2.1
24309	1.34921	2.1
24310	0.946131	2.1
24311	0.946132	1.02873
24312	1.25479	0.992929
24313	1.38357	0.516206
24314	1.38357	0.05
24315	0.946132	0.133568
24316	0.946131	1.98293
24317	1.31034	0.05
24318	1.37127	0.682954
24319	1.37127	0.05
24320	0.946131	0.137049
24321	1.23923	0.05
24322	1.41169	0.05
24323	0.94613	0.0570494
24324	1.12703	0.05
24325	0.946131	0.05
24326	0.946119	2.1
24327	1.41915	2.1
24328	1.02679	2.1
24329	0.946107	2.1
24330	1.41917	2.1
24331	0.946133	2.1
24332	1.36581	2.1
24333	0.946131	2.1
24334	1.36581	2.1
24335	0.946131	2.1
24336	0.946132	2.1
24337	1.04384	2.1
24338	1.34973	2.1
24339	1.34967	2.1
24340	0.851555	2.1
24341	0.946132	0.05
24342	1.3855	0.05
24343	1.08603	0.05
24344	1.08602	0.05
24345	1.16234	0.05
24346	0.946133	0.05
24347	1.29348	0.0658505
24348	1.20111	0.05
24349	1.20112	0.0543766
24350	1.3855	0.0569638
24351	0.946128	2.1
24352	1.30798	2.1
24353	0.946129	2.1
24354	1.34828	2.1
24355	1.30797	2.1
24356	1.24932	0.05
24357	1.40549	0.05

24358	0.946117	0.05
24359	1.19615	0.05
24360	0.946106	0.05
24361	0.946131	2.1
24362	1.29926	2.1
24363	1.25144	2.1
24364	0.946131	2.1
24365	1.29925	2.1
24366	0.946132	2.1
24367	1.39826	2.1
24368	0.946133	2.1
24369	0.946133	2.1
24370	1.29926	2.1
24371	0.946119	0.05
24372	1.33614	0.05
24373	0.946118	0.05
24374	1.41377	0.05
24375	1.33612	0.05
24376	0.946133	1.61654
24377	1.3933	2.1
24378	1.34999	0.547568
24379	0.946133	1.75362
24380	1.3933	1.05912
24381	0.946131	2.04603
24382	1.36139	2.1
24383	0.946132	1.10812
24384	0.946131	2.1
24385	1.39329	1.67191
24386	1.37449	1.34365
24387	1.27925	0.134972
24388	0.919796	0.163949
24389	1.01176	0.381232
24390	0.94613	0.958809
24391	0.946133	2.1
24392	1.27679	2.1
24393	0.946133	2.04329
24394	0.946132	2.1
24395	1.39731	2.1
24396	0.946119	2.1
24397	1.40765	1.95478
24398	1.1981	2.1
24399	0.946131	1.91694
24400	1.40764	2.1
24401	0.946127	2.1
24402	1.3557	2.1
24403	0.946127	2.1
24404	0.946128	2.1
24405	1.13485	2.1
24406	1.4008	2.1
24407	1.1587	2.1
24408	0.946131	2.1
24409	0.946131	2.1
24410	1.1587	2.1
24411	1.32082	0.05
24412	1.29215	0.05
24413	0.94613	0.05
24414	0.946131	0.05
24415	1.02366	0.05

24416	1.27697	0.0612691
24417	1.2929	0.0745915
24418	0.946132	0.0500836
24419	0.946132	0.05
24420	1.01451	0.05
24421	0.946132	2.1
24422	1.27252	2.1
24423	0.946132	2.1
24424	1.37869	2.1
24425	1.27252	2.1
24426	1.15627	2.1
24427	1.35703	2.1
24428	0.946132	2.1
24429	1.03783	2.1
24430	0.946132	2.1
24431	0.946128	2.1
24432	1.37869	2.1
24433	0.946131	2.1
24434	1.35357	2.1
24435	1.37869	2.1
24436	0.946132	2.1
24437	0.946131	2.1
24438	1.33471	2.1
24439	0.946131	2.1
24440	1.36373	2.1
24441	1.41342	0.56504
24442	1.06832	0.774891
24443	0.946128	1.65699
24444	0.94613	0.20037
24445	1.06832	1.27295
24446	1.26929	2.1
24447	0.946131	1.86419
24448	0.946128	2.1
24449	1.41022	2.1
24450	1.37034	2.1
24451	1.37759	0.954723
24452	1.07612	1.26549
24453	0.946126	1.03009
24454	0.946129	1.24122
24455	1.07612	1.09245
24456	0.946131	0.727408
24457	0.94613	0.05
24458	1.23848	0.050644
24459	1.38897	0.05
24460	0.946131	0.05
24461	0.946132	0.20025
24462	0.946133	0.05
24463	1.34369	0.05
24464	1.40332	0.05
24465	0.946132	0.05
24466	0.946131	2.1
24467	1.24023	2.1
24468	1.38244	2.1
24469	1.38244	2.1
24470	0.946131	2.1
24471	0.946132	2.1
24472	1.33468	2.1
24473	1.41619	2.1

24474	1.41619	2.1
24475	0.946131	2.1
24476	1.1908	1.22479
24477	0.946128	0.980192
24478	0.946126	1.65285
24479	1.26654	1.65857
24480	1.39514	2.02214
24481	0.93608	2.1
24482	1.04105	2.1
24483	1.39551	2.1
24484	1.52471	2.1
24485	1.04072	2.1
24486	1.33174	0.05
24487	0.946128	0.05
24488	0.94613	0.05
24489	1.17801	0.05
24490	0.94613	0.05
24491	0.946132	2.1
24492	0.946132	2.1
24493	1.29065	2.1
24494	1.29065	2.1
24495	0.975266	2.1
24496	0.946131	2.1
24497	1.26025	2.1
24498	0.955601	2.1
24499	0.946131	2.1
24500	1.26025	2.1
24501	0.94613	0.05
24502	1.23146	0.05
24503	0.946127	0.05
24504	1.32827	0.05
24505	1.23146	0.05
24506	1.25008	0.387408
24507	1.05018	0.409969
24508	0.94613	0.277184
24509	0.932744	0.670951
24510	1.04207	0.443025
24511	0.946132	0.112807
24512	1.10023	0.837212
24513	1.36748	0.0606383
24514	1.36748	0.319875
24515	0.946133	0.246618
24516	1.06023	0.05
24517	1.08019	0.05
24518	0.94613	0.0502763
24519	1.06023	0.05
24520	0.94613	0.05
24521	1.16727	0.0690693
24522	1.35856	0.0827344
24523	0.946132	0.156195
24524	1.16727	0.0866288
24525	0.946132	0.078973
24526	0.946133	2.1
24527	0.946131	2.1
24528	1.21691	2.1
24529	1.39558	2.1
24530	0.946131	2.1
24531	1.09905	0.05

24532	0.946132	0.119482
24533	1.3365	0.05
24534	1.35381	0.05
24535	0.946132	0.144462
24536	0.94613	0.05
24537	0.946128	0.05
24538	1.40332	0.05
24539	1.39041	0.05
24540	0.94613	0.05
24541	0.946132	0.166464
24542	1.3659	2.1
24543	1.39041	0.362128
24544	1.39041	1.54419
24545	0.946132	1.48545
24546	0.840292	2.1
24547	0.946133	1.93087
24548	0.851541	2.1
24549	1.28464	2.04564
24550	1.28806	2.00179
24551	1.12591	0.587824
24552	1.05033	0.783991
24553	0.946132	0.665005
24554	0.946132	0.959735
24555	1.13537	0.848916
24556	1.21177	0.998056
24557	0.946132	1.24369
24558	0.946132	2.1
24559	1.41134	1.20801
24560	1.27079	1.46309
24561	0.946131	0.05
24562	1.35403	0.05
24563	0.94613	0.05
24564	0.94613	0.05
24565	0.946131	0.05
24566	0.946131	0.05
24567	0.946132	0.05
24568	0.94613	0.05
24569	1.37052	0.05
24570	0.946131	0.05
24571	0.946133	0.05
24572	0.946132	0.05
24573	0.946133	0.05
24574	1.24249	0.05
24575	0.946132	0.05
24576	1.20608	2.1
24577	0.946132	2.1
24578	0.946131	2.1
24579	0.946132	2.1
24580	0.946132	2.1
24581	0.946131	2.1
24582	0.946128	2.1
24583	0.946129	2.1
24584	1.41232	2.1
24585	0.94613	2.1
24586	0.946132	0.05
24587	1.32449	0.05
24588	0.946131	0.05
24589	0.946131	0.05

24590	1.32449	0.05
24591	0.946116	1.69566
24592	1.41563	2.1
24593	0.946118	2.1
24594	0.946105	2.1
24595	0.946131	1.92779
24596	1.26563	2.1
24597	1.32819	2.1
24598	0.946131	0.111679
24599	0.946131	2.1
24600	0.946131	1.03007
24601	0.946131	2.06083
24602	0.946133	1.62281
24603	1.30382	2.1
24604	0.946131	1.42779
24605	0.946131	1.3533
24606	1.30382	1.95876
24607	0.946133	0.05
24608	1.15803	0.05
24609	1.28895	0.05
24610	0.954071	0.05
24611	0.946132	0.05
24612	1.0264	0.134821
24613	0.946132	1.563
24614	0.946132	0.693976
24615	0.946131	1.09065
24616	0.946132	0.747265
24617	1.35977	0.05
24618	0.946132	0.0661699
24619	0.946129	0.05
24620	0.94613	0.05
24621	0.946129	0.05
24622	0.876631	0.187645
24623	0.946133	0.386891
24624	0.946132	0.929634
24625	0.946132	1.36757
24626	0.876606	1.04627
24627	0.946131	0.05
24628	1.24979	0.05
24629	0.94613	0.05
24630	0.94613	0.05
24631	0.94613	0.05
24632	0.921791	0.05
24633	0.946131	0.05
24634	0.921762	0.05
24635	0.946131	0.05
24636	0.946131	0.05
24637	0.810944	0.05
24638	0.810945	0.05
24639	0.810959	0.05
24640	0.946133	0.05
24641	1.25797	0.05
24642	0.946132	0.05
24643	0.946132	0.05
24644	1.25797	0.05
24645	1.13748	0.0670752
24646	0.851512	0.229659
24647	0.94613	0.753083

24648	1.02373	0.0662303
24649	0.946131	0.407552
24650	0.946122	2.1
24651	1.3852	2.1
24652	0.946124	2.1
24653	0.946118	2.1
24654	1.38521	2.1
24655	0.946131	0.516409
24656	0.946131	1.15891
24657	1.30448	0.857122
24658	0.946129	0.591998
24659	0.94613	0.539197
24660	0.946133	2.1
24661	0.94613	2.1
24662	0.946131	2.1
24663	0.946131	2.1
24664	1.36503	2.1
24665	0.810947	0.05
24666	0.810947	0.05
24667	0.810934	0.05
24668	3.34066	0.05
24669	0.946133	0.333031
24670	1.4185	0.219509
24671	0.946132	0.131117
24672	0.946133	0.14145
24673	0.946132	0.208993
24674	0.94613	2.1
24675	0.946128	2.1
24676	1.03925	2.1
24677	0.944743	2.1
24678	0.947518	2.1
24679	0.946132	2.1
24680	1.20573	2.1
24681	0.94613	2.1
24682	0.94613	2.1
24683	1.20573	2.1
24684	1.20056	0.05
24685	0.946132	0.05
24686	0.946131	0.05
24687	0.946131	0.05
24688	0.946131	0.05
24689	0.94613	2.1
24690	0.946128	2.1
24691	1.38302	2.1
24692	0.94613	2.1
24693	0.94613	2.1
24694	0.827866	0.05
24695	0.913552	0.05
24696	0.716526	0.05
24697	0.82784	0.05
24698	1.20634	0.05
24699	0.946131	0.05
24700	0.94613	0.05
24701	1.39502	0.05
24702	0.88146	0.05
24703	0.946132	2.1
24704	0.946132	2.1
24705	0.946132	2.1

24706	0.946132	2.1
24707	1.39053	2.1
24708	0.946131	2.1
24709	1.40571	2.1
24710	0.946131	2.1
24711	0.946131	2.1
24712	0.946132	2.1
24713	0.94613	0.05
24714	1.2582	0.0739879
24715	0.946127	0.05
24716	0.946129	0.0643305
24717	0.946129	0.05
24718	0.946129	1.03562
24719	0.946132	0.998679
24720	0.946131	0.524563
24721	0.946131	1.12321
24722	1.29054	0.587595
24723	0.810944	0.05
24724	0.810941	0.05
24725	0.810931	0.05
24726	0.946132	0.523837
24727	0.946132	0.363784
24728	0.946132	1.38214
24729	0.946132	1.08797
24730	0.927608	0.60142
24731	0.946128	2.1
24732	0.946133	2.1
24733	0.946131	2.1
24734	0.94613	2.1
24735	1.38813	2.1
24736	0.94613	0.664314
24737	0.946128	2.1
24738	0.946132	0.59848
24739	1.41666	2.1
24740	0.94613	2.1
24741	0.946118	0.05
24742	1.34151	0.05
24743	0.946116	0.05
24744	0.946131	0.05
24745	1.34149	0.05
24746	0.946132	2.1
24747	1.31889	2.1
24748	0.946132	2.1
24749	0.946132	2.1
24750	1.31889	2.1
24751	0.864997	0.05
24752	0.928981	0.05
24753	0.872219	0.05
24754	0.946127	0.0913762
24755	0.946131	0.055552
24756	1.37709	0.05
24757	0.946131	0.0891354
24758	0.946129	0.0615703
24759	1.21882	0.05
24760	0.938168	0.05
24761	0.938161	0.05
24762	1.06327	0.05
24763	1.33457	0.05

24764	0.946133	0.924397
24765	0.946132	0.76468
24766	0.946133	0.474563
24767	0.946132	1.19694
24768	1.14444	0.418974
24769	1.12628	0.05
24770	0.946132	0.05
24771	0.946131	0.05
24772	1.12628	0.05
24773	0.946131	0.05
24774	0.946132	2.1
24775	0.946132	2.1
24776	0.946133	2.1
24777	0.946132	2.1
24778	1.29322	2.1
24779	0.946131	2.1
24780	0.946132	2.1
24781	0.94613	2.1
24782	0.946131	2.1
24783	1.28441	2.1
24784	0.946118	2.1
24785	0.946119	2.1
24786	0.946117	2.1
24787	1.28075	2.1
24788	0.946105	2.1
24789	0.810945	2.1
24790	0.810942	2.1
24791	0.81093	2.1
24792	0.946127	2.1
24793	0.946127	2.1
24794	1.28228	2.1
24795	0.946129	2.1
24796	0.946132	2.1
24797	1.14042	2.1
24798	0.946132	2.1
24799	0.946133	2.1
24800	1.14042	2.1
24801	0.946133	0.05
24802	0.946132	0.05
24803	1.28632	0.05
24804	1.28632	0.05
24805	0.946133	0.05
24806	0.946131	1.52492
24807	0.946131	1.12149
24808	0.946131	1.36985
24809	1.31817	1.54814
24810	0.946131	1.11732
24811	1.35048	2.1
24812	0.946132	2.1
24813	0.946132	2.1
24814	0.946132	2.1
24815	0.946131	2.1
24816	0.946131	2.1
24817	1.35943	2.1
24818	0.94613	2.1
24819	0.946132	2.1
24820	0.946131	2.1
24821	0.990713	2.1

24822	0.946132	2.1
24823	0.946132	2.1
24824	0.946133	2.1
24825	0.946132	2.1
24826	0.946133	1.40436
24827	1.01433	1.28245
24828	0.877931	1.57479
24829	0.96573	0.779225
24830	0.946131	0.845015
24831	0.946131	1.07954
24832	0.946133	0.660773
24833	1.39575	0.178573
24834	0.946131	0.271373
24835	0.94613	0.255111
24836	0.810943	0.101089
24837	0.810942	0.112545
24838	0.709589	0.0754803
24839	0.924071	0.0574967
24840	1.04293	0.05
24841	1.15747	0.05
24842	0.946131	0.059211
24843	1.04071	0.05
24844	0.810946	0.103257
24845	0.810943	0.0641646
24846	0.810959	0.0636745
24847	0.946128	1.37664
24848	1.39696	1.95028
24849	0.94613	0.494792
24850	0.94613	0.457253
24851	1.39696	1.53461
24852	0.946131	0.393411
24853	0.946131	1.92641
24854	0.946131	1.65466
24855	0.946131	0.992629
24856	1.29735	1.8878
24857	0.946128	1.39615
24858	0.946133	0.0883975
24859	0.94613	2.1
24860	0.946131	1.29991
24861	1.33266	0.310642
24862	0.946131	2.1
24863	0.94613	0.05
24864	0.946131	2.1
24865	1.40609	1.21648
24866	0.946131	1.96385
24867	0.946131	1.99921
24868	0.946133	2.04712
24869	0.946129	2.09795
24870	1.23339	2.1
24871	0.946131	1.89116
24872	0.946132	2.1
24873	0.946133	2.1
24874	0.946132	2.1
24875	1.29198	2.1
24876	0.946132	2.1
24877	0.946131	2.1
24878	1.07297	2.1
24879	0.946127	2.1

24880	0.94613	2.1
24881	1.07297	2.1
24882	0.946125	2.1
24883	0.946126	2.1
24884	0.946126	2.1
24885	1.3864	2.1
24886	0.946119	2.1
24887	0.946128	0.05
24888	1.09411	0.05
24889	0.946131	0.0579415
24890	0.946131	0.05
24891	1.09411	0.05
24892	0.946131	0.05
24893	1.25837	0.05
24894	0.946131	0.05
24895	0.946131	0.05
24896	1.25837	0.05
24897	0.810945	2.1
24898	0.810943	2.1
24899	0.810933	2.1
24900	0.94613	0.05
24901	0.946131	0.146404
24902	0.946132	0.05
24903	0.946129	0.05
24904	0.946131	0.0610497
24905	0.946131	0.05
24906	1.28911	0.05
24907	0.946129	0.05
24908	0.946133	0.05
24909	0.946131	0.05
24910	0.94613	0.0520431
24911	1.38954	0.0784765
24912	0.946132	0.05
24913	0.94613	0.0676589
24914	0.946131	0.0542475
24915	0.946131	0.586043
24916	0.946131	0.168582
24917	0.946131	0.110098
24918	1.37981	0.122372
24919	0.946131	0.125362
24920	0.946131	2.1
24921	1.26958	2.1
24922	0.946127	2.1
24923	0.946129	2.1
24924	1.26957	2.1
24925	0.94613	2.1
24926	1.16889	2.1
24927	0.946126	2.1
24928	0.946128	2.1
24929	1.16889	2.1
24930	0.946133	0.0672855
24931	1.13511	0.05
24932	0.946132	0.0849856
24933	0.946133	0.05
24934	0.946132	0.0541064
24935	1.27575	0.963702
24936	0.946129	2.1
24937	0.946131	1.84791

24938	0.94613	2.1
24939	0.946131	2.03552
24940	0.946131	2.1
24941	0.946128	2.1
24942	1.41712	2.1
24943	1.41712	2.1
24944	0.94613	2.1
24945	0.946129	0.05
24946	0.946132	0.05
24947	0.946131	0.05
24948	0.946131	0.05
24949	0.946131	0.05
24950	0.979045	2.06451
24951	0.946131	0.973777
24952	0.617549	2.1
24953	0.555844	2.1
24954	1.04071	0.910553
24955	0.946132	1.89532
24956	0.946132	1.33459
24957	0.946131	2.1
24958	1.22618	1.12336
24959	0.946132	0.500151
24960	0.946131	2.1
24961	1.13976	2.1
24962	0.946129	2.1
24963	0.946131	2.1
24964	1.13976	2.1
24965	0.810945	0.273732
24966	0.810943	0.0816565
24967	0.810932	0.510715
24968	1.25358	0.809411
24969	0.94613	0.138069
24970	0.946128	0.112879
24971	1.25358	1.92159
24972	1.07811	0.333404
24973	0.946129	2.1
24974	1.09197	2.1
24975	0.946131	2.1
24976	0.946131	2.1
24977	1.09197	2.1
24978	0.594912	2.1
24979	0.993703	2.1
24980	1.10173	2.1
24981	0.9916	2.1
24982	0.946131	2.1
24983	0.946128	2.1
24984	0.946131	2.1
24985	0.94613	2.1
24986	0.946129	2.1
24987	0.946132	2.1
24988	1.40335	2.1
24989	0.946133	2.1
24990	0.946133	2.1
24991	1.40335	2.1
24992	0.946132	0.934421
24993	0.946132	2.1
24994	0.946132	1.12831
24995	0.946132	1.3347

24996	1.411	1.9443
24997	0.611675	0.05
24998	0.946129	0.05
24999	1.31678	0.05
25000	0.946124	0.05
25001	0.946127	0.05
25002	0.946128	0.05
25003	0.946132	0.0592615
25004	0.946131	0.05
25005	0.946132	0.0606591
25006	0.946132	0.05
25007	0.946132	0.05
25008	0.810946	0.05
25009	0.810943	0.05
25010	0.81096	0.05
25011	1.39301	0.05
25012	0.946132	0.05
25013	0.625812	0.05
25014	0.946132	0.05
25015	0.625812	0.05
25016	0.810946	1.48219
25017	0.810945	2.09685
25018	0.810931	2.1
25019	0.810943	2.1
25020	0.810944	2.1
25021	0.810958	2.1
25022	0.946131	0.0533703
25023	0.946131	0.05
25024	0.946128	0.0581168
25025	0.946129	0.05
25026	0.94613	0.05
25027	0.946133	0.151115
25028	0.946133	0.313255
25029	0.98537	0.05
25030	0.985371	0.05
25031	0.946132	0.423528
25032	0.946129	0.05647
25033	1.38752	0.05
25034	0.946131	0.05
25035	0.946131	0.05
25036	1.38752	0.0534962
25037	0.946119	0.0528774
25038	1.33036	0.05
25039	0.946119	0.05
25040	0.946132	0.05
25041	1.33035	0.05
25042	1.3868	2.1
25043	0.946132	2.1
25044	0.946132	2.1
25045	0.946133	2.1
25046	0.946132	2.1
25047	0.810947	2.1
25048	0.810946	2.1
25049	0.81096	2.1
25050	0.810946	2.1
25051	0.810946	2.1
25052	0.810933	2.1
25053	0.946133	2.1

25054	1.35342	2.1
25055	0.946133	2.1
25056	0.946132	2.1
25057	1.35342	2.1
25058	0.946132	2.1
25059	0.946132	2.1
25060	1.28569	2.1
25061	0.946132	2.1
25062	0.946133	2.1
25063	0.946132	2.1
25064	1.32547	2.1
25065	0.946132	2.1
25066	0.946132	2.1
25067	1.32547	2.1
25068	0.94613	0.05
25069	0.946133	0.05
25070	1.39664	0.05
25071	1.39664	0.05
25072	0.946131	0.05
25073	0.946131	1.51771
25074	0.94613	2.1
25075	0.946132	0.673055
25076	0.946131	2.1
25077	1.41833	2.033
25078	0.946131	0.05
25079	0.946131	0.05
25080	0.946131	0.05
25081	1.26155	0.05
25082	0.946132	0.05
25083	0.810946	2.1
25084	0.810946	2.1
25085	0.810959	2.1
25086	0.94613	1.10238
25087	0.946132	0.392155
25088	1.40779	0.116995
25089	1.40779	0.236575
25090	0.94613	0.303686
25091	0.94613	0.05
25092	0.946129	0.05
25093	1.36376	0.05
25094	1.36376	0.05
25095	0.946131	0.05
25096	0.946128	2.1
25097	0.946127	2.1
25098	0.946131	2.1
25099	1.40756	2.1
25100	0.94613	2.1
25101	0.946131	2.00244
25102	0.946132	2.1
25103	0.94613	1.99934
25104	0.946131	2.06708
25105	1.31947	2.1
25106	0.946131	2.1
25107	0.946131	2.1
25108	0.946129	2.1
25109	0.946131	2.1
25110	1.25768	2.1
25111	0.946132	0.104121

25112	0.94613	0.104303
25113	0.946131	0.0744716
25114	0.946131	0.0765768
25115	0.946131	0.0909117
25116	0.810943	0.335
25117	0.810942	1.16212
25118	0.81093	2.04441
25119	0.946128	0.967458
25120	0.946133	0.413972
25121	0.946131	1.15278
25122	0.946131	0.792558
25123	0.94613	0.97199
25124	0.946132	1.01951
25125	0.946131	0.409198
25126	0.946132	1.1134
25127	0.946132	0.842052
25128	0.946132	1.04564
25129	0.946119	2.1
25130	0.946119	2.1
25131	1.31523	2.1
25132	0.946132	2.1
25133	0.946107	2.1
25134	0.946133	2.1
25135	0.946132	0.430687
25136	0.946132	0.422807
25137	1.28803	2.1
25138	0.946132	2.1
25139	1.37658	1.20045
25140	0.946132	1.01148
25141	0.946133	1.55368
25142	0.946132	1.26366
25143	0.946132	1.41215
25144	0.946132	1.17371
25145	0.94613	1.13253
25146	0.94613	1.69088
25147	1.15541	0.927031
25148	0.92943	1.45851
25149	0.946126	0.05
25150	0.946129	0.05
25151	1.33849	0.05
25152	1.33849	0.05
25153	0.946129	0.05
25154	0.810946	0.05
25155	0.810944	0.05
25156	0.810959	0.05
25157	1.39027	0.05
25158	0.946131	0.05
25159	0.946131	0.05
25160	1.39027	0.05
25161	0.946131	0.05
25162	1.40924	2.06373
25163	0.946131	1.36951
25164	0.946132	2.06944
25165	0.946132	1.15521
25166	0.946132	1.77415
25167	0.946131	2.1
25168	0.94613	2.1
25169	0.946128	2.1

25170	0.946128	2.1
25171	1.41744	2.1
25172	0.946132	0.309143
25173	0.946128	0.284071
25174	0.94613	1.01671
25175	1.37675	0.334712
25176	0.94613	1.08499
25177	0.946132	0.764345
25178	0.946132	0.156137
25179	0.946132	0.0845468
25180	0.946132	0.152918
25181	0.946131	0.125144
25182	0.946132	0.820494
25183	0.946132	0.298189
25184	0.946133	1.3325
25185	1.10003	0.142283
25186	0.946132	1.00077
25187	0.946132	0.05
25188	0.946131	0.05
25189	0.946132	0.05
25190	0.946131	0.05
25191	0.946132	0.05
25192	0.946132	0.05
25193	0.946131	0.05
25194	0.946132	0.05
25195	0.946132	0.05
25196	0.946131	0.05
25197	1.24094	0.05
25198	0.946126	0.05
25199	0.946128	0.05
25200	0.946129	0.05
25201	0.946129	0.05
25202	1.2929	0.05
25203	0.946131	0.05
25204	0.946131	0.05
25205	0.946131	0.05
25206	0.946131	0.05
25207	0.810946	0.05
25208	0.810945	0.05
25209	0.810959	0.05
25210	0.810946	1.99567
25211	0.810943	2.03605
25212	0.810933	2.1
25213	0.946128	2.1
25214	1.18315	2.1
25215	0.94613	2.1
25216	0.94613	2.1
25217	1.18316	2.1
25218	0.946126	0.05
25219	1.3324	0.05
25220	0.946127	0.05
25221	0.94613	0.05
25222	0.946128	0.05
25223	0.94613	2.1
25224	0.946128	2.1
25225	1.39523	2.1
25226	1.39523	2.1
25227	0.946131	2.1

25228	1.36541	2.1
25229	0.946132	2.1
25230	0.946132	2.1
25231	0.946132	2.1
25232	0.946132	2.1
25233	0.946131	2.1
25234	0.94613	0.797386
25235	0.946132	1.67685
25236	0.946131	0.0742197
25237	1.39509	1.60166
25238	0.946132	2.1
25239	0.946132	2.1
25240	1.29319	2.1
25241	0.946132	2.1
25242	0.946133	2.1
25243	0.946132	0.05
25244	0.946131	0.05
25245	0.946132	0.05
25246	1.01196	0.05
25247	0.946132	0.05
25248	0.946116	2.1
25249	0.946117	2.1
25250	1.38153	2.1
25251	1.38155	2.1
25252	0.946105	2.1
25253	0.946132	1.87572
25254	0.946131	1.95977
25255	0.946132	1.73108
25256	1.28684	1.73224
25257	0.946132	1.42256
25258	1.10185	1.61666
25259	0.94613	1.96661
25260	0.946128	2.1
25261	0.94613	2.1
25262	0.94613	1.94416
25263	0.810947	0.05
25264	0.810945	0.05
25265	0.810932	0.05
25266	0.946119	0.0548524
25267	1.16717	0.05
25268	0.946117	0.0569624
25269	0.94613	0.05
25270	1.16715	0.05
25271	0.94612	0.11781
25272	0.946119	0.0668386
25273	0.946119	0.0840459
25274	0.946132	0.05
25275	1.20882	0.05
25276	1.07043	0.149971
25277	0.823268	0.108426
25278	0.810959	0.129277
25279	0.946132	1.65531
25280	1.14879	0.433978
25281	0.94613	1.37381
25282	0.946131	1.64212
25283	1.14879	0.754293
25284	1.23837	2.1
25285	0.94612	2.1

25286	0.946118	2.1
25287	0.946131	2.1
25288	0.946105	2.1
25289	0.810946	0.05
25290	0.810946	0.05
25291	0.810959	0.05
25292	0.810947	1.30982
25293	0.810947	0.05
25294	0.810934	0.05
25295	0.946128	2.1
25296	0.946131	2.1
25297	0.946128	2.1
25298	0.946129	2.1
25299	0.94613	2.1
25300	0.94613	2.1
25301	0.946131	2.1
25302	0.946131	2.1
25303	0.946131	2.1
25304	0.946131	2.1
25305	0.946131	1.91503
25306	1.38585	2.1
25307	0.946128	1.46943
25308	0.94613	1.89763
25309	1.38585	1.94327
25310	0.946133	0.05
25311	0.946133	0.05
25312	0.946132	0.05
25313	0.946132	0.05
25314	0.946132	0.05
25315	0.918871	0.05
25316	0.810248	0.05
25317	0.838037	0.05
25318	0.946126	0.05
25319	0.943647	0.05
25320	1.13836	0.05
25321	1.03802	0.05
25322	0.946129	0.05
25323	0.946133	0.893199
25324	0.946132	0.606153
25325	0.946132	2.1
25326	1.1699	0.329573
25327	0.946132	0.368124
25328	0.946131	2.1
25329	0.946132	2.1
25330	1.37199	2.1
25331	1.37199	2.1
25332	0.946132	2.1
25333	0.946132	0.7463
25334	0.946133	0.30567
25335	0.946133	1.1164
25336	0.946132	0.445416
25337	1.38623	0.301176
25338	0.946132	2.1
25339	0.946132	2.1
25340	0.946132	2.1
25341	0.946132	2.1
25342	1.40098	2.1
25343	1.20787	0.158004

25344	1.41894	0.225697
25345	0.946133	0.05
25346	1.01777	0.339911
25347	0.946132	2.1
25348	0.946132	2.1
25349	0.946133	2.1
25350	1.40199	2.1
25351	0.946132	2.1
25352	0.810946	1.07223
25353	0.810947	1.88418
25354	0.810934	1.28355
25355	0.946126	0.656691
25356	0.94613	1.50199
25357	0.946125	1.44623
25358	1.40247	0.980386
25359	0.946128	1.00264
25360	1.25703	1.58587
25361	0.87821	0.955841
25362	0.878238	0.630044
25363	0.946119	1.97766
25364	0.946132	2.1
25365	0.946132	0.178544
25366	0.946132	0.273788
25367	0.946131	1.63908
25368	0.946132	0.501519
25369	1.28543	0.518286
25370	0.946115	2.1
25371	1.41638	2.1
25372	0.946116	2.1
25373	0.946104	2.1
25374	0.946129	2.1
25375	0.946132	2.1
25376	1.41432	2.1
25377	0.946132	2.1
25378	0.946132	2.1
25379	0.946132	2.1
25380	0.810947	0.05
25381	0.810947	0.05
25382	0.709567	0.05
25383	0.851542	0.05
25384	1.01217	0.05
25385	0.697993	0.05
25386	0.946133	0.05
25387	0.851488	0.05
25388	0.946129	2.1
25389	0.94613	2.1
25390	1.2254	1.89679
25391	0.946131	2.1
25392	0.946131	2.1
25393	0.700785	2.1
25394	0.915893	1.94958
25395	0.838013	1.66595
25396	0.760188	0.855266
25397	1.19601	0.05
25398	0.946133	0.0744994
25399	0.946132	0.0969667
25400	0.946132	0.05
25401	0.946132	0.080484

25402	0.946132	0.787098
25403	0.946133	2.1
25404	0.946132	1.50315
25405	0.946133	2.1
25406	1.36956	2.1
25407	0.946131	0.465368
25408	0.94613	0.05
25409	0.946133	0.244857
25410	1.34268	0.05
25411	0.946131	0.05
25412	0.946132	2.1
25413	1.25603	2.1
25414	0.946132	2.1
25415	0.946132	2.1
25416	1.25603	2.1
25417	0.810946	0.935305
25418	0.810943	0.958229
25419	0.810933	0.79105
25420	0.496545	0.05
25421	0.805767	0.448955
25422	0.936671	0.249915
25423	0.530063	0.189956
25424	1.05954	0.227286
25425	0.810946	0.05
25426	0.810943	0.05
25427	0.810959	0.05
25428	1.16699	0.05
25429	0.946131	0.05
25430	0.946129	0.05
25431	0.946131	0.05
25432	0.946131	0.05
25433	0.810943	1.43229
25434	0.810942	1.69227
25435	0.810933	1.50113
25436	0.946132	1.42685
25437	1.04832	1.28893
25438	0.870714	1.53774
25439	0.957819	1.9482
25440	0.94613	0.946179
25441	1.82931	2.1
25442	0.946132	0.934148
25443	0.946129	0.750352
25444	0.94613	1.13792
25445	0.946131	0.974274
25446	0.94613	0.681547
25447	0.946119	0.97223
25448	0.94612	1.10858
25449	0.94612	0.562145
25450	0.946132	0.994623
25451	1.36118	0.715784
25452	0.810947	0.05
25453	0.810947	0.0508686
25454	0.810934	0.05
25455	0.946132	2.1
25456	0.946132	2.1
25457	0.946132	2.1
25458	1.39754	2.1
25459	0.946131	2.1

25460	0.946129	2.1
25461	0.946133	2.1
25462	0.946131	2.1
25463	0.946131	2.1
25464	1.41732	2.1
25465	0.946132	0.738999
25466	0.946128	1.94028
25467	0.94613	1.06817
25468	0.94613	1.25628
25469	1.25277	1.57566
25470	0.94613	1.42926
25471	0.946128	2.01653
25472	1.21261	0.438441
25473	0.94613	1.4529
25474	0.94613	0.669065
25475	1.39407	2.1
25476	0.946133	2.1
25477	0.94613	2.1
25478	0.946131	2.1
25479	0.946131	2.1
25480	0.946119	1.36544
25481	0.946119	1.6297
25482	0.946119	1.82236
25483	1.1537	1.57745
25484	0.946106	0.819061
25485	0.946131	2.1
25486	1.11822	2.1
25487	0.946129	2.1
25488	0.946131	2.1
25489	1.11822	2.1
25490	1.46806	0.05
25491	0.498881	0.05
25492	0.996658	0.05
25493	1.2179	0.05
25494	0.991519	0.05
25495	0.946131	0.05
25496	0.946131	0.05
25497	1.28673	0.05
25498	0.946131	0.05
25499	0.946131	0.05
25500	1.33178	0.05
25501	0.946132	0.05
25502	0.946132	0.05
25503	0.946132	0.05
25504	0.946132	0.05
25505	0.946128	0.0523382
25506	0.972061	0.05
25507	0.946129	0.0873964
25508	0.946129	0.0751609
25509	0.972059	0.0674186
25510	0.946129	2.1
25511	0.94613	2.1
25512	0.94613	2.1
25513	0.94613	2.1
25514	0.94613	2.1
25515	0.946131	2.1
25516	0.946127	2.1
25517	0.946131	2.1

25518	0.946129	2.1
25519	1.4073	2.1
25520	0.946131	0.05
25521	1.05016	0.05
25522	0.946132	0.05
25523	0.946131	0.05
25524	0.946132	0.05
25525	0.946128	0.05
25526	1.13398	0.05
25527	0.946131	0.05
25528	0.946131	0.05
25529	0.94613	0.05
25530	0.946131	0.05
25531	0.946131	0.05
25532	1.38911	0.05
25533	1.38911	0.05
25534	0.946132	0.05
25535	0.946131	0.05
25536	0.94613	0.05
25537	0.946131	0.05
25538	0.946131	0.05
25539	0.946131	0.05
25540	0.946131	0.05
25541	1.23923	0.05
25542	0.94613	0.05
25543	0.946131	0.05
25544	0.946131	0.05
25545	1.40511	0.05
25546	0.946131	0.05
25547	0.946131	0.05
25548	0.946132	0.05
25549	0.946131	0.05
25550	1.12968	2.1
25551	0.946132	2.1
25552	0.946132	2.1
25553	0.946131	2.1
25554	0.946132	2.1
25555	0.946132	1.70107
25556	1.4172	2.1
25557	0.94613	1.38539
25558	0.94613	1.90588
25559	1.4172	2.1
25560	0.946132	2.1
25561	0.946132	2.1
25562	1.31323	2.1
25563	0.946133	2.1
25564	0.946132	2.1
25565	0.946131	2.1
25566	0.94613	0.219523
25567	1.36546	2.1
25568	1.36546	2.08475
25569	0.946131	1.92863
25570	0.946132	0.311185
25571	0.946133	0.233079
25572	0.946132	0.05
25573	1.36899	0.05
25574	0.946133	0.05
25575	0.946131	0.05

25576	0.946132	0.05
25577	1.03543	0.05
25578	1.1412	0.05
25579	0.941275	0.05
25580	1.28952	0.05
25581	0.94613	0.05
25582	0.946129	0.05
25583	0.946132	0.05
25584	0.94613	0.05
25585	0.94613	2.1
25586	0.94613	2.1
25587	0.946129	2.1
25588	0.94613	2.1
25589	1.35966	2.1
25590	0.810946	2.1
25591	0.810942	2.1
25592	0.810932	2.1
25593	0.94613	2.1
25594	1.40372	2.1
25595	0.946131	2.1
25596	0.94613	2.1
25597	1.40372	2.1
25598	0.946133	2.1
25599	0.946131	2.1
25600	0.94613	2.1
25601	0.946131	2.1
25602	0.946131	2.1
25603	1.31453	2.1
25604	0.94613	2.1
25605	0.946128	2.1
25606	0.94613	2.1
25607	0.94613	2.1
25608	0.946131	0.05
25609	1.12018	0.05
25610	0.94613	0.05
25611	0.94613	0.05
25612	0.94613	0.05
25613	0.946131	0.05
25614	1.16373	0.05
25615	0.946131	0.05
25616	0.94613	0.05
25617	0.946131	0.05
25618	0.946126	0.0703502
25619	0.946132	0.0651314
25620	0.946128	0.05
25621	0.946129	0.125459
25622	0.946129	0.108485
25623	0.946132	0.05
25624	0.946133	0.05
25625	0.946132	0.05
25626	0.946132	0.05
25627	0.946133	0.05
25628	0.946132	1.05555
25629	0.946129	1.67772
25630	0.94613	0.860915
25631	1.36399	1.15563
25632	0.946131	0.907499
25633	0.946132	2.1

25634	0.946132	2.1
25635	0.946133	2.1
25636	1.38302	2.1
25637	0.946133	2.1
25638	0.810943	2.1
25639	0.810944	2.1
25640	0.810945	2.1
25641	0.946132	2.1
25642	0.946131	2.1
25643	0.946132	2.1
25644	1.30188	2.1
25645	0.946132	2.1
25646	0.810946	0.859624
25647	0.72985	1.75304
25648	0.810934	1.2647
25649	0.856243	0.427434
25650	0.946129	0.502143
25651	0.946132	0.558341
25652	1.19686	0.221525
25653	0.851527	0.874682
25654	0.946131	2.1
25655	0.94613	2.1
25656	0.94613	2.1
25657	0.94613	2.1
25658	1.31961	2.1
25659	0.946131	2.1
25660	0.946131	2.1
25661	0.94613	2.1
25662	0.946131	2.1
25663	1.3605	2.1
25664	1.30116	2.1
25665	0.946133	0.28736
25666	0.946131	2.1
25667	0.946131	1.87472
25668	0.94613	2.1
25669	0.946132	0.05
25670	1.3035	0.05
25671	0.94613	0.05
25672	0.946131	0.05
25673	0.946131	0.05
25674	0.946132	2.1
25675	1.37869	2.1
25676	1.2051	2.1
25677	0.946132	2.1
25678	1.2051	2.1
25679	0.810946	2.1
25680	0.810945	2.1
25681	0.810946	2.1
25682	1.34623	2.1
25683	0.946131	2.1
25684	0.946131	2.1
25685	1.34623	2.1
25686	1.22993	2.1
25687	1.32299	0.05
25688	0.946132	0.05
25689	0.946132	0.05
25690	0.946132	0.05
25691	0.946131	0.05

25692	0.94613	0.05
25693	1.40574	0.05
25694	0.946128	0.0692692
25695	0.946131	0.11128
25696	1.40574	0.0751201
25697	0.946132	2.1
25698	1.41331	2.1
25699	0.94613	2.1
25700	0.946131	2.1
25701	1.41331	2.1
25702	0.94613	2.1
25703	1.37609	2.1
25704	0.946129	2.1
25705	0.946131	2.1
25706	1.37609	2.1
25707	1.039	0.334276
25708	0.946131	2.03662
25709	0.944514	1.27993
25710	0.946132	1.87185
25711	0.946132	1.5306
25712	1.23726	0.417922
25713	0.946128	1.79472
25714	0.946128	1.2424
25715	0.946129	1.74103
25716	0.94613	1.40627
25717	0.946129	2.1
25718	0.946131	2.1
25719	0.946131	2.1
25720	1.39214	2.1
25721	0.94613	2.1
25722	0.946118	0.194729
25723	0.946118	0.05
25724	1.41078	0.05
25725	1.41076	0.05
25726	0.946131	0.05
25727	0.946131	0.946073
25728	0.946131	0.589707
25729	0.946132	0.908396
25730	0.94613	0.559713
25731	0.946131	0.432726
25732	0.946132	0.05
25733	0.946132	0.05
25734	0.946133	0.05
25735	1.28924	0.05
25736	0.946132	0.05
25737	0.946133	0.110967
25738	1.09812	0.05
25739	0.946132	0.0699455
25740	0.946132	0.136894
25741	1.09812	0.126389
25742	0.946132	0.497582
25743	0.946132	0.178886
25744	0.946132	0.429991
25745	0.946132	0.386757
25746	1.15388	0.341918
25747	0.946132	0.10247
25748	0.974688	0.05
25749	0.946132	0.052445

25750	0.946132	0.0945498
25751	0.974687	0.0829255
25752	1.0644	1.34326
25753	0.946133	1.76024
25754	0.946132	1.23781
25755	0.946133	1.20515
25756	1.26547	1.35473
25757	1.02905	0.938458
25758	0.911537	1.03806
25759	0.838038	1.20391
25760	0.946131	0.05
25761	0.94613	0.05
25762	0.946132	0.05
25763	0.946131	0.05
25764	1.33922	0.05
25765	0.946132	0.05
25766	0.946132	0.05
25767	1.40926	0.05
25768	1.40927	0.05
25769	0.946132	0.05
25770	0.946129	2.1
25771	0.946131	2.08726
25772	0.946131	1.07994
25773	0.946131	0.674852
25774	1.40496	2.05166
25775	0.94613	1.51248
25776	0.94613	0.781381
25777	0.946127	1.29387
25778	0.946128	0.712488
25779	1.38914	0.755725
25780	2.675	0.05
25781	1.22266	0.05
25782	0.946131	0.05
25783	0.946132	0.05
25784	0.946132	0.05
25785	1.22266	0.05
25786	0.946124	0.05
25787	0.946128	0.05
25788	0.946126	0.05
25789	1.21428	0.05
25790	0.946127	0.05
25791	0.946131	0.05
25792	0.946131	0.05
25793	0.946132	0.05
25794	0.946129	0.05
25795	0.940118	0.05
25796	0.810943	2.1
25797	0.810945	2.1
25798	0.810958	2.1
25799	0.946133	0.05
25800	1.11539	0.05
25801	0.946132	0.05
25802	0.946132	0.05
25803	0.946133	0.05
25804	0.946133	2.1
25805	1.41195	2.1
25806	0.946133	2.1
25807	0.946132	2.1

25808	0.946133	2.1
25809	1.15968	0.05
25810	0.946131	0.05
25811	0.946131	0.05
25812	0.946131	0.05
25813	0.946131	0.05
25814	0.94613	2.1
25815	0.946128	2.1
25816	0.946132	1.19142
25817	0.94613	2.1
25818	0.94613	1.81127
25819	0.946131	2.04054
25820	1.04211	2.00426
25821	0.850155	0.819296
25822	0.946131	1.71318
25823	0.946131	1.24854
25824	0.946131	0.0556916
25825	0.94613	0.05
25826	0.946129	0.0568671
25827	0.946131	0.05
25828	0.946131	0.05
25829	1.40193	0.05
25830	1.19756	0.05
25831	0.561936	0.05
25832	0.808778	0.05
25833	1.19756	0.05
25834	0.946132	0.05
25835	0.946132	0.05
25836	1.33916	0.05
25837	1.33916	0.05
25838	0.946133	0.05
25839	0.946127	0.0700957
25840	0.946127	0.05
25841	0.946131	0.0663477
25842	0.946128	0.0539947
25843	0.946129	0.0586378
25844	0.589428	0.11932
25845	0.851501	0.05
25846	0.868924	0.0941771
25847	0.877703	0.0822927
25848	0.85153	0.0946366
25849	0.946132	2.02625
25850	1.25372	2.1
25851	0.946132	2.1
25852	0.946132	2.1
25853	1.25372	2.1
25854	0.946132	0.527211
25855	0.946128	2.1
25856	0.94613	0.908294
25857	0.94613	1.58978
25858	1.2778	1.98233
25859	0.946118	0.5199
25860	1.35629	0.538613
25861	0.946117	0.606969
25862	0.946131	0.456066
25863	0.946105	0.279319
25864	0.946132	0.136199
25865	0.946132	2.04278

25866	1.38646	1.37693
25867	1.38646	1.50547
25868	0.946132	1.46655
25869	0.946128	2.1
25870	0.946128	0.278271
25871	1.39659	2.1
25872	1.39659	2.1
25873	0.946129	2.01251
25874	0.946132	1.73881
25875	0.946131	1.28966
25876	0.946132	1.5646
25877	0.946132	1.51328
25878	1.15139	1.2213
25879	0.946133	2.1
25880	0.946132	2.1
25881	0.946132	2.1
25882	0.946132	2.1
25883	1.36151	2.1
25884	0.946127	2.1
25885	0.946131	2.1
25886	0.946128	2.1
25887	0.94613	2.1
25888	1.38397	2.1
25889	0.946131	0.0636681
25890	1.31112	0.0586075
25891	0.946127	0.100803
25892	0.946128	0.168901
25893	1.31112	0.081584
25894	1.29778	0.05
25895	0.883417	0.05
25896	1.32613	0.05
25897	0.94613	0.05
25898	0.94613	0.05
25899	0.946132	2.1
25900	0.94613	2.1
25901	0.946131	2.1
25902	0.946131	2.1
25903	1.36523	2.1
25904	0.946132	0.05
25905	1.01653	0.05
25906	0.946132	0.05
25907	0.946131	0.05
25908	0.946131	0.05
25909	0.94612	2.1
25910	1.08301	1.96541
25911	0.946119	2.1
25912	0.946132	2.1
25913	1.08299	2.1
25914	0.810942	2.1
25915	0.810944	1.64375
25916	0.810957	2.1
25917	0.946126	0.05
25918	0.946132	0.05
25919	0.946129	0.05
25920	1.3492	0.05
25921	0.94613	0.05
25922	0.946131	0.0512429
25923	1.24766	0.05

25924	0.946128	0.0848405
25925	0.946131	0.15754
25926	1.24766	0.0916145
25927	0.946132	0.05
25928	0.946131	0.05
25929	0.946131	0.05
25930	1.23351	0.05
25931	0.946132	0.05
25932	0.946131	0.05
25933	0.946132	0.05
25934	0.946131	0.05
25935	1.35657	0.05
25936	0.946131	0.05
25937	0.946132	0.116068
25938	1.3689	0.0601061
25939	0.946132	0.100469
25940	0.946132	0.114526
25941	1.3689	0.094165
25942	0.946133	0.0632702
25943	1.35822	0.05
25944	0.946133	0.0506557
25945	0.946132	0.0577987
25946	1.35822	0.0517879
25947	0.946131	0.331752
25948	0.946131	1.3635
25949	0.946131	1.37753
25950	0.946131	0.783278
25951	1.4101	1.58365
25952	0.810944	1.62892
25953	0.810939	1.6675
25954	0.810955	1.10139
25955	0.946132	2.1
25956	1.37733	2.1
25957	0.946132	2.1
25958	0.946131	2.1
25959	0.946131	2.1
25960	1.32582	2.1
25961	0.946132	2.1
25962	0.946132	2.1
25963	0.946132	2.1
25964	0.946132	2.1
25965	0.946132	2.1
25966	0.946132	2.1
25967	1.31401	2.1
25968	1.31401	2.1
25969	0.946131	2.1
25970	1.3469	1.51116
25971	0.946132	1.837
25972	0.946132	1.88941
25973	0.946133	1.03973
25974	0.946132	1.24628
25975	0.946126	2.1
25976	0.946127	2.1
25977	0.94613	2.1
25978	0.946128	2.1
25979	1.41206	2.1
25980	0.946127	2.1
25981	1.40109	2.1

25982	0.946128	2.1
25983	0.946129	2.1
25984	1.40109	2.1
25985	1.24448	0.05
25986	0.94613	0.05
25987	0.946132	0.05
25988	0.946129	0.05
25989	0.946131	0.05
25990	1.11015	0.05
25991	0.946133	0.0765956
25992	0.946132	0.05
25993	0.946133	0.05
25994	0.946132	0.05
25995	0.946132	2.1
25996	0.946131	2.1
25997	0.946131	2.1
25998	1.38775	2.1
25999	0.946131	2.1
26000	0.946133	0.221227
26001	0.946131	1.15885
26002	1.30598	2.0998
26003	1.30598	1.78581
26004	0.946131	1.59723
26005	0.946132	2.1
26006	0.946132	2.1
26007	0.946132	2.1
26008	1.40774	2.1
26009	0.946132	2.1
26010	0.798396	0.05
26011	0.946133	0.70409
26012	0.946128	1.46364
26013	0.946131	1.22923
26014	0.946131	1.22605
26015	1.28918	1.23368
26016	0.946132	0.0809653
26017	1.40668	0.0638534
26018	0.94613	0.112538
26019	0.94613	0.0743624
26020	0.94613	0.0950566
26021	0.810946	2.1
26022	0.810945	2.1
26023	0.810933	2.1
26024	0.946131	2.1
26025	1.30338	2.1
26026	0.94613	2.1
26027	1.30339	2.1
26028	0.946131	2.1
26029	0.946127	0.834751
26030	0.87161	1.37499
26031	0.946128	1.0079
26032	0.946129	1.16945
26033	0.827876	0.679457
26034	0.946131	2.1
26035	1.40796	2.1
26036	0.94613	2.1
26037	0.94613	2.1
26038	1.40796	2.1
26039	0.946132	0.196975

26040	0.946132	0.318061
26041	0.946132	0.179237
26042	0.946132	0.131474
26043	1.41724	0.111744
26044	1.34254	0.05
26045	0.946132	0.138974
26046	0.946132	0.0935522
26047	0.946132	0.0872169
26048	0.946133	0.0763489
26049	0.946133	1.99878
26050	0.946132	1.76469
26051	0.946132	1.66878
26052	1.26501	2.01953
26053	0.946132	1.48755
26054	0.946131	0.05
26055	1.35169	0.05
26056	0.946131	0.05
26057	0.946131	0.05
26058	1.35169	0.05
26059	0.676844	0.05
26060	0.946131	0.05
26061	0.94613	0.05
26062	1.13242	0.05
26063	1.13242	0.05
26064	0.946131	0.05
26065	0.946128	1.05657
26066	1.28828	2.1
26067	0.946131	1.18976
26068	0.946131	1.35818
26069	1.28828	1.66504
26070	0.946131	0.0787493
26071	1.2996	0.0523398
26072	0.946133	0.0780117
26073	0.946131	0.0586047
26074	0.946131	0.0613313
26075	0.946132	2.1
26076	1.40638	2.1
26077	0.946131	2.1
26078	0.946132	2.1
26079	1.40638	2.1
26080	2.84909	0.05
26081	1.35726	0.05
26082	0.946131	0.05
26083	0.946131	0.05
26084	0.946131	0.05
26085	0.946131	0.05
26086	1.39852	2.1
26087	0.946132	2.1
26088	0.94613	2.1
26089	0.94613	2.1
26090	0.94613	2.1
26091	0.946126	0.05
26092	0.946131	0.05
26093	0.946128	0.05
26094	1.31666	0.05
26095	0.946128	0.05
26096	0.946126	0.05
26097	0.946131	0.05

26098	0.946129	0.05
26099	1.32275	0.05
26100	0.946129	0.05
26101	0.946131	2.1
26102	0.946132	2.1
26103	1.41125	2.1
26104	0.946131	2.1
26105	0.946131	2.1
26106	0.946131	2.1
26107	0.946132	2.1
26108	1.31904	2.1
26109	0.946132	2.1
26110	0.946132	2.1
26111	0.946129	0.05
26112	0.946132	0.05
26113	0.94613	0.05
26114	1.02426	0.05
26115	0.9311	0.05
26116	0.946128	1.88961
26117	0.946133	1.32364
26118	0.946131	1.7108
26119	1.40092	1.83004
26120	0.946131	1.49689
26121	0.946117	0.609133
26122	0.946118	2.1
26123	0.946118	0.228527
26124	1.40017	2.1
26125	0.946105	2.0095
26126	0.946132	0.0673069
26127	1.03547	0.05
26128	0.946132	0.0597704
26129	0.946133	0.0875571
26130	0.946132	0.0807535
26131	0.946133	2.1
26132	0.946132	2.1
26133	0.946133	2.1
26134	0.946133	2.1
26135	1.36694	2.1
26136	0.946132	0.05
26137	1.22878	0.05
26138	0.946132	0.05
26139	0.946132	0.05
26140	1.22878	0.05
26141	1.11014	0.05
26142	0.913078	0.05
26143	1.11015	0.05
26144	0.49154	0.05
26145	0.946127	2.1
26146	0.946131	2.1
26147	0.946128	2.1
26148	0.946129	2.1
26149	1.38752	2.1
26150	0.946132	2.1
26151	1.37277	2.1
26152	0.946132	2.1
26153	0.946131	2.1
26154	1.37277	2.1
26155	0.94613	0.0641342

26156	1.16834	0.05
26157	0.946129	0.066915
26158	0.94613	0.05
26159	0.94613	0.05
26160	0.946127	0.0630975
26161	0.946131	0.05
26162	1.35594	0.05
26163	0.946128	0.0672032
26164	0.94613	0.05
26165	0.946128	1.39728
26166	0.946128	0.05
26167	1.38464	0.139749
26168	1.38463	0.05
26169	0.94613	0.0895987
26170	0.946129	1.4027
26171	0.946131	0.0586887
26172	0.94613	0.0865222
26173	1.39751	0.05
26174	0.946131	0.19353
26175	1.25837	0.05
26176	1.21134	0.05
26177	0.700013	0.05
26178	0.700041	0.05
26179	1.40583	2.1
26180	0.946132	2.1
26181	0.946132	2.1
26182	0.946133	2.1
26183	0.946132	2.1
26184	0.827865	2.1
26185	0.946131	2.1
26186	0.946131	2.06051
26187	0.946131	2.1
26188	0.946131	2.03584
26189	0.946132	0.05
26190	1.21087	0.05
26191	0.94613	0.05
26192	0.94613	0.05
26193	0.946131	0.05
26194	0.94613	0.373283
26195	1.17452	0.706642
26196	0.946132	0.395727
26197	0.94613	0.680754
26198	0.94613	0.249103
26199	0.946119	0.192311
26200	1.27374	0.280196
26201	0.946119	0.192834
26202	0.946131	0.267514
26203	0.946106	0.142226
26204	0.946133	2.1
26205	0.946132	2.1
26206	0.946133	2.1
26207	0.946132	2.1
26208	1.39621	2.1
26209	1.34352	2.1
26210	0.946132	2.1
26211	0.946132	2.1
26212	0.946132	2.1
26213	0.946132	2.1

26214	0.946132	0.300654
26215	0.946128	2.1
26216	0.94613	1.16197
26217	0.94613	1.93361
26218	1.41273	2.1
26219	1.23536	2.1
26220	0.946131	1.85236
26221	0.946132	2.1
26222	0.946132	2.1
26223	0.946132	2.1
26224	0.946132	2.1
26225	1.41131	2.1
26226	0.946131	2.1
26227	0.946131	2.1
26228	0.946131	2.1
26229	0.946133	0.328121
26230	0.946132	2.01896
26231	0.946133	0.098191
26232	1.41894	0.0920252
26233	0.946132	0.05
26234	0.946128	0.0959653
26235	1.4099	1.76483
26236	0.946131	0.255048
26237	0.946131	2.08034
26238	0.946131	0.05
26239	0.810947	2.1
26240	0.810945	2.1
26241	0.810933	2.1
26242	0.946131	0.172898
26243	1.25765	2.1
26244	0.946128	0.896571
26245	0.946129	0.157317
26246	1.25765	0.947517
26247	1.21347	2.1
26248	0.946129	2.1
26249	0.94613	2.1
26250	1.21347	2.1
26251	0.94613	2.1
26252	0.94613	0.05
26253	0.946132	0.05
26254	1.2493	0.05
26255	1.2493	0.05
26256	0.94613	0.05
26257	0.946132	0.877485
26258	0.946128	1.32596
26259	0.946131	0.74638
26260	0.94613	1.18612
26261	1.37938	1.00045
26262	0.946133	0.05
26263	0.946132	0.05
26264	0.946133	0.05
26265	0.946132	0.05
26266	0.946133	0.05
26267	0.946129	0.27658
26268	1.26426	0.135614
26269	0.946131	0.292317
26270	0.946131	0.313053
26271	1.26426	0.260189

26272	0.872447	1.89459
26273	0.946132	1.19455
26274	0.946132	1.77074
26275	0.946132	1.88162
26276	0.946133	1.55276
26277	0.946117	1.21879
26278	0.946116	1.05249
26279	0.946113	1.03747
26280	0.946129	1.14058
26281	1.27211	0.77496
26282	0.94613	0.05
26283	0.946129	0.0546121
26284	1.40029	0.05
26285	1.40028	0.0544882
26286	0.94613	0.141033
26287	0.810942	2.1
26288	0.810943	2.1
26289	0.810932	2.1
26290	1.29521	2.1
26291	0.946129	2.1
26292	0.946131	2.1
26293	1.29521	2.1
26294	1.29521	2.1
26295	0.946132	0.0628177
26296	0.946129	0.05
26297	1.24596	0.05
26298	0.946131	0.0658871
26299	0.946131	0.0513486
26300	0.946131	2.1
26301	1.35616	2.1
26302	0.946133	2.1
26303	0.94613	2.1
26304	1.35616	2.1
26305	0.879548	0.530633
26306	1.01272	0.591745
26307	0.946133	0.930783
26308	0.946133	0.30279
26309	0.946132	0.730827
26310	0.946132	0.883119
26311	0.918009	0.336108
26312	0.91801	1.45218
26313	0.946132	0.588938
26314	0.946132	1.08397
26315	0.946132	0.05
26316	1.28644	0.05
26317	0.946131	0.05
26318	0.946131	0.05
26319	0.946132	0.05
26320	0.946131	0.05
26321	1.40103	0.05
26322	0.946131	0.05
26323	0.946131	0.05
26324	0.946131	0.05
26325	0.946128	0.05
26326	1.03327	0.05
26327	0.946129	0.05
26328	0.946131	0.05
26329	0.94613	0.05

26330	0.946132	0.178974
26331	0.946133	0.05
26332	1.40265	0.05
26333	1.40265	0.051818
26334	0.946133	0.0592938
26335	0.946131	0.05
26336	0.946131	0.05
26337	0.946131	0.05
26338	0.856655	0.05
26339	0.856616	0.05
26340	0.946132	0.103902
26341	0.946132	0.137405
26342	0.946132	0.359684
26343	1.15844	0.0619924
26344	0.946131	0.318635
26345	0.946131	2.1
26346	1.4031	2.1
26347	1.40304	2.1
26348	0.851554	2.1
26349	0.946131	0.05
26350	0.94613	0.05
26351	1.24417	0.05
26352	1.24416	0.05
26353	0.946131	0.05
26354	0.946132	0.106307
26355	1.20992	0.0914642
26356	0.946132	0.0769059
26357	0.946132	0.235207
26358	1.20992	0.142644
26359	0.946128	0.778478
26360	0.94613	1.46292
26361	0.94613	0.8619
26362	0.94613	1.47514
26363	1.23426	1.54081
26364	0.810943	0.355364
26365	0.810946	0.0617509
26366	0.810933	0.0813722
26367	0.810945	2.1
26368	0.810945	0.185476
26369	0.810932	1.55416
26370	1.25104	1.83938
26371	0.946131	2.1
26372	0.946131	0.491567
26373	1.25104	2.01078
26374	1.25104	0.524539
26375	1.25488	2.1
26376	0.946132	2.1
26377	0.946132	2.1
26378	0.946132	2.1
26379	0.946131	2.1
26380	0.946131	0.05
26381	0.946126	0.05
26382	0.946128	0.05
26383	1.32131	0.05
26384	0.946128	0.05
26385	0.946133	2.1
26386	0.946132	0.41691
26387	0.946132	2.1

26388	0.946133	2.1
26389	1.41123	2.1
26390	0.946132	0.05
26391	0.946133	0.05
26392	0.946132	0.05
26393	1.32163	0.05
26394	0.946133	0.05
26395	0.946132	2.1
26396	0.946132	2.1
26397	1.38651	2.1
26398	1.38651	2.1
26399	0.946132	2.1
26400	0.94613	0.05
26401	1.3639	0.05
26402	0.946128	0.05
26403	0.946131	0.05
26404	1.3639	0.05
26405	0.946133	0.386345
26406	1.40724	0.0687624
26407	0.946132	0.0590988
26408	0.946133	0.0842119
26409	1.40724	0.096722
26410	1.21099	0.877146
26411	0.946128	1.76219
26412	0.946127	2.1
26413	0.946128	2.09848
26414	0.946129	1.46344
26415	0.946132	0.189438
26416	1.35946	0.13363
26417	0.946131	0.479091
26418	0.946131	0.359191
26419	1.35946	0.304656
26420	1.34794	0.155464
26421	0.517994	1.42641
26422	0.946132	0.260459
26423	0.946132	0.613987
26424	0.517965	0.708815
26425	1.23224	0.396349
26426	0.94613	1.99796
26427	0.946131	1.32821
26428	0.946131	0.92396
26429	0.946131	0.732586
26430	1.37511	0.700507
26431	0.946132	1.11635
26432	0.946133	2.05401
26433	0.946133	1.91714
26434	0.946132	1.11103
26435	0.851106	0.25256
26436	0.946129	0.279167
26437	0.946131	0.527699
26438	0.946131	1.14858
26439	0.851066	0.857396
26440	0.946131	0.05
26441	0.946131	0.05
26442	0.94613	0.05
26443	1.31357	0.05
26444	0.946131	0.05
26445	0.946133	0.957553

26446	0.946132	1.23436
26447	0.946133	0.645144
26448	0.946132	1.08793
26449	1.38149	0.858259
26450	0.810944	0.37329
26451	0.810945	1.34455
26452	0.810958	2.1
26453	0.946118	0.05
26454	0.946118	0.05
26455	1.37835	0.05
26456	1.37837	0.05
26457	0.946106	0.05
26458	0.946128	0.05
26459	0.946131	0.05
26460	1.34831	0.05
26461	1.34831	0.05
26462	0.94613	0.05
26463	0.946132	0.05
26464	0.946132	0.05
26465	0.909981	0.05
26466	1.00099	0.05
26467	0.946131	0.05
26468	0.946132	0.630509
26469	0.946133	1.41847
26470	0.946133	0.918938
26471	0.946132	1.17314
26472	1.35328	1.12601
26473	0.946132	0.05
26474	0.946132	0.05
26475	1.35386	0.05
26476	1.35385	0.05
26477	0.946133	0.05
26478	0.946121	0.05
26479	0.946132	0.05
26480	0.900167	0.05
26481	1.01189	0.05
26482	0.946132	0.05
26483	0.946132	2.1
26484	1.41585	1.1889
26485	0.946131	2.02861
26486	0.946131	1.25489
26487	0.94613	1.83379
26488	0.810946	2.1
26489	0.810946	2.1
26490	0.810933	2.1
26491	1.34447	2.1
26492	0.946131	2.1
26493	0.946131	2.1
26494	1.34447	2.1
26495	1.19886	2.1
26496	0.946132	0.05
26497	0.946132	0.05
26498	0.946132	0.05
26499	1.35065	0.05
26500	0.946132	0.05
26501	0.946131	0.11312
26502	1.3022	0.0966175
26503	0.94613	0.395071

26504	0.946131	0.245058
26505	1.3022	0.205321
26506	0.946127	0.05
26507	1.38683	0.05
26508	0.946127	0.0592764
26509	0.946128	0.0649161
26510	1.38683	0.05
26511	0.810946	0.05
26512	0.810947	0.05
26513	0.810959	0.05
26514	0.946132	0.05
26515	0.946131	0.05
26516	0.946132	0.05
26517	0.946132	0.05
26518	0.946132	0.05
26519	0.946127	1.10285
26520	1.35968	2.04951
26521	0.946131	0.966998
26522	0.946129	0.636914
26523	1.35969	1.71295
26524	0.946133	2.1
26525	1.35862	1.72894
26526	0.946132	2.1
26527	0.946132	1.02276
26528	1.35862	1.68671
26529	0.946132	0.935603
26530	0.946132	0.611589
26531	0.946132	1.47776
26532	0.946131	0.338534
26533	0.946132	0.533821
26534	0.946131	0.05
26535	0.946131	0.05
26536	0.946128	0.0538445
26537	0.946129	0.05
26538	1.41232	0.05
26539	0.946128	1.44272
26540	1.29331	2.1
26541	0.946131	1.57674
26542	0.946131	2.1
26543	1.29331	2.06769
26544	0.946133	0.193733
26545	0.946132	0.239592
26546	0.946132	0.191848
26547	0.946132	0.135922
26548	1.41556	0.117605
26549	0.94612	0.162372
26550	0.946119	0.134083
26551	0.946119	0.161761
26552	0.946132	0.0980737
26553	0.946107	0.104418
26554	0.946129	0.05
26555	1.1509	0.05
26556	0.946126	0.05
26557	0.946129	0.05
26558	0.94613	0.05
26559	0.946131	0.05
26560	1.19263	0.05
26561	0.946132	0.05

26562	0.946132	0.05
26563	0.946132	0.05
26564	0.94613	0.14994
26565	1.36939	0.201451
26566	0.946128	0.214705
26567	0.946131	0.499153
26568	1.36939	0.183867
26569	0.946133	0.947135
26570	0.827865	1.78405
26571	0.946132	0.910425
26572	0.946132	1.40286
26573	1.40398	1.33404
26574	0.946127	0.0988069
26575	0.946127	0.221509
26576	0.94613	0.05
26577	1.41498	0.138721
26578	0.946128	0.0521314
26579	0.94613	0.05
26580	0.946131	0.05
26581	0.946129	0.05
26582	0.94613	0.05
26583	0.94613	0.05
26584	0.946118	0.05
26585	0.946119	0.05
26586	0.946116	0.05
26587	1.36569	0.05
26588	0.946105	0.05
26589	0.946132	0.05
26590	1.23175	0.05
26591	0.94613	0.05
26592	0.946133	0.05
26593	0.946131	0.05
26594	1.25698	0.05
26595	0.946126	0.05
26596	0.946129	0.05
26597	0.946128	0.05
26598	0.946129	0.05
26599	0.810943	2.1
26600	0.810944	2.1
26601	0.810932	2.1
26602	0.946132	0.05
26603	1.09841	0.05
26604	0.946131	0.0773974
26605	0.946132	0.0602028
26606	1.09841	0.054774
26607	0.810936	0.565459
26608	0.810935	0.515025
26609	0.810934	1.60575
26610	1.23851	2.1
26611	0.946132	0.556503
26612	0.946131	1.30796
26613	1.23851	2.1
26614	1.23851	2.1
26615	0.946132	0.05
26616	0.946131	0.05
26617	0.946132	0.05
26618	0.946132	0.05
26619	0.946132	0.05

26620	0.939141	1.17844
26621	0.953118	1.61452
26622	0.946126	0.986073
26623	0.946129	1.12843
26624	1.03306	1.20392
26625	0.946132	0.05
26626	1.30927	0.05
26627	0.946132	0.05
26628	0.946132	0.05
26629	1.30928	0.05
26630	0.94613	2.1
26631	0.946131	2.1
26632	1.24511	2.1
26633	0.94613	2.1
26634	0.94613	2.1
26635	0.946133	0.05
26636	1.34152	0.05
26637	0.946131	0.05
26638	0.946131	0.05
26639	1.34152	0.05
26640	0.946132	0.05
26641	1.41383	0.05
26642	0.946132	0.05
26643	0.946132	0.05
26644	1.41383	0.05
26645	0.946132	0.149469
26646	0.946131	0.0755779
26647	1.41392	0.05
26648	0.946132	0.15814
26649	0.946132	0.0985782
26650	0.946132	0.254975
26651	0.946132	0.166419
26652	1.24333	0.0771168
26653	0.946133	0.338374
26654	0.946133	0.201544
26655	0.946124	0.05
26656	0.94613	0.05
26657	0.946126	0.05
26658	1.3065	0.05
26659	0.946127	0.05
26660	0.810946	0.05
26661	0.810943	0.05
26662	0.81096	0.05
26663	0.946131	0.05
26664	1.40271	0.05
26665	0.946131	0.05
26666	0.946131	0.05
26667	1.40271	0.05
26668	0.946132	0.05
26669	0.94613	0.05
26670	0.946131	0.05
26671	0.94613	0.05
26672	0.946131	0.05
26673	0.946133	0.236441
26674	1.30107	0.267895
26675	0.946132	0.240877
26676	0.946132	0.298471
26677	0.946133	0.290958

26678	0.810946	2.1
26679	0.810944	2.1
26680	0.810958	2.1
26681	0.946133	1.63607
26682	0.946131	1.46803
26683	0.946131	2.1
26684	1.30812	2.1
26685	0.946131	1.67602
26686	0.94613	0.448517
26687	0.975002	1.24315
26688	0.917259	2.1
26689	1.00902	1.71851
26690	0.94613	0.377132
26691	0.946131	2.1
26692	0.946127	2.1
26693	1.398	2.1
26694	0.946129	2.1
26695	0.946129	2.1
26696	0.946133	2.1
26697	0.946132	2.1
26698	0.946132	2.1
26699	0.946132	2.1
26700	1.3033	2.1
26701	1.09897	0.05
26702	0.946131	0.308107
26703	0.946132	0.160565
26704	0.946129	0.14466
26705	0.946131	0.193276
26706	0.946131	2.1
26707	0.946132	2.1
26708	1.39858	2.1
26709	0.946131	2.1
26710	0.946131	2.1
26711	0.946119	0.362686
26712	1.08878	0.46772
26713	0.946119	1.30646
26714	1.0888	0.47866
26715	0.946107	1.28158
26716	0.946132	0.05
26717	1.10733	0.05
26718	0.946132	0.05
26719	0.946131	0.05
26720	0.946132	0.05
26721	0.946128	0.05
26722	1.33671	0.05
26723	0.94613	0.05
26724	0.94613	0.05
26725	0.94613	0.05
26726	0.810943	2.1
26727	0.810944	2.1
26728	0.810945	2.1
26729	1.35117	2.1
26730	0.946129	2.1
26731	0.94613	2.1
26732	1.35117	2.1
26733	1.27174	2.1
26734	0.946132	2.1
26735	0.946133	2.1

26736	0.997296	1.40641
26737	0.946133	2.041
26738	0.946132	1.78483
26739	0.810943	2.1
26740	0.810944	2.1
26741	0.810945	2.1
26742	0.810946	0.739701
26743	0.810943	2.0895
26744	0.810933	1.46791
26745	0.946127	1.22021
26746	0.946131	0.812365
26747	0.946128	1.18399
26748	0.94613	1.25541
26749	0.946129	1.39364
26750	0.946131	0.05
26751	0.946131	0.05
26752	0.946129	0.05
26753	0.946131	0.05
26754	0.94613	0.05
26755	0.946133	0.0767378
26756	0.946132	0.0931585
26757	0.946132	0.0621605
26758	0.946132	0.0706284
26759	0.946133	0.0797009
26760	0.946131	0.791075
26761	0.94613	0.42695
26762	0.946129	0.942417
26763	0.946131	0.484463
26764	0.946131	0.647405
26765	0.946132	0.748378
26766	0.946129	2.1
26767	0.94613	2.06291
26768	0.946131	1.57237
26769	1.39356	2.04709
26770	0.810946	2.1
26771	0.810947	2.1
26772	0.810934	2.1
26773	0.946125	0.05
26774	0.946127	0.05
26775	1.33798	0.05
26776	1.33797	0.05
26777	0.946128	0.05
26778	0.946131	0.05
26779	0.946131	0.05
26780	1.23146	0.05
26781	1.23146	0.05
26782	0.946131	0.05
26783	0.946129	2.1
26784	0.94613	2.1
26785	0.946131	2.1
26786	0.94613	2.1
26787	1.30082	2.1
26788	0.946131	0.05
26789	0.946132	0.05
26790	0.946131	0.05
26791	1.20416	0.05
26792	0.946132	0.05
26793	0.946132	0.05

26794	0.946131	0.05
26795	0.946131	0.05
26796	1.18711	0.05
26797	0.946132	0.05
26798	0.946132	0.05
26799	0.946132	0.05
26800	0.946133	0.05
26801	0.946132	0.05
26802	0.946133	0.05
26803	0.946131	0.05
26804	0.946132	0.05
26805	0.94613	0.05
26806	0.946131	0.05
26807	0.946131	0.05
26808	0.946133	0.05
26809	0.946132	0.05
26810	0.946133	0.05
26811	0.946132	0.05
26812	0.946132	0.05
26813	0.946132	2.1
26814	0.946132	2.1
26815	0.946132	2.1
26816	1.36422	2.1
26817	0.946132	2.1
26818	0.946131	0.734337
26819	0.946132	1.83031
26820	0.946132	1.20576
26821	0.946132	1.20531
26822	1.4185	1.65502
26823	0.946133	0.05
26824	1.37794	0.05
26825	0.946132	0.05
26826	0.946133	0.05
26827	0.946132	0.05
26828	1.4045	2.1
26829	0.946132	2.1
26830	0.946132	2.1
26831	0.946132	2.1
26832	0.946132	2.1
26833	0.946131	2.1
26834	0.946132	2.1
26835	1.34285	2.1
26836	1.34285	2.1
26837	0.946132	2.1
26838	0.946131	2.1
26839	0.946132	2.1
26840	1.40739	2.1
26841	1.40739	2.1
26842	0.946131	2.1
26843	0.946128	2.1
26844	0.946132	2.1
26845	0.94613	2.1
26846	0.94613	2.1
26847	1.3164	2.1
26848	0.946132	2.1
26849	1.3484	2.1
26850	0.946132	2.05804
26851	0.946132	2.1

26852	1.3484	2.1
26853	0.946132	2.1
26854	0.946132	2.1
26855	0.946133	2.1
26856	0.946133	2.1
26857	1.41507	2.1
26858	0.946132	2.1
26859	1.32608	2.1
26860	0.946131	2.1
26861	0.946131	2.1
26862	0.946132	2.1
26863	0.94613	0.663956
26864	1.34646	2.09902
26865	0.946129	0.977638
26866	0.94613	0.584927
26867	1.34646	1.41223
26868	0.946131	0.053503
26869	0.946131	2.1
26870	0.94613	0.580884
26871	1.33728	0.05
26872	0.946131	0.215641
26873	0.946127	2.1
26874	0.946131	0.855976
26875	0.946128	2.1
26876	0.946129	2.1
26877	1.4064	2.04694
26878	0.946133	2.1
26879	0.946131	2.1
26880	0.946131	2.1
26881	0.946131	2.1
26882	0.94613	2.1
26883	1.34912	2.1
26884	0.946131	2.1
26885	0.946129	2.1
26886	0.946131	2.1
26887	0.94613	2.1
26888	0.946132	2.1
26889	1.40447	2.1
26890	0.946132	2.1
26891	0.946133	2.1
26892	1.40447	2.1
26893	1.33607	2.1
26894	0.946131	2.1
26895	0.946127	2.1
26896	0.94613	2.1
26897	0.946129	2.1
26898	0.946117	0.092709
26899	1.32664	1.12194
26900	0.946115	0.20259
26901	0.946105	1.53152
26902	0.94613	0.05
26903	0.946118	2.1
26904	0.946114	2.1
26905	1.35886	2.1
26906	1.35884	2.1
26907	0.946129	2.1
26908	0.946132	2.1
26909	1.2571	2.1

26910	0.946132	2.1
26911	0.946132	2.1
26912	1.2571	2.1
26913	0.946133	2.1
26914	1.35663	2.1
26915	0.946132	2.1
26916	0.946132	2.1
26917	1.35663	2.1
26918	0.946133	0.59547
26919	0.946129	1.89171
26920	0.946131	1.35454
26921	0.946131	1.19187
26922	1.30881	1.95221
26923	0.946132	2.1
26924	0.94613	2.1
26925	1.34652	2.1
26926	0.94613	2.1
26927	0.94613	2.1
26928	0.946132	2.1
26929	0.946133	1.86941
26930	1.36183	1.13366
26931	1.36183	1.51066
26932	0.946132	1.81151
26933	0.946132	0.0703293
26934	1.38353	0.0504056
26935	0.94613	0.0728517
26936	0.94613	0.0788204
26937	1.38354	0.0570396
26938	0.946128	0.0806437
26939	1.24887	0.05
26940	0.94613	0.067738
26941	0.94613	0.134328
26942	1.24887	0.0788727
26943	0.946129	0.843635
26944	0.946126	1.58145
26945	0.946124	0.940082
26946	0.946128	1.0569
26947	1.32056	1.21758
26948	0.946129	0.977483
26949	0.946128	1.65676
26950	0.946126	0.889515
26951	0.946129	1.15328
26952	1.20351	1.16952
26953	0.946131	1.73529
26954	0.946131	1.73402
26955	0.946127	2.1
26956	1.25591	2.03986
26957	0.94613	0.72739
26958	0.946132	1.57319
26959	0.946133	1.36213
26960	0.946132	1.91616
26961	1.15436	1.13298
26962	0.930306	1.0989
26963	0.946132	2.1
26964	0.946132	2.1
26965	0.946132	2.1
26966	0.946133	2.1
26967	0.946132	2.1

26968	0.946129	1.36081
26969	0.946132	1.02703
26970	0.946126	1.58908
26971	1.13245	0.571407
26972	0.946129	1.01862
26973	0.94613	2.1
26974	0.946132	2.1
26975	0.946129	1.98601
26976	0.946131	2.1
26977	0.946131	2.1
26978	1.41279	2.1
26979	1.76254	2.1
26980	0.600454	2.08019
26981	0.858991	2.08596
26982	0.940722	2.1
26983	1.28439	1.4012
26984	0.946131	1.79938
26985	0.94613	1.81002
26986	0.946131	2.1
26987	0.946131	2.1
26988	0.946132	0.83119
26989	1.39335	0.0971051
26990	0.946132	0.768254
26991	0.946132	0.163156
26992	0.946132	0.58065
26993	1.33879	0.05
26994	0.946131	0.0598416
26995	0.94613	0.199457
26996	1.33879	0.05
26997	0.946131	0.0887852
26998	0.946126	0.130691
26999	0.946131	0.164635
27000	1.37342	0.103168
27001	1.37343	0.0844351
27002	0.946128	0.494216
27003	1.39168	0.05
27004	0.946131	0.05
27005	0.946131	0.05
27006	0.946131	0.05
27007	0.946131	0.05
27008	0.946131	0.0598539
27009	1.412 0.05	
27010	0.946127	0.05
27011	0.946128	0.05
27012	0.946128	0.0531648
27013	1.1387	0.05
27014	0.943364	0.133528
27015	0.946132	0.0669103
27016	0.946132	0.05
27017	0.946133	0.073366
27018	1.21085	0.05
27019	0.88324	0.475166
27020	0.946129	0.205308
27021	0.946131	0.260512
27022	0.946131	0.173186
27023	1.33587	0.05
27024	0.946125	0.05
27025	0.946129	0.05

27026	0.946126	0.05
27027	0.946127	0.05
27028	1.27512	0.05
27029	0.946132	0.05
27030	0.946132	0.05
27031	0.946133	0.05
27032	0.946132	0.05
27033	0.946128	0.05
27034	1.30498	0.05
27035	0.946131	0.05
27036	0.94613	0.05
27037	1.30497	0.05
27038	0.946131	0.05
27039	1.25626	0.05
27040	0.946131	0.05
27041	0.946131	0.05
27042	1.25626	0.05
27043	0.946131	0.05
27044	0.94613	0.05
27045	1.35316	0.05
27046	0.946131	0.05
27047	0.946131	0.05
27048	0.94613	0.063086
27049	1.28727	0.05
27050	0.946128	0.05
27051	0.946131	0.05
27052	0.94613	0.0563691
27053	0.946131	0.05
27054	1.39502	0.05
27055	0.946132	0.05
27056	0.946132	0.05
27057	0.946132	0.05
27058	1.36173	0.05
27059	0.946131	0.196952
27060	0.946126	0.0924845
27061	0.946127	0.112273
27062	0.946128	0.0829706
27063	0.946129	0.886303
27064	0.946133	0.462552
27065	0.946131	0.957994
27066	0.946131	1.05117
27067	0.946131	0.983349
27068	0.946128	0.05
27069	0.946132	0.05
27070	1.24678	0.05
27071	0.94613	0.05
27072	0.94613	0.05
27073	0.946129	0.05
27074	0.94613	0.05
27075	1.25215	0.05
27076	0.946126	0.05
27077	0.946129	0.05
27078	0.946129	0.40988
27079	1.29689	0.330138
27080	0.946131	0.442061
27081	0.946131	0.295543
27082	0.946131	0.182106
27083	0.946128	0.05

27084	0.94613	0.05
27085	0.946131	0.05
27086	0.94613	0.05
27087	0.946131	0.05
27088	0.946127	0.05
27089	0.946128	0.05
27090	0.94613	0.05
27091	0.946129	0.05
27092	0.946129	0.05
27093	0.946133	0.05
27094	0.946132	0.05
27095	0.946132	0.05
27096	1.29957	0.05
27097	0.946133	0.05
27098	0.946132	0.05
27099	0.946133	0.05
27100	0.946132	0.05
27101	1.26201	0.05
27102	0.946132	0.05
27103	0.946133	0.05
27104	1.40427	0.05
27105	0.946132	0.05
27106	0.946132	0.05
27107	0.946133	0.05
27108	0.946132	0.05
27109	1.35123	0.05
27110	0.946132	0.05
27111	0.946132	0.05
27112	0.946131	0.05
27113	0.946128	0.05
27114	0.946129	0.05
27115	0.946131	0.05
27116	0.94613	0.05
27117	0.946131	0.05
27118	0.946127	0.112815
27119	1.23351	0.0857451
27120	0.946131	0.117415
27121	0.946129	0.0844553
27122	0.94613	0.0822559
27123	0.946131	0.05
27124	0.946131	0.05
27125	0.94613	0.05
27126	1.18119	0.05
27127	0.946131	0.05
27128	0.946132	0.05
27129	1.30563	0.05
27130	0.946133	0.05
27131	0.946132	0.05
27132	0.946133	0.05
27133	0.946132	0.05
27134	0.946132	0.05
27135	0.946133	0.698833
27136	1.20256	0.0599856
27137	0.946133	0.0616717
27138	0.94613	0.132516
27139	1.33007	0.0636237
27140	0.946128	0.215256
27141	0.946132	0.0702575

27142	0.946131	0.0928408
27143	0.946131	2.1
27144	0.946131	2.1
27145	0.946128	2.1
27146	0.946129	2.1
27147	0.94613	2.1
27148	0.810942	2.1
27149	0.810942	2.1
27150	0.81093	2.1
27151	0.946132	2.1
27152	1.39823	2.1
27153	0.946132	2.1
27154	0.946131	2.1
27155	0.946132	2.1
27156	0.946131	2.1
27157	0.946131	2.1
27158	0.946131	2.1
27159	1.37713	2.1
27160	0.946131	2.1
27161	0.946133	2.1
27162	0.946131	2.1
27163	1.40594	2.1
27164	1.40594	2.1
27165	0.94613	2.1
27166	0.946133	2.1
27167	0.946131	2.1
27168	1.38155	2.1
27169	1.38155	2.1
27170	0.946131	2.1
27171	0.946132	0.61289
27172	0.946133	2.1
27173	0.946132	0.966738
27174	0.946132	1.18619
27175	1.33694	1.78159
27176	0.946131	2.1
27177	1.228	2.1
27178	0.946129	2.1
27179	0.946131	2.1
27180	1.228	2.1
27181	0.946132	1.12043
27182	0.946132	1.64708
27183	0.946131	2.03199
27184	0.946132	1.27769
27185	1.35943	1.94979
27186	0.946133	0.05
27187	0.946133	0.05
27188	1.02377	0.05
27189	1.02377	0.05
27190	0.946132	0.05
27191	0.94613	0.0981091
27192	0.946131	0.0757305
27193	0.946132	0.124924
27194	1.29322	0.0568955
27195	0.946131	0.117868
27196	0.946131	2.1
27197	0.946131	0.05
27198	0.946132	2.1
27199	1.41492	0.05

27200	0.946131	0.500655
27201	0.946132	2.1
27202	0.946131	2.1
27203	0.946132	2.1
27204	1.35028	2.1
27205	0.946132	2.1
27206	0.946132	2.1
27207	0.946132	2.1
27208	0.946131	2.1
27209	1.32461	2.1
27210	0.946132	2.1
27211	0.946131	2.1
27212	0.946132	2.1
27213	1.40369	2.1
27214	0.94613	2.1
27215	0.946131	2.1
27216	0.946132	0.05
27217	0.979597	0.05
27218	0.946131	0.05
27219	0.946132	0.05
27220	0.946132	0.05
27221	0.946131	2.1
27222	0.946131	2.1
27223	0.94613	2.1
27224	0.946131	2.1
27225	1.41897	2.1
27226	1.37791	2.1
27227	0.94613	2.1
27228	0.946129	2.1
27229	0.946131	2.1
27230	0.946131	2.1
27231	1.39384	2.1
27232	0.946131	2.1
27233	0.946128	2.1
27234	0.946129	2.1
27235	0.946129	2.1
27236	0.810945	1.64145
27237	0.810944	2.1
27238	0.810958	1.7843
27239	0.946128	2.1
27240	0.946132	2.1
27241	0.94613	2.1
27242	1.3335	2.1
27243	0.946131	2.1
27244	0.94613	0.0733897
27245	0.946129	2.1
27246	0.946132	0.05
27247	1.41192	1.72208
27248	0.94613	0.050112
27249	0.946132	0.770532
27250	0.946133	0.158737
27251	0.946132	0.167704
27252	1.39428	0.108915
27253	0.946133	0.116445
27254	0.94613	1.96517
27255	0.946128	2.1
27256	0.946132	0.971872
27257	0.94613	1.90776

27258	1.40684	2.1
27259	0.946131	2.1
27260	0.946131	2.1
27261	0.946131	2.1
27262	0.946131	2.1
27263	1.39021	2.1
27264	0.946131	1.13229
27265	1.31649	2.1
27266	0.946131	0.792667
27267	0.946131	0.723491
27268	1.31649	1.48298
27269	0.810946	2.1
27270	0.810946	2.1
27271	0.810958	2.1
27272	1.16687	2.1
27273	0.946131	2.1
27274	0.946131	2.1
27275	1.16687	2.1
27276	1.16687	2.1
27277	0.946128	1.03692
27278	1.3564	1.00173
27279	0.946131	1.10125
27280	0.946131	1.01331
27281	0.94613	0.576739
27282	0.946128	2.1
27283	1.35828	2.1
27284	0.94613	2.1
27285	0.946131	2.1
27286	0.94613	2.1
27287	0.946133	0.10842
27288	1.36477	0.115416
27289	0.946132	0.149196
27290	0.946132	0.254079
27291	1.36477	0.139398
27292	0.946132	0.750853
27293	1.31857	2.07707
27294	0.946132	1.25106
27295	0.946132	1.58739
27296	1.31857	1.2488
27297	0.94613	0.235054
27298	1.35059	0.072322
27299	0.946128	0.0799809
27300	0.94613	0.125405
27301	1.35059	0.0993449
27302	0.946126	0.0611782
27303	1.35559	0.05
27304	0.946127	0.05
27305	0.946128	0.05
27306	1.35559	0.05
27307	0.946131	0.05
27308	1.33035	0.05
27309	0.946127	0.05
27310	0.946128	0.05
27311	1.33035	0.05
27312	0.94613	0.05
27313	1.3665	0.05
27314	0.946129	0.05
27315	0.946131	0.05

27316	1.3665	0.05
27317	0.94613	0.05
27318	1.32828	0.05
27319	0.946129	0.05
27320	0.94613	0.05
27321	0.946131	0.05
27322	0.946131	2.1
27323	0.946131	2.1
27324	1.32949	2.1
27325	1.32949	2.1
27326	0.946131	2.1
27327	0.946131	1.92345
27328	0.94613	0.05
27329	0.946133	0.477864
27330	1.38357	0.05
27331	0.946131	0.15512
27332	0.946131	2.1
27333	0.946128	2.1
27334	1.36919	2.1
27335	0.946131	2.1
27336	0.94613	2.1
27337	0.946131	1.77564
27338	1.3771	2.1
27339	0.946129	2.1
27340	0.94613	2.1
27341	0.946131	2.08243
27342	0.946131	1.23842
27343	0.94613	0.837979
27344	0.946129	0.150676
27345	1.30029	0.605869
27346	0.94613	0.367786
27347	1.21174	0.05
27348	0.946132	0.05
27349	0.946132	0.05
27350	0.946132	0.05
27351	0.946133	0.05
27352	0.810946	2.1
27353	0.810945	2.1
27354	0.810946	2.1
27355	1.1796	2.1
27356	0.946131	2.1
27357	0.946132	2.1
27358	1.1796	2.1
27359	1.1796	2.1
27360	0.946131	2.1
27361	0.946131	2.1
27362	0.946133	2.1
27363	0.946131	2.1
27364	1.40615	2.1
27365	0.810945	2.1
27366	0.810944	2.1
27367	0.810945	2.1
27368	1.40239	2.1
27369	0.94613	2.1
27370	1.35561	2.1
27371	0.946133	2.1
27372	1.35562	2.1
27373	1.41169	0.05

27374	0.946131	0.0727949
27375	0.946127	0.164088
27376	1.41169	0.05
27377	0.946128	0.0909236
27378	0.946119	2.1
27379	0.946119	2.1
27380	0.946119	2.1
27381	0.946107	2.1
27382	1.41917	2.1
27383	0.810946	1.45256
27384	0.810943	1.7477
27385	0.810934	2.0924
27386	0.946127	2.1
27387	1.1995	2.1
27388	0.946126	2.1
27389	0.946128	2.1
27390	1.1995	2.1
27391	0.810944	2.1
27392	0.810947	2.1
27393	0.81096	2.1
27394	0.946133	0.05
27395	1.3495	0.05
27396	0.946132	0.05
27397	0.946132	0.05
27398	0.946133	0.05
27399	0.946132	2.1
27400	0.946132	2.1
27401	0.946133	2.1
27402	1.39529	2.1
27403	0.946133	2.1
27404	0.946133	2.1
27405	1.28525	2.1
27406	0.946132	2.1
27407	0.946133	2.1
27408	1.28525	2.1
27409	0.810946	2.1
27410	0.810945	2.1
27411	0.810946	2.1
27412	1.33931	2.1
27413	0.946132	2.1
27414	0.946132	2.1
27415	1.33931	2.1
27416	1.24616	2.1
27417	0.946133	0.0607046
27418	0.946133	0.0656291
27419	0.946132	0.05
27420	0.946132	0.0570694
27421	0.946133	0.0507321
27422	0.946128	2.1
27423	1.40225	2.1
27424	0.946128	2.1
27425	0.94613	2.1
27426	0.94613	2.1
27427	0.946113	0.05
27428	0.946118	0.05
27429	0.946114	0.05
27430	1.40551	0.05
27431	0.946103	0.05

27432	0.94613	2.1
27433	0.946132	2.1
27434	0.946129	2.1
27435	0.946131	2.1
27436	1.37811	2.1
27437	0.946132	2.1
27438	1.39826	2.1
27439	0.946133	2.1
27440	0.946132	2.1
27441	1.39826	2.1
27442	0.946118	2.1
27443	1.28298	2.1
27444	0.946117	2.1
27445	0.946105	2.1
27446	1.283 2.1	
27447	0.946118	0.05
27448	1.41375	0.05
27449	0.946117	0.05
27450	0.946131	0.05
27451	0.946106	0.05
27452	0.946132	0.202009
27453	0.946129	0.0967676
27454	1.28436	0.0770303
27455	0.946131	0.215712
27456	0.946131	0.124537
27457	0.810947	0.05
27458	0.810946	0.05
27459	0.81096	0.05
27460	0.946132	0.05
27461	1.34338	0.05
27462	0.961435	0.05
27463	0.961435	0.05
27464	0.946132	0.05
27465	1.3028	2.07756
27466	0.946131	2.07124
27467	0.946127	2.1
27468	0.946128	2.1
27469	0.946129	2.09644
27470	0.946132	0.214495
27471	1.37973	0.0910415
27472	0.946132	0.246363
27473	0.946132	0.0803496
27474	0.946132	0.103506
27475	0.946131	0.577612
27476	0.94613	0.371594
27477	0.946129	0.583443
27478	0.946131	0.325498
27479	1.41798	0.31208
27480	0.946118	0.358007
27481	0.946118	0.20673
27482	0.946117	0.363054
27483	0.946131	0.227709
27484	0.946105	0.217516
27485	0.946131	0.679406
27486	0.946131	0.417706
27487	0.94613	0.677167
27488	0.946131	0.403608
27489	0.946131	0.325761

27490	0.946133	0.836779
27491	1.40274	2.1
27492	0.946133	0.304074
27493	0.946132	2.1
27494	1.40274	2.1
27495	0.946131	1.429
27496	1.39731	2.1
27497	0.946132	0.806455
27498	0.94613	1.304
27499	1.39731	2.016
27500	0.94612	2.1
27501	0.94612	1.73386
27502	0.946119	1.61055
27503	0.946132	1.69835
27504	1.40764	2.1
27505	0.946128	2.1
27506	0.946132	2.1
27507	0.94613	2.1
27508	0.94613	2.1
27509	1.31213	2.1
27510	0.946127	2.1
27511	0.94613	2.1
27512	0.946127	2.1
27513	0.946128	2.1
27514	1.36653	2.1
27515	0.946127	2.1
27516	1.3557	2.1
27517	0.946128	2.1
27518	0.946128	2.1
27519	1.3557	2.1
27520	0.946133	2.1
27521	0.946132	2.1
27522	0.946133	2.1
27523	0.946132	2.1
27524	1.40081	2.1
27525	0.946128	2.1
27526	0.946133	2.1
27527	0.946131	2.1
27528	0.946131	2.1
27529	1.32282	2.1
27530	0.810945	2.1
27531	0.810943	2.1
27532	0.810933	2.1
27533	1.27676	2.1
27534	0.94613	2.1
27535	0.946128	2.1
27536	0.946131	2.1
27537	0.946131	2.1
27538	0.946131	0.418984
27539	0.946127	0.0605247
27540	1.41328	0.206142
27541	1.41327	0.0945075
27542	0.946128	0.134499
27543	0.946132	0.05
27544	1.32083	0.05
27545	0.946132	0.05
27546	0.946133	0.05
27547	1.32082	0.05

27548	0.946128	0.05
27549	1.29215	0.05
27550	0.94613	0.05
27551	0.94613	0.05
27552	1.29215	0.05
27553	0.946132	0.05
27554	1.27696	0.05
27555	0.94613	0.05
27556	0.94613	0.0601403
27557	1.27696	0.05
27558	0.94612	0.05
27559	1.29289	0.05
27560	0.946119	0.05
27561	0.946132	0.0508697
27562	1.29287	0.05
27563	0.946128	2.1
27564	1.37869	2.1
27565	0.946131	2.1
27566	0.94613	2.1
27567	0.946131	2.1
27568	0.946133	2.1
27569	0.946132	2.1
27570	0.946132	2.1
27571	1.35703	2.1
27572	0.946133	2.1
27573	0.946132	2.1
27574	0.946132	2.1
27575	0.946133	2.1
27576	1.37017	2.1
27577	0.946133	2.1
27578	0.946119	0.05
27579	0.94612	0.05
27580	1.38583	0.05
27581	1.38585	0.05
27582	0.946107	0.05
27583	0.946126	0.05
27584	0.946127	0.05
27585	1.22904	0.05
27586	1.22904	0.05
27587	0.946128	0.05
27588	1.3279	0.179271
27589	0.946131	1.47897
27590	0.94613	0.843281
27591	0.946131	1.1855
27592	0.946131	0.808702
27593	0.946132	2.1
27594	0.946132	2.1
27595	0.946132	2.1
27596	0.946131	2.1
27597	1.40932	2.1
27598	1.26868	0.05
27599	0.946131	0.05
27600	0.94613	0.05
27601	0.946131	0.05
27602	0.946131	0.05
27603	0.810943	2.1
27604	0.810945	2.1
27605	0.810932	2.1

27606	0.810943	0.86233
27607	0.810944	0.0752428
27608	0.810957	0.0678985
27609	1.31539	0.139951
27610	0.946132	1.86914
27611	0.946128	0.05
27612	1.3154	0.180147
27613	1.17856	0.0533487
27614	1.21997	0.787948
27615	0.94613	1.93976
27616	0.946129	1.5622
27617	0.94613	2.05654
27618	0.94613	1.89683
27619	0.946131	0.999235
27620	0.946131	0.367817
27621	0.946129	1.25223
27622	0.94613	0.951686
27623	0.946131	1.23881
27624	0.946127	0.241531
27625	0.946131	0.130939
27626	0.946127	1.92692
27627	0.946129	0.569312
27628	1.41342	0.24761
27629	0.946131	0.159315
27630	0.946131	1.43156
27631	0.946128	1.88453
27632	0.946131	0.451684
27633	1.29149	1.35008
27634	1.41022	2.1
27635	0.94613	0.831426
27636	0.946128	2.1
27637	0.94613	2.1
27638	0.94613	2.1
27639	0.946128	0.05
27640	0.94613	0.0938546
27641	1.27864	0.05
27642	1.27864	0.05
27643	0.946131	0.0975346
27644	0.946129	0.629784
27645	0.946125	1.63721
27646	0.946124	1.28701
27647	0.946127	0.982124
27648	1.37759	1.39457
27649	0.946129	0.750341
27650	0.94613	1.63991
27651	0.946126	1.11588
27652	0.946129	1.03428
27653	1.38259	1.28508
27654	0.946131	0.05
27655	1.34118	0.05
27656	0.946132	0.0530631
27657	0.946132	0.0522976
27658	1.34118	0.05
27659	0.946131	0.05
27660	1.25017	0.05
27661	0.946128	0.05
27662	0.946131	0.05
27663	0.946131	0.05

27664	0.946131	2.1
27665	0.946128	2.1
27666	0.946133	2.1
27667	1.3937	2.1
27668	0.94613	2.1
27669	0.946131	0.77551
27670	0.94613	0.05
27671	1.40404	0.05
27672	1.40404	0.05
27673	0.946131	0.0507743
27674	0.946132	0.441628
27675	0.946132	0.05
27676	1.38897	0.05
27677	1.38897	0.05
27678	0.946132	0.05
27679	0.94613	0.05
27680	0.946128	0.0827541
27681	1.40332	0.05
27682	1.40331	0.062307
27683	0.94613	0.267025
27684	0.946132	0.131056
27685	0.946132	2.1
27686	0.946133	1.16875
27687	1.41619	2.1
27688	0.946132	1.34127
27689	0.946132	2.1
27690	0.946132	2.1
27691	0.946132	2.1
27692	1.38244	2.1
27693	0.946132	2.1
27694	0.810946	1.21556
27695	0.810943	0.502419
27696	0.810934	0.241797
27697	0.810947	1.17778
27698	0.810943	0.259441
27699	0.810934	0.284165
27700	1.12786	1.19476
27701	0.946132	2.1
27702	0.946132	1.89925
27703	0.946132	1.45289
27704	0.946133	1.40211
27705	0.946132	1.81551
27706	0.946132	1.88987
27707	0.946132	1.45198
27708	1.27806	1.835
27709	0.946133	1.38316
27710	0.946132	2.1
27711	0.946132	2.1
27712	0.946133	2.1
27713	0.946132	2.1
27714	0.946133	2.1
27715	1.39514	1.71218
27716	0.946129	1.18334
27717	0.946124	1.55874
27718	0.946127	0.805922
27719	0.946127	1.62372
27720	0.94613	2.1
27721	0.946129	2.1

27722	0.946132	2.1
27723	1.3991	2.1
27724	0.94613	2.1
27725	0.946132	0.12459
27726	0.946133	2.1
27727	0.946132	2.07222
27728	1.36835	1.11711
27729	0.946132	0.997046
27730	0.946132	2.1
27731	0.946132	2.1
27732	0.946133	2.1
27733	0.946132	2.1
27734	1.39047	2.1
27735	0.946132	0.05
27736	0.946128	0.05
27737	0.94613	0.05
27738	1.33174	0.05
27739	0.946131	0.05
27740	0.946128	0.05
27741	0.946131	0.05
27742	0.94613	0.05
27743	0.94613	0.05
27744	0.94613	0.05
27745	0.946119	2.1
27746	0.946119	2.1
27747	1.29064	2.1
27748	0.946132	2.1
27749	0.946107	2.1
27750	0.946133	2.1
27751	0.94613	2.1
27752	1.30584	2.1
27753	0.946131	2.1
27754	0.946131	2.1
27755	0.946131	2.1
27756	0.946132	2.1
27757	0.94613	2.1
27758	0.946131	2.1
27759	1.39549	2.1
27760	0.913386	1.19996
27761	0.978873	0.982055
27762	0.946126	0.933187
27763	0.94613	1.15543
27764	1.00473	0.566623
27765	0.946132	0.592534
27766	0.946133	0.462714
27767	0.946132	0.340793
27768	0.946132	0.770975
27769	1.38894	0.264218
27770	0.946128	0.393882
27771	0.946131	0.310878
27772	0.94613	0.300598
27773	0.94613	0.39918
27774	1.23865	0.20673
27775	0.946126	1.23001
27776	0.946129	1.23866
27777	0.946124	0.969848
27778	0.946127	1.17555
27779	1.40248	0.920116

27780	0.946133	0.716803
27781	0.946132	0.729703
27782	0.946132	0.399148
27783	0.946133	0.756949
27784	1.1391	0.428696
27785	0.827862	0.507482
27786	0.946133	0.806076
27787	0.946131	1.247
27788	0.946131	0.989249
27789	0.94613	0.804977
27790	0.946128	0.247505
27791	0.946132	0.534705
27792	0.94613	0.664553
27793	1.21388	0.134664
27794	0.94613	0.613962
27795	0.810943	0.0579078
27796	0.810945	0.122298
27797	0.810932	0.111388
27798	0.946131	0.31895
27799	0.946131	0.780198
27800	0.946133	1.20629
27801	1.36748	0.212671
27802	0.946132	0.054585
27803	0.946128	0.06911
27804	0.946131	0.0509053
27805	0.94613	0.0717255
27806	1.35856	0.05
27807	0.94613	0.0553325
27808	0.946132	2.1
27809	0.946131	2.1
27810	0.946133	2.1
27811	0.946132	2.1
27812	0.946131	2.1
27813	0.946133	2.1
27814	0.946131	2.1
27815	1.39558	2.1
27816	1.39558	2.1
27817	0.94613	2.1
27818	0.946131	0.05
27819	0.94613	0.05
27820	0.946129	0.05
27821	1.41388	0.05
27822	0.946131	0.05
27823	0.946133	0.05
27824	0.946132	0.05
27825	0.946132	0.05
27826	0.946133	0.05
27827	0.93452	0.05
27828	0.946129	2.1
27829	1.38897	2.1
27830	0.946131	2.1
27831	0.946131	2.1
27832	1.38897	2.1
27833	0.946133	2.05665
27834	0.946132	1.74641
27835	0.946133	1.96289
27836	0.946132	0.866125
27837	1.38773	1.36908

27838	1.41512	0.05
27839	0.946131	0.05
27840	0.946129	0.05
27841	0.946133	0.05
27842	0.946131	0.05
27843	0.946132	0.351827
27844	0.946132	2.1
27845	0.946132	0.638318
27846	0.946133	1.76262
27847	0.946133	2.1
27848	0.946126	0.200895
27849	0.946132	0.995107
27850	0.946129	0.258894
27851	0.946129	0.874477
27852	0.946129	1.48535
27853	0.939895	2.09467
27854	1.03386	2.1
27855	0.94613	2.1
27856	0.946131	2.02934
27857	1.03386	2.08411
27858	0.946131	2.1
27859	1.39309	2.1
27860	0.946133	2.1
27861	0.946131	2.1
27862	1.39309	2.1
27863	0.946133	2.1
27864	1.24853	2.1
27865	0.946132	2.1
27866	0.946132	2.1
27867	1.24853	2.1
27868	0.946133	2.1
27869	1.39699	0.520346
27870	0.946132	1.86464
27871	0.946132	2.1
27872	1.39699	1.03366
27873	0.946132	2.1
27874	0.946131	2.1
27875	0.946133	2.1
27876	0.946131	2.1
27877	1.38969	2.1
27878	1.28464	2.0972
27879	0.946133	2.1
27880	0.94613	2.1
27881	0.946131	1.97672
27882	0.946131	1.98738
27883	0.946119	0.770193
27884	0.94612	0.932393
27885	0.946119	0.657145
27886	0.946132	0.822572
27887	1.3749	0.528527
27888	0.810942	2.1
27889	0.810943	2.1
27890	0.810957	2.1
27891	1.41134	2.0995
27892	0.946132	0.613733
27893	0.946132	2.01654
27894	0.946132	1.01661
27895	0.946132	1.96502

27896	1.31338	2.1
27897	0.946132	1.0082
27898	0.946132	2.1
27899	0.946133	1.59046
27900	0.946132	1.99935
27901	0.946103	0.05
27902	0.752448	0.05
27903	0.946132	0.05
27904	0.946131	0.05
27905	1.20617	0.05
27906	0.946132	0.970047
27907	1.08054	0.407497
27908	1.25736	0.650988
27909	0.946132	0.982456
27910	0.946131	0.784993
27911	0.946128	2.1
27912	1.33275	1.08893
27913	1.1896	2.1
27914	0.946131	2.1
27915	1.18962	2.1
27916	1.21955	0.05
27917	0.946131	0.05
27918	1.33587	0.05
27919	0.741946	0.05
27920	1.07387	0.05
27921	0.945844	0.357216
27922	1.3292	2.1
27923	1.59472	1.90893
27924	0.946131	0.655551
27925	1.04043	0.717992
27926	1.13529	0.05
27927	0.946132	0.05
27928	1.24893	0.05
27929	1.12675	0.05
27930	0.936652	0.05
27931	1.36968	2.1
27932	0.946131	2.1
27933	0.946132	2.1
27934	1.36968	2.1
27935	1.258	2.1
27936	3.17458	0.05
27937	0.946132	2.07216
27938	1.35022	2.1
27939	1.35022	2.1
27940	0.94613	2.1
27941	1.12356	2.1
27942	0.946131	2.1
27943	1.22401	2.1
27944	1.22401	2.1
27945	0.94613	2.1
27946	0.952455	2.1
27947	1.2107	2.1
27948	0.946132	2.1
27949	1.33047	2.1
27950	0.946131	2.1
27951	0.946132	2.1
27952	0.61957	2.1
27953	0.8636	2.1

27954	0.946132	2.1
27955	1.19294	2.1
27956	0.946128	2.1
27957	1.36607	2.1
27958	0.94613	2.1
27959	1.39416	2.1
27960	1.36607	2.1
27961	1.21474	2.1
27962	0.946131	2.1
27963	0.946131	2.1
27964	1.16288	2.1
27965	1.08542	2.1
27966	0.851526	0.563796
27967	1.30374	2.1
27968	1.01708	2.1
27969	0.946114	0.909414
27970	0.96976	1.53856
27971	0.946132	0.05
27972	1.49997	0.05
27973	0.771735	0.05
27974	0.771764	0.05
27975	0.946133	0.05
27976	0.700863	1.16447
27977	1.42222	2.1
27978	0.946131	2.1
27979	0.946128	1.6139
27980	1.42509	2.1
27981	1.14298	1.88949
27982	0.837014	2.1
27983	0.946131	2.1
27984	1.29426	2.1
27985	0.836985	2.1
27986	0.946129	2.1
27987	1.22089	0.091744
27988	0.946132	1.12948
27989	0.712486	0.175608
27990	0.6234	0.096995
27991	0.946131	2.1
27992	1.40271	2.1
27993	1.32241	2.1
27994	0.946133	2.1
27995	1.32241	2.1
27996	0.769373	1.23657
27997	0.946129	2.1
27998	1.41302	2.1
27999	1.28254	2.1
28000	0.946131	2.1
28001	1.28254	2.1
28002	0.851543	2.00755
28003	1.23056	2.1
28004	1.24356	2.1
28005	0.851543	2.09865
28006	0.909417	2.1
28007	1.13808	0.151953
28008	0.946133	0.115018
28009	0.946131	0.05
28010	1.13808	0.951091
28011	1.13808	0.203525

28012	0.930941	1.19966
28013	1.37808	0.852027
28014	1.17573	0.43517
28015	0.946124	1.19603
28016	1.02404	0.594833
28017	0.946116	2.1
28018	1.3144	0.890275
28019	1.3144	2.1
28020	0.946118	2.1
28021	1.13089	2.1
28022	0.946128	0.05
28023	0.946101	0.05
28024	1.2606	0.05
28025	1.26055	0.05
28026	0.580823	0.360262
28027	0.946132	1.18684
28028	0.580825	0.409199
28029	1.23224	0.442335
28030	0.946128	2.1
28031	1.3719	2.1
28032	0.94613	2.1
28033	1.3719	2.1
28034	0.94613	2.1
28035	0.946132	1.45715
28036	1.35025	0.303126
28037	1.35025	0.694285
28038	0.946132	1.96691
28039	1.1794	1.10655
28040	0.946133	2.1
28041	1.25598	2.1
28042	1.29249	2.1
28043	1.38154	2.1
28044	0.851541	2.1
28045	1.31508	2.1
28046	1.37558	2.1
28047	0.946129	2.1
28048	0.867663	2.1
28049	0.94613	1.49023
28050	1.35426	1.1373
28051	0.84872	0.820308
28052	0.946129	1.03884
28053	1.17195	2.00934
28054	0.946133	2.1
28055	1.39009	2.1
28056	1.14342	2.1
28057	0.946133	2.1
28058	1.14343	2.1
28059	1.38819	2.1
28060	0.946133	2.1
28061	1.4116	2.1
28062	0.946131	2.1
28063	0.946131	2.1
28064	0.946127	2.1
28065	1.32244	2.1
28066	1.32244	2.1
28067	0.946131	2.1
28068	1.2176	2.1
28069	1.75823	2.1

28070	1.11239	2.1
28071	2.17979	2.1
28072	1.65533	2.1
28073	3.41356	2.1
28074	1.94841	2.1
28075	1.91767	2.1
28076	1.76726	2.1
28077	1.70302	2.1
28078	3.76137	2.1
28079	1.94841	2.1
28080	1.33962	2.1
28081	1.31365	2.1
28082	1.70302	2.1
28083	3.2123	2.1
28084	1.76726	2.1
28085	1.70302	2.1
28086	1.47581	2.1
28087	1.7457	2.1
28088	3.22764	2.1
28089	1.20478	2.1
28090	1.70302	2.1
28091	1.27956	2.1
28092	1.18569	2.1
28093	1.63979	2.1
28094	1.51949	2.1
28095	1.53082	2.1
28096	1.92911	2.1
28097	1.32068	2.1
28098	1.74454	2.1
28099	3.27536	2.1
28100	1.08172	2.1
28101	1.74454	2.1
28102	1.52603	2.1
28103	1.52603	2.1
28104	1.32068	2.1
28105	1.32068	2.1
28106	1.89226	2.1
28107	2.37364	2.1
28108	1.89226	2.1
28109	2.42769	2.1
28110	4.31995	2.1
28111	2.37365	2.1
28112	1.5362	2.1
28113	2.4277	2.1
28114	1.56328	2.1
28115	3.9639	2.1
28116	1.89225	2.1
28117	2.42768	2.1
28118	1.89213	2.1
28119	1.89225	2.1
28120	2.42756	2.1
28121	2.42768	2.1
28122	2.42768	2.1
28123	1.58163	2.1
28124	1.56315	2.1
28125	1.56327	2.1
28126	2.42757	2.1
28127	2.42768	2.1

28128	1.01641	2.1
28129	2.15005	2.1
28130	1.00099	2.1
28131	2.15114	2.1
28132	3.15104	2.1
28133	0.994813	2.1
28134	2.15114	2.1
28135	2.17159	2.1
28136	2.17159	2.1
28137	1.00099	2.1
28138	1.00099	2.1
28139	1.29608	2.1
28140	1.29608	2.1
28141	0.942035	2.1
28142	0.942035	2.09904
28143	1.29198	2.1
28144	0.942033	2.1
28145	0.946132	2.1
28146	0.946132	2.1
28147	0.946132	2.1
28148	0.942033	2.1
28149	0.946133	2.02291
28150	1.29198	2.1
28151	1.99985	2.1
28152	1.14207	2.1
28153	1.83584	2.1
28154	1.00232	2.1
28155	3.00217	2.1
28156	1.99867	0.05
28157	2.0815	0.05
28158	1.56806	0.05
28159	1.28828	0.05
28160	3.58519	0.05
28161	1.89226	0.05
28162	1.89226	0.05
28163	1.89226	0.05
28164	1.89226	0.05
28165	1.89226	0.05
28166	1.89226	0.05
28167	1.89226	0.05
28168	1.89226	0.05
28169	2.23193	0.05
28170	2.48978	0.05
28171	4.38204	0.05
28172	1.85293	0.823278
28173	1.64279	0.05
28174	1.76268	0.196657
28175	1.54214	0.05
28176	3.39507	0.05
28177	1.6428	0.0944302
28178	1.17302	0.05
28179	1.17302	1.02332
28180	1.28419	0.116085
28181	1.54214	0.05
28182	1.65331	0.0622043
28183	1.76268	1.03806
28184	1.54214	0.118836
28185	1.65324	0.106989

28186	1.42491	0.05
28187	3.19538	0.05
28188	0.651043	0.176911
28189	0.651043	0.828904
28190	0.647925	0.0732521
28191	0.647925	0.346178
28192	0.521747	0.0985832
28193	0.58651	0.138068
28194	0.77107	0.732049
28195	0.712457	0.142332
28196	0.712457	0.05
28197	0.521747	0.0597854
28198	0.58651	0.0719005
28199	0.77107	0.660812
28200	1.2873	2.1
28201	1.48751	2.1
28202	1.59659	2.1
28203	1.77596	2.01398
28204	3.06326	2.1
28205	1.07976	2.1
28206	1.59659	2.1
28207	1.30237	2.1
28208	1.77723	2.1
28209	2.89897	2.1
28210	1.77723	2.1
28211	1.77596	2.1
28212	1.82614	2.1
28213	1.59659	2.1
28214	2.0055	2.1
28215	1.77596	2.1
28216	2.64523	0.868885
28217	2.73982	0.05
28218	2.50057	0.512402
28219	2.61606	0.05
28220	5.26129	0.0596672
28221	2.73982	0.122555
28222	2.68987	0.0755996
28223	2.61607	0.121658
28224	2.57611	0.05
28225	5.30593	0.0550879
28226	1.89226	0.0705625
28227	1.89226	0.355134
28228	1.89226	0.216391
28229	2.02178	0.172137
28230	3.78452	0.24687
28231	1.89226	0.05
28232	1.89226	0.082776
28233	1.89226	1.05656
28234	1.89226	0.05
28235	1.89226	0.828124
28236	1.89226	0.05
28237	2.35204	2.1
28238	1.50108	2.1
28239	1.3715	2.1
28240	2.18469	2.1
28241	3.68577	2.1
28242	2.62514	2.1
28243	2.1847	2.1

28244	2.0487	2.1
28245	2.45213	2.1
28246	4.63682	2.1
28247	1.25075	2.1
28248	2.18469	2.1
28249	1.3715	2.1
28250	1.3715	2.1
28251	2.0487	2.1
28252	2.0487	2.1
28253	1.28667	2.1
28254	1.11285	2.1
28255	1.03529	2.1
28256	1.03529	2.1
28257	1.30711	2.1
28258	1.30711	2.1
28259	2.35053	2.1
28260	1.89225	2.1
28261	1.89225	2.1
28262	2.51216	2.1
28263	4.24278	2.1
28264	1.89225	2.1
28265	1.89225	2.1
28266	1.89225	2.1
28267	1.89225	2.1
28268	3.7845	2.1
28269	1.92025	2.1
28270	1.32119	2.1
28271	1.44777	2.1
28272	1.33834	2.1
28273	1.92025	2.1
28274	1.81082	2.1
28275	1.89226	2.1
28276	2.67272	2.1
28277	2.51218	2.1
28278	2.51218	2.1
28279	1.89226	2.1
28280	1.89226	2.1
28281	1.89226	2.1
28282	1.89226	2.1
28283	1.89226	2.1
28284	1.89226	2.1
28285	3.78452	2.1
28286	1.92027	2.1
28287	1.44778	2.1
28288	1.61121	2.1
28289	2.01555	2.1
28290	3.53147	2.1
28291	1.90727	0.05
28292	2.29851	0.0645709
28293	2.71643	0.0589538
28294	2.17695	0.05
28295	4.47547	0.05
28296	1.90728	0.05
28297	1.56339	0.05
28298	1.58143	0.05
28299	2.17696	0.05
28300	3.74035	0.05
28301	1.91368	0.05

28302	2.17696	0.0571978
28303	2.39986	0.05
28304	1.75904	0.05
28305	3.936	0.05
28306	1.18793	0.05
28307	2.74744	0.05
28308	1.38716	0.05
28309	2.57465	0.05
28310	3.93537	0.05
28311	1.3148	0.05
28312	2.74744	0.05
28313	2.70805	0.05
28314	1.38716	0.05
28315	2.70805	0.05
28316	1.38716	0.05
28317	1.20608	2.1
28318	0.975871	2.1
28319	0.946132	2.1
28320	0.946132	2.1
28321	0.946132	2.1
28322	0.946132	2.1
28323	0.975871	2.1
28324	0.975871	2.1
28325	1.20608	2.1
28326	0.946132	2.1
28327	0.946133	2.1
28328	0.975871	2.1
28329	2.8198	1.80284
28330	2.29967	2.1
28331	2.09542	2.1
28332	2.63784	0.351859
28333	4.93751	2.1
28334	2.09542	2.1
28335	1.39523	2.1
28336	1.21119	2.1
28337	1.39523	2.1
28338	2.11563	2.1
28339	2.29967	2.1
28340	1.88584	2.1
28341	2.63784	0.202033
28342	2.11262	1.96839
28343	1.91132	1.60604
28344	4.02153	2.1
28345	1.88584	2.1
28346	1.21119	2.1
28347	1.12606	2.1
28348	1.91132	2.1
28349	3.1225	2.1
28350	2.18077	2.1
28351	1.78167	2.1
28352	2.2947	0.432329
28353	1.93139	1.37043
28354	4.07637	0.648814
28355	1.5709	1.16967
28356	1.03235	2.1
28357	2.05933	1.95502
28358	1.78168	0.95302
28359	1.31	0.143153

28360	1.03235	0.200278
28361	2.2947	1.06662
28362	2.31785	2.1
28363	2.40627	2.1
28364	2.08288	0.833129
28365	4.37758	2.1
28366	1.95737	1.71089
28367	1.31	2.1
28368	2.08289	0.211034
28369	1.50108	0.686041
28370	3.39289	0.484829
28371	2.49676	2.1
28372	2.24781	2.1
28373	2.5276	1.6442
28374	2.7352	2.1
28375	5.02436	2.1
28376	2.49676	2.1
28377	2.43549	2.1
28378	2.6398	2.1
28379	2.7352	2.1
28380	5.13656	2.1
28381	1.89226	2.1
28382	2.7352	2.1
28383	1.89226	2.1
28384	2.6398	2.1
28385	1.89226	2.1
28386	2.6398	2.1
28387	2.16843	0.05
28388	1.00185	0.05
28389	1.90039	2.1
28390	3.1547	0.05
28391	2.54064	0.05
28392	2.46752	2.1
28393	1.90039	2.1
28394	2.26044	2.1
28395	4.36791	0.122881
28396	1.65815	2.1
28397	2.48652	2.1
28398	1.65815	2.1
28399	1.9004	2.1
28400	2.21711	2.1
28401	2.5034	2.1
28402	2.37167	2.1
28403	2.59965	2.1
28404	2.62812	2.1
28405	2.40308	2.1
28406	4.99979	2.1
28407	2.50137	2.1
28408	2.59965	2.1
28409	2.62812	2.1
28410	2.5214	2.1
28411	5.12948	2.1
28412	2.62814	2.1
28413	2.4031	2.1
28414	2.43509	2.1
28415	2.65066	2.1
28416	5.06323	2.1
28417	2.62814	2.1

28418	2.52142	2.1
28419	2.53008	2.1
28420	2.65066	2.1
28421	5.15822	2.1
28422	2.0815	2.1
28423	1.92067	2.1
28424	1.59611	2.1
28425	1.89226	2.1
28426	1.62453	2.1
28427	1.92067	2.1
28428	1.89226	2.1
28429	1.89226	2.1
28430	1.89226	2.1
28431	1.89226	2.1
28432	3.78453	2.1
28433	0.946131	2.1
28434	0.946131	2.1
28435	0.929501	2.1
28436	0.910761	2.1
28437	0.9461	2.1
28438	0.9461	2.1
28439	0.910792	2.1
28440	0.910792	2.1
28441	0.946131	2.1
28442	0.929501	2.1
28443	0.910761	2.1
28444	0.946131	2.1
28445	2.0815	2.1
28446	1.81661	2.1
28447	2.1341	2.1
28448	2.0815	2.1
28449	1.86921	2.1
28450	1.81661	2.1
28451	1.89226	2.1
28452	1.89226	2.1
28453	1.89226	2.1
28454	1.89226	2.1
28455	1.89226	2.1
28456	1.89226	2.1
28457	0.946131	2.1
28458	0.946131	2.1
28459	0.949104	2.1
28460	0.9295	2.1
28461	0.946132	2.1
28462	0.946132	2.1
28463	0.929499	2.1
28464	0.929499	2.1
28465	0.946131	2.1
28466	0.949105	2.1
28467	0.946131	2.1
28468	0.929501	2.1
28469	1.66104	2.1
28470	2.34044	2.1
28471	2.08889	2.1
28472	1.33264	2.1
28473	3.67308	2.1
28474	2.0889	2.1
28475	1.08011	2.1

28476	1.08011	2.1
28477	1.33264	2.1
28478	1.87598	2.1
28479	2.12851	2.1
28480	2.27472	2.1
28481	1.89226	1.69797
28482	2.27703	2.1
28483	1.89226	0.582793
28484	4.16699	1.18263
28485	1.89226	2.1
28486	1.89226	1.50722
28487	1.89226	1.56771
28488	1.89226	0.91098
28489	3.78452	1.95213
28490	1.00007	2.08223
28491	1.32316	0.802341
28492	1.29417	0.228005
28493	1.29417	1.90221
28494	1.02051	0.23641
28495	1.02051	1.67528
28496	2.2846	2.1
28497	1.89226	1.1742
28498	2.27703	1.5422
28499	2.27703	2.1
28500	1.89226	0.207576
28501	1.89226	2.1
28502	1.89226	2.1
28503	1.89226	2.09199
28504	1.89226	0.522313
28505	1.89226	0.165845
28506	3.78452	0.657743
28507	1.02051	2.1
28508	1.32316	2.04522
28509	1.03373	0.383486
28510	1.34066	0.884318
28511	2.35689	2.1
28512	1.703	2.1
28513	1.89224	2.1
28514	1.52266	2.1
28515	1.89224	2.1
28516	1.23344	2.1
28517	1.53282	2.1
28518	1.89223	2.1
28519	1.89223	2.1
28520	1.89223	2.1
28521	1.89223	2.1
28522	3.78447	2.1
28523	0.796845	2.1
28524	0.796845	2.1
28525	0.812146	2.1
28526	0.812146	2.1
28527	1.08006	2.1
28528	1.02679	2.1
28529	0.551538	2.1
28530	0.528929	2.1
28531	0.551529	2.1
28532	1.08005	2.1
28533	0.528935	2.1

28534	1.0268	2.1
28535	1.5001	2.1
28536	1.70302	2.1
28537	2.0726	2.1
28538	1.70302	2.1
28539	1.99601	2.1
28540	1.70303	2.1
28541	1.89226	2.1
28542	1.89226	2.1
28543	1.89226	2.1
28544	1.89226	2.1
28545	1.89226	2.1
28546	1.89226	2.1
28547	1.17285	2.1
28548	2.16015	2.1
28549	2.23563	2.1
28550	2.23563	2.1
28551	1.10309	2.1
28552	1.10309	2.1
28553	0.946128	2.1
28554	0.946128	2.1
28555	1.21847	0.303812
28556	1.2884	2.1
28557	0.94613	2.1
28558	0.94613	2.1
28559	1.21847	2.1
28560	1.21847	0.178401
28561	0.946131	2.1
28562	1.21847	2.1
28563	0.94613	2.1
28564	1.2884	2.1
28565	1.83922	2.09275
28566	2.5768	2.1
28567	1.83488	2.1
28568	1.82846	0.05
28569	2.43693	2.1
28570	2.43693	2.1
28571	2.36134	2.1
28572	1.81656	2.1
28573	1.86454	2.1
28574	1.6697	2.1
28575	2.55618	2.1
28576	2.36134	2.1
28577	2.55618	2.1
28578	1.9323	2.1
28579	1.9302	2.1
28580	1.81656	2.1
28581	2.66983	2.1
28582	2.55618	2.1
28583	1.89226	0.589325
28584	1.70302	0.200663
28585	1.94935	0.678924
28586	1.70302	0.411039
28587	1.95569	0.219924
28588	1.70302	0.0795293
28589	2.82465	2.1
28590	2.41707	2.1
28591	2.24481	2.1

28592	2.68958	2.1
28593	5.10665	2.1
28594	1.41232	2.1
28595	1.34479	2.1
28596	0.946129	2.1
28597	0.946129	2.1
28598	0.94613	2.1
28599	0.94613	2.1
28600	1.41232	2.1
28601	1.41232	2.1
28602	1.34479	2.1
28603	0.94613	2.1
28604	0.946131	2.1
28605	1.41232	2.1
28606	1.86859	0.05
28607	1.89226	0.05
28608	1.89226	0.05
28609	1.89226	0.05
28610	1.45875	0.05
28611	1.45875	0.05
28612	2.1769	0.256352
28613	2.50259	0.218908
28614	1.94955	0.0704223
28615	2.25959	0.357675
28616	4.45214	0.555547
28617	1.89226	0.382648
28618	1.89226	0.300521
28619	1.89226	0.185464
28620	1.89226	0.528082
28621	3.78452	0.164737
28622	1.89226	0.393166
28623	1.89226	0.97413
28624	1.89226	0.475015
28625	1.89226	0.536218
28626	3.78452	1.45932
28627	2.50259	0.297615
28628	2.49445	0.248689
28629	2.25959	0.220909
28630	2.20843	0.204151
28631	4.71102	0.118506
28632	1.94955	0.0947765
28633	2.25959	0.0896483
28634	1.24689	0.05
28635	1.79906	0.0791536
28636	3.74861	0.05
28637	1.89226	0.278618
28638	1.89226	0.310273
28639	1.89226	0.0825401
28640	1.89226	0.132538
28641	3.78452	0.511795
28642	1.89226	0.285449
28643	1.89226	0.166982
28644	1.89226	0.114911
28645	1.89226	0.0979007
28646	3.78452	0.0836346
28647	2.25959	0.0650968
28648	2.20843	0.0750503
28649	1.79906	0.0693417

28650	1.98391	0.11044
28651	4.00748	0.0977926
28652	1.89226	2.1
28653	1.89226	2.1
28654	1.89226	2.1
28655	1.89226	2.1
28656	3.78452	2.1
28657	1.89226	2.1
28658	1.89226	2.1
28659	1.89226	2.1
28660	1.89226	2.1
28661	3.78452	2.1
28662	1.64718	2.1
28663	1.23085	2.1
28664	1.46028	2.1
28665	1.39564	2.1
28666	1.68372	2.1
28667	1.64718	2.1
28668	1.64718	2.1
28669	1.64981	2.1
28670	1.70303	2.1
28671	1.6991	2.1
28672	3.3199	2.1
28673	1.89226	2.1
28674	1.89226	2.1
28675	1.89226	2.1
28676	1.89226	2.1
28677	3.78452	2.1
28678	1.89226	2.1
28679	1.89226	2.1
28680	1.89226	2.1
28681	1.89226	2.1
28682	3.78452	2.1
28683	0.666701	2.1
28684	0.65896	2.1
28685	0.920797	2.1
28686	0.895441	2.1
28687	0.775871	2.1
28688	0.687493	2.1
28689	0.777393	2.1
28690	0.777393	2.1
28691	0.677856	2.1
28692	0.90095	2.1
28693	0.621435	2.1
28694	0.858124	2.1
28695	1.70303	2.1
28696	1.70731	2.1
28697	1.79663	2.1
28698	1.68783	2.1
28699	3.17337	2.1
28700	1.22724	2.1
28701	2.0021	2.1
28702	1.90427	2.1
28703	1.08698	2.1
28704	3.08907	2.1
28705	2.41689	2.1
28706	2.00209	2.1
28707	1.90427	2.1

28708	2.35245	2.1
28709	4.35454	2.1
28710	1.90427	2.1
28711	0.956294	2.1
28712	0.956294	2.1
28713	1.08698	2.1
28714	1.81488	2.1
28715	1.94556	2.1
28716	1.90427	2.1
28717	2.35245	2.1
28718	2.28979	2.1
28719	1.81488	2.1
28720	4.16733	2.1
28721	1.6272	2.1
28722	1.70302	2.1
28723	1.70303	2.1
28724	1.70302	2.1
28725	1.91167	2.1
28726	1.91167	2.1
28727	1.49063	2.1
28728	1.89226	2.1
28729	1.89226	2.1
28730	1.89226	2.1
28731	1.6272	2.1
28732	1.6272	2.1
28733	2.25988	1.02874
28734	1.89226	0.05
28735	1.89226	0.751844
28736	2.23152	0.309672
28737	4.12379	0.123821
28738	2.25988	0.507092
28739	1.16361	1.44173
28740	1.15853	2.1
28741	2.23153	0.0694984
28742	3.39513	1.16236
28743	1.89226	0.611659
28744	2.23153	0.133659
28745	2.21022	0.965314
28746	2.23153	0.36267
28747	1.87095	0.0537406
28748	1.89226	0.05
28749	1.16239	1.61155
28750	2.23153	0.05
28751	1.15853	0.874994
28752	1.15853	0.0865467
28753	2.21022	0.966257
28754	2.21022	0.079468
28755	1.78381	0.05
28756	1.70302	0.05
28757	1.15453	0.05
28758	1.35007	0.05
28759	1.82021	0.05
28760	2.12851	0.05
28761	1.89226	0.05
28762	1.89226	0.05
28763	1.89226	0.05
28764	1.89226	0.05
28765	1.89226	0.05

28766	1.89226	0.05
28767	1.89258	0.05
28768	1.089	0.05
28769	1.22586	0.05
28770	1.79877	0.05
28771	2.88777	0.05
28772	1.91564	0.05
28773	1.79877	0.05
28774	1.96356	0.05
28775	1.70302	0.05
28776	2.37828	0.05
28777	2.04859	0.05
28778	1.89226	0.05
28779	1.89226	0.05
28780	1.89226	0.05
28781	1.89226	0.05
28782	3.78453	0.05
28783	1.89226	0.05
28784	1.89226	0.05
28785	1.89226	0.05
28786	1.89226	0.05
28787	1.89226	0.05
28788	1.89226	0.05
28789	2.28593	2.1
28790	1.89226	2.1
28791	2.2731	2.1
28792	1.89226	0.557849
28793	4.17819	1.87891
28794	1.89226	2.1
28795	1.89226	2.1
28796	1.89226	1.35863
28797	1.89226	1.00197
28798	3.78452	1.86633
28799	1.03933	2.1
28800	1.34355	0.805192
28801	1.35048	0.341745
28802	1.35048	2.1
28803	1.02975	0.211617
28804	1.02975	2.00854
28805	2.25105	2.1
28806	1.89226	2.01821
28807	2.27311	2.1
28808	2.27311	2.1
28809	1.89226	1.188
28810	1.89226	2.1
28811	1.89226	2.1
28812	1.89226	2.1
28813	1.89226	1.5766
28814	1.89226	0.0975696
28815	3.78452	0.788073
28816	1.02975	2.1
28817	1.34355	2.1
28818	1.01121	1.05763
28819	1.33079	0.79249
28820	2.35476	2.1
28821	1.24526	0.05
28822	2.36406	0.05
28823	2.81021	0.05

28824	1.7237	0.05
28825	4.08776	0.05
28826	1.89226	2.1
28827	1.89226	2.1
28828	1.89226	2.1
28829	1.89226	2.1
28830	3.78453	2.1
28831	1.79028	2.1
28832	1.89238	2.1
28833	1.65533	2.1
28834	1.77821	2.1
28835	3.5477	2.1
28836	1.89238	2.1
28837	1.7465	0.05
28838	1.77821	2.1
28839	1.63557	0.0522664
28840	3.52795	1.98382
28841	1.89226	2.1
28842	1.89226	0.05
28843	1.89226	2.1
28844	1.89226	0.05
28845	3.78452	0.634461
28846	1.89226	2.1
28847	1.89226	2.1
28848	1.89226	2.1
28849	1.89226	2.1
28850	3.78452	2.1
28851	1.65532	2.1
28852	1.77821	2.1
28853	1.53278	2.1
28854	1.67571	2.1
28855	3.33103	2.1
28856	1.77821	2.1
28857	1.63557	0.0530907
28858	1.67571	2.1
28859	1.52615	0.0701328
28860	3.31128	1.84717
28861	1.89226	2.1
28862	1.89226	0.05
28863	1.89226	2.1
28864	1.89226	0.0625871
28865	3.78452	1.97495
28866	2.83129	2.1
28867	1.89226	0.05
28868	1.89226	0.982331
28869	2.6491	2.0543
28870	4.54136	0.592322
28871	2.83129	2.1
28872	1.47278	2.1
28873	1.3909	2.1
28874	2.6491	2.1
28875	4.12188	2.1
28876	1.89225	2.1
28877	2.65393	2.1
28878	1.89225	2.1
28879	2.69591	2.1
28880	4.58816	2.1
28881	1.91703	2.1

28882	2.00209	2.1
28883	2.65393	2.1
28884	2.65393	2.1
28885	2.69591	2.1
28886	2.00209	2.1
28887	1.89226	2.1
28888	2.69593	2.1
28889	1.93967	2.1
28890	1.89226	2.1
28891	2.74334	2.1
28892	2.69593	2.1
28893	2.74335	2.1
28894	2.0021	2.1
28895	2.76367	2.1
28896	2.69593	2.1
28897	2.06984	2.1
28898	2.0021	2.1
28899	2.24481	2.1
28900	2.58921	2.1
28901	2.68958	2.1
28902	2.68958	2.1
28903	2.11984	2.1
28904	2.11984	2.1
28905	2.11984	2.1
28906	2.58921	2.1
28907	2.46488	2.1
28908	1.98339	2.1
28909	4.5726	2.1
28910	2.7777	2.1
28911	2.58921	2.1
28912	2.46488	2.1
28913	2.70401	2.1
28914	5.29322	2.1
28915	2.17741	2.1
28916	2.54197	2.1
28917	2.54018	2.1
28918	2.18608	2.1
28919	4.72805	2.1
28920	2.17742	2.1
28921	1.58054	2.1
28922	1.59169	2.1
28923	2.18608	2.1
28924	3.76662	2.1
28925	2.54018	2.1
28926	2.18608	2.1
28927	2.19104	2.1
28928	2.5392	2.1
28929	4.72528	2.1
28930	1.60497	2.1
28931	2.18608	2.1
28932	1.59169	2.1
28933	1.59169	2.1
28934	2.19105	2.1
28935	2.19105	2.1
28936	2.58661	2.1
28937	1.06203	2.1
28938	2.7043	2.1
28939	2.21397	2.1

28940	1.55236	2.1
28941	1.06203	2.1
28942	1.89226	1.44922
28943	1.89226	2.0099
28944	1.89226	0.38179
28945	1.89226	2.02681
28946	3.78453	1.47771
28947	1.08195	1.74488
28948	0.990019	2.1
28949	1.31976	1.96861
28950	1.4539	0.441216
28951	2.40171	2.1
28952	1.89226	1.13213
28953	1.89226	2.1
28954	1.89226	1.07648
28955	1.89226	0.169636
28956	3.78453	1.3411
28957	1.89226	1.88898
28958	1.89226	1.45412
28959	1.89226	0.625365
28960	1.89226	2.1
28961	3.78453	1.25945
28962	1.43895	2.1
28963	1.31976	2.07542
28964	1.70066	0.263886
28965	1.33783	2.1
28966	1.43895	2.1
28967	1.07612	2.1
28968	0.65988	1.98168
28969	0.850329	1.40009
28970	0.919598	0.748276
28971	0.726952	1.68409
28972	0.788106	0.301797
28973	0.788106	2.1
28974	0.789175	2.1
28975	0.789175	0.130011
28976	0.850329	0.829983
28977	0.919598	0.620131
28978	0.659879	1.76223
28979	0.726952	1.33574
28980	1.34389	2.1
28981	1.41279	1.98994
28982	1.15564	2.1
28983	1.15564	2.1
28984	1.55998	2.0619
28985	1.55998	2.1
28986	1.89226	1.79633
28987	1.89226	2.08868
28988	1.89226	2.1
28989	1.89226	1.14984
28990	1.89226	2.1
28991	1.89226	1.27978
28992	1.55998	2.1
28993	1.41279	2.1
28994	1.72901	2.09495
28995	1.62414	2.1
28996	3.1418	2.1
28997	1.89226	1.92126

28998	1.89226	2.1
28999	1.89226	2.1
29000	1.89226	1.08935
29001	3.78452	2.07399
29002	1.89226	0.429952
29003	1.89226	2.1
29004	1.89226	1.57249
29005	1.89226	2.1
29006	1.89226	0.819873
29007	1.89226	1.30961
29008	1.62414	2.1
29009	1.01243	2.1
29010	1.70932	2.1
29011	1.41279	1.7597
29012	1.30896	1.95842
29013	1.01243	1.38619
29014	1.9719	2.1
29015	1.10725	2.1
29016	1.12411	2.1
29017	1.98691	2.1
29018	3.09601	2.1
29019	1.97189	2.1
29020	2.47932	2.1
29021	2.49092	2.1
29022	1.98691	2.1
29023	4.46282	2.1
29024	1.1486	2.1
29025	1.98691	2.1
29026	1.12411	2.1
29027	1.12411	2.1
29028	2.01602	2.1
29029	2.01602	2.1
29030	2.49093	2.1
29031	1.98691	2.1
29032	2.01601	2.1
29033	2.51651	2.1
29034	4.50694	2.1
29035	1.93147	0.05
29036	2.51593	0.05
29037	1.35146	0.05
29038	1.35146	0.05
29039	2.48665	0.05
29040	1.90664	0.05
29041	1.63979	2.1
29042	1.7457	2.1
29043	1.66046	2.1
29044	1.56558	2.1
29045	3.31128	2.1
29046	0.819895	2.1
29047	0.782792	2.1
29048	0.60239	2.1
29049	0.54831	2.1
29050	0.688347	2.1
29051	0.688347	2.1
29052	0.679857	2.1
29053	0.679857	2.1
29054	0.782792	2.1
29055	0.54831	2.1

29056	0.60239	2.1
29057	0.819895	2.1
29058	1.66046	2.1
29059	1.42485	2.1
29060	1.56558	2.1
29061	1.56558	2.1
29062	1.52995	2.1
29063	1.52995	2.1
29064	2.58101	0.05
29065	2.09803	2.1
29066	2.408 0.05	
29067	1.86893	2.1
29068	4.44994	1.61289
29069	2.36062	2.1
29070	1.25104	2.1
29071	1.25104	2.1
29072	1.39703	2.1
29073	1.86893	2.1
29074	2.01492	2.1
29075	2.408 0.05	
29076	1.86893	2.1
29077	2.22575	0.0606317
29078	1.70677	2.1
29079	4.09468	1.64875
29080	0.737605	2.1
29081	0.737605	2.1
29082	0.741302	2.1
29083	0.741302	2.1
29084	0.55189	2.1
29085	0.625517	2.1
29086	0.934468	2.1
29087	0.853389	2.1
29088	0.853389	2.1
29089	0.55189	2.1
29090	0.625517	2.1
29091	0.934467	2.1
29092	2.35053	2.1
29093	1.09615	2.1
29094	1.25998	2.1
29095	2.51216	2.1
29096	3.61051	2.1
29097	1.33636	2.1
29098	1.25609	2.1
29099	0.698455	2.1
29100	0.629995	2.1
29101	0.986092	2.1
29102	0.986092	2.1
29103	0.980263	2.1
29104	0.980263	2.1
29105	1.33636	2.1
29106	0.698455	2.1
29107	0.629995	2.1
29108	1.25609	2.1
29109	1.89226	0.675333
29110	1.89226	0.939803
29111	1.89226	1.04307
29112	1.89226	0.805564
29113	3.78452	0.793997

29114	1.74321	0.511352
29115	1.89226	0.546632
29116	1.91755	0.652085
29117	1.91755	0.618551
29118	1.89226	0.600259
29119	1.89226	0.612033
29120	0.946132	0.817373
29121	0.946132	0.698713
29122	0.949798	0.321944
29123	1.08054	0.539353
29124	1.08054	0.41409
29125	1.08054	0.526811
29126	0.946132	0.720027
29127	0.946132	0.71028
29128	0.946132	0.663003
29129	0.949798	0.292041
29130	1.08054	0.442866
29131	0.946131	0.995493
29132	1.89226	1.04689
29133	1.89226	0.614574
29134	1.89226	0.543555
29135	1.89226	0.63029
29136	1.89226	0.712602
29137	1.89226	0.82307
29138	2.39378	0.05
29139	2.29234	0.05
29140	2.29234	0.05
29141	2.7933	0.05
29142	1.18422	0.05
29143	1.68518	0.05
29144	2.79329	0.05
29145	1.89226	0.05
29146	2.29233	0.05
29147	1.89226	0.05
29148	4.18459	0.05
29149	0.857046	0.05
29150	0.857037	0.05
29151	0.94613	0.05
29152	0.94613	0.05
29153	0.851539	0.05
29154	0.851539	0.05
29155	1.0315	0.05
29156	0.952867	0.05
29157	0.851486	0.05
29158	0.952869	0.05
29159	1.03156	0.05
29160	0.851528	0.05
29161	1.61981	2.1
29162	1.93096	2.1
29163	2.00776	2.1
29164	1.62218	2.1
29165	3.62757	2.1
29166	2.00776	2.1
29167	1.68541	2.1
29168	1.83215	2.1
29169	1.62218	2.1
29170	2.21773	2.1
29171	2.00776	2.1

29172	1.89188	0.05
29173	1.70303	0.05
29174	1.89226	0.05
29175	1.72465	0.05
29176	1.89226	0.05
29177	1.70552	0.05
29178	2.48659	0.05
29179	2.0815	0.05
29180	1.89226	0.05
29181	2.08111	0.05
29182	1.89226	0.05
29183	2.08111	0.05
29184	2.35332	0.05
29185	1.83284	0.05
29186	2.28483	0.05
29187	2.71955	0.05
29188	4.63815	0.05
29189	1.89227	2.1
29190	1.89227	2.1
29191	1.89227	2.1
29192	1.89227	2.1
29193	3.78453	2.1
29194	1.33506	2.1
29195	1.17129	2.1
29196	1.33506	2.1
29197	1.38415	2.1
29198	1.13664	2.1
29199	1.18573	2.1
29200	1.89226	2.1
29201	2.68785	2.1
29202	1.89226	2.1
29203	2.6692	2.1
29204	4.58011	2.1
29205	1.89226	2.1
29206	1.89226	2.1
29207	1.89226	2.1
29208	1.89226	2.1
29209	3.78453	2.1
29210	1.33506	2.1
29211	1.13664	2.1
29212	1.27575	2.1
29213	1.09627	2.1
29214	2.43133	2.1
29215	1.89226	2.1
29216	2.65349	2.1
29217	2.66919	2.1
29218	2.66919	2.1
29219	1.89226	2.1
29220	1.89226	2.1
29221	1.89226	2.1
29222	1.89226	2.1
29223	1.89226	2.1
29224	1.89226	2.1
29225	3.78453	2.1
29226	1.16834	2.1
29227	1.16834	2.1
29228	0.946132	2.1
29229	0.946132	2.1

29230	1.26978	2.1
29231	1.16834	2.1
29232	0.94613	2.1
29233	0.94613	2.1
29234	0.94613	2.1
29235	1.26978	2.1
29236	0.94613	2.1
29237	1.16834	2.1
29238	2.14698	2.1
29239	1.89226	2.1
29240	1.89226	2.1
29241	1.89226	2.1
29242	2.40719	2.1
29243	2.40719	2.1
29244	1.89226	2.1
29245	1.89226	2.1
29246	1.89226	2.1
29247	1.89226	2.1
29248	3.78453	2.1
29249	1.26977	2.1
29250	1.26977	2.1
29251	0.946133	2.1
29252	0.946133	2.1
29253	1.37467	2.1
29254	1.26978	2.1
29255	0.946131	2.1
29256	0.946131	2.1
29257	1.37467	2.1
29258	0.94613	2.1
29259	0.946131	2.1
29260	1.26978	2.1
29261	1.2036	2.1
29262	1.34912	2.1
29263	0.946131	2.1
29264	0.946131	2.1
29265	1.34912	2.1
29266	1.34912	2.1
29267	0.946129	2.1
29268	0.946129	2.1
29269	1.34912	2.1
29270	0.94613	2.1
29271	0.946131	2.1
29272	1.2036	2.1
29273	2.47164	0.0997967
29274	1.89019	0.05
29275	1.79109	0.081239
29276	2.41632	0.05
29277	4.26273	0.0935488
29278	1.54197	0.0543365
29279	2.41632	0.05
29280	1.79109	0.05
29281	1.79109	0.0635797
29282	2.20929	0.05
29283	2.20929	0.0562397
29284	1.70302	0.587345
29285	1.48104	0.913416
29286	1.99365	1.08882
29287	1.32897	0.498624

29288	1.70302	1.12119
29289	1.14034	0.519283
29290	1.70302	0.657447
29291	1.99365	0.656048
29292	1.99297	0.941496
29293	2.36337	0.289736
29294	4.04804	0.508394
29295	1.51569	0.441392
29296	2.49093	0.340477
29297	1.6026	0.561009
29298	1.43229	0.202123
29299	2.0815	0.669929
29300	1.83402	0.24336
29301	2.54665	2.1
29302	2.34044	2.1
29303	2.08889	2.1
29304	2.37653	2.1
29305	4.71697	2.1
29306	2.54665	2.1
29307	2.45212	2.1
29308	2.31012	2.1
29309	2.37653	2.1
29310	4.82865	2.1
29311	2.08889	2.1
29312	2.37653	2.1
29313	2.23366	2.1
29314	1.87598	2.1
29315	4.25251	2.1
29316	2.31012	2.1
29317	2.37653	2.1
29318	2.23367	2.1
29319	2.17384	2.1
29320	4.55037	2.1
29321	0.946131	2.1
29322	0.946131	2.1
29323	1.30338	2.1
29324	1.23186	2.1
29325	0.94613	2.1
29326	0.94613	2.1
29327	1.23187	2.1
29328	1.23187	2.1
29329	0.946131	2.1
29330	1.23186	2.1
29331	1.30338	2.1
29332	0.946131	2.1
29333	1.89226	2.1
29334	2.46373	2.1
29335	2.34725	2.1
29336	2.46373	2.1
29337	1.77579	2.1
29338	1.89226	2.1
29339	2.61901	2.1
29340	2.45899	2.1
29341	2.64067	2.1
29342	2.52708	2.1
29343	5.14609	2.1
29344	2.41795	2.1
29345	2.0815	2.1

29346	2.52708	2.1
29347	1.92277	2.1
29348	4.31368	2.1
29349	2.64067	2.1
29350	2.52708	2.1
29351	2.58638	2.1
29352	2.51039	2.1
29353	5.11346	2.1
29354	2.52708	2.1
29355	2.13642	2.1
29356	2.51039	2.1
29357	2.16633	2.1
29358	4.69341	2.1
29359	1.61028	1.48452
29360	1.65712	0.189615
29361	1.35002	0.0691923
29362	1.35002	1.57103
29363	1.88637	0.179631
29364	1.88637	2.1
29365	2.57663	2.1
29366	2.24227	2.1
29367	2.48354	2.1
29368	2.14317	2.1
29369	4.7198	2.1
29370	2.24227	2.1
29371	1.77353	2.1
29372	2.14317	2.1
29373	1.69956	2.1
29374	3.9167	2.1
29375	2.48353	2.1
29376	2.14317	2.1
29377	2.39282	2.1
29378	1.79824	2.1
29379	4.53599	2.1
29380	2.14317	2.1
29381	1.69956	2.1
29382	1.91427	2.1
29383	1.6347	2.1
29384	3.77787	2.1
29385	1.89226	0.897932
29386	1.89226	1.17063
29387	1.89226	1.10951
29388	1.89226	0.911044
29389	3.78453	1.70008
29390	1.89226	0.898587
29391	1.89226	0.676206
29392	1.89226	0.827585
29393	1.89226	0.699996
29394	3.78452	0.312595
29395	1.89226	0.671644
29396	1.89652	1.47351
29397	1.89226	0.212331
29398	1.89226	0.928461
29399	1.19352	0.765295
29400	1.19352	2.1
29401	1.89226	1.02444
29402	1.89653	0.382263
29403	1.89226	1.5891

29404	1.89226	1.12521
29405	1.89226	0.747032
29406	1.89226	0.761918
29407	1.89226	1.34945
29408	1.75326	0.713808
29409	1.89226	2.1
29410	1.12948	1.481
29411	1.89226	0.686731
29412	1.12948	0.322666
29413	2.61064	2.1
29414	1.89226	2.1
29415	1.89226	2.1
29416	1.89226	2.1
29417	2.6085	2.1
29418	2.6085	2.1
29419	2.6085	2.1
29420	1.89226	2.1
29421	1.89226	2.1
29422	1.89226	2.1
29423	2.62161	2.1
29424	2.62161	2.1
29425	2.16535	2.1
29426	1.44603	2.1
29427	2.79784	2.1
29428	1.84756	2.1
29429	4.01292	2.1
29430	2.65262	2.1
29431	1.03391	2.1
29432	2.20239	2.1
29433	1.74416	2.1
29434	1.49213	2.1
29435	1.03391	2.1
29436	2.29066	2.1
29437	2.49174	2.1
29438	2.56502	2.1
29439	2.33802	2.1
29440	4.85568	2.1
29441	2.39663	2.1
29442	2.49174	2.1
29443	2.56502	2.1
29444	2.45929	2.1
29445	4.96165	2.1
29446	2.56502	2.1
29447	2.33802	2.1
29448	2.37168	2.1
29449	2.59967	2.1
29450	4.9367	2.1
29451	2.56502	2.1
29452	2.45929	2.1
29453	2.50138	2.1
29454	2.59967	2.1
29455	5.0664	2.1
29456	2.27009	0.99018
29457	1.26247	2.1
29458	1.86812	2.1
29459	2.837	1.58695
29460	4.13822	2.1
29461	1.89226	1.32703

29462	1.89226	2.1
29463	1.89226	2.1
29464	1.89226	2.1
29465	3.78453	2.1
29466	1.75835	2.1
29467	1.72866	2.1
29468	1.65313	2.1
29469	1.64822	2.1
29470	3.3818	2.1
29471	1.72866	2.1
29472	1.58401	2.1
29473	1.64822	2.1
29474	1.52441	2.1
29475	3.25307	2.1
29476	1.89226	2.1
29477	1.89226	2.1
29478	1.89226	2.1
29479	1.89226	2.1
29480	3.78452	2.1
29481	1.89226	2.1
29482	1.89226	2.1
29483	1.89226	1.35488
29484	1.89226	2.1
29485	3.78453	2.1
29486	1.65313	2.1
29487	1.64822	2.1
29488	1.55779	2.1
29489	1.57968	2.1
29490	3.23281	2.1
29491	1.64822	2.1
29492	1.52441	2.1
29493	1.57967	2.1
29494	1.474	2.1
29495	3.10408	2.1
29496	1.89226	2.1
29497	1.89226	2.1
29498	1.89226	2.1
29499	1.89226	2.1
29500	3.78452	2.1
29501	1.86067	1.21479
29502	1.58407	2.1
29503	2.07965	0.484302
29504	1.83123	1.73877
29505	3.66372	0.941523
29506	1.89226	0.57328
29507	1.89226	2.1
29508	1.89226	0.911595
29509	1.89226	1.97906
29510	3.78452	1.31616
29511	1.89226	0.79594
29512	1.89226	2.1
29513	1.89226	0.341401
29514	1.89226	2.1
29515	1.89226	0.181168
29516	1.89226	2.1
29517	1.83123	0.589768
29518	1.03472	2.1
29519	1.91068	2.0063

29520	1.58406	1.05519
29521	1.36133	1.01519
29522	1.03472	0.608299
29523	2.07965	0.96609
29524	1.83123	1.87003
29525	2.28385	0.765999
29526	2.09114	1.45867
29527	4.1708	1.78343
29528	1.89226	1.39303
29529	1.89226	2.1
29530	1.89226	0.474494
29531	1.89226	1.79351
29532	3.78452	0.856178
29533	1.89226	0.712982
29534	1.89226	2.1
29535	1.89226	0.759249
29536	1.89226	2.1
29537	3.78452	1.11544
29538	1.83123	0.603641
29539	1.36133	2.1
29540	2.09114	0.456682
29541	1.69436	1.88844
29542	3.45248	1.08355
29543	1.89226	2.1
29544	1.89226	2.1
29545	1.89226	2.1
29546	1.89226	2.1
29547	3.78453	2.1
29548	1.53278	2.1
29549	1.67571	2.1
29550	1.40182	2.1
29551	1.55686	2.1
29552	3.07752	2.1
29553	1.67571	2.1
29554	1.52615	0.0524756
29555	1.55686	2.1
29556	1.38963	0.05
29557	3.06534	1.93435
29558	1.89226	2.1
29559	1.89226	0.0699492
29560	1.89226	2.1
29561	1.89226	0.05
29562	3.78452	1.63972
29563	1.89226	2.1
29564	1.89226	2.1
29565	1.89226	2.1
29566	1.89226	2.1
29567	3.78453	2.1
29568	1.40182	2.1
29569	1.55686	2.1
29570	1.24509	2.1
29571	1.38426	2.1
29572	2.78608	2.1
29573	1.55686	2.1
29574	1.38963	0.097923
29575	1.2064	2.1
29576	1.2064	0.258516
29577	1.38426	2.1

29578	1.56749	0.970191
29579	0.810946	2.1
29580	0.810946	0.0873841
29581	0.810945	2.1
29582	0.810945	2.1
29583	0.810945	2.1
29584	0.810945	1.96654
29585	1.89226	0.15995
29586	1.35169	0.808871
29587	1.08132	2.1
29588	1.08132	0.05
29589	1.08132	1.99182
29590	1.28418	0.136886
29591	1.59382	0.0907776
29592	1.78289	0.174769
29593	1.51021	0.05
29594	3.10403	0.0754539
29595	1.76897	1.18515
29596	1.59382	0.05
29597	1.78289	0.164554
29598	1.94302	0.193297
29599	3.53684	0.22196
29600	1.89226	0.05
29601	1.89226	0.0991024
29602	1.89226	0.1492
29603	1.89226	0.05
29604	3.78453	0.05
29605	1.89227	0.0898663
29606	1.89227	0.0527956
29607	1.89227	0.05
29608	1.89227	0.069071
29609	1.89227	0.05
29610	1.89227	0.0824461
29611	1.78286	0.18329
29612	1.51019	0.05
29613	1.79979	0.0967175
29614	2.02511	0.0912918
29615	3.5353	0.11055
29616	1.78286	0.162804
29617	1.94299	0.163441
29618	2.17831	0.712488
29619	2.02511	0.0767883
29620	3.9681	0.0858669
29621	1.89224	0.129375
29622	1.89224	0.05
29623	1.89224	0.0664955
29624	1.89224	0.169289
29625	3.78448	0.11941
29626	1.89224	0.0757253
29627	1.89224	0.0517932
29628	1.89224	0.05
29629	1.89224	0.0717304
29630	1.89224	0.0560592
29631	1.89224	0.0939787
29632	1.83198	0.130108
29633	1.0961	0.05
29634	1.39448	0.146563
29635	1.99568	0.0729798

29636	3.09178	0.119475
29637	1.25503	0.0812088
29638	1.99568	0.0517353
29639	2.3661	0.153166
29640	1.70302	0.05
29641	3.55833	0.05
29642	2.69899	0.05
29643	1.85796	0.05
29644	2.11407	0.05
29645	1.36769	0.05
29646	3.97203	0.05
29647	1.89226	0.05
29648	1.89226	0.05
29649	1.89226	0.05
29650	1.89226	0.05
29651	3.78452	0.05
29652	1.89225	0.05
29653	2.63355	0.05
29654	2.09529	0.05
29655	2.09529	0.05
29656	1.89225	0.05
29657	1.89225	0.05
29658	1.89226	0.05
29659	1.89226	0.05
29660	1.89226	0.05
29661	1.89226	0.05
29662	1.89226	0.05
29663	1.89226	0.05
29664	2.12204	0.05
29665	1.89226	0.05
29666	2.05798	0.05
29667	2.01954	0.05
29668	1.70302	0.05
29669	1.3865	0.05
29670	1.28326	0.05
29671	1.33326	0.590346
29672	1.19052	0.05
29673	1.25369	0.203874
29674	2.53694	0.103727
29675	1.33327	0.571898
29676	1.27339	2.1
29677	1.25369	0.15903
29678	1.19733	2.1
29679	2.52708	2.1
29680	1.19052	0.05
29681	1.25369	0.210283
29682	1.10938	0.05
29683	1.17614	0.872082
29684	2.36307	0.05
29685	1.25369	0.12582
29686	1.19733	2.1
29687	1.17614	1.11855
29688	1.11868	2.1
29689	2.37237	2.1
29690	1.10747	0.05
29691	1.47688	0.05
29692	1.97732	0.05
29693	1.62206	0.05

29694	3.09894	0.05
29695	1.89226	0.05
29696	1.89226	0.05
29697	1.89226	0.05
29698	1.89226	0.05
29699	1.89226	0.05
29700	1.89226	0.05
29701	1.62206	0.05
29702	1.70302	0.05
29703	1.22962	0.05
29704	1.1893	0.05
29705	2.15177	0.05
29706	2.08121	0.05
29707	1.97732	0.05
29708	2.08121	0.05
29709	1.62206	0.05
29710	1.62206	0.05
29711	2.41464	0.05
29712	2.41464	0.05
29713	1.89226	0.05
29714	1.89226	0.05
29715	1.89226	0.05
29716	1.89226	0.05
29717	3.78452	0.05
29718	0.946133	0.05
29719	0.946133	0.05
29720	0.946132	0.05
29721	0.946132	0.05
29722	0.946132	0.05
29723	0.946132	0.05
29724	0.946133	0.05
29725	0.946133	0.05
29726	0.946132	0.05
29727	0.946132	0.05
29728	0.946132	0.05
29729	0.946132	0.05
29730	1.83157	0.224581
29731	2.19989	0.167367
29732	2.20664	0.0562729
29733	1.81487	0.0715369
29734	4.03146	0.152308
29735	2.41055	2.1
29736	2.19989	2.1
29737	2.20665	2.08218
29738	2.38023	1.58115
29739	4.61044	2.02534
29740	2.19989	2.1
29741	2.22558	2.1
29742	2.38023	2.0537
29743	2.07065	0.158868
29744	4.42547	1.98284
29745	2.19988	0.328815
29746	1.70303	0.05
29747	1.89939	0.104188
29748	1.86357	0.0780726
29749	3.78536	0.103021
29750	1.04921	2.1
29751	1.30711	2.1

29752	1.53076	2.1
29753	1.22927	2.1
29754	2.57997	2.1
29755	1.53076	2.1
29756	1.46568	2.1
29757	1.56056	2.1
29758	1.22927	2.1
29759	1.86206	2.1
29760	1.53076	2.1
29761	1.18353	2.1
29762	2.28593	2.1
29763	1.16979	2.1
29764	2.2731	2.1
29765	3.45571	2.1
29766	0.854776	2.1
29767	0.854776	2.1
29768	0.855641	2.1
29769	0.855641	2.1
29770	1.12552	2.1
29771	1.13655	2.1
29772	0.57216	2.1
29773	0.584893	2.1
29774	0.57216	2.1
29775	1.12552	2.1
29776	0.584893	2.1
29777	1.13655	2.1
29778	1.54462	2.1
29779	2.76309	2.1
29780	1.34869	2.1
29781	2.48573	2.1
29782	4.03035	2.1
29783	1.35328	1.07127
29784	1.0383	0.900998
29785	1.03318	1.67066
29786	0.704834	1.56736
29787	1.0257	1.52746
29788	1.0257	0.932934
29789	1.03241	0.574504
29790	1.03241	1.74458
29791	1.35328	1.20921
29792	1.03319	1.7064
29793	1.0383	1.44791
29794	0.704835	1.31734
29795	2.27126	0.05
29796	2.77291	2.1
29797	1.81771	0.345496
29798	2.6111	0.287282
29799	4.88236	0.335024
29800	2.77291	2.1
29801	1.89227	1.72043
29802	1.89227	0.78442
29803	1.89227	2.1
29804	2.6111	0.294096
29805	2.6111	2.1
29806	2.22061	0.134894
29807	2.41522	0.118496
29808	2.41522	0.05
29809	2.6111	2.01119

29810	1.75765	0.05
29811	1.95353	2.1
29812	2.27502	0.237395
29813	1.89227	1.61103
29814	1.89227	0.310879
29815	2.8189	0.125885
29816	4.71116	0.18433
29817	1.89226	0.155609
29818	1.89226	1.68834
29819	1.89226	0.516275
29820	1.89226	0.229245
29821	1.89226	1.11297
29822	1.89226	0.508197
29823	1.89226	1.85404
29824	1.89226	1.74744
29825	1.89226	1.51266
29826	1.89226	1.17987
29827	3.78453	1.79348
29828	1.89226	1.74328
29829	2.57079	0.61765
29830	2.5932	1.30912
29831	1.89226	1.1177
29832	4.48546	1.12801
29833	0.589396	1.22573
29834	0.641454	0.799159
29835	0.814062	1.5142
29836	0.726518	1.2094
29837	0.675126	1.39086
29838	0.675125	1.32427
29839	0.692847	1.52446
29840	0.692847	1.23735
29841	0.641454	1.03773
29842	0.814062	1.39175
29843	0.726518	1.54397
29844	0.589396	1.23755
29845	1.89226	1.64044
29846	1.89226	1.18584
29847	1.89226	1.5547
29848	1.89226	1.88328
29849	1.89226	1.27475
29850	1.89226	1.84714
29851	1.16906	0.245765
29852	1.2966	1.23905
29853	0.946132	1.05354
29854	0.946132	1.80989
29855	1.16906	1.06547
29856	1.16906	0.439342
29857	0.946132	1.28118
29858	0.946132	1.42326
29859	1.16906	1.48986
29860	0.946131	1.3744
29861	1.2966	1.17954
29862	0.946132	1.75561
29863	0.641455	1.25947
29864	0.567191	0.691466
29865	0.807594	1.59517
29866	0.814062	1.23146
29867	0.673735	1.57309

29868	0.673734	1.10722
29869	0.707519	1.26866
29870	0.707519	1.65572
29871	0.807594	1.46006
29872	0.56719	1.77572
29873	0.641455	1.47939
29874	0.814063	1.85263
29875	1.89225	2.1
29876	1.89225	2.1
29877	1.89225	2.1
29878	1.89225	2.1
29879	3.7845	2.1
29880	1.89225	2.1
29881	1.89225	2.1
29882	1.89225	2.1
29883	1.89225	2.1
29884	1.89225	2.1
29885	1.89225	2.1
29886	1.89226	2.1
29887	1.89226	2.1
29888	1.89226	2.1
29889	1.89226	2.1
29890	3.78452	2.1
29891	1.89226	2.1
29892	1.89226	2.1
29893	1.89226	2.1
29894	1.89226	2.1
29895	1.89226	2.1
29896	1.89226	2.1
29897	1.89226	2.1
29898	1.89226	2.1
29899	1.89226	2.1
29900	1.89226	2.1
29901	3.78452	2.1
29902	1.89226	2.1
29903	1.38928	2.1
29904	1.89226	2.1
29905	1.51378	2.1
29906	1.89061	2.1
29907	1.89226	2.1
29908	1.89226	2.1
29909	1.89226	2.1
29910	1.89226	2.1
29911	1.89226	2.1
29912	3.78453	2.1
29913	1.04572	2.1
29914	0.994213	2.1
29915	0.912466	2.1
29916	0.912466	2.1
29917	0.947743	2.1
29918	1.00388	2.1
29919	0.900913	2.1
29920	0.900913	2.1
29921	0.900915	2.1
29922	0.946131	2.1
29923	0.946131	2.1
29924	0.999196	2.1
29925	1.89226	2.1

29926	1.89226	2.1
29927	1.89226	2.1
29928	1.89226	2.1
29929	3.78453	2.1
29930	0.946161	2.1
29931	1.1354	2.1
29932	0.850847	2.1
29933	0.850847	2.1
29934	1.01445	2.1
29935	1.02106	2.1
29936	0.946131	2.1
29937	0.946131	2.1
29938	1.13537	2.1
29939	1.01444	2.1
29940	0.850877	2.1
29941	0.946131	2.1
29942	1.80183	2.1
29943	1.89226	2.1
29944	1.89226	2.1
29945	1.89226	2.1
29946	1.79606	2.1
29947	1.79606	2.1
29948	1.24001	2.06423
29949	1.41822	1.637
29950	1.95665	0.797986
29951	1.77342	1.50849
29952	3.19666	1.27555
29953	1.95665	1.28516
29954	1.44859	1.93752
29955	1.41821	1.80567
29956	1.41821	1.86186
29957	1.95543	1.19341
29958	1.95543	1.5648
29959	1.95665	1.17654
29960	1.77342	1.61526
29961	2.33611	1.4177
29962	2.49694	0.70069
29963	4.29276	1.6709
29964	1.95665	1.62872
29965	1.95544	1.56424
29966	2.4728	0.418515
29967	2.49693	1.30993
29968	4.42945	1.24115
29969	2.24937	2.1
29970	2.49053	2.1
29971	2.16323	2.1
29972	2.36377	2.1
29973	4.61314	2.1
29974	2.49053	2.1
29975	2.58639	2.1
29976	2.36377	2.1
29977	2.47395	2.1
29978	4.95016	2.1
29979	2.45054	2.1
29980	2.36377	2.1
29981	1.92911	2.1
29982	2.181	2.1
29983	4.29288	2.1

29984	2.36377	2.1
29985	2.47395	2.1
29986	2.181 2.1	
29987	2.29881	2.1
29988	4.66258	2.1
29989	1.57885	2.1
29990	1.13339	2.1
29991	1.48594	2.1
29992	1.13322	2.1
29993	2.71207	2.1
29994	1.48596	2.1
29995	1.20761	2.1
29996	1.20761	2.1
29997	1.13323	2.1
29998	1.46568	2.1
29999	1.3913	2.1
30000	1.89226	0.338201
30001	1.89226	1.44045
30002	1.89226	0.372796
30003	1.89226	1.35907
30004	1.89226	0.111396
30005	1.89226	0.478554
30006	1.11712	0.792497
30007	0.946129	1.23641
30008	0.686934	0.56644
30009	0.946133	0.410273
30010	0.9461	0.756851
30011	1.10729	2.1
30012	0.686963	1.32676
30013	0.686963	0.190096
30014	1.10945	0.906772
30015	0.80397	2.1
30016	0.946131	0.415537
30017	0.946131	1.30878
30018	1.89226	2.1
30019	1.89226	1.78346
30020	1.89226	1.4921
30021	1.89226	2.06921
30022	3.78452	2.01802
30023	1.22615	2.1
30024	1.3439	2.1
30025	1.47868	2.1
30026	1.55998	2.07396
30027	2.78613	2.1
30028	1.04973	2.1
30029	1.47868	2.1
30030	1.20149	2.1
30031	1.61721	2.1
30032	2.68017	2.1
30033	1.89226	2.1
30034	1.89226	2.1
30035	1.89226	2.1
30036	1.89226	0.843946
30037	3.78452	2.1
30038	0.946133	0.310455
30039	0.946133	2.1
30040	0.946128	2.1
30041	0.946128	2.1

30042	0.946131	0.201842
30043	0.946131	1.99585
30044	0.946131	2.1
30045	0.946131	0.95913
30046	0.94613	2.1
30047	0.94613	1.9564
30048	0.946131	2.1
30049	0.946131	1.91152
30050	0.798126	2.1
30051	0.798126	2.1
30052	0.805719	2.1
30053	0.805719	2.1
30054	0.779992	2.1
30055	0.864507	2.1
30056	0.739338	2.1
30057	0.808607	2.1
30058	0.808606	2.1
30059	0.864506	2.1
30060	0.739339	2.1
30061	0.779993	2.1
30062	0.946128	2.1
30063	0.946128	2.1
30064	1.18959	1.78107
30065	1.00304	2.1
30066	1.00304	2.1
30067	1.00304	2.1
30068	0.94613	2.1
30069	0.94613	1.91045
30070	0.94613	2.1
30071	1.18959	2.1
30072	1.00304	2.1
30073	0.94613	2.1
30074	2.19163	2.1
30075	1.89226	2.1
30076	2.16541	2.1
30077	1.89226	1.79779
30078	4.08389	2.1
30079	1.89226	2.1
30080	1.89226	1.88839
30081	1.89226	2.1
30082	1.89226	0.96226
30083	3.78452	2.1
30084	0.964782	2.1
30085	1.30151	0.833914
30086	1.30318	0.492641
30087	1.30318	2.1
30088	0.949134	0.344614
30089	0.949134	2.1
30090	2.15005	2.1
30091	1.89226	2.1
30092	2.16541	2.1
30093	2.16541	2.1
30094	1.89226	1.92677
30095	1.89226	2.1
30096	1.89226	2.1
30097	1.89226	2.1
30098	1.89226	2.1
30099	1.89226	0.151678

30100	3.78452	1.3062
30101	0.949134	2.1
30102	1.30151	2.1
30103	0.949134	2.1
30104	1.32143	0.802326
30105	2.25064	2.1
30106	0.982859	0.05
30107	1.28326	0.05
30108	1.19052	0.05
30109	1.19052	0.111225
30110	1.10476	0.05
30111	1.10476	0.0802078
30112	1.37265	0.330839
30113	1.38247	0.393989
30114	1.36636	0.901216
30115	1.3294	0.087088
30116	2.71187	0.154141
30117	1.89226	0.569265
30118	1.89226	0.0632955
30119	1.89226	0.158659
30120	1.89226	0.168505
30121	1.89226	0.16133
30122	1.89226	0.42525
30123	1.36636	1.27607
30124	1.3294	0.0588919
30125	1.30001	0.254118
30126	1.36416	0.884298
30127	2.69356	0.498318
30128	1.89226	1.07128
30129	1.89226	0.0782191
30130	1.89226	0.135995
30131	1.89226	0.717845
30132	1.89226	0.0687931
30133	1.89226	0.294746
30134	2.83701	0.37098
30135	1.89226	0.237034
30136	1.89226	0.844246
30137	2.79312	0.062829
30138	4.68539	0.0914305
30139	1.44288	2.1
30140	0.948677	2.1
30141	1.84076	2.1
30142	1.42884	2.1
30143	2.78943	2.1
30144	1.89226	2.1
30145	1.89226	2.1
30146	1.89226	2.1
30147	1.89226	2.1
30148	3.78452	2.1
30149	0.909402	2.1
30150	0.909402	2.1
30151	1.3063	2.1
30152	1.56757	2.1
30153	1.00038	2.1
30154	1.5235	2.1
30155	0.946127	2.1
30156	0.946127	2.1
30157	0.946128	2.1

30158	1.26957	2.1
30159	1.00038	2.1
30160	0.946129	2.1
30161	1.17256	2.1
30162	2.2846	2.1
30163	1.1844	2.1
30164	2.28904	2.1
30165	3.469	2.1
30166	0.867886	2.1
30167	0.867886	2.1
30168	0.867277	2.1
30169	0.867277	2.1
30170	1.14296	2.1
30171	1.14452	2.1
30172	0.591766	2.1
30173	0.592199	2.1
30174	0.591766	2.1
30175	1.14296	2.1
30176	0.5922	2.1
30177	1.14452	2.1
30178	1.27408	0.05
30179	1.56339	0.0511746
30180	1.52522	0.05
30181	1.34447	0.05
30182	2.90786	0.05
30183	1.27408	0.05
30184	1.01121	0.05
30185	0.991868	0.05
30186	1.34447	0.05
30187	2.35569	0.05
30188	0.944818	0.0529875
30189	0.883008	0.05
30190	0.66957	0.05
30191	0.616883	0.05
30192	0.755679	0.05
30193	0.755679	0.0588142
30194	0.791912	0.0746145
30195	0.701943	0.05
30196	0.901541	0.05
30197	0.634847	0.05
30198	0.82781	0.05
30199	0.655405	0.0519066
30200	1.73819	2.1
30201	1.70303	2.1
30202	1.70303	2.1
30203	1.70303	2.1
30204	1.94516	2.1
30205	1.94517	2.1
30206	0.94994	2.1
30207	2.07469	2.1
30208	1.08506	2.1
30209	2.0815	2.1
30210	3.12902	2.1
30211	0.946131	2.1
30212	0.946131	2.1
30213	0.946131	2.1
30214	0.946131	2.1
30215	0.946128	2.1

30216	0.946128	2.1
30217	0.946133	2.1
30218	0.946133	2.1
30219	0.946131	2.1
30220	0.946131	2.1
30221	0.94613	2.1
30222	0.94613	2.1
30223	2.51796	2.1
30224	1.64193	2.1
30225	2.51172	2.1
30226	1.68893	2.1
30227	4.20689	2.1
30228	2.51172	2.1
30229	1.70002	2.1
30230	1.70002	2.1
30231	1.68893	2.1
30232	2.48689	2.1
30233	2.4758	2.1
30234	2.27963	0.05
30235	2.21582	0.05
30236	2.60486	0.05
30237	2.5105	0.05
30238	4.79013	0.05
30239	2.21582	0.05
30240	2.02069	0.05
30241	2.5105	0.05
30242	2.28615	0.05
30243	4.53119	0.05
30244	2.60486	0.05
30245	2.5105	0.05
30246	2.55791	0.05
30247	2.47141	0.05
30248	5.06842	0.05
30249	2.5105	0.05
30250	2.28615	0.05
30251	2.47141	0.05
30252	2.23337	0.05
30253	4.74387	0.05
30254	2.06984	2.1
30255	0.967499	2.1
30256	2.12435	2.1
30257	2.0021	2.1
30258	1.08975	2.1
30259	0.967499	2.1
30260	1.89226	0.05
30261	1.89226	0.05
30262	1.89226	0.05
30263	1.89226	0.05
30264	1.89226	0.05
30265	1.89226	0.05
30266	1.89226	2.1
30267	1.89226	2.1
30268	1.89226	2.1
30269	1.89226	2.1
30270	3.78452	2.1
30271	1.89227	2.1
30272	1.89227	2.1
30273	1.89227	2.1

30274	1.89227	2.1
30275	3.78453	2.1
30276	0.55882	2.1
30277	0.696462	2.1
30278	1.07003	2.1
30279	0.937179	2.1
30280	0.818077	2.1
30281	0.818077	2.1
30282	0.815565	2.1
30283	0.815565	2.1
30284	0.696462	2.1
30285	1.07003	2.1
30286	0.93718	2.1
30287	0.55882	2.1
30288	2.53857	2.1
30289	1.87436	2.1
30290	2.27541	2.1
30291	2.27541	2.1
30292	2.14005	2.1
30293	2.14005	2.1
30294	1.89226	2.1
30295	1.89226	2.1
30296	1.89226	2.1
30297	1.89226	2.1
30298	3.78452	2.1
30299	1.89227	2.1
30300	1.89227	2.1
30301	1.89227	2.1
30302	1.89227	2.1
30303	3.78453	2.1
30304	1.39292	2.1
30305	2.14006	2.1
30306	2.41083	2.1
30307	1.68761	2.1
30308	3.80376	2.1
30309	2.53857	2.1
30310	2.14005	2.1
30311	2.41083	2.1
30312	2.82043	2.1
30313	4.9494	2.1
30314	1.90439	0.05
30315	1.51835	0.05
30316	1.20473	0.05
30317	0.947693	0.05
30318	2.85208	0.05
30319	2.586	2.1
30320	1.46262	2.1
30321	2.14009	2.1
30322	2.78101	2.1
30323	4.72609	2.1
30324	1.89224	2.1
30325	2.78101	2.1
30326	2.586	2.1
30327	2.586	2.1
30328	1.89224	2.1
30329	1.89224	2.1
30330	0.994293	2.1
30331	1.19	2.1

30332	1.09483	2.1
30333	1.09483	2.1
30334	1.06827	2.1
30335	1.06827	2.1
30336	1.89226	2.1
30337	1.89226	2.1
30338	1.89226	2.1
30339	1.89226	2.1
30340	3.78452	2.1
30341	0.946132	2.1
30342	0.946132	2.1
30343	1.31912	2.1
30344	1.3572	2.1
30345	0.946131	2.1
30346	0.946131	2.1
30347	1.31912	2.1
30348	1.31912	2.1
30349	0.946131	2.1
30350	1.31912	2.1
30351	1.35721	2.1
30352	0.946131	2.1
30353	2.24577	0.157984
30354	1.09408	0.05
30355	1.60239	0.0771225
30356	1.09408	0.131535
30357	2.39404	0.0659596
30358	2.07382	0.05
30359	1.70303	0.05
30360	1.3216	0.05
30361	1.23104	0.05
30362	1.05475	0.05
30363	2.60408	0.05
30364	1.89226	0.0974874
30365	1.89226	0.0898872
30366	1.89226	0.0540497
30367	1.89226	0.0852436
30368	3.78452	0.11137
30369	1.89226	0.05
30370	2.3845	0.05
30371	2.6875	0.05
30372	1.89226	0.05
30373	2.6875	0.05
30374	1.89226	0.05
30375	1.46759	2.1
30376	1.17585	2.1
30377	1.85189	2.1
30378	1.39223	2.1
30379	2.85981	2.1
30380	1.85189	2.1
30381	1.71255	2.1
30382	1.71255	2.1
30383	1.39223	2.1
30384	2.28511	2.1
30385	1.96479	2.1
30386	1.89227	0.567751
30387	1.89227	0.901096
30388	1.89227	1.20877
30389	1.89227	0.434041

30390	1.89227	1.49427
30391	1.89227	0.728963
30392	1.89226	0.635647
30393	1.89226	0.80528
30394	1.89226	1.19994
30395	1.89226	0.335354
30396	1.89226	1.51971
30397	1.89226	0.55592
30398	1.89226	0.596677
30399	1.89226	1.07643
30400	1.89226	1.14367
30401	1.89226	0.332015
30402	1.89226	1.61513
30403	1.89226	0.674888
30404	1.89226	0.344306
30405	2.77788	0.481659
30406	2.21062	0.737219
30407	2.21062	0.227551
30408	1.89226	1.11037
30409	1.89226	0.358394
30410	0.868548	0.261213
30411	0.610104	0.457416
30412	0.796678	0.358887
30413	0.865021	0.204882
30414	1.0883	0.298738
30415	0.904938	0.425972
30416	2.21062	0.357258
30417	1.49548	0.368646
30418	1.2068	0.755902
30419	1.54764	0.376278
30420	0.790005	0.247071
30421	1.89226	2.1
30422	2.67457	2.1
30423	1.89226	2.1
30424	2.56352	2.1
30425	4.56683	2.1
30426	2.67456	2.1
30427	2.20011	2.1
30428	2.56351	2.1
30429	2.11083	2.1
30430	4.78539	2.1
30431	2.56348	2.1
30432	2.1108	2.1
30433	2.45763	2.1
30434	2.00986	2.1
30435	4.56843	2.1
30436	0.946133	0.05
30437	0.946133	0.05
30438	0.946132	0.05
30439	0.946132	0.05
30440	0.946132	0.05
30441	0.946132	0.05
30442	0.946132	0.05
30443	0.946132	0.05
30444	0.946132	0.05
30445	0.946132	0.05
30446	0.946133	0.05
30447	0.946133	0.05

30448	1.45927	0.451462
30449	1.25133	1.2656
30450	1.00082	0.887968
30451	1.41689	1.18366
30452	1.0432	0.601521
30453	1.45927	1.11807
30454	1.41689	0.855623
30455	1.29579	1.56115
30456	1.34874	1.1918
30457	1.25133	1.2924
30458	2.76563	1.80804
30459	0.675555	1.42225
30460	0.674371	1.28753
30461	0.625664	1.14803
30462	0.523133	1.1008
30463	0.676509	1.29448
30464	0.676509	1.60328
30465	0.62471	1.26485
30466	0.62471	0.934753
30467	0.675556	0.825007
30468	0.523132	0.607647
30469	0.674373	0.703023
30470	0.625663	0.889764
30471	1.89226	0.940947
30472	1.89226	0.890722
30473	1.89226	0.733895
30474	1.89226	0.810316
30475	3.78453	1.20266
30476	0.851517	1.39935
30477	0.946133	0.867532
30478	0.946132	1.18085
30479	0.946132	1.42774
30480	0.946133	0.485767
30481	0.851517	1.87895
30482	0.946132	1.14464
30483	0.946132	0.712999
30484	0.851516	1.00433
30485	0.851516	0.630407
30486	0.946133	1.48726
30487	0.946133	1.06891
30488	0.569995	0.773329
30489	0.816895	1.2996
30490	1.33893	1.28341
30491	1.18795	0.926531
30492	1.18795	0.835134
30493	0.569995	1.48222
30494	0.816895	1.43936
30495	1.33893	1.32739
30496	0.859572	1.55179
30497	0.859572	1.09864
30498	1.04936	1.19944
30499	1.04936	0.798719
30500	1.89226	2.1
30501	1.89226	2.1
30502	2.55618	2.1
30503	2.55618	2.1
30504	2.66983	2.1
30505	2.00591	2.1

30506	1.89226	0.697925
30507	2.64586	2.1
30508	2.83331	2.1
30509	2.83331	0.504922
30510	1.89226	2.1
30511	1.89226	0.187578
30512	2.64586	2.1
30513	2.0815	2.1
30514	1.99985	2.1
30515	1.94005	2.1
30516	2.83332	2.1
30517	2.74859	2.1
30518	1.98061	2.1
30519	1.68356	2.1
30520	1.83879	2.1
30521	1.55987	2.1
30522	3.54048	2.1
30523	1.68356	2.1
30524	1.20969	2.1
30525	1.20969	2.1
30526	1.30751	2.1
30527	1.55987	2.1
30528	1.65769	2.1
30529	1.83879	2.1
30530	1.55987	2.1
30531	1.68443	2.1
30532	1.4449	2.1
30533	3.2443	2.1
30534	0.660368	2.1
30535	0.660368	2.1
30536	0.666922	2.1
30537	0.666922	2.1
30538	0.560377	2.1
30539	0.604842	2.1
30540	0.779935	2.1
30541	0.722448	2.1
30542	0.722446	2.1
30543	0.560377	2.1
30544	0.604844	2.1
30545	0.779934	2.1
30546	1.89227	2.1
30547	1.89227	2.1
30548	1.89227	2.1
30549	1.89227	2.1
30550	3.78453	2.1
30551	1.42896	2.1
30552	1.23037	2.1
30553	1.42896	2.1
30554	1.474 2.1	
30555	1.2016	2.1
30556	1.24665	2.1
30557	1.89226	2.1
30558	2.7188	2.1
30559	1.89226	2.1
30560	2.70702	2.1
30561	4.61106	2.1
30562	1.89226	2.1
30563	1.89226	2.1

30564	1.89226	2.1
30565	1.89226	2.1
30566	3.78453	2.1
30567	0.644603	2.1
30568	0.644603	2.1
30569	0.648272	2.1
30570	0.648272	2.1
30571	0.585644	2.1
30572	0.600802	2.1
30573	0.692073	2.1
30574	0.714478	2.1
30575	0.585644	2.1
30576	0.692073	2.1
30577	0.714478	2.1
30578	0.600802	2.1
30579	1.35351	2.1
30580	1.35351	2.1
30581	0.946131	2.1
30582	0.946131	2.1
30583	1.34392	2.1
30584	1.35351	2.1
30585	0.946132	2.1
30586	0.946132	2.1
30587	0.946132	2.1
30588	1.34392	2.1
30589	0.946132	2.1
30590	1.35351	2.1
30591	1.87609	2.1
30592	1.79155	2.1
30593	1.79155	2.1
30594	1.89226	2.1
30595	1.89226	2.1
30596	1.99211	2.1
30597	1.89226	2.1
30598	1.71706	2.1
30599	1.71706	2.1
30600	1.79155	2.1
30601	1.89226	2.1
30602	1.96675	2.1
30603	1.46869	2.1
30604	1.89226	2.1
30605	1.89226	2.1
30606	1.89226	2.1
30607	1.5001	2.1
30608	1.5001	2.1
30609	1.89226	2.1
30610	1.89226	2.1
30611	1.89226	2.1
30612	1.89226	2.1
30613	1.89226	2.1
30614	1.89226	2.1
30615	1.21408	2.1
30616	2.23563	2.1
30617	2.34021	2.1
30618	2.34021	2.1
30619	1.17285	2.1
30620	1.17285	2.1
30621	1.89226	2.1

30622	1.89226	2.1
30623	1.47937	2.1
30624	1.89226	2.1
30625	1.14822	2.1
30626	1.46869	2.1
30627	1.89226	2.1
30628	1.89226	2.1
30629	1.89226	2.1
30630	1.89226	2.1
30631	1.89226	2.1
30632	1.89226	2.1
30633	1.25716	2.1
30634	2.34021	2.1
30635	2.4542	2.1
30636	2.4542	2.1
30637	1.21408	2.1
30638	1.21408	2.1
30639	1.66824	2.1
30640	2.44883	2.1
30641	1.93003	2.1
30642	2.24795	2.1
30643	3.88907	2.1
30644	1.89319	2.1
30645	2.24795	2.1
30646	2.82724	2.1
30647	2.31318	2.1
30648	2.30868	2.1
30649	1.93003	2.1
30650	1.89226	1.98684
30651	1.42589	2.1
30652	1.42589	2.1
30653	1.80501	2.1
30654	1.89226	2.1
30655	2.27137	2.1
30656	1.21067	0.05
30657	2.39166	0.0905989
30658	1.73577	0.063515
30659	1.73577	0.0606365
30660	1.53584	0.0525615
30661	1.53584	0.05
30662	1.35025	2.1
30663	2.0599	1.29383
30664	1.51084	0.855611
30665	2.27218	0.0644794
30666	3.57074	1.04746
30667	2.0599	0.578934
30668	2.51818	1.97059
30669	2.27218	0.05
30670	2.66532	1.23259
30671	4.72522	0.276313
30672	1.67115	1.8588
30673	2.27218	0.946191
30674	2.39605	1.09817
30675	2.39605	0.629421
30676	1.51084	1.59155
30677	1.51084	1.15329
30678	2.27218	0.450352
30679	2.66532	2.0013

30680	2.39605	0.0574122
30681	2.76829	0.349945
30682	5.06137	0.379619
30683	2.08684	1.00748
30684	2.36033	1.79891
30685	1.89224	0.55552
30686	1.89224	1.29887
30687	4.25257	0.863082
30688	2.41473	0.05
30689	1.00307	0.05
30690	2.01365	0.05
30691	1.00307	0.0760306
30692	3.05113	0.05
30693	2.04056	0.0560715
30694	1.11099	2.1
30695	2.25103	2.1
30696	2.22227	2.1
30697	2.22227	2.1
30698	1.14431	2.1
30699	1.14431	2.1
30700	1.07485	2.1
30701	2.22229	2.1
30702	2.19163	2.1
30703	2.19163	2.1
30704	1.111 2.1	
30705	1.111 2.1	
30706	1.0547	2.1
30707	1.19886	2.1
30708	0.946131	2.1
30709	0.946131	2.1
30710	0.946131	2.1
30711	0.946131	2.1
30712	1.19886	2.1
30713	1.19886	2.1
30714	1.0547	2.1
30715	0.946131	2.1
30716	0.946132	2.1
30717	1.19886	2.1
30718	1.89417	2.1
30719	2.64424	0.05
30720	1.76585	2.1
30721	2.47695	0.293201
30722	4.37112	1.52833
30723	1.89226	2.1
30724	1.89226	1.19464
30725	1.89226	2.1
30726	1.89226	0.346743
30727	3.78453	2.1
30728	0.930281	2.1
30729	1.0547	2.1
30730	0.946131	2.1
30731	0.946131	2.1
30732	0.930309	2.1
30733	0.930309	2.1
30734	1.07052	2.1
30735	1.07052	2.1
30736	0.93028	2.1
30737	0.946131	2.1

30738	1.0547	2.1
30739	0.946131	2.1
30740	1.00015	0.05
30741	1.00015	2.08586
30742	1.01918	2.1
30743	1.01918	0.172511
30744	1.17437	0.201555
30745	1.23847	0.05
30746	0.882926	2.1
30747	0.780859	2.08131
30748	0.780859	0.275104
30749	1.17438	0.541941
30750	0.882925	0.960506
30751	1.23848	0.775915
30752	1.89226	2.1
30753	1.89226	0.225964
30754	1.89226	1.19682
30755	1.89226	2.1
30756	1.89226	1.11348
30757	1.89226	2.1
30758	1.22083	2.1
30759	1.29579	2.1
30760	1.26236	2.1
30761	1.06191	2.1
30762	1.42128	2.1
30763	1.22083	2.1
30764	1.42128	2.1
30765	1.45806	2.1
30766	1.43502	2.1
30767	1.29579	2.1
30768	1.5605	2.1
30769	1.42128	2.1
30770	1.02813	2.1
30771	1.12121	2.1
30772	1.40557	2.1
30773	1.35298	2.1
30774	1.1314	2.1
30775	1.28679	2.1
30776	1.1715	2.1
30777	1.1715	2.1
30778	0.975656	2.1
30779	1.24908	2.1
30780	0.98956	2.1
30781	1.2681	2.1
30782	0.946132	2.1
30783	0.946132	2.1
30784	1.31889	2.1
30785	1.31889	2.1
30786	1.16546	2.1
30787	1.31889	2.1
30788	0.946133	2.1
30789	0.946133	2.1
30790	0.946133	2.1
30791	1.31889	2.1
30792	0.946132	2.1
30793	1.16546	2.1
30794	2.51518	0.05
30795	2.37584	0.05

30796	1.11712	0.05
30797	1.31242	0.05
30798	3.68826	0.05
30799	2.51518	0.05
30800	1.26311	0.05
30801	2.48977	0.05
30802	2.48977	0.05
30803	1.31242	0.05
30804	1.31242	0.05
30805	1.89226	0.144803
30806	1.89226	1.34286
30807	1.89226	0.05
30808	1.89226	1.28778
30809	3.78452	0.15304
30810	2.04737	0.05
30811	1.77122	1.44409
30812	1.94498	0.05
30813	1.65174	1.27116
30814	3.71621	0.059948
30815	1.41077	2.1
30816	1.29729	2.1
30817	1.77123	0.876632
30818	1.77123	2.1
30819	1.65175	1.58412
30820	1.29129	1.80126
30821	1.89226	0.77582
30822	1.89226	2.1
30823	1.89226	2.1
30824	1.89226	0.597498
30825	1.89226	2.1
30826	1.89226	1.99366
30827	1.89226	0.05
30828	1.89226	1.2231
30829	1.89226	0.05
30830	1.89226	0.587251
30831	3.78451	0.05
30832	1.94498	0.05
30833	1.65174	1.62007
30834	1.7968	0.05
30835	1.49831	0.453117
30836	3.44329	0.0704162
30837	1.49831	0.384954
30838	1.29729	2.1
30839	1.49831	2.1
30840	1.65175	0.773199
30841	1.16858	2.1
30842	1.32201	1.81264
30843	1.89226	0.224087
30844	1.89226	2.1
30845	1.89226	0.375473
30846	1.89226	2.1
30847	3.78452	2.1
30848	1.89226	0.05
30849	1.89226	0.05
30850	1.89226	0.05
30851	1.89226	0.05
30852	1.89226	0.05
30853	1.89226	0.05

30854	1.65324	0.564572
30855	1.26409	0.05
30856	1.42491	0.05
30857	1.42491	0.71664
30858	1.52268	0.05
30859	1.52268	0.681946
30860	1.52268	1.80194
30861	1.26409	0.113995
30862	1.32697	0.140894
30863	1.05724	0.05
30864	2.57991	0.0711753
30865	1.89226	0.05
30866	1.96548	0.05
30867	2.0178	0.05
30868	1.96548	0.05
30869	1.94458	0.05
30870	1.89226	0.05
30871	1.99366	0.05
30872	2.0178	0.05
30873	1.89226	0.05
30874	2.0178	0.05
30875	1.75999	0.05
30876	1.89226	0.05
30877	0.946126	2.1
30878	0.946126	2.1
30879	1.28228	2.1
30880	1.19444	2.1
30881	0.946127	2.1
30882	0.946127	2.1
30883	1.19444	2.1
30884	1.19444	2.1
30885	0.946128	2.1
30886	1.19444	2.1
30887	1.28228	2.1
30888	0.946128	2.1
30889	1.89226	2.1
30890	1.89226	2.1
30891	1.89226	2.1
30892	1.89226	2.1
30893	3.78452	2.1
30894	2.14104	2.1
30895	1.89226	2.1
30896	2.19948	2.1
30897	1.89226	2.1
30898	1.89226	2.1
30899	2.19948	2.1
30900	1.89226	2.1
30901	2.38888	2.1
30902	2.23932	2.1
30903	2.38888	2.1
30904	1.74269	2.1
30905	1.89226	2.1
30906	1.89226	2.1
30907	1.89226	2.1
30908	1.89226	2.1
30909	1.89226	2.1
30910	3.78452	2.1
30911	0.946132	2.1

30912	0.946132	2.1
30913	1.07052	2.1
30914	1.07052	2.1
30915	0.946132	2.1
30916	0.946132	2.1
30917	1.05132	2.1
30918	1.07052	2.1
30919	1.05132	2.1
30920	0.946132	2.1
30921	1.07052	2.1
30922	0.946133	2.1
30923	2.40171	2.1
30924	1.89226	2.1
30925	1.89226	2.1
30926	1.89226	2.1
30927	2.42723	2.1
30928	2.42723	2.1
30929	1.89226	2.1
30930	1.89226	2.04137
30931	1.89226	2.1
30932	1.89226	2.1
30933	3.78452	2.1
30934	1.63661	2.1
30935	2.3532	1.75497
30936	2.33287	1.86867
30937	2.33287	2.1
30938	1.64158	1.7337
30939	1.64158	2.1
30940	2.38918	2.1
30941	1.89226	2.1
30942	1.89226	2.1
30943	1.89226	2.1
30944	2.40171	2.1
30945	2.40171	2.1
30946	1.89226	2.1
30947	1.89226	2.1
30948	1.89226	2.1
30949	1.89226	2.1
30950	1.89226	2.1
30951	1.89226	2.1
30952	1.64158	2.1
30953	2.3532	2.1
30954	1.6488	2.1
30955	2.37364	2.1
30956	4.002 2.1	
30957	2.54752	0.137047
30958	1.42445	0.160372
30959	1.86697	0.213238
30960	1.86697	0.126516
30961	2.06666	0.21051
30962	2.06666	0.134186
30963	2.50872	0.055326
30964	2.06666	0.0811537
30965	1.90439	0.0596489
30966	1.51835	0.073035
30967	3.97105	0.06345
30968	0.755161	0.0817349
30969	0.755161	0.0750284

30970	0.793994	0.073165
30971	0.793994	0.0917852
30972	0.712225	0.0765414
30973	0.515826	0.0852723
30974	1.03333	0.0825682
30975	0.759175	0.0811297
30976	0.759173	0.0687991
30977	0.515827	0.05
30978	0.712226	0.0635476
30979	1.03333	0.0727557
30980	1.69633	0.05
30981	1.89226	0.05
30982	1.62477	0.05
30983	1.89226	0.05
30984	1.62477	0.05
30985	1.89226	0.05
30986	1.89226	0.05
30987	1.89226	0.080902
30988	1.89226	0.0675419
30989	1.89226	0.0579903
30990	3.78452	0.0587753
30991	2.28622	0.05
30992	1.09667	0.05
30993	1.17506	0.05
30994	2.24411	0.05
30995	1.17506	0.05
30996	2.24411	0.05
30997	2.24741	2.1
30998	1.70303	2.1
30999	2.49168	2.1
31000	1.57936	2.1
31001	2.53729	2.1
31002	1.70303	2.1
31003	2.24741	2.1
31004	2.47557	2.1
31005	1.89227	2.1
31006	1.89227	2.1
31007	4.36784	2.1
31008	2.27016	0.05
31009	1.10476	0.05
31010	1.44103	0.05
31011	1.10476	0.05
31012	2.26791	0.05
31013	1.93165	0.05
31014	2.27016	0.05
31015	1.89226	0.05
31016	1.89226	0.05
31017	2.4065	0.05
31018	4.29876	0.05
31019	2.27016	0.05
31020	1.44103	0.05
31021	1.53336	0.05
31022	2.4065	0.05
31023	3.84753	0.05
31024	2.7542	0.05
31025	1.89226	0.05
31026	2.35027	0.05
31027	1.89226	0.05

31028	2.35027	0.05
31029	1.89226	0.05
31030	2.3301	2.1
31031	2.36071	2.1
31032	2.07629	2.1
31033	2.01105	2.1
31034	4.37176	2.1
31035	1.86512	2.1
31036	2.01105	2.1
31037	2.01105	2.1
31038	2.3301	2.1
31039	1.57459	2.1
31040	1.89364	2.1
31041	2.52671	2.1
31042	2.01105	2.1
31043	1.78251	2.1
31044	1.70302	2.1
31045	3.98362	2.1
31046	1.227	2.1
31047	2.01105	2.1
31048	1.45869	2.1
31049	1.41713	2.1
31050	1.70302	2.1
31051	1.64994	2.1
31052	2.00989	2.1
31053	1.28938	2.1
31054	1.8769	2.1
31055	1.20969	2.1
31056	3.21958	2.1
31057	1.8769	2.1
31058	1.15864	2.1
31059	1.15864	2.1
31060	1.20969	2.1
31061	1.77353	2.1
31062	1.82458	2.1
31063	1.7986	2.1
31064	2.48717	2.1
31065	2.50161	2.1
31066	1.75869	2.1
31067	4.30021	2.1
31068	2.50161	2.1
31069	1.75251	2.1
31070	1.81678	2.1
31071	1.75869	2.1
31072	2.5597	2.1
31073	2.50161	2.1
31074	1.91334	0.256826
31075	1.85439	1.0263
31076	1.91334	0.362678
31077	1.70303	0.96571
31078	1.70303	0.24422
31079	1.49692	0.564993
31080	2.39986	0.05
31081	1.89226	0.0725843
31082	1.89226	0.0538464
31083	2.50756	0.05
31084	4.39982	0.05
31085	2.39986	0.0542511

31086	1.70303	0.05
31087	1.75564	0.05
31088	1.75904	0.0575362
31089	2.50272	0.05
31090	2.50756	0.0557502
31091	1.89226	0.0538232
31092	2.50756	0.05
31093	2.47857	0.05
31094	2.50756	0.0784627
31095	1.86327	0.05
31096	1.89226	0.05
31097	1.22241	0.0587768
31098	1.20827	0.0573733
31099	0.772416	0.05
31100	0.555208	0.05
31101	1.004	0.05
31102	1.004	0.0615634
31103	0.93504	0.0914881
31104	0.744364	0.05
31105	1.2089	0.05
31106	0.654935	0.05
31107	0.773927	0.0547899
31108	1.03618	0.0554717
31109	1.89227	0.05
31110	1.89227	0.05
31111	1.89227	0.05
31112	1.89227	0.05
31113	3.78453	0.05
31114	1.89226	0.05
31115	1.89226	0.05
31116	1.89226	0.05
31117	1.89226	0.05
31118	3.78452	0.05
31119	1.82643	0.05
31120	2.7084	0.05
31121	2.12654	0.05
31122	2.65614	0.05
31123	1.82643	0.05
31124	2.35603	0.05
31125	1.29385	0.05
31126	0.946133	0.05
31127	1.05492	0.05
31128	0.946132	0.05
31129	1.05492	0.05
31130	1.29385	0.05
31131	0.946133	0.05
31132	0.946133	0.05
31133	1.29385	0.05
31134	1.29385	0.05
31135	0.946132	0.05
31136	0.946132	0.05
31137	0.946132	0.05
31138	1.27329	0.05
31139	1.29385	0.05
31140	0.946129	0.05
31141	1.29385	0.05
31142	1.27328	0.05
31143	0.946131	0.05

31144	0.946131	0.05
31145	1.27328	0.05
31146	1.27328	0.05
31147	0.94613	0.05
31148	0.94613	0.05
31149	1.89226	1.94447
31150	1.89226	2.02551
31151	1.89226	0.825956
31152	1.89226	1.32723
31153	3.78453	1.60479
31154	1.89226	1.6679
31155	1.89226	2.00735
31156	1.89226	0.638628
31157	1.89226	1.66761
31158	3.78453	1.17044
31159	1.21488	1.965
31160	1.14605	2.1
31161	1.62974	1.49838
31162	1.52399	1.1773
31163	2.84463	1.99658
31164	0.577078	1.3557
31165	0.81251	2.1
31166	0.821003	1.65549
31167	0.543413	2.04048
31168	0.683929	1.40111
31169	0.722433	2.1
31170	0.730674	2.05485
31171	0.730674	1.19926
31172	0.857766	1.69135
31173	0.862119	1.37901
31174	0.600938	2.03783
31175	0.596094	1.78244
31176	1.89226	1.20897
31177	1.89226	1.83484
31178	1.89226	0.702894
31179	1.89226	1.51715
31180	3.78453	1.27984
31181	1.89226	1.10219
31182	1.89226	2.1
31183	1.89226	0.949046
31184	1.89226	1.32311
31185	3.78453	1.62827
31186	1.62974	2.06843
31187	1.56958	2.1
31188	2.2362	1.16251
31189	2.11424	1.59308
31190	3.86595	1.62355
31191	2.2362	1.24508
31192	1.85414	2.1
31193	2.28211	0.563418
31194	1.78367	0.849442
31195	2.2362	1.93444
31196	1.73256	2.08582
31197	0.851539	1.53631
31198	0.851539	0.152359
31199	1.14454	1.08102
31200	0.938988	1.99687
31201	0.851485	1.47607

31202	0.920593	0.116759
31203	0.851541	0.05
31204	0.851541	0.361777
31205	1.04073	0.121056
31206	1.16342	0.176275
31207	0.825315	0.537339
31208	0.825316	0.190447
31209	1.89226	2.1
31210	1.89226	2.1
31211	1.89226	2.1
31212	1.89226	2.1
31213	3.78453	2.1
31214	2.57016	2.1
31215	1.74789	2.1
31216	2.57016	2.1
31217	2.522	2.1
31218	1.61727	2.1
31219	1.56911	2.1
31220	1.28075	2.1
31221	1.406	2.1
31222	0.946131	2.1
31223	0.946131	2.1
31224	1.28075	2.1
31225	1.00333	2.1
31226	0.94613	2.1
31227	0.94613	2.1
31228	1.041	2.1
31229	1.041	2.1
31230	0.943484	2.1
31231	1.27717	2.1
31232	1.89226	2.1
31233	1.89226	2.1
31234	1.89226	2.1
31235	1.89226	2.1
31236	1.89226	2.1
31237	1.89226	2.1
31238	2.57016	2.1
31239	1.61727	2.1
31240	2.74196	2.1
31241	1.59094	2.1
31242	4.16109	2.1
31243	1.89226	2.1
31244	2.0815	2.1
31245	2.2034	2.1
31246	1.82493	2.1
31247	1.87847	2.1
31248	2.16142	2.1
31249	1.89226	2.1
31250	1.89226	2.1
31251	1.89226	2.1
31252	1.89226	2.1
31253	3.78453	2.1
31254	0.985252	2.1
31255	0.985252	2.1
31256	0.946132	2.1
31257	0.946132	2.1
31258	1.0655	2.1
31259	0.985255	2.1

31260	0.94613	2.1
31261	0.94613	2.1
31262	0.94613	2.1
31263	1.0655	2.1
31264	0.94613	2.1
31265	0.985255	2.1
31266	1.89226	2.1
31267	1.89226	2.1
31268	1.89226	2.1
31269	1.89226	2.1
31270	3.78453	2.1
31271	1.06549	2.1
31272	1.06549	2.1
31273	0.946133	2.1
31274	0.946133	2.1
31275	1.16834	2.1
31276	1.0655	2.1
31277	0.946131	2.1
31278	0.946131	2.1
31279	0.946131	2.1
31280	1.16834	2.1
31281	0.94613	2.1
31282	1.0655	2.1
31283	0.966631	2.1
31284	1.07349	2.1
31285	0.946131	2.1
31286	0.946131	2.1
31287	1.07349	2.1
31288	1.07349	2.1
31289	0.946129	2.1
31290	0.946129	2.1
31291	1.07349	2.1
31292	0.946131	2.1
31293	0.94613	2.1
31294	0.966631	2.1
31295	2.38055	0.25259
31296	1.89226	1.28557
31297	1.89226	1.06843
31298	1.46724	0.0665711
31299	3.35949	0.344611
31300	1.42249	2.1
31301	2.05499	2.1
31302	1.39708	2.1
31303	2.09033	2.1
31304	3.45207	2.1
31305	2.05499	2.1
31306	2.57425	2.1
31307	2.51287	2.1
31308	2.47753	2.1
31309	2.09033	2.1
31310	2.05499	2.1
31311	1.39327	2.1
31312	2.09033	2.1
31313	2.16217	2.1
31314	2.16217	2.1
31315	1.39708	2.1
31316	1.39708	2.1
31317	2.16217	2.1

31318	2.57426	2.1
31319	2.73006	2.1
31320	2.73006	2.1
31321	2.09033	2.1
31322	2.09033	2.1
31323	1.89226	0.05
31324	1.89226	0.05
31325	1.89226	0.05
31326	1.89226	0.05
31327	3.78452	0.05
31328	0.838749	0.05
31329	0.838749	0.05
31330	0.85824	0.05
31331	0.85824	0.05
31332	0.544644	0.05
31333	0.854718	0.05
31334	0.842271	0.05
31335	1.19141	0.05
31336	0.842272	0.05
31337	0.544643	0.05
31338	0.854718	0.05
31339	1.19141	0.05
31340	1.34214	0.05
31341	1.34214	0.05
31342	0.893236	0.05
31343	0.893236	0.05
31344	0.893265	0.05
31345	1.28924	0.05
31346	0.946133	0.05
31347	0.946133	0.05
31348	0.946132	0.05
31349	0.893265	0.05
31350	0.946133	0.05
31351	1.28924	0.05
31352	2.7013	0.05
31353	1.89226	0.05
31354	1.9216	0.05
31355	1.9216	0.05
31356	1.89226	0.05
31357	1.89226	0.05
31358	1.26397	2.1
31359	1.52995	2.1
31360	1.30116	2.1
31361	1.04084	2.1
31362	2.57079	2.1
31363	1.47013	2.1
31364	2.17459	2.1
31365	2.10668	2.1
31366	1.38328	2.1
31367	3.55787	2.1
31368	2.55006	2.1
31369	2.17459	2.1
31370	2.10668	2.1
31371	2.49168	2.1
31372	4.66626	2.1
31373	0.613622	2.1
31374	0.691639	2.1
31375	1.00105	2.1

31376	1.05334	2.1
31377	0.839874	2.1
31378	0.839874	2.1
31379	0.852814	2.1
31380	0.852814	2.1
31381	0.613622	2.1
31382	1.00105	2.1
31383	1.05334	2.1
31384	0.691639	2.1
31385	1.00105	2.1
31386	1.05334	2.1
31387	1.20844	2.1
31388	1.24584	2.1
31389	1.12717	2.1
31390	1.12717	2.1
31391	1.13462	2.1
31392	1.13462	2.1
31393	1.20845	2.1
31394	1.00105	2.1
31395	1.05334	2.1
31396	1.24584	2.1
31397	2.22558	2.1
31398	1.85981	2.1
31399	2.18943	1.80136
31400	1.89142	0.0728354
31401	4.0854	1.29686
31402	2.02641	2.1
31403	1.85981	2.0252
31404	2.18943	1.7515
31405	1.67346	1.21026
31406	3.88622	1.52948
31407	1.85981	0.05
31408	1.10736	0.05
31409	1.27421	0.05
31410	1.67346	0.05
31411	1.27421	0.05
31412	1.67346	0.996427
31413	1.85981	0.0651439
31414	1.27362	0.072588
31415	1.89142	0.15247
31416	1.27421	0.05
31417	3.13343	0.05
31418	1.81893	0.05
31419	1.64985	0.05
31420	1.23477	0.05
31421	2.25405	0.05
31422	3.48882	0.05
31423	2.23933	0.05
31424	1.89226	0.05
31425	1.89226	0.05
31426	2.78053	0.05
31427	1.89226	0.05
31428	2.78053	0.05
31429	0.946131	0.05
31430	0.946131	0.05
31431	0.946132	0.05
31432	0.946132	0.05
31433	0.946131	0.05

31434	0.946131	0.05
31435	0.946132	0.05
31436	0.946132	0.05
31437	0.946131	0.05
31438	0.946131	0.05
31439	0.946132	0.05
31440	0.946132	0.05
31441	1.89226	2.1
31442	2.48596	2.1
31443	1.89226	2.1
31444	2.51506	2.1
31445	4.40732	2.1
31446	2.48596	2.1
31447	1.63823	2.1
31448	2.51507	2.1
31449	1.67687	2.1
31450	4.1533	2.1
31451	1.89226	2.1
31452	2.51506	2.1
31453	1.92044	2.1
31454	1.89226	2.1
31455	2.54325	2.1
31456	2.51506	2.1
31457	2.51507	2.1
31458	1.5704	2.1
31459	1.70505	2.1
31460	1.67687	2.1
31461	2.54325	2.1
31462	2.51507	2.1
31463	2.0815	2.1
31464	1.70303	2.1
31465	1.70303	2.1
31466	1.80354	2.1
31467	1.98172	2.1
31468	2.09868	2.1
31469	1.27944	2.1
31470	1.79028	2.1
31471	1.13179	2.1
31472	1.65533	2.1
31473	2.93477	2.1
31474	1.89226	2.1
31475	1.89226	2.1
31476	1.89226	2.1
31477	1.89226	2.1
31478	1.89226	2.1
31479	1.89226	2.1
31480	1.98171	2.1
31481	1.89226	2.1
31482	1.89226	2.1
31483	1.89226	2.1
31484	1.65798	2.1
31485	1.65797	2.1
31486	1.00133	2.1
31487	1.65532	2.1
31488	1.53278	2.1
31489	1.53278	2.1
31490	1.13179	2.1
31491	1.13179	2.1

31492	0.810946	2.1
31493	0.810946	2.1
31494	0.810944	2.1
31495	0.810944	2.1
31496	0.810946	2.1
31497	0.810946	2.1
31498	1.89226	2.1
31499	1.35169	2.1
31500	1.08132	2.1
31501	1.08132	2.1
31502	1.08132	2.1
31503	1.99528	0.05
31504	1.70303	0.05
31505	2.08152	0.05
31506	1.70303	0.05
31507	2.33877	0.05
31508	1.93967	0.05
31509	1.89226	0.05
31510	1.89226	0.05
31511	1.89226	0.05
31512	1.89226	0.05
31513	3.78452	0.05
31514	1.26259	0.05
31515	2.17205	0.05
31516	2.32468	0.05
31517	2.32468	0.05
31518	1.28055	0.05
31519	1.28055	0.05
31520	0.946131	0.05
31521	0.946131	0.05
31522	0.997642	0.05
31523	0.997642	0.05
31524	0.946131	0.05
31525	0.946131	0.05
31526	0.989846	0.05
31527	0.997642	0.05
31528	0.946132	0.05
31529	0.989846	0.05
31530	0.997641	0.05
31531	0.946131	0.05
31532	1.89226	0.05
31533	1.89226	0.05
31534	1.89226	0.05
31535	1.89226	0.05
31536	3.78452	0.05
31537	1.28055	0.05
31538	2.17205	0.05
31539	1.14305	0.05
31540	1.92737	0.05
31541	3.3151	0.05
31542	0.954908	0.05
31543	0.9705	0.05
31544	0.946131	0.05
31545	0.946131	0.05
31546	0.946131	0.05
31547	0.946131	0.05
31548	0.9705	0.05
31549	0.9705	0.05

31550	0.954908	0.05
31551	0.946131	0.05
31552	0.946131	0.05
31553	0.9705	0.05
31554	0.835187	0.05
31555	0.954896	0.05
31556	0.946118	0.05
31557	0.946118	0.05
31558	0.835186	0.05
31559	0.835186	0.05
31560	1.06583	0.05
31561	1.06583	0.05
31562	0.835198	0.05
31563	0.946131	0.05
31564	0.954883	0.05
31565	0.946106	0.05
31566	1.89227	2.1
31567	1.89227	2.1
31568	1.89227	2.1
31569	1.89227	2.1
31570	1.89227	2.1
31571	1.89227	2.1
31572	0.885265	2.1
31573	0.885265	2.1
31574	0.871467	2.1
31575	0.871467	2.1
31576	1.11883	2.1
31577	0.787875	2.1
31578	0.996487	2.1
31579	0.637907	2.1
31580	0.996487	2.1
31581	1.11883	2.1
31582	0.787875	2.1
31583	0.637906	2.1
31584	1.89226	2.1
31585	2.39258	2.1
31586	2.39258	2.1
31587	2.54729	2.1
31588	1.89226	2.1
31589	2.04697	2.1
31590	1.89226	2.1
31591	2.0815	2.1
31592	2.0815	2.1
31593	2.48371	2.1
31594	2.0815	2.1
31595	2.51469	2.1
31596	1.00035	0.05
31597	1.55755	0.05
31598	1.03534	0.05
31599	1.61333	0.05
31600	2.59289	0.05
31601	1.55755	0.05
31602	1.85217	2.1
31603	1.61333	0.05
31604	1.92875	1.43302
31605	3.48629	0.05
31606	1.09962	0.106805
31607	1.61334	0.05

31608	1.70868	0.883302
31609	1.70868	0.05
31610	1.03535	1.43297
31611	1.03535	0.05
31612	1.61334	0.05
31613	1.92876	1.84861
31614	1.70868	0.05
31615	2.05274	2.1
31616	3.63744	0.05
31617	1.82233	0.0811302
31618	2.05768	0.359086
31619	1.89226	0.53562
31620	1.65406	0.0842638
31621	1.89226	0.442129
31622	1.65406	0.0927645
31623	2.26047	0.118862
31624	2.0815	0.740516
31625	1.89226	0.672499
31626	2.01156	0.134204
31627	1.89226	0.517517
31628	2.01156	0.129442
31629	1.89226	2.1
31630	1.89226	2.1
31631	1.89226	2.1
31632	1.89226	2.1
31633	3.78453	2.1
31634	2.70055	2.1
31635	2.28367	2.1
31636	2.56456	2.1
31637	2.33565	2.1
31638	4.84823	2.1
31639	2.28367	2.1
31640	1.5791	2.1
31641	1.96966	2.1
31642	1.09506	2.1
31643	3.37873	2.1
31644	1.89226	2.1
31645	1.89226	2.1
31646	1.89226	2.1
31647	1.89226	2.1
31648	3.78452	2.1
31649	0.94612	2.1
31650	0.94612	2.1
31651	0.946119	2.1
31652	0.946119	2.1
31653	0.946119	2.1
31654	0.946119	2.1
31655	0.946119	2.1
31656	0.946119	2.1
31657	0.946132	2.1
31658	0.946132	2.1
31659	0.946107	2.1
31660	0.946107	2.1
31661	2.56452	2.1
31662	1.57307	2.1
31663	2.10299	2.1
31664	2.1115	2.1
31665	2.52196	2.1

31666	2.52196	2.1
31667	1.89226	2.1
31668	1.89226	2.1
31669	1.89226	2.1
31670	1.89226	2.1
31671	3.78452	2.1
31672	2.37665	2.1
31673	1.89226	2.1
31674	2.28084	2.1
31675	1.89226	2.1
31676	4.26891	2.1
31677	0.946129	2.1
31678	0.946129	2.1
31679	0.946133	2.1
31680	0.946133	2.1
31681	0.946131	2.1
31682	0.946131	2.1
31683	0.946131	2.1
31684	0.946131	2.1
31685	0.946131	2.1
31686	0.946131	2.1
31687	0.946131	2.1
31688	0.946131	2.1
31689	1.7207	0.05
31690	1.65815	0.05
31691	1.33593	0.05
31692	1.31508	0.05
31693	2.99408	0.05
31694	0.676047	0.05
31695	0.676047	0.05
31696	0.684362	0.05
31697	0.684362	0.05
31698	0.547994	0.05
31699	0.702871	0.05
31700	0.829076	0.05
31701	0.657538	0.05
31702	0.657538	0.05
31703	0.547994	0.05
31704	0.829077	0.05
31705	0.702871	0.05
31706	1.9518	2.1
31707	1.30648	2.1
31708	1.28846	2.1
31709	1.92033	2.1
31710	3.22681	2.1
31711	1.89226	2.1
31712	1.89226	2.1
31713	1.89226	2.1
31714	1.89226	2.1
31715	3.78453	2.1
31716	0.946128	2.1
31717	0.946128	2.1
31718	1.10113	2.1
31719	1.09587	2.1
31720	0.946131	2.1
31721	0.946131	2.1
31722	1.09587	2.1
31723	1.09587	2.1

31724	0.946131	2.1
31725	1.09587	2.1
31726	1.10112	2.1
31727	0.94613	2.1
31728	1.26923	2.1
31729	1.92033	2.1
31730	1.28846	2.1
31731	1.28846	2.1
31732	1.89452	2.1
31733	1.89452	2.1
31734	1.89226	2.1
31735	1.89226	2.1
31736	1.89226	2.1
31737	1.89226	2.1
31738	3.78453	2.1
31739	1.89226	2.1
31740	2.19174	2.1
31741	2.15616	2.1
31742	2.19174	2.1
31743	1.85669	2.1
31744	1.89226	2.1
31745	1.89226	2.1
31746	1.89226	2.1
31747	1.89226	2.1
31748	1.89226	2.1
31749	3.78452	2.1
31750	1.89226	2.1
31751	1.89226	2.1
31752	1.89226	2.1
31753	1.89226	2.1
31754	3.78452	2.1
31755	1.51223	2.1
31756	1.41562	2.1
31757	1.5139	2.1
31758	1.42284	2.1
31759	1.6033	2.1
31760	1.51223	2.1
31761	1.51223	2.1
31762	1.25953	2.1
31763	1.38085	2.1
31764	1.6033	2.1
31765	2.89309	2.1
31766	0.946131	2.1
31767	0.946131	2.1
31768	0.946131	2.1
31769	0.946131	2.1
31770	0.946132	2.1
31771	0.946132	2.1
31772	0.946129	2.1
31773	0.946129	2.1
31774	0.946131	2.1
31775	0.946131	2.1
31776	0.94613	2.1
31777	0.94613	2.1
31778	1.89226	2.1
31779	1.89226	2.1
31780	1.89226	2.1
31781	1.89226	2.1

31782	3.78452	2.1
31783	0.707806	2.1
31784	0.697816	2.1
31785	0.823593	2.1
31786	0.801649	2.1
31787	0.74182	2.1
31788	0.74182	2.1
31789	0.757645	2.1
31790	0.757645	2.1
31791	0.823592	2.1
31792	0.697818	2.1
31793	0.707807	2.1
31794	0.801647	2.1
31795	1.52534	2.1
31796	1.6033	2.1
31797	1.64719	2.1
31798	1.38085	2.1
31799	1.64719	2.1
31800	1.38085	2.1
31801	2.67041	2.1
31802	1.89226	0.778183
31803	1.89226	2.1
31804	1.89226	2.1
31805	2.487	2.1
31806	2.487	2.1
31807	2.70097	2.1
31808	1.70303	2.1
31809	1.71163	2.1
31810	2.487	2.1
31811	4.46723	2.1
31812	1.14984	2.1
31813	1.2435	2.1
31814	0.778	2.1
31815	0.697616	2.1
31816	0.967211	2.1
31817	0.967211	2.1
31818	0.973907	2.1
31819	0.973907	2.1
31820	0.697616	2.1
31821	1.14984	2.1
31822	0.778	2.1
31823	1.2435	2.1
31824	2.4158	2.1
31825	1.26438	2.1
31826	1.676	2.1
31827	2.77505	2.1
31828	4.0918	2.1
31829	2.58258	2.1
31830	1.89226	2.1
31831	1.89226	2.1
31832	1.89226	2.1
31833	2.47032	2.1
31834	2.47032	2.1
31835	1.23516	2.1
31836	1.18734	2.1
31837	0.946131	2.1
31838	0.946131	2.1
31839	1.18734	2.1

31840	1.18734	2.1
31841	0.94613	2.1
31842	0.94613	2.1
31843	1.18734	2.1
31844	0.946131	2.1
31845	0.946131	2.1
31846	1.23516	2.1
31847	2.8239	2.1
31848	1.39361	2.1
31849	1.12342	2.1
31850	2.57275	2.1
31851	3.96637	2.1
31852	0.937929	0.842372
31853	0.937929	0.344454
31854	1.39734	0.132516
31855	1.39734	0.126865
31856	1.38914	0.179506
31857	0.94613	0.567584
31858	0.937902	0.647776
31859	0.94613	0.05
31860	0.94613	0.302781
31861	0.94613	0.358823
31862	1.38914	0.16259
31863	0.937902	0.128354
31864	1.89226	0.11435
31865	2.17765	0.793198
31866	1.84577	0.659719
31867	1.84577	0.227095
31868	1.89226	0.454258
31869	1.89226	0.144034
31870	2.11262	1.54611
31871	1.57766	1.53273
31872	2.22689	2.1
31873	1.91132	0.05
31874	2.19624	2.1
31875	1.79689	0.05
31876	1.99656	0.05
31877	1.57766	1.05548
31878	1.39067	2.1
31879	1.73802	0.149145
31880	3.31568	0.986762
31881	1.89226	0.274474
31882	1.89226	0.05489
31883	1.89226	0.087569
31884	1.89226	0.349295
31885	1.89226	0.05
31886	1.89226	0.104491
31887	1.84936	0.356301
31888	2.0815	0.05
31889	2.0815	0.0906468
31890	2.47217	0.229775
31891	1.04528	0.05
31892	1.62774	0.117911
31893	1.89226	0.0964252
31894	1.89226	0.05
31895	1.89226	0.0635297
31896	1.89226	0.147245
31897	1.89226	0.05

31898	1.89226	0.0746222
31899	1.69055	0.05
31900	2.68518	0.0539705
31901	1.69055	0.05
31902	2.25055	0.0643306
31903	2.39587	0.05
31904	3.01801	0.05
31905	1.89226	0.05
31906	1.89226	0.05
31907	1.89226	0.05
31908	1.89226	0.05
31909	3.78452	0.05
31910	1.24499	2.0583
31911	1.70302	2.1
31912	1.41207	2.1
31913	1.10574	0.170588
31914	2.66056	2.0788
31915	1.70303	2.1
31916	2.39605	1.5788
31917	2.72674	1.48845
31918	2.46904	0.54714
31919	1.84557	2.01163
31920	1.67115	1.32048
31921	2.46904	0.0639977
31922	2.76829	2.07257
31923	2.83666	0.05
31924	2.83666	0.558518
31925	2.39605	0.242519
31926	2.39605	2.04811
31927	1.07323	0.05
31928	1.07323	2.1
31929	1.06715	2.1
31930	1.18052	1.04982
31931	1.30067	0.242089
31932	1.26542	0.622613
31933	0.914516	2.1
31934	0.839633	2.1
31935	0.936958	1.25768
31936	1.26751	1.01972
31937	0.847764	0.994268
31938	1.35395	1.0733
31939	2.69069	0.126099
31940	1.03074	0.0621875
31941	1.74098	0.05
31942	1.97397	0.0612141
31943	3.71495	0.0646397
31944	2.44554	2.1
31945	1.89226	2.1
31946	1.89226	2.1
31947	1.89226	2.1
31948	2.40645	2.1
31949	2.40645	2.1
31950	1.89226	2.1
31951	1.89226	2.1
31952	1.89226	2.1
31953	1.89226	2.1
31954	3.78452	2.1
31955	1.82117	2.1

31956	2.55786	2.1
31957	2.51431	2.1
31958	2.51431	2.1
31959	1.85054	2.1
31960	1.84375	2.1
31961	2.38467	2.1
31962	1.89226	2.1
31963	1.89226	2.1
31964	1.89226	2.1
31965	2.44554	2.1
31966	2.44554	2.1
31967	1.89226	2.1
31968	1.89226	2.1
31969	1.89226	2.1
31970	1.89226	2.1
31971	1.89226	2.1
31972	1.89226	2.1
31973	2.02397	2.1
31974	2.55786	2.1
31975	1.77775	2.1
31976	2.61404	2.1
31977	4.33561	2.1
31978	1.89226	1.58547
31979	1.89226	1.55066
31980	1.89226	0.83555
31981	1.89226	1.11503
31982	3.78453	1.44789
31983	1.89226	1.76595
31984	2.80184	1.87398
31985	1.89226	1.17651
31986	1.89226	1.76574
31987	2.03045	1.93689
31988	2.03045	2.1
31989	2.52263	1.60137
31990	1.27921	2.1
31991	1.58429	2.1
31992	1.73571	2.1
31993	2.37121	1.9009
31994	2.52263	1.68067
31995	0.764974	2.1
31996	0.650577	2.1
31997	0.712428	2.1
31998	0.562516	2.1
31999	0.67264	2.1
32000	0.67264	2.1
32001	0.654851	2.1
32002	0.654851	2.1
32003	0.562515	2.1
32004	0.650579	2.1
32005	0.712427	2.1
32006	0.764976	2.1
32007	1.89227	2.1
32008	1.89227	2.1
32009	1.89227	2.1
32010	1.89227	2.1
32011	3.78453	2.1
32012	1.89226	2.1
32013	1.89226	2.1

32014	1.89226	2.1
32015	1.89226	2.1
32016	3.78453	2.1
32017	1.49248	2.1
32018	1.24959	2.1
32019	1.28211	2.1
32020	1.26432	2.1
32021	1.51027	2.1
32022	1.49248	2.1
32023	1.49248	2.1
32024	1.19017	2.1
32025	1.24977	2.1
32026	1.51027	2.1
32027	2.74225	2.1
32028	1.89226	2.1
32029	1.89226	2.1
32030	1.89226	2.1
32031	1.89226	2.1
32032	3.78453	2.1
32033	1.89226	2.1
32034	1.89226	2.1
32035	1.89226	2.1
32036	1.89226	2.1
32037	3.78453	2.1
32038	0.624796	2.1
32039	0.618472	2.1
32040	0.770147	2.1
32041	0.755132	2.1
32042	0.681445	2.1
32043	0.681445	2.1
32044	0.69216	2.1
32045	0.69216	2.1
32046	0.618472	2.1
32047	0.770147	2.1
32048	0.624796	2.1
32049	0.755132	2.1
32050	1.54029	2.1
32051	1.51027	2.1
32052	1.54029	2.1
32053	1.30682	2.1
32054	1.24977	2.1
32055	1.24977	2.1
32056	0.994293	2.1
32057	2.17159	2.1
32058	2.21149	2.1
32059	2.21149	2.1
32060	0.994814	2.1
32061	0.994814	2.1
32062	0.990481	2.1
32063	2.21149	2.1
32064	2.2671	2.1
32065	2.2671	2.1
32066	0.994293	2.1
32067	0.994293	2.1
32068	2.41579	2.1
32069	1.09847	2.1
32070	1.70302	2.1
32071	2.45069	2.1

32072	3.61169	2.1
32073	2.48689	2.1
32074	1.67969	2.1
32075	1.67969	2.1
32076	1.70002	2.1
32077	2.43826	2.1
32078	2.45859	2.1
32079	2.43827	2.1
32080	1.62408	2.1
32081	1.62408	2.1
32082	1.67969	2.1
32083	2.36729	2.1
32084	2.4229	2.1
32085	2.47199	2.1
32086	1.0823	2.1
32087	1.0607	2.1
32088	2.44276	2.1
32089	3.52506	2.1
32090	2.47199	2.1
32091	1.89226	2.1
32092	1.89226	2.1
32093	2.44277	2.1
32094	4.33503	2.1
32095	1.89226	2.1
32096	1.89226	2.1
32097	1.89226	2.1
32098	1.89226	2.1
32099	3.78453	2.1
32100	1.89226	2.1
32101	1.89226	2.1
32102	1.89226	2.1
32103	1.89226	2.1
32104	1.89226	2.1
32105	1.89226	2.1
32106	1.02986	2.1
32107	2.44276	2.1
32108	1.0607	2.1
32109	1.0607	2.1
32110	2.40055	2.1
32111	2.40055	2.1
32112	1.89226	2.1
32113	2.44277	2.1
32114	2.40056	2.1
32115	2.44277	2.1
32116	1.85005	2.1
32117	1.89226	2.1
32118	1.89226	2.1
32119	1.89226	2.1
32120	1.89226	2.1
32121	1.89226	2.1
32122	3.78453	2.1
32123	1.89226	2.1
32124	1.89226	2.1
32125	1.89226	2.1
32126	1.89226	2.1
32127	3.78452	2.1
32128	2.79502	0.05
32129	2.63448	0.610697

32130	2.68986	0.05
32131	2.53868	0.190569
32132	5.32434	0.05
32133	2.63449	0.072192
32134	2.39282	2.1
32135	2.53869	0.156385
32136	2.3085	2.1
32137	4.94299	1.81408
32138	2.68986	0.05
32139	2.53868	0.192726
32140	2.5761	0.05
32141	2.43404	0.194464
32142	5.1239	0.05
32143	2.53869	0.0986912
32144	2.3085	2.1
32145	2.43405	0.110708
32146	2.22575	2.1
32147	4.74255	1.86793
32148	1.89226	2.1
32149	1.89226	2.1
32150	1.89226	2.1
32151	1.89226	2.1
32152	3.78453	2.1
32153	1.32391	2.1
32154	1.44951	2.1
32155	1.44951	2.1
32156	1.89226	2.1
32157	1.89226	2.1
32158	2.20203	2.1
32159	0.507656	2.1
32160	0.578945	2.1
32161	1.05568	2.1
32162	0.959007	2.1
32163	0.76264	2.1
32164	0.76264	2.1
32165	0.775313	2.1
32166	0.775313	2.1
32167	0.578945	2.1
32168	1.05568	2.1
32169	0.959007	2.1
32170	0.507656	2.1
32171	1.89226	0.05
32172	2.4065	0.05
32173	2.36561	0.05
32174	2.4065	0.05
32175	1.85138	0.05
32176	1.89226	0.05
32177	1.40274	0.05
32178	2.4065	0.05
32179	1.53336	0.05
32180	1.53336	0.05
32181	2.36562	0.05
32182	2.36562	0.05
32183	0.946115	0.05
32184	0.946115	0.05
32185	1.06496	0.05
32186	1.18279	0.05
32187	0.946117	0.05

32188	0.946117	0.05
32189	1.06496	0.05
32190	1.06496	0.05
32191	0.94613	0.05
32192	1.06497	0.05
32193	0.946105	0.05
32194	1.18278	0.05
32195	1.00213	0.05
32196	2.36559	0.05
32197	1.40272	0.05
32198	1.40272	0.05
32199	2.12992	0.05
32200	2.12992	0.05
32201	0.95646	2.1
32202	2.07201	2.1
32203	1.01808	2.1
32204	2.14876	2.1
32205	3.09009	2.1
32206	1.89226	2.1
32207	1.89226	2.1
32208	1.89226	2.1
32209	1.89226	2.1
32210	3.78453	2.1
32211	1.89227	2.1
32212	1.89227	2.1
32213	1.89227	2.1
32214	1.89227	2.1
32215	1.89227	2.1
32216	1.89227	2.1
32217	2.71886	2.1
32218	1.60497	2.1
32219	1.43935	2.1
32220	2.55151	2.1
32221	4.15648	2.1
32222	2.71886	2.1
32223	1.89226	2.1
32224	1.89226	2.1
32225	2.55151	2.1
32226	4.44377	2.1
32227	1.89226	2.1
32228	1.89226	2.1
32229	1.89226	2.1
32230	1.89226	2.1
32231	3.78453	2.1
32232	1.89226	2.1
32233	1.89226	2.1
32234	1.89226	2.1
32235	1.89226	2.1
32236	3.78453	2.1
32237	1.58942	0.107071
32238	1.92622	0.265571
32239	2.52852	0.238216
32240	2.19457	0.103162
32241	4.12079	0.107673
32242	1.92649	0.106742
32243	1.44426	0.05
32244	1.87529	0.05
32245	2.19457	0.111359

32246	3.63883	0.0856914
32247	1.87529	0.0510167
32248	2.19457	0.102948
32249	2.7378	0.0966633
32250	2.29852	0.0531955
32251	4.49309	0.05
32252	1.35976	2.1
32253	1.89452	2.1
32254	1.39616	2.1
32255	1.1423	2.1
32256	1.8996	2.1
32257	1.52068	2.1
32258	1.89226	2.1
32259	1.89226	2.1
32260	1.89226	2.1
32261	1.89226	2.1
32262	1.89226	2.1
32263	1.89226	2.1
32264	1.89226	2.1
32265	2.15617	2.1
32266	2.0953	2.1
32267	2.15617	2.1
32268	1.83139	2.1
32269	1.89226	2.1
32270	1.16817	2.1
32271	1.8996	2.1
32272	1.23614	2.1
32273	1.48338	2.1
32274	1.80218	2.1
32275	2.16263	2.1
32276	1.89226	2.1
32277	1.89226	2.1
32278	1.89226	2.1
32279	1.89226	2.1
32280	1.89226	2.1
32281	1.89226	2.1
32282	1.89226	2.1
32283	2.0953	2.1
32284	2.03074	2.1
32285	2.0953	2.1
32286	1.8277	2.1
32287	1.89226	2.1
32288	1.74455	2.1
32289	2.181	2.1
32290	1.74455	2.1
32291	1.92911	2.1
32292	1.98195	2.1
32293	2.16651	2.1
32294	2.181	2.1
32295	2.29881	2.1
32296	1.98195	2.1
32297	1.80729	2.1
32298	4.28075	2.1
32299	1.74455	2.1
32300	1.98195	2.1
32301	1.52603	2.1
32302	1.76603	2.1
32303	3.51058	2.1

32304	1.76603	2.1
32305	1.94506	2.1
32306	1.76603	2.1
32307	1.98195	2.1
32308	1.86795	2.1
32309	2.08387	2.1
32310	0.984305	2.1
32311	1.9	2.1
32312	2.86619	2.1
32313	1.86189	2.1
32314	3.76189	2.1
32315	1.4621	2.1
32316	1.89226	2.1
32317	2.81374	2.1
32318	1.89226	2.1
32319	3.35437	2.1
32320	0.946133	2.1
32321	0.946133	2.1
32322	0.946132	2.1
32323	0.946132	2.1
32324	0.946132	2.1
32325	0.946132	2.1
32326	0.946133	2.1
32327	0.946133	2.1
32328	0.946132	2.1
32329	0.946132	2.1
32330	0.946132	2.1
32331	0.946132	2.1
32332	1.64268	2.1
32333	1.93673	2.1
32334	2.25284	2.1
32335	1.97548	2.1
32336	3.89552	2.1
32337	1.86206	2.1
32338	1.93673	2.1
32339	2.25284	2.1
32340	2.20075	2.1
32341	4.1149	2.1
32342	2.25284	2.1
32343	2.24781	2.1
32344	1.97548	2.1
32345	1.97548	2.1
32346	2.49676	2.1
32347	2.49676	2.1
32348	2.25284	2.1
32349	2.43549	2.1
32350	2.44467	2.1
32351	2.20075	2.1
32352	2.49676	2.1
32353	2.25284	2.1
32354	2.57928	2.1
32355	2.32606	2.1
32356	2.59522	2.1
32357	2.39022	2.1
32358	4.9695	2.1
32359	2.32606	2.1
32360	1.77677	2.1
32361	2.39022	2.1

32362	2.28669	2.1
32363	4.16699	2.1
32364	2.59522	2.1
32365	2.39022	2.1
32366	2.61901	2.1
32367	2.45899	2.1
32368	5.00923	2.1
32369	2.39022	2.1
32370	2.11575	2.1
32371	2.41795	2.1
32372	2.0815	2.1
32373	4.67085	2.1
32374	1.89226	2.1
32375	2.24723	2.1
32376	1.95405	2.1
32377	1.89226	2.1
32378	2.30902	2.1
32379	2.24723	2.1
32380	1.89226	2.1
32381	1.89226	2.1
32382	1.89226	2.1
32383	1.89226	2.1
32384	3.78453	2.1
32385	0.946131	2.1
32386	0.946131	2.1
32387	1.14643	2.1
32388	1.10673	2.1
32389	1.10673	2.1
32390	1.10673	2.1
32391	0.946133	2.1
32392	0.946133	2.1
32393	0.946131	2.1
32394	1.14643	2.1
32395	1.10673	2.1
32396	0.946131	2.1
32397	1.89226	2.1
32398	2.30902	2.1
32399	1.96635	2.1
32400	1.89226	2.1
32401	2.38311	2.1
32402	2.30902	2.1
32403	1.89226	2.1
32404	1.89226	2.1
32405	1.89226	2.1
32406	1.89226	2.1
32407	1.89226	2.1
32408	1.89226	2.1
32409	0.946131	2.1
32410	0.946131	2.1
32411	1.19156	2.1
32412	1.14643	2.1
32413	1.14643	2.1
32414	1.14643	2.1
32415	0.946133	2.1
32416	0.946133	2.1
32417	0.946131	2.1
32418	1.19156	2.1
32419	0.946131	2.1

32420	1.14643	2.1
32421	1.89226	1.5511
32422	1.89226	0.61366
32423	1.89226	0.472917
32424	1.89226	1.46758
32425	1.89226	0.73877
32426	1.89226	1.7771
32427	1.89226	1.11047
32428	1.89226	0.781413
32429	1.89226	0.673005
32430	1.9645	1.05574
32431	3.78452	1.19864
32432	2.39489	2.1
32433	2.15715	2.1
32434	2.07821	2.1
32435	2.33541	2.1
32436	4.49256	2.1
32437	2.39489	2.1
32438	2.34898	2.1
32439	2.30729	2.1
32440	2.33541	2.1
32441	4.6844	2.1
32442	2.07821	2.1
32443	2.33541	2.1
32444	2.26415	2.1
32445	1.99632	2.1
32446	4.33174	2.1
32447	2.30729	2.1
32448	2.33542	2.1
32449	2.26416	2.1
32450	2.26618	2.1
32451	4.60159	2.1
32452	1.19837	1.72586
32453	2.79151	0.107194
32454	2.25636	0.977433
32455	2.25636	0.404076
32456	1.94072	0.780297
32457	1.94072	0.515517
32458	2.53725	0.05
32459	1.16699	0.05
32460	1.69864	0.05
32461	2.0815	0.05
32462	3.58273	0.05
32463	1.91646	2.1
32464	2.51686	2.1
32465	2.49872	2.1
32466	1.85819	2.1
32467	4.41518	2.1
32468	0.929098	2.1
32469	0.8993	2.1
32470	1.24359	2.1
32471	1.24936	2.1
32472	1.06832	2.1
32473	1.06832	2.1
32474	1.08034	2.1
32475	1.08034	2.1
32476	0.899298	2.1
32477	1.24359	2.1

32478	1.24936	2.1
32479	0.929097	2.1
32480	2.0815	0.05
32481	1.89226	0.05
32482	2.0815	0.05
32483	1.82811	0.05
32484	1.94982	0.05
32485	1.71246	0.05
32486	2.3256	2.1
32487	1.89226	2.1
32488	1.89226	2.1
32489	1.89226	2.1
32490	2.38467	2.1
32491	2.38467	2.1
32492	1.89226	2.1
32493	1.89226	2.1
32494	1.89226	2.1
32495	1.89226	2.1
32496	1.89226	2.1
32497	1.89226	2.1
32498	1.89763	2.1
32499	2.61405	2.1
32500	2.15035	2.1
32501	2.68796	2.1
32502	1.42219	2.1
32503	1.77775	2.1
32504	2.26306	2.1
32505	1.89226	2.1
32506	1.89226	2.1
32507	1.89226	2.1
32508	2.3256	2.1
32509	2.3256	2.1
32510	1.89226	2.1
32511	1.89226	2.1
32512	1.89226	2.1
32513	1.89226	2.1
32514	1.89226	2.1
32515	1.89226	2.1
32516	1.92887	2.1
32517	2.41915	2.1
32518	2.50142	2.1
32519	2.50142	2.1
32520	1.90388	2.1
32521	1.89763	2.1
32522	1.89224	2.1
32523	1.89224	2.1
32524	1.89224	2.1
32525	1.89224	2.1
32526	3.78448	2.1
32527	2.25315	2.1
32528	1.05843	2.1
32529	2.38995	2.1
32530	1.31541	2.1
32531	3.56856	2.1
32532	0.517552	2.1
32533	0.68548	2.1
32534	1.27675	2.1
32535	1.13076	2.1

32536	0.913611	2.1
32537	0.913612	2.1
32538	0.902633	2.1
32539	0.902633	2.1
32540	0.68547	2.1
32541	1.27673	2.1
32542	1.13078	2.1
32543	0.517559	2.1
32544	1.89226	2.1
32545	1.89226	2.1
32546	1.89226	2.1
32547	1.89226	2.1
32548	3.78453	2.1
32549	0.962936	2.1
32550	0.962936	2.1
32551	0.996141	2.1
32552	0.996141	2.1
32553	0.830609	2.1
32554	0.657714	2.1
32555	1.30136	2.1
32556	1.19499	2.1
32557	1.30136	2.1
32558	0.830609	2.1
32559	1.19499	2.1
32560	0.657713	2.1
32561	1.60057	0.05
32562	1.39615	0.05
32563	1.06474	0.05
32564	1.39615	0.05
32565	1.64655	0.05
32566	2.04424	0.05
32567	1.89226	0.05
32568	1.89226	0.0561054
32569	1.89226	0.05
32570	1.89226	0.05
32571	1.89226	0.05
32572	1.89226	0.05
32573	2.56322	0.05
32574	1.89226	0.05
32575	1.89226	0.05
32576	1.89226	0.05
32577	2.18047	0.05
32578	2.18047	0.05
32579	1.89226	0.134054
32580	1.89226	0.15443
32581	1.89226	0.252958
32582	1.65573	0.0835487
32583	1.89226	0.262887
32584	1.65573	0.0879456
32585	1.05353	0.05
32586	1.83158	0.05
32587	1.50282	0.05
32588	1.20669	0.05
32589	2.88511	0.05
32590	1.44807	2.1
32591	1.93592	2.1
32592	1.80703	2.1
32593	1.27806	2.1

32594	3.21398	2.1
32595	2.0815	2.1
32596	1.87465	2.1
32597	1.87465	2.1
32598	1.50566	2.1
32599	2.0815	2.1
32600	1.69263	2.1
32601	1.89226	2.1
32602	1.89226	2.1
32603	1.89226	2.1
32604	1.89226	2.1
32605	1.89226	2.1
32606	1.89226	2.1
32607	1.62632	2.1
32608	0.994294	2.1
32609	0.994294	2.1
32610	1.27806	2.1
32611	1.81921	2.1
32612	2.10776	2.1
32613	2.0815	2.1
32614	1.70303	2.1
32615	1.70303	2.1
32616	1.85749	2.1
32617	1.90451	2.1
32618	2.07724	2.1
32619	1.89226	2.1
32620	1.89226	2.1
32621	1.89226	2.1
32622	1.89226	2.1
32623	1.89226	2.1
32624	1.89226	2.1
32625	2.69773	2.1
32626	1.88129	2.1
32627	2.55196	2.1
32628	2.26469	2.1
32629	4.94367	2.1
32630	1.88129	2.1
32631	1.25889	2.1
32632	1.26614	2.1
32633	1.05914	2.1
32634	2.26469	2.1
32635	1.88129	2.1
32636	2.55196	2.1
32637	2.0588	2.1
32638	2.6536	2.1
32639	2.15287	2.1
32640	4.70484	2.1
32641	0.8516	2.1
32642	0.8516	2.1
32643	0.849002	2.1
32644	0.849002	2.1
32645	0.629445	2.1
32646	0.671197	2.1
32647	1.0294	2.1
32648	1.07644	2.1
32649	1.07644	2.1
32650	0.671197	2.1
32651	0.629446	2.1

32652	1.0294	2.1
32653	2.02115	2.09238
32654	1.18382	2.1
32655	2.29107	0.14682
32656	1.46903	1.42268
32657	3.47488	0.75099
32658	2.60763	1.14092
32659	2.29107	1.78495
32660	1.82141	1.16794
32661	1.82141	2.1
32662	1.46903	2.02228
32663	2.29107	2.03482
32664	1.64007	2.1
32665	2.2024	2.1
32666	1.44044	2.1
32667	2.02634	2.1
32668	3.6664	2.1
32669	1.25565	2.1
32670	2.02635	2.1
32671	1.87228	2.1
32672	1.87228	2.1
32673	1.44045	2.1
32674	1.44045	2.1
32675	1.89226	0.05
32676	1.78503	0.05
32677	1.78503	0.05
32678	1.40048	0.05
32679	2.0815	0.05
32680	1.62972	0.05
32681	1.27875	2.1
32682	1.1483	2.08619
32683	1.40014	2.1
32684	1.40014	2.1
32685	1.04598	2.1
32686	1.04598	2.1
32687	2.63443	1.29487
32688	2.26444	2.1
32689	2.79392	0.143601
32690	2.4863	2.1
32691	5.12073	1.89553
32692	1.83158	0.05
32693	1.18508	0.05
32694	1.20669	0.05
32695	1.81488	0.05
32696	1.20669	0.05
32697	1.81488	0.05
32698	1.08134	0.05
32699	1.70303	0.05
32700	1.24027	0.05
32701	1.30359	0.05
32702	1.89939	0.05
32703	1.70303	0.05
32704	1.89226	2.1
32705	2.69027	2.1
32706	1.89226	2.1
32707	2.2078	2.1
32708	1.89226	2.1
32709	2.2078	2.1

32710	1.89226	2.1
32711	2.78977	2.1
32712	1.89226	2.1
32713	2.69027	2.1
32714	1.89226	2.1
32715	2.69027	2.1
32716	1.89226	2.1
32717	1.89226	2.1
32718	1.89226	1.99281
32719	1.89226	2.1
32720	3.78452	2.1
32721	1.43687	2.1
32722	1.71059	2.1
32723	2.53014	2.1
32724	2.6521	2.1
32725	4.24073	2.1
32726	2.6521	2.1
32727	1.66762	2.1
32728	2.50299	2.1
32729	2.50299	2.1
32730	1.71059	2.1
32731	1.71059	2.1
32732	1.89226	2.1
32733	1.89226	2.1
32734	1.89226	2.1
32735	1.89226	2.1
32736	1.89226	2.1
32737	1.89226	2.1
32738	2.10662	0.263207
32739	2.77417	0.142557
32740	2.78873	1.09824
32741	2.11661	0.193785
32742	4.89078	0.147434
32743	2.10662	0.720244
32744	1.22857	0.05
32745	1.25849	0.982337
32746	2.11661	0.302522
32747	3.34518	0.761454
32748	2.78873	0.780182
32749	2.11661	0.201137
32750	2.12475	0.347524
32751	2.79029	0.173712
32752	4.9069	0.328654
32753	1.2858	0.705369
32754	2.11661	0.286909
32755	1.25849	0.565275
32756	1.25849	0.470411
32757	2.12475	0.289686
32758	2.12475	0.359087
32759	1.13537	2.1
32760	1.20714	2.1
32761	0.946129	2.1
32762	0.946129	2.1
32763	0.946131	2.1
32764	0.946131	2.1
32765	1.29521	2.1
32766	1.20713	2.1
32767	1.13537	2.1

32768	0.946131	2.1
32769	0.94613	2.1
32770	1.29521	2.1
32771	1.00533	2.1
32772	0.876055	2.1
32773	0.946128	2.1
32774	0.946128	2.1
32775	0.913908	2.1
32776	0.913908	2.1
32777	1.17081	2.1
32778	0.896413	2.1
32779	0.955905	2.1
32780	0.893715	2.1
32781	1.20327	2.1
32782	0.998544	2.1
32783	1.89226	1.86679
32784	1.89226	2.04412
32785	1.89226	0.279746
32786	1.89226	1.2226
32787	3.78452	0.84731
32788	0.540975	1.19454
32789	0.726949	2.1
32790	0.712198	2.1
32791	0.506675	1.01369
32792	0.611916	1.04596
32793	0.611916	2.1
32794	0.621708	1.93973
32795	0.621708	0.920762
32796	0.726951	1.46359
32797	0.712196	1.09352
32798	0.506674	1.74831
32799	0.540976	1.55409
32800	1.89226	1.1305
32801	1.89226	2.1
32802	1.89226	1.13198
32803	1.89226	1.22182
32804	3.78453	1.80912
32805	1.89226	0.988391
32806	1.89226	1.18921
32807	1.89226	0.209686
32808	1.89226	2.1
32809	1.89226	0.288307
32810	1.89226	2.1
32811	0.919594	1.01094
32812	0.726949	1.88073
32813	0.898318	1.14447
32814	0.712198	1.83175
32815	0.817559	0.493368
32816	0.817559	2.1
32817	0.814234	0.568778
32818	0.814234	2.01555
32819	0.919597	0.897003
32820	0.898316	0.395229
32821	0.726951	1.97263
32822	0.712196	1.44095
32823	1.79663	0.589886
32824	1.12088	2.1
32825	1.54699	1.0787

32826	1.17475	1.27014
32827	1.79663	2.1
32828	1.42439	2.1
32829	2.28928	0.931662
32830	1.23077	1.64928
32831	1.65458	0.628492
32832	2.66533	1.79004
32833	3.94385	1.30464
32834	1.89226	2.1
32835	1.89226	2.1
32836	1.89226	2.1
32837	1.89226	2.1
32838	3.78452	2.1
32839	1.38426	2.1
32840	1.15067	2.1
32841	1.38426	2.1
32842	1.01595	2.1
32843	1.24509	2.1
32844	1.01595	2.1
32845	2.62097	2.1
32846	1.89226	2.1
32847	2.72561	2.1
32848	2.72561	2.1
32849	1.89226	2.1
32850	1.89226	2.1
32851	1.90103	0.05
32852	1.41902	2.1
32853	1.77249	0.0547108
32854	1.30272	2.1
32855	3.20375	0.949556
32856	1.77249	0.05
32857	1.19548	2.1
32858	1.19548	0.274103
32859	1.30272	2.1
32860	1.65815	0.05
32861	1.76539	2.1
32862	2.62161	2.1
32863	1.89226	2.1
32864	1.89226	2.1
32865	1.89226	2.1
32866	2.6446	2.1
32867	2.6446	2.1
32868	2.64459	2.1
32869	1.89226	2.1
32870	1.89226	2.1
32871	1.89226	2.1
32872	2.58257	2.1
32873	2.58257	2.1
32874	1.89226	0.127628
32875	1.89226	0.911195
32876	2.31301	0.829955
32877	2.76309	0.180219
32878	1.89226	0.649631
32879	2.26047	0.158327
32880	1.89226	0.131366
32881	2.31301	0.915303
32882	2.17765	0.759929
32883	2.17765	0.21183

32884	1.89226	0.600097
32885	1.89226	0.156177
32886	2.18516	2.1
32887	1.85196	2.1
32888	1.78595	2.1
32889	2.14606	2.1
32890	3.99802	2.1
32891	2.18516	2.1
32892	2.21189	2.1
32893	2.18937	2.1
32894	2.14606	2.1
32895	4.35796	2.1
32896	1.60735	2.1
32897	2.14606	2.1
32898	2.15714	2.1
32899	1.70302	2.1
32900	3.70055	2.1
32901	2.18937	2.1
32902	2.14606	2.1
32903	2.0927	2.1
32904	2.162	2.1
32905	4.30806	2.1
32906	2.17326	2.1
32907	2.58517	2.1
32908	2.55318	2.1
32909	2.1665	2.1
32910	4.75167	2.1
32911	2.17326	2.1
32912	1.53556	2.1
32913	1.56253	2.1
32914	2.1665	2.1
32915	3.70206	2.1
32916	2.55317	2.1
32917	2.1665	2.1
32918	2.17741	2.1
32919	2.54197	2.1
32920	4.70847	2.1
32921	2.17742	2.1
32922	2.1665	2.1
32923	2.18452	2.1
32924	1.58054	2.1
32925	1.56253	2.1
32926	1.56253	2.1
32927	1.71051	0.0531367
32928	1.89807	0.05
32929	1.19755	0.05
32930	1.19755	0.05
32931	2.25632	0.0591932
32932	2.25632	0.0560641
32933	0.876046	0.05
32934	1.27293	0.05
32935	0.946132	0.05
32936	0.946132	0.05
32937	0.946131	0.05
32938	0.869312	0.05
32939	1.12767	0.05
32940	0.946132	0.05
32941	1.22225	0.05

32942	1.37968	0.05
32943	0.851555	0.05
32944	0.851555	0.05
32945	0.946133	2.03932
32946	0.946133	2.1
32947	0.946132	2.1
32948	0.946132	1.39846
32949	0.946133	2.02966
32950	0.946133	2.097
32951	0.946132	2.1
32952	0.946132	2.1
32953	0.946132	2.1
32954	0.946132	2.1
32955	0.946133	2.1
32956	0.946133	2.1
32957	1.35152	2.1
32958	0.948936	2.1
32959	1.90166	2.1
32960	1.70303	2.1
32961	2.90858	2.1
32962	1.89226	2.1
32963	1.89226	2.1
32964	1.89226	2.1
32965	1.89226	2.1
32966	3.78452	2.1
32967	1.90166	2.1
32968	1.70303	2.1
32969	2.52454	2.1
32970	2.13919	2.1
32971	3.99529	2.1
32972	1.89226	1.5559
32973	1.89226	2.1
32974	1.89226	2.1
32975	1.89226	2.1
32976	3.78452	2.1
32977	1.89226	2.1
32978	1.89226	2.1
32979	1.89226	2.1
32980	1.89226	2.1
32981	1.89226	1.46314
32982	1.89226	2.1
32983	2.13918	2.1
32984	0.992674	2.1
32985	2.3397	2.1
32986	1.8023	2.1
32987	1.47769	2.1
32988	0.992674	2.1
32989	1.89226	0.05
32990	1.89226	0.05
32991	1.89226	0.080318
32992	1.89226	0.05
32993	3.78452	0.05
32994	1.89226	0.05
32995	2.54752	0.05
32996	2.63974	0.05
32997	1.89226	0.05
32998	4.53201	0.05
32999	0.498412	0.05

33000	0.500955	0.05
33001	0.57955	0.05
33002	0.563873	0.05
33003	0.529107	0.05
33004	0.529107	0.05
33005	0.53572	0.05
33006	0.53572	0.05
33007	0.500956	0.05
33008	0.579548	0.05
33009	0.563871	0.05
33010	0.498414	0.05
33011	1.89223	0.0568698
33012	1.89223	0.05
33013	1.89223	0.05
33014	1.89223	0.05
33015	1.89223	0.05
33016	1.89223	0.05
33017	1.28752	0.05
33018	1.31985	0.05
33019	0.946119	0.05
33020	0.946119	0.05
33021	1.28752	0.05
33022	1.28752	0.05
33023	0.946119	0.05
33024	0.946119	0.0530914
33025	1.28753	0.05
33026	0.946132	0.05
33027	1.31984	0.05
33028	0.946107	0.05
33029	0.500948	0.05
33030	0.479465	0.05
33031	0.562335	0.05
33032	0.579542	0.05
33033	0.528436	0.05
33034	0.528436	0.05
33035	0.530571	0.05
33036	0.530571	0.05
33037	0.56234	0.05
33038	0.479473	0.05
33039	0.500943	0.05
33040	0.579533	0.05
33041	1.89226	2.1
33042	1.89226	2.1
33043	1.89226	2.1
33044	1.89226	2.1
33045	3.78452	2.1
33046	1.50767	2.1
33047	1.14496	2.1
33048	1.50767	2.1
33049	1.58539	2.1
33050	1.07653	2.1
33051	1.15425	2.1
33052	1.89226	2.1
33053	2.43826	2.1
33054	2.43826	2.1
33055	2.48688	2.1
33056	1.89226	2.1
33057	1.94088	2.1

33058	1.89226	2.1
33059	1.89226	2.1
33060	1.89226	2.1
33061	1.89226	2.1
33062	3.78452	2.1
33063	1.50767	2.1
33064	1.07653	2.1
33065	1.41077	2.1
33066	0.991407	2.1
33067	2.49907	2.1
33068	1.21913	2.1
33069	1.21913	2.1
33070	0.946128	2.1
33071	0.946128	2.1
33072	1.21913	2.1
33073	1.18364	2.1
33074	0.946131	2.1
33075	0.946131	2.1
33076	1.18364	2.1
33077	0.946129	2.1
33078	1.21913	2.1
33079	0.94613	2.1
33080	1.89226	1.16826
33081	1.89226	2.1
33082	1.89226	1.46591
33083	1.89226	2.1
33084	3.78452	2.1
33085	1.93823	1.26027
33086	1.60976	2.1
33087	1.93823	2.1
33088	2.20045	1.20398
33089	1.36999	2.1
33090	1.63222	2.1
33091	1.38302	2.1
33092	1.38302	2.1
33093	0.946132	2.1
33094	0.946132	2.1
33095	1.27258	2.1
33096	1.38302	2.1
33097	0.946133	2.1
33098	0.946133	2.1
33099	0.946133	2.1
33100	1.27258	2.1
33101	0.946132	2.1
33102	1.38302	2.1
33103	1.89226	0.423513
33104	1.89226	2.1
33105	1.89226	1.26379
33106	1.89226	2.1
33107	3.78452	2.1
33108	1.93823	0.348838
33109	1.36999	2.1
33110	1.70423	0.287416
33111	1.14195	2.1
33112	3.08018	2.1
33113	1.27258	2.1
33114	1.27258	2.1
33115	0.946132	2.1

33116	0.946132	2.1
33117	1.17233	2.1
33118	1.27258	2.1
33119	0.946132	2.1
33120	0.946132	2.1
33121	1.17233	2.1
33122	0.946132	2.1
33123	0.946132	2.1
33124	1.27258	2.1
33125	2.70575	2.1
33126	1.89226	0.846486
33127	2.71429	2.1
33128	2.71429	2.1
33129	1.89226	2.1
33130	1.89226	1.14859
33131	0.946133	1.2655
33132	0.946133	2.1
33133	1.28713	2.1
33134	1.28713	2.1
33135	0.946131	2.1
33136	0.946131	1.21798
33137	1.28713	2.1
33138	1.36503	2.1
33139	0.946131	2.1
33140	1.36503	2.1
33141	1.28713	2.1
33142	0.946131	2.1
33143	1.89226	2.1
33144	1.89226	1.91152
33145	1.89226	2.1
33146	1.89226	2.1
33147	1.89226	2.1
33148	1.89226	2.00734
33149	1.22615	2.1
33150	1.04147	2.1
33151	1.3439	2.1
33152	1.3439	2.1
33153	0.969118	2.1
33154	0.969118	2.1
33155	0.980841	2.1
33156	0.992505	2.1
33157	1.34289	2.1
33158	1.70303	2.1
33159	2.56081	2.1
33160	1.89225	2.1
33161	2.66785	2.1
33162	2.79506	2.1
33163	2.79506	2.1
33164	1.89225	2.1
33165	1.89225	2.1
33166	0.978778	2.1
33167	0.978778	2.1
33168	0.993303	2.1
33169	0.993303	2.1
33170	0.603538	2.1
33171	0.638158	2.1
33172	1.33393	2.1
33173	1.39753	2.1

33174	1.33391	2.1
33175	0.603535	2.1
33176	0.638164	2.1
33177	1.39754	2.1
33178	1.33393	2.1
33179	1.33393	2.1
33180	0.946132	2.1
33181	0.946132	2.1
33182	1.33393	2.1
33183	1.27365	2.1
33184	0.946132	2.1
33185	0.946132	2.1
33186	0.946132	2.1
33187	1.27365	2.1
33188	1.33393	2.1
33189	0.946131	2.1
33190	1.89226	2.1
33191	1.89226	2.1
33192	1.89226	2.1
33193	1.89226	2.1
33194	3.78452	2.1
33195	1.89226	2.1
33196	1.89226	2.1
33197	1.89226	2.1
33198	1.89226	2.1
33199	3.78453	2.1
33200	0.979364	2.1
33201	0.979364	2.1
33202	0.89618	2.1
33203	0.89618	2.1
33204	0.896178	2.1
33205	0.929411	2.1
33206	0.946133	2.1
33207	0.946133	2.1
33208	0.946131	2.1
33209	0.89618	2.1
33210	0.946131	2.1
33211	0.929413	2.1
33212	1.89226	2.1
33213	1.89226	2.1
33214	1.89226	2.1
33215	1.89226	2.1
33216	1.89226	2.1
33217	1.89226	2.1
33218	1.89225	2.1
33219	1.89225	2.1
33220	1.89225	2.1
33221	1.89225	2.1
33222	3.7845	2.1
33223	1.89225	2.1
33224	1.89225	2.1
33225	1.89225	2.1
33226	1.89225	2.1
33227	1.89225	2.1
33228	1.89225	2.1
33229	0.946128	0.0643459
33230	0.946128	0.05
33231	0.946132	0.0563262

33232	0.946132	0.05
33233	0.94613	0.0569824
33234	0.94613	0.0731274
33235	0.946129	0.05
33236	0.946129	0.05
33237	0.94613	0.05
33238	0.94613	0.05
33239	0.94613	0.05
33240	0.94613	0.05
33241	1.89227	1.36972
33242	1.89227	1.35704
33243	1.89227	1.1084
33244	1.89227	0.801757
33245	3.78453	1.15448
33246	1.89226	1.39989
33247	2.43717	1.85373
33248	2.63259	1.14365
33249	1.89226	0.534989
33250	4.52485	1.4119
33251	0.616808	1.13756
33252	0.792277	1.33882
33253	1.04986	0.969746
33254	0.72303	1.26892
33255	0.702675	0.16574
33256	0.76862	1.97107
33257	0.809154	1.65609
33258	0.809154	0.95418
33259	0.808757	1.29408
33260	1.07789	0.543152
33261	0.772568	0.941183
33262	0.598878	1.45545
33263	1.89226	1.39443
33264	1.89226	1.25864
33265	1.89226	1.03731
33266	1.89226	0.963451
33267	3.78453	1.2987
33268	1.36428	1.95375
33269	1.31629	1.18874
33270	0.946132	1.3
33271	0.946132	1.21889
33272	1.36428	0.693556
33273	1.36428	1.99331
33274	0.946132	1.29602
33275	0.946132	1.7948
33276	1.36428	1.86174
33277	0.946132	0.861272
33278	1.31629	1.63459
33279	0.946132	0.571254
33280	1.578	1.40385
33281	2.85336	0.700998
33282	2.11368	0.445173
33283	2.14375	1.11095
33284	1.93852	1.18939
33285	1.93852	1.50995
33286	2.34806	0.178779
33287	1.42913	0.05
33288	1.61539	0.455822
33289	2.45322	0.0821373

33290	3.88235	0.178424
33291	2.34806	0.102004
33292	1.89226	0.068324
33293	1.89226	0.371472
33294	2.45322	0.117668
33295	4.34548	0.0608145
33296	1.89226	0.0801368
33297	1.89226	0.05
33298	1.89226	0.147706
33299	1.89226	0.125186
33300	3.78452	0.186982
33301	1.89226	0.0629689
33302	1.89226	0.05
33303	1.89226	0.101105
33304	1.89226	0.139272
33305	3.78452	0.0569329
33306	1.25972	0.134884
33307	2.45322	0.05
33308	2.58823	0.295704
33309	1.79398	0.071512
33310	4.2472	0.0577441
33311	1.89226	0.241583
33312	2.45322	0.0589582
33313	2.58823	0.0841177
33314	2.45322	0.0561103
33315	2.02727	0.063017
33316	1.89226	0.05
33317	1.89226	0.0675927
33318	1.89226	0.0662478
33319	1.89226	0.211509
33320	1.89226	0.054955
33321	3.78452	0.05
33322	1.89226	0.0521101
33323	1.89226	0.060854
33324	1.89226	0.05
33325	1.89226	0.05
33326	1.89226	0.213272
33327	1.89226	0.0762739
33328	1.70048	2.1
33329	2.1572	0.05
33330	1.45477	2.1
33331	1.93973	0.0625791
33332	3.64021	1.1345
33333	1.17614	2.1
33334	1.93973	0.0915116
33335	1.69661	2.1
33336	1.69661	0.1777
33337	1.45477	2.1
33338	1.45477	0.302244
33339	1.94343	2.1
33340	2.53782	1.75238
33341	2.59903	2.1
33342	2.59903	0.356115
33343	1.87598	2.1
33344	1.87598	0.878306
33345	0.946131	0.0911283
33346	0.946131	1.32734
33347	1.26891	2.1

33348	1.26891	0.05
33349	0.94613	1.78775
33350	0.94613	0.953705
33351	1.26891	1.67239
33352	1.29952	0.394619
33353	1.29952	0.11334
33354	0.946131	0.0543017
33355	1.26891	0.734272
33356	0.946131	0.947014
33357	2.00551	2.1
33358	2.59903	2.02824
33359	2.65811	2.1
33360	2.65811	0.997876
33361	1.94343	2.1
33362	1.94343	1.69931
33363	2.65811	0.165822
33364	1.89226	2.1
33365	1.89226	0.151828
33366	1.89226	1.41536
33367	2.59903	0.687455
33368	2.59903	2.1
33369	1.89227	2.1
33370	1.89227	2.1
33371	1.89227	2.1
33372	1.89227	2.1
33373	3.78453	2.1
33374	1.15471	2.1
33375	0.993889	2.1
33376	1.78919	2.1
33377	1.77325	2.1
33378	2.78308	2.1
33379	1.89226	2.1
33380	1.89226	2.1
33381	1.89226	2.1
33382	1.89226	2.1
33383	3.78453	2.1
33384	1.78919	2.1
33385	1.77325	2.1
33386	2.45969	2.1
33387	2.70835	2.1
33388	4.49753	2.1
33389	0.939314	2.1
33390	0.939315	2.1
33391	1.01133	2.1
33392	1.01133	2.1
33393	0.596471	2.1
33394	1.20813	2.1
33395	0.886619	2.1
33396	1.35417	2.1
33397	1.35417	2.1
33398	1.20813	2.1
33399	0.886621	2.1
33400	0.59647	2.1
33401	0.946131	2.1
33402	0.946131	2.1
33403	0.94613	2.1
33404	0.94613	2.1
33405	0.946133	2.1

33406	0.946133	2.1
33407	0.946128	2.1
33408	0.946128	2.1
33409	0.946131	2.1
33410	0.946131	2.1
33411	0.946131	2.1
33412	0.946131	2.1
33413	1.03939	2.1
33414	1.03939	2.1
33415	0.946132	1.13576
33416	0.946132	2.01662
33417	0.946132	2.08595
33418	0.946132	2.1
33419	1.23851	1.42885
33420	1.03939	2.1
33421	1.03939	2.1
33422	0.946132	2.1
33423	0.946133	2.1
33424	1.23851	2.1
33425	0.902503	2.1
33426	0.946129	2.1
33427	0.946132	1.46591
33428	0.946132	2.1
33429	0.902472	2.1
33430	0.902472	2.1
33431	1.08736	1.74946
33432	0.994093	2.1
33433	0.946133	2.1
33434	0.902504	2.1
33435	1.03939	2.1
33436	0.946132	2.1
33437	1.35306	1.19395
33438	1.29982	0.32827
33439	1.7216	0.352883
33440	1.89183	0.32807
33441	3.07466	0.403234
33442	0.485098	0.444294
33443	0.582401	0.471189
33444	0.649908	0.772398
33445	0.8608	0.565089
33446	0.587739	0.169465
33447	0.58774	0.627594
33448	0.644567	0.472349
33449	0.644567	1.3643
33450	0.58241	0.825933
33451	0.86081	0.265604
33452	0.649898	1.01772
33453	0.485093	0.45388
33454	2.25936	2.1
33455	2.71441	2.1
33456	2.63824	2.1
33457	2.20652	2.1
33458	4.92092	2.1
33459	2.25936	2.1
33460	1.57627	2.1
33461	1.5493	2.1
33462	2.20652	2.1
33463	3.78279	2.1

33464	2.63824	2.1
33465	2.20652	2.1
33466	2.17326	2.1
33467	2.58517	2.1
33468	4.79169	2.1
33469	2.17326	2.1
33470	2.20652	2.1
33471	2.19278	2.1
33472	1.53556	2.1
33473	1.5493	2.1
33474	1.5493	2.1
33475	1.97465	2.1
33476	2.13925	2.1
33477	2.04483	2.1
33478	1.62784	2.1
33479	3.76709	2.1
33480	1.89226	2.1
33481	1.89226	2.1
33482	1.89226	2.1
33483	1.89226	2.1
33484	1.89226	2.1
33485	1.89226	2.1
33486	0.724037	2.1
33487	0.81392	2.1
33488	0.96796	2.1
33489	1.02242	2.1
33490	0.882091	2.1
33491	0.882091	2.1
33492	0.899789	2.1
33493	0.899789	2.1
33494	0.724036	2.1
33495	0.96796	2.1
33496	1.02242	2.1
33497	0.81392	2.1
33498	1.13537	2.1
33499	1.63054	2.1
33500	0.946131	2.1
33501	0.946131	2.1
33502	0.946131	2.1
33503	0.946131	2.1
33504	1.258	2.1
33505	1.63054	2.1
33506	1.22632	2.1
33507	1.22632	2.1
33508	0.946131	2.1
33509	1.258	2.1
33510	1.89226	2.1
33511	1.89226	2.1
33512	1.89226	2.1
33513	1.89226	2.1
33514	1.89226	2.1
33515	1.89226	2.1
33516	0.809739	2.1
33517	0.862674	2.1
33518	1.1995	2.1
33519	1.33899	2.1
33520	1.06324	2.1
33521	1.06324	2.1

33522	0.981932	2.1
33523	1.19563	2.1
33524	0.809727	2.1
33525	1.14092	2.1
33526	1.33902	2.1
33527	0.863494	2.1
33528	2.23742	2.1
33529	1.84902	2.1
33530	1.88088	2.1
33531	2.20392	2.1
33532	4.08644	2.1
33533	1.3592	2.1
33534	1.84902	2.1
33535	1.88088	2.1
33536	1.3305	2.1
33537	1.88088	2.1
33538	1.3305	2.1
33539	1.84902	2.1
33540	1.15008	0.0509751
33541	1.3305	2.1
33542	1.70539	2.1
33543	2.9991	2.1
33544	1.84901	2.1
33545	2.0264	2.1
33546	2.20391	2.1
33547	1.70539	2.1
33548	3.87541	2.1
33549	1.03852	2.1
33550	1.38247	0.211967
33551	1.36635	0.730207
33552	1.01537	2.05416
33553	2.39784	1.59946
33554	2.0815	2.1
33555	1.91744	2.1
33556	1.91744	1.85532
33557	2.06496	2.1
33558	1.89227	1.98676
33559	2.03474	2.1
33560	1.89226	0.364124
33561	1.89226	1.9053
33562	1.89226	0.353213
33563	1.89226	2.1
33564	1.89226	0.212609
33565	1.89226	2.07752
33566	1.36635	0.904384
33567	1.02923	2.1
33568	1.02923	1.17084
33569	1.01537	2.1
33570	1.36416	0.696185
33571	1.3503	2.1
33572	1.89226	2.1
33573	1.89359	2.1
33574	1.89359	2.1
33575	1.91744	2.1
33576	1.89226	2.07408
33577	1.91611	2.1
33578	0.946128	2.1
33579	0.946128	2.06539

33580	0.946133	0.756408
33581	0.946133	0.30468
33582	0.94613	0.328599
33583	0.94613	2.00453
33584	0.946131	2.1
33585	0.946131	0.525696
33586	0.946131	1.73286
33587	0.946131	1.80062
33588	0.946131	1.69333
33589	0.946131	1.80877
33590	2.689 2.1	
33591	2.0815	2.1
33592	2.72786	2.1
33593	2.48295	2.1
33594	5.02397	2.1
33595	2.72786	2.1
33596	1.86424	2.1
33597	2.7188	2.1
33598	2.08981	2.1
33599	4.81768	2.1
33600	2.08982	2.1
33601	0.986987	2.1
33602	2.08982	2.1
33603	1.87249	2.1
33604	1.01964	2.1
33605	0.906115	2.1
33606	1.44247	2.1
33607	1.43872	2.1
33608	1.17054	2.1
33609	1.17054	2.1
33610	1.43872	2.1
33611	1.26045	2.1
33612	2.0815	0.05
33613	2.0815	0.05
33614	2.0815	0.05
33615	1.81468	0.05
33616	1.89065	0.05
33617	1.55625	0.05
33618	1.89062	0.05
33619	1.89224	0.05
33620	1.89224	0.05
33621	2.27073	0.05
33622	1.86856	0.05
33623	2.24232	0.05
33624	1.21419	2.1
33625	2.79716	2.1
33626	2.50005	2.1
33627	2.50005	2.1
33628	1.5008	2.1
33629	1.5008	2.1
33630	1.60139	0.05
33631	1.7103	0.05
33632	1.04159	0.05
33633	1.2489	0.05
33634	2.75189	0.05
33635	0.732812	0.05
33636	0.732812	0.05
33637	0.751986	0.05

33638	0.751986	0.05
33639	0.860346	0.05
33640	0.667961	0.05
33641	0.85515	0.05
33642	0.624452	0.05
33643	0.62445	0.05
33644	0.667962	0.05
33645	0.855148	0.05
33646	0.860348	0.05
33647	1.89226	0.0785921
33648	2.50872	0.0980066
33649	2.77909	0.082907
33650	1.89226	0.0892378
33651	2.77909	0.0828307
33652	1.89226	0.0895593
33653	2.15825	0.05
33654	2.50872	0.05
33655	1.90439	0.05
33656	1.90439	0.0540234
33657	2.77909	0.05
33658	2.77909	0.0639741
33659	1.12468	2.1
33660	1.43872	2.1
33661	1.64926	2.1
33662	1.2724	2.1
33663	2.77395	2.1
33664	1.64926	2.1
33665	1.38438	2.1
33666	1.47244	2.1
33667	1.2724	2.1
33668	1.8493	2.1
33669	1.64926	2.1
33670	1.36416	0.978109
33671	1.0652	2.1
33672	1.0652	1.51596
33673	1.02923	2.1
33674	1.37496	1.01726
33675	1.33898	2.1
33676	1.89227	2.1
33677	1.90433	2.1
33678	1.90433	2.1
33679	1.89359	2.1
33680	1.89227	2.1
33681	1.88153	2.1
33682	0.946129	2.1
33683	0.946129	2.1
33684	0.946133	1.16625
33685	0.946133	0.62464
33686	0.946131	0.711129
33687	0.946131	2.1
33688	0.946131	2.1
33689	0.946131	0.859763
33690	0.946131	1.86115
33691	0.946131	1.94339
33692	0.946131	1.77508
33693	0.946131	1.85075
33694	1.37496	1.16545
33695	1.07519	2.1

33696	1.07519	1.69672
33697	1.0652	2.1
33698	1.39725	1.12785
33699	1.38726	2.1
33700	1.89227	2.1
33701	1.9052	2.1
33702	1.9052	2.1
33703	1.90433	2.1
33704	1.89227	2.1
33705	1.8914	2.1
33706	0.946129	2.1
33707	0.946129	2.1
33708	0.946133	1.40128
33709	0.946133	0.754223
33710	0.946131	0.851113
33711	0.946131	2.1
33712	0.946131	2.1
33713	0.946131	0.944719
33714	0.946131	2.00709
33715	0.946131	2.07417
33716	0.946131	1.94324
33717	0.946131	2.00406
33718	1.91316	0.05
33719	1.89226	0.05
33720	1.89226	0.05
33721	1.89226	0.05
33722	1.99366	0.05
33723	1.99366	0.05
33724	1.93083	0.05
33725	1.89226	0.05
33726	1.51379	0.05
33727	1.89226	0.05
33728	1.53051	0.05
33729	1.91316	0.05
33730	2.16731	2.1
33731	1.21373	2.1
33732	1.47804	2.1
33733	2.37601	2.1
33734	3.64535	2.1
33735	1.7211	2.1
33736	2.37601	2.1
33737	1.47804	2.1
33738	1.47804	2.1
33739	2.56843	2.1
33740	2.56843	2.1
33741	2.58731	2.1
33742	1.59094	2.1
33743	1.59094	2.1
33744	1.76816	2.1
33745	2.41707	2.1
33746	2.5943	2.1
33747	0.718346	2.1
33748	0.795468	2.1
33749	1.20854	2.1
33750	1.1224	2.1
33751	0.961203	2.1
33752	0.961203	2.1
33753	0.95667	2.1

33754	0.95667	2.1
33755	0.718346	2.1
33756	1.1224	2.1
33757	0.795469	2.1
33758	1.20854	2.1
33759	1.84076	2.1
33760	1.42884	2.1
33761	2.24274	2.1
33762	1.85415	2.1
33763	3.69491	2.1
33764	1.89226	2.1
33765	1.89226	2.1
33766	1.89226	2.1
33767	1.89226	2.1
33768	3.78452	2.1
33769	2.24273	2.1
33770	2.24412	2.1
33771	1.85415	2.1
33772	1.85415	2.1
33773	2.68473	2.1
33774	2.68473	2.1
33775	1.89226	2.1
33776	1.89226	2.1
33777	1.89226	2.1
33778	1.89226	2.1
33779	1.89226	2.1
33780	1.89226	2.1
33781	1.89226	2.06779
33782	1.89226	2.1
33783	1.89226	2.1
33784	1.89226	2.1
33785	3.78451	2.1
33786	1.85415	2.1
33787	1.10961	2.1
33788	2.24411	2.1
33789	1.51067	2.1
33790	3.35373	2.1
33791	1.70302	2.1
33792	1.89226	2.1
33793	1.76399	2.1
33794	1.43577	2.1
33795	2.08149	2.1
33796	1.70302	2.1
33797	1.89226	2.1
33798	1.89226	2.1
33799	1.89226	2.1
33800	1.89226	2.1
33801	1.89226	2.1
33802	1.89226	2.1
33803	1.0593	2.1
33804	1.17289	2.1
33805	1.5379	2.1
33806	1.46757	2.1
33807	2.71079	2.1
33808	0.931235	2.1
33809	1.1151	2.1
33810	0.975236	2.1
33811	0.975236	2.1

33812	1.31147	2.1
33813	0.762882	2.1
33814	1.13152	2.1
33815	0.505799	2.1
33816	1.31149	2.1
33817	1.07629	2.1
33818	0.884817	2.1
33819	0.505793	2.1
33820	2.69027	2.1
33821	1.60441	2.1
33822	2.69027	2.1
33823	1.64771	2.1
33824	2.78977	2.1
33825	1.72731	2.1
33826	0.77878	0.05
33827	0.843226	0.05
33828	0.845997	0.05
33829	0.926091	0.05
33830	0.77878	0.05
33831	0.926093	0.05
33832	1.3922	1.45071
33833	1.3922	1.14128
33834	0.872156	2.1
33835	0.872156	2.09531
33836	0.872157	2.1
33837	0.946132	0.906203
33838	0.946129	0.71046
33839	1.31822	2.1
33840	0.94613	2.03502
33841	0.94613	2.06437
33842	1.31823	1.89077
33843	0.872157	2.06671
33844	1.81534	0.22936
33845	1.89226	0.19717
33846	1.59602	0.273436
33847	1.59602	0.538855
33848	1.89226	0.05
33849	1.89226	0.0822675
33850	1.89227	0.182011
33851	1.89227	0.245587
33852	1.89227	0.254994
33853	1.89227	0.134332
33854	3.78453	0.12873
33855	1.89226	0.241139
33856	1.89226	0.0792081
33857	1.89226	0.126166
33858	1.89226	0.104779
33859	3.78453	0.267638
33860	2.30348	0.05
33861	1.4185	0.0565293
33862	1.82551	0.0652604
33863	1.02011	0.05
33864	3.24401	0.05
33865	2.46702	0.05
33866	1.17129	0.05
33867	1.82551	0.05
33868	1.82551	0.05
33869	1.73525	0.05

33870	1.73525	0.05
33871	1.89227	0.152234
33872	1.89227	0.0713648
33873	1.89227	0.0716204
33874	1.89227	0.0817795
33875	1.89227	0.128031
33876	1.89227	0.145205
33877	2.80034	2.1
33878	2.45767	2.1
33879	2.67994	2.1
33880	2.35395	2.1
33881	5.1376	2.1
33882	2.45766	2.1
33883	1.8769	2.1
33884	2.00989	2.1
33885	2.00989	2.1
33886	2.35395	2.1
33887	2.35395	2.1
33888	2.57662	2.1
33889	2.35395	2.1
33890	2.57662	2.1
33891	2.67993	2.1
33892	2.24227	2.1
33893	2.34558	2.1
33894	1.02874	2.1
33895	1.02874	2.1
33896	1.03084	2.1
33897	1.03084	2.1
33898	0.938448	2.1
33899	0.886762	2.1
33900	1.17698	2.1
33901	1.12114	2.1
33902	1.12113	2.1
33903	0.886764	2.1
33904	0.93845	2.1
33905	1.17697	2.1
33906	1.91969	2.1
33907	2.5508	2.1
33908	1.88273	2.1
33909	2.76609	2.1
33910	4.68578	2.1
33911	2.83423	2.1
33912	1.89226	2.1
33913	2.76609	2.1
33914	1.89226	2.1
33915	4.65835	2.1
33916	1.56685	0.17881
33917	1.11712	0.0812711
33918	1.07999	1.28322
33919	1.51448	0.41183
33920	2.6316	0.469931
33921	1.56685	0.0589663
33922	1.67652	0.48768
33923	1.5955	0.765256
33924	1.51448	0.517161
33925	3.19099	0.382628
33926	1.89226	0.969232
33927	1.89226	0.05

33928	1.89226	0.51173
33929	1.89226	0.475735
33930	3.78453	0.770616
33931	1.89226	0.382652
33932	1.89226	0.107059
33933	1.89226	0.389479
33934	1.89226	0.670566
33935	1.89226	0.0557071
33936	1.89226	0.0703456
33937	1.0562	0.582062
33938	1.51448	0.192706
33939	1.07999	0.360156
33940	1.07999	0.121571
33941	1.46441	0.470674
33942	1.46441	0.195113
33943	1.5955	1.17948
33944	1.51448	0.287133
33945	1.46441	0.454655
33946	1.52909	0.378755
33947	3.04357	0.591879
33948	1.89226	0.279475
33949	1.89226	0.382299
33950	1.89226	0.409024
33951	1.89226	0.107237
33952	3.78453	0.108004
33953	1.89227	0.179289
33954	1.89227	0.05
33955	1.89227	0.745935
33956	1.89227	0.234079
33957	3.78453	0.374274
33958	1.89994	0.0638153
33959	1.70303	0.0708098
33960	1.68576	0.05
33961	1.89608	0.05
33962	3.8237	0.05
33963	1.89994	0.0770912
33964	2.31116	1.59699
33965	1.9386	0.113944
33966	1.88653	0.51299
33967	3.74885	0.542158
33968	2.31117	2.1
33969	2.20665	1.65183
33970	2.09617	1.46199
33971	2.41055	2.1
33972	2.09617	1.39943
33973	2.41055	2.1
33974	1.10777	0.05
33975	1.0523	0.0997865
33976	0.912098	0.0602772
33977	0.757533	0.0512403
33978	0.89287	0.125253
33979	1.07571	0.05
33980	0.77485	0.0729003
33981	0.98645	0.05
33982	0.989171	0.05
33983	0.875089	0.05
33984	0.979686	0.0744936
33985	0.979686	0.241817

33986	2.52442	2.1
33987	1.49998	2.1
33988	2.51692	2.1
33989	1.56957	2.1
33990	4.09399	2.1
33991	1.01298	2.1
33992	1.01298	2.1
33993	1.03079	2.1
33994	1.03079	2.1
33995	0.820964	2.1
33996	0.784786	2.1
33997	1.25898	2.1
33998	1.25846	2.1
33999	1.25898	2.1
34000	0.820964	2.1
34001	1.25846	2.1
34002	0.784786	2.1
34003	1.89226	0.05
34004	1.89226	0.05
34005	2.6297	0.05
34006	1.89226	0.05
34007	3.78452	0.05
34008	1.89226	0.05
34009	1.89226	0.05
34010	1.89226	0.05
34011	1.89226	0.05
34012	1.89226	0.05
34013	1.89226	0.05
34014	2.19397	0.05
34015	1.89226	0.05
34016	2.34112	0.05
34017	2.34112	0.05
34018	1.89226	0.05
34019	1.89226	0.05
34020	2.23366	2.1
34021	1.87598	2.1
34022	1.64716	2.1
34023	2.06048	2.1
34024	3.93645	2.1
34025	2.23367	2.1
34026	2.17384	2.1
34027	2.04818	2.1
34028	2.06048	2.1
34029	4.23432	2.1
34030	1.9855	2.1
34031	2.06048	2.1
34032	1.93163	2.1
34033	1.50737	2.1
34034	3.56785	2.1
34035	2.04818	2.1
34036	2.06048	2.1
34037	2.27465	2.1
34038	1.92264	2.1
34039	3.98312	2.1
34040	2.08149	0.132782
34041	1.89226	1.64206
34042	1.89226	0.998221
34043	1.33153	0.114006

34044	1.68038	1.95004
34045	1.18244	0.219911
34046	1.89226	0.58086
34047	1.89226	1.86111
34048	1.89226	1.63674
34049	1.89226	0.288996
34050	3.78452	0.695475
34051	2.69069	0.122378
34052	1.89226	0.368777
34053	1.89226	0.431258
34054	1.97397	0.119082
34055	1.89226	0.383562
34056	1.97397	0.105961
34057	1.89226	1.87435
34058	2.38056	0.253412
34059	1.89226	0.308862
34060	1.89226	1.06298
34061	3.78452	0.76497
34062	0.904435	2.1
34063	0.904435	2.1
34064	0.925315	2.1
34065	0.925315	2.1
34066	1.13312	2.1
34067	1.08109	2.1
34068	0.70697	2.1
34069	0.696633	2.1
34070	0.70697	2.1
34071	1.13312	2.1
34072	0.696633	2.1
34073	1.08109	2.1
34074	1.45506	2.1
34075	2.26623	2.1
34076	2.38363	2.1
34077	2.38363	2.1
34078	1.41394	2.1
34079	1.41394	2.1
34080	0.946131	2.1
34081	0.946131	2.1
34082	1.13311	2.1
34083	1.13311	2.1
34084	0.946127	2.1
34085	0.946127	2.1
34086	1.13312	2.1
34087	1.19182	2.1
34088	1.19181	2.1
34089	0.946129	2.1
34090	1.13312	2.1
34091	0.94613	2.1
34092	1.89226	0.232128
34093	1.89226	2.1
34094	1.89226	0.551756
34095	1.89226	2.05742
34096	1.89226	0.578939
34097	1.89226	2.05979
34098	1.05168	2.1
34099	0.696527	2.1
34100	0.907442	0.927588
34101	0.907442	1.35956

34102	0.953145	0.648966
34103	0.782285	2.1
34104	0.773928	2.1
34105	0.773928	0.998105
34106	0.696538	1.39804
34107	0.851554	1.57499
34108	1.04071	1.66006
34109	1.12335	1.7211
34110	1.89226	0.589347
34111	1.89226	0.1448
34112	1.89226	0.184382
34113	1.89226	0.0657137
34114	3.78453	0.0878051
34115	1.89226	0.503313
34116	1.89226	0.140945
34117	1.89226	0.23669
34118	1.89226	0.108677
34119	3.78453	0.360822
34120	2.50912	0.119204
34121	1.89226	0.0760103
34122	1.89226	0.0959907
34123	2.63195	0.0510156
34124	4.40139	0.0547611
34125	2.50912	0.121212
34126	1.3598	0.0578482
34127	1.70303	0.0595359
34128	2.36874	0.05
34129	3.82055	0.0746154
34130	1.89227	0.174864
34131	1.89227	0.0707213
34132	1.89227	0.0718358
34133	1.89227	0.05
34134	3.78453	0.123223
34135	1.89227	0.223681
34136	1.89227	0.0941453
34137	1.89227	0.0864271
34138	1.89227	0.05
34139	3.78453	0.0570275
34140	1.89226	0.082295
34141	2.67088	0.05
34142	2.63195	0.05
34143	2.63195	0.05
34144	1.89226	0.05
34145	1.89226	0.0820087
34146	1.30202	0.05
34147	1.28304	0.05
34148	0.809902	0.05
34149	0.888801	0.05
34150	1.13262	0.05
34151	0.968276	0.05
34152	1.0733	0.05
34153	1.0733	0.05
34154	1.35419	0.05
34155	0.801222	0.05
34156	0.899732	0.0592528
34157	1.23967	0.05
34158	1.31595	2.1
34159	0.869501	2.1

34160	2.65473	2.1
34161	1.88782	2.1
34162	3.20377	2.1
34163	1.89227	2.1
34164	1.89227	2.1
34165	1.89227	2.1
34166	1.89227	2.1
34167	3.78453	2.1
34168	2.65473	2.1
34169	1.89226	2.1
34170	1.88781	2.1
34171	1.88782	2.1
34172	2.0815	2.1
34173	2.0815	2.1
34174	1.89226	2.1
34175	1.89226	2.1
34176	1.89226	2.1
34177	1.89226	2.1
34178	1.89226	2.1
34179	1.89226	2.1
34180	1.89226	2.1
34181	1.89226	2.1
34182	1.89226	2.1
34183	1.89226	2.1
34184	1.89226	2.1
34185	1.89226	2.1
34186	2.39057	2.1
34187	1.89226	1.82477
34188	1.89226	0.824334
34189	1.89226	2.1
34190	2.14243	0.676671
34191	2.14243	2.1
34192	1.89226	0.0822059
34193	1.89226	2.1
34194	1.89226	0.365227
34195	1.89226	0.765861
34196	1.89226	0.94469
34197	1.89226	2.04073
34198	1.89226	0.103674
34199	1.89226	2.1
34200	1.89226	0.260276
34201	1.89226	0.634829
34202	1.89226	0.348742
34203	1.89226	1.95076
34204	2.56323	0.05
34205	1.36324	0.05
34206	1.05296	0.05
34207	2.18048	0.05
34208	3.54372	0.05
34209	0.946128	2.1
34210	0.946128	2.1
34211	1.08733	2.1
34212	1.08733	2.1
34213	0.946131	2.1
34214	0.946131	2.1
34215	1.09197	2.1
34216	1.08732	2.1
34217	0.946131	2.1

34218	1.09197	2.1
34219	1.08732	2.1
34220	0.946131	2.1
34221	1.35111	1.14713
34222	1.51327	0.801644
34223	1.64493	0.833989
34224	1.31058	1.13796
34225	2.99605	1.39459
34226	1.8124	0.709017
34227	1.51327	1.06981
34228	1.64493	1.28772
34229	1.31358	0.678932
34230	1.8124	1.19131
34231	1.48105	0.585315
34232	1.64493	0.872762
34233	1.31058	0.970984
34234	1.22297	0.885546
34235	1.70101	0.765811
34236	2.8679	0.621225
34237	1.70101	0.955357
34238	1.99297	0.729143
34239	1.64493	1.11019
34240	1.64493	0.561575
34241	1.99297	1.15028
34242	1.8124	0.427458
34243	2.0815	2.1
34244	1.80258	2.1
34245	1.80258	2.1
34246	1.83506	2.1
34247	2.0815	2.1
34248	2.1212	2.1
34249	2.0815	2.1
34250	1.82129	2.1
34251	1.82129	2.1
34252	1.80445	2.1
34253	2.0815	2.1
34254	2.06092	2.1
34255	1.89224	2.1
34256	1.703	2.1
34257	1.97839	2.1
34258	1.703	2.1
34259	1.97402	2.1
34260	1.703	2.1
34261	1.89226	0.510739
34262	1.89226	1.01609
34263	1.89226	0.35801
34264	2.75351	0.622323
34265	3.78453	0.7298
34266	1.89226	0.507971
34267	3.02887	1.09637
34268	2.03149	0.285628
34269	2.03149	0.467872
34270	1.89226	0.786613
34271	1.89226	1.22623
34272	2.17979	2.1
34273	1.65533	2.1
34274	2.57212	2.1
34275	2.20346	2.1

34276	4.22745	2.1
34277	1.89227	2.1
34278	2.20347	2.1
34279	1.89227	2.1
34280	2.57212	2.1
34281	1.89227	2.1
34282	2.57212	2.1
34283	2.05274	2.08956
34284	2.16783	2.1
34285	2.24429	1.32847
34286	2.38404	2.1
34287	4.41212	2.1
34288	2.16783	2.1
34289	2.05967	2.1
34290	2.38404	2.1
34291	2.24695	2.1
34292	4.41478	2.1
34293	2.24429	1.08469
34294	2.38404	2.1
34295	2.51819	1.57888
34296	2.60723	2.1
34297	4.85152	2.1
34298	2.38404	2.1
34299	2.24695	2.1
34300	2.60723	2.1
34301	2.45247	2.1
34302	4.85418	2.1
34303	1.89227	2.1
34304	1.89227	2.1
34305	1.89227	2.1
34306	1.89227	2.1
34307	3.78453	2.1
34308	1.89226	2.1
34309	1.89226	2.1
34310	1.89226	2.1
34311	1.89226	2.1
34312	3.78453	2.1
34313	0.618473	2.1
34314	0.619945	2.1
34315	0.797692	2.1
34316	0.770147	2.1
34317	0.68852	2.1
34318	0.68852	2.1
34319	0.701573	2.1
34320	0.701573	2.1
34321	0.619945	2.1
34322	0.797691	2.1
34323	0.618473	2.1
34324	0.770147	2.1
34325	0.797692	2.1
34326	0.770148	2.1
34327	0.694787	2.1
34328	0.653411	2.1
34329	0.722102	2.1
34330	0.722102	2.1
34331	0.729001	2.1
34332	0.729001	2.1
34333	0.797692	2.1

34334	0.694787	2.1
34335	0.770148	2.1
34336	0.653412	2.1
34337	1.89227	2.1
34338	1.89227	2.1
34339	1.89227	2.1
34340	1.89227	2.1
34341	3.78453	2.1
34342	1.89226	2.1
34343	1.89226	2.1
34344	1.89226	2.1
34345	1.89226	2.1
34346	3.78453	2.1
34347	1.23989	2.1
34348	1.59538	2.1
34349	1.66647	2.1
34350	1.26599	2.1
34351	2.90636	2.1
34352	1.66647	2.1
34353	1.38957	2.1
34354	1.47446	2.1
34355	1.40337	2.1
34356	1.66647	2.1
34357	1.59538	2.1
34358	2.80619	2.1
34359	2.0815	2.1
34360	2.0815	2.1
34361	2.0815	2.1
34362	2.73721	2.1
34363	2.74192	2.1
34364	2.73721	2.1
34365	2.0815	2.1
34366	2.0815	2.1
34367	2.0815	2.1
34368	2.689	2.1
34369	2.69166	2.1
34370	2.62369	2.1
34371	2.19717	2.1
34372	2.60405	2.1
34373	2.20566	2.1
34374	4.80122	2.1
34375	2.19717	2.1
34376	1.37392	2.1
34377	2.20566	2.1
34378	1.45408	2.1
34379	3.65125	2.1
34380	2.60405	2.1
34381	2.20566	2.1
34382	2.57466	2.1
34383	2.21715	2.1
34384	4.8212	2.1
34385	2.20566	2.1
34386	1.54249	2.1
34387	1.54249	2.1
34388	1.45408	2.1
34389	2.21715	2.1
34390	2.12874	2.1
34391	1.60647	2.1

34392	1.31346	2.1
34393	1.82649	2.1
34394	1.51067	2.1
34395	3.13995	2.1
34396	1.89226	2.1
34397	1.89226	2.1
34398	1.89226	2.1
34399	1.89226	2.1
34400	3.78452	2.1
34401	1.89226	2.1
34402	2.6096	2.1
34403	2.8067	2.1
34404	2.8067	2.1
34405	1.89226	2.1
34406	1.89226	2.1
34407	1.82649	2.1
34408	1.3596	2.1
34409	2.17708	2.1
34410	1.70302	2.1
34411	3.40243	2.1
34412	1.89226	2.1
34413	1.89226	2.1
34414	1.89226	1.7875
34415	1.89226	2.1
34416	3.78452	2.1
34417	2.27067	2.1
34418	2.43549	2.1
34419	2.63979	2.1
34420	2.41828	2.1
34421	2.27067	2.1
34422	2.04916	2.1
34423	1.30047	2.1
34424	2.04916	2.1
34425	2.27068	2.1
34426	1.5586	2.1
34427	3.57115	2.1
34428	2.27068	2.1
34429	1.84214	2.1
34430	1.80224	2.1
34431	1.5586	2.1
34432	2.51432	2.1
34433	2.27068	2.1
34434	1.63719	2.1
34435	2.15714	2.1
34436	2.07821	2.1
34437	1.52204	2.1
34438	3.67919	2.1
34439	2.07821	2.1
34440	1.40118	2.1
34441	1.40118	2.1
34442	1.52204	2.1
34443	1.99632	2.1
34444	2.11718	2.1
34445	0.946131	2.1
34446	0.946131	2.1
34447	1.06957	2.1
34448	1.06957	2.1
34449	0.946132	2.1

34450	0.946132	1.94077
34451	1.411	2.1
34452	1.06957	2.1
34453	0.946132	2.1
34454	1.411	2.1
34455	1.06957	2.1
34456	0.946132	2.1
34457	1.99875	0.606626
34458	2.57079	0.387617
34459	2.59319	1.27331
34460	1.9105	0.155549
34461	4.59194	0.590005
34462	1.99875	0.519241
34463	1.02247	0.0591856
34464	1.07017	0.113826
34465	1.15841	0.120131
34466	1.91051	0.0932129
34467	1.99875	0.129371
34468	2.59319	1.20417
34469	1.9105	0.197071
34470	2.33812	0.461805
34471	2.59319	0.482298
34472	1.59457	0.654204
34473	1.59457	0.701586
34474	2.50906	0.476762
34475	2.0467	0.165358
34476	2.03357	0.265213
34477	2.53176	0.14801
34478	4.54263	0.213328
34479	1.294	0.458375
34480	1.66636	0.289311
34481	2.03357	0.210605
34482	1.03079	0.123835
34483	3.32757	0.272262
34484	1.68108	0.204785
34485	2.53176	0.131526
34486	2.36417	0.104951
34487	2.71666	0.093575
34488	1.68108	0.539414
34489	2.03357	0.349953
34490	1.89226	0.05
34491	2.59302	0.0576731
34492	2.2727	0.05
34493	2.2727	0.05
34494	1.89226	0.075359
34495	1.89226	0.05
34496	1.89226	0.0979637
34497	1.89226	0.0712089
34498	1.89226	0.05
34499	1.89226	0.05
34500	3.78452	0.0933762
34501	1.89225	0.155789
34502	1.89225	0.05
34503	1.89225	0.05
34504	1.89225	0.05
34505	1.89225	0.05
34506	1.89225	0.050819
34507	0.934114	0.05

34508	0.934114	0.05
34509	0.944544	0.05
34510	0.944544	0.05
34511	0.721493	0.05
34512	0.58215	0.05
34513	1.2965	0.05
34514	1.13634	0.05
34515	1.13635	0.05
34516	0.582148	0.05
34517	1.29651	0.05
34518	0.721491	0.05
34519	1.89226	0.05
34520	1.89226	0.05
34521	1.89226	0.05
34522	1.89226	0.05
34523	3.78452	0.05
34524	0.946131	0.0608843
34525	0.946131	0.0642142
34526	0.946131	0.05
34527	0.946131	0.0655671
34528	0.946131	0.05
34529	0.946131	0.0574584
34530	0.946131	0.0615266
34531	0.946131	0.0828069
34532	0.946131	0.0527666
34533	0.946131	0.0519848
34534	0.946131	0.0602803
34535	0.946131	0.0602631
34536	1.89226	0.05
34537	1.89226	0.05
34538	2.78602	0.05
34539	1.89226	0.05
34540	3.78453	0.05
34541	1.52601	2.1
34542	1.76601	2.1
34543	1.27839	2.1
34544	1.56095	2.1
34545	3.0444	2.1
34546	1.76601	2.1
34547	1.86793	2.1
34548	1.56095	2.1
34549	1.68152	2.1
34550	3.44753	2.1
34551	1.27841	2.1
34552	1.56097	2.1
34553	1.0577	2.1
34554	1.36861	2.1
34555	2.64702	2.1
34556	1.56097	2.1
34557	1.68154	2.1
34558	1.36861	2.1
34559	1.53296	2.1
34560	3.05015	2.1
34561	1.89226	2.1
34562	1.89226	2.1
34563	1.89226	2.1
34564	1.89226	2.1
34565	3.78453	2.1

34566	0.983075	2.1
34567	0.788361	2.1
34568	0.724726	2.1
34569	0.724726	2.1
34570	0.983027	2.1
34571	0.724734	2.1
34572	0.788364	2.1
34573	0.788364	2.1
34574	0.788335	2.1
34575	0.94613	2.1
34576	0.724757	2.1
34577	0.946131	2.1
34578	1.15789	2.1
34579	2.11135	2.1
34580	2.25826	2.1
34581	1.28845	2.1
34582	3.41615	2.1
34583	1.89226	2.1
34584	1.89226	2.1
34585	1.89226	2.1
34586	1.89226	2.1
34587	3.78453	2.1
34588	1.57667	2.1
34589	1.70002	2.1
34590	1.70002	2.1
34591	1.89226	2.1
34592	1.89226	2.1
34593	2.05244	2.1
34594	2.25826	2.1
34595	1.42214	2.1
34596	1.43675	2.1
34597	1.28845	2.1
34598	2.40656	2.1
34599	2.25826	2.1
34600	1.58066	2.1
34601	1.74247	2.1
34602	1.85097	2.1
34603	1.61155	2.1
34604	3.43163	2.1
34605	0.785612	2.1
34606	0.789638	2.1
34607	0.882561	2.1
34608	0.763529	2.1
34609	0.686921	2.1
34610	0.86048	2.1
34611	0.871812	2.1
34612	0.871812	2.1
34613	0.830183	2.1
34614	0.882572	2.1
34615	0.902315	2.1
34616	0.785602	2.1
34617	1.01972	0.05
34618	0.936657	0.05
34619	0.946131	0.05
34620	0.946131	0.05
34621	0.927018	0.05
34622	0.927018	0.05
34623	1.1376	0.05

34624	0.953663	0.05
34625	1.1257	0.05
34626	1.04073	0.05
34627	1.00358	0.05
34628	0.851528	0.05
34629	1.73576	0.0688005
34630	2.00244	0.223862
34631	1.17798	0.0977715
34632	1.25796	0.0699926
34633	2.99373	0.0638148
34634	2.00244	0.209501
34635	1.10719	0.107693
34636	2.03236	0.316372
34637	2.03236	0.16354
34638	1.25797	0.264892
34639	1.25797	0.0946016
34640	1.89226	2.1
34641	1.89226	2.1
34642	1.89226	2.1
34643	1.89226	2.1
34644	3.78452	2.1
34645	2.3174	2.1
34646	1.50005	2.1
34647	1.90428	2.1
34648	2.64564	2.1
34649	4.22168	2.1
34650	1.89226	2.1
34651	1.89226	2.1
34652	1.89226	2.1
34653	1.89226	2.1
34654	1.89226	2.1
34655	1.89226	2.1
34656	1.32749	0.05
34657	1.70302	0.05
34658	2.08149	0.05
34659	1.70302	0.05
34660	2.19634	0.05
34661	1.93083	0.05
34662	1.89226	1.5128
34663	1.89226	2.06008
34664	1.89226	0.352239
34665	1.89226	2.1
34666	3.78453	0.973914
34667	1.89226	1.43122
34668	1.89226	2.0513
34669	1.89226	0.825556
34670	1.89226	1.50169
34671	3.78452	1.83682
34672	2.17499	0.952392
34673	1.79663	1.9703
34674	2.1538	0.207624
34675	1.818	0.812194
34676	2.17499	1.76665
34677	1.83919	2.1
34678	0.898316	2.07439
34679	1.0769	1.14122
34680	0.968276	2.1
34681	0.773495	1.6477

34682	0.921126	1.95976
34683	0.921126	2.1
34684	0.92927	1.82841
34685	0.92927	1.83254
34686	1.0769	1.78127
34687	0.968276	1.2277
34688	0.898316	2.05242
34689	0.773495	1.57002
34690	1.89226	0.907352
34691	1.89226	2.1
34692	1.89226	1.27786
34693	1.89226	1.17002
34694	3.78453	2.06494
34695	1.89226	1.43445
34696	1.89226	2.1
34697	1.89226	0.613668
34698	1.89226	1.73341
34699	3.78452	1.18303
34700	2.17499	1.94187
34701	2.1538	2.1
34702	2.53574	0.994208
34703	2.54157	0.697533
34704	4.71073	1.87365
34705	1.0769	1.68412
34706	1.26787	1.12107
34707	1.16641	1.43077
34708	0.968276	2.1
34709	1.11625	1.40697
34710	1.11625	1.89172
34711	1.11989	2.04007
34712	1.11989	0.533801
34713	1.26787	1.21101
34714	1.16641	2.1
34715	1.0769	2.1
34716	0.968277	1.86786
34717	1.89226	0.08488
34718	1.89226	0.0821958
34719	1.89226	0.090053
34720	1.89226	0.081418
34721	3.78452	0.0915693
34722	1.91739	0.0576752
34723	2.20045	0.06102
34724	1.79005	0.0680167
34725	2.33802	0.0660921
34726	4.25539	0.0553266
34727	2.52754	0.05
34728	1.89226	0.0676027
34729	2.69841	0.05
34730	2.69841	0.064089
34731	1.89226	0.05
34732	1.89226	0.0619261
34733	1.89226	0.114767
34734	1.89226	0.124296
34735	1.89226	0.0995424
34736	1.89226	0.262596
34737	3.78452	0.11356
34738	1.93544	0.115172
34739	2.33802	0.0974353

34740	1.55063	0.05
34741	2.1769	0.0634606
34742	3.88865	0.117845
34743	2.20929	0.05
34744	2.55162	0.05
34745	2.27807	0.05
34746	1.48831	0.05
34747	4.48736	0.05
34748	2.27807	0.05
34749	2.72723	0.05
34750	2.55162	0.05
34751	2.55162	0.05
34752	2.43157	0.05
34753	2.43157	0.05
34754	2.27807	0.05
34755	1.51612	0.05
34756	1.96048	0.05
34757	1.73022	0.05
34758	1.70302	0.05
34759	1.61016	0.05
34760	2.27807	0.05
34761	2.08048	0.05
34762	1.98522	0.05
34763	2.43157	0.05
34764	1.83798	0.05
34765	2.27807	0.05
34766	1.70302	0.05
34767	2.68507	0.05
34768	2.23856	0.0557119
34769	2.52174	0.05
34770	2.35331	0.0669758
34771	2.65099	0.05
34772	1.70303	0.0546847
34773	0.986848	0.05
34774	2.41566	0.05
34775	1.70303	0.05
34776	1.88139	0.05
34777	1.64955	0.05
34778	1.89226	2.1
34779	2.34978	1.84613
34780	1.89226	2.1
34781	2.68773	1.42683
34782	1.89226	2.1
34783	2.68773	1.82207
34784	1.89226	2.1
34785	1.89226	1.39902
34786	1.89226	1.60538
34787	1.89226	2.1
34788	3.78453	2.08243
34789	1.00825	0.558669
34790	1.09847	0.859335
34791	0.939987	0.772706
34792	0.783516	1.93084
34793	0.838041	1.28872
34794	0.838041	1.93297
34795	1.49693	2.04398
34796	1.26602	1.25248
34797	1.10875	0.613941

34798	1.05187	2.1
34799	1.28743	1.41379
34800	0.946132	0.886901
34801	0.946132	1.49972
34802	0.946132	2.1
34803	0.946132	2.1
34804	0.946132	0.613665
34805	1.31823	2.1
34806	0.946132	1.39015
34807	0.946132	2.1
34808	0.946131	2.1
34809	0.946131	2.1
34810	0.946132	1.93156
34811	1.31823	2.09201
34812	1.51688	0.05
34813	1.79978	0.084219
34814	1.1685	0.05
34815	1.1685	0.0597305
34816	1.85857	0.0578567
34817	1.51018	0.0877051
34818	0.946116	0.05
34819	0.946116	0.0671382
34820	0.94612	0.05
34821	0.94612	0.0538582
34822	0.946118	0.05
34823	0.946118	0.0673357
34824	0.946118	0.05
34825	0.946118	0.077738
34826	0.946131	0.05
34827	0.946131	0.0812781
34828	0.946105	0.0540655
34829	0.946105	0.0595577
34830	1.89224	2.1
34831	1.89224	2.1
34832	1.89224	2.1
34833	1.89224	2.1
34834	1.89224	2.1
34835	1.89224	2.1
34836	1.38678	2.1
34837	1.3349	2.1
34838	0.946119	2.1
34839	0.946119	2.1
34840	1.38678	2.1
34841	1.38678	2.1
34842	0.946119	2.1
34843	0.946119	2.1
34844	1.38676	2.1
34845	0.946107	2.1
34846	0.946132	2.1
34847	1.33491	2.1
34848	1.06455	2.1
34849	1.03932	2.1
34850	1.20649	2.1
34851	1.18773	2.1
34852	2.27105	2.1
34853	1.89226	2.1
34854	1.89226	2.1
34855	1.89226	2.1

34856	1.89226	2.1
34857	1.89226	2.1
34858	1.89226	2.1
34859	1.36918	2.1
34860	1.18774	2.1
34861	1.31144	2.1
34862	1.20651	2.1
34863	1.31144	2.1
34864	1.20651	2.1
34865	2.29066	2.1
34866	1.76476	2.1
34867	1.79825	2.1
34868	2.33801	2.1
34869	4.08891	2.1
34870	1.79825	2.1
34871	2.33801	2.1
34872	2.37168	2.1
34873	1.49325	2.1
34874	4.16993	2.1
34875	1.13641	2.1
34876	1.43213	1.12145
34877	1.44085	2.1
34878	1.18301	2.1
34879	2.61513	2.1
34880	1.89226	2.1
34881	2.23875	2.1
34882	2.23875	2.1
34883	2.10287	2.1
34884	1.89226	2.1
34885	1.75639	2.1
34886	1.89226	1.33572
34887	1.89226	2.1
34888	1.89226	1.55244
34889	1.89226	2.1
34890	1.89226	1.21838
34891	1.89226	2.1
34892	1.44085	2.1
34893	1.25173	2.1
34894	1.25173	1.99761
34895	1.18301	2.1
34896	1.4617	1.71272
34897	1.39298	2.1
34898	1.89227	2.1
34899	2.23875	2.1
34900	2.42504	2.1
34901	1.89227	2.1
34902	4.13101	2.1
34903	1.89226	1.23979
34904	1.89226	2.1
34905	1.89226	1.14332
34906	1.89226	2.1
34907	1.89226	1.0084
34908	1.89226	2.1
34909	1.89226	2.1
34910	2.41297	2.1
34911	2.70685	2.1
34912	2.70685	2.1
34913	1.89226	2.1

34914	1.89226	2.1
34915	1.89226	2.1
34916	1.89226	2.1
34917	1.89226	2.1
34918	1.89226	2.1
34919	1.89226	2.1
34920	1.89226	2.1
34921	1.89226	2.1
34922	1.89226	2.1
34923	1.89226	2.1
34924	1.89226	2.1
34925	3.78453	2.1
34926	1.89226	2.1
34927	1.89226	2.1
34928	1.89226	2.1
34929	1.89226	2.1
34930	1.89226	2.1
34931	1.89226	2.1
34932	1.69044	0.05
34933	1.57979	0.05
34934	2.27963	0.05
34935	2.21582	0.05
34936	3.85942	0.05
34937	2.21582	0.05
34938	1.30722	0.05
34939	2.02069	0.05
34940	2.02069	0.05
34941	1.57979	0.05
34942	1.57979	0.05
34943	1.67825	2.1
34944	1.89226	2.1
34945	1.89226	2.1
34946	1.89226	2.1
34947	1.91894	2.1
34948	1.91894	2.1
34949	0.946133	2.1
34950	0.946133	2.1
34951	0.946132	2.1
34952	0.946132	2.1
34953	0.946132	2.1
34954	0.946132	2.1
34955	0.946133	2.1
34956	0.946133	2.1
34957	0.946133	2.1
34958	0.946133	2.1
34959	0.946132	2.1
34960	0.946132	2.1
34961	0.942609	2.1
34962	0.942609	2.1
34963	1.329 2.1	
34964	1.329 2.1	
34965	1.32547	2.1
34966	0.94261	2.1
34967	0.946132	2.1
34968	0.946132	2.1
34969	0.946132	2.1
34970	1.32547	2.1
34971	0.946132	2.1

34972	0.94261	2.1
34973	1.55831	2.1
34974	1.07496	2.1
34975	1.80327	0.278079
34976	1.34851	0.595595
34977	2.87824	0.981696
34978	2.14346	0.584461
34979	2.77937	0.158725
34980	2.79173	0.859815
34981	2.17129	0.109619
34982	4.95066	0.0978574
34983	2.14346	0.69433
34984	1.34124	0.05
34985	1.39818	0.59773
34986	2.1713	0.18524
34987	3.51253	0.497766
34988	2.79173	0.462902
34989	2.21882	0.0726055
34990	2.17129	0.124365
34991	2.17129	0.149478
34992	2.81448	0.200591
34993	2.81448	0.232217
34994	1.10941	0.109577
34995	1.08565	0.136354
34996	0.699089	0.0910693
34997	0.740176	0.36624
34998	0.908594	0.0678945
34999	0.908594	0.0809869
35000	0.91723	0.685202
35001	0.91723	0.11637
35002	1.10941	0.125125
35003	0.740176	0.0893955
35004	0.699089	0.104432
35005	1.08565	0.0710528
35006	1.89227	0.197675
35007	1.89227	0.0776511
35008	1.89227	0.05
35009	1.89227	0.05
35010	3.78453	0.0613391
35011	2.40032	0.1094
35012	2.19526	0.0594423
35013	1.12907	0.05
35014	1.44253	0.0696415
35015	3.32433	0.05
35016	2.19526	0.05
35017	2.71093	0.0688626
35018	1.29826	0.05
35019	2.0815	0.05
35020	4.18911	0.0500908
35021	1.89226	0.05
35022	1.89226	0.0535856
35023	1.89226	0.05
35024	1.89226	0.05
35025	3.78452	0.05
35026	2.30822	0.05
35027	1.89226	0.05
35028	2.79329	0.05
35029	2.79329	0.05

35030	1.89226	0.0573993
35031	1.89226	0.05
35032	0.946131	2.1
35033	0.946131	0.866593
35034	1.38414	2.1
35035	1.38414	0.10287
35036	0.94613	2.1
35037	0.94613	2.1
35038	1.38415	0.685585
35039	1.41833	0.118357
35040	0.946132	1.96262
35041	1.41833	1.9074
35042	1.38414	1.73776
35043	0.946131	2.07164
35044	1.89226	2.1
35045	2.6536	2.1
35046	1.89226	2.1
35047	2.72238	2.1
35048	4.61465	2.1
35049	0.946132	2.1
35050	0.946132	2.1
35051	1.38657	2.1
35052	1.38657	2.1
35053	1.38657	2.1
35054	1.36119	2.1
35055	0.946131	2.1
35056	0.946131	2.1
35057	0.946132	2.1
35058	1.38657	2.1
35059	0.946132	2.1
35060	1.36119	2.1
35061	1.89226	2.1
35062	2.4717	2.1
35063	2.5026	2.1
35064	2.4717	2.1
35065	1.92316	2.1
35066	1.89226	2.1
35067	1.89226	2.1
35068	1.89226	2.1
35069	1.89226	2.1
35070	1.89226	2.1
35071	3.78453	2.1
35072	0.946128	2.1
35073	0.946128	2.1
35074	1.3419	2.1
35075	1.3419	2.1
35076	0.946131	2.1
35077	0.946131	2.1
35078	1.36607	2.1
35079	1.3419	2.1
35080	0.946131	2.1
35081	1.36607	2.1
35082	1.3419	2.1
35083	0.94613	2.1
35084	1.89226	2.1
35085	2.50259	2.1
35086	2.53563	2.1
35087	2.50259	2.1

35088	1.92529	2.1
35089	1.89226	2.1
35090	0.946132	2.1
35091	0.946132	2.1
35092	0.946131	2.1
35093	0.946131	2.1
35094	0.946132	2.1
35095	0.946132	2.1
35096	0.946131	2.1
35097	0.946131	2.1
35098	0.946131	2.1
35099	0.946131	2.1
35100	0.946131	2.1
35101	0.946131	2.1
35102	2.0815	2.1
35103	1.86506	2.1
35104	1.86506	2.1
35105	1.82567	2.1
35106	2.0815	2.1
35107	2.03335	2.1
35108	2.0815	2.1
35109	2.0815	2.1
35110	2.0815	2.1
35111	1.88668	2.1
35112	1.83221	2.1
35113	1.57275	2.1
35114	2.34061	0.05
35115	1.89226	0.05
35116	2.23254	0.05
35117	1.89226	0.05
35118	2.23254	0.05
35119	1.89226	0.05
35120	1.8619	0.05
35121	1.89226	0.05
35122	2.34061	0.05
35123	1.89226	0.05
35124	2.34061	0.05
35125	1.89226	0.05
35126	1.89227	0.05
35127	1.89227	0.05
35128	1.89227	0.05
35129	1.89227	0.05
35130	1.89227	0.05
35131	1.89227	0.05
35132	1.89227	0.05
35133	1.89227	0.05
35134	1.89227	0.05
35135	1.89227	0.05
35136	1.89227	0.05
35137	1.89227	0.05
35138	1.06365	0.05
35139	0.503305	0.05
35140	0.794912	0.05
35141	1.42752	0.05
35142	1.34826	0.05
35143	1.07163	0.05
35144	0.533113	0.05
35145	0.761277	0.05

35146	0.848403	0.05
35147	0.876191	0.05
35148	0.963372	0.05
35149	0.963372	0.05
35150	1.55571	0.05
35151	2.76574	0.05
35152	2.92453	0.05
35153	1.70301	0.05
35154	2.33089	0.05
35155	1.06905	0.05
35156	2.28979	2.1
35157	1.81488	2.1
35158	1.63722	2.1
35159	2.22627	2.1
35160	4.04115	2.1
35161	1.98246	2.1
35162	2.15715	2.1
35163	2.22627	2.1
35164	2.22627	2.1
35165	1.63719	2.1
35166	1.63719	2.1
35167	2.1644	0.05
35168	1.903 0.05	
35169	1.23639	0.05
35170	1.42704	0.05
35171	3.61546	0.05
35172	1.89226	0.05
35173	1.89226	0.05
35174	1.89226	0.05
35175	1.89226	0.05
35176	3.78451	0.05
35177	2.61343	0.05
35178	1.89226	0.05
35179	2.08149	0.05
35180	1.74357	0.05
35181	2.08149	0.05
35182	1.74357	0.05
35183	1.63483	0.05
35184	1.8631	0.05
35185	1.8631	0.05
35186	1.70301	0.05
35187	2.0815	0.05
35188	1.94319	0.05
35189	2.0815	0.05
35190	1.89226	0.05
35191	1.89226	0.05
35192	2.05236	0.05
35193	2.23014	0.05
35194	2.41882	0.05
35195	2.14993	2.1
35196	1.87217	2.1
35197	2.34488	2.1
35198	2.5254	2.1
35199	4.39757	2.1
35200	0.949995	2.1
35201	0.630972	2.1
35202	0.936084	2.1
35203	1.17244	2.1

35204	0.922394	2.1
35205	0.922394	2.1
35206	0.88102	2.1
35207	0.88102	2.1
35208	0.949998	2.1
35209	1.17244	2.1
35210	0.936081	2.1
35211	0.630973	2.1
35212	1.89226	2.1
35213	1.89226	2.1
35214	1.89226	2.1
35215	1.89226	2.1
35216	3.78452	2.1
35217	1.89226	2.07458
35218	1.89226	2.1
35219	1.89226	2.06634
35220	1.89226	2.1
35221	1.89226	2.01634
35222	1.89226	2.1
35223	2.34488	2.1
35224	2.5254	2.04192
35225	1.89226	2.07906
35226	1.89226	2.1
35227	4.41766	2.1
35228	1.89226	2.1
35229	1.89226	2.1
35230	1.89226	2.1
35231	1.89226	2.1
35232	3.78452	2.1
35233	1.89226	1.87813
35234	1.89226	2.1
35235	1.89226	1.87653
35236	1.89226	2.05684
35237	1.89226	1.91627
35238	1.89226	2.07725
35239	1.5605	2.1
35240	1.15806	2.1
35241	1.31565	2.1
35242	1.65642	2.1
35243	2.87615	2.1
35244	1.46643	2.1
35245	1.65642	2.1
35246	1.31565	2.1
35247	1.31565	2.1
35248	1.74246	2.1
35249	1.74246	2.1
35250	1.89226	2.1
35251	1.89226	2.1
35252	1.89226	2.1
35253	1.89226	2.1
35254	1.89226	2.1
35255	1.89226	2.1
35256	1.15099	2.1
35257	1.15099	2.1
35258	0.939316	2.1
35259	0.939316	2.1
35260	0.946127	2.1
35261	0.946127	2.1

35262	1.14418	2.1
35263	0.939289	2.1
35264	1.14417	2.1
35265	0.946129	2.1
35266	0.939287	2.1
35267	0.94613	2.1
35268	1.89226	2.1
35269	1.89226	2.1
35270	1.89226	2.1
35271	1.89226	2.1
35272	3.78452	2.1
35273	1.93164	0.05
35274	2.64898	0.05
35275	1.43213	0.05
35276	1.43213	0.05
35277	2.63206	0.05
35278	2.13254	0.05
35279	1.89226	2.1
35280	1.89226	2.1
35281	1.89226	2.1
35282	1.89226	2.1
35283	3.78452	2.1
35284	2.71655	2.1
35285	1.43323	2.1
35286	1.28413	2.1
35287	2.53614	2.1
35288	3.96937	2.1
35289	1.89226	2.1
35290	1.89226	2.1
35291	1.89226	2.1
35292	1.89226	2.1
35293	1.89226	2.1
35294	1.89226	2.1
35295	1.33356	2.1
35296	2.82297	2.1
35297	2.771 2.1	
35298	2.771 2.1	
35299	1.46037	2.1
35300	1.46037	2.1
35301	2.56034	2.1
35302	1.18474	2.1
35303	1.15113	2.1
35304	2.53724	2.1
35305	3.72198	2.1
35306	2.56034	2.1
35307	1.89226	2.1
35308	1.89226	2.1
35309	2.53725	2.1
35310	4.42951	2.1
35311	1.89226	2.1
35312	1.89226	2.1
35313	1.89226	2.1
35314	1.89226	2.1
35315	3.78453	2.1
35316	1.89226	2.1
35317	1.89226	2.1
35318	1.89226	2.1
35319	1.89226	2.1

35320	3.78452	2.1
35321	1.1229	2.1
35322	2.53724	2.1
35323	1.15113	2.1
35324	1.15113	2.1
35325	2.51559	2.1
35326	2.51559	2.1
35327	1.89226	2.1
35328	2.53724	2.1
35329	2.51559	2.1
35330	2.53724	2.1
35331	1.87061	2.1
35332	1.89226	2.1
35333	1.89226	2.1
35334	1.89226	2.1
35335	1.89226	2.1
35336	1.89226	2.1
35337	3.78453	2.1
35338	1.89226	2.1
35339	1.89226	2.1
35340	1.89226	2.1
35341	1.89226	2.1
35342	3.78452	2.1
35343	1.98143	1.35909
35344	2.81559	0.0975006
35345	1.65411	0.276383
35346	2.26675	0.984409
35347	4.24818	0.442186
35348	0.810656	2.1
35349	0.810656	2.1
35350	0.821888	2.1
35351	0.821888	2.1
35352	1.1235	2.1
35353	1.07439	2.1
35354	0.535693	2.1
35355	0.509042	2.1
35356	0.535693	2.1
35357	1.1235	2.1
35358	0.509043	2.1
35359	1.07439	2.1
35360	1.10378	2.1
35361	2.247 2.1	
35362	2.27472	2.1
35363	2.27472	2.1
35364	1.07139	2.1
35365	1.07139	2.1
35366	2.08081	2.1
35367	1.40395	2.1
35368	1.34245	2.1
35369	2.00239	2.1
35370	3.40634	2.1
35371	1.89226	2.1
35372	1.89226	2.1
35373	1.89226	2.1
35374	1.89226	2.1
35375	3.78453	2.1
35376	0.946128	2.1
35377	0.946128	2.1

35378	1.13785	2.1
35379	1.11496	2.1
35380	0.946131	2.1
35381	0.946131	2.1
35382	1.11495	2.1
35383	1.11495	2.1
35384	0.94613	2.1
35385	1.11495	2.1
35386	1.13785	2.1
35387	0.946131	2.1
35388	0.975888	2.1
35389	1.00118	2.1
35390	0.671218	2.1
35391	0.653229	2.1
35392	0.825372	2.1
35393	0.825372	2.1
35394	0.829038	2.1
35395	0.829038	2.1
35396	0.975901	2.1
35397	0.653238	2.1
35398	0.671209	2.1
35399	1.00117	2.1
35400	1.89224	2.1
35401	1.89224	2.1
35402	1.89224	2.1
35403	1.89224	2.1
35404	3.78448	2.1
35405	0.946115	2.1
35406	0.946115	2.1
35407	1.11494	2.1
35408	1.10111	2.1
35409	0.946118	2.1
35410	0.946118	2.1
35411	1.10111	2.1
35412	1.10111	2.1
35413	1.10112	2.1
35414	0.94613	2.1
35415	0.946105	2.1
35416	1.11492	2.1
35417	1.04028	0.05
35418	2.27126	0.05
35419	2.0815	0.05
35420	1.82249	0.191887
35421	1.33723	0.05
35422	1.18684	0.133002
35423	0.812616	0.05
35424	0.812616	0.05
35425	0.997777	0.05
35426	1.08122	0.05
35427	0.94613	0.05
35428	0.94613	0.05
35429	0.812648	0.05
35430	0.932779	0.05
35431	0.812648	0.05
35432	0.94613	0.05
35433	0.849335	0.05
35434	0.94613	0.05
35435	1.80327	0.821784

35436	1.56016	0.530589
35437	1.57731	1.98045
35438	1.34851	1.95215
35439	2.03207	0.622359
35440	1.80327	2.04241
35441	2.03207	1.90061
35442	1.56016	1.69422
35443	2.18076	0.872964
35444	1.78167	1.052
35445	3.81374	1.78478
35446	1.37597	2.01295
35447	1.60058	2.1
35448	1.70302	0.568819
35449	1.85448	1.21381
35450	3.15028	1.08246
35451	1.60058	0.74391
35452	1.4054	2.1
35453	1.66202	0.820991
35454	1.70302	2.1
35455	3.09979	1.34875
35456	1.70302	1.64074
35457	1.85448	1.85031
35458	2.21317	1.08594
35459	2.0961	1.11063
35460	4.01757	1.7441
35461	1.66202	1.33389
35462	1.70302	2.1
35463	2.09611	0.577324
35464	2.00892	1.78268
35465	3.6468	1.06107
35466	1.2966	0.05
35467	2.25495	0.05
35468	1.2966	0.05
35469	2.21117	0.05
35470	1.67687	0.05
35471	2.6167	0.05
35472	1.89227	0.05
35473	1.89227	0.05
35474	1.89227	0.05
35475	1.89227	0.05
35476	3.78453	0.05
35477	2.44375	0.05
35478	1.89227	0.05
35479	2.81853	0.05
35480	2.81853	0.05
35481	1.89227	0.05
35482	1.89227	0.05
35483	1.89226	0.972961
35484	1.75853	1.07534
35485	2.37636	0.638698
35486	2.37636	1.22431
35487	1.89226	0.57648
35488	1.89226	1.10624
35489	1.65814	0.05
35490	1.10187	2.1
35491	1.10187	0.469362
35492	1.19547	2.1
35493	1.56263	0.05

35494	1.65624	2.1
35495	1.56264	0.05
35496	1.01479	2.1
35497	1.01479	1.41147
35498	1.10187	2.1
35499	1.47561	0.05
35500	1.5627	2.1
35501	1.89225	2.1
35502	1.89225	2.1
35503	1.89225	2.1
35504	1.89225	2.1
35505	3.7845	2.1
35506	2.54386	2.1
35507	1.91546	2.1
35508	2.46008	2.1
35509	1.82815	2.1
35510	4.37554	2.1
35511	1.91546	2.1
35512	1.04909	2.1
35513	1.82815	2.1
35514	0.983141	2.1
35515	2.8986	2.1
35516	1.89225	2.1
35517	1.89225	2.1
35518	1.89225	2.1
35519	1.89225	2.1
35520	3.78449	2.1
35521	1.89226	2.1
35522	1.89226	2.1
35523	1.89226	2.1
35524	1.89226	2.1
35525	1.89226	2.1
35526	1.89226	2.1
35527	2.46009	2.1
35528	1.7513	2.1
35529	1.82817	2.1
35530	1.82817	2.1
35531	2.38127	2.1
35532	2.38127	2.1
35533	2.55006	2.1
35534	2.64569	2.1
35535	2.60128	2.1
35536	2.49168	2.1
35537	5.13737	2.1
35538	2.51605	2.1
35539	2.64569	2.1
35540	2.60128	2.1
35541	2.48891	2.1
35542	5.1346	2.1
35543	2.60128	2.1
35544	2.49168	2.1
35545	2.41689	2.1
35546	2.55248	2.1
35547	5.04416	2.1
35548	2.60128	2.1
35549	2.48891	2.1
35550	2.45922	2.1
35551	2.55249	2.1

35552	5.04139	2.1
35553	1.89227	2.1
35554	1.89227	2.1
35555	1.89227	2.1
35556	1.89227	2.1
35557	1.89227	2.1
35558	1.89227	2.1
35559	0.96063	2.1
35560	0.96063	2.1
35561	0.958806	2.1
35562	0.958806	2.1
35563	1.28892	2.1
35564	0.749042	2.1
35565	1.17409	2.1
35566	0.630516	2.1
35567	1.17409	2.1
35568	1.28892	2.1
35569	0.630515	2.1
35570	0.749042	2.1
35571	0.92511	2.1
35572	0.92511	2.1
35573	1.19511	2.1
35574	1.19511	2.1
35575	0.946132	2.1
35576	1.17409	2.1
35577	0.925081	2.1
35578	0.946129	2.1
35579	0.946131	2.07667
35580	0.946131	2.1
35581	1.17409	2.1
35582	0.925083	2.1
35583	1.89226	0.843374
35584	1.89226	1.18344
35585	1.89226	0.580157
35586	1.89226	1.28128
35587	1.89226	0.42802
35588	1.89226	1.086
35589	1.89226	1.7675
35590	1.89226	1.94517
35591	1.89226	1.47104
35592	1.89226	2.03139
35593	3.78453	1.67227
35594	0.946132	2.1
35595	0.946132	2.1
35596	1.28892	2.1
35597	1.28892	2.1
35598	0.946132	2.1
35599	1.28892	2.1
35600	0.946133	2.1
35601	1.17409	2.1
35602	0.946132	2.1
35603	0.946132	2.1
35604	1.17409	2.1
35605	1.28892	2.1
35606	0.625229	2.1
35607	0.890321	2.1
35608	1.21636	2.1
35609	1.13022	2.1

35610	1.07094	2.1
35611	1.03541	2.1
35612	0.942207	2.1
35613	0.942207	2.1
35614	0.898766	2.1
35615	1.26626	2.1
35616	1.0853	2.1
35617	0.600358	2.1
35618	0.719012	2.1
35619	0.719012	2.1
35620	1.17957	2.1
35621	1.17957	2.1
35622	0.719012	2.1
35623	0.952456	2.1
35624	0.946129	2.1
35625	0.946129	2.1
35626	0.946131	2.1
35627	0.952454	2.1
35628	0.946131	2.1
35629	0.719011	2.1
35630	0.94613	2.1
35631	0.94613	2.1
35632	0.94613	2.1
35633	0.94613	2.1
35634	0.946131	2.1
35635	0.946131	2.1
35636	0.946129	2.1
35637	0.946129	2.1
35638	0.94613	2.1
35639	0.94613	2.1
35640	0.94613	2.1
35641	0.94613	2.1
35642	1.1587	2.1
35643	0.973158	2.1
35644	0.750023	2.1
35645	0.536443	2.1
35646	0.84054	2.1
35647	0.84054	2.1
35648	0.854602	2.1
35649	0.854602	2.1
35650	1.1587	2.1
35651	0.750023	2.1
35652	0.973158	2.1
35653	0.536443	2.1
35654	1.89227	0.183418
35655	1.89227	2.1
35656	1.89227	2.09881
35657	1.89227	0.502742
35658	3.78453	1.80695
35659	1.89226	2.1
35660	1.89226	2.1
35661	1.89226	2.07363
35662	1.89226	2.1
35663	3.78453	2.1
35664	1.7632	2.1
35665	1.61149	2.1
35666	1.98749	2.1
35667	2.11615	2.1

35668	3.75068	2.1
35669	1.7632	2.1
35670	1.61149	2.1
35671	1.97733	0.405172
35672	2.11615	2.1
35673	3.74052	2.1
35674	1.89226	2.02008
35675	1.89226	0.809824
35676	1.89226	0.268006
35677	1.89226	2.1
35678	3.78453	0.89433
35679	1.89226	1.75943
35680	1.89226	2.1
35681	1.89226	2.1
35682	1.89226	2.1
35683	3.78453	2.1
35684	1.98749	2.1
35685	2.11615	2.1
35686	2.43151	2.1
35687	2.27541	2.1
35688	4.41899	2.1
35689	1.97733	0.508817
35690	2.11615	2.1
35691	2.43151	2.1
35692	2.33986	0.480056
35693	4.40883	2.1
35694	2.17892	0.05
35695	2.80057	0.449221
35696	1.88349	0.297606
35697	2.63062	0.0709766
35698	4.80954	0.12526
35699	1.89226	1.4445
35700	1.89226	0.05
35701	1.89226	0.261899
35702	1.89226	0.05
35703	3.78452	0.0579727
35704	2.0155	1.32088
35705	1.97652	0.0655569
35706	1.8247	0.212449
35707	1.80645	0.05
35708	3.80122	0.05
35709	1.97652	0.211381
35710	1.79681	0.15241
35711	1.80645	0.05
35712	1.59244	0.0595478
35713	3.56896	0.0542311
35714	1.89226	0.18761
35715	1.89226	0.987234
35716	1.89226	0.0582081
35717	1.89226	0.0814163
35718	3.78452	0.0738551
35719	1.89223	0.233109
35720	1.89223	0.0888623
35721	1.89223	0.846186
35722	1.89223	0.05
35723	3.78447	0.062558
35724	1.82467	0.20005
35725	1.80643	0.05

35726	1.55159	0.115379
35727	1.53969	0.05
35728	3.36437	0.05
35729	1.80643	0.0734061
35730	1.59242	0.115545
35731	1.5397	0.05
35732	1.40538	0.05
35733	3.13211	0.05
35734	1.89224	0.198724
35735	1.89224	0.15492
35736	1.89224	0.05
35737	1.89224	0.0680483
35738	3.78447	0.0995647
35739	1.37427	0.05
35740	2.37086	0.05
35741	2.37972	0.05
35742	1.94703	0.05
35743	2.19299	0.05
35744	1.91813	0.05
35745	1.07566	2.1
35746	1.12954	2.1
35747	0.946131	2.1
35748	0.946131	2.1
35749	1.12954	2.1
35750	1.12954	2.1
35751	0.946131	2.1
35752	0.946131	2.1
35753	1.12954	2.1
35754	0.946131	2.1
35755	0.946131	2.1
35756	1.07566	2.1
35757	2.25908	2.1
35758	1.89226	2.1
35759	1.89226	2.1
35760	1.89226	2.1
35761	2.37567	2.1
35762	2.37567	2.1
35763	1.37597	2.00044
35764	1.70302	0.355153
35765	1.24162	2.1
35766	1.12219	2.1
35767	1.37072	1.15037
35768	1.70302	2.1
35769	1.37072	2.1
35770	1.70302	1.19891
35771	1.54168	1.98118
35772	2.21318	1.10438
35773	3.39238	1.95509
35774	1.89226	0.05
35775	1.89226	0.05
35776	1.89226	0.05
35777	1.89226	0.05
35778	3.78452	0.05
35779	1.89227	0.05
35780	1.89227	0.05
35781	1.89227	0.05
35782	1.89227	0.05
35783	3.78453	0.05

35784	1.89226	0.05
35785	1.89226	0.05
35786	1.89226	0.05
35787	1.89226	0.05
35788	3.78453	0.05
35789	1.89226	0.05
35790	2.21654	0.05
35791	2.72007	0.05
35792	2.72007	0.05
35793	1.89226	0.05
35794	1.89226	0.05
35795	0.946128	0.05
35796	0.946128	0.05
35797	0.946132	0.05
35798	0.946132	0.05
35799	0.94613	0.05
35800	0.94613	0.05
35801	0.946131	0.05
35802	0.946131	0.05
35803	0.94613	0.05
35804	0.94613	0.05
35805	0.94613	0.05
35806	0.94613	0.05
35807	1.89226	0.05
35808	1.89226	0.05
35809	1.89226	0.05
35810	1.89226	0.05
35811	3.78453	0.05
35812	1.33457	0.05
35813	0.946131	0.05
35814	1.08045	0.05
35815	0.946132	0.05
35816	1.08045	0.05
35817	1.08045	0.05
35818	0.946132	0.05
35819	0.946132	0.05
35820	1.33457	0.05
35821	1.08045	0.05
35822	0.946132	0.05
35823	0.946132	0.05
35824	1.53347	1.11196
35825	1.13092	2.1
35826	1.52938	2.01256
35827	1.93568	1.70156
35828	3.06285	2.1
35829	1.89226	0.0789935
35830	2.72959	0.285077
35831	2.82996	0.409559
35832	2.82996	0.05
35833	1.89226	0.734635
35834	1.89226	0.0725039
35835	2.7296	0.207523
35836	2.57663	2.1
35837	2.48354	0.418224
35838	2.48354	2.1
35839	2.82996	0.314381
35840	2.82996	2.1
35841	2.63449	0.223824

35842	2.7296	0.224416
35843	2.39282	1.72011
35844	2.39282	2.1
35845	2.48354	2.1
35846	2.72521	2.1
35847	1.89224	2.1
35848	1.89224	1.97391
35849	1.89224	0.526968
35850	1.89224	2.1
35851	3.78447	1.63294
35852	1.89226	2.1
35853	2.10425	2.1
35854	2.5142	2.1
35855	2.5142	2.1
35856	1.89226	2.1
35857	1.89226	2.1
35858	0.946133	0.501772
35859	0.946133	1.31787
35860	0.946128	2.1
35861	0.946128	2.1
35862	0.946131	2.1
35863	0.946131	0.553788
35864	0.94613	0.0859849
35865	0.94613	2.1
35866	0.946131	1.94316
35867	0.946131	2.0579
35868	0.946131	1.93619
35869	0.946131	1.91699
35870	1.89226	0.386068
35871	1.89226	0.959027
35872	1.89226	0.502868
35873	1.89226	1.18072
35874	1.89226	0.36907
35875	1.89226	0.928972
35876	1.94446	0.474085
35877	2.50167	1.32309
35878	1.28418	0.66082
35879	1.94446	0.882883
35880	2.05205	0.666987
35881	2.05205	0.94404
35882	1.27835	2.1
35883	1.08341	2.1
35884	0.946132	2.1
35885	0.946132	2.1
35886	0.851542	2.1
35887	0.851542	2.1
35888	0.977356	2.1
35889	1.18368	2.1
35890	1.18378	2.1
35891	0.851555	2.1
35892	0.977346	2.1
35893	0.851476	2.1
35894	1.07369	2.1
35895	2.5813	2.1
35896	1.95053	2.1
35897	1.95053	2.1
35898	1.90196	2.1
35899	1.90196	2.1

35900	1.89226	2.1
35901	1.89226	2.1
35902	1.89226	2.1
35903	1.89226	2.1
35904	3.78452	2.1
35905	1.89224	2.1
35906	1.89224	2.1
35907	1.89224	2.1
35908	1.89224	2.1
35909	1.89224	2.1
35910	1.89224	2.1
35911	1.97004	0.0681163
35912	1.8072	1.39767
35913	1.5866	1.00797
35914	1.79478	0.121882
35915	3.60198	0.428064
35916	1.23267	2.1
35917	1.8072	0.363574
35918	1.58661	0.100318
35919	0.947921	2.1
35920	2.75512	1.2803
35921	1.89226	0.433252
35922	1.89226	2.1
35923	1.89226	0.548997
35924	1.89226	1.9684
35925	1.89226	0.304294
35926	1.89226	2.1
35927	1.89226	1.43946
35928	1.89226	0.149829
35929	1.89226	0.163746
35930	1.89226	1.19811
35931	3.78452	0.55679
35932	1.4263	1.45342
35933	1.79478	0.222923
35934	1.67651	0.217943
35935	1.79478	1.68105
35936	1.46834	0.0512479
35937	1.5866	0.433853
35938	1.89226	0.303489
35939	1.89226	1.07597
35940	1.89226	0.143258
35941	1.89226	0.579251
35942	1.89226	0.148263
35943	1.89226	1.51096
35944	2.23956	1.70527
35945	1.81847	1.80275
35946	2.10991	1.00669
35947	2.75317	1.1899
35948	4.34947	1.43596
35949	1.60578	0.05
35950	1.94974	0.05
35951	1.68062	0.05
35952	1.14721	0.05
35953	3.2864	0.05
35954	1.3946	0.05
35955	1.692	0.05
35956	2.0154	0.05
35957	2.0154	0.05

35958	1.11926	0.05
35959	1.11926	0.05
35960	1.90347	0.05
35961	1.83626	0.05
35962	1.84583	0.05
35963	1.89434	0.05
35964	3.79781	0.05
35965	1.89226	1.55014
35966	1.89226	1.16001
35967	1.89226	0.574504
35968	1.89226	1.38683
35969	3.78452	1.10626
35970	1.20986	1.61024
35971	1.20986	1.35205
35972	0.912635	1.27709
35973	0.912635	1.71601
35974	0.912667	0.832366
35975	1.17637	1.78809
35976	0.946125	1.77233
35977	0.946125	1.87473
35978	0.946128	1.89504
35979	0.912664	1.21709
35980	0.946127	2.06614
35981	1.17637	1.39213
35982	1.89226	0.05
35983	1.65573	0.05
35984	1.65573	0.05
35985	1.89226	0.05
35986	1.89226	0.05
35987	2.12879	0.05
35988	1.89226	0.05
35989	1.89226	0.05
35990	1.89226	0.05
35991	1.89226	0.05
35992	1.89226	0.05
35993	1.89226	0.05
35994	2.40928	0.05
35995	1.89226	0.05
35996	1.95959	0.05
35997	1.70302	0.05
35998	3.47338	0.05
35999	1.89226	0.05
36000	1.89226	0.05
36001	1.89226	0.05
36002	1.89226	0.05
36003	3.78452	0.05
36004	1.64985	0.05
36005	2.47846	0.05
36006	2.25405	0.05
36007	1.80768	0.05
36008	4.06173	0.05
36009	1.98846	0.05
36010	0.912729	0.05
36011	1.05417	0.05
36012	1.70303	0.05
36013	2.82107	0.05
36014	2.47846	0.05
36015	1.70303	0.05

36016	2.86918	0.05
36017	2.05973	0.05
36018	2.51808	0.05
36019	1.80768	0.05
36020	0.946131	0.05
36021	0.946131	0.05
36022	0.946132	0.05
36023	0.946132	0.05
36024	0.946131	0.05
36025	0.946131	0.05
36026	0.946133	0.05
36027	0.946133	0.05
36028	0.946131	0.05
36029	0.946131	0.05
36030	0.946132	0.05
36031	0.946132	0.05
36032	0.946133	0.05
36033	0.946133	0.05
36034	0.946132	0.05
36035	0.946132	0.05
36036	0.946133	0.05
36037	0.946133	0.05
36038	0.946132	0.05
36039	0.946132	0.05
36040	0.946132	0.05
36041	0.946132	0.05
36042	0.946133	0.05
36043	0.946133	0.05
36044	1.89227	0.05
36045	1.89227	0.05
36046	1.89227	0.05
36047	1.89227	0.05
36048	1.89227	0.0531398
36049	1.89227	0.05
36050	1.1656	2.1
36051	1.21847	2.1
36052	0.836006	2.1
36053	0.806007	2.1
36054	1.00653	2.1
36055	1.00653	2.1
36056	1.01794	2.1
36057	1.01794	2.1
36058	1.1656	2.1
36059	0.806007	2.1
36060	0.836006	2.1
36061	1.21847	2.1
36062	2.82263	2.1
36063	2.3312	2.1
36064	2.25936	2.1
36065	2.71441	2.1
36066	5.0456	2.1
36067	2.25936	2.1
36068	1.61201	2.1
36069	1.57627	2.1
36070	1.61201	2.1
36071	2.29545	2.1
36072	2.3312	2.1
36073	1.21269	0.05

36074	1.48867	0.05
36075	1.68264	0.05
36076	1.9898	0.05
36077	3.17131	0.05
36078	2.0487	2.1
36079	2.45212	2.1
36080	2.31012	2.1
36081	1.94095	2.1
36082	4.39307	2.1
36083	1.02435	2.1
36084	0.970469	2.1
36085	0.625375	2.1
36086	0.567166	2.1
36087	0.796854	2.1
36088	0.796854	2.1
36089	0.79466	2.1
36090	0.79466	2.1
36091	0.970471	2.1
36092	0.567165	2.1
36093	0.625374	2.1
36094	1.02435	2.1
36095	2.31012	2.1
36096	1.94095	2.1
36097	1.4825	2.1
36098	2.17384	2.1
36099	4.11479	2.1
36100	0.946187	2.1
36101	0.803763	2.1
36102	0.581357	2.1
36103	0.510356	2.1
36104	0.745632	2.1
36105	0.745632	2.1
36106	0.734566	2.1
36107	0.734563	2.1
36108	0.896142	2.1
36109	0.510366	2.1
36110	0.946173	2.1
36111	0.552965	2.1
36112	1.89226	0.994522
36113	1.89226	1.62898
36114	1.89226	1.86574
36115	1.89226	1.96323
36116	1.89226	1.15159
36117	1.89226	1.72334
36118	1.50743	1.54721
36119	1.0417	2.1
36120	1.50743	2.0691
36121	1.62974	1.15856
36122	1.14605	1.14294
36123	1.14605	2.1
36124	1.89226	0.792193
36125	1.89226	1.96282
36126	1.89226	0.413736
36127	1.89226	1.70208
36128	1.89226	0.540971
36129	1.89226	1.91126
36130	1.89226	1.86517
36131	1.89226	1.53561

36132	1.89226	0.318783
36133	1.89226	1.92992
36134	3.78453	1.14539
36135	1.77186	1.73358
36136	1.35668	2.0673
36137	1.70254	0.93934
36138	1.38756	2.01158
36139	1.77186	1.9097
36140	1.37814	2.1
36141	0.734857	2.08543
36142	1.00549	1.83879
36143	0.835261	1.13398
36144	1.08357	2.07003
36145	0.97665	1.54686
36146	0.795559	2.1
36147	0.891385	1.78863
36148	0.891385	0.403219
36149	0.874231	1.19099
36150	1.08356	1.43265
36151	0.734863	1.50725
36152	0.79449	1.85278
36153	1.89226	1.49151
36154	1.89226	0.185665
36155	2.42671	0.467559
36156	1.98783	1.09832
36157	4.31897	0.508679
36158	0.579854	0.171611
36159	0.965582	0.480919
36160	0.634403	1.02785
36161	1.17796	0.320381
36162	1.00592	0.229647
36163	0.797259	2.02137
36164	0.905246	1.14838
36165	0.905246	0.255592
36166	0.695715	0.260435
36167	0.637106	0.707546
36168	1.24374	0.813707
36169	0.95617	0.582963
36170	2.0815	0.248951
36171	1.60521	1.1371
36172	1.29556	0.449283
36173	2.32205	1.73945
36174	1.28445	0.103519
36175	2.0815	0.916785
36176	2.3085	2.1
36177	2.21147	2.1
36178	2.3085	2.1
36179	2.39282	2.1
36180	1.98899	2.1
36181	2.0733	2.1
36182	2.3275	2.1
36183	1.63471	2.1
36184	1.98899	2.1
36185	1.56801	2.1
36186	3.62369	2.1
36187	2.3085	2.1
36188	1.98899	2.1
36189	2.22575	2.1

36190	1.90502	2.1
36191	4.21353	2.1
36192	1.90502	2.1
36193	1.56801	2.1
36194	1.90502	2.1
36195	1.98899	2.1
36196	1.49733	2.1
36197	1.58129	2.1
36198	1.89226	1.11173
36199	2.6491	1.27862
36200	2.49266	2.1
36201	2.6491	0.794933
36202	1.73582	1.45834
36203	1.89226	0.0650131
36204	1.31854	2.1
36205	2.6491	1.99469
36206	1.3909	2.1
36207	1.3909	2.1
36208	2.49266	2.1
36209	2.49266	2.1
36210	1.89226	0.962068
36211	2.49266	1.02941
36212	2.38634	2.1
36213	2.49266	0.537637
36214	1.78595	0.8013
36215	1.89226	0.055894
36216	1.23042	2.1
36217	2.49266	1.60773
36218	1.31854	2.1
36219	1.31854	2.1
36220	2.38635	2.1
36221	2.38635	2.1
36222	2.83487	2.1
36223	1.89226	2.1
36224	2.594	2.1
36225	2.594	2.1
36226	1.89226	2.1
36227	1.89226	2.1
36228	1.89226	2.1
36229	1.89226	2.1
36230	1.89226	2.1
36231	1.89226	2.1
36232	3.78452	2.1
36233	1.09518	2.1
36234	1.28158	2.1
36235	1.36844	2.1
36236	1.59972	2.1
36237	2.65003	2.1
36238	1.89226	2.1
36239	1.89226	2.1
36240	1.89226	2.1
36241	1.89226	2.1
36242	3.78452	2.1
36243	1.36844	2.1
36244	1.59972	2.1
36245	1.67352	2.1
36246	1.94291	2.1
36247	3.31135	2.1

36248	1.52938	2.1
36249	1.93568	2.01834
36250	2.36221	0.559848
36251	1.94723	2.1
36252	3.89158	1.85367
36253	1.94724	1.14621
36254	1.26593	2.1
36255	1.75367	2.1
36256	2.4322	1.51627
36257	3.7009	2.1
36258	2.36221	1.00938
36259	2.43219	2.0997
36260	1.94723	1.50986
36261	1.94723	1.63461
36262	2.8202	1.73722
36263	2.8202	1.91444
36264	2.51604	2.1
36265	2.18533	2.1
36266	2.17228	2.1
36267	2.4889	2.1
36268	4.67423	2.1
36269	2.17228	2.1
36270	1.62183	2.1
36271	1.62316	2.1
36272	1.62183	2.1
36273	2.18666	2.1
36274	2.18533	2.1
36275	2.17227	2.1
36276	2.4889	2.1
36277	2.45922	2.1
36278	2.15686	2.1
36279	4.64576	2.1
36280	2.17228	2.1
36281	1.62316	2.1
36282	1.62241	2.1
36283	2.15686	2.1
36284	3.78002	2.1
36285	1.89226	0.959119
36286	1.89226	0.995826
36287	1.89226	0.770742
36288	1.89226	0.724455
36289	3.78453	1.32825
36290	1.89226	1.06217
36291	1.89226	0.976921
36292	1.89226	0.668334
36293	1.89226	0.199266
36294	3.78452	0.436342
36295	1.89226	1.02011
36296	1.89226	0.548249
36297	1.89226	0.974434
36298	1.89226	0.820277
36299	1.89226	0.671069
36300	1.89226	0.715426
36301	1.89226	0.936967
36302	1.89226	1.14881
36303	2.53413	0.2076
36304	1.89226	0.287101
36305	3.78453	0.265889

36306	1.38048	0.334588
36307	1.38048	0.472656
36308	0.490332	0.459553
36309	0.490332	0.385232
36310	0.924679	0.0541453
36311	0.946131	1.7331
36312	0.946131	1.33185
36313	0.490332	0.05
36314	0.924678	0.0614008
36315	0.490332	0.05
36316	0.946131	0.753586
36317	0.946131	0.312823
36318	1.89227	0.216068
36319	1.89227	0.874448
36320	1.89227	0.604386
36321	1.89227	1.11739
36322	1.89227	0.21724
36323	1.89227	0.431768
36324	1.30803	2.1
36325	2.55151	2.1
36326	1.43935	2.1
36327	1.43935	2.1
36328	2.34922	2.1
36329	2.34922	2.1
36330	0.946131	2.1
36331	0.946131	2.1
36332	1.27575	2.1
36333	1.17461	2.1
36334	0.94613	2.1
36335	0.94613	2.1
36336	1.17461	2.1
36337	1.17461	2.1
36338	0.946131	2.1
36339	1.17461	2.1
36340	0.946132	2.1
36341	1.27575	2.1
36342	1.89226	2.1
36343	1.89226	2.1
36344	1.89226	2.1
36345	1.89226	2.1
36346	1.89226	2.1
36347	1.89226	2.1
36348	0.946133	2.1
36349	0.946133	2.1
36350	0.946132	2.1
36351	0.946132	2.1
36352	0.946133	2.1
36353	0.946133	2.1
36354	0.946132	2.1
36355	0.946132	2.1
36356	0.946132	2.1
36357	0.946132	2.1
36358	0.946133	2.1
36359	0.946133	2.1
36360	1.07043	2.1
36361	1.17461	2.1
36362	0.654016	2.1
36363	0.5829	2.1

36364	0.870481	2.1
36365	0.870481	2.1
36366	0.887027	2.1
36367	0.887027	2.1
36368	1.07043	2.1
36369	0.5829	2.1
36370	0.654016	2.1
36371	1.17461	2.1
36372	1.89226	2.1
36373	1.89226	2.1
36374	1.89226	2.1
36375	1.89226	2.1
36376	3.78453	2.1
36377	1.89226	2.1
36378	1.89226	2.1
36379	1.89226	2.1
36380	1.89226	2.1
36381	3.78453	2.1
36382	1.26929	2.1
36383	2.19313	2.1
36384	1.09847	2.1
36385	1.69916	2.1
36386	2.96844	2.1
36387	1.84124	2.1
36388	2.24937	2.1
36389	1.69915	2.1
36390	1.75691	2.1
36391	3.94852	2.1
36392	1.53082	2.1
36393	2.04422	2.1
36394	1.53082	2.1
36395	1.69915	2.1
36396	1.92911	2.1
36397	2.09744	2.1
36398	1.39263	0.323502
36399	1.0171	0.05
36400	1.1211	0.0767034
36401	1.46482	0.115778
36402	2.48192	0.107985
36403	1.39263	0.176004
36404	1.57962	0.540145
36405	1.65238	1.71334
36406	1.46482	0.08677
36407	3.04443	0.17502
36408	1.89226	0.474303
36409	1.89226	0.05
36410	1.89226	0.0770798
36411	1.89226	0.164926
36412	3.78453	0.142043
36413	1.89227	0.05
36414	1.89227	0.050919
36415	1.89227	0.0940517
36416	1.89227	0.05
36417	3.78453	0.05
36418	1.28418	0.153948
36419	1.46482	0.11974
36420	1.1211	0.0888632
36421	1.1211	0.370335

36422	1.59382	0.0517821
36423	1.59382	0.224917
36424	1.76897	1.27211
36425	1.46482	0.0920194
36426	1.59382	0.165659
36427	1.46482	0.480535
36428	1.78138	0.12343
36429	1.65238	0.368785
36430	1.89226	0.0801569
36431	1.89226	0.131714
36432	1.89226	0.0565632
36433	1.89226	0.374417
36434	1.89226	0.05
36435	1.89226	0.376716
36436	1.89226	0.0959917
36437	1.89226	0.05
36438	1.89226	0.05
36439	1.89226	0.0716375
36440	1.89226	0.0516796
36441	1.89226	0.0870537
36442	1.96832	2.1
36443	2.40858	2.1
36444	1.75211	0.309551
36445	2.20797	2.1
36446	4.1607	2.09857
36447	1.75211	0.430354
36448	2.20797	2.1
36449	1.51592	2.1
36450	2.02064	2.1
36451	3.77275	2.1
36452	2.71793	0.233076
36453	2.67099	0.08336
36454	2.55105	0.16032
36455	2.71297	0.05
36456	5.26898	0.112881
36457	2.36417	0.299145
36458	2.67098	0.0918234
36459	2.55104	0.169466
36460	1.83418	0.0501337
36461	4.91522	0.113468
36462	2.55105	0.140895
36463	2.71297	0.0543022
36464	2.58032	0.106704
36465	2.28922	0.05
36466	5.13137	0.0820745
36467	2.55104	0.146473
36468	2.23787	0.0519742
36469	1.67265	0.0669839
36470	2.28922	0.05
36471	4.22369	0.0619767
36472	0.851542	0.576104
36473	0.851542	0.536081
36474	0.981419	0.230975
36475	0.854774	0.371057
36476	0.949431	0.280089
36477	0.753763	0.380791
36478	0.946131	0.560554
36479	0.946131	0.511769

36480	1.04073	0.495644
36481	0.981433	0.167682
36482	0.851528	0.739862
36483	0.854828	0.377266
36484	1.89226	0.898841
36485	1.89226	0.445934
36486	1.89226	0.42606
36487	1.89226	0.478861
36488	1.89226	0.725578
36489	1.89226	0.806881
36490	0.814227	0.319776
36491	0.814227	0.324397
36492	0.946117	0.154659
36493	1.07285	0.180725
36494	0.879132	0.1538
36495	0.923293	0.169583
36496	0.946119	0.333682
36497	0.946119	0.360841
36498	0.946131	0.28248
36499	0.814236	0.0904509
36500	0.946106	0.455802
36501	0.870405	0.228963
36502	1.89224	0.600601
36503	1.89224	0.26291
36504	1.89224	0.267412
36505	1.89224	0.298325
36506	1.89224	0.506266
36507	1.89224	0.56042
36508	1.29388	1.2083
36509	1.04164	2.1
36510	1.64834	2.01799
36511	1.82955	1.16047
36512	2.94222	2.03497
36513	1.4934	0.173549
36514	2.41522	0.0633354
36515	2.20387	0.05
36516	2.20387	2.1
36517	1.75765	0.0613074
36518	1.75765	1.96012
36519	2.41522	0.0662664
36520	2.77303	2.1
36521	2.20387	2.1
36522	2.58627	2.1
36523	5.00149	2.1
36524	1.22222	0.286127
36525	2.20387	0.52759
36526	1.96831	0.0742425
36527	1.96831	2.1
36528	1.4934	0.102769
36529	1.4934	2.1
36530	1.96832	2.1
36531	2.58627	2.1
36532	2.40858	0.999642
36533	2.40858	2.1
36534	2.20387	1.5151
36535	2.20387	2.1
36536	1.5605	2.1
36537	1.53775	2.1

36538	1.55398	2.1
36539	1.45806	2.1
36540	1.65642	2.1
36541	1.5605	2.1
36542	0.768876	2.1
36543	0.79033	2.1
36544	0.828211	2.1
36545	0.871233	2.1
36546	0.803843	2.1
36547	0.803843	2.1
36548	0.814698	2.1
36549	0.814698	2.1
36550	0.79033	2.1
36551	0.871233	2.1
36552	0.768876	2.1
36553	0.828212	2.1
36554	1.80252	2.1
36555	2.09715	1.0322
36556	1.9394	2.1
36557	1.50853	2.1
36558	3.60567	2.1
36559	1.96416	0.322259
36560	2.09715	0.968238
36561	1.88484	2.1
36562	1.57916	0.587941
36563	3.67631	1.21738
36564	1.89226	0.75449
36565	1.89226	0.05
36566	1.89226	0.317262
36567	1.89226	0.936157
36568	3.78453	0.0894203
36569	1.89226	0.718359
36570	1.89226	0.994424
36571	1.89226	2.1
36572	1.89226	0.05
36573	1.89226	2.1
36574	1.89226	0.05
36575	1.5852	2.1
36576	1.50853	2.1
36577	1.2955	2.1
36578	1.44472	0.446347
36579	2.95325	2.1
36580	1.53065	2.05914
36581	1.57916	0.0610883
36582	1.44472	0.369743
36583	1.78861	0.397581
36584	1.23527	0.969764
36585	1.23527	0.27643
36586	1.89227	0.05
36587	1.89227	0.131814
36588	1.89227	1.20641
36589	1.89227	0.507633
36590	3.78453	0.183467
36591	1.89226	0.413573
36592	1.89226	0.536897
36593	1.89226	2.1
36594	1.89226	0.379388
36595	1.89226	2.02311

36596	1.89226	0.05
36597	1.89227	1.24243
36598	1.89227	0.708492
36599	1.89227	0.754935
36600	1.89227	0.54763
36601	3.78453	0.604796
36602	0.943708	0.557394
36603	0.752219	0.37045
36604	0.946132	0.575105
36605	0.946132	0.61569
36606	0.946133	0.205016
36607	0.946133	1.15136
36608	0.752218	1.22589
36609	0.752218	0.713643
36610	0.752218	0.617932
36611	0.946132	0.330355
36612	0.946133	0.929539
36613	0.943708	0.617121
36614	0.943992	2.1
36615	0.943992	2.1
36616	1.23507	1.1526
36617	1.48209	2.1
36618	1.0384	2.1
36619	1.47953	2.1
36620	0.946128	1.09731
36621	0.946128	2.1
36622	0.946129	2.1
36623	1.23293	2.1
36624	1.0384	2.1
36625	0.946129	2.1
36626	1.89226	2.1
36627	2.46586	2.1
36628	2.80218	1.08424
36629	2.80218	2.1
36630	1.89226	1.16815
36631	1.89226	2.1
36632	1.32455	2.1
36633	1.89128	2.1
36634	1.32328	2.1
36635	1.97299	2.1
36636	3.29754	2.1
36637	1.89128	2.1
36638	2.4255	2.1
36639	1.973	2.1
36640	2.63807	2.1
36641	4.3985	2.1
36642	1.34008	2.1
36643	1.97299	2.1
36644	2.10766	2.1
36645	2.10766	2.1
36646	1.32328	2.1
36647	1.32328	2.1
36648	2.41055	2.1
36649	2.37491	2.1
36650	2.40754	2.1
36651	2.38023	2.1
36652	4.78546	2.1
36653	2.23743	2.1

36654	2.37491	2.1
36655	2.40754	2.1
36656	2.20392	2.1
36657	4.61234	2.1
36658	2.37491	2.1
36659	2.02641	2.1
36660	2.20392	2.1
36661	2.18943	2.1
36662	4.40132	2.1
36663	2.37491	2.1
36664	2.22558	2.1
36665	2.38023	2.1
36666	2.18943	2.1
36667	4.60049	2.1
36668	1.89226	0.05
36669	1.89226	0.05
36670	1.89226	0.05
36671	1.89226	0.05
36672	1.89226	0.05
36673	1.89226	0.05
36674	1.03599	0.05
36675	0.706698	0.05
36676	1.41141	0.05
36677	1.17573	0.05
36678	1.11957	0.05
36679	0.765034	0.05
36680	1.05913	0.05
36681	1.45054	0.05
36682	1.04847	0.05
36683	1.04847	0.05
36684	1.15323	0.05
36685	1.05825	0.05
36686	1.89226	0.05
36687	1.89226	0.05
36688	1.89226	0.05
36689	1.89226	0.05
36690	1.89226	0.05
36691	1.89226	0.05
36692	2.22575	0.05
36693	1.70677	2.1
36694	1.90157	0.05
36695	1.5534	2.1
36696	3.77914	1.34097
36697	1.70678	2.1
36698	0.968594	2.1
36699	0.968594	2.1
36700	1.10378	2.1
36701	1.5534	2.1
36702	1.68859	2.1
36703	2.31429	0.05
36704	1.5534	2.1
36705	1.90103	0.05
36706	1.41902	2.1
36707	3.45443	1.37496
36708	2.53709	0.05
36709	1.209	0.05
36710	1.05417	0.05
36711	2.6513	0.05

36712	3.70547	0.05
36713	1.02675	0.05
36714	1.16384	0.05
36715	2.22062	0.05
36716	2.48862	0.05
36717	3.38446	0.05
36718	1.89226	0.05
36719	1.89226	0.05
36720	1.89226	0.05
36721	1.89226	0.05
36722	1.89226	0.05
36723	1.89226	0.05
36724	1.89226	1.44238
36725	1.89226	2.1
36726	1.89226	2.1
36727	1.89226	1.80772
36728	1.89226	2.1
36729	1.89226	1.65549
36730	1.89226	1.13352
36731	1.89226	1.84027
36732	1.89226	2.01492
36733	1.89226	1.35605
36734	1.89226	1.90426
36735	1.89226	1.16365
36736	1.89227	1.4094
36737	1.89227	1.91754
36738	1.75032	2.0308
36739	1.75032	1.49311
36740	1.89227	1.90617
36741	1.89227	1.2999
36742	1.89226	0.05
36743	1.89226	0.0603933
36744	1.89226	0.05
36745	1.89226	0.070876
36746	1.89226	0.0571635
36747	1.89226	0.05
36748	0.946132	0.05
36749	0.946132	0.05
36750	0.798215	0.05
36751	0.848163	0.05
36752	0.848163	0.05
36753	0.946131	0.05
36754	0.946132	0.05
36755	0.798216	0.05
36756	0.996105	0.05
36757	0.996105	0.05
36758	0.79819	0.05
36759	0.79819	0.05
36760	1.07763	0.05
36761	2.28622	0.05
36762	2.16109	0.05
36763	1.17506	0.05
36764	3.36385	0.05
36765	1.89226	2.1
36766	1.6663	2.1
36767	1.6663	2.1
36768	1.71706	2.1
36769	1.89226	2.1

36770	1.94302	2.1
36771	1.89226	2.1
36772	1.62801	2.1
36773	1.62801	2.1
36774	1.6663	2.1
36775	1.89226	2.1
36776	1.93055	2.1
36777	1.48734	2.1
36778	1.99609	2.1
36779	1.52245	2.1
36780	2.04137	2.1
36781	3.51854	2.1
36782	1.99609	2.1
36783	2.28563	2.1
36784	2.04136	2.1
36785	2.35019	2.1
36786	4.34628	2.1
36787	1.50714	2.1
36788	2.04137	2.1
36789	2.04945	2.1
36790	2.04945	2.1
36791	1.52245	2.1
36792	1.52245	2.1
36793	2.04136	2.1
36794	2.35019	2.1
36795	2.04945	2.1
36796	2.38681	2.1
36797	4.39964	2.1
36798	2.50924	0.05
36799	1.38305	2.1
36800	2.56918	2.1
36801	1.44535	2.1
36802	3.95222	2.1
36803	1.89226	2.1
36804	1.89226	2.1
36805	1.89226	2.1
36806	1.89226	2.1
36807	3.78453	2.1
36808	1.40615	2.1
36809	1.38851	2.1
36810	0.748607	2.1
36811	0.724468	2.1
36812	1.06362	2.1
36813	1.06362	2.1
36814	1.067 2.1	
36815	1.067 2.1	
36816	1.40615	2.1
36817	0.748607	2.1
36818	0.724468	2.1
36819	1.38851	2.1
36820	2.56918	2.1
36821	1.53377	2.1
36822	1.53706	2.1
36823	1.44535	2.1
36824	2.66088	2.1
36825	2.56918	2.1
36826	1.89226	2.1
36827	1.89226	2.1

36828	1.89226	2.1
36829	1.89226	2.1
36830	3.78453	2.1
36831	1.40239	2.1
36832	1.68466	2.1
36833	1.26091	2.1
36834	1.54641	2.1
36835	2.94881	2.1
36836	1.68466	2.1
36837	1.65324	0.154472
36838	1.54641	2.1
36839	1.52268	0.154983
36840	3.19965	2.1
36841	1.05776	2.1
36842	1.54641	2.1
36843	1.36595	2.1
36844	1.36595	2.1
36845	1.26091	2.1
36846	1.26091	2.1
36847	1.54641	2.1
36848	1.32697	0.147531
36849	1.52268	0.222581
36850	1.52268	2.1
36851	1.36595	0.247983
36852	1.36595	2.1
36853	1.18647	1.69241
36854	0.988454	2.1
36855	1.4867	2.05545
36856	1.63892	1.67377
36857	2.67317	2.1
36858	1.4867	2.1
36859	1.63892	1.90192
36860	2.09605	0.439253
36861	1.97645	2.07101
36862	3.58275	1.46196
36863	1.29389	2.1
36864	1.97645	1.72003
36865	1.4867	1.76437
36866	1.4867	2.1
36867	1.82955	1.11693
36868	1.82955	2.1
36869	1.13454	1.03238
36870	0.983594	2.1
36871	1.16809	2.1
36872	1.06836	2.1
36873	1.08332	2.1
36874	0.983594	1.36856
36875	1.48751	2.1
36876	1.48047	1.83987
36877	1.77596	1.94458
36878	1.75506	1.89835
36879	3.25643	2.03741
36880	1.48047	1.77013
36881	1.25334	2.1
36882	1.75505	1.47301
36883	1.55831	0.25208
36884	3.03878	1.95378
36885	1.77596	2.0548

36886	1.75506	2.1
36887	2.00551	2.1
36888	2.00083	0.629892
36889	3.77679	2.1
36890	1.75506	2.04135
36891	1.80328	0.450605
36892	1.55831	1.97625
36893	1.55831	2.1
36894	2.00083	0.387164
36895	2.00083	1.18613
36896	2.58627	2.1
36897	2.79047	2.1
36898	2.40858	2.1
36899	2.64748	2.1
36900	5.23375	2.1
36901	1.26755	2.1
36902	1.71717	2.1
36903	1.03824	2.1
36904	1.48901	2.1
36905	2.75656	2.1
36906	1.89226	2.1
36907	1.89226	2.1
36908	1.89226	2.1
36909	1.89226	2.1
36910	1.89226	2.1
36911	1.89226	2.1
36912	1.36541	2.1
36913	1.22156	2.1
36914	0.946132	2.1
36915	0.946132	2.1
36916	1.36541	2.1
36917	1.36541	2.1
36918	0.946132	2.1
36919	0.946132	2.1
36920	1.36541	2.1
36921	0.946133	2.1
36922	0.946132	2.1
36923	1.22156	2.1
36924	1.18901	2.1
36925	1.61149	2.1
36926	1.98748	2.1
36927	1.534 2.1	
36928	3.1765	2.1
36929	1.89226	2.1
36930	1.89226	2.1
36931	1.89226	2.1
36932	1.89226	2.1
36933	3.78452	2.1
36934	1.98748	2.1
36935	1.534 2.1	
36936	2.27541	2.1
36937	1.98748	2.1
36938	1.87436	2.1
36939	1.87436	2.1
36940	2.2988	2.1
36941	2.2604	2.1
36942	2.20831	2.1
36943	2.02802	2.1

36944	4.32683	2.1
36945	2.26041	2.1
36946	1.98957	2.1
36947	2.02803	2.1
36948	1.75021	2.1
36949	4.0176	2.1
36950	2.34607	2.1
36951	2.02803	2.1
36952	1.86795	2.1
36953	1.81448	2.1
36954	3.89597	2.1
36955	2.02803	2.1
36956	1.75021	2.1
36957	1.81448	2.1
36958	1.57887	2.1
36959	3.6069	2.1
36960	0.946131	0.05
36961	0.946131	2.1
36962	1.1235	2.1
36963	1.1235	2.1
36964	0.946131	1.79137
36965	0.946131	0.584701
36966	1.1235	2.1
36967	1.13736	2.1
36968	1.13736	0.835896
36969	0.946131	1.72451
36970	1.1235	1.91509
36971	0.946131	1.88045
36972	1.89226	2.1
36973	1.89226	2.1
36974	1.89226	0.437642
36975	1.89226	0.328928
36976	3.78452	0.32117
36977	0.563266	2.1
36978	0.563266	0.864471
36979	0.565606	0.12265
36980	0.565606	2.1
36981	0.647086	2.08985
36982	0.624219	2.05594
36983	0.500035	1.01279
36984	0.481785	1.01069
36985	0.500034	0.169445
36986	0.647087	0.202359
36987	0.624219	1.95117
36988	0.481785	0.963247
36989	0.915788	0.05
36990	0.915788	0.0832065
36991	1.02732	0.479088
36992	1.02732	0.190593
36993	0.660831	0.27023
36994	1.11155	0.05
36995	0.713295	0.0643364
36996	1.26874	0.35434
36997	0.713295	0.172121
36998	0.660831	0.132657
36999	1.11155	0.149043
37000	1.26874	0.113592
37001	1.89226	0.467373

37002	1.85091	0.0718626
37003	2.11193	0.241116
37004	1.85091	0.422298
37005	2.15328	0.0768443
37006	1.89226	0.079018
37007	0.946131	0.070723
37008	0.946131	0.102571
37009	1.18275	0.100289
37010	1.05596	0.170469
37011	0.946127	0.121291
37012	0.946127	0.05
37013	1.18275	0.151458
37014	1.18275	0.091785
37015	0.946128	0.395121
37016	1.18275	0.0613091
37017	0.946129	0.132867
37018	1.05596	0.05
37019	1.60936	2.1
37020	2.35846	2.1
37021	2.06424	2.1
37022	2.57651	2.1
37023	4.42269	2.1
37024	1.89226	2.1
37025	1.89226	2.1
37026	1.89226	2.1
37027	1.89226	2.1
37028	3.78453	2.1
37029	1.89226	2.1
37030	1.89226	2.1
37031	1.89226	2.1
37032	1.89226	2.1
37033	1.89226	2.1
37034	1.89226	2.1
37035	1.95053	2.1
37036	2.06424	2.1
37037	1.95053	2.1
37038	1.30584	2.1
37039	2.5	2.1
37040	1.8553	2.1
37041	2.49999	2.1
37042	2.57651	2.1
37043	2.49999	2.1
37044	2.06423	2.1
37045	2.80738	2.1
37046	2.37162	2.1
37047	1.89226	2.1
37048	1.89226	2.1
37049	1.89226	2.1
37050	1.89226	2.1
37051	1.89226	2.1
37052	1.89226	2.1
37053	1.49295	0.0549278
37054	1.03824	0.05
37055	1.51396	0.122613
37056	2.08514	0.0651325
37057	3.12338	0.0616877
37058	1.49798	0.0548433
37059	2.32017	0.05

37060	2.68236	0.100339
37061	2.08514	0.0778431
37062	4.4053	0.05
37063	1.89226	0.05
37064	1.89226	0.05
37065	1.89226	0.105798
37066	1.89226	0.0639993
37067	3.78452	0.0742276
37068	1.89226	0.0893947
37069	1.89226	0.05
37070	1.89226	0.0742757
37071	1.89226	0.0586315
37072	1.89226	0.0554174
37073	1.89226	0.05
37074	1.51396	0.05
37075	2.08514	0.05
37076	2.55393	0.084216
37077	2.02901	0.0621611
37078	4.11415	0.05
37079	1.89226	0.05
37080	1.89226	0.05
37081	1.89226	0.0774328
37082	1.89226	0.0714169
37083	3.78451	0.05
37084	1.89226	0.05
37085	1.89226	0.074411
37086	1.89226	0.05
37087	1.89226	0.05
37088	1.89226	0.0820108
37089	1.89226	0.0719669
37090	0.946131	0.05
37091	0.946131	0.05
37092	0.946132	0.05
37093	0.946132	0.05
37094	0.946132	0.05
37095	0.946132	0.05
37096	0.946132	0.05
37097	0.946132	0.05
37098	0.946132	0.05
37099	0.946132	0.05
37100	0.946132	0.05
37101	0.946132	0.05
37102	1.89226	2.1
37103	1.89226	2.1
37104	1.89226	2.1
37105	1.89226	2.1
37106	3.78452	2.1
37107	1.28367	2.1
37108	1.82707	2.1
37109	1.11025	2.1
37110	1.6365	2.1
37111	2.92016	2.1
37112	1.82707	2.1
37113	1.8247	0.1333
37114	1.8247	2.1
37115	2.01549	0.567607
37116	1.6365	2.1
37117	1.82729	0.58932

37118	1.89226	2.1
37119	1.89226	0.139696
37120	1.89226	0.0829525
37121	1.89226	2.1
37122	1.89226	0.100701
37123	1.89226	2.1
37124	1.63648	2.1
37125	1.82467	0.0788191
37126	1.35877	2.1
37127	1.55159	0.0932421
37128	3.18345	1.70395
37129	1.89223	2.1
37130	1.89223	0.0784599
37131	1.89223	2.1
37132	1.89223	0.225394
37133	3.78447	2.1
37134	1.59972	2.1
37135	1.28037	2.1
37136	1.63702	2.1
37137	1.28158	2.1
37138	1.63581	2.1
37139	1.28037	2.1
37140	1.89226	2.1
37141	1.89226	2.1
37142	1.89226	2.1
37143	1.89226	2.1
37144	1.89226	2.1
37145	1.89226	2.1
37146	1.89226	2.1
37147	1.89226	2.1
37148	1.89226	2.1
37149	1.89226	2.1
37150	3.78451	2.1
37151	1.28037	2.1
37152	1.05071	2.1
37153	1.63581	2.1
37154	1.44287	2.1
37155	2.72324	2.1
37156	1.59972	2.1
37157	1.63581	2.1
37158	1.94291	2.1
37159	2.00118	2.1
37160	3.57872	2.1
37161	1.89226	2.1
37162	1.89226	2.1
37163	1.89226	2.1
37164	1.89226	2.1
37165	3.78452	2.1
37166	0.946131	2.1
37167	0.946131	2.1
37168	0.946127	2.1
37169	0.946127	2.1
37170	0.94613	2.1
37171	0.94613	2.1
37172	0.946128	2.1
37173	0.946128	2.1
37174	0.946128	2.1
37175	0.946128	2.1

37176	0.946129	2.1
37177	0.946129	2.1
37178	0.865056	2.1
37179	0.865056	2.1
37180	0.873224	2.1
37181	0.873224	2.1
37182	0.721437	2.1
37183	0.920378	2.1
37184	0.817903	2.1
37185	1.00059	2.1
37186	1.00059	2.1
37187	0.920376	2.1
37188	0.817904	2.1
37189	0.721437	2.1
37190	3.09157	2.1
37191	1.71255	2.1
37192	2.0815	2.1
37193	1.9918	2.1
37194	2.51364	2.1
37195	2.39396	2.1
37196	2.0815	2.1
37197	1.05051	2.1
37198	2.56724	2.1
37199	1.63991	2.1
37200	3.47929	2.1
37201	1.89226	0.05
37202	2.2973	0.109929
37203	1.90705	0.0839278
37204	1.90705	0.05
37205	1.89226	0.0607422
37206	1.89226	0.05
37207	1.89226	0.05
37208	1.90705	0.0921459
37209	1.45321	0.09514
37210	1.45321	0.05
37211	1.89226	0.0627484
37212	1.89226	0.05
37213	0.901146	2.1
37214	0.901146	2.1
37215	1.15227	2.1
37216	1.21279	2.1
37217	0.991265	2.1
37218	1.15003	2.1
37219	0.946132	2.1
37220	0.946132	2.1
37221	0.946132	2.1
37222	1.09997	2.1
37223	0.991266	2.1
37224	0.946132	2.1
37225	1.89226	2.1
37226	1.89226	2.1
37227	1.89226	2.1
37228	1.89226	2.1
37229	1.89226	2.1
37230	1.89226	2.1
37231	1.62229	2.1
37232	1.06053	2.1
37233	1.19698	2.1

37234	1.70106	2.1
37235	2.81928	2.1
37236	0.946132	2.1
37237	0.946132	2.1
37238	1.17527	2.1
37239	1.09997	2.1
37240	1.09997	2.1
37241	1.09997	2.1
37242	0.946133	2.1
37243	0.946133	2.1
37244	1.17527	2.1
37245	0.946132	2.1
37246	0.946133	2.1
37247	1.09997	2.1
37248	1.89226	2.1
37249	1.89226	2.1
37250	1.89226	2.1
37251	1.89226	2.1
37252	1.89226	2.1
37253	1.89226	2.1
37254	0.905416	2.1
37255	0.850527	2.1
37256	0.660601	2.1
37257	0.598494	2.1
37258	0.750137	2.1
37259	0.750137	2.1
37260	0.753774	2.1
37261	0.753774	2.1
37262	0.660599	2.1
37263	0.905418	2.1
37264	0.598493	2.1
37265	0.850529	2.1
37266	2.43509	2.1
37267	2.65066	2.1
37268	2.66602	2.1
37269	2.46339	2.1
37270	5.10111	2.1
37271	2.53008	2.1
37272	2.65066	2.1
37273	2.66602	2.1
37274	2.52852	2.1
37275	5.1961	2.1
37276	2.66602	2.1
37277	2.46339	2.1
37278	2.47932	2.1
37279	2.67393	2.1
37280	5.14535	2.1
37281	2.66602	2.1
37282	2.52853	2.1
37283	2.51686	2.1
37284	2.67393	2.1
37285	5.18288	2.1
37286	1.89226	0.985316
37287	1.89226	0.05
37288	1.89226	0.239282
37289	1.89226	0.05
37290	3.78452	0.0719328
37291	1.55161	0.161522

37292	1.53972	0.05
37293	1.32259	0.219851
37294	1.35082	0.05
37295	2.8623	0.05
37296	1.53972	0.124894
37297	1.4054	0.14328
37298	1.35083	0.05
37299	1.24913	0.0551928
37300	2.78885	0.060112
37301	1.89226	0.143808
37302	1.89226	0.900754
37303	1.89226	0.128027
37304	1.89226	0.0595902
37305	3.78452	0.105257
37306	1.89226	0.11219
37307	1.89226	0.0810314
37308	1.89226	1.04106
37309	1.89226	0.0774545
37310	1.89226	0.301262
37311	1.89226	0.05
37312	1.14599	0.0967842
37313	1.35082	0.05
37314	1.14599	0.0584654
37315	1.32259	1.18268
37316	1.19993	0.05
37317	1.37652	1.0596
37318	1.35083	0.0649968
37319	1.24913	0.370236
37320	1.19993	0.05
37321	1.11787	0.05
37322	2.44906	0.05
37323	1.89226	0.494038
37324	1.89226	0.420567
37325	1.89226	0.0775834
37326	1.89226	0.05
37327	3.78452	0.134885
37328	1.51697	0.05
37329	2.57264	0.05
37330	1.30203	0.05
37331	2.41701	0.05
37332	3.93398	0.05
37333	1.17464	0.05
37334	2.41701	0.05
37335	2.32462	0.05
37336	2.32462	0.05
37337	1.30203	0.05
37338	1.30203	0.05
37339	2.08149	2.1
37340	1.9839	2.1
37341	1.71398	2.1
37342	1.99182	0.402508
37343	3.71121	2.1
37344	2.16061	2.1
37345	2.01399	0.0764533
37346	1.99182	1.46329
37347	1.83895	1.12849
37348	4.00581	0.564341
37349	1.90444	2.1

37350	1.99182	1.09328
37351	1.71717	2.1
37352	1.805	2.1
37353	3.70899	2.1
37354	1.99182	1.92419
37355	1.83896	0.578507
37356	1.805	2.1
37357	1.65243	0.156539
37358	3.64425	0.268838
37359	1.18076	2.1
37360	1.27697	2.1
37361	1.05025	2.1
37362	1.27697	2.1
37363	1.25	2.1
37364	1.47671	2.1
37365	1.89226	2.1
37366	1.89226	2.1
37367	1.89226	2.1
37368	1.89226	2.1
37369	3.78452	2.1
37370	2.64124	2.1
37371	1.89226	2.1
37372	1.89226	2.1
37373	1.89226	2.1
37374	2.46904	2.1
37375	2.46904	2.1
37376	1.89226	2.1
37377	1.89226	2.1
37378	2.7408	2.1
37379	2.82858	2.1
37380	4.72084	2.1
37381	1.87875	2.1
37382	1.37704	2.1
37383	1.77042	2.1
37384	2.27801	2.1
37385	3.64916	2.1
37386	1.77042	2.1
37387	2.27801	2.1
37388	2.58523	2.1
37389	1.90178	2.1
37390	4.35565	2.1
37391	1.28667	2.1
37392	1.30711	2.1
37393	1.53077	2.1
37394	1.55635	2.1
37395	2.81743	2.1
37396	1.55635	2.1
37397	1.28228	2.1
37398	1.64268	2.1
37399	1.93673	2.1
37400	3.19903	2.1
37401	1.53077	2.1
37402	1.93673	2.1
37403	1.55635	2.1
37404	1.55635	2.1
37405	1.86206	2.1
37406	1.86206	2.1
37407	1.64892	2.1

37408	1.60532	2.1
37409	1.85282	2.1
37410	1.96716	2.1
37411	3.61608	2.1
37412	1.60531	2.1
37413	1.32414	2.1
37414	1.96716	2.1
37415	1.75823	2.1
37416	3.2913	2.1
37417	1.91219	2.1
37418	1.96716	2.1
37419	2.0815	2.1
37420	2.51396	2.1
37421	4.30843	2.1
37422	1.96716	2.1
37423	2.17979	2.1
37424	1.75823	2.1
37425	1.75823	2.1
37426	2.28541	2.1
37427	2.28541	2.1
37428	1.89226	1.30679
37429	1.89226	1.9719
37430	1.89226	1.31304
37431	1.89226	1.18948
37432	3.78453	1.43765
37433	1.89226	1.61207
37434	1.89226	1.59276
37435	1.89226	1.30517
37436	1.89226	1.70526
37437	1.89226	1.3712
37438	1.89226	1.7605
37439	1.61016	1.34168
37440	1.45303	1.87235
37441	1.62812	1.50182
37442	1.86379	1.46362
37443	3.23828	1.52118
37444	1.61016	1.7913
37445	1.78716	1.98327
37446	2.0815	1.09554
37447	1.6774	1.81495
37448	3.54454	1.75884
37449	1.89226	1.68057
37450	1.89226	1.55275
37451	1.89226	0.94814
37452	1.89226	1.68506
37453	3.78453	1.37911
37454	1.89226	1.74607
37455	1.89226	1.71109
37456	1.89226	1.52619
37457	1.89226	2.06043
37458	1.89226	0.854636
37459	1.89226	1.29007
37460	0.807591	1.82526
37461	0.814059	1.47702
37462	1.00217	1.87071
37463	0.931896	1.55261
37464	0.850586	1.59846
37465	0.850586	1.98545

37466	0.8889	1.27308
37467	0.8889	1.78271
37468	0.807592	1.13653
37469	1.00217	1.80802
37470	0.814061	1.6109
37471	0.931894	2.03408
37472	2.0815	1.31992
37473	1.6774	2.1
37474	2.00435	1.43981
37475	2.36104	1.35034
37476	3.86809	1.50921
37477	1.00532	0.05
37478	1.00532	0.05
37479	1.00183	0.05
37480	1.00183	0.05
37481	1.26377	0.05
37482	1.34921	0.05
37483	0.664872	0.05
37484	0.743381	0.05
37485	0.664872	0.05
37486	1.26377	0.05
37487	1.34921	0.05
37488	0.743381	0.05
37489	1.26826	0.05
37490	0.946131	2.1
37491	1.02404	2.1
37492	0.94613	2.1
37493	1.30281	2.1
37494	0.930941	2.1
37495	0.930941	2.1
37496	1.318 2.1	
37497	1.58161	2.1
37498	0.94613	2.1
37499	0.94613	2.09826
37500	1.56338	2.1
37501	1.02404	2.1
37502	2.81374	2.1
37503	1.89226	2.1
37504	1.89226	2.1
37505	1.89226	2.1
37506	3.78453	2.1
37507	0.946133	2.1
37508	0.946133	2.1
37509	0.946132	2.1
37510	0.946132	2.1
37511	0.946132	2.1
37512	0.946132	2.1
37513	0.946133	2.1
37514	0.946133	2.1
37515	0.946132	2.1
37516	0.946132	2.1
37517	0.946133	2.1
37518	0.946133	2.1
37519	1.89226	1.7613
37520	1.89226	2.1
37521	1.89226	1.99654
37522	1.89226	2.1
37523	3.78452	2.1

37524	1.89226	2.1
37525	1.89226	2.1
37526	1.89226	2.1
37527	1.89226	2.04147
37528	1.89226	2.1
37529	1.89226	2.04919
37530	0.946133	2.05449
37531	0.946133	2.1
37532	0.946132	2.01754
37533	0.946132	2.1
37534	0.946132	2.1
37535	0.946132	2.1
37536	0.946133	2.1
37537	0.946133	2.06377
37538	0.946132	2.1
37539	0.946132	2.09415
37540	0.946132	2.1
37541	0.946132	2.1
37542	2.56953	2.1
37543	2.64148	2.1
37544	2.48631	2.1
37545	2.62213	2.1
37546	5.19166	2.1
37547	2.64148	2.1
37548	2.57929	2.1
37549	2.62213	2.1
37550	2.59522	2.1
37551	5.20142	2.1
37552	2.48631	2.1
37553	2.62213	2.1
37554	2.41176	2.1
37555	2.59262	2.1
37556	5.03389	2.1
37557	2.62213	2.1
37558	2.59522	2.1
37559	2.59262	2.1
37560	2.61901	2.1
37561	5.24114	2.1
37562	0.946132	0.05
37563	0.946132	0.05
37564	0.946133	0.05
37565	0.946133	0.05
37566	0.946132	0.05
37567	0.946132	0.05
37568	0.946133	0.05
37569	0.946133	0.05
37570	0.946132	0.05
37571	0.946132	0.05
37572	0.946133	0.05
37573	0.946133	0.05
37574	1.5681	0.05
37575	1.01248	0.05
37576	1.10749	0.05
37577	0.570913	0.05
37578	1.15589	0.05
37579	1.15588	0.05
37580	0.977549	0.05
37581	0.977549	0.05

37582	1.01249	0.05
37583	0.654445	0.05
37584	1.36795	0.05
37585	1.10748	0.05
37586	1.73883	0.05
37587	1.87638	0.05
37588	1.85219	0.05
37589	1.93113	2.1
37590	3.7407	0.05
37591	1.87638	0.05
37592	1.73883	2.1
37593	1.93113	2.1
37594	1.80426	2.1
37595	3.66996	2.1
37596	2.02912	0.05
37597	1.44951	0.05
37598	1.50103	0.05
37599	2.02248	0.05
37600	3.47199	0.05
37601	1.89226	0.05
37602	1.89226	0.05
37603	1.89226	0.05
37604	1.89226	0.05
37605	3.78453	0.05
37606	2.57881	0.05
37607	1.89226	0.05
37608	1.89226	0.05
37609	1.89226	0.05
37610	2.6428	0.05
37611	2.6428	0.05
37612	1.01379	0.05
37613	0.985937	0.05
37614	0.769295	0.05
37615	0.715547	0.05
37616	0.853042	0.05
37617	0.853042	0.05
37618	0.864117	0.05
37619	0.864114	0.05
37620	0.715556	0.05
37621	1.03047	0.05
37622	0.731721	0.05
37623	0.985921	0.05
37624	1.89227	0.05
37625	1.89227	0.05
37626	1.89227	0.05
37627	1.89227	0.0613802
37628	3.78453	0.05
37629	0.946129	0.05
37630	0.946129	0.05
37631	1.28161	0.05
37632	1.3214	0.05
37633	0.94613	0.05
37634	0.94613	0.05
37635	1.28161	0.05
37636	1.28161	0.05
37637	1.28161	0.05
37638	0.94613	0.05
37639	0.946131	0.05

37640	1.3214	0.05
37641	0.946132	0.117531
37642	0.946132	0.0880849
37643	0.706045	0.05
37644	1.16718	0.05
37645	0.706017	0.05
37646	0.706017	0.251157
37647	1.4073	0.05
37648	1.4073	0.05
37649	0.94613	0.059037
37650	1.16719	0.0594107
37651	0.706046	0.0680333
37652	0.946131	0.0524526
37653	1.89226	0.25706
37654	1.89226	0.05
37655	1.89226	0.05
37656	1.89226	0.266447
37657	1.89226	0.05
37658	1.89226	0.262054
37659	0.946119	0.151369
37660	0.946119	0.133125
37661	0.946115	0.0815938
37662	0.946115	0.05
37663	0.946117	0.08385
37664	0.946117	0.22049
37665	0.946117	0.106423
37666	0.946117	0.0553984
37667	0.94613	0.0614077
37668	0.94613	0.0994131
37669	0.946105	0.0981941
37670	0.946105	0.0744441
37671	0.946132	0.514206
37672	0.946132	0.311105
37673	0.946131	0.372271
37674	0.946131	0.13574
37675	0.946132	0.736834
37676	0.946132	0.393013
37677	0.946132	0.282732
37678	0.827865	0.164247
37679	0.946132	0.161399
37680	0.946132	0.284096
37681	0.946132	0.372367
37682	0.827865	0.296716
37683	1.89226	1.38198
37684	1.89226	0.871827
37685	1.89226	1.53087
37686	1.89226	0.775345
37687	1.89226	1.94845
37688	1.89226	1.16849
37689	0.93094	0.731491
37690	0.93094	1.04409
37691	1.04649	0.752079
37692	0.614468	0.303051
37693	0.950358	0.426063
37694	0.799012	0.394425
37695	0.682742	0.820147
37696	0.682742	0.775388
37697	1.04073	1.51632

37698	1.15596	1.17151
37699	0.614478	1.18263
37700	0.851524	0.984159
37701	2.22575	2.1
37702	1.90502	2.1
37703	2.10893	2.1
37704	1.80258	2.1
37705	4.01395	2.1
37706	1.90502	2.1
37707	1.40863	2.1
37708	1.49733	2.1
37709	1.49733	2.1
37710	1.80258	2.1
37711	1.80258	2.1
37712	2.10893	2.1
37713	1.68356	2.1
37714	1.80258	2.1
37715	1.80258	2.1
37716	1.98061	2.1
37717	1.98061	2.1
37718	1.80258	2.1
37719	1.40863	2.1
37720	1.68356	2.1
37721	1.30751	2.1
37722	3.11009	2.1
37723	1.35374	1.97861
37724	1.35374	2.03099
37725	0.746096	1.37738
37726	0.746096	1.34927
37727	1.1537	1.41067
37728	0.746095	1.87994
37729	0.946132	1.70905
37730	0.946132	0.989618
37731	0.946132	1.04269
37732	0.746095	1.26222
37733	0.946131	0.820748
37734	1.1537	1.07837
37735	2.15247	2.1
37736	1.70303	2.1
37737	1.41207	2.1
37738	1.43052	2.1
37739	3.22793	2.1
37740	0.940246	2.1
37741	0.728442	2.1
37742	0.650037	2.1
37743	0.539874	2.1
37744	0.694756	2.1
37745	0.694756	2.1
37746	0.776346	2.1
37747	0.656772	2.1
37748	0.733146	2.1
37749	0.563769	2.1
37750	0.66316	2.1
37751	0.899692	2.1
37752	1.21223	2.1
37753	1.22418	2.1
37754	1.43052	2.1
37755	1.43052	2.1

37756	1.24353	2.1
37757	1.46181	2.1
37758	1.89226	0.05
37759	2.39894	0.05
37760	1.89226	0.05
37761	2.37417	0.05
37762	1.89226	0.05
37763	2.37417	0.05
37764	1.12767	0.05
37765	1.19947	0.05
37766	0.94613	0.05
37767	0.94613	0.05
37768	1.12766	0.05
37769	0.946128	0.05
37770	1.19947	0.05
37771	0.946132	0.05
37772	1.19947	0.05
37773	1.19947	0.05
37774	0.94613	0.05
37775	0.94613	0.05
37776	2.22621	0.05
37777	1.14533	2.1
37778	1.1131	2.1
37779	0.88238	2.1
37780	0.868521	2.1
37781	1.01153	2.1
37782	1.01153	2.1
37783	1.00232	2.1
37784	1.00232	2.1
37785	1.14533	2.1
37786	0.882378	2.1
37787	0.868519	2.1
37788	1.11311	2.1
37789	0.910183	1.89334
37790	1.07811	0.828927
37791	0.94613	0.0743404
37792	0.94613	0.169117
37793	0.910212	0.233335
37794	0.910212	2.00001
37795	1.11403	0.0765796
37796	1.11403	1.96909
37797	0.910183	0.516898
37798	0.94613	1.13364
37799	0.94613	1.15343
37800	1.07811	1.1489
37801	1.87228	1.08939
37802	2.81189	2.1
37803	1.6197	2.1
37804	2.56716	2.1
37805	4.43944	2.1
37806	0.946132	1.18318
37807	0.946132	2.1
37808	0.946132	0.12423
37809	0.946132	0.368629
37810	0.946131	0.954255
37811	0.946131	2.1
37812	0.946132	0.198927
37813	0.946132	1.36626

37814	0.946132	0.900633
37815	0.946132	1.01119
37816	0.946132	1.03774
37817	0.946132	1.16277
37818	1.28724	2.1
37819	2.56716	2.1
37820	2.3014	2.08124
37821	2.3014	2.1
37822	1.6197	2.1
37823	1.6197	2.1
37824	0.946132	2.1
37825	0.946132	2.1
37826	0.946132	0.119921
37827	0.946132	0.868338
37828	0.946132	1.12499
37829	0.946132	2.1
37830	0.946133	2.1
37831	0.946133	0.119123
37832	0.946133	1.4611
37833	0.946133	1.59306
37834	0.946132	1.56244
37835	0.946132	1.70988
37836	1.08039	2.1
37837	1.24445	2.1
37838	1.2873	2.1
37839	1.48751	2.1
37840	2.53175	2.1
37841	0.946125	0.386325
37842	0.946125	2.1
37843	1.12552	2.1
37844	1.12552	2.1
37845	0.946124	2.05743
37846	0.946124	2.1
37847	1.12552	2.1
37848	1.11114	2.1
37849	1.11113	2.1
37850	0.946118	2.1
37851	1.12552	2.1
37852	0.946131	2.1
37853	1.89225	0.770309
37854	1.89225	1.13779
37855	1.89225	2.1
37856	1.89225	2.1
37857	1.89225	0.213241
37858	1.89225	2.1
37859	0.987703	0.576281
37860	1.33078	2.05043
37861	1.31565	1.05851
37862	1.31565	2.1
37863	1.01121	1.08178
37864	1.01121	2.1
37865	2.19163	2.1
37866	1.89226	2.1
37867	1.89226	2.1
37868	1.89226	2.01618
37869	2.22229	2.1
37870	2.22229	2.07188
37871	1.89226	2.1

37872	1.89226	0.16241
37873	1.89226	0.104097
37874	1.89226	2.1
37875	1.89226	0.154045
37876	1.89226	2.1
37877	0.57141	2.1
37878	0.57141	0.241539
37879	0.574036	0.956744
37880	0.574036	2.1
37881	0.651591	2.1
37882	0.657827	0.883467
37883	0.482391	1.46438
37884	0.493855	2.07024
37885	0.482391	0.29605
37886	0.651591	0.428037
37887	0.493855	1.91977
37888	0.657828	1.85379
37889	1.88428	2.1
37890	2.03975	2.1
37891	1.97316	2.1
37892	1.8266	2.1
37893	3.86635	2.1
37894	2.23053	2.1
37895	1.42255	2.1
37896	1.3868	2.1
37897	1.8266	2.1
37898	3.24915	2.1
37899	1.97316	2.1
37900	1.8266	2.1
37901	1.77128	2.1
37902	1.91767	2.1
37903	3.74427	2.1
37904	1.77128	2.1
37905	1.8266	2.1
37906	1.77942	2.1
37907	1.33962	2.1
37908	1.3868	2.1
37909	1.3868	2.1
37910	0.944111	2.1
37911	0.944111	2.1
37912	1.26036	2.1
37913	1.16968	2.1
37914	1.03853	2.1
37915	1.16646	2.1
37916	0.946129	2.1
37917	0.946129	2.1
37918	0.94613	2.1
37919	1.25768	2.1
37920	1.03853	2.1
37921	0.946131	2.1
37922	1.82441	0.05
37923	1.33061	0.05
37924	1.13272	0.05
37925	1.60249	0.05
37926	2.95712	0.05
37927	1.82441	0.05
37928	2.08757	0.05
37929	1.81292	0.05

37930	1.60249	0.05
37931	3.63733	0.05
37932	1.81292	0.05
37933	1.332 0.05	
37934	1.60249	0.05
37935	1.60249	0.05
37936	1.5344	0.05
37937	1.5344	0.05
37938	1.10107	2.1
37939	2.51559	2.1
37940	1.1229	2.1
37941	1.1229	2.1
37942	2.49456	2.1
37943	2.49456	2.1
37944	1.89226	2.1
37945	2.51559	2.1
37946	2.49457	2.1
37947	2.51559	2.1
37948	1.87124	2.1
37949	1.89226	2.1
37950	1.89226	2.1
37951	1.89226	2.1
37952	1.89226	2.1
37953	1.89226	2.1
37954	1.89226	2.1
37955	1.89226	2.1
37956	1.89226	2.1
37957	1.89226	2.1
37958	1.89226	2.1
37959	1.89226	2.1
37960	1.89226	2.1
37961	1.89226	2.1
37962	1.23599	2.1
37963	1.24728	2.1
37964	0.550534	2.1
37965	0.541151	2.1
37966	0.893754	2.1
37967	0.893754	2.1
37968	0.894678	2.1
37969	0.894678	2.1
37970	1.23599	2.1
37971	0.541149	2.1
37972	0.550533	2.1
37973	1.24728	2.1
37974	0.946132	2.1
37975	0.946132	2.1
37976	1.23599	2.1
37977	1.24728	2.1
37978	0.946132	2.1
37979	0.946132	2.1
37980	1.23599	2.1
37981	1.23599	2.1
37982	1.23599	2.1
37983	0.946132	2.1
37984	0.946132	2.1
37985	1.24728	2.1
37986	1.89226	2.1
37987	1.89226	2.1

37988	1.89226	2.1
37989	1.89226	2.1
37990	1.89226	2.1
37991	1.89226	2.1
37992	0.946128	2.1
37993	0.946128	2.1
37994	0.946132	2.1
37995	0.946132	2.1
37996	0.94613	2.1
37997	0.94613	2.1
37998	0.946129	2.1
37999	0.946129	2.1
38000	0.94613	2.1
38001	0.94613	2.1
38002	0.94613	2.1
38003	0.94613	2.1
38004	2.58696	0.0506668
38005	2.57073	0.0890112
38006	2.12585	0.0550357
38007	2.07607	0.12118
38008	4.69658	0.0969806
38009	1.89226	0.0877683
38010	1.89226	0.11981
38011	1.89226	0.0749232
38012	1.89226	0.136412
38013	3.78452	0.0806796
38014	1.89226	0.122815
38015	1.89226	0.232973
38016	1.89226	0.143203
38017	1.89226	0.204641
38018	3.78453	0.200074
38019	2.57074	0.0911269
38020	2.32635	0.178985
38021	2.07608	0.127451
38022	1.73623	0.124062
38023	4.30697	0.10397
38024	2.12582	0.05
38025	1.51971	0.0622582
38026	1.57255	0.05
38027	2.07604	0.0638459
38028	1.62233	0.05
38029	2.12582	0.0557422
38030	1.89223	0.0630649
38031	1.89223	0.110763
38032	1.89223	0.104565
38033	1.89223	0.0779103
38034	1.89223	0.0843324
38035	1.89223	0.065146
38036	0.946119	0.153548
38037	0.946119	0.123398
38038	0.946119	0.0865044
38039	0.946119	0.159138
38040	0.946119	0.172281
38041	0.946119	0.161233
38042	0.94612	0.120636
38043	0.94612	0.116622
38044	0.946132	0.132923
38045	0.946132	0.109402

38046	0.946107	0.13402
38047	0.946107	0.13152
38048	0.811456	0.0779953
38049	0.811456	0.140184
38050	0.806521	0.0638878
38051	0.806521	0.135395
38052	0.868104	0.148118
38053	0.579949	0.101814
38054	0.759859	0.0636422
38055	1.03802	0.0635878
38056	0.579957	0.0914456
38057	0.759868	0.0844654
38058	1.03801	0.0941808
38059	0.868093	0.107444
38060	2.7408	2.1
38061	2.82858	2.1
38062	2.57368	2.1
38063	2.52633	2.1
38064	5.3549	2.1
38065	2.7408	2.1
38066	2.31353	2.1
38067	2.14063	2.1
38068	2.52632	2.1
38069	4.83985	2.1
38070	2.57368	2.1
38071	2.52633	2.1
38072	2.3301	2.1
38073	2.36071	2.1
38074	4.88704	2.1
38075	1.86512	2.1
38076	2.52632	2.1
38077	2.14063	2.1
38078	2.14063	2.1
38079	2.3301	2.1
38080	2.3301	2.1
38081	1.86431	2.1
38082	1.89227	2.1
38083	1.68992	2.1
38084	1.68992	2.1
38085	1.89227	2.1
38086	1.89227	2.1
38087	2.0815	2.1
38088	1.89226	2.1
38089	2.04022	2.1
38090	1.86431	2.1
38091	2.07081	2.1
38092	1.89226	2.1
38093	1.91686	1.47259
38094	1.65683	2.1
38095	1.86871	2.1
38096	1.65683	1.64712
38097	2.13075	2.1
38098	1.89226	0.233105
38099	1.89226	2.1
38100	1.89226	0.12868
38101	1.89226	0.602131
38102	1.89226	1.98409
38103	3.78452	1.16055

38104	0.851541	2.1
38105	0.851541	2.1
38106	0.952803	2.1
38107	0.851573	2.1
38108	0.946129	2.1
38109	0.946129	2.0313
38110	0.929184	2.1
38111	0.757482	2.1
38112	0.836274	2.1
38113	0.851528	2.1
38114	0.952816	2.1
38115	1.04073	2.1
38116	1.70303	0.89432
38117	1.70302	0.939311
38118	1.5544	2.09065
38119	1.70302	0.916366
38120	1.74957	1.03678
38121	1.91686	0.144456
38122	1.89226	0.288557
38123	1.89226	1.13714
38124	1.89226	1.82205
38125	1.89226	0.495447
38126	1.89226	0.537164
38127	1.89226	0.0664364
38128	1.89226	2.1
38129	1.89226	2.1
38130	2.03472	2.1
38131	1.89226	2.1
38132	1.99827	2.1
38133	1.85836	2.00189
38134	1.00509	2.1
38135	1.55998	2.1
38136	1.83347	2.1
38137	1.45246	2.1
38138	3.01244	2.1
38139	1.23839	2.1
38140	0.884251	2.1
38141	0.946133	2.1
38142	0.946133	2.1
38143	0.884226	2.1
38144	0.884226	2.1
38145	0.946158	2.1
38146	0.946159	2.1
38147	1.23839	2.1
38148	0.946131	2.1
38149	0.946131	2.1
38150	0.884254	2.1
38151	1.89226	2.1
38152	1.89226	2.1
38153	1.89226	2.1
38154	1.89226	2.1
38155	1.89226	2.1
38156	1.89226	2.1
38157	2.1499	2.1
38158	1.45244	2.1
38159	1.83345	2.1
38160	1.83345	2.1
38161	1.87214	2.1

38162	1.87214	2.1
38163	1.89224	2.1
38164	1.89224	2.1
38165	1.89224	2.1
38166	1.89224	2.1
38167	3.78448	2.1
38168	1.49155	0.622242
38169	2.0815	2.06516
38170	1.77515	1.9055
38171	1.72377	2.1
38172	3.50659	1.58958
38173	1.69876	0.05
38174	2.0815	2.1
38175	1.9133	0.121333
38176	1.68189	0.05
38177	2.17628	2.1
38178	1.94335	2.1
38179	2.65129	0.05
38180	1.15317	0.05
38181	2.57085	0.05
38182	1.209	0.05
38183	2.57085	0.05
38184	1.209	0.05
38185	1.89226	0.05
38186	1.89226	0.05
38187	1.89226	0.05
38188	1.89226	0.05
38189	3.78452	0.05
38190	2.48861	0.05
38191	1.15252	0.05
38192	2.60515	0.05
38193	2.60515	0.05
38194	1.16384	0.05
38195	1.16384	0.05
38196	1.59903	0.423098
38197	1.26865	0.0986142
38198	1.30486	1.29532
38199	1.70302	1.012
38200	2.98573	0.43951
38201	1.59903	0.279931
38202	1.79478	0.450101
38203	1.79478	0.205756
38204	1.97005	0.722328
38205	1.50365	0.146649
38206	1.64192	1.27237
38207	1.89226	0.109042
38208	1.89226	0.507196
38209	1.89226	0.506234
38210	1.89226	0.196815
38211	3.78453	0.173878
38212	1.89226	0.237368
38213	1.89226	0.499171
38214	1.89226	0.399996
38215	1.89226	0.135066
38216	1.89226	0.485117
38217	1.89226	0.88705
38218	0.761672	0.142176
38219	0.896406	0.213387

38220	0.603509	0.184008
38221	0.574071	0.700909
38222	0.695159	0.595444
38223	0.695159	0.0619725
38224	0.762564	1.56033
38225	0.770159	0.0950952
38226	0.803021	0.420301
38227	0.572529	1.15872
38228	0.696466	0.099554
38229	0.899733	0.121708
38230	1.66034	0.05
38231	1.79478	0.66824
38232	1.56685	0.177293
38233	1.68512	1.65805
38234	1.67652	0.361531
38235	1.67652	0.629064
38236	1.89226	0.729907
38237	1.89226	0.3976
38238	1.89226	0.698721
38239	1.89226	0.355746
38240	1.89226	0.852994
38241	1.89226	0.519332
38242	0.946133	0.120097
38243	0.946133	0.260689
38244	0.946132	1.08192
38245	0.946132	0.506206
38246	0.946132	0.652291
38247	0.946132	0.0766961
38248	0.946133	1.62445
38249	0.946133	0.137833
38250	0.946133	0.713723
38251	0.946133	0.388667
38252	0.946132	0.183711
38253	0.946132	0.148161
38254	1.80424	2.1
38255	1.45158	2.1
38256	1.9098	2.1
38257	1.56003	2.1
38258	3.36138	2.1
38259	1.90982	2.1
38260	1.71606	2.1
38261	2.15314	2.1
38262	1.70302	2.1
38263	3.89051	2.1
38264	1.96416	1.14464
38265	1.23706	1.10762
38266	1.00053	0.194727
38267	1.38287	0.153075
38268	1.57916	0.598395
38269	1.9615	0.881682
38270	0.827865	0.154968
38271	0.946132	0.110948
38272	0.946132	0.101374
38273	0.946132	0.502711
38274	0.946132	0.685671
38275	0.946132	0.196657
38276	0.946131	1.01121
38277	0.946131	0.231825

38278	0.827865	0.899812
38279	0.946132	0.0609271
38280	0.946132	0.0820723
38281	0.946132	0.157263
38282	1.89227	2.1
38283	1.89227	2.1
38284	1.89227	2.1
38285	1.89227	2.1
38286	3.78453	2.1
38287	2.3819	2.1
38288	1.58089	2.1
38289	1.58089	2.1
38290	1.6287	2.1
38291	2.33368	2.1
38292	2.3815	2.1
38293	0.946133	2.1
38294	0.946133	2.1
38295	0.946132	2.1
38296	0.946132	2.1
38297	0.946132	2.1
38298	0.946132	2.1
38299	0.946132	2.1
38300	0.946132	2.1
38301	0.946133	2.1
38302	0.946133	2.1
38303	0.946132	2.1
38304	0.946132	2.1
38305	1.89226	2.1
38306	1.89226	2.1
38307	1.89226	2.1
38308	1.89226	2.1
38309	3.78453	2.1
38310	2.7874	2.1
38311	2.33368	2.1
38312	2.71897	2.1
38313	2.27495	2.1
38314	5.05266	2.1
38315	0.970991	2.1
38316	0.970991	2.1
38317	0.956929	2.1
38318	0.956929	2.1
38319	0.732928	2.1
38320	0.790444	2.1
38321	1.16684	2.1
38322	1.13748	2.1
38323	1.13748	2.1
38324	0.732928	2.1
38325	0.790444	2.1
38326	1.16684	2.1
38327	0.946132	2.1
38328	0.946132	2.1
38329	0.946131	2.1
38330	0.946131	2.1
38331	0.946132	2.1
38332	0.946132	2.1
38333	0.946132	2.1
38334	0.946132	2.1
38335	0.946132	2.1

38336	0.946132	2.1
38337	0.946132	2.1
38338	0.946132	2.1
38339	1.10747	0.05
38340	2.30331	0.0619829
38341	1.47954	0.05
38342	2.63789	0.05
38343	3.78285	0.05
38344	1.65215	0.05
38345	2.63789	0.05
38346	2.82465	0.05
38347	2.82465	0.05
38348	1.47954	0.05
38349	1.47954	0.05
38350	1.13456	2.1
38351	1.08334	2.1
38352	1.20674	0.485527
38353	1.17885	2.1
38354	2.29008	2.04366
38355	1.20674	1.78718
38356	1.17885	2.1
38357	1.28649	0.79012
38358	1.28516	1.84273
38359	2.4919	1.87567
38360	1.89226	2.1
38361	1.89226	2.1
38362	1.89226	2.1
38363	1.89226	2.1
38364	1.89226	2.1
38365	1.89226	2.1
38366	2.50288	1.81424
38367	2.02115	2.1
38368	2.7717	1.65833
38369	2.29107	0.439098
38370	4.79395	2.1
38371	1.27339	2.1
38372	1.10817	2.1
38373	1.19733	2.1
38374	1.032 2.1	
38375	2.30539	2.1
38376	1.19733	2.1
38377	0.953637	2.1
38378	0.953637	2.1
38379	1.032 2.1	
38380	1.11868	2.1
38381	1.19704	2.1
38382	2.01024	0.850969
38383	1.11938	0.989121
38384	1.9186	1.341
38385	2.76298	0.793825
38386	3.92884	1.10172
38387	0.851541	0.05
38388	0.851541	0.05
38389	0.957577	0.05
38390	0.848952	0.05
38391	1.08043	0.05
38392	0.851541	0.05
38393	0.851484	0.05

38394	0.957578	0.05
38395	1.20045	0.05
38396	0.726289	0.05
38397	0.946131	0.05
38398	0.946131	0.05
38399	1.50524	2.1
38400	2.38363	2.1
38401	2.49018	2.1
38402	2.49018	2.1
38403	1.45506	2.1
38404	1.45506	2.1
38405	2.49017	2.1
38406	1.89226	2.1
38407	1.89226	2.1
38408	1.89226	2.1
38409	2.38363	2.1
38410	2.38363	2.1
38411	1.54711	2.1
38412	2.49018	2.1
38413	2.56975	2.1
38414	2.56975	2.1
38415	1.50524	2.1
38416	1.50524	2.1
38417	2.56975	2.1
38418	1.89226	2.1
38419	1.89226	2.1
38420	1.89226	2.1
38421	2.49017	2.1
38422	2.49017	2.1
38423	1.89226	0.05
38424	1.89226	0.05
38425	1.89226	0.05
38426	1.89226	0.05
38427	1.89226	0.05
38428	1.89226	0.05
38429	1.89226	0.05
38430	1.89226	0.05
38431	1.89226	0.0591741
38432	1.89226	0.05
38433	1.89226	0.0594466
38434	1.89226	0.05
38435	1.89226	0.05
38436	1.89226	0.05
38437	2.8358	0.05
38438	1.89226	0.0532566
38439	3.78453	0.05
38440	1.60565	0.05
38441	1.89226	1.18565
38442	1.89226	0.613185
38443	1.40013	0.184037
38444	1.89226	0.843534
38445	1.40013	0.285641
38446	1.1766	2.1
38447	1.29435	2.1
38448	1.35735	2.1
38449	1.53509	2.1
38450	2.65169	2.1
38451	0.69701	2.1

38452	0.69701	2.1
38453	0.711303	2.1
38454	0.711303	2.1
38455	0.761138	2.1
38456	0.612149	2.1
38457	0.647174	2.1
38458	0.767548	2.1
38459	0.767548	2.1
38460	0.761139	2.1
38461	0.612149	2.1
38462	0.647174	2.1
38463	1.29764	1.24427
38464	1.29579	1.81227
38465	1.34875	1.64081
38466	1.06717	1.66755
38467	1.29764	1.46104
38468	1.01606	1.25877
38469	0.756635	1.1143
38470	0.648821	1.25675
38471	0.675557	1.6778
38472	0.674374	1.53188
38473	0.74214	1.72722
38474	0.74214	1.32403
38475	0.688868	1.0817
38476	0.688868	1.47922
38477	0.675557	0.913042
38478	0.756634	1.11053
38479	0.648821	0.7188
38480	0.674374	0.957525
38481	1.51327	1.40397
38482	1.48105	0.878423
38483	1.29764	1.50668
38484	1.29764	1.0889
38485	1.48105	1.5526
38486	1.05655	0.808619
38487	1.13537	2.1
38488	1.07326	2.1
38489	0.94613	2.1
38490	0.94613	2.1
38491	0.946132	2.1
38492	0.946132	2.1
38493	1.13537	2.1
38494	1.08927	2.1
38495	1.13537	2.1
38496	0.946131	2.1
38497	0.946131	2.1
38498	0.95947	2.1
38499	1.46892	2.1
38500	1.89226	2.1
38501	1.89226	2.1
38502	1.89226	2.1
38503	1.88031	2.1
38504	1.88031	2.1
38505	1.89226	2.1
38506	1.89226	2.1
38507	1.89226	2.1
38508	1.89226	2.1
38509	3.78453	2.1

38510	2.18254	2.1
38511	1.89226	2.1
38512	2.50005	2.1
38513	1.89226	2.1
38514	4.39231	2.1
38515	1.88031	2.1
38516	2.0815	2.1
38517	2.0815	2.1
38518	1.92078	2.1
38519	2.0815	2.1
38520	1.9218	2.1
38521	1.89227	2.1
38522	1.89227	2.1
38523	1.89227	2.1
38524	1.89227	2.1
38525	1.89227	2.1
38526	1.89227	2.1
38527	1.98693	2.1
38528	1.82857	2.1
38529	2.32	2.1
38530	2.36972	2.1
38531	4.14857	2.1
38532	1.82857	2.1
38533	1.58688	2.1
38534	2.36971	2.1
38535	2.19093	2.1
38536	4.0195	2.1
38537	0.946132	2.1
38538	0.946132	2.1
38539	1.18486	2.1
38540	1.18486	2.1
38541	1.18486	2.1
38542	0.946132	2.1
38543	0.946132	2.1
38544	1.16	2.1
38545	0.946133	2.1
38546	0.946133	2.1
38547	1.16	2.1
38548	1.18486	2.1
38549	1.07798	1.91865
38550	1.08101	1.26913
38551	0.910786	2.1
38552	0.910786	2.1
38553	0.851537	2.1
38554	0.901377	1.23225
38555	1.14652	2.1
38556	1.04072	2.1
38557	0.946129	2.1
38558	0.946129	2.1
38559	1.0019	2.1
38560	1.04263	2.1
38561	1.89226	2.1
38562	1.89226	2.1
38563	1.89226	2.1
38564	1.89226	2.1
38565	3.78452	2.1
38566	1.89226	2.1
38567	2.30146	2.1

38568	1.89226	2.1
38569	2.18412	2.1
38570	4.19372	2.1
38571	2.30146	2.1
38572	1.39962	2.1
38573	2.18411	2.1
38574	1.28765	2.1
38575	3.5891	2.1
38576	1.89226	2.1
38577	1.89226	2.1
38578	1.89226	2.1
38579	1.89226	2.1
38580	1.89226	2.1
38581	1.89226	2.1
38582	1.89226	2.1
38583	1.89226	2.1
38584	1.89226	2.1
38585	1.89226	2.1
38586	3.78452	2.1
38587	2.18412	2.1
38588	1.20045	2.1
38589	1.20045	2.1
38590	1.28765	2.1
38591	2.08808	2.1
38592	2.17528	2.1
38593	0.946131	2.1
38594	0.946131	2.1
38595	0.946128	2.1
38596	0.946128	2.1
38597	0.946127	2.1
38598	0.946127	2.1
38599	0.946131	2.1
38600	0.946131	2.1
38601	0.946129	2.1
38602	0.946129	2.1
38603	0.94613	2.1
38604	0.94613	2.1
38605	1.89226	0.457807
38606	1.89226	1.57053
38607	1.89226	1.39471
38608	1.89226	0.484863
38609	3.78452	0.241697
38610	1.89227	0.254472
38611	1.89227	1.9334
38612	1.89227	0.168776
38613	1.89227	1.29286
38614	1.89227	0.180746
38615	1.89227	1.66284
38616	1.89226	1.72053
38617	1.89226	0.629632
38618	1.89226	0.585182
38619	2.29245	0.587597
38620	3.78452	1.20115
38621	1.89226	1.57904
38622	1.89226	0.46149
38623	1.89226	0.436646
38624	1.89226	1.07873
38625	1.89226	0.149736

38626	1.89226	0.28874
38627	1.28095	1.88329
38628	1.81846	2.05414
38629	2.10991	0.996928
38630	1.34695	1.71699
38631	3.39086	1.85503
38632	2.10991	1.31245
38633	1.36457	1.80654
38634	1.67421	2.06844
38635	1.34695	1.89905
38636	2.43717	1.42617
38637	2.10991	1.60268
38638	2.69541	2.1
38639	1.48572	2.1
38640	2.63553	2.1
38641	1.45385	2.1
38642	4.14926	2.1
38643	2.63553	2.1
38644	1.44207	2.1
38645	1.44207	2.1
38646	1.45385	2.1
38647	2.588 2.1	
38648	2.59978	2.1
38649	1.89227	2.1
38650	1.89227	2.1
38651	1.89227	2.1
38652	1.89227	2.1
38653	3.78453	2.1
38654	1.89226	2.1
38655	1.89226	2.1
38656	1.89226	2.1
38657	1.89226	2.1
38658	3.78453	2.1
38659	2.15929	2.1
38660	1.38276	2.1
38661	1.43617	2.1
38662	1.35614	2.1
38663	2.23932	2.1
38664	2.15929	2.1
38665	2.15929	2.1
38666	2.25168	2.1
38667	2.3558	2.1
38668	2.23932	2.1
38669	4.51509	2.1
38670	1.89226	2.1
38671	1.89226	2.1
38672	1.89226	2.1
38673	1.89226	2.1
38674	3.78453	2.1
38675	1.89226	2.1
38676	1.89226	2.1
38677	1.89226	2.1
38678	1.89226	2.1
38679	1.89226	2.1
38680	1.89226	2.1
38681	2.23932	2.1
38682	1.43854	2.1
38683	1.49173	2.1

38684	1.38276	2.1
38685	2.34829	2.1
38686	2.23932	2.1
38687	2.3558	2.1
38688	2.34829	2.1
38689	2.40685	2.1
38690	2.23932	2.1
38691	2.52332	2.1
38692	2.3558	2.1
38693	1.89226	0.05
38694	1.87361	0.05
38695	2.25082	0.05
38696	1.87361	0.05
38697	2.26947	0.05
38698	1.89226	0.05
38699	1.89226	0.05
38700	1.89226	0.05
38701	1.89226	0.05
38702	1.89226	0.05
38703	1.89226	0.05
38704	1.89226	0.05
38705	2.41314	0.05
38706	1.89226	0.05
38707	1.89226	0.05
38708	1.89226	0.05
38709	2.12204	0.05
38710	2.12204	0.05
38711	1.89226	0.05
38712	2.25082	0.05
38713	2.28189	0.05
38714	2.25082	0.05
38715	1.92333	0.05
38716	1.89226	0.05
38717	1.89226	0.05
38718	1.89226	0.05
38719	1.89226	0.05
38720	1.89226	0.05
38721	1.89226	0.05
38722	1.89226	0.05
38723	2.42521	0.05
38724	1.89226	0.05
38725	1.89226	0.05
38726	1.89226	0.05
38727	2.41314	0.05
38728	2.41314	0.05
38729	1.39725	1.57924
38730	1.07745	2.1
38731	1.07745	1.78611
38732	1.07519	2.1
38733	1.41336	1.15175
38734	1.41111	2.1
38735	1.89226	2.1
38736	1.92039	2.1
38737	1.92039	2.1
38738	1.9052	2.1
38739	1.89226	2.1
38740	1.87708	2.1
38741	0.946128	2.1

38742	0.946128	2.1
38743	0.946133	1.37691
38744	0.946133	0.929127
38745	0.94613	0.943762
38746	0.94613	2.1
38747	0.946131	2.1
38748	0.946131	0.99332
38749	0.946131	2.03257
38750	0.946131	2.06453
38751	0.946131	1.98227
38752	0.946131	2.03765
38753	1.41336	1.58438
38754	1.08911	2.1
38755	1.08911	1.98641
38756	1.07745	2.1
38757	1.42381	1.52934
38758	1.41215	2.1
38759	1.89227	2.1
38760	1.95833	2.1
38761	1.95833	2.1
38762	1.92039	2.1
38763	1.89227	2.1
38764	1.85433	2.1
38765	0.946129	2.1
38766	0.946129	2.1
38767	0.946133	1.83223
38768	0.946133	1.01283
38769	0.946131	1.00189
38770	0.946131	2.1
38771	0.946131	2.1
38772	0.946131	1.28111
38773	0.946131	2.1
38774	0.946131	2.1
38775	0.946131	2.1
38776	0.946131	2.1
38777	1.41943	2.1
38778	2.74398	2.1
38779	1.18238	2.1
38780	2.53897	2.1
38781	3.9584	2.1
38782	2.74398	2.1
38783	1.89226	2.1
38784	1.89226	2.1
38785	1.89226	2.1
38786	2.53897	2.1
38787	2.53897	2.1
38788	1.89227	0.972748
38789	1.89227	0.419944
38790	1.89227	1.04352
38791	1.89227	0.30755
38792	3.78453	0.459706
38793	1.89226	0.37626
38794	2.08134	0.148574
38795	2.00632	0.139918
38796	2.00632	0.138657
38797	1.89226	0.277135
38798	1.89226	0.271899
38799	1.89227	0.610961

38800	1.89227	0.188392
38801	1.89227	0.385678
38802	1.89227	0.158874
38803	3.78453	0.381068
38804	1.89227	0.907218
38805	1.89227	0.15757
38806	1.89227	0.166556
38807	1.89227	0.285964
38808	1.89227	0.455771
38809	1.89227	0.82598
38810	2.50912	0.135892
38811	1.89226	0.236643
38812	1.89226	0.0702442
38813	2.31261	0.164011
38814	2.31261	0.135782
38815	1.89226	0.29265
38816	2.50912	0.13583
38817	1.19999	0.0688461
38818	1.44056	0.0530525
38819	1.24405	0.077007
38820	2.50912	0.0912753
38821	2.31261	0.125506
38822	2.69224	0.320497
38823	2.28373	0.175803
38824	1.94788	1.4285
38825	2.4106	0.248002
38826	4.69434	1.03175
38827	2.69223	0.05
38828	2.63783	0.753716
38829	2.34737	1.58672
38830	2.4106	0.452293
38831	5.04843	0.607933
38832	1.89226	2.1
38833	1.99753	2.1
38834	1.87366	2.1
38835	1.99753	2.1
38836	1.76839	2.1
38837	1.89226	2.1
38838	1.89226	2.1
38839	1.89226	2.1
38840	1.89226	2.1
38841	1.89226	2.1
38842	3.78452	2.1
38843	0.946132	2.1
38844	0.946132	2.1
38845	1.02744	2.1
38846	1.02744	2.1
38847	0.946132	2.1
38848	0.946132	2.1
38849	1.00989	2.1
38850	1.02744	2.1
38851	1.00989	2.1
38852	0.946133	2.1
38853	1.02744	2.1
38854	0.946132	2.1
38855	1.70302	2.1
38856	1.87366	2.1
38857	1.71618	2.1

38858	1.87366	2.1
38859	1.73105	2.1
38860	1.89226	2.1
38861	1.89226	2.1
38862	1.89226	2.1
38863	1.89226	2.1
38864	1.89226	2.1
38865	1.89226	2.1
38866	1.89226	2.0745
38867	1.96624	2.1
38868	1.89226	2.1
38869	1.89226	2.1
38870	1.89226	2.1
38871	2.01977	2.1
38872	2.01977	2.1
38873	1.1032	2.03568
38874	1.15114	2.1
38875	1.14986	0.0682849
38876	1.19242	2.1
38877	2.301	2.1
38878	1.89226	1.42776
38879	1.89226	2.1
38880	1.89226	0.0769434
38881	1.89226	2.1
38882	3.78453	1.71347
38883	0.946132	2.1
38884	0.946132	2.1
38885	0.946132	2.1
38886	0.946132	2.1
38887	0.946133	2.1
38888	0.946133	2.1
38889	0.946132	2.1
38890	0.946132	2.1
38891	0.946132	2.1
38892	0.946132	2.1
38893	0.946133	2.1
38894	0.946133	2.1
38895	1.19243	2.1
38896	0.953928	2.1
38897	1.18671	2.1
38898	1.15114	2.1
38899	0.9895	2.1
38900	0.953928	2.1
38901	1.19611	0.0782185
38902	1.19242	2.1
38903	1.24693	0.187825
38904	1.24693	2.1
38905	1.14986	0.275249
38906	1.14986	2.1
38907	1.89225	0.0952341
38908	1.89225	2.1
38909	1.89225	2.1
38910	1.89225	1.01562
38911	1.89225	2.1
38912	1.89225	1.02377
38913	1.89225	2.1
38914	1.89225	2.1
38915	1.89225	2.1

38916	1.89225	2.1
38917	1.89225	2.1
38918	1.89225	2.1
38919	0.561532	2.1
38920	0.561532	2.1
38921	0.566382	2.1
38922	0.566382	2.1
38923	0.494747	2.1
38924	0.531705	2.1
38925	0.596208	2.1
38926	0.623464	2.1
38927	0.623469	2.1
38928	0.531708	2.1
38929	0.596205	2.1
38930	0.494743	2.1
38931	1.25742	2.1
38932	1.18783	2.1
38933	0.946131	2.1
38934	0.946131	2.1
38935	1.25742	2.1
38936	1.25742	2.1
38937	0.946131	2.1
38938	0.946131	2.1
38939	1.25742	2.1
38940	0.946131	2.1
38941	0.946131	2.1
38942	1.18783	2.1
38943	1.35048	2.1
38944	1.25742	2.1
38945	0.946132	2.1
38946	0.946132	2.1
38947	1.35048	2.1
38948	1.35048	2.1
38949	0.946132	2.1
38950	0.946132	2.1
38951	1.35044	2.1
38952	0.946106	2.1
38953	1.25745	2.1
38954	0.946158	2.1
38955	1.13525	2.1
38956	2.70095	2.1
38957	2.51483	2.1
38958	2.51483	2.1
38959	1.21593	2.1
38960	1.21593	2.1
38961	1.93142	0.0544207
38962	2.8053	0.05
38963	1.559	0.232078
38964	2.60359	0.05
38965	4.53501	0.101627
38966	2.80531	0.0621077
38967	1.89226	0.05
38968	2.60359	0.05
38969	1.89226	0.123371
38970	4.49586	0.05
38971	1.22938	0.103314
38972	2.60359	0.05
38973	2.37434	0.25967

38974	2.37434	0.05
38975	1.55901	0.615228
38976	1.55901	0.05
38977	1.18717	0.142138
38978	1.18717	0.05
38979	0.946132	0.05
38980	0.946132	0.05
38981	0.946133	0.0794007
38982	0.946133	0.147459
38983	1.3018	0.05
38984	1.18717	0.188746
38985	1.18714	0.0510204
38986	0.946107	0.0675426
38987	1.30183	0.05
38988	0.946158	0.05
38989	1.89226	2.1
38990	2.80196	2.1
38991	2.67272	2.1
38992	2.67272	2.1
38993	1.89226	2.1
38994	1.89226	2.1
38995	1.89226	2.1
38996	1.89226	2.1
38997	1.89226	2.1
38998	1.89226	2.1
38999	1.89226	2.1
39000	1.89226	2.1
39001	1.74471	2.1
39002	2.01555	2.1
39003	1.61121	2.1
39004	1.61121	2.1
39005	2.13878	2.1
39006	2.13878	2.1
39007	1.89226	2.1
39008	1.89226	2.1
39009	1.89226	2.1
39010	1.89226	2.1
39011	1.89226	2.1
39012	1.89226	2.1
39013	1.09766	2.1
39014	1.04263	2.1
39015	0.881794	2.1
39016	0.894188	2.1
39017	0.795402	2.1
39018	0.99524	2.1
39019	1.00647	2.1
39020	1.00647	2.1
39021	1.15401	2.1
39022	0.881789	2.1
39023	0.850526	2.1
39024	1.04263	2.1
39025	1.4054	0.933108
39026	1.04788	2.1
39027	1.70302	1.17332
39028	1.41375	2.1
39029	2.82706	2.1
39030	1.70302	0.70503
39031	1.38776	2.1

39032	1.59457	2.1
39033	1.41375	2.1
39034	1.77261	0.660366
39035	1.70302	2.1
39036	1.89226	2.1
39037	1.89226	2.1
39038	1.89226	2.1
39039	1.89226	2.1
39040	3.78453	2.1
39041	2.18042	2.1
39042	1.74738	2.1
39043	2.08433	2.1
39044	1.66347	2.1
39045	3.83171	2.1
39046	1.74738	2.1
39047	1.1181	2.1
39048	1.66347	2.1
39049	1.06543	2.1
39050	2.81281	2.1
39051	1.89226	2.1
39052	1.89226	2.1
39053	1.89226	2.1
39054	1.89226	2.1
39055	3.78453	2.1
39056	1.89227	2.1
39057	1.89227	2.1
39058	1.89227	2.1
39059	1.89227	2.1
39060	3.78453	2.1
39061	2.08433	2.1
39062	1.66347	2.1
39063	1.97092	2.1
39064	1.57431	2.1
39065	3.65864	2.1
39066	1.66347	2.1
39067	1.01474	2.1
39068	1.01474	2.1
39069	1.06544	2.1
39070	1.57431	2.1
39071	1.625 2.1	
39072	1.89226	2.1
39073	1.89226	2.1
39074	1.89226	2.1
39075	1.89226	2.1
39076	1.89226	2.1
39077	1.89226	2.1
39078	1.08467	2.1
39079	1.87228	2.1
39080	1.73305	2.1
39081	1.73305	2.1
39082	1.25565	2.1
39083	1.25565	2.1
39084	0.695361	0.0638993
39085	0.799996	0.16014
39086	1.13751	0.178233
39087	1.40945	0.26056
39088	1.40944	0.43326
39089	0.799999	0.05

39090	1.13751	0.135374
39091	0.695363	0.05
39092	1.01059	0.114652
39093	1.01059	0.155802
39094	0.926908	0.288758
39095	0.926909	0.05
39096	1.05481	2.1
39097	1.78069	2.1
39098	1.08074	2.1
39099	1.83284	2.1
39100	2.86143	2.1
39101	1.78069	2.1
39102	2.22044	2.1
39103	1.83284	2.1
39104	2.28696	1.12261
39105	4.06766	2.1
39106	1.07196	2.1
39107	1.64955	2.1
39108	2.02665	2.1
39109	2.0284	2.1
39110	1.08074	2.1
39111	1.08074	2.1
39112	1.64955	2.1
39113	2.28696	1.81403
39114	1.92033	2.1
39115	2.1004	2.1
39116	3.74988	2.1
39117	2.83787	0.0848867
39118	1.70303	1.38718
39119	1.70303	0.101929
39120	1.83195	0.188213
39121	2.64075	0.307166
39122	2.70231	1.27609
39123	1.2087	2.07196
39124	1.52909	0.182603
39125	1.43063	0.123309
39126	1.10586	1.99086
39127	2.63494	0.815514
39128	1.89227	1.67654
39129	2.10732	2.1
39130	2.10732	2.1
39131	2.2365	2.1
39132	1.89227	1.86559
39133	2.02145	2.1
39134	1.89226	0.28577
39135	1.89226	2.05131
39136	1.89226	0.492921
39137	1.89226	1.8091
39138	1.89226	0.204101
39139	1.89226	2.06065
39140	0.51926	2.07909
39141	0.552927	2.0589
39142	0.691235	0.320842
39143	0.715314	0.176881
39144	0.619684	0.116139
39145	0.619684	2.1
39146	0.624477	2.1
39147	0.624477	0.105

39148	0.519262	0.943634
39149	0.691234	0.928589
39150	0.715313	1.17816
39151	0.552928	0.883674
39152	1.89226	2.1
39153	1.87321	2.1
39154	1.87321	2.1
39155	1.89658	2.1
39156	2.0815	2.1
39157	2.14789	2.1
39158	0.946128	2.1
39159	0.946128	2.1
39160	0.946133	0.197045
39161	0.946133	0.25957
39162	0.94613	0.147785
39163	0.94613	2.1
39164	0.946131	0.423961
39165	0.946131	2.1
39166	0.946131	0.666949
39167	0.946131	0.719187
39168	0.946131	1.06574
39169	0.946131	1.02542
39170	1.99297	0.932805
39171	2.19514	0.19523
39172	2.46153	0.551998
39173	2.187 0.427093	
39174	4.45451	0.485967
39175	2.0815	0.34981
39176	2.33402	0.2202
39177	2.46154	0.595844
39178	2.83046	0.11778
39179	4.94496	0.257819
39180	2.46153	0.476569
39181	2.187 0.30834	
39182	2.27351	0.667901
39183	2.63356	0.160132
39184	4.73504	0.374046
39185	2.46154	0.530111
39186	2.83447	0.098109
39187	2.74513	0.209659
39188	2.5731	0.42863
39189	2.63357	0.179775
39190	2.46154	0.339445
39191	2.80398	2.1
39192	2.60406	2.1
39193	2.73988	2.1
39194	2.57466	2.1
39195	5.34394	2.1
39196	1.89226	1.38357
39197	1.89226	0.583754
39198	1.89226	0.151555
39199	1.89226	1.49296
39200	1.89226	0.293689
39201	1.89226	2.04594
39202	1.89224	1.64023
39203	1.89224	0.655537
39204	1.89224	0.436959
39205	1.89224	1.84048

39206	1.89224	0.687876
39207	1.89224	1.9955
39208	1.40245	1.11138
39209	1.40246	1.88461
39210	0.946117	1.37116
39211	0.946117	1.92992
39212	1.17635	0.842478
39213	1.40246	1.94171
39214	0.946112	0.829806
39215	0.946112	1.91507
39216	0.946127	1.40342
39217	1.17637	0.848397
39218	0.946102	2.00322
39219	1.40244	1.15292
39220	2.53269	2.1
39221	1.70302	2.1
39222	1.56194	2.1
39223	2.17516	2.1
39224	4.19807	2.1
39225	1.89226	2.1
39226	1.89226	2.06769
39227	1.89226	2.1
39228	1.89226	2.1
39229	3.78453	2.1
39230	2.57276	2.1
39231	1.89226	2.1
39232	1.89226	2.1
39233	1.89226	2.1
39234	2.32525	2.1
39235	2.32525	2.1
39236	1.15171	2.1
39237	2.17517	2.1
39238	1.41995	2.1
39239	1.41995	2.1
39240	1.80252	2.1
39241	1.80252	2.1
39242	1.89226	2.1
39243	1.89226	2.1
39244	1.89226	2.1
39245	1.89226	1.39179
39246	3.78453	2.1
39247	0.946133	2.1
39248	0.946133	2.1
39249	1.16263	2.1
39250	1.04687	2.1
39251	0.946133	2.1
39252	0.946133	2.1
39253	1.04687	2.1
39254	1.04687	2.1
39255	0.946133	2.1
39256	1.04687	2.1
39257	0.946132	2.1
39258	1.16263	2.1
39259	0.682281	1.80272
39260	0.669233	2.0861
39261	1.21859	2.08659
39262	1.31629	1.48346
39263	0.916158	1.11677

39264	0.916158	2.1
39265	0.971658	1.76613
39266	0.971658	1.92035
39267	0.669234	1.93014
39268	1.31629	1.50886
39269	0.682283	1.83345
39270	1.21858	1.9916
39271	0.669232	1.59025
39272	0.623495	1.93567
39273	1.36428	1.74338
39274	1.31629	1.12754
39275	0.946475	0.748792
39276	0.946476	2.1
39277	0.99331	1.71816
39278	0.99331	1.75951
39279	1.36428	1.62017
39280	0.623497	1.15823
39281	0.669234	1.72672
39282	1.31629	1.3112
39283	2.02069	0.05
39284	1.46253	0.05
39285	2.51479	0.05
39286	1.87136	0.05
39287	3.89203	0.05
39288	1.46253	0.05
39289	1.38536	0.05
39290	1.38536	0.05
39291	1.05579	0.05
39292	1.73281	0.05
39293	1.46916	0.05
39294	2.51479	0.05
39295	1.87136	0.05
39296	2.23338	0.05
39297	2.15603	0.05
39298	4.55146	0.05
39299	0.822553	0.05
39300	0.822563	0.05
39301	0.842935	0.05
39302	0.842935	0.05
39303	0.744203	0.05
39304	0.675346	0.05
39305	0.874795	0.05
39306	1.00453	0.05
39307	1.00454	0.05
39308	0.782417	0.05
39309	0.675337	0.05
39310	0.858007	0.05
39311	0.946132	1.42197
39312	1.24847	0.925557
39313	0.946132	1.27237
39314	1.16806	1.44615
39315	1.24847	0.520193
39316	1.24847	1.8778
39317	0.946133	0.417442
39318	0.946133	1.74112
39319	0.946133	1.19047
39320	0.946133	0.67136
39321	1.16805	1.47418

39322	1.24847	0.868617
39323	1.2364	1.42116
39324	0.946129	1.16107
39325	0.946133	1.37693
39326	1.24847	1.1208
39327	1.2364	0.875693
39328	1.2364	1.65739
39329	0.946131	0.606398
39330	0.946131	2.00621
39331	0.946131	1.35197
39332	0.946131	0.939654
39333	1.2364	1.74602
39334	1.24847	1.01867
39335	1.89226	1.45184
39336	1.89226	1.28539
39337	1.89226	0.846678
39338	1.89226	0.951604
39339	1.89226	1.39378
39340	1.89226	1.56062
39341	1.89226	1.32494
39342	1.89226	0.987342
39343	1.89226	0.80828
39344	1.89226	1.33608
39345	1.89226	1.01217
39346	1.89226	1.50906
39347	1.69014	1.17069
39348	1.09194	1.35497
39349	1.13537	1.49792
39350	1.13537	1.42441
39351	1.58481	0.770049
39352	1.58481	1.04286
39353	1.58147	0.957546
39354	1.13537	1.10615
39355	1.69015	1.23239
39356	1.01179	0.695878
39357	1.69015	1.18137
39358	1.01179	1.31449
39359	0.946132	1.63325
39360	0.946132	1.04344
39361	1.28543	0.230877
39362	1.07341	1.75756
39363	1.07341	1.62684
39364	1.07341	0.052516
39365	0.946132	0.36796
39366	0.946132	0.330777
39367	0.946133	0.531586
39368	1.28543	0.463562
39369	1.07341	0.426412
39370	0.946132	0.875642
39371	1.89226	0.667479
39372	1.89226	0.132795
39373	1.89226	0.0979173
39374	1.89226	2.00299
39375	1.89226	0.0935938
39376	1.89226	2.1
39377	0.805744	2.1
39378	0.988659	1.0419
39379	0.782044	0.0920397

39380	0.578223	0.532031
39381	0.77823	0.110951
39382	0.778231	2.1
39383	0.788653	0.0724537
39384	0.788653	1.67597
39385	0.988662	0.86262
39386	0.782042	0.851066
39387	0.805745	0.964911
39388	0.578222	0.677764
39389	1.89226	0.573383
39390	1.89226	0.544902
39391	1.89226	0.119276
39392	1.89226	2.01841
39393	1.89226	0.0531985
39394	1.89226	2.1
39395	1.97732	1.53548
39396	1.97819	0.364029
39397	1.56408	0.129458
39398	1.56408	2.03143
39399	2.33986	0.277409
39400	2.33986	2.1
39401	1.84516	2.1
39402	2.21152	2.1
39403	1.90702	2.1
39404	1.54599	2.1
39405	3.75751	2.1
39406	1.89224	2.1
39407	1.89224	2.1
39408	1.89224	2.1
39409	1.89224	2.1
39410	1.89224	2.1
39411	1.89224	2.1
39412	1.47672	2.1
39413	1.54601	2.1
39414	1.27697	2.1
39415	1.54601	2.1
39416	1.638 2.1	
39417	1.90705	2.1
39418	0.946132	2.1
39419	0.946132	2.1
39420	0.946131	2.1
39421	0.946131	2.1
39422	0.946131	2.1
39423	0.946131	2.1
39424	0.946132	2.1
39425	0.946132	2.1
39426	0.946132	2.1
39427	0.946132	2.1
39428	0.946131	2.1
39429	0.946131	2.1
39430	0.946128	2.1
39431	0.946128	2.1
39432	1.32062	2.1
39433	1.4164	2.1
39434	0.946129	2.1
39435	0.946129	2.1
39436	1.32062	2.1
39437	1.32062	2.1

39438	1.32062	2.1
39439	0.94613	2.1
39440	1.41639	2.1
39441	0.946129	2.1
39442	1.98979	0.05
39443	1.65977	0.05
39444	2.21772	0.05
39445	2.21772	0.05
39446	1.48866	0.05
39447	1.48866	0.05
39448	0.973129	0.05
39449	0.973129	0.05
39450	0.980954	0.05
39451	0.980954	0.05
39452	0.845218	0.05
39453	1.13981	0.05
39454	0.829886	0.05
39455	1.10886	0.05
39456	1.10886	0.05
39457	1.13982	0.05
39458	0.845219	0.05
39459	0.829886	0.05
39460	0.946132	1.89531
39461	0.946132	2.1
39462	1.3352	2.1
39463	1.41432	2.1
39464	0.946133	2.1
39465	0.946133	0.46628
39466	1.3352	2.1
39467	1.3352	2.1
39468	1.3352	2.1
39469	0.946132	2.1
39470	1.41432	2.1
39471	0.946133	2.1
39472	2.70097	2.1
39473	1.88175	2.1
39474	1.51482	2.1
39475	1.88175	2.1
39476	2.37784	2.1
39477	2.82864	2.1
39478	1.89227	0.05
39479	1.89227	0.05
39480	1.89227	0.05
39481	1.89227	0.05
39482	3.78453	0.05
39483	1.89226	0.05
39484	2.13295	0.05
39485	2.43203	0.05
39486	2.43203	0.05
39487	1.89226	0.05
39488	1.89226	0.05
39489	0.946133	0.05
39490	0.946133	0.05
39491	0.946132	0.05
39492	0.946132	0.05
39493	0.946132	0.05
39494	0.946132	0.05
39495	0.946133	0.05

39496	0.946133	0.05
39497	0.946132	0.05
39498	0.946132	0.05
39499	0.946132	0.05
39500	0.946132	0.05
39501	1.54987	2.1
39502	1.24128	2.1
39503	1.24128	2.1
39504	1.50738	2.1
39505	1.75702	2.1
39506	2.02312	2.1
39507	1.89289	2.1
39508	1.92264	2.1
39509	1.72994	2.1
39510	1.75702	2.1
39511	3.67966	2.1
39512	1.75702	2.1
39513	0.994291	2.1
39514	0.994291	2.1
39515	1.24128	2.1
39516	1.4964	2.1
39517	1.74339	2.1
39518	1.72993	2.1
39519	1.49641	2.1
39520	1.49641	2.1
39521	1.75702	2.1
39522	1.3939	2.1
39523	1.65452	2.1
39524	1.19964	2.1
39525	1.53296	2.1
39526	1.19964	2.1
39527	1.36861	2.1
39528	1.42624	2.1
39529	1.59521	2.1
39530	1.19964	2.1
39531	1.42624	2.1
39532	1.07156	2.1
39533	1.36226	2.1
39534	2.5619	2.1
39535	2.14058	1.19014
39536	1.93569	2.1
39537	2.36221	1.55764
39538	2.5753	1.46332
39539	4.50279	2.06669
39540	2.14057	1.90563
39541	2.20831	2.1
39542	2.64725	1.14394
39543	2.57529	1.83204
39544	4.78782	1.81609
39545	2.64725	1.40919
39546	2.57529	2.01607
39547	1.89226	1.12423
39548	1.89226	0.665324
39549	4.53951	1.6383
39550	2.0815	2.1
39551	1.86137	2.1
39552	1.86137	2.1
39553	1.86159	2.1

39554	2.0815	2.1
39555	2.10235	2.1
39556	1.33552	2.1
39557	2.32417	2.1
39558	1.18872	2.1
39559	1.93101	2.1
39560	3.26653	2.1
39561	1.89226	2.1
39562	1.89226	2.1
39563	1.89226	2.1
39564	1.89226	2.1
39565	1.89226	2.1
39566	1.89226	2.1
39567	0.955191	2.1
39568	1.03372	2.1
39569	0.946131	2.1
39570	0.946131	2.1
39571	0.930654	2.1
39572	0.930654	2.1
39573	1.04233	2.1
39574	1.05247	2.1
39575	0.955341	2.1
39576	0.946131	2.1
39577	1.02375	2.1
39578	1.13537	2.1
39579	1.1549	2.1
39580	1.93101	2.1
39581	1.91108	2.1
39582	1.52487	2.1
39583	1.3076	2.1
39584	1.06985	2.1
39585	1.89226	2.1
39586	1.89226	2.1
39587	1.89226	2.1
39588	1.89226	2.1
39589	1.89226	2.1
39590	1.89226	2.1
39591	1.58713	2.1
39592	2.6845	2.1
39593	1.58742	2.1
39594	2.74201	2.1
39595	4.27191	2.1
39596	2.68449	2.1
39597	1.89226	2.1
39598	2.74201	2.1
39599	1.89226	2.1
39600	4.57675	2.1
39601	1.57523	2.1
39602	2.74201	2.1
39603	2.8146	2.1
39604	2.8146	2.1
39605	1.58742	2.1
39606	1.58742	2.1
39607	0.946131	2.1
39608	0.946131	2.1
39609	1.371	2.1
39610	1.371	2.1
39611	0.946127	2.1

39612	0.946127	2.1
39613	1.37101	2.1
39614	1.4073	2.1
39615	0.94613	2.1
39616	1.4073	2.1
39617	1.371 2.1	
39618	0.946129	2.1
39619	1.44801	2.08122
39620	1.06821	2.05958
39621	2.00542	1.97079
39622	2.27703	2.1
39623	3.34524	1.89801
39624	1.70301	0.05
39625	1.89226	0.177844
39626	1.65809	0.0596226
39627	1.89226	0.14388
39628	1.62758	0.0604713
39629	1.85744	0.128866
39630	1.89226	0.05
39631	1.89226	0.17134
39632	1.89226	0.0974955
39633	1.89226	0.0858829
39634	3.78452	0.108487
39635	1.89226	0.169555
39636	1.89226	0.0643056
39637	1.89226	0.0845844
39638	1.89226	0.234936
39639	3.78452	0.120588
39640	0.851541	0.209209
39641	0.851541	0.172814
39642	0.91193	0.0951315
39643	0.824837	0.118921
39644	0.854559	0.101242
39645	0.851534	0.398935
39646	0.81851	0.175606
39647	0.717219	0.0544122
39648	0.871743	0.147441
39649	0.837985	0.107876
39650	0.82902	0.113338
39651	0.82902	0.179734
39652	1.89226	0.153543
39653	1.92659	0.124494
39654	2.03235	0.145033
39655	1.89226	0.293407
39656	2.03235	0.0852467
39657	1.89226	0.150199
39658	0.585557	0.362267
39659	0.585557	0.172726
39660	1.22612	0.189595
39661	1.09436	0.344404
39662	0.851537	0.514816
39663	0.526987	0.210973
39664	0.983879	0.142917
39665	1.04072	0.310854
39666	0.585526	0.220648
39667	0.792325	0.248397
39668	0.946129	0.28407
39669	0.946129	0.261555

39670	1.68152	2.1
39671	1.81445	2.1
39672	1.68152	2.1
39673	1.86792	2.1
39674	1.65888	2.1
39675	1.84529	2.1
39676	1.81445	2.1
39677	1.57885	2.1
39678	1.65889	2.1
39679	1.48594	2.1
39680	3.23773	2.1
39681	1.68154	2.1
39682	1.65891	2.1
39683	1.53296	2.1
39684	1.56299	2.1
39685	3.24453	2.1
39686	1.56299	2.1
39687	1.48596	2.1
39688	1.56299	2.1
39689	1.65891	2.1
39690	1.46568	2.1
39691	1.5616	2.1
39692	2.68785	2.1
39693	2.08831	2.1
39694	2.66919	2.1
39695	2.1024	2.1
39696	4.79025	2.1
39697	2.08832	2.1
39698	1.15478	2.1
39699	1.15478	2.1
39700	1.09062	2.1
39701	2.10241	2.1
39702	2.03825	2.1
39703	2.65348	2.1
39704	2.1024	2.1
39705	2.12313	2.1
39706	2.12313	2.1
39707	2.66919	2.1
39708	2.66919	2.1
39709	0.813169	2.1
39710	0.813169	2.1
39711	0.825788	2.1
39712	0.825788	2.1
39713	0.612902	2.1
39714	0.577387	2.1
39715	1.0512	2.1
39716	1.06157	2.1
39717	1.06157	2.1
39718	0.612902	2.1
39719	0.577387	2.1
39720	1.0512	2.1
39721	1.27227	2.1
39722	1.91724	2.1
39723	1.32591	2.1
39724	1.99837	2.1
39725	3.24315	2.1
39726	1.89223	2.1
39727	1.89223	2.1

39728	1.89223	2.1
39729	1.89223	2.1
39730	3.78446	2.1
39731	1.89224	2.1
39732	1.89224	1.21565
39733	1.89224	2.1
39734	1.89224	2.1
39735	3.78447	2.1
39736	1.91724	2.1
39737	2.321	2.1
39738	1.99838	2.1
39739	2.4149	2.1
39740	4.33214	2.1
39741	1.34817	2.1
39742	1.9984	2.1
39743	2.06238	2.1
39744	2.06238	2.1
39745	1.32593	2.1
39746	1.32593	2.1
39747	1.89226	2.1
39748	1.89226	2.1
39749	1.89226	2.1
39750	1.89226	2.1
39751	1.89226	2.1
39752	1.89226	2.1
39753	1.89226	2.1
39754	1.89226	2.1
39755	1.89226	2.1
39756	1.89226	1.5842
39757	3.78452	2.1
39758	1.9984	2.1
39759	2.41494	2.1
39760	2.06239	2.1
39761	2.5194	2.1
39762	4.47732	2.1
39763	1.19901	1.01069
39764	1.16661	2.1
39765	1.56859	2.1
39766	1.61029	1.2593
39767	2.7676	2.1
39768	1.56859	2.1
39769	1.88637	0.209183
39770	1.61029	0.115692
39771	1.61029	2.1
39772	1.856	0.130326
39773	1.856	2.1
39774	1.856	0.820894
39775	1.57916	2.1
39776	1.56859	1.33031
39777	1.56859	2.1
39778	1.57916	2.1
39779	1.23475	2.1
39780	0.890924	0.05
39781	1.07608	0.0549705
39782	0.72877	0.0995954
39783	0.910106	0.0924554
39784	1.00196	0.0581937
39785	1.09519	0.298385

39786	0.839967	0.0500044
39787	0.719043	0.05
39788	0.893859	0.05
39789	0.942408	0.153779
39790	0.873497	0.0573678
39791	0.873497	0.205613
39792	1.53046	0.05
39793	1.89994	0.0591812
39794	1.049	0.05
39795	1.049	0.05
39796	1.52512	0.05
39797	1.89994	0.05
39798	1.65573	1.06167
39799	1.89226	0.424928
39800	1.89226	0.393235
39801	2.73329	1.24243
39802	3.548	1.21131
39803	1.65573	0.554357
39804	2.42671	0.790913
39805	2.73329	0.383103
39806	1.89226	0.183634
39807	2.73329	0.985781
39808	1.89226	0.703893
39809	0.941078	0.0638488
39810	1.01599	0.083092
39811	0.818936	0.056227
39812	0.651554	0.0895833
39813	0.904729	0.0848775
39814	0.665756	0.0855148
39815	0.843423	0.11358
39816	0.918649	0.117617
39817	0.855806	0.171063
39818	0.814384	0.05
39819	0.844437	0.125081
39820	0.844437	0.0982609
39821	2.22558	1.2399
39822	1.95785	0.110493
39823	2.07065	0.268364
39824	1.89142	0.242453
39825	2.07065	1.77048
39826	1.89142	1.70559
39827	0.640813	0.124136
39828	0.951654	0.340771
39829	0.797442	0.0884601
39830	0.535364	0.161072
39831	0.525269	0.237316
39832	0.620874	0.170653
39833	0.916953	0.205501
39834	0.969374	0.202921
39835	0.788925	0.629837
39836	0.69596	0.05
39837	0.743154	0.230461
39838	0.743154	0.326758
39839	1.89226	2.1
39840	1.89226	1.74625
39841	1.89226	0.548814
39842	1.89226	2.1
39843	3.78452	1.17666

39844	1.32541	1.58015
39845	1.13722	1.09045
39846	0.91103	2.1
39847	0.881123	2.1
39848	1.03363	2.1
39849	1.03364	1.96436
39850	1.1505	1.82778
39851	1.1505	2.1
39852	1.32539	2.1
39853	1.07662	2.1
39854	0.881121	2.06286
39855	0.962303	1.93027
39856	1.89226	1.05751
39857	1.89226	2.1
39858	1.89226	2.1
39859	1.89226	1.40087
39860	3.78453	2.1
39861	1.89226	0.465396
39862	1.89226	2.1
39863	1.89226	1.77273
39864	1.89226	1.39213
39865	1.89226	1.81297
39866	1.89226	1.70545
39867	1.27606	1.80653
39868	1.07664	1.4008
39869	0.905895	2.1
39870	0.696099	2.1
39871	0.984902	2.1
39872	0.984905	1.90543
39873	1.01878	2.1
39874	1.01878	0.648604
39875	1.27604	2.1
39876	1.06044	2.1
39877	1.13192	2.1
39878	0.696111	2.1
39879	1.89226	0.102994
39880	1.89226	0.05
39881	1.89226	0.05
39882	1.89226	0.161316
39883	1.89226	0.05
39884	1.89226	0.141815
39885	1.52615	0.0851534
39886	1.01294	0.05
39887	1.01294	0.511975
39888	1.18549	0.05
39889	1.38963	0.05
39890	1.56218	0.05
39891	1.89226	0.0727807
39892	2.40801	0.05
39893	2.58101	0.126066
39894	2.58101	0.185934
39895	1.89226	0.0514492
39896	1.89226	0.05
39897	2.24071	2.1
39898	2.79317	2.1
39899	1.99136	2.1
39900	2.6167	2.1
39901	4.85741	2.1

39902	2.79317	2.1
39903	1.89226	0.197386
39904	1.89226	2.1
39905	1.89226	0.714417
39906	2.6167	2.1
39907	2.6167	0.306143
39908	1.08354	2.1
39909	1.08354	2.1
39910	1.10222	2.1
39911	1.10222	2.1
39912	1.2275	2.1
39913	1.30835	2.1
39914	0.995678	2.1
39915	0.877412	2.1
39916	0.877411	2.1
39917	1.2275	2.1
39918	0.995678	2.1
39919	1.30835	2.1
39920	1.05793	0.569223
39921	1.46441	0.0979697
39922	1.0562	0.392133
39923	1.0562	0.18353
39924	1.41787	0.37962
39925	1.41787	0.208112
39926	1.41787	0.239693
39927	1.52909	0.714743
39928	1.46441	0.209442
39929	1.46441	0.533704
39930	1.43063	0.111085
39931	1.43063	0.278528
39932	1.89226	0.715442
39933	1.89226	0.153832
39934	1.89226	0.163127
39935	1.89226	0.649875
39936	3.78453	0.464311
39937	1.89227	0.529743
39938	1.89227	0.279774
39939	1.89227	0.254247
39940	1.89227	0.148337
39941	1.89227	0.206186
39942	1.89227	0.17658
39943	1.00763	0.272288
39944	1.41787	0.19146
39945	1.05793	0.272218
39946	1.05793	0.379991
39947	1.37265	0.235943
39948	1.37265	0.311655
39949	1.37265	0.308495
39950	1.43063	0.294912
39951	1.41787	0.2323
39952	1.41787	0.804211
39953	1.38247	0.0926119
39954	1.38247	0.521406
39955	0.946132	0.281761
39956	0.946132	0.218677
39957	0.946132	0.176449
39958	0.946132	0.05
39959	0.946131	0.05

39960	0.946131	0.184946
39961	0.946132	0.474541
39962	0.946132	0.175185
39963	0.946132	0.105137
39964	0.946132	0.0834172
39965	0.946132	0.159082
39966	0.946132	0.114576
39967	1.89226	0.423824
39968	1.89226	0.0866509
39969	1.89226	0.21348
39970	1.89226	0.287566
39971	1.89226	0.165514
39972	1.89226	0.300929
39973	1.19645	0.997241
39974	1.89183	0.253977
39975	1.91106	0.626985
39976	1.35306	0.261699
39977	1.75446	0.603291
39978	1.19645	0.240963
39979	2.37576	0.138287
39980	1.90139	2.1
39981	2.1845	2.1
39982	1.86568	2.05201
39983	4.24145	2.1
39984	2.29435	2.1
39985	1.40602	2.1
39986	1.40602	0.493679
39987	1.55351	2.1
39988	1.86568	2.1
39989	2.01317	2.1
39990	2.18452	2.1
39991	1.8657	0.05
39992	2.01399	0.497573
39993	1.70141	2.1
39994	3.87969	0.841157
39995	0.774999	2.1
39996	0.774999	0.667866
39997	0.778721	1.97092
39998	0.778721	2.1
39999	0.628348	2.1
40000	0.703016	2.1
40001	0.932848	1.39577
40002	0.850704	2.1
40003	0.850704	2.1
40004	0.628348	2.1
40005	0.703015	1.0361
40006	0.932848	2.1
40007	1.65243	0.159926
40008	1.34412	0.05204
40009	1.45569	1.11721
40010	1.1315	1.23766
40011	2.78393	0.257184
40012	1.48901	2.1
40013	1.805	2.1
40014	1.48901	2.1
40015	1.71717	2.1
40016	1.60635	1.16986
40017	1.83451	2.1

40018	1.805	2.1
40019	1.65243	0.118693
40020	1.60635	2.07717
40021	1.45569	0.0841141
40022	3.25879	0.114943
40023	1.27875	2.1
40024	1.60635	2.1
40025	1.27875	2.1
40026	1.48901	2.1
40027	1.40014	2.1
40028	1.6104	2.1
40029	0.715199	0.159537
40030	0.715199	2.1
40031	0.712714	2.1
40032	0.712714	0.05
40033	0.727843	0.05
40034	0.619713	0.0565425
40035	0.803177	2.1
40036	0.70007	1.93829
40037	0.700069	0.141723
40038	0.619713	0.072432
40039	0.803177	0.13182
40040	0.727844	0.111877
40041	1.14357	2.1
40042	2.3506	2.09997
40043	1.14357	2.1
40044	2.68773	2.08512
40045	1.57829	2.1
40046	2.68773	2.08556
40047	2.00551	2.1
40048	2.65811	1.96039
40049	2.07203	2.1
40050	2.72117	0.05
40051	4.72667	2.1
40052	0.946131	0.0875863
40053	0.946131	2.1
40054	1.32905	2.1
40055	1.32905	0.0647427
40056	0.94613	0.96233
40057	0.94613	1.82815
40058	1.32905	1.64176
40059	1.36058	1.2455
40060	1.36058	0.135814
40061	0.946131	0.0683949
40062	1.32905	1.23587
40063	0.946131	1.84648
40064	1.21649	0.05
40065	1.21649	2.1
40066	1.21459	2.1
40067	1.21459	2.1
40068	1.39508	1.38342
40069	1.36057	0.530003
40070	1.03601	2.1
40071	1.07438	2.1
40072	1.07439	1.71796
40073	1.39509	1.87632
40074	1.036	1.9001
40075	1.36056	1.36842

40076	2.79015	0.232853
40077	2.72114	2.1
40078	1.89225	0.643123
40079	1.89225	2.1
40080	1.89225	2.02007
40081	2.72114	0.129453
40082	0.876258	2.1
40083	0.892889	2.1
40084	1.34352	2.1
40085	1.27985	2.1
40086	1.07462	2.1
40087	1.07462	2.1
40088	1.09812	2.1
40089	1.09812	2.1
40090	0.892888	2.1
40091	1.34352	2.1
40092	0.876256	2.1
40093	1.27985	2.1
40094	1.39725	1.95727
40095	1.30029	0.135567
40096	1.31415	0.401685
40097	1.41336	1.60571
40098	2.71366	0.996728
40099	1.89226	0.203622
40100	1.89226	0.962779
40101	1.89226	1.82538
40102	1.89226	0.182825
40103	3.78453	0.454224
40104	0.946133	0.711297
40105	0.946133	0.338632
40106	1.38709	0.0805097
40107	1.39437	0.491824
40108	0.946132	0.083791
40109	0.946132	0.276779
40110	1.39437	0.842121
40111	1.39437	0.0803937
40112	0.946132	0.129227
40113	1.39437	0.0686378
40114	1.38709	0.255191
40115	0.946132	0.322226
40116	0.706681	1.98751
40117	0.711908	1.72903
40118	0.657077	0.190356
40119	0.661003	0.614843
40120	0.684188	0.173997
40121	0.684188	1.60844
40122	0.683496	2.1
40123	0.683496	0.271219
40124	0.711908	0.800263
40125	0.661003	0.798461
40126	0.657076	0.998987
40127	0.706681	0.889502
40128	0.946132	1.86777
40129	0.946132	1.03735
40130	0.946132	0.318336
40131	0.946132	0.1393
40132	0.946132	0.150467
40133	0.946132	1.1357

40134	0.946133	0.394183
40135	0.946133	1.87867
40136	0.946133	0.472979
40137	0.946133	0.465492
40138	0.946132	0.75791
40139	0.946132	0.807805
40140	2.79029	0.0718057
40141	1.89227	1.08723
40142	2.78873	0.197677
40143	2.78873	0.139742
40144	1.89383	0.95257
40145	1.89227	0.330932
40146	1.43669	2.1
40147	2.11984	2.1
40148	2.24481	2.1
40149	2.24481	2.1
40150	1.20311	2.1
40151	1.20311	2.1
40152	0.49703	2.1
40153	0.601553	2.1
40154	1.05992	2.1
40155	0.991694	2.1
40156	0.787558	2.1
40157	0.787558	2.1
40158	0.805689	2.1
40159	0.805689	2.1
40160	0.991694	2.1
40161	0.49703	2.1
40162	1.05992	2.1
40163	0.601553	2.1
40164	2.45905	2.1
40165	1.89226	2.1
40166	1.89226	2.1
40167	1.89226	2.1
40168	2.47395	2.1
40169	2.47395	2.1
40170	1.89226	2.1
40171	1.89226	2.06177
40172	1.89226	2.1
40173	1.89226	1.69337
40174	3.78452	2.1
40175	1.38427	2.1
40176	2.31203	1.18371
40177	2.28743	1.21454
40178	2.28743	2.1
40179	1.63384	0.964758
40180	1.63384	2.1
40181	2.42723	2.1
40182	1.89226	2.1
40183	1.89226	2.1
40184	1.89226	2.1
40185	2.45905	2.1
40186	2.45905	2.1
40187	0.946131	0.216818
40188	0.946131	2.1
40189	0.946131	2.1
40190	0.946131	2.1
40191	0.94613	1.40481

40192	0.94613	2.1
40193	0.946132	2.1
40194	0.946132	2.1
40195	0.946131	2.1
40196	0.946131	2.1
40197	0.946131	2.1
40198	0.946131	2.1
40199	0.989441	2.1
40200	0.989441	2.1
40201	0.993918	2.1
40202	0.993918	2.1
40203	1.16644	2.1
40204	1.15601	1.15421
40205	0.818308	2.1
40206	0.816922	2.1
40207	0.818307	2.1
40208	1.16644	2.1
40209	1.15601	2.1
40210	0.816922	2.1
40211	0.996778	0.05
40212	2.44375	0.05
40213	0.996778	0.05
40214	1.92547	0.05
40215	1.56091	0.05
40216	2.4896	0.05
40217	1.56091	0.05
40218	2.44375	0.05
40219	1.90456	0.05
40220	1.90456	0.05
40221	2.81853	0.05
40222	2.47487	0.05
40223	1.32143	2.1
40224	1.43294	2.1
40225	1.37444	0.261929
40226	1.51691	2.1
40227	2.80738	1.98261
40228	1.89226	1.67935
40229	1.89226	2.1
40230	1.89226	0.399255
40231	1.89226	1.50999
40232	3.78453	1.18444
40233	1.89226	1.89974
40234	1.89226	2.1
40235	1.89226	1.01269
40236	1.89226	2.1
40237	3.78453	2.1
40238	1.43294	1.8131
40239	1.3268	2.1
40240	1.51691	1.63065
40241	1.44091	2.1
40242	2.87385	2.1
40243	1.37444	2.1
40244	1.51691	2.1
40245	1.4714	0.301886
40246	1.65434	1.73023
40247	3.02878	1.79802
40248	1.89226	2.1
40249	1.89226	2.06279

40250	1.89226	0.922021
40251	1.89226	1.83268
40252	3.78452	1.79006
40253	1.89226	1.49016
40254	1.89226	2.1
40255	1.89226	1.10622
40256	1.89226	2.1
40257	3.78453	2.1
40258	1.51691	2.0782
40259	1.44091	2.1
40260	1.65434	1.12258
40261	1.60647	2.1
40262	3.09526	2.1
40263	1.05013	2.1
40264	1.05013	2.1
40265	0.94613	2.1
40266	0.94613	2.1
40267	1.04959	2.1
40268	1.05014	2.1
40269	0.946129	2.1
40270	0.946129	2.1
40271	0.946131	2.1
40272	1.04958	2.1
40273	0.946131	2.1
40274	1.05013	2.1
40275	1.04959	2.1
40276	1.04959	2.1
40277	0.946131	2.1
40278	0.946131	2.1
40279	1.04765	2.1
40280	1.04959	2.1
40281	0.94613	2.1
40282	0.94613	2.1
40283	0.946131	2.1
40284	1.04765	2.1
40285	0.946131	2.1
40286	1.04959	2.1
40287	1.85744	2.1
40288	1.89226	2.1
40289	1.89226	2.1
40290	1.89226	2.1
40291	1.94839	2.1
40292	1.94839	2.1
40293	0.97419	2.1
40294	0.822054	2.1
40295	0.946123	2.1
40296	0.946123	2.1
40297	1.04202	2.1
40298	0.84898	2.1
40299	0.933307	2.1
40300	0.933307	2.1
40301	0.933285	2.1
40302	1.05018	2.1
40303	0.851528	2.1
40304	0.876787	2.1
40305	1.87528	0.0542596
40306	1.21137	0.05
40307	1.57893	0.05

40308	1.21137	0.0900541
40309	1.81182	0.05
40310	1.44426	0.0645587
40311	0.993833	0.05
40312	1.21137	0.0556529
40313	1.57893	0.0657505
40314	1.31774	0.05
40315	2.5291	0.05
40316	1.87528	0.0594745
40317	1.57893	0.05
40318	1.90728	0.05
40319	2.29851	0.0631446
40320	3.87744	0.0599026
40321	1.90728	0.05
40322	1.31774	0.05
40323	1.56339	0.05
40324	1.31774	0.0599862
40325	1.82458	0.05
40326	1.57893	0.05
40327	1.89226	0.45298
40328	1.89226	0.994283
40329	1.89226	0.790845
40330	1.89226	1.06714
40331	1.89226	0.832853
40332	1.89226	1.1226
40333	1.89226	0.843304
40334	1.89226	1.17931
40335	1.89226	1.35866
40336	1.89226	0.886743
40337	3.78452	1.1264
40338	1.89226	0.93331
40339	2.17251	0.816146
40340	2.45575	0.641119
40341	2.45575	0.798238
40342	1.89226	0.683977
40343	1.89226	0.85545
40344	1.08625	0.965301
40345	1.22788	0.724962
40346	0.54447	0.578808
40347	0.794861	0.689911
40348	0.967729	0.808655
40349	0.967729	0.909992
40350	0.913386	0.719055
40351	0.913386	0.558491
40352	1.08625	0.842155
40353	0.544469	0.670872
40354	0.794862	0.863166
40355	1.22788	0.710904
40356	0.946128	0.997823
40357	0.946128	1.28997
40358	0.946132	0.867453
40359	0.946132	0.873953
40360	0.94613	0.881551
40361	0.94613	1.00412
40362	0.94613	1.30558
40363	0.94613	0.695221
40364	0.94613	0.854094
40365	0.94613	0.869379

40366	0.94613	0.921452
40367	0.94613	1.04475
40368	0.946127	0.695967
40369	0.946127	0.726046
40370	1.08625	0.598123
40371	0.958779	0.750826
40372	1.08625	0.63603
40373	1.08625	0.681008
40374	0.946128	0.61957
40375	0.946128	0.724385
40376	0.946129	0.758894
40377	0.958777	0.780853
40378	1.08625	0.731135
40379	0.946129	0.814641
40380	2.16338	0.05
40381	1.94002	0.05
40382	1.98836	0.05
40383	2.60614	0.05
40384	1.5456	0.05
40385	2.16338	0.05
40386	1.19952	0.05
40387	2.34061	0.05
40388	1.21031	0.05
40389	2.1711	0.05
40390	3.54013	0.05
40391	1.13624	2.1
40392	1.70302	2.1
40393	1.8267	2.1
40394	1.11545	2.1
40395	2.85455	2.1
40396	1.82671	2.1
40397	1.91767	2.1
40398	1.81563	2.1
40399	1.91767	2.1
40400	1.71362	2.1
40401	1.83277	2.1
40402	1.66064	2.1
40403	0.996776	2.1
40404	0.996776	2.1
40405	1.11545	2.1
40406	1.54278	2.1
40407	1.66145	2.1
40408	1.66064	2.1
40409	1.7457	2.1
40410	1.81563	2.1
40411	1.81563	2.1
40412	1.54278	2.1
40413	1.54278	2.1
40414	2.71672	2.1
40415	2.70402	2.1
40416	2.64996	2.1
40417	2.69443	2.1
40418	5.39845	2.1
40419	2.71672	2.1
40420	2.54198	2.1
40421	2.54019	2.1
40422	2.69443	2.1
40423	5.23641	2.1

40424	2.64996	2.1
40425	2.69443	2.1
40426	2.68213	2.1
40427	2.61243	2.1
40428	5.30686	2.1
40429	2.54019	2.1
40430	2.69443	2.1
40431	2.68213	2.1
40432	2.5392	2.1
40433	5.23363	2.1
40434	1.89226	2.1
40435	2.34725	2.1
40436	2.22414	2.1
40437	2.34725	2.1
40438	1.76914	2.1
40439	1.89226	2.1
40440	0.946131	2.1
40441	0.946131	2.1
40442	1.11207	2.1
40443	1.03798	2.1
40444	0.94613	2.1
40445	0.94613	2.1
40446	1.03798	2.1
40447	1.03798	2.1
40448	0.946131	2.1
40449	1.03798	2.1
40450	0.946131	2.1
40451	1.11207	2.1
40452	1.89226	0.05
40453	1.89226	0.05
40454	1.89226	0.05
40455	1.89226	0.05
40456	1.89226	0.05
40457	1.89226	0.05
40458	1.89226	0.05
40459	1.89226	0.05
40460	1.89226	0.05
40461	1.89226	0.05
40462	1.89226	0.05
40463	1.89226	0.05
40464	1.89226	0.05
40465	1.89226	0.05
40466	1.89226	0.05
40467	1.89226	0.05
40468	1.89226	0.05
40469	1.89226	0.05
40470	1.44085	2.1
40471	1.31825	0.167223
40472	1.31923	0.602529
40473	1.31825	1.84321
40474	1.43311	0.358209
40475	1.43213	1.37899
40476	1.89226	0.202768
40477	1.89226	1.26043
40478	1.89226	0.346856
40479	1.89226	1.46814
40480	1.89226	0.155615
40481	1.89226	1.73919

40482	0.946133	0.609795
40483	0.946133	0.278151
40484	1.38969	0.0915632
40485	1.39587	0.224227
40486	0.946132	0.0945277
40487	0.946132	0.298561
40488	1.39587	0.645258
40489	1.39587	0.0624596
40490	0.946132	0.129284
40491	1.39587	0.0885229
40492	1.38969	0.243605
40493	0.946132	0.369302
40494	1.44085	2.1
40495	1.31923	0.100015
40496	1.33026	0.637931
40497	1.4617	1.73353
40498	2.78093	1.30932
40499	1.89226	0.152633
40500	1.89226	1.03158
40501	1.89226	0.212782
40502	1.89226	1.34949
40503	1.89226	0.113567
40504	1.89226	1.69347
40505	0.946133	0.396969
40506	0.946133	0.187108
40507	1.39587	0.12196
40508	1.40724	0.05
40509	0.946132	0.05
40510	0.946132	0.171727
40511	1.40724	0.349224
40512	1.40724	0.12526
40513	0.946132	0.13235
40514	1.40724	0.0853428
40515	0.946133	0.372624
40516	1.39587	0.165951
40517	1.15818	1.99048
40518	1.81846	1.80187
40519	1.52782	1.14654
40520	1.52782	2.1
40521	1.28095	0.85542
40522	1.28095	2.1
40523	2.56709	2.1
40524	1.4349	0.0726708
40525	1.4349	0.986033
40526	1.75471	1.19004
40527	2.29909	1.2744
40528	2.61889	2.1
40529	2.5671	2.1
40530	2.82858	2.1
40531	2.57368	2.1
40532	2.29909	1.56608
40533	5.12767	2.1
40534	2.29909	2.1
40535	1.09148	0.0690439
40536	1.09148	0.927931
40537	1.4349	0.579795
40538	1.99776	0.463329
40539	2.34119	1.59842

40540	2.57368	2.1
40541	1.99777	0.824847
40542	1.99777	2.1
40543	2.29909	2.1
40544	2.36071	2.1
40545	2.66203	2.1
40546	1.89226	0.360262
40547	1.89226	0.984406
40548	1.89226	0.633329
40549	1.89226	0.354173
40550	3.78453	0.411666
40551	0.946128	0.84875
40552	0.946128	1.81374
40553	0.946133	0.535641
40554	0.946133	0.850595
40555	0.946131	1.06142
40556	0.946131	1.32507
40557	0.946131	1.56835
40558	0.946131	0.929427
40559	0.94613	1.71396
40560	0.94613	1.06979
40561	0.946131	0.853394
40562	0.946131	1.33825
40563	1.89226	1.06592
40564	1.89226	1.27475
40565	1.89226	0.370827
40566	1.89226	1.27412
40567	3.78453	1.2986
40568	1.89226	1.37755
40569	1.89226	0.785584
40570	1.89226	0.454168
40571	1.89226	0.849713
40572	3.78453	1.00155
40573	1.89226	0.87035
40574	2.61682	1.84431
40575	1.89226	1.6087
40576	1.89226	1.73341
40577	1.89226	0.751079
40578	1.89226	1.71283
40579	1.89226	0.403014
40580	1.89226	0.446027
40581	1.89226	0.536988
40582	1.89226	0.666369
40583	3.78452	0.868933
40584	1.89226	0.354864
40585	1.89226	0.946576
40586	1.89226	0.328619
40587	1.89226	0.75049
40588	1.89226	0.625848
40589	1.89226	1.19981
40590	1.89227	0.42843
40591	1.89227	0.141792
40592	1.89227	0.901714
40593	1.89227	0.638058
40594	1.89227	0.439123
40595	1.89227	0.398235
40596	0.80447	1.04942
40597	0.534241	0.793014

40598	0.995175	0.909258
40599	0.92204	0.707075
40600	0.922042	0.159507
40601	0.534241	0.298132
40602	0.804468	0.638939
40603	0.995176	0.158642
40604	0.71543	0.307733
40605	0.71543	0.082942
40606	0.813988	0.351573
40607	0.813988	0.13844
40608	0.922045	0.146283
40609	0.942899	0.459136
40610	0.946132	0.288031
40611	0.946132	0.194019
40612	1.05169	0.05
40613	0.936661	0.342561
40614	0.922045	0.484431
40615	0.946133	0.147025
40616	1.01665	0.154132
40617	1.13008	0.05
40618	0.851529	0.135416
40619	0.851529	0.283386
40620	1.41908	2.1
40621	1.77723	2.1
40622	1.90485	2.1
40623	1.90485	2.1
40624	1.30237	2.1
40625	1.30237	2.1
40626	1.77723	2.1
40627	2.0055	2.1
40628	1.90485	2.1
40629	2.17158	2.1
40630	3.91035	2.1
40631	0.850906	2.1
40632	0.850906	2.1
40633	0.85668	2.1
40634	0.85668	2.1
40635	0.998045	2.1
40636	0.952425	2.1
40637	0.74367	2.1
40638	0.709541	2.1
40639	0.74367	2.1
40640	0.998045	2.1
40641	0.709541	2.1
40642	0.952425	2.1
40643	1.90485	2.1
40644	2.28563	2.1
40645	2.26282	2.1
40646	2.17158	2.1
40647	1.99609	2.1
40648	1.90485	2.08843
40649	2.00098	0.05
40650	1.90462	0.05
40651	1.60057	0.05
40652	1.79224	0.0523628
40653	3.69686	0.05
40654	2.65654	0.05
40655	1.79224	0.05

40656	2.22332	0.05
40657	2.22332	0.05
40658	2.24163	0.05
40659	2.24163	0.05
40660	1.89226	0.0571282
40661	1.89226	0.0542655
40662	1.89226	0.05
40663	1.89226	0.091014
40664	3.78452	0.0571309
40665	1.89226	0.05
40666	1.89226	0.0696068
40667	1.89226	0.0521291
40668	1.89226	0.0597032
40669	1.89226	0.0644606
40670	1.89226	0.0668754
40671	2.24163	0.0556379
40672	1.03645	0.0513984
40673	1.79224	0.064262
40674	1.79224	0.0517886
40675	1.54283	0.0643224
40676	1.54283	0.05
40677	0.946132	0.0601057
40678	0.946132	0.0553187
40679	0.946129	0.075398
40680	0.946129	0.0634447
40681	0.94613	0.0646257
40682	0.94613	0.0647007
40683	0.946131	0.0666744
40684	0.946131	0.0595464
40685	0.94613	0.0697087
40686	0.94613	0.0774534
40687	0.946131	0.0549911
40688	0.946131	0.0557255
40689	1.89226	2.1
40690	1.89226	2.1
40691	1.89226	2.1
40692	1.89226	2.1
40693	3.78453	2.1
40694	1.52441	2.1
40695	1.30347	2.1
40696	1.52441	2.1
40697	1.58401	2.1
40698	1.2627	2.1
40699	1.3223	2.1
40700	1.89226	2.1
40701	2.72787	2.1
40702	2.72787	2.1
40703	2.74762	2.1
40704	1.89226	2.1
40705	1.91202	2.1
40706	1.89227	2.1
40707	1.89227	2.1
40708	1.89227	2.1
40709	1.89227	2.1
40710	3.78453	2.1
40711	0.56414	2.1
40712	0.561557	2.1
40713	0.527284	2.1

40714	0.541143	2.1
40715	0.527276	2.1
40716	0.631685	2.1
40717	1.52441	2.1
40718	1.00232	2.1
40719	0.784495	2.1
40720	0.842304	2.1
40721	0.721569	2.1
40722	1.36393	2.1
40723	1.36393	2.1
40724	0.946132	2.1
40725	0.946132	2.1
40726	1.36393	2.1
40727	1.3594	2.1
40728	0.946132	2.1
40729	0.946132	2.1
40730	0.946107	2.1
40731	1.35936	2.1
40732	1.36397	2.1
40733	0.946158	2.1
40734	1.89226	2.1
40735	1.89226	2.1
40736	1.89226	2.1
40737	1.89226	2.1
40738	3.78453	2.1
40739	2.20802	2.1
40740	1.05677	2.1
40741	2.30479	2.1
40742	0.978699	2.1
40743	3.18672	2.1
40744	0.94613	2.1
40745	0.94613	2.1
40746	0.669866	2.1
40747	0.669866	2.1
40748	0.681588	2.1
40749	0.669865	2.1
40750	0.946133	2.1
40751	0.946133	2.1
40752	0.94613	2.1
40753	0.681589	2.1
40754	0.946131	2.1
40755	0.669867	2.1
40756	2.73514	2.1
40757	1.78301	2.1
40758	1.90618	2.1
40759	2.83464	2.1
40760	4.64132	2.1
40761	2.73514	2.1
40762	1.89226	2.1
40763	1.89226	2.1
40764	2.83464	2.1
40765	4.6274	2.1
40766	2.03915	2.1
40767	2.26616	2.1
40768	2.23549	2.1
40769	2.01704	2.1
40770	4.2832	2.1
40771	2.03916	2.1

40772	1.55766	2.1
40773	1.53728	2.1
40774	2.01704	2.1
40775	3.5747	2.1
40776	2.23551	2.1
40777	2.01705	2.1
40778	1.99823	2.1
40779	2.21189	2.1
40780	4.22894	2.1
40781	1.51788	2.1
40782	2.01705	2.1
40783	1.53729	2.1
40784	1.53729	2.1
40785	1.99823	2.1
40786	1.99823	2.1
40787	2.588	2.1
40788	1.45581	2.1
40789	1.45581	2.1
40790	1.44207	2.1
40791	2.54949	2.1
40792	2.53574	2.1
40793	0.98641	2.1
40794	0.98641	2.1
40795	1.00371	2.1
40796	1.00371	2.1
40797	0.749992	2.1
40798	0.727904	2.1
40799	1.26221	2.1
40800	1.27474	2.1
40801	1.26221	2.1
40802	0.749993	2.1
40803	0.727904	2.1
40804	1.27474	2.1
40805	1.23105	0.251109
40806	1.35435	1.94509
40807	1.89226	1.07501
40808	1.89226	0.582617
40809	1.89226	1.67617
40810	1.7043	0.914127
40811	1.89226	2.08298
40812	1.89226	0.907479
40813	1.89226	0.286001
40814	1.89226	2.03926
40815	3.78453	1.63118
40816	1.89226	0.202762
40817	1.89226	2.03169
40818	1.89226	0.870494
40819	1.89226	0.927125
40820	1.89226	1.53371
40821	1.89226	1.628
40822	0.946131	0.336725
40823	1.40945	0.235673
40824	0.94613	0.344872
40825	0.94613	1.65231
40826	0.946129	1.7248
40827	0.946129	0.650173
40828	0.946132	1.81204
40829	1.40945	0.187086

40830	0.94613	0.694201
40831	0.94613	0.626198
40832	0.946131	0.465189
40833	0.94613	0.335573
40834	0.946133	0.505998
40835	0.946133	0.445617
40836	0.946132	1.39694
40837	0.946132	1.96084
40838	0.946132	1.9352
40839	0.946132	0.635666
40840	0.946133	1.40687
40841	0.946133	0.479463
40842	0.946132	1.18529
40843	0.946132	1.09399
40844	0.946133	0.678708
40845	0.946133	0.74871
40846	1.89226	0.833402
40847	1.89226	2.1
40848	1.89226	1.61854
40849	1.89226	1.30288
40850	1.89226	1.94904
40851	1.89226	1.71062
40852	1.89226	2.1
40853	1.89226	2.1
40854	1.89226	2.1
40855	1.89226	2.1
40856	1.89226	2.1
40857	1.89226	2.1
40858	1.89226	2.1
40859	1.89226	2.1
40860	1.89226	2.1
40861	1.89226	2.1
40862	3.78453	2.1
40863	1.66647	2.1
40864	1.15399	2.1
40865	1.25443	2.1
40866	1.26599	2.1
40867	1.58697	2.1
40868	1.66647	2.1
40869	1.66647	2.1
40870	1.47446	2.1
40871	1.55433	2.1
40872	1.74241	2.1
40873	3.2208	2.1
40874	1.89227	2.1
40875	1.89227	2.1
40876	1.89227	2.1
40877	1.89227	2.1
40878	3.78453	2.1
40879	0.946132	2.1
40880	0.946132	2.1
40881	0.946132	2.1
40882	0.946132	2.1
40883	0.946132	2.1
40884	0.946132	2.1
40885	0.946133	2.1
40886	0.946133	2.1
40887	0.946132	2.1

40888	0.946132	2.1
40889	0.946133	2.1
40890	0.946133	2.1
40891	0.625067	2.1
40892	0.61907	2.1
40893	0.896913	2.1
40894	0.946199	2.1
40895	0.806287	2.1
40896	0.677685	2.1
40897	0.750657	2.1
40898	0.750657	2.1
40899	0.646004	2.1
40900	0.890236	2.1
40901	0.61049	2.1
40902	0.888979	2.1
40903	1.6223	2.1
40904	1.98034	2.1
40905	1.75003	2.1
40906	1.55433	2.1
40907	1.75003	2.1
40908	1.55433	2.1
40909	1.88602	2.1
40910	2.08149	2.1
40911	2.07991	2.1
40912	1.88473	2.1
40913	2.08149	2.1
40914	1.88602	2.1
40915	1.88458	2.1
40916	2.08149	2.1
40917	1.7069	2.1
40918	1.56284	2.1
40919	2.08149	2.1
40920	1.88458	2.1
40921	1.5955	2.1
40922	1.89226	2.1
40923	1.91975	2.1
40924	1.62299	2.1
40925	1.89226	2.1
40926	1.5955	2.1
40927	1.89226	2.1
40928	1.89226	2.1
40929	1.89226	2.1
40930	1.89226	2.1
40931	1.89226	1.70483
40932	1.89226	2.1
40933	1.89226	2.1
40934	1.89226	2.1
40935	1.89226	2.1
40936	1.89226	2.1
40937	1.89226	2.1
40938	1.89226	2.1
40939	1.62299	2.1
40940	1.89226	2.1
40941	1.71752	2.1
40942	1.44824	2.1
40943	1.89226	2.1
40944	1.62299	2.1
40945	1.89226	0.49038

40946	2.8317	0.212858
40947	1.89226	0.403216
40948	2.64055	0.0970069
40949	4.53281	0.389441
40950	2.8317	0.401655
40951	1.83151	0.0750564
40952	2.01001	0.158053
40953	2.01001	0.70675
40954	2.64055	0.0617287
40955	2.64055	0.166822
40956	0.946132	0.0700008
40957	0.946132	0.145957
40958	1.16167	0.0788193
40959	1.16167	0.142269
40960	1.16167	0.113486
40961	1.32027	0.056551
40962	0.946131	0.0656307
40963	0.946131	0.149264
40964	0.94613	0.0725912
40965	1.16167	0.05
40966	0.946131	0.0754677
40967	1.32027	0.0825118
40968	2.32335	0.161472
40969	1.83151	0.0764168
40970	2.38525	0.111674
40971	2.64055	0.0875428
40972	1.57621	0.0618655
40973	1.83151	0.0621677
40974	2.25677	2.1
40975	1.88158	2.1
40976	1.86856	2.1
40977	2.0815	2.1
40978	1.70303	2.1
40979	1.88158	2.1
40980	2.50381	2.1
40981	1.89227	2.1
40982	2.25677	2.1
40983	2.25677	2.1
40984	1.89227	2.1
40985	1.89227	2.1
40986	1.89226	0.05
40987	1.50899	0.05
40988	2.13659	0.05
40989	1.50899	0.0808355
40990	2.51986	0.05
40991	1.89226	0.0626065
40992	0.946131	0.0586675
40993	0.946131	0.05
40994	1.06829	0.05
40995	0.895375	0.05
40996	0.94613	0.05
40997	0.94613	0.0632404
40998	1.33922	0.05
40999	0.895376	0.05
41000	0.946131	0.05
41001	1.33922	0.05
41002	0.946131	0.05
41003	1.06829	0.05

41004	0.841467	0.111007
41005	0.578428	0.185096
41006	1.18079	0.117369
41007	0.920237	0.179983
41008	0.772317	0.0682407
41009	0.856555	0.230647
41010	0.910271	0.120363
41011	0.910271	0.272095
41012	0.975752	0.134557
41013	0.536929	0.0765781
41014	0.81952	0.220394
41015	1.15252	0.105152
41016	1.89226	2.1
41017	2.51431	2.1
41018	1.89226	2.1
41019	1.89226	2.1
41020	2.78815	2.1
41021	2.78815	2.1
41022	2.0815	2.1
41023	2.0516	2.1
41024	2.0815	2.1
41025	3.06699	2.1
41026	2.76576	2.1
41027	2.8002	2.1
41028	1.94112	2.1
41029	1.89226	2.1
41030	2.20358	2.1
41031	1.89226	2.1
41032	4.09584	2.1
41033	1.09154	2.1
41034	2.62647	2.1
41035	2.20358	2.1
41036	2.20358	2.1
41037	1.52314	2.1
41038	1.52314	2.1
41039	1.31323	2.1
41040	1.31323	2.1
41041	0.946132	2.1
41042	0.946132	2.1
41043	0.946132	2.1
41044	0.946132	2.1
41045	1.31323	2.1
41046	1.10179	2.1
41047	1.31323	2.1
41048	0.946132	2.1
41049	1.10179	2.1
41050	0.946133	2.1
41051	1.89226	0.320616
41052	1.89226	1.34594
41053	1.89226	1.22772
41054	1.89226	1.81107
41055	1.89226	0.252263
41056	1.89226	1.33041
41057	1.35126	2.01256
41058	1.35126	2.1
41059	0.903285	1.40352
41060	0.903285	1.8941
41061	1.30841	1.84921

41062	0.903313	2.1
41063	0.946132	1.75684
41064	0.946132	1.18812
41065	0.946132	1.67721
41066	0.903312	1.73045
41067	1.30841	1.60665
41068	0.946133	1.56447
41069	2.62097	2.1
41070	2.72561	2.1
41071	2.39016	2.1
41072	2.51062	2.1
41073	5.11577	2.1
41074	2.51062	2.1
41075	2.58286	0.151261
41076	2.40107	2.1
41077	2.40107	1.04519
41078	2.72561	2.1
41079	2.72561	1.64242
41080	2.39016	2.1
41081	2.51062	2.1
41082	2.20242	2.1
41083	2.34107	2.1
41084	4.73124	2.1
41085	2.51062	2.1
41086	2.40107	0.141091
41087	2.34107	2.1
41088	2.25197	0.216786
41089	4.74215	2.1
41090	2.75218	2.1
41091	1.89226	2.1
41092	2.3349	2.1
41093	2.3349	2.1
41094	1.89226	2.1
41095	1.89226	2.1
41096	1.46129	2.1
41097	0.996308	2.1
41098	1.88649	2.1
41099	1.70302	2.1
41100	2.95419	2.1
41101	1.89227	2.1
41102	1.89227	2.1
41103	1.89227	2.1
41104	1.89227	2.1
41105	3.78453	2.1
41106	0.646558	2.1
41107	0.646557	2.1
41108	0.60697	2.1
41109	0.60697	2.1
41110	0.587917	2.1
41111	0.519747	2.1
41112	0.586443	2.1
41113	0.733782	2.1
41114	0.733793	2.1
41115	0.587924	2.1
41116	0.519739	2.1
41117	0.586436	2.1
41118	2.1642	2.1
41119	1.42867	2.1

41120	2.28847	2.1
41121	1.68299	2.1
41122	3.71714	2.1
41123	1.89226	2.1
41124	1.89226	2.1
41125	1.89226	2.1
41126	1.89226	2.1
41127	3.78453	2.1
41128	0.946131	2.1
41129	0.946131	2.1
41130	1.24994	2.1
41131	1.24994	2.1
41132	1.11582	2.1
41133	1.24994	2.1
41134	0.946133	2.1
41135	0.946133	2.1
41136	0.946131	2.1
41137	1.24994	2.1
41138	0.94613	2.1
41139	1.11582	2.1
41140	2.28847	2.1
41141	1.53629	2.1
41142	1.57498	2.1
41143	1.47417	2.1
41144	2.42221	2.1
41145	2.28847	2.1
41146	1.89226	2.1
41147	1.89226	2.1
41148	1.89226	2.1
41149	1.89226	2.1
41150	3.78453	2.1
41151	1.89226	2.1
41152	2.49988	2.1
41153	2.71233	2.1
41154	2.71233	2.1
41155	1.89226	2.1
41156	1.89226	2.1
41157	0.924534	2.1
41158	0.924534	2.1
41159	0.990395	2.1
41160	0.990395	2.1
41161	0.946131	2.1
41162	0.946131	2.1
41163	0.924505	2.1
41164	0.968797	2.1
41165	0.924505	2.1
41166	0.946131	2.1
41167	0.968797	2.1
41168	0.946131	2.1
41169	0.924533	2.1
41170	0.924533	2.1
41171	0.948264	2.1
41172	0.945345	2.1
41173	0.851541	2.1
41174	0.851541	2.1
41175	0.958934	2.1
41176	1.02617	2.1
41177	0.936668	2.1

41178	1.04829	2.1
41179	0.924505	2.1
41180	0.946131	2.1
41181	2.28431	0.481412
41182	1.48035	0.05
41183	1.59971	0.0815424
41184	1.48035	0.0627861
41185	2.33818	0.102566
41186	2.21882	0.0821187
41187	2.28431	0.0891127
41188	1.89226	0.0618666
41189	1.89226	0.208835
41190	2.3968	0.05
41191	4.28906	0.05
41192	2.28431	0.190935
41193	1.70303	0.05
41194	1.5428	0.05
41195	1.59971	0.05
41196	2.31152	0.177434
41197	2.3968	0.199518
41198	1.99823	2.1
41199	2.21189	2.1
41200	2.18937	2.1
41201	1.979 2.1	
41202	4.19089	2.1
41203	0.999114	2.1
41204	0.9895	2.1
41205	0.758942	2.1
41206	0.749358	2.1
41207	0.874236	2.1
41208	0.874236	2.1
41209	0.874236	2.1
41210	0.874236	2.1
41211	0.9895	2.1
41212	0.749358	2.1
41213	0.758942	2.1
41214	0.999114	2.1
41215	2.18937	2.1
41216	1.979 2.1	
41217	1.95416	2.1
41218	2.162 2.1	
41219	4.14099	2.1
41220	1.95416	2.1
41221	1.49871	2.1
41222	1.47712	2.1
41223	1.49871	2.1
41224	1.9574	2.1
41225	1.979 2.1	
41226	1.68508	0.05
41227	1.64984	0.05
41228	2.67711	0.05
41229	2.76407	0.05
41230	4.41391	0.05
41231	1.84227	0.05
41232	2.76407	0.05
41233	2.76407	0.05
41234	1.85007	0.05
41235	2.72769	0.05

41236	1.80812	0.05
41237	1.94516	2.1
41238	1.89226	2.1
41239	1.89226	2.1
41240	1.89226	2.1
41241	1.98853	2.1
41242	1.98852	2.1
41243	1.28095	2.1
41244	2.0815	2.1
41245	2.29123	2.1
41246	2.06833	2.1
41247	1.08212	2.1
41248	0.976848	2.1
41249	1.89226	2.1
41250	1.89226	2.1
41251	1.89226	2.1
41252	1.89226	2.1
41253	1.89226	2.1
41254	1.89226	2.1
41255	1.13537	2.1
41256	1.18233	2.1
41257	0.946132	2.1
41258	0.946132	2.1
41259	0.946132	2.1
41260	0.946132	2.1
41261	0.994264	2.1
41262	1.18233	2.1
41263	1.13537	2.1
41264	0.946131	2.1
41265	0.946132	2.1
41266	0.994264	2.1
41267	0.953204	2.1
41268	0.953204	2.1
41269	0.966515	2.1
41270	0.966515	2.1
41271	1.30583	2.1
41272	1.14561	2.1
41273	0.640475	2.1
41274	0.774103	2.1
41275	1.30584	2.1
41276	0.774101	2.1
41277	0.640474	2.1
41278	1.14562	2.1
41279	0.946131	2.1
41280	0.946131	2.1
41281	0.946131	2.1
41282	0.946131	2.1
41283	0.946128	2.1
41284	0.946128	2.1
41285	0.946133	2.1
41286	0.946133	2.1
41287	0.94613	2.1
41288	0.94613	2.1
41289	0.946131	2.1
41290	0.946131	2.1
41291	0.946132	0.05
41292	0.946132	0.05
41293	0.946131	0.05

41294	0.946131	0.05
41295	0.946131	0.05
41296	0.946131	0.05
41297	0.946132	0.05
41298	0.946132	0.05
41299	0.946131	0.05
41300	0.946131	0.05
41301	0.946132	0.05
41302	0.946132	0.05
41303	0.946131	0.05
41304	0.946131	0.05
41305	0.946131	0.05
41306	0.946131	0.05
41307	0.94613	0.05
41308	0.94613	0.05
41309	0.946132	0.05
41310	0.946132	0.05
41311	0.946131	0.05
41312	0.946131	0.05
41313	0.946131	0.05
41314	0.946131	0.05
41315	2.57882	0.05
41316	1.48676	0.05
41317	1.48797	0.05
41318	2.64281	0.05
41319	4.12957	0.05
41320	2.56323	0.05
41321	2.64281	0.05
41322	2.51807	0.05
41323	1.36324	0.05
41324	1.48797	0.05
41325	1.48797	0.05
41326	1.04367	2.1
41327	2.42504	2.1
41328	2.65164	2.1
41329	1.49479	2.1
41330	3.91983	2.1
41331	2.15622	2.09153
41332	1.70539	2.1
41333	2.02369	2.1
41334	2.39039	2.1
41335	4.17992	2.1
41336	0.966979	2.1
41337	1.7054	2.1
41338	2.0237	2.1
41339	1.31502	2.1
41340	2.99067	2.1
41341	2.02369	2.1
41342	2.65637	2.1
41343	2.39039	1.84962
41344	2.39039	2.1
41345	2.29332	1.87344
41346	2.29332	2.1
41347	2.0237	2.1
41348	1.72116	2.1
41349	1.58465	2.1
41350	1.31502	2.1
41351	2.29332	2.1

41352	2.0237	2.1
41353	1.0372	0.05
41354	2.32462	0.05
41355	2.2291	0.05
41356	2.2291	0.05
41357	1.17464	0.05
41358	1.17464	0.05
41359	2.68289	0.05
41360	1.07805	0.05
41361	1.70302	0.05
41362	2.06653	0.05
41363	3.5779	0.05
41364	1.89226	0.05
41365	1.89226	0.05
41366	1.89226	0.05
41367	1.89226	0.05
41368	3.78453	0.05
41369	1.89226	0.05
41370	1.89226	0.05
41371	1.89226	0.05
41372	1.89226	0.05
41373	1.89226	0.05
41374	1.89226	0.05
41375	1.89225	0.05
41376	1.89225	0.05
41377	1.89225	0.05
41378	1.89225	0.05
41379	1.89225	0.05
41380	1.89225	0.05
41381	0.94613	0.05
41382	0.94613	0.05
41383	0.94613	0.05
41384	0.94613	0.05
41385	0.946128	0.05
41386	0.946128	0.05
41387	0.946132	0.05
41388	0.946132	0.05
41389	0.94613	0.05
41390	0.94613	0.05
41391	0.94613	0.05
41392	0.94613	0.05
41393	1.29417	2.1
41394	1.32732	2.1
41395	1.32316	0.77322
41396	1.34973	2.1
41397	2.65048	2.1
41398	1.89226	1.44416
41399	1.89226	2.1
41400	1.89226	0.817429
41401	1.89226	2.1
41402	3.78453	1.82931
41403	1.89226	2.1
41404	1.89226	2.1
41405	1.89226	1.82446
41406	1.89226	2.1
41407	3.78453	2.1
41408	1.32732	2.1
41409	1.13006	2.1

41410	1.13358	2.1
41411	1.11117	2.1
41412	1.34973	2.1
41413	1.32732	2.1
41414	1.32316	2.02798
41415	1.34973	2.1
41416	1.34066	1.09086
41417	1.36601	2.1
41418	2.68918	2.1
41419	1.89226	2.02989
41420	1.89226	2.1
41421	1.89226	0.190595
41422	1.89226	2.1
41423	3.78453	2.00789
41424	1.89226	2.1
41425	1.89226	2.1
41426	1.89226	2.1
41427	1.89226	2.1
41428	1.89226	2.1
41429	1.89226	2.1
41430	0.624134	2.1
41431	0.624134	2.1
41432	0.624334	2.1
41433	0.624334	2.1
41434	0.56503	2.1
41435	0.573605	2.1
41436	0.674864	2.1
41437	0.683007	2.1
41438	0.683007	2.1
41439	0.573605	2.1
41440	0.565029	2.1
41441	0.674864	2.1
41442	2.16633	2.1
41443	1.40846	2.1
41444	2.13977	2.1
41445	1.40771	2.1
41446	3.57404	2.1
41447	2.13977	2.1
41448	1.29932	2.1
41449	1.29932	2.1
41450	1.40771	2.1
41451	1.98957	2.1
41452	2.09796	2.1
41453	2.6044	0.0844376
41454	1.85548	0.191491
41455	1.89226	0.054889
41456	1.89226	0.300089
41457	3.74774	0.134336
41458	1.89226	0.0542543
41459	1.89226	0.328717
41460	1.89226	0.0745863
41461	1.89226	0.142373
41462	3.78453	0.103986
41463	1.89226	2.1
41464	2.64107	2.1
41465	1.89226	2.1
41466	2.78977	2.1
41467	1.89226	2.1

41468	2.78977	2.1
41469	1.89226	2.1
41470	1.89226	2.1
41471	1.89226	2.1
41472	1.89226	2.1
41473	1.89226	2.1
41474	1.89226	2.1
41475	2.43173	2.1
41476	1.41238	2.1
41477	2.0815	2.1
41478	1.98048	2.1
41479	1.62014	2.1
41480	1.50086	2.1
41481	0.946129	0.0951999
41482	0.946129	0.0729362
41483	1.27636	0.119004
41484	1.1984	0.05
41485	0.946131	0.05
41486	0.946131	0.0568438
41487	1.27636	0.115111
41488	1.27636	0.05
41489	0.946131	0.0877598
41490	1.27636	0.05
41491	0.946131	0.0531019
41492	1.1984	0.05
41493	1.99569	0.0723485
41494	2.55272	0.207802
41495	1.77403	0.05
41496	1.70303	0.0525248
41497	3.05772	0.129311
41498	2.55121	0.0966918
41499	0.946128	0.0622347
41500	0.946128	0.0964885
41501	1.38752	0.132751
41502	1.27636	0.05
41503	0.94613	0.05
41504	0.94613	0.0588018
41505	1.38752	0.132492
41506	1.38752	0.053533
41507	0.946131	0.0611353
41508	1.38752	0.0735326
41509	0.946131	0.123272
41510	1.27636	0.05
41511	2.77504	0.123189
41512	1.99569	0.0567767
41513	2.29794	0.0601064
41514	1.99569	0.0522921
41515	2.85497	0.05
41516	2.55272	0.05
41517	1.53284	2.1
41518	1.89226	2.1
41519	1.89226	2.1
41520	1.89226	2.1
41521	1.54763	2.1
41522	1.54763	2.1
41523	0.946131	2.1
41524	0.946131	2.1
41525	0.946127	2.1

41526	0.946127	2.1
41527	0.946127	2.1
41528	0.946127	2.1
41529	0.946131	2.1
41530	0.946131	2.1
41531	0.94613	2.1
41532	0.94613	2.1
41533	0.946129	2.1
41534	0.946129	2.1
41535	0.751754	2.1
41536	0.751754	2.1
41537	0.771562	2.1
41538	0.771561	2.1
41539	1.0268	2.1
41540	0.95482	2.1
41541	0.528937	2.1
41542	0.496512	2.1
41543	0.528936	2.1
41544	1.0268	2.1
41545	0.954821	2.1
41546	0.49651	2.1
41547	2.64075	0.0543404
41548	1.35181	1.14646
41549	1.35181	0.05
41550	1.83823	0.14363
41551	2.28373	0.215906
41552	2.88566	1.48869
41553	0.493018	0.845329
41554	0.675903	0.581398
41555	1.14187	0.183644
41556	0.973937	0.136424
41557	0.821166	0.361978
41558	0.821166	1.19
41559	0.828674	2.1
41560	0.828674	0.05
41561	0.973937	0.652583
41562	0.493018	0.62967
41563	0.675903	0.278893
41564	1.14187	0.426973
41565	1.89226	2.1
41566	2.11667	2.1
41567	1.91184	2.1
41568	1.89226	2.1
41569	2.13625	2.1
41570	2.11667	2.1
41571	1.89226	2.1
41572	1.89226	2.1
41573	1.89226	2.1
41574	1.89226	2.1
41575	3.78453	2.1
41576	0.907729	2.1
41577	0.907729	2.1
41578	1.04459	2.1
41579	0.890955	2.1
41580	0.988181	2.1
41581	0.841856	2.1
41582	0.946133	2.1
41583	0.946133	2.1

41584	1.04072	2.1
41585	1.1064	2.1
41586	0.89099	2.1
41587	0.851541	2.1
41588	1.89225	2.1
41589	2.13623	2.1
41590	1.91044	2.1
41591	1.89225	2.1
41592	2.15442	2.1
41593	2.13623	2.1
41594	1.89225	2.1
41595	1.89225	2.1
41596	1.89225	2.1
41597	1.89225	2.1
41598	1.89225	2.1
41599	1.89225	2.1
41600	0.907723	2.1
41601	0.907723	2.1
41602	1.07536	2.1
41603	1.07814	2.1
41604	0.998499	2.1
41605	1.03575	2.1
41606	0.946126	2.1
41607	0.946126	2.1
41608	1.04072	2.1
41609	1.14025	2.1
41610	0.851528	2.1
41611	0.898284	2.1
41612	1.89226	0.05
41613	1.89226	0.05
41614	1.89226	0.05
41615	1.89226	0.05
41616	1.89226	0.05
41617	1.89226	0.05
41618	2.53008	2.1
41619	1.94534	2.1
41620	1.98702	2.1
41621	1.98858	2.1
41622	2.52852	2.1
41623	2.53008	2.1
41624	1.03267	2.1
41625	1.12591	2.1
41626	1.2959	2.1
41627	1.28992	2.1
41628	1.10851	2.1
41629	1.33078	2.1
41630	1.10661	2.1
41631	1.10661	2.1
41632	0.982208	2.1
41633	1.28992	2.1
41634	1.03267	2.1
41635	1.23262	2.1
41636	2.2712	2.1
41637	1.89226	2.1
41638	1.95954	2.1
41639	1.95954	2.1
41640	1.89226	2.1
41641	1.89226	2.1

41642	1.89226	2.1
41643	1.89226	2.1
41644	1.89226	2.1
41645	1.89226	2.1
41646	3.78453	2.1
41647	1.33887	2.1
41648	1.25395	2.1
41649	0.88327	2.1
41650	0.676665	2.1
41651	0.84881	2.1
41652	0.987503	2.1
41653	1.13243	2.1
41654	1.13243	2.1
41655	1.15338	2.1
41656	0.849966	2.1
41657	0.793074	2.1
41658	1.11957	2.1
41659	0.946129	2.1
41660	0.946129	2.1
41661	1.28254	2.1
41662	1.1356	2.1
41663	1.1356	2.1
41664	1.1356	2.1
41665	0.946131	2.1
41666	0.946131	2.1
41667	0.946131	2.1
41668	1.28254	2.1
41669	1.1356	2.1
41670	0.946131	2.1
41671	1.89227	2.1
41672	1.89227	2.1
41673	1.89227	2.1
41674	1.89227	2.1
41675	3.78453	2.1
41676	0.936287	0.05
41677	1.01399	0.05
41678	0.959415	0.05
41679	0.959415	0.05
41680	0.910786	0.05
41681	1.38301	0.05
41682	1.0286	0.05
41683	0.535742	0.05
41684	0.505202	0.05
41685	0.937685	0.05
41686	1.11066	0.05
41687	1.46907	0.05
41688	1.00546	0.05
41689	1.01524	0.05
41690	0.94613	0.05
41691	0.94613	0.05
41692	1.05018	0.05
41693	0.914025	0.05
41694	0.913512	0.05
41695	0.851542	0.05
41696	1.05385	0.05
41697	1.05494	0.05
41698	0.91404	0.05
41699	0.91404	0.05

41700	2.03253	1.05452
41701	1.82325	0.05
41702	1.93938	0.526624
41703	1.73756	0.05
41704	3.77009	0.05
41705	1.82325	0.0529913
41706	1.47562	0.939862
41707	1.73756	0.05
41708	1.3868	0.05
41709	3.21317	0.05
41710	1.93938	0.600232
41711	1.73756	0.0595901
41712	1.85293	0.622336
41713	1.64279	0.05
41714	3.59049	0.05
41715	1.73756	0.137558
41716	1.3868	0.606164
41717	1.64279	0.05
41718	1.28419	0.123542
41719	3.02174	0.0702573
41720	1.35048	2.1
41721	1.37999	2.1
41722	1.34355	0.873863
41723	1.37854	2.1
41724	2.72354	2.1
41725	1.89226	1.2977
41726	1.89226	2.1
41727	1.89226	0.684911
41728	1.89226	1.96071
41729	3.78452	0.989633
41730	1.89226	2.1
41731	1.89226	2.1
41732	1.89226	0.730663
41733	1.89226	2.1
41734	3.78453	2.1
41735	1.37999	2.1
41736	1.18059	2.1
41737	1.17117	2.1
41738	1.17262	2.1
41739	1.37855	2.1
41740	1.37999	2.1
41741	1.34355	2.09124
41742	1.37854	2.1
41743	1.33079	1.03075
41744	1.3741	2.1
41745	2.71765	2.09448
41746	1.89226	1.93289
41747	1.89226	2.1
41748	1.89226	0.293231
41749	1.89226	2.1
41750	3.78453	1.92852
41751	1.89226	1.5636
41752	1.89226	2.1
41753	1.89226	1.79573
41754	1.89226	2.1
41755	3.78453	2.1
41756	0.640305	2.1
41757	0.640305	2.1

41758	0.643507	1.08673
41759	0.643507	2.1
41760	0.590294	2.1
41761	0.594539	2.1
41762	0.689273	2.04665
41763	0.687048	2.1
41764	0.687048	2.1
41765	0.594538	2.1
41766	0.590294	2.1
41767	0.689273	2.1
41768	2.01399	0.0759521
41769	1.70141	0.22507
41770	1.83896	2.1
41771	1.52482	1.94082
41772	3.53881	1.08946
41773	1.70141	0.987434
41774	1.0752	2.1
41775	1.0752	0.303117
41776	1.25669	1.73504
41777	1.52482	1.44213
41778	1.70632	2.1
41779	1.83896	1.59371
41780	1.52482	0.0730229
41781	1.65244	0.361854
41782	1.34412	1.8226
41783	3.17725	0.433095
41784	1.26782	0.05
41785	2.90353	0.05
41786	2.54203	0.05
41787	2.41979	0.05
41788	1.77007	0.05
41789	1.703 0.05	
41790	0.946118	0.05
41791	0.946118	0.05
41792	1.38643	0.05
41793	1.38644	0.05
41794	0.946117	0.05
41795	0.946117	0.05
41796	1.38644	0.05
41797	1.15545	0.05
41798	0.946131	0.05
41799	1.15546	0.05
41800	1.38642	0.05
41801	0.946105	0.05
41802	2.42256	0.103088
41803	2.63056	0.0520974
41804	2.43174	0.060704
41805	2.25035	0.05
41806	4.8543	0.05
41807	2.58031	0.0948877
41808	2.63056	0.0544859
41809	2.43174	0.0553275
41810	2.32127	0.05
41811	5.01205	0.0700136
41812	2.43174	0.0585793
41813	2.25035	0.05
41814	2.11465	0.05
41815	2.13042	0.05

41816	4.54639	0.05
41817	2.43174	0.0540699
41818	2.32127	0.05
41819	1.92997	0.05
41820	2.13041	0.05
41821	4.36171	0.05
41822	1.89227	2.1
41823	1.89227	2.1
41824	1.89227	2.1
41825	1.89227	2.1
41826	3.78453	2.1
41827	0.568724	2.1
41828	0.568724	2.1
41829	0.57678	2.1
41830	0.57678	2.1
41831	0.523796	2.1
41832	0.548137	2.1
41833	0.597368	2.1
41834	0.637877	2.1
41835	0.597367	2.1
41836	0.523796	2.1
41837	0.637877	2.1
41838	0.548137	2.1
41839	1.32674	2.1
41840	1.32674	2.1
41841	0.946132	2.1
41842	0.946132	2.1
41843	1.3195	2.1
41844	1.32674	2.1
41845	0.946132	2.1
41846	0.946132	2.1
41847	0.946132	2.1
41848	1.3195	2.1
41849	1.32674	2.1
41850	0.946133	2.1
41851	1.89227	2.1
41852	1.89227	2.1
41853	1.89227	2.1
41854	1.89227	2.1
41855	3.78453	2.1
41856	1.19473	2.1
41857	0.987132	2.1
41858	0.987132	2.1
41859	1.04759	2.1
41860	1.0894	2.1
41861	1.14986	2.1
41862	1.3195	2.1
41863	1.3195	2.1
41864	0.946132	2.1
41865	0.946132	2.1
41866	1.31185	2.1
41867	1.3195	2.1
41868	0.946132	2.1
41869	0.946132	2.1
41870	1.31185	2.1
41871	0.946133	2.1
41872	0.946132	2.1
41873	1.3195	2.1

41874	1.11256	0.707409
41875	1.70101	0.560695
41876	1.22297	0.681133
41877	1.22297	0.716989
41878	1.7252	0.754619
41879	1.7252	0.775032
41880	1.99298	0.716308
41881	1.70101	0.713129
41882	1.7252	0.854685
41883	2.18701	0.37222
41884	3.71818	0.545527
41885	1.03218	0.564464
41886	1.7252	0.450257
41887	1.11256	0.579583
41888	1.11256	0.599931
41889	1.7457	0.603276
41890	1.7457	0.611883
41891	1.7457	0.676786
41892	2.187 0.263266	
41893	1.7252	0.511639
41894	1.7252	0.611787
41895	2.27351	0.456387
41896	2.27351	0.550261
41897	1.95544	1.07702
41898	1.35897	2.06245
41899	1.44859	1.51475
41900	1.44859	1.8187
41901	1.80709	1.18804
41902	1.80709	1.73351
41903	1.80709	0.887818
41904	1.17822	1.94603
41905	1.35897	1.49168
41906	1.35897	1.98888
41907	1.52782	0.769366
41908	1.52782	1.69655
41909	1.11978	1.69376
41910	0.903546	1.20007
41911	0.977719	1.81271
41912	1.2364	1.19893
41913	1.03812	0.869123
41914	1.03812	1.9986
41915	1.05938	0.65727
41916	1.05938	1.94922
41917	1.11978	1.37551
41918	1.2364	0.74757
41919	0.97772	1.59411
41920	0.903546	1.29281
41921	1.80709	1.94019
41922	1.81847	1.41024
41923	1.96029	1.90462
41924	1.52782	1.6244
41925	2.23956	1.2208
41926	1.80709	1.40893
41927	1.89226	0.080026
41928	2.32962	0.05
41929	2.5992	0.05
41930	1.89226	0.05
41931	2.5992	0.0723471

41932	1.89226	0.0766808
41933	2.55791	0.05
41934	2.47141	0.05
41935	2.13515	0.05
41936	2.10939	0.05
41937	4.66731	0.05
41938	2.47141	0.05
41939	2.23337	0.05
41940	2.10939	0.05
41941	2.03438	0.05
41942	4.34276	0.05
41943	2.13512	0.05
41944	1.71027	0.05
41945	1.71027	0.05
41946	2.10936	0.05
41947	1.60137	0.05
41948	2.00046	0.05
41949	1.71027	0.05
41950	2.03435	0.05
41951	1.71027	0.05
41952	2.10936	0.05
41953	1.72067	0.05
41954	2.11976	0.05
41955	1.177 0.05622	
41956	1.36405	0.688692
41957	2.25635	0.734028
41958	2.60151	0.0685357
41959	3.6204	0.311567
41960	0.658869	0.05
41961	0.781697	0.05
41962	0.637037	0.05
41963	0.541148	0.05
41964	0.654682	0.05
41965	0.654682	0.05
41966	0.641224	0.0639063
41967	0.641224	0.05
41968	0.637039	0.05
41969	0.781696	0.05
41970	0.658867	0.05
41971	0.541149	0.05
41972	1.89226	0.05
41973	1.59152	0.05
41974	1.89226	0.05
41975	1.82125	0.05
41976	1.89226	0.05
41977	1.82125	0.05
41978	2.18967	0.05
41979	1.89227	0.05
41980	2.72752	0.05
41981	2.72752	0.05
41982	1.89227	0.05
41983	1.89227	0.05
41984	2.18967	0.05
41985	1.92547	0.05
41986	1.92547	0.05
41987	2.72752	0.05
41988	1.30572	0.05
41989	2.10777	0.05

41990	2.39663	2.1
41991	1.92293	2.1
41992	1.96889	2.1
41993	2.45928	2.1
41994	4.36552	2.1
41995	1.02605	2.1
41996	1.92293	2.1
41997	1.9689	2.1
41998	1.04679	2.1
41999	2.99495	2.1
42000	1.96889	2.1
42001	2.45928	2.1
42002	2.50138	2.1
42003	1.99782	2.1
42004	4.47027	2.1
42005	1.9689	2.1
42006	1.03449	2.1
42007	1.07572	2.1
42008	1.04679	2.1
42009	1.99783	2.1
42010	1.9689	2.1
42011	1.72162	0.778097
42012	1.89186	0.329462
42013	2.29771	0.536177
42014	1.80149	0.150811
42015	4.01933	0.391824
42016	1.16482	0.832306
42017	1.97003	0.242679
42018	1.72162	0.162862
42019	1.72162	0.321871
42020	1.29313	0.367472
42021	1.29313	0.659172
42022	2.29771	0.62711
42023	2.84159	0.101981
42024	2.19622	0.218254
42025	1.97366	0.20086
42026	2.50906	0.314001
42027	2.29771	0.358358
42028	1.29313	0.726164
42029	2.4483	0.231083
42030	2.14223	0.208019
42031	2.14228	0.290977
42032	1.294	0.390124
42033	1.294	0.529218
42034	1.21495	0.05
42035	1.70303	0.05
42036	1.7924	0.05
42037	1.64002	0.05
42038	3.11963	0.05
42039	2.81021	0.05
42040	1.89226	0.05
42041	1.89226	0.05
42042	1.89226	0.05
42043	2.36406	0.05
42044	2.36406	0.05
42045	1.89226	0.05
42046	1.89226	0.05
42047	1.89226	0.05

42048	1.89226	0.05
42049	1.89226	0.05
42050	1.89226	0.05
42051	2.29976	0.05
42052	1.64002	0.05
42053	1.8959	0.05
42054	1.90873	0.05
42055	1.85594	0.05
42056	1.85594	0.05
42057	1.89226	0.05
42058	1.89226	0.05
42059	1.89226	0.05
42060	1.89226	0.05
42061	3.78453	0.05
42062	1.89226	0.677934
42063	2.52823	2.1
42064	2.83441	1.74828
42065	2.83441	2.1
42066	1.89226	1.2277
42067	1.89226	2.0586
42068	1.04522	1.85538
42069	1.04522	1.57147
42070	1.12831	1.88362
42071	1.18364	2.1
42072	0.640525	1.80766
42073	0.924882	1.67436
42074	1.27877	1.94954
42075	1.43363	0.484663
42076	0.93474	1.72577
42077	1.58926	1.53266
42078	1.29573	1.97954
42079	0.664247	2.00053
42080	2.02191	2.1
42081	1.89226	2.1
42082	1.89226	2.1
42083	1.89226	2.1
42084	2.58639	2.1
42085	2.58639	2.1
42086	1.16759	2.1
42087	2.11828	2.1
42088	1.60936	2.1
42089	2.35846	2.1
42090	3.52605	2.1
42091	1.89226	2.1
42092	1.89226	2.1
42093	1.89226	2.1
42094	1.89226	2.1
42095	3.78453	2.1
42096	2.82765	0.05
42097	1.89809	0.05
42098	2.15971	0.0519395
42099	1.89809	0.05
42100	2.29233	0.05
42101	2.03071	0.05
42102	1.90664	0.05
42103	2.15971	0.05
42104	1.28956	0.05
42105	1.28956	0.05

42106	2.64414	0.05
42107	2.02706	0.05
42108	2.82765	0.05
42109	1.89226	0.05
42110	2.39968	0.05
42111	2.39968	0.05
42112	1.89226	0.05
42113	1.89226	0.0504211
42114	0.978643	2.1
42115	0.703968	2.1
42116	0.946129	2.1
42117	0.946129	2.1
42118	0.703939	2.1
42119	0.703939	2.1
42120	0.946159	2.1
42121	0.946159	2.1
42122	0.978642	2.1
42123	0.94613	2.1
42124	0.94613	2.1
42125	0.703968	2.1
42126	1.89226	2.1
42127	2.17309	1.22863
42128	2.17309	2.1
42129	1.47475	2.06186
42130	1.89226	2.1
42131	1.19393	1.53901
42132	1.89226	2.1
42133	1.89226	2.1
42134	1.89226	2.1
42135	1.89226	2.1
42136	3.78452	2.1
42137	1.17426	2.1
42138	0.978644	2.1
42139	0.946129	2.1
42140	0.946129	2.1
42141	0.946131	2.1
42142	0.946131	2.1
42143	0.978643	2.1
42144	0.978642	2.1
42145	1.17426	2.1
42146	0.946131	2.1
42147	0.978642	2.1
42148	0.94613	2.1
42149	1.89226	2.1
42150	1.89226	1.26837
42151	1.89226	2.1
42152	2.17309	1.43395
42153	2.56346	2.1
42154	2.94389	1.88724
42155	0.946131	2.1
42156	0.946131	2.1
42157	0.946128	2.1
42158	0.946128	2.1
42159	0.946131	2.1
42160	0.946131	2.1
42161	0.946128	2.1
42162	0.946128	2.1
42163	0.94613	2.1

42164	0.94613	2.1
42165	0.946129	2.1
42166	0.946129	2.1
42167	1.89226	0.769103
42168	1.89226	0.646008
42169	1.89226	0.997661
42170	1.89226	0.676094
42171	3.78453	0.304786
42172	1.89226	0.95853
42173	1.89226	0.575957
42174	1.89226	1.03683
42175	1.89226	1.11397
42176	1.89226	0.691638
42177	1.89226	0.658584
42178	1.89226	0.620397
42179	1.30733	0.996032
42180	1.89226	1.21344
42181	1.92403	0.77581
42182	3.38635	0.458566
42183	1.89226	0.687764
42184	3.02887	1.10249
42185	2.0815	0.236825
42186	1.88422	0.775179
42187	2.0815	0.789755
42188	1.88422	1.72664
42189	1.89226	1.20431
42190	1.70221	0.736677
42191	1.89226	1.63713
42192	1.20068	1.18986
42193	1.89226	0.99821
42194	1.20068	0.684075
42195	1.01393	1.00845
42196	1.20067	0.76156
42197	1.89226	1.35565
42198	2.44958	1.55743
42199	1.01393	0.543243
42200	1.89226	0.39764
42201	1.89226	2.1
42202	1.89226	2.1
42203	1.89226	2.1
42204	1.89226	2.1
42205	1.89226	2.1
42206	1.89226	2.1
42207	1.89226	2.1
42208	1.89226	0.386851
42209	1.89226	0.793099
42210	1.89226	2.1
42211	1.89226	1.34781
42212	1.89226	2.1
42213	1.89226	2.1
42214	1.89226	2.1
42215	1.89226	2.1
42216	1.89226	2.1
42217	1.89226	2.1
42218	1.89226	2.1
42219	1.91161	2.1
42220	2.45782	2.1
42221	1.39653	2.1

42222	2.44652	2.1
42223	4.35812	2.1
42224	2.73092	2.1
42225	1.89226	0.267951
42226	1.89226	2.1
42227	1.89226	0.488746
42228	2.44652	2.1
42229	2.44652	0.269015
42230	1.89226	2.1
42231	1.89226	0.661557
42232	1.89226	1.39139
42233	1.89226	2.1
42234	1.89226	1.63644
42235	1.89226	2.1
42236	2.64924	2.1
42237	1.31109	2.1
42238	1.31109	2.1
42239	1.49958	2.1
42240	1.95039	2.1
42241	2.14451	2.1
42242	0.825157	2.1
42243	0.634856	2.1
42244	0.816836	2.1
42245	0.816836	2.1
42246	0.556743	2.1
42247	0.639142	2.1
42248	0.985106	2.1
42249	0.795045	2.1
42250	0.967461	2.1
42251	0.552494	2.1
42252	0.676829	2.1
42253	0.98803	2.1
42254	2.28564	2.1
42255	2.34084	1.58835
42256	2.3502	2.1
42257	2.42908	2.1
42258	4.69104	2.1
42259	2.34084	2.1
42260	2.18077	2.1
42261	2.42908	2.1
42262	2.2947	0.941396
42263	4.63554	2.1
42264	2.3502	2.1
42265	2.42908	2.1
42266	2.38681	2.1
42267	2.50438	1.67825
42268	4.85458	2.1
42269	2.42908	2.1
42270	2.2947	1.69976
42271	2.50438	1.36465
42272	2.40627	2.1
42273	4.79908	1.76914
42274	1.89226	0.229826
42275	2.20711	1.64163
42276	1.89226	0.226013
42277	1.89226	1.9176
42278	2.02693	0.214022
42279	2.02693	1.94526

42280	1.2269	2.1
42281	2.20711	1.299
42282	2.0815	1.04058
42283	1.11506	2.1
42284	3.22109	2.1
42285	0.545802	2.01514
42286	0.547489	2.1
42287	0.85914	1.37445
42288	1.11641	1.56991
42289	0.899974	1.73862
42290	0.849893	2.1
42291	0.747885	2.03118
42292	0.747885	1.10121
42293	0.557184	2.1
42294	0.887039	2.1
42295	1.08948	2.1
42296	0.59815	2.1
42297	0.974139	2.1
42298	1.33385	2.1
42299	1.02368	2.1
42300	1.02368	2.1
42301	1.00833	2.1
42302	1.00833	2.1
42303	1.89226	0.474649
42304	1.89226	0.0845239
42305	1.89226	0.0825499
42306	1.89226	0.05
42307	3.78453	0.05
42308	1.74651	0.108661
42309	1.44605	0.05
42310	1.63557	0.0623085
42311	1.31993	0.05
42312	3.08162	0.05
42313	1.89226	0.0756307
42314	1.89226	0.0947819
42315	1.89226	0.0751282
42316	1.89226	0.05
42317	3.78452	0.05
42318	1.63557	0.0645503
42319	1.31992	0.05
42320	1.52615	0.0836846
42321	1.18549	0.05
42322	2.82106	0.05
42323	1.89226	0.05
42324	2.58101	0.05
42325	2.73797	0.216325
42326	2.73797	0.05
42327	1.89226	0.0832873
42328	1.89226	0.05
42329	1.91762	0.178781
42330	1.44354	0.13023
42331	1.33622	0.134171
42332	1.33622	0.10823
42333	2.20248	0.277177
42334	2.20248	0.191131
42335	1.11712	2.1
42336	1.11712	2.1
42337	1.11421	2.1

42338	1.11421	2.1
42339	0.833871	2.1
42340	0.926528	2.1
42341	1.3048	2.1
42342	1.40335	2.1
42343	0.926527	2.1
42344	1.40335	2.1
42345	1.3048	2.1
42346	0.833871	2.1
42347	0.879657	2.1
42348	0.937695	2.1
42349	0.946129	2.1
42350	0.946129	2.1
42351	0.851541	2.1
42352	0.851541	2.1
42353	1.1126	2.1
42354	1.04519	2.1
42355	0.851486	2.1
42356	0.980501	2.1
42357	0.898251	2.1
42358	0.851528	2.1
42359	1.37756	2.1
42360	2.73081	2.1
42361	2.44311	2.1
42362	2.44311	2.1
42363	1.66428	2.1
42364	1.66428	2.1
42365	1.89226	2.1
42366	1.89226	2.1
42367	1.89226	2.1
42368	1.89226	2.1
42369	3.78452	2.1
42370	1.65781	2.1
42371	1.89837	2.1
42372	1.46713	2.1
42373	1.98027	2.1
42374	3.63808	2.1
42375	2.29054	2.1
42376	2.26369	0.103
42377	1.98027	2.1
42378	2.15617	0.0640078
42379	4.24396	1.89759
42380	1.89226	2.1
42381	1.89226	0.102094
42382	1.89226	2.1
42383	1.89226	0.0951535
42384	3.78452	2.1
42385	0.946131	2.1
42386	0.946131	2.1
42387	0.946128	2.1
42388	0.946128	2.1
42389	0.946129	2.1
42390	0.946129	2.1
42391	0.94613	2.1
42392	0.94613	2.1
42393	0.94613	2.1
42394	0.94613	2.1
42395	0.94613	2.1

42396	0.94613	2.1
42397	0.812186	2.1
42398	0.812186	2.1
42399	0.819781	2.1
42400	0.819781	2.1
42401	0.913532	2.1
42402	0.990134	2.1
42403	0.733564	2.1
42404	0.641833	2.1
42405	0.641832	2.1
42406	0.913533	2.1
42407	0.733564	2.1
42408	0.990134	2.1
42409	0.994262	0.144987
42410	0.994262	2.1
42411	0.997354	2.1
42412	0.997354	0.147609
42413	1.00775	0.0533971
42414	1.07808	0.0640803
42415	0.913533	2.1
42416	0.990135	2.1
42417	0.913532	1.68209
42418	1.00775	1.52585
42419	0.990134	1.63789
42420	1.07808	1.43874
42421	0.946131	2.1
42422	0.946131	0.291818
42423	0.946128	2.1
42424	0.946128	0.156972
42425	0.946128	0.0731584
42426	0.946128	0.0721797
42427	0.94613	2.1
42428	0.94613	2.1
42429	0.946129	2.1
42430	0.946129	2.1
42431	0.946129	2.06114
42432	0.946129	2.09401
42433	0.816232	2.1
42434	0.760038	2.1
42435	0.845999	2.1
42436	0.845999	2.1
42437	1.0631	2.1
42438	1.28131	2.1
42439	0.474065	2.1
42440	0.591905	2.1
42441	0.601756	2.1
42442	1.23903	2.1
42443	0.988592	2.1
42444	0.49051	2.1
42445	2.57021	2.1
42446	1.89226	2.1
42447	2.68172	2.1
42448	1.89226	1.90321
42449	4.46247	2.1
42450	1.89226	2.1
42451	1.89226	1.76654
42452	1.89226	1.90839
42453	1.89226	1.73688

42454	3.78452	1.92638
42455	1.12573	2.1
42456	1.65504	1.60517
42457	1.38848	2.1
42458	1.38848	2.1
42459	1.30567	1.53368
42460	1.30567	2.1
42461	2.82413	2.1
42462	1.89226	2.1
42463	2.68172	2.1
42464	2.68172	2.1
42465	1.89226	2.1
42466	1.89226	2.1
42467	1.89226	2.1
42468	1.89226	2.09267
42469	1.89226	2.1
42470	1.89226	0.833938
42471	3.78452	2.00338
42472	1.30567	2.1
42473	1.65504	2.03406
42474	1.52724	2.1
42475	1.89249	2.1
42476	3.18228	2.1
42477	2.57386	0.05
42478	1.89226	0.0676459
42479	1.89226	0.0693793
42480	2.66753	0.05
42481	1.89226	0.05
42482	2.66753	0.05
42483	1.89226	0.05
42484	1.89226	0.0900326
42485	2.2973	0.0873079
42486	3.12479	0.05
42487	1.89226	0.0661749
42488	2.57385	0.05
42489	0.489293	2.1
42490	0.672698	2.1
42491	0.59038	2.1
42492	0.738357	2.1
42493	0.62269	2.1
42494	0.62269	2.1
42495	0.640388	2.1
42496	0.640388	2.1
42497	0.59038	2.1
42498	0.489293	2.1
42499	0.738357	2.1
42500	0.672698	2.1
42501	1.89226	2.1
42502	1.89226	2.1
42503	1.89226	2.1
42504	1.89226	1.51976
42505	3.78452	2.1
42506	2.82858	2.1
42507	1.89226	1.45924
42508	2.5671	2.1
42509	1.89226	2.1
42510	2.5671	2.1
42511	1.89226	2.1

42512	2.06581	0.05
42513	2.47308	0.05
42514	1.89433	0.05
42515	1.67571	0.05
42516	3.87274	0.05
42517	2.55791	0.05
42518	2.10276	0.05
42519	2.47308	0.05
42520	2.47308	0.05
42521	1.83415	0.05
42522	1.90922	0.05
42523	1.18421	0.05
42524	2.03277	0.05
42525	1.18421	0.05
42526	2.01275	0.05
42527	1.42818	0.05
42528	2.25673	0.05
42529	0.882904	0.05
42530	0.958492	0.05
42531	0.905756	0.05
42532	0.905756	0.05
42533	1.06352	0.05
42534	0.797654	0.05
42535	1.11319	0.05
42536	0.716795	0.05
42537	0.820721	0.05
42538	0.731963	0.05
42539	1.13143	0.05
42540	1.10528	0.05
42541	1.89226	0.05
42542	1.89226	0.05
42543	1.89226	0.05
42544	1.89226	0.05
42545	1.89226	0.05
42546	1.89226	0.05
42547	1.01241	0.05
42548	0.703929	0.05
42549	0.946133	0.05
42550	0.946133	0.05
42551	1.04072	0.05
42552	1.163 0.05	
42553	0.639952	0.0600993
42554	0.851543	0.05
42555	0.946132	0.05
42556	0.920761	0.05
42557	0.711038	0.05
42558	0.711038	0.05
42559	1.89226	0.05
42560	1.89226	0.05
42561	1.89226	0.05
42562	1.89226	0.05
42563	1.89226	0.05
42564	1.89226	0.05
42565	1.89226	0.05
42566	1.89226	0.05
42567	2.41075	0.05
42568	1.89226	0.05
42569	3.78451	0.05

42570	0.968476	2.04198
42571	1.20674	0.690232
42572	1.13456	0.427162
42573	1.13456	2.1
42574	1.02074	0.287226
42575	1.02074	2.1
42576	1.02074	2.1
42577	1.20674	2.05338
42578	1.07866	0.863303
42579	1.28649	0.876287
42580	2.2854	2.08076
42581	1.94221	0.052577
42582	1.36717	0.05
42583	1.45535	0.0538724
42584	1.93771	0.05
42585	3.39756	0.0524471
42586	1.94222	0.0889578
42587	2.25035	0.05
42588	2.41806	0.0537655
42589	2.42256	0.05
42590	1.93772	0.0616833
42591	1.94222	0.0529884
42592	1.49906	0.05
42593	1.93771	0.05
42594	1.45535	0.05
42595	1.45535	0.05
42596	1.91484	0.05
42597	1.91484	0.05
42598	1.91484	0.05
42599	2.25035	0.05
42600	1.93772	0.05
42601	1.93772	0.05
42602	2.11465	0.05
42603	2.11465	0.05
42604	2.61735	0.05
42605	1.5082	0.05
42606	2.36481	0.0540555
42607	1.58597	0.05
42608	2.36481	0.05
42609	1.46653	0.05
42610	2.36482	0.0631231
42611	1.08133	0.0589748
42612	1.5082	0.05
42613	2.01868	0.0738218
42614	3.52688	0.0623658
42615	0.94613	0.05
42616	0.94613	0.05
42617	0.946131	0.0503736
42618	0.946131	0.0716753
42619	0.946129	0.0682095
42620	0.946129	0.05
42621	0.946132	0.05
42622	0.946132	0.0526057
42623	0.94613	0.05
42624	0.94613	0.05
42625	0.946131	0.05
42626	0.946131	0.05
42627	2.53725	0.05

42628	1.89227	0.05
42629	1.89227	0.05
42630	2.27073	0.05
42631	1.89227	0.05
42632	2.27073	0.05
42633	1.89227	0.05
42634	1.89227	0.0726537
42635	1.89227	0.0787399
42636	1.89227	0.05
42637	1.89227	0.05707
42638	1.89227	0.05
42639	1.86766	2.1
42640	2.83487	2.1
42641	2.84318	2.1
42642	2.59401	2.1
42643	2.11684	2.1
42644	1.86766	2.1
42645	1.10638	2.1
42646	2.11684	2.1
42647	1.29111	2.1
42648	2.37439	2.1
42649	3.40796	2.1
42650	2.56843	2.1
42651	1.89226	2.1
42652	1.89226	2.1
42653	2.71932	2.1
42654	4.46069	2.1
42655	1.89226	2.1
42656	1.89226	2.1
42657	1.89226	2.1
42658	1.89226	2.1
42659	3.78452	2.1
42660	0.6416	2.1
42661	0.697585	2.1
42662	0.670706	2.1
42663	0.598927	2.1
42664	0.644313	2.1
42665	0.644313	2.1
42666	0.652199	2.1
42667	0.652199	2.1
42668	0.697587	2.1
42669	0.670705	2.1
42670	0.598926	2.1
42671	0.641601	2.1
42672	1.89226	2.1
42673	1.89226	2.1
42674	1.89226	2.1
42675	1.89226	2.1
42676	1.89226	2.1
42677	1.89226	2.1
42678	0.697585	2.1
42679	0.730617	2.1
42680	0.712082	2.1
42681	0.670706	2.1
42682	0.69854	2.1
42683	0.69854	2.1
42684	0.702783	2.1
42685	0.702783	2.1

42686	0.730618	2.1
42687	0.712081	2.1
42688	0.697586	2.1
42689	0.670705	2.1
42690	1.89226	2.1
42691	1.89226	2.1
42692	1.89226	1.07202
42693	1.89226	2.1
42694	1.89226	0.781585
42695	1.89226	2.1
42696	2.0815	2.1
42697	1.12076	2.1
42698	1.38662	2.1
42699	2.01244	2.1
42700	3.41508	2.1
42701	1.89226	2.1
42702	2.54324	2.1
42703	1.91981	2.1
42704	1.89226	2.1
42705	2.57079	2.1
42706	2.54324	2.1
42707	2.54325	2.1
42708	1.94779	2.1
42709	1.76879	2.1
42710	1.73811	2.1
42711	2.5708	2.1
42712	2.54325	2.1
42713	1.89226	2.1
42714	2.57079	2.1
42715	1.91894	2.1
42716	1.89226	2.1
42717	2.59747	2.1
42718	2.57079	2.1
42719	2.5708	2.1
42720	1.82268	2.1
42721	1.79738	2.1
42722	1.77071	2.1
42723	2.59747	2.1
42724	2.5708	2.1
42725	1.89226	0.05
42726	1.89226	1.252
42727	1.89226	0.05
42728	1.89226	1.41798
42729	3.78451	0.0557724
42730	2.21582	0.05
42731	1.94412	1.09098
42732	2.13428	0.05
42733	1.86292	0.14034
42734	4.07839	0.05
42735	1.94412	0.2813
42736	1.5854	2.1
42737	1.86293	0.219214
42738	1.50767	2.1
42739	3.45179	2.1
42740	1.89226	0.326369
42741	1.89226	2.1
42742	1.89226	1.74519
42743	1.89226	2.1

42744	3.78452	2.1
42745	1.89226	0.05
42746	1.89226	1.76224
42747	1.89226	0.05
42748	1.89226	1.05207
42749	3.78452	0.0598136
42750	2.13428	0.05
42751	1.86293	0.180633
42752	2.04737	0.05
42753	1.77122	1.05946
42754	3.9055	0.05
42755	1.86293	0.0739831
42756	1.50767	2.1
42757	1.41077	1.41548
42758	1.41077	2.1
42759	1.77123	1.59777
42760	1.86813	2.1
42761	0.810943	0.973913
42762	0.810943	2.1
42763	0.810945	0.483365
42764	0.810945	1.00129
42765	0.810958	2.1
42766	0.810958	2.1
42767	1.89226	2.1
42768	1.35168	2.1
42769	1.08132	1.68595
42770	1.08132	2.1
42771	1.0813	2.1
42772	0.745979	2.1
42773	0.745979	2.1
42774	0.778781	2.1
42775	0.778781	2.1
42776	0.59549	2.1
42777	0.649658	2.1
42778	0.994784	2.1
42779	0.875102	2.1
42780	0.875102	2.1
42781	0.59549	2.1
42782	0.649658	2.1
42783	0.994784	2.1
42784	1.57887	2.1
42785	1.75021	2.1
42786	1.13341	2.1
42787	1.13341	2.1
42788	1.19098	2.1
42789	1.63644	2.1
42790	2.5761	0.05
42791	2.43404	0.181616
42792	2.44813	0.05
42793	2.31751	0.151789
42794	4.88218	0.05
42795	2.43405	0.054766
42796	2.22575	2.1
42797	2.31751	0.126875
42798	2.10893	2.1
42799	4.54298	1.72352
42800	2.44813	0.05
42801	2.31751	0.144084

42802	2.30908	0.05
42803	2.19815	0.108165
42804	4.64628	0.05
42805	2.31751	0.0816544
42806	2.10893	2.1
42807	2.19815	0.0706726
42808	1.98061	2.1
42809	4.30708	1.4802
42810	0.94613	2.1
42811	0.94613	2.1
42812	1.40372	2.1
42813	1.40372	2.1
42814	1.40372	2.1
42815	1.37167	2.1
42816	0.946132	2.1
42817	0.946132	2.1
42818	0.946131	2.1
42819	1.40372	2.1
42820	0.94613	2.1
42821	1.37167	2.1
42822	2.80744	2.1
42823	2.06984	2.1
42824	2.83418	2.1
42825	2.74335	2.1
42826	2.16067	2.1
42827	2.06984	2.1
42828	1.077 0.05	
42829	0.809116	0.05
42830	0.600419	0.05
42831	0.790277	0.05
42832	0.927647	0.05
42833	0.758598	0.05
42834	0.85749	0.05
42835	0.85749	0.05
42836	1.02417	0.05
42837	0.600411	0.05
42838	0.751701	0.05
42839	1.07702	0.05
42840	1.89226	2.1
42841	1.89226	2.1
42842	1.89226	2.1
42843	1.89226	2.1
42844	1.89226	2.1
42845	1.89226	2.1
42846	1.89226	2.1
42847	1.89226	2.1
42848	1.89226	2.1
42849	1.89226	2.1
42850	3.78453	2.1
42851	1.89226	2.1
42852	1.89226	2.1
42853	1.89226	2.1
42854	1.89226	2.1
42855	1.89226	2.1
42856	1.89226	2.1
42857	0.946131	2.1
42858	0.946131	2.1
42859	0.946131	2.1

42860	0.946131	2.1
42861	0.946133	2.1
42862	0.946133	2.1
42863	0.946128	2.1
42864	0.946128	2.1
42865	0.946131	2.1
42866	0.946131	2.1
42867	0.94613	2.1
42868	0.94613	2.1
42869	2.17159	2.1
42870	2.00083	2.0757
42871	2.21305	2.1
42872	2.00551	2.1
42873	2.20837	2.1
42874	2.00083	2.02748
42875	2.20837	2.07162
42876	1.80328	1.03296
42877	2.22962	2.1
42878	2.00083	1.65504
42879	2.03207	0.670188
42880	1.80328	0.444742
42881	2.28564	2.1
42882	2.20837	1.75802
42883	2.30406	2.1
42884	2.17159	2.1
42885	2.34084	2.1
42886	2.20837	2.1
42887	1.09523	0.472909
42888	1.09523	2.06561
42889	1.09934	2.1
42890	1.09934	2.1
42891	1.01603	0.345602
42892	1.09038	1.94431
42893	1.10419	1.06309
42894	1.17042	2.1
42895	1.17042	2.1
42896	1.09038	1.90552
42897	1.10419	1.3903
42898	1.01603	2.1
42899	2.60152	0.0746081
42900	0.976334	0.276543
42901	2.22344	0.368883
42902	2.22344	0.0956947
42903	1.36405	0.436853
42904	1.36405	0.05
42905	1.89226	2.1
42906	1.70302	2.1
42907	1.69019	2.1
42908	1.70302	2.1
42909	1.73933	2.1
42910	1.70303	1.75467
42911	1.89226	2.1
42912	1.89226	0.96532
42913	1.89226	2.1
42914	1.89226	2.1
42915	3.78452	2.1
42916	1.89226	2.06299
42917	1.59036	2.1

42918	1.65683	2.1
42919	1.59036	2.1
42920	1.95873	2.1
42921	1.89226	0.897121
42922	0.946131	0.584018
42923	0.946131	1.11401
42924	0.946127	2.1
42925	0.946127	2.1
42926	0.946127	2.1
42927	0.946127	0.233269
42928	0.946131	2.1
42929	0.946131	1.12205
42930	0.946129	1.52073
42931	0.946129	1.58182
42932	0.94613	1.83009
42933	0.94613	1.7836
42934	0.851541	2.1
42935	0.851541	2.1
42936	0.851483	2.1
42937	1.15713	2.1
42938	0.851539	2.1
42939	0.851539	2.1
42940	0.942127	2.1
42941	1.14455	2.1
42942	0.838683	2.1
42943	0.946131	2.1
42944	0.957503	2.1
42945	0.957504	2.1
42946	1.89226	0.388485
42947	1.89226	0.05
42948	1.89226	0.16922
42949	1.89226	2.1
42950	3.78452	0.0527897
42951	1.89226	0.05
42952	1.89226	0.05
42953	1.89226	0.0974919
42954	1.89226	0.05
42955	1.89226	0.05
42956	1.89226	0.05
42957	1.89226	0.05
42958	1.89226	0.05
42959	1.89226	0.05
42960	1.89226	0.05
42961	3.78453	0.05
42962	1.89226	0.05
42963	1.89226	2.1
42964	1.89226	2.1
42965	1.89226	0.918713
42966	3.78453	2.1
42967	1.22575	0.197058
42968	1.05438	0.25625
42969	0.946131	0.372806
42970	0.946131	0.160824
42971	0.946129	1.99437
42972	1.22575	0.0558291
42973	1.05438	0.576706
42974	0.946132	0.05
42975	1.05438	0.644314

42976	1.05438	0.686301
42977	0.946131	0.129053
42978	0.946131	0.591206
42979	2.4515	0.0630043
42980	1.13726	0.05
42981	2.10875	0.05
42982	1.49515	0.05
42983	2.10875	0.05
42984	1.49515	0.05
42985	0.831477	0.05
42986	0.747575	0.05
42987	1.22575	0.054583
42988	1.28174	0.05
42989	1.28174	0.05
42990	0.831476	0.05
42991	1.22575	0.05
42992	0.747575	0.05
42993	1.03561	0.05
42994	1.03561	0.05
42995	1.02162	0.05
42996	1.02162	0.05
42997	1.89227	2.1
42998	2.56348	0.211765
42999	1.89227	2.1
43000	2.45151	1.11451
43001	1.89227	2.1
43002	2.45151	0.157452
43003	1.40013	0.181098
43004	1.89226	1.57335
43005	1.89226	0.522168
43006	0.946643	0.105765
43007	1.89226	1.10612
43008	0.946643	0.223132
43009	1.89226	0.05
43010	1.89226	0.05
43011	1.89226	0.05
43012	1.89226	0.05
43013	3.78453	0.05
43014	1.89226	0.05
43015	1.89226	0.05
43016	1.89226	0.05
43017	1.89226	0.05
43018	3.78453	0.05
43019	0.946131	0.05
43020	0.946131	0.05
43021	0.946128	0.05
43022	0.946128	0.05
43023	0.94613	0.05
43024	0.94613	0.05
43025	0.946129	0.05
43026	0.946129	0.05
43027	0.94613	0.05
43028	0.94613	0.05
43029	0.94613	0.05
43030	0.94613	0.05
43031	1.89226	0.05
43032	1.89226	0.05
43033	1.17244	0.05

43034	1.89226	0.05
43035	0.975291	0.05
43036	1.57408	0.05
43037	0.946132	0.05
43038	0.946132	0.05
43039	0.946131	0.05
43040	0.946131	0.05
43041	0.946132	0.05
43042	0.946132	0.05
43043	0.946131	0.05
43044	0.946131	0.05
43045	0.946131	0.05
43046	0.946131	0.05
43047	0.946132	0.05
43048	0.946132	0.05
43049	2.74744	0.05
43050	1.89226	0.05
43051	2.70805	0.05
43052	1.89226	0.05
43053	2.70805	0.05
43054	1.89226	0.05
43055	2.74744	0.05
43056	1.89226	0.05
43057	2.57465	0.05
43058	1.89226	0.05
43059	4.6397	0.05
43060	1.89226	0.05
43061	1.89226	0.0755671
43062	1.89226	0.0562707
43063	1.89226	0.282666
43064	3.78452	0.0669795
43065	1.89226	0.05
43066	1.89226	0.149604
43067	1.89226	0.05
43068	1.89226	0.05
43069	1.89226	0.276356
43070	1.89226	0.184308
43071	2.16206	0.05
43072	2.67861	0.05
43073	3.11996	0.05
43074	1.70303	0.05
43075	2.89015	0.05
43076	1.486 0.05	
43077	2.12839	0.05
43078	2.83631	0.05
43079	3.08096	0.05
43080	1.84157	0.05
43081	3.08096	0.05
43082	1.84157	0.05
43083	1.89225	0.776542
43084	1.89225	0.203567
43085	1.89225	0.31023
43086	1.89225	1.04872
43087	1.89225	0.197763
43088	1.89225	0.708401
43089	1.89226	0.14155
43090	1.89226	0.173032
43091	1.89226	0.705904

43092	1.89226	0.0885228
43093	3.78452	0.104443
43094	1.89226	0.0604058
43095	2.49041	0.05
43096	1.89226	0.0578385
43097	1.89226	0.05
43098	2.27073	0.05
43099	2.27073	0.05
43100	1.09904	0.05
43101	0.677147	0.05
43102	1.3365	0.05
43103	0.95759	0.05
43104	0.957591	0.05
43105	0.677148	0.05
43106	1.09904	0.05
43107	1.3365	0.05
43108	0.996015	0.05
43109	0.996015	0.05
43110	1.01764	0.05
43111	1.01764	0.05
43112	2.77349	0.05
43113	2.72723	0.05
43114	2.43156	0.05
43115	2.4564	0.05
43116	5.20505	0.05
43117	2.77349	0.05
43118	2.68508	0.05
43119	2.33761	0.05
43120	2.4564	0.05
43121	5.1111	0.05
43122	2.43156	0.05
43123	2.70208	0.05
43124	1.85779	0.05
43125	2.10695	0.05
43126	4.77572	0.05
43127	2.33761	0.05
43128	2.70208	0.05
43129	1.85779	0.05
43130	1.61597	0.05
43131	4.19542	0.05
43132	0.946132	0.797049
43133	0.946132	1.49009
43134	0.746729	1.63803
43135	0.746729	2.06811
43136	0.946132	2.09489
43137	0.915958	1.57955
43138	0.946133	0.747713
43139	0.74673	2.1
43140	0.946133	1.63285
43141	0.946133	1.62591
43142	0.915958	1.63263
43143	0.746729	1.50013
43144	1.50743	2.1
43145	2.45501	2.1
43146	2.29118	2.1
43147	2.29118	2.1
43148	1.75482	2.1
43149	1.75482	2.1

43150	2.45501	2.1
43151	2.76927	0.0964195
43152	2.29118	2.1
43153	2.64523	0.177429
43154	5.10024	1.81514
43155	1.25559	2.1
43156	2.29118	2.1
43157	2.0815	2.1
43158	2.26387	2.1
43159	1.35668	2.1
43160	1.47667	2.1
43161	2.28481	2.1
43162	2.64523	0.283461
43163	2.50057	2.1
43164	2.50057	0.471694
43165	2.29118	2.1
43166	2.29118	0.894498
43167	0.946129	0.0502048
43168	0.946129	0.05
43169	1.23929	0.05
43170	1.15246	0.05
43171	0.946131	0.05
43172	0.946131	0.0673915
43173	1.15246	0.05
43174	1.15246	0.05
43175	0.946131	0.05
43176	1.15246	0.054774
43177	0.946131	0.05
43178	1.23928	0.0514264
43179	0.946128	0.0641224
43180	0.946128	0.0568619
43181	1.15246	0.05
43182	0.942814	0.0594405
43183	0.946131	0.05
43184	0.946131	0.0773261
43185	0.961347	0.0520351
43186	0.942811	0.05
43187	0.946131	0.05
43188	0.961347	0.05
43189	0.946131	0.05
43190	1.15246	0.05
43191	2.32334	0.0643434
43192	1.57621	0.05
43193	1.90335	0.05
43194	1.24538	0.05
43195	3.47956	0.05
43196	0.965762	0.05
43197	0.965762	0.05
43198	1.00245	0.05
43199	1.00245	0.05
43200	0.92786	0.05
43201	0.705297	0.05
43202	0.856414	0.05
43203	1.28968	0.05
43204	1.00306	0.05
43205	0.705303	0.05
43206	1.28967	0.05
43207	0.975478	0.05

43208	3.05308	2.1
43209	1.76816	2.1
43210	1.76816	2.1
43211	1.69129	2.1
43212	2.58731	2.1
43213	2.51043	2.1
43214	1.89226	0.05
43215	2.71464	0.05
43216	2.34627	0.05
43217	1.89226	0.05
43218	2.34627	0.05
43219	1.89226	0.05
43220	2.34627	0.05
43221	2.71464	0.05
43222	1.98252	0.05
43223	1.98252	0.05
43224	1.51026	0.05
43225	2.34627	0.05
43226	2.38698	1.18134
43227	2.35142	2.1
43228	2.60343	1.11911
43229	2.6636	1.0585
43230	4.99041	1.49496
43231	1.07811	2.1
43232	1.1952	2.1
43233	1.17571	2.02596
43234	1.30171	0.836167
43235	1.18322	0.740361
43236	1.18322	2.1
43237	1.18769	2.1
43238	1.18769	1.14627
43239	1.1952	2.1
43240	1.30171	2.1
43241	1.17571	2.1
43242	1.07811	2.09998
43243	1.89226	1.44781
43244	2.6636	1.95636
43245	1.89226	0.184448
43246	2.60343	1.23082
43247	1.89226	1.52008
43248	2.60343	2.1
43249	1.5344	0.05
43250	1.4516	0.05
43251	1.17816	0.05
43252	1.21056	0.05
43253	2.71257	0.05
43254	1.89226	2.06536
43255	1.89226	0.657592
43256	1.89226	1.24592
43257	1.89226	1.38267
43258	3.78452	1.18909
43259	1.89226	1.36505
43260	1.89226	0.184785
43261	1.89226	0.660314
43262	1.89226	0.448884
43263	1.65573	1.02981
43264	1.65573	0.642516
43265	1.89226	1.2

43266	1.89226	1.49094
43267	1.89226	2.1
43268	2.64212	0.510371
43269	3.78452	1.64651
43270	1.89226	2.1
43271	1.89226	2.1
43272	1.89226	2.1
43273	1.89226	2.1
43274	3.78452	2.1
43275	2.7367	1.28893
43276	2.07947	2.1
43277	2.47319	2.1
43278	1.85057	2.1
43279	4.55267	2.1
43280	2.07948	2.1
43281	1.3294	2.1
43282	1.85057	2.1
43283	1.09962	2.1
43284	3.1791	2.1
43285	1.89226	2.1
43286	1.89226	2.1
43287	1.89226	2.1
43288	1.89226	2.1
43289	3.78453	2.1
43290	0.94613	2.1
43291	0.94613	1.77592
43292	0.94613	2.1
43293	0.94613	2.1
43294	0.946128	2.1
43295	0.946128	2.1
43296	0.946132	1.5241
43297	0.946132	1.68018
43298	0.94613	2.1
43299	0.94613	2.1
43300	0.946131	2.1
43301	0.946131	2.1
43302	1.00875	2.1
43303	1.00875	0.652646
43304	1.01676	2.1
43305	1.01676	1.04901
43306	0.925282	2.1
43307	0.804877	2.1
43308	1.2366	1.86222
43309	1.10023	1.90301
43310	0.804878	2.1
43311	1.10023	2.1
43312	1.2366	2.1
43313	0.925284	2.1
43314	1.89226	2.1
43315	1.89226	2.1
43316	1.89226	2.1
43317	1.89226	2.1
43318	3.78453	2.1
43319	1.89226	2.1
43320	1.89226	2.1
43321	1.89226	2.1
43322	1.89226	2.1
43323	1.89226	2.1

43324	1.89226	2.1
43325	1.09766	2.1
43326	1.14842	2.1
43327	1.02909	2.1
43328	1.07659	2.1
43329	1.16718	2.1
43330	0.954189	2.1
43331	1.06653	2.1
43332	1.06653	2.1
43333	1.20738	2.1
43334	1.02909	2.1
43335	1.09767	2.1
43336	0.815951	2.1
43337	2.00793	2.1
43338	1.14048	2.1
43339	1.19086	2.1
43340	1.13723	2.1
43341	2.00793	2.1
43342	2.11772	2.1
43343	1.89226	2.1
43344	1.89226	2.1
43345	1.89226	2.1
43346	1.89226	2.1
43347	1.89226	2.1
43348	1.89226	2.1
43349	1.89226	2.1
43350	1.89226	2.1
43351	1.89226	2.1
43352	1.89226	2.1
43353	1.89226	2.1
43354	1.89226	2.1
43355	1.1779	2.1
43356	1.26166	2.1
43357	1.0875	2.1
43358	1.00397	2.1
43359	1.13286	2.1
43360	1.13286	2.1
43361	1.13277	2.1
43362	1.13277	2.1
43363	1.26166	2.1
43364	1.08749	2.1
43365	1.1779	2.1
43366	1.00396	2.1
43367	1.0875	2.1
43368	1.00397	2.1
43369	0.685749	2.1
43370	0.595432	2.1
43371	0.83976	2.1
43372	0.83976	2.1
43373	0.843169	2.1
43374	0.843169	2.1
43375	1.0875	2.1
43376	0.685749	2.1
43377	1.00397	2.1
43378	0.595432	2.1
43379	1.89226	2.1
43380	1.86443	2.1
43381	2.4988	2.1

43382	2.0815	2.1
43383	2.28173	2.1
43384	1.86443	2.1
43385	1.89226	2.1
43386	1.89226	2.1
43387	1.89226	2.1
43388	1.89226	2.1
43389	1.89226	2.1
43390	1.89226	2.1
43391	0.946131	2.1
43392	0.946131	2.1
43393	0.969491	2.1
43394	0.949105	2.1
43395	0.949103	2.1
43396	0.949103	2.1
43397	0.946133	2.1
43398	0.946133	2.1
43399	0.946131	2.1
43400	0.969492	2.1
43401	0.946131	2.1
43402	0.949105	2.1
43403	1.89226	2.1
43404	2.07428	2.1
43405	1.93464	2.1
43406	1.89226	2.1
43407	2.11667	2.1
43408	2.07428	2.1
43409	0.946132	2.1
43410	0.946132	2.1
43411	0.946131	2.1
43412	0.946131	2.1
43413	0.946132	2.1
43414	0.946132	2.1
43415	0.946132	2.1
43416	0.946132	2.1
43417	0.946132	2.1
43418	0.946132	2.1
43419	0.946132	2.1
43420	0.946132	2.1
43421	1.97992	2.1
43422	1.89226	2.1
43423	1.89226	2.1
43424	1.93898	2.1
43425	1.93898	2.1
43426	1.89226	2.1
43427	2.12475	0.133274
43428	2.79029	0.34104
43429	2.7803	1.04675
43430	2.13052	0.10015
43431	4.92081	0.121985
43432	1.06238	0.456935
43433	1.06526	0.14594
43434	0.642901	0.163088
43435	0.655055	1.03751
43436	0.856391	0.159115
43437	0.856391	0.131979
43438	0.86104	1.85326
43439	0.86104	0.125739

43440	1.06526	0.180334
43441	0.655055	0.0827886
43442	0.642901	0.172739
43443	1.06238	0.165421
43444	2.7803	0.966015
43445	2.13053	0.05
43446	2.14346	0.334092
43447	2.77937	0.182202
43448	4.9099	0.392924
43449	2.14346	0.441853
43450	1.31011	0.0536187
43451	1.34124	0.110829
43452	1.31011	0.162382
43453	2.16166	0.130984
43454	2.13053	0.138618
43455	1.89226	2.1
43456	2.58176	2.1
43457	2.01422	2.1
43458	1.89226	2.1
43459	2.70373	2.1
43460	2.58176	2.1
43461	1.89226	2.1
43462	1.89226	2.1
43463	1.89226	2.1
43464	1.89226	2.1
43465	1.89226	2.1
43466	1.89226	2.1
43467	0.946128	2.1
43468	0.946128	2.1
43469	1.38521	2.1
43470	1.38521	2.1
43471	1.38521	2.1
43472	1.35186	2.1
43473	0.946131	2.1
43474	0.946131	2.1
43475	0.94613	2.1
43476	1.38521	2.1
43477	0.946131	2.1
43478	1.35186	2.1
43479	0.946133	2.1
43480	0.946133	2.1
43481	0.946132	2.1
43482	0.946132	2.1
43483	0.946132	2.1
43484	0.946132	2.1
43485	0.946132	2.1
43486	0.946132	2.1
43487	0.946132	2.1
43488	0.946132	2.1
43489	0.946133	2.1
43490	0.946133	2.1
43491	2.33374	2.1
43492	1.89226	2.1
43493	1.89226	2.1
43494	1.89226	2.1
43495	2.60377	2.1
43496	2.60377	2.1
43497	2.6771	0.05

43498	1.36416	0.05
43499	1.64983	0.05
43500	1.64983	0.05
43501	2.44131	0.05
43502	2.44131	0.05
43503	2.58321	0.0846801
43504	1.89226	0.161172
43505	2.44283	0.05
43506	2.44283	0.0681707
43507	1.89226	0.390036
43508	1.89226	0.85283
43509	2.58321	0.21067
43510	2.44283	0.0825264
43511	1.49358	0.05
43512	1.57702	0.0708881
43513	3.93641	0.0570116
43514	0.951913	0.05
43515	0.81556	0.05
43516	0.946132	0.05
43517	0.946132	0.05
43518	1.04072	0.05
43519	1.12182	0.05
43520	0.856745	0.05
43521	0.851542	0.05
43522	0.923163	0.05
43523	0.923163	0.05
43524	1.04304	0.05
43525	0.85098	0.05
43526	2.70125	0.749516
43527	2.37734	1.11969
43528	2.63495	1.02599
43529	2.30539	2.049
43530	5.01229	1.91324
43531	1.89226	1.94163
43532	1.89226	2.1
43533	1.89226	1.46339
43534	1.89226	2.1
43535	3.78452	1.13259
43536	1.89226	2.1
43537	1.89226	2.1
43538	1.89226	2.1
43539	1.89226	2.1
43540	3.78452	2.1
43541	2.37734	2.0725
43542	1.79346	2.1
43543	2.53594	1.71469
43544	1.70302	2.1
43545	4.27682	2.1
43546	2.63495	0.532098
43547	2.30539	0.925004
43548	2.49445	0.608822
43549	2.12089	0.57415
43550	4.75584	0.415246
43551	1.89226	1.10006
43552	1.89226	2.0098
43553	1.89226	1.14384
43554	1.89226	2.06564
43555	3.78452	2.1

43556	1.89226	1.96057
43557	1.89226	2.1
43558	1.89226	2.1
43559	1.89226	2.1
43560	1.89226	1.25382
43561	1.89226	1.48385
43562	2.53594	0.525565
43563	1.60017	1.13599
43564	1.57079	0.790633
43565	1.70302	0.55636
43566	2.15772	0.712956
43567	2.33299	0.401165
43568	2.09114	2.1
43569	2.34898	2.1
43570	2.30729	2.1
43571	2.06556	2.1
43572	4.41454	2.1
43573	2.09114	2.1
43574	1.59654	2.1
43575	1.57823	2.1
43576	2.06556	2.1
43577	3.6621	2.1
43578	2.30729	2.1
43579	2.06556	2.1
43580	2.03917	2.1
43581	2.26617	2.1
43582	4.33173	2.1
43583	2.03917	2.1
43584	2.06556	2.1
43585	2.045 2.1	
43586	1.55767	2.1
43587	1.57823	2.1
43588	1.57823	2.1
43589	1.89225	2.1
43590	1.09726	2.1
43591	1.61196	2.1
43592	1.28597	2.1
43593	1.28597	0.05
43594	1.45633	2.1
43595	1.45633	2.1
43596	1.9544	0.05
43597	1.61196	2.1
43598	1.45633	2.1
43599	1.53735	2.1
43600	3.14931	2.1
43601	1.89226	2.1
43602	1.89226	2.1
43603	1.89226	2.1
43604	1.89226	2.1
43605	3.78453	2.1
43606	0.810946	2.1
43607	0.810946	2.1
43608	0.810947	2.1
43609	0.810947	2.1
43610	0.810933	2.1
43611	0.810933	2.1
43612	1.89226	2.1
43613	1.3517	2.1

43614	1.08132	2.1
43615	1.08132	2.1
43616	1.08133	2.1
43617	0.851539	2.1
43618	0.851539	2.1
43619	0.972053	2.1
43620	1.05632	2.1
43621	0.94613	2.1
43622	0.94613	2.1
43623	1.0279	2.1
43624	0.94991	2.1
43625	1.0279	2.1
43626	0.946131	2.1
43627	0.865643	2.1
43628	0.94613	2.1
43629	1.89226	2.1
43630	1.89226	2.1
43631	1.89226	2.1
43632	1.89226	2.1
43633	3.78452	2.1
43634	1.03882	2.1
43635	1.91121	2.1
43636	1.41833	2.1
43637	2.34547	2.1
43638	3.32954	2.1
43639	2.3979	2.1
43640	1.89226	2.1
43641	1.89226	2.1
43642	1.89226	2.1
43643	2.0558	2.1
43644	2.0558	2.1
43645	1.89226	2.1
43646	1.89226	1.89427
43647	1.89226	2.1
43648	1.89226	2.1
43649	3.78452	2.1
43650	1.05816	2.1
43651	1.05816	2.1
43652	0.842045	2.1
43653	1.05252	2.1
43654	1.36057	2.1
43655	1.14339	2.1
43656	0.873397	2.1
43657	0.585073	2.1
43658	0.873388	2.1
43659	1.15129	2.1
43660	0.69143	2.1
43661	1.1434	2.1
43662	1.93338	2.1
43663	1.85883	2.1
43664	1.51714	2.1
43665	1.85883	2.1
43666	1.54795	2.1
43667	1.89226	2.1
43668	1.89226	2.1
43669	1.89226	2.1
43670	1.89226	2.1
43671	1.89226	2.1

43672	3.78452	2.1
43673	0.844726	2.1
43674	0.844726	2.1
43675	0.960524	2.1
43676	1.16288	2.1
43677	0.851541	2.1
43678	0.851541	2.1
43679	0.929147	2.1
43680	1.15533	2.1
43681	0.844697	2.1
43682	0.946131	2.1
43683	0.851632	2.1
43684	0.946131	2.1
43685	1.89226	2.1
43686	1.70303	2.1
43687	1.74006	2.1
43688	1.70303	2.1
43689	1.99471	2.1
43690	1.93338	2.1
43691	0.946131	2.1
43692	0.946131	2.1
43693	0.946131	2.1
43694	0.946131	2.1
43695	0.94613	2.1
43696	0.94613	2.1
43697	0.946132	2.1
43698	0.946132	2.1
43699	0.946131	2.1
43700	0.946131	2.1
43701	0.946131	2.1
43702	0.946131	2.1
43703	1.58158	2.1
43704	1.89226	2.1
43705	1.89226	2.1
43706	1.89226	2.1
43707	1.68939	2.1
43708	1.68939	2.1
43709	0.946132	0.05
43710	0.946132	0.05
43711	1.12988	0.05
43712	1.30783	0.05
43713	1.30783	0.05
43714	0.946133	0.05
43715	0.946132	0.05
43716	1.12988	0.05
43717	1.30783	0.05
43718	1.30783	0.05
43719	0.946133	0.05
43720	0.946133	0.05
43721	1.89227	0.05
43722	1.89227	0.05
43723	1.89227	0.05
43724	1.89227	0.05
43725	1.89227	0.05
43726	1.89227	0.05
43727	1.89226	0.05
43728	1.89226	0.05
43729	1.89226	0.05

43730	1.89226	0.05
43731	1.89226	0.05
43732	1.89226	0.05
43733	0.946131	0.05
43734	0.946131	0.05
43735	1.30783	0.05
43736	1.37715	0.05
43737	1.37715	0.05
43738	0.946133	0.05
43739	1.30783	0.05
43740	0.946129	0.05
43741	1.37715	0.05
43742	1.37715	0.05
43743	0.946131	0.05
43744	0.946131	0.05
43745	1.17056	0.05
43746	0.591757	0.05
43747	0.522025	0.05
43748	0.929727	0.05
43749	0.929728	0.05
43750	1.17056	0.05
43751	0.591756	0.05
43752	0.522025	0.05
43753	0.889054	0.05
43754	0.889054	0.05
43755	0.803535	0.05
43756	0.803535	0.05
43757	1.49814	2.1
43758	2.37439	2.1
43759	2.63923	2.1
43760	2.63923	2.1
43761	1.29111	2.1
43762	1.29111	2.1
43763	0.946132	2.1
43764	0.946132	2.1
43765	1.18719	2.1
43766	1.18719	2.1
43767	0.94613	2.1
43768	0.94613	2.1
43769	1.1872	2.1
43770	1.31961	2.1
43771	0.946131	2.1
43772	1.31961	2.1
43773	1.1872	2.1
43774	0.94613	2.1
43775	2.49445	0.144248
43776	2.12089	0.181301
43777	2.20843	0.165979
43778	1.82805	0.270445
43779	4.32932	0.19472
43780	1.89226	0.816317
43781	1.89226	1.12962
43782	1.89226	0.456898
43783	1.89226	0.534272
43784	3.78452	0.254235
43785	0.94613	0.540584
43786	0.94613	0.528621
43787	0.946128	0.149006

43788	0.946128	0.654071
43789	0.946131	0.754379
43790	0.946131	0.659827
43791	0.946127	0.58081
43792	0.946127	0.345504
43793	0.946129	0.523479
43794	0.946129	0.229507
43795	0.946129	0.496934
43796	0.946129	0.431799
43797	1.82804	0.16679
43798	1.60017	0.304309
43799	1.87679	0.294302
43800	2.12088	0.0852941
43801	1.35608	0.323159
43802	1.60017	0.15903
43803	2.20843	0.0637068
43804	1.3216	0.0613056
43805	1.44426	0.0572026
43806	1.82805	0.05
43807	1.80399	0.083437
43808	2.20843	0.0784799
43809	1.89226	0.142434
43810	1.89226	0.196296
43811	1.89226	0.5395
43812	1.89226	0.269512
43813	1.89226	0.201025
43814	1.89226	0.127933
43815	1.89226	0.214625
43816	1.89226	0.0965376
43817	1.89226	0.156121
43818	1.89226	0.213252
43819	1.89226	0.384356
43820	1.89226	0.126233
43821	0.627705	0.05
43822	0.634941	0.0882114
43823	0.629317	0.05
43824	0.783422	0.05
43825	0.629328	0.0554249
43826	0.448764	0.0663911
43827	1.35608	0.0550283
43828	1.00805	0.0593249
43829	0.814295	0.05
43830	0.598375	0.0858135
43831	1.04461	0.0512831
43832	1.09795	2.07213
43833	1.00775	2.1
43834	1.61202	2.1
43835	1.63419	1.99125
43836	2.64194	1.87866
43837	1.44801	1.79473
43838	1.61201	2.1
43839	2.35614	1.97124
43840	2.27703	2.06349
43841	3.88904	2.05462
43842	2.35615	1.79743
43843	1.63419	1.73003
43844	2.29852	1.68473
43845	1.63419	1.72199

43846	2.27634	1.75641
43847	1.61202	2.05395
43848	0.973793	2.1
43849	2.2671	2.1
43850	2.32981	2.1
43851	2.32981	2.1
43852	0.990481	2.1
43853	0.990481	2.1
43854	1.50877	2.1
43855	1.11193	2.1
43856	2.29667	2.1
43857	1.94158	2.1
43858	3.45035	2.1
43859	2.29667	2.1
43860	1.89227	2.1
43861	1.94158	2.1
43862	1.94158	2.1
43863	1.89227	2.1
43864	1.89227	2.1
43865	2.51379	2.1
43866	1.30026	2.1
43867	1.84502	2.1
43868	2.72099	2.1
43869	4.35882	2.1
43870	1.89226	2.1
43871	2.721	2.1
43872	2.5138	2.1
43873	2.5138	2.1
43874	1.89226	2.1
43875	1.89226	2.1
43876	1.89226	0.05
43877	1.89226	0.05
43878	2.59101	0.05
43879	1.89226	0.05
43880	3.78453	0.05
43881	0.851541	0.05
43882	0.851541	0.05
43883	1.42502	0.05
43884	0.948679	0.05
43885	0.946132	0.05
43886	0.946132	0.05
43887	0.853617	0.05
43888	0.853618	0.05
43889	0.851475	0.05
43890	0.948666	0.05
43891	1.166	0.05
43892	0.851554	0.05
43893	2.51818	0.884417
43894	2.60723	2.1
43895	2.66532	0.654515
43896	2.76315	2.1
43897	5.27255	2.1
43898	2.60723	2.1
43899	2.45247	2.1
43900	2.76315	2.1
43901	2.55197	2.1
43902	5.1592	2.1
43903	1.23768	0.252971

43904	1.11433	0.492588
43905	1.32037	1.58158
43906	1.32037	0.217716
43907	1.0142	0.776373
43908	1.0142	0.122576
43909	0.886711	0.967706
43910	1.16806	0.963149
43911	0.999083	1.97277
43912	0.710926	1.43804
43913	0.937757	1.58921
43914	0.937757	1.19712
43915	0.941224	0.472109
43916	0.941224	2.01704
43917	1.16805	1.27931
43918	0.999084	2.04142
43919	0.886709	1.6963
43920	0.710928	1.39652
43921	2.29332	2.1
43922	1.54904	2.1
43923	2.0815	2.1
43924	2.44199	2.1
43925	4.00418	2.1
43926	1.53873	0.05
43927	1.53422	0.185684
43928	1.39661	0.05
43929	1.42387	0.109287
43930	2.9626	0.0565097
43931	1.53423	0.185623
43932	1.44091	2.1
43933	1.42387	0.0954362
43934	1.35215	2.1
43935	2.86479	2.07349
43936	1.39661	0.05
43937	1.42387	0.134836
43938	1.28325	0.05
43939	1.33326	0.434736
43940	2.70713	0.05
43941	1.42387	0.0780032
43942	1.35215	2.1
43943	1.33327	0.505907
43944	1.27339	2.1
43945	2.69726	2.1
43946	2.58442	2.1
43947	1.1468	2.1
43948	1.18775	2.1
43949	2.60047	2.1
43950	3.74728	2.1
43951	2.58442	2.1
43952	1.89226	2.1
43953	1.89226	2.1
43954	2.60048	2.1
43955	4.49274	2.1
43956	1.89226	2.1
43957	1.89226	2.1
43958	1.89226	2.1
43959	1.89226	2.1
43960	3.78453	2.1
43961	1.89226	2.1

43962	1.89226	2.1
43963	1.89226	2.1
43964	1.89226	2.1
43965	3.78452	2.1
43966	1.22262	2.1
43967	2.60047	2.1
43968	1.18775	2.1
43969	1.18775	2.1
43970	2.61433	2.1
43971	2.61433	2.1
43972	1.89226	2.1
43973	2.60048	2.1
43974	2.61434	2.1
43975	2.60048	2.1
43976	1.90612	2.1
43977	1.89226	2.1
43978	1.89226	2.1
43979	1.89226	2.1
43980	1.89226	2.1
43981	1.89226	2.1
43982	3.78453	2.1
43983	1.89226	2.1
43984	1.89226	2.1
43985	1.89226	2.1
43986	1.89226	2.1
43987	3.78452	2.1
43988	2.12556	2.1
43989	0.991113	2.1
43990	1.27725	2.1
43991	2.31081	2.1
43992	3.40281	2.1
43993	1.89226	2.1
43994	2.31082	2.1
43995	2.12556	2.1
43996	2.12556	2.1
43997	1.89226	2.1
43998	1.89226	2.1
43999	1.22544	2.1
44000	1.12649	2.1
44001	0.775254	2.1
44002	0.643133	2.1
44003	0.917924	2.1
44004	0.934955	2.1
44005	0.975579	2.1
44006	0.975579	2.1
44007	1.23113	2.1
44008	0.685272	2.1
44009	0.651719	2.1
44010	1.1791	2.1
44011	1.89226	2.1
44012	2.5138	2.1
44013	2.31082	2.1
44014	2.31082	2.1
44015	1.89226	2.1
44016	1.89226	2.1
44017	1.89226	2.1
44018	1.89226	2.1
44019	1.89226	2.1

44020	1.89226	2.1
44021	3.78453	2.1
44022	0.922224	2.1
44023	0.922224	2.1
44024	1.23555	2.1
44025	1.19715	2.1
44026	1.01445	2.1
44027	1.16061	2.1
44028	0.946132	2.1
44029	0.946132	2.1
44030	0.946131	2.1
44031	1.2051	2.1
44032	1.13537	2.1
44033	1.01445	2.1
44034	0.946133	2.1
44035	0.946133	2.1
44036	1.20648	2.1
44037	1.20648	2.1
44038	1.06454	2.1
44039	1.20648	2.1
44040	0.946133	2.1
44041	0.946133	2.1
44042	1.20649	2.1
44043	0.946133	2.1
44044	1.06454	2.1
44045	0.946132	2.1
44046	0.946132	2.1
44047	0.946132	2.1
44048	0.946132	2.1
44049	0.946132	2.1
44050	0.946132	2.1
44051	0.946132	2.1
44052	0.946133	2.1
44053	0.946133	2.1
44054	0.946133	2.1
44055	0.946133	2.1
44056	0.946132	2.1
44057	0.946132	2.1
44058	1.33078	2.1
44059	1.37409	2.1
44060	1.31565	0.131789
44061	1.37114	2.1
44062	2.68973	2.1
44063	1.89225	2.02013
44064	1.89225	2.1
44065	1.89225	0.153358
44066	1.89225	1.97575
44067	3.7845	1.04731
44068	1.89225	1.91962
44069	1.89225	2.1
44070	1.89225	1.09362
44071	1.89225	2.1
44072	3.7845	2.1
44073	1.37409	2.06521
44074	1.20408	2.1
44075	1.18612	2.1
44076	1.18907	2.1
44077	1.37114	2.07092

44078	1.37409	2.1
44079	1.31566	2.1
44080	1.37115	2.1
44081	1.30318	0.150916
44082	1.37433	2.1
44083	2.68998	1.99941
44084	1.89226	2.1
44085	1.89226	2.1
44086	1.89226	0.222917
44087	1.89226	2.1
44088	3.78453	1.36533
44089	1.89226	1.53979
44090	1.89226	2.1
44091	1.89226	2.1
44092	1.89226	2.1
44093	1.89226	1.38963
44094	1.89226	2.1
44095	0.647001	2.1
44096	0.647001	2.1
44097	0.651851	1.43312
44098	0.651851	2.1
44099	0.602046	2.1
44100	0.613277	2.1
44101	0.685576	1.95533
44102	0.687164	2.1
44103	0.687164	2.1
44104	0.613276	2.1
44105	0.602046	2.1
44106	0.685576	2.1
44107	0.946128	0.584779
44108	0.946128	0.0633709
44109	1.15307	1.85114
44110	1.19317	1.07491
44111	0.94613	1.30886
44112	0.94613	0.05
44113	1.15307	1.21183
44114	1.15307	1.77215
44115	0.946131	1.05273
44116	1.15307	0.526457
44117	0.946131	0.365909
44118	1.19317	0.19482
44119	1.18278	2.1
44120	2.38635	1.03506
44121	1.23042	2.1
44122	1.23042	2.04872
44123	2.30614	2.1
44124	2.30614	2.1
44125	0.946129	0.250371
44126	0.946129	0.138561
44127	1.12994	0.650478
44128	1.15307	0.397298
44129	0.946131	0.637901
44130	0.946131	0.05
44131	1.12994	0.340861
44132	1.12994	0.370849
44133	1.12994	0.389698
44134	0.946131	0.362328
44135	0.946131	0.169135

44136	1.15307	0.124105
44137	2.25988	1.45707
44138	2.30614	0.153864
44139	2.28696	2.1
44140	1.16361	2.1
44141	1.18278	2.1
44142	1.18278	1.99257
44143	2.3771	2.1
44144	1.90121	2.1
44145	1.81191	2.1
44146	2.46904	2.1
44147	4.37025	2.1
44148	1.15676	2.1
44149	1.23452	2.1
44150	0.823592	2.1
44151	0.737606	2.1
44152	0.988113	2.1
44153	0.988113	2.1
44154	0.984014	2.1
44155	0.984014	2.1
44156	1.15676	2.1
44157	0.737605	2.1
44158	0.823592	2.1
44159	1.23452	2.1
44160	1.30318	2.1
44161	1.37433	2.1
44162	1.30151	0.781568
44163	1.39067	2.1
44164	2.67584	2.00001
44165	1.89226	2.00162
44166	1.89226	2.1
44167	1.89226	0.651535
44168	1.89226	1.62217
44169	3.78453	1.12213
44170	1.89226	0.619494
44171	1.89226	2.1
44172	1.89226	0.761454
44173	1.89226	2.1
44174	1.89226	1.38964
44175	1.89226	2.1
44176	0.656819	2.1
44177	0.656819	1.5679
44178	0.661755	1.48132
44179	0.661755	2.1
44180	0.613277	2.1
44181	0.63141	2.1
44182	0.687164	1.50838
44183	0.695336	2.1
44184	0.695337	2.1
44185	0.63141	2.1
44186	0.687164	2.1
44187	0.613276	2.1
44188	1.30151	2.1
44189	1.39067	2.1
44190	1.32143	0.73661
44191	1.43294	2.1
44192	2.73445	2.1
44193	1.89226	2.05017

44194	1.89226	2.1
44195	1.89226	0.316859
44196	1.89226	2.1
44197	3.78452	1.42715
44198	1.89226	1.64978
44199	1.89226	1.47272
44200	1.89226	1.43266
44201	1.89226	2.1
44202	1.89226	2.1
44203	1.89226	2.1
44204	1.43294	1.51732
44205	1.26282	2.1
44206	1.3268	1.62347
44207	1.3268	2.1
44208	1.39067	2.05803
44209	1.39067	2.1
44210	1.5511	0.05
44211	1.00665	0.05
44212	1.28239	0.05
44213	1.88631	0.05
44214	2.89296	0.05
44215	1.89227	0.05
44216	1.89227	0.05
44217	1.89227	0.05
44218	1.89227	0.05
44219	1.89227	0.05
44220	1.89227	0.05
44221	2.08848	0.05
44222	1.89226	0.05
44223	1.89226	0.05
44224	2.37947	0.05
44225	4.27173	0.05
44226	1.01456	0.05
44227	0.943157	0.05
44228	0.641198	0.05
44229	0.724757	0.05
44230	0.830923	0.05
44231	0.830923	0.05
44232	0.836991	0.05
44233	0.836991	0.05
44234	1.01456	0.05
44235	0.724757	0.05
44236	0.641197	0.05
44237	0.943157	0.05
44238	1.89227	0.05
44239	1.89227	0.05
44240	1.89227	0.05
44241	1.89227	0.05
44242	3.78453	0.05
44243	0.946129	0.05
44244	0.946129	0.05
44245	1.28941	0.05
44246	1.18974	0.05
44247	0.94613	0.05
44248	0.94613	0.05
44249	1.28941	0.05
44250	1.28941	0.05
44251	1.28941	0.05

44252	0.946131	0.05
44253	0.94613	0.05
44254	1.18974	0.05
44255	0.710636	2.1
44256	0.780249	2.1
44257	0.579033	2.1
44258	0.478812	2.1
44259	0.621822	2.1
44260	0.621822	2.1
44261	0.63724	2.1
44262	0.63724	2.1
44263	0.780251	2.1
44264	0.579032	2.1
44265	0.710637	2.1
44266	0.478811	2.1
44267	1.05499	2.1
44268	1.00521	2.1
44269	1.24445	1.99228
44270	1.23082	0.66872
44271	2.28581	2.06958
44272	1.48751	2.1
44273	1.23083	1.72936
44274	1.4941	2.1
44275	1.24445	1.61664
44276	1.48047	1.07661
44277	1.23083	1.32478
44278	0.621185	1.86725
44279	0.621185	2.06103
44280	0.620899	0.975187
44281	0.620899	2.1
44282	0.502515	1.73258
44283	0.626672	1.13183
44284	0.615412	0.852193
44285	0.740234	2.1
44286	0.740233	0.794806
44287	0.626672	0.524628
44288	0.615413	1.13949
44289	0.502516	1.4369
44290	1.95844	2.1
44291	2.6562	2.1
44292	1.95844	2.1
44293	2.20883	2.1
44294	2.38225	2.1
44295	2.63264	2.1
44296	1.89226	2.1
44297	1.89226	2.1
44298	1.89226	2.1
44299	1.89226	2.1
44300	3.78453	2.1
44301	1.95844	2.1
44302	2.38225	2.1
44303	1.71082	2.1
44304	2.14647	2.1
44305	4.1049	2.1
44306	0.946131	2.1
44307	0.946131	2.1
44308	0.946131	2.1
44309	0.946131	2.1

44310	0.946131	2.1
44311	0.946131	2.1
44312	0.946131	2.1
44313	0.946131	2.1
44314	0.946131	2.1
44315	0.946131	2.1
44316	0.946131	2.1
44317	0.946131	2.1
44318	2.12597	2.1
44319	1.2295	2.1
44320	1.28014	2.1
44321	2.19723	2.1
44322	3.40611	2.1
44323	2.12597	2.1
44324	2.79242	2.1
44325	2.73375	2.1
44326	2.66249	2.1
44327	2.19723	2.1
44328	2.12597	2.1
44329	1.13716	2.1
44330	1.09861	2.1
44331	0.666692	2.1
44332	0.640071	2.1
44333	0.891617	2.1
44334	0.891617	2.1
44335	0.885614	2.1
44336	0.885614	2.1
44337	1.13716	2.1
44338	0.666692	2.1
44339	0.640071	2.1
44340	1.09861	2.1
44341	1.89226	0.05
44342	1.70303	0.05
44343	1.89226	0.05
44344	2.64598	0.05
44345	1.81091	0.05
44346	2.53222	0.05
44347	2.32422	0.05
44348	1.89226	0.05
44349	2.78336	0.05
44350	2.78336	0.05
44351	1.89226	0.05
44352	1.89226	0.05
44353	0.946132	0.05
44354	0.946132	0.05
44355	0.946131	0.05
44356	0.946131	0.05
44357	0.946132	0.05
44358	0.946132	0.05
44359	0.946131	0.05
44360	0.946131	0.05
44361	0.946131	0.05
44362	0.946131	0.05
44363	0.946131	0.05
44364	0.946131	0.05
44365	0.946131	0.05
44366	0.946131	0.05
44367	0.946132	0.05

44368	0.946132	0.05
44369	0.946132	0.05
44370	0.946132	0.05
44371	0.946131	0.05
44372	0.946131	0.05
44373	0.946131	0.05
44374	0.946131	0.05
44375	0.946131	0.05
44376	0.946131	0.05
44377	2.24323	0.738497
44378	1.89226	2.03006
44379	2.75021	1.46189
44380	2.75021	1.76819
44381	1.89226	0.492124
44382	1.89226	0.940201
44383	2.30908	0.05
44384	2.19815	0.09711
44385	2.1609	0.05
44386	1.83796	0.501425
44387	4.35904	0.05
44388	2.19815	0.0509406
44389	2.02268	2.1
44390	2.17869	2.1
44391	1.9559	0.437714
44392	2.0815	2.1
44393	1.83424	0.292842
44394	2.1609	0.05
44395	1.88877	0.550573
44396	1.98413	0.05
44397	1.87355	0.169134
44398	4.03445	0.0705117
44399	1.87355	0.120415
44400	2.02268	2.1
44401	1.87355	2.1
44402	2.10488	0.291664
44403	1.68443	2.1
44404	1.89448	0.85308
44405	0.946131	0.0523665
44406	0.946131	0.05
44407	1.40574	0.0682747
44408	1.12373	0.0769249
44409	0.946129	0.0652188
44410	0.946129	0.05
44411	1.40574	0.0829308
44412	1.40574	0.0573488
44413	0.946131	0.0572626
44414	1.40574	0.0831253
44415	1.12373	0.0679946
44416	0.946131	0.05
44417	2.14277	0.0734251
44418	2.81148	0.0905455
44419	1.42762	0.05
44420	1.42762	0.05
44421	2.96261	0.0551185
44422	2.24746	0.058641
44423	1.89226	0.813519
44424	1.89226	0.8029
44425	1.89226	0.651564

44426	1.89226	1.01771
44427	3.78453	1.45925
44428	1.89226	0.515248
44429	1.89226	0.827648
44430	1.89226	0.782279
44431	1.89226	0.869412
44432	3.78452	0.448174
44433	0.68433	0.462178
44434	0.946127	1.17428
44435	0.543921	1.41166
44436	0.946131	0.683583
44437	0.946131	0.794751
44438	0.946131	2.00715
44439	0.543918	1.0549
44440	0.543918	1.03633
44441	0.684332	0.838021
44442	0.54392	2.066
44443	0.946129	0.331424
44444	0.946129	0.780082
44445	1.89226	0.804843
44446	1.08784	1.21394
44447	1.19352	0.169826
44448	1.89226	0.972769
44449	1.19352	1.86412
44450	1.89226	1.63858
44451	0.635711	0.543077
44452	0.946132	1.21183
44453	0.946132	1.52937
44454	0.56474	0.938292
44455	0.750045	1.18429
44456	1.25657	2.1
44457	0.635682	0.821734
44458	0.635682	0.501578
44459	0.891954	0.665636
44460	1.32751	2.02057
44461	0.564766	0.507528
44462	0.564766	1.24594
44463	0.858345	0.780582
44464	0.946128	1.32042
44465	0.635712	0.726505
44466	0.946133	0.743162
44467	0.977198	1.06332
44468	0.944521	2.08368
44469	0.635682	1.22065
44470	0.635682	0.177428
44471	1.02924	0.918117
44472	0.705625	2.1
44473	0.851528	0.210312
44474	0.851528	2.02335
44475	2.18903	2.1
44476	2.10182	2.1
44477	2.10182	2.1
44478	1.89226	2.1
44479	1.89226	2.1
44480	1.64983	2.1
44481	1.89226	2.1
44482	1.83867	2.1
44483	1.83867	2.1

44484	1.89164	2.1
44485	2.0815	2.1
44486	2.17638	2.1
44487	2.57957	0.819372
44488	2.3506	2.08413
44489	2.69056	0.254854
44490	2.49439	1.72563
44491	5.04116	0.455241
44492	2.3506	0.788009
44493	1.8911	2.1
44494	2.49439	0.884988
44495	2.06689	2.1
44496	4.41748	2.1
44497	2.69056	0.715243
44498	2.49439	1.86893
44499	2.77227	0.149879
44500	2.63443	1.47329
44501	5.32499	1.80252
44502	2.49439	0.722084
44503	2.06689	2.1
44504	2.63443	0.212651
44505	2.26444	2.1
44506	4.70131	1.91336
44507	2.46327	2.1
44508	2.54347	2.1
44509	2.52869	2.1
44510	2.16534	2.1
44511	2.82661	2.1
44512	2.46327	2.1
44513	0.946131	2.1
44514	0.946131	2.1
44515	1.27174	2.1
44516	1.27174	2.1
44517	1.27174	2.1
44518	0.94613	2.1
44519	0.946128	2.1
44520	1.4133	2.1
44521	0.946129	2.1
44522	0.946129	2.1
44523	1.27174	2.1
44524	1.4133	2.1
44525	1.42757	2.1
44526	1.7457	2.1
44527	1.66047	2.1
44528	1.7457	2.1
44529	1.45754	2.1
44530	1.54278	2.1
44531	1.42757	2.1
44532	1.52996	2.1
44533	1.66047	2.1
44534	1.66047	2.1
44535	1.26397	2.1
44536	1.26397	2.1
44537	0.968536	2.1
44538	1.02461	2.1
44539	1.57575	2.1
44540	1.62391	2.1
44541	2.59245	2.1

44542	0.946131	2.1
44543	0.946131	2.1
44544	0.946132	2.1
44545	0.946132	2.1
44546	0.946132	2.1
44547	0.946132	2.1
44548	0.946132	2.1
44549	0.946132	2.1
44550	0.946132	2.1
44551	0.946132	2.1
44552	0.946132	2.1
44553	0.946132	2.1
44554	2.23765	2.1
44555	1.62391	2.1
44556	2.22154	2.1
44557	1.57575	2.1
44558	2.2697	2.1
44559	1.62391	2.1
44560	1.89226	2.1
44561	1.89226	2.1
44562	1.89226	2.1
44563	1.89226	2.1
44564	1.89226	2.1
44565	1.89226	2.1
44566	1.89226	2.1
44567	1.89226	2.1
44568	1.89226	2.1
44569	1.89226	2.1
44570	1.89226	2.1
44571	1.89226	2.1
44572	2.2697	2.1
44573	1.46129	2.1
44574	2.25873	2.1
44575	1.62391	2.1
44576	2.09611	2.1
44577	1.46129	2.1
44578	1.89226	2.1
44579	2.07088	2.1
44580	1.89226	2.1
44581	2.31884	2.1
44582	4.21111	2.1
44583	1.89226	2.1
44584	2.31884	2.1
44585	2.11713	2.1
44586	1.89226	2.1
44587	2.54371	2.1
44588	2.31884	2.1
44589	2.54371	2.1
44590	1.24041	2.1
44591	2.55208	2.1
44592	2.31884	2.1
44593	1.23712	2.1
44594	1.00388	2.1
44595	2.72769	0.05
44596	2.57507	0.05
44597	1.89226	0.05
44598	1.89226	0.05
44599	4.46733	0.05

44600	1.89226	0.05
44601	2.57506	0.05
44602	1.89226	0.05
44603	2.30076	0.05
44604	1.89226	0.05
44605	2.30076	0.05
44606	1.21171	0.05
44607	1.48589	0.05
44608	2.09166	0.05
44609	1.78381	0.05
44610	3.2697	0.05
44611	2.09166	0.05
44612	1.70302	0.05
44613	1.48589	0.05
44614	1.18027	0.05
44615	2.26571	0.05
44616	1.79969	0.05
44617	1.89226	0.05
44618	1.89226	0.05
44619	1.89226	0.05
44620	1.89226	0.05
44621	1.89226	0.05
44622	1.89226	0.05
44623	0.946131	0.05
44624	0.946131	0.05
44625	0.946128	0.05
44626	0.946128	0.05
44627	0.946129	0.05
44628	0.946129	0.05
44629	0.94613	0.05
44630	0.94613	0.05
44631	0.94613	0.05
44632	0.94613	0.05
44633	0.94613	0.05
44634	0.94613	0.05
44635	1.89226	1.79177
44636	1.89226	1.91797
44637	1.89226	2.1
44638	1.89226	1.24609
44639	1.89226	2.1
44640	1.89226	1.15044
44641	1.89226	1.84011
44642	1.89226	1.87253
44643	1.89226	2.07771
44644	1.89226	1.30857
44645	1.89226	2.06457
44646	1.89226	1.25958
44647	1.89226	1.38654
44648	1.89226	1.52564
44649	1.89226	1.98535
44650	1.89226	0.78539
44651	1.89226	1.99794
44652	1.89226	0.783577
44653	1.79103	0.381549
44654	0.968249	2.1
44655	1.44807	1.99179
44656	2.17984	0.10736
44657	3.2391	2.1

44658	1.29735	1.35209
44659	1.08992	1.35979
44660	0.945147	2.1
44661	0.724033	2.1
44662	1.00723	2.1
44663	1.00723	1.72984
44664	1.01416	0.188535
44665	1.01416	2.1
44666	1.29735	1.93485
44667	0.94515	2.03764
44668	0.724034	2.02585
44669	1.08992	2.1
44670	1.39214	2.1
44671	1.39214	2.1
44672	0.946129	2.1
44673	0.946129	2.1
44674	1.33728	2.1
44675	1.39214	2.1
44676	0.94613	2.1
44677	0.94613	2.1
44678	0.94613	2.1
44679	1.33728	2.1
44680	0.94613	2.1
44681	1.39214	2.1
44682	2.78428	2.1
44683	2.28373	2.1
44684	2.67456	2.1
44685	2.20011	2.1
44686	4.95829	2.1
44687	1.90165	0.05
44688	2.24206	0.05
44689	1.66137	0.05
44690	2.07108	0.0763368
44691	3.90343	0.05
44692	1.89224	0.05
44693	1.89224	0.05
44694	1.89224	0.05
44695	1.89224	0.05
44696	3.78448	0.05
44697	2.82159	0.05
44698	1.89226	0.05
44699	2.50138	0.0870933
44700	1.89226	0.05
44701	4.39365	0.05
44702	1.66139	0.05
44703	1.86399	0.107821
44704	1.56005	0.05
44705	1.90324	0.05
44706	3.56463	0.05
44707	0.946133	0.05
44708	0.946133	0.0515087
44709	0.946132	0.05
44710	0.946132	0.0539879
44711	0.946133	0.05
44712	0.946133	0.0613513
44713	0.946132	0.05
44714	0.946132	0.0683546
44715	0.946133	0.05

44716	0.946133	0.05
44717	0.946132	0.05
44718	0.946132	0.05
44719	1.5176	0.401805
44720	1.97964	0.16561
44721	2.32889	0.29696
44722	1.91762	0.233005
44723	3.84649	0.215725
44724	2.3376	0.265196
44725	1.97963	0.169043
44726	2.32889	0.393104
44727	2.58303	0.165735
44728	4.66649	0.304599
44729	2.32889	0.264953
44730	2.20248	0.145649
44731	1.91762	0.132869
44732	1.91762	0.109348
44733	2.5615	0.306035
44734	2.5615	0.228036
44735	2.32889	0.366289
44736	2.58303	0.136611
44737	2.71793	0.310545
44738	2.56149	0.138456
44739	5.04681	0.194022
44740	1.89226	0.618379
44741	2.53785	1.93488
44742	1.89226	0.280406
44743	2.60895	0.249842
44744	1.89226	1.176
44745	2.60895	0.974938
44746	0.493248	0.232132
44747	1.30448	1.38853
44748	1.26893	1.9621
44749	0.653226	1.91055
44750	0.653227	1.05651
44751	0.493247	2.01114
44752	1.26893	2.06238
44753	1.30448	0.407893
44754	0.832232	2.02681
44755	0.832233	2.1
44756	0.929944	0.198508
44757	0.929944	0.830259
44758	1.89226	2.1
44759	1.47937	2.1
44760	1.50143	2.1
44761	1.47937	2.1
44762	1.91432	2.1
44763	1.89226	2.1
44764	1.89226	2.1
44765	1.89226	2.1
44766	1.89226	2.1
44767	1.89226	2.1
44768	1.89226	2.1
44769	1.89226	2.04032
44770	1.33419	2.1
44771	2.4542	2.1
44772	2.59834	2.1
44773	2.59834	2.1

44774	1.25716	2.1
44775	1.25716	2.1
44776	1.70303	2.1
44777	1.50143	2.1
44778	1.60878	2.1
44779	1.50143	2.1
44780	1.93599	2.1
44781	1.89226	2.1
44782	0.946131	1.85756
44783	0.946131	2.1
44784	0.946127	2.1
44785	0.946127	2.1
44786	0.946127	2.1
44787	0.946127	1.13965
44788	0.946131	2.1
44789	0.946131	2.1
44790	0.94613	2.1
44791	0.94613	2.1
44792	0.946129	2.1
44793	0.946129	2.1
44794	1.43912	2.1
44795	2.59834	2.1
44796	2.78093	2.1
44797	2.78093	2.1
44798	1.33419	2.1
44799	1.33419	2.1
44800	1.89226	2.1
44801	2.26322	2.1
44802	2.55682	2.1
44803	2.55682	2.1
44804	1.89226	2.1
44805	1.89226	2.1
44806	2.58032	0.108978
44807	2.28922	0.05
44808	1.88424	0.0551416
44809	2.32127	0.05
44810	4.46456	0.05
44811	1.67265	0.0849212
44812	2.28922	0.0523547
44813	1.88424	0.05
44814	1.11314	0.05
44815	3.55689	0.05
44816	1.43751	0.05
44817	2.32127	0.05
44818	1.92998	0.05
44819	2.37671	0.05
44820	1.43751	0.0725456
44821	1.88424	0.0744409
44822	2.36729	2.1
44823	1.56714	2.1
44824	1.56714	2.1
44825	1.62408	2.1
44826	2.28373	2.1
44827	2.34067	2.1
44828	0.937268	2.1
44829	0.937268	2.1
44830	0.94636	2.1
44831	0.94636	2.1

44832	0.76004	2.1
44833	0.783572	2.1
44834	1.14187	2.1
44835	1.10006	2.1
44836	1.10006	2.1
44837	0.76004	2.1
44838	0.783572	2.1
44839	1.14187	2.1
44840	1.28753	2.1
44841	1.36838	2.1
44842	1.62617	2.1
44843	1.82857	2.1
44844	3.1161	2.1
44845	1.82856	2.1
44846	1.17996	2.1
44847	1.53417	2.1
44848	1.56618	2.1
44849	1.36838	2.1
44850	1.36838	2.1
44851	1.35065	0.842401
44852	1.42659	0.0682461
44853	2.26675	0.327936
44854	2.26675	0.294674
44855	1.62137	0.193333
44856	2.53747	0.327886
44857	1.89226	0.05
44858	1.89226	0.05
44859	1.89226	0.05
44860	1.89226	0.05
44861	3.78452	0.05
44862	1.36569	0.05
44863	1.36569	0.05
44864	0.946131	0.05
44865	0.946131	0.05
44866	1.16041	0.05
44867	1.36569	0.05
44868	0.946129	0.05
44869	0.946129	0.05
44870	1.1604	0.05
44871	0.94613	0.05
44872	0.946131	0.05
44873	1.36569	0.05
44874	2.73139	0.05
44875	1.25265	0.05
44876	1.25265	0.05
44877	1.64799	0.05
44878	2.32081	0.05
44879	2.71615	0.05
44880	0.946133	0.05
44881	0.946133	0.05
44882	0.946132	0.05
44883	0.946132	0.05
44884	0.946132	0.05
44885	0.946132	0.05
44886	0.946133	0.05
44887	0.946133	0.05
44888	0.946132	0.05
44889	0.946132	0.05

44890	0.946133	0.05
44891	0.946133	0.05
44892	0.946131	0.05
44893	0.946131	0.05
44894	0.94613	0.05
44895	0.94613	0.05
44896	0.946132	0.05
44897	0.946132	0.05
44898	0.946129	0.05
44899	0.946129	0.05
44900	0.946131	0.05
44901	0.946131	0.05
44902	0.946131	0.05
44903	0.946131	0.05
44904	1.89226	0.281495
44905	2.48503	2.1
44906	2.64586	2.1
44907	2.64586	0.459171
44908	1.89226	2.1
44909	1.89226	0.144765
44910	2.48504	2.1
44911	1.99985	2.1
44912	1.83584	2.1
44913	1.83584	2.1
44914	2.64586	2.1
44915	2.64586	2.1
44916	1.89225	1.10084
44917	2.33095	2.1
44918	2.48501	2.1
44919	2.48501	0.46999
44920	1.89225	2.1
44921	1.89225	0.150539
44922	2.33096	2.1
44923	1.83583	2.1
44924	1.68436	2.1
44925	1.68436	2.1
44926	2.48502	2.1
44927	2.48502	2.1
44928	1.14807	2.1
44929	1.76545	2.1
44930	1.21292	2.1
44931	1.83936	2.1
44932	2.97837	2.1
44933	1.89226	2.1
44934	1.89226	2.1
44935	1.89226	2.1
44936	1.89226	2.1
44937	3.78451	2.1
44938	1.89226	2.1
44939	1.89226	1.1131
44940	1.89226	2.1
44941	1.89226	2.1
44942	3.78452	2.1
44943	1.76545	2.1
44944	2.12608	2.1
44945	1.83937	2.1
44946	2.22529	2.1
44947	3.99074	2.1

44948	0.780222	2.1
44949	0.780222	2.1
44950	0.784871	2.1
44951	0.784871	2.1
44952	0.95863	2.1
44953	0.919681	2.1
44954	0.636145	2.1
44955	0.606461	2.1
44956	0.636144	2.1
44957	0.958631	2.1
44958	0.60646	2.1
44959	0.919683	2.1
44960	1.89226	2.1
44961	1.89226	2.1
44962	1.89226	2.1
44963	1.89226	2.1
44964	3.78452	2.1
44965	1.89226	2.1
44966	1.89226	2.1
44967	1.89226	2.1
44968	1.89226	1.21955
44969	3.78452	2.1
44970	1.03789	2.1
44971	1.03789	2.1
44972	1.04231	2.1
44973	1.04231	2.1
44974	1.16052	2.1
44975	1.11265	1.58235
44976	0.958634	2.1
44977	0.919685	2.1
44978	0.958634	2.1
44979	1.16052	2.1
44980	0.919685	2.1
44981	1.11265	2.1
44982	1.43837	2.1
44983	2.57085	2.1
44984	1.39332	2.1
44985	2.58228	2.1
44986	3.96417	2.1
44987	1.89226	2.1
44988	1.89226	2.1
44989	1.89226	2.1
44990	1.89226	2.1
44991	1.89226	2.1
44992	1.89226	2.1
44993	1.89226	2.1
44994	1.89226	2.1
44995	1.89226	2.1
44996	1.89226	2.1
44997	3.78452	2.1
44998	2.57085	2.1
44999	1.89226	2.1
45000	2.58229	2.1
45001	1.89226	2.1
45002	4.46312	2.1
45003	1.32368	2.1
45004	2.58228	2.1
45005	2.55352	2.1

45006	2.55352	2.1
45007	1.39332	2.1
45008	1.39332	2.1
45009	1.89226	2.1
45010	1.89226	2.1
45011	1.89226	2.1
45012	1.89226	2.1
45013	1.89226	2.1
45014	1.89226	2.1
45015	1.89226	2.1
45016	1.89226	2.1
45017	1.89226	2.1
45018	1.89226	2.1
45019	1.89226	2.1
45020	1.89226	2.1
45021	2.55353	2.1
45022	1.89226	2.1
45023	2.58228	2.1
45024	1.89226	2.1
45025	1.89226	2.1
45026	2.58228	2.1
45027	1.40574	1.69481
45028	1.05175	1.20925
45029	1.71613	1.74455
45030	1.99961	1.21154
45031	3.12187	1.50444
45032	1.71613	1.7275
45033	2.50733	1.24738
45034	1.99961	1.98115
45035	1.99961	1.74149
45036	2.25746	1.1327
45037	2.25746	1.1921
45038	2.25746	1.14271
45039	1.89526	1.21934
45040	1.71613	1.68389
45041	1.71613	0.727691
45042	1.89526	1.2184
45043	1.27558	0.730778
45044	1.07536	2.1
45045	1.07536	2.1
45046	1.07034	2.1
45047	1.07034	2.1
45048	0.766879	2.1
45049	0.81527	2.1
45050	1.38896	2.1
45051	1.33043	2.1
45052	0.815264	2.1
45053	1.38895	2.1
45054	0.766884	2.1
45055	1.33044	2.1
45056	1.89225	2.1
45057	1.89225	2.1
45058	1.89225	2.1
45059	1.89225	2.1
45060	1.89225	2.1
45061	1.89225	2.1
45062	1.64059	2.1
45063	1.89225	2.1

45064	1.565	2.1
45065	1.565	2.1
45066	1.89225	2.1
45067	1.89225	2.1
45068	1.89226	2.1
45069	1.89226	2.1
45070	1.89226	2.1
45071	1.89226	2.1
45072	1.89226	2.1
45073	1.89226	2.1
45074	1.67906	2.1
45075	1.89226	2.1
45076	1.6406	2.1
45077	1.6406	2.1
45078	1.89226	2.1
45079	1.89226	2.1
45080	2.18106	1.74027
45081	2.20831	2.1
45082	2.64725	1.12935
45083	2.63102	1.50638
45084	4.82831	1.67112
45085	2.18106	1.46364
45086	2.09605	2.1
45087	2.5601	1.61185
45088	2.63102	1.49781
45089	4.74116	2.1
45090	2.64725	1.37813
45091	1.89226	1.56871
45092	1.87603	2.1
45093	2.63102	1.36929
45094	1.89226	1.84056
45095	2.64725	1.02951
45096	0.946132	1.67153
45097	1.28005	1.32414
45098	0.946132	2.1
45099	1.31551	1.0865
45100	1.28005	1.70806
45101	1.28005	2.09979
45102	0.946132	1.12501
45103	0.946132	1.60069
45104	0.946132	1.2103
45105	0.946132	1.39371
45106	1.28005	1.71218
45107	1.31551	1.76349
45108	2.0815	2.1
45109	1.59318	2.1
45110	2.0815	2.1
45111	1.40161	2.1
45112	2.49782	2.1
45113	1.68553	2.1
45114	0.819885	2.1
45115	0.935082	2.1
45116	0.867363	2.1
45117	0.867363	2.1
45118	0.715431	2.1
45119	0.902777	2.1
45120	0.738933	2.1
45121	0.980053	2.1

45122	0.936716	2.1
45123	0.917651	2.1
45124	0.775853	2.1
45125	0.74037	2.1
45126	2.78977	2.1
45127	2.38239	2.1
45128	2.78977	2.1
45129	1.96468	2.1
45130	2.64107	2.1
45131	1.80733	2.1
45132	1.80141	2.1
45133	2.28512	2.1
45134	2.28512	2.1
45135	1.8519	2.1
45136	2.64107	2.1
45137	2.20785	2.1
45138	0.946133	0.243168
45139	0.946133	0.128378
45140	0.946132	0.12722
45141	0.946132	0.0979155
45142	0.946132	0.133296
45143	0.946132	0.328057
45144	0.946132	0.135157
45145	0.946132	0.0883534
45146	0.946132	0.123335
45147	0.946132	0.134994
45148	0.946133	0.135392
45149	0.946133	0.209792
45150	1.89226	0.0781618
45151	1.89226	0.0738871
45152	1.89226	0.05
45153	1.89226	0.0733535
45154	1.89226	0.0577198
45155	1.89226	0.131519
45156	1.89226	0.0867611
45157	1.89226	0.0838856
45158	1.89226	0.0788956
45159	1.89226	0.182573
45160	3.78453	0.128289
45161	1.89226	0.125296
45162	1.89226	0.164918
45163	1.89226	0.0510938
45164	1.89226	0.256363
45165	1.89226	0.0857734
45166	1.89226	0.251605
45167	2.12925	0.05
45168	2.51397	0.05
45169	2.22488	0.05
45170	1.86113	0.05
45171	4.3751	0.05
45172	2.82754	0.05
45173	2.51397	0.05
45174	2.22489	0.05
45175	2.47291	0.05
45176	4.98688	0.05
45177	1.89226	0.05
45178	1.89226	0.05
45179	1.89226	0.05

45180	1.89226	0.05
45181	3.78453	0.05
45182	1.89226	0.05
45183	1.89226	0.05
45184	1.89226	0.05
45185	1.89226	0.05
45186	3.78452	0.05
45187	0.90848	0.05
45188	1.11244	0.05
45189	0.930566	0.05
45190	0.73385	0.05
45191	0.921298	0.05
45192	0.921298	0.05
45193	0.924994	0.05
45194	0.924994	0.05
45195	0.908478	0.05
45196	0.733851	0.05
45197	1.11244	0.05
45198	0.930567	0.05
45199	2.22489	0.05
45200	2.0815	0.05
45201	2.0815	0.05
45202	2.30674	0.05
45203	1.99867	0.05
45204	2.33358	0.05
45205	1.89226	0.05
45206	1.89226	0.05
45207	1.89226	0.05
45208	1.89226	0.05
45209	1.89226	0.05
45210	1.89226	0.05
45211	0.946131	0.05
45212	0.946131	0.05
45213	0.946128	0.05
45214	0.946128	0.05
45215	0.946129	0.05
45216	0.946129	0.05
45217	0.94613	0.05
45218	0.94613	0.05
45219	0.94613	0.05
45220	0.94613	0.05
45221	0.94613	0.05
45222	0.94613	0.05
45223	2.28904	2.1
45224	1.89226	1.95276
45225	2.2846	2.09275
45226	2.2846	2.1
45227	1.89226	0.434933
45228	1.89226	2.1
45229	1.89226	1.06858
45230	1.89226	1.38079
45231	1.89226	2.1
45232	1.89226	2.1
45233	1.89226	0.257269
45234	1.89226	2.05131
45235	1.04037	0.481535
45236	1.34066	2.04628
45237	1.34955	0.531587

45238	1.34955	2.1
45239	1.03373	0.914615
45240	1.03373	2.1
45241	0.946131	0.125904
45242	0.946131	2.1
45243	1.14452	2.1
45244	1.14452	2.1
45245	0.946131	1.61106
45246	0.946131	1.48749
45247	1.14296	2.1
45248	1.14452	2.1
45249	1.14296	1.56023
45250	0.946131	2.1
45251	1.14452	2.1
45252	0.946132	2.1
45253	1.89226	0.692057
45254	1.89226	2.1
45255	1.89226	1.91926
45256	1.89226	2.1
45257	1.89226	0.142083
45258	1.89226	2.1
45259	0.597454	0.654392
45260	0.597454	2.1
45261	0.597972	0.534675
45262	0.597972	2.1
45263	0.675238	2.1
45264	0.674776	1.00833
45265	0.519667	1.02439
45266	0.520188	1.43005
45267	0.519667	0.338558
45268	0.675238	0.507005
45269	0.520188	1.59917
45270	0.674777	1.62537
45271	1.89226	0.05
45272	1.82125	0.05
45273	1.89226	0.05
45274	1.84314	0.05
45275	1.89226	0.0568191
45276	1.84314	0.05
45277	1.89226	0.05
45278	1.84314	0.05
45279	1.89226	0.05
45280	1.65794	0.05
45281	1.89226	0.05
45282	1.65794	0.05
45283	1.348	2.08236
45284	1.43485	2.1
45285	1.79929	0.914227
45286	1.75309	1.87151
45287	3.14728	1.86657
45288	1.348	1.45862
45289	1.63892	2.00691
45290	1.59157	2.1
45291	1.18647	2.0527
45292	1.75309	1.54
45293	1.348	1.90807
45294	1.79929	1.73462
45295	1.75309	2.1

45296	2.18106	1.38152
45297	2.20831	1.34233
45298	3.98034	2.00394
45299	2.18106	1.1437
45300	1.63892	2.1
45301	1.75309	1.24346
45302	1.75309	2.1
45303	2.09605	1.13622
45304	2.09605	2.1
45305	2.66076	0.101797
45306	1.70302	0.05
45307	2.41575	0.0594675
45308	1.70302	0.05
45309	3.08598	0.05
45310	2.3661	0.05
45311	1.71053	0.05
45312	2.41575	0.107331
45313	1.20143	0.05
45314	1.20143	0.05
45315	2.2123	0.05
45316	1.66739	0.05
45317	2.25633	0.0511269
45318	2.68302	0.0734569
45319	1.71051	0.05
45320	1.71051	0.05
45321	2.74192	0.05
45322	2.1961	0.05
45323	1.32553	1.6913
45324	2.34875	0.0723915
45325	2.19139	2.1
45326	2.19139	0.0618733
45327	1.56172	2.1
45328	1.56172	0.0593095
45329	1.89226	2.1
45330	1.89226	0.0838835
45331	1.89226	2.1
45332	1.89226	0.410711
45333	3.78453	1.97303
45334	2.667 2.1	
45335	1.26097	2.1
45336	1.26097	2.1
45337	1.5683	2.1
45338	2.42949	2.1
45339	2.73682	2.1
45340	1.13213	0.845074
45341	2.19139	0.0738263
45342	2.0815	2.05283
45343	1.77615	0.05
45344	1.37767	2.04685
45345	1.19297	0.05
45346	0.946132	0.18294
45347	0.946132	2.1
45348	0.946131	2.1
45349	0.946131	0.637961
45350	0.946131	0.402848
45351	0.946131	0.0671574
45352	0.946132	2.1
45353	0.946132	2.1

45354	0.946132	1.88944
45355	0.946132	1.99198
45356	0.946131	1.71681
45357	0.946131	1.74949
45358	2.02064	2.1
45359	2.3606	2.1
45360	1.47478	2.1
45361	2.22997	2.1
45362	4.25061	2.1
45363	2.36059	2.1
45364	2.56952	2.1
45365	2.22997	2.1
45366	2.48631	2.1
45367	4.79949	2.1
45368	1.84261	2.1
45369	2.22997	2.1
45370	1.65706	2.1
45371	1.78837	2.1
45372	3.88703	2.1
45373	2.22997	2.1
45374	2.48631	2.1
45375	2.19528	2.1
45376	2.41175	2.1
45377	4.64172	2.1
45378	1.6466	2.1
45379	1.89226	2.1
45380	1.35972	2.1
45381	1.35972	2.1
45382	1.89226	2.1
45383	1.89226	2.1
45384	1.89226	2.1
45385	1.89226	2.1
45386	1.89226	2.1
45387	1.89226	2.1
45388	3.78453	2.1
45389	2.44797	2.1
45390	1.07214	2.1
45391	1.31589	2.1
45392	1.1211	2.1
45393	2.44797	2.1
45394	2.25318	2.1
45395	1.95954	2.1
45396	1.89226	2.1
45397	1.6466	2.1
45398	1.6466	2.1
45399	1.89226	2.1
45400	1.89226	2.1
45401	0.946133	2.1
45402	0.946133	2.1
45403	0.946132	2.1
45404	0.946132	2.1
45405	0.946132	2.1
45406	0.946132	2.1
45407	0.946133	2.1
45408	0.946133	2.1
45409	0.946132	2.1
45410	0.946132	2.1
45411	0.946133	2.1

45412	0.946133	2.1
45413	2.44797	2.1
45414	1.36904	2.1
45415	1.70303	2.1
45416	2.37586	2.1
45417	4.00507	2.1
45418	0.631553	0.05
45419	1.24489	0.05
45420	1.11597	0.05
45421	0.496799	0.05
45422	0.872272	0.05
45423	0.872272	0.05
45424	0.869415	0.05
45425	0.869415	0.05
45426	0.4968	0.05
45427	0.631553	0.05
45428	1.11597	0.05
45429	1.24489	0.05
45430	1.84253	2.1
45431	2.17384	2.1
45432	2.04818	2.1
45433	1.65925	2.1
45434	3.83309	2.1
45435	2.04818	2.1
45436	1.65925	2.1
45437	1.48162	2.1
45438	1.92264	2.1
45439	3.58189	2.1
45440	2.3305	0.05
45441	1.89226	0.0755763
45442	2.52753	0.0583427
45443	2.52753	0.079213
45444	1.89226	0.0549942
45445	1.89226	0.0807791
45446	1.89226	0.0719261
45447	1.89226	0.347938
45448	1.89226	0.159188
45449	1.89226	0.215825
45450	1.89226	0.126494
45451	1.89226	0.247897
45452	1.34563	0.05
45453	2.1769	0.0725708
45454	1.94955	0.0799912
45455	1.94955	0.293336
45456	1.55063	0.060726
45457	1.55063	0.17251
45458	2.09455	0.05
45459	1.89226	0.0992576
45460	2.3305	0.0585331
45461	2.3305	0.0730467
45462	1.89226	0.0551148
45463	1.89226	0.0768681
45464	1.89226	0.0822838
45465	1.89226	0.112101
45466	1.89226	0.174179
45467	1.89226	0.085098
45468	1.89226	0.113189
45469	1.89226	0.178896

45470	0.773392	0.05
45471	0.612765	0.409953
45472	0.79504	0.102962
45473	0.575323	0.252184
45474	1.0819	0.05
45475	1.06106	0.19479
45476	1.04675	0.0541454
45477	0.86858	0.146103
45478	0.796873	0.0934385
45479	0.557469	0.05
45480	0.807663	0.105096
45481	2.2024	2.1
45482	2.34106	2.1
45483	2.02634	2.1
45484	2.20125	2.1
45485	4.36739	2.1
45486	2.34106	2.1
45487	2.25195	0.18693
45488	2.20125	2.1
45489	2.13132	0.103415
45490	4.47237	2.1
45491	2.02635	2.1
45492	2.22465	2.1
45493	1.68504	2.1
45494	2.0815	2.1
45495	3.92673	2.1
45496	2.22465	2.1
45497	2.13133	0.0850162
45498	2.0815	2.1
45499	2.23579	0.549396
45500	4.44862	2.1
45501	1.89226	1.00695
45502	1.89226	1.30108
45503	1.89226	1.74902
45504	1.89226	1.05587
45505	1.89226	1.57084
45506	1.89226	0.928355
45507	1.93967	0.05
45508	1.70302	0.05
45509	1.21883	0.05
45510	1.70302	0.05
45511	1.19889	0.05
45512	1.70302	0.05
45513	0.946131	0.05
45514	0.946131	0.05
45515	0.946131	0.05
45516	0.946131	0.05
45517	0.94613	0.05
45518	0.94613	0.05
45519	0.946131	0.05
45520	0.946131	0.05
45521	0.946131	0.05
45522	0.946131	0.05
45523	0.946131	0.05
45524	0.946131	0.05
45525	0.883128	0.05
45526	0.883128	0.05
45527	0.827519	0.05

45528	0.82752	0.05
45529	1.16234	0.05
45530	1.19063	0.05
45531	0.631294	0.05
45532	0.548311	0.05
45533	0.631293	0.05
45534	1.16234	0.05
45535	1.19063	0.05
45536	0.54831	0.05
45537	1.89226	0.05
45538	1.89226	0.05
45539	1.89226	0.05
45540	1.89226	0.05
45541	3.78453	0.05
45542	2.33328	0.05
45543	1.87991	0.05
45544	1.87991	0.05
45545	2.82938	0.05
45546	1.84457	0.05
45547	2.79403	0.05
45548	2.82937	0.05
45549	2.70881	0.05
45550	1.8799	0.05
45551	1.82739	0.05
45552	4.58874	0.05
45553	1.89226	0.05
45554	1.89226	0.05
45555	1.89226	0.05
45556	1.89226	0.05
45557	3.78452	0.05
45558	1.17897	0.05
45559	1.82739	0.05
45560	1.17897	0.05
45561	1.76199	0.05
45562	1.23885	0.05
45563	1.80989	0.05
45564	0.946131	0.05
45565	0.946131	0.05
45566	0.946131	0.05
45567	0.946131	0.05
45568	0.946129	0.05
45569	0.946129	0.05
45570	0.946133	0.05
45571	0.946133	0.05
45572	0.946131	0.05
45573	0.946131	0.05
45574	0.946131	0.05
45575	0.946131	0.05
45576	0.946126	0.169569
45577	0.946126	2.1
45578	1.24887	2.1
45579	1.24887	2.1
45580	0.946127	2.1
45581	0.946127	0.450651
45582	1.24887	2.1
45583	1.28511	2.1
45584	1.2851	2.1
45585	0.946128	2.1

45586	1.24887	2.1
45587	0.946128	2.1
45588	1.89226	2.1
45589	1.89226	2.05058
45590	1.89226	2.1
45591	1.89226	0.223288
45592	3.78452	1.57498
45593	0.592142	2.1
45594	0.592142	2.1
45595	0.60496	2.1
45596	0.60496	2.1
45597	0.694237	2.1
45598	0.608714	0.804228
45599	0.562863	2.1
45600	0.502864	2.1
45601	0.562863	2.1
45602	0.694237	2.1
45603	0.608714	2.1
45604	0.502864	2.1
45605	1.89226	1.55805
45606	1.89226	2.1
45607	1.89226	2.1
45608	1.89226	2.1
45609	3.78453	2.1
45610	1.55779	2.1
45611	1.57968	2.1
45612	1.47186	2.1
45613	1.51725	2.1
45614	3.05154	2.1
45615	1.57967	2.1
45616	1.474	2.1
45617	1.51725	2.1
45618	1.42895	2.1
45619	3.00863	2.1
45620	1.89226	2.1
45621	1.89226	2.1
45622	1.89226	2.1
45623	1.89226	2.1
45624	3.78452	2.1
45625	1.89226	2.1
45626	1.89226	2.1
45627	1.89226	1.4722
45628	1.89226	2.1
45629	3.78453	2.1
45630	1.47186	2.1
45631	1.51725	2.1
45632	1.38859	2.1
45633	1.45332	2.1
45634	2.92519	2.1
45635	1.51725	2.1
45636	1.42895	2.1
45637	1.45332	2.1
45638	1.38414	2.1
45639	2.88228	2.1
45640	1.89226	2.1
45641	1.89226	2.1
45642	1.89226	2.1
45643	1.89226	2.1

45644	3.78452	2.1
45645	1.34497	0.05
45646	1.36747	0.05
45647	1.18919	0.05
45648	2.08149	0.05
45649	1.23556	0.05
45650	2.13702	0.05
45651	1.10106	0.05
45652	2.08001	0.05
45653	1.34497	0.05
45654	2.31605	0.05
45655	1.34497	0.05
45656	2.31605	0.05
45657	2.31605	0.05
45658	2.0815	0.05
45659	1.9419	0.05
45660	2.46665	0.05
45661	4.52922	0.05
45662	1.20349	0.05
45663	2.08149	0.05
45664	1.36948	0.05
45665	1.34737	0.05
45666	2.08149	0.05
45667	2.08149	0.05
45668	0.946132	0.05
45669	0.946132	0.05
45670	1.37715	0.05
45671	0.823199	0.05
45672	1.21598	0.05
45673	0.946132	0.05
45674	1.37715	0.05
45675	0.946132	0.05
45676	1.21598	0.05
45677	0.823198	0.05
45678	0.946133	0.05
45679	0.946133	0.05
45680	1.89226	0.05
45681	1.89226	0.05
45682	1.89226	0.05
45683	1.89226	0.05
45684	3.78453	0.05
45685	1.89227	0.05
45686	1.89227	0.05
45687	1.89227	0.05
45688	1.89227	0.05
45689	3.78453	0.05
45690	0.946132	0.05
45691	0.946132	0.05
45692	1.21599	0.05
45693	1.21599	0.05
45694	0.966749	0.05
45695	0.946133	0.05
45696	1.21598	0.05
45697	0.946132	0.05
45698	0.966748	0.05
45699	1.21598	0.05
45700	0.946133	0.05
45701	0.946133	0.05

45702	0.946133	0.05
45703	0.946133	0.05
45704	1.1134	0.05
45705	1.11555	0.05
45706	1.11555	0.05
45707	0.946132	0.05
45708	0.946132	0.05
45709	1.1134	0.05
45710	1.11555	0.05
45711	1.11555	0.05
45712	0.946132	0.05
45713	0.946132	0.05
45714	0.946133	0.05
45715	0.946133	0.05
45716	1.11555	0.05
45717	0.978467	0.05
45718	0.978468	0.05
45719	0.946133	0.05
45720	1.11555	0.05
45721	0.946132	0.05
45722	0.978468	0.05
45723	0.978468	0.05
45724	0.946132	0.05
45725	0.946132	0.05
45726	0.851542	2.1
45727	0.851542	2.1
45728	1.14168	2.1
45729	1.27897	2.1
45730	1.02817	2.1
45731	1.13536	2.1
45732	0.946131	2.1
45733	0.861207	2.1
45734	0.94613	2.1
45735	0.94613	2.1
45736	0.868751	2.1
45737	1.15181	2.1
45738	0.946132	2.1
45739	0.946132	2.05691
45740	0.946128	2.09151
45741	0.946128	1.05029
45742	0.94613	2.1
45743	0.94613	2.09632
45744	0.946131	1.95539
45745	0.946131	1.14089
45746	0.94613	1.13874
45747	0.94613	2.1
45748	0.94613	1.79689
45749	0.94613	2.1
45750	2.53273	0.05
45751	1.89226	0.05
45752	1.89226	0.05
45753	1.89226	0.05
45754	1.89226	0.05
45755	1.89226	0.05
45756	1.89226	2.1
45757	1.89226	2.1
45758	1.89226	2.1
45759	1.89226	2.1

45760	3.78452	2.1
45761	1.89226	2.1
45762	2.54394	2.1
45763	1.89226	2.1
45764	2.42221	2.1
45765	4.43621	2.1
45766	2.54394	2.1
45767	1.62743	2.1
45768	2.42221	2.1
45769	1.5437	2.1
45770	4.08764	2.1
45771	1.89226	2.1
45772	1.89226	2.1
45773	1.89226	2.1
45774	1.89226	2.1
45775	3.78452	2.1
45776	1.89226	2.1
45777	1.89226	2.1
45778	1.89226	2.1
45779	1.89226	2.1
45780	3.78452	2.1
45781	1.89226	2.1
45782	2.30146	2.1
45783	2.42221	2.1
45784	1.89226	2.1
45785	1.89226	2.1
45786	2.42221	2.1
45787	2.30146	2.1
45788	2.42221	2.1
45789	1.39962	2.1
45790	1.39962	2.1
45791	1.5437	2.1
45792	2.44553	2.1
45793	1.89226	2.1
45794	1.89226	2.1
45795	1.89226	2.1
45796	1.89226	2.1
45797	3.78452	2.1
45798	1.62196	2.1
45799	2.00695	2.1
45800	1.95288	2.1
45801	1.70303	2.1
45802	3.53316	2.1
45803	1.80703	2.1
45804	2.00695	2.1
45805	1.92991	2.1
45806	2.00695	2.1
45807	1.85888	2.1
45808	1.93592	2.1
45809	1.89226	2.1
45810	1.89226	2.1
45811	1.89226	2.1
45812	1.89226	2.1
45813	1.89226	2.1
45814	1.89226	2.1
45815	1.89226	2.1
45816	1.89226	2.1
45817	1.89226	2.1

45818	1.89226	2.1
45819	3.78453	2.1
45820	1.95288	2.1
45821	1.50524	2.1
45822	1.83564	2.1
45823	1.70303	2.1
45824	2.00749	2.1
45825	1.84671	2.1
45826	1.62632	2.1
45827	1.82498	2.1
45828	1.92991	2.1
45829	1.92991	2.1
45830	1.47928	2.1
45831	1.47751	2.1
45832	0.946128	2.1
45833	0.946128	2.1
45834	0.946132	2.1
45835	0.946132	2.1
45836	0.94613	2.1
45837	0.94613	2.1
45838	0.946129	2.1
45839	0.946129	2.1
45840	0.94613	2.1
45841	0.94613	2.1
45842	0.94613	2.1
45843	0.94613	2.1
45844	1.89226	2.1
45845	1.89226	2.1
45846	1.89226	2.1
45847	1.89226	2.1
45848	1.89226	2.1
45849	1.89226	2.1
45850	2.27351	0.502697
45851	2.74068	0.138638
45852	2.72278	0.220246
45853	2.63356	0.381874
45854	2.36273	0.207103
45855	2.27351	0.354926
45856	2.36273	0.34634
45857	2.83112	0.115057
45858	2.81212	0.180958
45859	2.74068	0.287243
45860	2.43416	0.173694
45861	2.36273	0.273029
45862	1.89227	0.237099
45863	2.83112	0.106376
45864	2.74069	0.208113
45865	2.74069	0.0930357
45866	1.89227	0.321674
45867	1.89227	0.141324
45868	2.40887	2.1
45869	1.4494	2.1
45870	1.52164	2.1
45871	2.47534	2.1
45872	3.93051	2.1
45873	1.89226	2.1
45874	2.47534	2.1
45875	2.40887	2.1

45876	2.40887	2.1
45877	1.89226	2.1
45878	1.89226	2.1
45879	1.60861	2.1
45880	2.47534	2.1
45881	1.52164	2.1
45882	1.52164	2.1
45883	2.55301	2.1
45884	2.55301	2.1
45885	0.946128	2.1
45886	0.946128	2.1
45887	1.23767	2.1
45888	1.2765	2.1
45889	1.23767	2.1
45890	1.23767	2.1
45891	0.946131	2.1
45892	0.946131	2.1
45893	0.946131	2.1
45894	1.2765	2.1
45895	1.23767	2.1
45896	0.946131	2.1
45897	1.89226	2.1
45898	1.87511	2.1
45899	1.87511	2.1
45900	1.95852	2.1
45901	2.0815	2.1
45902	2.22169	2.1
45903	1.89226	2.1
45904	1.89226	2.1
45905	1.89226	2.1
45906	1.89226	2.1
45907	1.89226	2.1
45908	1.89226	2.1
45909	2.0815	2.1
45910	1.80067	2.1
45911	1.80067	2.1
45912	2.05518	2.1
45913	1.89226	2.1
45914	2.16509	2.1
45915	1.87153	2.1
45916	2.65897	2.1
45917	1.58724	2.1
45918	2.38924	2.1
45919	4.26077	2.1
45920	1.89226	2.1
45921	1.89226	2.1
45922	1.89226	2.1
45923	1.89226	2.1
45924	1.89226	2.1
45925	1.89226	2.1
45926	1.89226	0.555578
45927	2.21022	0.05
45928	2.19769	0.589998
45929	2.21022	0.187515
45930	1.87973	0.0527911
45931	1.89226	0.05
45932	1.18376	0.816243
45933	2.21022	0.05

45934	1.16239	0.561021
45935	1.16239	0.05
45936	2.19769	0.535663
45937	2.19769	0.05
45938	0.946128	0.268953
45939	0.946128	0.0908523
45940	1.09362	0.730988
45941	1.09884	0.0933072
45942	0.94613	0.149275
45943	0.94613	0.05
45944	1.09362	0.557726
45945	1.09362	0.069572
45946	0.946131	0.397457
45947	1.09362	0.368957
45948	0.946131	0.0773816
45949	1.09884	0.05
45950	1.19323	0.628895
45951	2.19769	0.05
45952	1.18376	0.378834
45953	1.18376	0.05
45954	2.18724	0.402794
45955	2.18724	0.05
45956	2.0815	2.1
45957	0.922714	2.1
45958	2.0815	2.1
45959	1.84882	2.1
45960	1.20787	2.1
45961	1.08321	2.1
45962	2.6238	0.05
45963	1.89226	0.05
45964	1.89226	0.05
45965	2.4866	0.05
45966	1.89226	0.05
45967	2.4866	0.05
45968	2.66753	0.05
45969	1.89226	0.05
45970	1.89226	0.05
45971	2.6238	0.05
45972	1.89226	0.05
45973	2.6238	0.05
45974	0.946129	2.1
45975	0.946129	2.1
45976	1.04676	2.1
45977	1.04676	2.1
45978	0.946131	2.1
45979	0.946131	2.1
45980	1.08732	2.1
45981	1.04676	2.1
45982	1.08732	2.1
45983	0.946131	2.1
45984	1.04676	2.1
45985	0.946131	2.1
45986	1.20958	2.07679
45987	1.73802	0.21847
45988	1.39067	0.69557
45989	1.39067	0.0937734
45990	1.50969	0.663718
45991	1.50969	0.488613

45992	1.00382	1.13358
45993	1.50969	0.05
45994	1.20958	0.771912
45995	1.20958	0.05
45996	1.32039	1.17672
45997	1.32039	0.144714
45998	2.0815	2.1
45999	2.51396	2.1
46000	2.19826	2.1
46001	2.54175	2.1
46002	4.79599	2.1
46003	2.54174	2.1
46004	2.17978	2.1
46005	2.54174	2.1
46006	2.2854	2.1
46007	2.57212	2.1
46008	2.31578	2.1
46009	2.27103	2.1
46010	2.54175	2.1
46011	2.4255	2.1
46012	2.82251	2.1
46013	4.96725	2.1
46014	2.60614	0.05
46015	2.75374	0.05
46016	1.94002	0.05
46017	2.08392	0.05
46018	4.69006	0.05
46019	2.75373	0.05
46020	2.6875	0.05
46021	2.08392	0.05
46022	1.99944	0.05
46023	4.77142	0.05
46024	1.1211	0.05
46025	2.08392	0.05
46026	1.17787	0.05
46027	1.94002	0.05
46028	1.32178	0.05
46029	2.08392	0.05
46030	2.08392	0.05
46031	1.99944	0.05
46032	1.32178	0.05
46033	1.25929	0.05
46034	3.34321	0.05
46035	1.22266	0.05
46036	0.779473	0.05
46037	0.94613	0.05
46038	0.94613	0.05
46039	1.04072	0.05
46040	1.1407	0.05
46041	1.10042	0.05
46042	0.851542	0.05
46043	0.942046	0.05
46044	0.942046	0.05
46045	1.04114	0.05
46046	0.826602	0.05
46047	2.37468	2.1
46048	1.89226	2.1
46049	1.89226	2.1

46050	1.89226	2.1
46051	2.27917	2.1
46052	2.27917	2.1
46053	1.13959	2.1
46054	1.09451	2.1
46055	0.946131	2.1
46056	0.946131	2.1
46057	1.09451	2.1
46058	1.09451	2.1
46059	0.94613	2.1
46060	0.94613	2.1
46061	1.09451	2.1
46062	0.946131	2.1
46063	0.946131	2.1
46064	1.13959	2.1
46065	2.52454	0.736242
46066	2.12221	2.1
46067	2.67624	0.757443
46068	2.33865	2.1
46069	4.86319	2.1
46070	1.89226	2.1
46071	1.89226	2.1
46072	1.89226	2.0276
46073	1.89226	2.1
46074	3.78452	2.1
46075	1.89226	2.1
46076	1.89226	2.1
46077	1.89226	2.1
46078	1.89226	2.1
46079	3.78452	2.1
46080	2.12221	2.1
46081	1.47769	2.1
46082	2.33865	2.1
46083	1.62898	2.1
46084	3.81634	2.1
46085	2.67624	2.1
46086	2.37734	2.06444
46087	2.33865	2.1
46088	2.33865	2.1
46089	2.70125	2.1
46090	2.70125	2.1
46091	1.89226	2.1
46092	1.89226	2.1
46093	1.89226	2.1
46094	1.89226	2.1
46095	1.89226	0.806234
46096	1.89226	1.3754
46097	1.89226	2.1
46098	1.89226	2.1
46099	1.89226	2.1
46100	1.89226	2.1
46101	1.89226	2.1
46102	1.89226	2.1
46103	2.37733	2.1
46104	1.95927	2.1
46105	1.82516	2.06461
46106	1.82516	2.1
46107	2.33865	2.1

46108	2.33865	2.1
46109	2.57466	2.1
46110	2.21715	2.1
46111	2.55653	2.1
46112	2.24775	2.1
46113	4.77367	2.1
46114	0.937095	2.1
46115	0.937095	2.1
46116	0.958028	2.1
46117	0.958028	2.1
46118	0.828386	2.1
46119	0.771244	2.1
46120	1.10858	2.1
46121	1.12388	2.1
46122	1.12388	2.1
46123	0.828386	2.1
46124	0.771244	2.1
46125	1.10858	2.1
46126	2.55653	2.1
46127	2.32606	2.1
46128	2.24775	2.1
46129	2.24775	2.1
46130	2.57928	2.1
46131	2.57928	2.1
46132	0.990481	2.1
46133	0.990481	2.1
46134	1.00094	2.1
46135	1.00094	2.1
46136	0.888385	2.1
46137	0.828386	2.1
46138	1.16303	2.1
46139	1.12388	2.1
46140	1.16303	2.1
46141	0.888385	2.1
46142	1.12388	2.1
46143	0.828387	2.1
46144	0.962524	2.1
46145	0.962524	2.1
46146	0.7976	2.1
46147	0.7976	2.1
46148	0.797628	2.1
46149	0.814 2.1	
46150	0.946125	2.1
46151	0.946125	2.1
46152	0.946118	2.1
46153	0.797623	2.1
46154	0.814006	2.1
46155	0.946131	2.1
46156	1.8978	0.05
46157	2.23892	0.05
46158	1.71469	0.05
46159	4.13672	0.05
46160	2.64136	0.05
46161	2.23892	0.05
46162	2.37578	0.125824
46163	1.77489	2.1
46164	4.6147	0.128943
46165	1.02284	2.1

46166	0.898796	0.307289
46167	0.948554	2.1
46168	0.948554	2.1
46169	0.878797	2.1
46170	0.796197	2.1
46171	1.09145	1.94078
46172	0.963109	1.83174
46173	1.12114	2.1
46174	0.782739	2.1
46175	1.11084	2.1
46176	0.850742	2.1
46177	2.48024	2.1
46178	2.405	2.1
46179	2.24585	2.1
46180	2.34795	2.1
46181	4.75295	2.1
46182	1.93794	2.1
46183	2.405	2.1
46184	2.24585	2.1
46185	1.67594	2.1
46186	4.08094	2.1
46187	1.89226	2.1
46188	1.89226	0.223488
46189	1.89226	2.1
46190	1.89226	2.01557
46191	3.78452	2.1
46192	1.89226	2.1
46193	1.89226	2.1
46194	1.89226	2.1
46195	1.89226	2.1
46196	3.78452	2.1
46197	2.24585	2.1
46198	2.34795	2.1
46199	2.21155	2.1
46200	2.06626	2.1
46201	4.4142	2.1
46202	2.24585	2.1
46203	1.41498	2.1
46204	1.41498	2.1
46205	1.67594	2.1
46206	2.06625	2.1
46207	2.32721	2.1
46208	1.89226	2.1
46209	1.89226	1.98179
46210	1.89226	2.1
46211	1.89226	1.2178
46212	1.89226	2.1
46213	1.89226	0.667543
46214	1.89226	2.1
46215	1.89226	2.1
46216	1.89226	2.1
46217	1.89226	2.1
46218	3.78452	2.1
46219	1.89226	0.05
46220	1.89226	0.05
46221	1.89226	0.05
46222	1.89226	0.05
46223	3.78452	0.05

46224	1.89226	0.05
46225	2.53655	0.05
46226	1.89226	0.05
46227	1.89226	0.05
46228	1.89226	0.05
46229	1.89226	0.05
46230	1.89227	2.1
46231	1.89227	2.1
46232	1.89227	2.1
46233	1.89227	2.1
46234	1.89227	2.1
46235	1.89227	2.1
46236	0.91339	2.1
46237	0.91339	2.1
46238	1.2158	2.1
46239	1.20629	2.1
46240	1.00473	2.1
46241	1.15749	2.1
46242	0.946132	2.1
46243	0.946132	2.1
46244	0.946133	2.1
46245	1.17513	2.1
46246	1.00473	2.1
46247	0.946132	2.1
46248	1.89226	2.1
46249	1.89226	2.1
46250	1.89226	2.1
46251	1.89226	2.1
46252	1.89226	2.1
46253	1.89226	2.1
46254	1.89226	2.1
46255	2.6066	2.1
46256	2.35026	2.1
46257	2.35026	2.1
46258	1.89226	2.1
46259	1.89226	2.1
46260	2.04056	0.0572726
46261	2.77366	0.0684682
46262	1.24682	0.05
46263	1.24682	0.0666116
46264	2.93651	0.05
46265	2.14277	0.0656746
46266	2.68524	1.85288
46267	2.81847	1.7694
46268	1.89226	0.978124
46269	1.89226	1.70975
46270	4.57751	1.24397
46271	2.68524	1.48332
46272	1.89226	1.94537
46273	1.89226	1.98002
46274	2.50732	1.60828
46275	1.89226	1.78946
46276	2.50732	1.88166
46277	1.89226	1.11642
46278	1.89226	1.91136
46279	1.89226	1.66738
46280	1.89226	1.98916
46281	1.89226	1.23283

46282	1.89226	1.90511
46283	1.89226	1.95853
46284	1.89226	1.53736
46285	1.89226	1.73331
46286	1.89226	2.08586
46287	1.89226	0.876259
46288	1.89226	1.22757
46289	1.61016	1.28059
46290	1.07467	1.65409
46291	1.24474	1.1451
46292	1.24474	1.98893
46293	1.45303	1.27252
46294	1.45303	2.00999
46295	1.61016	1.69906
46296	1.24474	2.1
46297	1.61016	1.7981
46298	1.77186	1.43214
46299	1.37813	1.36413
46300	1.37813	1.78459
46301	1.73305	2.1
46302	2.0815	2.1
46303	1.97658	2.1
46304	1.75791	2.1
46305	1.87228	2.1
46306	1.66515	2.1
46307	2.0815	2.1
46308	1.93938	0.506969
46309	2.03253	0.204152
46310	2.2142	2.1
46311	1.97658	0.31037
46312	2.15324	2.1
46313	1.73305	2.1
46314	1.97658	2.1
46315	1.60428	2.1
46316	1.8589	2.1
46317	3.58085	2.1
46318	1.97657	2.1
46319	1.93938	0.252326
46320	1.51316	2.1
46321	1.85293	0.326514
46322	3.82951	2.1
46323	1.70303	0.05
46324	1.96087	0.05
46325	1.42308	0.05
46326	1.96087	0.05
46327	1.25023	0.05
46328	1.70303	0.05
46329	0.946132	0.05
46330	0.946132	0.05
46331	0.946131	0.05
46332	0.946131	0.05
46333	0.946132	0.05
46334	0.946132	0.05
46335	0.946132	0.05
46336	0.946132	0.05
46337	0.946132	0.05
46338	0.946132	0.05
46339	0.946132	0.05

46340	0.946132	0.05
46341	1.89226	0.05
46342	1.89226	0.0957111
46343	1.89226	0.0600907
46344	1.89226	0.0961545
46345	3.78452	0.0628967
46346	1.89226	0.05
46347	1.89226	0.0546329
46348	1.89226	0.05
46349	1.89226	0.0638418
46350	1.89226	0.05
46351	1.89226	0.05
46352	1.89226	0.05
46353	1.89226	0.0598801
46354	1.89226	0.0701216
46355	1.89226	0.0566927
46356	1.89226	0.0501337
46357	1.89226	0.05
46358	1.89226	0.05
46359	1.89226	0.05
46360	2.48255	0.05
46361	1.89226	0.05
46362	3.78452	0.05
46363	1.89226	2.1
46364	2.42758	2.1
46365	1.9209	2.1
46366	1.89226	2.1
46367	2.45622	2.1
46368	2.42758	2.1
46369	2.42758	2.1
46370	1.49987	2.1
46371	1.61028	2.1
46372	1.58164	2.1
46373	2.45622	2.1
46374	2.42758	2.1
46375	0.94613	2.1
46376	0.94613	2.1
46377	1.24298	2.1
46378	1.24298	2.1
46379	1.24298	2.1
46380	1.22811	2.1
46381	0.946131	2.1
46382	0.946131	2.1
46383	0.94613	2.1
46384	1.24298	2.1
46385	0.94613	2.1
46386	1.22811	2.1
46387	2.48596	2.1
46388	2.45622	2.1
46389	1.63823	2.1
46390	1.4304	2.1
46391	1.45739	2.1
46392	2.45622	2.1
46393	2.4169	2.1
46394	2.55249	2.1
46395	2.51125	2.1
46396	2.35245	2.1
46397	4.90494	2.1

46398	2.45922	2.1
46399	2.55249	2.1
46400	2.51126	2.1
46401	2.43226	2.1
46402	4.98474	2.1
46403	2.51125	2.1
46404	2.35245	2.1
46405	2.28979	2.1
46406	2.47228	2.1
46407	4.82473	2.1
46408	2.51126	2.1
46409	2.43226	2.1
46410	2.40668	2.1
46411	2.47228	2.1
46412	4.90453	2.1
46413	2.8045	2.1
46414	1.93707	2.1
46415	2.14087	2.1
46416	2.14087	2.1
46417	2.61595	2.1
46418	2.61595	2.1
46419	1.89226	2.1
46420	1.89226	2.1
46421	1.89226	2.1
46422	1.89226	2.1
46423	1.89226	2.1
46424	1.89226	1.58483
46425	1.22368	2.1
46426	1.32339	2.1
46427	0.957125	2.1
46428	0.750074	2.1
46429	1.08438	2.1
46430	1.08438	2.1
46431	1.13094	2.1
46432	0.970797	2.1
46433	1.17568	2.1
46434	0.764392	2.1
46435	0.944286	2.1
46436	1.27523	2.1
46437	1.89226	2.1
46438	1.89226	2.1
46439	1.89226	2.1
46440	1.89226	2.1
46441	3.78453	2.1
46442	2.8239	2.1
46443	1.89227	2.1
46444	2.57276	2.1
46445	2.57276	2.1
46446	1.89227	2.1
46447	1.89227	2.1
46448	1.18139	2.1
46449	1.85195	2.1
46450	1.78595	2.1
46451	1.08172	2.1
46452	2.93367	2.1
46453	1.60735	2.1
46454	1.20356	2.1
46455	0.967034	2.1

46456	1.08172	2.1
46457	1.67324	2.1
46458	1.77293	2.1
46459	1.89226	2.1
46460	1.89226	2.1
46461	1.8213	2.1
46462	1.82129	2.1
46463	2.14171	2.1
46464	2.40192	2.1
46465	0.810943	2.1
46466	0.810943	2.1
46467	0.798469	2.1
46468	0.788336	2.1
46469	0.804015	2.1
46470	0.810945	2.1
46471	1.947	2.1
46472	1.36506	2.1
46473	1.08686	2.1
46474	1.07208	2.1
46475	1.08132	2.1
46476	1.13537	0.05
46477	1.14754	1.86006
46478	0.946131	0.197788
46479	0.946131	2.1
46480	0.94613	0.13127
46481	0.94613	0.0501902
46482	1.25104	2.1
46483	1.14754	1.40363
46484	1.13537	0.566015
46485	0.946131	0.575017
46486	0.946131	1.13844
46487	1.25104	0.786715
46488	0.96542	0.05
46489	0.922284	1.03394
46490	0.946131	2.1
46491	0.946131	0.39911
46492	0.946131	0.304506
46493	0.946131	0.05
46494	1.13537	2.1
46495	0.924824	1.50853
46496	0.859476	0.560948
46497	0.842305	0.615019
46498	1.25996	0.552363
46499	1.04996	0.595561
46500	1.71007	0.05
46501	2.73283	0.05
46502	2.73283	0.05
46503	1.89226	0.05
46504	1.89226	0.05
46505	1.13262	0.05
46506	0.946131	0.05
46507	0.946131	0.05
46508	0.946131	0.05
46509	0.946131	0.05
46510	0.946132	0.05
46511	0.946132	0.05
46512	0.946131	0.05
46513	0.946131	0.05

46514	0.946131	0.05
46515	0.946131	0.05
46516	0.946132	0.05
46517	0.946132	0.05
46518	0.946131	0.05
46519	1.36642	0.05
46520	0.946125	0.05
46521	0.946125	0.05
46522	0.946128	0.05
46523	0.946128	0.05
46524	0.946126	0.05
46525	0.946128	0.05
46526	0.946128	0.05
46527	0.946128	0.05
46528	1.36641	0.05
46529	0.946128	0.05
46530	1.89226	0.05
46531	1.89226	0.05
46532	1.89226	0.05
46533	1.89226	0.05
46534	1.89226	0.05
46535	1.89226	0.05
46536	2.69784	2.1
46537	2.51651	2.1
46538	2.56999	2.1
46539	2.74623	2.1
46540	5.26783	2.1
46541	2.69784	2.1
46542	2.48718	2.1
46543	2.50161	2.1
46544	2.74623	2.1
46545	5.19945	2.1
46546	1.89226	2.1
46547	1.89226	2.1
46548	1.89226	2.1
46549	1.89226	2.1
46550	3.78453	2.1
46551	0.874264	2.1
46552	1.09879	2.1
46553	0.946131	2.1
46554	0.946131	2.1
46555	1.17062	2.1
46556	1.17062	2.1
46557	0.874293	2.1
46558	0.874293	2.1
46559	1.09879	2.1
46560	0.94613	2.1
46561	0.946131	2.1
46562	0.874265	2.1
46563	1.89226	2.1
46564	1.89226	2.1
46565	1.89226	2.1
46566	1.89226	2.1
46567	3.78453	2.1
46568	0.977382	2.1
46569	1.16424	2.1
46570	0.946133	2.1
46571	0.946133	2.1

46572	1.33012	2.1
46573	1.25589	2.1
46574	0.888498	2.1
46575	0.888498	2.1
46576	1.25375	2.1
46577	0.946131	2.1
46578	0.946131	2.1
46579	0.977384	2.1
46580	1.89226	2.1
46581	2.19757	2.1
46582	2.62906	2.1
46583	1.89226	2.1
46584	1.89226	2.1
46585	2.62906	2.1
46586	1.27944	1.22944
46587	1.27731	2.08178
46588	0.615701	1.8757
46589	0.615701	1.37681
46590	0.615673	0.05
46591	0.946133	1.79924
46592	0.948264	0.385793
46593	0.946132	1.27677
46594	0.94752	0.452079
46595	0.61706	0.0564705
46596	0.946132	0.91595
46597	0.946132	0.809308
46598	2.76604	2.1
46599	1.66728	2.1
46600	1.66728	2.1
46601	1.83036	2.1
46602	2.54515	2.1
46603	2.70823	2.1
46604	2.54515	2.1
46605	1.49958	2.1
46606	1.49958	2.1
46607	1.66728	2.1
46608	2.34465	2.1
46609	2.51235	2.1
46610	1.21128	2.1
46611	1.21128	2.1
46612	0.946131	2.1
46613	0.946131	2.1
46614	0.946131	2.1
46615	0.946131	2.1
46616	1.31401	2.1
46617	1.21128	2.1
46618	1.21128	2.1
46619	0.946131	2.1
46620	0.946131	2.1
46621	1.31401	2.1
46622	1.67374	2.1
46623	2.29586	2.1
46624	1.50033	2.1
46625	2.16598	2.1
46626	3.83972	2.1
46627	1.89226	2.1
46628	1.89226	2.1
46629	1.89226	2.1

46630	1.89226	2.1
46631	3.78452	2.1
46632	1.13537	2.1
46633	1.02188	2.1
46634	0.946132	2.1
46635	0.946132	2.1
46636	0.946132	2.1
46637	0.946132	2.1
46638	1.21128	2.1
46639	1.02188	2.1
46640	1.04672	2.1
46641	1.04672	2.1
46642	1.21128	2.1
46643	0.946132	2.1
46644	0.815411	2.1
46645	0.870464	2.1
46646	0.885437	2.1
46647	0.885437	2.1
46648	1.06395	2.1
46649	1.00483	2.1
46650	0.757125	2.1
46651	0.673956	2.1
46652	0.684461	2.1
46653	1.06979	2.1
46654	0.731403	2.1
46655	1.0559	2.1
46656	1.89226	2.1
46657	1.89226	2.1
46658	1.89226	2.1
46659	1.89226	2.1
46660	1.89226	2.1
46661	1.89226	2.1
46662	1.76897	1.39838
46663	1.76932	1.65816
46664	1.97455	2.1
46665	1.94302	0.23692
46666	3.71233	0.988184
46667	1.83347	2.1
46668	1.76932	1.84554
46669	1.97455	2.1
46670	1.76932	2.1
46671	1.76522	2.1
46672	1.55999	2.1
46673	1.89227	1.82156
46674	1.89227	2.1
46675	1.89227	2.1
46676	1.89227	0.966568
46677	3.78453	1.62267
46678	1.89226	1.63924
46679	1.89226	0.0964821
46680	1.89226	0.183634
46681	1.89226	0.772646
46682	3.78452	0.694525
46683	1.97452	2.1
46684	1.94299	0.175693
46685	2.17831	0.708669
46686	2.23195	0.900041
46687	4.17495	1.19141

46688	2.23196	1.18056
46689	2.1499	2.1
46690	1.97452	2.1
46691	1.97452	2.1
46692	2.09088	2.1
46693	1.83345	2.1
46694	0.94612	2.06898
46695	0.94612	2.1
46696	0.946119	0.78866
46697	0.946119	0.754978
46698	0.946119	0.798856
46699	0.946119	1.99871
46700	0.94612	1.11503
46701	0.94612	2.1
46702	0.946132	1.4519
46703	0.946132	1.19666
46704	0.946107	1.77657
46705	0.946107	1.69057
46706	1.89224	0.140157
46707	1.89224	0.602695
46708	1.89224	0.722636
46709	1.89224	0.133364
46710	3.78447	0.145812
46711	1.70302	0.126306
46712	1.056	0.0811725
46713	1.21829	0.194219
46714	1.89906	0.0976412
46715	3.07892	0.101447
46716	2.20248	0.131017
46717	1.56498	0.101344
46718	1.46539	0.0975991
46719	1.48787	0.07452
46720	2.37625	0.212776
46721	2.37625	0.141335
46722	1.14104	0.0817567
46723	1.00675	0.089326
46724	0.700689	0.0749225
46725	0.624389	0.0880509
46726	0.782149	0.05
46727	0.940197	0.0755702
46728	0.803437	0.0706797
46729	0.803437	0.0967508
46730	0.995423	0.0674092
46731	0.700699	0.05
46732	0.593895	0.155295
46733	1.00673	0.0769473
46734	1.18097	0.0976201
46735	1.15839	0.0889489
46736	0.995407	0.0875544
46737	0.865932	0.0803413
46738	1.17299	0.0608228
46739	0.957054	0.0754557
46740	1.08016	0.103675
46741	1.08016	0.149572
46742	0.995425	0.115314
46743	1.24161	0.055764
46744	0.796771	0.0590737
46745	1.15837	0.136022

46746	2.57264	0.05
46747	1.89226	0.05
46748	2.41701	0.05
46749	1.89226	0.05
46750	4.30928	0.05
46751	1.69055	0.05
46752	2.39587	0.05
46753	1.53463	0.05
46754	2.2053	0.05
46755	3.89585	0.05
46756	0.946129	0.05
46757	0.946129	0.05
46758	0.94613	0.05
46759	0.94613	0.05
46760	0.946132	0.05
46761	0.946132	0.05
46762	0.946126	0.05
46763	0.946126	0.05
46764	0.94613	0.05
46765	0.94613	0.05
46766	0.946129	0.05
46767	0.946129	0.05
46768	2.41701	0.05
46769	1.89226	0.05
46770	1.89226	0.05
46771	1.89226	0.05
46772	2.32462	0.05
46773	2.32462	0.05
46774	1.41942	0.05
46775	2.2053	0.05
46776	2.02097	0.05
46777	2.02097	0.05
46778	1.53463	0.05
46779	1.53463	0.05
46780	1.89226	0.05
46781	1.89226	0.05
46782	1.89226	0.05
46783	1.89226	0.05
46784	1.89226	0.05
46785	1.89226	0.05
46786	1.89226	0.798414
46787	1.89226	2.1
46788	1.89226	2.1
46789	1.89226	1.33989
46790	1.89226	1.9804
46791	1.89226	0.972822
46792	1.02816	0.714376
46793	1.02816	1.32896
46794	0.595141	1.59177
46795	0.595141	1.92941
46796	0.946132	1.04324
46797	0.595113	2.03445
46798	0.677174	1.9189
46799	0.946132	0.645876
46800	0.946132	1.47982
46801	0.946132	1.48297
46802	0.595113	1.44095
46803	0.677174	1.51248

46804	1.89226	1.26521
46805	1.89226	2.1
46806	1.89226	2.00729
46807	1.89226	1.40663
46808	1.89226	2.09021
46809	1.89226	1.63395
46810	0.946133	0.841345
46811	0.946133	0.43084
46812	0.946132	1.9931
46813	0.946132	2.0257
46814	0.946132	2.00298
46815	0.946132	0.949315
46816	0.946132	1.69835
46817	0.946132	0.4663
46818	0.946132	1.44094
46819	0.946132	1.73606
46820	0.946133	1.11161
46821	0.946133	1.14562
46822	0.946131	1.61154
46823	0.946131	1.59306
46824	0.946131	2.1
46825	0.946131	2.1
46826	0.946128	2.1
46827	0.946128	1.60928
46828	0.946133	2.1
46829	0.946133	1.46669
46830	0.94613	1.98959
46831	0.94613	2.09651
46832	0.946131	2.02437
46833	0.946131	1.86444
46834	2.0264	2.1
46835	1.67345	1.37966
46836	2.0264	1.84902
46837	1.70539	0.164486
46838	1.31611	0.89344
46839	1.31611	0.840959
46840	1.89226	0.0761376
46841	1.89226	0.07472
46842	1.89226	0.141416
46843	1.89226	0.0631882
46844	3.78452	0.0651652
46845	1.89226	0.138555
46846	1.89226	0.106244
46847	1.89226	0.0999624
46848	1.89226	0.190857
46849	1.89226	0.110468
46850	1.89226	0.232082
46851	1.02645	0.05
46852	0.810942	0.05
46853	0.882781	0.05
46854	0.882781	0.05
46855	2.80386	0.05
46856	1.56719	0.05
46857	1.22499	0.05
46858	1.08132	0.05
46859	1.36865	0.05
46860	0.810957	0.05
46861	0.810957	0.05

46862	0.946132	0.0511292
46863	0.946132	0.0719798
46864	0.946129	0.05
46865	0.946129	0.05
46866	0.946131	0.05
46867	0.946131	0.05
46868	0.946131	0.0678072
46869	0.946131	0.05
46870	0.94613	0.0508404
46871	0.94613	0.05
46872	0.946131	0.0759706
46873	0.946131	0.0529544
46874	1.89226	0.0731305
46875	1.89226	0.05
46876	1.89226	0.0941933
46877	1.89226	0.05
46878	3.78453	0.05
46879	1.30462	0.05
46880	1.79906	0.05
46881	1.01299	0.0692472
46882	1.44185	0.05
46883	1.3499	0.05
46884	1.79906	0.05
46885	1.89226	0.0606089
46886	1.89226	0.0696801
46887	1.89226	0.05
46888	1.89226	0.05
46889	1.89226	0.12641
46890	1.89226	0.261149
46891	1.89226	0.0618609
46892	1.89226	0.0639263
46893	1.89226	0.0900819
46894	1.89226	0.0713354
46895	1.89226	0.0644333
46896	1.89226	0.0567108
46897	1.3499	0.05
46898	1.79905	0.05
46899	1.29001	0.05
46900	1.36005	0.05
46901	1.88294	0.05
46902	1.79905	0.05
46903	1.89226	0.05
46904	2.75374	0.05
46905	2.60614	0.05
46906	1.89226	0.05
46907	2.60614	0.05
46908	1.89226	0.05
46909	0.946131	0.05
46910	0.946131	0.05
46911	1.37687	0.05
46912	1.37687	0.05
46913	0.946131	0.05
46914	1.34375	0.05
46915	1.37686	0.05
46916	0.946128	0.05
46917	1.37687	0.05
46918	1.34375	0.05
46919	0.94613	0.05

46920	0.94613	0.05
46921	1.44247	2.1
46922	1.12665	2.1
46923	1.50044	2.1
46924	1.73651	2.1
46925	2.94291	2.1
46926	1.73652	2.1
46927	1.64926	2.1
46928	1.44247	2.1
46929	1.44247	2.1
46930	1.64926	2.1
46931	1.43872	2.1
46932	1.50044	2.1
46933	1.73651	2.1
46934	2.00955	2.1
46935	1.87875	2.1
46936	3.50999	2.1
46937	1.64926	2.1
46938	1.73652	2.1
46939	2.00955	2.1
46940	1.8493	2.1
46941	3.65881	2.1
46942	1.89226	0.05
46943	2.28188	0.05
46944	1.99182	0.05
46945	2.28188	0.05
46946	1.6022	0.05
46947	1.89226	0.05
46948	1.89226	0.05
46949	1.89226	0.05
46950	1.89226	0.05
46951	1.89226	0.05
46952	1.89226	0.05
46953	1.89226	0.05
46954	1.89226	0.05
46955	1.89226	0.05
46956	2.19578	0.05
46957	1.89226	0.05
46958	2.81422	0.05
46959	2.42521	0.05
46960	1.89224	0.05
46961	1.99179	0.05
46962	1.454 0.05	
46963	1.99179	0.05
46964	1.35444	0.05
46965	1.89224	0.05
46966	1.89224	0.05
46967	1.89224	0.05
46968	1.89224	0.05
46969	1.89224	0.05
46970	1.89224	0.05
46971	1.89224	0.05
46972	1.703 0.05	
46973	2.19575	0.05
46974	1.9911	0.05
46975	2.19575	0.05
46976	1.67811	0.05
46977	1.89223	0.05

46978	1.69586	2.1
46979	2.55301	2.1
46980	1.60861	2.1
46981	1.60861	2.1
46982	2.65233	2.1
46983	2.65233	2.1
46984	0.946129	2.1
46985	0.946129	2.1
46986	1.2765	2.1
46987	1.32617	2.1
46988	1.2765	2.1
46989	1.2765	2.1
46990	0.946131	2.1
46991	0.946131	2.1
46992	0.946131	2.1
46993	1.32616	2.1
46994	0.946131	2.1
46995	1.2765	2.1
46996	1.36757	2.1
46997	1.32617	2.1
46998	0.891503	2.1
46999	0.847932	2.1
47000	1.10722	2.1
47001	1.10722	2.1
47002	1.10829	2.1
47003	1.10829	2.1
47004	1.36757	2.1
47005	0.891504	2.1
47006	0.847932	2.1
47007	1.32617	2.1
47008	0.946129	2.1
47009	0.946129	2.1
47010	1.36757	2.1
47011	1.32617	2.1
47012	1.32616	2.1
47013	1.32616	2.1
47014	0.946131	2.1
47015	0.946131	2.1
47016	1.36757	2.1
47017	0.946131	2.1
47018	0.946131	2.1
47019	1.32616	2.1
47020	1.89226	0.05
47021	1.89226	0.05
47022	1.89226	0.05
47023	1.89226	0.05
47024	3.78452	0.05
47025	2.36336	0.05
47026	1.89226	0.05
47027	2.67831	0.05
47028	2.67831	0.05
47029	1.89226	0.05
47030	1.89226	0.05
47031	1.89227	0.05
47032	1.89227	0.05
47033	1.89227	0.05
47034	1.89227	0.05
47035	3.78453	0.05

47036	1.89226	0.05
47037	1.89226	0.05
47038	1.89226	0.05
47039	1.89226	0.05
47040	3.78452	0.05
47041	2.36336	0.05
47042	2.0815	0.05
47043	2.0815	0.05
47044	2.41048	0.05
47045	1.66267	0.05
47046	2.13452	0.05
47047	1.89226	0.05
47048	1.89226	0.05
47049	1.89226	0.05
47050	1.89226	0.05
47051	1.89226	0.05
47052	1.89226	0.05
47053	1.89226	0.05
47054	1.89226	0.0966975
47055	1.89226	0.05
47056	1.89226	0.0909835
47057	1.89226	0.05
47058	1.89226	0.086609
47059	0.946128	0.122325
47060	0.946128	0.106183
47061	1.00122	0.0652292
47062	1.00122	0.0660186
47063	0.94613	0.158084
47064	1.01618	0.0891754
47065	0.946131	0.089493
47066	1.00122	0.0554465
47067	1.00122	0.0577777
47068	1.01618	0.0785418
47069	0.946131	0.128296
47070	0.946131	0.0913865
47071	1.89226	1.84037
47072	1.89226	0.668291
47073	1.89226	0.542376
47074	1.89226	2.1
47075	3.78452	0.961875
47076	1.89226	1.56083
47077	1.89226	2.1
47078	1.89226	2.1
47079	1.89226	1.84675
47080	1.89226	2.1
47081	1.89226	1.85113
47082	1.89226	0.239723
47083	1.89226	2.03423
47084	1.89226	1.83229
47085	1.89226	0.731552
47086	1.89226	1.83592
47087	1.89226	0.714538
47088	1.89226	0.606706
47089	1.89226	2.1
47090	1.89226	2.08459
47091	1.89226	1.06556
47092	1.89226	2.02348
47093	1.89226	1.15023

47094	2.71279	0.837179
47095	1.4155	0.715453
47096	2.28488	0.536942
47097	1.06122	0.704903
47098	3.70038	0.450072
47099	1.89226	1.16259
47100	1.89226	0.924275
47101	1.89226	0.744215
47102	1.89226	0.797451
47103	3.78452	1.43477
47104	0.851542	0.466513
47105	0.851542	0.379026
47106	0.851484	0.581355
47107	0.865567	0.858495
47108	0.981616	0.434161
47109	0.864767	0.573659
47110	0.851542	0.748109
47111	0.851542	0.493735
47112	0.851477	0.575889
47113	0.851477	0.782091
47114	0.865635	0.53852
47115	0.816472	0.356026
47116	1.68443	2.1
47117	1.44489	2.1
47118	1.54924	2.1
47119	1.34389	2.1
47120	3.02832	2.1
47121	1.44489	2.1
47122	1.03899	2.1
47123	1.03899	2.1
47124	1.12075	2.1
47125	1.34389	2.1
47126	1.42566	2.1
47127	1.54924	2.1
47128	1.34389	2.1
47129	1.44091	2.1
47130	1.25866	2.1
47131	2.7848	2.1
47132	0.573141	2.1
47133	0.573141	2.1
47134	0.575681	2.1
47135	0.575681	2.1
47136	0.481985	2.1
47137	0.519492	2.1
47138	0.671948	2.1
47139	0.629331	2.1
47140	0.629329	2.1
47141	0.481986	2.1
47142	0.519494	2.1
47143	0.671947	2.1
47144	1.89226	0.355362
47145	1.89226	1.20361
47146	1.89226	0.712041
47147	2.31925	0.619575
47148	3.78452	1.07139
47149	1.30916	2.1
47150	1.17426	2.1
47151	0.946129	2.1

47152	0.946129	2.1
47153	0.94613	2.1
47154	0.94613	2.1
47155	1.17426	2.1
47156	1.17426	2.1
47157	1.30916	2.1
47158	0.94613	2.1
47159	1.17426	2.1
47160	0.94613	2.1
47161	2.56346	2.1
47162	1.89226	1.34417
47163	1.89226	2.1
47164	1.89226	1.56207
47165	2.6074	2.1
47166	2.6074	1.57887
47167	1.89226	2.1
47168	1.89226	2.1
47169	1.89226	2.1
47170	1.89226	2.1
47171	3.78452	2.1
47172	1.33064	2.1
47173	1.84121	2.1
47174	0.946129	2.1
47175	0.946129	2.1
47176	0.946131	2.1
47177	0.946131	2.1
47178	1.30916	2.1
47179	1.8412	2.1
47180	1.33064	2.1
47181	0.94613	2.1
47182	1.30916	2.1
47183	0.946131	2.1
47184	1.19283	1.3823
47185	1.19283	1.13867
47186	0.946131	2.1
47187	0.946131	2.1
47188	0.946132	2.1
47189	0.946132	1.30368
47190	1.3037	2.1
47191	1.19283	1.19035
47192	1.19283	2.05582
47193	0.946131	2.05712
47194	0.946131	2.0195
47195	1.3037	2.08871
47196	1.89226	2.1
47197	1.89226	2.1
47198	1.89226	2.1
47199	1.89226	2.1
47200	1.89226	2.1
47201	1.89226	2.1
47202	2.17708	2.1
47203	1.70302	2.1
47204	2.28188	2.08807
47205	2.13621	2.1
47206	4.28253	2.1
47207	1.89226	1.80144
47208	1.89226	2.1
47209	1.89226	2.1

47210	1.89226	2.1
47211	3.78452	2.1
47212	0.946132	2.1
47213	0.946132	2.1
47214	0.946132	2.1
47215	0.946132	2.1
47216	0.946131	2.1
47217	0.946131	2.1
47218	0.946132	2.1
47219	0.946132	2.1
47220	0.946132	2.1
47221	0.946132	2.1
47222	0.946132	2.1
47223	0.946132	2.1
47224	1.74933	2.1
47225	1.11833	2.1
47226	1.90074	2.1
47227	1.669 2.1	
47228	1.335 2.1	
47229	1.11833	2.1
47230	2.28188	1.74991
47231	2.31804	2.1
47232	2.42758	2.05363
47233	2.14895	2.1
47234	4.43083	2.1
47235	1.89226	1.83611
47236	1.89226	2.1
47237	1.89226	1.41414
47238	1.89226	2.1
47239	3.78452	2.1
47240	1.89226	2.1
47241	1.89226	2.1
47242	1.89226	2.1
47243	1.89226	2.1
47244	1.89226	2.1
47245	1.89226	2.1
47246	0.864133	2.1
47247	0.864133	2.1
47248	0.967939	2.1
47249	0.793739	2.1
47250	0.650797	2.1
47251	0.757546	2.1
47252	1.08941	2.1
47253	1.10136	2.1
47254	1.10138	2.1
47255	0.796445	2.1
47256	0.866907	2.1
47257	0.650788	2.1
47258	2.63062	0.0821753
47259	1.89226	0.0743261
47260	1.89226	0.0527774
47261	1.89226	0.890467
47262	2.46557	0.05
47263	2.46557	1.15765
47264	1.16777	0.790454
47265	1.38859	2.1
47266	1.05042	2.1
47267	1.28909	2.1

47268	2.45686	2.1
47269	1.89227	0.889482
47270	1.89227	1.60541
47271	1.89227	0.0599657
47272	1.89227	2.1
47273	3.78453	0.958776
47274	2.15114	2.1
47275	1.89226	2.1
47276	2.15005	2.1
47277	2.15005	2.1
47278	1.89226	2.1
47279	1.89226	2.1
47280	1.89226	2.1
47281	1.89226	0.983919
47282	1.89226	2.1
47283	1.89226	2.1
47284	1.89226	0.395709
47285	1.89226	2.1
47286	0.973447	2.1
47287	1.32143	2.1
47288	1.37444	2.1
47289	1.37444	2.1
47290	0.949134	2.1
47291	0.949134	2.1
47292	0.946131	1.57679
47293	0.946131	2.1
47294	1.07557	2.1
47295	1.07557	2.1
47296	0.946131	2.1
47297	0.946131	2.1
47298	1.07557	2.1
47299	1.08579	2.1
47300	1.08579	2.1
47301	0.946131	2.1
47302	1.07557	2.1
47303	0.946131	2.1
47304	0.946131	0.141313
47305	0.946131	2.1
47306	0.946131	2.01259
47307	0.946131	2.1
47308	0.94613	0.837074
47309	0.94613	1.37449
47310	0.946131	2.1
47311	0.946131	2.1
47312	0.946131	1.8967
47313	0.946131	1.68767
47314	0.946131	1.87691
47315	0.946131	2.0823
47316	0.606115	2.1
47317	0.606115	2.1
47318	0.616308	2.1
47319	0.616308	2.1
47320	0.68722	0.752808
47321	0.735699	2.1
47322	0.514789	2.1
47323	0.486724	2.1
47324	0.514788	1.61412
47325	0.735699	1.94476

47326	0.486723	2.1
47327	0.687221	2.06504
47328	2.33761	0.609706
47329	2.51749	0.162863
47330	2.68092	0.337653
47331	2.58304	0.181983
47332	5.01853	0.24354
47333	2.50906	0.386998
47334	2.51749	0.188184
47335	2.68091	0.36698
47336	2.53176	0.117417
47337	5.18997	0.282654
47338	2.68092	0.342509
47339	2.58304	0.14885
47340	2.71793	0.307333
47341	2.67098	0.0978845
47342	5.39885	0.212077
47343	2.68091	0.37551
47344	2.53175	0.106461
47345	2.36417	0.252352
47346	2.67098	0.109856
47347	5.04508	0.175772
47348	2.76309	2.1
47349	1.89226	2.1
47350	1.89226	2.1
47351	1.89226	2.1
47352	2.48573	2.1
47353	2.48573	2.1
47354	1.35879	2.1
47355	1.55161	0.0741312
47356	1.10407	2.1
47357	1.32259	0.133938
47358	2.68138	2.1
47359	1.89226	2.1
47360	1.89226	0.123989
47361	1.89226	2.1
47362	1.89226	0.145053
47363	3.78452	1.88493
47364	1.19612	2.09648
47365	1.24694	2.1
47366	1.24844	0.1161
47367	1.29747	2.1
47368	2.49358	2.1
47369	1.89226	2.1
47370	1.89226	2.1
47371	1.89226	0.0680725
47372	1.89226	2.1
47373	3.78453	1.97655
47374	1.89226	2.1
47375	1.89226	2.1
47376	1.89226	2.1
47377	1.89226	2.1
47378	1.89226	2.1
47379	1.89226	2.1
47380	0.587463	2.1
47381	0.587463	2.1
47382	0.581977	2.1
47383	0.581977	2.1

47384	0.531709	2.1
47385	0.545972	2.1
47386	0.623469	2.1
47387	0.648733	2.1
47388	0.648733	2.1
47389	0.545971	2.1
47390	0.623469	2.1
47391	0.531709	2.1
47392	1.29417	0.151561
47393	1.29747	2.1
47394	1.27829	2.1
47395	1.24844	1.4391
47396	1.32732	2.1
47397	1.29747	1.13168
47398	1.89226	0.331088
47399	1.89226	2.1
47400	1.89226	0.108222
47401	1.89226	1.8569
47402	1.89226	1.16347
47403	1.89226	2.1
47404	1.89226	2.1
47405	1.89226	2.1
47406	1.89226	2.1
47407	1.89226	2.1
47408	1.89226	2.1
47409	1.89226	2.02525
47410	0.60346	2.1
47411	0.60346	2.1
47412	0.60086	2.06296
47413	0.60086	2.1
47414	0.545971	2.1
47415	0.555587	2.1
47416	0.648733	2.1
47417	0.663661	2.1
47418	0.663661	2.1
47419	0.555587	2.1
47420	0.648733	2.1
47421	0.545971	2.1
47422	1.34066	2.1
47423	1.36601	2.1
47424	1.34955	0.0897101
47425	1.37623	2.1
47426	2.71556	2.1
47427	1.89226	1.48392
47428	1.89226	2.1
47429	1.89226	0.0975017
47430	1.89226	2.03168
47431	3.78452	1.02447
47432	0.946132	2.1
47433	0.946132	2.1
47434	0.946132	1.163
47435	0.946132	2.1
47436	0.946132	2.1
47437	0.946132	2.1
47438	0.946131	2.04578
47439	0.946131	1.89615
47440	0.946132	2.1
47441	0.946132	2.1

47442	0.946132	2.1
47443	0.946132	2.1
47444	0.631351	1.99285
47445	0.631351	2.1
47446	0.632421	2.1
47447	0.632421	2.1
47448	0.573604	2.1
47449	0.580765	2.1
47450	0.683006	1.50542
47451	0.688118	2.1
47452	0.688118	2.1
47453	0.580765	2.1
47454	0.683007	2.1
47455	0.573604	2.1
47456	1.34955	1.93322
47457	1.37624	2.1
47458	1.35048	0.120638
47459	1.37999	2.1
47460	2.72954	1.9639
47461	0.946131	1.84638
47462	0.946131	1.15903
47463	0.946131	2.1
47464	0.946131	2.1
47465	0.94613	2.1
47466	0.94613	2.0556
47467	0.946133	0.59904
47468	0.946133	1.23735
47469	0.946131	1.68909
47470	0.946131	1.12355
47471	0.946132	1.01142
47472	0.946132	1.16049
47473	1.89226	2.1
47474	1.89226	2.1
47475	1.89226	2.1
47476	1.89226	2.1
47477	1.89226	2.1
47478	1.89226	2.1
47479	0.532189	2.1
47480	0.531141	2.1
47481	0.497784	2.1
47482	0.502536	2.1
47483	1.37999	2.1
47484	0.92433	2.1
47485	0.670081	2.1
47486	0.727155	2.1
47487	0.786438	2.04685
47488	0.589798	2.1
47489	0.497784	2.1
47490	1.89226	2.1
47491	2.72301	2.1
47492	2.4158	2.1
47493	2.4158	2.1
47494	1.89226	2.1
47495	1.89226	2.1
47496	1.89226	2.1
47497	1.89226	2.1
47498	1.89226	2.1
47499	1.89226	2.1

47500	1.89226	2.1
47501	1.89226	2.1
47502	1.36919	2.1
47503	1.12717	2.1
47504	1.38634	2.1
47505	1.56541	2.1
47506	2.75552	2.1
47507	1.89226	2.1
47508	1.89226	2.1
47509	1.89226	2.1
47510	1.89226	2.1
47511	1.89226	2.1
47512	1.89226	2.1
47513	0.907036	2.1
47514	0.782706	2.1
47515	0.838808	2.1
47516	0.693168	2.1
47517	0.794747	2.1
47518	0.794747	2.1
47519	0.805458	2.1
47520	0.805458	2.1
47521	0.907036	2.1
47522	0.838808	2.1
47523	0.693169	2.1
47524	0.782707	2.1
47525	0.94613	2.1
47526	0.94613	2.1
47527	1.188 2.1	
47528	1.28421	2.1
47529	1.18801	2.1
47530	1.18801	2.1
47531	0.946129	2.1
47532	0.946129	2.1
47533	1.28421	2.1
47534	0.94613	2.1
47535	1.188 2.1	
47536	0.94613	2.1
47537	1.89226	2.1
47538	1.89226	2.1
47539	1.89226	2.1
47540	1.89226	2.1
47541	1.89226	2.1
47542	1.89226	2.1
47543	1.16817	2.1
47544	1.06007	2.1
47545	1.19785	2.1
47546	1.2832	2.1
47547	2.36602	2.1
47548	0.960106	2.1
47549	2.0073	2.1
47550	1.51754	2.1
47551	2.56883	2.1
47552	3.52484	2.1
47553	1.06756	2.1
47554	1.57913	2.1
47555	1.87532	2.1
47556	1.38666	2.1
47557	2.94287	2.1

47558	2.15622	2.0913
47559	1.38668	2.1
47560	1.87534	2.1
47561	1.87534	2.1
47562	1.70539	2.1
47563	1.70539	2.1
47564	0.986095	2.1
47565	1.36226	2.1
47566	1.3331	2.1
47567	1.3331	2.1
47568	1.07156	2.1
47569	1.07156	2.1
47570	0.682081	1.43255
47571	0.68748	1.13175
47572	0.650003	0.110253
47573	0.645873	0.277381
47574	0.666344	0.104218
47575	0.666344	1.04442
47576	0.66161	1.89081
47577	0.66161	0.137795
47578	0.68748	0.435881
47579	0.645873	0.382397
47580	0.650004	0.515839
47581	0.682082	0.496679
47582	0.946132	1.24009
47583	0.946132	0.653405
47584	0.946132	0.153535
47585	0.946132	0.0718763
47586	0.946132	0.0753719
47587	0.946132	0.558386
47588	0.946133	0.225218
47589	0.946133	1.28536
47590	0.946132	0.216714
47591	0.946132	0.198312
47592	0.946133	0.431622
47593	0.946133	0.395842
47594	1.89227	0.916692
47595	2.79312	0.0669483
47596	2.77591	0.162787
47597	2.79312	0.251422
47598	1.87505	0.0814938
47599	1.89227	0.138667
47600	0.68748	1.66039
47601	0.698626	1.3076
47602	0.645873	0.181349
47603	0.650148	0.279771
47604	0.670561	0.173042
47605	0.670561	1.31784
47606	0.667067	1.96398
47607	0.667067	0.13806
47608	0.698627	0.63298
47609	0.650148	0.529004
47610	0.68748	0.698956
47611	0.645873	0.645406
47612	0.946132	1.49478
47613	0.946132	0.797653
47614	0.946132	0.33325
47615	0.946132	0.102886

47616	0.946132	0.105037
47617	0.946132	0.698679
47618	0.946133	0.40446
47619	0.946133	1.55233
47620	0.946133	0.298549
47621	0.946133	0.329757
47622	0.946132	0.654566
47623	0.946132	0.601626
47624	0.946133	0.613327
47625	0.946133	0.144852
47626	1.38709	0.340349
47627	1.38795	0.0701783
47628	0.946132	0.0753096
47629	0.946132	0.202039
47630	1.38709	0.69037
47631	1.38709	0.077336
47632	1.38709	0.165399
47633	0.946133	0.199961
47634	0.946132	0.447584
47635	1.38795	0.2268
47636	1.70301	0.05
47637	2.07659	0.05
47638	1.89226	0.05
47639	1.89226	0.05
47640	1.76332	0.05
47641	1.76331	0.05
47642	1.89226	0.05
47643	1.89226	0.05
47644	1.89226	0.05
47645	1.89226	0.05
47646	1.89226	0.05
47647	1.89226	0.05
47648	1.89226	0.05
47649	1.89226	0.05
47650	1.89226	0.05
47651	1.89226	0.05
47652	3.78452	0.05
47653	1.89226	0.05
47654	2.12492	0.05
47655	1.89226	0.05
47656	1.89226	0.05
47657	2.07659	0.05
47658	2.07659	0.05
47659	0.94613	0.05
47660	0.94613	0.05
47661	1.17498	0.05
47662	1.32446	0.05
47663	1.32446	0.05
47664	0.94613	0.05
47665	0.946127	0.05
47666	1.17498	0.05
47667	1.32446	0.05
47668	1.32446	0.05
47669	0.946128	0.05
47670	0.946128	0.05
47671	1.89226	2.1
47672	1.86472	2.1
47673	2.11049	2.1

47674	1.89226	2.1
47675	2.08295	2.1
47676	1.86472	2.1
47677	1.89226	2.1
47678	2.08295	2.1
47679	2.14023	2.1
47680	1.89226	2.1
47681	2.33091	2.1
47682	2.08295	2.1
47683	0.809215	2.1
47684	0.809215	2.1
47685	0.810915	2.1
47686	0.810915	2.1
47687	1.09581	2.1
47688	1.08271	2.1
47689	0.520996	2.1
47690	0.537426	2.1
47691	0.520997	2.1
47692	1.08271	2.1
47693	0.537426	2.1
47694	1.09581	2.1
47695	0.79674	2.1
47696	0.79674	2.1
47697	0.799279	2.1
47698	0.799279	2.1
47699	1.07502	2.1
47700	1.0827	2.1
47701	0.508206	2.1
47702	0.520996	2.1
47703	0.508206	2.1
47704	1.07502	2.1
47705	0.520996	2.1
47706	1.0827	2.1
47707	2.15714	2.1
47708	1.70302	2.1
47709	1.78732	2.1
47710	2.02219	2.1
47711	3.98971	2.1
47712	2.0927	2.1
47713	2.162	2.1
47714	2.11563	2.1
47715	2.02219	2.1
47716	4.18419	2.1
47717	1.62483	2.1
47718	2.02219	2.1
47719	1.93617	2.1
47720	1.52008	2.1
47721	3.54227	2.1
47722	2.11563	2.1
47723	2.02219	2.1
47724	2.29218	2.1
47725	2.03975	2.1
47726	4.06194	2.1
47727	2.007	2.1
47728	1.89226	2.1
47729	1.89226	2.1
47730	1.89226	2.1
47731	2.06776	2.1

47732	2.06776	2.1
47733	0.946129	2.1
47734	0.946129	2.1
47735	1.0035	2.1
47736	1.0035	2.1
47737	0.946131	2.1
47738	0.946131	2.1
47739	0.968797	2.1
47740	1.0035	2.1
47741	0.968771	2.1
47742	0.946105	2.1
47743	1.00353	2.1
47744	0.946157	2.1
47745	1.79103	2.1
47746	2.53286	0.139009
47747	2.60781	2.1
47748	2.219	0.641309
47749	2.17985	1.39689
47750	1.79103	0.110003
47751	2.219	1.71136
47752	2.66359	1.54368
47753	2.70084	2.1
47754	2.38698	1.22422
47755	2.53285	0.727161
47756	2.219	0.181571
47757	1.33179	1.36118
47758	0.946128	0.538146
47759	0.946133	1.09641
47760	1.26643	2.01992
47761	1.3318	0.744233
47762	1.3318	1.94192
47763	0.946131	0.249733
47764	0.946131	1.47774
47765	0.946131	0.37797
47766	0.946131	0.204638
47767	1.33179	0.949712
47768	1.26643	2.09559
47769	2.28979	2.1
47770	2.47227	2.1
47771	2.43451	2.1
47772	2.22627	2.1
47773	4.69855	2.1
47774	2.40667	2.1
47775	2.47228	2.1
47776	2.43451	2.1
47777	2.38069	2.1
47778	4.85296	2.1
47779	2.43451	2.1
47780	2.15715	2.1
47781	2.15715	2.1
47782	2.22627	2.1
47783	2.39489	2.1
47784	2.46402	2.1
47785	2.43451	2.1
47786	2.34899	2.1
47787	2.38069	2.1
47788	2.38069	2.1
47789	2.3949	2.1

47790	2.3949	2.1
47791	2.85425	1.54008
47792	1.70302	0.20756
47793	1.70302	0.05
47794	1.52254	0.0699674
47795	2.34181	0.0753623
47796	2.08499	0.305201
47797	1.89226	0.0566555
47798	1.89226	0.266773
47799	1.89226	0.05
47800	1.89226	0.073172
47801	1.89226	0.058339
47802	1.89226	0.79348
47803	2.34181	0.321176
47804	1.38287	0.790872
47805	1.38287	0.152683
47806	1.84132	0.142823
47807	1.96416	0.162295
47808	2.4907	0.358856
47809	0.931432	0.683587
47810	0.946135	0.0500928
47811	0.946133	0.05
47812	0.946133	0.05
47813	0.931464	0.300001
47814	0.931464	0.672949
47815	1.15573	0.05
47816	0.96378	1.17909
47817	0.931434	0.39191
47818	0.946131	0.05
47819	0.946131	0.05
47820	1.13808	0.05
47821	0.946132	0.430161
47822	0.946132	0.10385
47823	0.946132	0.261388
47824	0.946132	0.05
47825	0.946133	0.505149
47826	0.946133	0.0572302
47827	0.946132	0.956581
47828	0.946132	0.143446
47829	0.946133	0.180419
47830	0.946133	0.59969
47831	0.946132	0.05
47832	0.946132	0.05
47833	1.68074	2.1
47834	2.29459	2.1
47835	2.23361	2.1
47836	1.57234	2.1
47837	3.86693	2.1
47838	2.61243	2.1
47839	2.29458	2.1
47840	2.2336	2.1
47841	2.58234	2.1
47842	4.87692	2.1
47843	0.73506	2.1
47844	0.786166	2.1
47845	1.08729	2.1
47846	1.11679	2.1
47847	0.93134	2.1

47848	0.93134	2.1
47849	0.942113	2.1
47850	0.942113	2.1
47851	0.735065	2.1
47852	1.08729	2.1
47853	1.11679	2.1
47854	0.78616	2.1
47855	1.08729	2.1
47856	1.11679	2.1
47857	1.27502	2.1
47858	1.29116	2.1
47859	1.19259	2.1
47860	1.19259	2.1
47861	1.19923	2.1
47862	1.19923	2.1
47863	1.27503	2.1
47864	1.08729	2.1
47865	1.11679	2.1
47866	1.29115	2.1
47867	0.974715	0.757357
47868	1.43779	0.278948
47869	1.93915	0.34533
47870	1.53908	0.338587
47871	2.91387	0.311179
47872	1.53908	0.229312
47873	0.99845	0.251856
47874	1.5176	0.715919
47875	1.97963	0.205989
47876	3.05668	0.304502
47877	1.93915	0.328403
47878	1.97963	0.203454
47879	1.53908	0.256212
47880	1.53908	0.114413
47881	2.3376	0.749153
47882	2.3376	0.267923
47883	1.89226	2.1
47884	2.59747	2.1
47885	1.91802	2.1
47886	1.89226	2.1
47887	2.62322	2.1
47888	2.59747	2.1
47889	2.59747	2.1
47890	1.87119	2.1
47891	1.84844	2.1
47892	1.82268	2.1
47893	2.62323	2.1
47894	2.59747	2.1
47895	0.94613	2.1
47896	0.94613	2.1
47897	1.32697	2.1
47898	1.32697	2.1
47899	1.32697	2.1
47900	1.31161	2.1
47901	0.946132	2.1
47902	0.946132	2.1
47903	0.94613	2.1
47904	1.32697	2.1
47905	0.946131	2.1

47906	1.31161	2.1
47907	2.62323	2.1
47908	1.91704	2.1
47909	1.90191	2.1
47910	1.87119	2.1
47911	2.65395	2.1
47912	2.62323	2.1
47913	1.89226	2.1
47914	1.89226	2.1
47915	2.15958	2.1
47916	2.15958	2.1
47917	2.46558	2.1
47918	2.19826	2.1
47919	2.46558	2.1
47920	1.89226	2.1
47921	1.89226	2.1
47922	1.89226	2.1
47923	2.61064	2.1
47924	2.61064	2.1
47925	1.81759	0.476102
47926	1.89526	0.558451
47927	2.32219	1.18153
47928	1.881	0.277734
47929	1.81759	0.749493
47930	1.3764	0.193579
47931	1.81759	0.554262
47932	2.57079	0.372233
47933	2.32219	1.36927
47934	2.32219	0.428991
47935	1.99875	0.79664
47936	1.99875	0.200319
47937	0.536182	0.131278
47938	0.579206	0.0541682
47939	0.908796	0.466901
47940	0.999375	0.21594
47941	0.732091	0.298113
47942	0.732091	0.0515501
47943	0.755911	0.121841
47944	0.755911	0.324476
47945	0.999376	0.187402
47946	0.579206	0.266119
47947	0.908796	0.206649
47948	0.536182	0.181506
47949	0.94613	0.098888
47950	0.94613	0.130641
47951	1.31112	0.164677
47952	1.18275	0.0868977
47953	0.946126	0.106062
47954	0.946126	0.05
47955	1.31112	0.141411
47956	1.31112	0.0636702
47957	0.946128	0.114032
47958	1.31112	0.144943
47959	0.946128	0.0895022
47960	1.18275	0.05
47961	2.64617	0.25921
47962	1.27737	0.0636644
47963	1.83076	0.154344

47964	1.27737	0.198384
47965	3.03764	0.0990024
47966	2.3655	0.0520107
47967	1.12991	0.05
47968	1.22375	0.05
47969	1.35931	0.05
47970	1.04971	0.05
47971	1.0419	0.05
47972	1.15237	0.05
47973	1.19593	0.05
47974	1.30306	0.05
47975	1.12937	0.05
47976	1.31934	0.05
47977	1.15203	0.05
47978	1.15203	0.05
47979	2.65227	0.05
47980	1.46041	0.05
47981	2.22712	0.05
47982	2.04816	0.05
47983	2.22712	0.05
47984	2.04816	0.05
47985	1.89226	0.05
47986	1.89226	0.392055
47987	1.89226	0.0708662
47988	1.89226	0.058809
47989	3.78452	0.0777157
47990	1.24653	0.15161
47991	1.66554	0.317531
47992	1.53538	0.05
47993	1.53538	2.08419
47994	1.40915	0.05
47995	1.40915	2.05349
47996	1.66555	0.35506
47997	1.75835	2.1
47998	1.53538	2.1
47999	1.65313	2.1
48000	3.31868	2.1
48001	1.89226	0.400958
48002	1.89226	1.14109
48003	1.89226	0.057188
48004	1.89226	2.1
48005	3.78453	0.649276
48006	1.89226	0.05
48007	1.89226	0.0509206
48008	1.89226	0.05
48009	1.89226	0.835244
48010	3.78452	0.05
48011	0.658778	2.1
48012	0.658778	0.05
48013	0.661956	0.05
48014	0.661956	0.482142
48015	0.703822	0.408422
48016	0.767689	2.1
48017	0.623266	0.05
48018	0.553046	0.107867
48019	0.703804	0.208621
48020	0.55303	0.229762
48021	0.623282	0.102358

48022	0.767711	0.103478
48023	1.53538	2.1
48024	1.65313	2.1
48025	1.40765	0.506619
48026	1.55779	2.1
48027	3.06078	2.1
48028	1.89227	0.05
48029	1.89227	2.1
48030	1.89227	0.906217
48031	1.89227	1.20289
48032	3.78453	1.22812
48033	1.10517	2.1
48034	2.16532	2.1
48035	1.94374	2.1
48036	1.94001	2.1
48037	1.37756	2.1
48038	1.37756	2.1
48039	1.7453	0.05
48040	1.83284	0.05
48041	2.28483	0.05
48042	1.77804	0.05
48043	1.7453	0.05
48044	1.23851	0.05
48045	1.7453	0.05
48046	2.54752	0.05
48047	2.28483	0.05
48048	2.28483	0.05
48049	2.0189	0.05
48050	2.0189	0.05
48051	1.12457	0.05
48052	2.0189	0.05
48053	1.7453	0.05
48054	1.7453	0.05
48055	1.40707	0.05
48056	1.40707	0.05
48057	2.41176	2.1
48058	2.59262	2.1
48059	2.34085	2.1
48060	2.55827	2.1
48061	4.97003	2.1
48062	2.59262	2.1
48063	2.61901	2.1
48064	2.55827	2.1
48065	2.64067	2.1
48066	5.17728	2.1
48067	2.34084	2.1
48068	2.55827	2.1
48069	2.24937	2.1
48070	2.49053	2.1
48071	4.80764	2.1
48072	2.55826	2.1
48073	2.64067	2.1
48074	2.49053	2.1
48075	2.58639	2.1
48076	5.14465	2.1
48077	2.27918	0.05
48078	2.44132	0.05
48079	1.89226	0.05

48080	1.89226	0.05
48081	4.33359	0.05
48082	1.89226	0.05
48083	2.44132	0.05
48084	2.6771	0.05
48085	2.6771	0.05
48086	1.89226	0.05
48087	1.89226	0.05
48088	1.60329	0.05
48089	1.38246	0.05
48090	1.98182	0.05
48091	2.2217	0.05
48092	3.60417	0.05
48093	2.05362	0.05
48094	1.02663	0.05
48095	1.43255	0.05
48096	1.52072	0.05
48097	1.70302	0.05
48098	1.7685	0.05
48099	0.946131	0.05
48100	0.946131	0.05
48101	0.946131	0.05
48102	0.946131	0.05
48103	0.946131	0.05
48104	0.946131	0.05
48105	0.946131	0.05
48106	0.946131	0.05
48107	0.946131	0.05
48108	0.946131	0.05
48109	0.946131	0.05
48110	0.946131	0.05
48111	1.89226	0.05
48112	1.89226	0.05
48113	1.89226	0.05
48114	1.89226	0.05
48115	1.89226	0.05
48116	1.89226	0.05
48117	1.92645	0.177573
48118	2.83045	0.133366
48119	1.92225	0.236505
48120	2.01873	0.0859143
48121	2.51732	0.313715
48122	2.51732	0.110544
48123	2.51732	0.0843281
48124	2.23857	0.0687045
48125	1.92645	0.131917
48126	1.92645	0.0522269
48127	2.23857	0.199612
48128	1.50865	0.05
48129	2.02687	2.1
48130	2.39604	2.1
48131	2.29198	2.1
48132	2.73046	2.1
48133	4.68803	2.1
48134	1.21148	0.0797504
48135	1.87957	0.404688
48136	2.03555	0.0642745
48137	2.03555	0.182677

48138	1.09962	0.0784571
48139	1.09962	1.3389
48140	1.92558	0.055102
48141	2.05274	2.1
48142	2.2443	0.05
48143	2.24429	1.17768
48144	1.70869	0.05
48145	1.70868	1.96291
48146	0.818397	0.0584718
48147	0.818397	0.303558
48148	0.827911	0.05
48149	0.827908	0.440626
48150	1.00418	0.22101
48151	1.02054	0.146808
48152	0.692018	0.10819
48153	0.620899	0.098366
48154	0.65823	0.0536909
48155	1.00418	0.05
48156	0.620899	0.181383
48157	1.0262	0.0530513
48158	1.92558	0.888081
48159	2.51818	1.8578
48160	1.94816	0.086898
48161	2.01985	1.27899
48162	1.85389	0.0572127
48163	1.92558	1.14957
48164	0.903844	2.1
48165	0.903844	2.1
48166	0.946148	2.1
48167	0.946151	2.1
48168	0.946118	2.1
48169	0.903876	2.1
48170	0.946118	2.1
48171	1.10235	2.1
48172	0.946131	2.1
48173	0.946131	2.1
48174	1.10234	2.1
48175	0.903863	2.1
48176	1.63471	2.1
48177	1.08022	2.1
48178	1.56801	2.1
48179	1.03437	2.1
48180	2.66908	2.1
48181	1.56801	2.1
48182	0.97541	2.1
48183	0.97541	2.1
48184	1.03437	2.1
48185	1.49733	2.1
48186	1.55629	2.1
48187	1.89226	0.05
48188	1.89226	0.05
48189	1.89226	0.05
48190	1.89226	0.05
48191	3.78452	0.05
48192	0.946131	0.05
48193	1.16373	0.05
48194	0.946131	0.05
48195	0.734637	0.05

48196	0.946132	0.05
48197	0.946132	0.05
48198	1.16373	0.05
48199	0.734636	0.05
48200	0.946093	0.05
48201	0.946093	0.05
48202	0.734675	0.05
48203	0.734675	0.05
48204	1.98413	0.05
48205	1.87355	0.153911
48206	1.72774	0.05
48207	1.67865	0.383552
48208	3.60128	0.05
48209	1.87355	0.100095
48210	1.54924	2.1
48211	1.68443	2.1
48212	1.68443	0.206183
48213	1.67866	2.1
48214	1.67866	0.243909
48215	1.72774	0.05
48216	1.53422	0.136208
48217	1.67865	0.0539002
48218	1.67865	0.05
48219	1.53873	0.0744072
48220	1.53873	0.05
48221	1.67866	0.0700977
48222	1.54924	2.1
48223	1.53423	0.14793
48224	1.44091	2.1
48225	3.11957	1.95937
48226	1.89226	2.1
48227	1.89226	1.9288
48228	1.65573	2.1
48229	1.65573	2.1
48230	1.89226	2.1
48231	1.89226	2.1
48232	1.89226	2.1
48233	1.89614	1.33239
48234	1.7508	1.91219
48235	1.72666	1.97665
48236	1.89226	1.96764
48237	1.89226	2.07025
48238	1.89223	2.1
48239	1.89223	1.59302
48240	1.89223	1.72414
48241	1.89223	2.01029
48242	1.89223	1.82378
48243	1.89223	2.1
48244	1.89224	2.04163
48245	1.89224	0.848583
48246	1.89224	1.49639
48247	1.89224	1.55762
48248	1.89224	1.7247
48249	1.89224	1.91581
48250	2.09773	2.1
48251	2.06623	2.1
48252	2.43268	2.1
48253	2.33713	2.1

48254	1.703	2.1
48255	1.61372	2.1
48256	0.646929	2.1
48257	0.689776	2.1
48258	1.05897	2.1
48259	0.956364	2.1
48260	0.905594	2.1
48261	0.904315	2.1
48262	0.727014	2.1
48263	0.850757	2.1
48264	0.798445	2.1
48265	0.654101	2.1
48266	0.717539	2.1
48267	1.0478	2.1
48268	1.89223	2.1
48269	1.89223	1.7201
48270	1.89223	2.1
48271	1.6557	0.119186
48272	3.54794	1.96384
48273	0.946118	2.1
48274	0.946118	2.1
48275	0.946118	2.1
48276	0.946118	2.1
48277	0.946117	2.1
48278	0.946117	1.63321
48279	0.946119	2.1
48280	0.946119	2.1
48281	0.946105	2.1
48282	0.946105	2.1
48283	0.946131	2.1
48284	0.946131	2.1
48285	1.47671	2.1
48286	1.70303	2.1
48287	1.90705	2.1
48288	1.98945	2.1
48289	1.3454	2.1
48290	1.40353	2.1
48291	1.89226	2.1
48292	1.89226	2.1
48293	1.89226	2.1
48294	1.89226	2.1
48295	1.89226	2.1
48296	1.89226	1.41888
48297	2.39438	0.05
48298	2.69842	0.05
48299	2.45177	0.05
48300	2.28563	0.05
48301	4.84615	0.05
48302	1.28421	2.1
48303	1.35966	2.1
48304	0.961001	2.1
48305	0.860549	2.1
48306	1.1039	2.1
48307	1.1039	2.1
48308	1.11631	2.1
48309	1.11631	2.1
48310	1.35966	2.1
48311	0.961001	2.1

48312	0.860549	2.1
48313	1.28421	2.1
48314	1.89226	1.45314
48315	1.89226	2.08059
48316	1.89226	2.1
48317	1.89226	0.826235
48318	3.78452	1.59192
48319	1.48702	0.393681
48320	1.40656	0.474093
48321	0.626442	1.73044
48322	0.626442	1.40684
48323	0.626442	1.96136
48324	0.946132	0.452237
48325	1.05489	0.226754
48326	1.05012	2.01907
48327	0.946131	1.20354
48328	0.946131	1.29315
48329	0.626442	1.37364
48330	1.05012	1.0259
48331	1.89226	2.08048
48332	1.97212	0.635693
48333	1.89226	0.821142
48334	1.89226	1.53382
48335	3.78453	1.65419
48336	1.89226	2.1
48337	1.89226	0.945965
48338	1.89226	1.23172
48339	1.89226	1.98441
48340	3.78452	1.91215
48341	1.89226	0.874392
48342	1.89226	1.52758
48343	1.89226	1.9119
48344	1.89226	0.355057
48345	1.89226	1.95585
48346	1.89226	0.390273
48347	0.946161	0.221336
48348	0.946161	0.77471
48349	0.932156	1.5696
48350	0.932156	0.830114
48351	0.932184	1.30868
48352	0.946132	0.137054
48353	0.946133	1.565
48354	0.946133	0.373806
48355	0.946132	0.526875
48356	0.946132	0.638207
48357	0.932185	0.53462
48358	0.946133	0.341195
48359	0.946132	0.781738
48360	0.946132	0.349102
48361	0.946132	2.1
48362	0.946132	1.70615
48363	0.946132	1.56996
48364	0.946132	0.700623
48365	0.946133	0.935959
48366	0.946133	1.93903
48367	0.946132	1.32624
48368	0.946132	1.66269
48369	0.946133	0.885973

48370	0.946133	1.23063
48371	1.79397	0.079391
48372	1.00636	0.05
48373	1.31658	0.343642
48374	2.02924	0.0794189
48375	3.0356	0.101397
48376	1.89226	0.113443
48377	1.89226	0.05
48378	1.89226	0.105888
48379	1.89226	0.0542192
48380	1.89226	0.0742415
48381	1.89226	0.05
48382	0.946131	0.05
48383	0.946131	0.0676254
48384	1.24766	0.116253
48385	1.03691	0.0795821
48386	0.946129	0.0741968
48387	0.946129	0.05
48388	1.24766	0.126332
48389	1.24766	0.0704803
48390	0.946131	0.0821189
48391	1.24766	0.132921
48392	1.03691	0.0623216
48393	0.946131	0.05
48394	1.44824	0.0650996
48395	2.02924	0.05
48396	1.7065	0.0977389
48397	1.38604	0.275939
48398	2.32017	0.0730899
48399	2.32017	0.05
48400	1.89226	0.05
48401	1.89226	0.060834
48402	1.89226	0.05
48403	1.89226	0.05
48404	1.89226	0.14097
48405	1.89226	0.0798731
48406	0.478349	1.41574
48407	0.612092	2.1
48408	0.606115	2.1
48409	0.506213	2.1
48410	0.550677	2.1
48411	0.550677	1.41153
48412	0.567628	2.1
48413	0.567628	2.1
48414	0.506213	2.1
48415	0.478349	2.1
48416	0.606116	2.1
48417	0.612092	2.1
48418	1.52226	2.1
48419	1.54241	2.1
48420	1.23167	2.1
48421	1.23167	2.1
48422	1.75555	2.1
48423	1.75555	2.1
48424	1.75557	2.1
48425	1.54243	2.1
48426	1.9943	2.1
48427	1.85871	2.1

48428	3.53673	2.1
48429	1.85871	2.1
48430	1.06936	2.1
48431	1.88926	2.1
48432	1.54243	2.1
48433	1.41619	2.1
48434	1.06936	2.1
48435	1.28649	1.98902
48436	1.28516	2.1
48437	1.38986	0.128539
48438	1.42399	1.71406
48439	2.67502	1.59757
48440	1.42399	0.58627
48441	1.07283	2.1
48442	1.46528	2.1
48443	1.28517	1.13027
48444	1.25294	2.1
48445	1.07283	0.836763
48446	1.38986	2.1
48447	1.42399	2.1
48448	1.52885	0.506553
48449	1.60058	0.966423
48450	2.99043	1.58723
48451	0.715316	2.1
48452	0.715316	1.79185
48453	0.719126	0.103745
48454	0.719126	2.1
48455	0.722447	2.1
48456	0.626469	2.1
48457	0.711995	0.429929
48458	0.800291	0.762514
48459	0.80029	1.80877
48460	0.722447	1.35276
48461	0.711995	1.94842
48462	0.62647	1.97484
48463	2.05632	0.05
48464	1.70303	0.05
48465	2.3923	0.05
48466	2.61277	0.05
48467	1.44836	0.05
48468	1.58184	0.05
48469	1.89226	0.05
48470	1.89226	0.05
48471	1.89226	0.05
48472	1.89226	0.05
48473	3.78453	0.05
48474	1.44091	2.1
48475	1.1822	2.1
48476	1.25866	2.1
48477	1.25866	2.1
48478	1.35215	2.1
48479	1.35215	2.1
48480	1.27338	2.1
48481	1.1822	2.1
48482	1.27338	2.1
48483	1.35215	2.1
48484	1.10817	2.1
48485	1.18694	2.1

48486	0.946132	0.16004
48487	0.946132	0.107476
48488	0.946132	0.0742518
48489	0.946132	0.0562887
48490	0.946133	0.0744383
48491	0.946133	0.154805
48492	0.946132	0.103211
48493	0.946132	0.0514584
48494	0.946132	0.0915931
48495	0.946132	0.0931618
48496	0.946133	0.0909339
48497	0.946133	0.0923556
48498	2.6875	0.05
48499	2.40558	0.05
48500	2.1994	0.05
48501	1.70303	0.05
48502	4.68305	0.05
48503	0.915523	0.05
48504	0.915523	0.05
48505	0.861057	0.05
48506	0.946796	0.05
48507	0.838652	0.05
48508	0.654182	0.05
48509	1.14044	0.05
48510	0.80252	0.05
48511	0.836435	0.05
48512	0.608659	0.05
48513	1.22198	0.05
48514	0.863472	0.05
48515	2.1994	0.05
48516	0.981586	0.05
48517	1.59619	0.05
48518	1.70303	0.05
48519	1.24816	0.05
48520	1.38523	0.05
48521	2.71141	2.1
48522	1.70302	2.1
48523	2.56527	2.1
48524	1.88649	2.1
48525	2.41279	2.1
48526	1.70302	2.1
48527	1.89226	2.1
48528	1.89226	2.1
48529	1.89226	2.1
48530	1.89226	2.1
48531	3.78453	2.1
48532	1.89226	2.1
48533	1.89226	2.1
48534	1.89226	2.1
48535	1.89226	2.1
48536	1.89226	2.1
48537	1.89226	2.1
48538	2.18867	2.1
48539	0.983723	2.1
48540	2.42263	2.1
48541	1.8014	2.1
48542	1.62992	2.1
48543	0.983723	2.1

48544	2.71141	2.1
48545	1.89226	2.1
48546	2.31157	2.1
48547	2.31157	2.1
48548	1.89226	2.1
48549	1.89226	2.1
48550	1.89227	2.1
48551	1.89227	1.99954
48552	1.89227	2.03499
48553	1.89227	2.1
48554	3.78453	2.1
48555	1.89226	2.1
48556	1.89226	2.1
48557	1.89226	2.1
48558	1.89226	2.03822
48559	1.89226	2.1
48560	1.89226	2.02686
48561	1.89226	1.69785
48562	1.89226	1.37294
48563	1.89226	2.1
48564	1.89226	0.774992
48565	1.89226	1.49581
48566	1.89226	0.274798
48567	1.1427	2.02146
48568	1.14956	2.1
48569	1.70302	1.31794
48570	1.81267	2.00148
48571	2.88983	2.00094
48572	1.89226	0.982236
48573	1.89226	2.09145
48574	1.89226	0.899286
48575	1.89226	1.38986
48576	3.78453	1.4378
48577	1.89226	1.69199
48578	1.89226	1.86911
48579	1.89226	0.577236
48580	1.89226	1.4326
48581	3.78452	1.19501
48582	0.884197	2.1
48583	1.1125	1.50961
48584	1.13273	1.44245
48585	0.844557	1.76173
48586	0.981137	1.08022
48587	1.13971	1.94292
48588	0.980432	1.95027
48589	0.980432	1.20351
48590	1.15859	1.32476
48591	1.12206	0.773285
48592	0.898619	2.08727
48593	0.94426	1.57059
48594	2.21016	1.26828
48595	1.44091	2.1
48596	1.98408	1.21712
48597	1.4218	1.10972
48598	2.21016	2.1
48599	1.64788	2.1
48600	1.07526	0.801223
48601	1.91531	2.1

48602	1.49155	2.1
48603	1.58551	0.485861
48604	1.49155	0.226419
48605	1.58551	1.90679
48606	1.98979	0.05
48607	2.21773	0.05
48608	2.30533	0.05
48609	2.55895	0.05
48610	4.52305	0.05
48611	2.55896	0.05
48612	2.27963	0.05
48613	2.60486	0.05
48614	2.60486	0.05
48615	2.21773	0.05
48616	2.21773	0.05
48617	2.30533	0.05
48618	2.55895	0.05
48619	2.29539	0.05
48620	2.47308	0.05
48621	4.7784	0.05
48622	2.55896	0.05
48623	2.55792	0.05
48624	2.55792	0.05
48625	2.60486	0.05
48626	2.47308	0.05
48627	2.52003	0.05
48628	0.946133	1.92064
48629	0.946133	2.03865
48630	1.20851	1.36092
48631	1.20851	1.17562
48632	0.967411	1.15382
48633	1.20851	0.703738
48634	0.946132	2.1
48635	0.946132	1.87285
48636	0.946132	1.9955
48637	1.20851	1.2408
48638	0.967412	1.20879
48639	0.946133	1.85687
48640	1.89226	1.87724
48641	1.89226	1.01033
48642	1.89226	1.09298
48643	1.89226	1.05126
48644	1.89226	1.92514
48645	1.89226	1.92123
48646	1.89226	1.99805
48647	2.41701	1.77284
48648	2.71724	0.948964
48649	2.71724	1.68532
48650	1.89226	2.1
48651	1.89226	2.1
48652	0.946131	2.03343
48653	0.946131	2.1
48654	0.946128	1.7298
48655	0.946128	1.81
48656	0.94613	0.775271
48657	0.94613	1.16171
48658	0.94613	2.04295
48659	0.94613	2.08841

48660	0.94613	1.86514
48661	0.94613	1.07461
48662	0.94613	1.45855
48663	0.94613	2.1
48664	0.851541	0.05
48665	0.851541	0.05
48666	0.851481	0.05
48667	0.997219	0.05
48668	0.997112	0.05
48669	0.992853	0.05
48670	0.85154	0.05
48671	0.85154	0.05
48672	0.894824	0.05
48673	0.894824	0.05
48674	0.94613	0.05
48675	0.906485	0.05
48676	0.802947	2.1
48677	0.845556	2.1
48678	0.582339	2.1
48679	0.519756	2.1
48680	0.714508	2.1
48681	0.714508	2.1
48682	0.628932	2.1
48683	0.708518	2.1
48684	0.801358	2.1
48685	0.476016	2.1
48686	0.569477	2.1
48687	0.878553	2.1
48688	1.89226	2.1
48689	1.89226	2.1
48690	1.89226	2.1
48691	1.89226	2.1
48692	1.89226	2.1
48693	1.89226	2.1
48694	0.946128	2.1
48695	0.946128	2.1
48696	1.01537	2.1
48697	0.9662	2.1
48698	0.946131	2.1
48699	0.946131	2.1
48700	0.966198	2.1
48701	0.966198	2.1
48702	0.94613	2.1
48703	0.966198	2.1
48704	0.946131	2.1
48705	1.01537	2.1
48706	1.89226	2.1
48707	1.89226	2.1
48708	1.91883	2.1
48709	1.91883	2.1
48710	2.15905	2.1
48711	2.13249	2.1
48712	1.07953	2.1
48713	1.2107	2.1
48714	0.946132	2.1
48715	0.946132	2.1
48716	1.2107	2.1
48717	1.2107	2.1

48718	0.946132	2.1
48719	0.946132	2.1
48720	1.2107	2.1
48721	0.946133	2.1
48722	0.946132	2.1
48723	1.07953	2.1
48724	2.46752	2.1
48725	2.53733	2.1
48726	2.26044	2.1
48727	2.33222	2.1
48728	4.79974	2.1
48729	2.53733	2.1
48730	2.37576	0.187487
48731	2.33222	2.1
48732	2.1845	2.1
48733	4.70798	2.1
48734	2.48652	2.1
48735	2.33224	2.1
48736	2.08149	2.1
48737	1.9839	2.1
48738	4.45623	2.1
48739	2.33223	2.1
48740	2.18452	1.88884
48741	2.16061	2.1
48742	2.01399	0.233137
48743	4.34623	2.1
48744	1.4617	1.74022
48745	1.34701	2.1
48746	1.34701	2.0108
48747	1.25173	2.1
48748	1.5117	1.65787
48749	1.41642	2.1
48750	2.65164	2.1
48751	1.89227	2.1
48752	1.89227	2.1
48753	1.89227	2.1
48754	2.42504	2.1
48755	2.42504	2.1
48756	1.89226	1.10544
48757	1.89226	2.1
48758	1.89226	2.1
48759	1.89226	0.383116
48760	3.78452	1.69904
48761	1.5117	1.96486
48762	1.34701	2.1
48763	1.47001	2.1
48764	1.57961	0.767001
48765	2.92663	2.1
48766	1.89226	2.1
48767	1.89226	0.330528
48768	1.89226	0.734336
48769	1.89226	2.07868
48770	3.78452	1.88734
48771	0.412773	0.05
48772	0.570839	0.05
48773	0.50721	0.05
48774	0.513638	0.05
48775	0.518805	0.05

48776	0.41278	0.05
48777	1.5344	0.05
48778	0.939282	0.05
48779	0.720051	0.05
48780	0.691767	0.05
48781	0.761152	0.05
48782	1.89226	2.1
48783	1.89226	2.1
48784	1.89226	2.1
48785	1.89226	2.1
48786	1.89226	2.1
48787	1.89226	2.1
48788	1.24618	2.1
48789	2.10585	2.1
48790	1.08134	2.1
48791	1.93032	2.1
48792	3.1765	2.1
48793	1.98474	2.1
48794	2.57777	2.1
48795	1.93031	2.1
48796	2.19041	2.1
48797	3.99251	2.1
48798	1.89226	2.1
48799	1.89226	2.1
48800	1.89226	2.1
48801	1.89226	2.1
48802	3.78452	2.1
48803	1.67374	2.1
48804	2.19042	2.1
48805	1.67374	2.1
48806	2.09769	2.1
48807	2.29586	2.1
48808	2.7198	2.1
48809	1.89226	2.1
48810	1.89226	2.1
48811	1.89226	2.1
48812	1.89226	2.1
48813	1.89226	2.1
48814	1.89226	2.1
48815	0.946132	0.142555
48816	0.946132	0.107872
48817	0.947763	0.0814329
48818	0.909304	0.0978944
48819	0.946127	0.0749014
48820	0.947831	0.05
48821	0.907673	0.0585724
48822	0.907673	0.131322
48823	0.94613	0.076045
48824	0.947834	0.058155
48825	0.907671	0.237028
48826	0.94613	0.05
48827	0.906519	0.0533921
48828	0.906519	0.05
48829	0.946097	0.05
48830	0.946097	0.05
48831	0.947834	0.05
48832	0.906485	0.05
48833	0.946131	0.05

48834	0.946131	0.0566691
48835	0.94613	0.0682633
48836	0.906485	0.05
48837	0.946131	0.0635144
48838	0.947835	0.0518401
48839	2.58638	2.1
48840	2.51039	2.1
48841	2.47395	2.1
48842	2.41539	2.1
48843	5.00178	2.1
48844	2.51039	2.1
48845	2.16633	2.1
48846	2.4154	2.1
48847	2.13977	2.1
48848	4.58173	2.1
48849	2.47395	2.1
48850	2.41539	2.1
48851	2.2988	2.1
48852	2.2604	2.1
48853	4.7142	2.1
48854	2.4154	2.1
48855	2.13977	2.1
48856	2.26041	2.1
48857	1.98957	2.1
48858	4.40497	2.1
48859	2.15721	0.05
48860	2.31843	0.05
48861	1.93973	0.0899383
48862	2.13243	0.05
48863	4.25816	0.05
48864	2.31844	0.101243
48865	2.30908	0.05
48866	2.13243	0.05
48867	2.1609	0.05
48868	4.47934	0.05
48869	1.93973	0.212776
48870	2.13243	0.05
48871	1.69661	0.195383
48872	1.9339	0.05
48873	3.87363	0.05
48874	2.13243	0.0576412
48875	2.1609	0.05
48876	1.9339	0.05
48877	1.98414	0.0579501
48878	4.0948	0.05
48879	1.88857	0.05
48880	2.34107	0.05
48881	0.502431	0.05
48882	0.71416	0.05
48883	0.50243	0.05
48884	0.944284	0.05
48885	1.88856	2.1
48886	2.34107	2.1
48887	2.39662	2.1
48888	1.92293	2.1
48889	4.28519	2.1
48890	1.00486	2.1
48891	1.92293	2.1

48892	1.88857	2.1
48893	1.88857	2.1
48894	1.02605	2.1
48895	1.02605	2.1
48896	2.53728	2.1
48897	1.70303	2.1
48898	2.03734	2.1
48899	1.3624	2.1
48900	2.54706	2.1
48901	1.70303	2.1
48902	2.54706	2.1
48903	1.50876	2.1
48904	2.74067	2.1
48905	1.85391	2.1
48906	2.29667	2.1
48907	1.50876	2.1
48908	0.946133	2.1
48909	0.946133	2.1
48910	1.24191	2.1
48911	1.24191	2.1
48912	0.946132	2.1
48913	1.24191	2.1
48914	1.23779	2.1
48915	0.946132	2.1
48916	0.946132	2.1
48917	0.946132	2.1
48918	1.23779	2.1
48919	1.24191	2.1
48920	0.946131	2.1
48921	0.946131	2.1
48922	1.14833	2.1
48923	1.14833	2.1
48924	1.14834	2.1
48925	0.946132	2.1
48926	0.94613	2.1
48927	1.24191	2.1
48928	0.946131	2.1
48929	0.946131	2.1
48930	1.24191	2.1
48931	1.14833	2.1
48932	2.08149	2.1
48933	2.31116	2.1
48934	1.9701	1.20678
48935	2.18328	2.1
48936	4.49563	2.1
48937	2.08149	2.1
48938	2.14059	2.1
48939	1.72377	2.1
48940	2.18328	2.1
48941	3.88931	2.1
48942	2.14059	2.1
48943	2.40755	2.1
48944	2.31145	2.1
48945	2.23743	2.1
48946	2.31145	2.1
48947	2.23743	2.1
48948	2.31117	2.1
48949	2.40754	2.1

48950	2.41055	2.1
48951	2.31145	2.1
48952	2.41055	2.1
48953	2.31145	2.1
48954	2.5615	0.126617
48955	2.37625	0.093684
48956	2.20248	0.086968
48957	2.20248	0.0735775
48958	2.65967	0.211971
48959	2.65967	0.167166
48960	2.71793	0.239712
48961	2.65966	0.0836136
48962	2.56149	0.0664376
48963	2.56149	0.0705698
48964	2.71296	0.13747
48965	2.71296	0.157699
48966	1.31528	0.108126
48967	1.32983	0.0874661
48968	1.21128	0.097535
48969	1.18812	0.0859859
48970	1.24232	0.0637987
48971	1.24232	0.0767148
48972	1.26109	0.109533
48973	1.26109	0.164047
48974	1.21128	0.132104
48975	1.31528	0.0557145
48976	1.18812	0.0648321
48977	1.32983	0.144796
48978	2.63056	0.0616083
48979	2.58032	0.09433
48980	2.65966	0.05
48981	2.65966	0.0741386
48982	2.58032	0.121353
48983	2.71296	0.146065
48984	1.92039	2.1
48985	1.06226	2.1
48986	1.09252	2.1
48987	1.96052	2.1
48988	3.01291	2.1
48989	1.92039	2.1
48990	2.46338	2.1
48991	2.47522	2.1
48992	2.43508	2.1
48993	1.96052	2.1
48994	1.92039	2.1
48995	0.985949	2.1
48996	0.980261	2.1
48997	0.553625	2.1
48998	0.546262	2.1
48999	0.765671	2.1
49000	0.765671	2.1
49001	0.766539	2.1
49002	0.766539	2.1
49003	0.985949	2.1
49004	0.553625	2.1
49005	0.546261	2.1
49006	0.980261	2.1
49007	1.96052	2.1

49008	2.46338	2.1
49009	1.97189	2.1
49010	1.96052	2.1
49011	2.47932	2.1
49012	2.47932	2.1
49013	0.964608	0.05
49014	0.691524	0.05
49015	1.25462	0.05
49016	0.691524	0.05
49017	1.89226	0.05
49018	1.89226	0.05
49019	1.44894	0.05
49020	2.77655	0.05
49021	1.4237	0.05
49022	2.77701	0.05
49023	2.77701	0.05
49024	1.38847	2.1
49025	1.46031	2.01756
49026	1.65504	1.30062
49027	1.75396	1.0187
49028	3.11535	1.70236
49029	1.89226	1.83133
49030	1.89226	2.07064
49031	1.89226	1.4702
49032	1.89226	0.916547
49033	3.78452	1.85833
49034	1.89226	1.54568
49035	1.89226	2.1
49036	1.89226	0.37294
49037	1.89226	1.819
49038	3.78452	0.672569
49039	1.46031	1.20633
49040	1.34851	2.1
49041	1.75395	0.834157
49042	1.62287	1.37807
49043	3.08318	1.93278
49044	1.65504	1.85768
49045	1.75396	1.97485
49046	1.89249	2.08346
49047	1.9857	0.938363
49048	3.64073	1.86329
49049	1.89226	1.98326
49050	1.89226	1.84071
49051	1.89226	0.846642
49052	1.89226	1.03633
49053	3.78452	0.989264
49054	1.89226	1.03493
49055	1.89226	2.1
49056	1.89226	0.985899
49057	1.89226	1.75172
49058	3.78452	2.03346
49059	1.75395	1.6742
49060	1.62287	1.97691
49061	1.98569	0.684083
49062	1.86067	1.18294
49063	3.60856	1.05046
49064	1.35697	2.1
49065	2.02064	2.1

49066	1.89989	1.5036
49067	1.49571	2.1
49068	1.66753	2.03655
49069	1.36431	2.1
49070	0.744004	2.1
49071	0.743997	2.1
49072	0.717972	1.42498
49073	0.717972	2.1
49074	0.997982	2.1
49075	0.807796	2.1
49076	0.632235	1.83896
49077	0.488575	2.1
49078	0.849275	2.1
49079	0.488581	2.1
49080	0.724729	2.1
49081	0.997967	2.1
49082	2.5276	2.1
49083	1.70791	1.99558
49084	1.82872	0.36745
49085	1.56362	2.1
49086	2.5276	1.08637
49087	2.24781	2.1
49088	1.37591	2.1
49089	1.23217	2.01969
49090	1.00442	1.89597
49091	0.945282	0.529184
49092	1.15446	0.724161
49093	1.09123	2.1
49094	1.21506	2.06579
49095	1.21506	0.987792
49096	1.39452	1.0445
49097	1.13108	1.90281
49098	1.27876	1.23857
49099	0.929006	0.691928
49100	1.46505	0.05
49101	1.91484	0.05
49102	1.49906	0.05
49103	1.49906	0.05
49104	1.90196	0.05
49105	1.90196	0.05
49106	1.90196	0.05
49107	2.11465	0.05
49108	1.91484	0.05
49109	1.91484	0.05
49110	2.04016	0.05
49111	2.04016	0.05
49112	0.912201	0.05
49113	0.950978	0.05
49114	0.665307	0.05
49115	0.732528	0.05
49116	0.829512	0.05
49117	0.829512	0.05
49118	0.815217	0.05
49119	0.815217	0.05
49120	0.912202	0.05
49121	0.665305	0.05
49122	0.732527	0.05
49123	0.95098	0.05

49124	1.90196	0.05
49125	2.08757	0.05
49126	1.9626	0.05
49127	2.04016	0.05
49128	1.82441	0.05
49129	1.90196	0.05
49130	1.13048	2.1
49131	0.821162	2.1
49132	0.946131	2.1
49133	0.946131	2.1
49134	1.04567	2.1
49135	0.882347	2.1
49136	0.897276	2.1
49137	0.897276	2.1
49138	0.863109	2.1
49139	1.01055	2.1
49140	0.940305	2.1
49141	0.942166	2.1
49142	2.23933	0.05
49143	0.976217	0.05
49144	2.78053	0.05
49145	2.78053	0.05
49146	0.976217	0.05
49147	1.65984	0.05
49148	1.54168	2.1
49149	2.01197	1.89058
49150	1.69136	2.1
49151	2.13284	0.634309
49152	3.67452	2.1
49153	0.988805	2.1
49154	1.78069	2.1
49155	1.69136	2.1
49156	1.69136	2.1
49157	1.05481	2.1
49158	1.05481	2.1
49159	1.69136	2.1
49160	2.13284	1.25882
49161	1.78069	2.1
49162	2.22044	1.76422
49163	3.91353	2.1
49164	2.69379	0.875595
49165	2.32219	1.19988
49166	2.25746	1.90878
49167	2.25746	0.96758
49168	2.32219	1.16369
49169	1.89526	0.765321
49170	1.4556	2.1
49171	1.85028	2.1
49172	2.4909	0.355043
49173	2.03799	2.1
49174	2.73495	1.97893
49175	1.85028	2.1
49176	2.71938	0.34803
49177	1.85028	2.1
49178	3.36051	2.1
49179	2.73495	2.09849
49180	2.47584	2.1
49181	1.85028	1.43516

49182	2.47584	2.1
49183	1.04413	2.1
49184	2.1461	2.1
49185	1.74586	2.1
49186	1.44437	2.1
49187	1.04413	2.1
49188	1.89226	2.1
49189	1.89226	1.30789
49190	1.89226	2.1
49191	1.89226	1.98155
49192	3.78452	2.1
49193	1.52724	2.1
49194	1.89249	2.1
49195	1.68143	2.1
49196	2.02433	1.83684
49197	3.55157	2.1
49198	1.07444	2.1
49199	1.76545	2.1
49200	1.68142	2.1
49201	1.68142	2.1
49202	1.14807	2.1
49203	1.14807	2.1
49204	1.89226	2.1
49205	1.89226	2.1
49206	1.89226	2.1
49207	1.89226	2.1
49208	1.89226	2.1
49209	1.89226	1.92079
49210	1.89226	2.1
49211	1.89226	2.1
49212	1.89226	2.1
49213	1.89226	0.724411
49214	3.78452	2.1
49215	1.68143	2.1
49216	2.02433	2.08643
49217	1.76545	2.1
49218	2.12608	2.1
49219	3.78978	2.1
49220	2.62871	2.1
49221	1.49149	2.1
49222	1.36814	2.1
49223	2.44761	2.1
49224	3.9391	2.1
49225	1.89226	2.1
49226	1.89226	0.35481
49227	1.89226	2.1
49228	1.89226	2.1
49229	3.78452	2.1
49230	0.851542	2.1
49231	0.851542	2.1
49232	1.00897	2.1
49233	0.931224	2.1
49234	0.838952	2.1
49235	0.838952	2.1
49236	0.956848	2.1
49237	0.942474	2.1
49238	0.946132	2.1
49239	0.838952	2.1

49240	0.878743	2.1
49241	0.946132	2.1
49242	1.15541	2.1
49243	2.44761	1.23348
49244	1.36814	2.1
49245	1.36814	2.1
49246	2.19439	2.1
49247	2.19439	2.1
49248	1.89226	1.52292
49249	1.89226	2.1
49250	1.89226	2.1
49251	1.89226	0.538929
49252	3.78452	2.1
49253	1.89226	2.1
49254	1.67791	2.1
49255	1.5713	2.1
49256	1.67791	2.1
49257	1.78566	2.1
49258	1.89226	2.1
49259	1.89226	0.127201
49260	1.62911	2.1
49261	2.24602	2.00587
49262	1.89226	2.1
49263	1.98287	0.675916
49264	1.62911	0.932737
49265	1.89226	2.1
49266	1.89226	2.1
49267	1.89226	0.723352
49268	1.89226	2.1
49269	3.78452	1.79134
49270	1.26688	2.1
49271	1.11804	2.1
49272	0.852467	2.01427
49273	0.693314	2.1
49274	0.977459	2.1
49275	0.977459	2.1
49276	0.982741	1.93185
49277	0.982741	2.1
49278	1.26689	2.1
49279	0.852464	2.1
49280	0.693312	2.1
49281	1.11804	2.1
49282	0.946131	2.1
49283	0.946131	0.353434
49284	1.1794	0.426651
49285	1.1794	2.1
49286	0.991434	2.1
49287	1.1794	0.778223
49288	0.946131	0.149736
49289	0.946131	0.343487
49290	0.946131	2.02556
49291	1.1794	1.66889
49292	0.946131	2.01773
49293	0.991434	1.20368
49294	1.89226	0.368712
49295	1.89226	2.1
49296	1.89226	1.20004
49297	1.89226	1.58372

49298	3.78452	2.1
49299	2.53377	2.1
49300	1.53443	2.1
49301	2.0815	1.15909
49302	2.82979	2.1
49303	4.35462	2.1
49304	1.96192	0.372624
49305	1.23602	2.1
49306	2.16506	0.573319
49307	1.56841	1.75961
49308	3.40108	1.57106
49309	1.89226	2.01406
49310	1.89226	2.1
49311	1.89226	0.637703
49312	1.89226	2.1
49313	3.78452	1.86749
49314	0.946129	2.1
49315	0.946129	2.1
49316	1.13067	1.95526
49317	1.12952	2.1
49318	0.946129	2.1
49319	1.13089	2.1
49320	0.944978	1.97179
49321	0.944978	2.1
49322	0.946131	2.1
49323	1.13089	2.1
49324	0.944977	2.1
49325	0.946131	2.1
49326	0.710208	2.1
49327	0.710208	2.1
49328	0.718894	2.1
49329	0.718894	2.1
49330	0.559773	2.1
49331	0.579321	2.1
49332	0.886767	2.1
49333	0.849781	2.1
49334	0.849781	2.1
49335	0.559774	2.1
49336	0.579321	2.1
49337	0.886766	2.1
49338	1.63471	2.1
49339	1.69956	2.1
49340	1.08022	2.1
49341	1.08022	2.1
49342	1.11955	2.1
49343	1.67403	2.1
49344	1.03766	2.1
49345	2.02825	2.1
49346	2.02794	2.1
49347	2.0348	2.1
49348	1.07196	2.1
49349	1.07196	2.1
49350	1.9276	2.1
49351	2.1004	2.1
49352	2.1306	2.1
49353	2.1306	2.1
49354	1.92178	2.1
49355	1.92032	2.1

49356	0.986724	2.1
49357	2.03064	2.1
49358	2.01385	2.1
49359	2.02404	2.1
49360	1.03766	2.1
49361	1.03766	2.1
49362	1.92027	2.1
49363	2.1306	2.1
49364	2.15388	2.1
49365	2.15388	2.1
49366	1.92614	2.1
49367	1.9276	2.1
49368	1.09578	2.1
49369	0.963018	2.1
49370	0.782956	1.24954
49371	0.529693	2.1
49372	0.6994	1.74699
49373	0.862532	2.1
49374	0.898605	2.1
49375	0.898605	1.75536
49376	0.776756	2.1
49377	1.14805	2.1
49378	0.966521	2.1
49379	0.617676	2.1
49380	2.67185	2.1
49381	2.099 2.1	
49382	2.80548	2.1
49383	2.2727	2.1
49384	4.94455	2.1
49385	1.89226	2.1
49386	1.89226	2.1
49387	1.89226	2.1
49388	1.89226	2.1
49389	3.78453	2.1
49390	1.89226	2.1
49391	1.89226	2.1
49392	1.89226	2.1
49393	1.89226	2.1
49394	1.89226	2.1
49395	1.89226	2.1
49396	0.884801	2.1
49397	0.884801	2.1
49398	0.895199	2.1
49399	0.895199	2.1
49400	0.622774	2.1
49401	0.730501	2.1
49402	1.0495	2.1
49403	1.13635	2.1
49404	1.13635	2.1
49405	0.730503	2.1
49406	1.0495	2.1
49407	0.622775	2.1
49408	1.89226	2.1
49409	1.89226	2.1
49410	1.89226	2.1
49411	1.89226	2.1
49412	1.89226	2.1
49413	1.89226	2.1

49414	1.89226	2.1
49415	1.89226	2.1
49416	1.89226	2.1
49417	1.89226	2.1
49418	1.89226	2.1
49419	1.89226	2.1
49420	2.2727	2.1
49421	1.89586	2.1
49422	1.66913	2.1
49423	1.46101	2.1
49424	2.48082	2.1
49425	2.2727	2.1
49426	1.89226	2.1
49427	1.89226	2.1
49428	1.84641	2.1
49429	1.89226	2.1
49430	1.30868	2.1
49431	1.34118	1.80938
49432	1.89226	0.498865
49433	1.89226	0.970666
49434	1.89226	1.26693
49435	1.89226	0.540145
49436	1.89226	0.656176
49437	1.89226	0.206545
49438	1.89226	1.09543
49439	1.89226	0.0822987
49440	1.89226	0.395336
49441	1.89226	0.781594
49442	3.78452	0.655376
49443	0.946132	2.1
49444	0.946132	1.88302
49445	0.946131	2.1
49446	0.946131	2.1
49447	0.946132	2.1
49448	0.946132	1.52024
49449	0.946132	2.1
49450	0.946132	2.1
49451	0.946132	2.1
49452	0.946132	2.1
49453	0.946132	2.1
49454	0.946132	2.1
49455	1.89227	1.62702
49456	1.84641	2.01814
49457	2.29517	2.1
49458	1.84641	1.61937
49459	2.34102	1.8189
49460	1.89227	0.663409
49461	1.89226	0.26604
49462	1.89226	0.181248
49463	1.89226	0.44935
49464	1.89226	0.208081
49465	1.89226	0.178092
49466	1.89226	0.0745825
49467	1.89226	0.112015
49468	1.89226	0.313786
49469	1.89226	0.379942
49470	1.89226	0.159238
49471	1.89226	0.149867

49472	1.89226	0.056564
49473	1.89226	2.09562
49474	1.89226	1.2279
49475	1.89226	2.1
49476	1.89226	1.37063
49477	1.89226	1.84431
49478	1.89226	1.06092
49479	0.851544	0.05
49480	0.757542	0.05
49481	1.13421	0.05
49482	1.04072	0.05
49483	0.946132	0.05
49484	0.946132	0.05
49485	0.841686	0.05
49486	0.834428	0.05
49487	1.13649	0.05
49488	0.937687	0.05
49489	0.841713	0.05
49490	0.841713	0.05
49491	1.89226	0.0537114
49492	1.89226	0.0783482
49493	1.89226	0.0873734
49494	1.89226	0.05
49495	1.89226	0.0889217
49496	1.89226	0.05
49497	2.31988	1.3381
49498	2.00082	2.1
49499	2.4594	0.446414
49500	2.17638	2.1
49501	4.46022	1.23446
49502	2.00082	1.50902
49503	1.41829	2.1
49504	1.61207	2.03036
49505	1.43652	2.1
49506	2.17637	1.13713
49507	2.00082	2.1
49508	2.4594	0.423088
49509	2.17638	2.1
49510	2.57957	0.610895
49511	2.3506	2.1
49512	4.81	1.4171
49513	2.3506	1.32276
49514	1.57368	2.1
49515	1.89111	0.340638
49516	1.89111	2.1
49517	2.17638	0.324376
49518	2.17638	2.1
49519	1.32813	2.1
49520	1.7237	2.1
49521	1.32461	2.1
49522	1.81656	2.1
49523	3.14468	2.1
49524	1.55132	2.1
49525	2.0815	2.1
49526	1.81656	2.1
49527	2.19826	2.1
49528	3.65818	2.1
49529	0.775951	2.1

49530	0.775951	2.1
49531	0.794601	2.1
49532	0.794601	2.1
49533	0.945641	2.1
49534	0.90828	2.1
49535	0.662302	2.1
49536	0.662273	2.1
49537	0.662274	2.1
49538	0.945639	2.1
49539	0.662304	2.1
49540	0.908279	2.1
49541	1.03062	2.1
49542	1.03062	2.1
49543	1.05054	2.1
49544	1.05054	2.1
49545	1.13551	2.1
49546	1.21275	2.1
49547	0.945641	2.1
49548	0.90828	2.1
49549	0.945641	2.1
49550	1.21275	2.1
49551	0.90828	2.1
49552	1.13552	2.1
49553	1.89226	2.1
49554	1.79883	2.1
49555	1.95341	2.1
49556	1.89226	2.1
49557	1.85998	2.1
49558	1.79883	2.1
49559	1.89226	2.1
49560	1.89226	2.1
49561	1.89226	2.1
49562	1.89226	2.1
49563	3.78453	2.1
49564	0.851541	2.1
49565	0.851541	2.1
49566	0.970398	2.1
49567	0.954365	2.1
49568	0.855356	2.1
49569	0.855357	2.1
49570	0.946133	2.1
49571	0.946133	2.1
49572	1.04072	2.1
49573	0.970397	2.1
49574	0.950003	2.1
49575	0.851538	2.1
49576	1.89226	2.1
49577	1.85998	2.1
49578	1.95296	2.1
49579	1.89226	2.1
49580	1.92067	2.1
49581	1.85998	2.1
49582	0.946133	2.1
49583	0.946133	2.1
49584	0.946132	2.1
49585	0.946132	2.1
49586	0.946132	2.1
49587	0.946132	2.1

49588	0.946132	2.1
49589	0.946132	2.1
49590	0.946132	2.1
49591	0.946132	2.1
49592	0.946133	2.1
49593	0.946133	2.1
49594	1.82152	2.1
49595	1.89226	2.1
49596	1.89226	2.1
49597	2.11152	2.1
49598	1.7472	2.1
49599	1.89226	2.1
49600	2.02687	2.1
49601	2.29199	2.1
49602	1.2734	2.1
49603	1.41077	2.1
49604	1.47381	2.1
49605	2.29199	2.1
49606	1.46889	2.1
49607	2.29199	2.1
49608	1.86766	2.1
49609	2.59401	2.1
49610	4.15965	2.1
49611	1.58995	2.1
49612	1.74246	2.1
49613	1.46643	2.1
49614	1.46643	2.1
49615	1.85097	2.1
49616	1.85097	2.1
49617	1.58995	2.1
49618	1.66584	2.1
49619	1.89214	2.1
49620	1.91787	2.1
49621	3.48208	2.1
49622	1.18474	2.1
49623	1.8144	2.1
49624	1.1486	2.1
49625	1.1486	2.1
49626	2.13071	2.1
49627	2.1257	2.1
49628	2.0815	2.1
49629	2.51651	2.1
49630	1.73681	2.1
49631	2.01601	2.1
49632	2.21406	2.1
49633	2.56998	2.1
49634	1.19995	2.1
49635	1.06256	2.1
49636	0.620677	2.1
49637	0.598084	2.1
49638	0.831426	2.1
49639	0.946056	2.1
49640	0.827692	2.1
49641	0.827692	2.1
49642	1.08958	2.1
49643	0.630132	2.1
49644	0.577547	2.1
49645	1.06104	2.1

49646	2.0815	2.1
49647	2.66249	2.1
49648	2.78966	2.1
49649	2.47108	2.1
49650	2.33858	2.1
49651	2.0815	2.1
49652	1.53735	2.1
49653	1.42278	2.1
49654	1.23654	2.1
49655	1.34678	2.1
49656	2.76955	2.1
49657	1.89226	2.1
49658	1.89226	2.1
49659	1.89226	2.1
49660	1.89226	2.1
49661	3.78452	2.1
49662	1.23654	2.1
49663	1.34678	2.1
49664	1.19	2.1
49665	1.08576	2.1
49666	2.43254	2.1
49667	1.38776	2.1
49668	1.29366	2.1
49669	0.946131	2.1
49670	0.946131	2.1
49671	0.946131	2.1
49672	0.946131	2.1
49673	1.38775	2.1
49674	1.38775	2.1
49675	1.29366	2.1
49676	0.946132	2.1
49677	0.946131	2.1
49678	1.38775	2.1
49679	0.946131	2.1
49680	0.946131	2.1
49681	0.946129	2.1
49682	0.946129	2.1
49683	0.94613	2.1
49684	0.94613	2.1
49685	0.946131	2.1
49686	0.946131	2.1
49687	0.946131	2.1
49688	0.946131	2.1
49689	0.94613	2.1
49690	0.94613	2.1
49691	1.89226	0.05
49692	1.89226	0.907608
49693	1.89226	0.143331
49694	1.89226	0.178493
49695	1.89226	0.410344
49696	1.89226	1.06506
49697	1.89226	0.108356
49698	1.89226	2.01303
49699	1.89226	0.121729
49700	1.89226	0.186277
49701	1.89226	0.10343
49702	1.89226	1.03096
49703	1.89226	0.249624

49704	1.89226	1.2148
49705	1.89226	0.11233
49706	1.89226	0.526965
49707	1.89226	0.125657
49708	1.89226	1.27722
49709	0.92338	2.1
49710	0.92338	0.48343
49711	0.94428	0.602996
49712	0.94428	0.137357
49713	1.19528	1.23372
49714	1.30598	2.1
49715	0.714073	0.184953
49716	0.561679	1.66747
49717	1.19528	1.17366
49718	0.561678	1.18826
49719	0.714072	1.2678
49720	1.30598	1.34442
49721	2.61197	2.1
49722	1.89226	1.9413
49723	1.89226	1.49685
49724	1.89226	2.1
49725	2.39056	1.14695
49726	2.39056	2.1
49727	0.946132	2.04719
49728	0.946132	0.826583
49729	0.946132	0.05
49730	0.946132	0.125477
49731	0.946131	0.209531
49732	0.946131	2.04377
49733	0.946132	0.947419
49734	0.946132	0.125476
49735	0.946132	0.46406
49736	0.946132	0.348576
49737	0.946132	0.538736
49738	0.946132	0.600799
49739	1.89224	2.1
49740	1.89224	2.1
49741	1.89224	2.1
49742	1.89224	2.1
49743	3.78448	2.1
49744	1.97089	2.1
49745	1.57428	2.1
49746	1.84991	2.1
49747	1.49078	2.1
49748	3.42419	2.1
49749	0.621927	2.1
49750	0.621927	2.1
49751	0.630824	2.1
49752	0.630824	2.1
49753	0.483483	2.1
49754	0.507361	2.1
49755	0.787142	2.1
49756	0.745391	2.1
49757	0.745381	2.1
49758	0.483476	2.1
49759	0.507368	2.1
49760	0.787152	2.1
49761	1.89224	2.1

49762	1.89224	2.1
49763	1.89224	2.1
49764	1.89224	2.1
49765	1.89224	2.1
49766	1.89224	2.1
49767	1.89227	2.1
49768	1.89227	2.1
49769	1.89227	2.1
49770	1.89227	2.1
49771	1.89227	2.1
49772	1.89227	2.1
49773	1.84994	2.1
49774	1.41758	2.1
49775	1.4908	2.1
49776	1.4908	2.1
49777	1.74391	2.1
49778	1.74391	2.1
49779	1.89226	2.1
49780	1.89226	2.1
49781	1.89226	2.1
49782	1.89226	2.1
49783	3.78452	2.1
49784	1.01323	2.1
49785	1.01323	2.1
49786	1.01445	2.1
49787	1.01445	2.1
49788	0.839531	2.1
49789	0.875652	2.1
49790	1.19063	2.1
49791	1.15203	2.1
49792	1.15203	2.1
49793	0.839531	2.1
49794	0.875652	2.1
49795	1.19063	2.1
49796	1.89226	2.1
49797	2.75587	2.1
49798	2.81512	2.1
49799	2.81512	2.1
49800	1.89226	2.1
49801	1.89226	2.1
49802	1.89226	2.1
49803	1.89226	2.1
49804	1.89226	2.1
49805	1.89226	2.1
49806	3.78452	2.1
49807	0.974831	2.1
49808	0.974831	2.1
49809	0.976681	2.1
49810	0.976681	2.1
49811	0.803179	2.1
49812	0.83953	2.1
49813	1.11198	2.1
49814	1.15203	2.1
49815	1.11198	2.1
49816	0.803179	2.1
49817	1.15203	2.1
49818	0.83953	2.1
49819	1.37794	2.1

49820	1.37794	2.1
49821	0.946128	2.1
49822	0.946128	2.1
49823	1.3477	2.1
49824	1.37793	2.1
49825	0.946131	2.1
49826	0.946131	2.1
49827	0.946129	2.1
49828	1.3477	2.1
49829	1.37793	2.1
49830	0.946129	2.1
49831	2.38577	1.82827
49832	1.20247	2.1
49833	1.75182	2.1
49834	2.82546	1.86187
49835	4.1376	2.1
49836	2.82546	2.1
49837	1.89226	0.381681
49838	2.38577	2.07808
49839	2.38577	1.75138
49840	1.89226	2.1
49841	1.89226	1.62001
49842	1.035	2.1
49843	2.15429	2.1
49844	0.970998	2.1
49845	2.0815	2.1
49846	2.89672	2.1
49847	0.946131	0.05
49848	0.946131	0.05
49849	0.853249	0.05
49850	1.02715	0.05
49851	0.851537	0.05
49852	0.851537	0.05
49853	1.02663	0.05
49854	1.13139	0.05
49855	0.946128	0.05
49856	0.932039	0.05
49857	0.843985	0.05
49858	0.946129	0.05
49859	1.89226	0.05
49860	1.86408	0.05
49861	1.96548	0.05
49862	1.86408	0.05
49863	1.99366	0.05
49864	1.89226	0.05
49865	2.70702	2.1
49866	2.08981	2.1
49867	2.08693	2.1
49868	2.08693	2.1
49869	2.7188	2.1
49870	2.7188	2.1
49871	2.08982	2.1
49872	1.01964	2.1
49873	2.08693	2.1
49874	1.04615	2.1
49875	3.13597	2.1
49876	2.70702	2.1
49877	2.08693	2.1

49878	2.68784	2.1
49879	2.08831	2.1
49880	4.77477	2.1
49881	0.778233	2.1
49882	0.778233	2.1
49883	0.789001	2.1
49884	0.789001	2.1
49885	0.545307	2.1
49886	0.523075	2.1
49887	1.04347	2.1
49888	1.04416	2.1
49889	1.04416	2.1
49890	0.545308	2.1
49891	1.04347	2.1
49892	0.523075	2.1
49893	1.89226	0.472261
49894	1.89226	0.592533
49895	1.89226	0.177768
49896	1.89226	0.107427
49897	3.78453	0.158459
49898	1.39237	0.282415
49899	1.17933	1.65742
49900	0.755796	0.841549
49901	0.755796	0.962389
49902	0.755767	0.05
49903	0.94613	1.4241
49904	1.15918	0.13834
49905	0.94613	0.80176
49906	1.11632	0.174838
49907	0.92595	0.05
49908	0.94613	0.724811
49909	0.94613	0.445556
49910	1.89226	0.575511
49911	1.89226	0.238628
49912	1.89226	0.0952654
49913	1.89226	0.05
49914	3.78452	0.281695
49915	0.950067	0.0502669
49916	0.75071	0.0673361
49917	0.906516	0.0673455
49918	0.906516	0.05
49919	0.81586	0.05
49920	0.851541	0.209662
49921	0.962101	0.05
49922	0.851476	0.124773
49923	0.962105	0.05
49924	0.815808	0.05
49925	0.851529	0.106411
49926	0.851529	0.078225
49927	1.05032	0.05
49928	0.972326	0.0572259
49929	0.906515	0.05
49930	0.906515	0.05
49931	1.12245	0.05
49932	1.04072	0.05
49933	0.815857	0.05
49934	0.851539	0.145494
49935	1.04451	0.05

49936	0.815853	0.0588868
49937	0.919795	0.0907915
49938	0.919795	0.0517669
49939	1.5855	2.1
49940	2.14058	2.1
49941	1.9153	2.1
49942	1.79922	2.1
49943	3.72608	2.1
49944	1.58551	2.1
49945	1.24201	2.1
49946	0.960287	2.09927
49947	1.79922	2.1
49948	2.82752	2.1
49949	0.679602	2.1
49950	0.621009	2.1
49951	0.899614	2.1
49952	0.940441	2.1
49953	0.679602	2.1
49954	0.94044	2.1
49955	0.621009	2.1
49956	0.899615	2.1
49957	0.794027	2.1
49958	0.794027	2.1
49959	0.785189	2.1
49960	0.785189	2.1
49961	2.14058	2.1
49962	1.79922	2.1
49963	2.23742	2.1
49964	2.23742	2.1
49965	1.78404	2.1
49966	1.88088	2.1
49967	1.15177	2.1
49968	2.0815	2.1
49969	1.93765	2.1
49970	1.78342	2.1
49971	1.39511	2.1
49972	1.28406	2.1
49973	1.97719	2.1
49974	2.50057	0.156927
49975	2.35349	2.1
49976	2.35349	0.260165
49977	2.31686	2.1
49978	2.32857	0.568754
49979	1.93765	2.1
49980	2.35349	0.459803
49981	1.70048	2.1
49982	2.15721	0.05
49983	4.05397	1.22796
49984	0.850388	2.1
49985	0.850388	2.1
49986	0.840019	2.1
49987	0.840019	2.1
49988	1.13736	2.1
49989	1.13852	2.1
49990	0.573661	2.1
49991	0.551891	2.1
49992	0.573661	2.1
49993	1.13852	2.1

49994	0.551892	2.1
49995	1.13736	2.1
49996	0.860203	2.1
49997	0.860203	2.1
49998	0.855758	2.1
49999	0.855758	2.1
50000	1.1423	2.1
50001	1.13852	2.1
50002	0.58628	2.1
50003	0.573661	2.1
50004	0.586281	2.1
50005	1.1423	2.1
50006	0.573661	2.1
50007	1.13852	2.1
50008	1.28918	1.11053
50009	0.999086	0.970116
50010	1.0383	1.87223
50011	0.736019	1.54091
50012	1.00954	1.53732
50013	1.00954	1.03261
50014	1.01566	0.508106
50015	1.01566	1.87899
50016	1.28918	1.21523
50017	1.0383	1.91669
50018	0.999086	1.57732
50019	0.736019	1.36918
50020	2.20797	2.1
50021	2.64748	2.1
50022	2.50375	2.1
50023	2.50375	2.1
50024	2.40858	2.1
50025	2.40858	2.1
50026	2.64748	2.1
50027	2.74744	2.1
50028	2.50375	2.1
50029	2.6521	2.1
50030	5.29958	2.1
50031	2.20797	2.1
50032	2.50375	2.1
50033	2.02064	2.1
50034	2.3606	2.1
50035	4.52439	2.1
50036	2.36059	2.1
50037	2.6521	2.1
50038	2.56952	2.1
50039	2.56952	2.1
50040	2.50375	2.1
50041	2.50375	2.1
50042	2.49439	0.05
50043	1.89226	0.0784111
50044	2.81337	0.0742888
50045	1.89226	0.0816669
50046	4.38665	0.0802321
50047	1.89226	0.0947081
50048	1.89226	0.222926
50049	1.89226	0.244692
50050	1.89226	0.325932
50051	3.78452	0.190593

50052	1.89226	0.083111
50053	1.89226	0.127956
50054	1.89226	0.105996
50055	1.89226	0.15908
50056	3.78452	0.113974
50057	1.93948	0.264046
50058	1.98874	0.107138
50059	1.62494	0.248865
50060	2.14218	0.411628
50061	1.62494	0.05204
50062	2.14218	0.0740698
50063	1.47354	0.139009
50064	1.98874	0.0661282
50065	2.01865	0.138756
50066	1.62495	0.0630193
50067	2.01865	0.205298
50068	1.62495	0.0602317
50069	1.89226	1.39307
50070	1.89226	2.1
50071	1.89226	2.1
50072	1.89226	2.1
50073	3.78453	2.1
50074	2.05257	2.1
50075	1.94441	2.1
50076	1.88487	2.1
50077	1.82441	2.1
50078	3.82928	2.1
50079	1.94441	2.1
50080	1.74391	2.1
50081	1.82441	2.1
50082	1.65636	2.1
50083	3.60077	2.1
50084	1.89226	2.1
50085	1.89226	2.1
50086	1.89226	2.1
50087	1.89226	2.1
50088	3.78452	2.1
50089	1.89226	2.1
50090	1.89226	2.1
50091	1.89226	1.29582
50092	1.89226	2.1
50093	3.78453	2.1
50094	1.88487	2.1
50095	1.82441	2.1
50096	1.75835	2.1
50097	1.72866	2.1
50098	3.61354	2.1
50099	1.82441	2.1
50100	1.65636	2.1
50101	1.72866	2.1
50102	1.58401	2.1
50103	3.38503	2.1
50104	1.89226	2.1
50105	1.89226	2.1
50106	1.89226	2.1
50107	1.89226	2.1
50108	3.78452	2.1
50109	2.27432	2.1

50110	1.33339	2.1
50111	1.38905	2.1
50112	2.34471	2.1
50113	3.66337	2.1
50114	1.89226	2.1
50115	2.34471	2.1
50116	2.27432	2.1
50117	2.27432	2.1
50118	1.89226	2.1
50119	1.89226	2.1
50120	1.20443	2.1
50121	1.17235	2.1
50122	0.724695	2.1
50123	0.694522	2.1
50124	0.949994	2.1
50125	0.949993	2.1
50126	0.948955	2.1
50127	0.948955	2.1
50128	1.20443	2.1
50129	0.7247	2.1
50130	0.694517	2.1
50131	1.17234	2.1
50132	1.89225	2.1
50133	2.40885	2.1
50134	2.3447	2.1
50135	2.3447	2.1
50136	1.89225	2.1
50137	1.89225	2.1
50138	1.37138	2.1
50139	1.82597	2.1
50140	1.45027	2.1
50141	1.92772	2.1
50142	3.27624	2.1
50143	1.89226	2.1
50144	1.89226	2.1
50145	1.89226	2.1
50146	1.89226	2.1
50147	3.78452	2.1
50148	1.89226	2.1
50149	1.89226	1.82844
50150	1.89226	2.1
50151	1.89226	2.06753
50152	3.78452	2.1
50153	1.82597	2.1
50154	2.02312	0.130475
50155	1.92772	2.1
50156	2.15888	2.1
50157	3.98485	2.1
50158	1.53446	2.1
50159	1.92772	2.1
50160	2.03975	2.1
50161	2.03975	2.1
50162	1.45027	2.1
50163	1.45027	2.1
50164	1.89226	2.1
50165	1.89226	2.1
50166	1.89226	2.1
50167	1.89226	2.1

50168	1.89226	2.1
50169	1.89226	2.1
50170	1.89226	2.1
50171	1.89226	2.1
50172	1.89226	2.1
50173	1.89226	2.1
50174	3.78452	2.1
50175	1.92772	2.1
50176	2.15888	2.1
50177	2.03975	2.1
50178	2.3111	2.1
50179	4.19863	2.1
50180	1.89226	2.1
50181	1.89226	2.1
50182	1.89226	2.1
50183	1.89226	2.1
50184	3.78452	2.1
50185	1.78376	2.1
50186	1.3201	2.1
50187	1.78376	2.1
50188	1.86102	2.1
50189	1.2679	2.1
50190	1.34517	2.1
50191	1.89226	2.1
50192	2.52442	2.1
50193	1.89226	2.1
50194	2.51691	2.1
50195	4.41667	2.1
50196	1.89226	2.1
50197	1.89226	2.1
50198	1.89226	2.1
50199	1.89226	2.1
50200	3.78452	2.1
50201	1.78376	2.1
50202	1.2679	2.1
50203	1.71839	2.1
50204	1.23504	2.1
50205	3.0188	2.1
50206	1.89226	2.1
50207	2.51795	2.1
50208	2.51691	2.1
50209	2.51691	2.1
50210	1.89226	2.1
50211	1.89226	2.1
50212	1.89226	0.617084
50213	1.89226	1.43308
50214	1.89226	1.01251
50215	1.89226	0.927548
50216	3.78452	0.88445
50217	1.89227	0.690053
50218	1.89227	0.738732
50219	3.26088	1.14854
50220	2.79543	0.95018
50221	2.20733	0.631534
50222	1.89227	0.654054
50223	1.89226	1.2281
50224	1.89226	0.883402
50225	1.89226	0.670627

50226	1.89226	0.971852
50227	1.89226	0.916211
50228	1.89226	1.27325
50229	1.89227	0.670762
50230	2.20733	0.708651
50231	1.46973	1.16853
50232	1.89227	0.732384
50233	1.7848	1.1215
50234	2.20733	0.608778
50235	1.89226	2.1
50236	2.55682	2.1
50237	2.81593	2.1
50238	2.81593	2.1
50239	1.89226	2.1
50240	1.89226	2.1
50241	1.0607	2.1
50242	1.0607	2.1
50243	1.12617	2.1
50244	0.986957	2.1
50245	0.699328	2.1
50246	0.785567	2.1
50247	1.28855	2.1
50248	1.41913	2.1
50249	0.766737	2.04398
50250	1.34711	2.1
50251	1.24643	2.1
50252	0.687289	2.1
50253	3.11063	0.05
50254	1.89226	0.053504
50255	1.96536	0.05
50256	1.89226	0.05
50257	1.96536	0.05
50258	1.89226	0.05
50259	1.89226	0.213745
50260	1.89226	0.0710421
50261	1.89226	0.0891538
50262	1.89226	0.0699073
50263	3.78452	0.218377
50264	1.89226	0.0501488
50265	1.96256	0.05
50266	1.89226	0.05
50267	1.89226	0.05
50268	2.49192	0.05
50269	2.49192	0.05
50270	1.85716	0.05
50271	2.56871	0.05
50272	1.03043	0.05
50273	1.03043	0.05
50274	2.48125	0.05
50275	1.65452	0.05
50276	1.89226	2.1
50277	2.09622	2.1
50278	2.09622	2.1
50279	2.08508	2.1
50280	1.89226	2.1
50281	1.88112	2.1
50282	1.04811	2.1
50283	1.04811	2.1

50284	0.946131	2.1
50285	0.946131	2.1
50286	1.05014	2.1
50287	1.04811	2.1
50288	0.94613	2.1
50289	0.94613	2.1
50290	0.946131	2.1
50291	1.05013	2.1
50292	1.04811	2.1
50293	0.946131	2.1
50294	1.398	2.1
50295	2.10766	2.1
50296	2.3018	2.1
50297	2.3018	2.1
50298	1.34008	2.1
50299	1.34008	2.1
50300	1.1509	2.1
50301	1.1509	2.1
50302	0.946132	2.1
50303	0.946132	2.1
50304	0.946132	2.1
50305	0.946132	2.1
50306	1.05383	2.1
50307	1.1509	2.1
50308	1.1509	2.1
50309	0.946133	2.1
50310	0.946132	2.1
50311	1.05383	2.1
50312	0.931141	2.1
50313	0.931141	2.1
50314	0.971946	2.1
50315	0.971946	2.1
50316	1.28569	2.1
50317	1.1509	2.1
50318	0.699	2.1
50319	0.752185	2.1
50320	1.28568	2.1
50321	0.752186	2.1
50322	0.699001	2.1
50323	1.1509	2.1
50324	2.30181	2.1
50325	1.89226	2.1
50326	1.89226	2.1
50327	1.89226	2.1
50328	2.57137	2.1
50329	2.57137	2.1
50330	2.0815	2.1
50331	1.21962	2.1
50332	1.55173	2.1
50333	2.57849	2.1
50334	3.79001	2.1
50335	1.38779	2.1
50336	1.2923	2.1
50337	1.03974	2.1
50338	1.28832	2.1
50339	1.33566	2.1
50340	1.21116	2.1
50341	1.22343	2.1

50342	1.22343	2.1
50343	1.42288	2.1
50344	1.20138	2.1
50345	1.26025	2.1
50346	1.00644	2.1
50347	1.26066	2.1
50348	1.14271	2.1
50349	0.886635	2.1
50350	0.795277	2.1
50351	1.02966	2.1
50352	1.02967	2.1
50353	1.05216	2.1
50354	1.05216	2.1
50355	1.26068	2.1
50356	0.790042	2.1
50357	0.795269	2.1
50358	1.20135	2.1
50359	1.89226	0.143641
50360	1.89226	0.0960485
50361	1.89226	0.162467
50362	1.89226	0.0709182
50363	1.89226	0.217019
50364	1.89226	0.09349
50365	1.89227	0.0817367
50366	1.89227	0.0680202
50367	1.89227	0.105511
50368	1.89227	0.05
50369	1.89227	0.124599
50370	1.89227	0.0554425
50371	0.563873	0.05
50372	0.50988	0.05
50373	0.598693	0.0671663
50374	0.558819	0.05
50375	0.564855	0.05
50376	0.564855	0.05
50377	0.557838	0.0611312
50378	0.557838	0.05
50379	0.563873	0.05
50380	0.598694	0.05
50381	0.558819	0.05
50382	0.50988	0.05
50383	0.598693	0.063237
50384	0.558819	0.05
50385	0.572737	0.0979753
50386	0.534248	0.0687808
50387	0.566788	0.0655197
50388	0.566788	0.05
50389	0.566153	0.085824
50390	0.566153	0.0675035
50391	0.598694	0.0669534
50392	0.572737	0.0703593
50393	0.534248	0.060236
50394	0.558819	0.0564346
50395	1.85217	2.1
50396	1.93111	2.1
50397	1.92875	2.1
50398	2.02391	2.1
50399	3.85986	2.1

50400	1.93112	2.1
50401	1.80425	2.1
50402	2.02392	2.1
50403	1.90981	2.1
50404	3.84092	2.1
50405	1.92876	1.51915
50406	2.02393	2.1
50407	2.05274	2.1
50408	2.16783	2.1
50409	4.09659	2.1
50410	2.02393	2.1
50411	1.90982	2.1
50412	2.16784	2.1
50413	2.05967	2.1
50414	4.07766	2.1
50415	1.89226	1.71149
50416	1.89226	1.22882
50417	1.89226	1.63058
50418	1.89226	1.47478
50419	3.78453	0.957067
50420	1.89226	1.11748
50421	1.89226	1.73078
50422	1.89226	2.1
50423	2.44829	0.782602
50424	3.78453	2.00603
50425	1.89226	1.68266
50426	1.89226	1.85827
50427	1.89226	1.51043
50428	2.60452	0.854313
50429	3.78453	2.1
50430	1.58723	0.05
50431	1.88071	0.05
50432	2.97376	0.05
50433	2.13255	0.05
50434	4.43982	0.05
50435	2.20641	0.05
50436	1.33575	0.05
50437	2.15824	0.05
50438	2.15824	0.05
50439	1.2951	0.05
50440	1.2951	0.05
50441	0.825383	0.05
50442	0.825383	0.05
50443	0.856104	0.05
50444	0.856104	0.05
50445	0.952196	0.05
50446	0.602363	0.05
50447	0.667877	0.05
50448	1.07913	0.05
50449	0.602363	0.05
50450	0.667876	0.05
50451	1.07913	0.05
50452	0.952197	0.05
50453	2.57657	1.56595
50454	1.44437	2.1
50455	2.65378	2.1
50456	2.25076	2.1
50457	1.84739	2.1

50458	1.44437	2.1
50459	2.0189	0.05
50460	2.54752	0.05
50461	2.63975	0.05
50462	2.31049	0.05
50463	4.65865	0.05
50464	1.40707	0.05
50465	1.89605	0.05
50466	2.0189	0.05
50467	2.0189	0.05
50468	1.50438	0.05
50469	1.50438	0.05
50470	2.63971	0.05
50471	2.38246	0.05
50472	2.13402	0.05
50473	2.57503	0.05
50474	4.77373	0.05
50475	1.50436	0.05
50476	1.96803	0.05
50477	2.13402	0.05
50478	1.45446	0.05
50479	3.63838	0.05
50480	2.30793	0.05
50481	2.11032	0.05
50482	1.24082	0.05
50483	1.05689	0.05
50484	3.36482	0.05
50485	1.89226	0.05
50486	1.89226	0.0725881
50487	1.89226	0.0574437
50488	1.89226	0.0509423
50489	3.78453	0.0529748
50490	0.812967	0.05
50491	0.812967	0.05
50492	1.00181	0.05
50493	1.08141	0.05
50494	0.946132	0.05
50495	0.812938	0.05
50496	0.932815	0.05
50497	1.05577	0.05
50498	0.812938	0.05
50499	0.932812	0.05
50500	0.946133	0.05
50501	0.946133	0.05
50502	2.53897	2.1
50503	1.89226	2.1
50504	1.89226	2.1
50505	1.89226	2.1
50506	2.3245	2.1
50507	2.3245	2.1
50508	1.00255	1.7421
50509	1.06561	2.1
50510	1.05481	0.0704767
50511	1.11077	2.1
50512	2.12042	2.1
50513	1.89226	1.81799
50514	1.89226	2.1
50515	1.89226	0.0938594

50516	1.89226	2.1
50517	3.78453	1.8685
50518	1.89226	2.1
50519	2.77314	2.1
50520	2.81276	2.1
50521	2.81276	2.1
50522	1.89226	2.1
50523	1.89226	2.1
50524	1.11077	2.1
50525	1.15114	2.1
50526	1.10274	1.01914
50527	1.1032	2.1
50528	1.05481	2.1
50529	1.1032	0.949241
50530	1.89226	1.2776
50531	1.89226	2.1
50532	1.89226	0.05
50533	1.89226	2.1
50534	3.78452	1.46022
50535	0.721957	0.124572
50536	0.721957	0.126424
50537	0.946103	0.127866
50538	0.946103	0.0650897
50539	0.814237	0.0781267
50540	0.721929	0.0617465
50541	0.946132	0.133851
50542	0.946132	0.179759
50543	0.721928	0.183104
50544	0.946131	0.223959
50545	0.946132	0.274084
50546	0.814237	0.172265
50547	1.89226	0.396907
50548	1.89226	0.133093
50549	1.89226	0.13533
50550	1.89226	0.156234
50551	1.89226	0.292885
50552	1.89226	0.340713
50553	1.37196	0.097168
50554	2.83556	0.0669396
50555	1.5165	0.0829081
50556	1.5165	0.11168
50557	2.74923	0.068083
50558	2.74923	0.0993294
50559	0.946128	0.111813
50560	0.946128	0.156161
50561	0.827866	0.0677788
50562	0.946132	0.0889424
50563	0.94613	0.0624437
50564	0.94613	0.0708054
50565	0.94613	0.163615
50566	0.94613	0.124634
50567	0.94613	0.165075
50568	0.827864	0.217225
50569	0.94613	0.135315
50570	0.94613	0.278485
50571	1.36208	0.0513792
50572	2.74923	0.05
50573	1.37196	0.05

50574	1.37196	0.05
50575	2.53256	0.05
50576	2.53256	0.05
50577	1.07705	2.1
50578	1.07705	2.1
50579	1.08253	2.1
50580	1.08253	2.1
50581	0.762985	2.1
50582	0.781638	2.1
50583	1.37794	2.1
50584	1.40756	2.1
50585	1.37794	2.1
50586	0.762986	2.1
50587	0.781638	2.1
50588	1.40756	2.1
50589	2.69541	2.1
50590	2.75587	2.1
50591	1.48572	2.1
50592	1.48572	2.1
50593	1.52597	2.1
50594	2.73566	2.1
50595	2.0554	1.04395
50596	1.00387	0.535019
50597	2.01001	0.215912
50598	0.994002	0.184269
50599	3.01389	0.105544
50600	1.89226	2.1
50601	2.41579	2.1
50602	2.12209	2.1
50603	2.12209	2.1
50604	1.89226	2.1
50605	1.89226	2.1
50606	1.89226	2.1
50607	1.89226	2.1
50608	1.89226	2.1
50609	1.89226	2.1
50610	1.89226	2.1
50611	1.89226	2.1
50612	0.88209	2.1
50613	0.899471	2.1
50614	0.651501	2.1
50615	0.533531	2.1
50616	0.724105	2.1
50617	0.711243	2.1
50618	0.692622	2.1
50619	0.692622	2.1
50620	0.890668	2.1
50621	0.738066	2.1
50622	0.778223	2.1
50623	0.531121	2.1
50624	1.89226	2.1
50625	2.77504	2.1
50626	2.4158	2.1
50627	2.4158	2.1
50628	1.89226	2.1
50629	1.89226	2.1
50630	1.89226	2.1
50631	1.89226	2.1

50632	1.89226	2.1
50633	1.89226	2.1
50634	1.89226	2.1
50635	1.89226	2.1
50636	1.70303	2.1
50637	1.39832	2.1
50638	1.51749	2.1
50639	1.68783	2.1
50640	3.24844	2.1
50641	1.89226	2.1
50642	2.38311	2.1
50643	1.98321	2.1
50644	1.89226	2.1
50645	2.47406	2.1
50646	2.38311	2.1
50647	1.89226	2.1
50648	1.89226	2.1
50649	1.89226	2.1
50650	1.89226	2.1
50651	1.89226	2.1
50652	1.89226	2.1
50653	0.946131	2.1
50654	0.946131	2.1
50655	1.25167	2.1
50656	1.19156	2.1
50657	1.19156	2.1
50658	1.19156	2.1
50659	0.946133	2.1
50660	0.946133	2.1
50661	0.946131	2.1
50662	1.25167	2.1
50663	0.946131	2.1
50664	1.19156	2.1
50665	1.89226	2.1
50666	2.47406	2.1
50667	1.99996	2.1
50668	1.89226	2.1
50669	2.58176	2.1
50670	2.47406	2.1
50671	1.89226	2.1
50672	1.89226	2.1
50673	1.89226	2.1
50674	1.89226	2.1
50675	1.89226	2.1
50676	1.89226	2.1
50677	0.946131	2.1
50678	0.946131	2.1
50679	1.32241	2.1
50680	1.25167	2.1
50681	1.25167	2.1
50682	1.25167	2.1
50683	0.946133	2.1
50684	0.946133	2.1
50685	0.946131	2.1
50686	1.32241	2.1
50687	0.946131	2.1
50688	1.25167	2.1
50689	1.65151	2.1

50690	1.92471	2.1
50691	1.81834	2.1
50692	2.13272	2.1
50693	3.74306	2.1
50694	1.92472	2.1
50695	1.9943	2.1
50696	2.13273	2.1
50697	2.24475	2.1
50698	4.16947	2.1
50699	1.81834	2.1
50700	2.13272	2.1
50701	2.02687	2.1
50702	2.39605	2.1
50703	4.21439	2.1
50704	2.13273	2.1
50705	2.24475	2.1
50706	2.39605	2.1
50707	2.54903	2.1
50708	4.6408	2.1
50709	1.10534	2.1
50710	1.22993	2.1
50711	0.946132	2.1
50712	0.946132	2.1
50713	0.946132	2.1
50714	0.946132	2.1
50715	1.22993	2.1
50716	1.22993	2.1
50717	1.10534	2.1
50718	0.946131	2.1
50719	0.946132	2.1
50720	1.22993	2.1
50721	1.47221	2.1
50722	2.14647	2.1
50723	1.95567	2.1
50724	1.95567	2.1
50725	1.71082	2.1
50726	1.71082	2.1
50727	0.946131	2.1
50728	0.946131	2.1
50729	0.946131	2.1
50730	0.946131	2.1
50731	0.946132	2.1
50732	0.946132	2.1
50733	0.946131	2.1
50734	0.946131	2.1
50735	0.946131	2.1
50736	0.946131	2.1
50737	0.946132	2.1
50738	0.946132	2.1
50739	0.998086	2.1
50740	1.09429	2.1
50741	0.946125	2.1
50742	0.946125	2.1
50743	0.90732	2.1
50744	0.90732	2.1
50745	1.14414	2.1
50746	1.16553	2.1
50747	1.05018	2.1

50748	0.907332	2.1
50749	0.99482	2.1
50750	0.851529	2.1
50751	0.80534	2.1
50752	0.80534	2.1
50753	0.812206	2.1
50754	0.812206	2.1
50755	0.895134	2.1
50756	0.977828	2.1
50757	0.736099	2.1
50758	0.639718	2.1
50759	0.639722	2.1
50760	0.89514	2.1
50761	0.736095	2.1
50762	0.977821	2.1
50763	0.946125	2.1
50764	0.946125	2.1
50765	0.946125	2.1
50766	0.946125	2.1
50767	0.946125	2.1
50768	0.946125	2.1
50769	0.946124	2.1
50770	0.946124	2.1
50771	0.946131	2.1
50772	0.946131	2.1
50773	0.946118	2.1
50774	0.946118	2.1
50775	2.22622	0.05
50776	2.43347	0.05
50777	2.34108	0.05
50778	1.24587	2.1
50779	1.21674	2.1
50780	1.14533	2.1
50781	1.11311	2.1
50782	1.17874	2.1
50783	1.17874	2.1
50784	1.18024	2.1
50785	1.18024	2.1
50786	1.14533	2.1
50787	1.24587	2.1
50788	1.21674	2.1
50789	1.11311	2.1
50790	2.43347	2.1
50791	2.34107	2.1
50792	2.39663	2.1
50793	2.49174	2.1
50794	4.8301	2.1
50795	1.86206	2.1
50796	1.41058	2.1
50797	1.70302	2.1
50798	2.42083	2.1
50799	3.68129	2.1
50800	1.70302	2.1
50801	2.43549	2.1
50802	2.20075	2.1
50803	2.50358	2.1
50804	2.04916	2.1
50805	2.33113	2.1

50806	1.07624	2.1
50807	2.12621	2.1
50808	1.57297	2.1
50809	1.60383	2.1
50810	1.30047	2.1
50811	1.30047	2.1
50812	2.33379	2.1
50813	2.56149	1.22358
50814	2.36734	1.56385
50815	2.62796	1.45185
50816	4.92883	1.78788
50817	2.5615	1.36046
50818	2.57957	1.66586
50819	2.62796	1.07966
50820	2.69056	1.05338
50821	5.25206	1.84106
50822	2.39322	2.1
50823	2.62796	2.09077
50824	2.6849	2.1
50825	2.6849	2.08367
50826	2.36734	2.1
50827	2.36734	2.0599
50828	2.62796	1.87806
50829	2.69056	1.91223
50830	2.6849	0.244231
50831	2.77227	0.242545
50832	5.37546	0.729062
50833	1.89226	0.05
50834	1.89226	0.05
50835	1.89226	0.05
50836	1.89226	0.05
50837	1.89226	0.05
50838	1.89226	0.05
50839	1.06974	0.05
50840	1.06974	0.05
50841	1.02299	0.05
50842	1.02299	0.05
50843	1.35299	0.05
50844	1.26227	0.05
50845	0.646252	0.05
50846	0.830464	0.05
50847	0.830463	0.05
50848	1.35299	0.05
50849	1.26227	0.05
50850	0.646252	0.05
50851	2.52454	0.05
50852	1.89226	0.05
50853	2.70598	0.05
50854	1.89226	0.05
50855	4.59824	0.05
50856	1.89226	0.05
50857	1.89226	0.05
50858	1.89226	0.05
50859	1.89226	0.05
50860	1.89226	0.05
50861	1.89226	0.05
50862	1.89226	0.05
50863	1.89226	0.05

50864	1.89226	0.05
50865	1.89226	0.05
50866	1.89226	0.05
50867	1.89226	0.05
50868	1.7666	0.05
50869	2.70598	0.05
50870	2.65378	0.05
50871	2.65378	0.05
50872	1.66093	0.05
50873	1.66093	0.05
50874	2.70598	0.05
50875	1.89226	0.05
50876	1.89226	0.05
50877	1.89226	0.05
50878	2.65377	0.05
50879	2.65377	0.05
50880	0.946132	0.05
50881	0.946132	0.05
50882	0.946131	0.05
50883	0.946131	0.05
50884	0.946131	0.05
50885	0.946131	0.05
50886	0.946132	0.05
50887	0.946132	0.05
50888	0.946132	0.05
50889	0.946132	0.05
50890	0.946131	0.05
50891	0.946131	0.05
50892	1.69858	2.1
50893	2.18238	2.1
50894	2.506	2.1
50895	1.93586	2.1
50896	4.20458	2.1
50897	2.21772	2.1
50898	2.18238	2.1
50899	2.506	2.1
50900	2.59215	2.1
50901	4.72372	2.1
50902	2.50596	2.1
50903	1.93584	2.1
50904	2.16728	2.1
50905	2.7679	2.1
50906	4.67325	2.1
50907	1.53296	2.1
50908	1.56299	2.1
50909	1.42624	2.1
50910	1.52961	2.1
50911	2.98923	2.1
50912	1.56299	2.1
50913	1.46568	2.1
50914	1.52961	2.1
50915	1.51916	2.1
50916	3.08215	2.1
50917	1.36226	2.1
50918	1.52961	2.1
50919	1.36226	2.1
50920	1.42624	2.1
50921	1.55369	2.1

50922	1.61768	2.1
50923	1.52961	2.1
50924	1.51916	2.1
50925	1.55369	2.1
50926	1.64892	2.1
50927	3.07285	2.1
50928	2.13405	0.05
50929	2.57507	0.05
50930	2.39438	0.05
50931	1.93408	0.05
50932	4.52843	0.05
50933	2.13405	0.05
50934	1.27778	0.05
50935	1.2545	0.05
50936	1.45448	0.05
50937	1.93408	0.05
50938	2.13405	0.05
50939	2.39438	0.05
50940	1.93407	0.05
50941	1.60578	0.05
50942	1.87985	0.05
50943	4.00016	0.05
50944	1.0525	2.1
50945	1.05758	2.1
50946	1.43484	0.815188
50947	1.38506	1.91007
50948	2.48734	2.04543
50949	1.20455	2.1
50950	1.67352	2.1
50951	1.52852	2.1
50952	2.05164	2.1
50953	3.20203	2.1
50954	1.89226	2.1
50955	1.89226	2.1
50956	1.89226	2.1
50957	1.89226	2.1
50958	3.78452	2.1
50959	1.89226	2.1
50960	1.89226	2.1
50961	1.89226	2.1
50962	1.89226	2.1
50963	1.89226	2.1
50964	1.89226	2.1
50965	2.05165	2.1
50966	1.94291	2.1
50967	2.09398	2.1
50968	1.67352	2.1
50969	2.36337	2.1
50970	1.94291	2.1
50971	1.52851	2.1
50972	2.52049	2.1
50973	2.43433	2.1
50974	2.05164	2.1
50975	1.9112	2.1
50976	1.52851	2.1
50977	1.89226	2.1
50978	1.89226	2.1
50979	1.89226	2.1

50980	1.89226	2.1
50981	3.78451	2.1
50982	1.89226	2.1
50983	1.89226	2.1
50984	1.89226	2.1
50985	1.89226	2.1
50986	3.78452	2.1
50987	2.5205	2.1
50988	2.36337	2.1
50989	2.4605	2.1
50990	2.05165	2.1
50991	2.77222	2.1
50992	2.36337	2.1
50993	1.89226	2.1
50994	1.89226	2.1
50995	1.89226	2.1
50996	1.89226	2.1
50997	1.89226	2.1
50998	1.89226	2.1
50999	1.89226	2.1
51000	1.89226	2.1
51001	1.89226	2.1
51002	1.89226	2.1
51003	3.78452	2.1
51004	1.38905	2.1
51005	1.26166	2.1
51006	1.19216	2.1
51007	1.0875	2.1
51008	1.24393	2.1
51009	1.24393	2.1
51010	1.23261	2.1
51011	1.23261	2.1
51012	1.38905	2.1
51013	1.19216	2.1
51014	1.26166	2.1
51015	1.0875	2.1
51016	1.16233	2.1
51017	1.06028	2.1
51018	0.777395	2.1
51019	0.705508	2.1
51020	0.940018	2.1
51021	0.936465	2.1
51022	0.954216	2.1
51023	0.954216	2.1
51024	1.16125	2.1
51025	0.685505	2.1
51026	1.1157	2.1
51027	0.703534	2.1
51028	1.89226	2.1
51029	1.89226	2.1
51030	1.89226	2.1
51031	1.89226	2.1
51032	1.89226	2.1
51033	1.89226	2.1
51034	1.89226	2.1
51035	1.89226	2.1
51036	1.89226	2.1
51037	1.89226	2.1

51038	1.89226	2.1
51039	1.89226	2.1
51040	2.38433	2.1
51041	1.43456	2.1
51042	1.93881	2.1
51043	2.36952	2.1
51044	4.32313	2.1
51045	1.8493	2.1
51046	1.5927	2.1
51047	1.45535	2.1
51048	1.38438	2.1
51049	1.89657	2.1
51050	1.8493	2.1
51051	2.0815	2.1
51052	1.6697	2.1
51053	2.0657	2.1
51054	1.5927	2.1
51055	2.59749	2.1
51056	2.0815	2.1
51057	2.76597	2.1
51058	1.89226	2.1
51059	1.89226	2.1
51060	1.89226	2.1
51061	2.6126	2.1
51062	2.6126	2.1
51063	2.62513	2.1
51064	1.89226	2.1
51065	1.89226	2.1
51066	1.89226	2.1
51067	2.76598	2.1
51068	2.76598	2.1
51069	2.54665	2.1
51070	2.6126	2.1
51071	2.76598	2.1
51072	2.76598	2.1
51073	2.34044	2.1
51074	2.34044	2.1
51075	2.62514	2.1
51076	2.76598	2.1
51077	2.54666	2.1
51078	2.45212	2.1
51079	5.2181	2.1
51080	2.14063	2.1
51081	1.47521	2.1
51082	1.27679	2.1
51083	1.47521	2.1
51084	2.11511	2.1
51085	2.31353	2.1
51086	0.93256	2.1
51087	1.07032	2.1
51088	0.638396	2.1
51089	0.521342	2.1
51090	0.790674	2.1
51091	0.790674	2.1
51092	0.80098	2.1
51093	0.800981	2.1
51094	0.932535	2.1
51095	0.521328	2.1

51096	1.07034	2.1
51097	0.638414	2.1
51098	1.89226	2.1
51099	1.89226	1.71664
51100	1.89226	2.05401
51101	1.89226	2.1
51102	3.78453	2.1
51103	1.89226	2.1
51104	1.9842	2.1
51105	1.89226	2.1
51106	1.89226	2.1
51107	2.79705	2.1
51108	2.79705	2.1
51109	0.469955	2.1
51110	0.791665	2.1
51111	0.989115	1.96384
51112	0.763235	2.1
51113	0.776356	2.1
51114	0.805461	2.1
51115	0.776759	2.1
51116	0.776759	2.1
51117	0.799151	2.1
51118	0.940869	2.1
51119	0.78449	2.1
51120	0.489635	2.1
51121	1.89226	1.74859
51122	1.89226	2.1
51123	1.89226	1.54706
51124	1.89226	2.1
51125	1.89226	1.41271
51126	1.89226	2.1
51127	2.46058	1.98206
51128	2.28991	2.1
51129	1.8525	2.1
51130	1.87697	2.1
51131	2.28991	2.03698
51132	1.62397	2.1
51133	0.946131	0.05
51134	0.974033	0.05
51135	1.31667	0.05
51136	0.946131	0.05
51137	0.885497	0.05
51138	0.885497	0.05
51139	1.0468	0.05
51140	1.04678	0.05
51141	1.31667	0.05
51142	0.974019	0.05
51143	0.946131	0.05
51144	0.946131	0.05
51145	1.89226	0.05
51146	1.89226	0.05
51147	1.89226	0.05
51148	1.89226	0.05
51149	3.78452	0.05
51150	0.946125	0.05
51151	0.946125	0.05
51152	0.946131	0.05
51153	0.946131	0.05

51154	0.946128	0.05
51155	0.946128	0.05
51156	0.946128	0.05
51157	0.946128	0.05
51158	0.946128	0.05
51159	0.946128	0.05
51160	0.946128	0.05
51161	0.946128	0.05
51162	0.946131	0.05
51163	0.98453	0.05
51164	1.32276	0.05
51165	0.946131	0.05
51166	0.946132	0.05
51167	0.946132	0.05
51168	0.98453	0.05
51169	1.32276	0.05
51170	1.32276	0.05
51171	1.32276	0.05
51172	0.946132	0.05
51173	0.946132	0.05
51174	1.89227	2.1
51175	1.89227	2.1
51176	1.89227	2.1
51177	1.89227	2.1
51178	1.89227	2.1
51179	1.89227	2.1
51180	1.89226	2.1
51181	1.89226	2.1
51182	1.89226	2.1
51183	1.89226	2.1
51184	1.89226	2.1
51185	1.89226	2.1
51186	1.05556	2.1
51187	1.38859	2.1
51188	1.46996	2.1
51189	1.25364	2.1
51190	2.52552	2.1
51191	0.694296	2.1
51192	0.73498	2.1
51193	0.561881	2.1
51194	0.512189	2.1
51195	0.621304	2.1
51196	0.621304	2.1
51197	0.625865	2.1
51198	0.625865	2.1
51199	0.734979	2.1
51200	0.561881	2.1
51201	0.51219	2.1
51202	0.694296	2.1
51203	0.946126	2.1
51204	0.946126	2.1
51205	0.946126	2.1
51206	0.946126	2.1
51207	0.946126	2.1
51208	0.946126	2.1
51209	0.946126	2.1
51210	0.946126	2.1
51211	0.946132	2.1

51212	0.946132	2.1
51213	0.94612	2.1
51214	0.94612	2.1
51215	1.89225	2.1
51216	1.89225	2.1
51217	1.89225	2.1
51218	1.89225	2.1
51219	1.89225	2.1
51220	1.89225	2.1
51221	0.626814	2.1
51222	0.632155	2.1
51223	0.746234	2.1
51224	0.734974	2.1
51225	0.68202	2.1
51226	0.68202	2.1
51227	0.685108	2.1
51228	0.685108	2.1
51229	0.746239	2.1
51230	0.632159	2.1
51231	0.734969	2.1
51232	0.62681	2.1
51233	0.746235	2.1
51234	0.734975	2.1
51235	0.595081	2.1
51236	0.561877	2.1
51237	0.648584	2.1
51238	0.648584	2.1
51239	0.659528	2.1
51240	0.659528	2.1
51241	0.746239	2.1
51242	0.595085	2.1
51243	0.734969	2.1
51244	0.561874	2.1
51245	1.89249	2.1
51246	2.2137	2.1
51247	1.33125	2.1
51248	1.06499	2.1
51249	2.12571	2.1
51250	1.78918	2.1
51251	0.946131	2.1
51252	0.946131	2.1
51253	0.94613	2.1
51254	0.94613	2.1
51255	0.946132	2.1
51256	0.946132	2.1
51257	0.946129	2.1
51258	0.946129	2.1
51259	0.946131	2.1
51260	0.946131	2.1
51261	0.946131	2.1
51262	0.946131	2.1
51263	1.89227	2.1
51264	1.89227	2.1
51265	1.89227	2.1
51266	1.89227	2.1
51267	3.78453	2.1
51268	1.89226	2.1
51269	1.89226	2.1

51270	1.89226	2.1
51271	1.89226	2.1
51272	1.89226	2.1
51273	1.89226	2.1
51274	2.01388	2.1
51275	1.38757	2.1
51276	1.24229	2.1
51277	1.3309	2.1
51278	1.88011	2.1
51279	2.01388	2.1
51280	2.01388	2.1
51281	2.01555	2.1
51282	2.13879	2.1
51283	1.80766	2.1
51284	4.15267	2.1
51285	0.946133	2.1
51286	0.946133	2.1
51287	0.946132	2.1
51288	0.946132	2.1
51289	0.946132	2.1
51290	0.946132	2.1
51291	0.946133	2.1
51292	0.946133	2.1
51293	0.946132	2.1
51294	0.946132	2.1
51295	0.946132	2.1
51296	0.946132	2.1
51297	1.89226	2.1
51298	1.89226	2.1
51299	1.89226	2.1
51300	1.89226	2.1
51301	1.89226	2.1
51302	1.89226	2.1
51303	0.676745	2.1
51304	0.68382	2.1
51305	1.07048	2.1
51306	1.1327	2.1
51307	0.929324	2.1
51308	0.871438	2.1
51309	0.867362	2.1
51310	0.867362	2.1
51311	1.10666	2.1
51312	0.695036	2.1
51313	0.744098	2.1
51314	1.10144	2.1
51315	2.25168	2.1
51316	2.22499	2.1
51317	2.15929	2.1
51318	2.13879	2.1
51319	2.15929	2.1
51320	2.13879	2.1
51321	2.15314	2.1
51322	1.70302	2.1
51323	2.24694	2.1
51324	1.65245	2.1
51325	3.82722	2.1
51326	1.65245	2.1
51327	1.00371	2.1

51328	1.7437	2.1
51329	1.65748	2.1
51330	1.10459	2.1
51331	1.00371	2.1
51332	2.24694	2.1
51333	1.65246	2.1
51334	2.69773	2.1
51335	1.88129	2.1
51336	4.12824	2.1
51337	1.65246	2.1
51338	1.10459	2.1
51339	1.88129	2.1
51340	1.05914	2.1
51341	2.76496	2.1
51342	1.89226	2.1
51343	1.89226	2.1
51344	1.89226	2.1
51345	1.89226	2.1
51346	3.78452	2.1
51347	0.718577	2.1
51348	0.718577	2.1
51349	0.725249	2.1
51350	0.725249	2.1
51351	0.597916	2.1
51352	0.61752	2.1
51353	0.826306	2.1
51354	0.859194	2.1
51355	0.826306	2.1
51356	0.597916	2.1
51357	0.859194	2.1
51358	0.61752	2.1
51359	1.25898	2.1
51360	1.25898	2.1
51361	0.946128	2.1
51362	0.946128	2.1
51363	1.25897	2.1
51364	1.25585	2.1
51365	0.946131	2.1
51366	0.946131	2.1
51367	0.946129	2.1
51368	1.25586	2.1
51369	1.25898	2.1
51370	0.946129	2.1
51371	1.89226	2.1
51372	1.89226	2.1
51373	1.89226	2.1
51374	1.89226	2.1
51375	3.78452	2.1
51376	0.693258	2.1
51377	0.693258	2.1
51378	0.697356	2.1
51379	0.697356	2.1
51380	0.572479	2.1
51381	0.597916	2.1
51382	0.792698	2.1
51383	0.826306	2.1
51384	0.572478	2.1
51385	0.792697	2.1

51386	0.826307	2.1
51387	0.597917	2.1
51388	1.25586	2.1
51389	1.25586	2.1
51390	0.946128	2.1
51391	0.946128	2.1
51392	1.25585	2.1
51393	1.24344	2.1
51394	0.946131	2.1
51395	0.946131	2.1
51396	0.946129	2.1
51397	1.24344	2.1
51398	0.94613	2.1
51399	1.25586	2.1
51400	1.65151	2.1
51401	1.81835	2.1
51402	1.12064	2.1
51403	1.12064	2.1
51404	1.24965	2.1
51405	1.81835	2.1
51406	1.24965	2.1
51407	1.81835	2.1
51408	1.41077	2.1
51409	2.02687	2.1
51410	3.22911	2.1
51411	1.89122	1.27523
51412	1.38776	2.1
51413	2.37014	0.722812
51414	1.80818	2.1
51415	3.6994	2.06616
51416	2.15466	0.714197
51417	2.00083	2.1
51418	1.80818	1.97485
51419	1.80818	0.685615
51420	2.31988	2.02487
51421	2.31988	0.648177
51422	1.80818	0.815571
51423	1.18041	2.1
51424	2.00082	2.1
51425	1.43652	2.1
51426	3.18123	2.1
51427	1.89226	1.01068
51428	1.89226	1.76169
51429	1.89226	1.15605
51430	1.89226	0.987916
51431	3.78453	1.18796
51432	1.89226	1.75292
51433	1.89226	1.76826
51434	1.89226	1.11835
51435	1.89226	1.16901
51436	3.78452	1.87635
51437	0.80759	1.42068
51438	0.686119	1.75339
51439	0.972147	1.56358
51440	1.00217	1.74535
51441	0.821334	2.00939
51442	0.821334	1.76178
51443	0.866957	1.50842

51444	0.866957	1.41348
51445	0.972145	1.86123
51446	0.686121	2.1
51447	0.807592	1.93496
51448	1.00217	2.1
51449	0.972146	1.58316
51450	1.00217	1.70725
51451	1.26654	1.89546
51452	1.1908	1.58089
51453	1.05501	1.67631
51454	1.05501	2.1
51455	1.10794	1.50727
51456	1.10794	0.739388
51457	0.972146	0.92926
51458	1.26654	1.72695
51459	1.00217	1.53021
51460	1.1908	2.06149
51461	1.89226	1.48284
51462	1.89226	0.697314
51463	1.89226	0.987664
51464	1.89226	1.97221
51465	1.89226	0.437868
51466	1.89226	1.18965
51467	0.972147	1.61091
51468	0.867854	1.84438
51469	1.26132	1.20706
51470	1.26654	1.70069
51471	1.0424	1.87312
51472	1.0424	2.09379
51473	1.092	0.610551
51474	1.092	1.70704
51475	1.26132	1.79285
51476	0.867854	2.1
51477	0.972147	1.99948
51478	1.26654	2.1
51479	1.4375	0.05
51480	1.92998	0.05
51481	1.66953	0.05
51482	1.0953	0.05
51483	3.10703	0.05
51484	1.89226	2.1
51485	1.89226	2.1
51486	1.89226	2.1
51487	1.89226	2.1
51488	3.78452	2.1
51489	2.74034	2.1
51490	2.08808	2.1
51491	2.63859	2.1
51492	2.00343	2.1
51493	4.72667	2.1
51494	0.799825	2.1
51495	0.799825	2.1
51496	0.80211	2.1
51497	0.80211	2.1
51498	0.562428	2.1
51499	0.600223	2.1
51500	1.04404	2.1
51501	1.00171	2.1

51502	1.00171	2.1
51503	0.56243	2.1
51504	0.600225	2.1
51505	1.04404	2.1
51506	0.94613	2.1
51507	0.94613	2.1
51508	0.946128	2.1
51509	0.946128	2.1
51510	0.946127	2.1
51511	0.946127	2.1
51512	0.946131	2.1
51513	0.946131	2.1
51514	0.946129	2.1
51515	0.946129	2.1
51516	0.946129	2.1
51517	0.946129	2.1
51518	1.89226	2.1
51519	1.89226	2.1
51520	1.89226	2.1
51521	1.89226	2.1
51522	3.78452	2.1
51523	2.63859	2.1
51524	2.00343	2.1
51525	2.54388	2.1
51526	1.91548	2.1
51527	4.55407	2.1
51528	1.91548	2.1
51529	2.00342	2.1
51530	1.04909	2.1
51531	1.04909	2.1
51532	1.12486	2.1
51533	1.99124	2.1
51534	0.810942	2.1
51535	0.810942	2.1
51536	0.810942	2.1
51537	0.810942	2.1
51538	1.89226	2.1
51539	1.35169	2.1
51540	1.08131	2.1
51541	1.08132	2.1
51542	1.08132	2.1
51543	0.810944	2.1
51544	0.810944	2.1
51545	1.89226	0.05
51546	1.89226	0.632232
51547	1.89226	0.05
51548	1.89226	1.03979
51549	3.78452	0.0814901
51550	1.7968	0.05
51551	1.49831	0.530116
51552	1.59244	0.05
51553	1.3395	0.160443
51554	3.09074	0.05
51555	1.49831	0.192455
51556	1.16858	2.1
51557	1.33951	0.0986721
51558	1.0361	2.1
51559	2.53442	2.1

51560	1.89226	0.708131
51561	1.89226	0.174562
51562	1.89226	1.93188
51563	1.89226	2.1
51564	1.89226	2.1
51565	1.89226	2.1
51566	1.89223	0.05
51567	1.89223	0.435834
51568	1.89223	0.2366
51569	1.89223	1.115
51570	1.89223	0.05
51571	1.89223	0.303646
51572	1.59241	0.05
51573	1.18952	0.193758
51574	1.33949	0.204889
51575	1.33949	0.05
51576	1.40538	0.221997
51577	1.40538	0.05
51578	1.89226	0.05
51579	1.89226	0.100905
51580	1.89226	0.0545049
51581	1.89226	0.0832403
51582	1.89226	0.0618925
51583	1.89226	0.0988949
51584	1.89226	2.1
51585	1.89226	2.1
51586	1.89226	2.1
51587	1.89226	2.1
51588	3.78452	2.1
51589	1.89226	2.1
51590	1.89226	2.1
51591	1.89226	2.1
51592	1.89226	2.1
51593	3.78452	2.1
51594	2.01867	2.1
51595	1.46955	2.1
51596	1.58476	2.1
51597	1.38189	2.1
51598	2.22153	2.1
51599	2.01867	2.1
51600	2.01867	2.1
51601	2.52881	2.1
51602	2.21732	2.1
51603	2.21732	2.1
51604	2.22154	2.1
51605	2.22154	2.1
51606	1.89226	2.1
51607	1.89226	2.1
51608	1.89226	2.1
51609	1.89226	2.1
51610	1.89226	2.1
51611	1.89226	2.1
51612	1.89226	2.1
51613	1.89226	2.1
51614	1.89226	2.1
51615	1.89226	2.1
51616	1.89226	2.1
51617	1.89226	2.1

51618	2.22153	2.1
51619	1.63361	2.1
51620	1.77336	2.1
51621	1.46955	2.1
51622	2.52534	2.1
51623	2.22153	2.1
51624	2.34431	2.1
51625	1.62905	2.1
51626	1.49693	2.1
51627	2.19197	2.1
51628	3.82103	2.1
51629	1.89226	2.1
51630	1.89226	2.1
51631	1.89226	2.1
51632	1.89226	2.1
51633	3.78453	2.1
51634	0.946128	2.1
51635	0.946128	2.1
51636	1.22372	2.1
51637	1.17331	2.1
51638	0.946131	2.1
51639	0.946131	2.1
51640	1.17331	2.1
51641	1.17331	2.1
51642	0.946131	2.1
51643	1.17331	2.1
51644	1.22372	2.1
51645	0.94613	2.1
51646	1.04041	2.1
51647	1.09599	2.1
51648	0.748462	2.1
51649	0.701976	2.1
51650	0.896701	2.1
51651	0.896701	2.1
51652	0.901262	2.1
51653	0.901262	2.1
51654	1.04041	2.1
51655	0.701977	2.1
51656	0.748463	2.1
51657	1.09599	2.1
51658	1.89226	2.1
51659	1.89226	2.1
51660	1.89226	2.1
51661	1.89226	2.1
51662	3.78453	2.1
51663	0.946128	2.1
51664	0.946128	2.1
51665	1.17331	2.1
51666	1.13785	2.1
51667	0.94613	2.1
51668	0.94613	2.1
51669	1.13785	2.1
51670	1.13785	2.1
51671	1.13785	2.1
51672	0.94613	2.1
51673	0.94613	2.1
51674	1.17331	2.1
51675	2.50381	2.1

51676	1.89226	2.1
51677	1.89226	2.1
51678	2.73387	2.1
51679	4.39607	2.1
51680	1.80045	2.1
51681	1.99777	0.716707
51682	2.45134	2.1
51683	2.12463	2.1
51684	1.70302	2.1
51685	1.42655	2.1
51686	2.20231	2.1
51687	1.70302	2.1
51688	1.93128	2.1
51689	1.66768	2.1
51690	1.24499	1.05114
51691	1.70302	0.993086
51692	2.55572	0.05
51693	1.22811	0.05
51694	1.82584	0.05
51695	1.22811	0.05
51696	2.62764	0.05
51697	1.93147	0.05
51698	2.14513	0.05
51699	1.89226	0.05
51700	2.66481	0.05
51701	1.89226	0.05
51702	2.66481	0.05
51703	1.89226	0.05
51704	2.11649	2.1
51705	1.89226	2.1
51706	1.89226	2.1
51707	1.89226	2.1
51708	2.15426	2.1
51709	2.15426	2.1
51710	2.06776	2.1
51711	1.89226	2.1
51712	1.89226	2.1
51713	1.89226	2.1
51714	2.11649	2.1
51715	2.11649	2.1
51716	1.89226	2.1
51717	1.89226	2.1
51718	1.89226	2.1
51719	1.89226	2.1
51720	1.89226	2.1
51721	1.89226	2.1
51722	0.998134	2.1
51723	0.918904	2.1
51724	0.894708	2.1
51725	0.758742	2.1
51726	0.864276	2.1
51727	0.864276	2.1
51728	0.8926	2.1
51729	0.8926	2.1
51730	0.998134	2.1
51731	0.894709	2.1
51732	0.758742	2.1
51733	0.918904	2.1

51734	1.89226	2.1
51735	1.89226	2.1
51736	1.89226	2.1
51737	1.89226	2.1
51738	1.89226	2.1
51739	1.89226	2.1
51740	1.89226	2.1
51741	1.89226	2.1
51742	1.89226	2.1
51743	1.89226	2.1
51744	1.89226	2.1
51745	1.89226	2.1
51746	1.99627	2.1
51747	1.61047	2.1
51748	2.0815	2.1
51749	2.07569	2.1
51750	3.75063	2.1
51751	0.848077	2.1
51752	1.07748	2.1
51753	0.802498	2.1
51754	0.626808	2.1
51755	0.875552	2.1
51756	0.737527	2.1
51757	0.835602	2.1
51758	0.835602	2.1
51759	0.983245	2.1
51760	0.781108	2.1
51761	0.872306	2.1
51762	0.601497	2.1
51763	2.74745	2.1
51764	2.73988	2.1
51765	2.6521	2.1
51766	2.67792	2.1
51767	5.39198	2.1
51768	2.73988	2.1
51769	2.57466	2.1
51770	2.67791	2.1
51771	2.55653	2.1
51772	5.29641	2.1
51773	2.6521	2.1
51774	2.64148	2.1
51775	2.67792	2.1
51776	2.67792	2.1
51777	2.56953	2.1
51778	2.56953	2.1
51779	2.64148	2.1
51780	2.55653	2.1
51781	2.57929	2.1
51782	2.57929	2.1
51783	2.67792	2.1
51784	2.67792	2.1
51785	1.89226	0.229302
51786	1.89226	0.05
51787	1.89226	0.144566
51788	1.89226	0.05
51789	3.78453	0.05
51790	1.68703	0.23791
51791	2.50385	0.05

51792	1.45958	0.0668508
51793	2.0815	0.05
51794	3.93806	0.05
51795	2.50385	0.05
51796	2.57414	0.05
51797	2.0815	0.05
51798	2.39921	0.0545982
51799	4.91181	0.05
51800	1.89226	0.165575
51801	1.89226	0.05
51802	1.89226	0.05
51803	1.89226	0.05
51804	3.78453	0.05
51805	1.89226	0.300139
51806	1.89226	0.05
51807	1.89226	0.05
51808	1.89226	0.054318
51809	1.89226	0.05
51810	1.89226	0.138891
51811	1.23013	0.05
51812	2.0815	0.05
51813	1.90167	0.0620541
51814	1.71243	0.05
51815	1.45887	0.0690203
51816	1.31369	0.05
51817	2.0815	0.05
51818	2.39921	0.0509023
51819	1.7115	0.05
51820	2.2421	0.05
51821	4.09829	0.05
51822	1.89227	0.05
51823	1.89227	0.05
51824	1.89227	0.05
51825	1.89227	0.05
51826	3.78453	0.05
51827	2.39697	0.05
51828	1.85594	0.05
51829	1.91161	0.05
51830	2.49867	0.05
51831	4.35461	0.05
51832	1.12035	0.05
51833	1.85594	0.05
51834	1.91161	0.05
51835	1.15483	0.05
51836	3.01077	0.05
51837	1.89226	0.05
51838	1.89226	0.05
51839	1.89226	0.05
51840	1.89226	0.05
51841	1.89226	0.05
51842	1.89226	0.05
51843	1.89226	0.05
51844	1.89226	0.05
51845	1.89226	0.05
51846	1.89226	0.05
51847	1.89226	0.05
51848	1.89226	0.05
51849	0.936516	0.05

51850	1.03496	0.05
51851	1.22607	0.05
51852	1.28058	0.05
51853	1.06317	0.05
51854	1.06317	0.05
51855	1.19735	0.05
51856	1.0813	0.05
51857	0.858168	0.05
51858	1.32278	0.05
51859	0.957674	0.05
51860	1.25178	0.05
51861	1.92659	0.05
51862	0.998957	0.05
51863	0.998957	0.05
51864	1.15481	0.05
51865	1.6031	0.05
51866	1.74459	0.05
51867	0.946119	0.05
51868	0.946119	0.05
51869	0.946118	0.05
51870	0.946118	0.05
51871	0.946118	0.05
51872	0.946118	0.05
51873	0.946119	0.05
51874	0.946119	0.05
51875	0.946131	0.05
51876	0.946131	0.05
51877	0.946106	0.05
51878	0.946106	0.05
51879	0.946118	0.05
51880	0.946118	0.05
51881	0.946119	0.05
51882	0.946119	0.05
51883	0.946118	0.05
51884	0.946118	0.05
51885	0.946119	0.05
51886	0.946119	0.05
51887	0.946131	0.05
51888	0.946131	0.05
51889	0.946106	0.05
51890	0.946106	0.05
51891	2.47164	0.101011
51892	2.83689	0.05
51893	2.73872	0.120849
51894	2.41632	0.05
51895	5.21036	0.0522523
51896	0.946133	0.0703985
51897	0.946133	0.121214
51898	1.41845	0.0507842
51899	1.36936	0.0999919
51900	1.41845	0.05
51901	1.41845	0.05
51902	0.946132	0.119621
51903	0.946132	0.118727
51904	0.946132	0.0780611
51905	1.36936	0.0537361
51906	1.41845	0.0728351
51907	0.946133	0.0970689

51908	1.36936	0.05
51909	1.27581	0.0512024
51910	1.20816	0.05
51911	1.10464	0.0768788
51912	1.24454	0.05
51913	1.24454	0.05
51914	1.23943	0.0546856
51915	1.23943	0.0733977
51916	1.10465	0.05
51917	1.27581	0.05
51918	1.36936	0.05
51919	1.20816	0.05
51920	0.946131	2.1
51921	0.946131	2.1
51922	1.37277	2.1
51923	1.37277	2.1
51924	1.37277	2.1
51925	1.27186	2.1
51926	0.946131	2.1
51927	0.946131	2.1
51928	0.946131	2.1
51929	1.37277	2.1
51930	0.946131	2.1
51931	1.27186	2.1
51932	2.74554	2.1
51933	1.23712	2.1
51934	2.80992	2.1
51935	2.54371	2.1
51936	1.50333	2.1
51937	1.23712	2.1
51938	2.77314	2.1
51939	2.33316	2.1
51940	2.28748	2.1
51941	2.28748	2.1
51942	2.81275	2.1
51943	2.81275	2.1
51944	2.28749	2.1
51945	1.45887	2.1
51946	2.33317	2.1
51947	1.50022	2.1
51948	3.79204	2.1
51949	2.37364	2.1
51950	2.33317	2.1
51951	1.5362	2.1
51952	1.5362	2.1
51953	1.50022	2.1
51954	2.33317	2.1
51955	1.89226	0.0944788
51956	1.89226	0.103087
51957	1.89226	0.073322
51958	1.89226	0.107766
51959	1.89226	0.0813257
51960	1.89226	0.132209
51961	0.946128	0.05
51962	0.946128	0.0527437
51963	1.0644	0.05
51964	0.946133	0.05
51965	1.0644	0.05

51966	0.94613	0.05
51967	0.946131	0.0555554
51968	0.946131	0.05
51969	1.0644	0.05
51970	1.0644	0.05
51971	0.946131	0.05
51972	0.946131	0.05
51973	1.89226	1.67976
51974	1.89226	0.0699856
51975	1.89226	0.28325
51976	1.89226	0.05
51977	3.78452	0.0575681
51978	1.14599	1.44193
51979	1.19993	0.05
51980	1.00065	0.147785
51981	1.07497	0.05
51982	2.22096	0.0712066
51983	0.546551	0.0652011
51984	0.546551	0.253404
51985	0.54987	0.0754933
51986	0.54987	0.730281
51987	0.503067	0.05
51988	0.558937	0.0520739
51989	0.599966	0.0511585
51990	0.537484	0.202276
51991	0.503067	0.05
51992	0.537485	0.05
51993	0.599966	0.200236
51994	0.558936	0.05
51995	0.946132	0.0799921
51996	0.946132	0.3075
51997	0.94613	0.05
51998	0.94613	0.780257
51999	0.946131	0.137564
52000	0.946131	0.0636437
52001	0.946131	0.226353
52002	0.946131	0.32875
52003	0.946131	0.05
52004	0.946131	0.05
52005	0.946131	0.275583
52006	0.946131	0.188204
52007	2.19104	2.1
52008	2.5392	2.1
52009	2.53302	2.1
52010	2.19226	2.1
52011	4.73146	2.1
52012	1.09552	2.1
52013	1.09613	2.1
52014	0.802484	2.1
52015	0.807941	2.1
52016	0.95052	2.1
52017	0.95052	2.1
52018	0.952944	2.1
52019	0.952944	2.1
52020	1.09613	2.1
52021	0.807941	2.1
52022	0.802484	2.1
52023	1.09552	2.1

52024	2.533	2.1
52025	2.18531	2.1
52026	2.19224	2.1
52027	2.19224	2.1
52028	2.51603	2.1
52029	2.51603	2.1
52030	1.09266	2.1
52031	1.09612	2.1
52032	0.807935	2.1
52033	0.81091	2.1
52034	0.951897	2.1
52035	0.951897	2.1
52036	0.955134	2.1
52037	0.955134	2.1
52038	0.810915	2.1
52039	1.09266	2.1
52040	1.09611	2.1
52041	0.80793	2.1
52042	2.12741	0.489373
52043	2.79312	0.206758
52044	2.77591	1.025
52045	2.10714	0.198869
52046	4.90026	0.114914
52047	2.12741	0.256429
52048	1.20859	0.05
52049	1.21073	1.20634
52050	2.10714	0.335968
52051	3.31573	0.538588
52052	1.38708	0.391294
52053	1.38795	0.0906702
52054	1.05331	0.288839
52055	1.05357	0.295129
52056	1.22049	0.318236
52057	1.22049	0.343367
52058	1.22078	1.5491
52059	1.22078	0.0513135
52060	1.05331	0.210107
52061	1.38709	0.244991
52062	1.38795	0.20165
52063	1.05357	0.445878
52064	2.10662	0.754683
52065	2.10714	0.146446
52066	2.12499	0.211428
52067	1.22857	0.324023
52068	1.21073	0.12758
52069	1.21073	0.0527051
52070	1.82014	0.05
52071	2.08757	0.05
52072	1.81292	0.05
52073	1.67485	0.05
52074	3.63306	0.05
52075	1.82013	0.05
52076	1.21748	0.05
52077	1.29891	0.05
52078	1.4442	0.05
52079	1.67484	0.05
52080	1.82013	0.05
52081	1.81292	0.05

52082	1.4516	0.05
52083	1.39633	0.05
52084	1.67485	0.05
52085	1.5344	0.05
52086	1.81292	0.05
52087	1.67484	0.05
52088	1.00982	0.05
52089	0.994235	0.05
52090	1.21748	0.05
52091	1.45159	0.05
52092	1.67484	0.05
52093	1.55216	2.1
52094	1.34407	2.1
52095	1.34407	2.1
52096	1.52008	2.1
52097	1.81783	2.1
52098	1.99384	2.1
52099	1.90817	2.1
52100	2.03975	2.1
52101	1.97317	2.1
52102	1.81783	2.1
52103	3.85758	2.1
52104	0.599049	2.1
52105	0.660673	2.1
52106	0.785063	2.1
52107	0.924278	2.1
52108	0.760416	2.1
52109	0.760416	2.1
52110	0.719088	2.1
52111	0.694532	2.1
52112	0.522441	2.1
52113	0.796789	2.1
52114	0.655207	2.1
52115	0.886155	2.1
52116	1.97317	2.1
52117	1.81783	2.1
52118	1.65098	2.1
52119	1.91767	2.1
52120	3.7355	2.1
52121	1.15038	1.13359
52122	1.28649	2.08757
52123	1.38986	1.59409
52124	1.38986	2.1
52125	1.07866	1.79974
52126	1.07866	2.1
52127	0.664498	0.143022
52128	0.664498	2.1
52129	0.675122	2.1
52130	0.675122	2.1
52131	0.69493	0.626141
52132	0.764429	1.8547
52133	0.623438	1.68561
52134	0.575191	2.1
52135	0.764429	1.06032
52136	0.623438	0.801045
52137	0.575191	2.1
52138	0.69493	2.00371
52139	1.89226	0.79513

52140	1.89226	0.0645804
52141	1.89226	0.8435
52142	1.89226	0.082745
52143	3.78453	0.05
52144	2.50075	0.165164
52145	2.15276	0.0596935
52146	2.24902	0.86823
52147	2.30422	0.068816
52148	4.40178	0.05
52149	1.70424	0.283994
52150	1.34494	0.541085
52151	2.15276	0.05
52152	2.15276	0.28199
52153	2.0793	0.05
52154	1.63078	0.0923381
52155	1.89226	0.05
52156	1.89226	0.450097
52157	1.89226	1.69644
52158	1.89226	0.05
52159	1.89226	1.14007
52160	1.89226	0.212862
52161	1.89226	0.229896
52162	1.89226	0.169438
52163	1.89226	0.05
52164	1.89226	0.05
52165	3.78452	0.05
52166	2.24902	0.245588
52167	1.91497	0.05
52168	1.97135	0.431648
52169	1.73957	0.05
52170	3.98859	0.05
52171	1.73957	0.05
52172	1.34494	0.259135
52173	1.73957	1.65067
52174	1.69006	0.05
52175	1.3126	0.532007
52176	1.34848	0.163769
52177	0.946132	0.05
52178	0.946132	0.627327
52179	0.946132	0.05
52180	0.946132	1.71734
52181	0.946132	0.893099
52182	0.946132	0.23058
52183	0.946131	0.05
52184	0.946131	0.05
52185	0.946132	0.05
52186	0.946132	0.05
52187	0.946132	0.0827242
52188	0.946132	0.202897
52189	1.89226	2.1
52190	1.89226	1.97333
52191	1.89226	2.1
52192	1.89226	2.02929
52193	3.78453	2.1
52194	1.89226	2.1
52195	2.82624	1.75968
52196	1.89226	2.1
52197	2.02536	1.86285

52198	1.89226	2.06231
52199	2.02536	1.60961
52200	2.25027	0.05
52201	1.1706	0.05
52202	2.02733	0.05
52203	2.02733	0.05
52204	1.61462	0.05
52205	1.69832	0.05
52206	1.89226	0.05
52207	1.89226	0.05
52208	1.89226	0.05
52209	1.89226	0.05
52210	3.78452	0.05
52211	0.946131	0.05
52212	0.946131	0.05
52213	1.18182	0.05
52214	1.18182	0.05
52215	0.973819	0.05
52216	0.94613	0.05
52217	1.18182	0.05
52218	0.94613	0.05
52219	0.973819	0.05
52220	1.18182	0.05
52221	0.94613	0.05
52222	0.94613	0.05
52223	2.23944	2.1
52224	1.52903	2.1
52225	1.35977	2.1
52226	2.04847	2.1
52227	3.5775	2.1
52228	2.23944	2.1
52229	2.34795	2.1
52230	2.34795	2.1
52231	2.48025	2.1
52232	2.04847	2.1
52233	2.18077	2.1
52234	1.89226	2.1
52235	1.89226	2.1
52236	1.89226	2.1
52237	1.89226	2.1
52238	3.78452	2.1
52239	0.946127	2.1
52240	0.946127	2.1
52241	0.946131	2.1
52242	0.946131	2.1
52243	0.946128	2.1
52244	0.946128	2.1
52245	0.94613	2.1
52246	0.94613	2.1
52247	0.946129	2.1
52248	0.946129	2.1
52249	0.946129	2.1
52250	0.946129	2.1
52251	1.26956	2.1
52252	2.04847	2.1
52253	1.49576	2.1
52254	1.22377	2.1
52255	1.91172	2.1

52256	1.50198	2.1
52257	1.48568	2.1
52258	2.34795	2.1
52259	2.04847	2.1
52260	2.04847	2.1
52261	2.21155	2.1
52262	2.21155	2.1
52263	1.89226	2.1
52264	1.89226	2.1
52265	1.89226	2.1
52266	1.89226	2.1
52267	1.89226	2.1
52268	1.89226	2.1
52269	0.810943	2.1
52270	0.810943	2.1
52271	0.810944	2.1
52272	0.810944	2.1
52273	0.810957	2.1
52274	0.810957	2.1
52275	1.89226	2.1
52276	1.35168	2.1
52277	1.08132	2.1
52278	1.08132	2.1
52279	1.0813	2.1
52280	0.946132	0.05
52281	1.33855	0.05
52282	0.946131	0.05
52283	1.38204	0.05
52284	0.946131	0.05
52285	0.946131	0.05
52286	1.38204	0.05
52287	1.38204	0.05
52288	0.946131	0.05
52289	0.946131	0.05
52290	1.33855	0.05
52291	1.38204	0.05
52292	1.89226	0.05
52293	2.76407	0.05
52294	1.89226	0.05
52295	2.72769	0.05
52296	1.89226	0.05
52297	2.72769	0.05
52298	2.03914	0.05
52299	1.67727	0.05
52300	1.94831	0.05
52301	1.81591	0.05
52302	2.32098	0.05
52303	2.32098	0.05
52304	0.801647	0.05
52305	1.11086	0.05
52306	0.838634	0.05
52307	1.16049	0.05
52308	0.977891	0.05
52309	0.977891	0.05
52310	0.984246	0.05
52311	0.984246	0.05
52312	1.11086	0.05
52313	1.16049	0.05

52314	0.801647	0.05
52315	0.838634	0.05
52316	0.946132	0.05
52317	0.946132	0.05
52318	0.946131	0.05
52319	0.946131	0.05
52320	0.946131	0.05
52321	0.946131	0.05
52322	0.946132	0.05
52323	0.946132	0.05
52324	0.946131	0.05
52325	0.946131	0.05
52326	0.946131	0.05
52327	0.946131	0.05
52328	0.946131	0.05
52329	0.946131	0.05
52330	0.946131	0.05
52331	0.946131	0.05
52332	0.946131	0.05
52333	0.946131	0.05
52334	0.946131	0.05
52335	0.946131	0.05
52336	0.946131	0.05
52337	0.946131	0.05
52338	0.946131	0.05
52339	0.946131	0.05
52340	2.0815	2.1
52341	1.20787	2.1
52342	1.92277	2.1
52343	1.3227	2.1
52344	3.29401	2.1
52345	2.16633	2.1
52346	2.13642	2.1
52347	1.40846	2.1
52348	1.40846	2.1
52349	1.3227	2.1
52350	2.08058	2.1
52351	0.938106	2.1
52352	0.790258	2.1
52353	0.742975	2.1
52354	0.742975	2.1
52355	0.874389	2.1
52356	1.17364	2.1
52357	0.525948	2.1
52358	0.52513	2.1
52359	0.553732	2.1
52360	0.876996	2.1
52361	0.530954	2.1
52362	1.095	2.1
52363	1.00833	2.1
52364	1.70608	2.1
52365	1.33385	2.1
52366	1.33385	2.1
52367	1.07722	2.1
52368	1.07722	2.1
52369	0.953523	0.475508
52370	1.7457	0.343918
52371	1.03218	0.422566

52372	1.03218	0.431824
52373	1.79675	0.418865
52374	1.79675	0.433631
52375	1.13676	0.467559
52376	1.18136	0.210357
52377	0.87285	0.356798
52378	0.898375	0.479532
52379	1.03191	0.326235
52380	1.03191	0.189678
52381	1.0223	0.425732
52382	1.0223	0.486345
52383	1.18136	0.333253
52384	0.898374	0.277345
52385	0.87285	0.337748
52386	1.13676	0.314437
52387	2.36273	0.307881
52388	1.46965	0.186008
52389	1.79675	0.227023
52390	1.79675	0.13495
52391	2.43417	0.314778
52392	2.43417	0.191095
52393	1.4714	2.1
52394	1.65434	2.1
52395	1.62322	0.368532
52396	1.86068	1.98702
52397	3.27756	1.78762
52398	1.89226	2.1
52399	1.89226	2.1
52400	1.89226	0.430178
52401	1.89226	1.17164
52402	3.78453	1.37319
52403	1.89226	1.52519
52404	1.89226	2.1
52405	1.89226	0.849151
52406	1.89226	2.1
52407	3.78453	1.95779
52408	1.65434	1.57024
52409	1.60647	2.1
52410	1.86068	1.17842
52411	1.82649	2.1
52412	3.48083	2.1
52413	1.62322	2.1
52414	1.86068	2.1
52415	1.84368	0.403608
52416	2.18862	1.3812
52417	3.81184	1.71477
52418	1.89226	2.1
52419	1.89226	2.01626
52420	1.89226	0.178876
52421	1.89226	1.55338
52422	3.78453	1.60555
52423	1.89226	1.64549
52424	1.89226	2.1
52425	1.89226	0.955933
52426	1.89226	2.1
52427	3.78453	2.1
52428	1.86068	1.26504
52429	1.82649	2.1

52430	2.18862	0.872498
52431	2.1128	2.1
52432	4.01511	1.91994
52433	1.74351	2.1
52434	1.13854	2.1
52435	1.40233	2.1
52436	1.922 2.1	
52437	3.14584	2.1
52438	1.922 2.1	
52439	2.00775	2.1
52440	1.7435	2.1
52441	2.09224	2.1
52442	2.00775	2.1
52443	2.09295	2.1
52444	1.69858	2.1
52445	1.922 2.1	
52446	1.40233	2.1
52447	1.40233	2.1
52448	2.18238	2.1
52449	2.18238	2.1
52450	1.00387	2.1
52451	1.10886	2.1
52452	0.961001	2.1
52453	1.09119	2.1
52454	1.02868	2.1
52455	1.02868	2.1
52456	1.04118	2.1
52457	1.04118	2.1
52458	1.10886	2.1
52459	1.09119	2.1
52460	0.960999	2.1
52461	1.00388	2.1
52462	0.885958	0.302165
52463	0.885958	0.183301
52464	0.875826	0.150104
52465	0.875826	0.456851
52466	0.913965	0.492184
52467	0.598609	0.179462
52468	1.16317	0.2168
52469	0.868115	0.155453
52470	0.868115	0.324505
52471	0.598609	0.253986
52472	1.16317	0.202003
52473	0.913965	0.218978
52474	0.946131	0.322165
52475	0.946131	0.265697
52476	0.946131	0.447803
52477	0.946131	0.260721
52478	0.946131	0.380228
52479	0.946131	0.337097
52480	0.946131	0.269501
52481	0.946131	0.249732
52482	0.946131	0.34454
52483	0.946131	0.391297
52484	0.946131	0.298243
52485	0.946131	0.30322
52486	0.946133	0.05
52487	0.946133	0.05

52488	1.06246	0.05
52489	1.06246	0.05
52490	0.973851	0.05
52491	0.946133	0.05
52492	1.06246	0.05
52493	0.946132	0.05
52494	0.97385	0.05
52495	1.06246	0.05
52496	0.946133	0.05
52497	0.946133	0.05
52498	1.89226	0.05
52499	1.89226	0.05
52500	1.89226	0.05
52501	1.89226	0.05
52502	1.89226	0.05
52503	1.89226	0.05
52504	0.946131	0.05
52505	0.946131	0.05
52506	0.946127	0.05
52507	0.946127	0.05
52508	0.946131	0.05
52509	0.946131	0.05
52510	0.946127	0.05
52511	0.946127	0.05
52512	0.946128	0.05
52513	0.946128	0.05
52514	0.946129	0.05
52515	0.946129	0.05
52516	0.946132	0.05
52517	0.946132	0.05
52518	1.32446	0.05
52519	0.794912	0.05
52520	1.32429	0.05
52521	0.946131	0.05
52522	1.32446	0.05
52523	0.94613	0.05
52524	1.32429	0.05
52525	0.794915	0.05
52526	0.94613	0.05
52527	0.94613	0.05
52528	1.89226	0.05
52529	2.36365	0.05
52530	2.64857	0.05
52531	1.89226	0.05
52532	2.64857	0.05
52533	1.89226	0.05
52534	1.18463	2.1
52535	1.27174	2.1
52536	0.946129	2.1
52537	0.946129	2.1
52538	0.946131	2.1
52539	0.946131	2.1
52540	1.27174	2.1
52541	1.27174	2.1
52542	1.18463	2.1
52543	0.94613	2.1
52544	0.946131	2.1
52545	1.27174	2.1

52546	2.57997	2.1
52547	1.89226	2.1
52548	1.89226	2.1
52549	1.89226	2.1
52550	2.41915	2.1
52551	2.41915	2.1
52552	1.89226	2.1
52553	1.89226	2.1
52554	1.89226	2.1
52555	1.89226	2.1
52556	3.78452	2.1
52557	1.08807	2.1
52558	1.18463	2.1
52559	0.946129	2.1
52560	0.946129	2.1
52561	0.946131	2.1
52562	0.946131	2.1
52563	1.18463	2.1
52564	1.18463	2.1
52565	0.946131	2.1
52566	1.08807	2.1
52567	1.18463	2.1
52568	0.94613	2.1
52569	1.07017	2.1
52570	1.07017	2.1
52571	0.946131	2.1
52572	0.946131	2.1
52573	0.946132	2.1
52574	0.946132	2.1
52575	1.20957	2.1
52576	1.07017	2.1
52577	1.07017	2.1
52578	0.946132	2.1
52579	0.946131	2.1
52580	1.20957	2.1
52581	1.89226	2.1
52582	1.89226	2.1
52583	1.89226	2.1
52584	1.89226	2.1
52585	1.89226	2.1
52586	1.89226	2.1
52587	1.16181	0.05
52588	1.0227	0.05
52589	1.0227	0.05
52590	0.970959	0.05
52591	1.24295	0.05
52592	1.19121	0.05
52593	1.12076	2.1
52594	2.53614	2.1
52595	1.28413	2.1
52596	1.28413	2.1
52597	2.36423	2.1
52598	2.36423	2.1
52599	1.89226	2.1
52600	1.89226	2.1
52601	1.89226	2.1
52602	1.89226	2.1
52603	3.78452	2.1

52604	1.22557	2.1
52605	2.771	2.1
52606	2.73012	2.1
52607	2.73012	2.1
52608	1.33356	2.1
52609	1.33356	2.1
52610	0.998682	2.1
52611	2.36423	2.1
52612	1.12076	2.1
52613	1.12076	2.1
52614	2.13757	2.1
52615	2.13757	2.1
52616	1.89226	2.1
52617	1.89226	2.1
52618	1.89226	2.1
52619	1.89226	2.1
52620	1.89226	2.1
52621	1.89226	2.1
52622	1.13987	2.1
52623	2.73011	2.1
52624	2.70875	2.1
52625	2.70875	2.1
52626	1.22557	2.1
52627	1.22557	2.1
52628	1.89227	2.1
52629	2.79243	2.1
52630	2.6625	2.1
52631	2.6625	2.1
52632	1.89227	2.1
52633	1.89227	2.1
52634	1.89226	2.1
52635	2.55971	2.1
52636	1.89226	2.1
52637	1.89226	2.1
52638	2.68704	2.1
52639	2.68704	2.1
52640	2.22043	2.04066
52641	2.38975	1.85281
52642	2.28696	1.16345
52643	2.48682	0.714389
52644	4.67671	1.07182
52645	2.38976	1.10445
52646	2.31988	1.82216
52647	2.48683	0.682287
52648	2.4594	1.50816
52649	4.84915	1.50011
52650	2.28696	1.93594
52651	2.48682	1.72359
52652	2.33379	2.1
52653	2.56149	0.447621
52654	4.84845	1.52031
52655	2.48683	1.71138
52656	2.4594	1.65901
52657	2.5615	0.511535
52658	2.57957	1.29465
52659	5.0209	0.934044
52660	1.06659	2.1
52661	1.04291	0.771687

52662	1.36595	0.498355
52663	1.36595	2.1
52664	1.02761	2.1
52665	1.32697	0.338987
52666	0.946132	1.89926
52667	0.946132	1.77893
52668	1.36956	2.1
52669	1.1229	2.1
52670	1.1229	1.6972
52671	1.1229	2.1
52672	0.946132	0.884933
52673	0.946132	2.1
52674	0.946132	2.1
52675	1.36956	2.1
52676	1.1229	2.1
52677	0.946133	2.1
52678	0.946133	0.956092
52679	0.946133	0.814837
52680	0.946128	2.1
52681	0.946128	2.1
52682	0.946131	2.1
52683	0.946131	1.58778
52684	0.946131	0.183378
52685	0.946131	2.1
52686	0.94613	1.99749
52687	0.94613	1.8934
52688	0.946131	1.70604
52689	0.946131	1.70636
52690	0.966427	1.17035
52691	0.802265	2.05119
52692	0.803714	2.1
52693	0.808476	1.79951
52694	1.94371	2.1
52695	1.36851	2.1
52696	0.853864	2.1
52697	1.26635	1.76064
52698	1.09085	1.47209
52699	0.857653	2.1
52700	0.630243	1.88688
52701	1.20132	0.05
52702	1.24295	0.05
52703	1.24295	0.05
52704	1.16182	0.05
52705	1.28465	0.05
52706	1.20351	0.05
52707	0.600658	0.05
52708	0.642323	0.05
52709	0.54424	0.05
52710	0.576779	0.05
52711	0.590985	0.05
52712	0.590985	0.05
52713	0.586452	0.05
52714	0.586452	0.05
52715	0.642323	0.05
52716	0.576779	0.05
52717	0.600658	0.05
52718	0.54424	0.05
52719	1.28463	0.05

52720	1.24294	0.05
52721	1.12328	0.05
52722	1.15505	0.05
52723	2.39798	0.05
52724	1.98431	0.05
52725	2.18735	0.05
52726	1.98431	0.05
52727	1.70303	0.05
52728	1.70302	0.05
52729	1.37788	0.05
52730	1.89226	0.05
52731	2.37417	0.05
52732	1.89226	0.05
52733	2.18735	0.05
52734	1.89226	0.05
52735	2.18735	0.05
52736	1.89226	0.05
52737	1.89226	0.19444
52738	1.89226	0.05
52739	1.89226	0.0571422
52740	3.78452	0.0704028
52741	1.8613	0.05
52742	2.03576	0.177718
52743	1.61652	0.05
52744	1.83082	2.1
52745	3.65228	0.05
52746	2.03576	0.168883
52747	2.05257	2.1
52748	1.83082	2.1
52749	1.88487	2.1
52750	3.92064	2.1
52751	1.89226	0.181259
52752	1.89226	1.33712
52753	1.89226	0.05
52754	1.89226	2.1
52755	3.78453	0.495306
52756	0.946131	0.27854
52757	0.946131	0.05
52758	0.94613	0.05
52759	0.94613	0.240385
52760	0.946129	0.0553926
52761	0.946129	0.27256
52762	0.946132	0.0958968
52763	0.946132	0.05
52764	0.94613	0.05
52765	0.94613	0.05
52766	0.946131	0.05
52767	0.946131	0.05
52768	0.804708	2.1
52769	0.804708	0.05
52770	0.815274	0.354699
52771	0.815274	0.05
52772	0.83277	0.247046
52773	0.915407	2.1
52774	0.80826	0.0586538
52775	0.704574	0.05
52776	0.832771	0.0837197
52777	0.704573	0.107513

52778	0.808259	0.183238
52779	0.915409	0.154797
52780	1.83082	2.1
52781	1.88487	2.1
52782	1.66555	0.283841
52783	1.75835	2.1
52784	3.55042	2.1
52785	0.810947	0.05
52786	0.810947	1.30042
52787	0.810947	0.0525617
52788	0.810947	0.221109
52789	0.810934	0.503551
52790	0.810934	0.507809
52791	1.89227	2.1
52792	1.3517	0.831421
52793	1.08132	0.489671
52794	1.08132	1.20809
52795	1.08133	0.295655
52796	1.89226	2.1
52797	1.70303	2.1
52798	1.89226	2.1
52799	2.24712	2.1
52800	2.07363	2.1
52801	1.74617	2.1
52802	1.89226	2.1
52803	1.89226	1.49349
52804	1.89226	0.956897
52805	1.89226	2.1
52806	3.78452	1.7783
52807	1.50108	2.1
52808	1.43894	2.1
52809	1.8322	0.579752
52810	1.40967	2.1
52811	1.50108	2.1
52812	1.07854	2.1
52813	1.89226	0.65421
52814	1.89226	2.1
52815	1.89226	2.1
52816	1.89226	0.266986
52817	3.78452	2.0027
52818	1.89226	2.1
52819	1.89226	1.40214
52820	1.89226	1.4938
52821	1.89226	2.1
52822	3.78453	1.9322
52823	1.54457	2.1
52824	1.35097	1.95018
52825	1.70754	0.905118
52826	1.29629	2.1
52827	1.54457	2.1
52828	1.0305	2.1
52829	0.731763	2.1
52830	1.00043	2.1
52831	1.13883	1.01855
52832	0.939026	1.95233
52833	1.04003	1.01007
52834	0.853868	2.1
52835	0.927188	2.1

52836	0.927188	0.214408
52837	0.861565	1.77096
52838	1.13882	2.02431
52839	0.731769	2.04302
52840	0.89319	2.1
52841	1.46568	2.1
52842	1.20761	2.1
52843	1.51916	2.1
52844	1.36261	2.1
52845	2.72677	2.1
52846	1.51916	2.1
52847	1.60532	2.1
52848	1.36261	2.1
52849	1.36261	2.1
52850	1.64892	2.1
52851	1.64892	2.1
52852	1.3626	2.1
52853	0.98488	2.1
52854	1.60532	2.1
52855	1.32414	2.1
52856	2.68675	2.1
52857	1.78711	2.1
52858	1.14825	2.1
52859	2.16535	2.1
52860	1.6067	2.1
52861	3.3136	2.1
52862	1.89226	2.1
52863	1.89226	2.1
52864	1.89226	2.1
52865	1.89226	2.1
52866	3.78452	2.1
52867	2.34552	2.1
52868	1.53521	2.1
52869	1.53521	2.1
52870	1.60636	2.1
52871	1.92797	2.1
52872	1.99589	2.1
52873	1.34771	2.1
52874	1.34771	2.1
52875	0.946128	2.1
52876	0.946128	2.1
52877	1.3477	2.1
52878	1.31776	2.1
52879	0.946131	2.1
52880	0.946131	2.1
52881	0.94613	2.1
52882	1.31776	2.1
52883	0.946129	2.1
52884	1.3477	2.1
52885	1.89226	2.1
52886	1.89226	2.1
52887	1.89226	2.1
52888	1.89226	2.1
52889	3.78452	2.1
52890	0.92843	2.1
52891	0.672114	2.1
52892	0.89514	2.1
52893	0.89514	2.1

52894	0.720104	2.1
52895	0.755753	2.1
52896	0.789158	2.1
52897	0.967302	2.1
52898	1.06565	2.1
52899	0.7397	2.1
52900	0.758893	2.1
52901	0.94672	2.1
52902	1.89226	2.1
52903	2.588	2.1
52904	2.588	2.1
52905	2.63552	2.1
52906	1.89226	2.1
52907	1.93978	2.1
52908	1.49347	0.05
52909	2.67757	0.05
52910	1.80061	0.05
52911	2.27056	0.05
52912	4.07117	0.05
52913	1.1016	0.05
52914	2.27057	0.05
52915	1.49348	0.05
52916	1.81893	0.05
52917	1.49348	0.05
52918	1.81893	0.05
52919	1.81893	0.05
52920	2.82339	0.05
52921	2.27057	0.05
52922	2.25405	0.05
52923	4.52462	0.05
52924	1.89226	0.05
52925	1.89226	0.05
52926	1.89226	0.05
52927	1.89226	0.05
52928	1.89226	0.05
52929	1.89226	0.05
52930	1.89226	0.05
52931	1.89226	0.05
52932	1.89226	0.05
52933	1.89226	0.05
52934	3.78453	0.05
52935	1.89226	0.05
52936	1.89226	0.05
52937	1.89226	0.05
52938	1.89226	0.05
52939	3.78452	0.05
52940	1.89226	0.05
52941	1.89226	0.116533
52942	1.89226	0.05
52943	1.89226	0.05
52944	3.78451	0.05
52945	1.45633	2.1
52946	1.34678	2.1
52947	1.34678	2.1
52948	1.53735	2.1
52949	1.234	2.1
52950	1.42457	2.1
52951	1.89226	2.1

52952	1.89226	2.1
52953	1.89226	2.1
52954	1.89226	2.1
52955	1.89226	2.1
52956	1.89226	2.1
52957	1.09483	2.1
52958	1.34678	2.1
52959	1.234	2.1
52960	1.234	2.1
52961	1.19	2.1
52962	1.19	2.1
52963	0.946131	2.1
52964	0.946131	2.1
52965	0.946131	2.1
52966	0.946131	2.1
52967	0.946131	2.1
52968	0.946131	2.1
52969	0.946132	2.1
52970	0.946132	2.1
52971	0.946132	2.1
52972	0.946132	2.1
52973	0.946131	2.1
52974	0.946131	2.1
52975	2.82263	2.1
52976	1.89226	2.1
52977	2.71441	2.1
52978	2.71441	2.1
52979	1.89226	2.1
52980	1.89226	2.1
52981	1.1412	2.1
52982	1.1412	2.1
52983	1.14626	2.1
52984	1.14626	2.1
52985	1.28511	2.1
52986	1.34086	2.1
52987	0.992242	2.1
52988	0.946593	2.1
52989	0.992242	2.1
52990	1.34086	2.1
52991	0.946594	2.1
52992	1.28511	2.1
52993	1.18954	2.1
52994	1.18954	2.1
52995	1.21477	2.1
52996	1.21477	2.1
52997	1.41207	2.1
52998	1.34086	2.1
52999	1.01306	2.1
53000	0.992242	2.1
53001	1.01306	2.1
53002	1.41207	2.1
53003	0.992242	2.1
53004	1.34086	2.1
53005	1.09044	2.1
53006	1.44472	0.185974
53007	1.2955	2.1
53008	1.2955	1.94643
53009	1.22025	2.1

53010	1.22025	2.1
53011	0.810944	0.300148
53012	0.810944	2.1
53013	0.810946	0.670124
53014	0.810946	0.05
53015	0.810959	0.363019
53016	0.810959	0.202395
53017	1.89226	0.163245
53018	1.35167	0.36011
53019	1.08132	2.1
53020	1.08132	0.05
53021	1.0813	0.322651
53022	1.41894	0.29522
53023	1.28953	0.237527
53024	0.946132	0.0547756
53025	0.946132	1.61381
53026	0.946133	1.23758
53027	0.946133	0.20573
53028	1.41894	0.287064
53029	1.41894	1.32339
53030	1.28953	0.326088
53031	0.946132	1.03331
53032	0.946132	0.823108
53033	1.41894	0.151801
53034	1.89226	2.1
53035	2.15444	2.1
53036	1.93239	2.1
53037	1.89226	2.1
53038	2.19457	2.1
53039	2.15444	2.1
53040	1.89226	2.1
53041	1.89226	2.1
53042	1.89226	2.1
53043	1.89226	2.1
53044	1.89226	2.1
53045	1.89226	2.1
53046	0.938507	2.1
53047	0.938507	2.1
53048	1.07871	2.1
53049	1.15801	2.1
53050	1.03236	2.1
53051	1.14766	2.1
53052	0.946133	2.1
53053	0.946133	2.1
53054	0.946131	2.1
53055	1.07009	2.1
53056	0.946131	2.1
53057	1.03237	2.1
53058	1.89226	2.1
53059	2.19457	2.1
53060	1.94492	2.1
53061	1.89226	2.1
53062	2.24723	2.1
53063	2.19457	2.1
53064	0.946132	2.1
53065	0.946132	2.1
53066	0.946131	2.1
53067	0.946131	2.1

53068	0.946132	2.1
53069	0.946132	2.1
53070	0.946132	2.1
53071	0.946132	2.1
53072	0.946132	2.1
53073	0.946132	2.1
53074	0.946132	2.1
53075	0.946132	2.1
53076	2.21345	2.1
53077	1.89226	2.1
53078	1.89226	2.1
53079	2.14017	2.1
53080	2.14017	2.1
53081	1.89226	2.1
53082	1.89226	0.13219
53083	1.89226	1.03734
53084	1.89226	0.214755
53085	1.85439	0.928107
53086	2.23961	0.099548
53087	2.19479	0.317464
53088	1.2916	0.167483
53089	1.2916	0.123792
53090	0.946128	0.193172
53091	0.946128	0.150348
53092	1.2916	0.0835198
53093	0.946131	0.188516
53094	0.946126	0.827973
53095	1.1198	0.0597526
53096	1.2916	0.0819131
53097	1.1198	0.288259
53098	0.946128	0.57245
53099	0.946128	0.331083
53100	1.89226	0.416212
53101	2.71262	0.243475
53102	1.89226	0.216404
53103	2.49959	0.231082
53104	4.39185	0.607332
53105	2.71262	0.219174
53106	1.34592	0.154215
53107	2.49959	0.198865
53108	1.14721	0.109579
53109	3.84551	0.0909154
53110	0.946131	0.126759
53111	0.946131	0.157282
53112	1.11247	0.143167
53113	1.11247	0.121077
53114	1.24979	0.127745
53115	1.11247	0.0932376
53116	0.946129	0.193215
53117	0.946129	0.189701
53118	0.946131	0.0747019
53119	1.11247	0.05
53120	0.946131	0.0746135
53121	1.24979	0.10031
53122	0.946131	2.1
53123	0.946131	2.1
53124	0.94613	2.1
53125	0.94613	2.1

53126	0.946132	2.1
53127	0.946132	2.1
53128	0.946129	2.1
53129	0.946129	2.1
53130	0.946131	2.1
53131	0.946131	2.1
53132	0.946131	2.1
53133	0.946131	2.1
53134	2.77816	2.1
53135	1.89226	2.1
53136	1.89226	2.1
53137	1.89226	2.1
53138	3.78453	2.1
53139	1.89226	2.1
53140	1.89226	2.1
53141	1.89226	2.1
53142	1.89226	2.1
53143	1.89226	2.1
53144	1.89226	2.1
53145	1.89226	2.1
53146	1.89226	2.1
53147	1.89226	2.1
53148	1.89226	2.1
53149	3.78452	2.1
53150	1.55698	2.1
53151	1.89226	2.1
53152	1.89226	2.1
53153	1.89226	2.1
53154	1.8575	2.1
53155	1.8575	2.1
53156	1.89226	2.1
53157	1.89226	2.1
53158	1.89226	2.1
53159	1.89226	2.1
53160	1.89226	2.1
53161	1.89226	2.1
53162	1.89227	2.1
53163	1.89227	2.1
53164	1.89227	2.1
53165	1.89227	2.1
53166	1.89227	2.1
53167	1.89227	2.1
53168	1.35509	2.1
53169	2.06238	2.1
53170	2.12244	2.1
53171	2.12244	2.1
53172	1.34817	2.1
53173	1.34817	2.1
53174	0.946126	2.1
53175	0.946126	2.1
53176	0.946131	2.1
53177	0.946131	2.1
53178	0.946127	2.1
53179	0.946127	2.1
53180	0.94613	2.1
53181	0.94613	2.1
53182	0.946128	2.1
53183	0.946128	2.1

53184	0.946128	2.1
53185	0.946128	2.1
53186	1.89226	2.1
53187	1.89226	1.84682
53188	1.89226	2.1
53189	1.89226	2.1
53190	1.89226	2.1
53191	1.89226	2.1
53192	1.16742	2.1
53193	1.16742	2.1
53194	1.18122	2.1
53195	1.18122	2.1
53196	1.31745	2.1
53197	1.2597	2.1
53198	1.03119	2.1
53199	1.06122	2.1
53200	1.06122	2.1
53201	1.31745	2.1
53202	1.03119	2.1
53203	1.2597	2.1
53204	1.36849	2.1
53205	2.12244	2.1
53206	2.20092	2.1
53207	2.20092	2.1
53208	1.35509	2.1
53209	1.35509	2.1
53210	1.89226	2.1
53211	1.89226	2.1
53212	1.89226	2.1
53213	1.89226	2.1
53214	1.89226	2.1
53215	1.89226	2.1
53216	1.89226	2.1
53217	1.89226	1.87349
53218	1.89226	2.1
53219	1.89226	2.1
53220	3.78452	2.1
53221	1.21731	2.1
53222	1.21731	2.1
53223	1.23412	2.1
53224	1.23412	2.1
53225	1.39021	2.1
53226	1.31745	2.1
53227	1.06122	2.1
53228	1.10046	2.1
53229	1.10046	2.1
53230	1.39021	2.1
53231	1.06122	2.1
53232	1.31745	2.1
53233	2.16503	0.329003
53234	1.78252	1.14708
53235	1.77293	0.958428
53236	1.56839	2.1
53237	2.36957	0.0672713
53238	2.16503	2.1
53239	1.89223	0.365796
53240	1.89223	2.1
53241	1.89223	0.476962

53242	1.89223	1.38401
53243	3.78447	2.1
53244	2.3696	1.2098
53245	1.78254	1.54765
53246	2.63298	0.104073
53247	2.07312	2.05956
53248	4.44272	1.4172
53249	1.89226	1.66825
53250	1.89226	1.93469
53251	1.89226	1.49329
53252	1.89226	2.1
53253	1.89226	0.0958672
53254	1.89226	2.1
53255	1.89226	0.501951
53256	1.89226	0.0776097
53257	1.89226	0.241509
53258	1.89226	0.05
53259	3.78452	0.05
53260	2.26369	0.44877
53261	2.2168	0.05
53262	2.15617	0.0598024
53263	2.10864	0.05
53264	4.37234	0.05
53265	2.2168	0.110476
53266	1.94499	0.0711205
53267	1.94499	0.445236
53268	2.04737	0.104365
53269	2.10864	0.05
53270	2.21103	0.05
53271	1.89226	1.00685
53272	1.89226	0.0682846
53273	1.89226	0.339443
53274	1.89226	0.269925
53275	1.89226	0.225265
53276	1.89226	0.269337
53277	1.89226	0.136687
53278	1.89226	0.0735294
53279	1.89226	0.0888802
53280	1.89226	0.884687
53281	1.89226	0.05
53282	1.89226	0.788003
53283	1.02995	0.05
53284	1.02995	0.686731
53285	1.03212	0.0664388
53286	1.03212	0.131935
53287	0.988256	0.05
53288	1.05432	0.05
53289	1.00775	0.123322
53290	1.07809	0.154582
53291	1.00775	0.05
53292	0.988257	0.05
53293	1.07808	0.0611361
53294	1.05432	0.0764552
53295	2.10864	0.660429
53296	1.94499	0.120678
53297	1.97652	0.0515429
53298	1.79681	0.139592
53299	3.9215	0.141929

53300	0.810943	0.05
53301	0.810943	0.635851
53302	0.810943	0.0588122
53303	0.810943	0.0555401
53304	0.810958	0.05
53305	0.810958	0.05
53306	1.89226	0.123507
53307	1.35168	0.0612476
53308	1.08132	0.0627986
53309	1.08132	0.109108
53310	1.0813	0.116188
53311	0.657403	0.05
53312	1.35403	0.05
53313	1.24038	0.05
53314	0.499144	0.05
53315	1.24039	0.05
53316	1.35403	0.05
53317	0.657402	0.05
53318	0.499144	0.05
53319	0.915415	0.05
53320	0.915415	0.05
53321	0.937755	0.05
53322	0.937755	0.05
53323	1.72526	2.1
53324	1.17989	2.1
53325	1.96035	2.1
53326	1.47412	2.1
53327	3.14024	2.1
53328	1.89226	1.67711
53329	1.89226	2.1
53330	1.89226	2.1
53331	1.89226	0.901929
53332	3.78452	2.1
53333	0.946131	1.57556
53334	0.946131	1.63044
53335	1.25764	1.80946
53336	1.25764	2.1
53337	1.07087	1.11019
53338	1.25765	1.04256
53339	0.946128	0.948256
53340	0.946128	0.0929643
53341	0.94613	0.539664
53342	1.25765	0.956098
53343	1.07086	1.14842
53344	0.946129	1.19242
53345	1.96035	2.1
53346	1.32668	2.1
53347	2.29301	2.1
53348	1.70303	2.1
53349	3.56428	2.1
53350	1.89226	2.1
53351	1.89226	1.69947
53352	1.89226	0.370505
53353	1.89226	2.1
53354	3.78452	0.159744
53355	0.897738	0.0861229
53356	0.897738	0.0875488
53357	0.946102	0.0680059

53358	0.946102	0.05
53359	1.11247	0.0524918
53360	0.89771	0.05
53361	0.94613	0.0922876
53362	0.94613	0.0921384
53363	0.897709	0.106712
53364	0.946131	0.125491
53365	0.946131	0.185648
53366	1.11247	0.0788935
53367	1.89226	2.1
53368	1.89226	2.1
53369	1.89226	2.1
53370	1.89226	2.1
53371	1.89226	2.1
53372	1.89226	2.1
53373	2.15429	2.1
53374	1.89226	2.1
53375	1.89226	2.1
53376	1.89226	2.1
53377	2.27074	2.1
53378	2.27074	2.1
53379	0.993311	1.44401
53380	0.993311	2.02024
53381	0.904322	2.1
53382	0.904322	2.1
53383	0.803324	2.1
53384	1.36183	1.43534
53385	0.916274	2.1
53386	0.535808	1.63143
53387	0.535808	2.02395
53388	0.803322	2.09537
53389	0.916276	2.03839
53390	1.36183	2.09841
53391	1.89226	2.1
53392	1.89226	2.07638
53393	1.89226	2.1
53394	1.89226	2.1
53395	1.89226	2.1
53396	1.89226	2.1
53397	2.11955	2.1
53398	2.62097	2.1
53399	1.85542	2.1
53400	2.39016	2.1
53401	4.50971	2.1
53402	2.39016	2.1
53403	2.20242	2.1
53404	2.39016	2.1
53405	1.64008	2.1
53406	1.85542	2.1
53407	1.64008	2.1
53408	1.79667	2.1
53409	1.28267	2.1
53410	1.28267	2.1
53411	1.40117	2.1
53412	1.74514	2.1
53413	1.83994	2.1
53414	0.575914	2.1
53415	0.625292	2.1

53416	0.949144	2.1
53417	0.882782	2.1
53418	0.773697	2.1
53419	0.773697	2.1
53420	0.786497	2.1
53421	0.786494	2.1
53422	0.605477	2.1
53423	0.949148	2.1
53424	0.625291	2.1
53425	0.862374	2.1
53426	1.89226	2.1
53427	2.44918	2.1
53428	2.4616	2.1
53429	2.44918	2.1
53430	1.90468	2.1
53431	1.89226	2.1
53432	1.89226	2.1
53433	1.89226	2.1
53434	1.89226	2.1
53435	1.89226	2.1
53436	3.78453	2.1
53437	0.946128	2.1
53438	0.946128	2.1
53439	1.23631	2.1
53440	1.23631	2.1
53441	0.946131	2.1
53442	0.946131	2.1
53443	1.3147	2.1
53444	1.23631	2.1
53445	1.3147	2.1
53446	0.946131	2.1
53447	1.23631	2.1
53448	0.94613	2.1
53449	1.89226	2.1
53450	2.4616	2.1
53451	2.4717	2.1
53452	2.4616	2.1
53453	1.90237	2.1
53454	1.89226	2.1
53455	0.946132	2.1
53456	0.946132	2.1
53457	0.946131	2.1
53458	0.946131	2.1
53459	0.946132	2.1
53460	0.946132	2.1
53461	0.946131	2.1
53462	0.946131	2.1
53463	0.946131	2.1
53464	0.946131	2.1
53465	0.946131	2.1
53466	0.946131	2.1
53467	0.946128	2.1
53468	0.946128	2.1
53469	1.3147	2.1
53470	1.3147	2.1
53471	0.94613	2.1
53472	0.94613	2.1
53473	1.3419	2.1

53474	1.3147	2.1
53475	1.3419	2.1
53476	0.94613	2.1
53477	1.3147	2.1
53478	0.94613	2.1
53479	1.89226	2.1
53480	1.89226	2.1
53481	1.89226	2.1
53482	1.89226	2.1
53483	3.78452	2.1
53484	2.3333	2.1
53485	1.3864	2.1
53486	1.3864	2.1
53487	1.4628	2.1
53488	1.95763	2.1
53489	2.03403	2.1
53490	1.294	2.1
53491	1.294	2.1
53492	0.946128	2.1
53493	0.946128	2.1
53494	1.294	2.1
53495	1.27474	2.1
53496	0.946131	2.1
53497	0.946131	2.1
53498	0.946129	2.1
53499	1.27474	2.1
53500	1.294	2.1
53501	0.94613	2.1
53502	1.89226	2.1
53503	1.89226	2.1
53504	1.89226	2.1
53505	1.89226	2.1
53506	1.89226	2.1
53507	1.89226	2.1
53508	0.808088	2.1
53509	0.808088	2.1
53510	0.815623	2.1
53511	0.815623	2.1
53512	0.660053	2.1
53513	0.693199	2.1
53514	0.930512	2.1
53515	0.978817	2.1
53516	0.660053	2.1
53517	0.930513	2.1
53518	0.693198	2.1
53519	0.978817	2.1
53520	1.27474	2.1
53521	1.27474	2.1
53522	0.946128	2.1
53523	0.946128	2.1
53524	1.26221	2.1
53525	1.27474	2.1
53526	0.946131	2.1
53527	0.946131	2.1
53528	0.94613	2.1
53529	1.26221	2.1
53530	0.946129	2.1
53531	1.27474	2.1

53532	0.90207	0.05
53533	0.90207	0.05
53534	0.895719	0.05
53535	0.895719	0.05
53536	0.927855	0.05
53537	1.2493	0.05
53538	0.85723	0.05
53539	0.548484	0.05
53540	0.548485	0.05
53541	0.927855	0.05
53542	0.85723	0.05
53543	1.2493	0.05
53544	1.41319	0.293392
53545	1.22482	0.0674686
53546	1.46629	0.116332
53547	1.92622	0.305268
53548	3.15104	0.222081
53549	1.02305	0.0670568
53550	1.22482	0.101174
53551	1.80336	0.120227
53552	1.44426	0.0510196
53553	2.66908	0.05983
53554	1.3268	2.1
53555	0.999489	2.1
53556	1.44091	2.1
53557	1.14657	2.1
53558	2.4404	2.1
53559	1.89226	2.1
53560	1.89226	2.1
53561	1.89226	2.1
53562	1.89226	2.1
53563	3.78452	2.1
53564	1.89226	2.1
53565	2.28605	2.1
53566	2.43157	2.1
53567	2.43157	2.1
53568	1.89226	2.1
53569	1.89226	2.1
53570	1.60647	2.1
53571	1.44091	2.1
53572	1.31346	2.1
53573	1.31346	2.1
53574	1.14657	2.1
53575	1.44091	2.1
53576	1.89226	2.1
53577	1.89226	2.1
53578	1.89226	2.1
53579	1.89226	2.1
53580	3.78452	2.1
53581	0.946132	2.1
53582	0.946132	2.1
53583	1.3048	2.1
53584	1.3048	2.1
53585	1.3048	2.1
53586	1.21578	2.1
53587	0.946132	2.1
53588	0.946132	2.1
53589	1.3048	2.1

53590	0.946132	2.1
53591	0.946132	2.1
53592	1.21578	2.1
53593	1.89227	0.05
53594	1.89227	0.05
53595	1.89227	0.05
53596	1.89227	0.05
53597	3.78453	0.05
53598	1.6671	0.05
53599	1.87338	0.05
53600	1.6671	0.05
53601	2.02098	0.05
53602	1.38328	0.05
53603	1.73715	0.05
53604	1.89226	0.05
53605	1.89226	0.05
53606	1.89226	0.05
53607	1.89226	0.05
53608	3.78453	0.05
53609	1.6671	0.05
53610	1.38328	0.05
53611	1.35562	0.05
53612	1.01012	0.05
53613	2.67722	0.05
53614	0.819377	0.05
53615	0.647925	0.05
53616	1.17802	0.05
53617	1.33174	0.05
53618	0.98543	0.05
53619	0.98543	0.05
53620	0.994237	0.05
53621	0.994237	0.05
53622	1.17802	0.05
53623	0.647926	0.05
53624	1.33174	0.05
53625	0.819377	0.05
53626	1.27806	0.05
53627	1.68264	0.05
53628	1.44593	0.05
53629	1.878 0.05	
53630	3.15606	0.05
53631	1.878 0.05	
53632	1.9898	0.05
53633	1.878 0.05	
53634	1.68264	0.05
53635	2.30533	0.05
53636	2.10997	0.05
53637	0.814587	0.05
53638	0.814596	0.05
53639	0.82775	0.05
53640	0.82775	0.05
53641	1.0107	0.05
53642	0.91549	0.05
53643	0.741058	0.05
53644	0.714748	0.05
53645	0.714758	0.05
53646	1.03225	0.05
53647	0.704864	0.05

53648	0.915488	0.05
53649	1.90347	0.05
53650	2.06584	0.05
53651	1.878	0.05
53652	1.50236	0.05
53653	2.05749	0.05
53654	2.30533	0.05
53655	2.24475	2.1
53656	1.85871	2.1
53657	2.13711	2.1
53658	2.13711	2.1
53659	1.9943	2.1
53660	1.9943	2.1
53661	1.85871	2.1
53662	1.7871	2.1
53663	1.41619	2.1
53664	1.41619	2.1
53665	2.13711	2.1
53666	2.13711	2.1
53667	2.24475	2.1
53668	2.13711	2.1
53669	2.54902	2.1
53670	2.46327	2.1
53671	4.70802	2.1
53672	1.06913	2.1
53673	1.06913	2.1
53674	1.0821	2.1
53675	1.0821	2.1
53676	0.893552	2.1
53677	1.08267	2.1
53678	1.06855	2.1
53679	1.23163	2.1
53680	1.23163	2.1
53681	1.08267	2.1
53682	0.893551	2.1
53683	1.06855	2.1
53684	1.67046	1.01139
53685	2.4106	0.0733422
53686	2.05835	0.968044
53687	2.4106	1.34589
53688	1.59562	0.0720904
53689	1.94788	0.104314
53690	2.34737	1.14827
53691	2.4106	0.105369
53692	2.05834	1.12917
53693	1.99655	1.18425
53694	4.40715	0.447903
53695	1.67046	0.415944
53696	2.05835	0.054209
53697	1.588	1.04374
53698	1.34857	1.65741
53699	3.40692	0.469003
53700	1.73802	0.164894
53701	1.89668	0.754938
53702	1.99655	2.06963
53703	1.99655	0.1061
53704	1.79981	0.206135
53705	2.05834	0.0563053

53706	1.89226	1.16494
53707	1.89226	0.806802
53708	1.89226	0.108913
53709	1.89226	2.01383
53710	1.89226	0.15791
53711	1.89226	2.1
53712	1.89226	0.778321
53713	1.89226	2.1
53714	1.89226	1.92408
53715	1.89226	0.1347
53716	3.78453	1.7852
53717	0.719473	2.1
53718	0.916103	1.88825
53719	1.02744	0.865357
53720	0.850328	2.03468
53721	0.888094	0.651202
53722	0.888094	2.1
53723	0.878337	2.1
53724	0.878337	0.107983
53725	0.916103	1.26899
53726	1.02744	1.47319
53727	0.719473	1.86703
53728	0.850328	2.01785
53729	1.02744	1.22146
53730	0.850329	2.1
53731	1.0875	1.00974
53732	0.919597	1.292
53733	0.975841	0.334444
53734	0.975841	2.1
53735	0.971196	0.38544
53736	0.971196	2.04149
53737	1.02744	1.69835
53738	1.0875	0.966983
53739	0.850328	1.98467
53740	0.919598	1.72922
53741	1.89226	2.05265
53742	1.89226	1.69932
53743	1.89226	0.48421
53744	1.89226	1.77825
53745	3.78453	1.37088
53746	1.89226	1.64975
53747	1.89226	0.718655
53748	1.89226	0.147064
53749	1.89226	2.1
53750	1.89226	0.121181
53751	1.89226	2.1
53752	1.11103	1.78498
53753	0.916103	2.1
53754	1.21177	1.07087
53755	1.02744	1.21598
53756	1.07188	0.479411
53757	1.07188	2.1
53758	1.06659	0.449479
53759	1.06659	2.1
53760	1.11103	2.05077
53761	1.21177	1.61102
53762	0.916104	2.01806
53763	1.02744	2.07111

53764	2.54158	0.967192
53765	2.42354	1.40058
53766	2.17499	0.291501
53767	2.17499	2.1
53768	2.42354	1.92383
53769	2.05488	2.1
53770	1.89226	0.05
53771	1.89226	0.05
53772	1.89226	0.05
53773	1.89226	0.05
53774	3.78453	0.05
53775	1.89226	0.05
53776	1.89226	0.05
53777	1.89226	0.05
53778	1.89226	0.05
53779	1.89226	0.05
53780	1.89226	0.05
53781	1.89226	0.05
53782	1.89226	0.05
53783	1.89226	0.05
53784	1.89226	0.05
53785	1.89226	0.05
53786	1.89226	0.05
53787	1.89226	0.05
53788	1.89226	0.05
53789	1.89226	0.05
53790	1.89226	0.05
53791	3.78452	0.05
53792	0.802544	0.05
53793	0.946132	0.05
53794	0.946132	0.05
53795	0.787038	0.05
53796	0.787039	0.05
53797	0.787039	0.05
53798	0.961637	0.05
53799	0.961637	0.05
53800	0.787038	0.05
53801	0.802544	0.05
53802	0.946132	0.05
53803	0.946132	0.05
53804	1.89226	0.05
53805	1.60509	0.05
53806	1.89226	0.05
53807	1.89226	0.05
53808	1.3905	0.05
53809	1.3905	0.05
53810	0.946132	0.05
53811	0.946132	0.05
53812	0.946131	0.05
53813	0.946131	0.05
53814	0.946132	0.05
53815	0.946132	0.05
53816	0.946131	0.05
53817	0.946131	0.05
53818	0.946131	0.05
53819	0.946131	0.05
53820	0.946132	0.05
53821	0.946132	0.05

53822	0.946131	0.05
53823	0.946131	0.05
53824	0.946131	0.05
53825	0.946131	0.05
53826	0.946132	0.05
53827	0.946132	0.05
53828	0.946131	0.05
53829	0.946131	0.05
53830	0.946131	0.05
53831	0.946131	0.05
53832	0.946132	0.05
53833	0.946132	0.05
53834	1.42382	2.1
53835	1.32201	0.141869
53836	1.32096	0.525726
53837	1.42866	1.9222
53838	2.75067	1.13326
53839	1.89226	2.0437
53840	1.89226	0.104114
53841	1.89226	0.342282
53842	1.89226	1.14499
53843	1.89226	0.153507
53844	1.89226	0.660737
53845	1.89226	1.43215
53846	2.79029	0.134773
53847	2.7803	0.118901
53848	2.79029	0.318147
53849	1.88227	0.0692099
53850	1.89226	0.200891
53851	0.716065	1.78578
53852	0.714333	2.1
53853	0.660483	0.300384
53854	0.659126	0.728318
53855	0.687538	0.260078
53856	0.687538	1.7043
53857	0.685921	2.1
53858	0.685921	0.361562
53859	0.659126	0.742293
53860	0.716065	0.905752
53861	0.660483	1.19948
53862	0.714333	1.06302
53863	1.89226	1.69996
53864	1.89226	0.091744
53865	1.89226	0.447598
53866	1.89226	1.02157
53867	3.78453	0.900964
53868	0.946133	0.140064
53869	0.946133	0.624944
53870	1.39015	0.139239
53871	1.38969	0.05
53872	0.946132	0.05
53873	0.946132	0.164875
53874	1.38969	0.59658
53875	1.38969	0.191657
53876	1.38969	0.103409
53877	0.946132	0.153374
53878	0.946133	0.525417
53879	1.39015	0.172229

53880	2.72157	0.0574311
53881	1.22176	0.05
53882	1.59492	0.05
53883	1.22176	0.0808399
53884	2.40743	0.05
53885	2.03426	0.0685895
53886	0.946131	0.116271
53887	0.946131	0.0671916
53888	1.36079	0.0502744
53889	1.01713	0.0630018
53890	0.94613	0.0601907
53891	0.94613	0.120069
53892	1.36079	0.0608304
53893	1.36079	0.05
53894	0.946131	0.081203
53895	1.36079	0.0767759
53896	1.01713	0.0794438
53897	0.946131	0.0838074
53898	1.89226	0.10314
53899	1.89226	0.0671279
53900	1.89226	0.0502966
53901	1.89226	0.166608
53902	3.78453	0.0862539
53903	1.89226	0.0570617
53904	1.89226	0.0506366
53905	1.89226	0.05
53906	1.89226	0.107769
53907	1.89226	0.05
53908	1.89226	0.0771521
53909	0.946131	0.103456
53910	0.946131	0.0673239
53911	0.946131	0.060899
53912	1.36079	0.05
53913	0.94613	0.0609634
53914	0.94613	0.113035
53915	0.946133	0.0645814
53916	0.946133	0.05
53917	0.946131	0.0627858
53918	0.946131	0.0716479
53919	0.946132	0.05
53920	1.36079	0.0629326
53921	1.89226	0.0542298
53922	1.89226	0.151724
53923	1.89226	0.0617866
53924	1.89226	0.0980571
53925	1.89226	0.0632531
53926	1.89226	0.101268
53927	1.89227	0.0625796
53928	1.89227	0.05
53929	1.89227	0.05
53930	1.89227	0.0962893
53931	1.89227	0.05
53932	1.89227	0.0630666
53933	1.60428	2.1
53934	2.2367	2.1
53935	1.50097	2.1
53936	1.80125	2.1
53937	3.40553	2.1

53938	1.89095	2.1
53939	1.85293	0.175228
53940	1.80125	2.1
53941	1.76268	0.254937
53942	3.65418	2.1
53943	0.803177	2.1
53944	0.803177	2.1
53945	0.798644	2.1
53946	0.798644	2.1
53947	0.84233	2.1
53948	0.900624	2.1
53949	0.750483	2.1
53950	0.701197	2.1
53951	0.701197	2.1
53952	0.84233	2.1
53953	0.750483	2.1
53954	0.900625	2.1
53955	0.860925	0.321157
53956	0.860925	2.1
53957	0.862743	2.1
53958	0.862743	0.716469
53959	0.881338	0.146407
53960	0.826621	0.111781
53961	0.84233	2.1
53962	0.900625	2.1
53963	0.84233	2.1
53964	0.826621	2.0356
53965	0.900624	2.1
53966	0.881339	2.00921
53967	1.37938	0.983544
53968	1.03319	1.02446
53969	1.00512	1.31542
53970	0.639582	1.46827
53971	1.00463	1.35078
53972	1.00463	0.804383
53973	1.01434	0.82383
53974	1.01434	1.46233
53975	1.37938	1.14204
53976	1.00512	1.37536
53977	1.03319	1.20712
53978	0.639582	1.19994
53979	0.728713	0.05
53980	0.728713	0.05
53981	0.73425	0.0583905
53982	0.73425	1.90127
53983	0.525122	2.1
53984	0.605622	0.05
53985	0.948901	0.05
53986	0.857342	0.05
53987	0.85734	0.05
53988	0.525123	2.02631
53989	0.948899	0.05
53990	0.605624	0.05
53991	1.75449	0.421782
53992	1.4378	0.4218
53993	1.93916	0.686135
53994	2.22512	0.165324
53995	3.69364	0.520231

53996	1.75448	0.866892
53997	1.89186	0.250845
53998	2.29771	0.432638
53999	2.22512	0.27089
54000	4.05219	0.379969
54001	1.93916	0.691773
54002	2.51749	0.157041
54003	2.62357	0.264758
54004	2.22512	0.174422
54005	2.33761	0.620679
54006	1.93916	0.370203
54007	2.29771	0.505939
54008	2.51749	0.178634
54009	2.22512	0.19229
54010	2.22512	0.115915
54011	2.50905	0.665521
54012	2.50905	0.350605
54013	0.87382	0.05
54014	1.33662	0.05
54015	1.20756	0.05
54016	0.890467	0.05
54017	1.08859	0.05
54018	1.08859	0.05
54019	1.13084	0.05
54020	1.08793	0.05
54021	1.34722	0.05
54022	1.27184	0.05
54023	0.917678	0.05
54024	0.97821	0.05
54025	1.28396	0.05
54026	1.70302	0.05
54027	2.30076	0.05
54028	1.20417	0.05
54029	2.57506	0.05
54030	2.2656	0.05
54031	1.03642	1.00787
54032	1.03642	0.05
54033	1.07029	0.05
54034	1.07029	0.089986
54035	1.31531	0.501184
54036	1.23279	0.05
54037	0.941743	0.0668632
54038	0.791399	0.716941
54039	0.791399	0.05
54040	1.23279	0.05
54041	0.941744	0.0950241
54042	1.31531	0.180151
54043	0.937354	0.970519
54044	0.937354	0.05
54045	0.954794	0.05
54046	0.954794	0.05
54047	1.13563	0.05
54048	1.23279	0.307159
54049	0.791398	0.05
54050	0.659359	0.533862
54051	1.13563	0.05
54052	0.659359	0.05
54053	0.791399	0.05

54054	1.23279	0.05
54055	2.29852	0.650511
54056	1.89227	0.690635
54057	2.14485	0.325163
54058	2.14485	1.25032
54059	1.89227	0.363587
54060	1.89227	1.27688
54061	0.947863	0.482821
54062	1.19854	1.03026
54063	2.16841	0.669266
54064	1.76597	0.670289
54065	2.96451	0.515547
54066	1.89227	0.447111
54067	1.89227	0.561661
54068	1.89227	0.32028
54069	1.89227	1.06214
54070	1.89227	0.331697
54071	1.89227	1.08536
54072	2.46489	2.1
54073	1.98339	2.1
54074	1.81667	2.1
54075	2.36914	2.1
54076	4.35253	2.1
54077	2.46488	2.1
54078	2.70401	2.1
54079	2.64996	2.1
54080	2.36914	2.1
54081	5.07315	2.1
54082	1.81667	2.1
54083	2.29459	2.1
54084	2.36914	2.1
54085	2.36914	2.1
54086	1.68073	2.1
54087	1.68073	2.1
54088	2.64996	2.1
54089	2.29458	2.1
54090	2.29458	2.1
54091	2.36914	2.1
54092	2.61243	2.1
54093	2.68698	2.1
54094	1.89226	1.36392
54095	1.89226	0.826923
54096	1.89226	1.44456
54097	1.89226	1.06236
54098	1.89226	1.49427
54099	1.89226	1.08944
54100	1.89224	1.63623
54101	1.89224	0.794605
54102	1.65571	1.16413
54103	1.65571	0.885422
54104	1.89224	1.37621
54105	1.89224	1.13121
54106	1.89224	1.28772
54107	1.89224	1.08142
54108	1.89224	1.43119
54109	1.89224	0.902858
54110	1.89224	1.65391
54111	1.89224	1.06367

54112	1.98252	0.05
54113	2.20641	0.05
54114	1.05129	0.05
54115	1.2951	0.05
54116	3.27762	0.05
54117	1.85598	2.1
54118	1.88635	0.975453
54119	2.12346	0.763896
54120	2.10838	2.1
54121	3.97943	1.26306
54122	2.10838	1.9151
54123	1.87532	2.1
54124	1.85597	0.747112
54125	1.85597	2.1
54126	1.87532	1.98241
54127	1.57914	2.1
54128	2.12348	1.22867
54129	2.35141	2.1
54130	2.37191	1.40718
54131	2.10841	1.7562
54132	2.38698	0.778467
54133	2.12348	1.68895
54134	1.07811	2.1
54135	0.937672	2.1
54136	1.17571	2.054
54137	1.05421	0.428403
54138	1.07087	1.39951
54139	1.07087	2.1
54140	1.06145	2.1
54141	1.06145	1.06009
54142	1.17571	2.1
54143	1.07811	2.1
54144	1.05421	2.1
54145	0.937672	2.1
54146	1.67334	2.1
54147	2.76609	2.1
54148	2.74144	2.1
54149	2.74144	2.1
54150	1.71157	2.1
54151	1.71157	2.1
54152	2.76609	2.1
54153	1.89226	2.1
54154	1.89226	2.1
54155	1.89226	2.1
54156	2.74143	2.1
54157	2.74143	2.1
54158	1.10393	2.1
54159	1.10393	2.1
54160	1.10788	2.1
54161	1.10788	2.1
54162	1.38302	2.1
54163	1.37072	2.1
54164	0.836672	2.1
54165	0.841089	2.1
54166	1.38302	2.1
54167	0.84109	2.1
54168	0.836671	2.1
54169	1.37072	2.1

54170	1.38302	2.1
54171	1.38302	2.1
54172	0.946131	2.1
54173	0.946131	2.1
54174	0.946128	2.1
54175	0.946128	2.1
54176	1.38302	2.1
54177	1.37072	2.1
54178	1.38302	2.1
54179	0.946131	2.1
54180	0.94613	2.1
54181	1.37072	2.1
54182	1.28072	1.06728
54183	1.55779	2.1
54184	1.28072	2.1
54185	1.40765	2.1
54186	1.47186	2.1
54187	1.59879	2.1
54188	1.89227	1.30841
54189	1.89227	1.31947
54190	1.89227	0.119005
54191	1.89227	2.1
54192	1.89227	0.116524
54193	1.89227	2.1
54194	2.80057	0.150645
54195	1.89226	0.110055
54196	1.89226	0.0966208
54197	1.89226	1.05102
54198	2.63061	0.05
54199	2.63061	1.18883
54200	1.28072	0.728603
54201	1.38859	2.1
54202	1.47186	2.1
54203	1.47186	2.1
54204	1.16777	2.1
54205	1.16777	2.1
54206	1.89226	0.855183
54207	1.89226	1.32717
54208	1.89226	0.284437
54209	1.89226	2.1
54210	1.89226	0.110461
54211	1.89226	2.1
54212	1.83221	2.1
54213	2.0815	2.1
54214	2.0815	2.1
54215	2.08153	2.1
54216	1.86703	2.1
54217	1.87402	2.1
54218	1.86703	2.1
54219	2.08508	2.1
54220	2.08508	2.1
54221	2.29001	2.1
54222	1.89226	2.1
54223	2.08048	2.1
54224	2.7278	0.05
54225	1.10909	0.05
54226	1.50472	0.05
54227	1.10909	0.05

54228	2.69881	0.05
54229	2.30319	0.05
54230	1.23204	2.1
54231	1.92264	2.1
54232	1.48162	2.1
54233	1.48162	2.1
54234	1.72994	2.1
54235	1.72994	2.1
54236	0.864968	2.1
54237	0.696952	2.1
54238	0.61602	2.1
54239	0.479736	2.1
54240	0.66444	2.1
54241	0.66444	2.1
54242	0.680264	2.1
54243	0.680264	2.1
54244	0.696952	2.1
54245	0.479736	2.1
54246	0.61602	2.1
54247	0.864968	2.1
54248	1.89226	0.229121
54249	2.43219	2.08636
54250	1.89226	1.34649
54251	2.82019	1.4074
54252	1.89226	1.94022
54253	2.82019	1.6504
54254	1.89226	0.445545
54255	2.83701	2.1
54256	2.2701	1.68863
54257	2.2701	1.16751
54258	1.89226	1.76239
54259	1.89226	1.39377
54260	1.89226	1.70051
54261	1.89226	0.612111
54262	1.89226	0.746077
54263	1.89226	1.35796
54264	1.89226	1.51873
54265	1.89226	1.40045
54266	1.5795	1.78281
54267	1.44091	1.65543
54268	1.05267	2.05093
54269	1.05267	1.42796
54270	1.98408	1.83738
54271	1.98408	1.26746
54272	2.20624	2.1
54273	1.08831	2.1
54274	2.49053	2.1
54275	1.42486	2.1
54276	3.57883	2.1
54277	1.89226	2.1
54278	1.89226	2.1
54279	1.89226	2.1
54280	1.89226	2.1
54281	3.78453	2.1
54282	0.946132	2.1
54283	0.946132	2.1
54284	0.853591	2.1
54285	0.642207	2.1

54286	0.94613	2.1
54287	0.94613	2.1
54288	0.642209	2.1
54289	0.642209	2.1
54290	0.946131	2.1
54291	0.853591	2.1
54292	0.642207	2.1
54293	0.946132	2.1
54294	1.0506	2.1
54295	1.0506	2.1
54296	1.0879	2.1
54297	0.960424	2.1
54298	0.708687	2.1
54299	0.782927	2.1
54300	1.40041	2.1
54301	1.2518	2.1
54302	0.767748	2.1
54303	1.31801	2.1
54304	1.21412	2.1
54305	0.69461	2.1
54306	1.89226	2.1
54307	1.89226	2.1
54308	1.89226	2.1
54309	1.89226	2.1
54310	1.89226	2.1
54311	1.89226	2.1
54312	0.922223	2.1
54313	0.922223	2.1
54314	1.04548	2.1
54315	0.768229	2.1
54316	0.948441	2.1
54317	0.806483	2.1
54318	0.85362	2.1
54319	0.85362	2.1
54320	1.05942	2.1
54321	0.922187	2.1
54322	0.851554	2.1
54323	0.768264	2.1
54324	2.16728	2.1
54325	0.95455	2.1
54326	1.21372	2.1
54327	0.98227	2.1
54328	2.16728	2.1
54329	1.93584	2.1
54330	2.08849	0.05
54331	1.08732	0.05
54332	1.3357	0.05
54333	2.37948	0.05
54334	3.4668	0.05
54335	2.57882	0.05
54336	2.37948	0.05
54337	2.53055	0.05
54338	1.48676	0.05
54339	1.3357	0.05
54340	1.3357	0.05
54341	1.89226	2.1
54342	1.89226	2.1
54343	2.17471	2.1

54344	1.89226	2.1
54345	2.60085	2.1
54346	2.26306	2.1
54347	0.946131	2.1
54348	0.946131	2.1
54349	0.946131	2.1
54350	0.946131	2.1
54351	0.94613	2.1
54352	0.94613	2.1
54353	0.946132	2.1
54354	0.946132	2.1
54355	0.946131	2.1
54356	0.946131	2.1
54357	0.946131	2.1
54358	0.946131	2.1
54359	1.89226	2.1
54360	2.17471	2.1
54361	2.08196	2.1
54362	2.17471	2.1
54363	1.79952	2.1
54364	1.89226	2.1
54365	1.89226	2.1
54366	1.89226	2.1
54367	1.89226	2.1
54368	1.89226	2.1
54369	1.89226	2.1
54370	1.89226	2.1
54371	1.86016	2.1
54372	1.89226	2.1
54373	1.89226	2.1
54374	1.89226	2.1
54375	1.84826	2.1
54376	1.84826	2.1
54377	2.50136	2.1
54378	1.99781	2.1
54379	1.99729	2.1
54380	2.5214	2.1
54381	4.49865	2.1
54382	0.51724	2.1
54383	0.496076	2.1
54384	0.998906	2.1
54385	0.998645	2.1
54386	0.742249	2.1
54387	0.742249	2.1
54388	0.752734	2.1
54389	0.752734	2.1
54390	0.998639	2.1
54391	0.496073	2.1
54392	0.517243	2.1
54393	0.998913	2.1
54394	0.998653	2.1
54395	0.994293	2.1
54396	1.26071	2.1
54397	1.26504	2.1
54398	1.12529	2.1
54399	1.12529	2.1
54400	1.12971	2.1
54401	1.12971	2.1

54402	1.26504	2.1
54403	0.994292	2.1
54404	0.998651	2.1
54405	1.26071	2.1
54406	1.89226	2.1
54407	1.89226	2.1
54408	1.89226	2.1
54409	1.89226	2.1
54410	3.78452	2.1
54411	1.89226	2.1
54412	1.89226	2.1
54413	1.89226	2.1
54414	1.89226	2.1
54415	3.78452	2.1
54416	0.675872	2.1
54417	0.675641	2.1
54418	0.943072	2.1
54419	0.898317	2.1
54420	0.775717	2.1
54421	0.775717	2.1
54422	0.79824	2.1
54423	0.79824	2.1
54424	0.675642	2.1
54425	0.94307	2.1
54426	0.675874	2.1
54427	0.898315	2.1
54428	0.943071	2.1
54429	0.898316	2.1
54430	0.998133	2.1
54431	0.918903	2.1
54432	0.922369	2.1
54433	0.922369	2.1
54434	0.939606	2.1
54435	0.939606	2.1
54436	0.943071	2.1
54437	0.998133	2.1
54438	0.898316	2.1
54439	0.918903	2.1
54440	0.946131	2.1
54441	0.946131	2.1
54442	0.946131	2.1
54443	0.946131	2.1
54444	0.946132	2.1
54445	0.946132	2.1
54446	0.946129	2.1
54447	0.946129	2.1
54448	0.94613	2.1
54449	0.94613	2.1
54450	0.946131	2.1
54451	0.946131	2.1
54452	1.89226	2.1
54453	1.89226	2.1
54454	1.89226	2.1
54455	1.89226	2.1
54456	1.89226	2.1
54457	1.89226	2.1
54458	1.35129	2.1
54459	1.88614	2.1

54460	2.01867	2.1
54461	1.38189	2.1
54462	3.36995	2.1
54463	0.943071	2.1
54464	1.00934	2.1
54465	0.998134	2.1
54466	1.10866	2.1
54467	0.992617	2.1
54468	0.992617	2.1
54469	1.01485	2.1
54470	1.01485	2.1
54471	1.00934	2.1
54472	1.10866	2.1
54473	0.943072	2.1
54474	0.998134	2.1
54475	2.67272	2.1
54476	1.61859	2.1
54477	1.70303	2.1
54478	2.52175	2.1
54479	4.5326	2.1
54480	2.00954	2.1
54481	2.27801	2.1
54482	1.87875	2.1
54483	1.87875	2.1
54484	2.37837	2.1
54485	2.37837	2.1
54486	2.00955	2.1
54487	1.78	2.1
54488	2.21813	2.1
54489	1.8493	2.1
54490	2.37838	2.1
54491	2.00955	2.1
54492	2.37838	2.1
54493	2.27801	2.1
54494	2.58523	2.1
54495	2.71597	2.1
54496	4.9636	2.1
54497	2.37838	2.1
54498	2.71597	2.1
54499	1.72709	2.1
54500	2.0815	2.1
54501	2.59749	2.1
54502	2.78034	2.1
54503	0.94613	0.272826
54504	0.94613	0.428642
54505	0.946128	0.183618
54506	0.946128	0.112657
54507	0.94613	0.106308
54508	0.94613	0.179066
54509	0.946128	0.428092
54510	0.946128	0.0873816
54511	0.946129	0.180549
54512	0.946129	0.178621
54513	0.946129	0.262361
54514	0.946129	0.275328
54515	1.89226	0.963328
54516	1.89226	0.538125
54517	1.89226	0.876069

54518	1.89226	0.322303
54519	3.78452	0.383606
54520	0.946133	0.0974476
54521	0.946133	0.128453
54522	0.946132	0.0533476
54523	0.946132	0.0648868
54524	0.946132	0.0734447
54525	0.946132	0.0689434
54526	0.946133	0.12364
54527	0.946133	0.0928959
54528	0.946132	0.0770342
54529	0.946132	0.05
54530	0.946132	0.0800691
54531	0.946132	0.0896287
54532	1.89226	0.166259
54533	2.10495	0.0570146
54534	1.89226	0.0529319
54535	2.30932	0.0572708
54536	1.89226	0.177754
54537	2.30932	0.250354
54538	2.30932	0.0689689
54539	1.00366	0.05
54540	2.10496	0.05
54541	1.24728	0.05
54542	3.31298	0.05
54543	1.87557	2.1
54544	1.07167	2.1
54545	2.03045	2.1
54546	1.25185	2.1
54547	3.10212	2.1
54548	1.89226	2.1
54549	1.89226	2.1
54550	1.89226	2.1
54551	1.89226	2.1
54552	3.78453	2.1
54553	0.851541	2.1
54554	0.851541	2.1
54555	1.12236	2.1
54556	1.05783	2.1
54557	0.870663	2.1
54558	0.951856	2.1
54559	0.946132	2.1
54560	0.946132	2.1
54561	1.04073	2.1
54562	1.1296	2.1
54563	0.776062	2.1
54564	0.851527	2.1
54565	0.859395	2.1
54566	0.859395	2.1
54567	0.870165	2.1
54568	0.870165	2.1
54569	0.625924	2.1
54570	0.714333	2.1
54571	1.0821	2.1
54572	1.01523	2.1
54573	1.0821	2.1
54574	0.714333	2.1
54575	1.01523	2.1

54576	0.625924	2.1
54577	1.89226	2.1
54578	1.89226	2.1
54579	1.89226	2.1
54580	1.89226	2.1
54581	3.78453	2.1
54582	1.89226	2.1
54583	1.89226	2.1
54584	2.23164	2.1
54585	2.67802	2.1
54586	2.03663	2.1
54587	1.89226	2.1
54588	2.42221	2.1
54589	1.61933	2.1
54590	2.80896	2.1
54591	1.87043	2.1
54592	4.29262	2.1
54593	1.89226	2.1
54594	1.89226	2.1
54595	1.89226	2.1
54596	1.89226	2.1
54597	1.89226	2.1
54598	1.89226	2.1
54599	2.80892	2.1
54600	1.87041	2.1
54601	2.67182	2.1
54602	2.30891	2.1
54603	5.07665	2.1
54604	1.89224	2.1
54605	1.89224	2.1
54606	1.89224	2.1
54607	1.89224	2.1
54608	3.78448	2.1
54609	0.946118	2.1
54610	0.946118	2.1
54611	0.946118	2.1
54612	0.946118	2.1
54613	0.946115	2.1
54614	0.946115	2.1
54615	0.94612	2.1
54616	0.94612	2.1
54617	0.94613	2.1
54618	0.94613	2.1
54619	0.946105	2.1
54620	0.946105	2.1
54621	2.3089	2.1
54622	1.02783	2.1
54623	1.98533	2.1
54624	1.76762	2.1
54625	1.24553	2.1
54626	1.02783	2.1
54627	2.2049	0.05
54628	2.34673	0.05
54629	1.00763	0.05
54630	1.23746	0.05
54631	3.44236	0.05
54632	1.89226	0.05
54633	1.89226	0.0542194

54634	1.89226	0.05
54635	1.89226	0.05
54636	3.78452	0.05
54637	1.89226	0.0558977
54638	1.89226	0.05
54639	1.89226	0.05
54640	1.89226	0.0532138
54641	3.78453	0.0511911
54642	0.891618	0.05
54643	0.891618	0.05
54644	0.902158	0.05
54645	0.902158	0.05
54646	1.15396	0.05
54647	0.620408	0.05
54648	1.17337	0.05
54649	0.618733	0.05
54650	0.620408	0.05
54651	0.618733	0.05
54652	1.17337	0.05
54653	1.15396	0.05
54654	0.69978	0.05
54655	0.69978	0.05
54656	0.946102	0.05
54657	0.946102	0.05
54658	0.831702	0.05
54659	0.946129	0.05
54660	0.699752	0.05
54661	0.946132	0.05
54662	0.831703	0.05
54663	0.699751	0.05
54664	0.946131	0.05
54665	0.946131	0.05
54666	0.831675	0.05
54667	0.831675	0.05
54668	0.946163	0.05
54669	0.864397	0.05
54670	0.853471	0.05
54671	0.962623	0.05
54672	0.748555	0.05
54673	0.851543	0.05
54674	0.864714	0.05
54675	0.760411	0.05
54676	0.946133	0.05
54677	0.946133	0.05
54678	1.89226	0.17343
54679	1.89226	0.883717
54680	1.89226	0.522541
54681	1.89226	0.240757
54682	3.78452	0.484255
54683	1.89226	0.827655
54684	1.89226	0.480533
54685	1.89226	0.389286
54686	1.89226	0.968486
54687	3.78452	0.588848
54688	0.946132	0.593206
54689	0.946132	0.9301
54690	0.946132	0.342857
54691	0.946132	0.566708

54692	0.946132	0.241421
54693	0.946132	1.22603
54694	0.946133	0.421793
54695	0.946133	0.902952
54696	0.946133	0.357909
54697	0.946133	0.366273
54698	0.946132	0.715265
54699	0.946132	0.79991
54700	1.89226	0.79441
54701	2.39715	0.46209
54702	1.89226	0.754381
54703	1.89226	1.17871
54704	1.89226	0.298437
54705	1.89226	0.519149
54706	0.94613	0.856006
54707	0.94613	0.730076
54708	0.94613	0.537591
54709	0.94613	0.401368
54710	0.946131	0.428487
54711	0.946131	1.14562
54712	0.946129	0.78339
54713	0.946129	0.562726
54714	0.94613	0.406584
54715	0.94613	0.50713
54716	0.94613	0.836601
54717	0.94613	0.690807
54718	2.54204	2.1
54719	2.3148	2.1
54720	2.55328	2.1
54721	2.39646	2.1
54722	4.71126	2.1
54723	1.62611	2.1
54724	2.3148	2.1
54725	2.16986	2.1
54726	1.43745	2.1
54727	3.75225	2.1
54728	1.89226	2.1
54729	1.89226	2.1
54730	1.89226	2.1
54731	1.89226	2.1
54732	3.78453	2.1
54733	1.89226	2.1
54734	1.89226	2.1
54735	1.89226	2.1
54736	1.89226	2.1
54737	3.78453	2.1
54738	2.12125	2.1
54739	2.39642	2.1
54740	2.2941	2.1
54741	1.9933	2.1
54742	4.38972	2.1
54743	1.73784	2.1
54744	1.26892	2.1
54745	1.26892	2.1
54746	1.43743	2.1
54747	1.9933	2.1
54748	2.1618	2.1
54749	0.946118	2.1

54750	0.946118	2.1
54751	0.946118	2.1
54752	0.946118	2.1
54753	0.946119	2.1
54754	0.946119	2.1
54755	0.946118	2.1
54756	0.946118	2.1
54757	0.946131	2.1
54758	0.946131	2.1
54759	0.946106	2.1
54760	0.946106	2.1
54761	1.89224	2.1
54762	1.89224	2.1
54763	1.89224	2.1
54764	1.89224	2.1
54765	3.78448	2.1
54766	2.45922	2.1
54767	2.15686	2.1
54768	2.14259	2.1
54769	2.43225	2.1
54770	4.58911	2.1
54771	1.62027	2.1
54772	2.15686	2.1
54773	1.62241	2.1
54774	1.62241	2.1
54775	2.1426	2.1
54776	2.1426	2.1
54777	2.14259	2.1
54778	2.43225	2.1
54779	2.40667	2.1
54780	2.1281	2.1
54781	4.56035	2.1
54782	1.0713	2.1
54783	1.06405	2.1
54784	0.810136	2.1
54785	0.808433	2.1
54786	0.938508	2.1
54787	0.938508	2.1
54788	0.941223	2.1
54789	0.941223	2.1
54790	1.06405	2.1
54791	0.808433	2.1
54792	0.810135	2.1
54793	1.0713	2.1
54794	1.89226	0.107192
54795	1.89226	0.05
54796	1.89226	0.0899089
54797	1.89226	0.0562425
54798	3.78453	0.0604815
54799	1.89227	0.0539782
54800	1.89227	0.05
54801	1.89227	0.0792672
54802	1.89227	0.05
54803	3.78453	0.05
54804	1.19 0.05	
54805	1.12469	0.05
54806	1.01052	0.05
54807	1.08166	0.05

54808	2.20051	0.05
54809	0.594999	0.05
54810	0.540832	0.05
54811	0.514182	0.05
54812	0.567857	0.05
54813	0.554259	0.05
54814	0.554259	0.05
54815	0.55443	0.05
54816	0.55443	0.05
54817	0.540832	0.05
54818	0.514182	0.05
54819	0.567857	0.05
54820	0.594999	0.05
54821	1.38674	0.05
54822	0.946132	0.0540255
54823	1.36361	0.05
54824	0.946132	0.0565878
54825	1.36362	0.05
54826	1.36362	0.05
54827	0.946131	0.0540973
54828	0.946131	0.0678247
54829	1.38674	0.05
54830	1.36361	0.05
54831	0.946132	0.0521086
54832	0.946132	0.05
54833	1.89226	0.05
54834	2.68508	0.05
54835	2.77349	0.05
54836	1.89226	0.05
54837	2.77349	0.05
54838	1.89226	0.05
54839	2.15315	0.05
54840	1.96254	0.05
54841	1.51377	0.05
54842	1.89226	0.05
54843	1.47799	0.05
54844	1.89226	0.05
54845	2.49439	0.05
54846	1.89226	0.0717993
54847	2.15315	0.0756954
54848	1.89226	0.0535889
54849	2.15315	0.0512175
54850	1.89226	0.05
54851	1.89226	0.0838171
54852	1.89226	0.111521
54853	1.89226	0.075907
54854	1.89226	0.0655927
54855	1.89226	0.160095
54856	1.89226	0.132486
54857	1.89226	0.0667967
54858	1.89226	0.0638752
54859	1.89226	0.05
54860	1.89226	0.0858267
54861	1.89226	0.05
54862	1.89226	0.0538539
54863	1.375	0.05
54864	2.01865	0.0575535
54865	2.12047	0.05

54866	1.47354	0.05
54867	2.12047	0.0657149
54868	1.47354	0.0575001
54869	0.687499	0.05
54870	1.06023	0.05
54871	1.08019	0.05
54872	0.604229	0.05
54873	1.08018	0.05
54874	1.06023	0.05
54875	0.687502	0.05
54876	0.60423	0.05
54877	0.80646	0.05
54878	0.806461	0.05
54879	0.858001	0.05
54880	0.858001	0.05
54881	2.26414	2.1
54882	1.87342	2.1
54883	1.87342	2.1
54884	1.79667	2.1
54885	2.44045	2.1
54886	2.31924	2.1
54887	2.26414	2.1
54888	2.26616	2.1
54889	2.2355	2.1
54890	2.21858	2.1
54891	4.48474	2.1
54892	2.18516	2.1
54893	1.87344	2.1
54894	2.21859	2.1
54895	2.66233	2.1
54896	1.85196	2.1
54897	2.22236	2.1
54898	2.23551	2.1
54899	2.21859	2.1
54900	2.18516	2.1
54901	2.21189	2.1
54902	4.43049	2.1
54903	1.17171	2.1
54904	2.38924	2.1
54905	2.0815	2.1
54906	2.33348	2.1
54907	1.42851	2.1
54908	1.5959	2.1
54909	1.89226	2.1
54910	1.89226	2.1
54911	1.89226	2.1
54912	1.89226	2.1
54913	3.78452	2.1
54914	2.75425	2.1
54915	1.34366	2.1
54916	1.34366	2.1
54917	1.56512	2.1
54918	2.54186	2.1
54919	2.76332	2.1
54920	1.07751	2.1
54921	2.0815	2.1
54922	1.97259	2.1
54923	1.76455	2.1

54924	1.30191	2.1
54925	1.16461	2.1
54926	0.946131	2.1
54927	0.946131	2.1
54928	0.946129	2.1
54929	0.946129	2.1
54930	0.946131	2.1
54931	0.946131	2.1
54932	0.94613	2.1
54933	0.94613	2.1
54934	0.94613	2.1
54935	0.94613	2.1
54936	0.946131	2.1
54937	0.946131	2.1
54938	1.89226	0.0738555
54939	1.89226	0.05
54940	1.89226	0.05
54941	1.89226	0.05
54942	3.78453	0.05
54943	1.89226	0.0777241
54944	1.89226	0.05
54945	1.89226	0.05
54946	1.89226	0.05
54947	3.78453	0.0564333
54948	0.946132	0.05
54949	0.946132	0.05
54950	1.30327	0.05
54951	1.33544	0.05
54952	1.33544	0.05
54953	1.33544	0.05
54954	0.946132	0.05
54955	0.946132	0.05
54956	0.946132	0.05
54957	1.30327	0.05
54958	1.33544	0.05
54959	0.946132	0.05
54960	1.30327	0.05
54961	1.33544	0.05
54962	0.709426	0.05
54963	0.790129	0.05
54964	1.05886	0.05
54965	1.05886	0.05
54966	1.03454	0.05
54967	1.03454	0.05
54968	1.30327	0.05
54969	0.709426	0.05
54970	1.33544	0.05
54971	0.790128	0.05
54972	0.946132	0.05
54973	0.946132	0.05
54974	0.827866	0.05
54975	0.946133	0.05
54976	0.946132	0.05
54977	0.946132	0.05
54978	0.946133	0.05
54979	0.946133	0.05
54980	0.946132	0.05
54981	0.827866	0.05

54982	0.946133	0.05
54983	0.946133	0.05
54984	1.89227	0.05
54985	1.89227	0.05
54986	1.89227	0.05
54987	1.89227	0.05
54988	1.89227	0.05
54989	1.89227	0.05
54990	0.946133	0.05
54991	0.946133	0.05
54992	1.21602	0.05
54993	1.30327	0.05
54994	1.30327	0.05
54995	1.30327	0.05
54996	0.946132	0.05
54997	0.946132	0.05
54998	0.946132	0.05
54999	1.21602	0.05
55000	0.946133	0.05
55001	1.30327	0.05
55002	1.04227	0.05
55003	1.11706	0.05
55004	0.968413	0.05
55005	0.945482	0.05
55006	1.3204	0.05
55007	1.23215	0.05
55008	0.810791	0.05
55009	1.38978	0.05
55010	1.07728	0.05
55011	1.11704	0.05
55012	0.608052	0.05
55013	1.89226	0.05
55014	1.89226	0.05
55015	1.89226	0.05
55016	1.89226	0.05
55017	3.78453	0.05
55018	1.89226	0.05
55019	1.89226	0.05
55020	1.89226	0.05
55021	1.89226	0.05
55022	3.78451	0.05
55023	2.58476	0.05
55024	1.56598	0.05
55025	1.55045	0.05
55026	1.5124	0.05
55027	2.62281	0.05
55028	2.58476	0.05
55029	2.58477	0.05
55030	1.89226	0.05
55031	1.89226	0.05
55032	1.89226	0.05
55033	2.62282	0.05
55034	2.62282	0.05
55035	1.89224	0.05
55036	1.89224	0.05
55037	1.89224	0.05
55038	1.89224	0.05
55039	3.78448	0.05

55040	1.89223	0.05
55041	1.89223	0.05
55042	1.89223	0.05
55043	1.89223	0.05
55044	1.89223	0.05
55045	1.89223	0.05
55046	2.62278	0.05
55047	1.45053	0.05
55048	1.49678	0.05
55049	1.56596	0.05
55050	2.5536	0.05
55051	2.62278	0.05
55052	1.31139	0.05
55053	1.2768	0.05
55054	0.946118	0.05
55055	0.946118	0.05
55056	1.2768	0.05
55057	1.2768	0.05
55058	0.946116	0.05
55059	0.946116	0.05
55060	1.27682	0.05
55061	0.94613	0.05
55062	0.946105	0.05
55063	1.31138	0.05
55064	1.38506	1.77291
55065	1.53348	2.1
55066	1.17042	1.80818
55067	1.17042	2.04785
55068	1.73669	1.81512
55069	1.73669	2.04631
55070	1.73669	1.44503
55071	1.79928	0.812692
55072	1.38506	1.23727
55073	1.38506	2.06587
55074	1.79928	2.01828
55075	1.43484	2.1
55076	1.73669	1.34983
55077	1.93568	2.02913
55078	1.53347	1.0095
55079	1.53347	1.98361
55080	2.14058	1.12423
55081	2.14058	2.07856
55082	0.89964	0.806122
55083	1.10415	2.1
55084	0.868346	1.46063
55085	1.07029	2.1
55086	0.98687	1.60239
55087	0.98687	2.1
55088	0.985627	0.266241
55089	0.985627	2.1
55090	1.07029	1.9125
55091	1.10415	1.6054
55092	0.868344	1.97772
55093	0.899642	2.04852
55094	2.40667	2.1
55095	2.1281	2.1
55096	2.11176	2.1
55097	2.38069	2.1

55098	4.50878	2.1
55099	2.11176	2.1
55100	1.61687	2.1
55101	1.60965	2.1
55102	1.61687	2.1
55103	2.12088	2.1
55104	2.1281	2.1
55105	2.11176	2.1
55106	2.34898	2.1
55107	2.34898	2.1
55108	2.38069	2.1
55109	2.09114	2.1
55110	2.12285	2.1
55111	1.05588	2.1
55112	1.04557	2.1
55113	0.804824	2.1
55114	0.798269	2.1
55115	0.92615	2.1
55116	0.92615	2.1
55117	0.927999	2.1
55118	0.927999	2.1
55119	1.04557	2.1
55120	0.798269	2.1
55121	1.05588	2.1
55122	0.804824	2.1
55123	1.43548	0.0564471
55124	1.53873	0.0588724
55125	1.25063	0.05
55126	1.39662	0.05
55127	2.78937	0.05
55128	1.25063	0.081209
55129	1.39662	0.05
55130	1.10476	0.05
55131	1.28326	0.05
55132	2.53389	0.05
55133	1.51147	0.05
55134	1.86114	0.05
55135	1.86114	0.05
55136	2.12926	0.05
55137	1.3659	0.05
55138	1.63401	0.05
55139	1.89226	0.05
55140	1.89226	0.05
55141	1.89226	0.05
55142	1.89226	0.05
55143	1.89226	0.05
55144	1.89226	0.05
55145	1.89226	0.05
55146	1.89226	0.05
55147	2.46286	0.05
55148	1.89226	0.05
55149	3.78452	0.05
55150	1.89226	0.05
55151	1.89226	0.05
55152	1.89226	0.05
55153	1.89226	0.05
55154	1.89226	0.05
55155	1.89226	0.05

55156	1.89226	0.05
55157	1.89226	0.05
55158	2.28992	0.05
55159	1.89226	0.05
55160	3.78452	0.05
55161	1.13537	0.05
55162	0.886595	0.0811613
55163	0.946131	0.0736587
55164	0.946131	0.05
55165	0.94613	0.127355
55166	0.94613	0.05
55167	1.25069	0.07239
55168	0.841855	0.117084
55169	1.27436	0.05
55170	1.06196	0.05
55171	1.09758	0.05
55172	0.830306	0.05
55173	0.826931	0.319524
55174	0.826933	0.05
55175	0.764631	0.05
55176	0.764631	0.0691207
55177	1.00427	0.117792
55178	0.788034	0.05
55179	0.726852	0.05
55180	0.604206	0.213995
55181	0.828475	0.05
55182	0.604198	0.05
55183	0.8332	0.05
55184	1.00428	0.05
55185	1.89226	0.188522
55186	1.89226	0.05
55187	1.89226	0.05
55188	1.89226	0.0518189
55189	1.89226	0.05
55190	1.89226	0.0669887
55191	0.768789	2.1
55192	0.849132	0.05
55193	0.666423	2.1
55194	0.688993	0.230445
55195	0.868671	0.05
55196	0.89117	2.1
55197	0.705632	2.1
55198	1.80425	2.1
55199	1.73883	2.1
55200	1.45159	2.1
55201	1.45159	2.1
55202	1.37681	2.1
55203	1.73883	2.1
55204	1.39679	0.05
55205	2.08149	0.05
55206	1.89226	0.05
55207	2.32763	0.05
55208	1.89226	0.05
55209	2.31274	0.05
55210	1.89226	0.05
55211	1.89226	0.05
55212	1.89226	0.05
55213	1.89226	0.05

55214	1.89226	0.05
55215	1.89226	0.05
55216	1.89226	0.05
55217	1.89226	0.05
55218	1.89226	0.05
55219	1.89226	0.05
55220	1.89226	0.05
55221	1.89226	0.05
55222	1.95181	0.05
55223	1.89226	0.05
55224	1.89226	0.05
55225	1.89226	0.05
55226	3.78451	0.05
55227	0.946131	0.05
55228	0.946131	0.05
55229	0.946125	0.05
55230	0.946125	0.05
55231	0.946127	0.05
55232	0.946127	0.05
55233	0.946128	0.05
55234	0.946128	0.05
55235	0.946128	0.05
55236	0.946128	0.05
55237	0.946128	0.05
55238	0.946128	0.05
55239	1.89226	0.05
55240	1.89226	0.05
55241	1.89226	0.05
55242	1.89226	0.05
55243	1.89226	0.05
55244	1.89226	0.05
55245	1.89226	0.749047
55246	1.89226	0.618161
55247	1.89226	0.447986
55248	1.89226	0.257983
55249	3.78453	0.531368
55250	1.89226	0.696036
55251	1.89226	0.446449
55252	1.89226	0.544503
55253	1.89226	0.269154
55254	3.78452	0.477651
55255	1.21387	0.227302
55256	0.8164	0.0964349
55257	0.946133	0.27545
55258	0.946133	0.365432
55259	0.946102	0.0839773
55260	0.946102	0.417865
55261	0.816431	0.859973
55262	0.816431	0.327774
55263	0.816401	0.224719
55264	0.94613	0.134613
55265	0.946131	0.916822
55266	1.21388	0.272964
55267	0.946132	0.157671
55268	0.946132	0.196193
55269	0.946132	0.297014
55270	0.946132	0.307123
55271	0.946132	0.0954466

55272	0.946132	0.223963
55273	0.946132	0.610678
55274	0.946132	0.237069
55275	0.946131	0.0945131
55276	0.946131	0.25072
55277	0.946132	0.607438
55278	0.946132	0.244062
55279	1.89226	0.566192
55280	1.89226	0.109025
55281	1.89226	0.0927863
55282	1.89226	0.432972
55283	1.89226	0.144174
55284	1.89226	0.679693
55285	1.89226	0.657497
55286	1.89226	0.376684
55287	2.15871	0.0797654
55288	2.15871	0.420506
55289	1.81454	0.05
55290	1.54809	0.514485
55291	1.89226	1.01702
55292	1.89226	1.46628
55293	1.89226	1.08698
55294	1.89226	0.887522
55295	3.78453	1.25637
55296	1.89226	1.2244
55297	1.89226	0.743401
55298	1.89226	0.619751
55299	1.89226	1.15647
55300	1.89226	1.62732
55301	1.89226	1.2605
55302	2.29205	1.20418
55303	1.42633	1.01825
55304	1.69015	1.33853
55305	1.69015	1.28247
55306	2.10364	0.498887
55307	2.05344	0.592319
55308	2.29205	0.861953
55309	1.58147	0.993515
55310	2.16529	0.509756
55311	1.56339	0.299423
55312	2.29205	1.41413
55313	1.69015	1.25427
55314	1.89226	1.2734
55315	1.89226	1.09134
55316	1.89226	0.843817
55317	1.89226	1.05601
55318	3.78453	0.884911
55319	1.89226	1.07987
55320	1.89226	1.38591
55321	1.89226	1.22815
55322	1.89226	0.660318
55323	3.78452	1.42894
55324	2.29205	1.37677
55325	2.85335	0.314899
55326	2.55409	1.09461
55327	2.04345	0.573739
55328	2.76338	1.01935
55329	2.29205	0.685015

55330	2.29205	1.0932
55331	2.16529	1.20954
55332	2.72418	1.04658
55333	2.76338	0.170685
55334	5.01623	1.04503
55335	1.89227	0.05
55336	1.89227	0.05
55337	1.89227	0.05
55338	1.89227	0.05
55339	3.78453	0.05
55340	2.39588	0.05
55341	2.14918	0.05
55342	2.14918	0.05
55343	2.37907	0.05
55344	2.20531	0.05
55345	2.4352	0.05
55346	1.89226	0.05
55347	2.64327	0.05
55348	2.36175	0.05
55349	1.89226	0.05
55350	4.25402	0.05
55351	1.89226	0.05
55352	1.89226	0.05
55353	1.89226	0.05
55354	1.89226	0.05
55355	1.89226	0.05
55356	1.89226	0.05
55357	1.05395	0.05
55358	1.05395	0.05
55359	1.03114	0.05
55360	1.03114	0.05
55361	0.936689	0.05
55362	1.07459	0.05
55363	1.10265	0.05
55364	1.01049	0.05
55365	0.93669	0.05
55366	1.01049	0.05
55367	1.07459	0.05
55368	1.10265	0.05
55369	1.18088	0.05
55370	0.776509	0.05
55371	0.946132	0.05
55372	0.946132	0.05
55373	1.04225	0.05
55374	0.830392	0.05
55375	0.931352	0.05
55376	0.931352	0.05
55377	1.13006	0.05
55378	1.04074	0.05
55379	1.0628	0.05
55380	0.851529	0.05
55381	2.40032	2.1
55382	1.89226	2.1
55383	1.89226	2.1
55384	1.89226	2.1
55385	2.38918	2.1
55386	2.38918	2.1
55387	1.89226	2.1

55388	1.89226	2.1
55389	1.89226	2.1
55390	1.89226	2.1
55391	1.89226	2.1
55392	1.89226	2.1
55393	1.6585	2.1
55394	2.37364	2.1
55395	2.39622	2.1
55396	2.39622	2.1
55397	1.6488	2.1
55398	1.6488	2.1
55399	2.44603	2.1
55400	1.89224	2.1
55401	1.89224	2.1
55402	1.89224	2.1
55403	2.40029	2.1
55404	2.40029	2.1
55405	1.89224	2.1
55406	1.89224	2.1
55407	1.89224	2.1
55408	1.89224	2.1
55409	1.89224	2.1
55410	1.89224	2.1
55411	1.01946	2.1
55412	1.01946	2.1
55413	1.02497	2.1
55414	1.02497	2.1
55415	1.21519	2.1
55416	1.19809	2.1
55417	0.835303	2.1
55418	0.82924	2.1
55419	0.835314	2.1
55420	1.2152	2.1
55421	0.829228	2.1
55422	1.19808	2.1
55423	1.91027	2.1
55424	1.05809	2.1
55425	1.00659	2.1
55426	2.06727	2.1
55427	3.11819	2.1
55428	2.37166	2.1
55429	2.06726	2.1
55430	1.66994	2.1
55431	1.67623	2.1
55432	2.40308	2.1
55433	2.40308	2.1
55434	0.960194	2.1
55435	0.939664	2.1
55436	0.531131	2.1
55437	0.503298	2.1
55438	0.729927	2.1
55439	0.729927	2.1
55440	0.733565	2.1
55441	0.733565	2.1
55442	0.960195	2.1
55443	0.531132	2.1
55444	0.503297	2.1
55445	0.939663	2.1

55446	1.20154	2.1
55447	1.21754	2.1
55448	0.960195	2.1
55449	0.939665	2.1
55450	1.07745	2.1
55451	1.07745	2.1
55452	1.07976	2.1
55453	1.07976	2.1
55454	0.960194	2.1
55455	1.21754	2.1
55456	0.939662	2.1
55457	1.20155	2.1
55458	1.89224	2.1
55459	1.89224	2.1
55460	2.08609	2.1
55461	2.08609	2.1
55462	2.09527	2.1
55463	1.90142	2.1
55464	1.65815	2.1
55465	1.71398	2.1
55466	2.2001	2.1
55467	2.23791	2.1
55468	1.4502	2.1
55469	1.4502	2.1
55470	0.793587	2.1
55471	0.793587	2.1
55472	0.792408	2.1
55473	0.792408	2.1
55474	0.952221	2.1
55475	0.858585	2.1
55476	0.725101	2.1
55477	0.633774	2.1
55478	0.633775	2.1
55479	0.858585	2.1
55480	0.952221	2.1
55481	0.725101	2.1
55482	1.5735	2.1
55483	2.56975	2.1
55484	2.63166	2.1
55485	2.63166	2.1
55486	1.54711	2.1
55487	1.54711	2.1
55488	0.946131	2.1
55489	0.946131	2.1
55490	1.28487	2.1
55491	1.28487	2.1
55492	0.946127	2.1
55493	0.946127	2.1
55494	1.28488	2.1
55495	1.31583	2.1
55496	1.31583	2.1
55497	0.94613	2.1
55498	1.28487	2.1
55499	0.946129	2.1
55500	1.0596	2.1
55501	1.0596	2.1
55502	1.06939	2.1
55503	1.06939	2.1

55504	1.34225	2.1
55505	1.31583	2.1
55506	0.793563	2.1
55507	0.786748	2.1
55508	0.793563	2.1
55509	1.34225	2.1
55510	0.786748	2.1
55511	1.31583	2.1
55512	0.946131	2.1
55513	0.946131	2.1
55514	1.31582	2.1
55515	1.31582	2.1
55516	0.946127	2.1
55517	0.946127	2.1
55518	1.34225	2.1
55519	1.31583	2.1
55520	1.34225	2.1
55521	0.946129	2.1
55522	1.31583	2.1
55523	0.946129	2.1
55524	1.89226	2.1
55525	1.89226	2.1
55526	1.89226	2.1
55527	1.89226	2.1
55528	1.89226	2.1
55529	1.89226	2.1
55530	1.89226	2.1
55531	1.89226	2.1
55532	1.89226	2.1
55533	1.89226	2.1
55534	1.89226	2.1
55535	1.89226	2.1
55536	1.26992	2.1
55537	1.75003	2.1
55538	1.79883	2.1
55539	1.26732	2.1
55540	3.06875	2.1
55541	0.875017	2.1
55542	0.899414	2.1
55543	0.811147	2.1
55544	0.85053	2.1
55545	0.851513	2.1
55546	0.851513	2.1
55547	0.859048	2.1
55548	0.859048	2.1
55549	0.899413	2.1
55550	0.85053	2.1
55551	0.811147	2.1
55552	0.875016	2.1
55553	1.89227	2.1
55554	1.89227	2.1
55555	1.89227	2.1
55556	1.89227	2.1
55557	1.89227	2.1
55558	1.89227	2.1
55559	1.89226	2.1
55560	1.89226	2.1
55561	1.89226	2.1

55562	1.89226	2.1
55563	1.89226	2.1
55564	1.89226	2.1
55565	0.633661	2.1
55566	0.642005	2.1
55567	0.93285	2.1
55568	0.899413	2.1
55569	0.764487	2.1
55570	0.764487	2.1
55571	0.776931	2.1
55572	0.776931	2.1
55573	0.642004	2.1
55574	0.93285	2.1
55575	0.899414	2.1
55576	0.633661	2.1
55577	0.932851	2.1
55578	0.899414	2.1
55579	0.905419	2.1
55580	0.85053	2.1
55581	0.886336	2.1
55582	0.886336	2.1
55583	0.897045	2.1
55584	0.897045	2.1
55585	0.93285	2.1
55586	0.905419	2.1
55587	0.899414	2.1
55588	0.850531	2.1
55589	2.39322	2.1
55590	2.74149	1.32589
55591	2.70944	2.1
55592	2.6849	1.20409
55593	2.41776	2.1
55594	2.39322	0.176206
55595	2.41776	2.1
55596	2.80992	2.1
55597	2.77256	2.1
55598	2.74149	2.1
55599	2.44882	2.1
55600	2.41776	0.719879
55601	2.80992	2.1
55602	1.89226	1.80458
55603	2.74149	2.00236
55604	2.74149	2.1
55605	1.89226	0.471096
55606	1.89226	1.50533
55607	1.89226	0.05
55608	1.89226	0.05
55609	1.89226	0.05
55610	1.89226	0.05
55611	1.89226	0.05
55612	1.89226	0.05
55613	2.38796	0.05
55614	2.0815	0.05
55615	2.0815	0.05
55616	2.49452	0.05
55617	1.55994	0.05
55618	2.14721	0.05
55619	1.89227	0.05

55620	1.89227	0.05
55621	1.89227	0.05
55622	1.89227	0.05
55623	1.89227	0.05
55624	1.89227	0.05
55625	1.13387	0.05
55626	2.27494	0.05
55627	1.13387	0.05
55628	2.19489	0.05
55629	1.60867	0.05
55630	2.6994	0.05
55631	1.89226	0.05
55632	1.89226	0.05
55633	1.89226	0.05
55634	1.89226	0.05
55635	3.78453	0.05
55636	1.42381	1.92021
55637	1.10747	2.1
55638	1.10747	1.8448
55639	1.08911	2.1
55640	1.42866	1.39165
55641	1.4103	2.1
55642	1.89226	2.1
55643	1.88181	2.1
55644	1.88181	2.1
55645	1.76249	2.1
55646	2.0815	2.1
55647	1.97539	2.1
55648	0.946128	2.1
55649	0.946128	2.1
55650	0.946133	1.7337
55651	0.946133	0.700027
55652	0.94613	0.754409
55653	0.94613	2.1
55654	0.946131	2.1
55655	0.946131	1.17272
55656	0.946131	2.1
55657	0.946131	2.1
55658	0.946131	2.1
55659	0.946131	2.1
55660	1.42866	1.90219
55661	1.43213	1.74606
55662	1.10748	2.1
55663	1.10748	2.1
55664	1.13641	2.1
55665	1.43213	2.1
55666	2.0815	2.1
55667	2.10287	2.1
55668	2.10287	2.1
55669	2.0921	2.1
55670	1.89227	2.1
55671	1.83938	2.1
55672	0.810944	1.55167
55673	0.810944	0.845577
55674	0.810945	0.828935
55675	0.810945	2.1
55676	0.810933	1.9797
55677	0.810933	1.99958

55678	1.89226	2.1
55679	1.3517	2.1
55680	1.08132	1.99453
55681	1.08132	2.1
55682	1.08133	2.02383
55683	2.33097	2.1
55684	1.53822	2.1
55685	1.53822	2.1
55686	1.68437	2.1
55687	2.17996	2.1
55688	2.32612	2.1
55689	0.99189	2.1
55690	1.18948	0.0539105
55691	1.19756	0.0780716
55692	1.19756	2.1
55693	0.978851	2.1
55694	0.926995	2.1
55695	1.26924	0.05
55696	1.34642	0.0555779
55697	1.3228	1.48283
55698	0.990899	1.81061
55699	1.0627	2.0537
55700	1.33473	1.84777
55701	1.96196	2.1
55702	1.39702	2.1
55703	1.39702	2.1
55704	1.53822	2.1
55705	2.17372	2.1
55706	2.31011	2.1
55707	1.89226	0.8715
55708	1.89226	0.05
55709	1.89226	0.153734
55710	1.89226	0.05
55711	3.78453	0.05
55712	2.38475	0.790709
55713	1.73957	0.0635731
55714	1.88452	0.110618
55715	1.58695	0.05
55716	3.6241	0.0744361
55717	1.73958	0.05
55718	1.3126	1.04586
55719	1.58695	0.05
55720	1.14946	0.242434
55721	2.88903	0.05
55722	1.89226	0.05
55723	1.89226	0.994744
55724	1.89226	0.05
55725	1.89226	0.287534
55726	3.78453	0.05
55727	1.89225	0.102129
55728	1.89225	0.0551056
55729	1.89225	0.0853319
55730	1.89225	0.05
55731	3.7845	0.05
55732	1.88451	0.0732839
55733	1.58694	0.05
55734	1.74649	0.107006
55735	1.44604	0.05

55736	3.33055	0.05
55737	1.58694	0.05
55738	0.995727	0.13083
55739	0.995727	0.095055
55740	1.14945	0.106005
55741	1.44604	0.05
55742	1.59976	0.218445
55743	1.89225	0.05
55744	1.89225	0.109953
55745	1.89225	0.96332
55746	1.89225	0.05
55747	1.89225	0.899613
55748	1.89225	0.05
55749	2.01197	1.88042
55750	2.0961	1.80363
55751	2.13283	0.913653
55752	2.25399	0.427313
55753	4.22894	1.31835
55754	2.09611	1.013
55755	2.37014	1.16327
55756	2.29031	1.76705
55757	2.10084	2.09646
55758	2.25399	0.436457
55759	2.09611	1.98438
55760	2.13283	1.63559
55761	2.25399	2.1
55762	2.22044	1.5377
55763	2.38975	0.648719
55764	4.52258	1.37346
55765	2.38976	0.24238
55766	2.15466	1.2446
55767	2.31988	0.0748134
55768	2.31988	2.06291
55769	2.25399	0.174742
55770	2.25399	2.1
55771	2.58286	0.634955
55772	2.29193	0.05
55773	2.40107	1.04968
55774	2.13815	0.05
55775	4.72101	0.05
55776	2.29193	0.05
55777	1.90104	0.0632423
55778	2.13815	0.05
55779	1.77249	0.0852669
55780	4.03919	0.05
55781	2.25197	0.22475
55782	2.13815	0.05
55783	2.00625	0.0617124
55784	2.00625	0.05
55785	2.40107	0.0603318
55786	2.40107	0.05
55787	2.13815	0.05
55788	1.77249	0.0988917
55789	2.00625	0.05
55790	1.65815	0.0573706
55791	3.7963	0.05
55792	2.43417	0.243775
55793	2.06653	0.129518

55794	1.64587	0.158033
55795	1.65284	0.105188
55796	2.46898	0.231527
55797	2.46898	0.149589
55798	2.46895	0.148176
55799	1.89016	0.0739472
55800	1.87861	0.0959594
55801	1.87861	0.0569218
55802	2.47161	0.155327
55803	2.47161	0.0908592
55804	0.946132	0.393045
55805	0.946132	0.205565
55806	0.863311	0.475219
55807	1.34768	0.342539
55808	0.851542	0.423963
55809	0.851542	0.206212
55810	1.30451	0.280743
55811	1.45304	0.328421
55812	0.946132	0.244771
55813	1.20992	0.28579
55814	0.855162	0.449329
55815	0.946132	0.261106
55816	1.89226	0.203289
55817	1.89226	0.68245
55818	1.89226	0.432129
55819	1.89226	0.344393
55820	1.89226	0.452914
55821	1.89226	0.371314
55822	0.946132	0.173129
55823	0.946132	0.0592436
55824	0.946133	0.103795
55825	0.946133	0.0766864
55826	0.946132	0.108406
55827	0.946132	0.0776502
55828	0.946132	0.065789
55829	0.946132	0.0806525
55830	0.946132	0.134083
55831	0.946132	0.0789603
55832	0.946133	0.0983933
55833	0.946133	0.0970324
55834	2.35574	0.784418
55835	1.89226	0.0896992
55836	1.89226	2.1
55837	1.89226	0.0790203
55838	2.67456	1.38012
55839	2.67456	0.05
55840	0.583505	0.05
55841	0.761972	0.128893
55842	1.17787	1.04978
55843	1.33728	0.05
55844	0.965128	1.02248
55845	0.965128	0.0525806
55846	0.974714	0.359057
55847	0.974715	0.479324
55848	1.17787	0.101479
55849	0.583506	0.742615
55850	1.33728	0.05
55851	0.761974	0.0551607

55852	1.89226	0.742289
55853	1.89226	0.0608287
55854	1.89226	0.0715248
55855	1.89226	0.05
55856	1.89226	0.0719412
55857	1.89226	0.566434
55858	1.89226	0.05
55859	1.89226	0.05
55860	1.89226	0.05
55861	1.89226	0.0518997
55862	3.78453	0.05
55863	0.946132	0.05
55864	0.946132	0.05
55865	0.946132	0.0601007
55866	0.946132	0.05
55867	0.946133	0.155176
55868	0.946133	0.0619719
55869	0.946132	0.345025
55870	0.946132	0.05
55871	0.946133	0.05
55872	0.946133	0.294022
55873	0.946132	0.05
55874	0.946132	0.15183
55875	1.01779	2.1
55876	1.50685	2.1
55877	1.12064	2.1
55878	1.65151	2.1
55879	2.6275	2.1
55880	1.35733	2.1
55881	1.53507	2.1
55882	1.50683	2.1
55883	1.73626	2.1
55884	3.09359	2.1
55885	1.73626	2.1
55886	1.52226	2.1
55887	1.75555	2.1
55888	1.75555	2.1
55889	1.53507	2.1
55890	1.53507	2.1
55891	1.65151	2.1
55892	1.73628	2.1
55893	1.69528	2.1
55894	1.50685	2.1
55895	1.92471	2.1
55896	1.73628	2.1
55897	1.73629	2.1
55898	1.75558	2.1
55899	1.9943	2.1
55900	1.9943	2.1
55901	1.92471	2.1
55902	1.73629	2.1
55903	2.5601	1.13189
55904	1.97645	2.08649
55905	2.09605	1.04318
55906	2.09605	1.98065
55907	2.45853	1.18718
55908	2.45853	2.1
55909	2.33824	1.92413

55910	1.97646	1.74712
55911	2.45853	0.952487
55912	1.94984	2.1
55913	2.33824	1.89984
55914	1.82955	2.1
55915	1.22926	2.1
55916	0.946128	2.07203
55917	0.946133	2.1
55918	1.28005	1.56491
55919	1.22927	1.57762
55920	1.22926	1.9541
55921	0.946131	1.79555
55922	0.946131	1.60617
55923	0.946131	1.61711
55924	0.946131	1.43878
55925	1.28005	1.73469
55926	1.22926	1.64954
55927	2.35349	0.273886
55928	2.61606	0.0542266
55929	2.48209	0.209222
55930	2.48209	0.05
55931	2.50057	0.165682
55932	2.50057	0.05
55933	2.61607	0.156907
55934	2.57611	0.05
55935	2.4821	0.05
55936	2.44814	0.05
55937	5.06421	0.05
55938	2.15721	0.05
55939	2.48209	0.05
55940	2.31844	0.299267
55941	2.31844	0.05
55942	2.35349	0.304157
55943	2.35349	0.05
55944	2.44814	0.05
55945	2.30908	0.05
55946	2.44814	0.05
55947	2.31844	0.05
55948	2.4821	0.127455
55949	2.31844	0.0572652
55950	0.91766	0.05
55951	0.91766	0.05
55952	0.912463	0.05
55953	0.912463	0.0583472
55954	1.16525	0.05
55955	1.26377	0.05
55956	0.576692	0.05
55957	0.664871	0.05
55958	1.16525	0.05
55959	0.576692	0.05
55960	0.664871	0.05
55961	1.26377	0.0519128
55962	1.72901	2.1
55963	1.62414	2.1
55964	1.88971	2.1
55965	1.8411	1.75328
55966	3.57011	2.1
55967	1.89226	2.1

55968	1.89226	1.92641
55969	1.89226	0.957904
55970	1.89226	1.97765
55971	3.78452	1.11862
55972	1.89226	1.15092
55973	1.89226	2.1
55974	1.89226	2.07042
55975	1.89226	1.05954
55976	3.78452	2.1
55977	1.62414	2.1
55978	1.30896	2.1
55979	1.8411	1.60458
55980	1.52776	2.1
55981	3.15006	2.00795
55982	1.51448	2.1
55983	1.8411	2.1
55984	2.02311	2.1
55985	1.99309	0.545778
55986	3.86421	1.85062
55987	1.89226	1.05922
55988	1.89226	2.1
55989	1.89226	2.1
55990	1.89226	1.33974
55991	3.78452	2.1
55992	1.89226	2.1
55993	1.89226	1.81856
55994	1.89226	1.69228
55995	1.89226	2.1
55996	3.78452	2.07932
55997	1.8411	2.09993
55998	1.52776	2.1
55999	1.99309	2.1
56000	1.72526	2.1
56001	3.56636	2.1
56002	1.89226	2.1
56003	1.89226	2.09923
56004	1.89226	2.1
56005	1.89226	2.1
56006	3.78453	2.1
56007	1.0424	2.1
56008	1.12935	2.1
56009	0.929383	2.1
56010	0.929383	2.1
56011	1.24941	2.1
56012	1.12686	2.1
56013	0.946132	2.1
56014	0.946132	2.1
56015	1.12944	2.1
56016	0.851554	2.1
56017	1.05017	2.1
56018	0.929315	2.1
56019	0.898029	2.1
56020	0.973994	2.1
56021	0.946131	2.1
56022	0.946131	2.1
56023	1.0221	2.1
56024	1.0221	2.1
56025	0.898028	2.08172

56026	0.898028	2.1
56027	0.973994	2.1
56028	0.946131	2.1
56029	0.946131	2.1
56030	0.898029	2.1
56031	1.89226	2.08112
56032	1.89226	2.1
56033	1.89226	1.96419
56034	1.89226	2.1
56035	1.89226	1.99333
56036	1.89226	2.1
56037	1.13537	2.1
56038	0.879308	2.1
56039	0.946131	2.07771
56040	0.946131	2.09195
56041	1.13537	2.1
56042	0.85187	2.1
56043	0.94613	2.1
56044	0.94613	1.91469
56045	1.05777	2.1
56046	1.05777	2.1
56047	0.799318	2.1
56048	0.776452	2.1
56049	0.946116	2.1
56050	0.946116	2.1
56051	1.09531	2.1
56052	1.09531	2.1
56053	0.946118	2.1
56054	0.946118	2.1
56055	1.09135	2.1
56056	1.09531	2.1
56057	1.09134	2.1
56058	0.946105	2.1
56059	1.09532	2.1
56060	0.946131	2.1
56061	0.946129	2.1
56062	0.946129	2.1
56063	1.09137	2.1
56064	1.09137	2.1
56065	0.946131	2.1
56066	0.946131	2.1
56067	1.07713	2.1
56068	1.09137	2.1
56069	0.946131	2.1
56070	1.07713	2.1
56071	1.09137	2.1
56072	0.946131	2.1
56073	0.938916	2.1
56074	0.938916	2.1
56075	0.936995	2.1
56076	1.00447	2.1
56077	0.979819	2.1
56078	1.16821	2.1
56079	1.01263	2.1
56080	0.719167	2.1
56081	1.04536	2.1
56082	1.18901	2.1
56083	0.758057	2.1

56084	0.924111	2.1
56085	1.00532	2.1
56086	1.00532	2.1
56087	0.9925	2.1
56088	0.9925	2.1
56089	1.29322	2.1
56090	1.01985	2.1
56091	1.00365	2.1
56092	0.704601	2.1
56093	1.00365	2.1
56094	1.29322	2.1
56095	1.01985	2.1
56096	0.704602	2.1
56097	2.20011	2.1
56098	1.401	2.1
56099	1.401	2.1
56100	1.52008	2.1
56101	2.11084	2.1
56102	2.22991	2.1
56103	0.854068	2.1
56104	0.854068	2.1
56105	0.851357	2.1
56106	0.851357	2.1
56107	0.644681	2.1
56108	0.700493	2.1
56109	1.0554	2.1
56110	1.00493	2.1
56111	1.00495	2.1
56112	0.64469	2.1
56113	0.700483	2.1
56114	1.05539	2.1
56115	2.46665	0.05
56116	2.22713	0.05
56117	1.85877	0.05
56118	1.8967	0.05
56119	1.56002	0.05
56120	2.22713	0.05
56121	1.89225	2.1
56122	1.89225	2.1
56123	1.89225	2.1
56124	1.89225	2.1
56125	1.89225	2.1
56126	1.89225	2.1
56127	1.89225	2.1
56128	1.89225	2.1
56129	1.89225	2.1
56130	1.89225	2.1
56131	1.89225	2.1
56132	1.89225	2.1
56133	1.284	2.1
56134	1.86569	2.1
56135	1.95398	2.1
56136	1.31091	2.1
56137	3.23798	2.1
56138	0.932844	2.1
56139	0.976992	2.1
56140	0.960128	2.1
56141	0.905413	2.1

56142	0.938531	2.1
56143	0.938531	2.1
56144	0.943874	2.1
56145	0.943874	2.1
56146	0.976985	2.1
56147	0.960122	2.1
56148	0.93285	2.1
56149	0.905419	2.1
56150	1.89227	2.1
56151	1.89227	2.1
56152	1.89227	2.1
56153	1.89227	2.1
56154	3.78453	2.1
56155	1.89226	2.1
56156	1.89226	2.1
56157	1.89226	2.1
56158	1.89226	2.1
56159	1.89226	2.1
56160	1.89226	2.1
56161	0.65546	2.1
56162	0.665451	2.1
56163	1.00694	2.1
56164	0.976998	2.1
56165	0.816261	2.1
56166	0.816261	2.1
56167	0.826189	2.1
56168	0.826189	2.1
56169	1.00694	2.1
56170	0.665451	2.1
56171	0.976998	2.1
56172	0.65546	2.1
56173	2.01555	2.1
56174	2.01388	2.1
56175	1.92027	2.1
56176	1.92027	2.1
56177	2.01388	2.1
56178	1.954 2.1	
56179	2.75466	2.1
56180	1.89226	2.1
56181	1.89226	2.1
56182	2.54931	2.1
56183	4.44158	2.1
56184	2.47917	2.1
56185	1.92772	2.1
56186	1.80682	2.1
56187	2.54931	2.1
56188	4.47702	2.1
56189	0.946132	2.1
56190	0.946132	2.1
56191	1.17602	2.1
56192	1.27466	2.1
56193	0.946132	2.1
56194	0.946132	2.1
56195	1.17602	2.1
56196	1.17602	2.1
56197	0.946132	2.1
56198	1.17602	2.1
56199	0.946131	2.1

56200	1.27466	2.1
56201	2.35204	2.1
56202	2.54931	2.1
56203	2.40783	2.1
56204	1.50108	2.1
56205	1.64256	2.1
56206	1.64256	2.1
56207	1.89226	2.1
56208	1.89226	0.971118
56209	2.31012	2.1
56210	2.17534	2.1
56211	4.0676	2.1
56212	2.17533	2.1
56213	1.89226	0.798043
56214	2.17533	1.6751
56215	1.89226	0.971895
56216	1.66635	2.1
56217	1.38328	2.01196
56218	1.92027	2.1
56219	2.17597	2.1
56220	2.14619	2.1
56221	2.15388	2.1
56222	1.91087	2.1
56223	1.92027	2.1
56224	1.08622	2.1
56225	1.08622	2.1
56226	0.971184	2.1
56227	0.972761	2.1
56228	1.14845	2.1
56229	1.16321	2.1
56230	0.894769	2.1
56231	0.894967	2.1
56232	0.894775	2.1
56233	1.28563	2.1
56234	1.14844	2.1
56235	1.0167	2.1
56236	1.89226	1.32004
56237	1.89226	2.1
56238	1.89226	2.1
56239	1.89226	1.54874
56240	1.89226	2.1
56241	1.89226	1.80325
56242	0.937933	2.1
56243	0.937933	2.1
56244	1.26192	2.1
56245	1.26192	2.1
56246	0.937903	2.1
56247	1.25372	2.1
56248	0.946132	2.1
56249	0.946132	2.1
56250	0.946133	2.1
56251	1.25372	2.1
56252	0.946132	2.1
56253	0.937902	2.1
56254	0.946132	1.1283
56255	0.946132	0.632056
56256	0.946128	2.1
56257	0.946128	2.1

56258	0.94613	2.1
56259	0.94613	1.05193
56260	0.94613	0.372969
56261	0.94613	2.1
56262	0.946131	1.88631
56263	0.946131	1.97912
56264	0.94613	1.98554
56265	0.94613	1.97408
56266	0.946132	1.48833
56267	0.946132	0.944245
56268	1.2778	2.1
56269	1.00191	2.1
56270	1.00191	1.32882
56271	1.00191	2.1
56272	0.94613	2.1
56273	0.94613	1.49006
56274	0.946131	2.1
56275	1.2778	2.1
56276	1.00191	2.08301
56277	0.94613	2.05504
56278	1.50847	0.05
56279	2.0815	0.05
56280	1.56806	0.05
56281	2.0815	0.05
56282	1.52043	0.05
56283	1.9224	0.05
56284	1.06882	0.05
56285	0.69567	0.05
56286	0.773112	0.05
56287	1.09473	0.05
56288	0.8941	0.05
56289	0.8941	0.05
56290	0.937064	0.05
56291	0.981131	0.05
56292	0.763762	0.05
56293	0.722535	0.05
56294	1.2153	0.05
56295	1.07279	0.05
56296	1.89226	0.05
56297	1.89226	0.05
56298	1.89226	0.05
56299	1.89226	0.05
56300	1.89226	0.05
56301	1.89226	0.05
56302	1.89226	0.05
56303	1.89226	0.05
56304	1.89226	0.05
56305	1.89226	0.05
56306	3.78453	0.05
56307	2.51518	0.05
56308	1.89226	0.05
56309	1.89226	0.05
56310	1.89226	0.05
56311	2.37584	0.05
56312	2.37584	0.05
56313	1.25759	0.05
56314	0.946131	0.05
56315	0.946131	0.05

56316	1.24489	0.05
56317	0.946131	0.05
56318	0.946131	0.05
56319	1.25759	0.05
56320	1.25759	0.05
56321	1.25759	0.05
56322	1.24489	0.05
56323	0.946131	0.05
56324	0.946131	0.05
56325	1.08028	0.11085
56326	1.09362	0.158368
56327	0.596616	0.151929
56328	0.606432	0.345332
56329	0.844265	0.353328
56330	0.844265	0.05
56331	0.855786	1.16872
56332	0.855786	0.0732453
56333	1.08028	0.280947
56334	0.606433	0.0771709
56335	0.596616	0.0692242
56336	1.09362	0.0699968
56337	2.837	0.637994
56338	2.16055	0.05
56339	2.12741	0.521945
56340	2.79312	0.233098
56341	4.95367	0.522209
56342	2.12741	0.310979
56343	1.21286	0.05
56344	1.20859	0.601558
56345	1.21286	0.768836
56346	2.15628	0.149628
56347	2.16056	0.0580697
56348	1.89226	2.1
56349	1.89226	2.1
56350	1.89226	2.1
56351	1.89226	2.1
56352	3.78453	2.1
56353	0.946102	2.1
56354	0.946102	2.1
56355	0.850039	2.1
56356	0.850039	2.1
56357	0.914891	2.1
56358	0.85001	2.1
56359	0.946131	2.1
56360	0.946131	2.1
56361	0.94613	2.1
56362	0.91489	2.1
56363	0.850011	2.1
56364	0.946131	2.1
56365	0.71107	2.1
56366	0.784813	2.1
56367	1.28453	2.1
56368	1.20328	2.1
56369	0.992128	2.1
56370	0.992128	2.1
56371	0.995965	2.1
56372	0.995965	2.1
56373	0.784813	2.1

56374	1.28453	2.1
56375	0.711071	2.1
56376	1.20328	2.1
56377	1.89226	2.1
56378	1.89226	2.1
56379	1.89226	2.1
56380	1.89226	2.1
56381	3.78453	2.1
56382	0.946132	2.1
56383	0.946132	2.1
56384	0.914889	2.1
56385	0.914889	2.1
56386	0.985255	2.1
56387	0.914891	2.1
56388	0.946131	2.1
56389	0.946131	2.1
56390	0.946131	2.1
56391	0.985255	2.1
56392	0.94613	2.1
56393	0.91489	2.1
56394	0.784813	2.1
56395	0.871551	2.1
56396	1.37791	2.1
56397	1.28453	2.1
56398	1.07644	2.1
56399	1.07644	2.1
56400	1.07964	2.1
56401	1.07964	2.1
56402	0.871551	2.1
56403	1.37791	2.1
56404	0.784813	2.1
56405	1.28453	2.1
56406	1.73075	1.62383
56407	2.10582	0.454375
56408	1.89319	0.449927
56409	1.38766	2.03801
56410	3.27777	0.602282
56411	1.08715	1.9364
56412	1.54185	1.77749
56413	1.73074	1.41011
56414	1.73074	2.09395
56415	1.18948	0.923229
56416	1.18948	1.86316
56417	1.89319	0.39942
56418	1.41689	1.82038
56419	1.11619	0.629567
56420	1.38766	1.43287
56421	1.6052	0.459983
56422	1.89319	1.3907
56423	0.647894	1.64082
56424	0.594738	1.92374
56425	0.708445	1.66509
56426	0.770926	1.12581
56427	0.738326	1.71265
56428	0.738326	2.0329
56429	0.680494	1.57565
56430	0.680494	0.720403
56431	0.708443	1.00011

56432	0.647895	1.04007
56433	0.770925	1.14183
56434	0.59474	0.637907
56435	1.89111	1.56754
56436	1.06994	2.1
56437	2.06689	2.1
56438	1.37063	2.1
56439	3.13683	2.1
56440	2.06689	2.1
56441	1.39295	2.1
56442	1.56818	2.1
56443	1.37063	2.1
56444	2.26444	2.0674
56445	2.06689	2.1
56446	1.49808	2.1
56447	1.50201	2.1
56448	2.57784	2.1
56449	2.58615	2.1
56450	4.08424	2.1
56451	0.946133	2.1
56452	0.946133	2.1
56453	0.946132	2.1
56454	0.946132	2.1
56455	0.946132	2.1
56456	0.946132	2.1
56457	0.946132	2.1
56458	0.946132	2.1
56459	0.946133	2.1
56460	0.946133	2.1
56461	0.946132	2.1
56462	0.946132	2.1
56463	1.89226	2.1
56464	1.89226	2.1
56465	1.89226	2.1
56466	1.89226	2.1
56467	3.78452	2.1
56468	0.977201	2.1
56469	0.977201	2.1
56470	0.980489	2.1
56471	0.980489	2.1
56472	0.657973	2.1
56473	1.20669	2.1
56474	0.751002	2.1
56475	1.29307	2.1
56476	1.29308	2.1
56477	1.20669	2.1
56478	0.751003	2.1
56479	0.657972	2.1
56480	0.946131	2.1
56481	0.946131	2.1
56482	1.29308	2.1
56483	1.29308	2.1
56484	0.946132	2.1
56485	1.29308	2.1
56486	0.946132	2.1
56487	1.28892	2.1
56488	0.946132	2.1
56489	0.946132	2.1

56490	1.28892	2.1
56491	1.29308	2.1
56492	0.946132	2.1
56493	0.946132	1.90409
56494	0.946131	2.1
56495	0.946131	2.1
56496	0.946132	2.1
56497	0.946132	2.1
56498	0.946132	2.04735
56499	0.946132	1.88944
56500	0.946132	1.97457
56501	0.946132	2.1
56502	0.946132	2.1
56503	0.946132	2.1
56504	1.89226	2.1
56505	1.89226	2.1
56506	1.89226	2.1
56507	1.89226	2.1
56508	1.89226	2.1
56509	1.89226	2.0903
56510	0.94613	2.1
56511	0.94613	2.1
56512	1.20669	2.1
56513	1.20669	2.1
56514	1.20669	2.1
56515	0.946132	2.1
56516	0.946129	2.1
56517	1.29307	2.1
56518	0.946131	2.1
56519	0.946131	2.1
56520	1.29308	2.1
56521	1.20669	2.1
56522	1.89226	0.05
56523	1.89226	0.05
56524	1.89226	0.05
56525	1.89226	0.05
56526	3.78453	0.05
56527	0.946132	0.05
56528	0.946132	0.05
56529	0.946126	0.05
56530	0.946126	0.05
56531	0.946129	0.05
56532	0.946129	0.05
56533	0.946129	0.05
56534	0.946129	0.05
56535	0.946128	0.05
56536	0.946128	0.05
56537	0.946129	0.05
56538	0.946129	0.05
56539	0.725277	0.05
56540	0.588155	0.05
56541	1.19403	0.05
56542	1.27681	0.05
56543	0.918903	0.05
56544	0.918903	0.05
56545	0.946068	0.05
56546	0.946068	0.05
56547	1.19404	0.05

56548	0.588153	0.05
56549	0.725275	0.05
56550	1.27682	0.05
56551	1.19404	0.05
56552	1.27682	0.05
56553	0.946131	0.05
56554	0.946131	0.05
56555	1.19404	0.05
56556	1.19404	0.05
56557	0.946129	0.05
56558	0.946129	0.05
56559	1.19404	0.05
56560	0.94613	0.05
56561	1.27682	0.05
56562	0.946131	0.05
56563	1.89226	0.05
56564	1.89226	0.05
56565	1.89226	0.05
56566	1.89226	0.05
56567	1.89226	0.05
56568	1.89226	0.05
56569	1.4263	1.88758
56570	1.67651	0.0629392
56571	1.5955	0.261121
56572	1.31022	1.95451
56573	2.98674	0.926256
56574	1.18824	2.1
56575	1.28802	2.1
56576	0.946132	1.02944
56577	0.946132	1.90006
56578	0.946132	1.53531
56579	0.946132	2.1
56580	1.28802	0.671592
56581	1.28802	2.1
56582	1.18824	2.1
56583	0.946132	2.1
56584	0.946132	2.1
56585	1.28802	2.1
56586	1.89226	0.112576
56587	1.89226	1.66358
56588	1.89226	2.0266
56589	1.89226	0.102797
56590	3.78452	0.296065
56591	1.5955	0.606283
56592	1.52909	0.0939417
56593	1.31022	0.197578
56594	1.31022	2.08065
56595	1.2087	1.94633
56596	1.52909	1.93771
56597	1.11825	2.1
56598	1.18824	2.1
56599	0.946132	1.82527
56600	0.946132	1.70538
56601	0.946132	1.63307
56602	0.946132	2.1
56603	1.18824	1.09405
56604	1.18824	2.1
56605	0.946132	2.1

56606	1.11825	2.1
56607	1.18824	2.1
56608	0.946133	2.1
56609	0.810943	1.91258
56610	0.810943	1.48769
56611	0.810945	0.178085
56612	0.810945	1.73575
56613	1.89226	0.148257
56614	1.35168	0.380578
56615	1.08132	0.179469
56616	1.0813	0.749499
56617	1.08132	1.79637
56618	0.810959	0.551836
56619	0.810959	0.678596
56620	1.89226	0.0577362
56621	1.89226	0.05
56622	1.89226	0.05
56623	1.89226	0.05
56624	3.78453	0.05
56625	1.89226	0.05
56626	1.89226	0.05
56627	1.89226	0.05
56628	1.89226	0.05
56629	1.89226	0.05
56630	1.89226	0.05
56631	0.946131	0.05
56632	0.946131	0.05
56633	0.946126	0.05
56634	0.946126	0.05
56635	0.946128	0.05
56636	0.946128	0.05
56637	0.946128	0.05
56638	0.946128	0.05
56639	0.946128	0.05
56640	0.946128	0.05
56641	0.946128	0.05
56642	0.946128	0.05
56643	1.89226	0.0515337
56644	1.89226	0.05
56645	1.89226	0.05
56646	1.89226	0.05
56647	1.89226	0.05
56648	1.89226	0.05
56649	1.89226	0.05
56650	1.89226	0.05
56651	1.89226	0.0990759
56652	1.89226	0.05
56653	3.78453	0.05
56654	1.07176	0.05
56655	1.07176	0.05
56656	0.946126	0.05
56657	0.946126	0.05
56658	1.07176	0.05
56659	0.946129	0.05
56660	1.27474	0.05
56661	0.94613	0.05
56662	1.27474	0.05
56663	1.07176	0.05

56664	0.94613	0.05
56665	0.94613	0.05
56666	0.723486	0.05
56667	0.94613	0.05
56668	0.946126	0.05
56669	0.946126	0.05
56670	0.946129	0.05
56671	0.723484	0.05
56672	1.07176	0.05
56673	0.946129	0.05
56674	1.32398	0.05
56675	1.19835	0.05
56676	0.723471	0.05
56677	0.723471	0.05
56678	0.810946	0.0634692
56679	0.810946	0.05
56680	0.810946	0.0635353
56681	0.810946	0.05
56682	0.810933	0.05
56683	0.810933	0.05
56684	1.89226	0.05
56685	1.3517	0.05
56686	1.08132	0.05
56687	1.08132	0.05
56688	1.08133	0.05
56689	2.39923	2.1
56690	1.89226	2.1
56691	1.89226	2.1
56692	1.89226	2.1
56693	2.44606	2.1
56694	2.44606	2.1
56695	1.89226	2.1
56696	1.89226	2.1
56697	1.89226	2.1
56698	1.89226	2.1
56699	1.89226	2.1
56700	1.89226	2.1
56701	1.70302	2.1
56702	2.43041	2.1
56703	2.25275	2.1
56704	2.4747	2.1
56705	1.52079	2.1
56706	1.67063	2.1
56707	2.40644	2.1
56708	1.89226	2.1
56709	1.89226	2.1
56710	1.89226	2.1
56711	2.39923	2.1
56712	2.39923	2.1
56713	0.946131	1.90855
56714	0.946131	2.1
56715	0.946131	2.1
56716	0.946131	2.1
56717	0.94613	2.1
56718	0.94613	2.1
56719	0.946131	2.1
56720	0.946131	2.1
56721	0.946131	2.1

56722	0.946131	2.1
56723	0.946131	2.1
56724	0.946131	2.1
56725	1.04938	2.1
56726	1.04938	2.1
56727	1.111	2.1
56728	1.06978	2.1
56729	1.2748	2.1
56730	1.25472	2.1
56731	0.88782	2.1
56732	0.882987	2.1
56733	0.973779	2.1
56734	1.31997	2.1
56735	0.913905	2.1
56736	1.31312	2.1
56737	2.6536	2.1
56738	2.15287	2.1
56739	2.72238	2.1
56740	2.22887	2.1
56741	4.87525	2.1
56742	2.15288	2.1
56743	1.40719	2.1
56744	1.41839	2.1
56745	1.34239	2.1
56746	2.22887	2.1
56747	2.15288	2.1
56748	2.72237	2.1
56749	2.22887	2.1
56750	2.77314	2.1
56751	2.28748	2.1
56752	5.00985	2.1
56753	0.922802	2.1
56754	0.922802	2.1
56755	0.92107	2.1
56756	0.92107	2.1
56757	0.703593	2.1
56758	0.729436	2.1
56759	1.11444	2.1
56760	1.14374	2.1
56761	1.14374	2.1
56762	0.729436	2.1
56763	0.703593	2.1
56764	1.11444	2.1
56765	1.89226	2.1
56766	1.89226	2.1
56767	1.89226	2.1
56768	1.89226	2.1
56769	1.89226	2.1
56770	1.89226	2.1
56771	0.489927	2.1
56772	0.63008	2.1
56773	0.697385	2.1
56774	0.55524	2.1
56775	0.592141	2.1
56776	0.592141	2.1
56777	0.593179	2.1
56778	0.593179	2.1
56779	0.63008	2.1

56780	0.697385	2.1
56781	0.55524	2.1
56782	0.489927	2.1
56783	0.946133	2.1
56784	0.946133	2.1
56785	0.946132	1.79634
56786	0.946132	0.537939
56787	0.946133	1.30467
56788	0.946133	2.1
56789	0.946132	2.1
56790	0.946132	0.746034
56791	0.946132	1.89755
56792	0.946132	1.77818
56793	0.946132	1.50279
56794	0.946132	1.83342
56795	1.89226	2.1
56796	1.89226	2.1
56797	1.89226	2.1
56798	1.89226	2.1
56799	1.89226	2.1
56800	1.89226	2.1
56801	0.805746	2.1
56802	0.63008	2.1
56803	0.881598	2.1
56804	0.697385	2.1
56805	0.749417	2.1
56806	0.749417	2.1
56807	0.753714	2.1
56808	0.753714	2.1
56809	0.881598	2.1
56810	0.805746	2.1
56811	0.63008	2.1
56812	0.697385	2.1
56813	0.881599	2.1
56814	0.697385	2.1
56815	0.805746	2.1
56816	0.626731	1.47225
56817	0.755479	2.09233
56818	0.755479	2.1
56819	0.752851	1.41703
56820	0.752851	2.1
56821	0.881598	2.1
56822	0.805747	2.1
56823	0.697385	2.1
56824	0.626731	2.1
56825	2.65348	2.1
56826	2.12314	2.1
56827	2.63899	2.1
56828	2.13898	2.1
56829	4.76213	2.1
56830	0.763518	2.1
56831	0.765889	2.1
56832	0.84086	2.1
56833	0.84086	2.1
56834	0.632078	2.1
56835	0.597565	2.1
56836	1.08813	2.1
56837	0.985643	2.1

56838	0.982464	2.1
56839	0.66404	2.1
56840	1.0358	2.1
56841	0.598023	2.1
56842	1.29293	2.1
56843	1.29628	2.1
56844	1.19266	2.1
56845	1.19266	2.1
56846	1.07109	2.1
56847	1.13093	2.1
56848	1.34467	2.1
56849	1.35251	2.1
56850	1.34369	2.1
56851	1.12525	2.1
56852	1.28746	2.1
56853	1.13511	2.1
56854	2.19717	2.1
56855	2.16547	2.1
56856	1.37392	2.1
56857	1.37392	2.1
56858	1.2966	2.1
56859	2.11985	2.1
56860	1.89226	2.1
56861	1.89226	2.1
56862	1.89226	2.1
56863	1.89226	2.1
56864	1.89226	2.1
56865	1.89226	2.1
56866	1.65637	2.1
56867	1.41758	2.1
56868	1.65637	2.1
56869	1.74391	2.1
56870	1.35504	2.1
56871	1.44258	2.1
56872	1.40774	2.1
56873	1.40774	2.1
56874	0.946131	2.1
56875	0.946131	2.1
56876	1.40774	2.1
56877	1.38882	2.1
56878	0.946132	2.1
56879	0.946132	2.1
56880	0.946132	2.1
56881	1.38882	2.1
56882	0.946132	2.1
56883	1.40774	2.1
56884	1.89227	2.1
56885	1.89227	2.1
56886	1.89227	2.1
56887	1.89227	2.1
56888	3.78453	2.1
56889	0.603148	2.1
56890	0.600177	2.1
56891	0.558615	2.1
56892	0.580714	2.1
56893	0.558606	2.1
56894	0.67883	2.1
56895	1.65637	2.1

56896	1.07933	2.1
56897	0.842649	2.1
56898	0.905168	2.1
56899	0.774334	2.1
56900	1.38882	2.1
56901	1.38882	2.1
56902	0.946132	2.1
56903	0.946132	2.1
56904	1.38882	2.1
56905	1.37381	2.1
56906	0.946132	2.1
56907	0.946132	2.1
56908	0.946132	2.1
56909	1.37381	2.1
56910	0.946133	2.1
56911	1.38882	2.1
56912	2.3771	2.1
56913	1.9339	2.1
56914	1.70782	2.1
56915	1.9339	2.1
56916	2.42693	2.1
56917	2.83279	2.1
56918	2.34901	0.310991
56919	1.45833	0.427994
56920	1.86695	0.256263
56921	1.03101	0.449231
56922	3.32528	0.295912
56923	1.89226	0.0711695
56924	1.89226	0.0631033
56925	1.89226	0.05
56926	1.89226	0.05
56927	3.78453	0.05
56928	2.71753	0.05
56929	1.89226	0.0625208
56930	2.76228	0.05
56931	2.76228	0.05
56932	1.89226	0.066786
56933	1.89226	0.157211
56934	0.946131	0.05
56935	0.946131	0.05
56936	0.946132	0.05
56937	0.946132	0.05
56938	0.946132	0.05
56939	0.946132	0.05
56940	0.946132	0.05
56941	0.946132	0.05
56942	0.946132	0.05
56943	0.946132	0.05
56944	0.946132	0.0501258
56945	0.946132	0.05
56946	1.89227	0.05
56947	1.89227	0.05
56948	1.89227	0.05
56949	1.89227	0.05
56950	1.89227	0.05
56951	1.89227	0.05
56952	2.71753	0.05
56953	2.37907	0.05

56954	2.37907	0.05
56955	2.76228	0.05
56956	2.39588	0.05
56957	2.77909	0.05
56958	1.6571	0.595991
56959	1.11682	1.65537
56960	1.47132	0.525388
56961	1.93763	0.0514631
56962	3.12842	1.3439
56963	1.93762	0.177951
56964	2.12345	0.781862
56965	1.65709	0.0727724
56966	1.65709	1.82697
56967	2.12345	0.830389
56968	1.88635	2.08821
56969	1.47134	2.00129
56970	2.219	0.165563
56971	2.25734	2.1
56972	1.93765	0.349809
56973	1.79103	1.26423
56974	1.47134	0.0744775
56975	1.06174	0.613416
56976	1.19349	2.09243
56977	1.1095	1.92582
56978	0.968827	0.432318
56979	1.07892	0.348976
56980	1.07892	2.1
56981	1.0834	0.130364
56982	1.0834	0.975012
56983	1.1095	1.08531
56984	1.19349	0.832769
56985	0.968825	1.17508
56986	1.06174	0.9875
56987	2.40627	2.1
56988	2.08288	1.59433
56989	2.5444	0.457003
56990	2.29285	1.65217
56991	4.62729	0.941735
56992	2.08289	0.616096
56993	1.65703	0.944197
56994	1.71105	2.1
56995	1.50108	1.97137
56996	2.29286	1.95671
56997	2.08289	2.03337
56998	2.5444	0.977991
56999	2.29285	2.1
57000	2.71429	2.1
57001	2.50288	0.319133
57002	5.04728	2.1
57003	2.50288	0.412991
57004	2.00927	2.1
57005	2.02116	0.808488
57006	2.02116	2.1
57007	2.29286	0.410637
57008	2.29286	1.98214
57009	1.89226	2.1
57010	1.89226	2.1
57011	1.89226	1.75036

57012	1.89226	2.1
57013	3.78453	2.1
57014	1.17418	2.1
57015	1.29741	2.1
57016	1.04921	2.1
57017	1.19352	2.1
57018	2.3677	2.1
57019	1.29741	2.1
57020	1.27575	2.1
57021	1.19352	2.1
57022	1.19473	2.1
57023	2.46927	2.1
57024	1.89226	2.1
57025	1.89226	2.1
57026	1.89226	2.1
57027	1.89226	2.1
57028	1.89226	2.1
57029	1.89226	2.1
57030	0.561332	2.1
57031	0.561332	2.1
57032	0.567597	2.1
57033	0.567597	2.1
57034	0.597365	2.1
57035	0.544699	2.1
57036	0.596761	2.1
57037	0.531564	2.1
57038	0.5447	2.1
57039	0.531563	2.1
57040	0.596759	2.1
57041	0.597366	2.1
57042	0.946131	2.1
57043	0.946131	2.1
57044	0.946131	2.1
57045	0.946131	2.1
57046	0.946129	2.1
57047	0.946129	2.1
57048	0.946133	2.1
57049	0.946133	2.1
57050	0.946131	2.1
57051	0.946131	2.1
57052	0.946131	2.1
57053	0.946131	2.1
57054	1.10938	0.05
57055	1.17614	1.18022
57056	1.02478	0.05
57057	1.09056	0.616248
57058	2.19994	0.168478
57059	1.17614	1.29831
57060	1.11868	2.1
57061	1.03408	0.83174
57062	1.03408	2.1
57063	1.09056	0.773377
57064	1.17516	2.1
57065	1.89226	0.123393
57066	1.89226	0.0800809
57067	1.89226	0.12857
57068	1.89226	0.0634173
57069	1.89226	0.169977

57070	1.89226	0.0830462
57071	1.89226	0.0618072
57072	1.89226	0.100386
57073	1.89226	0.092699
57074	1.89226	0.05
57075	1.89226	0.130612
57076	1.89226	0.05
57077	0.579551	0.05
57078	0.563873	0.05
57079	0.611341	0.0579963
57080	0.598693	0.05
57081	0.589856	0.05
57082	0.589856	0.05
57083	0.588387	0.0534409
57084	0.588387	0.05
57085	0.57955	0.05
57086	0.611341	0.05
57087	0.563873	0.05
57088	0.598693	0.05
57089	0.611341	0.056133
57090	0.598693	0.05
57091	0.586048	0.0560413
57092	0.572737	0.0671935
57093	0.591824	0.0555047
57094	0.591824	0.05
57095	0.592254	0.0677665
57096	0.592254	0.0587804
57097	0.611341	0.0549444
57098	0.586048	0.0529277
57099	0.598693	0.0504147
57100	0.572737	0.0523805
57101	0.946119	0.056342
57102	0.946119	0.123757
57103	0.946119	0.0669668
57104	0.946119	0.129342
57105	0.94612	0.0654201
57106	0.94612	0.0523394
57107	0.946119	0.119952
57108	0.946119	0.152026
57109	0.946132	0.0817894
57110	0.946132	0.0791825
57111	0.946107	0.0949744
57112	0.946107	0.0947149
57113	1.89224	0.0695076
57114	1.89224	0.05
57115	1.89224	0.0834304
57116	1.89224	0.05
57117	1.89224	0.115779
57118	1.89224	0.0504688
57119	0.562335	0.05
57120	0.579543	0.05
57121	0.59499	0.05
57122	0.611333	0.05
57123	0.586619	0.05
57124	0.586619	0.05
57125	0.587049	0.05
57126	0.587049	0.05
57127	0.594999	0.05

57128	0.562342	0.05
57129	0.579535	0.05
57130	0.611325	0.05
57131	0.59499	0.05
57132	0.611333	0.05
57133	0.567849	0.05
57134	0.58604	0.0506485
57135	0.590978	0.05
57136	0.590978	0.05
57137	0.590052	0.0511255
57138	0.590052	0.05
57139	0.567857	0.05
57140	0.594998	0.05
57141	0.611325	0.05
57142	0.586032	0.05
57143	1.69661	0.0926541
57144	1.9339	0.05
57145	1.39379	0.0532408
57146	1.69107	0.05
57147	3.32768	0.05
57148	1.9339	0.109488
57149	1.98414	0.05
57150	1.69107	0.05
57151	1.72774	0.05
57152	3.66164	0.05
57153	1.39379	0.592989
57154	1.69107	0.05
57155	1.14397	0.05
57156	1.43548	0.05
57157	2.82927	0.05
57158	1.69107	0.05
57159	1.72774	0.050809
57160	1.53873	0.05
57161	1.53873	0.0834265
57162	1.43548	0.05
57163	1.62449	0.05
57164	2.58431	0.05
57165	1.34072	0.05
57166	1.80841	0.05
57167	1.34072	0.05
57168	2.51501	0.05
57169	2.04732	0.05
57170	1.89226	0.05
57171	1.89226	0.05
57172	1.89226	0.05
57173	1.89226	0.05
57174	1.89226	0.05
57175	1.89226	0.05
57176	0.946131	0.05
57177	0.946131	0.05
57178	1.3639	0.05
57179	1.1516	0.05
57180	0.946129	0.05
57181	0.946129	0.05
57182	1.36391	0.05
57183	1.36391	0.05
57184	0.946131	0.05
57185	1.3639	0.05

57186	1.1516	0.05
57187	0.946131	0.05
57188	0.946131	0.05
57189	0.946131	0.05
57190	0.94613	0.05
57191	0.94613	0.05
57192	0.946128	0.05
57193	0.946128	0.05
57194	0.946133	0.05
57195	0.946133	0.05
57196	0.946131	0.05
57197	0.946131	0.0502053
57198	0.946131	0.05
57199	0.946131	0.05
57200	1.89226	1.3243
57201	2.63634	2.1
57202	1.89226	1.49927
57203	1.83902	1.74128
57204	3.73128	1.36954
57205	1.47059	2.1
57206	2.02928	2.1
57207	1.47059	2.1
57208	1.65706	2.1
57209	1.98628	2.1
57210	2.17274	2.1
57211	2.43618	2.1
57212	2.41176	2.1
57213	1.98627	2.1
57214	2.34084	2.1
57215	4.39803	2.1
57216	1.47059	2.1
57217	1.82453	2.1
57218	1.98627	2.1
57219	1.98627	2.1
57220	1.26929	2.1
57221	1.26929	2.1
57222	1.98627	2.1
57223	2.34084	2.1
57224	1.47264	2.1
57225	2.24937	2.1
57226	4.23564	2.1
57227	1.33026	0.269769
57228	1.51171	1.58589
57229	1.51171	0.28556
57230	1.4617	1.81332
57231	1.35382	0.220203
57232	1.30382	2.1
57233	1.89226	0.138207
57234	1.89226	0.32159
57235	1.89226	0.122895
57236	1.89226	1.18105
57237	1.89226	0.0797965
57238	1.89226	1.49718
57239	1.39263	0.67182
57240	0.962704	0.05
57241	1.0171	0.0799924
57242	0.962704	0.16609
57243	1.40822	0.05

57244	1.35382	0.0797506
57245	0.755853	2.0979
57246	0.789808	0.600292
57247	0.696315	0.442452
57248	0.676912	0.0899673
57249	0.729751	0.0722837
57250	0.729751	0.517651
57251	0.722417	2.1
57252	0.722417	0.158276
57253	0.696315	0.351836
57254	0.789808	0.350858
57255	0.755853	0.515082
57256	0.676912	0.461432
57257	0.946132	0.30669
57258	0.946132	0.479073
57259	0.946132	0.109424
57260	0.946132	0.052431
57261	0.946131	0.05
57262	0.946131	0.274881
57263	0.946132	0.0996713
57264	0.946132	0.538903
57265	0.946132	0.1029
57266	0.946132	0.124424
57267	0.946132	0.163706
57268	0.946132	0.17119
57269	0.946133	0.107706
57270	0.946133	0.0516014
57271	0.946132	0.05
57272	0.946132	0.0604944
57273	0.946132	0.05
57274	0.946132	0.0708459
57275	0.946133	0.05
57276	0.946133	0.117319
57277	0.946132	0.0546166
57278	0.946132	0.060997
57279	0.946132	0.0701263
57280	0.946132	0.0929052
57281	1.89227	2.0233
57282	2.53014	2.05099
57283	1.89227	2.1
57284	1.98241	1.58755
57285	1.89227	2.1
57286	1.98241	1.75753
57287	1.89226	1.41327
57288	1.89226	2.1
57289	1.89226	2.04317
57290	1.89226	2.00178
57291	1.89226	2.1
57292	1.89226	2.1
57293	1.89226	1.27679
57294	1.89226	2.1
57295	1.89226	1.85685
57296	1.89226	1.82621
57297	3.78452	1.86575
57298	2.45304	0.765058
57299	1.89226	2.08381
57300	1.89226	1.80314
57301	1.89226	1.70642

57302	1.89226	1.3118
57303	1.89226	1.20207
57304	1.89227	2.0458
57305	1.89227	1.91778
57306	1.89227	2.1
57307	1.89227	1.43006
57308	1.89227	2.1
57309	1.89227	1.54185
57310	1.89226	1.72739
57311	1.89226	2.1
57312	1.89226	2.02275
57313	1.89226	2.02742
57314	1.89226	2.1
57315	1.89226	2.1
57316	2.09871	0.168979
57317	2.71891	0.260817
57318	1.31126	0.15115
57319	1.31126	0.345401
57320	2.55341	0.151294
57321	1.76597	0.598965
57322	1.89226	0.47818
57323	1.89226	0.552831
57324	1.89226	0.227375
57325	1.89226	1.01523
57326	1.89226	0.242871
57327	1.89226	1.06689
57328	0.946128	0.534166
57329	0.946128	0.216486
57330	0.946133	0.183576
57331	0.946133	0.119466
57332	0.946131	0.17819
57333	0.946131	0.435107
57334	0.94613	0.126275
57335	0.94613	0.237789
57336	0.946131	0.223523
57337	0.946131	0.216732
57338	0.946131	0.221354
57339	0.946131	0.234239
57340	2.71891	0.122949
57341	1.89226	0.140644
57342	2.09871	0.0852491
57343	2.09871	0.427081
57344	1.89226	0.072794
57345	1.89226	0.374645
57346	1.89227	0.482088
57347	1.89227	0.43066
57348	1.89227	0.268881
57349	1.89227	0.91486
57350	1.89227	0.278325
57351	1.89227	0.94091
57352	0.946129	0.421115
57353	0.946129	0.252408
57354	0.946133	0.169167
57355	0.946133	0.112015
57356	0.946131	0.165943
57357	0.946131	0.366871
57358	0.946131	0.118133
57359	0.946131	0.274055

57360	0.946131	0.193985
57361	0.946131	0.188059
57362	0.946131	0.207718
57363	0.946131	0.204228
57364	2.32462	0.05
57365	1.89226	0.05
57366	1.89226	0.05
57367	1.89226	0.05
57368	2.2291	0.05
57369	2.2291	0.05
57370	0.847669	0.05
57371	0.847669	0.05
57372	0.796966	0.05
57373	0.796966	0.05
57374	0.833552	0.05
57375	1.01049	0.05
57376	0.709709	0.05
57377	0.634147	0.05
57378	0.634148	0.05
57379	0.833549	0.05
57380	0.709711	0.05
57381	1.01049	0.05
57382	1.89226	0.05
57383	1.89226	0.05
57384	1.89226	0.05
57385	1.89226	0.05
57386	1.89226	0.05
57387	1.89226	0.05
57388	1.03611	0.05
57389	0.936655	0.05
57390	0.946132	0.05
57391	0.946132	0.05
57392	0.941918	0.05
57393	0.941918	0.05
57394	1.00308	0.05
57395	1.04117	0.05
57396	1.1406	0.05
57397	1.04074	0.05
57398	0.851529	0.05
57399	1.00311	0.05
57400	1.01814	0.05
57401	1.6671	0.05
57402	1.35561	0.05
57403	1.35561	0.05
57404	1.2683	0.05
57405	1.2683	0.05
57406	0.946128	0.05
57407	0.946128	0.05
57408	0.946129	0.05
57409	0.946129	0.05
57410	0.946132	0.05
57411	0.946132	0.05
57412	0.946126	0.05
57413	0.946126	0.05
57414	0.946129	0.05
57415	0.946129	0.05
57416	0.946129	0.05
57417	0.946129	0.05

57418	2.23338	0.05
57419	1.96	0.05
57420	2.03438	0.05
57421	1.87835	0.05
57422	4.11172	0.05
57423	1.96	0.05
57424	1.52661	0.05
57425	1.87834	0.05
57426	1.53175	0.05
57427	3.40495	0.05
57428	2.03435	0.05
57429	1.65813	0.05
57430	1.65813	0.05
57431	1.87832	0.05
57432	1.72067	0.05
57433	1.94086	0.05
57434	1.65813	0.05
57435	1.40572	0.05
57436	1.87832	0.05
57437	1.87832	0.05
57438	1.31154	0.05
57439	1.53173	0.05
57440	1.86662	2.1
57441	2.0815	2.1
57442	2.0815	2.1
57443	2.0815	2.1
57444	1.87246	2.1
57445	1.87362	2.1
57446	1.0273	2.1
57447	0.996347	2.1
57448	0.946129	2.1
57449	0.946129	2.1
57450	1.04427	2.1
57451	1.04196	2.1
57452	0.910674	2.1
57453	0.910674	2.1
57454	1.04072	2.1
57455	1.10931	2.1
57456	1.03132	2.1
57457	0.933698	2.1
57458	2.63789	0.05
57459	1.89226	0.180322
57460	2.3033	0.05
57461	2.3033	0.175611
57462	1.89226	0.05
57463	1.89226	0.181968
57464	1.89226	0.261282
57465	1.89226	0.196958
57466	1.89226	0.0799646
57467	1.89226	0.221516
57468	3.78452	0.203807
57469	1.51898	0.0705998
57470	1.36006	0.137861
57471	2.03143	0.12077
57472	2.03143	0.122251
57473	1.04026	0.146866
57474	1.04026	0.14304
57475	0.94613	0.050852

57476	0.94613	0.104305
57477	1.31894	0.05
57478	1.31894	0.0570792
57479	0.94613	0.05
57480	0.94613	0.123036
57481	1.31894	0.05
57482	1.41232	0.0996321
57483	0.946129	0.0560936
57484	1.41232	0.0621692
57485	1.31895	0.0537162
57486	0.946129	0.0528611
57487	1.89226	0.0567075
57488	1.89226	0.231927
57489	1.89226	0.05
57490	1.89226	0.167968
57491	1.89226	0.05
57492	1.89226	0.16967
57493	1.89804	0.05
57494	2.03143	0.05
57495	2.46286	0.0605832
57496	2.46287	0.05
57497	1.51898	0.0590861
57498	1.51898	0.05
57499	1.89226	2.1
57500	1.89226	2.1
57501	1.89226	2.1
57502	1.89226	2.1
57503	3.78452	2.1
57504	1.3864	2.1
57505	1.3864	2.1
57506	0.946132	2.1
57507	0.946132	2.1
57508	1.32905	2.1
57509	1.3864	2.1
57510	0.946132	2.1
57511	0.946132	2.1
57512	0.946132	2.1
57513	1.32905	2.1
57514	0.946133	2.1
57515	1.3864	2.1
57516	2.77279	2.1
57517	1.67883	2.1
57518	1.67883	2.1
57519	1.73415	2.1
57520	2.65811	2.1
57521	2.71343	2.1
57522	0.946131	2.1
57523	0.946131	2.1
57524	0.946128	2.1
57525	0.946128	2.1
57526	0.946127	2.1
57527	0.946127	2.1
57528	0.946131	2.1
57529	0.946131	2.1
57530	0.946129	2.1
57531	0.946129	2.1
57532	0.94613	2.1
57533	0.94613	2.1

57534	1.89226	2.1
57535	1.89226	2.1
57536	1.89226	2.1
57537	1.89226	2.1
57538	3.78452	2.1
57539	1.89226	2.1
57540	2.54394	2.1
57541	2.54394	2.1
57542	2.65811	2.1
57543	1.89226	2.1
57544	2.00643	2.1
57545	2.54394	2.1
57546	2.6581	2.1
57547	1.62743	2.1
57548	1.62743	2.1
57549	1.67883	2.1
57550	2.59533	2.1
57551	1.89226	2.1
57552	1.89226	2.1
57553	1.89226	2.1
57554	1.89226	2.1
57555	1.89226	2.1
57556	1.89226	2.1
57557	1.89226	1.58219
57558	1.89226	1.38207
57559	1.89226	2.1
57560	1.89226	0.889141
57561	1.89226	2.1
57562	1.89226	0.678526
57563	1.89226	1.71816
57564	1.89226	1.38934
57565	1.89226	2.1
57566	1.89226	1.18041
57567	1.89226	2.06905
57568	1.89226	1.02113
57569	1.89226	0.953102
57570	1.89226	0.348191
57571	1.89226	1.64596
57572	1.89226	0.279795
57573	1.89226	1.4863
57574	1.89226	0.223489
57575	2.68537	0.103089
57576	2.22575	0.147346
57577	2.47464	0.05
57578	1.79093	0.05
57579	4.70039	0.05
57580	2.81217	0.146419
57581	2.47464	0.05
57582	2.58286	0.103958
57583	2.29193	0.05
57584	5.0575	0.05
57585	2.47464	0.05
57586	2.20365	0.0689545
57587	2.29193	0.05
57588	1.90104	0.0509196
57589	4.37568	0.05
57590	1.89226	0.163462
57591	2.16957	2.1

57592	2.52823	1.03937
57593	2.52823	2.1
57594	1.89226	0.580905
57595	1.89226	1.88125
57596	1.36226	2.1
57597	1.55369	2.1
57598	1.33309	2.1
57599	1.62593	2.1
57600	2.98819	2.1
57601	0.802281	2.1
57602	0.802281	2.1
57603	0.835143	2.1
57604	0.835143	2.1
57605	0.824458	2.1
57606	0.92641	2.1
57607	0.776847	2.1
57608	0.812967	2.1
57609	0.812967	2.1
57610	0.926411	2.1
57611	0.776847	2.1
57612	0.824458	2.1
57613	1.33309	2.1
57614	1.62593	2.1
57615	1.32813	2.1
57616	1.7237	2.1
57617	2.95406	2.1
57618	0.814544	2.1
57619	0.875312	2.1
57620	0.910502	2.1
57621	0.910502	2.1
57622	0.886633	2.1
57623	0.975739	2.1
57624	0.792623	2.1
57625	0.840283	2.1
57626	0.796372	2.1
57627	1.06159	2.1
57628	0.81948	2.1
57629	0.933833	2.1
57630	2.63997	2.1
57631	1.29694	2.1
57632	1.28141	2.1
57633	2.62623	2.1
57634	3.92317	2.1
57635	2.63998	2.1
57636	1.89226	2.1
57637	1.89226	2.1
57638	2.62623	2.1
57639	4.5185	2.1
57640	1.89226	2.1
57641	1.89226	2.1
57642	1.89226	2.1
57643	1.89226	2.1
57644	3.78453	2.1
57645	1.89226	2.1
57646	1.89226	2.1
57647	1.89226	2.1
57648	1.89226	2.1
57649	1.89226	2.1

57650	1.89226	2.1
57651	1.25468	2.1
57652	2.62623	2.1
57653	1.28141	2.1
57654	1.28141	2.1
57655	2.60665	2.1
57656	2.60665	2.1
57657	1.89226	2.1
57658	2.62623	2.1
57659	2.60666	2.1
57660	2.62623	2.1
57661	1.87269	2.1
57662	1.89226	2.1
57663	1.89226	2.1
57664	1.89226	2.1
57665	1.89226	2.1
57666	1.89226	2.1
57667	3.78453	2.1
57668	1.89226	2.1
57669	1.89226	2.1
57670	1.89226	2.1
57671	1.89226	2.1
57672	1.89226	2.1
57673	1.89226	2.1
57674	2.30147	1.27093
57675	2.27703	2.09652
57676	1.89227	1.11429
57677	1.89227	1.94688
57678	4.1693	1.7813
57679	1.38617	1.09669
57680	1.24613	1.6451
57681	2.59886	0.857095
57682	2.6238	1.70592
57683	3.86993	1.03937
57684	0.946133	1.75484
57685	0.946133	0.751964
57686	0.946132	0.956529
57687	0.946132	2.1
57688	0.946133	2.09501
57689	0.946133	1.93213
57690	0.946132	0.733326
57691	0.946132	1.16995
57692	0.946133	1.57536
57693	0.946133	1.49037
57694	0.946132	1.24868
57695	0.946132	1.2935
57696	2.21773	2.1
57697	2.59216	2.1
57698	1.68541	2.1
57699	1.68541	2.1
57700	1.84814	2.1
57701	2.38046	2.1
57702	1.89966	2.1
57703	1.70303	2.1
57704	1.2028	2.1
57705	1.29897	2.1
57706	2.02873	2.1
57707	2.19093	2.1

57708	2.75218	2.1
57709	1.70303	2.1
57710	3.10254	2.1
57711	2.24783	2.1
57712	2.56839	2.1
57713	1.70303	2.1
57714	2.56839	2.1
57715	1.08276	2.1
57716	2.24061	2.1
57717	1.67491	2.1
57718	1.60948	2.1
57719	1.08276	2.1
57720	1.89226	0.93759
57721	1.89226	1.238
57722	1.89226	0.891832
57723	1.89226	0.859419
57724	3.78453	0.562921
57725	1.89226	0.893511
57726	1.89226	1.17936
57727	1.89226	0.706184
57728	1.89226	0.791347
57729	3.78452	1.60657
57730	1.89226	1.12332
57731	2.7535	0.404588
57732	1.89226	1.37302
57733	1.89226	1.17886
57734	1.89226	0.610473
57735	1.89226	0.636441
57736	1.89226	1.1939
57737	1.89226	0.873106
57738	1.89226	0.403251
57739	1.89226	1.1601
57740	1.89226	0.708515
57741	1.89226	1.54717
57742	1.89227	1.07674
57743	1.89227	0.937334
57744	1.89227	0.723274
57745	1.89227	0.623714
57746	3.78453	1.63876
57747	1.89226	1.01889
57748	2.53412	0.20293
57749	1.89226	1.751
57750	1.89226	0.404289
57751	1.89226	0.794777
57752	1.89226	0.311047
57753	2.21126	0.05
57754	1.89226	0.05
57755	2.57464	0.05
57756	1.89226	0.05
57757	2.57464	0.05
57758	1.89226	0.05
57759	1.89226	0.05
57760	1.89226	0.05
57761	1.89226	0.376006
57762	1.89226	0.05
57763	1.89226	0.204102
57764	1.89226	0.05
57765	1.1418	0.05

57766	0.653021	0.05
57767	1.37282	0.05
57768	0.931386	0.05
57769	1.34568	0.05
57770	1.17669	0.05
57771	0.666595	0.05
57772	0.786895	0.05
57773	0.933982	0.05
57774	1.05351	0.05
57775	1.00792	0.05
57776	1.00792	0.05
57777	1.89226	2.1
57778	1.89226	2.1
57779	1.89226	2.1
57780	1.89226	2.1
57781	3.78452	2.1
57782	1.89226	2.1
57783	1.89226	2.1
57784	1.89226	2.1
57785	1.89226	2.1
57786	1.89226	2.1
57787	1.89226	2.1
57788	1.65642	2.1
57789	1.30248	2.1
57790	1.38888	2.1
57791	1.25987	2.1
57792	1.70611	2.1
57793	1.65642	2.1
57794	1.65642	2.1
57795	1.81407	2.1
57796	2.19428	2.1
57797	1.92555	2.1
57798	3.8507	2.1
57799	1.89226	2.1
57800	1.89226	2.1
57801	1.89226	2.1
57802	1.89226	2.1
57803	3.78452	2.1
57804	1.89226	2.1
57805	1.89226	2.1
57806	1.89226	2.1
57807	1.89226	2.1
57808	1.89226	2.1
57809	1.89226	2.1
57810	0.705495	2.1
57811	0.809317	2.1
57812	1.1655	2.1
57813	1.09605	2.1
57814	0.983375	2.1
57815	0.797301	2.1
57816	0.906633	2.1
57817	0.906633	2.1
57818	0.85085	2.1
57819	1.16548	2.1
57820	0.705501	2.1
57821	1.00314	2.1
57822	2.19428	2.1
57823	2.31862	2.1

57824	2.27408	2.1
57825	2.45893	2.1
57826	4.46836	2.1
57827	1.89226	0.688045
57828	1.89226	1.07239
57829	1.89226	1.1746
57830	1.89226	0.647996
57831	1.89226	1.44173
57832	1.89226	1.06033
57833	2.0815	0.706438
57834	2.22136	0.748806
57835	1.5515	0.632205
57836	1.86323	0.378211
57837	2.74986	1.13143
57838	3.30238	0.69606
57839	1.89224	0.637738
57840	1.89224	1.00105
57841	1.89224	1.20577
57842	1.89224	0.547658
57843	1.89224	1.4933
57844	1.89224	0.939153
57845	1.89224	0.644216
57846	1.89224	1.27216
57847	1.89224	0.926873
57848	1.89224	0.446813
57849	1.89224	1.45712
57850	1.89224	0.980711
57851	1.17846	0.487809
57852	0.810935	0.602841
57853	0.93347	1.28554
57854	0.93347	0.452864
57855	0.810932	0.825064
57856	0.810932	0.547977
57857	2.22133	0.58546
57858	1.71919	0.519344
57859	1.32629	0.322986
57860	1.0813	0.818784
57861	1.57137	0.587167
57862	2.21149	2.1
57863	1.89226	2.1
57864	1.89226	2.1
57865	1.89226	2.1
57866	2.17159	2.1
57867	2.17159	2.1
57868	0.946131	0.0980857
57869	0.946131	2.1
57870	0.946131	2.1
57871	0.946131	2.1
57872	0.94613	0.842885
57873	0.94613	1.94682
57874	0.946132	2.1
57875	0.946132	2.1
57876	0.946131	1.78181
57877	0.946131	1.74313
57878	0.946131	2.1
57879	0.946131	1.99579
57880	1.12266	2.1
57881	1.4714	2.1

57882	1.62322	2.09656
57883	1.62322	2.00769
57884	1.02958	2.1
57885	1.02958	2.1
57886	2.2671	2.1
57887	1.89226	2.1
57888	1.89226	2.1
57889	1.89226	2.1
57890	2.21149	2.1
57891	2.21149	2.1
57892	1.89226	2.1
57893	1.89226	1.98697
57894	1.89226	2.1
57895	1.89226	1.09098
57896	1.89226	2.1
57897	1.89226	2.0979
57898	0.736914	2.1
57899	0.736914	2.1
57900	0.822839	2.1
57901	0.822836	2.1
57902	0.791298	0.520195
57903	1.00003	2.09727
57904	0.640254	2.1
57905	0.575379	2.1
57906	0.609001	1.9119
57907	1.00005	1.96694
57908	0.575371	2.1
57909	0.831908	1.86634
57910	1.89226	2.1
57911	1.89226	2.1
57912	1.89226	2.1
57913	1.89226	2.1
57914	1.89226	2.1
57915	1.89226	2.1
57916	1.89226	2.1
57917	1.89226	2.1
57918	1.89226	2.1
57919	1.89226	2.1
57920	1.89226	2.1
57921	1.89226	2.1
57922	0.730617	2.1
57923	0.71052	2.1
57924	0.7329	2.1
57925	0.712083	2.1
57926	0.701051	2.1
57927	0.701051	2.1
57928	0.721552	2.1
57929	0.721552	2.1
57930	0.710521	2.1
57931	0.732898	2.1
57932	0.730619	2.1
57933	0.712081	2.1
57934	0.7329	2.1
57935	0.712082	2.1
57936	0.559601	2.1
57937	0.495993	2.1
57938	0.603718	2.1
57939	0.603718	2.1

57940	0.625174	2.1
57941	0.625174	2.1
57942	0.732899	2.1
57943	0.559601	2.1
57944	0.712082	2.1
57945	0.495993	2.1
57946	0.94613	2.1
57947	0.94613	2.1
57948	0.94613	2.1
57949	0.94613	2.1
57950	0.946131	2.1
57951	0.946131	2.1
57952	0.946129	2.1
57953	0.946129	2.1
57954	0.94613	2.1
57955	0.94613	2.1
57956	0.94613	2.1
57957	0.94613	2.1
57958	1.89226	2.1
57959	1.89226	2.1
57960	1.89226	2.1
57961	1.89226	2.1
57962	1.89226	2.1
57963	1.89226	2.1
57964	1.42104	2.1
57965	1.4658	2.1
57966	1.51223	2.1
57967	1.42283	2.1
57968	2.93327	2.1
57969	0.732899	2.1
57970	0.756115	2.1
57971	0.629763	2.1
57972	0.559601	2.1
57973	0.646134	2.1
57974	0.646134	2.1
57975	0.669582	2.1
57976	0.669582	2.1
57977	0.756115	2.1
57978	0.629763	2.1
57979	0.732899	2.1
57980	0.559601	2.1
57981	2.566 2.1	
57982	1.20242	2.1
57983	2.64084	2.1
57984	2.18297	2.1
57985	1.66029	2.1
57986	1.20242	2.1
57987	0.946133	1.10791
57988	0.946133	1.50213
57989	0.946129	2.1
57990	0.946129	2.1
57991	0.946131	2.1
57992	0.946131	1.08574
57993	0.946131	0.0582214
57994	0.946131	2.1
57995	0.946131	0.942194
57996	0.946131	1.99168
57997	0.946131	1.46214

57998	0.946131	1.85297
57999	1.89226	0.05
58000	1.89226	0.05
58001	1.89226	0.05
58002	1.89226	0.05
58003	3.78453	0.05
58004	1.03073	0.05
58005	1.03073	0.05
58006	1.04066	0.05
58007	1.04066	0.05
58008	1.20802	0.05
58009	1.32689	0.05
58010	0.883301	0.05
58011	0.744506	0.05
58012	1.20802	0.05
58013	0.744506	0.05
58014	0.883301	0.05
58015	1.32689	0.05
58016	1.20802	0.05
58017	1.20802	0.05
58018	0.946131	0.05
58019	0.946131	0.05
58020	0.94613	0.05
58021	0.94613	0.05
58022	1.20802	0.05
58023	1.32689	0.05
58024	1.20802	0.05
58025	0.946131	0.05
58026	0.946131	0.05
58027	1.32689	0.05
58028	1.89226	0.05
58029	1.89226	0.05
58030	1.89226	0.05
58031	1.89226	0.05
58032	1.89226	0.05
58033	1.89226	0.05
58034	0.946119	0.05
58035	0.946119	0.05
58036	0.946118	0.05
58037	0.946118	0.05
58038	0.946118	0.05
58039	0.946118	0.05
58040	0.946119	0.05
58041	0.946119	0.05
58042	0.946131	0.05
58043	0.946131	0.05
58044	0.946106	0.05
58045	0.946106	0.05
58046	1.3939	2.1
58047	1.49641	2.1
58048	1.15096	2.1
58049	1.10101	2.1
58050	2.59742	2.1
58051	1.22106	2.1
58052	2.60663	2.1
58053	1.25467	2.1
58054	1.25467	2.1
58055	2.58377	2.1

58056	2.58377	2.1
58057	1.89225	2.1
58058	2.60664	2.1
58059	2.58377	2.1
58060	2.60664	2.1
58061	1.86938	2.1
58062	1.89225	2.1
58063	1.89225	2.1
58064	1.89225	2.1
58065	1.89225	2.1
58066	1.89225	2.1
58067	1.89225	2.1
58068	1.89225	2.1
58069	0.946121	2.1
58070	0.946121	2.1
58071	0.946126	2.1
58072	0.946126	2.1
58073	0.946124	2.1
58074	0.946124	2.1
58075	0.946123	2.1
58076	0.946123	2.1
58077	0.946117	2.1
58078	0.946117	2.1
58079	0.94613	2.1
58080	0.94613	2.1
58081	1.28017	2.1
58082	1.29189	2.1
58083	0.610534	2.1
58084	0.592373	2.1
58085	0.943764	2.1
58086	0.943764	2.1
58087	0.940501	2.1
58088	0.940501	2.1
58089	1.28017	2.1
58090	0.592371	2.1
58091	0.610533	2.1
58092	1.29189	2.1
58093	0.946133	2.1
58094	0.946133	2.1
58095	1.28017	2.1
58096	1.29189	2.1
58097	0.946132	2.1
58098	0.946132	2.1
58099	1.28017	2.1
58100	1.28017	2.1
58101	1.28017	2.1
58102	0.946132	2.1
58103	0.946133	2.1
58104	1.2919	2.1
58105	1.89226	2.1
58106	1.89226	2.1
58107	1.89226	2.1
58108	1.89226	2.1
58109	1.89226	2.1
58110	1.89226	2.1
58111	1.89226	2.1
58112	1.89226	2.1
58113	1.89226	2.1

58114	1.89226	2.1
58115	3.78452	2.1
58116	1.89226	2.1
58117	2.08196	2.1
58118	1.97854	2.1
58119	2.08196	2.1
58120	1.78884	2.1
58121	1.89226	2.1
58122	1.89226	2.1
58123	1.89226	2.1
58124	1.89226	2.1
58125	1.89226	2.1
58126	1.89226	2.1
58127	1.89226	2.1
58128	1.83688	2.1
58129	1.89226	2.1
58130	1.89226	2.1
58131	1.89226	2.1
58132	1.86015	2.1
58133	1.86015	2.1
58134	1.89226	2.1
58135	1.97854	2.1
58136	1.85883	2.1
58137	1.97854	2.1
58138	1.77255	2.1
58139	1.89226	2.1
58140	0.946131	2.1
58141	0.946131	2.1
58142	0.946131	2.1
58143	0.946131	2.1
58144	0.94613	2.1
58145	0.94613	2.1
58146	0.946132	2.1
58147	0.946132	2.1
58148	0.946131	2.1
58149	0.946131	2.1
58150	0.946131	2.1
58151	0.946131	2.1
58152	0.918412	2.1
58153	0.918412	2.1
58154	0.94893	2.1
58155	0.750211	2.1
58156	0.851541	2.1
58157	0.851541	2.1
58158	0.970566	2.1
58159	0.826567	2.1
58160	0.970554	2.1
58161	1.04071	2.1
58162	0.826632	2.1
58163	0.851554	2.1
58164	1.89226	0.05
58165	1.89226	0.05
58166	1.89226	0.05
58167	1.89226	0.05
58168	3.78453	0.05
58169	0.946131	0.05
58170	0.946131	0.05
58171	0.811232	0.05

58172	0.811232	0.05
58173	0.94613	0.05
58174	0.94613	0.05
58175	0.959701	0.05
58176	0.811233	0.05
58177	0.94613	0.05
58178	0.811233	0.05
58179	0.959702	0.05
58180	0.946131	0.05
58181	1.89226	0.05
58182	1.89226	0.05
58183	1.89226	0.05
58184	1.89226	0.05
58185	1.89226	0.05
58186	1.89226	0.05
58187	1.89227	0.05
58188	1.89227	0.05
58189	1.89227	0.05
58190	1.89227	0.05
58191	1.89227	0.05
58192	1.89227	0.05
58193	1.89226	0.05
58194	1.89226	0.05
58195	1.89226	0.05
58196	1.89226	0.05
58197	3.78453	0.05
58198	1.89226	0.05
58199	1.89226	0.05
58200	1.89226	0.05
58201	1.89226	0.05
58202	1.89226	0.05
58203	1.89226	0.05
58204	1.89226	2.1
58205	2.36204	2.1
58206	2.39853	2.1
58207	2.36204	2.1
58208	1.92876	2.1
58209	1.89226	2.1
58210	1.89226	2.1
58211	1.89226	2.1
58212	1.89226	2.1
58213	1.89226	0.600111
58214	3.78453	2.1
58215	0.946128	2.1
58216	0.946128	0.762018
58217	1.13182	2.1
58218	1.13182	2.1
58219	0.94613	2.04327
58220	0.94613	2.1
58221	1.1749	2.1
58222	1.13182	2.1
58223	1.1749	2.1
58224	0.94613	2.1
58225	1.13182	2.1
58226	0.94613	2.1
58227	1.89226	2.1
58228	2.39853	2.1
58229	2.44918	2.1

58230	2.39853	2.1
58231	1.94291	2.1
58232	1.89226	2.1
58233	0.946132	2.1
58234	0.946132	2.1
58235	0.946131	2.1
58236	0.946131	2.1
58237	0.946132	2.1
58238	0.946132	2.1
58239	0.946131	2.1
58240	0.946131	2.1
58241	0.946131	2.1
58242	0.946131	2.1
58243	0.946132	2.1
58244	0.946132	2.1
58245	0.946128	2.1
58246	0.946128	2.1
58247	1.1749	2.1
58248	1.1749	2.1
58249	0.946131	2.1
58250	0.946131	2.1
58251	1.23631	2.1
58252	1.1749	2.1
58253	1.23631	2.1
58254	0.94613	2.1
58255	1.1749	2.1
58256	0.946131	2.1
58257	1.89226	1.69213
58258	1.89226	2.1
58259	1.89226	2.1
58260	1.89226	2.1
58261	3.78453	2.1
58262	1.38859	2.1
58263	1.45333	2.1
58264	1.28909	2.1
58265	1.38091	2.1
58266	2.74242	2.1
58267	1.45332	2.1
58268	1.38414	2.1
58269	1.38091	2.1
58270	1.33506	2.1
58271	2.78838	2.1
58272	1.89226	2.1
58273	1.89226	2.1
58274	1.89226	2.1
58275	1.89226	2.1
58276	3.78452	2.1
58277	1.89226	2.1
58278	1.89226	2.1
58279	1.89226	2.1
58280	1.89226	2.1
58281	1.89226	2.1
58282	1.89226	2.1
58283	1.17418	2.1
58284	1.38091	2.1
58285	1.17418	2.1
58286	1.28909	2.1
58287	1.29741	2.1

58288	1.41232	2.1
58289	1.38091	2.1
58290	1.33506	2.1
58291	1.27575	2.1
58292	1.27575	2.1
58293	1.29741	2.1
58294	1.35671	2.1
58295	1.89226	2.1
58296	1.89226	2.1
58297	1.89226	2.1
58298	1.89226	2.1
58299	3.78452	2.1
58300	1.92737	0.05
58301	2.40223	0.05
58302	1.56178	0.05
58303	1.97605	0.05
58304	3.90343	0.05
58305	1.89226	0.05
58306	1.89226	0.051636
58307	1.89226	0.05
58308	1.89226	0.05
58309	3.78452	0.05
58310	1.89226	0.0519
58311	1.89226	0.0721777
58312	1.89226	0.0636551
58313	1.89226	0.0861847
58314	1.89226	0.05
58315	1.89226	0.056565
58316	1.13641	0.05
58317	1.13641	0.05
58318	1.12763	0.05
58319	1.12763	0.05
58320	1.29348	0.05
58321	1.06293	0.0573655
58322	1.20111	0.05
58323	0.988028	0.05
58324	0.988028	0.05
58325	1.06293	0.05
58326	1.29348	0.05
58327	1.20111	0.05
58328	1.56176	0.05
58329	1.46387	0.05
58330	1.97603	0.05
58331	1.97603	0.05
58332	1.08211	0.05
58333	1.08211	0.05
58334	1.89224	0.05
58335	1.89224	0.05
58336	1.89224	0.05
58337	1.89224	0.05
58338	1.89224	0.05
58339	1.89224	0.05
58340	1.89224	0.05
58341	1.89224	0.0609546
58342	1.89224	0.05
58343	1.89224	0.0584453
58344	3.78448	0.05
58345	0.820185	0.05

58346	0.818947	0.05
58347	0.911041	0.05
58348	0.695265	0.05
58349	1.46388	0.05
58350	1.23607	0.05
58351	0.927068	0.05
58352	1.02691	0.05
58353	1.12918	0.05
58354	0.846843	0.05
58355	0.911039	0.05
58356	1.29129	2.1
58357	1.45547	2.1
58358	1.67327	2.1
58359	1.66698	2.1
58360	1.20149	2.1
58361	1.20149	2.1
58362	0.946133	1.56919
58363	0.946133	2.1
58364	0.946129	2.1
58365	0.946129	2.1
58366	0.946131	2.1
58367	0.946131	1.45162
58368	0.946131	2.1
58369	0.946131	2.1
58370	0.946131	2.1
58371	0.946131	2.1
58372	0.946131	2.1
58373	0.946131	2.1
58374	1.89226	2.1
58375	1.89226	2.1
58376	1.89226	2.1
58377	1.89226	1.05196
58378	3.78452	2.1
58379	0.86875	2.1
58380	0.86875	2.1
58381	0.899002	2.1
58382	0.966245	2.1
58383	0.849364	1.35831
58384	1.01918	2.1
58385	0.764737	2.1
58386	0.89249	2.1
58387	0.764624	2.1
58388	0.967869	2.1
58389	0.808606	2.1
58390	0.864506	2.1
58391	0.773929	2.1
58392	0.773929	2.1
58393	0.781991	2.1
58394	0.836559	2.1
58395	0.983761	2.1
58396	0.834687	2.1
58397	0.680545	2.1
58398	0.640799	2.1
58399	0.70286	2.1
58400	0.935844	2.1
58401	0.629479	2.1
58402	0.823655	2.1
58403	0.946133	2.1

58404	0.946133	2.1
58405	0.946128	2.1
58406	0.946128	2.1
58407	0.94613	2.1
58408	0.94613	2.0638
58409	0.946131	2.1
58410	0.946131	2.1
58411	0.946131	2.1
58412	0.946131	2.1
58413	0.946131	2.1
58414	0.946131	2.1
58415	1.89226	2.1
58416	1.89226	1.17792
58417	1.89226	2.1
58418	1.89226	2.1
58419	1.89226	1.85877
58420	1.89226	2.1
58421	0.931638	2.1
58422	0.931638	2.1
58423	1.03559	2.1
58424	0.977442	2.1
58425	0.826487	0.905053
58426	0.993902	1.95331
58427	1.04922	2.1
58428	0.894324	2.1
58429	0.912984	2.1
58430	1.01156	2.1
58431	0.944692	2.1
58432	1.15456	2.1
58433	1.89226	0.05
58434	1.89226	0.05
58435	1.89226	0.05
58436	1.89226	0.05
58437	3.78453	0.05
58438	1.89226	0.05
58439	1.89226	0.05
58440	2.64262	0.05
58441	2.64262	0.05
58442	1.78052	0.05
58443	1.03015	0.05
58444	0.999721	0.05
58445	0.659011	0.05
58446	1.15564	0.05
58447	1.41388	0.05
58448	1.01583	0.05
58449	1.01583	0.05
58450	1.05706	0.05
58451	1.05706	0.05
58452	1.15564	0.05
58453	0.65901	0.05
58454	1.41388	0.05
58455	0.999721	0.05
58456	1.89226	2.1
58457	1.89226	2.1
58458	1.89226	2.1
58459	1.89226	2.1
58460	3.78453	2.1
58461	2.39022	2.1

58462	1.90734	2.1
58463	2.27634	2.1
58464	1.82666	2.1
58465	4.18368	2.1
58466	1.90734	2.1
58467	1.21564	2.1
58468	1.82666	2.1
58469	1.16938	2.1
58470	3.07672	2.1
58471	1.89226	2.1
58472	1.89226	2.1
58473	1.89226	2.1
58474	1.89226	2.1
58475	1.89226	2.1
58476	1.89226	2.1
58477	1.89227	2.1
58478	1.89227	2.1
58479	1.89227	2.1
58480	1.89227	2.1
58481	3.78453	2.1
58482	2.27634	2.1
58483	1.82666	2.1
58484	2.18042	2.1
58485	1.74738	2.1
58486	4.02372	2.1
58487	1.74738	2.1
58488	1.82666	2.1
58489	1.1181	2.1
58490	1.1181	2.1
58491	1.16938	2.1
58492	1.79865	2.1
58493	1.89226	2.1
58494	1.89226	2.1
58495	1.89226	2.1
58496	1.89226	2.1
58497	1.89226	2.1
58498	1.89226	2.1
58499	1.89226	1.35308
58500	1.89226	0.770346
58501	1.89226	1.00018
58502	1.89226	0.976838
58503	1.89226	1.53963
58504	1.89226	0.973045
58505	0.790736	0.984445
58506	1.08265	0.845164
58507	0.938909	1.26225
58508	0.638683	0.990909
58509	0.858588	1.14421
58510	0.858588	0.742383
58511	0.862743	1.38045
58512	0.862743	1.17287
58513	1.08265	1.28594
58514	0.938911	0.677549
58515	0.790735	1.25317
58516	0.638685	0.743486
58517	1.89226	0.733793
58518	1.89226	1.24053
58519	1.89226	1.11101

58520	1.89226	0.709379
58521	1.89226	1.4787
58522	1.89226	1.23194
58523	1.89226	0.94108
58524	1.89226	1.32892
58525	1.89226	0.854841
58526	1.89226	0.831832
58527	1.89226	1.28285
58528	1.89226	1.31032
58529	1.08265	0.611612
58530	1.36209	0.658231
58531	1.25233	0.709638
58532	0.938909	0.913851
58533	1.14195	0.608419
58534	1.14195	0.337442
58535	1.15904	1.33258
58536	1.15904	0.88362
58537	1.36209	1.09172
58538	1.25233	0.296134
58539	1.08265	1.04456
58540	0.938911	0.44565
58541	1.22077	0.573478
58542	0.915244	0.676474
58543	0.967835	1.00693
58544	0.742495	0.998457
58545	0.78648	0.895519
58546	0.985041	0.589459
58547	0.989098	1.37229
58548	0.989098	0.886098
58549	1.03206	0.640791
58550	0.967897	1.20369
58551	0.915404	1.18589
58552	0.706105	0.731521
58553	2.69662	0.05
58554	1.89226	0.05
58555	2.35499	0.05
58556	1.89226	0.05
58557	4.24725	0.05
58558	1.89227	0.05
58559	1.89227	0.05
58560	1.89227	0.05
58561	1.89227	0.05
58562	3.78453	0.05
58563	2.35499	0.05
58564	1.70302	0.05
58565	1.70302	0.05
58566	1.703	0.05
58567	1.97374	0.05
58568	1.97372	0.05
58569	0.946133	0.05
58570	0.946133	0.05
58571	0.946132	0.05
58572	0.946132	0.05
58573	0.946133	0.05
58574	0.946133	0.05
58575	0.946132	0.05
58576	0.946132	0.05
58577	0.946133	0.05

58578	0.946133	0.05
58579	0.946132	0.05
58580	0.946132	0.05
58581	0.6794	2.1
58582	0.6794	2.1
58583	0.670812	2.1
58584	0.62182	2.1
58585	0.648326	2.1
58586	0.792154	2.1
58587	0.719167	2.1
58588	0.494515	2.1
58589	0.649421	2.1
58590	0.804869	2.1
58591	0.485585	2.1
58592	0.627666	2.1
58593	1.79347	2.1
58594	1.03968	2.1
58595	1.70303	2.1
58596	0.933265	1.68128
58597	2.62068	1.92423
58598	1.89226	2.1
58599	1.89226	2.1
58600	1.89226	2.1
58601	1.89226	1.73296
58602	3.78452	2.1
58603	1.89226	2.1
58604	2.24604	2.1
58605	2.0554	2.1
58606	2.0554	2.1
58607	1.89226	2.1
58608	1.89226	2.1
58609	1.89226	2.1
58610	1.89226	0.170588
58611	1.89226	0.931768
58612	1.89226	2.1
58613	1.89226	1.20103
58614	1.89226	2.1
58615	2.0815	2.1
58616	2.64424	0.05
58617	2.80807	0.0991056
58618	3.232 2.1	
58619	1.89417	0.160123
58620	2.18012	2.1
58621	0.946131	2.1
58622	0.946131	0.16244
58623	0.946131	2.1
58624	0.946131	1.49099
58625	0.94613	1.20027
58626	0.94613	0.221679
58627	0.946132	2.1
58628	0.946132	2.1
58629	0.946131	2.1
58630	0.946131	2.1
58631	0.946131	2.1
58632	0.946131	2.1
58633	1.89227	0.05
58634	1.89227	0.05
58635	1.89227	0.05

58636	1.89227	0.05
58637	3.78453	0.05
58638	2.70881	0.05
58639	2.68687	0.05
58640	2.03046	0.05
58641	1.9682	0.05
58642	4.67701	0.05
58643	1.08518	0.05
58644	1.08518	0.05
58645	1.09033	0.05
58646	1.09033	0.05
58647	0.842273	0.05
58648	1.19141	0.05
58649	1.34343	0.05
58650	0.9841	0.05
58651	0.9841	0.05
58652	0.842273	0.05
58653	1.34344	0.05
58654	1.19141	0.05
58655	0.946133	0.05
58656	0.946133	0.05
58657	0.946132	0.05
58658	0.946132	0.05
58659	0.946132	0.05
58660	0.946132	0.05
58661	0.946132	0.05
58662	0.946132	0.05
58663	0.946132	0.05
58664	0.946132	0.05
58665	0.946133	0.05
58666	0.946133	0.05
58667	1.89227	0.05
58668	1.89227	0.05
58669	1.89227	0.05
58670	1.89227	0.05
58671	1.89227	0.05
58672	1.89227	0.05
58673	2.03046	0.05
58674	1.9682	0.05
58675	1.23886	0.05
58676	1.12971	0.05
58677	3.20706	0.05
58678	1.89226	0.131378
58679	1.89226	0.803757
58680	1.89226	0.0915006
58681	1.89226	0.471994
58682	3.78452	0.267012
58683	2.45569	0.05
58684	1.89226	0.107398
58685	2.22922	0.0597912
58686	2.0815	0.233888
58687	1.85074	0.05
58688	1.70303	0.122689
58689	2.72856	0.05
58690	1.89226	0.333477
58691	1.89226	0.05
58692	1.89226	0.160716
58693	3.78452	0.120441

58694	1.89226	0.186494
58695	1.89226	0.568322
58696	1.89226	0.105429
58697	1.89226	0.576671
58698	3.78452	0.265395
58699	0.946131	0.534727
58700	0.946131	0.097782
58701	0.946131	0.3576
58702	0.946131	0.0768281
58703	0.94613	0.298401
58704	0.94613	0.407535
58705	0.946132	0.0889354
58706	0.946132	0.109883
58707	0.946131	0.167885
58708	0.946131	0.189346
58709	0.946131	0.189098
58710	0.946131	0.169803
58711	2.0595	0.0563281
58712	2.58581	0.0587202
58713	1.48694	0.05
58714	1.48694	0.05
58715	2.60157	0.05
58716	2.02901	0.05
58717	1.89226	0.05
58718	1.89226	0.0682373
58719	1.89226	0.05
58720	1.89226	0.05
58721	1.89226	0.061025
58722	1.89226	0.0727137
58723	2.04728	0.05
58724	2.64161	0.05
58725	1.47606	0.05
58726	1.47606	0.05
58727	2.63069	0.05
58728	2.05947	0.05
58729	1.89224	0.0502303
58730	1.89224	0.05
58731	1.89224	0.0588238
58732	1.89224	0.05
58733	1.89224	0.05
58734	1.89224	0.05
58735	0.946114	0.05
58736	0.946114	0.05
58737	0.946118	0.0507119
58738	0.946118	0.05
58739	0.946114	0.0543049
58740	0.946114	0.05
58741	0.946118	0.05
58742	0.946118	0.05
58743	0.946128	0.0581598
58744	0.946128	0.0530677
58745	0.946103	0.05
58746	0.946103	0.05
58747	1.1233	2.1
58748	1.15009	2.1
58749	2.36036	2.1
58750	2.38225	2.1
58751	3.50554	2.1

58752	2.38224	2.1
58753	0.977775	2.1
58754	2.20473	2.1
58755	2.20473	2.1
58756	1.15009	2.1
58757	1.15009	2.1
58758	0.946119	2.1
58759	0.946119	2.1
58760	1.19111	2.1
58761	1.19111	2.1
58762	0.946118	2.1
58763	1.19111	2.1
58764	1.18016	2.1
58765	0.946118	1.53797
58766	0.94613	0.424811
58767	0.94613	2.1
58768	1.18015	2.1
58769	1.19109	2.1
58770	0.946118	2.1
58771	0.946118	2.1
58772	1.10235	2.1
58773	1.10235	2.1
58774	0.946118	2.1
58775	1.10235	2.1
58776	0.946115	2.1
58777	1.1911	2.1
58778	0.946129	2.1
58779	0.946129	2.1
58780	1.19109	2.1
58781	1.10234	2.1
58782	1.89226	2.1
58783	2.23932	2.1
58784	2.11568	2.1
58785	2.23932	2.1
58786	1.76862	2.1
58787	1.89226	2.1
58788	1.89226	2.1
58789	1.89226	2.1
58790	1.89226	2.1
58791	1.89226	2.1
58792	1.89226	2.1
58793	1.89226	2.1
58794	2.07838	2.1
58795	1.89226	2.1
58796	1.89226	2.1
58797	1.89226	2.1
58798	2.10264	2.1
58799	2.10264	2.1
58800	1.89226	2.1
58801	2.11568	2.1
58802	1.99753	2.1
58803	2.11568	2.1
58804	1.77411	2.1
58805	1.89226	2.1
58806	0.946128	2.1
58807	0.946128	2.1
58808	0.946133	2.1
58809	0.946133	2.1

58810	0.94613	2.1
58811	0.94613	2.1
58812	0.946131	2.1
58813	0.946131	2.1
58814	0.946131	2.1
58815	0.946131	2.1
58816	0.946131	2.1
58817	0.946131	2.1
58818	0.946132	2.1
58819	0.946132	2.1
58820	1.03919	2.1
58821	1.03919	2.1
58822	0.946131	2.1
58823	0.946131	2.1
58824	1.02744	2.1
58825	1.03919	2.1
58826	1.02744	2.1
58827	0.946132	2.1
58828	1.03919	2.1
58829	0.946132	2.1
58830	1.64834	2.06573
58831	1.82955	1.8262
58832	2.33824	1.19909
58833	2.19301	2.07309
58834	3.98658	1.72614
58835	1.40574	2.09772
58836	2.19301	1.61463
58837	1.64834	1.93712
58838	1.64834	1.90135
58839	1.99961	1.17717
58840	1.99961	1.51842
58841	2.33824	1.31729
58842	2.19301	2.07602
58843	2.68524	1.80467
58844	2.81847	1.57908
58845	5.02349	1.88687
58846	1.99961	2.06825
58847	2.19301	1.45056
58848	2.68524	1.48044
58849	2.50732	1.79367
58850	4.68485	1.60242
58851	1.89226	2.1
58852	1.89226	2.1
58853	1.89226	2.09326
58854	1.89226	2.1
58855	3.78453	2.1
58856	1.75142	2.1
58857	2.47026	2.1
58858	1.46811	2.1
58859	2.26652	2.1
58860	4.01794	2.1
58861	1.89226	2.1
58862	1.89226	2.1
58863	1.89226	2.1
58864	1.89226	2.1
58865	1.89226	2.1
58866	1.89226	2.1
58867	1.89226	2.1

58868	1.89226	2.1
58869	1.89226	2.1
58870	1.89226	2.1
58871	1.89226	2.1
58872	1.89226	2.1
58873	0.812082	2.1
58874	0.878759	2.1
58875	0.882782	2.1
58876	0.882782	2.1
58877	1.05572	2.1
58878	1.04037	2.1
58879	0.73849	2.1
58880	0.626857	2.1
58881	0.638677	2.1
58882	1.05581	2.1
58883	0.715695	2.1
58884	1.10492	2.1
58885	1.17058	2.1
58886	1.07863	2.1
58887	1.21113	2.1
58888	1.21113	2.1
58889	1.2811	2.1
58890	1.37319	2.1
58891	1.01788	2.1
58892	0.999492	2.1
58893	1.04352	2.1
58894	1.32112	2.1
58895	1.10492	2.1
58896	1.34699	2.1
58897	1.89226	2.1
58898	1.89226	2.1
58899	1.89226	2.1
58900	1.89226	2.1
58901	1.89226	2.1
58902	1.89226	2.1
58903	1.19813	0.05
58904	1.19813	0.05
58905	2.43432	0.05
58906	1.21716	0.05
58907	0.946124	0.05
58908	1.21715	0.05
58909	0.946118	0.05
58910	2.56627	2.1
58911	1.89226	2.1
58912	1.89226	2.1
58913	1.89226	2.1
58914	2.43434	2.1
58915	2.43434	2.1
58916	1.89227	0.05
58917	1.89227	0.05
58918	1.89227	0.05
58919	1.89227	0.05
58920	1.89227	0.05
58921	1.89227	0.05
58922	1.89226	0.05
58923	1.89226	0.05
58924	1.89226	0.05
58925	1.89226	0.05

58926	3.78453	0.05
58927	1.89226	0.05
58928	2.0764	0.05
58929	1.89226	0.05
58930	2.81674	0.05
58931	1.89226	0.05
58932	2.81674	0.05
58933	0.946132	0.05
58934	0.946132	0.05
58935	0.946131	0.05
58936	0.946131	0.05
58937	0.946132	0.05
58938	0.946132	0.05
58939	0.946132	0.05
58940	0.946132	0.05
58941	0.946132	0.05
58942	0.946132	0.05
58943	0.946132	0.05
58944	0.946132	0.05
58945	0.578684	1.37597
58946	0.578684	2.1
58947	0.589487	1.85623
58948	0.589487	2.1
58949	0.625461	2.1
58950	0.5378	2.1
58951	0.608714	2.1
58952	0.54271	1.83842
58953	0.608714	1.58482
58954	0.625461	0.941448
58955	0.54271	2.08108
58956	0.537801	1.94828
58957	0.946128	0.785502
58958	0.946128	2.08973
58959	0.946133	0.72816
58960	0.946133	2.1
58961	0.946131	1.12527
58962	0.946131	0.731947
58963	0.946131	0.731695
58964	0.946131	1.26268
58965	0.94613	1.16596
58966	0.94613	0.67567
58967	0.946131	1.25836
58968	0.946131	1.49941
58969	1.21743	2.07973
58970	1.25092	2.1
58971	1.38847	0.843817
58972	1.46031	0.975852
58973	2.67774	1.80092
58974	0.946128	0.797116
58975	0.946128	2.1
58976	0.946132	2.1
58977	0.946132	0.980076
58978	0.94613	1.53059
58979	0.94613	0.792581
58980	0.94613	1.23698
58981	0.94613	0.962279
58982	0.94613	0.889706
58983	0.94613	0.648283

58984	0.94613	1.66172
58985	0.94613	0.945853
58986	1.89226	0.635009
58987	1.89226	1.20317
58988	1.89226	0.159104
58989	1.89226	2.1
58990	1.89226	2.1
58991	1.89226	2.1
58992	0.539566	0.815907
58993	0.534046	2.1
58994	0.577914	1.99055
58995	0.471623	2.01346
58996	1.46031	0.429881
58997	0.945865	0.927742
58998	0.770603	0.409444
58999	0.737201	2.1
59000	0.714829	0.957801
59001	0.536085	1.721
59002	0.471617	1.44442
59003	1.89226	2.1
59004	1.89226	0.721575
59005	1.89226	0.681505
59006	1.89226	1.40233
59007	1.89226	1.52296
59008	1.89226	2.1
59009	1.89225	1.69794
59010	1.89225	0.262436
59011	1.65572	0.259887
59012	1.65572	0.752523
59013	1.89225	0.896283
59014	1.89225	1.82233
59015	1.89226	1.51074
59016	1.89226	0.771273
59017	1.89226	1.08948
59018	1.89226	0.0993417
59019	3.78453	1.63942
59020	1.89226	2.04541
59021	1.89226	0.378879
59022	1.89226	0.423581
59023	1.89226	1.20357
59024	1.89226	1.21495
59025	1.89226	2.09199
59026	1.89225	1.45087
59027	1.89225	0.128304
59028	1.89225	0.181547
59029	1.89225	0.603539
59030	1.89225	0.46763
59031	1.89225	1.53353
59032	0.763592	0.266953
59033	0.763592	1.0504
59034	0.987417	0.57019
59035	0.987417	0.647156
59036	0.763592	0.15897
59037	0.804881	0.0909634
59038	0.946126	0.622129
59039	0.946126	0.286331
59040	0.804879	0.0861929
59041	0.94613	0.427102

59042	0.946129	0.821238
59043	0.76359	0.191125
59044	1.46055	2.1
59045	2.04945	2.1
59046	2.04587	2.1
59047	2.04587	2.1
59048	1.50714	2.1
59049	1.50714	2.1
59050	2.04587	2.1
59051	2.38681	2.1
59052	2.42082	2.1
59053	2.42082	2.1
59054	2.04945	2.1
59055	2.04945	2.1
59056	0.872964	2.1
59057	0.872964	2.1
59058	0.884806	2.1
59059	0.884806	2.1
59060	1.0275	2.1
59061	1.02293	2.1
59062	0.711243	2.1
59063	0.730273	2.1
59064	0.711244	2.1
59065	1.0275	2.1
59066	0.730273	2.1
59067	1.02293	2.1
59068	2.04587	2.1
59069	2.47753	2.1
59070	2.42994	2.1
59071	2.42082	2.1
59072	2.05499	2.1
59073	2.04587	2.1
59074	0.946132	2.1
59075	0.946132	2.1
59076	1.13078	2.1
59077	1.07817	2.1
59078	0.946132	2.1
59079	0.946132	2.1
59080	1.07817	2.1
59081	1.07817	2.1
59082	0.946132	2.1
59083	1.07817	2.1
59084	1.13078	2.1
59085	0.946132	2.1
59086	1.89226	2.1
59087	1.89226	2.1
59088	1.89226	2.1
59089	1.89226	2.1
59090	1.89226	2.1
59091	1.89226	2.1
59092	1.89226	2.1
59093	1.89226	2.1
59094	1.89226	2.1
59095	1.89226	2.1
59096	1.89226	2.1
59097	1.89226	2.1
59098	1.89226	2.1
59099	1.89226	2.1

59100	1.8173	2.1
59101	2.66885	2.1
59102	2.7438	2.1
59103	2.7438	2.1
59104	1.89226	0.956986
59105	1.89226	0.539735
59106	1.89226	0.575262
59107	1.89226	0.975646
59108	3.78453	0.866
59109	1.89226	0.370659
59110	1.89226	0.397679
59111	1.89226	0.26299
59112	1.89226	1.61916
59113	1.89226	0.0790527
59114	1.89226	0.505188
59115	1.89226	0.197821
59116	1.97385	0.65071
59117	1.7531	0.331069
59118	1.89226	1.77493
59119	1.7531	0.0723052
59120	1.89226	0.362916
59121	0.946131	1.16561
59122	0.840904	0.243334
59123	0.684333	0.435397
59124	0.94613	0.735968
59125	0.972337	0.622793
59126	0.946278	1.75066
59127	0.684304	0.477235
59128	0.684304	0.780527
59129	0.999838	0.280236
59130	0.759605	1.72483
59131	0.851528	0.503806
59132	0.851528	1.21744
59133	1.89226	0.460936
59134	2.23818	1.13686
59135	1.79771	0.487061
59136	1.89226	1.57277
59137	1.79771	0.144653
59138	1.89226	0.537465
59139	2.79647	2.1
59140	2.01613	2.1
59141	1.46826	2.1
59142	2.54504	2.1
59143	4.56117	2.1
59144	1.89226	2.1
59145	1.89226	2.1
59146	1.89226	2.1
59147	1.89226	2.1
59148	1.89226	2.1
59149	1.89226	2.1
59150	1.89226	2.1
59151	2.66884	2.1
59152	2.56022	2.1
59153	2.66884	2.1
59154	1.78364	2.1
59155	1.89226	2.1
59156	1.14282	2.1
59157	1.24068	2.1

59158	0.880748	2.1
59159	0.834916	2.1
59160	1.05539	2.1
59161	1.05539	2.1
59162	1.04312	2.1
59163	1.04313	2.1
59164	1.2015	2.1
59165	0.83492	2.1
59166	0.789149	2.1
59167	1.24066	2.1
59168	1.89226	2.1
59169	1.89226	2.1
59170	1.89226	2.1
59171	1.89226	2.1
59172	3.78453	2.1
59173	0.946128	2.1
59174	0.946128	2.1
59175	1.28011	2.1
59176	1.22372	2.1
59177	0.946131	2.1
59178	0.946131	2.1
59179	1.22372	2.1
59180	1.22372	2.1
59181	1.22372	2.1
59182	0.94613	2.1
59183	0.946131	2.1
59184	1.28011	2.1
59185	2.28604	2.1
59186	1.34268	2.1
59187	2.43157	2.1
59188	1.50172	2.1
59189	3.77425	2.1
59190	2.6096	2.1
59191	2.43157	2.1
59192	1.66774	2.1
59193	1.66774	2.1
59194	1.50172	2.1
59195	2.43157	2.1
59196	2.16067	2.1
59197	1.08975	2.1
59198	2.19336	2.1
59199	2.06984	2.1
59200	1.21327	2.1
59201	1.08975	2.1
59202	2.28604	2.1
59203	1.21327	2.1
59204	2.29008	2.1
59205	2.16067	2.1
59206	1.34268	2.1
59207	1.21327	2.1
59208	1.89226	0.057556
59209	1.89226	0.05
59210	1.89226	0.0690238
59211	1.89226	0.0547215
59212	3.78452	0.05
59213	1.89804	0.05
59214	2.21656	0.05
59215	1.70537	0.0622808

59216	2.50023	0.05
59217	3.8982	0.05
59218	1.03136	0.05
59219	1.89486	0.05
59220	0.949364	0.05
59221	1.82283	0.05
59222	2.84422	0.05
59223	1.89226	0.05
59224	1.89226	0.05
59225	1.89226	0.0555077
59226	1.89226	0.0540283
59227	3.78452	0.0568006
59228	0.946132	0.0784592
59229	0.946132	0.083054
59230	0.946129	0.10519
59231	0.946129	0.05
59232	0.94613	0.05
59233	0.94613	0.107263
59234	0.946131	0.05
59235	0.946131	0.100397
59236	0.946131	0.115005
59237	0.946131	0.0932406
59238	0.946131	0.05
59239	0.946131	0.135277
59240	1.09466	0.0551163
59241	1.09466	0.05
59242	0.866512	0.0663449
59243	1.08352	0.05
59244	1.19189	0.05
59245	1.21883	0.055525
59246	0.971139	0.0526583
59247	0.873213	0.05
59248	0.873202	0.05
59249	1.00855	0.0500465
59250	0.923735	0.05
59251	1.21885	0.05
59252	1.89225	0.05
59253	2.40019	0.05
59254	1.89225	0.05
59255	1.89225	0.05
59256	1.89225	0.05
59257	1.89225	0.05
59258	1.89225	0.05
59259	1.89225	0.05
59260	2.05088	0.05
59261	1.89225	0.05
59262	1.89225	0.05
59263	1.89225	0.05
59264	2.03956	0.05
59265	2.03956	0.05
59266	1.89226	2.1
59267	2.40021	2.1
59268	2.38473	0.0594316
59269	2.40021	2.1
59270	1.87679	0.169357
59271	1.89226	2.1
59272	1.89226	2.1
59273	1.89226	0.0503409

59274	1.89226	2.1
59275	1.89226	0.05
59276	1.89226	2.1
59277	1.89226	0.05
59278	2.0692	2.1
59279	1.89226	0.343446
59280	1.89226	2.1
59281	1.89226	0.05
59282	2.05089	2.1
59283	2.05089	0.05
59284	1.89226	2.1
59285	1.89226	2.1
59286	1.89226	2.1
59287	1.89226	2.1
59288	3.78452	2.1
59289	1.89226	2.07333
59290	2.50051	2.1
59291	1.89226	2.1
59292	2.30076	2.1
59293	4.39277	2.1
59294	2.50052	2.1
59295	1.70377	2.1
59296	2.30076	2.1
59297	1.54231	2.1
59298	4.04283	2.1
59299	1.89226	2.1
59300	1.89226	2.1
59301	1.89226	2.1
59302	1.89226	2.1
59303	1.89226	2.1
59304	1.89226	2.1
59305	1.89226	2.08657
59306	1.89226	2.1
59307	1.89226	2.1
59308	1.89226	2.1
59309	3.78452	2.1
59310	2.07948	2.1
59311	2.30076	2.1
59312	1.3294	2.1
59313	1.3294	2.1
59314	1.54232	2.1
59315	2.29239	2.1
59316	0.810947	2.1
59317	0.810947	2.1
59318	0.810946	2.1
59319	0.810946	2.1
59320	1.89226	2.1
59321	1.35169	2.1
59322	1.08132	2.1
59323	1.08132	2.1
59324	1.08132	2.1
59325	0.810946	2.1
59326	0.810946	2.1
59327	1.34851	1.10385
59328	0.997471	2.1
59329	1.62287	0.49884
59330	1.27096	1.8782
59331	2.62034	1.05647

59332	1.89226	0.658556
59333	1.89226	2.1
59334	1.89226	0.974349
59335	1.89226	2.1
59336	3.78452	1.26915
59337	0.946128	2.1
59338	0.946128	2.1
59339	1.29331	0.408532
59340	1.29331	2.1
59341	1.29331	2.1
59342	1.10699	2.1
59343	0.946131	0.343571
59344	0.946131	2.09889
59345	0.94613	2.1
59346	1.29331	2.1
59347	1.10699	2.1
59348	0.946131	2.1
59349	1.62287	0.864939
59350	1.27096	2.02288
59351	1.86067	0.697834
59352	1.58407	1.94595
59353	3.20694	1.72425
59354	1.89226	1.32612
59355	1.89226	2.1
59356	1.89226	0.6
59357	1.89226	1.94524
59358	3.78452	1.29846
59359	2.62761	0.35078
59360	2.55849	0.529141
59361	2.12261	1.37425
59362	2.22939	0.670326
59363	4.78788	0.689402
59364	0.772522	1.89714
59365	0.839588	1.3216
59366	1.31126	0.0973825
59367	1.08787	0.090779
59368	1.01318	0.175803
59369	1.01318	2.00054
59370	0.900815	2.1
59371	1.10799	0.05
59372	0.772511	0.519592
59373	1.03473	0.473299
59374	1.31126	0.719492
59375	0.841464	1.05864
59376	1.89227	0.182393
59377	1.89227	2.09457
59378	1.89227	0.381096
59379	1.89227	1.64299
59380	1.89227	0.340349
59381	1.89227	1.9563
59382	1.89226	0.669846
59383	1.89226	0.60796
59384	1.89226	0.251189
59385	1.89226	0.702766
59386	1.89226	0.13816
59387	1.89226	0.564721
59388	2.12261	0.699744
59389	2.22939	0.134717

59390	1.97004	0.189027
59391	1.8072	0.962032
59392	4.03658	0.25811
59393	1.5847	1.87814
59394	1.8072	0.142304
59395	2.12262	0.718874
59396	2.12262	1.86883
59397	1.23267	0.450855
59398	1.23267	1.93044
59399	1.89227	0.173121
59400	1.89227	2.1
59401	1.89227	0.320418
59402	1.89227	1.82776
59403	1.89227	0.30583
59404	1.89227	2.03244
59405	1.89226	0.145421
59406	1.89226	0.525464
59407	1.89226	1.05276
59408	1.89226	0.145651
59409	3.78452	0.258392
59410	2.49093	2.1
59411	2.67393	2.1
59412	2.47932	2.1
59413	2.47932	2.1
59414	2.67994	2.1
59415	2.67994	2.1
59416	2.49873	2.1
59417	2.67393	2.1
59418	2.67993	2.1
59419	2.69807	2.1
59420	2.49873	2.1
59421	2.51686	2.1
59422	2.49093	2.1
59423	2.67994	2.1
59424	2.69784	2.1
59425	2.51651	2.1
59426	5.18877	2.1
59427	1.24359	2.1
59428	1.24936	2.1
59429	1.33997	2.1
59430	1.34892	2.1
59431	1.28805	2.1
59432	1.28805	2.1
59433	1.2955	2.1
59434	1.2955	2.1
59435	1.34892	2.1
59436	1.24359	2.1
59437	1.24936	2.1
59438	1.33997	2.1
59439	1.15506	2.09456
59440	1.15506	2.1
59441	0.946128	2.1
59442	0.946128	2.1
59443	0.946131	2.1
59444	1.15506	2.1
59445	1.06997	2.1
59446	0.946128	2.1
59447	1.06997	2.1

59448	1.15506	2.1
59449	0.946129	2.1
59450	0.946129	2.1
59451	1.70608	2.1
59452	2.13994	2.1
59453	1.33384	2.1
59454	1.43635	2.1
59455	3.14243	2.1
59456	2.13994	2.1
59457	2.31012	2.1
59458	1.43635	2.1
59459	1.23007	2.1
59460	3.74646	2.1
59461	2.46361	2.1
59462	1.89226	2.1
59463	1.89226	2.1
59464	1.89226	2.1
59465	2.39045	2.1
59466	2.39045	2.1
59467	1.89226	2.1
59468	1.89226	1.93548
59469	1.89226	2.1
59470	1.89226	1.13679
59471	3.78452	2.1
59472	1.52984	2.1
59473	2.25682	0.874007
59474	2.20831	0.617535
59475	2.20831	2.1
59476	1.61178	0.347563
59477	1.61178	2.1
59478	2.47395	2.1
59479	1.89226	2.1
59480	1.89226	2.1
59481	1.89226	2.1
59482	2.46362	2.1
59483	2.46362	2.1
59484	0.946131	0.210485
59485	0.946131	2.1
59486	0.946131	2.1
59487	0.946131	1.99043
59488	0.94613	1.11411
59489	0.94613	2.1
59490	0.946132	2.1
59491	0.946132	2.1
59492	0.946131	2.1
59493	0.946131	2.1
59494	0.946131	2.1
59495	0.946131	2.1
59496	0.982398	2.1
59497	0.982398	2.1
59498	0.931703	2.1
59499	0.97549	2.1
59500	1.11509	2.06834
59501	1.10017	0.770993
59502	0.774576	2.1
59503	0.789859	2.1
59504	0.716502	2.1
59505	1.12793	2.1

59506	1.14398	2.1
59507	0.817011	2.06064
59508	1.82499	2.1
59509	1.50524	2.1
59510	1.21304	2.1
59511	1.54191	2.1
59512	3.04715	2.1
59513	1.54191	2.1
59514	1.49823	2.1
59515	1.82499	2.1
59516	1.82499	2.1
59517	1.21147	2.1
59518	1.48977	2.1
59519	1.89226	2.1
59520	1.89226	2.1
59521	1.89226	2.1
59522	1.89226	2.1
59523	3.78452	2.1
59524	1.89226	2.1
59525	1.89226	2.1
59526	1.89226	2.1
59527	1.89226	2.1
59528	1.89226	2.1
59529	1.89226	2.1
59530	0.566674	2.1
59531	0.770957	2.1
59532	0.60652	2.1
59533	0.473557	2.1
59534	0.604441	2.1
59535	0.604441	2.1
59536	0.640073	2.1
59537	0.640073	2.1
59538	0.473557	2.1
59539	0.566674	2.1
59540	0.60652	2.1
59541	0.770956	2.1
59542	1.36203	2.1
59543	1.13335	2.1
59544	1.54191	2.1
59545	1.54191	2.1
59546	1.00965	2.1
59547	1.00965	2.1
59548	1.89226	2.1
59549	1.89226	2.1
59550	1.89226	2.1
59551	1.89226	2.1
59552	1.89226	2.1
59553	1.89226	2.1
59554	0.946131	2.1
59555	0.946131	2.1
59556	0.946131	2.1
59557	0.946131	2.1
59558	0.946132	2.1
59559	0.946132	2.1
59560	0.946131	2.1
59561	0.946131	2.1
59562	0.946132	2.1
59563	0.946132	2.1

59564	0.946131	2.1
59565	0.946131	2.1
59566	0.946132	0.05
59567	0.946132	0.05
59568	0.946133	0.05
59569	0.946133	0.05
59570	0.946132	0.05
59571	0.946132	0.05
59572	0.946132	0.05
59573	0.946132	0.05
59574	0.946132	0.05
59575	0.946132	0.05
59576	0.946133	0.05
59577	0.946133	0.05
59578	1.89226	0.05
59579	1.89226	0.05
59580	1.89226	0.05
59581	1.89226	0.05
59582	3.78453	0.05
59583	1.89226	0.05
59584	1.89226	0.05
59585	1.89226	0.05
59586	1.89226	0.05
59587	1.89226	0.05
59588	1.89226	0.05
59589	0.946132	0.05
59590	0.946132	0.05
59591	0.946131	0.05
59592	0.946131	0.05
59593	0.946132	0.05
59594	0.946132	0.05
59595	0.946132	0.05
59596	0.946132	0.05
59597	0.946132	0.05
59598	0.946132	0.05
59599	0.946132	0.05
59600	0.946132	0.05
59601	0.946129	0.05
59602	0.946129	0.05
59603	0.946128	0.05
59604	0.946128	0.05
59605	0.946132	0.05
59606	0.946132	0.05
59607	0.946126	0.05
59608	0.946126	0.05
59609	0.946129	0.05
59610	0.946129	0.05
59611	0.946129	0.05
59612	0.946129	0.05
59613	1.20674	0.05
59614	1.79877	0.05
59615	1.22586	0.05
59616	1.22586	0.05
59617	1.7919	0.05
59618	1.7919	0.05
59619	2.27622	0.05
59620	1.79877	0.05
59621	1.7919	0.05

59622	2.27102	0.05
59623	4.06979	0.05
59624	1.89226	0.05
59625	1.89226	0.05
59626	1.89226	0.05
59627	1.89226	0.05
59628	3.78452	0.05
59629	1.89226	0.05
59630	1.89226	0.05
59631	1.89226	0.05
59632	1.89226	0.05
59633	3.78453	0.05
59634	1.13259	0.05
59635	1.79187	0.05
59636	1.32741	0.05
59637	1.18784	0.05
59638	1.703 0.05	
59639	1.49575	0.05
59640	1.46201	0.05
59641	2.27099	0.05
59642	1.79187	0.05
59643	1.79187	0.05
59644	2.12923	0.05
59645	2.12923	0.05
59646	1.89224	0.05
59647	1.89224	0.05
59648	1.89224	0.05
59649	1.89224	0.05
59650	3.78447	0.05
59651	0.946119	0.05
59652	0.946119	0.05
59653	0.946118	0.05
59654	0.946118	0.05
59655	0.946118	0.05
59656	0.946118	0.05
59657	0.946119	0.05
59658	0.946119	0.05
59659	0.946131	0.05
59660	0.946131	0.05
59661	0.946106	0.05
59662	0.946106	0.05
59663	2.07526	0.0972388
59664	1.89226	0.138634
59665	1.89226	0.796127
59666	2.13445	0.184891
59667	4.02671	0.0710582
59668	1.89226	0.0739219
59669	1.89226	0.184017
59670	1.89226	0.320576
59671	1.89226	0.05
59672	1.89226	0.730349
59673	1.89226	0.0733133
59674	2.13446	0.134127
59675	1.00013	0.0903491
59676	1.1475	0.979841
59677	2.20046	0.18719
59678	3.20059	0.358765
59679	0.946133	0.135164

59680	0.946133	0.195652
59681	1.06722	0.181728
59682	1.10023	0.154343
59683	0.946131	0.213495
59684	0.946131	0.05
59685	1.10023	0.309488
59686	1.10023	0.0759187
59687	0.946131	0.30781
59688	1.10023	0.132708
59689	0.94613	0.296569
59690	1.06723	0.090364
59691	1.89226	0.0528992
59692	1.89226	0.062477
59693	1.89226	0.246713
59694	1.89226	0.159658
59695	3.78452	0.156058
59696	1.89226	0.17065
59697	1.89226	0.0970672
59698	1.89226	0.317799
59699	1.89226	0.201079
59700	1.89226	0.111086
59701	1.89226	0.0723621
59702	1.89226	2.1
59703	1.89226	0.05
59704	1.89226	0.20759
59705	1.89226	0.05
59706	1.89226	2.1
59707	0.828588	0.05
59708	0.828588	0.05
59709	0.841316	0.05
59710	0.841316	2.1
59711	0.528387	2.1
59712	0.565893	0.05
59713	1.10401	0.05
59714	1.10401	2.1
59715	0.528387	2.1
59716	1.89226	2.1
59717	1.36047	0.20759
59718	1.33973	2.1
59719	1.33973	2.1
59720	1.89226	0.05
59721	2.28951	0.05
59722	1.89226	0.934556
59723	2.80796	2.00506
59724	2.01584	1.92727
59725	2.01584	1.26202
59726	1.89226	1.76169
59727	1.89226	1.09954
59728	1.89226	0.66906
59729	1.89226	1.87011
59730	1.89226	1.61303
59731	1.89226	0.984956
59732	1.89226	1.65226
59733	1.89226	1.04838
59734	2.15223	0.928702
59735	1.30664	2.1
59736	1.96011	2.02624
59737	2.76516	0.94215

59738	4.11234	2.00464
59739	1.89226	0.571074
59740	1.89226	1.7708
59741	1.89226	1.42875
59742	1.89226	0.90737
59743	1.89226	1.49496
59744	1.89226	0.99012
59745	2.81143	2.1
59746	2.58517	2.1
59747	2.55318	2.1
59748	2.7542	2.1
59749	5.33937	2.1
59750	2.7777	2.1
59751	2.7542	2.1
59752	2.71672	2.1
59753	2.70401	2.1
59754	5.45821	2.1
59755	2.55318	2.1
59756	2.7542	2.1
59757	2.71672	2.1
59758	2.54198	2.1
59759	5.29618	2.1
59760	1.95416	2.1
59761	2.162 2.1	
59762	2.11563	2.1
59763	1.91813	2.1
59764	4.08013	2.1
59765	1.95417	2.1
59766	1.47712	2.1
59767	1.45182	2.1
59768	1.91813	2.1
59769	3.39525	2.1
59770	2.11562	2.1
59771	1.91813	2.1
59772	1.50979	2.1
59773	2.03974	2.1
59774	3.95787	2.1
59775	1.85605	2.1
59776	1.91813	2.1
59777	1.88885	2.1
59778	1.42255	2.1
59779	1.45182	2.1
59780	1.45182	2.1
59781	0.94613	0.486815
59782	0.94613	0.0627619
59783	0.946128	0.05
59784	0.946128	0.908841
59785	0.946127	0.193689
59786	0.946127	0.284499
59787	0.946131	0.05
59788	0.946131	0.05
59789	0.946129	0.131296
59790	0.946129	0.120377
59791	0.946128	0.0640421
59792	0.946128	0.094969
59793	0.543052	0.330168
59794	0.54608	0.05
59795	0.602293	0.05

59796	0.535325	0.064278
59797	1.05446	0.419166
59798	0.850496	0.162901
59799	0.699838	0.0752397
59800	0.679751	0.407234
59801	0.713797	0.0679593
59802	0.509794	0.05
59803	0.602304	0.05
59804	1.40017	0.258036
59805	1.40017	2.1
59806	0.94613	0.0613799
59807	0.94613	2.1
59808	1.40017	2.1
59809	1.33997	2.1
59810	0.946131	0.0928664
59811	0.946131	0.142819
59812	1.33997	2.09943
59813	0.946131	2.06827
59814	0.946131	2.02896
59815	1.40017	2.1
59816	1.89226	0.05
59817	1.89226	0.05
59818	1.89226	0.05
59819	1.89226	0.05
59820	3.78452	0.05
59821	0.946133	0.05
59822	0.946133	0.05
59823	0.946132	0.05
59824	0.946132	0.05
59825	0.946132	0.05
59826	0.946132	0.05
59827	0.946133	0.05
59828	0.946133	0.05
59829	0.946132	0.05
59830	0.946132	0.05
59831	0.946132	0.05
59832	0.946132	0.05
59833	1.89224	0.05
59834	1.89224	0.05
59835	1.89224	0.05
59836	1.89224	0.05
59837	1.89224	0.05
59838	1.89224	0.05
59839	0.94612	0.05
59840	0.94612	0.05
59841	0.946119	0.05
59842	0.946119	0.05
59843	0.946119	0.05
59844	0.946119	0.05
59845	0.94612	0.05
59846	0.94612	0.05
59847	0.946132	0.05
59848	0.946132	0.05
59849	0.946107	0.05
59850	0.946107	0.05
59851	2.63939	2.1
59852	1.2862	2.1
59853	1.29891	2.1

59854	2.64419	2.1
59855	3.93039	2.1
59856	2.6394	2.1
59857	1.89226	2.1
59858	1.89226	2.1
59859	2.64419	2.1
59860	4.53646	2.1
59861	1.89226	2.1
59862	1.89226	2.1
59863	1.89226	2.1
59864	1.89226	2.1
59865	3.78453	2.1
59866	1.89226	2.1
59867	1.89226	2.1
59868	1.89226	2.1
59869	1.89226	2.1
59870	1.89226	2.1
59871	1.89226	2.1
59872	1.31998	2.1
59873	1.32209	2.1
59874	0.649456	2.1
59875	0.648474	2.1
59876	0.984995	2.1
59877	0.984995	2.1
59878	0.985573	2.1
59879	0.985573	2.1
59880	1.31999	2.1
59881	0.648472	2.1
59882	0.649455	2.1
59883	1.32209	2.1
59884	0.946133	2.1
59885	0.946133	2.1
59886	1.31999	2.1
59887	1.3221	2.1
59888	0.946132	2.1
59889	0.946132	2.1
59890	1.31999	2.1
59891	1.31999	2.1
59892	1.31999	2.1
59893	0.946132	2.1
59894	0.946133	2.1
59895	1.3221	2.1
59896	0.946132	2.1
59897	0.946132	2.1
59898	0.946131	2.1
59899	0.946131	2.1
59900	0.946132	2.1
59901	0.946132	2.1
59902	0.946131	2.1
59903	0.946131	2.1
59904	0.946131	2.1
59905	0.946131	2.1
59906	0.946132	2.1
59907	0.946132	2.1
59908	0.946128	2.1
59909	0.946128	2.1
59910	0.946132	2.1
59911	0.946132	2.1

59912	0.946131	2.1
59913	0.946131	2.1
59914	0.94613	2.1
59915	0.94613	2.1
59916	0.94613	2.1
59917	0.94613	2.1
59918	0.946131	2.1
59919	0.946131	2.1
59920	1.89226	0.05
59921	1.89226	0.101877
59922	1.89226	0.0976812
59923	1.89226	0.0598836
59924	1.89226	0.05
59925	1.89226	0.05
59926	1.89226	0.0613404
59927	1.89226	0.490511
59928	1.89226	0.132372
59929	1.89226	0.283994
59930	3.78453	0.476204
59931	0.946133	0.0859034
59932	0.946133	0.0777592
59933	0.946132	0.05
59934	0.946132	0.05
59935	0.946133	0.100155
59936	0.946133	0.10253
59937	0.946132	0.0977457
59938	0.946132	0.05
59939	0.946132	0.0763357
59940	0.946132	0.124028
59941	0.946132	0.05
59942	0.946132	0.188172
59943	2.23638	0.05
59944	1.89226	0.05
59945	2.66014	0.066933
59946	2.66014	0.0863697
59947	1.89226	0.0733416
59948	1.89226	0.05
59949	1.11819	0.05
59950	1.33007	0.05
59951	0.660255	0.05
59952	0.91151	0.05
59953	0.911511	0.05
59954	1.33007	0.05
59955	1.11819	0.05
59956	0.660255	0.05
59957	0.985327	0.05
59958	0.985327	0.05
59959	1.005	0.05
59960	1.005	0.05
59961	1.89226	2.1
59962	1.89226	2.1
59963	1.89226	2.1
59964	1.89226	2.1
59965	3.78453	2.1
59966	1.59533	2.1
59967	2.68571	2.1
59968	1.28291	2.1
59969	2.46974	2.1

59970	4.06506	2.1
59971	2.68571	2.1
59972	1.89226	2.1
59973	1.89226	2.1
59974	1.89226	2.1
59975	2.46973	2.1
59976	2.46973	2.1
59977	1.89226	2.1
59978	1.89226	2.1
59979	1.89226	2.1
59980	1.89226	2.1
59981	1.89226	2.1
59982	1.89226	2.1
59983	1.89226	2.1
59984	1.89226	2.1
59985	1.89226	2.1
59986	1.89226	2.1
59987	1.89226	2.1
59988	1.89226	2.1
59989	0.883594	2.1
59990	0.883594	2.1
59991	0.876484	2.1
59992	0.876484	2.1
59993	1.10442	2.1
59994	1.23487	2.1
59995	0.641457	2.1
59996	0.52521	2.1
59997	1.10442	2.1
59998	0.52521	2.1
59999	0.641457	2.1
60000	1.23487	2.1
60001	0.946131	2.1
60002	0.946131	2.1
60003	0.946131	2.1
60004	0.946131	2.1
60005	0.946131	2.1
60006	0.946131	2.1
60007	0.946131	2.1
60008	0.946131	2.1
60009	0.946131	2.1
60010	0.946131	2.1
60011	0.946131	2.1
60012	0.946131	2.1
60013	2.04016	0.05
60014	2.13042	0.05
60015	2.11465	0.05
60016	2.11465	0.05
60017	1.95908	0.05
60018	1.95908	0.05
60019	1.66953	0.05
60020	2.13041	0.05
60021	1.95908	0.05
60022	2.21952	0.05
60023	1.66953	0.05
60024	1.92997	0.05
60025	2.04016	0.05
60026	1.95908	0.05
60027	1.82014	0.05

60028	2.08757	0.05
60029	3.86029	0.05
60030	0.834764	0.05
60031	0.722102	0.05
60032	0.979537	0.05
60033	0.910065	0.05
60034	0.840053	0.05
60035	0.840052	0.05
60036	0.861587	0.05
60037	0.861587	0.05
60038	0.910067	0.05
60039	0.722101	0.05
60040	0.834763	0.05
60041	0.979539	0.05
60042	1.89226	0.05
60043	1.89226	0.05
60044	1.89226	0.05
60045	1.89226	0.05
60046	1.89226	0.05
60047	1.89226	0.05
60048	1.89226	0.05
60049	1.89226	0.05
60050	1.89226	0.05
60051	1.89226	0.05
60052	3.78453	0.05
60053	1.89226	0.05
60054	1.89226	0.05
60055	1.89226	0.05
60056	1.89226	0.05
60057	1.89226	0.05
60058	1.89226	0.05
60059	1.20691	0.05
60060	2.27379	0.05
60061	2.58476	0.05
60062	1.5124	0.05
60063	3.79167	0.05
60064	1.89226	0.05
60065	2.2738	0.05
60066	2.58477	0.05
60067	1.89226	0.05
60068	4.47703	0.05
60069	0.952225	1.27878
60070	0.952225	1.93557
60071	0.909865	2.04106
60072	0.909865	2.1
60073	0.909895	2.1
60074	0.946132	1.44531
60075	0.946132	2.0209
60076	0.915958	1.32979
60077	0.946132	1.97484
60078	0.946132	1.99409
60079	0.909894	1.94528
60080	0.915958	2.01753
60081	0.983493	2.1
60082	2.40055	2.1
60083	1.02986	2.1
60084	1.02986	2.1
60085	2.34217	2.1

60086	2.34217	2.1
60087	1.89226	2.1
60088	2.40056	2.1
60089	2.34217	2.1
60090	2.40056	2.1
60091	1.83388	2.1
60092	1.89226	2.1
60093	1.89226	2.1
60094	1.89226	2.1
60095	1.89226	2.1
60096	1.89226	2.1
60097	1.89226	2.1
60098	1.89226	2.1
60099	1.89226	2.1
60100	1.89226	2.1
60101	1.89226	2.1
60102	1.89226	2.1
60103	1.89226	2.1
60104	1.89226	2.1
60105	0.946133	2.1
60106	0.946133	2.1
60107	1.17109	2.1
60108	1.13078	2.1
60109	0.946132	2.1
60110	0.946132	2.1
60111	1.13078	2.1
60112	1.13078	2.1
60113	1.13078	2.1
60114	0.946133	2.1
60115	0.946132	2.1
60116	1.17109	2.1
60117	1.89226	2.1
60118	1.89226	2.1
60119	1.89226	2.1
60120	1.89226	2.1
60121	1.89226	2.1
60122	1.89226	2.1
60123	2.33963	1.10775
60124	1.89227	0.382965
60125	1.74565	0.553488
60126	1.74565	0.675764
60127	1.89227	0.154754
60128	1.89227	0.286701
60129	1.89227	0.776764
60130	1.89227	0.432634
60131	1.89227	1.35392
60132	1.89227	0.683998
60133	3.78453	0.633906
60134	1.31398	0.325499
60135	1.10392	0.446543
60136	0.796878	0.295055
60137	0.524356	0.19699
60138	0.870614	0.321396
60139	0.945739	0.166217
60140	0.89208	0.392098
60141	0.89208	0.85003
60142	1.23425	0.274136
60143	0.774658	0.699352

60144	1.03182	0.23054
60145	0.557289	0.159844
60146	2.33963	0.907767
60147	1.89226	0.130354
60148	1.89226	1.04
60149	2.77245	0.291512
60150	4.23189	0.695689
60151	1.89226	1.17166
60152	1.89226	0.42613
60153	1.89226	1.11578
60154	1.89226	1.0282
60155	1.89226	0.50565
60156	1.89226	0.43561
60157	1.89226	0.075437
60158	1.89226	0.108793
60159	1.89226	0.063717
60160	1.89226	0.256083
60161	1.89226	0.0581143
60162	1.89226	0.215219
60163	1.89226	0.0591681
60164	1.89226	0.0637791
60165	1.89226	0.05
60166	1.89226	0.141507
60167	1.89226	0.05
60168	1.89226	0.100744
60169	0.946128	0.05
60170	0.946128	0.05
60171	0.963918	0.05
60172	1.37543	0.05
60173	0.946129	0.05
60174	0.946129	0.0520862
60175	1.37542	0.05
60176	1.37542	0.05
60177	0.94613	0.05
60178	1.37542	0.05
60179	0.963916	0.05
60180	0.94613	0.05
60181	0.946132	0.0720895
60182	0.946132	0.05
60183	0.946131	0.0528398
60184	0.946131	0.05
60185	0.946131	0.053052
60186	0.946131	0.0807646
60187	0.946132	0.05
60188	0.946132	0.05
60189	0.946131	0.05
60190	0.946131	0.05
60191	0.946131	0.05
60192	0.946131	0.05
60193	1.89226	0.0810004
60194	1.89226	0.110383
60195	1.89226	0.05
60196	1.89226	0.197749
60197	3.78452	0.0845343
60198	0.946127	0.109116
60199	0.946127	0.05
60200	0.946131	0.062225
60201	0.946131	0.05

60202	0.946128	0.0627997
60203	0.946128	0.101393
60204	0.94613	0.05
60205	0.94613	0.05
60206	0.946129	0.0500577
60207	0.946129	0.054497
60208	0.946129	0.0510039
60209	0.946129	0.0569918
60210	1.89226	0.267535
60211	1.89226	0.735569
60212	1.89226	2.1
60213	1.89226	0.064336
60214	3.78453	0.0820139
60215	1.89226	0.160003
60216	1.89226	0.05
60217	1.89226	0.05
60218	1.89226	0.0594236
60219	3.78453	0.115706
60220	0.946131	0.05
60221	0.946131	0.05
60222	0.946132	0.89719
60223	0.946132	0.123599
60224	0.946132	0.691161
60225	0.946132	0.0597497
60226	0.946132	0.687461
60227	0.946132	0.0788473
60228	0.946132	0.129851
60229	0.946132	0.278153
60230	0.946132	0.348886
60231	0.946132	0.0998585
60232	0.548169	0.05
60233	0.736603	0.05
60234	1.18322	0.05
60235	1.01139	0.05
60236	1.01139	0.05
60237	0.548168	0.05
60238	0.736603	0.05
60239	1.18322	0.05
60240	0.861589	0.05
60241	0.861589	0.05
60242	0.869798	0.05
60243	0.869798	0.05
60244	1.16037	0.05
60245	0.942012	0.05
60246	0.942012	0.05
60247	0.62356	0.05
60248	0.891932	0.05
60249	0.900255	0.05
60250	2.02279	0.05
60251	1.48677	0.05
60252	1.54724	0.05
60253	0.831456	0.05
60254	1.19775	0.05
60255	1.89226	1.43217
60256	2.76291	1.18555
60257	1.89226	1.53504
60258	1.89226	1.16202
60259	3.78452	1.33332

60260	1.89226	0.864935
60261	1.89226	1.37383
60262	1.89226	1.73034
60263	1.89226	0.998898
60264	1.89226	1.6649
60265	1.89226	0.885364
60266	1.89226	1.43263
60267	1.89226	1.00153
60268	1.89226	1.29846
60269	1.89226	1.08881
60270	3.78452	1.14353
60271	1.89226	0.760851
60272	1.89226	1.42813
60273	1.89226	1.61549
60274	1.89226	1.00971
60275	3.78453	1.2821
60276	0.946128	0.875976
60277	0.946128	1.21892
60278	0.946129	1.51938
60279	0.946129	1.23514
60280	0.946132	0.676414
60281	0.946132	1.26112
60282	0.946126	1.3341
60283	0.946126	1.02659
60284	0.946129	1.23976
60285	0.946129	1.21152
60286	0.946129	1.14623
60287	0.946129	1.06189
60288	2.80495	1.10789
60289	1.89225	0.894275
60290	1.89225	1.36489
60291	1.87794	1.3655
60292	1.89225	1.11733
60293	1.87794	1.11811
60294	2.25195	0.264302
60295	2.00624	0.0816514
60296	2.13132	0.119357
60297	1.90239	0.05
60298	4.13756	0.05
60299	0.804661	0.0725437
60300	0.804663	0.05
60301	0.889591	0.05
60302	0.889591	0.199973
60303	0.808324	0.0580761
60304	0.761765	0.0915032
60305	1.02822	0.152317
60306	0.879624	0.05
60307	0.879617	0.05
60308	0.800864	0.05
60309	0.978023	0.101408
60310	0.808329	0.05
60311	2.13133	0.165006
60312	2.00558	0.05
60313	2.09316	0.05
60314	2.09316	0.544575
60315	2.03253	0.05
60316	2.03253	1.23981
60317	0.84577	0.05

60318	0.845767	0.0703815
60319	0.843917	0.05
60320	0.843917	0.51659
60321	0.76177	0.05
60322	0.719345	0.0601249
60323	0.934435	0.05
60324	0.884197	0.05
60325	0.934439	0.05
60326	0.75627	0.05
60327	0.849424	0.05
60328	0.76177	0.05
60329	2.61855	0.05
60330	1.17793	0.05
60331	1.42965	0.05
60332	1.17793	0.05
60333	2.50057	0.05
60334	2.24885	0.05
60335	0.946133	0.05
60336	0.946133	0.05
60337	1.30928	0.05
60338	1.12443	0.05
60339	0.946132	0.05
60340	0.946132	0.05
60341	1.30928	0.05
60342	1.30928	0.05
60343	0.946132	0.05
60344	1.30928	0.05
60345	1.12443	0.05
60346	0.946133	0.05
60347	1.89226	0.05
60348	1.89226	0.05
60349	1.89226	0.05
60350	1.89226	0.05
60351	3.78452	0.05
60352	1.89226	0.05
60353	1.89226	0.05
60354	1.89226	0.05
60355	1.89226	0.05
60356	1.89226	0.05
60357	1.89226	0.05
60358	1.89226	0.05
60359	1.89226	0.05
60360	1.89226	0.05
60361	1.89226	0.0515938
60362	3.78452	0.05
60363	0.94613	0.05
60364	0.94613	0.05
60365	0.946131	0.05
60366	0.946131	0.05
60367	0.946128	0.05
60368	0.946128	0.05
60369	0.946133	0.05
60370	0.946133	0.05
60371	0.946131	0.05
60372	0.946131	0.05
60373	0.946131	0.05
60374	0.946131	0.05
60375	1.89226	2.1

60376	1.89226	2.1
60377	1.89226	2.1
60378	1.89226	2.1
60379	3.78453	2.1
60380	1.0465	2.1
60381	2.73926	2.1
60382	2.03721	2.1
60383	2.03721	2.1
60384	1.44709	2.1
60385	1.44709	2.1
60386	0.946131	2.1
60387	0.946131	2.1
60388	0.946131	2.1
60389	0.946131	2.1
60390	0.946132	2.1
60391	0.946132	2.1
60392	0.94613	2.1
60393	0.94613	2.1
60394	0.946131	2.1
60395	0.946131	2.1
60396	0.946131	2.1
60397	0.946131	2.1
60398	0.946132	2.1
60399	0.946132	2.1
60400	0.946131	2.1
60401	0.946131	2.1
60402	0.946132	2.1
60403	0.946132	2.1
60404	0.946132	2.1
60405	0.946132	2.1
60406	0.946132	2.1
60407	0.946132	2.1
60408	0.946132	2.1
60409	0.946132	2.1
60410	1.89226	2.1
60411	1.89226	2.1
60412	1.89226	2.1
60413	1.89226	2.1
60414	3.78452	2.1
60415	1.61969	0.0585294
60416	1.79374	0.05
60417	1.29382	0.05
60418	1.29382	0.05
60419	2.05759	0.05
60420	2.05759	0.05
60421	1.89226	0.0563614
60422	1.89226	0.05
60423	1.89226	0.05
60424	1.89226	0.0560735
60425	1.89226	0.05
60426	1.89226	0.0526925
60427	1.89226	0.121482
60428	1.89226	0.280429
60429	1.89226	0.133375
60430	1.89226	0.141978
60431	1.89226	0.108835
60432	1.89226	0.116348
60433	1.89226	1.46559

60434	1.89226	0.514034
60435	1.89226	0.546621
60436	1.89226	1.08989
60437	3.78453	1.44349
60438	0.946131	0.304889
60439	0.946131	0.382517
60440	0.946131	0.133471
60441	0.946131	0.346026
60442	0.946131	0.223547
60443	0.946131	0.143137
60444	0.946131	0.3347
60445	0.946131	0.528946
60446	0.946131	0.207775
60447	0.946131	0.205347
60448	0.946131	0.373819
60449	0.946131	0.268282
60450	2.45177	0.05
60451	2.68507	0.05
60452	2.3376	0.05
60453	2.22086	0.05
60454	4.78937	0.05
60455	2.45178	0.05
60456	2.35552	0.05
60457	1.68062	0.05
60458	1.81902	0.05
60459	4.13239	0.05
60460	2.3376	0.05
60461	1.55612	0.05
60462	1.69811	0.05
60463	2.31527	0.05
60464	1.79553	0.05
60465	2.3376	0.05
60466	1.55613	0.05
60467	1.06336	0.05
60468	1.97516	0.05
60469	1.88966	0.05
60470	1.06336	0.05
60471	1.68062	0.05
60472	1.89226	0.521325
60473	1.91634	0.340285
60474	2.03709	1.45364
60475	1.91634	0.585903
60476	1.83185	0.417728
60477	1.70303	0.057466
60478	1.89226	0.189732
60479	1.89226	0.275082
60480	1.89226	1.20126
60481	1.89226	0.351185
60482	1.89226	0.208671
60483	1.89226	0.05
60484	1.89226	1.99962
60485	2.03472	2.1
60486	2.22183	2.1
60487	2.03472	1.97481
60488	2.07936	2.1
60489	1.89226	0.692258
60490	1.89226	0.908006
60491	1.8519	0.116162

60492	1.92119	0.781599
60493	1.8519	0.48809
60494	1.96156	0.121813
60495	1.89226	0.05
60496	1.89226	0.22544
60497	1.89226	0.203035
60498	1.89226	1.06142
60499	1.89226	0.348063
60500	1.89226	0.107342
60501	1.89226	0.05
60502	1.89226	1.29996
60503	2.22183	1.85158
60504	2.39541	2.1
60505	2.22183	1.08066
60506	2.06585	1.67497
60507	1.89226	0.376086
60508	1.47001	2.1
60509	1.57962	0.54626
60510	1.65238	1.85874
60511	1.60502	2.1
60512	3.18464	2.1
60513	1.02415	2.1
60514	1.60503	2.1
60515	1.47001	2.1
60516	1.47001	2.1
60517	1.28228	2.1
60518	1.28228	2.1
60519	0.946133	2.1
60520	0.946133	2.1
60521	0.946132	2.1
60522	0.946132	1.99859
60523	0.946132	2.01861
60524	0.946132	2.1
60525	0.946133	2.1
60526	0.946133	2.1
60527	0.946132	2.1
60528	0.946132	2.1
60529	0.946133	2.1
60530	0.946133	2.1
60531	1.89226	0.512563
60532	1.89226	1.54386
60533	1.89226	2.1
60534	1.89226	0.170812
60535	3.78452	0.579619
60536	1.76897	1.60212
60537	1.60502	1.98556
60538	1.65238	0.768924
60539	1.65238	2.1
60540	1.76932	0.6574
60541	1.76932	2.1
60542	1.55998	2.1
60543	1.60503	2.1
60544	1.76932	2.1
60545	1.60503	2.1
60546	1.44657	2.1
60547	1.28228	2.1
60548	0.946133	2.1
60549	0.946133	2.1

60550	0.946132	2.1
60551	0.946132	1.19788
60552	0.946132	1.11125
60553	0.946132	2.1
60554	0.946133	2.1
60555	0.946133	2.1
60556	0.946132	2.1
60557	0.946132	2.1
60558	0.946132	2.1
60559	0.946132	2.1
60560	0.946128	2.01193
60561	0.946128	0.721481
60562	0.946133	0.118537
60563	0.946133	0.345901
60564	0.94613	0.127071
60565	0.94613	0.685089
60566	0.946131	2.01629
60567	0.946131	0.614069
60568	0.946131	0.547274
60569	0.946131	0.446091
60570	0.946131	0.821537
60571	0.946131	0.803492
60572	1.85779	0.05
60573	1.00763	0.05
60574	1.74874	0.05
60575	1.74873	0.05
60576	1.14467	0.05
60577	1.14466	0.05
60578	1.85779	0.05
60579	1.61597	0.05
60580	1.00053	0.05
60581	1.03018	0.05
60582	2.65819	0.05
60583	1.89226	0.0656134
60584	1.89226	0.05
60585	1.89226	0.103452
60586	1.89226	0.0553651
60587	3.78451	0.067627
60588	1.89226	0.0875098
60589	1.89226	0.05
60590	1.89226	0.05
60591	1.89226	0.05
60592	1.89226	0.05
60593	1.89226	0.05
60594	1.11983	0.05
60595	0.810945	0.05
60596	0.913906	0.05
60597	0.913906	0.05
60598	2.23303	0.05
60599	1.66057	0.05
60600	1.28724	0.05
60601	1.08132	0.05
60602	1.49316	0.05
60603	0.810958	0.05
60604	0.810958	0.05
60605	0.827861	0.05
60606	0.946126	0.05
60607	0.946132	0.0620142

60608	0.946132	0.05
60609	0.827863	0.05
60610	0.827863	0.05
60611	0.946129	0.05
60612	0.946129	0.05
60613	0.827863	0.05
60614	0.946128	0.05
60615	0.946129	0.05
60616	0.946129	0.05
60617	0.974718	0.05
60618	1.53671	0.05
60619	2.80542	0.05
60620	2.27259	0.05
60621	3.8093	0.05
60622	1.89226	0.05
60623	1.89226	0.103428
60624	1.89226	0.05
60625	1.89226	0.0881665
60626	3.78453	0.0591444
60627	0.946132	0.05
60628	0.946132	0.069205
60629	0.946131	0.0658677
60630	0.946131	0.05
60631	0.946132	0.0664325
60632	0.946132	0.0909886
60633	0.946131	0.05
60634	0.946131	0.05
60635	0.946132	0.05
60636	0.946132	0.0639701
60637	0.946131	0.05
60638	0.946131	0.05
60639	2.80542	0.05
60640	1.89226	0.05
60641	2.27259	0.05
60642	2.27259	0.05
60643	1.89226	0.05
60644	1.89226	0.05
60645	1.89226	0.05
60646	1.89226	0.0964892
60647	1.89226	0.05
60648	1.89226	0.0900339
60649	1.89226	0.05
60650	1.89226	0.0921084
60651	0.946132	0.0936892
60652	0.946132	0.05
60653	0.946131	0.0825399
60654	0.946131	0.05
60655	0.946132	0.0844888
60656	0.946132	0.0832653
60657	0.946131	0.05
60658	0.946131	0.05
60659	0.946131	0.05
60660	0.946131	0.0538189
60661	0.946131	0.05
60662	0.946131	0.05
60663	2.68213	2.1
60664	2.58234	2.1
60665	2.58234	2.1

60666	2.61243	2.1
60667	2.67035	2.1
60668	2.70044	2.1
60669	2.68213	2.1
60670	2.53921	2.1
60671	2.53303	2.1
60672	2.67035	2.1
60673	5.20956	2.1
60674	2.67033	2.1
60675	2.58232	2.1
60676	2.55004	2.1
60677	2.64567	2.1
60678	5.228 2.1	
60679	2.53301	2.1
60680	2.64567	2.1
60681	2.64567	2.1
60682	2.67033	2.1
60683	2.51603	2.1
60684	2.54069	2.1
60685	0.907497	2.1
60686	0.907497	2.1
60687	0.926641	2.1
60688	0.926641	2.1
60689	1.14989	2.1
60690	1.10046	2.1
60691	0.695334	2.1
60692	0.684247	2.1
60693	0.695333	2.1
60694	1.14989	2.1
60695	0.684246	2.1
60696	1.10046	2.1
60697	0.946126	2.1
60698	0.946126	2.1
60699	0.946131	2.1
60700	0.946131	2.1
60701	0.946127	2.1
60702	0.946127	2.1
60703	0.94613	2.1
60704	0.94613	2.1
60705	0.946128	2.1
60706	0.946128	2.1
60707	0.946128	2.1
60708	0.946128	2.1
60709	1.89226	2.1
60710	1.89226	2.1
60711	1.89226	2.1
60712	1.89226	2.09738
60713	1.89226	2.1
60714	1.89226	1.18025
60715	1.42174	2.1
60716	2.29978	2.1
60717	2.41233	2.1
60718	2.41233	2.1
60719	1.39067	2.1
60720	1.39067	2.1
60721	1.89226	2.1
60722	1.89226	2.1
60723	1.89226	2.1

60724	1.89226	2.1
60725	3.78452	2.1
60726	1.89226	2.1
60727	1.89226	2.1
60728	1.89226	2.1
60729	1.89226	2.1
60730	1.89226	2.1
60731	1.89226	2.1
60732	2.41234	2.1
60733	1.89226	2.1
60734	2.29979	2.1
60735	1.89226	2.1
60736	1.89226	2.1
60737	2.29979	2.1
60738	1.09506	0.05
60739	2.82921	0.05
60740	2.53251	0.05
60741	2.53251	0.05
60742	1.42901	0.05
60743	1.42901	0.05
60744	0.946132	0.05
60745	0.946132	0.05
60746	0.946128	0.05
60747	0.946128	0.05
60748	0.94613	0.0572285
60749	0.94613	0.0587504
60750	0.946131	0.05
60751	0.946131	0.05
60752	0.94613	0.05
60753	0.94613	0.05
60754	0.94613	0.0508736
60755	0.94613	0.0595964
60756	1.89226	0.0616414
60757	1.89226	0.0774121
60758	1.89226	0.0752326
60759	1.89226	0.108161
60760	3.78452	0.0730802
60761	2.53251	0.05
60762	1.89226	0.0669631
60763	2.82921	0.05
60764	1.89226	0.05
60765	1.89226	0.114945
60766	2.82921	0.1092
60767	1.89226	0.0582387
60768	1.89226	0.05
60769	1.89226	0.0685336
60770	1.89226	0.0687933
60771	1.89226	0.05
60772	1.89226	0.0500689
60773	2.53251	0.05
60774	1.89226	0.05
60775	2.06158	0.05
60776	1.89226	0.05
60777	4.42477	0.05
60778	1.23296	2.1
60779	2.55352	2.1
60780	2.47476	2.1
60781	2.47476	2.1

60782	1.32368	2.1
60783	1.32368	2.1
60784	1.89226	2.1
60785	1.89226	2.1
60786	1.89226	2.1
60787	1.89226	2.1
60788	1.89226	2.1
60789	1.89226	2.1
60790	1.89226	2.1
60791	1.89226	2.1
60792	1.89226	2.1
60793	1.89226	2.1
60794	1.89226	2.1
60795	1.89226	2.1
60796	2.47476	2.1
60797	1.89226	2.1
60798	1.89226	2.1
60799	1.89226	2.1
60800	2.55353	2.1
60801	2.55353	2.1
60802	1.05983	2.1
60803	2.47476	2.1
60804	2.37664	2.1
60805	2.37664	2.1
60806	1.23296	2.1
60807	1.23296	2.1
60808	0.946127	2.1
60809	0.946127	2.1
60810	0.946131	2.1
60811	0.946131	2.1
60812	0.946127	2.1
60813	0.946127	2.1
60814	0.946131	2.1
60815	0.946131	2.1
60816	0.946129	2.1
60817	0.946129	2.1
60818	0.946128	2.1
60819	0.946128	2.1
60820	1.89226	2.1
60821	1.89226	2.1
60822	1.89226	2.1
60823	1.89226	2.1
60824	3.78452	2.1
60825	2.37665	2.1
60826	1.89226	2.1
60827	1.89226	2.1
60828	1.89226	2.1
60829	2.47476	2.1
60830	2.47476	2.1
60831	1.89226	0.800747
60832	1.92119	0.0772888
60833	1.99586	0.627058
60834	1.92119	0.470245
60835	1.96693	0.0795428
60836	1.89226	0.052367
60837	1.89226	0.546838
60838	1.89226	0.108352
60839	1.89226	0.906358

60840	1.89226	0.254607
60841	1.89226	0.107928
60842	1.89226	0.05
60843	1.89226	0.868932
60844	2.39541	0.765455
60845	2.57183	1.73745
60846	2.39541	0.483278
60847	2.06868	0.480685
60848	1.89226	0.126973
60849	0.946133	0.287028
60850	0.946133	0.213909
60851	0.997928	0.147816
60852	1.03763	0.247094
60853	0.946131	0.474531
60854	0.946131	0.05
60855	1.03763	0.276226
60856	1.03763	0.079315
60857	1.03763	0.274654
60858	0.946131	0.440711
60859	0.94613	0.231114
60860	0.99793	0.0623938
60861	1.89226	0.0530854
60862	1.89226	0.449325
60863	1.89226	0.40614
60864	1.89226	0.05
60865	1.89226	0.813719
60866	1.89226	0.05
60867	0.946131	0.173139
60868	0.946131	0.1799
60869	1.36939	0.190404
60870	1.28592	0.538951
60871	0.946129	0.416055
60872	0.946129	0.05
60873	1.36939	0.25594
60874	1.36939	0.327977
60875	0.946131	0.180929
60876	1.36939	0.163522
60877	0.946131	0.147719
60878	1.28592	0.0834817
60879	1.89226	0.111893
60880	1.89226	0.478853
60881	1.89226	0.242833
60882	1.89226	0.229812
60883	3.78453	0.285632
60884	1.89226	0.485769
60885	1.89226	0.443394
60886	1.89226	0.244028
60887	1.89226	0.35666
60888	3.78452	0.340448
60889	1.91005	0.490175
60890	1.89226	0.655577
60891	2.60215	0.288756
60892	2.60215	0.445407
60893	1.89226	0.370847
60894	1.89226	0.790597
60895	0.946132	0.419665
60896	0.946132	0.356182
60897	0.946132	0.438956

60898	0.946132	0.264712
60899	0.946132	0.40501
60900	0.946132	0.492465
60901	0.946132	0.364379
60902	0.946132	0.185053
60903	0.946132	0.322484
60904	0.946132	0.32759
60905	0.946131	0.249418
60906	0.946131	0.330093
60907	1.89226	0.403358
60908	1.89226	0.534573
60909	1.89226	0.989028
60910	1.89226	0.492834
60911	1.89226	0.336125
60912	1.89226	0.835561
60913	0.780483	0.379428
60914	0.78928	0.85726
60915	0.818566	0.412089
60916	0.460387	0.839038
60917	1.82793	0.226248
60918	1.32039	0.632767
60919	0.613889	0.238968
60920	1.0592	0.692886
60921	1.48698	0.68764
60922	0.818555	0.436846
60923	1.11515	0.77365
60924	1.2802	1.10481
60925	2.20046	0.113389
60926	1.1475	0.585066
60927	1.1475	0.104575
60928	2.27062	0.361284
60929	2.27062	0.103654
60930	1.89226	0.66474
60931	2.20046	0.138392
60932	2.27062	0.201251
60933	2.20046	0.111148
60934	1.96242	0.207466
60935	1.89226	0.11332
60936	1.89226	0.128654
60937	1.89226	0.0764754
60938	1.89226	0.539379
60939	1.89226	0.127108
60940	3.78452	0.0968258
60941	1.89226	0.10547
60942	1.89226	0.0620132
60943	1.89226	0.447761
60944	1.89226	0.182142
60945	3.78452	0.203034
60946	1.17403	0.094716
60947	1.13531	0.209858
60948	0.6401	0.166442
60949	0.714566	0.428864
60950	0.915987	0.218849
60951	0.915987	0.05
60952	0.933891	0.948115
60953	0.933891	0.139424
60954	1.17403	0.289919
60955	0.714566	0.0900463

60956	0.6401	0.233769
60957	1.13531	0.123247
60958	0.946133	0.108887
60959	0.946133	0.154794
60960	1.17403	0.0939459
60961	1.13531	0.115188
60962	0.946131	0.120415
60963	0.946131	0.0664563
60964	1.17403	0.248849
60965	1.17403	0.05
60966	1.17403	0.138164
60967	0.94613	0.142165
60968	0.946131	0.30461
60969	1.13531	0.0797634
60970	1.89226	0.0699153
60971	1.89226	0.109385
60972	1.89226	0.0596767
60973	1.89226	0.05
60974	1.89226	0.242119
60975	1.89226	0.0852864
60976	0.946131	0.0910424
60977	0.946131	0.0692189
60978	0.946131	0.134752
60979	0.946131	0.127553
60980	0.946129	0.184739
60981	0.946129	0.0602737
60982	0.946133	0.246655
60983	0.946133	0.05
60984	0.946131	0.0996705
60985	0.946131	0.113904
60986	0.946131	0.0553793
60987	0.946131	0.0761638
60988	2.29413	2.1
60989	1.99332	2.1
60990	1.84774	2.1
60991	2.21582	2.1
60992	4.20915	2.1
60993	0.517359	2.1
60994	0.634469	2.1
60995	0.996661	2.1
60996	0.92387	2.1
60997	0.768098	2.1
60998	0.768098	2.1
60999	0.790242	2.1
61000	0.790242	2.1
61001	0.923871	2.1
61002	0.517359	2.1
61003	0.634469	2.1
61004	0.996661	2.1
61005	1.89226	2.1
61006	1.89226	2.1
61007	1.89226	2.1
61008	1.89226	2.1
61009	3.78453	2.1
61010	0.946132	2.1
61011	0.946132	2.1
61012	0.946131	2.1
61013	0.946131	2.1

61014	0.946132	2.1
61015	0.946132	2.1
61016	0.946131	2.1
61017	0.946131	2.1
61018	0.946132	2.1
61019	0.946132	2.1
61020	0.946131	2.1
61021	0.946131	2.1
61022	1.62708	2.1
61023	2.21582	2.1
61024	2.13925	2.1
61025	2.21582	2.1
61026	1.77117	2.1
61027	1.84774	2.1
61028	0.946132	2.1
61029	0.946132	2.1
61030	0.946131	2.1
61031	0.946131	2.1
61032	0.946132	2.1
61033	0.946132	2.1
61034	0.946131	2.1
61035	0.946131	2.1
61036	0.946131	2.1
61037	0.946131	2.1
61038	0.946131	2.1
61039	0.946131	2.1
61040	1.89226	1.97543
61041	1.89226	0.373464
61042	1.89226	0.262486
61043	1.89226	1.10396
61044	3.78453	1.48193
61045	1.89226	0.303408
61046	1.89226	1.57708
61047	1.89226	1.52853
61048	1.89226	2.1
61049	1.89226	0.163865
61050	1.89226	1.13221
61051	1.89226	0.33661
61052	1.89226	2.1
61053	1.89226	0.115215
61054	1.89226	1.87041
61055	1.89226	0.237719
61056	1.89226	2.1
61057	0.914369	0.959229
61058	0.914369	1.36591
61059	0.810945	2.1
61060	0.810945	1.17507
61061	2.26581	2.1
61062	1.66199	2.1
61063	1.49503	1.4221
61064	1.28816	2.1
61065	1.08133	1.96744
61066	0.810933	1.95226
61067	1.1212	1.94991
61068	1.89227	0.40503
61069	1.89227	0.284681
61070	1.89227	0.164807
61071	1.89227	0.504214

61072	3.78453	0.240574
61073	1.89226	0.160259
61074	1.89226	0.70059
61075	1.89226	0.388403
61076	1.89226	0.294138
61077	3.78453	0.438652
61078	1.89226	0.684956
61079	1.89226	0.343076
61080	1.89226	0.28396
61081	1.89226	0.854734
61082	3.78453	0.408185
61083	1.89226	0.2929
61084	1.89226	0.944175
61085	1.89226	0.479823
61086	2.25318	0.346161
61087	3.78453	0.748987
61088	1.89226	0.441543
61089	1.89226	0.864683
61090	1.89226	0.374629
61091	1.9637	0.319824
61092	3.78453	0.607476
61093	1.89226	0.592858
61094	1.89226	0.506112
61095	1.89226	0.259656
61096	1.89226	0.747839
61097	3.78453	0.408687
61098	1.89226	0.492869
61099	1.89226	1.27079
61100	1.89226	0.698451
61101	2.53384	0.560917
61102	3.78453	0.93026
61103	1.55405	0.46263
61104	1.03818	1.43101
61105	1.03818	0.0731841
61106	1.34857	0.171507
61107	1.46338	0.185688
61108	1.77377	1.32254
61109	1.86273	0.195096
61110	1.73802	0.222341
61111	1.50969	1.84443
61112	1.46337	1.20855
61113	3.20139	0.324305
61114	1.2377	1.18281
61115	1.46337	0.290399
61116	1.27408	0.319595
61117	1.32039	1.72648
61118	1.50969	0.705767
61119	1.50969	0.192115
61120	1.89226	2.1
61121	1.89226	2.1
61122	1.89226	2.1
61123	1.89226	2.1
61124	1.89226	2.1
61125	1.89226	2.1
61126	1.89169	2.1
61127	1.89226	2.1
61128	1.89226	2.1
61129	1.89226	2.1

61130	1.96624	2.1
61131	1.96624	2.1
61132	2.62871	2.1
61133	1.6201	2.1
61134	1.49149	2.1
61135	1.6201	2.1
61136	2.62559	2.1
61137	2.75419	2.1
61138	0.946129	1.15914
61139	0.946129	2.1
61140	0.946133	2.1
61141	0.946133	2.1
61142	0.946131	2.1
61143	0.946131	0.630095
61144	0.946131	2.1
61145	0.946131	2.1
61146	0.946131	2.1
61147	0.946131	2.1
61148	0.946131	2.1
61149	0.946131	2.1
61150	0.851543	2.1
61151	0.851543	2.1
61152	1.04043	2.1
61153	0.997939	2.1
61154	0.946132	2.1
61155	0.946132	2.1
61156	0.894185	2.1
61157	0.893901	2.1
61158	0.998267	2.1
61159	1.04074	2.1
61160	0.85127	2.1
61161	0.85153	2.1
61162	1.89226	2.1
61163	1.89226	2.1
61164	1.89226	2.1
61165	1.89226	2.1
61166	1.89226	2.1
61167	1.89226	2.1
61168	0.928691	2.1
61169	0.928691	2.1
61170	0.935332	2.1
61171	0.935332	2.1
61172	1.398	2.1
61173	1.14418	2.1
61174	0.479331	2.1
61175	0.719848	2.1
61176	1.398	2.1
61177	0.719848	2.1
61178	1.14417	2.1
61179	0.47933	2.1
61180	2.28835	2.1
61181	1.89226	2.1
61182	1.89226	2.1
61183	1.89226	2.1
61184	2.796	2.1
61185	2.796	2.1
61186	0.946131	2.1
61187	0.946131	2.1

61188	0.946131	2.1
61189	0.946131	2.1
61190	0.946128	2.1
61191	0.946128	2.1
61192	0.946133	2.1
61193	0.946133	2.1
61194	0.946131	2.1
61195	0.946131	2.1
61196	0.94613	2.1
61197	0.94613	2.1
61198	0.946131	2.1
61199	0.946131	2.1
61200	0.946131	2.1
61201	0.946131	2.1
61202	0.946131	2.1
61203	0.946131	2.1
61204	0.946131	2.1
61205	0.946131	2.1
61206	0.946131	2.1
61207	0.946131	2.1
61208	0.946131	2.1
61209	0.946131	2.1
61210	1.89226	2.1
61211	1.89226	2.1
61212	1.89226	2.1
61213	1.89226	2.1
61214	3.78452	2.1
61215	2.05207	0.0521524
61216	1.70302	0.151358
61217	1.33794	1.94853
61218	1.98194	0.41244
61219	3.36873	0.248859
61220	2.62761	0.258058
61221	1.98194	0.341515
61222	2.28009	1.3
61223	2.28009	0.10734
61224	2.22939	1.1064
61225	2.22939	0.111069
61226	1.89226	0.456576
61227	1.89226	0.072302
61228	1.89226	0.236988
61229	1.89226	0.112845
61230	1.89226	0.54797
61231	1.89226	0.375422
61232	1.89227	0.556042
61233	1.89227	0.16079
61234	1.89227	1.71254
61235	1.89227	0.788925
61236	1.89227	0.0652683
61237	1.89227	0.05
61238	0.888355	0.072918
61239	0.990973	0.513039
61240	0.71133	0.230207
61241	0.634324	0.8698
61242	0.806209	0.623617
61243	0.806209	0.05
61244	0.819087	1.44622
61245	0.819087	0.671981

61246	0.888355	0.76107
61247	0.634324	0.981723
61248	0.71133	0.374398
61249	0.990972	0.358806
61250	1.11469	1.15393
61251	0.985023	0.360771
61252	0.888354	0.131825
61253	0.990972	1.09869
61254	0.994754	0.513077
61255	0.994754	0.17076
61256	1.0083	1.15665
61257	1.0083	0.069628
61258	0.888354	0.16679
61259	0.985024	0.570646
61260	0.990971	0.189619
61261	1.11469	0.138425
61262	0.946132	0.852025
61263	0.946132	0.135459
61264	0.946132	0.209357
61265	0.946132	0.366554
61266	0.946132	0.393699
61267	0.946132	0.15731
61268	0.946133	1.4332
61269	0.946133	0.265398
61270	0.946133	0.597798
61271	0.946133	0.375123
61272	0.946132	0.368245
61273	0.946132	0.963437
61274	0.810944	0.231452
61275	0.810944	0.173306
61276	0.810947	0.551806
61277	0.810947	0.05
61278	1.89227	1.55815
61279	1.35168	0.236301
61280	1.08132	0.245988
61281	1.08131	0.338144
61282	1.08132	0.0643681
61283	0.810959	0.0927313
61284	0.810959	0.086762
61285	1.70303	2.1
61286	2.03975	2.1
61287	1.99324	2.1
61288	2.17615	2.1
61289	1.40549	2.1
61290	1.53446	2.1
61291	0.946133	2.1
61292	0.946133	2.1
61293	0.946129	2.1
61294	0.946129	2.1
61295	0.946131	2.1
61296	0.946131	2.1
61297	0.946131	2.1
61298	0.946131	2.1
61299	0.946131	2.1
61300	0.946131	2.1
61301	0.946131	2.1
61302	0.946131	2.1
61303	1.89226	2.1

61304	1.89226	2.1
61305	1.89226	2.1
61306	1.89226	2.1
61307	1.89226	2.1
61308	1.89226	2.1
61309	2.17615	2.1
61310	2.3111	2.1
61311	2.50288	2.1
61312	2.50288	2.1
61313	2.03975	2.1
61314	2.03975	2.1
61315	1.76401	2.1
61316	1.95852	2.1
61317	2.32134	2.1
61318	2.12306	2.1
61319	1.8513	2.1
61320	1.70303	2.1
61321	1.89226	2.1
61322	1.89226	2.1
61323	1.89226	2.1
61324	1.89226	2.1
61325	3.78452	2.1
61326	1.89226	2.1
61327	1.89226	1.59305
61328	1.89226	2.1
61329	1.89226	2.1
61330	3.78452	2.1
61331	1.2243	2.1
61332	1.2243	2.1
61333	1.24189	2.1
61334	1.24189	2.1
61335	1.37811	2.1
61336	1.25144	2.1
61337	1.08807	2.1
61338	1.17949	2.1
61339	1.17949	2.1
61340	1.37811	2.1
61341	1.08807	2.1
61342	1.25144	2.1
61343	1.89226	2.1
61344	1.89226	2.1
61345	1.89226	2.1
61346	1.89226	2.1
61347	1.89226	2.1
61348	1.89226	2.1
61349	1.89227	2.1
61350	1.89227	2.1
61351	1.89227	2.1
61352	1.89227	2.1
61353	1.89227	2.1
61354	1.89227	2.1
61355	1.89224	2.1
61356	1.89224	2.1
61357	1.89224	2.1
61358	1.89224	2.1
61359	3.78447	2.1
61360	1.89224	2.1
61361	1.89224	2.1

61362	1.89224	2.1
61363	1.89224	2.1
61364	1.89224	2.1
61365	1.89224	2.1
61366	1.89224	2.1
61367	1.89224	2.1
61368	1.89224	2.1
61369	1.89224	2.1
61370	3.78448	2.1
61371	1.89224	2.1
61372	1.703 2.1	
61373	1.89224	2.1
61374	1.72991	2.1
61375	1.07643	2.1
61376	1.36657	2.1
61377	1.44472	2.1
61378	2.41233	2.1
61379	2.51628	2.1
61380	2.51628	2.1
61381	1.42174	2.1
61382	1.42174	2.1
61383	1.89226	2.1
61384	1.89226	2.1
61385	1.89226	2.1
61386	1.89226	2.1
61387	1.89226	2.1
61388	1.89226	2.1
61389	1.89226	2.1
61390	1.89226	2.1
61391	1.89226	2.1
61392	1.89226	1.93443
61393	3.78452	2.1
61394	2.51628	2.1
61395	1.89226	2.1
61396	1.89226	2.1
61397	1.89226	2.1
61398	2.41234	2.1
61399	2.41234	2.1
61400	0.996254	2.1
61401	0.996254	2.1
61402	1.01153	2.1
61403	1.01153	2.1
61404	1.28542	2.1
61405	1.25814	2.1
61406	0.719184	2.1
61407	0.72236	2.1
61408	0.719183	2.1
61409	1.28542	2.1
61410	0.722359	2.1
61411	1.25814	2.1
61412	0.946126	2.1
61413	0.946126	2.1
61414	0.946131	2.1
61415	0.946131	2.1
61416	0.946127	2.1
61417	0.946127	2.1
61418	0.94613	2.1
61419	0.94613	2.1

61420	0.946128	2.1
61421	0.946128	2.1
61422	0.946128	2.1
61423	0.946128	2.1
61424	1.89226	2.1
61425	1.89226	2.1
61426	1.89226	2.1
61427	1.89226	2.1
61428	1.89226	2.1
61429	1.89226	2.1
61430	0.946132	2.1
61431	0.946132	2.1
61432	1.25814	2.1
61433	1.25814	2.1
61434	0.946131	2.1
61435	0.946131	2.1
61436	1.28543	2.1
61437	1.25814	2.1
61438	1.28543	2.1
61439	0.946132	2.1
61440	1.25814	2.1
61441	0.946132	2.1
61442	1.89226	1.22881
61443	1.89226	0.177923
61444	1.89226	0.951087
61445	1.89226	0.635264
61446	1.89226	0.428788
61447	1.89226	0.250817
61448	1.89226	0.971899
61449	2.27719	0.116946
61450	1.89226	0.179913
61451	1.89226	0.358717
61452	3.78452	0.307435
61453	1.89225	1.39124
61454	1.89225	0.230751
61455	1.89225	0.633396
61456	1.89225	0.819605
61457	3.78451	0.772741
61458	1.89226	0.12883
61459	1.89226	0.212252
61460	1.89226	0.551008
61461	1.89226	0.0747411
61462	3.78452	0.148053
61463	0.946132	0.0509185
61464	0.946132	0.146321
61465	0.946132	0.244535
61466	0.946132	0.136975
61467	0.946131	0.105394
61468	0.946131	0.0633476
61469	0.946132	0.202124
61470	0.946132	0.0727511
61471	0.946132	0.0852017
61472	0.946132	0.108794
61473	0.946132	0.0961934
61474	0.946132	0.0576905
61475	0.946126	0.175712
61476	0.946126	0.102248
61477	0.946132	1.30509

61478	0.946132	0.393596
61479	0.946128	0.142818
61480	0.946128	0.303138
61481	0.946129	0.232246
61482	0.946129	0.76393
61483	0.946129	0.399287
61484	0.946129	0.537149
61485	0.946129	0.25827
61486	0.946129	0.270665
61487	1.39858	2.1
61488	1.39858	2.1
61489	0.94613	2.1
61490	0.94613	2.1
61491	0.946132	2.1
61492	0.946132	2.1
61493	1.39858	2.1
61494	1.25002	2.1
61495	1.39858	2.1
61496	0.946131	2.1
61497	1.25002	2.1
61498	0.946131	2.1
61499	2.0815	2.1
61500	1.89226	2.1
61501	1.89226	2.1
61502	2.05298	2.1
61503	2.5593	2.1
61504	2.77667	2.1
61505	0.946133	2.1
61506	0.946133	2.1
61507	0.946132	2.1
61508	0.946132	2.1
61509	0.946132	2.1
61510	0.946132	2.1
61511	0.946133	2.1
61512	0.946133	2.1
61513	0.946132	2.1
61514	0.946132	2.1
61515	0.946132	2.1
61516	0.946132	2.1
61517	1.38908	2.1
61518	1.38908	2.1
61519	0.946132	2.1
61520	0.946132	2.1
61521	0.946132	2.1
61522	0.946132	2.1
61523	1.38908	2.1
61524	1.27965	2.1
61525	1.38908	2.1
61526	0.946132	2.1
61527	0.946133	2.1
61528	1.27965	2.1
61529	0.946133	2.1
61530	0.946133	2.1
61531	0.946132	2.1
61532	0.946132	2.1
61533	0.946132	2.1
61534	0.946132	2.1
61535	0.946133	2.1

61536	0.946133	2.1
61537	0.946132	2.1
61538	0.946132	2.1
61539	0.946133	2.1
61540	0.946133	2.1
61541	1.89227	1.42684
61542	1.89227	1.53925
61543	1.89227	1.01881
61544	1.89227	0.418655
61545	3.78453	1.14654
61546	1.33154	1.08368
61547	1.33154	1.17344
61548	0.946132	1.23826
61549	0.946132	0.973202
61550	1.19095	0.594797
61551	1.33154	1.23341
61552	0.946133	1.24779
61553	0.946133	1.42062
61554	0.946133	1.92943
61555	1.19095	0.766139
61556	0.946132	1.73458
61557	1.33154	1.09547
61558	1.0888	0.777705
61559	1.04745	0.732952
61560	0.946132	0.829629
61561	0.946132	0.822151
61562	1.04745	0.341928
61563	1.04745	1.17515
61564	0.946132	1.54439
61565	0.946132	0.446306
61566	1.04745	1.40597
61567	0.946133	0.479232
61568	0.946132	1.35198
61569	1.0888	0.773646
61570	1.89226	1.87263
61571	1.89226	0.458426
61572	1.89226	0.384004
61573	1.89226	0.718845
61574	1.89226	0.737178
61575	1.89226	1.15855
61576	1.89226	1.694
61577	1.87592	0.179617
61578	1.87592	0.42104
61579	2.3819	0.755068
61580	1.89226	1.20137
61581	2.39825	1.21127
61582	1.89226	0.581641
61583	2.0949	1.19988
61584	1.88741	0.235023
61585	1.89226	0.305032
61586	1.89226	0.359498
61587	1.88741	1.15043
61588	1.07508	1.28801
61589	1.63419	1.81897
61590	1.63419	1.50674
61591	1.09795	1.89153
61592	1.56137	1.29042
61593	1.02513	2.06589

61594	0.967672	0.518252
61595	1.56137	1.08163
61596	1.56137	0.553309
61597	1.07508	1.16328
61598	1.41319	0.414223
61599	0.926902	1.42242
61600	1.56137	1.06059
61601	1.63419	1.50104
61602	2.29852	1.67855
61603	2.14485	1.01224
61604	3.77904	0.81121
61605	1.41319	0.294522
61606	1.92622	0.315814
61607	1.56137	0.234185
61608	1.56137	1.0406
61609	2.14485	0.995191
61610	1.92622	0.644855
61611	1.89227	2.1
61612	1.89227	2.1
61613	1.89227	2.1
61614	1.89227	2.1
61615	3.78453	2.1
61616	2.56618	2.1
61617	2.08612	2.1
61618	2.48048	2.1
61619	1.99644	2.1
61620	4.5666	2.1
61621	2.08612	2.1
61622	1.30659	2.1
61623	1.99644	2.1
61624	1.25404	2.1
61625	3.34016	2.1
61626	1.89226	2.1
61627	1.89226	2.1
61628	1.89226	2.1
61629	1.89226	2.1
61630	1.89226	2.1
61631	1.89226	2.1
61632	1.89227	2.1
61633	1.89227	2.1
61634	1.89227	2.1
61635	1.89227	2.1
61636	3.78453	2.1
61637	2.48048	2.1
61638	1.99644	2.1
61639	2.39022	2.1
61640	1.90734	2.1
61641	4.38782	2.1
61642	1.90734	2.1
61643	1.99644	2.1
61644	1.21564	2.1
61645	1.21564	2.1
61646	1.25404	2.1
61647	1.94574	2.1
61648	0.810946	2.1
61649	0.810946	2.1
61650	0.810947	2.1
61651	0.810947	2.1

61652	1.89226	2.1
61653	1.3517	2.1
61654	1.08133	2.1
61655	1.08132	2.1
61656	1.08132	2.1
61657	0.810934	2.1
61658	0.810934	2.1
61659	1.89226	2.1
61660	1.89226	0.287411
61661	1.89226	0.995583
61662	1.89226	2.1
61663	3.78453	2.1
61664	1.89226	0.0533477
61665	1.89226	0.05
61666	1.89226	1.04993
61667	1.89226	0.05
61668	1.89226	0.261128
61669	1.89226	0.05
61670	0.946131	0.0791204
61671	0.946131	0.05
61672	0.94613	0.18896
61673	0.94613	0.141245
61674	0.946128	0.079066
61675	0.946128	0.05
61676	0.946133	0.05
61677	0.946133	0.248446
61678	0.946131	0.0561686
61679	0.946131	0.0563714
61680	0.946131	0.125181
61681	0.946131	0.0654225
61682	1.89226	2.1
61683	1.89226	0.169393
61684	1.89226	2.03957
61685	1.89226	1.21937
61686	1.89226	0.535823
61687	1.89226	0.322312
61688	2.56348	0.214206
61689	1.89226	2.1
61690	1.89226	2.1
61691	2.53234	0.79703
61692	4.45574	0.448928
61693	2.53234	0.05
61694	1.65583	0.05
61695	2.56348	0.05
61696	2.56348	0.05
61697	1.66295	0.05
61698	1.65583	0.05
61699	0.736602	0.05
61700	0.827916	0.05
61701	1.26617	0.05
61702	1.18322	0.05
61703	0.7366	0.05
61704	1.18321	0.05
61705	1.26617	0.05
61706	0.827918	0.05
61707	0.99929	0.05
61708	0.99929	0.05
61709	1.00348	0.05

61710	1.00348	0.05
61711	1.26617	0.05
61712	1.18322	0.0680161
61713	0.946131	2.1
61714	0.946131	0.314458
61715	0.946129	0.115622
61716	1.18321	0.05
61717	0.946133	2.1
61718	1.26617	1.17146
61719	1.26617	1.35681
61720	1.26617	0.05
61721	0.946131	2.1
61722	0.946131	0.440822
61723	2.23267	0.05
61724	1.89226	0.05
61725	2.07099	0.05
61726	1.89226	0.05
61727	2.07099	0.05
61728	1.89226	0.05
61729	0.946132	0.05
61730	1.35403	0.05
61731	1.24038	0.05
61732	0.946131	0.05
61733	0.946131	0.05
61734	0.946131	0.05
61735	1.24039	0.05
61736	1.35403	0.05
61737	1.24038	0.05
61738	1.24038	0.05
61739	0.946131	0.05
61740	0.946131	0.05
61741	1.89226	0.05
61742	1.89226	0.0852004
61743	1.89226	0.05
61744	1.89226	0.05
61745	1.89226	0.152518
61746	1.89226	0.124553
61747	1.89226	0.05
61748	1.89226	0.0900938
61749	1.89226	0.05
61750	1.89226	0.0527664
61751	3.78452	0.0789875
61752	1.56971	0.05
61753	2.80086	0.05
61754	2.59806	0.05
61755	1.93489	0.05
61756	2.59806	0.05
61757	1.93489	0.05
61758	0.827467	0.05
61759	1.26652	0.05
61760	1.13679	0.05
61761	0.639035	0.05
61762	1.11547	0.05
61763	1.30656	0.05
61764	0.869062	0.05
61765	0.696608	0.05
61766	1.07205	0.05
61767	0.953656	0.05

61768	0.970744	0.05
61769	0.970744	0.05
61770	2.38681	2.1
61771	2.50438	1.96127
61772	2.42082	2.1
61773	2.59106	2.1
61774	4.92521	2.1
61775	2.50438	1.69759
61776	2.40627	2.1
61777	2.59106	2.1
61778	2.5444	1.15255
61779	5.04879	2.1
61780	2.42082	2.1
61781	2.70575	2.1
61782	2.64778	2.1
61783	2.59106	2.1
61784	2.47753	2.1
61785	2.42082	1.98564
61786	2.70575	2.1
61787	2.5444	1.71894
61788	2.76095	2.1
61789	2.59106	2.1
61790	2.71429	2.1
61791	2.5444	2.09628
61792	1.89226	2.1
61793	2.38473	2.1
61794	2.36689	2.1
61795	2.38473	2.1
61796	1.87442	2.1
61797	1.89226	2.1
61798	1.89226	2.1
61799	1.89226	1.81992
61800	1.89226	2.1
61801	1.89226	2.0265
61802	1.89226	2.1
61803	1.89226	2.1
61804	2.14952	2.1
61805	1.89226	0.402807
61806	1.89226	2.1
61807	1.89226	0.311396
61808	2.0692	2.1
61809	2.0692	2.1
61810	1.89226	2.1
61811	2.36689	2.1
61812	2.36204	2.1
61813	2.36689	2.1
61814	1.88742	2.1
61815	1.89226	2.1
61816	0.946132	0.507426
61817	0.946132	2.1
61818	0.946131	2.1
61819	0.946131	2.1
61820	0.946132	2.1
61821	0.946132	2.1
61822	0.946131	2.1
61823	0.946131	2.1
61824	0.946132	2.1
61825	0.946132	2.1

61826	0.946131	2.1
61827	0.946131	2.1
61828	0.946128	2.1
61829	0.946128	2.1
61830	1.07476	2.1
61831	1.07476	2.1
61832	0.946131	2.07169
61833	0.946131	2.1
61834	1.13182	2.1
61835	1.07476	2.1
61836	1.13182	2.1
61837	0.946131	2.1
61838	1.07476	2.1
61839	0.94613	2.05574
61840	1.89227	0.177724
61841	1.89227	0.418268
61842	1.89227	0.510444
61843	1.89227	0.615625
61844	3.78453	0.334421
61845	1.89226	0.219232
61846	1.89226	0.394407
61847	1.89226	0.168314
61848	1.89226	0.820195
61849	3.78452	0.235228
61850	1.89226	0.356505
61851	1.89226	0.608319
61852	1.89226	1.0126
61853	1.89226	1.07381
61854	3.78452	0.37308
61855	1.89226	0.382281
61856	1.89226	0.626428
61857	1.89226	0.371561
61858	1.89226	0.552198
61859	1.89226	0.739488
61860	1.89226	0.879021
61861	2.58344	0.720594
61862	2.00267	0.107504
61863	2.14218	0.773839
61864	2.22674	1.14683
61865	2.14218	0.0562674
61866	2.22674	0.0834205
61867	2.83531	1.26951
61868	1.56043	0.354185
61869	2.22674	1.28463
61870	1.90912	1.63647
61871	2.22674	0.0757061
61872	1.90912	0.129726
61873	0.780217	0.307219
61874	1.11337	0.395324
61875	1.00133	1.21681
61876	0.804472	0.780545
61877	0.804472	1.04806
61878	0.780217	0.226119
61879	1.00133	0.100247
61880	1.11337	0.764166
61881	0.856668	0.721283
61882	0.856668	1.51679
61883	0.924883	0.248763

61884	0.924883	0.173845
61885	1.93107	2.1
61886	1.68657	2.1
61887	1.7237	2.1
61888	1.89226	2.1
61889	1.53436	2.1
61890	1.68657	2.1
61891	1.89226	2.1
61892	1.89226	2.1
61893	1.89226	2.1
61894	1.89226	2.1
61895	3.78453	2.1
61896	0.946131	2.1
61897	0.946131	2.1
61898	0.855092	2.1
61899	0.83953	2.1
61900	0.946131	2.1
61901	0.946131	2.1
61902	0.839531	2.1
61903	0.839531	2.1
61904	0.946131	2.1
61905	0.855093	2.1
61906	0.839531	2.1
61907	0.946131	2.1
61908	1.89226	2.1
61909	1.70302	2.1
61910	2.21419	2.1
61911	1.93107	2.1
61912	1.97872	2.1
61913	1.70302	2.1
61914	0.946133	2.1
61915	0.946133	2.1
61916	0.946132	2.1
61917	0.946132	2.1
61918	0.946132	2.1
61919	0.946132	2.1
61920	0.946132	2.1
61921	0.946132	2.1
61922	0.946133	2.1
61923	0.946133	2.1
61924	0.946132	2.1
61925	0.946132	2.1
61926	1.90081	2.1
61927	1.89226	2.1
61928	1.51379	2.1
61929	1.36813	2.1
61930	1.71019	2.1
61931	1.89226	2.1
61932	1.89227	1.42915
61933	1.89227	0.615939
61934	1.89227	0.671528
61935	1.89227	1.20004
61936	1.89227	1.48792
61937	1.89227	1.8395
61938	1.36428	1.22741
61939	1.37869	1.19033
61940	0.946132	0.792195
61941	0.946132	1.30983

61942	1.37869	0.527799
61943	1.37869	2.05842
61944	0.946133	1.30424
61945	0.946133	1.59075
61946	1.37869	0.539168
61947	0.946132	1.10413
61948	1.36428	1.41823
61949	0.946133	0.268424
61950	0.94612	1.09092
61951	0.94612	0.992932
61952	0.946119	1.08723
61953	0.946119	1.21344
61954	0.946119	0.36991
61955	0.946119	1.64139
61956	0.94612	1.33942
61957	0.94612	1.74154
61958	0.946132	1.02902
61959	0.946132	0.426212
61960	0.946107	0.490792
61961	0.946107	1.392
61962	1.89225	0.05
61963	1.89225	2.1
61964	1.63564	0.05
61965	1.42278	2.1
61966	1.42278	2.1
61967	1.57534	0.05
61968	1.53735	2.1
61969	1.68991	0.05
61970	1.57535	0.05
61971	1.04442	2.1
61972	1.04442	2.1
61973	1.22031	0.05
61974	1.42278	2.1
61975	1.59868	0.05
61976	0.946132	2.1
61977	0.946132	2.1
61978	0.946131	2.1
61979	0.946131	2.1
61980	0.946131	2.1
61981	0.946131	2.1
61982	0.946132	2.1
61983	0.946132	2.1
61984	0.946132	2.1
61985	0.946132	2.1
61986	0.946131	2.1
61987	0.946131	2.1
61988	1.89226	2.1
61989	1.89226	2.1
61990	1.89226	2.1
61991	1.89226	2.1
61992	1.89226	2.1
61993	1.89226	2.1
61994	2.03143	0.41033
61995	1.47111	2.04704
61996	2.28685	1.8624
61997	2.28685	1.97054
61998	1.36006	0.934768
61999	1.36006	0.934678

62000	1.89226	0.362115
62001	1.89226	1.76478
62002	1.89226	1.03084
62003	1.89226	1.51211
62004	1.89226	0.393472
62005	1.89226	0.930605
62006	1.89226	1.41169
62007	1.89226	2.1
62008	1.89226	2.1
62009	1.89226	2.1
62010	1.89226	1.63916
62011	1.89226	2.1
62012	0.902821	2.1
62013	0.902821	2.1
62014	0.889218	2.1
62015	0.889218	2.1
62016	0.675759	2.1
62017	1.05649	2.1
62018	0.735554	2.1
62019	1.14342	2.1
62020	1.14343	2.1
62021	1.05649	2.1
62022	0.735554	2.1
62023	0.675758	2.1
62024	2.46286	0.128875
62025	2.28685	0.317665
62026	2.67843	0.244996
62027	2.67843	0.223096
62028	2.03143	0.176429
62029	2.03143	0.177476
62030	1.89226	0.37351
62031	1.89226	0.29632
62032	1.89226	0.162474
62033	1.89226	0.190687
62034	3.78452	0.648735
62035	0.94613	0.796745
62036	0.94613	1.22079
62037	0.946128	2.07826
62038	0.946128	0.719609
62039	0.94613	2.1
62040	0.94613	0.939229
62041	0.946128	0.154803
62042	0.946128	0.810245
62043	0.946129	0.0892572
62044	0.946129	1.51243
62045	0.946129	0.155716
62046	0.946129	2.1
62047	2.67843	0.479155
62048	2.11297	2.07692
62049	2.52453	1.58526
62050	2.52453	2.1
62051	2.28685	0.98984
62052	2.28685	0.853461
62053	1.89226	0.0767376
62054	1.89226	1.84613
62055	1.89226	0.05
62056	1.89226	2.1
62057	3.78451	0.180914

62058	2.39524	0.05
62059	2.10188	1.72037
62060	2.29839	0.05
62061	2.02242	1.70792
62062	4.40028	0.0743578
62063	1.71839	2.1
62064	1.65261	2.1
62065	2.10189	1.32664
62066	2.10189	2.1
62067	2.02243	2.0585
62068	1.63893	1.99494
62069	0.946132	2.1
62070	0.946132	1.98412
62071	0.946129	2.1
62072	0.946129	2.1
62073	0.94613	2.1
62074	0.94613	2.1
62075	0.946131	1.06585
62076	0.946131	1.98407
62077	0.946131	2.1
62078	0.946131	2.1
62079	0.946131	2.1
62080	0.946131	2.1
62081	1.89226	0.05
62082	1.89226	2.1
62083	1.89226	0.05
62084	1.89226	1.1316
62085	3.78452	0.0731825
62086	2.2984	0.05
62087	2.02242	1.94462
62088	2.21582	0.05
62089	1.94412	0.991406
62090	4.24251	0.120854
62091	1.94412	0.920166
62092	1.5854	2.1
62093	2.02243	2.1
62094	2.02243	2.07329
62095	1.57431	1.59685
62096	1.65261	2.1
62097	0.810943	1.93484
62098	0.810943	2.1
62099	0.810945	2.1
62100	0.810945	2.1
62101	1.89226	1.03009
62102	1.3517	2.1
62103	1.08133	2.1
62104	1.08132	2.1
62105	1.08132	0.955394
62106	0.810933	2.1
62107	0.810933	2.1
62108	1.89226	0.05
62109	1.89226	0.05
62110	1.89226	0.05
62111	1.89226	0.05
62112	1.89226	0.05
62113	1.89226	0.05
62114	1.89226	0.05
62115	1.89226	0.05

62116	1.89226	0.05
62117	1.89226	0.05
62118	3.78452	0.05
62119	1.89226	0.05
62120	1.89226	0.05
62121	1.89226	0.05
62122	1.89226	0.05
62123	1.89226	0.05
62124	1.89226	0.05
62125	0.946128	0.05
62126	0.946128	0.05
62127	0.946132	0.05
62128	0.946132	0.05
62129	0.94613	0.05
62130	0.94613	0.05
62131	0.946129	0.05
62132	0.946129	0.05
62133	0.94613	0.05
62134	0.94613	0.05
62135	0.94613	0.05
62136	0.94613	0.05
62137	1.89226	0.05
62138	1.89226	0.05
62139	1.89226	0.05
62140	1.89226	0.05
62141	1.89226	0.05
62142	1.89226	0.05
62143	2.81478	2.1
62144	1.89226	2.1
62145	2.65557	2.1
62146	1.89226	2.1
62147	4.54783	2.1
62148	1.89226	2.1
62149	1.89226	2.1
62150	1.89226	2.1
62151	1.89226	2.1
62152	3.78452	2.1
62153	2.65557	2.1
62154	1.89226	2.1
62155	1.89226	2.1
62156	1.89226	2.1
62157	2.57997	2.1
62158	2.57997	2.1
62159	0.946131	2.1
62160	0.946131	2.1
62161	0.946128	2.1
62162	0.946128	2.1
62163	0.94613	2.1
62164	0.94613	2.1
62165	0.946129	2.1
62166	0.946129	2.1
62167	0.94613	2.1
62168	0.94613	2.1
62169	0.94613	2.1
62170	0.94613	2.1
62171	1.89226	0.809181
62172	2.27703	1.96701
62173	2.35615	2.03938

62174	2.35615	1.06583
62175	1.89226	2.02981
62176	1.89226	1.00846
62177	0.946132	0.752032
62178	1.17807	2.07836
62179	1.14926	0.739576
62180	0.946132	1.53705
62181	0.946133	1.47679
62182	0.946133	1.59052
62183	1.14926	0.949763
62184	1.14926	0.853727
62185	0.946132	1.43741
62186	0.946132	1.09314
62187	1.17807	1.60907
62188	1.14926	0.995984
62189	2.16841	0.797527
62190	1.35487	1.40855
62191	1.19854	1.12301
62192	1.19854	0.781388
62193	2.46078	1.12386
62194	2.46078	0.95196
62195	0.69308	0.691028
62196	1.3119	1.48145
62197	0.677433	1.57424
62198	1.23039	1.05451
62199	0.978178	1.57627
62200	0.978178	1.5488
62201	0.945294	0.870883
62202	0.945294	1.05129
62203	1.3119	1.17212
62204	1.23039	1.07275
62205	0.693081	1.13729
62206	0.677432	1.14015
62207	1.89226	0.846819
62208	1.89226	2.06605
62209	1.89226	1.98296
62210	1.89226	1.01121
62211	1.89226	1.94157
62212	1.89226	1.01682
62213	0.946132	0.751181
62214	0.946132	2.03572
62215	0.946132	0.530674
62216	0.946132	1.304
62217	0.946133	1.28359
62218	0.946133	1.55001
62219	0.946132	0.781235
62220	0.946132	0.639656
62221	0.946133	1.12645
62222	0.946133	0.869409
62223	0.946132	1.26086
62224	0.946132	0.993732
62225	2.60637	2.1
62226	1.89226	2.1
62227	1.89226	2.1
62228	1.89226	1.80673
62229	3.78452	2.1
62230	1.89226	0.64286
62231	1.8396	1.80706

62232	1.89226	2.06555
62233	1.89226	0.646449
62234	1.60993	2.1
62235	1.60993	0.6445
62236	0.946131	1.18543
62237	0.946131	2.00316
62238	0.946128	2.1
62239	0.946128	2.1
62240	0.946131	1.11016
62241	0.946131	2.05868
62242	0.946127	2.1
62243	0.946127	1.86421
62244	0.946129	2.1
62245	0.946129	2.1
62246	0.94613	2.1
62247	0.94613	2.1
62248	1.89226	2.1
62249	1.89226	1.89256
62250	1.89226	2.1
62251	1.89226	2.1
62252	1.89226	2.1
62253	1.89226	2.1
62254	1.89226	0.679427
62255	1.89226	1.6158
62256	1.89226	2.03076
62257	1.89226	0.671928
62258	1.89226	2.1
62259	1.89226	0.548209
62260	0.946131	1.29313
62261	0.946131	1.98476
62262	0.946128	2.1
62263	0.946128	2.04897
62264	0.946131	1.2104
62265	0.946131	1.93929
62266	0.946127	1.96099
62267	0.946127	2.1
62268	0.94613	2.1
62269	0.94613	2.09533
62270	0.946129	2.0712
62271	0.946129	2.08147
62272	0.946133	2.1
62273	0.946133	2.1
62274	1.28153	2.1
62275	1.26781	2.1
62276	0.946132	2.1
62277	0.946132	2.1
62278	1.28153	2.1
62279	1.28153	2.1
62280	0.946132	2.1
62281	1.28153	2.1
62282	0.946133	2.1
62283	1.26781	2.1
62284	1.89226	2.1
62285	1.89226	2.1
62286	1.89226	2.1
62287	1.89226	2.1
62288	1.89226	2.1
62289	1.89226	2.1

62290	2.5844	2.1
62291	1.09563	2.1
62292	1.1468	2.1
62293	1.09563	2.1
62294	2.6142	2.1
62295	2.56304	2.1
62296	0.946126	2.1
62297	0.946126	2.1
62298	1.2922	2.1
62299	1.28152	2.1
62300	0.946126	2.1
62301	0.946126	2.1
62302	1.2922	2.1
62303	1.2922	2.1
62304	1.29221	2.1
62305	0.946132	2.1
62306	0.946119	2.1
62307	1.28151	2.1
62308	1.89225	2.1
62309	1.89225	2.1
62310	1.89225	2.1
62311	1.89225	2.1
62312	1.89225	2.1
62313	1.89225	2.1
62314	0.946121	2.1
62315	0.946121	2.1
62316	0.946126	2.1
62317	0.946126	2.1
62318	0.946124	2.1
62319	0.946124	2.1
62320	0.946123	2.1
62321	0.946123	2.1
62322	0.94613	2.1
62323	0.94613	2.1
62324	0.946117	2.1
62325	0.946117	2.1
62326	1.89226	1.35204
62327	1.89226	1.14518
62328	1.89226	0.90319
62329	1.89226	2.1
62330	1.89226	0.133224
62331	1.89226	2.1
62332	1.89226	2.1
62333	1.89226	1.97152
62334	1.89226	0.417846
62335	1.89226	2.1
62336	1.89226	0.112272
62337	1.89226	2.1
62338	1.89226	2.1
62339	2.81281	2.1
62340	2.50369	1.15438
62341	2.50369	2.1
62342	1.89226	1.37937
62343	1.89226	2.1
62344	1.89226	2.1
62345	1.89226	2.1
62346	1.89226	2.1
62347	1.89226	2.1

62348	1.89226	2.1
62349	1.89226	2.1
62350	1.89226	2.1
62351	1.89226	2.1
62352	1.89226	2.1
62353	1.89226	2.1
62354	1.89226	2.1
62355	1.89226	2.1
62356	0.907036	2.1
62357	1.09714	2.1
62358	0.994609	2.1
62359	0.838808	2.1
62360	0.976536	2.1
62361	0.976536	2.1
62362	0.959412	2.1
62363	0.959412	2.1
62364	1.09714	2.1
62365	0.994609	2.1
62366	0.907036	2.1
62367	0.838808	2.1
62368	0.99461	2.1
62369	0.838808	2.1
62370	0.738212	2.1
62371	0.565634	2.1
62372	0.775921	2.1
62373	0.775921	2.1
62374	0.784322	2.1
62375	0.784322	2.1
62376	0.994609	2.1
62377	0.738212	2.1
62378	0.838808	2.1
62379	0.565635	2.1
62380	1.89226	2.1
62381	1.89226	2.1
62382	1.89226	2.1
62383	1.89226	2.1
62384	1.89226	2.1
62385	1.89226	2.1
62386	1.89226	2.1
62387	1.89226	2.1
62388	1.89226	2.1
62389	1.89226	2.1
62390	1.89226	2.1
62391	1.89226	2.1
62392	2.19428	2.1
62393	1.98922	2.1
62394	2.27322	2.1
62395	2.45893	2.1
62396	4.4675	2.1
62397	0.99461	2.1
62398	1.13661	2.1
62399	0.902272	2.1
62400	0.738212	2.1
62401	0.931874	2.1
62402	0.931874	2.1
62403	0.942953	2.1
62404	0.942953	2.1
62405	1.13658	2.1

62406	0.902248	2.1
62407	0.994637	2.1
62408	0.738233	2.1
62409	1.89226	2.08811
62410	1.89226	1.98009
62411	1.89226	2.1
62412	1.89226	2.09587
62413	1.89226	2.09315
62414	1.89226	1.9694
62415	1.89227	2.09962
62416	2.58246	1.22244
62417	1.89227	1.92078
62418	1.89227	1.94239
62419	3.78453	1.60486
62420	1.89226	2.1
62421	1.89226	1.96668
62422	1.65573	2.1
62423	1.65573	2.1
62424	1.89226	2.1
62425	1.89226	2.1
62426	1.89226	1.73838
62427	1.89226	1.37928
62428	1.89226	1.64751
62429	1.89226	1.72046
62430	1.89226	1.52413
62431	1.89226	1.62351
62432	1.89226	1.27641
62433	1.89226	1.54036
62434	1.89226	1.18703
62435	1.89226	1.63171
62436	1.89226	1.1934
62437	1.89226	1.63712
62438	0.946132	2.1
62439	0.946132	2.034
62440	1.01023	1.38374
62441	1.13651	2.06076
62442	1.01023	1.30121
62443	0.946133	1.8688
62444	1.0644	1.95961
62445	1.0644	2.1
62446	0.946132	2.09483
62447	0.946132	1.99481
62448	1.13651	1.89653
62449	0.946132	1.92206
62450	1.89226	0.270021
62451	1.89226	1.81476
62452	1.89226	1.06196
62453	1.89226	0.1643
62454	3.78451	0.44474
62455	1.00934	0.11439
62456	0.741561	0.0790533
62457	0.946131	0.209782
62458	0.946131	0.317456
62459	0.946131	0.744289
62460	0.741561	0.05
62461	1.00934	0.0619916
62462	0.946131	0.446671
62463	0.741586	0.164317

62464	0.741586	0.166407
62465	0.946106	0.167566
62466	0.946106	0.0800451
62467	1.14895	0.222239
62468	0.957449	0.381188
62469	0.934806	1.28023
62470	0.934806	0.94379
62471	1.13536	0.130171
62472	1.02829	1.7302
62473	0.946129	1.91119
62474	0.946129	0.219449
62475	0.946127	0.714689
62476	0.946127	0.876391
62477	0.946127	0.554999
62478	1.0283	0.75736
62479	1.89226	1.79726
62480	1.89226	0.374619
62481	1.89226	0.363959
62482	1.89226	1.40487
62483	3.78452	0.988315
62484	1.89226	0.127851
62485	1.89226	0.566421
62486	1.89226	0.460876
62487	1.89226	0.109278
62488	3.78453	0.169381
62489	1.89226	0.571722
62490	2.69068	0.119239
62491	1.89226	0.113131
62492	1.89226	0.437004
62493	3.78452	0.297032
62494	1.89226	0.38238
62495	1.89226	1.65204
62496	1.89226	1.62357
62497	1.89226	0.677439
62498	1.89226	1.32572
62499	1.89226	0.470324
62500	1.89226	2.1
62501	1.89226	2.1
62502	1.89226	2.1
62503	1.89226	2.1
62504	1.89226	2.1
62505	1.89226	2.1
62506	1.89226	2.1
62507	1.89226	2.1
62508	1.89226	2.1
62509	1.89226	2.1
62510	1.89226	2.1
62511	1.89226	2.1
62512	0.985109	2.1
62513	1.31144	2.1
62514	1.45864	2.1
62515	1.10667	2.1
62516	2.44375	2.1
62517	0.655718	2.1
62518	0.729316	2.1
62519	0.684594	2.1
62520	0.782707	2.1
62521	0.700849	2.1

62522	0.700849	2.1
62523	0.71306	2.1
62524	0.71306	2.1
62525	0.729318	2.1
62526	0.782705	2.1
62527	0.684592	2.1
62528	0.655719	2.1
62529	0.946128	2.1
62530	0.946128	2.1
62531	0.946133	2.1
62532	0.946133	2.1
62533	0.946131	2.1
62534	0.946131	2.1
62535	0.946131	2.1
62536	0.946131	2.1
62537	0.94613	2.1
62538	0.94613	2.1
62539	0.946131	2.1
62540	0.946131	2.1
62541	1.89226	2.1
62542	1.89226	2.1
62543	1.89226	2.1
62544	1.89226	2.1
62545	1.89226	2.1
62546	1.89226	2.1
62547	0.553334	2.1
62548	0.629936	2.1
62549	0.828211	2.1
62550	0.729319	2.1
62551	0.674025	2.1
62552	0.674025	2.1
62553	0.68523	2.1
62554	0.68523	2.1
62555	0.828211	2.1
62556	0.629935	2.1
62557	0.72932	2.1
62558	0.553334	2.1
62559	0.828208	2.1
62560	0.729316	2.1
62561	0.907037	2.1
62562	0.782707	2.1
62563	0.799048	2.1
62564	0.799048	2.1
62565	0.811867	2.1
62566	0.811867	2.1
62567	0.828209	2.1
62568	0.907035	2.1
62569	0.729318	2.1
62570	0.782706	2.1
62571	2.32981	2.1
62572	1.89226	2.1
62573	1.89226	2.1
62574	1.89226	2.1
62575	2.2671	2.1
62576	2.2671	2.1
62577	1.89226	2.1
62578	1.89226	2.1
62579	1.89226	2.1

62580	1.89226	1.7677
62581	1.89226	2.1
62582	1.89226	2.1
62583	1.3991	2.1
62584	1.81793	2.0561
62585	2.07226	2.1
62586	2.07226	1.75858
62587	1.24924	2.1
62588	1.24924	2.1
62589	0.946131	2.1
62590	0.946131	2.1
62591	1.16491	2.1
62592	1.16491	2.1
62593	0.946131	2.1
62594	0.946131	2.1
62595	1.16491	2.1
62596	1.19523	2.1
62597	0.946132	2.1
62598	1.19523	2.1
62599	1.16491	2.1
62600	0.946131	2.1
62601	1.89226	2.1
62602	1.89226	1.98219
62603	1.89226	2.1
62604	1.89226	0.24022
62605	3.78452	1.79247
62606	0.901204	2.1
62607	0.901204	2.1
62608	0.902504	2.1
62609	0.902504	2.1
62610	1.10416	2.01753
62611	1.03613	0.775779
62612	0.764922	2.1
62613	0.699552	2.1
62614	0.764921	2.1
62615	1.10416	2.1
62616	0.699551	2.1
62617	1.03613	1.86357
62618	1.89226	0.469189
62619	1.89226	1.09387
62620	1.89226	1.20194
62621	1.89226	0.570574
62622	3.78453	0.867322
62623	1.89226	0.968784
62624	1.89226	1.2884
62625	1.89226	0.909418
62626	1.89226	1.63367
62627	1.89226	0.935737
62628	1.89226	1.6385
62629	0.946131	1.06589
62630	0.946131	1.17802
62631	0.946131	0.676036
62632	0.946131	0.672434
62633	0.946131	0.709342
62634	0.946131	1.23433
62635	0.946131	1.2591
62636	0.946131	0.486755
62637	0.946131	0.646808

62638	0.946131	0.681654
62639	0.946131	0.772951
62640	0.946131	0.865742
62641	1.89226	0.976502
62642	1.89226	1.30495
62643	1.89226	0.951864
62644	1.89226	1.44994
62645	1.89226	0.94513
62646	1.89226	1.44355
62647	0.946127	0.954795
62648	0.946127	1.00302
62649	1.22788	0.803973
62650	1.35469	0.7996
62651	1.35469	0.960653
62652	1.35469	0.809952
62653	0.946128	1.01274
62654	0.946128	0.705099
62655	0.946129	0.817521
62656	1.22788	0.859225
62657	1.35469	0.902154
62658	0.946129	0.857475
62659	1.22788	0.970792
62660	1.35469	0.945186
62661	1.02602	0.573932
62662	0.794861	0.845808
62663	1.15301	0.922935
62664	1.15301	1.05491
62665	1.10089	0.567654
62666	1.10089	0.983234
62667	0.794861	0.878521
62668	1.22788	0.988119
62669	1.02602	0.997733
62670	1.35469	0.912621
62671	1.89226	0.05
62672	1.89226	0.156901
62673	1.89226	1.51618
62674	1.89226	0.100363
62675	3.78452	0.161508
62676	1.13536	1.93775
62677	0.942524	0.0585424
62678	0.946131	0.100983
62679	0.946131	0.05
62680	0.946127	0.165776
62681	0.946127	1.94539
62682	1.26282	0.05
62683	0.917471	1.41767
62684	1.21023	0.182873
62685	1.00851	0.173017
62686	1.17955	0.17757
62687	0.883746	0.107656
62688	1.89226	0.114948
62689	1.89226	2.1
62690	1.89226	0.777328
62691	1.89226	1.27211
62692	1.89226	1.12029
62693	1.89226	2.00309
62694	1.89226	0.0840162
62695	1.89226	0.954925

62696	1.89226	0.0840865
62697	1.89226	0.132565
62698	1.89226	0.110427
62699	1.89226	0.511933
62700	0.94613	0.713236
62701	0.94613	0.107651
62702	0.94613	0.125456
62703	0.94613	0.0925459
62704	0.946128	0.219624
62705	0.946128	0.762283
62706	0.946131	1.22125
62707	0.946131	0.0721929
62708	0.94613	0.202063
62709	0.94613	0.569453
62710	0.94613	0.281722
62711	0.94613	0.206444
62712	0.883758	1.99497
62713	1.03041	0.503482
62714	0.946131	0.385825
62715	0.946131	0.0745153
62716	0.851537	0.231846
62717	0.851537	1.99293
62718	1.23942	0.05
62719	1.14906	1.76472
62720	0.851484	0.248918
62721	0.985518	0.216054
62722	1.0156	0.587037
62723	0.851526	0.603297
62724	1.89226	0.166324
62725	1.89226	2.1
62726	1.89226	0.495781
62727	1.89226	1.30814
62728	1.89226	0.871932
62729	1.89226	2.06435
62730	1.89226	0.239054
62731	1.89226	0.740237
62732	1.89226	0.185009
62733	1.89226	0.358275
62734	1.89226	0.181586
62735	1.89226	0.692121
62736	1.89227	0.12835
62737	1.89227	0.234962
62738	1.89227	0.154899
62739	1.89227	0.461716
62740	3.78453	0.177719
62741	1.89226	0.20258
62742	1.89226	0.204705
62743	1.89226	0.31785
62744	1.89226	0.667574
62745	3.78452	0.395456
62746	0.946132	0.522954
62747	0.946132	0.664059
62748	0.946128	0.925131
62749	0.946128	0.528856
62750	0.94613	0.403349
62751	0.94613	0.505865
62752	0.94613	0.187586
62753	0.94613	0.582473

62754	0.946131	0.430295
62755	0.946131	0.854862
62756	0.94613	0.253027
62757	0.94613	0.399155
62758	1.89226	0.471341
62759	1.89226	0.386824
62760	1.89226	0.497987
62761	1.89226	0.278742
62762	1.89226	0.546493
62763	1.89226	0.294489
62764	1.98874	0.0654996
62765	2.33455	0.11597
62766	2.01865	0.222606
62767	2.07024	0.187312
62768	4.32329	0.270566
62769	1.98874	0.106584
62770	2.00266	0.119992
62771	2.14218	0.397871
62772	2.07024	0.724121
62773	3.9914	0.105017
62774	1.00133	0.209806
62775	0.804471	0.102679
62776	1.03512	0.493243
62777	0.995176	0.507101
62778	0.80447	0.914342
62779	0.995178	0.222319
62780	1.00133	0.184642
62781	1.03512	0.874442
62782	0.880587	0.720889
62783	0.880587	0.412606
62784	0.959002	0.102371
62785	0.959002	0.201295
62786	2.33455	0.121033
62787	1.99036	0.156746
62788	2.07024	0.183002
62789	2.58645	0.276464
62790	4.32491	0.093886
62791	1.89226	0.147495
62792	1.89226	0.123167
62793	1.89226	0.161288
62794	1.89226	0.0875832
62795	3.78453	0.0858725
62796	1.89226	0.394483
62797	1.89226	0.13614
62798	1.89226	0.20657
62799	1.89226	0.16775
62800	3.78453	0.367618
62801	1.89226	0.0936977
62802	1.89226	0.0515089
62803	1.89226	0.0642697
62804	1.89226	0.0797374
62805	1.89226	0.0626563
62806	1.89226	0.0790774
62807	1.89226	0.0854291
62808	2.1523	0.05
62809	2.53257	0.05
62810	2.53257	0.05
62811	1.89226	0.0824172

62812	1.89226	0.0732198
62813	0.946133	0.0629574
62814	0.946133	0.104538
62815	0.946132	0.05
62816	0.946132	0.0562224
62817	0.946132	0.05
62818	0.946132	0.05
62819	0.946132	0.108092
62820	0.946132	0.0520713
62821	0.946133	0.0710853
62822	0.946133	0.0741412
62823	0.946132	0.109105
62824	0.946132	0.0795752
62825	0.946132	0.0985325
62826	0.946132	0.180826
62827	0.946132	0.0769457
62828	0.946132	0.132029
62829	0.946132	0.0768251
62830	0.946132	0.0838805
62831	0.946132	0.185671
62832	0.946132	0.130877
62833	0.946132	0.101166
62834	0.946132	0.102213
62835	0.946131	0.139414
62836	0.946131	0.133781
62837	1.62772	0.05
62838	1.70303	0.05
62839	1.70303	0.05
62840	1.70303	0.05
62841	1.90116	0.05
62842	1.90116	0.05
62843	1.89226	0.05
62844	1.89226	0.0528358
62845	1.89226	0.0908576
62846	1.89226	0.119201
62847	1.89226	0.05
62848	1.89226	0.05
62849	1.89226	0.0633249
62850	1.89226	0.107231
62851	1.89226	0.0625298
62852	1.89226	0.120253
62853	3.78452	0.0939863
62854	1.89226	0.069187
62855	1.89226	0.0887429
62856	1.89226	0.0679845
62857	1.89226	0.0807792
62858	1.89226	0.0603559
62859	1.89226	0.0745846
62860	0.946132	0.06388
62861	0.946132	0.0609705
62862	0.946129	0.0561028
62863	0.946129	0.0582553
62864	0.94613	0.0631096
62865	0.94613	0.0672293
62866	0.946131	0.0555961
62867	0.946131	0.0508193
62868	0.94613	0.0519667
62869	0.94613	0.0611231

62870	0.946131	0.05
62871	0.946131	0.0772425
62872	1.89226	0.0603476
62873	1.89226	0.0837634
62874	1.89226	0.141241
62875	1.89226	0.136118
62876	1.89226	0.0514279
62877	1.89226	0.0505222
62878	1.89226	0.179818
62879	1.89226	0.949285
62880	1.89226	0.921645
62881	1.89226	0.106134
62882	3.78452	0.298466
62883	1.89226	0.412863
62884	1.89226	0.876166
62885	1.89226	0.207729
62886	1.89226	1.02494
62887	1.89226	0.265646
62888	1.89226	1.29804
62889	1.30165	0.374472
62890	0.946127	0.665289
62891	1.09659	0.237536
62892	0.946131	0.326647
62893	1.09659	0.225017
62894	1.09659	1.26801
62895	0.946128	1.58507
62896	0.946128	0.128797
62897	1.30165	0.339486
62898	1.09659	0.790141
62899	0.946129	0.3574
62900	0.946129	0.716577
62901	0.946128	0.584385
62902	0.946128	0.866977
62903	0.946133	0.269182
62904	0.946133	0.601915
62905	0.946131	0.197228
62906	0.946131	1.14693
62907	0.946131	0.918147
62908	0.946131	0.274189
62909	0.94613	0.280792
62910	0.94613	0.232366
62911	0.946131	0.310696
62912	0.946131	0.594693
62913	0.946133	0.71758
62914	0.946133	0.978554
62915	0.946132	0.426403
62916	0.946132	0.686247
62917	0.946132	0.312603
62918	0.946132	1.07306
62919	0.946132	1.04533
62920	0.946132	0.473855
62921	0.946132	0.5159
62922	0.946132	0.548199
62923	0.946133	0.719173
62924	0.946133	0.807101
62925	1.30165	0.967386
62926	0.972228	0.956851
62927	1.09659	0.569766

62928	0.656268	1.05057
62929	1.06217	0.429811
62930	1.06217	1.78658
62931	1.00665	0.230334
62932	1.00665	1.6409
62933	0.97223	0.25199
62934	0.656267	0.264227
62935	1.30165	0.518666
62936	1.09659	0.761633
62937	0.946132	1.66963
62938	0.946132	2.1
62939	1.19895	2.1
62940	1.19895	2.1
62941	0.94613	2.1
62942	0.94613	1.05115
62943	1.39356	2.1
62944	1.19895	2.1
62945	0.94613	2.1
62946	1.39356	2.1
62947	1.19895	2.1
62948	0.946131	2.1
62949	1.89226	1.33443
62950	1.89226	2.1
62951	1.89226	2.1
62952	1.89226	1.32001
62953	1.89226	2.1
62954	1.89226	1.24003
62955	0.946132	0.935156
62956	0.946132	0.385919
62957	0.946128	1.63706
62958	0.946128	2.1
62959	0.94613	1.67811
62960	0.94613	0.319755
62961	0.94613	0.512353
62962	0.94613	1.89009
62963	0.946131	0.480154
62964	0.946131	0.47839
62965	0.94613	1.19526
62966	0.94613	1.13286
62967	1.89226	0.05
62968	1.89226	0.05
62969	1.89226	0.05
62970	1.89226	0.05
62971	1.89226	0.05
62972	1.89226	0.05
62973	1.29881	0.05
62974	1.09985	0.05
62975	0.946126	0.05
62976	0.946126	0.05
62977	0.851539	0.05
62978	1.20421	0.05
62979	0.851483	0.05
62980	0.999426	0.05
62981	1.47172	0.05
62982	0.999413	0.05
62983	0.851552	0.05
62984	0.851552	0.05
62985	1.89226	0.05

62986	1.89226	0.05
62987	1.89226	0.05
62988	1.89226	0.05
62989	1.89226	0.05
62990	1.89226	0.05
62991	1.89226	0.05
62992	1.89226	0.05
62993	1.89226	0.05
62994	1.89226	0.05
62995	1.89226	0.05
62996	1.89226	0.05
62997	1.33915	2.1
62998	1.99297	2.1
62999	2.05216	2.1
63000	2.60163	2.1
63001	4.04513	2.1
63002	1.89226	2.1
63003	1.89226	2.1
63004	1.89226	2.1
63005	1.89226	2.1
63006	3.78452	2.1
63007	0.946132	2.1
63008	0.946132	2.1
63009	0.946133	2.1
63010	0.946133	2.1
63011	0.946132	2.1
63012	0.946132	1.86178
63013	0.946133	2.1
63014	0.946133	2.1
63015	0.946132	2.1
63016	0.946132	2.1
63017	0.946132	2.1
63018	0.946132	2.1
63019	1.89226	2.1
63020	1.89226	1.1414
63021	1.89226	1.43544
63022	1.89226	2.1
63023	3.78452	1.81967
63024	1.89227	1.38801
63025	1.89227	2.1
63026	1.89227	2.1
63027	1.89227	1.37189
63028	1.89227	2.1
63029	1.89227	1.56771
63030	2.29301	2.1
63031	1.70303	2.1
63032	2.46749	2.1
63033	2.30728	2.1
63034	4.53937	2.1
63035	1.89226	0.826477
63036	1.89226	2.1
63037	1.89226	2.1
63038	1.89226	1.31353
63039	3.78452	2.1
63040	1.89226	0.225838
63041	1.89226	2.1
63042	1.89226	2.1
63043	1.89226	2.1

63044	1.89226	0.666582
63045	1.89226	1.16931
63046	2.30728	2.1
63047	1.00757	1.81881
63048	2.0833	2.1
63049	1.65318	2.1
63050	1.417 2.1	
63051	1.00757	2.1
63052	2.46749	2.1
63053	2.0975	2.1
63054	2.79653	2.1
63055	2.44785	2.1
63056	4.91534	2.1
63057	1.89226	2.1
63058	1.89226	2.1
63059	1.89226	1.08679
63060	1.89226	2.1
63061	3.78452	1.98135
63062	1.89226	1.20559
63063	1.89226	2.1
63064	1.89226	2.1
63065	1.89226	1.77971
63066	1.89226	2.1
63067	1.89226	1.71304
63068	0.970559	2.1
63069	0.970559	2.1
63070	0.979305	2.1
63071	0.979305	2.1
63072	0.708502	2.1
63073	0.901117	2.1
63074	1.04875	2.1
63075	1.22392	2.1
63076	1.22392	2.1
63077	0.901117	2.1
63078	1.04875	2.1
63079	0.7085	2.1
63080	1.89226	1.61456
63081	1.89226	0.446528
63082	1.89226	0.970958
63083	1.89226	0.63171
63084	3.78452	1.64632
63085	1.89226	1.28362
63086	1.89226	0.340488
63087	1.89226	0.915816
63088	1.89226	0.418595
63089	3.78452	0.474655
63090	1.89226	0.0783467
63091	1.89226	0.05
63092	1.89226	0.0686787
63093	1.89226	0.0554138
63094	3.78453	0.0684436
63095	1.89226	0.14507
63096	1.89226	0.107743
63097	1.89226	0.06397
63098	1.89226	0.0535666
63099	1.89226	0.0721325
63100	1.89226	0.05
63101	1.89226	0.190142

63102	2.35441	0.297429
63103	1.89226	0.888717
63104	2.30931	0.0639068
63105	4.24667	0.103351
63106	1.11612	0.060422
63107	0.946131	0.0539927
63108	1.1772	0.112472
63109	0.946131	0.121646
63110	1.17721	0.233474
63111	1.11612	0.0793835
63112	0.94613	0.0765415
63113	0.94613	0.233469
63114	1.11612	0.0905121
63115	1.11612	0.0619378
63116	0.946131	0.0664352
63117	0.946131	0.0700302
63118	1.11612	0.05
63119	0.582675	0.05
63120	1.17721	0.05
63121	0.651969	0.05
63122	0.651969	0.05
63123	0.582674	0.05
63124	1.17721	0.05
63125	1.11612	0.05
63126	0.877955	0.05
63127	0.877955	0.05
63128	0.881925	0.05
63129	0.881925	0.05
63130	1.17721	0.0553152
63131	0.651969	0.05
63132	1.15466	0.05
63133	0.623642	0.05
63134	0.623642	0.05
63135	0.651969	0.05
63136	1.17721	0.0515716
63137	1.15466	0.05
63138	0.904714	0.0546548
63139	0.904714	0.05
63140	0.901914	0.05
63141	0.901914	0.05
63142	1.89226	1.16611
63143	1.89226	0.343037
63144	1.89226	0.154191
63145	1.89226	1.51703
63146	1.89226	0.0579096
63147	1.89226	2.049
63148	1.89226	1.18674
63149	1.89226	0.126123
63150	1.89226	0.111673
63151	1.89226	2.1
63152	1.89226	0.101301
63153	1.89226	2.1
63154	2.33986	2.03096
63155	1.97819	0.572639
63156	2.39166	0.0589236
63157	2.70141	2.07587
63158	4.73151	0.433786
63159	0.989095	0.691289

63160	1.19583	0.139298
63161	0.851194	0.14571
63162	0.581197	0.287325
63163	0.872704	0.05
63164	0.872704	1.19045
63165	0.904322	0.131485
63166	0.904322	0.716697
63167	1.19583	0.105904
63168	0.851193	0.108343
63169	0.989095	0.399964
63170	0.581197	0.259128
63171	1.89226	0.578125
63172	1.89226	0.36463
63173	1.89226	0.246111
63174	1.89226	1.63951
63175	1.89226	0.133772
63176	1.89226	2.07591
63177	1.89226	1.30329
63178	1.89226	0.135087
63179	1.89226	0.105414
63180	1.89226	2.1
63181	1.89226	0.071026
63182	1.89226	2.1
63183	2.39166	0.520283
63184	1.70239	1.02201
63185	2.13665	0.158059
63186	2.82684	0.227092
63187	4.52831	0.592974
63188	1.89226	0.05
63189	1.89226	0.05
63190	1.89226	0.05
63191	1.89226	0.05
63192	3.78453	0.05
63193	1.89226	0.05
63194	1.89226	0.05
63195	1.89226	0.05
63196	1.89226	0.05
63197	1.89226	0.05
63198	1.89226	0.05
63199	1.89226	0.05
63200	1.89226	0.05
63201	1.89226	0.05
63202	1.89226	0.05
63203	1.89226	0.05
63204	1.89226	0.05
63205	1.89226	0.05
63206	1.89226	0.05
63207	1.89226	0.1182
63208	1.89226	0.05
63209	1.89226	0.132658
63210	1.89226	0.05
63211	1.89226	0.05
63212	1.89226	0.05
63213	1.89226	0.0516941
63214	1.89226	0.05
63215	1.89226	0.0746394
63216	1.89226	0.05
63217	1.5169	0.0978894

63218	1.61969	0.05
63219	1.17262	0.05
63220	1.17262	0.0615009
63221	1.88868	0.05
63222	1.88868	0.0826593
63223	1.79981	0.0824315
63224	1.88868	0.05
63225	1.5169	0.05
63226	1.5169	0.0822444
63227	2.12891	0.05
63228	2.12891	0.122521
63229	1.89226	0.104065
63230	1.89226	0.0502659
63231	1.89226	0.05
63232	1.89226	0.0744286
63233	3.78452	0.0701223
63234	0.946132	0.05
63235	0.946132	0.059474
63236	0.946132	0.05
63237	0.946132	0.05
63238	0.946132	0.05
63239	0.946132	0.0647492
63240	0.946133	0.05
63241	0.946133	0.0769755
63242	0.946133	0.05
63243	0.946133	0.05
63244	0.946132	0.05
63245	0.946132	0.0511972
63246	1.88868	0.0892823
63247	2.05759	0.05
63248	1.61969	0.05
63249	1.61969	0.0697574
63250	2.29747	0.05
63251	2.29747	0.0655239
63252	2.12891	0.127642
63253	2.29747	0.05
63254	1.88868	0.0503421
63255	1.88868	0.0681015
63256	2.50894	0.0521038
63257	2.50894	0.0775207
63258	1.89226	0.05
63259	1.89226	0.0741096
63260	1.89226	0.05
63261	1.89226	0.0937662
63262	1.89226	0.05
63263	1.89226	0.10319
63264	0.946132	0.0636927
63265	0.946132	0.05
63266	0.946132	0.05
63267	0.946132	0.05
63268	0.946131	0.05
63269	0.946131	0.0885909
63270	0.946132	0.05
63271	0.946132	0.0808994
63272	0.946132	0.05
63273	0.946132	0.051045
63274	0.946132	0.0501746
63275	0.946132	0.0627113

63276	2.42758	1.73251
63277	2.14895	2.1
63278	2.56975	1.45729
63279	2.28142	2.1
63280	4.71869	2.1
63281	1.89226	1.12986
63282	1.89226	2.1
63283	1.89226	1.55317
63284	1.89226	2.1
63285	3.78452	2.1
63286	1.89226	2.1
63287	1.89226	2.1
63288	1.89226	2.1
63289	1.89226	2.1
63290	1.89226	2.1
63291	1.89226	2.1
63292	0.960916	2.1
63293	0.960916	2.1
63294	0.978118	2.1
63295	0.875674	2.1
63296	0.775661	2.1
63297	0.773613	2.1
63298	1.07631	2.1
63299	1.14266	2.1
63300	1.16927	2.1
63301	0.898612	2.1
63302	1.04757	2.1
63303	0.757536	2.1
63304	2.56975	1.01092
63305	2.28142	2.1
63306	2.64384	1.56343
63307	2.40621	2.1
63308	4.97596	2.1
63309	1.89226	1.07654
63310	1.89226	2.1
63311	1.89226	0.242347
63312	1.89226	2.1
63313	3.78452	2.1
63314	1.89226	2.1
63315	1.89226	2.1
63316	1.89226	2.1
63317	1.89226	2.1
63318	1.89226	2.1
63319	1.89226	2.1
63320	1.03328	2.1
63321	1.03328	2.1
63322	1.04321	2.1
63323	1.15424	2.1
63324	0.936183	2.1
63325	0.944491	2.1
63326	1.14266	2.1
63327	1.20517	2.1
63328	1.23323	2.1
63329	0.961268	2.1
63330	1.11215	2.1
63331	0.827247	2.1
63332	1.89226	2.1
63333	1.89226	2.1

63334	1.89226	2.1
63335	1.89226	2.1
63336	3.78453	2.1
63337	2.14647	2.1
63338	2.20589	2.1
63339	1.95567	2.1
63340	2.03224	2.1
63341	4.17871	2.1
63342	2.06035	2.1
63343	1.88452	0.0572161
63344	1.88452	2.1
63345	2.26997	0.117635
63346	2.03224	2.1
63347	2.42272	0.611393
63348	1.89226	2.1
63349	1.89226	0.05
63350	1.89226	0.05
63351	1.89226	2.1
63352	1.89226	0.05
63353	1.89226	2.1
63354	1.89225	2.1
63355	1.89225	2.1
63356	1.89225	2.1
63357	1.89225	2.1
63358	1.89225	2.1
63359	1.89225	2.1
63360	0.952418	2.1
63361	0.952418	2.1
63362	0.95883	2.1
63363	0.95883	2.1
63364	0.946182	2.1
63365	1.01611	2.1
63366	0.895134	2.1
63367	0.977828	2.1
63368	0.89514	2.1
63369	0.946189	2.1
63370	0.977821	2.1
63371	1.01611	2.1
63372	2.03223	2.1
63373	1.88451	0.05
63374	1.89236	2.1
63375	1.74649	0.05
63376	3.77687	1.13191
63377	0.81094	2.1
63378	0.81094	0.0859995
63379	0.810939	2.1
63380	0.810939	2.1
63381	0.810945	1.71009
63382	0.810945	1.57098
63383	1.89225	0.05
63384	1.35167	0.548985
63385	1.08131	2.1
63386	1.08131	0.05
63387	1.0813	1.61107
63388	1.89226	0.05
63389	1.89226	0.05
63390	1.89226	0.05
63391	1.89226	0.05

63392	1.89226	0.05
63393	1.89226	0.05
63394	1.01842	0.05
63395	0.936656	0.05
63396	0.946131	0.05
63397	0.946131	0.05
63398	1.11157	0.05
63399	1.04281	0.05
63400	0.925803	0.05
63401	0.925803	0.05
63402	0.851553	0.05
63403	1.11158	0.05
63404	1.05017	0.05
63405	0.925798	0.05
63406	1.89226	0.05
63407	1.89226	0.05
63408	1.89226	0.05
63409	1.89226	0.05
63410	1.89226	0.05
63411	1.89226	0.05
63412	1.23504	0.05
63413	1.23504	0.05
63414	0.946131	0.05
63415	0.946131	0.05
63416	1.37358	0.05
63417	1.23504	0.05
63418	0.946129	0.05
63419	0.946129	0.05
63420	1.37358	0.05
63421	0.946131	0.05
63422	0.94613	0.05
63423	1.23504	0.05
63424	1.00261	0.05
63425	1.00261	0.05
63426	1.02952	0.05
63427	1.02952	0.05
63428	0.850964	0.05
63429	0.658553	0.05
63430	1.37358	0.05
63431	1.23504	0.05
63432	1.37358	0.05
63433	0.850965	0.05
63434	1.23504	0.05
63435	0.658553	0.05
63436	0.946133	0.05
63437	0.946133	0.05
63438	0.946132	0.05
63439	0.946132	0.05
63440	0.946132	0.05
63441	0.946132	0.05
63442	0.946133	0.05
63443	0.946133	0.05
63444	0.946133	0.05
63445	0.946133	0.05
63446	0.946132	0.05
63447	0.946132	0.05
63448	1.89226	0.05
63449	1.89226	0.05

63450	1.89226	0.05
63451	1.89226	0.05
63452	3.78452	0.05
63453	1.89226	0.05
63454	1.89226	0.05
63455	1.89226	0.05
63456	1.89226	0.05
63457	3.78452	0.05
63458	1.89227	0.05
63459	1.89227	0.05
63460	1.89227	0.05
63461	1.89227	0.05
63462	1.89227	0.05
63463	1.89227	0.05
63464	0.94613	0.05
63465	0.94613	0.05
63466	0.946127	0.05
63467	0.946127	0.05
63468	0.94613	0.05
63469	0.94613	0.05
63470	0.946127	0.05
63471	0.946127	0.05
63472	0.946128	0.05
63473	0.946128	0.05
63474	0.946128	0.05
63475	0.946128	0.05
63476	0.946132	0.05
63477	0.946132	0.05
63478	0.946128	0.05
63479	0.946128	0.05
63480	0.94613	0.05
63481	0.94613	0.05
63482	0.946131	0.05
63483	0.946131	0.05
63484	0.94613	0.05
63485	0.94613	0.05
63486	0.94613	0.05
63487	0.94613	0.05
63488	1.89226	2.1
63489	1.89226	2.1
63490	1.89226	2.1
63491	1.89226	2.1
63492	3.78453	2.1
63493	2.72844	2.1
63494	1.89226	2.1
63495	2.26536	2.1
63496	2.26536	2.1
63497	1.89226	2.1
63498	1.89226	2.1
63499	1.89226	2.1
63500	1.89226	2.1
63501	1.89226	2.1
63502	1.89226	2.1
63503	1.89226	2.1
63504	1.89226	2.1
63505	1.89226	2.1
63506	1.89226	2.1
63507	1.89226	2.1

63508	1.89226	2.1
63509	1.89226	2.1
63510	1.89226	2.1
63511	2.72844	2.1
63512	2.26536	2.1
63513	2.28367	2.1
63514	1.5791	2.1
63515	4.54903	2.1
63516	0.810945	2.1
63517	0.810945	2.1
63518	0.810943	2.1
63519	0.810943	2.1
63520	1.89226	2.1
63521	1.3517	2.1
63522	1.08133	2.1
63523	1.08132	2.1
63524	1.08132	2.1
63525	0.810933	2.1
63526	0.810933	2.1
63527	1.89227	2.1
63528	1.89227	2.1
63529	1.89227	2.1
63530	1.89227	2.1
63531	3.78453	2.1
63532	2.71897	2.1
63533	2.27495	2.1
63534	2.648	2.1
63535	2.20832	2.1
63536	4.92729	2.1
63537	2.27495	2.1
63538	1.38455	2.1
63539	1.38455	2.1
63540	1.46586	2.1
63541	2.20831	2.1
63542	2.28962	2.1
63543	0.946133	2.1
63544	0.946133	2.1
63545	0.946132	2.1
63546	0.946132	2.1
63547	0.946132	2.1
63548	0.946132	2.1
63549	0.946132	2.1
63550	0.946132	2.1
63551	0.946132	2.1
63552	0.946132	2.1
63553	0.946133	2.1
63554	0.946133	2.1
63555	1.89226	2.1
63556	1.89226	2.1
63557	1.89226	2.1
63558	1.89226	2.1
63559	1.89226	2.1
63560	1.89226	2.1
63561	2.648	2.1
63562	2.08612	2.1
63563	2.08612	2.1
63564	2.20831	2.1
63565	2.56618	2.1

63566	2.68837	2.1
63567	2.08612	2.1
63568	2.20831	2.1
63569	1.30659	2.1
63570	1.30659	2.1
63571	1.38455	2.1
63572	2.16408	2.1
63573	0.810946	2.1
63574	0.810946	2.1
63575	0.810946	2.1
63576	0.810946	2.1
63577	1.89226	2.1
63578	1.3517	2.1
63579	1.08133	2.1
63580	1.08132	2.1
63581	1.08132	2.1
63582	0.810934	2.1
63583	0.810934	2.1
63584	1.89226	1.98269
63585	1.89226	1.5538
63586	1.89226	0.753212
63587	1.89226	2.1
63588	3.78452	1.25648
63589	1.89226	0.689005
63590	1.89226	2.1
63591	1.89226	2.1
63592	1.89226	1.34312
63593	3.78453	2.0856
63594	1.08733	2.1
63595	0.856615	2.1
63596	1.25964	1.52411
63597	1.12378	1.40218
63598	1.23551	1.04959
63599	0.969894	2.1
63600	0.985977	2.1
63601	0.985977	1.08986
63602	1.16169	1.86106
63603	1.21072	1.97408
63604	0.765175	2.01179
63605	0.863419	1.96667
63606	2.39934	2.1
63607	2.44427	1.58186
63608	2.64003	0.697241
63609	2.39077	2.1
63610	2.39934	1.99406
63611	2.10194	2.1
63612	1.89226	0.802723
63613	1.89226	2.1
63614	1.89226	1.87983
63615	1.89226	0.302106
63616	3.78452	1.92288
63617	0.946132	2.1
63618	0.946132	2.1
63619	0.946131	0.818025
63620	0.946131	0.987991
63621	0.946132	0.919826
63622	0.946132	2.1
63623	0.946132	2.1

63624	0.946132	1.11746
63625	0.946132	1.83037
63626	0.946132	2.00467
63627	0.946132	1.62019
63628	0.946132	1.97841
63629	1.19967	2.1
63630	0.946128	1.95164
63631	0.946133	0.913721
63632	1.32002	1.6535
63633	1.19967	2.08261
63634	1.19967	2.04317
63635	0.94613	2.1
63636	0.94613	0.248177
63637	0.946131	1.32543
63638	0.946131	1.58698
63639	1.19967	1.75825
63640	1.32001	1.73946
63641	1.89226	0.0917931
63642	1.89226	2.1
63643	1.89226	0.05
63644	1.89226	2.1
63645	3.78451	0.327488
63646	2.6402	0.05
63647	2.28552	2.1
63648	2.50958	0.05
63649	2.18948	1.07429
63650	4.79509	0.159862
63651	2.28552	1.41969
63652	1.86102	2.1
63653	2.18949	1.03869
63654	1.78376	2.1
63655	4.06928	2.1
63656	1.89226	1.53483
63657	1.89226	2.1
63658	1.89226	2.1
63659	1.89226	2.1
63660	3.78452	2.1
63661	1.89226	0.05
63662	1.89226	2.1
63663	1.89226	0.05
63664	1.89226	2.05958
63665	3.78452	0.0879973
63666	2.50958	0.05
63667	2.18948	1.07625
63668	2.39524	0.05
63669	2.10188	2.07219
63670	4.61146	0.05
63671	2.18949	0.419633
63672	1.78376	2.1
63673	1.71839	2.1
63674	1.71839	2.1
63675	2.10189	2.1
63676	2.16726	2.1
63677	0.810943	2.1
63678	0.810943	2.1
63679	0.810945	1.95078
63680	0.810945	1.98737
63681	0.810959	2.1

63682	0.810959	2.1
63683	1.89226	2.1
63684	1.35168	2.1
63685	1.08132	2.1
63686	1.08132	2.1
63687	1.0813	2.1
63688	1.89226	0.73081
63689	1.89226	1.87686
63690	1.89226	1.18897
63691	1.89226	1.22395
63692	1.89226	1.5118
63693	1.89226	1.86888
63694	1.28421	1.52375
63695	0.992041	0.623431
63696	1.07707	2.1
63697	0.789746	1.88717
63698	1.03815	2.03311
63699	1.03815	1.75023
63700	1.03581	1.91804
63701	1.03581	1.39872
63702	1.28421	1.69319
63703	1.07707	2.0555
63704	0.789748	1.72121
63705	0.992038	1.27579
63706	1.89226	0.730174
63707	1.89226	1.94783
63708	1.89226	1.35151
63709	1.89226	1.06912
63710	1.89226	1.512
63711	1.89226	1.26385
63712	1.89226	0.783117
63713	1.89226	1.39473
63714	1.89226	0.961758
63715	1.89226	1.20709
63716	1.89226	1.2982
63717	1.89226	1.65954
63718	0.92236	2.1
63719	0.655971	1.99433
63720	0.916772	2.0612
63721	0.913834	1.32047
63722	1.18074	1.89137
63723	0.922346	2.1
63724	2.15414	0.960271
63725	1.53528	1.80228
63726	1.22748	2.0352
63727	1.57442	1.07596
63728	0.874683	1.84946
63729	1.25935	2.1
63730	2.61433	2.1
63731	1.22262	2.1
63732	1.22262	2.1
63733	2.62813	2.1
63734	2.62813	2.1
63735	1.89226	2.1
63736	2.61434	2.1
63737	2.62814	2.1
63738	2.61434	2.1
63739	1.90607	2.1

63740	1.89226	2.1
63741	1.89226	2.1
63742	1.89226	2.1
63743	1.89226	2.1
63744	1.89226	2.1
63745	1.89226	2.1
63746	1.89226	2.1
63747	1.89226	2.1
63748	1.89226	2.1
63749	1.89226	2.1
63750	1.89226	2.1
63751	1.89226	2.1
63752	1.89226	2.1
63753	1.31969	2.1
63754	1.31406	2.1
63755	0.629677	2.1
63756	0.643104	2.1
63757	0.976621	2.1
63758	0.976621	2.1
63759	0.98055	2.1
63760	0.98055	2.1
63761	1.3197	2.1
63762	0.643102	2.1
63763	0.629675	2.1
63764	1.31407	2.1
63765	0.946133	2.1
63766	0.946133	2.1
63767	1.3197	2.1
63768	1.31407	2.1
63769	0.946132	2.1
63770	0.946132	2.1
63771	1.3197	2.1
63772	1.3197	2.1
63773	1.3197	2.1
63774	0.946133	2.1
63775	0.946132	2.1
63776	1.31407	2.1
63777	1.89226	2.1
63778	1.89226	2.1
63779	1.89226	2.1
63780	1.89226	2.1
63781	1.89226	2.1
63782	1.89226	2.1
63783	1.89226	2.1
63784	1.89226	2.1
63785	1.89226	2.1
63786	1.89226	2.1
63787	1.89226	2.1
63788	1.89226	2.1
63789	1.89226	0.05
63790	1.89226	0.05
63791	1.89226	0.05
63792	1.89226	0.05
63793	3.78452	0.05
63794	1.89226	0.05
63795	1.89226	0.05
63796	1.89226	0.05
63797	1.89226	0.05

63798	1.89226	0.05
63799	1.89226	0.05
63800	0.946131	0.05
63801	0.946131	0.05
63802	0.946128	0.05
63803	0.946128	0.05
63804	0.94613	0.05
63805	0.94613	0.05
63806	0.94613	0.05
63807	0.94613	0.05
63808	0.94613	0.05
63809	0.94613	0.05
63810	0.94613	0.05
63811	0.94613	0.05
63812	1.89226	0.05
63813	1.89226	0.05
63814	1.89226	0.05
63815	1.89226	0.05
63816	1.89226	0.05
63817	1.89226	0.05
63818	0.924614	0.05
63819	0.914275	0.05
63820	0.906003	0.05
63821	0.62479	0.05
63822	2.02733	0.05
63823	1.48332	0.05
63824	0.833106	0.05
63825	1.19364	0.05
63826	1.57482	0.05
63827	1.18103	0.05
63828	0.905988	0.05
63829	1.89226	2.1
63830	1.89226	2.1
63831	1.89226	2.1
63832	1.89226	2.1
63833	3.78452	2.1
63834	1.89226	2.1
63835	1.89226	2.1
63836	1.89226	2.1
63837	1.89226	2.1
63838	1.89226	2.1
63839	1.89226	2.1
63840	0.816805	2.1
63841	0.928516	2.1
63842	1.4045	2.1
63843	1.26268	2.1
63844	1.0881	2.1
63845	1.0881	2.1
63846	1.10309	2.1
63847	1.10309	2.1
63848	0.928518	2.1
63849	1.4045	2.1
63850	0.816807	2.1
63851	1.26267	2.1
63852	1.89226	2.1
63853	2.52535	2.1
63854	1.89226	2.1
63855	1.89226	2.1

63856	2.809	2.1
63857	2.809	2.1
63858	1.89226	2.1
63859	1.89226	2.1
63860	1.89226	2.1
63861	1.89226	2.1
63862	3.78452	2.1
63863	0.946127	2.1
63864	0.946127	2.1
63865	0.946131	2.1
63866	0.946131	2.1
63867	0.94613	2.1
63868	0.94613	2.1
63869	0.946128	2.1
63870	0.946128	2.1
63871	0.946129	2.1
63872	0.946129	2.1
63873	0.946129	2.1
63874	0.946129	2.1
63875	1.89224	1.47176
63876	1.89224	2.1
63877	1.89224	2.1
63878	1.89224	2.1
63879	3.78448	2.1
63880	2.39244	2.1
63881	2.27036	2.1
63882	2.25945	2.1
63883	2.27398	2.1
63884	4.52981	2.1
63885	2.27036	2.1
63886	1.97088	2.1
63887	1.86302	2.1
63888	1.84991	2.1
63889	4.12027	2.1
63890	1.89224	2.1
63891	1.89224	2.1
63892	1.89224	2.1
63893	1.89224	2.1
63894	3.78447	2.1
63895	1.89226	2.1
63896	1.89226	2.1
63897	1.89226	1.36913
63898	1.89226	2.1
63899	3.78453	2.1
63900	2.25948	2.1
63901	2.33611	2.1
63902	2.05257	2.1
63903	1.94441	2.1
63904	4.20389	2.1
63905	1.92514	2.1
63906	1.84993	2.1
63907	1.94441	2.1
63908	1.74391	2.1
63909	3.79434	2.1
63910	1.89226	2.1
63911	1.89226	2.1
63912	1.89226	2.1
63913	1.89226	2.1

63914	3.78452	2.1
63915	15.1381	0.189522
63916	15.1381	0.122867
63917	15.1381	0.161513
63918	15.1381	1.38846
63919	30.2762	0.0873435
63920	15.1381	0.250321
63921	15.1381	0.837149
63922	15.1381	0.268881
63923	15.1381	0.696036
63924	30.2762	0.796601
63925	15.1381	2.05327
63926	15.1381	2.09813
63927	15.1381	1.78665
63928	15.1381	2.1
63929	30.2762	2.1
63930	15.1381	0.097739
63931	15.1381	0.711757
63932	15.1381	0.497191
63933	15.1381	0.328054
63934	30.2762	0.0941141
63935	1.89225	2.1
63936	1.89225	2.1
63937	1.89225	2.1
63938	1.89225	2.1
63939	3.78449	2.1
63940	1.39909	2.1
63941	1.39909	2.1
63942	0.946123	2.1
63943	0.946123	2.1
63944	1.3291	2.1
63945	1.39908	2.1
63946	0.946126	2.1
63947	0.946126	2.1
63948	0.946118	2.1
63949	1.32909	2.1
63950	0.94613	2.1
63951	1.3991	2.1
63952	3.07801	2.1
63953	1.84322	2.1
63954	1.84322	2.1
63955	1.68788	2.1
63956	2.6582	2.1
63957	2.50287	2.1
63958	1.89225	2.1
63959	1.89225	2.1
63960	1.89225	2.1
63961	1.89225	2.1
63962	3.7845	2.1
63963	1.89226	2.1
63964	1.89226	2.1
63965	1.89226	2.1
63966	1.89226	2.1
63967	3.78452	2.1
63968	1.89226	2.1
63969	2.50051	2.1
63970	2.50051	2.1
63971	2.65822	2.1

63972	1.89226	2.1
63973	2.04997	2.1
63974	2.50052	2.1
63975	2.65822	2.1
63976	1.70377	2.1
63977	1.70377	2.1
63978	1.84324	2.1
63979	2.63998	2.1
63980	0.810947	2.1
63981	0.810947	2.1
63982	0.810946	2.1
63983	0.810946	2.1
63984	1.89226	2.1
63985	1.35169	2.1
63986	1.08132	2.1
63987	1.08132	2.1
63988	1.08132	2.1
63989	0.810946	2.1
63990	0.810946	2.1
63991	1.89226	2.1
63992	1.89226	2.1
63993	1.89226	2.1
63994	1.89226	2.1
63995	3.78453	2.1
63996	0.946164	2.1
63997	0.946164	2.1
63998	0.929842	2.1
63999	0.929842	2.1
64000	0.946132	2.1
64001	0.946132	2.1
64002	1.04101	2.1
64003	0.929874	2.1
64004	0.946132	2.1
64005	0.929874	2.1
64006	1.04101	2.1
64007	0.946132	2.1
64008	1.89226	2.1
64009	1.89226	2.1
64010	1.89226	2.1
64011	1.89226	2.1
64012	3.78453	2.1
64013	1.89226	2.1
64014	1.89226	2.1
64015	1.89226	2.1
64016	1.89226	2.1
64017	1.89226	2.1
64018	1.89226	2.1
64019	1.89226	2.1
64020	1.89226	2.1
64021	1.89226	2.1
64022	1.89226	2.1
64023	1.89226	2.1
64024	1.89226	2.1
64025	0.946131	2.1
64026	0.946131	2.1
64027	0.946131	2.1
64028	0.946131	2.1
64029	0.946132	2.1

64030	0.946132	2.1
64031	0.946131	2.1
64032	0.946131	2.1
64033	0.946131	2.1
64034	0.946131	2.1
64035	0.946132	2.1
64036	0.946132	2.1
64037	1.89226	1.35205
64038	1.89226	0.499507
64039	1.89226	0.0966671
64040	1.89226	0.695794
64041	3.78453	0.957279
64042	2.73497	0.05
64043	1.93823	0.962919
64044	1.93823	0.05
64045	2.20046	1.24548
64046	2.42279	0.05
64047	2.68501	2.05492
64048	1.89226	0.0996025
64049	1.89226	1.0795
64050	1.89226	2.08323
64051	1.89226	0.05
64052	1.89226	2.08098
64053	1.89226	0.05
64054	1.89226	0.139589
64055	1.89226	0.124625
64056	1.89226	0.238935
64057	1.89226	1.42141
64058	1.89226	0.105585
64059	1.89226	0.28835
64060	2.82984	0.258502
64061	2.15276	0.0866425
64062	2.15276	0.536989
64063	2.42278	0.138337
64064	2.50074	0.0652352
64065	2.77077	0.0925882
64066	2.42279	0.05
64067	1.93823	1.0952
64068	1.70423	0.05
64069	1.70423	0.244691
64070	2.15276	0.05
64071	2.38676	0.406193
64072	0.810946	0.0742782
64073	0.810946	1.46486
64074	0.810944	0.05
64075	0.810944	0.05
64076	0.810946	0.05
64077	0.810946	0.113754
64078	1.89226	1.16203
64079	1.35169	0.05
64080	1.08132	0.05
64081	1.08132	0.527954
64082	1.08132	0.0640508
64083	1.89226	2.1
64084	1.89226	2.1
64085	1.89226	2.1
64086	1.89226	2.1
64087	3.78452	2.1

64088	1.89226	2.1
64089	1.89226	2.1
64090	1.89226	2.1
64091	1.89226	2.1
64092	3.78453	2.1
64093	1.04144	2.1
64094	1.04144	2.1
64095	0.938972	2.1
64096	0.938972	2.1
64097	0.969661	2.1
64098	1.03285	2.1
64099	0.946133	2.1
64100	0.946133	2.1
64101	0.946131	2.1
64102	0.969663	2.1
64103	0.946131	2.1
64104	1.03284	2.1
64105	0.946131	2.1
64106	0.946131	2.1
64107	0.946128	2.1
64108	0.946128	2.1
64109	0.946127	2.1
64110	0.946127	2.1
64111	0.946131	2.1
64112	0.946131	2.1
64113	0.94613	2.1
64114	0.94613	2.1
64115	0.946129	2.1
64116	0.946129	2.1
64117	1.89226	2.1
64118	1.89226	2.1
64119	1.89226	2.1
64120	1.89226	2.1
64121	3.78452	2.1
64122	1.89226	2.1
64123	1.89226	2.1
64124	1.89226	2.1
64125	1.89226	2.1
64126	3.78453	2.1
64127	1.89226	2.1
64128	1.89226	2.1
64129	1.85882	2.1
64130	1.85882	2.1
64131	1.93932	2.1
64132	1.97276	2.1
64133	0.94613	2.1
64134	0.94613	2.1
64135	0.946128	2.1
64136	0.946128	2.1
64137	0.946127	2.1
64138	0.946127	2.1
64139	0.946131	2.1
64140	0.946131	2.1
64141	0.946129	2.1
64142	0.946129	2.1
64143	0.946129	2.1
64144	0.946129	2.1
64145	1.89225	2.1

64146	1.89225	2.1
64147	1.89225	2.1
64148	1.89225	2.1
64149	3.7845	2.1
64150	1.0086	2.1
64151	1.09028	2.1
64152	0.946125	2.1
64153	0.946125	2.1
64154	0.916906	2.1
64155	0.916906	2.1
64156	1.13051	2.1
64157	1.1505	2.1
64158	1.11557	2.1
64159	1.04071	2.1
64160	0.851542	2.1
64161	0.991196	2.1
64162	1.89225	2.1
64163	1.89225	2.1
64164	1.89225	2.1
64165	1.89225	2.1
64166	3.7845	2.1
64167	1.89225	2.1
64168	1.89225	2.1
64169	1.89225	2.1
64170	1.89225	2.1
64171	1.89225	2.1
64172	1.89225	2.1
64173	1.89226	2.1
64174	1.89226	2.1
64175	1.89226	2.1
64176	1.89226	2.1
64177	3.78452	2.1
64178	0.890962	2.1
64179	0.952547	2.1
64180	0.946132	2.1
64181	0.946132	2.1
64182	0.851543	2.1
64183	0.851543	2.1
64184	1.11558	2.1
64185	1.06304	2.1
64186	0.994315	2.1
64187	1.04072	2.1
64188	0.900943	2.1
64189	0.851542	2.1
64190	1.89226	2.1
64191	1.89226	2.1
64192	1.89226	2.1
64193	1.89226	2.1
64194	1.89226	2.1
64195	1.89226	2.1
64196	1.89226	2.1
64197	1.89226	2.1
64198	1.89226	2.1
64199	1.89226	2.1
64200	3.78452	2.1
64201	1.89226	2.1
64202	1.89226	2.1
64203	1.89226	2.1

64204	1.89226	2.1
64205	3.78452	2.1
64206	0.782015	2.1
64207	0.929718	2.1
64208	0.946132	2.1
64209	0.946132	2.1
64210	0.782016	2.1
64211	0.782016	2.1
64212	1.06706	2.1
64213	1.11785	2.1
64214	0.782015	2.1
64215	0.946133	2.1
64216	0.878931	2.1
64217	0.946132	2.1
64218	1.89226	2.1
64219	1.89226	2.1
64220	1.89226	2.1
64221	1.89226	2.1
64222	1.89226	2.1
64223	1.89226	2.1
64224	0.946131	2.1
64225	0.946131	2.1
64226	0.946129	2.1
64227	0.946129	2.1
64228	0.946131	2.1
64229	0.946131	2.1
64230	0.94613	2.1
64231	0.94613	2.1
64232	0.946131	2.1
64233	0.946131	2.1
64234	0.94613	2.1
64235	0.94613	2.1
64236	0.827866	2.1
64237	0.946131	2.1
64238	0.946129	2.1
64239	0.946129	2.1
64240	0.94613	2.1
64241	0.94613	2.1
64242	0.94613	2.1
64243	0.94613	2.1
64244	0.827865	2.1
64245	0.94613	2.1
64246	0.94613	2.1
64247	0.94613	2.1
64248	1.89226	2.1
64249	1.89226	2.1
64250	1.89226	2.1
64251	1.89226	2.1
64252	3.78452	2.1
64253	1.89226	2.1
64254	1.89226	2.1
64255	1.89226	2.1
64256	1.89226	2.1
64257	1.89226	2.1
64258	1.89226	2.1
64259	1.89226	2.1
64260	1.89226	2.1
64261	1.89226	2.1

64262	1.89226	2.1
64263	3.78453	2.1
64264	1.89226	2.1
64265	1.89226	2.1
64266	1.89226	2.1
64267	1.89226	2.1
64268	3.78452	2.1
64269	0.744377	2.1
64270	0.541919	2.1
64271	0.652022	2.1
64272	0.544198	2.1
64273	0.764633	2.1
64274	0.673254	2.1
64275	1.89226	2.1
64276	1.31149	2.1
64277	1.05423	2.1
64278	1.02189	2.1
64279	0.870602	2.1
64280	1.89226	2.1
64281	1.89226	2.1
64282	1.89226	2.1
64283	1.89226	2.1
64284	1.89226	2.1
64285	1.89226	2.1
64286	1.89226	2.1
64287	1.89226	2.1
64288	1.89226	2.1
64289	1.89226	2.1
64290	1.89226	2.1
64291	1.89226	2.1
64292	1.89226	2.1
64293	1.89226	2.1
64294	1.89226	2.1
64295	1.89226	2.1
64296	1.89226	2.1
64297	1.89226	2.1
64298	0.815314	2.1
64299	0.815313	2.1
64300	0.714029	2.1
64301	0.782951	2.1
64302	0.810944	2.1
64303	0.782954	2.1
64304	1.89226	2.1
64305	1.30999	2.1
64306	1.08132	2.1
64307	1.03962	2.1
64308	0.95209	2.1
64309	1.89226	2.1
64310	1.89226	2.1
64311	1.89226	2.1
64312	1.89226	2.1
64313	1.89226	2.1
64314	1.89226	2.1
64315	1.89226	2.1
64316	1.89226	2.1
64317	1.89226	2.1
64318	1.89226	2.1
64319	1.89226	2.1

64320	1.89226	2.1
64321	1.89226	2.1
64322	1.89226	2.1
64323	1.89226	2.1
64324	1.89226	2.1
64325	1.89226	2.1
64326	1.89226	2.1
64327	0.871558	2.1
64328	0.713078	2.1
64329	0.756858	2.1
64330	0.703674	2.1
64331	0.838025	2.1
64332	0.862196	2.1
64333	1.89226	2.1
64334	1.33194	2.1
64335	1.10834	2.1
64336	1.06163	2.1
64337	1.01785	2.1
64338	1.89226	2.1
64339	1.89226	2.1
64340	1.89226	2.1
64341	1.89226	2.1
64342	3.78452	2.1
64343	1.89226	2.1
64344	1.89226	2.1
64345	1.89226	2.1
64346	1.89226	2.1
64347	1.89226	2.1
64348	1.89226	2.1
64349	1.89226	2.1
64350	1.89226	2.1
64351	1.89226	2.1
64352	1.89226	2.1
64353	1.89226	2.1
64354	1.89226	2.1
64355	0.958406	2.1
64356	1.1383	2.1
64357	0.865636	2.1
64358	1.10093	2.1
64359	0.838024	2.1
64360	0.975154	2.1
64361	1.89226	2.1
64362	1.38553	2.1
64363	1.10834	2.1
64364	1.11522	2.1
64365	1.15271	2.1
64366	1.89227	2.1
64367	1.89227	2.1
64368	1.89227	2.1
64369	1.89227	2.1
64370	3.78453	2.1
64371	0.946132	2.1
64372	0.946132	2.1
64373	0.946132	2.1
64374	0.946132	2.1
64375	0.946132	2.1
64376	0.946132	2.1
64377	0.946133	2.1

64378	0.946133	2.1
64379	0.946132	2.1
64380	0.946132	2.1
64381	0.946133	2.1
64382	0.946133	2.1
64383	0.719271	2.1
64384	0.774968	2.1
64385	1.28401	2.1
64386	1.17415	2.1
64387	0.960999	2.1
64388	0.960999	2.1
64389	0.988115	2.1
64390	0.988115	2.1
64391	0.774968	2.1
64392	1.28401	2.1
64393	0.719271	2.1
64394	1.17415	2.1
64395	2.77811	2.1
64396	2.34829	2.1
64397	2.56802	2.1
64398	2.52333	2.1
64399	2.56802	2.1
64400	2.52333	2.1
64401	1.89227	2.1
64402	1.89227	2.1
64403	1.89227	2.1
64404	1.89227	2.1
64405	3.78453	2.1
64406	1.89226	2.1
64407	1.89226	2.1
64408	1.89226	2.1
64409	1.89226	2.1
64410	1.89226	2.1
64411	1.89226	2.1
64412	1.89226	2.1
64413	2.77811	2.1
64414	1.89226	2.1
64415	2.56802	2.1
64416	1.89226	2.1
64417	2.56802	2.1
64418	1.89226	0.0567769
64419	1.89226	2.1
64420	1.89226	0.05
64421	1.89226	2.1
64422	3.78452	0.208871
64423	2.41802	1.84364
64424	1.95764	2.1
64425	1.95764	2.1
64426	2.27727	2.1
64427	2.39311	2.1
64428	2.73961	2.1
64429	0.946132	2.1
64430	0.946132	2.1
64431	0.94613	2.1
64432	0.94613	2.1
64433	0.946131	2.1
64434	0.946131	2.1
64435	0.946131	2.1

64436	0.946131	2.1
64437	0.946131	2.1
64438	0.946131	2.1
64439	0.946131	2.1
64440	0.946131	2.1
64441	1.89226	0.0802946
64442	1.89226	2.06954
64443	1.89226	2.1
64444	1.89226	0.0659212
64445	1.89226	2.1
64446	1.89226	0.066357
64447	2.78856	0.05
64448	2.28552	2.09333
64449	2.28552	0.0550269
64450	2.3931	1.51728
64451	2.6402	0.05
64452	2.74778	0.90956
64453	2.39311	0.868915
64454	1.95764	2.1
64455	2.28552	1.96411
64456	1.86103	2.1
64457	4.24316	2.1
64458	1.89226	2.03119
64459	1.89226	2.1
64460	1.89226	2.1
64461	1.89226	2.1
64462	1.89226	2.1
64463	1.89226	2.1
64464	1.47412	2.1
64465	2.18862	1.8847
64466	2.07226	0.596018
64467	2.33645	1.51171
64468	4.26088	1.46625
64469	1.89226	0.402812
64470	1.89226	1.97
64471	1.89226	0.612449
64472	1.89226	2.05208
64473	1.89226	1.47008
64474	1.89226	2.1
64475	1.89226	1.22301
64476	1.89226	2.1
64477	1.89226	0.0555156
64478	1.89226	2.1
64479	3.78453	1.23715
64480	2.18862	1.15932
64481	2.1128	2.1
64482	2.33645	1.00408
64483	2.28188	2.1
64484	4.47051	2.1
64485	2.07226	2.1
64486	2.33645	2.03384
64487	2.20831	0.453864
64488	2.4646	0.861128
64489	4.53686	1.2747
64490	0.946131	0.328971
64491	0.946131	2.1
64492	0.946131	2.1
64493	0.946131	1.25124

64494	0.94613	2.1
64495	0.94613	0.968081
64496	0.946133	2.1
64497	0.946133	2.1
64498	0.946132	0.519353
64499	0.946132	0.315959
64500	0.946131	2.1
64501	0.946131	1.77386
64502	1.89226	1.95536
64503	1.89226	2.1
64504	1.89226	0.566064
64505	1.89226	2.1
64506	3.78453	1.72439
64507	2.33646	1.40427
64508	2.28189	2.1
64509	2.4646	0.508157
64510	2.42758	2.1
64511	4.74648	1.55025
64512	1.89226	2.1
64513	1.89226	2.1
64514	1.89226	2.1
64515	1.89226	2.1
64516	1.89226	2.1
64517	1.89226	2.1
64518	1.89226	2.1
64519	1.89226	2.1
64520	1.89226	2.1
64521	1.89226	2.1
64522	1.89226	2.1
64523	1.89226	2.1
64524	1.89226	2.1
64525	1.89226	2.1
64526	1.89226	2.1
64527	1.89226	2.1
64528	3.78453	2.1
64529	1.89226	2.1
64530	1.89226	2.1
64531	1.89226	2.1
64532	1.89226	2.1
64533	1.89226	2.1
64534	1.89226	2.1
64535	1.89226	2.1
64536	1.89226	2.1
64537	1.89226	2.1
64538	1.89226	2.1
64539	3.78452	2.1
64540	0.877238	2.1
64541	0.877238	2.1
64542	1.24746	2.1
64543	1.04764	2.1
64544	0.964969	2.1
64545	1.1521	2.1
64546	0.946132	2.1
64547	0.946132	2.1
64548	0.946132	2.1
64549	1.12659	2.1
64550	0.964969	2.1
64551	0.946131	2.1

64552	1.89226	2.1
64553	1.89226	2.1
64554	1.89226	2.1
64555	1.89226	2.1
64556	1.89226	2.1
64557	1.89226	2.1
64558	1.89226	2.1
64559	1.89226	2.1
64560	1.89226	2.1
64561	1.89226	2.1
64562	3.78453	2.1
64563	0.868144	2.1
64564	0.992762	2.1
64565	0.946131	2.1
64566	0.946131	2.1
64567	1.07072	2.1
64568	1.07072	2.1
64569	0.868171	2.1
64570	0.868171	2.1
64571	0.992762	2.1
64572	0.946131	2.1
64573	0.946131	2.1
64574	0.868144	2.1
64575	0.946132	2.1
64576	0.946132	2.1
64577	0.946131	2.1
64578	0.946131	2.1
64579	0.946132	2.1
64580	0.946132	2.1
64581	0.946132	2.1
64582	0.946132	2.1
64583	0.946132	2.1
64584	0.946132	2.1
64585	0.946132	2.1
64586	0.946132	2.1
64587	1.89227	2.1
64588	1.89227	2.1
64589	1.89227	2.1
64590	1.89227	2.1
64591	1.89227	2.1
64592	1.89227	2.1
64593	1.89226	2.1
64594	1.89226	2.1
64595	1.89226	2.1
64596	1.89226	2.1
64597	1.89226	2.1
64598	1.89226	2.1
64599	1.98552	2.1
64600	2.22298	2.1
64601	2.22298	2.1
64602	1.89226	2.1
64603	1.89226	2.1
64604	1.54525	2.1
64605	0.810946	2.1
64606	0.810946	2.1
64607	0.810947	2.1
64608	0.810947	2.1
64609	0.81096	2.1

64610	0.81096	2.1
64611	1.89226	2.1
64612	1.35168	2.1
64613	1.08132	2.1
64614	1.08132	2.1
64615	1.0813	2.1
64616	1.89226	2.1
64617	1.89226	2.1
64618	1.89226	2.1
64619	1.89226	2.1
64620	3.78452	2.1
64621	1.7753	2.1
64622	2.39703	2.1
64623	1.90577	2.1
64624	2.03445	2.1
64625	3.82335	2.1
64626	2.39703	2.1
64627	2.47949	0.131039
64628	2.26051	2.1
64629	2.36891	0.100497
64630	4.76594	2.1
64631	1.89226	2.1
64632	1.89226	0.105545
64633	1.89226	2.1
64634	1.89226	0.110702
64635	3.78452	1.75807
64636	1.89226	2.1
64637	1.89226	2.1
64638	1.89226	2.1
64639	1.89226	2.1
64640	1.89226	2.1
64641	1.89226	2.1
64642	0.883745	2.1
64643	0.973143	2.1
64644	0.988173	2.1
64645	0.988173	2.1
64646	0.941927	2.1
64647	1.02971	2.1
64648	0.854065	2.1
64649	0.82702	2.1
64650	0.847835	2.1
64651	0.981126	2.1
64652	1.02996	2.1
64653	1.10704	2.1
64654	2.28031	2.1
64655	2.26369	0.233985
64656	2.26369	2.1
64657	2.36891	0.698636
64658	2.30788	2.1
64659	2.42455	0.698967
64660	1.89226	2.1
64661	1.89226	0.246403
64662	1.89226	0.195712
64663	1.89226	2.1
64664	1.89226	0.345272
64665	1.89226	2.1
64666	2.71406	2.1
64667	1.83244	2.1

64668	1.83244	2.1
64669	2.07567	2.1
64670	2.52044	2.1
64671	2.76367	2.1
64672	1.89226	2.1
64673	1.89226	2.1
64674	1.89226	2.1
64675	1.89226	2.1
64676	3.78453	2.1
64677	1.89226	2.1
64678	1.89226	2.1
64679	1.89226	2.1
64680	1.89226	2.1
64681	1.89226	2.1
64682	1.89226	2.1
64683	2.70714	2.1
64684	2.3148	2.1
64685	2.3148	2.1
64686	2.52044	2.1
64687	2.54204	2.1
64688	2.74768	2.1
64689	2.52044	2.1
64690	2.3148	2.1
64691	1.83244	2.1
64692	1.83244	2.1
64693	1.62611	2.1
64694	2.3148	2.1
64695	0.946131	2.1
64696	0.946131	2.1
64697	0.946131	2.1
64698	0.946131	2.1
64699	0.946131	2.1
64700	0.946131	2.1
64701	0.946131	2.1
64702	0.946131	2.1
64703	0.946131	2.1
64704	0.946131	2.1
64705	0.946131	2.1
64706	0.946131	2.1
64707	1.89226	2.1
64708	1.89226	2.1
64709	1.89226	2.1
64710	1.89226	2.1
64711	3.78453	2.1
64712	1.89226	2.1
64713	1.89226	2.1
64714	1.89226	2.1
64715	1.89226	2.1
64716	1.89226	2.1
64717	1.89226	2.1
64718	1.89227	2.1
64719	1.89227	2.1
64720	1.89227	2.1
64721	1.89227	2.1
64722	3.78453	2.1
64723	1.89226	2.1
64724	1.89226	2.1
64725	1.89226	2.1

64726	1.89226	2.1
64727	3.78453	2.1
64728	0.915781	2.1
64729	0.863524	2.1
64730	0.717056	2.1
64731	0.830182	2.1
64732	0.865094	2.1
64733	0.918003	2.1
64734	1.89226	2.1
64735	1.29933	2.1
64736	1.11215	2.1
64737	1.05227	2.1
64738	0.966269	2.1
64739	1.89226	2.1
64740	1.89226	2.1
64741	1.89226	2.1
64742	1.89226	2.1
64743	1.89226	2.1
64744	1.89226	2.1
64745	1.89226	2.1
64746	1.89226	2.1
64747	1.89226	2.1
64748	1.89226	2.1
64749	1.89226	2.1
64750	1.89226	2.1
64751	1.89226	2.1
64752	1.89226	2.1
64753	1.89226	2.1
64754	1.89226	2.1
64755	1.89226	2.1
64756	1.89226	2.1
64757	1.01725	2.1
64758	0.997048	2.1
64759	0.818657	2.1
64760	0.960724	2.1
64761	0.838013	2.1
64762	1.04859	2.1
64763	1.89226	2.1
64764	1.41754	2.1
64765	1.17625	2.1
64766	1.0793	2.1
64767	1.18822	2.1
64768	2.16402	2.1
64769	1.89452	2.1
64770	1.50084	2.1
64771	2.09288	2.1
64772	3.9874	2.1
64773	2.16402	2.1
64774	2.13925	2.1
64775	2.04483	2.1
64776	2.09288	2.1
64777	4.23212	2.1
64778	1.89226	2.1
64779	1.89226	2.1
64780	1.89226	2.1
64781	1.89226	2.1
64782	3.78452	2.1
64783	1.89226	2.1

64784	1.89226	2.1
64785	1.89226	2.1
64786	1.89226	2.1
64787	1.89226	2.1
64788	1.89226	2.1
64789	1.9824	2.1
64790	2.09288	2.1
64791	1.69889	2.1
64792	1.6817	2.1
64793	2.00695	2.1
64794	2.00695	2.1
64795	1.93592	2.1
64796	2.09288	2.1
64797	2.00695	2.1
64798	2.09288	2.1
64799	1.9589	2.1
64800	2.04483	2.1
64801	0.946128	2.1
64802	0.946128	2.1
64803	0.946132	2.1
64804	0.946132	2.1
64805	0.94613	2.1
64806	0.94613	2.1
64807	0.946129	2.1
64808	0.946129	2.1
64809	0.94613	2.1
64810	0.94613	2.1
64811	0.94613	2.1
64812	0.94613	2.1
64813	0.946131	2.1
64814	0.946131	2.1
64815	0.946131	2.1
64816	0.946131	2.1
64817	0.946131	2.1
64818	0.946131	2.1
64819	0.946131	2.1
64820	0.946131	2.1
64821	0.946131	2.1
64822	0.946131	2.1
64823	0.946131	2.1
64824	0.946131	2.1
64825	1.89226	2.1
64826	2.48083	2.1
64827	2.6968	2.1
64828	2.6968	2.1
64829	1.89226	2.1
64830	1.89226	2.1
64831	1.89226	2.1
64832	1.89226	2.1
64833	1.89226	2.1
64834	1.89226	2.1
64835	3.78453	2.1
64836	1.89226	2.1
64837	1.89226	2.1
64838	1.89226	2.1
64839	1.89226	2.1
64840	1.89226	2.1
64841	1.89226	2.1

64842	1.1017	2.1
64843	1.1017	2.1
64844	1.10605	2.1
64845	1.01647	2.1
64846	0.868255	2.1
64847	0.883424	2.1
64848	1.24624	2.1
64849	1.35473	2.1
64850	0.942231	2.1
64851	1.31464	2.1
64852	0.910064	2.1
64853	1.27147	2.1
64854	1.89226	2.1
64855	1.89226	2.1
64856	1.89226	1.38078
64857	1.89226	2.1
64858	3.78453	2.1
64859	1.89226	2.1
64860	1.89226	2.1
64861	1.89226	2.1
64862	1.89226	2.1
64863	1.89226	2.1
64864	1.89226	2.1
64865	1.89226	2.1
64866	1.89226	2.1
64867	1.89226	2.1
64868	1.89226	2.1
64869	3.78453	2.1
64870	1.89226	2.1
64871	2.70015	2.1
64872	1.89226	2.1
64873	2.61064	2.1
64874	4.59241	2.1
64875	2.70015	2.1
64876	2.3372	2.1
64877	2.61064	2.1
64878	1.85184	2.1
64879	4.55198	2.1
64880	1.89226	2.1
64881	1.89226	2.1
64882	1.89226	2.1
64883	1.89226	2.1
64884	1.89226	2.1
64885	1.89226	2.1
64886	1.89227	2.1
64887	1.89227	2.1
64888	1.89227	2.1
64889	1.89227	2.1
64890	3.78453	2.1
64891	1.89226	2.1
64892	2.52194	2.1
64893	2.61064	2.1
64894	2.61064	2.1
64895	1.89226	2.1
64896	1.89226	2.1
64897	1.095 2.1	
64898	1.095 2.1	
64899	1.09189	2.1

64900	1.09189	2.1
64901	0.875334	2.1
64902	0.925919	2.1
64903	1.26097	2.1
64904	1.30532	2.1
64905	1.26097	2.1
64906	0.875334	2.1
64907	1.30532	2.1
64908	0.92592	2.1
64909	1.89226	2.1
64910	1.89226	2.1
64911	1.89226	2.1
64912	1.89226	2.1
64913	1.89226	2.1
64914	1.89226	2.1
64915	1.89226	2.1
64916	1.89226	2.1
64917	1.89226	2.1
64918	1.89226	2.1
64919	3.78453	2.1
64920	1.26097	2.1
64921	1.26097	2.1
64922	0.946132	2.1
64923	0.946132	2.1
64924	1.22176	2.1
64925	1.26097	2.1
64926	0.946132	2.1
64927	0.946132	2.1
64928	0.946132	2.1
64929	1.22176	2.1
64930	1.26097	2.1
64931	0.946132	2.1
64932	2.52194	2.1
64933	1.67381	2.1
64934	1.67381	2.1
64935	1.75067	2.1
64936	2.44352	2.1
64937	2.52038	2.1
64938	1.89226	2.1
64939	1.89226	2.1
64940	1.89226	2.1
64941	1.89226	2.1
64942	1.89226	2.1
64943	1.89226	2.1
64944	1.89227	2.1
64945	1.89227	2.1
64946	1.89227	2.1
64947	1.89227	2.1
64948	1.89227	2.1
64949	1.89227	2.1
64950	1.22176	2.1
64951	1.22176	2.1
64952	0.946132	2.1
64953	0.946132	2.1
64954	1.19095	2.1
64955	1.22176	2.1
64956	0.946133	2.1
64957	0.946133	2.1

64958	1.19095	2.1
64959	0.946132	2.1
64960	0.946133	2.1
64961	1.22176	2.1
64962	2.44352	2.1
64963	1.6287	2.1
64964	1.6287	2.1
64965	1.67381	2.1
64966	2.3819	2.1
64967	2.427	2.1
64968	1.89226	2.1
64969	1.89226	2.1
64970	1.89226	2.1
64971	1.89226	2.1
64972	1.89226	2.1
64973	1.89226	2.1
64974	1.89226	2.1
64975	1.89226	2.1
64976	1.89226	2.1
64977	1.89226	2.1
64978	3.78453	2.1
64979	1.89226	2.1
64980	1.89226	2.1
64981	1.89226	2.1
64982	1.89226	2.1
64983	1.89226	2.1
64984	1.89226	2.1
64985	1.89226	2.1
64986	1.89226	2.1
64987	1.89226	2.1
64988	1.89226	2.1
64989	1.89226	2.1
64990	1.89226	2.1
64991	0.851542	2.1
64992	0.851542	2.1
64993	1.1736	2.1
64994	1.29261	2.1
64995	0.852708	2.1
64996	1.18843	2.1
64997	0.946132	2.1
64998	0.946132	2.1
64999	0.946132	2.1
65000	1.07901	2.1
65001	1.04208	2.1
65002	0.936669	2.1
65003	1.89227	2.1
65004	1.89227	2.1
65005	1.89227	2.1
65006	1.89227	2.1
65007	3.78453	2.1
65008	1.89226	2.1
65009	1.89226	2.1
65010	1.89226	2.1
65011	1.89226	2.1
65012	3.78453	2.1
65013	1.89226	2.1
65014	1.89226	2.1
65015	1.89226	2.1

65016	1.89226	2.1
65017	1.89226	2.1
65018	1.89226	2.1
65019	0.946133	2.1
65020	0.946133	2.1
65021	1.28525	2.1
65022	1.28525	2.1
65023	1.07901	2.1
65024	1.28525	2.1
65025	0.946132	2.1
65026	0.946132	2.1
65027	0.946132	2.1
65028	1.28525	2.1
65029	0.946133	2.1
65030	1.07901	2.1
65031	1.89226	2.1
65032	1.89226	2.1
65033	1.89226	2.1
65034	1.89226	2.1
65035	1.89226	2.1
65036	1.89226	2.1
65037	1.89226	2.1
65038	1.89226	2.1
65039	1.89226	2.1
65040	1.89226	2.1
65041	1.89226	2.1
65042	1.89226	2.1
65043	1.24419	2.1
65044	1.09093	2.1
65045	1.22722	2.1
65046	1.18154	2.1
65047	1.27455	2.1
65048	1.08473	2.1
65049	1.15541	2.1
65050	1.15541	2.1
65051	1.29607	2.1
65052	1.23814	2.1
65053	1.13024	2.1
65054	1.12096	2.1
65055	1.18858	2.1
65056	1.13181	2.1
65057	0.988718	2.1
65058	0.802498	2.1
65059	0.994034	2.1
65060	1.15773	2.1
65061	1.0029	2.1
65062	1.0029	2.1
65063	1.23814	2.1
65064	0.997523	2.1
65065	1.11498	2.1
65066	0.899532	2.1
65067	1.89226	2.1
65068	1.89226	2.1
65069	1.89226	2.1
65070	1.89226	2.1
65071	1.89226	2.1
65072	1.89226	2.1
65073	1.89226	2.1

65074	1.89226	2.1
65075	1.89226	2.1
65076	1.89226	2.1
65077	1.89226	2.1
65078	1.89226	2.1
65079	0.993139	2.1
65080	0.834108	2.1
65081	1.01147	2.1
65082	1.01358	2.1
65083	1.20067	2.1
65084	0.993156	2.1
65085	2.4158	2.1
65086	1.69956	2.1
65087	1.3544	2.1
65088	1.60095	2.1
65089	1.11219	2.1
65090	1.89226	2.1
65091	1.89226	2.1
65092	1.89226	2.1
65093	1.89226	2.1
65094	3.78452	2.1
65095	1.89226	2.1
65096	1.89226	2.1
65097	1.89226	2.1
65098	1.89226	2.1
65099	1.89226	2.1
65100	1.89226	2.1
65101	1.89226	2.1
65102	2.83014	2.1
65103	2.32404	2.1
65104	2.32404	2.1
65105	1.89226	2.1
65106	1.89226	2.1
65107	1.37966	2.1
65108	1.13294	2.1
65109	0.881723	2.1
65110	0.582304	2.1
65111	0.977944	2.1
65112	0.977948	2.1
65113	1.0264	2.1
65114	1.0264	2.1
65115	1.37967	2.1
65116	1.03896	2.1
65117	1.19108	2.1
65118	0.582298	2.1
65119	1.89226	2.1
65120	1.89226	2.1
65121	1.89226	2.1
65122	1.89226	2.1
65123	3.78452	2.1
65124	0.946131	2.1
65125	0.946131	2.1
65126	0.946131	2.1
65127	0.946131	2.1
65128	0.946132	2.1
65129	0.946132	2.1
65130	0.946129	2.1
65131	0.946129	2.1

65132	0.946131	2.1
65133	0.946131	2.1
65134	0.94613	2.1
65135	0.94613	2.1
65136	1.89226	2.1
65137	1.89226	2.1
65138	1.89226	2.1
65139	1.89226	2.1
65140	3.78453	2.1
65141	1.89226	2.1
65142	1.89226	2.1
65143	1.89226	2.1
65144	1.89226	2.1
65145	1.89226	2.1
65146	1.89226	2.1
65147	2.65216	2.1
65148	1.93528	2.1
65149	1.78595	2.1
65150	2.48544	2.1
65151	4.42072	2.1
65152	1.89226	2.1
65153	1.89226	2.1
65154	1.89226	2.1
65155	1.89226	2.1
65156	1.89226	2.1
65157	1.89226	2.1
65158	1.89226	2.1
65159	1.89226	2.1
65160	1.89226	2.1
65161	1.89226	2.1
65162	3.78452	2.1
65163	1.66537	2.1
65164	2.48544	2.1
65165	1.78595	2.1
65166	1.78595	2.1
65167	2.36197	2.1
65168	2.36197	2.1
65169	2.80975	2.1
65170	2.48544	2.1
65171	2.36198	2.1
65172	2.65135	2.1
65173	5.13679	2.1
65174	1.89226	2.1
65175	1.89226	2.1
65176	1.89226	2.1
65177	1.89226	2.1
65178	1.89226	2.1
65179	1.89226	2.1
65180	0.946127	2.1
65181	0.946127	2.1
65182	0.946131	2.1
65183	0.946131	2.1
65184	0.946128	2.1
65185	0.946128	2.1
65186	0.94613	2.1
65187	0.94613	2.1
65188	0.946129	2.1
65189	0.946129	2.1

65190	0.946129	2.1
65191	0.946129	2.1
65192	1.11972	2.1
65193	1.18099	2.1
65194	0.832687	2.1
65195	0.764517	2.1
65196	0.974426	2.1
65197	0.974426	2.1
65198	0.971078	2.1
65199	0.971078	2.1
65200	1.11972	2.1
65201	0.764515	2.1
65202	0.832686	2.1
65203	1.18099	2.1
65204	2.36198	2.1
65205	2.48025	2.1
65206	2.48025	2.1
65207	2.65135	2.1
65208	2.23944	2.1
65209	2.41055	2.1
65210	0.946132	2.1
65211	0.946132	2.1
65212	0.946131	2.1
65213	0.946131	2.1
65214	0.946131	2.1
65215	0.946131	2.1
65216	0.946132	2.1
65217	0.946132	2.1
65218	0.946131	2.1
65219	0.946131	2.1
65220	0.946132	2.1
65221	0.946132	2.1
65222	0.810942	2.1
65223	0.810942	2.1
65224	0.810944	2.1
65225	0.810944	2.1
65226	1.89226	2.1
65227	1.3517	2.1
65228	1.08132	2.1
65229	1.08133	2.1
65230	1.08132	2.1
65231	0.810932	2.1
65232	0.810932	2.1
65233	1.89249	2.1
65234	1.9857	1.78483
65235	2.02433	1.8326
65236	2.15807	1.60234
65237	4.01003	1.82103
65238	1.89226	1.34426
65239	1.89226	1.94358
65240	1.89226	2.02192
65241	1.89226	0.929254
65242	3.78452	1.89275
65243	1.89226	1.79465
65244	1.89226	1.89295
65245	1.89226	0.481822
65246	1.89226	1.59252
65247	3.78452	0.820962

65248	1.98569	1.37399
65249	1.86067	1.90859
65250	2.15807	1.13377
65251	2.07965	1.19283
65252	4.06534	1.99837
65253	2.02433	2.0853
65254	2.15807	2.1
65255	2.12608	2.1
65256	2.3025	0.89342
65257	4.32683	2.1
65258	1.89226	2.1
65259	1.89226	1.6459
65260	1.89226	1.0077
65261	1.89226	0.933666
65262	3.78452	1.1486
65263	1.89226	1.09139
65264	1.89226	2.09724
65265	1.89226	0.98295
65266	1.89226	1.19824
65267	3.78452	2.00304
65268	2.15807	2.00378
65269	2.07965	1.9022
65270	2.3025	0.739505
65271	2.28384	1.04989
65272	4.38215	0.901035
65273	1.89226	0.506696
65274	1.89226	0.05
65275	1.89226	0.1667
65276	1.89226	0.05
65277	3.78453	0.0714146
65278	2.16905	0.0805903
65279	2.56369	0.05
65280	1.88394	0.107488
65281	2.41938	0.05
65282	4.44763	0.05
65283	2.56369	0.05
65284	2.80663	0.0880388
65285	2.41938	0.05
65286	2.69045	0.05
65287	5.25414	0.05
65288	1.89226	0.0506225
65289	1.89226	0.05
65290	1.89226	0.0530874
65291	1.89226	0.05
65292	3.78453	0.05
65293	1.89226	0.0737077
65294	1.89226	0.05
65295	1.89226	0.529177
65296	1.89226	0.0740036
65297	1.89226	0.511499
65298	1.89226	0.05
65299	1.01988	0.05
65300	1.01988	0.05
65301	1.03333	0.05
65302	1.03333	0.0661457
65303	1.13811	0.0678447
65304	1.20969	0.05
65305	0.941974	0.0644833

65306	0.843515	0.05
65307	0.843514	0.05
65308	1.13811	0.05
65309	0.941973	0.05
65310	1.20969	0.05
65311	2.41938	0.05
65312	2.57414	0.05
65313	2.57414	0.446928
65314	2.69045	0.219303
65315	2.27623	0.05
65316	2.39253	0.0757851
65317	1.89227	0.12596
65318	1.89227	0.05
65319	1.89227	0.05
65320	1.89227	0.0669557
65321	1.89227	0.05
65322	1.89227	0.05
65323	2.28385	1.39487
65324	2.09114	1.70143
65325	2.46968	0.458951
65326	2.32438	1.53067
65327	4.56082	0.641303
65328	1.89226	0.773428
65329	1.89226	1.98042
65330	1.89226	0.871611
65331	1.89226	1.72051
65332	3.78452	1.64903
65333	1.89226	0.836597
65334	1.89226	2.1
65335	1.89226	0.832336
65336	1.89226	1.42211
65337	3.78452	1.31154
65338	2.09114	0.481682
65339	1.5249	2.04641
65340	2.55684	0.544512
65341	1.86899	1.58692
65342	3.96011	1.33395
65343	2.46968	1.00318
65344	2.32439	1.93665
65345	2.62768	0.545044
65346	2.506	1.29815
65347	4.97568	2.04583
65348	1.89226	1.50963
65349	1.89226	1.73984
65350	1.89226	0.26028
65351	1.89226	1.37944
65352	3.78452	0.487123
65353	1.89226	0.597449
65354	1.89226	1.58001
65355	1.89226	1.18279
65356	1.89226	2.1
65357	1.89226	0.187185
65358	1.89226	2.1
65359	2.55684	1.06702
65360	2.16506	1.51773
65361	1.86901	1.89081
65362	1.86899	1.21168
65363	2.75664	1.01118

65364	2.75662	0.524905
65365	1.89224	0.05
65366	1.89224	0.0570494
65367	1.89224	0.05
65368	1.89224	1.0689
65369	3.78447	0.05
65370	2.24206	0.05
65371	2.38862	1.95389
65372	2.07107	0.05
65373	2.23097	0.62316
65374	4.47303	0.162549
65375	2.38863	2.01438
65376	2.25945	2.1
65377	2.25945	1.10994
65378	2.39244	2.1
65379	2.23098	0.804865
65380	2.36397	2.1
65381	1.89224	0.05
65382	1.89224	2.1
65383	1.89224	1.59335
65384	1.89224	0.89415
65385	1.89224	1.16549
65386	1.89224	1.17172
65387	1.89226	0.05
65388	1.89226	0.122071
65389	1.89226	0.0802075
65390	1.89226	0.05
65391	1.89226	0.05
65392	1.89226	0.05
65393	0.920975	2.1
65394	0.920979	0.0599077
65395	1.03015	0.128341
65396	1.03015	0.05
65397	0.992407	0.122202
65398	1.08759	2.1
65399	0.854346	0.0626545
65400	0.87241	0.05
65401	0.872397	0.0697363
65402	1.04334	0.0537265
65403	1.00965	0.202127
65404	1.0876	0.160922
65405	2.23101	2.05648
65406	2.25948	2.1
65407	2.03577	0.137965
65408	2.05257	2.1
65409	4.29524	2.1
65410	0.810947	0.05
65411	0.810947	1.3934
65412	0.810947	0.124742
65413	0.810947	0.05
65414	0.810934	0.286165
65415	0.810934	0.291577
65416	1.89227	2.1
65417	1.3517	0.648089
65418	1.08132	0.274144
65419	1.08132	1.37286
65420	1.08133	0.179508
65421	1.89226	1.72503

65422	1.89226	1.98402
65423	1.89226	0.891899
65424	1.89226	2.1
65425	1.89226	0.141647
65426	1.89226	2.1
65427	1.89226	2.1
65428	1.89226	2.1
65429	1.89226	1.29438
65430	1.89226	2.1
65431	1.89226	1.12045
65432	1.89226	2.1
65433	0.879432	2.1
65434	0.879432	1.54914
65435	0.900223	0.0869188
65436	0.900223	2.1
65437	0.54571	2.1
65438	0.743092	1.92058
65439	1.03656	2.1
65440	1.19234	0.37292
65441	1.19234	1.87624
65442	0.743092	2.09867
65443	1.03656	2.08458
65444	0.545711	2.05056
65445	1.89226	0.302471
65446	2.38467	1.99241
65447	2.69293	0.289565
65448	2.69293	2.1
65449	1.89226	0.144828
65450	1.89226	1.88155
65451	1.89226	1.04768
65452	1.89226	1.90671
65453	1.89226	0.449302
65454	1.89226	2.1
65455	3.78452	1.1189
65456	0.946129	2.1
65457	0.946129	2.1
65458	0.946133	0.1715
65459	0.946133	2.1
65460	0.946131	2.01367
65461	0.946131	2.1
65462	0.946131	0.17913
65463	0.946131	2.09522
65464	0.946131	2.07537
65465	0.946131	2.04113
65466	0.946131	1.9784
65467	0.946131	1.98029
65468	1.04846	0.115342
65469	1.04846	2.1
65470	1.05594	0.191803
65471	1.05594	2.1
65472	0.743092	2.1
65473	0.912061	1.52637
65474	1.19234	0.200884
65475	1.34647	0.304644
65476	0.912061	0.265662
65477	1.34646	1.05066
65478	1.19234	0.50655
65479	0.743093	0.610196

65480	1.89226	0.880894
65481	1.89226	2.1
65482	1.89226	0.0803746
65483	1.89226	0.933446
65484	3.78452	1.08768
65485	0.946128	2.1
65486	0.946128	1.97251
65487	0.946133	1.98454
65488	0.946133	0.423045
65489	0.94613	2.1
65490	0.94613	2.1
65491	0.946131	2.06498
65492	0.946131	0.730783
65493	0.946131	2.1
65494	0.946131	2.1
65495	0.946131	1.98672
65496	0.946131	1.95997
65497	1.89226	1.43335
65498	1.89226	2.1
65499	1.89226	0.540163
65500	1.89226	1.56139
65501	3.78452	1.87213
65502	1.89226	1.35958
65503	1.89226	1.47598
65504	1.89226	1.02478
65505	1.89226	2.1
65506	1.89226	0.0789028
65507	1.89226	2.1
65508	1.89226	1.73648
65509	1.89226	1.96864
65510	1.89226	0.999361
65511	1.89226	2.1
65512	1.89226	0.429806
65513	1.89226	2.1
65514	0.946132	2.1
65515	0.946132	1.52761
65516	1.17758	0.331678
65517	1.17759	2.1
65518	1.04514	2.1
65519	1.17758	0.981127
65520	0.946133	0.264095
65521	0.946133	0.585167
65522	0.946107	1.21365
65523	1.17755	1.17272
65524	1.04517	1.25737
65525	0.946158	1.07904
65526	1.89226	2.1
65527	2.10171	2.1
65528	1.95064	2.1
65529	2.10171	2.1
65530	1.7412	2.1
65531	1.89226	2.1
65532	1.89226	2.1
65533	1.89226	2.1
65534	1.89226	2.1
65535	1.89226	2.1
65536	1.89226	2.1
65537	1.89226	2.1

65538	1.89226	2.1
65539	1.89226	2.1
65540	1.89226	2.1
65541	1.89226	2.1
65542	3.78453	2.1
65543	0.946133	2.1
65544	0.946133	2.1
65545	0.946132	2.1
65546	0.946132	2.1
65547	0.946133	2.1
65548	0.946133	2.1
65549	0.946132	2.1
65550	0.946132	2.1
65551	0.946132	2.1
65552	0.946132	2.1
65553	0.946132	2.1
65554	0.946132	2.1
65555	1.70303	2.1
65556	1.95064	2.1
65557	1.74039	2.1
65558	1.95064	2.1
65559	1.69368	2.1
65560	1.89226	2.1
65561	1.89226	2.1
65562	1.89226	2.1
65563	1.89226	2.1
65564	1.89226	2.1
65565	3.78453	2.1
65566	0.946132	1.68331
65567	0.946132	2.1
65568	0.946132	2.1
65569	0.946132	2.1
65570	0.946133	2.1
65571	0.946133	2.03232
65572	0.946132	2.1
65573	0.946132	2.1
65574	0.946107	2.1
65575	0.946107	2.1
65576	0.946158	2.1
65577	0.946158	2.1
65578	0.946133	2.1
65579	0.946133	2.1
65580	0.946132	2.1
65581	0.946132	2.1
65582	0.946133	2.1
65583	0.946133	2.1
65584	0.946132	2.1
65585	0.946132	2.1
65586	0.946107	2.1
65587	0.946107	2.1
65588	0.946158	2.1
65589	0.946158	2.1
65590	1.89226	2.1
65591	1.89226	2.1
65592	1.89226	2.1
65593	1.89226	2.1
65594	1.89226	2.1
65595	1.89226	2.1

65596	1.89226	2.1
65597	1.89226	2.1
65598	1.89226	2.1
65599	1.89226	2.1
65600	1.89226	2.1
65601	1.89226	2.1
65602	2.5198	2.1
65603	2.45893	2.1
65604	2.27322	2.1
65605	2.27322	2.1
65606	2.63576	2.1
65607	2.63576	2.1
65608	1.24045	2.1
65609	1.11906	2.1
65610	1.13578	2.1
65611	0.916205	2.1
65612	1.12539	2.1
65613	1.08404	2.1
65614	1.08484	2.1
65615	1.08484	2.1
65616	1.22836	2.1
65617	0.919363	2.1
65618	1.16507	2.1
65619	0.924864	2.1
65620	1.89226	2.1
65621	1.89226	2.1
65622	1.89226	2.1
65623	1.89226	2.1
65624	1.89226	2.1
65625	1.89226	2.1
65626	1.89226	2.1
65627	1.89226	2.1
65628	1.89226	2.1
65629	1.89226	2.1
65630	1.89226	2.1
65631	1.89226	2.1
65632	2.6946	2.1
65633	2.63576	2.1
65634	2.5198	2.1
65635	2.5198	2.1
65636	2.8362	2.1
65637	2.8362	2.1
65638	1.3265	2.1
65639	1.24045	2.1
65640	0.983223	2.1
65641	0.954429	2.1
65642	1.06046	2.1
65643	1.10594	2.1
65644	1.1915	2.1
65645	1.1915	2.1
65646	1.31357	2.1
65647	1.09005	2.1
65648	1.29145	2.1
65649	0.995825	2.1
65650	1.89226	2.1
65651	1.89226	2.1
65652	1.89226	2.1
65653	1.89226	2.1

65654	3.78452	2.1
65655	1.89226	2.1
65656	1.89226	2.1
65657	1.89226	2.1
65658	1.89226	2.1
65659	3.78453	2.1
65660	1.91802	2.1
65661	2.27409	2.1
65662	2.48048	2.1
65663	2.22264	2.1
65664	1.91802	2.1
65665	1.66018	2.1
65666	1.13704	2.1
65667	1.24023	2.1
65668	1.22947	2.1
65669	1.31788	2.1
65670	1.23859	2.1
65671	1.23859	2.1
65672	1.23111	2.1
65673	1.23111	2.1
65674	1.24024	2.1
65675	1.31788	2.1
65676	1.13704	2.1
65677	1.22947	2.1
65678	1.89226	2.1
65679	1.89226	2.1
65680	1.89226	2.1
65681	1.89226	2.1
65682	1.89226	2.1
65683	1.89226	2.1
65684	1.89226	2.1
65685	1.89226	2.1
65686	1.89226	2.1
65687	1.89226	2.1
65688	3.78453	2.1
65689	1.05568	2.1
65690	0.959008	2.1
65691	1.32068	2.1
65692	1.24024	2.1
65693	1.15206	2.1
65694	1.15206	2.1
65695	1.14386	2.1
65696	1.14386	2.1
65697	1.05568	2.1
65698	1.32068	2.1
65699	0.959008	2.1
65700	1.24024	2.1
65701	1.32068	2.1
65702	1.24023	2.1
65703	1.31788	2.1
65704	1.4181	2.1
65705	1.31436	2.1
65706	1.31436	2.1
65707	1.3242	2.1
65708	1.3242	2.1
65709	1.32068	2.1
65710	1.4181	2.1
65711	1.24024	2.1

65712	1.31788	2.1
65713	1.89226	2.09282
65714	1.89226	0.05
65715	1.89226	0.406864
65716	1.89226	0.112954
65717	1.89226	0.508971
65718	1.89226	0.472856
65719	1.02925	0.05
65720	1.16678	0.0663622
65721	0.946132	0.05
65722	0.946132	0.505837
65723	0.935677	0.576813
65724	0.935677	0.05
65725	1.18902	0.205175
65726	1.21562	0.194172
65727	1.13433	0.0634087
65728	1.04071	0.0884701
65729	0.851553	0.0698807
65730	1.06075	0.0546856
65731	1.89226	0.227987
65732	1.89226	0.05
65733	1.89226	0.0807938
65734	1.89226	0.05
65735	1.89226	0.05
65736	1.89226	0.125971
65737	1.89226	0.777045
65738	1.89226	0.05
65739	1.89226	0.05
65740	1.89226	0.0554691
65741	3.78453	0.05
65742	0.946128	0.166791
65743	0.946128	0.0569118
65744	0.946133	0.0555082
65745	0.946133	0.05
65746	0.946131	0.05
65747	0.946131	0.05
65748	0.94613	0.275543
65749	0.94613	0.0511264
65750	0.946131	0.127701
65751	0.946131	0.14268
65752	0.946131	0.05
65753	0.946131	0.05
65754	0.946132	0.500844
65755	0.946132	0.05
65756	0.946132	0.510898
65757	0.946132	0.05
65758	0.946132	0.154666
65759	0.946132	0.05
65760	0.946131	0.108937
65761	0.946131	0.310064
65762	0.946132	0.05
65763	0.946132	0.56292
65764	0.946132	0.05
65765	0.946132	0.07936
65766	0.946131	2.1
65767	0.946131	1.39222
65768	0.946131	2.1
65769	0.946131	0.5301

65770	0.946131	0.671979
65771	0.946131	2.06335
65772	0.946131	0.487316
65773	0.946131	0.424808
65774	0.946131	1.15238
65775	0.946131	1.13125
65776	0.946131	1.15707
65777	0.946131	1.19321
65778	1.89226	1.36596
65779	1.89226	1.3218
65780	1.89226	0.622342
65781	1.89226	2.1
65782	1.89226	0.365137
65783	1.89226	2.1
65784	1.89226	1.05864
65785	1.89226	0.729394
65786	1.89226	0.941328
65787	1.89226	2.1
65788	1.89226	0.39118
65789	1.89226	2.1
65790	1.89226	1.57034
65791	1.89226	0.816832
65792	1.89226	0.306017
65793	1.89226	2.1
65794	1.89226	0.117314
65795	1.89226	2.1
65796	1.89226	2.1
65797	1.89226	2.1
65798	1.89226	0.319006
65799	1.89226	2.1
65800	3.78452	1.76952
65801	0.946131	2.1
65802	0.946131	0.451795
65803	1.17195	0.418005
65804	1.17195	2.1
65805	0.958489	2.1
65806	1.17195	1.38232
65807	0.946129	0.444168
65808	0.946129	1.65752
65809	0.946131	1.1621
65810	1.17195	1.1722
65811	0.958487	1.18671
65812	0.94613	1.18404
65813	2.69656	2.1
65814	2.17516	2.1
65815	2.41885	2.1
65816	2.41885	2.1
65817	2.46476	2.1
65818	2.46476	1.50077
65819	2.69657	2.1
65820	2.30233	0.116243
65821	2.30233	1.90047
65822	2.59476	1.25199
65823	2.46477	2.0488
65824	2.7572	1.95298
65825	1.89226	2.07926
65826	1.89226	0.0577117
65827	1.89226	0.0523457

65828	1.89226	0.0717315
65829	1.89226	0.0528565
65830	1.89226	0.186647
65831	1.89226	2.1
65832	1.89226	1.61444
65833	1.89226	2.1
65834	1.89226	0.0567977
65835	3.78452	1.76903
65836	2.46477	2.1
65837	2.17516	2.1
65838	1.80252	2.1
65839	2.09715	1.05687
65840	4.27231	2.1
65841	2.46477	2.1
65842	2.09715	0.946526
65843	2.30233	1.50468
65844	2.30233	1.25189
65845	1.96416	0.0602198
65846	2.09715	0.0669461
65847	1.89227	0.82178
65848	1.89227	0.05
65849	1.89227	0.0617244
65850	1.89227	0.0563238
65851	1.89227	0.235991
65852	1.89227	1.09738
65853	1.89226	2.1
65854	1.89226	0.05
65855	1.89226	0.818246
65856	1.89226	0.880026
65857	3.78452	1.0164
65858	0.946132	2.1
65859	0.946132	2.1
65860	0.946132	2.1
65861	0.946132	2.1
65862	0.946132	2.1
65863	0.946132	2.1
65864	0.946132	2.1
65865	0.946132	2.1
65866	0.946132	2.1
65867	0.946132	2.1
65868	0.946131	2.1
65869	0.946131	2.1
65870	1.89226	2.1
65871	1.89226	2.1
65872	1.89226	2.1
65873	1.89226	2.1
65874	3.78453	2.1
65875	1.89226	2.1
65876	1.89226	2.1
65877	1.89226	2.1
65878	1.89226	2.1
65879	1.89226	2.1
65880	1.89226	2.1
65881	1.89226	2.1
65882	1.63707	2.1
65883	1.63707	2.1
65884	1.38859	2.1
65885	1.89226	2.1

65886	1.64377	2.1
65887	0.94613	2.1
65888	0.94613	2.1
65889	0.94613	2.1
65890	0.94613	2.1
65891	0.946131	2.1
65892	0.946131	2.1
65893	0.946129	2.1
65894	0.946129	2.1
65895	0.94613	2.1
65896	0.94613	2.1
65897	0.94613	2.1
65898	0.94613	2.1
65899	2.15888	2.1
65900	1.99309	2.1
65901	2.16841	2.1
65902	2.16841	2.1
65903	2.02311	1.21994
65904	2.02311	0.973496
65905	1.89226	1.42979
65906	1.89226	1.96121
65907	1.89226	2.1
65908	1.89226	1.89942
65909	1.89226	2.1
65910	1.89226	0.784143
65911	1.89226	2.1
65912	1.89226	2.1
65913	1.89226	2.1
65914	1.89226	2.1
65915	1.89226	2.1
65916	1.89226	1.98886
65917	1.99309	2.1
65918	1.96035	2.1
65919	1.72526	2.1
65920	1.72526	2.1
65921	2.16841	2.1
65922	2.16841	2.1
65923	2.15888	2.1
65924	2.16841	2.1
65925	2.3111	2.1
65926	2.35938	2.1
65927	4.51826	2.1
65928	1.89226	2.1
65929	1.89226	1.06556
65930	1.89226	1.41676
65931	1.89226	0.05
65932	3.78452	2.1
65933	1.89226	2.1
65934	1.89226	2.1
65935	1.89226	2.1
65936	1.89226	0.641734
65937	3.78452	2.1
65938	2.16841	2.1
65939	1.96035	2.1
65940	2.35938	2.1
65941	2.18493	2.1
65942	4.31973	2.1
65943	1.89226	2.1

65944	1.89226	2.1
65945	1.89226	2.1
65946	1.89226	2.1
65947	3.78453	2.1
65948	1.89226	2.1
65949	1.89226	2.1
65950	1.89226	2.1
65951	1.89226	2.1
65952	3.78452	2.1
65953	0.946158	2.1
65954	0.946158	2.1
65955	0.818509	2.1
65956	0.818509	2.1
65957	0.933975	2.1
65958	0.818539	2.1
65959	0.946128	2.1
65960	0.946128	2.1
65961	0.94613	2.1
65962	0.933974	2.1
65963	0.818537	2.1
65964	0.946129	2.1
65965	0.946131	2.1
65966	0.946131	2.1
65967	0.946131	2.1
65968	0.946131	2.1
65969	0.946132	2.1
65970	0.946132	2.1
65971	0.946129	2.1
65972	0.946129	2.1
65973	0.946131	2.1
65974	0.946131	2.1
65975	0.94613	2.1
65976	0.94613	2.1
65977	1.89226	2.1
65978	1.89226	2.1
65979	1.89226	2.1
65980	1.89226	2.1
65981	3.78453	2.1
65982	1.89226	2.1
65983	1.89226	2.1
65984	1.89226	2.1
65985	1.89226	2.1
65986	3.78452	2.1
65987	1.89226	2.1
65988	1.8706	2.1
65989	1.8706	2.1
65990	1.68112	2.1
65991	2.0815	2.1
65992	1.88715	2.1
65993	0.94613	2.1
65994	0.94613	2.1
65995	0.94613	2.1
65996	0.94613	2.1
65997	0.946131	2.1
65998	0.946131	2.1
65999	0.946129	2.1
66000	0.946129	2.1
66001	0.94613	2.1

66002	0.94613	2.1
66003	0.94613	2.1
66004	0.94613	2.1
66005	1.76401	2.1
66006	2.60983	2.1
66007	2.50461	2.1
66008	2.35897	2.1
66009	1.90965	2.1
66010	1.76401	2.1
66011	1.89226	2.1
66012	1.89226	2.1
66013	1.89226	2.1
66014	1.89226	2.1
66015	3.78452	2.1
66016	1.89226	2.1
66017	1.89226	2.1
66018	1.89226	2.1
66019	1.89226	2.02582
66020	1.89226	2.1
66021	1.89226	2.1
66022	1.90965	2.1
66023	2.83834	2.1
66024	2.7538	2.1
66025	2.60983	2.1
66026	2.05361	2.1
66027	1.90965	2.1
66028	1.89226	2.1
66029	1.89226	2.1
66030	1.89226	2.1
66031	1.89226	2.1
66032	1.89226	2.1
66033	1.89226	2.1
66034	1.89226	2.1
66035	1.89226	2.1
66036	1.89226	2.1
66037	1.89226	2.09806
66038	3.78452	2.1
66039	2.83834	2.1
66040	1.89226	2.1
66041	2.60983	2.1
66042	2.60983	2.1
66043	1.89226	2.1
66044	1.89226	2.1
66045	1.89226	2.1
66046	1.89226	2.1
66047	1.89226	2.1
66048	1.89226	2.1
66049	1.89226	2.1
66050	1.89226	2.1
66051	1.89226	2.07132
66052	1.89226	2.1
66053	1.89226	2.1
66054	1.89226	1.73309
66055	1.89226	2.1
66056	1.89226	1.89237
66057	2.44785	2.1
66058	1.80223	2.1
66059	2.80894	2.1

66060	2.18296	2.1
66061	4.63081	2.1
66062	1.89226	2.1
66063	1.89226	2.1
66064	1.89226	2.1
66065	1.89226	2.1
66066	1.89226	2.1
66067	1.89226	2.1
66068	0.946131	1.25854
66069	0.946131	2.1
66070	0.946127	2.1
66071	0.946127	2.1
66072	0.946131	2.08098
66073	0.946131	2.1
66074	0.946128	2.1
66075	0.946128	2.1
66076	0.94613	2.1
66077	0.94613	2.1
66078	0.946129	2.1
66079	0.946129	2.1
66080	1.89226	2.1
66081	1.89226	2.1
66082	1.89226	2.1
66083	1.89226	2.1
66084	3.78452	2.1
66085	1.89226	2.1
66086	1.89226	2.1
66087	1.89226	2.1
66088	1.89226	2.1
66089	3.78453	2.1
66090	2.41083	2.1
66091	2.93937	2.1
66092	1.68761	2.1
66093	1.68761	2.1
66094	1.67248	2.1
66095	2.3957	2.1
66096	1.89226	2.1
66097	1.89226	2.1
66098	1.89226	2.1
66099	1.89226	2.1
66100	1.89226	2.1
66101	1.89226	2.1
66102	1.89227	2.1
66103	1.89227	2.1
66104	1.89227	2.1
66105	1.89227	2.1
66106	1.89227	2.1
66107	1.89227	2.1
66108	1.89224	0.0997562
66109	1.89224	0.05
66110	1.89224	0.682451
66111	1.89224	0.372521
66112	3.78448	0.120808
66113	1.89224	1.10474
66114	1.89224	0.162264
66115	1.89224	1.30129
66116	1.89224	0.05
66117	1.89224	1.57004

66118	1.89224	0.251242
66119	1.11152	0.0799738
66120	1.29346	0.37974
66121	0.978596	0.371265
66122	0.918324	1.20165
66123	1.06821	1.13114
66124	1.06821	0.05
66125	0.985654	1.52501
66126	1.15278	1.14601
66127	1.07693	0.897072
66128	0.902432	1.13177
66129	0.968939	1.33369
66130	1.34644	0.403431
66131	0.946132	0.107045
66132	0.946132	0.267334
66133	0.946132	0.308572
66134	0.946132	0.0877735
66135	0.946131	0.211517
66136	0.946131	0.05
66137	0.946132	0.932596
66138	0.946132	0.0614541
66139	0.946132	0.326184
66140	0.946132	0.168135
66141	0.946132	0.0501769
66142	0.946132	0.0805069
66143	0.946133	0.194833
66144	0.946133	0.0516876
66145	0.946132	0.326536
66146	0.946132	1.01615
66147	0.946132	0.444387
66148	0.946132	0.05
66149	0.946133	2.01358
66150	0.946133	0.0702532
66151	0.946132	0.793147
66152	0.946132	0.289298
66153	0.946132	0.0634431
66154	0.946132	0.0569602
66155	1.89224	0.599141
66156	1.89224	2.1
66157	1.89224	2.05201
66158	1.89224	2.1
66159	1.89224	1.97541
66160	1.89224	2.1
66161	1.89224	2.01779
66162	1.89224	2.1
66163	1.89224	2.1
66164	1.89224	2.1
66165	1.89224	2.1
66166	1.89224	2.1
66167	1.89226	1.35652
66168	1.89226	2.1
66169	1.89226	2.1
66170	1.89226	2.1
66171	3.78453	2.1
66172	1.11056	2.1
66173	1.21595	2.1
66174	1.12763	2.1
66175	1.12763	2.1

66176	1.23339	2.1
66177	1.35697	2.1
66178	1.03581	2.1
66179	0.95954	2.1
66180	0.89761	2.1
66181	1.26602	2.1
66182	1.12749	2.1
66183	1.45307	2.1
66184	2.71775	2.1
66185	1.89226	2.1
66186	1.89226	2.1
66187	1.89226	2.1
66188	2.47025	2.1
66189	2.47025	2.1
66190	0.94613	2.1
66191	0.94613	2.1
66192	0.94613	2.1
66193	0.94613	2.1
66194	0.946128	2.1
66195	0.946128	2.1
66196	0.946132	2.1
66197	0.946132	2.1
66198	0.94613	2.1
66199	0.94613	2.1
66200	0.94613	2.1
66201	0.94613	2.1
66202	1.89226	0.56105
66203	1.89226	2.1
66204	1.89226	2.1
66205	1.89226	1.20555
66206	1.89226	2.1
66207	1.89226	1.5902
66208	1.89226	0.861313
66209	1.89226	2.1
66210	1.89226	2.1
66211	1.89226	0.727448
66212	1.89226	2.1
66213	1.89226	1.0839
66214	0.946132	2.1
66215	0.946132	2.1
66216	0.946133	2.1
66217	0.946133	2.1
66218	0.946133	2.1
66219	0.946133	2.1
66220	0.946132	2.05549
66221	0.946132	2.1
66222	0.946133	2.1
66223	0.946133	2.1
66224	0.946132	2.1
66225	0.946132	2.1
66226	1.89226	1.12822
66227	1.89226	2.1
66228	1.89226	2.1
66229	1.89226	1.67929
66230	1.89226	2.1
66231	1.89226	1.7606
66232	0.946131	1.47693
66233	0.946131	0.931087

66234	0.946127	2.1
66235	0.946127	2.1
66236	0.946131	1.42066
66237	0.946131	2.1
66238	0.946127	2.1
66239	0.946127	1.51685
66240	0.94613	2.06736
66241	0.94613	2.04035
66242	0.946129	1.99393
66243	0.946129	1.97971
66244	1.94291	2.1
66245	2.00118	2.1
66246	2.36337	2.1
66247	2.41217	2.1
66248	4.35508	2.1
66249	1.89226	2.1
66250	1.89226	2.1
66251	1.89226	2.1
66252	1.89226	2.1
66253	3.78452	2.1
66254	1.89226	2.1
66255	1.89226	2.1
66256	1.89226	2.1
66257	1.89226	2.1
66258	3.78452	2.1
66259	1.06209	2.1
66260	1.06209	2.1
66261	1.05987	2.1
66262	1.05987	2.1
66263	0.920378	2.1
66264	1.12137	2.1
66265	1.00059	2.1
66266	1.20608	2.1
66267	1.20608	2.1
66268	1.12137	2.1
66269	1.00059	2.1
66270	0.920376	2.1
66271	2.77222	2.1
66272	2.41216	2.1
66273	2.77002	2.1
66274	2.36337	2.1
66275	2.81882	2.1
66276	2.41216	2.1
66277	1.89226	2.1
66278	1.89226	2.1
66279	1.89226	2.1
66280	1.89226	2.1
66281	1.89226	2.1
66282	1.89226	2.1
66283	1.89226	2.1
66284	1.89226	2.1
66285	1.89226	2.1
66286	1.89226	2.1
66287	3.78451	2.1
66288	2.41216	2.1
66289	2.24273	2.1
66290	2.81882	2.1
66291	2.68473	2.1

66292	5.09689	2.1
66293	1.89226	2.1
66294	1.89226	2.1
66295	1.89226	2.1
66296	1.89226	2.1
66297	3.78453	2.1
66298	2.17614	2.1
66299	2.65326	2.1
66300	1.93101	2.1
66301	2.58552	2.1
66302	4.58427	2.1
66303	2.65326	2.1
66304	1.89226	2.1
66305	1.89226	2.1
66306	1.89226	2.1
66307	2.58552	2.1
66308	2.58552	2.1
66309	1.89226	2.1
66310	1.89226	2.1
66311	1.89226	2.1
66312	1.89226	2.1
66313	3.78453	2.1
66314	1.89226	2.1
66315	1.89226	2.1
66316	1.89226	2.1
66317	1.89226	2.1
66318	3.78453	2.1
66319	1.93101	2.1
66320	2.58552	2.1
66321	1.46507	2.1
66322	2.52893	2.1
66323	4.45994	2.1
66324	1.26446	2.1
66325	1.26446	2.1
66326	0.946132	2.1
66327	0.946132	2.1
66328	0.94613	2.1
66329	0.94613	2.1
66330	1.29276	2.1
66331	1.26446	2.1
66332	1.26446	2.1
66333	0.94613	2.1
66334	0.94613	2.1
66335	1.29276	2.1
66336	1.89226	2.1
66337	1.89226	2.1
66338	1.89226	2.1
66339	1.89226	2.1
66340	3.78453	2.1
66341	1.89226	2.1
66342	1.89226	2.1
66343	1.89226	2.1
66344	1.89226	2.1
66345	3.78453	2.1
66346	1.83378	2.1
66347	2.52893	2.1
66348	1.75592	2.1
66349	2.49844	2.1

66350	4.28485	2.1
66351	1.24922	2.1
66352	1.24922	2.1
66353	0.946133	2.1
66354	0.946133	2.1
66355	0.946131	2.1
66356	0.946131	2.1
66357	1.26446	2.1
66358	1.24922	2.1
66359	1.24922	2.1
66360	0.946131	2.1
66361	1.26446	2.1
66362	0.94613	2.1
66363	0.946132	2.1
66364	0.946132	2.1
66365	0.946132	2.1
66366	0.946132	2.1
66367	0.946132	2.1
66368	0.946132	2.1
66369	0.946132	2.1
66370	0.946132	2.1
66371	0.946132	2.1
66372	0.946132	2.1
66373	0.946131	2.1
66374	0.946131	2.1
66375	1.89226	2.1
66376	1.89226	2.1
66377	1.89226	2.1
66378	1.89226	2.1
66379	1.89226	2.1
66380	1.89226	2.1
66381	1.0465	2.1
66382	1.0465	2.1
66383	1.06047	2.1
66384	1.06047	2.1
66385	1.25687	2.1
66386	1.24922	2.1
66387	0.87796	2.1
66388	0.857749	2.1
66389	0.857749	2.1
66390	1.25687	2.1
66391	0.877959	2.1
66392	1.24922	2.1
66393	2.49843	2.1
66394	1.89226	2.1
66395	2.51374	2.1
66396	1.89226	2.1
66397	4.406 2.1	
66398	1.89226	2.1
66399	1.89226	2.1
66400	1.89226	2.1
66401	1.89226	2.1
66402	1.89226	2.1
66403	1.89226	2.1
66404	1.89226	1.89507
66405	1.89226	2.1
66406	1.89226	2.1
66407	1.89226	2.04709

66408	1.89226	2.1
66409	1.89226	2.1
66410	1.89226	2.1
66411	1.89226	2.1
66412	1.89226	2.1
66413	1.89226	2.04181
66414	1.89226	2.1
66415	1.89226	2.1
66416	1.03835	2.1
66417	1.03835	2.1
66418	1.15388	2.1
66419	0.94267	2.1
66420	0.736433	2.1
66421	0.849239	2.1
66422	1.15014	2.1
66423	1.39058	2.1
66424	0.838639	2.1
66425	1.39056	2.1
66426	1.09399	2.1
66427	0.736444	2.1
66428	1.89226	1.56978
66429	1.89226	2.1
66430	1.89226	2.1
66431	1.89226	1.4954
66432	1.89226	2.1
66433	1.89226	1.74595
66434	0.94613	1.59044
66435	0.94613	1.65556
66436	0.946127	2.1
66437	0.946127	2.1
66438	0.946131	2.1
66439	0.946131	1.26342
66440	0.946126	2.1
66441	0.946126	1.86809
66442	0.946128	2.1
66443	0.946128	2.1
66444	0.946128	2.1
66445	0.946128	2.1
66446	1.89226	2.1
66447	1.89226	1.19192
66448	1.89226	0.351098
66449	1.89226	2.1
66450	3.78453	1.43244
66451	0.946133	1.77155
66452	0.946133	0.349191
66453	0.946128	2.05452
66454	0.946128	1.73821
66455	0.946131	1.80596
66456	0.946131	1.70934
66457	0.946131	0.120008
66458	0.946131	1.69569
66459	0.946131	1.2901
66460	0.946131	1.24413
66461	0.94613	1.42301
66462	0.94613	1.48307
66463	1.89226	0.722554
66464	1.89226	2.1
66465	1.89226	2.08015

66466	1.89226	1.09925
66467	1.89226	2.07473
66468	1.89226	1.32789
66469	1.89226	0.76758
66470	1.89226	1.9989
66471	1.89226	1.60726
66472	1.89226	1.17087
66473	1.89226	1.66415
66474	1.89226	1.67464
66475	1.89226	0.58002
66476	1.89226	2.1
66477	1.89226	1.3514
66478	1.89226	1.42246
66479	1.89226	1.43291
66480	1.89226	1.72747
66481	1.89226	0.783573
66482	2.67388	2.1
66483	2.23684	2.1
66484	2.23684	1.5784
66485	1.89226	2.1
66486	1.89226	1.75655
66487	1.89226	2.1
66488	1.89226	2.1
66489	1.89226	2.1
66490	1.89226	2.1
66491	1.89226	2.1
66492	1.89226	2.1
66493	1.72197	2.1
66494	2.51374	2.1
66495	2.58004	2.1
66496	2.58004	2.1
66497	1.7155	2.1
66498	1.7155	2.1
66499	2.51374	2.1
66500	1.89226	2.1
66501	1.89226	2.1
66502	1.89226	2.1
66503	2.58003	2.1
66504	2.58003	2.1
66505	0.946132	2.1
66506	0.946132	2.1
66507	0.946132	2.1
66508	0.946132	2.1
66509	0.946132	2.1
66510	0.946132	2.1
66511	0.946132	2.1
66512	0.946132	2.1
66513	0.946131	2.1
66514	0.946131	2.1
66515	0.946132	2.1
66516	0.946132	2.1
66517	1.89226	2.1
66518	1.89226	2.1
66519	1.89226	2.1
66520	1.89226	2.1
66521	3.78453	2.1
66522	1.08262	2.1
66523	1.08262	2.1

66524	1.09674	2.1
66525	1.09674	2.1
66526	1.34652	2.1
66527	1.29002	2.1
66528	0.860984	2.1
66529	0.889338	2.1
66530	1.34652	2.1
66531	0.889338	2.1
66532	0.860983	2.1
66533	1.29002	2.1
66534	1.34652	2.1
66535	1.34652	2.1
66536	0.946133	2.1
66537	0.946133	2.1
66538	0.946131	2.1
66539	0.946131	2.1
66540	1.34652	2.1
66541	1.29002	2.1
66542	1.34652	2.1
66543	0.946131	2.1
66544	0.94613	2.1
66545	1.29002	2.1
66546	1.89226	2.1
66547	1.89226	2.1
66548	1.89226	2.1
66549	1.89226	2.1
66550	1.89226	2.1
66551	1.89226	2.1
66552	1.89226	2.1
66553	1.89226	2.1
66554	1.89226	2.1
66555	1.89226	2.1
66556	1.89226	2.1
66557	1.89226	2.1
66558	0.946131	2.1
66559	0.946131	2.1
66560	0.946131	2.1
66561	0.946131	2.1
66562	0.946131	2.1
66563	0.946131	2.1
66564	0.946131	2.1
66565	0.946131	2.1
66566	0.946131	2.1
66567	0.946131	2.1
66568	0.946131	2.1
66569	0.946131	2.1
66570	1.89226	2.1
66571	1.89226	2.1
66572	1.89226	2.1
66573	1.89226	2.1
66574	1.89226	2.1
66575	1.89226	2.1
66576	1.04103	2.1
66577	1.13849	2.1
66578	0.943014	2.1
66579	0.943014	2.1
66580	0.943591	2.1
66581	0.943591	2.1

66582	1.03757	2.1
66583	1.04098	2.1
66584	1.03761	2.1
66585	0.946131	2.1
66586	0.943041	2.1
66587	0.946131	2.1
66588	0.946128	2.1
66589	0.946128	2.1
66590	0.946133	2.1
66591	0.946133	2.1
66592	0.946131	2.1
66593	0.946131	2.1
66594	0.946131	2.1
66595	0.946131	2.1
66596	0.946131	2.1
66597	0.946131	2.1
66598	0.94613	2.1
66599	0.94613	2.1
66600	0.946132	2.1
66601	0.946132	2.1
66602	0.946132	2.1
66603	0.946132	2.1
66604	0.946132	2.1
66605	0.946132	2.1
66606	0.946132	2.1
66607	0.946132	2.1
66608	0.946132	2.1
66609	0.946132	2.1
66610	0.946131	2.1
66611	0.946131	2.1
66612	1.89226	0.949999
66613	1.89226	2.07542
66614	1.89226	1.08373
66615	1.89226	1.12476
66616	3.78452	1.55646
66617	0.946132	1.29496
66618	0.946132	1.57343
66619	0.946131	1.02634
66620	0.946131	0.859168
66621	0.946132	1.09375
66622	0.946132	2.1
66623	0.946132	1.3049
66624	0.946132	1.30838
66625	0.946132	1.27776
66626	0.946132	1.29367
66627	0.946132	1.37623
66628	0.946132	1.55506
66629	0.935482	1.88936
66630	1.08999	1.77046
66631	1.24315	1.43017
66632	1.12559	1.76187
66633	1.21343	1.31315
66634	0.889397	2.1
66635	1.01265	0.613086
66636	1.01265	1.97896
66637	1.07332	1.21958
66638	1.34292	1.63667
66639	0.865649	1.73873

66640	0.840024	2.03327
66641	1.15433	1.67673
66642	1.02858	1.97374
66643	1.34082	1.51242
66644	1.08357	1.57389
66645	1.10938	1.03977
66646	1.33349	2.1
66647	1.12232	1.9654
66648	1.12232	0.756875
66649	1.21357	1.5559
66650	1.3408	1.63591
66651	1.02859	1.81693
66652	1.24211	1.96492
66653	1.89226	1.38837
66654	1.89226	0.412919
66655	1.89226	0.980034
66656	1.89226	2.1
66657	1.89226	0.380735
66658	1.89226	1.5817
66659	1.89226	0.714462
66660	1.89226	1.1881
66661	1.89226	0.781366
66662	1.89226	1.8853
66663	1.89226	1.29705
66664	1.89226	1.86068
66665	2.0815	1.70007
66666	2.79029	1.4795
66667	2.36793	1.2744
66668	2.58428	2.1
66669	2.38161	0.583513
66670	2.59922	1.82878
66671	1.89226	2.1
66672	1.89226	1.85623
66673	1.89226	2.1
66674	1.89226	2.1
66675	1.89226	2.1
66676	1.89226	2.1
66677	1.89226	1.8878
66678	1.89226	0.329225
66679	1.89226	0.875346
66680	1.89226	1.16433
66681	3.78452	1.17027
66682	1.89226	2.1
66683	1.89226	1.12483
66684	1.89226	1.98963
66685	1.89226	1.85498
66686	1.89226	1.93671
66687	1.89226	1.85483
66688	0.946132	1.23059
66689	0.946132	2.09744
66690	0.94613	2.1
66691	0.94613	2.08804
66692	0.946131	2.1
66693	0.946131	1.16309
66694	0.946131	2.07942
66695	0.946131	2.1
66696	0.946131	2.1
66697	0.946131	2.1

66698	0.946131	2.1
66699	0.946131	2.1
66700	1.85178	2.06623
66701	1.89226	1.01368
66702	2.0815	0.591733
66703	2.27054	1.39782
66704	1.70303	0.307922
66705	1.5217	0.606501
66706	1.89226	2.1
66707	1.89226	0.947267
66708	1.89226	1.82618
66709	1.89226	1.2448
66710	1.89226	2.1
66711	1.89226	1.71352
66712	2.58823	0.154318
66713	2.02924	0.0838645
66714	1.79398	0.05
66715	1.79398	0.05
66716	2.76708	0.344195
66717	2.76708	0.156305
66718	0.946133	0.0985735
66719	0.946133	0.102338
66720	1.29411	0.103269
66721	1.38353	0.0864907
66722	0.946131	0.0744738
66723	0.946131	0.0899303
66724	1.38354	0.141868
66725	1.38354	0.0560109
66726	0.946131	0.0860596
66727	1.38354	0.104882
66728	0.94613	0.136466
66729	1.29411	0.0687589
66730	1.89226	0.0570259
66731	1.89226	0.05
66732	1.89226	0.100902
66733	1.89226	0.0752378
66734	3.78452	0.0908553
66735	0.946131	0.05
66736	0.946131	0.0629727
66737	0.946131	0.115525
66738	0.946131	0.0565654
66739	0.946129	0.0560167
66740	0.946129	0.05
66741	0.946133	0.123108
66742	0.946133	0.064159
66743	0.946131	0.180999
66744	0.946131	0.05
66745	0.946131	0.05
66746	0.946131	0.05
66747	1.89226	0.05
66748	1.89226	0.05
66749	1.89226	0.0730094
66750	1.89226	0.0704957
66751	3.78452	0.05
66752	0.94613	0.052178
66753	0.94613	0.05
66754	0.946131	0.05
66755	0.946131	0.0647663

66756	0.946128	0.0535379
66757	0.946128	0.05
66758	0.946133	0.0624428
66759	0.946133	0.0533315
66760	0.946131	0.0801397
66761	0.946131	0.05
66762	0.946131	0.0813133
66763	0.946131	0.05
66764	0.946129	0.266539
66765	0.946129	0.126267
66766	1.13486	0.089192
66767	0.859753	0.203282
66768	0.851541	0.180388
66769	0.851541	0.150497
66770	1.34346	0.130566
66771	1.26212	0.101491
66772	0.851553	0.105391
66773	1.12403	0.131969
66774	0.851474	0.0850701
66775	0.956163	0.130353
66776	0.946132	0.0894132
66777	0.946132	0.0611867
66778	0.946133	0.0822924
66779	0.946133	0.05
66780	0.946132	0.089343
66781	0.946132	0.05
66782	0.946133	0.05
66783	0.946133	0.0764366
66784	0.946133	0.105729
66785	0.946133	0.0593204
66786	0.946132	0.05
66787	0.946132	0.0573784
66788	1.89226	0.0541047
66789	1.89226	0.084969
66790	1.89226	0.115151
66791	1.89226	0.0861353
66792	3.78453	0.0642919
66793	0.946128	0.302164
66794	0.946128	0.216771
66795	0.946132	0.206393
66796	0.946132	0.185897
66797	0.94613	0.21525
66798	0.94613	0.232611
66799	0.94613	0.247051
66800	0.94613	0.205459
66801	0.946131	0.27866
66802	0.946131	0.204918
66803	0.94613	0.205487
66804	0.94613	0.265019
66805	0.946132	0.0838868
66806	0.946132	0.05
66807	0.946133	0.0821063
66808	0.946133	0.05
66809	0.946132	0.0676149
66810	0.946132	0.05
66811	0.946133	0.0595947
66812	0.946133	0.05
66813	0.946132	0.105937

66814	0.946132	0.05
66815	0.946132	0.0581742
66816	0.946132	0.05
66817	1.89226	0.0745247
66818	1.89226	0.0522971
66819	1.89226	0.164147
66820	1.89226	0.05
66821	1.89226	0.152357
66822	1.89226	0.05
66823	1.89226	0.131993
66824	1.89226	0.120679
66825	1.89226	0.0673481
66826	1.89226	0.109717
66827	1.89226	0.0913371
66828	1.89226	0.143479
66829	1.89226	2.1
66830	1.89226	2.1
66831	1.89226	2.1
66832	1.89226	2.1
66833	3.78452	2.1
66834	1.07652	2.1
66835	1.07652	2.1
66836	0.928749	2.1
66837	0.928749	2.1
66838	0.946132	2.1
66839	0.946132	2.1
66840	1.05914	2.1
66841	0.928746	2.1
66842	1.05914	2.1
66843	0.946131	2.1
66844	0.94613	2.1
66845	0.928747	2.1
66846	1.89226	2.1
66847	1.89226	2.1
66848	1.89226	2.1
66849	1.89226	2.1
66850	1.89226	2.1
66851	1.89226	2.1
66852	1.89227	2.1
66853	1.89227	2.1
66854	1.89227	2.1
66855	1.89227	2.1
66856	1.89227	2.1
66857	1.89227	2.1
66858	1.89226	2.1
66859	1.89226	2.1
66860	1.89226	2.1
66861	1.89226	2.1
66862	1.89226	2.1
66863	1.89226	2.1
66864	2.11828	2.1
66865	1.89226	2.1
66866	2.35845	2.1
66867	1.89226	2.1
66868	4.25071	2.1
66869	1.89226	2.1
66870	1.89226	2.1
66871	1.89226	2.1

66872	1.89226	2.1
66873	3.78453	2.1
66874	1.89226	2.1
66875	1.89226	2.1
66876	1.89226	2.1
66877	1.89226	2.1
66878	1.89226	2.1
66879	1.89226	2.1
66880	1.89226	0.71576
66881	1.89226	1.8387
66882	1.65573	1.70332
66883	1.65573	0.945145
66884	1.89226	1.65577
66885	1.89226	0.862063
66886	1.89226	0.551686
66887	1.89226	1.58387
66888	1.89226	1.41572
66889	1.89226	0.880471
66890	1.89226	1.45923
66891	1.89226	0.929407
66892	1.89226	0.653602
66893	1.89226	1.61859
66894	1.89226	1.56444
66895	1.89226	0.866922
66896	1.89226	1.58187
66897	1.89226	0.867938
66898	1.89226	0.525631
66899	1.89226	1.61974
66900	1.89226	1.41278
66901	1.89226	0.790781
66902	1.89226	1.42894
66903	1.89226	0.827851
66904	0.946128	0.905733
66905	0.946128	0.684356
66906	0.946125	1.32135
66907	0.946125	1.43607
66908	0.94613	0.801847
66909	0.94613	1.99026
66910	0.946124	1.66204
66911	0.946124	0.837602
66912	0.946127	1.19864
66913	0.946127	1.19996
66914	0.946127	1.21076
66915	0.946127	1.24898
66916	1.89226	0.700641
66917	1.89226	1.39319
66918	1.89226	0.475481
66919	1.89226	0.271811
66920	3.78452	0.71594
66921	1.89226	1.16018
66922	1.89226	1.26248
66923	1.89226	0.53268
66924	1.89226	1.17158
66925	3.78453	1.04617
66926	1.05926	1.84134
66927	0.891358	2.1
66928	0.946129	1.42725
66929	0.946129	1.95323

66930	0.946101	1.2843
66931	0.946101	2.1
66932	0.891386	1.59071
66933	0.891386	1.78449
66934	0.891357	1.80304
66935	0.946129	1.63367
66936	0.94613	1.79505
66937	1.05926	1.76046
66938	0.946126	0.811764
66939	0.946126	1.18037
66940	0.94613	0.872079
66941	0.94613	1.36372
66942	0.946131	0.950504
66943	0.946131	1.30619
66944	0.946125	1.36163
66945	0.946125	0.275439
66946	0.946127	0.994563
66947	0.946127	0.737147
66948	0.946128	1.29895
66949	0.946128	1.12396
66950	1.89226	0.739261
66951	1.89226	0.304082
66952	1.89226	0.196289
66953	1.89226	1.1925
66954	1.89226	0.302624
66955	1.89226	1.57953
66956	1.89226	0.809359
66957	1.89226	1.51168
66958	1.89226	1.19501
66959	1.89226	1.61153
66960	1.89226	0.732316
66961	1.89226	1.58504
66962	1.16109	2.06147
66963	1.23243	2.1
66964	0.659819	1.60439
66965	0.659819	2.05343
66966	0.697965	1.03771
66967	1.00087	2.1
66968	0.891386	2.03903
66969	0.891386	1.66862
66970	0.946129	1.94824
66971	0.65979	1.40087
66972	0.891357	1.90729
66973	0.946129	1.77192
66974	1.89225	0.386363
66975	1.89225	1.14392
66976	1.89225	1.59184
66977	1.89225	0.226678
66978	1.89225	2.04807
66979	1.89225	0.623874
66980	1.79981	0.0926979
66981	2.02514	0.0849593
66982	2.33247	0.216608
66983	2.12891	0.05
66984	4.15405	0.059339
66985	2.48284	0.536904
66986	2.02514	0.0710631
66987	2.33247	0.118959

66988	2.02514	0.288293
66989	2.48567	0.1134
66990	2.17834	0.266162
66991	1.89226	0.0609252
66992	1.89226	0.16674
66993	1.89226	0.246487
66994	1.89226	0.0800984
66995	3.78453	0.080009
66996	1.89227	0.0700439
66997	1.89227	0.05
66998	1.89227	0.05
66999	1.89227	0.0998641
67000	1.89227	0.05
67001	1.89227	0.142015
67002	2.50894	0.05
67003	2.70118	0.127972
67004	2.12891	0.05
67005	2.12891	0.097882
67006	2.7125	0.0566976
67007	2.33247	0.0864579
67008	1.89226	0.21173
67009	1.89226	0.0775575
67010	1.89226	0.0687067
67011	1.89226	0.17917
67012	1.89226	0.0545186
67013	1.89226	0.124856
67014	0.946133	0.0774012
67015	0.946133	0.0919777
67016	0.946132	0.05
67017	0.946132	0.0652581
67018	0.946132	0.05
67019	0.946132	0.122211
67020	0.946133	0.05
67021	0.946133	0.10357
67022	0.946132	0.05
67023	0.946132	0.0835754
67024	0.946132	0.07361
67025	0.946132	0.0587071
67026	1.90098	0.05
67027	1.36463	0.05
67028	1.56656	0.05
67029	1.36463	0.05
67030	2.04386	0.05
67031	1.89227	0.05
67032	1.89226	0.05
67033	1.89226	0.05
67034	1.89226	0.05
67035	1.89226	0.0675406
67036	1.89226	0.05
67037	1.89226	0.05
67038	1.89226	0.077194
67039	1.89226	0.0595211
67040	1.89226	0.0739899
67041	1.89226	0.110027
67042	1.89226	0.0524999
67043	1.89226	0.0840401
67044	1.89226	0.0568996
67045	1.89226	0.05

67046	1.89226	0.0662984
67047	1.89226	0.0807973
67048	1.89226	0.05
67049	1.89226	0.0645183
67050	1.89226	0.05
67051	1.70303	0.05
67052	2.1292	0.05
67053	1.70303	0.05
67054	2.32302	0.05
67055	1.90098	0.05
67056	1.89226	0.05
67057	1.89226	0.05
67058	1.89226	0.05
67059	1.89226	0.05
67060	1.89226	0.05
67061	1.89226	0.05
67062	1.89226	0.0584959
67063	1.89226	0.0595388
67064	1.89226	0.0654011
67065	1.89226	0.0762881
67066	1.89226	0.0524452
67067	1.89226	0.064445
67068	1.89226	0.05
67069	1.89226	0.0530614
67070	1.89226	0.0553316
67071	1.89226	0.0579959
67072	1.89226	0.05
67073	1.89226	0.05
67074	1.89226	0.291181
67075	1.89226	1.50712
67076	1.89226	1.45304
67077	1.89226	0.484928
67078	3.78453	0.284539
67079	1.89226	1.18064
67080	1.89226	2.1
67081	1.89226	0.395188
67082	1.89226	2.1
67083	1.89226	1.31697
67084	1.89226	2.1
67085	1.89226	1.50969
67086	1.89226	0.442447
67087	1.89226	0.388324
67088	1.89226	0.717383
67089	3.78452	1.14545
67090	1.89224	1.46994
67091	1.89224	0.318087
67092	1.89224	0.798698
67093	1.89224	1.62135
67094	1.89224	0.383007
67095	1.89224	0.655603
67096	1.89224	1.19663
67097	1.89224	2.1
67098	1.89224	1.79134
67099	1.89224	2.1
67100	1.89224	0.387095
67101	1.89224	2.1
67102	1.31307	2.1
67103	1.31307	2.1

67104	0.888948	1.60172
67105	0.888948	1.19499
67106	1.2559	1.80603
67107	0.888979	2.1
67108	0.946114	2.1
67109	0.946114	1.09844
67110	0.946129	0.787159
67111	0.888989	1.51549
67112	0.946104	0.442029
67113	1.25588	1.12705
67114	1.89224	0.241558
67115	1.89224	1.70209
67116	1.89224	0.372344
67117	1.89224	1.45804
67118	1.89224	1.00465
67119	1.89224	2.01102
67120	1.79375	0.05
67121	1.47689	0.05
67122	1.97732	0.05
67123	2.26687	0.05
67124	3.74375	0.05
67125	2.05759	0.05
67126	2.26686	0.05
67127	1.79374	0.05
67128	1.79374	0.0509243
67129	2.49889	0.05
67130	2.49889	0.0577518
67131	1.89226	0.0579049
67132	1.89226	0.0500038
67133	1.89226	0.05
67134	1.89226	0.054878
67135	3.78452	0.0590162
67136	1.89227	0.05
67137	1.89227	0.05
67138	1.89227	0.05
67139	1.89227	0.05
67140	1.89227	0.05
67141	1.89227	0.05
67142	1.97732	0.05
67143	2.26687	0.05
67144	2.6607	0.05
67145	2.41464	0.05
67146	4.68151	0.05
67147	1.89226	0.05
67148	1.89226	0.0663361
67149	1.89226	0.0618449
67150	1.89226	0.05
67151	3.78452	0.05
67152	1.89226	0.05
67153	1.89226	0.05
67154	1.89226	0.05
67155	1.89226	0.0573845
67156	1.89226	0.05
67157	1.89226	0.0553569
67158	1.89226	0.624349
67159	1.89226	1.52613
67160	1.89226	0.924864
67161	1.89226	0.60781

67162	3.78452	0.760623
67163	1.89226	0.724946
67164	1.89226	1.81966
67165	1.89226	0.548986
67166	1.89226	1.40838
67167	1.89226	0.823087
67168	1.89226	1.69958
67169	1.89226	1.58549
67170	1.89226	0.497544
67171	1.89226	0.53981
67172	1.89226	1.38609
67173	1.89226	0.611384
67174	1.89226	1.36301
67175	1.89226	0.968332
67176	1.89226	0.536185
67177	1.89226	0.459541
67178	1.89226	1.42822
67179	1.89226	0.550136
67180	1.89226	1.4822
67181	1.89226	0.792413
67182	2.45282	1.28446
67183	1.89226	1.47988
67184	1.89226	1.83792
67185	1.89226	0.520807
67186	1.89226	1.19035
67187	1.89226	0.575173
67188	1.89226	1.51624
67189	1.89226	0.604859
67190	1.89226	0.763012
67191	1.89226	1.59311
67192	1.89226	2.00336
67193	1.87308	0.05
67194	1.88631	0.05
67195	1.63908	0.05
67196	1.70302	0.05
67197	2.08745	0.05
67198	2.16916	0.05
67199	2.41019	0.05
67200	2.733	0.05
67201	2.08121	0.05
67202	2.08121	0.05
67203	2.74362	0.05
67204	2.41464	0.05
67205	1.89226	0.05
67206	1.89226	0.05
67207	1.89226	0.05
67208	1.89226	0.05
67209	1.89226	0.05
67210	1.89226	0.05
67211	0.946133	0.05
67212	0.946133	0.05
67213	0.946132	0.05
67214	0.946132	0.05
67215	0.946132	0.05
67216	0.946132	0.05
67217	0.946133	0.05
67218	0.946133	0.05
67219	0.946132	0.05

67220	0.946132	0.05
67221	0.946133	0.05
67222	0.946133	0.05
67223	2.02912	0.05
67224	2.57016	0.05
67225	1.88631	0.05
67226	1.88631	0.05
67227	2.55301	0.05
67228	2.4102	0.05
67229	1.89226	0.05
67230	1.89226	0.05
67231	1.89226	0.05
67232	1.89226	0.05
67233	3.78452	0.05
67234	1.89227	0.05
67235	1.89227	0.05
67236	1.89227	0.05
67237	1.89227	0.05
67238	1.89227	0.05
67239	1.89227	0.05
67240	1.89226	2.1
67241	1.89226	2.1
67242	1.89226	2.1
67243	1.89226	2.1
67244	3.78452	2.1
67245	1.2515	2.1
67246	1.34788	2.1
67247	0.946132	2.1
67248	0.946132	2.1
67249	0.973043	2.1
67250	1.16766	2.1
67251	0.919222	2.1
67252	1.2159	2.1
67253	0.946132	2.1
67254	0.946132	2.1
67255	1.33746	2.1
67256	1.13537	2.1
67257	1.89226	2.1
67258	1.89226	2.1
67259	1.89226	2.1
67260	1.89226	2.1
67261	1.89226	2.1
67262	1.89226	2.1
67263	1.89226	2.1
67264	1.89226	2.1
67265	1.89226	2.1
67266	1.89226	2.1
67267	3.78452	2.1
67268	1.89227	2.1
67269	1.89227	1.916
67270	1.89227	2.1
67271	1.89227	2.1
67272	1.89227	2.1
67273	1.89227	2.1
67274	0.946132	2.1
67275	0.946132	2.1
67276	0.946129	2.1
67277	0.946129	2.1

67278	0.946131	2.1
67279	0.946131	2.1
67280	0.946131	2.1
67281	0.946131	2.1
67282	0.946131	2.1
67283	0.946131	2.1
67284	0.94613	2.1
67285	0.94613	2.1
67286	1.89226	1.28033
67287	1.89226	1.13913
67288	1.89226	1.23223
67289	1.89226	0.931487
67290	3.78453	1.11828
67291	0.946132	1.72728
67292	0.946132	1.05955
67293	0.946132	1.23946
67294	0.946132	1.1826
67295	0.946132	0.523289
67296	0.946132	1.82393
67297	0.946132	1.20066
67298	0.946132	1.27042
67299	0.946131	1.87101
67300	0.946131	0.900333
67301	0.946132	1.32234
67302	0.946132	1.12108
67303	1.89226	1.37631
67304	1.89226	0.646121
67305	1.89226	0.597938
67306	1.89226	0.897058
67307	1.89226	1.20249
67308	1.89226	1.36666
67309	1.89226	1.5514
67310	1.89226	0.8699
67311	1.89226	0.670305
67312	1.89226	0.855056
67313	3.78453	0.911691
67314	0.96614	1.12749
67315	0.96614	1.20026
67316	0.946131	0.395777
67317	0.946131	0.813593
67318	0.946131	0.901393
67319	0.96614	0.615806
67320	1.37736	1.23175
67321	0.946131	1.44508
67322	1.37736	1.69538
67323	0.96614	0.577197
67324	0.946131	1.41568
67325	0.946131	0.547434
67326	0.946129	1.37859
67327	0.946129	0.922513
67328	0.946128	1.12639
67329	0.946128	1.32563
67330	0.946132	0.504129
67331	0.946132	1.7907
67332	0.946126	1.26238
67333	0.946126	1.01338
67334	0.946129	0.62028
67335	0.946129	0.851816

67336	0.946129	0.550768
67337	0.946129	1.12429
67338	1.89226	0.05
67339	1.65723	0.05
67340	1.83117	0.05
67341	1.65723	0.05
67342	2.0662	0.05
67343	1.89226	0.05
67344	1.89226	0.05
67345	1.89226	0.05
67346	1.89226	0.05
67347	1.89226	0.05
67348	1.89226	0.05
67349	1.89226	0.05
67350	1.89226	0.05
67351	1.89226	0.05
67352	1.89226	0.0592786
67353	1.89226	0.0593672
67354	1.89226	0.05
67355	1.89226	0.05
67356	1.89226	0.05
67357	1.89226	0.05
67358	1.89226	0.05
67359	1.89226	0.05
67360	1.89226	0.05
67361	1.89226	0.05
67362	1.89226	0.05
67363	1.83117	0.05
67364	1.92807	0.05
67365	1.83117	0.05
67366	1.98916	0.05
67367	1.89226	0.05
67368	1.89226	0.05
67369	1.89226	0.05
67370	1.89226	0.05
67371	1.89226	0.05
67372	3.78453	0.05
67373	1.89226	0.05
67374	1.89226	0.0555209
67375	1.89226	0.0799182
67376	1.89226	0.0888163
67377	1.89226	0.05
67378	1.89226	0.05
67379	1.89226	0.05
67380	1.89226	0.05
67381	1.89226	0.0571412
67382	1.89226	0.0539264
67383	1.89226	0.05
67384	1.89226	0.05
67385	1.88037	0.05
67386	1.89227	0.05
67387	1.89227	0.05
67388	1.89227	0.05
67389	1.98599	0.05
67390	1.98599	0.05
67391	1.89226	0.05
67392	1.89226	0.05
67393	1.89226	0.0509307

67394	1.89226	0.0531232
67395	1.89226	0.05
67396	1.89226	0.05
67397	1.89226	0.05
67398	1.89226	0.0549916
67399	1.89226	0.0566033
67400	1.89226	0.0809903
67401	3.78452	0.05
67402	1.89226	0.05
67403	1.89226	0.0594303
67404	1.89226	0.05
67405	1.89226	0.05
67406	1.89226	0.05
67407	1.89226	0.05
67408	1.90116	0.05
67409	1.89227	0.05
67410	1.89226	0.05
67411	1.89227	0.05
67412	1.88036	0.05
67413	1.88037	0.05
67414	1.89226	0.05
67415	1.89226	0.05
67416	1.89226	0.0500215
67417	1.89226	0.05
67418	1.89226	0.05
67419	1.89226	0.05
67420	0.946132	0.0644116
67421	0.946132	0.0602025
67422	0.946128	0.0608304
67423	0.946128	0.0598223
67424	0.94613	0.064973
67425	0.94613	0.065064
67426	0.94613	0.0551356
67427	0.94613	0.0582409
67428	0.946131	0.065179
67429	0.946131	0.0728131
67430	0.94613	0.05
67431	0.94613	0.0768739
67432	1.89226	0.05
67433	1.89226	0.0555679
67434	1.89226	0.0536685
67435	1.89226	0.0537512
67436	1.89226	0.0565425
67437	1.89226	0.056175
67438	1.89226	0.223825
67439	1.89226	1.50408
67440	2.35239	1.42025
67441	2.27873	0.305385
67442	1.89226	1.32068
67443	1.83301	0.332263
67444	2.68449	0.480948
67445	1.89226	2.1
67446	1.89226	0.770855
67447	1.89226	1.04951
67448	3.78453	0.845701
67449	0.946132	2.04206
67450	0.946132	0.84812
67451	0.946129	0.635777

67452	0.946129	1.98147
67453	0.946131	1.98618
67454	0.946131	1.45406
67455	0.94613	0.807945
67456	0.94613	1.16135
67457	0.946131	1.5324
67458	0.946131	1.05377
67459	0.946131	1.1021
67460	0.946131	1.58792
67461	1.89226	0.294386
67462	1.89226	1.11711
67463	1.89226	1.5639
67464	1.89226	0.373007
67465	1.89226	1.47457
67466	1.89226	0.406565
67467	1.70303	0.267128
67468	1.89226	1.41657
67469	1.89226	0.277541
67470	1.89226	0.500867
67471	3.78453	0.39312
67472	1.89227	1.11594
67473	1.89227	1.32693
67474	1.89227	0.425937
67475	1.89227	1.89935
67476	3.78453	1.27547
67477	1.89226	0.918866
67478	1.89226	2.02623
67479	1.89226	1.13294
67480	1.89226	1.70292
67481	3.78452	1.52683
67482	1.89226	2.1
67483	1.89226	2.1
67484	1.89226	2.1
67485	1.89226	2.1
67486	3.78452	2.1
67487	0.946132	1.90572
67488	0.946132	2.1
67489	0.946129	2.1
67490	0.946129	2.1
67491	0.94613	2.1
67492	0.94613	2.09424
67493	0.946131	2.1
67494	0.946131	2.1
67495	0.946131	2.1
67496	0.946131	2.1
67497	0.946131	2.1
67498	0.946131	2.07247
67499	1.89226	2.1
67500	1.89226	2.01903
67501	1.89226	2.09977
67502	1.89226	2.1
67503	3.78452	2.1
67504	1.89226	1.62752
67505	1.89226	2.1
67506	1.89226	2.1
67507	1.89226	1.69728
67508	1.89226	2.1
67509	1.89226	1.63188

67510	1.89227	2.1
67511	2.48308	1.22483
67512	1.89227	1.72218
67513	1.89227	2.09893
67514	3.78453	2.1
67515	1.89226	1.99307
67516	1.89226	2.1
67517	1.89226	2.1
67518	1.89226	1.90252
67519	1.89226	2.1
67520	1.89226	1.76244
67521	1.89226	1.99376
67522	1.89226	1.95922
67523	1.89226	2.1
67524	1.89226	1.95334
67525	3.78453	2.1
67526	0.946131	1.99356
67527	0.946131	2.1
67528	0.946131	2.1
67529	0.946131	2.1
67530	0.946131	2.1
67531	0.946131	2.1
67532	0.946131	2.1
67533	0.946131	2.1
67534	0.946131	2.1
67535	0.946131	2.1
67536	0.946131	2.1
67537	0.946131	2.02959
67538	1.23719	2.1
67539	1.28439	2.02002
67540	0.94613	1.92653
67541	0.94613	2.05649
67542	1.28439	2.1
67543	1.6431	2.1
67544	0.946129	1.9502
67545	0.946129	1.83622
67546	1.28439	1.54132
67547	0.946131	1.33718
67548	1.21038	1.23471
67549	1.21038	1.55572
67550	1.89226	2.05492
67551	1.89226	1.84437
67552	1.89226	1.9369
67553	1.89226	1.91754
67554	1.89226	1.84977
67555	1.89226	1.84401
67556	1.89226	1.99331
67557	1.89226	2.07541
67558	1.89226	2.1
67559	1.89226	1.78042
67560	1.89226	2.1
67561	1.89226	1.61419
67562	1.89226	1.32164
67563	1.89226	0.155881
67564	1.89226	0.201388
67565	1.89226	0.559891
67566	1.89226	0.482796
67567	1.89226	1.27555

67568	0.946128	0.201489
67569	0.946128	0.627206
67570	0.946125	0.0622612
67571	0.946125	0.211125
67572	0.94613	0.0899135
67573	0.94613	0.208926
67574	0.946124	0.367124
67575	0.946124	0.719668
67576	0.946127	0.111037
67577	0.946127	0.0665385
67578	0.946127	0.310419
67579	0.946127	0.603921
67580	1.89226	0.0587284
67581	2.78671	0.0726428
67582	2.59302	0.0637947
67583	2.59302	0.0616331
67584	1.89226	0.142793
67585	1.89226	0.0900485
67586	1.89226	0.14412
67587	1.89226	0.220207
67588	1.89226	0.1076
67589	1.89226	0.108683
67590	1.89226	0.335078
67591	1.89226	0.223131
67592	1.89225	0.0921892
67593	1.89225	0.05
67594	1.89225	0.156708
67595	1.89225	0.103641
67596	1.89225	0.247388
67597	1.89225	0.0687936
67598	0.979536	0.0531526
67599	0.816597	0.0583483
67600	1.15446	0.05
67601	1.07423	0.05
67602	1.08452	0.0633448
67603	0.603522	0.05
67604	1.70302	0.05
67605	1.29344	0.05
67606	1.21806	0.05
67607	0.723758	0.05
67608	1.37562	0.0552642
67609	1.89226	0.05
67610	1.92807	0.05
67611	1.90843	0.05
67612	1.92807	0.05
67613	1.87263	0.05
67614	1.89226	0.05
67615	0.946132	0.05
67616	0.946132	0.05
67617	0.946131	0.05
67618	0.946131	0.05
67619	0.946132	0.05
67620	0.946132	0.05
67621	0.946132	0.05
67622	0.946132	0.05
67623	0.946132	0.05
67624	0.946132	0.0506259
67625	0.946132	0.05

67626	0.946132	0.05
67627	1.89226	0.05
67628	1.89226	0.086903
67629	1.89226	0.0570889
67630	1.89226	0.0875377
67631	3.78452	0.0583647
67632	1.89226	0.05
67633	1.89226	0.0626121
67634	1.89226	0.0623958
67635	1.89226	0.0605102
67636	1.89226	0.05
67637	1.89226	0.05
67638	1.70303	0.05
67639	1.90843	0.05
67640	1.77002	0.05
67641	1.90843	0.05
67642	1.75709	0.05
67643	1.89226	0.05
67644	1.89226	0.05
67645	1.89226	0.05
67646	1.89226	0.05
67647	1.89226	0.05
67648	3.78453	0.05
67649	1.89226	0.0500127
67650	1.89226	0.0902018
67651	1.89226	0.05
67652	1.89226	0.106976
67653	3.78452	0.0836876
67654	0.946131	0.05
67655	0.946131	0.05
67656	0.946131	0.05
67657	0.946131	0.05
67658	0.946129	0.0543139
67659	0.946129	0.0515256
67660	0.946133	0.05
67661	0.946133	0.05
67662	0.946131	0.0633854
67663	0.946131	0.05
67664	0.946131	0.05
67665	0.946131	0.0545451
67666	1.89226	0.0535427
67667	2.10275	0.353528
67668	1.76377	0.246433
67669	1.76377	0.0951576
67670	1.89226	0.158516
67671	1.89226	0.0645799
67672	1.89226	0.0534535
67673	1.89226	0.331411
67674	2.10275	0.297672
67675	2.55889	0.0777038
67676	1.89226	0.209386
67677	2.30274	0.0653069
67678	1.89226	0.143274
67679	1.89226	0.932437
67680	1.89226	0.863754
67681	1.89226	0.173334
67682	1.89226	0.673403
67683	1.89226	0.154278

67684	1.89226	0.107792
67685	1.89226	0.993127
67686	1.89226	0.775851
67687	1.89226	0.207761
67688	1.89226	0.587386
67689	1.89226	0.173458
67690	1.89226	0.05
67691	1.89226	0.308492
67692	1.89226	0.280825
67693	1.89226	0.0691585
67694	1.89226	0.198058
67695	1.89226	0.0604703
67696	1.89226	0.0513652
67697	1.89226	0.325442
67698	1.89226	0.259432
67699	1.89226	0.0843056
67700	1.89226	0.193116
67701	1.89226	0.0732362
67702	1.89226	0.150753
67703	1.89226	0.880541
67704	1.89226	0.793643
67705	1.89226	0.214883
67706	1.89226	0.690689
67707	1.89226	0.203384
67708	1.89226	0.636539
67709	1.89226	0.906926
67710	1.89226	0.163916
67711	1.89226	0.28286
67712	1.89226	0.14473
67713	1.89226	0.566962
67714	0.946131	0.05
67715	0.976399	0.05
67716	1.10124	0.05
67717	0.946131	0.132441
67718	0.887633	0.114721
67719	0.887633	0.0528981
67720	1.16933	0.05
67721	1.23163	0.05
67722	0.946131	0.063043
67723	0.946131	0.050975
67724	0.976399	0.05
67725	1.14992	0.05
67726	1.89226	0.066843
67727	1.89226	0.484839
67728	1.89226	0.0759002
67729	1.89226	0.118513
67730	3.78452	0.0874624
67731	1.89226	0.05
67732	1.89226	0.179359
67733	1.89226	0.05
67734	1.89226	0.0589029
67735	3.78453	0.05
67736	0.946132	0.05
67737	0.946132	0.0718478
67738	0.946131	0.05
67739	0.946131	0.073827
67740	0.946131	0.0733516
67741	0.946131	0.05

67742	0.946132	0.05
67743	0.946132	0.05
67744	0.946131	0.05
67745	0.946131	0.05
67746	0.946132	0.05
67747	0.946132	0.05
67748	0.946132	0.117538
67749	0.946132	0.05
67750	0.946131	0.0527246
67751	0.946131	0.185101
67752	0.946131	0.189128
67753	0.946131	0.0952254
67754	0.946132	0.0595956
67755	0.946132	0.05
67756	0.946131	0.0770076
67757	0.946131	0.0856702
67758	0.946132	0.0742715
67759	0.946132	0.0670199
67760	1.89226	0.152541
67761	1.89226	0.267723
67762	1.89226	0.0557057
67763	1.89226	0.387486
67764	3.78452	0.212491
67765	1.89226	0.892636
67766	1.89226	0.703629
67767	1.89226	0.635364
67768	1.89226	0.355847
67769	3.78453	0.396414
67770	1.89226	0.222142
67771	1.89226	0.836064
67772	1.89226	0.626594
67773	1.89226	0.990797
67774	1.89226	0.257763
67775	1.89226	0.438282
67776	1.89225	0.220644
67777	1.89225	0.373677
67778	1.89225	0.380722
67779	1.89225	0.879792
67780	1.89225	0.129146
67781	1.89225	0.284462
67782	1.89226	0.384683
67783	1.89226	0.521637
67784	1.89226	0.438419
67785	1.89226	0.761406
67786	1.89226	0.182228
67787	1.89226	0.304486
67788	1.89226	1.00061
67789	1.89226	0.347113
67790	1.89226	0.175254
67791	1.89226	0.0945418
67792	3.78453	0.23337
67793	1.39491	0.15771
67794	1.39491	0.154584
67795	0.924649	0.137758
67796	0.924649	0.157468
67797	0.946131	0.30518
67798	1.37343	0.0850483
67799	0.924678	0.05

67800	0.946131	1.02047
67801	0.924678	0.0978294
67802	1.37343	0.0835204
67803	0.946131	0.498003
67804	0.946131	0.496121
67805	1.89226	0.382632
67806	1.89226	0.0786637
67807	1.89226	0.180243
67808	1.89226	0.264057
67809	1.89226	0.0742388
67810	1.89226	0.108712
67811	0.946126	0.05
67812	0.975461	0.05
67813	0.946132	0.05
67814	1.12674	0.05
67815	0.946129	0.05
67816	0.946129	0.05
67817	1.12673	0.05
67818	1.12673	0.05
67819	0.946129	0.05
67820	0.946129	0.05
67821	0.975464	0.05
67822	1.12673	0.05
67823	1.89226	0.05
67824	1.89226	0.05
67825	1.89226	0.05
67826	1.89226	0.05
67827	1.89226	0.05
67828	1.89226	0.05
67829	0.946126	0.05
67830	0.946126	0.05
67831	0.946131	0.05
67832	0.946131	0.05
67833	0.946129	0.05
67834	0.946129	0.05
67835	0.946128	0.05
67836	0.946128	0.05
67837	0.946129	0.05
67838	0.946129	0.05
67839	0.946128	0.05
67840	0.946128	0.05
67841	1.89226	0.05
67842	1.89226	0.05
67843	1.89226	0.05
67844	1.89226	0.05
67845	3.78451	0.05
67846	1.89226	0.05
67847	1.89226	0.05
67848	1.89226	0.05
67849	1.89226	0.05
67850	3.78452	0.05
67851	0.946125	0.05
67852	0.946125	0.05
67853	0.946131	0.05
67854	0.946131	0.05
67855	0.946128	0.05
67856	0.946128	0.05
67857	0.946128	0.05

67858	0.946128	0.05
67859	0.946128	0.05
67860	0.946128	0.05
67861	0.946128	0.05
67862	0.946128	0.05
67863	1.89226	0.05
67864	1.89226	0.107269
67865	1.89226	0.0729876
67866	1.89226	0.05
67867	3.78452	0.0634037
67868	1.89226	0.05
67869	1.89226	0.0822579
67870	1.89226	0.0717063
67871	1.89226	0.0911453
67872	1.89226	0.05
67873	1.89226	0.0644051
67874	1.12492	0.0566757
67875	1.41201	0.0531335
67876	1.12082	0.0506737
67877	0.771416	0.0590491
67878	1.10725	0.05
67879	1.10725	0.058445
67880	1.07618	0.114856
67881	1.07618	0.05
67882	1.12492	0.0523582
67883	0.771415	0.0666228
67884	1.12082	0.0555674
67885	1.41201	0.0668452
67886	2.82401	0.05
67887	1.89226	0.0725713
67888	1.89226	0.05
67889	1.89226	0.0556228
67890	2.24983	0.05191
67891	2.24983	0.0625658
67892	0.946133	0.0776714
67893	0.946133	0.0650452
67894	0.946129	0.05
67895	0.946129	0.0552062
67896	0.946131	0.0690868
67897	0.946131	0.112495
67898	0.946131	0.0761729
67899	0.946131	0.05
67900	0.946131	0.0541475
67901	0.946131	0.0692983
67902	0.946131	0.0692849
67903	0.946131	0.0827884
67904	0.946132	0.0773377
67905	0.946132	0.0839052
67906	0.946133	0.0697035
67907	0.946133	0.063142
67908	0.946132	0.0738406
67909	0.946132	0.0951174
67910	0.946133	0.0559821
67911	0.946133	0.0637206
67912	0.946132	0.0711975
67913	0.946132	0.0549399
67914	0.946133	0.0829522
67915	0.946133	0.0631654

67916	1.89227	0.0692277
67917	1.89227	0.12472
67918	1.89227	0.134144
67919	1.89227	0.05
67920	1.89227	0.136473
67921	1.89227	0.05
67922	1.42982	0.05
67923	1.19944	0.05
67924	0.936311	0.05
67925	0.936311	0.05
67926	0.946132	0.05
67927	1.02993	0.05
67928	1.41513	0.05
67929	0.946133	0.05
67930	0.946133	0.05
67931	0.946133	0.05
67932	1.02993	0.05
67933	1.18181	0.05
67934	1.89226	0.0789756
67935	1.89226	0.165596
67936	1.89226	0.295459
67937	1.89226	0.0520192
67938	3.78453	0.0922529
67939	1.89226	0.05
67940	1.89226	0.05
67941	1.89226	0.05
67942	1.89226	0.05
67943	1.89226	0.05
67944	1.89226	0.05
67945	1.89226	0.05
67946	1.89226	0.05
67947	1.89226	0.05
67948	1.89226	0.05
67949	1.89226	0.05
67950	1.89226	0.05
67951	0.946131	0.05
67952	0.946131	0.0811012
67953	0.946132	0.159083
67954	0.946132	0.115564
67955	0.946132	0.0593323
67956	0.946132	0.0979248
67957	0.946132	0.170296
67958	0.946132	0.0540278
67959	0.946132	0.0744881
67960	0.946132	0.0901275
67961	0.946132	0.0781366
67962	0.946132	0.0549075
67963	1.89226	0.788493
67964	1.89226	0.891139
67965	1.89226	1.42354
67966	1.89226	0.451403
67967	1.89226	1.50055
67968	1.89226	0.47825
67969	1.89226	1.27882
67970	2.22749	0.138739
67971	1.89226	0.262831
67972	1.89226	0.438805
67973	3.78453	0.468844

67974	1.89226	1.01379
67975	1.89226	1.25886
67976	1.89226	1.63613
67977	1.89226	0.444985
67978	3.78453	0.813134
67979	1.89226	0.369261
67980	1.89226	0.52303
67981	1.89226	0.904771
67982	1.89226	0.202447
67983	1.89226	0.989859
67984	1.89226	0.231515
67985	0.94613	0.167008
67986	0.94613	0.784751
67987	0.94613	1.46436
67988	0.94613	0.608727
67989	0.946131	0.436431
67990	0.946131	0.181979
67991	0.946129	1.27436
67992	0.946129	0.434489
67993	0.94613	0.435887
67994	0.94613	0.478819
67995	0.94613	0.445552
67996	0.94613	0.328223
67997	1.89226	0.05
67998	1.89226	0.05
67999	1.89226	0.05
68000	1.89226	0.05
68001	1.89226	0.05
68002	1.89226	0.05
68003	1.89226	0.05
68004	1.89226	0.05
68005	1.89226	0.05
68006	1.89226	0.05
68007	3.78452	0.05
68008	1.89225	0.05
68009	1.89225	0.05
68010	1.89225	0.05
68011	1.89225	0.05
68012	1.89225	0.05
68013	1.89225	0.05
68014	1.89226	0.05
68015	1.89226	0.05
68016	1.89226	0.05
68017	1.89226	0.05
68018	1.89226	0.05
68019	1.89226	0.05
68020	0.946132	0.05
68021	0.946132	0.05
68022	0.946132	0.05
68023	0.946132	0.05
68024	0.946131	0.05
68025	0.946131	0.05
68026	0.946132	0.05
68027	0.946132	0.05
68028	0.946132	0.05
68029	0.946132	0.05
68030	0.946132	0.05
68031	0.946132	0.05

68032	1.89226	0.05
68033	1.89226	0.0528929
68034	1.89226	0.05
68035	1.89226	0.0747362
68036	1.89226	0.05
68037	1.89226	0.065129
68038	1.89226	0.05
68039	1.89226	0.05
68040	1.89226	0.05
68041	1.89226	0.054009
68042	1.89226	0.05
68043	1.89226	0.05
68044	1.89226	0.05
68045	1.89226	0.05
68046	1.89226	0.05
68047	1.89226	0.05
68048	1.89226	0.05
68049	1.89226	0.05
68050	1.89226	0.05
68051	1.89226	0.0531339
68052	1.89226	0.05
68053	1.89226	0.0629958
68054	3.78452	0.05
68055	1.89226	0.05
68056	1.89226	0.05
68057	1.89226	0.05
68058	1.89226	0.0595569
68059	1.89226	0.05
68060	1.89226	0.05
68061	1.89226	0.05
68062	1.89226	0.05
68063	1.89226	0.05
68064	1.89226	0.05
68065	3.78453	0.05
68066	1.89226	0.05
68067	2.48862	0.05
68068	1.89226	0.05
68069	2.22062	0.05
68070	1.89226	0.05
68071	2.22062	0.05
68072	1.89227	0.05
68073	1.89227	0.05
68074	1.89227	0.05
68075	1.89227	0.05
68076	1.89227	0.05
68077	1.89227	0.05
68078	0.946132	0.05
68079	0.946132	0.05
68080	0.946131	0.05
68081	0.946131	0.05
68082	0.94613	0.05
68083	0.94613	0.05
68084	0.946133	0.05
68085	0.946133	0.05
68086	0.946131	0.05
68087	0.946131	0.05
68088	0.946131	0.05
68089	0.946131	0.05

68090	1.89226	0.05
68091	1.89226	0.05
68092	1.89226	0.05
68093	1.89226	0.05
68094	3.78453	0.05
68095	1.89226	0.05
68096	1.89226	0.05
68097	1.89226	0.05
68098	1.89226	0.05
68099	3.78453	0.05
68100	1.89226	0.0592187
68101	1.89226	0.0525363
68102	1.89226	0.05
68103	1.89226	0.0946286
68104	1.89226	0.05
68105	1.89226	0.0625591
68106	1.89226	0.0999213
68107	1.89226	0.0846077
68108	1.89226	0.05
68109	1.89226	0.136795
68110	3.78452	0.0702543
68111	1.89226	0.05
68112	1.89226	0.120569
68113	1.89226	0.0620064
68114	1.89226	0.0623447
68115	3.78453	0.0753033
68116	2.57454	0.0579347
68117	1.89226	0.0856189
68118	1.3663	0.0551999
68119	1.89226	0.0578968
68120	3.25856	0.0659131
68121	1.89226	0.0698736
68122	1.89226	0.0640046
68123	1.89226	0.05
68124	1.89226	0.0906381
68125	3.78451	0.0555271
68126	1.89226	0.0601443
68127	1.89226	0.158089
68128	1.89226	0.0593979
68129	1.89226	0.0869693
68130	1.89226	0.0625797
68131	1.89226	0.0917774
68132	0.810946	0.0750567
68133	0.810946	0.0641043
68134	0.810946	0.0873288
68135	0.810946	0.147409
68136	0.810959	0.0718451
68137	0.810959	0.0534714
68138	1.89226	0.0811376
68139	1.35168	0.0652048
68140	1.08132	0.0500364
68141	1.08132	0.11699
68142	1.0813	0.0834942
68143	1.89226	0.051037
68144	1.89226	0.161311
68145	1.89226	0.221724
68146	1.89226	0.165565
68147	3.78453	0.125512

68148	1.89226	0.0968974
68149	1.89226	0.128548
68150	1.89226	0.0859179
68151	1.89226	0.312669
68152	1.89226	0.05
68153	1.89226	0.11258
68154	1.89226	0.128394
68155	1.89226	0.2449
68156	1.89226	0.349452
68157	1.89226	0.173438
68158	3.78452	0.163782
68159	1.89226	0.16264
68160	1.89226	0.193759
68161	1.89226	0.174192
68162	1.89226	0.569655
68163	3.78452	0.219586
68164	2.142	0.259938
68165	1.93949	0.0661179
68166	1.39296	0.228539
68167	2.55711	0.424489
68168	1.39296	0.05
68169	2.55711	0.0701876
68170	1.89226	0.115431
68171	2.5571	0.05
68172	2.142	0.11892
68173	1.89226	0.559346
68174	4.44936	0.132297
68175	0.946131	0.124683
68176	0.946131	0.110577
68177	1.27855	0.14887
68178	1.29171	0.145098
68179	1.29171	0.262368
68180	0.946127	0.194894
68181	1.27855	0.05
68182	0.946131	0.442233
68183	1.29172	0.174367
68184	1.29172	0.0864539
68185	0.946129	0.0831031
68186	0.946129	0.129355
68187	1.27855	0.183888
68188	1.29172	0.155868
68189	0.969743	0.276642
68190	1.07109	0.204489
68191	1.29172	0.767115
68192	1.07109	0.0808923
68193	0.969745	0.0639732
68194	1.27855	0.365816
68195	1.10873	0.349764
68196	1.10873	0.447733
68197	1.15273	0.0919434
68198	1.15273	0.100888
68199	1.89227	0.05
68200	1.89227	0.05
68201	1.89227	0.05
68202	1.89227	0.05
68203	2.1288	0.05
68204	2.1288	0.05
68205	1.89227	0.05

68206	1.89227	0.0653388
68207	1.89227	0.05
68208	1.89227	0.0579879
68209	3.78453	0.0598858
68210	1.89226	0.0785914
68211	1.89226	0.104676
68212	1.89226	0.0642432
68213	1.89226	0.0770025
68214	3.78453	0.0678051
68215	1.89226	0.05
68216	1.89226	0.0593526
68217	1.89226	0.05
68218	1.89226	0.05
68219	1.89226	0.05
68220	1.89226	0.0621541
68221	1.94432	0.05
68222	1.89226	0.05
68223	2.07911	0.05
68224	1.89226	0.05
68225	2.07911	0.05
68226	1.89226	0.05
68227	1.89226	0.05
68228	1.89226	0.05
68229	1.89226	0.05
68230	1.89226	0.05
68231	1.89226	0.05
68232	1.89226	0.05
68233	1.89226	0.182057
68234	1.89226	0.211485
68235	1.89226	0.369315
68236	1.89226	0.0942113
68237	1.89226	0.414106
68238	1.89226	0.107149
68239	1.89226	0.0638479
68240	1.89226	0.0649955
68241	1.89226	0.110293
68242	1.89226	0.05
68243	1.89226	0.127025
68244	1.89226	0.05
68245	1.86778	0.05
68246	2.72348	0.05
68247	1.89226	0.0637141
68248	1.89226	0.05
68249	3.76004	0.05
68250	1.89226	0.190527
68251	1.89226	0.224532
68252	1.89226	0.598851
68253	1.89226	0.0894848
68254	3.78451	0.166406
68255	1.89226	0.065455
68256	1.89226	0.0804825
68257	1.89226	0.123434
68258	1.89226	0.05
68259	1.89226	0.138415
68260	1.89226	0.05
68261	0.94613	0.05
68262	0.94613	0.05
68263	0.94613	0.113061

68264	0.94613	0.05
68265	0.946131	0.0554133
68266	0.946131	0.05
68267	0.946129	0.0751533
68268	0.946129	0.05
68269	0.94613	0.05
68270	0.94613	0.0594455
68271	0.94613	0.05
68272	0.94613	0.05
68273	0.946124	0.0870322
68274	0.946124	0.05
68275	0.94613	0.241746
68276	0.94613	0.0927295
68277	0.946128	0.0509032
68278	0.946128	0.0765397
68279	0.946126	0.216174
68280	0.946126	0.0857767
68281	0.946127	0.087627
68282	0.946127	0.0959442
68283	0.946127	0.0819804
68284	0.946127	0.0705962
68285	2.09166	0.05
68286	2.1285	0.05
68287	1.78381	0.05
68288	1.78381	0.05
68289	2.47689	0.05
68290	2.47689	0.05
68291	2.09167	0.05
68292	2.26572	0.05
68293	2.73393	0.05
68294	2.4769	0.05
68295	4.74261	0.05
68296	1.89226	0.05
68297	1.89226	0.05
68298	1.89226	0.05
68299	1.89226	0.05
68300	1.89226	0.05
68301	1.89226	0.05
68302	0.946131	0.05
68303	0.946131	0.05
68304	0.946129	0.05
68305	0.946129	0.05
68306	0.94613	0.05
68307	0.94613	0.05
68308	0.946131	0.05
68309	0.946131	0.05
68310	0.94613	0.05
68311	0.94613	0.05
68312	0.946131	0.05
68313	0.946131	0.05
68314	2.27622	0.05
68315	2.66786	0.05
68316	2.1285	0.05
68317	2.1285	0.05
68318	2.62461	0.05
68319	2.47689	0.05
68320	1.89226	0.05
68321	1.89226	0.05

68322	1.89226	0.05
68323	1.89226	0.05
68324	3.78453	0.05
68325	1.89226	0.05
68326	1.89226	0.05
68327	1.89226	0.05
68328	1.89226	0.05
68329	1.89226	0.05
68330	1.89226	0.05
68331	1.89226	0.42594
68332	1.89226	1.0241
68333	1.89226	0.473516
68334	1.89226	0.922823
68335	1.89226	0.483196
68336	1.89226	0.909267
68337	1.89226	1.15823
68338	1.89226	0.552554
68339	1.89226	0.477648
68340	1.89226	1.27722
68341	3.78452	0.7796
68342	1.89227	0.472765
68343	1.89227	1.10875
68344	1.89227	1.09435
68345	1.89227	0.671817
68346	3.78453	0.855202
68347	0.946127	1.11371
68348	0.946127	0.938437
68349	1.06438	1.44635
68350	0.946131	0.720441
68351	1.06438	1.08491
68352	0.94613	1.17302
68353	0.946128	1.02209
68354	0.946128	0.642578
68355	1.06438	1.08213
68356	1.06438	1.41279
68357	0.946129	1.07828
68358	0.946129	0.966436
68359	1.89226	0.479854
68360	1.89226	1.10127
68361	1.89226	0.701289
68362	1.89226	1.07672
68363	1.89226	0.740306
68364	1.89226	1.11469
68365	0.946133	0.830937
68366	0.946133	0.715127
68367	1.39771	1.11601
68368	0.946132	0.551985
68369	1.39771	1.11329
68370	1.39772	0.586151
68371	0.946133	0.80496
68372	0.946133	0.461448
68373	0.946132	1.11145
68374	1.39772	1.08525
68375	0.946132	0.97746
68376	0.946132	0.775818
68377	1.98182	0.05
68378	2.2217	0.05
68379	2.79889	0.05

68380	2.50097	0.05
68381	4.72267	0.05
68382	2.05362	0.05
68383	1.70302	0.05
68384	2.75109	0.05
68385	2.138 0.05	
68386	2.0815	0.05
68387	1.84751	0.05
68388	0.946131	0.05
68389	0.946131	0.05
68390	0.946131	0.05
68391	0.946131	0.05
68392	0.946131	0.05
68393	0.946131	0.05
68394	0.946132	0.05
68395	0.946132	0.05
68396	0.946131	0.05
68397	0.946131	0.05
68398	0.946132	0.05
68399	0.946132	0.05
68400	1.89226	0.05
68401	1.89226	0.05
68402	1.89226	0.05
68403	1.89226	0.05
68404	3.78451	0.05
68405	0.946126	0.05
68406	1.25048	0.05
68407	0.946131	0.05
68408	1.39945	0.05
68409	0.946127	0.05
68410	0.946127	0.05
68411	1.39944	0.05
68412	1.39944	0.05
68413	0.946129	0.05
68414	0.946129	0.05
68415	1.39944	0.05
68416	1.25048	0.05
68417	1.89226	0.05
68418	1.89226	0.05
68419	1.89226	0.05
68420	1.89226	0.05
68421	1.89226	0.05
68422	1.89226	0.05
68423	0.946126	0.05
68424	0.946126	0.05
68425	0.946131	0.05
68426	0.946131	0.05
68427	0.946128	0.05
68428	0.946128	0.05
68429	0.946129	0.05
68430	0.946129	0.05
68431	0.946129	0.05
68432	0.946129	0.05
68433	0.946128	0.05
68434	0.946128	0.05
68435	1.89226	0.05
68436	1.89226	0.05
68437	1.89226	0.05

68438	1.89226	0.05
68439	1.89226	0.05
68440	1.89226	0.05
68441	1.01393	0.05
68442	0.775245	0.05
68443	0.946126	0.05
68444	0.946126	0.05
68445	0.78305	0.05
68446	0.78305	0.05
68447	0.946159	0.05
68448	0.929267	0.05
68449	1.14956	0.05
68450	1.04073	0.05
68451	0.851526	0.05
68452	0.704781	0.05
68453	1.89226	0.05
68454	1.89226	0.05
68455	1.89226	0.05
68456	1.89226	0.05
68457	3.78451	0.05
68458	1.89226	0.05
68459	1.89226	0.05
68460	1.89226	0.05
68461	1.89226	0.05
68462	1.89226	0.05
68463	1.89226	0.05
68464	1.89226	0.05
68465	1.89226	0.05
68466	1.89226	0.05
68467	1.89226	0.05
68468	1.89226	0.05
68469	1.89226	0.05
68470	1.16317	0.05
68471	1.0292	0.05
68472	0.946126	0.05
68473	0.946126	0.05
68474	0.93562	0.05
68475	0.93562	0.05
68476	1.0418	0.05
68477	1.04181	0.05
68478	1.16317	0.05
68479	0.946129	0.05
68480	1.02919	0.05
68481	0.946128	0.05
68482	0.946131	0.05
68483	0.946131	0.05
68484	0.946126	0.05
68485	0.946126	0.05
68486	0.946129	0.05
68487	0.946129	0.05
68488	0.946128	0.05
68489	0.946128	0.05
68490	0.946129	0.05
68491	0.946129	0.05
68492	0.946128	0.05
68493	0.946128	0.05
68494	1.89226	0.05
68495	1.89226	0.05

68496	1.89226	0.05
68497	1.89226	0.05
68498	1.89226	0.05
68499	1.89226	0.05
68500	1.89226	0.05
68501	1.89226	0.05
68502	1.89226	0.05
68503	1.89226	0.05
68504	1.89226	0.05
68505	1.89226	0.05
68506	1.89226	0.05
68507	1.89226	0.05
68508	1.89226	0.05
68509	1.89226	0.05
68510	3.78453	0.05
68511	1.89226	0.05
68512	1.89226	0.05
68513	1.89226	0.05
68514	1.89226	0.05
68515	3.78452	0.05
68516	1.89225	0.05
68517	1.89225	0.05
68518	1.89225	0.05
68519	1.89225	0.05
68520	1.89225	0.05
68521	1.89225	0.05
68522	0.946132	0.05
68523	0.946132	0.05
68524	0.946133	0.05
68525	0.946133	0.05
68526	0.946132	0.05
68527	0.946132	0.05
68528	0.946132	0.05
68529	0.946132	0.05
68530	0.946133	0.05
68531	0.946133	0.05
68532	0.946132	0.05
68533	0.946132	0.05
68534	1.89226	0.05
68535	1.89226	0.05
68536	1.89226	0.05
68537	1.89226	0.05
68538	3.78453	0.05
68539	1.13537	0.05
68540	1.09222	0.05
68541	0.946126	0.05
68542	0.946126	0.05
68543	0.946128	0.05
68544	0.946128	0.05
68545	1.16317	0.05
68546	1.13034	0.05
68547	1.30155	0.05
68548	1.08462	0.05
68549	0.992915	0.05
68550	0.807642	0.05
68551	0.946131	0.05
68552	0.946131	0.05
68553	0.946125	0.05

68554	0.946125	0.05
68555	0.946128	0.05
68556	0.946128	0.05
68557	0.946128	0.05
68558	0.946128	0.05
68559	0.946128	0.05
68560	0.946128	0.05
68561	0.946128	0.05
68562	0.946128	0.05
68563	1.89226	0.05
68564	1.89226	0.05
68565	1.89226	0.05
68566	1.89226	0.05
68567	1.89226	0.05
68568	1.89226	0.05
68569	1.89224	0.05
68570	1.89224	0.05
68571	1.89224	0.05
68572	1.89224	0.05
68573	1.89224	0.05
68574	1.89224	0.05
68575	0.946086	0.05
68576	1.17919	0.05
68577	0.89738	0.05
68578	0.89738	0.05
68579	0.946116	0.05
68580	0.946116	0.05
68581	1.13048	0.05
68582	1.11844	0.05
68583	0.946128	0.05
68584	0.897362	0.05
68585	1.1282	0.05
68586	0.946103	0.05
68587	1.89223	0.05
68588	1.89223	0.05
68589	1.89223	0.05
68590	1.89223	0.05
68591	3.78446	0.05
68592	0.946118	0.05
68593	0.946118	0.05
68594	0.946118	0.05
68595	0.946118	0.05
68596	0.946119	0.05
68597	0.946119	0.05
68598	0.946118	0.05
68599	0.946118	0.05
68600	0.946131	0.05
68601	0.946131	0.05
68602	0.946106	0.05
68603	0.946106	0.05
68604	1.89227	0.592449
68605	1.89227	0.936511
68606	1.89227	0.651352
68607	1.89227	0.470333
68608	3.78453	0.458027
68609	1.89226	0.907668
68610	1.89226	0.276359
68611	1.89226	0.256817

68612	1.89226	0.346487
68613	1.89226	0.646072
68614	1.89226	0.829564
68615	1.89226	0.165965
68616	2.59378	0.108952
68617	2.30348	0.0838617
68618	2.30348	0.119189
68619	1.89226	0.167749
68620	1.89226	0.264019
68621	1.89226	0.608874
68622	1.89226	0.297576
68623	1.89226	0.323269
68624	1.89226	0.303154
68625	3.78453	0.593038
68626	1.89226	0.297789
68627	1.89226	0.203692
68628	1.89226	0.149272
68629	1.89226	0.206914
68630	1.89226	0.379086
68631	1.89226	0.546857
68632	0.904406	0.107017
68633	1.01667	0.138366
68634	1.11974	0.111043
68635	0.994417	0.120851
68636	1.02013	0.112747
68637	0.628203	0.167512
68638	1.70302	0.138302
68639	1.49898	0.106292
68640	1.14559	0.114579
68641	0.900571	0.117421
68642	1.58886	0.14142
68643	2.81102	0.05
68644	2.3923	0.05
68645	2.05632	0.05
68646	2.43514	0.05
68647	4.82744	0.05
68648	1.89226	0.05
68649	1.89226	0.05
68650	1.89226	0.05
68651	1.89226	0.05
68652	1.89226	0.05
68653	1.89226	0.05
68654	1.89226	0.05
68655	1.89226	0.05
68656	1.89226	0.05
68657	1.89226	0.05
68658	3.78451	0.05
68659	2.50803	0.05
68660	1.903	0.05
68661	1.903	0.05
68662	2.19162	0.05
68663	2.09934	0.05
68664	2.39063	0.05
68665	1.00243	0.05
68666	0.731731	0.05
68667	0.876616	0.05
68668	1.00451	0.05
68669	0.986901	0.05

68670	0.986901	0.05
68671	0.887729	0.05
68672	0.887732	0.05
68673	0.876605	0.05
68674	0.769283	0.05
68675	1.00245	0.05
68676	1.18712	0.05
68677	0.946131	0.05
68678	0.946131	0.05
68679	0.946131	0.05
68680	0.946131	0.05
68681	0.946131	0.05
68682	0.946131	0.05
68683	0.946131	0.05
68684	0.946131	0.05
68685	0.946131	0.05
68686	0.946131	0.05
68687	0.946131	0.05
68688	0.946131	0.05
68689	1.89226	0.05
68690	1.89226	0.05
68691	1.89226	0.05
68692	1.89226	0.05
68693	1.89226	0.05
68694	1.89226	0.05
68695	1.89226	0.05
68696	1.89226	0.05
68697	1.89226	0.05
68698	1.89226	0.05
68699	3.78453	0.05
68700	1.89226	0.05
68701	1.89226	0.05
68702	1.89226	0.05
68703	1.89226	0.0575131
68704	3.78453	0.05
68705	1.89226	0.05
68706	1.89226	0.05
68707	1.89226	0.05
68708	1.89226	0.05
68709	1.89226	0.05
68710	1.89226	0.05
68711	0.946132	0.05
68712	0.946132	0.05
68713	0.946131	0.05
68714	0.946131	0.05
68715	0.946131	0.05
68716	0.946131	0.05
68717	0.946132	0.05
68718	0.946132	0.05
68719	0.946131	0.05
68720	0.946131	0.05
68721	0.946131	0.05
68722	0.946131	0.05
68723	0.946131	0.05
68724	0.946131	0.05
68725	0.946131	0.05
68726	0.946131	0.05
68727	0.94613	0.05

68728	0.94613	0.05
68729	0.946132	0.05
68730	0.946132	0.05
68731	0.946131	0.05
68732	0.946131	0.05
68733	0.946131	0.05
68734	0.946131	0.05
68735	1.89226	0.05
68736	1.89226	0.05
68737	1.89226	0.05
68738	1.89226	0.05
68739	1.89226	0.05
68740	1.89226	0.05
68741	1.89226	0.05
68742	1.89226	0.05
68743	1.89226	0.05
68744	1.89226	0.05
68745	3.78453	0.05
68746	1.89226	0.05
68747	1.89226	0.05
68748	1.89226	0.05
68749	1.89226	0.05
68750	1.89226	0.05
68751	1.89226	0.05
68752	0.946132	0.05
68753	0.946132	0.05
68754	0.946131	0.05
68755	0.946131	0.05
68756	0.946132	0.05
68757	0.946132	0.05
68758	0.946131	0.05
68759	0.946131	0.05
68760	0.946132	0.05
68761	0.946132	0.05
68762	0.946131	0.05
68763	0.946131	0.05
68764	0.946131	0.05
68765	0.946131	0.05
68766	0.946132	0.05
68767	0.946132	0.05
68768	0.946132	0.05
68769	0.946132	0.05
68770	0.946131	0.05
68771	0.946131	0.05
68772	0.946132	0.05
68773	0.946132	0.05
68774	0.946131	0.05
68775	0.946131	0.05
68776	1.89226	0.140096
68777	1.89226	0.151492
68778	1.89226	0.192547
68779	1.89226	0.105709
68780	3.78452	0.0966119
68781	1.89226	0.621653
68782	1.89226	0.141763
68783	1.89226	0.273798
68784	1.89226	0.313365
68785	3.78452	0.627215

68786	1.89226	0.154701
68787	1.89226	0.187764
68788	1.89226	0.267918
68789	1.89226	0.12232
68790	3.78452	0.1212
68791	1.89226	0.164865
68792	2.75945	0.0510396
68793	1.89226	0.0841815
68794	2.35026	0.0751749
68795	4.24252	0.212957
68796	0.946131	0.0886359
68797	0.946131	0.134037
68798	0.946131	0.0543812
68799	0.946131	0.073383
68800	0.946132	0.0527081
68801	0.946132	0.0623759
68802	0.946129	0.145868
68803	0.946129	0.0786999
68804	0.946131	0.0970025
68805	0.946131	0.107441
68806	0.94613	0.169236
68807	0.94613	0.103994
68808	1.89226	0.204805
68809	1.89226	0.159956
68810	1.89226	0.130169
68811	1.89226	0.22397
68812	1.89226	0.149941
68813	1.89226	0.296764
68814	0.946129	0.0796814
68815	0.946129	0.0957989
68816	0.946133	0.0751402
68817	0.946133	0.0689925
68818	0.946131	0.0642077
68819	0.946131	0.0670256
68820	0.946131	0.0973071
68821	0.946131	0.0714762
68822	0.946131	0.0921515
68823	0.946131	0.0891817
68824	0.946131	0.121579
68825	0.946131	0.0925799
68826	1.89226	0.05
68827	2.35026	0.05
68828	1.32126	0.05
68829	1.89226	0.05
68830	1.77925	0.05
68831	2.35026	0.05
68832	1.89226	0.05
68833	1.89226	0.05
68834	1.89226	0.05
68835	1.89226	0.05
68836	1.89226	0.05
68837	1.89226	0.05
68838	1.89226	0.05
68839	1.89226	0.05
68840	1.89226	0.05
68841	1.89226	0.05
68842	3.78453	0.05
68843	1.89226	0.05

68844	1.89226	0.05
68845	1.89226	0.05
68846	1.89226	0.05
68847	1.89226	0.05
68848	1.89226	0.05
68849	0.946131	0.05
68850	0.946131	0.05
68851	0.946131	0.05
68852	0.946131	0.05
68853	0.946132	0.05
68854	0.946132	0.05
68855	0.946131	0.05
68856	0.946131	0.05
68857	0.946132	0.05
68858	0.946132	0.05
68859	0.946131	0.05
68860	0.946131	0.05
68861	1.89226	0.05
68862	1.89226	0.05
68863	1.89226	0.05
68864	1.89226	0.05
68865	1.89226	0.05
68866	1.89226	0.05
68867	1.89226	0.05
68868	1.89226	0.0505696
68869	1.89226	0.05
68870	1.89226	0.05
68871	3.78453	0.05
68872	1.89227	0.051684
68873	1.89227	0.0537413
68874	1.89227	0.05
68875	1.89227	0.0780045
68876	3.78453	0.05
68877	1.89226	0.05
68878	1.89226	0.05
68879	1.89226	0.05
68880	1.89226	0.05
68881	3.78452	0.05
68882	0.946132	0.05
68883	0.946132	0.05
68884	0.946131	0.05
68885	0.946131	0.05
68886	0.946131	0.05
68887	0.946131	0.05
68888	0.946133	0.05
68889	0.946133	0.05
68890	0.946132	0.05
68891	0.946132	0.05
68892	0.946131	0.05
68893	0.946131	0.05
68894	1.89227	0.05
68895	1.89227	0.0573313
68896	1.89227	0.05
68897	1.89227	0.05
68898	1.89227	0.05
68899	1.89227	0.05
68900	2.20491	0.05
68901	1.81656	0.05

68902	1.81656	0.05
68903	1.89226	0.05
68904	1.89226	0.05
68905	1.89226	0.05
68906	1.89226	0.079946
68907	1.89226	0.115331
68908	1.89226	0.0803792
68909	1.89226	0.0827336
68910	3.78452	0.0759719
68911	1.89226	0.273598
68912	1.89226	0.208655
68913	1.89226	0.164939
68914	1.89226	0.299392
68915	3.78453	0.274385
68916	1.89226	0.22383
68917	1.89226	0.21564
68918	1.89226	0.189461
68919	1.89226	0.248224
68920	1.89226	0.226066
68921	1.89226	0.303877
68922	1.89226	0.0579386
68923	2.11032	0.05
68924	2.30793	0.05
68925	1.89226	0.05
68926	2.30793	0.05
68927	1.89226	0.0570589
68928	1.89226	0.110786
68929	1.89226	0.128601
68930	1.89226	0.0853809
68931	1.89226	0.100379
68932	1.89226	0.126864
68933	1.89226	0.152884
68934	0.810943	0.118311
68935	0.810943	0.0964392
68936	0.810943	0.118878
68937	0.810943	0.0983619
68938	0.810933	0.0995806
68939	0.810933	0.119995
68940	1.89226	0.0873078
68941	1.3517	0.115452
68942	1.08132	0.105297
68943	1.08132	0.135199
68944	1.08133	0.108752
68945	1.89226	0.05
68946	1.89226	0.05
68947	1.89226	0.05
68948	1.89226	0.05
68949	3.78453	0.05
68950	1.89226	0.05
68951	1.89226	0.05
68952	1.89226	0.05
68953	1.89226	0.05
68954	1.89226	0.05
68955	1.89226	0.05
68956	0.810945	0.05
68957	0.810945	0.05
68958	0.870829	0.05
68959	0.870829	0.05

68960	0.990502	0.05
68961	0.810933	0.05
68962	2.82777	0.05
68963	1.53126	0.05
68964	1.32076	0.05
68965	1.201	0.05
68966	1.08133	0.05
68967	1.89226	0.05
68968	1.89226	0.05
68969	1.89226	0.05
68970	1.89226	0.05
68971	1.89226	0.05
68972	1.89226	0.05
68973	0.946132	0.05
68974	0.946132	0.05
68975	0.946133	0.0577262
68976	0.946133	0.05
68977	0.946132	0.05
68978	0.946132	0.05
68979	0.946132	0.05
68980	0.946132	0.05
68981	0.946133	0.05
68982	0.946133	0.05
68983	0.946132	0.05
68984	0.946132	0.05
68985	0.946133	0.05
68986	0.946133	0.05
68987	0.946132	0.05
68988	0.946132	0.05
68989	0.946132	0.05
68990	0.946132	0.05
68991	0.946132	0.05
68992	0.946132	0.05
68993	0.946133	0.05
68994	0.946133	0.05
68995	0.946132	0.05
68996	0.946132	0.05
68997	1.89226	0.05
68998	1.89226	0.05
68999	1.89226	0.05
69000	1.89226	0.05
69001	1.89226	0.05
69002	1.89226	0.05
69003	1.89226	0.05
69004	1.89226	0.0945719
69005	1.89226	0.05
69006	1.89226	0.0698577
69007	3.78452	0.0874713
69008	1.89226	0.0899953
69009	1.89226	0.104591
69010	1.89226	0.0708535
69011	1.89226	0.261764
69012	3.78452	0.0798539
69013	1.89226	0.05
69014	1.89226	0.0915269
69015	1.89226	0.05
69016	1.89226	0.05
69017	1.89226	0.0788547

69018	1.89226	0.0818418
69019	0.946128	0.05
69020	0.946128	0.05
69021	0.946132	0.05
69022	0.946132	0.05
69023	0.946129	0.05
69024	0.946129	0.05
69025	0.946131	0.05
69026	0.946131	0.05
69027	0.94613	0.05
69028	0.94613	0.05
69029	0.946131	0.05
69030	0.946131	0.05
69031	0.946129	0.0573542
69032	0.946129	0.05
69033	0.946133	0.05
69034	0.946133	0.05
69035	0.946131	0.0547661
69036	0.946131	0.062534
69037	0.946131	0.05
69038	0.946131	0.05
69039	0.946131	0.0518901
69040	0.946131	0.05
69041	0.946131	0.05
69042	0.946131	0.05
69043	1.89226	0.05
69044	1.89226	0.05
69045	1.89226	0.05
69046	1.89226	0.0527601
69047	3.78452	0.05
69048	1.89225	0.05
69049	1.89225	0.05
69050	1.89225	0.05
69051	1.89225	0.05
69052	1.89225	0.05
69053	1.89225	0.05
69054	1.89226	0.05
69055	1.89226	0.05
69056	1.89226	0.05
69057	1.89226	0.05
69058	3.78452	0.05
69059	1.89226	0.05
69060	1.89226	0.11863
69061	1.89226	0.0515111
69062	1.89226	0.05
69063	3.78453	0.0509758
69064	1.89226	0.05
69065	1.89226	0.05
69066	1.89226	0.05
69067	1.89226	0.05
69068	3.78452	0.05
69069	1.22164	0.05
69070	1.22164	0.05
69071	0.806032	0.05
69072	0.806032	0.05
69073	1.08154	0.05
69074	0.946126	0.05
69075	0.946129	0.05

69076	0.806065	0.05
69077	0.806063	0.05
69078	1.08155	0.05
69079	0.946128	0.05
69080	0.946128	0.05
69081	1.28554	0.05
69082	1.28554	0.05
69083	0.946126	0.05
69084	0.946126	0.05
69085	1.28554	0.05
69086	0.946129	0.05
69087	1.08155	0.05
69088	0.94613	0.05
69089	1.08155	0.05
69090	1.28554	0.05
69091	0.94613	0.05
69092	0.94613	0.05
69093	0.946132	0.05
69094	0.946132	0.05
69095	0.946132	0.05
69096	0.946132	0.0509296
69097	0.946132	0.117912
69098	0.946132	0.05
69099	0.946133	0.05
69100	0.946133	0.05
69101	0.946132	0.05
69102	0.946132	0.05
69103	0.946133	0.05
69104	0.946133	0.05
69105	1.89226	0.27315
69106	1.89226	0.0912569
69107	1.89226	0.27201
69108	1.89226	0.0945222
69109	3.78453	0.215969
69110	1.89226	0.202561
69111	1.89226	0.0589385
69112	1.89226	0.0668903
69113	1.89226	0.0799317
69114	1.89226	0.0714406
69115	1.89226	0.0854184
69116	1.89226	0.05
69117	1.89226	0.05
69118	1.89226	0.05
69119	1.89226	0.05
69120	1.89226	0.05
69121	1.89226	0.05
69122	1.89226	0.0528527
69123	1.89226	0.05
69124	1.89226	0.05
69125	1.89226	0.05
69126	1.89226	0.05
69127	1.89226	0.05
69128	0.946132	0.0809935
69129	0.946132	0.101493
69130	0.946132	0.05
69131	0.946132	0.081536
69132	0.946132	0.05
69133	0.946132	0.0504941

69134	0.946132	0.0849011
69135	0.946132	0.0568613
69136	0.946131	0.05
69137	0.946131	0.0594058
69138	0.946132	0.0500048
69139	0.946132	0.0562298
69140	1.89226	0.0652403
69141	1.89226	0.05
69142	1.89226	0.0606604
69143	1.89226	0.05
69144	3.78453	0.05
69145	0.851541	0.05
69146	0.72883	0.05
69147	0.991276	0.05
69148	0.851485	0.05
69149	1.00003	0.05
69150	1.00003	0.05
69151	0.809788	0.05
69152	0.809788	0.05
69153	0.890738	0.05
69154	0.991264	0.05
69155	0.851554	0.05
69156	0.851554	0.05
69157	0.8628	0.05
69158	0.78387	0.05
69159	0.830233	0.05
69160	0.830233	0.05
69161	0.808312	0.05
69162	0.817238	0.05
69163	1.70303	0.05
69164	1.24116	0.05
69165	0.995299	0.05
69166	1.0989	0.05
69167	1.02714	0.05
69168	1.89226	0.185827
69169	1.89226	0.238019
69170	1.89226	0.0871021
69171	1.89226	1.296
69172	3.78451	0.121564
69173	0.946131	0.168605
69174	0.946131	0.05
69175	0.946131	0.19447
69176	0.946131	0.0982581
69177	0.94613	0.168338
69178	0.94613	0.05
69179	0.946132	0.05
69180	0.946132	0.05
69181	0.946131	0.0581146
69182	0.946131	0.120492
69183	0.946131	0.05
69184	0.946131	0.05
69185	1.89226	0.05
69186	1.89226	2.02426
69187	1.89226	1.04781
69188	1.89226	1.3813
69189	3.78453	1.33393
69190	1.89226	2.1
69191	1.89226	2.1

69192	1.89226	2.1
69193	1.89226	0.887324
69194	1.89226	2.1
69195	1.89226	0.912463
69196	0.946126	0.05
69197	1.17111	0.0934528
69198	1.3393	0.0859571
69199	0.946131	0.0878473
69200	0.946127	0.0766386
69201	0.946127	0.05
69202	1.3393	0.05
69203	1.17111	0.0811767
69204	1.3393	0.0642979
69205	1.3393	0.05
69206	0.946129	0.05
69207	0.946129	0.05
69208	0.946126	2.06372
69209	0.946126	0.062908
69210	0.946132	2.1
69211	0.946132	2.1
69212	0.94613	2.1
69213	0.94613	0.495647
69214	0.946129	2.00558
69215	0.946129	0.771932
69216	0.946129	0.05
69217	0.946129	2.1
69218	0.94613	1.14634
69219	0.94613	0.769786
69220	0.946131	0.10452
69221	0.946131	0.0783498
69222	0.94613	0.0709426
69223	0.94613	0.0815882
69224	0.946128	0.102385
69225	0.946128	0.114291
69226	0.946132	0.075396
69227	0.946132	0.0786435
69228	0.94613	0.103994
69229	0.94613	0.0635389
69230	0.94613	0.0819127
69231	0.94613	0.0938957
69232	0.94613	1.29449
69233	0.94613	0.542947
69234	0.94613	0.476972
69235	0.94613	0.31206
69236	0.946128	1.21299
69237	0.946128	1.57236
69238	0.946131	0.290472
69239	0.946131	0.20911
69240	0.94613	0.948086
69241	0.94613	0.186317
69242	0.94613	0.463744
69243	0.94613	1.12708
69244	1.89226	1.55345
69245	1.89226	0.687632
69246	1.89226	0.938597
69247	1.89226	2.079
69248	3.78453	1.11794
69249	1.89226	0.304401

69250	1.89226	0.470508
69251	1.89226	0.101432
69252	1.89226	0.345025
69253	1.89226	0.11436
69254	1.89226	0.470181
69255	1.89226	0.174907
69256	1.89226	0.702343
69257	1.89226	0.111581
69258	1.89226	0.23243
69259	1.89226	0.155721
69260	1.89226	0.318153
69261	1.89227	0.0598672
69262	2.28622	0.0763826
69263	2.16109	0.0598478
69264	1.89227	0.100319
69265	4.17849	0.0589284
69266	15.1381	0.0917847
69267	15.1381	0.660282
69268	15.1381	0.151327
69269	15.1381	0.225609
69270	30.2762	0.0758854
69271	15.1381	0.0646171
69272	15.1381	0.612828
69273	15.1381	0.05
69274	15.1381	0.193829
69275	30.2762	0.0524635
69276	15.1381	0.203876
69277	15.1381	0.620863
69278	15.1381	0.05
69279	15.1381	0.570502
69280	30.2762	0.299871
69281	15.1381	0.0700704
69282	15.1381	0.781118
69283	15.1381	0.05
69284	15.1381	0.158176
69285	30.2762	0.237247
69286	15.138	0.831817
69287	15.138	1.93759
69288	15.138	0.743744
69289	15.138	2.08806
69290	30.276	1.62923
69291	15.138	1.97564
69292	15.138	2.1
69293	15.138	2.09939
69294	15.138	2.1
69295	30.276	2.1
69296	15.138	1.73213
69297	15.138	1.35639
69298	15.138	0.790125
69299	15.138	2.1
69300	30.2759	1.81909
69301	15.138	1.55552
69302	15.138	2.1
69303	15.138	2.1
69304	15.138	1.41609
69305	30.2759	1.87478
69306	15.138	2.1
69307	15.138	2.1

69308	15.138	2.1
69309	15.138	2.1
69310	30.276	2.1
69311	15.1381	2.1
69312	15.1381	2.1
69313	15.1381	2.1
69314	15.1381	2.1
69315	30.2762	2.1
69316	15.1381	2.1
69317	15.1381	2.1
69318	15.1381	2.1
69319	15.1381	2.1
69320	30.2762	2.1
69321	15.138	1.92803
69322	15.138	2.1
69323	15.138	2.1
69324	15.138	2.1
69325	30.276	2.1
69326	15.138	2.1
69327	15.138	2.1
69328	15.138	2.1
69329	15.138	2.1
69330	30.276	2.1
69331	15.1381	1.41501
69332	15.1381	2.1
69333	15.1381	2.03152
69334	15.1381	1.77267
69335	30.2762	1.94672
69336	15.1381	2.08725
69337	15.1381	2.1
69338	15.1381	1.98307
69339	15.1381	2.1
69340	30.2762	2.1
69341	15.1381	2.1
69342	15.1381	2.1
69343	15.1381	2.1
69344	15.1381	2.1
69345	30.2762	2.1
69346	15.138	0.148909
69347	15.138	0.216506
69348	15.138	0.109983
69349	15.138	0.731625
69350	30.276	0.0888658
69351	1.89226	2.1
69352	1.89226	2.1
69353	1.89226	2.1
69354	1.89226	2.1
69355	3.78453	2.1
69356	1.89226	2.1
69357	1.89226	2.1
69358	1.89226	2.1
69359	1.89226	2.1
69360	1.89226	2.1
69361	1.89226	2.1
69362	1.89226	2.1
69363	1.89226	2.1
69364	1.89226	2.1
69365	1.89226	2.1

69366	1.89226	2.1
69367	1.89226	2.1
69368	1.89226	2.1
69369	1.89226	2.1
69370	1.89226	2.1
69371	1.89226	2.1
69372	3.78453	2.1
69373	1.89226	2.1
69374	1.89226	2.1
69375	1.89226	2.1
69376	1.89226	2.1
69377	1.89226	2.1
69378	1.89226	2.1
69379	0.946127	2.1
69380	0.946127	2.1
69381	0.946131	2.1
69382	0.946131	2.1
69383	0.946131	2.1
69384	0.946131	2.1
69385	0.946128	2.1
69386	0.946128	2.1
69387	0.94613	2.1
69388	0.94613	2.1
69389	0.946129	2.1
69390	0.946129	2.1
69391	1.89226	2.1
69392	1.89226	2.1
69393	1.89226	2.1
69394	1.89226	2.1
69395	1.89226	2.1
69396	1.89226	2.1
69397	1.89226	2.1
69398	1.89226	2.1
69399	1.89226	2.1
69400	1.89226	2.1
69401	3.78452	2.1
69402	1.89226	2.1
69403	1.89226	2.1
69404	1.89226	2.1
69405	1.89226	2.1
69406	1.89226	2.1
69407	1.89226	2.1
69408	0.946129	2.1
69409	0.946129	2.1
69410	0.946133	2.1
69411	0.946133	2.1
69412	0.946131	2.1
69413	0.946131	2.1
69414	0.946131	2.1
69415	0.946131	2.1
69416	0.946131	2.1
69417	0.946131	2.1
69418	0.946131	2.1
69419	0.946131	2.1
69420	0.946132	2.1
69421	0.946132	2.1
69422	0.946132	2.1
69423	0.946132	2.1

69424	0.946132	2.1
69425	0.946132	2.1
69426	0.946133	2.1
69427	0.946133	2.1
69428	0.946132	2.1
69429	0.946132	2.1
69430	0.946133	2.1
69431	0.946133	2.1
69432	1.89226	2.1
69433	1.89226	2.1
69434	1.89226	2.1
69435	1.89226	2.1
69436	3.78452	2.1
69437	1.89226	2.1
69438	1.89226	2.1
69439	1.89226	2.1
69440	1.89226	2.1
69441	1.89226	2.1
69442	1.89226	2.1
69443	0.946129	2.1
69444	0.946129	2.1
69445	0.946133	2.1
69446	0.946133	2.1
69447	0.946131	2.1
69448	0.946131	2.1
69449	0.946131	2.1
69450	0.946131	2.1
69451	0.946131	2.1
69452	0.946131	2.1
69453	0.946131	2.1
69454	0.946131	2.1
69455	0.946132	2.1
69456	0.946132	2.1
69457	0.946132	2.1
69458	0.946132	2.1
69459	0.946132	2.1
69460	0.946132	2.1
69461	0.946133	2.1
69462	0.946133	2.1
69463	0.946133	2.1
69464	0.946133	2.1
69465	0.946132	2.1
69466	0.946132	2.1
69467	1.89226	2.1
69468	1.89226	2.1
69469	1.89226	2.1
69470	1.89226	2.1
69471	3.78453	2.1
69472	0.946128	2.1
69473	0.946128	2.1
69474	0.946132	2.1
69475	0.946132	2.1
69476	0.94613	2.1
69477	0.94613	2.1
69478	0.94613	2.1
69479	0.94613	2.1
69480	0.94613	2.1
69481	0.94613	2.1

69482	0.94613	2.1
69483	0.94613	2.1
69484	0.946127	2.1
69485	0.946127	2.1
69486	0.946131	2.1
69487	0.946131	2.1
69488	0.94613	2.1
69489	0.94613	2.1
69490	0.946128	2.1
69491	0.946128	2.1
69492	0.946129	2.1
69493	0.946129	2.1
69494	0.946129	2.1
69495	0.946129	2.1
69496	0.94613	2.1
69497	0.94613	2.1
69498	0.94613	2.1
69499	0.94613	2.1
69500	0.946131	2.1
69501	0.946131	2.1
69502	0.946129	2.1
69503	0.946129	2.1
69504	0.94613	2.1
69505	0.94613	2.1
69506	0.94613	2.1
69507	0.94613	2.1
69508	1.89226	2.1
69509	1.89226	2.1
69510	1.89226	2.1
69511	1.89226	2.1
69512	3.78453	2.1
69513	1.89226	2.1
69514	1.89226	2.1
69515	1.89226	2.1
69516	1.89226	2.1
69517	1.89226	2.1
69518	1.89226	2.1
69519	1.89226	2.1
69520	1.89226	2.1
69521	1.89226	2.1
69522	1.89226	2.1
69523	1.89226	2.1
69524	1.89226	2.1
69525	0.946131	2.1
69526	0.946131	2.1
69527	0.946131	2.1
69528	0.946131	2.1
69529	0.946132	2.1
69530	0.946132	2.1
69531	0.946129	2.1
69532	0.946129	2.1
69533	0.946131	2.1
69534	0.946131	2.1
69535	0.94613	2.1
69536	0.94613	2.1
69537	1.89224	2.1
69538	1.89224	2.1
69539	1.89224	2.1

69540	1.89224	2.1
69541	3.78448	2.1
69542	1.89224	2.1
69543	1.89224	2.1
69544	1.89224	2.1
69545	1.89224	2.1
69546	3.78447	2.1
69547	1.89223	2.1
69548	2.38995	2.1
69549	2.25315	2.1
69550	2.25315	2.1
69551	1.89223	2.1
69552	1.89223	2.1
69553	1.89224	2.1
69554	1.89224	2.1
69555	1.89224	2.1
69556	1.89224	2.1
69557	1.89224	2.1
69558	1.89224	2.1
69559	1.89226	2.1
69560	1.89226	2.1
69561	1.89226	2.1
69562	1.89226	2.1
69563	3.78453	2.1
69564	1.89226	2.1
69565	1.89226	2.1
69566	1.89226	2.1
69567	1.89226	2.1
69568	1.89226	2.1
69569	1.89226	2.1
69570	1.89226	2.1
69571	2.60272	2.1
69572	2.38998	2.1
69573	2.38998	2.1
69574	1.89226	2.1
69575	1.89226	2.1
69576	0.94613	2.1
69577	0.94613	2.1
69578	0.94613	2.1
69579	0.94613	2.1
69580	0.946131	2.1
69581	0.946131	2.1
69582	0.946129	2.1
69583	0.946129	2.1
69584	0.94613	2.1
69585	0.94613	2.1
69586	0.94613	2.1
69587	0.94613	2.1
69588	1.89226	2.1
69589	1.89226	2.1
69590	1.89226	2.1
69591	1.89226	2.1
69592	3.78452	2.1
69593	1.89226	2.1
69594	1.89226	2.1
69595	1.89226	2.1
69596	1.89226	2.1
69597	3.78452	2.1

69598	1.89226	2.1
69599	1.89226	2.1
69600	1.89226	2.1
69601	1.89226	2.1
69602	1.89226	2.1
69603	1.89226	2.1
69604	0.946132	2.1
69605	0.946132	2.1
69606	0.946132	2.1
69607	0.946132	2.1
69608	0.946131	2.1
69609	0.946131	2.1
69610	0.946132	2.1
69611	0.946132	2.1
69612	0.946132	2.1
69613	0.946132	2.1
69614	0.946132	2.1
69615	0.946132	2.1
69616	1.89226	2.1
69617	1.89226	2.1
69618	1.89226	2.1
69619	1.89226	2.1
69620	3.78452	2.1
69621	1.89226	2.1
69622	1.89226	2.1
69623	1.89226	2.1
69624	1.89226	2.1
69625	3.78452	2.1
69626	0.946129	2.1
69627	0.946129	2.1
69628	0.946133	2.1
69629	0.946133	2.1
69630	0.946131	2.1
69631	0.946131	2.1
69632	0.946131	2.1
69633	0.946131	2.1
69634	0.946131	2.1
69635	0.946131	2.1
69636	0.946131	2.1
69637	0.946131	2.1
69638	0.946132	2.1
69639	0.946132	2.1
69640	0.946132	2.1
69641	0.946132	2.1
69642	0.946132	2.1
69643	0.946132	2.1
69644	0.946133	2.1
69645	0.946133	2.1
69646	0.946133	2.1
69647	0.946133	2.1
69648	0.946132	2.1
69649	0.946132	2.1
69650	1.89224	2.1
69651	1.89224	2.1
69652	1.89224	2.1
69653	1.89224	2.1
69654	3.78447	2.1
69655	1.89223	2.1

69656	1.89223	2.1
69657	1.89223	2.1
69658	1.89223	2.1
69659	1.89223	2.1
69660	1.89223	2.1
69661	1.89224	2.1
69662	1.89224	2.1
69663	1.89224	2.1
69664	1.89224	2.1
69665	3.78447	2.1
69666	1.89224	2.1
69667	1.89224	2.1
69668	1.89224	2.1
69669	1.89224	2.1
69670	3.78448	2.1
69671	1.89226	2.1
69672	1.89226	2.1
69673	1.89226	2.1
69674	1.89226	2.1
69675	3.78452	2.1
69676	1.89226	2.1
69677	1.89226	2.1
69678	1.89226	2.1
69679	1.89226	2.1
69680	1.89226	2.1
69681	1.89226	2.1
69682	1.89226	2.1
69683	1.89226	2.1
69684	1.89226	2.1
69685	1.89226	2.1
69686	1.89226	2.1
69687	1.89226	2.1
69688	0.810946	2.1
69689	0.810946	2.1
69690	0.810946	2.1
69691	0.810946	2.1
69692	0.810933	2.1
69693	0.810933	2.1
69694	1.89226	2.1
69695	1.3517	2.1
69696	1.08132	2.1
69697	1.08132	2.1
69698	1.08133	2.1
69699	1.89226	2.1
69700	1.89226	2.1
69701	1.89226	2.1
69702	1.89226	2.1
69703	3.78453	2.1
69704	1.89226	2.1
69705	1.89226	2.1
69706	1.89226	2.1
69707	1.89226	2.1
69708	3.78452	2.1
69709	1.89226	2.1
69710	1.89226	2.1
69711	1.89226	2.1
69712	1.89226	2.1
69713	1.89226	2.1

69714	1.89226	2.1
69715	0.94613	2.1
69716	0.94613	2.1
69717	0.94613	2.1
69718	0.94613	2.1
69719	0.946131	2.1
69720	0.946131	2.1
69721	0.946129	2.1
69722	0.946129	2.1
69723	0.94613	2.1
69724	0.94613	2.1
69725	0.94613	2.1
69726	0.94613	2.1
69727	1.89226	2.1
69728	1.89226	2.1
69729	1.89226	2.1
69730	1.89226	2.1
69731	3.78453	2.1
69732	1.89226	2.1
69733	1.89226	2.1
69734	1.89226	2.1
69735	1.89226	2.1
69736	3.78452	2.1
69737	1.89226	2.1
69738	1.89226	2.1
69739	1.89226	2.1
69740	1.89226	2.1
69741	3.78452	2.1
69742	1.89226	2.1
69743	1.89226	2.1
69744	1.89226	2.1
69745	1.89226	2.1
69746	1.89226	2.1
69747	1.89226	2.1
69748	1.89226	2.1
69749	1.89226	2.1
69750	1.89226	2.1
69751	1.89226	2.1
69752	3.78452	2.1
69753	1.89226	2.1
69754	1.89226	2.1
69755	1.89226	2.1
69756	1.89226	2.1
69757	3.78452	2.1
69758	0.946128	2.1
69759	0.946128	2.1
69760	0.946133	2.1
69761	0.946133	2.1
69762	0.94613	2.1
69763	0.94613	2.1
69764	0.946131	2.1
69765	0.946131	2.1
69766	0.946131	2.1
69767	0.946131	2.1
69768	0.946131	2.1
69769	0.946131	2.1
69770	0.946132	2.1
69771	0.946132	2.1

69772	0.946132	2.1
69773	0.946132	2.1
69774	0.946131	2.1
69775	0.946131	2.1
69776	0.946132	2.1
69777	0.946132	2.1
69778	0.946132	2.1
69779	0.946132	2.1
69780	0.946132	2.1
69781	0.946132	2.1
69782	1.89226	2.1
69783	1.89226	2.1
69784	1.89226	2.1
69785	1.89226	2.1
69786	3.78452	2.1
69787	1.89226	2.1
69788	1.89226	2.1
69789	1.89226	2.1
69790	1.89226	2.1
69791	3.78452	2.1
69792	0.946129	2.1
69793	0.946129	2.1
69794	0.946133	2.1
69795	0.946133	2.1
69796	0.946131	2.1
69797	0.946131	2.1
69798	0.946131	2.1
69799	0.946131	2.1
69800	0.946131	2.1
69801	0.946131	2.1
69802	0.946131	2.1
69803	0.946131	2.1
69804	0.946132	2.1
69805	0.946132	2.1
69806	0.946132	2.1
69807	0.946132	2.1
69808	0.946132	2.1
69809	0.946132	2.1
69810	0.946133	2.1
69811	0.946133	2.1
69812	0.946132	2.1
69813	0.946132	2.1
69814	0.946133	2.1
69815	0.946133	2.1
69816	1.89226	2.1
69817	1.89226	2.1
69818	1.89226	2.1
69819	1.89226	2.1
69820	1.89226	2.1
69821	1.89226	2.1
69822	0.946128	2.1
69823	0.946128	2.1
69824	0.946133	2.1
69825	0.946133	2.1
69826	0.946131	2.1
69827	0.946131	2.1
69828	0.94613	2.1
69829	0.94613	2.1

69830	0.946131	2.1
69831	0.946131	2.1
69832	0.946131	2.1
69833	0.946131	2.1
69834	1.89226	2.1
69835	1.89226	2.1
69836	1.89226	2.1
69837	1.89226	2.1
69838	1.89226	2.1
69839	1.89226	2.1
69840	1.89226	2.1
69841	1.89226	2.1
69842	1.89226	2.1
69843	1.89226	2.1
69844	3.78453	2.1
69845	1.89226	2.1
69846	1.89226	2.1
69847	1.89226	2.1
69848	1.89226	2.1
69849	1.89226	2.1
69850	1.89226	2.1
69851	0.946127	2.1
69852	0.946127	2.1
69853	0.946131	2.1
69854	0.946131	2.1
69855	0.946131	2.1
69856	0.946131	2.1
69857	0.946127	2.1
69858	0.946127	2.1
69859	0.946129	2.1
69860	0.946129	2.1
69861	0.94613	2.1
69862	0.94613	2.1
69863	0.946131	2.1
69864	0.946131	2.1
69865	0.946131	2.1
69866	0.946131	2.1
69867	0.946133	2.1
69868	0.946133	2.1
69869	0.946128	2.1
69870	0.946128	2.1
69871	0.94613	2.1
69872	0.94613	2.1
69873	0.946131	2.1
69874	0.946131	2.1
69875	1.89226	2.1
69876	1.89226	2.1
69877	1.89226	2.1
69878	1.89226	2.1
69879	1.89226	2.1
69880	1.89226	2.1
69881	1.89226	2.1
69882	1.89226	2.1
69883	1.89226	2.1
69884	1.89226	2.1
69885	3.78452	2.1
69886	1.89226	2.1
69887	1.89226	2.1

69888	1.89226	2.1
69889	1.89226	2.1
69890	3.78452	2.1
69891	1.89226	2.1
69892	1.89226	2.1
69893	1.89226	2.1
69894	1.89226	2.1
69895	1.89226	2.1
69896	1.89226	2.1
69897	1.89226	2.1
69898	1.89226	2.1
69899	1.89226	2.1
69900	1.89226	2.1
69901	3.78453	2.1
69902	1.89226	2.1
69903	1.89226	2.1
69904	1.89226	2.1
69905	1.89226	2.1
69906	3.78452	2.1
69907	1.89226	2.1
69908	1.89226	2.1
69909	1.89226	2.1
69910	1.89226	2.1
69911	3.78452	2.1
69912	1.89226	2.1
69913	1.89226	2.1
69914	1.89226	2.1
69915	1.89226	2.1
69916	1.89226	2.1
69917	1.89226	2.1
69918	0.946131	2.1
69919	0.946131	2.1
69920	0.946131	2.1
69921	0.946131	2.1
69922	0.946131	2.1
69923	0.946131	2.1
69924	0.946131	2.1
69925	0.946131	2.1
69926	0.946131	2.1
69927	0.946131	2.1
69928	0.946131	2.1
69929	0.946131	2.1
69930	1.89226	2.1
69931	1.89226	2.1
69932	1.89226	2.1
69933	1.89226	2.1
69934	1.89226	2.1
69935	1.89226	2.1
69936	1.89226	2.1
69937	1.89226	2.1
69938	1.89226	2.1
69939	1.89226	2.1
69940	1.89226	2.1
69941	1.89226	2.1
69942	0.946131	2.1
69943	0.946131	2.1
69944	0.946131	2.1
69945	0.946131	2.1

69946	0.946131	2.1
69947	0.946131	2.1
69948	0.946131	2.1
69949	0.946131	2.1
69950	0.946131	2.1
69951	0.946131	2.1
69952	0.946131	2.1
69953	0.946131	2.1
69954	1.89226	2.1
69955	1.89226	2.1
69956	1.89226	2.1
69957	1.89226	2.1
69958	1.89226	2.1
69959	1.89226	2.1
69960	0.946128	2.1
69961	0.946128	2.1
69962	0.946133	2.1
69963	0.946133	2.1
69964	0.946131	2.1
69965	0.946131	2.1
69966	0.946131	2.1
69967	0.946131	2.1
69968	0.946131	2.1
69969	0.946131	2.1
69970	0.94613	2.1
69971	0.94613	2.1
69972	1.89226	2.1
69973	1.89226	2.1
69974	1.89226	2.1
69975	1.89226	2.1
69976	1.89226	2.1
69977	1.89226	2.1
69978	0.946131	2.1
69979	0.946131	2.1
69980	0.946131	2.1
69981	0.946131	2.1
69982	0.946132	2.1
69983	0.946132	2.1
69984	0.946131	2.1
69985	0.946131	2.1
69986	0.946132	2.1
69987	0.946132	2.1
69988	0.946131	2.1
69989	0.946131	2.1
69990	1.89226	2.1
69991	1.89226	2.1
69992	1.89226	2.1
69993	1.89226	2.1
69994	1.89226	2.1
69995	1.89226	2.1
69996	1.89226	2.1
69997	1.89226	2.1
69998	1.89226	2.1
69999	1.89226	2.1
70000	3.78452	2.1
70001	1.89226	2.1
70002	1.89226	2.1
70003	1.89226	2.1

70004	1.89226	2.1
70005	3.78452	2.1
70006	0.946131	2.1
70007	0.946131	2.1
70008	0.946131	2.1
70009	0.946131	2.1
70010	0.946132	2.1
70011	0.946132	2.1
70012	0.946131	2.1
70013	0.946131	2.1
70014	0.946132	2.1
70015	0.946132	2.1
70016	0.946131	2.1
70017	0.946131	2.1
70018	1.89226	2.1
70019	1.89226	2.1
70020	1.89226	2.1
70021	1.89226	2.1
70022	1.89226	2.1
70023	1.89226	2.1
70024	1.89226	2.1
70025	1.89226	2.1
70026	1.89226	2.1
70027	1.89226	2.1
70028	3.78452	2.1
70029	1.89226	2.1
70030	1.89226	2.1
70031	1.89226	2.1
70032	1.89226	2.1
70033	3.78452	2.1
70034	0.946131	2.1
70035	0.946131	2.1
70036	0.946131	2.1
70037	0.946131	2.1
70038	0.946131	2.1
70039	0.946131	2.1
70040	0.946131	2.1
70041	0.946131	2.1
70042	0.946131	2.1
70043	0.946131	2.1
70044	0.946131	2.1
70045	0.946131	2.1
70046	1.89226	2.1
70047	1.89226	2.1
70048	1.89226	2.1
70049	1.89226	2.1
70050	3.78453	2.1
70051	1.89226	2.1
70052	1.89226	2.1
70053	1.89226	2.1
70054	1.89226	2.1
70055	1.89226	2.1
70056	1.89226	2.1
70057	1.89226	2.1
70058	1.89226	2.1
70059	1.89226	2.1
70060	1.89226	2.1
70061	3.78453	2.1

70062	1.89226	2.1
70063	1.89226	2.1
70064	1.89226	2.1
70065	1.89226	2.1
70066	3.78453	2.1
70067	1.89226	2.1
70068	1.89226	2.1
70069	1.89226	2.1
70070	1.89226	2.1
70071	3.78453	2.1
70072	0.946132	2.1
70073	0.946132	2.1
70074	0.946132	2.1
70075	0.946132	2.1
70076	0.946132	2.1
70077	0.946132	2.1
70078	0.946132	2.1
70079	0.946132	2.1
70080	0.946132	2.1
70081	0.946132	2.1
70082	0.946131	2.1
70083	0.946131	2.1
70084	0.946131	2.1
70085	0.946131	2.1
70086	0.946131	2.1
70087	0.946131	2.1
70088	0.946132	2.1
70089	0.946132	2.1
70090	0.946131	2.1
70091	0.946131	2.1
70092	0.946132	2.1
70093	0.946132	2.1
70094	0.946131	2.1
70095	0.946131	2.1
70096	1.89226	2.1
70097	1.89226	2.1
70098	1.89226	2.1
70099	1.89226	2.1
70100	3.78453	2.1
70101	1.89226	2.1
70102	1.89226	2.1
70103	1.89226	2.1
70104	1.89226	2.1
70105	3.78453	2.1
70106	0.946131	2.1
70107	0.946131	2.1
70108	0.946131	2.1
70109	0.946131	2.1
70110	0.946131	2.1
70111	0.946131	2.1
70112	0.946131	2.1
70113	0.946131	2.1
70114	0.946131	2.1
70115	0.946131	2.1
70116	0.946131	2.1
70117	0.946131	2.1
70118	1.89226	2.1
70119	1.89226	2.1

70120	1.89226	2.1
70121	1.89226	2.1
70122	3.78452	2.1
70123	0.946132	2.1
70124	0.946132	2.1
70125	0.946131	2.1
70126	0.946131	2.1
70127	0.946132	2.1
70128	0.946132	2.1
70129	0.946131	2.1
70130	0.946131	2.1
70131	0.946131	2.1
70132	0.946131	2.1
70133	0.946131	2.1
70134	0.946131	2.1
70135	1.89226	2.1
70136	1.89226	2.1
70137	1.89226	2.1
70138	1.89226	2.1
70139	1.89226	2.1
70140	1.89226	2.1
70141	0.946132	2.1
70142	0.946132	2.1
70143	0.946132	2.1
70144	0.946132	2.1
70145	0.946132	2.1
70146	0.946132	2.1
70147	0.946132	2.1
70148	0.946132	2.1
70149	0.946132	2.1
70150	0.946132	2.1
70151	0.946131	2.1
70152	0.946131	2.1
70153	0.946131	2.1
70154	0.946131	2.1
70155	0.946131	2.1
70156	0.946131	2.1
70157	0.946132	2.1
70158	0.946132	2.1
70159	0.946131	2.1
70160	0.946131	2.1
70161	0.946132	2.1
70162	0.946132	2.1
70163	0.946131	2.1
70164	0.946131	2.1
70165	0.946132	2.1
70166	0.946132	2.1
70167	0.946131	2.1
70168	0.946131	2.1
70169	0.946132	2.1
70170	0.946132	2.1
70171	0.946131	2.1
70172	0.946131	2.1
70173	0.946132	2.1
70174	0.946132	2.1
70175	0.946131	2.1
70176	0.946131	2.1
70177	1.89226	2.1

70178	1.89226	2.1
70179	1.89226	2.1
70180	1.89226	2.1
70181	3.78453	2.1
70182	1.89226	2.1
70183	1.89226	2.1
70184	1.89226	2.1
70185	1.89226	2.1
70186	3.78453	2.1
70187	1.89226	2.1
70188	1.89226	2.1
70189	1.89226	2.1
70190	1.89226	2.1
70191	1.89226	2.1
70192	1.89226	2.1
70193	1.89226	2.1
70194	1.89226	2.1
70195	1.89226	2.1
70196	1.89226	2.1
70197	3.78453	2.1
70198	1.89226	2.1
70199	1.89226	2.1
70200	1.89226	2.1
70201	1.89226	2.1
70202	3.78453	2.1
70203	1.89226	2.1
70204	1.89226	2.1
70205	1.89226	2.1
70206	1.89226	2.1
70207	1.89226	2.1
70208	1.89226	2.1
70209	1.89226	2.1
70210	1.89226	2.1
70211	1.89226	2.1
70212	1.89226	2.1
70213	1.89226	2.1
70214	1.89226	2.1
70215	1.89226	2.1
70216	1.89226	2.1
70217	1.89226	2.1
70218	1.89226	2.1
70219	3.78453	2.1
70220	1.89226	2.1
70221	1.89226	2.1
70222	1.89226	2.1
70223	1.89226	2.1
70224	1.89226	2.1
70225	1.89226	2.1
70226	1.89226	2.1
70227	1.89226	2.1
70228	1.89226	2.1
70229	1.89226	2.1
70230	1.89226	2.1
70231	1.89226	2.1
70232	1.89226	2.1
70233	1.89226	1.42271
70234	1.89226	2.1
70235	1.89226	0.670884

70236	3.78453	1.99606
70237	1.89226	2.1
70238	1.89226	0.337602
70239	1.89226	0.11263
70240	1.89226	2.1
70241	1.89226	0.118139
70242	1.89226	2.1
70243	1.89226	2.1
70244	1.89226	2.1
70245	1.89226	2.1
70246	1.89226	2.1
70247	1.89226	2.1
70248	1.89226	2.1
70249	1.89226	2.1
70250	1.89226	2.1
70251	1.89226	2.1
70252	1.89226	2.1
70253	3.78453	2.1
70254	1.89226	2.1
70255	1.89226	1.78977
70256	1.89226	2.1
70257	1.89226	1.21673
70258	1.89226	2.1
70259	1.89226	1.68843
70260	1.89226	2.1
70261	1.89226	1.20801
70262	1.89226	2.1
70263	1.89226	2.1
70264	1.89226	1.9909
70265	1.89226	1.15307
70266	1.89226	2.1
70267	1.89226	2.1
70268	1.89226	2.1
70269	1.89226	2.1
70270	1.89226	2.1
70271	1.89226	2.1
70272	1.89226	2.1
70273	1.89226	2.1
70274	1.89226	2.1
70275	1.89226	2.1
70276	3.78452	2.1
70277	1.89226	2.1
70278	1.89226	1.39166
70279	1.89226	2.1
70280	1.89226	0.12906
70281	3.78452	2.1
70282	0.946131	2.1
70283	0.946131	0.1052
70284	0.946131	1.8441
70285	0.946131	2.1
70286	0.94613	0.534176
70287	0.94613	1.8773
70288	0.946132	2.1
70289	0.946132	2.1
70290	0.946131	1.85145
70291	0.946131	1.3535
70292	0.946131	1.89575
70293	0.946131	1.57393

70294	1.89226	2.1
70295	1.89226	2.1
70296	1.89226	2.1
70297	1.89226	2.1
70298	1.89226	2.1
70299	1.89226	2.1
70300	1.89226	2.1
70301	1.89226	2.1
70302	1.89226	2.1
70303	1.89226	2.1
70304	3.78452	2.1
70305	1.89226	2.1
70306	1.89226	0.741683
70307	1.89226	2.1
70308	1.89226	0.05
70309	3.78452	1.00308
70310	0.946131	1.19671
70311	0.946131	2.1
70312	0.946131	2.1
70313	0.946131	1.13277
70314	0.94613	0.389137
70315	0.94613	0.117842
70316	0.946132	2.1
70317	0.946132	2.1
70318	0.946131	2.05362
70319	0.946131	2.02962
70320	0.946131	1.83208
70321	0.946131	0.994899
70322	1.89226	2.1
70323	1.89226	2.1
70324	1.89226	2.1
70325	1.89226	2.1
70326	3.78452	2.1
70327	1.89226	2.1
70328	1.89226	2.1
70329	1.89226	2.1
70330	1.89226	2.1
70331	3.78453	2.1
70332	1.89226	2.1
70333	1.89226	2.1
70334	1.89226	2.1
70335	1.89226	0.374516
70336	3.78452	1.92468
70337	1.89226	2.1
70338	1.89226	2.1
70339	1.89226	2.1
70340	1.89226	1.50452
70341	1.89226	2.1
70342	1.89226	2.1
70343	1.89226	2.1
70344	1.89226	2.1
70345	1.89226	2.1
70346	1.89226	2.1
70347	3.78452	2.1
70348	1.89226	2.1
70349	1.89226	2.1
70350	1.89226	2.1
70351	1.89226	2.1

70352	3.78453	2.1
70353	1.89226	2.1
70354	1.89226	0.726727
70355	1.89226	2.1
70356	1.89226	0.150927
70357	3.78453	2.1
70358	1.89226	2.1
70359	1.89226	0.361465
70360	1.89226	2.1
70361	1.89226	1.40852
70362	1.89226	2.1
70363	1.89226	0.11766
70364	1.89226	2.1
70365	1.89226	2.1
70366	1.89226	2.1
70367	1.89226	1.7052
70368	3.78452	2.1
70369	1.89226	2.1
70370	1.89226	2.1
70371	1.89226	2.1
70372	1.89226	1.99206
70373	3.78453	2.1
70374	1.89226	2.1
70375	1.89226	2.06245
70376	1.89226	2.1
70377	1.89226	0.260179
70378	3.78453	1.29415
70379	1.89226	2.1
70380	1.89226	1.33721
70381	1.89226	1.89789
70382	1.89226	0.885527
70383	1.89226	2.1
70384	1.89226	2.08696
70385	0.946128	1.99686
70386	0.946128	2.1
70387	0.946132	2.1
70388	0.946132	2.1
70389	0.946129	2.1
70390	0.946129	2.1
70391	0.94613	2.1
70392	0.94613	2.1
70393	0.94613	2.1
70394	0.94613	2.1
70395	0.94613	2.1
70396	0.94613	2.1
70397	1.89226	2.1
70398	1.89226	2.1
70399	1.89226	2.1
70400	1.89226	2.1
70401	1.89226	2.1
70402	1.89226	2.08648
70403	1.89226	2.1
70404	1.89226	1.01867
70405	1.89226	1.6705
70406	1.89226	2.1
70407	1.89226	0.0697058
70408	1.89226	2.1
70409	0.946127	2.1

70410	0.946127	1.57792
70411	0.946131	0.655584
70412	0.946131	2.1
70413	0.946128	2.05518
70414	0.946128	0.472527
70415	0.94613	2.1
70416	0.94613	2.1
70417	0.946129	1.99011
70418	0.946129	2.02999
70419	0.946129	2.1
70420	0.946129	2.1
70421	1.89226	2.1
70422	1.89226	2.1
70423	1.89226	2.1
70424	1.89226	2.1
70425	3.78452	2.1
70426	1.89226	2.1
70427	1.89226	2.1
70428	1.89226	2.1
70429	1.89226	2.1
70430	1.89226	2.1
70431	1.89226	2.1
70432	1.89226	2.1
70433	1.89226	0.909206
70434	1.89226	1.78374
70435	1.89226	2.1
70436	1.89226	0.102859
70437	1.89226	2.1
70438	1.89226	0.543197
70439	1.89226	2.1
70440	1.89226	2.1
70441	1.89226	1.50849
70442	1.89226	2.1
70443	1.89226	2.00601
70444	0.946131	2.1
70445	0.946131	2.1
70446	0.946131	2.1
70447	0.946131	2.1
70448	0.946131	2.1
70449	0.946131	2.1
70450	0.946132	2.1
70451	0.946132	2.1
70452	0.946132	2.1
70453	0.946132	2.1
70454	0.946131	2.1
70455	0.946131	2.1
70456	1.89226	2.1
70457	1.89226	2.1
70458	1.89226	2.1
70459	1.89226	2.1
70460	1.89226	2.1
70461	1.89226	2.1
70462	1.89226	2.1
70463	1.89226	0.475731
70464	1.89226	1.51142
70465	1.89226	2.1
70466	1.89226	0.05
70467	1.89226	2.1

70468	0.946131	2.1
70469	0.946131	1.19804
70470	0.946131	0.812768
70471	0.946131	2.1
70472	0.94613	0.180547
70473	0.94613	0.240622
70474	0.946132	2.1
70475	0.946132	2.1
70476	0.946131	2.1
70477	0.946131	2.1
70478	0.946131	2.1
70479	0.946131	1.82436
70480	1.89226	2.1
70481	1.89226	2.1
70482	1.89226	2.1
70483	1.89226	2.1
70484	3.78453	2.1
70485	1.89226	2.1
70486	1.89226	2.1
70487	1.89226	2.1
70488	1.89226	2.1
70489	1.89226	2.1
70490	1.89226	2.1
70491	1.89226	2.1
70492	1.89226	1.76633
70493	1.89226	2.1
70494	1.89226	0.128378
70495	3.78452	1.29848
70496	1.89226	2.1
70497	1.89226	1.65243
70498	1.89226	2.1
70499	1.89226	0.526464
70500	3.78452	2.0499
70501	0.946131	1.76903
70502	0.946131	2.1
70503	0.946131	2.1
70504	0.946131	2.1
70505	0.946131	2.1
70506	0.946131	2.1
70507	0.946131	2.1
70508	0.946131	2.1
70509	0.946131	2.1
70510	0.946131	2.1
70511	0.946131	2.1
70512	0.946131	2.1
70513	0.946128	2.1
70514	0.946128	2.1
70515	0.946132	2.1
70516	0.946132	2.1
70517	0.946129	2.1
70518	0.946129	2.1
70519	0.94613	2.1
70520	0.94613	2.1
70521	0.94613	2.1
70522	0.94613	2.1
70523	0.94613	2.1
70524	0.94613	2.1
70525	1.89226	2.1

70526	1.89226	0.499959
70527	1.89226	0.638259
70528	1.89226	2.1
70529	1.89226	0.05
70530	1.89226	2.1
70531	0.810945	2.1
70532	0.810945	1.0219
70533	0.810944	0.913806
70534	0.810944	0.900373
70535	1.89226	2.1
70536	1.35169	2.1
70537	1.08132	1.21481
70538	1.08132	1.9537
70539	1.08132	2.1
70540	0.810945	1.7618
70541	0.810945	2.1
70542	0.946131	2.1
70543	0.946131	2.1
70544	0.946131	2.1
70545	0.946131	2.1
70546	0.946131	2.1
70547	0.946131	2.1
70548	0.946131	2.1
70549	0.946131	2.1
70550	0.946131	2.1
70551	0.946131	2.1
70552	0.946131	2.1
70553	0.946131	2.1
70554	1.89226	2.1
70555	1.89226	2.1
70556	1.89226	2.1
70557	1.89226	2.1
70558	1.89226	2.1
70559	1.89226	2.1
70560	1.89226	2.1
70561	1.89226	2.1
70562	1.89226	2.1
70563	1.89226	2.1
70564	1.89226	2.1
70565	1.89226	2.1
70566	0.946131	2.1
70567	0.946131	2.1
70568	0.946131	2.1
70569	0.946131	2.1
70570	0.946131	2.1
70571	0.946131	2.1
70572	0.946131	2.1
70573	0.946131	2.1
70574	0.946131	2.1
70575	0.946131	2.1
70576	0.946131	2.1
70577	0.946131	2.1
70578	1.89226	2.1
70579	1.89226	2.1
70580	1.89226	2.1
70581	1.89226	2.1
70582	1.89226	2.1
70583	1.89226	2.1

70584	1.89226	2.1
70585	1.89226	2.1
70586	1.89226	2.1
70587	1.89226	2.1
70588	1.89226	2.1
70589	1.89226	2.1
70590	1.89226	2.1
70591	1.89226	2.1
70592	1.89226	2.1
70593	1.89226	2.1
70594	1.89226	2.1
70595	1.89226	2.1
70596	0.946132	2.1
70597	0.946132	2.1
70598	0.946131	2.1
70599	0.946131	2.1
70600	0.946132	2.1
70601	0.946132	2.1
70602	0.946132	2.1
70603	0.946132	2.1
70604	0.946132	2.1
70605	0.946132	2.1
70606	0.946132	2.1
70607	0.946132	2.1
70608	0.946131	2.1
70609	0.946131	2.1
70610	0.946131	2.1
70611	0.946131	2.1
70612	0.946131	2.1
70613	0.946131	2.1
70614	0.946131	2.1
70615	0.946131	2.1
70616	0.946131	2.1
70617	0.946131	2.1
70618	0.946131	2.1
70619	0.946131	2.1
70620	0.946132	2.1
70621	0.946132	2.1
70622	0.946131	2.1
70623	0.946131	2.1
70624	0.946132	2.1
70625	0.946132	2.1
70626	0.946131	2.1
70627	0.946131	2.1
70628	0.946131	2.1
70629	0.946131	2.1
70630	0.946131	2.1
70631	0.946131	2.1
70632	1.89226	2.1
70633	1.89226	2.1
70634	1.89226	2.1
70635	1.89226	2.1
70636	1.89226	2.1
70637	1.89226	2.1
70638	1.37713	2.1
70639	1.27093	2.1
70640	0.946132	2.1
70641	0.946132	2.1

70642	0.946132	2.1
70643	0.946132	2.1
70644	1.37713	2.1
70645	1.37713	2.1
70646	1.27093	2.1
70647	0.946131	2.1
70648	0.946132	2.1
70649	1.37713	2.1
70650	0.946131	2.1
70651	0.946131	2.1
70652	0.946131	2.1
70653	0.946131	2.1
70654	0.946132	2.1
70655	0.946132	2.1
70656	0.946131	2.1
70657	0.946131	2.1
70658	0.946131	2.1
70659	0.946131	2.1
70660	0.946132	2.1
70661	0.946132	2.1
70662	0.946132	2.1
70663	0.946132	2.1
70664	0.946131	2.1
70665	0.946131	2.1
70666	0.946132	2.1
70667	0.946132	2.1
70668	0.946131	2.1
70669	0.946131	2.1
70670	0.946131	2.1
70671	0.946131	2.1
70672	0.946132	2.1
70673	0.946132	2.1
70674	1.89226	2.1
70675	1.89226	2.1
70676	1.89226	2.1
70677	1.89226	2.1
70678	1.89226	2.1
70679	1.89226	2.1
70680	1.89226	2.1
70681	1.89226	2.1
70682	1.89226	2.1
70683	1.89226	2.1
70684	1.89226	2.1
70685	1.89226	2.1
70686	1.89226	2.1
70687	1.89226	2.1
70688	1.89226	2.1
70689	1.89226	0.823192
70690	3.78452	2.1
70691	1.89226	2.1
70692	1.89226	2.0955
70693	1.89226	2.1
70694	1.89226	1.72965
70695	1.89226	2.1
70696	1.89226	2.1
70697	1.89226	2.1
70698	1.89226	2.1
70699	1.89226	2.1

70700	1.89226	2.1
70701	3.78452	2.1
70702	1.89226	2.1
70703	1.89226	2.1
70704	1.89226	2.1
70705	1.89226	2.1
70706	3.78453	2.1
70707	1.89226	2.1
70708	1.89226	1.08928
70709	1.89226	1.89321
70710	1.89226	2.1
70711	1.89226	0.205114
70712	1.89226	2.1
70713	1.89226	0.256411
70714	1.89226	2.1
70715	1.89226	2.1
70716	1.89226	1.8462
70717	1.89226	2.1
70718	1.89226	2.09007
70719	1.89226	2.1
70720	1.89226	2.1
70721	1.89226	2.1
70722	1.89226	2.1
70723	3.78452	2.1
70724	1.89226	2.1
70725	1.89226	2.1
70726	1.89226	2.1
70727	1.89226	2.1
70728	1.89226	2.1
70729	1.89226	2.1
70730	1.89226	2.1
70731	1.89226	2.05324
70732	1.89226	1.96301
70733	1.89226	2.1
70734	1.89226	0.317132
70735	1.89226	2.1
70736	1.89226	1.38023
70737	1.89226	2.1
70738	1.89226	2.1
70739	1.89226	2.04589
70740	1.89226	2.1
70741	1.89226	2.1
70742	0.946131	2.1
70743	0.946131	2.1
70744	0.946131	2.1
70745	0.946131	2.1
70746	0.946131	2.1
70747	0.946131	2.1
70748	0.946132	2.1
70749	0.946132	2.1
70750	0.946131	2.1
70751	0.946131	2.1
70752	0.946132	2.1
70753	0.946132	2.1
70754	1.89226	2.1
70755	1.89226	2.1
70756	1.89226	2.1
70757	1.89226	2.1

70758	1.89226	2.1
70759	1.89226	2.1
70760	1.89226	2.1
70761	1.89226	1.45572
70762	1.89226	1.35335
70763	1.89226	2.1
70764	1.89226	0.0627955
70765	1.89226	2.1
70766	0.946131	2.1
70767	0.946131	1.55107
70768	0.946131	1.34982
70769	0.946131	2.1
70770	0.94613	0.983919
70771	0.94613	0.603403
70772	0.946132	2.1
70773	0.946132	2.1
70774	0.946131	2.00646
70775	0.946131	2.1
70776	0.946131	2.1
70777	0.946131	2.04414
70778	1.89226	2.1
70779	1.89226	1.63668
70780	1.89226	0.833438
70781	1.89226	2.1
70782	3.78453	2.1
70783	2.81189	2.1
70784	1.89226	2.1
70785	1.89226	2.1
70786	1.89226	2.1
70787	2.56715	2.1
70788	2.56715	2.1
70789	1.89226	2.1
70790	1.89226	2.1
70791	1.89226	2.1
70792	1.89226	2.1
70793	3.78452	2.1
70794	1.89226	1.78477
70795	1.89226	2.1
70796	1.89226	2.1
70797	1.89226	2.1
70798	3.78452	2.1
70799	1.89226	1.19334
70800	1.89226	2.1
70801	1.89226	2.02699
70802	1.89226	2.1
70803	1.89226	1.82992
70804	1.89226	2.1
70805	1.1507	2.1
70806	1.1507	2.1
70807	0.946133	2.1
70808	0.946133	2.1
70809	0.946131	2.1
70810	0.946131	2.1
70811	1.28358	2.1
70812	1.1507	2.1
70813	1.1507	2.1
70814	0.946131	2.1
70815	0.94613	2.1

70816	1.28358	2.1
70817	1.89226	2.1
70818	1.89226	2.1
70819	1.89226	2.1
70820	1.89226	2.1
70821	1.89226	2.1
70822	1.89226	2.1
70823	0.946131	2.1
70824	0.946131	2.1
70825	0.946131	1.82708
70826	0.946131	2.1
70827	0.946128	2.1
70828	0.946128	2.1
70829	0.946133	2.1
70830	0.946133	2.08765
70831	0.946131	2.1
70832	0.946131	2.1
70833	0.94613	2.1
70834	0.94613	2.1
70835	1.89226	0.265167
70836	1.89226	2.1
70837	1.89226	2.1
70838	1.89226	1.21263
70839	3.78453	1.83601
70840	1.89226	2.1
70841	1.89226	2.1
70842	1.89226	1.76713
70843	1.89226	2.1
70844	3.78452	2.1
70845	1.89226	2.1
70846	1.89226	2.1
70847	1.89226	2.1
70848	1.89226	2.1
70849	3.78452	2.1
70850	1.89226	2.1
70851	1.89226	2.1
70852	1.89226	1.30367
70853	1.89226	2.1
70854	3.78452	2.1
70855	0.946132	2.1
70856	0.946132	2.09945
70857	0.946132	0.155017
70858	0.946132	1.4625
70859	0.946132	1.38514
70860	0.946132	2.1
70861	0.946132	2.1
70862	0.946132	0.137897
70863	0.946132	1.90908
70864	0.946132	1.96817
70865	0.946131	1.86185
70866	0.946131	1.95391
70867	1.89226	2.1
70868	1.89226	2.1
70869	1.89226	2.1
70870	1.89226	2.1
70871	1.89226	2.1
70872	1.89226	2.1
70873	1.89226	1.54059

70874	1.89226	2.1
70875	1.89226	2.1
70876	1.89226	2.1
70877	1.89226	2.1
70878	1.89226	2.1
70879	1.89226	2.1
70880	1.89226	2.1
70881	1.89226	2.1
70882	1.89226	2.1
70883	3.78453	2.1
70884	1.89226	2.1
70885	1.89226	2.1
70886	1.89226	2.1
70887	1.89226	2.1
70888	3.78452	2.1
70889	1.89226	2.1
70890	1.89226	2.1
70891	1.89226	2.1
70892	1.89226	2.1
70893	1.89226	2.1
70894	1.89226	2.1
70895	1.89226	2.1
70896	1.89226	2.1
70897	1.89226	2.1
70898	1.89226	2.1
70899	3.78453	2.1
70900	1.89226	2.1
70901	1.89226	2.1
70902	1.89226	2.1
70903	1.89226	2.1
70904	1.89226	2.1
70905	1.89226	2.1
70906	1.89226	2.1
70907	1.89226	2.1
70908	1.89226	2.1
70909	1.89226	2.1
70910	3.78452	2.1
70911	1.89226	2.1
70912	1.89226	2.1
70913	1.89226	2.1
70914	1.89226	2.1
70915	3.78452	2.1
70916	1.89226	2.1
70917	1.89226	2.1
70918	1.89226	2.1
70919	1.89226	2.1
70920	3.78453	2.1
70921	1.89224	2.1
70922	1.89224	2.1
70923	1.89224	2.1
70924	1.89224	2.1
70925	3.78448	2.1
70926	1.89224	2.1
70927	1.89224	2.1
70928	1.89224	2.1
70929	1.89224	2.1
70930	1.89224	2.1
70931	1.89224	2.1

70932	1.89224	2.1
70933	1.89224	2.1
70934	1.89224	2.1
70935	1.89224	2.1
70936	3.78447	2.1
70937	0.946119	2.1
70938	0.946119	2.1
70939	0.946119	2.1
70940	0.946119	2.1
70941	0.946119	2.1
70942	0.946119	2.1
70943	0.946119	2.1
70944	0.946119	2.1
70945	0.946106	2.1
70946	0.946106	2.1
70947	0.946131	2.1
70948	0.946131	2.1
70949	1.89226	2.1
70950	1.89226	2.1
70951	1.89226	2.1
70952	1.89226	2.1
70953	3.78453	2.1
70954	0.810943	2.1
70955	0.810943	2.1
70956	0.810945	2.1
70957	0.810945	2.1
70958	0.810932	2.1
70959	0.810932	2.1
70960	1.89226	2.1
70961	1.3517	2.1
70962	1.08132	2.1
70963	1.08132	2.1
70964	1.08133	2.1
70965	1.89226	2.1
70966	1.89226	2.1
70967	1.89226	2.1
70968	1.89226	2.1
70969	1.89226	2.1
70970	1.89226	2.1
70971	0.946131	2.1
70972	0.946131	2.1
70973	0.946131	2.1
70974	0.946131	2.1
70975	0.946131	2.1
70976	0.946131	2.1
70977	0.946131	2.1
70978	0.946131	2.1
70979	0.946131	2.1
70980	0.946131	2.1
70981	0.946131	2.1
70982	0.946131	2.1
70983	1.89226	2.1
70984	1.89226	2.1
70985	1.89226	2.1
70986	1.89226	2.1
70987	3.78453	2.1
70988	1.89226	2.1
70989	1.89226	2.1

70990	1.89226	2.1
70991	1.89226	2.1
70992	3.78452	2.1
70993	1.89226	2.1
70994	1.89226	2.1
70995	1.89226	2.1
70996	1.89226	2.1
70997	3.78452	2.1
70998	1.89226	2.1
70999	1.89226	2.1
71000	1.89226	2.1
71001	1.89226	2.1
71002	3.78453	2.1
71003	1.89226	2.1
71004	1.89226	2.1
71005	1.89226	2.1
71006	1.89226	2.1
71007	1.89226	2.1
71008	1.89226	2.1
71009	1.89226	2.1
71010	1.89226	2.1
71011	1.89226	2.1
71012	1.89226	2.1
71013	1.89226	2.1
71014	1.89226	2.1
71015	1.89226	2.1
71016	1.89226	2.1
71017	1.89226	2.1
71018	1.89226	2.1
71019	3.78452	2.1
71020	1.89226	2.1
71021	1.89226	2.1
71022	1.89226	2.1
71023	1.89226	2.1
71024	1.89226	2.1
71025	1.89226	2.1
71026	1.89226	2.1
71027	1.89226	2.1
71028	1.89226	2.1
71029	1.89226	2.1
71030	1.89226	2.1
71031	1.89226	2.1
71032	0.946128	2.1
71033	0.946128	2.1
71034	0.946133	2.1
71035	0.946133	2.1
71036	0.946131	2.1
71037	0.946131	2.1
71038	0.946131	2.1
71039	0.946131	2.1
71040	0.94613	2.1
71041	0.94613	2.1
71042	0.946131	2.1
71043	0.946131	2.1
71044	1.89226	2.1
71045	1.89226	2.1
71046	1.89226	2.1
71047	1.89226	2.1

71048	1.89226	2.1
71049	1.89226	2.1
71050	1.89226	2.1
71051	1.89226	2.1
71052	1.89226	2.1
71053	1.89226	2.1
71054	1.89226	2.1
71055	1.89226	2.1
71056	2.76309	2.1
71057	1.89226	2.1
71058	2.57778	2.1
71059	1.89226	2.1
71060	4.47004	2.1
71061	0.946128	2.1
71062	0.946128	2.1
71063	0.946133	2.1
71064	0.946133	2.1
71065	0.946131	2.1
71066	0.946131	2.1
71067	0.946131	2.1
71068	0.946131	2.1
71069	0.946131	2.1
71070	0.946131	2.1
71071	0.94613	2.1
71072	0.94613	2.1
71073	0.946132	2.1
71074	0.946132	2.1
71075	0.946132	2.1
71076	0.946132	2.1
71077	0.946132	2.1
71078	0.946132	2.1
71079	0.946132	2.1
71080	0.946132	2.1
71081	0.946132	2.1
71082	0.946132	2.1
71083	0.946131	2.1
71084	0.946131	2.1
71085	1.89227	2.00618
71086	1.89227	0.476417
71087	1.89227	0.234725
71088	1.89227	2.1
71089	3.78453	0.936484
71090	1.89226	0.12038
71091	1.89226	1.8408
71092	1.89226	1.15748
71093	1.89226	0.789237
71094	3.78452	1.09867
71095	1.89226	2.09789
71096	1.89226	0.241867
71097	1.89226	1.65847
71098	1.89226	1.75709
71099	1.89226	0.477292
71100	1.89226	0.504342
71101	0.946131	0.382759
71102	0.946131	0.274267
71103	0.946131	2.1
71104	0.946131	2.1
71105	0.946133	2.1

71106	0.946133	0.711152
71107	0.946129	0.674741
71108	0.946129	2.1
71109	0.946131	1.17789
71110	0.946131	1.32427
71111	0.946131	1.07078
71112	0.946131	0.788155
71113	1.89226	0.18761
71114	1.89226	1.96705
71115	1.89226	1.90892
71116	1.89226	0.378425
71117	1.89226	1.81527
71118	1.89226	0.61557
71119	1.89226	1.13967
71120	1.89226	0.452225
71121	1.89226	0.136411
71122	1.89226	1.63676
71123	3.78452	0.358276
71124	1.89226	1.50024
71125	1.89226	0.823682
71126	1.89226	1.68907
71127	1.89226	0.287284
71128	1.89226	1.95008
71129	1.89226	0.569442
71130	0.94613	0.337435
71131	0.94613	0.530185
71132	0.946131	2.1
71133	0.946131	1.9198
71134	0.946133	0.749287
71135	0.946133	1.98081
71136	0.946128	2.03078
71137	0.946128	0.478194
71138	0.946131	1.47598
71139	0.946131	1.5387
71140	0.946131	0.827015
71141	0.946131	0.921569
71142	1.89226	0.470914
71143	1.89226	2.1
71144	1.89226	1.99134
71145	1.89226	1.03047
71146	1.89226	1.98783
71147	1.89226	1.1187
71148	1.89226	0.584965
71149	1.89226	1.8917
71150	1.89226	1.32267
71151	1.89226	1.2436
71152	1.89226	1.3534
71153	1.89226	1.54752
71154	0.946133	1.54438
71155	0.946133	0.343335
71156	0.946129	2.1
71157	0.946129	1.38824
71158	0.946131	2.1
71159	0.946131	1.13062
71160	0.946131	1.54437
71161	0.946131	1.0678
71162	0.946131	1.42075
71163	0.946131	1.31676

71164	0.946131	1.24842
71165	0.946131	1.37571
[truncated: 361,165 more chars]
