# Supplementary material for: On the Use of Bone Remodelling Models to Estimate the Density Distribution of Bones. Uniqueness of the Solution
Source: PLoS One. 2016 Feb 9;11(2):e0148603. doi: 10.1371/journal.pone.0148603 (PMC4747586; doi:10.1371/journal.pone.0148603)
Supplement: S5 File — (PDF) [file pone.0148603.s005.pdf]

N° Elem	Volume (mm3)	Density (g/cm3)
1	1.32415	2.1
2	1.75823	2.1
3	0.984099	2.1
4	1.46442	2.1
5	1.53082	2.1
6	1.32068	2.1
7	1.91404	0.05
8	1.17036	0.05
9	0.929209	0.05
10	1.27254	0.05
11	2.0926	0.05
12	2.20407	0.05
13	1.21488	0.05
14	1.26024	0.05
15	0.794454	0.05
16	1.95087	0.05
17	0.953925	2.1
18	0.98949	2.1
19	1.27875	2.1
20	1.04598	2.1
21	0.594005	2.1
22	0.578861	2.1
23	1.09506	2.1
24	1.45327	2.1
25	2.58396	2.1
26	1.58581	2.1
27	1.5858	2.1
28	0.636946	2.1
29	0.94202	2.1
30	0.946119	2.09776
31	0.636953	2.1
32	0.946107	2.1
33	1.09505	2.1
34	1.27389	2.09224
35	1.00232	2.1
36	1.00231	2.1
37	1.83583	2.1
38	1.00231	2.1
39	1.28828	0.05
40	1.81696	0.05
41	1.28828	0.05
42	1.28828	0.05
43	1.0044	0.05
44	0.85148	0.05
45	0.851539	0.05
46	1.57767	0.05
47	1.52227	0.05
48	0.730657	0.05
49	0.94613	0.05
50	1.28228	2.1
51	1.64268	2.1
52	1.10101	2.1
53	1.3939	2.1
54	0.959471	2.1
55	1.10101	2.1

56	1.2873	2.1
57	1.59659	2.1
58	1.80841	0.669052
59	1.80841	1.99959
60	0.946129	0.812933
61	1.36991	0.333172
62	1.32262	2.08647
63	1.32261	1.20051
64	1.38464	1.23081
65	0.962877	0.700405
66	0.962878	0.214295
67	1.82129	0.259398
68	1.82129	0.843156
69	0.94613	0.702117
70	1.39751	0.767518
71	0.946131	0.47176
72	1.34493	0.153156
73	1.39751	0.206759
74	0.837577	0.209188
75	0.823478	0.196085
76	1.10889	0.281312
77	1.01686	0.222836
78	1.1942	0.283344
79	0.836129	0.168705
80	0.937165	0.167622
81	0.969438	0.177667
82	0.895386	0.118008
83	1.30554	0.220051
84	1.15798	0.144018
85	1.22578	0.252096
86	1.15255	0.134035
87	0.9765	0.145504
88	2.02178	0.294572
89	1.63755	0.250348
90	0.727713	0.173535
91	0.810945	0.210129
92	1.00559	0.139862
93	0.879098	0.153038
94	1.07852	0.223482
95	0.657764	0.0922511
96	0.829406	0.183052
97	0.720201	0.161995
98	0.946131	0.0514157
99	1.11827	0.064484
100	1.50754	0.0561957
101	1.64835	0.163892
102	1.71903	0.210559
103	0.851554	0.0504642
104	1.75626	0.197175
105	2.1646	0.256129
106	1.5563	0.205961
107	1.5563	0.284813
108	2.10876	0.349501
109	1.05438	0.315824
110	0.946133	0.153962
111	1.05438	0.559798
112	1.20256	0.205147
113	1.47879	1.16679

114	1.47879	1.3443
115	0.77815	1.43768
116	0.947114	2.1
117	1.31257	2.1
118	1.09235	2.1
119	0.946132	2.1
120	1.54246	2.1
121	1.54246	2.1
122	1.09235	2.1
123	0.946131	2.1
124	1.29507	2.1
125	1.0353	2.1
126	1.03529	2.1
127	1.28667	2.1
128	1.44777	2.1
129	1.61121	2.1
130	1.22176	0.0772359
131	0.946129	0.125423
132	1.08848	0.05
133	0.946131	0.121053
134	1.61201	0.0638805
135	1.61201	0.05
136	1.19993	0.0975222
137	1.35822	0.0758717
138	1.1539	0.05
139	1.00765	0.05
140	1.28228	2.1
141	2.65339	2.1
142	1.28228	2.1
143	1.55998	2.1
144	1.00509	2.1
145	0.93845	2.1
146	1.27674	2.1
147	0.810958	2.1
148	1.91381	2.1
149	1.1861	2.1
150	1.24886	2.1
151	2.02842	2.1
152	0.991436	0.05
153	1.34588	0.05
154	2.02729	0.05
155	0.72904	0.05
156	1.06139	0.05
157	0.835368	0.05
158	1.63819	0.05
159	1.10745	0.05
160	1.79925	0.05
161	1.00085	0.05
162	0.946133	2.1
163	1.41123	2.1
164	1.8327	2.1
165	1.8327	2.1
166	0.946132	2.07922
167	1.41123	2.1
168	1.3676	2.1
169	1.11048	2.1
170	1.11048	2.1
171	1.65815	2.1

172	1.9004	2.1
173	1.88691	2.1
174	1.82232	2.1
175	1.46591	2.1
176	1.09115	2.1
177	1.02051	1.68819
178	1.03373	2.03305
179	1.10307	2.1
180	1.05556	2.1
181	1.82533	0.729491
182	1.19496	0.539205
183	1.28597	2.1
184	1.20421	2.1
185	2.23644	2.1
186	1.08378	2.1
187	0.983724	2.1
188	0.602075	2.1
189	1.45876	2.1
190	1.45875	2.1
191	0.602103	2.1
192	1.11822	2.1
193	0.946133	2.1
194	0.602103	2.1
195	1.20421	2.1
196	1.26371	0.33353
197	1.55179	0.148449
198	1.14042	0.0990354
199	1.56876	0.115576
200	2.27496	0.251479
201	1.13748	0.195158
202	1.02884	0.391333
203	1.25124	0.326196
204	0.82703	0.201128
205	0.607775	0.350172
206	1.49242	0.307355
207	1.35674	0.193398
208	1.6697	2.1
209	1.81655	2.1
210	0.900108	1.88428
211	0.851537	1.32511
212	0.703076	2.00818
213	0.639143	1.7586
214	1.42358	1.91771
215	1.4976	2.02567
216	0.86284	1.83949
217	1.2965	1.88046
218	1.27835	2.1
219	1.00012	1.10873
220	1.74332	2.05472
221	1.08576	2.1
222	1.19 2.1	
223	1.08576	2.1
224	1.20854	2.1
225	0.946131	2.1
226	0.946131	2.1
227	1.75985	2.1
228	1.75985	2.1
229	1.41232	2.1

230	1.29365	2.1
231	0.973559	2.1
232	1.23698	0.05
233	1.45055	0.05
234	1.26947	0.05
235	0.856218	0.05
236	1.75303	0.05
237	1.14938	0.05
238	0.984711	0.05
239	2.05771	0.05
240	0.625171	0.05
241	0.965675	0.05
242	1.17631	0.05
243	1.4677	0.05
244	1.35403	0.05
245	0.975149	0.05
246	0.946131	0.0508232
247	1.80893	0.05
248	1.80893	0.05
249	0.975149	0.05
250	0.946132	0.05
251	1.9503	0.05
252	2.80207	0.05
253	1.76411	0.05
254	2.77469	0.05
255	1.9503	0.05
256	1.26784	0.05
257	2.31093	0.0551633
258	0.851541	0.0799914
259	1.39521	0.05
260	1.45613	0.0563269
261	0.851541	0.0812601
262	0.996013	0.05
263	1.07989	0.0673858
264	0.946131	0.0686447
265	1.08213	0.0514589
266	1.74289	0.05
267	1.75838	0.0522403
268	2.11163	0.0725585
269	1.23773	2.1
270	1.25398	2.1
271	1.0304	2.1
272	1.32368	2.1
273	1.11712	0.80085
274	1.07999	0.490339
275	1.1766	2.1
276	1.35735	2.1
277	1.089 0.05	
278	1.81193	0.05
279	1.11081	0.05
280	1.78069	0.05
281	0.717512	0.05
282	0.810946	0.05
283	0.703901	0.05
284	1.09028	0.05
285	0.958031	0.05
286	0.892585	0.05
287	0.873431	0.05

288	0.997755	0.05
289	1.85479	0.05
290	0.983029	0.112554
291	1.27922	0.12274
292	1.64346	0.430175
293	1.27922	0.177274
294	1.09795	2.06589
295	1.07508	1.56281
296	1.02975	2.03853
297	1.01121	2.1
298	1.72369	0.05
299	1.12035	0.05
300	2.13511	0.05
301	0.9705	0.05
302	0.946131	0.05
303	0.86182	0.05
304	1.51379	0.05
305	1.51379	0.05
306	0.946132	0.05
307	0.861849	0.05
308	1.34591	0.357575
309	1.14721	0.221967
310	1.15539	2.1
311	1.90938	2.1
312	2.19436	2.1
313	1.41563	2.1
314	0.785641	2.1
315	0.736384	2.1
316	1.46489	2.1
317	1.46488	2.1
318	0.736372	2.1
319	0.946132	2.1
320	1.90941	2.1
321	1.18907	2.1
322	1.20409	2.1
323	0.973559	2.1
324	1.29461	2.1
325	0.946129	2.1
326	0.94613	2.1
327	1.78751	2.1
328	1.78751	2.1
329	1.38885	2.1
330	1.34479	2.1
331	1.07283	2.1
332	1.6869	2.1
333	1.52544	2.1
334	1.07797	2.1
335	1.13819	2.09915
336	0.776179	2.1
337	1.61092	2.1
338	1.36155	2.1
339	0.77618	2.1
340	1.2933	2.1
341	1.15946	2.1
342	0.69857	2.1
343	1.87607	2.1
344	1.07612	2.1
345	1.31976	1.26131

346	1.01243	1.97542
347	1.41279	1.94099
348	1.35146	0.0715069
349	1.90664	0.0650027
350	1.35145	0.0751009
351	1.22811	0.097505
352	1.93147	0.0834824
353	1.42486	2.1
354	1.56558	2.1
355	1.0476	0.05
356	2.31605	0.05
357	1.89367	0.05
358	0.804706	0.05
359	0.783919	0.05
360	1.3116	0.05
361	1.03891	0.05
362	0.960473	0.05
363	1.08462	0.05
364	0.785994	0.05
365	1.1318	0.05
366	1.29576	0.05
367	1.3946	0.05
368	1.11924	0.05
369	0.834322	2.1
370	1.0256	2.1
371	1.74321	0.857019
372	1.41798	0.777372
373	0.871608	0.744887
374	1.47253	0.719136
375	1.47252	0.761038
376	0.817064	0.751904
377	0.817064	0.760947
378	0.946129	0.765852
379	1.63413	0.685604
380	1.9194	0.05
381	1.93562	0.05
382	2.06307	0.05
383	1.46021	0.05
384	0.955002	1.16465
385	0.833409	1.54557
386	0.838039	1.12072
387	1.48702	1.04494
388	1.1697	1.02903
389	1.23549	1.09581
390	1.18373	1.13207
391	2.02254	0.927754
392	1.68867	1.09393
393	0.875481	0.759416
394	0.821635	1.28877
395	0.858878	0.675884
396	1.30245	0.680779
397	1.07476	0.682993
398	1.11907	0.712252
399	1.10453	0.752476
400	1.70233	0.719162
401	1.70233	0.769478
402	2.6558	1.06486
403	1.7818	2.1

404	1.87059	2.1
405	1.34412	0.05
406	1.98068	0.05
407	2.56369	0.05
408	1.49512	0.05
409	1.35977	0.203853
410	1.34254	0.261982
411	1.75618	0.217423
412	1.75618	0.185784
413	0.946129	0.356411
414	0.946129	0.268463
415	1.17665	0.260653
416	1.01976	0.21961
417	1.27376	0.151971
418	1.35978	0.190978
419	1.68741	0.160916
420	1.68741	0.137378
421	0.946132	0.262681
422	0.946133	0.200116
423	1.14241	0.192562
424	0.996831	0.162371
425	0.996828	0.193949
426	1.12774	0.242766
427	1.13664	2.1
428	1.09627	2.1
429	2.8814	2.1
430	3.49751	2.1
431	1.79109	0.317251
432	1.54197	0.250786
433	2.75153	1.65703
434	1.93019	1.34273
435	1.51569	0.71876
436	1.55145	0.954267
437	1.78595	2.1
438	3.74452	2.1
439	1.66537	2.1
440	3.55161	2.1
441	1.35002	0.699682
442	1.19901	0.69664
443	1.61028	1.95858
444	1.35002	0.716064
445	1.65712	0.151031
446	1.10585	0.178278
447	0.961347	0.153759
448	0.810944	0.121747
449	1.16148	0.231382
450	1.08066	0.363932
451	0.934577	0.169467
452	1.05164	0.161835
453	0.79216	0.17249
454	2.99194	2.1
455	1.2832	2.1
456	2.97842	2.1
457	1.39517	2.1
458	1.03391	2.1
459	1.74416	2.1
460	1.05071	2.1
461	0.923782	2.1

462	1.55369	2.1
463	1.86444	2.1
464	1.01617	2.1
465	1.27174	2.1
466	1.20573	2.1
467	1.01616	2.1
468	1.13621	2.09204
469	1.75366	2.09023
470	2.27009	1.92994
471	1.6813	2.02253
472	1.47904	0.05
473	2.62714	0.05
474	1.80085	0.0649939
475	1.80085	0.0763395
476	2.74104	0.073234
477	1.37052	0.0634729
478	0.946131	0.05
479	1.37052	0.0623235
480	1.31357	0.05
481	1.7837	0.182708
482	1.7837	0.160507
483	0.900397	0.0839309
484	1.74487	0.230397
485	2.88672	2.1
486	2.88249	2.1
487	1.0961	0.177478
488	1.20143	0.0608595
489	1.66739	0.121005
490	1.85795	0.05
491	1.45789	0.05
492	2.11407	0.05
493	0.713524	0.05
494	1.58391	0.05
495	1.58391	0.05
496	1.04765	0.05
497	1.24979	0.05
498	0.713551	0.05
499	0.946127	0.05
500	1.36768	0.05
501	1.14433	0.05
502	1.66923	0.05
503	0.699479	0.05
504	0.70351	0.05
505	0.758416	0.05
506	1.07678	0.05
507	0.909874	0.05
508	0.940218	0.05
509	0.876604	0.05
510	1.27148	0.05
511	2.42348	0.05
512	1.19837	0.05
513	0.764764	0.05
514	0.829259	0.05
515	0.740584	0.05
516	1.2772	0.05
517	0.964465	0.05
518	1.06231	0.05
519	1.06924	0.05

520	1.17566	0.05
521	1.3063	0.05
522	2.51253	0.0569859
523	2.42267	0.0532517
524	1.89226	0.0529519
525	1.46492	0.05
526	0.946132	0.0652867
527	0.653151	0.0538545
528	0.653124	0.0677738
529	1.53995	0.05
530	1.53995	0.05
531	0.936806	0.0500153
532	1.25626	0.05
533	1.1893	0.0708949
534	1.62206	0.05
535	1.04921	2.1
536	1.22927	2.1
537	0.801765	2.1
538	0.774917	2.1
539	0.844623	2.1
540	0.737481	2.1
541	3.24379	2.1
542	2.48573	2.1
543	1.40967	1.11996
544	1.47203	1.35217
545	2.07659	0.816077
546	1.40967	1.23154
547	2.06638	0.851068
548	1.17418	2.1
549	1.04921	2.1
550	1.74593	2.1
551	1.74593	2.1
552	0.946132	2.1
553	1.38651	2.1
554	1.20761	1.95987
555	1.20761	2.1
556	1.30555	2.1
557	1.46812	0.439188
558	2.41602	0.684726
559	1.46812	0.602197
560	1.46812	0.532244
561	0.734059	0.561861
562	0.946132	1.44436
563	1.21261	0.93834
564	1.34225	0.74237
565	0.734084	1.04674
566	1.82077	0.838267
567	1.82077	0.653226
568	1.13751	0.292267
569	0.946131	0.644728
570	1.13751	0.474728
571	1.13748	0.306954
572	0.784392	0.571068
573	1.4906	0.412219
574	1.4906	0.202782
575	1.17879	1.52078
576	1.28291	1.35443
577	0.626495	2.1

578	1.45334	2.1
579	1.45334	2.1
580	0.626494	2.1
581	1.3852	2.1
582	0.946116	2.1
583	0.626499	2.1
584	1.47203	0.936873
585	1.42639	2.1
586	1.42639	2.1
587	0.795469	2.1
588	0.795469	2.1
589	0.946132	2.1
590	0.850877	2.1
591	1.37098	2.1
592	1.87194	2.1
593	1.76988	2.1
594	3.16295	0.05
595	3.26725	0.05
596	1.13322	2.1
597	1.20761	2.1
598	0.686933	0.19891
599	0.493248	0.05
600	1.2053	0.636168
601	0.947149	0.124857
602	1.27591	0.109689
603	1.75732	0.176484
604	0.493261	0.198944
605	1.34337	0.40448
606	2.73207	0.155621
607	1.19857	0.125306
608	0.671688	0.0751716
609	0.946134	0.3448
610	1.36603	0.111794
611	2.07817	0.115838
612	1.82573	0.182668
613	0.671699	0.119686
614	1.47772	0.0820664
615	0.975349	0.295351
616	1.22615	2.1
617	1.47868	2.1
618	2.29351	2.1
619	1.47642	2.1
620	2.94443	2.09901
621	1.80454	2.1
622	0.949135	2.1
623	0.949134	2.1
624	1.10476	0.116992
625	0.982857	0.188269
626	1.10938	0.124711
627	1.19052	1.22425
628	1.01012	0.05
629	1.35562	0.05
630	0.691742	0.05
631	2.39596	0.05
632	2.35604	0.05
633	0.974081	0.05
634	3.22444	0.05
635	1.3294	0.273023

636	1.37265	0.599043
637	1.3294	0.902393
638	1.51113	2.1
639	1.43643	2.1
640	1.72726	2.1
641	1.634	2.1
642	0.755566	2.1
643	1.29319	2.1
644	0.755567	2.1
645	0.946131	2.1
646	1.29319	2.1
647	0.973563	2.1
648	0.904398	2.1
649	0.904406	2.1
650	1.1538	2.1
651	1.10437	2.1
652	0.935418	2.1
653	1.16649	2.1
654	0.975667	2.1
655	1.17952	2.1
656	1.95053	2.1
657	0.948676	2.1
658	1.44287	2.1
659	0.746095	2.1
660	1.51224	2.1
661	1.27354	2.1
662	0.746066	2.1
663	1.20573	2.1
664	1.12442	2.1
665	0.671487	2.1
666	1.04355	2.1
667	0.948675	2.1
668	0.967673	1.018
669	1.12931	0.05
670	1.38212	0.05
671	2.40113	0.05
672	1.08054	0.05
673	1.04072	0.05
674	0.691062	0.05
675	1.58052	0.05
676	1.33502	0.05
677	0.946131	0.05
678	0.691062	0.05
679	1.02663	0.05
680	1.80569	0.05
681	1.26062	0.05
682	1.18203	0.05
683	0.946131	0.05
684	0.795065	0.05
685	1.58753	0.05
686	1.58753	0.05
687	0.946131	0.05
688	0.795093	0.05
689	0.805057	2.1
690	0.812415	2.1
691	1.23868	0.0647383
692	1.34448	0.100087
693	1.67201	2.1

694	1.61202	2.1
695	1.41103	2.1
696	1.41103	2.1
697	0.841088	2.1
698	1.38302	2.1
699	0.841089	2.1
700	0.869097	2.1
701	1.38302	2.1
702	0.949941	2.1
703	0.94994	2.1
704	1.88608	2.1
705	0.854942	2.1
706	1.68893	2.1
707	1.70002	2.1
708	2.02107	0.114827
709	2.04982	0.114658
710	3.11063	0.153055
711	2.03805	0.110906
712	1.67638	2.1
713	0.967492	2.1
714	2.00209	2.1
715	0.967491	2.1
716	0.870742	2.1
717	1.30266	0.05
718	0.651334	0.05
719	1.69413	0.05
720	1.69413	0.05
721	0.946132	0.05
722	1.03079	0.05
723	1.31468	0.05
724	0.946132	0.05
725	0.919055	0.05
726	1.16815	0.05
727	2.79004	0.05
728	1.2136	0.05
729	2.62935	0.05
730	0.947692	0.201377
731	1.51835	0.175927
732	0.947693	0.125425
733	0.947692	0.142853
734	0.980841	2.1
735	1.43577	2.1
736	0.980841	2.1
737	1.67858	2.1
738	1.34124	0.336778
739	1.39818	0.249861
740	1.2065	2.1
741	0.946133	2.1
742	1.2079	2.1
743	1.65229	2.1
744	1.65229	2.1
745	0.946132	2.1
746	1.2079	2.1
747	1.39053	2.1
748	1.20651	2.1
749	1.36919	2.1
750	1.12717	2.1
751	1.09483	2.1

752	0.994294	2.1
753	0.97356	2.1
754	1.06827	2.1
755	1.40571	2.1
756	1.29259	2.1
757	0.946131	2.1
758	1.7787	2.1
759	1.7787	2.1
760	1.29259	2.1
761	0.946132	2.1
762	1.09408	0.762979
763	1.03824	0.335535
764	0.851541	0.354556
765	1.23608	0.352654
766	0.851539	0.343296
767	1.5021	0.333363
768	1.54839	0.350802
769	1.12373	0.33521
770	1.22617	0.330018
771	1.42762	0.472251
772	2.24746	0.438882
773	1.05475	0.073775
774	1.46845	0.0615023
775	1.05475	0.05
776	0.929726	0.0602952
777	1.52072	0.05
778	1.82488	0.05
779	1.02271	0.0623444
780	1.19225	0.05
781	1.43071	0.05
782	0.94613	0.0543684
783	1.05475	0.05
784	1.39223	2.1
785	1.71255	2.1
786	0.946132	0.483144
787	1.10531	0.41465
788	1.46355	0.372525
789	1.46355	0.354395
790	0.78086	0.81741
791	1.10531	0.477378
792	1.1391	0.404643
793	1.56166	0.534883
794	2.21062	0.456541
795	1.0361	2.1
796	1.73577	2.1
797	1.73577	2.1
798	0.946116	2.1
799	1.28174	2.1
800	0.946118	2.1
801	1.22883	2.1
802	1.28172	2.1
803	1.03609	2.1
804	0.553567	0.05
805	1.60581	0.05
806	1.60581	0.05
807	0.553594	0.05
808	0.966748	0.05
809	0.966748	0.05

810	0.946133	0.05
811	1.05602	0.05
812	2.38525	0.05
813	1.14503	0.05
814	1.24678	0.05
815	0.56262	0.05
816	1.24678	0.05
817	0.978467	0.05
818	1.66262	0.05
819	1.66262	0.05
820	0.562633	0.05
821	1.09522	0.05
822	2.49356	0.05
823	0.592547	0.05
824	1.04321	1.62093
825	1.25133	1.647
826	1.70508	1.30453
827	1.81043	1.25227
828	0.494934	1.1122
829	0.601047	0.855581
830	0.993428	1.27972
831	0.946573	1.14627
832	1.35039	0.923129
833	0.816895	1.20005
834	1.5063	1.08238
835	0.816895	0.911593
836	0.946132	0.984189
837	1.9067	1.40496
838	2.14504	1.41028
839	0.816895	1.11388
840	2.29624	1.00146
841	2.76933	0.471879
842	1.254	0.956462
843	0.946128	2.1
844	1.38813	2.1
845	1.79998	2.1
846	1.79998	2.1
847	0.94613	2.1
848	1.38813	2.1
849	1.35798	2.1
850	1.27809	2.1
851	1.35798	2.1
852	1.68994	2.1
853	1.68994	2.1
854	0.946132	2.1
855	0.946132	2.1
856	1.18067	2.1
857	0.958256	2.1
858	0.958255	2.1
859	0.958257	2.1
860	0.958255	2.1
861	1.06457	2.1
862	1.14195	2.1
863	1.70423	2.1
864	1.14195	2.1
865	1.64285	2.1
866	2.02057	2.1
867	0.946132	2.1

868	1.44186	2.1
869	0.946133	2.1
870	1.16366	2.1
871	1.17233	2.1
872	1.31259	2.1
873	1.2016	2.1
874	1.17129	2.1
875	1.05724	0.546429
876	1.32697	2.09825
877	1.05724	1.0216
878	1.04292	2.1
879	3.39037	2.1
880	2.57275	2.1
881	0.629212	2.1
882	2.32525	2.1
883	1.76949	0.308142
884	1.65809	0.303704
885	1.27665	2.1
886	1.19861	2.1
887	1.20045	2.1
888	1.1635	2.1
889	0.953633	0.170569
890	0.953634	0.0915019
891	1.21408	2.1
892	1.25716	2.1
893	2.01791	2.1
894	1.89318	2.1
895	0.947921	2.1
896	0.939049	2.1
897	0.947921	2.1
898	0.740753	2.1
899	0.916104	2.1
900	1.16941	2.1
901	1.72038	2.1
902	2.04441	2.1
903	0.973182	2.1
904	1.11751	2.1
905	0.611089	2.1
906	1.53584	0.0674318
907	0.767923	0.176764
908	1.38925	0.123489
909	1.38926	0.118243
910	0.946131	0.207395
911	0.961347	0.158582
912	1.19583	0.10276
913	0.946131	0.166202
914	1.3663	0.232501
915	1.21067	0.0606793
916	1.00166	0.062637
917	1.40707	0.0669934
918	1.50435	0.0677656
919	1.47954	1.09059
920	1.1233	2.1
921	2.36036	1.97446
922	1.04024	0.829221
923	0.739729	1.03051
924	1.45533	0.658744
925	1.45532	0.568992

926	0.946118	0.792358
927	1.15163	0.435384
928	1.15165	0.878905
929	0.739748	0.695613
930	1.04025	1.67193
931	1.47608	0.152201
932	0.946131	0.239382
933	1.02028	0.202798
934	0.946129	0.225212
935	1.78223	0.154229
936	1.78223	0.147785
937	1.34153	0.196766
938	1.38683	0.194389
939	1.3407	0.131184
940	1.3407	0.122513
941	2.04729	0.141106
942	0.851529	0.199593
943	1.26671	0.151788
944	0.946116	0.190151
945	1.4869	0.129668
946	1.29553	0.124806
947	1.03645	0.183843
948	1.20731	0.167514
949	0.73665	2.1
950	0.693596	2.1
951	2.11152	2.1
952	1.73627	2.1
953	1.06191	2.1
954	1.52226	2.1
955	1.76869	2.1
956	1.1881	2.1
957	1.32349	2.1
958	1.51041	2.1
959	1.61591	2.1
960	0.946132	2.1
961	0.946132	2.1
962	1.13537	2.1
963	3.0048	2.1
964	0.985109	2.1
965	1.68899	2.1
966	3.23261	2.1
967	1.08831	2.1
968	2.20623	2.1
969	0.91749	2.1
970	1.15515	2.1
971	0.550438	2.1
972	1.66294	2.1
973	1.08257	2.1
974	0.87851	2.1
975	2.09046	2.1
976	0.738711	2.1
977	1.31242	0.05
978	1.26311	0.05
979	1.67652	0.05
980	0.968594	2.1
981	0.968594	2.1
982	0.981099	0.05
983	1.32418	0.05

984	2.67175	0.05
985	2.09678	0.05
986	1.2511	0.05
987	0.912698	0.05
988	0.810946	0.05
989	0.810959	0.05
990	1.67479	0.05
991	1.65677	0.05
992	1.28469	0.05
993	1.48817	0.05
994	1.08131	0.05
995	1.04349	1.29701
996	1.42492	0.554968
997	1.05724	0.393946
998	1.65723	0.113263
999	1.95313	0.0969624
1000	1.9293	0.102229
1001	2.02729	0.0847038
1002	0.963393	1.6199
1003	0.963392	1.10226
1004	2.90171	2.1
1005	2.65447	2.1
1006	1.64158	2.1
1007	1.6488	2.1
1008	1.15991	0.160258
1009	0.946132	0.229721
1010	1.58199	0.173697
1011	1.58199	0.200832
1012	1.03333	0.239529
1013	1.25436	0.177905
1014	1.25436	0.212313
1015	0.946132	0.210924
1016	1.3771	0.191264
1017	0.687745	0.172893
1018	1.37709	0.156712
1019	0.812386	0.201219
1020	1.50172	0.189513
1021	1.50172	0.125271
1022	0.687758	0.204101
1023	1.17506	0.120551
1024	2.00539	0.140709
1025	2.2441	0.202863
1026	1.09667	0.124513
1027	1.43577	2.1
1028	2.24741	2.1
1029	1.26103	2.1
1030	0.851544	2.1
1031	1.5543	2.1
1032	1.59756	2.1
1033	0.851543	2.1
1034	1.44673	2.1
1035	1.31947	2.1
1036	1.1237	2.1
1037	1.26103	2.1
1038	1.089 0.05	
1039	0.946128	0.0597453
1040	0.965824	0.0535709
1041	0.94613	0.0615289

1042	1.51344	0.05
1043	1.51344	0.05
1044	1.13508	0.05
1045	1.32449	0.05
1046	1.089	0.05
1047	3.33706	0.126748
1048	2.7542	0.155581
1049	1.27256	0.25323
1050	0.954172	0.19689
1051	1.55527	0.187142
1052	1.05765	0.30656
1053	1.57326	0.249634
1054	1.38251	0.120671
1055	0.94613	0.238429
1056	2.38829	0.139278
1057	2.00539	0.147721
1058	1.53798	0.121934
1059	1.20969	2.1
1060	1.15864	2.1
1061	1.7986	2.1
1062	1.74533	2.1
1063	0.944827	1.4526
1064	0.946131	1.15943
1065	0.851543	1.31529
1066	1.52816	1.14064
1067	1.5314	1.52939
1068	1.39266	1.06881
1069	0.851528	1.08575
1070	1.39589	1.2547
1071	1.7348	1.07622
1072	1.85439	1.00244
1073	1.86367	1.1872
1074	1.90905	0.780143
1075	1.22176	0.0887795
1076	1.59492	0.0864166
1077	2.97929	2.1
1078	2.1905	2.1
1079	1.24128	2.1
1080	0.994293	2.1
1081	2.49767	0.05
1082	2.37105	0.05
1083	1.10834	2.0338
1084	1.53214	0.373245
1085	1.417	2.1
1086	2.13599	0.521132
1087	1.61727	2.1
1088	1.75004	2.1
1089	1.96301	2.1
1090	0.966631	2.1
1091	1.37791	2.1
1092	1.47302	2.1
1093	1.47302	2.1
1094	0.871519	2.1
1095	0.94613	2.1
1096	0.87155	2.1
1097	2.38731	2.1
1098	2.12399	0.877922
1099	1.74865	0.846227

1100	1.46724	0.390984
1101	2.38056	0.453453
1102	1.46724	0.39782
1103	0.818887	0.562165
1104	0.810944	1.19315
1105	0.805227	0.563382
1106	1.06558	0.583638
1107	0.841487	0.521123
1108	1.08456	0.624515
1109	0.903083	0.706331
1110	1.08929	0.05
1111	1.68454	0.05
1112	1.08929	0.05
1113	1.07658	0.05
1114	0.904439	0.05
1115	1.58501	0.05
1116	1.19729	0.05
1117	1.05427	0.05
1118	1.47764	0.05
1119	0.810959	0.05
1120	1.60946	0.792889
1121	2.28887	0.754663
1122	0.946133	0.817214
1123	1.14444	0.548489
1124	1.67964	0.464544
1125	1.67964	0.517047
1126	0.755334	0.818638
1127	1.14444	0.674801
1128	1.29054	0.598564
1129	1.37762	0.562089
1130	0.946132	0.723682
1131	0.533645	0.56514
1132	1.48361	0.463581
1133	1.48361	0.557673
1134	0.533645	0.66459
1135	0.872823	0.708573
1136	1.14444	0.556568
1137	1.37762	0.504049
1138	2.10101	0.474782
1139	1.02311	0.623948
1140	1.04083	2.1
1141	1.04084	2.1
1142	1.04084	2.1
1143	1.30116	2.1
1144	1.23477	0.05
1145	1.81893	0.05
1146	1.23477	0.05
1147	1.23477	0.05
1148	1.11967	0.05
1149	0.946131	0.05
1150	1.11966	0.05
1151	1.12628	0.05
1152	0.629641	0.05
1153	1.6163	0.05
1154	1.6163	0.05
1155	1.11117	2.1
1156	1.13006	2.1
1157	1.15149	2.1

1158	1.13179	2.1
1159	0.585368	1.10792
1160	0.986036	1.10076
1161	1.94788	0.276208
1162	1.34857	0.692691
1163	2.31806	2.1
1164	2.06363	2.1
1165	1.00423	2.1
1166	0.829065	2.1
1167	0.551816	2.1
1168	0.99406	2.1
1169	1.98339	2.1
1170	1.68073	2.1
1171	2.558	2.1
1172	0.711814	2.1
1173	2.97316	2.1
1174	2.41579	2.1
1175	1.15248	0.05
1176	1.14305	0.05
1177	1.15483	0.05
1178	1.90981	0.05
1179	0.998957	0.05
1180	1.83742	0.05
1181	1.27581	2.1
1182	0.94613	2.1
1183	1.6315	2.1
1184	1.6315	2.1
1185	0.946132	2.1
1186	1.28441	2.1
1187	1.28441	2.1
1188	1.00365	2.1
1189	0.968536	2.1
1190	1.57575	2.1
1191	1.27581	2.1
1192	1.99297	2.1
1193	1.32784	2.1
1194	1.4259	2.1
1195	2.27952	2.1
1196	1.5685	2.1
1197	1.54231	2.1
1198	3.54638	2.1
1199	1.3294	2.1
1200	2.98237	2.1
1201	2.01633	0.428712
1202	2.0985	0.448227
1203	2.26047	0.241797
1204	1.7913	0.181217
1205	1.09504	2.1
1206	1.74126	2.1
1207	1.05983	2.1
1208	2.37664	2.1
1209	2.28084	2.1
1210	2.19948	2.1
1211	1.33593	0.05
1212	1.31508	0.05
1213	1.30647	2.1
1214	1.28846	2.1
1215	1.2955	2.1

1216	1.09044	2.1
1217	1.4099	2.1
1218	1.14984	2.1
1219	0.946132	2.09637
1220	1.70727	2.1
1221	1.70727	2.1
1222	1.14984	2.1
1223	0.946132	2.1
1224	0.911818	2.1
1225	2.12209	2.1
1226	2.4158	2.1
1227	1.20867	2.1
1228	2.84085	2.1
1229	1.42284	2.1
1230	2.45689	2.1
1231	1.41562	2.1
1232	0.918358	2.1
1233	1.11222	2.1
1234	0.999083	2.1
1235	0.696781	2.1
1236	1.30869	2.1
1237	1.66132	2.1
1238	0.696807	2.1
1239	0.745315	2.1
1240	1.23296	2.1
1241	0.780548	2.1
1242	1.93242	1.0908
1243	1.27362	1.94598
1244	2.77828	1.902
1245	1.02653	0.646348
1246	0.808576	0.677034
1247	0.744012	1.22184
1248	0.605174	0.48724
1249	1.16501	0.386261
1250	0.803432	0.443298
1251	0.961016	0.390632
1252	1.10425	0.508335
1253	1.3756	0.899489
1254	2.17765	0.373709
1255	1.84578	0.323895
1256	1.32801	0.189304
1257	1.76377	0.202537
1258	0.946131	0.213739
1259	1.3022	0.3498
1260	0.694814	0.450674
1261	1.53028	0.21677
1262	1.53028	0.188238
1263	1.3022	0.317318
1264	0.922889	0.320781
1265	1.57766	2.1
1266	2.06911	2.1
1267	1.57766	2.1
1268	1.37721	0.05
1269	1.72174	0.05
1270	1.4468	0.05
1271	1.37721	0.05
1272	1.73972	0.105785
1273	1.62324	0.117299

1274	0.946125	0.109552
1275	1.34259	0.0739911
1276	1.04187	0.0641481
1277	1.05019	0.0825034
1278	1.2361	0.100095
1279	1.69055	0.0774967
1280	2.25056	0.0735993
1281	1.10574	1.84297
1282	1.21632	2.06171
1283	1.10574	2.08775
1284	1.46181	2.1
1285	0.946131	1.7974
1286	1.74111	1.93938
1287	1.74111	1.64328
1288	0.94613	1.52727
1289	1.26891	0.617932
1290	1.26891	0.675721
1291	1.23452	1.68632
1292	1.00255	1.95913
1293	1.72286	0.153565
1294	1.12233	0.103642
1295	1.30682	0.205722
1296	1.97397	0.238734
1297	1.67425	2.1
1298	1.77775	2.1
1299	1.73571	1.98969
1300	0.426265	2.05626
1301	1.31817	1.93147
1302	1.81042	2.08251
1303	1.85456	2.07871
1304	0.466532	1.90106
1305	1.28025	1.90611
1306	0.964467	1.83013
1307	1.27921	2.09027
1308	2.11851	1.87418
1309	2.37121	1.96707
1310	1.49357	0.135098
1311	1.42486	2.1
1312	1.00278	2.1
1313	1.00278	2.1
1314	1.12503	2.1
1315	0.568697	2.1
1316	0.555211	2.1
1317	0.982272	2.1
1318	1.57558	2.1
1319	0.851539	2.1
1320	1.07307	2.1
1321	1.58292	2.1
1322	1.50731	2.1
1323	0.851541	2.1
1324	1.00304	2.1
1325	1.36003	2.1
1326	1.13127	2.1
1327	0.672234	2.1
1328	0.763044	2.1
1329	0.966979	2.1
1330	1.31502	2.1
1331	1.67969	2.1

1332	1.62408	2.1
1333	0.661955	2.1
1334	1.35048	2.1
1335	1.40447	2.1
1336	1.40447	2.1
1337	0.607961	2.1
1338	0.946131	2.1
1339	0.607963	2.1
1340	1.01531	2.1
1341	1.01531	2.1
1342	1.91802	2.1
1343	0.777749	2.1
1344	1.22585	0.05
1345	1.20672	0.05
1346	1.03166	0.169453
1347	0.553805	2.1
1348	2.00551	2.1
1349	2.07203	2.1
1350	0.589479	2.1
1351	1.22352	2.1
1352	0.984227	2.1
1353	0.802455	2.1
1354	1.40942	2.1
1355	1.6662	2.1
1356	0.802485	2.1
1357	0.936669	2.1
1358	1.31127	0.280078
1359	0.946132	0.367807
1360	1.09729	0.220575
1361	0.946133	0.402637
1362	1.68703	0.244163
1363	1.68703	0.188757
1364	1.3689	0.290739
1365	1.26426	0.301245
1366	1.26923	2.1
1367	1.23614	2.1
1368	0.828371	2.1
1369	2.04556	2.1
1370	0.772648	2.1
1371	0.791059	2.1
1372	0.809958	2.1
1373	1.70012	2.1
1374	0.267032	2.1
1375	1.67604	2.1
1376	0.803823	2.1
1377	0.267305	2.1
1378	0.80246	2.1
1379	0.874582	2.1
1380	1.00188	2.1
1381	0.709588	2.1
1382	1.50679	2.1
1383	1.22456	2.09994
1384	0.870622	2.1
1385	1.05754	2.09994
1386	1.1689	2.1
1387	1.54556	2.09582
1388	1.89226	2.08029
1389	0.995311	2.1

1390	1.26195	2.1
1391	1.9	2.1
1392	0.845055	2.1
1393	0.737836	2.1
1394	0.823707	2.1
1395	0.626587	2.1
1396	1.16109	2.1
1397	0.85517	2.1
1398	0.932036	2.1
1399	1.08321	2.1
1400	1.43863	2.1
1401	2.72297	2.1
1402	2.90798	2.1
1403	1.47818	1.20675
1404	1.47819	1.55974
1405	0.694357	1.12487
1406	0.694382	1.29792
1407	0.946132	0.595906
1408	0.694382	1.04634
1409	1.22641	0.747288
1410	1.31155	1.49465
1411	2.45282	1.32319
1412	0.787843	0.709208
1413	0.808984	0.445036
1414	1.14232	1.40597
1415	0.795037	1.26301
1416	0.887382	1.12405
1417	1.14493	0.901266
1418	0.78393	0.59057
1419	1.09129	1.11975
1420	0.791482	1.02996
1421	1.1137	1.52177
1422	1.11819	1.09204
1423	0.960173	1.30027
1424	1.09447	1.55903
1425	0.874365	1.02629
1426	1.2474	0.198087
1427	1.75038	0.384615
1428	1.75038	0.443511
1429	0.94613	0.859789
1430	1.30076	0.421029
1431	1.12817	1.22596
1432	0.94613	0.645505
1433	1.30076	0.530755
1434	1.51841	0.511711
1435	1.51841	0.617319
1436	0.946132	1.3639
1437	1.39575	0.581856
1438	0.970358	1.66055
1439	0.946133	0.997132
1440	1.39575	0.835063
1441	1.06486	0.294502
1442	1.06486	0.285148
1443	1.38622	0.341246
1444	1.04521	0.05
1445	1.00809	0.0506574
1446	1.28952	0.0503046
1447	0.849316	0.05

1448	0.946132	0.05
1449	0.849344	0.0735363
1450	1.53631	0.05
1451	1.53631	0.050376
1452	1.77843	0.05
1453	1.69863	0.05
1454	1.91646	2.1
1455	1.8582	2.1
1456	2.8349	0.05
1457	1.36307	0.05
1458	1.23886	0.05
1459	1.12971	0.05
1460	1.59662	0.05
1461	1.59662	0.05
1462	0.640304	0.05
1463	0.640333	0.05
1464	0.886276	0.05
1465	0.886276	0.05
1466	1.35065	0.05
1467	2.09002	0.05
1468	1.35543	0.05
1469	1.94688	2.1
1470	1.97221	2.1
1471	0.719563	2.1
1472	1.20358	2.1
1473	0.874264	2.1
1474	1.27676	2.1
1475	1.46557	2.1
1476	1.46557	2.1
1477	0.68546	2.1
1478	0.94613	2.1
1479	0.685489	2.1
1480	1.39615	0.100598
1481	1.05404	0.0592736
1482	1.06474	0.0991448
1483	1.00713	0.0791721
1484	0.714535	0.0937807
1485	0.714563	0.133084
1486	1.41646	0.0572911
1487	1.52582	0.05
1488	0.721694	0.0827656
1489	0.93665	0.0813962
1490	1.21067	0.0589982
1491	1.21067	0.0576064
1492	1.73577	0.101574
1493	1.17799	0.0516992
1494	3.21432	0.237373
1495	2.41452	0.223369
1496	1.36208	0.19236
1497	2.53256	0.229874
1498	0.834593	0.172293
1499	2.5853	0.201339
1500	1.77259	0.17479
1501	0.615724	0.364241
1502	1.11374	0.469826
1503	0.615726	0.296793
1504	0.946129	0.645051
1505	0.615714	0.527616

1506	1.75489	0.47691
1507	1.75489	0.378442
1508	1.23146	0.225002
1509	2.2772	0.276206
1510	0.74463	0.231606
1511	1.3546	0.293766
1512	1.80252	0.192101
1513	2.38304	0.224186
1514	1.19151	0.324081
1515	1.1231	0.358561
1516	1.82345	0.277885
1517	1.79805	0.246177
1518	0.761337	0.471413
1519	0.946127	0.356347
1520	0.557726	0.301326
1521	1.05353	0.116719
1522	1.05353	0.128272
1523	1.68599	0.183986
1524	1.05353	0.134917
1525	1.40588	2.1
1526	1.09373	2.1
1527	1.18382	0.91997
1528	1.46903	0.72953
1529	1.25103	2.1
1530	1.10378	2.1
1531	1.58941	2.1
1532	1.29639	2.1
1533	1.14874	0.05
1534	0.899817	0.05
1535	0.666898	0.05
1536	1.76519	0.05
1537	1.17289	0.05
1538	1.03526	0.05
1539	1.97708	0.05
1540	1.31711	0.05
1541	0.992517	0.05
1542	1.70193	0.05
1543	1.13852	0.05
1544	1.23943	1.57678
1545	1.40014	2.1
1546	1.04598	2.1
1547	1.1483	2.1
1548	1.11433	1.21664
1549	1.32037	0.422386
1550	1.00381	1.42221
1551	1.11435	0.239918
1552	0.973502	0.779593
1553	0.946128	1.93513
1554	1.83697	0.86951
1555	1.83697	2.1
1556	1.31721	1.84234
1557	1.38614	1.08113
1558	1.39696	0.927545
1559	1.31721	1.58977
1560	1.0756	1.86967
1561	1.08542	0.910599
1562	0.946127	2.09985
1563	1.81052	0.584967

1564	1.81052	2.08711
1565	1.24315	2.09869
1566	1.39696	0.40364
1567	1.35969	1.84803
1568	1.24315	1.71788
1569	1.0756	0.231026
1570	1.20669	0.124404
1571	1.18508	0.15507
1572	1.71059	2.08151
1573	1.66762	2.1
1574	2.50853	2.1
1575	1.8236	2.1
1576	1.78872	2.1
1577	1.41721	2.1
1578	1.01335	1.92418
1579	1.42439	0.619633
1580	0.946129	2.03122
1581	1.33266	1.5584
1582	1.79776	1.84341
1583	1.79776	2.09642
1584	0.946131	0.912227
1585	1.33266	1.96337
1586	1.41123	2.04959
1587	1.25346	2.1
1588	0.946132	0.703876
1589	0.827287	1.11315
1590	1.57878	0.69232
1591	1.57878	1.07342
1592	0.827287	0.520421
1593	1.07341	0.771928
1594	1.33266	0.901017
1595	1.25346	1.7956
1596	1.61149	2.1
1597	1.15644	1.97167
1598	1.27558	1.04515
1599	1.3764	0.583369
1600	1.89526	1.41761
1601	1.37641	0.506111
1602	1.2064	2.1
1603	1.38426	2.1
1604	1.01595	2.1
1605	1.15067	2.1
1606	1.82276	2.1
1607	1.82276	2.1
1608	0.946131	2.09745
1609	1.40609	2.09709
1610	0.946132	2.1
1611	1.29143	2.1
1612	1.40609	2.1
1613	1.30272	2.1
1614	1.19548	2.1
1615	2.95451	2.1
1616	1.46124	2.1
1617	2.93215	2.1
1618	1.42105	2.1
1619	1.75477	0.45326
1620	2.31301	0.453487
1621	2.10275	0.211404

1622	1.80099	0.22074
1623	0.97541	2.1
1624	1.49733	2.1
1625	0.97541	2.1
1626	1.30751	2.1
1627	1.28614	0.05
1628	1.74759	0.0695044
1629	1.19756	0.05
1630	1.71053	0.0806018
1631	1.19757	0.0881674
1632	1.28954	0.05
1633	2.02703	0.0548502
1634	2.29453	0.05
1635	1.28265	0.05
1636	0.793267	0.05
1637	1.19974	0.0907812
1638	0.509551	0.05
1639	1.04072	0.0572439
1640	1.35302	0.0583727
1641	1.53133	0.118562
1642	0.566153	0.0620938
1643	1.09634	0.0907508
1644	1.12792	0.0906566
1645	1.68908	2.1
1646	1.68908	2.1
1647	0.768212	2.1
1648	0.768213	2.1
1649	0.946132	2.1
1650	0.768212	2.1
1651	1.32461	2.1
1652	1.87291	2.1
1653	2.64921	2.1
1654	1.53642	2.1
1655	2.26537	2.1
1656	1.53642	2.1
1657	1.79874	2.1
1658	1.79874	2.1
1659	0.726635	2.08354
1660	0.726636	2.1
1661	0.946128	2.09058
1662	0.726634	2.09052
1663	1.23339	2.06998
1664	0.992674	2.1
1665	1.8023	2.1
1666	1.12324	0.05
1667	1.29163	0.05
1668	0.996831	0.168588
1669	1.0019	0.159288
1670	0.993831	0.0846462
1671	1.07283	2.1
1672	1.04788	2.1
1673	1.44489	2.1
1674	1.04788	2.1
1675	1.07652	2.1
1676	0.991406	2.1
1677	1.37 2.1	
1678	1.14195	2.1
1679	1.47904	0.0529363

1680	1.26593	2.02076
1681	1.75366	1.93912
1682	1.57621	0.0964597
1683	1.24538	0.0570543
1684	0.969118	2.1
1685	0.946133	2.0929
1686	1.69387	2.1
1687	1.69387	2.1
1688	0.94613	2.1
1689	1.28713	2.1
1690	1.28713	2.1
1691	1.23877	2.1
1692	0.969118	2.1
1693	1.22615	2.1
1694	0.98084	2.1
1695	1.01843	2.1
1696	1.50876	2.1
1697	1.85391	2.1
1698	1.84322	2.1
1699	1.70377	2.1
1700	1.78919	2.1
1701	2.66787	2.1
1702	1.41549	2.1
1703	1.41549	2.1
1704	0.86707	2.1
1705	0.896172	2.1
1706	0.946126	2.1
1707	0.867074	2.1
1708	0.946118	2.1
1709	1.54468	0.0525429
1710	1.54468	0.0560436
1711	2.27259	0.0673827
1712	0.946131	0.118423
1713	1.25837	0.0913998
1714	0.663171	0.137409
1715	1.68931	0.0667425
1716	1.68931	0.0568691
1717	1.25837	0.0972636
1718	1.09411	0.0898457
1719	1.73571	0.0519136
1720	2.34894	0.0629986
1721	0.946128	0.0857786
1722	1.30497	0.069782
1723	0.772312	0.0991212
1724	1.66895	0.0520579
1725	1.66895	0.05
1726	1.30497	0.0676091
1727	1.13629	0.0585226
1728	1.22852	0.440921
1729	1.578 0.414452	
1730	2.07781	2.09744
1731	1.5478	2.08995
1732	1.70048	2.1
1733	1.42329	2.1
1734	1.05481	2.09457
1735	1.1032	1.97792
1736	1.19267	2.1
1737	1.77324	2.1

1738	1.01121	0.0739441
1739	0.991867	0.124771
1740	2.0744	2.09847
1741	1.75142	2.1
1742	1.35418	2.09852
1743	1.40041	2.1
1744	1.50045	2.1
1745	1.37704	2.1
1746	1.50045	2.1
1747	1.87875	2.1
1748	1.35308	0.299847
1749	1.29983	0.272433
1750	1.62784	2.1
1751	1.44807	2.1
1752	1.00965	2.1
1753	2.69264	2.1
1754	1.04075	2.1
1755	0.946131	2.1
1756	0.946132	2.1
1757	1.52704	2.1
1758	1.83246	2.1
1759	1.56757	2.1
1760	1.16687	2.1
1761	2.04857	2.1
1762	1.32707	2.1
1763	1.25519	0.462025
1764	1.32101	1.08097
1765	1.25055	2.06761
1766	0.827853	1.75255
1767	1.11835	0.982129
1768	1.07281	2.1
1769	1.54302	1.7254
1770	0.724064	2.0307
1771	1.32709	2.1
1772	3.44123	2.1
1773	2.46889	0.918617
1774	0.730076	0.0572527
1775	1.25809	0.147795
1776	0.730077	0.0858973
1777	0.946129	0.102188
1778	1.72777	0.05
1779	1.72777	0.141846
1780	0.73005	0.104834
1781	1.7522	0.0627508
1782	2.51618	0.05
1783	0.628177	0.0529531
1784	1.1068	0.180042
1785	0.747435	0.0800587
1786	0.946133	0.140617
1787	1.84668	0.0507099
1788	1.8563	0.14344
1789	0.628177	0.136461
1790	1.45501	0.15152
1791	2.19809	0.127921
1792	1.45501	0.0591649
1793	1.48974	0.05
1794	1.7709	2.1
1795	2.63288	2.1

1796	1.5362	2.1
1797	1.56327	2.1
1798	3.03945	0.05
1799	0.899979	0.05
1800	0.869386	0.05
1801	1.85864	0.05
1802	1.78028	0.05
1803	1.28719	0.05
1804	1.09532	0.05
1805	0.881898	0.05
1806	1.28911	0.05
1807	1.39644	0.05
1808	0.688829	0.05
1809	2.14611	0.05
1810	1.80313	0.05
1811	0.68883	0.05
1812	0.946131	0.05
1813	1.25334	0.05
1814	1.88987	0.05
1815	1.94432	0.05
1816	1.37592	0.05
1817	1.11691	2.1
1818	1.02923	2.1
1819	0.45824	0.05
1820	0.646512	0.05
1821	2.46292	0.05
1822	0.493451	0.05
1823	2.00826	2.1
1824	2.49784	2.1
1825	1.17054	2.1
1826	1.01485	2.1
1827	1.26045	2.1
1828	1.17054	2.1
1829	1.44247	2.1
1830	2.02687	0.05
1831	1.66365	0.05
1832	1.64656	0.05
1833	1.56597	0.05
1834	1.21419	2.1
1835	1.5008	2.1
1836	1.10711	2.1
1837	1.44287	2.1
1838	1.44287	2.1
1839	0.750368	2.1
1840	1.25876	2.1
1841	0.82542	2.1
1842	1.04074	2.1
1843	1.25874	2.1
1844	1.04159	0.05
1845	1.2489	0.05
1846	0.980547	0.119865
1847	1.62235	0.0945028
1848	1.15991	0.124709
1849	0.980547	0.0972421
1850	0.94613	0.151432
1851	1.54662	0.111844
1852	1.54661	0.130554
1853	1.07912	0.16264

1854	1.1032	0.114468
1855	1.1032	0.131622
1856	0.94613	0.129411
1857	1.12468	2.1
1858	1.2724	2.1
1859	1.06521	2.1
1860	1.0752	2.1
1861	1.94936	0.0878196
1862	1.62772	0.0715761
1863	1.94733	0.070059
1864	1.42901	0.0591257
1865	1.33022	2.1
1866	1.70839	2.1
1867	1.75048	2.1
1868	1.41201	2.1
1869	1.52314	2.1
1870	1.44039	2.1
1871	1.51113	2.1
1872	2.58638	2.1
1873	0.987247	0.121051
1874	1.38622	0.325652
1875	0.858359	0.134725
1876	1.19967	0.187545
1877	1.06414	0.132353
1878	0.946132	0.320739
1879	0.94613	0.128209
1880	0.687079	0.538732
1881	0.946132	0.281054
1882	0.822611	0.313518
1883	1.70242	0.151596
1884	1.70242	0.280977
1885	0.946132	0.543663
1886	1.11155	0.18121
1887	0.946132	0.682568
1888	1.11155	0.404478
1889	1.37981	0.471082
1890	1.18348	0.172393
1891	2.44179	0.249545
1892	2.00403	0.431444
1893	1.10961	2.1
1894	1.85415	2.1
1895	1.44825	2.1
1896	0.932272	2.1
1897	0.810942	2.1
1898	1.00189	2.1
1899	1.11779	2.1
1900	1.3359	2.1
1901	0.965279	2.1
1902	0.833779	2.1
1903	1.05337	2.1
1904	0.526656	2.1
1905	1.44599	2.1
1906	1.44599	2.1
1907	1.24853	2.1
1908	0.946132	2.1
1909	1.24853	2.1
1910	0.724122	2.1
1911	1.91547	2.1

1912	2.49705	2.1
1913	1.05931	2.1
1914	1.1729	2.1
1915	1.34526	2.1
1916	1.5379	2.1
1917	1.90855	2.1
1918	2.08597	2.1
1919	1.68165	2.1
1920	1.45887	2.1
1921	1.50022	2.1
1922	1.4748	1.94947
1923	2.63645	1.81236
1924	1.74431	1.86878
1925	1.25288	1.65978
1926	1.25289	1.44595
1927	0.970025	1.5815
1928	0.747542	2.00507
1929	0.810959	1.49518
1930	1.15369	1.51677
1931	0.977472	1.51929
1932	1.12695	1.52937
1933	0.996762	1.57737
1934	1.28249	0.501903
1935	0.946132	0.55757
1936	0.528621	0.600186
1937	1.59186	0.362417
1938	1.44072	0.370472
1939	0.633488	0.491202
1940	0.598506	0.58199
1941	1.51618	0.520984
1942	1.19999	0.341364
1943	1.46511	0.310267
1944	1.44056	0.269122
1945	0.912754	0.264145
1946	1.4725	0.192534
1947	1.4725	0.183214
1948	0.912753	0.268824
1949	1.23351	0.235092
1950	1.23351	0.262464
1951	0.946131	0.307672
1952	1.17129	0.190366
1953	1.82551	0.17172
1954	1.27575	1.90243
1955	0.946129	1.99443
1956	0.946129	2.04032
1957	0.946128	1.90485
1958	0.792754	2.0406
1959	0.94613	1.86651
1960	0.792752	1.89445
1961	2.08054	2.00209
1962	1.20091	2.0421
1963	2.1669	1.95378
1964	1.24383	1.71027
1965	0.904669	1.9083
1966	0.83804	1.57322
1967	1.90486	1.56478
1968	1.29162	1.53859
1969	1.37383	1.69659

1970	1.86533	1.62747
1971	1.76472	1.71542
1972	1.60428	1.66224
1973	2.43993	1.89049
1974	1.37833	2.1
1975	1.18632	2.1
1976	0.946132	2.1
1977	1.27544	2.1
1978	1.05085	2.1
1979	0.961178	2.1
1980	0.859844	2.1
1981	1.0499	2.1
1982	1.75592	2.1
1983	2.08668	2.1
1984	1.75592	2.1
1985	1.4402	2.1
1986	1.72285	2.1
1987	0.83312	0.05
1988	1.07295	0.05
1989	1.37607	0.05
1990	1.32097	0.05
1991	1.11332	0.05
1992	1.18568	0.05
1993	0.877482	0.05
1994	1.4458	0.05
1995	2.34112	0.05
1996	2.68021	0.05
1997	0.681677	0.05
1998	0.946131	0.05
1999	0.681649	0.05
2000	1.83115	0.05
2001	1.68029	0.05
2002	1.05482	0.05
2003	1.31485	0.05
2004	2.08247	0.709917
2005	1.33153	0.573333
2006	0.935062	0.926132
2007	0.806958	1.41114
2008	0.827683	0.85037
2009	1.01041	0.875581
2010	0.741832	0.753748
2011	1.15668	0.934915
2012	0.908649	0.915073
2013	0.59561	0.370206
2014	1.19028	0.510611
2015	0.59561	0.449475
2016	0.946132	0.614075
2017	0.595623	0.702011
2018	1.58164	0.521882
2019	1.58164	0.495403
2020	0.946131	2.1
2021	1.55201	2.1
2022	1.55201	2.1
2023	0.946127	2.1
2024	1.13312	2.1
2025	1.13312	2.1
2026	1.08108	2.1
2027	1.04973	2.1

2028	1.20149	2.1
2029	1.86902	1.71823
2030	0.613447	2.1
2031	1.22618	1.84437
2032	1.38663	1.98853
2033	1.38662	2.1
2034	0.613447	1.97608
2035	1.22617	2.04963
2036	0.773899	2.08183
2037	1.23515	1.95565
2038	1.87823	1.70431
2039	1.70303	0.64603
2040	1.4714	2.1
2041	1.74067	2.1
2042	0.735699	2.1
2043	1.73461	2.1
2044	1.47224	2.1
2045	1.22827	2.1
2046	1.1634	2.1
2047	1.36471	2.1
2048	0.735701	2.1
2049	2.8386	1.52005
2050	2.39056	2.1
2051	2.44128	2.06948
2052	1.54102	2.1
2053	1.54102	2.1
2054	0.936141	2.1
2055	1.40594	2.1
2056	1.07121	2.06967
2057	0.936142	2.1
2058	1.07121	2.1
2059	1.22691	0.0687367
2060	1.42907	0.0525294
2061	2.18048	0.05
2062	0.947662	0.05
2063	2.18394	2.1
2064	1.21292	2.1
2065	2.18394	2.1
2066	2.19064	2.1
2067	1.27229	2.1
2068	1.20859	0.474704
2069	1.21073	0.325222
2070	1.73899	2.1
2071	1.3164	2.1
2072	1.61245	2.1
2073	1.41444	2.1
2074	2.55116	2.1
2075	1.26193	2.1
2076	1.89553	2.1
2077	0.916438	2.1
2078	1.26113	2.1
2079	0.810934	2.1
2080	2.00419	2.1
2081	1.27872	2.1
2082	1.35306	2.1
2083	1.96749	2.1
2084	1.07955	2.1
2085	1.31854	2.1

2086	1.29505	0.318894
2087	1.29504	0.370561
2088	0.612469	0.529043
2089	0.990053	0.378941
2090	1.04072	0.505836
2091	0.985266	0.350723
2092	0.990039	0.336757
2093	1.06892	0.491012
2094	1.57158	0.246463
2095	1.895	0.307794
2096	1.05698	0.251264
2097	0.925053	2.1
2098	0.624712	2.1
2099	1.42465	2.1
2100	1.49014	2.1
2101	0.687198	2.1
2102	1.22058	2.1
2103	0.851488	2.1
2104	0.588128	2.1
2105	1.12452	2.1
2106	1.02783	2.1
2107	1.7083	2.1
2108	1.06977	0.212927
2109	0.818569	0.133465
2110	1.14386	0.1739
2111	0.983791	0.133669
2112	1.12214	0.230576
2113	1.0915	0.158452
2114	0.810934	0.182354
2115	0.764114	0.379573
2116	1.87956	0.287605
2117	1.87956	0.324533
2118	0.764141	0.424003
2119	1.37675	0.261355
2120	0.764141	0.392102
2121	0.946132	0.323287
2122	1.9637	0.274349
2123	2.10107	0.252196
2124	1.67814	0.407441
2125	3.02886	0.303852
2126	0.908982	0.0707699
2127	0.621727	0.163691
2128	0.525963	0.105118
2129	0.946128	0.174127
2130	1.60979	0.0743684
2131	1.45596	0.117605
2132	0.556172	0.162102
2133	2.06779	0.070733
2134	2.17911	0.0828812
2135	1.00205	0.05
2136	1.30685	0.0731977
2137	1.16534	0.0827168
2138	1.65532	2.1
2139	2.20346	2.1
2140	0.993889	2.1
2141	1.09328	2.1
2142	2.09372	2.1
2143	2.10036	2.1

2144	1.33727	0.472527
2145	2.53383	0.349489
2146	0.893379	0.516726
2147	1.52828	0.430428
2148	1.09599	0.05
2149	1.09599	0.05
2150	1.50524	2.1
2151	1.54711	2.1
2152	1.41469	2.1
2153	1.99459	2.03528
2154	1.79805	2.1
2155	1.79805	2.1
2156	0.707344	2.1
2157	1.30318	2.1
2158	0.707344	2.1
2159	0.946129	2.1
2160	1.30318	2.1
2161	2.40442	2.1
2162	1.89226	2.1
2163	1.40794	2.09449
2164	0.898721	2.09229
2165	0.791921	1.7891
2166	0.810933	2.09436
2167	1.10904	2.06172
2168	0.991102	2.09096
2169	0.808502	1.71059
2170	1.1398	2.06986
2171	1.31346	2.1
2172	1.11833	2.1
2173	1.669	2.1
2174	1.25715	2.1
2175	1.3199	2.1
2176	1.63092	2.1
2177	1.63092	2.1
2178	0.946131	2.1
2179	0.946131	2.1
2180	1.13534	2.1
2181	0.979853	2.1
2182	2.19994	2.1
2183	0.725844	2.1
2184	2.19994	2.1
2185	2.35055	2.1
2186	1.52204	2.1
2187	1.40118	2.1
2188	3.42285	2.1
2189	2.05217	2.1
2190	1.50319	2.1
2191	0.947516	2.03565
2192	0.903572	2.04199
2193	0.76996	2.0714
2194	1.90959	2.1
2195	1.18897	2.1
2196	1.24166	2.1
2197	2.26785	2.09223
2198	2.05217	2.1
2199	2.73306	2.1
2200	1.42729	0.05
2201	1.387	0.0588454

2202	1.59458	0.348424
2203	1.91051	0.397223
2204	1.68108	0.20276
2205	2.03357	0.283409
2206	0.928982	0.0667567
2207	1.55687	0.0678075
2208	1.55687	0.05
2209	0.92898	0.0551049
2210	1.3495	0.0781221
2211	1.3495	0.0552997
2212	0.946133	0.0833953
2213	1.85795	0.05
2214	2.2727	0.0601746
2215	1.20324	2.06215
2216	1.07784	0.192319
2217	0.957792	0.128331
2218	1.2745	0.123589
2219	1.4659	0.143323
2220	0.946129	0.208289
2221	0.95778	0.196704
2222	0.851553	0.258531
2223	1.74103	0.145447
2224	2.28994	0.156493
2225	2.08213	0.115995
2226	2.1556	0.128708
2227	2.20372	0.105057
2228	0.838027	0.05
2229	1.11373	0.05
2230	1.2909	0.05
2231	0.998424	0.05
2232	1.40621	0.05
2233	1.19478	0.05
2234	1.11783	0.05
2235	1.6695	0.05
2236	1.7498	0.05
2237	0.477652	0.05
2238	1.77291	0.05
2239	0.590448	0.05
2240	1.07668	0.05
2241	0.810946	0.05
2242	1.1946	0.05
2243	2.03914	0.05
2244	1.25585	0.05
2245	2.02622	0.05
2246	1.39091	0.05
2247	1.31871	0.05
2248	1.15789	2.1
2249	1.15755	2.1
2250	1.82268	2.1
2251	1.87119	2.1
2252	3.10636	2.1
2253	2.96285	2.1
2254	1.73443	0.702929
2255	2.33812	0.696582
2256	1.59457	0.377123
2257	1.73443	0.930339
2258	2.00665	2.1
2259	1.99841	2.1

2260	1.19901	2.1
2261	1.16661	2.1
2262	2.15883	0.106672
2263	1.20951	0.107678
2264	2.49192	0.14575
2265	0.65112	0.0967566
2266	2.45317	0.05
2267	1.73091	0.05
2268	2.0795	0.05
2269	1.45891	0.05
2270	1.6531	0.05
2271	1.78304	0.05
2272	0.775132	0.05
2273	1.35051	0.05
2274	1.0461	0.05
2275	1.37837	0.05
2276	1.00259	0.05
2277	1.25796	0.0626739
2278	1.10719	0.0770998
2279	1.51067	2.1
2280	2.65632	2.07503
2281	1.30521	2.1
2282	2.456 2.1	
2283	3.19218	2.1
2284	2.67387	2.06446
2285	0.988862	2.1
2286	1.917 2.1	
2287	0.946132	2.1
2288	1.16202	2.1
2289	1.53871	2.1
2290	1.53871	2.1
2291	0.946132	2.1
2292	1.16202	2.1
2293	1.32282	2.1
2294	2.46356	2.1
2295	2.32404	2.1
2296	1.37562	0.0540514
2297	0.985546	0.05
2298	1.28983	0.05
2299	1.05566	0.05
2300	0.895312	0.05
2301	1.53325	0.05
2302	1.04342	0.05
2303	1.80881	0.05
2304	0.876865	0.05
2305	0.5689	0.05
2306	1.09506	0.05
2307	1.51789	2.1
2308	1.49872	2.1
2309	0.949364	0.0983027
2310	2.01233	0.100466
2311	1.55063	0.104079
2312	1.57783	0.240732
2313	1.57783	0.258403
2314	0.674605	0.303553
2315	0.98537	0.130899
2316	0.674633	0.104833
2317	0.98537	0.115411

2318	1.26706	0.295028
2319	0.980664	0.442631
2320	1.03298	0.158386
2321	1.84936	0.343391
2322	1.51696	0.107251
2323	1.97074	0.0980724
2324	1.03298	0.0835965
2325	1.51324	0.0788893
2326	1.51324	0.0822485
2327	0.758454	0.0799986
2328	1.28632	0.0708833
2329	0.758482	0.0541419
2330	0.758482	0.0627645
2331	0.985372	0.0784394
2332	1.14105	0.05
2333	1.59787	0.246483
2334	2.46244	0.31689
2335	1.70367	0.146409
2336	2.13757	1.625
2337	1.98142	1.60963
2338	1.35878	0.05
2339	2.39947	0.05
2340	1.24554	0.05
2341	1.7522	0.05
2342	1.49693	1.65692
2343	1.49652	1.70986
2344	1.6211	1.74846
2345	1.45938	1.79047
2346	0.737431	1.96109
2347	1.18643	1.80105
2348	1.04072	1.99984
2349	0.9459	1.87818
2350	0.66364	1.94966
2351	0.985109	1.52102
2352	0.638368	1.89434
2353	0.83804	1.43551
2354	0.998024	1.36173
2355	0.914471	1.17906
2356	1.14281	1.43225
2357	0.833972	1.50483
2358	0.991865	0.05
2359	1.42208	0.05
2360	1.70718	2.1
2361	1.79888	2.1
2362	1.16851	0.344965
2363	1.16851	0.310415
2364	1.51021	0.44997
2365	0.946132	0.246684
2366	1.14897	0.147887
2367	0.946132	0.275808
2368	1.77177	0.257813
2369	1.77177	0.155405
2370	1.33038	0.218851
2371	1.38752	0.229534
2372	1.1726	0.15687
2373	1.51688	0.241989
2374	1.06455	2.1
2375	0.98511	2.1

2376	1.31144	2.1
2377	1.79825	2.1
2378	1.79825	2.1
2379	1.73044	2.1
2380	1.59589	0.05
2381	1.59589	0.05
2382	2.78009	2.1
2383	1.68761	2.1
2384	3.05511	2.1
2385	2.25318	2.1
2386	0.578998	2.1
2387	1.03512	2.1
2388	2.26156	2.1
2389	1.44859	0.245968
2390	1.35897	0.591648
2391	1.18301	2.1
2392	1.25173	2.1
2393	1.98851	2.1
2394	2.70685	2.1
2395	0.682342	2.1
2396	1.52491	2.1
2397	1.37119	2.1
2398	0.682313	2.1
2399	1.35342	2.1
2400	0.947555	2.1
2401	0.614107	2.1
2402	1.23007	2.1
2403	1.5735	2.1
2404	1.58713	2.1
2405	1.57979	0.05
2406	1.30723	0.05
2407	0.935658	0.547397
2408	0.8952	0.413199
2409	1.27352	1.35852
2410	1.0985	0.914933
2411	1.07163	0.729086
2412	1.15823	0.691895
2413	0.836451	0.645099
2414	2.08051	0.329455
2415	1.92601	1.56216
2416	2.0644	0.317846
2417	1.37259	2.1
2418	1.37259	2.1
2419	0.752217	2.1
2420	1.28569	2.1
2421	0.752186	2.1
2422	0.839124	2.1
2423	1.28568	2.1
2424	1.04199	2.1
2425	1.04199	2.1
2426	2.59268	2.1
2427	1.04199	2.1
2428	1.5098	2.1
2429	1.5098	2.1
2430	0.520997	2.1
2431	1.29634	2.1
2432	0.520996	2.1
2433	0.73446	2.1

2434	1.29634	2.1
2435	0.983724	2.1
2436	1.23914	2.1
2437	2.65095	2.1
2438	1.52851	2.1
2439	2.399	2.1
2440	1.68137	2.1
2441	1.11285	2.1
2442	1.04921	2.1
2443	1.30711	2.1
2444	1.04921	2.1
2445	1.07496	0.618867
2446	1.55831	0.28166
2447	1.07497	0.338218
2448	1.07497	0.827156
2449	1.27391	2.1
2450	2.5615	2.1
2451	1.2739	2.1
2452	1.75253	2.09607
2453	1.12907	0.0603611
2454	1.9194	0.0605983
2455	1.96617	0.0559998
2456	1.01225	2.1
2457	0.947979	2.1
2458	1.01225	2.09825
2459	1.06561	2.1
2460	1.01225	2.1
2461	3.56876	2.1
2462	3.68767	2.1
2463	1.52301	2.1
2464	1.61013	2.1
2465	1.43726	2.1
2466	2.04275	2.1
2467	0.995734	2.1
2468	1.16199	2.1
2469	1.73704	0.05
2470	1.04019	0.05
2471	0.956294	2.1
2472	1.81488	2.1
2473	1.63719	2.1
2474	1.50103	0.05
2475	1.75289	0.05
2476	1.31491	0.05
2477	1.03567	0.05
2478	0.672257	0.05
2479	1.04072	0.05
2480	0.746903	0.05
2481	1.43317	0.05
2482	1.39986	0.05
2483	1.06385	0.05
2484	1.1624	0.05
2485	1.61667	0.05
2486	1.27258	0.05
2487	2.559	0.05
2488	1.66093	0.05
2489	1.10719	0.0840736
2490	2.03235	0.0855392
2491	1.17105	0.151341

2492	1.10719	0.0952565
2493	1.3028	2.1
2494	0.946131	2.1
2495	0.946127	2.09694
2496	1.52912	2.1
2497	1.52912	2.1
2498	0.946128	2.05944
2499	0.949997	2.08924
2500	1.31565	2.1
2501	1.46643	2.1
2502	1.5482	2.1
2503	1.47102	2.1
2504	1.47102	2.1
2505	0.774101	2.1
2506	1.30583	2.1
2507	0.774103	2.1
2508	0.939287	2.1
2509	1.30584	2.1
2510	0.958661	2.1
2511	2.28835	2.1
2512	1.43213	0.05
2513	2.13255	0.05
2514	1.43213	0.05
2515	1.10476	0.05
2516	1.93165	0.05
2517	1.03235	0.927974
2518	1.31	0.995117
2519	1.22082	2.1
2520	1.35827	2.1
2521	0.790936	2.1
2522	0.716644	2.1
2523	1.43257	2.1
2524	1.43257	2.1
2525	0.716615	2.1
2526	0.946131	2.1
2527	0.730157	2.1
2528	1.47212	2.1
2529	1.47212	2.1
2530	0.946131	2.1
2531	0.790792	2.1
2532	1.41149	2.1
2533	0.790791	2.1
2534	1.01006	2.1
2535	1.53045	0.482089
2536	1.53045	0.651737
2537	0.946132	1.33978
2538	1.40779	0.548948
2539	1.06879	1.58181
2540	0.990713	0.942882
2541	1.06879	1.07164
2542	1.06486	0.306459
2543	1.18347	0.28602
2544	1.38622	0.323934
2545	1.18347	0.188005
2546	1.7304	0.297317
2547	1.7304	0.40125
2548	0.946131	0.755799
2549	1.26874	0.385634

2550	1.13337	1.39143
2551	1.13337	0.615989
2552	1.40779	0.638439
2553	1.695 2.1	
2554	1.77071	2.1
2555	0.649313	2.1
2556	0.692829	2.1
2557	1.40395	2.1
2558	1.34243	2.1
2559	0.813292	0.130967
2560	0.632691	0.415176
2561	1.75766	0.340018
2562	2.25727	1.84091
2563	1.74206	0.104834
2564	0.826073	0.122536
2565	1.30022	0.121927
2566	1.45641	0.0856722
2567	0.946131	0.129972
2568	0.752245	0.0829824
2569	0.960952	0.163326
2570	0.743477	0.110862
2571	1.04355	0.0702873
2572	1.6253	0.102143
2573	0.960577	0.100388
2574	1.34852	0.554242
2575	1.03235	0.588587
2576	1.78168	0.175152
2577	1.2966	0.05
2578	2.21116	0.05
2579	1.6395	0.05
2580	1.6395	0.05
2581	0.94613	0.05
2582	1.22187	0.05
2583	0.962737	0.05
2584	0.962735	0.05
2585	1.36376	0.05
2586	1.2966	0.05
2587	1.2966	0.05
2588	1.19669	0.524815
2589	2.37636	0.666903
2590	1.08894	0.987777
2591	1.62551	0.792104
2592	2.51472	1.02094
2593	1.89226	0.65064
2594	0.980038	0.961138
2595	1.10187	2.1
2596	1.01479	2.1
2597	1.81663	2.1
2598	1.83247	2.1
2599	0.921851	0.245649
2600	0.983148	2.1
2601	1.82816	2.1
2602	1.26103	2.1
2603	2.34818	2.1
2604	1.23152	2.1
2605	2.15738	2.1
2606	1.23152	2.1
2607	1.51042	2.1

2608	0.842995	2.1
2609	1.22257	2.1
2610	0.810959	2.1
2611	1.68242	2.1
2612	0.95701	2.1
2613	1.12389	2.1
2614	1.95255	2.1
2615	1.57974	2.1
2616	1.94632	2.1
2617	0.81534	2.1
2618	1.13996	2.1
2619	1.18958	0.05
2620	1.25929	0.05
2621	3.02933	1.17473
2622	2.37434	0.638424
2623	1.10609	2.05668
2624	1.64132	1.35759
2625	1.64132	0.990478
2626	0.946132	0.441681
2627	1.40029	1.33586
2628	1.08946	0.344979
2629	1.08946	0.440191
2630	1.18717	0.138941
2631	2.71421	0.0936817
2632	1.08189	0.05
2633	0.94613	0.05
2634	1.37109	0.0583697
2635	1.17925	0.05
2636	0.851541	0.0546627
2637	0.942512	0.0564328
2638	1.18541	0.05
2639	0.851526	0.0589293
2640	1.0819	0.05
2641	1.86009	0.0642836
2642	0.946132	0.05
2643	1.67409	0.05
2644	1.67409	0.05
2645	0.946131	0.05
2646	1.31716	0.05
2647	1.30307	0.05
2648	0.946131	0.05
2649	1.18601	0.05
2650	3.15646	2.1
2651	0.985109	2.1
2652	3.37048	2.1
2653	1.10667	2.1
2654	1.77065	1.20189
2655	1.45837	1.03926
2656	1.77063	0.810412
2657	2.58104	0.766859
2658	1.37652	2.1
2659	1.54168	2.1
2660	1.00224	0.05
2661	1.05851	0.05
2662	1.39093	0.05
2663	1.43358	0.05
2664	0.575741	0.05
2665	0.518161	0.05

2666	1.04071	0.05
2667	1.13092	1.77687
2668	1.17042	2.02374
2669	1.53347	1.67534
2670	1.52938	1.76587
2671	1.00613	0.858309
2672	1.81618	2.1
2673	1.81618	1.03972
2674	0.946127	2.1
2675	1.36479	2.1
2676	0.94613	0.578147
2677	1.31724	1.71017
2678	1.3648	1.67207
2679	1.00613	0.966103
2680	0.781375	0.136115
2681	1.13431	0.170452
2682	1.03547	0.163843
2683	1.40668	0.179121
2684	0.94613	0.164429
2685	0.713285	0.211355
2686	1.72885	0.193701
2687	1.72885	0.212748
2688	0.713298	0.133876
2689	3.30075	0.125239
2690	2.81337	0.157273
2691	1.39296	0.238206
2692	0.830164	2.1
2693	1.50493	2.1
2694	1.50494	2.1
2695	0.830136	2.1
2696	1.28298	2.1
2697	1.0521	2.1
2698	0.830147	2.1
2699	2.0799	2.1
2700	1.52115	2.09982
2701	2.5142	2.1
2702	1.31253	0.429001
2703	1.94446	0.924745
2704	1.18817	0.643153
2705	0.598348	0.480837
2706	1.69315	0.532562
2707	1.69315	0.732061
2708	0.598377	0.48882
2709	0.598347	0.494763
2710	0.94613	0.471313
2711	1.28418	0.823046
2712	2.05205	1.08248
2713	1.28418	0.873932
2714	1.0737	2.1
2715	1.81024	2.1
2716	2.73834	2.1
2717	1.71369	2.1
2718	1.71368	2.1
2719	0.946118	2.1
2720	1.29063	2.1
2721	1.36917	2.1
2722	0.94613	2.1
2723	1.29062	2.1

2724	0.947921	2.1
2725	1.58661	1.82213
2726	1.37658	1.58815
2727	1.2364	1.4688
2728	1.66685	0.373175
2729	1.66685	1.92262
2730	0.946133	0.254341
2731	0.946133	0.880697
2732	1.11978	1.54895
2733	1.09194	0.358223
2734	1.21858	1.70934
2735	1.37658	1.20192
2736	1.64903	0.393624
2737	1.64903	2.08066
2738	0.946132	0.424402
2739	0.946132	1.05153
2740	1.05496	1.13088
2741	1.22851	0.296527
2742	1.22852	1.91711
2743	1.58482	0.199181
2744	1.60578	0.0848457
2745	1.06336	0.0789059
2746	1.68062	0.0876777
2747	1.14721	0.0611049
2748	1.11924	0.05
2749	1.18421	0.05
2750	2.01276	0.05
2751	1.23502	1.81401
2752	1.34926	1.93773
2753	0.546751	1.5429
2754	0.790446	1.75679
2755	1.04072	1.68183
2756	0.555868	1.44238
2757	0.930305	1.50324
2758	1.09344	1.51227
2759	1.18351	1.50125
2760	1.97215	1.58402
2761	1.07416	1.64373
2762	1.33204	1.37411
2763	1.22038	0.907938
2764	0.597449	0.83532
2765	0.821422	1.22626
2766	1.04071	1.19526
2767	0.608026	0.642514
2768	0.929427	1.05474
2769	1.64929	0.05
2770	1.68541	0.05
2771	0.496093	0.05
2772	1.3385	0.05
2773	0.790798	0.05
2774	1.22971	0.05
2775	0.898853	0.05
2776	2.14361	0.05
2777	2.10813	0.05
2778	1.05417	0.05
2779	1.80768	0.05
2780	1.09725	2.1
2781	1.41131	2.1

2782	1.1656	2.1
2783	0.946129	2.1
2784	1.68365	2.1
2785	1.68365	2.1
2786	1.1656	2.1
2787	0.94613	2.1
2788	1.21269	0.05
2789	1.48867	0.05
2790	1.27806	0.05
2791	1.68264	0.05
2792	1.37813	1.70766
2793	1.50743	1.15704
2794	1.04121	1.35454
2795	1.12434	0.479271
2796	1.39553	0.525254
2797	1.44721	1.78611
2798	0.946132	0.67684
2799	1.23943	0.817385
2800	1.05292	1.07034
2801	1.28607	0.439671
2802	1.16915	0.339704
2803	1.76306	2.04135
2804	1.42266	1.90207
2805	1.36844	2.1
2806	1.36844	2.1
2807	1.67352	2.1
2808	1.11256	1.18881
2809	1.03217	0.949333
2810	1.18422	0.05
2811	1.04159	0.05
2812	1.60139	0.05
2813	1.04159	0.05
2814	0.988805	2.1
2815	1.05481	2.1
2816	1.52938	2.00961
2817	1.94724	1.76565
2818	0.896336	0.0679669
2819	0.911484	0.0712493
2820	1.18315	0.05
2821	1.10515	0.05
2822	1.04067	0.05
2823	1.12758	0.0578257
2824	0.904106	0.0605451
2825	1.6901	0.05
2826	1.68671	0.05
2827	1.99908	0.05
2828	0.772426	0.52965
2829	0.810945	1.76388
2830	0.667562	1.17046
2831	0.496617	0.315163
2832	0.847689	0.924687
2833	0.914378	1.36096
2834	0.934003	0.797197
2835	1.07459	0.810749
2836	1.40225	2.1
2837	1.07043	2.1
2838	0.94613	2.1
2839	1.63072	2.1

2840	1.63072	2.1
2841	1.07043	2.1
2842	0.946131	2.1
2843	1.53082	2.1
2844	1.69916	2.1
2845	1.75211	1.46266
2846	1.96831	2.1
2847	1.75211	2.07805
2848	0.946127	0.625813
2849	0.817065	0.582948
2850	1.41798	0.572032
2851	1.41798	0.468079
2852	0.791656	0.614583
2853	0.791656	0.639012
2854	1.41798	0.586475
2855	1.58331	0.552687
2856	1.41776	0.421788
2857	0.791647	0.415132
2858	1.4512	0.380213
2859	1.45119	0.392799
2860	0.75821	0.487967
2861	0.758248	0.455811
2862	0.946104	0.468818
2863	1.36482	0.439991
2864	1.29389	1.98421
2865	1.04164	1.9012
2866	1.40574	1.75178
2867	1.64833	1.8047
2868	1.71183	0.151886
2869	1.71183	0.266907
2870	0.946131	0.592277
2871	1.35721	0.260311
2872	0.946131	0.97181
2873	0.946131	0.634662
2874	1.30076	0.572552
2875	1.2474	0.193273
2876	2.85225	0.357392
2877	2.71441	0.156887
2878	1.2474	0.13174
2879	1.38622	0.33962
2880	0.858359	0.123409
2881	1.3682	0.161012
2882	1.57364	0.124573
2883	0.729695	0.320205
2884	0.729723	0.111575
2885	0.822611	0.664399
2886	0.946133	0.329638
2887	0.729723	0.187769
2888	0.974704	0.336961
2889	0.974702	0.332566
2890	1.81603	2.1
2891	1.96348	2.1
2892	1.43612	0.524818
2893	1.43612	0.859427
2894	0.634295	0.872582
2895	0.93796	0.898698
2896	0.946133	1.13362
2897	0.634326	0.455512

2898	0.946133	0.664675
2899	1.2654	0.345208
2900	1.26865	0.554898
2901	2.2649	0.531991
2902	1.00864	0.498048
2903	1.09689	0.355873
2904	0.832021	0.370175
2905	1.53229	0.317778
2906	1.1145	0.366537
2907	0.929583	0.362291
2908	1.613	0.286281
2909	0.644729	0.345889
2910	1.03471	2.1
2911	2.7739	2.1
2912	1.36133	2.08633
2913	2.50215	2.1
2914	2.52195	2.1
2915	1.0465	2.1
2916	1.42673	2.1
2917	1.42673	2.1
2918	0.946132	2.1
2919	1.31904	2.1
2920	0.986499	2.1
2921	0.946132	2.1
2922	1.31904	2.1
2923	0.966978	2.1
2924	1.42237	0.05
2925	2.60925	0.062763
2926	0.753659	0.0856221
2927	1.77241	0.0537272
2928	1.77241	0.0675138
2929	0.946129	0.0904257
2930	0.75366	0.0520822
2931	1.22144	0.0795505
2932	0.94613	0.0827631
2933	1.50732	0.0540247
2934	1.18514	0.0656704
2935	0.902187	0.0790457
2936	1.89858	0.05
2937	1.24294	0.0568672
2938	1.67211	0.0627526
2939	1.41786	0.05
2940	0.810959	0.0732969
2941	1.50732	0.058706
2942	2.44288	0.0755281
2943	1.40969	0.05
2944	2.06654	0.05
2945	1.40969	0.05
2946	1.40882	0.05
2947	1.94063	0.0587475
2948	0.867921	0.05
2949	1.29289	0.05
2950	0.867918	0.05
2951	0.946128	0.0505066
2952	0.867944	0.0514972
2953	1.66589	0.05
2954	1.66589	0.05
2955	0.968595	2.1

2956	1.5534	2.1
2957	1.27793	2.1
2958	1.13433	2.1
2959	1.3439	0.05
2960	2.53709	0.05
2961	0.571998	0.05
2962	0.64837	0.05
2963	1.99755	0.05
2964	1.99854	0.05
2965	0.764229	2.1
2966	1.61842	2.1
2967	1.61842	2.1
2968	1.18315	2.1
2969	1.1995	2.1
2970	1.18315	2.1
2971	0.764256	2.1
2972	1.52364	2.1
2973	2.36631	2.1
2974	0.641912	2.0428
2975	1.7181	2.1
2976	1.459 2.1	
2977	0.634845	2.00421
2978	1.18315	2.1
2979	1.17615	2.1
2980	0.571367	2.09941
2981	1.3324	0.142555
2982	0.798214	0.147721
2983	0.946128	0.154731
2984	0.627252	0.137075
2985	1.50337	0.132743
2986	1.50337	0.0986291
2987	0.62725	0.1694
2988	1.07763	0.0928109
2989	1.07763	0.125836
2990	2.16109	0.131999
2991	1.07763	0.122031
2992	1.12486	2.1
2993	1.11827	2.1
2994	1.0491	2.1
2995	1.09034	2.1
2996	1.09431	2.1
2997	0.946131	2.1
2998	0.748607	2.1
2999	1.44002	2.1
3000	1.44002	2.1
3001	0.748635	2.1
3002	0.782505	2.1
3003	1.40615	2.1
3004	1.19539	2.1
3005	1.29389	1.91502
3006	1.4867	1.32432
3007	1.50103	0.05
3008	1.63025	0.05
3009	2.72109	0.05
3010	0.915755	0.05
3011	0.946132	0.05
3012	0.915755	0.05
3013	1.70236	0.05

3014	1.70236	0.05
3015	1.26155	0.05
3016	1.35657	0.05
3017	1.83151	0.05
3018	1.83151	0.05
3019	2.52309	0.05
3020	0.983607	2.1
3021	1.06838	0.820806
3022	0.983609	2.1
3023	0.983596	2.06688
3024	2.74384	0.0945182
3025	1.6261	0.0678316
3026	1.15477	2.1
3027	1.02136	2.1
3028	1.83561	2.1
3029	1.83561	2.1
3030	0.946131	2.1
3031	1.39523	2.1
3032	1.29313	2.1
3033	1.29313	2.1
3034	1.38651	2.1
3035	1.06313	2.1
3036	0.974888	2.1
3037	1.0894	2.1
3038	0.974888	2.1
3039	1.82282	2.1
3040	1.82282	2.1
3041	0.946131	2.1
3042	1.37372	2.1
3043	1.32374	2.1
3044	1.32374	2.1
3045	1.39523	2.1
3046	1.40882	0.0529112
3047	0.861998	0.0614448
3048	1.24448	0.0682607
3049	0.861999	0.0579288
3050	0.946129	0.0833827
3051	0.862011	0.0782293
3052	1.67537	0.0666172
3053	1.67537	0.05
3054	1.72399	0.05
3055	2.58579	0.05
3056	1.72399	0.05
3057	1.50951	0.05
3058	2.48897	0.0527759
3059	1.26755	2.1
3060	1.03824	2.1
3061	1.27875	2.1
3062	1.48901	2.1
3063	1.18902	2.1
3064	1.11764	2.1
3065	1.87436	2.1
3066	0.95345	2.00761
3067	0.724191	1.77752
3068	0.86668	1.81435
3069	0.811523	1.8887
3070	1.04074	1.50846
3071	0.843097	1.68247

3072	0.833434	1.85855
3073	0.965727	1.79874
3074	0.85346	1.7714
3075	1.00185	0.902733
3076	1.00184	1.74445
3077	0.980651	1.88149
3078	0.980664	1.68079
3079	0.475737	1.43767
3080	0.719696	1.44353
3081	0.946109	1.07346
3082	0.946116	1.23055
3083	0.719693	1.35079
3084	0.946117	1.23004
3085	1.14879	1.7989
3086	0.719683	1.69731
3087	0.900977	1.35043
3088	0.972364	1.45552
3089	1.22961	1.41847
3090	0.405384	1.3104
3091	1.34406	2.1
3092	1.2187	2.1
3093	0.963571	0.502762
3094	0.946131	1.26593
3095	1.57246	1.17661
3096	1.57246	2.1
3097	0.946131	1.22352
3098	1.1235	2.1
3099	1.1235	2.08149
3100	1.07439	2.09647
3101	1.19612	1.39179
3102	1.24844	1.47552
3103	1.00007	2.01285
3104	1.02429	2.1
3105	1.10459	2.1
3106	1.06724	0.20514
3107	0.67519	0.505236
3108	0.49762	0.237741
3109	1.43435	0.170859
3110	0.833315	0.374874
3111	0.58135	0.271925
3112	1.14749	0.701667
3113	1.85091	1.72066
3114	1.2802	0.529841
3115	1.27737	1.29161
3116	2.3655	0.949369
3117	1.30584	2.1
3118	2.06424	2.1
3119	0.946131	0.110534
3120	1.04257	0.090281
3121	0.94613	0.238017
3122	1.67201	0.0937646
3123	1.67201	0.0588241
3124	1.27696	0.0734011
3125	1.34118	0.05
3126	1.3413	0.0679186
3127	2.36238	0.0580747
3128	0.6036	0.100247
3129	1.11745	0.110312

3130	1.46126	0.081067
3131	1.39612	0.0603494
3132	0.670678	0.0997469
3133	1.03402	0.109256
3134	1.11271	0.101129
3135	0.838028	0.0544149
3136	0.807019	0.0795359
3137	1.27787	0.05
3138	0.994705	0.051307
3139	1.20014	0.05
3140	0.964793	0.0733365
3141	0.863868	0.0618932
3142	1.3413	0.05
3143	1.79549	0.0550977
3144	1.35877	2.1
3145	1.63648	2.1
3146	1.08171	2.1
3147	1.27839	2.1
3148	1.52601	2.1
3149	1.27841	2.1
3150	1.71255	2.1
3151	2.16307	2.1
3152	1.91592	2.1
3153	1.78508	2.1
3154	0.946128	2.1
3155	1.41199	2.1
3156	0.946128	2.1
3157	0.901088	2.1
3158	1.55244	2.1
3159	1.59475	2.1
3160	1.40921	2.1
3161	0.960106	2.1
3162	1.40921	2.1
3163	2.0073	2.1
3164	2.2973	0.109537
3165	1.90705	0.116332
3166	1.53671	0.0767469
3167	1.86035	0.0718271
3168	1.33747	2.1
3169	1.14007	2.1
3170	1.3212	2.1
3171	1.66867	0.0673098
3172	1.30203	0.05
3173	1.27696	2.1
3174	1.05025	2.1
3175	1.05025	2.1
3176	1.3704	2.1
3177	1.15677	2.1
3178	0.946129	2.1
3179	1.65879	2.1
3180	1.65879	2.1
3181	1.15676	2.1
3182	0.94613	2.1
3183	0.867217	1.5285
3184	1.28684	1.39071
3185	1.58866	1.19462
3186	1.58866	0.808126
3187	0.867246	1.80729

3188	1.28684	1.23982
3189	1.16906	1.44487
3190	1.13438	1.18046
3191	1.37224	1.41337
3192	1.61519	1.52615
3193	0.883939	1.84171
3194	1.40092	1.58039
3195	1.80383	1.7865
3196	1.80383	1.58742
3197	0.883935	1.87512
3198	1.40092	1.50911
3199	1.28684	1.75108
3200	1.76788	1.16459
3201	2.57368	1.3273
3202	1.37224	1.84353
3203	1.65411	0.837271
3204	1.77042	2.1
3205	1.77042	2.1
3206	1.90446	2.1
3207	1.28667	2.1
3208	1.55635	2.1
3209	1.30646	0.33196
3210	2.31836	0.0939683
3211	1.30645	0.0900887
3212	1.86429	0.154134
3213	1.48676	0.0935644
3214	0.743379	0.110183
3215	1.41848	0.100881
3216	1.41848	0.091739
3217	0.946131	0.107156
3218	0.812649	0.0895847
3219	1.34921	0.101226
3220	0.812649	0.1019
3221	0.949366	0.095445
3222	1.32974	0.082018
3223	0.794204	0.05
3224	0.634352	2.1
3225	0.838025	2.08833
3226	1.08802	2.04047
3227	0.838038	2.0135
3228	1.18314	2.0413
3229	1.12495	2.0539
3230	1.22774	2.03168
3231	1.04343	2.04802
3232	0.921111	0.05
3233	2.1609	0.05
3234	1.09987	0.05
3235	2.5043	0.05
3236	1.05492	0.05
3237	0.590344	0.05
3238	1.05492	0.05
3239	1.25215	0.05
3240	1.71674	0.05
3241	1.71674	0.05
3242	0.590331	0.05
3243	1.17078	0.05
3244	0.625542	0.05
3245	0.724673	2.1

3246	1.08011	2.1
3247	1.87598	2.1
3248	1.50738	2.1
3249	1.44951	0.107586
3250	1.50102	0.103757
3251	1.8042	0.390686
3252	0.687769	0.744506
3253	1.66483	0.474753
3254	1.66483	0.546638
3255	0.946132	0.700156
3256	0.687798	0.477482
3257	1.20885	0.722089
3258	0.687799	0.61893
3259	0.886063	0.232082
3260	0.798751	0.252763
3261	0.584813	0.207269
3262	1.49483	0.147173
3263	0.888005	0.191848
3264	1.03589	0.166121
3265	1.70435	0.209155
3266	0.710414	2.1
3267	0.917609	2.06394
3268	0.810947	2.07775
3269	0.81096	2.053
3270	1.48658	1.90283
3271	1.53653	2.00307
3272	1.19329	2.07115
3273	0.946153	2.00643
3274	1.50798	2.03939
3275	1.85017	2.1
3276	1.19768	2.1
3277	0.864304	2.1
3278	0.792906	2.09155
3279	0.989025	1.99157
3280	1.19768	1.83961
3281	1.54548	1.98245
3282	1.18798	2.06797
3283	1.31875	1.85214
3284	1.05725	1.99849
3285	2.17365	0.05
3286	2.48106	0.05
3287	1.87451	0.05
3288	1.27189	1.44326
3289	2.79399	1.86743
3290	0.859565	1.42566
3291	0.926174	1.28279
3292	0.604744	1.2099
3293	1.41832	0.606705
3294	1.05494	0.986942
3295	0.874749	0.974997
3296	1.50067	0.800202
3297	0.917817	1.64084
3298	0.63591	1.6636
3299	1.72849	1.38562
3300	1.72849	1.01909
3301	0.635943	1.26368
3302	1.39699	1.5996
3303	0.967411	1.95598

3304	0.635943	1.41485
3305	1.39908	1.06765
3306	1.26971	1.31195
3307	1.02969	1.70019
3308	1.15326	1.91323
3309	2.30741	1.78556
3310	0.93545	1.28357
3311	0.802747	0.949759
3312	1.15466	1.55966
3313	0.859164	1.65088
3314	1.02616	1.73013
3315	1.23149	1.47136
3316	0.83804	1.09223
3317	1.13266	1.92758
3318	1.64142	1.89399
3319	1.21223	2.1
3320	1.43051	2.1
3321	1.32418	1.14783
3322	0.621319	2.03946
3323	1.47134	2.08817
3324	1.79103	0.875927
3325	3.25951	0.05
3326	3.16764	0.05
3327	1.65583	0.176974
3328	1.4732	0.096196
3329	1.76476	2.1
3330	1.80114	2.09256
3331	0.754121	2.1
3332	0.94613	1.72571
3333	0.754119	2.1
3334	1.45636	2.06061
3335	1.45636	1.08997
3336	1.30029	1.97863
3337	0.910183	1.95277
3338	1.29099	2.1
3339	1.08039	2.1
3340	1.05498	2.1
3341	1.24445	2.1
3342	1.2873	2.1
3343	0.987703	2.1
3344	0.964782	2.1
3345	1.73645	0.05
3346	0.871279	0.0588201
3347	0.884744	0.0714168
3348	1.85193	0.05
3349	1.68681	0.05
3350	1.33689	0.0554187
3351	1.23493	0.05
3352	0.892482	0.0681109
3353	2.95185	0.05
3354	2.10496	0.05
3355	1.87334	2.1
3356	0.94411	2.1
3357	0.754196	2.1
3358	1.43122	2.1
3359	1.58684	2.1
3360	0.838027	2.1
3361	1.1536	2.1

3362	1.24878	2.1
3363	1.23125	2.1
3364	2.86862	2.1
3365	1.57636	2.1
3366	0.998451	0.349332
3367	1.5176	0.273891
3368	1.13271	0.05
3369	1.60248	0.05
3370	1.22083	2.1
3371	1.79996	2.1
3372	1.19087	2.1
3373	2.00342	2.1
3374	1.3715	2.1
3375	2.79227	2.1
3376	2.7873	2.1
3377	0.987265	0.05
3378	1.62715	2.1
3379	1.70117	2.1
3380	1.27806	0.05
3381	1.45246	2.1
3382	1.45244	2.1
3383	1.87214	2.1
3384	0.995796	2.1
3385	1.68189	0.882134
3386	1.49155	2.1
3387	1.58054	2.1
3388	1.59169	2.1
3389	0.736364	0.05
3390	0.70236	0.05
3391	2.24367	0.05
3392	2.57085	0.05
3393	1.70952	0.05
3394	2.60516	0.05
3395	1.45158	2.1
3396	1.00371	2.1
3397	1.65748	2.1
3398	1.23706	0.162937
3399	1.55834	1.16584
3400	1.72671	0.673379
3401	0.597656	0.480607
3402	1.39137	1.18268
3403	0.855578	0.122424
3404	1.48398	0.562463
3405	0.745117	0.143859
3406	1.23706	0.363793
3407	1.57916	0.772288
3408	0.893025	0.229072
3409	3.22433	0.277551
3410	2.47324	1.7748
3411	1.63852	2.1
3412	1.63852	2.1
3413	0.946132	2.1
3414	1.19095	2.1
3415	0.946133	2.1
3416	1.16684	2.1
3417	1.19095	2.1
3418	1.06698	0.158377
3419	1.48692	0.128617

3420	1.08334	2.00982
3421	1.07283	2.1
3422	1.28517	1.49664
3423	1.2746	0.05
3424	1.30723	0.05
3425	2.02069	0.05
3426	1.05579	0.05
3427	1.59475	2.1
3428	2.40113	2.09875
3429	1.58875	2.1
3430	1.6881	2.1
3431	1.6881	2.1
3432	0.819953	2.1
3433	0.819954	2.1
3434	0.946131	2.1
3435	0.946131	2.09574
3436	1.34782	2.1
3437	1.65619	2.04944
3438	1.65619	2.03112
3439	0.79437	2.07283
3440	1.25	2.04986
3441	0.94613	2.081
3442	0.946131	2.04109
3443	0.794371	2.08389
3444	1.58875	2.08034
3445	2.5	2.05051
3446	1.04147	2.1
3447	0.946131	0.777194
3448	1.79686	2.1
3449	1.79686	2.1
3450	1.25144	0.751269
3451	1.35714	2.06156
3452	1.38585	1.8962
3453	1.25144	2.0846
3454	1.15564	2.1
3455	1.34389	2.1
3456	0.946133	0.678359
3457	1.74353	2.04814
3458	1.74353	2.1
3459	1.14553	0.640797
3460	1.38585	1.54912
3461	1.30382	1.13668
3462	1.14553	1.97228
3463	1.15564	2.1
3464	1.90618	2.1
3465	2.11056	2.1
3466	1.032	2.1
3467	0.953636	2.1
3468	1.23131	1.33741
3469	1.39763	1.07973
3470	2.01024	0.904421
3471	1.9186	0.982175
3472	1.83658	0.080058
3473	1.81656	0.0846921
3474	2.2049	0.104608
3475	2.16081	0.0851117
3476	1.12219	0.107116
3477	0.599771	0.127345

3478	1.61401	0.0785684
3479	1.05912	0.10344
3480	0.951648	0.103862
3481	1.89328	0.0828076
3482	0.913682	0.122698
3483	1.00763	0.0895351
3484	1.29129	2.1
3485	1.37138	2.1
3486	1.41986	0.05
3487	1.41986	0.0685103
3488	0.944166	0.0572003
3489	1.41789	0.05
3490	0.944167	0.05
3491	0.944166	0.05
3492	0.946132	0.05
3493	1.98713	0.05
3494	1.23626	0.05
3495	0.932791	0.05
3496	0.810251	0.05
3497	0.986748	0.0516529
3498	0.898155	0.0578504
3499	0.92624	0.05
3500	1.1349	0.05
3501	0.934572	0.05
3502	1.23104	0.661389
3503	1.23104	0.817493
3504	0.799994	0.41393
3505	0.94613	0.677169
3506	0.802822	0.407511
3507	1.40944	0.614676
3508	0.800035	0.697873
3509	1.41223	0.610984
3510	1.41223	0.447133
3511	1.6	0.366063
3512	1.60565	0.416762
3513	1.23042	2.1
3514	1.0645	2.1
3515	1.00319	2.1
3516	1.29435	2.1
3517	1.2243	2.1
3518	1.2243	2.1
3519	1.05655	1.51388
3520	1.29764	1.73612
3521	2.55252	2.1
3522	1.15676	2.08653
3523	0.855295	2.07907
3524	0.810959	2.09137
3525	1.65857	2.08859
3526	1.00849	2.05194
3527	1.16021	2.08658
3528	1.75869	2.08847
3529	1.13775	2.08028
3530	1.20091	2.09575
3531	1.61582	2.1
3532	1.88031	2.1
3533	0.968536	2.1
3534	0.946131	2.1
3535	1.61481	2.1

3536	1.61481	2.1
3537	1.09547	2.1
3538	1.18486	2.1
3539	1.37609	2.1
3540	1.09546	2.1
3541	1.02461	2.1
3542	1.83223	2.1
3543	1.74002	2.1
3544	1.70939	2.1
3545	1.7094	2.1
3546	0.726287	2.1
3547	1.01712	2.09563
3548	1.30318	2.1
3549	0.946131	2.1
3550	0.87155	2.1
3551	1.77093	2.00745
3552	2.02356	1.97666
3553	1.32449	2.1
3554	1.10003	2.1
3555	1.5161	2.1
3556	1.5161	2.1
3557	0.946132	2.1
3558	1.09206	2.1
3559	0.946132	2.1
3560	1.04404	2.1
3561	1.09206	2.1
3562	0.635628	1.20934
3563	1.14623	0.355169
3564	1.73197	0.501889
3565	1.73197	1.09617
3566	0.529655	0.627348
3567	1.14623	0.585632
3568	0.992085	0.718264
3569	1.16915	1.30601
3570	2.29245	0.407539
3571	0.584573	0.559279
3572	0.956756	0.227908
3573	1.52471	0.411366
3574	1.51409	1.73164
3575	0.584575	0.394233
3576	0.94613	0.2274
3577	1.14622	0.3078
3578	1.28096	1.17947
3579	1.34695	1.5527
3580	1.04193	2.1
3581	1.05378	2.1
3582	0.798209	2.1
3583	1.32007	2.04126
3584	1.64327	0.05
3585	2.64674	0.373402
3586	2.38936	0.05
3587	2.88475	0.05
3588	1.07745	2.1
3589	1.08911	2.1
3590	1.49127	2.1
3591	1.49127	2.1
3592	0.709714	2.1
3593	1.37199	2.1

3594	0.82899	2.1
3595	0.709714	2.1
3596	0.946132	2.1
3597	1.00133	2.1
3598	1.40182	2.1
3599	1.53278	2.1
3600	1.40182	2.1
3601	1.31764	0.05
3602	1.00691	0.05
3603	1.24094	0.05
3604	0.64868	0.05
3605	0.946133	0.05
3606	0.64868	0.05
3607	1.71762	0.05
3608	1.71762	0.05
3609	1.41662	0.05
3610	0.975477	0.05
3611	1.73584	0.05
3612	2.48188	0.05
3613	2.2045	0.295943
3614	2.08134	0.249729
3615	0.993366	1.43093
3616	0.993368	1.41672
3617	1.14187	0.238266
3618	0.946132	0.608093
3619	0.946133	0.744484
3620	1.68951	1.06331
3621	1.68951	0.905812
3622	1.34612	0.637897
3623	1.28953	0.469842
3624	1.34611	1.76901
3625	1.31892	1.73118
3626	0.946131	1.07111
3627	1.80988	1.72202
3628	1.80988	2.1
3629	1.31892	1.50882
3630	0.946131	1.60896
3631	2.13214	2.1
3632	1.94691	2.1
3633	1.52661	0.05
3634	1.53175	0.05
3635	0.956005	0.05
3636	0.955991	0.05
3637	1.40572	0.05
3638	0.955992	0.05
3639	1.51084	2.1
3640	1.67115	2.1
3641	1.73143	2.1
3642	1.55234	2.1
3643	1.69377	2.1
3644	2.51483	2.1
3645	1.25987	2.1
3646	1.4472	2.1
3647	1.83151	0.342495
3648	2.01001	0.641283
3649	0.993998	0.301536
3650	1.83151	0.217396
3651	1.40915	1.29434

3652	1.24653	1.20934
3653	1.74472	2.1
3654	1.74472	2.1
3655	1.74244	2.1
3656	1.04788	2.1
3657	1.28522	2.1
3658	1.03785	2.1
3659	0.896519	2.1
3660	1.60428	2.1
3661	1.73305	2.1
3662	1.39072	0.125386
3663	2.27501	0.282912
3664	1.39073	0.171477
3665	1.39073	0.243496
3666	1.06838	2.09708
3667	0.968462	1.10559
3668	1.06837	1.86989
3669	1.13455	1.131
3670	1.23527	1.1905
3671	2.71686	0.565268
3672	1.86881	0.882674
3673	1.10586	2.09535
3674	1.03852	2.1
3675	1.03899	2.1
3676	0.963973	2.1
3677	1.00319	2.1
3678	1.00319	2.1
3679	0.987132	2.1
3680	0.974889	2.1
3681	0.987131	2.1
3682	1.7677	2.1
3683	1.7677	2.1
3684	0.946131	2.1
3685	1.31185	2.1
3686	0.946132	2.1
3687	1.30203	2.1
3688	1.31185	2.1
3689	1.82958	2.1
3690	1.82958	2.1
3691	0.946132	2.1
3692	1.40199	2.1
3693	0.946133	2.1
3694	1.36994	2.1
3695	1.40199	2.1
3696	0.97489	2.1
3697	1.05939	1.88178
3698	1.01922	1.96409
3699	0.810941	1.73643
3700	1.05372	1.85117
3701	0.754065	1.99796
3702	0.926131	1.95888
3703	1.35905	1.81304
3704	0.810931	1.82105
3705	1.75647	1.05067
3706	1.68265	2.1
3707	1.41995	2.1
3708	1.36457	1.56007
3709	0.933392	1.25426

3710	1.42255	2.1
3711	1.3868	2.1
3712	1.08074	2.1
3713	1.07196	2.1
3714	1.13537	0.407954
3715	1.01179	0.30806
3716	1.60196	0.281056
3717	1.72798	0.293088
3718	1.3715	0.300002
3719	1.56409	1.02945
3720	1.56409	0.183552
3721	1.97819	0.96668
3722	1.47954	0.693616
3723	0.798655	0.251292
3724	1.47952	0.445427
3725	2.30327	0.349592
3726	1.49111	2.1
3727	1.96013	2.03011
3728	1.54599	2.1
3729	2.06632	2.1
3730	1.54601	2.1
3731	1.10557	0.102731
3732	1.17624	0.206915
3733	1.60497	2.1
3734	1.61587	2.1
3735	1.65977	0.05
3736	1.69044	0.05
3737	1.00659	2.1
3738	1.06226	2.1
3739	1.15171	2.1
3740	1.50853	2.1
3741	1.80252	2.1
3742	1.41432	2.1
3743	1.04687	2.1
3744	0.940878	2.1
3745	1.52031	2.1
3746	1.52031	2.1
3747	0.940877	2.1
3748	0.946132	2.1
3749	1.50853	2.1
3750	0.963973	2.1
3751	1.70562	0.05
3752	2.43203	0.05
3753	1.36004	0.05
3754	1.06648	0.05
3755	1.66009	0.05
3756	1.66009	0.05
3757	0.766427	0.05
3758	0.766452	0.05
3759	0.946132	0.05
3760	1.98012	0.05
3761	1.20357	2.1
3762	1.20356	2.1
3763	1.67324	2.1
3764	1.52008	2.1
3765	1.19964	2.1
3766	1.36861	2.1
3767	1.19964	2.1

3768	2.85415	2.05514
3769	1.43687	2.08676
3770	0.935852	2.1
3771	1.09972	2.0834
3772	0.810933	2.04735
3773	2.25974	2.06269
3774	1.74149	2.05749
3775	1.36984	2.07749
3776	1.87602	2.07422
3777	0.890748	2.05545
3778	1.20587	1.91612
3779	0.810956	1.81498
3780	2.03174	1.82063
3781	1.34403	1.86978
3782	1.58825	1.87053
3783	1.63096	1.8941
3784	2.53735	1.90123
3785	1.98241	1.91093
3786	2.53014	2.03534
3787	1.1775	2.0419
3788	1.06538	1.74059
3789	0.946132	0.982122
3790	1.4101	2.0271
3791	1.75162	2.1
3792	1.75162	1.00813
3793	0.946132	1.4741
3794	1.4101	1.23317
3795	1.28765	1.03887
3796	1.1427	0.503853
3797	1.1427	0.502552
3798	1.3109	2.1
3799	1.02721	1.98109
3800	0.846447	1.8698
3801	0.862076	2.1
3802	0.644098	1.83248
3803	0.581308	2.00588
3804	1.02306	1.99726
3805	0.322641	0.952384
3806	0.589346	1.1709
3807	2.25572	1.86089
3808	1.31372	2.1
3809	1.18873	2.1
3810	1.78225	2.1
3811	1.764 2.1	
3812	1.17503	2.0571
3813	1.06821	2.0765
3814	1.06821	1.87344
3815	1.7777	1.79638
3816	1.9096	0.240988
3817	1.70239	0.242537
3818	1.16182	0.05
3819	1.20132	0.05
3820	1.23475	2.1
3821	1.56859	2.1
3822	2.46263	2.1
3823	0.528744	2.1
3824	2.93337	2.1
3825	2.42504	2.1

3826	1.38646	0.301253
3827	1.61963	0.569921
3828	1.88016	0.254259
3829	0.622283	0.151274
3830	1.03243	0.50363
3831	0.710307	1.51174
3832	0.798124	0.253091
3833	0.931073	1.16327
3834	0.892862	1.01189
3835	1.06791	0.318709
3836	0.552787	0.241693
3837	0.946132	0.0930262
3838	1.36665	1.58382
3839	1.36665	0.50891
3840	0.506966	0.185451
3841	0.506964	0.129081
3842	1.36665	0.345863
3843	0.653469	0.87275
3844	1.36665	0.108316
3845	1.21339	0.133615
3846	1.21339	0.393068
3847	0.552757	0.604351
3848	0.552786	0.169088
3849	1.21336	0.194218
3850	1.70744	1.74616
3851	1.0775	0.0969091
3852	1.59527	0.523065
3853	1.48312	0.24515
3854	2.01868	0.219715
3855	1.18947	0.0855014
3856	1.48312	0.250605
3857	0.785626	0.117152
3858	1.19601	0.132357
3859	0.785626	0.12316
3860	0.946132	0.136466
3861	0.7856	0.188225
3862	1.59087	0.12954
3863	1.51158	0.118392
3864	1.74098	0.1128
3865	2.39202	0.118737
3866	0.870489	0.231257
3867	1.34534	0.283285
3868	0.870488	0.206834
3869	0.946133	0.320779
3870	0.870489	0.425544
3871	1.67086	0.287627
3872	1.67086	0.26641
3873	2.11311	2.1
3874	1.98765	1.94073
3875	0.946133	1.09987
3876	1.30881	2.06557
3877	1.72211	2.09835
3878	1.72211	1.66067
3879	0.94613	1.99197
3880	1.30881	1.76302
3881	1.35943	1.58959
3882	1.01294	0.436619
3883	1.01294	0.404427

3884	1.38963	2.08664
3885	1.01294	0.81863
3886	1.60056	0.612968
3887	1.60056	2.01255
3888	0.946132	2.09488
3889	1.204	1.92749
3890	0.946132	0.296058
3891	1.11288	1.31746
3892	1.204	0.840371
3893	1.14599	2.07675
3894	1.00065	2.04525
3895	1.74685	2.09877
3896	1.74685	2.1
3897	0.946128	2.09617
3898	1.38463	2.00417
3899	1.2275	2.1
3900	1.2275	2.1
3901	1.30835	2.1
3902	2.04407	2.1
3903	2.50425	2.1
3904	1.19647	0.279072
3905	1.19647	0.34275
3906	1.19645	0.420372
3907	1.75446	0.261944
3908	1.1315	1.3913
3909	1.1315	1.02132
3910	1.45569	1.02489
3911	1.1315	0.790727
3912	1.14357	2.08218
3913	1.25793	2.09346
3914	1.14357	2.06954
3915	2.08181	1.99522
3916	1.4714	2.1
3917	0.71295	2.1
3918	1.68285	2.1
3919	1.68285	2.1
3920	1.25603	2.1
3921	1.13976	2.1
3922	1.25603	2.1
3923	0.71295	2.1
3924	1.61095	2.1
3925	2.51207	2.1
3926	1.63762	2.1
3927	2.74451	2.1
3928	0.671255	2.02609
3929	1.87601	2.1
3930	1.87601	2.1
3931	0.671226	2.01632
3932	1.25603	2.1
3933	1.29123	2.09408
3934	0.671226	2.09329
3935	1.63723	2.1
3936	0.872928	0.0531495
3937	1.47904	0.05
3938	1.18793	0.05
3939	2.57464	0.05
3940	1.14986	2.09973
3941	1.19611	2.08633

3942	0.96029	1.1946
3943	1.7961	2.1
3944	0.928721	2.1
3945	1.34352	2.1
3946	1.37935	2.1
3947	1.37935	2.1
3948	0.892888	2.1
3949	0.946131	2.1
3950	0.892888	2.1
3951	1.75422	2.1
3952	1.05556	2.1
3953	1.27362	0.227951
3954	1.27421	0.169195
3955	1.3003	1.23704
3956	1.31415	1.30354
3957	1.01431	2.1
3958	0.673951	2.1
3959	1.63384	2.1
3960	1.63661	2.1
3961	1.52245	2.1
3962	1.50714	2.1
3963	0.996778	0.05
3964	1.56091	0.05
3965	1.06543	2.1
3966	2.66689	2.1
3967	1.01474	2.1
3968	2.76422	2.1
3969	1.45321	0.107279
3970	0.974716	0.0720655
3971	0.810943	0.081734
3972	0.909982	0.149498
3973	0.937793	0.094896
3974	1.05138	0.0679398
3975	1.25044	0.0713538
3976	0.92095	0.0816638
3977	0.830414	0.104191
3978	2.1402	0.0905667
3979	1.75938	2.1
3980	1.05556	2.1
3981	1.78558	2.1
3982	1.379 2.1	
3983	0.970214	0.292395
3984	0.9702	0.286306
3985	1.08894	1.00844
3986	2.17251	1.04685
3987	1.51569	0.628587
3988	1.06012	0.560296
3989	0.98966	0.495661
3990	1.55926	0.378544
3991	1.92645	0.466682
3992	1.37427	0.05
3993	2.16338	0.05
3994	1.5456	0.05
3995	1.5456	0.05
3996	1.94002	0.05
3997	1.33135	0.05
3998	1.31948	0.05
3999	2.23255	0.05

4000	2.34061	0.05
4001	1.52903	2.1
4002	3.38327	2.1
4003	1.35977	2.1
4004	2.22414	2.1
4005	1.6812	0.0725262
4006	2.33397	0.0816452
4007	1.16699	0.121418
4008	0.946131	0.116487
4009	1.58115	0.113449
4010	1.58115	0.102024
4011	0.519726	0.182029
4012	0.946131	0.144508
4013	0.519697	0.116424
4014	1.26868	0.0838204
4015	1.16699	0.0900352
4016	1.5951	0.0736299
4017	1.5951	0.06507
4018	0.840568	0.116076
4019	0.946131	0.0946978
4020	0.840598	0.0862258
4021	1.30047	2.1
4022	1.5586	2.1
4023	1.31825	1.52217
4024	1.31924	1.43854
4025	1.15818	1.86221
4026	1.52782	0.563386
4027	1.15818	1.10863
4028	1.15818	1.36243
4029	0.99518	0.05
4030	0.94613	0.05
4031	0.922045	0.05
4032	1.29322	0.0525849
4033	1.29321	0.05
4034	1.29321	0.0845509
4035	0.922056	0.05
4036	1.06848	0.0807401
4037	1.84409	0.0559968
4038	1.9186	1.00104
4039	2.72244	0.750257
4040	0.946132	0.875913
4041	1.36122	0.62916
4042	1.79658	0.599692
4043	1.79658	0.567113
4044	0.946133	1.0686
4045	1.36122	0.786037
4046	1.38149	0.625898
4047	1.59106	0.575495
4048	0.946116	0.796955
4049	1.29052	0.565279
4050	1.76641	0.510852
4051	1.76641	0.540554
4052	0.885309	0.947963
4053	1.29054	0.708865
4054	1.36118	0.597375
4055	1.59103	0.615994
4056	2.27816	0.457086
4057	1.56164	0.561633

4058	1.41114	0.05
4059	2.04128	0.05
4060	0.986723	2.1
4061	1.03645	0.133742
4062	1.79224	0.121052
4063	1.2627	2.1
4064	1.23036	2.1
4065	1.45257	2.1
4066	1.41469	2.1
4067	1.41469	2.1
4068	2.60636	2.1
4069	1.45789	0.05
4070	0.851541	0.05
4071	1.57877	0.05
4072	1.53134	0.05
4073	0.851479	0.05
4074	1.08982	0.05
4075	0.716284	0.05
4076	0.946131	0.05
4077	1.73193	0.05
4078	2.57309	0.05
4079	1.36307	0.05
4080	2.42175	0.05
4081	1.72948	0.05
4082	0.978698	2.1
4083	0.978699	2.1
4084	2.08398	2.1
4085	0.978698	2.1
4086	1.44096	2.1
4087	1.44096	2.1
4088	0.638164	2.1
4089	0.681588	2.1
4090	0.946133	2.1
4091	0.638165	2.1
4092	0.94613	2.1
4093	1.55432	2.1
4094	0.901147	2.1
4095	0.85777	2.1
4096	1.36073	2.1
4097	1.44004	2.1
4098	0.946133	2.1
4099	1.09982	2.1
4100	1.27562	2.1
4101	1.55432	2.1
4102	1.62229	2.1
4103	1.0101	2.1
4104	2.28459	2.0932
4105	2.50554	1.97738
4106	0.946131	1.28361
4107	1.25276	1.98664
4108	1.73721	2.01432
4109	1.73721	1.56553
4110	0.93406	1.90091
4111	1.25277	1.78383
4112	1.4185	1.71345
4113	1.53064	1.56217
4114	0.946133	1.66777
4115	0.609462	2.01249

4116	1.65123	2.00451
4117	1.65123	1.78781
4118	0.609466	2.05742
4119	1.00792	1.94647
4120	1.25277	1.85959
4121	1.53064	1.86945
4122	2.15223	1.36969
4123	1.30664	1.81933
4124	1.10674	2.1
4125	1.22862	2.1
4126	1.49358	0.079763
4127	2.0207	0.0694361
4128	2.09374	2.1
4129	1.88175	2.1
4130	1.10938	0.740117
4131	1.02478	1.79877
4132	1.13272	0.05
4133	1.13272	0.05
4134	1.03935	1.06635
4135	0.810945	1.32964
4136	0.527573	0.690769
4137	0.925011	1.09885
4138	1.03193	0.635835
4139	1.38587	0.758263
4140	0.703469	0.710978
4141	0.912282	1.02704
4142	1.75505	2.1
4143	1.78529	2.1
4144	1.60017	0.515881
4145	1.35608	0.164262
4146	1.01479	2.1
4147	1.47561	1.87955
4148	1.01479	2.1
4149	1.28418	1.93924
4150	2.62605	2.1
4151	1.71233	2.1
4152	3.22222	2.1
4153	1.97526	2.1
4154	0.949712	0.101693
4155	1.78986	0.0988021
4156	2.72879	0.0887486
4157	2.66428	0.092453
4158	1.68108	0.168745
4159	1.67265	0.144034
4160	2.24877	0.18451
4161	1.15558	0.21522
4162	1.39708	2.1
4163	1.39327	2.1
4164	1.69788	0.05
4165	1.1273	0.05
4166	1.17302	1.5894
4167	1.04349	1.27999
4168	0.979853	2.1
4169	1.19094	2.1
4170	1.39408	2.1
4171	1.61857	2.1
4172	1.66523	2.1
4173	0.946132	2.1

4174	0.838554	2.1
4175	1.05151	2.1
4176	1.18901	2.1
4177	1.18901	2.1
4178	1.61149	2.1
4179	1.22857	0.34155
4180	1.25848	0.3687
4181	1.09154	2.1
4182	2.20358	2.1
4183	1.23013	2.1
4184	1.55698	2.1
4185	1.55828	2.09019
4186	1.52391	2.09987
4187	0.60346	1.97056
4188	1.06336	2.09783
4189	0.870714	1.87453
4190	0.543109	1.98215
4191	1.10367	1.9273
4192	1.20686	1.98557
4193	1.20684	2.04883
4194	2.5118	1.95034
4195	1.20684	2.0814
4196	1.52468	1.99005
4197	1.52469	1.99004
4198	0.532308	1.76025
4199	0.903301	1.99131
4200	0.946119	1.76856
4201	0.532315	1.71182
4202	0.946107	1.81443
4203	1.46129	2.1
4204	0.804766	2.1
4205	1.4809	2.1
4206	1.4809	2.1
4207	1.11822	2.1
4208	1.16744	2.1
4209	1.11822	2.1
4210	0.804737	2.1
4211	1.04193	2.1
4212	0.983723	2.1
4213	1.8014	2.1
4214	1.1729	2.1
4215	1.03951	2.1
4216	1.03951	2.1
4217	1.17583	2.1
4218	1.42866	2.1
4219	1.78503	2.1
4220	1.17822	0.789265
4221	1.89121	2.1
4222	1.44472	2.1
4223	1.62143	2.1
4224	1.43837	2.1
4225	1.01121	0.0608582
4226	0.962703	1.00189
4227	0.946129	0.606033
4228	1.10941	0.367017
4229	0.946131	0.677841
4230	1.60326	0.747488
4231	1.60326	0.419386

4232	1.14215	0.54907
4233	1.40724	0.555627
4234	0.962703	0.704758
4235	1.53336	0.05
4236	1.40272	0.05
4237	1.64984	0.05
4238	1.67345	0.05
4239	1.08212	2.1
4240	1.40905	2.1
4241	0.946132	0.05
4242	0.946132	0.05
4243	0.81268	0.05
4244	0.946131	0.05
4245	0.812681	0.05
4246	0.946131	0.05
4247	0.946132	0.05
4248	0.997755	0.05
4249	1.94585	0.05
4250	1.2925	0.05
4251	2.52454	0.05
4252	1.69182	0.05
4253	1.69182	0.05
4254	0.92361	0.05
4255	1.35316	0.05
4256	1.26227	0.05
4257	0.946131	0.05
4258	1.35316	0.05
4259	1.48676	0.107301
4260	1.33917	0.0889443
4261	0.757292	2.1
4262	1.02415	2.1
4263	1.48495	2.1
4264	0.97587	2.1
4265	0.946132	2.1
4266	0.747365	2.1
4267	1.55432	2.1
4268	1.55432	2.1
4269	0.946132	2.1
4270	0.747394	2.1
4271	1.17464	0.05
4272	1.0372	0.05
4273	2.16553	0.05
4274	2.00616	0.05
4275	1.12184	0.05
4276	1.33177	0.05
4277	0.936674	0.05
4278	0.946129	0.05
4279	0.851552	0.0550435
4280	1.60205	0.05
4281	1.77391	0.05
4282	1.03327	0.0767687
4283	1.41512	0.083153
4284	1.03327	0.0651249
4285	0.946133	0.0916703
4286	0.946131	0.100322
4287	1.80077	0.0811967
4288	1.80077	0.0677893
4289	1.54848	2.1

4290	1.29931	2.1
4291	1.04482	0.111795
4292	2.08069	0.127379
4293	0.946127	0.113633
4294	0.515539	0.154642
4295	0.515566	0.211071
4296	1.38423	0.114783
4297	1.38423	0.103957
4298	0.972058	0.163131
4299	0.927737	0.162248
4300	0.94971	0.0863088
4301	2.19682	0.113123
4302	0.946132	0.168612
4303	1.09841	0.119479
4304	0.522382	0.21543
4305	1.5962	0.0968323
4306	1.72791	0.079507
4307	1.07428	0.140414
4308	1.05215	0.125291
4309	1.54632	1.77834
4310	1.15693	1.81261
4311	1.41238	2.1
4312	1.41238	2.1
4313	1.77966	2.1
4314	1.77966	2.1
4315	0.946129	2.1
4316	1.40525	2.1
4317	0.946131	2.1
4318	0.94613	2.1
4319	1.32054	2.1
4320	1.41238	2.1
4321	1.94026	2.1
4322	1.0171	0.624174
4323	1.1211	0.543878
4324	0.993022	2.1
4325	0.773813	2.1
4326	1.4073	2.1
4327	1.4073	2.1
4328	0.94613	2.1
4329	0.787615	2.1
4330	0.94613	2.1
4331	0.787615	2.1
4332	1.764	2.1
4333	1.90964	2.1
4334	1.05787	2.1
4335	1.403	0.95967
4336	0.744189	1.29943
4337	2.32834	2.1
4338	2.34986	2.1
4339	1.17106	0.337366
4340	1.94826	0.05
4341	2.40606	0.05
4342	1.26387	0.053799
4343	0.954013	0.0556215
4344	0.946131	0.0673074
4345	1.71	0.05
4346	1.51926	0.05
4347	0.953997	0.05

4348	0.851554	0.0582776
4349	1.96502	0.05
4350	1.72954	0.05
4351	2.89117	0.05
4352	0.971958	0.0565286
4353	0.985575	0.0713772
4354	1.41943	0.05
4355	1.20021	0.05
4356	1.29599	0.05291
4357	1.15687	0.05
4358	0.810958	0.0645501
4359	1.12919	0.05
4360	1.98859	2.1
4361	1.82044	2.1
4362	1.90488	2.1
4363	0.851542	2.1
4364	1.02973	2.1
4365	1.39286	2.1
4366	1.44873	2.1
4367	0.946132	2.1
4368	1.11826	2.1
4369	1.26842	2.1
4370	2.09461	2.1
4371	1.76952	0.05
4372	1.6473	0.05
4373	0.946131	0.05
4374	1.36486	0.05
4375	1.04243	0.05
4376	1.05017	0.05
4377	1.24059	0.05
4378	2.11646	0.05
4379	1.50368	0.05
4380	2.38796	0.05
4381	1.50368	0.05
4382	1.71844	0.05
4383	1.71844	0.05
4384	0.914836	0.05
4385	1.24416	0.05
4386	0.914805	0.05
4387	1.24416	0.05
4388	1.38911	0.05
4389	1.46859	0.05
4390	1.35348	0.05
4391	0.946131	0.05
4392	1.1226	0.05
4393	0.857078	0.05
4394	0.9746	0.05
4395	1.37624	0.05
4396	1.15131	0.05
4397	1.139 0.05	
4398	1.90456	0.05
4399	2.09692	0.05
4400	1.16655	2.1
4401	1.23017	2.1
4402	2.14592	2.1
4403	1.23017	2.1
4404	1.0752	2.1
4405	1.52482	1.37744

4406	1.09252	2.1
4407	1.10725	2.1
4408	1.14305	0.05
4409	0.851541	0.0667997
4410	1.48073	0.05
4411	1.45607	0.05
4412	0.946131	0.0703537
4413	0.932823	0.05
4414	1.45828	0.05
4415	0.851528	0.0507503
4416	1.14305	0.05
4417	1.92737	0.0574426
4418	1.08211	0.0616061
4419	1.69831	0.05
4420	1.69831	0.05
4421	0.946131	0.05
4422	1.26868	0.05
4423	1.23377	0.05
4424	1.23377	0.05
4425	0.94723	0.05
4426	1.64322	0.05
4427	1.64322	0.05
4428	0.946131	1.51854
4429	1.32068	0.05
4430	0.946129	0.05
4431	1.18789	1.83462
4432	1.32068	0.0560054
4433	1.32813	2.1
4434	1.32461	2.1
4435	1.42729	0.05
4436	2.47846	0.05
4437	1.00024	0.0826237
4438	1.23923	0.05
4439	0.946131	0.0882044
4440	0.712304	0.05
4441	1.77501	0.0810381
4442	1.77501	0.05
4443	0.593593	0.120222
4444	1.23923	0.105006
4445	1.23175	0.0749466
4446	0.94613	0.128239
4447	0.713646	0.0803061
4448	1.75735	0.113336
4449	1.75735	0.05
4450	0.713633	0.17504
4451	1.37157	0.05
4452	1.47169	0.05
4453	2.21808	0.065342
4454	1.47169	0.05
4455	1.04759	2.1
4456	0.987132	2.1
4457	1.11181	2.04848
4458	2.61682	1.9526
4459	1.11181	2.07219
4460	1.06461	1.58895
4461	1.69733	2.1
4462	2.79705	2.1
4463	1.08245	2.1

4464	1.40583	2.1
4465	1.39852	2.1
4466	2.0239	2.1
4467	2.0239	2.1
4468	0.780455	2.1
4469	0.946132	2.1
4470	0.780455	2.1
4471	1.01885	2.1
4472	0.564217	2.1
4473	0.733461	2.1
4474	0.537126	2.1
4475	0.683838	2.1
4476	0.779662	2.1
4477	0.634141	2.1
4478	0.685337	2.1
4479	1.58848	2.09745
4480	1.17129	0.130738
4481	0.663168	0.196758
4482	1.55587	0.157461
4483	1.31342	0.143359
4484	0.596868	0.201082
4485	1.1666	0.206696
4486	0.867261	0.217084
4487	0.946132	0.240608
4488	1.28843	0.143679
4489	1.31248	0.179844
4490	2.59921	0.161608
4491	1.32634	0.118093
4492	1.35564	0.05
4493	1.52661	0.05
4494	1.12323	0.05
4495	1.17701	1.18784
4496	1.36405	1.10752
4497	1.19837	1.12389
4498	2.25636	1.05478
4499	1.0823	0.068615
4500	1.31774	0.0795721
4501	1.0823	0.0520965
4502	1.01121	0.10253
4503	1.27408	0.05
4504	0.946131	0.190856
4505	0.566074	0.169136
4506	0.79576	0.170276
4507	1.24596	0.179631
4508	1.47567	0.181792
4509	1.47567	0.0952107
4510	0.56605	0.177654
4511	1.6567	0.125739
4512	0.967792	0.121481
4513	1.00366	0.121491
4514	1.00366	0.130832
4515	1.56409	0.05
4516	1.56409	0.05
4517	0.774911	0.05
4518	1.09483	0.05
4519	0.774912	0.05
4520	1.09483	0.05
4521	1.24417	0.05

4522	1.13387	0.05
4523	1.13387	0.05
4524	1.30572	0.05
4525	2.18966	0.05
4526	0.98575	0.05
4527	1.54982	0.05
4528	2.48833	0.05
4529	1.54982	0.05
4530	1.28954	0.0672728
4531	1.64002	0.05
4532	1.64002	0.05
4533	1.85594	0.05
4534	1.12968	2.1
4535	1.23536	2.1
4536	1.4882	2.1
4537	1.4882	2.1
4538	0.876836	2.1
4539	0.946131	2.1
4540	0.876835	2.1
4541	1.43166	2.1
4542	2.25936	2.1
4543	1.0305	2.1
4544	0.992095	2.1
4545	1.12968	2.1
4546	1.53813	2.1
4547	1.53812	2.1
4548	0.583653	2.1
4549	0.94613	2.1
4550	0.583651	2.1
4551	0.964033	2.1
4552	0.964034	2.1
4553	1.54457	2.1
4554	3.54109	0.0565674
4555	0.99974	0.079274
4556	0.78814	0.0936211
4557	1.65729	0.0575924
4558	1.1283	0.0733326
4559	1.09407	0.0780024
4560	1.55091	0.059606
4561	0.870773	0.0865657
4562	1.19967	0.0665605
4563	1.56521	0.067107
4564	1.30896	1.94221
4565	1.17989	2.1
4566	1.72526	2.1
4567	0.851537	1.07347
4568	1.58944	2.0837
4569	1.56915	2.0855
4570	0.891039	1.03826
4571	1.4172	1.80275
4572	0.963818	1.55402
4573	0.851474	1.86945
4574	1.17989	2.1
4575	1.56261	2.1
4576	1.56261	2.1
4577	0.761574	2.1
4578	1.31323	2.1
4579	0.761573	2.1

4580	0.946133	2.1
4581	1.31323	2.1
4582	1.16759	2.1
4583	1.16759	2.1
4584	2.11829	2.1
4585	1.16759	2.1
4586	1.10748	0.05
4587	1.47688	0.0523044
4588	0.946133	0.05
4589	0.953319	0.072832
4590	0.946132	0.0565439
4591	1.51168	0.05
4592	1.51168	0.0627751
4593	1.25797	0.0532281
4594	1.19984	0.05
4595	1.95729	2.1
4596	2.34853	2.1
4597	1.94343	2.1
4598	2.00551	2.1
4599	1.20067	1.05247
4600	1.87244	1.3514
4601	1.4178	2.1
4602	1.33538	2.1
4603	0.946131	2.1
4604	1.22895	2.1
4605	1.12433	2.1
4606	0.956757	2.1
4607	0.91826	2.1
4608	1.44221	2.1
4609	1.14206	2.1
4610	1.80514	1.0755
4611	0.890304	1.85951
4612	1.1362	1.05204
4613	1.32819	1.30653
4614	1.46311	2.03574
4615	0.8654	1.35198
4616	1.1699	1.63406
4617	1.00267	1.68049
4618	1.02882	1.14086
4619	1.00832	2.1
4620	0.974137	2.1
4621	1.02368	2.1
4622	0.974138	2.1
4623	0.995734	2.02198
4624	1.44605	0.541583
4625	1.18549	0.586641
4626	1.33621	0.270262
4627	1.5176	0.278047
4628	1.91762	0.265379
4629	1.33621	0.250529
4630	1.6638	0.253797
4631	1.66774	2.1
4632	1.11833	2.1
4633	0.926499	2.1
4634	1.51229	2.1
4635	1.51229	2.1
4636	0.926528	2.1
4637	1.40335	2.1

4638	1.03544	2.1
4639	0.926527	2.1
4640	1.85305	2.1
4641	1.5812	2.1
4642	1.15529	2.1
4643	1.31086	2.1
4644	0.999206	2.1
4645	1.46714	2.1
4646	1.73087	2.1
4647	0.772299	2.1
4648	1.24341	2.1
4649	0.997867	2.1
4650	0.772311	2.1
4651	0.936657	2.1
4652	1.35986	2.1
4653	0.959414	2.1
4654	1.36541	2.1
4655	1.49268	2.1
4656	1.49268	2.1
4657	0.832137	2.1
4658	0.946132	2.1
4659	0.832138	2.1
4660	1.64523	2.1
4661	1.11764	2.1
4662	1.05776	2.1
4663	0.972465	2.1
4664	0.972464	2.1
4665	1.98425	2.1
4666	0.969822	2.1
4667	1.30566	2.1
4668	1.52724	2.1
4669	2.32237	0.105882
4670	2.30274	0.116517
4671	2.27761	0.0584558
4672	3.33012	0.0638862
4673	1.24001	0.38552
4674	1.41821	0.380252
4675	0.978584	2.1
4676	1.18076	2.1
4677	1.07645	2.1
4678	0.877351	1.51767
4679	0.946132	2.1
4680	0.877353	1.89913
4681	1.58444	2.1
4682	1.49614	2.1
4683	1.28355	2.1
4684	1.13491	2.1
4685	1.56875	0.162501
4686	0.499592	0.05
4687	0.946131	0.05
4688	1.20538	0.05
4689	1.09913	0.05
4690	1.8049	0.05
4691	1.8049	0.05
4692	0.499606	0.05
4693	2.41076	0.05
4694	0.860202	0.05
4695	0.906798	0.05

4696	1.8626	0.05
4697	1.33986	0.05
4698	1.2992	0.05
4699	1.58702	0.05
4700	1.05939	0.05
4701	1.02074	2.0899
4702	1.07866	2.09964
4703	1.5082	0.0768247
4704	1.5522	0.0597078
4705	1.65903	0.118548
4706	1.86766	2.1
4707	2.11684	2.1
4708	0.946132	2.1
4709	1.6585	2.1
4710	1.6585	2.1
4711	0.94613	2.1
4712	1.1872	2.1
4713	1.1872	2.1
4714	1.05842	2.1
4715	1.20455	2.1
4716	1.34141	2.1
4717	1.34141	2.1
4718	1.42416	2.1
4719	1.75818	2.1
4720	2.63715	2.02707
4721	1.80454	2.1
4722	2.26198	2.1
4723	0.696255	2.1
4724	0.624075	2.1
4725	1.50905	1.07135
4726	1.50905	2.06572
4727	0.624075	2.1
4728	1.31857	1.00971
4729	0.946131	2.06226
4730	0.624075	2.01415
4731	0.914628	2.1
4732	1.14721	2.1
4733	1.16153	2.1
4734	1.47664	2.1
4735	1.54791	2.1
4736	1.19098	2.1
4737	1.13341	2.1
4738	1.00503	0.174814
4739	1.00503	1.03449
4740	1.22655	2.1
4741	0.999489	2.1
4742	1.3268	2.1
4743	0.946132	2.1
4744	1.60061	2.1
4745	1.60061	2.1
4746	1.08034	2.1
4747	1.40372	2.1
4748	1.14302	2.1
4749	1.08034	2.1
4750	0.999491	2.1
4751	1.17152	0.212673
4752	1.17152	0.13435
4753	1.17152	0.149231

4754	2.05423	0.166751
4755	1.40984	2.03568
4756	1.2489	2.1
4757	0.885089	2.1
4758	0.810931	2.1
4759	1.84128	2.1
4760	1.11206	2.1
4761	1.21926	2.1
4762	1.99084	2.1
4763	0.976334	0.707691
4764	0.599363	0.637271
4765	1.02621	0.334464
4766	0.462959	0.565997
4767	1.42612	0.253854
4768	0.299682	0.376074
4769	1.51379	2.1
4770	0.719531	2.1
4771	1.3702	2.1
4772	1.33293	2.1
4773	0.851539	2.1
4774	0.62998	2.1
4775	0.851543	2.1
4776	0.629966	2.1
4777	1.43132	2.1
4778	1.13328	0.61534
4779	1.93212	0.318177
4780	1.62345	1.00398
4781	1.38511	1.1656
4782	1.40013	0.568747
4783	1.04132	0.517127
4784	0.83473	1.08996
4785	0.868732	0.951914
4786	0.810595	1.28115
4787	0.729919	0.915779
4788	0.992428	0.917643
4789	0.577107	0.766693
4790	1.18464	0.935902
4791	0.791199	0.957932
4792	0.810943	0.05
4793	0.860723	0.05
4794	1.33348	0.05
4795	1.57467	0.05
4796	0.981032	0.05
4797	0.731569	0.05
4798	0.770312	0.05
4799	1.46055	2.1
4800	1.42249	2.1
4801	1.75999	0.21513
4802	1.6868	0.253634
4803	1.17042	1.1891
4804	1.01485	2.1
4805	1.19952	0.05
4806	1.40592	0.05
4807	1.19952	0.05
4808	2.20145	0.05
4809	1.33649	0.227531
4810	1.11612	0.637737
4811	0.957588	0.457557

4812	0.946126	0.665136
4813	1.50648	0.216964
4814	1.50648	0.345341
4815	0.946128	0.456842
4816	2.10671	0.0538625
4817	1.91518	0.126517
4818	2.23223	0.188422
4819	1.53556	2.1
4820	1.56253	2.1
4821	1.27806	0.05
4822	1.44593	0.05
4823	1.03818	0.831418
4824	1.03818	0.739514
4825	1.38969	0.284442
4826	1.85548	0.168863
4827	2.6044	0.287933
4828	1.85548	0.171296
4829	1.49346	1.67468
4830	1.83192	1.69654
4831	0.834043	1.72543
4832	0.810943	1.0881
4833	0.887113	1.26367
4834	0.893827	1.05541
4835	0.991061	1.08712
4836	0.91939	1.18775
4837	0.793307	1.31997
4838	1.75291	1.22066
4839	1.35435	1.24862
4840	1.16989	0.198738
4841	1.36717	0.166152
4842	2.12276	0.0967416
4843	2.30493	0.146602
4844	2.47857	0.160148
4845	1.92269	0.0879183
4846	1.66426	0.140944
4847	0.956698	2.0847
4848	0.956698	2.1
4849	0.857957	2.1
4850	1.4706	2.1
4851	1.65706	2.1
4852	1.47059	2.1
4853	1.04713	0.0696597
4854	0.946131	0.114275
4855	1.47374	0.109486
4856	1.47374	0.0744907
4857	0.951675	0.103155
4858	1.2582	0.12585
4859	1.2582	0.0987393
4860	0.94613	0.130491
4861	1.4468	0.05
4862	1.90335	0.0628477
4863	1.14886	2.1
4864	2.59689	2.1
4865	1.02747	2.1
4866	0.946131	2.1
4867	0.934061	2.1
4868	1.63979	2.1
4869	1.65206	2.1

4870	1.48	2.1
4871	1.27467	2.1
4872	2.20449	2.1
4873	1.1333	0.05
4874	1.95334	2.1
4875	2.20984	2.1
4876	1.46389	0.0590672
4877	1.4639	0.0681879
4878	1.4639	0.0879473
4879	0.946132	0.12033
4880	1.5144	0.0775104
4881	1.5144	0.100071
4882	0.946132	0.123252
4883	1.35732	0.0782866
4884	1.10321	0.110323
4885	0.946132	0.106607
4886	0.980549	0.0928179
4887	1.32819	2.1
4888	1.30171	1.52955
4889	1.68377	1.5159
4890	1.68377	2.1
4891	0.946132	1.36193
4892	0.946132	1.95578
4893	1.1952	2.00643
4894	0.990019	1.6647
4895	1.08195	1.95333
4896	1.2269	2.08921
4897	1.19219	2.05825
4898	1.01485	2.1
4899	1.57125	0.0561081
4900	2.10696	0.0562872
4901	1.29666	0.05
4902	1.57125	0.0594157
4903	1.47712	2.1
4904	1.45182	2.1
4905	1.17816	0.05
4906	1.4516	0.05
4907	1.17816	0.05
4908	1.17816	0.05
4909	0.596954	0.746543
4910	1.65133	0.737406
4911	0.583859	0.525032
4912	1.04072	1.44273
4913	1.28795	0.580793
4914	1.59423	0.695547
4915	0.483559	0.708831
4916	1.74088	0.556448
4917	1.09545	1.21374
4918	1.02519	1.72805
4919	2.47579	0.729218
4920	1.54732	0.860229
4921	2.17663	0.822878
4922	1.75006	1.02503
4923	1.32354	1.17964
4924	1.09962	2.1
4925	1.85057	2.1
4926	1.91046	2.1
4927	2.17037	2.1

4928	1.99528	0.0514984
4929	1.97969	0.05
4930	3.82765	2.1
4931	1.85767	2.1
4932	2.77042	2.1
4933	1.37236	0.05
4934	2.23254	0.05
4935	1.10089	0.05
4936	0.994294	2.1
4937	1.81921	2.1
4938	1.00073	2.1
4939	0.681043	2.1
4940	0.946131	2.1
4941	0.681073	2.1
4942	1.51804	2.1
4943	1.60653	2.1
4944	1.30188	2.1
4945	0.919622	2.1
4946	1.00965	2.1
4947	1.93821	2.1
4948	2.60377	2.1
4949	1.36416	0.05
4950	1.36416	0.05
4951	1.36416	0.05
4952	2.44132	0.05
4953	2.15871	0.240236
4954	2.22171	0.183928
4955	1.70747	0.151776
4956	1.49515	0.121256
4957	0.773007	0.163002
4958	0.946125	0.16139
4959	0.946131	0.229089
4960	0.773034	0.12123
4961	1.15844	0.157628
4962	1.98104	0.103751
4963	2.44283	0.127135
4964	1.05417	0.05
4965	1.1596	0.05
4966	0.829921	0.05
4967	0.946131	0.05
4968	0.951911	0.05
4969	1.39027	0.05
4970	0.829946	0.05
4971	1.51223	0.05
4972	1.51223	0.05
4973	1.65984	0.05
4974	1.90382	0.05
4975	1.93228	0.803643
4976	2.47924	1.02656
4977	1.73904	0.823111
4978	1.40547	0.599609
4979	2.37589	0.671999
4980	0.662605	1.04919
4981	2.33293	0.47647
4982	1.72249	2.1
4983	1.68478	2.1
4984	1.00514	0.449315
4985	1.4537	2.1

4986	1.92698	2.1
4987	1.46041	0.05
4988	2.04817	0.05
4989	1.65954	2.1
4990	1.42342	2.1
4991	1.04405	0.05
4992	1.17056	0.05
4993	1.11339	0.05
4994	0.946133	0.05
4995	0.929724	0.05
4996	1.35424	0.05
4997	1.35424	0.05
4998	0.929713	0.05
4999	1.85945	0.05
5000	1.38905	2.1
5001	1.39056	2.1
5002	1.52852	2.1
5003	1.06472	2.1
5004	0.749096	2.1
5005	1.46076	2.1
5006	1.29263	2.1
5007	0.94613	2.1
5008	0.69097	2.1
5009	0.982284	2.1
5010	0.67419	2.1
5011	1.52852	2.1
5012	1.91121	2.1
5013	1.00775	2.04577
5014	1.61201	2.0059
5015	0.529987	2.1
5016	0.506125	2.1
5017	1.44696	2.1
5018	2.32981	2.1
5019	1.32784	2.1
5020	1.94158	2.1
5021	1.31595	2.1
5022	0.663922	2.1
5023	1.44663	2.1
5024	1.44663	2.1
5025	1.13976	2.1
5026	0.970788	2.1
5027	1.13976	2.1
5028	0.663921	2.1
5029	0.966114	2.1
5030	1.05498	2.02128
5031	1.38086	2.1
5032	0.946127	2.1
5033	0.922513	2.1
5034	1.49903	2.1
5035	1.49903	2.1
5036	0.92251	2.1
5037	1.06105	2.1
5038	1.36049	2.1
5039	1.38086	2.1
5040	1.52534	2.1
5041	1.04101	2.1
5042	1.30861	0.05
5043	0.949112	0.05

5044	0.894275	0.0539705
5045	1.10232	0.05
5046	0.830889	0.05
5047	1.26554	0.05
5048	0.948347	0.05
5049	0.81096	0.05
5050	1.18338	0.05
5051	0.883764	0.0569204
5052	1.54793	0.05
5053	1.02867	0.05
5054	1.8573	0.05
5055	1.06065	0.05
5056	0.64842	0.05
5057	1.99319	0.05
5058	1.9476	0.0507913
5059	2.51436	0.05
5060	1.68337	0.05
5061	1.92321	0.05
5062	0.810942	0.05
5063	0.921001	0.05
5064	0.99758	0.05
5065	0.960785	0.05
5066	1.01565	0.05
5067	0.908624	0.05
5068	0.912835	0.05
5069	1.18351	0.05
5070	1.40239	2.1
5071	1.26091	2.1
5072	0.947979	2.1
5073	0.946131	2.1
5074	1.81959	2.1
5075	1.81959	2.1
5076	0.94613	2.1
5077	1.38415	2.05807
5078	1.38414	2.1
5079	1.33266	2.1
5080	0.946132	2.1
5081	1.76225	2.1
5082	1.76225	2.1
5083	1.27599	2.1
5084	1.38158	2.1
5085	1.3268	2.1
5086	1.27599	2.1
5087	0.947979	2.1
5088	1.0142	0.453475
5089	1.01422	0.341733
5090	1.11435	0.306732
5091	1.42186	0.820169
5092	1.24001	0.913033
5093	1.77342	1.11181
5094	1.42186	1.37867
5095	1.99817	0.74874
5096	1.27361	2.1
5097	1.68914	2.1
5098	1.30116	2.1
5099	1.32819	2.01889
5100	1.68322	2.04293
5101	1.68322	2.1

5102	0.94613	1.88474
5103	0.94613	2.09338
5104	1.14666	2.1
5105	1.07612	2.02524
5106	1.31151	2.1
5107	1.22095	2.1
5108	1.57256	2.1
5109	1.6679	2.1
5110	0.946132	2.09717
5111	0.946133	2.1
5112	1.13537	2.1
5113	1.07855	2.1
5114	1.07855	2.1
5115	1.43895	1.80408
5116	1.38481	0.05
5117	1.72385	0.05
5118	0.550441	0.05
5119	0.79807	0.05
5120	1.11627	0.05
5121	1.30349	0.05
5122	0.94613	0.05
5123	0.686176	0.05
5124	1.11627	0.05
5125	1.73363	0.05
5126	1.73363	0.05
5127	0.686139	0.05
5128	1.24932	0.05
5129	0.899582	0.05
5130	2.00472	0.05
5131	1.17121	0.05
5132	1.2874	0.05
5133	2.0194	0.05
5134	0.88601	0.05
5135	1.77409	0.05
5136	1.73293	0.05
5137	2.74045	0.05
5138	1.83787	0.05
5139	1.45448	0.0702635
5140	1.27778	0.058051
5141	1.1192	2.1
5142	1.25953	2.1
5143	1.93611	2.1
5144	1.92104	2.1
5145	1.15506	0.05
5146	1.15506	0.05
5147	1.23943	1.94439
5148	0.851543	2.1
5149	1.6061	2.1
5150	1.80058	2.1
5151	0.869471	2.1
5152	1.3931	2.1
5153	1.06454	2.1
5154	0.93667	2.1
5155	2.16916	2.1
5156	3.10246	2.1
5157	1.91539	2.0913
5158	1.91539	2.06933
5159	0.620263	2.04463

5160	0.620265	2.08779
5161	0.946132	2.03149
5162	0.620265	2.03485
5163	1.30226	1.97916
5164	1.87292	2.1
5165	1.18769	2.09607
5166	2.60452	2.08789
5167	1.26574	2.1
5168	2.46679	2.09458
5169	1.36459	2.1
5170	1.26865	1.63763
5171	1.1744	1.06711
5172	1.15841	0.342094
5173	1.02247	0.20192
5174	1.93649	2.1
5175	1.64718	2.1
5176	1.59654	2.1
5177	1.57823	2.1
5178	1.00665	0.0904565
5179	1.28239	0.105468
5180	0.887895	2.1
5181	1.22083	2.1
5182	1.42127	2.1
5183	1.07374	2.1
5184	1.00503	1.13238
5185	1.23082	0.65225
5186	2.78663	2.1
5187	2.20883	2.1
5188	2.54588	2.1
5189	0.946128	2.1
5190	1.13716	2.1
5191	1.58724	2.1
5192	1.58724	2.1
5193	0.946131	2.1
5194	1.13716	2.1
5195	1.39621	2.1
5196	1.02438	2.1
5197	1.49543	0.05
5198	2.78336	0.05
5199	1.27148	0.05
5200	1.0906	0.05
5201	1.04072	0.05
5202	0.63574	0.05
5203	1.45506	0.05
5204	1.5863	0.05
5205	0.928352	0.05
5206	0.647687	0.05
5207	2.2375	1.47807
5208	1.50998	1.5873
5209	2.75021	1.31717
5210	1.91912	0.948666
5211	1.99872	0.932101
5212	0.918127	1.6998
5213	1.03031	1.1949
5214	0.951169	1.06698
5215	1.27975	0.914132
5216	1.12735	1.01294
5217	1.15741	1.07108

5218	1.24998	1.10696
5219	1.65953	0.27544
5220	0.967555	0.501577
5221	0.967542	0.305721
5222	1.03824	0.215365
5223	1.48693	0.185619
5224	1.48693	0.152707
5225	2.02901	0.158534
5226	0.946127	0.268093
5227	1.07139	0.245887
5228	0.946126	0.248304
5229	1.84644	0.185651
5230	1.84644	0.182829
5231	1.38683	0.215857
5232	1.40574	0.216845
5233	1.36866	0.0960396
5234	0.649325	0.162658
5235	1.93631	2.1
5236	1.50145	2.1
5237	1.6287	2.1
5238	1.31302	2.1
5239	1.18041	2.1
5240	1.18041	2.1
5241	0.974196	2.1
5242	0.946131	2.1
5243	1.74169	2.1
5244	1.74169	2.1
5245	1.23163	2.1
5246	1.27451	2.1
5247	1.41331	2.1
5248	1.23163	2.1
5249	1.28037	2.1
5250	1.28158	2.1
5251	1.28037	2.1
5252	2.28015	0.05
5253	2.06561	0.05
5254	2.61343	0.05
5255	1.11712	0.05
5256	1.63689	0.249281
5257	1.01393	0.212514
5258	0.996775	2.1
5259	1.54277	2.1
5260	1.26397	2.1
5261	1.46129	2.1
5262	1.62391	2.1
5263	1.08848	0.05
5264	1.11833	2.1
5265	1.24041	2.1
5266	2.31884	2.1
5267	1.335 2.1	
5268	1.48589	0.05
5269	1.77131	0.05
5270	1.8422	1.16096
5271	2.47453	1.59349
5272	1.23726	1.52115
5273	1.21996	1.48793
5274	1.65512	1.45251
5275	1.65512	1.37027

5276	0.802107	1.8051
5277	0.946129	1.40933
5278	0.802137	1.37323
5279	1.3279	1.08953
5280	1.23726	1.07174
5281	1.79759	0.978925
5282	1.79759	0.882134
5283	0.767577	1.56283
5284	0.946131	1.02106
5285	0.767577	0.981642
5286	1.03534	0.433924
5287	1.09962	2.01262
5288	0.929217	2.09123
5289	1.70126	2.09583
5290	0.991407	2.1
5291	1.41077	2.1
5292	0.991406	2.1
5293	1.62966	2.1
5294	1.62966	2.1
5295	0.946128	2.1
5296	1.18364	2.1
5297	0.946131	2.1
5298	1.14186	2.1
5299	1.18364	2.1
5300	1.16858	2.1
5301	1.66137	0.798262
5302	1.90165	0.510402
5303	1.66139	0.133811
5304	1.26893	0.136865
5305	1.15918	0.100882
5306	0.653228	0.131156
5307	0.946133	0.05836
5308	1.77488	0.239455
5309	1.77489	0.198684
5310	0.653228	0.109116
5311	1.56043	0.165696
5312	1.60894	0.174459
5313	1.56043	0.0859409
5314	1.06848	0.14449
5315	1.04307	2.1
5316	1.14188	2.1
5317	1.09195	2.1
5318	0.615121	2.1
5319	1.50867	2.1
5320	1.27152	2.1
5321	0.615094	2.1
5322	1.07297	2.1
5323	1.1372	2.1
5324	0.553608	2.1
5325	1.23019	2.1
5326	0.992675	2.1
5327	1.40984	2.04298
5328	2.55682	2.1
5329	3.66069	2.1
5330	3.58726	2.1
5331	1.4375	0.078881
5332	1.88424	0.104087
5333	1.56714	2.1

5334	1.6721	2.1
5335	1.18474	2.1
5336	1.2295	2.1
5337	1.28754	2.1
5338	1.36838	2.1
5339	2.78661	2.1
5340	2.64055	2.1
5341	1.35065	0.932606
5342	2.26675	0.557477
5343	1.35065	0.843148
5344	1.21558	0.582505
5345	1.49709	0.05
5346	1.4971	0.05
5347	0.85472	0.05
5348	0.854718	0.05
5349	0.946129	0.05
5350	0.854717	0.05
5351	1.1604	0.05
5352	1.25265	0.05
5353	2.32081	0.05
5354	1.15371	0.05
5355	1.14945	2.1
5356	0.995725	2.1
5357	1.00307	0.211192
5358	1.10907	0.154761
5359	2.30315	0.157402
5360	1.10908	0.174027
5361	1.27558	1.087
5362	1.71613	1.72219
5363	1.32977	2.1
5364	0.815276	2.1
5365	1.41697	2.1
5366	1.41697	2.1
5367	0.815276	2.1
5368	1.38897	2.1
5369	0.946131	2.1
5370	0.815275	2.1
5371	1.44684	2.1
5372	1.21489	1.6806
5373	1.22476	1.59247
5374	0.870366	1.03648
5375	0.862572	0.825986
5376	0.835435	0.667157
5377	1.6993	0.518744
5378	1.23618	0.584783
5379	1.18624	0.533294
5380	1.53058	0.663949
5381	2.16108	0.748109
5382	2.21589	0.64279
5383	1.04037	2.08927
5384	1.03933	2.09559
5385	1.57753	0.0912836
5386	1.87644	0.119392
5387	1.24728	0.117335
5388	1.30394	0.0829712
5389	1.3538	2.1
5390	1.69858	2.1
5391	1.1726	0.186811

5392	0.94612	0.131175
5393	1.09805	0.0774259
5394	0.946118	0.174158
5395	1.72576	0.132914
5396	1.72575	0.068482
5397	1.34152	0.11689
5398	1.33034	0.118787
5399	0.784151	2.1
5400	0.946131	2.1
5401	0.784151	2.1
5402	1.47963	2.1
5403	1.47963	2.1
5404	1.3335	2.1
5405	0.930281	2.1
5406	1.29435	2.1
5407	0.883308	2.1
5408	1.31589	2.1
5409	1.67846	2.1
5410	0.993598	0.05
5411	2.23193	0.05
5412	0.9936	0.05
5413	0.615629	0.05
5414	1.65925	2.1
5415	2.05992	2.1
5416	1.65925	2.1
5417	4.08735	1.62705
5418	2.72856	1.25268
5419	0.869602	2.04683
5420	3.42833	0.804603
5421	1.14397	1.60884
5422	1.34563	0.0839352
5423	1.08189	0.0707554
5424	0.782128	1.97953
5425	1.0022	2.07421
5426	0.900427	1.99058
5427	1.51499	2.00411
5428	1.05588	1.95831
5429	1.13739	2.07626
5430	1.37004	2.00011
5431	1.14603	1.9898
5432	0.851541	1.51482
5433	0.946799	1.91043
5434	1.14854	1.82135
5435	1.33793	1.63363
5436	0.94613	1.81913
5437	1.24336	1.83548
5438	0.851473	1.85284
5439	2.08792	1.69906
5440	1.01869	1.80373
5441	0.986953	0.0521339
5442	0.896238	0.090014
5443	0.906574	0.0897285
5444	0.852145	0.0652599
5445	2.14191	0.0519537
5446	1.15979	0.060796
5447	1.18327	0.0546975
5448	2.32742	0.063341
5449	2.35961	0.0908477

5450	2.38126	0.0545202
5451	1.13632	0.054761
5452	1.17897	0.05
5453	1.76199	0.05
5454	1.00573	2.1
5455	0.946127	1.77544
5456	1.7077	2.1
5457	1.7077	2.1
5458	0.946127	2.08299
5459	1.24887	2.1
5460	1.24887	2.1
5461	1.22441	2.1
5462	1.08542	2.1
5463	1.21743	2.03722
5464	1.12572	2.1
5465	1.05499	0.867854
5466	1.00521	0.850852
5467	2.86191	0.05
5468	2.27972	0.05
5469	1.01169	2.1
5470	1.03373	2.1
5471	1.39319	2.1
5472	0.636837	2.1
5473	1.63908	2.09556
5474	1.45713	1.9553
5475	1.04072	2.1
5476	1.25025	1.9803
5477	1.38914	2.08223
5478	0.636807	2.09655
5479	1.50211	2.1
5480	1.36555	2.1
5481	2.77545	1.91209
5482	0.682805	2.1
5483	1.50267	1.95975
5484	1.50267	2.1
5485	0.797749	2.1
5486	1.38773	1.95823
5487	1.38773	2.1
5488	0.682776	2.1
5489	2.01245	0.05
5490	0.925918	0.05
5491	0.75689	0.05
5492	1.25823	0.05
5493	1.16505	0.05
5494	0.838779	0.05
5495	1.14722	0.05
5496	0.756849	0.05
5497	1.52298	0.05
5498	2.11034	0.05
5499	1.92174	0.05
5500	1.92174	0.05
5501	0.576083	0.05
5502	1.23146	0.05
5503	1.26637	0.05
5504	1.23146	0.05
5505	1.26637	0.05
5506	0.946133	0.590046
5507	1.37034	0.453119

5508	1.84145	0.482346
5509	1.84145	0.378841
5510	0.946132	0.733959
5511	1.37034	0.494342
5512	1.41724	0.485043
5513	1.0685	0.359397
5514	1.14547	0.378337
5515	1.24976	2.1
5516	1.30682	2.1
5517	2.12095	2.1
5518	1.46392	2.1
5519	1.46392	2.1
5520	0.935738	2.1
5521	1.32949	2.1
5522	1.07017	2.1
5523	0.935766	2.1
5524	1.07017	2.1
5525	1.78095	2.1
5526	1.05619	0.294108
5527	1.05793	0.226268
5528	1.77677	2.1
5529	2.04766	2.1
5530	1.02524	2.1
5531	1.18803	2.1
5532	1.70047	2.1
5533	1.556 2.1	
5534	1.24201	2.1
5535	1.3592	2.1
5536	2.11824	0.05
5537	2.20248	0.0836563
5538	3.43878	0.0521776
5539	3.12466	0.05
5540	2.09351	2.1
5541	0.946128	2.1
5542	1.51269	2.1
5543	1.51269	2.1
5544	0.946131	2.1
5545	1.01306	2.1
5546	1.04676	2.1
5547	1.01306	2.1
5548	1.07444	2.1
5549	2.17465	2.1
5550	1.14808	2.1
5551	1.39067	2.06943
5552	1.20958	1.98984
5553	0.662832	0.0641642
5554	1.93512	0.0688577
5555	1.15471	2.1
5556	1.75119	2.1
5557	1.75118	2.1
5558	0.946131	2.1
5559	1.28606	2.1
5560	1.27087	2.1
5561	0.946131	2.1
5562	1.28606	2.1
5563	1.58916	0.05
5564	0.927822	0.0771075
5565	2.24367	0.05

5566	1.8734	0.05
5567	3.51668	0.05
5568	2.68986	0.05
5569	2.32395	2.1
5570	1.39564	2.1
5571	2.10862	2.1
5572	1.36763	2.1
5573	0.970963	0.05
5574	0.97096	0.05
5575	1.11897	0.132913
5576	1.26885	0.193195
5577	1.11605	0.143813
5578	2.06612	0.116426
5579	1.717	0.130294
5580	0.59502	0.186755
5581	0.825755	0.105379
5582	0.709624	0.212999
5583	1.65741	0.114269
5584	0.307486	0.106417
5585	1.4785	0.123055
5586	0.983143	2.1
5587	1.08247	2.1
5588	1.42353	2.1
5589	1.42353	2.1
5590	0.781667	2.1
5591	0.797634	2.1
5592	0.946131	2.1
5593	0.781638	2.1
5594	0.946131	2.1
5595	0.98315	2.1
5596	1.083	2.1
5597	1.61975	0.05
5598	1.11946	0.05
5599	1.18977	0.0517372
5600	0.996484	0.0603405
5601	2.24343	0.05
5602	1.4787	0.05
5603	1.66127	0.05
5604	1.86028	0.05
5605	1.09128	0.05
5606	1.03115	0.05
5607	0.838025	0.05
5608	1.3736	0.05
5609	1.44009	0.05
5610	0.92917	0.05
5611	1.03889	0.05
5612	0.956974	0.05
5613	3.23468	0.05
5614	0.953922	0.0693737
5615	0.957104	0.0652299
5616	1.66231	0.05
5617	1.26778	0.05
5618	1.47615	0.05
5619	1.28621	0.0561691
5620	1.08043	0.0562203
5621	2.35026	2.1
5622	2.35025	2.1
5623	2.60659	2.1

5624	1.57627	2.1
5625	1.5493	2.1
5626	1.24682	0.398504
5627	2.14277	0.395886
5628	1.24682	0.545959
5629	1.00307	0.332344
5630	2.04056	0.314321
5631	1.45408	2.1
5632	1.54249	2.1
5633	1.24474	1.59217
5634	1.37813	1.71173
5635	0.924158	0.05
5636	0.916623	0.05
5637	1.35344	0.05
5638	1.00859	0.05
5639	1.34786	0.05
5640	0.952457	0.05
5641	0.85404	0.05
5642	1.06341	2.1
5643	1.09194	2.1
5644	1.93707	2.1
5645	2.14087	2.1
5646	1.93707	2.1
5647	1.07519	2.1
5648	1.07519	2.1
5649	1.62518	2.1
5650	1.3592	2.1
5651	1.13183	2.1
5652	1.20357	2.1
5653	1.4858	2.1
5654	1.23694	2.1
5655	1.44011	2.1
5656	1.23989	2.1
5657	2.21604	2.1
5658	1.85574	1.09992
5659	1.55649	2.1
5660	1.48861	0.704973
5661	1.15968	0.05
5662	0.946132	0.05
5663	0.663056	0.05
5664	1.70837	0.05
5665	1.70837	0.05
5666	0.946131	0.05
5667	0.663026	0.05
5668	2.09604	0.05
5669	0.81094	0.05
5670	1.06559	0.05
5671	0.810956	0.05
5672	1.15474	0.05
5673	0.990051	0.05
5674	0.894971	0.05
5675	1.32536	0.05
5676	0.946133	2.1
5677	1.33125	2.1
5678	1.75823	2.1
5679	1.75823	2.1
5680	0.946133	2.1
5681	1.33125	2.1

5682	1.37312	2.1
5683	1.27985	2.1
5684	1.37312	2.1
5685	1.70684	2.1
5686	1.70684	2.1
5687	0.946132	2.1
5688	0.946132	2.1
5689	1.25081	2.1
5690	0.956293	2.1
5691	0.956294	2.1
5692	1.36035	2.1
5693	1.41944	2.1
5694	0.830638	2.1
5695	0.747568	2.1
5696	0.888498	2.1
5697	1.04071	2.1
5698	1.17128	2.1
5699	1.92339	2.1
5700	2.91208	2.1
5701	1.82208	0.585382
5702	1.89653	0.245548
5703	1.82588	1.34943
5704	1.26703	0.263101
5705	1.26703	0.65975
5706	0.6157	0.801781
5707	1.26706	1.47385
5708	0.61567	0.132121
5709	0.615671	0.14697
5710	1.26706	0.282098
5711	1.89158	0.187257
5712	1.23134	0.158252
5713	2.49497	0.05
5714	1.68103	0.05
5715	2.0554	2.1
5716	1.72647	2.1
5717	1.67375	2.1
5718	1.65037	2.1
5719	1.04349	1.36079
5720	1.69055	0.05
5721	1.53463	0.05
5722	1.19022	1.79038
5723	1.89226	1.2223
5724	1.15009	1.24376
5725	1.7054	1.12069
5726	1.31612	0.356809
5727	1.31611	0.957772
5728	1.67345	0.909179
5729	1.06577	0.206575
5730	1.40193	0.125378
5731	1.72411	0.108255
5732	1.46123	0.128896
5733	0.808808	0.192177
5734	0.897714	0.131759
5735	0.851554	0.231934
5736	1.3499	0.05
5737	1.29001	0.05
5738	1.22083	2.1
5739	1.06191	2.1

5740	1.05758	0.922662
5741	1.348	1.81054
5742	1.43485	0.906513
5743	1.34799	1.71833
5744	1.36307	0.05
5745	2.28188	0.05
5746	1.7926	0.05
5747	1.38957	2.1
5748	1.47446	2.1
5749	0.953638	2.1
5750	1.11868	2.1
5751	0.953636	2.1
5752	1.03408	2.1
5753	1.52443	0.05
5754	1.38539	0.05
5755	0.946132	0.05
5756	1.12747	0.05
5757	1.04393	0.0505854
5758	1.05017	0.05
5759	1.20529	0.05
5760	1.3663	0.24435
5761	1.89226	0.219774
5762	1.17885	0.183976
5763	0.867881	0.139057
5764	1.32917	0.104432
5765	1.32917	0.0698194
5766	0.946127	0.225081
5767	1.19583	0.134365
5768	1.00122	0.0651766
5769	0.946128	0.190506
5770	1.71405	0.23816
5771	0.871815	2.06703
5772	1.16435	2.1
5773	0.686619	2.09688
5774	1.63102	2.1
5775	1.06589	2.09302
5776	1.05375	2.1
5777	1.9023	2.1
5778	1.27394	0.499936
5779	1.88345	0.435298
5780	2.05636	0.422297
5781	0.672977	0.49532
5782	1.41274	0.460895
5783	1.65606	0.449491
5784	0.869329	0.49372
5785	1.2207	0.479648
5786	1.35631	0.478186
5787	0.908776	0.516305
5788	0.955081	0.390192
5789	1.2243	2.1
5790	1.23169	2.1
5791	1.52228	2.1
5792	1.23169	2.1
5793	0.990892	0.222569
5794	1.08376	0.161613
5795	1.79348	0.159899
5796	1.50043	0.126221
5797	1.32599	0.328507

5798	1.37924	0.18132
5799	0.838014	0.178958
5800	0.87737	0.447958
5801	0.947086	0.286714
5802	1.27199	0.323426
5803	1.04918	0.222512
5804	1.04851	0.490653
5805	1.1699	0.357437
5806	0.810934	0.42781
5807	1.83602	0.521177
5808	2.21909	0.355401
5809	0.619441	0.431922
5810	2.03755	0.293703
5811	1.83472	0.389147
5812	1.20416	0.474578
5813	1.26691	0.264763
5814	0.655795	0.473312
5815	0.94613	0.380157
5816	2.35169	0.467152
5817	3.54705	2.08406
5818	2.60741	2.08579
5819	1.59019	0.05
5820	1.24526	0.05
5821	1.59019	0.05
5822	2.36406	0.05
5823	1.16776	2.09403
5824	1.67311	2.1
5825	1.67311	2.01601
5826	0.94613	1.72316
5827	1.38645	2.1
5828	1.13563	0.669779
5829	1.13563	1.71511
5830	1.23279	1.46441
5831	1.05042	2.1
5832	1.17418	2.1
5833	1.28909	2.1
5834	0.973448	2.1
5835	1.02958	2.1
5836	3.3005	2.1
5837	2.86325	2.1
5838	1.35879	2.1
5839	1.69332	2.1
5840	1.69332	2.1
5841	0.946128	2.1
5842	1.39659	2.1
5843	1.12035	2.1
5844	1.12035	2.1
5845	1.24286	2.1
5846	1.10407	2.1
5847	1.14599	2.1
5848	1.32259	2.06512
5849	0.788592	0.05
5850	2.4588	2.1
5851	0.909581	1.97779
5852	1.35656	1.85475
5853	0.986895	1.9067
5854	2.30602	1.77227
5855	1.46873	1.85234

5856	1.55584	1.81586
5857	2.1547	1.83475
5858	1.87794	1.89511
5859	0.808286	1.99925
5860	0.928464	1.94522
5861	0.810959	1.91477
5862	1.50962	1.66629
5863	1.1053	1.81614
5864	1.0712	1.85922
5865	1.40504	1.86523
5866	1.87794	1.3938
5867	2.80496	1.48673
5868	1.41479	1.87817
5869	1.38634	2.1
5870	1.38634	2.1
5871	1.67762	2.1
5872	0.946131	2.1
5873	1.188 2.1	
5874	1.62585	2.1
5875	1.62585	2.1
5876	0.946129	2.1
5877	1.18801	2.1
5878	1.38397	2.1
5879	1.04546	2.1
5880	1.16817	2.1
5881	1.06007	2.1
5882	1.19785	2.1
5883	1.43652	2.1
5884	1.06994	2.1
5885	1.89111	2.1
5886	1.07144	2.1
5887	1.51754	2.1
5888	2.0873	2.1
5889	0.758799	2.1
5890	1.59518	2.1
5891	1.59518	2.1
5892	0.946132	2.1
5893	0.758771	2.1
5894	1.06957	2.1
5895	0.758771	2.1
5896	1.33916	2.1
5897	2.03045	1.62157
5898	2.80184	1.78526
5899	1.76788	1.77914
5900	1.27442	2.05607
5901	1.06756	2.1
5902	0.966979	2.1
5903	1.7054	2.1
5904	1.07156	2.1
5905	0.986096	2.1
5906	1.32813	2.1
5907	1.3331	2.1
5908	2.08369	0.105129
5909	2.23818	0.764551
5910	1.10335	0.367671
5911	0.622314	0.177501
5912	1.72451	0.187177
5913	1.72451	0.503875

5914	0.622283	0.25743
5915	0.622314	0.26426
5916	0.946131	0.367462
5917	1.31254	0.990849
5918	1.15704	0.846128
5919	1.24463	0.24676
5920	2.2067	0.529076
5921	1.30001	0.985137
5922	1.29175	1.15713
5923	1.1233	0.05
5924	1.8689	0.05
5925	1.29494	0.05
5926	1.11895	0.05
5927	0.851539	0.05
5928	0.851474	0.05
5929	1.58352	0.05
5930	1.58228	0.05
5931	0.851527	0.05
5932	2.6389	0.05
5933	1.77548	0.05
5934	1.24555	2.1
5935	2.0512	2.1
5936	1.46101	2.1
5937	2.60635	2.1
5938	1.10476	0.05
5939	1.44103	0.05
5940	0.652391	2.1
5941	0.618322	2.1
5942	2.22915	2.1
5943	1.39067	2.1
5944	1.99737	2.1
5945	1.42174	2.1
5946	0.946132	0.382011
5947	1.29735	1.77106
5948	1.61765	2.1
5949	1.61765	0.657229
5950	0.946132	1.85528
5951	1.29735	1.11625
5952	1.26643	1.18821
5953	1.01335	0.804203
5954	1.01335	0.167871
5955	0.947669	2.1
5956	2.17167	2.09685
5957	2.59733	1.96951
5958	1.50954	2.034
5959	1.02638	1.79312
5960	0.555785	1.97818
5961	1.91101	1.9563
5962	2.09809	1.83113
5963	0.641974	2.02971
5964	1.2022	1.95804
5965	1.36359	1.8993
5966	1.8128	1.92194
5967	2.34348	0.19297
5968	1.66343	0.133799
5969	0.974717	0.382485
5970	1.53908	0.295954
5971	1.27737	1.40814

5972	1.09408	0.876395
5973	2.07382	0.751778
5974	1.10466	1.77523
5975	1.05654	1.5305
5976	1.23498	1.0524
5977	1.48104	1.40062
5978	1.23498	1.00843
5979	1.17261	2.1
5980	1.18059	2.1
5981	2.43005	2.1
5982	1.04546	2.1
5983	2.81222	2.1
5984	1.16817	2.1
5985	1.75766	0.869908
5986	1.47021	1.23425
5987	1.07236	0.259237
5988	1.81759	0.724307
5989	1.42913	0.384883
5990	1.00636	0.473499
5991	1.00636	0.310778
5992	1.79397	0.308612
5993	0.946131	0.456933
5994	1.03691	0.509224
5995	0.851539	0.454473
5996	1.46001	0.472621
5997	1.37878	0.520798
5998	0.933258	0.46036
5999	1.32423	0.468246
6000	1.32614	0.05
6001	1.11356	0.05
6002	1.32614	0.05
6003	1.28895	0.05
6004	1.66895	0.05
6005	1.66895	0.05
6006	0.946132	0.05
6007	1.29576	0.05
6008	1.1318	0.05
6009	1.37621	1.74683
6010	1.94071	1.6899
6011	1.10517	2.1
6012	1.97098	2.1
6013	0.811 2.1	
6014	0.956127	2.1
6015	1.18647	1.9966
6016	0.988455	1.58348
6017	1.12457	0.0766695
6018	1.7453	0.0961064
6019	0.92492	2.1
6020	1.24353	2.1
6021	1.84536	0.554285
6022	1.67662	0.457714
6023	1.38247	0.05
6024	1.02663	0.05
6025	0.946132	0.493719
6026	1.41723	0.395256
6027	1.72976	0.437786
6028	1.72976	0.328551
6029	0.946131	0.658959

6030	1.41723	0.462545
6031	1.25866	0.46441
6032	1.34254	0.282069
6033	1.25866	0.362841
6034	1.65507	0.292009
6035	1.65507	0.247129
6036	0.946132	0.495719
6037	0.946132	0.378979
6038	1.11929	0.366386
6039	0.961779	0.292599
6040	0.961779	0.299661
6041	0.946132	2.1
6042	1.69361	2.1
6043	1.69361	2.1
6044	0.94613	2.1
6045	1.36523	2.1
6046	1.36523	2.1
6047	1.19802	2.1
6048	0.974196	2.1
6049	1.09518	2.1
6050	0.946131	2.1
6051	1.7161	2.1
6052	1.7161	2.1
6053	0.94613	2.1
6054	1.297	2.1
6055	1.297	2.1
6056	1.14599	2.1
6057	0.974197	2.1
6058	0.974197	2.1
6059	1.80778	2.1
6060	2.20473	2.1
6061	1.80777	2.1
6062	1.3515	2.1
6063	0.583237	2.1
6064	1.39359	2.1
6065	1.39359	2.1
6066	1.07296	2.1
6067	0.946114	2.1
6068	1.07297	2.1
6069	0.903862	2.1
6070	0.956572	2.1
6071	1.03437	2.1
6072	0.975409	2.1
6073	1.31167	0.05
6074	2.70246	0.05
6075	1.12018	0.05
6076	0.655836	0.05
6077	0.655837	0.05
6078	1.81557	0.05
6079	1.81557	0.05
6080	0.655836	0.05
6081	0.946131	0.0583574
6082	1.01847	0.05
6083	1.61334	0.05
6084	0.810946	0.0508585
6085	0.8311	0.0600268
6086	1.2466	0.05
6087	1.20286	0.05

6088	0.97508	0.0526786
6089	0.870805	0.05
6090	0.760087	0.0627674
6091	0.853389	2.1
6092	0.820894	2.1
6093	0.928291	2.1
6094	1.06922	2.1
6095	1.32829	2.1
6096	1.12173	2.1
6097	1.16615	2.1
6098	0.957748	2.1
6099	0.612491	2.00591
6100	0.548141	2.1
6101	0.979614	2.1
6102	0.658184	2.09435
6103	0.678456	2.00361
6104	0.529544	1.99852
6105	0.739013	2.09911
6106	0.694037	2.08678
6107	0.475716	2.03684
6108	1.58794	1.99872
6109	1.58794	1.86915
6110	0.475743	1.94349
6111	1.08301	2.07147
6112	0.980664	2.09397
6113	0.475737	2.05685
6114	1.32709	2.1
6115	1.59396	2.1
6116	1.19719	0.175635
6117	1.34921	0.1815
6118	1.69061	0.168209
6119	1.69061	0.120409
6120	0.946132	0.223107
6121	1.34921	0.180311
6122	1.28753	0.177085
6123	0.95895	0.132271
6124	1.01051	0.149889
6125	1.12468	0.16738
6126	1.22588	0.217302
6127	1.34254	0.192941
6128	1.74561	0.179755
6129	1.74561	0.143982
6130	0.946128	0.245903
6131	1.34253	0.193487
6132	1.3492	0.197787
6133	1.01051	0.138735
6134	1.922 2.1	
6135	1.922 2.1	
6136	2.12556	2.1
6137	0.946131	2.1
6138	1.06278	2.1
6139	1.47631	2.1
6140	1.47631	2.1
6141	0.946129	2.1
6142	1.06278	2.1
6143	1.35966	2.1
6144	0.991985	2.1
6145	0.738507	1.09781

6146	0.536935	1.77478
6147	0.799004	1.16033
6148	1.04281	0.907643
6149	0.879197	0.867501
6150	1.06538	0.926383
6151	0.715942	1.09787
6152	1.00636	0.324505
6153	1.03824	0.234146
6154	1.70147	0.238352
6155	1.21223	2.1
6156	1.01243	2.1
6157	1.01242	2.1
6158	1.06935	2.1
6159	1.54241	2.1
6160	0.998971	0.05
6161	1.60312	0.05
6162	1.44836	0.05
6163	2.05632	0.05
6164	1.44836	0.05
6165	0.973765	0.05
6166	0.946131	0.05
6167	0.851541	0.05
6168	1.73854	0.05
6169	1.91752	0.05
6170	1.49622	0.05
6171	1.23351	0.05
6172	3.33289	2.1
6173	1.86606	2.1
6174	2.71233	2.1
6175	0.788816	2.1
6176	0.963972	2.1
6177	1.25866	2.1
6178	1.10817	2.1
6179	1.41908	2.1
6180	1.48734	2.1
6181	1.40822	0.153858
6182	1.40822	0.152731
6183	2.09871	0.240069
6184	0.946133	0.196121
6185	1.14926	0.108525
6186	0.946133	0.204664
6187	1.78098	0.129275
6188	1.78098	0.0933432
6189	1.35822	0.16177
6190	1.3689	0.162116
6191	1.22176	0.0697995
6192	2.03426	0.138182
6193	0.946131	0.2073
6194	1.01713	0.143083
6195	0.704112	0.21844
6196	1.36237	0.135596
6197	1.36237	0.111538
6198	1.01713	0.187533
6199	1.04936	0.181587
6200	1.87377	2.1
6201	1.60501	2.1
6202	0.981584	0.05
6203	1.69435	0.05

6204	0.85154	2.1
6205	1.47841	2.1
6206	1.4952	2.1
6207	1.31941	2.1
6208	1.04023	2.1
6209	1.1995	2.1
6210	0.914791	2.1
6211	1.1427	1.83127
6212	1.64787	0.756572
6213	1.07526	1.62163
6214	1.07527	0.887129
6215	1.07527	1.15978
6216	1.58551	1.52657
6217	2.77362	1.19955
6218	2.17974	0.821231
6219	2.71723	1.19747
6220	1.29868	0.0667433
6221	1.3594	0.0614981
6222	1.04786	0.05
6223	0.527876	0.0564118
6224	1.4085	0.058875
6225	1.48538	0.05
6226	0.76829	0.05
6227	1.18514	0.0587162
6228	0.617954	0.05
6229	0.864161	0.05
6230	1.18835	0.05
6231	1.16817	2.1
6232	1.04263	2.1
6233	1.50524	2.1
6234	1.85355	2.1
6235	1.23963	2.1
6236	1.06279	2.1
6237	0.94613	2.1
6238	1.35229	2.1
6239	1.15947	2.1
6240	0.965593	2.1
6241	0.851553	2.1
6242	2.21296	2.1
6243	1.11764	2.1
6244	2.46399	2.1
6245	1.39292	2.1
6246	1.34701	2.1
6247	1.34701	2.1
6248	1.47001	2.1
6249	0.963166	0.05
6250	0.893445	0.05
6251	1.21056	0.05
6252	1.67375	2.1
6253	2.0977	2.1
6254	1.09154	2.1
6255	1.09154	2.1
6256	1.57122	0.751268
6257	1.60976	0.183679
6258	1.69533	0.139738
6259	1.6951	0.111674
6260	1.5831	0.0960985
6261	1.20969	2.1

6262	1.12076	2.1
6263	1.49808	2.1
6264	1.50201	2.1
6265	1.00278	2.1
6266	1.22811	0.0943181
6267	1.11545	2.1
6268	1.09646	2.1
6269	1.03107	0.05
6270	1.44293	0.05
6271	1.66752	2.1
6272	1.11023	2.1
6273	2.57831	1.97586
6274	2.20191	0.889239
6275	2.18051	1.98287
6276	1.48035	0.281278
6277	1.0961	0.153291
6278	1.63241	0.222941
6279	1.0961	0.365974
6280	1.20959	2.1
6281	2.86136	2.1
6282	1.64933	2.1
6283	1.47217	2.1
6284	0.915178	2.1
6285	1.19074	2.1
6286	0.897276	2.1
6287	0.915177	2.1
6288	0.987037	2.1
6289	1.04288	2.1
6290	2.39323	2.1
6291	0.976218	0.05
6292	2.23933	0.05
6293	0.976215	0.05
6294	1.07384	0.05
6295	1.07652	2.1
6296	1.55678	2.1
6297	1.54168	2.1
6298	1.69136	2.1
6299	1.64391	1.27274
6300	1.76477	1.54685
6301	0.611486	1.50136
6302	0.683421	0.837078
6303	0.946131	1.51974
6304	1.1019	1.2568
6305	1.12946	1.05157
6306	1.54226	1.79031
6307	1.19496	0.991385
6308	1.96199	0.997211
6309	2.0775	1.47086
6310	1.22297	1.06312
6311	1.3469	1.11559
6312	1.25366	1.38904
6313	1.65443	1.38773
6314	1.65443	1.48637
6315	0.946132	1.68678
6316	0.946132	1.76119
6317	1.12873	1.63644
6318	1.07468	1.34249
6319	1.2854	0.667651

6320	1.3469	1.23379
6321	1.68616	1.15896
6322	1.68616	0.943391
6323	0.946132	1.70301
6324	0.946132	1.60079
6325	1.16109	1.44645
6326	1.17879	1.07979
6327	1.17879	1.13461
6328	1.45304	1.51144
6329	2.21058	2.1
6330	2.81552	2.1
6331	1.45535	0.119534
6332	1.49906	0.0877445
6333	1.04413	2.1
6334	1.74586	2.1
6335	1.34526	2.1
6336	1.17402	1.05094
6337	1.74564	0.742749
6338	1.52724	2.1
6339	1.68142	2.1
6340	1.09651	2.1
6341	1.49149	2.1
6342	1.36814	2.1
6343	1.46783	2.1
6344	2.18116	2.09923
6345	1.23602	2.09057
6346	1.74538	1.98924
6347	0.851539	2.1
6348	1.45775	2.1
6349	1.28919	2.1
6350	0.851483	2.1
6351	1.26102	2.1
6352	0.869889	2.1
6353	0.85151	2.1
6354	1.23451	2.09851
6355	1.23602	2.1
6356	1.11955	2.1
6357	1.08022	2.1
6358	0.977776	2.1
6359	1.27896	2.1
6360	1.64268	2.1
6361	1.97548	2.1
6362	1.72527	2.06203
6363	0.946132	2.1
6364	1.69702	2.1
6365	1.69702	2.1
6366	1.13635	2.1
6367	1.40274	2.1
6368	1.24041	2.1
6369	1.13635	2.1
6370	1.78427	0.05
6371	1.68338	0.05
6372	1.00681	0.05
6373	1.12988	0.05
6374	0.946132	0.05
6375	0.957543	0.05
6376	1.40515	0.05
6377	1.40515	0.05

6378	0.797932	0.05
6379	1.30187	0.05
6380	1.14846	0.05
6381	0.665137	0.176381
6382	1.09575	0.214141
6383	0.596894	0.177393
6384	1.04072	0.27381
6385	0.663171	0.269983
6386	1.47218	0.219226
6387	1.32889	0.176301
6388	1.32639	0.0842705
6389	1.94887	0.107746
6390	1.32639	0.0906409
6391	1.19323	0.141302
6392	2.19794	0.179239
6393	0.754756	0.0924633
6394	1.11015	0.104422
6395	0.754755	0.0861601
6396	0.946133	0.129536
6397	0.754768	0.115498
6398	1.59986	0.104357
6399	1.59986	0.0746751
6400	1.84082	2.1
6401	1.79044	2.1
6402	1.75922	2.1
6403	1.86766	2.1
6404	1.58995	2.1
6405	1.58995	2.1
6406	1.74351	2.1
6407	1.04442	2.1
6408	1.42278	2.1
6409	1.08576	2.1
6410	0.994294	2.1
6411	1.44884	2.1
6412	1.71036	2.08444
6413	0.714101	1.65199
6414	1.17541	2.1
6415	1.00872	1.0056
6416	0.714072	1.91931
6417	0.936677	1.82578
6418	0.966979	2.1
6419	1.4908	2.1
6420	1.03108	0.151523
6421	0.949712	0.0869332
6422	0.810946	0.14515
6423	0.88209	0.147485
6424	0.646702	0.0975983
6425	0.972663	0.0620541
6426	0.862301	0.0815106
6427	0.868143	0.0865147
6428	1.11094	0.0897928
6429	1.35809	0.0835004
6430	1.32455	2.1
6431	1.32328	2.1
6432	1.69586	2.1
6433	1.78301	2.1
6434	1.67906	2.1
6435	1.60635	2.1

6436	1.57234	2.1
6437	1.47012	2.1
6438	1.22811	0.108997
6439	1.08732	0.13001
6440	2.08849	0.10998
6441	1.08732	0.122375
6442	1.30635	0.05
6443	0.77315	0.05
6444	1.25732	0.05
6445	2.43845	2.1
6446	2.38577	2.08809
6447	0.946132	1.31991
6448	1.19288	2.1
6449	1.54508	2.1
6450	1.54508	1.84474
6451	0.94515	2.1
6452	1.19288	2.03055
6453	1.29735	1.98058
6454	1.12087	1.25478
6455	1.54699	1.59634
6456	1.43635	2.1
6457	1.33385	2.1
6458	1.43635	2.1
6459	1.02368	2.1
6460	1.035 2.1	
6461	1.00128	2.1
6462	1.04009	2.1
6463	1.14052	2.1
6464	0.443552	2.1
6465	0.984351	2.1
6466	0.591443	2.1
6467	0.929561	2.1
6468	1.52079	2.1
6469	1.035 2.1	
6470	3.5282	2.1
6471	1.88288	2.1
6472	2.73877	2.02153
6473	0.906059	2.1
6474	1.23107	0.05
6475	0.906712	0.05
6476	0.841322	0.0597355
6477	1.53005	0.05
6478	1.10007	0.05
6479	1.38033	0.05
6480	1.03442	0.05
6481	0.787223	0.05
6482	1.42589	0.05
6483	2.35665	0.05
6484	1.17793	0.122959
6485	1.81356	0.11812
6486	1.42964	0.122603
6487	1.95641	0.106856
6488	1.39139	0.105363
6489	1.39139	0.129617
6490	0.564533	0.0869655
6491	1.20016	0.123399
6492	0.755765	0.071744
6493	0.755766	0.0788126

6494	1.20016	0.0987755
6495	1.91011	0.0671478
6496	1.4695	0.0693148
6497	1.70048	2.1
6498	1.93765	2.1
6499	0.741307	2.1
6500	0.780371	2.1
6501	0.946133	0.388705
6502	1.28918	1.01446
6503	1.5111	1.87473
6504	1.5111	0.567101
6505	0.946131	1.3951
6506	1.28918	0.742613
6507	1.16805	0.433318
6508	1.01179	0.417683
6509	0.946132	0.531985
6510	1.35328	1.12198
6511	1.69633	1.52317
6512	1.69633	0.680996
6513	0.946133	1.382
6514	1.35328	0.862496
6515	1.28918	0.522634
6516	1.01179	0.744127
6517	1.58147	1.0714
6518	1.27737	0.536799
6519	2.17223	0.05
6520	0.697168	0.05
6521	1.62495	0.108922
6522	1.62495	0.156674
6523	1.93948	0.237579
6524	0.601592	2.1
6525	1.93528	2.1
6526	2.13757	2.1
6527	1.67561	2.1
6528	2.70875	2.1
6529	1.93528	2.1
6530	3.98475	2.1
6531	1.12376	2.1
6532	1.19016	2.1
6533	1.67323	0.141838
6534	1.74019	0.122722
6535	1.84719	0.143738
6536	1.00764	0.109663
6537	1.23475	2.1
6538	1.06757	2.1
6539	1.57916	2.1
6540	1.06758	2.1
6541	1.28014	2.1
6542	1.33339	2.1
6543	1.2679	2.1
6544	1.23504	2.1
6545	1.50108	2.1
6546	1.3715	2.1
6547	0.58831	0.596057
6548	0.865971	0.505367
6549	0.813704	0.69759
6550	0.658856	0.53806
6551	0.495869	0.694026

6552	1.13845	0.650966
6553	0.780804	0.630479
6554	0.642023	0.692725
6555	0.707748	0.564114
6556	1.64476	0.464482
6557	1.64476	0.540986
6558	0.99611	0.573167
6559	1.35639	0.443361
6560	1.3564	0.512262
6561	0.946129	0.511308
6562	1.99222	0.543794
6563	1.4777	2.1
6564	1.03968	2.1
6565	1.82516	2.1
6566	0.851539	2.1
6567	1.63693	2.1
6568	1.59416	2.1
6569	0.878566	2.1
6570	1.40796	2.1
6571	1.13172	2.1
6572	0.851527	2.1
6573	1.14366	2.1
6574	0.823822	0.194103
6575	0.882001	0.200564
6576	1.09756	0.0728049
6577	1.11857	0.118159
6578	1.0934	0.115867
6579	0.960104	0.179363
6580	0.982001	0.281551
6581	1.74319	0.130027
6582	1.03043	0.141961
6583	0.967194	0.101203
6584	1.16938	2.1
6585	2.48377	2.1
6586	1.1181	2.1
6587	2.57349	2.1
6588	1.0465	2.1
6589	1.44709	2.1
6590	0.946132	2.1
6591	1.29302	2.1
6592	1.73502	2.1
6593	1.73502	2.1
6594	0.946132	2.1
6595	1.29302	2.1
6596	1.38813	2.1
6597	1.03933	2.1
6598	1.01976	0.263297
6599	1.11764	0.314341
6600	0.961779	0.389957
6601	1.0685	0.319878
6602	1.08426	1.70524
6603	0.99695	1.9122
6604	2.25593	1.57446
6605	1.47992	1.70473
6606	1.88989	1.46174
6607	1.65813	1.59319
6608	1.20278	1.69859
6609	0.981842	1.96428

6610	0.802655	2.09591
6611	0.953489	2.01078
6612	0.961719	2.00145
6613	0.975614	1.97841
6614	0.991617	1.96691
6615	0.940748	1.95073
6616	2.25019	2.0243
6617	2.30712	1.81465
6618	1.98823	1.99605
6619	0.522844	1.93202
6620	0.946122	1.94802
6621	0.946132	1.95712
6622	0.593846	1.89558
6623	1.26501	1.84494
6624	1.21171	0.05
6625	0.851542	0.0643769
6626	0.940327	0.0678979
6627	0.940356	0.0711155
6628	1.52117	0.05
6629	1.58072	0.05
6630	1.35169	0.05
6631	1.13889	0.05
6632	1.35007	0.05
6633	1.35007	0.05
6634	1.78381	0.05
6635	0.946132	0.0658476
6636	1.06627	0.0571283
6637	0.946131	0.0707618
6638	1.73005	0.05
6639	1.73005	0.05
6640	1.32449	0.05
6641	1.35169	0.05
6642	1.42446	0.303859
6643	1.86697	0.260141
6644	1.13413	0.273925
6645	1.42446	0.231774
6646	1.33575	0.104464
6647	1.20473	0.120843
6648	1.87517	0.05
6649	1.87517	0.05
6650	0.488712	0.05
6651	1.13242	0.05
6652	0.488713	0.05
6653	0.488713	0.05
6654	1.23146	0.05
6655	1.57932	0.05
6656	1.01698	0.05
6657	1.42085	0.05
6658	0.246725	0.05
6659	0.996283	0.05
6660	1.36965	0.05
6661	0.86361	0.05
6662	0.810945	0.05
6663	1.72938	0.0610228
6664	1.21649	0.0621893
6665	1.1865	0.05
6666	1.62387	0.05
6667	1.05302	0.05

6668	1.10049	0.855701
6669	0.946129	2.1
6670	1.70184	1.45248
6671	1.70184	2.1
6672	1.12538	2.1
6673	1.35969	1.25121
6674	1.28828	2.09936
6675	1.12538	2.08627
6676	0.99747	1.93691
6677	1.34851	0.942996
6678	0.923724	2.1
6679	1.47154	2.09485
6680	1.47154	2.1
6681	1.10698	2.1
6682	1.28828	2.04954
6683	1.10698	2.1
6684	0.923695	2.1
6685	0.99747	1.93901
6686	1.65677	2.1
6687	1.77677	2.1
6688	1.05689	0.174788
6689	2.11031	0.157761
6690	1.07367	0.119271
6691	0.656239	0.177921
6692	1.4563	0.149431
6693	1.4563	0.134608
6694	0.656238	0.177694
6695	0.946133	0.161068
6696	0.812937	0.192347
6697	0.946131	0.201605
6698	1.05689	0.129542
6699	1.34009	2.1
6700	1.398 2.1	
6701	3.29047	2.1
6702	2.53897	2.1
6703	1.24509	2.1
6704	3.06162	2.1
6705	1.01595	2.1
6706	1.52661	2.1
6707	1.5266	2.1
6708	0.946131	2.1
6709	1.31049	2.1
6710	1.16225	2.1
6711	1.05978	2.1
6712	1.16225	2.1
6713	1.06561	2.1
6714	0.953927	2.1
6715	1.15114	2.1
6716	1.1807	0.331616
6717	0.507259	0.281734
6718	1.47709	0.262553
6719	1.36192	0.236018
6720	0.487293	0.26504
6721	1.65569	0.31425
6722	0.679725	0.217517
6723	0.721928	0.274772
6724	1.03931	0.260738
6725	1.0775	0.134376

6726	1.0775	0.393387
6727	2.30207	0.664626
6728	1.01814	0.629068
6729	2.33963	0.558926
6730	2.42589	0.555752
6731	1.33962	2.1
6732	1.27956	2.1
6733	2.14624	0.05
6734	1.6812	0.05
6735	1.25698	0.05
6736	1.35725	0.05
6737	1.86387	0.05
6738	1.86387	0.05
6739	0.750365	0.05
6740	0.946128	0.05
6741	0.750365	0.05
6742	1.72271	0.05
6743	1.72272	0.05
6744	2.53736	0.0596004
6745	1.07327	2.1
6746	1.05376	2.1
6747	1.00387	1.1547
6748	2.0554	1.45745
6749	1.00387	0.833558
6750	1.00387	0.485914
6751	1.27119	2.1
6752	1.51748	2.1
6753	3.15271	2.1
6754	3.45801	2.1
6755	1.99871	2.1
6756	1.41186	2.1
6757	1.07624	2.1
6758	1.5954	2.1
6759	1.19323	1.20541
6760	1.21286	0.744585
6761	0.946114	2.1
6762	1.38395	2.1
6763	1.73389	2.1
6764	1.7339	2.1
6765	0.946115	2.1
6766	1.38397	2.1
6767	1.29604	2.1
6768	1.04544	2.1
6769	1.60578	0.0750067
6770	1.93407	0.0816974
6771	1.0525	0.780574
6772	1.0525	1.0364
6773	1.0525	2.00856
6774	1.38506	1.58766
6775	2.34737	0.05
6776	1.3531	0.05
6777	1.2925	0.05
6778	0.871466	0.05
6779	1.26623	0.05
6780	0.700631	0.05
6781	1.88774	0.05
6782	1.14167	0.05
6783	1.18606	0.05

6784	2.1238	0.05
6785	1.42762	0.766454
6786	1.67791	0.05
6787	1.47118	0.05
6788	0.946128	2.1
6789	1.31641	2.1
6790	1.75933	2.1
6791	1.75933	2.1
6792	0.946131	2.1
6793	1.31641	2.1
6794	1.38905	2.1
6795	1.38437	2.1
6796	1.51829	2.1
6797	0.947114	2.1
6798	1.13335	2.1
6799	1.47521	2.1
6800	1.14911	2.1
6801	1.53822	2.1
6802	1.39702	2.1
6803	0.739166	2.1
6804	0.706056	2.1
6805	0.964033	2.1
6806	1.58993	2.09736
6807	2.51892	2.08068
6808	0.595905	0.05
6809	1.18155	0.05
6810	1.73364	0.05
6811	0.952735	0.05
6812	2.10381	0.05
6813	1.12115	0.05
6814	1.27826	0.05
6815	2.74021	0.05
6816	1.78416	2.1
6817	1.78416	2.1
6818	0.946132	2.1
6819	1.41126	2.1
6820	1.21275	2.1
6821	0.946133	2.1
6822	1.41126	2.1
6823	1.33125	2.1
6824	1.33125	2.1
6825	1.78918	2.1
6826	1.0465	2.1
6827	1.99715	2.1
6828	1.17683	2.1
6829	1.25889	2.1
6830	0.860834	0.05
6831	0.946131	0.05
6832	1.05008	0.05
6833	0.970444	0.05
6834	1.19401	0.05
6835	1.25989	0.05
6836	0.702942	0.05
6837	1.40591	0.05
6838	1.72168	0.05
6839	0.446352	0.05
6840	1.04072	0.05
6841	0.711038	0.05

6842	0.980737	0.05
6843	1.05099	0.05
6844	1.26003	0.05
6845	0.495934	0.05
6846	1.03114	0.05
6847	1.09911	0.05
6848	1.24825	0.05
6849	2.19827	0.05
6850	2.0953	0.05
6851	2.30437	0.05
6852	0.775923	0.05
6853	2.2283	0.05
6854	1.19583	2.1
6855	1.14496	2.1
6856	1.62183	2.1
6857	1.62316	2.1
6858	1.02305	0.23475
6859	1.21136	0.121131
6860	1.44426	0.190677
6861	1.21137	0.0859357
6862	1.20143	0.212815
6863	1.15009	0.352082
6864	1.24965	2.1
6865	1.41077	2.1
6866	1.00052	0.103436
6867	1.79553	0.103874
6868	1.06336	0.0764282
6869	1.00053	0.0803704
6870	1.18041	2.1
6871	1.80818	2.1
6872	1.73571	1.60242
6873	1.94429	1.73318
6874	1.4375	0.05
6875	1.44421	0.05
6876	1.66953	0.05
6877	1.09529	0.05
6878	0.952395	2.1
6879	0.717337	2.1
6880	1.03609	2.1
6881	1.33949	2.09465
6882	1.2774	0.218497
6883	2.24983	0.198441
6884	1.28727	0.166505
6885	0.583592	0.141495
6886	0.583591	0.177707
6887	1.82859	0.212905
6888	1.82859	0.224972
6889	0.583593	0.232959
6890	0.946128	0.136454
6891	0.692233	0.314176
6892	1.12226	0.256377
6893	2.57454	0.243572
6894	0.810944	0.207146
6895	0.810945	0.176338
6896	1.12226	0.241737
6897	1.45867	0.240294
6898	1.22403	0.203473
6899	1.15262	0.224264

6900	1.08132	0.217507
6901	0.846671	0.176265
6902	0.480954	0.268608
6903	0.917975	0.255801
6904	0.917975	0.307652
6905	0.720957	0.299483
6906	0.795998	0.238231
6907	0.69316	0.294177
6908	0.641306	0.308496
6909	0.690817	0.201958
6910	0.961899	2.1
6911	2.49579	2.1
6912	2.31106	2.1
6913	1.70482	0.0895251
6914	1.93156	0.114756
6915	1.23868	0.142053
6916	1.73878	0.133061
6917	1.26267	2.1
6918	1.26441	2.1
6919	1.58095	2.1
6920	1.58095	2.1
6921	0.946132	2.1
6922	0.946132	2.1
6923	1.11077	2.1
6924	1.62905	2.1
6925	1.49692	2.1
6926	3.49966	2.1
6927	2.39213	2.1
6928	2.12603	2.1
6929	0.946129	2.1
6930	0.461661	2.1
6931	1.52841	2.1
6932	1.39091	2.1
6933	0.550679	2.1
6934	0.509894	2.1
6935	1.49508	2.1
6936	2.05862	2.1
6937	2.47851	2.1
6938	1.07213	2.1
6939	1.09148	1.80253
6940	1.99776	2.03643
6941	1.38333	2.01395
6942	1.04412	2.1
6943	1.04412	2.1
6944	1.1893	0.0545894
6945	0.946132	0.0685596
6946	0.965737	0.0993055
6947	0.946132	0.0744489
6948	1.54061	0.065688
6949	1.54061	0.100736
6950	1.22878	0.0777624
6951	1.25797	0.0793217
6952	1.00665	0.0766439
6953	1.00665	0.0935965
6954	1.80369	0.0633462
6955	0.946128	0.0939012
6956	1.04424	0.119886
6957	0.85154	0.0922923

6958	1.39322	0.0852118
6959	1.35464	0.119687
6960	0.939857	0.102011
6961	1.28272	0.103985
6962	1.19323	0.149348
6963	1.03498	0.065073
6964	2.22029	0.0799459
6965	0.540005	0.0637492
6966	0.970382	0.107244
6967	2.14514	0.0762102
6968	1.31542	0.0734412
6969	1.05575	0.11823
6970	0.810943	0.142373
6971	1.31542	0.058919
6972	1.70493	0.079049
6973	1.22582	0.105179
6974	1.31681	0.114172
6975	1.40775	0.0839365
6976	0.928633	0.167363
6977	1.2545	0.0926576
6978	2.66481	0.102404
6979	1.32051	0.0625208
6980	1.32051	0.117601
6981	2.66305	2.1
6982	1.3551	2.1
6983	2.45325	2.1
6984	1.3685	2.1
6985	1.51748	2.1
6986	1.78941	2.1
6987	1.02963	0.05
6988	1.28643	0.05
6989	0.86131	0.05
6990	0.851539	0.0510937
6991	1.46585	0.05
6992	1.38822	0.05
6993	0.996402	0.05
6994	0.851553	0.05
6995	1.73156	0.05
6996	1.91695	0.05
6997	1.92535	0.05
6998	2.57287	0.05
6999	1.77743	0.05
7000	1.02836	0.263234
7001	1.36362	0.278622
7002	1.36936	0.216406
7003	1.78684	0.19354
7004	1.78685	0.213465
7005	0.946132	0.341336
7006	1.27581	0.308945
7007	0.946132	0.3911
7008	1.02836	0.181807
7009	1.55398	2.1
7010	1.07167	2.1
7011	1.87557	2.1
7012	0.751639	2.1
7013	1.50884	2.1
7014	1.33793	2.1
7015	0.751664	2.1

7016	1.37277	2.1
7017	0.979515	2.1
7018	0.676508	2.1
7019	1.09578	2.1
7020	0.953925	2.1
7021	0.94613	2.1
7022	1.64707	2.1
7023	1.64707	2.1
7024	1.16658	2.1
7025	1.40638	2.1
7026	1.18682	2.1
7027	1.16658	2.1
7028	0.543765	0.160475
7029	1.57462	0.147872
7030	1.57462	0.119767
7031	1.00083	0.149301
7032	1.16834	0.185658
7033	1.16834	0.168012
7034	0.889626	0.175195
7035	1.25944	0.172294
7036	0.824061	0.183034
7037	1.9397	0.105468
7038	1.21893	0.131774
7039	1.91364	0.123859
7040	1.33672	0.163935
7041	0.928069	0.226868
7042	1.41484	0.171022
7043	0.538379	0.141401
7044	1.41484	0.148515
7045	1.35594	0.180938
7046	2.11414	0.157895
7047	2.11414	0.101425
7048	0.538378	0.160272
7049	1.38532	0.110401
7050	0.962877	1.40708
7051	1.07497	0.558306
7052	1.00613	0.815512
7053	0.962878	0.280132
7054	0.711447	2.1
7055	1.40008	0.0691894
7056	2.51674	0.0847437
7057	1.40008	0.0623973
7058	1.7263	0.05
7059	1.40008	0.0760958
7060	1.9216	0.05
7061	2.7013	0.05
7062	2.58584	0.05
7063	0.979296	0.05
7064	1.46459	1.227
7065	0.899254	0.894938
7066	1.15038	2.1
7067	1.24687	2.1
7068	1.16738	2.1
7069	0.942323	2.1
7070	1.65573	2.01008
7071	0.844791	2.1
7072	0.54674	2.09782
7073	0.795811	2.1

7074	1.08131	2.1
7075	1.03553	2.1
7076	0.758334	2.1
7077	0.708665	2.04861
7078	1.22626	2.01659
7079	0.835439	2.01168
7080	0.861131	1.95795
7081	0.68296	2.06464
7082	1.65224	2.03156
7083	2.82624	2.0777
7084	1.0151	1.80319
7085	1.15905	1.9393
7086	1.71076	1.80612
7087	1.4529	1.50414
7088	0.494895	2.07011
7089	0.946132	1.9805
7090	0.613132	1.91285
7091	0.863631	1.95736
7092	1.78391	2.1
7093	1.1706	0.05
7094	2.02734	0.05
7095	1.1706	0.05
7096	0.681532	0.05
7097	1.50316	0.05
7098	1.50316	0.05
7099	0.681534	0.05
7100	0.973819	0.05
7101	0.97382	0.05
7102	0.94613	0.05
7103	1.1706	0.05
7104	1.67727	0.05
7105	1.60329	0.05
7106	1.3227	2.1
7107	1.40846	2.1
7108	1.07889	2.1
7109	1.07721	2.1
7110	0.953523	0.570413
7111	1.79675	0.622503
7112	2.09859	0.889238
7113	1.21309	2.1
7114	1.54567	0.817056
7115	1.19722	0.236359
7116	1.82793	0.175733
7117	1.1599	0.204159
7118	1.73621	0.152282
7119	0.933474	0.290586
7120	1.51477	0.217785
7121	1.51477	0.256583
7122	0.93347	0.305332
7123	1.27374	0.215863
7124	1.27376	0.260552
7125	0.946105	0.254479
7126	1.1972	0.20084
7127	0.851541	0.05
7128	1.36971	0.05
7129	1.27832	0.05
7130	0.853489	0.05
7131	0.973847	0.05

7132	0.976308	0.05
7133	0.851528	0.05
7134	2.72654	0.05
7135	1.77867	0.05
7136	3.23032	2.1
7137	2.78208	2.1
7138	1.0227	0.05
7139	1.02269	0.05
7140	1.02269	0.05
7141	1.24293	0.05
7142	0.831695	2.1
7143	0.67513	2.1
7144	0.956294	2.1
7145	1.08623	2.1
7146	1.05556	2.1
7147	1.38859	2.1
7148	1.13179	2.1
7149	1.02438	2.1
7150	1.06659	2.1
7151	1.36595	2.1
7152	1.06659	2.1
7153	1.06659	2.1
7154	0.946131	2.02019
7155	0.875912	2.1
7156	1.65971	2.1
7157	1.65971	2.08846
7158	0.875912	2.1
7159	1.1229	2.1
7160	1.41273	2.1
7161	1.54699	2.05432
7162	1.93655	1.73319
7163	1.42307	2.00787
7164	1.97907	2.0342
7165	0.996774	2.1
7166	1.15505	0.05
7167	1.28463	0.05
7168	1.86353	0.0696202
7169	2.71398	0.0634272
7170	1.56787	0.297269
7171	1.66295	0.22419
7172	1.73432	1.1212
7173	1.05019	0.05
7174	1.42117	0.05
7175	0.888299	0.05
7176	1.38127	0.05
7177	1.72993	0.05
7178	0.664129	0.05
7179	0.99302	2.1
7180	0.993023	2.1
7181	1.13491	2.1
7182	0.876316	2.1
7183	1.2588	2.1
7184	0.895613	2.1
7185	2.07526	2.1
7186	1.23369	2.1
7187	1.25146	2.1
7188	2.15282	2.1
7189	1.22239	2.0999

7190	1.10962	2.1
7191	1.77402	2.09421
7192	1.0305	2.1
7193	1.50108	2.04766
7194	1.20761	2.1
7195	1.3626	2.1
7196	1.14825	2.1
7197	1.78711	2.1
7198	1.17933	2.1
7199	1.14824	2.1
7200	1.09651	0.110579
7201	1.23851	0.108953
7202	1.83284	0.131897
7203	1.23851	0.0709506
7204	1.53521	2.1
7205	1.46279	2.1
7206	1.9233	0.05
7207	1.2966	0.05
7208	2.11443	0.05
7209	1.30265	0.05
7210	1.13528	0.13989
7211	0.946131	0.234399
7212	1.41169	0.179669
7213	1.33879	0.101275
7214	0.946128	0.197249
7215	1.80435	0.154096
7216	1.80435	0.0580214
7217	1.234	2.1
7218	1.45633	2.1
7219	1.234	2.1
7220	1.98448	2.1
7221	2.02612	2.1
7222	1.23527	1.33996
7223	1.44472	1.86424
7224	0.993366	2.1
7225	1.22025	2.06444
7226	0.993368	1.53017
7227	2.43669	2.1
7228	2.56707	2.1
7229	1.80348	0.718395
7230	1.74406	0.360353
7231	1.90937	0.633457
7232	1.49357	0.147641
7233	2.5832	0.215104
7234	1.82274	0.347059
7235	1.6957	0.297431
7236	2.22495	0.291524
7237	2.49959	0.299039
7238	2.01183	0.0509849
7239	1.37427	0.05
7240	1.15069	2.1
7241	1.39379	2.1
7242	1.69661	1.89091
7243	1.39379	1.99535
7244	1.39654	2.1
7245	1.93505	2.1
7246	1.5525	2.1
7247	1.5525	2.1

7248	0.615065	2.1
7249	1.38908	2.1
7250	0.615065	2.1
7251	0.77849	2.1
7252	1.38908	2.1
7253	1.56839	2.0979
7254	1.09142	2.1
7255	2.07313	2.08209
7256	0.998288	0.05
7257	1.31481	0.0573436
7258	1.88175	0.05
7259	2.23267	0.05
7260	1.06935	2.1
7261	1.06936	2.1
7262	1.17989	2.09752
7263	1.00757	2.07325
7264	1.65318	2.1
7265	1.4185	0.223085
7266	2.22495	0.259551
7267	1.02011	0.15515
7268	0.59676	0.208995
7269	1.43606	0.178546
7270	1.43606	0.159154
7271	0.89771	0.187904
7272	1.13511	0.216925
7273	0.59676	0.144545
7274	0.897709	0.199756
7275	1.79542	0.182664
7276	2.38566	2.08447
7277	1.63838	1.40586
7278	1.63837	1.75273
7279	0.916275	2.05025
7280	1.36183	1.58666
7281	1.19282	2.07036
7282	0.916275	1.92158
7283	1.19283	2.0062
7284	2.01582	2.1
7285	1.00215	0.05
7286	2.60643	2.1
7287	2.15971	2.1
7288	1.28267	2.1
7289	1.29954	2.1
7290	3.40327	2.1
7291	3.49128	2.1
7292	1.3864	2.1
7293	1.3201	2.1
7294	1.45666	0.05
7295	1.55798	0.05
7296	0.857202	0.05
7297	1.24374	0.05
7298	1.04157	0.05
7299	0.85723	0.05
7300	1.02722	0.05
7301	1.01361	0.05
7302	0.974077	0.05
7303	1.01814	0.05
7304	0.974077	0.05
7305	1.43987	0.05

7306	1.43986	0.05
7307	0.927881	0.05
7308	0.946132	0.05
7309	0.927849	0.05
7310	0.927852	0.05
7311	1.2493	0.05
7312	1.22482	0.445443
7313	1.41319	0.707553
7314	1.22482	0.287199
7315	0.999492	2.1
7316	1.14657	2.1
7317	1.3617	0.05
7318	0.983269	0.05
7319	1.33174	0.05
7320	1.62816	0.05
7321	1.62817	0.05
7322	0.81938	0.05
7323	1.33174	0.05
7324	1.01332	0.05
7325	0.874788	0.05
7326	1.16725	2.1
7327	1.63991	2.1
7328	1.08848	0.05
7329	1.15354	0.05
7330	1.12665	2.1
7331	1.58742	2.1
7332	1.57523	2.1
7333	1.32201	1.48041
7334	1.32097	1.4483
7335	0.851542	0.141366
7336	0.877188	0.0850792
7337	0.797489	0.144016
7338	1.44721	0.0768911
7339	1.61761	0.0550719
7340	0.943799	0.130071
7341	1.36079	0.097022
7342	1.16361	2.1
7343	1.15852	2.09714
7344	1.07624	2.1
7345	1.07624	2.1
7346	0.946132	0.616988
7347	1.37938	0.977926
7348	1.78653	1.21926
7349	1.78653	0.649078
7350	0.946131	1.27107
7351	1.37938	0.851338
7352	1.35328	0.589246
7353	1.44847	0.570208
7354	0.946133	0.757652
7355	1.38149	0.796418
7356	1.81474	0.895279
7357	1.81474	0.609609
7358	0.946133	1.2015
7359	1.38149	0.82096
7360	1.37938	0.627434
7361	1.44847	0.778327
7362	1.93665	0.54186
7363	1.5515	0.571449

7364	1.05025	2.1
7365	1.05025	2.1
7366	1.71468	2.1
7367	1.04759	2.1
7368	1.04646	0.05
7369	1.00739	0.05
7370	1.37086	0.05
7371	1.12547	0.05
7372	1.03853	0.05
7373	1.34824	0.05
7374	0.799786	0.05
7375	2.10105	0.05
7376	2.72007	0.05
7377	0.589918	0.05
7378	0.589918	0.05
7379	1.1509	0.05
7380	0.619832	0.05
7381	1.1509	0.05
7382	0.705903	0.05
7383	1.53703	0.05
7384	1.53703	0.05
7385	0.705904	0.05
7386	0.946133	0.05
7387	0.946132	0.05
7388	0.946132	0.05
7389	0.588467	0.05
7390	1.1078	0.05
7391	0.408061	0.05
7392	1.38703	0.05
7393	1.68328	0.05
7394	1.87031	0.05
7395	1.86268	0.0940517
7396	1.27472	0.0739377
7397	0.947862	0.607687
7398	1.19854	1.01855
7399	0.946129	0.803457
7400	0.963111	0.474253
7401	0.946131	1.0291
7402	1.39055	0.462539
7403	1.39055	0.344344
7404	1.26426	0.538156
7405	1.07242	0.566994
7406	1.31126	0.27564
7407	1.76597	0.528043
7408	0.94613	1.38774
7409	1.27214	0.855635
7410	1.90751	0.91489
7411	1.90751	0.774882
7412	0.767114	0.988137
7413	1.27214	1.13153
7414	1.40248	1.01872
7415	1.41479	1.31577
7416	2.22625	1.3543
7417	0.913373	1.41475
7418	1.0158	0.81769
7419	1.61259	0.856057
7420	1.69051	0.669167
7421	0.560949	1.04881

7422	0.898559	1.23367
7423	1.28011	1.07484
7424	1.51026	0.0653498
7425	1.98252	0.0863969
7426	1.05129	0.0794862
7427	1.2951	0.089289
7428	1.46041	0.05
7429	1.7155	2.1
7430	1.72196	2.1
7431	0.933885	2.1
7432	0.986723	2.1
7433	1.28072	1.73871
7434	1.40765	2.1
7435	1.28072	2.09666
7436	1.61687	2.1
7437	1.60965	2.1
7438	1.21424	2.1
7439	2.07297	2.1
7440	1.19943	2.1
7441	2.38784	2.1
7442	1.10278	0.0976759
7443	1.43678	0.114523
7444	1.1142	0.131414
7445	1.18034	0.118547
7446	0.94613	0.143792
7447	0.752356	0.113311
7448	0.752329	0.137157
7449	1.46634	0.103163
7450	1.31789	0.106883
7451	0.958159	0.158523
7452	1.22752	0.148882
7453	1.50472	0.115667
7454	1.98974	2.1
7455	2.54186	2.1
7456	0.76512	2.1
7457	2.08777	2.1
7458	1.7753	2.1
7459	3.07372	2.1
7460	1.9756	2.1
7461	1.48162	2.1
7462	1.23204	2.1
7463	0.946131	1.0757
7464	1.13505	2.06518
7465	1.47432	2.09943
7466	1.47432	1.53074
7467	0.876831	1.9078
7468	1.13505	1.78665
7469	1.2161	1.65811
7470	1.05267	1.56197
7471	1.05267	1.34297
7472	1.5795	1.31834
7473	0.98186	2.1
7474	1.56735	2.1
7475	0.859099	2.1
7476	1.69858	2.1
7477	1.93586	2.1
7478	1.09233	2.1
7479	1.08732	0.119515

7480	1.3357	0.117638
7481	1.84826	2.1
7482	0.924132	2.1
7483	1.39188	2.1
7484	1.43685	2.1
7485	0.851541	2.1
7486	1.16251	2.1
7487	0.946131	2.1
7488	1.05685	2.1
7489	2.13781	2.1
7490	1.98859	2.1
7491	1.99731	2.1
7492	1.51379	2.1
7493	1.48796	2.1
7494	0.851542	2.1
7495	0.802558	2.1
7496	1.09244	2.1
7497	1.33891	2.1
7498	0.844958	2.1
7499	0.710864	2.1
7500	1.26087	2.1
7501	1.03679	2.1
7502	1.51026	0.0609996
7503	1.05248	0.461759
7504	0.827258	0.433649
7505	1.05248	0.450738
7506	1.28435	0.448429
7507	1.50953	0.451294
7508	1.50953	0.138603
7509	0.827298	0.472972
7510	1.00366	0.117587
7511	2.10495	0.220498
7512	1.17885	2.1
7513	1.25185	2.1
7514	1.02784	2.1
7515	1.76764	2.1
7516	1.23746	0.101442
7517	1.24082	0.113918
7518	1.46506	0.0610348
7519	1.33061	0.05
7520	0.768407	0.267067
7521	0.972058	0.15796
7522	1.16536	0.155245
7523	1.13325	0.110281
7524	0.978906	0.657319
7525	1.02291	0.179821
7526	0.838015	0.162774
7527	1.01052	0.173983
7528	1.08166	0.196465
7529	1.42607	0.05
7530	1.47354	0.0653568
7531	1.21703	2.1
7532	1.29897	2.1
7533	1.83293	2.1
7534	1.29897	2.1
7535	1.24688	0.118556
7536	0.782563	2.1
7537	0.946129	2.1

7538	0.782591	2.1
7539	1.49487	2.1
7540	1.49487	2.1
7541	1.37713	2.1
7542	0.900337	2.1
7543	1.31284	2.1
7544	0.924468	2.1
7545	1.40602	2.1
7546	1.25669	2.1
7547	0.986901	2.1
7548	0.958565	2.1
7549	1.37266	2.1
7550	1.64146	2.1
7551	0.49345	2.1
7552	1.08276	2.1
7553	1.08276	2.1
7554	1.26929	2.1
7555	1.09847	2.1
7556	0.827081	0.215199
7557	1.07236	0.366298
7558	1.25063	1.17312
7559	1.43548	0.197313
7560	1.25063	0.211831
7561	1.3659	0.05
7562	1.72441	0.05
7563	1.3659	0.05
7564	1.4677	0.05
7565	1.86114	0.05
7566	1.40104	0.05
7567	0.962675	0.05
7568	0.946131	0.0542584
7569	1.74134	0.05
7570	1.74134	0.05
7571	0.962676	0.05
7572	0.946131	0.05
7573	1.05548	0.0706623
7574	0.902187	0.0885491
7575	2.14178	0.05
7576	1.74998	0.061825
7577	1.38541	0.0687862
7578	1.64468	0.05
7579	0.949317	0.0823923
7580	2.06654	0.0531692
7581	0.839626	0.0716112
7582	0.810945	0.0837139
7583	1.29203	0.05
7584	1.18091	0.0558004
7585	1.06872	0.068953
7586	1.11956	0.05
7587	0.926911	0.0880832
7588	0.901716	0.0820315
7589	0.909146	0.0933868
7590	1.33776	0.05423
7591	1.02048	0.074032
7592	1.08365	0.0813783
7593	1.21042	0.0566508
7594	0.865634	0.0946602
7595	0.590354	2.1

7596	0.61173	2.1
7597	3.46825	2.1
7598	3.28404	2.1
7599	1.56004	0.10101
7600	1.69344	0.317423
7601	1.7151	0.888294
7602	0.946131	0.335421
7603	1.40265	0.225352
7604	1.13537	1.31905
7605	0.965708	0.44595
7606	1.13537	0.580778
7607	1.29681	0.523322
7608	1.40915	0.310578
7609	1.61652	1.82077
7610	1.33171	2.09722
7611	1.37681	2.1
7612	0.81094	0.05
7613	0.598606	0.05
7614	0.723111	0.05
7615	1.04562	0.05
7616	0.979052	0.05
7617	0.798185	0.05
7618	0.880117	0.05
7619	0.979341	0.20365
7620	0.800229	0.15743
7621	1.39126	0.192435
7622	1.13005	0.20922
7623	0.913877	0.223253
7624	1.26959	0.180938
7625	0.699741	0.217091
7626	1.19496	0.992337
7627	1.13392	0.05
7628	1.11913	0.05
7629	1.06866	0.05
7630	0.615546	0.05
7631	0.831006	0.05
7632	1.53286	0.05
7633	0.690977	0.05
7634	1.01956	0.05
7635	2.33818	0.05
7636	1.6585	2.1
7637	1.67061	2.1
7638	1.41414	2.1
7639	1.16756	2.1
7640	1.00898	0.05
7641	0.966964	2.1
7642	2.85443	2.1
7643	0.966977	2.1
7644	2.08427	2.1
7645	2.94901	2.1
7646	1.10735	0.353297
7647	1.65815	2.1
7648	1.4502	2.1
7649	1.45026	2.1
7650	1.53446	2.1
7651	0.946129	0.768649
7652	1.81075	2.1
7653	1.81075	1.07696

7654	0.946131	1.65671
7655	1.37075	2.1
7656	1.37075	1.15615
7657	1.34245	1.81816
7658	0.973502	1.11542
7659	1.08542	2.08529
7660	1.5814	0.05
7661	1.43836	0.05
7662	0.946132	0.05
7663	1.13747	0.05
7664	1.04429	0.05
7665	1.05017	0.05
7666	1.24732	0.05
7667	1.13387	0.05
7668	2.19489	0.05
7669	1.10748	2.1
7670	1.25006	2.1
7671	1.58834	2.1
7672	1.58834	2.1
7673	0.94613	2.1
7674	1.16548	2.1
7675	0.946132	2.1
7676	1.08998	2.1
7677	1.16548	2.1
7678	0.995732	2.07638
7679	0.657014	2.1
7680	0.957173	2.1
7681	1.01485	2.1
7682	1.12468	2.1
7683	1.43871	2.1
7684	1.23716	2.1
7685	1.89277	0.588324
7686	2.06653	0.412127
7687	0.967544	0.303783
7688	1.89016	0.345364
7689	2.22165	2.1
7690	0.83514	2.1
7691	1.76565	2.1
7692	1.76565	2.1
7693	0.94613	2.1
7694	0.835112	2.1
7695	1.23426	2.1
7696	1.36653	2.1
7697	0.895892	1.77629
7698	0.859278	1.8235
7699	0.791461	1.78979
7700	1.86249	1.76949
7701	1.14607	1.85283
7702	1.19904	1.76847
7703	1.84898	1.79561
7704	1.03452	1.81128
7705	0.946131	1.51142
7706	0.940472	0.712975
7707	1.53478	2.1
7708	1.84175	1.85567
7709	1.55687	1.55193
7710	1.17787	1.2347
7711	1.60589	0.332779

7712	2.35574	0.411545
7713	1.3305	0.687533
7714	1.15008	1.12213
7715	1.12457	0.0511481
7716	1.12457	0.0635082
7717	1.01778	2.1
7718	1.35733	2.1
7719	1.50683	2.1
7720	1.12064	2.1
7721	1.42716	0.854453
7722	1.42717	1.01814
7723	1.45927	1.42632
7724	1.04321	1.59887
7725	1.32634	0.17765
7726	2.27022	0.154717
7727	1.19352	0.129265
7728	1.32634	0.119998
7729	1.40924	1.7249
7730	1.22927	1.12393
7731	1.69237	1.62027
7732	1.69237	2.08536
7733	0.946132	0.982775
7734	0.946133	1.5942
7735	1.16912	1.50991
7736	1.0417	1.27212
7737	1.14605	1.67942
7738	1.1233	2.1
7739	1.96882	1.53193
7740	1.03804	0.0728256
7741	1.15338	0.0576881
7742	1.15338	0.0566225
7743	2.33051	0.0633747
7744	1.81507	2.1
7745	2.42949	2.1
7746	0.725879	2.1
7747	1.88394	2.09703
7748	2.2699	1.03485
7749	2.78952	2.1
7750	1.88394	1.24352
7751	1.79606	2.1
7752	1.20091	2.0778
7753	1.9614	2.07876
7754	1.19901	2.07783
7755	3.07915	2.1
7756	1.32592	2.1
7757	2.86461	2.1
7758	1.34817	2.1
7759	1.44859	2.1
7760	2.03969	2.1
7761	0.946132	2.1
7762	1.50709	2.1
7763	1.50709	2.1
7764	0.946132	2.1
7765	1.29322	2.1
7766	1.29322	2.1
7767	1.01985	2.1
7768	0.968537	2.1
7769	1.401 2.1	

7770	1.28936	2.1
7771	1.56002	0.05
7772	1.92548	0.05
7773	1.46041	0.05
7774	2.22712	0.05
7775	1.56001	0.05
7776	1.50524	2.1
7777	1.21304	2.1
7778	1.60665	1.93149
7779	1.85178	1.33959
7780	2.17534	2.09398
7781	2.31012	2.1
7782	2.17533	1.98479
7783	0.986724	2.1
7784	2.01895	2.1
7785	2.00433	2.1
7786	1.45752	0.122557
7787	1.12695	0.120963
7788	0.652605	2.1
7789	1.5133	2.1
7790	1.5133	2.1
7791	0.652603	2.1
7792	1.228	2.1
7793	0.946132	2.1
7794	0.652604	2.1
7795	0.946132	1.95079
7796	0.716815	2.1
7797	1.62203	2.04939
7798	1.62203	2.1
7799	0.716814	2.1
7800	1.00191	2.09417
7801	1.33694	2.06454
7802	1.38216	2.1
7803	2.53338	2.09945
7804	1.46932	0.05
7805	1.50848	0.05
7806	1.50108	1.41538
7807	1.18382	0.446138
7808	2.02115	0.230881
7809	0.921823	0.502042
7810	2.08356	0.05
7811	1.543	0.05
7812	1.00763	0.218597
7813	1.4185	0.23394
7814	1.08028	0.201852
7815	0.946131	0.391029
7816	1.56599	0.183594
7817	1.56599	0.389324
7818	1.08028	0.188052
7819	0.946131	0.0957361
7820	1.37518	2.1
7821	1.63453	2.1
7822	1.04627	1.42686
7823	1.04626	1.49104
7824	1.35111	1.5362
7825	1.22297	1.41426
7826	1.64499	2.1
7827	1.87598	2.1

7828	1.06994	2.1
7829	1.37063	2.1
7830	1.99943	0.05
7831	2.38807	0.05
7832	0.99972	0.0709432
7833	1.41388	0.0820605
7834	1.66179	0.0691425
7835	1.66179	0.05
7836	0.946129	0.0953473
7837	1.41388	0.0771919
7838	1.19404	0.0765232
7839	1.31023	2.08274
7840	1.20871	2.08183
7841	1.69731	2.1
7842	1.67971	2.1
7843	0.998971	0.05
7844	1.32988	0.05
7845	0.701079	0.05
7846	0.946131	0.05
7847	0.582785	0.05
7848	1.48592	0.05
7849	1.48592	0.05
7850	1.23351	0.05
7851	0.835199	0.05
7852	0.92427	0.05
7853	1.68262	0.05
7854	2.46702	0.05
7855	1.08715	2.03173
7856	1.01606	1.87049
7857	1.29579	1.94042
7858	1.01606	1.93076
7859	1.63823	2.1
7860	1.67687	2.1
7861	1.11048	2.1
7862	1.39477	2.1
7863	0.991114	2.1
7864	2.90978	0.340353
7865	0.906811	2.06339
7866	0.872849	0.435803
7867	1.17091	1.34568
7868	0.685763	0.846873
7869	1.6386	1.44147
7870	1.06907	0.460325
7871	1.05516	1.73855
7872	1.9189	0.911978
7873	1.04615	0.110867
7874	1.2966	2.1
7875	1.37392	2.1
7876	1.35504	2.1
7877	1.30347	2.1
7878	1.50877	2.1
7879	1.11193	2.1
7880	1.40984	2.08261
7881	1.67682	2.08835
7882	1.00371	2.1
7883	1.15412	2.1
7884	1.41638	2.1
7885	1.03796	2.1

7886	0.946117	2.1
7887	1.50823	2.1
7888	1.50822	2.1
7889	0.966923	2.1
7890	0.946131	2.1
7891	2.07593	2.1
7892	1.9339	2.1
7893	1.1817	0.264807
7894	0.729178	0.357496
7895	1.5079	0.265683
7896	1.26991	0.341378
7897	0.994049	0.366852
7898	1.17452	0.252648
7899	1.05711	0.315795
7900	1.04071	0.306935
7901	1.80299	0.353513
7902	1.60417	0.334808
7903	0.943405	0.05
7904	1.01129	0.05
7905	1.42972	0.05
7906	1.15904	0.05
7907	0.680409	0.05
7908	1.2816	0.05
7909	0.806176	0.05
7910	1.18954	0.05
7911	1.26055	0.05
7912	1.06313	2.1
7913	1.19352	2.1
7914	1.55766	2.1
7915	1.53729	2.1
7916	0.993021	1.73128
7917	1.09056	2.1
7918	1.03408	2.1
7919	0.993023	2.1
7920	0.659901	2.1
7921	0.632905	2.1
7922	1.34072	0.13193
7923	1.17793	0.116696
7924	2.24885	0.129883
7925	0.946132	0.121033
7926	1.12443	0.131835
7927	0.904207	0.120895
7928	1.51237	0.0951006
7929	1.51237	0.116348
7930	1.12443	0.130909
7931	1.29215	0.128093
7932	1.6943	2.06381
7933	2.43858	2.07454
7934	2.0589	1.85668
7935	1.67204	2.09038
7936	1.67204	2.04633
7937	0.555875	1.96706
7938	0.91951	2.09857
7939	0.946131	1.85846
7940	0.555904	1.94014
7941	0.946131	1.97047
7942	1.6943	2.1
7943	1.04744	1.82704

7944	1.50938	2.1
7945	1.60861	2.1
7946	1.92406	0.0518472
7947	1.18716	0.05
7948	0.962705	0.833225
7949	1.35382	1.51594
7950	1.43632	0.05
7951	1.09386	0.05
7952	1.92547	0.05
7953	0.994164	0.05
7954	0.903974	1.94654
7955	0.928829	1.74022
7956	0.810958	1.62867
7957	1.92853	1.44143
7958	1.598 1.49184	
7959	1.28967	1.61929
7960	1.44993	1.64256
7961	0.946132	0.765322
7962	0.882984	0.679261
7963	0.882955	0.890764
7964	1.56071	0.295312
7965	1.56071	0.263152
7966	1.35946	0.546485
7967	1.08421	0.610151
7968	1.41943	0.05
7969	1.26265	0.05
7970	1.03235	0.506419
7971	1.50778	2.1
7972	1.70387	2.1
7973	1.65608	2.1
7974	1.39076	2.1
7975	1.6104	2.1
7976	1.37456	2.1
7977	1.51898	0.406293
7978	1.9726	0.205257
7979	2.13757	1.00474
7980	1.74856	1.08071
7981	0.635949	0.620173
7982	2.46448	1.01884
7983	1.23224	1.06224
7984	1.12786	1.59612
7985	1.82721	1.12002
7986	1.82721	0.759364
7987	0.532892	1.94959
7988	0.946131	1.66904
7989	0.53292	1.57734
7990	1.41394	2.1
7991	1.45506	2.1
7992	1.39523	2.1
7993	1.09006	2.1
7994	1.80264	0.733623
7995	1.80264	2.07235
7996	0.946131	0.674759
7997	1.34268	0.126137
7998	0.946132	1.93348
7999	1.23732	1.14654
8000	1.34268	1.10253
8001	1.2064	2.1

8002	1.58164	2.1
8003	1.73853	2.1
8004	1.01243	1.75778
8005	0.910672	1.5106
8006	1.47793	1.74952
8007	1.47793	2.09857
8008	0.910702	1.54006
8009	1.30382	1.12294
8010	1.08478	0.898664
8011	0.910702	1.99775
8012	2.60709	1.17951
8013	1.01243	1.64744
8014	1.85516	0.731341
8015	2.52823	0.420101
8016	1.24001	0.52956
8017	1.89793	0.638563
8018	1.73584	0.05
8019	1.24613	1.61007
8020	1.37074	1.36438
8021	1.23763	1.39371
8022	1.04072	1.96412
8023	0.75499	1.61587
8024	1.72329	1.36655
8025	1.46195	1.22204
8026	0.946132	1.5765
8027	0.747438	1.73988
8028	1.24613	1.42237
8029	2.59886	0.847926
8030	1.87279	2.1
8031	1.07977	2.1
8032	1.29606	2.1
8033	1.45178	2.1
8034	1.45177	2.1
8035	0.92406	2.1
8036	0.946131	2.1
8037	0.924047	2.1
8038	2.04529	2.1
8039	1.04544	2.1
8040	1.08276	2.1
8041	1.67491	2.1
8042	0.961826	1.50413
8043	0.810942	0.465833
8044	0.826622	1.2751
8045	0.914378	1.40118
8046	0.89022	1.11746
8047	0.922383	1.13738
8048	0.988165	0.85887
8049	1.31357	0.056352
8050	0.946131	0.05
8051	1.31357	0.0591467
8052	1.10564	0.05
8053	1.6797	0.0988758
8054	1.6797	0.0915332
8055	0.739506	0.05
8056	1.33957	0.0964736
8057	1.33957	0.154037
8058	1.33957	0.130088
8059	2.34223	0.160111

8060	1.5515	0.674604
8061	2.22137	0.494641
8062	0.94612	0.443877
8063	1.13908	0.45828
8064	1.45423	0.380138
8065	1.45422	0.489594
8066	0.795519	0.900606
8067	1.1391	0.499442
8068	1.11065	0.445977
8069	1.12266	2.1
8070	1.24925	2.1
8071	1.20242	2.1
8072	2.18297	2.1
8073	3.00107	2.05271
8074	2.58297	0.68313
8075	0.884783	2.1
8076	0.851538	1.06232
8077	0.713587	2.07268
8078	1.47949	1.13913
8079	1.66673	2.093
8080	0.696199	2.1
8081	0.9383	1.93147
8082	1.29149	1.52566
8083	1.32266	2.1
8084	2.11428	0.05
8085	2.17439	0.05
8086	1.40489	0.05
8087	1.21071	0.05
8088	0.946118	0.05
8089	1.0746	0.05
8090	0.973119	0.05
8091	1.31333	0.05
8092	1.08723	0.05
8093	0.994292	2.1
8094	1.4964	2.1
8095	1.15096	2.1
8096	1.10101	2.1
8097	1.50438	2.1
8098	1.67825	2.1
8099	2.02103	2.1
8100	2.01978	2.1
8101	1.50931	0.05
8102	1.50931	0.05
8103	0.65023	0.05
8104	1.34831	0.05
8105	0.811233	0.05
8106	1.34831	0.05
8107	0.94613	0.05
8108	1.00754	1.58123
8109	0.714132	2.02667
8110	1.28877	1.3605
8111	1.39905	1.40819
8112	1.03282	1.42957
8113	0.997769	1.5313
8114	1.11743	1.5538
8115	1.86863	1.11434
8116	3.30723	1.50223
8117	1.66635	1.9897

8118	1.35609	0.05
8119	1.38212	0.05
8120	1.95597	2.1
8121	1.64257	2.1
8122	2.93316	2.1
8123	3.13417	2.1
8124	0.733947	2.1
8125	1.15259	2.1
8126	1.07976	2.1
8127	1.30237	2.1
8128	1.99943	0.05
8129	0.907431	0.0504727
8130	0.878161	0.0600975
8131	1.07544	0.05
8132	1.03401	0.05
8133	0.991975	0.05
8134	1.03635	0.05
8135	0.788549	0.05
8136	1.44984	0.05
8137	1.84007	0.0521922
8138	2.31127	0.05
8139	1.27362	2.1
8140	1.27737	0.868324
8141	1.87783	0.540197
8142	2.35499	0.05
8143	2.69662	0.05
8144	2.24065	0.05
8145	0.996082	2.1
8146	0.996083	2.1
8147	1.44859	2.1
8148	1.03968	2.08927
8149	1.60018	0.947104
8150	1.73082	1.54431
8151	0.946132	1.66407
8152	1.49742	1.77482
8153	1.49742	0.731408
8154	1.00501	1.16006
8155	1.41585	1.94434
8156	1.41585	1.21789
8157	0.946131	1.89905
8158	1.03697	1.21292
8159	2.95786	2.1
8160	2.32185	2.1
8161	1.65783	2.02572
8162	1.772 2.1	
8163	0.946131	2.06929
8164	1.40404	1.7692
8165	1.13537	2.1
8166	1.04559	2.1
8167	1.16091	2.1
8168	2.63751	2.1
8169	1.68455	0.05
8170	1.9682	0.05
8171	2.16834	0.114703
8172	2.45569	0.185863
8173	0.946128	0.131035
8174	1.36428	0.200315
8175	0.663978	0.271912

8176	1.58218	0.138422
8177	1.58219	0.120825
8178	1.36428	0.190207
8179	0.881886	0.188995
8180	1.73849	2.1
8181	1.35668	2.1
8182	1.1233	2.1
8183	1.15009	2.1
8184	1.16482	0.254157
8185	1.29313	0.277547
8186	1.47608	0.138296
8187	2.05949	0.151515
8188	0.946119	0.0976649
8189	1.02974	0.11593
8190	0.946119	0.103544
8191	1.66758	0.073674
8192	1.66757	0.079757
8193	1.32082	0.0869674
8194	1.29287	0.0828059
8195	1.36005	2.1
8196	1.47109	2.1
8197	2.45958	2.1
8198	2.2975	2.1
8199	0.953521	0.807298
8200	0.953521	0.555229
8201	1.67054	2.1
8202	1.67054	2.1
8203	0.946131	2.1
8204	1.38154	2.1
8205	1.13326	2.1
8206	1.13326	2.1
8207	1.23513	2.1
8208	1.50998	1.85063
8209	2.47982	0.0825383
8210	1.45091	0.0764102
8211	0.958953	0.05
8212	0.79239	0.106209
8213	2.11929	0.16819
8214	1.26511	0.169269
8215	1.27733	0.0822167
8216	2.18127	0.107481
8217	1.30169	0.0670618
8218	1.19259	0.230058
8219	0.963395	1.78256
8220	0.999947	2.09827
8221	1.62241	2.1
8222	1.62027	2.1
8223	0.981586	0.05
8224	0.981585	0.05
8225	0.721961	2.1
8226	0.891321	2.1
8227	1.04442	2.1
8228	1.66554	2.1
8229	2.43434	2.1
8230	1.73944	0.05
8231	1.88032	0.05
8232	0.711391	0.05
8233	1.40837	0.05

8234	0.946133	0.05
8235	0.92252	0.05
8236	1.13964	0.05
8237	1.83394	0.05
8238	2.81674	0.05
8239	1.10049	1.57503
8240	1.25092	0.250813
8241	0.760299	0.784138
8242	1.48017	0.956861
8243	1.48017	0.940602
8244	0.760271	0.646934
8245	1.35861	0.951342
8246	0.946129	0.805844
8247	0.760269	0.714126
8248	2.26155	2.1
8249	2.15634	2.1
8250	1.39824	2.1
8251	1.07817	2.1
8252	0.946132	2.1
8253	1.53028	2.1
8254	1.53028	2.1
8255	1.00807	2.1
8256	0.946133	2.1
8257	2.06126	0.199712
8258	1.97385	0.955407
8259	1.75754	0.102226
8260	2.01613	2.1
8261	1.97995	2.1
8262	1.47696	2.1
8263	1.50172	2.1
8264	1.5177	0.05
8265	1.44146	0.05
8266	1.8807	0.05
8267	2.07068	0.05
8268	1.84267	0.05
8269	1.08975	2.1
8270	1.21327	2.1
8271	1.10106	0.05
8272	1.34496	0.05
8273	2.08452	0.188814
8274	1.70537	0.0626391
8275	2.62464	2.1
8276	1.5726	2.1
8277	1.5726	2.1
8278	0.94613	2.1
8279	1.15038	2.1
8280	0.946132	2.1
8281	1.03974	2.1
8282	1.15038	2.1
8283	0.99747	1.98016
8284	1.03472	2.09363
8285	1.58407	1.69987
8286	1.21148	2.1
8287	1.35025	2.1
8288	1.3428	0.216429
8289	1.00775	2.1
8290	1.00775	2.1
8291	1.3879	2.1

8292	1.39958	2.1
8293	0.853042	2.1
8294	1.06997	2.1
8295	0.853042	2.1
8296	0.853042	2.1
8297	1.12491	2.1
8298	1.17879	2.1
8299	1.07162	2.08781
8300	1.61179	2.1
8301	1.76016	2.1
8302	0.946132	0.545906
8303	1.23449	0.397078
8304	1.70392	0.404201
8305	1.70392	0.358696
8306	0.94613	0.601474
8307	1.23449	0.434462
8308	1.41556	0.430532
8309	1.14547	0.387442
8310	1.1721	0.298501
8311	0.96478	0.35127
8312	1.41843	0.367278
8313	1.2358	0.313396
8314	1.70678	0.306313
8315	1.70678	0.31022
8316	0.946118	0.50092
8317	1.23582	0.422399
8318	0.946105	0.508827
8319	0.964769	0.261226
8320	1.1357	0.27196
8321	0.964767	0.262487
8322	0.619856	0.05
8323	1.72366	0.05
8324	1.72367	0.05
8325	0.619831	0.05
8326	1.19262	0.05
8327	0.619829	0.05
8328	0.946129	0.05
8329	1.57459	2.1
8330	1.86512	2.1
8331	0.93841	2.1
8332	1.57459	2.1
8333	1.10476	0.05
8334	1.00013	1.01959
8335	2.13446	0.687411
8336	0.759781	2.1
8337	1.96011	1.51293
8338	0.946129	0.8783
8339	1.32056	1.53903
8340	1.75702	1.0551
8341	1.75702	1.67106
8342	0.946124	1.45469
8343	1.32056	1.26371
8344	1.38258	1.03195
8345	1.96011	1.50611
8346	2.31099	1.29827
8347	0.97356	2.1
8348	1.38885	2.1
8349	1.3771	2.1

8350	0.946131	2.1
8351	1.84843	2.1
8352	1.84843	2.1
8353	1.3771	2.1
8354	0.946131	2.1
8355	1.00278	2.1
8356	1.05447	2.1
8357	1.05446	2.1
8358	1.24913	0.736783
8359	1.05447	2.1
8360	1.80882	2.1
8361	1.80882	2.1
8362	0.946129	2.1
8363	1.33997	2.1
8364	0.946131	2.0832
8365	1.28831	2.1
8366	1.33997	2.1
8367	1.44391	0.05
8368	1.44391	0.05
8369	0.850964	0.05
8370	0.850963	0.05
8371	0.946132	0.05
8372	0.921299	0.05
8373	1.37358	0.05
8374	1.46297	0.05
8375	1.46298	0.05
8376	0.823988	0.05
8377	0.921287	0.05
8378	0.94612	0.05
8379	0.823995	0.05
8380	0.946107	0.05
8381	1.44437	2.1
8382	1.06203	2.1
8383	2.21397	2.1
8384	0.637677	2.1
8385	1.8892	2.1
8386	2.075 2.1	
8387	1.32051	0.052993
8388	2.23637	0.0900221
8389	1.10904	0.201276
8390	1.11819	0.122935
8391	0.94613	0.220503
8392	0.735845	0.113777
8393	1.4914	0.196088
8394	1.4914	0.0789581
8395	0.735831	0.314424
8396	1.61683	2.1
8397	1.61683	2.1
8398	0.946131	2.1
8399	1.3281	2.1
8400	1.10441	2.1
8401	1.10442	2.1
8402	1.23487	2.1
8403	1.09914	2.1
8404	1.20691	0.05
8405	2.27379	0.05
8406	1.1369	0.056272
8407	1.26868	0.0629518

8408	1.54422	0.0541856
8409	1.54422	0.05
8410	0.861357	0.0811253
8411	0.94613	0.0641648
8412	0.861359	0.0580421
8413	1.2858	0.289923
8414	1.31011	0.507426
8415	1.294	0.22788
8416	1.03079	0.346717
8417	1.81979	1.89956
8418	1.57829	2.02005
8419	1.19023	1.5925
8420	1.19022	1.75932
8421	1.46458	1.78094
8422	1.46458	1.92667
8423	0.789173	2.01183
8424	1.34386	1.92553
8425	0.909896	2.02631
8426	0.946131	1.87367
8427	0.909893	1.90723
8428	1.27725	2.1
8429	1.68451	2.1
8430	2.26155	2.1
8431	2.34217	2.1
8432	1.55485	0.490737
8433	0.90854	0.401405
8434	0.858256	0.323117
8435	1.47042	0.258009
8436	1.62771	0.266988
8437	0.777398	0.516506
8438	0.982525	0.33701
8439	1.23865	0.305609
8440	2.09928	0.35499
8441	0.946128	0.134181
8442	0.474856	0.108055
8443	0.474855	0.150885
8444	1.58747	0.069877
8445	1.58747	0.058597
8446	0.963917	0.116847
8447	1.09841	0.102685
8448	2.23904	2.1
8449	2.60055	1.83168
8450	1.02599	2.1
8451	2.17837	2.1
8452	2.07122	2.1
8453	2.7309	2.02344
8454	2.08482	2.1
8455	1.18322	0.261927
8456	1.35381	0.995177
8457	1.01139	0.547577
8458	0.946133	1.14632
8459	1.59089	0.885194
8460	1.59089	0.352082
8461	0.946133	0.752028
8462	1.09634	0.110047
8463	2.02279	0.123479
8464	1.03108	0.140701
8465	0.808978	1.50802

8466	1.07317	1.42736
8467	0.831695	1.36522
8468	1.22583	1.1856
8469	1.10215	1.2714
8470	1.07508	1.30571
8471	1.22204	1.24622
8472	1.04679	1.98648
8473	1.92535	0.05
8474	1.11081	0.05
8475	0.946131	0.12638
8476	0.714824	0.125649
8477	0.714795	0.117308
8478	1.4231	0.116224
8479	1.4231	0.144237
8480	0.828617	0.124856
8481	1.30928	0.141208
8482	1.49894	2.1
8483	1.55702	2.1
8484	0.946131	2.1
8485	1.10689	2.1
8486	0.946227	2.1
8487	0.946131	2.1
8488	1.21754	2.1
8489	1.504 2.1	
8490	1.504 2.1	
8491	0.723573	2.1
8492	1.24512	2.1
8493	0.723544	2.1
8494	0.946131	2.1
8495	1.24512	2.1
8496	1.29383	0.120809
8497	1.29383	0.0756358
8498	1.61969	0.123298
8499	0.946132	0.066606
8500	1.12818	0.0609925
8501	0.946132	0.256007
8502	1.80922	0.0696725
8503	1.80922	0.05
8504	1.41383	0.0658827
8505	1.34153	0.05
8506	1.47689	0.0553741
8507	1.79375	0.0703127
8508	1.24333	0.3408
8509	0.768989	0.28634
8510	1.24333	0.256931
8511	1.0027	0.400228
8512	1.47705	0.342742
8513	1.47705	0.144779
8514	0.768977	0.319613
8515	1.28436	0.528736
8516	0.928583	0.602577
8517	1.28436	0.229358
8518	1.24333	0.586224
8519	1.5991	0.551678
8520	1.5991	0.108889
8521	0.928583	0.577953
8522	1.85717	0.152736
8523	2.48665	0.223172

8524	1.18423	0.05
8525	1.9493	2.08959
8526	1.8519	1.98241
8527	1.68802	2.0812
8528	1.0302	0.107843
8529	0.900473	0.0950406
8530	1.99663	0.1003
8531	2.61301	0.0831515
8532	0.745772	0.157699
8533	0.946131	0.108206
8534	1.48881	0.109702
8535	1.48881	0.0853067
8536	0.745745	0.137216
8537	0.809786	0.141353
8538	1.3065	0.133137
8539	0.946132	0.0844557
8540	0.768357	0.0915056
8541	0.768387	0.100368
8542	1.76897	0.0542902
8543	1.76896	0.05
8544	1.40271	0.0698608
8545	1.00777	0.0704771
8546	1.37427	0.05
8547	1.1967	0.660299
8548	0.946132	2.1
8549	1.59397	2.1
8550	1.59397	2.1
8551	0.946131	2.1
8552	1.14989	2.1
8553	1.14989	2.1
8554	1.10046	2.1
8555	1.09506	0.05
8556	2.53251	0.05
8557	1.9212	1.5321
8558	1.99586	0.982152
8559	0.913967	0.187702
8560	1.55028	0.0998306
8561	1.55028	0.13053
8562	0.913966	0.203278
8563	1.16318	0.102286
8564	1.16318	0.138381
8565	0.946132	0.132804
8566	1.20478	2.1
8567	1.09662	2.1
8568	1.03472	2.1
8569	1.84774	2.1
8570	1.7984	2.1
8571	2.1221	2.1
8572	1.68223	2.0918
8573	1.68224	2.05274
8574	0.514872	1.9294
8575	0.888991	2.0304
8576	0.946127	1.77541
8577	0.514845	1.95561
8578	0.946129	1.95699
8579	1.10127	1.85871
8580	2.61623	1.86244
8581	0.983581	0.214833

8582	0.869663	0.152167
8583	1.1818	0.166151
8584	1.10406	0.137396
8585	1.07133	0.226646
8586	1.09405	0.166609
8587	0.838014	0.195276
8588	0.842998	1.095
8589	1.03818	0.85056
8590	1.46338	0.156291
8591	1.903 2.1	
8592	1.23942	2.1
8593	0.965933	2.1
8594	0.810075	2.1
8595	1.40571	2.1
8596	1.66219	2.1
8597	0.810047	2.1
8598	0.936678	2.1
8599	1.45808	2.1
8600	1.6384	2.1
8601	1.54699	2.1
8602	0.719877	2.1
8603	1.33256	2.1
8604	0.753547	2.1
8605	1.04073	2.1
8606	1.25822	2.1
8607	0.851542	2.1
8608	0.714102	2.1
8609	1.45886	2.1
8610	1.80171	2.1
8611	0.597337	2.1
8612	0.957713	2.1
8613	1.3033	2.1
8614	0.596613	0.250283
8615	1.1386	0.351779
8616	0.596612	0.263117
8617	0.946133	0.461634
8618	0.596652	0.429477
8619	1.64092	0.358809
8620	1.64092	0.280567
8621	1.991 2.1	
8622	1.23013	2.1
8623	2.77816	2.1
8624	2.74107	0.474034
8625	2.07681	0.371571
8626	1.47764	0.05
8627	1.72677	0.0502889
8628	1.52161	2.1
8629	1.28526	2.1
8630	1.90502	2.1
8631	1.87359	0.115528
8632	1.93489	0.121145
8633	2.70895	2.1
8634	2.83141	2.1
8635	1.41766	0.166354
8636	1.11337	0.154028
8637	1.41765	0.255474
8638	1.29172	0.178627
8639	1.76324	0.18747

8640	1.76324	0.23076
8641	0.946131	0.13649
8642	1.52428	2.1
8643	1.71459	2.1
8644	1.93852	0.342675
8645	1.07119	1.00965
8646	1.29698	0.953496
8647	1.42203	0.33323
8648	1.42203	1.43684
8649	0.946132	0.369159
8650	0.946132	0.653689
8651	0.96926	0.863003
8652	0.769913	1.76085
8653	0.946119	1.39513
8654	1.37867	0.893461
8655	1.94028	0.822552
8656	0.94612	0.551094
8657	0.946132	1.2307
8658	1.37865	0.77074
8659	2.14234	0.893816
8660	3.06459	0.89858
8661	1.44421	0.05
8662	1.33924	0.05
8663	1.33671	0.05
8664	1.10733	0.05
8665	0.68396	0.0614482
8666	1.76008	0.05
8667	1.76008	0.05
8668	0.68393	0.05
8669	0.946131	0.0506592
8670	3.79242	2.1
8671	2.46293	2.1
8672	3.56947	2.1
8673	1.35487	1.32791
8674	1.38616	1.53519
8675	0.954048	0.112843
8676	1.36663	0.095939
8677	0.909487	0.104778
8678	0.887288	2.03404
8679	0.810947	1.40672
8680	0.810934	1.81123
8681	1.07046	2.00093
8682	1.13979	2.02627
8683	0.927838	1.6124
8684	0.919995	1.88721
8685	1.61447	2.1
8686	2.56305	2.1
8687	0.943414	2.1
8688	1.17016	2.1
8689	1.7238	2.1
8690	1.89334	2.1
8691	0.946128	2.02619
8692	1.25184	2.1
8693	1.55639	2.1
8694	3.12098	2.05448
8695	3.57307	1.18444
8696	2.81281	2.1
8697	0.851542	2.1

8698	1.27689	2.1
8699	1.52568	2.1
8700	0.839689	2.1
8701	1.36471	2.1
8702	0.827865	2.1
8703	0.93667	2.1
8704	0.900861	2.1
8705	0.943152	2.1
8706	1.00667	2.1
8707	1.18141	2.1
8708	1.15298	2.1
8709	1.20335	2.1
8710	1.00713	2.1
8711	0.597537	2.1
8712	1.0577	2.1
8713	0.967342	0.557895
8714	0.838027	0.828675
8715	0.893587	0.504667
8716	1.06436	0.564403
8717	0.850048	0.475828
8718	1.22026	0.618097
8719	0.988077	0.549658
8720	1.18769	0.20757
8721	1.3991	2.1
8722	1.52984	2.1
8723	1.35469	1.58214
8724	1.25083	1.43727
8725	1.6594	1.72603
8726	1.6594	1.60569
8727	0.946129	1.22327
8728	1.02602	1.12301
8729	0.94613	0.911357
8730	1.29724	0.078117
8731	1.14501	0.201512
8732	1.13078	0.159861
8733	1.48381	0.155597
8734	1.45921	0.140434
8735	0.851539	0.217122
8736	0.890836	0.219166
8737	0.946131	0.267943
8738	1.60428	2.1
8739	1.50097	2.1
8740	0.714507	0.0547008
8741	1.51396	0.0629445
8742	1.51396	0.0668129
8743	0.946132	0.0527725
8744	0.813862	0.0618614
8745	1.41461	0.0565508
8746	0.813862	0.062071
8747	0.921311	2.1
8748	1.25084	1.30405
8749	1.30165	0.873422
8750	1.60636	1.44375
8751	1.60636	1.04668
8752	0.94613	0.904655
8753	0.972232	0.841109
8754	0.946131	0.774473
8755	2.35003	2.1

8756	0.92546	2.1
8757	1.00713	2.1
8758	0.810947	2.1
8759	0.810934	2.1
8760	1.056	2.1
8761	0.926811	2.1
8762	0.766408	2.1
8763	1.34292	2.1
8764	1.57932	0.05
8765	2.67595	0.05
8766	1.77737	0.05
8767	1.77738	0.05
8768	0.789633	0.05
8769	1.22903	0.05
8770	0.789663	0.05
8771	0.789662	0.05
8772	1.33797	0.05
8773	0.946128	2.1
8774	1.47351	2.1
8775	1.47351	2.1
8776	0.94613	2.1
8777	1.30082	2.1
8778	1.30082	2.1
8779	0.996486	2.1
8780	0.946127	2.1
8781	1.72122	2.1
8782	1.72122	2.1
8783	0.94613	2.1
8784	1.36653	2.1
8785	1.36653	2.1
8786	1.02608	2.1
8787	0.709123	1.69761
8788	0.946127	1.40955
8789	1.41342	0.555067
8790	1.818	1.72727
8791	1.818	0.971205
8792	0.946127	0.333312
8793	1.41342	1.17514
8794	1.35071	1.28957
8795	1.8993	0.304753
8796	1.4748	2.03682
8797	1.18711	0.05
8798	0.946131	0.05
8799	1.18711	0.05
8800	1.20416	0.05
8801	1.91358	0.0542953
8802	1.91358	0.0581495
8803	0.477691	0.05
8804	2.16798	0.05
8805	2.22307	0.05
8806	0.758971	0.05
8807	1.80694	0.05
8808	1.80694	0.05
8809	0.946132	0.05
8810	0.75897	0.05
8811	1.25124	0.05
8812	0.946131	0.05
8813	1.76837	2.1

8814	1.76837	2.1
8815	0.946131	2.1
8816	1.36422	2.1
8817	0.946131	2.1
8818	1.14184	2.1
8819	1.36422	2.1
8820	0.793303	0.05
8821	1.79584	0.05
8822	1.35333	1.96537
8823	1.19967	1.51353
8824	1.55088	1.72972
8825	1.64246	2.00262
8826	0.946132	1.37702
8827	0.886293	1.91308
8828	1.25018	1.9843
8829	1.5795	1.80367
8830	2.15414	1.38384
8831	0.94613	0.55209
8832	1.37758	1.69222
8833	1.71567	0.975113
8834	1.71567	2.04208
8835	0.946125	1.42272
8836	1.37759	1.19922
8837	1.28421	0.751077
8838	0.933959	1.13965
8839	0.946133	0.05
8840	1.7813	0.05
8841	1.7813	0.05
8842	1.05704	0.05
8843	1.37794	0.05
8844	1.37794	0.05
8845	0.946132	0.05
8846	0.946132	0.05
8847	1.68197	0.05
8848	1.68197	0.05
8849	1.01367	0.05
8850	1.25017	0.05
8851	1.25017	0.05
8852	0.946132	0.05
8853	0.838953	2.1
8854	1.4045	2.1
8855	1.56388	2.1
8856	1.3182	2.1
8857	0.928518	2.1
8858	1.04073	2.1
8859	0.835676	2.1
8860	1.47507	2.1
8861	1.47506	2.1
8862	0.797664	2.1
8863	1.34285	2.1
8864	0.929875	2.1
8865	0.797663	2.1
8866	0.946133	2.1
8867	1.83628	1.2601
8868	1.83627	1.7212
8869	0.946131	1.65711
8870	1.36748	0.652574
8871	0.946133	1.22221

8872	1.21139	0.74319
8873	1.36748	0.502529
8874	1.06795	2.1
8875	1.08192	2.1
8876	0.827866	2.1
8877	1.02548	2.1
8878	0.95006	2.1
8879	1.53933	2.1
8880	0.586511	2.1
8881	0.868145	2.1
8882	1.28401	2.1
8883	1.37719	2.1
8884	1.37719	2.1
8885	0.77497	2.1
8886	0.946132	2.1
8887	0.774968	2.1
8888	1.7053	1.8056
8889	1.7053	2.1
8890	0.94613	2.1
8891	1.25715	2.1
8892	0.946132	1.41269
8893	1.19655	2.09876
8894	1.25716	2.1
8895	0.851542	2.1
8896	0.80043	2.1
8897	1.27239	2.1
8898	1.02719	2.1
8899	0.946131	2.1
8900	0.981318	2.1
8901	1.06628	2.1
8902	1.26022	2.1
8903	0.946132	2.1
8904	0.946132	2.1
8905	1.76447	2.1
8906	1.76447	2.1
8907	1.35357	2.1
8908	1.35703	2.1
8909	0.946131	2.1
8910	1.50538	2.1
8911	1.50538	2.1
8912	1.10311	2.1
8913	1.3484	2.1
8914	1.10312	2.1
8915	0.966428	2.1
8916	0.946133	2.1
8917	1.4008	2.1
8918	1.71908	2.1
8919	1.71908	2.1
8920	0.946133	2.1
8921	1.40081	2.1
8922	1.26441	2.1
8923	0.851543	2.1
8924	0.961732	2.1
8925	1.54576	2.1
8926	1.35364	2.1
8927	0.946133	2.1
8928	1.02913	2.1
8929	1.2735	2.1

8930	1.32608	2.1
8931	0.967643	2.1
8932	0.94613	2.1
8933	1.44873	2.1
8934	1.44873	2.1
8935	0.967641	2.1
8936	0.946131	2.1
8937	1.40488	2.1
8938	1.24272	2.1
8939	0.946131	2.1
8940	1.78483	2.1
8941	1.78483	2.1
8942	1.24272	2.1
8943	0.946131	2.1
8944	0.94613	2.09241
8945	1.5627	0.428782
8946	1.5627	1.86872
8947	1.03656	2.09502
8948	1.31649	0.273517
8949	1.19233	1.6423
8950	1.03656	1.41742
8951	0.912059	2.1
8952	1.47954	0.852302
8953	1.47954	2.04203
8954	0.912061	2.1
8955	1.34647	0.732387
8956	1.04514	2.04902
8957	0.912061	1.89569
8958	0.761972	0.0827572
8959	0.946132	0.608505
8960	0.761974	0.324201
8961	1.70225	1.4455
8962	1.70225	0.787115
8963	1.33728	0.617965
8964	1.01188	0.523085
8965	0.94613	1.849
8966	0.746361	1.57805
8967	1.6369	2.05746
8968	1.62454	1.79848
8969	0.736738	0.985638
8970	0.958487	1.62951
8971	1.40641	1.89493
8972	1.2284	2.1
8973	0.92609	2.1
8974	0.595102	2.1
8975	1.80871	2.1
8976	1.1516	2.1
8977	0.962474	2.1
8978	2.24024	2.1
8979	0.946131	2.1
8980	1.73689	2.1
8981	1.73689	2.1
8982	0.946132	2.1
8983	1.30492	2.1
8984	1.30492	2.1
8985	1.17949	2.1
8986	0.946131	2.1
8987	1.8566	2.1

8988	1.8566	2.1
8989	1.22392	2.1
8990	1.39826	2.1
8991	1.40447	2.1
8992	1.22392	2.1
8993	0.946131	2.1
8994	1.74134	2.1
8995	1.74134	2.1
8996	1.09148	2.1
8997	1.40447	2.1
8998	1.283	2.1
8999	1.09148	2.1
9000	1.33607	2.1
9001	1.41022	2.1
9002	1.80016	2.1
9003	1.80015	2.1
9004	0.946127	2.1
9005	0.946129	2.1
9006	1.20541	2.1
9007	1.13078	2.1
9008	1.33607	2.1
9009	1.54624	2.1
9010	1.85553	2.1
9011	0.920606	2.1
9012	0.946131	2.1
9013	1.01271	2.1
9014	1.32664	1.90995
9015	0.954692	2.07136
9016	0.946116	1.71682
9017	1.47771	1.90971
9018	1.4777	2.1
9019	0.95468	1.95577
9020	0.94613	1.98096
9021	1.31381	1.11841
9022	1.14005	0.755379
9023	0.946128	0.754499
9024	1.69433	0.494885
9025	1.69433	1.55585
9026	1.14005	0.684582
9027	0.94613	0.558953
9028	1.521	2.1
9029	1.411	2.1
9030	0.94612	2.1
9031	1.35115	2.1
9032	1.04127	2.1
9033	1.05017	2.1
9034	1.03564	2.1
9035	0.607156	2.1
9036	1.54549	2.1
9037	1.36715	2.1
9038	0.607127	2.09991
9039	1.2571	2.1
9040	0.998701	2.1
9041	0.546436	2.1
9042	0.946133	2.1
9043	1.75286	2.1
9044	1.75286	2.1
9045	1.12206	2.1

9046	1.34237	2.1
9047	1.35663	2.1
9048	1.12206	2.1
9049	0.919455	2.1
9050	1.66517	2.1
9051	1.99822	2.1
9052	1.228 2.1	
9053	1.35662	2.1
9054	1.228 2.1	
9055	1.01143	2.1
9056	0.851543	1.53893
9057	0.986856	2.09792
9058	1.42762	1.87827
9059	1.23095	2.1
9060	0.946132	2.09347
9061	1.00659	1.99046
9062	1.1779	1.98635
9063	1.40025	2.1
9064	1.40025	2.1
9065	0.889308	2.1
9066	1.34652	2.1
9067	0.943042	2.1
9068	0.943041	2.1
9069	1.34652	2.1
9070	1.39514	1.20256
9071	1.30809	1.03036
9072	1.75711	0.86908
9073	1.7571	2.07236
9074	0.946125	0.377981
9075	0.946127	1.21717
9076	1.18396	1.21639
9077	1.4844	1.24201
9078	1.4844	1.53094
9079	0.803322	1.92772
9080	0.946133	0.997912
9081	0.803324	2.06905
9082	0.803324	1.7679
9083	1.36183	1.72533
9084	0.946131	0.132715
9085	1.01462	0.117934
9086	0.946132	0.357748
9087	1.59749	0.184777
9088	1.59749	0.0909638
9089	1.16009	0.128358
9090	1.38354	0.0830634
9091	0.851539	1.031
9092	1.36005	1.40066
9093	1.5532	1.09869
9094	1.80402	1.47141
9095	0.851537	1.53045
9096	0.902632	1.37144
9097	1.44437	1.16545
9098	0.633098	2.1
9099	1.00307	0.392873
9100	0.94613	0.707875
9101	1.16623	0.458086
9102	0.946128	0.805049
9103	1.64588	0.6848

9104	1.64588	0.454719
9105	1.35059	0.630231
9106	1.24142	0.721641
9107	0.946131	0.094619
9108	1.13343	0.0815924
9109	0.946127	0.11556
9110	1.63367	0.078354
9111	1.63367	0.0694468
9112	1.33035	0.0915212
9113	1.24945	0.100739
9114	1.78733	1.68774
9115	1.70856	1.73599
9116	0.637995	1.26998
9117	0.712454	1.29187
9118	0.946132	1.35912
9119	0.637964	1.16948
9120	1.13021	1.26785
9121	0.94613	0.072239
9122	1.20509	0.0798407
9123	0.946127	0.0685019
9124	1.70545	0.0627219
9125	1.70545	0.0848024
9126	1.28508	0.0629896
9127	1.3665	0.0841843
9128	1.58612	2.0859
9129	1.51845	2.09882
9130	0.797374	2.1
9131	1.20057	2.09873
9132	0.946132	2.09508
9133	0.946132	2.0896
9134	0.899819	2.09945
9135	1.50471	0.980479
9136	1.50472	0.747776
9137	0.59387	0.867224
9138	0.593844	0.503767
9139	0.946126	1.06619
9140	0.966137	0.620377
9141	1.13245	0.635839
9142	1.20801	0.59365
9143	0.946131	1.09193
9144	1.47644	0.752956
9145	0.733223	0.570489
9146	0.563609	0.739711
9147	1.69218	0.711049
9148	1.6173	0.67819
9149	1.8126	1.90229
9150	1.8126	1.92923
9151	0.678938	1.94256
9152	0.678935	1.92128
9153	0.946133	2.03928
9154	0.946132	1.93646
9155	0.678936	2.03834
9156	1.21997	1.83221
9157	1.28439	1.83504
9158	1.65282	1.88399
9159	1.86735	1.85789
9160	0.851539	1.93229
9161	0.94613	1.67244

9162	0.87688	1.64567
9163	0.804877	0.356082
9164	1.40747	0.418342
9165	1.41312	0.167927
9166	0.885349	0.232286
9167	1.25405	0.426057
9168	1.34463	0.261549
9169	0.804878	0.258132
9170	0.810299	0.0843257
9171	1.04072	0.154984
9172	1.25601	0.126616
9173	1.04943	0.0836
9174	0.900294	0.169731
9175	1.38777	0.117312
9176	1.33131	0.05
9177	1.39168	0.05
9178	0.946132	0.05
9179	0.747712	0.05
9180	1.61944	0.05
9181	1.61944	0.05
9182	0.946132	0.05
9183	0.747712	0.05
9184	1.41201	0.113429
9185	1.12082	0.0881804
9186	0.946128	0.106328
9187	1.79415	0.13049
9188	1.79415	0.114228
9189	1.12082	0.083331
9190	0.94613	0.0944897
9191	0.711721	0.112825
9192	1.12316	0.129786
9193	0.707038	0.10849
9194	1.04072	0.152166
9195	0.704409	0.155223
9196	1.49036	0.132458
9197	1.27689	0.0974662
9198	0.793044	0.478501
9199	0.810886	0.725245
9200	0.885957	0.509676
9201	1.38254	0.461131
9202	1.35991	0.449139
9203	1.01375	0.498771
9204	1.03169	0.577208
9205	0.946131	0.578915
9206	1.3279	0.737254
9207	1.42284	0.615102
9208	1.42284	0.547058
9209	0.851192	1.12499
9210	0.946131	0.827855
9211	0.851165	0.703397
9212	0.748062	0.05
9213	1.27511	0.05
9214	0.74806	0.05
9215	0.94613	0.05
9216	0.748059	0.05
9217	1.86293	0.05
9218	1.86293	0.05
9219	0.851542	0.0815594

9220	1.38186	0.0645262
9221	0.851542	0.0901174
9222	1.67869	0.05
9223	1.66579	0.05
9224	1.25626	0.061111
9225	1.23046	0.0530359
9226	1.46023	0.05
9227	1.27083	0.05
9228	0.946131	0.05
9229	0.999304	0.05
9230	0.946803	0.05
9231	0.851554	0.05
9232	0.999227	0.05
9233	0.750097	0.05
9234	1.91815	0.05
9235	0.946132	0.05
9236	0.768117	0.05
9237	1.78003	0.05
9238	1.99572	0.05
9239	0.650453	0.05
9240	0.946132	0.05
9241	1.23845	0.05
9242	0.946131	0.0502154
9243	1.75477	0.05
9244	1.75477	0.05
9245	1.33393	0.05
9246	1.36697	0.05
9247	0.749018	0.675894
9248	1.02366	0.583319
9249	1.43924	0.688805
9250	1.00018	0.479479
9251	1.06438	0.480813
9252	0.946127	0.513886
9253	0.74906	0.548982
9254	0.946131	0.52822
9255	1.26488	0.05
9256	0.946131	0.05
9257	0.94613	0.05
9258	1.49051	0.05
9259	1.59837	0.05
9260	0.907797	0.05
9261	1.04455	0.05
9262	1.25214	0.05
9263	0.68452	0.05
9264	1.25215	0.05
9265	1.24677	0.05
9266	1.8144	0.05
9267	1.81441	0.05
9268	0.684518	0.05
9269	0.851542	0.366778
9270	1.52275	0.303389
9271	1.45223	0.316278
9272	1.0269	0.344593
9273	1.29689	0.333692
9274	1.32822	0.370318
9275	0.851553	0.409886
9276	1.21756	0.05
9277	0.946131	0.05

9278	0.946127	0.05
9279	1.78014	0.05
9280	1.78014	0.05
9281	1.32076	0.05
9282	1.40551	0.05
9283	0.711214	0.0680398
9284	1.82057	0.0510299
9285	1.82057	0.05
9286	0.946129	0.0725071
9287	1.22716	0.05
9288	1.30463	0.0634131
9289	0.94613	0.0616515
9290	0.568927	0.05
9291	0.946132	0.05
9292	0.568927	0.05
9293	1.99266	0.05
9294	1.99266	0.05
9295	1.26201	0.05
9296	1.29957	0.05
9297	1.21614	0.0563112
9298	0.851484	0.0557755
9299	0.851543	0.067065
9300	1.85815	0.05
9301	1.81937	0.05
9302	0.697705	0.05
9303	0.946132	0.0610875
9304	0.593295	0.103084
9305	1.53642	0.0871782
9306	1.53642	0.07103
9307	0.908276	0.115383
9308	0.593296	0.0688231
9309	0.593295	0.0834932
9310	0.94613	0.0980726
9311	0.867593	0.226409
9312	1.42107	0.158351
9313	1.42107	0.160468
9314	0.867621	0.218316
9315	1.05516	0.194984
9316	0.867624	0.164349
9317	0.946129	0.219721
9318	0.766681	0.0782515
9319	1.18119	0.0971241
9320	1.57018	0.0729639
9321	1.57018	0.0566228
9322	0.766652	0.104775
9323	1.18119	0.0901032
9324	1.15564	0.0862101
9325	0.946131	0.126802
9326	1.239	0.0973959
9327	0.851541	0.141548
9328	0.851487	0.0946053
9329	1.40021	0.126812
9330	1.38373	0.0758301
9331	0.851554	0.151633
9332	1.12266	0.287142
9333	0.946131	0.191499
9334	1.08234	0.249182
9335	1.2882	0.124661

9336	1.49963	0.412692
9337	1.46188	0.355324
9338	0.851555	0.252292
9339	1.33007	0.291393
9340	1.08054	0.25628
9341	0.946129	0.416152
9342	0.91151	0.223336
9343	1.49914	0.261213
9344	1.49914	0.138432
9345	0.911471	0.537782
9346	0.946132	0.709514
9347	1.35943	2.05178
9348	1.68117	1.50064
9349	1.68117	2.1
9350	0.946132	1.86846
9351	1.35943	1.49468
9352	1.26787	1.02466
9353	1.81612	2.1
9354	1.81612	1.61043
9355	0.946131	1.5604
9356	1.41492	1.20992
9357	0.946132	2.1
9358	1.25037	2.1
9359	1.41492	2.1
9360	1.30541	0.05
9361	1.23646	0.05
9362	0.94613	0.05
9363	1.77304	0.05
9364	1.77304	0.05
9365	1.23646	0.05
9366	0.946132	0.05
9367	0.946131	2.1
9368	1.26689	2.1
9369	1.66806	2.1
9370	1.66806	2.1
9371	0.94613	2.1
9372	1.26689	2.1
9373	1.3473	2.1
9374	1.39384	2.1
9375	1.4181	2.1
9376	1.86581	2.1
9377	1.86581	2.1
9378	0.946128	2.1
9379	0.946129	2.1
9380	1.32068	2.1
9381	1.20328	2.1
9382	1.39384	2.1
9383	1.651	2.1
9384	1.651	2.1
9385	0.946129	2.1
9386	0.946131	2.1
9387	1.12913	2.1
9388	1.21474	2.1
9389	0.946132	2.1
9390	0.94613	2.1
9391	1.79724	2.1
9392	1.79724	2.1
9393	1.40987	2.1

9394	1.3335	2.1
9395	1.65642	2.1
9396	1.65642	2.1
9397	0.946132	2.1
9398	1.19063	2.1
9399	0.946133	2.1
9400	1.15203	2.1
9401	1.19063	2.1
9402	0.946131	1.86434
9403	1.77815	2.1
9404	1.77815	2.1
9405	0.946131	2.09313
9406	1.31745	2.1
9407	1.31745	2.1
9408	1.2597	2.1
9409	0.946131	0.760375
9410	1.85184	1.97054
9411	1.85184	2.1
9412	0.946128	1.5655
9413	1.40684	2.1
9414	1.40684	1.96001
9415	1.34049	2.00351
9416	0.946132	1.7874
9417	1.71333	0.532468
9418	1.71333	2.09967
9419	1.1848	1.71146
9420	1.34297	0.421691
9421	1.31649	0.679073
9422	1.1848	0.918598
9423	1.36604	0.132233
9424	0.954559	0.133963
9425	1.36604	0.134673
9426	1.41766	0.123047
9427	1.83756	0.145762
9428	1.83756	0.249129
9429	0.946131	0.119911
9430	0.946127	0.213176
9431	1.14874	0.155819
9432	0.946127	0.239695
9433	1.66393	0.200637
9434	1.66393	0.133308
9435	1.35559	0.202726
9436	1.25447	0.213013
9437	0.94613	0.0668591
9438	1.20732	0.0755995
9439	0.946129	0.0683051
9440	1.75073	0.0535668
9441	1.75073	0.0838481
9442	1.3665	0.0558923
9443	1.33035	0.0844472
9444	1.73256	2.1
9445	1.73256	2.1
9446	0.946131	2.1
9447	1.34921	2.1
9448	1.19462	2.1
9449	1.19462	2.1
9450	1.32949	2.1
9451	1.75754	0.357231

9452	1.75754	1.20685
9453	0.946132	1.35303
9454	1.3201	1.04661
9455	0.946132	0.337923
9456	1.25479	0.469125
9457	1.3201	0.359246
9458	1.8087	2.07828
9459	1.8087	0.534794
9460	0.946131	0.755392
9461	1.38357	0.371515
9462	0.946133	2.06856
9463	1.31034	1.34878
9464	1.38357	1.32862
9465	1.12702	0.136316
9466	0.946131	0.225191
9467	1.23923	0.185946
9468	1.4117	0.111505
9469	0.946131	0.225541
9470	1.70479	0.159462
9471	1.70479	0.0662709
9472	0.946119	2.1
9473	1.55309	2.1
9474	1.55309	2.1
9475	0.946119	2.1
9476	1.08006	2.1
9477	1.08005	2.1
9478	1.02681	2.1
9479	1.36581	2.1
9480	1.93075	2.1
9481	0.946133	2.1
9482	0.772538	2.1
9483	0.946131	2.1
9484	0.946131	2.1
9485	1.33748	2.1
9486	1.44991	2.1
9487	1.31183	2.1
9488	0.946132	2.1
9489	0.944104	2.1
9490	0.915236	2.1
9491	1.05017	2.1
9492	0.944148	2.1
9493	0.946132	0.094007
9494	1.60171	0.0511651
9495	1.60171	0.05
9496	0.946131	0.127177
9497	1.16234	0.0570197
9498	1.3855	0.0586008
9499	0.946132	0.07151
9500	0.946133	0.105869
9501	1.73285	0.0561657
9502	1.73285	0.05
9503	0.946133	0.112248
9504	1.3855	0.0563374
9505	1.29348	0.0810926
9506	0.946132	0.0840373
9507	1.34828	2.1
9508	1.20943	2.1
9509	0.946129	2.1

9510	1.71013	2.1
9511	1.71013	2.1
9512	1.20943	2.1
9513	0.94613	2.1
9514	1.19613	0.05
9515	0.946118	0.05
9516	0.946117	0.05
9517	1.70868	0.05
9518	1.70869	0.05
9519	1.40551	0.05
9520	1.2493	0.05
9521	0.946131	2.1
9522	1.73123	2.1
9523	1.73123	2.1
9524	0.946132	2.1
9525	1.37811	2.1
9526	1.37811	2.1
9527	1.25144	2.1
9528	0.946132	2.1
9529	1.75139	2.1
9530	1.75139	2.1
9531	1.23374	2.1
9532	1.29926	2.1
9533	1.39827	2.1
9534	1.23374	2.1
9535	1.41375	0.05
9536	1.25697	0.05
9537	0.946118	0.05
9538	1.80377	0.05
9539	1.80377	0.05
9540	1.25699	0.05
9541	0.946106	0.05
9542	0.946133	2.03414
9543	1.81806	1.31933
9544	1.81806	2.1
9545	0.946133	2.07309
9546	1.37089	2.1
9547	1.37089	1.21839
9548	1.34999	2.00753
9549	0.946131	2.1
9550	1.80855	1.83038
9551	1.80855	2.1
9552	1.31184	2.1
9553	1.3933	1.71521
9554	1.36139	2.08984
9555	1.31184	2.0966
9556	1.01175	2.00513
9557	0.94613	1.04944
9558	0.919796	2.01956
9559	1.50484	0.701126
9560	1.80584	1.84508
9561	1.53512	1.38613
9562	1.14539	1.29857
9563	0.946133	2.1
9564	1.72797	2.1
9565	1.72797	2.1
9566	1.2111	2.1
9567	1.39731	2.09838

9568	1.27679	2.1
9569	1.21111	2.1
9570	0.946119	1.0241
9571	1.67673	1.70931
9572	1.67673	2.1
9573	0.946118	1.60463
9574	1.21519	2.1
9575	1.2152	2.0712
9576	1.19808	2.1
9577	0.946127	2.1
9578	1.54442	2.1
9579	1.54442	2.1
9580	1.04806	2.1
9581	1.13485	2.1
9582	1.3557	2.1
9583	1.04805	2.1
9584	0.946129	2.1
9585	1.32282	2.1
9586	1.77749	2.1
9587	1.77749	2.1
9588	0.946131	2.1
9589	1.32282	2.1
9590	1.4008	2.1
9591	0.946128	0.109048
9592	1.02366	0.12852
9593	0.94613	0.114499
9594	1.66684	0.0833847
9595	1.66684	0.0982171
9596	1.29215	0.108025
9597	1.32082	0.105308
9598	0.946133	0.07607
9599	1.01451	0.105515
9600	0.946132	0.0796893
9601	1.62374	0.0708812
9602	1.62374	0.0692269
9603	1.2929	0.0728345
9604	1.27697	0.0639402
9605	1.37869	2.1
9606	1.17215	2.1
9607	0.946132	2.1
9608	1.70508	2.1
9609	1.70508	2.1
9610	1.17215	2.1
9611	0.946132	2.1
9612	1.03784	2.1
9613	0.946132	2.1
9614	0.946132	2.1
9615	1.56718	2.1
9616	1.56717	2.1
9617	1.35703	2.1
9618	1.15628	2.1
9619	1.35356	2.1
9620	1.28976	2.1
9621	0.946131	2.1
9622	1.78612	2.1
9623	1.78612	2.1
9624	1.28976	2.1
9625	0.94613	2.1

9626	0.946132	2.1
9627	1.36373	2.1
9628	1.92382	2.1
9629	0.946131	2.1
9630	1.92382	2.1
9631	1.33471	2.1
9632	0.946132	2.1
9633	0.94613	0.217882
9634	1.29149	0.760686
9635	1.75878	0.412071
9636	1.75878	2.1
9637	0.946128	1.12186
9638	1.29149	0.354738
9639	1.41342	0.386952
9640	1.41022	2.1
9641	1.37034	2.1
9642	1.83443	2.1
9643	1.83443	2.1
9644	0.946128	2.1
9645	0.946131	2.1
9646	1.26928	2.1
9647	0.946128	0.72381
9648	1.38259	1.71814
9649	1.81405	1.08887
9650	1.81405	1.90675
9651	0.946126	1.55181
9652	1.38259	1.24356
9653	1.37759	0.854115
9654	1.68131	0.317689
9655	1.68131	1.15599
9656	0.946131	0.608905
9657	1.38896	0.250984
9658	1.17438	2.04586
9659	1.17438	0.802548
9660	1.23848	0.982238
9661	1.80088	1.14492
9662	1.80088	0.653218
9663	0.946132	0.648187
9664	1.40332	1.23228
9665	1.28185	0.586407
9666	1.28185	0.389258
9667	1.34369	0.219095
9668	1.71939	2.1
9669	1.71939	2.1
9670	0.946131	2.1
9671	1.28308	2.1
9672	0.946133	2.1
9673	1.24024	2.1
9674	1.28309	2.1
9675	1.8525	2.1
9676	1.8525	2.1
9677	0.946132	2.1
9678	1.38244	2.1
9679	0.946132	2.1
9680	1.33469	2.1
9681	1.38244	2.1
9682	1.26654	1.13246
9683	1.39514	1.0646

9684	1.71556	0.999709
9685	1.71556	2.02096
9686	0.946126	0.602652
9687	0.946129	1.4137
9688	1.1908	1.26688
9689	1.49995	2.1
9690	1.63875	2.1
9691	0.93608	2.1
9692	0.937208	2.1
9693	0.943708	2.1
9694	0.936134	2.1
9695	1.14341	2.1
9696	1.17802	0.05
9697	1.3542	0.05
9698	1.73981	0.05
9699	1.73981	0.05
9700	0.94613	0.05
9701	1.3542	0.05
9702	1.33174	0.05
9703	1.59452	2.1
9704	1.59452	2.1
9705	0.946132	2.1
9706	1.25	2.1
9707	0.975266	2.1
9708	0.946132	2.1
9709	1.25	2.1
9710	0.946131	2.1
9711	1.48685	2.1
9712	1.48685	2.1
9713	0.946132	2.1
9714	1.17273	2.1
9715	1.17273	2.1
9716	0.955602	2.1
9717	1.32827	0.101026
9718	1.11166	0.0884428
9719	0.946127	0.095357
9720	1.6136	0.118164
9721	1.6136	0.0832926
9722	1.11166	0.0796165
9723	0.946129	0.0957816
9724	0.851541	0.431272
9725	1.22107	0.363706
9726	1.64594	0.29059
9727	1.5271	0.305964
9728	0.94613	0.621014
9729	1.22112	0.397813
9730	1.25002	0.337958
9731	1.65795	1.26805
9732	1.65795	2.1
9733	0.946132	2.1
9734	1.2366	2.1
9735	0.946132	0.934047
9736	1.10023	2.05197
9737	1.2366	1.97931
9738	1.35856	0.05
9739	0.946132	0.05
9740	1.35856	0.05
9741	1.08019	0.05

9742	1.49261	0.05
9743	1.49261	0.05
9744	0.94613	0.05
9745	1.29322	0.05
9746	0.946132	0.05
9747	1.29322	0.05
9748	1.35856	0.05
9749	1.70565	0.05
9750	1.70565	0.05
9751	0.946132	0.05
9752	1.66635	2.1
9753	1.66635	2.1
9754	0.946133	2.1
9755	1.39558	2.1
9756	1.14793	2.1
9757	1.14793	2.1
9758	1.21691	2.1
9759	1.35381	0.11213
9760	1.3365	1.16343
9761	1.09905	0.741959
9762	0.946132	1.05335
9763	1.74418	0.818564
9764	1.74418	0.336199
9765	0.946132	0.714331
9766	1.84759	2.1
9767	1.84759	1.72529
9768	0.94613	2.01612
9769	1.3904	2.1
9770	1.34522	0.655192
9771	1.34522	1.78084
9772	1.40331	1.51731
9773	1.86047	2.1
9774	1.86047	2.1
9775	0.946132	2.1
9776	1.41619	2.1
9777	0.946132	2.00558
9778	1.3659	2.1
9779	1.41619	2.1
9780	1.28464	2.06878
9781	1.14496	1.92294
9782	1.57806	1.94945
9783	1.7753	2.01969
9784	0.851541	2.01987
9785	0.946131	2.02062
9786	0.872786	2.01503
9787	0.85062	0.387954
9788	1.52635	0.565545
9789	1.63785	0.387679
9790	1.69972	0.989227
9791	0.946132	1.20722
9792	1.37493	0.494041
9793	1.23854	0.446996
9794	1.41134	2.09684
9795	1.27079	1.89572
9796	1.736	1.17171
9797	1.736	2.1
9798	0.946132	0.44571
9799	0.946133	1.44049

9800	1.21177	1.7816
9801	0.946131	0.053202
9802	0.946129	0.05
9803	0.94613	0.0528842
9804	0.946131	0.05
9805	0.946131	0.05
9806	1.35403	0.05
9807	0.946131	0.0560439
9808	1.37052	0.148108
9809	0.946132	0.506687
9810	0.94613	0.22574
9811	0.946133	0.0992838
9812	0.946131	0.12153
9813	0.946131	0.31124
9814	0.946131	0.119742
9815	1.24249	0.180956
9816	0.946132	0.192811
9817	0.946133	0.133366
9818	0.946132	0.0702947
9819	0.946132	0.128596
9820	0.946132	0.210635
9821	0.946132	0.104557
9822	0.946132	2.1
9823	0.946132	2.1
9824	0.946131	2.1
9825	0.946132	2.1
9826	0.946132	2.1
9827	0.946132	2.1
9828	1.20608	2.1
9829	1.41232	2.1
9830	0.946128	2.1
9831	0.946129	2.1
9832	0.94613	2.1
9833	0.94613	2.1
9834	0.94613	2.1
9835	0.94613	2.1
9836	0.946132	0.0692356
9837	0.946131	0.0639641
9838	0.946131	0.0744294
9839	0.946132	0.05
9840	0.946132	0.05
9841	0.946131	0.062821
9842	0.946132	0.0536985
9843	0.946116	1.83219
9844	0.94612	2.1
9845	0.946118	2.0916
9846	0.946124	2.1
9847	0.946118	2.09466
9848	1.41561	2.1
9849	0.946131	2.09917
9850	0.945846	2.1
9851	1.03508	2.1
9852	0.77579	2.1
9853	0.658908	2.1
9854	1.17474	2.1
9855	0.94303	2.1
9856	0.849189	2.1
9857	0.94298	2.1

9858	1.03963	2.1
9859	0.946131	1.99652
9860	0.946131	1.41348
9861	0.946131	2.1
9862	0.946131	1.30007
9863	0.946131	0.875906
9864	0.946131	1.77309
9865	1.32819	1.8933
9866	0.946133	0.775748
9867	0.946129	2.05466
9868	0.946128	2.01679
9869	0.94613	1.57797
9870	0.946131	2.1
9871	0.946131	1.27761
9872	0.946131	1.71076
9873	0.954072	0.05
9874	1.28895	0.05
9875	0.946133	0.05
9876	1.15803	0.05
9877	1.29689	0.05
9878	1.29689	0.05
9879	0.946132	0.05
9880	0.946132	0.966984
9881	0.946132	0.936273
9882	0.946132	0.751486
9883	0.946132	1.00956
9884	0.946132	1.56847
9885	0.946131	1.31401
9886	1.0264	1.28895
9887	0.946126	0.275332
9888	0.946132	0.249836
9889	0.946131	0.194828
9890	0.94613	0.251198
9891	0.946129	0.345719
9892	0.94613	0.269337
9893	1.35977	0.251738
9894	0.946132	0.178507
9895	0.946133	0.118299
9896	0.876632	0.375023
9897	0.946132	0.088185
9898	1.01566	0.146359
9899	1.01566	0.114139
9900	0.876606	0.15139
9901	0.946131	0.05
9902	0.946128	0.05
9903	0.946128	0.05
9904	0.94613	0.05
9905	0.94613	0.05
9906	1.24979	0.05
9907	0.94613	0.05
9908	0.946131	0.05
9909	0.946131	0.05
9910	0.921762	0.05
9911	0.9705	0.05
9912	0.9705	0.05
9913	0.946131	0.05
9914	0.921791	0.05
9915	0.810944	0.1087

9916	0.810945	0.113876
9917	0.810959	0.0836051
9918	2.42267	0.054497
9919	1.35168	0.0657775
9920	1.08132	0.0868888
9921	1.08132	0.0646119
9922	1.08131	0.0788147
9923	0.946133	0.0664761
9924	0.946132	0.0910113
9925	0.946132	0.0626503
9926	0.946133	0.0931988
9927	0.946133	0.0559726
9928	0.946132	0.077509
9929	0.946133	0.108164
9930	0.946131	0.526864
9931	0.946131	0.944373
9932	0.94613	0.545014
9933	0.851514	0.456948
9934	0.946131	0.764206
9935	0.965268	0.51794
9936	0.851513	0.537379
9937	0.946122	2.1
9938	0.946123	2.1
9939	0.946126	2.1
9940	0.946124	2.1
9941	0.946124	2.1
9942	0.946118	2.1
9943	0.94613	2.1
9944	0.946131	0.0686875
9945	1.30448	0.0533662
9946	0.946132	0.138985
9947	0.946129	0.0752964
9948	0.946131	0.135699
9949	0.946131	0.0703399
9950	0.94613	0.138953
9951	0.946133	2.07703
9952	0.946128	2.1
9953	0.946128	2.1
9954	0.94613	2.1
9955	1.36503	2.1
9956	0.946131	2.1
9957	0.946131	2.1
9958	0.810947	0.05
9959	0.810947	0.05
9960	2.48233	0.05
9961	1.3517	0.05
9962	1.08132	0.05
9963	1.08132	0.05
9964	1.08133	0.05
9965	0.810934	0.05
9966	1.48874	0.05
9967	2.48233	0.05
9968	2.2369	0.05
9969	0.946133	0.790704
9970	0.946132	0.157589
9971	0.946132	0.896208
9972	0.946133	0.407645
9973	0.946133	0.883484

9974	1.41851	0.436942
9975	0.946132	0.481599
9976	1.03925	2.1
9977	0.946131	2.1
9978	0.94613	2.1
9979	0.946128	2.1
9980	0.946133	2.1
9981	0.944743	2.1
9982	1.13703	2.1
9983	0.946132	2.1
9984	0.946128	2.1
9985	0.946128	2.1
9986	0.946131	2.1
9987	0.94613	2.1
9988	0.94613	2.1
9989	0.94613	2.1
9990	0.946131	0.05
9991	0.946132	0.05
9992	0.946131	0.05
9993	0.946132	0.05
9994	0.946132	0.05
9995	0.946131	0.05
9996	1.20057	0.05
9997	0.946129	2.1
9998	0.94613	2.1
9999	0.94613	2.1
10000	0.946128	2.1
10001	0.946132	2.1
10002	0.94613	2.1
10003	1.38302	2.1
10004	0.856567	0.05
10005	1.14319	0.05
10006	0.716526	0.05
10007	1.20631	0.05
10008	0.626936	0.05
10009	0.946131	0.05
10010	0.94613	0.05
10011	1.39502	0.05
10012	0.881445	0.05
10013	0.946128	0.05
10014	0.94613	0.05
10015	0.881447	0.05
10016	0.946117	0.05
10017	0.946116	0.05
10018	0.88146	0.05
10019	0.946132	2.1
10020	1.39053	2.1
10021	0.946132	2.1
10022	0.946132	2.1
10023	0.946132	2.1
10024	0.946132	2.1
10025	0.946131	2.1
10026	0.946131	2.1
10027	0.946131	2.1
10028	0.946131	2.1
10029	0.946132	2.1
10030	0.946132	2.1
10031	1.40571	2.1

10032	0.946132	2.1
10033	0.94613	0.122257
10034	0.946127	0.136938
10035	0.946128	0.118296
10036	0.946131	0.129212
10037	0.946127	0.112204
10038	1.25819	0.125256
10039	0.946129	0.126615
10040	0.946129	0.518949
10041	1.29054	0.56349
10042	0.94613	0.449717
10043	0.94613	0.546742
10044	0.946131	1.18795
10045	0.946131	0.68331
10046	0.94613	0.547273
10047	0.810944	0.05
10048	0.810941	0.05
10049	1.61558	0.05
10050	1.3517	0.05
10051	1.08131	0.05
10052	1.08132	0.05
10053	1.08133	0.05
10054	0.810931	0.05
10055	1.02036	1.53161
10056	0.946132	1.11777
10057	1.02036	1.10768
10058	0.946132	0.59974
10059	1.1576	0.914088
10060	0.964656	1.09516
10061	0.927608	0.699666
10062	0.946128	2.1
10063	1.38813	2.1
10064	0.946129	2.1
10065	0.946131	2.1
10066	0.946131	2.1
10067	0.94613	2.1
10068	0.946131	2.1
10069	0.946131	2.1
10070	0.94613	2.1
10071	0.94613	2.1
10072	1.41665	2.1
10073	0.946132	2.1
10074	0.946131	2.1
10075	0.94613	2.1
10076	0.946118	0.187379
10077	0.946118	0.167766
10078	0.946116	0.185741
10079	0.946126	0.131222
10080	0.94612	0.145404
10081	0.946131	0.159865
10082	0.946105	0.14934
10083	0.946132	2.1
10084	0.946132	2.1
10085	0.946132	2.1
10086	0.946131	2.1
10087	0.946132	2.1
10088	0.946132	2.1
10089	0.946132	2.1

10090	0.864997	0.05
10091	0.786573	0.05
10092	0.978561	0.05
10093	1.47385	0.05
10094	1.68652	0.05
10095	1.27803	0.05
10096	1.19771	0.05
10097	1.27595	0.05
10098	0.946127	0.45699
10099	1.3771	0.336037
10100	0.946127	0.335165
10101	0.946131	0.535193
10102	0.946129	0.30827
10103	0.946129	0.464049
10104	0.946129	0.440736
10105	0.95697	0.05
10106	1.48133	0.05
10107	1.24499	0.05
10108	1.5295	0.05
10109	0.938161	0.05
10110	0.808717	0.05
10111	1.3542	0.05
10112	0.946133	0.549517
10113	1.14444	0.532004
10114	0.946132	0.445976
10115	0.946132	0.540899
10116	0.946133	1.06934
10117	0.946132	0.640732
10118	0.946132	0.537851
10119	0.946131	0.05
10120	0.946132	0.0625867
10121	0.946131	0.05
10122	0.946132	0.05
10123	0.946131	0.0680249
10124	0.946132	0.05
10125	0.946132	0.05
10126	0.946132	2.1
10127	0.946133	2.1
10128	0.946133	2.1
10129	0.946132	2.1
10130	1.29322	2.1
10131	0.946132	2.1
10132	0.946132	2.1
10133	0.946131	2.1
10134	0.946132	2.1
10135	0.946131	2.1
10136	0.946132	2.1
10137	1.28441	2.1
10138	0.946131	2.1
10139	0.946131	2.1
10140	0.946113	2.1
10141	0.946118	2.1
10142	0.946118	2.09283
10143	1.28073	2.1
10144	0.946117	2.1
10145	0.946131	2.1
10146	0.946105	2.1
10147	0.810945	2.1

10148	0.810942	2.1
10149	2.75904	2.1
10150	1.3517	2.1
10151	1.08131	2.1
10152	1.08133	2.1
10153	1.08132	2.1
10154	0.81093	2.1
10155	0.946127	2.1
10156	1.37952	2.1
10157	1.86965	2.1
10158	0.946127	2.1
10159	1.86965	2.1
10160	1.28228	2.1
10161	1.37952	2.1
10162	0.946128	2.1
10163	0.946132	2.1
10164	1.14042	2.1
10165	0.946132	2.1
10166	1.14042	2.1
10167	1.09974	2.1
10168	1.09974	2.1
10169	0.946132	2.1
10170	0.946132	0.0916343
10171	0.946132	0.0648101
10172	0.946133	0.0934275
10173	0.946132	0.0663024
10174	0.946132	0.0843857
10175	0.946132	0.0712608
10176	0.946133	0.0915841
10177	1.31817	1.86419
10178	0.946131	1.52032
10179	0.946131	1.81261
10180	0.946131	1.76988
10181	0.946131	1.28807
10182	0.946131	1.61342
10183	0.946131	1.62528
10184	0.946132	2.1
10185	0.946132	2.1
10186	0.946132	2.1
10187	0.946132	2.1
10188	0.946132	2.1
10189	0.946132	2.1
10190	1.35048	2.1
10191	0.946131	2.1
10192	0.946131	2.1
10193	0.94613	2.1
10194	0.946133	2.1
10195	0.946133	2.1
10196	1.35943	2.1
10197	0.946131	2.1
10198	0.946133	2.1
10199	0.946132	2.1
10200	0.946132	2.1
10201	0.946133	2.1
10202	0.946133	2.1
10203	0.946133	2.1
10204	0.990712	2.1
10205	0.965725	1.98883

10206	0.946129	1.26646
10207	0.946133	1.36492
10208	1.21721	1.30124
10209	0.877931	0.787742
10210	0.946131	1.58217
10211	0.946131	1.10692
10212	0.946132	0.498139
10213	0.946131	0.40247
10214	0.946131	0.934063
10215	0.946133	0.481009
10216	0.946128	1.42254
10217	0.946131	0.828544
10218	1.39575	0.746207
10219	0.810943	0.347474
10220	0.810942	0.36071
10221	2.58794	0.241539
10222	1.21652	0.228288
10223	0.946154	0.300053
10224	0.979952	0.29359
10225	1.0813	0.303097
10226	0.810959	0.328334
10227	0.924071	0.250972
10228	1.16453	0.177558
10229	1.28586	0.178591
10230	0.946131	0.277424
10231	1.1227	0.171955
10232	1.16462	0.217352
10233	0.851553	0.307138
10234	0.709578	0.238775
10235	0.810943	0.390163
10236	0.810959	0.263661
10237	1.82615	0.200553
10238	1.21651	0.212828
10239	1.08132	0.259066
10240	0.946155	0.244067
10241	0.979937	0.313253
10242	0.946128	1.76116
10243	0.946132	2.1
10244	0.946132	0.78255
10245	0.94613	2.02613
10246	0.94613	2.07302
10247	0.94613	0.461883
10248	0.94613	1.25365
10249	0.946131	1.75467
10250	1.29735	2.04789
10251	0.946131	2.1
10252	0.946131	0.872967
10253	0.946131	1.85193
10254	0.946131	0.969937
10255	0.946131	1.78385
10256	0.946128	2.09968
10257	1.33266	1.25625
10258	0.94613	1.36302
10259	0.946131	2.0922
10260	0.94613	0.86213
10261	0.946131	1.75151
10262	0.946131	1.51998
10263	0.946131	2.1

10264	0.946131	2.1
10265	0.946131	2.05662
10266	1.40608	2.1
10267	0.946131	2.1
10268	0.946131	2.1
10269	0.946131	2.1
10270	0.946131	1.87717
10271	0.946131	2.08703
10272	0.946131	1.9783
10273	1.2334	1.99994
10274	0.946129	2.02636
10275	0.946131	2.01155
10276	0.946131	2.07054
10277	0.946133	2.1
10278	0.946133	2.1
10279	0.946132	2.07136
10280	1.29198	2.1
10281	0.946132	2.0942
10282	0.946132	2.1
10283	0.946132	2.1
10284	0.946131	2.1
10285	0.946128	2.1
10286	0.946128	2.1
10287	0.946131	2.1
10288	0.946127	2.1
10289	0.94613	2.1
10290	0.946129	2.1
10291	0.946123	2.1
10292	0.946126	2.1
10293	0.946125	2.1
10294	1.38639	2.1
10295	0.946126	2.1
10296	0.946132	2.1
10297	0.946119	2.1
10298	0.946128	0.14701
10299	0.946132	0.127571
10300	0.946131	0.161006
10301	0.94613	0.0759619
10302	0.94613	0.0945935
10303	0.946131	0.120401
10304	0.94613	0.101703
10305	0.946131	0.135513
10306	0.946131	0.108609
10307	0.946131	0.148593
10308	0.946131	0.0625194
10309	0.946131	0.0773734
10310	0.946131	0.103538
10311	0.946131	0.0845943
10312	0.810945	2.1
10313	0.810943	2.1
10314	0.810933	2.1
10315	2.24688	2.1
10316	1.3517	2.1
10317	1.08132	2.1
10318	1.08132	2.1
10319	1.08133	2.1
10320	1.25809	0.146437
10321	0.946131	0.516286

10322	1.25809	0.156524
10323	0.946129	0.44784
10324	0.94613	0.149753
10325	0.946131	0.433733
10326	0.946131	0.242605
10327	0.946131	0.05
10328	0.946131	0.05
10329	0.946129	0.05
10330	1.28911	0.05
10331	0.946131	0.05
10332	0.946131	0.05
10333	0.946131	0.05
10334	0.94613	0.170821
10335	0.946131	0.13944
10336	0.94613	0.115396
10337	0.946128	0.169399
10338	0.946132	0.113705
10339	1.38954	0.145163
10340	0.946131	0.152851
10341	0.946131	0.167718
10342	0.946131	0.373601
10343	0.946131	0.584726
10344	1.37981	0.267518
10345	0.946131	0.998235
10346	0.946131	0.446195
10347	0.946131	0.51049
10348	0.946131	2.1
10349	0.946127	2.1
10350	0.946127	2.1
10351	0.946131	2.1
10352	0.946127	2.1
10353	0.946129	2.1
10354	0.946128	2.1
10355	0.94613	2.1
10356	0.946126	2.1
10357	0.946127	2.1
10358	0.946131	2.1
10359	0.946126	2.1
10360	0.946128	2.1
10361	0.946128	2.1
10362	0.946133	0.296188
10363	0.946132	0.2121
10364	0.946132	0.2364
10365	0.946132	0.254094
10366	0.946132	0.279187
10367	1.13511	0.191485
10368	0.946132	0.233561
10369	0.946132	1.87613
10370	0.946129	1.83505
10371	0.94613	1.5757
10372	0.946131	1.8788
10373	0.946131	1.97844
10374	0.94613	2.0376
10375	1.27575	1.98211
10376	0.946129	2.1
10377	0.946131	2.1
10378	0.946131	2.1
10379	0.946128	2.1

10380	0.946133	2.1
10381	0.946131	2.1
10382	0.94613	2.1
10383	1.15581	0.05
10384	0.946132	0.05
10385	0.946131	0.05
10386	0.94613	0.05
10387	1.15581	0.05
10388	0.94613	0.05
10389	0.946131	0.05
10390	0.611407	1.2637
10391	0.946131	0.805563
10392	1.37335	1.07508
10393	1.15086	1.46084
10394	0.617549	1.29689
10395	0.851553	1.42315
10396	1.14592	1.43573
10397	1.22618	1.9395
10398	0.946132	1.0073
10399	0.946132	1.95929
10400	0.946132	1.23388
10401	0.946131	1.43806
10402	0.946132	1.75393
10403	0.946132	1.68456
10404	0.946131	2.1
10405	0.94613	2.1
10406	0.946131	2.1
10407	0.946133	2.1
10408	0.946129	2.1
10409	0.946131	2.1
10410	0.946131	2.1
10411	0.810945	0.931165
10412	0.810943	2.04827
10413	2.50715	1.71109
10414	1.3517	1.41884
10415	1.08133	1.32506
10416	1.08132	2.02782
10417	1.08132	0.320008
10418	0.810932	1.17922
10419	1.07811	2.05261
10420	0.94613	1.24373
10421	0.946128	2.0594
10422	1.25358	0.680253
10423	0.946131	1.50801
10424	1.25358	1.23168
10425	0.94613	1.27271
10426	0.946129	2.1
10427	1.09197	2.1
10428	0.946131	2.1
10429	1.09197	2.1
10430	1.09532	2.1
10431	1.09532	2.1
10432	0.946131	2.1
10433	1.58788	2.1
10434	1.34255	2.1
10435	0.594912	2.1
10436	0.58894	2.1
10437	0.946129	2.1

10438	1.16679	2.1
10439	1.04071	2.1
10440	0.535418	2.1
10441	0.946131	2.1
10442	0.946128	2.1
10443	0.946131	2.09558
10444	1.20221	2.1
10445	0.946128	2.1
10446	0.94613	2.1
10447	1.20221	2.1
10448	0.946132	2.1
10449	0.946133	2.1
10450	0.946132	2.1
10451	0.946132	2.1
10452	0.946133	2.1
10453	0.946133	2.1
10454	0.946132	2.1
10455	0.946132	1.96061
10456	0.946132	2.08778
10457	0.946133	2.1
10458	0.946132	2.06788
10459	1.411	2.09199
10460	0.946132	2.08846
10461	0.946133	2.08938
10462	1.05575	0.0645478
10463	0.563734	0.0722507
10464	1.05575	0.0749122
10465	0.773833	0.05
10466	0.751686	0.0535807
10467	0.75884	0.0599682
10468	0.815612	0.0507644
10469	0.793465	0.0665653
10470	0.768563	0.095618
10471	0.946129	0.0708747
10472	0.946125	0.0516525
10473	0.946126	0.0724493
10474	0.94613	0.0585571
10475	0.946124	0.0812759
10476	1.31678	0.05
10477	0.946128	0.0617667
10478	0.946132	0.281988
10479	1.29914	0.198549
10480	0.946132	0.201503
10481	0.946132	0.213721
10482	0.946132	0.302149
10483	1.29914	0.155393
10484	0.946132	0.227767
10485	0.810946	0.268083
10486	0.810943	0.261938
10487	2.21082	0.148956
10488	1.35168	0.163662
10489	1.08132	0.198234
10490	1.08132	0.207395
10491	1.0813	0.252489
10492	0.81096	0.243384
10493	0.625813	0.05
10494	0.946132	0.05
10495	0.946131	0.05

10496	0.946132	0.05
10497	0.946131	0.05
10498	1.39301	0.05
10499	0.625812	0.05
10500	0.810946	2.02662
10501	0.810945	2.1
10502	0.810931	2.09994
10503	2.70043	2.1
10504	1.3517	2.1
10505	1.08131	2.05381
10506	1.08131	2.1
10507	1.08133	2.08982
10508	0.810943	2.1
10509	0.810944	2.1
10510	0.810958	2.1
10511	2.44802	2.1
10512	1.35168	2.1
10513	1.08132	2.1
10514	1.08132	2.1
10515	1.0813	2.1
10516	0.946131	0.13534
10517	0.946129	0.112521
10518	0.946128	0.0998193
10519	0.946131	0.174004
10520	1.3492	0.13362
10521	1.3492	0.136573
10522	0.94613	0.105721
10523	0.946132	0.362267
10524	0.946132	0.16277
10525	0.946133	0.377658
10526	0.946133	0.148235
10527	0.946132	0.522223
10528	0.946132	0.151073
10529	0.946132	0.431958
10530	0.946129	0.360433
10531	0.946133	0.252875
10532	0.946131	0.365143
10533	0.94613	0.244244
10534	0.946131	0.401946
10535	0.946131	0.318028
10536	0.946131	0.303346
10537	0.946119	0.199151
10538	0.946119	0.132992
10539	0.946119	0.208212
10540	0.946125	0.121773
10541	0.94612	0.225879
10542	0.946132	0.162833
10543	0.946107	0.154917
10544	0.946133	2.1
10545	0.946132	2.1
10546	0.946132	2.1
10547	0.946132	2.1
10548	0.946132	2.1
10549	0.946133	2.1
10550	1.3868	2.1
10551	0.810947	2.1
10552	0.810946	2.1
10553	0.81096	2.1

10554	2.66094	2.1
10555	1.35168	2.1
10556	1.08132	2.1
10557	1.08132	2.1
10558	1.0813	2.1
10559	0.810946	2.1
10560	0.810946	2.1
10561	0.810933	2.1
10562	2.75737	2.1
10563	1.3517	2.1
10564	1.08132	2.1
10565	1.08132	2.1
10566	1.08133	2.1
10567	0.946133	2.1
10568	0.946132	2.1
10569	0.946132	2.1
10570	0.946132	2.1
10571	0.946133	2.1
10572	0.946132	2.1
10573	0.946133	2.1
10574	0.946133	2.1
10575	0.946133	2.1
10576	0.946132	2.1
10577	0.946132	2.1
10578	0.946133	2.1
10579	0.946132	2.1
10580	1.28569	2.1
10581	0.946132	2.04559
10582	0.946133	2.1
10583	0.946133	2.1
10584	0.946132	2.09041
10585	0.946132	2.1
10586	0.946132	2.1
10587	0.946133	2.1
10588	0.946132	0.05
10589	0.946131	0.05
10590	0.94613	0.05
10591	0.946133	0.05
10592	0.946128	0.05
10593	0.946131	0.05
10594	0.946131	0.0534263
10595	0.946131	2.1
10596	0.94613	2.1
10597	0.946131	2.1
10598	0.94613	2.1
10599	1.41833	2.00598
10600	0.946131	2.1
10601	0.946131	2.1
10602	1.26155	0.05
10603	0.946131	0.05
10604	0.946131	0.05
10605	0.946132	0.05
10606	0.946132	0.05
10607	0.946131	0.05
10608	0.946132	0.05
10609	0.810946	2.1
10610	0.810946	2.1
10611	0.810959	2.1

10612	2.47938	2.1
10613	1.35168	2.1
10614	1.08132	2.1
10615	1.08132	2.1
10616	1.08131	2.1
10617	0.946131	0.47323
10618	0.94613	0.364656
10619	0.94613	0.851758
10620	0.946132	0.340285
10621	0.946128	1.28137
10622	0.94613	0.717059
10623	0.94613	0.796827
10624	0.94613	0.05
10625	0.946131	0.05
10626	0.94613	0.0869123
10627	0.946129	0.0602289
10628	0.946132	0.0635787
10629	0.94613	0.0603221
10630	0.946131	0.0530414
10631	0.946132	2.1
10632	0.946131	2.1
10633	0.946128	2.1
10634	1.40756	2.1
10635	0.946131	2.1
10636	0.946129	2.1
10637	0.94613	2.1
10638	0.946131	2.09816
10639	0.946131	2.00512
10640	0.946131	2.1
10641	0.946132	2.1
10642	1.31947	2.08943
10643	0.946131	2.1
10644	0.946131	2.09984
10645	0.946131	2.1
10646	1.25768	2.1
10647	0.946132	2.1
10648	0.946132	2.1
10649	0.946129	2.1
10650	0.946131	2.1
10651	0.94613	2.1
10652	0.946132	0.052238
10653	0.946129	0.05
10654	0.946129	0.0886876
10655	0.94613	0.0740728
10656	1.31716	0.0696761
10657	1.31716	0.05876
10658	0.946131	0.0837963
10659	0.810943	1.26491
10660	0.810942	2.1
10661	0.81093	2.06366
10662	2.37052	2.1
10663	1.3517	2.07516
10664	1.08132	1.4554
10665	1.08132	2.1
10666	1.08133	1.91881
10667	0.946128	0.726585
10668	1.10335	0.360467
10669	0.94613	0.340781

10670	0.946131	0.803248
10671	0.946131	0.416976
10672	1.10335	0.70224
10673	0.94613	0.786321
10674	0.946132	1.04276
10675	1.18818	0.67285
10676	0.946132	0.622813
10677	0.946132	1.0564
10678	0.946132	0.703918
10679	1.18818	1.1266
10680	0.946132	1.17888
10681	0.946125	2.1
10682	0.94612	2.1
10683	0.946119	2.1
10684	0.946119	2.1
10685	0.946119	2.1
10686	0.946132	2.1
10687	1.31522	2.1
10688	1.28803	2.1
10689	0.946132	2.00774
10690	0.946132	2.1
10691	0.946133	2.1
10692	0.946133	1.84723
10693	0.946133	2.1
10694	0.946132	2.1
10695	0.946133	1.04483
10696	0.946132	1.60756
10697	0.946132	1.25617
10698	0.946132	0.340654
10699	0.946133	0.232393
10700	0.946132	0.258078
10701	1.37658	0.551321
10702	1.02239	1.60834
10703	0.946128	1.25069
10704	0.946132	1.48137
10705	1.02239	1.07731
10706	0.94613	1.06794
10707	0.96283	1.55651
10708	0.92943	1.08525
10709	0.946132	0.05
10710	0.946131	0.05
10711	0.946126	0.05
10712	0.946129	0.05
10713	0.946128	0.05
10714	0.946128	0.05
10715	0.946129	0.05
10716	0.810946	0.05
10717	0.810944	0.05
10718	2.22493	0.05
10719	1.21651	0.05
10720	1.0813	0.05
10721	0.946154	0.05
10722	0.979951	0.05
10723	0.70959	0.05
10724	0.946132	0.0512815
10725	0.946131	0.0823681
10726	0.946131	0.0566519
10727	0.946133	0.05

10728	0.946131	0.087863
10729	0.946132	0.05
10730	0.946132	0.0679229
10731	0.946132	1.91464
10732	0.946131	1.63924
10733	0.946132	2.06505
10734	0.946132	1.13797
10735	0.946132	0.798979
10736	0.946132	1.20691
10737	1.40924	1.72372
10738	0.946131	2.1
10739	0.946128	2.1
10740	0.946127	2.1
10741	0.94613	2.1
10742	1.41743	2.1
10743	0.946128	2.1
10744	0.946129	2.1
10745	1.37675	1.20475
10746	0.946128	1.27185
10747	0.94613	0.589398
10748	0.94613	1.45995
10749	0.94613	0.816857
10750	0.946129	0.856801
10751	0.94613	1.16073
10752	0.946132	0.194698
10753	0.946132	0.372019
10754	0.946132	0.582864
10755	1.35721	0.217958
10756	0.946132	0.952806
10757	0.946132	0.527179
10758	1.35721	0.45612
10759	0.946133	0.574338
10760	0.946133	0.399587
10761	0.946132	0.51099
10762	1.10003	0.292342
10763	0.946133	0.309534
10764	0.946132	0.434456
10765	0.946132	0.391307
10766	0.946132	0.0847377
10767	0.946131	0.0544107
10768	0.946131	0.0668287
10769	0.946131	0.0946427
10770	1.0616	0.0539875
10771	1.0616	0.0617266
10772	0.946132	0.0823176
10773	0.946132	0.104918
10774	0.946131	0.0705953
10775	0.946131	0.084388
10776	0.946131	0.118589
10777	1.09402	0.0706824
10778	1.09402	0.0784498
10779	0.946131	0.111808
10780	0.946132	0.052992
10781	0.946126	0.0543711
10782	1.24093	0.05
10783	0.946129	0.0673049
10784	0.946129	0.059281
10785	0.946128	0.054027

10786	0.946129	0.0555109
10787	0.946131	0.0873712
10788	0.946131	0.08894
10789	1.2929	0.0745777
10790	0.946131	0.117208
10791	0.946131	0.0931589
10792	0.946131	0.0887804
10793	0.946131	0.091236
10794	0.810946	0.05
10795	0.810945	0.05
10796	0.810959	0.05
10797	2.41233	0.05
10798	1.35168	0.05
10799	1.08132	0.05
10800	1.08132	0.05
10801	1.0813	0.05
10802	0.810946	1.90338
10803	0.810943	2.1
10804	0.750103	2.07094
10805	2.22778	2.08886
10806	1.27059	2.07023
10807	1.00021	1.99272
10808	1.02049	2.09907
10809	1.08133	2.04098
10810	0.946128	2.08389
10811	0.946132	2.1
10812	0.946132	2.1
10813	0.94613	2.1
10814	0.94613	2.1
10815	0.94613	2.1
10816	0.946131	2.1
10817	0.946126	0.314291
10818	0.946131	0.264527
10819	0.946127	0.392975
10820	1.3324	0.221964
10821	0.946127	0.22694
10822	0.946128	0.323689
10823	0.946128	0.445937
10824	0.94613	2.1
10825	0.946131	2.1
10826	0.94613	2.1
10827	0.946128	2.1
10828	0.946133	2.1
10829	0.946131	2.1
10830	0.946131	2.1
10831	0.946133	2.1
10832	0.946132	2.1
10833	0.946133	2.1
10834	0.946133	2.1
10835	0.946132	2.1
10836	0.946132	2.1
10837	1.36541	2.1
10838	0.946131	1.06581
10839	0.94613	2.1
10840	0.946131	1.63312
10841	0.94613	0.939358
10842	1.39509	1.8725
10843	0.946131	0.335396

10844	0.946131	1.93548
10845	0.946133	2.1
10846	0.946133	2.1
10847	0.946132	2.1
10848	0.946132	2.1
10849	0.946133	2.1
10850	0.946132	2.1
10851	1.29319	2.1
10852	1.01196	0.0661165
10853	0.946131	0.127272
10854	0.946132	0.0656255
10855	0.946132	0.0882164
10856	0.946132	0.123349
10857	0.946132	0.107722
10858	0.946132	0.100947
10859	0.946112	2.1
10860	0.946118	2.1
10861	0.946116	2.1
10862	0.946117	2.1
10863	0.946118	2.1
10864	0.94613	2.1
10865	0.946105	2.1
10866	1.28684	1.29349
10867	0.946131	1.45344
10868	0.946132	1.34064
10869	0.946132	1.63531
10870	0.946132	1.81974
10871	0.946132	1.46726
10872	0.946132	1.69337
10873	0.94613	2.00538
10874	0.94613	2.05621
10875	0.946128	1.97126
10876	0.946131	1.76587
10877	0.946131	1.91731
10878	0.94613	1.96908
10879	1.10185	2.00285
10880	0.810947	0.05
10881	0.810945	0.05
10882	1.57348	0.05
10883	1.3517	0.05
10884	1.08131	0.05
10885	1.08132	0.05
10886	1.08133	0.05
10887	0.810932	0.05
10888	0.946119	0.23472
10889	0.946116	0.196771
10890	0.946117	0.241358
10891	0.946121	0.164361
10892	0.946118	0.187876
10893	0.94613	0.196487
10894	0.946105	0.188966
10895	0.94612	0.402776
10896	0.946122	0.245381
10897	0.946119	0.343941
10898	0.946119	0.424755
10899	1.20884	0.272288
10900	0.946132	0.352676
10901	0.946107	0.32092

10902	2.67849	1.67302
10903	1.17709	1.53364
10904	0.605418	1.72307
10905	0.286984	1.58843
10906	0.172654	1.62196
10907	0.810946	1.09095
10908	0.940878	0.927085
10909	0.941235	0.814806
10910	1.26696	0.694381
10911	1.56837	0.753887
10912	1.13737	0.83425
10913	1.34115	0.739545
10914	1.24889	0.802738
10915	0.946132	1.84142
10916	0.946129	1.02076
10917	0.946128	1.05572
10918	0.94613	1.35927
10919	0.94613	1.06592
10920	0.946131	1.28273
10921	0.94613	1.24424
10922	0.946116	2.1
10923	0.94612	2.1
10924	0.946118	2.1
10925	0.946123	2.1
10926	0.946118	2.1
10927	0.946131	2.1
10928	1.23836	2.1
10929	0.810946	0.05
10930	0.810946	0.05
10931	0.810959	0.05
10932	2.80677	0.05
10933	1.35168	0.05
10934	1.08132	0.05
10935	1.08132	0.05
10936	1.08131	0.05
10937	0.810947	0.146989
10938	0.810947	0.699435
10939	2.41574	0.38218
10940	1.21653	0.394156
10941	1.08133	0.263636
10942	0.946153	1.01485
10943	0.979949	0.665667
10944	0.709567	0.159714
10945	0.94613	2.08986
10946	0.946131	2.0944
10947	0.946128	2.1
10948	1.34782	2.0977
10949	0.946128	2.0991
10950	0.946129	2.1
10951	1.34782	2.1
10952	0.946132	2.01166
10953	0.946132	2.05852
10954	0.94613	2.07721
10955	1.16023	2.05537
10956	0.946131	2.07939
10957	0.946131	2.1
10958	1.16023	2.09483
10959	0.946131	0.961848

10960	0.946127	2.1
10961	0.946127	2.1
10962	0.946131	1.74565
10963	0.946128	2.1
10964	0.94613	1.82227
10965	0.946129	1.98098
10966	0.946133	0.159447
10967	0.946132	0.120186
10968	0.946132	0.135859
10969	0.946133	0.178956
10970	1.20045	0.121402
10971	1.20045	0.128355
10972	0.946132	0.170478
10973	0.918871	0.05
10974	0.971961	0.05
10975	1.37324	0.05
10976	1.78255	0.05
10977	1.25155	0.05
10978	1.42172	0.05
10979	1.40554	0.05
10980	1.08862	0.05
10981	1.03803	0.05
10982	0.946132	0.05
10983	0.946126	0.05
10984	0.943647	0.05
10985	0.948611	0.05
10986	0.946129	0.05
10987	0.946129	0.05
10988	1.1699	1.00178
10989	0.946132	0.480451
10990	0.946133	1.11559
10991	0.946133	0.524168
10992	0.946132	0.70848
10993	0.946133	0.863719
10994	0.946132	1.47829
10995	0.946132	2.1
10996	0.946131	2.1
10997	0.946131	2.1
10998	0.946132	2.1
10999	0.946131	2.1
11000	0.946131	2.1
11001	0.946132	2.1
11002	0.946132	0.611085
11003	1.38623	0.500597
11004	0.946132	0.343395
11005	0.946132	0.406948
11006	0.946133	0.621067
11007	0.946132	0.408515
11008	0.946133	0.637692
11009	0.946132	2.1
11010	1.40098	2.1
11011	0.946131	2.1
11012	0.946131	2.1
11013	0.946132	2.1
11014	0.946132	2.1
11015	0.946132	2.1
11016	1.01777	0.548309
11017	0.946132	0.278621

11018	0.946133	1.2434
11019	1.68068	0.453653
11020	1.68068	1.20197
11021	1.41894	0.29087
11022	1.20787	0.0786432
11023	0.946132	0.191724
11024	0.946133	2.1
11025	0.946133	2.1
11026	0.946132	2.1
11027	1.40199	2.1
11028	0.946133	2.1
11029	0.946132	2.1
11030	0.946132	2.1
11031	0.810946	1.56506
11032	0.810947	0.927
11033	2.37827	1.73383
11034	1.3517	1.49742
11035	1.08132	1.48785
11036	1.08132	1.77749
11037	1.08133	1.53191
11038	0.810934	1.48915
11039	0.946124	2.01428
11040	0.94613	1.61619
11041	1.40248	1.38038
11042	0.946131	1.30279
11043	0.946125	0.771733
11044	0.946127	1.64436
11045	0.946128	1.0264
11046	1.25703	1.74767
11047	0.87821	1.29454
11048	1.18912	1.74157
11049	0.946119	1.28887
11050	0.87825	1.45103
11051	1.1891	1.6559
11052	0.946107	1.87548
11053	0.946132	1.97017
11054	1.28543	1.26573
11055	0.946132	0.941247
11056	0.946132	1.30274
11057	0.946131	1.17484
11058	0.946132	0.319914
11059	0.946132	0.293386
11060	0.946115	2.1
11061	0.946119	2.1
11062	0.946116	2.1
11063	0.946123	2.1
11064	0.946118	2.1
11065	1.41636	2.1
11066	0.946129	2.1
11067	0.946132	2.08407
11068	0.946132	2.1
11069	0.946132	2.1
11070	0.946131	2.1
11071	0.946131	2.1
11072	1.41432	2.1
11073	0.946132	2.1
11074	0.810947	0.05
11075	0.810947	0.05

11076	2.44433	0.05
11077	1.21654	0.05
11078	0.946154	0.05
11079	0.97995	0.05
11080	1.08133	0.05
11081	0.810934	0.05
11082	0.851542	0.05
11083	0.835698	0.05
11084	1.22216	0.05
11085	0.946133	0.05
11086	1.01217	0.05
11087	1.09996	0.05
11088	0.851529	0.05
11089	0.946132	1.77714
11090	0.946132	1.99797
11091	0.946129	2.07688
11092	0.94613	1.93031
11093	0.946131	2.02295
11094	0.946131	2.02688
11095	1.2254	2.04182
11096	0.800897	1.83279
11097	0.838029	2.09658
11098	0.838013	1.87008
11099	0.93679	1.36688
11100	1.20217	1.4854
11101	1.16923	1.68885
11102	0.94948	1.83039
11103	0.958118	1.95992
11104	0.36751	1.43432
11105	0.800535	1.72399
11106	0.272087	0.994161
11107	0.428084	1.10954
11108	0.986335	1.73092
11109	0.946132	0.27063
11110	0.946133	0.330497
11111	1.19601	0.262932
11112	0.946132	0.326795
11113	0.946132	0.35128
11114	0.946133	0.34281
11115	0.946133	0.275761
11116	0.946132	1.99405
11117	1.36956	2.1
11118	0.946132	2.1
11119	0.946132	2.00887
11120	0.946132	2.1
11121	0.946133	2.06797
11122	0.946132	2.08845
11123	0.946132	0.835178
11124	0.946131	2.07418
11125	0.946131	1.03356
11126	1.34268	0.240478
11127	0.946133	2.02559
11128	0.946132	0.823596
11129	0.946131	1.2288
11130	0.946132	2.0887
11131	0.946131	2.1
11132	0.946131	2.1
11133	0.946132	2.1

11134	0.946132	2.1
11135	0.946132	2.1
11136	0.946132	2.1
11137	0.810946	1.1733
11138	0.810943	0.911303
11139	2.51472	1.09159
11140	1.3517	1.03067
11141	1.08132	0.862552
11142	1.08132	1.12742
11143	1.08133	1.11176
11144	0.810933	1.15705
11145	0.902316	0.509304
11146	1.04517	0.545164
11147	0.974924	0.377996
11148	0.530063	0.67963
11149	1.1272	0.535053
11150	0.68207	0.55854
11151	0.477061	0.596345
11152	0.810946	0.08538
11153	0.810943	0.139133
11154	0.810959	0.0972198
11155	1.99211	0.0789433
11156	1.35168	0.0800843
11157	1.08132	0.0924366
11158	1.08132	0.0881961
11159	1.08131	0.113881
11160	0.94613	0.143375
11161	0.946131	0.136934
11162	0.946132	0.113155
11163	0.946132	0.136967
11164	0.946129	0.21582
11165	0.946131	0.139949
11166	1.16699	0.129595
11167	0.810943	1.89456
11168	0.810942	1.77995
11169	1.6943	2.02131
11170	1.27059	2.03542
11171	1.00021	2.01823
11172	1.02049	1.97393
11173	1.08133	1.91092
11174	0.750103	1.85675
11175	0.957813	1.99206
11176	0.946128	2.02971
11177	0.946132	1.78565
11178	1.04832	2.05596
11179	0.870714	1.7027
11180	0.94613	1.89109
11181	0.94613	1.94847
11182	1.86374	2.09896
11183	2.0815	1.84557
11184	1.15918	0.112149
11185	0.946129	0.0694218
11186	1.15918	0.132086
11187	0.946131	0.0847626
11188	0.946131	0.127691
11189	0.946131	0.100562
11190	0.94613	0.0902544
11191	0.946119	0.496645

11192	1.3612	0.654643
11193	0.946125	0.511061
11194	0.946119	0.58371
11195	0.94612	1.1859
11196	0.946132	0.778758
11197	0.946107	0.581444
11198	0.810947	0.144224
11199	0.810947	0.202364
11200	2.50398	0.135788
11201	1.3517	0.135681
11202	1.08132	0.121866
11203	1.08133	0.153964
11204	1.08132	0.142525
11205	0.810934	0.135045
11206	0.946132	2.1
11207	0.946132	2.1
11208	0.946132	2.1
11209	1.39754	2.1
11210	0.946132	2.1
11211	0.946132	2.1
11212	0.946131	2.1
11213	0.946129	2.1
11214	1.41732	2.1
11215	0.946129	2.1
11216	0.946131	2.1
11217	0.946131	2.1
11218	0.946131	2.1
11219	0.946131	2.1
11220	0.946132	1.19724
11221	1.25276	1.70973
11222	0.946131	1.82773
11223	0.94613	1.29504
11224	0.94613	1.72823
11225	0.94613	1.47263
11226	0.946131	1.49642
11227	1.21261	0.836799
11228	0.94613	1.07649
11229	0.94613	1.68987
11230	0.946128	0.915697
11231	0.946131	1.35122
11232	0.94613	1.52201
11233	0.946129	1.34399
11234	0.946128	2.1
11235	0.946133	2.1
11236	0.946132	2.1
11237	0.946131	2.1
11238	0.94613	2.1
11239	0.946131	2.1
11240	1.39407	2.1
11241	0.946116	1.83561
11242	0.946119	1.93402
11243	0.946119	1.4764
11244	1.15369	1.83483
11245	0.946119	1.35168
11246	0.946131	1.76199
11247	0.946106	1.73625
11248	0.946131	2.1
11249	0.946131	2.1

11250	0.946131	2.1
11251	0.946133	2.1
11252	0.946129	2.1
11253	0.946131	2.1
11254	0.946131	2.1
11255	1.19564	0.05
11256	0.498881	0.05
11257	1.33964	0.05
11258	0.498878	0.05
11259	0.923583	0.05
11260	0.831259	0.05
11261	0.553735	0.05
11262	0.946132	0.05
11263	0.946132	0.05
11264	0.946131	0.05
11265	0.946131	0.05
11266	0.946132	0.05
11267	0.946131	0.05
11268	1.28673	0.05
11269	0.946132	0.089026
11270	0.946132	0.0955479
11271	1.33178	0.0835268
11272	0.946132	0.102105
11273	0.946132	0.100224
11274	0.946131	0.0964641
11275	0.946131	0.0884236
11276	0.946128	0.21662
11277	0.946132	0.147982
11278	0.946129	0.237027
11279	0.946131	0.090083
11280	0.946131	0.116575
11281	0.946129	0.136708
11282	0.94613	0.116581
11283	0.946131	2.1
11284	0.946131	2.1
11285	0.946129	2.1
11286	1.40525	2.1
11287	0.94613	2.1
11288	0.94613	2.1
11289	1.40525	2.1
11290	0.946131	2.1
11291	0.946126	2.1
11292	0.946127	2.1
11293	0.946127	2.1
11294	1.4073	2.1
11295	0.946129	2.1
11296	0.946129	2.1
11297	0.946131	0.077152
11298	0.946131	0.0595187
11299	0.946132	0.0823337
11300	0.946131	0.05
11301	0.946131	0.0519492
11302	1.05016	0.0532375
11303	0.946132	0.0767783
11304	0.946128	0.0824686
11305	0.946132	0.0661697
11306	0.946131	0.0883883
11307	0.946129	0.05

11308	0.94613	0.0553393
11309	1.13398	0.0559568
11310	0.94613	0.0923438
11311	0.946132	0.05
11312	0.946132	0.05
11313	0.946131	0.05
11314	0.946131	0.05
11315	0.946132	0.05
11316	0.946131	0.05
11317	0.946132	0.05
11318	0.946131	0.0883849
11319	0.94613	0.05
11320	0.946131	0.0621543
11321	0.94613	0.0957371
11322	1.38645	0.0501349
11323	1.38645	0.0628673
11324	0.946131	0.0727575
11325	0.946131	0.193547
11326	0.946131	0.116971
11327	0.94613	0.230294
11328	1.23923	0.09992
11329	0.94613	0.0732517
11330	0.946131	0.201267
11331	0.946131	0.29514
11332	0.946132	0.05
11333	0.946131	0.05
11334	0.946131	0.05
11335	0.946132	0.05
11336	0.946132	0.05
11337	0.946132	0.05
11338	1.40511	0.05
11339	0.946132	2.1
11340	0.946132	2.09996
11341	0.946132	2.1
11342	0.946132	2.1
11343	0.946132	2.1
11344	0.946131	2.1
11345	1.12968	2.1
11346	0.946132	1.54782
11347	0.946128	2.1
11348	0.946128	2.08494
11349	0.94613	1.95903
11350	0.94613	2.1
11351	0.94613	1.93814
11352	0.94613	1.97614
11353	0.946133	2.1
11354	0.946133	2.1
11355	0.946132	2.1
11356	0.946132	2.1
11357	0.946132	2.1
11358	0.946133	2.1
11359	1.31323	2.1
11360	0.94613	2.1
11361	0.946131	2.09321
11362	0.946131	2.09387
11363	0.94613	2.02475
11364	0.946131	2.1
11365	0.946131	2.1

11366	0.946131	2.1
11367	0.946132	2.08469
11368	0.946132	0.859776
11369	0.946132	2.1
11370	1.36899	2.00508
11371	0.946132	0.188437
11372	0.946132	1.67725
11373	0.946133	1.18972
11374	1.03543	0.05
11375	0.946131	0.05
11376	0.946131	0.05
11377	0.946132	0.05
11378	0.94613	0.05
11379	0.950987	0.05
11380	0.941275	0.05
11381	0.946131	0.10406
11382	0.94613	0.112237
11383	1.28952	0.114033
11384	0.946132	0.0983663
11385	0.94613	0.150796
11386	0.946131	0.132743
11387	0.946131	0.106834
11388	0.94613	2.1
11389	1.35966	2.1
11390	0.946131	2.1
11391	0.946131	2.1
11392	0.946129	2.1
11393	0.94613	2.1
11394	0.94613	2.1
11395	0.810946	2.1
11396	0.810942	2.04414
11397	0.810932	2.1
11398	2.6655	2.1
11399	1.3517	2.1
11400	1.08132	2.1
11401	1.08131	2.04498
11402	1.08133	2.1
11403	0.94613	2.1
11404	0.946131	2.1
11405	0.94613	2.1
11406	0.946128	2.1
11407	0.946131	2.1
11408	0.94613	2.1
11409	0.94613	2.1
11410	0.946128	2.1
11411	0.946128	2.1
11412	0.946133	2.1
11413	1.25375	2.1
11414	0.94613	2.1
11415	0.946131	2.1
11416	1.25375	2.1
11417	0.94613	2.1
11418	0.94613	2.1
11419	0.946132	2.1
11420	0.946132	2.1
11421	0.946128	2.1
11422	0.94613	2.1
11423	1.31453	2.1

11424	0.946131	0.0581315
11425	0.946129	0.05
11426	0.94613	0.0604419
11427	0.94613	0.05
11428	0.94613	0.05
11429	1.12018	0.05
11430	0.94613	0.0626271
11431	0.946131	0.085392
11432	0.946129	0.05
11433	0.946131	0.0918631
11434	1.16373	0.05
11435	0.94613	0.05
11436	0.94613	0.084731
11437	0.946131	0.113681
11438	1.36811	0.313068
11439	0.946132	0.504726
11440	1.36811	0.269505
11441	0.946129	0.457044
11442	0.94613	0.338507
11443	0.946129	0.466816
11444	0.946129	0.406663
11445	0.946132	0.135659
11446	0.946133	0.0796653
11447	0.946132	0.103417
11448	0.946133	0.136552
11449	1.35732	0.0883831
11450	1.35732	0.100207
11451	0.946133	0.114162
11452	1.36399	0.623342
11453	0.946129	1.23952
11454	0.94613	0.671737
11455	0.946131	1.19766
11456	0.94613	1.78169
11457	0.946131	1.04334
11458	0.946131	1.3571
11459	0.946133	2.1
11460	0.946132	2.1
11461	0.946132	2.1
11462	1.38302	2.1
11463	0.946133	2.1
11464	0.946132	2.1
11465	0.946133	2.1
11466	0.810943	2.1
11467	0.810944	2.1
11468	0.810945	2.1
11469	2.80542	2.1
11470	1.35169	2.1
11471	1.08132	2.1
11472	1.08132	2.1
11473	1.08132	2.1
11474	1.30189	2.1
11475	0.946131	2.1
11476	0.946132	2.1
11477	0.946131	2.1
11478	0.946131	2.1
11479	0.946131	2.1
11480	0.946132	2.1
11481	0.810946	1.0358

11482	0.810947	0.776215
11483	2.33293	0.659352
11484	1.24357	0.643337
11485	1.08132	0.829889
11486	0.973183	1.04689
11487	1.00023	0.865825
11488	0.810934	1.01608
11489	1.06584	0.542342
11490	0.946129	0.606393
11491	0.84731	0.377186
11492	1.16646	0.581543
11493	0.946126	1.06892
11494	1.2831	0.431377
11495	0.851527	0.690793
11496	0.946131	2.1
11497	0.946128	2.1
11498	0.946128	2.1
11499	0.94613	2.1
11500	1.31961	2.1
11501	0.94613	2.1
11502	0.94613	2.1
11503	0.946131	2.1
11504	1.3605	2.1
11505	0.946133	2.1
11506	0.946132	2.1
11507	0.94613	2.1
11508	0.946131	2.1
11509	0.946131	2.1
11510	0.946128	2.1
11511	0.946133	1.87557
11512	0.946132	2.1
11513	0.946131	1.87152
11514	0.946131	1.5567
11515	0.946131	2.02836
11516	1.30116	2.09281
11517	0.946132	0.05
11518	0.946129	0.05
11519	0.94613	0.05
11520	1.3035	0.05
11521	0.946131	0.05
11522	0.94613	0.05
11523	0.946131	0.05
11524	0.946132	2.1
11525	1.2051	2.1
11526	1.2051	2.1
11527	0.946132	2.1
11528	1.37869	2.1
11529	0.946132	2.1
11530	0.946131	2.1
11531	0.810946	2.1
11532	0.810945	2.1
11533	2.69247	2.1
11534	1.35169	2.1
11535	1.08132	2.1
11536	1.08132	2.1
11537	1.08132	2.1
11538	0.810946	2.1
11539	1.22993	2.1

11540	0.946131	2.1
11541	0.946131	2.1
11542	1.34623	2.1
11543	0.946131	2.1
11544	1.34623	2.1
11545	0.946131	2.1
11546	0.946131	0.05
11547	0.946132	0.0523363
11548	0.946132	0.05
11549	0.946132	0.05
11550	0.946131	0.05
11551	0.946132	0.0513983
11552	1.32299	0.05
11553	0.94613	0.182341
11554	0.946131	0.204348
11555	0.946128	0.182331
11556	0.946132	0.161608
11557	0.946133	0.163323
11558	0.946131	0.190786
11559	0.946131	0.17377
11560	0.946132	2.1
11561	0.946129	2.1
11562	0.946128	2.1
11563	0.94613	2.1
11564	0.94613	2.1
11565	0.946131	2.1
11566	0.94613	2.1
11567	0.94613	2.1
11568	0.94613	2.1
11569	0.946131	2.1
11570	0.946132	2.1
11571	0.946129	2.1
11572	0.946131	2.1
11573	0.946131	2.1
11574	0.946132	1.51513
11575	0.946131	1.47252
11576	1.13731	1.15951
11577	0.94775	1.55647
11578	0.944514	1.85969
11579	0.946132	1.83891
11580	1.039	1.7732
11581	0.946131	1.50797
11582	0.946128	1.30053
11583	0.94613	0.957227
11584	0.946131	1.38958
11585	0.946128	1.74741
11586	0.946129	1.73738
11587	1.23726	1.65547
11588	0.946132	2.1
11589	0.946132	2.1
11590	0.946129	2.1
11591	1.39214	2.1
11592	0.946131	2.1
11593	0.946131	2.1
11594	0.94613	2.1
11595	0.946125	1.1801
11596	0.946119	0.465692
11597	0.946118	0.598412

11598	0.946118	0.310762
11599	0.94612	1.52791
11600	0.946106	0.666086
11601	0.946131	0.715027
11602	1.26893	0.107679
11603	0.946131	0.0780954
11604	1.26893	0.129748
11605	0.94613	0.110687
11606	0.946131	0.111724
11607	0.946131	0.106612
11608	0.946131	0.211537
11609	0.946133	0.05
11610	0.946133	0.05
11611	0.946132	0.0583972
11612	1.28924	0.05
11613	0.946133	0.0629587
11614	0.946132	0.05
11615	0.946132	0.05
11616	0.946133	0.539861
11617	0.946132	0.547366
11618	0.946132	0.555024
11619	0.946133	0.412264
11620	0.946133	0.451482
11621	0.946132	0.510191
11622	0.946133	0.461794
11623	0.946132	1.42501
11624	0.946133	0.552667
11625	0.946133	1.09106
11626	0.946132	1.45289
11627	1.15388	0.80139
11628	0.946132	1.28268
11629	0.946133	0.945538
11630	0.946132	0.446064
11631	0.946133	0.414479
11632	0.946132	0.46116
11633	0.946133	0.335753
11634	0.946133	0.372588
11635	0.946132	0.423646
11636	0.946132	0.377026
11637	1.0644	1.21548
11638	1.12486	1.71566
11639	1.08674	1.74212
11640	0.946132	1.37919
11641	0.946132	1.79065
11642	0.946133	1.60413
11643	0.946132	1.56785
11644	0.808978	1.27486
11645	0.911537	1.28325
11646	0.932832	1.30522
11647	1.14604	1.35739
11648	1.56953	1.24132
11649	1.25866	1.20561
11650	1.18488	1.33344
11651	1.341	1.15725
11652	0.946131	0.13104
11653	0.946131	0.0639495
11654	0.946131	0.0909593
11655	0.94613	0.134865

11656	1.33922	0.0731942
11657	0.946131	0.0998607
11658	0.946131	0.087188
11659	0.946132	0.0553931
11660	0.946133	0.05
11661	0.946132	0.0712693
11662	0.946132	0.0549574
11663	0.946132	0.0728752
11664	0.946133	0.0584857
11665	0.946132	0.0567648
11666	0.946129	0.677354
11667	0.946133	2.1
11668	0.946133	1.48243
11669	0.946131	1.44656
11670	1.40496	2.1
11671	0.946131	1.20237
11672	0.946131	2.09559
11673	0.94613	1.86437
11674	0.946127	0.760387
11675	0.946127	1.66169
11676	0.94613	1.93269
11677	1.38913	1.21738
11678	0.946128	1.84556
11679	0.946128	1.45066
11680	1.98863	0.05
11681	2.59915	0.05
11682	1.40338	0.05
11683	0.946131	0.05
11684	1.22266	0.05
11685	1.40338	0.05
11686	0.946132	0.05
11687	0.946132	0.05
11688	1.22266	0.05
11689	0.94613	0.05
11690	0.94613	0.05
11691	0.946124	0.0839808
11692	1.21428	0.05
11693	0.946126	0.0797451
11694	0.946127	0.0703047
11695	0.946127	0.0559239
11696	1.03411	0.05
11697	0.946131	0.121039
11698	1.03411	0.05
11699	0.946129	0.113121
11700	1.14258	0.05
11701	0.952143	0.10691
11702	0.940118	0.0844232
11703	0.810943	2.1
11704	0.810945	2.1
11705	0.810958	2.1
11706	2.82603	2.1
11707	1.35168	2.1
11708	1.08132	2.1
11709	1.08132	2.1
11710	1.0813	2.1
11711	0.946133	0.0613375
11712	0.946132	0.056373
11713	0.946132	0.0738586

11714	0.946132	0.05
11715	0.946132	0.0585731
11716	1.11539	0.0579507
11717	0.946133	0.0743738
11718	0.946133	2.1
11719	0.946132	2.1
11720	0.946133	2.1
11721	0.946133	2.1
11722	0.946132	2.1
11723	1.41195	2.1
11724	0.946133	2.1
11725	0.946131	0.05
11726	0.946131	0.05
11727	0.946131	0.05
11728	0.946131	0.05
11729	0.946131	0.05
11730	0.946131	0.05
11731	1.15968	0.05
11732	0.946131	1.47549
11733	0.94613	1.63867
11734	0.94613	2.0029
11735	1.34386	1.71956
11736	0.946132	1.93718
11737	0.94613	2.01327
11738	1.34386	1.88069
11739	0.946131	1.24687
11740	0.935202	1.00689
11741	0.946131	1.81398
11742	1.25054	1.29611
11743	0.850155	1.76326
11744	0.946131	1.7606
11745	0.935205	1.60191
11746	0.946131	0.28087
11747	1.40193	0.204524
11748	0.946132	0.207618
11749	0.946132	0.218487
11750	0.946129	0.323803
11751	1.40193	0.172823
11752	0.946131	0.244098
11753	0.808779	0.184955
11754	0.561937	0.127517
11755	1.40193	0.125469
11756	1.40193	0.119577
11757	0.561936	0.159806
11758	0.561937	0.100993
11759	1.40193	0.124614
11760	0.946132	0.05
11761	0.946132	0.05
11762	0.946132	0.0561712
11763	0.946132	0.05
11764	0.946133	0.0530649
11765	0.946132	0.05
11766	0.946133	0.05
11767	0.946127	0.205977
11768	0.946131	0.206567
11769	0.946131	0.106311
11770	0.946127	0.134378
11771	1.19583	0.0760676

11772	1.19583	0.096867
11773	0.946129	0.157192
11774	0.589428	0.216688
11775	1.08737	0.189744
11776	1.10961	0.247389
11777	0.872901	0.151059
11778	0.541327	0.169525
11779	0.541312	0.129514
11780	0.85153	0.195858
11781	0.946132	2.05305
11782	0.946131	2.1
11783	0.946131	2.1
11784	0.946132	2.08836
11785	0.946132	2.1
11786	0.946132	2.1
11787	0.946132	2.1
11788	0.946132	1.71992
11789	1.2778	2.06979
11790	0.946131	2.1
11791	0.946131	1.78525
11792	0.94613	2.08432
11793	0.94613	1.90619
11794	0.94613	1.96168
11795	0.946118	0.598827
11796	0.946112	0.499366
11797	0.946118	0.471733
11798	0.946119	0.558587
11799	0.946117	0.495987
11800	1.35631	0.454742
11801	0.946105	0.512497
11802	0.946133	2.1
11803	0.946133	2.1
11804	0.946132	2.1
11805	0.946132	2.1
11806	0.946133	1.76311
11807	0.946132	2.1
11808	0.946132	2.1
11809	0.94613	2.1
11810	0.946131	2.09929
11811	0.946128	2.09887
11812	0.946128	2.065
11813	0.94613	2.1
11814	0.946129	2.1
11815	0.946129	2.1
11816	0.946132	1.55057
11817	1.15139	1.34279
11818	0.946132	1.27369
11819	0.946132	1.61063
11820	0.946132	1.52311
11821	0.946132	1.66617
11822	0.946132	1.51283
11823	0.946133	2.1
11824	1.36151	2.1
11825	0.946132	2.1
11826	0.946132	2.1
11827	0.946132	2.1
11828	0.946132	2.1
11829	0.946133	2.1

11830	0.946127	2.1
11831	1.38397	2.1
11832	0.94613	2.1
11833	0.946131	2.1
11834	0.946128	2.1
11835	0.94613	2.1
11836	0.946129	2.1
11837	0.946131	0.278506
11838	0.946127	0.32436
11839	0.946127	0.284196
11840	0.946132	0.305174
11841	0.946131	0.290067
11842	0.946128	0.295459
11843	0.946129	0.290342
11844	1.0268	0.05
11845	1.23823	0.05
11846	1.2171	0.05
11847	0.883446	0.05
11848	1.23827	0.05
11849	1.32614	0.05
11850	0.94613	0.05
11851	0.946132	2.1
11852	0.946129	2.1
11853	0.946129	2.1
11854	0.94613	2.1
11855	1.36523	2.1
11856	0.946131	2.1
11857	0.94613	2.1
11858	0.946132	0.0866777
11859	0.946131	0.0603847
11860	0.946132	0.0955907
11861	0.946131	0.05
11862	0.946131	0.0526165
11863	1.01653	0.05
11864	0.946131	0.101641
11865	0.94612	2.1
11866	0.946121	2.07033
11867	0.946119	2.1
11868	0.946119	2.0891
11869	0.946119	2.1
11870	0.946132	2.1
11871	0.946107	2.1
11872	0.810942	2.1
11873	0.810944	2.00662
11874	2.60324	2.1
11875	1.21651	2.1
11876	1.0813	2.0993
11877	0.946153	2.00691
11878	0.979948	2.1
11879	0.709588	2.1
11880	1.3492	0.197563
11881	0.946132	0.216428
11882	0.946131	0.177661
11883	0.94613	0.21338
11884	0.946129	0.297828
11885	0.946129	0.222216
11886	0.94613	0.237974
11887	0.946131	0.223242

11888	0.94613	0.262297
11889	0.946128	0.227632
11890	0.946133	0.237627
11891	0.946133	0.23377
11892	0.946131	0.244735
11893	0.946131	0.234064
11894	1.23351	0.05
11895	0.946131	0.05
11896	0.946131	0.05
11897	0.946132	0.05
11898	0.946132	0.05
11899	0.946131	0.05
11900	0.946132	0.05
11901	1.35657	0.05
11902	0.946132	0.05
11903	0.946131	0.05
11904	0.946132	0.05
11905	0.946132	0.05
11906	0.946131	0.05
11907	0.946131	0.05
11908	0.946132	0.290101
11909	0.946132	0.212574
11910	0.946132	0.294168
11911	0.946131	0.154209
11912	0.946131	0.219721
11913	0.946132	0.18944
11914	0.946132	0.179968
11915	0.946133	0.171252
11916	0.946132	0.11254
11917	0.946133	0.171628
11918	0.946132	0.0748759
11919	0.946132	0.127559
11920	0.946132	0.105181
11921	0.946133	0.0966907
11922	0.946131	0.786363
11923	1.4101	1.78819
11924	0.946131	2.1
11925	0.946131	0.47794
11926	0.946131	1.46604
11927	0.946131	0.543505
11928	0.946131	1.61141
11929	0.810944	1.30871
11930	0.810939	1.36507
11931	0.709586	0.869689
11932	2.71261	0.879528
11933	1.21651	1.13186
11934	0.946151	0.776476
11935	0.979943	1.22161
11936	1.0813	1.39308
11937	0.946132	2.1
11938	0.946131	2.1
11939	0.946132	2.1
11940	0.946131	2.1
11941	0.946131	2.1
11942	1.37733	2.1
11943	0.946131	2.1
11944	0.946133	2.1
11945	0.946132	2.1

11946	0.946132	2.1
11947	0.946133	2.1
11948	0.946133	2.1
11949	0.946132	2.1
11950	1.32582	2.1
11951	0.946132	2.1
11952	0.946132	2.1
11953	0.946132	2.1
11954	0.946132	2.1
11955	0.946132	2.1
11956	0.946132	2.1
11957	0.946131	2.1
11958	0.946133	1.80677
11959	0.946132	1.52124
11960	0.946132	1.52905
11961	0.946132	1.40229
11962	0.946132	1.53283
11963	0.946133	1.4387
11964	1.3469	1.46831
11965	0.946126	2.09784
11966	0.94613	2.1
11967	0.946131	2.1
11968	0.946127	2.1
11969	1.41206	2.1
11970	0.946128	2.1
11971	0.946128	2.1
11972	0.946127	2.1
11973	0.94613	2.1
11974	0.946131	2.03137
11975	0.94613	2.1
11976	0.946128	2.02205
11977	0.946129	2.1
11978	0.946129	2.1
11979	0.946131	0.141704
11980	0.94613	0.130735
11981	1.24448	0.11272
11982	0.946129	0.1969
11983	0.946131	0.142291
11984	0.94613	0.142807
11985	0.946131	0.142405
11986	0.946132	0.201295
11987	0.946133	0.186083
11988	1.11015	0.161376
11989	0.946133	0.316719
11990	0.946132	0.217816
11991	0.946132	0.207419
11992	0.946132	0.211393
11993	1.38775	2.1
11994	0.946131	2.1
11995	0.946131	2.1
11996	0.946132	2.1
11997	0.946132	2.1
11998	0.946131	2.1
11999	0.946131	2.1
12000	0.946129	2.1
12001	0.946129	2.1
12002	0.946133	2.0997
12003	0.946131	2.1

12004	0.946131	1.71658
12005	0.946131	2.1
12006	0.946131	2.1
12007	0.946132	2.1
12008	0.946133	2.1
12009	0.946132	2.1
12010	1.40774	2.1
12011	0.946132	2.1
12012	0.946133	2.1
12013	0.946132	2.1
12014	0.610968	0.05
12015	0.688233	0.05
12016	0.715226	0.05
12017	0.792409	0.05
12018	1.13418	0.05
12019	0.956667	0.05
12020	0.757321	0.05
12021	0.968845	0.05
12022	0.595679	0.05
12023	0.946133	1.0606
12024	1.28917	1.24629
12025	0.946131	1.59378
12026	0.94613	0.928934
12027	0.946131	1.31816
12028	0.946131	1.21171
12029	0.946131	1.20701
12030	0.946132	0.157665
12031	0.946128	0.184047
12032	0.94613	0.206089
12033	1.40668	0.193674
12034	0.946131	0.237893
12035	0.946131	0.159568
12036	0.94613	0.181063
12037	0.810946	2.1
12038	0.810945	2.1
12039	2.78163	2.1
12040	1.3517	2.1
12041	1.08132	2.1
12042	1.08133	2.1
12043	1.08132	2.1
12044	0.810933	2.1
12045	0.946131	2.1
12046	1.39081	2.1
12047	0.94613	2.1
12048	1.30339	2.1
12049	1.30339	2.1
12050	1.39081	2.1
12051	0.946131	2.1
12052	0.946127	0.557178
12053	0.821631	0.466541
12054	0.946131	0.425074
12055	0.946131	0.557362
12056	0.946128	0.421207
12057	0.996109	0.544679
12058	0.827876	0.540177
12059	0.946131	2.1
12060	0.946129	2.1
12061	0.946129	2.1

12062	0.94613	2.1
12063	0.94613	2.1
12064	0.94613	2.1
12065	0.94613	2.1
12066	0.946132	0.377353
12067	1.41723	0.412208
12068	0.946132	0.372832
12069	0.946133	0.318952
12070	0.946132	0.616759
12071	0.946132	0.450897
12072	0.946133	0.472062
12073	0.946133	0.366451
12074	0.946132	0.315309
12075	0.946133	0.26115
12076	0.946133	0.324384
12077	0.946132	0.481887
12078	0.946132	0.369831
12079	1.34254	0.34409
12080	1.26501	1.37133
12081	0.946132	1.68752
12082	0.946133	1.56389
12083	0.946133	1.85994
12084	0.946132	1.86552
12085	0.946133	1.65147
12086	0.946132	1.89256
12087	0.946131	0.0727649
12088	0.946131	0.0686869
12089	0.946131	0.077881
12090	0.946131	0.05
12091	0.946131	0.05
12092	0.946131	0.0647346
12093	0.946131	0.0558855
12094	0.878843	0.05
12095	0.82561	0.05
12096	0.586974	0.05
12097	1.05343	0.05
12098	0.80631	0.05
12099	0.782663	0.05
12100	0.789405	0.05
12101	0.902491	0.05
12102	1.05344	0.05
12103	0.94613	0.05
12104	0.946131	0.05
12105	0.946131	0.0640263
12106	0.94613	0.05
12107	0.946131	0.05
12108	0.946131	0.05
12109	0.946131	0.05
12110	0.946128	2.1
12111	0.946133	2.09793
12112	0.946133	1.07914
12113	0.94613	2.1
12114	0.946131	1.24966
12115	0.946131	1.95119
12116	0.946131	2.03174
12117	0.946131	0.297106
12118	0.946131	0.240054
12119	0.94613	0.228321

12120	0.946128	0.262913
12121	0.946133	0.25809
12122	1.2996	0.212375
12123	0.946131	0.252052
12124	0.946132	2.1
12125	0.946132	2.1
12126	0.946132	2.1
12127	0.946132	2.1
12128	0.946131	2.1
12129	0.946132	2.1
12130	0.946132	2.1
12131	1.99211	0.0545119
12132	2.55024	0.05
12133	0.946131	0.0656766
12134	0.946131	0.0664215
12135	0.946131	0.0586947
12136	0.946131	0.0660524
12137	0.946131	0.0894237
12138	0.946131	0.0683569
12139	1.35726	0.0629695
12140	0.946128	2.1
12141	0.946132	2.06834
12142	0.946131	2.1
12143	0.94613	2.08913
12144	0.94613	2.1
12145	0.94613	2.1
12146	1.39852	2.1
12147	1.31666	0.05
12148	0.946131	0.05
12149	0.946128	0.05
12150	0.94613	0.05
12151	0.946128	0.05
12152	0.946128	0.05
12153	0.946128	0.05
12154	1.32275	0.05
12155	0.946131	0.0644357
12156	0.946129	0.05
12157	0.946128	0.05
12158	0.946129	0.05
12159	0.946128	0.05
12160	0.946129	0.05
12161	0.946132	2.1
12162	0.946131	2.1
12163	0.946131	2.1
12164	0.946132	2.1
12165	0.94613	2.1
12166	0.946131	2.1
12167	1.41125	2.1
12168	0.946132	2.1
12169	0.946132	2.1
12170	0.946131	2.1
12171	0.946132	2.1
12172	0.946132	2.1
12173	0.946132	2.1
12174	1.31904	2.1
12175	1.02425	0.05
12176	0.946132	0.05
12177	0.94613	0.05

12178	0.946131	0.05
12179	1.1534	0.05
12180	0.961161	0.05
12181	0.9311	0.05
12182	1.40092	1.38728
12183	0.946133	1.52233
12184	0.946132	1.46945
12185	0.946131	1.79425
12186	0.946131	1.53471
12187	0.94613	1.51707
12188	0.946131	1.78855
12189	0.946113	2.1
12190	0.946119	2.09974
12191	0.946117	2.1
12192	1.40015	2.1
12193	0.946118	2.09949
12194	0.946131	2.1
12195	0.946105	2.1
12196	0.946132	0.178753
12197	0.946133	0.18005
12198	0.946132	0.227061
12199	1.03547	0.210757
12200	0.946133	0.183796
12201	0.946133	0.226512
12202	0.946132	0.149679
12203	0.946133	2.1
12204	1.36694	2.1
12205	0.946133	2.1
12206	0.946132	2.1
12207	0.946133	2.1
12208	0.946133	2.1
12209	0.946132	2.1
12210	0.946132	0.0827168
12211	0.946133	0.110484
12212	0.946132	0.0830973
12213	0.946133	0.110437
12214	0.946133	0.0756042
12215	0.946132	0.0966612
12216	0.946132	0.109098
12217	0.491552	0.124068
12218	0.913078	0.15266
12219	0.946132	0.205442
12220	0.491552	0.106376
12221	0.946132	0.188024
12222	0.49154	0.164934
12223	1.53168	0.15553
12224	1.53168	0.112066
12225	0.946127	2.1
12226	1.38752	2.1
12227	0.946129	2.1
12228	0.94613	2.1
12229	0.946128	2.1
12230	0.946129	2.1
12231	0.946129	2.1
12232	0.946132	2.1
12233	0.946132	2.1
12234	0.946132	2.1
12235	0.946132	2.1

12236	0.946132	2.1
12237	0.946131	2.1
12238	0.946132	2.1
12239	0.94613	0.289207
12240	0.94613	0.201192
12241	0.94613	0.218432
12242	0.946131	0.228793
12243	0.946129	0.279888
12244	1.16834	0.160096
12245	0.94613	0.231141
12246	0.946127	0.320163
12247	1.35594	0.205418
12248	0.946131	0.235153
12249	0.946128	0.332469
12250	0.946127	0.162355
12251	0.946129	0.287381
12252	0.94613	0.255548
12253	0.94613	2.06645
12254	0.946131	0.65329
12255	0.946128	0.879487
12256	0.946128	0.296306
12257	0.946131	2.05553
12258	0.94613	1.36958
12259	0.94613	1.27949
12260	0.946132	0.77523
12261	0.946132	0.348105
12262	0.946129	0.699304
12263	1.39751	0.550553
12264	0.94613	0.599823
12265	0.946131	0.313139
12266	0.946131	0.344753
12267	0.946132	0.103537
12268	0.700041	0.0844224
12269	0.700013	0.112714
12270	1.7697	0.0542469
12271	1.7697	0.05
12272	1.21134	0.0784796
12273	0.946131	0.0795366
12274	1.25837	0.0726534
12275	0.946133	2.1
12276	0.946132	2.06194
12277	0.946132	2.06238
12278	0.946132	2.08094
12279	0.946132	2.1
12280	0.946133	2.1
12281	1.40583	2.1
12282	0.946131	1.84985
12283	0.946131	2.03516
12284	0.946131	1.83497
12285	0.946131	2.1
12286	0.946131	2.03996
12287	0.827865	2.04966
12288	0.946131	2.06854
12289	0.946132	0.05
12290	0.946129	0.05
12291	0.946129	0.05
12292	0.946131	0.05
12293	0.94613	0.05

12294	1.21087	0.05
12295	0.946131	0.05
12296	0.94613	0.300866
12297	0.946131	0.237258
12298	0.94613	0.211163
12299	0.946128	0.294137
12300	0.946132	0.210703
12301	1.17452	0.254047
12302	0.94613	0.264189
12303	0.946119	0.250489
12304	0.946114	0.209054
12305	0.946119	0.178648
12306	0.946119	0.244105
12307	0.946119	0.179792
12308	1.27376	0.210158
12309	0.946106	0.216603
12310	0.946133	2.1
12311	1.39621	2.1
12312	0.946132	2.1
12313	0.946132	2.1
12314	0.946133	2.1
12315	0.946132	2.1
12316	0.946132	2.1
12317	0.946132	2.1
12318	0.946132	2.1
12319	0.946131	2.1
12320	0.946131	2.1
12321	0.946132	2.1
12322	0.946132	2.1
12323	1.34352	2.1
12324	0.946132	1.93642
12325	1.41272	2.09262
12326	0.946131	2.1
12327	0.94613	1.82161
12328	0.94613	2.08858
12329	0.94613	1.92352
12330	0.94613	2.02622
12331	0.946132	2.1
12332	0.946131	2.05136
12333	0.946132	2.1
12334	0.946132	2.06571
12335	0.946132	2.07615
12336	0.946132	2.1
12337	1.23536	2.1
12338	0.946132	2.1
12339	0.946131	2.1
12340	0.946131	2.1
12341	0.946132	2.1
12342	0.946132	2.1
12343	1.41131	2.1
12344	0.946131	2.1
12345	1.41894	1.01431
12346	0.946132	0.897134
12347	0.946133	0.530049
12348	0.946132	1.01438
12349	0.946132	1.67612
12350	0.946133	0.765401
12351	0.946132	0.801526

12352	0.946128	0.651057
12353	0.946133	1.98627
12354	0.946131	1.30952
12355	0.946129	2.1
12356	0.94613	1.80268
12357	1.4099	1.99806
12358	0.946131	1.78181
12359	0.810947	2.1
12360	0.810945	2.08713
12361	0.810933	2.1
12362	2.62883	2.1
12363	1.3517	2.1
12364	1.08132	2.1
12365	1.08132	2.09254
12366	1.08133	2.1
12367	0.946131	0.759305
12368	0.946126	2.02537
12369	0.946127	2.06336
12370	0.94613	1.43971
12371	0.946128	2.1
12372	0.946129	1.54221
12373	0.946129	1.40839
12374	1.21347	2.07765
12375	0.946129	2.1
12376	0.94613	2.07948
12377	1.21347	2.1
12378	1.33064	2.1
12379	0.94613	2.1
12380	1.33064	2.1
12381	0.94613	0.05
12382	0.946129	0.05
12383	0.94613	0.05
12384	0.946132	0.05
12385	0.946126	0.05
12386	0.946129	0.05
12387	0.94613	0.05
12388	0.946132	0.56893
12389	1.37938	0.948236
12390	0.946131	1.0173
12391	0.94613	0.65277
12392	0.946131	1.46957
12393	0.94613	1.02434
12394	0.94613	0.804338
12395	0.946133	0.05
12396	0.946133	0.05
12397	1.2968	0.05
12398	0.946132	0.05
12399	0.946133	0.05
12400	1.2968	0.05
12401	0.946133	0.05
12402	1.43643	0.05
12403	0.946129	0.680941
12404	0.946133	0.446454
12405	0.946131	0.742004
12406	0.94613	0.275984
12407	0.946131	0.399817
12408	0.946131	0.366695
12409	0.946131	0.345481

12410	0.872447	1.61219
12411	0.946132	1.24839
12412	0.872447	1.16408
12413	0.827867	1.48851
12414	0.946132	1.46792
12415	0.827867	1.71274
12416	0.872447	1.66819
12417	0.946117	0.893104
12418	1.27213	0.700024
12419	0.946124	0.640499
12420	0.946119	0.860815
12421	0.946113	0.933379
12422	0.946129	0.930497
12423	0.946104	0.82062
12424	0.94613	1.65936
12425	0.946131	2.1
12426	0.94613	1.88708
12427	0.946129	2.1
12428	0.946132	0.479963
12429	0.946131	1.63441
12430	0.94613	1.62767
12431	0.810942	2.1
12432	0.810943	2.1
12433	2.80212	2.1
12434	1.3517	2.1
12435	1.08133	2.1
12436	1.08132	2.1
12437	1.08132	2.1
12438	0.810932	2.1
12439	1.29521	2.1
12440	0.946129	2.1
12441	0.946131	2.1
12442	1.40106	2.1
12443	0.94613	2.1
12444	1.40106	2.1
12445	0.946131	2.1
12446	0.946132	0.481542
12447	1.24596	0.298387
12448	0.94613	0.369747
12449	0.946131	0.453372
12450	0.946131	0.194603
12451	0.946131	0.443545
12452	0.946131	0.39724
12453	0.946131	2.1
12454	0.946131	2.1
12455	0.946131	2.1
12456	0.946128	2.1
12457	0.946133	2.1
12458	0.94613	2.1
12459	0.946131	2.1
12460	0.879548	0.173464
12461	1.21527	0.0779338
12462	0.967506	0.0818387
12463	0.946132	0.210748
12464	0.946133	0.143251
12465	0.967511	0.140613
12466	0.946132	0.285507
12467	0.946132	0.390887

12468	0.946132	0.203686
12469	0.974253	0.204088
12470	0.974253	0.440052
12471	0.91801	0.25771
12472	0.918009	0.280697
12473	0.946132	0.382374
12474	0.946132	0.0591828
12475	0.946131	0.05
12476	0.946131	0.0595194
12477	0.946133	0.05
12478	0.946132	0.05
12479	1.28644	0.05
12480	0.946132	0.0544933
12481	0.946131	0.0634977
12482	0.946131	0.05
12483	0.946131	0.0658815
12484	0.946131	0.05
12485	0.946131	0.05
12486	1.40103	0.05
12487	0.946131	0.056733
12488	0.946128	0.134337
12489	0.946132	0.0971908
12490	0.946129	0.158571
12491	1.03327	0.0773071
12492	0.946129	0.0776445
12493	0.946129	0.130528
12494	0.94613	0.169558
12495	0.946134	0.608877
12496	0.946133	1.04698
12497	0.946132	0.331869
12498	0.946133	1.01756
12499	0.946132	0.155182
12500	0.946132	0.31186
12501	0.946133	0.247825
12502	0.856654	0.05
12503	0.946131	0.05
12504	0.946131	0.05
12505	0.946132	0.05
12506	0.856616	0.05
12507	1.03565	0.05
12508	1.03565	0.05
12509	1.15844	0.204173
12510	0.946132	0.273869
12511	0.946132	0.146671
12512	0.946132	0.193312
12513	0.946132	0.15911
12514	0.946132	0.213541
12515	0.946131	0.19885
12516	1.4582	2.1
12517	1.35067	2.1
12518	0.946131	2.1
12519	1.04106	2.1
12520	0.938748	2.1
12521	0.943015	2.1
12522	1.05017	2.1
12523	0.938793	2.1
12524	0.946131	0.05
12525	0.946132	0.05

12526	0.946131	0.0614613
12527	0.94613	0.05
12528	0.946133	0.05
12529	0.946131	0.05
12530	0.946131	0.05
12531	0.946132	0.797817
12532	0.946133	1.0053
12533	0.946132	0.847182
12534	0.946133	0.891243
12535	0.946133	0.821459
12536	0.946132	0.882081
12537	0.946133	0.899607
12538	0.946128	1.92093
12539	0.946132	2.0727
12540	0.946132	1.73152
12541	0.94613	1.99032
12542	1.23426	1.93297
12543	0.94613	2.01465
12544	0.946131	1.96703
12545	0.810943	0.253291
12546	0.810946	0.868997
12547	2.60388	0.378167
12548	1.3517	0.338625
12549	1.08133	0.267182
12550	1.08132	0.838544
12551	1.08132	0.248178
12552	0.810933	0.0850885
12553	0.810945	2.09314
12554	0.810945	1.84362
12555	2.72377	2.1
12556	1.3517	2.1
12557	1.08133	2.09771
12558	1.08132	1.94485
12559	1.08132	2.1
12560	0.810932	2.1
12561	1.25104	1.32911
12562	0.946131	2.04427
12563	0.946131	1.58861
12564	1.36188	2.1
12565	0.946131	2.07654
12566	1.36188	2.1
12567	0.946132	2.1
12568	0.946132	2.05704
12569	0.946132	2.05947
12570	0.946132	2.05812
12571	0.946132	2.0653
12572	0.946132	2.05463
12573	0.946132	2.09425
12574	1.25488	2.0902
12575	1.32132	0.05
12576	0.946126	0.0727889
12577	0.946127	0.05
12578	0.946128	0.0647054
12579	0.946128	0.0775098
12580	0.946128	0.0591932
12581	0.946128	0.0655474
12582	0.946133	2.1
12583	1.41123	2.1

12584	0.946134	2.1
12585	0.946133	2.1
12586	0.946132	2.03857
12587	0.946133	2.1
12588	0.946132	2.1
12589	1.32163	0.05
12590	0.946133	0.05344
12591	0.946133	0.05
12592	0.946132	0.05
12593	0.946132	0.05
12594	0.946132	0.05
12595	0.946133	0.0561891
12596	0.946133	2.1
12597	0.946133	2.1
12598	0.946132	2.1
12599	0.946132	2.1
12600	0.946132	2.1
12601	0.946133	2.1
12602	0.946132	2.1
12603	0.94613	0.149367
12604	0.946131	0.137917
12605	0.946128	0.147881
12606	0.946133	0.119693
12607	0.946133	0.119328
12608	0.946131	0.116446
12609	0.946131	0.119942
12610	0.946133	1.13536
12611	0.946132	0.605093
12612	0.946132	1.20815
12613	0.946134	0.669833
12614	0.946133	1.51013
12615	0.946133	0.921181
12616	0.946132	0.880639
12617	0.94613	1.85193
12618	0.946128	1.98747
12619	0.946127	1.72414
12620	0.94613	1.38905
12621	0.946131	1.79094
12622	0.946128	1.86767
12623	1.21099	1.90582
12624	0.946132	0.676944
12625	0.946131	0.596024
12626	0.946131	0.746711
12627	0.946133	0.231245
12628	0.946133	0.28065
12629	0.946131	0.352894
12630	0.946132	0.302826
12631	1.34794	0.853059
12632	0.517994	0.953777
12633	0.517965	0.631725
12634	0.946132	1.0353
12635	0.9198	1.79641
12636	0.946132	1.31937
12637	0.9198	1.29195
12638	0.946132	1.40236
12639	0.94613	1.47152
12640	0.946131	0.72693
12641	0.946131	1.42533

12642	0.946131	1.86933
12643	0.946131	1.38433
12644	1.23224	1.01317
12645	0.946132	1.8057
12646	0.946132	1.90953
12647	0.946133	1.73816
12648	0.946132	1.14746
12649	0.946132	1.49559
12650	0.946133	1.74865
12651	1.37511	1.67867
12652	0.946133	0.721403
12653	0.946129	0.834473
12654	0.851104	1.47951
12655	0.946131	0.220884
12656	1.0412	0.384814
12657	1.0412	0.2702
12658	0.851066	0.91612
12659	1.31357	0.0999869
12660	0.946131	0.220986
12661	0.94613	0.120891
12662	0.946131	0.0985293
12663	0.94613	0.0816498
12664	0.946131	0.159129
12665	0.946131	0.0722873
12666	0.946133	0.473223
12667	1.38149	0.783593
12668	0.946132	0.739164
12669	0.946132	0.563569
12670	0.946133	1.37866
12671	0.946132	0.922122
12672	0.946133	0.661759
12673	0.810944	1.54186
12674	0.810945	2.1
12675	0.810958	2.09313
12676	2.65782	2.1
12677	1.35168	2.09774
12678	1.08132	1.8454
12679	1.08132	2.1
12680	1.0813	1.97802
12681	0.946113	0.05
12682	0.946119	0.05
12683	0.946118	0.05
12684	0.946118	0.05
12685	0.946119	0.05
12686	0.946131	0.05
12687	0.946106	0.05
12688	0.946132	0.05
12689	0.946132	0.05
12690	0.946128	0.05
12691	0.946131	0.05
12692	0.94613	0.05
12693	0.94613	0.05
12694	0.94613	0.05
12695	1.00099	0.0533808
12696	0.946132	0.101297
12697	1.17876	0.052579
12698	0.982282	0.0724365
12699	0.909981	0.109491

12700	0.946132	0.0879729
12701	0.946131	0.0855952
12702	0.946132	0.654826
12703	1.35328	1.06523
12704	0.946132	1.45561
12705	0.946132	0.597463
12706	0.946133	1.41243
12707	0.946132	1.02343
12708	0.946133	1.06297
12709	0.946133	0.05
12710	0.946133	0.05
12711	0.946132	0.05
12712	0.946132	0.05
12713	0.946132	0.05
12714	0.946132	0.05
12715	0.946133	0.05
12716	0.900195	0.05
12717	0.946132	0.05
12718	0.900167	0.05
12719	1.41963	0.05
12720	0.900195	0.05
12721	0.946133	0.05
12722	1.35385	0.05
12723	0.946132	1.85485
12724	0.946129	1.90318
12725	0.946129	2.0215
12726	0.946131	1.86884
12727	0.946131	2.0402
12728	1.41585	1.58187
12729	0.94613	2.00538
12730	0.810946	2.1
12731	0.810946	2.1
12732	2.68895	2.1
12733	1.3517	2.1
12734	1.08133	2.1
12735	1.08132	2.1
12736	1.08132	2.1
12737	0.810933	2.1
12738	1.19886	2.1
12739	0.946131	2.1
12740	0.946131	2.1
12741	1.34447	2.1
12742	0.946131	2.1
12743	1.34447	2.1
12744	0.946131	2.1
12745	0.946133	0.05
12746	0.946133	0.05
12747	0.946132	0.062081
12748	1.35065	0.05
12749	0.946132	0.0665802
12750	0.946133	0.0554404
12751	0.946132	0.05
12752	0.946131	0.452908
12753	0.946131	0.384705
12754	0.94613	0.539023
12755	0.946131	0.183329
12756	0.946131	0.214951
12757	0.946131	0.286428

12758	0.946131	0.234596
12759	0.946127	0.194902
12760	0.946131	0.189567
12761	0.946127	0.192318
12762	0.94613	0.148733
12763	0.946131	0.157083
12764	0.946128	0.181276
12765	0.946129	0.164475
12766	0.810946	0.05
12767	0.810947	0.05
12768	0.810959	0.05
12769	2.4391	0.05
12770	1.35168	0.05
12771	1.08132	0.05
12772	1.08132	0.05
12773	1.08131	0.05
12774	0.946132	0.05
12775	0.946132	0.05
12776	0.946132	0.05
12777	1.40837	0.05
12778	0.946132	0.05
12779	0.946132	0.05
12780	1.40837	0.05
12781	0.946127	2.07672
12782	0.946132	2.1
12783	0.946131	0.342285
12784	0.946127	2.09292
12785	0.946131	1.31473
12786	0.946129	1.23079
12787	0.94613	1.61613
12788	0.946133	2.00105
12789	0.946132	1.74883
12790	0.946132	1.80388
12791	0.946132	1.43631
12792	0.946132	1.87409
12793	0.946132	1.36622
12794	0.946133	2.01506
12795	0.946132	0.440967
12796	1.24344	0.151404
12797	0.946132	0.132801
12798	0.946132	0.816581
12799	0.946132	0.233938
12800	1.24344	0.236205
12801	0.946132	0.768842
12802	0.946131	0.187565
12803	0.946128	0.148907
12804	0.946128	0.0928356
12805	0.946131	0.20757
12806	1.41232	0.0998262
12807	0.946129	0.101571
12808	0.94613	0.126205
12809	0.946128	2.1
12810	0.946133	2.1
12811	0.946133	1.82413
12812	0.946131	2.1
12813	0.946131	1.8687
12814	0.946131	2.1
12815	0.94613	2.1

12816	0.946133	0.397327
12817	1.41556	0.390353
12818	0.946133	0.346114
12819	0.946133	0.331167
12820	0.946132	0.611898
12821	0.946132	0.419473
12822	0.946132	0.498728
12823	0.94612	0.35354
12824	1.41843	0.342766
12825	0.946114	0.33713
12826	0.94612	0.307015
12827	0.946119	0.549076
12828	1.41845	0.344148
12829	0.946107	0.46395
12830	0.946129	0.05
12831	0.946129	0.05
12832	0.94613	0.05
12833	0.946132	0.05
12834	0.946126	0.05
12835	1.1509	0.05
12836	0.94613	0.05
12837	0.946131	0.05
12838	0.946132	0.05
12839	0.946132	0.05
12840	0.946132	0.05
12841	0.946132	0.05
12842	1.19263	0.05
12843	0.946132	0.05
12844	0.94613	0.617714
12845	0.946131	0.921547
12846	0.946128	0.660675
12847	0.946133	1.04007
12848	0.946133	0.702893
12849	0.946131	0.737375
12850	0.946131	0.874551
12851	0.946133	1.11757
12852	1.22848	1.38648
12853	1.00336	1.36697
12854	0.827866	1.20711
12855	0.946132	1.63004
12856	0.827865	1.43934
12857	0.946133	1.34049
12858	0.946131	2.1
12859	0.94613	0.830071
12860	0.946127	2.08851
12861	1.41498	2.09547
12862	0.94613	0.644663
12863	0.946128	1.84394
12864	0.946128	1.40118
12865	0.94613	0.05
12866	0.94613	0.05
12867	0.94613	0.05
12868	1.37358	0.05
12869	0.946129	0.05
12870	0.94613	0.05
12871	1.37357	0.05
12872	0.946111	0.05
12873	0.946118	0.05

12874	0.946118	0.05
12875	1.36568	0.05
12876	0.946116	0.05
12877	0.94613	0.05
12878	0.946105	0.05
12879	0.946132	0.221636
12880	0.946131	0.155735
12881	0.94613	0.290982
12882	1.23175	0.126139
12883	0.946131	0.108045
12884	0.946131	0.237388
12885	0.946131	0.336847
12886	0.946132	0.05
12887	0.946126	0.05
12888	0.946128	0.05
12889	0.946129	0.05
12890	0.946129	0.0606475
12891	0.946128	0.05
12892	1.25698	0.05
12893	0.810943	2.1
12894	0.810944	2.1
12895	2.81171	2.1
12896	1.3517	2.1
12897	1.08132	2.1
12898	1.08133	2.1
12899	1.08132	2.1
12900	0.810932	2.1
12901	0.946132	0.173489
12902	0.946131	0.140152
12903	0.946131	0.183075
12904	0.946132	0.0811226
12905	0.946132	0.100522
12906	0.946132	0.124527
12907	0.946131	0.107652
12908	0.810936	2.04982
12909	0.810935	2.1
12910	2.80914	2.1
12911	1.35167	2.1
12912	1.0813	2.1
12913	1.0813	2.1
12914	1.0813	1.83937
12915	0.810934	2.09826
12916	1.23851	2.1
12917	0.946132	2.09778
12918	0.946131	2.1
12919	1.40459	2.03418
12920	0.946132	2.1
12921	1.40459	2.1
12922	0.946132	2.1
12923	1.19974	0.110319
12924	0.946131	0.335218
12925	1.19974	0.118171
12926	0.946132	0.265194
12927	0.946132	0.0828653
12928	0.946132	0.315609
12929	0.946132	0.150613
12930	0.939141	0.847314
12931	1.14375	1.00667

12932	1.03306	0.951056
12933	0.946132	1.04085
12934	0.946126	1.16745
12935	0.946129	1.15883
12936	0.94613	0.992493
12937	0.946132	0.119347
12938	0.946131	0.135538
12939	0.946132	0.120403
12940	0.946132	0.126339
12941	0.946132	0.106789
12942	0.946132	0.117912
12943	0.946132	0.120525
12944	0.946131	2.1
12945	0.94613	2.1
12946	0.94613	2.1
12947	0.946131	2.1
12948	0.946129	2.1
12949	0.94613	2.1
12950	1.24512	2.1
12951	0.946133	0.109118
12952	0.946129	0.0723045
12953	0.946131	0.179789
12954	0.946132	0.0585398
12955	0.946131	0.120236
12956	0.946131	0.0958874
12957	0.946131	0.0837744
12958	0.946132	0.0777814
12959	0.946133	0.0739124
12960	0.946132	0.0885173
12961	0.946132	0.0527748
12962	0.946133	0.112408
12963	0.946132	0.0650022
12964	0.946132	0.0867756
12965	0.946132	0.559052
12966	1.41392	0.390528
12967	0.946132	0.402925
12968	0.946132	0.632701
12969	0.946132	0.287572
12970	0.946132	0.55084
12971	0.946132	0.505429
12972	0.946132	0.872573
12973	1.24333	0.674411
12974	0.946132	0.64163
12975	0.946133	0.996872
12976	0.946132	0.440485
12977	0.946132	0.934105
12978	0.946133	0.76273
12979	1.30649	0.164627
12980	0.94613	0.197225
12981	0.94613	0.153879
12982	0.946128	0.172994
12983	0.946126	0.218546
12984	0.946127	0.191368
12985	0.946127	0.193714
12986	0.709578	0.269567
12987	0.810943	0.24554
12988	2.53303	0.133915
12989	1.21651	0.13394

12990	0.979951	0.175964
12991	1.08132	0.150164
12992	0.946141	0.254759
12993	0.81096	0.221085
12994	0.946131	0.10159
12995	0.946131	0.106116
12996	0.946131	0.110375
12997	0.946131	0.0612739
12998	0.946131	0.0732294
12999	0.946131	0.10533
13000	0.946131	0.0842048
13001	0.946132	0.05
13002	0.946129	0.05
13003	0.946129	0.05
13004	0.94613	0.05
13005	1.20634	0.05
13006	1.20634	0.05
13007	0.946131	0.05
13008	0.946133	0.188588
13009	0.946132	0.0923559
13010	0.946132	0.0911162
13011	0.946132	0.202763
13012	0.946132	0.0836173
13013	1.30107	0.143624
13014	0.946133	0.177517
13015	0.810946	2.1
13016	0.810944	2.1
13017	2.73936	2.1
13018	1.35168	2.1
13019	1.0813	2.1
13020	1.08132	2.1
13021	1.08132	2.1
13022	0.810958	2.1
13023	0.946129	2.08158
13024	0.946129	2.1
13025	0.946133	1.77549
13026	1.30812	2.07933
13027	0.946131	1.96984
13028	0.946131	2.05002
13029	0.946131	2.04727
13030	1.00901	1.56545
13031	0.94613	1.95803
13032	0.94613	0.845711
13033	1.17001	1.69804
13034	0.917259	1.05674
13035	0.94613	1.3845
13036	0.94613	1.45449
13037	0.946126	2.1
13038	0.946127	2.1
13039	0.946131	2.1
13040	0.946127	2.1
13041	0.946131	2.1
13042	0.946129	2.1
13043	1.398	2.1
13044	0.946133	2.1
13045	1.3033	2.1
13046	0.946132	2.1
13047	0.946132	2.1

13048	0.946132	2.1
13049	0.946132	2.1
13050	0.946133	2.1
13051	0.946131	0.447937
13052	0.946131	0.473821
13053	1.09897	0.352046
13054	0.946129	0.647528
13055	0.946131	0.49104
13056	0.94613	0.474757
13057	0.94613	0.452274
13058	0.946132	2.1
13059	0.946131	2.1
13060	0.946131	2.1
13061	0.946132	2.1
13062	0.94613	2.1
13063	0.946131	2.1
13064	1.39858	2.1
13065	1.07117	0.47515
13066	1.08878	0.548239
13067	0.946119	0.97282
13068	1.08878	1.03107
13069	0.946119	0.285961
13070	0.946132	0.7907
13071	1.07116	0.536895
13072	0.946132	0.0652427
13073	0.946132	0.053525
13074	0.946132	0.0679024
13075	0.946132	0.05
13076	0.946132	0.05
13077	1.10733	0.05
13078	0.946132	0.0567403
13079	0.946128	0.0761449
13080	0.946132	0.0573624
13081	0.94613	0.0806944
13082	0.946129	0.05
13083	0.94613	0.05
13084	1.3367	0.05
13085	0.94613	0.0690839
13086	0.810943	2.1
13087	0.810944	2.1
13088	2.70234	2.1
13089	1.21653	2.1
13090	1.08132	2.1
13091	0.946155	2.1
13092	0.979951	2.1
13093	0.709578	2.1
13094	1.27174	2.1
13095	0.946129	2.1
13096	0.94613	2.1
13097	1.35117	2.1
13098	0.94613	2.1
13099	1.35117	2.1
13100	0.94613	2.1
13101	0.946133	1.50607
13102	0.946133	1.65926
13103	0.946132	1.98322
13104	0.946133	1.44719
13105	0.946132	2.08502

13106	0.946133	1.96943
13107	0.997296	2.02965
13108	0.810943	2.1
13109	0.810944	2.1
13110	0.810945	2.1
13111	2.78833	2.1
13112	1.35169	2.1
13113	1.08132	2.1
13114	1.08132	2.1
13115	1.08132	2.1
13116	0.810946	2.1
13117	0.810943	0.761617
13118	0.810933	1.68015
13119	2.70049	1.85999
13120	1.3517	1.3088
13121	1.08132	2.08479
13122	1.08132	0.29171
13123	1.08133	1.23427
13124	0.946127	1.49056
13125	1.35469	1.6841
13126	0.94613	1.67739
13127	0.946131	1.51986
13128	0.946128	1.50685
13129	1.35469	1.71158
13130	0.946129	1.71012
13131	0.946131	0.251239
13132	1.2722	0.200271
13133	0.946132	0.199248
13134	0.946132	0.206984
13135	0.946129	0.284698
13136	1.2722	0.173575
13137	0.94613	0.221663
13138	0.946133	0.0586492
13139	0.946132	0.0667256
13140	0.946132	0.076365
13141	0.946132	0.0749338
13142	1.41461	0.079487
13143	1.41461	0.069129
13144	0.946133	0.0687333
13145	0.946131	1.32315
13146	1.30165	0.583195
13147	0.946132	0.522265
13148	0.946132	1.47537
13149	0.946129	0.74823
13150	1.30165	1.21748
13151	0.946131	1.55894
13152	0.946132	1.94438
13153	0.946129	2.09988
13154	0.94613	1.99989
13155	1.39356	2.1
13156	0.946131	2.03795
13157	0.946131	2.04848
13158	0.94613	2.09333
13159	0.810946	2.1
13160	0.810947	2.1
13161	0.810934	2.1
13162	2.35003	2.1
13163	1.3517	2.1

13164	1.08132	2.1
13165	1.08132	2.1
13166	1.08133	2.1
13167	0.946131	0.05
13168	0.946131	0.05
13169	0.946125	0.0551556
13170	0.946127	0.05
13171	0.946129	0.05
13172	0.946128	0.05
13173	0.946128	0.05
13174	0.946131	0.05
13175	0.946131	0.05
13176	0.946131	0.05
13177	0.946131	0.05
13178	0.946132	0.05
13179	0.946132	0.05
13180	0.946131	0.05
13181	0.946129	2.1
13182	0.946132	2.1
13183	0.946132	2.1
13184	0.94613	2.1
13185	1.30082	2.1
13186	0.94613	2.1
13187	0.946131	2.1
13188	1.20416	0.05
13189	0.946132	0.0781344
13190	0.946131	0.05
13191	0.946132	0.05
13192	0.946131	0.05
13193	0.946131	0.0524084
13194	0.946132	0.05
13195	1.18711	0.05
13196	0.946131	0.078087
13197	0.946131	0.05
13198	0.946132	0.05
13199	0.946131	0.05
13200	0.946131	0.0702217
13201	0.946132	0.05
13202	0.946133	0.05
13203	0.946133	0.05
13204	0.946132	0.05
13205	1.30212	0.05
13206	0.946133	0.05
13207	0.946132	0.05
13208	1.30212	0.05
13209	0.946131	0.05
13210	0.946131	0.05
13211	0.946131	0.05
13212	0.946132	0.05
13213	1.31467	0.05
13214	1.31467	0.05
13215	0.946131	0.05
13216	0.946133	0.05
13217	0.946132	0.05
13218	0.946132	0.05
13219	0.946132	0.05
13220	1.25124	0.05
13221	1.25124	0.05

13222	0.946132	0.05
13223	0.946132	2.1
13224	0.946132	2.1
13225	0.946132	2.1
13226	1.36422	2.1
13227	0.946132	2.1
13228	0.946131	2.1
13229	0.946132	2.1
13230	0.946131	1.10438
13231	1.41851	1.72325
13232	0.946132	1.97718
13233	0.946132	1.12534
13234	0.946132	1.67974
13235	0.946132	1.32378
13236	0.946132	1.45587
13237	0.946133	0.05
13238	0.946132	0.05
13239	0.946132	0.05
13240	0.946132	0.05
13241	0.946132	0.05
13242	1.37794	0.05
13243	0.946132	0.05
13244	0.946132	2.1
13245	0.946132	2.1
13246	0.946131	2.1
13247	0.946131	2.1
13248	0.946132	2.1
13249	0.946132	2.1
13250	1.4045	2.1
13251	0.946133	2.1
13252	0.946132	2.1
13253	0.946131	2.1
13254	0.946132	2.1
13255	0.946131	2.1
13256	0.946131	2.1
13257	0.946132	2.1
13258	0.946132	2.1
13259	0.946132	2.1
13260	0.946131	2.1
13261	0.946132	2.1
13262	0.946131	2.1
13263	0.946131	2.1
13264	0.946131	2.1
13265	0.946128	2.1
13266	1.31641	2.1
13267	0.946129	2.1
13268	0.94613	2.1
13269	0.94613	2.1
13270	0.94613	2.1
13271	0.94613	2.1
13272	0.946132	2.1
13273	0.946131	2.1
13274	0.946131	2.1
13275	0.946132	2.1
13276	0.946132	2.1
13277	0.946132	2.1
13278	0.946132	2.1
13279	0.946132	2.1

13280	1.41507	2.1
13281	0.946132	2.1
13282	0.946132	2.1
13283	0.946133	2.1
13284	0.946133	2.1
13285	0.946132	2.1
13286	0.946132	2.1
13287	0.946131	2.1
13288	0.946131	2.1
13289	0.946133	2.1
13290	0.946132	2.1
13291	1.32608	2.1
13292	0.946132	2.1
13293	0.94613	2.03593
13294	0.94613	2.1
13295	0.94613	0.313209
13296	0.946131	2.06315
13297	0.946129	1.34047
13298	0.94613	0.701852
13299	0.94613	0.972418
13300	1.33728	2
13301	0.946131	1.94232
13302	0.94613	1.00808
13303	0.946132	2.02713
13304	0.946132	2.1
13305	0.946131	1.72003
13306	0.946131	1.79736
13307	0.946127	2.1
13308	1.40641	2.09108
13309	0.946129	2.0969
13310	0.94613	2.1
13311	0.946128	1.90166
13312	0.946129	2.1
13313	0.946129	2.1
13314	0.946129	2.1
13315	0.946128	2.1
13316	0.946133	2.1
13317	1.37467	2.1
13318	0.946131	2.1
13319	0.946131	2.1
13320	1.37467	2.1
13321	0.946131	2.1
13322	0.946131	2.1
13323	0.946132	2.1
13324	0.946132	2.1
13325	0.946129	2.1
13326	0.946131	2.1
13327	1.34912	2.1
13328	0.946132	2.1
13329	0.946133	2.1
13330	0.946133	2.1
13331	0.946132	2.1
13332	0.946132	2.1
13333	0.946133	2.1
13334	0.946132	2.1
13335	0.946127	2.1
13336	0.946131	2.1
13337	0.946132	2.1

13338	0.946131	2.1
13339	0.946127	2.1
13340	0.94613	2.1
13341	1.33607	2.1
13342	0.946117	0.211458
13343	0.946117	1.06688
13344	0.946115	1.02137
13345	0.946124	2.07416
13346	0.946119	0.599119
13347	1.32662	0.896727
13348	0.94613	0.55784
13349	0.946119	2.1
13350	0.946114	2.1
13351	0.946118	2.1
13352	0.946114	2.1
13353	0.946118	2.1
13354	0.946104	2.1
13355	0.946129	2.1
13356	0.946132	2.1
13357	0.946133	2.1
13358	0.946133	2.1
13359	0.946133	2.1
13360	0.946132	2.1
13361	0.946132	2.1
13362	0.946133	2.1
13363	0.946133	2.1
13364	0.946132	2.1
13365	0.946132	2.1
13366	0.946133	2.1
13367	0.946132	2.1
13368	0.946132	2.1
13369	0.946132	2.1
13370	0.946133	1.67354
13371	1.30881	2.01582
13372	0.946132	2.1
13373	0.946131	1.51703
13374	0.946131	1.85749
13375	0.946131	1.5755
13376	0.946131	1.82982
13377	0.946128	2.1
13378	0.946128	2.1
13379	0.946132	2.1
13380	0.94613	2.1
13381	0.94613	2.1
13382	0.94613	2.1
13383	1.34652	2.1
13384	0.946133	1.40349
13385	0.946133	1.27514
13386	0.946132	1.91192
13387	0.946133	1.18633
13388	0.946132	2.01756
13389	0.946132	1.68119
13390	0.946132	1.9005
13391	0.946132	0.162818
13392	0.946128	0.0892525
13393	0.94613	0.183057
13394	0.946131	0.0674163
13395	0.94613	0.216812

13396	0.94613	0.137469
13397	0.94613	0.123556
13398	0.946128	0.570867
13399	0.946132	0.624765
13400	0.94613	0.581463
13401	0.94613	0.482484
13402	0.946131	0.494587
13403	0.94613	0.562179
13404	0.94613	0.527786
13405	0.946129	0.755429
13406	1.32056	1.26215
13407	0.946131	1.46553
13408	0.94613	0.874472
13409	0.946124	1.39215
13410	0.946128	1.11001
13411	0.946127	1.06221
13412	0.946129	0.788178
13413	1.20351	1.11816
13414	0.946132	1.11836
13415	0.946132	0.95695
13416	0.946126	1.2863
13417	0.946129	1.14519
13418	0.946129	0.992611
13419	0.946127	2.03134
13420	0.946127	2.08921
13421	0.946131	1.76107
13422	1.25592	2.03663
13423	0.946127	1.863
13424	0.946129	1.97699
13425	0.94613	1.99824
13426	1.02332	1.97462
13427	0.946133	1.74018
13428	0.946132	1.44881
13429	1.02332	1.72278
13430	0.946132	1.06775
13431	0.96196	1.72081
13432	0.930306	1.44566
13433	0.946133	1.98008
13434	0.946133	2.01375
13435	0.946132	2.08321
13436	1.20057	2.03369
13437	0.946132	2.06819
13438	0.946133	2.09056
13439	1.20057	2.07591
13440	0.946128	1.49407
13441	0.946129	0.891847
13442	0.946129	1.18355
13443	1.13245	0.629064
13444	0.946126	0.531619
13445	0.946128	1.06053
13446	0.946129	0.852974
13447	0.946131	1.71437
13448	0.946131	1.94902
13449	0.94613	2.04104
13450	1.25	1.90267
13451	0.946129	2.04421
13452	0.946131	2.07381
13453	1.25	2.03382

13454	0.540425	1.96477
13455	0.748493	1.93948
13456	1.46266	2.05275
13457	1.20046	2.01102
13458	0.600454	1.99319
13459	1.41283	2.02681
13460	0.582567	1.95792
13461	0.946131	1.9126
13462	0.946131	1.88209
13463	0.946132	1.74028
13464	0.946132	1.90882
13465	0.94613	1.88459
13466	0.946131	2.05022
13467	1.28439	2.01405
13468	0.946132	0.576842
13469	0.946131	0.373529
13470	0.946131	0.603848
13471	0.946132	0.414265
13472	0.946132	0.748772
13473	1.39335	0.363547
13474	0.946132	0.449705
13475	0.946131	0.206394
13476	0.946131	0.43699
13477	0.94613	0.228198
13478	0.946132	0.180486
13479	0.946131	0.380228
13480	0.946131	0.190949
13481	0.946131	0.249439
13482	0.946131	0.228506
13483	0.94613	0.178856
13484	0.946126	0.295364
13485	0.946131	0.136071
13486	0.946125	0.524524
13487	0.946127	0.152148
13488	0.946128	0.317374
13489	0.946131	0.05
13490	0.946131	0.0595974
13491	0.946131	0.05
13492	0.946131	0.05
13493	0.946131	0.05
13494	0.946131	0.0555106
13495	1.39168	0.05
13496	0.946131	0.124137
13497	0.946126	0.0588215
13498	0.946127	0.0924227
13499	0.946131	0.0847583
13500	0.94613	0.107714
13501	1.412	0.0817493
13502	0.946128	0.09193
13503	0.948901	0.262675
13504	0.943364	0.266935
13505	1.03768	0.226792
13506	0.946132	0.392441
13507	0.946133	0.321942
13508	1.03767	0.302624
13509	0.946132	0.283945
13510	1.00902	0.496148
13511	0.88324	0.451138

13512	0.971584	0.375226
13513	0.946132	0.466246
13514	0.946129	0.785119
13515	0.946131	0.549286
13516	0.971572	0.533586
13517	0.946131	0.05
13518	0.946125	0.0512368
13519	1.33587	0.05
13520	0.946126	0.0665843
13521	0.946127	0.0523222
13522	0.946126	0.05
13523	0.946128	0.0517052
13524	0.946132	0.0696371
13525	0.946132	0.0696811
13526	1.27512	0.0521345
13527	0.946133	0.100296
13528	0.946132	0.0714905
13529	0.946133	0.0652189
13530	0.946133	0.0784179
13531	0.946128	0.0964987
13532	0.946133	0.0869083
13533	0.946131	0.101141
13534	0.94613	0.05
13535	0.946131	0.061462
13536	0.94613	0.0786508
13537	0.946131	0.067357
13538	0.946131	0.0817923
13539	0.946131	0.0703266
13540	0.946131	0.0849541
13541	0.946131	0.05
13542	0.946131	0.0516515
13543	0.946131	0.0659408
13544	0.946131	0.056974
13545	0.94613	0.05
13546	0.946131	0.05
13547	0.946131	0.05
13548	0.94613	0.05
13549	0.946132	0.05
13550	0.946131	0.05
13551	1.35316	0.05
13552	0.94613	0.149517
13553	0.94613	0.117684
13554	0.946128	0.128254
13555	0.946132	0.115862
13556	0.946132	0.135594
13557	1.28727	0.121483
13558	0.94613	0.0999545
13559	0.946131	0.05
13560	0.946132	0.05
13561	0.946132	0.05
13562	1.39502	0.05
13563	0.946132	0.05
13564	0.946132	0.05
13565	0.946132	0.05
13566	0.946125	0.359079
13567	0.946131	0.317713
13568	0.94613	0.251086
13569	0.946129	0.330914

13570	0.946126	0.521682
13571	0.946127	0.386035
13572	1.36173	0.35043
13573	0.946129	0.603655
13574	1.02363	0.442328
13575	0.94613	0.438858
13576	0.946131	0.60872
13577	0.946131	0.446586
13578	1.02363	0.607208
13579	0.946131	0.595589
13580	0.946128	0.05
13581	1.24678	0.05
13582	0.94613	0.05
13583	0.94613	0.05
13584	0.94613	0.05
13585	0.946131	0.05
13586	0.94613	0.05
13587	0.946129	0.05
13588	1.25215	0.05
13589	0.946132	0.05
13590	0.946126	0.05
13591	0.946129	0.05
13592	0.94613	0.05
13593	0.946129	0.05
13594	0.946129	0.400881
13595	0.946133	0.352155
13596	0.946133	0.288055
13597	0.946131	0.362437
13598	0.946131	0.31907
13599	1.29689	0.282404
13600	0.946131	0.316556
13601	0.946128	0.0782159
13602	0.946132	0.05
13603	0.946132	0.059568
13604	0.94613	0.0862889
13605	1.22716	0.05
13606	1.22716	0.0560332
13607	0.946131	0.0693116
13608	0.946127	0.09958
13609	0.946131	0.0619492
13610	0.946131	0.0783645
13611	0.946128	0.111003
13612	1.30463	0.0637473
13613	1.30463	0.0736637
13614	0.946129	0.09
13615	1.29958	0.05
13616	0.946132	0.05
13617	0.946132	0.05
13618	0.946132	0.05
13619	0.946132	0.05
13620	0.946132	0.05
13621	0.946133	0.05
13622	1.26201	0.05
13623	0.946133	0.05
13624	0.946132	0.05
13625	0.946132	0.05
13626	0.946132	0.05
13627	0.946133	0.05

13628	0.946132	0.05
13629	0.946133	0.081071
13630	0.946132	0.057549
13631	0.946132	0.0860036
13632	0.946133	0.05
13633	0.946132	0.05
13634	1.40427	0.05
13635	0.946133	0.0780569
13636	0.946132	0.0797248
13637	0.946132	0.0543966
13638	0.946132	0.0835261
13639	0.946132	0.05
13640	0.946132	0.05
13641	1.35123	0.05
13642	0.946131	0.0804566
13643	0.946128	0.124949
13644	0.946132	0.079992
13645	0.946132	0.0980131
13646	0.946129	0.142592
13647	1.22144	0.0822449
13648	1.22144	0.0919878
13649	0.946131	0.116178
13650	0.946127	0.289115
13651	0.946132	0.234141
13652	0.946131	0.214051
13653	0.946127	0.263267
13654	0.946131	0.237127
13655	1.23351	0.207335
13656	0.94613	0.238291
13657	1.18119	0.102056
13658	0.946131	0.149699
13659	0.946132	0.0955655
13660	0.946132	0.123788
13661	0.94613	0.166328
13662	0.946131	0.134062
13663	0.946131	0.138375
13664	0.946132	0.190252
13665	0.946132	0.159261
13666	0.946133	0.23266
13667	1.30563	0.133944
13668	0.946132	0.145809
13669	0.946132	0.186964
13670	0.946133	0.23744
13671	1.20256	0.424261
13672	0.946132	2.0046
13673	0.946133	1.32693
13674	0.946132	0.681778
13675	0.946132	0.415443
13676	0.946132	1.67979
13677	0.946133	1.06346
13678	0.94613	0.878623
13679	0.94613	0.659197
13680	0.946128	1.00834
13681	1.33007	0.522697
13682	0.946129	0.435909
13683	0.94613	0.922769
13684	0.946131	1.34226
13685	0.946127	2.1

13686	0.946127	2.1
13687	0.946131	2.1
13688	1.30136	2.1
13689	0.946128	2.1
13690	0.946129	2.1
13691	1.30136	2.1
13692	0.810942	2.1
13693	0.810942	2.1
13694	0.81093	2.1
13695	2.3208	2.1
13696	1.3517	2.1
13697	1.08132	2.1
13698	1.08132	2.1
13699	1.08133	2.1
13700	0.946132	2.1
13701	0.946132	2.1
13702	0.946132	2.1
13703	0.946132	2.1
13704	0.946132	2.1
13705	1.39823	2.1
13706	0.946132	2.1
13707	1.37713	2.1
13708	0.946131	2.1
13709	0.946131	2.1
13710	0.946131	2.1
13711	0.946131	2.1
13712	0.946131	2.1
13713	0.946131	2.1
13714	0.946128	2.1
13715	0.946128	2.1
13716	0.946133	2.1
13717	0.946131	2.1
13718	0.946131	2.1
13719	0.946131	2.1
13720	0.94613	2.1
13721	0.946129	2.1
13722	0.946129	2.1
13723	0.946133	2.1
13724	0.946131	2.1
13725	0.946131	2.1
13726	0.946131	2.1
13727	0.946131	2.1
13728	0.946132	1.40717
13729	1.33694	2.02481
13730	0.946133	2.1
13731	0.946133	1.42377
13732	0.946132	2.02374
13733	0.946132	1.66695
13734	0.946133	1.82214
13735	0.946131	2.1
13736	0.94613	2.1
13737	0.946131	2.1
13738	0.946133	2.1
13739	0.946129	2.1
13740	0.946131	2.1
13741	0.946131	2.1
13742	0.946132	1.88878
13743	1.35943	2.00843

13744	0.946132	2.1
13745	0.946132	1.19542
13746	0.946131	1.72464
13747	0.946132	1.25662
13748	0.946132	1.88556
13749	0.946132	0.05
13750	0.946132	0.05
13751	0.946133	0.05
13752	0.946133	0.05
13753	0.946132	0.05
13754	0.946132	0.05
13755	0.946132	0.05
13756	1.29322	0.05
13757	0.946131	0.05
13758	0.946132	0.05
13759	0.946129	0.05
13760	0.946131	0.05
13761	0.946131	0.05
13762	0.946131	0.05
13763	0.946132	1.82354
13764	0.946132	2.1
13765	0.946131	1.72553
13766	1.41492	1.22729
13767	0.946132	2.1
13768	0.946132	2.05922
13769	0.946131	2.1
13770	0.946132	2.1
13771	0.946132	2.1
13772	0.946132	2.1
13773	1.35028	2.1
13774	0.946132	2.1
13775	0.946132	2.1
13776	0.946132	2.1
13777	0.946132	1.96216
13778	0.946132	2.09965
13779	0.946132	2.08542
13780	1.32461	2.05001
13781	0.946131	2.1
13782	0.946132	2.08
13783	0.946132	2.09991
13784	0.946131	2.1
13785	0.946131	2.1
13786	0.946131	2.1
13787	0.946132	2.1
13788	0.946129	2.1
13789	0.94613	2.1
13790	1.40369	2.1
13791	0.946132	0.109185
13792	0.946131	0.0729055
13793	0.946131	0.124037
13794	0.979598	0.0583567
13795	0.946131	0.0556755
13796	0.946131	0.104346
13797	0.946132	0.134867
13798	0.946131	2.1
13799	1.41897	2.1
13800	0.946132	2.1
13801	0.946132	2.1

13802	0.94613	2.1
13803	0.946131	2.1
13804	0.946131	2.1
13805	0.946131	2.1
13806	0.94613	2.1
13807	0.946132	2.1
13808	0.946132	2.1
13809	0.946129	2.1
13810	0.946131	2.1
13811	1.37791	2.1
13812	0.946127	2.1
13813	0.946131	2.1
13814	0.946131	2.1
13815	0.94613	2.1
13816	0.946128	2.1
13817	0.946129	2.1
13818	1.39384	2.1
13819	0.810945	2.05541
13820	0.810944	1.04829
13821	0.810958	1.73627
13822	2.70852	1.56512
13823	1.35168	1.1296
13824	1.08132	1.91653
13825	1.08132	0.364762
13826	1.0813	1.32219
13827	1.3335	2.1
13828	0.946132	2.1
13829	0.94613	2.1
13830	0.946132	2.1
13831	0.946131	2.1
13832	0.94613	2.1
13833	0.946131	2.1
13834	0.946132	2.1
13835	0.946131	1.97027
13836	0.94613	2.1
13837	1.41192	2.1
13838	0.946132	2.00059
13839	0.946131	2.1
13840	0.94613	2.09888
13841	0.946133	1.58858
13842	0.946133	0.73741
13843	0.946132	1.50182
13844	1.39428	1.11582
13845	0.946132	0.215956
13846	0.946132	0.784201
13847	0.946133	0.565029
13848	0.94613	0.9237
13849	0.946129	2.1
13850	0.94613	1.97169
13851	0.946128	1.63055
13852	1.40684	2.1
13853	0.94613	1.82911
13854	0.94613	2.1
13855	0.946131	2.08259
13856	0.946131	2.1
13857	0.946131	2.1
13858	0.946131	2.1
13859	1.39021	2.1

13860	0.946131	2.1
13861	0.946131	2.1
13862	0.946131	2.05193
13863	0.946131	2.07854
13864	0.946131	0.782844
13865	0.946131	2.07432
13866	0.946131	2.1
13867	0.946131	0.592378
13868	0.946131	0.914719
13869	0.810946	2.1
13870	0.810946	2.1
13871	2.69264	2.1
13872	1.35168	2.1
13873	1.0813	2.1
13874	1.08132	2.1
13875	1.08132	2.1
13876	0.810958	2.1
13877	1.16687	2.1
13878	0.946131	2.1
13879	0.946131	2.1
13880	1.34632	2.1
13881	0.946131	2.1
13882	1.34632	2.1
13883	0.946131	2.1
13884	0.946128	0.514109
13885	0.946133	0.419467
13886	0.946133	0.358123
13887	0.946131	0.49919
13888	0.946131	0.358047
13889	1.3564	0.441611
13890	0.94613	0.469181
13891	0.946128	2.1
13892	0.946132	2.1
13893	0.94613	2.1
13894	0.94613	2.1
13895	0.946131	2.1
13896	1.35827	2.1
13897	0.94613	2.1
13898	0.946133	0.929922
13899	0.946132	1.32062
13900	0.946132	0.996401
13901	0.946133	1.30151
13902	0.946133	1.02288
13903	0.946132	1.06539
13904	0.946133	1.19442
13905	0.946132	2.1
13906	0.946132	1.90862
13907	0.946132	0.374717
13908	0.946131	2.09749
13909	0.946132	0.859253
13910	0.946132	1.52799
13911	0.946132	1.37516
13912	0.94613	0.565593
13913	0.94613	0.390541
13914	0.946128	0.60794
13915	0.946132	0.344303
13916	0.946132	0.60217
13917	0.94613	0.425412

13918	0.946131	0.425744
13919	0.946126	0.177228
13920	0.946131	0.127517
13921	0.946127	0.189585
13922	0.946129	0.112182
13923	0.94613	0.174755
13924	0.946128	0.135351
13925	0.946128	0.133779
13926	0.946131	0.0811147
13927	0.946127	0.0734975
13928	0.946127	0.0818219
13929	0.946131	0.0672231
13930	0.946131	0.0706925
13931	0.946128	0.061598
13932	0.946129	0.0843275
13933	0.94613	0.069736
13934	0.946131	0.0821746
13935	0.946129	0.0657833
13936	0.946132	0.0966905
13937	0.946132	0.0602522
13938	0.946131	0.06026
13939	0.94613	0.103613
13940	0.94613	0.103036
13941	0.946131	0.0660743
13942	0.946129	0.0976388
13943	0.946132	0.0631944
13944	0.946132	0.0988259
13945	1.32827	0.0756871
13946	0.946131	0.0949642
13947	0.946132	2.1
13948	0.946132	2.1
13949	0.946131	2.1
13950	0.946131	2.1
13951	0.946132	2.1
13952	0.946131	2.1
13953	0.946131	2.1
13954	0.946132	0.727264
13955	0.946132	2.0927
13956	0.946131	0.677906
13957	1.38356	0.347693
13958	0.946133	2.072
13959	0.946131	1.11727
13960	0.946131	1.48608
13961	0.94613	2.1
13962	0.946131	2.1
13963	0.946131	2.1
13964	0.946128	2.1
13965	0.946133	2.1
13966	0.946131	2.1
13967	1.36918	2.1
13968	0.946131	1.914
13969	0.94613	2.1
13970	0.946129	2.0962
13971	0.946133	2.1
13972	0.946132	2.1
13973	1.3771	2.1
13974	0.946131	2.1
13975	1.30029	1.2851

13976	0.94613	0.617084
13977	0.946129	1.93174
13978	0.946132	1.68327
13979	0.946132	0.135532
13980	0.946131	1.05672
13981	0.94613	0.786695
13982	0.946133	0.05
13983	0.946132	0.05
13984	0.946132	0.05
13985	0.946132	0.05
13986	0.946132	0.05
13987	0.946132	0.05
13988	1.21174	0.05
13989	0.810946	2.1
13990	0.810945	2.1
13991	2.63045	2.1
13992	1.35169	2.1
13993	1.08132	2.1
13994	1.08132	2.1
13995	1.08132	2.1
13996	0.810946	2.1
13997	1.1796	2.1
13998	0.946131	2.1
13999	0.946132	2.1
14000	1.31522	2.1
14001	0.946132	2.1
14002	1.31522	2.1
14003	0.946132	2.1
14004	0.946131	2.1
14005	1.40615	2.1
14006	0.946129	2.1
14007	0.946128	2.1
14008	0.946133	2.1
14009	0.946131	2.1
14010	0.94613	2.1
14011	0.810945	2.1
14012	0.810944	2.1
14013	2.80479	2.1
14014	1.35169	2.1
14015	1.08132	2.1
14016	1.08132	2.1
14017	1.08132	2.1
14018	0.810945	2.1
14019	1.35562	2.1
14020	0.94613	2.1
14021	1.35561	2.1
14022	0.946133	2.1
14023	0.946131	2.1
14024	1.40239	2.1
14025	0.946131	2.1
14026	0.946125	0.233726
14027	0.946131	0.417243
14028	0.946127	0.228363
14029	0.946129	0.228864
14030	0.946128	0.427511
14031	0.946129	0.252775
14032	0.946128	0.282754
14033	0.946119	2.1

14034	0.946113	2.1
14035	0.94612	2.1
14036	0.946119	2.1
14037	1.41915	2.1
14038	0.946107	2.1
14039	0.946132	2.1
14040	0.810946	1.84678
14041	0.810943	2.1
14042	0.810934	2.04178
14043	2.31411	2.09043
14044	1.35171	2.06595
14045	1.08132	1.93774
14046	1.08132	2.09616
14047	1.08133	2.00535
14048	0.946127	2.1
14049	0.94613	2.1
14050	0.94613	2.1
14051	0.946131	2.1
14052	0.946126	2.1
14053	0.946128	2.1
14054	0.946128	2.1
14055	0.810944	2.1
14056	0.810947	2.1
14057	0.81096	2.1
14058	2.78019	2.1
14059	1.35168	2.1
14060	1.08132	2.1
14061	1.08132	2.1
14062	1.08131	2.1
14063	0.946133	0.0781838
14064	0.946132	0.0677146
14065	0.946132	0.077775
14066	0.946133	0.0646314
14067	0.946132	0.0880827
14068	1.3495	0.0585155
14069	0.946133	0.0841863
14070	0.946133	2.1
14071	0.946132	2.1
14072	0.946132	2.1
14073	1.39529	2.1
14074	0.946133	2.1
14075	0.946132	2.1
14076	0.946133	2.1
14077	0.946133	2.1
14078	0.946132	2.1
14079	0.946132	2.1
14080	0.946132	2.1
14081	0.946132	2.1
14082	0.946133	2.1
14083	0.946132	2.1
14084	0.810946	2.1
14085	0.810945	2.1
14086	2.67861	2.1
14087	1.35169	2.1
14088	1.08132	2.1
14089	1.08132	2.1
14090	1.08132	2.1
14091	0.810946	2.1

14092	1.24616	2.1
14093	0.946132	2.1
14094	0.946132	2.1
14095	1.33931	2.1
14096	0.946132	2.1
14097	1.33931	2.1
14098	0.946133	2.1
14099	0.946133	0.112813
14100	0.946133	0.0515927
14101	0.946132	0.0633215
14102	0.946133	0.104014
14103	1.3855	0.05
14104	1.3855	0.0686347
14105	0.946133	0.0953688
14106	0.946128	2.1
14107	0.946132	2.1
14108	0.946128	2.1
14109	0.94613	2.1
14110	0.946131	2.1
14111	1.40225	2.1
14112	0.94613	2.1
14113	1.40548	0.05
14114	0.946118	0.05
14115	0.946114	0.05
14116	0.946112	0.05
14117	0.946117	0.05
14118	0.946128	0.05
14119	0.946103	0.05
14120	0.94613	2.1
14121	0.946132	2.1
14122	0.946131	2.1
14123	0.946132	2.1
14124	1.37811	2.1
14125	0.946131	2.1
14126	0.946131	2.1
14127	0.946132	2.1
14128	0.946133	2.1
14129	0.946133	2.1
14130	0.946132	2.1
14131	0.946133	2.1
14132	0.946132	2.1
14133	0.946132	2.1
14134	0.946118	2.1
14135	0.946113	2.1
14136	0.946118	2.1
14137	0.946119	2.1
14138	0.946117	2.1
14139	0.946105	2.1
14140	0.946131	2.1
14141	0.946118	0.05
14142	0.946118	0.05
14143	0.946117	0.05
14144	0.946113	0.05
14145	0.94612	0.05
14146	1.41377	0.05
14147	0.946106	0.05
14148	0.946132	1.09949
14149	1.28436	0.776792

14150	0.946131	0.810844
14151	0.946131	1.11328
14152	0.946131	0.330581
14153	0.94613	1.12601
14154	0.946131	0.901289
14155	0.810947	0.327103
14156	0.810946	0.355253
14157	2.68675	0.20723
14158	1.35168	0.223975
14159	1.08132	0.274399
14160	1.08132	0.266975
14161	1.0813	0.337944
14162	0.81096	0.300907
14163	0.946132	0.210942
14164	1.34338	0.175865
14165	0.946132	0.188606
14166	1.34338	0.229035
14167	0.946132	0.246113
14168	1.34337	0.200774
14169	0.946132	0.206534
14170	0.946127	2.08588
14171	0.946131	2.07802
14172	0.946127	2.06884
14173	0.946131	1.99254
14174	0.946131	2.05592
14175	0.946128	2.05323
14176	1.30281	2.08628
14177	0.946132	0.471451
14178	0.946132	0.350277
14179	0.946132	0.388905
14180	0.946131	0.40234
14181	0.946132	0.459004
14182	1.37973	0.295223
14183	0.946132	0.370825
14184	0.946131	0.655427
14185	1.41798	0.509762
14186	0.946132	0.457809
14187	0.946132	0.526049
14188	0.946129	0.85784
14189	0.946131	0.552086
14190	0.946131	0.797992
14191	0.946118	0.54902
14192	1.41776	0.42954
14193	0.946113	0.423808
14194	0.946119	0.431955
14195	0.946117	0.701516
14196	1.41778	0.434969
14197	0.946105	0.689212
14198	0.946131	0.763547
14199	1.41798	0.852365
14200	0.946132	0.934541
14201	0.946132	0.674597
14202	0.94613	1.013
14203	1.41799	0.626746
14204	0.946131	0.709277
14205	0.946133	2.1
14206	0.946132	2.1
14207	0.946132	1.65484

14208	0.946132	2.1
14209	0.946133	1.86502
14210	0.946132	2.1
14211	0.946132	2.1
14212	0.946131	2.1
14213	0.946131	2.1
14214	0.94613	1.00163
14215	0.946129	2.1
14216	0.946132	1.34134
14217	0.94613	1.92434
14218	0.946131	2.07764
14219	0.94612	1.32495
14220	0.946126	2.1
14221	0.946119	1.37906
14222	0.94612	1.86632
14223	1.40766	2.06823
14224	0.946132	0.535085
14225	0.946107	2.09803
14226	0.946128	2.1
14227	1.31213	2.1
14228	0.946129	2.1
14229	0.94613	2.1
14230	0.94613	2.1
14231	0.94613	2.1
14232	0.94613	2.1
14233	0.946127	2.1
14234	0.946131	2.1
14235	0.94613	2.1
14236	0.94613	2.1
14237	1.36653	2.1
14238	0.946128	2.1
14239	0.946128	2.1
14240	0.946127	2.1
14241	0.94613	2.1
14242	0.946131	2.1
14243	0.94613	2.1
14244	0.946128	2.1
14245	0.946128	2.1
14246	0.946129	2.1
14247	0.946133	2.1
14248	1.40081	2.1
14249	0.946132	2.1
14250	0.946132	2.1
14251	0.946133	2.1
14252	0.946132	2.1
14253	0.946133	2.1
14254	0.946128	2.1
14255	1.32282	2.1
14256	0.946129	2.1
14257	0.94613	2.1
14258	0.946131	2.1
14259	0.946131	2.1
14260	0.946131	2.1
14261	0.810945	2.1
14262	0.810943	2.1
14263	0.810933	2.1
14264	2.44097	2.1
14265	1.3517	2.1

14266	1.08132	2.1
14267	1.08132	2.1
14268	1.08133	2.1
14269	0.946131	2.1
14270	0.94613	2.1
14271	0.946133	2.1
14272	0.946133	2.1
14273	0.946128	2.1
14274	0.946131	2.1
14275	1.27676	2.1
14276	0.946126	1.33236
14277	0.946126	2.03867
14278	0.946131	1.407
14279	0.946127	2.03445
14280	0.94613	0.315296
14281	0.946128	1.29416
14282	0.946128	1.21908
14283	0.946132	0.0876448
14284	0.946133	0.112053
14285	0.946132	0.0898832
14286	0.946132	0.085075
14287	0.946132	0.0712607
14288	0.946133	0.0965826
14289	0.946132	0.0890002
14290	0.946128	0.108475
14291	0.946131	0.129341
14292	0.94613	0.110496
14293	0.946129	0.104068
14294	0.94613	0.0883566
14295	0.94613	0.110965
14296	0.94613	0.107549
14297	0.946132	0.05
14298	0.946128	0.0569903
14299	0.94613	0.127137
14300	0.946131	0.05
14301	0.94613	0.0718873
14302	0.94613	0.112174
14303	0.946131	0.05
14304	0.94612	0.0578945
14305	0.946119	0.0873455
14306	0.946119	0.0640315
14307	0.946124	0.0612959
14308	0.946119	0.0541086
14309	0.946132	0.0698013
14310	0.946107	0.0612204
14311	0.946128	2.1
14312	0.946133	2.1
14313	0.946131	2.1
14314	0.94613	2.1
14315	0.946131	2.1
14316	1.37869	2.1
14317	0.946131	2.1
14318	1.35703	2.1
14319	0.946132	2.1
14320	0.946132	2.1
14321	0.946132	2.1
14322	0.946132	2.1
14323	0.946132	2.1

14324	0.946133	2.1
14325	0.946133	2.1
14326	0.946133	2.1
14327	0.946132	2.1
14328	1.37017	2.1
14329	0.946133	2.1
14330	0.946132	2.1
14331	0.946133	2.1
14332	0.946114	0.05
14333	0.946119	0.05
14334	0.946119	0.05
14335	0.94612	0.05
14336	0.946119	0.05
14337	0.946132	0.05
14338	0.946107	0.05
14339	0.946131	0.05
14340	0.946131	0.05
14341	0.946126	0.0603298
14342	0.946127	0.05
14343	0.94613	0.05
14344	0.946129	0.05
14345	0.946128	0.05
14346	0.946131	1.10065
14347	0.946131	0.893687
14348	0.946132	0.678898
14349	0.946132	0.965198
14350	0.94613	1.54981
14351	0.946131	1.34861
14352	1.3279	1.2743
14353	0.946132	2.1
14354	1.40932	2.1
14355	0.946132	2.1
14356	0.946132	2.1
14357	0.946132	2.1
14358	0.946131	2.1
14359	0.946132	2.1
14360	0.946131	0.10212
14361	0.946131	0.103023
14362	0.946132	0.0827189
14363	0.946132	0.103984
14364	0.94613	0.145412
14365	0.946131	0.109452
14366	1.26868	0.0996669
14367	0.810943	2.1
14368	0.810945	2.1
14369	0.810932	2.1
14370	2.82321	2.1
14371	1.3517	2.1
14372	1.08132	2.1
14373	1.08132	2.1
14374	1.08133	2.1
14375	0.810943	1.08489
14376	0.810944	0.545894
14377	2.63079	1.1992
14378	1.35168	0.870161
14379	1.0813	0.853975
14380	1.08132	0.452842
14381	1.08132	2.00578

14382	0.810957	1.05157
14383	1.17856	0.108151
14384	0.946132	0.769331
14385	0.946128	0.467905
14386	1.3154	2.00994
14387	0.94613	0.817116
14388	1.31539	1.0719
14389	0.94613	1.40863
14390	0.94613	1.76827
14391	0.94613	1.65765
14392	0.946131	1.34202
14393	0.946131	1.71931
14394	0.946129	1.86057
14395	0.94613	1.96333
14396	1.21997	1.90394
14397	0.946131	1.55088
14398	1.25083	1.39833
14399	0.946132	1.314
14400	0.946132	1.60326
14401	0.946129	1.23572
14402	1.25083	1.72283
14403	0.946131	1.74933
14404	0.946127	1.68517
14405	1.41342	0.405863
14406	0.94613	1.32855
14407	0.946131	1.4198
14408	0.946127	0.446222
14409	0.946129	0.817945
14410	0.946129	0.639372
14411	0.946131	1.93496
14412	1.29149	1.58794
14413	0.946133	2.1
14414	0.946133	0.779129
14415	0.946128	2.00114
14416	0.946131	0.484547
14417	0.94613	0.961516
14418	0.94613	2.1
14419	0.94613	2.09838
14420	0.946132	2.1
14421	0.946132	2.09938
14422	0.946128	2.04345
14423	0.94613	2.1
14424	1.41022	2.1
14425	0.946132	0.128081
14426	0.946132	0.0964594
14427	0.946128	0.119242
14428	0.94613	0.0946837
14429	0.94613	0.142978
14430	0.94613	0.0857377
14431	0.946131	0.173825
14432	0.946129	0.700189
14433	1.37758	1.6358
14434	0.946131	2.08828
14435	0.94613	0.597839
14436	0.946124	1.52491
14437	0.946127	0.768597
14438	0.946127	1.36025
14439	0.946129	0.716147

14440	1.38259	1.41273
14441	0.946132	1.85307
14442	0.946132	0.743185
14443	0.946126	1.50197
14444	0.946129	0.989299
14445	0.94613	1.18
14446	0.946131	0.0624698
14447	0.946132	0.05
14448	0.946132	0.189348
14449	0.946132	0.05
14450	0.946132	0.0953723
14451	0.946132	0.0678907
14452	0.946132	0.05
14453	0.946131	0.05
14454	0.94613	0.05
14455	0.94613	0.05
14456	0.946133	0.05
14457	0.946128	0.05
14458	1.25017	0.05
14459	0.946131	0.05
14460	0.946131	2.1
14461	0.946131	2.1
14462	0.946131	2.1
14463	1.3937	2.1
14464	0.946133	2.1
14465	0.946131	2.1
14466	0.94613	2.1
14467	0.946131	1.95947
14468	0.946131	0.717403
14469	0.946131	0.894427
14470	0.94613	0.42653
14471	0.946133	2.00115
14472	0.946132	1.16269
14473	0.946131	1.01493
14474	0.946132	1.04939
14475	0.946132	1.04625
14476	0.946132	0.715185
14477	0.946132	0.943087
14478	0.946131	0.347391
14479	0.946132	0.260426
14480	0.946132	0.289801
14481	0.946129	1.41496
14482	0.94613	2.1
14483	0.94613	2.01401
14484	0.946128	2.1
14485	0.946132	0.490267
14486	0.946131	1.67113
14487	0.94613	1.81347
14488	0.946132	2.1
14489	0.946132	2.1
14490	0.946132	2.1
14491	1.41619	2.1
14492	0.946133	1.95584
14493	0.946133	2.1
14494	0.946132	2.1
14495	0.946132	2.1
14496	0.946132	2.1
14497	0.946132	2.1

14498	1.38244	2.1
14499	0.946132	2.1
14500	0.946131	2.1
14501	0.946132	2.1
14502	0.810946	0.741933
14503	0.810943	1.36604
14504	0.810934	0.698619
14505	2.42741	0.363896
14506	1.35171	0.504912
14507	1.08132	0.616087
14508	1.08132	0.710691
14509	1.08133	0.742481
14510	0.810947	0.652497
14511	0.810943	1.35141
14512	2.44179	0.370553
14513	1.35171	0.537465
14514	1.08132	0.563194
14515	1.08132	0.564185
14516	1.08133	0.638471
14517	0.810934	0.599271
14518	0.946132	1.8766
14519	0.946132	1.79693
14520	0.946133	1.31837
14521	0.946133	1.77431
14522	0.946132	1.98261
14523	0.946132	1.80219
14524	1.12786	1.72142
14525	1.27806	0.907863
14526	0.946132	1.5654
14527	0.946133	1.10652
14528	0.946133	1.63433
14529	0.946132	1.88937
14530	0.946132	1.46198
14531	0.946133	1.78445
14532	0.946132	2.1
14533	0.946132	2.1
14534	0.946132	2.1
14535	1.37098	2.1
14536	0.946133	2.1
14537	0.946132	2.1
14538	1.37098	2.1
14539	0.946125	1.19707
14540	0.946129	0.78593
14541	0.946131	1.99081
14542	0.94613	0.452566
14543	0.946124	0.400383
14544	0.946127	0.591835
14545	1.39514	1.18639
14546	0.946132	2.1
14547	0.946131	2.1
14548	0.94613	2.1
14549	1.39909	2.1
14550	0.946132	2.1
14551	0.946131	2.1
14552	0.94613	2.1
14553	0.946133	2.1
14554	0.946133	1.76337
14555	0.946132	2.1

14556	1.36835	2.1
14557	0.946132	1.89055
14558	0.946133	2.1
14559	0.946132	2.05436
14560	0.946132	2.1
14561	0.946133	2.1
14562	0.946133	2.1
14563	0.946132	2.1
14564	1.39047	2.1
14565	0.946132	2.1
14566	0.946132	2.1
14567	1.33174	0.05
14568	0.946128	0.05
14569	0.94613	0.05
14570	0.94613	0.05
14571	0.94613	0.05
14572	0.94613	0.05
14573	0.946131	0.05
14574	0.946128	0.05
14575	1.36004	0.05
14576	0.946129	0.05
14577	0.94613	0.05
14578	0.94613	0.05
14579	1.36003	0.05
14580	0.94613	0.05
14581	0.946124	2.1
14582	0.946119	2.1
14583	0.946119	2.1
14584	0.946119	2.1
14585	0.94612	2.1
14586	0.946132	2.1
14587	1.29062	2.1
14588	0.946128	2.1
14589	0.946128	2.1
14590	0.946133	2.1
14591	0.94613	2.1
14592	0.946131	2.1
14593	0.946131	2.1
14594	1.30584	2.1
14595	0.946131	2.1
14596	0.946131	2.1
14597	0.946131	2.1
14598	0.946132	2.1
14599	1.39549	2.1
14600	0.946131	2.1
14601	0.946131	2.1
14602	0.913386	0.944342
14603	1.17466	0.743399
14604	1.00473	0.679913
14605	0.946132	0.827342
14606	0.946126	1.1337
14607	0.94613	0.95546
14608	0.946129	0.916542
14609	0.946132	0.366169
14610	1.38894	0.368032
14611	0.946132	0.271347
14612	0.946132	0.307709
14613	0.946132	1.03702

14614	0.946132	0.40436
14615	0.946133	0.393547
14616	0.946128	0.32991
14617	1.23865	0.324094
14618	0.946129	0.247066
14619	0.94613	0.274175
14620	0.94613	0.481084
14621	0.94613	0.319537
14622	0.94613	0.350495
14623	0.946126	0.782371
14624	1.40248	0.748695
14625	0.946129	0.688181
14626	0.94613	0.839469
14627	0.946124	1.04937
14628	0.946127	0.95789
14629	0.946128	0.809826
14630	0.946133	0.360307
14631	1.1391	0.457445
14632	0.946132	0.389338
14633	0.946132	0.332226
14634	0.946132	1.11792
14635	0.946133	0.451656
14636	0.946132	0.430508
14637	1.17156	1.19818
14638	0.946133	1.17606
14639	1.33893	1.03693
14640	0.827864	1.01429
14641	0.995232	1.0784
14642	0.827864	1.14539
14643	0.94613	1.08029
14644	1.21387	0.429406
14645	0.946132	0.415992
14646	0.946131	0.213031
14647	0.94613	0.303887
14648	0.94613	0.281887
14649	0.94613	0.323857
14650	0.94613	0.310806
14651	0.810943	0.131892
14652	0.810945	0.0937928
14653	2.42463	0.0874692
14654	1.3517	0.0914092
14655	1.08132	0.106793
14656	1.08132	0.123816
14657	1.08133	0.103393
14658	0.810932	0.127417
14659	0.946133	1.88902
14660	0.946132	1.41332
14661	0.946131	1.35988
14662	1.36748	1.2291
14663	0.946133	1.61627
14664	0.946131	1.06882
14665	0.946132	0.318952
14666	1.35856	0.05
14667	0.946131	0.05
14668	0.94613	0.05
14669	0.94613	0.05
14670	0.946131	0.05
14671	0.94613	0.05

14672	0.94613	0.05
14673	0.946132	2.1
14674	0.946131	2.1
14675	0.946131	2.1
14676	0.946131	2.1
14677	1.41149	2.1
14678	1.41149	2.1
14679	0.946131	2.1
14680	0.946128	2.1
14681	0.946128	2.1
14682	0.946133	2.1
14683	0.946131	2.1
14684	0.946131	2.1
14685	0.946131	2.1
14686	0.94613	2.1
14687	1.41388	0.089464
14688	0.94613	0.131023
14689	0.946132	0.0854448
14690	0.946132	0.116416
14691	0.946129	0.153087
14692	0.946131	0.115905
14693	0.946131	0.126725
14694	1.02798	0.0641884
14695	0.946132	0.161951
14696	1.02798	0.0672946
14697	0.946133	0.143252
14698	1.14931	0.0596321
14699	0.957746	0.136545
14700	0.93452	0.109772
14701	0.946129	2.1
14702	0.946134	2.1
14703	0.946133	2.1
14704	0.946131	2.1
14705	0.946131	2.1
14706	0.946131	2.1
14707	0.946131	2.1
14708	0.946133	2.0712
14709	0.946132	1.62435
14710	0.946132	1.90448
14711	0.946132	2.05415
14712	1.38773	1.74634
14713	0.946132	1.98338
14714	0.946133	1.84573
14715	0.946131	0.174755
14716	0.946131	0.175287
14717	1.41512	0.159513
14718	0.946133	0.236905
14719	0.946131	0.22667
14720	0.946131	0.199957
14721	0.946131	0.182606
14722	1.35381	1.11081
14723	0.946132	2.1
14724	1.35381	1.133
14725	0.946133	2.1
14726	0.946132	1.17863
14727	0.946132	2.1
14728	0.946133	2.08909
14729	1.3365	1.16758

14730	0.946132	1.96758
14731	1.3365	1.05064
14732	0.946129	1.80237
14733	0.94613	1.23622
14734	0.946128	1.85376
14735	0.946129	1.6134
14736	0.939895	1.95608
14737	1.14285	2.093
14738	0.952366	2.09392
14739	0.946131	2.03512
14740	0.94613	2.09882
14741	0.946131	2.0659
14742	0.94613	2.06867
14743	0.946131	2.1
14744	0.946131	2.1
14745	0.946131	2.1
14746	0.946129	2.1
14747	0.946133	2.1
14748	0.946131	2.1
14749	0.946131	2.1
14750	0.946133	2.1
14751	0.946132	2.1
14752	0.946132	2.1
14753	0.946133	2.1
14754	0.946132	2.1
14755	0.946132	2.1
14756	0.946133	2.1
14757	0.946133	2.09774
14758	0.946132	1.72823
14759	0.946132	2.08544
14760	0.946133	2.0242
14761	0.946132	2.1
14762	0.946132	2.1
14763	0.946132	1.75899
14764	0.946132	2.1
14765	0.946131	2.1
14766	0.946131	2.1
14767	0.946131	2.1
14768	1.38969	2.1
14769	0.946131	2.1
14770	0.946132	2.1
14771	0.946128	2.05463
14772	0.946133	1.83884
14773	0.946132	1.89579
14774	0.946131	1.85212
14775	0.94613	1.94509
14776	0.946131	1.96768
14777	1.28464	1.96729
14778	0.946119	0.445558
14779	1.37492	0.590558
14780	0.946125	0.952904
14781	0.946119	0.303281
14782	0.946119	1.20453
14783	0.946132	0.420918
14784	0.946107	0.639115
14785	0.810942	2.1
14786	0.810943	2.1
14787	0.709587	2.1

14788	2.64488	2.1
14789	1.21651	2.1
14790	0.946151	2.1
14791	0.979948	2.1
14792	1.0813	2.1
14793	0.946133	1.76105
14794	0.946132	2.1
14795	0.946132	2.1
14796	0.946133	0.585966
14797	0.946132	0.376727
14798	0.946132	0.652208
14799	1.41134	1.46237
14800	0.946133	1.9839
14801	0.946132	1.61424
14802	0.946133	2.03017
14803	0.946133	1.44724
14804	0.946132	1.13946
14805	0.946133	1.70143
14806	1.31338	1.81778
14807	2.07612	0.371077
14808	0.946102	0.05
14809	0.752448	0.05
14810	1.20617	0.05
14811	0.94613	0.05
14812	0.75242	0.05
14813	0.946131	0.05
14814	0.752419	0.05
14815	0.946132	1.03195
14816	1.25736	0.911256
14817	1.25736	0.915559
14818	0.946132	0.869374
14819	1.08054	0.78742
14820	1.25736	0.897443
14821	0.946132	0.962011
14822	0.946128	2.1
14823	1.1896	2.1
14824	1.18959	2.1
14825	0.946131	2.09201
14826	1.33271	2.1
14827	0.946105	2.1
14828	0.946156	2.1
14829	0.741946	0.05
14830	1.33587	0.05
14831	0.741947	0.05
14832	1.21954	0.05
14833	0.94613	0.05
14834	0.741946	0.05
14835	1.81347	0.05
14836	1.81347	0.05
14837	0.653322	0.05
14838	1.36935	0.05
14839	0.661882	0.05
14840	1.04753	0.05
14841	0.507464	0.05
14842	0.945844	1.02807
14843	1.59507	2.1
14844	1.04043	1.07239
14845	0.946131	2.1

14846	1.32891	2.04959
14847	0.946131	1.92674
14848	0.946131	1.65927
14849	1.28866	1.79417
14850	1.12682	0.0982822
14851	0.851543	0.137227
14852	1.04072	0.102209
14853	0.946132	0.174279
14854	1.13437	0.0862133
14855	1.04167	0.0968085
14856	0.936652	0.103017
14857	1.258 2.1	
14858	0.946131	2.1
14859	0.946132	2.1
14860	1.36968	2.1
14861	0.946132	2.1
14862	1.36968	2.1
14863	0.946131	2.1
14864	2.30046	0.168072
14865	2.82969	0.190861
14866	0.946132	2.08222
14867	1.35022	2.1
14868	1.12356	2.06735
14869	0.94613	2.1
14870	1.35022	2.1
14871	0.946131	2.1
14872	0.94613	2.1
14873	0.946131	2.1
14874	1.22401	2.1
14875	0.952456	2.1
14876	0.94613	2.1
14877	1.22401	2.1
14878	0.946131	2.1
14879	0.946131	2.1
14880	1.33047	2.1
14881	0.946132	2.1
14882	1.33047	2.1
14883	0.946131	2.1
14884	1.33047	2.1
14885	0.946132	2.1
14886	1.2107	2.1
14887	0.61957	2.05767
14888	1.80666	2.1
14889	1.53743	2.1
14890	0.619572	2.00176
14891	1.32547	2.1
14892	1.16166	2.09745
14893	1.04074	2.09904
14894	0.557621	2.09774
14895	0.946128	2.1
14896	1.36607	2.1
14897	0.94613	2.1
14898	1.36607	2.1
14899	1.39416	2.1
14900	0.94613	2.1
14901	0.94613	2.1
14902	1.23969	2.1
14903	0.946131	2.1

14904	0.946131	2.1
14905	1.13537	2.1
14906	1.05719	2.1
14907	0.806848	2.1
14908	1.30251	2.1
14909	0.851526	1.57109
14910	1.14176	2.1
14911	0.93666	1.49977
14912	0.946114	2.1
14913	1.15832	2.07858
14914	0.924624	2.07362
14915	0.967609	1.94119
14916	0.771764	0.122536
14917	1.26652	0.139083
14918	0.946133	0.142367
14919	1.17957	0.0751231
14920	0.771735	0.11147
14921	0.946122	0.14057
14922	0.946133	0.123241
14923	0.924325	1.47389
14924	0.656739	1.81291
14925	0.637876	1.89022
14926	0.530092	1.64181
14927	0.914415	1.53292
14928	0.821311	1.91843
14929	0.630589	1.86586
14930	0.738823	1.80996
14931	0.839109	1.86048
14932	1.55029	0.05
14933	1.13537	1.80072
14934	0.946131	2.09229
14935	0.946128	1.98184
14936	1.30162	2.1
14937	0.952476	2.1
14938	1.29289	2.1
14939	0.939783	2.1
14940	0.94612	2.1
14941	0.946131	2.1
14942	1.07998	2.1
14943	0.836985	2.1
14944	1.1604	2.1
14945	0.94613	2.1
14946	0.837016	2.1
14947	0.712461	0.584891
14948	1.36399	0.619244
14949	1.8724	0.426351
14950	1.8724	0.299936
14951	0.623426	1.18363
14952	1.36399	0.542379
14953	1.22089	0.56336
14954	0.946132	0.562634
14955	0.946131	2.1
14956	1.32241	2.1
14957	1.32241	2.1
14958	0.946133	2.1
14959	1.40271	2.1
14960	0.946131	2.1
14961	0.946131	2.1

14962	1.1989	1.28486
14963	0.954106	1.68271
14964	0.820068	1.28451
14965	0.563139	1.58301
14966	1.06448	1.55889
14967	0.892472	1.69149
14968	0.862598	1.32276
14969	0.745851	1.74218
14970	1.13336	1.57447
14971	0.946129	2.1
14972	1.28254	2.1
14973	1.28254	2.1
14974	0.946131	2.1
14975	1.41302	2.1
14976	0.946131	2.1
14977	0.946131	2.1
14978	0.851543	2.02458
14979	1.21961	2.1
14980	0.851484	1.98062
14981	0.851543	2.1
14982	1.15705	2.1
14983	0.946133	2.1
14984	0.909417	2.07726
14985	1.13808	0.918441
14986	0.946133	0.204031
14987	0.946131	1.02354
14988	1.30194	0.186452
14989	0.946131	0.3315
14990	1.30194	0.126791
14991	0.946131	0.183412
14992	0.930941	1.09965
14993	1.20185	0.862356
14994	1.02404	0.82659
14995	0.946124	1.31296
14996	1.3563	0.939144
14997	0.946127	1.33406
14998	0.946128	1.09281
14999	0.946116	2.1
15000	1.3144	2.1
15001	1.13088	2.1
15002	0.946118	2.09295
15003	1.31438	2.1
15004	0.946105	2.1
15005	0.946131	2.1
15006	1.76972	0.0688666
15007	1.42091	0.05
15008	0.946128	0.0739395
15009	0.946101	0.0799088
15010	0.863552	0.104503
15011	0.94613	0.0605572
15012	1.21232	0.0773772
15013	0.94613	0.109148
15014	1.2137	0.41993
15015	1.23224	0.843082
15016	1.86512	0.560602
15017	1.86512	0.321331
15018	0.580825	1.50716
15019	1.2137	0.931595

15020	0.946133	0.910463
15021	0.580822	0.807352
15022	0.946128	2.1
15023	1.40586	2.1
15024	0.94613	2.1
15025	1.3719	2.1
15026	1.3719	2.1
15027	1.40586	2.1
15028	0.94613	2.1
15029	0.946132	2.09372
15030	1.35025	1.86143
15031	1.1794	2.07244
15032	0.946132	0.582651
15033	1.35025	1.532
15034	0.946131	1.53689
15035	0.946132	1.44276
15036	0.946133	2.1
15037	1.60149	2.1
15038	1.67058	2.1
15039	0.851542	2.1
15040	1.20296	2.1
15041	1.17501	2.1
15042	0.946132	2.1
15043	1.04993	2.1
15044	0.851541	2.1
15045	1.48219	2.1
15046	0.874565	2.09983
15047	0.946129	2.1
15048	1.22049	2.1
15049	0.946131	2.1
15050	0.946131	2.1
15051	0.94613	2.07631
15052	0.848719	1.99874
15053	1.17195	2.02751
15054	0.946129	0.751471
15055	1.35426	1.49268
15056	0.94613	1.70802
15057	0.94613	1.75262
15058	0.946133	2.1
15059	1.14342	2.1
15060	1.14342	2.1
15061	0.946133	2.1
15062	1.39009	2.1
15063	0.946132	2.1
15064	0.946132	2.1
15065	1.38819	2.1
15066	0.946133	2.1
15067	1.38819	2.1
15068	0.946131	2.1
15069	1.4116	2.1
15070	0.946131	2.1
15071	1.38819	2.1
15072	0.946127	2.1
15073	1.32244	2.1
15074	1.2176	2.1
15075	0.946131	2.1
15076	1.32244	2.1
15077	0.94613	2.1

15078	0.946129	2.1
15079	1.32415	2.1
15080	1.11239	2.1
15081	0.984099	2.1
15082	1.65532	2.1
15083	1.53082	2.1
15084	1.32068	2.1
15085	1.32068	2.1
15086	1.08172	2.1
15087	3.76293	0.05
15088	1.36335	0.05
15089	0.953925	2.1
15090	0.989497	2.1
15091	0.98949	2.1
15092	1.06341	2.1
15093	1.27875	2.1
15094	1.04598	2.1
15095	0.603154	2.1
15096	0.582457	2.1
15097	0.582457	2.1
15098	0.568994	2.1
15099	1.5791	2.1
15100	1.09506	2.1
15101	1.45327	2.1
15102	1.88406	2.1
15103	1.88404	2.1
15104	1.27389	2.08739
15105	1.14206	2.1
15106	1.00232	2.1
15107	1.00231	2.1
15108	1.68436	2.1
15109	1.4677	0.05
15110	1.28828	0.05
15111	1.28828	0.05
15112	1.46932	0.05
15113	1.28228	2.1
15114	1.64268	2.09726
15115	0.959473	2.1
15116	1.10101	2.1
15117	1.07976	2.1
15118	1.2873	2.1
15119	1.70303	0.166762
15120	1.40066	0.351828
15121	1.5563	0.125139
15122	1.13727	0.352082
15123	1.03529	2.1
15124	1.11285	2.1
15125	1.03529	2.1
15126	1.28667	2.1
15127	1.32119	2.1
15128	1.44777	2.1
15129	1.44778	2.1
15130	1.61121	2.1
15131	1.09372	0.05
15132	1.17664	0.05
15133	1.17664	0.05
15134	0.872931	0.05
15135	1.02415	2.1

15136	1.28228	2.1
15137	2.14692	2.1
15138	3.90469	2.1
15139	1.00509	2.1
15140	1.28228	2.1
15141	3.32936	0.05
15142	1.1333	0.05
15143	1.11048	2.1
15144	0.979854	2.1
15145	1.65815	2.1
15146	1.00185	2.1
15147	1.84887	2.1
15148	1.59597	2.1
15149	1.78468	2.1
15150	1.87508	2.1
15151	1.82716	2.1
15152	1.33429	2.1
15153	1.34626	2.1
15154	0.797142	2.1
15155	1.00007	1.85997
15156	1.02051	2.1
15157	1.02051	2.04438
15158	1.03373	2.1
15159	1.05786	2.1
15160	0.887631	2.1
15161	0.992773	2.1
15162	1.28462	2.1
15163	2.35274	1.60022
15164	1.82533	0.655477
15165	1.82533	1.3017
15166	1.19496	0.515211
15167	1.28597	0.05
15168	1.09725	2.1
15169	1.60948	2.1
15170	1.20421	2.1
15171	1.16566	2.1
15172	1.88522	2.1
15173	1.81948	0.14475
15174	1.14042	0.121492
15175	1.21558	0.246381
15176	1.56876	0.191966
15177	1.6697	2.1
15178	1.81655	2.1
15179	1.81655	2.1
15180	1.5956	2.1
15181	0.906059	2.1
15182	1.63045	2.1
15183	0.9533	1.56669
15184	1.1475	1.11469
15185	1.72231	2.09929
15186	1.94936	2.07197
15187	1.06826	2.1
15188	1.08576	2.1
15189	1.06826	2.1
15190	0.973559	2.1
15191	1.62737	0.05
15192	0.965676	0.05
15193	1.45055	0.05

15194	1.17631	0.05
15195	1.92535	0.05
15196	1.9503	0.05
15197	1.99828	0.05
15198	1.70302	0.05
15199	1.65139	0.05
15200	1.26784	0.0551176
15201	1.35923	0.0619153
15202	1.70302	0.0725795
15203	1.33033	2.1
15204	1.12481	2.1
15205	1.29452	2.1
15206	1.25399	2.1
15207	1.12481	2.1
15208	0.918358	2.1
15209	1.39332	2.1
15210	1.32368	2.1
15211	1.11712	1.44193
15212	1.07999	0.808613
15213	1.07999	0.937233
15214	1.05619	0.531902
15215	1.1766	2.1
15216	1.35735	2.1
15217	1.42456	0.05
15218	1.089	0.05
15219	1.11081	0.05
15220	2.88521	0.05
15221	1.09754	0.05
15222	1.85479	0.05
15223	1.27922	0.642094
15224	1.03556	0.371206
15225	1.07508	2.02388
15226	1.09795	2.08582
15227	0.967672	1.36339
15228	1.07508	1.89022
15229	1.03933	2.07883
15230	1.02975	2.1
15231	1.02975	2.1
15232	1.01121	2.1
15233	1.24526	0.05
15234	1.72368	0.05
15235	1.89607	0.05
15236	2.13512	0.05
15237	1.34591	0.303579
15238	1.14721	0.343536
15239	1.15539	2.1
15240	1.90938	2.06118
15241	1.90941	2.1
15242	1.89242	2.1
15243	1.18907	2.1
15244	1.20408	2.1
15245	1.20409	2.1
15246	1.22655	2.1
15247	0.701447	2.1
15248	1.57567	2.1
15249	1.38806	2.1
15250	2.09407	2.1
15251	1.07612	1.3629

15252	0.990019	1.25163
15253	1.15564	2.08732
15254	1.01243	1.97429
15255	1.28956	0.0559926
15256	1.35146	0.0673347
15257	1.35145	0.0723917
15258	1.22811	0.0865643
15259	1.09662	2.1
15260	1.42486	2.1
15261	1.10106	0.05
15262	1.90814	0.05
15263	1.3946	0.05
15264	1.11926	0.05
15265	0.725839	2.1
15266	0.925068	2.1
15267	0.925074	2.1
15268	1.43282	2.1
15269	1.91756	0.893852
15270	1.74321	0.987849
15271	1.74321	0.686274
15272	1.63413	0.762344
15273	1.9194	0.057969
15274	1.62246	0.05
15275	1.90456	0.05
15276	1.06578	0.05
15277	1.46019	0.05
15278	1.17036	0.05
15279	1.70233	1.01247
15280	1.53515	0.801708
15281	2.03494	2.1
15282	1.78441	2.1
15283	1.75992	2.1
15284	1.94351	2.1
15285	1.51057	0.05
15286	0.991435	0.05
15287	1.98068	0.05
15288	1.64283	0.05
15289	2.96126	0.05
15290	2.20692	0.05
15291	1.95054	0.05
15292	1.21325	0.05
15293	0.996828	0.246523
15294	1.01976	0.211773
15295	1.17129	2.1
15296	1.13664	2.1
15297	1.13664	2.1
15298	1.09627	2.1
15299	2.65123	2.1
15300	3.23062	2.1
15301	3.23062	2.1
15302	3.92038	2.1
15303	1.89019	0.244898
15304	1.79109	0.28266
15305	1.79109	0.207572
15306	1.54197	0.217771
15307	3.41824	2.1
15308	1.93553	0.930976
15309	2.26215	2.1

15310	2.0103	0.780576
15311	1.51569	0.970536
15312	1.01683	0.828896
15313	1.78595	2.1
15314	1.66537	2.1
15315	3.73426	2.1
15316	3.53905	2.1
15317	1.66537	2.1
15318	1.52903	2.1
15319	3.53905	2.1
15320	3.3837	2.1
15321	1.19901	1.35473
15322	1.35002	1.92006
15323	1.35002	0.124926
15324	1.11683	0.217232
15325	1.75326	1.18103
15326	0.712404	0.163346
15327	2.97768	2.1
15328	2.98949	2.1
15329	1.2832	2.1
15330	1.39518	2.1
15331	2.98949	2.1
15332	2.99339	2.1
15333	1.39517	2.1
15334	1.46124	2.1
15335	1.28534	2.1
15336	1.03391	2.1
15337	1.75366	1.66794
15338	1.26247	1.92666
15339	1.13621	2.03804
15340	2.43994	2.1
15341	1.47904	0.05
15342	1.80085	0.0775139
15343	1.80085	0.05
15344	1.64656	0.125766
15345	2.87331	2.1
15346	2.82863	2.1
15347	2.82859	2.1
15348	2.84673	2.1
15349	1.0961	0.0720559
15350	1.39448	0.18328
15351	1.21366	0.21881
15352	1.20143	0.113944
15353	1.85795	0.05
15354	1.36768	0.05
15355	1.36769	0.05
15356	1.45789	0.05
15357	1.32605	0.05
15358	1.27148	0.05
15359	1.70218	0.05
15360	1.3063	0.0620479
15361	1.45603	0.05
15362	1.89226	0.0866279
15363	1.2742	0.05
15364	2.10112	0.05
15365	1.10748	0.05
15366	1.1893	0.0519051
15367	1.04921	2.1

15368	1.22927	2.1
15369	1.22927	2.1
15370	1.46568	2.1
15371	0.811186	2.1
15372	0.792162	2.1
15373	0.792162	2.1
15374	0.758749	2.1
15375	1.03581	2.1
15376	0.737481	2.1
15377	2.24071	2.1
15378	1.34868	2.1
15379	1.47203	0.796776
15380	1.40967	0.814363
15381	1.40967	1.00062
15382	1.27916	0.87924
15383	1.17418	2.1
15384	1.04921	2.1
15385	1.28291	0.698001
15386	1.3213	0.476201
15387	1.46812	0.507588
15388	2.42522	0.802143
15389	1.17879	0.918323
15390	1.28291	0.962034
15391	1.28291	1.04952
15392	1.13438	0.918646
15393	1.75004	2.1
15394	1.78247	2.1
15395	1.78247	2.1
15396	1.76421	2.1
15397	2.25975	0.05
15398	3.66111	0.05
15399	3.6611	0.05
15400	3.44043	0.05
15401	1.13339	2.1
15402	1.13322	2.1
15403	1.13323	2.1
15404	1.20761	2.1
15405	1.34337	0.133655
15406	1.90912	0.34488
15407	1.04399	0.139906
15408	2.00578	0.405991
15409	1.04973	2.1
15410	1.22615	2.1
15411	1.93384	2.1
15412	2.62816	1.85985
15413	1.13127	2.1
15414	1.47642	2.1
15415	2.62816	2.1
15416	3.31755	1.58299
15417	1.47642	2.1
15418	1.80454	2.1
15419	0.964782	2.1
15420	0.949135	2.1
15421	0.949134	2.1
15422	0.949134	2.1
15423	1.10476	1.55253
15424	0.982858	0.230749
15425	1.10938	1.11961

15426	0.982858	1.14388
15427	1.01012	0.05
15428	0.974081	0.05
15429	1.82643	0.05
15430	1.29585	0.05
15431	1.00763	0.590803
15432	1.3294	0.759106
15433	1.3294	0.288281
15434	1.30001	1.01167
15435	1.59151	2.1
15436	1.99443	2.1
15437	1.07369	2.1
15438	1.30584	2.1
15439	1.05071	2.1
15440	0.948676	2.1
15441	1.13842	2.1
15442	1.42884	2.1
15443	1.59019	0.05
15444	1.38212	0.05
15445	1.34102	0.05
15446	1.02663	0.05
15447	0.797011	2.1
15448	0.81301	2.1
15449	0.81301	2.1
15450	0.811187	2.1
15451	0.991869	0.0934623
15452	1.23868	0.0998413
15453	1.67201	2.1
15454	1.61201	2.1
15455	1.61202	2.1
15456	1.57627	2.1
15457	0.94994	2.1
15458	1.77867	2.1
15459	0.854945	2.1
15460	0.973905	2.1
15461	1.64193	2.1
15462	1.68893	2.1
15463	1.68893	2.1
15464	1.70002	2.1
15465	2.08149	0.219507
15466	1.69456	0.147228
15467	1.67399	0.05
15468	1.83922	2.1
15469	1.91703	2.1
15470	0.967492	2.1
15471	1.13317	2.1
15472	0.980766	2.1
15473	2.06158	0.05
15474	0.986498	0.05
15475	1.76289	0.05
15476	1.17248	0.05
15477	1.51794	0.05
15478	1.30266	0.05
15479	1.03165	0.168065
15480	0.947692	0.196471
15481	1.20473	0.133561
15482	0.947693	0.135512
15483	1.54357	2.1

15484	0.980841	2.1
15485	1.34124	0.139724
15486	1.39818	0.380471
15487	1.39818	0.130498
15488	1.48035	0.518569
15489	1.03932	2.1
15490	1.2065	2.1
15491	1.20651	2.1
15492	1.12717	2.1
15493	1.09483	2.1
15494	0.994293	2.1
15495	0.994293	2.1
15496	0.97356	2.1
15497	0.995252	0.751124
15498	1.66294	0.788096
15499	1.82775	0.456524
15500	1.42762	0.482879
15501	1.04713	0.0585813
15502	1.05475	0.0773508
15503	1.29002	0.05
15504	1.05475	0.05
15505	1.17585	2.1
15506	1.39223	2.1
15507	1.39223	2.1
15508	1.71255	2.1
15509	1.56166	0.59805
15510	1.37762	0.421214
15511	1.16858	2.1
15512	1.0361	2.1
15513	1.23966	0.05
15514	1.10718	0.05
15515	1.99957	0.05
15516	0.658392	0.05
15517	1.36904	0.05
15518	1.12524	0.05
15519	1.04321	1.6779
15520	1.04627	1.66056
15521	1.91351	1.33751
15522	1.63379	1.08238
15523	1.79718	1.242
15524	0.59634	1.06874
15525	0.958257	2.1
15526	1.06457	2.1
15527	0.958255	2.1
15528	1.03933	2.1
15529	1.14195	2.1
15530	1.49438	2.09832
15531	1.49438	2.1
15532	1.31259	2.1
15533	1.23036	2.1
15534	1.2016	2.1
15535	1.2016	2.1
15536	1.17129	2.1
15537	1.05724	2.09682
15538	1.04291	2.1
15539	1.12341	2.1
15540	2.32525	2.1
15541	3.39038	2.1

15542	2.09374	2.1
15543	1.76185	0.29303
15544	1.7934	0.298917
15545	1.65809	0.29569
15546	1.54852	0.299443
15547	1.38766	2.1
15548	1.28765	2.1
15549	1.26599	2.1
15550	1.21397	2.1
15551	1.28765	2.1
15552	1.20045	2.1
15553	1.21398	2.1
15554	1.15234	2.1
15555	0.953634	0.322351
15556	1.39132	0.110945
15557	1.17285	2.1
15558	1.21408	2.1
15559	1.21408	2.1
15560	0.982848	2.1
15561	2.00101	2.1
15562	2.00973	2.1
15563	2.20314	2.1
15564	1.89318	2.1
15565	1.23267	2.1
15566	0.947921	2.1
15567	1.18872	2.1
15568	0.740752	2.1
15569	1.92269	0.137755
15570	1.53584	0.0768952
15571	1.53584	0.0899767
15572	1.21067	0.0513084
15573	1.40707	0.0512908
15574	1.50437	0.0584223
15575	1.50435	0.05
15576	1.45446	0.0572502
15577	1.47954	1.00319
15578	2.08687	1.69287
15579	2.08687	2.07558
15580	1.1233	2.08753
15581	1.04025	1.34858
15582	1.36005	2.00911
15583	1.47606	0.12438
15584	1.3407	0.143207
15585	0.758743	2.1
15586	0.716917	2.1
15587	0.716922	2.1
15588	0.672964	2.1
15589	2.40014	2.1
15590	1.96848	2.1
15591	1.96848	2.1
15592	1.53555	2.1
15593	1.06191	2.1
15594	1.29579	2.1
15595	1.29579	2.1
15596	1.71289	2.1
15597	1.78584	2.1
15598	2.15211	2.1
15599	2.00427	2.1

15600	3.41615	2.1
15601	1.66408	2.1
15602	1.73954	2.1
15603	2.91663	2.1
15604	3.65818	2.1
15605	1.93286	2.1
15606	1.08831	2.1
15607	1.31242	0.05
15608	1.11712	0.05
15609	1.26311	0.05
15610	1.31242	0.05
15611	1.10378	2.1
15612	0.968594	2.1
15613	1.48389	0.05
15614	1.49613	0.05
15615	1.04349	0.899113
15616	1.26409	0.572788
15617	1.26409	0.495124
15618	1.05724	1.42833
15619	1.65723	0.124667
15620	1.83116	0.115076
15621	2.06284	0.106709
15622	2.00512	0.0982162
15623	1.83116	0.119027
15624	2.03139	0.104566
15625	2.20564	0.0998359
15626	1.90048	0.0849449
15627	0.963392	2.1
15628	1.01346	0.955243
15629	2.98092	2.1
15630	2.70295	2.1
15631	2.70295	2.1
15632	2.48828	2.1
15633	1.63661	2.1
15634	1.64158	2.1
15635	1.64158	2.1
15636	1.6488	2.1
15637	1.17506	0.124571
15638	1.09667	0.106214
15639	2.00539	0.341918
15640	1.09667	0.150899
15641	1.54946	2.1
15642	1.43577	2.1
15643	1.26103	2.1
15644	1.49808	2.1
15645	1.089 0.05	
15646	1.22586	0.05
15647	2.35027	0.233277
15648	1.37549	0.1441
15649	3.33706	0.1373
15650	1.69894	0.115289
15651	2.00539	0.155574
15652	1.53798	0.113829
15653	1.28938	2.1
15654	1.20969	2.1
15655	1.20969	2.1
15656	1.15864	2.1
15657	1.7986	2.1

15658	1.75869	2.1
15659	1.58281	2.1
15660	1.7392	2.1
15661	1.30733	1.22163
15662	1.5419	1.53257
15663	1.50883	1.04138
15664	1.85439	0.985349
15665	1.73531	0.980415
15666	1.70225	0.968329
15667	1.22176	0.05
15668	1.59492	0.0945843
15669	1.59492	0.0702341
15670	2.15542	0.102702
15671	3.24379	2.1
15672	2.64775	2.1
15673	3.16707	2.1
15674	1.93032	2.1
15675	1.50738	2.1
15676	1.24128	2.1
15677	1.24128	2.1
15678	0.994293	2.1
15679	2.90808	0.05
15680	2.45797	0.05
15681	1.85501	0.05
15682	2.90809	0.05
15683	0.892835	1.99095
15684	1.55871	2.1
15685	1.04615	0.440115
15686	1.66643	1.26368
15687	1.70044	2.1
15688	1.80224	2.1
15689	1.70186	0.595695
15690	3.30123	1.78739
15691	1.74789	2.1
15692	1.77446	2.1
15693	1.77901	2.1
15694	1.57995	2.1
15695	1.81518	2.1
15696	2.17716	2.1
15697	2.17716	2.1
15698	2.65123	2.1
15699	2.01899	0.940008
15700	1.77479	0.854553
15701	1.19122	0.452951
15702	1.46724	0.382639
15703	1.70944	0.05
15704	1.08929	0.05
15705	1.68454	0.05
15706	1.08929	0.05
15707	1.51067	1.03816
15708	1.06729	0.676416
15709	1.37762	0.577482
15710	1.13992	0.412793
15711	1.26397	2.1
15712	1.04083	2.1
15713	1.00278	2.1
15714	1.04084	2.1
15715	1.1016	0.05

15716	1.23477	0.05
15717	1.23477	0.05
15718	1.64985	0.05
15719	1.11117	2.1
15720	1.13006	2.1
15721	1.13006	2.1
15722	1.14721	2.1
15723	1.21946	2.1
15724	1.0186	2.1
15725	1.13178	2.1
15726	1.00133	2.1
15727	1.67046	0.0613103
15728	0.986036	0.291521
15729	1.67046	0.251331
15730	1.34857	1.77715
15731	2.46016	2.1
15732	2.07161	2.1
15733	2.10732	2.1
15734	2.08371	2.1
15735	1.35602	2.1
15736	0.829065	2.1
15737	1.81667	2.1
15738	0.99406	2.1
15739	1.81667	2.1
15740	1.68073	2.1
15741	2.05736	2.1
15742	2.97311	2.1
15743	2.14011	2.1
15744	1.23411	2.1
15745	1.13632	0.05
15746	1.40862	0.05
15747	1.28055	0.05
15748	1.14305	0.05
15749	1.12035	0.05
15750	1.15483	0.05
15751	1.94099	0.05
15752	1.82126	0.05
15753	1.15481	0.05
15754	0.998957	0.05
15755	2.10078	0.05
15756	1.81617	0.05
15757	1.27581	2.1
15758	0.968536	2.1
15759	1.33916	2.1
15760	1.27581	2.1
15761	1.32784	2.1
15762	1.4259	2.1
15763	1.70377	2.1
15764	1.54231	2.1
15765	3.77569	2.1
15766	3.42809	2.1
15767	1.54231	2.1
15768	1.51298	2.1
15769	3.55866	2.1
15770	2.86136	2.1
15771	1.83301	0.423898
15772	1.26371	0.360229
15773	1.98301	0.465851

15774	2.0609	0.403464
15775	2.26047	0.23832
15776	2.00537	0.201881
15777	1.7913	0.177082
15778	1.48864	0.158032
15779	1.09504	2.1
15780	1.39827	2.1
15781	2.28084	2.1
15782	1.05983	2.1
15783	1.33593	0.05
15784	1.31508	0.05
15785	1.31508	0.05
15786	1.09599	0.05
15787	1.30647	2.1
15788	1.28846	2.1
15789	1.28846	2.1
15790	1.26923	2.1
15791	1.2955	2.1
15792	1.09044	2.1
15793	2.12209	2.1
15794	1.26438	2.1
15795	0.911819	2.1
15796	1.38752	2.1
15797	2.57704	2.1
15798	2.71736	2.1
15799	1.42284	2.1
15800	1.41562	2.1
15801	2.69131	2.1
15802	2.36178	2.1
15803	1.41562	2.1
15804	1.39564	2.1
15805	1.32368	2.1
15806	0.908053	2.1
15807	0.908053	2.1
15808	0.629213	2.1
15809	1.8758	1.96897
15810	1.27362	2.04382
15811	1.3756	0.795868
15812	2.28749	1.45634
15813	1.84577	0.360955
15814	2.17765	0.358549
15815	1.38969	0.319259
15816	1.84578	0.325318
15817	1.32801	0.205432
15818	1.76377	0.189949
15819	1.22845	2.1
15820	1.57766	2.06182
15821	1.57766	2.1
15822	1.39067	2.1
15823	1.15743	0.05
15824	1.37721	0.05
15825	1.69055	0.0524456
15826	1.14858	0.05
15827	1.38333	2.1
15828	1.10575	1.33181
15829	1.22418	2.1
15830	1.10574	2.1
15831	1.91509	0.121159

15832	1.02001	0.10526
15833	1.03074	0.245919
15834	1.19122	0.20298
15835	1.67887	2.1
15836	1.67597	2.1
15837	1.84984	2.1
15838	1.77775	2.1
15839	1.73571	2.06357
15840	1.27921	2.06919
15841	2.11851	1.86161
15842	1.27921	2.04862
15843	1.42486	2.1
15844	1.12503	2.1
15845	1.12503	2.1
15846	1.30116	2.1
15847	0.568995	2.1
15848	0.557348	2.1
15849	0.557348	2.1
15850	0.53907	2.1
15851	0.633575	2.1
15852	1.33756	2.1
15853	1.51074	2.1
15854	1.93384	2.03364
15855	0.655202	2.1
15856	0.731934	2.1
15857	0.731934	2.1
15858	1.09061	2.1
15859	0.966979	2.1
15860	1.31502	2.1
15861	1.31502	2.1
15862	1.72116	2.1
15863	1.70002	2.1
15864	1.67969	2.1
15865	1.67969	2.1
15866	1.62408	2.1
15867	1.01531	2.1
15868	1.66018	2.1
15869	1.01531	2.1
15870	0.886968	2.1
15871	1.22585	0.05
15872	1.20674	0.05
15873	1.20672	0.05
15874	1.02962	0.05
15875	0.956467	2.1
15876	2.00551	2.1
15877	0.553801	2.1
15878	0.617006	2.1
15879	1.26923	2.1
15880	1.23614	2.1
15881	1.23614	2.1
15882	1.16817	2.1
15883	2.14482	2.1
15884	1.72637	2.07452
15885	1.09368	2.1
15886	1.26195	2.1
15887	0.760546	2.1
15888	1.84094	2.1
15889	2.62868	2.1

15890	2.79784	2.1
15891	2.79784	2.1
15892	3.00129	2.1
15893	1.27593	0.930141
15894	1.38877	1.79061
15895	1.06486	0.346607
15896	1.2474	0.179486
15897	1.76234	0.05
15898	1.8685	0.05
15899	1.69863	0.05
15900	1.29666	0.05
15901	1.91646	2.1
15902	1.8582	2.1
15903	1.8582	2.1
15904	1.7986	2.1
15905	3.44043	0.05
15906	1.94764	0.05
15907	1.94764	0.05
15908	1.36307	0.05
15909	1.32075	0.05
15910	1.12971	0.05
15911	2.06319	0.05
15912	1.94982	0.05
15913	1.77255	0.05
15914	0.979297	0.05
15915	1.59997	2.1
15916	1.93506	2.1
15917	1.94409	2.1
15918	1.9597	2.1
15919	0.57899	2.1
15920	0.953036	2.1
15921	0.953049	2.1
15922	1.53503	2.1
15923	1.39615	0.068026
15924	1.06474	0.0815172
15925	0.947564	0.0887308
15926	1.45506	0.0952396
15927	1.17799	0.05
15928	1.21067	0.0732635
15929	1.35945	0.208169
15930	2.05648	0.265872
15931	1.36208	0.238415
15932	2.15229	0.180752
15933	2.89288	0.166036
15934	1.95719	0.175201
15935	2.25169	0.138393
15936	1.52756	0.155735
15937	1.19322	0.279227
15938	1.23146	0.235131
15939	2.45024	0.413219
15940	0.794122	0.24989
15941	1.6343	0.240261
15942	1.34161	0.168751
15943	1.05353	0.623543
15944	1.50795	0.152338
15945	1.05353	0.1288
15946	1.20669	0.178438
15947	1.59289	2.1

15948	0.989543	2.1
15949	1.40588	2.1
15950	0.991427	2.1
15951	0.921822	1.76434
15952	1.46903	0.342648
15953	1.46903	2.07154
15954	1.8214	0.195983
15955	1.39702	2.1
15956	1.25103	2.1
15957	1.25103	2.1
15958	1.10378	2.1
15959	1.71813	2.1
15960	1.39595	2.1
15961	1.386 2.1	
15962	1.13008	2.1
15963	1.31711	0.05
15964	1.70191	0.05
15965	0.893249	0.05
15966	1.42669	0.05
15967	1.23943	2.1
15968	1.1483	2.1
15969	1.04598	2.1
15970	1.1483	2.1
15971	1.00381	0.503149
15972	1.11433	0.184167
15973	0.973501	1.04233
15974	1.0756	0.925532
15975	1.20669	0.131781
15976	1.18508	0.170141
15977	1.18508	0.13582
15978	0.983027	0.141132
15979	1.43687	2.08407
15980	1.71059	2.1
15981	1.71059	2.1
15982	1.66762	2.1
15983	2.8415	2.1
15984	2.40555	2.1
15985	1.91546	2.1
15986	1.61385	2.1
15987	2.01569	2.1
15988	1.33183	2.1
15989	1.23218	2.1
15990	1.23999	2.1
15991	1.01335	0.454131
15992	1.12088	0.648034
15993	1.25346	2.09014
15994	1.15644	2.1
15995	1.3764	1.36441
15996	1.27558	0.937969
15997	1.2064	2.1
15998	1.15067	2.1
15999	1.01595	2.1
16000	1.15067	2.1
16001	1.41902	2.1
16002	1.30272	2.1
16003	1.30272	2.1
16004	1.19548	2.1
16005	2.99339	2.1

16006	2.98041	2.1
16007	1.46124	2.1
16008	1.42105	2.1
16009	2.98041	2.1
16010	2.86339	2.1
16011	1.42105	2.1
16012	1.42284	2.1
16013	2.31301	0.417762
16014	1.80273	0.420957
16015	2.17765	0.419275
16016	2.31301	0.373146
16017	1.76378	0.212523
16018	2.10275	0.190985
16019	2.10275	0.213606
16020	2.26047	0.214356
16021	0.97541	2.1
16022	1.40863	2.1
16023	1.40863	2.1
16024	1.30751	2.1
16025	1.20143	0.12655
16026	1.19756	0.139611
16027	1.43707	0.05
16028	1.28954	0.0505195
16029	3.34254	0.0837888
16030	1.92916	0.05
16031	1.80089	0.0568704
16032	1.1323	0.05
16033	1.47321	0.227657
16034	1.23756	0.179313
16035	1.87291	2.1
16036	1.53642	2.1
16037	1.53642	2.1
16038	1.5791	2.1
16039	1.0446	2.1
16040	0.992674	2.1
16041	1.12324	0.05
16042	1.29163	0.05
16043	1.29163	0.05
16044	1.15371	0.05
16045	0.996831	0.129022
16046	1.00192	0.154015
16047	1.0019	0.126622
16048	0.958937	0.148563
16049	1.07283	2.1
16050	1.25294	2.1
16051	1.25294	2.1
16052	1.04788	2.1
16053	1.14496	2.1
16054	1.07652	2.1
16055	1.07652	2.1
16056	0.991406	2.1
16057	1.60976	2.1
16058	1.37	2.1
16059	1.37	2.1
16060	1.14195	2.1
16061	1.26593	1.98263
16062	1.75366	2.1
16063	1.57621	0.0896243

16064	1.24538	0.0952004
16065	0.969118	2.1
16066	1.04147	2.1
16067	0.969118	2.1
16068	1.22615	2.1
16069	0.98084	2.1
16070	1.01843	2.1
16071	1.09435	2.1
16072	1.50876	2.1
16073	1.5976	2.1
16074	1.84322	2.1
16075	1.84324	2.1
16076	1.70377	2.1
16077	1.20709	2.1
16078	2.54729	2.1
16079	0.974716	0.05
16080	1.54468	0.0763624
16081	1.39021	0.05
16082	1.70303	0.0699998
16083	1.22852	1.62586
16084	1.578	0.595756
16085	1.578	1.70182
16086	1.93852	0.397066
16087	2.2506	2.1
16088	1.39302	2.07499
16089	1.5478	2.1
16090	1.2269	2.1
16091	1.70048	2.1
16092	1.45477	2.1
16093	1.30929	2.1
16094	1.15069	2.1
16095	1.00255	2.1
16096	1.05481	0.640273
16097	1.05481	2.1
16098	1.1032	0.705775
16099	0.993888	2.1
16100	1.19294	2.1
16101	1.01121	0.0766639
16102	0.991866	0.0781931
16103	2.20052	1.97228
16104	1.7409	2.1
16105	1.75142	2.1
16106	1.46811	2.1
16107	1.7409	2.08189
16108	1.23267	2.1
16109	1.46811	2.1
16110	1.18872	2.1
16111	1.12665	2.1
16112	1.50045	2.1
16113	1.50045	2.1
16114	1.37704	2.1
16115	1.35308	0.79297
16116	1.29983	0.182255
16117	1.29983	0.928681
16118	0.970212	0.211768
16119	1.82272	2.1
16120	1.62784	2.1
16121	1.62784	2.1

16122	1.87691	2.1
16123	3.65294	2.1
16124	2.33374	2.1
16125	2.12888	2.1
16126	1.62229	2.1
16127	1.41496	2.1
16128	1.32707	2.05535
16129	2.18871	1.87322
16130	1.4235	0.96865
16131	1.46015	0.073458
16132	1.25635	0.0617174
16133	1.45501	0.178282
16134	1.3543	0.204617
16135	1.38139	2.1
16136	1.98159	2.1
16137	2.15868	2.1
16138	4.04308	2.1
16139	1.5362	2.1
16140	1.56328	2.1
16141	1.56327	2.1
16142	1.58163	2.1
16143	1.88987	0.05
16144	1.08761	0.05
16145	0.862154	0.05
16146	1.77688	0.05
16147	1.14238	2.1
16148	1.11691	2.1
16149	1.01537	2.1
16150	1.02923	2.1
16151	0.977425	0.05
16152	1.15211	0.05
16153	2.0815	2.1
16154	1.18621	2.1
16155	1.01485	2.1
16156	1.17054	2.1
16157	1.17054	2.1
16158	1.12665	2.1
16159	1.87213	0.05
16160	1.82244	0.05
16161	1.45039	0.05
16162	1.7226	0.05
16163	1.841 0.05	
16164	1.79011	0.05
16165	1.8792	0.05
16166	1.45053	0.05
16167	1.08947	2.1
16168	1.5008	2.1
16169	1.5008	2.1
16170	1.83186	2.1
16171	1.04159	0.05
16172	1.2489	0.05
16173	1.2489	0.05
16174	1.33592	0.05
16175	1.51973	0.0937122
16176	0.980547	0.105901
16177	1.51974	0.116816
16178	1.15991	0.153986
16179	1.34965	2.1

16180	1.2724	2.1
16181	1.2724	2.1
16182	1.38438	2.1
16183	1.02923	2.1
16184	1.06521	2.1
16185	1.06521	2.1
16186	1.0752	2.1
16187	2.03139	0.0932799
16188	1.92951	0.0891394
16189	1.74852	0.0703296
16190	1.62772	0.0744389
16191	1.73655	0.0784749
16192	1.76809	0.0727813
16193	1.62772	0.0629002
16194	1.1432	0.0564504
16195	1.09235	2.1
16196	1.48081	2.1
16197	1.46712	2.1
16198	1.89321	2.1
16199	1.94548	2.1
16200	1.5636	2.1
16201	1.5636	2.1
16202	1.21124	2.1
16203	1.52314	2.1
16204	2.0219	2.1
16205	1.51113	2.1
16206	2.0219	2.1
16207	0.987247	0.359217
16208	1.18347	0.176316
16209	1.89226	0.490548
16210	1.61441	0.156758
16211	1.42884	2.1
16212	1.10961	2.1
16213	1.56991	2.1
16214	1.05337	2.1
16215	1.05337	2.1
16216	1.91547	2.1
16217	1.05931	2.1
16218	1.1729	2.1
16219	1.34526	2.1
16220	1.0593	2.1
16221	1.0856	2.1
16222	1.90854	2.1
16223	1.91674	2.1
16224	1.11345	2.1
16225	1.75067	2.1
16226	1.50022	2.1
16227	1.50022	2.1
16228	1.53619	2.1
16229	1.4748	2.02819
16230	1.74431	1.77682
16231	2.02581	1.42736
16232	1.25288	1.46456
16233	1.01814	0.51425
16234	1.2881	0.390805
16235	1.2881	0.317932
16236	1.54433	0.266258
16237	1.19999	0.27448

16238	1.44056	0.197235
16239	1.02011	0.163469
16240	1.17129	0.211043
16241	1.13775	1.9179
16242	3.34815	1.95376
16243	1.60428	1.86922
16244	1.85928	1.8236
16245	1.75592	2.1
16246	1.25989	2.1
16247	1.75592	2.1
16248	1.71549	2.1
16249	1.37634	2.1
16250	1.64693	2.1
16251	1.64693	2.1
16252	1.64193	2.1
16253	1.18351	0.05
16254	2.19398	0.05
16255	2.16272	0.616188
16256	1.19837	0.573425
16257	1.04973	2.1
16258	1.20149	2.1
16259	1.60574	2.1
16260	1.11164	1.06757
16261	1.87823	0.984032
16262	1.11164	1.71667
16263	0.772882	2.1
16264	1.4714	2.1
16265	2.14242	2.03106
16266	1.12336	2.1
16267	2.8386	1.62789
16268	2.13346	2.1
16269	1.06788	0.0680544
16270	0.947662	0.0633209
16271	1.05297	0.05
16272	1.42907	0.0598155
16273	2.17465	2.1
16274	2.18394	2.1
16275	1.14808	2.1
16276	1.21292	2.1
16277	1.21292	2.1
16278	1.27229	2.1
16279	1.20859	0.717727
16280	1.21073	0.460141
16281	1.21073	0.485557
16282	1.22857	0.386626
16283	1.79158	2.1
16284	1.73899	2.1
16285	1.34012	2.1
16286	1.39991	2.1
16287	1.73898	2.1
16288	1.61245	2.1
16289	1.40136	2.1
16290	1.57234	2.1
16291	1.82162	2.1
16292	3.09875	2.1
16293	1.10264	2.1
16294	1.02339	2.1
16295	1.02339	2.1

16296	1.23042	2.1
16297	2.82266	0.342385
16298	1.05698	0.206777
16299	1.96384	0.319508
16300	1.10244	0.276541
16301	0.929061	2.1
16302	1.47007	2.1
16303	0.985303	2.1
16304	1.24554	2.1
16305	1.05993	2.1
16306	2.02074	2.1
16307	2.10106	0.299854
16308	1.18171	0.184618
16309	1.68111	0.319689
16310	1.84226	0.349958
16311	1.82131	0.131994
16312	1.32371	0.0735459
16313	1.41902	0.0692082
16314	1.00205	0.05
16315	1.65532	2.1
16316	1.18977	2.1
16317	1.15471	2.1
16318	0.993889	2.1
16319	2.08521	2.1
16320	2.09885	2.1
16321	2.09497	2.1
16322	2.10604	2.1
16323	0.881028	0.358834
16324	1.52828	0.422588
16325	1.40574	0.05
16326	1.09599	0.05
16327	1.45506	2.1
16328	1.50524	2.1
16329	1.50524	2.1
16330	1.54711	2.1
16331	1.41469	2.1
16332	2.40442	2.1
16333	1.40794	2.1
16334	1.89226	2.1
16335	1.31346	2.1
16336	1.51067	2.1
16337	1.31363	2.1
16338	1.11833	2.1
16339	2.3373	2.1
16340	2.19994	2.1
16341	2.19994	2.1
16342	1.09616	2.1
16343	1.63719	2.1
16344	1.52204	2.1
16345	1.52205	2.1
16346	1.40118	2.1
16347	2.5946	2.1
16348	4.07065	2.1
16349	1.33916	2.1
16350	2.05217	2.1
16351	1.67023	2.1
16352	2.05217	2.1
16353	1.59458	0.561087

16354	1.02247	0.405092
16355	1.68108	0.282068
16356	1.03079	0.19885
16357	1.1643	0.0627947
16358	1.85795	0.0666073
16359	1.70302	0.168894
16360	1.42076	0.130098
16361	1.91351	0.140438
16362	1.01148	0.123994
16363	2.52402	0.05
16364	0.752625	0.05
16365	0.886968	2.1
16366	1.28845	2.1
16367	1.28845	2.1
16368	1.27766	2.1
16369	1.82268	2.1
16370	1.87118	2.1
16371	1.87119	2.1
16372	1.91704	2.1
16373	3.13938	2.1
16374	2.97839	2.1
16375	2.97839	2.1
16376	2.87331	2.1
16377	1.73443	1.22318
16378	1.59457	0.690428
16379	2.01827	2.1
16380	2.04586	2.1
16381	2.04586	2.1
16382	2.00865	2.1
16383	1.19901	2.07798
16384	1.16661	2.1
16385	1.13215	0.191794
16386	1.96256	0.128567
16387	2.34043	0.05
16388	2.04154	0.05
16389	1.58991	0.05
16390	1.45893	0.05
16391	2.56818	0.05
16392	1.70532	0.05
16393	1.17798	0.0569394
16394	1.25796	0.0662159
16395	1.25797	0.0704394
16396	1.10719	0.0796997
16397	1.51067	2.1
16398	1.60707	2.1
16399	2.06946	2.09171
16400	3.19218	2.1
16401	2.19965	2.1
16402	1.30521	2.1
16403	2.23684	1.99277
16404	1.43363	2.06508
16405	1.51007	2.1
16406	2.46356	2.1
16407	1.90428	2.1
16408	1.13618	2.1
16409	2.13276	0.0508908
16410	0.998345	0.0548699
16411	1.2861	0.05

16412	1.20457	0.05
16413	1.51789	2.1
16414	1.49872	2.1
16415	1.49872	2.1
16416	1.47712	2.1
16417	1.87129	0.0574946
16418	0.949364	0.0864472
16419	1.83142	0.0630356
16420	1.55063	0.0937387
16421	1.03298	0.107598
16422	0.980664	0.194103
16423	1.51696	0.0777952
16424	1.34927	0.14635
16425	1.70302	0.329735
16426	1.65952	0.229507
16427	1.76658	0.188388
16428	0.986848	0.137326
16429	2.13757	1.67037
16430	1.98142	0.727579
16431	1.98142	1.45131
16432	1.65411	0.726498
16433	1.1323	0.0684097
16434	1.46015	0.0626622
16435	2.15376	1.58996
16436	1.64622	1.7122
16437	0.991867	0.05
16438	1.58074	0.0566465
16439	1.69368	2.1
16440	1.76403	2.1
16441	1.76403	2.1
16442	1.83318	2.1
16443	1.28418	0.565208
16444	1.16851	0.411293
16445	1.1685	0.265197
16446	1.1726	0.229713
16447	1.06455	2.1
16448	1.18773	2.1
16449	0.98511	2.1
16450	1.18774	2.1
16451	1.76476	2.1
16452	1.79825	2.1
16453	1.79825	2.1
16454	0.961895	2.1
16455	1.59589	0.05
16456	2.25975	0.05
16457	2.76339	2.1
16458	3.0551	2.1
16459	1.68761	2.1
16460	1.59561	2.1
16461	2.17257	2.1
16462	1.05845	2.1
16463	1.03512	2.1
16464	2.19011	2.1
16465	1.44859	2.1
16466	1.35897	0.139531
16467	1.35897	2.00536
16468	1.17822	0.29044
16469	1.13641	2.1

16470	1.18301	2.1
16471	1.18301	2.1
16472	1.25173	2.1
16473	2.41297	2.1
16474	1.36463	2.1
16475	1.54711	2.1
16476	1.5735	2.1
16477	1.5735	2.1
16478	1.58713	2.1
16479	1.69044	0.05
16480	1.57979	0.05
16481	1.57979	0.05
16482	1.14387	0.05
16483	1.04199	2.1
16484	1.96491	2.1
16485	1.04199	2.1
16486	1.46892	2.1
16487	0.983724	2.1
16488	1.70579	2.1
16489	1.88522	2.1
16490	1.23914	2.1
16491	1.79424	2.1
16492	1.52851	2.1
16493	1.04921	2.1
16494	1.11285	2.1
16495	1.25334	0.495078
16496	1.07496	0.184384
16497	1.07497	2.04326
16498	1.34852	0.403822
16499	1.27391	2.1
16500	2.20307	2.1
16501	1.81311	2.1
16502	2.10305	2.1
16503	1.21059	0.0677417
16504	1.44253	0.095547
16505	1.9194	0.0533328
16506	1.21159	0.0781617
16507	0.947979	2.1
16508	1.01225	2.01612
16509	0.947979	2.1
16510	1.01225	2.1
16511	1.00255	2.1
16512	1.01225	2.1
16513	1.01225	2.1
16514	1.06561	2.1
16515	3.50562	2.1
16516	3.61336	2.1
16517	3.61336	2.1
16518	3.73635	2.1
16519	1.61245	2.1
16520	1.52301	2.1
16521	1.57612	2.1
16522	1.83035	2.1
16523	1.52301	2.1
16524	1.24789	2.1
16525	1.85156	2.1
16526	1.89072	2.1
16527	0.995735	2.1

16528	1.57738	2.1
16529	1.87141	0.05
16530	1.73388	0.05
16531	1.61344	0.05
16532	0.922676	0.05
16533	1.56614	2.1
16534	0.956294	2.1
16535	1.82589	2.1
16536	1.63719	2.1
16537	1.12399	0.05
16538	1.50103	0.05
16539	1.56976	0.05
16540	1.03567	0.05
16541	1.21776	0.05
16542	1.89708	0.05
16543	1.16323	0.05
16544	1.63533	0.05
16545	2.29397	0.05
16546	3.01596	0.05
16547	1.80145	0.05
16548	1.7666	0.05
16549	1.91621	0.071377
16550	1.10719	0.0751962
16551	1.57828	0.358514
16552	1.17105	0.112505
16553	1.15806	2.1
16554	1.31565	2.1
16555	1.31565	2.1
16556	1.46643	2.1
16557	1.47507	2.1
16558	1.87858	2.1
16559	0.958661	2.1
16560	1.87858	2.1
16561	1.44293	0.05
16562	1.43213	0.0555452
16563	1.43213	0.05
16564	1.10476	0.05
16565	1.03235	1.93113
16566	1.31	0.265705
16567	1.31	1.97528
16568	1.50108	0.28295
16569	1.32215	2.1
16570	1.10611	2.1
16571	1.10611	2.1
16572	0.904978	2.1
16573	1.06486	0.329637
16574	1.18347	0.181294
16575	1.70535	2.1
16576	1.94779	2.1
16577	1.77071	2.1
16578	1.82268	2.1
16579	0.61701	2.1
16580	0.672495	2.1
16581	0.672494	2.1
16582	0.713174	2.1
16583	1.40395	2.1
16584	1.34245	2.1
16585	1.34243	2.1

16586	1.30646	2.1
16587	1.06204	0.247145
16588	0.632691	0.59108
16589	1.75766	0.801521
16590	1.14287	1.79753
16591	1.52101	0.0953485
16592	1.71414	0.0963382
16593	1.86411	0.10773
16594	1.6253	0.094179
16595	1.04355	0.101503
16596	1.0443	0.0989816
16597	1.34852	2.09855
16598	1.56016	0.164458
16599	1.56016	0.288202
16600	1.03235	0.16588
16601	1.2966	0.05
16602	1.82354	0.05
16603	1.2966	0.05
16604	1.67687	0.05
16605	1.19669	0.72375
16606	1.82979	0.785885
16607	1.58972	1.06947
16608	1.09219	0.900084
16609	1.42077	0.895171
16610	1.89226	0.787277
16611	1.19547	2.1
16612	1.10187	2.1
16613	1.10188	2.1
16614	1.01479	2.1
16615	1.81663	2.1
16616	1.81533	2.1
16617	1.83247	2.1
16618	1.51511	2.1
16619	0.983148	2.1
16620	1.7513	2.1
16621	1.85017	2.1
16622	1.26103	2.1
16623	1.23152	2.1
16624	1.65508	2.1
16625	1.23152	2.1
16626	2.15082	2.1
16627	1.57974	2.1
16628	1.07289	2.1
16629	0.81534	2.1
16630	1.51007	2.1
16631	1.00898	0.05
16632	1.18959	0.05
16633	1.32178	0.05
16634	1.25929	0.05
16635	2.17892	0.389033
16636	1.22938	0.497756
16637	3.02933	0.985198
16638	2.34619	0.527493
16639	1.47679	0.0571874
16640	1.0819	0.0780586
16641	3.10558	2.1
16642	3.31457	2.1
16643	0.985109	2.1

16644	1.10667	2.1
16645	3.31457	2.1
16646	3.54443	2.1
16647	1.10667	2.1
16648	1.25987	2.1
16649	1.77063	1.11345
16650	1.51065	0.696314
16651	1.24688	2.1
16652	1.37652	2.1
16653	1.37652	2.1
16654	1.54168	2.1
16655	1.17042	1.10254
16656	1.13092	1.64892
16657	1.13092	1.81866
16658	1.52938	2.08591
16659	1.11787	0.934542
16660	1.00613	2.1
16661	0.781373	0.207246
16662	1.6885	0.205234
16663	2.49439	0.0934475
16664	1.42657	0.144677
16665	1.77903	2.1
16666	2.63606	2.1
16667	2.10425	2.1
16668	1.21425	2.1
16669	1.31253	1.14308
16670	1.28418	0.832274
16671	1.28418	1.20948
16672	1.58972	1.13717
16673	0.858948	2.1
16674	1.90196	2.1
16675	1.81024	2.1
16676	1.90194	2.1
16677	1.4263	1.69144
16678	0.947921	1.86585
16679	1.22852	0.388753
16680	1.09194	0.239796
16681	1.60578	0.0661231
16682	1.14721	0.0733696
16683	1.06336	0.110336
16684	1.14721	0.0874934
16685	1.11924	0.05
16686	1.53814	0.05
16687	1.18421	0.05
16688	1.8458	0.05
16689	1.7565	1.93502
16690	1.09344	1.06308
16691	1.09344	1.16542
16692	1.16971	1.94252
16693	1.71465	0.05
16694	1.19412	0.05
16695	1.64985	0.05
16696	1.05417	0.05
16697	1.2127	0.05
16698	1.48867	0.05
16699	1.27806	0.05
16700	1.21269	0.05
16701	1.37813	1.03547

16702	1.0417	1.05532
16703	1.88626	0.241419
16704	1.14977	0.74156
16705	1.78803	2.1
16706	1.33793	2.07696
16707	1.42266	2.09968
16708	1.26865	2.01989
16709	1.09518	2.1
16710	1.36844	2.1
16711	1.20455	2.1
16712	1.36844	2.1
16713	1.22297	1.11698
16714	1.11256	1.41702
16715	1.11256	0.928912
16716	1.03217	1.16065
16717	1.18422	0.05
16718	1.4282	0.05
16719	1.04159	0.05
16720	1.4282	0.05
16721	0.988805	2.1
16722	1.05481	2.1
16723	1.52938	1.21753
16724	1.26593	1.77665
16725	1.53082	2.1
16726	1.09847	2.1
16727	1.75211	2.02993
16728	1.22222	2.1
16729	1.75211	1.37371
16730	1.51592	2.1
16731	1.63413	0.540819
16732	1.26662	0.562474
16733	1.42495	0.380593
16734	1.4356	0.425614
16735	1.29389	1.63425
16736	1.04164	1.98794
16737	1.40574	1.79661
16738	1.04164	1.65627
16739	2.22344	0.635713
16740	1.45945	0.147355
16741	0.987248	0.369806
16742	1.2474	0.186852
16743	0.974702	0.456183
16744	1.43777	0.287283
16745	1.71289	2.1
16746	1.91529	2.1
16747	1.91529	2.1
16748	2.01827	2.1
16749	1.87111	0.689288
16750	1.00864	0.297674
16751	1.26865	0.431264
16752	1.18769	0.686794
16753	1.24167	2.1
16754	1.36133	1.97551
16755	2.28168	2.1
16756	3.98524	2.1
16757	1.36133	2.1
16758	1.69436	1.75366
16759	2.21926	2.1

16760	1.70302	2.1
16761	1.50732	0.0520797
16762	1.42237	0.0599273
16763	1.18659	0.0679389
16764	1.50732	0.0742371
16765	1.19785	0.05
16766	1.40969	0.05
16767	1.28339	0.0582306
16768	1.40882	0.063549
16769	0.968595	2.1
16770	1.41903	2.1
16771	1.12567	2.1
16772	1.15898	2.1
16773	1.27241	2.1
16774	0.995796	2.1
16775	1.89983	0.05
16776	1.05417	0.05
16777	0.571999	0.05
16778	0.743597	0.05
16779	1.89353	0.05
16780	0.924068	0.05
16781	1.52851	2.1
16782	1.26969	2.1
16783	1.07763	0.202874
16784	1.82301	0.128669
16785	1.07763	0.106518
16786	1.17506	0.107346
16787	1.20045	2.1
16788	1.12486	2.1
16789	1.15234	2.1
16790	1.11598	2.1
16791	1.12486	2.1
16792	1.0491	2.1
16793	1.11598	2.1
16794	1.1047	2.1
16795	1.05902	2.1
16796	1.14361	2.1
16797	1.14361	2.1
16798	1.26851	2.1
16799	1.29389	1.33087
16800	0.988456	1.21991
16801	1.83688	0.05
16802	1.70302	0.05
16803	1.49386	0.05
16804	1.83151	0.05
16805	1.01346	0.930511
16806	0.983607	0.964712
16807	0.983596	2.1
16808	1.08332	2.00849
16809	2.36848	0.110404
16810	2.97066	0.102422
16811	1.12352	0.0648616
16812	2.5311	0.0586079
16813	1.09062	2.1
16814	0.962182	2.1
16815	0.962181	2.1
16816	1.2258	2.1
16817	0.974888	2.1

16818	1.06312	2.1
16819	1.73583	0.05
16820	1.72399	0.05
16821	1.724	0.0532093
16822	1.50951	0.0560852
16823	1.26755	2.1
16824	1.03824	2.1
16825	1.27875	2.1
16826	1.03824	2.1
16827	1.18902	2.1
16828	1.534	2.1
16829	1.11764	2.1
16830	1.534	2.1
16831	1.52008	2.1
16832	1.34406	2.1
16833	1.34406	2.1
16834	1.2187	2.1
16835	0.963571	1.26264
16836	1.19612	0.912744
16837	0.963571	2.05399
16838	1.00007	2.1
16839	0.903333	2.1
16840	1.12724	2.1
16841	1.23771	2.1
16842	1.17683	2.1
16843	1.20059	0.556551
16844	0.772023	0.2725
16845	1.14749	0.832895
16846	1.2802	0.715578
16847	1.85091	1.96885
16848	2.11193	1.7503
16849	1.2802	0.623423
16850	1.42913	0.530024
16851	2.11193	1.167
16852	1.27737	1.00132
16853	1.30584	2.1
16854	1.60936	2.1
16855	1.3413	0.0907837
16856	1.53336	0.057752
16857	2.60679	0.0582787
16858	1.3413	0.05
16859	1.35877	2.1
16860	1.11024	2.1
16861	1.27839	2.1
16862	1.08171	2.1
16863	1.27841	2.1
16864	1.0577	2.1
16865	1.71255	2.1
16866	1.10611	2.1
16867	0.960106	2.1
16868	1.40921	2.1
16869	1.90705	0.109077
16870	2.79564	0.102123
16871	1.45321	0.116139
16872	1.90705	0.104876
16873	0.974718	0.0756723
16874	1.53671	0.0634993
16875	1.69039	0.0683278

16876	1.78229	0.0548028
16877	1.0101	2.1
16878	1.3167	2.1
16879	1.19699	2.1
16880	1.3212	2.1
16881	1.60811	0.0969316
16882	1.43224	0.0635865
16883	1.35401	0.0604693
16884	1.17464	0.05
16885	1.27696	2.1
16886	1.05025	2.1
16887	1.05025	2.1
16888	1.18076	2.1
16889	1.37224	1.66524
16890	1.13438	1.61538
16891	1.76788	1.65276
16892	1.73444	1.38352
16893	1.37704	2.1
16894	1.77042	2.1
16895	1.77042	2.1
16896	1.09778	2.1
16897	1.28667	2.1
16898	1.28228	2.1
16899	1.30646	0.162202
16900	1.68867	0.11676
16901	1.30645	0.67728
16902	0.986494	0.307253
16903	1.6253	0.0914933
16904	1.48676	0.0915181
16905	1.48676	0.083918
16906	1.32974	0.0803079
16907	0.634347	0.05
16908	1.15142	0.05
16909	1.11395	0.05
16910	1.18068	0.05
16911	1.36904	0.05
16912	0.764563	0.05
16913	2.09213	0.05
16914	1.56631	2.1
16915	1.08011	2.1
16916	1.80283	2.1
16917	1.50738	2.1
16918	1.44951	0.103563
16919	1.50102	0.0935242
16920	1.50102	0.0854403
16921	1.39615	0.0890037
16922	1.02653	0.343576
16923	2.98258	0.343363
16924	2.0815	0.05
16925	1.39525	0.05
16926	1.82176	0.05
16927	0.977607	0.05
16928	1.96133	1.81917
16929	1.27189	1.07292
16930	0.826032	1.11358
16931	2.12831	0.961267
16932	1.77798	2.05513
16933	1.02969	1.79069

16934	1.06463	1.65905
16935	1.49219	2.01388
16936	1.10003	2.1
16937	1.21223	2.1
16938	1.00513	1.11337
16939	1.43788	2.0995
16940	1.47134	0.888094
16941	0.968249	0.939252
16942	3.22585	0.113777
16943	3.36933	0.0530748
16944	3.36932	0.111956
16945	3.03865	0.05
16946	1.66295	0.332581
16947	1.65583	0.219363
16948	1.65583	0.338949
16949	1.4732	0.222817
16950	1.73704	0.05
16951	1.76476	2.1
16952	2.13345	1.95304
16953	1.55816	2.1
16954	1.55816	2.06607
16955	1.02601	2.1
16956	1.08039	2.1
16957	1.05498	1.93367
16958	1.08039	2.1
16959	1.2873	2.1
16960	1.01121	2.1
16961	0.987703	2.1
16962	0.98771	2.1
16963	0.964782	2.1
16964	1.35719	2.1
16965	2.61092	2.1
16966	2.6607	2.1
16967	3.41693	2.1
16968	1.23125	2.1
16969	1.73772	2.1
16970	0.99845	0.249994
16971	1.5176	0.343057
16972	1.13271	0.05
16973	1.332	0.05
16974	1.63159	2.1
16975	1.61342	2.1
16976	1.03679	2.1
16977	1.19087	2.1
16978	1.97172	2.1
16979	2.68634	2.1
16980	1.19086	2.1
16981	1.50091	2.1
16982	2.84677	2.1
16983	2.77894	2.1
16984	2.77894	2.1
16985	2.74206	2.1
16986	1.3284	2.1
16987	1.83524	2.1
16988	1.65853	2.1
16989	1.56741	2.1
16990	1.00509	2.1
16991	1.45246	2.1

16992	1.26193	2.1
16993	1.45244	2.1
16994	1.61963	0.440779
16995	1.68189	0.365188
16996	1.68189	1.62646
16997	1.49155	1.14862
16998	1.58054	2.1
16999	1.59169	2.1
17000	1.59169	2.1
17001	1.60497	2.1
17002	0.743595	0.05
17003	0.702359	0.05
17004	1.15317	0.05
17005	2.24367	0.05
17006	1.15252	0.05
17007	2.60354	0.05
17008	1.45158	2.1
17009	1.56004	2.1
17010	1.64527	2.1
17011	1.00371	2.1
17012	1.38287	0.438651
17013	0.893024	0.658856
17014	1.23527	1.48005
17015	1.23706	0.688938
17016	2.20993	0.465106
17017	1.54858	1.69996
17018	0.798666	0.15539
17019	1.22487	0.150738
17020	1.33158	0.121968
17021	1.85902	0.122959
17022	1.08334	2.1
17023	1.17885	1.94498
17024	1.17885	1.4571
17025	1.07283	1.69588
17026	1.30723	0.05
17027	1.62507	0.05
17028	1.62507	0.05
17029	1.08854	0.05
17030	1.59475	2.1
17031	1.58874	2.09783
17032	1.58875	2.08935
17033	1.35787	2.04435
17034	1.04147	2.1
17035	1.15564	2.1
17036	1.78301	2.1
17037	1.90618	2.1
17038	1.71555	2.1
17039	2.13143	2.1
17040	1.10817	2.1
17041	1.032	2.1
17042	1.032	2.1
17043	0.953636	2.1
17044	1.27916	1.15106
17045	1.11938	0.920944
17046	1.11937	0.893188
17047	1.9186	0.926978
17048	1.19967	0.0730366
17049	1.63489	0.0805757

17050	1.00763	0.0984077
17051	1.81656	0.105785
17052	1.31814	0.0720857
17053	2.98183	0.0779942
17054	1.20149	2.1
17055	1.29129	2.1
17056	1.29129	2.1
17057	1.37138	2.1
17058	1.23104	0.61192
17059	1.75236	0.955656
17060	1.6	0.234308
17061	1.60565	0.384049
17062	1.60565	0.351269
17063	1.40013	0.454658
17064	1.23042	2.1
17065	1.18278	2.1
17066	1.10853	2.1
17067	1.04724	2.1
17068	1.1766	2.1
17069	1.00319	2.1
17070	1.29435	2.1
17071	1.00319	2.1
17072	1.05655	1.70949
17073	1.01606	1.72778
17074	1.80391	2.1
17075	2.7699	2.1
17076	1.52073	2.1
17077	2.06835	2.1
17078	2.00202	2.1
17079	2.04906	2.1
17080	0.968536	2.1
17081	1.02461	2.1
17082	1.59784	2.1
17083	1.78249	2.1
17084	1.81409	2.1
17085	1.79844	2.1
17086	1.77093	2.01589
17087	2.02356	1.79673
17088	1.32449	2.1
17089	1.10003	2.1
17090	1.16915	0.481086
17091	1.05937	0.407591
17092	1.28096	2.0924
17093	1.34696	1.94304
17094	1.34695	2.09987
17095	1.36457	1.87229
17096	1.06478	2.1
17097	1.06239	2.1
17098	1.06239	2.1
17099	1.09629	2.1
17100	0.798209	2.02935
17101	1.8532	2.1
17102	2.10112	0.05
17103	2.8704	0.05
17104	2.8704	0.05
17105	2.92457	0.05
17106	1.0752	2.1
17107	1.07745	2.1

17108	1.07745	2.1
17109	1.08911	2.1
17110	1.40182	2.1
17111	1.00133	2.1
17112	1.40182	2.1
17113	1.24509	2.1
17114	2.22243	0.05
17115	1.07303	0.05
17116	1.29736	0.05
17117	1.73584	0.05
17118	2.0815	0.324389
17119	1.07999	0.19598
17120	2.1692	2.1
17121	1.99992	2.1
17122	1.99992	2.1
17123	1.78327	2.1
17124	1.52661	0.05
17125	0.956003	0.05
17126	1.53172	0.05
17127	0.955991	0.05
17128	1.35025	2.1
17129	1.51084	2.1
17130	1.51084	2.1
17131	1.67115	2.1
17132	2.00977	2.1
17133	1.63632	2.1
17134	1.63632	2.1
17135	2.66128	2.1
17136	1.13525	2.1
17137	2.37566	2.1
17138	1.25987	2.1
17139	1.4472	2.1
17140	1.4472	2.1
17141	1.66018	2.1
17142	0.994001	0.645085
17143	1.83151	0.604115
17144	1.83151	0.192705
17145	1.57621	0.202265
17146	1.40915	0.392524
17147	1.24653	1.93919
17148	1.24653	0.251137
17149	1.10609	2.07493
17150	1.61121	2.1
17151	1.74472	2.1
17152	1.74472	2.1
17153	1.0368	2.1
17154	1.04788	2.1
17155	1.28522	2.1
17156	1.28522	2.1
17157	1.54196	2.1
17158	1.23387	2.1
17159	0.896519	2.1
17160	1.60428	2.1
17161	1.08467	2.1
17162	1.56875	0.276085
17163	1.39072	0.206623
17164	1.39073	0.146608
17165	1.6	0.266877

17166	1.01346	2.09232
17167	1.06838	0.975159
17168	0.968463	1.17881
17169	1.06837	1.07218
17170	3.22433	0.0818956
17171	2.05993	1.96567
17172	2.01836	0.219777
17173	1.66154	1.96177
17174	1.20871	2.09956
17175	1.10586	2.1
17176	1.10586	2.09867
17177	1.03853	2.1
17178	1.12076	2.1
17179	1.03899	2.1
17180	1.03899	2.1
17181	0.963973	2.1
17182	0.987132	2.1
17183	1.0894	2.1
17184	2.37823	1.89638
17185	1.75647	1.25781
17186	1.64625	2.1
17187	1.56195	2.1
17188	1.41995	2.1
17189	1.15171	2.1
17190	1.36457	2.1
17191	1.33847	1.56359
17192	1.33847	2.06542
17193	0.869601	1.23335
17194	1.42255	2.1
17195	1.3868	2.1
17196	1.3868	2.1
17197	1.33962	2.1
17198	1.05481	2.1
17199	1.08074	2.1
17200	1.08074	2.1
17201	0.857557	2.1
17202	1.13537	1.57943
17203	1.09194	0.162042
17204	1.01179	1.90086
17205	1.13537	0.23889
17206	1.15645	1.95062
17207	1.56409	0.196088
17208	1.56409	0.73077
17209	1.16239	1.00621
17210	1.10746	0.377176
17211	1.47952	0.487315
17212	1.14733	2.01458
17213	3.77068	2.1
17214	1.38497	2.1
17215	1.54599	2.1
17216	1.54601	2.1
17217	1.27697	2.1
17218	1.01393	0.313923
17219	1.10558	0.749896
17220	0.995012	0.16692
17221	1.17624	0.680656
17222	1.60497	2.1
17223	1.61588	2.1

17224	1.61587	2.1
17225	1.62182	2.1
17226	1.48866	0.05
17227	1.65977	0.05
17228	1.65977	0.05
17229	1.69044	0.05
17230	0.961892	2.1
17231	1.00659	2.1
17232	1.0066	2.1
17233	1.06226	2.1
17234	1.15171	2.1
17235	1.50853	2.1
17236	1.50853	2.1
17237	1.2955	2.1
17238	1.3204	0.05
17239	2.13295	0.05
17240	2.75524	0.05
17241	1.18156	0.05
17242	1.73729	2.1
17243	1.20356	2.1
17244	1.62483	2.1
17245	1.52008	2.1
17246	1.19964	2.1
17247	1.0577	2.1
17248	1.19964	2.1
17249	1.07156	2.1
17250	1.98241	2.03656
17251	1.43687	1.98717
17252	1.27863	2.1
17253	1.19915	2.06408
17254	0.97269	2.05133
17255	1.0739	1.81508
17256	1.1427	1.98857
17257	1.22476	0.368327
17258	1.06584	1.85958
17259	1.22626	1.57924
17260	1.31388	2.1
17261	1.16932	2.1
17262	1.28819	2.1
17263	1.0499	2.1
17264	1.55894	2.1
17265	1.78709	2.1
17266	1.75986	2.1
17267	1.764 2.1	
17268	1.44801	2.05878
17269	1.28186	2.05013
17270	1.20797	1.79922
17271	1.06821	1.97137
17272	1.87287	0.228874
17273	2.08014	0.248726
17274	1.63225	0.248827
17275	1.39501	0.25005
17276	1.20132	0.05
17277	1.16182	0.05
17278	1.08848	0.05
17279	1.20132	0.05
17280	1.23475	2.1
17281	1.16661	2.1

17282	2.31317	2.1
17283	2.50145	2.1
17284	1.04367	2.1
17285	2.23875	2.1
17286	0.953632	0.213842
17287	1.38646	0.318046
17288	1.38646	0.352837
17289	1.61963	0.354699
17290	1.62934	1.5917
17291	1.88626	1.35635
17292	1.0775	0.833825
17293	1.119	0.433767
17294	1.08133	0.227829
17295	1.48312	0.195993
17296	1.57125	0.118999
17297	1.74098	0.121919
17298	1.94372	1.9089
17299	1.22237	1.93791
17300	1.18549	0.838782
17301	1.01294	2.01304
17302	1.01294	2.06977
17303	1.2064	2.1
17304	1.14599	2.1
17305	1.00065	2.03179
17306	1.76357	2.1
17307	2.23468	2.1
17308	2.21735	2.1
17309	3.32769	2.1
17310	1.19647	0.702902
17311	1.35307	0.234228
17312	1.19645	0.28007
17313	1.43778	0.210307
17314	1.34412	1.20324
17315	1.1315	2.1
17316	1.1315	1.44419
17317	1.23943	1.05127
17318	1.57829	2.06433
17319	1.22537	2.09528
17320	1.14357	2.06732
17321	1.32772	1.99022
17322	1.4259	2.1
17323	1.34245	2.1
17324	1.47953	2.1
17325	1.70303	2.1
17326	2.21127	0.0606752
17327	1.47904	0.0655917
17328	1.18793	0.05
17329	2.21126	0.0641159
17330	1.1032	2.0808
17331	1.14986	1.05405
17332	1.14986	2.1
17333	1.19611	0.727674
17334	1.91883	2.1
17335	1.83019	2.1
17336	1.83019	2.1
17337	1.82462	2.1
17338	1.27362	0.160738
17339	1.27421	0.30471

17340	1.27421	0.172866
17341	1.10736	0.351936
17342	1.3003	0.637357
17343	1.31415	1.41911
17344	1.31415	0.788745
17345	1.32201	1.57944
17346	1.21124	2.1
17347	0.81568	2.1
17348	0.81568	2.1
17349	0.551816	2.1
17350	1.67119	2.1
17351	1.63384	2.1
17352	1.63384	2.1
17353	1.63661	2.1
17354	1.48734	2.1
17355	1.52245	2.1
17356	1.52245	2.1
17357	1.50714	2.1
17358	0.996778	0.055391
17359	1.56091	0.05
17360	1.56091	0.05
17361	1.90457	0.05
17362	1.1181	2.1
17363	1.06543	2.1
17364	2.66828	2.1
17365	2.76932	2.1
17366	1.06543	2.1
17367	1.01474	2.1
17368	2.76932	2.1
17369	2.86704	2.1
17370	1.29724	0.0848317
17371	1.96938	0.069336
17372	1.82462	2.1
17373	1.84553	2.1
17374	1.05556	2.1
17375	1.25363	2.1
17376	1.84552	2.1
17377	1.53208	2.1
17378	1.12826	2.1
17379	1.39075	2.1
17380	0.9702	0.791299
17381	1.16481	0.293029
17382	1.08894	1.06606
17383	1.91756	1.05895
17384	1.50865	0.471823
17385	1.06012	0.379549
17386	1.5456	0.05
17387	1.37427	0.05
17388	1.1211	0.05
17389	1.5456	0.05
17390	1.33135	0.05
17391	1.07956	0.05
17392	2.23255	0.05
17393	1.21031	0.05
17394	1.52903	2.1
17395	1.35977	2.1
17396	3.3837	2.1
17397	3.20581	2.1

17398	1.35977	2.1
17399	1.15414	2.1
17400	3.20582	2.1
17401	2.07596	2.1
17402	1.6812	0.081577
17403	1.03939	0.0664613
17404	1.30047	2.1
17405	1.5586	2.1
17406	1.5586	2.1
17407	1.84214	2.1
17408	1.31825	0.844592
17409	1.31923	1.88789
17410	1.31924	0.843786
17411	1.33026	1.79307
17412	1.15818	0.444401
17413	1.17822	0.602356
17414	1.15818	2.00545
17415	1.28096	1.32765
17416	1.06848	0.0632684
17417	1.89639	0.0834255
17418	1.9186	1.05509
17419	1.77065	0.771754
17420	1.59103	0.642476
17421	1.56164	0.427572
17422	1.09697	0.05
17423	2.38719	0.05
17424	2.22123	0.05
17425	2.46059	0.05
17426	1.45506	0.134175
17427	1.03645	0.139414
17428	1.30347	2.1
17429	1.2627	2.1
17430	1.2627	2.1
17431	1.23036	2.1
17432	1.41469	2.1
17433	1.45257	2.1
17434	1.46907	0.05
17435	1.70302	0.05
17436	1.49196	0.05
17437	1.36307	0.05
17438	1.05677	2.1
17439	0.978698	2.1
17440	0.978699	2.1
17441	2.01822	2.1
17442	1.47446	2.1
17443	1.55432	2.1
17444	1.55432	2.1
17445	1.06053	2.1
17446	1.86813	1.89503
17447	1.21892	1.97481
17448	1.53064	1.28241
17449	1.30664	1.37977
17450	1.09629	2.1
17451	1.19244	2.1
17452	1.19244	2.1
17453	1.37635	2.1
17454	1.49358	0.101144
17455	1.57702	0.0670789

17456	2.09374	2.1
17457	1.88175	2.1
17458	1.88175	2.1
17459	1.51085	2.1
17460	1.10938	0.176739
17461	1.02478	1.60839
17462	1.33061	0.05
17463	1.13272	0.05
17464	1.75505	2.1
17465	1.78529	2.1
17466	1.6353	2.1
17467	1.59307	2.1
17468	1.60017	0.363076
17469	1.35608	0.427733
17470	1.35608	0.150187
17471	1.04713	0.162929
17472	1.01479	1.91325
17473	1.3868	1.45796
17474	1.3868	1.489
17475	1.28418	2.1
17476	2.15949	2.1
17477	2.90429	2.1
17478	1.51902	2.1
17479	1.54755	2.1
17480	2.90429	2.1
17481	3.49966	2.1
17482	1.70842	2.1
17483	1.97526	2.1
17484	0.949712	0.0713723
17485	1.92784	0.0721835
17486	1.34093	0.0717971
17487	2.53426	0.0943374
17488	1.92783	0.0685949
17489	3.89373	0.0630595
17490	2.53426	0.078047
17491	2.35961	0.0785759
17492	1.68108	0.21297
17493	1.15558	0.182178
17494	1.67265	0.187828
17495	1.27608	0.157708
17496	1.42249	2.1
17497	1.39708	2.1
17498	1.39708	2.1
17499	1.39327	2.1
17500	1.97046	0.05
17501	1.11688	0.05
17502	1.28418	1.17229
17503	1.17302	2.1
17504	1.17302	1.37019
17505	1.04349	2.1
17506	0.979853	2.1
17507	1.26016	2.1
17508	1.18901	2.1
17509	1.26016	2.1
17510	1.22857	0.32371
17511	1.25848	0.374724
17512	1.25848	0.244337
17513	1.2858	0.503354

17514	1.09154	2.1
17515	2.01915	2.1
17516	1.23013	2.1
17517	1.55698	2.1
17518	1.55698	2.1
17519	1.8575	2.1
17520	2.01854	2.1
17521	1.20686	2.05162
17522	1.20684	2.02762
17523	1.77796	2.09646
17524	1.46129	2.1
17525	1.04193	2.1
17526	1.10051	2.1
17527	0.983723	2.1
17528	1.1729	2.1
17529	0.98505	2.1
17530	0.985052	2.1
17531	1.03951	2.1
17532	1.03949	2.1
17533	1.17583	2.1
17534	1.42866	2.1
17535	1.97261	2.1
17536	1.73274	2.1
17537	1.53629	2.1
17538	1.98182	2.1
17539	1.72511	2.1
17540	1.42174	2.1
17541	1.44472	2.1
17542	1.72511	2.1
17543	1.45411	2.1
17544	1.44472	2.1
17545	1.43836	2.1
17546	0.962703	0.465614
17547	1.0171	0.893853
17548	1.53336	0.05
17549	1.40274	0.05
17550	1.40272	0.05
17551	1.00213	0.05
17552	1.50892	0.05
17553	1.64984	0.05
17554	1.67464	0.05
17555	1.67481	0.05
17556	1.08212	2.1
17557	1.28095	2.1
17558	1.46956	2.1
17559	1.70303	2.1
17560	1.2925	0.05
17561	1.84716	0.05
17562	1.48676	0.108009
17563	1.48797	0.101705
17564	1.33917	0.0839939
17565	1.22691	0.0873852
17566	0.528744	2.1
17567	1.13728	2.1
17568	1.13728	2.1
17569	1.99843	2.1
17570	1.17464	0.05
17571	1.0372	0.05

17572	2.0815	0.05
17573	0.933462	0.05
17574	1.54931	2.1
17575	1.5485	2.1
17576	1.4077	2.1
17577	1.29931	2.1
17578	1.14931	0.12257
17579	2.02223	0.112871
17580	1.04482	0.0877557
17581	0.94971	0.113564
17582	1.54632	1.77503
17583	1.15693	1.76562
17584	1.66763	2.1
17585	1.41238	2.1
17586	1.41238	2.1
17587	1.48068	2.1
17588	1.0171	0.371472
17589	1.1211	0.773155
17590	1.1211	0.334028
17591	1.28418	0.604226
17592	0.993021	2.1
17593	1.764 2.1	
17594	0.993023	2.1
17595	1.05787	2.1
17596	1.90914	0.47179
17597	1.12229	2.05233
17598	1.02025	0.938073
17599	0.585368	2.06705
17600	1.99323	2.1
17601	2.34988	2.1
17602	2.34988	2.1
17603	2.36987	2.1
17604	1.23107	0.05
17605	1.90664	0.0516776
17606	1.53496	0.05
17607	1.70302	0.054283
17608	1.98859	2.1
17609	1.61057	2.1
17610	1.79844	2.1
17611	2.10811	2.1
17612	1.4618	2.1
17613	1.96722	2.1
17614	1.95191	2.1
17615	2.13418	2.1
17616	1.50368	0.05
17617	1.61417	0.05
17618	1.15008	0.05
17619	1.19685	0.05
17620	1.90456	0.05
17621	1.90899	0.05
17622	2.08864	0.05
17623	2.10447	0.05
17624	1.16653	2.1
17625	1.23017	2.1
17626	1.0752	1.6364
17627	1.34412	1.13234
17628	1.06226	2.1
17629	1.09252	2.1

17630	1.09252	2.1
17631	0.88579	2.1
17632	1.56178	0.05
17633	1.14305	0.0596855
17634	1.56175	0.05
17635	1.08211	0.05
17636	1.32813	2.1
17637	1.32461	2.1
17638	1.32461	2.1
17639	1.32455	2.1
17640	1.42729	0.0721017
17641	1.18716	0.05
17642	1.47169	0.121959
17643	1.24687	0.0561071
17644	1.09627	2.1
17645	1.04759	2.1
17646	1.04759	2.1
17647	0.987132	2.1
17648	1.11181	1.87718
17649	1.80662	2.08639
17650	1.8066	2.05556
17651	1.06461	1.71615
17652	1.56091	2.1
17653	1.9842	2.1
17654	1.73525	0.133682
17655	1.40557	0.134084
17656	2.26906	0.143616
17657	1.31248	0.192137
17658	1.66832	0.126677
17659	1.32634	0.112257
17660	1.03314	0.05
17661	1.24682	0.05
17662	1.38536	0.05
17663	1.52661	0.05
17664	1.177	1.17516
17665	1.36405	0.8169
17666	1.19837	1.27696
17667	1.17701	0.521943
17668	0.993831	0.0647485
17669	1.0823	0.0717326
17670	1.0823	0.05
17671	1.01121	0.061963
17672	1.49653	0.0901865
17673	1.89583	0.114484
17674	0.67242	0.0871929
17675	1.36047	0.109479
17676	1.00366	0.0962022
17677	1.24728	0.110681
17678	1.13387	0.05
17679	1.60867	0.05
17680	1.30572	0.05
17681	1.54982	0.05
17682	0.788592	0.05
17683	1.82961	0.05
17684	1.54982	0.05
17685	1.82961	0.05
17686	1.26062	0.05
17687	1.64002	0.05

17688	1.12035	0.05
17689	1.64002	0.05
17690	1.1673	2.1
17691	1.75367	2.1
17692	0.964034	2.1
17693	1.0305	2.0976
17694	1.30896	2.0193
17695	1.52776	2.02984
17696	1.52776	2.1
17697	1.17989	2.09029
17698	1.16759	2.1
17699	1.8575	2.1
17700	1.16759	2.1
17701	1.60936	2.1
17702	1.10747	0.05
17703	1.47688	0.0806764
17704	1.40794	2.1
17705	1.95729	1.97692
17706	1.95729	2.1
17707	3.18156	1.89666
17708	1.87598	2.1
17709	1.94343	2.1
17710	1.94343	2.1
17711	2.00551	2.1
17712	1.20067	1.43326
17713	1.70744	1.26559
17714	1.87244	1.50492
17715	1.32075	1.25313
17716	1.64955	2.1
17717	1.44221	2.1
17718	1.31109	2.1
17719	1.14206	2.1
17720	1.60452	1.93939
17721	1.12111	0.823197
17722	1.00832	2.1
17723	0.974137	2.1
17724	0.974137	2.1
17725	0.98505	2.1
17726	1.02368	2.1
17727	0.985051	2.1
17728	0.995734	0.787935
17729	1.31993	0.650445
17730	1.31993	0.493547
17731	1.18549	2.00405
17732	1.5176	0.306582
17733	1.33622	0.21809
17734	1.33621	0.266899
17735	1.02782	0.212362
17736	1.66774	2.1
17737	1.85305	2.1
17738	1.85305	2.1
17739	2.07088	2.1
17740	1.78873	2.1
17741	1.47533	2.1
17742	1.23152	2.1
17743	0.99922	2.1
17744	1.48493	2.1
17745	1.1394	2.1

17746	1.19179	2.1
17747	1.64289	2.1
17748	1.47858	2.1
17749	1.89687	2.1
17750	0.972464	2.1
17751	1.79473	2.1
17752	1.06971	2.1
17753	1.21089	2.1
17754	1.12572	2.1
17755	1.30566	2.1
17756	1.30566	2.1
17757	1.52724	2.1
17758	2.32237	0.10148
17759	2.20248	0.0882003
17760	2.79564	0.11336
17761	2.32237	0.101536
17762	1.59354	0.0589314
17763	3.098	0.0562067
17764	3.09801	0.0600193
17765	3.45132	0.0515263
17766	1.24001	1.90155
17767	1.41822	0.301323
17768	1.41821	2.1
17769	1.44859	0.161883
17770	1.34539	2.1
17771	1.06991	2.1
17772	1.18076	2.1
17773	0.978586	2.1
17774	0.999189	0.05
17775	2.41076	0.05
17776	0.968475	2.09494
17777	1.02074	2.1
17778	1.02074	2.1
17779	1.07866	2.1
17780	0.817702	0.0605304
17781	1.5082	0.0625246
17782	1.48376	0.0829753
17783	1.18947	0.0842311
17784	1.10638	2.1
17785	1.86766	2.1
17786	1.19785	2.1
17787	1.34141	2.1
17788	1.34141	2.1
17789	0.991985	2.1
17790	2.35516	1.02014
17791	1.24815	2.03083
17792	1.80454	2.1
17793	1.22633	2.1
17794	0.696255	2.1
17795	1.19379	2.05318
17796	1.14721	2.1
17797	1.16153	2.1
17798	1.16153	2.1
17799	1.17261	2.1
17800	1.20814	2.1
17801	1.54791	2.1
17802	1.54791	2.1
17803	1.60477	2.1

17804	1.29931	2.1
17805	1.19098	2.1
17806	1.19098	2.1
17807	1.13341	2.1
17808	1.00503	1.86923
17809	1.25334	0.277234
17810	1.22655	2.1
17811	1.26282	2.1
17812	1.26282	2.1
17813	0.999489	2.1
17814	1.54197	0.167758
17815	1.17152	0.175959
17816	1.17152	0.173019
17817	1.49627	0.152578
17818	1.36405	0.74485
17819	0.976334	0.64363
17820	1.12495	2.1
17821	1.54096	2.1
17822	1.75263	2.1
17823	1.49114	2.1
17824	0.842998	1.71201
17825	1.60244	0.265309
17826	1.48911	1.09572
17827	2.18685	0.798267
17828	1.75236	0.889067
17829	1.54143	1.25596
17830	1.40013	0.431863
17831	0.946646	0.523192
17832	1.50714	2.1
17833	1.46055	2.1
17834	1.46055	2.1
17835	1.42249	2.1
17836	2.12839	0.157236
17837	1.75211	0.154918
17838	1.76297	0.242738
17839	1.70736	0.143139
17840	1.19952	0.05
17841	1.56475	0.05
17842	1.74312	0.0649847
17843	1.48973	0.102071
17844	1.91518	0.15831
17845	1.16534	0.134136
17846	1.53556	2.1
17847	1.56253	2.1
17848	1.56253	2.1
17849	1.58054	2.1
17850	1.27806	0.05
17851	1.44593	0.05
17852	1.44593	0.05
17853	1.3946	0.05
17854	1.34857	0.478768
17855	1.03818	1.94083
17856	1.38969	0.170094
17857	1.85548	0.25783
17858	1.03108	0.162007
17859	1.85548	0.170636
17860	1.49346	1.59181
17861	1.83192	1.92757

17862	1.75291	1.10582
17863	1.35435	1.56291
17864	0.921851	0.194343
17865	1.09646	0.192608
17866	1.2183	0.17195
17867	1.36717	0.157019
17868	2.14179	0.097852
17869	1.79805	0.12635
17870	1.3428	0.15508
17871	2.30493	0.156172
17872	1.81545	0.150461
17873	1.92269	0.106644
17874	2.30492	0.207899
17875	1.3663	0.195619
17876	1.22418	2.1
17877	0.956698	2.03153
17878	1.4706	2.1
17879	0.953295	2.1
17880	1.47059	2.1
17881	1.26929	2.1
17882	1.24538	0.0593033
17883	1.4468	0.0593599
17884	2.86762	2.1
17885	2.32911	2.1
17886	2.10864	2.1
17887	1.94548	2.1
17888	1.83134	2.1
17889	2.19519	2.1
17890	2.19853	2.1
17891	2.63855	2.1
17892	1.08213	0.0538497
17893	1.46389	0.0680672
17894	1.62235	0.0914074
17895	0.980549	0.102813
17896	1.08195	1.76924
17897	0.99002	2.06598
17898	1.2269	2.1
17899	1.0838	2.1
17900	0.889491	2.09799
17901	1.19587	2.08497
17902	1.18801	0.0564891
17903	1.57125	0.0592243
17904	1.47712	2.1
17905	1.45182	2.1
17906	1.45182	2.1
17907	1.42255	2.1
17908	1.17816	0.05
17909	1.00982	0.05
17910	1.21056	0.05
17911	1.17816	0.05
17912	3.80902	0.592951
17913	0.976991	0.526278
17914	1.09962	2.1
17915	1.60976	2.1
17916	1.87029	2.1
17917	2.33806	2.1
17918	2.12549	2.1
17919	2.21472	2.1

17920	2.16221	0.05
17921	1.99528	0.05
17922	1.99528	0.05
17923	1.97969	0.05
17924	3.60854	2.1
17925	4.00847	2.1
17926	2.70372	2.1
17927	1.253 2.1	
17928	1.65739	2.1
17929	0.994294	2.1
17930	1.36203	2.1
17931	1.00965	2.1
17932	2.33374	2.1
17933	1.36209	2.1
17934	1.64983	0.05
17935	1.36416	0.05
17936	2.19766	0.05
17937	1.36416	0.05
17938	2.15871	0.293891
17939	1.81454	0.192693
17940	1.54607	0.103546
17941	1.57702	0.14775
17942	0.948748	0.05
17943	1.04522	0.05
17944	1.60994	0.05
17945	1.90382	0.05
17946	1.90382	0.05
17947	1.57999	0.05
17948	1.73905	0.548427
17949	1.26535	1.28125
17950	2.12552	0.77252
17951	1.06891	0.572546
17952	1.13999	1.27465
17953	2.29812	0.769172
17954	2.83329	0.569961
17955	2.16272	0.415439
17956	1.71277	2.1
17957	1.68401	2.1
17958	1.68401	2.1
17959	1.69368	2.1
17960	1.06472	2.1
17961	1.56017	2.1
17962	1.2765	2.1
17963	1.99541	2.1
17964	1.46041	0.05
17965	2.04817	0.05
17966	2.04817	0.05
17967	2.33181	0.05
17968	2.06353	2.1
17969	1.35449	2.1
17970	1.52045	2.1
17971	1.45437	2.1
17972	1.04405	0.05
17973	1.85945	0.05
17974	1.85945	0.05
17975	2.22679	0.05
17976	1.33339	2.1
17977	1.19376	2.1

17978	1.33266	2.1
17979	1.44244	2.1
17980	1.20455	2.1
17981	1.52852	2.1
17982	1.03882	2.1
17983	1.52852	2.1
17984	1.44801	2.0186
17985	1.00775	2.07276
17986	0.53907	2.1
17987	0.506124	2.1
17988	2.39045	2.1
17989	0.973794	2.1
17990	1.11193	2.1
17991	1.32784	2.1
17992	1.31595	2.1
17993	0.966115	2.1
17994	1.25953	2.1
17995	1.38086	2.1
17996	1.38086	2.1
17997	1.04101	2.1
17998	1.99319	0.05
17999	1.23107	0.05
18000	3.3606	0.05
18001	1.23112	0.05
18002	1.40239	2.1
18003	1.26091	2.1
18004	1.26091	2.1
18005	1.05776	2.1
18006	1.23768	0.232236
18007	1.0142	1.27013
18008	1.11435	1.24463
18009	1.01422	0.332869
18010	1.24001	0.447444
18011	1.42186	1.01586
18012	1.42186	0.630587
18013	1.47204	0.72747
18014	1.27361	2.1
18015	1.56642	2.1
18016	1.71676	2.1
18017	2.16815	2.1
18018	1.07855	1.85507
18019	1.07612	1.75179
18020	1.703 0.05	
18021	1.46853	0.05
18022	1.45448	0.052021
18023	1.27778	0.0609356
18024	0.991986	2.1
18025	1.1192	2.1
18026	1.1192	2.1
18027	1.25953	2.1
18028	2.08506	2.1
18029	1.75162	2.1
18030	1.94523	2.1
18031	1.88405	1.5482
18032	1.15506	0.05
18033	1.1233	0.05
18034	2.09736	2.1
18035	2.98303	2.1

18036	2.71184	2.1
18037	3.51614	2.1
18038	1.18769	2.09216
18039	1.24053	2.09645
18040	1.24053	2.1
18041	1.45327	2.1
18042	1.26865	2.07353
18043	1.1744	1.72913
18044	1.1744	1.89597
18045	1.11712	1.24167
18046	1.15841	0.158909
18047	1.02247	0.204461
18048	1.93649	2.1
18049	1.81191	2.1
18050	1.64718	2.1
18051	1.47521	2.1
18052	1.59654	2.1
18053	1.57823	2.1
18054	1.57823	2.1
18055	1.55768	2.1
18056	1.00665	0.110903
18057	1.28239	0.0870167
18058	1.28239	0.119929
18059	1.44951	0.0940076
18060	1.22083	2.1
18061	0.957622	2.1
18062	0.887896	2.1
18063	1.27387	2.1
18064	1.00521	1.07263
18065	1.00503	0.768577
18066	1.95844	2.1
18067	1.05042	2.1
18068	2.78662	2.1
18069	2.22399	2.1
18070	2.3356	0.05
18071	1.49543	0.05
18072	1.96854	0.05
18073	1.21681	0.05
18074	2.17803	1.40254
18075	1.50998	1.87408
18076	2.35239	0.925254
18077	2.26987	0.811301
18078	1.03824	0.210571
18079	1.51396	0.216986
18080	1.51396	0.145224
18081	1.48693	0.163104
18082	1.08784	1.08553
18083	1.36865	0.719554
18084	0.712405	1.65727
18085	1.08784	1.02768
18086	2.02522	2.1
18087	1.6738	2.1
18088	1.35175	2.1
18089	1.50094	2.1
18090	1.6738	2.1
18091	1.62871	2.1
18092	1.35084	2.1
18093	1.34276	2.1

18094	1.18041	2.1
18095	1.43652	2.1
18096	0.974196	2.1
18097	1.28037	2.1
18098	1.28037	2.1
18099	1.05071	2.1
18100	2.44737	0.05
18101	1.4672	0.05
18102	1.22883	0.05
18103	2.08149	0.05
18104	1.82208	1.37645
18105	1.59826	0.210809
18106	1.59826	0.619815
18107	1.01393	0.29425
18108	1.42757	2.1
18109	0.996775	2.1
18110	1.42757	2.1
18111	1.26397	2.1
18112	1.02461	2.1
18113	1.46129	2.1
18114	1.11833	2.1
18115	1.335 2.1	
18116	2.07087	2.1
18117	1.24041	2.1
18118	1.335 2.1	
18119	1.55398	2.1
18120	1.21171	0.05
18121	1.48589	0.05
18122	1.32886	0.05
18123	1.8027	0.05
18124	1.53515	1.54896
18125	1.60428	1.33051
18126	1.00035	0.05
18127	1.03534	0.356747
18128	1.03535	2.1
18129	1.09962	2.1
18130	0.62132	2.06823
18131	1.21033	2.1
18132	1.30326	2.1
18133	2.6823	2.1
18134	0.991407	2.1
18135	1.29729	2.1
18136	1.29729	2.1
18137	1.16858	2.1
18138	1.66137	0.394481
18139	1.23012	0.371758
18140	1.66139	0.846646
18141	1.41821	0.647703
18142	1.56043	0.15443
18143	1.06848	0.198275
18144	1.56043	0.312035
18145	1.90911	0.249232
18146	0.982847	2.1
18147	1.05863	2.1
18148	1.05863	2.1
18149	1.22352	2.1
18150	1.23019	2.0728
18151	1.60509	2.08757

18152	1.19122	2.1
18153	1.47769	2.1
18154	2.18868	2.1
18155	1.40984	2.09733
18156	3.55197	2.1
18157	3.57227	2.1
18158	3.57227	2.1
18159	3.48926	2.1
18160	1.4375	0.103083
18161	1.11314	0.088275
18162	1.62408	2.1
18163	1.74438	2.1
18164	1.72387	2.1
18165	1.50018	2.1
18166	1.1486	2.1
18167	1.18474	2.1
18168	1.18474	2.1
18169	1.2295	2.1
18170	1.28754	2.1
18171	1.36838	2.1
18172	1.36838	2.1
18173	1.21703	2.1
18174	2.74206	2.1
18175	2.70795	2.1
18176	2.70795	2.1
18177	2.55918	2.1
18178	1.35065	0.986999
18179	1.65411	0.505242
18180	1.48572	0.825815
18181	1.28393	0.548112
18182	1.25265	0.05
18183	1.70944	0.05
18184	1.31259	2.1
18185	1.14945	2.1
18186	1.14945	2.06693
18187	0.995725	2.1
18188	1.00307	0.195542
18189	2.05786	0.211655
18190	1.56357	0.135582
18191	1.10907	0.176662
18192	1.27558	1.71819
18193	1.05175	1.33092
18194	1.2685	2.1
18195	1.41369	2.1
18196	1.4137	2.1
18197	1.49654	2.1
18198	1.21489	2.05373
18199	1.14605	1.33803
18200	1.22476	2.1
18201	1.21489	0.530841
18202	2.51472	0.870767
18203	2.52091	0.813507
18204	2.52091	0.725936
18205	1.79171	0.622392
18206	1.03373	2.0991
18207	1.04037	2.1
18208	1.04038	2.1
18209	1.03933	2.1

18210	1.74123	0.0841073
18211	1.41902	0.0699961
18212	1.89777	0.0915014
18213	1.89899	0.0977651
18214	1.24728	0.083399
18215	1.30394	0.0874212
18216	1.30394	0.0622755
18217	1.16535	0.0989835
18218	1.09914	2.1
18219	1.54259	2.1
18220	1.40233	2.1
18221	1.69858	2.1
18222	1.53555	2.1
18223	1.09859	2.1
18224	1.09859	2.1
18225	0.725879	2.1
18226	1.07213	2.1
18227	1.31589	2.1
18228	1.36904	2.1
18229	1.69711	2.1
18230	1.51028	0.05
18231	0.993598	0.05
18232	0.61563	0.05
18233	1.26311	0.05
18234	1.19496	2.1
18235	1.65925	2.1
18236	1.65925	2.1
18237	1.48162	2.1
18238	2.75738	1.43136
18239	1.24699	1.01401
18240	4.08729	1.6455
18241	2.35271	1.04775
18242	1.55063	0.0704842
18243	1.34563	0.0740469
18244	1.34563	0.0562471
18245	1.08189	0.0605488
18246	1.09662	0.0559664
18247	2.35961	0.106459
18248	0.986952	0.050703
18249	0.813251	0.05
18250	1.17897	0.05
18251	1.67685	0.05
18252	1.00573	2.03985
18253	1.08542	1.2845
18254	1.00573	2.1
18255	1.12572	2.1
18256	1.05499	2.1
18257	1.00521	1.02271
18258	2.22679	0.05
18259	2.86746	0.05
18260	2.86746	0.05
18261	1.99958	0.05
18262	0.996517	2.1
18263	1.01169	2.1
18264	1.01169	2.1
18265	1.26346	2.1
18266	1.68274	2.1
18267	1.75505	2.1

18268	2.28748	2.03253
18269	1.36555	2.06351
18270	3.47888	0.05
18271	0.692788	0.05
18272	1.0685	0.347964
18273	1.14547	0.406896
18274	1.19017	2.1
18275	1.24976	2.1
18276	1.24976	2.1
18277	1.30682	2.1
18278	2.2153	2.1
18279	1.85457	2.1
18280	2.03267	2.1
18281	1.51042	2.1
18282	1.05619	0.572201
18283	1.05793	0.317092
18284	1.05793	0.395437
18285	1.00763	0.255609
18286	1.77677	2.1
18287	1.02524	2.1
18288	1.02524	2.1
18289	1.18803	2.1
18290	1.68669	2.1
18291	1.71162	2.1
18292	1.556	2.1
18293	1.39523	2.1
18294	0.960288	2.03937
18295	1.24201	2.1
18296	1.24202	2.1
18297	1.3592	2.1
18298	2.14045	0.05
18299	1.62054	0.05
18300	2.20248	0.0773628
18301	2.34699	0.0665557
18302	3.45132	0.05
18303	3.38241	0.05
18304	3.38241	0.05
18305	2.96127	0.05
18306	2.02612	2.1
18307	2.09351	2.1
18308	2.09351	2.1
18309	2.17465	2.1
18310	1.07445	2.1
18311	1.14808	2.1
18312	1.39067	2.1
18313	1.20958	2.0994
18314	1.20958	2.1
18315	1.00382	2.01422
18316	0.986496	0.16844
18317	1.27142	0.588016
18318	1.39857	0.454622
18319	2.26482	0.457928
18320	2.24367	0.05
18321	1.9234	0.05
18322	1.59076	0.05
18323	1.185	0.05
18324	3.86179	0.05
18325	3.0638	0.05

18326	2.7574	0.05
18327	1.83266	0.05
18328	2.59418	2.1
18329	2.23048	2.1
18330	1.39564	2.1
18331	1.36763	2.1
18332	2.23048	2.1
18333	2.02522	2.1
18334	1.36763	2.1
18335	1.35175	2.1
18336	1.16181	0.05
18337	0.97096	0.05
18338	2.05665	0.127668
18339	0.483583	0.118037
18340	0.601928	0.0953885
18341	3.88545	0.124267
18342	1.04909	2.1
18343	0.983143	2.1
18344	1.10469	2.1
18345	1.10516	2.1
18346	1.10517	2.1
18347	1.09951	2.1
18348	2.33985	2.1
18349	2.35026	2.1
18350	2.35025	2.1
18351	1.19462	2.1
18352	1.57627	2.1
18353	1.5493	2.1
18354	1.5493	2.1
18355	1.53556	2.1
18356	1.42763	0.389104
18357	1.24682	0.440595
18358	1.24682	0.300055
18359	1.00307	0.369479
18360	1.37392	2.1
18361	1.45408	2.1
18362	1.45408	2.1
18363	1.54249	2.1
18364	1.24474	1.5141
18365	1.07467	1.52968
18366	1.37813	1.57318
18367	1.24474	1.62325
18368	1.06341	2.1
18369	1.09194	2.1
18370	1.09194	2.1
18371	1.11117	2.1
18372	1.1658	2.1
18373	1.93707	2.1
18374	1.93707	2.1
18375	1.47729	2.1
18376	1.25669	2.1
18377	1.07519	2.1
18378	1.65417	2.1
18379	1.50956	2.1
18380	1.37231	2.1
18381	1.27285	2.1
18382	1.23612	2.1
18383	1.1899	2.1

18384	1.08172	2.1
18385	1.20357	2.1
18386	1.5664	2.1
18387	1.7472	2.1
18388	1.48434	2.1
18389	1.23989	2.1
18390	1.58836	2.1
18391	1.38766	2.1
18392	1.23989	2.1
18393	1.26599	2.1
18394	2.56248	2.1
18395	2.08673	2.08665
18396	1.87216	2.09559
18397	1.60476	0.695079
18398	1.84355	2.1
18399	1.25971	2.07922
18400	1.44141	2.05043
18401	1.25521	0.44343
18402	1.19837	0.05
18403	2.31936	0.05
18404	1.38153	2.1
18405	2.41734	2.1
18406	2.70856	2.1
18407	3.48385	2.1
18408	1.89226	0.820434
18409	1.64617	0.594435
18410	1.89112	0.213806
18411	1.23135	0.167193
18412	1.23134	0.197344
18413	1.34927	0.198673
18414	3.60096	0.05
18415	1.68103	0.05
18416	2.25644	2.1
18417	1.91955	2.1
18418	1.91955	2.1
18419	1.47688	2.1
18420	1.67375	2.1
18421	1.50033	2.1
18422	1.5098	2.1
18423	1.46908	2.1
18424	1.69055	0.05
18425	1.53463	0.05
18426	1.53463	0.05
18427	1.41943	0.05
18428	1.19022	1.81746
18429	1.95981	1.23859
18430	1.15009	1.91502
18431	1.31612	1.55473
18432	1.31611	0.861736
18433	1.10735	0.5014
18434	1.18601	0.05
18435	1.3499	0.05
18436	1.3499	0.05
18437	1.29001	0.05
18438	1.22083	2.1
18439	1.06191	2.1
18440	1.348	0.643276
18441	1.05758	1.35927

18442	1.34799	1.75114
18443	1.18647	1.72474
18444	2.92457	0.05
18445	1.99182	0.05
18446	2.25313	0.05
18447	1.30859	0.05
18448	1.30682	2.1
18449	1.38957	2.1
18450	1.38957	2.1
18451	1.47446	2.1
18452	0.953638	2.1
18453	1.03408	2.1
18454	1.17885	0.233447
18455	1.3663	0.169181
18456	1.08265	0.183066
18457	1.71948	0.232191
18458	1.29501	0.573602
18459	0.955093	0.499074
18460	0.955081	0.409668
18461	1.88345	0.436256
18462	1.2243	2.1
18463	1.23169	2.1
18464	2.04726	0.567001
18465	0.842869	0.372032
18466	3.18156	2.1
18467	3.60786	1.79477
18468	3.60786	2.09185
18469	2.38566	1.6445
18470	1.24526	0.05
18471	1.59019	0.05
18472	1.16776	1.84057
18473	1.05042	2.1
18474	1.17418	2.1
18475	1.05042	2.1
18476	0.949135	2.1
18477	0.973448	2.1
18478	0.973447	2.1
18479	1.02958	2.1
18480	3.57768	2.1
18481	3.04246	2.1
18482	3.04246	2.1
18483	2.47824	2.1
18484	1.35879	2.1
18485	1.10407	2.1
18486	1.14599	2.08113
18487	1.10407	2.09311
18488	1.87794	1.69564
18489	1.53429	1.46466
18490	1.12717	2.1
18491	1.38634	2.1
18492	1.38634	2.1
18493	1.13127	2.1
18494	1.04546	2.1
18495	1.06007	2.1
18496	1.06007	2.1
18497	1.19785	2.1
18498	1.43652	2.1
18499	1.71741	2.1

18500	1.70921	2.1
18501	1.06994	2.1
18502	0.960106	2.1
18503	1.48077	2.1
18504	1.48077	2.1
18505	2.5946	2.1
18506	2.03045	1.86373
18507	1.76788	1.90739
18508	2.03045	2.05156
18509	0.633098	2.09565
18510	1.06756	2.1
18511	1.38667	2.1
18512	0.966979	2.1
18513	1.38669	2.1
18514	1.07156	2.1
18515	0.986095	2.1
18516	1.32813	2.1
18517	0.986095	2.1
18518	1.98697	0.36924
18519	1.12016	0.686683
18520	1.24463	0.589193
18521	1.1967	0.556808
18522	1.30001	0.532131
18523	1.29175	1.17224
18524	1.29175	0.570997
18525	1.3003	1.27116
18526	1.5606	0.05
18527	2.32098	0.05
18528	2.57888	0.05
18529	2.97463	0.05
18530	1.24555	2.1
18531	1.46101	2.1
18532	1.90723	2.1
18533	2.29126	2.1
18534	1.46101	2.1
18535	1.84089	2.1
18536	2.5519	2.1
18537	2.91663	2.1
18538	1.10476	0.05
18539	1.44103	0.05
18540	1.44103	0.05
18541	1.53336	0.05
18542	0.672964	2.1
18543	0.633884	2.1
18544	0.633884	2.1
18545	0.603154	2.1
18546	2.29663	2.1
18547	2.06892	2.1
18548	1.3685	2.1
18549	1.39067	2.1
18550	2.06892	2.1
18551	1.78208	2.1
18552	1.39067	2.1
18553	1.42174	2.1
18554	1.01335	1.66398
18555	1.08195	0.699203
18556	1.86463	1.98932
18557	1.39827	1.9626

18558	0.861515	2.04099
18559	2.10074	2.1
18560	2.09511	1.15314
18561	2.04853	0.547286
18562	2.21488	0.838158
18563	1.38287	1.12092
18564	0.974717	0.331768
18565	0.998451	0.267628
18566	1.42281	1.56388
18567	1.82595	1.43069
18568	1.50458	0.841896
18569	1.09408	0.807949
18570	1.23498	1.38802
18571	1.05654	1.20689
18572	1.17261	2.1
18573	1.18058	2.1
18574	1.18059	2.1
18575	1.18907	2.1
18576	2.38998	2.1
18577	2.77437	2.1
18578	1.04546	2.1
18579	1.16817	2.1
18580	2.77437	2.1
18581	2.97768	2.1
18582	1.16817	2.1
18583	1.2832	2.1
18584	1.75766	0.134607
18585	1.4934	1.92964
18586	1.4934	0.277336
18587	1.20324	2.03867
18588	1.07236	0.727395
18589	1.37641	0.511639
18590	1.42913	0.415359
18591	1.93373	0.38708
18592	1.42629	0.321025
18593	1.00636	0.299614
18594	0.933959	1.88267
18595	2.22873	0.957431
18596	2.22873	1.69703
18597	2.13757	0.720112
18598	1.10517	2.1
18599	1.86737	2.1
18600	0.811 2.1	
18601	1.19179	2.1
18602	1.18647	1.83519
18603	0.988453	2.05684
18604	1.12457	0.0985288
18605	1.23851	0.0810057
18606	0.92492	2.1
18607	1.45656	2.1
18608	1.61251	0.584325
18609	1.56696	0.581422
18610	1.90245	0.438072
18611	1.46046	0.426039
18612	1.60329	0.05
18613	1.38247	0.05
18614	1.38247	0.05
18615	1.23198	0.05

18616	1.09518	2.1
18617	1.28159	2.1
18618	0.977777	2.1
18619	1.80778	2.1
18620	1.80777	2.1
18621	1.16655	2.1
18622	1.3515	2.1
18623	0.956573	2.1
18624	1.08022	2.1
18625	1.03437	2.1
18626	1.03437	2.1
18627	0.975409	2.1
18628	1.47369	0.05
18629	1.31167	0.0505744
18630	1.01847	0.05
18631	2.75562	0.05
18632	1.13443	2.1
18633	1.32709	2.1
18634	1.01051	0.200861
18635	0.958947	0.167624
18636	1.7211	2.1
18637	1.922 2.1	
18638	1.922 2.1	
18639	0.991113	2.1
18640	1.00636	0.332219
18641	1.31658	0.33
18642	1.44627	0.245291
18643	1.03824	0.233725
18644	1.21223	2.1
18645	1.01242	2.1
18646	1.01243	2.1
18647	0.956698	2.1
18648	1.23167	2.1
18649	1.06935	2.1
18650	1.29215	0.05
18651	0.998971	0.05
18652	1.50103	0.05
18653	1.44836	0.05
18654	3.02513	2.1
18655	3.7335	2.1
18656	2.49988	2.1
18657	1.24948	2.1
18658	0.963972	2.1
18659	1.1822	2.1
18660	1.1822	2.1
18661	1.10817	2.1
18662	1.30237	2.1
18663	1.41908	2.1
18664	1.41908	2.1
18665	1.48734	2.1
18666	1.31127	0.190785
18667	1.40822	0.222183
18668	1.40822	0.108904
18669	1.22176	0.127629
18670	1.82525	2.1
18671	1.59394	2.1
18672	1.22731	0.05
18673	0.981584	0.05

18674	1.1427	0.359231
18675	1.44091	0.621448
18676	1.49154	1.30251
18677	1.07526	1.03291
18678	1.07527	0.649572
18679	0.96029	1.31731
18680	2.40568	1.08335
18681	3.46486	1.08374
18682	2.417	1.14893
18683	1.52054	0.857304
18684	1.51638	0.0750092
18685	1.04786	0.0621897
18686	1.51404	0.0689942
18687	1.3461	0.0597085
18688	1.27696	0.05
18689	0.736457	0.05
18690	1.16817	2.1
18691	1.04263	2.1
18692	1.13449	2.1
18693	1.50524	2.1
18694	2.1182	2.1
18695	2.49001	2.1
18696	1.11764	2.1
18697	1.39292	2.1
18698	2.49001	2.1
18699	2.76339	2.1
18700	1.39292	2.1
18701	1.68761	2.1
18702	1.25173	2.1
18703	1.34701	2.1
18704	1.02415	2.1
18705	1.34701	2.1
18706	1.332	0.05
18707	0.989764	0.05
18708	0.963167	0.05
18709	1.21056	0.05
18710	1.67375	2.1
18711	1.17965	2.1
18712	1.09154	2.1
18713	1.52314	2.1
18714	1.54433	0.2003
18715	1.81969	0.173482
18716	1.3598	0.14424
18717	1.71041	0.128036
18718	1.70934	0.113695
18719	1.33871	0.105206
18720	1.79485	0.0961493
18721	1.51404	0.0862853
18722	1.30751	2.1
18723	1.20969	2.1
18724	1.20969	2.1
18725	1.12076	2.1
18726	1.49808	2.1
18727	1.50201	2.1
18728	1.50201	2.1
18729	1.31594	2.1
18730	1.2187	2.1
18731	1.11545	2.1

18732	1.227	2.1
18733	1.09645	2.1
18734	1.05902	0.05
18735	1.41595	1.60639
18736	1.35696	2.1
18737	1.29446	1.95852
18738	0.857957	2.1
18739	1.21125	0.05
18740	2.43115	2.1
18741	2.1151	0.284681
18742	2.0815	1.50781
18743	1.1723	1.96771
18744	1.48035	0.125795
18745	1.59971	0.45244
18746	1.33668	0.280317
18747	1.0961	0.241857
18748	1.46235	2.1
18749	0.882195	2.1
18750	2.46624	2.1
18751	2.72161	2.1
18752	2.96094	2.1
18753	2.25644	2.1
18754	1.25923	0.05
18755	0.976218	0.05
18756	1.10319	0.05
18757	1.82583	0.05
18758	0.888955	2.1
18759	1.30817	2.1
18760	1.30817	2.1
18761	1.60787	2.1
18762	0.988805	2.1
18763	1.54168	2.1
18764	1.19496	0.676233
18765	1.12224	1.46039
18766	1.93597	1.40035
18767	1.31113	1.14132
18768	1.17879	1.29763
18769	1.07468	1.30992
18770	2.0523	2.1
18771	2.41385	2.1
18772	2.41385	2.1
18773	3.0355	2.1
18774	1.36717	0.127418
18775	1.45535	0.108089
18776	1.45535	0.0904514
18777	1.49906	0.0764472
18778	1.37785	2.1
18779	1.04413	2.1
18780	1.06729	0.671422
18781	1.54305	0.592671
18782	1.07444	2.1
18783	1.52724	2.1
18784	1.49151	2.1
18785	1.36814	2.1
18786	1.36814	2.1
18787	1.15541	2.1
18788	1.19379	2.1
18789	1.78657	1.88247

18790	1.6079	2.1
18791	2.59469	1.53656
18792	1.35545	0.951711
18793	1.23602	1.98279
18794	1.23452	2.1
18795	1.56842	2.05669
18796	1.15864	2.1
18797	1.11955	2.1
18798	1.11955	2.1
18799	1.08022	2.1
18800	1.64268	2.1
18801	1.26247	2.1
18802	1.16912	2.1
18803	2.1095	1.19752
18804	1.75549	0.05
18805	1.7491	0.05
18806	1.59833	0.05
18807	1.68338	0.05
18808	1.14846	0.05
18809	1.06515	0.05
18810	1.2721	0.108549
18811	1.32639	0.100294
18812	1.32639	0.181201
18813	1.19323	0.158661
18814	1.81034	2.1
18815	1.9034	2.1
18816	2.08245	2.1
18817	1.84887	2.1
18818	1.41448	2.1
18819	1.77326	2.1
18820	1.76872	2.1
18821	1.86766	2.1
18822	1.46643	2.1
18823	1.58995	2.1
18824	1.58995	2.1
18825	1.13854	2.1
18826	1.23654	2.1
18827	1.04442	2.1
18828	1.23654	2.1
18829	1.08576	2.1
18830	0.966979	2.1
18831	1.41758	2.1
18832	1.03108	0.0933945
18833	1.94412	0.105951
18834	1.32455	2.1
18835	1.32328	2.1
18836	1.32328	2.1
18837	1.34008	2.1
18838	1.60861	2.1
18839	1.69586	2.1
18840	1.69586	2.1
18841	1.78301	2.1
18842	1.7513	2.1
18843	1.67906	2.1
18844	1.67906	2.1
18845	1.60635	2.1
18846	1.68073	2.1
18847	1.57234	2.1

18848	1.57233	2.1
18849	1.47012	2.1
18850	1.22811	0.131296
18851	1.79683	0.0941126
18852	1.53417	0.101824
18853	1.08732	0.107855
18854	1.8903	2.09139
18855	1.20247	2.08966
18856	1.12087	1.69749
18857	1.54699	2.08821
18858	1.43635	2.1
18859	1.02368	2.1
18860	1.43635	2.1
18861	1.23007	2.1
18862	1.035 2.1	
18863	1.07889	2.1
18864	3.0355	2.1
18865	3.78805	2.1
18866	2.57183	2.1
18867	1.27835	2.05776
18868	1.29813	0.128639
18869	1.42965	0.117771
18870	1.69832	0.120144
18871	2.18359	0.10858
18872	1.42964	0.128859
18873	1.65723	0.124625
18874	1.98508	0.114352
18875	2.06284	0.104183
18876	1.50705	0.059067
18877	1.51153	0.086312
18878	1.36037	0.0720304
18879	1.09767	0.098178
18880	1.70048	2.1
18881	1.15177	2.1
18882	0.713174	2.1
18883	0.763575	2.1
18884	0.763575	2.1
18885	0.797011	2.1
18886	1.01179	0.425377
18887	1.27737	0.536528
18888	3.16531	0.05
18889	1.0141	0.05
18890	1.47354	0.148767
18891	1.62495	0.14212
18892	1.62495	0.119046
18893	1.39296	0.220058
18894	0.998682	2.1
18895	1.93528	2.1
18896	2.71383	2.1
18897	1.13987	2.1
18898	1.93528	2.1
18899	1.78595	2.1
18900	3.98933	2.1
18901	3.73426	2.1
18902	1.02438	2.1
18903	1.12376	2.1
18904	1.12375	2.1
18905	1.19016	2.1

18906	1.37157	0.117624
18907	1.60814	0.114987
18908	1.70303	0.151658
18909	1.01219	0.128898
18910	1.06757	2.1
18911	1.23475	2.1
18912	1.2295	2.1
18913	1.28014	2.1
18914	1.28014	2.1
18915	1.33339	2.1
18916	1.3201	2.1
18917	1.2679	2.1
18918	1.2679	2.1
18919	1.23504	2.1
18920	1.50108	2.1
18921	1.3715	2.1
18922	1.3715	2.1
18923	1.25075	2.1
18924	1.99222	0.681172
18925	1.4155	0.531142
18926	1.4777	2.1
18927	1.52613	2.1
18928	1.91031	2.1
18929	1.03968	2.1
18930	2.04289	0.142815
18931	0.967192	0.114346
18932	1.03043	0.0915903
18933	1.65452	0.141633
18934	1.21564	2.1
18935	1.16938	2.1
18936	2.47056	2.1
18937	2.56853	2.1
18938	1.16938	2.1
18939	1.1181	2.1
18940	2.56853	2.1
18941	2.66828	2.1
18942	1.0465	2.1
18943	1.44709	2.1
18944	1.44709	2.1
18945	1.96491	2.1
18946	1.01976	0.322371
18947	0.961779	0.288056
18948	1.11764	0.317604
18949	0.961779	0.289065
18950	1.21171	0.05
18951	1.35007	0.05
18952	1.03102	0.253481
18953	1.42446	0.268353
18954	1.42446	0.240209
18955	1.03166	0.214009
18956	1.2951	0.100766
18957	1.33575	0.11465
18958	1.33575	0.119527
18959	1.20473	0.129917
18960	1.10049	0.587258
18961	0.99747	0.943516
18962	1.54249	2.1
18963	1.65677	2.1

18964	1.65677	2.1
18965	1.77677	2.1
18966	1.73525	0.14478
18967	1.05689	0.183746
18968	1.26051	0.122699
18969	1.16259	0.115448
18970	1.34009	2.1
18971	1.398	2.1
18972	1.398	2.1
18973	1.50437	2.1
18974	2.3245	2.1
18975	1.18237	2.1
18976	1.24509	2.1
18977	1.01595	2.1
18978	3.29047	2.1
18979	2.79168	2.1
18980	1.06561	2.1
18981	1.11077	2.1
18982	1.11077	2.1
18983	0.953927	2.1
18984	1.30509	0.287459
18985	1.04685	0.301806
18986	1.04685	0.219964
18987	0.834593	0.231476
18988	1.0775	0.13814
18989	1.27362	0.127393
18990	1.71762	0.589738
18991	2.5736	0.533337
18992	2.04717	0.68575
18993	1.45521	0.423781
18994	1.33962	2.1
18995	1.27956	2.1
18996	1.27956	2.1
18997	1.20478	2.1
18998	3.65841	0.05
18999	1.18656	0.05
19000	1.72272	0.0593476
19001	1.6812	0.05
19002	1.09951	2.1
19003	1.08231	2.1
19004	1.08231	2.1
19005	1.06478	2.1
19006	2.16196	1.70623
19007	1.00387	1.29381
19008	1.00387	0.50084
19009	0.993999	0.456593
19010	1.04101	2.1
19011	1.27119	2.1
19012	1.27119	2.1
19013	1.51748	2.1
19014	3.00129	2.1
19015	3.27303	2.1
19016	3.27303	2.1
19017	3.60855	2.1
19018	2.22399	2.1
19019	1.71995	2.1
19020	1.54793	2.1
19021	1.227	2.1

19022	1.07624	2.1
19023	1.264	2.1
19024	1.19323	1.868
19025	1.21286	1.23738
19026	1.21286	1.19935
19027	1.20859	0.70379
19028	1.60578	0.111245
19029	1.27778	0.0821177
19030	1.0525	2.1
19031	1.05758	0.753736
19032	1.0525	0.713515
19033	1.17042	1.46721
19034	1.62536	0.05
19035	3.71662	0.05
19036	1.78169	0.05
19037	1.91434	0.05
19038	2.12331	0.05
19039	1.33406	0.05
19040	1.38437	2.1
19041	1.51829	2.1
19042	1.51829	2.1
19043	1.6697	2.1
19044	0.947114	2.1
19045	1.13335	2.1
19046	1.13335	2.1
19047	1.00965	2.1
19048	1.47521	2.1
19049	1.27679	2.1
19050	1.14911	2.1
19051	0.93841	2.1
19052	1.68437	2.1
19053	1.53822	2.1
19054	1.53822	2.1
19055	1.39702	2.1
19056	0.726453	2.1
19057	0.754101	2.1
19058	0.754101	2.1
19059	0.69776	2.1
19060	0.964033	2.1
19061	1.62397	2.1
19062	1.70302	2.081
19063	1.78637	2.06476
19064	3.68158	0.05
19065	2.11685	0.05
19066	1.33125	2.1
19067	1.15471	2.1
19068	1.0465	2.1
19069	1.33125	2.1
19070	1.17683	2.1
19071	1.25889	2.1
19072	1.25889	2.1
19073	1.34239	2.1
19074	1.40591	0.05
19075	0.79348	0.05
19076	1.04019	0.05
19077	0.999187	0.05
19078	1.30218	0.05
19079	1.83339	0.05

19080	1.88574	0.05
19081	1.5177	0.05
19082	1.23504	2.1
19083	1.19583	2.1
19084	1.19583	2.1
19085	1.14496	2.1
19086	1.62183	2.1
19087	1.62316	2.1
19088	1.62316	2.1
19089	1.62241	2.1
19090	1.02305	0.193809
19091	1.21136	0.177647
19092	0.993832	0.0822133
19093	1.21137	0.113385
19094	1.12064	2.1
19095	1.24965	2.1
19096	1.24965	2.1
19097	1.41077	2.1
19098	1.00052	0.135024
19099	1.49842	0.102007
19100	1.49843	0.0811223
19101	1.10801	0.0676565
19102	1.54196	2.1
19103	1.18041	2.1
19104	1.73571	1.85205
19105	1.37224	1.93188
19106	1.4375	0.0626547
19107	1.09529	0.0524309
19108	1.44421	0.05
19109	1.0953	0.05
19110	1.0753	2.1
19111	0.815365	2.1
19112	0.815365	2.1
19113	0.597537	2.1
19114	1.03609	2.1
19115	1.18952	2.1
19116	1.54283	0.21743
19117	1.16719	0.191746
19118	1.16719	0.267701
19119	1.89226	0.234355
19120	2.55919	2.1
19121	2.37492	2.1
19122	2.37492	2.1
19123	2.07435	2.1
19124	1.36016	0.10682
19125	1.70302	0.111138
19126	1.85252	0.120877
19127	1.2111	0.201464
19128	1.62905	2.1
19129	1.49692	2.1
19130	1.49692	2.1
19131	1.40395	2.1
19132	2.19083	2.1
19133	1.30542	2.1
19134	1.58736	2.1
19135	2.16579	2.1
19136	2.0815	2.1
19137	1.17935	2.1

19138	1.53803	2.1
19139	1.09148	2.03314
19140	1.81301	2.1
19141	1.38333	1.67259
19142	1.04412	2.1
19143	1.44437	2.1
19144	1.4675	0.0609954
19145	1.00665	0.0727036
19146	1.50951	0.0807439
19147	0.983105	0.0761018
19148	1.80252	0.123721
19149	0.540005	0.0865585
19150	1.2545	0.141278
19151	2.14514	0.0967355
19152	1.32051	0.0748374
19153	1.82302	0.0641516
19154	2.71057	2.1
19155	2.51107	2.1
19156	1.34817	2.1
19157	1.3551	2.1
19158	2.51107	2.1
19159	2.29663	2.1
19160	1.3551	2.1
19161	1.3685	2.1
19162	1.51748	2.1
19163	1.23389	2.1
19164	0.901928	0.05
19165	1.70302	0.05
19166	1.66787	0.05
19167	1.92535	0.05
19168	1.13571	0.224864
19169	1.02836	0.249295
19170	1.55398	2.1
19171	1.54179	2.1
19172	1.89608	2.1
19173	1.07167	2.1
19174	3.3488	0.116929
19175	0.550044	0.102163
19176	0.962877	0.397957
19177	1.00065	0.790095
19178	1.00613	0.417479
19179	1.07497	0.468064
19180	1.00486	0.05
19181	1.00486	0.05
19182	0.726453	2.1
19183	1.32634	0.061221
19184	1.40008	0.0905401
19185	1.40008	0.05
19186	2.42267	0.05
19187	1.9216	0.05
19188	1.28066	0.05
19189	1.79102	2.09751
19190	1.18219	0.737619
19191	1.18219	2.03832
19192	0.709123	0.655997
19193	1.07866	2.1
19194	1.15038	2.1
19195	1.15038	2.1

19196	1.24687	2.1
19197	1.83613	2.07593
19198	1.99157	2.05389
19199	1.45789	0.05
19200	1.1706	0.05
19201	1.74746	0.05
19202	1.1706	0.05
19203	1.96383	0.05
19204	1.67727	0.05
19205	1.67727	0.05
19206	1.60329	0.05
19207	1.18803	2.1
19208	1.3227	2.1
19209	1.3227	2.1
19210	1.40844	2.1
19211	1.07889	2.1
19212	1.07721	2.1
19213	1.07721	2.1
19214	1.00832	2.1
19215	0.953523	0.624218
19216	1.62875	0.52105
19217	1.07427	0.18537
19218	1.19722	0.210657
19219	1.1972	0.153463
19220	1.1599	0.176473
19221	2.97463	0.05
19222	1.9477	0.05
19223	1.95222	0.05
19224	1.75083	0.05
19225	3.46787	2.1
19226	3.02896	2.1
19227	3.02896	2.1
19228	2.46145	2.1
19229	0.970962	0.05
19230	1.0227	0.05
19231	1.12328	0.05
19232	1.02269	0.05
19233	0.904978	2.1
19234	0.725887	2.1
19235	0.725887	2.1
19236	0.601592	2.1
19237	0.956294	2.1
19238	1.13179	2.1
19239	0.956294	2.1
19240	1.13179	2.1
19241	1.05556	2.1
19242	1.13179	2.1
19243	1.38859	2.1
19244	1.13179	2.1
19245	1.06659	2.1
19246	1.05776	2.1
19247	1.06659	2.1
19248	1.04291	2.1
19249	1.54699	1.42081
19250	1.42307	1.6764
19251	1.5654	2.04587
19252	1.97906	2.1
19253	1.15354	0.05

19254	1.15505	0.05
19255	1.26455	0.113172
19256	2.50042	0.0654263
19257	2.50042	0.116194
19258	3.22585	0.0629777
19259	1.19258	0.297098
19260	1.34562	0.243171
19261	1.49515	0.352082
19262	1.66295	0.22248
19263	1.02395	2.1
19264	1.51067	2.1
19265	1.35821	2.09603
19266	2.10671	2.1
19267	1.0305	2.05586
19268	1.07855	2.03149
19269	1.20761	2.1
19270	0.98488	2.1
19271	1.4162	2.1
19272	1.14825	2.1
19273	1.29415	2.1
19274	1.6067	2.1
19275	1.23851	0.133772
19276	1.09651	0.1091
19277	1.60636	2.1
19278	1.53521	2.1
19279	1.53521	2.1
19280	1.46279	2.1
19281	1.28044	0.05
19282	1.64147	0.0579234
19283	1.18422	0.05
19284	1.68208	0.06289
19285	1.09726	2.1
19286	1.234	2.1
19287	1.234	2.1
19288	1.09483	2.1
19289	1.89318	2.1
19290	1.98448	2.1
19291	1.98448	2.1
19292	2.02612	2.1
19293	1.22026	2.08369
19294	1.23527	1.88396
19295	1.09044	2.06929
19296	0.993366	1.49636
19297	1.22026	2.02844
19298	0.993368	1.14061
19299	2.61357	2.1
19300	2.48208	2.1
19301	2.48208	2.1
19302	2.62868	2.1
19303	1.85439	0.768847
19304	1.81776	0.480666
19305	1.81776	0.356831
19306	2.1587	0.354679
19307	1.8914	0.66163
19308	2.23961	0.414705
19309	1.49357	0.120477
19310	2.23961	0.270449
19311	1.78014	0.355931

19312	1.67635	0.315716
19313	1.66219	0.28568
19314	1.56036	0.262804
19315	1.14721	0.277589
19316	2.22495	0.349669
19317	1.88509	0.05
19318	1.95249	0.05
19319	2.11959	0.05
19320	1.37427	0.05
19321	1.39379	2.06077
19322	1.17614	1.92805
19323	1.39379	2.1
19324	1.14397	1.52633
19325	1.09651	2.1
19326	1.42843	2.1
19327	1.7069	2.1
19328	2.31293	2.1
19329	1.56839	2.1
19330	1.78252	1.82384
19331	1.78254	1.37253
19332	1.09142	2.08355
19333	0.99829	0.05
19334	1.09372	0.05
19335	1.88175	0.0505009
19336	0.898452	0.05
19337	1.06936	2.1
19338	1.4162	2.1
19339	1.17989	2.1
19340	1.47411	2.1
19341	1.25376	2.1
19342	1.00757	2.09239
19343	1.4185	0.190721
19344	1.02011	0.202932
19345	2.22495	0.220813
19346	1.79542	0.20207
19347	1.79542	0.169887
19348	1.19352	0.161215
19349	2.38566	2.1
19350	1.83255	1.80577
19351	1.62667	2.1
19352	1.17879	1.91956
19353	2.79168	2.1
19354	2.28162	2.1
19355	2.28162	2.1
19356	1.90905	2.1
19357	1.40117	2.1
19358	1.28268	2.1
19359	1.41096	2.1
19360	1.22978	2.1
19361	3.20034	2.1
19362	3.42052	2.1
19363	3.42052	2.1
19364	3.50562	2.1
19365	1.46279	2.1
19366	1.3864	2.1
19367	1.3864	2.1
19368	1.3201	2.1
19369	1.35561	0.05

19370	0.974077	0.05
19371	0.967673	0.766882
19372	1.22482	0.642086
19373	1.02305	0.266174
19374	1.22482	0.40355
19375	0.999492	2.1
19376	1.14657	2.1
19377	1.14657	2.1
19378	1.31346	2.1
19379	1.84417	0.05
19380	1.31777	0.05
19381	1.19796	0.05
19382	0.691742	0.05
19383	1.16725	2.1
19384	1.9679	2.1
19385	1.08848	0.05
19386	1.15356	0.05
19387	1.58713	2.1
19388	1.58742	2.1
19389	1.58742	2.1
19390	1.49662	2.1
19391	1.32201	0.810294
19392	1.32097	1.66093
19393	1.32097	0.838735
19394	1.31825	1.73295
19395	1.12028	2.1
19396	1.15852	2.1
19397	1.15852	2.1
19398	1.16239	2.1
19399	1.07624	2.1
19400	1.30047	2.1
19401	1.44847	0.573903
19402	1.55292	0.518292
19403	1.21125	0.05
19404	1.15262	2.1
19405	1.05025	2.1
19406	1.55351	2.1
19407	1.5329	0.05
19408	2.26758	0.05
19409	1.94968	0.05
19410	1.65747	0.05
19411	1.42549	0.05
19412	1.57656	0.05
19413	2.11157	0.690687
19414	1.56531	0.637377
19415	1.69279	0.540878
19416	1.06204	0.714138
19417	0.947863	0.929572
19418	1.19854	1.07417
19419	0.947862	0.37672
19420	1.31126	0.405492
19421	1.3425	1.6616
19422	1.29976	1.34704
19423	1.05129	0.0772098
19424	1.51026	0.0878298
19425	1.05129	0.0837467
19426	1.2951	0.0965343
19427	1.7155	2.1

19428	1.72196	2.1
19429	1.72196	2.1
19430	1.77867	2.1
19431	0.964755	2.1
19432	1.14142	2.1
19433	1.03766	2.1
19434	0.986723	2.1
19435	1.28072	2.1
19436	1.10609	2.1
19437	1.28072	1.77037
19438	1.16776	2.1
19439	1.61687	2.1
19440	1.60965	2.1
19441	1.60965	2.1
19442	1.59654	2.1
19443	1.26512	2.1
19444	1.23964	2.1
19445	2.07997	2.1
19446	2.60048	2.1
19447	1.23732	2.1
19448	1.3372	2.1
19449	2.62253	2.1
19450	2.47056	2.1
19451	0.92782	0.0858314
19452	1.40335	0.0946132
19453	1.26301	0.107019
19454	1.52668	0.104668
19455	0.99818	0.1075
19456	1.51165	0.130879
19457	1.66213	0.100228
19458	1.55246	0.104556
19459	2.31255	2.1
19460	1.34366	2.1
19461	1.90577	2.1
19462	0.969739	2.1
19463	3.4245	2.1
19464	2.8415	2.1
19465	1.97559	2.1
19466	1.82362	2.1
19467	1.48162	2.1
19468	1.23204	2.1
19469	1.23204	2.1
19470	0.95947	2.1
19471	1.05267	2.07895
19472	1.44091	1.15494
19473	1.05267	1.43829
19474	1.5795	1.97157
19475	0.738711	2.1
19476	1.28549	2.1
19477	1.15694	2.1
19478	1.72061	2.1
19479	1.69858	2.1
19480	0.954563	2.1
19481	0.859087	2.1
19482	0.788886	2.1
19483	1.08732	0.134579
19484	1.3357	0.122663
19485	1.3357	0.125412

19486	1.48676	0.110344
19487	2.15079	2.1
19488	2.12414	2.1
19489	2.12414	2.1
19490	2.04664	2.1
19491	1.98859	2.1
19492	0.992159	2.1
19493	1.48064	2.1
19494	1.2163	2.1
19495	1.26337	2.1
19496	1.63159	2.1
19497	1.00366	0.326137
19498	1.65452	0.186392
19499	1.10408	2.1
19500	1.37704	2.1
19501	1.50224	2.1
19502	1.42866	2.1
19503	1.43939	2.1
19504	1.02784	2.1
19505	1.00763	0.0971564
19506	1.23746	0.099037
19507	1.23747	0.10712
19508	1.13642	0.113789
19509	1.49906	0.0628438
19510	1.46506	0.0526913
19511	1.46506	0.05
19512	1.33061	0.05
19513	1.08166	0.160879
19514	1.01052	0.146584
19515	1.02836	0.176463
19516	1.08166	0.161419
19517	1.25334	0.05
19518	1.23749	0.05
19519	1.37501	0.0640341
19520	1.47354	0.0878222
19521	1.30167	2.1
19522	1.29897	2.1
19523	1.51042	2.1
19524	1.07684	2.1
19525	1.07684	2.1
19526	0.765121	2.1
19527	1.5535	2.1
19528	1.40602	2.1
19529	1.40603	2.1
19530	1.25669	2.1
19531	1.08276	2.1
19532	1.60948	2.1
19533	1.26929	2.1
19534	1.09847	2.1
19535	0.827082	0.176713
19536	1.15841	0.191531
19537	1.25063	0.460284
19538	1.14397	0.45173
19539	1.25063	1.77873
19540	1.10476	0.255427
19541	1.14872	0.05
19542	1.3659	0.05
19543	1.36589	0.05

19544	1.4677	0.05
19545	0.590354	2.1
19546	0.655202	2.1
19547	3.48926	2.1
19548	3.32709	2.1
19549	3.32709	2.1
19550	3.13938	2.1
19551	1.24458	0.880317
19552	1.29681	0.624219
19553	1.40915	1.04324
19554	1.17891	1.79594
19555	1.33171	0.05
19556	1.37681	1.68632
19557	1.37681	2.1
19558	1.4516	2.1
19559	2.68241	0.05
19560	1.61031	0.05
19561	1.6488	2.1
19562	1.6585	2.1
19563	1.65848	2.1
19564	1.67061	2.1
19565	1.15152	2.1
19566	1.32548	2.1
19567	1.43677	2.1
19568	1.10264	2.1
19569	1.01472	2.1
19570	0.966964	2.1
19571	2.867	2.1
19572	2.94897	2.1
19573	1.65815	2.1
19574	1.4502	2.1
19575	1.4502	2.1
19576	1.26755	2.1
19577	1.37138	2.1
19578	1.45026	2.1
19579	1.45026	2.1
19580	1.53446	2.1
19581	0.973502	2.07859
19582	1.08542	2.1
19583	1.13387	0.05
19584	1.71031	0.05
19585	1.08911	2.1
19586	1.10748	2.1
19587	1.21823	2.1
19588	1.25005	2.1
19589	0.657014	2.1
19590	1.38139	2.1
19591	1.01485	2.1
19592	1.26045	2.1
19593	1.12468	2.1
19594	1.26045	2.1
19595	1.89278	0.499801
19596	1.06433	0.391877
19597	1.87861	0.405073
19598	0.967544	0.316447
19599	1.50319	2.1
19600	3.47245	2.1
19601	2.2354	1.13178

19602	1.16701	0.337472
19603	1.3592	2.1
19604	1.3305	1.53437
19605	1.3305	2.1
19606	1.15008	0.822645
19607	1.12457	0.05
19608	1.40707	0.0567827
19609	1.01778	2.1
19610	1.35733	2.1
19611	1.01779	2.1
19612	1.12064	2.1
19613	1.30694	0.473974
19614	1.42716	1.37822
19615	1.42717	1.61773
19616	1.04321	1.56559
19617	1.19352	0.144795
19618	1.32634	0.19427
19619	1.14605	1.93436
19620	1.0417	1.6751
19621	1.19676	0.0722061
19622	1.03804	0.0806946
19623	2.09455	0.0592586
19624	1.15338	0.0664527
19625	2.21119	2.1
19626	1.26097	2.1
19627	1.88394	1.74067
19628	1.24106	0.951412
19629	2.98143	2.1
19630	2.56248	2.1
19631	1.88394	2.1
19632	1.68703	0.762443
19633	1.5846	2.1
19634	1.94799	2.1
19635	1.20091	2.06097
19636	1.40393	2.09805
19637	3.09031	2.1
19638	2.90243	2.1
19639	1.27227	2.1
19640	1.32592	2.1
19641	2.90247	2.1
19642	2.71057	2.1
19643	1.32594	2.1
19644	1.34817	2.1
19645	1.4092	2.1
19646	1.44859	2.1
19647	1.52008	2.1
19648	1.401	2.1
19649	1.40099	2.1
19650	1.28936	2.1
19651	1.56002	0.05
19652	1.08811	0.05
19653	1.46041	0.05
19654	1.56002	0.05
19655	1.50524	2.1
19656	1.21304	2.1
19657	1.21304	2.1
19658	0.947115	2.1
19659	1.60665	2.03349

19660	1.85179	1.77666
19661	1.85178	1.79263
19662	2.06277	1.1532
19663	2.17534	2.1
19664	1.23007	2.1
19665	2.17533	2.09633
19666	1.66635	1.98659
19667	2.00869	2.1
19668	0.986724	2.1
19669	2.00475	2.1
19670	1.99394	2.1
19671	1.67244	0.122971
19672	1.39604	0.110158
19673	1.39604	0.124228
19674	0.954046	0.0968816
19675	0.988863	2.1
19676	1.9864	2.1
19677	1.9864	2.1
19678	3.38645	2.1
19679	1.46932	0.05
19680	1.50847	0.05
19681	1.50848	0.05
19682	1.42705	0.05
19683	1.50108	2.1
19684	1.51621	0.291139
19685	1.94317	0.413951
19686	1.18382	0.1671
19687	2.05091	0.05
19688	1.88593	0.05
19689	1.54299	0.05
19690	0.987265	0.05
19691	1.27766	2.1
19692	1.5178	2.1
19693	1.5178	2.1
19694	1.81518	2.1
19695	1.04626	1.46346
19696	1.31057	1.50169
19697	1.31057	1.32325
19698	1.22297	1.60847
19699	1.67844	2.1
19700	1.62862	2.1
19701	1.62112	2.1
19702	1.87598	2.1
19703	1.17844	2.1
19704	1.37063	2.1
19705	1.37063	2.1
19706	1.68723	2.1
19707	1.99943	0.067149
19708	1.17631	0.05
19709	1.4263	1.96837
19710	1.31023	2.1
19711	1.31023	2.0789
19712	1.20871	2.1
19713	1.50356	2.1
19714	1.69311	2.1
19715	1.69599	2.1
19716	1.6801	2.1
19717	1.5843	0.05

19718	0.840233	0.05
19719	1.95358	0.05
19720	1.40216	0.05
19721	1.39078	2.05129
19722	1.18948	2.04155
19723	1.01606	1.88884
19724	1.18948	2.00734
19725	1.63823	2.1
19726	1.67687	2.1
19727	1.67687	2.1
19728	1.52855	2.1
19729	1.11048	2.1
19730	1.25346	2.1
19731	2.42984	1.22353
19732	3.41731	1.50912
19733	1.2258	2.1
19734	1.2966	2.1
19735	1.2966	2.1
19736	1.37392	2.1
19737	1.41758	2.1
19738	1.35504	2.1
19739	1.35504	2.1
19740	1.30347	2.1
19741	1.50877	2.1
19742	1.11193	2.1
19743	1.40984	2.05764
19744	1.54537	2.07532
19745	1.71638	2.08303
19746	1.77795	2.0066
19747	2.07593	2.1
19748	1.93387	2.1
19749	1.9339	2.1
19750	1.75669	2.1
19751	1.80299	0.430499
19752	1.31251	0.341105
19753	1.31249	0.420137
19754	1.13411	0.329816
19755	1.06313	2.1
19756	1.04921	2.1
19757	1.55766	2.1
19758	1.53728	2.1
19759	1.53729	2.1
19760	1.51789	2.1
19761	0.993021	2.1
19762	1.02478	2.1
19763	1.03408	2.1
19764	1.09056	2.1
19765	0.697755	2.1
19766	0.632901	2.1
19767	1.34072	0.113615
19768	1.80841	0.1192
19769	1.80841	0.111219
19770	1.17793	0.118799
19771	1.75032	2.04454
19772	1.13076	2.09813
19773	2.11851	2.1
19774	1.73255	2.01179
19775	1.73255	2.1

19776	1.11181	1.92787
19777	1.43772	2.1
19778	1.67381	2.1
19779	1.52164	2.1
19780	1.60861	2.1
19781	1.30588	0.05
19782	1.75241	0.05
19783	1.33026	1.83339
19784	0.962705	1.5157
19785	0.99678	0.05
19786	1.30572	0.05
19787	1.41943	0.05
19788	1.2683	0.05
19789	1.14145	0.05
19790	1.01361	0.05
19791	1.29943	2.1
19792	1.3483	2.1
19793	1.34832	2.1
19794	1.83488	2.1
19795	1.71065	2.1
19796	1.66178	2.1
19797	1.39076	2.1
19798	1.37456	2.1
19799	1.66126	2.1
19800	1.562	2.1
19801	1.37456	2.1
19802	1.36065	2.1
19803	1.04026	0.415685
19804	1.51898	0.394783
19805	1.67088	0.235278
19806	1.9726	0.1723
19807	1.74852	0.916659
19808	1.95512	1.10522
19809	1.16165	0.956339
19810	1.06584	0.734677
19811	1.39327	2.1
19812	1.41394	2.1
19813	1.41394	2.1
19814	1.45506	2.1
19815	1.39523	2.1
19816	1.21119	2.1
19817	1.09005	2.1
19818	0.921311	2.1
19819	1.58164	2.1
19820	1.77574	2.1
19821	1.73184	2.1
19822	1.59423	2.1
19823	1.8214	2.1
19824	3.10544	0.566069
19825	1.01243	1.81973
19826	1.30896	1.80579
19827	2.16956	0.874479
19828	1.29608	0.166302
19829	1.38617	1.51125
19830	1.49536	1.34078
19831	2.35239	0.845716
19832	1.24613	1.6224
19833	1.94351	2.1

19834	2.05361	2.1
19835	2.05358	2.1
19836	2.38994	2.1
19837	1.13533	2.1
19838	1.08276	2.1
19839	1.55611	0.230958
19840	1.33957	0.159238
19841	1.33957	0.131367
19842	1.69011	0.303995
19843	1.5515	0.558127
19844	1.59106	0.469242
19845	1.02958	2.1
19846	1.12266	2.1
19847	1.12266	2.1
19848	1.24925	2.1
19849	1.80223	2.1
19850	1.20242	2.1
19851	2.13665	0.893113
19852	1.3924	0.784001
19853	0.904894	2.06578
19854	1.82633	2.1
19855	1.94138	0.05
19856	1.34009	0.05
19857	1.15096	2.1
19858	0.994292	2.1
19859	1.10101	2.1
19860	1.15096	2.1
19861	1.50438	2.1
19862	1.67825	2.1
19863	1.67825	2.1
19864	1.91894	2.1
19865	2.04664	2.1
19866	1.92063	2.1
19867	1.72857	2.1
19868	1.89757	2.1
19869	1.61843	0.05
19870	1.49431	0.05
19871	2.33925	2.1
19872	1.80683	2.1
19873	1.64257	2.1
19874	1.50108	2.1
19875	2.82561	2.1
19876	2.97847	2.1
19877	2.97848	2.1
19878	3.20034	2.1
19879	0.66055	2.1
19880	1.95919	2.1
19881	1.07976	2.1
19882	1.30237	2.1
19883	1.99943	0.05
19884	1.31802	0.05
19885	1.53336	0.0718932
19886	1.31802	0.0510387
19887	1.27737	0.473778
19888	1.44848	0.473919
19889	2.35499	0.05
19890	1.30041	0.05
19891	2.35499	0.05

19892	1.65381	0.05
19893	0.996083	2.1
19894	1.28754	2.1
19895	0.996081	2.1
19896	1.44859	2.1
19897	1.24763	2.05259
19898	1.03697	2.09065
19899	0.951273	1.302
19900	1.60018	1.6808
19901	2.0815	2.1
19902	1.20488	2.1
19903	2.95785	2.1
19904	2.52245	2.1
19905	1.68455	0.05
19906	1.12971	0.05
19907	1.1952	0.116113
19908	2.0815	0.174731
19909	1.3963	2.1
19910	1.4934	2.1
19911	1.47973	2.1
19912	1.25559	2.1
19913	1.1233	2.1
19914	1.15009	2.1
19915	1.15009	2.1
19916	0.977775	2.1
19917	1.16482	0.679405
19918	1.29313	0.249192
19919	1.29313	0.418489
19920	1.294	0.250717
19921	1.48693	0.135796
19922	1.47608	0.15539
19923	1.36005	2.04484
19924	1.47109	2.1
19925	1.47109	2.1
19926	1.3515	2.1
19927	2.48828	2.1
19928	2.32378	2.1
19929	2.32378	2.1
19930	2.1692	2.1
19931	1.03217	0.731921
19932	0.953521	0.918993
19933	1.4515	0.101614
19934	3.72406	0.0784198
19935	0.963395	2.06063
19936	1.01346	1.13844
19937	1.62241	2.1
19938	1.62027	2.1
19939	1.62027	2.1
19940	1.61687	2.1
19941	1.2593	0.05
19942	0.981586	0.05
19943	0.721961	2.1
19944	0.888955	2.1
19945	1.22031	0.05
19946	1.27566	2.1
19947	2.50066	2.1
19948	1.19814	2.1
19949	1.42278	0.05

19950	2.02657	0.05
19951	1.0756	0.231054
19952	1.10049	0.258249
19953	2.26155	2.1
19954	2.15634	2.1
19955	2.15634	2.1
19956	2.01613	2.1
19957	1.94233	1.02391
19958	1.18127	0.753255
19959	1.36865	0.140088
19960	1.75755	0.393391
19961	2.01613	2.1
19962	1.78958	2.1
19963	1.97995	2.1
19964	1.79198	2.1
19965	1.20841	2.1
19966	1.6519	2.1
19967	1.80207	2.1
19968	1.66774	2.1
19969	1.17935	0.05
19970	1.88071	0.0584852
19971	1.58723	0.05
19972	1.7361	0.05
19973	0.794165	2.1
19974	1.21327	2.1
19975	1.21327	2.1
19976	1.34268	2.1
19977	1.34497	0.05
19978	0.793303	0.05
19979	1.12323	0.05
19980	1.34496	0.05
19981	0.928226	0.0559861
19982	1.89804	0.120239
19983	2.60141	0.05
19984	2.60142	2.1
19985	2.60772	2.09666
19986	0.99747	2.1
19987	1.27096	1.64863
19988	1.27096	1.08675
19989	1.03472	1.706
19990	1.09962	2.08309
19991	1.21148	2.1
19992	1.21148	2.1
19993	1.35025	2.1
19994	1.09795	2.0979
19995	1.00775	2.1
19996	1.07162	2.1
19997	1.60665	2.02947
19998	1.52984	2.1
19999	1.61179	2.1
20000	1.70057	2.1
20001	1.76063	2.1
20002	1.14547	0.328268
20003	0.964782	0.293492
20004	1.17208	0.293438
20005	0.964769	0.257972
20006	1.04268	2.1
20007	1.57459	2.1

20008	1.57459	2.1
20009	1.32449	2.1
20010	2.07527	0.688604
20011	1.00013	0.740003
20012	0.813711	0.05
20013	1.05677	2.1
20014	1.30664	1.49717
20015	1.96011	1.82861
20016	1.7151	1.24929
20017	1.61309	1.31225
20018	1.18954	2.1
20019	1.05447	2.1
20020	1.05446	0.95476
20021	1.11787	0.877078
20022	1.44437	2.1
20023	1.84739	2.1
20024	1.84739	2.1
20025	1.06203	2.1
20026	1.78391	2.1
20027	1.89267	2.1
20028	1.9212	2.1
20029	2.02997	2.1
20030	1.32051	0.165521
20031	1.47169	0.0880048
20032	1.20691	0.05
20033	1.72271	0.05
20034	1.2858	0.169179
20035	1.31011	0.851121
20036	1.31011	0.129714
20037	1.34124	0.625368
20038	1.294	0.450045
20039	1.03079	0.303753
20040	1.83192	1.88741
20041	1.81979	2.00367
20042	1.81979	1.98735
20043	1.57829	2.07204
20044	1.35435	1.4963
20045	1.19023	1.79827
20046	0.991115	2.1
20047	1.27725	2.1
20048	1.38204	2.1
20049	1.48013	2.1
20050	0.983493	2.1
20051	2.26155	2.1
20052	1.02311	0.426492
20053	1.55485	0.42497
20054	1.71033	0.310945
20055	2.07319	0.29794
20056	2.29074	1.10812
20057	1.50822	1.85315
20058	2.08147	2.1
20059	1.1585	2.1
20060	3.74092	1.91259
20061	2.40799	2.1
20062	2.05349	2.1
20063	1.92656	2.1
20064	1.09634	0.161063
20065	1.45502	0.276524

20066	1.17261	0.129371
20067	1.29383	0.123351
20068	1.29383	0.0740449
20069	1.47689	0.105183
20070	1.85717	0.445524
20071	1.53798	0.177513
20072	1.9493	2.1
20073	2.0371	2.09059
20074	1.8519	2.1
20075	1.92119	2.02118
20076	1.00764	0.101379
20077	1.0302	0.0896649
20078	1.0302	0.0882616
20079	0.90047	0.084825
20080	1.49155	0.150329
20081	2.26403	0.0809935
20082	2.06158	0.05
20083	1.09506	0.05
20084	1.9212	1.95679
20085	1.99586	1.57067
20086	1.99586	1.66113
20087	2.07527	1.01328
20088	1.20478	2.1
20089	1.09662	2.1
20090	1.55961	2.1
20091	1.03472	2.1
20092	1.54983	2.1
20093	2.02953	2.1
20094	1.84502	2.1
20095	2.1221	2.1
20096	1.02969	1.99893
20097	2.23523	2.08425
20098	1.2377	0.05
20099	1.03818	0.0959926
20100	1.978	2.1
20101	1.78159	2.1
20102	1.45762	2.1
20103	1.34234	2.1
20104	2.01624	2.1
20105	2.52072	2.1
20106	1.23013	2.1
20107	2.5593	2.1
20108	3.06464	0.940386
20109	2.63697	0.404953
20110	2.63697	0.776525
20111	1.87111	0.338238
20112	1.04574	0.05
20113	2.13428	0.05
20114	1.61715	2.1
20115	1.44137	2.1
20116	1.44867	2.1
20117	1.18283	2.1
20118	1.50584	0.0720873
20119	1.45466	0.0911586
20120	1.4959	0.0946709
20121	1.93489	0.080112
20122	2.60772	2.1
20123	2.68868	2.1

20124	2.68868	2.1
20125	2.82561	2.1
20126	1.49654	2.1
20127	1.38672	2.1
20128	1.36813	2.1
20129	1.98396	2.1
20130	1.93852	1.33952
20131	2.14237	0.527725
20132	1.44421	0.05
20133	1.21749	0.05
20134	1.09572	0.05
20135	1.11081	0.05
20136	2.32363	2.1
20137	1.24375	2.1
20138	3.79242	2.1
20139	3.46787	2.1
20140	1.19854	1.25365
20141	1.35487	1.29175
20142	1.35486	1.47512
20143	1.38616	1.36759
20144	1.67265	0.133933
20145	0.909487	0.104088
20146	2.53562	2.1
20147	1.09564	2.1
20148	2.9068	2.1
20149	3.57307	0.945256
20150	2.50369	2.03749
20151	1.47348	2.1
20152	1.24925	2.1
20153	1.3991	2.1
20154	1.3991	2.1
20155	1.52984	2.1
20156	1.60428	2.1
20157	1.50097	2.1
20158	1.50097	2.1
20159	1.40239	2.1
20160	2.19621	2.1
20161	2.35003	2.1
20162	1.57932	0.05
20163	1.61194	0.05
20164	2.0815	0.05
20165	1.18539	0.05
20166	1.5795	0.880804
20167	1.53064	1.33735
20168	1.89227	0.05
20169	3.01037	0.05
20170	2.08149	2.08175
20171	2.06678	2.0287
20172	1.89226	0.05
20173	3.47888	0.05
20174	1.89226	0.0795833
20175	3.65842	0.05
20176	3.88546	0.121852
20177	1.89226	0.194817
20178	1.11239	2.1
20179	1.29133	0.05
20180	0.889309	0.05
20181	0.881934	0.05

20182	1.88406	2.1
20183	0.636946	2.1
20184	0.636945	2.0843
20185	1.28073	2.1
20186	1.28075	2.1
20187	0.942007	2.1
20188	1.68436	2.1
20189	1.4677	0.05
20190	0.851542	0.05
20191	1.39063	0.05
20192	0.851539	0.05
20193	1.11597	0.05
20194	1.35403	0.05
20195	0.959473	2.1
20196	1.07976	2.1
20197	0.946129	2.1
20198	0.946129	0.469958
20199	1.38464	1.98428
20200	1.36991	1.02491
20201	0.946131	1.16814
20202	0.94613	1.20117
20203	1.34493	0.553189
20204	1.36991	0.379741
20205	1.36991	0.134033
20206	0.946131	0.215323
20207	0.78392	0.221615
20208	0.80282	0.174719
20209	1.10351	0.218743
20210	0.80846	0.193976
20211	0.838026	0.223817
20212	1.08558	0.178247
20213	0.718203	0.188469
20214	0.501866	0.0959584
20215	0.85426	0.168352
20216	1.24249	0.0511446
20217	1.37052	0.0793361
20218	0.851541	0.05
20219	1.0092	0.116335
20220	0.851554	0.0675049
20221	1.49826	0.346207
20222	1.13727	0.307029
20223	0.778149	0.208577
20224	1.20257	0.266865
20225	0.946133	0.211708
20226	0.778149	0.348337
20227	0.77815	0.312925
20228	0.946132	2.1
20229	1.17602	2.1
20230	0.946132	2.1
20231	1.31257	2.1
20232	1.17602	2.1
20233	1.35821	0.0908859
20234	1.19993	0.05
20235	0.946131	0.05
20236	0.946131	0.0994268
20237	1.08848	0.0862664
20238	1.00509	2.1
20239	1.19629	2.1

20240	0.867593	2.1
20241	0.757921	2.1
20242	0.729116	0.05
20243	0.89162	0.05
20244	1.16811	0.05
20245	1.3676	2.1
20246	1.2638	2.07723
20247	0.946132	2.1
20248	0.946132	2.1
20249	1.2638	2.1
20250	1.00185	2.1
20251	1.60948	2.1
20252	0.602075	2.1
20253	0.942611	2.1
20254	0.946132	2.1
20255	0.942611	2.1
20256	1.11822	2.1
20257	1.21558	0.101036
20258	0.607788	0.164326
20259	0.827029	0.203666
20260	1.00691	0.412815
20261	0.607789	0.159809
20262	0.607775	0.312724
20263	1.23248	1.75619
20264	0.902653	2.04192
20265	0.639143	2.03618
20266	0.851545	1.61358
20267	0.605153	1.94855
20268	1.06826	2.1
20269	1.29365	2.1
20270	1.41232	2.1
20271	0.946131	2.1
20272	1.20854	2.1
20273	0.946131	2.1
20274	0.909005	0.05
20275	1.16672	0.05
20276	0.810959	0.05
20277	0.946131	0.0507219
20278	1.40104	0.05
20279	0.946131	0.0500004
20280	1.35403	0.05
20281	1.40104	0.05
20282	1.92535	0.05
20283	2.14534	0.05
20284	1.77532	0.05
20285	0.851541	0.0586633
20286	1.05585	0.0676134
20287	1.27098	0.0572817
20288	0.870925	0.0676465
20289	1.15547	0.0688122
20290	1.47583	0.05
20291	1.35007	0.05
20292	0.740095	0.05
20293	0.644912	0.05
20294	0.819489	0.05
20295	0.983028	0.0967058
20296	0.861849	0.05
20297	1.40511	0.05

20298	0.86182	0.05
20299	0.9705	0.05
20300	1.40511	0.05
20301	1.15539	2.1
20302	0.946119	2.1
20303	0.736382	2.1
20304	0.736384	2.1
20305	1.41561	2.1
20306	0.785651	2.1
20307	1.34479	2.1
20308	1.38885	2.1
20309	0.94613	2.1
20310	1.29461	2.1
20311	0.94613	2.1
20312	0.776179	2.1
20313	0.858611	2.1
20314	0.946128	2.1
20315	1.04073	2.1
20316	1.16399	2.1
20317	0.990019	2.1
20318	1.15564	2.07461
20319	1.28956	0.0713181
20320	1.35145	0.098744
20321	1.09662	2.1
20322	1.8155	0.05
20323	0.811459	0.05
20324	0.731982	0.05
20325	0.900554	0.05
20326	0.946127	0.788785
20327	0.817066	0.747508
20328	0.817064	0.808678
20329	1.41798	0.741823
20330	0.871606	0.789767
20331	1.30265	0.05
20332	0.849402	1.70536
20333	0.868582	1.10881
20334	0.909137	1.19799
20335	0.807016	1.13831
20336	0.83393	0.569251
20337	0.9034	0.73856
20338	1.53515	0.716617
20339	1.17665	0.278115
20340	0.946132	0.395512
20341	0.946129	0.273399
20342	1.35977	0.271726
20343	1.34254	0.25232
20344	1.14241	0.194956
20345	0.946132	0.295286
20346	0.946132	0.194927
20347	1.27376	0.199585
20348	1.35978	0.185563
20349	1.01976	0.182637
20350	1.23498	0.680886
20351	1.35002	0.75296
20352	1.11683	1.505
20353	0.88359	0.123036
20354	0.751374	1.09723
20355	0.791714	0.156405

20356	1.6067	2.1
20357	0.923782	2.1
20358	1.44689	2.1
20359	0.94613	2.1
20360	0.946131	2.1
20361	1.5261	2.1
20362	1.26247	2.09324
20363	1.80085	0.0902601
20364	1.7693	0.122688
20365	0.900422	0.0502526
20366	1.31357	0.0736843
20367	0.94613	0.05
20368	0.900424	0.105061
20369	0.900397	0.0502476
20370	1.39448	0.0555569
20371	1.36769	0.05
20372	0.713524	0.05
20373	0.713554	0.05
20374	0.946124	0.05
20375	1.24979	0.05
20376	1.04765	0.05
20377	0.70054	0.05
20378	0.776991	0.05
20379	0.738905	0.05
20380	1.32605	0.05
20381	0.784957	0.05
20382	0.803955	0.05
20383	0.819486	0.05
20384	1.79306	0.05
20385	1.18275	0.05
20386	1.25626	0.05
20387	0.936806	0.05
20388	0.653124	0.0519723
20389	0.946132	0.05
20390	0.653151	0.05
20391	1.10748	0.0770734
20392	1.95244	2.1
20393	1.40967	1.49514
20394	1.27916	1.22784
20395	0.946132	1.97153
20396	0.946132	2.1
20397	1.30555	2.1
20398	1.38651	2.1
20399	0.946132	2.1
20400	1.23571	0.385871
20401	1.21261	0.825337
20402	1.34225	0.882843
20403	0.946132	1.4173
20404	0.734059	0.540693
20405	0.734084	0.894564
20406	0.78438	0.251114
20407	1.13748	0.438813
20408	0.946131	0.63722
20409	0.78438	0.240711
20410	0.784392	0.43853
20411	0.626495	2.1
20412	0.694635	2.1
20413	0.946125	2.1

20414	0.694632	2.1
20415	1.38521	2.1
20416	0.795469	2.1
20417	0.850877	2.1
20418	1.37098	2.1
20419	0.946131	2.1
20420	0.79547	2.1
20421	0.946132	0.160212
20422	1.30448	0.0755456
20423	0.628361	0.0585891
20424	0.686964	0.16028
20425	0.493261	0.0626449
20426	1.90912	0.26178
20427	0.946127	0.170181
20428	1.36604	0.0876498
20429	0.671687	0.0795149
20430	0.946128	0.290665
20431	0.671699	0.104263
20432	1.04973	2.1
20433	0.982858	0.235748
20434	0.974081	0.05
20435	1.69993	0.05
20436	1.00763	0.265309
20437	0.755566	2.1
20438	0.946129	2.1
20439	1.04184	2.1
20440	1.13509	2.1
20441	0.755566	2.1
20442	1.114 2.1	
20443	0.810946	2.1
20444	0.786259	2.1
20445	1.43455	2.1
20446	1.05071	2.1
20447	0.746095	2.1
20448	0.82737	2.1
20449	0.946126	2.1
20450	1.04071	2.1
20451	1.0852	2.1
20452	1.59019	0.05
20453	0.621972	0.05
20454	1.14475	0.05
20455	0.691062	0.05
20456	1.20057	0.05
20457	0.846868	0.05
20458	1.26062	0.05
20459	0.795093	0.05
20460	1.20056	0.05
20461	0.795065	0.05
20462	1.18203	0.05
20463	1.20057	0.05
20464	0.991869	0.0710569
20465	0.841088	2.1
20466	0.946131	2.1
20467	0.869097	2.1
20468	0.946131	2.1
20469	0.841089	2.1
20470	1.77867	2.1
20471	1.99296	0.0939711

20472	1.91703	2.1
20473	0.651334	0.05
20474	1.31467	0.05
20475	0.651332	0.05
20476	0.651332	0.05
20477	1.03079	0.05
20478	0.808519	0.05
20479	1.41417	0.05
20480	1.03165	0.16762
20481	1.43577	2.1
20482	1.39053	2.1
20483	1.07006	2.1
20484	0.946132	2.1
20485	0.946133	2.1
20486	1.07006	2.1
20487	1.12717	2.1
20488	0.994293	2.1
20489	0.946132	2.1
20490	1.31912	2.1
20491	0.946131	2.1
20492	1.40571	2.1
20493	1.31912	2.1
20494	1.12292	0.318998
20495	1.07755	0.374674
20496	0.851539	0.348992
20497	0.946131	0.32574
20498	0.929834	0.336361
20499	1.83919	0.434953
20500	1.04713	0.0643881
20501	0.929726	0.0507099
20502	1.50985	0.05
20503	0.946127	0.05
20504	1.0227	0.0574011
20505	1.2582	0.05
20506	1.1391	0.345128
20507	0.780831	0.568198
20508	0.78086	0.39456
20509	0.946133	0.540127
20510	0.780831	0.375381
20511	1.37762	0.462201
20512	0.946116	2.1
20513	1.22882	2.1
20514	1.40015	2.1
20515	1.40017	2.1
20516	0.946105	2.1
20517	0.553567	0.05
20518	1.19263	0.05
20519	0.946132	0.05
20520	0.553594	0.05
20521	1.19263	0.05
20522	1.18237	0.05
20523	0.946133	0.05
20524	0.978467	0.05
20525	0.56262	0.05
20526	0.946132	0.05
20527	0.562633	0.05
20528	0.900175	0.05
20529	1.04627	1.81999

20530	0.549928	0.989266
20531	1.17765	0.715368
20532	1.11479	1.52267
20533	0.398261	1.13688
20534	0.927608	1.13706
20535	1.38467	1.01137
20536	1.04824	0.675298
20537	1.33893	0.713739
20538	1.55775	1.06294
20539	0.816895	1.2259
20540	1.96057	1.22574
20541	1.35798	2.1
20542	1.29262	2.1
20543	0.94613	2.1
20544	0.94613	2.1
20545	1.29261	2.1
20546	1.18067	2.1
20547	0.946131	2.1
20548	0.946132	2.1
20549	1.27809	2.1
20550	1.35799	2.1
20551	1.03933	2.1
20552	1.49438	2.1
20553	0.946132	2.1
20554	1.13537	2.1
20555	1.41666	2.1
20556	1.74237	2.1
20557	0.946132	2.1
20558	1.04291	0.863069
20559	1.63802	2.1
20560	1.24714	2.1
20561	0.842013	2.1
20562	0.810959	2.1
20563	0.767923	0.146812
20564	1.19583	0.123045
20565	0.767921	0.0982119
20566	0.767921	0.0668633
20567	0.961348	0.128831
20568	2.08687	1.9878
20569	0.739729	0.701089
20570	1.04342	0.627099
20571	0.739757	0.607147
20572	0.946129	0.732943
20573	1.04341	1.05162
20574	1.38683	0.18926
20575	1.34153	0.16307
20576	0.946129	0.199756
20577	0.946131	0.200448
20578	1.02028	0.190042
20579	1.47606	0.115508
20580	1.20739	0.155966
20581	0.957684	0.124061
20582	0.946116	0.162201
20583	0.851528	0.188116
20584	0.957685	0.180483
20585	1.04504	2.1
20586	0.902938	2.1
20587	0.946132	2.1

20588	1.07566	2.1
20589	1.4558	2.1
20590	1.93286	2.1
20591	1.27241	2.1
20592	0.828671	2.1
20593	0.810933	2.1
20594	1.31335	0.05
20595	1.40305	0.05
20596	0.912698	0.05
20597	0.810946	0.05
20598	1.11608	0.05
20599	1.26409	1.14757
20600	0.946132	0.203458
20601	1.27376	0.207851
20602	0.946132	0.200923
20603	1.03333	0.211946
20604	1.27376	0.205961
20605	0.94613	0.232832
20606	0.812384	0.202113
20607	0.687744	0.147676
20608	0.946132	0.228226
20609	0.687758	0.201917
20610	1.09667	0.131255
20611	1.56977	2.1
20612	0.851544	2.1
20613	1.01136	2.1
20614	0.851481	2.1
20615	0.946131	2.1
20616	0.705409	2.1
20617	1.32449	0.05
20618	1.13508	0.05
20619	0.94613	0.053028
20620	0.94613	0.05
20621	0.965822	0.05
20622	1.95301	0.112623
20623	0.851538	0.302108
20624	1.29262	0.26024
20625	0.95417	0.172775
20626	0.851541	0.310672
20627	0.94613	0.272233
20628	1.02727	0.976927
20629	1.37675	1.28306
20630	0.955646	1.04783
20631	0.95114	1.61885
20632	0.851528	1.14388
20633	1.76513	1.40684
20634	0.87155	2.1
20635	0.94613	2.1
20636	0.871519	2.1
20637	0.966631	2.1
20638	1.37791	2.1
20639	1.19122	0.347232
20640	0.818971	0.784408
20641	0.628799	0.447087
20642	0.861027	0.591018
20643	0.800034	0.05
20644	0.91846	0.05
20645	0.765645	0.05

20646	1.13709	0.645438
20647	1.29054	0.607903
20648	0.755336	0.710669
20649	0.755334	0.642143
20650	0.946133	0.791848
20651	0.755336	0.511039
20652	1.14444	0.619943
20653	0.872823	0.653881
20654	0.533645	0.627027
20655	0.946132	0.552772
20656	0.533645	0.426322
20657	1.02311	0.42937
20658	1.00278	2.1
20659	1.1016	0.05
20660	0.629615	0.05
20661	1.12628	0.05
20662	0.946131	0.05
20663	0.629615	0.05
20664	0.629641	0.05
20665	1.67046	1.06638
20666	1.81667	2.1
20667	1.7145	2.1
20668	0.94613	2.1
20669	1.29322	2.1
20670	1.00365	2.1
20671	0.946131	2.1
20672	1.29322	2.1
20673	1.27581	2.1
20674	1.33916	2.1
20675	1.32784	2.1
20676	1.74787	2.1
20677	2.28084	2.1
20678	2.98092	2.1
20679	0.946132	2.06513
20680	1.2435	2.1
20681	0.946132	2.1
20682	1.4099	2.1
20683	1.2435	2.1
20684	1.26438	2.1
20685	0.745287	2.1
20686	0.696827	2.1
20687	0.696781	2.1
20688	1.41195	2.1
20689	0.946131	2.1
20690	1.8758	1.53882
20691	0.832979	0.511695
20692	0.75054	0.441325
20693	0.810958	0.435704
20694	0.922889	0.55493
20695	0.694844	0.204933
20696	0.694814	0.385066
20697	0.946131	0.313188
20698	0.694844	0.29034
20699	1.02369	2.1
20700	1.4468	0.05
20701	0.946125	0.131549
20702	0.93487	0.0901983
20703	1.23608	0.135169

20704	1.34257	0.0735332
20705	0.851525	0.111215
20706	1.03298	0.05
20707	1.22418	2.09908
20708	0.946131	2.08694
20709	1.41833	1.92817
20710	1.23452	1.16363
20711	0.946131	0.582677
20712	1.41833	1.57702
20713	1.13077	0.184304
20714	0.942874	1.91398
20715	0.946132	1.8698
20716	0.504366	1.86415
20717	0.593647	2.09259
20718	0.898829	1.87771
20719	1.27921	2.04901
20720	1.12503	2.1
20721	1.22539	2.1
20722	1.10332	2.1
20723	0.851541	2.1
20724	0.94613	2.1
20725	0.945109	2.1
20726	0.607962	2.1
20727	0.946133	2.1
20728	0.607961	2.1
20729	0.661956	2.1
20730	1.35048	2.1
20731	1.66018	2.1
20732	0.956467	2.1
20733	0.851542	2.1
20734	0.890734	2.1
20735	0.802455	2.1
20736	1.35943	2.1
20737	1.04798	2.1
20738	1.26426	0.273371
20739	1.3689	0.17639
20740	0.946133	0.22622
20741	0.946133	0.290596
20742	1.09729	0.266684
20743	0.765259	2.1
20744	0.810945	2.1
20745	0.810957	2.1
20746	1.10284	2.07675
20747	1.09368	2.1
20748	0.772601	2.1
20749	0.788011	2.1
20750	0.810934	2.1
20751	0.694357	1.14971
20752	1.22641	1.13548
20753	0.946132	1.95017
20754	0.946132	0.435134
20755	0.946133	0.614359
20756	1.42754	1.67177
20757	0.776414	0.648776
20758	0.806525	1.33067
20759	0.575339	1.02624
20760	0.97154	0.692062
20761	0.8217	1.4179

20762	0.838039	1.37589
20763	0.94613	1.2051
20764	0.946132	0.803078
20765	1.39575	0.462535
20766	1.39575	0.610256
20767	1.12818	0.686451
20768	0.946132	1.63942
20769	0.946133	1.30129
20770	1.06879	0.658882
20771	1.06879	0.972477
20772	0.970358	0.956505
20773	1.2474	0.282334
20774	1.01919	0.05
20775	0.94613	0.0693925
20776	1.28952	0.05
20777	1.00809	0.05
20778	0.849344	0.0533181
20779	0.640304	0.0530121
20780	1.35065	0.05
20781	0.946132	0.05
20782	0.640333	0.05
20783	0.946132	0.05
20784	0.685489	2.1
20785	0.94613	2.1
20786	0.68546	2.1
20787	0.874264	2.1
20788	1.27676	2.1
20789	0.946129	0.104079
20790	1.09024	0.0693365
20791	0.714563	0.0657745
20792	1.12335	0.0731481
20793	1.07931	0.0700958
20794	1.17799	0.05
20795	1.8795	0.179462
20796	2.15229	0.17612
20797	1.13859	0.396122
20798	0.946132	0.747586
20799	1.11374	0.418967
20800	1.1386	0.493187
20801	0.615713	0.518679
20802	1.19322	0.196912
20803	1.3377	0.159715
20804	0.670801	0.212187
20805	0.94613	0.524649
20806	0.66617	0.386485
20807	1.36173	0.362179
20808	0.980473	0.341382
20809	1.39132	0.330792
20810	0.864653	0.05
20811	1.23837	0.05
20812	0.810959	0.05
20813	1.1483	1.7647
20814	1.00381	1.92599
20815	0.946128	1.79938
20816	1.39696	1.07662
20817	0.946131	0.773538
20818	0.946131	1.35416
20819	1.38613	1.64466

20820	0.973501	1.95449
20821	0.946127	1.36277
20822	1.35968	1.60716
20823	0.946131	0.924609
20824	0.946129	1.85872
20825	1.39696	1.80677
20826	1.12088	2.1
20827	1.41122	2.1
20828	1.14464	0.856711
20829	0.946131	2.06795
20830	0.946131	1.99451
20831	1.14464	2.05354
20832	1.33266	1.59117
20833	1.07341	0.228923
20834	0.827287	1.94899
20835	0.946132	1.78482
20836	0.827287	2.0398
20837	1.15644	1.85113
20838	1.27558	0.591441
20839	1.15067	2.1
20840	0.946131	2.1
20841	1.29143	2.05545
20842	1.36281	2.1
20843	1.36281	2.1
20844	0.946131	2.1
20845	1.40863	2.1
20846	1.20143	0.05
20847	1.19755	0.05
20848	0.566153	0.05
20849	0.946131	0.075218
20850	1.07979	0.05
20851	1.00463	0.0891619
20852	0.566153	0.121044
20853	0.768212	2.1
20854	1.32461	2.1
20855	1.13268	2.1
20856	1.13268	2.1
20857	0.946133	2.09887
20858	1.53642	2.1
20859	1.5791	2.1
20860	0.726635	2.1
20861	1.2334	2.0949
20862	1.29198	2.1
20863	1.29198	2.06944
20864	0.946131	2.01974
20865	0.956585	2.1
20866	1.25294	2.1
20867	0.946133	2.1
20868	1.35287	2.1
20869	1.23877	2.1
20870	0.946131	2.1
20871	1.35287	2.1
20872	1.01843	2.1
20873	3.77569	2.1
20874	0.86707	2.1
20875	0.867066	2.1
20876	1.38639	2.1
20877	1.38639	2.1

20878	0.896168	2.1
20879	0.974716	0.05
20880	1.09411	0.0873891
20881	0.663171	0.0566278
20882	0.663171	0.100955
20883	0.946132	0.0979598
20884	0.66317	0.0722608
20885	1.47104	0.05
20886	1.13629	0.0655403
20887	0.772342	0.05
20888	0.772312	0.0738387
20889	0.94613	0.0704379
20890	0.77234	0.0660205
20891	1.43154	2.1
20892	1.37704	2.1
20893	1.40025	2.1
20894	1.3063	2.1
20895	0.946132	2.1
20896	1.04075	2.1
20897	0.946131	2.1
20898	0.946118	2.1
20899	0.750011	1.72832
20900	1.0971	2.04063
20901	0.889667	1.8752
20902	0.709598	2.03097
20903	2.39737	2.1
20904	1.19974	0.0592584
20905	0.94613	0.132939
20906	1.25809	0.05
20907	1.19974	0.109997
20908	0.73005	0.195088
20909	1.50764	0.05
20910	1.25809	0.0711403
20911	0.946133	0.168486
20912	1.10357	0.103079
20913	1.25809	0.135245
20914	0.628177	0.207929
20915	1.3543	0.106313
20916	0.793711	0.05
20917	1.18455	0.05
20918	1.03966	0.05
20919	0.946131	0.05
20920	0.767453	0.05
20921	0.612292	0.05
20922	0.946131	0.05
20923	1.24128	0.05
20924	1.08761	0.05
20925	0.762059	0.05
20926	1.005 2.1	
20927	1.17054	2.1
20928	1.12665	2.1
20929	0.750368	2.1
20930	0.851542	2.1
20931	0.975436	2.1
20932	0.975442	2.1
20933	0.675367	2.1
20934	1.51973	0.113114
20935	0.94613	0.129925

20936	1.38954	0.134145
20937	0.946131	0.126949
20938	1.07912	0.135629
20939	1.38954	0.126555
20940	2.0219	2.1
20941	1.18347	0.1275
20942	0.946132	0.638497
20943	0.94613	0.319626
20944	1.00202	0.232721
20945	1.00201	0.261959
20946	0.946132	0.334719
20947	0.946132	0.795093
20948	1.37981	0.475854
20949	1.26874	0.373421
20950	1.26874	0.397202
20951	0.946132	0.477143
20952	2.8687	0.156719
20953	1.42884	2.1
20954	1.05188	2.1
20955	0.625307	2.1
20956	0.810956	2.1
20957	0.526656	2.1
20958	0.526684	2.1
20959	0.724122	2.1
20960	0.946132	2.1
20961	0.526684	2.1
20962	1.05337	2.1
20963	1.0593	2.1
20964	1.47981	2.1
20965	1.74431	1.88012
20966	0.885612	1.95433
20967	0.810946	1.3502
20968	0.845185	1.5686
20969	1.38622	0.450674
20970	0.723194	0.478153
20971	0.554302	0.55616
20972	0.709597	0.414025
20973	0.909507	0.399401
20974	0.912754	0.265829
20975	1.15174	0.193239
20976	0.946131	0.268291
20977	0.912753	0.256071
20978	1.15174	0.256414
20979	1.02011	0.15205
20980	0.792754	1.96226
20981	0.946129	2.059
20982	0.792754	1.94828
20983	1.27575	1.90028
20984	0.94613	1.95795
20985	1.60132	1.98252
20986	0.922455	1.98265
20987	1.18713	1.5907
20988	0.926957	1.69472
20989	1.66525	1.71649
20990	0.946132	2.1
20991	0.851541	2.1
20992	0.961191	2.1
20993	1.27534	2.1

20994	0.851554	2.1
20995	1.0499	2.1
20996	1.01776	0.05
20997	0.97622	0.05
20998	0.810934	0.05
20999	2.6802	0.05
21000	1.31485	0.05
21001	1.09699	0.05
21002	0.681649	0.05
21003	0.790369	0.05
21004	0.946131	0.05
21005	0.838026	1.20868
21006	0.567833	0.64531
21007	0.891492	0.900409
21008	0.986984	0.427486
21009	0.946132	0.912375
21010	1.19028	0.452487
21011	0.986984	0.478743
21012	0.595623	0.563834
21013	0.946131	2.1
21014	1.36502	2.1
21015	1.08109	2.1
21016	0.946129	2.1
21017	1.36503	2.1
21018	0.773897	2.08752
21019	0.946133	1.94362
21020	0.613447	2.09014
21021	0.613449	2.04863
21022	0.946131	2.0889
21023	1.11163	2.04317
21024	0.966114	2.1
21025	0.735699	2.1
21026	0.662147	2.1
21027	1.04072	2.1
21028	0.946131	2.1
21029	0.862978	2.1
21030	1.48839	2.02939
21031	0.936141	2.0524
21032	0.946131	2.1
21033	0.936142	2.1
21034	1.40595	2.1
21035	0.946131	2.09978
21036	1.05297	0.05
21037	3.09035	2.1
21038	1.19952	2.1
21039	0.907198	2.1
21040	0.849144	2.1
21041	0.612469	0.300509
21042	0.551237	0.27064
21043	0.985256	0.436657
21044	0.551209	0.298163
21045	0.85153	0.597463
21046	0.624712	2.1
21047	0.900018	2.1
21048	0.851542	2.1
21049	0.864787	2.1
21050	1.27669	2.1
21051	0.946711	0.227328

21052	0.810947	0.272794
21053	0.841547	0.185502
21054	0.764114	0.373717
21055	1.26692	0.353893
21056	0.946132	0.385624
21057	1.37675	0.321201
21058	1.26692	0.336921
21059	1.37545	0.406225
21060	1.49637	0.104924
21061	0.709598	0.170606
21062	0.751252	0.161717
21063	1.36811	0.152852
21064	0.635625	0.172995
21065	1.31713	0.0554713
21066	1.18977	2.1
21067	0.893377	0.476866
21068	0.707344	2.1
21069	0.946131	2.1
21070	1.20221	2.1
21071	1.20221	2.1
21072	0.707345	2.1
21073	1.26786	2.1
21074	0.988048	2.09022
21075	0.751293	2.08202
21076	0.632008	2.08869
21077	1.51067	2.1
21078	1.13534	2.1
21079	0.946133	2.1
21080	0.946131	2.1
21081	1.25716	2.1
21082	1.3199	2.1
21083	1.09616	2.1
21084	1.36677	2.07284
21085	1.34752	2.09311
21086	0.838041	2.04557
21087	1.67023	2.1
21088	1.02247	0.305933
21089	1.03079	0.176724
21090	0.928982	0.0882981
21091	1.13635	0.05
21092	0.946132	0.0803105
21093	0.92898	0.0636574
21094	1.13635	0.0700382
21095	1.1643	0.05
21096	0.851541	0.219683
21097	1.39939	0.170631
21098	0.946129	0.228809
21099	1.07775	0.187754
21100	1.14503	0.203699
21101	1.78573	0.119498
21102	1.29827	0.107983
21103	0.872373	0.05
21104	1.01162	0.05
21105	0.83804	0.05
21106	1.48851	0.05
21107	1.47385	0.05
21108	0.843858	0.05
21109	0.938626	0.05

21110	0.89567	0.05
21111	1.59457	0.58178
21112	2.09666	0.0921076
21113	0.775132	0.05
21114	0.927005	0.05
21115	0.854224	0.05
21116	0.775142	0.05
21117	0.946106	0.05
21118	1.80784	2.1
21119	2.04593	2.1
21120	1.32282	2.1
21121	0.952138	2.1
21122	0.946132	2.1
21123	0.946132	2.1
21124	0.952138	2.1
21125	1.46988	2.1
21126	0.80465	0.05
21127	1.14155	0.05
21128	0.810931	0.05
21129	1.66203	0.108435
21130	0.674605	0.294644
21131	0.946132	0.605978
21132	1.26706	0.409892
21133	0.674633	0.145772
21134	0.946132	1.19941
21135	0.980664	0.252268
21136	1.34927	0.150467
21137	0.758454	0.0971404
21138	0.946133	0.0959013
21139	0.985371	0.0766248
21140	1.28632	0.0760183
21141	0.946133	0.142415
21142	1.46868	0.19971
21143	1.7522	0.05
21144	0.737431	2.01731
21145	0.663679	2.02184
21146	1.16541	1.7973
21147	1.3179	1.90659
21148	1.04081	1.93551
21149	0.887866	1.69716
21150	0.806961	1.09607
21151	0.929061	1.47509
21152	1.28418	0.314336
21153	1.38752	0.266209
21154	1.33038	0.178921
21155	0.946132	0.155195
21156	0.946132	0.215601
21157	1.14897	0.212056
21158	1.1685	0.15889
21159	1.18774	2.1
21160	0.961899	2.1
21161	1.48841	2.1
21162	1.79105	2.1
21163	3.51614	2.1
21164	0.682342	2.1
21165	0.783184	2.1
21166	0.946132	2.1
21167	0.868396	2.1

21168	1.21813	2.1
21169	0.848305	0.46719
21170	0.94559	0.500734
21171	0.805691	0.364487
21172	0.752217	2.1
21173	0.946132	2.1
21174	0.839123	2.1
21175	0.946131	2.1
21176	0.752187	2.1
21177	1.96491	2.1
21178	0.520997	2.1
21179	0.946132	2.1
21180	0.73446	2.1
21181	0.946133	2.1
21182	0.520995	2.1
21183	1.88522	2.1
21184	1.8361	2.1
21185	1.11285	2.1
21186	1.25334	0.973569
21187	2.00756	2.1
21188	1.44252	0.0649577
21189	1.00255	2.08361
21190	1.64516	2.1
21191	1.12399	0.05
21192	1.13542	0.05
21193	1.09038	0.05
21194	0.746903	0.05
21195	0.672241	0.05
21196	0.871774	0.05
21197	1.73513	0.112075
21198	0.949995	2.1
21199	1.17244	2.09393
21200	0.946127	2.1
21201	1.3028	2.09129
21202	1.17244	2.09164
21203	0.774101	2.1
21204	0.946127	2.1
21205	0.939289	2.1
21206	0.946129	2.1
21207	0.774101	2.1
21208	1.87858	2.1
21209	1.44293	0.05
21210	1.43213	0.05
21211	0.946131	2.1
21212	0.716614	2.1
21213	0.716644	2.1
21214	1.35828	2.1
21215	0.790935	2.1
21216	0.730157	2.1
21217	1.41149	2.1
21218	0.730186	2.1
21219	0.730186	2.1
21220	0.946131	2.1
21221	0.946132	1.61335
21222	0.946132	1.33615
21223	0.990713	0.66775
21224	1.40779	0.919999
21225	0.946132	1.09309

21226	1.06486	0.327311
21227	0.946131	1.31462
21228	0.946133	0.696556
21229	1.40779	0.509073
21230	1.26874	0.567624
21231	0.946131	0.668342
21232	1.02588	0.706324
21233	0.826073	0.101554
21234	1.41233	0.100403
21235	0.861269	0.0997304
21236	1.04073	0.151541
21237	1.27111	0.164748
21238	1.56016	1.61539
21239	1.60866	0.05
21240	0.94613	0.05
21241	0.946129	0.05
21242	1.36376	0.05
21243	1.22187	0.05
21244	0.94613	0.05
21245	1.82581	0.609136
21246	1.19705	1.00426
21247	1.7513	2.1
21248	1.85017	2.1
21249	1.72116	2.1
21250	1.12058	2.1
21251	0.899991	2.1
21252	0.616288	2.1
21253	1.07289	2.1
21254	1.7092	0.745408
21255	0.946132	0.499108
21256	0.946133	0.479471
21257	1.18717	0.348028
21258	1.40029	0.850242
21259	0.946132	0.177398
21260	0.94613	0.0671545
21261	1.18547	0.0502777
21262	0.960326	0.0631581
21263	0.960345	0.0505599
21264	0.942557	0.0644315
21265	1.18601	0.05
21266	0.946132	0.0531474
21267	1.30307	0.05
21268	1.08169	0.05
21269	1.08169	0.05
21270	1.31716	0.0537261
21271	1.51065	0.654014
21272	0.877642	0.05
21273	0.617384	0.05
21274	0.575741	0.05
21275	0.972448	0.05
21276	1.11334	0.05
21277	1.13092	2.1
21278	0.946127	1.70291
21279	1.31724	2.1
21280	1.39751	0.60192
21281	1.39751	1.48639
21282	0.946128	1.56801
21283	0.946132	0.185961

21284	0.713284	0.147747
21285	1.40668	0.188554
21286	1.03547	0.173647
21287	0.713298	0.172156
21288	1.88774	0.142719
21289	0.830164	2.1
21290	1.05211	2.1
21291	0.946119	2.1
21292	0.946107	2.1
21293	1.283	2.1
21294	2.63609	2.1
21295	1.28418	0.460432
21296	0.946128	0.684151
21297	1.10335	0.449673
21298	0.598377	0.824972
21299	1.18818	0.472555
21300	1.10335	0.465209
21301	1.58972	1.08066
21302	1.90194	2.1
21303	0.946118	2.1
21304	0.946115	2.1
21305	0.950969	2.1
21306	1.36918	2.1
21307	0.950955	2.1
21308	1.4263	2.1
21309	1.11978	0.260457
21310	0.946132	1.025
21311	0.946133	1.47872
21312	1.37658	0.258814
21313	1.2364	0.440895
21314	1.05496	0.508448
21315	0.946132	1.36425
21316	0.946132	1.15979
21317	1.21858	0.396421
21318	1.37658	0.820993
21319	1.09194	1.10979
21320	1.14721	0.0655272
21321	1.53814	0.05
21322	0.546751	0.938848
21323	0.492063	1.20456
21324	1.16889	1.97491
21325	1.00349	1.7695
21326	0.863559	1.05919
21327	1.38102	1.51547
21328	0.597449	1.2841
21329	0.537748	0.535678
21330	1.16297	1.49639
21331	0.999366	0.899707
21332	0.896554	0.770507
21333	0.566962	0.05
21334	0.946129	0.05
21335	0.452729	0.05
21336	0.696552	0.05
21337	0.852524	0.05
21338	1.57992	0.05
21339	1.64985	0.05
21340	0.946128	2.1
21341	1.21847	2.1

21342	0.946129	2.1
21343	1.41131	2.1
21344	1.21847	2.1
21345	1.21269	0.05
21346	1.0417	1.9558
21347	1.15821	1.38105
21348	0.946132	0.635589
21349	0.946132	1.92179
21350	1.37967	0.871883
21351	0.851529	1.17896
21352	1.20455	2.1
21353	1.4282	0.05
21354	1.26593	2.08928
21355	0.838026	0.0736881
21356	0.985096	0.0612423
21357	0.83804	0.0725847
21358	0.822395	1.27628
21359	0.420272	0.272135
21360	0.946131	2.1
21361	1.17461	2.1
21362	0.94613	2.1
21363	1.40225	2.1
21364	1.17461	2.1
21365	1.09847	2.1
21366	1.22222	2.07374
21367	1.41798	0.470284
21368	0.791658	0.60742
21369	0.791656	0.662294
21370	0.946129	0.609997
21371	0.817064	0.59652
21372	0.946114	0.529382
21373	0.75824	0.494557
21374	0.75821	0.542339
21375	1.41778	0.43418
21376	0.791635	0.498759
21377	1.04164	1.7869
21378	0.946131	0.956858
21379	1.11172	0.692585
21380	1.30076	0.494649
21381	1.35721	0.600296
21382	1.11172	0.562957
21383	1.87218	0.165169
21384	0.987248	0.166892
21385	0.729695	0.701803
21386	0.974389	0.302668
21387	1.18002	0.286594
21388	0.822612	0.30747
21389	1.35721	0.299769
21390	0.634295	0.556554
21391	0.634326	0.376164
21392	1.13245	1.16074
21393	1.13245	0.579541
21394	0.937961	0.397651
21395	1.18769	0.465763
21396	0.854949	0.295068
21397	0.967357	0.352925
21398	0.810934	0.284592
21399	3.91082	2.1

21400	0.946132	2.1
21401	0.946132	2.1
21402	1.05383	2.1
21403	1.05383	2.1
21404	0.986498	2.1
21405	1.50732	0.05
21406	0.753659	0.0686136
21407	1.22144	0.082608
21408	1.30463	0.0658163
21409	0.753659	0.0788371
21410	1.30463	0.0790723
21411	0.886189	0.0794956
21412	1.04321	0.0717174
21413	0.909928	0.0663823
21414	1.18659	0.0575625
21415	1.19785	0.05
21416	1.40969	0.0544109
21417	1.24094	0.05
21418	0.946126	0.0621352
21419	1.2929	0.05
21420	1.24093	0.05
21421	0.867944	0.05
21422	1.41903	2.1
21423	2.47881	0.05
21424	1.12943	0.05
21425	0.764229	2.1
21426	0.764256	2.1
21427	0.94613	2.1
21428	0.94613	2.1
21429	1.1995	2.1
21430	1.83423	2.1
21431	0.634816	2.08976
21432	0.877837	2.1
21433	0.94613	2.09813
21434	1.04073	2.1
21435	1.06485	2.1
21436	0.94613	0.166251
21437	0.62725	0.144491
21438	0.798212	0.166154
21439	1.33241	0.108222
21440	0.62725	0.145389
21441	1.82301	0.0756896
21442	1.40615	2.1
21443	0.782504	2.1
21444	0.748635	2.1
21445	0.946131	2.1
21446	0.748607	2.1
21447	0.988456	1.97609
21448	1.64833	0.05
21449	1.35657	0.05
21450	1.26155	0.05
21451	0.915755	0.05
21452	0.915755	0.05
21453	0.946132	0.05
21454	1.49386	0.05
21455	1.01346	2.1
21456	0.946131	2.1
21457	0.946132	2.1

21458	1.38651	2.1
21459	1.39523	2.1
21460	0.946132	2.1
21461	1.06312	2.1
21462	0.946131	2.1
21463	0.946129	2.1
21464	1.39523	2.1
21465	1.37372	2.1
21466	0.946131	2.1
21467	1.2929	0.0605191
21468	0.94613	0.103441
21469	1.24448	0.0563156
21470	1.2929	0.0731229
21471	0.862011	0.066451
21472	1.73583	0.05
21473	1.724 0.05	
21474	1.03824	2.1
21475	1.534 2.1	
21476	0.725492	1.89707
21477	0.771967	2.0186
21478	0.61731	1.46285
21479	0.475743	1.94679
21480	0.719694	1.71715
21481	1.14878	1.30784
21482	0.946117	1.92846
21483	0.94613	1.89206
21484	0.946105	1.93772
21485	0.482666	1.43165
21486	0.609172	1.44665
21487	0.946131	2.1
21488	1.39509	0.986651
21489	1.07439	2.1
21490	0.946131	2.08491
21491	1.39509	2.09939
21492	0.963571	1.762
21493	0.591011	0.425958
21494	1.1357	0.226581
21495	2.11193	1.32707
21496	1.60936	2.1
21497	1.34118	0.0783642
21498	1.27696	0.0729709
21499	0.94613	0.0881768
21500	0.94613	0.0692182
21501	1.04257	0.05
21502	1.53336	0.05
21503	1.0631	0.0669645
21504	1.04072	0.118439
21505	0.670678	0.0886424
21506	0.709531	0.0983314
21507	0.891275	0.108967
21508	0.683583	0.108362
21509	0.902478	0.0683015
21510	0.807671	0.079996
21511	1.11024	2.1
21512	1.08171	2.1
21513	1.16725	2.1
21514	0.946128	2.1
21515	1.13537	2.1

21516	1.40525	2.1
21517	1.28365	2.1
21518	1.04523	2.1
21519	0.960106	2.1
21520	0.946128	2.1
21521	1.23452	2.1
21522	0.946129	2.1
21523	1.3704	2.1
21524	1.23452	2.1
21525	1.16906	1.0065
21526	0.946131	1.78533
21527	0.867246	1.28145
21528	0.867217	1.56899
21529	0.946131	1.43141
21530	1.13438	1.47636
21531	1.28684	1.59856
21532	0.946132	1.87508
21533	0.883935	1.56306
21534	0.883938	1.99977
21535	0.946133	1.61409
21536	1.73444	1.15342
21537	1.21835	2.1
21538	1.28228	2.1
21539	1.8643	0.351425
21540	0.743379	0.0981182
21541	1.34921	0.0919915
21542	0.743381	0.0993067
21543	0.743381	0.104393
21544	0.946131	0.108095
21545	0.915528	2.0563
21546	0.982742	2.08293
21547	0.975965	2.05032
21548	0.891132	0.05
21549	1.27533	0.05
21550	0.946132	0.05
21551	1.25215	0.05
21552	0.590343	0.05
21553	0.946133	0.05
21554	0.590331	0.05
21555	1.62375	2.1
21556	0.687769	0.627745
21557	1.14374	0.608703
21558	1.20885	0.503677
21559	0.946132	0.683965
21560	1.14374	0.834378
21561	1.09308	0.212598
21562	1.02055	0.214451
21563	0.810934	0.157923
21564	0.917609	2.03875
21565	1.13094	2.08048
21566	0.70959	1.94722
21567	1.11223	2.1
21568	0.83051	1.95558
21569	0.810946	2.06299
21570	0.810959	1.97852
21571	1.5773	0.05
21572	1.96133	1.18252
21573	1.0214	1.22574

21574	0.762783	1.72203
21575	0.838036	0.931567
21576	0.63591	1.80862
21577	0.967411	1.19209
21578	0.946132	2.04858
21579	0.946132	2.03347
21580	1.39699	1.88601
21581	1.61642	1.94015
21582	0.860954	1.15476
21583	0.957411	1.81106
21584	0.660856	1.55387
21585	1.10003	2.1
21586	0.968249	2.09697
21587	0.910184	2.05427
21588	1.30029	0.964557
21589	0.754119	1.82025
21590	0.754121	1.97636
21591	0.94613	1.93535
21592	1.08039	2.1
21593	0.810942	0.0684458
21594	1.15153	0.0639643
21595	1.06968	0.0568212
21596	1.38456	2.1
21597	1.04118	2.1
21598	0.838027	2.1
21599	1.04073	2.1
21600	0.754209	2.1
21601	1.332	0.05
21602	1.26193	2.1
21603	1.15317	0.05
21604	3.86179	0.05
21605	1.56005	2.1
21606	0.683035	0.212281
21607	0.931463	0.305476
21608	0.556019	0.609399
21609	0.929172	0.567682
21610	0.756874	0.138805
21611	1.23527	0.541974
21612	2.05961	0.608853
21613	0.946132	2.1
21614	1.16684	2.1
21615	1.3937	2.1
21616	1.3937	2.1
21617	0.946132	2.1
21618	1.17885	2.1
21619	1.62507	0.05
21620	1.58874	2.1
21621	0.819953	2.1
21622	1.16023	2.1
21623	1.34782	2.09686
21624	1.16023	2.09761
21625	0.819955	2.1
21626	0.79437	2.0874
21627	0.794373	2.08875
21628	1.20056	2.02451
21629	1.25	2.05059
21630	1.20056	2.06664
21631	1.35787	2.08086

21632	0.946131	2.06675
21633	1.38585	2.01883
21634	0.946128	1.32907
21635	0.946129	1.91567
21636	1.35714	2.04122
21637	1.04147	2.1
21638	0.946133	1.55222
21639	1.30382	1.32668
21640	0.946131	0.614673
21641	0.946131	1.32905
21642	1.38585	1.64356
21643	1.22305	0.95543
21644	1.00763	0.083688
21645	0.813199	0.12865
21646	0.810948	0.11461
21647	1.15928	0.108886
21648	0.944166	0.05
21649	0.94613	0.05
21650	0.946133	0.0700627
21651	1.4179	0.0517862
21652	0.946132	0.05
21653	1.36965	0.0624012
21654	1.05444	0.05
21655	0.690302	0.0530016
21656	0.838038	0.05
21657	0.802821	0.418651
21658	1.40944	0.630935
21659	0.946126	0.760092
21660	0.799998	0.342994
21661	0.800035	0.528769
21662	1.1766	2.1
21663	1.00319	2.1
21664	1.01606	1.59625
21665	0.872158	2.0908
21666	1.04884	2.05112
21667	0.679494	2.0864
21668	0.946131	2.1
21669	1.37609	2.1
21670	0.94613	2.1
21671	0.946131	2.1
21672	1.18486	2.1
21673	0.726287	2.1
21674	0.946131	2.1
21675	0.726286	2.1
21676	1.30318	2.1
21677	1.01712	2.1
21678	0.946132	2.1
21679	1.04404	2.1
21680	1.37017	2.1
21681	1.37017	2.1
21682	0.946133	2.1
21683	0.992084	1.27692
21684	0.946131	0.800985
21685	0.529655	1.9744
21686	0.529686	1.25935
21687	0.946131	0.942062
21688	1.05937	1.64882
21689	1.14622	0.452636

21690	0.946131	0.501178
21691	0.584575	1.9362
21692	0.584575	0.283282
21693	0.946129	0.285031
21694	0.709714	2.1
21695	0.946132	2.1
21696	0.709714	2.1
21697	1.37199	2.1
21698	0.82899	2.1
21699	1.00133	2.1
21700	0.778424	0.05
21701	0.946132	0.05
21702	1.24094	0.05
21703	1.00691	0.05
21704	0.64868	0.05
21705	1.29736	0.05
21706	1.24767	0.262409
21707	1.28953	0.39605
21708	1.34612	1.04892
21709	0.946133	0.691617
21710	1.14187	1.07452
21711	0.946132	0.519468
21712	0.946129	0.971361
21713	1.4099	2.1
21714	0.946131	1.85414
21715	1.34612	1.31041
21716	1.4099	1.69744
21717	0.956003	0.05
21718	1.40572	0.05
21719	3.54443	2.1
21720	0.994001	0.207933
21721	1.03679	2.1
21722	1.08467	2.1
21723	1.56875	0.138708
21724	0.968463	2.1
21725	0.974889	2.1
21726	0.946131	2.1
21727	1.30203	2.1
21728	1.40199	2.1
21729	1.40199	2.1
21730	0.946132	2.1
21731	0.946132	2.1
21732	1.36994	2.1
21733	1.37372	2.1
21734	1.37372	2.1
21735	0.946132	2.1
21736	0.928979	1.29062
21737	0.565509	1.51354
21738	1.60196	0.287663
21739	1.16239	0.227208
21740	1.10746	0.330941
21741	1.15412	2.1
21742	1.15171	2.1
21743	0.946133	2.1
21744	0.940877	2.1
21745	0.940878	2.1
21746	1.41432	2.1
21747	1.04687	2.1

21748	2.75524	0.05
21749	0.946132	0.05
21750	0.766452	0.05
21751	0.766427	0.05
21752	1.36004	0.05
21753	1.06648	0.05
21754	1.62483	2.1
21755	1.0577	2.1
21756	1.39158	2.0717
21757	0.94255	2.09895
21758	1.05029	2.07637
21759	1.03735	1.92062
21760	0.931055	2.04043
21761	1.03797	1.93112
21762	1.43687	1.88683
21763	1.28765	1.35659
21764	1.1811	1.17686
21765	0.946132	1.27461
21766	0.946132	1.56457
21767	1.1811	1.11201
21768	0.67805	1.06036
21769	1.50998	1.74615
21770	1.16661	2.1
21771	1.36749	2.1
21772	0.810945	0.958218
21773	1.14745	0.473564
21774	0.865588	0.263561
21775	1.36665	0.170815
21776	0.506964	0.128634
21777	0.506966	2.02744
21778	0.552786	0.112397
21779	0.946133	0.140782
21780	1.21336	0.393612
21781	0.552786	0.138645
21782	0.552757	2.1
21783	0.653468	0.252619
21784	1.36665	1.98905
21785	1.27922	0.133295
21786	1.08133	0.209494
21787	1.05348	0.117365
21788	0.946132	0.200364
21789	1.19601	0.120255
21790	1.13276	0.128497
21791	0.7856	0.13258
21792	1.57125	0.113208
21793	1.19601	0.22466
21794	0.946131	0.520044
21795	1.34534	0.294532
21796	1.19601	0.257624
21797	0.870489	0.28992
21798	1.49404	2.1
21799	1.35943	1.48162
21800	1.10427	1.96383
21801	0.94613	1.44747
21802	0.946131	1.76486
21803	1.10427	1.71928
21804	1.2064	1.16675
21805	0.946132	1.00083

21806	1.11288	2.09429
21807	1.34268	0.178646
21808	1.34268	0.774255
21809	0.946132	0.557512
21810	0.946128	2.1
21811	0.946128	2.067
21812	1.30835	2.1
21813	1.38463	2.1
21814	0.946129	2.1
21815	1.43778	0.503117
21816	1.23943	1.63689
21817	1.4748	2.04266
21818	0.71295	2.1
21819	0.712951	2.1
21820	0.946132	2.1
21821	0.946133	2.1
21822	1.13976	2.1
21823	1.71109	2.1
21824	1.32426	2.1
21825	0.671255	2.09345
21826	1.29123	2.09958
21827	0.946132	2.09849
21828	0.946132	2.08766
21829	1.25603	2.09705
21830	2.21126	0.0550025
21831	0.892889	2.1
21832	0.946129	2.1
21833	0.892888	2.1
21834	0.92872	2.1
21835	1.34352	2.1
21836	0.622784	0.171776
21837	1.02947	0.104632
21838	0.810957	0.0987465
21839	1.91756	1.14398
21840	1.06012	0.63983
21841	1.09569	0.333458
21842	1.5456	0.05
21843	1.1211	0.05
21844	1.21031	0.05
21845	1.03939	0.0637909
21846	0.519697	0.0892909
21847	0.933889	0.1814
21848	0.519726	0.155493
21849	1.16699	0.141075
21850	0.933889	0.153132
21851	0.840599	0.0569367
21852	0.94613	0.129753
21853	0.840568	0.0964767
21854	1.26868	0.0936279
21855	1.16699	0.0862348
21856	1.17822	1.93274
21857	0.922044	0.05
21858	1.29322	0.05
21859	0.946132	0.05
21860	0.995178	0.05
21861	0.922056	0.05
21862	1.76232	0.0960502
21863	1.77065	0.730633

21864	1.38149	0.545294
21865	0.959301	0.943825
21866	0.946133	0.684887
21867	0.946132	0.753379
21868	0.959301	0.630953
21869	1.3612	0.566543
21870	0.885314	0.804141
21871	0.885309	0.648817
21872	0.94613	0.779476
21873	0.8853	0.560594
21874	1.56164	0.518683
21875	1.45506	0.128672
21876	1.45257	2.1
21877	0.851541	0.05
21878	1.29018	0.05
21879	0.851541	0.05
21880	1.21087	0.05
21881	1.24979	0.05
21882	1.56983	0.05
21883	1.60151	0.05
21884	1.78952	2.1
21885	0.638164	2.1
21886	0.638163	2.1
21887	1.39754	2.1
21888	1.39754	2.1
21889	0.681589	2.1
21890	1.34993	2.1
21891	1.04525	2.1
21892	0.946133	2.1
21893	1.04072	2.1
21894	0.857805	2.1
21895	1.06053	2.1
21896	1.49066	2.09465
21897	1.4185	1.31216
21898	0.934059	1.85107
21899	0.93406	1.37406
21900	0.94613	1.76425
21901	0.93406	1.64554
21902	1.25277	1.64459
21903	1.00792	2.0223
21904	0.609466	1.77903
21905	0.946132	1.94347
21906	0.609463	1.84976
21907	1.30664	1.89023
21908	0.810943	1.40002
21909	1.05741	0.97831
21910	1.3868	2.1
21911	1.15558	0.134016
21912	1.13537	2.1
21913	0.946132	2.1
21914	0.946132	2.1
21915	1.08266	2.1
21916	1.54934	2.1
21917	1.26016	2.1
21918	2.01915	2.1
21919	0.60346	1.88145
21920	0.651529	1.90978
21921	1.15581	2.05254

21922	1.13037	1.89426
21923	1.04071	1.8692
21924	1.77796	2.02967
21925	0.532308	1.78652
21926	0.532308	1.33185
21927	1.15369	1.99735
21928	1.1537	1.81957
21929	0.903288	1.81807
21930	0.804766	2.1
21931	0.804739	2.1
21932	0.946128	2.1
21933	0.946131	2.1
21934	1.16745	2.1
21935	1.04193	2.1
21936	0.98505	2.1
21937	1.40724	0.76409
21938	1.14216	0.476123
21939	0.946131	0.377801
21940	0.946131	0.535803
21941	1.10941	0.545007
21942	0.81268	0.05
21943	0.946131	0.05
21944	1.28673	0.05
21945	1.28673	0.05
21946	0.812681	0.05
21947	1.84716	0.05
21948	1.84716	0.05
21949	0.92361	0.05
21950	0.94613	0.05
21951	0.923582	0.05
21952	1.26227	0.05
21953	0.923581	0.05
21954	0.747394	2.1
21955	1.32582	2.1
21956	0.747365	2.1
21957	0.97587	2.1
21958	1.32582	2.1
21959	1.04182	0.05
21960	0.961743	0.05
21961	0.946126	0.0577076
21962	1.47463	0.05
21963	1.12183	0.05
21964	0.851552	0.05
21965	1.33177	0.0707044
21966	0.946131	0.119416
21967	1.41512	0.079064
21968	1.33178	0.0835725
21969	0.946131	0.0813363
21970	0.927735	0.218893
21971	0.97206	0.106518
21972	0.515566	0.166657
21973	0.946129	0.152584
21974	0.515538	0.126959
21975	1.04482	0.0761762
21976	0.975535	0.123369
21977	0.522411	0.0795724
21978	0.522382	0.135808
21979	0.925346	0.138478

21980	0.575817	0.0993832
21981	0.946129	2.1
21982	1.14256	2.1
21983	1.32054	2.1
21984	1.40525	2.1
21985	1.14256	2.1
21986	1.59475	2.1
21987	0.773813	2.1
21988	1.4073	2.1
21989	0.773813	2.1
21990	0.773813	2.1
21991	1.4073	2.1
21992	0.993021	2.1
21993	1.50466	0.05
21994	0.851539	0.0664028
21995	1.20299	0.05
21996	0.946131	0.0552036
21997	1.54464	0.0527005
21998	1.20309	0.0501922
21999	2.00701	0.05
22000	0.810946	0.0720017
22001	1.07678	0.0636143
22002	0.900128	0.0571994
22003	1.26842	2.1
22004	1.11206	2.1
22005	0.946132	2.1
22006	1.04074	2.1
22007	0.948809	2.1
22008	0.946131	0.05
22009	0.929617	0.05
22010	1.24057	0.05
22011	1.36485	0.05
22012	0.851554	0.05
22013	1.96823	0.05
22014	0.914836	0.05
22015	0.94613	0.05
22016	1.38911	0.05
22017	0.914805	0.05
22018	0.946131	0.05
22019	0.946131	0.05
22020	1.04072	0.05
22021	1.26837	0.05
22022	0.970054	0.05
22023	0.923958	0.05
22024	1.16653	2.1
22025	1.34412	2.1
22026	0.851541	0.05
22027	1.38645	0.0516521
22028	0.891637	0.05
22029	0.851487	0.05
22030	0.890871	0.0509356
22031	1.56178	0.05
22032	0.946128	0.05
22033	1.26868	0.05
22034	0.946131	0.056363
22035	1.18789	0.311927
22036	1.26868	0.05
22037	1.26868	0.0560054

22038	0.946128	0.0560054
22039	1.18716	0.05
22040	0.946131	0.0723125
22041	0.59358	0.0505258
22042	1.23923	0.0844
22043	1.00024	0.05
22044	0.593593	0.0693123
22045	0.946131	0.1063
22046	0.713645	0.0925862
22047	1.23175	0.130255
22048	1.23923	0.0559107
22049	0.713633	0.101032
22050	1.24687	0.05
22051	1.80662	2.04284
22052	2.15762	2.1
22053	0.780455	2.1
22054	0.946131	2.1
22055	0.780455	2.1
22056	1.40583	2.1
22057	1.39852	2.1
22058	0.565619	2.1
22059	1.1428	2.1
22060	0.663168	0.224629
22061	1.02163	0.160354
22062	1.04072	0.226121
22063	0.663169	0.195453
22064	1.13511	0.20088
22065	1.85277	0.118136
22066	1.17701	1.00593
22067	0.993831	0.0631828
22068	1.0823	0.0782301
22069	0.795761	0.184685
22070	1.24596	0.123273
22071	0.566075	0.185525
22072	0.94613	0.208106
22073	0.56605	0.190299
22074	0.774911	0.05
22075	0.946131	0.05
22076	1.24417	0.05
22077	0.774911	0.05
22078	0.946132	0.05
22079	1.54982	0.05
22080	1.82961	0.05
22081	1.12035	0.05
22082	0.876835	2.1
22083	0.946131	2.1
22084	0.876836	2.1
22085	1.12968	2.1
22086	1.23536	2.1
22087	2.15082	2.1
22088	0.58365	2.1
22089	0.946133	2.1
22090	0.583653	2.1
22091	0.992097	2.1
22092	1.12968	2.1
22093	1.0305	2.1
22094	0.82613	0.0914287
22095	0.879644	0.0800213

22096	1.00688	0.0783631
22097	1.52776	2.1
22098	0.851537	1.69088
22099	1.00851	1.11712
22100	0.946128	0.929167
22101	0.851552	1.24558
22102	1.4895	1.76958
22103	0.761574	2.1
22104	0.946132	2.1
22105	1.01095	2.1
22106	1.01095	2.1
22107	0.761572	2.1
22108	1.8575	2.1
22109	1.19984	0.05
22110	1.25797	0.0643938
22111	0.946132	0.0874696
22112	0.946132	0.0610326
22113	0.95332	0.0767141
22114	0.946131	2.1
22115	0.851542	2.1
22116	1.03172	2.1
22117	1.22889	2.1
22118	1.0313	2.1
22119	1.15977	1.9924
22120	0.918875	1.41161
22121	0.8654	1.95376
22122	0.865374	1.54848
22123	0.946132	1.67241
22124	0.985051	2.1
22125	1.31993	2.01208
22126	1.33622	0.261016
22127	0.959992	0.230883
22128	0.926499	2.1
22129	1.03544	2.1
22130	0.946132	2.1
22131	0.946132	2.1
22132	1.40335	2.1
22133	0.772299	2.1
22134	0.851529	2.1
22135	0.857243	2.1
22136	1.38155	2.1
22137	1.05462	2.1
22138	0.832138	2.1
22139	0.946132	2.1
22140	0.832137	2.1
22141	0.959414	2.1
22142	1.36541	2.1
22143	1.91894	2.1
22144	1.04661	2.1
22145	1.28355	2.1
22146	0.877353	2.1
22147	0.877353	2.1
22148	0.94613	2.1
22149	1.20537	0.05
22150	1.09914	0.05
22151	0.94613	0.05
22152	0.499593	0.05
22153	0.499606	0.05

22154	1.0959	0.05
22155	0.838027	0.05
22156	0.883644	0.05
22157	0.95019	0.0709337
22158	1.10638	2.1
22159	0.946132	2.1
22160	1.41744	2.1
22161	1.05842	2.1
22162	0.94613	2.1
22163	1.41744	2.1
22164	0.991985	2.1
22165	3.31755	2.0973
22166	1.05556	2.1
22167	0.624075	2.04383
22168	0.814554	2.03186
22169	0.946131	2.03438
22170	0.814554	2.07181
22171	1.31857	2.05665
22172	1.26282	2.1
22173	0.946132	2.1
22174	1.14302	2.1
22175	0.946132	2.1
22176	0.946132	2.1
22177	1.40372	2.1
22178	1.24688	0.140149
22179	0.89889	2.1
22180	1.17286	2.1
22181	0.71741	2.1
22182	0.491096	0.572478
22183	1.05432	0.298215
22184	0.719531	2.1
22185	1.25145	2.1
22186	0.864686	2.1
22187	0.867974	2.1
22188	1.21734	2.1
22189	0.405517	0.681779
22190	0.818878	0.92589
22191	0.502457	0.05
22192	1.05868	0.05
22193	0.912018	0.05
22194	1.40591	0.05
22195	0.957584	0.447613
22196	0.946131	0.658622
22197	1.11612	0.439735
22198	1.3365	0.458896
22199	0.946128	0.708251
22200	1.16534	0.11926
22201	1.38969	0.172261
22202	0.891597	1.3571
22203	0.640032	1.80058
22204	0.956758	1.36367
22205	1.3428	0.177466
22206	0.953295	2.1
22207	0.946131	0.115026
22208	1.16167	0.0782907
22209	0.94613	0.114792
22210	0.951675	0.119345
22211	1.16167	0.103641

22212	1.24538	0.05
22213	1.13482	2.1
22214	1.38775	2.1
22215	0.934061	2.1
22216	0.91408	2.1
22217	1.06099	2.1
22218	0.980549	0.0794124
22219	0.946132	0.0955132
22220	1.10321	0.111325
22221	0.99126	0.0988461
22222	0.991261	0.109464
22223	1.35732	0.103174
22224	1.1952	2.1
22225	0.946132	1.6399
22226	0.946132	2.1
22227	1.32819	1.9255
22228	1.30171	1.9601
22229	1.29667	0.0570578
22230	1.00982	0.05
22231	0.407589	0.547054
22232	1.18266	1.56642
22233	1.04037	0.553398
22234	0.956694	0.882646
22235	0.552639	0.810629
22236	0.810943	1.90505
22237	1.43144	1.04778
22238	0.918074	1.06607
22239	1.60976	2.1
22240	4.00847	2.1
22241	2.3995	0.05
22242	1.36203	2.1
22243	0.919619	2.1
22244	1.30188	2.1
22245	0.681073	2.1
22246	0.681043	2.1
22247	1.04687	2.1
22248	3.32085	2.1
22249	1.80396	0.05
22250	0.773007	0.162625
22251	0.773034	0.109123
22252	1.22141	0.175456
22253	1.22141	0.156569
22254	0.946131	0.234087
22255	2.02071	0.0868489
22256	0.951912	0.05
22257	1.39027	0.05
22258	0.94613	0.05
22259	0.829922	0.05
22260	0.829946	0.05
22261	1.24902	1.27373
22262	2.8333	0.865401
22263	0.946131	0.05
22264	0.929725	0.05
22265	1.1134	0.05
22266	1.17056	0.05
22267	0.929713	0.05
22268	0.749096	2.1
22269	1.31961	2.1

22270	0.870191	2.1
22271	1.04071	2.1
22272	1.1877	2.1
22273	1.03882	2.1
22274	1.44801	2.0317
22275	3.55198	2.1
22276	1.11193	2.1
22277	0.663922	2.1
22278	0.663922	2.1
22279	0.946129	2.1
22280	0.946131	2.1
22281	0.97079	2.1
22282	1.36049	2.1
22283	1.06105	2.1
22284	0.92251	2.1
22285	0.94613	2.1
22286	0.922511	2.1
22287	1.04101	2.1
22288	0.810944	0.0525387
22289	0.994109	0.05
22290	0.623143	0.05
22291	0.864244	0.0573588
22292	1.14627	0.05
22293	0.64586	0.05
22294	0.721422	0.05
22295	0.870885	0.05
22296	0.855175	0.05
22297	0.946131	2.08141
22298	1.38157	2.1
22299	1.33266	1.89888
22300	0.946131	2.1
22301	1.38158	2.1
22302	0.946132	2.1
22303	1.3268	2.1
22304	0.946132	2.1
22305	0.946132	2.1
22306	1.38158	2.1
22307	1.42186	1.89583
22308	1.47204	1.68798
22309	1.14665	2.1
22310	0.946132	1.98785
22311	0.94613	2.1
22312	1.30116	2.09346
22313	1.32819	2.1
22314	1.25388	2.1
22315	0.887808	2.09993
22316	0.946132	2.1
22317	1.23536	2.1
22318	1.34309	2.1
22319	1.07612	2.1
22320	0.728982	0.05
22321	1.3157	0.05
22322	0.946131	0.05
22323	0.686176	0.05
22324	1.3035	0.05
22325	0.686139	0.05
22326	0.906806	0.05
22327	0.794122	0.05

22328	1.17173	0.05
22329	1.8194	0.05
22330	0.851543	2.1
22331	1.19345	2.1
22332	0.946133	2.1
22333	0.946132	2.1
22334	1.56179	2.1
22335	0.620263	2.08198
22336	1.30226	2.07845
22337	1.2334	2.00735
22338	1.2334	1.94762
22339	0.946133	1.89787
22340	1.24053	2.09773
22341	1.4828	2.1
22342	0.957622	2.1
22343	1.00521	1.77692
22344	1.49463	2.1
22345	1.39621	2.1
22346	1.09861	2.1
22347	0.946131	2.1
22348	0.946131	2.1
22349	1.09861	2.1
22350	2.16987	0.05
22351	0.572182	0.05
22352	1.15802	0.05
22353	0.63574	0.05
22354	1.18897	0.05
22355	0.996733	0.05
22356	1.82402	1.419
22357	0.969602	1.16997
22358	0.794777	1.66935
22359	1.02626	1.32391
22360	1.51396	0.138844
22361	1.40574	0.211867
22362	1.38683	0.195592
22363	0.946126	0.236427
22364	0.946128	0.21191
22365	1.07138	0.203779
22366	0.946131	2.1
22367	1.41331	2.1
22368	0.94613	2.1
22369	0.94613	2.1
22370	1.27451	2.1
22371	0.974196	2.1
22372	1.2049	0.05
22373	1.42757	2.1
22374	1.02461	2.1
22375	2.07087	2.1
22376	1.92515	1.15165
22377	0.802138	1.21936
22378	0.946128	1.90271
22379	0.802107	1.53942
22380	1.23726	1.49742
22381	1.21997	1.42206
22382	0.767577	0.762538
22383	0.946131	1.73221
22384	0.767577	1.1668
22385	1.3279	1.10561

22386	1.23726	1.00617
22387	1.29729	2.1
22388	0.946128	2.1
22389	1.14186	2.1
22390	1.39214	2.1
22391	1.39214	2.1
22392	0.946129	2.1
22393	1.23012	0.455395
22394	0.653228	0.0690338
22395	0.946133	0.095227
22396	1.15918	0.0809236
22397	1.26893	0.0951846
22398	0.653228	0.0719489
22399	1.06848	0.197442
22400	0.615121	2.1
22401	0.841488	2.09655
22402	0.946131	2.1
22403	1.04071	2.1
22404	0.965717	2.1
22405	1.77893	2.036
22406	1.11314	0.0723953
22407	1.65411	0.824136
22408	0.85472	0.05
22409	1.1604	0.05
22410	1.19141	0.05
22411	1.19141	0.05
22412	0.94613	0.05
22413	1.70944	0.05
22414	1.89082	0.122296
22415	1.05175	1.06593
22416	0.815276	2.1
22417	0.843283	2.1
22418	0.946131	2.1
22419	0.843284	2.1
22420	1.38897	2.1
22421	1.01019	0.681735
22422	1.05197	0.66587
22423	0.810959	0.63629
22424	1.33037	0.136134
22425	1.3415	0.094856
22426	0.946118	0.0870919
22427	0.946131	0.118942
22428	1.09803	0.112791
22429	0.930281	2.1
22430	1.3335	2.1
22431	0.784151	2.1
22432	0.78415	2.1
22433	0.946132	2.1
22434	1.6299	0.05
22435	0.995795	2.1
22436	1.84846	1.21198
22437	0.929873	1.95556
22438	0.889245	2.00033
22439	0.808072	1.98311
22440	0.851541	1.81738
22441	1.51956	1.82783
22442	0.94613	1.82299
22443	0.851553	1.82961

22444	0.946787	1.86856
22445	1.15544	1.74112
22446	1.30289	0.0712911
22447	1.28323	0.0649571
22448	0.85024	0.0551662
22449	1.09662	0.071312
22450	1.67687	0.05
22451	0.946127	2.1
22452	1.40496	2.1
22453	1.22442	2.1
22454	0.946128	2.1
22455	1.40496	2.1
22456	1.00573	2.1
22457	0.636837	2.09816
22458	0.988642	2.09858
22459	0.573141	2.08167
22460	0.946128	2.08183
22461	0.786538	2.08024
22462	2.28748	1.86975
22463	0.682805	2.1
22464	0.946132	2.1
22465	0.682776	2.08269
22466	0.946132	2.09987
22467	0.79775	2.1
22468	1.05403	0.05
22469	0.888273	0.05
22470	0.876373	0.05
22471	0.576083	0.05
22472	0.946132	0.05
22473	0.576057	0.05
22474	0.576058	0.05
22475	0.946131	0.05
22476	1.41724	0.384773
22477	1.31678	0.654175
22478	0.946132	0.644868
22479	0.946132	0.542376
22480	1.31678	0.504257
22481	0.935738	2.1
22482	0.946132	2.1
22483	0.935765	2.1
22484	1.32949	2.1
22485	0.946131	2.1
22486	1.02525	2.1
22487	0.946128	2.1
22488	1.41207	2.1
22489	1.04675	2.1
22490	0.946131	2.1
22491	1.41207	2.1
22492	0.946131	2.1
22493	0.946132	2.1
22494	1.41125	2.1
22495	1.41125	2.1
22496	1.27087	2.1
22497	0.924071	0.20279
22498	0.608893	0.150099
22499	0.528907	0.202991
22500	1.78249	0.180095
22501	0.720094	0.206646

22502	0.781667	2.1
22503	0.781638	2.1
22504	1.40756	2.1
22505	1.40756	2.1
22506	0.797635	2.1
22507	1.11946	0.05
22508	1.32068	0.05
22509	0.88757	0.0633232
22510	1.27891	0.05
22511	0.810957	0.0568096
22512	0.914656	0.05
22513	0.640819	0.05
22514	0.745643	0.0500421
22515	0.865108	0.0726316
22516	1.06883	0.0603587
22517	0.806001	0.0793518
22518	1.19462	2.1
22519	1.42763	0.358847
22520	1.24682	0.277121
22521	0.78392	0.05
22522	1.02874	0.05
22523	0.884512	0.05
22524	1.1658	2.1
22525	0.663026	0.05
22526	1.21174	0.05
22527	0.663056	0.05
22528	1.15968	0.05
22529	1.21174	0.05
22530	0.993976	0.05
22531	0.945495	0.05
22532	0.671202	0.05
22533	1.37312	2.1
22534	1.28499	2.1
22535	0.946133	2.1
22536	0.946132	2.1
22537	1.28499	2.1
22538	1.25081	2.1
22539	0.946132	2.1
22540	0.946132	2.1
22541	1.27985	2.1
22542	1.37312	2.1
22543	0.830638	2.1
22544	1.05358	2.1
22545	1.22209	2.1
22546	1.09874	2.1
22547	0.74758	2.1
22548	2.50011	1.1486
22549	0.6157	0.21337
22550	0.674634	0.153672
22551	0.946128	1.77278
22552	0.674633	0.185327
22553	0.946131	0.492885
22554	1.96192	1.59757
22555	1.31612	0.62687
22556	1.10735	0.412162
22557	0.936676	0.166954
22558	0.808779	0.182727
22559	0.808808	0.20845

22560	1.00245	0.187546
22561	1.26179	0.160249
22562	1.05758	2.04763
22563	1.03408	2.1
22564	0.946132	0.0534487
22565	0.914368	0.05
22566	1.20524	0.05
22567	1.12743	0.05
22568	0.851555	0.05
22569	1.17885	0.175879
22570	0.867881	0.141838
22571	1.00122	0.116729
22572	0.867883	0.0918145
22573	0.867881	0.0554686
22574	1.19583	0.132102
22575	1.17743	2.07804
22576	0.878684	2.05627
22577	0.687087	2.09785
22578	0.955079	0.392429
22579	0.672977	0.52692
22580	0.735937	0.466859
22581	0.851531	0.534446
22582	0.672957	0.485444
22583	0.923974	0.493615
22584	1.2243	2.1
22585	0.808463	0.251301
22586	0.971078	0.316349
22587	1.06161	0.182195
22588	0.810947	0.439823
22589	0.886074	0.484947
22590	0.786829	0.464809
22591	0.55062	0.420149
22592	0.574458	0.360402
22593	0.946129	0.429316
22594	1.42526	0.356768
22595	1.15962	0.364248
22596	1.24526	0.05
22597	0.94613	0.622084
22598	0.946128	1.71823
22599	1.23279	1.88872
22600	1.38645	1.82915
22601	0.94613	1.4345
22602	1.05042	2.1
22603	0.946128	2.1
22604	0.946129	2.1
22605	1.24286	2.1
22606	1.39659	2.1
22607	0.94613	2.1
22608	1.10407	2.1
22609	1.32809	1.88524
22610	0.993999	1.97701
22611	0.810959	1.86108
22612	0.983643	1.92548
22613	0.869552	2.01559
22614	0.747801	1.86463
22615	1.53429	1.26395
22616	1.13127	2.1
22617	1.38397	2.1

22618	1.08366	2.1
22619	0.946129	2.1
22620	0.946131	2.1
22621	1.08366	2.1
22622	1.06007	2.1
22623	1.79641	2.1
22624	0.758799	2.1
22625	1.28442	2.1
22626	1.06957	2.1
22627	0.946133	2.1
22628	1.28442	2.1
22629	1.76788	1.85681
22630	1.38669	2.1
22631	0.986095	2.1
22632	1.38478	0.117931
22633	0.946132	0.415694
22634	1.24344	0.181915
22635	0.622283	0.899342
22636	1.10335	0.257253
22637	1.24344	0.371838
22638	1.1967	0.256331
22639	0.946131	0.05
22640	0.901005	0.05
22641	1.05751	0.05
22642	1.37771	0.05
22643	0.851527	0.05
22644	1.26643	0.539633
22645	1.08992	1.79387
22646	0.946132	0.918267
22647	0.946132	1.10788
22648	1.08992	1.19332
22649	1.53534	2.09385
22650	1.58825	1.77538
22651	1.0447	1.9945
22652	0.593818	1.83657
22653	0.89883	1.97212
22654	0.734088	1.85637
22655	0.998451	0.371944
22656	1.64156	0.85522
22657	1.05654	1.0338
22658	1.37641	0.257206
22659	1.74553	0.318202
22660	1.31112	0.441962
22661	0.74518	0.561313
22662	0.851539	0.50418
22663	0.851554	0.446038
22664	0.851475	0.462766
22665	0.946132	0.05
22666	1.28895	0.05
22667	1.11356	0.05
22668	0.946132	0.05
22669	0.946132	0.05
22670	1.84214	2.1
22671	1.23851	0.0687167
22672	1.25866	0.339027
22673	1.28655	0.595569
22674	0.946131	0.547003
22675	0.946132	0.497336

22676	1.28655	0.451437
22677	1.11929	0.39045
22678	0.946132	0.552759
22679	0.946132	0.385237
22680	1.34254	0.379031
22681	1.25866	0.351355
22682	0.946132	2.1
22683	1.27451	2.1
22684	1.19803	2.1
22685	0.94613	2.1
22686	1.27451	2.1
22687	0.946131	2.1
22688	1.36523	2.1
22689	1.14599	2.1
22690	0.946129	2.1
22691	1.36523	2.1
22692	1.09518	2.1
22693	0.977777	2.1
22694	0.583237	2.1
22695	0.583267	2.1
22696	0.903872	2.1
22697	0.946129	2.1
22698	0.583258	2.1
22699	1.58179	0.05
22700	0.946131	0.05
22701	1.35123	0.05
22702	0.655837	0.05
22703	1.12018	0.05
22704	1.35123	0.05
22705	0.62967	0.05
22706	0.860497	0.0566559
22707	0.816639	0.05
22708	0.746716	2.1
22709	0.810947	2.1
22710	0.810959	2.1
22711	0.653422	2.07567
22712	0.289127	1.93347
22713	0.475716	2.05682
22714	0.980651	1.8637
22715	0.946119	2.08876
22716	0.946132	2.09622
22717	1.08299	2.09992
22718	1.34539	2.1
22719	1.28753	0.118016
22720	0.946132	0.222384
22721	0.946132	0.175344
22722	1.19719	0.182003
22723	0.946133	0.17641
22724	0.958947	0.13626
22725	1.3492	0.143466
22726	0.946132	0.239764
22727	0.946128	0.212533
22728	1.22588	0.203084
22729	0.946129	0.199023
22730	0.991113	2.1
22731	1.35966	2.1
22732	0.961 2.1	
22733	0.946129	2.1

22734	0.946131	2.1
22735	0.961001	2.1
22736	0.727096	1.6658
22737	0.810946	0.83524
22738	0.810959	1.06047
22739	1.31658	0.235871
22740	1.23167	2.1
22741	1.44836	0.05
22742	1.50103	0.05
22743	1.23351	0.05
22744	1.35657	0.05
22745	0.851541	0.05
22746	0.93668	0.05
22747	0.946131	0.05
22748	3.7335	2.1
22749	1.1822	2.1
22750	1.31127	0.167321
22751	1.3689	0.153232
22752	1.35822	0.0832758
22753	0.946133	0.116066
22754	0.946132	0.163633
22755	1.14926	0.147205
22756	1.40822	0.0805516
22757	1.04935	0.156354
22758	0.704112	0.110795
22759	0.704112	0.152927
22760	0.946131	0.18159
22761	0.704112	0.16208
22762	1.37721	0.05
22763	0.85154	2.1
22764	1.06949	2.1
22765	0.851541	2.1
22766	0.946128	2.1
22767	1.1175	2.1
22768	1.44091	2.1
22769	0.96029	1.68471
22770	3.46486	0.980422
22771	0.603286	0.0731661
22772	0.432283	0.05
22773	0.851541	0.0636885
22774	1.09355	0.05
22775	0.851534	0.0682307
22776	1.04263	2.1
22777	0.851541	2.1
22778	0.965604	2.1
22779	0.94613	2.1
22780	1.23953	2.1
22781	0.869615	2.1
22782	1.02415	2.1
22783	0.983032	2.1
22784	1.35385	1.32676
22785	1.59971	0.124787
22786	0.915178	2.1
22787	0.962435	2.1
22788	1.3116	2.1
22789	1.38302	2.1
22790	1.13227	2.1
22791	1.25923	0.05

22792	0.988805	2.1
22793	0.611486	1.2779
22794	1.1019	0.803776
22795	1.11124	1.77999
22796	0.716713	0.878317
22797	0.946131	1.42185
22798	1.22297	0.848316
22799	1.12873	1.66863
22800	0.946131	1.68734
22801	0.946132	1.69335
22802	1.3469	1.74859
22803	1.25366	1.87133
22804	1.1611	1.30446
22805	0.946132	1.64066
22806	0.946132	1.40877
22807	1.2854	1.59833
22808	1.3469	1.73191
22809	1.07468	1.3739
22810	1.79317	2.1
22811	1.07444	2.1
22812	1.69436	2.0907
22813	0.851539	2.1
22814	0.870925	2.1
22815	0.851541	2.1
22816	0.870951	2.1
22817	1.26097	2.1
22818	1.26247	2.1
22819	0.946132	2.1
22820	1.24041	2.1
22821	0.946132	2.1
22822	0.946132	2.1
22823	1.40274	2.1
22824	0.946131	0.05
22825	0.797945	0.05
22826	1.12988	0.05
22827	1.00681	0.05
22828	0.797932	0.05
22829	0.977483	0.188214
22830	0.943362	0.357464
22831	0.9891	0.176543
22832	1.1703	0.229025
22833	0.663171	0.231064
22834	1.40882	0.0862387
22835	1.32639	0.136677
22836	1.24448	0.0907021
22837	0.946132	0.151399
22838	1.11015	0.084026
22839	1.24448	0.113336
22840	0.754768	0.106064
22841	1.13854	2.1
22842	1.23654	2.1
22843	0.714101	0.847583
22844	0.851538	1.71936
22845	0.79261	2.05494
22846	1.30598	1.98887
22847	1.05338	1.7274
22848	1.41758	2.1
22849	0.83317	0.104779

22850	1.00058	0.105332
22851	0.810958	0.105743
22852	1.63791	0.135699
22853	1.55116	2.1
22854	1.29735	1.57606
22855	0.945147	2.09908
22856	0.94515	1.79779
22857	0.94613	2.03469
22858	0.945149	1.97651
22859	1.02368	2.1
22860	1.12787	2.1
22861	1.04007	2.1
22862	3.78805	2.1
22863	0.816891	0.0588067
22864	0.943996	0.0536246
22865	0.870002	0.05
22866	0.564533	0.0702524
22867	0.564536	0.0839505
22868	0.946128	0.125754
22869	0.564535	0.110494
22870	0.946128	0.0936647
22871	1.15177	2.1
22872	1.16806	0.498919
22873	0.999083	1.08094
22874	0.946131	0.675114
22875	0.946131	0.829892
22876	0.999084	0.708479
22877	1.28918	0.510189
22878	1.0383	1.14002
22879	0.946133	0.732341
22880	0.946132	0.843198
22881	1.0383	0.672663
22882	1.27737	1.42491
22883	1.39296	0.239879
22884	0.998682	2.1
22885	3.98933	2.1
22886	1.05328	0.114534
22887	1.23475	2.1
22888	0.900856	0.696159
22889	0.94001	0.587733
22890	0.707748	0.520521
22891	0.707751	0.555258
22892	0.946128	0.512405
22893	0.70775	0.519378
22894	0.996109	0.517307
22895	1.68551	2.1
22896	0.851539	2.1
22897	0.962688	2.1
22898	0.94613	2.1
22899	1.04073	2.1
22900	1.44824	2.1
22901	0.794313	0.205629
22902	0.838031	0.216315
22903	1.11076	0.187181
22904	1.38813	2.1
22905	1.17204	2.1
22906	0.946132	2.1
22907	0.946131	2.1

22908	1.17204	2.1
22909	0.961779	0.246896
22910	1.0685	0.38003
22911	0.810947	2.00782
22912	1.2899	1.50134
22913	0.890404	1.49906
22914	0.909981	2.08632
22915	0.810946	1.86174
22916	0.890402	1.83067
22917	0.522844	2.03221
22918	0.593846	2.01374
22919	1.30226	1.74902
22920	1.30226	1.86949
22921	0.946132	1.82941
22922	1.03537	0.0542826
22923	1.50035	0.05
22924	0.940356	0.0636868
22925	0.946131	0.0538894
22926	1.03441	0.0548949
22927	1.21171	0.05
22928	1.35169	0.05
22929	1.32449	0.05
22930	0.946131	0.0563936
22931	0.946131	0.0507821
22932	1.06627	0.0503423
22933	1.03102	0.274815
22934	0.488712	0.05
22935	0.946131	0.05
22936	1.23146	0.05
22937	1.13242	0.05
22938	0.946131	0.05
22939	0.409199	0.05
22940	0.429959	0.05
22941	1.08357	0.050056
22942	0.80926	0.05
22943	0.879791	0.05
22944	0.946129	2.06124
22945	1.28828	2.1
22946	0.946131	2.03394
22947	0.946131	2.09945
22948	1.35969	2.08659
22949	1.10049	1.96131
22950	0.923724	2.1
22951	0.923695	2.1
22952	0.94613	2.1
22953	0.94613	2.1
22954	1.28828	2.1
22955	1.73525	0.131381
22956	0.656239	0.190103
22957	1.2996	0.143958
22958	0.812939	0.206388
22959	0.65624	0.178099
22960	1.2996	0.171893
22961	1.67372	2.1
22962	0.946131	2.1
22963	0.946132	2.1
22964	1.05978	2.1
22965	1.31049	2.1

22966	0.946132	2.1
22967	1.11077	2.1
22968	0.579725	0.360158
22969	0.373532	0.235339
22970	1.13537	0.368095
22971	1.20726	0.254884
22972	0.946131	0.263811
22973	1.45694	0.476809
22974	0.750367	0.05
22975	0.946126	0.0532153
22976	0.750365	0.05
22977	1.25698	0.05
22978	1.35726	0.05
22979	1.6812	0.05
22980	1.78665	1.78616
22981	1.46568	2.1
22982	1.29606	2.1
22983	1.25298	2.1
22984	0.946115	2.1
22985	0.946129	2.1
22986	1.25296	2.1
22987	1.27778	0.0618242
22988	1.17042	2.1
22989	1.25858	0.05
22990	0.93106	0.05
22991	0.810959	0.05
22992	1.38905	2.1
22993	1.19216	2.1
22994	0.946131	2.1
22995	0.946131	2.1
22996	1.19216	2.1
22997	1.58339	2.09389
22998	0.81094	0.05
22999	0.845129	0.05
23000	0.918535	0.05
23001	0.946132	2.1
23002	0.946132	2.1
23003	1.31904	2.1
23004	1.31904	2.1
23005	1.21275	2.1
23006	1.15471	2.1
23007	1.33125	2.1
23008	0.702954	0.05
23009	0.942973	0.05
23010	0.94613	0.05
23011	0.641772	0.05
23012	0.702942	0.05
23013	0.991865	0.05
23014	0.639952	0.05
23015	1.17333	0.05
23016	0.946132	0.05
23017	0.490975	0.05
23018	0.495934	0.05
23019	1.19904	0.05
23020	1.30488	0.05
23021	1.02305	0.124806
23022	1.49842	0.0815042
23023	1.54196	2.1

23024	1.37224	1.88944
23025	1.0953	0.05
23026	1.18952	2.1
23027	1.68851	0.254702
23028	0.946131	0.143921
23029	1.12491	0.142214
23030	0.583591	0.137045
23031	1.28727	0.18
23032	1.12491	0.144485
23033	0.884641	0.291211
23034	0.810944	0.219277
23035	0.917973	0.197427
23036	0.846671	0.187232
23037	0.596968	0.180804
23038	0.716347	0.232881
23039	1.36255	0.0984558
23040	1.11077	2.1
23041	0.946132	2.1
23042	0.946132	2.1
23043	1.26267	2.1
23044	1.26441	2.1
23045	1.85238	2.1
23046	1.36693	2.1
23047	0.616123	2.1
23048	0.481844	2.1
23049	0.709599	2.1
23050	0.815889	2.1
23051	1.1502	2.1
23052	1.63937	1.98044
23053	1.25797	0.0637513
23054	1.22878	0.0874271
23055	0.946132	0.107428
23056	0.946132	0.0845789
23057	0.965737	0.0900433
23058	1.1893	0.0900288
23059	1.22877	0.0861061
23060	0.735217	0.11567
23061	0.85154	0.12346
23062	0.851553	0.108137
23063	0.851473	0.109854
23064	1.58916	0.0652241
23065	1.25545	0.0628694
23066	0.810942	0.123607
23067	0.919314	0.136997
23068	0.928633	0.115689
23069	2.14514	0.0742845
23070	1.23389	2.1
23071	0.946128	0.0506527
23072	0.969504	0.05
23073	0.851539	0.05
23074	1.41503	0.05
23075	0.958517	0.05
23076	0.990615	0.05
23077	1.7943	0.05
23078	0.946133	0.34671
23079	1.27581	0.360901
23080	0.946132	0.357183
23081	1.36362	0.314727

23082	1.36936	0.318173
23083	1.71903	2.1
23084	0.751639	2.1
23085	0.77464	2.1
23086	0.946133	2.1
23087	1.04073	2.1
23088	1.23551	2.1
23089	0.94613	2.1
23090	1.18682	2.1
23091	0.946131	2.1
23092	0.94613	2.1
23093	1.40638	2.1
23094	0.483346	0.193276
23095	0.432527	0.116821
23096	0.946132	0.204988
23097	0.483312	0.154608
23098	0.946132	0.158906
23099	0.878798	0.193864
23100	0.810944	0.18973
23101	1.14604	0.166784
23102	0.946129	0.183058
23103	1.35594	0.146969
23104	0.538378	0.14805
23105	0.946131	0.192408
23106	0.538378	0.168184
23107	1.00065	1.95611
23108	0.962878	0.905997
23109	1.32634	0.0572506
23110	1.28066	0.05
23111	0.785651	2.1
23112	1.20327	2.1
23113	1.47058	2.0223
23114	0.551834	1.47861
23115	1.04072	2.05123
23116	0.506719	1.91557
23117	1.12786	1.97822
23118	1.45789	0.05
23119	0.681532	0.05
23120	1.21087	0.05
23121	0.94613	0.05
23122	0.681534	0.05
23123	1.21087	0.05
23124	1.98618	0.565757
23125	1.71742	1.46286
23126	1.07427	0.252021
23127	1.1972	0.193504
23128	0.933474	0.253624
23129	1.1745	0.266703
23130	0.94612	0.251371
23131	0.933485	0.262931
23132	1.17449	0.255941
23133	0.851541	0.05
23134	1.12774	0.05
23135	0.946129	0.05
23136	0.851486	0.05
23137	1.12517	0.05
23138	1.12328	0.05
23139	1.13179	2.1

23140	1.02438	2.1
23141	1.05776	2.1
23142	1.41273	2.04654
23143	1.1229	2.1
23144	0.875912	2.07411
23145	0.946132	2.1
23146	0.875912	2.1
23147	1.42307	2.1
23148	1.15354	0.05
23149	0.848382	0.05
23150	1.2685	2.1
23151	0.938104	2.1
23152	0.793166	2.1
23153	1.07855	2.1
23154	0.98488	2.1
23155	1.4162	2.1
23156	1.09651	0.0980364
23157	1.98502	0.052401
23158	1.41168	0.105299
23159	1.33879	0.163199
23160	0.946127	0.236568
23161	1.13528	0.130937
23162	0.946128	0.171775
23163	1.09726	2.1
23164	1.22026	1.63198
23165	1.09044	2.1
23166	2.23961	0.336718
23167	1.14721	0.225902
23168	1.17614	2.1
23169	0.615065	2.1
23170	0.946131	2.1
23171	0.778488	2.1
23172	0.94613	2.1
23173	0.615066	2.1
23174	1.78254	2.1
23175	0.898452	0.05
23176	1.47411	2.1
23177	0.59676	0.246858
23178	0.59676	0.160731
23179	0.94613	0.25269
23180	1.13511	0.194787
23181	0.946131	0.185776
23182	0.916275	2.07581
23183	0.946132	2.0259
23184	0.916274	1.69127
23185	1.36182	1.91922
23186	0.946131	2.00826
23187	0.857202	0.05
23188	0.941918	0.05
23189	0.943349	0.05
23190	1.2493	0.05
23191	0.946133	0.05
23192	1.01814	0.05
23193	0.927881	0.05
23194	1.11845	0.05
23195	1.2493	0.05
23196	1.11845	0.05
23197	0.946129	0.05

23198	0.967673	0.467129
23199	1.01332	0.05
23200	0.946133	0.05
23201	0.81938	0.05
23202	0.819377	0.05
23203	0.946133	0.05
23204	1.22474	0.114039
23205	1.15321	0.0873962
23206	0.797489	0.0892075
23207	0.946131	0.12071
23208	0.797461	0.100542
23209	1.35328	0.51907
23210	1.03318	1.14865
23211	0.946131	0.71166
23212	0.94613	0.769656
23213	1.03319	0.682622
23214	1.37938	0.535989
23215	1.00512	1.05495
23216	0.946133	0.687709
23217	0.946132	0.763198
23218	1.00512	0.668493
23219	1.5515	0.866797
23220	1.55351	2.1
23221	0.94261	0.05
23222	0.828156	0.05
23223	0.916495	0.05
23224	3.06309	0.05
23225	0.619832	0.05
23226	0.705903	0.05
23227	1.2968	0.05
23228	0.946133	0.05
23229	0.705904	0.05
23230	1.2968	0.05
23231	1.01971	0.05
23232	0.60302	0.05
23233	1.07242	0.50682
23234	1.26426	0.334086
23235	0.946131	0.477112
23236	0.946131	0.526873
23237	0.963109	0.525168
23238	0.947862	0.296923
23239	1.40248	1.15556
23240	0.767143	1.09405
23241	0.767114	1.30502
23242	0.946129	1.19578
23243	0.767143	0.985004
23244	1.22637	1.16367
23245	1.2281	1.2016
23246	0.590909	1.19247
23247	0.490832	1.26204
23248	0.804174	1.28109
23249	0.747368	1.1192
23250	1.05129	0.0641054
23251	1.10609	2.0633
23252	1.3639	0.11768
23253	0.776117	0.102644
23254	0.752329	0.118614
23255	1.04073	0.156323

23256	0.67713	0.137746
23257	3.4245	2.1
23258	1.18526	2.1
23259	1.2161	1.26436
23260	0.876834	1.83926
23261	0.876831	1.20236
23262	0.946132	1.77138
23263	0.876834	1.57191
23264	0.954563	2.1
23265	0.924132	2.1
23266	1.25075	2.1
23267	1.01652	2.1
23268	0.92413	2.1
23269	1.37579	2.1
23270	0.992159	2.1
23271	1.26092	2.1
23272	0.929435	2.1
23273	0.844958	2.1
23274	0.79598	2.1
23275	0.852559	2.1
23276	0.946131	0.482445
23277	1.28435	0.223764
23278	0.827261	0.399645
23279	0.946128	0.513902
23280	0.827298	0.503939
23281	1.65452	0.092552
23282	1.79929	2.1
23283	0.791947	0.24457
23284	0.902072	0.39388
23285	0.882672	0.190614
23286	1.21703	2.1
23287	0.900338	2.1
23288	1.37712	2.1
23289	0.782591	2.1
23290	0.782562	2.1
23291	0.94613	2.1
23292	1.34749	2.1
23293	0.61359	2.1
23294	1.14397	1.43031
23295	1.02963	0.05
23296	1.36589	0.05
23297	0.946132	0.0543518
23298	1.28644	0.05
23299	0.946131	0.05
23300	1.40104	0.05
23301	1.28644	0.05
23302	0.810946	0.0896554
23303	1.09178	0.0812884
23304	1.26956	0.070452
23305	0.810943	0.0779839
23306	0.885633	0.0799358
23307	1.00844	0.0705027
23308	0.825671	0.0908681
23309	0.792093	0.0844071
23310	0.925037	0.081331
23311	0.946131	1.89346
23312	0.946131	0.283857
23313	1.18052	0.703895

23314	1.40265	0.573367
23315	0.946132	1.06631
23316	1.17891	0.580604
23317	0.734248	0.05
23318	0.810943	0.05
23319	0.731621	0.05
23320	0.799857	0.190499
23321	0.834507	0.268368
23322	0.810932	0.190289
23323	1.01316	0.05
23324	1.23884	0.05
23325	1.88438	2.1
23326	0.946129	2.1
23327	1.38614	2.08498
23328	1.34245	1.8589
23329	0.946131	1.24816
23330	1.38614	1.77331
23331	0.946132	0.05
23332	0.910733	0.05
23333	1.24726	0.05
23334	1.13742	0.05
23335	0.851555	0.05
23336	1.50368	0.05
23337	0.94613	2.1
23338	1.08998	2.1
23339	1.36899	2.00246
23340	1.36898	2.1
23341	0.946131	2.1
23342	1.26045	2.1
23343	1.06432	0.559686
23344	1.89016	0.369312
23345	0.83514	2.1
23346	1.36653	2.1
23347	1.23426	2.1
23348	0.94613	2.1
23349	0.835112	2.1
23350	1.14567	1.79825
23351	1.12096	1.86512
23352	0.881106	1.70988
23353	1.17787	0.651318
23354	1.29738	2.09364
23355	0.940472	1.37432
23356	1.03452	1.42503
23357	0.946131	1.25226
23358	2.7489	0.558958
23359	1.01778	2.1
23360	1.04321	1.0663
23361	1.19352	0.127406
23362	1.16912	1.62681
23363	0.946132	1.19352
23364	0.946132	1.7226
23365	1.40924	1.74538
23366	1.22927	1.93052
23367	2.09455	0.0608331
23368	3.00575	2.1
23369	1.08928	2.1
23370	1.94799	2.09865
23371	1.4092	2.1

23372	0.946132	2.1
23373	1.16	2.1
23374	1.01985	2.1
23375	0.946133	2.1
23376	1.16	2.1
23377	1.15371	0.05
23378	1.56002	0.05
23379	1.23007	2.0856
23380	1.9992	2.1
23381	0.652605	2.1
23382	0.937903	2.1
23383	0.946132	2.1
23384	0.937902	2.1
23385	1.228	2.1
23386	1.33694	1.9818
23387	1.00191	2.1
23388	0.716814	2.0131
23389	0.94613	2.09447
23390	0.716816	2.07041
23391	1.70388	1.34118
23392	0.946129	0.188732
23393	1.09362	0.172543
23394	0.946131	0.296348
23395	1.4185	0.213682
23396	1.09362	0.114537
23397	1.31057	1.64071
23398	1.17631	0.05
23399	1.19404	0.05
23400	0.94613	0.101012
23401	0.946129	0.0669577
23402	0.99972	0.0757106
23403	0.94613	0.0772575
23404	0.835199	0.05
23405	1.23351	0.05
23406	0.582785	0.05
23407	0.701079	0.05
23408	0.946132	0.05
23409	2.17385	0.05
23410	1.18948	1.98738
23411	1.25346	2.1
23412	1.18586	0.357009
23413	0.880876	0.413237
23414	0.68632	0.792772
23415	0.946118	2.1
23416	0.966936	2.1
23417	0.946117	2.1
23418	1.41636	2.1
23419	1.03798	2.1
23420	0.729178	0.311436
23421	0.721888	0.34551
23422	0.946132	0.310661
23423	0.656286	0.319748
23424	1.13898	0.313958
23425	1.02351	0.05
23426	1.48803	0.05
23427	0.946131	0.05
23428	1.04921	2.1
23429	1.02478	1.77514

23430	0.993023	2.1
23431	1.80841	0.126754
23432	1.29215	0.0985525
23433	0.904206	0.113852
23434	0.904207	0.131606
23435	0.946132	0.13765
23436	0.904207	0.118115
23437	1.07347	2.1
23438	0.555875	1.83904
23439	0.555904	1.75942
23440	1.30841	2.02108
23441	1.30841	1.99729
23442	0.91951	1.99302
23443	1.33026	0.822037
23444	1.43289	0.05
23445	1.15703	1.6733
23446	0.886594	1.92257
23447	1.05129	1.73034
23448	1.08421	0.361821
23449	1.35946	0.241886
23450	0.882955	0.640903
23451	0.946131	0.547531
23452	0.882985	0.459527
23453	0.583501	0.589226
23454	0.532921	0.700619
23455	0.94613	1.90033
23456	0.532892	1.61266
23457	1.23224	1.67955
23458	1.12786	1.63463
23459	0.946131	2.08957
23460	1.23732	0.30629
23461	1.40609	2.1
23462	1.40609	1.29962
23463	0.946131	1.01343
23464	0.910672	1.53235
23465	1.08478	0.750831
23466	0.94613	0.505658
23467	0.94613	1.3177
23468	1.30382	1.11847
23469	3.10544	1.63296
23470	0.679509	1.9251
23471	1.15966	1.61417
23472	0.75499	1.96522
23473	1.37511	1.67623
23474	1.01776	1.67284
23475	2.35239	0.805956
23476	0.92406	2.1
23477	0.946118	2.1
23478	0.92406	2.1
23479	1.07979	2.1
23480	1.29604	2.1
23481	1.29897	2.1
23482	0.902645	0.415677
23483	0.729493	1.8058
23484	0.906844	0.921157
23485	0.739518	0.05
23486	1.10564	0.0761022
23487	0.946131	0.05

23488	0.739519	0.05
23489	0.739506	0.05
23490	1.79907	0.261281
23491	1.59106	0.608295
23492	1.11067	0.339231
23493	0.795516	0.608036
23494	0.795519	0.417508
23495	0.946132	0.551251
23496	0.795506	0.434155
23497	1.80223	2.1
23498	1.95572	2.07073
23499	1.16237	0.894779
23500	1.07518	2.1
23501	0.696199	1.59288
23502	0.94613	1.914
23503	0.696199	1.67913
23504	1.6379	0.05
23505	0.946118	0.05
23506	0.851529	0.05
23507	1.08716	0.05
23508	0.973118	0.05
23509	0.851528	0.05
23510	1.15096	2.1
23511	0.65023	0.05
23512	0.94613	0.05
23513	0.650205	0.05
23514	0.650205	0.05
23515	0.811233	0.05
23516	0.778721	1.97886
23517	0.810945	1.23685
23518	0.997518	1.45877
23519	0.838028	0.0616296
23520	0.952893	0.05
23521	0.838037	0.0524616
23522	1.58166	0.05
23523	1.44848	1.12798
23524	1.30041	0.05
23525	1.03697	0.700114
23526	0.946132	1.93019
23527	1.0277	1.12405
23528	0.946131	1.98658
23529	1.00501	1.41486
23530	1.0277	1.70302
23531	1.64988	2.1
23532	0.946131	2.1
23533	0.94613	2.03453
23534	1.27702	2.1
23535	1.55163	2.1
23536	0.897905	2.1
23537	1.12971	0.05
23538	1.35715	0.107822
23539	0.881884	0.319329
23540	0.664007	0.131479
23541	0.663978	0.208422
23542	0.94613	0.191534
23543	0.664006	0.18957
23544	1.48693	0.128373
23545	1.29289	0.0746971

23546	1.32081	0.0841799
23547	0.946119	0.109928
23548	0.946132	0.0904506
23549	1.02972	0.0873852
23550	0.946131	2.1
23551	0.946127	2.1
23552	1.23513	2.1
23553	1.38154	2.1
23554	0.94613	2.1
23555	1.25933	0.0872074
23556	0.917954	0.0936558
23557	0.868589	0.05
23558	3.0957	2.1
23559	0.711391	0.05
23560	0.711391	0.05
23561	0.991303	0.05
23562	1.37322	0.05
23563	0.729145	0.05
23564	2.55648	0.05
23565	1.0756	1.90854
23566	0.760299	1.11249
23567	0.763592	0.570884
23568	0.946126	0.820802
23569	0.76359	0.561788
23570	1.35862	1.02723
23571	0.946133	2.1
23572	1.00807	2.1
23573	0.946132	2.1
23574	1.39823	2.1
23575	1.07817	2.1
23576	1.44379	0.201339
23577	1.17934	0.05
23578	1.13451	0.145512
23579	0.94613	2.1
23580	1.03974	2.1
23581	1.36835	2.1
23582	1.36835	2.1
23583	0.94613	2.1
23584	1.27096	2.09509
23585	0.853042	2.1
23586	0.946131	2.1
23587	1.13658	2.1
23588	1.06997	2.1
23589	0.94613	2.1
23590	1.41556	0.36399
23591	1.21708	0.54664
23592	0.94613	0.571251
23593	0.946131	0.472929
23594	1.21708	0.464997
23595	0.964782	0.364069
23596	0.946119	0.514374
23597	1.23447	0.458376
23598	0.946118	0.521258
23599	1.41844	0.419201
23600	1.23446	0.472032
23601	1.1357	0.297027
23602	0.619856	0.05
23603	1.1509	0.05

23604	0.946126	0.05
23605	1.19262	0.05
23606	1.1509	0.05
23607	1.04268	2.1
23608	2.07527	0.951662
23609	1.38259	0.833575
23610	0.980054	1.42032
23611	0.946124	0.976365
23612	0.946127	1.18018
23613	0.980056	1.03456
23614	1.62916	1.60247
23615	0.946131	2.1
23616	1.40571	2.1
23617	0.946131	2.1
23618	1.38885	2.1
23619	1.40571	2.1
23620	1.11787	2.1
23621	0.946129	2.1
23622	1.28831	2.1
23623	1.41498	2.09781
23624	1.41498	2.1
23625	0.946131	2.1
23626	0.850964	0.05
23627	0.921299	0.05
23628	1.37358	0.05
23629	0.946132	0.05
23630	0.850964	0.05
23631	0.823988	0.05
23632	0.823984	0.05
23633	1.36568	0.05
23634	1.36569	0.05
23635	0.921275	0.05
23636	1.84739	2.1
23637	1.47169	0.0547198
23638	0.946131	0.156879
23639	0.735844	0.127081
23640	1.11819	0.202201
23641	1.10904	0.0873897
23642	0.735831	0.133233
23643	0.946131	2.1
23644	0.946132	2.1
23645	1.23487	2.1
23646	1.3281	2.1
23647	0.946131	2.1
23648	1.72271	0.05
23649	0.861359	0.05
23650	0.946131	0.0917238
23651	0.861357	0.0628855
23652	1.1369	0.064426
23653	1.26868	0.0581774
23654	0.789173	1.99168
23655	0.789143	2.03865
23656	0.946133	1.69204
23657	1.34386	1.89601
23658	0.789144	1.94032
23659	0.983493	2.1
23660	1.18944	0.329191
23661	1.05121	0.378092

23662	0.777398	0.390821
23663	0.946132	0.522824
23664	0.777423	0.51904
23665	1.09841	0.102335
23666	0.963919	0.0807839
23667	0.474855	0.106914
23668	0.946131	0.111912
23669	0.474855	0.102892
23670	3.40074	2.09916
23671	1.29453	2.1
23672	1.01139	0.40139
23673	0.946133	1.15673
23674	1.35381	0.182016
23675	1.18322	0.819914
23676	0.946133	1.2362
23677	1.45502	0.134959
23678	0.970088	1.31278
23679	0.960333	1.54785
23680	0.838036	1.41984
23681	1.30927	0.11453
23682	0.828617	0.127567
23683	0.714795	0.125191
23684	0.946132	0.131482
23685	0.714824	0.121122
23686	0.946131	2.1
23687	0.851542	2.1
23688	1.38204	2.1
23689	1.24512	2.1
23690	1.04086	2.1
23691	0.723573	2.1
23692	0.946131	2.1
23693	0.982454	2.1
23694	0.982454	2.1
23695	0.723544	2.1
23696	1.17261	0.0745161
23697	1.34153	0.0692922
23698	1.41383	0.0538413
23699	0.946132	0.100942
23700	0.946133	0.0791395
23701	1.12818	0.142537
23702	1.29383	0.05
23703	0.946133	0.390614
23704	1.0027	0.365137
23705	0.768989	0.221361
23706	0.946133	0.418621
23707	0.768977	0.395628
23708	0.946132	0.562823
23709	1.24333	0.349477
23710	0.928583	0.550719
23711	0.946132	0.587889
23712	0.928583	0.598919
23713	1.53798	0.112069
23714	2.95436	0.0617666
23715	1.30649	0.0896289
23716	0.809789	0.162625
23717	0.745744	0.155791
23718	0.745774	0.138601
23719	0.946128	0.144022

23720	1.00777	0.0648468
23721	1.40271	0.0510334
23722	0.768387	0.0828044
23723	0.946132	0.0744933
23724	0.922045	0.0684322
23725	0.946132	2.1
23726	1.39021	2.1
23727	1.10046	2.1
23728	0.946132	2.1
23729	1.39021	2.1
23730	2.06158	0.05
23731	0.913967	0.175415
23732	1.30107	0.149406
23733	0.946132	0.185728
23734	0.913966	0.139024
23735	1.30107	0.14308
23736	1.64117	2.1
23737	0.514872	1.95534
23738	0.514846	1.58593
23739	1.30811	2.06947
23740	1.30812	1.75651
23741	0.888989	1.92103
23742	1.99279	1.93894
23743	0.910271	0.253999
23744	0.838027	0.317494
23745	0.928444	0.194157
23746	1.2377	0.913267
23747	0.851537	2.1
23748	0.899142	2.1
23749	0.810075	2.1
23750	1.3771	2.1
23751	1.03281	2.1
23752	0.719877	2.1
23753	0.901838	2.1
23754	1.01748	2.1
23755	1.12315	2.1
23756	0.64787	2.1
23757	1.173 2.1	
23758	0.876903	2.1
23759	0.597337	2.1
23760	1.00291	2.1
23761	0.597309	2.1
23762	1.09897	0.287449
23763	0.946132	0.550176
23764	1.1386	0.275169
23765	1.09897	0.359389
23766	0.596652	0.38783
23767	2.5593	2.1
23768	0.946133	0.173079
23769	1.29172	0.169424
23770	1.11337	0.115406
23771	0.946131	0.161545
23772	0.946131	0.123826
23773	0.96926	0.532614
23774	0.946132	0.886349
23775	0.946132	1.1001
23776	1.07118	0.329935
23777	1.29698	0.432854

23778	1.37867	0.874499
23779	0.946119	1.72541
23780	0.94612	1.84765
23781	1.33154	0.700089
23782	0.946107	1.06548
23783	2.17757	0.97323
23784	0.946131	0.0607967
23785	0.68393	0.05
23786	0.68396	0.05
23787	1.33671	0.05
23788	1.10733	0.05
23789	2.31367	2.1
23790	0.989326	1.91873
23791	0.689952	2.07785
23792	0.854787	1.83244
23793	3.73635	2.1
23794	1.41489	2.1
23795	1.25544	2.0329
23796	0.946128	2.1
23797	0.946129	2.1
23798	1.13536	2.1
23799	2.10284	1.67778
23800	0.851542	2.1
23801	0.828523	2.1
23802	0.946132	2.1
23803	0.946132	2.1
23804	1.50873	2.1
23805	0.989048	2.0997
23806	0.925055	2.1
23807	0.838041	2.09801
23808	0.807714	0.653645
23809	0.656831	0.383545
23810	0.997603	0.54747
23811	0.946131	1.41946
23812	1.02602	1.26077
23813	0.946129	1.42257
23814	1.35469	1.10942
23815	1.25083	1.12077
23816	0.851541	0.235485
23817	0.851483	0.214643
23818	0.851539	0.24136
23819	1.19785	0.207276
23820	1.07615	0.218854
23821	0.714507	0.0634622
23822	1.41461	0.0573263
23823	0.714507	0.062541
23824	0.714507	0.0615759
23825	0.946133	0.0632993
23826	0.946131	1.21527
23827	0.972232	0.883316
23828	0.94613	1.38683
23829	1.25083	0.788356
23830	1.30165	0.937584
23831	1.05332	2.1
23832	0.574768	2.1
23833	1.94541	0.05
23834	0.789633	0.05
23835	0.946127	0.05

23836	1.33798	0.05
23837	1.22903	0.05
23838	0.946128	0.05
23839	0.946128	2.1
23840	1.11882	2.1
23841	0.996487	2.1
23842	0.94613	2.1
23843	1.11883	2.1
23844	0.946127	2.1
23845	1.30082	2.1
23846	1.02608	2.1
23847	0.946128	2.1
23848	1.30081	2.1
23849	1.3507	1.85086
23850	1.19583	0.195086
23851	0.946127	1.7283
23852	0.94613	1.16436
23853	1.19583	1.43333
23854	0.477652	0.05
23855	1.20416	0.05
23856	0.946131	0.05
23857	0.477652	0.05
23858	0.477691	0.05
23859	1.34536	0.05
23860	0.758971	0.05
23861	1.25124	0.05
23862	1.31467	0.05
23863	0.758971	0.05
23864	1.31467	0.05
23865	0.946131	2.1
23866	1.14184	2.1
23867	1.35028	2.1
23868	1.35028	2.1
23869	0.946131	2.1
23870	1.13537	2.04674
23871	0.946132	1.53448
23872	0.946132	2.04879
23873	1.23031	1.92066
23874	1.27555	1.93895
23875	1.53064	1.98608
23876	1.28421	0.758886
23877	1.07707	1.22819
23878	0.946125	0.845723
23879	0.946127	1.19585
23880	1.07707	0.971582
23881	0.946133	0.05
23882	1.3495	0.05
23883	0.946132	0.05
23884	1.05704	0.05
23885	1.3495	0.05
23886	0.946132	0.05
23887	1.37794	0.05
23888	0.946132	0.05
23889	1.01367	0.05
23890	1.37794	0.05
23891	0.928516	2.1
23892	0.946133	2.1
23893	0.928518	2.1

23894	1.14105	2.1
23895	1.26406	2.1
23896	0.797664	2.1
23897	0.946132	2.1
23898	0.797663	2.1
23899	1.34285	2.1
23900	0.929875	2.1
23901	0.946131	2.08403
23902	1.21139	0.927902
23903	1.41492	1.22312
23904	1.41492	0.638847
23905	0.946131	0.426474
23906	0.946131	2.1
23907	0.689388	2.1
23908	0.94365	2.1
23909	0.777345	2.1
23910	0.709599	2.1
23911	0.774968	2.1
23912	0.946132	2.1
23913	0.77497	2.1
23914	0.868145	2.1
23915	1.28401	2.1
23916	0.94613	2.06874
23917	1.19655	2.1
23918	1.39428	1.14415
23919	1.39428	2.0817
23920	0.946131	2.09771
23921	1.1848	2.1
23922	1.07591	2.1
23923	0.946131	2.1
23924	0.946131	2.1
23925	0.874788	2.1
23926	1.35703	2.1
23927	1.35357	2.1
23928	0.946132	2.1
23929	1.26022	2.1
23930	0.946132	2.1
23931	0.946131	2.1
23932	0.966426	2.1
23933	0.946133	2.1
23934	0.946131	2.1
23935	1.3484	2.1
23936	1.26441	2.1
23937	1.2079	2.1
23938	0.946133	2.1
23939	0.946132	2.1
23940	1.2079	2.1
23941	1.2736	2.1
23942	1.25774	2.1
23943	0.946133	2.1
23944	0.851555	2.1
23945	0.96172	2.1
23946	0.946128	2.1
23947	1.06879	2.1
23948	0.94613	2.1
23949	1.32608	2.1
23950	1.06879	2.1
23951	0.946132	2.1

23952	1.32608	2.1
23953	0.946131	2.1
23954	1.40488	2.1
23955	1.32608	2.1
23956	0.94613	1.50731
23957	1.19234	0.947707
23958	0.946129	1.15395
23959	0.94613	1.64227
23960	1.31649	1.75281
23961	0.912059	1.83661
23962	1.04514	1.77223
23963	0.946132	1.78351
23964	0.946132	2.05933
23965	1.34646	2.02115
23966	1.01187	0.418623
23967	1.33728	1.51829
23968	0.761974	0.665132
23969	0.761974	0.544436
23970	0.94613	0.53535
23971	1.40641	2.08804
23972	0.955752	0.79635
23973	0.736738	2.00793
23974	0.94613	1.54886
23975	0.736739	1.77344
23976	0.861471	2.1
23977	1.34556	2.1
23978	0.810957	2.1
23979	0.946131	2.1
23980	1.37811	2.1
23981	1.17949	2.1
23982	0.946132	2.1
23983	1.37811	2.1
23984	0.946131	2.1
23985	1.40447	2.1
23986	0.946132	2.1
23987	0.946132	2.1
23988	1.39826	2.1
23989	0.946131	2.1
23990	1.283	2.1
23991	0.94613	2.1
23992	0.946131	2.1
23993	1.40447	2.1
23994	1.20541	2.1
23995	0.946131	2.1
23996	0.946127	2.1
23997	1.33607	2.1
23998	1.41021	2.1
23999	1.01271	2.1
24000	0.946131	2.1
24001	0.920606	2.1
24002	1.13078	2.1
24003	1.60331	2.1
24004	0.946118	1.4374
24005	1.09718	2.1
24006	0.946116	2.07529
24007	1.32662	1.87383
24008	1.0972	2.00818
24009	0.94613	0.656619

24010	1.32666	1.5694
24011	0.946128	0.838642
24012	1.31381	0.552933
24013	1.32666	0.607766
24014	0.94612	2.1
24015	0.940748	2.1
24016	1.03561	2.1
24017	1.35111	2.1
24018	0.851527	2.1
24019	0.607156	2.1
24020	0.789158	2.1
24021	0.946129	2.1
24022	1.04071	2.1
24023	1.13144	2.1
24024	0.946133	2.1
24025	1.35663	2.1
24026	0.946132	2.1
24027	0.946133	2.1
24028	1.34237	2.1
24029	0.919455	2.1
24030	1.01143	2.1
24031	0.946128	2.1
24032	0.946131	2.1
24033	1.62796	2.1
24034	1.17796	1.76512
24035	1.23024	2.07946
24036	0.946132	1.75711
24037	0.85153	1.99446
24038	0.986867	1.96771
24039	0.889308	2.1
24040	0.94613	2.1
24041	0.889338	2.1
24042	0.946131	2.1
24043	0.889337	2.1
24044	1.18396	1.03742
24045	0.94613	0.45597
24046	0.946125	1.83318
24047	1.39514	0.830375
24048	1.30809	1.29459
24049	0.803322	2.03743
24050	0.925893	1.89514
24051	1.36183	1.62611
24052	0.925892	1.6212
24053	0.946133	1.68509
24054	1.38354	0.142132
24055	1.16009	0.129487
24056	0.946132	0.119456
24057	0.946132	0.115078
24058	1.01462	0.0811357
24059	1.18854	0.935182
24060	0.888265	1.55217
24061	0.745095	1.10476
24062	0.709597	1.35278
24063	0.936541	1.25477
24064	1.24142	0.701171
24065	1.35059	0.469759
24066	0.946128	0.451655
24067	0.94613	0.612105

24068	1.16623	0.623494
24069	1.24945	0.0838562
24070	1.33035	0.0783547
24071	0.946127	0.106156
24072	0.946128	0.096247
24073	1.13343	0.137034
24074	0.637995	1.17432
24075	1.10455	1.07375
24076	1.22641	1.68731
24077	1.22641	1.70952
24078	0.946132	1.36053
24079	1.3665	0.0775916
24080	1.28507	0.0849553
24081	0.946127	0.112753
24082	0.946129	0.072396
24083	1.2051	0.131556
24084	0.797374	2.09891
24085	0.797376	2.1
24086	1.13113	2.08103
24087	1.20057	2.09175
24088	1.12049	2.09697
24089	0.59387	0.983576
24090	1.13245	0.50929
24091	0.966134	1.124
24092	0.593842	0.534297
24093	0.946128	0.869814
24094	0.641455	0.521437
24095	0.916507	0.482117
24096	1.04072	1.11734
24097	0.415585	0.430581
24098	0.507244	0.830074
24099	0.678938	2.03844
24100	1.24154	2.00548
24101	1.25	1.8823
24102	1.24154	1.94116
24103	1.25	1.96671
24104	0.851009	1.71631
24105	0.946131	1.99099
24106	0.851539	1.80844
24107	1.21997	1.75096
24108	1.45109	1.74967
24109	0.804877	0.588109
24110	1.00062	0.212163
24111	0.851536	0.484916
24112	0.909673	0.230753
24113	0.946129	0.4802
24114	1.20494	0.0785218
24115	1.09088	0.132393
24116	0.887634	0.159154
24117	0.81027	0.0771563
24118	0.900294	0.110007
24119	0.747713	0.05
24120	0.975467	0.05
24121	0.747712	0.05
24122	1.39168	0.05
24123	0.975467	0.05
24124	0.94613	0.0975338
24125	1.32827	0.0951493

24126	0.946128	0.0820579
24127	1.41201	0.105951
24128	1.32827	0.0935017
24129	0.981121	0.110029
24130	0.936311	0.190348
24131	1.00715	0.107219
24132	1.15914	0.140013
24133	0.704409	0.133284
24134	0.713444	0.68846
24135	0.954538	0.563917
24136	0.944508	0.52469
24137	0.851165	0.921741
24138	0.946131	1.43578
24139	0.851192	0.811764
24140	0.946131	0.779069
24141	1.3279	0.707308
24142	1.33587	0.05
24143	0.946126	0.051958
24144	1.27512	0.05
24145	1.33587	0.05
24146	0.748059	0.05
24147	1.17451	0.0554869
24148	1.04299	0.05
24149	0.851542	0.0682826
24150	0.946131	0.0630696
24151	0.892109	0.0587048
24152	0.946131	0.05
24153	0.851541	0.05
24154	1.2178	0.05
24155	1.2179	0.05
24156	0.946793	0.05
24157	0.709599	0.05
24158	0.987426	0.05
24159	1.39502	0.05
24160	1.13624	0.05
24161	0.73176	0.05
24162	1.36697	0.05
24163	1.33393	0.05
24164	0.946131	0.0505216
24165	0.946132	0.05
24166	1.23845	0.05
24167	0.50702	0.629167
24168	1.06442	0.460246
24169	0.946131	0.634731
24170	1.02363	0.512249
24171	1.13537	0.05
24172	1.25049	0.05
24173	0.94613	0.05
24174	1.14993	0.05
24175	1.38056	0.05
24176	0.946126	0.05
24177	1.24678	0.05
24178	0.684521	0.05
24179	0.946124	0.05
24180	0.684518	0.05
24181	0.851542	0.35854
24182	0.925459	0.314217
24183	0.946131	0.35845

24184	0.851474	0.373233
24185	0.923305	0.374186
24186	1.4055	0.05
24187	1.32077	0.05
24188	0.946127	0.05
24189	1.21757	0.05
24190	0.946129	0.05
24191	0.711214	0.05
24192	1.30463	0.0640415
24193	0.711185	0.0533084
24194	0.711185	0.0613036
24195	1.22716	0.0607249
24196	1.29958	0.05
24197	1.26201	0.05
24198	0.568927	0.05
24199	0.568927	0.05
24200	0.946132	0.05
24201	0.851542	0.0662339
24202	1.44968	0.05
24203	0.851543	0.0550175
24204	1.35123	0.0544253
24205	1.40427	0.05
24206	0.593295	0.0886582
24207	0.946129	0.0952434
24208	1.22144	0.0789544
24209	0.908278	0.102626
24210	1.22144	0.0939232
24211	0.867593	0.221267
24212	1.23351	0.167687
24213	0.946131	0.217327
24214	1.05516	0.205294
24215	1.23351	0.188843
24216	1.15564	0.0570735
24217	0.946131	0.111998
24218	0.766652	0.0757438
24219	0.766681	0.0657085
24220	0.946131	0.0897458
24221	0.946131	0.120237
24222	0.897851	0.1066
24223	1.1751	0.139358
24224	1.00273	0.102816
24225	0.851554	0.114211
24226	0.883272	0.150914
24227	1.17111	0.346756
24228	0.851541	0.186658
24229	1.01665	0.333949
24230	0.851555	0.150627
24231	0.946131	0.349204
24232	0.911508	0.217836
24233	1.08054	0.385683
24234	1.33007	0.143122
24235	0.911471	0.273821
24236	1.26787	1.8059
24237	1.16641	1.86459
24238	0.946132	1.37563
24239	0.946133	1.56132
24240	1.16641	1.73498
24241	0.946131	2.1

24242	1.25037	0.856491
24243	1.34733	2.1
24244	1.34733	2.1
24245	0.946131	2.1
24246	0.946131	0.05
24247	1.41377	0.05
24248	0.94613	0.05
24249	1.30541	0.05
24250	1.41377	0.05
24251	1.3473	2.1
24252	1.11804	2.1
24253	0.94613	2.1
24254	0.946131	2.1
24255	1.11805	2.1
24256	1.32068	2.1
24257	0.946131	2.1
24258	0.946128	2.1
24259	1.39384	2.1
24260	1.4181	2.1
24261	1.12913	2.1
24262	0.94613	2.1
24263	0.946129	2.1
24264	1.20328	2.1
24265	1.39385	2.1
24266	1.3335	2.1
24267	1.40987	2.1
24268	0.94613	2.1
24269	1.21474	2.1
24270	0.94613	2.1
24271	0.946132	2.1
24272	1.15203	2.1
24273	1.41192	2.1
24274	1.41192	2.1
24275	0.946132	2.1
24276	0.946131	2.1
24277	1.40684	2.1
24278	1.2597	2.1
24279	0.946131	2.1
24280	1.40684	2.1
24281	0.946131	2.09026
24282	1.39113	2.0838
24283	1.34049	2.06037
24284	0.946131	1.91804
24285	1.39113	1.9927
24286	0.946132	1.31138
24287	1.31649	0.367179
24288	0.946132	0.40798
24289	0.946132	0.94056
24290	1.34297	1.1583
24291	0.946131	0.142097
24292	1.41766	0.149019
24293	0.954559	0.237645
24294	0.946131	0.16436
24295	0.946131	0.11121
24296	1.25447	0.207452
24297	1.35559	0.138976
24298	0.946127	0.159515
24299	0.946128	0.205587

24300	1.14874	0.198187
24301	1.33035	0.0682937
24302	1.3665	0.0822204
24303	0.946129	0.109095
24304	0.94613	0.0657704
24305	1.20732	0.126655
24306	0.946131	2.1
24307	0.946131	2.1
24308	1.32949	2.1
24309	1.34921	2.1
24310	0.946131	2.1
24311	0.946132	0.787886
24312	1.25479	1.27678
24313	1.38357	0.440486
24314	1.38357	0.326361
24315	0.946132	0.192584
24316	0.946131	2.1
24317	1.31034	0.380563
24318	1.37127	2.09977
24319	1.37127	1.25746
24320	0.946131	1.39423
24321	1.23923	0.097304
24322	1.41169	0.161923
24323	0.94613	0.227381
24324	1.12703	0.113503
24325	0.946131	0.157628
24326	0.946119	2.1
24327	1.41915	2.1
24328	1.02679	2.1
24329	0.946107	2.1
24330	1.41917	2.1
24331	0.946133	2.1
24332	1.36581	2.1
24333	0.946131	2.1
24334	1.36581	2.1
24335	0.946131	2.1
24336	0.946132	2.1
24337	1.04384	2.1
24338	1.34973	2.1
24339	1.34967	2.1
24340	0.851555	2.1
24341	0.946132	0.0782921
24342	1.3855	0.0597999
24343	1.08603	0.0788533
24344	1.08602	0.0604043
24345	1.16234	0.071462
24346	0.946133	0.0809049
24347	1.29348	0.0672031
24348	1.20111	0.0809559
24349	1.20112	0.0775366
24350	1.3855	0.0836466
24351	0.946128	2.1
24352	1.30798	2.1
24353	0.946129	2.1
24354	1.34828	2.1
24355	1.30797	2.1
24356	1.24932	0.05
24357	1.40549	0.05

24358	0.946117	0.05
24359	1.19615	0.05
24360	0.946106	0.05
24361	0.946131	2.1
24362	1.29926	2.1
24363	1.25144	2.1
24364	0.946131	2.1
24365	1.29925	2.1
24366	0.946132	2.1
24367	1.39826	2.1
24368	0.946133	2.1
24369	0.946133	2.1
24370	1.29926	2.1
24371	0.946119	0.05
24372	1.33614	0.05
24373	0.946118	0.05
24374	1.41377	0.05
24375	1.33612	0.05
24376	0.946133	1.68585
24377	1.3933	2.09069
24378	1.34999	0.750735
24379	0.946133	1.67795
24380	1.3933	1.63685
24381	0.946131	2.05818
24382	1.36139	2.1
24383	0.946132	1.75126
24384	0.946131	2.09414
24385	1.39329	2.08375
24386	1.37449	1.84796
24387	1.27925	0.524625
24388	0.919796	0.988076
24389	1.01176	1.47114
24390	0.94613	1.24225
24391	0.946133	2.1
24392	1.27679	2.1
24393	0.946133	2.1
24394	0.946132	2.1
24395	1.39731	2.1
24396	0.946119	2.1
24397	1.40765	1.92946
24398	1.1981	2.1
24399	0.946131	2.05706
24400	1.40764	2.1
24401	0.946127	2.1
24402	1.3557	2.1
24403	0.946127	2.1
24404	0.946128	2.1
24405	1.13485	2.1
24406	1.4008	2.1
24407	1.1587	2.1
24408	0.946131	2.1
24409	0.946131	2.1
24410	1.1587	2.1
24411	1.32082	0.0868269
24412	1.29215	0.0994479
24413	0.94613	0.122462
24414	0.946131	0.114763
24415	1.02366	0.103763

24416	1.27697	0.0665465
24417	1.2929	0.0753222
24418	0.946132	0.0993898
24419	0.946132	0.069162
24420	1.01451	0.0663737
24421	0.946132	2.1
24422	1.27252	2.1
24423	0.946132	2.1
24424	1.37869	2.1
24425	1.27252	2.1
24426	1.15627	2.1
24427	1.35703	2.1
24428	0.946132	2.1
24429	1.03783	2.1
24430	0.946132	2.1
24431	0.946128	2.1
24432	1.37869	2.1
24433	0.946131	2.1
24434	1.35357	2.1
24435	1.37869	2.1
24436	0.946132	2.1
24437	0.946131	2.1
24438	1.33471	2.1
24439	0.946131	2.1
24440	1.36373	2.1
24441	1.41342	0.445516
24442	1.06832	0.928103
24443	0.946128	0.506504
24444	0.94613	0.245409
24445	1.06832	0.577947
24446	1.26929	2.1
24447	0.946131	2.1
24448	0.946128	2.1
24449	1.41022	2.1
24450	1.37034	2.1
24451	1.37759	0.79612
24452	1.07612	1.462
24453	0.946126	0.951539
24454	0.946129	1.1883
24455	1.07612	1.02601
24456	0.946131	2.06635
24457	0.94613	0.499487
24458	1.23848	1.22301
24459	1.38897	0.939555
24460	0.946131	1.06803
24461	0.946132	0.763668
24462	0.946133	0.532947
24463	1.34369	0.15626
24464	1.40332	0.577306
24465	0.946132	0.193422
24466	0.946131	2.1
24467	1.24023	2.1
24468	1.38244	2.1
24469	1.38244	2.1
24470	0.946131	2.1
24471	0.946132	2.1
24472	1.33468	2.1
24473	1.41619	2.1

24474	1.41619	2.1
24475	0.946131	2.1
24476	1.1908	1.19761
24477	0.946128	0.797169
24478	0.946126	1.5425
24479	1.26654	1.4983
24480	1.39514	1.85292
24481	0.93608	2.1
24482	1.04105	2.1
24483	1.39551	2.1
24484	1.52471	2.1
24485	1.04072	2.1
24486	1.33174	0.05
24487	0.946128	0.05
24488	0.94613	0.05
24489	1.17801	0.05
24490	0.94613	0.05
24491	0.946132	2.1
24492	0.946132	2.1
24493	1.29065	2.1
24494	1.29065	2.1
24495	0.975266	2.1
24496	0.946131	2.1
24497	1.26025	2.1
24498	0.955601	2.1
24499	0.946131	2.1
24500	1.26025	2.1
24501	0.94613	0.0979154
24502	1.23146	0.0865505
24503	0.946127	0.09022
24504	1.32827	0.0865155
24505	1.23146	0.0937303
24506	1.25008	0.336086
24507	1.05018	0.418212
24508	0.94613	0.369486
24509	0.932744	0.540244
24510	1.04207	0.420045
24511	0.946132	1.47127
24512	1.10023	2.1
24513	1.36748	0.832584
24514	1.36748	1.95773
24515	0.946133	1.97884
24516	1.06023	0.05
24517	1.08019	0.05
24518	0.94613	0.05
24519	1.06023	0.05
24520	0.94613	0.05
24521	1.16727	0.05
24522	1.35856	0.05
24523	0.946132	0.05
24524	1.16727	0.05
24525	0.946132	0.05
24526	0.946133	2.1
24527	0.946131	2.1
24528	1.21691	2.1
24529	1.39558	2.1
24530	0.946131	2.1
24531	1.09905	0.442846

24532	0.946132	1.11399
24533	1.3365	0.447115
24534	1.35381	0.706722
24535	0.946132	1.27807
24536	0.94613	0.787764
24537	0.946128	2.04043
24538	1.40332	1.47305
24539	1.39041	1.86382
24540	0.94613	1.58692
24541	0.946132	2.08059
24542	1.3659	2.1
24543	1.39041	2.1
24544	1.39041	2.1
24545	0.946132	2.1
24546	0.840292	2.03725
24547	0.946133	2.01865
24548	0.851541	2.06281
24549	1.28464	2.02237
24550	1.28806	2.01784
24551	1.12591	0.360084
24552	1.05033	0.662055
24553	0.946132	0.459096
24554	0.946132	0.751263
24555	1.13537	0.503483
24556	1.21177	1.25159
24557	0.946132	1.25084
24558	0.946132	2.08849
24559	1.41134	0.864764
24560	1.27079	1.59321
24561	0.946131	0.05
24562	1.35403	0.05
24563	0.94613	0.0586191
24564	0.94613	0.05
24565	0.946131	0.05
24566	0.946131	0.10085
24567	0.946132	0.108552
24568	0.94613	0.32456
24569	1.37052	0.11738
24570	0.946131	0.110143
24571	0.946133	0.0871086
24572	0.946132	0.132685
24573	0.946133	0.103843
24574	1.24249	0.0815999
24575	0.946132	0.118935
24576	1.20608	2.1
24577	0.946132	2.1
24578	0.946131	2.1
24579	0.946132	2.1
24580	0.946132	2.1
24581	0.946131	2.1
24582	0.946128	2.1
24583	0.946129	2.1
24584	1.41232	2.1
24585	0.94613	2.1
24586	0.946132	0.05
24587	1.32449	0.05
24588	0.946131	0.0655587
24589	0.946131	0.056906

24590	1.32449	0.0507316
24591	0.946116	2.09553
24592	1.41563	2.1
24593	0.946118	2.1
24594	0.946105	2.1
24595	0.946131	2.1
24596	1.26563	2.1
24597	1.32819	2.04107
24598	0.946131	0.896447
24599	0.946131	2.05636
24600	0.946131	1.60766
24601	0.946131	1.94767
24602	0.946133	1.56453
24603	1.30382	2.1
24604	0.946131	1.54671
24605	0.946131	1.49567
24606	1.30382	1.55212
24607	0.946133	0.05
24608	1.15803	0.05
24609	1.28895	0.05
24610	0.954071	0.05
24611	0.946132	0.05
24612	1.0264	0.673843
24613	0.946132	1.53889
24614	0.946132	1.10524
24615	0.946131	1.22498
24616	0.946132	1.02681
24617	1.35977	0.188855
24618	0.946132	0.325941
24619	0.946129	0.291607
24620	0.94613	0.280545
24621	0.946129	0.249579
24622	0.876631	0.111049
24623	0.946133	0.112803
24624	0.946132	0.0991163
24625	0.946132	0.262966
24626	0.876606	0.11297
24627	0.946131	0.05
24628	1.24979	0.05
24629	0.94613	0.05
24630	0.94613	0.05
24631	0.94613	0.05
24632	0.921791	0.05
24633	0.946131	0.05
24634	0.921762	0.05
24635	0.946131	0.05
24636	0.946131	0.05
24637	0.810944	0.0760374
24638	0.810945	0.0901536
24639	0.810959	0.0790632
24640	0.946133	0.0632062
24641	1.25797	0.0883326
24642	0.946132	0.114877
24643	0.946132	0.0640546
24644	1.25797	0.0820977
24645	1.13748	0.34099
24646	0.851512	0.615617
24647	0.94613	0.96626

24648	1.02373	0.335103
24649	0.946131	0.635294
24650	0.946122	2.1
24651	1.3852	2.1
24652	0.946124	2.1
24653	0.946118	2.1
24654	1.38521	2.1
24655	0.946131	0.0857093
24656	0.946131	0.0659105
24657	1.30448	0.0906906
24658	0.946129	0.131465
24659	0.94613	0.0554177
24660	0.946133	2.1
24661	0.94613	2.1
24662	0.946131	2.1
24663	0.946131	2.1
24664	1.36503	2.1
24665	0.810947	0.05
24666	0.810947	0.05
24667	0.810934	0.05
24668	3.34066	0.05
24669	0.946133	0.791066
24670	1.4185	0.185172
24671	0.946132	0.342418
24672	0.946133	0.481627
24673	0.946132	0.459977
24674	0.94613	2.1
24675	0.946128	2.1
24676	1.03925	2.1
24677	0.944743	2.1
24678	0.947518	2.1
24679	0.946132	2.1
24680	1.20573	2.1
24681	0.94613	2.1
24682	0.94613	2.1
24683	1.20573	2.1
24684	1.20056	0.05
24685	0.946132	0.05
24686	0.946131	0.05
24687	0.946131	0.05
24688	0.946131	0.05
24689	0.94613	2.1
24690	0.946128	2.1
24691	1.38302	2.1
24692	0.94613	2.1
24693	0.94613	2.1
24694	0.827866	0.05
24695	0.913552	0.05
24696	0.716526	0.05
24697	0.82784	0.05
24698	1.20634	0.05
24699	0.946131	0.05
24700	0.94613	0.05
24701	1.39502	0.05
24702	0.88146	0.05
24703	0.946132	2.1
24704	0.946132	2.1
24705	0.946132	2.1

24706	0.946132	2.1
24707	1.39053	2.1
24708	0.946131	2.1
24709	1.40571	2.1
24710	0.946131	2.1
24711	0.946131	2.1
24712	0.946132	2.1
24713	0.94613	0.108371
24714	1.2582	0.14095
24715	0.946127	0.106358
24716	0.946129	0.129726
24717	0.946129	0.112968
24718	0.946129	0.907252
24719	0.946132	0.810176
24720	0.946131	0.555523
24721	0.946131	0.845316
24722	1.29054	0.528258
24723	0.810944	0.05
24724	0.810941	0.05
24725	0.810931	0.05
24726	0.946132	0.706673
24727	0.946132	0.6927
24728	0.946132	1.5128
24729	0.946132	0.928392
24730	0.927608	0.515033
24731	0.946128	2.1
24732	0.946133	2.1
24733	0.946131	2.1
24734	0.94613	2.1
24735	1.38813	2.1
24736	0.94613	2.1
24737	0.946128	2.1
24738	0.946132	2.1
24739	1.41666	2.1
24740	0.94613	2.1
24741	0.946118	0.156736
24742	1.34151	0.125693
24743	0.946116	0.174276
24744	0.946131	0.15468
24745	1.34149	0.143956
24746	0.946132	2.1
24747	1.31889	2.1
24748	0.946132	2.1
24749	0.946132	2.1
24750	1.31889	2.1
24751	0.864997	0.05
24752	0.928981	0.05
24753	0.872219	0.05
24754	0.946127	0.556691
24755	0.946131	0.445308
24756	1.37709	0.340156
24757	0.946131	0.542147
24758	0.946129	0.446748
24759	1.21882	0.05
24760	0.938168	0.05
24761	0.938161	0.05
24762	1.06327	0.05
24763	1.33457	0.05

24764	0.946133	0.810327
24765	0.946132	0.658997
24766	0.946133	0.567104
24767	0.946132	0.861009
24768	1.14444	0.504391
24769	1.12628	0.05
24770	0.946132	0.051839
24771	0.946131	0.0654406
24772	1.12628	0.05
24773	0.946131	0.05
24774	0.946132	2.1
24775	0.946132	2.1
24776	0.946133	2.1
24777	0.946132	2.1
24778	1.29322	2.1
24779	0.946131	2.1
24780	0.946132	2.1
24781	0.94613	2.1
24782	0.946131	2.1
24783	1.28441	2.1
24784	0.946118	2.1
24785	0.946119	2.1
24786	0.946117	2.1
24787	1.28075	2.1
24788	0.946105	2.1
24789	0.810945	2.1
24790	0.810942	2.1
24791	0.81093	2.1
24792	0.946127	2.1
24793	0.946127	2.1
24794	1.28228	2.1
24795	0.946129	2.1
24796	0.946132	2.1
24797	1.14042	2.1
24798	0.946132	2.1
24799	0.946133	2.1
24800	1.14042	2.1
24801	0.946133	0.0970129
24802	0.946132	0.0918555
24803	1.28632	0.0652379
24804	1.28632	0.05
24805	0.946133	0.0777843
24806	0.946131	1.69853
24807	0.946131	1.50878
24808	0.946131	1.54624
24809	1.31817	1.70449
24810	0.946131	1.39556
24811	1.35048	2.1
24812	0.946132	2.1
24813	0.946132	2.1
24814	0.946132	2.1
24815	0.946131	2.1
24816	0.946131	2.1
24817	1.35943	2.1
24818	0.94613	2.1
24819	0.946132	2.1
24820	0.946131	2.1
24821	0.990713	2.1

24822	0.946132	2.1
24823	0.946132	2.1
24824	0.946133	2.1
24825	0.946132	2.1
24826	0.946133	1.54341
24827	1.01433	1.35432
24828	0.877931	1.78787
24829	0.96573	0.91752
24830	0.946131	0.994065
24831	0.946131	1.44094
24832	0.946133	1.02712
24833	1.39575	0.638853
24834	0.946131	0.799913
24835	0.94613	0.774758
24836	0.810943	0.432145
24837	0.810942	0.443943
24838	0.709589	0.322309
24839	0.924071	0.290243
24840	1.04293	0.217945
24841	1.15747	0.161491
24842	0.946131	0.294482
24843	1.04071	0.238289
24844	0.810946	0.371962
24845	0.810943	0.328918
24846	0.810959	0.287297
24847	0.946128	1.17539
24848	1.39696	1.84276
24849	0.94613	0.691681
24850	0.94613	0.59632
24851	1.39696	1.37288
24852	0.946131	0.648631
24853	0.946131	1.87558
24854	0.946131	1.26477
24855	0.946131	0.964844
24856	1.29735	1.73173
24857	0.946128	1.99859
24858	0.946133	0.700663
24859	0.94613	2.04973
24860	0.946131	1.78767
24861	1.33266	1.42567
24862	0.946131	2.1
24863	0.94613	1.90522
24864	0.946131	2.1
24865	1.40609	2.1
24866	0.946131	2.1
24867	0.946131	2.03803
24868	0.946133	2.04905
24869	0.946129	2.07558
24870	1.23339	2.0838
24871	0.946131	2.03581
24872	0.946132	2.09898
24873	0.946133	2.0991
24874	0.946132	2.1
24875	1.29198	2.1
24876	0.946132	2.09092
24877	0.946131	2.1
24878	1.07297	2.1
24879	0.946127	2.1

24880	0.94613	2.1
24881	1.07297	2.1
24882	0.946125	2.1
24883	0.946126	2.1
24884	0.946126	2.1
24885	1.3864	2.1
24886	0.946119	2.1
24887	0.946128	0.10536
24888	1.09411	0.0863106
24889	0.946131	0.133086
24890	0.946131	0.117592
24891	1.09411	0.0974397
24892	0.946131	0.0885586
24893	1.25837	0.0703622
24894	0.946131	0.112652
24895	0.946131	0.0993618
24896	1.25837	0.0790255
24897	0.810945	2.1
24898	0.810943	2.1
24899	0.810933	2.1
24900	0.94613	0.151674
24901	0.946131	0.556206
24902	0.946132	0.2008
24903	0.946129	0.217207
24904	0.946131	0.4889
24905	0.946131	0.05
24906	1.28911	0.05
24907	0.946129	0.05
24908	0.946133	0.05
24909	0.946131	0.05
24910	0.94613	0.146315
24911	1.38954	0.144178
24912	0.946132	0.142006
24913	0.94613	0.147423
24914	0.946131	0.147529
24915	0.946131	0.93128
24916	0.946131	0.577455
24917	0.946131	0.422899
24918	1.37981	0.438959
24919	0.946131	0.515383
24920	0.946131	2.1
24921	1.26958	2.1
24922	0.946127	2.1
24923	0.946129	2.1
24924	1.26957	2.1
24925	0.94613	2.1
24926	1.16889	2.1
24927	0.946126	2.1
24928	0.946128	2.1
24929	1.16889	2.1
24930	0.946133	0.312239
24931	1.13511	0.208926
24932	0.946132	0.318281
24933	0.946133	0.239661
24934	0.946132	0.23899
24935	1.27575	1.48225
24936	0.946129	1.98558
24937	0.946131	1.8705

24938	0.94613	1.98749
24939	0.946131	1.90531
24940	0.946131	2.1
24941	0.946128	2.1
24942	1.41712	2.1
24943	1.41712	2.1
24944	0.94613	2.1
24945	0.946129	0.05
24946	0.946132	0.05
24947	0.946131	0.05
24948	0.946131	0.05
24949	0.946131	0.05
24950	0.979045	1.96013
24951	0.946131	1.38971
24952	0.617549	2.01211
24953	0.555844	1.99415
24954	1.04071	1.4386
24955	0.946132	1.98489
24956	0.946132	1.56095
24957	0.946131	2.03609
24958	1.22618	1.74916
24959	0.946132	1.50324
24960	0.946131	2.1
24961	1.13976	2.1
24962	0.946129	2.1
24963	0.946131	2.1
24964	1.13976	2.1
24965	0.810945	0.446409
24966	0.810943	0.907905
24967	0.810932	1.1911
24968	1.25358	1.616
24969	0.94613	0.715873
24970	0.946128	1.27809
24971	1.25358	1.76072
24972	1.07811	1.49162
24973	0.946129	2.1
24974	1.09197	2.1
24975	0.946131	2.1
24976	0.946131	2.1
24977	1.09197	2.1
24978	0.594912	2.1
24979	0.993703	2.1
24980	1.10173	2.1
24981	0.9916	2.1
24982	0.946131	2.1
24983	0.946128	2.1
24984	0.946131	2.1
24985	0.94613	2.1
24986	0.946129	2.1
24987	0.946132	2.1
24988	1.40335	2.1
24989	0.946133	2.1
24990	0.946133	2.1
24991	1.40335	2.1
24992	0.946132	2.03492
24993	0.946132	2.1
24994	0.946132	2.051
24995	0.946132	2.05404

24996	1.411	2.09407
24997	0.611675	0.0821951
24998	0.946129	0.0969452
24999	1.31678	0.0503898
25000	0.946124	0.0946716
25001	0.946127	0.0576614
25002	0.946128	0.0914381
25003	0.946132	0.354637
25004	0.946131	0.230585
25005	0.946132	0.370097
25006	0.946132	0.242997
25007	0.946132	0.247005
25008	0.810946	0.286633
25009	0.810943	0.307893
25010	0.81096	0.260256
25011	1.39301	0.05
25012	0.946132	0.05
25013	0.625812	0.05
25014	0.946132	0.05
25015	0.625812	0.05
25016	0.810946	1.98333
25017	0.810945	2.06402
25018	0.810931	2.1
25019	0.810943	2.1
25020	0.810944	2.1
25021	0.810958	2.1
25022	0.946131	0.138995
25023	0.946131	0.115768
25024	0.946128	0.162595
25025	0.946129	0.127166
25026	0.94613	0.107884
25027	0.946133	0.277078
25028	0.946133	0.476403
25029	0.98537	0.138794
25030	0.985371	0.0927861
25031	0.946132	0.38432
25032	0.946129	0.396323
25033	1.38752	0.274784
25034	0.946131	0.255998
25035	0.946131	0.324054
25036	1.38752	0.299086
25037	0.946119	0.218813
25038	1.33036	0.137899
25039	0.946119	0.139207
25040	0.946132	0.168832
25041	1.33035	0.151741
25042	1.3868	2.1
25043	0.946132	2.1
25044	0.946132	2.1
25045	0.946133	2.1
25046	0.946132	2.1
25047	0.810947	2.1
25048	0.810946	2.1
25049	0.81096	2.1
25050	0.810946	2.1
25051	0.810946	2.1
25052	0.810933	2.1
25053	0.946133	2.1

25054	1.35342	2.1
25055	0.946133	2.1
25056	0.946132	2.1
25057	1.35342	2.1
25058	0.946132	2.1
25059	0.946132	2.1
25060	1.28569	2.1
25061	0.946132	2.1
25062	0.946133	2.1
25063	0.946132	2.07434
25064	1.32547	2.1
25065	0.946132	2.07998
25066	0.946132	2.1
25067	1.32547	2.1
25068	0.94613	0.05
25069	0.946133	0.05
25070	1.39664	0.05
25071	1.39664	0.05
25072	0.946131	0.05
25073	0.946131	2.03339
25074	0.94613	2.1
25075	0.946132	1.71767
25076	0.946131	2.1
25077	1.41833	2.1
25078	0.946131	0.05
25079	0.946131	0.05
25080	0.946131	0.05
25081	1.26155	0.05
25082	0.946132	0.05
25083	0.810946	2.1
25084	0.810946	2.1
25085	0.810959	2.1
25086	0.94613	1.44002
25087	0.946132	0.833965
25088	1.40779	0.523971
25089	1.40779	0.685215
25090	0.94613	0.845028
25091	0.94613	0.0722519
25092	0.946129	0.0842591
25093	1.36376	0.05
25094	1.36376	0.05
25095	0.946131	0.0620131
25096	0.946128	2.1
25097	0.946127	2.1
25098	0.946131	2.1
25099	1.40756	2.1
25100	0.94613	2.1
25101	0.946131	2.08494
25102	0.946132	2.1
25103	0.94613	2.08781
25104	0.946131	2.1
25105	1.31947	2.1
25106	0.946131	2.1
25107	0.946131	2.1
25108	0.946129	2.1
25109	0.946131	2.1
25110	1.25768	2.1
25111	0.946132	0.087979

25112	0.94613	0.069323
25113	0.946131	0.0752223
25114	0.946131	0.0549484
25115	0.946131	0.0918341
25116	0.810943	1.23725
25117	0.810942	1.89814
25118	0.81093	2.06112
25119	0.946128	0.822019
25120	0.946133	0.400482
25121	0.946131	0.973891
25122	0.946131	0.514152
25123	0.94613	0.844922
25124	0.946132	1.12788
25125	0.946131	0.730587
25126	0.946132	1.1701
25127	0.946132	0.977565
25128	0.946132	1.22778
25129	0.946119	2.1
25130	0.946119	2.1
25131	1.31523	2.1
25132	0.946132	2.1
25133	0.946107	2.1
25134	0.946133	2.1
25135	0.946132	1.79798
25136	0.946132	2.02169
25137	1.28803	2.1
25138	0.946132	2.1
25139	1.37658	1.1265
25140	0.946132	0.40029
25141	0.946133	1.47938
25142	0.946132	0.424873
25143	0.946132	1.07553
25144	0.946132	1.4925
25145	0.94613	1.1119
25146	0.94613	1.88004
25147	1.15541	0.910625
25148	0.92943	1.54398
25149	0.946126	0.05
25150	0.946129	0.05
25151	1.33849	0.05
25152	1.33849	0.05
25153	0.946129	0.05
25154	0.810946	0.05
25155	0.810944	0.05
25156	0.810959	0.05
25157	1.39027	0.05
25158	0.946131	0.0716709
25159	0.946131	0.0907385
25160	1.39027	0.05
25161	0.946131	0.0584208
25162	1.40924	1.85288
25163	0.946131	1.31553
25164	0.946132	1.8203
25165	0.946132	0.977439
25166	0.946132	1.51442
25167	0.946131	2.1
25168	0.94613	2.1
25169	0.946128	2.1

25170	0.946128	2.1
25171	1.41744	2.1
25172	0.946132	0.654635
25173	0.946128	1.12463
25174	0.94613	1.1766
25175	1.37675	0.789543
25176	0.94613	1.29857
25177	0.946132	1.10192
25178	0.946132	0.573268
25179	0.946132	0.371768
25180	0.946132	0.518295
25181	0.946131	0.511819
25182	0.946132	0.721591
25183	0.946132	0.331127
25184	0.946133	1.154
25185	1.10003	0.283427
25186	0.946132	0.696432
25187	0.946132	0.0663069
25188	0.946131	0.0740554
25189	0.946132	0.0613026
25190	0.946131	0.0737999
25191	0.946132	0.0706528
25192	0.946132	0.0932409
25193	0.946131	0.0956963
25194	0.946132	0.0802226
25195	0.946132	0.0956774
25196	0.946131	0.0918415
25197	1.24094	0.05
25198	0.946126	0.0653125
25199	0.946128	0.0518419
25200	0.946129	0.0610207
25201	0.946129	0.054676
25202	1.2929	0.0635948
25203	0.946131	0.112824
25204	0.946131	0.0862211
25205	0.946131	0.101728
25206	0.946131	0.0938291
25207	0.810946	0.05
25208	0.810945	0.05
25209	0.810959	0.05
25210	0.810946	1.98855
25211	0.810943	2.0002
25212	0.810933	2.07121
25213	0.946128	2.1
25214	1.18315	2.1
25215	0.94613	2.09977
25216	0.94613	2.1
25217	1.18316	2.1
25218	0.946126	0.325693
25219	1.3324	0.215493
25220	0.946127	0.339788
25221	0.94613	0.221435
25222	0.946128	0.271575
25223	0.94613	2.1
25224	0.946128	2.1
25225	1.39523	2.1
25226	1.39523	2.1
25227	0.946131	2.1

25228	1.36541	2.1
25229	0.946132	2.1
25230	0.946132	2.1
25231	0.946132	2.1
25232	0.946132	2.1
25233	0.946131	2.09604
25234	0.94613	1.47049
25235	0.946132	1.91249
25236	0.946131	0.296956
25237	1.39509	1.58887
25238	0.946132	2.1
25239	0.946132	2.1
25240	1.29319	2.1
25241	0.946132	2.1
25242	0.946133	2.1
25243	0.946132	0.0780132
25244	0.946131	0.141355
25245	0.946132	0.0897292
25246	1.01196	0.0725887
25247	0.946132	0.12859
25248	0.946116	2.1
25249	0.946117	2.1
25250	1.38153	2.1
25251	1.38155	2.1
25252	0.946105	2.1
25253	0.946132	1.76136
25254	0.946131	1.89035
25255	0.946132	1.68222
25256	1.28684	1.67944
25257	0.946132	1.53283
25258	1.10185	1.77182
25259	0.94613	1.92162
25260	0.946128	2.05716
25261	0.94613	2.00709
25262	0.94613	1.92295
25263	0.810947	0.05
25264	0.810945	0.05
25265	0.810932	0.05
25266	0.946119	0.214301
25267	1.16717	0.152826
25268	0.946117	0.211289
25269	0.94613	0.163673
25270	1.16715	0.162295
25271	0.94612	0.346525
25272	0.946119	0.364504
25273	0.946119	0.308012
25274	0.946132	0.238468
25275	1.20882	0.275058
25276	1.07043	0.808025
25277	0.823268	0.844073
25278	0.810959	0.789975
25279	0.946132	1.62872
25280	1.14879	0.884188
25281	0.94613	1.57055
25282	0.946131	1.56049
25283	1.14879	0.756392
25284	1.23837	2.1
25285	0.94612	2.1

25286	0.946118	2.1
25287	0.946131	2.1
25288	0.946105	2.1
25289	0.810946	0.05
25290	0.810946	0.05
25291	0.810959	0.05
25292	0.810947	1.48813
25293	0.810947	0.241353
25294	0.810934	0.253682
25295	0.946128	2.1
25296	0.946131	2.1
25297	0.946128	2.09336
25298	0.946129	2.1
25299	0.94613	2.1
25300	0.94613	2.08846
25301	0.946131	2.08236
25302	0.946131	2.05351
25303	0.946131	2.09125
25304	0.946131	2.08645
25305	0.946131	1.89981
25306	1.38585	2.1
25307	0.946128	1.9382
25308	0.94613	1.93374
25309	1.38585	1.96604
25310	0.946133	0.151471
25311	0.946133	0.1469
25312	0.946132	0.129726
25313	0.946132	0.152566
25314	0.946132	0.143126
25315	0.918871	0.05
25316	0.810248	0.05
25317	0.838037	0.05
25318	0.946126	0.05
25319	0.943647	0.05
25320	1.13836	0.05
25321	1.03802	0.05
25322	0.946129	0.05
25323	0.946133	1.50256
25324	0.946132	1.0354
25325	0.946132	2.07354
25326	1.1699	0.846942
25327	0.946132	0.857119
25328	0.946131	2.1
25329	0.946132	2.1
25330	1.37199	2.1
25331	1.37199	2.1
25332	0.946132	2.1
25333	0.946132	0.792169
25334	0.946133	0.520477
25335	0.946133	0.824918
25336	0.946132	0.595031
25337	1.38623	0.459889
25338	0.946132	2.1
25339	0.946132	2.1
25340	0.946132	2.1
25341	0.946132	2.1
25342	1.40098	2.1
25343	1.20787	0.174628

25344	1.41894	0.506557
25345	0.946133	0.301802
25346	1.01777	0.370113
25347	0.946132	2.1
25348	0.946132	2.1
25349	0.946133	2.1
25350	1.40199	2.1
25351	0.946132	2.1
25352	0.810946	1.46209
25353	0.810947	1.9607
25354	0.810934	1.60353
25355	0.946126	1.14424
25356	0.94613	1.63532
25357	0.946125	1.74163
25358	1.40247	1.03643
25359	0.946128	1.21735
25360	1.25703	1.72252
25361	0.87821	1.37993
25362	0.878238	0.920854
25363	0.946119	1.93283
25364	0.946132	2.02999
25365	0.946132	0.364067
25366	0.946132	0.317418
25367	0.946131	0.833233
25368	0.946132	0.200706
25369	1.28543	0.262439
25370	0.946115	2.1
25371	1.41638	2.1
25372	0.946116	2.1
25373	0.946104	2.1
25374	0.946129	2.1
25375	0.946132	2.1
25376	1.41432	2.1
25377	0.946132	2.1
25378	0.946132	2.1
25379	0.946132	2.1
25380	0.810947	0.05
25381	0.810947	0.05
25382	0.709567	0.05
25383	0.851542	0.05
25384	1.01217	0.05
25385	0.697993	0.05
25386	0.946133	0.05
25387	0.851488	0.05
25388	0.946129	2.03317
25389	0.94613	2.08468
25390	1.2254	1.86758
25391	0.946131	2.04275
25392	0.946131	1.98419
25393	0.700785	2.09876
25394	0.915893	2.00972
25395	0.838013	1.87273
25396	0.760188	1.29068
25397	1.19601	0.193757
25398	0.946133	0.336513
25399	0.946132	0.329869
25400	0.946132	0.270544
25401	0.946132	0.327423

25402	0.946132	1.87258
25403	0.946133	2.1
25404	0.946132	2.01031
25405	0.946133	2.07431
25406	1.36956	2.08734
25407	0.946131	2.09912
25408	0.94613	0.218061
25409	0.946133	2.08227
25410	1.34268	0.455135
25411	0.946131	1.03058
25412	0.946132	2.1
25413	1.25603	2.1
25414	0.946132	2.1
25415	0.946132	2.1
25416	1.25603	2.1
25417	0.810946	1.25141
25418	0.810943	1.24661
25419	0.810933	1.14349
25420	0.496545	0.393445
25421	0.805767	0.68374
25422	0.936671	0.578012
25423	0.530063	0.565501
25424	1.05954	0.551689
25425	0.810946	0.130202
25426	0.810943	0.118399
25427	0.810959	0.100855
25428	1.16699	0.105688
25429	0.946131	0.197634
25430	0.946129	0.171108
25431	0.946131	0.15003
25432	0.946131	0.142656
25433	0.810943	1.8527
25434	0.810942	1.92055
25435	0.810933	1.82573
25436	0.946132	1.84864
25437	1.04832	1.80918
25438	0.870714	2.0074
25439	0.957819	2.02153
25440	0.94613	1.8489
25441	1.82931	2.1
25442	0.946132	0.0962162
25443	0.946129	0.0600727
25444	0.94613	0.190404
25445	0.946131	0.216464
25446	0.94613	0.0627848
25447	0.946119	0.886899
25448	0.94612	0.93004
25449	0.94612	0.550794
25450	0.946132	0.756584
25451	1.36118	0.587655
25452	0.810947	0.134128
25453	0.810947	0.108627
25454	0.810934	0.128067
25455	0.946132	2.1
25456	0.946132	2.1
25457	0.946132	2.1
25458	1.39754	2.1
25459	0.946131	2.1

25460	0.946129	2.1
25461	0.946133	2.1
25462	0.946131	2.1
25463	0.946131	2.1
25464	1.41732	2.1
25465	0.946132	1.06671
25466	0.946128	1.68933
25467	0.94613	1.22613
25468	0.94613	1.44075
25469	1.25277	1.51748
25470	0.94613	1.53596
25471	0.946128	1.77992
25472	1.21261	0.870286
25473	0.94613	1.46173
25474	0.94613	1.16769
25475	1.39407	2.1
25476	0.946133	2.1
25477	0.94613	2.1
25478	0.946131	2.1
25479	0.946131	2.1
25480	0.946119	1.66058
25481	0.946119	1.80204
25482	0.946119	1.96788
25483	1.1537	1.81292
25484	0.946106	1.58231
25485	0.946131	2.1
25486	1.11822	2.1
25487	0.946129	2.1
25488	0.946131	2.1
25489	1.11822	2.1
25490	1.46806	0.05
25491	0.498881	0.05
25492	0.996658	0.05
25493	1.2179	0.05
25494	0.991519	0.05
25495	0.946131	0.05
25496	0.946131	0.05
25497	1.28673	0.05
25498	0.946131	0.05
25499	0.946131	0.05
25500	1.33178	0.0674529
25501	0.946132	0.100007
25502	0.946132	0.0918703
25503	0.946132	0.093019
25504	0.946132	0.0958008
25505	0.946128	0.135495
25506	0.972061	0.0934123
25507	0.946129	0.155498
25508	0.946129	0.143173
25509	0.972059	0.10265
25510	0.946129	2.1
25511	0.94613	2.1
25512	0.94613	2.1
25513	0.94613	2.1
25514	0.94613	2.1
25515	0.946131	2.1
25516	0.946127	2.1
25517	0.946131	2.1

25518	0.946129	2.1
25519	1.4073	2.1
25520	0.946131	0.0575995
25521	1.05016	0.05
25522	0.946132	0.0700783
25523	0.946131	0.065207
25524	0.946132	0.0621401
25525	0.946128	0.0644648
25526	1.13398	0.0513545
25527	0.946131	0.0856227
25528	0.946131	0.0739205
25529	0.94613	0.069276
25530	0.946131	0.05
25531	0.946131	0.05
25532	1.38911	0.05
25533	1.38911	0.05
25534	0.946132	0.05
25535	0.946131	0.072835
25536	0.94613	0.0611196
25537	0.946131	0.0721368
25538	0.946131	0.0642918
25539	0.946131	0.072395
25540	0.946131	0.160262
25541	1.23923	0.109036
25542	0.94613	0.19959
25543	0.946131	0.11183
25544	0.946131	0.128985
25545	1.40511	0.05
25546	0.946131	0.05
25547	0.946131	0.05
25548	0.946132	0.05
25549	0.946131	0.05
25550	1.12968	2.1
25551	0.946132	2.1
25552	0.946132	2.1
25553	0.946131	2.1
25554	0.946132	2.1
25555	0.946132	1.76612
25556	1.4172	2.1
25557	0.94613	1.84273
25558	0.94613	1.86693
25559	1.4172	1.99353
25560	0.946132	2.1
25561	0.946132	2.1
25562	1.31323	2.1
25563	0.946133	2.1
25564	0.946132	2.1
25565	0.946131	2.1
25566	0.94613	2.06338
25567	1.36546	2.1
25568	1.36546	2.1
25569	0.946131	2.1
25570	0.946132	1.90188
25571	0.946133	2.1
25572	0.946132	0.256234
25573	1.36899	1.47909
25574	0.946133	1.01605
25575	0.946131	0.05

25576	0.946132	0.05
25577	1.03543	0.05
25578	1.1412	0.05
25579	0.941275	0.05
25580	1.28952	0.085144
25581	0.94613	0.112684
25582	0.946129	0.120866
25583	0.946132	0.0938153
25584	0.94613	0.113727
25585	0.94613	2.1
25586	0.94613	2.1
25587	0.946129	2.1
25588	0.94613	2.1
25589	1.35966	2.1
25590	0.810946	2.1
25591	0.810942	2.1
25592	0.810932	2.1
25593	0.94613	2.1
25594	1.40372	2.1
25595	0.946131	2.1
25596	0.94613	2.1
25597	1.40372	2.1
25598	0.946133	2.1
25599	0.946131	2.1
25600	0.94613	2.1
25601	0.946131	2.1
25602	0.946131	2.1
25603	1.31453	2.1
25604	0.94613	2.1
25605	0.946128	2.1
25606	0.94613	2.1
25607	0.94613	2.1
25608	0.946131	0.05
25609	1.12018	0.05
25610	0.94613	0.0543145
25611	0.94613	0.0540588
25612	0.94613	0.05
25613	0.946131	0.0602288
25614	1.16373	0.05
25615	0.946131	0.091214
25616	0.94613	0.0532592
25617	0.946131	0.0551418
25618	0.946126	0.327287
25619	0.946132	0.505678
25620	0.946128	0.380709
25621	0.946129	0.502417
25622	0.946129	0.579799
25623	0.946132	0.107645
25624	0.946133	0.109795
25625	0.946132	0.106407
25626	0.946132	0.104753
25627	0.946133	0.112748
25628	0.946132	1.20239
25629	0.946129	1.70855
25630	0.94613	1.09719
25631	1.36399	1.1959
25632	0.946131	1.25815
25633	0.946132	2.1

25634	0.946132	2.1
25635	0.946133	2.1
25636	1.38302	2.1
25637	0.946133	2.1
25638	0.810943	2.1
25639	0.810944	2.1
25640	0.810945	2.1
25641	0.946132	2.1
25642	0.946131	2.1
25643	0.946132	2.1
25644	1.30188	2.1
25645	0.946132	2.1
25646	0.810946	1.0335
25647	0.72985	1.63348
25648	0.810934	1.13777
25649	0.856243	0.513572
25650	0.946129	0.731034
25651	0.946132	0.508476
25652	1.19686	0.380212
25653	0.851527	0.646246
25654	0.946131	2.1
25655	0.94613	2.1
25656	0.94613	2.1
25657	0.94613	2.1
25658	1.31961	2.1
25659	0.946131	2.1
25660	0.946131	2.1
25661	0.94613	2.1
25662	0.946131	2.1
25663	1.3605	2.1
25664	1.30116	2.1
25665	0.946133	1.60248
25666	0.946131	2.1
25667	0.946131	2.01802
25668	0.94613	2.09326
25669	0.946132	0.05
25670	1.3035	0.05
25671	0.94613	0.05
25672	0.946131	0.05
25673	0.946131	0.05
25674	0.946132	2.1
25675	1.37869	2.1
25676	1.2051	2.1
25677	0.946132	2.1
25678	1.2051	2.1
25679	0.810946	2.1
25680	0.810945	2.1
25681	0.810946	2.1
25682	1.34623	2.1
25683	0.946131	2.1
25684	0.946131	2.1
25685	1.34623	2.1
25686	1.22993	2.1
25687	1.32299	0.05
25688	0.946132	0.05
25689	0.946132	0.0513498
25690	0.946132	0.05
25691	0.946131	0.05

25692	0.94613	0.158804
25693	1.40574	0.149764
25694	0.946128	0.202816
25695	0.946131	0.194561
25696	1.40574	0.189676
25697	0.946132	2.1
25698	1.41331	2.1
25699	0.94613	2.1
25700	0.946131	2.1
25701	1.41331	2.1
25702	0.94613	2.1
25703	1.37609	2.1
25704	0.946129	2.1
25705	0.946131	2.1
25706	1.37609	2.1
25707	1.039	0.987846
25708	0.946131	1.86018
25709	0.944514	1.57711
25710	0.946132	1.74108
25711	0.946132	1.582
25712	1.23726	0.925216
25713	0.946128	1.72767
25714	0.946128	1.5391
25715	0.946129	1.65193
25716	0.94613	1.46806
25717	0.946129	2.1
25718	0.946131	2.1
25719	0.946131	2.1
25720	1.39214	2.1
25721	0.94613	2.1
25722	0.946118	1.99618
25723	0.946118	0.644081
25724	1.41078	0.957956
25725	1.41076	0.565158
25726	0.946131	0.894787
25727	0.946131	0.132341
25728	0.946131	0.0906719
25729	0.946132	0.100343
25730	0.94613	0.148385
25731	0.946131	0.0970112
25732	0.946132	0.0694692
25733	0.946132	0.05
25734	0.946133	0.05
25735	1.28924	0.05
25736	0.946132	0.0531946
25737	0.946133	0.475176
25738	1.09812	0.348015
25739	0.946132	0.551457
25740	0.946132	0.459478
25741	1.09812	0.504733
25742	0.946132	1.26073
25743	0.946132	1.12401
25744	0.946132	1.11366
25745	0.946132	1.00072
25746	1.15388	1.07549
25747	0.946132	0.407408
25748	0.974688	0.263918
25749	0.946132	0.4332

25750	0.946132	0.37079
25751	0.974687	0.399007
25752	1.0644	1.54343
25753	0.946133	1.7529
25754	0.946132	1.53634
25755	0.946133	1.54507
25756	1.26547	1.5446
25757	1.02905	1.11418
25758	0.911537	1.24255
25759	0.838038	1.25205
25760	0.946131	0.101365
25761	0.94613	0.095131
25762	0.946132	0.0935177
25763	0.946131	0.0874802
25764	1.33922	0.0834577
25765	0.946132	0.0846073
25766	0.946132	0.0689007
25767	1.40926	0.0571141
25768	1.40927	0.05
25769	0.946132	0.0618384
25770	0.946129	2.09552
25771	0.946131	2.0139
25772	0.946131	2.0048
25773	0.946131	1.26075
25774	1.40496	2.08754
25775	0.94613	1.81805
25776	0.94613	1.68736
25777	0.946127	1.73825
25778	0.946128	1.45156
25779	1.38914	1.47288
25780	2.675 0.05	
25781	1.22266	0.05
25782	0.946131	0.05
25783	0.946132	0.05
25784	0.946132	0.05
25785	1.22266	0.05
25786	0.946124	0.100102
25787	0.946128	0.05
25788	0.946126	0.05
25789	1.21428	0.05
25790	0.946127	0.0871582
25791	0.946131	0.0581457
25792	0.946131	0.136583
25793	0.946132	0.0587882
25794	0.946129	0.0830986
25795	0.940118	0.134174
25796	0.810943	2.1
25797	0.810945	2.1
25798	0.810958	2.1
25799	0.946133	0.0643726
25800	1.11539	0.05
25801	0.946132	0.0569763
25802	0.946132	0.0595716
25803	0.946133	0.0604774
25804	0.946133	2.1
25805	1.41195	2.1
25806	0.946133	2.1
25807	0.946132	2.1

25808	0.946133	2.1
25809	1.15968	0.05
25810	0.946131	0.05
25811	0.946131	0.05
25812	0.946131	0.05
25813	0.946131	0.05
25814	0.94613	1.98146
25815	0.946128	2.00419
25816	0.946132	1.60458
25817	0.94613	1.97865
25818	0.94613	1.81414
25819	0.946131	1.81999
25820	1.04211	1.80426
25821	0.850155	1.25537
25822	0.946131	1.72
25823	0.946131	1.4553
25824	0.946131	0.34553
25825	0.94613	0.254522
25826	0.946129	0.365266
25827	0.946131	0.26083
25828	0.946131	0.259294
25829	1.40193	0.129464
25830	1.19756	0.140069
25831	0.561936	0.180026
25832	0.808778	0.160524
25833	1.19756	0.153231
25834	0.946132	0.0571673
25835	0.946132	0.0545401
25836	1.33916	0.05
25837	1.33916	0.05
25838	0.946133	0.05
25839	0.946127	0.197855
25840	0.946127	0.146376
25841	0.946131	0.180095
25842	0.946128	0.127068
25843	0.946129	0.130404
25844	0.589428	0.212738
25845	0.851501	0.198034
25846	0.868924	0.204372
25847	0.877703	0.168511
25848	0.85153	0.193312
25849	0.946132	2.04728
25850	1.25372	2.1
25851	0.946132	2.07362
25852	0.946132	2.1
25853	1.25372	2.1
25854	0.946132	1.47584
25855	0.946128	2.0526
25856	0.94613	1.70675
25857	0.94613	1.91923
25858	1.2778	1.95297
25859	0.946118	0.591113
25860	1.35629	0.488585
25861	0.946117	0.581164
25862	0.946131	0.494362
25863	0.946105	0.495374
25864	0.946132	1.88412
25865	0.946132	2.1

25866	1.38646	2.1
25867	1.38646	2.1
25868	0.946132	2.1
25869	0.946128	2.1
25870	0.946128	2.0762
25871	1.39659	2.1
25872	1.39659	2.1
25873	0.946129	2.1
25874	0.946132	1.71357
25875	0.946131	1.57115
25876	0.946132	1.71817
25877	0.946132	1.67613
25878	1.15139	1.60585
25879	0.946133	2.1
25880	0.946132	2.1
25881	0.946132	2.1
25882	0.946132	2.1
25883	1.36151	2.1
25884	0.946127	2.1
25885	0.946131	2.1
25886	0.946128	2.1
25887	0.94613	2.1
25888	1.38397	2.1
25889	0.946131	0.276703
25890	1.31112	0.308976
25891	0.946127	0.322046
25892	0.946128	0.308185
25893	1.31112	0.296029
25894	1.29778	0.05
25895	0.883417	0.05
25896	1.32613	0.05
25897	0.94613	0.05
25898	0.94613	0.05
25899	0.946132	2.1
25900	0.94613	2.1
25901	0.946131	2.1
25902	0.946131	2.1
25903	1.36523	2.1
25904	0.946132	0.0620526
25905	1.01653	0.0506736
25906	0.946132	0.0878405
25907	0.946131	0.0791165
25908	0.946131	0.0651375
25909	0.94612	2.1
25910	1.08301	2.05717
25911	0.946119	2.1
25912	0.946132	2.1
25913	1.08299	2.0893
25914	0.810942	2.1
25915	0.810944	2.1
25916	0.810957	2.1
25917	0.946126	0.245548
25918	0.946132	0.290646
25919	0.946129	0.247381
25920	1.3492	0.221368
25921	0.94613	0.241863
25922	0.946131	0.222256
25923	1.24766	0.221752

25924	0.946128	0.259015
25925	0.946131	0.25444
25926	1.24766	0.252542
25927	0.946132	0.05
25928	0.946131	0.05
25929	0.946131	0.05
25930	1.23351	0.05
25931	0.946132	0.05
25932	0.946131	0.05
25933	0.946132	0.05
25934	0.946131	0.05
25935	1.35657	0.05
25936	0.946131	0.05
25937	0.946132	0.229498
25938	1.3689	0.161555
25939	0.946132	0.21452
25940	0.946132	0.210551
25941	1.3689	0.16941
25942	0.946133	0.138101
25943	1.35822	0.0784685
25944	0.946133	0.117466
25945	0.946132	0.115632
25946	1.35822	0.0829506
25947	0.946131	0.335412
25948	0.946131	1.17124
25949	0.946131	0.793738
25950	0.946131	0.642549
25951	1.4101	1.18003
25952	0.810944	1.58696
25953	0.810939	1.48808
25954	0.810955	1.25196
25955	0.946132	2.1
25956	1.37733	2.1
25957	0.946132	2.1
25958	0.946131	2.1
25959	0.946131	2.1
25960	1.32582	2.1
25961	0.946132	2.1
25962	0.946132	2.1
25963	0.946132	2.1
25964	0.946132	2.1
25965	0.946132	2.1
25966	0.946132	2.1
25967	1.31401	2.1
25968	1.31401	2.1
25969	0.946131	2.1
25970	1.3469	1.54776
25971	0.946132	1.78709
25972	0.946132	1.77884
25973	0.946133	1.28931
25974	0.946132	1.44652
25975	0.946126	2.1
25976	0.946127	2.1
25977	0.94613	2.1
25978	0.946128	2.1
25979	1.41206	2.1
25980	0.946127	2.1
25981	1.40109	2.1

25982	0.946128	2.1
25983	0.946129	2.1
25984	1.40109	2.1
25985	1.24448	0.0965226
25986	0.94613	0.182775
25987	0.946132	0.131527
25988	0.946129	0.166305
25989	0.946131	0.152978
25990	1.11015	0.141945
25991	0.946133	0.298243
25992	0.946132	0.190578
25993	0.946133	0.245424
25994	0.946132	0.229461
25995	0.946132	2.1
25996	0.946131	2.1
25997	0.946131	2.1
25998	1.38775	2.1
25999	0.946131	2.1
26000	0.946133	1.81002
26001	0.946131	2.1
26002	1.30598	2.1
26003	1.30598	2.1
26004	0.946131	2.1
26005	0.946132	2.1
26006	0.946132	2.1
26007	0.946132	2.1
26008	1.40774	2.1
26009	0.946132	2.1
26010	0.798396	0.05
26011	0.946133	0.39737
26012	0.946128	1.35727
26013	0.946131	0.778664
26014	0.946131	1.10473
26015	1.28918	1.05361
26016	0.946132	0.159298
26017	1.40668	0.13036
26018	0.94613	0.128934
26019	0.94613	0.167493
26020	0.94613	0.171907
26021	0.810946	2.1
26022	0.810945	2.1
26023	0.810933	2.1
26024	0.946131	2.1
26025	1.30338	2.1
26026	0.94613	2.1
26027	1.30339	2.1
26028	0.946131	2.1
26029	0.946127	0.532181
26030	0.87161	0.474601
26031	0.946128	0.547194
26032	0.946129	0.527067
26033	0.827876	0.553294
26034	0.946131	2.1
26035	1.40796	2.1
26036	0.94613	2.1
26037	0.94613	2.1
26038	1.40796	2.1
26039	0.946132	0.543104

26040	0.946132	0.662107
26041	0.946132	0.523388
26042	0.946132	0.482464
26043	1.41724	0.425444
26044	1.34254	0.251938
26045	0.946132	0.452858
26046	0.946132	0.400118
26047	0.946132	0.375576
26048	0.946133	0.346065
26049	0.946133	1.90554
26050	0.946132	1.85268
26051	0.946132	1.79163
26052	1.26501	1.93734
26053	0.946132	1.76409
26054	0.946131	0.0514868
26055	1.35169	0.05
26056	0.946131	0.0710586
26057	0.946131	0.0588983
26058	1.35169	0.0509804
26059	0.676844	0.05
26060	0.946131	0.05
26061	0.94613	0.0524208
26062	1.13242	0.05
26063	1.13242	0.05
26064	0.946131	0.05
26065	0.946128	1.85155
26066	1.28828	2.08626
26067	0.946131	1.7932
26068	0.946131	1.9616
26069	1.28828	2.02657
26070	0.946131	0.307735
26071	1.2996	0.236861
26072	0.946133	0.292153
26073	0.946131	0.264464
26074	0.946131	0.246577
26075	0.946132	2.1
26076	1.40638	2.1
26077	0.946131	2.1
26078	0.946132	2.1
26079	1.40638	2.1
26080	2.84909	0.05
26081	1.35726	0.05
26082	0.946131	0.0852169
26083	0.946131	0.0748487
26084	0.946131	0.0690803
26085	0.946131	0.0662033
26086	1.39852	2.1
26087	0.946132	2.1
26088	0.94613	2.1
26089	0.94613	2.1
26090	0.94613	2.1
26091	0.946126	0.05
26092	0.946131	0.05
26093	0.946128	0.05
26094	1.31666	0.05
26095	0.946128	0.05
26096	0.946126	0.05
26097	0.946131	0.05

26098	0.946129	0.0519221
26099	1.32275	0.05
26100	0.946129	0.05
26101	0.946131	2.1
26102	0.946132	2.1
26103	1.41125	2.1
26104	0.946131	2.1
26105	0.946131	2.1
26106	0.946131	2.1
26107	0.946132	2.1
26108	1.31904	2.1
26109	0.946132	2.1
26110	0.946132	2.1
26111	0.946129	0.05
26112	0.946132	0.05
26113	0.94613	0.05
26114	1.02426	0.05
26115	0.9311	0.05
26116	0.946128	1.7933
26117	0.946133	1.61346
26118	0.946131	1.63846
26119	1.40092	1.81072
26120	0.946131	1.61051
26121	0.946117	2.1
26122	0.946118	2.1
26123	0.946118	2.09336
26124	1.40017	2.1
26125	0.946105	2.1
26126	0.946132	0.177962
26127	1.03547	0.158162
26128	0.946132	0.129487
26129	0.946133	0.186129
26130	0.946132	0.166988
26131	0.946133	2.1
26132	0.946132	2.1
26133	0.946133	2.1
26134	0.946133	2.1
26135	1.36694	2.1
26136	0.946132	0.0808581
26137	1.22878	0.105187
26138	0.946132	0.123345
26139	0.946132	0.082552
26140	1.22878	0.0976057
26141	1.11014	0.126956
26142	0.913078	0.122815
26143	1.11015	0.164717
26144	0.49154	0.161832
26145	0.946127	2.1
26146	0.946131	2.1
26147	0.946128	2.1
26148	0.946129	2.1
26149	1.38752	2.1
26150	0.946132	2.1
26151	1.37277	2.1
26152	0.946132	2.1
26153	0.946131	2.1
26154	1.37277	2.1
26155	0.94613	0.348585

26156	1.16834	0.189589
26157	0.946129	0.342292
26158	0.94613	0.237462
26159	0.94613	0.236769
26160	0.946127	0.37747
26161	0.946131	0.249415
26162	1.35594	0.202322
26163	0.946128	0.37971
26164	0.94613	0.272459
26165	0.946128	2.09964
26166	0.946128	0.539291
26167	1.38464	1.97864
26168	1.38463	1.38879
26169	0.94613	1.38417
26170	0.946129	1.09899
26171	0.946131	0.604284
26172	0.94613	0.367915
26173	1.39751	0.286149
26174	0.946131	0.369349
26175	1.25837	0.0739809
26176	1.21134	0.0591767
26177	0.700013	0.0813294
26178	0.700041	0.077651
26179	1.40583	2.09675
26180	0.946132	2.1
26181	0.946132	2.1
26182	0.946133	2.1
26183	0.946132	2.1
26184	0.827865	2.1
26185	0.946131	2.1
26186	0.946131	2.0349
26187	0.946131	2.09794
26188	0.946131	2.034
26189	0.946132	0.05
26190	1.21087	0.05
26191	0.94613	0.05
26192	0.94613	0.05
26193	0.946131	0.05
26194	0.94613	0.272961
26195	1.17452	0.239909
26196	0.946132	0.273211
26197	0.94613	0.252549
26198	0.94613	0.261962
26199	0.946119	0.222576
26200	1.27374	0.210913
26201	0.946119	0.220954
26202	0.946131	0.214785
26203	0.946106	0.213409
26204	0.946133	2.1
26205	0.946132	2.1
26206	0.946133	2.1
26207	0.946132	2.1
26208	1.39621	2.1
26209	1.34352	2.1
26210	0.946132	2.1
26211	0.946132	2.1
26212	0.946132	2.1
26213	0.946132	2.1

26214	0.946132	1.3265
26215	0.946128	2.07426
26216	0.94613	1.71643
26217	0.94613	1.95152
26218	1.41273	2.02676
26219	1.23536	2.1
26220	0.946131	2.08047
26221	0.946132	2.1
26222	0.946132	2.1
26223	0.946132	2.1
26224	0.946132	2.1
26225	1.41131	2.1
26226	0.946131	2.1
26227	0.946131	2.1
26228	0.946131	2.1
26229	0.946133	0.548527
26230	0.946132	2.06041
26231	0.946133	0.83284
26232	1.41894	0.610251
26233	0.946132	1.01357
26234	0.946128	1.79294
26235	1.4099	2.1
26236	0.946131	1.98883
26237	0.946131	2.01296
26238	0.946131	1.80696
26239	0.810947	2.1
26240	0.810945	2.1
26241	0.810933	2.1
26242	0.946131	0.752795
26243	1.25765	2.06391
26244	0.946128	1.16859
26245	0.946129	1.29182
26246	1.25765	1.62756
26247	1.21347	2.1
26248	0.946129	2.1
26249	0.94613	2.1
26250	1.21347	2.1
26251	0.94613	2.1
26252	0.94613	0.05
26253	0.946132	0.05
26254	1.2493	0.05
26255	1.2493	0.05
26256	0.94613	0.05
26257	0.946132	0.744536
26258	0.946128	1.23127
26259	0.946131	0.567757
26260	0.94613	0.968141
26261	1.37938	0.787344
26262	0.946133	0.05
26263	0.946132	0.05
26264	0.946133	0.05
26265	0.946132	0.05
26266	0.946133	0.05
26267	0.946129	0.429648
26268	1.26426	0.296603
26269	0.946131	0.451643
26270	0.946131	0.421643
26271	1.26426	0.327798

26272	0.872447	1.87458
26273	0.946132	1.59825
26274	0.946132	1.93803
26275	0.946132	1.87077
26276	0.946133	1.75441
26277	0.946117	1.25449
26278	0.946116	0.833317
26279	0.946113	1.11663
26280	0.946129	1.07232
26281	1.27211	0.757336
26282	0.94613	0.825824
26283	0.946129	1.8608
26284	1.40029	1.40042
26285	1.40028	1.58023
26286	0.94613	1.5813
26287	0.810942	2.1
26288	0.810943	2.1
26289	0.810932	2.1
26290	1.29521	2.1
26291	0.946129	2.1
26292	0.946131	2.1
26293	1.29521	2.1
26294	1.29521	2.1
26295	0.946132	0.51159
26296	0.946129	0.313713
26297	1.24596	0.28623
26298	0.946131	0.525586
26299	0.946131	0.397851
26300	0.946131	2.1
26301	1.35616	2.1
26302	0.946133	2.1
26303	0.94613	2.1
26304	1.35616	2.1
26305	0.879548	0.237865
26306	1.01272	0.106381
26307	0.946133	0.400807
26308	0.946133	0.132921
26309	0.946132	0.163265
26310	0.946132	0.417019
26311	0.918009	0.220221
26312	0.91801	0.488153
26313	0.946132	0.26447
26314	0.946132	0.359714
26315	0.946132	0.05
26316	1.28644	0.05
26317	0.946131	0.0579852
26318	0.946131	0.05
26319	0.946132	0.05
26320	0.946131	0.05
26321	1.40103	0.05
26322	0.946131	0.0606994
26323	0.946131	0.0515419
26324	0.946131	0.05
26325	0.946128	0.114914
26326	1.03327	0.0832367
26327	0.946129	0.132874
26328	0.946131	0.0856721
26329	0.94613	0.106175

26330	0.946132	0.606539
26331	0.946133	0.448503
26332	1.40265	0.312005
26333	1.40265	0.296334
26334	0.946133	0.213585
26335	0.946131	0.05
26336	0.946131	0.05
26337	0.946131	0.05
26338	0.856655	0.05
26339	0.856616	0.05
26340	0.946132	0.181957
26341	0.946132	0.23278
26342	0.946132	0.408566
26343	1.15844	0.14316
26344	0.946131	0.357157
26345	0.946131	2.1
26346	1.4031	2.1
26347	1.40304	2.1
26348	0.851554	2.1
26349	0.946131	0.05
26350	0.94613	0.0602019
26351	1.24417	0.05
26352	1.24416	0.05
26353	0.946131	0.05
26354	0.946132	0.808901
26355	1.20992	0.847884
26356	0.946132	0.989739
26357	0.946132	0.864188
26358	1.20992	0.936014
26359	0.946128	1.88369
26360	0.94613	2.0673
26361	0.94613	1.89965
26362	0.94613	1.99372
26363	1.23426	2.01452
26364	0.810943	0.526421
26365	0.810946	0.137323
26366	0.810933	0.0872888
26367	0.810945	2.1
26368	0.810945	2.07412
26369	0.810932	2.1
26370	1.25104	2.1
26371	0.946131	2.1
26372	0.946131	2.01741
26373	1.25104	2.1
26374	1.25104	2.06797
26375	1.25488	2.05655
26376	0.946132	2.06757
26377	0.946132	2.05152
26378	0.946132	2.08065
26379	0.946131	2.08479
26380	0.946131	0.0513652
26381	0.946126	0.0847412
26382	0.946128	0.0514032
26383	1.32131	0.0572599
26384	0.946128	0.06913
26385	0.946133	2.1
26386	0.946132	2.03307
26387	0.946132	2.1

26388	0.946133	2.1
26389	1.41123	2.1
26390	0.946132	0.05
26391	0.946133	0.0570408
26392	0.946132	0.071269
26393	1.32163	0.05
26394	0.946133	0.0645927
26395	0.946132	2.1
26396	0.946132	2.1
26397	1.38651	2.1
26398	1.38651	2.1
26399	0.946132	2.1
26400	0.94613	0.126352
26401	1.3639	0.113349
26402	0.946128	0.144747
26403	0.946131	0.118894
26404	1.3639	0.109259
26405	0.946133	1.43166
26406	1.40724	0.761607
26407	0.946132	0.623676
26408	0.946133	0.947299
26409	1.40724	0.872051
26410	1.21099	1.40911
26411	0.946128	1.80286
26412	0.946127	2.00017
26413	0.946128	1.92233
26414	0.946129	1.71715
26415	0.946132	0.315129
26416	1.35946	0.253026
26417	0.946131	0.60957
26418	0.946131	0.391967
26419	1.35946	0.280993
26420	1.34794	0.673936
26421	0.517994	1.64873
26422	0.946132	0.848398
26423	0.946132	0.945919
26424	0.517965	1.20418
26425	1.23224	0.858266
26426	0.94613	1.92808
26427	0.946131	1.62653
26428	0.946131	1.29093
26429	0.946131	1.22472
26430	1.37511	1.25303
26431	0.946132	1.51445
26432	0.946133	1.91197
26433	0.946133	1.81542
26434	0.946132	1.51485
26435	0.851106	0.401275
26436	0.946129	0.573142
26437	0.946131	0.302704
26438	0.946131	1.63372
26439	0.851066	0.618348
26440	0.946131	0.108008
26441	0.946131	0.13472
26442	0.94613	0.146805
26443	1.31357	0.100714
26444	0.946131	0.104435
26445	0.946133	0.855966

26446	0.946132	1.14667
26447	0.946133	0.548452
26448	0.946132	0.87036
26449	1.38149	0.666748
26450	0.810944	1.49298
26451	0.810945	1.955
26452	0.810958	2.09232
26453	0.946118	0.05
26454	0.946118	0.05
26455	1.37835	0.05
26456	1.37837	0.05
26457	0.946106	0.05
26458	0.946128	0.05
26459	0.946131	0.05
26460	1.34831	0.05
26461	1.34831	0.05
26462	0.94613	0.05
26463	0.946132	0.0672598
26464	0.946132	0.121676
26465	0.909981	0.0732492
26466	1.00099	0.0637586
26467	0.946131	0.0990787
26468	0.946132	0.436458
26469	0.946133	1.28258
26470	0.946133	0.574469
26471	0.946132	0.935517
26472	1.35328	0.888318
26473	0.946132	0.05
26474	0.946132	0.05
26475	1.35386	0.05
26476	1.35385	0.05
26477	0.946133	0.05
26478	0.946121	0.05
26479	0.946132	0.05
26480	0.900167	0.05
26481	1.01189	0.05
26482	0.946132	0.05
26483	0.946132	2.07128
26484	1.41585	1.68942
26485	0.946131	2.08291
26486	0.946131	1.7419
26487	0.94613	2.07207
26488	0.810946	2.1
26489	0.810946	2.1
26490	0.810933	2.1
26491	1.34447	2.1
26492	0.946131	2.1
26493	0.946131	2.1
26494	1.34447	2.1
26495	1.19886	2.1
26496	0.946132	0.0792216
26497	0.946132	0.05
26498	0.946132	0.05
26499	1.35065	0.05
26500	0.946132	0.0696363
26501	0.946131	0.23851
26502	1.3022	0.200876
26503	0.94613	0.400329

26504	0.946131	0.305014
26505	1.3022	0.221354
26506	0.946127	0.160976
26507	1.38683	0.141521
26508	0.946127	0.191913
26509	0.946128	0.1799
26510	1.38683	0.170968
26511	0.810946	0.05
26512	0.810947	0.05
26513	0.810959	0.05
26514	0.946132	0.05
26515	0.946131	0.05
26516	0.946132	0.05
26517	0.946132	0.05
26518	0.946132	0.05
26519	0.946127	1.2962
26520	1.35968	1.96689
26521	0.946131	1.09989
26522	0.946129	1.10761
26523	1.35969	1.76089
26524	0.946133	2.0358
26525	1.35862	1.45092
26526	0.946132	2.0551
26527	0.946132	1.66782
26528	1.35862	1.72796
26529	0.946132	0.712003
26530	0.946132	0.203108
26531	0.946132	0.88697
26532	0.946131	0.232084
26533	0.946132	0.344071
26534	0.946131	0.176281
26535	0.946131	0.1183
26536	0.946128	0.203385
26537	0.946129	0.0841582
26538	1.41232	0.0932861
26539	0.946128	2.09571
26540	1.29331	2.1
26541	0.946131	2.0964
26542	0.946131	2.1
26543	1.29331	2.1
26544	0.946133	0.542313
26545	0.946132	0.602788
26546	0.946132	0.542401
26547	0.946132	0.483587
26548	1.41556	0.422461
26549	0.94612	0.495882
26550	0.946119	0.460668
26551	0.946119	0.502043
26552	0.946132	0.403446
26553	0.946107	0.399082
26554	0.946129	0.05
26555	1.1509	0.05
26556	0.946126	0.05
26557	0.946129	0.05
26558	0.94613	0.05
26559	0.946131	0.05
26560	1.19263	0.05
26561	0.946132	0.05

26562	0.946132	0.05
26563	0.946132	0.05
26564	0.94613	0.653494
26565	1.36939	1.08094
26566	0.946128	0.915812
26567	0.946131	0.788961
26568	1.36939	0.891192
26569	0.946133	1.1622
26570	0.827865	1.55069
26571	0.946132	1.20202
26572	0.946132	1.45848
26573	1.40398	1.37053
26574	0.946127	0.925569
26575	0.946127	2.08519
26576	0.94613	0.687947
26577	1.41498	1.86469
26578	0.946128	1.30269
26579	0.94613	0.05
26580	0.946131	0.05
26581	0.946129	0.05
26582	0.94613	0.05
26583	0.94613	0.05
26584	0.946118	0.0508873
26585	0.946119	0.05
26586	0.946116	0.05
26587	1.36569	0.05
26588	0.946105	0.05
26589	0.946132	0.211762
26590	1.23175	0.144259
26591	0.94613	0.223704
26592	0.946133	0.133251
26593	0.946131	0.159567
26594	1.25698	0.05
26595	0.946126	0.0604228
26596	0.946129	0.05
26597	0.946128	0.05
26598	0.946129	0.05
26599	0.810943	2.1
26600	0.810944	2.1
26601	0.810932	2.1
26602	0.946132	0.116331
26603	1.09841	0.0890395
26604	0.946131	0.145688
26605	0.946132	0.125468
26606	1.09841	0.100256
26607	0.810936	1.79534
26608	0.810935	2.05979
26609	0.810934	2.1
26610	1.23851	2.1
26611	0.946132	1.9994
26612	0.946131	2.1
26613	1.23851	2.1
26614	1.23851	2.1
26615	0.946132	0.103392
26616	0.946131	0.329732
26617	0.946132	0.116849
26618	0.946132	0.196888
26619	0.946132	0.399011

26620	0.939141	1.16493
26621	0.953118	1.1767
26622	0.946126	1.05334
26623	0.946129	1.06244
26624	1.03306	1.08303
26625	0.946132	0.108375
26626	1.30927	0.124089
26627	0.946132	0.138699
26628	0.946132	0.113621
26629	1.30928	0.116097
26630	0.94613	2.1
26631	0.946131	2.1
26632	1.24511	2.1
26633	0.94613	2.1
26634	0.94613	2.1
26635	0.946133	0.114662
26636	1.34152	0.0737078
26637	0.946131	0.0841622
26638	0.946131	0.0845382
26639	1.34152	0.0741084
26640	0.946132	0.0942245
26641	1.41383	0.0887895
26642	0.946132	0.0930701
26643	0.946132	0.0570842
26644	1.41383	0.0549883
26645	0.946132	0.654088
26646	0.946131	0.496418
26647	1.41392	0.37915
26648	0.946132	0.661641
26649	0.946132	0.511108
26650	0.946132	0.926702
26651	0.946132	0.762977
26652	1.24333	0.635268
26653	0.946133	1.00532
26654	0.946133	0.826914
26655	0.946124	0.187451
26656	0.94613	0.222657
26657	0.946126	0.214052
26658	1.3065	0.168762
26659	0.946127	0.216085
26660	0.810946	0.277007
26661	0.810943	0.199552
26662	0.81096	0.264334
26663	0.946131	0.0769739
26664	1.40271	0.0748612
26665	0.946131	0.109144
26666	0.946131	0.0950216
26667	1.40271	0.082881
26668	0.946132	0.05
26669	0.94613	0.05
26670	0.946131	0.05
26671	0.94613	0.05
26672	0.946131	0.05
26673	0.946133	0.171973
26674	1.30107	0.0814608
26675	0.946132	0.219943
26676	0.946132	0.133655
26677	0.946133	0.198778

26678	0.810946	2.1
26679	0.810944	2.1
26680	0.810958	2.1
26681	0.946133	1.96218
26682	0.946131	1.95104
26683	0.946131	2.1
26684	1.30812	2.06364
26685	0.946131	2.02611
26686	0.94613	1.14324
26687	0.975002	1.459
26688	0.917259	2.0312
26689	1.00902	1.79098
26690	0.94613	1.14829
26691	0.946131	2.1
26692	0.946127	2.1
26693	1.398	2.1
26694	0.946129	2.1
26695	0.946129	2.1
26696	0.946133	2.1
26697	0.946132	2.1
26698	0.946132	2.1
26699	0.946132	2.1
26700	1.3033	2.1
26701	1.09897	0.291702
26702	0.946131	0.594876
26703	0.946132	0.466812
26704	0.946129	0.503578
26705	0.946131	0.510547
26706	0.946131	2.1
26707	0.946132	2.1
26708	1.39858	2.1
26709	0.946131	2.1
26710	0.946131	2.1
26711	0.946119	0.383571
26712	1.08878	0.410699
26713	0.946119	1.23231
26714	1.0888	0.415267
26715	0.946107	1.02953
26716	0.946132	0.05
26717	1.10733	0.05
26718	0.946132	0.058667
26719	0.946131	0.0520179
26720	0.946132	0.0518705
26721	0.946128	0.0502919
26722	1.33671	0.05
26723	0.94613	0.0666294
26724	0.94613	0.0602392
26725	0.94613	0.0585048
26726	0.810943	2.1
26727	0.810944	2.1
26728	0.810945	2.1
26729	1.35117	2.1
26730	0.946129	2.1
26731	0.94613	2.1
26732	1.35117	2.1
26733	1.27174	2.1
26734	0.946132	2.07686
26735	0.946133	2.03964

26736	0.997296	1.81661
26737	0.946133	2.00665
26738	0.946132	1.95847
26739	0.810943	2.1
26740	0.810944	2.1
26741	0.810945	2.1
26742	0.810946	1.54497
26743	0.810943	1.97386
26744	0.810933	1.54096
26745	0.946127	1.60678
26746	0.946131	1.55083
26747	0.946128	1.57801
26748	0.94613	1.68821
26749	0.946129	1.68362
26750	0.946131	0.306661
26751	0.946131	0.239034
26752	0.946129	0.315762
26753	0.946131	0.228003
26754	0.94613	0.236393
26755	0.946133	0.0681135
26756	0.946132	0.0699406
26757	0.946132	0.0729107
26758	0.946132	0.0661056
26759	0.946133	0.0754465
26760	0.946131	1.39908
26761	0.94613	0.770447
26762	0.946129	1.44721
26763	0.946131	1.08381
26764	0.946131	1.4214
26765	0.946132	2.01451
26766	0.946129	2.1
26767	0.94613	2.0994
26768	0.946131	2.05237
26769	1.39356	2.09335
26770	0.810946	2.1
26771	0.810947	2.1
26772	0.810934	2.1
26773	0.946125	0.05
26774	0.946127	0.0541511
26775	1.33798	0.05
26776	1.33797	0.05
26777	0.946128	0.05
26778	0.946131	0.05
26779	0.946131	0.05
26780	1.23146	0.05
26781	1.23146	0.05
26782	0.946131	0.05
26783	0.946129	2.1
26784	0.94613	2.1
26785	0.946131	2.1
26786	0.94613	2.1
26787	1.30082	2.1
26788	0.946131	0.05
26789	0.946132	0.05
26790	0.946131	0.0507394
26791	1.20416	0.05
26792	0.946132	0.05
26793	0.946132	0.05

26794	0.946131	0.05
26795	0.946131	0.0566453
26796	1.18711	0.05
26797	0.946132	0.05
26798	0.946132	0.05
26799	0.946132	0.05
26800	0.946133	0.05
26801	0.946132	0.05
26802	0.946133	0.05
26803	0.946131	0.05
26804	0.946132	0.05
26805	0.94613	0.05
26806	0.946131	0.05
26807	0.946131	0.05
26808	0.946133	0.05
26809	0.946132	0.05
26810	0.946133	0.05
26811	0.946132	0.05
26812	0.946132	0.05
26813	0.946132	2.1
26814	0.946132	2.1
26815	0.946132	2.1
26816	1.36422	2.1
26817	0.946132	2.1
26818	0.946131	0.770864
26819	0.946132	1.6447
26820	0.946132	1.11893
26821	0.946132	1.29364
26822	1.4185	1.47479
26823	0.946133	0.0526356
26824	1.37794	0.05
26825	0.946132	0.05
26826	0.946133	0.05
26827	0.946132	0.05
26828	1.4045	2.1
26829	0.946132	2.1
26830	0.946132	2.1
26831	0.946132	2.1
26832	0.946132	2.1
26833	0.946131	2.1
26834	0.946132	2.1
26835	1.34285	2.1
26836	1.34285	2.1
26837	0.946132	2.1
26838	0.946131	2.1
26839	0.946132	2.1
26840	1.40739	2.1
26841	1.40739	2.1
26842	0.946131	2.1
26843	0.946128	2.1
26844	0.946132	2.1
26845	0.94613	2.1
26846	0.94613	2.1
26847	1.3164	2.1
26848	0.946132	2.1
26849	1.3484	2.1
26850	0.946132	2.1
26851	0.946132	2.1

26852	1.3484	2.1
26853	0.946132	2.1
26854	0.946132	2.1
26855	0.946133	2.1
26856	0.946133	2.1
26857	1.41507	2.1
26858	0.946132	2.1
26859	1.32608	2.1
26860	0.946131	2.1
26861	0.946131	2.1
26862	0.946132	2.1
26863	0.94613	0.852565
26864	1.34646	1.87052
26865	0.946129	0.930794
26866	0.94613	0.743711
26867	1.34646	1.1578
26868	0.946131	0.992717
26869	0.946131	2.1
26870	0.94613	1.94557
26871	1.33728	1.46101
26872	0.946131	1.85241
26873	0.946127	2.1
26874	0.946131	1.87265
26875	0.946128	2.1
26876	0.946129	2.1
26877	1.4064	2.09974
26878	0.946133	2.1
26879	0.946131	2.1
26880	0.946131	2.1
26881	0.946131	2.1
26882	0.94613	2.1
26883	1.34912	2.1
26884	0.946131	2.1
26885	0.946129	2.1
26886	0.946131	2.1
26887	0.94613	2.1
26888	0.946132	2.1
26889	1.40447	2.1
26890	0.946132	2.1
26891	0.946133	2.1
26892	1.40447	2.1
26893	1.33607	2.1
26894	0.946131	2.1
26895	0.946127	2.1
26896	0.94613	2.1
26897	0.946129	2.1
26898	0.946117	0.493499
26899	1.32664	1.50759
26900	0.946115	1.13594
26901	0.946105	1.11503
26902	0.94613	0.674203
26903	0.946118	2.1
26904	0.946114	2.1
26905	1.35886	2.1
26906	1.35884	2.1
26907	0.946129	2.1
26908	0.946132	2.1
26909	1.2571	2.1

26910	0.946132	2.1
26911	0.946132	2.1
26912	1.2571	2.1
26913	0.946133	2.1
26914	1.35663	2.1
26915	0.946132	2.1
26916	0.946132	2.1
26917	1.35663	2.1
26918	0.946133	0.963151
26919	0.946129	1.861
26920	0.946131	1.51141
26921	0.946131	1.57289
26922	1.30881	1.81924
26923	0.946132	2.1
26924	0.94613	2.1
26925	1.34652	2.1
26926	0.94613	2.1
26927	0.94613	2.1
26928	0.946132	2.03287
26929	0.946133	1.85535
26930	1.36183	1.56146
26931	1.36183	1.73096
26932	0.946132	1.89935
26933	0.946132	0.202048
26934	1.38353	0.0973073
26935	0.94613	0.104269
26936	0.94613	0.147232
26937	1.38354	0.122067
26938	0.946128	0.507368
26939	1.24887	0.435169
26940	0.94613	0.622296
26941	0.94613	0.538721
26942	1.24887	0.562635
26943	0.946129	0.828833
26944	0.946126	1.36747
26945	0.946124	0.855753
26946	0.946128	1.01334
26947	1.32056	1.10301
26948	0.946129	0.990912
26949	0.946128	1.25853
26950	0.946126	0.915709
26951	0.946129	1.06202
26952	1.20351	1.06605
26953	0.946131	1.95994
26954	0.946131	1.96788
26955	0.946127	2.08823
26956	1.25591	2.04037
26957	0.94613	1.87375
26958	0.946132	1.69637
26959	0.946133	1.52068
26960	0.946132	1.96087
26961	1.15436	1.4217
26962	0.930306	1.38789
26963	0.946132	2.08238
26964	0.946132	2.08476
26965	0.946132	2.00765
26966	0.946133	2.07808
26967	0.946132	2.06359

26968	0.946129	1.40905
26969	0.946132	0.904936
26970	0.946126	1.70147
26971	1.13245	0.636981
26972	0.946129	0.996193
26973	0.94613	2.05598
26974	0.946132	2.04662
26975	0.946129	1.94225
26976	0.946131	2.05514
26977	0.946131	1.99729
26978	1.41279	2.01698
26979	1.76254	2.05571
26980	0.600454	1.96284
26981	0.858991	1.9714
26982	0.940722	1.99224
26983	1.28439	1.71659
26984	0.946131	1.91743
26985	0.94613	1.88757
26986	0.946131	2.00466
26987	0.946131	1.97175
26988	0.946132	0.865728
26989	1.39335	0.335647
26990	0.946132	0.912996
26991	0.946132	0.491344
26992	0.946132	0.769448
26993	1.33879	0.147826
26994	0.946131	0.284243
26995	0.94613	0.469429
26996	1.33879	0.146892
26997	0.946131	0.270096
26998	0.946126	0.306683
26999	0.946131	0.252895
27000	1.37342	0.179357
27001	1.37343	0.131886
27002	0.946128	0.429517
27003	1.39168	0.05
27004	0.946131	0.05
27005	0.946131	0.0595113
27006	0.946131	0.05
27007	0.946131	0.05
27008	0.946131	0.100269
27009	1.412	0.0775424
27010	0.946127	0.0674463
27011	0.946128	0.0706791
27012	0.946128	0.0630135
27013	1.1387	0.184944
27014	0.943364	0.391445
27015	0.946132	0.277503
27016	0.946132	0.305443
27017	0.946133	0.308999
27018	1.21085	0.348596
27019	0.88324	0.7084
27020	0.946129	0.57133
27021	0.946131	0.56132
27022	0.946131	0.498633
27023	1.33587	0.05
27024	0.946125	0.0650878
27025	0.946129	0.05

27026	0.946126	0.0582203
27027	0.946127	0.05
27028	1.27512	0.055293
27029	0.946132	0.0989508
27030	0.946132	0.0844151
27031	0.946133	0.0853262
27032	0.946132	0.0718386
27033	0.946128	0.068217
27034	1.30498	0.0560716
27035	0.946131	0.0901209
27036	0.94613	0.0748248
27037	1.30497	0.064824
27038	0.946131	0.0567597
27039	1.25626	0.05
27040	0.946131	0.0722229
27041	0.946131	0.0618468
27042	1.25626	0.0551952
27043	0.946131	0.05
27044	0.94613	0.05
27045	1.35316	0.05
27046	0.946131	0.05
27047	0.946131	0.05
27048	0.94613	0.14824
27049	1.28727	0.155519
27050	0.946128	0.0959578
27051	0.946131	0.12853
27052	0.94613	0.104163
27053	0.946131	0.05
27054	1.39502	0.05
27055	0.946132	0.05
27056	0.946132	0.05
27057	0.946132	0.05
27058	1.36173	0.234838
27059	0.946131	0.487864
27060	0.946126	0.409284
27061	0.946127	0.404021
27062	0.946128	0.342279
27063	0.946129	0.59964
27064	0.946133	0.448554
27065	0.946131	0.629559
27066	0.946131	0.558327
27067	0.946131	0.621655
27068	0.946128	0.05
27069	0.946132	0.05
27070	1.24678	0.05
27071	0.94613	0.05
27072	0.94613	0.05
27073	0.946129	0.05
27074	0.94613	0.05
27075	1.25215	0.05
27076	0.946126	0.05
27077	0.946129	0.05
27078	0.946129	0.40307
27079	1.29689	0.335865
27080	0.946131	0.398504
27081	0.946131	0.334771
27082	0.946131	0.30828
27083	0.946128	0.0607528

27084	0.94613	0.0623695
27085	0.946131	0.0581434
27086	0.94613	0.0627111
27087	0.946131	0.0646969
27088	0.946127	0.0772524
27089	0.946128	0.0814829
27090	0.94613	0.0740381
27091	0.946129	0.0824488
27092	0.946129	0.0833488
27093	0.946133	0.05
27094	0.946132	0.05
27095	0.946132	0.05
27096	1.29957	0.05
27097	0.946133	0.05
27098	0.946132	0.05
27099	0.946133	0.05
27100	0.946132	0.05
27101	1.26201	0.05
27102	0.946132	0.05
27103	0.946133	0.0541592
27104	1.40427	0.05
27105	0.946132	0.0724083
27106	0.946132	0.0663422
27107	0.946133	0.0599551
27108	0.946132	0.0531782
27109	1.35123	0.05
27110	0.946132	0.0744493
27111	0.946132	0.0681055
27112	0.946131	0.056823
27113	0.946128	0.100295
27114	0.946129	0.104557
27115	0.946131	0.092064
27116	0.94613	0.108361
27117	0.946131	0.103754
27118	0.946127	0.287203
27119	1.23351	0.229353
27120	0.946131	0.281565
27121	0.946129	0.244427
27122	0.94613	0.233523
27123	0.946131	0.121648
27124	0.946131	0.175443
27125	0.94613	0.128807
27126	1.18119	0.115698
27127	0.946131	0.149487
27128	0.946132	0.168699
27129	1.30563	0.131746
27130	0.946133	0.192266
27131	0.946132	0.139296
27132	0.946133	0.166374
27133	0.946132	0.369602
27134	0.946132	0.730671
27135	0.946133	1.74076
27136	1.20256	0.456541
27137	0.946133	0.588154
27138	0.94613	1.02022
27139	1.33007	0.596329
27140	0.946128	1.02141
27141	0.946132	0.586987

27142	0.946131	0.719209
27143	0.946131	2.1
27144	0.946131	2.1
27145	0.946128	2.1
27146	0.946129	2.1
27147	0.94613	2.1
27148	0.810942	2.1
27149	0.810942	2.1
27150	0.81093	2.1
27151	0.946132	2.1
27152	1.39823	2.1
27153	0.946132	2.1
27154	0.946131	2.1
27155	0.946132	2.1
27156	0.946131	2.1
27157	0.946131	2.1
27158	0.946131	2.1
27159	1.37713	2.1
27160	0.946131	2.1
27161	0.946133	2.1
27162	0.946131	2.1
27163	1.40594	2.1
27164	1.40594	2.1
27165	0.94613	2.1
27166	0.946133	2.1
27167	0.946131	2.1
27168	1.38155	2.1
27169	1.38155	2.1
27170	0.946131	2.1
27171	0.946132	1.23991
27172	0.946133	1.98508
27173	0.946132	1.51082
27174	0.946132	1.66457
27175	1.33694	1.82166
27176	0.946131	2.1
27177	1.228	2.1
27178	0.946129	2.1
27179	0.946131	2.1
27180	1.228	2.1
27181	0.946132	0.981866
27182	0.946132	1.742
27183	0.946131	1.85846
27184	0.946132	1.43928
27185	1.35943	1.83226
27186	0.946133	0.05
27187	0.946133	0.05
27188	1.02377	0.05
27189	1.02377	0.05
27190	0.946132	0.05
27191	0.94613	0.05
27192	0.946131	0.05
27193	0.946132	0.05
27194	1.29322	0.05
27195	0.946131	0.05
27196	0.946131	2.1
27197	0.946131	1.24412
27198	0.946132	2.1
27199	1.41492	2.00757

27200	0.946131	2.1
27201	0.946132	2.1
27202	0.946131	2.1
27203	0.946132	2.1
27204	1.35028	2.1
27205	0.946132	2.1
27206	0.946132	2.1
27207	0.946132	2.09595
27208	0.946131	2.09666
27209	1.32461	2.0998
27210	0.946132	2.09267
27211	0.946131	2.1
27212	0.946132	2.1
27213	1.40369	2.1
27214	0.94613	2.1
27215	0.946131	2.1
27216	0.946132	0.0849514
27217	0.979597	0.0649152
27218	0.946131	0.104878
27219	0.946132	0.0666535
27220	0.946132	0.0803742
27221	0.946131	2.1
27222	0.946131	2.1
27223	0.94613	2.1
27224	0.946131	2.1
27225	1.41897	2.1
27226	1.37791	2.1
27227	0.94613	2.1
27228	0.946129	2.1
27229	0.946131	2.1
27230	0.946131	2.1
27231	1.39384	2.1
27232	0.946131	2.1
27233	0.946128	2.1
27234	0.946129	2.1
27235	0.946129	2.1
27236	0.810945	1.65159
27237	0.810944	2.1
27238	0.810958	1.35165
27239	0.946128	2.1
27240	0.946132	2.1
27241	0.94613	2.1
27242	1.3335	2.1
27243	0.946131	2.1
27244	0.94613	2.05396
27245	0.946129	2.1
27246	0.946132	1.79735
27247	1.41192	2.1
27248	0.94613	2.08681
27249	0.946132	0.826725
27250	0.946133	1.44891
27251	0.946132	0.356207
27252	1.39428	0.644178
27253	0.946133	0.303784
27254	0.94613	2.05981
27255	0.946128	2.06586
27256	0.946132	2.00687
27257	0.94613	1.99649

27258	1.40684	2.1
27259	0.946131	2.1
27260	0.946131	2.1
27261	0.946131	2.1
27262	0.946131	2.1
27263	1.39021	2.1
27264	0.946131	0.757272
27265	1.31649	1.84175
27266	0.946131	0.578013
27267	0.946131	0.511915
27268	1.31649	0.92071
27269	0.810946	2.1
27270	0.810946	2.1
27271	0.810958	2.1
27272	1.16687	2.1
27273	0.946131	2.1
27274	0.946131	2.1
27275	1.16687	2.1
27276	1.16687	2.1
27277	0.946128	0.495138
27278	1.3564	0.4301
27279	0.946131	0.496052
27280	0.946131	0.447828
27281	0.94613	0.461028
27282	0.946128	2.1
27283	1.35828	2.1
27284	0.94613	2.1
27285	0.946131	2.1
27286	0.94613	2.1
27287	0.946133	0.989366
27288	1.36477	1.32779
27289	0.946132	1.29509
27290	0.946132	1.04725
27291	1.36477	1.21866
27292	0.946132	1.255
27293	1.31857	1.90552
27294	0.946132	1.46481
27295	0.946132	1.69879
27296	1.31857	1.43979
27297	0.94613	0.573957
27298	1.35059	0.385305
27299	0.946128	0.387004
27300	0.94613	0.458297
27301	1.35059	0.42188
27302	0.946126	0.173767
27303	1.35559	0.12391
27304	0.946127	0.132371
27305	0.946128	0.144173
27306	1.35559	0.126434
27307	0.946131	0.0775047
27308	1.33035	0.0733705
27309	0.946127	0.0827157
27310	0.946128	0.0552052
27311	1.33035	0.0685995
27312	0.94613	0.0745147
27313	1.3665	0.0887041
27314	0.946129	0.0984344
27315	0.946131	0.05

27316	1.3665	0.0879386
27317	0.94613	0.091785
27318	1.32828	0.0711214
27319	0.946129	0.0845828
27320	0.94613	0.0760469
27321	0.946131	0.0721712
27322	0.946131	2.1
27323	0.946131	2.1
27324	1.32949	2.1
27325	1.32949	2.1
27326	0.946131	2.1
27327	0.946131	2.1
27328	0.94613	0.21921
27329	0.946133	2.08239
27330	1.38357	0.987906
27331	0.946131	1.4335
27332	0.946131	2.1
27333	0.946128	2.1
27334	1.36919	2.1
27335	0.946131	2.1
27336	0.94613	2.1
27337	0.946131	2.09999
27338	1.3771	2.1
27339	0.946129	2.1
27340	0.94613	2.1
27341	0.946131	2.1
27342	0.946131	1.88822
27343	0.94613	0.513809
27344	0.946129	0.537887
27345	1.30029	1.04161
27346	0.94613	0.796686
27347	1.21174	0.05
27348	0.946132	0.05
27349	0.946132	0.05
27350	0.946132	0.05
27351	0.946133	0.05
27352	0.810946	2.1
27353	0.810945	2.1
27354	0.810946	2.1
27355	1.1796	2.1
27356	0.946131	2.1
27357	0.946132	2.1
27358	1.1796	2.1
27359	1.1796	2.1
27360	0.946131	2.1
27361	0.946131	2.1
27362	0.946133	2.1
27363	0.946131	2.1
27364	1.40615	2.1
27365	0.810945	2.1
27366	0.810944	2.1
27367	0.810945	2.1
27368	1.40239	2.1
27369	0.94613	2.1
27370	1.35561	2.1
27371	0.946133	2.1
27372	1.35562	2.1
27373	1.41169	0.168954

27374	0.946131	0.339755
27375	0.946127	0.457318
27376	1.41169	0.173898
27377	0.946128	0.321358
27378	0.946119	2.1
27379	0.946119	2.1
27380	0.946119	2.1
27381	0.946107	2.1
27382	1.41917	2.1
27383	0.810946	1.85878
27384	0.810943	1.92745
27385	0.810934	2.04104
27386	0.946127	2.1
27387	1.1995	2.1
27388	0.946126	2.1
27389	0.946128	2.1
27390	1.1995	2.1
27391	0.810944	2.1
27392	0.810947	2.1
27393	0.81096	2.1
27394	0.946133	0.109604
27395	1.3495	0.0626726
27396	0.946132	0.0890807
27397	0.946132	0.0723151
27398	0.946133	0.076225
27399	0.946132	2.1
27400	0.946132	2.1
27401	0.946133	2.1
27402	1.39529	2.1
27403	0.946133	2.1
27404	0.946133	2.1
27405	1.28525	2.1
27406	0.946132	2.1
27407	0.946133	2.1
27408	1.28525	2.1
27409	0.810946	2.1
27410	0.810945	2.1
27411	0.810946	2.1
27412	1.33931	2.1
27413	0.946132	2.1
27414	0.946132	2.1
27415	1.33931	2.1
27416	1.24616	2.1
27417	0.946133	0.0994301
27418	0.946133	0.0727404
27419	0.946132	0.107365
27420	0.946132	0.0692067
27421	0.946133	0.0897535
27422	0.946128	2.1
27423	1.40225	2.1
27424	0.946128	2.1
27425	0.94613	2.1
27426	0.94613	2.1
27427	0.946113	0.05
27428	0.946118	0.05
27429	0.946114	0.05
27430	1.40551	0.05
27431	0.946103	0.05

27432	0.94613	2.1
27433	0.946132	2.1
27434	0.946129	2.1
27435	0.946131	2.1
27436	1.37811	2.1
27437	0.946132	2.1
27438	1.39826	2.1
27439	0.946133	2.1
27440	0.946132	2.1
27441	1.39826	2.1
27442	0.946118	2.1
27443	1.28298	2.1
27444	0.946117	2.1
27445	0.946105	2.1
27446	1.283 2.1	
27447	0.946118	0.05
27448	1.41375	0.05
27449	0.946117	0.0501565
27450	0.946131	0.05
27451	0.946106	0.05
27452	0.946132	1.13497
27453	0.946129	0.811677
27454	1.28436	0.763566
27455	0.946131	1.16321
27456	0.946131	0.916311
27457	0.810947	0.363278
27458	0.810946	0.365811
27459	0.81096	0.313251
27460	0.946132	0.242673
27461	1.34338	0.205769
27462	0.961435	0.179154
27463	0.961435	0.203423
27464	0.946132	0.216074
27465	1.3028	2.00878
27466	0.946131	2.02648
27467	0.946127	2.0767
27468	0.946128	2.08141
27469	0.946129	2.04214
27470	0.946132	0.541073
27471	1.37973	0.332224
27472	0.946132	0.547374
27473	0.946132	0.365885
27474	0.946132	0.378719
27475	0.946131	1.02366
27476	0.94613	0.754545
27477	0.946129	1.01492
27478	0.946131	0.673671
27479	1.41798	0.62445
27480	0.946118	0.748235
27481	0.946118	0.548055
27482	0.946117	0.75577
27483	0.946131	0.546694
27484	0.946105	0.549404
27485	0.946131	1.13133
27486	0.946131	0.876369
27487	0.94613	1.12013
27488	0.946131	0.761779
27489	0.946131	0.727585

27490	0.946133	1.93901
27491	1.40274	2.1
27492	0.946133	1.71045
27493	0.946132	2.1
27494	1.40274	2.1
27495	0.946131	1.7805
27496	1.39731	2.1
27497	0.946132	1.05543
27498	0.94613	1.9326
27499	1.39731	2.07539
27500	0.94612	2.09947
27501	0.94612	1.81831
27502	0.946119	1.91433
27503	0.946132	1.19685
27504	1.40764	2.04983
27505	0.946128	2.1
27506	0.946132	2.1
27507	0.94613	2.1
27508	0.94613	2.1
27509	1.31213	2.1
27510	0.946127	2.1
27511	0.94613	2.1
27512	0.946127	2.1
27513	0.946128	2.1
27514	1.36653	2.1
27515	0.946127	2.1
27516	1.3557	2.1
27517	0.946128	2.1
27518	0.946128	2.1
27519	1.3557	2.1
27520	0.946133	2.1
27521	0.946132	2.1
27522	0.946133	2.1
27523	0.946132	2.1
27524	1.40081	2.1
27525	0.946128	2.1
27526	0.946133	2.1
27527	0.946131	2.1
27528	0.946131	2.1
27529	1.32282	2.1
27530	0.810945	2.1
27531	0.810943	2.1
27532	0.810933	2.1
27533	1.27676	2.1
27534	0.94613	2.1
27535	0.946128	2.1
27536	0.946131	2.1
27537	0.946131	2.1
27538	0.946131	0.659592
27539	0.946127	1.3265
27540	1.41328	1.21088
27541	1.41327	1.27176
27542	0.946128	1.25481
27543	0.946132	0.070993
27544	1.32083	0.0919325
27545	0.946132	0.112253
27546	0.946133	0.0930141
27547	1.32082	0.0844739

27548	0.946128	0.0906999
27549	1.29215	0.109416
27550	0.94613	0.131685
27551	0.94613	0.105694
27552	1.29215	0.100746
27553	0.946132	0.05
27554	1.27696	0.05
27555	0.94613	0.0590255
27556	0.94613	0.0626776
27557	1.27696	0.05
27558	0.94612	0.05
27559	1.29289	0.0677431
27560	0.946119	0.083138
27561	0.946132	0.0849585
27562	1.29287	0.0630739
27563	0.946128	2.1
27564	1.37869	2.1
27565	0.946131	2.1
27566	0.94613	2.1
27567	0.946131	2.1
27568	0.946133	2.1
27569	0.946132	2.1
27570	0.946132	2.1
27571	1.35703	2.1
27572	0.946133	2.1
27573	0.946132	2.1
27574	0.946132	2.1
27575	0.946133	2.1
27576	1.37017	2.1
27577	0.946133	2.1
27578	0.946119	0.05
27579	0.94612	0.05
27580	1.38583	0.05
27581	1.38585	0.05
27582	0.946107	0.05
27583	0.946126	0.05
27584	0.946127	0.0600067
27585	1.22904	0.05
27586	1.22904	0.05
27587	0.946128	0.05
27588	1.3279	0.665309
27589	0.946131	1.5009
27590	0.94613	1.19044
27591	0.946131	1.27057
27592	0.946131	1.0234
27593	0.946132	2.1
27594	0.946132	2.1
27595	0.946132	2.1
27596	0.946131	2.1
27597	1.40932	2.1
27598	1.26868	0.0734086
27599	0.946131	0.138134
27600	0.94613	0.113851
27601	0.946131	0.111676
27602	0.946131	0.100992
27603	0.810943	2.1
27604	0.810945	2.1
27605	0.810932	2.1

27606	0.810943	2.07762
27607	0.810944	0.967422
27608	0.810957	1.10405
27609	1.31539	0.987623
27610	0.946132	2.1
27611	0.946128	0.834845
27612	1.3154	1.65223
27613	1.17856	0.818199
27614	1.21997	1.27349
27615	0.94613	1.8697
27616	0.946129	1.75516
27617	0.94613	1.89096
27618	0.94613	1.79468
27619	0.946131	1.53942
27620	0.946131	1.33106
27621	0.946129	1.54876
27622	0.94613	1.61628
27623	0.946131	1.74842
27624	0.946127	0.996471
27625	0.946131	0.159735
27626	0.946127	1.80911
27627	0.946129	0.89365
27628	1.41342	0.5549
27629	0.946131	0.341617
27630	0.946131	1.4973
27631	0.946128	1.44606
27632	0.946131	0.50805
27633	1.29149	0.826744
27634	1.41022	2.1
27635	0.94613	2.05281
27636	0.946128	2.1
27637	0.94613	2.1
27638	0.94613	2.1
27639	0.946128	0.100272
27640	0.94613	0.17264
27641	1.27864	0.081096
27642	1.27864	0.0713411
27643	0.946131	0.111546
27644	0.946129	0.415836
27645	0.946125	1.50613
27646	0.946124	0.782357
27647	0.946127	0.741751
27648	1.37759	1.19986
27649	0.946129	0.626417
27650	0.94613	1.47806
27651	0.946126	0.844553
27652	0.946129	0.921902
27653	1.38259	1.14445
27654	0.946131	0.0802071
27655	1.34118	0.05
27656	0.946132	0.055756
27657	0.946132	0.0649017
27658	1.34118	0.05
27659	0.946131	0.05
27660	1.25017	0.05
27661	0.946128	0.05
27662	0.946131	0.05
27663	0.946131	0.05

27664	0.946131	2.1
27665	0.946128	2.1
27666	0.946133	2.1
27667	1.3937	2.1
27668	0.94613	2.1
27669	0.946131	2.09505
27670	0.94613	0.687092
27671	1.40404	1.72002
27672	1.40404	1.05734
27673	0.946131	1.16533
27674	0.946132	1.63277
27675	0.946132	0.489881
27676	1.38897	0.298748
27677	1.38897	0.214264
27678	0.946132	0.363422
27679	0.94613	0.868787
27680	0.946128	1.99139
27681	1.40332	1.27567
27682	1.40331	1.61518
27683	0.94613	1.75505
27684	0.946132	2.00782
27685	0.946132	2.1
27686	0.946133	2.1
27687	1.41619	2.1
27688	0.946132	2.1
27689	0.946132	2.1
27690	0.946132	2.1
27691	0.946132	2.1
27692	1.38244	2.1
27693	0.946132	2.1
27694	0.810946	1.48986
27695	0.810943	0.830284
27696	0.810934	0.694583
27697	0.810947	1.33121
27698	0.810943	0.59363
27699	0.810934	0.659124
27700	1.12786	1.5967
27701	0.946132	2.02144
27702	0.946132	1.93196
27703	0.946132	1.76206
27704	0.946133	1.78247
27705	0.946132	1.75171
27706	0.946132	1.85241
27707	0.946132	1.6487
27708	1.27806	1.75629
27709	0.946133	1.70057
27710	0.946132	2.1
27711	0.946132	2.1
27712	0.946133	2.1
27713	0.946132	2.1
27714	0.946133	2.1
27715	1.39514	1.47044
27716	0.946129	1.20406
27717	0.946124	1.50657
27718	0.946127	0.50166
27719	0.946127	1.43962
27720	0.94613	2.1
27721	0.946129	2.1

27722	0.946132	2.1
27723	1.3991	2.1
27724	0.94613	2.1
27725	0.946132	1.56224
27726	0.946133	2.1
27727	0.946132	2.08284
27728	1.36835	2.1
27729	0.946132	2.08338
27730	0.946132	2.1
27731	0.946132	2.1
27732	0.946133	2.1
27733	0.946132	2.1
27734	1.39047	2.1
27735	0.946132	0.05
27736	0.946128	0.05
27737	0.94613	0.05
27738	1.33174	0.05
27739	0.946131	0.05
27740	0.946128	0.05
27741	0.946131	0.05
27742	0.94613	0.05
27743	0.94613	0.05
27744	0.94613	0.05
27745	0.946119	2.1
27746	0.946119	2.1
27747	1.29064	2.1
27748	0.946132	2.1
27749	0.946107	2.1
27750	0.946133	2.1
27751	0.94613	2.1
27752	1.30584	2.1
27753	0.946131	2.1
27754	0.946131	2.1
27755	0.946131	2.1
27756	0.946132	2.1
27757	0.94613	2.1
27758	0.946131	2.1
27759	1.39549	2.1
27760	0.913386	1.25459
27761	0.978873	0.935777
27762	0.946126	1.07586
27763	0.94613	1.18816
27764	1.00473	0.742477
27765	0.946132	0.544199
27766	0.946133	0.468668
27767	0.946132	0.362249
27768	0.946132	0.558556
27769	1.38894	0.309167
27770	0.946128	0.427688
27771	0.946131	0.372584
27772	0.94613	0.349937
27773	0.94613	0.396793
27774	1.23865	0.285224
27775	0.946126	1.21216
27776	0.946129	0.912877
27777	0.946124	1.01978
27778	0.946127	1.03778
27779	1.40248	0.771804

27780	0.946133	0.631718
27781	0.946132	0.672864
27782	0.946132	0.380533
27783	0.946133	0.575613
27784	1.1391	0.391103
27785	0.827862	0.964222
27786	0.946133	1.12314
27787	0.946131	1.30147
27788	0.946131	1.35797
27789	0.94613	1.11482
27790	0.946128	0.28432
27791	0.946132	0.55697
27792	0.94613	0.607627
27793	1.21388	0.21177
27794	0.94613	0.478173
27795	0.810943	0.134161
27796	0.810945	0.180471
27797	0.810932	0.168667
27798	0.946131	1.83059
27799	0.946131	1.23058
27800	0.946133	1.54578
27801	1.36748	0.571487
27802	0.946132	0.37073
27803	0.946128	0.05
27804	0.946131	0.05
27805	0.94613	0.05
27806	1.35856	0.05
27807	0.94613	0.05
27808	0.946132	2.1
27809	0.946131	2.1
27810	0.946133	2.1
27811	0.946132	2.1
27812	0.946131	2.1
27813	0.946133	2.1
27814	0.946131	2.1
27815	1.39558	2.1
27816	1.39558	2.1
27817	0.94613	2.1
27818	0.946131	0.110968
27819	0.94613	0.159309
27820	0.946129	0.114144
27821	1.41388	0.103809
27822	0.946131	0.132395
27823	0.946133	0.0782978
27824	0.946132	0.170188
27825	0.946132	0.0881187
27826	0.946133	0.103531
27827	0.93452	0.167782
27828	0.946129	2.1
27829	1.38897	2.1
27830	0.946131	2.1
27831	0.946131	2.1
27832	1.38897	2.1
27833	0.946133	2.00307
27834	0.946132	1.91733
27835	0.946133	1.96443
27836	0.946132	1.69146
27837	1.38773	1.7986

27838	1.41512	0.132698
27839	0.946131	0.240658
27840	0.946129	0.183543
27841	0.946133	0.191164
27842	0.946131	0.201194
27843	0.946132	1.57976
27844	0.946132	2.1
27845	0.946132	1.73267
27846	0.946133	2.1
27847	0.946133	2.1
27848	0.946126	1.18568
27849	0.946132	1.95804
27850	0.946129	1.36547
27851	0.946129	1.95679
27852	0.946129	2.06558
27853	0.939895	2.03878
27854	1.03386	2.09339
27855	0.94613	2.03655
27856	0.946131	2.04559
27857	1.03386	2.06274
27858	0.946131	2.1
27859	1.39309	2.1
27860	0.946133	2.1
27861	0.946131	2.1
27862	1.39309	2.1
27863	0.946133	2.1
27864	1.24853	2.1
27865	0.946132	2.1
27866	0.946132	2.1
27867	1.24853	2.1
27868	0.946133	2.02413
27869	1.39699	1.20279
27870	0.946132	1.95801
27871	0.946132	2.1
27872	1.39699	1.31073
27873	0.946132	2.1
27874	0.946131	2.1
27875	0.946133	2.1
27876	0.946131	2.1
27877	1.38969	2.1
27878	1.28464	1.97712
27879	0.946133	2.00747
27880	0.94613	2.04559
27881	0.946131	1.94262
27882	0.946131	1.96384
27883	0.946119	0.586376
27884	0.94612	0.861918
27885	0.946119	0.435474
27886	0.946132	0.53669
27887	1.3749	0.4241
27888	0.810942	2.1
27889	0.810943	2.1
27890	0.810957	2.1
27891	1.41134	2.00921
27892	0.946132	0.546111
27893	0.946132	2.01625
27894	0.946132	0.79989
27895	0.946132	1.78423

27896	1.31338	1.96676
27897	0.946132	1.39209
27898	0.946132	1.95289
27899	0.946133	1.59837
27900	0.946132	1.81441
27901	0.946103	0.05
27902	0.752448	0.05
27903	0.946132	0.05
27904	0.946131	0.05
27905	1.20617	0.05
27906	0.946132	1.08924
27907	1.08054	0.755274
27908	1.25736	0.915806
27909	0.946132	1.09121
27910	0.946131	0.892002
27911	0.946128	2.1
27912	1.33275	2.0954
27913	1.1896	2.1
27914	0.946131	2.1
27915	1.18962	2.1
27916	1.21955	0.05
27917	0.946131	0.05
27918	1.33587	0.05
27919	0.741946	0.05
27920	1.07387	0.05
27921	0.945844	1.15122
27922	1.3292	2.1
27923	1.59472	2.05474
27924	0.946131	1.59352
27925	1.04043	1.84679
27926	1.13529	0.0749433
27927	0.946132	0.0787436
27928	1.24893	0.0958147
27929	1.12675	0.102033
27930	0.936652	0.110751
27931	1.36968	2.1
27932	0.946131	2.1
27933	0.946132	2.1
27934	1.36968	2.1
27935	1.258 2.1	
27936	3.17458	0.10267
27937	0.946132	2.07866
27938	1.35022	2.1
27939	1.35022	2.1
27940	0.94613	2.08878
27941	1.12356	2.09945
27942	0.946131	2.1
27943	1.22401	2.1
27944	1.22401	2.1
27945	0.94613	2.1
27946	0.952455	2.1
27947	1.2107	2.1
27948	0.946132	2.1
27949	1.33047	2.1
27950	0.946131	2.1
27951	0.946132	2.1
27952	0.61957	2.06374
27953	0.8636	2.1

27954	0.946132	2.07152
27955	1.19294	2.09792
27956	0.946128	2.1
27957	1.36607	2.1
27958	0.94613	2.1
27959	1.39416	2.1
27960	1.36607	2.1
27961	1.21474	2.1
27962	0.946131	2.1
27963	0.946131	2.1
27964	1.16288	2.1
27965	1.08542	2.1
27966	0.851526	1.2388
27967	1.30374	2.1
27968	1.01708	2.1
27969	0.946114	1.78268
27970	0.96976	1.97378
27971	0.946132	0.0744192
27972	1.49997	0.100708
27973	0.771735	0.0985577
27974	0.771764	0.110236
27975	0.946133	0.149944
27976	0.700863	1.89368
27977	1.42222	2.1
27978	0.946131	2.1
27979	0.946128	2.09546
27980	1.42509	2.1
27981	1.14298	2.1
27982	0.837014	2.1
27983	0.946131	2.1
27984	1.29426	2.1
27985	0.836985	2.1
27986	0.946129	2.1
27987	1.22089	0.38519
27988	0.946132	1.11839
27989	0.712486	0.52298
27990	0.6234	0.518639
27991	0.946131	2.1
27992	1.40271	2.1
27993	1.32241	2.1
27994	0.946133	2.1
27995	1.32241	2.1
27996	0.769373	1.35087
27997	0.946129	2.1
27998	1.41302	2.1
27999	1.28254	2.1
28000	0.946131	2.1
28001	1.28254	2.1
28002	0.851543	2.00162
28003	1.23056	2.1
28004	1.24356	2.1
28005	0.851543	2.02633
28006	0.909417	2.07513
28007	1.13808	0.195062
28008	0.946133	0.506917
28009	0.946131	0.300911
28010	1.13808	1.0039
28011	1.13808	0.310152

28012	0.930941	1.40006
28013	1.37808	1.00391
28014	1.17573	0.776962
28015	0.946124	1.23326
28016	1.02404	0.880975
28017	0.946116	2.1
28018	1.3144	2.09636
28019	1.3144	2.1
28020	0.946118	2.1
28021	1.13089	2.1
28022	0.946128	0.0764914
28023	0.946101	0.0557267
28024	1.2606	0.0757083
28025	1.26055	0.0540002
28026	0.580823	0.648371
28027	0.946132	1.3993
28028	0.580825	0.764071
28029	1.23224	0.881738
28030	0.946128	2.1
28031	1.3719	2.1
28032	0.94613	2.1
28033	1.3719	2.1
28034	0.94613	2.1
28035	0.946132	1.72205
28036	1.35025	0.404953
28037	1.35025	0.984173
28038	0.946132	1.82272
28039	1.1794	1.53577
28040	0.946133	2.1
28041	1.25598	2.1
28042	1.29249	2.1
28043	1.38154	2.1
28044	0.851541	2.1
28045	1.31508	2.1
28046	1.37558	2.1
28047	0.946129	2.1
28048	0.867663	2.1
28049	0.94613	1.57632
28050	1.35426	0.547864
28051	0.84872	0.617796
28052	0.946129	1.5237
28053	1.17195	1.72915
28054	0.946133	2.1
28055	1.39009	2.1
28056	1.14342	2.1
28057	0.946133	2.1
28058	1.14343	2.1
28059	1.38819	2.1
28060	0.946133	2.1
28061	1.4116	2.1
28062	0.946131	2.1
28063	0.946131	2.1
28064	0.946127	2.1
28065	1.32244	2.1
28066	1.32244	2.1
28067	0.946131	2.1
28068	1.2176	2.1
28069	1.75823	2.1

28070	1.11239	2.1
28071	2.17979	2.1
28072	1.65533	2.1
28073	3.41356	2.1
28074	1.94841	2.1
28075	1.91767	2.1
28076	1.76726	2.1
28077	1.70302	2.1
28078	3.76137	2.1
28079	1.94841	2.1
28080	1.33962	2.1
28081	1.31365	2.1
28082	1.70302	2.1
28083	3.2123	2.1
28084	1.76726	2.1
28085	1.70302	2.1
28086	1.47581	2.1
28087	1.7457	2.1
28088	3.22764	2.1
28089	1.20478	2.1
28090	1.70302	2.1
28091	1.27956	2.1
28092	1.18569	2.1
28093	1.63979	2.1
28094	1.51949	2.1
28095	1.53082	2.1
28096	1.92911	2.1
28097	1.32068	2.1
28098	1.74454	2.1
28099	3.27536	2.1
28100	1.08172	2.1
28101	1.74454	2.1
28102	1.52603	2.1
28103	1.52603	2.1
28104	1.32068	2.1
28105	1.32068	2.1
28106	1.89226	2.1
28107	2.37364	2.1
28108	1.89226	2.1
28109	2.42769	2.1
28110	4.31995	2.1
28111	2.37365	2.1
28112	1.5362	2.1
28113	2.4277	2.1
28114	1.56328	2.1
28115	3.9639	2.1
28116	1.89225	2.1
28117	2.42768	2.1
28118	1.89213	2.1
28119	1.89225	2.1
28120	2.42756	2.1
28121	2.42768	2.1
28122	2.42768	2.1
28123	1.58163	2.1
28124	1.56315	2.1
28125	1.56327	2.1
28126	2.42757	2.1
28127	2.42768	2.1

28128	1.01641	2.1
28129	2.15005	2.1
28130	1.00099	2.1
28131	2.15114	2.1
28132	3.15104	2.1
28133	0.994813	2.1
28134	2.15114	2.1
28135	2.17159	2.1
28136	2.17159	2.1
28137	1.00099	2.1
28138	1.00099	2.1
28139	1.29608	2.1
28140	1.29608	2.1
28141	0.942035	2.1
28142	0.942035	2.08977
28143	1.29198	2.1
28144	0.942033	2.1
28145	0.946132	2.1
28146	0.946132	2.1
28147	0.946132	2.1
28148	0.942033	2.1
28149	0.946133	2.08308
28150	1.29198	2.1
28151	1.99985	2.1
28152	1.14207	2.1
28153	1.83584	2.1
28154	1.00232	2.1
28155	3.00217	2.1
28156	1.99867	0.05
28157	2.0815	0.05
28158	1.56806	0.05
28159	1.28828	0.05
28160	3.58519	0.05
28161	1.89226	0.05
28162	1.89226	0.0537861
28163	1.89226	0.05
28164	1.89226	0.0581744
28165	1.89226	0.05
28166	1.89226	0.0539494
28167	1.89226	0.05
28168	1.89226	0.05
28169	2.23193	0.05
28170	2.48978	0.05
28171	4.38204	0.05
28172	1.85293	2.08726
28173	1.64279	0.43196
28174	1.76268	2.09918
28175	1.54214	0.412202
28176	3.39507	1.14383
28177	1.6428	0.409454
28178	1.17302	1.38604
28179	1.17302	1.978
28180	1.28419	1.01791
28181	1.54214	0.357433
28182	1.65331	1.06253
28183	1.76268	2.1
28184	1.54214	0.643376
28185	1.65324	2.07028

28186	1.42491	0.763839
28187	3.19538	1.06488
28188	0.651043	1.84349
28189	0.651043	1.06671
28190	0.647925	0.577861
28191	0.647925	1.7829
28192	0.521747	1.17951
28193	0.58651	1.31852
28194	0.77107	0.238502
28195	0.712457	0.429251
28196	0.712457	0.5789
28197	0.521747	0.452582
28198	0.58651	0.500942
28199	0.77107	0.564844
28200	1.2873	2.1
28201	1.48751	2.1
28202	1.59659	2.1
28203	1.77596	2.08753
28204	3.06326	2.1
28205	1.07976	2.1
28206	1.59659	2.1
28207	1.30237	2.1
28208	1.77723	2.1
28209	2.89897	2.1
28210	1.77723	2.1
28211	1.77596	2.1
28212	1.82614	2.1
28213	1.59659	2.1
28214	2.0055	2.1
28215	1.77596	2.1
28216	2.64523	2.07518
28217	2.73982	0.55616
28218	2.50057	2.06139
28219	2.61606	0.477644
28220	5.26129	1.27822
28221	2.73982	0.273341
28222	2.68987	0.650218
28223	2.61607	0.528979
28224	2.57611	0.792269
28225	5.30593	0.0655277
28226	1.89226	0.138645
28227	1.89226	0.180488
28228	1.89226	0.16885
28229	2.02178	0.10166
28230	3.78452	0.148384
28231	1.89226	0.610897
28232	1.89226	0.562347
28233	1.89226	1.56243
28234	1.89226	0.338134
28235	1.89226	1.38688
28236	1.89226	0.246873
28237	2.35204	2.1
28238	1.50108	2.1
28239	1.3715	2.1
28240	2.18469	2.1
28241	3.68577	2.1
28242	2.62514	2.1
28243	2.1847	2.1

28244	2.0487	2.1
28245	2.45213	2.1
28246	4.63682	2.1
28247	1.25075	2.1
28248	2.18469	2.1
28249	1.3715	2.1
28250	1.3715	2.1
28251	2.0487	2.1
28252	2.0487	2.1
28253	1.28667	2.1
28254	1.11285	2.1
28255	1.03529	2.1
28256	1.03529	2.1
28257	1.30711	2.1
28258	1.30711	2.1
28259	2.35053	2.1
28260	1.89225	2.1
28261	1.89225	2.1
28262	2.51216	2.1
28263	4.24278	2.1
28264	1.89225	2.1
28265	1.89225	2.1
28266	1.89225	2.1
28267	1.89225	2.1
28268	3.7845	2.1
28269	1.92025	2.1
28270	1.32119	2.1
28271	1.44777	2.1
28272	1.33834	2.1
28273	1.92025	2.1
28274	1.81082	2.1
28275	1.89226	2.1
28276	2.67272	2.1
28277	2.51218	2.1
28278	2.51218	2.1
28279	1.89226	2.1
28280	1.89226	2.1
28281	1.89226	2.1
28282	1.89226	2.1
28283	1.89226	2.1
28284	1.89226	2.1
28285	3.78452	2.1
28286	1.92027	2.1
28287	1.44778	2.1
28288	1.61121	2.1
28289	2.01555	2.1
28290	3.53147	2.1
28291	1.90727	0.05
28292	2.29851	0.111325
28293	2.71643	0.082947
28294	2.17695	0.0583599
28295	4.47547	0.0508086
28296	1.90728	0.0611848
28297	1.56339	0.05
28298	1.58143	0.05
28299	2.17696	0.074186
28300	3.74035	0.0520153
28301	1.91368	0.05

28302	2.17696	0.0773184
28303	2.39986	0.0538153
28304	1.75904	0.05
28305	3.936	0.05
28306	1.18793	0.05
28307	2.74744	0.05
28308	1.38716	0.05
28309	2.57465	0.0747037
28310	3.93537	0.05
28311	1.3148	0.05
28312	2.74744	0.0542425
28313	2.70805	0.05
28314	1.38716	0.05
28315	2.70805	0.0569778
28316	1.38716	0.05
28317	1.20608	2.1
28318	0.975871	2.1
28319	0.946132	2.1
28320	0.946132	2.1
28321	0.946132	2.1
28322	0.946132	2.1
28323	0.975871	2.1
28324	0.975871	2.1
28325	1.20608	2.1
28326	0.946132	2.1
28327	0.946133	2.1
28328	0.975871	2.1
28329	2.8198	2.1
28330	2.29967	2.1
28331	2.09542	2.1
28332	2.63784	2.02044
28333	4.93751	2.1
28334	2.09542	2.1
28335	1.39523	2.1
28336	1.21119	2.1
28337	1.39523	2.1
28338	2.11563	2.1
28339	2.29967	2.1
28340	1.88584	2.1
28341	2.63784	1.84036
28342	2.11262	2.1
28343	1.91132	2.1
28344	4.02153	2.1
28345	1.88584	2.1
28346	1.21119	2.1
28347	1.12606	2.1
28348	1.91132	2.1
28349	3.1225	2.1
28350	2.18077	2.09072
28351	1.78167	1.9891
28352	2.2947	0.807788
28353	1.93139	1.60288
28354	4.07637	1.04801
28355	1.5709	0.990831
28356	1.03235	1.80644
28357	2.05933	1.62698
28358	1.78168	0.755994
28359	1.31	0.299127

28360	1.03235	0.791787
28361	2.2947	1.44599
28362	2.31785	2.00169
28363	2.40627	2.1
28364	2.08288	0.704212
28365	4.37758	2.02576
28366	1.95737	1.88453
28367	1.31	1.84877
28368	2.08289	0.258114
28369	1.50108	0.262929
28370	3.39289	0.404038
28371	2.49676	2.1
28372	2.24781	2.1
28373	2.5276	2.09965
28374	2.7352	2.1
28375	5.02436	2.1
28376	2.49676	2.1
28377	2.43549	2.1
28378	2.6398	2.1
28379	2.7352	2.1
28380	5.13656	2.1
28381	1.89226	2.1
28382	2.7352	2.1
28383	1.89226	2.1
28384	2.6398	2.1
28385	1.89226	2.1
28386	2.6398	2.1
28387	2.16843	0.05
28388	1.00185	0.05
28389	1.90039	2.1
28390	3.1547	0.05
28391	2.54064	0.05
28392	2.46752	2.1
28393	1.90039	2.1
28394	2.26044	2.1
28395	4.36791	2.00734
28396	1.65815	2.1
28397	2.48652	2.1
28398	1.65815	2.1
28399	1.9004	2.1
28400	2.21711	2.1
28401	2.5034	2.1
28402	2.37167	2.1
28403	2.59965	2.1
28404	2.62812	2.1
28405	2.40308	2.1
28406	4.99979	2.1
28407	2.50137	2.1
28408	2.59965	2.1
28409	2.62812	2.1
28410	2.5214	2.1
28411	5.12948	2.1
28412	2.62814	2.1
28413	2.4031	2.1
28414	2.43509	2.1
28415	2.65066	2.1
28416	5.06323	2.1
28417	2.62814	2.1

28418	2.52142	2.1
28419	2.53008	2.1
28420	2.65066	2.1
28421	5.15822	2.1
28422	2.0815	2.1
28423	1.92067	2.1
28424	1.59611	2.1
28425	1.89226	2.1
28426	1.62453	2.1
28427	1.92067	2.1
28428	1.89226	2.1
28429	1.89226	2.1
28430	1.89226	2.1
28431	1.89226	2.1
28432	3.78453	2.1
28433	0.946131	2.1
28434	0.946131	2.1
28435	0.929501	2.1
28436	0.910761	2.1
28437	0.9461	2.1
28438	0.9461	2.1
28439	0.910792	2.1
28440	0.910792	2.1
28441	0.946131	2.1
28442	0.929501	2.1
28443	0.910761	2.1
28444	0.946131	2.1
28445	2.0815	2.1
28446	1.81661	2.1
28447	2.1341	2.1
28448	2.0815	2.1
28449	1.86921	2.1
28450	1.81661	2.1
28451	1.89226	2.1
28452	1.89226	2.1
28453	1.89226	2.1
28454	1.89226	2.1
28455	1.89226	2.1
28456	1.89226	2.1
28457	0.946131	2.1
28458	0.946131	2.1
28459	0.949104	2.1
28460	0.9295	2.1
28461	0.946132	2.1
28462	0.946132	2.1
28463	0.929499	2.1
28464	0.929499	2.1
28465	0.946131	2.1
28466	0.949105	2.1
28467	0.946131	2.1
28468	0.929501	2.1
28469	1.66104	2.1
28470	2.34044	2.1
28471	2.08889	2.1
28472	1.33264	2.1
28473	3.67308	2.1
28474	2.0889	2.1
28475	1.08011	2.1

28476	1.08011	2.1
28477	1.33264	2.1
28478	1.87598	2.1
28479	2.12851	2.1
28480	2.27472	2.1
28481	1.89226	1.94305
28482	2.27703	2.1
28483	1.89226	1.81998
28484	4.16699	2.07669
28485	1.89226	2.03033
28486	1.89226	1.43191
28487	1.89226	1.95262
28488	1.89226	1.37011
28489	3.78452	1.54
28490	1.00007	2.01188
28491	1.32316	0.965334
28492	1.29417	0.997051
28493	1.29417	1.98896
28494	1.02051	1.59883
28495	1.02051	1.89618
28496	2.2846	2.1
28497	1.89226	2.07061
28498	2.27703	2.07158
28499	2.27703	2.1
28500	1.89226	1.79799
28501	1.89226	2.1
28502	1.89226	2.1
28503	1.89226	2.03695
28504	1.89226	1.71121
28505	1.89226	0.496034
28506	3.78452	1.02537
28507	1.02051	2.1
28508	1.32316	1.9947
28509	1.03373	1.67032
28510	1.34066	1.51808
28511	2.35689	2.06254
28512	1.703	2.1
28513	1.89224	2.1
28514	1.52266	2.1
28515	1.89224	2.1
28516	1.23344	2.1
28517	1.53282	2.1
28518	1.89223	2.1
28519	1.89223	2.1
28520	1.89223	2.1
28521	1.89223	2.1
28522	3.78447	2.1
28523	0.796845	2.1
28524	0.796845	2.1
28525	0.812146	2.1
28526	0.812146	2.1
28527	1.08006	2.1
28528	1.02679	2.1
28529	0.551538	2.1
28530	0.528929	2.1
28531	0.551529	2.1
28532	1.08005	2.1
28533	0.528935	2.1

28534	1.0268	2.1
28535	1.5001	2.1
28536	1.70302	2.1
28537	2.0726	2.1
28538	1.70302	2.1
28539	1.99601	2.1
28540	1.70303	2.1
28541	1.89226	2.1
28542	1.89226	2.1
28543	1.89226	2.1
28544	1.89226	2.1
28545	1.89226	2.1
28546	1.89226	2.1
28547	1.17285	2.1
28548	2.16015	2.1
28549	2.23563	2.1
28550	2.23563	2.1
28551	1.10309	2.1
28552	1.10309	2.1
28553	0.946128	2.1
28554	0.946128	2.1
28555	1.21847	2.1
28556	1.2884	2.1
28557	0.94613	2.1
28558	0.94613	2.1
28559	1.21847	2.1
28560	1.21847	2.1
28561	0.946131	2.1
28562	1.21847	2.1
28563	0.94613	2.1
28564	1.2884	2.1
28565	1.83922	2.1
28566	2.5768	2.1
28567	1.83488	2.1
28568	1.82846	0.05
28569	2.43693	2.1
28570	2.43693	2.1
28571	2.36134	2.1
28572	1.81656	2.1
28573	1.86454	2.1
28574	1.6697	2.1
28575	2.55618	2.1
28576	2.36134	2.1
28577	2.55618	2.1
28578	1.9323	2.1
28579	1.9302	2.1
28580	1.81656	2.1
28581	2.66983	2.1
28582	2.55618	2.1
28583	1.89226	1.0635
28584	1.70302	1.41267
28585	1.94935	1.72587
28586	1.70302	1.29013
28587	1.95569	1.25398
28588	1.70302	0.822167
28589	2.82465	2.1
28590	2.41707	2.1
28591	2.24481	2.1

28592	2.68958	2.1
28593	5.10665	2.1
28594	1.41232	2.1
28595	1.34479	2.1
28596	0.946129	2.1
28597	0.946129	2.1
28598	0.94613	2.1
28599	0.94613	2.1
28600	1.41232	2.1
28601	1.41232	2.1
28602	1.34479	2.1
28603	0.94613	2.1
28604	0.946131	2.1
28605	1.41232	2.1
28606	1.86859	0.05
28607	1.89226	0.05
28608	1.89226	0.0522392
28609	1.89226	0.05
28610	1.45875	0.05
28611	1.45875	0.05
28612	2.1769	0.113859
28613	2.50259	0.184443
28614	1.94955	0.105886
28615	2.25959	0.0898439
28616	4.45214	0.131109
28617	1.89226	0.267683
28618	1.89226	0.309508
28619	1.89226	0.15569
28620	1.89226	0.323059
28621	3.78452	0.188737
28622	1.89226	0.385658
28623	1.89226	0.705384
28624	1.89226	0.320908
28625	1.89226	0.459363
28626	3.78452	0.803772
28627	2.50259	0.254727
28628	2.49445	0.178211
28629	2.25959	0.0892343
28630	2.20843	0.293495
28631	4.71102	0.1741
28632	1.94955	0.110347
28633	2.25959	0.0803109
28634	1.24689	0.05
28635	1.79906	0.106074
28636	3.74861	0.0817717
28637	1.89226	0.163584
28638	1.89226	0.195507
28639	1.89226	0.105006
28640	1.89226	0.131004
28641	3.78452	0.233342
28642	1.89226	0.205595
28643	1.89226	0.287638
28644	1.89226	0.124895
28645	1.89226	0.192695
28646	3.78452	0.133926
28647	2.25959	0.0802666
28648	2.20843	0.171807
28649	1.79906	0.0990204

28650	1.98391	0.0989948
28651	4.00748	0.115488
28652	1.89226	2.1
28653	1.89226	2.1
28654	1.89226	2.1
28655	1.89226	2.1
28656	3.78452	2.1
28657	1.89226	2.1
28658	1.89226	2.1
28659	1.89226	2.1
28660	1.89226	2.1
28661	3.78452	2.1
28662	1.64718	2.1
28663	1.23085	2.1
28664	1.46028	2.1
28665	1.39564	2.1
28666	1.68372	2.1
28667	1.64718	2.1
28668	1.64718	2.1
28669	1.64981	2.1
28670	1.70303	2.1
28671	1.6991	2.1
28672	3.3199	2.1
28673	1.89226	2.1
28674	1.89226	2.1
28675	1.89226	2.1
28676	1.89226	2.1
28677	3.78452	2.1
28678	1.89226	2.1
28679	1.89226	2.1
28680	1.89226	2.1
28681	1.89226	2.1
28682	3.78452	2.1
28683	0.666701	2.1
28684	0.65896	2.1
28685	0.920797	2.1
28686	0.895441	2.1
28687	0.775871	2.1
28688	0.687493	2.1
28689	0.777393	2.1
28690	0.777393	2.1
28691	0.677856	2.1
28692	0.90095	2.1
28693	0.621435	2.1
28694	0.858124	2.1
28695	1.70303	2.1
28696	1.70731	2.1
28697	1.79663	2.1
28698	1.68783	2.1
28699	3.17337	2.1
28700	1.22724	2.1
28701	2.0021	2.1
28702	1.90427	2.1
28703	1.08698	2.1
28704	3.08907	2.1
28705	2.41689	2.1
28706	2.00209	2.1
28707	1.90427	2.1

28708	2.35245	2.1
28709	4.35454	2.1
28710	1.90427	2.1
28711	0.956294	2.1
28712	0.956294	2.1
28713	1.08698	2.1
28714	1.81488	2.1
28715	1.94556	2.1
28716	1.90427	2.1
28717	2.35245	2.1
28718	2.28979	2.1
28719	1.81488	2.1
28720	4.16733	2.1
28721	1.6272	2.1
28722	1.70302	2.1
28723	1.70303	2.1
28724	1.70302	2.1
28725	1.91167	2.1
28726	1.91167	2.1
28727	1.49063	2.1
28728	1.89226	2.1
28729	1.89226	2.1
28730	1.89226	2.1
28731	1.6272	2.1
28732	1.6272	2.1
28733	2.25988	1.65092
28734	1.89226	0.414464
28735	1.89226	0.859051
28736	2.23152	1.18856
28737	4.12379	0.832083
28738	2.25988	1.85285
28739	1.16361	2.09419
28740	1.15853	2.1
28741	2.23153	1.52848
28742	3.39513	2.08095
28743	1.89226	0.58677
28744	2.23153	0.839666
28745	2.21022	1.31946
28746	2.23153	0.719793
28747	1.87095	0.786573
28748	1.89226	0.268615
28749	1.16239	2.08813
28750	2.23153	0.964492
28751	1.15853	2.03488
28752	1.15853	1.41945
28753	2.21022	2.03466
28754	2.21022	1.28432
28755	1.78381	0.05
28756	1.70302	0.05
28757	1.15453	0.05
28758	1.35007	0.05
28759	1.82021	0.05
28760	2.12851	0.05
28761	1.89226	0.05
28762	1.89226	0.0752176
28763	1.89226	0.0541681
28764	1.89226	0.083024
28765	1.89226	0.05

28766	1.89226	0.0720229
28767	1.89258	0.05
28768	1.089 0.05	
28769	1.22586	0.05
28770	1.79877	0.05
28771	2.88777	0.05
28772	1.91564	0.05
28773	1.79877	0.05
28774	1.96356	0.05
28775	1.70302	0.05
28776	2.37828	0.05
28777	2.04859	0.05
28778	1.89226	0.05
28779	1.89226	0.074235
28780	1.89226	0.05
28781	1.89226	0.0846896
28782	3.78453	0.0586795
28783	1.89226	0.05
28784	1.89226	0.0682361
28785	1.89226	0.05
28786	1.89226	0.0736431
28787	1.89226	0.05
28788	1.89226	0.0661793
28789	2.28593	2.1
28790	1.89226	2.08284
28791	2.2731	2.1
28792	1.89226	1.9536
28793	4.17819	2.1
28794	1.89226	2.1
28795	1.89226	2.07224
28796	1.89226	2.01847
28797	1.89226	1.39969
28798	3.78452	1.76047
28799	1.03933	2.1
28800	1.34355	1.02306
28801	1.35048	0.916321
28802	1.35048	2.09469
28803	1.02975	1.10824
28804	1.02975	2.06897
28805	2.25105	2.1
28806	1.89226	2.1
28807	2.27311	2.1
28808	2.27311	2.1
28809	1.89226	2.08055
28810	1.89226	2.1
28811	1.89226	2.1
28812	1.89226	2.03214
28813	1.89226	2.06434
28814	1.89226	0.314076
28815	3.78452	1.20568
28816	1.02975	2.1
28817	1.34355	2.00936
28818	1.01121	2.03106
28819	1.33079	1.1994
28820	2.35476	2.09959
28821	1.24526	0.05
28822	2.36406	0.05
28823	2.81021	0.05

28824	1.7237	0.05
28825	4.08776	0.05
28826	1.89226	2.1
28827	1.89226	2.1
28828	1.89226	2.1
28829	1.89226	2.1
28830	3.78453	2.1
28831	1.79028	2.1
28832	1.89238	2.1
28833	1.65533	2.1
28834	1.77821	2.1
28835	3.5477	2.1
28836	1.89238	2.1
28837	1.7465	2.0987
28838	1.77821	2.1
28839	1.63557	2.05006
28840	3.52795	2.1
28841	1.89226	2.1
28842	1.89226	1.91185
28843	1.89226	2.1
28844	1.89226	2.04034
28845	3.78452	2.1
28846	1.89226	2.1
28847	1.89226	2.1
28848	1.89226	2.1
28849	1.89226	2.1
28850	3.78452	2.1
28851	1.65532	2.1
28852	1.77821	2.1
28853	1.53278	2.1
28854	1.67571	2.1
28855	3.33103	2.1
28856	1.77821	2.1
28857	1.63557	2.09398
28858	1.67571	2.1
28859	1.52615	2.08802
28860	3.31128	2.1
28861	1.89226	2.1
28862	1.89226	2.09026
28863	1.89226	2.1
28864	1.89226	1.91894
28865	3.78452	2.1
28866	2.83129	2.1
28867	1.89226	1.54033
28868	1.89226	2.07677
28869	2.6491	2.1
28870	4.54136	2.07949
28871	2.83129	2.1
28872	1.47278	2.1
28873	1.3909	2.1
28874	2.6491	2.1
28875	4.12188	2.1
28876	1.89225	2.1
28877	2.65393	2.1
28878	1.89225	2.1
28879	2.69591	2.1
28880	4.58816	2.1
28881	1.91703	2.1

28882	2.00209	2.1
28883	2.65393	2.1
28884	2.65393	2.1
28885	2.69591	2.1
28886	2.00209	2.1
28887	1.89226	2.1
28888	2.69593	2.1
28889	1.93967	2.1
28890	1.89226	2.1
28891	2.74334	2.1
28892	2.69593	2.1
28893	2.74335	2.1
28894	2.0021	2.1
28895	2.76367	2.1
28896	2.69593	2.1
28897	2.06984	2.1
28898	2.0021	2.1
28899	2.24481	2.1
28900	2.58921	2.1
28901	2.68958	2.1
28902	2.68958	2.1
28903	2.11984	2.1
28904	2.11984	2.1
28905	2.11984	2.1
28906	2.58921	2.1
28907	2.46488	2.1
28908	1.98339	2.1
28909	4.5726	2.1
28910	2.7777	2.1
28911	2.58921	2.1
28912	2.46488	2.1
28913	2.70401	2.1
28914	5.29322	2.1
28915	2.17741	2.1
28916	2.54197	2.1
28917	2.54018	2.1
28918	2.18608	2.1
28919	4.72805	2.1
28920	2.17742	2.1
28921	1.58054	2.1
28922	1.59169	2.1
28923	2.18608	2.1
28924	3.76662	2.1
28925	2.54018	2.1
28926	2.18608	2.1
28927	2.19104	2.1
28928	2.5392	2.1
28929	4.72528	2.1
28930	1.60497	2.1
28931	2.18608	2.1
28932	1.59169	2.1
28933	1.59169	2.1
28934	2.19105	2.1
28935	2.19105	2.1
28936	2.58661	2.1
28937	1.06203	2.1
28938	2.7043	2.1
28939	2.21397	2.1

28940	1.55236	2.1
28941	1.06203	2.1
28942	1.89226	1.61239
28943	1.89226	1.60576
28944	1.89226	0.354613
28945	1.89226	2.00574
28946	3.78453	1.30077
28947	1.08195	1.3634
28948	0.990019	2.1
28949	1.31976	1.98047
28950	1.4539	0.637799
28951	2.40171	1.99632
28952	1.89226	0.702321
28953	1.89226	2.08926
28954	1.89226	1.58404
28955	1.89226	0.250135
28956	3.78453	1.30472
28957	1.89226	1.92555
28958	1.89226	1.39839
28959	1.89226	0.932216
28960	1.89226	2.04832
28961	3.78453	1.64371
28962	1.43895	2.02914
28963	1.31976	1.82668
28964	1.70066	0.980377
28965	1.33783	2.09484
28966	1.43895	1.94092
28967	1.07612	2.1
28968	0.65988	1.91234
28969	0.850329	1.58867
28970	0.919598	0.500315
28971	0.726952	1.73856
28972	0.788106	0.340519
28973	0.788106	2.1
28974	0.789175	2.05775
28975	0.789175	0.243637
28976	0.850329	0.706767
28977	0.919598	0.764612
28978	0.659879	1.58128
28979	0.726952	1.49763
28980	1.34389	2.1
28981	1.41279	2.02722
28982	1.15564	2.1
28983	1.15564	2.1
28984	1.55998	2.09282
28985	1.55998	2.1
28986	1.89226	1.93357
28987	1.89226	2.03068
28988	1.89226	2.1
28989	1.89226	1.47521
28990	1.89226	2.1
28991	1.89226	1.38612
28992	1.55998	2.1
28993	1.41279	2.06363
28994	1.72901	2.09274
28995	1.62414	2.09307
28996	3.1418	2.09676
28997	1.89226	2.03204

28998	1.89226	2.0807
28999	1.89226	2.09283
29000	1.89226	1.36015
29001	3.78452	2.03777
29002	1.89226	1.14672
29003	1.89226	2.03225
29004	1.89226	1.91312
29005	1.89226	1.9307
29006	1.89226	1.50477
29007	1.89226	1.36926
29008	1.62414	2.09935
29009	1.01243	2.01625
29010	1.70932	2.09103
29011	1.41279	1.99524
29012	1.30896	2.02248
29013	1.01243	1.92533
29014	1.9719	2.1
29015	1.10725	2.1
29016	1.12411	2.1
29017	1.98691	2.1
29018	3.09601	2.1
29019	1.97189	2.1
29020	2.47932	2.1
29021	2.49092	2.1
29022	1.98691	2.1
29023	4.46282	2.1
29024	1.1486	2.1
29025	1.98691	2.1
29026	1.12411	2.1
29027	1.12411	2.1
29028	2.01602	2.1
29029	2.01602	2.1
29030	2.49093	2.1
29031	1.98691	2.1
29032	2.01601	2.1
29033	2.51651	2.1
29034	4.50694	2.1
29035	1.93147	0.0977269
29036	2.51593	0.054292
29037	1.35146	0.087235
29038	1.35146	0.0705635
29039	2.48665	0.0789223
29040	1.90664	0.0638532
29041	1.63979	2.1
29042	1.7457	2.1
29043	1.66046	2.1
29044	1.56558	2.1
29045	3.31128	2.1
29046	0.819895	2.1
29047	0.782792	2.1
29048	0.60239	2.1
29049	0.54831	2.1
29050	0.688347	2.1
29051	0.688347	2.1
29052	0.679857	2.1
29053	0.679857	2.1
29054	0.782792	2.1
29055	0.54831	2.1

29056	0.60239	2.1
29057	0.819895	2.1
29058	1.66046	2.1
29059	1.42485	2.1
29060	1.56558	2.1
29061	1.56558	2.1
29062	1.52995	2.1
29063	1.52995	2.1
29064	2.58101	2.07746
29065	2.09803	2.1
29066	2.408	2.02274
29067	1.86893	2.1
29068	4.44994	2.1
29069	2.36062	2.1
29070	1.25104	2.1
29071	1.25104	2.1
29072	1.39703	2.1
29073	1.86893	2.1
29074	2.01492	2.1
29075	2.408	1.97991
29076	1.86893	2.1
29077	2.22575	2.09669
29078	1.70677	2.1
29079	4.09468	2.1
29080	0.737605	2.1
29081	0.737605	2.1
29082	0.741302	2.1
29083	0.741302	2.1
29084	0.55189	2.1
29085	0.625517	2.1
29086	0.934468	2.1
29087	0.853389	2.1
29088	0.853389	2.1
29089	0.55189	2.1
29090	0.625517	2.1
29091	0.934467	2.1
29092	2.35053	2.1
29093	1.09615	2.1
29094	1.25998	2.1
29095	2.51216	2.1
29096	3.61051	2.1
29097	1.33636	2.1
29098	1.25609	2.1
29099	0.698455	2.1
29100	0.629995	2.1
29101	0.986092	2.1
29102	0.986092	2.1
29103	0.980263	2.1
29104	0.980263	2.1
29105	1.33636	2.1
29106	0.698455	2.1
29107	0.629995	2.1
29108	1.25609	2.1
29109	1.89226	1.17323
29110	1.89226	1.19905
29111	1.89226	1.41639
29112	1.89226	1.18066
29113	3.78452	1.28314

29114	1.74321	0.935433
29115	1.89226	0.889793
29116	1.91755	0.990148
29117	1.91755	1.01957
29118	1.89226	0.94616
29119	1.89226	1.00342
29120	0.946132	0.849717
29121	0.946132	0.963534
29122	0.949798	0.658958
29123	1.08054	0.752131
29124	1.08054	0.726348
29125	1.08054	0.751084
29126	0.946132	0.965491
29127	0.946132	0.736246
29128	0.946132	0.763596
29129	0.949798	0.645007
29130	1.08054	0.743106
29131	0.946131	0.848513
29132	1.89226	1.34632
29133	1.89226	0.958939
29134	1.89226	1.0511
29135	1.89226	1.02929
29136	1.89226	1.1402
29137	1.89226	1.12051
29138	2.39378	0.0598682
29139	2.29234	0.05
29140	2.29234	0.05
29141	2.7933	0.05
29142	1.18422	0.05
29143	1.68518	0.05
29144	2.79329	0.05
29145	1.89226	0.05
29146	2.29233	0.05
29147	1.89226	0.05
29148	4.18459	0.05
29149	0.857046	0.05
29150	0.857037	0.05
29151	0.94613	0.05
29152	0.94613	0.05
29153	0.851539	0.05
29154	0.851539	0.05
29155	1.0315	0.05
29156	0.952867	0.05
29157	0.851486	0.05
29158	0.952869	0.05
29159	1.03156	0.05
29160	0.851528	0.05
29161	1.61981	2.1
29162	1.93096	2.1
29163	2.00776	2.1
29164	1.62218	2.1
29165	3.62757	2.1
29166	2.00776	2.1
29167	1.68541	2.1
29168	1.83215	2.1
29169	1.62218	2.1
29170	2.21773	2.1
29171	2.00776	2.1

29172	1.89188	0.05
29173	1.70303	0.05
29174	1.89226	0.05
29175	1.72465	0.05
29176	1.89226	0.05
29177	1.70552	0.05
29178	2.48659	0.0505706
29179	2.0815	0.0616714
29180	1.89226	0.0674151
29181	2.08111	0.0519472
29182	1.89226	0.0678795
29183	2.08111	0.0563304
29184	2.35332	0.261535
29185	1.83284	0.138932
29186	2.28483	0.175582
29187	2.71955	0.157712
29188	4.63815	0.154614
29189	1.89227	2.1
29190	1.89227	2.1
29191	1.89227	2.1
29192	1.89227	2.1
29193	3.78453	2.1
29194	1.33506	2.1
29195	1.17129	2.1
29196	1.33506	2.1
29197	1.38415	2.1
29198	1.13664	2.1
29199	1.18573	2.1
29200	1.89226	2.1
29201	2.68785	2.1
29202	1.89226	2.1
29203	2.6692	2.1
29204	4.58011	2.1
29205	1.89226	2.1
29206	1.89226	2.1
29207	1.89226	2.1
29208	1.89226	2.1
29209	3.78453	2.1
29210	1.33506	2.1
29211	1.13664	2.1
29212	1.27575	2.1
29213	1.09627	2.1
29214	2.43133	2.1
29215	1.89226	2.1
29216	2.65349	2.1
29217	2.66919	2.1
29218	2.66919	2.1
29219	1.89226	2.1
29220	1.89226	2.1
29221	1.89226	2.1
29222	1.89226	2.1
29223	1.89226	2.1
29224	1.89226	2.1
29225	3.78453	2.1
29226	1.16834	2.1
29227	1.16834	2.1
29228	0.946132	2.1
29229	0.946132	2.1

29230	1.26978	2.1
29231	1.16834	2.1
29232	0.94613	2.1
29233	0.94613	2.1
29234	0.94613	2.1
29235	1.26978	2.1
29236	0.94613	2.1
29237	1.16834	2.1
29238	2.14698	2.1
29239	1.89226	2.1
29240	1.89226	2.1
29241	1.89226	2.1
29242	2.40719	2.1
29243	2.40719	2.1
29244	1.89226	2.1
29245	1.89226	2.1
29246	1.89226	2.1
29247	1.89226	2.1
29248	3.78453	2.1
29249	1.26977	2.1
29250	1.26977	2.1
29251	0.946133	2.1
29252	0.946133	2.1
29253	1.37467	2.1
29254	1.26978	2.1
29255	0.946131	2.1
29256	0.946131	2.1
29257	1.37467	2.1
29258	0.94613	2.1
29259	0.946131	2.1
29260	1.26978	2.1
29261	1.2036	2.1
29262	1.34912	2.1
29263	0.946131	2.1
29264	0.946131	2.1
29265	1.34912	2.1
29266	1.34912	2.1
29267	0.946129	2.1
29268	0.946129	2.1
29269	1.34912	2.1
29270	0.94613	2.1
29271	0.946131	2.1
29272	1.2036	2.1
29273	2.47164	0.393719
29274	1.89019	0.244521
29275	1.79109	0.36711
29276	2.41632	0.244534
29277	4.26273	0.368741
29278	1.54197	0.304777
29279	2.41632	0.196129
29280	1.79109	0.25025
29281	1.79109	0.308442
29282	2.20929	0.215994
29283	2.20929	0.288622
29284	1.70302	0.911217
29285	1.48104	1.11271
29286	1.99365	1.27145
29287	1.32897	0.811914

29288	1.70302	1.3108
29289	1.14034	0.858966
29290	1.70302	0.928552
29291	1.99365	0.917528
29292	1.99297	1.18716
29293	2.36337	0.656163
29294	4.04804	0.855606
29295	1.51569	0.729934
29296	2.49093	0.662576
29297	1.6026	0.794334
29298	1.43229	0.564502
29299	2.0815	0.880943
29300	1.83402	0.616844
29301	2.54665	2.1
29302	2.34044	2.1
29303	2.08889	2.1
29304	2.37653	2.1
29305	4.71697	2.1
29306	2.54665	2.1
29307	2.45212	2.1
29308	2.31012	2.1
29309	2.37653	2.1
29310	4.82865	2.1
29311	2.08889	2.1
29312	2.37653	2.1
29313	2.23366	2.1
29314	1.87598	2.1
29315	4.25251	2.1
29316	2.31012	2.1
29317	2.37653	2.1
29318	2.23367	2.1
29319	2.17384	2.1
29320	4.55037	2.1
29321	0.946131	2.1
29322	0.946131	2.1
29323	1.30338	2.1
29324	1.23186	2.1
29325	0.94613	2.1
29326	0.94613	2.1
29327	1.23187	2.1
29328	1.23187	2.1
29329	0.946131	2.1
29330	1.23186	2.1
29331	1.30338	2.1
29332	0.946131	2.1
29333	1.89226	2.1
29334	2.46373	2.1
29335	2.34725	2.1
29336	2.46373	2.1
29337	1.77579	2.1
29338	1.89226	2.1
29339	2.61901	2.1
29340	2.45899	2.1
29341	2.64067	2.1
29342	2.52708	2.1
29343	5.14609	2.1
29344	2.41795	2.1
29345	2.0815	2.1

29346	2.52708	2.1
29347	1.92277	2.1
29348	4.31368	2.1
29349	2.64067	2.1
29350	2.52708	2.1
29351	2.58638	2.1
29352	2.51039	2.1
29353	5.11346	2.1
29354	2.52708	2.1
29355	2.13642	2.1
29356	2.51039	2.1
29357	2.16633	2.1
29358	4.69341	2.1
29359	1.61028	1.65051
29360	1.65712	0.222483
29361	1.35002	0.174153
29362	1.35002	1.82084
29363	1.88637	0.202152
29364	1.88637	2.02094
29365	2.57663	2.1
29366	2.24227	2.1
29367	2.48354	2.1
29368	2.14317	2.1
29369	4.7198	2.1
29370	2.24227	2.1
29371	1.77353	2.1
29372	2.14317	2.1
29373	1.69956	2.1
29374	3.9167	2.1
29375	2.48353	2.1
29376	2.14317	2.1
29377	2.39282	2.1
29378	1.79824	2.1
29379	4.53599	2.1
29380	2.14317	2.1
29381	1.69956	2.1
29382	1.91427	2.1
29383	1.6347	2.1
29384	3.77787	2.1
29385	1.89226	0.226187
29386	1.89226	0.189208
29387	1.89226	0.0986311
29388	1.89226	0.12776
29389	3.78453	0.128829
29390	1.89226	0.153281
29391	1.89226	0.162316
29392	1.89226	0.134495
29393	1.89226	0.128339
29394	3.78452	0.158853
29395	1.89226	0.170969
29396	1.89652	0.224623
29397	1.89226	0.197243
29398	1.89226	0.828837
29399	1.19352	0.128662
29400	1.19352	0.274133
29401	1.89226	0.2047
29402	1.89653	0.174951
29403	1.89226	0.582513

29404	1.89226	1.05487
29405	1.89226	0.129871
29406	1.89226	0.136768
29407	1.89226	0.194287
29408	1.75326	0.202276
29409	1.89226	0.595401
29410	1.12948	0.204626
29411	1.89226	0.0903581
29412	1.12948	0.0953322
29413	2.61064	2.1
29414	1.89226	2.1
29415	1.89226	2.1
29416	1.89226	2.1
29417	2.6085	2.1
29418	2.6085	2.1
29419	2.6085	2.1
29420	1.89226	2.1
29421	1.89226	2.1
29422	1.89226	2.1
29423	2.62161	2.1
29424	2.62161	2.1
29425	2.16535	2.1
29426	1.44603	2.1
29427	2.79784	2.1
29428	1.84756	2.1
29429	4.01292	2.1
29430	2.65262	2.1
29431	1.03391	2.1
29432	2.20239	2.1
29433	1.74416	2.1
29434	1.49213	2.1
29435	1.03391	2.1
29436	2.29066	2.1
29437	2.49174	2.1
29438	2.56502	2.1
29439	2.33802	2.1
29440	4.85568	2.1
29441	2.39663	2.1
29442	2.49174	2.1
29443	2.56502	2.1
29444	2.45929	2.1
29445	4.96165	2.1
29446	2.56502	2.1
29447	2.33802	2.1
29448	2.37168	2.1
29449	2.59967	2.1
29450	4.9367	2.1
29451	2.56502	2.1
29452	2.45929	2.1
29453	2.50138	2.1
29454	2.59967	2.1
29455	5.0664	2.1
29456	2.27009	1.52491
29457	1.26247	2.06142
29458	1.86812	2.023
29459	2.837	1.77507
29460	4.13822	1.95262
29461	1.89226	2.1

29462	1.89226	2.1
29463	1.89226	2.1
29464	1.89226	2.1
29465	3.78453	2.1
29466	1.75835	2.1
29467	1.72866	2.1
29468	1.65313	2.1
29469	1.64822	2.1
29470	3.3818	2.1
29471	1.72866	2.1
29472	1.58401	2.1
29473	1.64822	2.1
29474	1.52441	2.1
29475	3.25307	2.1
29476	1.89226	2.1
29477	1.89226	2.1
29478	1.89226	2.1
29479	1.89226	2.1
29480	3.78452	2.1
29481	1.89226	2.1
29482	1.89226	2.1
29483	1.89226	2.1
29484	1.89226	2.1
29485	3.78453	2.1
29486	1.65313	2.1
29487	1.64822	2.1
29488	1.55779	2.1
29489	1.57968	2.1
29490	3.23281	2.1
29491	1.64822	2.1
29492	1.52441	2.1
29493	1.57967	2.1
29494	1.474 2.1	
29495	3.10408	2.1
29496	1.89226	2.1
29497	1.89226	2.1
29498	1.89226	2.1
29499	1.89226	2.1
29500	3.78452	2.1
29501	1.86067	1.32899
29502	1.58407	2.0923
29503	2.07965	0.321909
29504	1.83123	1.58965
29505	3.66372	0.953042
29506	1.89226	1.07804
29507	1.89226	2.09316
29508	1.89226	0.471038
29509	1.89226	2.04894
29510	3.78452	1.5335
29511	1.89226	2.03197
29512	1.89226	2.09936
29513	1.89226	1.67013
29514	1.89226	2.1
29515	1.89226	1.61926
29516	1.89226	2.1
29517	1.83123	0.689963
29518	1.03472	2.0997
29519	1.91068	1.95615

29520	1.58406	1.74818
29521	1.36133	1.89973
29522	1.03472	1.76634
29523	2.07965	0.610058
29524	1.83123	1.83365
29525	2.28385	0.775569
29526	2.09114	1.66366
29527	4.1708	1.20967
29528	1.89226	0.989949
29529	1.89226	2.05315
29530	1.89226	0.697761
29531	1.89226	1.65256
29532	3.78452	1.33915
29533	1.89226	1.95617
29534	1.89226	2.09709
29535	1.89226	1.08634
29536	1.89226	2.1
29537	3.78452	2.04119
29538	1.83123	0.653333
29539	1.36133	2.09633
29540	2.09114	0.963075
29541	1.69436	1.74451
29542	3.45248	1.63607
29543	1.89226	2.1
29544	1.89226	2.1
29545	1.89226	2.1
29546	1.89226	2.1
29547	3.78453	2.1
29548	1.53278	2.1
29549	1.67571	2.1
29550	1.40182	2.1
29551	1.55686	2.1
29552	3.07752	2.1
29553	1.67571	2.1
29554	1.52615	2.08158
29555	1.55686	2.1
29556	1.38963	1.90178
29557	3.06534	2.1
29558	1.89226	2.1
29559	1.89226	2.01414
29560	1.89226	2.1
29561	1.89226	1.9142
29562	3.78452	2.1
29563	1.89226	2.1
29564	1.89226	2.1
29565	1.89226	2.1
29566	1.89226	2.1
29567	3.78453	2.1
29568	1.40182	2.1
29569	1.55686	2.1
29570	1.24509	2.1
29571	1.38426	2.1
29572	2.78608	2.1
29573	1.55686	2.1
29574	1.38963	2.0837
29575	1.2064	2.1
29576	1.2064	2.1
29577	1.38426	2.1

29578	1.56749	2.1
29579	0.810946	2.1
29580	0.810946	2.07953
29581	0.810945	2.1
29582	0.810945	2.1
29583	0.810945	2.1
29584	0.810945	2.1
29585	1.89226	2.08884
29586	1.35169	2.1
29587	1.08132	2.1
29588	1.08132	2.04254
29589	1.08132	2.1
29590	1.28418	0.509744
29591	1.59382	0.720892
29592	1.78289	0.92615
29593	1.51021	0.491515
29594	3.10403	0.597903
29595	1.76897	1.96677
29596	1.59382	0.731173
29597	1.78289	1.09525
29598	1.94302	1.51526
29599	3.53684	1.51734
29600	1.89226	0.718451
29601	1.89226	1.16073
29602	1.89226	1.4296
29603	1.89226	0.681696
29604	3.78453	0.879249
29605	1.89227	0.71502
29606	1.89227	0.3917
29607	1.89227	0.41223
29608	1.89227	0.558645
29609	1.89227	0.475428
29610	1.89227	0.612187
29611	1.78286	0.748615
29612	1.51019	0.399474
29613	1.79979	0.445355
29614	2.02511	0.578947
29615	3.5353	0.540206
29616	1.78286	0.872179
29617	1.94299	1.24425
29618	2.17831	1.74577
29619	2.02511	0.662519
29620	3.9681	0.825348
29621	1.89224	1.1723
29622	1.89224	0.534524
29623	1.89224	0.631078
29624	1.89224	0.996498
29625	3.78448	0.87217
29626	1.89224	0.558628
29627	1.89224	0.351222
29628	1.89224	0.392199
29629	1.89224	0.552047
29630	1.89224	0.380849
29631	1.89224	0.548149
29632	1.83198	0.233803
29633	1.0961	0.0917649
29634	1.39448	0.150091
29635	1.99568	0.197927

29636	3.09178	0.17199
29637	1.25503	0.0935648
29638	1.99568	0.167367
29639	2.3661	0.237435
29640	1.70302	0.115058
29641	3.55833	0.118831
29642	2.69899	0.05
29643	1.85796	0.05
29644	2.11407	0.05
29645	1.36769	0.05
29646	3.97203	0.05
29647	1.89226	0.0623302
29648	1.89226	0.0503306
29649	1.89226	0.05
29650	1.89226	0.05
29651	3.78452	0.0606813
29652	1.89225	0.05
29653	2.63355	0.05
29654	2.09529	0.05
29655	2.09529	0.05
29656	1.89225	0.05
29657	1.89225	0.05
29658	1.89226	0.0691056
29659	1.89226	0.0825876
29660	1.89226	0.0573297
29661	1.89226	0.105842
29662	1.89226	0.057312
29663	1.89226	0.101
29664	2.12204	0.05
29665	1.89226	0.0781016
29666	2.05798	0.05
29667	2.01954	0.05
29668	1.70302	0.0538531
29669	1.3865	0.0611906
29670	1.28326	0.870205
29671	1.33326	2.1
29672	1.19052	1.18283
29673	1.25369	2.0981
29674	2.53694	1.9921
29675	1.33327	2.1
29676	1.27339	2.1
29677	1.25369	2.09964
29678	1.19733	2.1
29679	2.52708	2.1
29680	1.19052	1.09313
29681	1.25369	2.1
29682	1.10938	0.934721
29683	1.17614	2.1
29684	2.36307	1.93828
29685	1.25369	2.1
29686	1.19733	2.1
29687	1.17614	2.1
29688	1.11868	2.1
29689	2.37237	2.1
29690	1.10747	0.073952
29691	1.47688	0.0999044
29692	1.97732	0.05
29693	1.62206	0.0601671

29694	3.09894	0.0780906
29695	1.89226	0.105496
29696	1.89226	0.0869143
29697	1.89226	0.0933853
29698	1.89226	0.1306
29699	1.89226	0.0588916
29700	1.89226	0.0558654
29701	1.62206	0.0794218
29702	1.70302	0.074304
29703	1.22962	0.0843372
29704	1.1893	0.105441
29705	2.15177	0.0663927
29706	2.08121	0.070108
29707	1.97732	0.0658447
29708	2.08121	0.0649627
29709	1.62206	0.0751856
29710	1.62206	0.116352
29711	2.41464	0.0529137
29712	2.41464	0.0726012
29713	1.89226	0.111767
29714	1.89226	0.146602
29715	1.89226	0.0644915
29716	1.89226	0.0700738
29717	3.78452	0.0739173
29718	0.946133	0.0885992
29719	0.946133	0.0929449
29720	0.946132	0.109591
29721	0.946132	0.110177
29722	0.946132	0.100586
29723	0.946132	0.116255
29724	0.946133	0.0712913
29725	0.946133	0.0916838
29726	0.946132	0.0694164
29727	0.946132	0.0897478
29728	0.946132	0.117939
29729	0.946132	0.0903702
29730	1.83157	1.7003
29731	2.19989	1.51914
29732	2.20664	0.64515
29733	1.81487	0.667483
29734	4.03146	1.29821
29735	2.41055	2.1
29736	2.19989	2.1
29737	2.20665	2.07563
29738	2.38023	1.98015
29739	4.61044	2.0839
29740	2.19989	2.1
29741	2.22558	2.1
29742	2.38023	2.07316
29743	2.07065	0.973283
29744	4.42547	2.09969
29745	2.19988	1.85933
29746	1.70303	1.08931
29747	1.89939	0.769832
29748	1.86357	0.524681
29749	3.78536	0.878139
29750	1.04921	2.1
29751	1.30711	2.1

29752	1.53076	2.1
29753	1.22927	2.1
29754	2.57997	2.1
29755	1.53076	2.1
29756	1.46568	2.1
29757	1.56056	2.1
29758	1.22927	2.1
29759	1.86206	2.1
29760	1.53076	2.1
29761	1.18353	2.1
29762	2.28593	2.1
29763	1.16979	2.1
29764	2.2731	2.1
29765	3.45571	2.1
29766	0.854776	2.1
29767	0.854776	2.1
29768	0.855641	2.1
29769	0.855641	2.1
29770	1.12552	2.1
29771	1.13655	2.1
29772	0.57216	2.1
29773	0.584893	2.1
29774	0.57216	2.1
29775	1.12552	2.1
29776	0.584893	2.1
29777	1.13655	2.1
29778	1.54462	2.1
29779	2.76309	2.1
29780	1.34869	2.1
29781	2.48573	2.1
29782	4.03035	2.1
29783	1.35328	0.802464
29784	1.0383	0.769947
29785	1.03318	1.21855
29786	0.704834	1.32794
29787	1.0257	1.24204
29788	1.0257	0.736727
29789	1.03241	0.6586
29790	1.03241	1.31271
29791	1.35328	1.13856
29792	1.03319	1.26588
29793	1.0383	1.16738
29794	0.704835	1.18259
29795	2.27126	0.654848
29796	2.77291	2.1
29797	1.81771	1.83882
29798	2.6111	1.90421
29799	4.88236	1.92991
29800	2.77291	2.1
29801	1.89227	2.1
29802	1.89227	2.01305
29803	1.89227	2.1
29804	2.6111	1.95664
29805	2.6111	2.1
29806	2.22061	1.80003
29807	2.41522	1.96229
29808	2.41522	1.17604
29809	2.6111	2.1

29810	1.75765	1.12751
29811	1.95353	2.1
29812	2.27502	0.497907
29813	1.89227	1.39099
29814	1.89227	0.651919
29815	2.8189	0.480859
29816	4.71116	0.487914
29817	1.89226	0.565623
29818	1.89226	1.55063
29819	1.89226	0.980598
29820	1.89226	0.781011
29821	1.89226	1.10111
29822	1.89226	0.854992
29823	1.89226	1.77009
29824	1.89226	1.64054
29825	1.89226	1.63797
29826	1.89226	1.23172
29827	3.78453	1.69396
29828	1.89226	1.76321
29829	2.57079	0.839899
29830	2.5932	1.48205
29831	1.89226	1.26415
29832	4.48546	1.33704
29833	0.589396	1.34266
29834	0.641454	0.97242
29835	0.814062	1.57572
29836	0.726518	1.33964
29837	0.675126	1.50039
29838	0.675125	1.40554
29839	0.692847	1.57024
29840	0.692847	1.37382
29841	0.641454	1.12589
29842	0.814062	1.46322
29843	0.726518	1.60544
29844	0.589396	1.39864
29845	1.89226	1.72835
29846	1.89226	1.23817
29847	1.89226	1.65367
29848	1.89226	1.75647
29849	1.89226	1.51038
29850	1.89226	1.74214
29851	1.16906	0.617398
29852	1.2966	1.3802
29853	0.946132	1.24776
29854	0.946132	1.81645
29855	1.16906	1.21294
29856	1.16906	0.708464
29857	0.946132	1.45961
29858	0.946132	1.64414
29859	1.16906	1.41614
29860	0.946131	1.38862
29861	1.2966	1.38885
29862	0.946132	1.6301
29863	0.641455	1.32694
29864	0.567191	0.973184
29865	0.807594	1.65797
29866	0.814062	1.3809
29867	0.673735	1.6108

29868	0.673734	1.22171
29869	0.707519	1.35819
29870	0.707519	1.68115
29871	0.807594	1.50264
29872	0.56719	1.64974
29873	0.641455	1.55525
29874	0.814063	1.77711
29875	1.89225	2.1
29876	1.89225	2.1
29877	1.89225	2.1
29878	1.89225	2.1
29879	3.7845	2.1
29880	1.89225	2.1
29881	1.89225	2.1
29882	1.89225	2.1
29883	1.89225	2.1
29884	1.89225	2.1
29885	1.89225	2.1
29886	1.89226	2.1
29887	1.89226	2.1
29888	1.89226	2.1
29889	1.89226	2.1
29890	3.78452	2.1
29891	1.89226	2.1
29892	1.89226	2.1
29893	1.89226	2.1
29894	1.89226	2.1
29895	1.89226	2.1
29896	1.89226	2.1
29897	1.89226	2.1
29898	1.89226	2.1
29899	1.89226	2.1
29900	1.89226	2.1
29901	3.78452	2.1
29902	1.89226	2.1
29903	1.38928	2.1
29904	1.89226	2.1
29905	1.51378	2.1
29906	1.89061	2.1
29907	1.89226	2.1
29908	1.89226	2.1
29909	1.89226	2.08824
29910	1.89226	2.08737
29911	1.89226	2.1
29912	3.78453	2.1
29913	1.04572	2.1
29914	0.994213	2.1
29915	0.912466	2.09351
29916	0.912466	2.09924
29917	0.947743	2.09527
29918	1.00388	2.1
29919	0.900913	2.08777
29920	0.900913	2.1
29921	0.900915	2.1
29922	0.946131	2.1
29923	0.946131	2.1
29924	0.999196	2.1
29925	1.89226	2.06362

29926	1.89226	2.1
29927	1.89226	2.1
29928	1.89226	2.07876
29929	3.78453	2.09601
29930	0.946161	2.1
29931	1.1354	2.1
29932	0.850847	2.1
29933	0.850847	2.1
29934	1.01445	2.1
29935	1.02106	2.1
29936	0.946131	2.1
29937	0.946131	2.1
29938	1.13537	2.1
29939	1.01444	2.1
29940	0.850877	2.1
29941	0.946131	2.1
29942	1.80183	2.1
29943	1.89226	2.08422
29944	1.89226	2.08016
29945	1.89226	2.1
29946	1.79606	2.08135
29947	1.79606	2.1
29948	1.24001	2.0256
29949	1.41822	0.703226
29950	1.95665	0.252389
29951	1.77342	1.6052
29952	3.19666	0.660994
29953	1.95665	1.23217
29954	1.44859	1.78015
29955	1.41821	1.89102
29956	1.41821	1.90539
29957	1.95543	1.42446
29958	1.95543	0.952943
29959	1.95665	0.268674
29960	1.77342	1.474
29961	2.33611	1.43408
29962	2.49694	0.425313
29963	4.29276	1.6853
29964	1.95665	1.60459
29965	1.95544	0.507269
29966	2.4728	0.127763
29967	2.49693	1.55017
29968	4.42945	0.298209
29969	2.24937	2.1
29970	2.49053	2.1
29971	2.16323	2.1
29972	2.36377	2.1
29973	4.61314	2.1
29974	2.49053	2.1
29975	2.58639	2.1
29976	2.36377	2.1
29977	2.47395	2.1
29978	4.95016	2.1
29979	2.45054	2.1
29980	2.36377	2.1
29981	1.92911	2.1
29982	2.181 2.1	
29983	4.29288	2.1

29984	2.36377	2.1
29985	2.47395	2.1
29986	2.181 2.1	
29987	2.29881	2.1
29988	4.66258	2.1
29989	1.57885	2.1
29990	1.13339	2.1
29991	1.48594	2.1
29992	1.13322	2.1
29993	2.71207	2.1
29994	1.48596	2.1
29995	1.20761	2.1
29996	1.20761	2.1
29997	1.13323	2.1
29998	1.46568	2.1
29999	1.3913	2.1
30000	1.89226	0.05
30001	1.89226	0.152903
30002	1.89226	0.100407
30003	1.89226	0.393085
30004	1.89226	0.0554397
30005	1.89226	0.122413
30006	1.11712	0.100867
30007	0.946129	0.395626
30008	0.686934	0.05
30009	0.946133	0.05
30010	0.9461	0.05
30011	1.10729	0.635994
30012	0.686963	0.176323
30013	0.686963	0.05
30014	1.10945	0.0965129
30015	0.80397	0.441582
30016	0.946131	0.05
30017	0.946131	0.0767322
30018	1.89226	2.1
30019	1.89226	1.99686
30020	1.89226	2.07963
30021	1.89226	2.09563
30022	3.78452	2.0907
30023	1.22615	2.1
30024	1.3439	2.1
30025	1.47868	2.1
30026	1.55998	2.09799
30027	2.78613	2.1
30028	1.04973	2.1
30029	1.47868	2.1
30030	1.20149	2.1
30031	1.61721	2.1
30032	2.68017	2.1
30033	1.89226	2.1
30034	1.89226	2.1
30035	1.89226	2.1
30036	1.89226	2.06627
30037	3.78452	2.1
30038	0.946133	1.74216
30039	0.946133	2.09976
30040	0.946128	2.1
30041	0.946128	2.1

30042	0.946131	1.62867
30043	0.946131	2.09112
30044	0.946131	2.1
30045	0.946131	2.05416
30046	0.94613	2.1
30047	0.94613	2.09411
30048	0.946131	2.1
30049	0.946131	2.09882
30050	0.798126	2.1
30051	0.798126	2.1
30052	0.805719	2.1
30053	0.805719	2.1
30054	0.779992	2.1
30055	0.864507	2.1
30056	0.739338	2.1
30057	0.808607	2.1
30058	0.808606	2.1
30059	0.864506	2.1
30060	0.739339	2.1
30061	0.779993	2.1
30062	0.946128	2.1
30063	0.946128	2.1
30064	1.18959	2.1
30065	1.00304	2.1
30066	1.00304	2.1
30067	1.00304	2.1
30068	0.94613	2.1
30069	0.94613	2.1
30070	0.94613	2.1
30071	1.18959	2.1
30072	1.00304	2.1
30073	0.94613	2.1
30074	2.19163	2.1
30075	1.89226	2.1
30076	2.16541	2.1
30077	1.89226	2.1
30078	4.08389	2.1
30079	1.89226	2.1
30080	1.89226	1.9592
30081	1.89226	2.1
30082	1.89226	1.25006
30083	3.78452	2.08824
30084	0.964782	2.1
30085	1.30151	1.05387
30086	1.30318	0.964726
30087	1.30318	2.1
30088	0.949134	1.32841
30089	0.949134	2.1
30090	2.15005	2.1
30091	1.89226	2.1
30092	2.16541	2.1
30093	2.16541	2.1
30094	1.89226	2.1
30095	1.89226	2.1
30096	1.89226	2.1
30097	1.89226	2.04843
30098	1.89226	2.1
30099	1.89226	0.576965

30100	3.78452	1.91917
30101	0.949134	2.1
30102	1.30151	2.03835
30103	0.949134	2.1
30104	1.32143	1.10744
30105	2.25064	2.1
30106	0.982859	0.233522
30107	1.28326	0.840305
30108	1.19052	0.255985
30109	1.19052	1.12311
30110	1.10476	0.239764
30111	1.10476	1.14891
30112	1.37265	0.687566
30113	1.38247	1.61823
30114	1.36636	1.73578
30115	1.3294	0.735891
30116	2.71187	1.22605
30117	1.89226	1.70854
30118	1.89226	0.78792
30119	1.89226	0.812724
30120	1.89226	1.62806
30121	1.89226	0.805752
30122	1.89226	1.61932
30123	1.36636	1.89161
30124	1.3294	0.943988
30125	1.30001	1.01171
30126	1.36416	1.82527
30127	2.69356	1.52227
30128	1.89226	1.86971
30129	1.89226	0.863324
30130	1.89226	1.0157
30131	1.89226	1.90496
30132	1.89226	0.942149
30133	1.89226	1.8053
30134	2.83701	0.313519
30135	1.89226	0.840579
30136	1.89226	1.01861
30137	2.79312	0.303798
30138	4.68539	0.65948
30139	1.44288	2.1
30140	0.948677	2.1
30141	1.84076	2.1
30142	1.42884	2.1
30143	2.78943	2.1
30144	1.89226	2.1
30145	1.89226	2.1
30146	1.89226	2.1
30147	1.89226	2.1
30148	3.78452	2.1
30149	0.909402	2.1
30150	0.909402	2.1
30151	1.3063	2.1
30152	1.56757	2.1
30153	1.00038	2.1
30154	1.5235	2.1
30155	0.946127	2.1
30156	0.946127	2.1
30157	0.946128	2.1

30158	1.26957	2.1
30159	1.00038	2.1
30160	0.946129	2.1
30161	1.17256	2.1
30162	2.2846	2.1
30163	1.1844	2.1
30164	2.28904	2.1
30165	3.469	2.1
30166	0.867886	2.1
30167	0.867886	2.1
30168	0.867277	2.1
30169	0.867277	2.1
30170	1.14296	2.1
30171	1.14452	2.1
30172	0.591766	2.1
30173	0.592199	2.1
30174	0.591766	2.1
30175	1.14296	2.1
30176	0.5922	2.1
30177	1.14452	2.1
30178	1.27408	0.0604207
30179	1.56339	0.13131
30180	1.52522	0.0523069
30181	1.34447	0.05
30182	2.90786	0.05
30183	1.27408	0.10207
30184	1.01121	0.0718269
30185	0.991868	0.05
30186	1.34447	0.0578645
30187	2.35569	0.0647476
30188	0.944818	0.0718331
30189	0.883008	0.0730821
30190	0.66957	0.05
30191	0.616883	0.05
30192	0.755679	0.05
30193	0.755679	0.15282
30194	0.791912	0.0744291
30195	0.701943	0.05
30196	0.901541	0.0573023
30197	0.634847	0.0675373
30198	0.82781	0.062337
30199	0.655405	0.0905916
30200	1.73819	2.1
30201	1.70303	2.1
30202	1.70303	2.1
30203	1.70303	2.1
30204	1.94516	2.1
30205	1.94517	2.1
30206	0.94994	2.1
30207	2.07469	2.1
30208	1.08506	2.1
30209	2.0815	2.1
30210	3.12902	2.1
30211	0.946131	2.1
30212	0.946131	2.1
30213	0.946131	2.1
30214	0.946131	2.1
30215	0.946128	2.1

30216	0.946128	2.1
30217	0.946133	2.1
30218	0.946133	2.1
30219	0.946131	2.1
30220	0.946131	2.1
30221	0.94613	2.1
30222	0.94613	2.1
30223	2.51796	2.1
30224	1.64193	2.1
30225	2.51172	2.1
30226	1.68893	2.1
30227	4.20689	2.1
30228	2.51172	2.1
30229	1.70002	2.1
30230	1.70002	2.1
30231	1.68893	2.1
30232	2.48689	2.1
30233	2.4758	2.1
30234	2.27963	0.05
30235	2.21582	0.05
30236	2.60486	0.05
30237	2.5105	0.05
30238	4.79013	0.05
30239	2.21582	0.05
30240	2.02069	0.05
30241	2.5105	0.05
30242	2.28615	0.05
30243	4.53119	0.05
30244	2.60486	0.05
30245	2.5105	0.05
30246	2.55791	0.05
30247	2.47141	0.05
30248	5.06842	0.05
30249	2.5105	0.05
30250	2.28615	0.05
30251	2.47141	0.05
30252	2.23337	0.05
30253	4.74387	0.05
30254	2.06984	2.1
30255	0.967499	2.1
30256	2.12435	2.1
30257	2.0021	2.1
30258	1.08975	2.1
30259	0.967499	2.1
30260	1.89226	0.05
30261	1.89226	0.05
30262	1.89226	0.05
30263	1.89226	0.05
30264	1.89226	0.05
30265	1.89226	0.05
30266	1.89226	2.1
30267	1.89226	2.1
30268	1.89226	2.1
30269	1.89226	2.1
30270	3.78452	2.1
30271	1.89227	2.1
30272	1.89227	2.1
30273	1.89227	2.1

30274	1.89227	2.1
30275	3.78453	2.1
30276	0.55882	2.1
30277	0.696462	2.1
30278	1.07003	2.1
30279	0.937179	2.1
30280	0.818077	2.1
30281	0.818077	2.1
30282	0.815565	2.1
30283	0.815565	2.1
30284	0.696462	2.1
30285	1.07003	2.1
30286	0.93718	2.1
30287	0.55882	2.1
30288	2.53857	2.1
30289	1.87436	2.1
30290	2.27541	2.1
30291	2.27541	2.1
30292	2.14005	2.1
30293	2.14005	2.1
30294	1.89226	2.1
30295	1.89226	2.1
30296	1.89226	2.1
30297	1.89226	2.1
30298	3.78452	2.1
30299	1.89227	2.1
30300	1.89227	2.1
30301	1.89227	2.1
30302	1.89227	2.1
30303	3.78453	2.1
30304	1.39292	2.1
30305	2.14006	2.1
30306	2.41083	2.1
30307	1.68761	2.1
30308	3.80376	2.1
30309	2.53857	2.1
30310	2.14005	2.1
30311	2.41083	2.1
30312	2.82043	2.1
30313	4.9494	2.1
30314	1.90439	0.171199
30315	1.51835	0.165998
30316	1.20473	0.142628
30317	0.947693	0.192384
30318	2.85208	0.171128
30319	2.586	2.1
30320	1.46262	2.1
30321	2.14009	2.1
30322	2.78101	2.1
30323	4.72609	2.1
30324	1.89224	2.1
30325	2.78101	2.1
30326	2.586	2.1
30327	2.586	2.1
30328	1.89224	2.1
30329	1.89224	2.1
30330	0.994293	2.1
30331	1.19	2.1

30332	1.09483	2.1
30333	1.09483	2.1
30334	1.06827	2.1
30335	1.06827	2.1
30336	1.89226	2.1
30337	1.89226	2.1
30338	1.89226	2.1
30339	1.89226	2.1
30340	3.78452	2.1
30341	0.946132	2.1
30342	0.946132	2.1
30343	1.31912	2.1
30344	1.3572	2.1
30345	0.946131	2.1
30346	0.946131	2.1
30347	1.31912	2.1
30348	1.31912	2.1
30349	0.946131	2.1
30350	1.31912	2.1
30351	1.35721	2.1
30352	0.946131	2.1
30353	2.24577	0.438983
30354	1.09408	0.520014
30355	1.60239	0.509892
30356	1.09408	0.517942
30357	2.39404	0.446904
30358	2.07382	0.477828
30359	1.70303	0.063073
30360	1.3216	0.0847616
30361	1.23104	0.05
30362	1.05475	0.0905162
30363	2.60408	0.0603108
30364	1.89226	0.103124
30365	1.89226	0.164295
30366	1.89226	0.0980356
30367	1.89226	0.129667
30368	3.78452	0.156642
30369	1.89226	0.0567247
30370	2.3845	0.0603129
30371	2.6875	0.0693141
30372	1.89226	0.0721858
30373	2.6875	0.05
30374	1.89226	0.0525143
30375	1.46759	2.1
30376	1.17585	2.1
30377	1.85189	2.1
30378	1.39223	2.1
30379	2.85981	2.1
30380	1.85189	2.1
30381	1.71255	2.1
30382	1.71255	2.1
30383	1.39223	2.1
30384	2.28511	2.1
30385	1.96479	2.1
30386	1.89227	0.529891
30387	1.89227	0.816499
30388	1.89227	1.09455
30389	1.89227	0.414488

30390	1.89227	1.29205
30391	1.89227	0.504783
30392	1.89226	0.621919
30393	1.89226	0.730191
30394	1.89226	1.00796
30395	1.89226	0.401896
30396	1.89226	1.22856
30397	1.89226	0.480808
30398	1.89226	0.535193
30399	1.89226	0.993568
30400	1.89226	1.0166
30401	1.89226	0.374096
30402	1.89226	1.39271
30403	1.89226	0.491639
30404	1.89226	0.377706
30405	2.77788	0.402518
30406	2.21062	0.560239
30407	2.21062	0.301841
30408	1.89226	0.998606
30409	1.89226	0.354637
30410	0.868548	0.356688
30411	0.610104	0.47724
30412	0.796678	0.357602
30413	0.865021	0.295192
30414	1.0883	0.363895
30415	0.904938	0.448023
30416	2.21062	0.399969
30417	1.49548	0.377896
30418	1.2068	0.70271
30419	1.54764	0.344284
30420	0.790005	0.326361
30421	1.89226	2.1
30422	2.67457	2.1
30423	1.89226	2.1
30424	2.56352	2.1
30425	4.56683	2.1
30426	2.67456	2.1
30427	2.20011	2.1
30428	2.56351	2.1
30429	2.11083	2.1
30430	4.78539	2.1
30431	2.56348	2.1
30432	2.1108	2.1
30433	2.45763	2.1
30434	2.00986	2.1
30435	4.56843	2.1
30436	0.946133	0.05
30437	0.946133	0.05
30438	0.946132	0.05
30439	0.946132	0.05
30440	0.946132	0.05
30441	0.946132	0.05
30442	0.946132	0.05
30443	0.946132	0.05
30444	0.946132	0.05
30445	0.946132	0.05
30446	0.946133	0.05
30447	0.946133	0.05

30448	1.45927	1.4252
30449	1.25133	1.80829
30450	1.00082	1.82168
30451	1.41689	1.72287
30452	1.0432	1.56482
30453	1.45927	1.69564
30454	1.41689	1.70632
30455	1.29579	1.81577
30456	1.34874	1.62139
30457	1.25133	1.88287
30458	2.76563	1.93992
30459	0.675555	1.6803
30460	0.674371	1.63912
30461	0.625664	1.71419
30462	0.523133	1.71955
30463	0.676509	1.78696
30464	0.676509	1.77011
30465	0.62471	1.60417
30466	0.62471	1.60543
30467	0.675556	1.49172
30468	0.523132	1.31263
30469	0.674373	1.32131
30470	0.625663	1.50082
30471	1.89226	1.39163
30472	1.89226	1.16906
30473	1.89226	0.593602
30474	1.89226	1.05222
30475	3.78453	1.16996
30476	0.851517	1.17407
30477	0.946133	1.01535
30478	0.946132	1.21684
30479	0.946132	1.31288
30480	0.946133	0.781137
30481	0.851517	1.78499
30482	0.946132	1.37722
30483	0.946132	1.12145
30484	0.851516	1.10633
30485	0.851516	0.886912
30486	0.946133	1.511
30487	0.946133	1.16194
30488	0.569995	0.778607
30489	0.816895	1.34545
30490	1.33893	1.30367
30491	1.18795	0.836521
30492	1.18795	0.830486
30493	0.569995	1.49568
30494	0.816895	1.57282
30495	1.33893	1.23982
30496	0.859572	1.69577
30497	0.859572	0.881596
30498	1.04936	1.07709
30499	1.04936	0.895332
30500	1.89226	2.1
30501	1.89226	2.1
30502	2.55618	2.1
30503	2.55618	2.1
30504	2.66983	2.1
30505	2.00591	2.1

30506	1.89226	2.1
30507	2.64586	2.1
30508	2.83331	2.1
30509	2.83331	2.1
30510	1.89226	2.1
30511	1.89226	2.1
30512	2.64586	2.1
30513	2.0815	2.1
30514	1.99985	2.1
30515	1.94005	2.1
30516	2.83332	2.1
30517	2.74859	2.1
30518	1.98061	2.1
30519	1.68356	2.1
30520	1.83879	2.1
30521	1.55987	2.1
30522	3.54048	2.1
30523	1.68356	2.1
30524	1.20969	2.1
30525	1.20969	2.1
30526	1.30751	2.1
30527	1.55987	2.1
30528	1.65769	2.1
30529	1.83879	2.1
30530	1.55987	2.1
30531	1.68443	2.1
30532	1.4449	2.1
30533	3.2443	2.1
30534	0.660368	2.1
30535	0.660368	2.1
30536	0.666922	2.1
30537	0.666922	2.1
30538	0.560377	2.1
30539	0.604842	2.1
30540	0.779935	2.1
30541	0.722448	2.1
30542	0.722446	2.1
30543	0.560377	2.1
30544	0.604844	2.1
30545	0.779934	2.1
30546	1.89227	2.1
30547	1.89227	2.1
30548	1.89227	2.1
30549	1.89227	2.1
30550	3.78453	2.1
30551	1.42896	2.1
30552	1.23037	2.1
30553	1.42896	2.1
30554	1.474 2.1	
30555	1.2016	2.1
30556	1.24665	2.1
30557	1.89226	2.1
30558	2.7188	2.1
30559	1.89226	2.1
30560	2.70702	2.1
30561	4.61106	2.1
30562	1.89226	2.1
30563	1.89226	2.1

30564	1.89226	2.1
30565	1.89226	2.1
30566	3.78453	2.1
30567	0.644603	2.1
30568	0.644603	2.1
30569	0.648272	2.1
30570	0.648272	2.1
30571	0.585644	2.1
30572	0.600802	2.1
30573	0.692073	2.1
30574	0.714478	2.1
30575	0.585644	2.1
30576	0.692073	2.1
30577	0.714478	2.1
30578	0.600802	2.1
30579	1.35351	2.1
30580	1.35351	2.1
30581	0.946131	2.1
30582	0.946131	2.1
30583	1.34392	2.1
30584	1.35351	2.1
30585	0.946132	2.1
30586	0.946132	2.1
30587	0.946132	2.1
30588	1.34392	2.1
30589	0.946132	2.1
30590	1.35351	2.1
30591	1.87609	2.1
30592	1.79155	2.1
30593	1.79155	2.1
30594	1.89226	2.1
30595	1.89226	2.1
30596	1.99211	2.1
30597	1.89226	2.1
30598	1.71706	2.1
30599	1.71706	2.1
30600	1.79155	2.1
30601	1.89226	2.1
30602	1.96675	2.1
30603	1.46869	2.1
30604	1.89226	2.1
30605	1.89226	2.1
30606	1.89226	2.1
30607	1.5001	2.1
30608	1.5001	2.1
30609	1.89226	2.1
30610	1.89226	2.1
30611	1.89226	2.1
30612	1.89226	2.1
30613	1.89226	2.1
30614	1.89226	2.1
30615	1.21408	2.1
30616	2.23563	2.1
30617	2.34021	2.1
30618	2.34021	2.1
30619	1.17285	2.1
30620	1.17285	2.1
30621	1.89226	2.1

30622	1.89226	2.1
30623	1.47937	2.1
30624	1.89226	2.1
30625	1.14822	2.1
30626	1.46869	2.1
30627	1.89226	2.1
30628	1.89226	2.1
30629	1.89226	2.1
30630	1.89226	2.1
30631	1.89226	2.1
30632	1.89226	2.1
30633	1.25716	2.1
30634	2.34021	2.1
30635	2.4542	2.1
30636	2.4542	2.1
30637	1.21408	2.1
30638	1.21408	2.1
30639	1.66824	2.1
30640	2.44883	2.1
30641	1.93003	2.1
30642	2.24795	2.1
30643	3.88907	2.1
30644	1.89319	2.1
30645	2.24795	2.1
30646	2.82724	2.1
30647	2.31318	2.1
30648	2.30868	2.1
30649	1.93003	2.1
30650	1.89226	2.1
30651	1.42589	2.1
30652	1.42589	2.1
30653	1.80501	2.1
30654	1.89226	2.1
30655	2.27137	2.1
30656	1.21067	0.0564518
30657	2.39166	0.133733
30658	1.73577	0.058577
30659	1.73577	0.0661548
30660	1.53584	0.103439
30661	1.53584	0.0938458
30662	1.35025	2.1
30663	2.0599	1.64118
30664	1.51084	2.1
30665	2.27218	0.646129
30666	3.57074	2.03132
30667	2.0599	1.41733
30668	2.51818	2.1
30669	2.27218	0.528873
30670	2.66532	2.06209
30671	4.72522	1.69695
30672	1.67115	2.1
30673	2.27218	1.30325
30674	2.39605	2.1
30675	2.39605	0.999511
30676	1.51084	2.1
30677	1.51084	1.96465
30678	2.27218	0.990175
30679	2.66532	2.1

30680	2.39605	0.631997
30681	2.76829	2.06145
30682	5.06137	1.11203
30683	2.08684	1.49732
30684	2.36033	2.00331
30685	1.89224	1.1967
30686	1.89224	1.93318
30687	4.25257	1.41842
30688	2.41473	0.161134
30689	1.00307	0.208528
30690	2.01365	0.174554
30691	1.00307	0.222185
30692	3.05113	0.161019
30693	2.04056	0.242074
30694	1.11099	2.1
30695	2.25103	2.1
30696	2.22227	2.1
30697	2.22227	2.1
30698	1.14431	2.1
30699	1.14431	2.1
30700	1.07485	2.1
30701	2.22229	2.1
30702	2.19163	2.1
30703	2.19163	2.1
30704	1.111 2.1	
30705	1.111 2.1	
30706	1.0547	2.1
30707	1.19886	2.1
30708	0.946131	2.1
30709	0.946131	2.1
30710	0.946131	2.1
30711	0.946131	2.1
30712	1.19886	2.1
30713	1.19886	2.1
30714	1.0547	2.1
30715	0.946131	2.1
30716	0.946132	2.1
30717	1.19886	2.1
30718	1.89417	2.1
30719	2.64424	1.35386
30720	1.76585	2.1
30721	2.47695	2.05551
30722	4.37112	2.1
30723	1.89226	2.1
30724	1.89226	2.1
30725	1.89226	2.1
30726	1.89226	2.07484
30727	3.78453	2.1
30728	0.930281	2.1
30729	1.0547	2.1
30730	0.946131	2.1
30731	0.946131	2.1
30732	0.930309	2.1
30733	0.930309	2.1
30734	1.07052	2.1
30735	1.07052	2.1
30736	0.93028	2.1
30737	0.946131	2.1

30738	1.0547	2.1
30739	0.946131	2.1
30740	1.00015	1.06495
30741	1.00015	2.1
30742	1.01918	2.1
30743	1.01918	1.97379
30744	1.17437	2.01736
30745	1.23847	0.91605
30746	0.882926	2.1
30747	0.780859	2.1
30748	0.780859	2.06247
30749	1.17438	2.08188
30750	0.882925	2.09778
30751	1.23848	2.09641
30752	1.89226	2.1
30753	1.89226	2.02724
30754	1.89226	2.1
30755	1.89226	2.1
30756	1.89226	2.1
30757	1.89226	2.1
30758	1.22083	2.1
30759	1.29579	2.1
30760	1.26236	2.1
30761	1.06191	2.1
30762	1.42128	2.1
30763	1.22083	2.1
30764	1.42128	2.1
30765	1.45806	2.1
30766	1.43502	2.1
30767	1.29579	2.1
30768	1.5605	2.1
30769	1.42128	2.1
30770	1.02813	2.1
30771	1.12121	2.1
30772	1.40557	2.1
30773	1.35298	2.1
30774	1.1314	2.1
30775	1.28679	2.1
30776	1.1715	2.1
30777	1.1715	2.1
30778	0.975656	2.1
30779	1.24908	2.1
30780	0.98956	2.1
30781	1.2681	2.1
30782	0.946132	2.1
30783	0.946132	2.1
30784	1.31889	2.1
30785	1.31889	2.1
30786	1.16546	2.1
30787	1.31889	2.1
30788	0.946133	2.1
30789	0.946133	2.1
30790	0.946133	2.1
30791	1.31889	2.1
30792	0.946132	2.1
30793	1.16546	2.1
30794	2.51518	0.05
30795	2.37584	0.05

30796	1.11712	0.05
30797	1.31242	0.05
30798	3.68826	0.05
30799	2.51518	0.05
30800	1.26311	0.05
30801	2.48977	0.05
30802	2.48977	0.05
30803	1.31242	0.05
30804	1.31242	0.05
30805	1.89226	1.08071
30806	1.89226	2.1
30807	1.89226	0.918932
30808	1.89226	2.1
30809	3.78452	1.97413
30810	2.04737	0.664133
30811	1.77122	2.1
30812	1.94498	0.893551
30813	1.65174	2.1
30814	3.71621	2.03226
30815	1.41077	2.1
30816	1.29729	2.1
30817	1.77123	2.1
30818	1.77123	2.1
30819	1.65175	2.1
30820	1.29129	2.1
30821	1.89226	2.1
30822	1.89226	2.1
30823	1.89226	2.1
30824	1.89226	2.1
30825	1.89226	2.1
30826	1.89226	2.1
30827	1.89226	0.751746
30828	1.89226	2.1
30829	1.89226	0.884504
30830	1.89226	2.1
30831	3.78451	1.83433
30832	1.94498	0.738139
30833	1.65174	2.1
30834	1.7968	1.16243
30835	1.49831	2.1
30836	3.44329	1.93931
30837	1.49831	2.1
30838	1.29729	2.1
30839	1.49831	2.1
30840	1.65175	2.1
30841	1.16858	2.1
30842	1.32201	2.1
30843	1.89226	2.1
30844	1.89226	2.1
30845	1.89226	2.1
30846	1.89226	2.1
30847	3.78452	2.1
30848	1.89226	0.05
30849	1.89226	0.05
30850	1.89226	0.05
30851	1.89226	0.05
30852	1.89226	0.05
30853	1.89226	0.05

30854	1.65324	2.06065
30855	1.26409	0.389427
30856	1.42491	0.383201
30857	1.42491	2.06714
30858	1.52268	0.933808
30859	1.52268	2.04448
30860	1.52268	2.1
30861	1.26409	0.713663
30862	1.32697	2.06492
30863	1.05724	0.675436
30864	2.57991	1.52666
30865	1.89226	0.103772
30866	1.96548	0.100191
30867	2.0178	0.104315
30868	1.96548	0.103422
30869	1.94458	0.112536
30870	1.89226	0.109143
30871	1.99366	0.095883
30872	2.0178	0.0899421
30873	1.89226	0.0943171
30874	2.0178	0.0934021
30875	1.75999	0.103519
30876	1.89226	0.0947481
30877	0.946126	2.1
30878	0.946126	2.1
30879	1.28228	2.1
30880	1.19444	2.1
30881	0.946127	2.1
30882	0.946127	2.1
30883	1.19444	2.1
30884	1.19444	2.1
30885	0.946128	2.1
30886	1.19444	2.1
30887	1.28228	2.1
30888	0.946128	2.1
30889	1.89226	2.1
30890	1.89226	2.1
30891	1.89226	2.1
30892	1.89226	2.1
30893	3.78452	2.1
30894	2.14104	2.1
30895	1.89226	2.1
30896	2.19948	2.1
30897	1.89226	2.1
30898	1.89226	2.1
30899	2.19948	2.1
30900	1.89226	2.1
30901	2.38888	2.1
30902	2.23932	2.1
30903	2.38888	2.1
30904	1.74269	2.1
30905	1.89226	2.1
30906	1.89226	2.1
30907	1.89226	2.1
30908	1.89226	2.1
30909	1.89226	2.1
30910	3.78452	2.1
30911	0.946132	2.1

30912	0.946132	2.1
30913	1.07052	2.1
30914	1.07052	2.1
30915	0.946132	2.1
30916	0.946132	2.1
30917	1.05132	2.1
30918	1.07052	2.1
30919	1.05132	2.1
30920	0.946132	2.1
30921	1.07052	2.1
30922	0.946133	2.1
30923	2.40171	2.1
30924	1.89226	2.1
30925	1.89226	2.1
30926	1.89226	2.1
30927	2.42723	2.1
30928	2.42723	2.1
30929	1.89226	2.1
30930	1.89226	2.1
30931	1.89226	2.1
30932	1.89226	2.09867
30933	3.78452	2.1
30934	1.63661	2.1
30935	2.3532	2.08929
30936	2.33287	2.09709
30937	2.33287	2.1
30938	1.64158	2.09995
30939	1.64158	2.1
30940	2.38918	2.1
30941	1.89226	2.1
30942	1.89226	2.1
30943	1.89226	2.1
30944	2.40171	2.1
30945	2.40171	2.1
30946	1.89226	2.1
30947	1.89226	2.1
30948	1.89226	2.1
30949	1.89226	2.1
30950	1.89226	2.1
30951	1.89226	2.1
30952	1.64158	2.1
30953	2.3532	2.1
30954	1.6488	2.1
30955	2.37364	2.1
30956	4.002 2.1	
30957	2.54752	0.204412
30958	1.42445	0.267328
30959	1.86697	0.259424
30960	1.86697	0.229321
30961	2.06666	0.253338
30962	2.06666	0.230512
30963	2.50872	0.159796
30964	2.06666	0.222534
30965	1.90439	0.186213
30966	1.51835	0.185088
30967	3.97105	0.183075
30968	0.755161	0.238962
30969	0.755161	0.212701

30970	0.793994	0.186245
30971	0.793994	0.208235
30972	0.712225	0.229476
30973	0.515826	0.212209
30974	1.03333	0.217209
30975	0.759175	0.191857
30976	0.759173	0.207127
30977	0.515827	0.188641
30978	0.712226	0.200322
30979	1.03333	0.203649
30980	1.69633	0.205725
30981	1.89226	0.206933
30982	1.62477	0.186964
30983	1.89226	0.213772
30984	1.62477	0.198343
30985	1.89226	0.217893
30986	1.89226	0.418005
30987	1.89226	0.537495
30988	1.89226	0.5277
30989	1.89226	0.467564
30990	3.78452	0.483943
30991	2.28622	0.214418
30992	1.09667	0.132297
30993	1.17506	0.136346
30994	2.24411	0.187767
30995	1.17506	0.164806
30996	2.24411	0.234317
30997	2.24741	2.1
30998	1.70303	2.1
30999	2.49168	2.1
31000	1.57936	2.1
31001	2.53729	2.1
31002	1.70303	2.1
31003	2.24741	2.1
31004	2.47557	2.1
31005	1.89227	2.1
31006	1.89227	2.1
31007	4.36784	2.1
31008	2.27016	0.05
31009	1.10476	0.05
31010	1.44103	0.05
31011	1.10476	0.0533228
31012	2.26791	0.05
31013	1.93165	0.05
31014	2.27016	0.05
31015	1.89226	0.0629766
31016	1.89226	0.05
31017	2.4065	0.0500116
31018	4.29876	0.05
31019	2.27016	0.05
31020	1.44103	0.05
31021	1.53336	0.05
31022	2.4065	0.05
31023	3.84753	0.05
31024	2.7542	0.302207
31025	1.89226	0.38527
31026	2.35027	0.354844
31027	1.89226	0.352636

31028	2.35027	0.324463
31029	1.89226	0.325712
31030	2.3301	2.1
31031	2.36071	2.1
31032	2.07629	2.1
31033	2.01105	2.1
31034	4.37176	2.1
31035	1.86512	2.1
31036	2.01105	2.1
31037	2.01105	2.1
31038	2.3301	2.1
31039	1.57459	2.1
31040	1.89364	2.1
31041	2.52671	2.1
31042	2.01105	2.1
31043	1.78251	2.1
31044	1.70302	2.1
31045	3.98362	2.1
31046	1.227	2.1
31047	2.01105	2.1
31048	1.45869	2.1
31049	1.41713	2.1
31050	1.70302	2.1
31051	1.64994	2.1
31052	2.00989	2.1
31053	1.28938	2.1
31054	1.8769	2.1
31055	1.20969	2.1
31056	3.21958	2.1
31057	1.8769	2.1
31058	1.15864	2.1
31059	1.15864	2.1
31060	1.20969	2.1
31061	1.77353	2.1
31062	1.82458	2.1
31063	1.7986	2.1
31064	2.48717	2.1
31065	2.50161	2.1
31066	1.75869	2.1
31067	4.30021	2.1
31068	2.50161	2.1
31069	1.75251	2.1
31070	1.81678	2.1
31071	1.75869	2.1
31072	2.5597	2.1
31073	2.50161	2.1
31074	1.91334	0.685757
31075	1.85439	1.16668
31076	1.91334	0.489364
31077	1.70303	1.14836
31078	1.70303	0.510444
31079	1.49692	1.09693
31080	2.39986	0.05
31081	1.89226	0.118954
31082	1.89226	0.0986671
31083	2.50756	0.05
31084	4.39982	0.05
31085	2.39986	0.0745363

31086	1.70303	0.05
31087	1.75564	0.05
31088	1.75904	0.105807
31089	2.50272	0.0719435
31090	2.50756	0.0991467
31091	1.89226	0.102945
31092	2.50756	0.0517637
31093	2.47857	0.0872788
31094	2.50756	0.115318
31095	1.86327	0.0923571
31096	1.89226	0.095784
31097	1.22241	0.0976854
31098	1.20827	0.118028
31099	0.772416	0.0880653
31100	0.555208	0.13702
31101	1.004	0.0899683
31102	1.004	0.110902
31103	0.93504	0.136421
31104	0.744364	0.1141
31105	1.2089	0.126692
31106	0.654935	0.126907
31107	0.773927	0.0894406
31108	1.03618	0.104443
31109	1.89227	0.05
31110	1.89227	0.05
31111	1.89227	0.05
31112	1.89227	0.05
31113	3.78453	0.05
31114	1.89226	0.05
31115	1.89226	0.05
31116	1.89226	0.05
31117	1.89226	0.05
31118	3.78452	0.05
31119	1.82643	0.05
31120	2.7084	0.05
31121	2.12654	0.05
31122	2.65614	0.05
31123	1.82643	0.05
31124	2.35603	0.05
31125	1.29385	0.05
31126	0.946133	0.05
31127	1.05492	0.05
31128	0.946132	0.05
31129	1.05492	0.05
31130	1.29385	0.05
31131	0.946133	0.05
31132	0.946133	0.05
31133	1.29385	0.05
31134	1.29385	0.05
31135	0.946132	0.05
31136	0.946132	0.05
31137	0.946132	0.05
31138	1.27329	0.05
31139	1.29385	0.05
31140	0.946129	0.05
31141	1.29385	0.05
31142	1.27328	0.05
31143	0.946131	0.05

31144	0.946131	0.05
31145	1.27328	0.05
31146	1.27328	0.05
31147	0.94613	0.05
31148	0.94613	0.05
31149	1.89226	1.9284
31150	1.89226	2.00017
31151	1.89226	0.607136
31152	1.89226	1.15129
31153	3.78453	1.5663
31154	1.89226	1.62311
31155	1.89226	1.85072
31156	1.89226	0.461128
31157	1.89226	1.60758
31158	3.78453	0.892955
31159	1.21488	1.78459
31160	1.14605	2.05637
31161	1.62974	1.31139
31162	1.52399	0.659884
31163	2.84463	1.72818
31164	0.577078	0.939649
31165	0.81251	2.07385
31166	0.821003	1.25923
31167	0.543413	1.88424
31168	0.683929	1.05519
31169	0.722433	2.1
31170	0.730674	2.00289
31171	0.730674	0.941483
31172	0.857766	1.19118
31173	0.862119	0.952412
31174	0.600938	1.92716
31175	0.596094	1.30861
31176	1.89226	1.19034
31177	1.89226	1.71012
31178	1.89226	0.33848
31179	1.89226	1.35031
31180	3.78453	1.05673
31181	1.89226	0.783719
31182	1.89226	2.0771
31183	1.89226	0.770909
31184	1.89226	1.23041
31185	3.78453	1.5932
31186	1.62974	2.01754
31187	1.56958	2.02469
31188	2.2362	0.462803
31189	2.11424	1.20016
31190	3.86595	1.20191
31191	2.2362	0.716858
31192	1.85414	2.07496
31193	2.28211	0.270705
31194	1.78367	0.324739
31195	2.2362	1.65736
31196	1.73256	1.93496
31197	0.851539	0.548369
31198	0.851539	0.587428
31199	1.14454	2.01548
31200	0.938988	1.89761
31201	0.851485	0.547417

31202	0.920593	1.39897
31203	0.851541	0.435955
31204	0.851541	1.74615
31205	1.04073	0.672079
31206	1.16342	0.943214
31207	0.825315	1.20208
31208	0.825316	0.677206
31209	1.89226	2.1
31210	1.89226	2.1
31211	1.89226	2.1
31212	1.89226	2.1
31213	3.78453	2.1
31214	2.57016	2.1
31215	1.74789	2.1
31216	2.57016	2.1
31217	2.522	2.1
31218	1.61727	2.1
31219	1.56911	2.1
31220	1.28075	2.1
31221	1.406	2.1
31222	0.946131	2.1
31223	0.946131	2.1
31224	1.28075	2.1
31225	1.00333	2.1
31226	0.94613	2.1
31227	0.94613	2.1
31228	1.041	2.1
31229	1.041	2.1
31230	0.943484	2.1
31231	1.27717	2.1
31232	1.89226	2.1
31233	1.89226	2.0938
31234	1.89226	2.09682
31235	1.89226	2.1
31236	1.89226	2.09892
31237	1.89226	2.1
31238	2.57016	2.1
31239	1.61727	2.1
31240	2.74196	2.1
31241	1.59094	2.1
31242	4.16109	2.1
31243	1.89226	2.09519
31244	2.0815	2.1
31245	2.2034	2.1
31246	1.82493	2.09212
31247	1.87847	2.1
31248	2.16142	2.1
31249	1.89226	2.1
31250	1.89226	2.1
31251	1.89226	2.1
31252	1.89226	2.1
31253	3.78453	2.1
31254	0.985252	2.1
31255	0.985252	2.1
31256	0.946132	2.1
31257	0.946132	2.1
31258	1.0655	2.1
31259	0.985255	2.1

31260	0.94613	2.1
31261	0.94613	2.1
31262	0.94613	2.1
31263	1.0655	2.1
31264	0.94613	2.1
31265	0.985255	2.1
31266	1.89226	2.1
31267	1.89226	2.1
31268	1.89226	2.1
31269	1.89226	2.1
31270	3.78453	2.1
31271	1.06549	2.1
31272	1.06549	2.1
31273	0.946133	2.1
31274	0.946133	2.1
31275	1.16834	2.1
31276	1.0655	2.1
31277	0.946131	2.1
31278	0.946131	2.1
31279	0.946131	2.1
31280	1.16834	2.1
31281	0.94613	2.1
31282	1.0655	2.1
31283	0.966631	2.1
31284	1.07349	2.1
31285	0.946131	2.1
31286	0.946131	2.1
31287	1.07349	2.1
31288	1.07349	2.1
31289	0.946129	2.1
31290	0.946129	2.1
31291	1.07349	2.1
31292	0.946131	2.1
31293	0.94613	2.1
31294	0.966631	2.1
31295	2.38055	0.668745
31296	1.89226	1.1544
31297	1.89226	1.09777
31298	1.46724	0.439688
31299	3.35949	0.738742
31300	1.42249	2.1
31301	2.05499	2.1
31302	1.39708	2.1
31303	2.09033	2.1
31304	3.45207	2.1
31305	2.05499	2.1
31306	2.57425	2.1
31307	2.51287	2.1
31308	2.47753	2.1
31309	2.09033	2.1
31310	2.05499	2.1
31311	1.39327	2.1
31312	2.09033	2.1
31313	2.16217	2.1
31314	2.16217	2.1
31315	1.39708	2.1
31316	1.39708	2.1
31317	2.16217	2.1

31318	2.57426	2.1
31319	2.73006	2.1
31320	2.73006	2.1
31321	2.09033	2.1
31322	2.09033	2.1
31323	1.89226	0.0750425
31324	1.89226	0.05
31325	1.89226	0.05
31326	1.89226	0.0615858
31327	3.78452	0.0646402
31328	0.838749	0.05
31329	0.838749	0.05
31330	0.85824	0.05
31331	0.85824	0.05
31332	0.544644	0.05
31333	0.854718	0.05
31334	0.842271	0.05
31335	1.19141	0.05
31336	0.842272	0.05
31337	0.544643	0.05
31338	0.854718	0.05
31339	1.19141	0.05
31340	1.34214	0.05
31341	1.34214	0.05
31342	0.893236	0.0517882
31343	0.893236	0.05
31344	0.893265	0.05
31345	1.28924	0.05
31346	0.946133	0.05
31347	0.946133	0.05
31348	0.946132	0.05
31349	0.893265	0.05
31350	0.946133	0.05
31351	1.28924	0.05
31352	2.7013	0.05
31353	1.89226	0.0522356
31354	1.9216	0.0556243
31355	1.9216	0.05
31356	1.89226	0.0529474
31357	1.89226	0.05
31358	1.26397	2.1
31359	1.52995	2.1
31360	1.30116	2.1
31361	1.04084	2.1
31362	2.57079	2.1
31363	1.47013	2.1
31364	2.17459	2.1
31365	2.10668	2.1
31366	1.38328	2.1
31367	3.55787	2.1
31368	2.55006	2.1
31369	2.17459	2.1
31370	2.10668	2.1
31371	2.49168	2.1
31372	4.66626	2.1
31373	0.613622	2.1
31374	0.691639	2.1
31375	1.00105	2.1

31376	1.05334	2.1
31377	0.839874	2.1
31378	0.839874	2.1
31379	0.852814	2.1
31380	0.852814	2.1
31381	0.613622	2.1
31382	1.00105	2.1
31383	1.05334	2.1
31384	0.691639	2.1
31385	1.00105	2.1
31386	1.05334	2.1
31387	1.20844	2.1
31388	1.24584	2.1
31389	1.12717	2.1
31390	1.12717	2.1
31391	1.13462	2.1
31392	1.13462	2.1
31393	1.20845	2.1
31394	1.00105	2.1
31395	1.05334	2.1
31396	1.24584	2.1
31397	2.22558	2.1
31398	1.85981	2.1
31399	2.18943	2.00186
31400	1.89142	0.794059
31401	4.0854	2.07963
31402	2.02641	2.1
31403	1.85981	2.1
31404	2.18943	1.94419
31405	1.67346	2.01279
31406	3.88622	1.99373
31407	1.85981	0.776428
31408	1.10736	0.393111
31409	1.27421	0.654729
31410	1.67346	0.684237
31411	1.27421	0.486777
31412	1.67346	1.89859
31413	1.85981	1.21119
31414	1.27362	0.772836
31415	1.89142	0.457019
31416	1.27421	0.420109
31417	3.13343	0.572754
31418	1.81893	0.05
31419	1.64985	0.05
31420	1.23477	0.05
31421	2.25405	0.0630245
31422	3.48882	0.0558979
31423	2.23933	0.05
31424	1.89226	0.054457
31425	1.89226	0.054282
31426	2.78053	0.05
31427	1.89226	0.05
31428	2.78053	0.05
31429	0.946131	0.0589714
31430	0.946131	0.0567762
31431	0.946132	0.0860977
31432	0.946132	0.10112
31433	0.946131	0.110529

31434	0.946131	0.0945949
31435	0.946132	0.0584056
31436	0.946132	0.0775376
31437	0.946131	0.0719191
31438	0.946131	0.11037
31439	0.946132	0.05
31440	0.946132	0.0872655
31441	1.89226	2.1
31442	2.48596	2.1
31443	1.89226	2.1
31444	2.51506	2.1
31445	4.40732	2.1
31446	2.48596	2.1
31447	1.63823	2.1
31448	2.51507	2.1
31449	1.67687	2.1
31450	4.1533	2.1
31451	1.89226	2.1
31452	2.51506	2.1
31453	1.92044	2.1
31454	1.89226	2.1
31455	2.54325	2.1
31456	2.51506	2.1
31457	2.51507	2.1
31458	1.5704	2.1
31459	1.70505	2.1
31460	1.67687	2.1
31461	2.54325	2.1
31462	2.51507	2.1
31463	2.0815	2.1
31464	1.70303	2.1
31465	1.70303	2.1
31466	1.80354	2.1
31467	1.98172	2.1
31468	2.09868	2.1
31469	1.27944	2.1
31470	1.79028	2.1
31471	1.13179	2.1
31472	1.65533	2.1
31473	2.93477	2.1
31474	1.89226	2.1
31475	1.89226	2.1
31476	1.89226	2.1
31477	1.89226	2.1
31478	1.89226	2.1
31479	1.89226	2.1
31480	1.98171	2.1
31481	1.89226	2.1
31482	1.89226	2.1
31483	1.89226	2.1
31484	1.65798	2.1
31485	1.65797	2.1
31486	1.00133	2.1
31487	1.65532	2.1
31488	1.53278	2.1
31489	1.53278	2.1
31490	1.13179	2.1
31491	1.13179	2.1

31492	0.810946	2.1
31493	0.810946	2.1
31494	0.810944	2.1
31495	0.810944	2.1
31496	0.810946	2.1
31497	0.810946	2.1
31498	1.89226	2.1
31499	1.35169	2.1
31500	1.08132	2.1
31501	1.08132	2.1
31502	1.08132	2.1
31503	1.99528	0.0529315
31504	1.70303	0.0583516
31505	2.08152	0.05
31506	1.70303	0.0744385
31507	2.33877	0.05
31508	1.93967	0.0745167
31509	1.89226	0.0603826
31510	1.89226	0.120239
31511	1.89226	0.0720585
31512	1.89226	0.0754586
31513	3.78452	0.0816763
31514	1.26259	0.05
31515	2.17205	0.0699436
31516	2.32468	0.05
31517	2.32468	0.05286
31518	1.28055	0.05
31519	1.28055	0.0539668
31520	0.946131	0.0703288
31521	0.946131	0.05
31522	0.997642	0.05
31523	0.997642	0.05
31524	0.946131	0.05
31525	0.946131	0.0745377
31526	0.989846	0.05
31527	0.997642	0.05
31528	0.946132	0.05
31529	0.989846	0.05
31530	0.997641	0.0501361
31531	0.946131	0.0521561
31532	1.89226	0.0756024
31533	1.89226	0.0704737
31534	1.89226	0.05
31535	1.89226	0.106114
31536	3.78452	0.0688876
31537	1.28055	0.05
31538	2.17205	0.0652154
31539	1.14305	0.05
31540	1.92737	0.05
31541	3.3151	0.05
31542	0.954908	0.05
31543	0.9705	0.05
31544	0.946131	0.05
31545	0.946131	0.05
31546	0.946131	0.05
31547	0.946131	0.05
31548	0.9705	0.05
31549	0.9705	0.05

31550	0.954908	0.05
31551	0.946131	0.05
31552	0.946131	0.05
31553	0.9705	0.05
31554	0.835187	0.05
31555	0.954896	0.05
31556	0.946118	0.05
31557	0.946118	0.05
31558	0.835186	0.05
31559	0.835186	0.05
31560	1.06583	0.05
31561	1.06583	0.05
31562	0.835198	0.05
31563	0.946131	0.05
31564	0.954883	0.05
31565	0.946106	0.05
31566	1.89227	2.1
31567	1.89227	2.1
31568	1.89227	2.1
31569	1.89227	2.1
31570	1.89227	2.1
31571	1.89227	2.1
31572	0.885265	2.1
31573	0.885265	2.1
31574	0.871467	2.1
31575	0.871467	2.1
31576	1.11883	2.1
31577	0.787875	2.1
31578	0.996487	2.1
31579	0.637907	2.1
31580	0.996487	2.1
31581	1.11883	2.1
31582	0.787875	2.1
31583	0.637906	2.1
31584	1.89226	2.1
31585	2.39258	2.1
31586	2.39258	2.1
31587	2.54729	2.1
31588	1.89226	2.1
31589	2.04697	2.1
31590	1.89226	2.1
31591	2.0815	2.1
31592	2.0815	2.1
31593	2.48371	2.1
31594	2.0815	2.1
31595	2.51469	2.1
31596	1.00035	0.05
31597	1.55755	0.88482
31598	1.03534	1.15839
31599	1.61333	1.68202
31600	2.59289	0.466392
31601	1.55755	0.88482
31602	1.85217	2.1
31603	1.61333	2.09252
31604	1.92875	2.1
31605	3.48629	2.1
31606	1.09962	1.11829
31607	1.61334	0.986129

31608	1.70868	2.09317
31609	1.70868	1.8994
31610	1.03535	2.1
31611	1.03535	1.1982
31612	1.61334	1.08741
31613	1.92876	2.1
31614	1.70868	1.4105
31615	2.05274	2.1
31616	3.63744	1.94946
31617	1.82233	0.26571
31618	2.05768	0.466555
31619	1.89226	0.535287
31620	1.65406	0.30764
31621	1.89226	0.519546
31622	1.65406	0.317367
31623	2.26047	0.312525
31624	2.0815	0.635651
31625	1.89226	0.681892
31626	2.01156	0.376389
31627	1.89226	0.622244
31628	2.01156	0.368645
31629	1.89226	2.1
31630	1.89226	2.1
31631	1.89226	2.1
31632	1.89226	2.1
31633	3.78453	2.1
31634	2.70055	2.1
31635	2.28367	2.1
31636	2.56456	2.1
31637	2.33565	2.1
31638	4.84823	2.1
31639	2.28367	2.1
31640	1.5791	2.1
31641	1.96966	2.1
31642	1.09506	2.1
31643	3.37873	2.1
31644	1.89226	2.1
31645	1.89226	2.1
31646	1.89226	2.09998
31647	1.89226	2.1
31648	3.78452	2.1
31649	0.94612	2.1
31650	0.94612	2.1
31651	0.946119	2.1
31652	0.946119	2.1
31653	0.946119	2.1
31654	0.946119	2.1
31655	0.946119	2.1
31656	0.946119	2.1
31657	0.946132	2.1
31658	0.946132	2.1
31659	0.946107	2.1
31660	0.946107	2.1
31661	2.56452	2.1
31662	1.57307	2.1
31663	2.10299	2.1
31664	2.1115	2.1
31665	2.52196	2.1

31666	2.52196	2.1
31667	1.89226	2.1
31668	1.89226	2.1
31669	1.89226	2.1
31670	1.89226	2.1
31671	3.78452	2.1
31672	2.37665	2.1
31673	1.89226	2.1
31674	2.28084	2.1
31675	1.89226	2.1
31676	4.26891	2.1
31677	0.946129	2.1
31678	0.946129	2.1
31679	0.946133	2.1
31680	0.946133	2.1
31681	0.946131	2.1
31682	0.946131	2.1
31683	0.946131	2.1
31684	0.946131	2.1
31685	0.946131	2.1
31686	0.946131	2.1
31687	0.946131	2.1
31688	0.946131	2.1
31689	1.7207	0.05
31690	1.65815	0.05
31691	1.33593	0.05
31692	1.31508	0.05
31693	2.99408	0.05
31694	0.676047	0.05
31695	0.676047	0.05
31696	0.684362	0.05
31697	0.684362	0.05
31698	0.547994	0.05
31699	0.702871	0.05
31700	0.829076	0.05
31701	0.657538	0.05
31702	0.657538	0.05
31703	0.547994	0.05
31704	0.829077	0.05
31705	0.702871	0.05
31706	1.9518	2.1
31707	1.30648	2.1
31708	1.28846	2.1
31709	1.92033	2.1
31710	3.22681	2.1
31711	1.89226	2.1
31712	1.89226	2.1
31713	1.89226	2.1
31714	1.89226	2.1
31715	3.78453	2.1
31716	0.946128	2.1
31717	0.946128	2.1
31718	1.10113	2.1
31719	1.09587	2.1
31720	0.946131	2.1
31721	0.946131	2.1
31722	1.09587	2.1
31723	1.09587	2.1

31724	0.946131	2.1
31725	1.09587	2.1
31726	1.10112	2.1
31727	0.94613	2.1
31728	1.26923	2.1
31729	1.92033	2.1
31730	1.28846	2.1
31731	1.28846	2.1
31732	1.89452	2.1
31733	1.89452	2.1
31734	1.89226	2.1
31735	1.89226	2.1
31736	1.89226	2.1
31737	1.89226	2.1
31738	3.78453	2.1
31739	1.89226	2.1
31740	2.19174	2.1
31741	2.15616	2.1
31742	2.19174	2.1
31743	1.85669	2.1
31744	1.89226	2.1
31745	1.89226	2.1
31746	1.89226	2.1
31747	1.89226	2.1
31748	1.89226	2.1
31749	3.78452	2.1
31750	1.89226	2.1
31751	1.89226	2.1
31752	1.89226	2.1
31753	1.89226	2.1
31754	3.78452	2.1
31755	1.51223	2.1
31756	1.41562	2.1
31757	1.5139	2.1
31758	1.42284	2.1
31759	1.6033	2.1
31760	1.51223	2.1
31761	1.51223	2.1
31762	1.25953	2.1
31763	1.38085	2.1
31764	1.6033	2.1
31765	2.89309	2.1
31766	0.946131	2.1
31767	0.946131	2.1
31768	0.946131	2.1
31769	0.946131	2.1
31770	0.946132	2.1
31771	0.946132	2.1
31772	0.946129	2.1
31773	0.946129	2.1
31774	0.946131	2.1
31775	0.946131	2.1
31776	0.94613	2.1
31777	0.94613	2.1
31778	1.89226	2.1
31779	1.89226	2.1
31780	1.89226	2.1
31781	1.89226	2.1

31782	3.78452	2.1
31783	0.707806	2.1
31784	0.697816	2.1
31785	0.823593	2.1
31786	0.801649	2.1
31787	0.74182	2.1
31788	0.74182	2.1
31789	0.757645	2.1
31790	0.757645	2.1
31791	0.823592	2.1
31792	0.697818	2.1
31793	0.707807	2.1
31794	0.801647	2.1
31795	1.52534	2.1
31796	1.6033	2.1
31797	1.64719	2.1
31798	1.38085	2.1
31799	1.64719	2.1
31800	1.38085	2.1
31801	2.67041	2.1
31802	1.89226	2.0775
31803	1.89226	2.1
31804	1.89226	2.1
31805	2.487	2.1
31806	2.487	2.1
31807	2.70097	2.1
31808	1.70303	2.1
31809	1.71163	2.1
31810	2.487	2.1
31811	4.46723	2.1
31812	1.14984	2.1
31813	1.2435	2.1
31814	0.778	2.1
31815	0.697616	2.1
31816	0.967211	2.1
31817	0.967211	2.1
31818	0.973907	2.1
31819	0.973907	2.1
31820	0.697616	2.1
31821	1.14984	2.1
31822	0.778	2.1
31823	1.2435	2.1
31824	2.4158	2.1
31825	1.26438	2.1
31826	1.676	2.1
31827	2.77505	2.1
31828	4.0918	2.1
31829	2.58258	2.1
31830	1.89226	2.1
31831	1.89226	2.1
31832	1.89226	2.1
31833	2.47032	2.1
31834	2.47032	2.1
31835	1.23516	2.1
31836	1.18734	2.1
31837	0.946131	2.1
31838	0.946131	2.1
31839	1.18734	2.1

31840	1.18734	2.1
31841	0.94613	2.1
31842	0.94613	2.1
31843	1.18734	2.1
31844	0.946131	2.1
31845	0.946131	2.1
31846	1.23516	2.1
31847	2.8239	2.1
31848	1.39361	2.1
31849	1.12342	2.1
31850	2.57275	2.1
31851	3.96637	2.1
31852	0.937929	1.54322
31853	0.937929	0.965194
31854	1.39734	0.737109
31855	1.39734	0.656518
31856	1.38914	0.879485
31857	0.94613	1.29402
31858	0.937902	1.13641
31859	0.94613	0.407026
31860	0.94613	0.647197
31861	0.94613	0.935773
31862	1.38914	0.81829
31863	0.937902	0.691376
31864	1.89226	0.219423
31865	2.17765	0.658453
31866	1.84577	0.557285
31867	1.84577	0.324553
31868	1.89226	0.496341
31869	1.89226	0.275096
31870	2.11262	2.05735
31871	1.57766	2.08268
31872	2.22689	2.1
31873	1.91132	1.51797
31874	2.19624	2.1
31875	1.79689	1.13568
31876	1.99656	1.2844
31877	1.57766	2.07875
31878	1.39067	2.1
31879	1.73802	1.25514
31880	3.31568	2.03934
31881	1.89226	0.310045
31882	1.89226	0.130597
31883	1.89226	0.212896
31884	1.89226	0.504327
31885	1.89226	0.0816284
31886	1.89226	0.17345
31887	1.84936	0.258947
31888	2.0815	0.0754464
31889	2.0815	0.15122
31890	2.47217	0.172854
31891	1.04528	0.0617141
31892	1.62774	0.11435
31893	1.89226	0.195864
31894	1.89226	0.0949012
31895	1.89226	0.134681
31896	1.89226	0.241083
31897	1.89226	0.0802354

31898	1.89226	0.144831
31899	1.69055	0.05
31900	2.68518	0.0507146
31901	1.69055	0.05
31902	2.25055	0.0690409
31903	2.39587	0.05
31904	3.01801	0.0596072
31905	1.89226	0.0846244
31906	1.89226	0.0936233
31907	1.89226	0.0586618
31908	1.89226	0.0528825
31909	3.78452	0.0806825
31910	1.24499	2.1
31911	1.70302	2.1
31912	1.41207	2.1
31913	1.10574	1.96452
31914	2.66056	2.1
31915	1.70303	2.1
31916	2.39605	1.97605
31917	2.72674	2.1
31918	2.46904	1.34447
31919	1.84557	2.1
31920	1.67115	2.00671
31921	2.46904	0.482584
31922	2.76829	2.1
31923	2.83666	0.318615
31924	2.83666	1.8982
31925	2.39605	0.560418
31926	2.39605	2.08556
31927	1.07323	0.720696
31928	1.07323	2.1
31929	1.06715	2.1
31930	1.18052	1.8462
31931	1.30067	0.621263
31932	1.26542	0.914967
31933	0.914516	2.1
31934	0.839633	2.1
31935	0.936958	2.00142
31936	1.26751	2.01885
31937	0.847764	2.06988
31938	1.35395	2.06207
31939	2.69069	0.299525
31940	1.03074	0.241731
31941	1.74098	0.204961
31942	1.97397	0.237531
31943	3.71495	0.237005
31944	2.44554	2.1
31945	1.89226	2.1
31946	1.89226	2.1
31947	1.89226	2.1
31948	2.40645	2.1
31949	2.40645	2.1
31950	1.89226	2.1
31951	1.89226	2.1
31952	1.89226	2.1
31953	1.89226	2.1
31954	3.78452	2.1
31955	1.82117	2.1

31956	2.55786	2.1
31957	2.51431	2.1
31958	2.51431	2.1
31959	1.85054	2.1
31960	1.84375	2.1
31961	2.38467	2.1
31962	1.89226	2.1
31963	1.89226	2.1
31964	1.89226	2.1
31965	2.44554	2.1
31966	2.44554	2.1
31967	1.89226	2.1
31968	1.89226	2.1
31969	1.89226	2.1
31970	1.89226	2.1
31971	1.89226	2.1
31972	1.89226	2.1
31973	2.02397	2.1
31974	2.55786	2.1
31975	1.77775	2.1
31976	2.61404	2.1
31977	4.33561	2.1
31978	1.89226	1.6397
31979	1.89226	1.61708
31980	1.89226	1.29135
31981	1.89226	1.30693
31982	3.78453	1.64117
31983	1.89226	1.85284
31984	2.80184	1.77132
31985	1.89226	1.57158
31986	1.89226	1.78328
31987	2.03045	1.88488
31988	2.03045	1.94149
31989	2.52263	1.67622
31990	1.27921	2.01239
31991	1.58429	2.05709
31992	1.73571	1.94523
31993	2.37121	1.8154
31994	2.52263	1.6781
31995	0.764974	2.1
31996	0.650577	2.1
31997	0.712428	2.1
31998	0.562516	2.1
31999	0.67264	2.1
32000	0.67264	2.1
32001	0.654851	2.1
32002	0.654851	2.1
32003	0.562515	2.1
32004	0.650579	2.1
32005	0.712427	2.1
32006	0.764976	2.1
32007	1.89227	2.1
32008	1.89227	2.1
32009	1.89227	2.1
32010	1.89227	2.1
32011	3.78453	2.1
32012	1.89226	2.1
32013	1.89226	2.1

32014	1.89226	2.1
32015	1.89226	2.1
32016	3.78453	2.1
32017	1.49248	2.1
32018	1.24959	2.1
32019	1.28211	2.1
32020	1.26432	2.1
32021	1.51027	2.1
32022	1.49248	2.1
32023	1.49248	2.1
32024	1.19017	2.1
32025	1.24977	2.1
32026	1.51027	2.1
32027	2.74225	2.1
32028	1.89226	2.1
32029	1.89226	2.1
32030	1.89226	2.1
32031	1.89226	2.1
32032	3.78453	2.1
32033	1.89226	2.1
32034	1.89226	2.1
32035	1.89226	2.1
32036	1.89226	2.1
32037	3.78453	2.1
32038	0.624796	2.1
32039	0.618472	2.1
32040	0.770147	2.1
32041	0.755132	2.1
32042	0.681445	2.1
32043	0.681445	2.1
32044	0.69216	2.1
32045	0.69216	2.1
32046	0.618472	2.1
32047	0.770147	2.1
32048	0.624796	2.1
32049	0.755132	2.1
32050	1.54029	2.1
32051	1.51027	2.1
32052	1.54029	2.1
32053	1.30682	2.1
32054	1.24977	2.1
32055	1.24977	2.1
32056	0.994293	2.1
32057	2.17159	2.1
32058	2.21149	2.1
32059	2.21149	2.1
32060	0.994814	2.1
32061	0.994814	2.1
32062	0.990481	2.1
32063	2.21149	2.1
32064	2.2671	2.1
32065	2.2671	2.1
32066	0.994293	2.1
32067	0.994293	2.1
32068	2.41579	2.1
32069	1.09847	2.1
32070	1.70302	2.1
32071	2.45069	2.1

32072	3.61169	2.1
32073	2.48689	2.1
32074	1.67969	2.1
32075	1.67969	2.1
32076	1.70002	2.1
32077	2.43826	2.1
32078	2.45859	2.1
32079	2.43827	2.1
32080	1.62408	2.1
32081	1.62408	2.1
32082	1.67969	2.1
32083	2.36729	2.1
32084	2.4229	2.1
32085	2.47199	2.1
32086	1.0823	2.1
32087	1.0607	2.1
32088	2.44276	2.1
32089	3.52506	2.1
32090	2.47199	2.1
32091	1.89226	2.1
32092	1.89226	2.1
32093	2.44277	2.1
32094	4.33503	2.1
32095	1.89226	2.1
32096	1.89226	2.1
32097	1.89226	2.1
32098	1.89226	2.1
32099	3.78453	2.1
32100	1.89226	2.1
32101	1.89226	2.1
32102	1.89226	2.1
32103	1.89226	2.1
32104	1.89226	2.1
32105	1.89226	2.1
32106	1.02986	2.1
32107	2.44276	2.1
32108	1.0607	2.1
32109	1.0607	2.1
32110	2.40055	2.1
32111	2.40055	2.1
32112	1.89226	2.1
32113	2.44277	2.1
32114	2.40056	2.1
32115	2.44277	2.1
32116	1.85005	2.1
32117	1.89226	2.1
32118	1.89226	2.1
32119	1.89226	2.1
32120	1.89226	2.1
32121	1.89226	2.1
32122	3.78453	2.1
32123	1.89226	2.1
32124	1.89226	2.1
32125	1.89226	2.1
32126	1.89226	2.1
32127	3.78452	2.1
32128	2.79502	0.748491
32129	2.63448	2.09669

32130	2.68986	0.610168
32131	2.53868	2.1
32132	5.32434	1.65539
32133	2.63449	2.09285
32134	2.39282	2.1
32135	2.53869	2.1
32136	2.3085	2.1
32137	4.94299	2.1
32138	2.68986	0.536262
32139	2.53868	2.1
32140	2.5761	0.858892
32141	2.43404	2.08303
32142	5.1239	1.55513
32143	2.53869	2.1
32144	2.3085	2.1
32145	2.43405	2.0899
32146	2.22575	2.1
32147	4.74255	2.1
32148	1.89226	2.1
32149	1.89226	2.1
32150	1.89226	2.1
32151	1.89226	2.1
32152	3.78453	2.1
32153	1.32391	2.1
32154	1.44951	2.1
32155	1.44951	2.1
32156	1.89226	2.1
32157	1.89226	2.1
32158	2.20203	2.1
32159	0.507656	2.1
32160	0.578945	2.1
32161	1.05568	2.1
32162	0.959007	2.1
32163	0.76264	2.1
32164	0.76264	2.1
32165	0.775313	2.1
32166	0.775313	2.1
32167	0.578945	2.1
32168	1.05568	2.1
32169	0.959007	2.1
32170	0.507656	2.1
32171	1.89226	0.05
32172	2.4065	0.053427
32173	2.36561	0.05
32174	2.4065	0.0580552
32175	1.85138	0.05
32176	1.89226	0.0531502
32177	1.40274	0.05
32178	2.4065	0.05
32179	1.53336	0.05
32180	1.53336	0.05
32181	2.36562	0.05
32182	2.36562	0.05
32183	0.946115	0.0549714
32184	0.946115	0.05
32185	1.06496	0.05
32186	1.18279	0.05
32187	0.946117	0.05

32188	0.946117	0.0573924
32189	1.06496	0.05
32190	1.06496	0.05
32191	0.94613	0.05
32192	1.06497	0.05
32193	0.946105	0.05
32194	1.18278	0.05
32195	1.00213	0.05
32196	2.36559	0.05
32197	1.40272	0.05
32198	1.40272	0.05
32199	2.12992	0.05
32200	2.12992	0.05
32201	0.95646	2.1
32202	2.07201	2.1
32203	1.01808	2.1
32204	2.14876	2.1
32205	3.09009	2.1
32206	1.89226	2.1
32207	1.89226	2.1
32208	1.89226	2.1
32209	1.89226	2.1
32210	3.78453	2.1
32211	1.89227	2.1
32212	1.89227	2.1
32213	1.89227	2.1
32214	1.89227	2.1
32215	1.89227	2.1
32216	1.89227	2.1
32217	2.71886	2.1
32218	1.60497	2.1
32219	1.43935	2.1
32220	2.55151	2.1
32221	4.15648	2.1
32222	2.71886	2.1
32223	1.89226	2.1
32224	1.89226	2.1
32225	2.55151	2.1
32226	4.44377	2.1
32227	1.89226	2.1
32228	1.89226	2.1
32229	1.89226	2.1
32230	1.89226	2.1
32231	3.78453	2.1
32232	1.89226	2.1
32233	1.89226	2.1
32234	1.89226	2.1
32235	1.89226	2.1
32236	3.78453	2.1
32237	1.58942	0.263667
32238	1.92622	0.409091
32239	2.52852	0.335727
32240	2.19457	0.261069
32241	4.12079	0.257731
32242	1.92649	0.213667
32243	1.44426	0.154528
32244	1.87529	0.165741
32245	2.19457	0.23749

32246	3.63883	0.208175
32247	1.87529	0.12429
32248	2.19457	0.204292
32249	2.7378	0.177976
32250	2.29852	0.134391
32251	4.49309	0.129281
32252	1.35976	2.1
32253	1.89452	2.1
32254	1.39616	2.1
32255	1.1423	2.1
32256	1.8996	2.1
32257	1.52068	2.1
32258	1.89226	2.1
32259	1.89226	2.1
32260	1.89226	2.1
32261	1.89226	2.1
32262	1.89226	2.1
32263	1.89226	2.1
32264	1.89226	2.1
32265	2.15617	2.1
32266	2.0953	2.1
32267	2.15617	2.1
32268	1.83139	2.1
32269	1.89226	2.1
32270	1.16817	2.1
32271	1.8996	2.1
32272	1.23614	2.1
32273	1.48338	2.1
32274	1.80218	2.1
32275	2.16263	2.1
32276	1.89226	2.1
32277	1.89226	2.1
32278	1.89226	2.1
32279	1.89226	2.1
32280	1.89226	2.1
32281	1.89226	2.1
32282	1.89226	2.1
32283	2.0953	2.1
32284	2.03074	2.1
32285	2.0953	2.1
32286	1.8277	2.1
32287	1.89226	2.1
32288	1.74455	2.1
32289	2.181	2.1
32290	1.74455	2.1
32291	1.92911	2.1
32292	1.98195	2.1
32293	2.16651	2.1
32294	2.181	2.1
32295	2.29881	2.1
32296	1.98195	2.1
32297	1.80729	2.1
32298	4.28075	2.1
32299	1.74455	2.1
32300	1.98195	2.1
32301	1.52603	2.1
32302	1.76603	2.1
32303	3.51058	2.1

32304	1.76603	2.1
32305	1.94506	2.1
32306	1.76603	2.1
32307	1.98195	2.1
32308	1.86795	2.1
32309	2.08387	2.1
32310	0.984305	2.1
32311	1.9	2.1
32312	2.86619	2.1
32313	1.86189	2.09949
32314	3.76189	2.1
32315	1.4621	2.1
32316	1.89226	2.1
32317	2.81374	2.1
32318	1.89226	2.1
32319	3.35437	2.1
32320	0.946133	2.09691
32321	0.946133	2.1
32322	0.946132	2.09565
32323	0.946132	2.1
32324	0.946132	2.1
32325	0.946132	2.1
32326	0.946133	2.1
32327	0.946133	2.09766
32328	0.946132	2.1
32329	0.946132	2.09547
32330	0.946132	2.1
32331	0.946132	2.1
32332	1.64268	2.1
32333	1.93673	2.1
32334	2.25284	2.1
32335	1.97548	2.1
32336	3.89552	2.1
32337	1.86206	2.1
32338	1.93673	2.1
32339	2.25284	2.1
32340	2.20075	2.1
32341	4.1149	2.1
32342	2.25284	2.1
32343	2.24781	2.1
32344	1.97548	2.1
32345	1.97548	2.1
32346	2.49676	2.1
32347	2.49676	2.1
32348	2.25284	2.1
32349	2.43549	2.1
32350	2.44467	2.1
32351	2.20075	2.1
32352	2.49676	2.1
32353	2.25284	2.1
32354	2.57928	2.1
32355	2.32606	2.1
32356	2.59522	2.1
32357	2.39022	2.1
32358	4.9695	2.1
32359	2.32606	2.1
32360	1.77677	2.1
32361	2.39022	2.1

32362	2.28669	2.1
32363	4.16699	2.1
32364	2.59522	2.1
32365	2.39022	2.1
32366	2.61901	2.1
32367	2.45899	2.1
32368	5.00923	2.1
32369	2.39022	2.1
32370	2.11575	2.1
32371	2.41795	2.1
32372	2.0815	2.1
32373	4.67085	2.1
32374	1.89226	2.1
32375	2.24723	2.1
32376	1.95405	2.1
32377	1.89226	2.1
32378	2.30902	2.1
32379	2.24723	2.1
32380	1.89226	2.1
32381	1.89226	2.1
32382	1.89226	2.1
32383	1.89226	2.1
32384	3.78453	2.1
32385	0.946131	2.1
32386	0.946131	2.1
32387	1.14643	2.1
32388	1.10673	2.1
32389	1.10673	2.1
32390	1.10673	2.1
32391	0.946133	2.1
32392	0.946133	2.1
32393	0.946131	2.1
32394	1.14643	2.1
32395	1.10673	2.1
32396	0.946131	2.1
32397	1.89226	2.1
32398	2.30902	2.1
32399	1.96635	2.1
32400	1.89226	2.1
32401	2.38311	2.1
32402	2.30902	2.1
32403	1.89226	2.1
32404	1.89226	2.1
32405	1.89226	2.1
32406	1.89226	2.1
32407	1.89226	2.1
32408	1.89226	2.1
32409	0.946131	2.1
32410	0.946131	2.1
32411	1.19156	2.1
32412	1.14643	2.1
32413	1.14643	2.1
32414	1.14643	2.1
32415	0.946133	2.1
32416	0.946133	2.1
32417	0.946131	2.1
32418	1.19156	2.1
32419	0.946131	2.1

32420	1.14643	2.1
32421	1.89226	1.69861
32422	1.89226	0.739646
32423	1.89226	1.0539
32424	1.89226	1.7437
32425	1.89226	0.697938
32426	1.89226	1.44022
32427	1.89226	0.949636
32428	1.89226	0.801885
32429	1.89226	0.411316
32430	1.9645	0.906844
32431	3.78452	1.33239
32432	2.39489	2.1
32433	2.15715	2.1
32434	2.07821	2.1
32435	2.33541	2.1
32436	4.49256	2.1
32437	2.39489	2.1
32438	2.34898	2.1
32439	2.30729	2.1
32440	2.33541	2.1
32441	4.6844	2.1
32442	2.07821	2.1
32443	2.33541	2.1
32444	2.26415	2.1
32445	1.99632	2.1
32446	4.33174	2.1
32447	2.30729	2.1
32448	2.33542	2.1
32449	2.26416	2.1
32450	2.26618	2.1
32451	4.60159	2.1
32452	1.19837	1.75393
32453	2.79151	0.464813
32454	2.25636	1.24147
32455	2.25636	0.852965
32456	1.94072	1.20313
32457	1.94072	0.896383
32458	2.53725	0.05
32459	1.16699	0.0533714
32460	1.69864	0.051271
32461	2.0815	0.0584366
32462	3.58273	0.0531815
32463	1.91646	2.1
32464	2.51686	2.1
32465	2.49872	2.1
32466	1.85819	2.1
32467	4.41518	2.1
32468	0.929098	2.1
32469	0.8993	2.1
32470	1.24359	2.1
32471	1.24936	2.1
32472	1.06832	2.1
32473	1.06832	2.1
32474	1.08034	2.1
32475	1.08034	2.1
32476	0.899298	2.1
32477	1.24359	2.1

32478	1.24936	2.1
32479	0.929097	2.1
32480	2.0815	0.05
32481	1.89226	0.05
32482	2.0815	0.05
32483	1.82811	0.05
32484	1.94982	0.05
32485	1.71246	0.05
32486	2.3256	2.1
32487	1.89226	2.1
32488	1.89226	2.1
32489	1.89226	2.1
32490	2.38467	2.1
32491	2.38467	2.1
32492	1.89226	2.1
32493	1.89226	2.1
32494	1.89226	2.1
32495	1.89226	2.1
32496	1.89226	2.1
32497	1.89226	2.1
32498	1.89763	2.1
32499	2.61405	2.1
32500	2.15035	2.1
32501	2.68796	2.1
32502	1.42219	2.1
32503	1.77775	2.1
32504	2.26306	2.1
32505	1.89226	2.1
32506	1.89226	2.1
32507	1.89226	2.1
32508	2.3256	2.1
32509	2.3256	2.1
32510	1.89226	2.1
32511	1.89226	2.1
32512	1.89226	2.1
32513	1.89226	2.1
32514	1.89226	2.1
32515	1.89226	2.1
32516	1.92887	2.1
32517	2.41915	2.1
32518	2.50142	2.1
32519	2.50142	2.1
32520	1.90388	2.1
32521	1.89763	2.1
32522	1.89224	2.1
32523	1.89224	2.1
32524	1.89224	2.1
32525	1.89224	2.1
32526	3.78448	2.1
32527	2.25315	2.1
32528	1.05843	2.1
32529	2.38995	2.1
32530	1.31541	2.1
32531	3.56856	2.1
32532	0.517552	2.1
32533	0.68548	2.1
32534	1.27675	2.1
32535	1.13076	2.1

32536	0.913611	2.1
32537	0.913612	2.1
32538	0.902633	2.1
32539	0.902633	2.1
32540	0.68547	2.1
32541	1.27673	2.1
32542	1.13078	2.1
32543	0.517559	2.1
32544	1.89226	2.1
32545	1.89226	2.1
32546	1.89226	2.1
32547	1.89226	2.1
32548	3.78453	2.1
32549	0.962936	2.1
32550	0.962936	2.1
32551	0.996141	2.1
32552	0.996141	2.1
32553	0.830609	2.1
32554	0.657714	2.1
32555	1.30136	2.1
32556	1.19499	2.1
32557	1.30136	2.1
32558	0.830609	2.1
32559	1.19499	2.1
32560	0.657713	2.1
32561	1.60057	0.097984
32562	1.39615	0.0889691
32563	1.06474	0.0837518
32564	1.39615	0.135244
32565	1.64655	0.0758646
32566	2.04424	0.116236
32567	1.89226	0.0854603
32568	1.89226	0.138813
32569	1.89226	0.0991307
32570	1.89226	0.124728
32571	1.89226	0.107122
32572	1.89226	0.136505
32573	2.56322	0.0705457
32574	1.89226	0.141512
32575	1.89226	0.0878933
32576	1.89226	0.116531
32577	2.18047	0.0803461
32578	2.18047	0.105031
32579	1.89226	0.494995
32580	1.89226	0.471611
32581	1.89226	0.549258
32582	1.65573	0.385269
32583	1.89226	0.604663
32584	1.65573	0.440601
32585	1.05353	0.529026
32586	1.83158	0.678818
32587	1.50282	0.344719
32588	1.20669	0.239918
32589	2.88511	0.258479
32590	1.44807	2.1
32591	1.93592	2.1
32592	1.80703	2.1
32593	1.27806	2.1

32594	3.21398	2.1
32595	2.0815	2.1
32596	1.87465	2.1
32597	1.87465	2.1
32598	1.50566	2.1
32599	2.0815	2.1
32600	1.69263	2.1
32601	1.89226	2.1
32602	1.89226	2.1
32603	1.89226	2.1
32604	1.89226	2.1
32605	1.89226	2.1
32606	1.89226	2.1
32607	1.62632	2.1
32608	0.994294	2.1
32609	0.994294	2.1
32610	1.27806	2.1
32611	1.81921	2.1
32612	2.10776	2.1
32613	2.0815	2.1
32614	1.70303	2.1
32615	1.70303	2.1
32616	1.85749	2.1
32617	1.90451	2.1
32618	2.07724	2.1
32619	1.89226	2.1
32620	1.89226	2.1
32621	1.89226	2.1
32622	1.89226	2.1
32623	1.89226	2.1
32624	1.89226	2.1
32625	2.69773	2.1
32626	1.88129	2.1
32627	2.55196	2.1
32628	2.26469	2.1
32629	4.94367	2.1
32630	1.88129	2.1
32631	1.25889	2.1
32632	1.26614	2.1
32633	1.05914	2.1
32634	2.26469	2.1
32635	1.88129	2.1
32636	2.55196	2.1
32637	2.0588	2.1
32638	2.6536	2.1
32639	2.15287	2.1
32640	4.70484	2.1
32641	0.8516	2.1
32642	0.8516	2.1
32643	0.849002	2.1
32644	0.849002	2.1
32645	0.629445	2.1
32646	0.671197	2.1
32647	1.0294	2.1
32648	1.07644	2.1
32649	1.07644	2.1
32650	0.671197	2.1
32651	0.629446	2.1

32652	1.0294	2.1
32653	2.02115	2.04683
32654	1.18382	1.82986
32655	2.29107	0.224952
32656	1.46903	0.565835
32657	3.47488	0.459158
32658	2.60763	1.55897
32659	2.29107	1.4804
32660	1.82141	1.0245
32661	1.82141	1.61731
32662	1.46903	1.66894
32663	2.29107	1.82092
32664	1.64007	2.1
32665	2.2024	2.1
32666	1.44044	2.1
32667	2.02634	2.1
32668	3.6664	2.1
32669	1.25565	2.1
32670	2.02635	2.1
32671	1.87228	2.1
32672	1.87228	2.1
32673	1.44045	2.1
32674	1.44045	2.1
32675	1.89226	0.05
32676	1.78503	0.05
32677	1.78503	0.05
32678	1.40048	0.05
32679	2.0815	0.05
32680	1.62972	0.05
32681	1.27875	2.1
32682	1.1483	2.1
32683	1.40014	2.1
32684	1.40014	2.1
32685	1.04598	2.1
32686	1.04598	2.1
32687	2.63443	1.7513
32688	2.26444	2.07068
32689	2.79392	0.633301
32690	2.4863	2.1
32691	5.12073	2.0059
32692	1.83158	0.803721
32693	1.18508	0.234817
32694	1.20669	0.187939
32695	1.81488	0.482744
32696	1.20669	0.328519
32697	1.81488	0.473367
32698	1.08134	0.21996
32699	1.70303	0.547639
32700	1.24027	0.369061
32701	1.30359	0.2224
32702	1.89939	0.577289
32703	1.70303	0.586751
32704	1.89226	2.09577
32705	2.69027	2.1
32706	1.89226	2.1
32707	2.2078	2.0806
32708	1.89226	2.1
32709	2.2078	2.0849

32710	1.89226	2.1
32711	2.78977	2.1
32712	1.89226	2.1
32713	2.69027	2.09981
32714	1.89226	2.1
32715	2.69027	2.1
32716	1.89226	2.09184
32717	1.89226	2.07583
32718	1.89226	1.94289
32719	1.89226	2.09508
32720	3.78452	2.07254
32721	1.43687	1.99849
32722	1.71059	2.09576
32723	2.53014	2.02924
32724	2.6521	2.06339
32725	4.24073	2.04804
32726	2.6521	2.06393
32727	1.66762	2.1
32728	2.50299	2.0823
32729	2.50299	2.09746
32730	1.71059	2.09566
32731	1.71059	2.09827
32732	1.89226	2.09518
32733	1.89226	2.09769
32734	1.89226	2.1
32735	1.89226	2.07087
32736	1.89226	2.1
32737	1.89226	2.07558
32738	2.10662	0.278397
32739	2.77417	0.567458
32740	2.78873	0.718098
32741	2.11661	0.256729
32742	4.89078	0.427931
32743	2.10662	0.356383
32744	1.22857	0.105008
32745	1.25849	0.304083
32746	2.11661	0.340911
32747	3.34518	0.256725
32748	2.78873	0.823358
32749	2.11661	0.270412
32750	2.12475	0.382707
32751	2.79029	0.634991
32752	4.9069	0.440499
32753	1.2858	0.258409
32754	2.11661	0.325469
32755	1.25849	0.245006
32756	1.25849	0.434609
32757	2.12475	0.234058
32758	2.12475	0.398656
32759	1.13537	2.1
32760	1.20714	2.1
32761	0.946129	2.1
32762	0.946129	2.1
32763	0.946131	2.1
32764	0.946131	2.1
32765	1.29521	2.1
32766	1.20713	2.1
32767	1.13537	2.1

32768	0.946131	2.1
32769	0.94613	2.1
32770	1.29521	2.1
32771	1.00533	2.1
32772	0.876055	2.1
32773	0.946128	2.1
32774	0.946128	2.1
32775	0.913908	2.1
32776	0.913908	2.1
32777	1.17081	2.1
32778	0.896413	2.1
32779	0.955905	2.1
32780	0.893715	2.1
32781	1.20327	2.1
32782	0.998544	2.1
32783	1.89226	1.88128
32784	1.89226	1.99656
32785	1.89226	0.278887
32786	1.89226	0.968587
32787	3.78452	0.453353
32788	0.540975	1.34841
32789	0.726949	2.1
32790	0.712198	2.0644
32791	0.506675	0.818149
32792	0.611916	1.01234
32793	0.611916	2.1
32794	0.621708	1.88326
32795	0.621708	0.874387
32796	0.726951	1.01313
32797	0.712196	0.666546
32798	0.506674	1.55019
32799	0.540976	1.67009
32800	1.89226	0.873443
32801	1.89226	2.1
32802	1.89226	1.67015
32803	1.89226	0.962117
32804	3.78453	1.70403
32805	1.89226	0.621337
32806	1.89226	1.25689
32807	1.89226	0.508311
32808	1.89226	2.05968
32809	1.89226	0.504084
32810	1.89226	2.1
32811	0.919594	0.796406
32812	0.726949	1.79308
32813	0.898318	1.10572
32814	0.712198	1.67776
32815	0.817559	0.66829
32816	0.817559	2.1
32817	0.814234	0.728545
32818	0.814234	1.92479
32819	0.919597	0.421295
32820	0.898316	0.311764
32821	0.726951	1.74962
32822	0.712196	1.20598
32823	1.79663	0.542665
32824	1.12088	2.07468
32825	1.54699	1.74278

32826	1.17475	1.52117
32827	1.79663	2.05678
32828	1.42439	1.97474
32829	2.28928	1.19668
32830	1.23077	0.992568
32831	1.65458	0.241925
32832	2.66533	1.87849
32833	3.94385	0.900286
32834	1.89226	2.1
32835	1.89226	2.1
32836	1.89226	2.1
32837	1.89226	2.1
32838	3.78452	2.1
32839	1.38426	2.1
32840	1.15067	2.1
32841	1.38426	2.1
32842	1.01595	2.1
32843	1.24509	2.1
32844	1.01595	2.1
32845	2.62097	2.1
32846	1.89226	2.1
32847	2.72561	2.1
32848	2.72561	2.1
32849	1.89226	2.1
32850	1.89226	2.1
32851	1.90103	1.95257
32852	1.41902	2.1
32853	1.77249	1.97681
32854	1.30272	2.1
32855	3.20375	2.1
32856	1.77249	1.90462
32857	1.19548	2.1
32858	1.19548	2.1
32859	1.30272	2.1
32860	1.65815	2.0501
32861	1.76539	2.1
32862	2.62161	2.1
32863	1.89226	2.1
32864	1.89226	2.1
32865	1.89226	2.1
32866	2.6446	2.1
32867	2.6446	2.1
32868	2.64459	2.1
32869	1.89226	2.1
32870	1.89226	2.1
32871	1.89226	2.1
32872	2.58257	2.1
32873	2.58257	2.1
32874	1.89226	0.301742
32875	1.89226	0.832709
32876	2.31301	0.79047
32877	2.76309	0.380145
32878	1.89226	0.741732
32879	2.26047	0.364409
32880	1.89226	0.274662
32881	2.31301	0.793997
32882	2.17765	0.700563
32883	2.17765	0.382368

32884	1.89226	0.65536
32885	1.89226	0.335793
32886	2.18516	2.1
32887	1.85196	2.1
32888	1.78595	2.1
32889	2.14606	2.1
32890	3.99802	2.1
32891	2.18516	2.1
32892	2.21189	2.1
32893	2.18937	2.1
32894	2.14606	2.1
32895	4.35796	2.1
32896	1.60735	2.1
32897	2.14606	2.1
32898	2.15714	2.1
32899	1.70302	2.1
32900	3.70055	2.1
32901	2.18937	2.1
32902	2.14606	2.1
32903	2.0927	2.1
32904	2.162 2.1	
32905	4.30806	2.1
32906	2.17326	2.1
32907	2.58517	2.1
32908	2.55318	2.1
32909	2.1665	2.1
32910	4.75167	2.1
32911	2.17326	2.1
32912	1.53556	2.1
32913	1.56253	2.1
32914	2.1665	2.1
32915	3.70206	2.1
32916	2.55317	2.1
32917	2.1665	2.1
32918	2.17741	2.1
32919	2.54197	2.1
32920	4.70847	2.1
32921	2.17742	2.1
32922	2.1665	2.1
32923	2.18452	2.1
32924	1.58054	2.1
32925	1.56253	2.1
32926	1.56253	2.1
32927	1.71051	0.05
32928	1.89807	0.0577299
32929	1.19755	0.0553513
32930	1.19755	0.05
32931	2.25632	0.0898686
32932	2.25632	0.05
32933	0.876046	0.0593503
32934	1.27293	0.0550612
32935	0.946132	0.173975
32936	0.946132	0.0926253
32937	0.946131	0.0695325
32938	0.869312	0.0682914
32939	1.12767	0.134807
32940	0.946132	0.112629
32941	1.22225	0.150431

32942	1.37968	0.0635182
32943	0.851555	0.158662
32944	0.851555	0.0710833
32945	0.946133	2.06249
32946	0.946133	2.1
32947	0.946132	2.07229
32948	0.946132	1.95934
32949	0.946133	2.0348
32950	0.946133	2.07345
32951	0.946132	2.1
32952	0.946132	2.07131
32953	0.946132	2.09934
32954	0.946132	2.07985
32955	0.946133	2.06058
32956	0.946133	2.08479
32957	1.35152	2.1
32958	0.948936	2.1
32959	1.90166	2.1
32960	1.70303	2.1
32961	2.90858	2.1
32962	1.89226	2.1
32963	1.89226	2.1
32964	1.89226	2.1
32965	1.89226	2.1
32966	3.78452	2.1
32967	1.90166	2.1
32968	1.70303	2.1
32969	2.52454	2.1
32970	2.13919	2.1
32971	3.99529	2.1
32972	1.89226	2.09343
32973	1.89226	2.1
32974	1.89226	2.1
32975	1.89226	2.1
32976	3.78452	2.1
32977	1.89226	2.1
32978	1.89226	2.1
32979	1.89226	2.1
32980	1.89226	2.1
32981	1.89226	2.09353
32982	1.89226	2.1
32983	2.13918	2.1
32984	0.992674	2.1
32985	2.3397	2.1
32986	1.8023	2.1
32987	1.47769	2.1
32988	0.992674	2.1
32989	1.89226	0.273545
32990	1.89226	0.257034
32991	1.89226	0.369025
32992	1.89226	0.195432
32993	3.78452	0.278272
32994	1.89226	0.264177
32995	2.54752	0.13925
32996	2.63974	0.201468
32997	1.89226	0.188443
32998	4.53201	0.185605
32999	0.498412	0.194301

33000	0.500955	0.174752
33001	0.57955	0.243585
33002	0.563873	0.210812
33003	0.529107	0.206
33004	0.529107	0.176166
33005	0.53572	0.25796
33006	0.53572	0.189357
33007	0.500956	0.199687
33008	0.579548	0.195941
33009	0.563871	0.20014
33010	0.498414	0.203186
33011	1.89223	0.319213
33012	1.89223	0.189533
33013	1.89223	0.243052
33014	1.89223	0.273564
33015	1.89223	0.24399
33016	1.89223	0.274701
33017	1.28752	0.141308
33018	1.31985	0.183038
33019	0.946119	0.182863
33020	0.946119	0.248611
33021	1.28752	0.183656
33022	1.28752	0.142828
33023	0.946119	0.180137
33024	0.946119	0.306315
33025	1.28753	0.184535
33026	0.946132	0.183824
33027	1.31984	0.196967
33028	0.946107	0.193675
33029	0.500948	0.178053
33030	0.479465	0.164506
33031	0.562335	0.226271
33032	0.579542	0.192745
33033	0.528436	0.18936
33034	0.528436	0.166341
33035	0.530571	0.177689
33036	0.530571	0.234689
33037	0.56234	0.188619
33038	0.479473	0.189895
33039	0.500943	0.193756
33040	0.579533	0.189042
33041	1.89226	2.1
33042	1.89226	2.1
33043	1.89226	2.1
33044	1.89226	2.1
33045	3.78452	2.1
33046	1.50767	2.1
33047	1.14496	2.1
33048	1.50767	2.1
33049	1.58539	2.1
33050	1.07653	2.1
33051	1.15425	2.1
33052	1.89226	2.1
33053	2.43826	2.1
33054	2.43826	2.1
33055	2.48688	2.1
33056	1.89226	2.1
33057	1.94088	2.1

33058	1.89226	2.1
33059	1.89226	2.1
33060	1.89226	2.1
33061	1.89226	2.1
33062	3.78452	2.1
33063	1.50767	2.1
33064	1.07653	2.1
33065	1.41077	2.1
33066	0.991407	2.1
33067	2.49907	2.1
33068	1.21913	2.1
33069	1.21913	2.1
33070	0.946128	2.1
33071	0.946128	2.1
33072	1.21913	2.1
33073	1.18364	2.1
33074	0.946131	2.1
33075	0.946131	2.1
33076	1.18364	2.1
33077	0.946129	2.1
33078	1.21913	2.1
33079	0.94613	2.1
33080	1.89226	2.1
33081	1.89226	2.1
33082	1.89226	2.1
33083	1.89226	2.1
33084	3.78452	2.1
33085	1.93823	2.1
33086	1.60976	2.1
33087	1.93823	2.1
33088	2.20045	2.1
33089	1.36999	2.1
33090	1.63222	2.1
33091	1.38302	2.1
33092	1.38302	2.1
33093	0.946132	2.1
33094	0.946132	2.1
33095	1.27258	2.1
33096	1.38302	2.1
33097	0.946133	2.1
33098	0.946133	2.1
33099	0.946133	2.1
33100	1.27258	2.1
33101	0.946132	2.1
33102	1.38302	2.1
33103	1.89226	2.1
33104	1.89226	2.1
33105	1.89226	2.1
33106	1.89226	2.1
33107	3.78452	2.1
33108	1.93823	2.1
33109	1.36999	2.1
33110	1.70423	2.1
33111	1.14195	2.1
33112	3.08018	2.1
33113	1.27258	2.1
33114	1.27258	2.1
33115	0.946132	2.1

33116	0.946132	2.1
33117	1.17233	2.1
33118	1.27258	2.1
33119	0.946132	2.1
33120	0.946132	2.1
33121	1.17233	2.1
33122	0.946132	2.1
33123	0.946132	2.1
33124	1.27258	2.1
33125	2.70575	2.1
33126	1.89226	1.65931
33127	2.71429	2.1
33128	2.71429	2.1
33129	1.89226	2.1
33130	1.89226	2.07807
33131	0.946133	2.08141
33132	0.946133	2.1
33133	1.28713	2.1
33134	1.28713	2.1
33135	0.946131	2.1
33136	0.946131	2.06792
33137	1.28713	2.1
33138	1.36503	2.1
33139	0.946131	2.1
33140	1.36503	2.1
33141	1.28713	2.1
33142	0.946131	2.1
33143	1.89226	2.1
33144	1.89226	1.95905
33145	1.89226	2.1
33146	1.89226	2.1
33147	1.89226	2.1
33148	1.89226	2.09376
33149	1.22615	2.1
33150	1.04147	2.1
33151	1.3439	2.1
33152	1.3439	2.1
33153	0.969118	2.1
33154	0.969118	2.1
33155	0.980841	2.1
33156	0.992505	2.1
33157	1.34289	2.1
33158	1.70303	2.1
33159	2.56081	2.1
33160	1.89225	2.1
33161	2.66785	2.1
33162	2.79506	2.1
33163	2.79506	2.1
33164	1.89225	2.1
33165	1.89225	2.1
33166	0.978778	2.1
33167	0.978778	2.1
33168	0.993303	2.1
33169	0.993303	2.1
33170	0.603538	2.1
33171	0.638158	2.1
33172	1.33393	2.1
33173	1.39753	2.1

33174	1.33391	2.1
33175	0.603535	2.1
33176	0.638164	2.1
33177	1.39754	2.1
33178	1.33393	2.1
33179	1.33393	2.1
33180	0.946132	2.1
33181	0.946132	2.1
33182	1.33393	2.1
33183	1.27365	2.1
33184	0.946132	2.1
33185	0.946132	2.1
33186	0.946132	2.1
33187	1.27365	2.1
33188	1.33393	2.1
33189	0.946131	2.1
33190	1.89226	2.1
33191	1.89226	2.1
33192	1.89226	2.1
33193	1.89226	2.1
33194	3.78452	2.1
33195	1.89226	2.1
33196	1.89226	2.1
33197	1.89226	2.1
33198	1.89226	2.1
33199	3.78453	2.1
33200	0.979364	2.1
33201	0.979364	2.1
33202	0.89618	2.1
33203	0.89618	2.1
33204	0.896178	2.1
33205	0.929411	2.1
33206	0.946133	2.1
33207	0.946133	2.1
33208	0.946131	2.1
33209	0.89618	2.1
33210	0.946131	2.1
33211	0.929413	2.1
33212	1.89226	2.1
33213	1.89226	2.1
33214	1.89226	2.1
33215	1.89226	2.1
33216	1.89226	2.1
33217	1.89226	2.1
33218	1.89225	2.1
33219	1.89225	2.1
33220	1.89225	2.1
33221	1.89225	2.1
33222	3.7845	2.1
33223	1.89225	2.1
33224	1.89225	2.1
33225	1.89225	2.1
33226	1.89225	2.1
33227	1.89225	2.1
33228	1.89225	2.1
33229	0.946128	0.173237
33230	0.946128	0.114615
33231	0.946132	0.145462

33232	0.946132	0.0909143
33233	0.94613	0.149071
33234	0.94613	0.174811
33235	0.946129	0.1041
33236	0.946129	0.0793483
33237	0.94613	0.127973
33238	0.94613	0.12851
33239	0.94613	0.11777
33240	0.94613	0.114947
33241	1.89227	1.24654
33242	1.89227	1.30783
33243	1.89227	0.572802
33244	1.89227	0.457063
33245	3.78453	0.798212
33246	1.89226	1.16592
33247	2.43717	1.74369
33248	2.63259	0.907101
33249	1.89226	0.482299
33250	4.52485	1.2959
33251	0.616808	0.776645
33252	0.792277	0.940799
33253	1.04986	0.529648
33254	0.72303	1.15335
33255	0.702675	0.20078
33256	0.76862	2.05952
33257	0.809154	1.61982
33258	0.809154	0.50824
33259	0.808757	0.938246
33260	1.07789	0.27627
33261	0.772568	0.634047
33262	0.598878	1.18222
33263	1.89226	1.1545
33264	1.89226	1.3584
33265	1.89226	0.758263
33266	1.89226	0.607488
33267	3.78453	1.19642
33268	1.36428	1.3976
33269	1.31629	1.10865
33270	0.946132	0.905613
33271	0.946132	0.907099
33272	1.36428	0.598213
33273	1.36428	1.89349
33274	0.946132	1.3117
33275	0.946132	1.69428
33276	1.36428	1.54324
33277	0.946132	0.774648
33278	1.31629	1.47365
33279	0.946132	0.814848
33280	1.578	1.36744
33281	2.85336	0.331247
33282	2.11368	0.273231
33283	2.14375	1.13951
33284	1.93852	0.654068
33285	1.93852	1.19583
33286	2.34806	0.219177
33287	1.42913	0.239048
33288	1.61539	0.304424
33289	2.45322	0.259321

33290	3.88235	0.273894
33291	2.34806	0.137161
33292	1.89226	0.185614
33293	1.89226	0.412638
33294	2.45322	0.295965
33295	4.34548	0.167523
33296	1.89226	0.175734
33297	1.89226	0.113581
33298	1.89226	0.19653
33299	1.89226	0.255059
33300	3.78452	0.221726
33301	1.89226	0.24012
33302	1.89226	0.1661
33303	1.89226	0.256136
33304	1.89226	0.315099
33305	3.78452	0.219582
33306	1.25972	0.164802
33307	2.45322	0.165108
33308	2.58823	0.315921
33309	1.79398	0.247116
33310	4.2472	0.174635
33311	1.89226	0.282362
33312	2.45322	0.0981663
33313	2.58823	0.201591
33314	2.45322	0.249625
33315	2.02727	0.113463
33316	1.89226	0.161762
33317	1.89226	0.0916947
33318	1.89226	0.140237
33319	1.89226	0.236131
33320	1.89226	0.190763
33321	3.78452	0.105703
33322	1.89226	0.165349
33323	1.89226	0.21387
33324	1.89226	0.199669
33325	1.89226	0.105467
33326	1.89226	0.312806
33327	1.89226	0.157673
33328	1.70048	2.1
33329	2.1572	1.65197
33330	1.45477	2.1
33331	1.93973	2.02092
33332	3.64021	2.1
33333	1.17614	2.1
33334	1.93973	1.95102
33335	1.69661	2.1
33336	1.69661	1.88348
33337	1.45477	2.1
33338	1.45477	1.95144
33339	1.94343	2.1
33340	2.53782	1.98456
33341	2.59903	2.1
33342	2.59903	1.35091
33343	1.87598	2.1
33344	1.87598	1.85081
33345	0.946131	1.23384
33346	0.946131	1.97537
33347	1.26891	2.1

33348	1.26891	0.647987
33349	0.94613	2.03501
33350	0.94613	1.69904
33351	1.26891	1.83874
33352	1.29952	1.08272
33353	1.29952	0.200342
33354	0.946131	0.343658
33355	1.26891	1.0915
33356	0.946131	1.19934
33357	2.00551	2.1
33358	2.59903	2.04258
33359	2.65811	2.1
33360	2.65811	1.85053
33361	1.94343	2.1
33362	1.94343	2.07203
33363	2.65811	1.26259
33364	1.89226	2.08088
33365	1.89226	0.605005
33366	1.89226	1.69279
33367	2.59903	0.907552
33368	2.59903	2.06172
33369	1.89227	2.1
33370	1.89227	2.1
33371	1.89227	2.1
33372	1.89227	2.1
33373	3.78453	2.1
33374	1.15471	2.1
33375	0.993889	2.1
33376	1.78919	2.1
33377	1.77325	2.1
33378	2.78308	2.1
33379	1.89226	2.1
33380	1.89226	2.1
33381	1.89226	2.1
33382	1.89226	2.1
33383	3.78453	2.1
33384	1.78919	2.1
33385	1.77325	2.1
33386	2.45969	2.1
33387	2.70835	2.1
33388	4.49753	2.1
33389	0.939314	2.1
33390	0.939315	2.1
33391	1.01133	2.1
33392	1.01133	2.1
33393	0.596471	2.1
33394	1.20813	2.1
33395	0.886619	2.1
33396	1.35417	2.1
33397	1.35417	2.1
33398	1.20813	2.1
33399	0.886621	2.1
33400	0.59647	2.1
33401	0.946131	2.1
33402	0.946131	2.1
33403	0.94613	2.1
33404	0.94613	2.1
33405	0.946133	2.1

33406	0.946133	2.1
33407	0.946128	2.1
33408	0.946128	2.1
33409	0.946131	2.1
33410	0.946131	2.1
33411	0.946131	2.1
33412	0.946131	2.1
33413	1.03939	2.1
33414	1.03939	2.1
33415	0.946132	2.08758
33416	0.946132	2.1
33417	0.946132	2.1
33418	0.946132	2.1
33419	1.23851	2.09956
33420	1.03939	2.1
33421	1.03939	2.1
33422	0.946132	2.1
33423	0.946133	2.1
33424	1.23851	2.1
33425	0.902503	2.1
33426	0.946129	2.1
33427	0.946132	2.09055
33428	0.946132	2.1
33429	0.902472	2.1
33430	0.902472	2.1
33431	1.08736	2.09973
33432	0.994093	2.1
33433	0.946133	2.1
33434	0.902504	2.1
33435	1.03939	2.1
33436	0.946132	2.1
33437	1.35306	1.13537
33438	1.29982	0.368794
33439	1.7216	0.258595
33440	1.89183	0.29606
33441	3.07466	0.321067
33442	0.485098	0.342156
33443	0.582401	0.351692
33444	0.649908	0.531792
33445	0.8608	0.414985
33446	0.587739	0.211414
33447	0.58774	0.585669
33448	0.644567	0.331589
33449	0.644567	1.23801
33450	0.58241	0.399663
33451	0.86081	0.225415
33452	0.649898	0.698746
33453	0.485093	0.377056
33454	2.25936	2.1
33455	2.71441	2.1
33456	2.63824	2.1
33457	2.20652	2.1
33458	4.92092	2.1
33459	2.25936	2.1
33460	1.57627	2.1
33461	1.5493	2.1
33462	2.20652	2.1
33463	3.78279	2.1

33464	2.63824	2.1
33465	2.20652	2.1
33466	2.17326	2.1
33467	2.58517	2.1
33468	4.79169	2.1
33469	2.17326	2.1
33470	2.20652	2.1
33471	2.19278	2.1
33472	1.53556	2.1
33473	1.5493	2.1
33474	1.5493	2.1
33475	1.97465	2.1
33476	2.13925	2.1
33477	2.04483	2.1
33478	1.62784	2.1
33479	3.76709	2.1
33480	1.89226	2.1
33481	1.89226	2.1
33482	1.89226	2.1
33483	1.89226	2.1
33484	1.89226	2.1
33485	1.89226	2.1
33486	0.724037	2.1
33487	0.81392	2.1
33488	0.96796	2.1
33489	1.02242	2.1
33490	0.882091	2.1
33491	0.882091	2.1
33492	0.899789	2.1
33493	0.899789	2.1
33494	0.724036	2.1
33495	0.96796	2.1
33496	1.02242	2.1
33497	0.81392	2.1
33498	1.13537	2.1
33499	1.63054	2.1
33500	0.946131	2.1
33501	0.946131	2.1
33502	0.946131	2.1
33503	0.946131	2.1
33504	1.258 2.1	
33505	1.63054	2.1
33506	1.22632	2.1
33507	1.22632	2.1
33508	0.946131	2.1
33509	1.258 2.1	
33510	1.89226	2.1
33511	1.89226	2.1
33512	1.89226	2.1
33513	1.89226	2.1
33514	1.89226	2.1
33515	1.89226	2.1
33516	0.809739	2.1
33517	0.862674	2.1
33518	1.1995	2.1
33519	1.33899	2.1
33520	1.06324	2.1
33521	1.06324	2.1

33522	0.981932	2.1
33523	1.19563	2.1
33524	0.809727	2.1
33525	1.14092	2.1
33526	1.33902	2.1
33527	0.863494	2.1
33528	2.23742	2.1
33529	1.84902	2.1
33530	1.88088	2.1
33531	2.20392	2.1
33532	4.08644	2.1
33533	1.3592	2.1
33534	1.84902	2.1
33535	1.88088	2.1
33536	1.3305	2.1
33537	1.88088	2.1
33538	1.3305	2.1
33539	1.84902	2.1
33540	1.15008	1.00461
33541	1.3305	2.1
33542	1.70539	2.1
33543	2.9991	2.1
33544	1.84901	2.1
33545	2.0264	2.1
33546	2.20391	2.1
33547	1.70539	2.1
33548	3.87541	2.1
33549	1.03852	2.1
33550	1.38247	1.64913
33551	1.36635	1.73944
33552	1.01537	2.09931
33553	2.39784	2.04601
33554	2.0815	2.1
33555	1.91744	2.1
33556	1.91744	2.08217
33557	2.06496	2.1
33558	1.89227	2.08793
33559	2.03474	2.1
33560	1.89226	1.74729
33561	1.89226	2.08678
33562	1.89226	1.76405
33563	1.89226	2.1
33564	1.89226	1.53383
33565	1.89226	2.09962
33566	1.36635	1.89172
33567	1.02923	2.09927
33568	1.02923	2.00801
33569	1.01537	2.1
33570	1.36416	1.84269
33571	1.3503	2.1
33572	1.89226	2.1
33573	1.89359	2.1
33574	1.89359	2.09823
33575	1.91744	2.1
33576	1.89226	2.09436
33577	1.91611	2.1
33578	0.946128	2.09962
33579	0.946128	2.09188

33580	0.946133	1.86789
33581	0.946133	1.73545
33582	0.94613	1.71545
33583	0.94613	2.09282
33584	0.946131	2.1
33585	0.946131	1.84554
33586	0.946131	2.07087
33587	0.946131	2.07798
33588	0.946131	2.06697
33589	0.946131	2.07747
33590	2.689	2.1
33591	2.0815	2.1
33592	2.72786	2.1
33593	2.48295	2.1
33594	5.02397	2.1
33595	2.72786	2.1
33596	1.86424	2.1
33597	2.7188	2.1
33598	2.08981	2.1
33599	4.81768	2.1
33600	2.08982	2.1
33601	0.986987	2.1
33602	2.08982	2.1
33603	1.87249	2.1
33604	1.01964	2.1
33605	0.906115	2.1
33606	1.44247	2.1
33607	1.43872	2.1
33608	1.17054	2.1
33609	1.17054	2.1
33610	1.43872	2.1
33611	1.26045	2.1
33612	2.0815	0.05
33613	2.0815	0.05
33614	2.0815	0.05
33615	1.81468	0.05
33616	1.89065	0.05
33617	1.55625	0.05
33618	1.89062	0.05
33619	1.89224	0.05
33620	1.89224	0.05
33621	2.27073	0.05
33622	1.86856	0.05
33623	2.24232	0.05
33624	1.21419	2.1
33625	2.79716	2.1
33626	2.50005	2.1
33627	2.50005	2.1
33628	1.5008	2.1
33629	1.5008	2.1
33630	1.60139	0.05
33631	1.7103	0.05
33632	1.04159	0.05
33633	1.2489	0.05
33634	2.75189	0.05
33635	0.732812	0.05
33636	0.732812	0.05
33637	0.751986	0.05

33638	0.751986	0.05
33639	0.860346	0.05
33640	0.667961	0.05
33641	0.85515	0.05
33642	0.624452	0.05
33643	0.62445	0.05
33644	0.667962	0.05
33645	0.855148	0.05
33646	0.860348	0.05
33647	1.89226	0.177944
33648	2.50872	0.170204
33649	2.77909	0.158643
33650	1.89226	0.198158
33651	2.77909	0.149906
33652	1.89226	0.193987
33653	2.15825	0.166547
33654	2.50872	0.157021
33655	1.90439	0.131335
33656	1.90439	0.184262
33657	2.77909	0.133828
33658	2.77909	0.184782
33659	1.12468	2.1
33660	1.43872	2.1
33661	1.64926	2.1
33662	1.2724	2.1
33663	2.77395	2.1
33664	1.64926	2.1
33665	1.38438	2.1
33666	1.47244	2.1
33667	1.2724	2.1
33668	1.8493	2.1
33669	1.64926	2.1
33670	1.36416	1.96996
33671	1.0652	2.1
33672	1.0652	2.03167
33673	1.02923	2.1
33674	1.37496	1.93433
33675	1.33898	2.1
33676	1.89227	2.1
33677	1.90433	2.1
33678	1.90433	2.1
33679	1.89359	2.1
33680	1.89227	2.09986
33681	1.88153	2.1
33682	0.946129	2.1
33683	0.946129	2.09894
33684	0.946133	1.95553
33685	0.946133	1.91745
33686	0.946131	1.90858
33687	0.946131	2.09944
33688	0.946131	2.1
33689	0.946131	1.91869
33690	0.946131	2.07087
33691	0.946131	2.07748
33692	0.946131	2.04599
33693	0.946131	2.05206
33694	1.37496	2.00148
33695	1.07519	2.1

33696	1.07519	2.0563
33697	1.0652	2.1
33698	1.39725	1.98745
33699	1.38726	2.1
33700	1.89227	2.1
33701	1.9052	2.1
33702	1.9052	2.1
33703	1.90433	2.1
33704	1.89227	2.1
33705	1.8914	2.1
33706	0.946129	2.1
33707	0.946129	2.1
33708	0.946133	2.01011
33709	0.946133	1.94512
33710	0.946131	1.94176
33711	0.946131	2.1
33712	0.946131	2.1
33713	0.946131	1.97406
33714	0.946131	2.08755
33715	0.946131	2.09221
33716	0.946131	2.06734
33717	0.946131	2.07132
33718	1.91316	0.0840533
33719	1.89226	0.0775529
33720	1.89226	0.0826731
33721	1.89226	0.081087
33722	1.99366	0.084251
33723	1.99366	0.0800152
33724	1.93083	0.0681443
33725	1.89226	0.0629307
33726	1.51379	0.06058
33727	1.89226	0.0607765
33728	1.53051	0.0672301
33729	1.91316	0.0618779
33730	2.16731	2.1
33731	1.21373	2.1
33732	1.47804	2.1
33733	2.37601	2.1
33734	3.64535	2.1
33735	1.7211	2.1
33736	2.37601	2.1
33737	1.47804	2.1
33738	1.47804	2.1
33739	2.56843	2.1
33740	2.56843	2.1
33741	2.58731	2.1
33742	1.59094	2.1
33743	1.59094	2.1
33744	1.76816	2.1
33745	2.41707	2.1
33746	2.5943	2.1
33747	0.718346	2.1
33748	0.795468	2.1
33749	1.20854	2.1
33750	1.1224	2.1
33751	0.961203	2.1
33752	0.961203	2.1
33753	0.95667	2.1

33754	0.95667	2.1
33755	0.718346	2.1
33756	1.1224	2.1
33757	0.795469	2.1
33758	1.20854	2.1
33759	1.84076	2.1
33760	1.42884	2.1
33761	2.24274	2.1
33762	1.85415	2.1
33763	3.69491	2.1
33764	1.89226	2.1
33765	1.89226	2.1
33766	1.89226	2.1
33767	1.89226	2.1
33768	3.78452	2.1
33769	2.24273	2.1
33770	2.24412	2.1
33771	1.85415	2.1
33772	1.85415	2.1
33773	2.68473	2.1
33774	2.68473	2.1
33775	1.89226	2.1
33776	1.89226	2.1
33777	1.89226	2.1
33778	1.89226	2.09092
33779	1.89226	2.1
33780	1.89226	2.08822
33781	1.89226	2.08382
33782	1.89226	2.1
33783	1.89226	2.1
33784	1.89226	2.0969
33785	3.78451	2.1
33786	1.85415	2.1
33787	1.10961	2.1
33788	2.24411	2.1
33789	1.51067	2.1
33790	3.35373	2.1
33791	1.70302	2.1
33792	1.89226	2.1
33793	1.76399	2.1
33794	1.43577	2.1
33795	2.08149	2.1
33796	1.70302	2.1
33797	1.89226	2.1
33798	1.89226	2.1
33799	1.89226	2.1
33800	1.89226	2.1
33801	1.89226	2.1
33802	1.89226	2.1
33803	1.0593	2.1
33804	1.17289	2.1
33805	1.5379	2.1
33806	1.46757	2.1
33807	2.71079	2.1
33808	0.931235	2.1
33809	1.1151	2.1
33810	0.975236	2.1
33811	0.975236	2.1

33812	1.31147	2.1
33813	0.762882	2.1
33814	1.13152	2.1
33815	0.505799	2.1
33816	1.31149	2.1
33817	1.07629	2.1
33818	0.884817	2.1
33819	0.505793	2.1
33820	2.69027	2.1
33821	1.60441	2.1
33822	2.69027	2.1
33823	1.64771	2.1
33824	2.78977	2.1
33825	1.72731	2.1
33826	0.77878	0.05
33827	0.843226	0.05
33828	0.845997	0.05
33829	0.926091	0.05
33830	0.77878	0.05
33831	0.926093	0.05
33832	1.3922	1.64617
33833	1.3922	1.44543
33834	0.872156	1.96619
33835	0.872156	1.90379
33836	0.872157	1.98425
33837	0.946132	1.3247
33838	0.946129	1.38846
33839	1.31822	2.02247
33840	0.94613	1.85583
33841	0.94613	1.8688
33842	1.31823	1.80746
33843	0.872157	1.90633
33844	1.81534	0.41681
33845	1.89226	0.290563
33846	1.59602	0.454939
33847	1.59602	0.540859
33848	1.89226	0.213114
33849	1.89226	0.274504
33850	1.89227	0.335867
33851	1.89227	0.455278
33852	1.89227	0.477765
33853	1.89227	0.334341
33854	3.78453	0.347904
33855	1.89226	0.432883
33856	1.89226	0.25454
33857	1.89226	0.321993
33858	1.89226	0.390147
33859	3.78453	0.416525
33860	2.30348	0.181561
33861	1.4185	0.226454
33862	1.82551	0.26326
33863	1.02011	0.192652
33864	3.24401	0.19379
33865	2.46702	0.166306
33866	1.17129	0.213055
33867	1.82551	0.175656
33868	1.82551	0.194487
33869	1.73525	0.169305

33870	1.73525	0.208652
33871	1.89227	0.384941
33872	1.89227	0.267902
33873	1.89227	0.268255
33874	1.89227	0.311042
33875	1.89227	0.338161
33876	1.89227	0.380843
33877	2.80034	2.1
33878	2.45767	2.1
33879	2.67994	2.1
33880	2.35395	2.1
33881	5.1376	2.1
33882	2.45766	2.1
33883	1.8769	2.1
33884	2.00989	2.1
33885	2.00989	2.1
33886	2.35395	2.1
33887	2.35395	2.1
33888	2.57662	2.1
33889	2.35395	2.1
33890	2.57662	2.1
33891	2.67993	2.1
33892	2.24227	2.1
33893	2.34558	2.1
33894	1.02874	2.1
33895	1.02874	2.1
33896	1.03084	2.1
33897	1.03084	2.1
33898	0.938448	2.1
33899	0.886762	2.1
33900	1.17698	2.1
33901	1.12114	2.1
33902	1.12113	2.1
33903	0.886764	2.1
33904	0.93845	2.1
33905	1.17697	2.1
33906	1.91969	2.1
33907	2.5508	2.1
33908	1.88273	1.90137
33909	2.76609	2.1
33910	4.68578	2.1
33911	2.83423	2.1
33912	1.89226	2.1
33913	2.76609	2.1
33914	1.89226	2.1
33915	4.65835	2.1
33916	1.56685	0.204125
33917	1.11712	0.355334
33918	1.07999	1.18474
33919	1.51448	0.395973
33920	2.6316	0.451384
33921	1.56685	0.107526
33922	1.67652	0.930289
33923	1.5955	1.07298
33924	1.51448	0.299942
33925	3.19099	0.490664
33926	1.89226	1.03422
33927	1.89226	0.31107

33928	1.89226	0.335239
33929	1.89226	1.25591
33930	3.78453	0.639389
33931	1.89226	0.420863
33932	1.89226	0.351794
33933	1.89226	0.673737
33934	1.89226	1.36028
33935	1.89226	0.245126
33936	1.89226	0.321008
33937	1.0562	0.459547
33938	1.51448	0.268999
33939	1.07999	0.413692
33940	1.07999	0.240754
33941	1.46441	0.463702
33942	1.46441	0.236658
33943	1.5955	1.12643
33944	1.51448	0.283926
33945	1.46441	0.422629
33946	1.52909	1.22735
33947	3.04357	0.73914
33948	1.89226	0.329166
33949	1.89226	1.34888
33950	1.89226	1.2908
33951	1.89226	0.440159
33952	3.78453	0.742995
33953	1.89227	0.324016
33954	1.89227	0.246303
33955	1.89227	0.466509
33956	1.89227	0.514502
33957	3.78453	0.358867
33958	1.89994	0.740526
33959	1.70303	1.1151
33960	1.68576	0.366768
33961	1.89608	0.391046
33962	3.8237	0.715961
33963	1.89994	1.28518
33964	2.31116	2.09545
33965	1.9386	0.869853
33966	1.88653	1.56865
33967	3.74885	1.77905
33968	2.31117	2.1
33969	2.20665	1.98324
33970	2.09617	2.04538
33971	2.41055	2.1
33972	2.09617	1.9014
33973	2.41055	2.1
33974	1.10777	0.751239
33975	1.0523	1.29315
33976	0.912098	0.884405
33977	0.757533	0.766103
33978	0.89287	1.55837
33979	1.07571	0.300293
33980	0.77485	1.09807
33981	0.98645	0.473863
33982	0.989171	0.35618
33983	0.875089	0.400095
33984	0.979686	1.11783
33985	0.979686	1.72883

33986	2.52442	2.1
33987	1.49998	2.1
33988	2.51692	2.1
33989	1.56957	2.1
33990	4.09399	2.1
33991	1.01298	2.1
33992	1.01298	2.1
33993	1.03079	2.1
33994	1.03079	2.1
33995	0.820964	2.1
33996	0.784786	2.1
33997	1.25898	2.1
33998	1.25846	2.1
33999	1.25898	2.1
34000	0.820964	2.1
34001	1.25846	2.1
34002	0.784786	2.1
34003	1.89226	0.05
34004	1.89226	0.05
34005	2.6297	0.05
34006	1.89226	0.05
34007	3.78452	0.05
34008	1.89226	0.05
34009	1.89226	0.05
34010	1.89226	0.05
34011	1.89226	0.05
34012	1.89226	0.05
34013	1.89226	0.05
34014	2.19397	0.05
34015	1.89226	0.05
34016	2.34112	0.05
34017	2.34112	0.05
34018	1.89226	0.05
34019	1.89226	0.05
34020	2.23366	2.1
34021	1.87598	2.1
34022	1.64716	2.1
34023	2.06048	2.1
34024	3.93645	2.1
34025	2.23367	2.1
34026	2.17384	2.1
34027	2.04818	2.1
34028	2.06048	2.1
34029	4.23432	2.1
34030	1.9855	2.1
34031	2.06048	2.1
34032	1.93163	2.1
34033	1.50737	2.1
34034	3.56785	2.1
34035	2.04818	2.1
34036	2.06048	2.1
34037	2.27465	2.1
34038	1.92264	2.1
34039	3.98312	2.1
34040	2.08149	0.616914
34041	1.89226	1.36518
34042	1.89226	1.21885
34043	1.33153	0.575308

34044	1.68038	1.5966
34045	1.18244	0.699077
34046	1.89226	0.90371
34047	1.89226	1.63554
34048	1.89226	1.5571
34049	1.89226	0.708171
34050	3.78452	1.00641
34051	2.69069	0.491186
34052	1.89226	0.626545
34053	1.89226	0.638243
34054	1.97397	0.486777
34055	1.89226	0.6321
34056	1.97397	0.47462
34057	1.89226	1.63025
34058	2.38056	0.67006
34059	1.89226	0.727747
34060	1.89226	1.10351
34061	3.78452	0.949598
34062	0.904435	2.1
34063	0.904435	2.1
34064	0.925315	2.1
34065	0.925315	2.1
34066	1.13312	2.1
34067	1.08109	2.1
34068	0.70697	2.1
34069	0.696633	2.1
34070	0.70697	2.1
34071	1.13312	2.1
34072	0.696633	2.1
34073	1.08109	2.1
34074	1.45506	2.1
34075	2.26623	2.1
34076	2.38363	2.1
34077	2.38363	2.1
34078	1.41394	2.1
34079	1.41394	2.1
34080	0.946131	2.1
34081	0.946131	2.1
34082	1.13311	2.1
34083	1.13311	2.1
34084	0.946127	2.1
34085	0.946127	2.1
34086	1.13312	2.1
34087	1.19182	2.1
34088	1.19181	2.1
34089	0.946129	2.1
34090	1.13312	2.1
34091	0.94613	2.1
34092	1.89226	1.11341
34093	1.89226	2.00143
34094	1.89226	1.21374
34095	1.89226	2.00592
34096	1.89226	1.37592
34097	1.89226	2.01734
34098	1.05168	2.09969
34099	0.696527	2.08753
34100	0.907442	1.79487
34101	0.907442	1.95098

34102	0.953145	1.71121
34103	0.782285	2.1
34104	0.773928	2.09752
34105	0.773928	1.88961
34106	0.696538	1.93214
34107	0.851554	1.92651
34108	1.04071	1.98714
34109	1.12335	1.99079
34110	1.89226	0.604672
34111	1.89226	0.21981
34112	1.89226	0.316595
34113	1.89226	0.174496
34114	3.78453	0.194948
34115	1.89226	0.51201
34116	1.89226	0.225157
34117	1.89226	0.360601
34118	1.89226	0.214048
34119	3.78453	0.363698
34120	2.50912	0.200592
34121	1.89226	0.146858
34122	1.89226	0.20797
34123	2.63195	0.138933
34124	4.40139	0.148611
34125	2.50912	0.195252
34126	1.3598	0.143377
34127	1.70303	0.160887
34128	2.36874	0.114545
34129	3.82055	0.166106
34130	1.89227	0.250907
34131	1.89227	0.158074
34132	1.89227	0.175386
34133	1.89227	0.118815
34134	3.78453	0.2133
34135	1.89227	0.28559
34136	1.89227	0.175489
34137	1.89227	0.216658
34138	1.89227	0.132494
34139	3.78453	0.154041
34140	1.89226	0.167813
34141	2.67088	0.098147
34142	2.63195	0.0947782
34143	2.63195	0.124426
34144	1.89226	0.119228
34145	1.89226	0.155993
34146	1.30202	0.111112
34147	1.28304	0.122425
34148	0.809902	0.110703
34149	0.888801	0.110339
34150	1.13262	0.0802686
34151	0.968276	0.0966296
34152	1.0733	0.12588
34153	1.0733	0.122914
34154	1.35419	0.104402
34155	0.801222	0.0772354
34156	0.899732	0.133408
34157	1.23967	0.0979624
34158	1.31595	2.1
34159	0.869501	2.1

34160	2.65473	2.1
34161	1.88782	2.1
34162	3.20377	2.1
34163	1.89227	2.1
34164	1.89227	2.1
34165	1.89227	2.1
34166	1.89227	2.1
34167	3.78453	2.1
34168	2.65473	2.1
34169	1.89226	2.1
34170	1.88781	2.1
34171	1.88782	2.1
34172	2.0815	2.1
34173	2.0815	2.1
34174	1.89226	2.09746
34175	1.89226	2.1
34176	1.89226	2.1
34177	1.89226	2.1
34178	1.89226	2.1
34179	1.89226	2.1
34180	1.89226	2.0986
34181	1.89226	2.1
34182	1.89226	2.1
34183	1.89226	2.1
34184	1.89226	2.1
34185	1.89226	2.09722
34186	2.39057	2.1
34187	1.89226	2.1
34188	1.89226	2.05046
34189	1.89226	2.1
34190	2.14243	2.05182
34191	2.14243	2.1
34192	1.89226	0.935089
34193	1.89226	2.1
34194	1.89226	1.90305
34195	1.89226	2.02047
34196	1.89226	1.87697
34197	1.89226	2.05523
34198	1.89226	1.25674
34199	1.89226	2.1
34200	1.89226	2.00457
34201	1.89226	2.04604
34202	1.89226	1.93713
34203	1.89226	2.07555
34204	2.56323	0.0798276
34205	1.36324	0.0741917
34206	1.05296	0.0629388
34207	2.18048	0.103833
34208	3.54372	0.0656731
34209	0.946128	2.1
34210	0.946128	2.1
34211	1.08733	2.1
34212	1.08733	2.1
34213	0.946131	2.1
34214	0.946131	2.1
34215	1.09197	2.1
34216	1.08732	2.1
34217	0.946131	2.1

34218	1.09197	2.1
34219	1.08732	2.1
34220	0.946131	2.1
34221	1.35111	1.53059
34222	1.51327	1.18766
34223	1.64493	1.22343
34224	1.31058	1.51183
34225	2.99605	1.54614
34226	1.8124	1.07576
34227	1.51327	1.28844
34228	1.64493	1.45707
34229	1.31358	0.969729
34230	1.8124	1.44376
34231	1.48105	0.979795
34232	1.64493	1.20903
34233	1.31058	1.39127
34234	1.22297	1.36146
34235	1.70101	1.06934
34236	2.8679	1.10519
34237	1.70101	1.17997
34238	1.99297	1.0706
34239	1.64493	1.29022
34240	1.64493	0.877521
34241	1.99297	1.37475
34242	1.8124	0.819268
34243	2.0815	2.1
34244	1.80258	2.1
34245	1.80258	2.1
34246	1.83506	2.1
34247	2.0815	2.1
34248	2.1212	2.1
34249	2.0815	2.1
34250	1.82129	2.1
34251	1.82129	2.1
34252	1.80445	2.1
34253	2.0815	2.1
34254	2.06092	2.1
34255	1.89224	2.1
34256	1.703 2.1	
34257	1.97839	2.1
34258	1.703 2.1	
34259	1.97402	2.1
34260	1.703 2.1	
34261	1.89226	0.231919
34262	1.89226	0.36224
34263	1.89226	0.32721
34264	2.75351	0.239082
34265	3.78453	0.291392
34266	1.89226	0.314173
34267	3.02887	0.304342
34268	2.03149	0.259363
34269	2.03149	0.361691
34270	1.89226	0.230587
34271	1.89226	0.324516
34272	2.17979	2.1
34273	1.65533	2.1
34274	2.57212	2.1
34275	2.20346	2.1

34276	4.22745	2.1
34277	1.89227	2.1
34278	2.20347	2.1
34279	1.89227	2.1
34280	2.57212	2.1
34281	1.89227	2.1
34282	2.57212	2.1
34283	2.05274	2.1
34284	2.16783	2.1
34285	2.24429	2.1
34286	2.38404	2.1
34287	4.41212	2.1
34288	2.16783	2.1
34289	2.05967	2.1
34290	2.38404	2.1
34291	2.24695	2.1
34292	4.41478	2.1
34293	2.24429	2.1
34294	2.38404	2.1
34295	2.51819	2.1
34296	2.60723	2.1
34297	4.85152	2.1
34298	2.38404	2.1
34299	2.24695	2.1
34300	2.60723	2.1
34301	2.45247	2.1
34302	4.85418	2.1
34303	1.89227	2.1
34304	1.89227	2.1
34305	1.89227	2.1
34306	1.89227	2.1
34307	3.78453	2.1
34308	1.89226	2.1
34309	1.89226	2.1
34310	1.89226	2.1
34311	1.89226	2.1
34312	3.78453	2.1
34313	0.618473	2.1
34314	0.619945	2.1
34315	0.797692	2.1
34316	0.770147	2.1
34317	0.68852	2.1
34318	0.68852	2.1
34319	0.701573	2.1
34320	0.701573	2.1
34321	0.619945	2.1
34322	0.797691	2.1
34323	0.618473	2.1
34324	0.770147	2.1
34325	0.797692	2.1
34326	0.770148	2.1
34327	0.694787	2.1
34328	0.653411	2.1
34329	0.722102	2.1
34330	0.722102	2.1
34331	0.729001	2.1
34332	0.729001	2.1
34333	0.797692	2.1

34334	0.694787	2.1
34335	0.770148	2.1
34336	0.653412	2.1
34337	1.89227	2.1
34338	1.89227	2.1
34339	1.89227	2.1
34340	1.89227	2.1
34341	3.78453	2.1
34342	1.89226	2.1
34343	1.89226	2.1
34344	1.89226	2.1
34345	1.89226	2.1
34346	3.78453	2.1
34347	1.23989	2.1
34348	1.59538	2.1
34349	1.66647	2.1
34350	1.26599	2.1
34351	2.90636	2.1
34352	1.66647	2.1
34353	1.38957	2.1
34354	1.47446	2.1
34355	1.40337	2.1
34356	1.66647	2.1
34357	1.59538	2.1
34358	2.80619	2.1
34359	2.0815	2.1
34360	2.0815	2.1
34361	2.0815	2.1
34362	2.73721	2.1
34363	2.74192	2.1
34364	2.73721	2.1
34365	2.0815	2.1
34366	2.0815	2.1
34367	2.0815	2.1
34368	2.689	2.1
34369	2.69166	2.1
34370	2.62369	2.1
34371	2.19717	2.1
34372	2.60405	2.1
34373	2.20566	2.1
34374	4.80122	2.1
34375	2.19717	2.1
34376	1.37392	2.1
34377	2.20566	2.1
34378	1.45408	2.1
34379	3.65125	2.1
34380	2.60405	2.1
34381	2.20566	2.1
34382	2.57466	2.1
34383	2.21715	2.1
34384	4.8212	2.1
34385	2.20566	2.1
34386	1.54249	2.1
34387	1.54249	2.1
34388	1.45408	2.1
34389	2.21715	2.1
34390	2.12874	2.1
34391	1.60647	2.1

34392	1.31346	2.1
34393	1.82649	2.1
34394	1.51067	2.1
34395	3.13995	2.1
34396	1.89226	2.1
34397	1.89226	2.1
34398	1.89226	2.1
34399	1.89226	2.1
34400	3.78452	2.1
34401	1.89226	2.1
34402	2.6096	2.1
34403	2.8067	2.1
34404	2.8067	2.1
34405	1.89226	2.1
34406	1.89226	2.1
34407	1.82649	2.1
34408	1.3596	2.1
34409	2.17708	2.1
34410	1.70302	2.1
34411	3.40243	2.1
34412	1.89226	2.1
34413	1.89226	2.1
34414	1.89226	2.1
34415	1.89226	2.1
34416	3.78452	2.1
34417	2.27067	2.1
34418	2.43549	2.1
34419	2.63979	2.1
34420	2.41828	2.1
34421	2.27067	2.1
34422	2.04916	2.1
34423	1.30047	2.1
34424	2.04916	2.1
34425	2.27068	2.1
34426	1.5586	2.1
34427	3.57115	2.1
34428	2.27068	2.1
34429	1.84214	2.1
34430	1.80224	2.1
34431	1.5586	2.1
34432	2.51432	2.1
34433	2.27068	2.1
34434	1.63719	2.1
34435	2.15714	2.1
34436	2.07821	2.1
34437	1.52204	2.1
34438	3.67919	2.1
34439	2.07821	2.1
34440	1.40118	2.1
34441	1.40118	2.1
34442	1.52204	2.1
34443	1.99632	2.1
34444	2.11718	2.1
34445	0.946131	2.1
34446	0.946131	2.1
34447	1.06957	2.1
34448	1.06957	2.1
34449	0.946132	2.1

34450	0.946132	2.06372
34451	1.411	2.1
34452	1.06957	2.1
34453	0.946132	2.1
34454	1.411	2.1
34455	1.06957	2.1
34456	0.946132	2.1
34457	1.99875	0.87778
34458	2.57079	0.675502
34459	2.59319	1.49048
34460	1.9105	0.495755
34461	4.59194	0.799679
34462	1.99875	0.803797
34463	1.02247	0.266747
34464	1.07017	0.373582
34465	1.15841	0.389616
34466	1.91051	0.359433
34467	1.99875	0.402976
34468	2.59319	1.38515
34469	1.9105	0.521206
34470	2.33812	0.757456
34471	2.59319	0.810126
34472	1.59457	0.787908
34473	1.59457	0.894841
34474	2.50906	0.431984
34475	2.0467	0.200274
34476	2.03357	0.229892
34477	2.53176	0.185544
34478	4.54263	0.221807
34479	1.294	0.396004
34480	1.66636	0.281658
34481	2.03357	0.214748
34482	1.03079	0.197369
34483	3.32757	0.261267
34484	1.68108	0.218402
34485	2.53176	0.198344
34486	2.36417	0.162482
34487	2.71666	0.161483
34488	1.68108	0.527165
34489	2.03357	0.320847
34490	1.89226	0.132698
34491	2.59302	0.111043
34492	2.2727	0.111766
34493	2.2727	0.107257
34494	1.89226	0.144034
34495	1.89226	0.121897
34496	1.89226	0.251891
34497	1.89226	0.174516
34498	1.89226	0.169745
34499	1.89226	0.108408
34500	3.78452	0.224437
34501	1.89225	0.234582
34502	1.89225	0.0782266
34503	1.89225	0.0982839
34504	1.89225	0.127137
34505	1.89225	0.112385
34506	1.89225	0.148779
34507	0.934114	0.127372

34508	0.934114	0.0840195
34509	0.944544	0.0928055
34510	0.944544	0.0980799
34511	0.721493	0.0773445
34512	0.58215	0.073756
34513	1.2965	0.0951925
34514	1.13634	0.0904344
34515	1.13635	0.0739962
34516	0.582148	0.0699399
34517	1.29651	0.0959395
34518	0.721491	0.0875015
34519	1.89226	0.118073
34520	1.89226	0.0845319
34521	1.89226	0.0850412
34522	1.89226	0.063312
34523	3.78452	0.0638437
34524	0.946131	0.377014
34525	0.946131	0.421026
34526	0.946131	0.309438
34527	0.946131	0.400466
34528	0.946131	0.29915
34529	0.946131	0.337322
34530	0.946131	0.428004
34531	0.946131	0.459272
34532	0.946131	0.348342
34533	0.946131	0.36217
34534	0.946131	0.388473
34535	0.946131	0.362668
34536	1.89226	0.05
34537	1.89226	0.05
34538	2.78602	0.05
34539	1.89226	0.05
34540	3.78453	0.05
34541	1.52601	2.1
34542	1.76601	2.1
34543	1.27839	2.1
34544	1.56095	2.1
34545	3.0444	2.1
34546	1.76601	2.1
34547	1.86793	2.1
34548	1.56095	2.1
34549	1.68152	2.1
34550	3.44753	2.1
34551	1.27841	2.1
34552	1.56097	2.1
34553	1.0577	2.1
34554	1.36861	2.1
34555	2.64702	2.1
34556	1.56097	2.1
34557	1.68154	2.1
34558	1.36861	2.1
34559	1.53296	2.1
34560	3.05015	2.1
34561	1.89226	2.1
34562	1.89226	2.1
34563	1.89226	2.1
34564	1.89226	2.1
34565	3.78453	2.1

34566	0.983075	2.1
34567	0.788361	2.1
34568	0.724726	2.1
34569	0.724726	2.1
34570	0.983027	2.1
34571	0.724734	2.1
34572	0.788364	2.1
34573	0.788364	2.1
34574	0.788335	2.1
34575	0.94613	2.1
34576	0.724757	2.1
34577	0.946131	2.1
34578	1.15789	2.1
34579	2.11135	2.1
34580	2.25826	2.1
34581	1.28845	2.1
34582	3.41615	2.1
34583	1.89226	2.1
34584	1.89226	2.1
34585	1.89226	2.1
34586	1.89226	2.1
34587	3.78453	2.1
34588	1.57667	2.1
34589	1.70002	2.1
34590	1.70002	2.1
34591	1.89226	2.1
34592	1.89226	2.1
34593	2.05244	2.1
34594	2.25826	2.1
34595	1.42214	2.1
34596	1.43675	2.1
34597	1.28845	2.1
34598	2.40656	2.1
34599	2.25826	2.1
34600	1.58066	2.1
34601	1.74247	2.1
34602	1.85097	2.1
34603	1.61155	2.1
34604	3.43163	2.1
34605	0.785612	2.1
34606	0.789638	2.1
34607	0.882561	2.1
34608	0.763529	2.1
34609	0.686921	2.1
34610	0.86048	2.1
34611	0.871812	2.1
34612	0.871812	2.1
34613	0.830183	2.1
34614	0.882572	2.1
34615	0.902315	2.1
34616	0.785602	2.1
34617	1.01972	0.05
34618	0.936657	0.05
34619	0.946131	0.05
34620	0.946131	0.05
34621	0.927018	0.05
34622	0.927018	0.05
34623	1.1376	0.05

34624	0.953663	0.05
34625	1.1257	0.05
34626	1.04073	0.05
34627	1.00358	0.05
34628	0.851528	0.05
34629	1.73576	0.0755903
34630	2.00244	0.115855
34631	1.17798	0.05
34632	1.25796	0.0602022
34633	2.99373	0.05
34634	2.00244	0.0643519
34635	1.10719	0.060816
34636	2.03236	0.0789049
34637	2.03236	0.209073
34638	1.25797	0.05
34639	1.25797	0.0889518
34640	1.89226	2.1
34641	1.89226	2.1
34642	1.89226	2.1
34643	1.89226	2.1
34644	3.78452	2.1
34645	2.3174	2.1
34646	1.50005	2.1
34647	1.90428	2.1
34648	2.64564	2.1
34649	4.22168	2.1
34650	1.89226	2.1
34651	1.89226	2.1
34652	1.89226	2.1
34653	1.89226	2.1
34654	1.89226	2.1
34655	1.89226	2.1
34656	1.32749	0.0516918
34657	1.70302	0.05
34658	2.08149	0.0501548
34659	1.70302	0.05
34660	2.19634	0.05
34661	1.93083	0.05
34662	1.89226	1.82785
34663	1.89226	1.84184
34664	1.89226	0.486439
34665	1.89226	2.07209
34666	3.78453	1.02883
34667	1.89226	1.3803
34668	1.89226	2.01349
34669	1.89226	0.669128
34670	1.89226	1.00573
34671	3.78452	1.70453
34672	2.17499	0.38874
34673	1.79663	1.98654
34674	2.1538	0.488306
34675	1.818	0.82428
34676	2.17499	1.81551
34677	1.83919	2.1
34678	0.898316	1.77061
34679	1.0769	0.790688
34680	0.968276	2.06303
34681	0.773495	1.88199

34682	0.921126	1.97464
34683	0.921126	1.9968
34684	0.92927	1.567
34685	0.92927	1.93175
34686	1.0769	1.79094
34687	0.968276	1.58586
34688	0.898316	1.89711
34689	0.773495	1.67296
34690	1.89226	0.714267
34691	1.89226	2.09661
34692	1.89226	1.68637
34693	1.89226	0.985475
34694	3.78453	1.87944
34695	1.89226	1.5754
34696	1.89226	2.06605
34697	1.89226	0.373612
34698	1.89226	1.80656
34699	3.78452	0.862399
34700	2.17499	1.71032
34701	2.1538	2.1
34702	2.53574	0.933516
34703	2.54157	0.427114
34704	4.71073	1.83137
34705	1.0769	1.53188
34706	1.26787	0.915756
34707	1.16641	1.77843
34708	0.968276	2.01783
34709	1.11625	1.83561
34710	1.11625	1.84786
34711	1.11989	1.98835
34712	1.11989	0.57017
34713	1.26787	1.48104
34714	1.16641	1.98732
34715	1.0769	2.02583
34716	0.968277	1.86899
34717	1.89226	0.207188
34718	1.89226	0.248221
34719	1.89226	0.210072
34720	1.89226	0.199418
34721	3.78452	0.170937
34722	1.91739	0.178884
34723	2.20045	0.0501084
34724	1.79005	0.0751312
34725	2.33802	0.113706
34726	4.25539	0.12247
34727	2.52754	0.107707
34728	1.89226	0.125706
34729	2.69841	0.108229
34730	2.69841	0.134498
34731	1.89226	0.0925293
34732	1.89226	0.114453
34733	1.89226	0.186385
34734	1.89226	0.206358
34735	1.89226	0.158759
34736	1.89226	0.203091
34737	3.78452	0.206793
34738	1.93544	0.0988933
34739	2.33802	0.138728

34740	1.55063	0.127024
34741	2.1769	0.0509433
34742	3.88865	0.0947637
34743	2.20929	0.245058
34744	2.55162	0.172086
34745	2.27807	0.272789
34746	1.48831	0.175715
34747	4.48736	0.187696
34748	2.27807	0.280489
34749	2.72723	0.176266
34750	2.55162	0.207114
34751	2.55162	0.265666
34752	2.43157	0.182508
34753	2.43157	0.252546
34754	2.27807	0.212706
34755	1.51612	0.149408
34756	1.96048	0.159332
34757	1.73022	0.151955
34758	1.70302	0.216099
34759	1.61016	0.187526
34760	2.27807	0.216925
34761	2.08048	0.140257
34762	1.98522	0.157255
34763	2.43157	0.19007
34764	1.83798	0.160897
34765	2.27807	0.195896
34766	1.70302	0.272438
34767	2.68507	0.216624
34768	2.23856	0.274439
34769	2.52174	0.194552
34770	2.35331	0.317215
34771	2.65099	0.241782
34772	1.70303	0.25514
34773	0.986848	0.146659
34774	2.41566	0.170348
34775	1.70303	0.163067
34776	1.88139	0.163458
34777	1.64955	0.159401
34778	1.89226	2.00251
34779	2.34978	1.80034
34780	1.89226	1.98442
34781	2.68773	1.71227
34782	1.89226	2.00102
34783	2.68773	1.80358
34784	1.89226	1.99779
34785	1.89226	1.68844
34786	1.89226	1.74233
34787	1.89226	1.94752
34788	3.78453	1.92221
34789	1.00825	1.0786
34790	1.09847	1.15481
34791	0.939987	1.20906
34792	0.783516	1.7581
34793	0.838041	1.46799
34794	0.838041	1.76029
34795	1.49693	1.85985
34796	1.26602	1.40939
34797	1.10875	1.06051

34798	1.05187	1.93176
34799	1.28743	1.46199
34800	0.946132	1.46149
34801	0.946132	1.69499
34802	0.946132	1.99681
34803	0.946132	1.93478
34804	0.946132	1.3001
34805	1.31823	2.00166
34806	0.946132	1.68041
34807	0.946132	1.92905
34808	0.946131	1.95943
34809	0.946131	2.03004
34810	0.946132	1.8645
34811	1.31823	1.92566
34812	1.51688	0.248488
34813	1.79978	0.389831
34814	1.1685	0.261121
34815	1.1685	0.355221
34816	1.85857	0.256355
34817	1.51018	0.379997
34818	0.946116	0.334755
34819	0.946116	0.359497
34820	0.94612	0.208531
34821	0.94612	0.229427
34822	0.946118	0.218185
34823	0.946118	0.330902
34824	0.946118	0.24857
34825	0.946118	0.358982
34826	0.946131	0.267486
34827	0.946131	0.278234
34828	0.946105	0.297948
34829	0.946105	0.299908
34830	1.89224	2.1
34831	1.89224	2.1
34832	1.89224	2.1
34833	1.89224	2.1
34834	1.89224	2.1
34835	1.89224	2.1
34836	1.38678	2.1
34837	1.3349	2.1
34838	0.946119	2.1
34839	0.946119	2.1
34840	1.38678	2.1
34841	1.38678	2.1
34842	0.946119	2.1
34843	0.946119	2.1
34844	1.38676	2.1
34845	0.946107	2.1
34846	0.946132	2.1
34847	1.33491	2.1
34848	1.06455	2.1
34849	1.03932	2.1
34850	1.20649	2.1
34851	1.18773	2.1
34852	2.27105	2.1
34853	1.89226	2.1
34854	1.89226	2.1
34855	1.89226	2.1

34856	1.89226	2.1
34857	1.89226	2.1
34858	1.89226	2.1
34859	1.36918	2.1
34860	1.18774	2.1
34861	1.31144	2.1
34862	1.20651	2.1
34863	1.31144	2.1
34864	1.20651	2.1
34865	2.29066	2.1
34866	1.76476	2.1
34867	1.79825	2.1
34868	2.33801	2.1
34869	4.08891	2.1
34870	1.79825	2.1
34871	2.33801	2.1
34872	2.37168	2.1
34873	1.49325	2.1
34874	4.16993	2.1
34875	1.13641	2.1
34876	1.43213	2.0389
34877	1.44085	2.1
34878	1.18301	2.1
34879	2.61513	2.1
34880	1.89226	2.1
34881	2.23875	2.1
34882	2.23875	2.1
34883	2.10287	2.1
34884	1.89226	2.1
34885	1.75639	2.1
34886	1.89226	2.05225
34887	1.89226	2.1
34888	1.89226	2.07254
34889	1.89226	2.1
34890	1.89226	2.05168
34891	1.89226	2.1
34892	1.44085	2.1
34893	1.25173	2.1
34894	1.25173	2.09539
34895	1.18301	2.1
34896	1.4617	2.08703
34897	1.39298	2.1
34898	1.89227	2.1
34899	2.23875	2.1
34900	2.42504	2.1
34901	1.89227	2.1
34902	4.13101	2.1
34903	1.89226	2.0686
34904	1.89226	2.1
34905	1.89226	2.04565
34906	1.89226	2.1
34907	1.89226	2.03776
34908	1.89226	2.1
34909	1.89226	2.1
34910	2.41297	2.1
34911	2.70685	2.1
34912	2.70685	2.1
34913	1.89226	2.1

34914	1.89226	2.1
34915	1.89226	2.1
34916	1.89226	2.1
34917	1.89226	2.1
34918	1.89226	2.1
34919	1.89226	2.1
34920	1.89226	2.1
34921	1.89226	2.1
34922	1.89226	2.1
34923	1.89226	2.1
34924	1.89226	2.1
34925	3.78453	2.1
34926	1.89226	2.1
34927	1.89226	2.1
34928	1.89226	2.1
34929	1.89226	2.1
34930	1.89226	2.1
34931	1.89226	2.1
34932	1.69044	0.05
34933	1.57979	0.05
34934	2.27963	0.05
34935	2.21582	0.05
34936	3.85942	0.05
34937	2.21582	0.05
34938	1.30722	0.05
34939	2.02069	0.05
34940	2.02069	0.05
34941	1.57979	0.05
34942	1.57979	0.05
34943	1.67825	2.1
34944	1.89226	2.1
34945	1.89226	2.1
34946	1.89226	2.1
34947	1.91894	2.1
34948	1.91894	2.1
34949	0.946133	2.1
34950	0.946133	2.1
34951	0.946132	2.1
34952	0.946132	2.1
34953	0.946132	2.1
34954	0.946132	2.1
34955	0.946133	2.1
34956	0.946133	2.1
34957	0.946133	2.1
34958	0.946133	2.1
34959	0.946132	2.1
34960	0.946132	2.1
34961	0.942609	2.1
34962	0.942609	2.1
34963	1.329 2.1	
34964	1.329 2.1	
34965	1.32547	2.1
34966	0.94261	2.1
34967	0.946132	2.1
34968	0.946132	2.1
34969	0.946132	2.1
34970	1.32547	2.1
34971	0.946132	2.1

34972	0.94261	2.1
34973	1.55831	2.1
34974	1.07496	2.09477
34975	1.80327	0.280251
34976	1.34851	0.345147
34977	2.87824	0.679942
34978	2.14346	0.484564
34979	2.77937	0.668261
34980	2.79173	0.942621
34981	2.17129	0.409322
34982	4.95066	0.527642
34983	2.14346	0.581874
34984	1.34124	0.193407
34985	1.39818	0.300575
34986	2.1713	0.427546
34987	3.51253	0.349042
34988	2.79173	0.848424
34989	2.21882	0.414742
34990	2.17129	0.466811
34991	2.17129	0.629002
34992	2.81448	0.510499
34993	2.81448	0.644303
34994	1.10941	0.404631
34995	1.08565	0.491674
34996	0.699089	0.20542
34997	0.740176	0.288575
34998	0.908594	0.215136
34999	0.908594	0.378883
35000	0.91723	0.624888
35001	0.91723	0.230442
35002	1.10941	0.331906
35003	0.740176	0.320044
35004	0.699089	0.312384
35005	1.08565	0.324764
35006	1.89227	0.0792614
35007	1.89227	0.0976064
35008	1.89227	0.0674587
35009	1.89227	0.0532277
35010	3.78453	0.0770771
35011	2.40032	0.147168
35012	2.19526	0.076
35013	1.12907	0.0618435
35014	1.44253	0.104523
35015	3.32433	0.0814183
35016	2.19526	0.0689367
35017	2.71093	0.117164
35018	1.29826	0.0814475
35019	2.0815	0.0557645
35020	4.18911	0.0881511
35021	1.89226	0.0876718
35022	1.89226	0.0863957
35023	1.89226	0.05
35024	1.89226	0.0578275
35025	3.78452	0.0608999
35026	2.30822	0.0609201
35027	1.89226	0.0518371
35028	2.79329	0.05
35029	2.79329	0.05

35030	1.89226	0.0706396
35031	1.89226	0.0674736
35032	0.946131	2.1
35033	0.946131	2.00599
35034	1.38414	2.1
35035	1.38414	1.97758
35036	0.94613	2.1
35037	0.94613	2.1
35038	1.38415	2.02142
35039	1.41833	1.37015
35040	0.946132	2.1
35041	1.41833	2.1
35042	1.38414	2.1
35043	0.946131	2.1
35044	1.89226	2.1
35045	2.6536	2.1
35046	1.89226	2.1
35047	2.72238	2.1
35048	4.61465	2.1
35049	0.946132	2.1
35050	0.946132	2.1
35051	1.38657	2.1
35052	1.38657	2.1
35053	1.38657	2.1
35054	1.36119	2.1
35055	0.946131	2.1
35056	0.946131	2.1
35057	0.946132	2.1
35058	1.38657	2.1
35059	0.946132	2.1
35060	1.36119	2.1
35061	1.89226	2.1
35062	2.4717	2.1
35063	2.5026	2.1
35064	2.4717	2.1
35065	1.92316	2.1
35066	1.89226	2.1
35067	1.89226	2.1
35068	1.89226	2.1
35069	1.89226	2.1
35070	1.89226	2.1
35071	3.78453	2.1
35072	0.946128	2.1
35073	0.946128	2.1
35074	1.3419	2.1
35075	1.3419	2.1
35076	0.946131	2.1
35077	0.946131	2.1
35078	1.36607	2.1
35079	1.3419	2.1
35080	0.946131	2.1
35081	1.36607	2.1
35082	1.3419	2.1
35083	0.94613	2.1
35084	1.89226	2.1
35085	2.50259	2.1
35086	2.53563	2.1
35087	2.50259	2.1

35088	1.92529	2.1
35089	1.89226	2.1
35090	0.946132	2.1
35091	0.946132	2.1
35092	0.946131	2.1
35093	0.946131	2.1
35094	0.946132	2.1
35095	0.946132	2.1
35096	0.946131	2.1
35097	0.946131	2.1
35098	0.946131	2.1
35099	0.946131	2.1
35100	0.946131	2.1
35101	0.946131	2.1
35102	2.0815	2.1
35103	1.86506	2.1
35104	1.86506	2.1
35105	1.82567	2.1
35106	2.0815	2.1
35107	2.03335	2.1
35108	2.0815	2.1
35109	2.0815	2.1
35110	2.0815	2.1
35111	1.88668	2.1
35112	1.83221	2.1
35113	1.57275	2.1
35114	2.34061	0.05
35115	1.89226	0.05
35116	2.23254	0.05
35117	1.89226	0.05
35118	2.23254	0.05
35119	1.89226	0.05
35120	1.8619	0.05
35121	1.89226	0.05
35122	2.34061	0.05
35123	1.89226	0.05
35124	2.34061	0.05
35125	1.89226	0.05
35126	1.89227	0.05
35127	1.89227	0.05
35128	1.89227	0.05
35129	1.89227	0.05
35130	1.89227	0.05
35131	1.89227	0.05
35132	1.89227	0.05
35133	1.89227	0.05
35134	1.89227	0.05
35135	1.89227	0.05
35136	1.89227	0.05
35137	1.89227	0.05
35138	1.06365	0.05
35139	0.503305	0.05
35140	0.794912	0.05
35141	1.42752	0.05
35142	1.34826	0.05
35143	1.07163	0.05
35144	0.533113	0.05
35145	0.761277	0.05

35146	0.848403	0.05
35147	0.876191	0.05
35148	0.963372	0.05
35149	0.963372	0.05
35150	1.55571	0.05
35151	2.76574	0.05
35152	2.92453	0.05
35153	1.70301	0.05
35154	2.33089	0.05
35155	1.06905	0.05
35156	2.28979	2.1
35157	1.81488	2.1
35158	1.63722	2.1
35159	2.22627	2.1
35160	4.04115	2.1
35161	1.98246	2.1
35162	2.15715	2.1
35163	2.22627	2.1
35164	2.22627	2.1
35165	1.63719	2.1
35166	1.63719	2.1
35167	2.1644	0.05
35168	1.903 0.05	
35169	1.23639	0.05
35170	1.42704	0.05
35171	3.61546	0.05
35172	1.89226	0.05
35173	1.89226	0.05
35174	1.89226	0.05
35175	1.89226	0.05
35176	3.78451	0.05
35177	2.61343	0.05
35178	1.89226	0.05
35179	2.08149	0.05
35180	1.74357	0.05
35181	2.08149	0.05
35182	1.74357	0.05
35183	1.63483	0.05
35184	1.8631	0.05
35185	1.8631	0.05
35186	1.70301	0.05
35187	2.0815	0.05
35188	1.94319	0.05
35189	2.0815	0.05
35190	1.89226	0.05
35191	1.89226	0.05
35192	2.05236	0.05
35193	2.23014	0.05
35194	2.41882	0.05
35195	2.14993	2.1
35196	1.87217	2.1
35197	2.34488	2.1
35198	2.5254	2.09624
35199	4.39757	2.09848
35200	0.949995	2.1
35201	0.630972	2.1
35202	0.936084	2.1
35203	1.17244	2.1

35204	0.922394	2.1
35205	0.922394	2.1
35206	0.88102	2.1
35207	0.88102	2.1
35208	0.949998	2.1
35209	1.17244	2.1
35210	0.936081	2.1
35211	0.630973	2.1
35212	1.89226	2.1
35213	1.89226	2.1
35214	1.89226	2.1
35215	1.89226	2.1
35216	3.78452	2.1
35217	1.89226	2.09111
35218	1.89226	2.1
35219	1.89226	2.09236
35220	1.89226	2.1
35221	1.89226	2.08585
35222	1.89226	2.1
35223	2.34488	2.0893
35224	2.5254	2.06839
35225	1.89226	2.05267
35226	1.89226	2.09305
35227	4.41766	2.09931
35228	1.89226	2.09667
35229	1.89226	2.1
35230	1.89226	2.09342
35231	1.89226	2.09749
35232	3.78452	2.08114
35233	1.89226	2.05316
35234	1.89226	2.09085
35235	1.89226	2.05535
35236	1.89226	2.07411
35237	1.89226	2.07198
35238	1.89226	2.08327
35239	1.5605	2.1
35240	1.15806	2.1
35241	1.31565	2.1
35242	1.65642	2.1
35243	2.87615	2.1
35244	1.46643	2.1
35245	1.65642	2.1
35246	1.31565	2.1
35247	1.31565	2.1
35248	1.74246	2.1
35249	1.74246	2.1
35250	1.89226	2.1
35251	1.89226	2.1
35252	1.89226	2.1
35253	1.89226	2.1
35254	1.89226	2.1
35255	1.89226	2.1
35256	1.15099	2.1
35257	1.15099	2.1
35258	0.939316	2.1
35259	0.939316	2.1
35260	0.946127	2.1
35261	0.946127	2.1

35262	1.14418	2.1
35263	0.939289	2.1
35264	1.14417	2.1
35265	0.946129	2.1
35266	0.939287	2.1
35267	0.94613	2.1
35268	1.89226	2.1
35269	1.89226	2.1
35270	1.89226	2.1
35271	1.89226	2.1
35272	3.78452	2.1
35273	1.93164	0.051709
35274	2.64898	0.05
35275	1.43213	0.05
35276	1.43213	0.0577695
35277	2.63206	0.05
35278	2.13254	0.0515509
35279	1.89226	2.1
35280	1.89226	2.1
35281	1.89226	2.1
35282	1.89226	2.1
35283	3.78452	2.1
35284	2.71655	2.1
35285	1.43323	2.1
35286	1.28413	2.1
35287	2.53614	2.1
35288	3.96937	2.1
35289	1.89226	2.1
35290	1.89226	2.1
35291	1.89226	2.1
35292	1.89226	2.1
35293	1.89226	2.1
35294	1.89226	2.1
35295	1.33356	2.1
35296	2.82297	2.1
35297	2.771 2.1	
35298	2.771 2.1	
35299	1.46037	2.1
35300	1.46037	2.1
35301	2.56034	2.1
35302	1.18474	2.1
35303	1.15113	2.1
35304	2.53724	2.1
35305	3.72198	2.1
35306	2.56034	2.1
35307	1.89226	2.1
35308	1.89226	2.1
35309	2.53725	2.1
35310	4.42951	2.1
35311	1.89226	2.1
35312	1.89226	2.1
35313	1.89226	2.1
35314	1.89226	2.1
35315	3.78453	2.1
35316	1.89226	2.1
35317	1.89226	2.1
35318	1.89226	2.1
35319	1.89226	2.1

35320	3.78452	2.1
35321	1.1229	2.1
35322	2.53724	2.1
35323	1.15113	2.1
35324	1.15113	2.1
35325	2.51559	2.1
35326	2.51559	2.1
35327	1.89226	2.1
35328	2.53724	2.1
35329	2.51559	2.1
35330	2.53724	2.1
35331	1.87061	2.1
35332	1.89226	2.1
35333	1.89226	2.1
35334	1.89226	2.1
35335	1.89226	2.1
35336	1.89226	2.1
35337	3.78453	2.1
35338	1.89226	2.1
35339	1.89226	2.1
35340	1.89226	2.1
35341	1.89226	2.1
35342	3.78452	2.1
35343	1.98143	1.57226
35344	2.81559	0.445874
35345	1.65411	0.660327
35346	2.26675	1.32168
35347	4.24818	0.902098
35348	0.810656	2.1
35349	0.810656	2.1
35350	0.821888	2.1
35351	0.821888	2.1
35352	1.1235	2.1
35353	1.07439	2.1
35354	0.535693	2.1
35355	0.509042	2.1
35356	0.535693	2.1
35357	1.1235	2.1
35358	0.509043	2.1
35359	1.07439	2.1
35360	1.10378	2.1
35361	2.247	2.1
35362	2.27472	2.1
35363	2.27472	2.1
35364	1.07139	2.1
35365	1.07139	2.1
35366	2.08081	2.1
35367	1.40395	2.1
35368	1.34245	2.1
35369	2.00239	2.1
35370	3.40634	2.1
35371	1.89226	2.1
35372	1.89226	2.1
35373	1.89226	2.1
35374	1.89226	2.1
35375	3.78453	2.1
35376	0.946128	2.1
35377	0.946128	2.1

35378	1.13785	2.1
35379	1.11496	2.1
35380	0.946131	2.1
35381	0.946131	2.1
35382	1.11495	2.1
35383	1.11495	2.1
35384	0.94613	2.1
35385	1.11495	2.1
35386	1.13785	2.1
35387	0.946131	2.1
35388	0.975888	2.1
35389	1.00118	2.1
35390	0.671218	2.1
35391	0.653229	2.1
35392	0.825372	2.1
35393	0.825372	2.1
35394	0.829038	2.1
35395	0.829038	2.1
35396	0.975901	2.1
35397	0.653238	2.1
35398	0.671209	2.1
35399	1.00117	2.1
35400	1.89224	2.1
35401	1.89224	2.1
35402	1.89224	2.1
35403	1.89224	2.1
35404	3.78448	2.1
35405	0.946115	2.1
35406	0.946115	2.1
35407	1.11494	2.1
35408	1.10111	2.1
35409	0.946118	2.1
35410	0.946118	2.1
35411	1.10111	2.1
35412	1.10111	2.1
35413	1.10112	2.1
35414	0.94613	2.1
35415	0.946105	2.1
35416	1.11492	2.1
35417	1.04028	0.639804
35418	2.27126	0.596472
35419	2.0815	0.343501
35420	1.82249	1.72721
35421	1.33723	0.438226
35422	1.18684	1.55081
35423	0.812616	0.107502
35424	0.812616	0.10143
35425	0.997777	0.100075
35426	1.08122	0.0994158
35427	0.94613	0.100092
35428	0.94613	0.110005
35429	0.812648	0.103543
35430	0.932779	0.0967923
35431	0.812648	0.0867662
35432	0.94613	0.0842
35433	0.849335	0.0722995
35434	0.94613	0.0849929
35435	1.80327	0.690313

35436	1.56016	0.482819
35437	1.57731	1.96
35438	1.34851	1.717
35439	2.03207	1.38274
35440	1.80327	1.96948
35441	2.03207	1.80021
35442	1.56016	1.59478
35443	2.18076	1.80272
35444	1.78167	0.559167
35445	3.81374	1.5548
35446	1.37597	2.09376
35447	1.60058	2.1
35448	1.70302	0.677216
35449	1.85448	1.13779
35450	3.15028	1.14648
35451	1.60058	1.1739
35452	1.4054	2.07917
35453	1.66202	0.538727
35454	1.70302	2.07933
35455	3.09979	1.75967
35456	1.70302	1.92187
35457	1.85448	1.69326
35458	2.21317	1.76688
35459	2.0961	0.763732
35460	4.01757	1.4154
35461	1.66202	1.0185
35462	1.70302	2.09727
35463	2.09611	0.434737
35464	2.00892	2.05304
35465	3.6468	1.19607
35466	1.2966	0.05
35467	2.25495	0.05
35468	1.2966	0.05
35469	2.21117	0.05
35470	1.67687	0.05
35471	2.6167	0.05
35472	1.89227	0.0919746
35473	1.89227	0.0520367
35474	1.89227	0.0562463
35475	1.89227	0.1075
35476	3.78453	0.0688809
35477	2.44375	0.05
35478	1.89227	0.0728371
35479	2.81853	0.0549597
35480	2.81853	0.0552806
35481	1.89227	0.05
35482	1.89227	0.05
35483	1.89226	0.93689
35484	1.75853	0.804221
35485	2.37636	0.765303
35486	2.37636	1.10699
35487	1.89226	0.622924
35488	1.89226	0.968307
35489	1.65814	1.89854
35490	1.10187	2.1
35491	1.10187	2.1
35492	1.19547	2.1
35493	1.56263	2.02505

35494	1.65624	2.1
35495	1.56264	1.76087
35496	1.01479	2.1
35497	1.01479	2.1
35498	1.10187	2.1
35499	1.47561	1.92587
35500	1.5627	2.1
35501	1.89225	2.1
35502	1.89225	2.1
35503	1.89225	2.1
35504	1.89225	2.1
35505	3.7845	2.1
35506	2.54386	2.1
35507	1.91546	2.1
35508	2.46008	2.1
35509	1.82815	2.1
35510	4.37554	2.1
35511	1.91546	2.1
35512	1.04909	2.1
35513	1.82815	2.1
35514	0.983141	2.1
35515	2.8986	2.1
35516	1.89225	2.1
35517	1.89225	2.1
35518	1.89225	2.1
35519	1.89225	2.1
35520	3.78449	2.1
35521	1.89226	2.1
35522	1.89226	2.1
35523	1.89226	2.1
35524	1.89226	2.1
35525	1.89226	2.1
35526	1.89226	2.1
35527	2.46009	2.1
35528	1.7513	2.1
35529	1.82817	2.1
35530	1.82817	2.1
35531	2.38127	2.1
35532	2.38127	2.1
35533	2.55006	2.1
35534	2.64569	2.1
35535	2.60128	2.1
35536	2.49168	2.1
35537	5.13737	2.1
35538	2.51605	2.1
35539	2.64569	2.1
35540	2.60128	2.1
35541	2.48891	2.1
35542	5.1346	2.1
35543	2.60128	2.1
35544	2.49168	2.1
35545	2.41689	2.1
35546	2.55248	2.1
35547	5.04416	2.1
35548	2.60128	2.1
35549	2.48891	2.1
35550	2.45922	2.1
35551	2.55249	2.1

35552	5.04139	2.1
35553	1.89227	2.1
35554	1.89227	2.1
35555	1.89227	2.1
35556	1.89227	2.1
35557	1.89227	2.1
35558	1.89227	2.1
35559	0.96063	2.1
35560	0.96063	2.1
35561	0.958806	2.1
35562	0.958806	2.1
35563	1.28892	2.1
35564	0.749042	2.1
35565	1.17409	2.1
35566	0.630516	2.1
35567	1.17409	2.1
35568	1.28892	2.1
35569	0.630515	2.1
35570	0.749042	2.1
35571	0.92511	2.1
35572	0.92511	2.1
35573	1.19511	2.1
35574	1.19511	2.06167
35575	0.946132	2.1
35576	1.17409	2.1
35577	0.925081	2.1
35578	0.946129	2.07947
35579	0.946131	2.1
35580	0.946131	2.1
35581	1.17409	2.1
35582	0.925083	2.1
35583	1.89226	1.75853
35584	1.89226	2.03043
35585	1.89226	1.49852
35586	1.89226	1.9482
35587	1.89226	1.592
35588	1.89226	1.96793
35589	1.89226	1.97089
35590	1.89226	2.08019
35591	1.89226	2.02134
35592	1.89226	2.09326
35593	3.78453	2.04718
35594	0.946132	2.1
35595	0.946132	2.1
35596	1.28892	2.1
35597	1.28892	2.1
35598	0.946132	2.1
35599	1.28892	2.1
35600	0.946133	2.1
35601	1.17409	2.1
35602	0.946132	2.1
35603	0.946132	2.1
35604	1.17409	2.1
35605	1.28892	2.1
35606	0.625229	2.1
35607	0.890321	2.1
35608	1.21636	2.1
35609	1.13022	2.1

35610	1.07094	2.1
35611	1.03541	2.1
35612	0.942207	2.1
35613	0.942207	2.1
35614	0.898766	2.1
35615	1.26626	2.1
35616	1.0853	2.1
35617	0.600358	2.1
35618	0.719012	2.1
35619	0.719012	2.1
35620	1.17957	2.1
35621	1.17957	2.1
35622	0.719012	2.1
35623	0.952456	2.1
35624	0.946129	2.1
35625	0.946129	2.1
35626	0.946131	2.1
35627	0.952454	2.1
35628	0.946131	2.1
35629	0.719011	2.1
35630	0.94613	2.1
35631	0.94613	2.1
35632	0.94613	2.1
35633	0.94613	2.1
35634	0.946131	2.1
35635	0.946131	2.1
35636	0.946129	2.1
35637	0.946129	2.1
35638	0.94613	2.1
35639	0.94613	2.1
35640	0.94613	2.1
35641	0.94613	2.1
35642	1.1587	2.1
35643	0.973158	2.1
35644	0.750023	2.1
35645	0.536443	2.1
35646	0.84054	2.1
35647	0.84054	2.1
35648	0.854602	2.1
35649	0.854602	2.1
35650	1.1587	2.1
35651	0.750023	2.1
35652	0.973158	2.1
35653	0.536443	2.1
35654	1.89227	1.6727
35655	1.89227	2.1
35656	1.89227	2.1
35657	1.89227	1.97417
35658	3.78453	2.09899
35659	1.89226	2.1
35660	1.89226	2.1
35661	1.89226	2.1
35662	1.89226	2.1
35663	3.78453	2.1
35664	1.7632	2.1
35665	1.61149	2.1
35666	1.98749	2.1
35667	2.11615	2.1

35668	3.75068	2.1
35669	1.7632	2.1
35670	1.61149	2.1
35671	1.97733	2.01948
35672	2.11615	2.1
35673	3.74052	2.1
35674	1.89226	2.1
35675	1.89226	1.89155
35676	1.89226	1.56508
35677	1.89226	2.1
35678	3.78453	2.04804
35679	1.89226	2.1
35680	1.89226	2.1
35681	1.89226	2.1
35682	1.89226	2.1
35683	3.78453	2.1
35684	1.98749	2.1
35685	2.11615	2.1
35686	2.43151	2.1
35687	2.27541	2.1
35688	4.41899	2.1
35689	1.97733	1.92367
35690	2.11615	2.1
35691	2.43151	2.1
35692	2.33986	2.01275
35693	4.40883	2.1
35694	2.17892	0.231808
35695	2.80057	1.86697
35696	1.88349	0.667084
35697	2.63062	0.631138
35698	4.80954	0.670154
35699	1.89226	2.09267
35700	1.89226	0.671123
35701	1.89226	2.0695
35702	1.89226	0.384948
35703	3.78452	1.39371
35704	2.0155	2.09865
35705	1.97652	0.670906
35706	1.8247	2.05458
35707	1.80645	0.404087
35708	3.80122	1.18184
35709	1.97652	0.447472
35710	1.79681	1.01334
35711	1.80645	0.399407
35712	1.59244	0.7828
35713	3.56896	0.267768
35714	1.89226	0.512889
35715	1.89226	1.39024
35716	1.89226	0.337441
35717	1.89226	0.796932
35718	3.78452	0.175065
35719	1.89223	2.06984
35720	1.89223	0.605658
35721	1.89223	2.07043
35722	1.89223	0.399574
35723	3.78447	1.15519
35724	1.82467	2.05738
35725	1.80643	0.466123

35726	1.55159	2.03458
35727	1.53969	0.334682
35728	3.36437	1.17681
35729	1.80643	0.425005
35730	1.59242	0.949269
35731	1.5397	0.377182
35732	1.40538	0.971753
35733	3.13211	0.302757
35734	1.89224	0.450544
35735	1.89224	0.98428
35736	1.89224	0.42009
35737	1.89224	0.904282
35738	3.78447	0.333713
35739	1.37427	0.05
35740	2.37086	0.05
35741	2.37972	0.05
35742	1.94703	0.05
35743	2.19299	0.05
35744	1.91813	0.05
35745	1.07566	2.1
35746	1.12954	2.1
35747	0.946131	2.1
35748	0.946131	2.1
35749	1.12954	2.1
35750	1.12954	2.1
35751	0.946131	2.1
35752	0.946131	2.1
35753	1.12954	2.1
35754	0.946131	2.1
35755	0.946131	2.1
35756	1.07566	2.1
35757	2.25908	2.1
35758	1.89226	2.1
35759	1.89226	2.1
35760	1.89226	2.1
35761	2.37567	2.1
35762	2.37567	2.1
35763	1.37597	2.02764
35764	1.70302	0.937256
35765	1.24162	2.1
35766	1.12219	2.07079
35767	1.37072	2.08815
35768	1.70302	2.1
35769	1.37072	2.1
35770	1.70302	1.65942
35771	1.54168	2.1
35772	2.21318	1.67575
35773	3.39238	2.07498
35774	1.89226	0.05
35775	1.89226	0.05
35776	1.89226	0.05
35777	1.89226	0.05
35778	3.78452	0.05
35779	1.89227	0.05
35780	1.89227	0.05
35781	1.89227	0.05
35782	1.89227	0.05
35783	3.78453	0.05

35784	1.89226	0.05
35785	1.89226	0.05
35786	1.89226	0.05
35787	1.89226	0.05
35788	3.78453	0.05
35789	1.89226	0.05
35790	2.21654	0.05
35791	2.72007	0.05
35792	2.72007	0.05
35793	1.89226	0.05
35794	1.89226	0.05
35795	0.946128	0.05
35796	0.946128	0.05
35797	0.946132	0.05
35798	0.946132	0.05
35799	0.94613	0.05
35800	0.94613	0.05
35801	0.946131	0.05
35802	0.946131	0.05
35803	0.94613	0.05
35804	0.94613	0.05
35805	0.94613	0.05
35806	0.94613	0.05
35807	1.89226	0.05
35808	1.89226	0.05
35809	1.89226	0.05
35810	1.89226	0.05
35811	3.78453	0.05
35812	1.33457	0.05
35813	0.946131	0.05
35814	1.08045	0.05
35815	0.946132	0.05
35816	1.08045	0.05
35817	1.08045	0.05
35818	0.946132	0.05
35819	0.946132	0.05
35820	1.33457	0.05
35821	1.08045	0.05
35822	0.946132	0.05
35823	0.946132	0.05
35824	1.53347	1.19943
35825	1.13092	2.09537
35826	1.52938	1.93781
35827	1.93568	1.55727
35828	3.06285	1.94836
35829	1.89226	0.786205
35830	2.72959	2.1
35831	2.82996	2.09976
35832	2.82996	1.0726
35833	1.89226	2.1
35834	1.89226	0.826294
35835	2.7296	2.1
35836	2.57663	2.1
35837	2.48354	2.1
35838	2.48354	2.1
35839	2.82996	2.1
35840	2.82996	2.1
35841	2.63449	2.1

35842	2.7296	2.1
35843	2.39282	2.1
35844	2.39282	2.1
35845	2.48354	2.1
35846	2.72521	2.1
35847	1.89224	2.1
35848	1.89224	1.94001
35849	1.89224	1.85532
35850	1.89224	2.1
35851	3.78447	2.03446
35852	1.89226	2.1
35853	2.10425	2.1
35854	2.5142	2.1
35855	2.5142	2.1
35856	1.89226	2.1
35857	1.89226	2.1
35858	0.946133	1.42652
35859	0.946133	1.91034
35860	0.946128	2.1
35861	0.946128	2.1
35862	0.946131	2.1
35863	0.946131	1.48253
35864	0.94613	1.49087
35865	0.94613	2.1
35866	0.946131	2.07668
35867	0.946131	2.08942
35868	0.946131	2.09191
35869	0.946131	2.09231
35870	1.89226	0.75014
35871	1.89226	1.13642
35872	1.89226	0.711658
35873	1.89226	1.21729
35874	1.89226	0.7029
35875	1.89226	1.19996
35876	1.94446	0.860389
35877	2.50167	1.37145
35878	1.28418	0.778391
35879	1.94446	1.21663
35880	2.05205	1.07183
35881	2.05205	1.19366
35882	1.27835	2.1
35883	1.08341	2.1
35884	0.946132	2.1
35885	0.946132	2.1
35886	0.851542	2.1
35887	0.851542	2.1
35888	0.977356	2.1
35889	1.18368	2.1
35890	1.18378	2.1
35891	0.851555	2.1
35892	0.977346	2.1
35893	0.851476	2.1
35894	1.07369	2.1
35895	2.5813	2.1
35896	1.95053	2.1
35897	1.95053	2.1
35898	1.90196	2.1
35899	1.90196	2.1

35900	1.89226	2.1
35901	1.89226	2.1
35902	1.89226	2.1
35903	1.89226	2.1
35904	3.78452	2.1
35905	1.89224	2.1
35906	1.89224	2.1
35907	1.89224	2.1
35908	1.89224	2.1
35909	1.89224	2.1
35910	1.89224	2.1
35911	1.97004	0.240163
35912	1.8072	1.73778
35913	1.5866	1.47136
35914	1.79478	0.588523
35915	3.60198	1.02803
35916	1.23267	2.1
35917	1.8072	1.73993
35918	1.58661	1.26222
35919	0.947921	2.1
35920	2.75512	2.04753
35921	1.89226	1.78598
35922	1.89226	2.1
35923	1.89226	2.02535
35924	1.89226	2.1
35925	1.89226	1.93858
35926	1.89226	2.1
35927	1.89226	1.78299
35928	1.89226	0.812667
35929	1.89226	0.659035
35930	1.89226	1.99753
35931	3.78452	1.28543
35932	1.4263	1.77941
35933	1.79478	0.753389
35934	1.67651	0.729049
35935	1.79478	1.87673
35936	1.46834	0.764101
35937	1.5866	1.71196
35938	1.89226	0.848079
35939	1.89226	2.01973
35940	1.89226	1.2213
35941	1.89226	1.77055
35942	1.89226	0.929463
35943	1.89226	1.82929
35944	2.23956	1.52403
35945	1.81847	1.59984
35946	2.10991	0.673044
35947	2.75317	0.463856
35948	4.34947	1.19993
35949	1.60578	0.0989217
35950	1.94974	0.096805
35951	1.68062	0.139944
35952	1.14721	0.0846619
35953	3.2864	0.0953019
35954	1.3946	0.05
35955	1.692 0.05	
35956	2.0154	0.05
35957	2.0154	0.05

35958	1.11926	0.05
35959	1.11926	0.05
35960	1.90347	0.05
35961	1.83626	0.05
35962	1.84583	0.05
35963	1.89434	0.05
35964	3.79781	0.05
35965	1.89226	1.81847
35966	1.89226	1.32406
35967	1.89226	0.874091
35968	1.89226	1.54136
35969	3.78452	1.20606
35970	1.20986	1.61016
35971	1.20986	1.07272
35972	0.912635	1.23961
35973	0.912635	1.42034
35974	0.912667	0.754554
35975	1.17637	1.54614
35976	0.946125	1.72976
35977	0.946125	1.83908
35978	0.946128	1.61121
35979	0.912664	1.28497
35980	0.946127	1.82661
35981	1.17637	1.34657
35982	1.89226	0.05
35983	1.65573	0.05
35984	1.65573	0.05
35985	1.89226	0.05
35986	1.89226	0.05
35987	2.12879	0.05
35988	1.89226	0.05
35989	1.89226	0.05
35990	1.89226	0.0565674
35991	1.89226	0.05
35992	1.89226	0.0555957
35993	1.89226	0.05
35994	2.40928	0.05
35995	1.89226	0.05
35996	1.95959	0.05
35997	1.70302	0.05
35998	3.47338	0.05
35999	1.89226	0.05
36000	1.89226	0.05
36001	1.89226	0.05
36002	1.89226	0.05
36003	3.78452	0.05
36004	1.64985	0.05
36005	2.47846	0.0870047
36006	2.25405	0.0723027
36007	1.80768	0.0778973
36008	4.06173	0.0598988
36009	1.98846	0.05
36010	0.912729	0.0612656
36011	1.05417	0.05
36012	1.70303	0.0639281
36013	2.82107	0.065136
36014	2.47846	0.0915459
36015	1.70303	0.119452

36016	2.86918	0.127283
36017	2.05973	0.122035
36018	2.51808	0.0921568
36019	1.80768	0.0893909
36020	0.946131	0.107551
36021	0.946131	0.0937228
36022	0.946132	0.112985
36023	0.946132	0.161827
36024	0.946131	0.176821
36025	0.946131	0.144848
36026	0.946133	0.103218
36027	0.946133	0.0882456
36028	0.946131	0.138012
36029	0.946131	0.17821
36030	0.946132	0.132334
36031	0.946132	0.144202
36032	0.946133	0.0721319
36033	0.946133	0.0704553
36034	0.946132	0.108098
36035	0.946132	0.0969448
36036	0.946133	0.102642
36037	0.946133	0.0976059
36038	0.946132	0.0624514
36039	0.946132	0.0964985
36040	0.946132	0.0807671
36041	0.946132	0.1155
36042	0.946133	0.0607598
36043	0.946133	0.0930665
36044	1.89227	0.179997
36045	1.89227	0.183803
36046	1.89227	0.196024
36047	1.89227	0.150178
36048	1.89227	0.244498
36049	1.89227	0.187936
36050	1.1656	2.1
36051	1.21847	2.1
36052	0.836006	2.1
36053	0.806007	2.1
36054	1.00653	2.1
36055	1.00653	2.1
36056	1.01794	2.1
36057	1.01794	2.1
36058	1.1656	2.1
36059	0.806007	2.1
36060	0.836006	2.1
36061	1.21847	2.1
36062	2.82263	2.1
36063	2.3312	2.1
36064	2.25936	2.1
36065	2.71441	2.1
36066	5.0456	2.1
36067	2.25936	2.1
36068	1.61201	2.1
36069	1.57627	2.1
36070	1.61201	2.1
36071	2.29545	2.1
36072	2.3312	2.1
36073	1.21269	0.05

36074	1.48867	0.05
36075	1.68264	0.05
36076	1.9898	0.05
36077	3.17131	0.05
36078	2.0487	2.1
36079	2.45212	2.1
36080	2.31012	2.1
36081	1.94095	2.1
36082	4.39307	2.1
36083	1.02435	2.1
36084	0.970469	2.1
36085	0.625375	2.1
36086	0.567166	2.1
36087	0.796854	2.1
36088	0.796854	2.1
36089	0.79466	2.1
36090	0.79466	2.1
36091	0.970471	2.1
36092	0.567165	2.1
36093	0.625374	2.1
36094	1.02435	2.1
36095	2.31012	2.1
36096	1.94095	2.1
36097	1.4825	2.1
36098	2.17384	2.1
36099	4.11479	2.1
36100	0.946187	2.1
36101	0.803763	2.1
36102	0.581357	2.1
36103	0.510356	2.1
36104	0.745632	2.1
36105	0.745632	2.1
36106	0.734566	2.1
36107	0.734563	2.1
36108	0.896142	2.1
36109	0.510366	2.1
36110	0.946173	2.1
36111	0.552965	2.1
36112	1.89226	0.8491
36113	1.89226	1.47755
36114	1.89226	1.64735
36115	1.89226	1.8055
36116	1.89226	0.883442
36117	1.89226	1.71308
36118	1.50743	1.37867
36119	1.0417	2.02652
36120	1.50743	1.81051
36121	1.62974	0.875471
36122	1.14605	0.805261
36123	1.14605	1.99793
36124	1.89226	0.641625
36125	1.89226	1.78848
36126	1.89226	0.463089
36127	1.89226	1.64833
36128	1.89226	0.536146
36129	1.89226	1.86091
36130	1.89226	1.71185
36131	1.89226	1.35775

36132	1.89226	0.659734
36133	1.89226	1.65982
36134	3.78453	1.10614
36135	1.77186	1.57113
36136	1.35668	1.83804
36137	1.70254	0.929598
36138	1.38756	1.81721
36139	1.77186	1.62674
36140	1.37814	2.02578
36141	0.734857	1.94879
36142	1.00549	1.68129
36143	0.835261	0.80878
36144	1.08357	1.91676
36145	0.97665	1.18483
36146	0.795559	2.09712
36147	0.891385	1.59295
36148	0.891385	0.506977
36149	0.874231	0.836729
36150	1.08356	1.0835
36151	0.734863	1.30401
36152	0.79449	1.64913
36153	1.89226	1.63323
36154	1.89226	0.212732
36155	2.42671	0.331439
36156	1.98783	1.24925
36157	4.31897	1.05339
36158	0.579854	0.59484
36159	0.965582	0.791321
36160	0.634403	0.868228
36161	1.17796	0.327629
36162	1.00592	0.283435
36163	0.797259	2.0884
36164	0.905246	1.42286
36165	0.905246	0.349595
36166	0.695715	0.386746
36167	0.637106	0.371897
36168	1.24374	1.18656
36169	0.95617	0.582499
36170	2.0815	0.734359
36171	1.60521	1.5743
36172	1.29556	0.728019
36173	2.32205	1.76667
36174	1.28445	0.589978
36175	2.0815	1.45445
36176	2.3085	2.1
36177	2.21147	2.1
36178	2.3085	2.1
36179	2.39282	2.1
36180	1.98899	2.1
36181	2.0733	2.1
36182	2.3275	2.1
36183	1.63471	2.1
36184	1.98899	2.1
36185	1.56801	2.1
36186	3.62369	2.1
36187	2.3085	2.1
36188	1.98899	2.1
36189	2.22575	2.1

36190	1.90502	2.1
36191	4.21353	2.1
36192	1.90502	2.1
36193	1.56801	2.1
36194	1.90502	2.1
36195	1.98899	2.1
36196	1.49733	2.1
36197	1.58129	2.1
36198	1.89226	2.03539
36199	2.6491	2.1
36200	2.49266	2.1
36201	2.6491	2.0578
36202	1.73582	2.1
36203	1.89226	1.51079
36204	1.31854	2.1
36205	2.6491	2.1
36206	1.3909	2.1
36207	1.3909	2.1
36208	2.49266	2.1
36209	2.49266	2.1
36210	1.89226	1.89344
36211	2.49266	2.06846
36212	2.38634	2.1
36213	2.49266	1.92892
36214	1.78595	2.06646
36215	1.89226	1.28234
36216	1.23042	2.1
36217	2.49266	2.08671
36218	1.31854	2.1
36219	1.31854	2.1
36220	2.38635	2.1
36221	2.38635	2.1
36222	2.83487	2.1
36223	1.89226	2.1
36224	2.594	2.1
36225	2.594	2.1
36226	1.89226	2.1
36227	1.89226	2.1
36228	1.89226	2.1
36229	1.89226	2.1
36230	1.89226	2.1
36231	1.89226	2.1
36232	3.78452	2.1
36233	1.09518	2.1
36234	1.28158	2.1
36235	1.36844	2.1
36236	1.59972	2.1
36237	2.65003	2.1
36238	1.89226	2.1
36239	1.89226	2.1
36240	1.89226	2.1
36241	1.89226	2.1
36242	3.78452	2.1
36243	1.36844	2.1
36244	1.59972	2.1
36245	1.67352	2.1
36246	1.94291	2.1
36247	3.31135	2.1

36248	1.52938	1.93519
36249	1.93568	1.74035
36250	2.36221	0.814321
36251	1.94723	2.07818
36252	3.89158	1.77967
36253	1.94724	1.28805
36254	1.26593	2.08
36255	1.75367	1.98428
36256	2.4322	1.63784
36257	3.7009	1.93301
36258	2.36221	0.780565
36259	2.43219	1.97707
36260	1.94723	1.6929
36261	1.94723	1.49421
36262	2.8202	1.73439
36263	2.8202	1.55671
36264	2.51604	2.1
36265	2.18533	2.1
36266	2.17228	2.1
36267	2.4889	2.1
36268	4.67423	2.1
36269	2.17228	2.1
36270	1.62183	2.1
36271	1.62316	2.1
36272	1.62183	2.1
36273	2.18666	2.1
36274	2.18533	2.1
36275	2.17227	2.1
36276	2.4889	2.1
36277	2.45922	2.1
36278	2.15686	2.1
36279	4.64576	2.1
36280	2.17228	2.1
36281	1.62316	2.1
36282	1.62241	2.1
36283	2.15686	2.1
36284	3.78002	2.1
36285	1.89226	1.41909
36286	1.89226	1.36484
36287	1.89226	1.1658
36288	1.89226	1.14502
36289	3.78453	1.28513
36290	1.89226	1.15878
36291	1.89226	1.17731
36292	1.89226	0.938426
36293	1.89226	0.391475
36294	3.78452	0.933175
36295	1.89226	1.36969
36296	1.89226	0.496298
36297	1.89226	1.16132
36298	1.89226	1.06181
36299	1.89226	0.79913
36300	1.89226	0.935282
36301	1.89226	1.18194
36302	1.89226	1.33057
36303	2.53413	0.330728
36304	1.89226	0.466091
36305	3.78453	1.02791

36306	1.38048	0.52408
36307	1.38048	0.943238
36308	0.490332	1.00731
36309	0.490332	0.637986
36310	0.924679	0.16802
36311	0.946131	1.54744
36312	0.946131	1.41705
36313	0.490332	0.135233
36314	0.924678	0.213422
36315	0.490332	0.152329
36316	0.946131	0.784866
36317	0.946131	0.548666
36318	1.89227	0.336089
36319	1.89227	0.943501
36320	1.89227	0.291758
36321	1.89227	1.24455
36322	1.89227	0.389688
36323	1.89227	0.828657
36324	1.30803	2.1
36325	2.55151	2.1
36326	1.43935	2.1
36327	1.43935	2.1
36328	2.34922	2.1
36329	2.34922	2.1
36330	0.946131	2.1
36331	0.946131	2.1
36332	1.27575	2.1
36333	1.17461	2.1
36334	0.94613	2.1
36335	0.94613	2.1
36336	1.17461	2.1
36337	1.17461	2.1
36338	0.946131	2.1
36339	1.17461	2.1
36340	0.946132	2.1
36341	1.27575	2.1
36342	1.89226	2.1
36343	1.89226	2.1
36344	1.89226	2.1
36345	1.89226	2.1
36346	1.89226	2.1
36347	1.89226	2.1
36348	0.946133	2.1
36349	0.946133	2.1
36350	0.946132	2.1
36351	0.946132	2.1
36352	0.946133	2.1
36353	0.946133	2.1
36354	0.946132	2.1
36355	0.946132	2.1
36356	0.946132	2.1
36357	0.946132	2.1
36358	0.946133	2.1
36359	0.946133	2.1
36360	1.07043	2.1
36361	1.17461	2.1
36362	0.654016	2.1
36363	0.5829	2.1

36364	0.870481	2.1
36365	0.870481	2.1
36366	0.887027	2.1
36367	0.887027	2.1
36368	1.07043	2.1
36369	0.5829	2.1
36370	0.654016	2.1
36371	1.17461	2.1
36372	1.89226	2.1
36373	1.89226	2.1
36374	1.89226	2.1
36375	1.89226	2.1
36376	3.78453	2.1
36377	1.89226	2.1
36378	1.89226	2.1
36379	1.89226	2.1
36380	1.89226	2.1
36381	3.78453	2.1
36382	1.26929	2.1
36383	2.19313	2.1
36384	1.09847	2.1
36385	1.69916	2.1
36386	2.96844	2.1
36387	1.84124	2.1
36388	2.24937	2.1
36389	1.69915	2.1
36390	1.75691	2.1
36391	3.94852	2.1
36392	1.53082	2.1
36393	2.04422	2.1
36394	1.53082	2.1
36395	1.69915	2.1
36396	1.92911	2.1
36397	2.09744	2.1
36398	1.39263	1.34465
36399	1.0171	0.561819
36400	1.1211	0.787349
36401	1.46482	1.08303
36402	2.48192	0.929612
36403	1.39263	1.4308
36404	1.57962	1.94978
36405	1.65238	2.1
36406	1.46482	1.22057
36407	3.04443	1.68089
36408	1.89226	1.94612
36409	1.89226	0.840742
36410	1.89226	1.18239
36411	1.89226	1.63919
36412	3.78453	1.5048
36413	1.89227	0.559497
36414	1.89227	0.803804
36415	1.89227	1.06094
36416	1.89227	0.539555
36417	3.78453	0.660946
36418	1.28418	0.646961
36419	1.46482	0.95264
36420	1.1211	0.615341
36421	1.1211	1.16893

36422	1.59382	0.595227
36423	1.59382	1.11544
36424	1.76897	2.02769
36425	1.46482	1.04545
36426	1.59382	1.43369
36427	1.46482	1.82853
36428	1.78138	1.2461
36429	1.65238	1.70943
36430	1.89226	1.01405
36431	1.89226	1.33147
36432	1.89226	0.846063
36433	1.89226	1.672
36434	1.89226	0.868861
36435	1.89226	1.78601
36436	1.89226	0.936488
36437	1.89226	0.468202
36438	1.89226	0.516615
36439	1.89226	0.727549
36440	1.89226	0.58971
36441	1.89226	0.764096
36442	1.96832	2.1
36443	2.40858	2.1
36444	1.75211	2.04807
36445	2.20797	2.1
36446	4.1607	2.1
36447	1.75211	2.08022
36448	2.20797	2.1
36449	1.51592	2.1
36450	2.02064	2.1
36451	3.77275	2.1
36452	2.71793	0.286485
36453	2.67099	0.156055
36454	2.55105	0.201776
36455	2.71297	0.141475
36456	5.26898	0.182618
36457	2.36417	0.290655
36458	2.67098	0.175037
36459	2.55104	0.219711
36460	1.83418	0.131177
36461	4.91522	0.17921
36462	2.55105	0.202507
36463	2.71297	0.143837
36464	2.58032	0.177185
36465	2.28922	0.110639
36466	5.13137	0.155131
36467	2.55104	0.221445
36468	2.23787	0.134785
36469	1.67265	0.147216
36470	2.28922	0.123095
36471	4.22369	0.147634
36472	0.851542	0.677848
36473	0.851542	0.739905
36474	0.981419	0.558332
36475	0.854774	0.58495
36476	0.949431	0.575781
36477	0.753763	0.586511
36478	0.946131	0.739587
36479	0.946131	0.625866

36480	1.04073	0.63294
36481	0.981433	0.512446
36482	0.851528	0.701136
36483	0.854828	0.626744
36484	1.89226	1.19943
36485	1.89226	0.712084
36486	1.89226	0.77148
36487	1.89226	0.779237
36488	1.89226	0.944966
36489	1.89226	0.968806
36490	0.814227	0.522107
36491	0.814227	0.589836
36492	0.946117	0.471102
36493	1.07285	0.443647
36494	0.879132	0.451497
36495	0.923293	0.432817
36496	0.946119	0.588449
36497	0.946119	0.508253
36498	0.946131	0.501223
36499	0.814236	0.400802
36500	0.946106	0.552901
36501	0.870405	0.503541
36502	1.89224	0.917801
36503	1.89224	0.537318
36504	1.89224	0.59309
36505	1.89224	0.612194
36506	1.89224	0.758827
36507	1.89224	0.798878
36508	1.29388	1.50899
36509	1.04164	1.90556
36510	1.64834	1.87278
36511	1.82955	1.45876
36512	2.94222	1.81927
36513	1.4934	1.95979
36514	2.41522	1.84538
36515	2.20387	1.13016
36516	2.20387	2.1
36517	1.75765	1.36742
36518	1.75765	2.1
36519	2.41522	1.86662
36520	2.77303	2.1
36521	2.20387	2.1
36522	2.58627	2.1
36523	5.00149	2.1
36524	1.22222	2.0023
36525	2.20387	2.08339
36526	1.96831	1.28745
36527	1.96831	2.1
36528	1.4934	1.47065
36529	1.4934	2.1
36530	1.96832	2.1
36531	2.58627	2.1
36532	2.40858	2.0865
36533	2.40858	2.1
36534	2.20387	2.1
36535	2.20387	2.1
36536	1.5605	2.1
36537	1.53775	2.1

36538	1.55398	2.1
36539	1.45806	2.1
36540	1.65642	2.1
36541	1.5605	2.1
36542	0.768876	2.1
36543	0.79033	2.1
36544	0.828211	2.1
36545	0.871233	2.1
36546	0.803843	2.1
36547	0.803843	2.1
36548	0.814698	2.1
36549	0.814698	2.1
36550	0.79033	2.1
36551	0.871233	2.1
36552	0.768876	2.1
36553	0.828212	2.1
36554	1.80252	2.1
36555	2.09715	2.08168
36556	1.9394	2.1
36557	1.50853	2.1
36558	3.60567	2.1
36559	1.96416	1.88445
36560	2.09715	2.04753
36561	1.88484	2.1
36562	1.57916	1.15337
36563	3.67631	2.03635
36564	1.89226	2.00426
36565	1.89226	0.385173
36566	1.89226	1.15182
36567	1.89226	1.37731
36568	3.78453	1.08862
36569	1.89226	2.0528
36570	1.89226	2.09506
36571	1.89226	2.1
36572	1.89226	1.51496
36573	1.89226	2.1
36574	1.89226	1.10522
36575	1.5852	2.1
36576	1.50853	2.1
36577	1.2955	2.1
36578	1.44472	2.05318
36579	2.95325	2.1
36580	1.53065	2.1
36581	1.57916	0.905221
36582	1.44472	2.00496
36583	1.78861	1.97183
36584	1.23527	2.00025
36585	1.23527	1.86693
36586	1.89227	0.876657
36587	1.89227	1.11527
36588	1.89227	2.01204
36589	1.89227	0.526512
36590	3.78453	1.10937
36591	1.89226	2.06745
36592	1.89226	2.08055
36593	1.89226	2.1
36594	1.89226	2.0025
36595	1.89226	2.1

36596	1.89226	1.28014
36597	1.89227	1.28801
36598	1.89227	0.745095
36599	1.89227	0.660565
36600	1.89227	0.431031
36601	3.78453	0.44385
36602	0.943708	0.441766
36603	0.752219	0.33098
36604	0.946132	0.429604
36605	0.946132	0.433955
36606	0.946133	0.257696
36607	0.946133	0.920273
36608	0.752218	1.23323
36609	0.752218	0.54035
36610	0.752218	0.395952
36611	0.946132	0.318567
36612	0.946133	0.688448
36613	0.943708	0.482224
36614	0.943992	2.1
36615	0.943992	2.1
36616	1.23507	2.1
36617	1.48209	2.1
36618	1.0384	2.1
36619	1.47953	2.1
36620	0.946128	2.09913
36621	0.946128	2.1
36622	0.946129	2.1
36623	1.23293	2.1
36624	1.0384	2.1
36625	0.946129	2.1
36626	1.89226	2.1
36627	2.46586	2.1
36628	2.80218	2.08493
36629	2.80218	2.1
36630	1.89226	2.09123
36631	1.89226	2.1
36632	1.32455	2.1
36633	1.89128	2.1
36634	1.32328	2.1
36635	1.97299	2.1
36636	3.29754	2.1
36637	1.89128	2.1
36638	2.4255	2.1
36639	1.973 2.1	
36640	2.63807	2.1
36641	4.3985	2.1
36642	1.34008	2.1
36643	1.97299	2.1
36644	2.10766	2.1
36645	2.10766	2.1
36646	1.32328	2.1
36647	1.32328	2.1
36648	2.41055	2.1
36649	2.37491	2.1
36650	2.40754	2.1
36651	2.38023	2.1
36652	4.78546	2.1
36653	2.23743	2.1

36654	2.37491	2.1
36655	2.40754	2.1
36656	2.20392	2.1
36657	4.61234	2.1
36658	2.37491	2.1
36659	2.02641	2.1
36660	2.20392	2.1
36661	2.18943	2.1
36662	4.40132	2.1
36663	2.37491	2.1
36664	2.22558	2.1
36665	2.38023	2.1
36666	2.18943	2.1
36667	4.60049	2.1
36668	1.89226	0.0785243
36669	1.89226	0.153493
36670	1.89226	0.109817
36671	1.89226	0.108978
36672	1.89226	0.100943
36673	1.89226	0.101145
36674	1.03599	0.0729371
36675	0.706698	0.0688927
36676	1.41141	0.0918737
36677	1.17573	0.0750384
36678	1.11957	0.0605617
36679	0.765034	0.0625167
36680	1.05913	0.066308
36681	1.45054	0.079441
36682	1.04847	0.0663259
36683	1.04847	0.0799348
36684	1.15323	0.069992
36685	1.05825	0.0551945
36686	1.89226	0.109265
36687	1.89226	0.123482
36688	1.89226	0.12413
36689	1.89226	0.102996
36690	1.89226	0.127985
36691	1.89226	0.107392
36692	2.22575	2.06756
36693	1.70677	2.1
36694	1.90157	2.04613
36695	1.5534	2.1
36696	3.77914	2.1
36697	1.70678	2.1
36698	0.968594	2.1
36699	0.968594	2.1
36700	1.10378	2.1
36701	1.5534	2.1
36702	1.68859	2.1
36703	2.31429	1.91052
36704	1.5534	2.1
36705	1.90103	2.05581
36706	1.41902	2.1
36707	3.45443	2.1
36708	2.53709	0.05
36709	1.209	0.05
36710	1.05417	0.05
36711	2.6513	0.05

36712	3.70547	0.05
36713	1.02675	0.05
36714	1.16384	0.05
36715	2.22062	0.05
36716	2.48862	0.05
36717	3.38446	0.05
36718	1.89226	0.05
36719	1.89226	0.05
36720	1.89226	0.05
36721	1.89226	0.05
36722	1.89226	0.05
36723	1.89226	0.05
36724	1.89226	1.87906
36725	1.89226	2.1
36726	1.89226	2.1
36727	1.89226	1.94141
36728	1.89226	2.1
36729	1.89226	1.92604
36730	1.89226	1.75086
36731	1.89226	2.02245
36732	1.89226	2.07096
36733	1.89226	1.79605
36734	1.89226	2.06263
36735	1.89226	1.72856
36736	1.89227	1.75542
36737	1.89227	1.98862
36738	1.75032	2.04255
36739	1.75032	1.73965
36740	1.89227	2.02776
36741	1.89227	1.68093
36742	1.89226	0.3858
36743	1.89226	0.496854
36744	1.89226	0.340001
36745	1.89226	0.392804
36746	1.89226	0.39891
36747	1.89226	0.459862
36748	0.946132	0.200396
36749	0.946132	0.182605
36750	0.798215	0.176379
36751	0.848163	0.168317
36752	0.848163	0.195489
36753	0.946131	0.198527
36754	0.946132	0.163072
36755	0.798216	0.168844
36756	0.996105	0.183003
36757	0.996105	0.150331
36758	0.79819	0.17636
36759	0.79819	0.210601
36760	1.07763	0.200905
36761	2.28622	0.194129
36762	2.16109	0.132037
36763	1.17506	0.142747
36764	3.36385	0.157243
36765	1.89226	2.1
36766	1.6663	2.1
36767	1.6663	2.1
36768	1.71706	2.1
36769	1.89226	2.1

36770	1.94302	2.1
36771	1.89226	2.1
36772	1.62801	2.1
36773	1.62801	2.1
36774	1.6663	2.1
36775	1.89226	2.1
36776	1.93055	2.1
36777	1.48734	2.1
36778	1.99609	2.1
36779	1.52245	2.1
36780	2.04137	2.1
36781	3.51854	2.1
36782	1.99609	2.1
36783	2.28563	2.1
36784	2.04136	2.1
36785	2.35019	2.1
36786	4.34628	2.1
36787	1.50714	2.1
36788	2.04137	2.1
36789	2.04945	2.1
36790	2.04945	2.1
36791	1.52245	2.1
36792	1.52245	2.1
36793	2.04136	2.1
36794	2.35019	2.1
36795	2.04945	2.1
36796	2.38681	2.1
36797	4.39964	2.1
36798	2.50924	0.05
36799	1.38305	2.1
36800	2.56918	2.1
36801	1.44535	2.1
36802	3.95222	2.1
36803	1.89226	2.1
36804	1.89226	2.1
36805	1.89226	2.1
36806	1.89226	2.1
36807	3.78453	2.1
36808	1.40615	2.1
36809	1.38851	2.1
36810	0.748607	2.1
36811	0.724468	2.1
36812	1.06362	2.1
36813	1.06362	2.1
36814	1.067 2.1	
36815	1.067 2.1	
36816	1.40615	2.1
36817	0.748607	2.1
36818	0.724468	2.1
36819	1.38851	2.1
36820	2.56918	2.1
36821	1.53377	2.1
36822	1.53706	2.1
36823	1.44535	2.1
36824	2.66088	2.1
36825	2.56918	2.1
36826	1.89226	2.1
36827	1.89226	2.1

36828	1.89226	2.1
36829	1.89226	2.1
36830	3.78453	2.1
36831	1.40239	2.1
36832	1.68466	2.1
36833	1.26091	2.1
36834	1.54641	2.1
36835	2.94881	2.1
36836	1.68466	2.1
36837	1.65324	2.08221
36838	1.54641	2.1
36839	1.52268	2.07918
36840	3.19965	2.1
36841	1.05776	2.1
36842	1.54641	2.1
36843	1.36595	2.1
36844	1.36595	2.1
36845	1.26091	2.1
36846	1.26091	2.1
36847	1.54641	2.1
36848	1.32697	2.06923
36849	1.52268	2.08208
36850	1.52268	2.1
36851	1.36595	2.0864
36852	1.36595	2.1
36853	1.18647	1.40072
36854	0.988454	2.09975
36855	1.4867	1.91926
36856	1.63892	1.30199
36857	2.67317	2.02617
36858	1.4867	2.05323
36859	1.63892	1.5441
36860	2.09605	0.702178
36861	1.97645	1.90818
36862	3.58275	1.3437
36863	1.29389	1.91528
36864	1.97645	1.47681
36865	1.4867	1.65805
36866	1.4867	1.98088
36867	1.82955	1.20022
36868	1.82955	1.92332
36869	1.13454	1.06939
36870	0.983594	2.1
36871	1.16809	2.1
36872	1.06836	2.06085
36873	1.08332	2.09728
36874	0.983594	1.90757
36875	1.48751	2.1
36876	1.48047	1.84831
36877	1.77596	2.0734
36878	1.75506	2.01854
36879	3.25643	2.06872
36880	1.48047	1.92489
36881	1.25334	2.05539
36882	1.75505	1.97954
36883	1.55831	0.503443
36884	3.03878	1.78657
36885	1.77596	2.096

36886	1.75506	2.08858
36887	2.00551	2.1
36888	2.00083	1.39431
36889	3.77679	2.0933
36890	1.75506	2.08233
36891	1.80328	0.536858
36892	1.55831	1.94116
36893	1.55831	2.07764
36894	2.00083	0.985231
36895	2.00083	1.63432
36896	2.58627	2.1
36897	2.79047	2.1
36898	2.40858	2.1
36899	2.64748	2.1
36900	5.23375	2.1
36901	1.26755	2.1
36902	1.71717	2.1
36903	1.03824	2.1
36904	1.48901	2.1
36905	2.75656	2.1
36906	1.89226	2.1
36907	1.89226	2.1
36908	1.89226	2.1
36909	1.89226	2.1
36910	1.89226	2.1
36911	1.89226	2.1
36912	1.36541	2.1
36913	1.22156	2.1
36914	0.946132	2.1
36915	0.946132	2.1
36916	1.36541	2.1
36917	1.36541	2.1
36918	0.946132	2.1
36919	0.946132	2.1
36920	1.36541	2.1
36921	0.946133	2.1
36922	0.946132	2.1
36923	1.22156	2.1
36924	1.18901	2.1
36925	1.61149	2.1
36926	1.98748	2.1
36927	1.534 2.1	
36928	3.1765	2.1
36929	1.89226	2.1
36930	1.89226	2.1
36931	1.89226	2.1
36932	1.89226	2.1
36933	3.78452	2.1
36934	1.98748	2.1
36935	1.534 2.1	
36936	2.27541	2.1
36937	1.98748	2.1
36938	1.87436	2.1
36939	1.87436	2.1
36940	2.2988	2.1
36941	2.2604	2.1
36942	2.20831	2.1
36943	2.02802	2.1

36944	4.32683	2.1
36945	2.26041	2.1
36946	1.98957	2.1
36947	2.02803	2.1
36948	1.75021	2.1
36949	4.0176	2.1
36950	2.34607	2.1
36951	2.02803	2.1
36952	1.86795	2.1
36953	1.81448	2.1
36954	3.89597	2.1
36955	2.02803	2.1
36956	1.75021	2.1
36957	1.81448	2.1
36958	1.57887	2.1
36959	3.6069	2.1
36960	0.946131	1.66112
36961	0.946131	2.1
36962	1.1235	2.05685
36963	1.1235	2.1
36964	0.946131	1.81118
36965	0.946131	1.45708
36966	1.1235	2.1
36967	1.13736	2.1
36968	1.13736	2.08187
36969	0.946131	2.09632
36970	1.1235	2.1
36971	0.946131	2.1
36972	1.89226	2.08867
36973	1.89226	2.1
36974	1.89226	1.07059
36975	1.89226	0.59052
36976	3.78452	0.6001
36977	0.563266	2.1
36978	0.563266	1.74456
36979	0.565606	0.77003
36980	0.565606	2.1
36981	0.647086	2.07881
36982	0.624219	2.05813
36983	0.500035	1.4647
36984	0.481785	1.1767
36985	0.500034	0.425202
36986	0.647087	0.392802
36987	0.624219	1.93435
36988	0.481785	1.50784
36989	0.915788	0.21278
36990	0.915788	0.303114
36991	1.02732	0.713913
36992	1.02732	0.487257
36993	0.660831	0.512275
36994	1.11155	0.224463
36995	0.713295	0.269221
36996	1.26874	0.648265
36997	0.713295	0.391539
36998	0.660831	0.34128
36999	1.11155	0.377769
37000	1.26874	0.36106
37001	1.89226	0.728077

37002	1.85091	0.951853
37003	2.11193	1.02012
37004	1.85091	0.87638
37005	2.15328	0.751866
37006	1.89226	0.642023
37007	0.946131	0.465973
37008	0.946131	0.519111
37009	1.18275	0.61345
37010	1.05596	0.72628
37011	0.946127	0.640011
37012	0.946127	0.395379
37013	1.18275	0.546403
37014	1.18275	0.635795
37015	0.946128	0.564786
37016	1.18275	0.563702
37017	0.946129	0.574695
37018	1.05596	0.58731
37019	1.60936	2.1
37020	2.35846	2.1
37021	2.06424	2.1
37022	2.57651	2.1
37023	4.42269	2.1
37024	1.89226	2.1
37025	1.89226	2.1
37026	1.89226	2.1
37027	1.89226	2.1
37028	3.78453	2.1
37029	1.89226	2.1
37030	1.89226	2.1
37031	1.89226	2.1
37032	1.89226	2.1
37033	1.89226	2.1
37034	1.89226	2.1
37035	1.95053	2.1
37036	2.06424	2.1
37037	1.95053	2.1
37038	1.30584	2.1
37039	2.5	2.1
37040	1.8553	2.1
37041	2.49999	2.1
37042	2.57651	2.1
37043	2.49999	2.1
37044	2.06423	2.1
37045	2.80738	2.1
37046	2.37162	2.1
37047	1.89226	2.1
37048	1.89226	2.1
37049	1.89226	2.1
37050	1.89226	2.1
37051	1.89226	2.1
37052	1.89226	2.1
37053	1.49295	0.145293
37054	1.03824	0.15879
37055	1.51396	0.16708
37056	2.08514	0.157516
37057	3.12338	0.155109
37058	1.49798	0.0978127
37059	2.32017	0.065924

37060	2.68236	0.141562
37061	2.08514	0.120215
37062	4.4053	0.0971118
37063	1.89226	0.0662813
37064	1.89226	0.119564
37065	1.89226	0.116044
37066	1.89226	0.101825
37067	3.78452	0.100371
37068	1.89226	0.159922
37069	1.89226	0.195591
37070	1.89226	0.167748
37071	1.89226	0.17757
37072	1.89226	0.163254
37073	1.89226	0.167313
37074	1.51396	0.103974
37075	2.08514	0.0979004
37076	2.55393	0.116216
37077	2.02901	0.132556
37078	4.11415	0.109052
37079	1.89226	0.0651469
37080	1.89226	0.05
37081	1.89226	0.0978334
37082	1.89226	0.111004
37083	3.78451	0.0626412
37084	1.89226	0.0985322
37085	1.89226	0.156742
37086	1.89226	0.118887
37087	1.89226	0.100959
37088	1.89226	0.131446
37089	1.89226	0.120972
37090	0.946131	0.125034
37091	0.946131	0.144127
37092	0.946132	0.271917
37093	0.946132	0.169361
37094	0.946132	0.182239
37095	0.946132	0.150888
37096	0.946132	0.195463
37097	0.946132	0.261821
37098	0.946132	0.139142
37099	0.946132	0.187219
37100	0.946132	0.204989
37101	0.946132	0.169219
37102	1.89226	2.1
37103	1.89226	2.1
37104	1.89226	2.1
37105	1.89226	2.1
37106	3.78452	2.1
37107	1.28367	2.1
37108	1.82707	2.1
37109	1.11025	2.1
37110	1.6365	2.1
37111	2.92016	2.1
37112	1.82707	2.1
37113	1.8247	2.05938
37114	1.8247	2.1
37115	2.01549	2.09856
37116	1.6365	2.1
37117	1.82729	2.09927

37118	1.89226	2.1
37119	1.89226	2.0661
37120	1.89226	2.02769
37121	1.89226	2.1
37122	1.89226	2.06206
37123	1.89226	2.1
37124	1.63648	2.1
37125	1.82467	2.06957
37126	1.35877	2.1
37127	1.55159	2.028
37128	3.18345	2.1
37129	1.89223	2.1
37130	1.89223	2.07361
37131	1.89223	2.1
37132	1.89223	2.073
37133	3.78447	2.1
37134	1.59972	2.1
37135	1.28037	2.1
37136	1.63702	2.1
37137	1.28158	2.1
37138	1.63581	2.1
37139	1.28037	2.1
37140	1.89226	2.1
37141	1.89226	2.1
37142	1.89226	2.1
37143	1.89226	2.1
37144	1.89226	2.1
37145	1.89226	2.1
37146	1.89226	2.1
37147	1.89226	2.1
37148	1.89226	2.1
37149	1.89226	2.1
37150	3.78451	2.1
37151	1.28037	2.1
37152	1.05071	2.1
37153	1.63581	2.1
37154	1.44287	2.1
37155	2.72324	2.1
37156	1.59972	2.1
37157	1.63581	2.1
37158	1.94291	2.1
37159	2.00118	2.1
37160	3.57872	2.1
37161	1.89226	2.1
37162	1.89226	2.1
37163	1.89226	2.1
37164	1.89226	2.1
37165	3.78452	2.1
37166	0.946131	2.1
37167	0.946131	2.1
37168	0.946127	2.1
37169	0.946127	2.1
37170	0.94613	2.1
37171	0.94613	2.1
37172	0.946128	2.1
37173	0.946128	2.1
37174	0.946128	2.1
37175	0.946128	2.1

37176	0.946129	2.1
37177	0.946129	2.1
37178	0.865056	2.1
37179	0.865056	2.1
37180	0.873224	2.1
37181	0.873224	2.1
37182	0.721437	2.1
37183	0.920378	2.1
37184	0.817903	2.1
37185	1.00059	2.1
37186	1.00059	2.1
37187	0.920376	2.1
37188	0.817904	2.1
37189	0.721437	2.1
37190	3.09157	2.1
37191	1.71255	2.1
37192	2.0815	2.1
37193	1.9918	2.1
37194	2.51364	2.1
37195	2.39396	2.1
37196	2.0815	2.1
37197	1.05051	2.1
37198	2.56724	2.1
37199	1.63991	2.1
37200	3.47929	2.1
37201	1.89226	0.0945216
37202	2.2973	0.17126
37203	1.90705	0.162881
37204	1.90705	0.116252
37205	1.89226	0.14895
37206	1.89226	0.095584
37207	1.89226	0.0860187
37208	1.90705	0.160479
37209	1.45321	0.1638
37210	1.45321	0.115163
37211	1.89226	0.14667
37212	1.89226	0.0949805
37213	0.901146	2.1
37214	0.901146	2.1
37215	1.15227	2.1
37216	1.21279	2.1
37217	0.991265	2.1
37218	1.15003	2.1
37219	0.946132	2.1
37220	0.946132	2.1
37221	0.946132	2.1
37222	1.09997	2.1
37223	0.991266	2.1
37224	0.946132	2.1
37225	1.89226	2.1
37226	1.89226	2.1
37227	1.89226	2.1
37228	1.89226	2.1
37229	1.89226	2.1
37230	1.89226	2.1
37231	1.62229	2.1
37232	1.06053	2.1
37233	1.19698	2.1

37234	1.70106	2.1
37235	2.81928	2.1
37236	0.946132	2.1
37237	0.946132	2.1
37238	1.17527	2.1
37239	1.09997	2.1
37240	1.09997	2.1
37241	1.09997	2.1
37242	0.946133	2.1
37243	0.946133	2.1
37244	1.17527	2.1
37245	0.946132	2.1
37246	0.946133	2.1
37247	1.09997	2.1
37248	1.89226	2.1
37249	1.89226	2.1
37250	1.89226	2.1
37251	1.89226	2.1
37252	1.89226	2.1
37253	1.89226	2.1
37254	0.905416	2.1
37255	0.850527	2.1
37256	0.660601	2.1
37257	0.598494	2.1
37258	0.750137	2.1
37259	0.750137	2.1
37260	0.753774	2.1
37261	0.753774	2.1
37262	0.660599	2.1
37263	0.905418	2.1
37264	0.598493	2.1
37265	0.850529	2.1
37266	2.43509	2.1
37267	2.65066	2.1
37268	2.66602	2.1
37269	2.46339	2.1
37270	5.10111	2.1
37271	2.53008	2.1
37272	2.65066	2.1
37273	2.66602	2.1
37274	2.52852	2.1
37275	5.1961	2.1
37276	2.66602	2.1
37277	2.46339	2.1
37278	2.47932	2.1
37279	2.67393	2.1
37280	5.14535	2.1
37281	2.66602	2.1
37282	2.52853	2.1
37283	2.51686	2.1
37284	2.67393	2.1
37285	5.18288	2.1
37286	1.89226	2.0911
37287	1.89226	0.602351
37288	1.89226	2.07038
37289	1.89226	0.513783
37290	3.78452	1.40372
37291	1.55161	2.08458

37292	1.53972	0.548695
37293	1.32259	2.0526
37294	1.35082	0.415777
37295	2.8623	1.19116
37296	1.53972	0.474796
37297	1.4054	0.961991
37298	1.35083	0.421237
37299	1.24913	0.651596
37300	2.78885	0.377636
37301	1.89226	0.507551
37302	1.89226	1.31594
37303	1.89226	0.531696
37304	1.89226	0.636023
37305	3.78452	0.193865
37306	1.89226	2.06142
37307	1.89226	0.721153
37308	1.89226	2.07312
37309	1.89226	0.760478
37310	1.89226	2.07773
37311	1.89226	1.10632
37312	1.14599	2.05781
37313	1.35082	0.580554
37314	1.14599	1.21395
37315	1.32259	2.05949
37316	1.19993	0.555204
37317	1.37652	2.05863
37318	1.35083	0.458284
37319	1.24913	1.18147
37320	1.19993	0.744279
37321	1.11787	0.962727
37322	2.44906	0.343364
37323	1.89226	0.672242
37324	1.89226	1.16305
37325	1.89226	0.826266
37326	1.89226	0.544124
37327	3.78452	0.356687
37328	1.51697	0.05
37329	2.57264	0.059648
37330	1.30203	0.05
37331	2.41701	0.05
37332	3.93398	0.05
37333	1.17464	0.05
37334	2.41701	0.05
37335	2.32462	0.05
37336	2.32462	0.05
37337	1.30203	0.05
37338	1.30203	0.05
37339	2.08149	2.1
37340	1.9839	2.1
37341	1.71398	2.1
37342	1.99182	2.09796
37343	3.71121	2.1
37344	2.16061	2.1
37345	2.01399	0.718824
37346	1.99182	2.1
37347	1.83895	2.07249
37348	4.00581	2.08016
37349	1.90444	2.1

37350	1.99182	2.1
37351	1.71717	2.1
37352	1.805	2.1
37353	3.70899	2.1
37354	1.99182	2.1
37355	1.83896	1.10006
37356	1.805	2.1
37357	1.65243	1.16023
37358	3.64425	2.05872
37359	1.18076	2.1
37360	1.27697	2.1
37361	1.05025	2.1
37362	1.27697	2.1
37363	1.25	2.1
37364	1.47671	2.1
37365	1.89226	2.1
37366	1.89226	2.1
37367	1.89226	2.1
37368	1.89226	2.1
37369	3.78452	2.1
37370	2.64124	2.1
37371	1.89226	2.1
37372	1.89226	2.1
37373	1.89226	2.1
37374	2.46904	2.1
37375	2.46904	2.1
37376	1.89226	2.1
37377	1.89226	2.1
37378	2.7408	2.1
37379	2.82858	2.1
37380	4.72084	2.1
37381	1.87875	2.1
37382	1.37704	2.1
37383	1.77042	2.1
37384	2.27801	2.1
37385	3.64916	2.1
37386	1.77042	2.1
37387	2.27801	2.1
37388	2.58523	2.1
37389	1.90178	2.1
37390	4.35565	2.1
37391	1.28667	2.1
37392	1.30711	2.1
37393	1.53077	2.1
37394	1.55635	2.1
37395	2.81743	2.1
37396	1.55635	2.1
37397	1.28228	2.1
37398	1.64268	2.1
37399	1.93673	2.1
37400	3.19903	2.1
37401	1.53077	2.1
37402	1.93673	2.1
37403	1.55635	2.1
37404	1.55635	2.1
37405	1.86206	2.1
37406	1.86206	2.1
37407	1.64892	2.1

37408	1.60532	2.1
37409	1.85282	2.1
37410	1.96716	2.1
37411	3.61608	2.1
37412	1.60531	2.1
37413	1.32414	2.1
37414	1.96716	2.1
37415	1.75823	2.1
37416	3.2913	2.1
37417	1.91219	2.1
37418	1.96716	2.1
37419	2.0815	2.1
37420	2.51396	2.1
37421	4.30843	2.1
37422	1.96716	2.1
37423	2.17979	2.1
37424	1.75823	2.1
37425	1.75823	2.1
37426	2.28541	2.1
37427	2.28541	2.1
37428	1.89226	1.33724
37429	1.89226	1.6998
37430	1.89226	1.36762
37431	1.89226	1.13755
37432	3.78453	1.34188
37433	1.89226	1.5986
37434	1.89226	1.52552
37435	1.89226	1.4896
37436	1.89226	1.56703
37437	1.89226	1.48208
37438	1.89226	1.56151
37439	1.61016	1.4752
37440	1.45303	1.68403
37441	1.62812	1.4836
37442	1.86379	1.48103
37443	3.23828	1.47001
37444	1.61016	1.65534
37445	1.78716	1.78276
37446	2.0815	1.08261
37447	1.6774	1.5422
37448	3.54454	1.57932
37449	1.89226	1.54173
37450	1.89226	1.41876
37451	1.89226	1.04436
37452	1.89226	1.39128
37453	3.78453	1.41055
37454	1.89226	1.68636
37455	1.89226	1.58746
37456	1.89226	1.65364
37457	1.89226	1.81574
37458	1.89226	1.18001
37459	1.89226	1.26484
37460	0.807591	1.73321
37461	0.814059	1.48151
37462	1.00217	1.79788
37463	0.931896	1.61471
37464	0.850586	1.66499
37465	0.850586	1.7813

37466	0.8889	1.46917
37467	0.8889	1.6999
37468	0.807592	1.14226
37469	1.00217	1.71347
37470	0.814061	1.6281
37471	0.931894	1.8896
37472	2.0815	1.34985
37473	1.6774	1.87984
37474	2.00435	1.38505
37475	2.36104	1.35429
37476	3.86809	1.39166
37477	1.00532	0.0888803
37478	1.00532	0.0930733
37479	1.00183	0.101806
37480	1.00183	0.083855
37481	1.26377	0.0841506
37482	1.34921	0.0849827
37483	0.664872	0.0918042
37484	0.743381	0.0900819
37485	0.664872	0.0897484
37486	1.26377	0.0967781
37487	1.34921	0.0909636
37488	0.743381	0.101149
37489	1.26826	0.05
37490	0.946131	2.06928
37491	1.02404	2.01143
37492	0.94613	2.09858
37493	1.30281	2.0485
37494	0.930941	2.09556
37495	0.930941	2.07315
37496	1.318	2.04045
37497	1.58161	2.02168
37498	0.94613	2.05389
37499	0.94613	2.01424
37500	1.56338	2.0354
37501	1.02404	2.02637
37502	2.81374	2.04604
37503	1.89226	2.09951
37504	1.89226	2.06665
37505	1.89226	2.0802
37506	3.78453	2.08463
37507	0.946133	2.09871
37508	0.946133	2.05943
37509	0.946132	2.07121
37510	0.946132	2.09424
37511	0.946132	2.09395
37512	0.946132	2.08456
37513	0.946133	2.08858
37514	0.946133	2.06024
37515	0.946132	2.08556
37516	0.946132	2.06414
37517	0.946133	2.09992
37518	0.946133	2.08742
37519	1.89226	1.90295
37520	1.89226	2.07317
37521	1.89226	1.95773
37522	1.89226	2.01165
37523	3.78452	2.01563

37524	1.89226	2.06113
37525	1.89226	2.04886
37526	1.89226	2.08522
37527	1.89226	1.99146
37528	1.89226	2.08502
37529	1.89226	1.99191
37530	0.946133	2.01153
37531	0.946133	2.06916
37532	0.946132	2.01509
37533	0.946132	2.07524
37534	0.946132	2.07579
37535	0.946132	2.05994
37536	0.946133	2.0703
37537	0.946133	2.01354
37538	0.946132	2.06084
37539	0.946132	2.03119
37540	0.946132	2.08069
37541	0.946132	2.05374
37542	2.56953	2.1
37543	2.64148	2.1
37544	2.48631	2.1
37545	2.62213	2.1
37546	5.19166	2.1
37547	2.64148	2.1
37548	2.57929	2.1
37549	2.62213	2.1
37550	2.59522	2.1
37551	5.20142	2.1
37552	2.48631	2.1
37553	2.62213	2.1
37554	2.41176	2.1
37555	2.59262	2.1
37556	5.03389	2.1
37557	2.62213	2.1
37558	2.59522	2.1
37559	2.59262	2.1
37560	2.61901	2.1
37561	5.24114	2.1
37562	0.946132	0.05
37563	0.946132	0.05
37564	0.946133	0.05
37565	0.946133	0.05
37566	0.946132	0.05
37567	0.946132	0.05
37568	0.946133	0.05
37569	0.946133	0.05
37570	0.946132	0.05
37571	0.946132	0.05
37572	0.946133	0.05
37573	0.946133	0.05
37574	1.5681	0.05
37575	1.01248	0.05
37576	1.10749	0.05
37577	0.570913	0.05
37578	1.15589	0.05
37579	1.15588	0.05
37580	0.977549	0.05
37581	0.977549	0.05

37582	1.01249	0.05
37583	0.654445	0.05
37584	1.36795	0.05
37585	1.10748	0.05
37586	1.73883	0.05
37587	1.87638	0.05
37588	1.85219	0.05
37589	1.93113	2.1
37590	3.7407	0.05
37591	1.87638	0.05
37592	1.73883	2.1
37593	1.93113	2.1
37594	1.80426	2.1
37595	3.66996	2.1
37596	2.02912	0.121865
37597	1.44951	0.11187
37598	1.50103	0.0831509
37599	2.02248	0.107276
37600	3.47199	0.0941752
37601	1.89226	0.107788
37602	1.89226	0.146088
37603	1.89226	0.105802
37604	1.89226	0.103936
37605	3.78453	0.113533
37606	2.57881	0.10198
37607	1.89226	0.117428
37608	1.89226	0.115873
37609	1.89226	0.12312
37610	2.6428	0.0998285
37611	2.6428	0.110268
37612	1.01379	0.106377
37613	0.985937	0.100141
37614	0.769295	0.0931634
37615	0.715547	0.0844527
37616	0.853042	0.0964134
37617	0.853042	0.138224
37618	0.864117	0.10394
37619	0.864114	0.0843373
37620	0.715556	0.0979524
37621	1.03047	0.0906499
37622	0.731721	0.106429
37623	0.985921	0.107445
37624	1.89227	0.130909
37625	1.89227	0.118059
37626	1.89227	0.0889121
37627	1.89227	0.140089
37628	3.78453	0.109745
37629	0.946129	0.130508
37630	0.946129	0.115898
37631	1.28161	0.0825694
37632	1.3214	0.0932698
37633	0.94613	0.100329
37634	0.94613	0.144741
37635	1.28161	0.101004
37636	1.28161	0.0839737
37637	1.28161	0.0880062
37638	0.94613	0.0990326
37639	0.946131	0.111836

37640	1.3214	0.105883
37641	0.946132	0.380582
37642	0.946132	0.332019
37643	0.706045	0.295951
37644	1.16718	0.260586
37645	0.706017	0.28565
37646	0.706017	0.476464
37647	1.4073	0.255545
37648	1.4073	0.23808
37649	0.94613	0.311554
37650	1.16719	0.308099
37651	0.706046	0.345101
37652	0.946131	0.329465
37653	1.89226	0.481283
37654	1.89226	0.477504
37655	1.89226	0.296391
37656	1.89226	0.69846
37657	1.89226	0.294966
37658	1.89226	0.606356
37659	0.946119	0.340192
37660	0.946119	0.306265
37661	0.946115	0.315329
37662	0.946115	0.24302
37663	0.946117	0.320166
37664	0.946117	0.533095
37665	0.946117	0.27667
37666	0.946117	0.239649
37667	0.94613	0.239837
37668	0.94613	0.322552
37669	0.946105	0.383739
37670	0.946105	0.289255
37671	0.946132	1.53463
37672	0.946132	1.13586
37673	0.946131	1.4806
37674	0.946131	0.81822
37675	0.946132	1.73725
37676	0.946132	1.52723
37677	0.946132	1.04605
37678	0.827865	0.952156
37679	0.946132	0.987007
37680	0.946132	1.26699
37681	0.946132	1.17132
37682	0.827865	1.12883
37683	1.89226	1.56291
37684	1.89226	1.23958
37685	1.89226	1.60964
37686	1.89226	1.23109
37687	1.89226	1.81335
37688	1.89226	1.65385
37689	0.93094	1.01305
37690	0.93094	1.11472
37691	1.04649	0.924567
37692	0.614468	0.800898
37693	0.950358	0.812158
37694	0.799012	0.717137
37695	0.682742	0.937924
37696	0.682742	1.0175
37697	1.04073	1.37353

37698	1.15596	1.1126
37699	0.614478	1.3654
37700	0.851524	1.02453
37701	2.22575	2.1
37702	1.90502	2.1
37703	2.10893	2.1
37704	1.80258	2.1
37705	4.01395	2.1
37706	1.90502	2.1
37707	1.40863	2.1
37708	1.49733	2.1
37709	1.49733	2.1
37710	1.80258	2.1
37711	1.80258	2.1
37712	2.10893	2.1
37713	1.68356	2.1
37714	1.80258	2.1
37715	1.80258	2.1
37716	1.98061	2.1
37717	1.98061	2.1
37718	1.80258	2.1
37719	1.40863	2.1
37720	1.68356	2.1
37721	1.30751	2.1
37722	3.11009	2.1
37723	1.35374	1.90031
37724	1.35374	1.8343
37725	0.746096	1.53453
37726	0.746096	1.47068
37727	1.1537	1.56558
37728	0.746095	1.81125
37729	0.946132	1.79132
37730	0.946132	1.37814
37731	0.946132	1.07672
37732	0.746095	1.50406
37733	0.946131	1.16375
37734	1.1537	1.40006
37735	2.15247	2.1
37736	1.70303	2.1
37737	1.41207	2.1
37738	1.43052	2.1
37739	3.22793	2.1
37740	0.940246	2.1
37741	0.728442	2.1
37742	0.650037	2.1
37743	0.539874	2.1
37744	0.694756	2.1
37745	0.694756	2.1
37746	0.776346	2.1
37747	0.656772	2.1
37748	0.733146	2.1
37749	0.563769	2.1
37750	0.66316	2.1
37751	0.899692	2.1
37752	1.21223	2.1
37753	1.22418	2.1
37754	1.43052	2.1
37755	1.43052	2.1

37756	1.24353	2.1
37757	1.46181	2.1
37758	1.89226	0.177187
37759	2.39894	0.0831564
37760	1.89226	0.149518
37761	2.37417	0.0723102
37762	1.89226	0.202684
37763	2.37417	0.119687
37764	1.12767	0.0705242
37765	1.19947	0.108999
37766	0.94613	0.221883
37767	0.94613	0.0889308
37768	1.12766	0.0702813
37769	0.946128	0.0676482
37770	1.19947	0.102895
37771	0.946132	0.142127
37772	1.19947	0.174732
37773	1.19947	0.0850255
37774	0.94613	0.156951
37775	0.94613	0.0614693
37776	2.22621	0.05
37777	1.14533	2.1
37778	1.1131	2.1
37779	0.88238	2.1
37780	0.868521	2.1
37781	1.01153	2.1
37782	1.01153	2.1
37783	1.00232	2.1
37784	1.00232	2.1
37785	1.14533	2.1
37786	0.882378	2.1
37787	0.868519	2.1
37788	1.11311	2.1
37789	0.910183	2.088
37790	1.07811	1.90927
37791	0.94613	0.773076
37792	0.94613	1.47177
37793	0.910212	1.44573
37794	0.910212	2.09269
37795	1.11403	0.865103
37796	1.11403	1.94455
37797	0.910183	1.78577
37798	0.94613	1.82045
37799	0.94613	1.86653
37800	1.07811	1.84872
37801	1.87228	2.08933
37802	2.81189	2.1
37803	1.6197	2.1
37804	2.56716	2.1
37805	4.43944	2.1
37806	0.946132	2.08165
37807	0.946132	2.1
37808	0.946132	1.40468
37809	0.946132	2.0053
37810	0.946131	2.03789
37811	0.946131	2.1
37812	0.946132	1.51202
37813	0.946132	2.08214

37814	0.946132	2.0969
37815	0.946132	2.09692
37816	0.946132	2.09715
37817	0.946132	2.0975
37818	1.28724	2.1
37819	2.56716	2.1
37820	2.3014	2.1
37821	2.3014	2.1
37822	1.6197	2.1
37823	1.6197	2.1
37824	0.946132	2.1
37825	0.946132	2.1
37826	0.946132	1.66491
37827	0.946132	2.0731
37828	0.946132	2.05706
37829	0.946132	2.1
37830	0.946133	2.1
37831	0.946133	1.78953
37832	0.946133	2.06633
37833	0.946133	2.04936
37834	0.946132	2.07893
37835	0.946132	2.07236
37836	1.08039	2.1
37837	1.24445	2.09794
37838	1.2873	2.1
37839	1.48751	2.1
37840	2.53175	2.1
37841	0.946125	1.96583
37842	0.946125	2.1
37843	1.12552	2.1
37844	1.12552	2.1
37845	0.946124	2.08001
37846	0.946124	2.09459
37847	1.12552	2.1
37848	1.11114	2.1
37849	1.11113	2.1
37850	0.946118	2.1
37851	1.12552	2.1
37852	0.946131	2.1
37853	1.89225	1.90992
37854	1.89225	1.53566
37855	1.89225	2.08743
37856	1.89225	2.1
37857	1.89225	1.10794
37858	1.89225	2.1
37859	0.987703	1.83025
37860	1.33078	2.02939
37861	1.31565	1.87975
37862	1.31565	1.96625
37863	1.01121	1.91849
37864	1.01121	2.09166
37865	2.19163	2.1
37866	1.89226	2.1
37867	1.89226	2.1
37868	1.89226	2.1
37869	2.22229	2.1
37870	2.22229	2.1
37871	1.89226	2.1

37872	1.89226	0.53705
37873	1.89226	0.472282
37874	1.89226	2.1
37875	1.89226	1.04908
37876	1.89226	2.1
37877	0.57141	2.1
37878	0.57141	1.48978
37879	0.574036	2.03047
37880	0.574036	2.1
37881	0.651591	2.04072
37882	0.657827	1.58539
37883	0.482391	2.06901
37884	0.493855	2.09017
37885	0.482391	1.0451
37886	0.651591	1.0035
37887	0.493855	2.03035
37888	0.657828	2.00917
37889	1.88428	2.1
37890	2.03975	2.1
37891	1.97316	2.1
37892	1.8266	2.1
37893	3.86635	2.1
37894	2.23053	2.1
37895	1.42255	2.1
37896	1.3868	2.1
37897	1.8266	2.1
37898	3.24915	2.1
37899	1.97316	2.1
37900	1.8266	2.1
37901	1.77128	2.1
37902	1.91767	2.1
37903	3.74427	2.1
37904	1.77128	2.1
37905	1.8266	2.1
37906	1.77942	2.1
37907	1.33962	2.1
37908	1.3868	2.1
37909	1.3868	2.1
37910	0.944111	2.1
37911	0.944111	2.1
37912	1.26036	2.1
37913	1.16968	2.1
37914	1.03853	2.1
37915	1.16646	2.1
37916	0.946129	2.1
37917	0.946129	2.1
37918	0.94613	2.1
37919	1.25768	2.1
37920	1.03853	2.1
37921	0.946131	2.1
37922	1.82441	0.05
37923	1.33061	0.05
37924	1.13272	0.05
37925	1.60249	0.05
37926	2.95712	0.05
37927	1.82441	0.05
37928	2.08757	0.05
37929	1.81292	0.05

37930	1.60249	0.05
37931	3.63733	0.05
37932	1.81292	0.05
37933	1.332	0.05
37934	1.60249	0.05
37935	1.60249	0.05
37936	1.5344	0.05
37937	1.5344	0.05
37938	1.10107	2.1
37939	2.51559	2.1
37940	1.1229	2.1
37941	1.1229	2.1
37942	2.49456	2.1
37943	2.49456	2.1
37944	1.89226	2.1
37945	2.51559	2.1
37946	2.49457	2.1
37947	2.51559	2.1
37948	1.87124	2.1
37949	1.89226	2.1
37950	1.89226	2.1
37951	1.89226	2.1
37952	1.89226	2.1
37953	1.89226	2.1
37954	1.89226	2.1
37955	1.89226	2.1
37956	1.89226	2.1
37957	1.89226	2.1
37958	1.89226	2.1
37959	1.89226	2.1
37960	1.89226	2.1
37961	1.89226	2.1
37962	1.23599	2.1
37963	1.24728	2.1
37964	0.550534	2.1
37965	0.541151	2.1
37966	0.893754	2.1
37967	0.893754	2.1
37968	0.894678	2.1
37969	0.894678	2.1
37970	1.23599	2.1
37971	0.541149	2.1
37972	0.550533	2.1
37973	1.24728	2.1
37974	0.946132	2.1
37975	0.946132	2.1
37976	1.23599	2.1
37977	1.24728	2.1
37978	0.946132	2.1
37979	0.946132	2.1
37980	1.23599	2.1
37981	1.23599	2.1
37982	1.23599	2.1
37983	0.946132	2.1
37984	0.946132	2.1
37985	1.24728	2.1
37986	1.89226	2.1
37987	1.89226	2.1

37988	1.89226	2.1
37989	1.89226	2.1
37990	1.89226	2.1
37991	1.89226	2.1
37992	0.946128	2.1
37993	0.946128	2.1
37994	0.946132	2.1
37995	0.946132	2.1
37996	0.94613	2.1
37997	0.94613	2.1
37998	0.946129	2.1
37999	0.946129	2.1
38000	0.94613	2.1
38001	0.94613	2.1
38002	0.94613	2.1
38003	0.94613	2.1
38004	2.58696	0.0730178
38005	2.57073	0.129648
38006	2.12585	0.104923
38007	2.07607	0.0916684
38008	4.69658	0.101271
38009	1.89226	0.161588
38010	1.89226	0.131456
38011	1.89226	0.106649
38012	1.89226	0.201307
38013	3.78452	0.142929
38014	1.89226	0.134139
38015	1.89226	0.235214
38016	1.89226	0.205365
38017	1.89226	0.159594
38018	3.78453	0.178683
38019	2.57074	0.132575
38020	2.32635	0.133501
38021	2.07608	0.0959459
38022	1.73623	0.159392
38023	4.30697	0.118273
38024	2.12582	0.107895
38025	1.51971	0.103951
38026	1.57255	0.090988
38027	2.07604	0.127265
38028	1.62233	0.0876744
38029	2.12582	0.121024
38030	1.89223	0.109759
38031	1.89223	0.200754
38032	1.89223	0.13188
38033	1.89223	0.152015
38034	1.89223	0.133418
38035	1.89223	0.155231
38036	0.946119	0.21275
38037	0.946119	0.195472
38038	0.946119	0.130359
38039	0.946119	0.166693
38040	0.946119	0.171327
38041	0.946119	0.208181
38042	0.94612	0.201283
38043	0.94612	0.130028
38044	0.946132	0.17684
38045	0.946132	0.173014

38046	0.946107	0.175618
38047	0.946107	0.175984
38048	0.811456	0.137964
38049	0.811456	0.17086
38050	0.806521	0.107548
38051	0.806521	0.152837
38052	0.868104	0.147611
38053	0.579949	0.171486
38054	0.759859	0.109123
38055	1.03802	0.13056
38056	0.579957	0.137722
38057	0.759868	0.136517
38058	1.03801	0.143945
38059	0.868093	0.143822
38060	2.7408	2.1
38061	2.82858	2.1
38062	2.57368	2.1
38063	2.52633	2.1
38064	5.3549	2.1
38065	2.7408	2.1
38066	2.31353	2.1
38067	2.14063	2.1
38068	2.52632	2.1
38069	4.83985	2.1
38070	2.57368	2.1
38071	2.52633	2.1
38072	2.3301	2.1
38073	2.36071	2.1
38074	4.88704	2.1
38075	1.86512	2.1
38076	2.52632	2.1
38077	2.14063	2.1
38078	2.14063	2.1
38079	2.3301	2.1
38080	2.3301	2.1
38081	1.86431	2.1
38082	1.89227	2.1
38083	1.68992	2.1
38084	1.68992	2.1
38085	1.89227	2.1
38086	1.89227	2.1
38087	2.0815	2.1
38088	1.89226	2.1
38089	2.04022	2.1
38090	1.86431	2.1
38091	2.07081	2.1
38092	1.89226	2.1
38093	1.91686	2.05757
38094	1.65683	2.1
38095	1.86871	2.1
38096	1.65683	2.09317
38097	2.13075	2.1
38098	1.89226	1.73138
38099	1.89226	2.1
38100	1.89226	1.68203
38101	1.89226	1.99058
38102	1.89226	2.1
38103	3.78452	2.05652

38104	0.851541	2.1
38105	0.851541	2.1
38106	0.952803	2.1
38107	0.851573	2.1
38108	0.946129	2.1
38109	0.946129	2.1
38110	0.929184	2.1
38111	0.757482	2.1
38112	0.836274	2.1
38113	0.851528	2.1
38114	0.952816	2.1
38115	1.04073	2.1
38116	1.70303	1.85131
38117	1.70302	2.05479
38118	1.5544	2.09964
38119	1.70302	1.95772
38120	1.74957	2.02596
38121	1.91686	1.30975
38122	1.89226	1.71406
38123	1.89226	2.07301
38124	1.89226	2.09535
38125	1.89226	1.92852
38126	1.89226	1.99883
38127	1.89226	1.18286
38128	1.89226	2.1
38129	1.89226	2.1
38130	2.03472	2.1
38131	1.89226	2.1
38132	1.99827	2.1
38133	1.85836	2.09715
38134	1.00509	2.1
38135	1.55998	2.1
38136	1.83347	2.1
38137	1.45246	2.1
38138	3.01244	2.1
38139	1.23839	2.1
38140	0.884251	2.1
38141	0.946133	2.1
38142	0.946133	2.1
38143	0.884226	2.1
38144	0.884226	2.1
38145	0.946158	2.1
38146	0.946159	2.1
38147	1.23839	2.1
38148	0.946131	2.1
38149	0.946131	2.1
38150	0.884254	2.1
38151	1.89226	2.1
38152	1.89226	2.1
38153	1.89226	2.1
38154	1.89226	2.1
38155	1.89226	2.1
38156	1.89226	2.1
38157	2.1499	2.1
38158	1.45244	2.1
38159	1.83345	2.1
38160	1.83345	2.1
38161	1.87214	2.1

38162	1.87214	2.1
38163	1.89224	2.1
38164	1.89224	2.1
38165	1.89224	2.1
38166	1.89224	2.1
38167	3.78448	2.1
38168	1.49155	1.99741
38169	2.0815	2.1
38170	1.77515	1.99392
38171	1.72377	2.1
38172	3.50659	1.98942
38173	1.69876	0.423147
38174	2.0815	2.09748
38175	1.9133	1.4123
38176	1.68189	0.710926
38177	2.17628	2.0655
38178	1.94335	2.1
38179	2.65129	0.05
38180	1.15317	0.05
38181	2.57085	0.05
38182	1.209	0.05
38183	2.57085	0.05
38184	1.209	0.05
38185	1.89226	0.05
38186	1.89226	0.05
38187	1.89226	0.05
38188	1.89226	0.05
38189	3.78452	0.05
38190	2.48861	0.05
38191	1.15252	0.05
38192	2.60515	0.05
38193	2.60515	0.05
38194	1.16384	0.05
38195	1.16384	0.05
38196	1.59903	0.512987
38197	1.26865	0.95939
38198	1.30486	1.59249
38199	1.70302	0.616226
38200	2.98573	0.912692
38201	1.59903	0.278209
38202	1.79478	0.832644
38203	1.79478	0.761077
38204	1.97005	0.298244
38205	1.50365	0.428644
38206	1.64192	0.992958
38207	1.89226	0.264543
38208	1.89226	0.832528
38209	1.89226	0.90127
38210	1.89226	0.37528
38211	3.78453	0.308961
38212	1.89226	1.14021
38213	1.89226	0.491666
38214	1.89226	1.15617
38215	1.89226	0.38322
38216	1.89226	0.928565
38217	1.89226	0.690375
38218	0.761672	0.239566
38219	0.896406	0.331958

38220	0.603509	0.68059
38221	0.574071	0.784452
38222	0.695159	0.874968
38223	0.695159	0.193718
38224	0.762564	1.26334
38225	0.770159	0.75605
38226	0.803021	0.639967
38227	0.572529	0.792219
38228	0.696466	0.463559
38229	0.899733	0.447078
38230	1.66034	0.165385
38231	1.79478	0.839151
38232	1.56685	0.316729
38233	1.68512	1.46912
38234	1.67652	0.380768
38235	1.67652	0.718674
38236	1.89226	0.918484
38237	1.89226	0.408895
38238	1.89226	0.521434
38239	1.89226	0.99675
38240	1.89226	0.547244
38241	1.89226	1.00462
38242	0.946133	0.25605
38243	0.946133	0.332487
38244	0.946132	0.729363
38245	0.946132	0.63172
38246	0.946132	0.625429
38247	0.946132	0.141138
38248	0.946133	1.44681
38249	0.946133	0.472643
38250	0.946133	0.516651
38251	0.946133	0.631604
38252	0.946132	0.250582
38253	0.946132	0.296249
38254	1.80424	2.1
38255	1.45158	2.1
38256	1.9098	2.1
38257	1.56003	2.1
38258	3.36138	2.1
38259	1.90982	2.1
38260	1.71606	2.1
38261	2.15314	2.1
38262	1.70302	2.1
38263	3.89051	2.1
38264	1.96416	1.82089
38265	1.23706	0.627437
38266	1.00053	1.02083
38267	1.38287	0.476464
38268	1.57916	0.822327
38269	1.9615	0.769406
38270	0.827865	0.364745
38271	0.946132	0.202123
38272	0.946132	0.463832
38273	0.946132	0.663985
38274	0.946132	0.459785
38275	0.946132	0.378455
38276	0.946131	0.491281
38277	0.946131	0.716071

38278	0.827865	0.744371
38279	0.946132	0.3009
38280	0.946132	0.256006
38281	0.946132	0.218305
38282	1.89227	2.1
38283	1.89227	2.1
38284	1.89227	2.1
38285	1.89227	2.1
38286	3.78453	2.1
38287	2.3819	2.1
38288	1.58089	2.1
38289	1.58089	2.1
38290	1.6287	2.1
38291	2.33368	2.1
38292	2.3815	2.1
38293	0.946133	2.1
38294	0.946133	2.1
38295	0.946132	2.1
38296	0.946132	2.1
38297	0.946132	2.1
38298	0.946132	2.1
38299	0.946132	2.1
38300	0.946132	2.1
38301	0.946133	2.1
38302	0.946133	2.1
38303	0.946132	2.1
38304	0.946132	2.1
38305	1.89226	2.1
38306	1.89226	2.1
38307	1.89226	2.1
38308	1.89226	2.1
38309	3.78453	2.1
38310	2.7874	2.1
38311	2.33368	2.1
38312	2.71897	2.1
38313	2.27495	2.1
38314	5.05266	2.1
38315	0.970991	2.1
38316	0.970991	2.1
38317	0.956929	2.1
38318	0.956929	2.1
38319	0.732928	2.1
38320	0.790444	2.1
38321	1.16684	2.1
38322	1.13748	2.1
38323	1.13748	2.1
38324	0.732928	2.1
38325	0.790444	2.1
38326	1.16684	2.1
38327	0.946132	2.1
38328	0.946132	2.1
38329	0.946131	2.1
38330	0.946131	2.1
38331	0.946132	2.1
38332	0.946132	2.1
38333	0.946132	2.1
38334	0.946132	2.1
38335	0.946132	2.1

38336	0.946132	2.1
38337	0.946132	2.1
38338	0.946132	2.1
38339	1.10747	0.209383
38340	2.30331	0.284044
38341	1.47954	0.170553
38342	2.63789	0.194509
38343	3.78285	0.209121
38344	1.65215	0.116356
38345	2.63789	0.15453
38346	2.82465	0.144874
38347	2.82465	0.167737
38348	1.47954	0.117993
38349	1.47954	0.143648
38350	1.13456	2.0753
38351	1.08334	2.1
38352	1.20674	0.483231
38353	1.17885	2.08694
38354	2.29008	2.03237
38355	1.20674	1.77232
38356	1.17885	2.1
38357	1.28649	0.739982
38358	1.28516	2.00664
38359	2.4919	1.94797
38360	1.89226	2.06647
38361	1.89226	2.08473
38362	1.89226	2.09004
38363	1.89226	2.05957
38364	1.89226	2.08626
38365	1.89226	2.05285
38366	2.50288	1.6986
38367	2.02115	1.8726
38368	2.7717	2.01397
38369	2.29107	0.552021
38370	4.79395	1.88454
38371	1.27339	2.1
38372	1.10817	2.1
38373	1.19733	2.1
38374	1.032 2.1	
38375	2.30539	2.1
38376	1.19733	2.1
38377	0.953637	2.1
38378	0.953637	2.1
38379	1.032 2.1	
38380	1.11868	2.1
38381	1.19704	2.1
38382	2.01024	0.831844
38383	1.11938	0.727343
38384	1.9186	0.983815
38385	2.76298	0.837587
38386	3.92884	0.784978
38387	0.851541	0.121157
38388	0.851541	0.105246
38389	0.957577	0.0971929
38390	0.848952	0.10002
38391	1.08043	0.123581
38392	0.851541	0.129853
38393	0.851484	0.101334

38394	0.957578	0.0865777
38395	1.20045	0.116358
38396	0.726289	0.0840195
38397	0.946131	0.105218
38398	0.946131	0.121966
38399	1.50524	2.1
38400	2.38363	2.1
38401	2.49018	2.1
38402	2.49018	2.1
38403	1.45506	2.1
38404	1.45506	2.1
38405	2.49017	2.1
38406	1.89226	2.1
38407	1.89226	2.1
38408	1.89226	2.1
38409	2.38363	2.1
38410	2.38363	2.1
38411	1.54711	2.1
38412	2.49018	2.1
38413	2.56975	2.1
38414	2.56975	2.1
38415	1.50524	2.1
38416	1.50524	2.1
38417	2.56975	2.1
38418	1.89226	2.1
38419	1.89226	2.1
38420	1.89226	2.1
38421	2.49017	2.1
38422	2.49017	2.1
38423	1.89226	0.0742417
38424	1.89226	0.0850712
38425	1.89226	0.133615
38426	1.89226	0.0588637
38427	1.89226	0.134268
38428	1.89226	0.05
38429	1.89226	0.132674
38430	1.89226	0.131596
38431	1.89226	0.247573
38432	1.89226	0.0912948
38433	1.89226	0.254582
38434	1.89226	0.0827434
38435	1.89226	0.05
38436	1.89226	0.0622391
38437	2.8358	0.05
38438	1.89226	0.165702
38439	3.78453	0.05
38440	1.60565	0.397689
38441	1.89226	1.01774
38442	1.89226	0.890406
38443	1.40013	0.588704
38444	1.89226	0.782691
38445	1.40013	0.560699
38446	1.1766	2.1
38447	1.29435	2.1
38448	1.35735	2.1
38449	1.53509	2.1
38450	2.65169	2.1
38451	0.69701	2.1

38452	0.69701	2.1
38453	0.711303	2.1
38454	0.711303	2.1
38455	0.761138	2.1
38456	0.612149	2.1
38457	0.647174	2.1
38458	0.767548	2.1
38459	0.767548	2.1
38460	0.761139	2.1
38461	0.612149	2.1
38462	0.647174	2.1
38463	1.29764	1.59805
38464	1.29579	1.88394
38465	1.34875	1.8289
38466	1.06717	1.7512
38467	1.29764	1.772
38468	1.01606	1.64295
38469	0.756635	1.46042
38470	0.648821	1.58184
38471	0.675557	1.79146
38472	0.674374	1.75424
38473	0.74214	1.80022
38474	0.74214	1.58803
38475	0.688868	1.46813
38476	0.688868	1.74097
38477	0.675557	1.42672
38478	0.756634	1.52973
38479	0.648821	1.3167
38480	0.674374	1.4942
38481	1.51327	1.51291
38482	1.48105	1.2492
38483	1.29764	1.58723
38484	1.29764	1.28325
38485	1.48105	1.66609
38486	1.05655	1.17618
38487	1.13537	2.1
38488	1.07326	2.1
38489	0.94613	2.1
38490	0.94613	2.1
38491	0.946132	2.1
38492	0.946132	2.1
38493	1.13537	2.1
38494	1.08927	2.1
38495	1.13537	2.1
38496	0.946131	2.1
38497	0.946131	2.1
38498	0.95947	2.1
38499	1.46892	2.1
38500	1.89226	2.1
38501	1.89226	2.1
38502	1.89226	2.1
38503	1.88031	2.1
38504	1.88031	2.1
38505	1.89226	2.1
38506	1.89226	2.1
38507	1.89226	2.1
38508	1.89226	2.1
38509	3.78453	2.1

38510	2.18254	2.1
38511	1.89226	2.1
38512	2.50005	2.1
38513	1.89226	2.1
38514	4.39231	2.1
38515	1.88031	2.1
38516	2.0815	2.1
38517	2.0815	2.1
38518	1.92078	2.1
38519	2.0815	2.1
38520	1.9218	2.1
38521	1.89227	2.1
38522	1.89227	2.1
38523	1.89227	2.1
38524	1.89227	2.1
38525	1.89227	2.1
38526	1.89227	2.1
38527	1.98693	2.1
38528	1.82857	2.1
38529	2.32	2.1
38530	2.36972	2.1
38531	4.14857	2.1
38532	1.82857	2.1
38533	1.58688	2.1
38534	2.36971	2.1
38535	2.19093	2.1
38536	4.0195	2.1
38537	0.946132	2.1
38538	0.946132	2.1
38539	1.18486	2.1
38540	1.18486	2.1
38541	1.18486	2.1
38542	0.946132	2.1
38543	0.946132	2.1
38544	1.16	2.1
38545	0.946133	2.1
38546	0.946133	2.1
38547	1.16	2.1
38548	1.18486	2.1
38549	1.07798	2.05629
38550	1.08101	1.94576
38551	0.910786	2.1
38552	0.910786	2.1
38553	0.851537	2.09297
38554	0.901377	1.93043
38555	1.14652	2.09867
38556	1.04072	2.1
38557	0.946129	2.0975
38558	0.946129	2.09676
38559	1.0019	2.09031
38560	1.04263	2.09855
38561	1.89226	2.1
38562	1.89226	2.1
38563	1.89226	2.1
38564	1.89226	2.1
38565	3.78452	2.1
38566	1.89226	2.1
38567	2.30146	2.1

38568	1.89226	2.1
38569	2.18412	2.1
38570	4.19372	2.1
38571	2.30146	2.1
38572	1.39962	2.1
38573	2.18411	2.1
38574	1.28765	2.1
38575	3.5891	2.1
38576	1.89226	2.1
38577	1.89226	2.1
38578	1.89226	2.1
38579	1.89226	2.1
38580	1.89226	2.1
38581	1.89226	2.1
38582	1.89226	2.1
38583	1.89226	2.1
38584	1.89226	2.1
38585	1.89226	2.1
38586	3.78452	2.1
38587	2.18412	2.1
38588	1.20045	2.1
38589	1.20045	2.1
38590	1.28765	2.1
38591	2.08808	2.1
38592	2.17528	2.1
38593	0.946131	2.1
38594	0.946131	2.1
38595	0.946128	2.1
38596	0.946128	2.1
38597	0.946127	2.1
38598	0.946127	2.1
38599	0.946131	2.1
38600	0.946131	2.1
38601	0.946129	2.1
38602	0.946129	2.1
38603	0.94613	2.1
38604	0.94613	2.1
38605	1.89226	0.387227
38606	1.89226	1.70525
38607	1.89226	1.75958
38608	1.89226	0.312099
38609	3.78452	0.361183
38610	1.89227	0.631256
38611	1.89227	1.94004
38612	1.89227	0.511453
38613	1.89227	1.63252
38614	1.89227	0.5502
38615	1.89227	1.82811
38616	1.89226	1.7119
38617	1.89226	0.524433
38618	1.89226	0.308828
38619	2.29245	0.648226
38620	3.78452	1.76549
38621	1.89226	1.74493
38622	1.89226	0.237453
38623	1.89226	0.330218
38624	1.89226	1.77291
38625	1.89226	0.340972

38626	1.89226	0.453037
38627	1.28095	1.75595
38628	1.81846	1.77135
38629	2.10991	0.758928
38630	1.34695	1.4867
38631	3.39086	1.50442
38632	2.10991	1.13897
38633	1.36457	1.50423
38634	1.67421	1.80663
38635	1.34695	1.7469
38636	2.43717	1.08001
38637	2.10991	1.5815
38638	2.69541	2.1
38639	1.48572	2.1
38640	2.63553	2.1
38641	1.45385	2.1
38642	4.14926	2.1
38643	2.63553	2.1
38644	1.44207	2.1
38645	1.44207	2.1
38646	1.45385	2.1
38647	2.588	2.1
38648	2.59978	2.1
38649	1.89227	2.1
38650	1.89227	2.1
38651	1.89227	2.1
38652	1.89227	2.1
38653	3.78453	2.1
38654	1.89226	2.1
38655	1.89226	2.1
38656	1.89226	2.1
38657	1.89226	2.1
38658	3.78453	2.1
38659	2.15929	2.1
38660	1.38276	2.1
38661	1.43617	2.1
38662	1.35614	2.1
38663	2.23932	2.1
38664	2.15929	2.1
38665	2.15929	2.1
38666	2.25168	2.1
38667	2.3558	2.1
38668	2.23932	2.1
38669	4.51509	2.1
38670	1.89226	2.1
38671	1.89226	2.1
38672	1.89226	2.1
38673	1.89226	2.1
38674	3.78453	2.1
38675	1.89226	2.1
38676	1.89226	2.1
38677	1.89226	2.1
38678	1.89226	2.1
38679	1.89226	2.1
38680	1.89226	2.1
38681	2.23932	2.1
38682	1.43854	2.1
38683	1.49173	2.1

38684	1.38276	2.1
38685	2.34829	2.1
38686	2.23932	2.1
38687	2.3558	2.1
38688	2.34829	2.1
38689	2.40685	2.1
38690	2.23932	2.1
38691	2.52332	2.1
38692	2.3558	2.1
38693	1.89226	0.05
38694	1.87361	0.0513022
38695	2.25082	0.05
38696	1.87361	0.0597134
38697	2.26947	0.05
38698	1.89226	0.0575417
38699	1.89226	0.0605931
38700	1.89226	0.0833355
38701	1.89226	0.0557434
38702	1.89226	0.104771
38703	1.89226	0.0544391
38704	1.89226	0.101908
38705	2.41314	0.05
38706	1.89226	0.0600593
38707	1.89226	0.05
38708	1.89226	0.0749545
38709	2.12204	0.05
38710	2.12204	0.0734808
38711	1.89226	0.05
38712	2.25082	0.05
38713	2.28189	0.05
38714	2.25082	0.0621426
38715	1.92333	0.05
38716	1.89226	0.0590976
38717	1.89226	0.0615215
38718	1.89226	0.0738756
38719	1.89226	0.0526106
38720	1.89226	0.102693
38721	1.89226	0.0538453
38722	1.89226	0.096029
38723	2.42521	0.05
38724	1.89226	0.0539808
38725	1.89226	0.05
38726	1.89226	0.0667043
38727	2.41314	0.05
38728	2.41314	0.0648567
38729	1.39725	2.03775
38730	1.07745	2.1
38731	1.07745	2.07343
38732	1.07519	2.1
38733	1.41336	2.01994
38734	1.41111	2.1
38735	1.89226	2.1
38736	1.92039	2.1
38737	1.92039	2.1
38738	1.9052	2.1
38739	1.89226	2.1
38740	1.87708	2.1
38741	0.946128	2.1

38742	0.946128	2.1
38743	0.946133	2.03415
38744	0.946133	1.97776
38745	0.94613	1.97694
38746	0.94613	2.1
38747	0.946131	2.1
38748	0.946131	2.00371
38749	0.946131	2.09147
38750	0.946131	2.0941
38751	0.946131	2.07797
38752	0.946131	2.08089
38753	1.41336	2.06475
38754	1.08911	2.1
38755	1.08911	2.08646
38756	1.07745	2.1
38757	1.42381	2.05226
38758	1.41215	2.1
38759	1.89227	2.1
38760	1.95833	2.1
38761	1.95833	2.1
38762	1.92039	2.1
38763	1.89227	2.1
38764	1.85433	2.1
38765	0.946129	2.1
38766	0.946129	2.1
38767	0.946133	2.06462
38768	0.946133	2.01301
38769	0.946131	2.01196
38770	0.946131	2.1
38771	0.946131	2.1
38772	0.946131	2.03804
38773	0.946131	2.09974
38774	0.946131	2.1
38775	0.946131	2.09119
38776	0.946131	2.09276
38777	1.41943	2.1
38778	2.74398	2.1
38779	1.18238	2.1
38780	2.53897	2.1
38781	3.9584	2.1
38782	2.74398	2.1
38783	1.89226	2.1
38784	1.89226	2.1
38785	1.89226	2.1
38786	2.53897	2.1
38787	2.53897	2.1
38788	1.89227	0.816819
38789	1.89227	0.384718
38790	1.89227	1.0829
38791	1.89227	0.407664
38792	3.78453	0.51731
38793	1.89226	0.377499
38794	2.08134	0.236565
38795	2.00632	0.269421
38796	2.00632	0.244283
38797	1.89226	0.386771
38798	1.89226	0.315702
38799	1.89227	0.566144

38800	1.89227	0.309103
38801	1.89227	0.591254
38802	1.89227	0.27828
38803	3.78453	0.42959
38804	1.89227	0.86877
38805	1.89227	0.273495
38806	1.89227	0.311138
38807	1.89227	0.337293
38808	1.89227	0.702987
38809	1.89227	0.665489
38810	2.50912	0.24882
38811	1.89226	0.298035
38812	1.89226	0.187121
38813	2.31261	0.233042
38814	2.31261	0.214111
38815	1.89226	0.287183
38816	2.50912	0.253183
38817	1.19999	0.185528
38818	1.44056	0.177955
38819	1.24405	0.171249
38820	2.50912	0.224642
38821	2.31261	0.225606
38822	2.69224	1.16458
38823	2.28373	0.256908
38824	1.94788	0.927663
38825	2.4106	0.445029
38826	4.69434	0.4742
38827	2.69223	1.03093
38828	2.63783	1.55631
38829	2.34737	2.0813
38830	2.4106	0.637061
38831	5.04843	1.50701
38832	1.89226	2.1
38833	1.99753	2.1
38834	1.87366	2.1
38835	1.99753	2.1
38836	1.76839	2.1
38837	1.89226	2.1
38838	1.89226	2.1
38839	1.89226	2.1
38840	1.89226	2.1
38841	1.89226	2.1
38842	3.78452	2.1
38843	0.946132	2.1
38844	0.946132	2.1
38845	1.02744	2.1
38846	1.02744	2.1
38847	0.946132	2.1
38848	0.946132	2.1
38849	1.00989	2.1
38850	1.02744	2.1
38851	1.00989	2.1
38852	0.946133	2.1
38853	1.02744	2.1
38854	0.946132	2.1
38855	1.70302	2.1
38856	1.87366	2.1
38857	1.71618	2.1

38858	1.87366	2.1
38859	1.73105	2.1
38860	1.89226	2.1
38861	1.89226	2.1
38862	1.89226	2.1
38863	1.89226	2.1
38864	1.89226	2.1
38865	1.89226	2.1
38866	1.89226	2.1
38867	1.96624	2.1
38868	1.89226	2.1
38869	1.89226	2.1
38870	1.89226	2.1
38871	2.01977	2.1
38872	2.01977	2.1
38873	1.1032	2.09598
38874	1.15114	2.1
38875	1.14986	1.20516
38876	1.19242	2.1
38877	2.301	2.1
38878	1.89226	1.84855
38879	1.89226	2.1
38880	1.89226	1.21226
38881	1.89226	2.1
38882	3.78453	2.09916
38883	0.946132	2.1
38884	0.946132	2.1
38885	0.946132	2.1
38886	0.946132	2.1
38887	0.946133	2.1
38888	0.946133	2.1
38889	0.946132	2.1
38890	0.946132	2.1
38891	0.946132	2.1
38892	0.946132	2.1
38893	0.946133	2.1
38894	0.946133	2.1
38895	1.19243	2.1
38896	0.953928	2.1
38897	1.18671	2.1
38898	1.15114	2.1
38899	0.9895	2.1
38900	0.953928	2.1
38901	1.19611	1.13331
38902	1.19242	2.1
38903	1.24693	1.82025
38904	1.24693	2.1
38905	1.14986	1.51595
38906	1.14986	2.1
38907	1.89225	1.16123
38908	1.89225	2.1
38909	1.89225	2.1
38910	1.89225	1.91351
38911	1.89225	2.1
38912	1.89225	2.08323
38913	1.89225	2.1
38914	1.89225	2.1
38915	1.89225	2.1

38916	1.89225	2.1
38917	1.89225	2.1
38918	1.89225	2.1
38919	0.561532	2.1
38920	0.561532	2.1
38921	0.566382	2.1
38922	0.566382	2.1
38923	0.494747	2.1
38924	0.531705	2.1
38925	0.596208	2.1
38926	0.623464	2.1
38927	0.623469	2.1
38928	0.531708	2.1
38929	0.596205	2.1
38930	0.494743	2.1
38931	1.25742	2.1
38932	1.18783	2.1
38933	0.946131	2.1
38934	0.946131	2.1
38935	1.25742	2.1
38936	1.25742	2.1
38937	0.946131	2.1
38938	0.946131	2.1
38939	1.25742	2.1
38940	0.946131	2.1
38941	0.946131	2.1
38942	1.18783	2.1
38943	1.35048	2.1
38944	1.25742	2.1
38945	0.946132	2.1
38946	0.946132	2.1
38947	1.35048	2.1
38948	1.35048	2.1
38949	0.946132	2.1
38950	0.946132	2.1
38951	1.35044	2.1
38952	0.946106	2.1
38953	1.25745	2.1
38954	0.946158	2.1
38955	1.13525	2.1
38956	2.70095	2.1
38957	2.51483	2.1
38958	2.51483	2.1
38959	1.21593	2.1
38960	1.21593	2.1
38961	1.93142	1.39185
38962	2.8053	0.403637
38963	1.559	0.681981
38964	2.60359	0.213045
38965	4.53501	0.447928
38966	2.80531	0.33774
38967	1.89226	0.395213
38968	2.60359	0.258992
38969	1.89226	1.35985
38970	4.49586	0.391499
38971	1.22938	0.582666
38972	2.60359	0.212359
38973	2.37434	1.24163

38974	2.37434	0.601673
38975	1.55901	1.12447
38976	1.55901	0.231698
38977	1.18717	1.27404
38978	1.18717	0.189223
38979	0.946132	0.125827
38980	0.946132	0.580804
38981	0.946133	0.426743
38982	0.946133	1.27572
38983	1.3018	0.142943
38984	1.18717	0.809267
38985	1.18714	0.39296
38986	0.946107	0.444785
38987	1.30183	0.452148
38988	0.946158	0.482139
38989	1.89226	2.1
38990	2.80196	2.1
38991	2.67272	2.1
38992	2.67272	2.1
38993	1.89226	2.1
38994	1.89226	2.1
38995	1.89226	2.1
38996	1.89226	2.1
38997	1.89226	2.1
38998	1.89226	2.1
38999	1.89226	2.1
39000	1.89226	2.1
39001	1.74471	2.1
39002	2.01555	2.1
39003	1.61121	2.1
39004	1.61121	2.1
39005	2.13878	2.1
39006	2.13878	2.1
39007	1.89226	2.1
39008	1.89226	2.1
39009	1.89226	2.1
39010	1.89226	2.1
39011	1.89226	2.1
39012	1.89226	2.1
39013	1.09766	2.1
39014	1.04263	2.1
39015	0.881794	2.1
39016	0.894188	2.1
39017	0.795402	2.1
39018	0.99524	2.1
39019	1.00647	2.1
39020	1.00647	2.1
39021	1.15401	2.1
39022	0.881789	2.1
39023	0.850526	2.1
39024	1.04263	2.1
39025	1.4054	2.01832
39026	1.04788	2.1
39027	1.70302	2.06694
39028	1.41375	2.1
39029	2.82706	2.1
39030	1.70302	2.01587
39031	1.38776	2.1

39032	1.59457	2.09567
39033	1.41375	2.1
39034	1.77261	1.89392
39035	1.70302	2.1
39036	1.89226	2.1
39037	1.89226	2.1
39038	1.89226	2.1
39039	1.89226	2.1
39040	3.78453	2.1
39041	2.18042	2.1
39042	1.74738	2.1
39043	2.08433	2.1
39044	1.66347	2.1
39045	3.83171	2.1
39046	1.74738	2.1
39047	1.1181	2.1
39048	1.66347	2.1
39049	1.06543	2.1
39050	2.81281	2.1
39051	1.89226	2.1
39052	1.89226	2.1
39053	1.89226	2.1
39054	1.89226	2.1
39055	3.78453	2.1
39056	1.89227	2.1
39057	1.89227	2.1
39058	1.89227	2.1
39059	1.89227	2.1
39060	3.78453	2.1
39061	2.08433	2.1
39062	1.66347	2.1
39063	1.97092	2.1
39064	1.57431	2.1
39065	3.65864	2.1
39066	1.66347	2.1
39067	1.01474	2.1
39068	1.01474	2.1
39069	1.06544	2.1
39070	1.57431	2.1
39071	1.625 2.1	
39072	1.89226	2.1
39073	1.89226	2.1
39074	1.89226	2.1
39075	1.89226	2.1
39076	1.89226	2.1
39077	1.89226	2.1
39078	1.08467	2.1
39079	1.87228	2.1
39080	1.73305	2.1
39081	1.73305	2.1
39082	1.25565	2.1
39083	1.25565	2.1
39084	0.695361	0.27291
39085	0.799996	0.2871
39086	1.13751	0.451804
39087	1.40945	0.472566
39088	1.40944	0.52819
39089	0.799999	0.274124

39090	1.13751	0.295804
39091	0.695363	0.224328
39092	1.01059	0.31352
39093	1.01059	0.504196
39094	0.926908	0.50338
39095	0.926909	0.211111
39096	1.05481	2.1
39097	1.78069	2.1
39098	1.08074	2.1
39099	1.83284	2.1
39100	2.86143	2.1
39101	1.78069	2.1
39102	2.22044	2.1
39103	1.83284	2.1
39104	2.28696	1.98277
39105	4.06766	2.1
39106	1.07196	2.1
39107	1.64955	2.1
39108	2.02665	2.1
39109	2.0284	2.1
39110	1.08074	2.1
39111	1.08074	2.1
39112	1.64955	2.1
39113	2.28696	2.09545
39114	1.92033	2.1
39115	2.1004	2.1
39116	3.74988	2.1
39117	2.83787	0.607837
39118	1.70303	1.10741
39119	1.70303	0.397366
39120	1.83195	0.147003
39121	2.64075	0.243252
39122	2.70231	0.767618
39123	1.2087	2.09972
39124	1.52909	1.30229
39125	1.43063	1.14261
39126	1.10586	2.09499
39127	2.63494	1.9289
39128	1.89227	2.09781
39129	2.10732	2.1
39130	2.10732	2.1
39131	2.2365	2.1
39132	1.89227	2.1
39133	2.02145	2.1
39134	1.89226	1.36942
39135	1.89226	2.1
39136	1.89226	1.91247
39137	1.89226	2.0959
39138	1.89226	1.64888
39139	1.89226	2.09691
39140	0.51926	2.1
39141	0.552927	2.09812
39142	0.691235	1.22976
39143	0.715314	1.38514
39144	0.619684	1.37017
39145	0.619684	2.1
39146	0.624477	2.1
39147	0.624477	1.32166

39148	0.519262	2.01727
39149	0.691234	2.00841
39150	0.715313	2.0178
39151	0.552928	2.0182
39152	1.89226	2.1
39153	1.87321	2.1
39154	1.87321	2.1
39155	1.89658	2.1
39156	2.0815	2.1
39157	2.14789	2.1
39158	0.946128	2.1
39159	0.946128	2.1
39160	0.946133	1.43678
39161	0.946133	1.75136
39162	0.94613	1.53014
39163	0.94613	2.1
39164	0.946131	1.62163
39165	0.946131	2.1
39166	0.946131	1.95398
39167	0.946131	1.94567
39168	0.946131	2.06383
39169	0.946131	2.07448
39170	1.99297	1.14807
39171	2.19514	0.570142
39172	2.46153	0.827656
39173	2.187	0.740225
39174	4.45451	0.752637
39175	2.0815	0.671643
39176	2.33402	0.572162
39177	2.46154	0.861833
39178	2.83046	0.457086
39179	4.94496	0.634672
39180	2.46153	0.761803
39181	2.187	0.633446
39182	2.27351	0.955251
39183	2.63356	0.498996
39184	4.73504	0.705432
39185	2.46154	0.796559
39186	2.83447	0.41514
39187	2.74513	0.540587
39188	2.5731	0.697702
39189	2.63357	0.514864
39190	2.46154	0.6402
39191	2.80398	2.1
39192	2.60406	2.1
39193	2.73988	2.1
39194	2.57466	2.1
39195	5.34394	2.1
39196	1.89226	1.80646
39197	1.89226	0.762927
39198	1.89226	0.577667
39199	1.89226	1.77031
39200	1.89226	0.665127
39201	1.89226	1.92807
39202	1.89224	1.92081
39203	1.89224	0.813552
39204	1.89224	0.795688
39205	1.89224	1.93219

39206	1.89224	0.830428
39207	1.89224	1.82113
39208	1.40245	1.37992
39209	1.40246	1.47572
39210	0.946117	1.41996
39211	0.946117	1.66429
39212	1.17635	0.821973
39213	1.40246	1.69123
39214	0.946112	0.923235
39215	0.946112	1.93859
39216	0.946127	1.48668
39217	1.17637	0.979198
39218	0.946102	1.86002
39219	1.40244	1.31206
39220	2.53269	2.1
39221	1.70302	2.1
39222	1.56194	2.1
39223	2.17516	2.1
39224	4.19807	2.1
39225	1.89226	2.1
39226	1.89226	2.1
39227	1.89226	2.1
39228	1.89226	2.1
39229	3.78453	2.1
39230	2.57276	2.1
39231	1.89226	2.1
39232	1.89226	2.1
39233	1.89226	2.1
39234	2.32525	2.1
39235	2.32525	2.1
39236	1.15171	2.1
39237	2.17517	2.1
39238	1.41995	2.1
39239	1.41995	2.1
39240	1.80252	2.1
39241	1.80252	2.1
39242	1.89226	2.1
39243	1.89226	2.1
39244	1.89226	2.1
39245	1.89226	2.09903
39246	3.78453	2.1
39247	0.946133	2.1
39248	0.946133	2.1
39249	1.16263	2.1
39250	1.04687	2.1
39251	0.946133	2.1
39252	0.946133	2.1
39253	1.04687	2.1
39254	1.04687	2.1
39255	0.946133	2.1
39256	1.04687	2.1
39257	0.946132	2.1
39258	1.16263	2.1
39259	0.682281	1.63742
39260	0.669233	1.69941
39261	1.21859	1.71891
39262	1.31629	1.26941
39263	0.916158	0.734346

39264	0.916158	1.983
39265	0.971658	1.73659
39266	0.971658	1.77208
39267	0.669234	1.76067
39268	1.31629	1.40053
39269	0.682283	1.67209
39270	1.21858	1.80501
39271	0.669232	1.51111
39272	0.623495	1.51602
39273	1.36428	1.41999
39274	1.31629	1.1271
39275	0.946475	0.64718
39276	0.946476	1.90546
39277	0.99331	1.6767
39278	0.99331	1.60465
39279	1.36428	1.30527
39280	0.623497	0.990829
39281	0.669234	1.4498
39282	1.31629	1.18649
39283	2.02069	0.05
39284	1.46253	0.05
39285	2.51479	0.05
39286	1.87136	0.05
39287	3.89203	0.05
39288	1.46253	0.05
39289	1.38536	0.05
39290	1.38536	0.05
39291	1.05579	0.05
39292	1.73281	0.05
39293	1.46916	0.05
39294	2.51479	0.05
39295	1.87136	0.05
39296	2.23338	0.05
39297	2.15603	0.05
39298	4.55146	0.05
39299	0.822553	0.05
39300	0.822563	0.05
39301	0.842935	0.05
39302	0.842935	0.05
39303	0.744203	0.05
39304	0.675346	0.05
39305	0.874795	0.05
39306	1.00453	0.05
39307	1.00454	0.05
39308	0.782417	0.05
39309	0.675337	0.05
39310	0.858007	0.05
39311	0.946132	1.20544
39312	1.24847	0.250009
39313	0.946132	0.993659
39314	1.16806	1.10047
39315	1.24847	0.310091
39316	1.24847	1.99091
39317	0.946133	0.196045
39318	0.946133	1.56415
39319	0.946133	0.484477
39320	0.946133	0.336057
39321	1.16805	0.912286

39322	1.24847	0.342531
39323	1.2364	1.57092
39324	0.946129	0.337085
39325	0.946133	1.0657
39326	1.24847	0.566665
39327	1.2364	0.277473
39328	1.2364	1.67996
39329	0.946131	0.14621
39330	0.946131	2.09541
39331	0.946131	1.21127
39332	0.946131	0.292143
39333	1.2364	1.63011
39334	1.24847	0.489924
39335	1.89226	0.748941
39336	1.89226	1.45786
39337	1.89226	0.674626
39338	1.89226	0.410836
39339	1.89226	1.3793
39340	1.89226	1.45411
39341	1.89226	1.17088
39342	1.89226	0.696761
39343	1.89226	0.263593
39344	1.89226	1.20007
39345	1.89226	0.578256
39346	1.89226	1.55496
39347	1.69014	1.10577
39348	1.09194	1.13976
39349	1.13537	1.39826
39350	1.13537	1.20941
39351	1.58481	0.741659
39352	1.58481	0.439083
39353	1.58147	1.01367
39354	1.13537	0.421633
39355	1.69015	1.14837
39356	1.01179	0.646946
39357	1.69015	0.447101
39358	1.01179	0.60119
39359	0.946132	1.30735
39360	0.946132	0.621049
39361	1.28543	0.218081
39362	1.07341	1.32932
39363	1.07341	1.10888
39364	1.07341	0.0678282
39365	0.946132	0.556447
39366	0.946132	0.237871
39367	0.946133	0.236504
39368	1.28543	0.336855
39369	1.07341	0.338482
39370	0.946132	0.380276
39371	1.89226	1.66521
39372	1.89226	0.772267
39373	1.89226	0.82319
39374	1.89226	1.99979
39375	1.89226	0.599793
39376	1.89226	2.07596
39377	0.805744	2.1
39378	0.988659	2.02594
39379	0.782044	0.840418

39380	0.578223	1.36402
39381	0.77823	1.28768
39382	0.778231	2.1
39383	0.788653	0.839479
39384	0.788653	1.98886
39385	0.988662	1.61379
39386	0.782042	1.70319
39387	0.805745	1.94985
39388	0.578222	1.91258
39389	1.89226	0.771702
39390	1.89226	1.51875
39391	1.89226	1.30401
39392	1.89226	1.95049
39393	1.89226	0.570768
39394	1.89226	1.99953
39395	1.97732	1.92861
39396	1.97819	0.738506
39397	1.56408	0.839446
39398	1.56408	2.03614
39399	2.33986	0.877347
39400	2.33986	2.06128
39401	1.84516	2.1
39402	2.21152	2.1
39403	1.90702	2.1
39404	1.54599	2.1
39405	3.75751	2.1
39406	1.89224	2.1
39407	1.89224	2.1
39408	1.89224	2.1
39409	1.89224	2.1
39410	1.89224	2.1
39411	1.89224	2.1
39412	1.47672	2.1
39413	1.54601	2.1
39414	1.27697	2.1
39415	1.54601	2.1
39416	1.638	2.1
39417	1.90705	2.1
39418	0.946132	2.1
39419	0.946132	2.1
39420	0.946131	2.1
39421	0.946131	2.1
39422	0.946131	2.1
39423	0.946131	2.1
39424	0.946132	2.1
39425	0.946132	2.1
39426	0.946132	2.1
39427	0.946132	2.1
39428	0.946131	2.1
39429	0.946131	2.1
39430	0.946128	2.1
39431	0.946128	2.1
39432	1.32062	2.1
39433	1.4164	2.1
39434	0.946129	2.1
39435	0.946129	2.1
39436	1.32062	2.1
39437	1.32062	2.1

39438	1.32062	2.1
39439	0.94613	2.1
39440	1.41639	2.1
39441	0.946129	2.1
39442	1.98979	0.05
39443	1.65977	0.05
39444	2.21772	0.05
39445	2.21772	0.05
39446	1.48866	0.05
39447	1.48866	0.05
39448	0.973129	0.05
39449	0.973129	0.05
39450	0.980954	0.05
39451	0.980954	0.05
39452	0.845218	0.05
39453	1.13981	0.05
39454	0.829886	0.05
39455	1.10886	0.05
39456	1.10886	0.05
39457	1.13982	0.05
39458	0.845219	0.05
39459	0.829886	0.05
39460	0.946132	2.1
39461	0.946132	2.1
39462	1.3352	2.1
39463	1.41432	2.1
39464	0.946133	2.1
39465	0.946133	2.06559
39466	1.3352	2.1
39467	1.3352	2.1
39468	1.3352	2.1
39469	0.946132	2.1
39470	1.41432	2.1
39471	0.946133	2.1
39472	2.70097	2.1
39473	1.88175	2.1
39474	1.51482	2.1
39475	1.88175	2.1
39476	2.37784	2.1
39477	2.82864	2.1
39478	1.89227	0.0630056
39479	1.89227	0.05
39480	1.89227	0.05
39481	1.89227	0.05
39482	3.78453	0.05
39483	1.89226	0.05
39484	2.13295	0.05
39485	2.43203	0.05
39486	2.43203	0.05
39487	1.89226	0.05
39488	1.89226	0.05
39489	0.946133	0.05
39490	0.946133	0.05
39491	0.946132	0.05
39492	0.946132	0.05
39493	0.946132	0.05
39494	0.946132	0.05
39495	0.946133	0.05

39496	0.946133	0.05
39497	0.946132	0.05
39498	0.946132	0.05
39499	0.946132	0.05
39500	0.946132	0.05
39501	1.54987	2.1
39502	1.24128	2.1
39503	1.24128	2.1
39504	1.50738	2.1
39505	1.75702	2.1
39506	2.02312	2.1
39507	1.89289	2.1
39508	1.92264	2.1
39509	1.72994	2.1
39510	1.75702	2.1
39511	3.67966	2.1
39512	1.75702	2.1
39513	0.994291	2.1
39514	0.994291	2.1
39515	1.24128	2.1
39516	1.4964	2.1
39517	1.74339	2.1
39518	1.72993	2.1
39519	1.49641	2.1
39520	1.49641	2.1
39521	1.75702	2.1
39522	1.3939	2.1
39523	1.65452	2.1
39524	1.19964	2.1
39525	1.53296	2.1
39526	1.19964	2.1
39527	1.36861	2.1
39528	1.42624	2.1
39529	1.59521	2.1
39530	1.19964	2.1
39531	1.42624	2.1
39532	1.07156	2.1
39533	1.36226	2.1
39534	2.5619	2.1
39535	2.14058	0.903291
39536	1.93569	2.07358
39537	2.36221	1.69922
39538	2.5753	1.40761
39539	4.50279	1.93617
39540	2.14057	1.90662
39541	2.20831	2.04059
39542	2.64725	0.630811
39543	2.57529	1.85251
39544	4.78782	1.73926
39545	2.64725	1.05078
39546	2.57529	1.94256
39547	1.89226	1.15197
39548	1.89226	0.378898
39549	4.53951	1.63333
39550	2.0815	2.1
39551	1.86137	2.1
39552	1.86137	2.1
39553	1.86159	2.1

39554	2.0815	2.1
39555	2.10235	2.1
39556	1.33552	2.1
39557	2.32417	2.1
39558	1.18872	2.1
39559	1.93101	2.1
39560	3.26653	2.1
39561	1.89226	2.1
39562	1.89226	2.1
39563	1.89226	2.1
39564	1.89226	2.1
39565	1.89226	2.1
39566	1.89226	2.1
39567	0.955191	2.1
39568	1.03372	2.1
39569	0.946131	2.1
39570	0.946131	2.1
39571	0.930654	2.1
39572	0.930654	2.1
39573	1.04233	2.1
39574	1.05247	2.1
39575	0.955341	2.1
39576	0.946131	2.1
39577	1.02375	2.1
39578	1.13537	2.1
39579	1.1549	2.1
39580	1.93101	2.1
39581	1.91108	2.1
39582	1.52487	2.1
39583	1.3076	2.1
39584	1.06985	2.1
39585	1.89226	2.1
39586	1.89226	2.1
39587	1.89226	2.1
39588	1.89226	2.1
39589	1.89226	2.1
39590	1.89226	2.1
39591	1.58713	2.1
39592	2.6845	2.1
39593	1.58742	2.1
39594	2.74201	2.1
39595	4.27191	2.1
39596	2.68449	2.1
39597	1.89226	2.1
39598	2.74201	2.1
39599	1.89226	2.1
39600	4.57675	2.1
39601	1.57523	2.1
39602	2.74201	2.1
39603	2.8146	2.1
39604	2.8146	2.1
39605	1.58742	2.1
39606	1.58742	2.1
39607	0.946131	2.1
39608	0.946131	2.1
39609	1.371 2.1	
39610	1.371 2.1	
39611	0.946127	2.1

39612	0.946127	2.1
39613	1.37101	2.1
39614	1.4073	2.1
39615	0.94613	2.1
39616	1.4073	2.1
39617	1.371 2.1	
39618	0.946129	2.1
39619	1.44801	1.82583
39620	1.06821	1.96181
39621	2.00542	1.8036
39622	2.27703	1.98553
39623	3.34524	1.83438
39624	1.70301	0.198195
39625	1.89226	0.17154
39626	1.65809	0.188041
39627	1.89226	0.199512
39628	1.62758	0.157463
39629	1.85744	0.165703
39630	1.89226	0.136332
39631	1.89226	0.187716
39632	1.89226	0.146789
39633	1.89226	0.127974
39634	3.78452	0.128388
39635	1.89226	0.190009
39636	1.89226	0.137036
39637	1.89226	0.120118
39638	1.89226	0.161004
39639	3.78452	0.157341
39640	0.851541	0.183785
39641	0.851541	0.183945
39642	0.91193	0.168649
39643	0.824837	0.171501
39644	0.854559	0.189178
39645	0.851534	0.160034
39646	0.81851	0.168646
39647	0.717219	0.188269
39648	0.871743	0.161963
39649	0.837985	0.160163
39650	0.82902	0.173324
39651	0.82902	0.181552
39652	1.89226	0.184446
39653	1.92659	0.0694107
39654	2.03235	0.0813509
39655	1.89226	0.242887
39656	2.03235	0.057318
39657	1.89226	0.191777
39658	0.585557	0.189304
39659	0.585557	0.169676
39660	1.22612	0.140238
39661	1.09436	0.145956
39662	0.851537	0.205518
39663	0.526987	0.137449
39664	0.983879	0.0967115
39665	1.04072	0.263729
39666	0.585526	0.0615242
39667	0.792325	0.0604638
39668	0.946129	0.194494
39669	0.946129	0.17684

39670	1.68152	2.1
39671	1.81445	2.1
39672	1.68152	2.1
39673	1.86792	2.1
39674	1.65888	2.1
39675	1.84529	2.1
39676	1.81445	2.1
39677	1.57885	2.1
39678	1.65889	2.1
39679	1.48594	2.1
39680	3.23773	2.1
39681	1.68154	2.1
39682	1.65891	2.1
39683	1.53296	2.1
39684	1.56299	2.1
39685	3.24453	2.1
39686	1.56299	2.1
39687	1.48596	2.1
39688	1.56299	2.1
39689	1.65891	2.1
39690	1.46568	2.1
39691	1.5616	2.1
39692	2.68785	2.1
39693	2.08831	2.1
39694	2.66919	2.1
39695	2.1024	2.1
39696	4.79025	2.1
39697	2.08832	2.1
39698	1.15478	2.1
39699	1.15478	2.1
39700	1.09062	2.1
39701	2.10241	2.1
39702	2.03825	2.1
39703	2.65348	2.1
39704	2.1024	2.1
39705	2.12313	2.1
39706	2.12313	2.1
39707	2.66919	2.1
39708	2.66919	2.1
39709	0.813169	2.1
39710	0.813169	2.1
39711	0.825788	2.1
39712	0.825788	2.1
39713	0.612902	2.1
39714	0.577387	2.1
39715	1.0512	2.1
39716	1.06157	2.1
39717	1.06157	2.1
39718	0.612902	2.1
39719	0.577387	2.1
39720	1.0512	2.1
39721	1.27227	2.1
39722	1.91724	2.1
39723	1.32591	2.1
39724	1.99837	2.1
39725	3.24315	2.1
39726	1.89223	2.1
39727	1.89223	2.1

39728	1.89223	2.1
39729	1.89223	2.1
39730	3.78446	2.1
39731	1.89224	2.1
39732	1.89224	2.08045
39733	1.89224	2.1
39734	1.89224	2.1
39735	3.78447	2.1
39736	1.91724	2.1
39737	2.321	2.1
39738	1.99838	2.1
39739	2.4149	2.1
39740	4.33214	2.1
39741	1.34817	2.1
39742	1.9984	2.1
39743	2.06238	2.1
39744	2.06238	2.1
39745	1.32593	2.1
39746	1.32593	2.1
39747	1.89226	2.1
39748	1.89226	2.1
39749	1.89226	2.1
39750	1.89226	2.1
39751	1.89226	2.1
39752	1.89226	2.1
39753	1.89226	2.1
39754	1.89226	2.1
39755	1.89226	2.1
39756	1.89226	2.08297
39757	3.78452	2.1
39758	1.9984	2.1
39759	2.41494	2.1
39760	2.06239	2.1
39761	2.5194	2.1
39762	4.47732	2.1
39763	1.19901	1.62998
39764	1.16661	2.1
39765	1.56859	2.1
39766	1.61029	1.73676
39767	2.7676	2.09769
39768	1.56859	2.1
39769	1.88637	0.958652
39770	1.61029	0.964574
39771	1.61029	2.08151
39772	1.856	1.01973
39773	1.856	2.08497
39774	1.856	2.0427
39775	1.57916	2.1
39776	1.56859	2.08975
39777	1.56859	2.1
39778	1.57916	2.1
39779	1.23475	2.1
39780	0.890924	0.471464
39781	1.07608	0.411972
39782	0.72877	0.845212
39783	0.910106	0.715111
39784	1.00196	1.0275
39785	1.09519	0.933752

39786	0.839967	0.518638
39787	0.719043	0.375069
39788	0.893859	0.529914
39789	0.942408	1.19634
39790	0.873497	0.702214
39791	0.873497	1.59075
39792	1.53046	0.348032
39793	1.89994	0.54254
39794	1.049	0.379511
39795	1.049	0.297603
39796	1.52512	0.252158
39797	1.89994	0.738583
39798	1.65573	1.67813
39799	1.89226	0.338714
39800	1.89226	0.171192
39801	2.73329	1.07267
39802	3.548	1.56106
39803	1.65573	0.201685
39804	2.42671	0.690993
39805	2.73329	0.206348
39806	1.89226	0.196429
39807	2.73329	0.180949
39808	1.89226	0.234083
39809	0.941078	0.394227
39810	1.01599	0.723304
39811	0.818936	0.446182
39812	0.651554	0.782702
39813	0.904729	0.725969
39814	0.665756	0.661178
39815	0.843423	1.18334
39816	0.918649	0.742111
39817	0.855806	0.772356
39818	0.814384	0.231567
39819	0.844437	1.01406
39820	0.844437	0.9315
39821	2.22558	2.04243
39822	1.95785	0.452835
39823	2.07065	1.0039
39824	1.89142	1.16467
39825	2.07065	1.81885
39826	1.89142	1.85949
39827	0.640813	0.869208
39828	0.951654	0.743825
39829	0.797442	0.260521
39830	0.535364	0.72862
39831	0.525269	0.821745
39832	0.620874	0.400258
39833	0.916953	0.482163
39834	0.969374	0.614833
39835	0.788925	0.860627
39836	0.69596	0.304172
39837	0.743154	0.852595
39838	0.743154	0.914753
39839	1.89226	2.01336
39840	1.89226	1.70264
39841	1.89226	1.09802
39842	1.89226	2.06708
39843	3.78452	1.66819

39844	1.32541	1.71143
39845	1.13722	1.29531
39846	0.91103	2.1
39847	0.881123	2.03186
39848	1.03363	2.08131
39849	1.03364	1.90469
39850	1.1505	1.7343
39851	1.1505	2.03678
39852	1.32539	2.01455
39853	1.07662	2.06148
39854	0.881121	1.90954
39855	0.962303	1.91036
39856	1.89226	1.77699
39857	1.89226	2.1
39858	1.89226	2.09368
39859	1.89226	1.88261
39860	3.78453	2.06532
39861	1.89226	1.01664
39862	1.89226	2.02118
39863	1.89226	1.87264
39864	1.89226	1.45153
39865	1.89226	1.86799
39866	1.89226	1.55693
39867	1.27606	1.94096
39868	1.07664	1.804
39869	0.905895	2.09995
39870	0.696099	2.09978
39871	0.984902	2.09621
39872	0.984905	1.98577
39873	1.01878	2.09454
39874	1.01878	1.60467
39875	1.27604	2.04975
39876	1.06044	2.03661
39877	1.13192	2.04905
39878	0.696111	2.0475
39879	1.89226	1.97172
39880	1.89226	0.669721
39881	1.89226	0.251984
39882	1.89226	2.00141
39883	1.89226	0.685029
39884	1.89226	1.87957
39885	1.52615	2.08351
39886	1.01294	0.933554
39887	1.01294	2.09457
39888	1.18549	0.353971
39889	1.38963	1.85044
39890	1.56218	0.714351
39891	1.89226	1.01845
39892	2.40801	1.88177
39893	2.58101	2.0631
39894	2.58101	1.08668
39895	1.89226	2.07987
39896	1.89226	0.414542
39897	2.24071	2.1
39898	2.79317	2.1
39899	1.99136	2.1
39900	2.6167	2.1
39901	4.85741	2.1

39902	2.79317	2.1
39903	1.89226	2.08252
39904	1.89226	2.1
39905	1.89226	2.09773
39906	2.6167	2.1
39907	2.6167	2.08233
39908	1.08354	2.1
39909	1.08354	2.1
39910	1.10222	2.1
39911	1.10222	2.1
39912	1.2275	2.1
39913	1.30835	2.1
39914	0.995678	2.1
39915	0.877412	2.1
39916	0.877411	2.1
39917	1.2275	2.1
39918	0.995678	2.1
39919	1.30835	2.1
39920	1.05793	0.348717
39921	1.46441	0.370587
39922	1.0562	0.265711
39923	1.0562	0.359295
39924	1.41787	0.327986
39925	1.41787	0.345882
39926	1.41787	0.436572
39927	1.52909	1.30698
39928	1.46441	0.430623
39929	1.46441	1.12377
39930	1.43063	0.491613
39931	1.43063	1.08662
39932	1.89226	1.36121
39933	1.89226	0.508828
39934	1.89226	0.488124
39935	1.89226	1.77494
39936	3.78453	0.990591
39937	1.89227	0.343278
39938	1.89227	0.497522
39939	1.89227	0.276221
39940	1.89227	0.530396
39941	1.89227	0.297826
39942	1.89227	0.411639
39943	1.00763	0.181248
39944	1.41787	0.523108
39945	1.05793	0.179254
39946	1.05793	0.523086
39947	1.37265	0.19373
39948	1.37265	0.468633
39949	1.37265	0.463222
39950	1.43063	1.38469
39951	1.41787	0.497248
39952	1.41787	1.25049
39953	1.38247	0.569137
39954	1.38247	1.22574
39955	0.946132	1.39902
39956	0.946132	1.62176
39957	0.946132	0.502669
39958	0.946132	0.638025
39959	0.946131	0.622947

39960	0.946131	1.62194
39961	0.946132	1.56797
39962	0.946132	0.701212
39963	0.946132	1.12297
39964	0.946132	1.14527
39965	0.946132	1.20781
39966	0.946132	1.18122
39967	1.89226	0.216499
39968	1.89226	0.711501
39969	1.89226	0.219936
39970	1.89226	0.673487
39971	1.89226	0.21122
39972	1.89226	0.550839
39973	1.19645	0.933659
39974	1.89183	0.233305
39975	1.91106	0.50093
39976	1.35306	0.277594
39977	1.75446	0.427363
39978	1.19645	0.252283
39979	2.37576	2.03273
39980	1.90139	2.1
39981	2.1845	2.1
39982	1.86568	2.1
39983	4.24145	2.1
39984	2.29435	2.1
39985	1.40602	2.1
39986	1.40602	2.09599
39987	1.55351	2.1
39988	1.86568	2.1
39989	2.01317	2.1
39990	2.18452	2.1
39991	1.8657	1.4656
39992	2.01399	1.96001
39993	1.70141	2.1
39994	3.87969	2.07903
39995	0.774999	2.1
39996	0.774999	2.08818
39997	0.778721	2.1
39998	0.778721	2.1
39999	0.628348	2.1
40000	0.703016	2.1
40001	0.932848	2.1
40002	0.850704	2.1
40003	0.850704	2.1
40004	0.628348	2.1
40005	0.703015	2.1
40006	0.932848	2.1
40007	1.65243	1.01304
40008	1.34412	0.736246
40009	1.45569	1.88846
40010	1.1315	1.86927
40011	2.78393	0.867503
40012	1.48901	2.1
40013	1.805	2.1
40014	1.48901	2.1
40015	1.71717	2.1
40016	1.60635	2.1
40017	1.83451	2.1

40018	1.805	2.1
40019	1.65243	0.96105
40020	1.60635	2.1
40021	1.45569	1.6563
40022	3.25879	2.02878
40023	1.27875	2.1
40024	1.60635	2.1
40025	1.27875	2.1
40026	1.48901	2.1
40027	1.40014	2.1
40028	1.6104	2.1
40029	0.715199	2.03832
40030	0.715199	2.1
40031	0.712714	2.1
40032	0.712714	1.2753
40033	0.727843	1.07919
40034	0.619713	1.28
40035	0.803177	2.1
40036	0.70007	2.1
40037	0.700069	2.1
40038	0.619713	2.09449
40039	0.803177	2.04761
40040	0.727844	2.06294
40041	1.14357	2.06991
40042	2.3506	1.98929
40043	1.14357	2.05015
40044	2.68773	1.94285
40045	1.57829	2.04105
40046	2.68773	1.93251
40047	2.00551	2.1
40048	2.65811	2.06018
40049	2.07203	2.1
40050	2.72117	1.03511
40051	4.72667	2.1
40052	0.946131	1.43591
40053	0.946131	2.1
40054	1.32905	2.1
40055	1.32905	0.843609
40056	0.94613	1.16401
40057	0.94613	1.9854
40058	1.32905	1.88336
40059	1.36058	1.6957
40060	1.36058	0.274155
40061	0.946131	0.174914
40062	1.32905	1.63844
40063	0.946131	1.78791
40064	1.21649	1.19742
40065	1.21649	2.1
40066	1.21459	2.1
40067	1.21459	2.1
40068	1.39508	1.74538
40069	1.36057	1.60961
40070	1.03601	2.1
40071	1.07438	2.1
40072	1.07439	2.08104
40073	1.39509	2.09183
40074	1.036	2.09425
40075	1.36056	2.09346

40076	2.79015	0.721942
40077	2.72114	2.1
40078	1.89225	0.997539
40079	1.89225	2.1
40080	1.89225	2.01784
40081	2.72114	1.86846
40082	0.876258	2.1
40083	0.892889	2.1
40084	1.34352	2.1
40085	1.27985	2.1
40086	1.07462	2.1
40087	1.07462	2.1
40088	1.09812	2.1
40089	1.09812	2.1
40090	0.892888	2.1
40091	1.34352	2.1
40092	0.876256	2.1
40093	1.27985	2.1
40094	1.39725	2.03744
40095	1.30029	1.20602
40096	1.31415	1.42761
40097	1.41336	2.01349
40098	2.71366	1.84182
40099	1.89226	1.27346
40100	1.89226	1.97588
40101	1.89226	2.02984
40102	1.89226	1.24257
40103	3.78453	1.80726
40104	0.946133	1.3165
40105	0.946133	1.21395
40106	1.38709	0.564399
40107	1.39437	0.663441
40108	0.946132	0.578094
40109	0.946132	1.20173
40110	1.39437	1.31597
40111	1.39437	0.631993
40112	0.946132	0.91935
40113	1.39437	0.906694
40114	1.38709	0.913666
40115	0.946132	0.919041
40116	0.706681	2.06773
40117	0.711908	2.04163
40118	0.657077	1.31467
40119	0.661003	1.56098
40120	0.684188	1.33966
40121	0.684188	2.04127
40122	0.683496	2.07832
40123	0.683496	1.46068
40124	0.711908	1.9035
40125	0.661003	1.89305
40126	0.657076	1.85618
40127	0.706681	1.8646
40128	0.946132	2.05477
40129	0.946132	2.00532
40130	0.946132	1.4534
40131	0.946132	1.1697
40132	0.946132	1.18475
40133	0.946132	2.01472

40134	0.946133	1.53514
40135	0.946133	2.06124
40136	0.946133	1.80087
40137	0.946133	1.85703
40138	0.946132	1.80569
40139	0.946132	1.83724
40140	2.79029	0.61729
40141	1.89227	1.46212
40142	2.78873	0.837198
40143	2.78873	1.16273
40144	1.89383	0.775584
40145	1.89227	1.14565
40146	1.43669	2.1
40147	2.11984	2.1
40148	2.24481	2.1
40149	2.24481	2.1
40150	1.20311	2.1
40151	1.20311	2.1
40152	0.49703	2.1
40153	0.601553	2.1
40154	1.05992	2.1
40155	0.991694	2.1
40156	0.787558	2.1
40157	0.787558	2.1
40158	0.805689	2.1
40159	0.805689	2.1
40160	0.991694	2.1
40161	0.49703	2.1
40162	1.05992	2.1
40163	0.601553	2.1
40164	2.45905	2.1
40165	1.89226	2.1
40166	1.89226	2.1
40167	1.89226	2.1
40168	2.47395	2.1
40169	2.47395	2.1
40170	1.89226	2.1
40171	1.89226	2.09748
40172	1.89226	2.1
40173	1.89226	2.03762
40174	3.78452	2.1
40175	1.38427	2.1
40176	2.31203	1.99443
40177	2.28743	2.01472
40178	2.28743	2.1
40179	1.63384	2.00924
40180	1.63384	2.1
40181	2.42723	2.1
40182	1.89226	2.1
40183	1.89226	2.1
40184	1.89226	2.1
40185	2.45905	2.1
40186	2.45905	2.1
40187	0.946131	1.88138
40188	0.946131	2.1
40189	0.946131	2.1
40190	0.946131	2.1
40191	0.94613	2.06822

40192	0.94613	2.07199
40193	0.946132	2.1
40194	0.946132	2.1
40195	0.946131	2.1
40196	0.946131	2.1
40197	0.946131	2.1
40198	0.946131	2.1
40199	0.989441	2.1
40200	0.989441	2.1
40201	0.993918	2.1
40202	0.993918	2.1
40203	1.16644	2.1
40204	1.15601	2.07817
40205	0.818308	2.1
40206	0.816922	2.1
40207	0.818307	2.1
40208	1.16644	2.1
40209	1.15601	2.1
40210	0.816922	2.1
40211	0.996778	0.05
40212	2.44375	0.05
40213	0.996778	0.0502627
40214	1.92547	0.05
40215	1.56091	0.05
40216	2.4896	0.05
40217	1.56091	0.05
40218	2.44375	0.05
40219	1.90456	0.05
40220	1.90456	0.05
40221	2.81853	0.05
40222	2.47487	0.05
40223	1.32143	2.06232
40224	1.43294	2.1
40225	1.37444	0.605296
40226	1.51691	2.09655
40227	2.80738	2.02134
40228	1.89226	1.73352
40229	1.89226	2.1
40230	1.89226	0.818422
40231	1.89226	1.90071
40232	3.78453	1.26848
40233	1.89226	2.08184
40234	1.89226	2.1
40235	1.89226	1.56098
40236	1.89226	2.1
40237	3.78453	2.1
40238	1.43294	2.07615
40239	1.3268	2.1
40240	1.51691	2.02694
40241	1.44091	2.1
40242	2.87385	2.1
40243	1.37444	2.04799
40244	1.51691	2.1
40245	1.4714	0.658269
40246	1.65434	1.9522
40247	3.02878	1.78457
40248	1.89226	2.04606
40249	1.89226	2.09468

40250	1.89226	0.89255
40251	1.89226	1.98405
40252	3.78452	1.75378
40253	1.89226	1.90236
40254	1.89226	2.1
40255	1.89226	1.64591
40256	1.89226	2.1
40257	3.78453	2.1
40258	1.51691	2.09562
40259	1.44091	2.1
40260	1.65434	1.65958
40261	1.60647	2.1
40262	3.09526	2.1
40263	1.05013	2.1
40264	1.05013	2.1
40265	0.94613	2.1
40266	0.94613	2.1
40267	1.04959	2.1
40268	1.05014	2.1
40269	0.946129	2.1
40270	0.946129	2.1
40271	0.946131	2.1
40272	1.04958	2.1
40273	0.946131	2.1
40274	1.05013	2.1
40275	1.04959	2.1
40276	1.04959	2.1
40277	0.946131	2.1
40278	0.946131	2.1
40279	1.04765	2.1
40280	1.04959	2.1
40281	0.94613	2.1
40282	0.94613	2.1
40283	0.946131	2.1
40284	1.04765	2.1
40285	0.946131	2.1
40286	1.04959	2.1
40287	1.85744	2.1
40288	1.89226	2.1
40289	1.89226	2.1
40290	1.89226	2.1
40291	1.94839	2.1
40292	1.94839	2.1
40293	0.97419	2.1
40294	0.822054	2.1
40295	0.946123	2.1
40296	0.946123	2.1
40297	1.04202	2.1
40298	0.84898	2.1
40299	0.933307	2.1
40300	0.933307	2.1
40301	0.933285	2.1
40302	1.05018	2.1
40303	0.851528	2.1
40304	0.876787	2.1
40305	1.87528	0.135979
40306	1.21137	0.0931034
40307	1.57893	0.0944916

40308	1.21137	0.160171
40309	1.81182	0.0973752
40310	1.44426	0.153237
40311	0.993833	0.0680846
40312	1.21137	0.113299
40313	1.57893	0.0993037
40314	1.31774	0.0672258
40315	2.5291	0.0740324
40316	1.87528	0.108793
40317	1.57893	0.0771558
40318	1.90728	0.0757648
40319	2.29851	0.12372
40320	3.87744	0.106995
40321	1.90728	0.0608994
40322	1.31774	0.0667382
40323	1.56339	0.05
40324	1.31774	0.0860937
40325	1.82458	0.05
40326	1.57893	0.0940367
40327	1.89226	1.07095
40328	1.89226	1.24209
40329	1.89226	1.12726
40330	1.89226	1.35924
40331	1.89226	1.12499
40332	1.89226	1.35573
40333	1.89226	1.44811
40334	1.89226	1.43922
40335	1.89226	1.66255
40336	1.89226	1.40851
40337	3.78452	1.60456
40338	1.89226	1.32724
40339	2.17251	1.28501
40340	2.45575	1.23246
40341	2.45575	1.21231
40342	1.89226	1.23798
40343	1.89226	1.2257
40344	1.08625	1.2489
40345	1.22788	1.17989
40346	0.54447	1.08384
40347	0.794861	1.17844
40348	0.967729	1.34888
40349	0.967729	1.24484
40350	0.913386	1.15355
40351	0.913386	1.0376
40352	1.08625	1.33611
40353	0.544469	1.04219
40354	0.794862	1.30623
40355	1.22788	1.22781
40356	0.946128	1.34408
40357	0.946128	1.57865
40358	0.946132	1.30575
40359	0.946132	1.40363
40360	0.94613	1.30244
40361	0.94613	1.36298
40362	0.94613	1.57338
40363	0.94613	1.29304
40364	0.94613	1.24236
40365	0.94613	1.2501

40366	0.94613	1.22685
40367	0.94613	1.36032
40368	0.946127	1.04267
40369	0.946127	1.17203
40370	1.08625	1.1134
40371	0.958779	1.12687
40372	1.08625	1.02548
40373	1.08625	1.08765
40374	0.946128	1.12401
40375	0.946128	1.17706
40376	0.946129	1.13233
40377	0.958777	1.14479
40378	1.08625	1.15793
40379	0.946129	1.16531
40380	2.16338	0.05
40381	1.94002	0.05
40382	1.98836	0.05
40383	2.60614	0.05
40384	1.5456	0.05
40385	2.16338	0.05
40386	1.19952	0.05
40387	2.34061	0.05
40388	1.21031	0.05
40389	2.1711	0.05
40390	3.54013	0.05
40391	1.13624	2.1
40392	1.70302	2.1
40393	1.8267	2.1
40394	1.11545	2.1
40395	2.85455	2.1
40396	1.82671	2.1
40397	1.91767	2.1
40398	1.81563	2.1
40399	1.91767	2.1
40400	1.71362	2.1
40401	1.83277	2.1
40402	1.66064	2.1
40403	0.996776	2.1
40404	0.996776	2.1
40405	1.11545	2.1
40406	1.54278	2.1
40407	1.66145	2.1
40408	1.66064	2.1
40409	1.7457	2.1
40410	1.81563	2.1
40411	1.81563	2.1
40412	1.54278	2.1
40413	1.54278	2.1
40414	2.71672	2.1
40415	2.70402	2.1
40416	2.64996	2.1
40417	2.69443	2.1
40418	5.39845	2.1
40419	2.71672	2.1
40420	2.54198	2.1
40421	2.54019	2.1
40422	2.69443	2.1
40423	5.23641	2.1

40424	2.64996	2.1
40425	2.69443	2.1
40426	2.68213	2.1
40427	2.61243	2.1
40428	5.30686	2.1
40429	2.54019	2.1
40430	2.69443	2.1
40431	2.68213	2.1
40432	2.5392	2.1
40433	5.23363	2.1
40434	1.89226	2.1
40435	2.34725	2.1
40436	2.22414	2.1
40437	2.34725	2.1
40438	1.76914	2.1
40439	1.89226	2.1
40440	0.946131	2.1
40441	0.946131	2.1
40442	1.11207	2.1
40443	1.03798	2.1
40444	0.94613	2.1
40445	0.94613	2.1
40446	1.03798	2.1
40447	1.03798	2.1
40448	0.946131	2.1
40449	1.03798	2.1
40450	0.946131	2.1
40451	1.11207	2.1
40452	1.89226	0.199725
40453	1.89226	0.179092
40454	1.89226	0.210115
40455	1.89226	0.151356
40456	1.89226	0.241382
40457	1.89226	0.17987
40458	1.89226	0.221778
40459	1.89226	0.224501
40460	1.89226	0.263272
40461	1.89226	0.175648
40462	1.89226	0.291317
40463	1.89226	0.201842
40464	1.89226	0.129644
40465	1.89226	0.124057
40466	1.89226	0.146892
40467	1.89226	0.104624
40468	1.89226	0.162189
40469	1.89226	0.122037
40470	1.44085	2.09844
40471	1.31825	1.46424
40472	1.31923	1.88426
40473	1.31825	2.06996
40474	1.43311	1.77689
40475	1.43213	2.03242
40476	1.89226	1.51376
40477	1.89226	2.03759
40478	1.89226	1.49124
40479	1.89226	2.0485
40480	1.89226	1.3099
40481	1.89226	2.05952

40482	0.946133	1.57063
40483	0.946133	1.24541
40484	1.38969	0.693378
40485	1.39587	0.921426
40486	0.946132	0.707297
40487	0.946132	1.20588
40488	1.39587	1.58987
40489	1.39587	0.8305
40490	0.946132	0.969899
40491	1.39587	0.963154
40492	1.38969	0.978051
40493	0.946132	1.00981
40494	1.44085	2.1
40495	1.31923	1.37216
40496	1.33026	1.86125
40497	1.4617	2.07859
40498	2.78093	2.02638
40499	1.89226	1.41292
40500	1.89226	2.02175
40501	1.89226	1.47991
40502	1.89226	2.06508
40503	1.89226	1.32082
40504	1.89226	2.08123
40505	0.946133	1.4658
40506	0.946133	1.21899
40507	1.39587	0.863365
40508	1.40724	0.635655
40509	0.946132	0.66381
40510	0.946132	1.19676
40511	1.40724	1.49407
40512	1.40724	0.838801
40513	0.946132	0.989352
40514	1.40724	0.985527
40515	0.946133	1.14277
40516	1.39587	1.04245
40517	1.15818	1.81728
40518	1.81846	1.47683
40519	1.52782	0.775007
40520	1.52782	2.06178
40521	1.28095	0.506065
40522	1.28095	1.81718
40523	2.56709	2.1
40524	1.4349	1.61741
40525	1.4349	2.09416
40526	1.75471	2.1
40527	2.29909	2.09039
40528	2.61889	2.1
40529	2.5671	2.1
40530	2.82858	2.1
40531	2.57368	2.1
40532	2.29909	2.1
40533	5.12767	2.1
40534	2.29909	2.1
40535	1.09148	1.19538
40536	1.09148	2.09282
40537	1.4349	2.02676
40538	1.99776	2.06107
40539	2.34119	2.08274

40540	2.57368	2.1
40541	1.99777	2.08724
40542	1.99777	2.1
40543	2.29909	2.1
40544	2.36071	2.1
40545	2.66203	2.1
40546	1.89226	1.16083
40547	1.89226	1.54258
40548	1.89226	1.53
40549	1.89226	1.12224
40550	3.78453	1.15349
40551	0.946128	1.78793
40552	0.946128	1.95109
40553	0.946133	1.53628
40554	0.946133	1.75002
40555	0.946131	1.73889
40556	0.946131	1.87586
40557	0.946131	1.89856
40558	0.946131	1.77088
40559	0.94613	1.92541
40560	0.94613	1.78503
40561	0.946131	1.67773
40562	0.946131	1.84444
40563	1.89226	1.57294
40564	1.89226	1.61664
40565	1.89226	1.11097
40566	1.89226	1.73456
40567	3.78453	1.69579
40568	1.89226	1.71093
40569	1.89226	1.4825
40570	1.89226	1.23693
40571	1.89226	1.6328
40572	3.78453	1.6522
40573	1.89226	1.48881
40574	2.61682	1.9072
40575	1.89226	1.8462
40576	1.89226	1.89434
40577	1.89226	1.37145
40578	1.89226	1.86001
40579	1.89226	0.100693
40580	1.89226	0.05
40581	1.89226	0.0668902
40582	1.89226	0.0673171
40583	3.78452	0.115216
40584	1.89226	0.111758
40585	1.89226	0.0791027
40586	1.89226	0.0606081
40587	1.89226	0.0880603
40588	1.89226	0.110215
40589	1.89226	0.108557
40590	1.89227	0.0516462
40591	1.89227	0.0558844
40592	1.89227	0.0776669
40593	1.89227	0.0815829
40594	1.89227	0.05
40595	1.89227	0.05
40596	0.80447	0.11487
40597	0.534241	0.134901

40598	0.995175	0.15247
40599	0.92204	0.0704574
40600	0.922042	0.0719711
40601	0.534241	0.0746094
40602	0.804468	0.119317
40603	0.995176	0.0565618
40604	0.71543	0.141309
40605	0.71543	0.0669656
40606	0.813988	0.0665427
40607	0.813988	0.0720492
40608	0.922045	0.05
40609	0.942899	0.0692925
40610	0.946132	0.05
40611	0.946132	0.05
40612	1.05169	0.05
40613	0.936661	0.0538431
40614	0.922045	0.05
40615	0.946133	0.05
40616	1.01665	0.05
40617	1.13008	0.0590416
40618	0.851529	0.05
40619	0.851529	0.05
40620	1.41908	2.1
40621	1.77723	2.1
40622	1.90485	2.1
40623	1.90485	2.1
40624	1.30237	2.1
40625	1.30237	2.1
40626	1.77723	2.1
40627	2.0055	2.1
40628	1.90485	2.1
40629	2.17158	2.1
40630	3.91035	2.1
40631	0.850906	2.1
40632	0.850906	2.1
40633	0.85668	2.1
40634	0.85668	2.1
40635	0.998045	2.1
40636	0.952425	2.1
40637	0.74367	2.1
40638	0.709541	2.1
40639	0.74367	2.1
40640	0.998045	2.1
40641	0.709541	2.1
40642	0.952425	2.1
40643	1.90485	2.1
40644	2.28563	2.1
40645	2.26282	2.1
40646	2.17158	2.1
40647	1.99609	2.1
40648	1.90485	2.1
40649	2.00098	0.128559
40650	1.90462	0.0900502
40651	1.60057	0.104799
40652	1.79224	0.14017
40653	3.69686	0.108446
40654	2.65654	0.135425
40655	1.79224	0.0963618

40656	2.22332	0.098715
40657	2.22332	0.118858
40658	2.24163	0.115848
40659	2.24163	0.121374
40660	1.89226	0.126942
40661	1.89226	0.114172
40662	1.89226	0.0966326
40663	1.89226	0.135733
40664	3.78452	0.12603
40665	1.89226	0.0888723
40666	1.89226	0.174772
40667	1.89226	0.110393
40668	1.89226	0.14489
40669	1.89226	0.110182
40670	1.89226	0.144889
40671	2.24163	0.165189
40672	1.03645	0.143227
40673	1.79224	0.153523
40674	1.79224	0.148444
40675	1.54283	0.172608
40676	1.54283	0.161455
40677	0.946132	0.135466
40678	0.946132	0.131403
40679	0.946129	0.141601
40680	0.946129	0.133485
40681	0.94613	0.118254
40682	0.94613	0.13373
40683	0.946131	0.150688
40684	0.946131	0.15163
40685	0.94613	0.133631
40686	0.94613	0.1664
40687	0.946131	0.130758
40688	0.946131	0.110878
40689	1.89226	2.1
40690	1.89226	2.1
40691	1.89226	2.1
40692	1.89226	2.1
40693	3.78453	2.1
40694	1.52441	2.1
40695	1.30347	2.1
40696	1.52441	2.1
40697	1.58401	2.1
40698	1.2627	2.1
40699	1.3223	2.1
40700	1.89226	2.1
40701	2.72787	2.1
40702	2.72787	2.1
40703	2.74762	2.1
40704	1.89226	2.1
40705	1.91202	2.1
40706	1.89227	2.1
40707	1.89227	2.1
40708	1.89227	2.1
40709	1.89227	2.1
40710	3.78453	2.1
40711	0.56414	2.1
40712	0.561557	2.1
40713	0.527284	2.1

40714	0.541143	2.1
40715	0.527276	2.1
40716	0.631685	2.1
40717	1.52441	2.1
40718	1.00232	2.1
40719	0.784495	2.1
40720	0.842304	2.1
40721	0.721569	2.1
40722	1.36393	2.1
40723	1.36393	2.1
40724	0.946132	2.1
40725	0.946132	2.1
40726	1.36393	2.1
40727	1.3594	2.1
40728	0.946132	2.1
40729	0.946132	2.1
40730	0.946107	2.1
40731	1.35936	2.1
40732	1.36397	2.1
40733	0.946158	2.1
40734	1.89226	2.1
40735	1.89226	2.1
40736	1.89226	2.1
40737	1.89226	2.1
40738	3.78453	2.1
40739	2.20802	2.1
40740	1.05677	2.1
40741	2.30479	2.1
40742	0.978699	2.1
40743	3.18672	2.1
40744	0.94613	2.1
40745	0.94613	2.1
40746	0.669866	2.1
40747	0.669866	2.1
40748	0.681588	2.1
40749	0.669865	2.1
40750	0.946133	2.1
40751	0.946133	2.1
40752	0.94613	2.1
40753	0.681589	2.1
40754	0.946131	2.1
40755	0.669867	2.1
40756	2.73514	2.1
40757	1.78301	2.1
40758	1.90618	2.1
40759	2.83464	2.1
40760	4.64132	2.1
40761	2.73514	2.1
40762	1.89226	2.1
40763	1.89226	2.1
40764	2.83464	2.1
40765	4.6274	2.1
40766	2.03915	2.1
40767	2.26616	2.1
40768	2.23549	2.1
40769	2.01704	2.1
40770	4.2832	2.1
40771	2.03916	2.1

40772	1.55766	2.1
40773	1.53728	2.1
40774	2.01704	2.1
40775	3.5747	2.1
40776	2.23551	2.1
40777	2.01705	2.1
40778	1.99823	2.1
40779	2.21189	2.1
40780	4.22894	2.1
40781	1.51788	2.1
40782	2.01705	2.1
40783	1.53729	2.1
40784	1.53729	2.1
40785	1.99823	2.1
40786	1.99823	2.1
40787	2.588	2.1
40788	1.45581	2.1
40789	1.45581	2.1
40790	1.44207	2.1
40791	2.54949	2.1
40792	2.53574	2.1
40793	0.98641	2.1
40794	0.98641	2.1
40795	1.00371	2.1
40796	1.00371	2.1
40797	0.749992	2.1
40798	0.727904	2.1
40799	1.26221	2.1
40800	1.27474	2.1
40801	1.26221	2.1
40802	0.749993	2.1
40803	0.727904	2.1
40804	1.27474	2.1
40805	1.23105	0.705584
40806	1.35435	1.68179
40807	1.89226	1.36113
40808	1.89226	0.929927
40809	1.89226	1.41999
40810	1.7043	1.00179
40811	1.89226	1.85357
40812	1.89226	1.3827
40813	1.89226	0.933753
40814	1.89226	1.84692
40815	3.78453	1.6981
40816	1.89226	0.757008
40817	1.89226	1.83717
40818	1.89226	1.31382
40819	1.89226	1.29629
40820	1.89226	1.49809
40821	1.89226	1.46737
40822	0.946131	0.714004
40823	1.40945	0.646607
40824	0.94613	0.926052
40825	0.94613	1.5258
40826	0.946129	1.54052
40827	0.946129	1.00047
40828	0.946132	1.48023
40829	1.40945	0.600966

40830	0.94613	0.957911
40831	0.94613	1.04423
40832	0.946131	0.950497
40833	0.94613	0.716831
40834	0.946133	0.90679
40835	0.946133	0.875133
40836	0.946132	1.51702
40837	0.946132	1.761
40838	0.946132	1.75002
40839	0.946132	1.13525
40840	0.946133	1.42405
40841	0.946133	0.884961
40842	0.946132	1.35189
40843	0.946132	1.40687
40844	0.946133	0.955153
40845	0.946133	1.18206
40846	1.89226	1.25346
40847	1.89226	1.90598
40848	1.89226	1.70461
40849	1.89226	1.5512
40850	1.89226	1.82083
40851	1.89226	1.69273
40852	1.89226	2.1
40853	1.89226	2.1
40854	1.89226	2.1
40855	1.89226	2.1
40856	1.89226	2.1
40857	1.89226	2.1
40858	1.89226	2.1
40859	1.89226	2.1
40860	1.89226	2.1
40861	1.89226	2.1
40862	3.78453	2.1
40863	1.66647	2.1
40864	1.15399	2.1
40865	1.25443	2.1
40866	1.26599	2.1
40867	1.58697	2.1
40868	1.66647	2.1
40869	1.66647	2.1
40870	1.47446	2.1
40871	1.55433	2.1
40872	1.74241	2.1
40873	3.2208	2.1
40874	1.89227	2.1
40875	1.89227	2.1
40876	1.89227	2.1
40877	1.89227	2.1
40878	3.78453	2.1
40879	0.946132	2.1
40880	0.946132	2.1
40881	0.946132	2.1
40882	0.946132	2.1
40883	0.946132	2.1
40884	0.946132	2.1
40885	0.946133	2.1
40886	0.946133	2.1
40887	0.946132	2.1

40888	0.946132	2.1
40889	0.946133	2.1
40890	0.946133	2.1
40891	0.625067	2.1
40892	0.61907	2.1
40893	0.896913	2.1
40894	0.946199	2.1
40895	0.806287	2.1
40896	0.677685	2.1
40897	0.750657	2.1
40898	0.750657	2.1
40899	0.646004	2.1
40900	0.890236	2.1
40901	0.61049	2.1
40902	0.888979	2.1
40903	1.6223	2.1
40904	1.98034	2.1
40905	1.75003	2.1
40906	1.55433	2.1
40907	1.75003	2.1
40908	1.55433	2.1
40909	1.88602	2.1
40910	2.08149	2.1
40911	2.07991	2.1
40912	1.88473	2.1
40913	2.08149	2.1
40914	1.88602	2.1
40915	1.88458	2.1
40916	2.08149	2.1
40917	1.7069	2.1
40918	1.56284	2.1
40919	2.08149	2.1
40920	1.88458	2.1
40921	1.5955	2.1
40922	1.89226	2.1
40923	1.91975	2.09857
40924	1.62299	2.1
40925	1.89226	2.1
40926	1.5955	2.1
40927	1.89226	2.1
40928	1.89226	2.1
40929	1.89226	2.02195
40930	1.89226	2.1
40931	1.89226	2.01322
40932	1.89226	2.1
40933	1.89226	2.1
40934	1.89226	2.1
40935	1.89226	2.09844
40936	1.89226	2.1
40937	1.89226	2.08221
40938	1.89226	2.1
40939	1.62299	2.1
40940	1.89226	2.1
40941	1.71752	2.1
40942	1.44824	2.1
40943	1.89226	2.1
40944	1.62299	2.1
40945	1.89226	0.99976

40946	2.8317	0.630563
40947	1.89226	0.882594
40948	2.64055	0.581532
40949	4.53281	0.867714
40950	2.8317	0.726329
40951	1.83151	0.443864
40952	2.01001	0.498651
40953	2.01001	0.896421
40954	2.64055	0.350275
40955	2.64055	0.69705
40956	0.946132	0.253197
40957	0.946132	0.325012
40958	1.16167	0.287588
40959	1.16167	0.265324
40960	1.16167	0.217737
40961	1.32027	0.216014
40962	0.946131	0.297602
40963	0.946131	0.309091
40964	0.94613	0.246031
40965	1.16167	0.190022
40966	0.946131	0.257068
40967	1.32027	0.220559
40968	2.32335	0.23244
40969	1.83151	0.233241
40970	2.38525	0.193205
40971	2.64055	0.26871
40972	1.57621	0.171811
40973	1.83151	0.199015
40974	2.25677	2.1
40975	1.88158	2.1
40976	1.86856	2.1
40977	2.0815	2.1
40978	1.70303	2.1
40979	1.88158	2.1
40980	2.50381	2.1
40981	1.89227	2.1
40982	2.25677	2.1
40983	2.25677	2.1
40984	1.89227	2.1
40985	1.89227	2.1
40986	1.89226	0.0998692
40987	1.50899	0.0997944
40988	2.13659	0.0976415
40989	1.50899	0.144377
40990	2.51986	0.0783992
40991	1.89226	0.128357
40992	0.946131	0.150435
40993	0.946131	0.109893
40994	1.06829	0.0940288
40995	0.895375	0.0820842
40996	0.94613	0.0910381
40997	0.94613	0.149744
40998	1.33922	0.0956763
40999	0.895376	0.0789289
41000	0.946131	0.109298
41001	1.33922	0.106816
41002	0.946131	0.111478
41003	1.06829	0.120339

41004	0.841467	0.189792
41005	0.578428	0.224909
41006	1.18079	0.181114
41007	0.920237	0.226079
41008	0.772317	0.147002
41009	0.856555	0.301685
41010	0.910271	0.189306
41011	0.910271	0.302189
41012	0.975752	0.186984
41013	0.536929	0.156527
41014	0.81952	0.25885
41015	1.15252	0.183188
41016	1.89226	2.1
41017	2.51431	2.1
41018	1.89226	2.1
41019	1.89226	2.1
41020	2.78815	2.1
41021	2.78815	2.1
41022	2.0815	2.1
41023	2.0516	2.1
41024	2.0815	2.1
41025	3.06699	2.1
41026	2.76576	2.1
41027	2.8002	2.1
41028	1.94112	2.1
41029	1.89226	2.1
41030	2.20358	2.1
41031	1.89226	2.1
41032	4.09584	2.1
41033	1.09154	2.1
41034	2.62647	2.1
41035	2.20358	2.1
41036	2.20358	2.1
41037	1.52314	2.1
41038	1.52314	2.1
41039	1.31323	2.1
41040	1.31323	2.1
41041	0.946132	2.1
41042	0.946132	2.1
41043	0.946132	2.1
41044	0.946132	2.1
41045	1.31323	2.1
41046	1.10179	2.1
41047	1.31323	2.1
41048	0.946132	2.1
41049	1.10179	2.1
41050	0.946133	2.1
41051	1.89226	1.17531
41052	1.89226	1.8291
41053	1.89226	1.75696
41054	1.89226	1.93283
41055	1.89226	1.05483
41056	1.89226	1.72835
41057	1.35126	1.97624
41058	1.35126	2.04368
41059	0.903285	1.71841
41060	0.903285	1.86166
41061	1.30841	1.84337

41062	0.903313	2.02113
41063	0.946132	1.91703
41064	0.946132	1.67676
41065	0.946132	1.82195
41066	0.903312	1.86417
41067	1.30841	1.7954
41068	0.946133	1.78117
41069	2.62097	2.1
41070	2.72561	2.1
41071	2.39016	2.1
41072	2.51062	2.1
41073	5.11577	2.1
41074	2.51062	2.1
41075	2.58286	2.08321
41076	2.40107	2.1
41077	2.40107	2.1
41078	2.72561	2.1
41079	2.72561	2.1
41080	2.39016	2.1
41081	2.51062	2.1
41082	2.20242	2.1
41083	2.34107	2.1
41084	4.73124	2.1
41085	2.51062	2.1
41086	2.40107	2.081
41087	2.34107	2.1
41088	2.25197	2.1
41089	4.74215	2.1
41090	2.75218	2.1
41091	1.89226	2.1
41092	2.3349	2.1
41093	2.3349	2.1
41094	1.89226	2.1
41095	1.89226	2.1
41096	1.46129	2.1
41097	0.996308	2.1
41098	1.88649	2.1
41099	1.70302	2.1
41100	2.95419	2.1
41101	1.89227	2.1
41102	1.89227	2.1
41103	1.89227	2.1
41104	1.89227	2.1
41105	3.78453	2.1
41106	0.646558	2.1
41107	0.646557	2.1
41108	0.60697	2.1
41109	0.60697	2.1
41110	0.587917	2.1
41111	0.519747	2.1
41112	0.586443	2.1
41113	0.733782	2.1
41114	0.733793	2.1
41115	0.587924	2.1
41116	0.519739	2.1
41117	0.586436	2.1
41118	2.1642	2.1
41119	1.42867	2.1

41120	2.28847	2.1
41121	1.68299	2.1
41122	3.71714	2.1
41123	1.89226	2.1
41124	1.89226	2.1
41125	1.89226	2.1
41126	1.89226	2.1
41127	3.78453	2.1
41128	0.946131	2.1
41129	0.946131	2.1
41130	1.24994	2.1
41131	1.24994	2.1
41132	1.11582	2.1
41133	1.24994	2.1
41134	0.946133	2.1
41135	0.946133	2.1
41136	0.946131	2.1
41137	1.24994	2.1
41138	0.94613	2.1
41139	1.11582	2.1
41140	2.28847	2.1
41141	1.53629	2.1
41142	1.57498	2.1
41143	1.47417	2.1
41144	2.42221	2.1
41145	2.28847	2.1
41146	1.89226	2.1
41147	1.89226	2.1
41148	1.89226	2.1
41149	1.89226	2.1
41150	3.78453	2.1
41151	1.89226	2.1
41152	2.49988	2.1
41153	2.71233	2.1
41154	2.71233	2.1
41155	1.89226	2.1
41156	1.89226	2.1
41157	0.924534	2.1
41158	0.924534	2.1
41159	0.990395	2.1
41160	0.990395	2.1
41161	0.946131	2.1
41162	0.946131	2.1
41163	0.924505	2.1
41164	0.968797	2.1
41165	0.924505	2.1
41166	0.946131	2.1
41167	0.968797	2.1
41168	0.946131	2.1
41169	0.924533	2.1
41170	0.924533	2.1
41171	0.948264	2.1
41172	0.945345	2.1
41173	0.851541	2.1
41174	0.851541	2.1
41175	0.958934	2.1
41176	1.02617	2.1
41177	0.936668	2.1

41178	1.04829	2.1
41179	0.924505	2.1
41180	0.946131	2.1
41181	2.28431	0.477675
41182	1.48035	0.203297
41183	1.59971	0.310649
41184	1.48035	0.393738
41185	2.33818	0.269311
41186	2.21882	0.342616
41187	2.28431	0.439611
41188	1.89226	0.549638
41189	1.89226	0.733925
41190	2.3968	0.374715
41191	4.28906	0.457166
41192	2.28431	0.445628
41193	1.70303	0.209816
41194	1.5428	0.227393
41195	1.59971	0.3194
41196	2.31152	0.285247
41197	2.3968	0.357516
41198	1.99823	2.1
41199	2.21189	2.1
41200	2.18937	2.1
41201	1.979 2.1	
41202	4.19089	2.1
41203	0.999114	2.1
41204	0.9895	2.1
41205	0.758942	2.1
41206	0.749358	2.1
41207	0.874236	2.1
41208	0.874236	2.1
41209	0.874236	2.1
41210	0.874236	2.1
41211	0.9895	2.1
41212	0.749358	2.1
41213	0.758942	2.1
41214	0.999114	2.1
41215	2.18937	2.1
41216	1.979 2.1	
41217	1.95416	2.1
41218	2.162 2.1	
41219	4.14099	2.1
41220	1.95416	2.1
41221	1.49871	2.1
41222	1.47712	2.1
41223	1.49871	2.1
41224	1.9574	2.1
41225	1.979 2.1	
41226	1.68508	0.05
41227	1.64984	0.05
41228	2.67711	0.05
41229	2.76407	0.05
41230	4.41391	0.05
41231	1.84227	0.05
41232	2.76407	0.05
41233	2.76407	0.05
41234	1.85007	0.05
41235	2.72769	0.05

41236	1.80812	0.05
41237	1.94516	2.1
41238	1.89226	2.1
41239	1.89226	2.1
41240	1.89226	2.1
41241	1.98853	2.1
41242	1.98852	2.1
41243	1.28095	2.1
41244	2.0815	2.1
41245	2.29123	2.1
41246	2.06833	2.1
41247	1.08212	2.1
41248	0.976848	2.1
41249	1.89226	2.1
41250	1.89226	2.1
41251	1.89226	2.1
41252	1.89226	2.1
41253	1.89226	2.1
41254	1.89226	2.1
41255	1.13537	2.1
41256	1.18233	2.1
41257	0.946132	2.1
41258	0.946132	2.1
41259	0.946132	2.1
41260	0.946132	2.1
41261	0.994264	2.1
41262	1.18233	2.1
41263	1.13537	2.1
41264	0.946131	2.1
41265	0.946132	2.1
41266	0.994264	2.1
41267	0.953204	2.1
41268	0.953204	2.1
41269	0.966515	2.1
41270	0.966515	2.1
41271	1.30583	2.1
41272	1.14561	2.1
41273	0.640475	2.1
41274	0.774103	2.1
41275	1.30584	2.1
41276	0.774101	2.1
41277	0.640474	2.1
41278	1.14562	2.1
41279	0.946131	2.1
41280	0.946131	2.1
41281	0.946131	2.1
41282	0.946131	2.1
41283	0.946128	2.1
41284	0.946128	2.1
41285	0.946133	2.1
41286	0.946133	2.1
41287	0.94613	2.1
41288	0.94613	2.1
41289	0.946131	2.1
41290	0.946131	2.1
41291	0.946132	0.05
41292	0.946132	0.05
41293	0.946131	0.05

41294	0.946131	0.05
41295	0.946131	0.05
41296	0.946131	0.05
41297	0.946132	0.05
41298	0.946132	0.05
41299	0.946131	0.05
41300	0.946131	0.05
41301	0.946132	0.05
41302	0.946132	0.05
41303	0.946131	0.05
41304	0.946131	0.05
41305	0.946131	0.05
41306	0.946131	0.05
41307	0.94613	0.05
41308	0.94613	0.05
41309	0.946132	0.05
41310	0.946132	0.05
41311	0.946131	0.05
41312	0.946131	0.05
41313	0.946131	0.05
41314	0.946131	0.05
41315	2.57882	0.103513
41316	1.48676	0.113335
41317	1.48797	0.0949965
41318	2.64281	0.112222
41319	4.12957	0.0989268
41320	2.56323	0.088346
41321	2.64281	0.1074
41322	2.51807	0.0845786
41323	1.36324	0.0897737
41324	1.48797	0.0913062
41325	1.48797	0.106266
41326	1.04367	2.1
41327	2.42504	2.1
41328	2.65164	2.1
41329	1.49479	2.1
41330	3.91983	2.1
41331	2.15622	2.1
41332	1.70539	2.1
41333	2.02369	2.1
41334	2.39039	2.1
41335	4.17992	2.1
41336	0.966979	2.1
41337	1.7054	2.1
41338	2.0237	2.1
41339	1.31502	2.1
41340	2.99067	2.1
41341	2.02369	2.1
41342	2.65637	2.09894
41343	2.39039	2.09389
41344	2.39039	2.1
41345	2.29332	2.09408
41346	2.29332	2.1
41347	2.0237	2.1
41348	1.72116	2.1
41349	1.58465	2.1
41350	1.31502	2.1
41351	2.29332	2.1

41352	2.0237	2.1
41353	1.0372	0.05
41354	2.32462	0.05
41355	2.2291	0.05
41356	2.2291	0.05
41357	1.17464	0.05
41358	1.17464	0.05
41359	2.68289	0.05
41360	1.07805	0.05
41361	1.70302	0.05
41362	2.06653	0.05
41363	3.5779	0.05
41364	1.89226	0.120776
41365	1.89226	0.182486
41366	1.89226	0.164581
41367	1.89226	0.0990597
41368	3.78453	0.131656
41369	1.89226	0.060746
41370	1.89226	0.0810759
41371	1.89226	0.0783275
41372	1.89226	0.0672185
41373	1.89226	0.0743188
41374	1.89226	0.0636871
41375	1.89225	0.0623506
41376	1.89225	0.0733454
41377	1.89225	0.0747404
41378	1.89225	0.062941
41379	1.89225	0.0758127
41380	1.89225	0.0654334
41381	0.94613	0.103739
41382	0.94613	0.127765
41383	0.94613	0.186505
41384	0.94613	0.144209
41385	0.946128	0.115993
41386	0.946128	0.133204
41387	0.946132	0.186208
41388	0.946132	0.101843
41389	0.94613	0.122998
41390	0.94613	0.142985
41391	0.94613	0.131905
41392	0.94613	0.112029
41393	1.29417	2.1
41394	1.32732	2.1
41395	1.32316	1.02768
41396	1.34973	2.1
41397	2.65048	2.1
41398	1.89226	1.5148
41399	1.89226	2.1
41400	1.89226	1.37255
41401	1.89226	2.1
41402	3.78453	2.07742
41403	1.89226	2.1
41404	1.89226	2.1
41405	1.89226	2.1
41406	1.89226	2.1
41407	3.78453	2.1
41408	1.32732	2.1
41409	1.13006	2.1

41410	1.13358	2.1
41411	1.11117	2.1
41412	1.34973	2.1
41413	1.32732	2.1
41414	1.32316	2.01345
41415	1.34973	2.1
41416	1.34066	1.65395
41417	1.36601	2.1
41418	2.68918	2.1
41419	1.89226	2.02991
41420	1.89226	2.1
41421	1.89226	0.511703
41422	1.89226	2.1
41423	3.78453	2.09241
41424	1.89226	2.1
41425	1.89226	2.1
41426	1.89226	2.1
41427	1.89226	2.1
41428	1.89226	2.1
41429	1.89226	2.1
41430	0.624134	2.1
41431	0.624134	2.1
41432	0.624334	2.1
41433	0.624334	2.1
41434	0.56503	2.1
41435	0.573605	2.1
41436	0.674864	2.1
41437	0.683007	2.1
41438	0.683007	2.1
41439	0.573605	2.1
41440	0.565029	2.1
41441	0.674864	2.1
41442	2.16633	2.1
41443	1.40846	2.1
41444	2.13977	2.1
41445	1.40771	2.1
41446	3.57404	2.1
41447	2.13977	2.1
41448	1.29932	2.1
41449	1.29932	2.1
41450	1.40771	2.1
41451	1.98957	2.1
41452	2.09796	2.1
41453	2.6044	0.182863
41454	1.85548	0.223119
41455	1.89226	0.121954
41456	1.89226	0.316138
41457	3.74774	0.217151
41458	1.89226	0.133652
41459	1.89226	0.364567
41460	1.89226	0.17985
41461	1.89226	0.232023
41462	3.78453	0.178913
41463	1.89226	2.1
41464	2.64107	2.1
41465	1.89226	2.1
41466	2.78977	2.1
41467	1.89226	2.1

41468	2.78977	2.1
41469	1.89226	2.1
41470	1.89226	2.1
41471	1.89226	2.1
41472	1.89226	2.09658
41473	1.89226	2.1
41474	1.89226	2.09781
41475	2.43173	2.09643
41476	1.41238	2.1
41477	2.0815	2.1
41478	1.98048	2.1
41479	1.62014	2.1
41480	1.50086	2.1
41481	0.946129	0.578761
41482	0.946129	0.497376
41483	1.27636	0.403363
41484	1.1984	0.319096
41485	0.946131	0.32624
41486	0.946131	0.492018
41487	1.27636	0.604719
41488	1.27636	0.363553
41489	0.946131	0.42355
41490	1.27636	0.420701
41491	0.946131	0.4432
41492	1.1984	0.43051
41493	1.99569	0.206207
41494	2.55272	0.415033
41495	1.77403	0.233756
41496	1.70303	0.292035
41497	3.05772	0.24149
41498	2.55121	0.311704
41499	0.946128	0.431054
41500	0.946128	0.499073
41501	1.38752	0.367208
41502	1.27636	0.278898
41503	0.94613	0.284885
41504	0.94613	0.428146
41505	1.38752	0.51725
41506	1.38752	0.314396
41507	0.946131	0.381824
41508	1.38752	0.377095
41509	0.946131	0.40173
41510	1.27636	0.382802
41511	2.77504	0.346959
41512	1.99569	0.175834
41513	2.29794	0.215285
41514	1.99569	0.275362
41515	2.85497	0.200857
41516	2.55272	0.255789
41517	1.53284	2.1
41518	1.89226	2.1
41519	1.89226	2.1
41520	1.89226	2.1
41521	1.54763	2.1
41522	1.54763	2.1
41523	0.946131	2.1
41524	0.946131	2.1
41525	0.946127	2.1

41526	0.946127	2.1
41527	0.946127	2.1
41528	0.946127	2.1
41529	0.946131	2.1
41530	0.946131	2.1
41531	0.94613	2.1
41532	0.94613	2.1
41533	0.946129	2.1
41534	0.946129	2.1
41535	0.751754	2.1
41536	0.751754	2.1
41537	0.771562	2.1
41538	0.771561	2.1
41539	1.0268	2.1
41540	0.95482	2.1
41541	0.528937	2.1
41542	0.496512	2.1
41543	0.528936	2.1
41544	1.0268	2.1
41545	0.954821	2.1
41546	0.49651	2.1
41547	2.64075	0.264883
41548	1.35181	1.06568
41549	1.35181	0.31758
41550	1.83823	0.387679
41551	2.28373	0.416248
41552	2.88566	1.15711
41553	0.493018	1.18788
41554	0.675903	0.904992
41555	1.14187	0.299815
41556	0.973937	0.0680723
41557	0.821166	0.368672
41558	0.821166	1.27003
41559	0.828674	1.65847
41560	0.828674	0.256742
41561	0.973937	0.550955
41562	0.493018	0.57958
41563	0.675903	0.501699
41564	1.14187	0.545381
41565	1.89226	2.1
41566	2.11667	2.1
41567	1.91184	2.1
41568	1.89226	2.1
41569	2.13625	2.1
41570	2.11667	2.1
41571	1.89226	2.1
41572	1.89226	2.1
41573	1.89226	2.1
41574	1.89226	2.1
41575	3.78453	2.1
41576	0.907729	2.1
41577	0.907729	2.1
41578	1.04459	2.1
41579	0.890955	2.1
41580	0.988181	2.1
41581	0.841856	2.1
41582	0.946133	2.1
41583	0.946133	2.1

41584	1.04072	2.1
41585	1.1064	2.1
41586	0.89099	2.1
41587	0.851541	2.1
41588	1.89225	2.1
41589	2.13623	2.1
41590	1.91044	2.1
41591	1.89225	2.1
41592	2.15442	2.1
41593	2.13623	2.1
41594	1.89225	2.1
41595	1.89225	2.1
41596	1.89225	2.1
41597	1.89225	2.1
41598	1.89225	2.1
41599	1.89225	2.1
41600	0.907723	2.1
41601	0.907723	2.1
41602	1.07536	2.1
41603	1.07814	2.1
41604	0.998499	2.1
41605	1.03575	2.1
41606	0.946126	2.1
41607	0.946126	2.1
41608	1.04072	2.1
41609	1.14025	2.1
41610	0.851528	2.1
41611	0.898284	2.1
41612	1.89226	0.0578432
41613	1.89226	0.133908
41614	1.89226	0.0923949
41615	1.89226	0.0883598
41616	1.89226	0.0789727
41617	1.89226	0.0735465
41618	2.53008	2.1
41619	1.94534	2.1
41620	1.98702	2.1
41621	1.98858	2.1
41622	2.52852	2.1
41623	2.53008	2.1
41624	1.03267	2.1
41625	1.12591	2.1
41626	1.2959	2.1
41627	1.28992	2.1
41628	1.10851	2.1
41629	1.33078	2.1
41630	1.10661	2.1
41631	1.10661	2.1
41632	0.982208	2.1
41633	1.28992	2.1
41634	1.03267	2.1
41635	1.23262	2.1
41636	2.2712	2.1
41637	1.89226	2.1
41638	1.95954	2.1
41639	1.95954	2.1
41640	1.89226	2.1
41641	1.89226	2.1

41642	1.89226	2.1
41643	1.89226	2.1
41644	1.89226	2.1
41645	1.89226	2.1
41646	3.78453	2.1
41647	1.33887	2.1
41648	1.25395	2.1
41649	0.88327	2.1
41650	0.676665	2.1
41651	0.84881	2.1
41652	0.987503	2.1
41653	1.13243	2.1
41654	1.13243	2.1
41655	1.15338	2.1
41656	0.849966	2.1
41657	0.793074	2.1
41658	1.11957	2.1
41659	0.946129	2.1
41660	0.946129	2.1
41661	1.28254	2.1
41662	1.1356	2.1
41663	1.1356	2.1
41664	1.1356	2.1
41665	0.946131	2.1
41666	0.946131	2.1
41667	0.946131	2.1
41668	1.28254	2.1
41669	1.1356	2.1
41670	0.946131	2.1
41671	1.89227	2.1
41672	1.89227	2.1
41673	1.89227	2.1
41674	1.89227	2.1
41675	3.78453	2.1
41676	0.936287	0.05
41677	1.01399	0.05
41678	0.959415	0.05
41679	0.959415	0.05
41680	0.910786	0.05
41681	1.38301	0.05
41682	1.0286	0.05
41683	0.535742	0.05
41684	0.505202	0.05
41685	0.937685	0.05
41686	1.11066	0.05
41687	1.46907	0.05
41688	1.00546	0.05
41689	1.01524	0.05
41690	0.94613	0.05
41691	0.94613	0.05
41692	1.05018	0.05
41693	0.914025	0.05
41694	0.913512	0.05
41695	0.851542	0.05
41696	1.05385	0.05
41697	1.05494	0.05
41698	0.91404	0.05
41699	0.91404	0.05

41700	2.03253	2.07453
41701	1.82325	0.475583
41702	1.93938	2.1
41703	1.73756	0.364388
41704	3.77009	1.40176
41705	1.82325	0.36565
41706	1.47562	1.9495
41707	1.73756	0.418464
41708	1.3868	1.55676
41709	3.21317	0.563783
41710	1.93938	2.1
41711	1.73756	0.487097
41712	1.85293	2.07834
41713	1.64279	0.419505
41714	3.59049	1.12223
41715	1.73756	0.337266
41716	1.3868	1.51558
41717	1.64279	0.489536
41718	1.28419	1.85751
41719	3.02174	0.630054
41720	1.35048	2.09957
41721	1.37999	2.1
41722	1.34355	0.989544
41723	1.37854	2.1
41724	2.72354	2.1
41725	1.89226	1.62592
41726	1.89226	2.1
41727	1.89226	0.893012
41728	1.89226	2.07956
41729	3.78452	1.91493
41730	1.89226	2.1
41731	1.89226	2.1
41732	1.89226	2.06628
41733	1.89226	2.1
41734	3.78453	2.1
41735	1.37999	2.1
41736	1.18059	2.1
41737	1.17117	2.1
41738	1.17262	2.1
41739	1.37855	2.1
41740	1.37999	2.1
41741	1.34355	2.01329
41742	1.37854	2.1
41743	1.33079	1.56431
41744	1.3741	2.1
41745	2.71765	2.08417
41746	1.89226	1.95413
41747	1.89226	2.1
41748	1.89226	0.47455
41749	1.89226	2.1
41750	3.78453	2.02283
41751	1.89226	2.1
41752	1.89226	2.1
41753	1.89226	2.1
41754	1.89226	2.1
41755	3.78453	2.1
41756	0.640305	2.1
41757	0.640305	2.1

41758	0.643507	2.1
41759	0.643507	2.1
41760	0.590294	2.1
41761	0.594539	2.1
41762	0.689273	2.1
41763	0.687048	2.1
41764	0.687048	2.1
41765	0.594538	2.1
41766	0.590294	2.1
41767	0.689273	2.1
41768	2.01399	0.965328
41769	1.70141	1.65144
41770	1.83896	2.09918
41771	1.52482	2.085
41772	3.53881	1.85221
41773	1.70141	2.06596
41774	1.0752	2.1
41775	1.0752	2.024
41776	1.25669	2.1
41777	1.52482	2.1
41778	1.70632	2.1
41779	1.83896	1.85583
41780	1.52482	1.10862
41781	1.65244	1.3662
41782	1.34412	2.05393
41783	3.17725	1.50972
41784	1.26782	0.05
41785	2.90353	0.05
41786	2.54203	0.05
41787	2.41979	0.0604354
41788	1.77007	0.05
41789	1.703	0.0571013
41790	0.946118	0.0835151
41791	0.946118	0.0605532
41792	1.38643	0.0612337
41793	1.38644	0.05
41794	0.946117	0.0669298
41795	0.946117	0.0860207
41796	1.38644	0.05
41797	1.15545	0.0617405
41798	0.946131	0.0632664
41799	1.15546	0.0591746
41800	1.38642	0.0632297
41801	0.946105	0.0638602
41802	2.42256	0.168517
41803	2.63056	0.119103
41804	2.43174	0.135562
41805	2.25035	0.111789
41806	4.8543	0.11889
41807	2.58031	0.165933
41808	2.63056	0.126125
41809	2.43174	0.13433
41810	2.32127	0.0983056
41811	5.01205	0.136902
41812	2.43174	0.126028
41813	2.25035	0.101915
41814	2.11465	0.099429
41815	2.13042	0.0800643

41816	4.54639	0.103582
41817	2.43174	0.126119
41818	2.32127	0.0922044
41819	1.92997	0.0897268
41820	2.13041	0.0824949
41821	4.36171	0.0921105
41822	1.89227	2.1
41823	1.89227	2.1
41824	1.89227	2.1
41825	1.89227	2.1
41826	3.78453	2.1
41827	0.568724	2.1
41828	0.568724	2.1
41829	0.57678	2.1
41830	0.57678	2.1
41831	0.523796	2.1
41832	0.548137	2.1
41833	0.597368	2.1
41834	0.637877	2.1
41835	0.597367	2.1
41836	0.523796	2.1
41837	0.637877	2.1
41838	0.548137	2.1
41839	1.32674	2.1
41840	1.32674	2.1
41841	0.946132	2.1
41842	0.946132	2.1
41843	1.3195	2.1
41844	1.32674	2.1
41845	0.946132	2.1
41846	0.946132	2.1
41847	0.946132	2.1
41848	1.3195	2.1
41849	1.32674	2.1
41850	0.946133	2.1
41851	1.89227	2.1
41852	1.89227	2.1
41853	1.89227	2.1
41854	1.89227	2.1
41855	3.78453	2.1
41856	1.19473	2.1
41857	0.987132	2.1
41858	0.987132	2.1
41859	1.04759	2.1
41860	1.0894	2.1
41861	1.14986	2.1
41862	1.3195	2.1
41863	1.3195	2.1
41864	0.946132	2.1
41865	0.946132	2.1
41866	1.31185	2.1
41867	1.3195	2.1
41868	0.946132	2.1
41869	0.946132	2.1
41870	1.31185	2.1
41871	0.946133	2.1
41872	0.946132	2.1
41873	1.3195	2.1

41874	1.11256	1.17811
41875	1.70101	0.890748
41876	1.22297	1.11795
41877	1.22297	1.09947
41878	1.7252	1.14224
41879	1.7252	1.1225
41880	1.99298	1.0205
41881	1.70101	0.969511
41882	1.7252	1.20309
41883	2.18701	0.714476
41884	3.71818	0.927761
41885	1.03218	0.986323
41886	1.7252	0.777973
41887	1.11256	0.964725
41888	1.11256	0.942183
41889	1.7457	0.961864
41890	1.7457	0.940345
41891	1.7457	1.03032
41892	2.187	0.614464
41893	1.7252	0.824684
41894	1.7252	0.908005
41895	2.27351	0.773246
41896	2.27351	0.829741
41897	1.95544	0.304045
41898	1.35897	2.03126
41899	1.44859	0.852399
41900	1.44859	1.87816
41901	1.80709	0.295577
41902	1.80709	1.5422
41903	1.80709	0.220521
41904	1.17822	1.68551
41905	1.35897	0.363314
41906	1.35897	1.97572
41907	1.52782	0.208167
41908	1.52782	1.42955
41909	1.11978	1.62382
41910	0.903546	0.333409
41911	0.977719	1.83067
41912	1.2364	0.484252
41913	1.03812	0.221499
41914	1.03812	2.03682
41915	1.05938	0.18301
41916	1.05938	2.0891
41917	1.11978	0.317605
41918	1.2364	0.149655
41919	0.97772	1.19654
41920	0.903546	0.415963
41921	1.80709	2.01314
41922	1.81847	0.861627
41923	1.96029	1.77188
41924	1.52782	1.37406
41925	2.23956	0.495716
41926	1.80709	1.26767
41927	1.89226	0.298891
41928	2.32962	0.182875
41929	2.5992	0.186866
41930	1.89226	0.206845
41931	2.5992	0.264502

41932	1.89226	0.286619
41933	2.55791	0.05
41934	2.47141	0.05
41935	2.13515	0.05
41936	2.10939	0.05
41937	4.66731	0.05
41938	2.47141	0.05
41939	2.23337	0.05
41940	2.10939	0.05
41941	2.03438	0.05
41942	4.34276	0.05
41943	2.13512	0.05
41944	1.71027	0.05
41945	1.71027	0.05
41946	2.10936	0.05
41947	1.60137	0.05
41948	2.00046	0.05
41949	1.71027	0.05
41950	2.03435	0.05
41951	1.71027	0.05
41952	2.10936	0.05
41953	1.72067	0.05
41954	2.11976	0.05
41955	1.177	0.505932
41956	1.36405	0.930265
41957	2.25635	1.1049
41958	2.60151	0.35895
41959	3.6204	0.823085
41960	0.658869	0.0668791
41961	0.781697	0.0703298
41962	0.637037	0.0608055
41963	0.541148	0.0615262
41964	0.654682	0.05
41965	0.654682	0.15058
41966	0.641224	0.0574286
41967	0.641224	0.0716023
41968	0.637039	0.05
41969	0.781696	0.0530973
41970	0.658867	0.0716964
41971	0.541149	0.0660598
41972	1.89226	0.244763
41973	1.59152	0.163946
41974	1.89226	0.153555
41975	1.82125	0.153878
41976	1.89226	0.279044
41977	1.82125	0.253534
41978	2.18967	0.05
41979	1.89227	0.0646075
41980	2.72752	0.0503932
41981	2.72752	0.0540833
41982	1.89227	0.05
41983	1.89227	0.05
41984	2.18967	0.05
41985	1.92547	0.05
41986	1.92547	0.05
41987	2.72752	0.05
41988	1.30572	0.05
41989	2.10777	0.05

41990	2.39663	2.1
41991	1.92293	2.1
41992	1.96889	2.1
41993	2.45928	2.1
41994	4.36552	2.1
41995	1.02605	2.1
41996	1.92293	2.1
41997	1.9689	2.1
41998	1.04679	2.1
41999	2.99495	2.1
42000	1.96889	2.1
42001	2.45928	2.1
42002	2.50138	2.1
42003	1.99782	2.1
42004	4.47027	2.1
42005	1.9689	2.1
42006	1.03449	2.1
42007	1.07572	2.1
42008	1.04679	2.1
42009	1.99783	2.1
42010	1.9689	2.1
42011	1.72162	0.661119
42012	1.89186	0.317882
42013	2.29771	0.368739
42014	1.80149	0.168922
42015	4.01933	0.346906
42016	1.16482	0.664513
42017	1.97003	0.212903
42018	1.72162	0.185465
42019	1.72162	0.344106
42020	1.29313	0.287897
42021	1.29313	0.45795
42022	2.29771	0.517806
42023	2.84159	0.144585
42024	2.19622	0.246404
42025	1.97366	0.234028
42026	2.50906	0.25299
42027	2.29771	0.277873
42028	1.29313	0.608932
42029	2.4483	0.222769
42030	2.14223	0.205628
42031	2.14228	0.337593
42032	1.294	0.264458
42033	1.294	0.379505
42034	1.21495	0.05
42035	1.70303	0.05
42036	1.7924	0.05
42037	1.64002	0.05
42038	3.11963	0.05
42039	2.81021	0.05
42040	1.89226	0.05
42041	1.89226	0.05
42042	1.89226	0.05
42043	2.36406	0.05
42044	2.36406	0.05
42045	1.89226	0.05
42046	1.89226	0.05
42047	1.89226	0.05

42048	1.89226	0.05
42049	1.89226	0.05
42050	1.89226	0.05
42051	2.29976	0.05
42052	1.64002	0.05
42053	1.8959	0.05
42054	1.90873	0.05
42055	1.85594	0.05
42056	1.85594	0.05
42057	1.89226	0.05
42058	1.89226	0.05
42059	1.89226	0.05
42060	1.89226	0.05
42061	3.78453	0.05
42062	1.89226	0.991507
42063	2.52823	2.1
42064	2.83441	1.83516
42065	2.83441	2.07248
42066	1.89226	1.40194
42067	1.89226	1.88454
42068	1.04522	1.52109
42069	1.04522	1.33653
42070	1.12831	1.87501
42071	1.18364	2.1
42072	0.640525	1.57933
42073	0.924882	1.11639
42074	1.27877	1.8182
42075	1.43363	0.790354
42076	0.93474	0.912206
42077	1.58926	1.38138
42078	1.29573	1.74892
42079	0.664247	1.93852
42080	2.02191	2.1
42081	1.89226	2.1
42082	1.89226	2.1
42083	1.89226	2.1
42084	2.58639	2.1
42085	2.58639	2.1
42086	1.16759	2.1
42087	2.11828	2.1
42088	1.60936	2.1
42089	2.35846	2.1
42090	3.52605	2.1
42091	1.89226	2.1
42092	1.89226	2.1
42093	1.89226	2.1
42094	1.89226	2.1
42095	3.78453	2.1
42096	2.82765	0.0501142
42097	1.89809	0.0580574
42098	2.15971	0.0500743
42099	1.89809	0.0696307
42100	2.29233	0.05
42101	2.03071	0.05
42102	1.90664	0.0796767
42103	2.15971	0.05
42104	1.28956	0.0709544
42105	1.28956	0.05

42106	2.64414	0.0573096
42107	2.02706	0.05
42108	2.82765	0.05
42109	1.89226	0.0686358
42110	2.39968	0.0744535
42111	2.39968	0.116796
42112	1.89226	0.0531436
42113	1.89226	0.05
42114	0.978643	2.1
42115	0.703968	2.1
42116	0.946129	2.1
42117	0.946129	2.1
42118	0.703939	2.1
42119	0.703939	2.1
42120	0.946159	2.1
42121	0.946159	2.1
42122	0.978642	2.1
42123	0.94613	2.1
42124	0.94613	2.1
42125	0.703968	2.1
42126	1.89226	2.09933
42127	2.17309	1.63809
42128	2.17309	2.07727
42129	1.47475	1.91901
42130	1.89226	2.07503
42131	1.19393	1.84533
42132	1.89226	2.1
42133	1.89226	2.1
42134	1.89226	2.1
42135	1.89226	2.06018
42136	3.78452	2.1
42137	1.17426	2.1
42138	0.978644	2.1
42139	0.946129	2.1
42140	0.946129	2.1
42141	0.946131	2.1
42142	0.946131	2.1
42143	0.978643	2.1
42144	0.978642	2.1
42145	1.17426	2.1
42146	0.946131	2.1
42147	0.978642	2.1
42148	0.94613	2.1
42149	1.89226	2.09973
42150	1.89226	1.60221
42151	1.89226	2.05072
42152	2.17309	1.6863
42153	2.56346	2.03705
42154	2.94389	1.81469
42155	0.946131	2.09693
42156	0.946131	2.0447
42157	0.946128	2.1
42158	0.946128	2.09653
42159	0.946131	2.09249
42160	0.946131	2.04384
42161	0.946128	2.1
42162	0.946128	2.1
42163	0.94613	2.08571

42164	0.94613	2.08104
42165	0.946129	2.08731
42166	0.946129	2.07346
42167	1.89226	1.22416
42168	1.89226	0.829829
42169	1.89226	0.677937
42170	1.89226	0.515915
42171	3.78453	0.825997
42172	1.89226	0.788155
42173	1.89226	0.599575
42174	1.89226	0.769377
42175	1.89226	1.31671
42176	1.89226	0.759446
42177	1.89226	1.06537
42178	1.89226	0.651845
42179	1.30733	1.1356
42180	1.89226	1.37097
42181	1.92403	1.06841
42182	3.38635	0.867723
42183	1.89226	0.783079
42184	3.02887	1.14739
42185	2.0815	1.06181
42186	1.88422	1.21506
42187	2.0815	1.06964
42188	1.88422	1.15105
42189	1.89226	1.09333
42190	1.70221	0.799443
42191	1.89226	1.01642
42192	1.20068	1.26086
42193	1.89226	0.674979
42194	1.20068	0.284512
42195	1.01393	1.07464
42196	1.20067	0.413982
42197	1.89226	0.435436
42198	2.44958	1.48444
42199	1.01393	0.193614
42200	1.89226	0.202878
42201	1.89226	2.1
42202	1.89226	2.1
42203	1.89226	2.1
42204	1.89226	2.1
42205	1.89226	2.1
42206	1.89226	2.1
42207	1.89226	2.1
42208	1.89226	2.09207
42209	1.89226	2.1
42210	1.89226	2.1
42211	1.89226	2.1
42212	1.89226	2.1
42213	1.89226	2.1
42214	1.89226	2.1
42215	1.89226	2.1
42216	1.89226	2.1
42217	1.89226	2.1
42218	1.89226	2.1
42219	1.91161	2.1
42220	2.45782	2.1
42221	1.39653	2.1

42222	2.44652	2.1
42223	4.35812	2.1
42224	2.73092	2.1
42225	1.89226	2.1
42226	1.89226	2.1
42227	1.89226	2.05888
42228	2.44652	2.1
42229	2.44652	2.00891
42230	1.89226	2.1
42231	1.89226	2.1
42232	1.89226	2.1
42233	1.89226	2.1
42234	1.89226	2.1
42235	1.89226	2.1
42236	2.64924	2.1
42237	1.31109	2.1
42238	1.31109	2.1
42239	1.49958	2.1
42240	1.95039	2.1
42241	2.14451	2.1
42242	0.825157	2.1
42243	0.634856	2.1
42244	0.816836	2.1
42245	0.816836	2.1
42246	0.556743	2.1
42247	0.639142	2.1
42248	0.985106	2.1
42249	0.795045	2.1
42250	0.967461	2.1
42251	0.552494	2.1
42252	0.676829	2.1
42253	0.98803	2.1
42254	2.28564	2.1
42255	2.34084	2.04965
42256	2.3502	2.1
42257	2.42908	2.1
42258	4.69104	2.1
42259	2.34084	2.07208
42260	2.18077	2.09782
42261	2.42908	2.1
42262	2.2947	1.15173
42263	4.63554	2.08323
42264	2.3502	2.1
42265	2.42908	2.1
42266	2.38681	2.1
42267	2.50438	2.06485
42268	4.85458	2.1
42269	2.42908	2.1
42270	2.2947	1.58372
42271	2.50438	1.97652
42272	2.40627	2.1
42273	4.79908	2.00452
42274	1.89226	1.2046
42275	2.20711	1.89179
42276	1.89226	1.15045
42277	1.89226	1.94421
42278	2.02693	1.14654
42279	2.02693	1.92693

42280	1.2269	2.08061
42281	2.20711	1.89428
42282	2.0815	1.91317
42283	1.11506	2.09967
42284	3.22109	2.06719
42285	0.545802	2.0156
42286	0.547489	2.06528
42287	0.85914	1.91581
42288	1.11641	2.00877
42289	0.899974	2.02916
42290	0.849893	2.07185
42291	0.747885	2.01558
42292	0.747885	1.8526
42293	0.557184	2.05883
42294	0.887039	2.05864
42295	1.08948	2.05555
42296	0.59815	2.06765
42297	0.974139	2.1
42298	1.33385	2.1
42299	1.02368	2.1
42300	1.02368	2.1
42301	1.00833	2.1
42302	1.00833	2.1
42303	1.89226	1.85953
42304	1.89226	0.506298
42305	1.89226	2.06179
42306	1.89226	0.373726
42307	3.78453	0.712112
42308	1.74651	2.09623
42309	1.44605	0.30069
42310	1.63557	2.05168
42311	1.31993	0.453871
42312	3.08162	0.796935
42313	1.89226	2.08585
42314	1.89226	0.766183
42315	1.89226	1.92757
42316	1.89226	0.760443
42317	3.78452	0.650732
42318	1.63557	2.08168
42319	1.31992	0.376488
42320	1.52615	2.09806
42321	1.18549	0.675964
42322	2.82106	1.17133
42323	1.89226	0.383398
42324	2.58101	1.95868
42325	2.73797	2.08356
42326	2.73797	0.866331
42327	1.89226	2.09851
42328	1.89226	0.426858
42329	1.91762	0.233673
42330	1.44354	0.205261
42331	1.33622	0.216189
42332	1.33622	0.184003
42333	2.20248	0.306638
42334	2.20248	0.229269
42335	1.11712	2.1
42336	1.11712	2.1
42337	1.11421	2.1

42338	1.11421	2.1
42339	0.833871	2.1
42340	0.926528	2.1
42341	1.3048	2.1
42342	1.40335	2.1
42343	0.926527	2.1
42344	1.40335	2.1
42345	1.3048	2.1
42346	0.833871	2.1
42347	0.879657	2.1
42348	0.937695	2.1
42349	0.946129	2.1
42350	0.946129	2.1
42351	0.851541	2.1
42352	0.851541	2.1
42353	1.1126	2.1
42354	1.04519	2.1
42355	0.851486	2.1
42356	0.980501	2.1
42357	0.898251	2.1
42358	0.851528	2.1
42359	1.37756	2.1
42360	2.73081	2.1
42361	2.44311	2.1
42362	2.44311	2.1
42363	1.66428	2.1
42364	1.66428	2.1
42365	1.89226	2.1
42366	1.89226	2.1
42367	1.89226	2.1
42368	1.89226	2.1
42369	3.78452	2.1
42370	1.65781	2.1
42371	1.89837	2.1
42372	1.46713	2.1
42373	1.98027	2.1
42374	3.63808	2.1
42375	2.29054	2.1
42376	2.26369	2.08708
42377	1.98027	2.1
42378	2.15617	2.03159
42379	4.24396	2.1
42380	1.89226	2.1
42381	1.89226	2.08762
42382	1.89226	2.1
42383	1.89226	2.07333
42384	3.78452	2.1
42385	0.946131	2.1
42386	0.946131	2.1
42387	0.946128	2.1
42388	0.946128	2.1
42389	0.946129	2.1
42390	0.946129	2.1
42391	0.94613	2.1
42392	0.94613	2.1
42393	0.94613	2.1
42394	0.94613	2.1
42395	0.94613	2.1

42396	0.94613	2.1
42397	0.812186	2.1
42398	0.812186	2.1
42399	0.819781	2.1
42400	0.819781	2.1
42401	0.913532	2.1
42402	0.990134	2.1
42403	0.733564	2.1
42404	0.641833	2.1
42405	0.641832	2.1
42406	0.913533	2.1
42407	0.733564	2.1
42408	0.990134	2.1
42409	0.994262	2.0862
42410	0.994262	2.1
42411	0.997354	2.1
42412	0.997354	2.06921
42413	1.00775	1.95805
42414	1.07808	2.00745
42415	0.913533	2.1
42416	0.990135	2.1
42417	0.913532	2.1
42418	1.00775	2.1
42419	0.990134	2.1
42420	1.07808	2.1
42421	0.946131	2.1
42422	0.946131	2.08685
42423	0.946128	2.1
42424	0.946128	2.09062
42425	0.946128	2.06836
42426	0.946128	1.97058
42427	0.94613	2.1
42428	0.94613	2.1
42429	0.946129	2.1
42430	0.946129	2.1
42431	0.946129	2.1
42432	0.946129	2.1
42433	0.816232	2.1
42434	0.760038	2.1
42435	0.845999	2.1
42436	0.845999	2.1
42437	1.0631	2.1
42438	1.28131	2.1
42439	0.474065	2.1
42440	0.591905	2.1
42441	0.601756	2.1
42442	1.23903	2.1
42443	0.988592	2.1
42444	0.49051	2.1
42445	2.57021	2.1
42446	1.89226	2.1
42447	2.68172	2.1
42448	1.89226	2.1
42449	4.46247	2.1
42450	1.89226	2.1
42451	1.89226	1.82808
42452	1.89226	2.08884
42453	1.89226	2.01678

42454	3.78452	2.07374
42455	1.12573	2.1
42456	1.65504	1.99929
42457	1.38848	2.1
42458	1.38848	2.1
42459	1.30567	2.0879
42460	1.30567	2.1
42461	2.82413	2.1
42462	1.89226	2.1
42463	2.68172	2.1
42464	2.68172	2.1
42465	1.89226	2.1
42466	1.89226	2.1
42467	1.89226	2.1
42468	1.89226	2.09329
42469	1.89226	2.1
42470	1.89226	1.60868
42471	3.78452	2.09646
42472	1.30567	2.1
42473	1.65504	2.08906
42474	1.52724	2.1
42475	1.89249	2.1
42476	3.18228	2.1
42477	2.57386	0.0879916
42478	1.89226	0.15247
42479	1.89226	0.15846
42480	2.66753	0.101292
42481	1.89226	0.143264
42482	2.66753	0.097696
42483	1.89226	0.0886244
42484	1.89226	0.173694
42485	2.2973	0.167369
42486	3.12479	0.108507
42487	1.89226	0.156923
42488	2.57385	0.100605
42489	0.489293	2.1
42490	0.672698	2.1
42491	0.59038	2.1
42492	0.738357	2.1
42493	0.62269	2.1
42494	0.62269	2.1
42495	0.640388	2.1
42496	0.640388	2.1
42497	0.59038	2.1
42498	0.489293	2.1
42499	0.738357	2.1
42500	0.672698	2.1
42501	1.89226	2.1
42502	1.89226	2.1
42503	1.89226	2.1
42504	1.89226	2.09826
42505	3.78452	2.1
42506	2.82858	2.1
42507	1.89226	2.1
42508	2.5671	2.1
42509	1.89226	2.1
42510	2.5671	2.1
42511	1.89226	2.1

42512	2.06581	0.05
42513	2.47308	0.05
42514	1.89433	0.05
42515	1.67571	0.05
42516	3.87274	0.05
42517	2.55791	0.05
42518	2.10276	0.05
42519	2.47308	0.05
42520	2.47308	0.05
42521	1.83415	0.05
42522	1.90922	0.05
42523	1.18421	0.05
42524	2.03277	0.05
42525	1.18421	0.05
42526	2.01275	0.05
42527	1.42818	0.05
42528	2.25673	0.05
42529	0.882904	0.05
42530	0.958492	0.05
42531	0.905756	0.05
42532	0.905756	0.05
42533	1.06352	0.05
42534	0.797654	0.05
42535	1.11319	0.05
42536	0.716795	0.05
42537	0.820721	0.05
42538	0.731963	0.05
42539	1.13143	0.05
42540	1.10528	0.05
42541	1.89226	0.05
42542	1.89226	0.05
42543	1.89226	0.05
42544	1.89226	0.05
42545	1.89226	0.05
42546	1.89226	0.05
42547	1.01241	0.05
42548	0.703929	0.05
42549	0.946133	0.05
42550	0.946133	0.05
42551	1.04072	0.05
42552	1.163 0.05	
42553	0.639952	0.05
42554	0.851543	0.05
42555	0.946132	0.05
42556	0.920761	0.05
42557	0.711038	0.05
42558	0.711038	0.05
42559	1.89226	0.05
42560	1.89226	0.05
42561	1.89226	0.05
42562	1.89226	0.05
42563	1.89226	0.05
42564	1.89226	0.05
42565	1.89226	0.05
42566	1.89226	0.05
42567	2.41075	0.05
42568	1.89226	0.05
42569	3.78451	0.05

42570	0.968476	2.06387
42571	1.20674	0.660665
42572	1.13456	0.633465
42573	1.13456	2.1
42574	1.02074	0.945388
42575	1.02074	2.0903
42576	1.02074	2.1
42577	1.20674	1.87237
42578	1.07866	1.79084
42579	1.28649	0.891572
42580	2.2854	2.04328
42581	1.94221	0.14202
42582	1.36717	0.12119
42583	1.45535	0.140189
42584	1.93771	0.109913
42585	3.39756	0.135341
42586	1.94222	0.167636
42587	2.25035	0.111851
42588	2.41806	0.128676
42589	2.42256	0.125405
42590	1.93772	0.137978
42591	1.94222	0.135308
42592	1.49906	0.0970784
42593	1.93771	0.0979134
42594	1.45535	0.104235
42595	1.45535	0.120421
42596	1.91484	0.0800026
42597	1.91484	0.0910403
42598	1.91484	0.0986604
42599	2.25035	0.102608
42600	1.93772	0.10665
42601	1.93772	0.12098
42602	2.11465	0.0838788
42603	2.11465	0.0911938
42604	2.61735	0.14097
42605	1.5082	0.150003
42606	2.36481	0.178022
42607	1.58597	0.17136
42608	2.36481	0.139522
42609	1.46653	0.129261
42610	2.36482	0.184708
42611	1.08133	0.222603
42612	1.5082	0.144241
42613	2.01868	0.234121
42614	3.52688	0.229871
42615	0.94613	0.195495
42616	0.94613	0.18552
42617	0.946131	0.293018
42618	0.946131	0.303451
42619	0.946129	0.304774
42620	0.946129	0.234341
42621	0.946132	0.184491
42622	0.946132	0.246758
42623	0.94613	0.258138
42624	0.94613	0.290644
42625	0.946131	0.242679
42626	0.946131	0.234618
42627	2.53725	0.103894

42628	1.89227	0.142224
42629	1.89227	0.137684
42630	2.27073	0.128645
42631	1.89227	0.117441
42632	2.27073	0.11097
42633	1.89227	0.213883
42634	1.89227	0.348852
42635	1.89227	0.360486
42636	1.89227	0.279823
42637	1.89227	0.281663
42638	1.89227	0.218057
42639	1.86766	2.1
42640	2.83487	2.1
42641	2.84318	2.1
42642	2.59401	2.1
42643	2.11684	2.1
42644	1.86766	2.1
42645	1.10638	2.1
42646	2.11684	2.1
42647	1.29111	2.1
42648	2.37439	2.1
42649	3.40796	2.1
42650	2.56843	2.1
42651	1.89226	2.1
42652	1.89226	2.1
42653	2.71932	2.1
42654	4.46069	2.1
42655	1.89226	2.1
42656	1.89226	2.1
42657	1.89226	2.1
42658	1.89226	2.1
42659	3.78452	2.1
42660	0.6416	2.1
42661	0.697585	2.1
42662	0.670706	2.1
42663	0.598927	2.1
42664	0.644313	2.1
42665	0.644313	2.1
42666	0.652199	2.1
42667	0.652199	2.1
42668	0.697587	2.1
42669	0.670705	2.1
42670	0.598926	2.1
42671	0.641601	2.1
42672	1.89226	2.1
42673	1.89226	2.1
42674	1.89226	2.1
42675	1.89226	2.1
42676	1.89226	2.1
42677	1.89226	2.1
42678	0.697585	2.1
42679	0.730617	2.1
42680	0.712082	2.1
42681	0.670706	2.1
42682	0.69854	2.1
42683	0.69854	2.1
42684	0.702783	2.1
42685	0.702783	2.1

42686	0.730618	2.1
42687	0.712081	2.1
42688	0.697586	2.1
42689	0.670705	2.1
42690	1.89226	2.1
42691	1.89226	2.1
42692	1.89226	2.05453
42693	1.89226	2.1
42694	1.89226	2.03764
42695	1.89226	2.1
42696	2.0815	2.1
42697	1.12076	2.1
42698	1.38662	2.1
42699	2.01244	2.1
42700	3.41508	2.1
42701	1.89226	2.1
42702	2.54324	2.1
42703	1.91981	2.1
42704	1.89226	2.1
42705	2.57079	2.1
42706	2.54324	2.1
42707	2.54325	2.1
42708	1.94779	2.1
42709	1.76879	2.1
42710	1.73811	2.1
42711	2.5708	2.1
42712	2.54325	2.1
42713	1.89226	2.1
42714	2.57079	2.1
42715	1.91894	2.1
42716	1.89226	2.1
42717	2.59747	2.1
42718	2.57079	2.1
42719	2.5708	2.1
42720	1.82268	2.1
42721	1.79738	2.1
42722	1.77071	2.1
42723	2.59747	2.1
42724	2.5708	2.1
42725	1.89226	1.30622
42726	1.89226	2.1
42727	1.89226	1.02864
42728	1.89226	2.1
42729	3.78451	1.93967
42730	2.21582	0.991292
42731	1.94412	2.1
42732	2.13428	0.990965
42733	1.86292	2.1
42734	4.07839	1.92365
42735	1.94412	2.1
42736	1.5854	2.1
42737	1.86293	2.1
42738	1.50767	2.1
42739	3.45179	2.1
42740	1.89226	2.1
42741	1.89226	2.1
42742	1.89226	2.1
42743	1.89226	2.1

42744	3.78452	2.1
42745	1.89226	0.916768
42746	1.89226	2.1
42747	1.89226	1.03309
42748	1.89226	2.1
42749	3.78452	2.04166
42750	2.13428	0.871494
42751	1.86293	2.1
42752	2.04737	0.942477
42753	1.77122	2.1
42754	3.9055	1.89814
42755	1.86293	2.1
42756	1.50767	2.1
42757	1.41077	2.1
42758	1.41077	2.1
42759	1.77123	2.1
42760	1.86813	2.1
42761	0.810943	2.1
42762	0.810943	2.1
42763	0.810945	2.1
42764	0.810945	2.1
42765	0.810958	2.1
42766	0.810958	2.1
42767	1.89226	2.1
42768	1.35168	2.1
42769	1.08132	2.1
42770	1.08132	2.1
42771	1.0813	2.1
42772	0.745979	2.1
42773	0.745979	2.1
42774	0.778781	2.1
42775	0.778781	2.1
42776	0.59549	2.1
42777	0.649658	2.1
42778	0.994784	2.1
42779	0.875102	2.1
42780	0.875102	2.1
42781	0.59549	2.1
42782	0.649658	2.1
42783	0.994784	2.1
42784	1.57887	2.1
42785	1.75021	2.1
42786	1.13341	2.1
42787	1.13341	2.1
42788	1.19098	2.1
42789	1.63644	2.1
42790	2.5761	0.786548
42791	2.43404	2.07936
42792	2.44813	0.570881
42793	2.31751	2.09613
42794	4.88218	1.58814
42795	2.43405	2.07722
42796	2.22575	2.1
42797	2.31751	2.09984
42798	2.10893	2.1
42799	4.54298	2.1
42800	2.44813	0.462149
42801	2.31751	2.09514

42802	2.30908	0.755032
42803	2.19815	2.06764
42804	4.64628	1.45116
42805	2.31751	2.09476
42806	2.10893	2.1
42807	2.19815	2.07978
42808	1.98061	2.1
42809	4.30708	2.1
42810	0.94613	2.1
42811	0.94613	2.1
42812	1.40372	2.1
42813	1.40372	2.1
42814	1.40372	2.1
42815	1.37167	2.1
42816	0.946132	2.1
42817	0.946132	2.1
42818	0.946131	2.1
42819	1.40372	2.1
42820	0.94613	2.1
42821	1.37167	2.1
42822	2.80744	2.1
42823	2.06984	2.1
42824	2.83418	2.1
42825	2.74335	2.1
42826	2.16067	2.1
42827	2.06984	2.1
42828	1.077 0.231067	
42829	0.809116	0.184461
42830	0.600419	0.261352
42831	0.790277	0.189722
42832	0.927647	0.17362
42833	0.758598	0.175973
42834	0.85749	0.238094
42835	0.85749	0.240276
42836	1.02417	0.196032
42837	0.600411	0.210509
42838	0.751701	0.202133
42839	1.07702	0.192988
42840	1.89226	2.1
42841	1.89226	2.1
42842	1.89226	2.1
42843	1.89226	2.1
42844	1.89226	2.1
42845	1.89226	2.1
42846	1.89226	2.1
42847	1.89226	2.1
42848	1.89226	2.1
42849	1.89226	2.1
42850	3.78453	2.1
42851	1.89226	2.1
42852	1.89226	2.1
42853	1.89226	2.1
42854	1.89226	2.1
42855	1.89226	2.1
42856	1.89226	2.1
42857	0.946131	2.1
42858	0.946131	2.1
42859	0.946131	2.1

42860	0.946131	2.1
42861	0.946133	2.1
42862	0.946133	2.1
42863	0.946128	2.1
42864	0.946128	2.1
42865	0.946131	2.1
42866	0.946131	2.1
42867	0.94613	2.1
42868	0.94613	2.1
42869	2.17159	2.1
42870	2.00083	2.00205
42871	2.21305	2.1
42872	2.00551	2.1
42873	2.20837	2.1
42874	2.00083	2.09922
42875	2.20837	2.09241
42876	1.80328	0.929391
42877	2.22962	2.03085
42878	2.00083	1.92582
42879	2.03207	1.08711
42880	1.80328	1.50188
42881	2.28564	2.1
42882	2.20837	1.96415
42883	2.30406	2.1
42884	2.17159	2.1
42885	2.34084	2.1
42886	2.20837	2.1
42887	1.09523	0.846711
42888	1.09523	1.98178
42889	1.09934	2.1
42890	1.09934	2.1
42891	1.01603	0.731714
42892	1.09038	1.95573
42893	1.10419	1.83129
42894	1.17042	2.1
42895	1.17042	2.03051
42896	1.09038	2.00821
42897	1.10419	1.89619
42898	1.01603	2.03166
42899	2.60152	0.366459
42900	0.976334	0.608092
42901	2.22344	0.708958
42902	2.22344	0.387772
42903	1.36405	0.826665
42904	1.36405	0.23618
42905	1.89226	2.1
42906	1.70302	2.1
42907	1.69019	2.1
42908	1.70302	2.1
42909	1.73933	2.1
42910	1.70303	2.08569
42911	1.89226	2.1
42912	1.89226	2.02852
42913	1.89226	2.1
42914	1.89226	2.1
42915	3.78452	2.1
42916	1.89226	2.0981
42917	1.59036	2.1

42918	1.65683	2.1
42919	1.59036	2.1
42920	1.95873	2.1
42921	1.89226	1.9948
42922	0.946131	1.85429
42923	0.946131	2.03742
42924	0.946127	2.1
42925	0.946127	2.1
42926	0.946127	2.1
42927	0.946127	1.70897
42928	0.946131	2.1
42929	0.946131	2.05609
42930	0.946129	2.1
42931	0.946129	2.1
42932	0.94613	2.0988
42933	0.94613	2.1
42934	0.851541	2.1
42935	0.851541	2.1
42936	0.851483	2.1
42937	1.15713	2.1
42938	0.851539	2.1
42939	0.851539	2.1
42940	0.942127	2.1
42941	1.14455	2.1
42942	0.838683	2.1
42943	0.946131	2.1
42944	0.957503	2.1
42945	0.957504	2.1
42946	1.89226	1.41499
42947	1.89226	0.879212
42948	1.89226	0.751116
42949	1.89226	2.1
42950	3.78452	0.771623
42951	1.89226	0.125296
42952	1.89226	0.490112
42953	1.89226	0.470606
42954	1.89226	0.490845
42955	1.89226	0.165884
42956	1.89226	0.0778697
42957	1.89226	0.519472
42958	1.89226	0.375535
42959	1.89226	0.32858
42960	1.89226	0.774088
42961	3.78453	0.295602
42962	1.89226	0.980246
42963	1.89226	2.1
42964	1.89226	2.1
42965	1.89226	2.06476
42966	3.78453	2.1
42967	1.22575	0.404333
42968	1.05438	1.26971
42969	0.946131	0.740511
42970	0.946131	0.954127
42971	0.946129	1.92525
42972	1.22575	0.472536
42973	1.05438	0.530157
42974	0.946132	0.33522
42975	1.05438	0.767437

42976	1.05438	1.14199
42977	0.946131	0.586054
42978	0.946131	1.1287
42979	2.4515	0.3639
42980	1.13726	0.331728
42981	2.10875	0.226263
42982	1.49515	0.215033
42983	2.10875	0.349747
42984	1.49515	0.172467
42985	0.831477	0.118683
42986	0.747575	0.349051
42987	1.22575	0.352082
42988	1.28174	0.182612
42989	1.28174	0.285678
42990	0.831476	0.243454
42991	1.22575	0.352082
42992	0.747575	0.295245
42993	1.03561	0.352082
42994	1.03561	0.35704
42995	1.02162	0.181917
42996	1.02162	0.100098
42997	1.89227	2.09596
42998	2.56348	1.30344
42999	1.89227	2.1
43000	2.45151	2.09312
43001	1.89227	2.1
43002	2.45151	1.02931
43003	1.40013	0.58935
43004	1.89226	1.25153
43005	1.89226	0.926783
43006	0.946643	0.575139
43007	1.89226	0.982256
43008	0.946643	0.666153
43009	1.89226	0.05
43010	1.89226	0.0660292
43011	1.89226	0.05
43012	1.89226	0.0565761
43013	3.78453	0.0571466
43014	1.89226	0.100229
43015	1.89226	0.142814
43016	1.89226	0.0970544
43017	1.89226	0.186104
43018	3.78453	0.104127
43019	0.946131	0.157323
43020	0.946131	0.131148
43021	0.946128	0.0812774
43022	0.946128	0.0939006
43023	0.94613	0.142472
43024	0.94613	0.165866
43025	0.946129	0.0897317
43026	0.946129	0.0866096
43027	0.94613	0.123756
43028	0.94613	0.0693385
43029	0.94613	0.107271
43030	0.94613	0.163084
43031	1.89226	0.05
43032	1.89226	0.05
43033	1.17244	0.05

43034	1.89226	0.05
43035	0.975291	0.05
43036	1.57408	0.05
43037	0.946132	0.0864249
43038	0.946132	0.0574167
43039	0.946131	0.0895444
43040	0.946131	0.0561433
43041	0.946132	0.0783689
43042	0.946132	0.097332
43043	0.946131	0.0565134
43044	0.946131	0.0604629
43045	0.946131	0.0773686
43046	0.946131	0.0761902
43047	0.946132	0.065134
43048	0.946132	0.0934764
43049	2.74744	0.0757664
43050	1.89226	0.212285
43051	2.70805	0.108063
43052	1.89226	0.123138
43053	2.70805	0.13986
43054	1.89226	0.174737
43055	2.74744	0.0619917
43056	1.89226	0.0798012
43057	2.57465	0.0991286
43058	1.89226	0.182985
43059	4.6397	0.077147
43060	1.89226	0.219001
43061	1.89226	0.557177
43062	1.89226	0.392106
43063	1.89226	0.669206
43064	3.78452	0.451309
43065	1.89226	0.284513
43066	1.89226	0.751724
43067	1.89226	0.282113
43068	1.89226	0.204839
43069	1.89226	0.740169
43070	1.89226	0.678951
43071	2.16206	0.192458
43072	2.67861	0.171536
43073	3.11996	0.203876
43074	1.70303	0.108086
43075	2.89015	0.333547
43076	1.486	0.193443
43077	2.12839	0.112751
43078	2.83631	0.215421
43079	3.08096	0.155302
43080	1.84157	0.111559
43081	3.08096	0.298223
43082	1.84157	0.165266
43083	1.89225	1.67554
43084	1.89225	0.827664
43085	1.89225	1.01716
43086	1.89225	1.47479
43087	1.89225	0.881284
43088	1.89225	1.21807
43089	1.89226	0.611092
43090	1.89226	0.766309
43091	1.89226	1.15507

43092	1.89226	0.489279
43093	3.78452	0.51147
43094	1.89226	0.307592
43095	2.49041	0.285965
43096	1.89226	0.346954
43097	1.89226	0.168344
43098	2.27073	0.169075
43099	2.27073	0.285037
43100	1.09904	0.209217
43101	0.677147	0.141922
43102	1.3365	0.0981009
43103	0.95759	0.433663
43104	0.957591	0.196534
43105	0.677148	0.231511
43106	1.09904	0.17053
43107	1.3365	0.254404
43108	0.996015	0.23007
43109	0.996015	0.17199
43110	1.01764	0.461511
43111	1.01764	0.196485
43112	2.77349	0.271792
43113	2.72723	0.167572
43114	2.43156	0.293486
43115	2.4564	0.163686
43116	5.20505	0.247032
43117	2.77349	0.257703
43118	2.68508	0.149937
43119	2.33761	0.231989
43120	2.4564	0.158557
43121	5.1111	0.180885
43122	2.43156	0.199499
43123	2.70208	0.141319
43124	1.85779	0.222275
43125	2.10695	0.136137
43126	4.77572	0.150949
43127	2.33761	0.182153
43128	2.70208	0.136862
43129	1.85779	0.199472
43130	1.61597	0.129118
43131	4.19542	0.163004
43132	0.946132	1.20621
43133	0.946132	1.5504
43134	0.746729	1.6095
43135	0.746729	1.8491
43136	0.946132	1.87302
43137	0.915958	1.64039
43138	0.946133	1.18813
43139	0.74673	1.90682
43140	0.946133	1.57732
43141	0.946133	1.57622
43142	0.915958	1.63576
43143	0.746729	1.48814
43144	1.50743	2.1
43145	2.45501	2.1
43146	2.29118	2.1
43147	2.29118	2.1
43148	1.75482	2.1
43149	1.75482	2.1

43150	2.45501	2.1
43151	2.76927	2.05142
43152	2.29118	2.1
43153	2.64523	2.08005
43154	5.10024	2.1
43155	1.25559	2.1
43156	2.29118	2.1
43157	2.0815	2.1
43158	2.26387	2.1
43159	1.35668	2.1
43160	1.47667	2.1
43161	2.28481	2.1
43162	2.64523	2.08399
43163	2.50057	2.1
43164	2.50057	2.07485
43165	2.29118	2.1
43166	2.29118	2.08534
43167	0.946129	0.125521
43168	0.946129	0.110496
43169	1.23929	0.0782919
43170	1.15246	0.0981858
43171	0.946131	0.111546
43172	0.946131	0.143769
43173	1.15246	0.0978272
43174	1.15246	0.104834
43175	0.946131	0.0945923
43176	1.15246	0.120409
43177	0.946131	0.0933124
43178	1.23928	0.107545
43179	0.946128	0.167581
43180	0.946128	0.154072
43181	1.15246	0.121921
43182	0.942814	0.196349
43183	0.946131	0.119959
43184	0.946131	0.165794
43185	0.961347	0.109863
43186	0.942811	0.166031
43187	0.946131	0.130742
43188	0.961347	0.11451
43189	0.946131	0.111063
43190	1.15246	0.151974
43191	2.32334	0.107019
43192	1.57621	0.0922859
43193	1.90335	0.118175
43194	1.24538	0.0775478
43195	3.47956	0.0818862
43196	0.965762	0.0512964
43197	0.965762	0.0648549
43198	1.00245	0.05
43199	1.00245	0.05
43200	0.92786	0.05
43201	0.705297	0.05
43202	0.856414	0.06291
43203	1.28968	0.05
43204	1.00306	0.0512576
43205	0.705303	0.05
43206	1.28967	0.05
43207	0.975478	0.0546607

43208	3.05308	2.1
43209	1.76816	2.1
43210	1.76816	2.1
43211	1.69129	2.1
43212	2.58731	2.1
43213	2.51043	2.1
43214	1.89226	0.0851982
43215	2.71464	0.0849987
43216	2.34627	0.0790707
43217	1.89226	0.104489
43218	2.34627	0.068922
43219	1.89226	0.0974143
43220	2.34627	0.0765334
43221	2.71464	0.0766289
43222	1.98252	0.0648452
43223	1.98252	0.0999896
43224	1.51026	0.0659529
43225	2.34627	0.0869778
43226	2.38698	0.815436
43227	2.35142	2.09719
43228	2.60343	1.79252
43229	2.6636	0.858921
43230	4.99041	1.61783
43231	1.07811	2.1
43232	1.1952	2.1
43233	1.17571	1.98468
43234	1.30171	1.69367
43235	1.18322	1.80464
43236	1.18322	2.1
43237	1.18769	2.1
43238	1.18769	1.84499
43239	1.1952	2.08347
43240	1.30171	2.07362
43241	1.17571	2.0865
43242	1.07811	2.08568
43243	1.89226	1.75215
43244	2.6636	1.7109
43245	1.89226	0.471465
43246	2.60343	1.61556
43247	1.89226	1.01633
43248	2.60343	2.1
43249	1.5344	0.05
43250	1.4516	0.05
43251	1.17816	0.05
43252	1.21056	0.05
43253	2.71257	0.05
43254	1.89226	1.95889
43255	1.89226	0.946132
43256	1.89226	1.54072
43257	1.89226	1.63365
43258	3.78452	1.61943
43259	1.89226	1.52993
43260	1.89226	0.514322
43261	1.89226	1.03893
43262	1.89226	0.754181
43263	1.65573	1.12464
43264	1.65573	0.829767
43265	1.89226	1.47804

43266	1.89226	1.66852
43267	1.89226	1.98377
43268	2.64212	0.956377
43269	3.78452	1.60506
43270	1.89226	2.1
43271	1.89226	2.1
43272	1.89226	2.1
43273	1.89226	2.1
43274	3.78452	2.1
43275	2.7367	2.1
43276	2.07947	2.1
43277	2.47319	2.1
43278	1.85057	2.1
43279	4.55267	2.1
43280	2.07948	2.1
43281	1.3294	2.1
43282	1.85057	2.1
43283	1.09962	2.1
43284	3.1791	2.1
43285	1.89226	2.1
43286	1.89226	2.1
43287	1.89226	2.1
43288	1.89226	2.1
43289	3.78453	2.1
43290	0.94613	2.1
43291	0.94613	2.1
43292	0.94613	2.1
43293	0.94613	2.1
43294	0.946128	2.1
43295	0.946128	2.1
43296	0.946132	2.1
43297	0.946132	2.1
43298	0.94613	2.1
43299	0.94613	2.1
43300	0.946131	2.1
43301	0.946131	2.1
43302	1.00875	2.1
43303	1.00875	2.1
43304	1.01676	2.1
43305	1.01676	2.1
43306	0.925282	2.1
43307	0.804877	2.1
43308	1.2366	2.1
43309	1.10023	2.1
43310	0.804878	2.1
43311	1.10023	2.1
43312	1.2366	2.1
43313	0.925284	2.1
43314	1.89226	2.1
43315	1.89226	2.1
43316	1.89226	2.1
43317	1.89226	2.1
43318	3.78453	2.1
43319	1.89226	2.1
43320	1.89226	2.1
43321	1.89226	2.1
43322	1.89226	2.1
43323	1.89226	2.1

43324	1.89226	2.1
43325	1.09766	2.1
43326	1.14842	2.1
43327	1.02909	2.1
43328	1.07659	2.1
43329	1.16718	2.1
43330	0.954189	2.1
43331	1.06653	2.1
43332	1.06653	2.1
43333	1.20738	2.1
43334	1.02909	2.1
43335	1.09767	2.1
43336	0.815951	2.1
43337	2.00793	2.1
43338	1.14048	2.1
43339	1.19086	2.1
43340	1.13723	2.1
43341	2.00793	2.1
43342	2.11772	2.1
43343	1.89226	2.1
43344	1.89226	2.1
43345	1.89226	2.1
43346	1.89226	2.1
43347	1.89226	2.1
43348	1.89226	2.1
43349	1.89226	2.1
43350	1.89226	2.1
43351	1.89226	2.1
43352	1.89226	2.1
43353	1.89226	2.1
43354	1.89226	2.1
43355	1.1779	2.1
43356	1.26166	2.1
43357	1.0875	2.1
43358	1.00397	2.1
43359	1.13286	2.1
43360	1.13286	2.1
43361	1.13277	2.1
43362	1.13277	2.1
43363	1.26166	2.1
43364	1.08749	2.1
43365	1.1779	2.1
43366	1.00396	2.1
43367	1.0875	2.1
43368	1.00397	2.1
43369	0.685749	2.1
43370	0.595432	2.1
43371	0.83976	2.1
43372	0.83976	2.1
43373	0.843169	2.1
43374	0.843169	2.1
43375	1.0875	2.1
43376	0.685749	2.1
43377	1.00397	2.1
43378	0.595432	2.1
43379	1.89226	2.1
43380	1.86443	2.1
43381	2.4988	2.1

43382	2.0815	2.1
43383	2.28173	2.1
43384	1.86443	2.1
43385	1.89226	2.1
43386	1.89226	2.1
43387	1.89226	2.1
43388	1.89226	2.1
43389	1.89226	2.1
43390	1.89226	2.1
43391	0.946131	2.1
43392	0.946131	2.1
43393	0.969491	2.1
43394	0.949105	2.1
43395	0.949103	2.1
43396	0.949103	2.1
43397	0.946133	2.1
43398	0.946133	2.1
43399	0.946131	2.1
43400	0.969492	2.1
43401	0.946131	2.1
43402	0.949105	2.1
43403	1.89226	2.1
43404	2.07428	2.1
43405	1.93464	2.1
43406	1.89226	2.1
43407	2.11667	2.1
43408	2.07428	2.1
43409	0.946132	2.1
43410	0.946132	2.1
43411	0.946131	2.1
43412	0.946131	2.1
43413	0.946132	2.1
43414	0.946132	2.1
43415	0.946132	2.1
43416	0.946132	2.1
43417	0.946132	2.1
43418	0.946132	2.1
43419	0.946132	2.1
43420	0.946132	2.1
43421	1.97992	2.1
43422	1.89226	2.1
43423	1.89226	2.1
43424	1.93898	2.1
43425	1.93898	2.1
43426	1.89226	2.1
43427	2.12475	0.385941
43428	2.79029	0.666253
43429	2.7803	0.96587
43430	2.13052	0.369262
43431	4.92081	0.510876
43432	1.06238	0.504456
43433	1.06526	0.372566
43434	0.642901	0.154599
43435	0.655055	0.266353
43436	0.856391	0.201798
43437	0.856391	0.326191
43438	0.86104	1.41815
43439	0.86104	0.152683

43440	1.06526	0.299037
43441	0.655055	0.324339
43442	0.642901	0.281148
43443	1.06238	0.273615
43444	2.7803	0.887359
43445	2.13053	0.329933
43446	2.14346	0.457023
43447	2.77937	0.665465
43448	4.9099	0.548945
43449	2.14346	0.500687
43450	1.31011	0.153898
43451	1.34124	0.349694
43452	1.31011	0.509955
43453	2.16166	0.231843
43454	2.13053	0.352462
43455	1.89226	2.1
43456	2.58176	2.1
43457	2.01422	2.1
43458	1.89226	2.1
43459	2.70373	2.1
43460	2.58176	2.1
43461	1.89226	2.1
43462	1.89226	2.1
43463	1.89226	2.1
43464	1.89226	2.1
43465	1.89226	2.1
43466	1.89226	2.1
43467	0.946128	2.1
43468	0.946128	2.1
43469	1.38521	2.1
43470	1.38521	2.1
43471	1.38521	2.1
43472	1.35186	2.1
43473	0.946131	2.1
43474	0.946131	2.1
43475	0.94613	2.1
43476	1.38521	2.1
43477	0.946131	2.1
43478	1.35186	2.1
43479	0.946133	2.1
43480	0.946133	2.1
43481	0.946132	2.1
43482	0.946132	2.1
43483	0.946132	2.1
43484	0.946132	2.1
43485	0.946132	2.1
43486	0.946132	2.1
43487	0.946132	2.1
43488	0.946132	2.1
43489	0.946133	2.1
43490	0.946133	2.1
43491	2.33374	2.1
43492	1.89226	2.1
43493	1.89226	2.1
43494	1.89226	2.1
43495	2.60377	2.1
43496	2.60377	2.1
43497	2.6771	0.05

43498	1.36416	0.05
43499	1.64983	0.05
43500	1.64983	0.05
43501	2.44131	0.05
43502	2.44131	0.05
43503	2.58321	0.189806
43504	1.89226	0.214557
43505	2.44283	0.120492
43506	2.44283	0.14489
43507	1.89226	0.42701
43508	1.89226	0.533774
43509	2.58321	0.204227
43510	2.44283	0.123792
43511	1.49358	0.0976792
43512	1.57702	0.117671
43513	3.93641	0.108752
43514	0.951913	0.05
43515	0.81556	0.05
43516	0.946132	0.05
43517	0.946132	0.05
43518	1.04072	0.05
43519	1.12182	0.05
43520	0.856745	0.05
43521	0.851542	0.05
43522	0.923163	0.05
43523	0.923163	0.0594742
43524	1.04304	0.05
43525	0.85098	0.05
43526	2.70125	1.1178
43527	2.37734	2.02881
43528	2.63495	1.18486
43529	2.30539	1.69113
43530	5.01229	1.81251
43531	1.89226	1.97781
43532	1.89226	2.1
43533	1.89226	1.48806
43534	1.89226	2.09927
43535	3.78452	1.7557
43536	1.89226	2.1
43537	1.89226	2.1
43538	1.89226	2.1
43539	1.89226	2.1
43540	3.78452	2.1
43541	2.37734	2.1
43542	1.79346	2.1
43543	2.53594	1.57978
43544	1.70302	2.1
43545	4.27682	2.1
43546	2.63495	0.920881
43547	2.30539	1.09916
43548	2.49445	0.24918
43549	2.12089	0.994285
43550	4.75584	0.681695
43551	1.89226	1.22535
43552	1.89226	1.96341
43553	1.89226	1.03867
43554	1.89226	1.76858
43555	3.78452	1.97835

43556	1.89226	2.01129
43557	1.89226	2.04371
43558	1.89226	2.08695
43559	1.89226	2.1
43560	1.89226	1.52948
43561	1.89226	1.72818
43562	2.53594	0.773734
43563	1.60017	1.60685
43564	1.57079	1.01249
43565	1.70302	1.33408
43566	2.15772	1.0717
43567	2.33299	1.12686
43568	2.09114	2.1
43569	2.34898	2.1
43570	2.30729	2.1
43571	2.06556	2.1
43572	4.41454	2.1
43573	2.09114	2.1
43574	1.59654	2.1
43575	1.57823	2.1
43576	2.06556	2.1
43577	3.6621	2.1
43578	2.30729	2.1
43579	2.06556	2.1
43580	2.03917	2.1
43581	2.26617	2.1
43582	4.33173	2.1
43583	2.03917	2.1
43584	2.06556	2.1
43585	2.045 2.1	
43586	1.55767	2.1
43587	1.57823	2.1
43588	1.57823	2.1
43589	1.89225	2.1
43590	1.09726	2.1
43591	1.61196	2.1
43592	1.28597	2.1
43593	1.28597	0.05
43594	1.45633	2.1
43595	1.45633	2.1
43596	1.9544	0.05
43597	1.61196	2.1
43598	1.45633	2.1
43599	1.53735	2.1
43600	3.14931	2.1
43601	1.89226	2.1
43602	1.89226	2.1
43603	1.89226	2.1
43604	1.89226	2.1
43605	3.78453	2.1
43606	0.810946	2.1
43607	0.810946	2.1
43608	0.810947	2.1
43609	0.810947	2.1
43610	0.810933	2.1
43611	0.810933	2.1
43612	1.89226	2.1
43613	1.3517	2.1

43614	1.08132	2.1
43615	1.08132	2.1
43616	1.08133	2.1
43617	0.851539	2.1
43618	0.851539	2.1
43619	0.972053	2.1
43620	1.05632	2.1
43621	0.94613	2.1
43622	0.94613	2.1
43623	1.0279	2.1
43624	0.94991	2.1
43625	1.0279	2.1
43626	0.946131	2.1
43627	0.865643	2.1
43628	0.94613	2.1
43629	1.89226	2.1
43630	1.89226	2.1
43631	1.89226	2.1
43632	1.89226	2.1
43633	3.78452	2.1
43634	1.03882	2.1
43635	1.91121	2.1
43636	1.41833	2.1
43637	2.34547	2.1
43638	3.32954	2.1
43639	2.3979	2.1
43640	1.89226	2.1
43641	1.89226	2.1
43642	1.89226	2.1
43643	2.0558	2.1
43644	2.0558	2.1
43645	1.89226	2.1
43646	1.89226	2.07785
43647	1.89226	2.1
43648	1.89226	2.1
43649	3.78452	2.1
43650	1.05816	2.1
43651	1.05816	2.1
43652	0.842045	2.1
43653	1.05252	2.1
43654	1.36057	2.1
43655	1.14339	2.1
43656	0.873397	2.1
43657	0.585073	2.1
43658	0.873388	2.1
43659	1.15129	2.1
43660	0.69143	2.1
43661	1.1434	2.1
43662	1.93338	2.1
43663	1.85883	2.1
43664	1.51714	2.1
43665	1.85883	2.1
43666	1.54795	2.1
43667	1.89226	2.1
43668	1.89226	2.1
43669	1.89226	2.1
43670	1.89226	2.1
43671	1.89226	2.1

43672	3.78452	2.1
43673	0.844726	2.1
43674	0.844726	2.1
43675	0.960524	2.1
43676	1.16288	2.1
43677	0.851541	2.1
43678	0.851541	2.1
43679	0.929147	2.1
43680	1.15533	2.1
43681	0.844697	2.1
43682	0.946131	2.1
43683	0.851632	2.1
43684	0.946131	2.1
43685	1.89226	2.1
43686	1.70303	2.1
43687	1.74006	2.1
43688	1.70303	2.1
43689	1.99471	2.1
43690	1.93338	2.1
43691	0.946131	2.1
43692	0.946131	2.1
43693	0.946131	2.1
43694	0.946131	2.1
43695	0.94613	2.1
43696	0.94613	2.1
43697	0.946132	2.1
43698	0.946132	2.1
43699	0.946131	2.1
43700	0.946131	2.1
43701	0.946131	2.1
43702	0.946131	2.1
43703	1.58158	2.1
43704	1.89226	2.1
43705	1.89226	2.1
43706	1.89226	2.1
43707	1.68939	2.1
43708	1.68939	2.1
43709	0.946132	0.05
43710	0.946132	0.05
43711	1.12988	0.05
43712	1.30783	0.05
43713	1.30783	0.05
43714	0.946133	0.05
43715	0.946132	0.05
43716	1.12988	0.05
43717	1.30783	0.05
43718	1.30783	0.05
43719	0.946133	0.05
43720	0.946133	0.05
43721	1.89227	0.05
43722	1.89227	0.05
43723	1.89227	0.05
43724	1.89227	0.05
43725	1.89227	0.05
43726	1.89227	0.05
43727	1.89226	0.05
43728	1.89226	0.05
43729	1.89226	0.05

43730	1.89226	0.05
43731	1.89226	0.05
43732	1.89226	0.05
43733	0.946131	0.05
43734	0.946131	0.05
43735	1.30783	0.05
43736	1.37715	0.05
43737	1.37715	0.05
43738	0.946133	0.05
43739	1.30783	0.05
43740	0.946129	0.05
43741	1.37715	0.05
43742	1.37715	0.05
43743	0.946131	0.05
43744	0.946131	0.05
43745	1.17056	0.05
43746	0.591757	0.05
43747	0.522025	0.05
43748	0.929727	0.05
43749	0.929728	0.05
43750	1.17056	0.05
43751	0.591756	0.05
43752	0.522025	0.05
43753	0.889054	0.05
43754	0.889054	0.05
43755	0.803535	0.05
43756	0.803535	0.05
43757	1.49814	2.1
43758	2.37439	2.1
43759	2.63923	2.1
43760	2.63923	2.1
43761	1.29111	2.1
43762	1.29111	2.1
43763	0.946132	2.1
43764	0.946132	2.1
43765	1.18719	2.1
43766	1.18719	2.1
43767	0.94613	2.1
43768	0.94613	2.1
43769	1.1872	2.1
43770	1.31961	2.1
43771	0.946131	2.1
43772	1.31961	2.1
43773	1.1872	2.1
43774	0.94613	2.1
43775	2.49445	0.139196
43776	2.12089	0.592784
43777	2.20843	0.247649
43778	1.82805	0.244987
43779	4.32932	0.31246
43780	1.89226	0.680979
43781	1.89226	1.13478
43782	1.89226	0.456806
43783	1.89226	0.705125
43784	3.78452	0.462359
43785	0.94613	0.788761
43786	0.94613	0.857276
43787	0.946128	0.620031

43788	0.946128	1.06158
43789	0.946131	1.26215
43790	0.946131	1.04684
43791	0.946127	0.846567
43792	0.946127	0.778886
43793	0.946129	0.76475
43794	0.946129	0.651102
43795	0.946129	1.20169
43796	0.946129	0.896999
43797	1.82804	0.21943
43798	1.60017	0.699465
43799	1.87679	0.336094
43800	2.12088	0.394246
43801	1.35608	0.403487
43802	1.60017	0.452536
43803	2.20843	0.126736
43804	1.3216	0.106774
43805	1.44426	0.111701
43806	1.82805	0.162977
43807	1.80399	0.0832478
43808	2.20843	0.150087
43809	1.89226	0.215918
43810	1.89226	0.280548
43811	1.89226	0.389925
43812	1.89226	0.332088
43813	1.89226	0.284838
43814	1.89226	0.275543
43815	1.89226	0.393922
43816	1.89226	0.348543
43817	1.89226	0.249445
43818	1.89226	0.315667
43819	1.89226	0.479802
43820	1.89226	0.266599
43821	0.627705	0.0868523
43822	0.634941	0.141804
43823	0.629317	0.0916449
43824	0.783422	0.126657
43825	0.629328	0.133485
43826	0.448764	0.133565
43827	1.35608	0.18391
43828	1.00805	0.170775
43829	0.814295	0.142937
43830	0.598375	0.161643
43831	1.04461	0.138533
43832	1.09795	1.92471
43833	1.00775	2.05771
43834	1.61202	2.00926
43835	1.63419	1.98299
43836	2.64194	1.93971
43837	1.44801	1.71025
43838	1.61201	2.00724
43839	2.35614	1.75952
43840	2.27703	1.9639
43841	3.88904	1.91344
43842	2.35615	1.666
43843	1.63419	1.89847
43844	2.29852	1.66176
43845	1.63419	1.90096

43846	2.27634	1.71889
43847	1.61202	2.00915
43848	0.973793	2.1
43849	2.2671	2.1
43850	2.32981	2.1
43851	2.32981	2.1
43852	0.990481	2.1
43853	0.990481	2.1
43854	1.50877	2.1
43855	1.11193	2.1
43856	2.29667	2.1
43857	1.94158	2.1
43858	3.45035	2.1
43859	2.29667	2.1
43860	1.89227	2.1
43861	1.94158	2.1
43862	1.94158	2.1
43863	1.89227	2.1
43864	1.89227	2.1
43865	2.51379	2.1
43866	1.30026	2.1
43867	1.84502	2.1
43868	2.72099	2.1
43869	4.35882	2.1
43870	1.89226	2.1
43871	2.721	2.1
43872	2.5138	2.1
43873	2.5138	2.1
43874	1.89226	2.1
43875	1.89226	2.1
43876	1.89226	0.0538793
43877	1.89226	0.0647761
43878	2.59101	0.05
43879	1.89226	0.0879062
43880	3.78453	0.0512509
43881	0.851541	0.0624896
43882	0.851541	0.05
43883	1.42502	0.05
43884	0.948679	0.05
43885	0.946132	0.0579497
43886	0.946132	0.0680017
43887	0.853617	0.05
43888	0.853618	0.05
43889	0.851475	0.0565499
43890	0.948666	0.0618188
43891	1.166	0.0555538
43892	0.851554	0.0581869
43893	2.51818	2.09833
43894	2.60723	2.1
43895	2.66532	2.05973
43896	2.76315	2.1
43897	5.27255	2.1
43898	2.60723	2.1
43899	2.45247	2.1
43900	2.76315	2.1
43901	2.55197	2.1
43902	5.1592	2.1
43903	1.23768	0.236439

43904	1.11433	0.418536
43905	1.32037	1.70799
43906	1.32037	0.895222
43907	1.0142	0.887676
43908	1.0142	0.569719
43909	0.886711	0.568311
43910	1.16806	0.502361
43911	0.999083	1.83376
43912	0.710926	1.50111
43913	0.937757	1.57483
43914	0.937757	0.782536
43915	0.941224	0.392031
43916	0.941224	1.7699
43917	1.16805	1.27886
43918	0.999084	1.9356
43919	0.886709	1.44288
43920	0.710928	1.33591
43921	2.29332	2.1
43922	1.54904	2.1
43923	2.0815	2.1
43924	2.44199	2.1
43925	4.00418	2.1
43926	1.53873	0.806004
43927	1.53422	2.1
43928	1.39661	1.20863
43929	1.42387	2.09542
43930	2.9626	1.90329
43931	1.53423	2.1
43932	1.44091	2.1
43933	1.42387	2.09921
43934	1.35215	2.1
43935	2.86479	2.1
43936	1.39661	1.26424
43937	1.42387	2.09851
43938	1.28325	0.830726
43939	1.33326	2.1
43940	2.70713	1.88158
43941	1.42387	2.09657
43942	1.35215	2.1
43943	1.33327	2.1
43944	1.27339	2.1
43945	2.69726	2.1
43946	2.58442	2.1
43947	1.1468	2.1
43948	1.18775	2.1
43949	2.60047	2.1
43950	3.74728	2.1
43951	2.58442	2.1
43952	1.89226	2.1
43953	1.89226	2.1
43954	2.60048	2.1
43955	4.49274	2.1
43956	1.89226	2.1
43957	1.89226	2.1
43958	1.89226	2.1
43959	1.89226	2.1
43960	3.78453	2.1
43961	1.89226	2.1

43962	1.89226	2.1
43963	1.89226	2.1
43964	1.89226	2.1
43965	3.78452	2.1
43966	1.22262	2.1
43967	2.60047	2.1
43968	1.18775	2.1
43969	1.18775	2.1
43970	2.61433	2.1
43971	2.61433	2.1
43972	1.89226	2.1
43973	2.60048	2.1
43974	2.61434	2.1
43975	2.60048	2.1
43976	1.90612	2.1
43977	1.89226	2.1
43978	1.89226	2.1
43979	1.89226	2.1
43980	1.89226	2.1
43981	1.89226	2.1
43982	3.78453	2.1
43983	1.89226	2.1
43984	1.89226	2.1
43985	1.89226	2.1
43986	1.89226	2.1
43987	3.78452	2.1
43988	2.12556	2.1
43989	0.991113	2.1
43990	1.27725	2.1
43991	2.31081	2.1
43992	3.40281	2.1
43993	1.89226	2.1
43994	2.31082	2.1
43995	2.12556	2.1
43996	2.12556	2.1
43997	1.89226	2.1
43998	1.89226	2.1
43999	1.22544	2.1
44000	1.12649	2.1
44001	0.775254	2.1
44002	0.643133	2.1
44003	0.917924	2.1
44004	0.934955	2.1
44005	0.975579	2.1
44006	0.975579	2.1
44007	1.23113	2.1
44008	0.685272	2.1
44009	0.651719	2.1
44010	1.1791	2.1
44011	1.89226	2.1
44012	2.5138	2.1
44013	2.31082	2.1
44014	2.31082	2.1
44015	1.89226	2.1
44016	1.89226	2.1
44017	1.89226	2.1
44018	1.89226	2.1
44019	1.89226	2.1

44020	1.89226	2.1
44021	3.78453	2.1
44022	0.922224	2.1
44023	0.922224	2.1
44024	1.23555	2.1
44025	1.19715	2.1
44026	1.01445	2.1
44027	1.16061	2.1
44028	0.946132	2.1
44029	0.946132	2.1
44030	0.946131	2.1
44031	1.2051	2.1
44032	1.13537	2.1
44033	1.01445	2.1
44034	0.946133	2.1
44035	0.946133	2.1
44036	1.20648	2.1
44037	1.20648	2.1
44038	1.06454	2.1
44039	1.20648	2.1
44040	0.946133	2.1
44041	0.946133	2.1
44042	1.20649	2.1
44043	0.946133	2.1
44044	1.06454	2.1
44045	0.946132	2.1
44046	0.946132	2.1
44047	0.946132	2.1
44048	0.946132	2.1
44049	0.946132	2.1
44050	0.946132	2.1
44051	0.946132	2.1
44052	0.946133	2.1
44053	0.946133	2.1
44054	0.946133	2.1
44055	0.946133	2.1
44056	0.946132	2.1
44057	0.946132	2.1
44058	1.33078	2.07252
44059	1.37409	2.1
44060	1.31565	0.448209
44061	1.37114	2.1
44062	2.68973	2.09548
44063	1.89225	1.88803
44064	1.89225	2.1
44065	1.89225	0.466338
44066	1.89225	2.08011
44067	3.7845	1.54343
44068	1.89225	2.1
44069	1.89225	2.1
44070	1.89225	2.05066
44071	1.89225	2.1
44072	3.7845	2.1
44073	1.37409	2.1
44074	1.20408	2.1
44075	1.18612	2.1
44076	1.18907	2.1
44077	1.37114	2.1

44078	1.37409	2.1
44079	1.31566	2.05319
44080	1.37115	2.1
44081	1.30318	0.656933
44082	1.37433	2.1
44083	2.68998	2.04015
44084	1.89226	2.06267
44085	1.89226	2.1
44086	1.89226	0.490097
44087	1.89226	2.1
44088	3.78453	1.81673
44089	1.89226	2.09295
44090	1.89226	2.1
44091	1.89226	2.1
44092	1.89226	2.1
44093	1.89226	2.1
44094	1.89226	2.1
44095	0.647001	2.1
44096	0.647001	2.1
44097	0.651851	2.1
44098	0.651851	2.1
44099	0.602046	2.1
44100	0.613277	2.1
44101	0.685576	2.1
44102	0.687164	2.1
44103	0.687164	2.1
44104	0.613276	2.1
44105	0.602046	2.1
44106	0.685576	2.1
44107	0.946128	1.46506
44108	0.946128	0.752178
44109	1.15307	2.08354
44110	1.19317	2.03784
44111	0.94613	2.0301
44112	0.94613	0.660912
44113	1.15307	1.68433
44114	1.15307	2.07311
44115	0.946131	1.80326
44116	1.15307	1.84265
44117	0.946131	1.77405
44118	1.19317	1.76065
44119	1.18278	2.1
44120	2.38635	2.05474
44121	1.23042	2.1
44122	1.23042	2.1
44123	2.30614	2.1
44124	2.30614	2.1
44125	0.946129	0.913484
44126	0.946129	0.785668
44127	1.12994	1.92841
44128	1.15307	1.80281
44129	0.946131	1.76221
44130	0.946131	0.480108
44131	1.12994	1.04682
44132	1.12994	1.86468
44133	1.12994	1.35289
44134	0.946131	1.34051
44135	0.946131	1.33751

44136	1.15307	1.37738
44137	2.25988	2.0966
44138	2.30614	1.84962
44139	2.28696	2.1
44140	1.16361	2.1
44141	1.18278	2.1
44142	1.18278	2.09214
44143	2.3771	2.1
44144	1.90121	2.1
44145	1.81191	2.1
44146	2.46904	2.1
44147	4.37025	2.1
44148	1.15676	2.1
44149	1.23452	2.1
44150	0.823592	2.1
44151	0.737606	2.1
44152	0.988113	2.1
44153	0.988113	2.1
44154	0.984014	2.1
44155	0.984014	2.1
44156	1.15676	2.1
44157	0.737605	2.1
44158	0.823592	2.1
44159	1.23452	2.1
44160	1.30318	2.1
44161	1.37433	2.1
44162	1.30151	0.67036
44163	1.39067	2.1
44164	2.67584	2.05346
44165	1.89226	1.9249
44166	1.89226	2.1
44167	1.89226	0.657077
44168	1.89226	1.97673
44169	3.78453	1.56983
44170	1.89226	1.82815
44171	1.89226	2.1
44172	1.89226	2.06936
44173	1.89226	2.1
44174	1.89226	2.06399
44175	1.89226	2.1
44176	0.656819	2.1
44177	0.656819	2.09885
44178	0.661755	2.1
44179	0.661755	2.1
44180	0.613277	2.1
44181	0.63141	2.1
44182	0.687164	2.08288
44183	0.695336	2.1
44184	0.695337	2.1
44185	0.63141	2.1
44186	0.687164	2.1
44187	0.613276	2.1
44188	1.30151	2.02334
44189	1.39067	2.1
44190	1.32143	0.84018
44191	1.43294	2.0956
44192	2.73445	2.06293
44193	1.89226	1.96574

44194	1.89226	2.1
44195	1.89226	0.451274
44196	1.89226	2.1
44197	3.78452	1.76036
44198	1.89226	2.03041
44199	1.89226	2.06106
44200	1.89226	2.08052
44201	1.89226	2.1
44202	1.89226	2.1
44203	1.89226	2.1
44204	1.43294	2.02068
44205	1.26282	2.1
44206	1.3268	2.09928
44207	1.3268	2.1
44208	1.39067	2.09892
44209	1.39067	2.1
44210	1.5511	0.0943393
44211	1.00665	0.116465
44212	1.28239	0.0813922
44213	1.88631	0.081601
44214	2.89296	0.0887291
44215	1.89227	0.0805111
44216	1.89227	0.135168
44217	1.89227	0.103488
44218	1.89227	0.0928262
44219	1.89227	0.117742
44220	1.89227	0.102692
44221	2.08848	0.114645
44222	1.89226	0.115542
44223	1.89226	0.0851694
44224	2.37947	0.118816
44225	4.27173	0.114266
44226	1.01456	0.0929054
44227	0.943157	0.0900592
44228	0.641198	0.0995484
44229	0.724757	0.093708
44230	0.830923	0.0999604
44231	0.830923	0.118936
44232	0.836991	0.0797188
44233	0.836991	0.0992658
44234	1.01456	0.0858451
44235	0.724757	0.0972516
44236	0.641197	0.0961341
44237	0.943157	0.106925
44238	1.89227	0.113329
44239	1.89227	0.140076
44240	1.89227	0.0941085
44241	1.89227	0.099259
44242	3.78453	0.101606
44243	0.946129	0.116199
44244	0.946129	0.112726
44245	1.28941	0.107317
44246	1.18974	0.112259
44247	0.94613	0.112947
44248	0.94613	0.124576
44249	1.28941	0.100996
44250	1.28941	0.116079
44251	1.28941	0.105676

44252	0.946131	0.112659
44253	0.94613	0.112902
44254	1.18974	0.121907
44255	0.710636	2.1
44256	0.780249	2.1
44257	0.579033	2.1
44258	0.478812	2.1
44259	0.621822	2.1
44260	0.621822	2.1
44261	0.63724	2.1
44262	0.63724	2.1
44263	0.780251	2.1
44264	0.579032	2.1
44265	0.710637	2.1
44266	0.478811	2.1
44267	1.05499	2.09889
44268	1.00521	2.05071
44269	1.24445	2.1
44270	1.23082	1.02448
44271	2.28581	2.05996
44272	1.48751	2.1
44273	1.23083	1.64701
44274	1.4941	2.08453
44275	1.24445	2.06328
44276	1.48047	1.85449
44277	1.23083	2.05983
44278	0.621185	1.9639
44279	0.621185	2.0484
44280	0.620899	1.85901
44281	0.620899	2.1
44282	0.502515	1.13184
44283	0.626672	0.752954
44284	0.615412	0.937116
44285	0.740234	2.1
44286	0.740233	0.808256
44287	0.626672	0.812809
44288	0.615413	1.04846
44289	0.502516	1.60643
44290	1.95844	2.1
44291	2.6562	2.1
44292	1.95844	2.1
44293	2.20883	2.1
44294	2.38225	2.1
44295	2.63264	2.1
44296	1.89226	2.1
44297	1.89226	2.1
44298	1.89226	2.1
44299	1.89226	2.1
44300	3.78453	2.1
44301	1.95844	2.1
44302	2.38225	2.1
44303	1.71082	2.1
44304	2.14647	2.1
44305	4.1049	2.1
44306	0.946131	2.1
44307	0.946131	2.1
44308	0.946131	2.1
44309	0.946131	2.1

44310	0.946131	2.1
44311	0.946131	2.1
44312	0.946131	2.1
44313	0.946131	2.1
44314	0.946131	2.1
44315	0.946131	2.1
44316	0.946131	2.1
44317	0.946131	2.1
44318	2.12597	2.1
44319	1.2295	2.1
44320	1.28014	2.1
44321	2.19723	2.1
44322	3.40611	2.1
44323	2.12597	2.1
44324	2.79242	2.1
44325	2.73375	2.1
44326	2.66249	2.1
44327	2.19723	2.1
44328	2.12597	2.1
44329	1.13716	2.1
44330	1.09861	2.1
44331	0.666692	2.1
44332	0.640071	2.1
44333	0.891617	2.1
44334	0.891617	2.1
44335	0.885614	2.1
44336	0.885614	2.1
44337	1.13716	2.1
44338	0.666692	2.1
44339	0.640071	2.1
44340	1.09861	2.1
44341	1.89226	0.05
44342	1.70303	0.05
44343	1.89226	0.05
44344	2.64598	0.05
44345	1.81091	0.05
44346	2.53222	0.05
44347	2.32422	0.05
44348	1.89226	0.05
44349	2.78336	0.05
44350	2.78336	0.05
44351	1.89226	0.05
44352	1.89226	0.05
44353	0.946132	0.05
44354	0.946132	0.05
44355	0.946131	0.05
44356	0.946131	0.05
44357	0.946132	0.05
44358	0.946132	0.05
44359	0.946131	0.05
44360	0.946131	0.05
44361	0.946131	0.05
44362	0.946131	0.05
44363	0.946131	0.05
44364	0.946131	0.05
44365	0.946131	0.05
44366	0.946131	0.0527779
44367	0.946132	0.05

44368	0.946132	0.0692707
44369	0.946132	0.0702933
44370	0.946132	0.0603845
44371	0.946131	0.05
44372	0.946131	0.05
44373	0.946131	0.0502647
44374	0.946131	0.0632706
44375	0.946131	0.0539785
44376	0.946131	0.05
44377	2.24323	1.0958
44378	1.89226	1.82693
44379	2.75021	1.59585
44380	2.75021	1.66414
44381	1.89226	0.904679
44382	1.89226	1.24678
44383	2.30908	0.83666
44384	2.19815	2.06898
44385	2.1609	0.665365
44386	1.83796	2.1
44387	4.35904	1.64167
44388	2.19815	2.06664
44389	2.02268	2.1
44390	2.17869	2.1
44391	1.9559	2.1
44392	2.0815	2.1
44393	1.83424	2.1
44394	2.1609	0.644437
44395	1.88877	2.1
44396	1.98413	0.993694
44397	1.87355	2.09039
44398	4.03445	1.77684
44399	1.87355	2.09307
44400	2.02268	2.1
44401	1.87355	2.1
44402	2.10488	2.1
44403	1.68443	2.1
44404	1.89448	2.1
44405	0.946131	0.243098
44406	0.946131	0.201681
44407	1.40574	0.226827
44408	1.12373	0.28251
44409	0.946129	0.264358
44410	0.946129	0.245009
44411	1.40574	0.223668
44412	1.40574	0.221721
44413	0.946131	0.223909
44414	1.40574	0.235897
44415	1.12373	0.265317
44416	0.946131	0.242055
44417	2.14277	0.329033
44418	2.81148	0.279653
44419	1.42762	0.2827
44420	1.42762	0.355074
44421	2.96261	0.288524
44422	2.24746	0.333406
44423	1.89226	0.237284
44424	1.89226	0.147371
44425	1.89226	0.0755705

44426	1.89226	0.133361
44427	3.78453	0.160113
44428	1.89226	0.0883047
44429	1.89226	0.311524
44430	1.89226	0.202173
44431	1.89226	0.0547105
44432	3.78452	0.0852359
44433	0.68433	0.0816916
44434	0.946127	0.134769
44435	0.543921	0.133767
44436	0.946131	0.0649419
44437	0.946131	0.229279
44438	0.946131	0.136432
44439	0.543918	1.53365
44440	0.543918	0.114595
44441	0.684332	0.0869486
44442	0.54392	0.670501
44443	0.946129	0.102455
44444	0.946129	1.20808
44445	1.89226	0.154044
44446	1.08784	0.666467
44447	1.19352	0.180266
44448	1.89226	0.211947
44449	1.19352	0.944438
44450	1.89226	0.191049
44451	0.635711	0.0685375
44452	0.946132	0.195922
44453	0.946132	0.163081
44454	0.56474	0.0652246
44455	0.750045	0.107511
44456	1.25657	0.705764
44457	0.635682	0.0904738
44458	0.635682	0.0941183
44459	0.891954	0.0732916
44460	1.32751	0.373094
44461	0.564766	0.0830732
44462	0.564766	0.472944
44463	0.858345	0.0863371
44464	0.946128	0.567935
44465	0.635712	0.097665
44466	0.946133	0.0785427
44467	0.977198	0.0856823
44468	0.944521	0.605515
44469	0.635682	0.695375
44470	0.635682	0.0636994
44471	1.02924	0.127594
44472	0.705625	0.879738
44473	0.851528	0.0780606
44474	0.851528	1.55697
44475	2.18903	2.1
44476	2.10182	2.1
44477	2.10182	2.1
44478	1.89226	2.1
44479	1.89226	2.1
44480	1.64983	2.1
44481	1.89226	2.1
44482	1.83867	2.1
44483	1.83867	2.1

44484	1.89164	2.1
44485	2.0815	2.1
44486	2.17638	2.1
44487	2.57957	0.837835
44488	2.3506	2.07389
44489	2.69056	0.384814
44490	2.49439	1.83383
44491	5.04116	1.11547
44492	2.3506	2.0131
44493	1.8911	2.1
44494	2.49439	1.48322
44495	2.06689	2.1
44496	4.41748	2.1
44497	2.69056	0.785991
44498	2.49439	2.04428
44499	2.77227	0.33543
44500	2.63443	1.89665
44501	5.32499	1.43018
44502	2.49439	1.34081
44503	2.06689	2.1
44504	2.63443	1.54421
44505	2.26444	2.1
44506	4.70131	2.07887
44507	2.46327	2.1
44508	2.54347	2.1
44509	2.52869	2.1
44510	2.16534	2.1
44511	2.82661	2.1
44512	2.46327	2.1
44513	0.946131	2.1
44514	0.946131	2.1
44515	1.27174	2.1
44516	1.27174	2.1
44517	1.27174	2.1
44518	0.94613	2.1
44519	0.946128	2.1
44520	1.4133	2.1
44521	0.946129	2.1
44522	0.946129	2.1
44523	1.27174	2.1
44524	1.4133	2.1
44525	1.42757	2.1
44526	1.7457	2.1
44527	1.66047	2.1
44528	1.7457	2.1
44529	1.45754	2.1
44530	1.54278	2.1
44531	1.42757	2.1
44532	1.52996	2.1
44533	1.66047	2.1
44534	1.66047	2.1
44535	1.26397	2.1
44536	1.26397	2.1
44537	0.968536	2.1
44538	1.02461	2.1
44539	1.57575	2.1
44540	1.62391	2.1
44541	2.59245	2.1

44542	0.946131	2.1
44543	0.946131	2.1
44544	0.946132	2.1
44545	0.946132	2.1
44546	0.946132	2.1
44547	0.946132	2.1
44548	0.946132	2.1
44549	0.946132	2.1
44550	0.946132	2.1
44551	0.946132	2.1
44552	0.946132	2.1
44553	0.946132	2.1
44554	2.23765	2.1
44555	1.62391	2.1
44556	2.22154	2.1
44557	1.57575	2.1
44558	2.2697	2.1
44559	1.62391	2.1
44560	1.89226	2.1
44561	1.89226	2.1
44562	1.89226	2.1
44563	1.89226	2.1
44564	1.89226	2.1
44565	1.89226	2.1
44566	1.89226	2.1
44567	1.89226	2.1
44568	1.89226	2.1
44569	1.89226	2.1
44570	1.89226	2.1
44571	1.89226	2.1
44572	2.2697	2.1
44573	1.46129	2.1
44574	2.25873	2.1
44575	1.62391	2.1
44576	2.09611	2.1
44577	1.46129	2.1
44578	1.89226	2.1
44579	2.07088	2.1
44580	1.89226	2.1
44581	2.31884	2.1
44582	4.21111	2.1
44583	1.89226	2.1
44584	2.31884	2.1
44585	2.11713	2.1
44586	1.89226	2.1
44587	2.54371	2.1
44588	2.31884	2.1
44589	2.54371	2.1
44590	1.24041	2.1
44591	2.55208	2.1
44592	2.31884	2.1
44593	1.23712	2.1
44594	1.00388	2.1
44595	2.72769	0.05
44596	2.57507	0.0677096
44597	1.89226	0.05
44598	1.89226	0.069339
44599	4.46733	0.0506479

44600	1.89226	0.0693579
44601	2.57506	0.0664862
44602	1.89226	0.05
44603	2.30076	0.0563072
44604	1.89226	0.05
44605	2.30076	0.0633458
44606	1.21171	0.05
44607	1.48589	0.05
44608	2.09166	0.05
44609	1.78381	0.05
44610	3.2697	0.05
44611	2.09166	0.05
44612	1.70302	0.05
44613	1.48589	0.05
44614	1.18027	0.05
44615	2.26571	0.05
44616	1.79969	0.05
44617	1.89226	0.05
44618	1.89226	0.076035
44619	1.89226	0.0801858
44620	1.89226	0.0518426
44621	1.89226	0.0815249
44622	1.89226	0.0544124
44623	0.946131	0.05
44624	0.946131	0.0870737
44625	0.946128	0.0767015
44626	0.946128	0.0527282
44627	0.946129	0.0803557
44628	0.946129	0.0808892
44629	0.94613	0.05
44630	0.94613	0.05
44631	0.94613	0.0593003
44632	0.94613	0.0669775
44633	0.94613	0.0644203
44634	0.94613	0.0583304
44635	1.89226	1.82175
44636	1.89226	1.85389
44637	1.89226	1.95512
44638	1.89226	1.57337
44639	1.89226	1.95411
44640	1.89226	1.55995
44641	1.89226	1.81719
44642	1.89226	1.81417
44643	1.89226	1.92507
44644	1.89226	1.59076
44645	1.89226	1.9332
44646	1.89226	1.60248
44647	1.89226	1.591
44648	1.89226	1.61436
44649	1.89226	1.82579
44650	1.89226	1.17566
44651	1.89226	1.85433
44652	1.89226	1.23658
44653	1.79103	1.33573
44654	0.968249	2.09995
44655	1.44807	2.08794
44656	2.17984	0.87361
44657	3.2391	2.04502

44658	1.29735	1.54084
44659	1.08992	1.56815
44660	0.945147	2.09983
44661	0.724033	2.1
44662	1.00723	2.1
44663	1.00723	1.87449
44664	1.01416	1.20946
44665	1.01416	2.09158
44666	1.29735	2.01547
44667	0.94515	2.00459
44668	0.724034	2.06987
44669	1.08992	2.08243
44670	1.39214	2.1
44671	1.39214	2.1
44672	0.946129	2.1
44673	0.946129	2.1
44674	1.33728	2.1
44675	1.39214	2.1
44676	0.94613	2.1
44677	0.94613	2.1
44678	0.94613	2.1
44679	1.33728	2.1
44680	0.94613	2.1
44681	1.39214	2.1
44682	2.78428	2.1
44683	2.28373	2.1
44684	2.67456	2.1
44685	2.20011	2.1
44686	4.95829	2.1
44687	1.90165	0.277224
44688	2.24206	0.81033
44689	1.66137	0.196912
44690	2.07108	1.80642
44691	3.90343	0.404567
44692	1.89224	0.643971
44693	1.89224	0.847346
44694	1.89224	0.32086
44695	1.89224	0.270195
44696	3.78448	0.312953
44697	2.82159	0.795477
44698	1.89226	0.661288
44699	2.50138	1.77782
44700	1.89226	0.339497
44701	4.39365	0.648952
44702	1.66139	0.177564
44703	1.86399	1.8861
44704	1.56005	0.490572
44705	1.90324	0.963404
44706	3.56463	0.609574
44707	0.946133	0.103715
44708	0.946133	1.01237
44709	0.946132	0.309734
44710	0.946132	0.794531
44711	0.946133	0.36426
44712	0.946133	0.993259
44713	0.946132	0.298343
44714	0.946132	0.36736
44715	0.946133	0.245809

44716	0.946133	0.305304
44717	0.946132	0.170441
44718	0.946132	0.259595
44719	1.5176	0.357545
44720	1.97964	0.198031
44721	2.32889	0.284072
44722	1.91762	0.254365
44723	3.84649	0.232801
44724	2.3376	0.264225
44725	1.97963	0.228502
44726	2.32889	0.345975
44727	2.58303	0.196799
44728	4.66649	0.297731
44729	2.32889	0.24771
44730	2.20248	0.205595
44731	1.91762	0.205463
44732	1.91762	0.185074
44733	2.5615	0.322088
44734	2.5615	0.248922
44735	2.32889	0.344723
44736	2.58303	0.182853
44737	2.71793	0.263673
44738	2.56149	0.201049
44739	5.04681	0.206988
44740	1.89226	0.1498
44741	2.53785	0.188421
44742	1.89226	0.107517
44743	2.60895	0.136754
44744	1.89226	0.093685
44745	2.60895	0.0961755
44746	0.493248	0.102996
44747	1.30448	0.195188
44748	1.26893	0.363352
44749	0.653226	0.0857325
44750	0.653227	0.152147
44751	0.493247	0.621266
44752	1.26893	0.308252
44753	1.30448	0.198853
44754	0.832232	0.289968
44755	0.832233	0.488762
44756	0.929944	0.0864804
44757	0.929944	0.30529
44758	1.89226	2.1
44759	1.47937	2.1
44760	1.50143	2.1
44761	1.47937	2.1
44762	1.91432	2.1
44763	1.89226	2.1
44764	1.89226	2.1
44765	1.89226	2.1
44766	1.89226	2.1
44767	1.89226	2.1
44768	1.89226	2.1
44769	1.89226	2.1
44770	1.33419	2.1
44771	2.4542	2.1
44772	2.59834	2.1
44773	2.59834	2.1

44774	1.25716	2.1
44775	1.25716	2.1
44776	1.70303	2.1
44777	1.50143	2.1
44778	1.60878	2.1
44779	1.50143	2.1
44780	1.93599	2.1
44781	1.89226	2.1
44782	0.946131	2.09385
44783	0.946131	2.1
44784	0.946127	2.1
44785	0.946127	2.1
44786	0.946127	2.1
44787	0.946127	2.07056
44788	0.946131	2.1
44789	0.946131	2.1
44790	0.94613	2.1
44791	0.94613	2.1
44792	0.946129	2.1
44793	0.946129	2.1
44794	1.43912	2.1
44795	2.59834	2.1
44796	2.78093	2.1
44797	2.78093	2.1
44798	1.33419	2.1
44799	1.33419	2.1
44800	1.89226	2.1
44801	2.26322	2.1
44802	2.55682	2.1
44803	2.55682	2.1
44804	1.89226	2.1
44805	1.89226	2.1
44806	2.58032	0.181925
44807	2.28922	0.111231
44808	1.88424	0.125426
44809	2.32127	0.103531
44810	4.46456	0.120057
44811	1.67265	0.170898
44812	2.28922	0.125095
44813	1.88424	0.103362
44814	1.11314	0.101609
44815	3.55689	0.115577
44816	1.43751	0.0897683
44817	2.32127	0.0969472
44818	1.92998	0.0767524
44819	2.37671	0.0830105
44820	1.43751	0.134195
44821	1.88424	0.128499
44822	2.36729	2.1
44823	1.56714	2.1
44824	1.56714	2.1
44825	1.62408	2.1
44826	2.28373	2.1
44827	2.34067	2.1
44828	0.937268	2.1
44829	0.937268	2.1
44830	0.94636	2.1
44831	0.94636	2.1

44832	0.76004	2.1
44833	0.783572	2.1
44834	1.14187	2.1
44835	1.10006	2.1
44836	1.10006	2.1
44837	0.76004	2.1
44838	0.783572	2.1
44839	1.14187	2.1
44840	1.28753	2.1
44841	1.36838	2.1
44842	1.62617	2.1
44843	1.82857	2.1
44844	3.1161	2.1
44845	1.82856	2.1
44846	1.17996	2.1
44847	1.53417	2.1
44848	1.56618	2.1
44849	1.36838	2.1
44850	1.36838	2.1
44851	1.35065	0.988541
44852	1.42659	0.284204
44853	2.26675	0.690912
44854	2.26675	0.522504
44855	1.62137	0.522043
44856	2.53747	0.655591
44857	1.89226	0.0917648
44858	1.89226	0.05
44859	1.89226	0.0510887
44860	1.89226	0.0757864
44861	3.78452	0.0723216
44862	1.36569	0.05
44863	1.36569	0.05
44864	0.946131	0.05
44865	0.946131	0.05
44866	1.16041	0.05
44867	1.36569	0.05
44868	0.946129	0.05
44869	0.946129	0.0500295
44870	1.1604	0.05
44871	0.94613	0.05
44872	0.946131	0.05
44873	1.36569	0.05
44874	2.73139	0.05
44875	1.25265	0.05
44876	1.25265	0.05
44877	1.64799	0.05
44878	2.32081	0.05
44879	2.71615	0.05
44880	0.946133	0.05
44881	0.946133	0.05
44882	0.946132	0.0731106
44883	0.946132	0.0588054
44884	0.946132	0.05
44885	0.946132	0.0579687
44886	0.946133	0.05
44887	0.946133	0.0661568
44888	0.946132	0.05
44889	0.946132	0.0561508

44890	0.946133	0.0526502
44891	0.946133	0.05
44892	0.946131	0.05
44893	0.946131	0.05
44894	0.94613	0.0986605
44895	0.94613	0.0685181
44896	0.946132	0.0775608
44897	0.946132	0.0510332
44898	0.946129	0.0638803
44899	0.946129	0.0969513
44900	0.946131	0.0629101
44901	0.946131	0.0504669
44902	0.946131	0.0519082
44903	0.946131	0.0660357
44904	1.89226	2.1
44905	2.48503	2.1
44906	2.64586	2.1
44907	2.64586	2.1
44908	1.89226	2.1
44909	1.89226	2.1
44910	2.48504	2.1
44911	1.99985	2.1
44912	1.83584	2.1
44913	1.83584	2.1
44914	2.64586	2.1
44915	2.64586	2.1
44916	1.89225	2.1
44917	2.33095	2.1
44918	2.48501	2.1
44919	2.48501	2.1
44920	1.89225	2.1
44921	1.89225	2.1
44922	2.33096	2.1
44923	1.83583	2.1
44924	1.68436	2.1
44925	1.68436	2.1
44926	2.48502	2.1
44927	2.48502	2.1
44928	1.14807	2.1
44929	1.76545	2.1
44930	1.21292	2.1
44931	1.83936	2.1
44932	2.97837	2.1
44933	1.89226	2.1
44934	1.89226	2.1
44935	1.89226	2.1
44936	1.89226	2.1
44937	3.78451	2.1
44938	1.89226	2.1
44939	1.89226	2.03505
44940	1.89226	2.1
44941	1.89226	2.1
44942	3.78452	2.1
44943	1.76545	2.1
44944	2.12608	2.1
44945	1.83937	2.1
44946	2.22529	2.1
44947	3.99074	2.1

44948	0.780222	2.1
44949	0.780222	2.1
44950	0.784871	2.1
44951	0.784871	2.1
44952	0.95863	2.1
44953	0.919681	2.1
44954	0.636145	2.1
44955	0.606461	2.1
44956	0.636144	2.1
44957	0.958631	2.1
44958	0.60646	2.1
44959	0.919683	2.1
44960	1.89226	2.1
44961	1.89226	2.1
44962	1.89226	2.1
44963	1.89226	2.1
44964	3.78452	2.1
44965	1.89226	2.1
44966	1.89226	2.1
44967	1.89226	2.1
44968	1.89226	2.04741
44969	3.78452	2.1
44970	1.03789	2.1
44971	1.03789	2.1
44972	1.04231	2.1
44973	1.04231	2.1
44974	1.16052	2.1
44975	1.11265	2.1
44976	0.958634	2.1
44977	0.919685	2.1
44978	0.958634	2.1
44979	1.16052	2.1
44980	0.919685	2.1
44981	1.11265	2.1
44982	1.43837	2.1
44983	2.57085	2.1
44984	1.39332	2.1
44985	2.58228	2.1
44986	3.96417	2.1
44987	1.89226	2.1
44988	1.89226	2.1
44989	1.89226	2.1
44990	1.89226	2.1
44991	1.89226	2.1
44992	1.89226	2.1
44993	1.89226	2.1
44994	1.89226	2.1
44995	1.89226	2.1
44996	1.89226	2.1
44997	3.78452	2.1
44998	2.57085	2.1
44999	1.89226	2.1
45000	2.58229	2.1
45001	1.89226	2.1
45002	4.46312	2.1
45003	1.32368	2.1
45004	2.58228	2.1
45005	2.55352	2.1

45006	2.55352	2.1
45007	1.39332	2.1
45008	1.39332	2.1
45009	1.89226	2.1
45010	1.89226	2.1
45011	1.89226	2.1
45012	1.89226	2.1
45013	1.89226	2.1
45014	1.89226	2.1
45015	1.89226	2.1
45016	1.89226	2.1
45017	1.89226	2.1
45018	1.89226	2.1
45019	1.89226	2.1
45020	1.89226	2.1
45021	2.55353	2.1
45022	1.89226	2.1
45023	2.58228	2.1
45024	1.89226	2.1
45025	1.89226	2.1
45026	2.58228	2.1
45027	1.40574	1.83192
45028	1.05175	1.42433
45029	1.71613	1.74498
45030	1.99961	1.5777
45031	3.12187	1.62256
45032	1.71613	1.71986
45033	2.50733	1.48009
45034	1.99961	1.87248
45035	1.99961	1.61983
45036	2.25746	1.5576
45037	2.25746	1.3517
45038	2.25746	1.3903
45039	1.89526	1.4613
45040	1.71613	1.75542
45041	1.71613	1.0356
45042	1.89526	1.53527
45043	1.27558	1.0277
45044	1.07536	2.1
45045	1.07536	2.1
45046	1.07034	2.1
45047	1.07034	2.1
45048	0.766879	2.1
45049	0.81527	2.1
45050	1.38896	2.1
45051	1.33043	2.1
45052	0.815264	2.1
45053	1.38895	2.1
45054	0.766884	2.1
45055	1.33044	2.1
45056	1.89225	2.1
45057	1.89225	2.1
45058	1.89225	2.1
45059	1.89225	2.1
45060	1.89225	2.1
45061	1.89225	2.1
45062	1.64059	2.1
45063	1.89225	2.1

45064	1.565	2.1
45065	1.565	2.1
45066	1.89225	2.1
45067	1.89225	2.1
45068	1.89226	2.1
45069	1.89226	2.1
45070	1.89226	2.1
45071	1.89226	2.1
45072	1.89226	2.1
45073	1.89226	2.1
45074	1.67906	2.1
45075	1.89226	2.1
45076	1.6406	2.1
45077	1.6406	2.1
45078	1.89226	2.1
45079	1.89226	2.1
45080	2.18106	1.74594
45081	2.20831	2.09216
45082	2.64725	0.73406
45083	2.63102	1.12328
45084	4.82831	1.48182
45085	2.18106	1.29266
45086	2.09605	2.1
45087	2.5601	1.54549
45088	2.63102	1.17332
45089	4.74116	2.03334
45090	2.64725	1.00787
45091	1.89226	1.37864
45092	1.87603	2.1
45093	2.63102	1.20982
45094	1.89226	1.92909
45095	2.64725	0.657722
45096	0.946132	1.35741
45097	1.28005	1.22779
45098	0.946132	2.04363
45099	1.31551	0.899112
45100	1.28005	1.60325
45101	1.28005	1.95371
45102	0.946132	1.12449
45103	0.946132	1.42921
45104	0.946132	1.12027
45105	0.946132	1.30649
45106	1.28005	1.59197
45107	1.31551	1.69906
45108	2.0815	2.1
45109	1.59318	2.1
45110	2.0815	2.1
45111	1.40161	2.1
45112	2.49782	2.1
45113	1.68553	2.1
45114	0.819885	2.1
45115	0.935082	2.1
45116	0.867363	2.1
45117	0.867363	2.1
45118	0.715431	2.1
45119	0.902777	2.1
45120	0.738933	2.1
45121	0.980053	2.1

45122	0.936716	2.1
45123	0.917651	2.1
45124	0.775853	2.1
45125	0.74037	2.1
45126	2.78977	2.1
45127	2.38239	2.1
45128	2.78977	2.1
45129	1.96468	2.1
45130	2.64107	2.1
45131	1.80733	2.1
45132	1.80141	2.1
45133	2.28512	2.1
45134	2.28512	2.1
45135	1.8519	2.1
45136	2.64107	2.1
45137	2.20785	2.1
45138	0.946133	0.832641
45139	0.946133	0.618506
45140	0.946132	0.767083
45141	0.946132	0.563144
45142	0.946132	0.770226
45143	0.946132	0.989803
45144	0.946132	0.578632
45145	0.946132	0.57303
45146	0.946132	0.638845
45147	0.946132	0.716147
45148	0.946133	0.66095
45149	0.946133	0.787999
45150	1.89226	0.325817
45151	1.89226	0.342426
45152	1.89226	0.256192
45153	1.89226	0.395477
45154	1.89226	0.278673
45155	1.89226	0.392621
45156	1.89226	0.418985
45157	1.89226	0.469538
45158	1.89226	0.360048
45159	1.89226	0.555653
45160	3.78453	0.47273
45161	1.89226	0.671462
45162	1.89226	0.497245
45163	1.89226	0.421724
45164	1.89226	0.811568
45165	1.89226	0.489831
45166	1.89226	0.668545
45167	2.12925	0.05
45168	2.51397	0.05
45169	2.22488	0.05
45170	1.86113	0.05
45171	4.3751	0.05
45172	2.82754	0.05
45173	2.51397	0.05
45174	2.22489	0.05
45175	2.47291	0.05
45176	4.98688	0.05
45177	1.89226	0.05
45178	1.89226	0.0853487
45179	1.89226	0.05

45180	1.89226	0.0812422
45181	3.78453	0.0703595
45182	1.89226	0.05
45183	1.89226	0.0790344
45184	1.89226	0.05
45185	1.89226	0.0815955
45186	3.78452	0.0541608
45187	0.90848	0.05
45188	1.11244	0.05
45189	0.930566	0.05
45190	0.73385	0.05
45191	0.921298	0.05
45192	0.921298	0.05
45193	0.924994	0.05
45194	0.924994	0.05
45195	0.908478	0.05
45196	0.733851	0.05
45197	1.11244	0.05
45198	0.930567	0.05
45199	2.22489	0.05
45200	2.0815	0.05
45201	2.0815	0.05
45202	2.30674	0.05
45203	1.99867	0.05
45204	2.33358	0.05
45205	1.89226	0.05
45206	1.89226	0.0748581
45207	1.89226	0.05
45208	1.89226	0.0647678
45209	1.89226	0.05
45210	1.89226	0.0595281
45211	0.946131	0.0744777
45212	0.946131	0.0548261
45213	0.946128	0.0684081
45214	0.946128	0.05
45215	0.946129	0.0686802
45216	0.946129	0.0792842
45217	0.94613	0.05
45218	0.94613	0.05
45219	0.94613	0.0570195
45220	0.94613	0.05
45221	0.94613	0.0515843
45222	0.94613	0.0633481
45223	2.28904	2.1
45224	1.89226	2.09087
45225	2.2846	2.09709
45226	2.2846	2.1
45227	1.89226	1.89961
45228	1.89226	2.1
45229	1.89226	1.9048
45230	1.89226	1.40411
45231	1.89226	2.1
45232	1.89226	2.0997
45233	1.89226	0.775255
45234	1.89226	2.09904
45235	1.04037	1.63632
45236	1.34066	2.07722
45237	1.34955	1.46509

45238	1.34955	2.06195
45239	1.03373	1.59779
45240	1.03373	2.1
45241	0.946131	1.82484
45242	0.946131	2.1
45243	1.14452	2.1
45244	1.14452	2.1
45245	0.946131	1.97605
45246	0.946131	2.01411
45247	1.14296	2.1
45248	1.14452	2.1
45249	1.14296	2.09952
45250	0.946131	2.1
45251	1.14452	2.1
45252	0.946132	2.1
45253	1.89226	1.62712
45254	1.89226	2.049
45255	1.89226	2.00348
45256	1.89226	2.1
45257	1.89226	0.742812
45258	1.89226	2.1
45259	0.597454	1.6084
45260	0.597454	2.00676
45261	0.597972	1.74936
45262	0.597972	2.1
45263	0.675238	2.06883
45264	0.674776	1.61349
45265	0.519667	1.72565
45266	0.520188	1.85731
45267	0.519667	0.822576
45268	0.675238	0.8682
45269	0.520188	1.77993
45270	0.674777	1.79125
45271	1.89226	0.237543
45272	1.82125	0.184945
45273	1.89226	0.170663
45274	1.84314	0.150349
45275	1.89226	0.330361
45276	1.84314	0.285258
45277	1.89226	0.190098
45278	1.84314	0.20017
45279	1.89226	0.163973
45280	1.65794	0.155074
45281	1.89226	0.201911
45282	1.65794	0.188685
45283	1.348	2.06224
45284	1.43485	2.1
45285	1.79929	0.529975
45286	1.75309	1.75873
45287	3.14728	1.89659
45288	1.348	1.1716
45289	1.63892	1.85019
45290	1.59157	1.98534
45291	1.18647	1.92278
45292	1.75309	1.20284
45293	1.348	1.87515
45294	1.79929	1.53326
45295	1.75309	2.1

45296	2.18106	1.21741
45297	2.20831	1.13283
45298	3.98034	1.91887
45299	2.18106	0.887855
45300	1.63892	1.95173
45301	1.75309	0.915038
45302	1.75309	2.06957
45303	2.09605	1.01647
45304	2.09605	2.06543
45305	2.66076	0.180722
45306	1.70302	0.0899231
45307	2.41575	0.10972
45308	1.70302	0.154953
45309	3.08598	0.0980593
45310	2.3661	0.139889
45311	1.71053	0.060836
45312	2.41575	0.141375
45313	1.20143	0.05
45314	1.20143	0.0751316
45315	2.2123	0.05
45316	1.66739	0.0825142
45317	2.25633	0.0723421
45318	2.68302	0.096597
45319	1.71051	0.05
45320	1.71051	0.0748335
45321	2.74192	0.05
45322	2.1961	0.0671749
45323	1.32553	2.1
45324	2.34875	1.8663
45325	2.19139	2.1
45326	2.19139	1.42317
45327	1.56172	2.1
45328	1.56172	1.19877
45329	1.89226	2.1
45330	1.89226	1.92041
45331	1.89226	2.1
45332	1.89226	2.0992
45333	3.78453	2.1
45334	2.667 2.1	
45335	1.26097	2.1
45336	1.26097	2.1
45337	1.5683	2.1
45338	2.42949	2.1
45339	2.73682	2.1
45340	1.13213	2.1
45341	2.19139	1.72204
45342	2.0815	2.1
45343	1.77615	1.18134
45344	1.37767	2.1
45345	1.19297	1.08219
45346	0.946132	1.97823
45347	0.946132	2.1
45348	0.946131	2.1
45349	0.946131	2.097
45350	0.946131	2.09315
45351	0.946131	1.70766
45352	0.946132	2.1
45353	0.946132	2.1

45354	0.946132	2.1
45355	0.946132	2.1
45356	0.946131	2.1
45357	0.946131	2.1
45358	2.02064	2.1
45359	2.3606	2.1
45360	1.47478	2.1
45361	2.22997	2.1
45362	4.25061	2.1
45363	2.36059	2.1
45364	2.56952	2.1
45365	2.22997	2.1
45366	2.48631	2.1
45367	4.79949	2.1
45368	1.84261	2.1
45369	2.22997	2.1
45370	1.65706	2.1
45371	1.78837	2.1
45372	3.88703	2.1
45373	2.22997	2.1
45374	2.48631	2.1
45375	2.19528	2.1
45376	2.41175	2.1
45377	4.64172	2.1
45378	1.6466	2.1
45379	1.89226	2.1
45380	1.35972	2.1
45381	1.35972	2.1
45382	1.89226	2.1
45383	1.89226	2.1
45384	1.89226	2.1
45385	1.89226	2.1
45386	1.89226	2.1
45387	1.89226	2.1
45388	3.78453	2.1
45389	2.44797	2.1
45390	1.07214	2.1
45391	1.31589	2.1
45392	1.1211	2.1
45393	2.44797	2.1
45394	2.25318	2.1
45395	1.95954	2.1
45396	1.89226	2.1
45397	1.6466	2.1
45398	1.6466	2.1
45399	1.89226	2.1
45400	1.89226	2.1
45401	0.946133	2.1
45402	0.946133	2.1
45403	0.946132	2.1
45404	0.946132	2.1
45405	0.946132	2.1
45406	0.946132	2.1
45407	0.946133	2.1
45408	0.946133	2.1
45409	0.946132	2.1
45410	0.946132	2.1
45411	0.946133	2.1

45412	0.946133	2.1
45413	2.44797	2.1
45414	1.36904	2.1
45415	1.70303	2.1
45416	2.37586	2.1
45417	4.00507	2.1
45418	0.631553	0.05
45419	1.24489	0.05
45420	1.11597	0.05
45421	0.496799	0.05
45422	0.872272	0.05
45423	0.872272	0.05
45424	0.869415	0.05
45425	0.869415	0.05
45426	0.4968	0.05
45427	0.631553	0.05
45428	1.11597	0.05
45429	1.24489	0.05
45430	1.84253	2.1
45431	2.17384	2.1
45432	2.04818	2.1
45433	1.65925	2.1
45434	3.83309	2.1
45435	2.04818	2.1
45436	1.65925	2.1
45437	1.48162	2.1
45438	1.92264	2.1
45439	3.58189	2.1
45440	2.3305	0.0878122
45441	1.89226	0.103366
45442	2.52753	0.0899884
45443	2.52753	0.124479
45444	1.89226	0.0759245
45445	1.89226	0.101728
45446	1.89226	0.114035
45447	1.89226	0.183522
45448	1.89226	0.15302
45449	1.89226	0.219661
45450	1.89226	0.108725
45451	1.89226	0.163386
45452	1.34563	0.0920823
45453	2.1769	0.0789037
45454	1.94955	0.078443
45455	1.94955	0.146091
45456	1.55063	0.0776194
45457	1.55063	0.137841
45458	2.09455	0.0679902
45459	1.89226	0.0845398
45460	2.3305	0.0734603
45461	2.3305	0.0944896
45462	1.89226	0.0604372
45463	1.89226	0.0773385
45464	1.89226	0.0873075
45465	1.89226	0.10697
45466	1.89226	0.121623
45467	1.89226	0.108389
45468	1.89226	0.115596
45469	1.89226	0.107268

45470	0.773392	0.059606
45471	0.612765	0.0811284
45472	0.79504	0.0761589
45473	0.575323	0.067207
45474	1.0819	0.069898
45475	1.06106	0.0708097
45476	1.04675	0.0549197
45477	0.86858	0.10277
45478	0.796873	0.0683762
45479	0.557469	0.0859473
45480	0.807663	0.0806411
45481	2.2024	2.1
45482	2.34106	2.1
45483	2.02634	2.1
45484	2.20125	2.1
45485	4.36739	2.1
45486	2.34106	2.1
45487	2.25195	2.1
45488	2.20125	2.1
45489	2.13132	2.08815
45490	4.47237	2.1
45491	2.02635	2.1
45492	2.22465	2.1
45493	1.68504	2.1
45494	2.0815	2.1
45495	3.92673	2.1
45496	2.22465	2.1
45497	2.13133	2.0919
45498	2.0815	2.1
45499	2.23579	2.1
45500	4.44862	2.1
45501	1.89226	1.23768
45502	1.89226	1.22431
45503	1.89226	1.5275
45504	1.89226	1.25426
45505	1.89226	1.44802
45506	1.89226	1.10371
45507	1.93967	0.0519244
45508	1.70302	0.067419
45509	1.21883	0.0515736
45510	1.70302	0.0768351
45511	1.19889	0.0520074
45512	1.70302	0.0771458
45513	0.946131	0.11739
45514	0.946131	0.0892604
45515	0.946131	0.0663356
45516	0.946131	0.078693
45517	0.94613	0.0781099
45518	0.94613	0.137441
45519	0.946131	0.0719073
45520	0.946131	0.0834456
45521	0.946131	0.0806935
45522	0.946131	0.0809858
45523	0.946131	0.0994261
45524	0.946131	0.087862
45525	0.883128	0.0605737
45526	0.883128	0.0752297
45527	0.827519	0.05

45528	0.82752	0.0556453
45529	1.16234	0.0554428
45530	1.19063	0.0860654
45531	0.631294	0.0611852
45532	0.548311	0.0583584
45533	0.631293	0.05
45534	1.16234	0.05
45535	1.19063	0.06315
45536	0.54831	0.0627259
45537	1.89226	0.0550112
45538	1.89226	0.115325
45539	1.89226	0.11466
45540	1.89226	0.0639963
45541	3.78453	0.0781409
45542	2.33328	0.05
45543	1.87991	0.05
45544	1.87991	0.05
45545	2.82938	0.05
45546	1.84457	0.05
45547	2.79403	0.05
45548	2.82937	0.05
45549	2.70881	0.05
45550	1.8799	0.05
45551	1.82739	0.05
45552	4.58874	0.05
45553	1.89226	0.122132
45554	1.89226	0.050793
45555	1.89226	0.0677234
45556	1.89226	0.105962
45557	3.78452	0.0930282
45558	1.17897	0.05
45559	1.82739	0.05
45560	1.17897	0.05
45561	1.76199	0.05
45562	1.23885	0.05
45563	1.80989	0.05
45564	0.946131	0.05
45565	0.946131	0.05
45566	0.946131	0.0818902
45567	0.946131	0.0748119
45568	0.946129	0.0782359
45569	0.946129	0.05
45570	0.946133	0.0646977
45571	0.946133	0.0805114
45572	0.946131	0.0648098
45573	0.946131	0.05
45574	0.946131	0.0525623
45575	0.946131	0.0633986
45576	0.946126	1.96738
45577	0.946126	2.1
45578	1.24887	2.1
45579	1.24887	2.1
45580	0.946127	2.1
45581	0.946127	2.03417
45582	1.24887	2.1
45583	1.28511	2.1
45584	1.2851	2.1
45585	0.946128	2.1

45586	1.24887	2.1
45587	0.946128	2.1
45588	1.89226	2.1
45589	1.89226	2.05386
45590	1.89226	2.1
45591	1.89226	0.889104
45592	3.78452	2.02228
45593	0.592142	2.08734
45594	0.592142	2.1
45595	0.60496	2.1
45596	0.60496	2.1
45597	0.694237	2.1
45598	0.608714	1.80347
45599	0.562863	2.1
45600	0.502864	2.1
45601	0.562863	2.1
45602	0.694237	2.1
45603	0.608714	2.1
45604	0.502864	2.1
45605	1.89226	2.1
45606	1.89226	2.1
45607	1.89226	2.1
45608	1.89226	2.1
45609	3.78453	2.1
45610	1.55779	2.1
45611	1.57968	2.1
45612	1.47186	2.1
45613	1.51725	2.1
45614	3.05154	2.1
45615	1.57967	2.1
45616	1.474	2.1
45617	1.51725	2.1
45618	1.42895	2.1
45619	3.00863	2.1
45620	1.89226	2.1
45621	1.89226	2.1
45622	1.89226	2.1
45623	1.89226	2.1
45624	3.78452	2.1
45625	1.89226	2.1
45626	1.89226	2.1
45627	1.89226	2.1
45628	1.89226	2.1
45629	3.78453	2.1
45630	1.47186	2.1
45631	1.51725	2.1
45632	1.38859	2.1
45633	1.45332	2.1
45634	2.92519	2.1
45635	1.51725	2.1
45636	1.42895	2.1
45637	1.45332	2.1
45638	1.38414	2.1
45639	2.88228	2.1
45640	1.89226	2.1
45641	1.89226	2.1
45642	1.89226	2.1
45643	1.89226	2.1

45644	3.78452	2.1
45645	1.34497	0.05
45646	1.36747	0.05
45647	1.18919	0.05
45648	2.08149	0.05
45649	1.23556	0.05
45650	2.13702	0.05
45651	1.10106	0.05
45652	2.08001	0.05
45653	1.34497	0.05
45654	2.31605	0.05
45655	1.34497	0.05
45656	2.31605	0.05
45657	2.31605	0.05
45658	2.0815	0.05
45659	1.9419	0.05
45660	2.46665	0.05
45661	4.52922	0.05
45662	1.20349	0.05
45663	2.08149	0.05
45664	1.36948	0.05
45665	1.34737	0.05
45666	2.08149	0.05
45667	2.08149	0.05
45668	0.946132	0.05
45669	0.946132	0.05
45670	1.37715	0.05
45671	0.823199	0.05
45672	1.21598	0.05
45673	0.946132	0.05
45674	1.37715	0.05
45675	0.946132	0.05
45676	1.21598	0.05
45677	0.823198	0.05
45678	0.946133	0.05
45679	0.946133	0.05
45680	1.89226	0.05
45681	1.89226	0.05
45682	1.89226	0.05
45683	1.89226	0.05
45684	3.78453	0.05
45685	1.89227	0.05
45686	1.89227	0.05
45687	1.89227	0.05
45688	1.89227	0.05
45689	3.78453	0.05
45690	0.946132	0.05
45691	0.946132	0.05
45692	1.21599	0.05
45693	1.21599	0.05
45694	0.966749	0.05
45695	0.946133	0.05
45696	1.21598	0.05
45697	0.946132	0.05
45698	0.966748	0.05
45699	1.21598	0.05
45700	0.946133	0.05
45701	0.946133	0.05

45702	0.946133	0.05
45703	0.946133	0.05
45704	1.1134	0.05
45705	1.11555	0.05
45706	1.11555	0.05
45707	0.946132	0.05
45708	0.946132	0.05
45709	1.1134	0.05
45710	1.11555	0.05
45711	1.11555	0.05
45712	0.946132	0.05
45713	0.946132	0.05
45714	0.946133	0.05
45715	0.946133	0.05
45716	1.11555	0.05
45717	0.978467	0.05
45718	0.978468	0.05
45719	0.946133	0.05
45720	1.11555	0.05
45721	0.946132	0.05
45722	0.978468	0.05
45723	0.978468	0.05
45724	0.946132	0.05
45725	0.946132	0.05
45726	0.851542	2.1
45727	0.851542	2.1
45728	1.14168	2.1
45729	1.27897	2.08738
45730	1.02817	2.1
45731	1.13536	2.1
45732	0.946131	2.1
45733	0.861207	2.1
45734	0.94613	2.1
45735	0.94613	2.1
45736	0.868751	2.1
45737	1.15181	2.1
45738	0.946132	2.1
45739	0.946132	2.02271
45740	0.946128	2.07256
45741	0.946128	1.49731
45742	0.94613	2.1
45743	0.94613	2.07358
45744	0.946131	1.97252
45745	0.946131	1.76074
45746	0.94613	1.86955
45747	0.94613	2.07055
45748	0.94613	1.96999
45749	0.94613	2.02954
45750	2.53273	0.05
45751	1.89226	0.05
45752	1.89226	0.05
45753	1.89226	0.05
45754	1.89226	0.05
45755	1.89226	0.05
45756	1.89226	2.1
45757	1.89226	2.1
45758	1.89226	2.1
45759	1.89226	2.1

45760	3.78452	2.1
45761	1.89226	2.1
45762	2.54394	2.1
45763	1.89226	2.1
45764	2.42221	2.1
45765	4.43621	2.1
45766	2.54394	2.1
45767	1.62743	2.1
45768	2.42221	2.1
45769	1.5437	2.1
45770	4.08764	2.1
45771	1.89226	2.1
45772	1.89226	2.1
45773	1.89226	2.1
45774	1.89226	2.1
45775	3.78452	2.1
45776	1.89226	2.1
45777	1.89226	2.1
45778	1.89226	2.1
45779	1.89226	2.1
45780	3.78452	2.1
45781	1.89226	2.1
45782	2.30146	2.1
45783	2.42221	2.1
45784	1.89226	2.1
45785	1.89226	2.1
45786	2.42221	2.1
45787	2.30146	2.1
45788	2.42221	2.1
45789	1.39962	2.1
45790	1.39962	2.1
45791	1.5437	2.1
45792	2.44553	2.1
45793	1.89226	2.1
45794	1.89226	2.1
45795	1.89226	2.1
45796	1.89226	2.1
45797	3.78452	2.1
45798	1.62196	2.1
45799	2.00695	2.1
45800	1.95288	2.1
45801	1.70303	2.1
45802	3.53316	2.1
45803	1.80703	2.1
45804	2.00695	2.1
45805	1.92991	2.1
45806	2.00695	2.1
45807	1.85888	2.1
45808	1.93592	2.1
45809	1.89226	2.1
45810	1.89226	2.1
45811	1.89226	2.1
45812	1.89226	2.1
45813	1.89226	2.1
45814	1.89226	2.1
45815	1.89226	2.1
45816	1.89226	2.1
45817	1.89226	2.1

45818	1.89226	2.1
45819	3.78453	2.1
45820	1.95288	2.1
45821	1.50524	2.1
45822	1.83564	2.1
45823	1.70303	2.1
45824	2.00749	2.1
45825	1.84671	2.1
45826	1.62632	2.1
45827	1.82498	2.1
45828	1.92991	2.1
45829	1.92991	2.1
45830	1.47928	2.1
45831	1.47751	2.1
45832	0.946128	2.1
45833	0.946128	2.1
45834	0.946132	2.1
45835	0.946132	2.1
45836	0.94613	2.1
45837	0.94613	2.1
45838	0.946129	2.1
45839	0.946129	2.1
45840	0.94613	2.1
45841	0.94613	2.1
45842	0.94613	2.1
45843	0.94613	2.1
45844	1.89226	2.1
45845	1.89226	2.1
45846	1.89226	2.1
45847	1.89226	2.1
45848	1.89226	2.1
45849	1.89226	2.1
45850	2.27351	0.835899
45851	2.74068	0.462153
45852	2.72278	0.555367
45853	2.63356	0.702247
45854	2.36273	0.543775
45855	2.27351	0.682761
45856	2.36273	0.692624
45857	2.83112	0.411607
45858	2.81212	0.492357
45859	2.74068	0.586921
45860	2.43416	0.486235
45861	2.36273	0.575324
45862	1.89227	0.575148
45863	2.83112	0.409346
45864	2.74069	0.512067
45865	2.74069	0.387727
45866	1.89227	0.676723
45867	1.89227	0.473844
45868	2.40887	2.1
45869	1.4494	2.1
45870	1.52164	2.1
45871	2.47534	2.1
45872	3.93051	2.1
45873	1.89226	2.1
45874	2.47534	2.1
45875	2.40887	2.1

45876	2.40887	2.1
45877	1.89226	2.1
45878	1.89226	2.1
45879	1.60861	2.1
45880	2.47534	2.1
45881	1.52164	2.1
45882	1.52164	2.1
45883	2.55301	2.1
45884	2.55301	2.1
45885	0.946128	2.1
45886	0.946128	2.1
45887	1.23767	2.1
45888	1.2765	2.1
45889	1.23767	2.1
45890	1.23767	2.1
45891	0.946131	2.1
45892	0.946131	2.1
45893	0.946131	2.1
45894	1.2765	2.1
45895	1.23767	2.1
45896	0.946131	2.1
45897	1.89226	2.1
45898	1.87511	2.1
45899	1.87511	2.1
45900	1.95852	2.1
45901	2.0815	2.1
45902	2.22169	2.1
45903	1.89226	2.1
45904	1.89226	2.1
45905	1.89226	2.1
45906	1.89226	2.1
45907	1.89226	2.1
45908	1.89226	2.1
45909	2.0815	2.1
45910	1.80067	2.1
45911	1.80067	2.1
45912	2.05518	2.1
45913	1.89226	2.1
45914	2.16509	2.1
45915	1.87153	2.1
45916	2.65897	2.1
45917	1.58724	2.1
45918	2.38924	2.1
45919	4.26077	2.1
45920	1.89226	2.1
45921	1.89226	2.1
45922	1.89226	2.1
45923	1.89226	2.1
45924	1.89226	2.1
45925	1.89226	2.1
45926	1.89226	0.401143
45927	2.21022	0.594144
45928	2.19769	0.863283
45929	2.21022	0.47455
45930	1.87973	0.475153
45931	1.89226	0.160231
45932	1.18376	1.83474
45933	2.21022	0.701502

45934	1.16239	1.69084
45935	1.16239	0.937854
45936	2.19769	1.68565
45937	2.19769	0.822732
45938	0.946128	0.16824
45939	0.946128	0.202514
45940	1.09362	0.483513
45941	1.09884	0.395256
45942	0.94613	0.400496
45943	0.94613	0.118313
45944	1.09362	0.226329
45945	1.09362	0.370799
45946	0.946131	0.24153
45947	1.09362	0.242649
45948	0.946131	0.151302
45949	1.09884	0.138191
45950	1.19323	1.18792
45951	2.19769	0.442679
45952	1.18376	1.07378
45953	1.18376	0.648404
45954	2.18724	1.08725
45955	2.18724	0.617541
45956	2.0815	2.1
45957	0.922714	2.1
45958	2.0815	2.1
45959	1.84882	2.1
45960	1.20787	2.1
45961	1.08321	2.1
45962	2.6238	0.0622882
45963	1.89226	0.0908633
45964	1.89226	0.097057
45965	2.4866	0.0636279
45966	1.89226	0.0927673
45967	2.4866	0.0678475
45968	2.66753	0.0781165
45969	1.89226	0.124557
45970	1.89226	0.128381
45971	2.6238	0.0845761
45972	1.89226	0.119551
45973	2.6238	0.0862858
45974	0.946129	2.1
45975	0.946129	2.1
45976	1.04676	2.1
45977	1.04676	2.1
45978	0.946131	2.1
45979	0.946131	2.1
45980	1.08732	2.1
45981	1.04676	2.1
45982	1.08732	2.1
45983	0.946131	2.1
45984	1.04676	2.1
45985	0.946131	2.1
45986	1.20958	2.09654
45987	1.73802	1.13213
45988	1.39067	1.97908
45989	1.39067	1.45109
45990	1.50969	1.94097
45991	1.50969	1.60215

45992	1.00382	1.93917
45993	1.50969	0.522888
45994	1.20958	1.89284
45995	1.20958	0.816828
45996	1.32039	1.92118
45997	1.32039	0.899736
45998	2.0815	2.1
45999	2.51396	2.1
46000	2.19826	2.1
46001	2.54175	2.1
46002	4.79599	2.1
46003	2.54174	2.1
46004	2.17978	2.1
46005	2.54174	2.1
46006	2.2854	2.1
46007	2.57212	2.1
46008	2.31578	2.1
46009	2.27103	2.1
46010	2.54175	2.1
46011	2.4255	2.1
46012	2.82251	2.1
46013	4.96725	2.1
46014	2.60614	0.05
46015	2.75374	0.05
46016	1.94002	0.05
46017	2.08392	0.05
46018	4.69006	0.05
46019	2.75373	0.05
46020	2.6875	0.05
46021	2.08392	0.05
46022	1.99944	0.05
46023	4.77142	0.05
46024	1.1211	0.05
46025	2.08392	0.05
46026	1.17787	0.05
46027	1.94002	0.05
46028	1.32178	0.05
46029	2.08392	0.05
46030	2.08392	0.05
46031	1.99944	0.05
46032	1.32178	0.05
46033	1.25929	0.05
46034	3.34321	0.05
46035	1.22266	0.05
46036	0.779473	0.05
46037	0.94613	0.05
46038	0.94613	0.05
46039	1.04072	0.05
46040	1.1407	0.05
46041	1.10042	0.05
46042	0.851542	0.05
46043	0.942046	0.05
46044	0.942046	0.05
46045	1.04114	0.05
46046	0.826602	0.05
46047	2.37468	2.1
46048	1.89226	2.1
46049	1.89226	2.1

46050	1.89226	2.1
46051	2.27917	2.1
46052	2.27917	2.1
46053	1.13959	2.1
46054	1.09451	2.1
46055	0.946131	2.1
46056	0.946131	2.1
46057	1.09451	2.1
46058	1.09451	2.1
46059	0.94613	2.1
46060	0.94613	2.1
46061	1.09451	2.1
46062	0.946131	2.1
46063	0.946131	2.1
46064	1.13959	2.1
46065	2.52454	2.01695
46066	2.12221	2.1
46067	2.67624	1.60836
46068	2.33865	2.1
46069	4.86319	2.1
46070	1.89226	2.0998
46071	1.89226	2.1
46072	1.89226	2.0848
46073	1.89226	2.1
46074	3.78452	2.1
46075	1.89226	2.1
46076	1.89226	2.1
46077	1.89226	2.1
46078	1.89226	2.1
46079	3.78452	2.1
46080	2.12221	2.1
46081	1.47769	2.1
46082	2.33865	2.1
46083	1.62898	2.1
46084	3.81634	2.1
46085	2.67624	2.1
46086	2.37734	2.1
46087	2.33865	2.1
46088	2.33865	2.1
46089	2.70125	2.1
46090	2.70125	2.1
46091	1.89226	2.095
46092	1.89226	2.1
46093	1.89226	2.1
46094	1.89226	2.1
46095	1.89226	1.69191
46096	1.89226	1.82917
46097	1.89226	2.1
46098	1.89226	2.1
46099	1.89226	2.1
46100	1.89226	2.1
46101	1.89226	2.1
46102	1.89226	2.1
46103	2.37733	2.1
46104	1.95927	2.1
46105	1.82516	2.0856
46106	1.82516	2.1
46107	2.33865	2.1

46108	2.33865	2.1
46109	2.57466	2.1
46110	2.21715	2.1
46111	2.55653	2.1
46112	2.24775	2.1
46113	4.77367	2.1
46114	0.937095	2.1
46115	0.937095	2.1
46116	0.958028	2.1
46117	0.958028	2.1
46118	0.828386	2.1
46119	0.771244	2.1
46120	1.10858	2.1
46121	1.12388	2.1
46122	1.12388	2.1
46123	0.828386	2.1
46124	0.771244	2.1
46125	1.10858	2.1
46126	2.55653	2.1
46127	2.32606	2.1
46128	2.24775	2.1
46129	2.24775	2.1
46130	2.57928	2.1
46131	2.57928	2.1
46132	0.990481	2.1
46133	0.990481	2.1
46134	1.00094	2.1
46135	1.00094	2.1
46136	0.888385	2.1
46137	0.828386	2.1
46138	1.16303	2.1
46139	1.12388	2.1
46140	1.16303	2.1
46141	0.888385	2.1
46142	1.12388	2.1
46143	0.828387	2.1
46144	0.962524	2.1
46145	0.962524	2.1
46146	0.7976	2.1
46147	0.7976	2.1
46148	0.797628	2.1
46149	0.814 2.1	
46150	0.946125	2.1
46151	0.946125	2.1
46152	0.946118	2.1
46153	0.797623	2.1
46154	0.814006	2.1
46155	0.946131	2.1
46156	1.8978	0.05
46157	2.23892	0.05
46158	1.71469	0.05
46159	4.13672	0.05
46160	2.64136	0.05
46161	2.23892	1.59809
46162	2.37578	1.99712
46163	1.77489	2.1
46164	4.6147	2.08916
46165	1.02284	2.1

46166	0.898796	2.06762
46167	0.948554	2.1
46168	0.948554	2.1
46169	0.878797	2.1
46170	0.796197	2.1
46171	1.09145	2.1
46172	0.963109	2.1
46173	1.12114	2.1
46174	0.782739	2.1
46175	1.11084	2.1
46176	0.850742	2.1
46177	2.48024	2.1
46178	2.405	2.1
46179	2.24585	2.1
46180	2.34795	2.1
46181	4.75295	2.1
46182	1.93794	2.1
46183	2.405	2.1
46184	2.24585	2.1
46185	1.67594	2.1
46186	4.08094	2.1
46187	1.89226	2.1
46188	1.89226	2.03436
46189	1.89226	2.1
46190	1.89226	2.1
46191	3.78452	2.1
46192	1.89226	2.1
46193	1.89226	2.1
46194	1.89226	2.1
46195	1.89226	2.1
46196	3.78452	2.1
46197	2.24585	2.1
46198	2.34795	2.1
46199	2.21155	2.1
46200	2.06626	2.1
46201	4.4142	2.1
46202	2.24585	2.1
46203	1.41498	2.1
46204	1.41498	2.1
46205	1.67594	2.1
46206	2.06625	2.1
46207	2.32721	2.1
46208	1.89226	2.1
46209	1.89226	2.1
46210	1.89226	2.1
46211	1.89226	2.0953
46212	1.89226	2.1
46213	1.89226	2.05285
46214	1.89226	2.1
46215	1.89226	2.1
46216	1.89226	2.1
46217	1.89226	2.1
46218	3.78452	2.1
46219	1.89226	0.118024
46220	1.89226	0.177433
46221	1.89226	0.222816
46222	1.89226	0.0894824
46223	3.78452	0.0952525

46224	1.89226	0.135505
46225	2.53655	0.0544326
46226	1.89226	0.0557316
46227	1.89226	0.0707612
46228	1.89226	0.123956
46229	1.89226	0.131033
46230	1.89227	2.1
46231	1.89227	2.1
46232	1.89227	2.1
46233	1.89227	2.1
46234	1.89227	2.1
46235	1.89227	2.1
46236	0.91339	2.1
46237	0.91339	2.1
46238	1.2158	2.1
46239	1.20629	2.1
46240	1.00473	2.1
46241	1.15749	2.1
46242	0.946132	2.1
46243	0.946132	2.1
46244	0.946133	2.1
46245	1.17513	2.1
46246	1.00473	2.1
46247	0.946132	2.1
46248	1.89226	2.1
46249	1.89226	2.1
46250	1.89226	2.1
46251	1.89226	2.1
46252	1.89226	2.1
46253	1.89226	2.1
46254	1.89226	2.1
46255	2.6066	2.1
46256	2.35026	2.1
46257	2.35026	2.1
46258	1.89226	2.1
46259	1.89226	2.1
46260	2.04056	0.269637
46261	2.77366	0.225676
46262	1.24682	0.212518
46263	1.24682	0.313418
46264	2.93651	0.230709
46265	2.14277	0.292587
46266	2.68524	1.706
46267	2.81847	1.56526
46268	1.89226	1.13227
46269	1.89226	1.51028
46270	4.57751	1.36516
46271	2.68524	1.58344
46272	1.89226	1.77439
46273	1.89226	1.74876
46274	2.50732	1.51738
46275	1.89226	1.62161
46276	2.50732	1.67247
46277	1.89226	1.22583
46278	1.89226	1.5886
46279	1.89226	1.46141
46280	1.89226	1.75509
46281	1.89226	1.19288

46282	1.89226	1.72163
46283	1.89226	1.79113
46284	1.89226	1.43701
46285	1.89226	1.69513
46286	1.89226	1.85132
46287	1.89226	1.144
46288	1.89226	1.27101
46289	1.61016	1.38043
46290	1.07467	1.57433
46291	1.24474	1.27153
46292	1.24474	1.81044
46293	1.45303	1.40756
46294	1.45303	1.81666
46295	1.61016	1.53542
46296	1.24474	1.89904
46297	1.61016	1.55194
46298	1.77186	1.39142
46299	1.37813	1.31583
46300	1.37813	1.52096
46301	1.73305	2.1
46302	2.0815	2.1
46303	1.97658	2.1
46304	1.75791	2.1
46305	1.87228	2.1
46306	1.66515	2.1
46307	2.0815	2.1
46308	1.93938	2.1
46309	2.03253	2.08656
46310	2.2142	2.1
46311	1.97658	2.09993
46312	2.15324	2.1
46313	1.73305	2.1
46314	1.97658	2.1
46315	1.60428	2.1
46316	1.8589	2.1
46317	3.58085	2.1
46318	1.97657	2.1
46319	1.93938	2.1
46320	1.51316	2.1
46321	1.85293	2.07986
46322	3.82951	2.1
46323	1.70303	0.0644163
46324	1.96087	0.0608686
46325	1.42308	0.0616645
46326	1.96087	0.0639296
46327	1.25023	0.0575679
46328	1.70303	0.0597879
46329	0.946132	0.0913324
46330	0.946132	0.0918569
46331	0.946131	0.0769997
46332	0.946131	0.0694113
46333	0.946132	0.0786119
46334	0.946132	0.117298
46335	0.946132	0.068152
46336	0.946132	0.10486
46337	0.946132	0.0838415
46338	0.946132	0.0714412
46339	0.946132	0.0855135

46340	0.946132	0.0899492
46341	1.89226	0.106285
46342	1.89226	0.074127
46343	1.89226	0.0751375
46344	1.89226	0.0889041
46345	3.78452	0.0897682
46346	1.89226	0.0591515
46347	1.89226	0.0560006
46348	1.89226	0.0579162
46349	1.89226	0.0658228
46350	1.89226	0.0555872
46351	1.89226	0.0609201
46352	1.89226	0.0602624
46353	1.89226	0.0672501
46354	1.89226	0.05
46355	1.89226	0.0644603
46356	1.89226	0.0525624
46357	1.89226	0.0881163
46358	1.89226	0.05
46359	1.89226	0.05
46360	2.48255	0.05
46361	1.89226	0.05
46362	3.78452	0.05
46363	1.89226	2.1
46364	2.42758	2.1
46365	1.9209	2.1
46366	1.89226	2.1
46367	2.45622	2.1
46368	2.42758	2.1
46369	2.42758	2.1
46370	1.49987	2.1
46371	1.61028	2.1
46372	1.58164	2.1
46373	2.45622	2.1
46374	2.42758	2.1
46375	0.94613	2.1
46376	0.94613	2.1
46377	1.24298	2.1
46378	1.24298	2.1
46379	1.24298	2.1
46380	1.22811	2.1
46381	0.946131	2.1
46382	0.946131	2.1
46383	0.94613	2.1
46384	1.24298	2.1
46385	0.94613	2.1
46386	1.22811	2.1
46387	2.48596	2.1
46388	2.45622	2.1
46389	1.63823	2.1
46390	1.4304	2.1
46391	1.45739	2.1
46392	2.45622	2.1
46393	2.4169	2.1
46394	2.55249	2.1
46395	2.51125	2.1
46396	2.35245	2.1
46397	4.90494	2.1

46398	2.45922	2.1
46399	2.55249	2.1
46400	2.51126	2.1
46401	2.43226	2.1
46402	4.98474	2.1
46403	2.51125	2.1
46404	2.35245	2.1
46405	2.28979	2.1
46406	2.47228	2.1
46407	4.82473	2.1
46408	2.51126	2.1
46409	2.43226	2.1
46410	2.40668	2.1
46411	2.47228	2.1
46412	4.90453	2.1
46413	2.8045	2.1
46414	1.93707	2.1
46415	2.14087	2.1
46416	2.14087	2.1
46417	2.61595	2.1
46418	2.61595	2.1
46419	1.89226	2.1
46420	1.89226	2.1
46421	1.89226	2.1
46422	1.89226	2.1
46423	1.89226	2.1
46424	1.89226	2.1
46425	1.22368	2.1
46426	1.32339	2.1
46427	0.957125	2.1
46428	0.750074	2.1
46429	1.08438	2.1
46430	1.08438	2.1
46431	1.13094	2.1
46432	0.970797	2.1
46433	1.17568	2.1
46434	0.764392	2.1
46435	0.944286	2.1
46436	1.27523	2.1
46437	1.89226	2.1
46438	1.89226	2.1
46439	1.89226	2.1
46440	1.89226	2.1
46441	3.78453	2.1
46442	2.8239	2.1
46443	1.89227	2.1
46444	2.57276	2.1
46445	2.57276	2.1
46446	1.89227	2.1
46447	1.89227	2.1
46448	1.18139	2.1
46449	1.85195	2.1
46450	1.78595	2.1
46451	1.08172	2.1
46452	2.93367	2.1
46453	1.60735	2.1
46454	1.20356	2.1
46455	0.967034	2.1

46456	1.08172	2.1
46457	1.67324	2.1
46458	1.77293	2.1
46459	1.89226	2.1
46460	1.89226	2.1
46461	1.8213	2.1
46462	1.82129	2.1
46463	2.14171	2.1
46464	2.40192	2.1
46465	0.810943	2.1
46466	0.810943	2.1
46467	0.798469	2.1
46468	0.788336	2.1
46469	0.804015	2.1
46470	0.810945	2.1
46471	1.947	2.1
46472	1.36506	2.1
46473	1.08686	2.1
46474	1.07208	2.1
46475	1.08132	2.1
46476	1.13537	1.17423
46477	1.14754	2.09832
46478	0.946131	2.00316
46479	0.946131	2.1
46480	0.94613	1.97908
46481	0.94613	1.17213
46482	1.25104	2.1
46483	1.14754	2.08268
46484	1.13537	2.04908
46485	0.946131	2.05562
46486	0.946131	2.08847
46487	1.25104	2.08044
46488	0.96542	0.83001
46489	0.922284	2.0434
46490	0.946131	2.1
46491	0.946131	2.02374
46492	0.946131	1.99405
46493	0.946131	0.845899
46494	1.13537	2.1
46495	0.924824	2.07832
46496	0.859476	2.04406
46497	0.842305	2.04362
46498	1.25996	2.03493
46499	1.04996	2.04305
46500	1.71007	0.05
46501	2.73283	0.05
46502	2.73283	0.05
46503	1.89226	0.05
46504	1.89226	0.05
46505	1.13262	0.05
46506	0.946131	0.05
46507	0.946131	0.05
46508	0.946131	0.05
46509	0.946131	0.05
46510	0.946132	0.05
46511	0.946132	0.05
46512	0.946131	0.05
46513	0.946131	0.05

46514	0.946131	0.05
46515	0.946131	0.05
46516	0.946132	0.05
46517	0.946132	0.05
46518	0.946131	0.05
46519	1.36642	0.05
46520	0.946125	0.05
46521	0.946125	0.05
46522	0.946128	0.05
46523	0.946128	0.05
46524	0.946126	0.05
46525	0.946128	0.05
46526	0.946128	0.05
46527	0.946128	0.05
46528	1.36641	0.05
46529	0.946128	0.05
46530	1.89226	0.05
46531	1.89226	0.05
46532	1.89226	0.05
46533	1.89226	0.05
46534	1.89226	0.05
46535	1.89226	0.05
46536	2.69784	2.1
46537	2.51651	2.1
46538	2.56999	2.1
46539	2.74623	2.1
46540	5.26783	2.1
46541	2.69784	2.1
46542	2.48718	2.1
46543	2.50161	2.1
46544	2.74623	2.1
46545	5.19945	2.1
46546	1.89226	2.1
46547	1.89226	2.1
46548	1.89226	2.1
46549	1.89226	2.1
46550	3.78453	2.1
46551	0.874264	2.1
46552	1.09879	2.1
46553	0.946131	2.1
46554	0.946131	2.1
46555	1.17062	2.1
46556	1.17062	2.1
46557	0.874293	2.1
46558	0.874293	2.1
46559	1.09879	2.1
46560	0.94613	2.1
46561	0.946131	2.1
46562	0.874265	2.1
46563	1.89226	2.1
46564	1.89226	2.1
46565	1.89226	2.1
46566	1.89226	2.1
46567	3.78453	2.1
46568	0.977382	2.1
46569	1.16424	2.1
46570	0.946133	2.1
46571	0.946133	2.1

46572	1.33012	2.1
46573	1.25589	2.1
46574	0.888498	2.1
46575	0.888498	2.1
46576	1.25375	2.1
46577	0.946131	2.1
46578	0.946131	2.1
46579	0.977384	2.1
46580	1.89226	2.1
46581	2.19757	2.1
46582	2.62906	2.1
46583	1.89226	2.1
46584	1.89226	2.1
46585	2.62906	2.1
46586	1.27944	0.316545
46587	1.27731	1.51419
46588	0.615701	1.32471
46589	0.615701	0.489238
46590	0.615673	0.130572
46591	0.946133	1.86524
46592	0.948264	0.184962
46593	0.946132	0.35351
46594	0.94752	0.182771
46595	0.61706	0.19002
46596	0.946132	0.3118
46597	0.946132	0.500643
46598	2.76604	2.1
46599	1.66728	2.1
46600	1.66728	2.1
46601	1.83036	2.1
46602	2.54515	2.1
46603	2.70823	2.1
46604	2.54515	2.1
46605	1.49958	2.1
46606	1.49958	2.1
46607	1.66728	2.1
46608	2.34465	2.1
46609	2.51235	2.1
46610	1.21128	2.1
46611	1.21128	2.1
46612	0.946131	2.1
46613	0.946131	2.1
46614	0.946131	2.1
46615	0.946131	2.1
46616	1.31401	2.1
46617	1.21128	2.1
46618	1.21128	2.1
46619	0.946131	2.1
46620	0.946131	2.1
46621	1.31401	2.1
46622	1.67374	2.1
46623	2.29586	2.1
46624	1.50033	2.1
46625	2.16598	2.1
46626	3.83972	2.1
46627	1.89226	2.1
46628	1.89226	2.1
46629	1.89226	2.1

46630	1.89226	2.1
46631	3.78452	2.1
46632	1.13537	2.1
46633	1.02188	2.1
46634	0.946132	2.1
46635	0.946132	2.1
46636	0.946132	2.1
46637	0.946132	2.1
46638	1.21128	2.1
46639	1.02188	2.1
46640	1.04672	2.1
46641	1.04672	2.1
46642	1.21128	2.1
46643	0.946132	2.1
46644	0.815411	2.1
46645	0.870464	2.1
46646	0.885437	2.1
46647	0.885437	2.1
46648	1.06395	2.1
46649	1.00483	2.1
46650	0.757125	2.1
46651	0.673956	2.1
46652	0.684461	2.1
46653	1.06979	2.1
46654	0.731403	2.1
46655	1.0559	2.1
46656	1.89226	2.1
46657	1.89226	2.1
46658	1.89226	2.1
46659	1.89226	2.1
46660	1.89226	2.1
46661	1.89226	2.1
46662	1.76897	2.02122
46663	1.76932	2.06731
46664	1.97455	2.1
46665	1.94302	1.78461
46666	3.71233	2.04661
46667	1.83347	2.1
46668	1.76932	2.08031
46669	1.97455	2.1
46670	1.76932	2.1
46671	1.76522	2.1
46672	1.55999	2.1
46673	1.89227	2.08065
46674	1.89227	2.1
46675	1.89227	2.1
46676	1.89227	2.03847
46677	3.78453	2.08747
46678	1.89226	2.06844
46679	1.89226	1.31984
46680	1.89226	1.69809
46681	1.89226	1.9989
46682	3.78452	2.00151
46683	1.97452	2.09894
46684	1.94299	1.52747
46685	2.17831	1.88872
46686	2.23195	2.02425
46687	4.17495	2.02984

46688	2.23196	2.0615
46689	2.1499	2.1
46690	1.97452	2.1
46691	1.97452	2.1
46692	2.09088	2.1
46693	1.83345	2.1
46694	0.94612	2.1
46695	0.94612	2.1
46696	0.946119	2.00205
46697	0.946119	1.99143
46698	0.946119	2.00044
46699	0.946119	2.09777
46700	0.94612	2.05742
46701	0.94612	2.1
46702	0.946132	2.07602
46703	0.946132	2.04433
46704	0.946107	2.0936
46705	0.946107	2.08925
46706	1.89224	1.40923
46707	1.89224	1.86541
46708	1.89224	2.00141
46709	1.89224	1.08679
46710	3.78447	1.33357
46711	1.70302	0.208956
46712	1.056	0.184005
46713	1.21829	0.27203
46714	1.89906	0.182163
46715	3.07892	0.195878
46716	2.20248	0.210731
46717	1.56498	0.177137
46718	1.46539	0.181219
46719	1.48787	0.166015
46720	2.37625	0.25854
46721	2.37625	0.202579
46722	1.14104	0.160587
46723	1.00675	0.181794
46724	0.700689	0.173008
46725	0.624389	0.174461
46726	0.782149	0.146225
46727	0.940197	0.151541
46728	0.803437	0.175775
46729	0.803437	0.186454
46730	0.995423	0.16533
46731	0.700699	0.142644
46732	0.593895	0.208266
46733	1.00673	0.162249
46734	1.18097	0.159685
46735	1.15839	0.170052
46736	0.995407	0.161161
46737	0.865932	0.17112
46738	1.17299	0.144773
46739	0.957054	0.151083
46740	1.08016	0.183509
46741	1.08016	0.219385
46742	0.995425	0.181002
46743	1.24161	0.141255
46744	0.796771	0.151059
46745	1.15837	0.194383

46746	2.57264	0.05
46747	1.89226	0.0537304
46748	2.41701	0.05
46749	1.89226	0.05
46750	4.30928	0.05
46751	1.69055	0.05
46752	2.39587	0.05
46753	1.53463	0.05
46754	2.2053	0.05
46755	3.89585	0.05
46756	0.946129	0.05
46757	0.946129	0.05
46758	0.94613	0.05
46759	0.94613	0.05
46760	0.946132	0.05
46761	0.946132	0.0511491
46762	0.946126	0.05
46763	0.946126	0.05
46764	0.94613	0.05
46765	0.94613	0.05
46766	0.946129	0.05
46767	0.946129	0.0500177
46768	2.41701	0.05
46769	1.89226	0.05
46770	1.89226	0.05
46771	1.89226	0.05
46772	2.32462	0.05
46773	2.32462	0.05
46774	1.41942	0.05
46775	2.2053	0.05
46776	2.02097	0.05
46777	2.02097	0.05
46778	1.53463	0.05
46779	1.53463	0.05
46780	1.89226	0.05
46781	1.89226	0.05
46782	1.89226	0.05
46783	1.89226	0.05
46784	1.89226	0.05
46785	1.89226	0.05
46786	1.89226	1.37603
46787	1.89226	1.95041
46788	1.89226	1.92604
46789	1.89226	1.65659
46790	1.89226	1.8226
46791	1.89226	1.45202
46792	1.02816	1.03968
46793	1.02816	1.30671
46794	0.595141	1.59523
46795	0.595141	1.6898
46796	0.946132	1.20844
46797	0.595113	1.80543
46798	0.677174	1.68754
46799	0.946132	1.01864
46800	0.946132	1.46827
46801	0.946132	1.52462
46802	0.595113	1.54172
46803	0.677174	1.39957

46804	1.89226	1.54639
46805	1.89226	1.98438
46806	1.89226	1.87208
46807	1.89226	1.64505
46808	1.89226	1.90497
46809	1.89226	1.70026
46810	0.946133	1.11703
46811	0.946133	0.974451
46812	0.946132	1.80772
46813	0.946132	1.82326
46814	0.946132	1.80754
46815	0.946132	1.33901
46816	0.946132	1.63499
46817	0.946132	0.989003
46818	0.946132	1.5506
46819	0.946132	1.69659
46820	0.946133	1.2263
46821	0.946133	1.40794
46822	0.946131	1.72849
46823	0.946131	1.7259
46824	0.946131	1.98112
46825	0.946131	1.97378
46826	0.946128	1.99159
46827	0.946128	1.73714
46828	0.946133	1.97909
46829	0.946133	1.67861
46830	0.94613	1.86405
46831	0.94613	1.93466
46832	0.946131	1.90005
46833	0.946131	1.81734
46834	2.0264	2.1
46835	1.67345	2.03039
46836	2.0264	2.05366
46837	1.70539	1.27117
46838	1.31611	1.65347
46839	1.31611	1.8635
46840	1.89226	0.502972
46841	1.89226	0.434739
46842	1.89226	0.646829
46843	1.89226	0.373771
46844	3.78452	0.396542
46845	1.89226	0.652995
46846	1.89226	0.511214
46847	1.89226	0.540137
46848	1.89226	0.712861
46849	1.89226	0.561216
46850	1.89226	0.760628
46851	1.02645	0.191191
46852	0.810942	0.232714
46853	0.882781	0.277657
46854	0.882781	0.247145
46855	2.80386	0.199487
46856	1.56719	0.235144
46857	1.22499	0.195724
46858	1.08132	0.267007
46859	1.36865	0.222959
46860	0.810957	0.254531
46861	0.810957	0.224233

46862	0.946132	0.369974
46863	0.946132	0.432046
46864	0.946129	0.264233
46865	0.946129	0.310103
46866	0.946131	0.249875
46867	0.946131	0.282419
46868	0.946131	0.438745
46869	0.946131	0.374731
46870	0.94613	0.352648
46871	0.94613	0.372515
46872	0.946131	0.489135
46873	0.946131	0.356602
46874	1.89226	0.481329
46875	1.89226	0.322084
46876	1.89226	0.550285
46877	1.89226	0.311048
46878	3.78453	0.329253
46879	1.30462	0.05
46880	1.79906	0.0802845
46881	1.01299	0.0719051
46882	1.44185	0.0656293
46883	1.3499	0.05
46884	1.79906	0.0520737
46885	1.89226	0.061623
46886	1.89226	0.103562
46887	1.89226	0.0528391
46888	1.89226	0.0988526
46889	1.89226	0.0640836
46890	1.89226	0.126814
46891	1.89226	0.0921355
46892	1.89226	0.115304
46893	1.89226	0.0910013
46894	1.89226	0.100314
46895	1.89226	0.0902469
46896	1.89226	0.10825
46897	1.3499	0.05
46898	1.79905	0.0708056
46899	1.29001	0.05
46900	1.36005	0.0621289
46901	1.88294	0.0741593
46902	1.79905	0.0620788
46903	1.89226	0.0554462
46904	2.75374	0.05
46905	2.60614	0.0596025
46906	1.89226	0.0643868
46907	2.60614	0.05
46908	1.89226	0.05
46909	0.946131	0.0532646
46910	0.946131	0.06026
46911	1.37687	0.0593095
46912	1.37687	0.0543672
46913	0.946131	0.0641738
46914	1.34375	0.0661407
46915	1.37686	0.05
46916	0.946128	0.0511283
46917	1.37687	0.05
46918	1.34375	0.0530396
46919	0.94613	0.0652848

46920	0.94613	0.0627286
46921	1.44247	2.1
46922	1.12665	2.1
46923	1.50044	2.1
46924	1.73651	2.1
46925	2.94291	2.1
46926	1.73652	2.1
46927	1.64926	2.1
46928	1.44247	2.1
46929	1.44247	2.1
46930	1.64926	2.1
46931	1.43872	2.1
46932	1.50044	2.1
46933	1.73651	2.1
46934	2.00955	2.1
46935	1.87875	2.1
46936	3.50999	2.1
46937	1.64926	2.1
46938	1.73652	2.1
46939	2.00955	2.1
46940	1.8493	2.1
46941	3.65881	2.1
46942	1.89226	0.05
46943	2.28188	0.05
46944	1.99182	0.05
46945	2.28188	0.0610215
46946	1.6022	0.05
46947	1.89226	0.0569999
46948	1.89226	0.0612275
46949	1.89226	0.073751
46950	1.89226	0.0526971
46951	1.89226	0.102608
46952	1.89226	0.0525124
46953	1.89226	0.0931069
46954	1.89226	0.05
46955	1.89226	0.0500603
46956	2.19578	0.05
46957	1.89226	0.0716587
46958	2.81422	0.05
46959	2.42521	0.0652482
46960	1.89224	0.05
46961	1.99179	0.05
46962	1.454 0.05	
46963	1.99179	0.0514652
46964	1.35444	0.05
46965	1.89224	0.05
46966	1.89224	0.0524475
46967	1.89224	0.0672574
46968	1.89224	0.0517339
46969	1.89224	0.0936613
46970	1.89224	0.05
46971	1.89224	0.0833228
46972	1.703 0.05	
46973	2.19575	0.05
46974	1.9911	0.05
46975	2.19575	0.0627227
46976	1.67811	0.05
46977	1.89223	0.0575784

46978	1.69586	2.1
46979	2.55301	2.1
46980	1.60861	2.1
46981	1.60861	2.1
46982	2.65233	2.1
46983	2.65233	2.1
46984	0.946129	2.1
46985	0.946129	2.1
46986	1.2765	2.1
46987	1.32617	2.1
46988	1.2765	2.1
46989	1.2765	2.1
46990	0.946131	2.1
46991	0.946131	2.1
46992	0.946131	2.1
46993	1.32616	2.1
46994	0.946131	2.1
46995	1.2765	2.1
46996	1.36757	2.1
46997	1.32617	2.1
46998	0.891503	2.1
46999	0.847932	2.1
47000	1.10722	2.1
47001	1.10722	2.1
47002	1.10829	2.1
47003	1.10829	2.1
47004	1.36757	2.1
47005	0.891504	2.1
47006	0.847932	2.1
47007	1.32617	2.1
47008	0.946129	2.1
47009	0.946129	2.1
47010	1.36757	2.1
47011	1.32617	2.1
47012	1.32616	2.1
47013	1.32616	2.1
47014	0.946131	2.1
47015	0.946131	2.1
47016	1.36757	2.1
47017	0.946131	2.1
47018	0.946131	2.1
47019	1.32616	2.1
47020	1.89226	0.0960624
47021	1.89226	0.0564
47022	1.89226	0.0566105
47023	1.89226	0.111542
47024	3.78452	0.0747631
47025	2.36336	0.05
47026	1.89226	0.0533557
47027	2.67831	0.05
47028	2.67831	0.05
47029	1.89226	0.05
47030	1.89226	0.05
47031	1.89227	0.0600558
47032	1.89227	0.127033
47033	1.89227	0.114892
47034	1.89227	0.0687038
47035	3.78453	0.0932637

47036	1.89226	0.05
47037	1.89226	0.106006
47038	1.89226	0.0992317
47039	1.89226	0.059587
47040	3.78452	0.079966
47041	2.36336	0.05
47042	2.0815	0.05
47043	2.0815	0.05
47044	2.41048	0.05
47045	1.66267	0.05
47046	2.13452	0.05
47047	1.89226	0.110202
47048	1.89226	0.0673989
47049	1.89226	0.10317
47050	1.89226	0.057242
47051	1.89226	0.114164
47052	1.89226	0.0776623
47053	1.89226	0.145019
47054	1.89226	0.150951
47055	1.89226	0.145005
47056	1.89226	0.185016
47057	1.89226	0.105022
47058	1.89226	0.151869
47059	0.946128	0.171738
47060	0.946128	0.169379
47061	1.00122	0.0980863
47062	1.00122	0.0965951
47063	0.94613	0.211469
47064	1.01618	0.0869124
47065	0.946131	0.201747
47066	1.00122	0.0572074
47067	1.00122	0.0560851
47068	1.01618	0.0630646
47069	0.946131	0.187354
47070	0.946131	0.174288
47071	1.89226	2.00304
47072	1.89226	1.46898
47073	1.89226	1.56621
47074	1.89226	2.04412
47075	3.78452	1.8223
47076	1.89226	1.92346
47077	1.89226	2.1
47078	1.89226	2.1
47079	1.89226	1.97198
47080	1.89226	2.1
47081	1.89226	1.98408
47082	1.89226	1.26599
47083	1.89226	1.97062
47084	1.89226	1.91131
47085	1.89226	1.34354
47086	1.89226	1.93118
47087	1.89226	1.37847
47088	1.89226	1.36125
47089	1.89226	2.01916
47090	1.89226	1.9887
47091	1.89226	1.48247
47092	1.89226	1.99297
47093	1.89226	1.55894

47094	2.71279	0.430638
47095	1.4155	0.532785
47096	2.28488	0.47392
47097	1.06122	0.49948
47098	3.70038	0.476569
47099	1.89226	0.666368
47100	1.89226	0.554587
47101	1.89226	0.497409
47102	1.89226	0.672174
47103	3.78452	0.626435
47104	0.851542	0.602551
47105	0.851542	0.587154
47106	0.851484	0.514967
47107	0.865567	0.587714
47108	0.981616	0.599137
47109	0.864767	0.588519
47110	0.851542	0.520325
47111	0.851542	0.582039
47112	0.851477	0.602177
47113	0.851477	0.595627
47114	0.865635	0.583178
47115	0.816472	0.591596
47116	1.68443	2.1
47117	1.44489	2.1
47118	1.54924	2.1
47119	1.34389	2.1
47120	3.02832	2.1
47121	1.44489	2.1
47122	1.03899	2.1
47123	1.03899	2.1
47124	1.12075	2.1
47125	1.34389	2.1
47126	1.42566	2.1
47127	1.54924	2.1
47128	1.34389	2.1
47129	1.44091	2.1
47130	1.25866	2.1
47131	2.7848	2.1
47132	0.573141	2.1
47133	0.573141	2.1
47134	0.575681	2.1
47135	0.575681	2.1
47136	0.481985	2.1
47137	0.519492	2.1
47138	0.671948	2.1
47139	0.629331	2.1
47140	0.629329	2.1
47141	0.481986	2.1
47142	0.519494	2.1
47143	0.671947	2.1
47144	1.89226	0.246997
47145	1.89226	0.395003
47146	1.89226	0.406819
47147	2.31925	0.237759
47148	3.78452	0.352054
47149	1.30916	2.08488
47150	1.17426	2.1
47151	0.946129	2.1

47152	0.946129	2.1
47153	0.94613	2.1
47154	0.94613	2.08242
47155	1.17426	2.1
47156	1.17426	2.1
47157	1.30916	2.1
47158	0.94613	2.1
47159	1.17426	2.1
47160	0.94613	2.1
47161	2.56346	2.07722
47162	1.89226	1.5953
47163	1.89226	2.0291
47164	1.89226	1.66251
47165	2.6074	2.02079
47166	2.6074	1.6641
47167	1.89226	2.08141
47168	1.89226	2.08775
47169	1.89226	2.1
47170	1.89226	2.02141
47171	3.78452	2.09819
47172	1.33064	2.07762
47173	1.84121	2.08584
47174	0.946129	2.1
47175	0.946129	2.09955
47176	0.946131	2.09975
47177	0.946131	2.0788
47178	1.30916	2.1
47179	1.8412	2.1
47180	1.33064	2.1
47181	0.94613	2.1
47182	1.30916	2.1
47183	0.946131	2.1
47184	1.19283	1.60586
47185	1.19283	1.38738
47186	0.946131	2.07687
47187	0.946131	2.01417
47188	0.946132	2.0281
47189	0.946132	1.55575
47190	1.3037	2.07569
47191	1.19283	1.4617
47192	1.19283	1.91787
47193	0.946131	1.92003
47194	0.946131	1.87938
47195	1.3037	1.94137
47196	1.89226	2.09967
47197	1.89226	2.01607
47198	1.89226	2.07404
47199	1.89226	2.0796
47200	1.89226	2.07936
47201	1.89226	2.08185
47202	2.17708	2.1
47203	1.70302	2.1
47204	2.28188	2.1
47205	2.13621	2.1
47206	4.28253	2.1
47207	1.89226	2.1
47208	1.89226	2.1
47209	1.89226	2.1

47210	1.89226	2.1
47211	3.78452	2.1
47212	0.946132	2.1
47213	0.946132	2.1
47214	0.946132	2.1
47215	0.946132	2.1
47216	0.946131	2.1
47217	0.946131	2.1
47218	0.946132	2.1
47219	0.946132	2.1
47220	0.946132	2.1
47221	0.946132	2.1
47222	0.946132	2.1
47223	0.946132	2.1
47224	1.74933	2.1
47225	1.11833	2.1
47226	1.90074	2.1
47227	1.669 2.1	
47228	1.335 2.1	
47229	1.11833	2.1
47230	2.28188	2.1
47231	2.31804	2.1
47232	2.42758	2.1
47233	2.14895	2.1
47234	4.43083	2.1
47235	1.89226	2.1
47236	1.89226	2.1
47237	1.89226	2.08862
47238	1.89226	2.1
47239	3.78452	2.1
47240	1.89226	2.1
47241	1.89226	2.1
47242	1.89226	2.1
47243	1.89226	2.1
47244	1.89226	2.1
47245	1.89226	2.1
47246	0.864133	2.1
47247	0.864133	2.1
47248	0.967939	2.1
47249	0.793739	2.1
47250	0.650797	2.1
47251	0.757546	2.1
47252	1.08941	2.1
47253	1.10136	2.1
47254	1.10138	2.1
47255	0.796445	2.1
47256	0.866907	2.1
47257	0.650788	2.1
47258	2.63062	1.84116
47259	1.89226	1.62357
47260	1.89226	1.03264
47261	1.89226	2.09137
47262	2.46557	0.851001
47263	2.46557	2.09413
47264	1.16777	2.09406
47265	1.38859	2.1
47266	1.05042	2.1
47267	1.28909	2.1

47268	2.45686	2.1
47269	1.89227	2.08998
47270	1.89227	2.1
47271	1.89227	1.71635
47272	1.89227	2.1
47273	3.78453	2.09941
47274	2.15114	2.1
47275	1.89226	2.1
47276	2.15005	2.1
47277	2.15005	2.1
47278	1.89226	2.1
47279	1.89226	2.1
47280	1.89226	2.1
47281	1.89226	1.55764
47282	1.89226	2.1
47283	1.89226	2.1
47284	1.89226	1.30365
47285	1.89226	2.1
47286	0.973447	2.1
47287	1.32143	2.06542
47288	1.37444	2.1
47289	1.37444	2.07486
47290	0.949134	2.1
47291	0.949134	2.1
47292	0.946131	2.1
47293	0.946131	2.1
47294	1.07557	2.1
47295	1.07557	2.1
47296	0.946131	2.1
47297	0.946131	2.1
47298	1.07557	2.1
47299	1.08579	2.1
47300	1.08579	2.1
47301	0.946131	2.1
47302	1.07557	2.1
47303	0.946131	2.1
47304	0.946131	1.16593
47305	0.946131	2.1
47306	0.946131	2.1
47307	0.946131	2.07445
47308	0.94613	1.19234
47309	0.94613	1.57352
47310	0.946131	2.1
47311	0.946131	2.1
47312	0.946131	2.06709
47313	0.946131	2.02902
47314	0.946131	2.07954
47315	0.946131	2.09233
47316	0.606115	2.1
47317	0.606115	2.08161
47318	0.616308	2.1
47319	0.616308	2.1
47320	0.68722	1.40391
47321	0.735699	2.00064
47322	0.514789	2.1
47323	0.486724	2.1
47324	0.514788	2.04756
47325	0.735699	2.08087

47326	0.486723	2.09388
47327	0.687221	2.07968
47328	2.33761	0.576393
47329	2.51749	0.194199
47330	2.68092	0.256196
47331	2.58304	0.217937
47332	5.01853	0.214359
47333	2.50906	0.321907
47334	2.51749	0.231097
47335	2.68091	0.301724
47336	2.53176	0.159327
47337	5.18997	0.286482
47338	2.68092	0.296659
47339	2.58304	0.207949
47340	2.71793	0.285988
47341	2.67098	0.156203
47342	5.39885	0.243151
47343	2.68091	0.362034
47344	2.53175	0.16977
47345	2.36417	0.229159
47346	2.67098	0.174339
47347	5.04508	0.206664
47348	2.76309	2.1
47349	1.89226	2.1
47350	1.89226	2.1
47351	1.89226	2.1
47352	2.48573	2.1
47353	2.48573	2.1
47354	1.35879	2.1
47355	1.55161	2.09019
47356	1.10407	2.1
47357	1.32259	2.05357
47358	2.68138	2.1
47359	1.89226	2.1
47360	1.89226	2.08937
47361	1.89226	2.1
47362	1.89226	2.06807
47363	3.78452	2.1
47364	1.19612	2.05294
47365	1.24694	2.1
47366	1.24844	0.999248
47367	1.29747	2.1
47368	2.49358	2.1
47369	1.89226	2.06877
47370	1.89226	2.1
47371	1.89226	0.756436
47372	1.89226	2.1
47373	3.78453	2.09615
47374	1.89226	2.1
47375	1.89226	2.1
47376	1.89226	2.1
47377	1.89226	2.1
47378	1.89226	2.1
47379	1.89226	2.1
47380	0.587463	2.1
47381	0.587463	2.1
47382	0.581977	2.1
47383	0.581977	2.1

47384	0.531709	2.1
47385	0.545972	2.1
47386	0.623469	2.1
47387	0.648733	2.1
47388	0.648733	2.1
47389	0.545971	2.1
47390	0.623469	2.1
47391	0.531709	2.1
47392	1.29417	1.58674
47393	1.29747	2.1
47394	1.27829	2.1
47395	1.24844	2.00881
47396	1.32732	2.1
47397	1.29747	2.06018
47398	1.89226	0.612791
47399	1.89226	2.1
47400	1.89226	1.59372
47401	1.89226	2.1
47402	1.89226	1.62425
47403	1.89226	2.1
47404	1.89226	2.1
47405	1.89226	2.1
47406	1.89226	2.1
47407	1.89226	2.1
47408	1.89226	2.1
47409	1.89226	2.1
47410	0.60346	2.1
47411	0.60346	2.1
47412	0.60086	2.1
47413	0.60086	2.1
47414	0.545971	2.1
47415	0.555587	2.1
47416	0.648733	2.1
47417	0.663661	2.1
47418	0.663661	2.1
47419	0.555587	2.1
47420	0.648733	2.1
47421	0.545971	2.1
47422	1.34066	2.08071
47423	1.36601	2.1
47424	1.34955	0.585736
47425	1.37623	2.1
47426	2.71556	2.1
47427	1.89226	1.55976
47428	1.89226	2.1
47429	1.89226	0.582162
47430	1.89226	2.1
47431	3.78452	1.7974
47432	0.946132	2.1
47433	0.946132	2.1
47434	0.946132	2.1
47435	0.946132	2.1
47436	0.946132	2.1
47437	0.946132	2.1
47438	0.946131	2.1
47439	0.946131	2.1
47440	0.946132	2.1
47441	0.946132	2.1

47442	0.946132	2.1
47443	0.946132	2.1
47444	0.631351	2.1
47445	0.631351	2.1
47446	0.632421	2.1
47447	0.632421	2.1
47448	0.573604	2.1
47449	0.580765	2.1
47450	0.683006	2.1
47451	0.688118	2.1
47452	0.688118	2.1
47453	0.580765	2.1
47454	0.683007	2.1
47455	0.573604	2.1
47456	1.34955	1.9712
47457	1.37624	2.1
47458	1.35048	0.91887
47459	1.37999	2.1
47460	2.72954	2.0652
47461	0.946131	2.1
47462	0.946131	1.6951
47463	0.946131	2.09517
47464	0.946131	2.1
47465	0.94613	2.1
47466	0.94613	2.1
47467	0.946133	0.949966
47468	0.946133	1.16141
47469	0.946131	2.07151
47470	0.946131	2.07017
47471	0.946132	2.03045
47472	0.946132	1.96953
47473	1.89226	2.1
47474	1.89226	2.1
47475	1.89226	2.1
47476	1.89226	2.1
47477	1.89226	2.1
47478	1.89226	2.1
47479	0.532189	2.1
47480	0.531141	2.1
47481	0.497784	2.1
47482	0.502536	2.1
47483	1.37999	2.1
47484	0.92433	2.1
47485	0.670081	2.1
47486	0.727155	2.1
47487	0.786438	2.1
47488	0.589798	2.1
47489	0.497784	2.1
47490	1.89226	2.1
47491	2.72301	2.1
47492	2.4158	2.1
47493	2.4158	2.1
47494	1.89226	2.1
47495	1.89226	2.1
47496	1.89226	2.1
47497	1.89226	2.1
47498	1.89226	2.1
47499	1.89226	2.1

47500	1.89226	2.1
47501	1.89226	2.1
47502	1.36919	2.1
47503	1.12717	2.1
47504	1.38634	2.1
47505	1.56541	2.1
47506	2.75552	2.1
47507	1.89226	2.1
47508	1.89226	2.1
47509	1.89226	2.1
47510	1.89226	2.1
47511	1.89226	2.1
47512	1.89226	2.1
47513	0.907036	2.1
47514	0.782706	2.1
47515	0.838808	2.1
47516	0.693168	2.1
47517	0.794747	2.1
47518	0.794747	2.1
47519	0.805458	2.1
47520	0.805458	2.1
47521	0.907036	2.1
47522	0.838808	2.1
47523	0.693169	2.1
47524	0.782707	2.1
47525	0.94613	2.1
47526	0.94613	2.1
47527	1.188 2.1	
47528	1.28421	2.1
47529	1.18801	2.1
47530	1.18801	2.1
47531	0.946129	2.1
47532	0.946129	2.1
47533	1.28421	2.1
47534	0.94613	2.1
47535	1.188 2.1	
47536	0.94613	2.1
47537	1.89226	2.1
47538	1.89226	2.1
47539	1.89226	2.1
47540	1.89226	2.1
47541	1.89226	2.1
47542	1.89226	2.1
47543	1.16817	2.1
47544	1.06007	2.1
47545	1.19785	2.1
47546	1.2832	2.1
47547	2.36602	2.1
47548	0.960106	2.1
47549	2.0073	2.1
47550	1.51754	2.1
47551	2.56883	2.1
47552	3.52484	2.1
47553	1.06756	2.1
47554	1.57913	2.1
47555	1.87532	2.1
47556	1.38666	2.1
47557	2.94287	2.1

47558	2.15622	2.1
47559	1.38668	2.1
47560	1.87534	2.1
47561	1.87534	2.1
47562	1.70539	2.1
47563	1.70539	2.1
47564	0.986095	2.1
47565	1.36226	2.1
47566	1.3331	2.1
47567	1.3331	2.1
47568	1.07156	2.1
47569	1.07156	2.1
47570	0.682081	1.96628
47571	0.68748	1.91704
47572	0.650003	0.981338
47573	0.645873	1.1395
47574	0.666344	1.01097
47575	0.666344	1.93062
47576	0.66161	1.98047
47577	0.66161	1.1428
47578	0.68748	1.57672
47579	0.645873	1.54598
47580	0.650004	1.49983
47581	0.682082	1.52406
47582	0.946132	1.95012
47583	0.946132	1.92423
47584	0.946132	1.05337
47585	0.946132	0.925714
47586	0.946132	0.908736
47587	0.946132	1.93502
47588	0.946133	1.13764
47589	0.946133	1.98902
47590	0.946132	1.53303
47591	0.946132	1.6183
47592	0.946133	1.54407
47593	0.946133	1.58622
47594	1.89227	1.08321
47595	2.79312	0.361768
47596	2.77591	0.566578
47597	2.79312	1.05439
47598	1.87505	0.51116
47599	1.89227	0.946378
47600	0.68748	2.00174
47601	0.698626	1.97412
47602	0.645873	1.14543
47603	0.650148	1.22381
47604	0.670561	1.1505
47605	0.670561	1.98331
47606	0.667067	2.01897
47607	0.667067	1.18843
47608	0.698627	1.72775
47609	0.650148	1.69558
47610	0.68748	1.69311
47611	0.645873	1.68427
47612	0.946132	2.00138
47613	0.946132	1.94257
47614	0.946132	1.24398
47615	0.946132	1.15556

47616	0.946132	1.12323
47617	0.946132	1.94567
47618	0.946133	1.34834
47619	0.946133	2.02681
47620	0.946133	1.50076
47621	0.946133	1.50258
47622	0.946132	1.54622
47623	0.946132	1.58246
47624	0.946133	1.25166
47625	0.946133	1.1556
47626	1.38709	0.580505
47627	1.38795	0.471114
47628	0.946132	0.486257
47629	0.946132	1.16138
47630	1.38709	1.23092
47631	1.38709	0.553924
47632	1.38709	0.830552
47633	0.946133	0.840214
47634	0.946132	0.798957
47635	1.38795	0.828933
47636	1.70301	0.05
47637	2.07659	0.05
47638	1.89226	0.05
47639	1.89226	0.05
47640	1.76332	0.05
47641	1.76331	0.05
47642	1.89226	0.05
47643	1.89226	0.05
47644	1.89226	0.05
47645	1.89226	0.05
47646	1.89226	0.05
47647	1.89226	0.05
47648	1.89226	0.05
47649	1.89226	0.05
47650	1.89226	0.05
47651	1.89226	0.05
47652	3.78452	0.05
47653	1.89226	0.05
47654	2.12492	0.05
47655	1.89226	0.05
47656	1.89226	0.05
47657	2.07659	0.05
47658	2.07659	0.05
47659	0.94613	0.05
47660	0.94613	0.05
47661	1.17498	0.05
47662	1.32446	0.05
47663	1.32446	0.05
47664	0.94613	0.05
47665	0.946127	0.05
47666	1.17498	0.05
47667	1.32446	0.05
47668	1.32446	0.05
47669	0.946128	0.05
47670	0.946128	0.05
47671	1.89226	2.1
47672	1.86472	2.1
47673	2.11049	2.1

47674	1.89226	2.1
47675	2.08295	2.1
47676	1.86472	2.1
47677	1.89226	2.1
47678	2.08295	2.1
47679	2.14023	2.1
47680	1.89226	2.1
47681	2.33091	2.1
47682	2.08295	2.1
47683	0.809215	2.1
47684	0.809215	2.1
47685	0.810915	2.1
47686	0.810915	2.1
47687	1.09581	2.1
47688	1.08271	2.1
47689	0.520996	2.1
47690	0.537426	2.1
47691	0.520997	2.1
47692	1.08271	2.1
47693	0.537426	2.1
47694	1.09581	2.1
47695	0.79674	2.1
47696	0.79674	2.1
47697	0.799279	2.1
47698	0.799279	2.1
47699	1.07502	2.1
47700	1.0827	2.1
47701	0.508206	2.1
47702	0.520996	2.1
47703	0.508206	2.1
47704	1.07502	2.1
47705	0.520996	2.1
47706	1.0827	2.1
47707	2.15714	2.1
47708	1.70302	2.1
47709	1.78732	2.1
47710	2.02219	2.1
47711	3.98971	2.1
47712	2.0927	2.1
47713	2.162	2.1
47714	2.11563	2.1
47715	2.02219	2.1
47716	4.18419	2.1
47717	1.62483	2.1
47718	2.02219	2.1
47719	1.93617	2.1
47720	1.52008	2.1
47721	3.54227	2.1
47722	2.11563	2.1
47723	2.02219	2.1
47724	2.29218	2.1
47725	2.03975	2.1
47726	4.06194	2.1
47727	2.007	2.1
47728	1.89226	2.1
47729	1.89226	2.1
47730	1.89226	2.1
47731	2.06776	2.1

47732	2.06776	2.1
47733	0.946129	2.1
47734	0.946129	2.1
47735	1.0035	2.1
47736	1.0035	2.1
47737	0.946131	2.1
47738	0.946131	2.1
47739	0.968797	2.1
47740	1.0035	2.1
47741	0.968771	2.1
47742	0.946105	2.1
47743	1.00353	2.1
47744	0.946157	2.1
47745	1.79103	2.03073
47746	2.53286	0.280242
47747	2.60781	2.1
47748	2.219	0.775827
47749	2.17985	1.24053
47750	1.79103	0.20188
47751	2.219	1.42654
47752	2.66359	1.74992
47753	2.70084	2.08893
47754	2.38698	1.21733
47755	2.53285	0.335092
47756	2.219	0.565793
47757	1.33179	1.1777
47758	0.946128	0.543867
47759	0.946133	1.07039
47760	1.26643	1.94054
47761	1.3318	0.513658
47762	1.3318	1.88706
47763	0.946131	0.617387
47764	0.946131	1.33821
47765	0.946131	0.244777
47766	0.946131	0.190987
47767	1.33179	0.850417
47768	1.26643	2.0106
47769	2.28979	2.1
47770	2.47227	2.1
47771	2.43451	2.1
47772	2.22627	2.1
47773	4.69855	2.1
47774	2.40667	2.1
47775	2.47228	2.1
47776	2.43451	2.1
47777	2.38069	2.1
47778	4.85296	2.1
47779	2.43451	2.1
47780	2.15715	2.1
47781	2.15715	2.1
47782	2.22627	2.1
47783	2.39489	2.1
47784	2.46402	2.1
47785	2.43451	2.1
47786	2.34899	2.1
47787	2.38069	2.1
47788	2.38069	2.1
47789	2.3949	2.1

47790	2.3949	2.1
47791	2.85425	2.09857
47792	1.70302	0.522764
47793	1.70302	1.24452
47794	1.52254	1.01646
47795	2.34181	1.28969
47796	2.08499	1.37614
47797	1.89226	1.22969
47798	1.89226	0.482612
47799	1.89226	0.597162
47800	1.89226	0.366601
47801	1.89226	0.420112
47802	1.89226	0.694147
47803	2.34181	1.93512
47804	1.38287	0.568034
47805	1.38287	1.35208
47806	1.84132	0.717879
47807	1.96416	1.12587
47808	2.4907	1.12744
47809	0.931432	1.19161
47810	0.946135	0.203407
47811	0.946133	0.110713
47812	0.946133	0.255305
47813	0.931464	0.402896
47814	0.931464	1.20116
47815	1.15573	0.142897
47816	0.96378	1.00119
47817	0.931434	0.506041
47818	0.946131	0.236206
47819	0.946131	0.207429
47820	1.13808	0.172639
47821	0.946132	0.582302
47822	0.946132	0.433664
47823	0.946132	0.937017
47824	0.946132	0.452793
47825	0.946133	0.558056
47826	0.946133	0.194705
47827	0.946132	0.634673
47828	0.946132	0.901095
47829	0.946133	0.499754
47830	0.946133	0.591034
47831	0.946132	0.224562
47832	0.946132	0.268665
47833	1.68074	2.1
47834	2.29459	2.1
47835	2.23361	2.1
47836	1.57234	2.1
47837	3.86693	2.1
47838	2.61243	2.1
47839	2.29458	2.1
47840	2.2336	2.1
47841	2.58234	2.1
47842	4.87692	2.1
47843	0.73506	2.1
47844	0.786166	2.1
47845	1.08729	2.1
47846	1.11679	2.1
47847	0.93134	2.1

47848	0.93134	2.1
47849	0.942113	2.1
47850	0.942113	2.1
47851	0.735065	2.1
47852	1.08729	2.1
47853	1.11679	2.1
47854	0.78616	2.1
47855	1.08729	2.1
47856	1.11679	2.1
47857	1.27502	2.1
47858	1.29116	2.1
47859	1.19259	2.1
47860	1.19259	2.1
47861	1.19923	2.1
47862	1.19923	2.1
47863	1.27503	2.1
47864	1.08729	2.1
47865	1.11679	2.1
47866	1.29115	2.1
47867	0.974715	0.555054
47868	1.43779	0.25177
47869	1.93915	0.309835
47870	1.53908	0.342903
47871	2.91387	0.288056
47872	1.53908	0.274055
47873	0.99845	0.278368
47874	1.5176	0.537575
47875	1.97963	0.237694
47876	3.05668	0.319059
47877	1.93915	0.287806
47878	1.97963	0.251006
47879	1.53908	0.274955
47880	1.53908	0.198517
47881	2.3376	0.573523
47882	2.3376	0.284376
47883	1.89226	2.1
47884	2.59747	2.1
47885	1.91802	2.1
47886	1.89226	2.1
47887	2.62322	2.1
47888	2.59747	2.1
47889	2.59747	2.1
47890	1.87119	2.1
47891	1.84844	2.1
47892	1.82268	2.1
47893	2.62323	2.1
47894	2.59747	2.1
47895	0.94613	2.1
47896	0.94613	2.1
47897	1.32697	2.1
47898	1.32697	2.1
47899	1.32697	2.1
47900	1.31161	2.1
47901	0.946132	2.1
47902	0.946132	2.1
47903	0.94613	2.1
47904	1.32697	2.1
47905	0.946131	2.1

47906	1.31161	2.1
47907	2.62323	2.1
47908	1.91704	2.1
47909	1.90191	2.1
47910	1.87119	2.1
47911	2.65395	2.1
47912	2.62323	2.1
47913	1.89226	2.1
47914	1.89226	2.1
47915	2.15958	2.1
47916	2.15958	2.1
47917	2.46558	2.1
47918	2.19826	2.1
47919	2.46558	2.1
47920	1.89226	2.1
47921	1.89226	2.1
47922	1.89226	2.1
47923	2.61064	2.1
47924	2.61064	2.1
47925	1.81759	0.792889
47926	1.89526	0.894542
47927	2.32219	1.38975
47928	1.881	0.610515
47929	1.81759	1.16753
47930	1.3764	0.58705
47931	1.81759	0.879956
47932	2.57079	0.70101
47933	2.32219	1.50678
47934	2.32219	0.698499
47935	1.99875	1.14958
47936	1.99875	0.497038
47937	0.536182	0.410155
47938	0.579206	0.227468
47939	0.908796	0.816672
47940	0.999375	0.509049
47941	0.732091	0.533465
47942	0.732091	0.248718
47943	0.755911	0.371402
47944	0.755911	0.692758
47945	0.999376	0.478589
47946	0.579206	0.495642
47947	0.908796	0.519337
47948	0.536182	0.507547
47949	0.94613	0.373687
47950	0.94613	0.389827
47951	1.31112	0.439984
47952	1.18275	0.451309
47953	0.946126	0.471116
47954	0.946126	0.306225
47955	1.31112	0.379631
47956	1.31112	0.405683
47957	0.946128	0.39982
47958	1.31112	0.406597
47959	0.946128	0.391382
47960	1.18275	0.395873
47961	2.64617	0.653669
47962	1.27737	0.756853
47963	1.83076	0.784445

47964	1.27737	0.739457
47965	3.03764	0.677198
47966	2.3655	0.604634
47967	1.12991	0.05
47968	1.22375	0.05
47969	1.35931	0.05
47970	1.04971	0.05
47971	1.0419	0.05
47972	1.15237	0.05
47973	1.19593	0.05
47974	1.30306	0.05
47975	1.12937	0.05
47976	1.31934	0.05
47977	1.15203	0.05
47978	1.15203	0.05
47979	2.65227	0.05
47980	1.46041	0.05
47981	2.22712	0.05
47982	2.04816	0.05
47983	2.22712	0.05
47984	2.04816	0.05
47985	1.89226	0.417206
47986	1.89226	2.09653
47987	1.89226	1.37186
47988	1.89226	1.37353
47989	3.78452	1.45767
47990	1.24653	1.96321
47991	1.66554	2.09735
47992	1.53538	1.38221
47993	1.53538	2.1
47994	1.40915	1.52746
47995	1.40915	2.1
47996	1.66555	2.09853
47997	1.75835	2.1
47998	1.53538	2.1
47999	1.65313	2.1
48000	3.31868	2.1
48001	1.89226	2.09661
48002	1.89226	2.1
48003	1.89226	1.49542
48004	1.89226	2.1
48005	3.78453	2.1
48006	1.89226	1.06593
48007	1.89226	1.67147
48008	1.89226	0.504675
48009	1.89226	2.1
48010	3.78452	1.18899
48011	0.658778	2.1
48012	0.658778	2.00732
48013	0.661956	1.32415
48014	0.661956	2.1
48015	0.703822	2.1
48016	0.767689	2.1
48017	0.623266	1.20455
48018	0.553046	2.05517
48019	0.703804	2.09954
48020	0.55303	2.09964
48021	0.623282	2.00209

48022	0.767711	2.01707
48023	1.53538	2.1
48024	1.65313	2.1
48025	1.40765	2.1
48026	1.55779	2.1
48027	3.06078	2.1
48028	1.89227	1.64975
48029	1.89227	2.1
48030	1.89227	2.1
48031	1.89227	2.1
48032	3.78453	2.1
48033	1.10517	2.1
48034	2.16532	2.1
48035	1.94374	2.1
48036	1.94001	2.1
48037	1.37756	2.1
48038	1.37756	2.1
48039	1.7453	0.111011
48040	1.83284	0.120679
48041	2.28483	0.151455
48042	1.77804	0.0950992
48043	1.7453	0.175417
48044	1.23851	0.108306
48045	1.7453	0.135017
48046	2.54752	0.120812
48047	2.28483	0.145702
48048	2.28483	0.104965
48049	2.0189	0.159826
48050	2.0189	0.118615
48051	1.12457	0.0872208
48052	2.0189	0.0837233
48053	1.7453	0.10402
48054	1.7453	0.0739342
48055	1.40707	0.111524
48056	1.40707	0.0826667
48057	2.41176	2.1
48058	2.59262	2.1
48059	2.34085	2.1
48060	2.55827	2.1
48061	4.97003	2.1
48062	2.59262	2.1
48063	2.61901	2.1
48064	2.55827	2.1
48065	2.64067	2.1
48066	5.17728	2.1
48067	2.34084	2.1
48068	2.55827	2.1
48069	2.24937	2.1
48070	2.49053	2.1
48071	4.80764	2.1
48072	2.55826	2.1
48073	2.64067	2.1
48074	2.49053	2.1
48075	2.58639	2.1
48076	5.14465	2.1
48077	2.27918	0.05
48078	2.44132	0.05
48079	1.89226	0.05

48080	1.89226	0.05
48081	4.33359	0.05
48082	1.89226	0.05
48083	2.44132	0.05
48084	2.6771	0.05
48085	2.6771	0.05
48086	1.89226	0.05
48087	1.89226	0.05
48088	1.60329	0.05
48089	1.38246	0.05
48090	1.98182	0.05
48091	2.2217	0.05
48092	3.60417	0.05
48093	2.05362	0.05
48094	1.02663	0.05
48095	1.43255	0.05
48096	1.52072	0.05
48097	1.70302	0.05
48098	1.7685	0.05
48099	0.946131	0.05
48100	0.946131	0.05
48101	0.946131	0.05
48102	0.946131	0.05
48103	0.946131	0.05
48104	0.946131	0.05
48105	0.946131	0.05
48106	0.946131	0.05
48107	0.946131	0.05
48108	0.946131	0.05
48109	0.946131	0.05
48110	0.946131	0.05
48111	1.89226	0.05
48112	1.89226	0.0545375
48113	1.89226	0.056322
48114	1.89226	0.05
48115	1.89226	0.0598458
48116	1.89226	0.05
48117	1.92645	0.504255
48118	2.83045	0.448306
48119	1.92225	0.533725
48120	2.01873	0.394723
48121	2.51732	0.612724
48122	2.51732	0.437074
48123	2.51732	0.346847
48124	2.23857	0.351419
48125	1.92645	0.408734
48126	1.92645	0.294161
48127	2.23857	0.489597
48128	1.50865	0.294749
48129	2.02687	2.1
48130	2.39604	2.1
48131	2.29198	2.1
48132	2.73046	2.1
48133	4.68803	2.1
48134	1.21148	1.72695
48135	1.87957	1.9094
48136	2.03555	1.95669
48137	2.03555	1.11067

48138	1.09962	1.7909
48139	1.09962	1.87393
48140	1.92558	0.811646
48141	2.05274	2.1
48142	2.2443	1.57227
48143	2.24429	2.1
48144	1.70869	0.79642
48145	1.70868	2.1
48146	0.818397	0.389503
48147	0.818397	2.1
48148	0.827911	2.09254
48149	0.827908	1.96473
48150	1.00418	0.88882
48151	1.02054	0.958853
48152	0.692018	1.99852
48153	0.620899	1.89636
48154	0.65823	1.14805
48155	1.00418	0.571268
48156	0.620899	1.03439
48157	1.0262	0.76251
48158	1.92558	1.81078
48159	2.51818	2.1
48160	1.94816	0.890535
48161	2.01985	2.1
48162	1.85389	0.908942
48163	1.92558	2.1
48164	0.903844	2.1
48165	0.903844	2.1
48166	0.946148	2.1
48167	0.946151	2.1
48168	0.946118	2.1
48169	0.903876	2.1
48170	0.946118	2.1
48171	1.10235	2.1
48172	0.946131	2.1
48173	0.946131	2.1
48174	1.10234	2.1
48175	0.903863	2.1
48176	1.63471	2.1
48177	1.08022	2.1
48178	1.56801	2.1
48179	1.03437	2.1
48180	2.66908	2.1
48181	1.56801	2.1
48182	0.97541	2.1
48183	0.97541	2.1
48184	1.03437	2.1
48185	1.49733	2.1
48186	1.55629	2.1
48187	1.89226	0.0585568
48188	1.89226	0.123608
48189	1.89226	0.0767399
48190	1.89226	0.10614
48191	3.78452	0.117581
48192	0.946131	0.0524474
48193	1.16373	0.0528713
48194	0.946131	0.0608317
48195	0.734637	0.0551888

48196	0.946132	0.0582183
48197	0.946132	0.0691215
48198	1.16373	0.05
48199	0.734636	0.05
48200	0.946093	0.0570232
48201	0.946093	0.05
48202	0.734675	0.05
48203	0.734675	0.0623584
48204	1.98413	0.94946
48205	1.87355	2.09531
48206	1.72774	0.724124
48207	1.67865	2.1
48208	3.60128	1.71761
48209	1.87355	2.09472
48210	1.54924	2.1
48211	1.68443	2.1
48212	1.68443	2.09969
48213	1.67866	2.1
48214	1.67866	2.1
48215	1.72774	0.546626
48216	1.53422	2.1
48217	1.67865	2.04716
48218	1.67865	0.937306
48219	1.53873	2.03476
48220	1.53873	0.902893
48221	1.67866	2.05896
48222	1.54924	2.1
48223	1.53423	2.1
48224	1.44091	2.1
48225	3.11957	2.1
48226	1.89226	2.1
48227	1.89226	1.99598
48228	1.65573	2.07315
48229	1.65573	2.09518
48230	1.89226	2.06518
48231	1.89226	2.09273
48232	1.89226	2.08017
48233	1.89614	1.78703
48234	1.7508	2.00551
48235	1.72666	2.04086
48236	1.89226	1.98604
48237	1.89226	2.02161
48238	1.89223	2.08192
48239	1.89223	1.80957
48240	1.89223	1.86456
48241	1.89223	2.04199
48242	1.89223	1.8998
48243	1.89223	2.08074
48244	1.89224	2.01034
48245	1.89224	1.28149
48246	1.89224	1.70622
48247	1.89224	1.92929
48248	1.89224	1.76546
48249	1.89224	2.0188
48250	2.09773	2.1
48251	2.06623	2.1
48252	2.43268	2.1
48253	2.33713	2.1

48254	1.703	2.1
48255	1.61372	2.1
48256	0.646929	2.1
48257	0.689776	2.1
48258	1.05897	2.1
48259	0.956364	2.1
48260	0.905594	2.1
48261	0.904315	2.1
48262	0.727014	2.1
48263	0.850757	2.1
48264	0.798445	2.1
48265	0.654101	2.1
48266	0.717539	2.1
48267	1.0478	2.1
48268	1.89223	2.1
48269	1.89223	2.1
48270	1.89223	2.1
48271	1.6557	1.94072
48272	3.54794	2.1
48273	0.946118	2.1
48274	0.946118	2.1
48275	0.946118	2.1
48276	0.946118	2.1
48277	0.946117	2.1
48278	0.946117	2.1
48279	0.946119	2.1
48280	0.946119	2.1
48281	0.946105	2.1
48282	0.946105	2.1
48283	0.946131	2.1
48284	0.946131	2.1
48285	1.47671	2.1
48286	1.70303	2.1
48287	1.90705	2.1
48288	1.98945	2.1
48289	1.3454	2.1
48290	1.40353	2.1
48291	1.89226	2.1
48292	1.89226	2.1
48293	1.89226	2.1
48294	1.89226	2.1
48295	1.89226	2.1
48296	1.89226	2.09914
48297	2.39438	0.15753
48298	2.69842	0.134631
48299	2.45177	0.20473
48300	2.28563	0.116868
48301	4.84615	0.150092
48302	1.28421	2.1
48303	1.35966	2.1
48304	0.961001	2.1
48305	0.860549	2.1
48306	1.1039	2.1
48307	1.1039	2.1
48308	1.11631	2.1
48309	1.11631	2.1
48310	1.35966	2.1
48311	0.961001	2.1

48312	0.860549	2.1
48313	1.28421	2.1
48314	1.89226	1.66257
48315	1.89226	1.90443
48316	1.89226	1.98586
48317	1.89226	1.3287
48318	3.78452	1.71737
48319	1.48702	0.911501
48320	1.40656	0.86636
48321	0.626442	1.58355
48322	0.626442	1.44791
48323	0.626442	1.75145
48324	0.946132	0.842487
48325	1.05489	0.805529
48326	1.05012	1.77215
48327	0.946131	1.32418
48328	0.946131	1.36241
48329	0.626442	1.40851
48330	1.05012	1.19155
48331	1.89226	1.89676
48332	1.97212	1.1074
48333	1.89226	1.30936
48334	1.89226	1.57792
48335	3.78453	1.60515
48336	1.89226	1.96345
48337	1.89226	1.40177
48338	1.89226	1.52504
48339	1.89226	1.86449
48340	3.78452	1.82687
48341	1.89226	1.16161
48342	1.89226	1.51054
48343	1.89226	1.76174
48344	1.89226	0.807182
48345	1.89226	1.8162
48346	1.89226	0.929334
48347	0.946161	0.687101
48348	0.946161	1.1437
48349	0.932156	1.47742
48350	0.932156	1.06844
48351	0.932184	1.26752
48352	0.946132	0.583851
48353	0.946133	1.52336
48354	0.946133	0.820172
48355	0.946132	0.909822
48356	0.946132	0.938916
48357	0.932185	0.926255
48358	0.946133	0.840778
48359	0.946132	1.26567
48360	0.946132	0.900363
48361	0.946132	1.974
48362	0.946132	1.69402
48363	0.946132	1.61456
48364	0.946132	1.14396
48365	0.946133	1.38325
48366	0.946133	1.8247
48367	0.946132	1.58817
48368	0.946132	1.71202
48369	0.946133	1.3058

48370	0.946133	1.44988
48371	1.79397	0.192764
48372	1.00636	0.207689
48373	1.31658	0.260541
48374	2.02924	0.211464
48375	3.0356	0.218176
48376	1.89226	0.210654
48377	1.89226	0.236056
48378	1.89226	0.233063
48379	1.89226	0.22581
48380	1.89226	0.20303
48381	1.89226	0.18099
48382	0.946131	0.317644
48383	0.946131	0.286048
48384	1.24766	0.338231
48385	1.03691	0.381597
48386	0.946129	0.370219
48387	0.946129	0.29882
48388	1.24766	0.316279
48389	1.24766	0.322748
48390	0.946131	0.315693
48391	1.24766	0.330405
48392	1.03691	0.349882
48393	0.946131	0.327409
48394	1.44824	0.155346
48395	2.02924	0.139524
48396	1.7065	0.177941
48397	1.38604	0.245778
48398	2.32017	0.172111
48399	2.32017	0.142013
48400	1.89226	0.138999
48401	1.89226	0.171741
48402	1.89226	0.150958
48403	1.89226	0.106006
48404	1.89226	0.201021
48405	1.89226	0.146233
48406	0.478349	2.07759
48407	0.612092	2.1
48408	0.606115	2.1
48409	0.506213	2.1
48410	0.550677	2.1
48411	0.550677	2.07841
48412	0.567628	2.1
48413	0.567628	2.1
48414	0.506213	2.1
48415	0.478349	2.1
48416	0.606116	2.1
48417	0.612092	2.1
48418	1.52226	2.1
48419	1.54241	2.1
48420	1.23167	2.1
48421	1.23167	2.1
48422	1.75555	2.1
48423	1.75555	2.1
48424	1.75557	2.1
48425	1.54243	2.1
48426	1.9943	2.1
48427	1.85871	2.1

48428	3.53673	2.1
48429	1.85871	2.1
48430	1.06936	2.1
48431	1.88926	2.1
48432	1.54243	2.1
48433	1.41619	2.1
48434	1.06936	2.1
48435	1.28649	1.96984
48436	1.28516	2.1
48437	1.38986	0.301006
48438	1.42399	1.89987
48439	2.67502	1.82338
48440	1.42399	1.20261
48441	1.07283	2.1
48442	1.46528	2.1
48443	1.28517	1.81879
48444	1.25294	2.1
48445	1.07283	1.95636
48446	1.38986	2.05146
48447	1.42399	2.1
48448	1.52885	1.07855
48449	1.60058	1.19815
48450	2.99043	1.55338
48451	0.715316	2.1
48452	0.715316	1.74459
48453	0.719126	0.865902
48454	0.719126	2.1
48455	0.722447	2.1
48456	0.626469	2.1
48457	0.711995	0.972801
48458	0.800291	0.955611
48459	0.80029	1.95353
48460	0.722447	1.91282
48461	0.711995	2.0275
48462	0.62647	2.02996
48463	2.05632	0.05
48464	1.70303	0.05
48465	2.3923	0.05
48466	2.61277	0.05
48467	1.44836	0.05
48468	1.58184	0.05
48469	1.89226	0.05
48470	1.89226	0.05
48471	1.89226	0.05
48472	1.89226	0.05
48473	3.78453	0.05
48474	1.44091	2.1
48475	1.1822	2.1
48476	1.25866	2.1
48477	1.25866	2.1
48478	1.35215	2.1
48479	1.35215	2.1
48480	1.27338	2.1
48481	1.1822	2.1
48482	1.27338	2.1
48483	1.35215	2.1
48484	1.10817	2.1
48485	1.18694	2.1

48486	0.946132	0.265967
48487	0.946132	0.210713
48488	0.946132	0.174349
48489	0.946132	0.136851
48490	0.946133	0.172188
48491	0.946133	0.250251
48492	0.946132	0.201898
48493	0.946132	0.139735
48494	0.946132	0.199669
48495	0.946132	0.17926
48496	0.946133	0.188665
48497	0.946133	0.20144
48498	2.6875	0.05
48499	2.40558	0.05
48500	2.1994	0.05
48501	1.70303	0.05
48502	4.68305	0.05
48503	0.915523	0.05
48504	0.915523	0.05
48505	0.861057	0.05
48506	0.946796	0.05
48507	0.838652	0.05
48508	0.654182	0.05
48509	1.14044	0.05
48510	0.80252	0.05
48511	0.836435	0.05
48512	0.608659	0.05
48513	1.22198	0.05
48514	0.863472	0.05
48515	2.1994	0.05
48516	0.981586	0.05
48517	1.59619	0.05
48518	1.70303	0.05
48519	1.24816	0.05
48520	1.38523	0.05
48521	2.71141	2.1
48522	1.70302	2.1
48523	2.56527	2.1
48524	1.88649	2.1
48525	2.41279	2.1
48526	1.70302	2.1
48527	1.89226	2.09896
48528	1.89226	2.1
48529	1.89226	2.1
48530	1.89226	2.1
48531	3.78453	2.1
48532	1.89226	2.1
48533	1.89226	2.1
48534	1.89226	2.1
48535	1.89226	2.1
48536	1.89226	2.1
48537	1.89226	2.0986
48538	2.18867	2.1
48539	0.983723	2.1
48540	2.42263	2.1
48541	1.8014	2.1
48542	1.62992	2.1
48543	0.983723	2.1

48544	2.71141	2.1
48545	1.89226	2.1
48546	2.31157	2.1
48547	2.31157	2.1
48548	1.89226	2.1
48549	1.89226	2.1
48550	1.89227	2.1
48551	1.89227	2.05654
48552	1.89227	2.08594
48553	1.89227	2.1
48554	3.78453	2.1
48555	1.89226	2.07474
48556	1.89226	2.1
48557	1.89226	2.1
48558	1.89226	2.05005
48559	1.89226	2.1
48560	1.89226	2.0493
48561	1.89226	1.76674
48562	1.89226	1.00096
48563	1.89226	2.07642
48564	1.89226	0.464795
48565	1.89226	1.60983
48566	1.89226	0.230322
48567	1.1427	1.99259
48568	1.14956	2.08459
48569	1.70302	0.690042
48570	1.81267	2.05247
48571	2.88983	1.90689
48572	1.89226	0.358474
48573	1.89226	2.00619
48574	1.89226	1.04335
48575	1.89226	1.31252
48576	3.78453	1.20001
48577	1.89226	1.62827
48578	1.89226	1.37244
48579	1.89226	0.213436
48580	1.89226	1.88093
48581	3.78452	0.874415
48582	0.884197	2.07505
48583	1.1125	1.17439
48584	1.13273	1.21846
48585	0.844557	1.67252
48586	0.981137	1.07913
48587	1.13971	1.23361
48588	0.980432	1.56892
48589	0.980432	1.24153
48590	1.15859	0.888632
48591	1.12206	0.359656
48592	0.898619	2.04157
48593	0.94426	1.04005
48594	2.21016	0.913642
48595	1.44091	2.1
48596	1.98408	1.31863
48597	1.4218	0.761162
48598	2.21016	2.00198
48599	1.64788	1.94875
48600	1.07526	0.7462
48601	1.91531	2.1

48602	1.49155	2.1
48603	1.58551	1.99156
48604	1.49155	1.63353
48605	1.58551	2.1
48606	1.98979	0.05
48607	2.21773	0.05
48608	2.30533	0.05
48609	2.55895	0.05
48610	4.52305	0.05
48611	2.55896	0.05
48612	2.27963	0.05
48613	2.60486	0.05
48614	2.60486	0.05
48615	2.21773	0.05
48616	2.21773	0.05
48617	2.30533	0.05
48618	2.55895	0.05
48619	2.29539	0.05
48620	2.47308	0.05
48621	4.7784	0.05
48622	2.55896	0.05
48623	2.55792	0.05
48624	2.55792	0.05
48625	2.60486	0.05
48626	2.47308	0.05
48627	2.52003	0.05
48628	0.946133	1.94806
48629	0.946133	2.02689
48630	1.20851	1.8525
48631	1.20851	1.70952
48632	0.967411	1.60376
48633	1.20851	1.47008
48634	0.946132	2.07792
48635	0.946132	1.98695
48636	0.946132	2.01525
48637	1.20851	1.7482
48638	0.967412	1.7493
48639	0.946133	1.96307
48640	1.89226	1.87464
48641	1.89226	1.4332
48642	1.89226	1.59183
48643	1.89226	1.44861
48644	1.89226	1.92952
48645	1.89226	1.90639
48646	1.89226	2.03858
48647	2.41701	1.79481
48648	2.71724	1.78885
48649	2.71724	1.85083
48650	1.89226	2.06031
48651	1.89226	2.05558
48652	0.946131	1.96339
48653	0.946131	2.03258
48654	0.946128	1.87173
48655	0.946128	1.80865
48656	0.94613	1.41021
48657	0.94613	1.53611
48658	0.94613	1.97817
48659	0.94613	1.97476

48660	0.94613	1.92357
48661	0.94613	1.59433
48662	0.94613	1.72061
48663	0.94613	1.96723
48664	0.851541	0.0900203
48665	0.851541	0.109657
48666	0.851481	0.0950679
48667	0.997219	0.102936
48668	0.997112	0.073023
48669	0.992853	0.0687069
48670	0.85154	0.0855195
48671	0.85154	0.0987668
48672	0.894824	0.0698867
48673	0.894824	0.0971998
48674	0.94613	0.11066
48675	0.906485	0.0719024
48676	0.802947	2.1
48677	0.845556	2.1
48678	0.582339	2.1
48679	0.519756	2.1
48680	0.714508	2.1
48681	0.714508	2.1
48682	0.628932	2.1
48683	0.708518	2.1
48684	0.801358	2.1
48685	0.476016	2.1
48686	0.569477	2.1
48687	0.878553	2.1
48688	1.89226	2.1
48689	1.89226	2.1
48690	1.89226	2.1
48691	1.89226	2.1
48692	1.89226	2.1
48693	1.89226	2.1
48694	0.946128	2.1
48695	0.946128	2.1
48696	1.01537	2.1
48697	0.9662	2.1
48698	0.946131	2.1
48699	0.946131	2.1
48700	0.966198	2.1
48701	0.966198	2.1
48702	0.94613	2.1
48703	0.966198	2.1
48704	0.946131	2.1
48705	1.01537	2.1
48706	1.89226	2.1
48707	1.89226	2.1
48708	1.91883	2.1
48709	1.91883	2.1
48710	2.15905	2.1
48711	2.13249	2.1
48712	1.07953	2.1
48713	1.2107	2.1
48714	0.946132	2.1
48715	0.946132	2.1
48716	1.2107	2.1
48717	1.2107	2.1

48718	0.946132	2.1
48719	0.946132	2.1
48720	1.2107	2.1
48721	0.946133	2.1
48722	0.946132	2.1
48723	1.07953	2.1
48724	2.46752	2.1
48725	2.53733	2.1
48726	2.26044	2.1
48727	2.33222	2.1
48728	4.79974	2.1
48729	2.53733	2.1
48730	2.37576	2.05418
48731	2.33222	2.1
48732	2.1845	2.1
48733	4.70798	2.1
48734	2.48652	2.1
48735	2.33224	2.1
48736	2.08149	2.1
48737	1.9839	2.1
48738	4.45623	2.1
48739	2.33223	2.1
48740	2.18452	2.09194
48741	2.16061	2.1
48742	2.01399	1.90215
48743	4.34623	2.1
48744	1.4617	2.08462
48745	1.34701	2.1
48746	1.34701	2.09561
48747	1.25173	2.1
48748	1.5117	2.08378
48749	1.41642	2.1
48750	2.65164	2.1
48751	1.89227	2.1
48752	1.89227	2.1
48753	1.89227	2.1
48754	2.42504	2.1
48755	2.42504	2.1
48756	1.89226	2.05082
48757	1.89226	2.1
48758	1.89226	2.1
48759	1.89226	1.96258
48760	3.78452	2.09608
48761	1.5117	2.1
48762	1.34701	2.1
48763	1.47001	2.1
48764	1.57961	2.0702
48765	2.92663	2.1
48766	1.89226	2.1
48767	1.89226	1.89857
48768	1.89226	2.06952
48769	1.89226	2.1
48770	3.78452	2.09972
48771	0.412773	0.05
48772	0.570839	0.05
48773	0.50721	0.05
48774	0.513638	0.05
48775	0.518805	0.05

48776	0.41278	0.05
48777	1.5344	0.05
48778	0.939282	0.05
48779	0.720051	0.05
48780	0.691767	0.05
48781	0.761152	0.05
48782	1.89226	2.1
48783	1.89226	2.1
48784	1.89226	2.1
48785	1.89226	2.1
48786	1.89226	2.1
48787	1.89226	2.1
48788	1.24618	2.1
48789	2.10585	2.1
48790	1.08134	2.1
48791	1.93032	2.1
48792	3.1765	2.1
48793	1.98474	2.1
48794	2.57777	2.1
48795	1.93031	2.1
48796	2.19041	2.1
48797	3.99251	2.1
48798	1.89226	2.1
48799	1.89226	2.1
48800	1.89226	2.1
48801	1.89226	2.1
48802	3.78452	2.1
48803	1.67374	2.1
48804	2.19042	2.1
48805	1.67374	2.1
48806	2.09769	2.1
48807	2.29586	2.1
48808	2.7198	2.1
48809	1.89226	2.1
48810	1.89226	2.1
48811	1.89226	2.1
48812	1.89226	2.1
48813	1.89226	2.1
48814	1.89226	2.1
48815	0.946132	0.285615
48816	0.946132	0.223198
48817	0.947763	0.222042
48818	0.909304	0.238903
48819	0.946127	0.178871
48820	0.947831	0.163114
48821	0.907673	0.210292
48822	0.907673	0.253586
48823	0.94613	0.19247
48824	0.947834	0.16826
48825	0.907671	0.306372
48826	0.94613	0.172653
48827	0.906519	0.130381
48828	0.906519	0.124347
48829	0.946097	0.134758
48830	0.946097	0.145144
48831	0.947834	0.121923
48832	0.906485	0.113156
48833	0.946131	0.133376

48834	0.946131	0.145936
48835	0.94613	0.181177
48836	0.906485	0.11055
48837	0.946131	0.15247
48838	0.947835	0.139926
48839	2.58638	2.1
48840	2.51039	2.1
48841	2.47395	2.1
48842	2.41539	2.1
48843	5.00178	2.1
48844	2.51039	2.1
48845	2.16633	2.1
48846	2.4154	2.1
48847	2.13977	2.1
48848	4.58173	2.1
48849	2.47395	2.1
48850	2.41539	2.1
48851	2.2988	2.1
48852	2.2604	2.1
48853	4.7142	2.1
48854	2.4154	2.1
48855	2.13977	2.1
48856	2.26041	2.1
48857	1.98957	2.1
48858	4.40497	2.1
48859	2.15721	1.71992
48860	2.31843	0.643671
48861	1.93973	2.02344
48862	2.13243	0.144081
48863	4.25816	0.728882
48864	2.31844	0.481886
48865	2.30908	1.01454
48866	2.13243	0.313685
48867	2.1609	0.561739
48868	4.47934	0.332128
48869	1.93973	1.9809
48870	2.13243	0.270957
48871	1.69661	1.85351
48872	1.9339	0.333081
48873	3.87363	0.937411
48874	2.13243	0.157351
48875	2.1609	0.642889
48876	1.9339	0.375681
48877	1.98414	0.936997
48878	4.0948	0.170713
48879	1.88857	0.05
48880	2.34107	0.05
48881	0.502431	0.05
48882	0.71416	0.05
48883	0.50243	0.05
48884	0.944284	0.05
48885	1.88856	2.1
48886	2.34107	2.1
48887	2.39662	2.1
48888	1.92293	2.1
48889	4.28519	2.1
48890	1.00486	2.1
48891	1.92293	2.1

48892	1.88857	2.1
48893	1.88857	2.1
48894	1.02605	2.1
48895	1.02605	2.1
48896	2.53728	2.1
48897	1.70303	2.1
48898	2.03734	2.1
48899	1.3624	2.1
48900	2.54706	2.1
48901	1.70303	2.1
48902	2.54706	2.1
48903	1.50876	2.1
48904	2.74067	2.1
48905	1.85391	2.1
48906	2.29667	2.1
48907	1.50876	2.1
48908	0.946133	2.1
48909	0.946133	2.1
48910	1.24191	2.1
48911	1.24191	2.1
48912	0.946132	2.1
48913	1.24191	2.1
48914	1.23779	2.1
48915	0.946132	2.1
48916	0.946132	2.1
48917	0.946132	2.1
48918	1.23779	2.1
48919	1.24191	2.1
48920	0.946131	2.1
48921	0.946131	2.1
48922	1.14833	2.1
48923	1.14833	2.1
48924	1.14834	2.1
48925	0.946132	2.1
48926	0.94613	2.1
48927	1.24191	2.1
48928	0.946131	2.1
48929	0.946131	2.1
48930	1.24191	2.1
48931	1.14833	2.1
48932	2.08149	2.1
48933	2.31116	2.1
48934	1.9701	1.99178
48935	2.18328	2.1
48936	4.49563	2.1
48937	2.08149	2.1
48938	2.14059	2.1
48939	1.72377	2.1
48940	2.18328	2.1
48941	3.88931	2.1
48942	2.14059	2.1
48943	2.40755	2.1
48944	2.31145	2.1
48945	2.23743	2.1
48946	2.31145	2.1
48947	2.23743	2.1
48948	2.31117	2.1
48949	2.40754	2.1

48950	2.41055	2.1
48951	2.31145	2.1
48952	2.41055	2.1
48953	2.31145	2.1
48954	2.5615	0.208185
48955	2.37625	0.164388
48956	2.20248	0.170511
48957	2.20248	0.159383
48958	2.65967	0.260931
48959	2.65967	0.211729
48960	2.71793	0.249259
48961	2.65966	0.159956
48962	2.56149	0.156059
48963	2.56149	0.156882
48964	2.71296	0.229199
48965	2.71296	0.214448
48966	1.31528	0.152645
48967	1.32983	0.154322
48968	1.21128	0.15354
48969	1.18812	0.163219
48970	1.24232	0.135728
48971	1.24232	0.139297
48972	1.26109	0.171544
48973	1.26109	0.230289
48974	1.21128	0.177783
48975	1.31528	0.131877
48976	1.18812	0.142031
48977	1.32983	0.196717
48978	2.63056	0.133194
48979	2.58032	0.165464
48980	2.65966	0.118665
48981	2.65966	0.142075
48982	2.58032	0.218101
48983	2.71296	0.196444
48984	1.92039	2.1
48985	1.06226	2.1
48986	1.09252	2.1
48987	1.96052	2.1
48988	3.01291	2.1
48989	1.92039	2.1
48990	2.46338	2.1
48991	2.47522	2.1
48992	2.43508	2.1
48993	1.96052	2.1
48994	1.92039	2.1
48995	0.985949	2.1
48996	0.980261	2.1
48997	0.553625	2.1
48998	0.546262	2.1
48999	0.765671	2.1
49000	0.765671	2.1
49001	0.766539	2.1
49002	0.766539	2.1
49003	0.985949	2.1
49004	0.553625	2.1
49005	0.546261	2.1
49006	0.980261	2.1
49007	1.96052	2.1

49008	2.46338	2.1
49009	1.97189	2.1
49010	1.96052	2.1
49011	2.47932	2.1
49012	2.47932	2.1
49013	0.964608	0.05
49014	0.691524	0.05
49015	1.25462	0.05
49016	0.691524	0.05
49017	1.89226	0.05
49018	1.89226	0.05
49019	1.44894	0.05
49020	2.77655	0.05
49021	1.4237	0.05
49022	2.77701	0.05
49023	2.77701	0.05
49024	1.38847	2.1
49025	1.46031	1.74092
49026	1.65504	1.87039
49027	1.75396	1.44228
49028	3.11535	1.8777
49029	1.89226	1.94329
49030	1.89226	1.74242
49031	1.89226	1.91658
49032	1.89226	0.726729
49033	3.78452	1.54248
49034	1.89226	1.50881
49035	1.89226	2.07213
49036	1.89226	0.365369
49037	1.89226	1.68046
49038	3.78452	0.725436
49039	1.46031	1.03453
49040	1.34851	2.04927
49041	1.75395	1.03684
49042	1.62287	1.56378
49043	3.08318	1.61137
49044	1.65504	2.06714
49045	1.75396	1.98705
49046	1.89249	2.09996
49047	1.9857	1.04737
49048	3.64073	1.97424
49049	1.89226	2.0777
49050	1.89226	1.3758
49051	1.89226	1.4167
49052	1.89226	1.11242
49053	3.78452	1.44038
49054	1.89226	0.747684
49055	1.89226	2.02314
49056	1.89226	0.847828
49057	1.89226	1.8448
49058	3.78452	1.60369
49059	1.75395	2.03573
49060	1.62287	1.95649
49061	1.98569	0.611571
49062	1.86067	0.801347
49063	3.60856	1.00374
49064	1.35697	2.1
49065	2.02064	2.1

49066	1.89989	2.1
49067	1.49571	2.1
49068	1.66753	2.1
49069	1.36431	2.1
49070	0.744004	2.1
49071	0.743997	2.1
49072	0.717972	2.1
49073	0.717972	2.1
49074	0.997982	2.1
49075	0.807796	2.1
49076	0.632235	2.1
49077	0.488575	2.1
49078	0.849275	2.1
49079	0.488581	2.1
49080	0.724729	2.1
49081	0.997967	2.1
49082	2.5276	2.09967
49083	1.70791	2.07816
49084	1.82872	1.69479
49085	1.56362	2.1
49086	2.5276	1.8853
49087	2.24781	2.1
49088	1.37591	2.1
49089	1.23217	2.08342
49090	1.00442	1.86934
49091	0.945282	1.30803
49092	1.15446	1.72888
49093	1.09123	2.1
49094	1.21506	2.09026
49095	1.21506	1.59084
49096	1.39452	1.99999
49097	1.13108	2.01106
49098	1.27876	2.02201
49099	0.929006	2.01217
49100	1.46505	0.0654695
49101	1.91484	0.068127
49102	1.49906	0.0686145
49103	1.49906	0.0845454
49104	1.90196	0.0529405
49105	1.90196	0.0648291
49106	1.90196	0.0660322
49107	2.11465	0.0722074
49108	1.91484	0.0706887
49109	1.91484	0.0872633
49110	2.04016	0.0557522
49111	2.04016	0.0667882
49112	0.912201	0.05
49113	0.950978	0.05
49114	0.665307	0.05
49115	0.732528	0.05
49116	0.829512	0.05
49117	0.829512	0.05
49118	0.815217	0.05
49119	0.815217	0.05279
49120	0.912202	0.05
49121	0.665305	0.05
49122	0.732527	0.0578599
49123	0.95098	0.05

49124	1.90196	0.0575793
49125	2.08757	0.05
49126	1.9626	0.05
49127	2.04016	0.05
49128	1.82441	0.05
49129	1.90196	0.05
49130	1.13048	2.1
49131	0.821162	2.1
49132	0.946131	2.1
49133	0.946131	2.1
49134	1.04567	2.1
49135	0.882347	2.1
49136	0.897276	2.1
49137	0.897276	2.1
49138	0.863109	2.1
49139	1.01055	2.1
49140	0.940305	2.1
49141	0.942166	2.1
49142	2.23933	0.05
49143	0.976217	0.05
49144	2.78053	0.05
49145	2.78053	0.05
49146	0.976217	0.05
49147	1.65984	0.05
49148	1.54168	2.1
49149	2.01197	2.04593
49150	1.69136	2.1
49151	2.13284	1.69646
49152	3.67452	2.1
49153	0.988805	2.1
49154	1.78069	2.1
49155	1.69136	2.1
49156	1.69136	2.1
49157	1.05481	2.1
49158	1.05481	2.1
49159	1.69136	2.1
49160	2.13284	2.00263
49161	1.78069	2.1
49162	2.22044	2.05118
49163	3.91353	2.1
49164	2.69379	1.16431
49165	2.32219	1.40005
49166	2.25746	1.81769
49167	2.25746	1.11
49168	2.32219	1.4813
49169	1.89526	1.03197
49170	1.4556	2.1
49171	1.85028	2.1
49172	2.4909	1.96116
49173	2.03799	2.1
49174	2.73495	2.1
49175	1.85028	2.1
49176	2.71938	1.59131
49177	1.85028	2.1
49178	3.36051	2.1
49179	2.73495	2.0979
49180	2.47584	2.1
49181	1.85028	2.09858

49182	2.47584	2.1
49183	1.04413	2.1
49184	2.1461	2.1
49185	1.74586	2.1
49186	1.44437	2.1
49187	1.04413	2.1
49188	1.89226	2.1
49189	1.89226	1.88552
49190	1.89226	2.1
49191	1.89226	2.08581
49192	3.78452	2.1
49193	1.52724	2.1
49194	1.89249	2.1
49195	1.68143	2.1
49196	2.02433	2.08141
49197	3.55157	2.1
49198	1.07444	2.1
49199	1.76545	2.1
49200	1.68142	2.1
49201	1.68142	2.1
49202	1.14807	2.1
49203	1.14807	2.1
49204	1.89226	2.1
49205	1.89226	2.1
49206	1.89226	2.1
49207	1.89226	2.1
49208	1.89226	2.1
49209	1.89226	2.1
49210	1.89226	2.1
49211	1.89226	2.1
49212	1.89226	2.1
49213	1.89226	1.89706
49214	3.78452	2.1
49215	1.68143	2.1
49216	2.02433	2.1
49217	1.76545	2.1
49218	2.12608	2.1
49219	3.78978	2.1
49220	2.62871	2.1
49221	1.49149	2.1
49222	1.36814	2.1
49223	2.44761	2.1
49224	3.9391	2.1
49225	1.89226	2.1
49226	1.89226	2.04081
49227	1.89226	2.1
49228	1.89226	2.1
49229	3.78452	2.1
49230	0.851542	2.1
49231	0.851542	2.1
49232	1.00897	2.1
49233	0.931224	2.1
49234	0.838952	2.1
49235	0.838952	2.1
49236	0.956848	2.1
49237	0.942474	2.1
49238	0.946132	2.1
49239	0.838952	2.1

49240	0.878743	2.1
49241	0.946132	2.1
49242	1.15541	2.1
49243	2.44761	2.09773
49244	1.36814	2.1
49245	1.36814	2.1
49246	2.19439	2.1
49247	2.19439	2.1
49248	1.89226	2.09724
49249	1.89226	2.1
49250	1.89226	2.1
49251	1.89226	2.07791
49252	3.78452	2.1
49253	1.89226	2.1
49254	1.67791	2.1
49255	1.5713	2.1
49256	1.67791	2.1
49257	1.78566	2.1
49258	1.89226	2.1
49259	1.89226	0.74718
49260	1.62911	2.1
49261	2.24602	1.87576
49262	1.89226	2.0372
49263	1.98287	1.73283
49264	1.62911	1.92131
49265	1.89226	2.1
49266	1.89226	2.1
49267	1.89226	1.91979
49268	1.89226	2.1
49269	3.78452	2.1
49270	1.26688	2.1
49271	1.11804	2.1
49272	0.852467	2.1
49273	0.693314	2.1
49274	0.977459	2.1
49275	0.977459	2.1
49276	0.982741	2.1
49277	0.982741	2.1
49278	1.26689	2.1
49279	0.852464	2.1
49280	0.693312	2.1
49281	1.11804	2.1
49282	0.946131	2.1
49283	0.946131	1.39578
49284	1.1794	0.683017
49285	1.1794	1.84472
49286	0.991434	2.09826
49287	1.1794	1.37829
49288	0.946131	1.43951
49289	0.946131	0.723419
49290	0.946131	2.06749
49291	1.1794	1.99265
49292	0.946131	2.07056
49293	0.991434	1.92269
49294	1.89226	1.46977
49295	1.89226	2.1
49296	1.89226	2.0967
49297	1.89226	2.08359

49298	3.78452	2.09959
49299	2.53377	2.1
49300	1.53443	2.1
49301	2.0815	2.09167
49302	2.82979	2.1
49303	4.35462	2.1
49304	1.96192	0.88294
49305	1.23602	2.1
49306	2.16506	1.72461
49307	1.56841	2.04048
49308	3.40108	2.02395
49309	1.89226	2.1
49310	1.89226	2.1
49311	1.89226	1.76025
49312	1.89226	2.1
49313	3.78452	2.1
49314	0.946129	2.1
49315	0.946129	2.1
49316	1.13067	2.1
49317	1.12952	2.1
49318	0.946129	2.1
49319	1.13089	2.1
49320	0.944978	2.1
49321	0.944978	2.1
49322	0.946131	2.1
49323	1.13089	2.1
49324	0.944977	2.1
49325	0.946131	2.1
49326	0.710208	2.1
49327	0.710208	2.1
49328	0.718894	2.1
49329	0.718894	2.1
49330	0.559773	2.1
49331	0.579321	2.1
49332	0.886767	2.1
49333	0.849781	2.1
49334	0.849781	2.1
49335	0.559774	2.1
49336	0.579321	2.1
49337	0.886766	2.1
49338	1.63471	2.1
49339	1.69956	2.1
49340	1.08022	2.1
49341	1.08022	2.1
49342	1.11955	2.1
49343	1.67403	2.1
49344	1.03766	2.1
49345	2.02825	2.1
49346	2.02794	2.1
49347	2.0348	2.1
49348	1.07196	2.1
49349	1.07196	2.1
49350	1.9276	2.1
49351	2.1004	2.1
49352	2.1306	2.1
49353	2.1306	2.1
49354	1.92178	2.1
49355	1.92032	2.1

49356	0.986724	2.1
49357	2.03064	2.1
49358	2.01385	2.1
49359	2.02404	2.1
49360	1.03766	2.1
49361	1.03766	2.1
49362	1.92027	2.1
49363	2.1306	2.1
49364	2.15388	2.1
49365	2.15388	2.1
49366	1.92614	2.1
49367	1.9276	2.1
49368	1.09578	2.1
49369	0.963018	2.1
49370	0.782956	2.01642
49371	0.529693	2.1
49372	0.6994	2.08999
49373	0.862532	2.1
49374	0.898605	2.1
49375	0.898605	2.05731
49376	0.776756	2.1
49377	1.14805	2.1
49378	0.966521	2.1
49379	0.617676	2.1
49380	2.67185	2.1
49381	2.099	2.1
49382	2.80548	2.1
49383	2.2727	2.1
49384	4.94455	2.1
49385	1.89226	2.1
49386	1.89226	2.1
49387	1.89226	2.1
49388	1.89226	2.1
49389	3.78453	2.1
49390	1.89226	2.1
49391	1.89226	2.1
49392	1.89226	2.1
49393	1.89226	2.1
49394	1.89226	2.1
49395	1.89226	2.1
49396	0.884801	2.1
49397	0.884801	2.1
49398	0.895199	2.1
49399	0.895199	2.1
49400	0.622774	2.1
49401	0.730501	2.1
49402	1.0495	2.1
49403	1.13635	2.1
49404	1.13635	2.1
49405	0.730503	2.1
49406	1.0495	2.1
49407	0.622775	2.1
49408	1.89226	2.1
49409	1.89226	2.1
49410	1.89226	2.1
49411	1.89226	2.1
49412	1.89226	2.1
49413	1.89226	2.1

49414	1.89226	2.1
49415	1.89226	2.1
49416	1.89226	2.1
49417	1.89226	2.1
49418	1.89226	2.1
49419	1.89226	2.1
49420	2.2727	2.1
49421	1.89586	2.1
49422	1.66913	2.1
49423	1.46101	2.1
49424	2.48082	2.1
49425	2.2727	2.1
49426	1.89226	2.1
49427	1.89226	2.1
49428	1.84641	2.1
49429	1.89226	2.1
49430	1.30868	2.1
49431	1.34118	2.08503
49432	1.89226	1.7177
49433	1.89226	2.00676
49434	1.89226	2.06079
49435	1.89226	1.89085
49436	1.89226	1.90927
49437	1.89226	1.3874
49438	1.89226	2.02829
49439	1.89226	1.24795
49440	1.89226	1.74178
49441	1.89226	1.96782
49442	3.78452	1.82913
49443	0.946132	2.1
49444	0.946132	2.07242
49445	0.946131	2.1
49446	0.946131	2.1
49447	0.946132	2.1
49448	0.946132	2.03567
49449	0.946132	2.1
49450	0.946132	2.1
49451	0.946132	2.1
49452	0.946132	2.1
49453	0.946132	2.1
49454	0.946132	2.1
49455	1.89227	2.06905
49456	1.84641	2.1
49457	2.29517	2.1
49458	1.84641	2.09279
49459	2.34102	2.08079
49460	1.89227	1.92449
49461	1.89226	1.16598
49462	1.89226	1.50733
49463	1.89226	1.7469
49464	1.89226	1.30143
49465	1.89226	1.30055
49466	1.89226	0.890464
49467	1.89226	1.12743
49468	1.89226	1.56825
49469	1.89226	1.748
49470	1.89226	1.30898
49471	1.89226	1.34731

49472	1.89226	0.96669
49473	1.89226	2.1
49474	1.89226	2.0537
49475	1.89226	2.1
49476	1.89226	2.02241
49477	1.89226	2.09052
49478	1.89226	1.94491
49479	0.851544	0.05
49480	0.757542	0.05
49481	1.13421	0.05
49482	1.04072	0.05
49483	0.946132	0.05
49484	0.946132	0.05
49485	0.841686	0.05
49486	0.834428	0.05
49487	1.13649	0.05
49488	0.937687	0.05
49489	0.841713	0.05
49490	0.841713	0.05
49491	1.89226	0.292832
49492	1.89226	0.326064
49493	1.89226	0.340324
49494	1.89226	0.249458
49495	1.89226	0.354528
49496	1.89226	0.262572
49497	2.31988	1.43921
49498	2.00082	2.1
49499	2.4594	0.923129
49500	2.17638	2.08013
49501	4.46022	1.90626
49502	2.00082	2.1
49503	1.41829	2.1
49504	1.61207	2.09863
49505	1.43652	2.1
49506	2.17637	2.08101
49507	2.00082	2.1
49508	2.4594	0.92856
49509	2.17638	2.09424
49510	2.57957	0.420142
49511	2.3506	2.08073
49512	4.81	1.68647
49513	2.3506	2.0641
49514	1.57368	2.1
49515	1.89111	1.86308
49516	1.89111	2.1
49517	2.17638	1.83158
49518	2.17638	2.1
49519	1.32813	2.1
49520	1.7237	2.1
49521	1.32461	2.1
49522	1.81656	2.1
49523	3.14468	2.1
49524	1.55132	2.1
49525	2.0815	2.1
49526	1.81656	2.1
49527	2.19826	2.1
49528	3.65818	2.1
49529	0.775951	2.1

49530	0.775951	2.1
49531	0.794601	2.1
49532	0.794601	2.1
49533	0.945641	2.1
49534	0.90828	2.1
49535	0.662302	2.1
49536	0.662273	2.1
49537	0.662274	2.1
49538	0.945639	2.1
49539	0.662304	2.1
49540	0.908279	2.1
49541	1.03062	2.1
49542	1.03062	2.1
49543	1.05054	2.1
49544	1.05054	2.1
49545	1.13551	2.1
49546	1.21275	2.1
49547	0.945641	2.1
49548	0.90828	2.1
49549	0.945641	2.1
49550	1.21275	2.1
49551	0.90828	2.1
49552	1.13552	2.1
49553	1.89226	2.1
49554	1.79883	2.1
49555	1.95341	2.1
49556	1.89226	2.1
49557	1.85998	2.1
49558	1.79883	2.1
49559	1.89226	2.1
49560	1.89226	2.1
49561	1.89226	2.1
49562	1.89226	2.1
49563	3.78453	2.1
49564	0.851541	2.1
49565	0.851541	2.1
49566	0.970398	2.1
49567	0.954365	2.1
49568	0.855356	2.1
49569	0.855357	2.1
49570	0.946133	2.1
49571	0.946133	2.1
49572	1.04072	2.1
49573	0.970397	2.1
49574	0.950003	2.1
49575	0.851538	2.1
49576	1.89226	2.1
49577	1.85998	2.1
49578	1.95296	2.1
49579	1.89226	2.1
49580	1.92067	2.1
49581	1.85998	2.1
49582	0.946133	2.1
49583	0.946133	2.1
49584	0.946132	2.1
49585	0.946132	2.1
49586	0.946132	2.1
49587	0.946132	2.1

49588	0.946132	2.1
49589	0.946132	2.1
49590	0.946132	2.1
49591	0.946132	2.1
49592	0.946133	2.1
49593	0.946133	2.1
49594	1.82152	2.1
49595	1.89226	2.1
49596	1.89226	2.1
49597	2.11152	2.1
49598	1.7472	2.1
49599	1.89226	2.1
49600	2.02687	2.1
49601	2.29199	2.1
49602	1.2734	2.1
49603	1.41077	2.1
49604	1.47381	2.1
49605	2.29199	2.1
49606	1.46889	2.1
49607	2.29199	2.1
49608	1.86766	2.1
49609	2.59401	2.1
49610	4.15965	2.1
49611	1.58995	2.1
49612	1.74246	2.1
49613	1.46643	2.1
49614	1.46643	2.1
49615	1.85097	2.1
49616	1.85097	2.1
49617	1.58995	2.1
49618	1.66584	2.1
49619	1.89214	2.1
49620	1.91787	2.1
49621	3.48208	2.1
49622	1.18474	2.1
49623	1.8144	2.1
49624	1.1486	2.1
49625	1.1486	2.1
49626	2.13071	2.1
49627	2.1257	2.1
49628	2.0815	2.1
49629	2.51651	2.1
49630	1.73681	2.1
49631	2.01601	2.1
49632	2.21406	2.1
49633	2.56998	2.1
49634	1.19995	2.1
49635	1.06256	2.1
49636	0.620677	2.1
49637	0.598084	2.1
49638	0.831426	2.1
49639	0.946056	2.1
49640	0.827692	2.1
49641	0.827692	2.1
49642	1.08958	2.1
49643	0.630132	2.1
49644	0.577547	2.1
49645	1.06104	2.1

49646	2.0815	2.1
49647	2.66249	2.1
49648	2.78966	2.1
49649	2.47108	2.1
49650	2.33858	2.1
49651	2.0815	2.1
49652	1.53735	2.1
49653	1.42278	2.1
49654	1.23654	2.1
49655	1.34678	2.1
49656	2.76955	2.1
49657	1.89226	2.1
49658	1.89226	2.1
49659	1.89226	2.1
49660	1.89226	2.1
49661	3.78452	2.1
49662	1.23654	2.1
49663	1.34678	2.1
49664	1.19	2.1
49665	1.08576	2.1
49666	2.43254	2.1
49667	1.38776	2.1
49668	1.29366	2.1
49669	0.946131	2.1
49670	0.946131	2.1
49671	0.946131	2.1
49672	0.946131	2.1
49673	1.38775	2.1
49674	1.38775	2.1
49675	1.29366	2.1
49676	0.946132	2.1
49677	0.946131	2.1
49678	1.38775	2.1
49679	0.946131	2.1
49680	0.946131	2.1
49681	0.946129	2.1
49682	0.946129	2.1
49683	0.94613	2.1
49684	0.94613	2.1
49685	0.946131	2.1
49686	0.946131	2.1
49687	0.946131	2.1
49688	0.946131	2.1
49689	0.94613	2.1
49690	0.94613	2.1
49691	1.89226	0.195403
49692	1.89226	1.81187
49693	1.89226	0.867142
49694	1.89226	0.830671
49695	1.89226	0.882513
49696	1.89226	1.18212
49697	1.89226	0.751868
49698	1.89226	2.1
49699	1.89226	1.50103
49700	1.89226	1.68141
49701	1.89226	1.38235
49702	1.89226	1.85986
49703	1.89226	0.360526

49704	1.89226	2.02171
49705	1.89226	0.956959
49706	1.89226	1.14834
49707	1.89226	0.83806
49708	1.89226	1.58945
49709	0.92338	2.1
49710	0.92338	2.01842
49711	0.94428	2.03994
49712	0.94428	1.24369
49713	1.19528	2.0541
49714	1.30598	2.1
49715	0.714073	1.39557
49716	0.561679	2.07457
49717	1.19528	2.08467
49718	0.561678	2.0935
49719	0.714072	2.09208
49720	1.30598	2.07779
49721	2.61197	2.1
49722	1.89226	2.1
49723	1.89226	2.08162
49724	1.89226	2.1
49725	2.39056	2.06097
49726	2.39056	2.1
49727	0.946132	2.1
49728	0.946132	2.02673
49729	0.946132	0.758994
49730	0.946132	1.7321
49731	0.946131	1.65826
49732	0.946131	2.1
49733	0.946132	2.02387
49734	0.946132	1.06356
49735	0.946132	1.81248
49736	0.946132	1.85541
49737	0.946132	1.95626
49738	0.946132	1.95317
49739	1.89224	2.1
49740	1.89224	2.1
49741	1.89224	2.1
49742	1.89224	2.1
49743	3.78448	2.1
49744	1.97089	2.1
49745	1.57428	2.1
49746	1.84991	2.1
49747	1.49078	2.1
49748	3.42419	2.1
49749	0.621927	2.1
49750	0.621927	2.1
49751	0.630824	2.1
49752	0.630824	2.1
49753	0.483483	2.1
49754	0.507361	2.1
49755	0.787142	2.1
49756	0.745391	2.1
49757	0.745381	2.1
49758	0.483476	2.1
49759	0.507368	2.1
49760	0.787152	2.1
49761	1.89224	2.1

49762	1.89224	2.1
49763	1.89224	2.1
49764	1.89224	2.1
49765	1.89224	2.1
49766	1.89224	2.1
49767	1.89227	2.1
49768	1.89227	2.1
49769	1.89227	2.1
49770	1.89227	2.1
49771	1.89227	2.1
49772	1.89227	2.1
49773	1.84994	2.1
49774	1.41758	2.1
49775	1.4908	2.1
49776	1.4908	2.1
49777	1.74391	2.1
49778	1.74391	2.1
49779	1.89226	2.1
49780	1.89226	2.1
49781	1.89226	2.1
49782	1.89226	2.1
49783	3.78452	2.1
49784	1.01323	2.1
49785	1.01323	2.1
49786	1.01445	2.1
49787	1.01445	2.1
49788	0.839531	2.1
49789	0.875652	2.1
49790	1.19063	2.1
49791	1.15203	2.1
49792	1.15203	2.1
49793	0.839531	2.1
49794	0.875652	2.1
49795	1.19063	2.1
49796	1.89226	2.1
49797	2.75587	2.1
49798	2.81512	2.1
49799	2.81512	2.1
49800	1.89226	2.1
49801	1.89226	2.1
49802	1.89226	2.1
49803	1.89226	2.1
49804	1.89226	2.1
49805	1.89226	2.1
49806	3.78452	2.1
49807	0.974831	2.1
49808	0.974831	2.1
49809	0.976681	2.1
49810	0.976681	2.1
49811	0.803179	2.1
49812	0.83953	2.1
49813	1.11198	2.1
49814	1.15203	2.1
49815	1.11198	2.1
49816	0.803179	2.1
49817	1.15203	2.1
49818	0.83953	2.1
49819	1.37794	2.1

49820	1.37794	2.1
49821	0.946128	2.1
49822	0.946128	2.1
49823	1.3477	2.1
49824	1.37793	2.1
49825	0.946131	2.1
49826	0.946131	2.1
49827	0.946129	2.1
49828	1.3477	2.1
49829	1.37793	2.1
49830	0.946129	2.1
49831	2.38577	2.06931
49832	1.20247	2.1
49833	1.75182	2.1
49834	2.82546	2.07453
49835	4.1376	2.1
49836	2.82546	2.09528
49837	1.89226	1.32317
49838	2.38577	2.07238
49839	2.38577	1.79447
49840	1.89226	2.04464
49841	1.89226	1.78115
49842	1.035	2.1
49843	2.15429	2.1
49844	0.970998	2.1
49845	2.0815	2.1
49846	2.89672	2.1
49847	0.946131	0.126586
49848	0.946131	0.113758
49849	0.853249	0.118228
49850	1.02715	0.108707
49851	0.851537	0.122657
49852	0.851537	0.127782
49853	1.02663	0.112851
49854	1.13139	0.118283
49855	0.946128	0.103946
49856	0.932039	0.0981907
49857	0.843985	0.0912268
49858	0.946129	0.103307
49859	1.89226	0.111424
49860	1.86408	0.108838
49861	1.96548	0.107612
49862	1.86408	0.110115
49863	1.99366	0.118398
49864	1.89226	0.118506
49865	2.70702	2.1
49866	2.08981	2.1
49867	2.08693	2.1
49868	2.08693	2.1
49869	2.7188	2.1
49870	2.7188	2.1
49871	2.08982	2.1
49872	1.01964	2.1
49873	2.08693	2.1
49874	1.04615	2.1
49875	3.13597	2.1
49876	2.70702	2.1
49877	2.08693	2.1

49878	2.68784	2.1
49879	2.08831	2.1
49880	4.77477	2.1
49881	0.778233	2.1
49882	0.778233	2.1
49883	0.789001	2.1
49884	0.789001	2.1
49885	0.545307	2.1
49886	0.523075	2.1
49887	1.04347	2.1
49888	1.04416	2.1
49889	1.04416	2.1
49890	0.545308	2.1
49891	1.04347	2.1
49892	0.523075	2.1
49893	1.89226	0.0580884
49894	1.89226	0.0889802
49895	1.89226	0.0771912
49896	1.89226	0.05
49897	3.78453	0.058161
49898	1.39237	0.087623
49899	1.17933	0.143365
49900	0.755796	0.0753395
49901	0.755796	0.110226
49902	0.755767	0.0615183
49903	0.94613	0.145417
49904	1.15918	0.068374
49905	0.94613	0.0713732
49906	1.11632	0.0838812
49907	0.92595	0.103447
49908	0.94613	0.0737532
49909	0.94613	0.0866709
49910	1.89226	0.0975645
49911	1.89226	0.0802499
49912	1.89226	0.05
49913	1.89226	0.0782439
49914	3.78452	0.0679454
49915	0.950067	0.0587451
49916	0.75071	0.0596659
49917	0.906516	0.05
49918	0.906516	0.05
49919	0.81586	0.05
49920	0.851541	0.0662813
49921	0.962101	0.05
49922	0.851476	0.0566121
49923	0.962105	0.05
49924	0.815808	0.060562
49925	0.851529	0.0644383
49926	0.851529	0.0508745
49927	1.05032	0.0684734
49928	0.972326	0.0653842
49929	0.906515	0.05
49930	0.906515	0.05
49931	1.12245	0.05
49932	1.04072	0.0512481
49933	0.815857	0.05
49934	0.851539	0.0668131
49935	1.04451	0.05

49936	0.815853	0.067515
49937	0.919795	0.0568024
49938	0.919795	0.0721407
49939	1.5855	2.1
49940	2.14058	2.1
49941	1.9153	2.1
49942	1.79922	2.1
49943	3.72608	2.1
49944	1.58551	2.1
49945	1.24201	2.1
49946	0.960287	2.1
49947	1.79922	2.1
49948	2.82752	2.1
49949	0.679602	2.1
49950	0.621009	2.1
49951	0.899614	2.1
49952	0.940441	2.1
49953	0.679602	2.1
49954	0.94044	2.1
49955	0.621009	2.1
49956	0.899615	2.1
49957	0.794027	2.1
49958	0.794027	2.1
49959	0.785189	2.1
49960	0.785189	2.1
49961	2.14058	2.1
49962	1.79922	2.1
49963	2.23742	2.1
49964	2.23742	2.1
49965	1.78404	2.1
49966	1.88088	2.1
49967	1.15177	2.1
49968	2.0815	2.1
49969	1.93765	2.1
49970	1.78342	2.1
49971	1.39511	2.1
49972	1.28406	2.1
49973	1.97719	2.1
49974	2.50057	2.05143
49975	2.35349	2.1
49976	2.35349	2.02863
49977	2.31686	2.1
49978	2.32857	2.06306
49979	1.93765	2.1
49980	2.35349	2.06434
49981	1.70048	2.1
49982	2.15721	1.77909
49983	4.05397	2.1
49984	0.850388	2.1
49985	0.850388	2.1
49986	0.840019	2.1
49987	0.840019	2.1
49988	1.13736	2.1
49989	1.13852	2.1
49990	0.573661	2.1
49991	0.551891	2.1
49992	0.573661	2.1
49993	1.13852	2.1

49994	0.551892	2.1
49995	1.13736	2.1
49996	0.860203	2.1
49997	0.860203	2.1
49998	0.855758	2.1
49999	0.855758	2.1
50000	1.1423	2.1
50001	1.13852	2.1
50002	0.58628	2.1
50003	0.573661	2.1
50004	0.586281	2.1
50005	1.1423	2.1
50006	0.573661	2.1
50007	1.13852	2.1
50008	1.28918	0.744794
50009	0.999086	0.683814
50010	1.0383	1.53324
50011	0.736019	1.42568
50012	1.00954	1.37105
50013	1.00954	0.732467
50014	1.01566	0.568341
50015	1.01566	1.51577
50016	1.28918	1.18088
50017	1.0383	1.63807
50018	0.999086	1.32103
50019	0.736019	1.29148
50020	2.20797	2.1
50021	2.64748	2.1
50022	2.50375	2.1
50023	2.50375	2.1
50024	2.40858	2.1
50025	2.40858	2.1
50026	2.64748	2.1
50027	2.74744	2.1
50028	2.50375	2.1
50029	2.6521	2.1
50030	5.29958	2.1
50031	2.20797	2.1
50032	2.50375	2.1
50033	2.02064	2.1
50034	2.3606	2.1
50035	4.52439	2.1
50036	2.36059	2.1
50037	2.6521	2.1
50038	2.56952	2.1
50039	2.56952	2.1
50040	2.50375	2.1
50041	2.50375	2.1
50042	2.49439	0.0930164
50043	1.89226	0.1294
50044	2.81337	0.155609
50045	1.89226	0.123848
50046	4.38665	0.126385
50047	1.89226	0.102413
50048	1.89226	0.163949
50049	1.89226	0.192522
50050	1.89226	0.136508
50051	3.78452	0.125537

50052	1.89226	0.127375
50053	1.89226	0.0895893
50054	1.89226	0.127677
50055	1.89226	0.12152
50056	3.78452	0.124845
50057	1.93948	0.236061
50058	1.98874	0.106892
50059	1.62494	0.143827
50060	2.14218	0.186271
50061	1.62494	0.11281
50062	2.14218	0.161354
50063	1.47354	0.141458
50064	1.98874	0.0870257
50065	2.01865	0.119888
50066	1.62495	0.109669
50067	2.01865	0.115665
50068	1.62495	0.0930062
50069	1.89226	2.1
50070	1.89226	2.1
50071	1.89226	2.1
50072	1.89226	2.1
50073	3.78453	2.1
50074	2.05257	2.1
50075	1.94441	2.1
50076	1.88487	2.1
50077	1.82441	2.1
50078	3.82928	2.1
50079	1.94441	2.1
50080	1.74391	2.1
50081	1.82441	2.1
50082	1.65636	2.1
50083	3.60077	2.1
50084	1.89226	2.1
50085	1.89226	2.1
50086	1.89226	2.1
50087	1.89226	2.1
50088	3.78452	2.1
50089	1.89226	2.1
50090	1.89226	2.1
50091	1.89226	2.1
50092	1.89226	2.1
50093	3.78453	2.1
50094	1.88487	2.1
50095	1.82441	2.1
50096	1.75835	2.1
50097	1.72866	2.1
50098	3.61354	2.1
50099	1.82441	2.1
50100	1.65636	2.1
50101	1.72866	2.1
50102	1.58401	2.1
50103	3.38503	2.1
50104	1.89226	2.1
50105	1.89226	2.1
50106	1.89226	2.1
50107	1.89226	2.1
50108	3.78452	2.1
50109	2.27432	2.1

50110	1.33339	2.1
50111	1.38905	2.1
50112	2.34471	2.1
50113	3.66337	2.1
50114	1.89226	2.1
50115	2.34471	2.1
50116	2.27432	2.1
50117	2.27432	2.1
50118	1.89226	2.1
50119	1.89226	2.1
50120	1.20443	2.1
50121	1.17235	2.1
50122	0.724695	2.1
50123	0.694522	2.1
50124	0.949994	2.1
50125	0.949993	2.1
50126	0.948955	2.1
50127	0.948955	2.1
50128	1.20443	2.1
50129	0.7247	2.1
50130	0.694517	2.1
50131	1.17234	2.1
50132	1.89225	2.1
50133	2.40885	2.1
50134	2.3447	2.1
50135	2.3447	2.1
50136	1.89225	2.1
50137	1.89225	2.1
50138	1.37138	2.1
50139	1.82597	2.1
50140	1.45027	2.1
50141	1.92772	2.1
50142	3.27624	2.1
50143	1.89226	2.1
50144	1.89226	2.1
50145	1.89226	2.1
50146	1.89226	2.1
50147	3.78452	2.1
50148	1.89226	2.1
50149	1.89226	2.09449
50150	1.89226	2.1
50151	1.89226	2.07903
50152	3.78452	2.1
50153	1.82597	2.1
50154	2.02312	1.53777
50155	1.92772	2.1
50156	2.15888	2.1
50157	3.98485	2.1
50158	1.53446	2.1
50159	1.92772	2.1
50160	2.03975	2.1
50161	2.03975	2.1
50162	1.45027	2.1
50163	1.45027	2.1
50164	1.89226	2.1
50165	1.89226	2.1
50166	1.89226	2.1
50167	1.89226	2.1

50168	1.89226	2.1
50169	1.89226	2.1
50170	1.89226	2.1
50171	1.89226	2.1
50172	1.89226	2.1
50173	1.89226	2.1
50174	3.78452	2.1
50175	1.92772	2.1
50176	2.15888	2.1
50177	2.03975	2.1
50178	2.3111	2.1
50179	4.19863	2.1
50180	1.89226	2.1
50181	1.89226	2.1
50182	1.89226	2.1
50183	1.89226	2.1
50184	3.78452	2.1
50185	1.78376	2.1
50186	1.3201	2.1
50187	1.78376	2.1
50188	1.86102	2.1
50189	1.2679	2.1
50190	1.34517	2.1
50191	1.89226	2.1
50192	2.52442	2.1
50193	1.89226	2.1
50194	2.51691	2.1
50195	4.41667	2.1
50196	1.89226	2.1
50197	1.89226	2.1
50198	1.89226	2.1
50199	1.89226	2.1
50200	3.78452	2.1
50201	1.78376	2.1
50202	1.2679	2.1
50203	1.71839	2.1
50204	1.23504	2.1
50205	3.0188	2.1
50206	1.89226	2.1
50207	2.51795	2.1
50208	2.51691	2.1
50209	2.51691	2.1
50210	1.89226	2.1
50211	1.89226	2.1
50212	1.89226	0.543829
50213	1.89226	0.769246
50214	1.89226	0.799087
50215	1.89226	0.59165
50216	3.78452	0.663039
50217	1.89227	0.578809
50218	1.89227	0.775734
50219	3.26088	0.670431
50220	2.79543	0.786602
50221	2.20733	0.716825
50222	1.89227	0.817384
50223	1.89226	0.767654
50224	1.89226	0.594723
50225	1.89226	0.570559

50226	1.89226	0.712026
50227	1.89226	0.556617
50228	1.89226	0.71031
50229	1.89227	0.565789
50230	2.20733	0.706942
50231	1.46973	0.666066
50232	1.89227	0.682712
50233	1.7848	0.669926
50234	2.20733	0.701307
50235	1.89226	2.1
50236	2.55682	2.1
50237	2.81593	2.1
50238	2.81593	2.1
50239	1.89226	2.1
50240	1.89226	2.1
50241	1.0607	2.1
50242	1.0607	2.1
50243	1.12617	2.1
50244	0.986957	2.1
50245	0.699328	2.1
50246	0.785567	2.1
50247	1.28855	2.1
50248	1.41913	2.1
50249	0.766737	2.1
50250	1.34711	2.1
50251	1.24643	2.1
50252	0.687289	2.1
50253	3.11063	0.242387
50254	1.89226	0.342961
50255	1.96536	0.295176
50256	1.89226	0.303028
50257	1.96536	0.257814
50258	1.89226	0.261494
50259	1.89226	0.70284
50260	1.89226	0.45172
50261	1.89226	0.566935
50262	1.89226	0.505693
50263	3.78452	0.700526
50264	1.89226	0.322031
50265	1.96256	0.151036
50266	1.89226	0.198525
50267	1.89226	0.311918
50268	2.49192	0.298383
50269	2.49192	0.182429
50270	1.85716	0.307021
50271	2.56871	0.165086
50272	1.03043	0.215053
50273	1.03043	0.239825
50274	2.48125	0.136064
50275	1.65452	0.241655
50276	1.89226	2.1
50277	2.09622	2.1
50278	2.09622	2.1
50279	2.08508	2.1
50280	1.89226	2.1
50281	1.88112	2.1
50282	1.04811	2.1
50283	1.04811	2.1

50284	0.946131	2.1
50285	0.946131	2.1
50286	1.05014	2.1
50287	1.04811	2.1
50288	0.94613	2.1
50289	0.94613	2.1
50290	0.946131	2.1
50291	1.05013	2.1
50292	1.04811	2.1
50293	0.946131	2.1
50294	1.398	2.1
50295	2.10766	2.1
50296	2.3018	2.1
50297	2.3018	2.1
50298	1.34008	2.1
50299	1.34008	2.1
50300	1.1509	2.1
50301	1.1509	2.1
50302	0.946132	2.1
50303	0.946132	2.1
50304	0.946132	2.1
50305	0.946132	2.1
50306	1.05383	2.1
50307	1.1509	2.1
50308	1.1509	2.1
50309	0.946133	2.1
50310	0.946132	2.1
50311	1.05383	2.1
50312	0.931141	2.1
50313	0.931141	2.1
50314	0.971946	2.1
50315	0.971946	2.1
50316	1.28569	2.1
50317	1.1509	2.1
50318	0.699	2.1
50319	0.752185	2.1
50320	1.28568	2.1
50321	0.752186	2.1
50322	0.699001	2.1
50323	1.1509	2.1
50324	2.30181	2.1
50325	1.89226	2.1
50326	1.89226	2.1
50327	1.89226	2.1
50328	2.57137	2.1
50329	2.57137	2.1
50330	2.0815	2.1
50331	1.21962	2.1
50332	1.55173	2.1
50333	2.57849	2.1
50334	3.79001	2.1
50335	1.38779	2.1
50336	1.2923	2.1
50337	1.03974	2.1
50338	1.28832	2.1
50339	1.33566	2.1
50340	1.21116	2.1
50341	1.22343	2.1

50342	1.22343	2.1
50343	1.42288	2.1
50344	1.20138	2.1
50345	1.26025	2.1
50346	1.00644	2.1
50347	1.26066	2.1
50348	1.14271	2.1
50349	0.886635	2.1
50350	0.795277	2.1
50351	1.02966	2.1
50352	1.02967	2.1
50353	1.05216	2.1
50354	1.05216	2.1
50355	1.26068	2.1
50356	0.790042	2.1
50357	0.795269	2.1
50358	1.20135	2.1
50359	1.89226	0.492012
50360	1.89226	0.386731
50361	1.89226	0.482751
50362	1.89226	0.341074
50363	1.89226	0.57742
50364	1.89226	0.403143
50365	1.89227	0.374302
50366	1.89227	0.331509
50367	1.89227	0.402782
50368	1.89227	0.27687
50369	1.89227	0.462689
50370	1.89227	0.314429
50371	0.563873	0.261249
50372	0.50988	0.227545
50373	0.598693	0.327248
50374	0.558819	0.277478
50375	0.564855	0.267906
50376	0.564855	0.232363
50377	0.557838	0.32119
50378	0.557838	0.273772
50379	0.563873	0.283323
50380	0.598694	0.276548
50381	0.558819	0.261541
50382	0.50988	0.269642
50383	0.598693	0.324214
50384	0.558819	0.275767
50385	0.572737	0.386671
50386	0.534248	0.344459
50387	0.566788	0.336145
50388	0.566788	0.286775
50389	0.566153	0.378304
50390	0.566153	0.336147
50391	0.598694	0.342355
50392	0.572737	0.336851
50393	0.534248	0.316903
50394	0.558819	0.317796
50395	1.85217	2.1
50396	1.93111	2.1
50397	1.92875	2.1
50398	2.02391	2.1
50399	3.85986	2.1

50400	1.93112	2.1
50401	1.80425	2.1
50402	2.02392	2.1
50403	1.90981	2.1
50404	3.84092	2.1
50405	1.92876	2.1
50406	2.02393	2.1
50407	2.05274	2.1
50408	2.16783	2.1
50409	4.09659	2.1
50410	2.02393	2.1
50411	1.90982	2.1
50412	2.16784	2.1
50413	2.05967	2.1
50414	4.07766	2.1
50415	1.89226	1.92121
50416	1.89226	1.6895
50417	1.89226	1.83532
50418	1.89226	1.83415
50419	3.78453	1.66197
50420	1.89226	1.60665
50421	1.89226	1.88336
50422	1.89226	2.02558
50423	2.44829	1.41681
50424	3.78453	1.89595
50425	1.89226	1.92964
50426	1.89226	1.94169
50427	1.89226	1.94099
50428	2.60452	1.69365
50429	3.78453	1.99781
50430	1.58723	0.05
50431	1.88071	0.0660987
50432	2.97376	0.05
50433	2.13255	0.0560192
50434	4.43982	0.05
50435	2.20641	0.102696
50436	1.33575	0.150783
50437	2.15824	0.122046
50438	2.15824	0.120601
50439	1.2951	0.12067
50440	1.2951	0.125129
50441	0.825383	0.170007
50442	0.825383	0.142695
50443	0.856104	0.124539
50444	0.856104	0.134017
50445	0.952196	0.162881
50446	0.602363	0.137973
50447	0.667877	0.153956
50448	1.07913	0.124978
50449	0.602363	0.153256
50450	0.667876	0.150195
50451	1.07913	0.150346
50452	0.952197	0.141968
50453	2.57657	2.1
50454	1.44437	2.1
50455	2.65378	2.1
50456	2.25076	2.1
50457	1.84739	2.1

50458	1.44437	2.1
50459	2.0189	0.129522
50460	2.54752	0.127423
50461	2.63975	0.182845
50462	2.31049	0.0947749
50463	4.65865	0.130752
50464	1.40707	0.0860858
50465	1.89605	0.0864596
50466	2.0189	0.102424
50467	2.0189	0.0730129
50468	1.50438	0.109732
50469	1.50438	0.079805
50470	2.63971	0.169661
50471	2.38246	0.0972825
50472	2.13402	0.127473
50473	2.57503	0.117051
50474	4.77373	0.122549
50475	1.50436	0.085586
50476	1.96803	0.0861153
50477	2.13402	0.119092
50478	1.45446	0.0665146
50479	3.63838	0.0863836
50480	2.30793	0.161457
50481	2.11032	0.138728
50482	1.24082	0.13737
50483	1.05689	0.171707
50484	3.36482	0.156995
50485	1.89226	0.205361
50486	1.89226	0.291217
50487	1.89226	0.301305
50488	1.89226	0.233421
50489	3.78453	0.248003
50490	0.812967	0.182553
50491	0.812967	0.192179
50492	1.00181	0.166734
50493	1.08141	0.160679
50494	0.946132	0.156051
50495	0.812938	0.202294
50496	0.932815	0.203359
50497	1.05577	0.161169
50498	0.812938	0.182183
50499	0.932812	0.183654
50500	0.946133	0.190635
50501	0.946133	0.172417
50502	2.53897	2.1
50503	1.89226	2.1
50504	1.89226	2.1
50505	1.89226	2.1
50506	2.3245	2.1
50507	2.3245	2.1
50508	1.00255	2.09567
50509	1.06561	2.1
50510	1.05481	1.32073
50511	1.11077	2.1
50512	2.12042	2.1
50513	1.89226	2.011
50514	1.89226	2.1
50515	1.89226	1.43102

50516	1.89226	2.1
50517	3.78453	2.1
50518	1.89226	2.1
50519	2.77314	2.1
50520	2.81276	2.1
50521	2.81276	2.1
50522	1.89226	2.1
50523	1.89226	2.1
50524	1.11077	2.1
50525	1.15114	2.1
50526	1.10274	2.08753
50527	1.1032	2.1
50528	1.05481	2.09686
50529	1.1032	1.94729
50530	1.89226	1.97946
50531	1.89226	2.1
50532	1.89226	0.739646
50533	1.89226	2.1
50534	3.78452	2.09167
50535	0.721957	0.380614
50536	0.721957	0.424891
50537	0.946103	0.388079
50538	0.946103	0.313437
50539	0.814237	0.341352
50540	0.721929	0.308105
50541	0.946132	0.434886
50542	0.946132	0.394599
50543	0.721928	0.430076
50544	0.946131	0.459379
50545	0.946132	0.491732
50546	0.814237	0.457167
50547	1.89226	0.71724
50548	1.89226	0.41172
50549	1.89226	0.451444
50550	1.89226	0.450905
50551	1.89226	0.599583
50552	1.89226	0.613057
50553	1.37196	0.374278
50554	2.83556	0.27961
50555	1.5165	0.329936
50556	1.5165	0.40533
50557	2.74923	0.302408
50558	2.74923	0.385076
50559	0.946128	0.415129
50560	0.946128	0.51493
50561	0.827866	0.315583
50562	0.946132	0.402751
50563	0.94613	0.307356
50564	0.94613	0.343872
50565	0.94613	0.519464
50566	0.94613	0.470924
50567	0.94613	0.457369
50568	0.827864	0.49932
50569	0.94613	0.478395
50570	0.94613	0.600996
50571	1.36208	0.292709
50572	2.74923	0.216712
50573	1.37196	0.250829

50574	1.37196	0.301012
50575	2.53256	0.229889
50576	2.53256	0.289897
50577	1.07705	2.1
50578	1.07705	2.1
50579	1.08253	2.1
50580	1.08253	2.1
50581	0.762985	2.1
50582	0.781638	2.1
50583	1.37794	2.1
50584	1.40756	2.1
50585	1.37794	2.1
50586	0.762986	2.1
50587	0.781638	2.1
50588	1.40756	2.1
50589	2.69541	2.1
50590	2.75587	2.1
50591	1.48572	2.1
50592	1.48572	2.1
50593	1.52597	2.1
50594	2.73566	2.1
50595	2.0554	1.1086
50596	1.00387	0.64429
50597	2.01001	0.886004
50598	0.994002	0.563376
50599	3.01389	0.592358
50600	1.89226	2.1
50601	2.41579	2.1
50602	2.12209	2.1
50603	2.12209	2.1
50604	1.89226	2.1
50605	1.89226	2.1
50606	1.89226	2.1
50607	1.89226	2.1
50608	1.89226	2.1
50609	1.89226	2.1
50610	1.89226	2.1
50611	1.89226	2.1
50612	0.88209	2.1
50613	0.899471	2.1
50614	0.651501	2.1
50615	0.533531	2.1
50616	0.724105	2.1
50617	0.711243	2.1
50618	0.692622	2.1
50619	0.692622	2.1
50620	0.890668	2.1
50621	0.738066	2.1
50622	0.778223	2.1
50623	0.531121	2.1
50624	1.89226	2.1
50625	2.77504	2.1
50626	2.4158	2.1
50627	2.4158	2.1
50628	1.89226	2.1
50629	1.89226	2.1
50630	1.89226	2.1
50631	1.89226	2.1

50632	1.89226	2.1
50633	1.89226	2.1
50634	1.89226	2.1
50635	1.89226	2.1
50636	1.70303	2.1
50637	1.39832	2.1
50638	1.51749	2.1
50639	1.68783	2.1
50640	3.24844	2.1
50641	1.89226	2.1
50642	2.38311	2.1
50643	1.98321	2.1
50644	1.89226	2.1
50645	2.47406	2.1
50646	2.38311	2.1
50647	1.89226	2.1
50648	1.89226	2.1
50649	1.89226	2.1
50650	1.89226	2.1
50651	1.89226	2.1
50652	1.89226	2.1
50653	0.946131	2.1
50654	0.946131	2.1
50655	1.25167	2.1
50656	1.19156	2.1
50657	1.19156	2.1
50658	1.19156	2.1
50659	0.946133	2.1
50660	0.946133	2.1
50661	0.946131	2.1
50662	1.25167	2.1
50663	0.946131	2.1
50664	1.19156	2.1
50665	1.89226	2.1
50666	2.47406	2.1
50667	1.99996	2.1
50668	1.89226	2.1
50669	2.58176	2.1
50670	2.47406	2.1
50671	1.89226	2.1
50672	1.89226	2.1
50673	1.89226	2.1
50674	1.89226	2.1
50675	1.89226	2.1
50676	1.89226	2.1
50677	0.946131	2.1
50678	0.946131	2.1
50679	1.32241	2.1
50680	1.25167	2.1
50681	1.25167	2.1
50682	1.25167	2.1
50683	0.946133	2.1
50684	0.946133	2.1
50685	0.946131	2.1
50686	1.32241	2.1
50687	0.946131	2.1
50688	1.25167	2.1
50689	1.65151	2.1

50690	1.92471	2.1
50691	1.81834	2.1
50692	2.13272	2.1
50693	3.74306	2.1
50694	1.92472	2.1
50695	1.9943	2.1
50696	2.13273	2.1
50697	2.24475	2.1
50698	4.16947	2.1
50699	1.81834	2.1
50700	2.13272	2.1
50701	2.02687	2.1
50702	2.39605	2.1
50703	4.21439	2.1
50704	2.13273	2.1
50705	2.24475	2.1
50706	2.39605	2.1
50707	2.54903	2.1
50708	4.6408	2.1
50709	1.10534	2.1
50710	1.22993	2.1
50711	0.946132	2.1
50712	0.946132	2.1
50713	0.946132	2.1
50714	0.946132	2.1
50715	1.22993	2.1
50716	1.22993	2.1
50717	1.10534	2.1
50718	0.946131	2.1
50719	0.946132	2.1
50720	1.22993	2.1
50721	1.47221	2.1
50722	2.14647	2.1
50723	1.95567	2.1
50724	1.95567	2.1
50725	1.71082	2.1
50726	1.71082	2.1
50727	0.946131	2.1
50728	0.946131	2.1
50729	0.946131	2.1
50730	0.946131	2.1
50731	0.946132	2.1
50732	0.946132	2.1
50733	0.946131	2.1
50734	0.946131	2.1
50735	0.946131	2.1
50736	0.946131	2.1
50737	0.946132	2.1
50738	0.946132	2.1
50739	0.998086	2.1
50740	1.09429	2.1
50741	0.946125	2.1
50742	0.946125	2.1
50743	0.90732	2.1
50744	0.90732	2.1
50745	1.14414	2.1
50746	1.16553	2.1
50747	1.05018	2.1

50748	0.907332	2.1
50749	0.99482	2.1
50750	0.851529	2.1
50751	0.80534	2.1
50752	0.80534	2.1
50753	0.812206	2.1
50754	0.812206	2.1
50755	0.895134	2.1
50756	0.977828	2.1
50757	0.736099	2.1
50758	0.639718	2.1
50759	0.639722	2.1
50760	0.89514	2.1
50761	0.736095	2.1
50762	0.977821	2.1
50763	0.946125	2.1
50764	0.946125	2.1
50765	0.946125	2.1
50766	0.946125	2.1
50767	0.946125	2.1
50768	0.946125	2.1
50769	0.946124	2.1
50770	0.946124	2.1
50771	0.946131	2.1
50772	0.946131	2.1
50773	0.946118	2.1
50774	0.946118	2.1
50775	2.22622	0.05
50776	2.43347	0.05
50777	2.34108	0.05
50778	1.24587	2.1
50779	1.21674	2.1
50780	1.14533	2.1
50781	1.11311	2.1
50782	1.17874	2.1
50783	1.17874	2.1
50784	1.18024	2.1
50785	1.18024	2.1
50786	1.14533	2.1
50787	1.24587	2.1
50788	1.21674	2.1
50789	1.11311	2.1
50790	2.43347	2.1
50791	2.34107	2.1
50792	2.39663	2.1
50793	2.49174	2.1
50794	4.8301	2.1
50795	1.86206	2.1
50796	1.41058	2.1
50797	1.70302	2.1
50798	2.42083	2.1
50799	3.68129	2.1
50800	1.70302	2.1
50801	2.43549	2.1
50802	2.20075	2.1
50803	2.50358	2.1
50804	2.04916	2.1
50805	2.33113	2.1

50806	1.07624	2.1
50807	2.12621	2.1
50808	1.57297	2.1
50809	1.60383	2.1
50810	1.30047	2.1
50811	1.30047	2.1
50812	2.33379	2.1
50813	2.56149	1.43087
50814	2.36734	2.0782
50815	2.62796	1.86946
50816	4.92883	2.05938
50817	2.5615	1.61376
50818	2.57957	1.49176
50819	2.62796	1.88768
50820	2.69056	0.943896
50821	5.25206	1.45669
50822	2.39322	2.1
50823	2.62796	2.0846
50824	2.6849	2.1
50825	2.6849	2.0714
50826	2.36734	2.1
50827	2.36734	2.06878
50828	2.62796	2.09373
50829	2.69056	1.85969
50830	2.6849	1.0905
50831	2.77227	0.681455
50832	5.37546	1.19756
50833	1.89226	0.05
50834	1.89226	0.05
50835	1.89226	0.05
50836	1.89226	0.05
50837	1.89226	0.05
50838	1.89226	0.05
50839	1.06974	0.05
50840	1.06974	0.05
50841	1.02299	0.05
50842	1.02299	0.05
50843	1.35299	0.05
50844	1.26227	0.05
50845	0.646252	0.05
50846	0.830464	0.05
50847	0.830463	0.05
50848	1.35299	0.05
50849	1.26227	0.05
50850	0.646252	0.05
50851	2.52454	0.05
50852	1.89226	0.05
50853	2.70598	0.05
50854	1.89226	0.05
50855	4.59824	0.05
50856	1.89226	0.05
50857	1.89226	0.05
50858	1.89226	0.05
50859	1.89226	0.05
50860	1.89226	0.05
50861	1.89226	0.05
50862	1.89226	0.05
50863	1.89226	0.05

50864	1.89226	0.05
50865	1.89226	0.05
50866	1.89226	0.05
50867	1.89226	0.05
50868	1.7666	0.05
50869	2.70598	0.05
50870	2.65378	0.05
50871	2.65378	0.05
50872	1.66093	0.05
50873	1.66093	0.05
50874	2.70598	0.05
50875	1.89226	0.05
50876	1.89226	0.05
50877	1.89226	0.05
50878	2.65377	0.05
50879	2.65377	0.05
50880	0.946132	0.05
50881	0.946132	0.05
50882	0.946131	0.05
50883	0.946131	0.05
50884	0.946131	0.05
50885	0.946131	0.05
50886	0.946132	0.05
50887	0.946132	0.05
50888	0.946132	0.05
50889	0.946132	0.05
50890	0.946131	0.05
50891	0.946131	0.05
50892	1.69858	2.1
50893	2.18238	2.1
50894	2.506	2.1
50895	1.93586	2.1
50896	4.20458	2.1
50897	2.21772	2.1
50898	2.18238	2.1
50899	2.506	2.1
50900	2.59215	2.1
50901	4.72372	2.1
50902	2.50596	2.1
50903	1.93584	2.1
50904	2.16728	2.1
50905	2.7679	2.1
50906	4.67325	2.1
50907	1.53296	2.1
50908	1.56299	2.1
50909	1.42624	2.1
50910	1.52961	2.1
50911	2.98923	2.1
50912	1.56299	2.1
50913	1.46568	2.1
50914	1.52961	2.1
50915	1.51916	2.1
50916	3.08215	2.1
50917	1.36226	2.1
50918	1.52961	2.1
50919	1.36226	2.1
50920	1.42624	2.1
50921	1.55369	2.1

50922	1.61768	2.1
50923	1.52961	2.1
50924	1.51916	2.1
50925	1.55369	2.1
50926	1.64892	2.1
50927	3.07285	2.1
50928	2.13405	0.119679
50929	2.57507	0.112942
50930	2.39438	0.166893
50931	1.93408	0.0926949
50932	4.52843	0.119641
50933	2.13405	0.111303
50934	1.27778	0.0698208
50935	1.2545	0.0852375
50936	1.45448	0.0826333
50937	1.93408	0.0831069
50938	2.13405	0.08316
50939	2.39438	0.147118
50940	1.93407	0.0918181
50941	1.60578	0.11547
50942	1.87985	0.107772
50943	4.00016	0.113261
50944	1.0525	2.1
50945	1.05758	2.09672
50946	1.43484	0.560549
50947	1.38506	1.92042
50948	2.48734	2.00157
50949	1.20455	2.1
50950	1.67352	2.1
50951	1.52852	2.1
50952	2.05164	2.1
50953	3.20203	2.1
50954	1.89226	2.1
50955	1.89226	2.1
50956	1.89226	2.1
50957	1.89226	2.1
50958	3.78452	2.1
50959	1.89226	2.1
50960	1.89226	2.1
50961	1.89226	2.1
50962	1.89226	2.1
50963	1.89226	2.1
50964	1.89226	2.1
50965	2.05165	2.1
50966	1.94291	2.1
50967	2.09398	2.1
50968	1.67352	2.1
50969	2.36337	2.1
50970	1.94291	2.1
50971	1.52851	2.1
50972	2.52049	2.1
50973	2.43433	2.1
50974	2.05164	2.1
50975	1.9112	2.1
50976	1.52851	2.1
50977	1.89226	2.1
50978	1.89226	2.1
50979	1.89226	2.1

50980	1.89226	2.1
50981	3.78451	2.1
50982	1.89226	2.1
50983	1.89226	2.1
50984	1.89226	2.1
50985	1.89226	2.1
50986	3.78452	2.1
50987	2.5205	2.1
50988	2.36337	2.1
50989	2.4605	2.1
50990	2.05165	2.1
50991	2.77222	2.1
50992	2.36337	2.1
50993	1.89226	2.1
50994	1.89226	2.1
50995	1.89226	2.1
50996	1.89226	2.1
50997	1.89226	2.1
50998	1.89226	2.1
50999	1.89226	2.1
51000	1.89226	2.1
51001	1.89226	2.1
51002	1.89226	2.1
51003	3.78452	2.1
51004	1.38905	2.1
51005	1.26166	2.1
51006	1.19216	2.1
51007	1.0875	2.1
51008	1.24393	2.1
51009	1.24393	2.1
51010	1.23261	2.1
51011	1.23261	2.1
51012	1.38905	2.1
51013	1.19216	2.1
51014	1.26166	2.1
51015	1.0875	2.1
51016	1.16233	2.1
51017	1.06028	2.1
51018	0.777395	2.1
51019	0.705508	2.1
51020	0.940018	2.1
51021	0.936465	2.1
51022	0.954216	2.1
51023	0.954216	2.1
51024	1.16125	2.1
51025	0.685505	2.1
51026	1.1157	2.1
51027	0.703534	2.1
51028	1.89226	2.1
51029	1.89226	2.1
51030	1.89226	2.1
51031	1.89226	2.1
51032	1.89226	2.1
51033	1.89226	2.1
51034	1.89226	2.1
51035	1.89226	2.1
51036	1.89226	2.1
51037	1.89226	2.1

51038	1.89226	2.1
51039	1.89226	2.1
51040	2.38433	2.1
51041	1.43456	2.1
51042	1.93881	2.1
51043	2.36952	2.1
51044	4.32313	2.1
51045	1.8493	2.1
51046	1.5927	2.1
51047	1.45535	2.1
51048	1.38438	2.1
51049	1.89657	2.1
51050	1.8493	2.1
51051	2.0815	2.1
51052	1.6697	2.1
51053	2.0657	2.1
51054	1.5927	2.1
51055	2.59749	2.1
51056	2.0815	2.1
51057	2.76597	2.1
51058	1.89226	2.1
51059	1.89226	2.1
51060	1.89226	2.1
51061	2.6126	2.1
51062	2.6126	2.1
51063	2.62513	2.1
51064	1.89226	2.1
51065	1.89226	2.1
51066	1.89226	2.1
51067	2.76598	2.1
51068	2.76598	2.1
51069	2.54665	2.1
51070	2.6126	2.1
51071	2.76598	2.1
51072	2.76598	2.1
51073	2.34044	2.1
51074	2.34044	2.1
51075	2.62514	2.1
51076	2.76598	2.1
51077	2.54666	2.1
51078	2.45212	2.1
51079	5.2181	2.1
51080	2.14063	2.1
51081	1.47521	2.1
51082	1.27679	2.1
51083	1.47521	2.1
51084	2.11511	2.1
51085	2.31353	2.1
51086	0.93256	2.1
51087	1.07032	2.1
51088	0.638396	2.1
51089	0.521342	2.1
51090	0.790674	2.1
51091	0.790674	2.1
51092	0.80098	2.1
51093	0.800981	2.1
51094	0.932535	2.1
51095	0.521328	2.1

51096	1.07034	2.1
51097	0.638414	2.1
51098	1.89226	2.1
51099	1.89226	2.04114
51100	1.89226	2.08875
51101	1.89226	2.1
51102	3.78453	2.09591
51103	1.89226	2.1
51104	1.9842	2.1
51105	1.89226	2.1
51106	1.89226	2.1
51107	2.79705	2.1
51108	2.79705	2.1
51109	0.469955	2.1
51110	0.791665	2.1
51111	0.989115	2.06218
51112	0.763235	2.07567
51113	0.776356	2.0653
51114	0.805461	2.1
51115	0.776759	2.08078
51116	0.776759	2.1
51117	0.799151	2.09067
51118	0.940869	2.09453
51119	0.78449	2.09359
51120	0.489635	2.0953
51121	1.89226	2.02653
51122	1.89226	2.06791
51123	1.89226	1.95283
51124	1.89226	2.1
51125	1.89226	1.94017
51126	1.89226	2.1
51127	2.46058	1.91751
51128	2.28991	2.07603
51129	1.8525	2.03936
51130	1.87697	2.07349
51131	2.28991	2.01759
51132	1.62397	2.06903
51133	0.946131	0.05
51134	0.974033	0.05
51135	1.31667	0.05
51136	0.946131	0.05
51137	0.885497	0.05
51138	0.885497	0.05
51139	1.0468	0.05
51140	1.04678	0.05
51141	1.31667	0.05
51142	0.974019	0.05
51143	0.946131	0.05
51144	0.946131	0.05
51145	1.89226	0.05
51146	1.89226	0.0775348
51147	1.89226	0.0610867
51148	1.89226	0.05
51149	3.78452	0.0666112
51150	0.946125	0.0825883
51151	0.946125	0.0676352
51152	0.946131	0.0881613
51153	0.946131	0.100341

51154	0.946128	0.0947978
51155	0.946128	0.088895
51156	0.946128	0.05
51157	0.946128	0.0747422
51158	0.946128	0.0769788
51159	0.946128	0.0676239
51160	0.946128	0.0798614
51161	0.946128	0.0740212
51162	0.946131	0.05
51163	0.98453	0.05
51164	1.32276	0.05
51165	0.946131	0.05
51166	0.946132	0.05
51167	0.946132	0.05
51168	0.98453	0.05
51169	1.32276	0.05
51170	1.32276	0.05
51171	1.32276	0.05
51172	0.946132	0.05
51173	0.946132	0.05
51174	1.89227	2.1
51175	1.89227	2.1
51176	1.89227	2.1
51177	1.89227	2.1
51178	1.89227	2.1
51179	1.89227	2.1
51180	1.89226	2.1
51181	1.89226	2.1
51182	1.89226	2.1
51183	1.89226	2.1
51184	1.89226	2.1
51185	1.89226	2.1
51186	1.05556	2.1
51187	1.38859	2.1
51188	1.46996	2.1
51189	1.25364	2.1
51190	2.52552	2.1
51191	0.694296	2.1
51192	0.73498	2.1
51193	0.561881	2.1
51194	0.512189	2.1
51195	0.621304	2.1
51196	0.621304	2.1
51197	0.625865	2.1
51198	0.625865	2.1
51199	0.734979	2.1
51200	0.561881	2.1
51201	0.51219	2.1
51202	0.694296	2.1
51203	0.946126	2.1
51204	0.946126	2.1
51205	0.946126	2.1
51206	0.946126	2.1
51207	0.946126	2.1
51208	0.946126	2.1
51209	0.946126	2.1
51210	0.946126	2.1
51211	0.946132	2.1

51212	0.946132	2.1
51213	0.94612	2.1
51214	0.94612	2.1
51215	1.89225	2.1
51216	1.89225	2.1
51217	1.89225	2.1
51218	1.89225	2.1
51219	1.89225	2.1
51220	1.89225	2.1
51221	0.626814	2.1
51222	0.632155	2.1
51223	0.746234	2.1
51224	0.734974	2.1
51225	0.68202	2.1
51226	0.68202	2.1
51227	0.685108	2.1
51228	0.685108	2.1
51229	0.746239	2.1
51230	0.632159	2.1
51231	0.734969	2.1
51232	0.62681	2.1
51233	0.746235	2.1
51234	0.734975	2.1
51235	0.595081	2.1
51236	0.561877	2.1
51237	0.648584	2.1
51238	0.648584	2.1
51239	0.659528	2.1
51240	0.659528	2.1
51241	0.746239	2.1
51242	0.595085	2.1
51243	0.734969	2.1
51244	0.561874	2.1
51245	1.89249	2.1
51246	2.2137	2.1
51247	1.33125	2.1
51248	1.06499	2.1
51249	2.12571	2.1
51250	1.78918	2.1
51251	0.946131	2.1
51252	0.946131	2.1
51253	0.94613	2.1
51254	0.94613	2.1
51255	0.946132	2.1
51256	0.946132	2.1
51257	0.946129	2.1
51258	0.946129	2.1
51259	0.946131	2.1
51260	0.946131	2.1
51261	0.946131	2.1
51262	0.946131	2.1
51263	1.89227	2.1
51264	1.89227	2.1
51265	1.89227	2.1
51266	1.89227	2.1
51267	3.78453	2.1
51268	1.89226	2.1
51269	1.89226	2.1

51270	1.89226	2.1
51271	1.89226	2.1
51272	1.89226	2.1
51273	1.89226	2.1
51274	2.01388	2.1
51275	1.38757	2.1
51276	1.24229	2.1
51277	1.3309	2.1
51278	1.88011	2.1
51279	2.01388	2.1
51280	2.01388	2.1
51281	2.01555	2.1
51282	2.13879	2.1
51283	1.80766	2.1
51284	4.15267	2.1
51285	0.946133	2.1
51286	0.946133	2.1
51287	0.946132	2.1
51288	0.946132	2.1
51289	0.946132	2.1
51290	0.946132	2.1
51291	0.946133	2.1
51292	0.946133	2.1
51293	0.946132	2.1
51294	0.946132	2.1
51295	0.946132	2.1
51296	0.946132	2.1
51297	1.89226	2.1
51298	1.89226	2.1
51299	1.89226	2.1
51300	1.89226	2.1
51301	1.89226	2.1
51302	1.89226	2.1
51303	0.676745	2.1
51304	0.68382	2.1
51305	1.07048	2.1
51306	1.1327	2.1
51307	0.929324	2.1
51308	0.871438	2.1
51309	0.867362	2.1
51310	0.867362	2.1
51311	1.10666	2.1
51312	0.695036	2.1
51313	0.744098	2.1
51314	1.10144	2.1
51315	2.25168	2.1
51316	2.22499	2.1
51317	2.15929	2.1
51318	2.13879	2.1
51319	2.15929	2.1
51320	2.13879	2.1
51321	2.15314	2.1
51322	1.70302	2.1
51323	2.24694	2.1
51324	1.65245	2.1
51325	3.82722	2.1
51326	1.65245	2.1
51327	1.00371	2.1

51328	1.7437	2.1
51329	1.65748	2.1
51330	1.10459	2.1
51331	1.00371	2.1
51332	2.24694	2.1
51333	1.65246	2.1
51334	2.69773	2.1
51335	1.88129	2.1
51336	4.12824	2.1
51337	1.65246	2.1
51338	1.10459	2.1
51339	1.88129	2.1
51340	1.05914	2.1
51341	2.76496	2.1
51342	1.89226	2.1
51343	1.89226	2.1
51344	1.89226	2.1
51345	1.89226	2.1
51346	3.78452	2.1
51347	0.718577	2.1
51348	0.718577	2.1
51349	0.725249	2.1
51350	0.725249	2.1
51351	0.597916	2.1
51352	0.61752	2.1
51353	0.826306	2.1
51354	0.859194	2.1
51355	0.826306	2.1
51356	0.597916	2.1
51357	0.859194	2.1
51358	0.61752	2.1
51359	1.25898	2.1
51360	1.25898	2.1
51361	0.946128	2.1
51362	0.946128	2.1
51363	1.25897	2.1
51364	1.25585	2.1
51365	0.946131	2.1
51366	0.946131	2.1
51367	0.946129	2.1
51368	1.25586	2.1
51369	1.25898	2.1
51370	0.946129	2.1
51371	1.89226	2.1
51372	1.89226	2.1
51373	1.89226	2.1
51374	1.89226	2.1
51375	3.78452	2.1
51376	0.693258	2.1
51377	0.693258	2.1
51378	0.697356	2.1
51379	0.697356	2.1
51380	0.572479	2.1
51381	0.597916	2.1
51382	0.792698	2.1
51383	0.826306	2.1
51384	0.572478	2.1
51385	0.792697	2.1

51386	0.826307	2.1
51387	0.597917	2.1
51388	1.25586	2.1
51389	1.25586	2.1
51390	0.946128	2.1
51391	0.946128	2.1
51392	1.25585	2.1
51393	1.24344	2.1
51394	0.946131	2.1
51395	0.946131	2.1
51396	0.946129	2.1
51397	1.24344	2.1
51398	0.94613	2.1
51399	1.25586	2.1
51400	1.65151	2.1
51401	1.81835	2.1
51402	1.12064	2.1
51403	1.12064	2.1
51404	1.24965	2.1
51405	1.81835	2.1
51406	1.24965	2.1
51407	1.81835	2.1
51408	1.41077	2.1
51409	2.02687	2.1
51410	3.22911	2.1
51411	1.89122	1.92174
51412	1.38776	2.1
51413	2.37014	1.36324
51414	1.80818	2.1
51415	3.6994	2.09621
51416	2.15466	1.11685
51417	2.00083	2.1
51418	1.80818	2.05974
51419	1.80818	1.68411
51420	2.31988	2.07063
51421	2.31988	1.46775
51422	1.80818	2.0528
51423	1.18041	2.1
51424	2.00082	2.1
51425	1.43652	2.1
51426	3.18123	2.1
51427	1.89226	1.12877
51428	1.89226	1.54022
51429	1.89226	1.23729
51430	1.89226	1.09948
51431	3.78453	1.13951
51432	1.89226	1.66005
51433	1.89226	1.68775
51434	1.89226	1.45321
51435	1.89226	1.25082
51436	3.78452	1.76032
51437	0.80759	1.47924
51438	0.686119	1.66399
51439	0.972147	1.67524
51440	1.00217	1.69449
51441	0.821334	1.89639
51442	0.821334	1.66158
51443	0.866957	1.63363

51444	0.866957	1.468
51445	0.972145	1.7559
51446	0.686121	2.00757
51447	0.807592	1.83036
51448	1.00217	2.01909
51449	0.972146	1.58501
51450	1.00217	1.55222
51451	1.26654	1.76231
51452	1.1908	1.70259
51453	1.05501	1.62632
51454	1.05501	1.94531
51455	1.10794	1.51401
51456	1.10794	1.03474
51457	0.972146	0.937655
51458	1.26654	1.6051
51459	1.00217	1.55054
51460	1.1908	1.88721
51461	1.89226	1.43813
51462	1.89226	1.05712
51463	1.89226	1.33043
51464	1.89226	1.7829
51465	1.89226	0.913743
51466	1.89226	1.23559
51467	0.972147	1.58271
51468	0.867854	1.79302
51469	1.26132	1.41002
51470	1.26654	1.52965
51471	1.0424	1.7553
51472	1.0424	1.86525
51473	1.092	1.03757
51474	1.092	1.67849
51475	1.26132	1.70072
51476	0.867854	2.07732
51477	0.972147	1.86114
51478	1.26654	2.0331
51479	1.4375	0.090529
51480	1.92998	0.0745792
51481	1.66953	0.0525936
51482	1.0953	0.0554146
51483	3.10703	0.0624715
51484	1.89226	2.1
51485	1.89226	2.1
51486	1.89226	2.1
51487	1.89226	2.1
51488	3.78452	2.1
51489	2.74034	2.1
51490	2.08808	2.1
51491	2.63859	2.1
51492	2.00343	2.1
51493	4.72667	2.1
51494	0.799825	2.1
51495	0.799825	2.1
51496	0.80211	2.1
51497	0.80211	2.1
51498	0.562428	2.1
51499	0.600223	2.1
51500	1.04404	2.1
51501	1.00171	2.1

51502	1.00171	2.1
51503	0.56243	2.1
51504	0.600225	2.1
51505	1.04404	2.1
51506	0.94613	2.1
51507	0.94613	2.1
51508	0.946128	2.1
51509	0.946128	2.1
51510	0.946127	2.1
51511	0.946127	2.1
51512	0.946131	2.1
51513	0.946131	2.1
51514	0.946129	2.1
51515	0.946129	2.1
51516	0.946129	2.1
51517	0.946129	2.1
51518	1.89226	2.1
51519	1.89226	2.1
51520	1.89226	2.1
51521	1.89226	2.1
51522	3.78452	2.1
51523	2.63859	2.1
51524	2.00343	2.1
51525	2.54388	2.1
51526	1.91548	2.1
51527	4.55407	2.1
51528	1.91548	2.1
51529	2.00342	2.1
51530	1.04909	2.1
51531	1.04909	2.1
51532	1.12486	2.1
51533	1.99124	2.1
51534	0.810942	2.1
51535	0.810942	2.1
51536	0.810942	2.1
51537	0.810942	2.1
51538	1.89226	2.1
51539	1.35169	2.1
51540	1.08131	2.1
51541	1.08132	2.1
51542	1.08132	2.1
51543	0.810944	2.1
51544	0.810944	2.1
51545	1.89226	0.863495
51546	1.89226	2.1
51547	1.89226	0.945053
51548	1.89226	2.1
51549	3.78452	1.82275
51550	1.7968	0.82207
51551	1.49831	2.1
51552	1.59244	0.866957
51553	1.3395	2.09184
51554	3.09074	1.86819
51555	1.49831	2.1
51556	1.16858	2.1
51557	1.33951	2.09238
51558	1.0361	2.1
51559	2.53442	2.1

51560	1.89226	2.1
51561	1.89226	2.1
51562	1.89226	2.1
51563	1.89226	2.1
51564	1.89226	2.1
51565	1.89226	2.1
51566	1.89223	0.8005
51567	1.89223	2.1
51568	1.89223	1.57288
51569	1.89223	2.093
51570	1.89223	1.00361
51571	1.89223	2.09543
51572	1.59241	0.725274
51573	1.18952	2.1
51574	1.33949	2.09197
51575	1.33949	1.1834
51576	1.40538	2.09233
51577	1.40538	0.8916
51578	1.89226	0.113734
51579	1.89226	0.13311
51580	1.89226	0.122598
51581	1.89226	0.136724
51582	1.89226	0.154693
51583	1.89226	0.164523
51584	1.89226	2.1
51585	1.89226	2.1
51586	1.89226	2.1
51587	1.89226	2.1
51588	3.78452	2.1
51589	1.89226	2.1
51590	1.89226	2.1
51591	1.89226	2.1
51592	1.89226	2.1
51593	3.78452	2.1
51594	2.01867	2.1
51595	1.46955	2.1
51596	1.58476	2.1
51597	1.38189	2.1
51598	2.22153	2.1
51599	2.01867	2.1
51600	2.01867	2.1
51601	2.52881	2.1
51602	2.21732	2.1
51603	2.21732	2.1
51604	2.22154	2.1
51605	2.22154	2.1
51606	1.89226	2.1
51607	1.89226	2.1
51608	1.89226	2.1
51609	1.89226	2.1
51610	1.89226	2.1
51611	1.89226	2.1
51612	1.89226	2.1
51613	1.89226	2.1
51614	1.89226	2.1
51615	1.89226	2.1
51616	1.89226	2.1
51617	1.89226	2.1

51618	2.22153	2.1
51619	1.63361	2.1
51620	1.77336	2.1
51621	1.46955	2.1
51622	2.52534	2.1
51623	2.22153	2.1
51624	2.34431	2.1
51625	1.62905	2.1
51626	1.49693	2.1
51627	2.19197	2.1
51628	3.82103	2.1
51629	1.89226	2.1
51630	1.89226	2.1
51631	1.89226	2.1
51632	1.89226	2.1
51633	3.78453	2.1
51634	0.946128	2.1
51635	0.946128	2.1
51636	1.22372	2.1
51637	1.17331	2.1
51638	0.946131	2.1
51639	0.946131	2.1
51640	1.17331	2.1
51641	1.17331	2.1
51642	0.946131	2.1
51643	1.17331	2.1
51644	1.22372	2.1
51645	0.94613	2.1
51646	1.04041	2.1
51647	1.09599	2.1
51648	0.748462	2.1
51649	0.701976	2.1
51650	0.896701	2.1
51651	0.896701	2.1
51652	0.901262	2.1
51653	0.901262	2.1
51654	1.04041	2.1
51655	0.701977	2.1
51656	0.748463	2.1
51657	1.09599	2.1
51658	1.89226	2.1
51659	1.89226	2.1
51660	1.89226	2.1
51661	1.89226	2.1
51662	3.78453	2.1
51663	0.946128	2.1
51664	0.946128	2.1
51665	1.17331	2.1
51666	1.13785	2.1
51667	0.94613	2.1
51668	0.94613	2.1
51669	1.13785	2.1
51670	1.13785	2.1
51671	1.13785	2.1
51672	0.94613	2.1
51673	0.94613	2.1
51674	1.17331	2.1
51675	2.50381	2.1

51676	1.89226	2.1
51677	1.89226	2.1
51678	2.73387	2.1
51679	4.39607	2.1
51680	1.80045	2.1
51681	1.99777	2.06464
51682	2.45134	2.1
51683	2.12463	2.1
51684	1.70302	2.1
51685	1.42655	2.1
51686	2.20231	2.1
51687	1.70302	2.1
51688	1.93128	2.1
51689	1.66768	2.1
51690	1.24499	2.04866
51691	1.70302	2.04767
51692	2.55572	0.0804083
51693	1.22811	0.125191
51694	1.82584	0.105461
51695	1.22811	0.0885472
51696	2.62764	0.110382
51697	1.93147	0.0933923
51698	2.14513	0.124469
51699	1.89226	0.234921
51700	2.66481	0.133251
51701	1.89226	0.145511
51702	2.66481	0.242783
51703	1.89226	0.253219
51704	2.11649	2.1
51705	1.89226	2.1
51706	1.89226	2.1
51707	1.89226	2.1
51708	2.15426	2.1
51709	2.15426	2.1
51710	2.06776	2.1
51711	1.89226	2.1
51712	1.89226	2.1
51713	1.89226	2.1
51714	2.11649	2.1
51715	2.11649	2.1
51716	1.89226	2.1
51717	1.89226	2.1
51718	1.89226	2.1
51719	1.89226	2.1
51720	1.89226	2.1
51721	1.89226	2.1
51722	0.998134	2.1
51723	0.918904	2.1
51724	0.894708	2.1
51725	0.758742	2.1
51726	0.864276	2.1
51727	0.864276	2.1
51728	0.8926	2.1
51729	0.8926	2.1
51730	0.998134	2.1
51731	0.894709	2.1
51732	0.758742	2.1
51733	0.918904	2.1

51734	1.89226	2.1
51735	1.89226	2.1
51736	1.89226	2.1
51737	1.89226	2.1
51738	1.89226	2.1
51739	1.89226	2.1
51740	1.89226	2.1
51741	1.89226	2.1
51742	1.89226	2.1
51743	1.89226	2.1
51744	1.89226	2.1
51745	1.89226	2.1
51746	1.99627	2.1
51747	1.61047	2.1
51748	2.0815	2.1
51749	2.07569	2.1
51750	3.75063	2.1
51751	0.848077	2.1
51752	1.07748	2.1
51753	0.802498	2.1
51754	0.626808	2.1
51755	0.875552	2.1
51756	0.737527	2.1
51757	0.835602	2.1
51758	0.835602	2.1
51759	0.983245	2.1
51760	0.781108	2.1
51761	0.872306	2.1
51762	0.601497	2.1
51763	2.74745	2.1
51764	2.73988	2.1
51765	2.6521	2.1
51766	2.67792	2.1
51767	5.39198	2.1
51768	2.73988	2.1
51769	2.57466	2.1
51770	2.67791	2.1
51771	2.55653	2.1
51772	5.29641	2.1
51773	2.6521	2.1
51774	2.64148	2.1
51775	2.67792	2.1
51776	2.67792	2.1
51777	2.56953	2.1
51778	2.56953	2.1
51779	2.64148	2.1
51780	2.55653	2.1
51781	2.57929	2.1
51782	2.57929	2.1
51783	2.67792	2.1
51784	2.67792	2.1
51785	1.89226	1.38543
51786	1.89226	0.878095
51787	1.89226	1.99565
51788	1.89226	0.297703
51789	3.78453	0.900656
51790	1.68703	1.36532
51791	2.50385	0.300497

51792	1.45958	0.628647
51793	2.0815	0.169098
51794	3.93806	0.430768
51795	2.50385	0.263126
51796	2.57414	0.673272
51797	2.0815	0.243434
51798	2.39921	1.60726
51799	4.91181	0.444116
51800	1.89226	0.856002
51801	1.89226	0.734707
51802	1.89226	0.268431
51803	1.89226	0.235822
51804	3.78453	0.373573
51805	1.89226	1.99655
51806	1.89226	0.202125
51807	1.89226	1.03082
51808	1.89226	1.03412
51809	1.89226	0.605691
51810	1.89226	1.33216
51811	1.23013	0.438404
51812	2.0815	0.13053
51813	1.90167	0.895007
51814	1.71243	0.440695
51815	1.45887	0.758222
51816	1.31369	0.236377
51817	2.0815	0.208517
51818	2.39921	1.73547
51819	1.7115	0.332249
51820	2.2421	0.698738
51821	4.09829	0.518148
51822	1.89227	0.155398
51823	1.89227	0.332471
51824	1.89227	0.621627
51825	1.89227	0.842568
51826	3.78453	0.120415
51827	2.39697	0.05
51828	1.85594	0.05
51829	1.91161	0.05
51830	2.49867	0.05
51831	4.35461	0.05
51832	1.12035	0.05
51833	1.85594	0.05
51834	1.91161	0.05
51835	1.15483	0.05
51836	3.01077	0.05
51837	1.89226	0.05
51838	1.89226	0.05
51839	1.89226	0.05
51840	1.89226	0.05
51841	1.89226	0.05
51842	1.89226	0.05
51843	1.89226	0.05
51844	1.89226	0.05
51845	1.89226	0.05
51846	1.89226	0.05
51847	1.89226	0.05
51848	1.89226	0.05
51849	0.936516	0.05

51850	1.03496	0.05
51851	1.22607	0.05
51852	1.28058	0.05
51853	1.06317	0.05
51854	1.06317	0.05
51855	1.19735	0.05
51856	1.0813	0.05
51857	0.858168	0.05
51858	1.32278	0.05
51859	0.957674	0.05
51860	1.25178	0.05
51861	1.92659	0.05
51862	0.998957	0.05
51863	0.998957	0.05
51864	1.15481	0.05
51865	1.6031	0.05
51866	1.74459	0.05
51867	0.946119	0.05
51868	0.946119	0.05
51869	0.946118	0.05
51870	0.946118	0.05
51871	0.946118	0.05
51872	0.946118	0.05
51873	0.946119	0.05
51874	0.946119	0.05
51875	0.946131	0.05
51876	0.946131	0.05
51877	0.946106	0.05
51878	0.946106	0.05
51879	0.946118	0.05
51880	0.946118	0.05
51881	0.946119	0.05
51882	0.946119	0.05
51883	0.946118	0.05
51884	0.946118	0.05
51885	0.946119	0.05
51886	0.946119	0.05
51887	0.946131	0.05
51888	0.946131	0.05
51889	0.946106	0.05
51890	0.946106	0.05
51891	2.47164	0.411088
51892	2.83689	0.24581
51893	2.73872	0.443164
51894	2.41632	0.250028
51895	5.21036	0.270427
51896	0.946133	0.335059
51897	0.946133	0.449516
51898	1.41845	0.271323
51899	1.36936	0.414987
51900	1.41845	0.265256
51901	1.41845	0.250041
51902	0.946132	0.456363
51903	0.946132	0.453443
51904	0.946132	0.369262
51905	1.36936	0.305988
51906	1.41845	0.335565
51907	0.946133	0.397475

51908	1.36936	0.186862
51909	1.27581	0.308391
51910	1.20816	0.215153
51911	1.10464	0.357446
51912	1.24454	0.197708
51913	1.24454	0.192723
51914	1.23943	0.322756
51915	1.23943	0.338522
51916	1.10465	0.291233
51917	1.27581	0.271941
51918	1.36936	0.284237
51919	1.20816	0.258055
51920	0.946131	2.1
51921	0.946131	2.1
51922	1.37277	2.1
51923	1.37277	2.1
51924	1.37277	2.1
51925	1.27186	2.1
51926	0.946131	2.1
51927	0.946131	2.1
51928	0.946131	2.1
51929	1.37277	2.1
51930	0.946131	2.1
51931	1.27186	2.1
51932	2.74554	2.1
51933	1.23712	2.1
51934	2.80992	2.1
51935	2.54371	2.1
51936	1.50333	2.1
51937	1.23712	2.1
51938	2.77314	2.1
51939	2.33316	2.1
51940	2.28748	2.1
51941	2.28748	2.1
51942	2.81275	2.1
51943	2.81275	2.1
51944	2.28749	2.1
51945	1.45887	2.1
51946	2.33317	2.1
51947	1.50022	2.1
51948	3.79204	2.1
51949	2.37364	2.1
51950	2.33317	2.1
51951	1.5362	2.1
51952	1.5362	2.1
51953	1.50022	2.1
51954	2.33317	2.1
51955	1.89226	0.465703
51956	1.89226	0.501218
51957	1.89226	0.400258
51958	1.89226	0.52069
51959	1.89226	0.412132
51960	1.89226	0.545505
51961	0.946128	0.276288
51962	0.946128	0.319773
51963	1.0644	0.211728
51964	0.946133	0.272445
51965	1.0644	0.202751

51966	0.94613	0.239689
51967	0.946131	0.339196
51968	0.946131	0.274424
51969	1.0644	0.253162
51970	1.0644	0.263288
51971	0.946131	0.279175
51972	0.946131	0.255076
51973	1.89226	2.1
51974	1.89226	0.927534
51975	1.89226	2.06658
51976	1.89226	0.309357
51977	3.78452	1.15923
51978	1.14599	2.0995
51979	1.19993	0.603114
51980	1.00065	2.04053
51981	1.07497	0.297441
51982	2.22096	1.66358
51983	0.546551	1.20417
51984	0.546551	0.660151
51985	0.54987	0.569226
51986	0.54987	1.69661
51987	0.503067	0.7493
51988	0.558937	0.792323
51989	0.599966	0.215607
51990	0.537484	0.297034
51991	0.503067	0.230636
51992	0.537485	0.213407
51993	0.599966	0.253138
51994	0.558936	0.285786
51995	0.946132	0.530242
51996	0.946132	1.22559
51997	0.94613	0.329451
51998	0.94613	1.02357
51999	0.946131	0.670995
52000	0.946131	0.78771
52001	0.946131	0.334686
52002	0.946131	0.274626
52003	0.946131	0.280616
52004	0.946131	0.0970732
52005	0.946131	0.448695
52006	0.946131	0.53913
52007	2.19104	2.1
52008	2.5392	2.1
52009	2.53302	2.1
52010	2.19226	2.1
52011	4.73146	2.1
52012	1.09552	2.1
52013	1.09613	2.1
52014	0.802484	2.1
52015	0.807941	2.1
52016	0.95052	2.1
52017	0.95052	2.1
52018	0.952944	2.1
52019	0.952944	2.1
52020	1.09613	2.1
52021	0.807941	2.1
52022	0.802484	2.1
52023	1.09552	2.1

52024	2.533	2.1
52025	2.18531	2.1
52026	2.19224	2.1
52027	2.19224	2.1
52028	2.51603	2.1
52029	2.51603	2.1
52030	1.09266	2.1
52031	1.09612	2.1
52032	0.807935	2.1
52033	0.81091	2.1
52034	0.951897	2.1
52035	0.951897	2.1
52036	0.955134	2.1
52037	0.955134	2.1
52038	0.810915	2.1
52039	1.09266	2.1
52040	1.09611	2.1
52041	0.80793	2.1
52042	2.12741	0.125595
52043	2.79312	0.371626
52044	2.77591	0.639037
52045	2.10714	0.207418
52046	4.90026	0.230361
52047	2.12741	0.187431
52048	1.20859	0.242044
52049	1.21073	0.556633
52050	2.10714	0.277588
52051	3.31573	0.317306
52052	1.38708	0.597231
52053	1.38795	0.479968
52054	1.05331	0.229473
52055	1.05357	0.193018
52056	1.22049	0.240422
52057	1.22049	0.535271
52058	1.22078	0.720675
52059	1.22078	0.180422
52060	1.05331	0.363362
52061	1.38709	0.381855
52062	1.38795	0.313016
52063	1.05357	0.3124
52064	2.10662	0.405288
52065	2.10714	0.164107
52066	2.12499	0.131405
52067	1.22857	0.364774
52068	1.21073	0.276565
52069	1.21073	0.133276
52070	1.82014	0.05
52071	2.08757	0.05
52072	1.81292	0.05
52073	1.67485	0.05
52074	3.63306	0.05
52075	1.82013	0.05
52076	1.21748	0.05
52077	1.29891	0.05
52078	1.4442	0.05
52079	1.67484	0.05
52080	1.82013	0.05
52081	1.81292	0.05

52082	1.4516	0.05
52083	1.39633	0.05
52084	1.67485	0.05
52085	1.5344	0.05
52086	1.81292	0.05
52087	1.67484	0.05
52088	1.00982	0.05
52089	0.994235	0.05
52090	1.21748	0.05
52091	1.45159	0.05
52092	1.67484	0.05
52093	1.55216	2.1
52094	1.34407	2.1
52095	1.34407	2.1
52096	1.52008	2.1
52097	1.81783	2.1
52098	1.99384	2.1
52099	1.90817	2.1
52100	2.03975	2.1
52101	1.97317	2.1
52102	1.81783	2.1
52103	3.85758	2.1
52104	0.599049	2.1
52105	0.660673	2.1
52106	0.785063	2.1
52107	0.924278	2.1
52108	0.760416	2.1
52109	0.760416	2.1
52110	0.719088	2.1
52111	0.694532	2.1
52112	0.522441	2.1
52113	0.796789	2.1
52114	0.655207	2.1
52115	0.886155	2.1
52116	1.97317	2.1
52117	1.81783	2.1
52118	1.65098	2.1
52119	1.91767	2.1
52120	3.7355	2.1
52121	1.15038	2.02873
52122	1.28649	1.96457
52123	1.38986	2.05518
52124	1.38986	2.02821
52125	1.07866	2.05814
52126	1.07866	2.07434
52127	0.664498	0.822767
52128	0.664498	2.1
52129	0.675122	2.1
52130	0.675122	2.1
52131	0.69493	1.04286
52132	0.764429	1.91848
52133	0.623438	2.08298
52134	0.575191	2.1
52135	0.764429	1.84551
52136	0.623438	1.50467
52137	0.575191	2.07662
52138	0.69493	2.0615
52139	1.89226	1.47569

52140	1.89226	0.530789
52141	1.89226	1.79892
52142	1.89226	0.830502
52143	3.78453	0.402059
52144	2.50075	1.91081
52145	2.15276	0.724116
52146	2.24902	1.7674
52147	2.30422	0.678828
52148	4.40178	0.279458
52149	1.70424	2.1
52150	1.34494	2.1
52151	2.15276	0.984756
52152	2.15276	2.09406
52153	2.0793	0.896999
52154	1.63078	1.57833
52155	1.89226	0.976331
52156	1.89226	2.1
52157	1.89226	2.1
52158	1.89226	0.723011
52159	1.89226	2.1
52160	1.89226	1.6477
52161	1.89226	1.829
52162	1.89226	0.934697
52163	1.89226	1.34653
52164	1.89226	0.363551
52165	3.78452	0.380023
52166	2.24902	1.81667
52167	1.91497	0.649219
52168	1.97135	2.09375
52169	1.73957	0.452362
52170	3.98859	0.68269
52171	1.73957	0.455437
52172	1.34494	2.1
52173	1.73957	2.1
52174	1.69006	0.546761
52175	1.3126	2.1
52176	1.34848	1.31968
52177	0.946132	0.558351
52178	0.946132	2.1
52179	0.946132	1.30662
52180	0.946132	2.1
52181	0.946132	2.1
52182	0.946132	2.1
52183	0.946131	0.47394
52184	0.946131	0.131665
52185	0.946132	1.05773
52186	0.946132	1.61918
52187	0.946132	1.48961
52188	0.946132	1.89332
52189	1.89226	2.1
52190	1.89226	2.04573
52191	1.89226	2.09781
52192	1.89226	2.02598
52193	3.78453	2.08065
52194	1.89226	2.03916
52195	2.82624	1.93001
52196	1.89226	2.04489
52197	2.02536	1.91446

52198	1.89226	2.03991
52199	2.02536	1.86228
52200	2.25027	0.05
52201	1.1706	0.05
52202	2.02733	0.05
52203	2.02733	0.05
52204	1.61462	0.05
52205	1.69832	0.05
52206	1.89226	0.05
52207	1.89226	0.05
52208	1.89226	0.05
52209	1.89226	0.05
52210	3.78452	0.05
52211	0.946131	0.05
52212	0.946131	0.05
52213	1.18182	0.05
52214	1.18182	0.05
52215	0.973819	0.05
52216	0.94613	0.05
52217	1.18182	0.05
52218	0.94613	0.05
52219	0.973819	0.05
52220	1.18182	0.05
52221	0.94613	0.05
52222	0.94613	0.05
52223	2.23944	2.1
52224	1.52903	2.1
52225	1.35977	2.1
52226	2.04847	2.1
52227	3.5775	2.1
52228	2.23944	2.1
52229	2.34795	2.1
52230	2.34795	2.1
52231	2.48025	2.1
52232	2.04847	2.1
52233	2.18077	2.1
52234	1.89226	2.1
52235	1.89226	2.1
52236	1.89226	2.1
52237	1.89226	2.1
52238	3.78452	2.1
52239	0.946127	2.1
52240	0.946127	2.1
52241	0.946131	2.1
52242	0.946131	2.1
52243	0.946128	2.1
52244	0.946128	2.1
52245	0.94613	2.1
52246	0.94613	2.1
52247	0.946129	2.1
52248	0.946129	2.1
52249	0.946129	2.1
52250	0.946129	2.1
52251	1.26956	2.1
52252	2.04847	2.1
52253	1.49576	2.1
52254	1.22377	2.1
52255	1.91172	2.1

52256	1.50198	2.1
52257	1.48568	2.1
52258	2.34795	2.1
52259	2.04847	2.1
52260	2.04847	2.1
52261	2.21155	2.1
52262	2.21155	2.1
52263	1.89226	2.1
52264	1.89226	2.1
52265	1.89226	2.1
52266	1.89226	2.1
52267	1.89226	2.1
52268	1.89226	2.1
52269	0.810943	2.1
52270	0.810943	2.1
52271	0.810944	2.1
52272	0.810944	2.1
52273	0.810957	2.1
52274	0.810957	2.1
52275	1.89226	2.1
52276	1.35168	2.1
52277	1.08132	2.1
52278	1.08132	2.1
52279	1.0813	2.1
52280	0.946132	0.05
52281	1.33855	0.0508508
52282	0.946131	0.05
52283	1.38204	0.0620791
52284	0.946131	0.0640663
52285	0.946131	0.0561727
52286	1.38204	0.05
52287	1.38204	0.05
52288	0.946131	0.05
52289	0.946131	0.0519159
52290	1.33855	0.05
52291	1.38204	0.0503634
52292	1.89226	0.0678048
52293	2.76407	0.0629624
52294	1.89226	0.05
52295	2.72769	0.0562981
52296	1.89226	0.05
52297	2.72769	0.05859
52298	2.03914	0.05
52299	1.67727	0.05
52300	1.94831	0.05
52301	1.81591	0.05
52302	2.32098	0.05
52303	2.32098	0.05
52304	0.801647	0.05
52305	1.11086	0.05
52306	0.838634	0.05
52307	1.16049	0.05
52308	0.977891	0.05
52309	0.977891	0.05
52310	0.984246	0.05
52311	0.984246	0.05
52312	1.11086	0.05
52313	1.16049	0.05

52314	0.801647	0.05
52315	0.838634	0.05
52316	0.946132	0.05
52317	0.946132	0.0617724
52318	0.946131	0.05
52319	0.946131	0.0707936
52320	0.946131	0.0729572
52321	0.946131	0.0643158
52322	0.946132	0.05
52323	0.946132	0.05
52324	0.946131	0.05
52325	0.946131	0.0568925
52326	0.946131	0.05
52327	0.946131	0.0556969
52328	0.946131	0.05
52329	0.946131	0.0733448
52330	0.946131	0.0715543
52331	0.946131	0.05
52332	0.946131	0.0741697
52333	0.946131	0.0711676
52334	0.946131	0.05
52335	0.946131	0.05
52336	0.946131	0.0542779
52337	0.946131	0.0625332
52338	0.946131	0.0618194
52339	0.946131	0.0557135
52340	2.0815	2.1
52341	1.20787	2.1
52342	1.92277	2.1
52343	1.3227	2.1
52344	3.29401	2.1
52345	2.16633	2.1
52346	2.13642	2.1
52347	1.40846	2.1
52348	1.40846	2.1
52349	1.3227	2.1
52350	2.08058	2.1
52351	0.938106	2.1
52352	0.790258	2.1
52353	0.742975	2.1
52354	0.742975	2.1
52355	0.874389	2.1
52356	1.17364	2.1
52357	0.525948	2.1
52358	0.52513	2.1
52359	0.553732	2.1
52360	0.876996	2.1
52361	0.530954	2.1
52362	1.095	2.1
52363	1.00833	2.1
52364	1.70608	2.1
52365	1.33385	2.1
52366	1.33385	2.1
52367	1.07722	2.1
52368	1.07722	2.1
52369	0.953523	0.86013
52370	1.7457	0.668415
52371	1.03218	0.771035

52372	1.03218	0.780063
52373	1.79675	0.759331
52374	1.79675	0.769417
52375	1.13676	0.791071
52376	1.18136	0.545081
52377	0.87285	0.684126
52378	0.898375	0.835567
52379	1.03191	0.651109
52380	1.03191	0.545298
52381	1.0223	0.747143
52382	1.0223	0.857687
52383	1.18136	0.642612
52384	0.898374	0.584111
52385	0.87285	0.650721
52386	1.13676	0.642973
52387	2.36273	0.642953
52388	1.46965	0.499904
52389	1.79675	0.564998
52390	1.79675	0.461758
52391	2.43417	0.670375
52392	2.43417	0.513952
52393	1.4714	2.07861
52394	1.65434	2.09579
52395	1.62322	0.717654
52396	1.86068	2.02432
52397	3.27756	1.76297
52398	1.89226	2.1
52399	1.89226	2.09847
52400	1.89226	0.822526
52401	1.89226	1.61786
52402	3.78453	1.26358
52403	1.89226	1.92443
52404	1.89226	2.1
52405	1.89226	1.09404
52406	1.89226	2.1
52407	3.78453	2.08457
52408	1.65434	1.9254
52409	1.60647	2.1
52410	1.86068	1.62778
52411	1.82649	2.1
52412	3.48083	2.1
52413	1.62322	2.09122
52414	1.86068	2.09689
52415	1.84368	0.98725
52416	2.18862	1.59534
52417	3.81184	1.51818
52418	1.89226	2.09831
52419	1.89226	2.01958
52420	1.89226	0.848739
52421	1.89226	1.73714
52422	3.78453	1.5155
52423	1.89226	1.77172
52424	1.89226	2.1
52425	1.89226	1.1672
52426	1.89226	2.1
52427	3.78453	2.09867
52428	1.86068	1.81433
52429	1.82649	2.1

52430	2.18862	1.07102
52431	2.1128	2.1
52432	4.01511	2.06274
52433	1.74351	2.1
52434	1.13854	2.1
52435	1.40233	2.1
52436	1.922 2.1	
52437	3.14584	2.1
52438	1.922 2.1	
52439	2.00775	2.1
52440	1.7435	2.1
52441	2.09224	2.1
52442	2.00775	2.1
52443	2.09295	2.1
52444	1.69858	2.1
52445	1.922 2.1	
52446	1.40233	2.1
52447	1.40233	2.1
52448	2.18238	2.1
52449	2.18238	2.1
52450	1.00387	2.1
52451	1.10886	2.1
52452	0.961001	2.1
52453	1.09119	2.1
52454	1.02868	2.1
52455	1.02868	2.1
52456	1.04118	2.1
52457	1.04118	2.1
52458	1.10886	2.1
52459	1.09119	2.1
52460	0.960999	2.1
52461	1.00388	2.1
52462	0.885958	0.19284
52463	0.885958	0.166781
52464	0.875826	0.130819
52465	0.875826	0.175339
52466	0.913965	0.170266
52467	0.598609	0.206731
52468	1.16317	0.131069
52469	0.868115	0.165115
52470	0.868115	0.173788
52471	0.598609	0.178457
52472	1.16317	0.16752
52473	0.913965	0.167171
52474	0.946131	0.264027
52475	0.946131	0.245969
52476	0.946131	0.196173
52477	0.946131	0.170799
52478	0.946131	0.197908
52479	0.946131	0.25466
52480	0.946131	0.246843
52481	0.946131	0.172944
52482	0.946131	0.211215
52483	0.946131	0.207538
52484	0.946131	0.222466
52485	0.946131	0.223499
52486	0.946133	0.05
52487	0.946133	0.05

52488	1.06246	0.05
52489	1.06246	0.05
52490	0.973851	0.05
52491	0.946133	0.05
52492	1.06246	0.05
52493	0.946132	0.05
52494	0.97385	0.05
52495	1.06246	0.05
52496	0.946133	0.05
52497	0.946133	0.05
52498	1.89226	0.05
52499	1.89226	0.05
52500	1.89226	0.05
52501	1.89226	0.05
52502	1.89226	0.05
52503	1.89226	0.05
52504	0.946131	0.05
52505	0.946131	0.05
52506	0.946127	0.05
52507	0.946127	0.05
52508	0.946131	0.05
52509	0.946131	0.05
52510	0.946127	0.05
52511	0.946127	0.05
52512	0.946128	0.05
52513	0.946128	0.05
52514	0.946129	0.05
52515	0.946129	0.05
52516	0.946132	0.05
52517	0.946132	0.05
52518	1.32446	0.05
52519	0.794912	0.05
52520	1.32429	0.05
52521	0.946131	0.05
52522	1.32446	0.05
52523	0.94613	0.05
52524	1.32429	0.05
52525	0.794915	0.05
52526	0.94613	0.05
52527	0.94613	0.05
52528	1.89226	0.05
52529	2.36365	0.05
52530	2.64857	0.05
52531	1.89226	0.05
52532	2.64857	0.05
52533	1.89226	0.05
52534	1.18463	2.1
52535	1.27174	2.1
52536	0.946129	2.1
52537	0.946129	2.1
52538	0.946131	2.1
52539	0.946131	2.1
52540	1.27174	2.1
52541	1.27174	2.1
52542	1.18463	2.1
52543	0.94613	2.1
52544	0.946131	2.1
52545	1.27174	2.1

52546	2.57997	2.1
52547	1.89226	2.1
52548	1.89226	2.1
52549	1.89226	2.1
52550	2.41915	2.1
52551	2.41915	2.1
52552	1.89226	2.1
52553	1.89226	2.1
52554	1.89226	2.1
52555	1.89226	2.1
52556	3.78452	2.1
52557	1.08807	2.1
52558	1.18463	2.1
52559	0.946129	2.1
52560	0.946129	2.1
52561	0.946131	2.1
52562	0.946131	2.1
52563	1.18463	2.1
52564	1.18463	2.1
52565	0.946131	2.1
52566	1.08807	2.1
52567	1.18463	2.1
52568	0.94613	2.1
52569	1.07017	2.1
52570	1.07017	2.1
52571	0.946131	2.1
52572	0.946131	2.1
52573	0.946132	2.1
52574	0.946132	2.1
52575	1.20957	2.1
52576	1.07017	2.1
52577	1.07017	2.1
52578	0.946132	2.1
52579	0.946131	2.1
52580	1.20957	2.1
52581	1.89226	2.1
52582	1.89226	2.1
52583	1.89226	2.1
52584	1.89226	2.1
52585	1.89226	2.1
52586	1.89226	2.1
52587	1.16181	0.05
52588	1.0227	0.05
52589	1.0227	0.05
52590	0.970959	0.05
52591	1.24295	0.05
52592	1.19121	0.05
52593	1.12076	2.1
52594	2.53614	2.1
52595	1.28413	2.1
52596	1.28413	2.1
52597	2.36423	2.1
52598	2.36423	2.1
52599	1.89226	2.1
52600	1.89226	2.1
52601	1.89226	2.1
52602	1.89226	2.1
52603	3.78452	2.1

52604	1.22557	2.1
52605	2.771	2.1
52606	2.73012	2.1
52607	2.73012	2.1
52608	1.33356	2.1
52609	1.33356	2.1
52610	0.998682	2.1
52611	2.36423	2.1
52612	1.12076	2.1
52613	1.12076	2.1
52614	2.13757	2.1
52615	2.13757	2.1
52616	1.89226	2.1
52617	1.89226	2.1
52618	1.89226	2.1
52619	1.89226	2.1
52620	1.89226	2.1
52621	1.89226	2.1
52622	1.13987	2.1
52623	2.73011	2.1
52624	2.70875	2.1
52625	2.70875	2.1
52626	1.22557	2.1
52627	1.22557	2.1
52628	1.89227	2.1
52629	2.79243	2.1
52630	2.6625	2.1
52631	2.6625	2.1
52632	1.89227	2.1
52633	1.89227	2.1
52634	1.89226	2.1
52635	2.55971	2.1
52636	1.89226	2.1
52637	1.89226	2.1
52638	2.68704	2.1
52639	2.68704	2.1
52640	2.22043	2.1
52641	2.38975	1.47141
52642	2.28696	1.90132
52643	2.48682	1.18821
52644	4.67671	1.70374
52645	2.38976	0.821068
52646	2.31988	2.01611
52647	2.48683	0.914351
52648	2.4594	1.73137
52649	4.84915	0.842622
52650	2.28696	2.09112
52651	2.48682	1.91934
52652	2.33379	2.1
52653	2.56149	0.85162
52654	4.84845	1.92697
52655	2.48683	2.01419
52656	2.4594	1.93174
52657	2.5615	0.544474
52658	2.57957	1.14359
52659	5.0209	0.778649
52660	1.06659	2.1
52661	1.04291	2.1

52662	1.36595	2.09833
52663	1.36595	2.1
52664	1.02761	2.1
52665	1.32697	2.09663
52666	0.946132	2.04685
52667	0.946132	2.02965
52668	1.36956	2.1
52669	1.1229	2.1
52670	1.1229	2.02721
52671	1.1229	2.1
52672	0.946132	1.9497
52673	0.946132	2.1
52674	0.946132	2.09763
52675	1.36956	2.09753
52676	1.1229	2.09946
52677	0.946133	2.1
52678	0.946133	1.41326
52679	0.946133	1.39417
52680	0.946128	2.09346
52681	0.946128	2.0675
52682	0.946131	2.03403
52683	0.946131	1.64161
52684	0.946131	1.11921
52685	0.946131	2.06335
52686	0.94613	1.92777
52687	0.94613	1.90221
52688	0.946131	1.90514
52689	0.946131	1.92448
52690	0.966427	1.44335
52691	0.802265	1.83673
52692	0.803714	2.05196
52693	0.808476	1.87731
52694	1.94371	2.0464
52695	1.36851	2.02852
52696	0.853864	2.08068
52697	1.26635	1.87915
52698	1.09085	1.67466
52699	0.857653	1.96605
52700	0.630243	1.93876
52701	1.20132	0.05
52702	1.24295	0.05
52703	1.24295	0.05
52704	1.16182	0.05
52705	1.28465	0.05
52706	1.20351	0.05
52707	0.600658	0.05
52708	0.642323	0.05
52709	0.54424	0.05
52710	0.576779	0.05
52711	0.590985	0.05
52712	0.590985	0.05
52713	0.586452	0.05
52714	0.586452	0.05
52715	0.642323	0.05
52716	0.576779	0.05
52717	0.600658	0.05
52718	0.54424	0.05
52719	1.28463	0.05

52720	1.24294	0.05
52721	1.12328	0.05
52722	1.15505	0.05
52723	2.39798	0.05
52724	1.98431	0.0925993
52725	2.18735	0.0537485
52726	1.98431	0.0567286
52727	1.70303	0.0683016
52728	1.70302	0.169951
52729	1.37788	0.156542
52730	1.89226	0.15063
52731	2.37417	0.0709846
52732	1.89226	0.114929
52733	2.18735	0.0503311
52734	1.89226	0.208647
52735	2.18735	0.150733
52736	1.89226	0.40617
52737	1.89226	2.07486
52738	1.89226	0.980465
52739	1.89226	1.50635
52740	3.78452	1.32656
52741	1.8613	1.85488
52742	2.03576	2.08282
52743	1.61652	0.98638
52744	1.83082	2.1
52745	3.65228	1.96468
52746	2.03576	2.08199
52747	2.05257	2.1
52748	1.83082	2.1
52749	1.88487	2.1
52750	3.92064	2.1
52751	1.89226	2.06953
52752	1.89226	2.1
52753	1.89226	1.60686
52754	1.89226	2.1
52755	3.78453	2.1
52756	0.946131	2.09633
52757	0.946131	0.960162
52758	0.94613	0.32476
52759	0.94613	1.83275
52760	0.946129	1.57157
52761	0.946129	2.0914
52762	0.946132	1.24472
52763	0.946132	0.323032
52764	0.94613	1.2324
52765	0.94613	1.43155
52766	0.946131	1.12812
52767	0.946131	1.17284
52768	0.804708	2.1
52769	0.804708	1.81774
52770	0.815274	2.09186
52771	0.815274	1.07744
52772	0.83277	2.09103
52773	0.915407	2.1
52774	0.80826	1.78409
52775	0.704574	1.03337
52776	0.832771	2.07992
52777	0.704573	2.08479

52778	0.808259	2.03469
52779	0.915409	2.04222
52780	1.83082	2.1
52781	1.88487	2.1
52782	1.66555	2.09592
52783	1.75835	2.1
52784	3.55042	2.1
52785	0.810947	1.64895
52786	0.810947	2.1
52787	0.810947	1.59595
52788	0.810947	2.08996
52789	0.810934	2.08762
52790	0.810934	2.08887
52791	1.89227	2.1
52792	1.3517	2.1
52793	1.08132	2.09692
52794	1.08132	2.1
52795	1.08133	2.1
52796	1.89226	2.0951
52797	1.70303	2.09868
52798	1.89226	2.1
52799	2.24712	2.1
52800	2.07363	2.1
52801	1.74617	2.09859
52802	1.89226	2.09925
52803	1.89226	1.77416
52804	1.89226	1.67456
52805	1.89226	2.08073
52806	3.78452	1.96847
52807	1.50108	2.07984
52808	1.43894	1.97373
52809	1.8322	1.59586
52810	1.40967	2.09507
52811	1.50108	2.00442
52812	1.07854	2.1
52813	1.89226	1.3937
52814	1.89226	2.07212
52815	1.89226	2.03975
52816	1.89226	1.06835
52817	3.78452	1.92785
52818	1.89226	2.1
52819	1.89226	1.96833
52820	1.89226	1.99429
52821	1.89226	2.1
52822	3.78453	2.05738
52823	1.54457	2.1
52824	1.35097	1.99644
52825	1.70754	1.93286
52826	1.29629	2.1
52827	1.54457	2.06963
52828	1.0305	2.1
52829	0.731763	2.08036
52830	1.00043	2.04684
52831	1.13883	1.35465
52832	0.939026	1.62643
52833	1.04003	1.303
52834	0.853868	2.1
52835	0.927188	2.05976

52836	0.927188	1.05423
52837	0.861565	1.75154
52838	1.13882	1.8751
52839	0.731769	1.97634
52840	0.89319	1.99904
52841	1.46568	2.1
52842	1.20761	2.1
52843	1.51916	2.1
52844	1.36261	2.1
52845	2.72677	2.1
52846	1.51916	2.1
52847	1.60532	2.1
52848	1.36261	2.1
52849	1.36261	2.1
52850	1.64892	2.1
52851	1.64892	2.1
52852	1.3626	2.1
52853	0.98488	2.1
52854	1.60532	2.1
52855	1.32414	2.1
52856	2.68675	2.1
52857	1.78711	2.1
52858	1.14825	2.1
52859	2.16535	2.1
52860	1.6067	2.1
52861	3.3136	2.1
52862	1.89226	2.1
52863	1.89226	2.1
52864	1.89226	2.1
52865	1.89226	2.1
52866	3.78452	2.1
52867	2.34552	2.1
52868	1.53521	2.1
52869	1.53521	2.1
52870	1.60636	2.1
52871	1.92797	2.1
52872	1.99589	2.1
52873	1.34771	2.1
52874	1.34771	2.1
52875	0.946128	2.1
52876	0.946128	2.1
52877	1.3477	2.1
52878	1.31776	2.1
52879	0.946131	2.1
52880	0.946131	2.1
52881	0.94613	2.1
52882	1.31776	2.1
52883	0.946129	2.1
52884	1.3477	2.1
52885	1.89226	2.1
52886	1.89226	2.1
52887	1.89226	2.1
52888	1.89226	2.1
52889	3.78452	2.1
52890	0.92843	2.1
52891	0.672114	2.1
52892	0.89514	2.1
52893	0.89514	2.1

52894	0.720104	2.1
52895	0.755753	2.1
52896	0.789158	2.1
52897	0.967302	2.1
52898	1.06565	2.1
52899	0.7397	2.1
52900	0.758893	2.1
52901	0.94672	2.1
52902	1.89226	2.1
52903	2.588	2.1
52904	2.588	2.1
52905	2.63552	2.1
52906	1.89226	2.1
52907	1.93978	2.1
52908	1.49347	0.05
52909	2.67757	0.0813873
52910	1.80061	0.0661687
52911	2.27056	0.0760134
52912	4.07117	0.05
52913	1.1016	0.05
52914	2.27057	0.0591323
52915	1.49348	0.0582141
52916	1.81893	0.0568836
52917	1.49348	0.05
52918	1.81893	0.05
52919	1.81893	0.05
52920	2.82339	0.0985796
52921	2.27057	0.0775097
52922	2.25405	0.0804296
52923	4.52462	0.0540117
52924	1.89226	0.0864982
52925	1.89226	0.127555
52926	1.89226	0.14075
52927	1.89226	0.0961532
52928	1.89226	0.149705
52929	1.89226	0.112001
52930	1.89226	0.05
52931	1.89226	0.10719
52932	1.89226	0.0647266
52933	1.89226	0.076476
52934	3.78453	0.05
52935	1.89226	0.126205
52936	1.89226	0.15234
52937	1.89226	0.0846605
52938	1.89226	0.202125
52939	3.78452	0.190697
52940	1.89226	0.149577
52941	1.89226	0.360676
52942	1.89226	0.179798
52943	1.89226	0.221322
52944	3.78451	0.152519
52945	1.45633	2.1
52946	1.34678	2.1
52947	1.34678	2.1
52948	1.53735	2.1
52949	1.234	2.1
52950	1.42457	2.1
52951	1.89226	2.1

52952	1.89226	2.1
52953	1.89226	2.1
52954	1.89226	2.1
52955	1.89226	2.1
52956	1.89226	2.1
52957	1.09483	2.1
52958	1.34678	2.1
52959	1.234	2.1
52960	1.234	2.1
52961	1.19	2.1
52962	1.19	2.1
52963	0.946131	2.1
52964	0.946131	2.1
52965	0.946131	2.1
52966	0.946131	2.1
52967	0.946131	2.1
52968	0.946131	2.1
52969	0.946132	2.1
52970	0.946132	2.1
52971	0.946132	2.1
52972	0.946132	2.1
52973	0.946131	2.1
52974	0.946131	2.1
52975	2.82263	2.1
52976	1.89226	2.1
52977	2.71441	2.1
52978	2.71441	2.1
52979	1.89226	2.1
52980	1.89226	2.1
52981	1.1412	2.1
52982	1.1412	2.1
52983	1.14626	2.1
52984	1.14626	2.1
52985	1.28511	2.1
52986	1.34086	2.1
52987	0.992242	2.1
52988	0.946593	2.1
52989	0.992242	2.1
52990	1.34086	2.1
52991	0.946594	2.1
52992	1.28511	2.1
52993	1.18954	2.1
52994	1.18954	2.1
52995	1.21477	2.1
52996	1.21477	2.1
52997	1.41207	2.1
52998	1.34086	2.1
52999	1.01306	2.1
53000	0.992242	2.1
53001	1.01306	2.1
53002	1.41207	2.1
53003	0.992242	2.1
53004	1.34086	2.1
53005	1.09044	2.1
53006	1.44472	1.94025
53007	1.2955	2.1
53008	1.2955	2.09999
53009	1.22025	2.1

53010	1.22025	2.1
53011	0.810944	2.04738
53012	0.810944	2.1
53013	0.810946	2.04451
53014	0.810946	0.74987
53015	0.810959	1.98244
53016	0.810959	1.9926
53017	1.89226	1.98238
53018	1.35167	2.02621
53019	1.08132	2.1
53020	1.08132	0.806162
53021	1.0813	2.01753
53022	1.41894	0.524154
53023	1.28953	0.133114
53024	0.946132	0.598627
53025	0.946132	1.97967
53026	0.946133	0.778833
53027	0.946133	0.187091
53028	1.41894	1.48374
53029	1.41894	1.10418
53030	1.28953	0.474575
53031	0.946132	0.525046
53032	0.946132	0.992348
53033	1.41894	0.595316
53034	1.89226	2.1
53035	2.15444	2.1
53036	1.93239	2.1
53037	1.89226	2.1
53038	2.19457	2.1
53039	2.15444	2.1
53040	1.89226	2.1
53041	1.89226	2.1
53042	1.89226	2.1
53043	1.89226	2.1
53044	1.89226	2.1
53045	1.89226	2.1
53046	0.938507	2.1
53047	0.938507	2.1
53048	1.07871	2.1
53049	1.15801	2.1
53050	1.03236	2.1
53051	1.14766	2.1
53052	0.946133	2.1
53053	0.946133	2.1
53054	0.946131	2.1
53055	1.07009	2.1
53056	0.946131	2.1
53057	1.03237	2.1
53058	1.89226	2.1
53059	2.19457	2.1
53060	1.94492	2.1
53061	1.89226	2.1
53062	2.24723	2.1
53063	2.19457	2.1
53064	0.946132	2.1
53065	0.946132	2.1
53066	0.946131	2.1
53067	0.946131	2.1

53068	0.946132	2.1
53069	0.946132	2.1
53070	0.946132	2.1
53071	0.946132	2.1
53072	0.946132	2.1
53073	0.946132	2.1
53074	0.946132	2.1
53075	0.946132	2.1
53076	2.21345	2.1
53077	1.89226	2.1
53078	1.89226	2.1
53079	2.14017	2.1
53080	2.14017	2.1
53081	1.89226	2.1
53082	1.89226	0.316748
53083	1.89226	1.18894
53084	1.89226	0.302654
53085	1.85439	1.10592
53086	2.23961	0.226318
53087	2.19479	0.722253
53088	1.2916	0.263574
53089	1.2916	0.26965
53090	0.946128	0.345352
53091	0.946128	0.284804
53092	1.2916	0.195085
53093	0.946131	0.297283
53094	0.946126	1.0268
53095	1.1198	0.176193
53096	1.2916	0.212475
53097	1.1198	0.351255
53098	0.946128	0.413464
53099	0.946128	0.380858
53100	1.89226	0.508681
53101	2.71262	0.365089
53102	1.89226	0.38359
53103	2.49959	0.465492
53104	4.39185	0.509805
53105	2.71262	0.355049
53106	1.34592	0.381873
53107	2.49959	0.434896
53108	1.14721	0.341164
53109	3.84551	0.32835
53110	0.946131	0.394656
53111	0.946131	0.433278
53112	1.11247	0.328365
53113	1.11247	0.296129
53114	1.24979	0.313793
53115	1.11247	0.367186
53116	0.946129	0.448482
53117	0.946129	0.341059
53118	0.946131	0.318251
53119	1.11247	0.261481
53120	0.946131	0.273799
53121	1.24979	0.316253
53122	0.946131	2.1
53123	0.946131	2.1
53124	0.94613	2.1
53125	0.94613	2.1

53126	0.946132	2.1
53127	0.946132	2.1
53128	0.946129	2.1
53129	0.946129	2.1
53130	0.946131	2.1
53131	0.946131	2.1
53132	0.946131	2.1
53133	0.946131	2.1
53134	2.77816	2.1
53135	1.89226	2.1
53136	1.89226	2.1
53137	1.89226	2.1
53138	3.78453	2.1
53139	1.89226	2.1
53140	1.89226	2.1
53141	1.89226	2.1
53142	1.89226	2.1
53143	1.89226	2.1
53144	1.89226	2.1
53145	1.89226	2.1
53146	1.89226	2.1
53147	1.89226	2.1
53148	1.89226	2.1
53149	3.78452	2.1
53150	1.55698	2.1
53151	1.89226	2.1
53152	1.89226	2.1
53153	1.89226	2.1
53154	1.8575	2.1
53155	1.8575	2.1
53156	1.89226	2.1
53157	1.89226	2.1
53158	1.89226	2.1
53159	1.89226	2.1
53160	1.89226	2.1
53161	1.89226	2.1
53162	1.89227	2.1
53163	1.89227	2.1
53164	1.89227	2.1
53165	1.89227	2.1
53166	1.89227	2.1
53167	1.89227	2.1
53168	1.35509	2.1
53169	2.06238	2.1
53170	2.12244	2.1
53171	2.12244	2.1
53172	1.34817	2.1
53173	1.34817	2.1
53174	0.946126	2.1
53175	0.946126	2.1
53176	0.946131	2.1
53177	0.946131	2.1
53178	0.946127	2.1
53179	0.946127	2.1
53180	0.94613	2.1
53181	0.94613	2.1
53182	0.946128	2.1
53183	0.946128	2.1

53184	0.946128	2.1
53185	0.946128	2.1
53186	1.89226	2.1
53187	1.89226	2.1
53188	1.89226	2.1
53189	1.89226	2.1
53190	1.89226	2.1
53191	1.89226	2.1
53192	1.16742	2.1
53193	1.16742	2.1
53194	1.18122	2.1
53195	1.18122	2.1
53196	1.31745	2.1
53197	1.2597	2.1
53198	1.03119	2.1
53199	1.06122	2.1
53200	1.06122	2.1
53201	1.31745	2.1
53202	1.03119	2.1
53203	1.2597	2.1
53204	1.36849	2.1
53205	2.12244	2.1
53206	2.20092	2.1
53207	2.20092	2.1
53208	1.35509	2.1
53209	1.35509	2.1
53210	1.89226	2.1
53211	1.89226	2.1
53212	1.89226	2.1
53213	1.89226	2.1
53214	1.89226	2.1
53215	1.89226	2.1
53216	1.89226	2.1
53217	1.89226	2.1
53218	1.89226	2.1
53219	1.89226	2.1
53220	3.78452	2.1
53221	1.21731	2.1
53222	1.21731	2.1
53223	1.23412	2.1
53224	1.23412	2.1
53225	1.39021	2.1
53226	1.31745	2.1
53227	1.06122	2.1
53228	1.10046	2.1
53229	1.10046	2.1
53230	1.39021	2.1
53231	1.06122	2.1
53232	1.31745	2.1
53233	2.16503	1.3957
53234	1.78252	1.73157
53235	1.77293	1.26786
53236	1.56839	2.09966
53237	2.36957	0.662902
53238	2.16503	2.1
53239	1.89223	1.43393
53240	1.89223	2.1
53241	1.89223	2.05918

53242	1.89223	2.08669
53243	3.78447	2.0953
53244	2.3696	1.66174
53245	1.78254	1.80672
53246	2.63298	0.311366
53247	2.07312	2.08184
53248	4.44272	1.5311
53249	1.89226	2.09315
53250	1.89226	2.07974
53251	1.89226	1.98459
53252	1.89226	2.1
53253	1.89226	1.26286
53254	1.89226	2.1
53255	1.89226	2.08803
53256	1.89226	0.703912
53257	1.89226	2.07261
53258	1.89226	0.366335
53259	3.78452	1.13549
53260	2.26369	2.08669
53261	2.2168	0.562142
53262	2.15617	2.0233
53263	2.10864	0.289936
53264	4.37234	1.1974
53265	2.2168	0.40898
53266	1.94499	0.861874
53267	1.94499	0.713138
53268	2.04737	0.764301
53269	2.10864	0.207868
53270	2.21103	0.977811
53271	1.89226	0.774521
53272	1.89226	0.73068
53273	1.89226	0.846463
53274	1.89226	0.449685
53275	1.89226	1.00934
53276	1.89226	0.315321
53277	1.89226	2.06424
53278	1.89226	0.768693
53279	1.89226	1.12274
53280	1.89226	2.03217
53281	1.89226	0.507927
53282	1.89226	2.06782
53283	1.02995	0.618886
53284	1.02995	2.06302
53285	1.03212	0.710289
53286	1.03212	1.96146
53287	0.988256	0.427545
53288	1.05432	0.333039
53289	1.00775	1.95843
53290	1.07809	2.00829
53291	1.00775	1.12426
53292	0.988257	1.13489
53293	1.07808	1.2995
53294	1.05432	1.18859
53295	2.10864	0.663755
53296	1.94499	0.963989
53297	1.97652	0.46351
53298	1.79681	0.932469
53299	3.9215	0.286878

53300	0.810943	0.764006
53301	0.810943	1.16004
53302	0.810943	0.490886
53303	0.810943	0.533237
53304	0.810958	0.201856
53305	0.810958	0.319222
53306	1.89226	0.811482
53307	1.35168	0.330408
53308	1.08132	0.74903
53309	1.08132	0.681197
53310	1.0813	0.263508
53311	0.657403	0.05
53312	1.35403	0.05
53313	1.24038	0.05
53314	0.499144	0.05
53315	1.24039	0.05
53316	1.35403	0.0566658
53317	0.657402	0.05
53318	0.499144	0.05
53319	0.915415	0.05
53320	0.915415	0.0631255
53321	0.937755	0.05
53322	0.937755	0.0606851
53323	1.72526	2.1
53324	1.17989	2.1
53325	1.96035	2.1
53326	1.47412	2.1
53327	3.14024	2.1
53328	1.89226	1.88604
53329	1.89226	2.1
53330	1.89226	2.1
53331	1.89226	1.21232
53332	3.78452	2.1
53333	0.946131	0.718048
53334	0.946131	1.44189
53335	1.25764	2.05757
53336	1.25764	2.07482
53337	1.07087	0.717581
53338	1.25765	1.47759
53339	0.946128	1.91192
53340	0.946128	0.68308
53341	0.94613	1.02241
53342	1.25765	1.44118
53343	1.07086	1.55203
53344	0.946129	1.40227
53345	1.96035	2.1
53346	1.32668	2.1
53347	2.29301	2.1
53348	1.70303	2.1
53349	3.56428	2.1
53350	1.89226	2.1
53351	1.89226	1.41323
53352	1.89226	1.7415
53353	1.89226	2.1
53354	3.78452	1.37635
53355	0.897738	0.297697
53356	0.897738	0.325593
53357	0.946102	0.257862

53358	0.946102	0.195003
53359	1.11247	0.25076
53360	0.89771	0.19574
53361	0.94613	0.251906
53362	0.94613	0.34026
53363	0.897709	0.308225
53364	0.946131	0.316347
53365	0.946131	0.371757
53366	1.11247	0.296908
53367	1.89226	2.1
53368	1.89226	2.02254
53369	1.89226	2.0862
53370	1.89226	2.06549
53371	1.89226	2.08778
53372	1.89226	2.07023
53373	2.15429	2.1
53374	1.89226	2.1
53375	1.89226	2.1
53376	1.89226	2.09661
53377	2.27074	2.1
53378	2.27074	2.09534
53379	0.993311	1.64233
53380	0.993311	1.9501
53381	0.904322	2.09998
53382	0.904322	2.03096
53383	0.803324	2.06492
53384	1.36183	1.57391
53385	0.916274	2.09829
53386	0.535808	1.84905
53387	0.535808	1.96011
53388	0.803322	1.97392
53389	0.916276	1.95767
53390	1.36183	1.95343
53391	1.89226	2.1
53392	1.89226	1.97495
53393	1.89226	2.08962
53394	1.89226	2.0949
53395	1.89226	2.09818
53396	1.89226	2.09947
53397	2.11955	2.1
53398	2.62097	2.1
53399	1.85542	2.1
53400	2.39016	2.1
53401	4.50971	2.1
53402	2.39016	2.1
53403	2.20242	2.1
53404	2.39016	2.1
53405	1.64008	2.1
53406	1.85542	2.1
53407	1.64008	2.1
53408	1.79667	2.1
53409	1.28267	2.1
53410	1.28267	2.1
53411	1.40117	2.1
53412	1.74514	2.1
53413	1.83994	2.1
53414	0.575914	2.1
53415	0.625292	2.1

53416	0.949144	2.1
53417	0.882782	2.1
53418	0.773697	2.1
53419	0.773697	2.1
53420	0.786497	2.1
53421	0.786494	2.1
53422	0.605477	2.1
53423	0.949148	2.1
53424	0.625291	2.1
53425	0.862374	2.1
53426	1.89226	2.1
53427	2.44918	2.1
53428	2.4616	2.1
53429	2.44918	2.1
53430	1.90468	2.1
53431	1.89226	2.1
53432	1.89226	2.1
53433	1.89226	2.1
53434	1.89226	2.1
53435	1.89226	2.1
53436	3.78453	2.1
53437	0.946128	2.1
53438	0.946128	2.1
53439	1.23631	2.1
53440	1.23631	2.1
53441	0.946131	2.1
53442	0.946131	2.1
53443	1.3147	2.1
53444	1.23631	2.1
53445	1.3147	2.1
53446	0.946131	2.1
53447	1.23631	2.1
53448	0.94613	2.1
53449	1.89226	2.1
53450	2.4616	2.1
53451	2.4717	2.1
53452	2.4616	2.1
53453	1.90237	2.1
53454	1.89226	2.1
53455	0.946132	2.1
53456	0.946132	2.1
53457	0.946131	2.1
53458	0.946131	2.1
53459	0.946132	2.1
53460	0.946132	2.1
53461	0.946131	2.1
53462	0.946131	2.1
53463	0.946131	2.1
53464	0.946131	2.1
53465	0.946131	2.1
53466	0.946131	2.1
53467	0.946128	2.1
53468	0.946128	2.1
53469	1.3147	2.1
53470	1.3147	2.1
53471	0.94613	2.1
53472	0.94613	2.1
53473	1.3419	2.1

53474	1.3147	2.1
53475	1.3419	2.1
53476	0.94613	2.1
53477	1.3147	2.1
53478	0.94613	2.1
53479	1.89226	2.1
53480	1.89226	2.1
53481	1.89226	2.1
53482	1.89226	2.1
53483	3.78452	2.1
53484	2.3333	2.1
53485	1.3864	2.1
53486	1.3864	2.1
53487	1.4628	2.1
53488	1.95763	2.1
53489	2.03403	2.1
53490	1.294	2.1
53491	1.294	2.1
53492	0.946128	2.1
53493	0.946128	2.1
53494	1.294	2.1
53495	1.27474	2.1
53496	0.946131	2.1
53497	0.946131	2.1
53498	0.946129	2.1
53499	1.27474	2.1
53500	1.294	2.1
53501	0.94613	2.1
53502	1.89226	2.1
53503	1.89226	2.1
53504	1.89226	2.1
53505	1.89226	2.1
53506	1.89226	2.1
53507	1.89226	2.1
53508	0.808088	2.1
53509	0.808088	2.1
53510	0.815623	2.1
53511	0.815623	2.1
53512	0.660053	2.1
53513	0.693199	2.1
53514	0.930512	2.1
53515	0.978817	2.1
53516	0.660053	2.1
53517	0.930513	2.1
53518	0.693198	2.1
53519	0.978817	2.1
53520	1.27474	2.1
53521	1.27474	2.1
53522	0.946128	2.1
53523	0.946128	2.1
53524	1.26221	2.1
53525	1.27474	2.1
53526	0.946131	2.1
53527	0.946131	2.1
53528	0.94613	2.1
53529	1.26221	2.1
53530	0.946129	2.1
53531	1.27474	2.1

53532	0.90207	0.05
53533	0.90207	0.05
53534	0.895719	0.05
53535	0.895719	0.05
53536	0.927855	0.05
53537	1.2493	0.05
53538	0.85723	0.05
53539	0.548484	0.05
53540	0.548485	0.05
53541	0.927855	0.05
53542	0.85723	0.05
53543	1.2493	0.05
53544	1.41319	0.461711
53545	1.22482	0.290466
53546	1.46629	0.327519
53547	1.92622	0.509
53548	3.15104	0.424144
53549	1.02305	0.201797
53550	1.22482	0.289852
53551	1.80336	0.291757
53552	1.44426	0.203607
53553	2.66908	0.215457
53554	1.3268	2.1
53555	0.999489	2.1
53556	1.44091	2.1
53557	1.14657	2.1
53558	2.4404	2.1
53559	1.89226	2.1
53560	1.89226	2.1
53561	1.89226	2.1
53562	1.89226	2.1
53563	3.78452	2.1
53564	1.89226	2.1
53565	2.28605	2.1
53566	2.43157	2.1
53567	2.43157	2.1
53568	1.89226	2.1
53569	1.89226	2.1
53570	1.60647	2.1
53571	1.44091	2.1
53572	1.31346	2.1
53573	1.31346	2.1
53574	1.14657	2.1
53575	1.44091	2.1
53576	1.89226	2.1
53577	1.89226	2.1
53578	1.89226	2.1
53579	1.89226	2.1
53580	3.78452	2.1
53581	0.946132	2.1
53582	0.946132	2.1
53583	1.3048	2.1
53584	1.3048	2.1
53585	1.3048	2.1
53586	1.21578	2.1
53587	0.946132	2.1
53588	0.946132	2.1
53589	1.3048	2.1

53590	0.946132	2.1
53591	0.946132	2.1
53592	1.21578	2.1
53593	1.89227	0.05
53594	1.89227	0.05
53595	1.89227	0.05
53596	1.89227	0.05
53597	3.78453	0.05
53598	1.6671	0.05
53599	1.87338	0.05
53600	1.6671	0.05
53601	2.02098	0.05
53602	1.38328	0.05
53603	1.73715	0.05
53604	1.89226	0.05
53605	1.89226	0.05
53606	1.89226	0.05
53607	1.89226	0.05
53608	3.78453	0.05
53609	1.6671	0.05
53610	1.38328	0.05
53611	1.35562	0.05
53612	1.01012	0.05
53613	2.67722	0.05
53614	0.819377	0.05
53615	0.647925	0.05
53616	1.17802	0.05
53617	1.33174	0.05
53618	0.98543	0.05
53619	0.98543	0.05
53620	0.994237	0.05
53621	0.994237	0.05
53622	1.17802	0.05
53623	0.647926	0.05
53624	1.33174	0.05
53625	0.819377	0.05
53626	1.27806	0.05
53627	1.68264	0.05
53628	1.44593	0.05
53629	1.878 0.05	
53630	3.15606	0.05
53631	1.878 0.05	
53632	1.9898	0.05
53633	1.878 0.05	
53634	1.68264	0.05
53635	2.30533	0.05
53636	2.10997	0.05
53637	0.814587	0.05
53638	0.814596	0.05
53639	0.82775	0.05
53640	0.82775	0.05
53641	1.0107	0.05
53642	0.91549	0.05
53643	0.741058	0.05
53644	0.714748	0.05
53645	0.714758	0.05
53646	1.03225	0.05
53647	0.704864	0.05

53648	0.915488	0.05
53649	1.90347	0.05
53650	2.06584	0.05
53651	1.878	0.05
53652	1.50236	0.05
53653	2.05749	0.05
53654	2.30533	0.05
53655	2.24475	2.1
53656	1.85871	2.1
53657	2.13711	2.1
53658	2.13711	2.1
53659	1.9943	2.1
53660	1.9943	2.1
53661	1.85871	2.1
53662	1.7871	2.1
53663	1.41619	2.1
53664	1.41619	2.1
53665	2.13711	2.1
53666	2.13711	2.1
53667	2.24475	2.1
53668	2.13711	2.1
53669	2.54902	2.1
53670	2.46327	2.1
53671	4.70802	2.1
53672	1.06913	2.1
53673	1.06913	2.1
53674	1.0821	2.1
53675	1.0821	2.1
53676	0.893552	2.1
53677	1.08267	2.1
53678	1.06855	2.1
53679	1.23163	2.1
53680	1.23163	2.1
53681	1.08267	2.1
53682	0.893551	2.1
53683	1.06855	2.1
53684	1.67046	0.415162
53685	2.4106	0.189505
53686	2.05835	1.43154
53687	2.4106	1.29348
53688	1.59562	0.493666
53689	1.94788	0.301266
53690	2.34737	1.98595
53691	2.4106	0.379275
53692	2.05834	1.01584
53693	1.99655	1.79248
53694	4.40715	1.00175
53695	1.67046	0.256103
53696	2.05835	0.183843
53697	1.588	1.11713
53698	1.34857	1.71797
53699	3.40692	0.375646
53700	1.73802	0.882904
53701	1.89668	0.987694
53702	1.99655	2.06877
53703	1.99655	0.406204
53704	1.79981	1.35124
53705	2.05834	0.330742

53706	1.89226	1.4866
53707	1.89226	0.458617
53708	1.89226	0.260373
53709	1.89226	1.96181
53710	1.89226	0.28694
53711	1.89226	2.1
53712	1.89226	0.871238
53713	1.89226	2.07819
53714	1.89226	1.93126
53715	1.89226	0.63041
53716	3.78453	1.7046
53717	0.719473	2.06605
53718	0.916103	1.94421
53719	1.02744	0.843431
53720	0.850328	1.72754
53721	0.888094	0.806645
53722	0.888094	2.1
53723	0.878337	2.048
53724	0.878337	0.615961
53725	0.916103	1.42165
53726	1.02744	1.66982
53727	0.719473	1.82962
53728	0.850328	1.9062
53729	1.02744	1.45269
53730	0.850329	1.98551
53731	1.0875	0.62849
53732	0.919597	1.18426
53733	0.975841	0.312427
53734	0.975841	2.1
53735	0.971196	0.313502
53736	0.971196	1.99638
53737	1.02744	1.75904
53738	1.0875	0.656143
53739	0.850328	1.79681
53740	0.919598	1.74648
53741	1.89226	2.02551
53742	1.89226	1.5541
53743	1.89226	0.326445
53744	1.89226	1.7637
53745	3.78453	1.2471
53746	1.89226	1.86126
53747	1.89226	0.678302
53748	1.89226	0.660587
53749	1.89226	1.96748
53750	1.89226	0.633287
53751	1.89226	2.09777
53752	1.11103	1.84224
53753	0.916103	2.04005
53754	1.21177	0.869189
53755	1.02744	0.89746
53756	1.07188	0.63753
53757	1.07188	2.1
53758	1.06659	0.652722
53759	1.06659	1.98548
53760	1.11103	1.90624
53761	1.21177	1.68089
53762	0.916104	1.80608
53763	1.02744	1.95541

53764	2.54158	0.59754
53765	2.42354	1.52201
53766	2.17499	0.403724
53767	2.17499	2.1
53768	2.42354	1.80334
53769	2.05488	2.1
53770	1.89226	0.05
53771	1.89226	0.0596121
53772	1.89226	0.05
53773	1.89226	0.0572115
53774	3.78453	0.05
53775	1.89226	0.05
53776	1.89226	0.05
53777	1.89226	0.05
53778	1.89226	0.05
53779	1.89226	0.05
53780	1.89226	0.05
53781	1.89226	0.0970429
53782	1.89226	0.125383
53783	1.89226	0.110838
53784	1.89226	0.0724181
53785	1.89226	0.147939
53786	1.89226	0.109818
53787	1.89226	0.0966913
53788	1.89226	0.161017
53789	1.89226	0.103456
53790	1.89226	0.165269
53791	3.78452	0.13875
53792	0.802544	0.05
53793	0.946132	0.05
53794	0.946132	0.05
53795	0.787038	0.05
53796	0.787039	0.05
53797	0.787039	0.05
53798	0.961637	0.05
53799	0.961637	0.05
53800	0.787038	0.05
53801	0.802544	0.05
53802	0.946132	0.05
53803	0.946132	0.05
53804	1.89226	0.05
53805	1.60509	0.05
53806	1.89226	0.05
53807	1.89226	0.05
53808	1.3905	0.05
53809	1.3905	0.05
53810	0.946132	0.0660287
53811	0.946132	0.05
53812	0.946131	0.0591319
53813	0.946131	0.0591628
53814	0.946132	0.059486
53815	0.946132	0.0656807
53816	0.946131	0.0541471
53817	0.946131	0.0587229
53818	0.946131	0.0542818
53819	0.946131	0.0565401
53820	0.946132	0.0503295
53821	0.946132	0.0506298

53822	0.946131	0.0779938
53823	0.946131	0.087907
53824	0.946131	0.0840187
53825	0.946131	0.0528256
53826	0.946132	0.0763153
53827	0.946132	0.0977472
53828	0.946131	0.0654917
53829	0.946131	0.0617046
53830	0.946131	0.0777347
53831	0.946131	0.05
53832	0.946132	0.0604362
53833	0.946132	0.0705886
53834	1.42382	2.08488
53835	1.32201	1.4168
53836	1.32096	1.69448
53837	1.42866	2.0707
53838	2.75067	1.96776
53839	1.89226	2.07979
53840	1.89226	1.1857
53841	1.89226	1.70404
53842	1.89226	2.03008
53843	1.89226	1.4142
53844	1.89226	1.94462
53845	1.89226	1.70766
53846	2.79029	0.657707
53847	2.7803	0.88326
53848	2.79029	1.29399
53849	1.88227	0.794088
53850	1.89226	1.16841
53851	0.716065	2.07369
53852	0.714333	2.09641
53853	0.660483	1.49506
53854	0.659126	1.75057
53855	0.687538	1.52177
53856	0.687538	2.07244
53857	0.685921	2.1
53858	0.685921	1.62937
53859	0.659126	1.95837
53860	0.716065	1.96781
53861	0.660483	1.94355
53862	0.714333	1.94665
53863	1.89226	2.07373
53864	1.89226	1.27021
53865	1.89226	1.66453
53866	1.89226	2.01427
53867	3.78453	1.89336
53868	0.946133	1.26762
53869	0.946133	1.56145
53870	1.39015	0.855712
53871	1.38969	0.631817
53872	0.946132	0.647658
53873	0.946132	1.25242
53874	1.38969	1.52857
53875	1.38969	0.836286
53876	1.38969	1.00069
53877	0.946132	1.02589
53878	0.946133	1.09423
53879	1.39015	1.02366

53880	2.72157	0.0979908
53881	1.22176	0.0838571
53882	1.59492	0.0795293
53883	1.22176	0.141706
53884	2.40743	0.0635465
53885	2.03426	0.134982
53886	0.946131	0.195158
53887	0.946131	0.144772
53888	1.36079	0.0991098
53889	1.01713	0.140217
53890	0.94613	0.137559
53891	0.94613	0.184498
53892	1.36079	0.125496
53893	1.36079	0.0893667
53894	0.946131	0.121424
53895	1.36079	0.121672
53896	1.01713	0.111133
53897	0.946131	0.121826
53898	1.89226	0.198274
53899	1.89226	0.149164
53900	1.89226	0.137397
53901	1.89226	0.26198
53902	3.78453	0.221526
53903	1.89226	0.135692
53904	1.89226	0.0915759
53905	1.89226	0.086579
53906	1.89226	0.150934
53907	1.89226	0.0824301
53908	1.89226	0.150502
53909	0.946131	0.194178
53910	0.946131	0.152035
53911	0.946131	0.131855
53912	1.36079	0.0912783
53913	0.94613	0.132958
53914	0.94613	0.175327
53915	0.946133	0.142327
53916	0.946133	0.0918664
53917	0.946131	0.151083
53918	0.946131	0.139468
53919	0.946132	0.13815
53920	1.36079	0.120267
53921	1.89226	0.137347
53922	1.89226	0.246339
53923	1.89226	0.169799
53924	1.89226	0.179374
53925	1.89226	0.163453
53926	1.89226	0.182604
53927	1.89227	0.13951
53928	1.89227	0.0828546
53929	1.89227	0.0916192
53930	1.89227	0.164086
53931	1.89227	0.0810931
53932	1.89227	0.155958
53933	1.60428	2.1
53934	2.2367	2.1
53935	1.50097	2.1
53936	1.80125	2.1
53937	3.40553	2.1

53938	1.89095	2.1
53939	1.85293	2.07976
53940	1.80125	2.1
53941	1.76268	2.09858
53942	3.65418	2.1
53943	0.803177	2.1
53944	0.803177	2.1
53945	0.798644	2.1
53946	0.798644	2.1
53947	0.84233	2.1
53948	0.900624	2.1
53949	0.750483	2.1
53950	0.701197	2.1
53951	0.701197	2.1
53952	0.84233	2.1
53953	0.750483	2.1
53954	0.900625	2.1
53955	0.860925	2.1
53956	0.860925	2.1
53957	0.862743	2.1
53958	0.862743	2.1
53959	0.881338	2.09833
53960	0.826621	2.07757
53961	0.84233	2.1
53962	0.900625	2.1
53963	0.84233	2.1
53964	0.826621	2.1
53965	0.900624	2.1
53966	0.881339	2.1
53967	1.37938	0.77668
53968	1.03319	0.913128
53969	1.00512	0.925425
53970	0.639582	1.17413
53971	1.00463	1.00772
53972	1.00463	0.710277
53973	1.01434	0.869229
53974	1.01434	1.10754
53975	1.37938	0.990259
53976	1.00512	1.04217
53977	1.03319	0.971509
53978	0.639582	1.03643
53979	0.728713	1.14287
53980	0.728713	1.11996
53981	0.73425	1.26421
53982	0.73425	2.08346
53983	0.525122	2.1
53984	0.605622	0.245119
53985	0.948901	0.245119
53986	0.857342	0.245119
53987	0.85734	0.270803
53988	0.525123	2.1
53989	0.948899	0.270803
53990	0.605624	0.270803
53991	1.75449	0.301467
53992	1.4378	0.347728
53993	1.93916	0.492744
53994	2.22512	0.203532
53995	3.69364	0.416759

53996	1.75448	0.826877
53997	1.89186	0.25247
53998	2.29771	0.277146
53999	2.22512	0.262172
54000	4.05219	0.282598
54001	1.93916	0.592055
54002	2.51749	0.18082
54003	2.62357	0.28753
54004	2.22512	0.233414
54005	2.33761	0.387328
54006	1.93916	0.270915
54007	2.29771	0.390923
54008	2.51749	0.21156
54009	2.22512	0.21174
54010	2.22512	0.189427
54011	2.50905	0.470861
54012	2.50905	0.342844
54013	0.87382	0.05
54014	1.33662	0.05
54015	1.20756	0.05
54016	0.890467	0.0609338
54017	1.08859	0.0624886
54018	1.08859	0.0539639
54019	1.13084	0.05
54020	1.08793	0.05
54021	1.34722	0.05
54022	1.27184	0.0516556
54023	0.917678	0.05
54024	0.97821	0.05
54025	1.28396	0.05
54026	1.70302	0.0624655
54027	2.30076	0.0524478
54028	1.20417	0.05
54029	2.57506	0.0572046
54030	2.2656	0.05
54031	1.03642	1.98712
54032	1.03642	0.467973
54033	1.07029	0.12505
54034	1.07029	0.706847
54035	1.31531	1.94032
54036	1.23279	0.706735
54037	0.941743	0.232914
54038	0.791399	0.951095
54039	0.791399	0.692751
54040	1.23279	0.66098
54041	0.941744	0.60995
54042	1.31531	0.630546
54043	0.937354	1.99644
54044	0.937354	0.408219
54045	0.954794	0.1507
54046	0.954794	0.686794
54047	1.13563	0.652028
54048	1.23279	1.92933
54049	0.791398	0.209669
54050	0.659359	0.748163
54051	1.13563	0.575881
54052	0.659359	0.647536
54053	0.791399	0.513209

54054	1.23279	0.534016
54055	2.29852	1.04779
54056	1.89227	1.15692
54057	2.14485	0.654581
54058	2.14485	1.58209
54059	1.89227	0.690417
54060	1.89227	1.5978
54061	0.947863	0.426904
54062	1.19854	1.09726
54063	2.16841	0.484932
54064	1.76597	0.680697
54065	2.96451	0.499806
54066	1.89227	0.78011
54067	1.89227	0.971491
54068	1.89227	0.552704
54069	1.89227	1.50147
54070	1.89227	0.607841
54071	1.89227	1.56342
54072	2.46489	2.1
54073	1.98339	2.1
54074	1.81667	2.1
54075	2.36914	2.1
54076	4.35253	2.1
54077	2.46488	2.1
54078	2.70401	2.1
54079	2.64996	2.1
54080	2.36914	2.1
54081	5.07315	2.1
54082	1.81667	2.1
54083	2.29459	2.1
54084	2.36914	2.1
54085	2.36914	2.1
54086	1.68073	2.1
54087	1.68073	2.1
54088	2.64996	2.1
54089	2.29458	2.1
54090	2.29458	2.1
54091	2.36914	2.1
54092	2.61243	2.1
54093	2.68698	2.1
54094	1.89226	1.61659
54095	1.89226	0.994718
54096	1.89226	1.39769
54097	1.89226	1.40898
54098	1.89226	1.35519
54099	1.89226	1.29735
54100	1.89224	1.82111
54101	1.89224	0.983955
54102	1.65571	1.1209
54103	1.65571	1.24233
54104	1.89224	1.21688
54105	1.89224	1.57462
54106	1.89224	1.42505
54107	1.89224	0.91879
54108	1.89224	1.26951
54109	1.89224	1.16604
54110	1.89224	1.37134
54111	1.89224	1.22221

54112	1.98252	0.0911982
54113	2.20641	0.0976802
54114	1.05129	0.0804146
54115	1.2951	0.123015
54116	3.27762	0.0894506
54117	1.85598	2.06491
54118	1.88635	1.071
54119	2.12346	0.805642
54120	2.10838	2.086
54121	3.97943	1.62107
54122	2.10838	2.06513
54123	1.87532	2.1
54124	1.85597	1.99898
54125	1.85597	2.1
54126	1.87532	2.08174
54127	1.57914	2.1
54128	2.12348	0.843594
54129	2.35141	2.05747
54130	2.37191	1.61143
54131	2.10841	1.89597
54132	2.38698	1.18255
54133	2.12348	1.94523
54134	1.07811	2.1
54135	0.937672	2.1
54136	1.17571	2.02989
54137	1.05421	1.67125
54138	1.07087	1.9919
54139	1.07087	2.1
54140	1.06145	2.1
54141	1.06145	1.91579
54142	1.17571	2.09591
54143	1.07811	2.09624
54144	1.05421	2.1
54145	0.937672	2.1
54146	1.67334	2.1
54147	2.76609	2.1
54148	2.74144	2.1
54149	2.74144	2.1
54150	1.71157	2.1
54151	1.71157	2.1
54152	2.76609	2.1
54153	1.89226	2.1
54154	1.89226	2.1
54155	1.89226	2.1
54156	2.74143	2.1
54157	2.74143	2.1
54158	1.10393	2.1
54159	1.10393	2.1
54160	1.10788	2.1
54161	1.10788	2.1
54162	1.38302	2.1
54163	1.37072	2.1
54164	0.836672	2.1
54165	0.841089	2.1
54166	1.38302	2.1
54167	0.84109	2.1
54168	0.836671	2.1
54169	1.37072	2.1

54170	1.38302	2.1
54171	1.38302	2.1
54172	0.946131	2.1
54173	0.946131	2.1
54174	0.946128	2.1
54175	0.946128	2.1
54176	1.38302	2.1
54177	1.37072	2.1
54178	1.38302	2.1
54179	0.946131	2.1
54180	0.94613	2.1
54181	1.37072	2.1
54182	1.28072	2.1
54183	1.55779	2.1
54184	1.28072	2.1
54185	1.40765	2.1
54186	1.47186	2.1
54187	1.59879	2.1
54188	1.89227	2.1
54189	1.89227	2.1
54190	1.89227	1.89549
54191	1.89227	2.1
54192	1.89227	1.93714
54193	1.89227	2.1
54194	2.80057	1.80927
54195	1.89226	1.87319
54196	1.89226	1.21313
54197	1.89226	2.09446
54198	2.63061	0.809399
54199	2.63061	2.09566
54200	1.28072	2.0965
54201	1.38859	2.1
54202	1.47186	2.1
54203	1.47186	2.1
54204	1.16777	2.1
54205	1.16777	2.1
54206	1.89226	2.09316
54207	1.89226	2.1
54208	1.89226	1.99887
54209	1.89226	2.1
54210	1.89226	1.91191
54211	1.89226	2.1
54212	1.83221	2.1
54213	2.0815	2.1
54214	2.0815	2.1
54215	2.08153	2.1
54216	1.86703	2.1
54217	1.87402	2.1
54218	1.86703	2.1
54219	2.08508	2.1
54220	2.08508	2.1
54221	2.29001	2.1
54222	1.89226	2.1
54223	2.08048	2.1
54224	2.7278	0.116135
54225	1.10909	0.127878
54226	1.50472	0.110812
54227	1.10909	0.145548

54228	2.69881	0.105293
54229	2.30319	0.160794
54230	1.23204	2.1
54231	1.92264	2.1
54232	1.48162	2.1
54233	1.48162	2.1
54234	1.72994	2.1
54235	1.72994	2.1
54236	0.864968	2.1
54237	0.696952	2.1
54238	0.61602	2.1
54239	0.479736	2.1
54240	0.66444	2.1
54241	0.66444	2.1
54242	0.680264	2.1
54243	0.680264	2.1
54244	0.696952	2.1
54245	0.479736	2.1
54246	0.61602	2.1
54247	0.864968	2.1
54248	1.89226	0.567457
54249	2.43219	1.96453
54250	1.89226	1.52343
54251	2.82019	1.44412
54252	1.89226	1.60191
54253	2.82019	1.14513
54254	1.89226	0.833333
54255	2.83701	1.96207
54256	2.2701	1.71707
54257	2.2701	1.17743
54258	1.89226	1.75978
54259	1.89226	1.32873
54260	1.89226	1.80828
54261	1.89226	0.515814
54262	1.89226	1.0259
54263	1.89226	0.794702
54264	1.89226	1.20338
54265	1.89226	0.710277
54266	1.5795	1.7777
54267	1.44091	1.26068
54268	1.05267	1.86288
54269	1.05267	0.943099
54270	1.98408	1.89892
54271	1.98408	0.989039
54272	2.20624	2.1
54273	1.08831	2.1
54274	2.49053	2.1
54275	1.42486	2.1
54276	3.57883	2.1
54277	1.89226	2.1
54278	1.89226	2.1
54279	1.89226	2.1
54280	1.89226	2.1
54281	3.78453	2.1
54282	0.946132	2.1
54283	0.946132	2.1
54284	0.853591	2.1
54285	0.642207	2.1

54286	0.94613	2.1
54287	0.94613	2.1
54288	0.642209	2.1
54289	0.642209	2.1
54290	0.946131	2.1
54291	0.853591	2.1
54292	0.642207	2.1
54293	0.946132	2.1
54294	1.0506	2.1
54295	1.0506	2.1
54296	1.0879	2.1
54297	0.960424	2.1
54298	0.708687	2.1
54299	0.782927	2.1
54300	1.40041	2.1
54301	1.2518	2.1
54302	0.767748	2.1
54303	1.31801	2.1
54304	1.21412	2.1
54305	0.69461	2.1
54306	1.89226	2.1
54307	1.89226	2.1
54308	1.89226	2.1
54309	1.89226	2.1
54310	1.89226	2.1
54311	1.89226	2.1
54312	0.922223	2.1
54313	0.922223	2.1
54314	1.04548	2.1
54315	0.768229	2.1
54316	0.948441	2.1
54317	0.806483	2.1
54318	0.85362	2.1
54319	0.85362	2.1
54320	1.05942	2.1
54321	0.922187	2.1
54322	0.851554	2.1
54323	0.768264	2.1
54324	2.16728	2.1
54325	0.95455	2.1
54326	1.21372	2.1
54327	0.98227	2.1
54328	2.16728	2.1
54329	1.93584	2.1
54330	2.08849	0.109875
54331	1.08732	0.130728
54332	1.3357	0.122899
54333	2.37948	0.114514
54334	3.4668	0.12095
54335	2.57882	0.102583
54336	2.37948	0.120525
54337	2.53055	0.116155
54338	1.48676	0.112993
54339	1.3357	0.120417
54340	1.3357	0.117633
54341	1.89226	2.1
54342	1.89226	2.1
54343	2.17471	2.1

54344	1.89226	2.1
54345	2.60085	2.1
54346	2.26306	2.1
54347	0.946131	2.1
54348	0.946131	2.1
54349	0.946131	2.1
54350	0.946131	2.1
54351	0.94613	2.1
54352	0.94613	2.1
54353	0.946132	2.1
54354	0.946132	2.1
54355	0.946131	2.1
54356	0.946131	2.1
54357	0.946131	2.1
54358	0.946131	2.1
54359	1.89226	2.1
54360	2.17471	2.1
54361	2.08196	2.1
54362	2.17471	2.1
54363	1.79952	2.1
54364	1.89226	2.1
54365	1.89226	2.1
54366	1.89226	2.1
54367	1.89226	2.1
54368	1.89226	2.1
54369	1.89226	2.1
54370	1.89226	2.1
54371	1.86016	2.1
54372	1.89226	2.1
54373	1.89226	2.1
54374	1.89226	2.1
54375	1.84826	2.1
54376	1.84826	2.1
54377	2.50136	2.1
54378	1.99781	2.1
54379	1.99729	2.1
54380	2.5214	2.1
54381	4.49865	2.1
54382	0.51724	2.1
54383	0.496076	2.1
54384	0.998906	2.1
54385	0.998645	2.1
54386	0.742249	2.1
54387	0.742249	2.1
54388	0.752734	2.1
54389	0.752734	2.1
54390	0.998639	2.1
54391	0.496073	2.1
54392	0.517243	2.1
54393	0.998913	2.1
54394	0.998653	2.1
54395	0.994293	2.1
54396	1.26071	2.1
54397	1.26504	2.1
54398	1.12529	2.1
54399	1.12529	2.1
54400	1.12971	2.1
54401	1.12971	2.1

54402	1.26504	2.1
54403	0.994292	2.1
54404	0.998651	2.1
54405	1.26071	2.1
54406	1.89226	2.1
54407	1.89226	2.1
54408	1.89226	2.1
54409	1.89226	2.1
54410	3.78452	2.1
54411	1.89226	2.1
54412	1.89226	2.1
54413	1.89226	2.1
54414	1.89226	2.1
54415	3.78452	2.1
54416	0.675872	2.1
54417	0.675641	2.1
54418	0.943072	2.1
54419	0.898317	2.1
54420	0.775717	2.1
54421	0.775717	2.1
54422	0.79824	2.1
54423	0.79824	2.1
54424	0.675642	2.1
54425	0.94307	2.1
54426	0.675874	2.1
54427	0.898315	2.1
54428	0.943071	2.1
54429	0.898316	2.1
54430	0.998133	2.1
54431	0.918903	2.1
54432	0.922369	2.1
54433	0.922369	2.1
54434	0.939606	2.1
54435	0.939606	2.1
54436	0.943071	2.1
54437	0.998133	2.1
54438	0.898316	2.1
54439	0.918903	2.1
54440	0.946131	2.1
54441	0.946131	2.1
54442	0.946131	2.1
54443	0.946131	2.1
54444	0.946132	2.1
54445	0.946132	2.1
54446	0.946129	2.1
54447	0.946129	2.1
54448	0.94613	2.1
54449	0.94613	2.1
54450	0.946131	2.1
54451	0.946131	2.1
54452	1.89226	2.1
54453	1.89226	2.1
54454	1.89226	2.1
54455	1.89226	2.1
54456	1.89226	2.1
54457	1.89226	2.1
54458	1.35129	2.1
54459	1.88614	2.1

54460	2.01867	2.1
54461	1.38189	2.1
54462	3.36995	2.1
54463	0.943071	2.1
54464	1.00934	2.1
54465	0.998134	2.1
54466	1.10866	2.1
54467	0.992617	2.1
54468	0.992617	2.1
54469	1.01485	2.1
54470	1.01485	2.1
54471	1.00934	2.1
54472	1.10866	2.1
54473	0.943072	2.1
54474	0.998134	2.1
54475	2.67272	2.1
54476	1.61859	2.1
54477	1.70303	2.1
54478	2.52175	2.1
54479	4.5326	2.1
54480	2.00954	2.1
54481	2.27801	2.1
54482	1.87875	2.1
54483	1.87875	2.1
54484	2.37837	2.1
54485	2.37837	2.1
54486	2.00955	2.1
54487	1.78 2.1	
54488	2.21813	2.1
54489	1.8493	2.1
54490	2.37838	2.1
54491	2.00955	2.1
54492	2.37838	2.1
54493	2.27801	2.1
54494	2.58523	2.1
54495	2.71597	2.1
54496	4.9636	2.1
54497	2.37838	2.1
54498	2.71597	2.1
54499	1.72709	2.1
54500	2.0815	2.1
54501	2.59749	2.1
54502	2.78034	2.1
54503	0.94613	0.99056
54504	0.94613	1.09742
54505	0.946128	0.817766
54506	0.946128	0.728696
54507	0.94613	0.711875
54508	0.94613	0.82996
54509	0.946128	1.11243
54510	0.946128	0.74536
54511	0.946129	0.894835
54512	0.946129	0.90585
54513	0.946129	0.910434
54514	0.946129	0.872266
54515	1.89226	1.59177
54516	1.89226	1.18472
54517	1.89226	1.64554

54518	1.89226	1.01358
54519	3.78452	1.09571
54520	0.946133	0.670465
54521	0.946133	0.701474
54522	0.946132	0.476775
54523	0.946132	0.513295
54524	0.946132	0.491411
54525	0.946132	0.532375
54526	0.946133	0.717996
54527	0.946133	0.658782
54528	0.946132	0.622184
54529	0.946132	0.271847
54530	0.946132	0.572358
54531	0.946132	0.536166
54532	1.89226	0.938306
54533	2.10495	0.313393
54534	1.89226	0.44826
54535	2.30932	0.510033
54536	1.89226	0.781315
54537	2.30932	0.880359
54538	2.30932	0.440854
54539	1.00366	0.193975
54540	2.10496	0.143164
54541	1.24728	0.179336
54542	3.31298	0.200134
54543	1.87557	2.1
54544	1.07167	2.1
54545	2.03045	2.1
54546	1.25185	2.1
54547	3.10212	2.1
54548	1.89226	2.1
54549	1.89226	2.1
54550	1.89226	2.1
54551	1.89226	2.1
54552	3.78453	2.1
54553	0.851541	2.1
54554	0.851541	2.1
54555	1.12236	2.1
54556	1.05783	2.1
54557	0.870663	2.1
54558	0.951856	2.1
54559	0.946132	2.1
54560	0.946132	2.1
54561	1.04073	2.1
54562	1.1296	2.1
54563	0.776062	2.1
54564	0.851527	2.1
54565	0.859395	2.1
54566	0.859395	2.1
54567	0.870165	2.1
54568	0.870165	2.1
54569	0.625924	2.1
54570	0.714333	2.1
54571	1.0821	2.1
54572	1.01523	2.1
54573	1.0821	2.1
54574	0.714333	2.1
54575	1.01523	2.1

54576	0.625924	2.1
54577	1.89226	2.1
54578	1.89226	2.1
54579	1.89226	2.1
54580	1.89226	2.1
54581	3.78453	2.1
54582	1.89226	2.1
54583	1.89226	2.1
54584	2.23164	2.1
54585	2.67802	2.1
54586	2.03663	2.1
54587	1.89226	2.1
54588	2.42221	2.1
54589	1.61933	2.1
54590	2.80896	2.1
54591	1.87043	2.1
54592	4.29262	2.1
54593	1.89226	2.1
54594	1.89226	2.1
54595	1.89226	2.1
54596	1.89226	2.1
54597	1.89226	2.1
54598	1.89226	2.1
54599	2.80892	2.1
54600	1.87041	2.1
54601	2.67182	2.1
54602	2.30891	2.1
54603	5.07665	2.1
54604	1.89224	2.1
54605	1.89224	2.1
54606	1.89224	2.1
54607	1.89224	2.1
54608	3.78448	2.1
54609	0.946118	2.1
54610	0.946118	2.1
54611	0.946118	2.1
54612	0.946118	2.1
54613	0.946115	2.1
54614	0.946115	2.1
54615	0.94612	2.1
54616	0.94612	2.1
54617	0.94613	2.1
54618	0.94613	2.1
54619	0.946105	2.1
54620	0.946105	2.1
54621	2.3089	2.1
54622	1.02783	2.1
54623	1.98533	2.1
54624	1.76762	2.1
54625	1.24553	2.1
54626	1.02783	2.1
54627	2.2049	0.115438
54628	2.34673	0.10866
54629	1.00763	0.103643
54630	1.23746	0.142366
54631	3.44236	0.110877
54632	1.89226	0.154061
54633	1.89226	0.219675

54634	1.89226	0.186593
54635	1.89226	0.196757
54636	3.78452	0.206694
54637	1.89226	0.229538
54638	1.89226	0.196806
54639	1.89226	0.202203
54640	1.89226	0.285678
54641	3.78453	0.214397
54642	0.891618	0.156495
54643	0.891618	0.14464
54644	0.902158	0.114771
54645	0.902158	0.127776
54646	1.15396	0.148202
54647	0.620408	0.134237
54648	1.17337	0.116471
54649	0.618733	0.147029
54650	0.620408	0.139076
54651	0.618733	0.131065
54652	1.17337	0.135692
54653	1.15396	0.143371
54654	0.69978	0.139167
54655	0.69978	0.133636
54656	0.946102	0.133467
54657	0.946102	0.134978
54658	0.831702	0.150167
54659	0.946129	0.151157
54660	0.699752	0.136717
54661	0.946132	0.122334
54662	0.831703	0.142159
54663	0.699751	0.120122
54664	0.946131	0.125979
54665	0.946131	0.133303
54666	0.831675	0.16821
54667	0.831675	0.172814
54668	0.946163	0.153457
54669	0.864397	0.156333
54670	0.853471	0.191661
54671	0.962623	0.154727
54672	0.748555	0.151717
54673	0.851543	0.156187
54674	0.864714	0.168134
54675	0.760411	0.156886
54676	0.946133	0.158221
54677	0.946133	0.173989
54678	1.89226	0.107606
54679	1.89226	0.30569
54680	1.89226	0.247552
54681	1.89226	0.111264
54682	3.78452	0.142987
54683	1.89226	0.415112
54684	1.89226	0.213295
54685	1.89226	0.239289
54686	1.89226	0.410559
54687	3.78452	0.314322
54688	0.946132	0.363828
54689	0.946132	0.477573
54690	0.946132	0.167298
54691	0.946132	0.224865

54692	0.946132	0.167182
54693	0.946132	0.50235
54694	0.946133	0.211823
54695	0.946133	0.427878
54696	0.946133	0.259784
54697	0.946133	0.271516
54698	0.946132	0.342522
54699	0.946132	0.422422
54700	1.89226	0.264808
54701	2.39715	0.117035
54702	1.89226	0.142887
54703	1.89226	0.196374
54704	1.89226	0.15526
54705	1.89226	0.197122
54706	0.94613	0.37659
54707	0.94613	0.401234
54708	0.94613	0.245607
54709	0.94613	0.197424
54710	0.946131	0.198505
54711	0.946131	0.357957
54712	0.946129	0.401289
54713	0.946129	0.230105
54714	0.94613	0.280045
54715	0.94613	0.269741
54716	0.94613	0.294971
54717	0.94613	0.289075
54718	2.54204	2.1
54719	2.3148	2.1
54720	2.55328	2.1
54721	2.39646	2.1
54722	4.71126	2.1
54723	1.62611	2.1
54724	2.3148	2.1
54725	2.16986	2.1
54726	1.43745	2.1
54727	3.75225	2.1
54728	1.89226	2.1
54729	1.89226	2.1
54730	1.89226	2.1
54731	1.89226	2.1
54732	3.78453	2.1
54733	1.89226	2.1
54734	1.89226	2.1
54735	1.89226	2.1
54736	1.89226	2.1
54737	3.78453	2.1
54738	2.12125	2.1
54739	2.39642	2.1
54740	2.2941	2.1
54741	1.9933	2.1
54742	4.38972	2.1
54743	1.73784	2.1
54744	1.26892	2.1
54745	1.26892	2.1
54746	1.43743	2.1
54747	1.9933	2.1
54748	2.1618	2.1
54749	0.946118	2.1

54750	0.946118	2.1
54751	0.946118	2.1
54752	0.946118	2.1
54753	0.946119	2.1
54754	0.946119	2.1
54755	0.946118	2.1
54756	0.946118	2.1
54757	0.946131	2.1
54758	0.946131	2.1
54759	0.946106	2.1
54760	0.946106	2.1
54761	1.89224	2.1
54762	1.89224	2.1
54763	1.89224	2.1
54764	1.89224	2.1
54765	3.78448	2.1
54766	2.45922	2.1
54767	2.15686	2.1
54768	2.14259	2.1
54769	2.43225	2.1
54770	4.58911	2.1
54771	1.62027	2.1
54772	2.15686	2.1
54773	1.62241	2.1
54774	1.62241	2.1
54775	2.1426	2.1
54776	2.1426	2.1
54777	2.14259	2.1
54778	2.43225	2.1
54779	2.40667	2.1
54780	2.1281	2.1
54781	4.56035	2.1
54782	1.0713	2.1
54783	1.06405	2.1
54784	0.810136	2.1
54785	0.808433	2.1
54786	0.938508	2.1
54787	0.938508	2.1
54788	0.941223	2.1
54789	0.941223	2.1
54790	1.06405	2.1
54791	0.808433	2.1
54792	0.810135	2.1
54793	1.0713	2.1
54794	1.89226	0.444117
54795	1.89226	0.266306
54796	1.89226	0.395737
54797	1.89226	0.298539
54798	3.78453	0.309617
54799	1.89227	0.321266
54800	1.89227	0.263746
54801	1.89227	0.382783
54802	1.89227	0.225809
54803	3.78453	0.304185
54804	1.19	0.240756
54805	1.12469	0.175833
54806	1.01052	0.20749
54807	1.08166	0.193859

54808	2.20051	0.192801
54809	0.594999	0.241623
54810	0.540832	0.206981
54811	0.514182	0.271769
54812	0.567857	0.223383
54813	0.554259	0.217012
54814	0.554259	0.199889
54815	0.55443	0.243372
54816	0.55443	0.263303
54817	0.540832	0.225495
54818	0.514182	0.212285
54819	0.567857	0.217455
54820	0.594999	0.213212
54821	1.38674	0.25418
54822	0.946132	0.326447
54823	1.36361	0.260638
54824	0.946132	0.323309
54825	1.36362	0.250124
54826	1.36362	0.19906
54827	0.946131	0.33283
54828	0.946131	0.369636
54829	1.38674	0.281129
54830	1.36361	0.230766
54831	0.946132	0.318039
54832	0.946132	0.29138
54833	1.89226	0.31622
54834	2.68508	0.195349
54835	2.77349	0.225543
54836	1.89226	0.256303
54837	2.77349	0.23829
54838	1.89226	0.265538
54839	2.15315	0.063865
54840	1.96254	0.0642277
54841	1.51377	0.0681457
54842	1.89226	0.0704891
54843	1.47799	0.0611545
54844	1.89226	0.0576508
54845	2.49439	0.0936432
54846	1.89226	0.103832
54847	2.15315	0.103898
54848	1.89226	0.0959395
54849	2.15315	0.109373
54850	1.89226	0.0834994
54851	1.89226	0.102216
54852	1.89226	0.0958359
54853	1.89226	0.0933533
54854	1.89226	0.095947
54855	1.89226	0.0866257
54856	1.89226	0.094762
54857	1.89226	0.0541501
54858	1.89226	0.0738727
54859	1.89226	0.0620803
54860	1.89226	0.0572685
54861	1.89226	0.0659875
54862	1.89226	0.0583856
54863	1.375	0.0644627
54864	2.01865	0.0584225
54865	2.12047	0.0533071

54866	1.47354	0.064831
54867	2.12047	0.05
54868	1.47354	0.0782684
54869	0.687499	0.05
54870	1.06023	0.05
54871	1.08019	0.05
54872	0.604229	0.05
54873	1.08018	0.05
54874	1.06023	0.05
54875	0.687502	0.05
54876	0.60423	0.05
54877	0.80646	0.05
54878	0.806461	0.05
54879	0.858001	0.05
54880	0.858001	0.0524779
54881	2.26414	2.1
54882	1.87342	2.1
54883	1.87342	2.1
54884	1.79667	2.1
54885	2.44045	2.1
54886	2.31924	2.1
54887	2.26414	2.1
54888	2.26616	2.1
54889	2.2355	2.1
54890	2.21858	2.1
54891	4.48474	2.1
54892	2.18516	2.1
54893	1.87344	2.1
54894	2.21859	2.1
54895	2.66233	2.1
54896	1.85196	2.1
54897	2.22236	2.1
54898	2.23551	2.1
54899	2.21859	2.1
54900	2.18516	2.1
54901	2.21189	2.1
54902	4.43049	2.1
54903	1.17171	2.1
54904	2.38924	2.1
54905	2.0815	2.1
54906	2.33348	2.1
54907	1.42851	2.1
54908	1.5959	2.1
54909	1.89226	2.1
54910	1.89226	2.1
54911	1.89226	2.1
54912	1.89226	2.1
54913	3.78452	2.1
54914	2.75425	2.1
54915	1.34366	2.1
54916	1.34366	2.1
54917	1.56512	2.1
54918	2.54186	2.1
54919	2.76332	2.1
54920	1.07751	2.1
54921	2.0815	2.1
54922	1.97259	2.1
54923	1.76455	2.1

54924	1.30191	2.1
54925	1.16461	2.1
54926	0.946131	2.1
54927	0.946131	2.1
54928	0.946129	2.1
54929	0.946129	2.1
54930	0.946131	2.1
54931	0.946131	2.1
54932	0.94613	2.1
54933	0.94613	2.1
54934	0.94613	2.1
54935	0.94613	2.1
54936	0.946131	2.1
54937	0.946131	2.1
54938	1.89226	0.154148
54939	1.89226	0.101778
54940	1.89226	0.100655
54941	1.89226	0.0792618
54942	3.78453	0.0804877
54943	1.89226	0.176454
54944	1.89226	0.118315
54945	1.89226	0.115616
54946	1.89226	0.0822155
54947	3.78453	0.137784
54948	0.946132	0.0942005
54949	0.946132	0.107107
54950	1.30327	0.0806893
54951	1.33544	0.0885177
54952	1.33544	0.0766871
54953	1.33544	0.065417
54954	0.946132	0.0957436
54955	0.946132	0.0774615
54956	0.946132	0.0743244
54957	1.30327	0.0615017
54958	1.33544	0.0885924
54959	0.946132	0.100326
54960	1.30327	0.0752362
54961	1.33544	0.073564
54962	0.709426	0.0683567
54963	0.790129	0.0699782
54964	1.05886	0.0533437
54965	1.05886	0.0650418
54966	1.03454	0.0939522
54967	1.03454	0.0751733
54968	1.30327	0.0669265
54969	0.709426	0.0528663
54970	1.33544	0.0921088
54971	0.790128	0.0699357
54972	0.946132	0.0629349
54973	0.946132	0.0747711
54974	0.827866	0.05
54975	0.946133	0.0645987
54976	0.946132	0.05
54977	0.946132	0.0555644
54978	0.946133	0.0567259
54979	0.946133	0.0830603
54980	0.946132	0.05
54981	0.827866	0.0636107

54982	0.946133	0.0877906
54983	0.946133	0.0725082
54984	1.89227	0.101345
54985	1.89227	0.0553208
54986	1.89227	0.0724637
54987	1.89227	0.0708959
54988	1.89227	0.0650309
54989	1.89227	0.063576
54990	0.946133	0.0603582
54991	0.946133	0.0665388
54992	1.21602	0.0500925
54993	1.30327	0.0559206
54994	1.30327	0.05
54995	1.30327	0.05
54996	0.946132	0.05
54997	0.946132	0.0634522
54998	0.946132	0.05
54999	1.21602	0.05
55000	0.946133	0.0676862
55001	1.30327	0.0553177
55002	1.04227	0.05
55003	1.11706	0.05
55004	0.968413	0.05
55005	0.945482	0.05
55006	1.3204	0.05
55007	1.23215	0.05
55008	0.810791	0.05
55009	1.38978	0.05
55010	1.07728	0.0534894
55011	1.11704	0.0546379
55012	0.608052	0.05
55013	1.89226	0.1507
55014	1.89226	0.0757126
55015	1.89226	0.0970935
55016	1.89226	0.112777
55017	3.78453	0.110261
55018	1.89226	0.0555368
55019	1.89226	0.0669349
55020	1.89226	0.0895883
55021	1.89226	0.05
55022	3.78451	0.0599251
55023	2.58476	0.0593931
55024	1.56598	0.05
55025	1.55045	0.05
55026	1.5124	0.05
55027	2.62281	0.05
55028	2.58476	0.05
55029	2.58477	0.0679061
55030	1.89226	0.0792418
55031	1.89226	0.0935291
55032	1.89226	0.0590717
55033	2.62282	0.0953249
55034	2.62282	0.0614678
55035	1.89224	0.10315
55036	1.89224	0.120919
55037	1.89224	0.164024
55038	1.89224	0.081342
55039	3.78448	0.106207

55040	1.89223	0.0955001
55041	1.89223	0.05
55042	1.89223	0.0673423
55043	1.89223	0.0586211
55044	1.89223	0.0728807
55045	1.89223	0.0658548
55046	2.62278	0.0635867
55047	1.45053	0.05
55048	1.49678	0.05
55049	1.56596	0.05
55050	2.5536	0.05
55051	2.62278	0.05
55052	1.31139	0.0695576
55053	1.2768	0.0513998
55054	0.946118	0.118155
55055	0.946118	0.0833414
55056	1.2768	0.0842503
55057	1.2768	0.0614641
55058	0.946116	0.115363
55059	0.946116	0.0700182
55060	1.27682	0.0730172
55061	0.94613	0.0849817
55062	0.946105	0.0781972
55063	1.31138	0.0759003
55064	1.38506	1.51599
55065	1.53348	2.0559
55066	1.17042	1.92076
55067	1.17042	1.90961
55068	1.73669	1.9108
55069	1.73669	1.84356
55070	1.73669	1.62715
55071	1.79928	0.602585
55072	1.38506	1.41076
55073	1.38506	1.92472
55074	1.79928	1.98105
55075	1.43484	2.01766
55076	1.73669	1.19991
55077	1.93568	1.98652
55078	1.53347	1.55567
55079	1.53347	1.87922
55080	2.14058	1.57755
55081	2.14058	1.93147
55082	0.89964	0.536664
55083	1.10415	2.09492
55084	0.868346	1.62905
55085	1.07029	2.00508
55086	0.98687	1.75807
55087	0.98687	2.1
55088	0.985627	0.306641
55089	0.985627	2.06486
55090	1.07029	1.92263
55091	1.10415	1.63073
55092	0.868344	1.90394
55093	0.899642	2.00091
55094	2.40667	2.1
55095	2.1281	2.1
55096	2.11176	2.1
55097	2.38069	2.1

55098	4.50878	2.1
55099	2.11176	2.1
55100	1.61687	2.1
55101	1.60965	2.1
55102	1.61687	2.1
55103	2.12088	2.1
55104	2.1281	2.1
55105	2.11176	2.1
55106	2.34898	2.1
55107	2.34898	2.1
55108	2.38069	2.1
55109	2.09114	2.1
55110	2.12285	2.1
55111	1.05588	2.1
55112	1.04557	2.1
55113	0.804824	2.1
55114	0.798269	2.1
55115	0.92615	2.1
55116	0.92615	2.1
55117	0.927999	2.1
55118	0.927999	2.1
55119	1.04557	2.1
55120	0.798269	2.1
55121	1.05588	2.1
55122	0.804824	2.1
55123	1.43548	0.325971
55124	1.53873	0.950171
55125	1.25063	0.395378
55126	1.39662	1.25483
55127	2.78937	0.347723
55128	1.25063	0.38719
55129	1.39662	1.29956
55130	1.10476	0.287573
55131	1.28326	0.803884
55132	2.53389	0.503835
55133	1.51147	0.05
55134	1.86114	0.05
55135	1.86114	0.05
55136	2.12926	0.05
55137	1.3659	0.05
55138	1.63401	0.05
55139	1.89226	0.05
55140	1.89226	0.0758513
55141	1.89226	0.0562675
55142	1.89226	0.05
55143	1.89226	0.0675139
55144	1.89226	0.0599391
55145	1.89226	0.0799569
55146	1.89226	0.113747
55147	2.46286	0.067535
55148	1.89226	0.138348
55149	3.78452	0.0689362
55150	1.89226	0.122857
55151	1.89226	0.234383
55152	1.89226	0.182379
55153	1.89226	0.181974
55154	1.89226	0.13158
55155	1.89226	0.140189

55156	1.89226	0.0938843
55157	1.89226	0.114279
55158	2.28992	0.0830452
55159	1.89226	0.143998
55160	3.78452	0.0828587
55161	1.13537	0.123114
55162	0.886595	0.688204
55163	0.946131	1.63586
55164	0.946131	0.701013
55165	0.94613	0.504522
55166	0.94613	0.0950853
55167	1.25069	1.63324
55168	0.841855	1.0101
55169	1.27436	0.458226
55170	1.06196	0.552099
55171	1.09758	0.755259
55172	0.830306	0.461225
55173	0.826931	2.02063
55174	0.826933	0.475641
55175	0.764631	0.148052
55176	0.764631	0.805859
55177	1.00427	1.94823
55178	0.788034	0.866238
55179	0.726852	0.0989956
55180	0.604206	0.658612
55181	0.828475	0.711982
55182	0.604198	0.716713
55183	0.8332	0.647319
55184	1.00428	0.642263
55185	1.89226	0.582047
55186	1.89226	0.488457
55187	1.89226	0.170613
55188	1.89226	0.765365
55189	1.89226	0.677278
55190	1.89226	0.884784
55191	0.768789	2.1
55192	0.849132	0.05
55193	0.666423	2.1
55194	0.688993	1.95257
55195	0.868671	0.05
55196	0.89117	2.1
55197	0.705632	2.1
55198	1.80425	2.1
55199	1.73883	2.1
55200	1.45159	2.1
55201	1.45159	2.1
55202	1.37681	2.1
55203	1.73883	2.1
55204	1.39679	0.05
55205	2.08149	0.05
55206	1.89226	0.05
55207	2.32763	0.05
55208	1.89226	0.05
55209	2.31274	0.05
55210	1.89226	0.05
55211	1.89226	0.0526602
55212	1.89226	0.05
55213	1.89226	0.05

55214	1.89226	0.05
55215	1.89226	0.05
55216	1.89226	0.05
55217	1.89226	0.05
55218	1.89226	0.05
55219	1.89226	0.05
55220	1.89226	0.05
55221	1.89226	0.05
55222	1.95181	0.05
55223	1.89226	0.05
55224	1.89226	0.05
55225	1.89226	0.05
55226	3.78451	0.05
55227	0.946131	0.05
55228	0.946131	0.05
55229	0.946125	0.05
55230	0.946125	0.05
55231	0.946127	0.05
55232	0.946127	0.05
55233	0.946128	0.05
55234	0.946128	0.05
55235	0.946128	0.05
55236	0.946128	0.05
55237	0.946128	0.05
55238	0.946128	0.05
55239	1.89226	0.05
55240	1.89226	0.0697683
55241	1.89226	0.053093
55242	1.89226	0.05
55243	1.89226	0.0504628
55244	1.89226	0.05
55245	1.89226	0.920553
55246	1.89226	0.511083
55247	1.89226	0.329524
55248	1.89226	0.265892
55249	3.78453	0.579433
55250	1.89226	0.921088
55251	1.89226	0.384937
55252	1.89226	0.403595
55253	1.89226	0.28912
55254	3.78452	0.318917
55255	1.21387	0.282975
55256	0.8164	0.20816
55257	0.946133	0.267848
55258	0.946133	0.340975
55259	0.946102	0.183391
55260	0.946102	0.379717
55261	0.816431	0.791171
55262	0.816431	0.318374
55263	0.816401	0.254933
55264	0.94613	0.211809
55265	0.946131	0.722572
55266	1.21388	0.300542
55267	0.946132	0.200462
55268	0.946132	0.230781
55269	0.946132	0.311968
55270	0.946132	0.272728
55271	0.946132	0.173142

55272	0.946132	0.273946
55273	0.946132	0.454794
55274	0.946132	0.24444
55275	0.946131	0.16684
55276	0.946131	0.251819
55277	0.946132	0.400022
55278	0.946132	0.278519
55279	1.89226	0.417332
55280	1.89226	0.172338
55281	1.89226	0.234538
55282	1.89226	0.442282
55283	1.89226	0.242019
55284	1.89226	0.382398
55285	1.89226	0.505416
55286	1.89226	0.323105
55287	2.15871	0.199539
55288	2.15871	0.388708
55289	1.81454	0.173372
55290	1.54809	0.4512
55291	1.89226	0.355526
55292	1.89226	1.56435
55293	1.89226	0.975975
55294	1.89226	0.407242
55295	3.78453	1.18468
55296	1.89226	1.20003
55297	1.89226	0.260802
55298	1.89226	0.235238
55299	1.89226	0.913277
55300	1.89226	1.55172
55301	1.89226	0.935241
55302	2.29205	1.15265
55303	1.42633	0.720385
55304	1.69015	1.14216
55305	1.69015	1.1599
55306	2.10364	0.446157
55307	2.05344	0.294562
55308	2.29205	0.343555
55309	1.58147	0.891446
55310	2.16529	0.474114
55311	1.56339	0.279055
55312	2.29205	1.26596
55313	1.69015	1.02266
55314	1.89226	1.05523
55315	1.89226	1.00798
55316	1.89226	0.293053
55317	1.89226	1.05085
55318	3.78453	0.430576
55319	1.89226	1.02758
55320	1.89226	1.21159
55321	1.89226	1.18255
55322	1.89226	0.377211
55323	3.78452	1.41273
55324	2.29205	1.11988
55325	2.85335	0.212202
55326	2.55409	1.10882
55327	2.04345	0.393086
55328	2.76338	0.610987
55329	2.29205	0.368186

55330	2.29205	0.795776
55331	2.16529	0.998951
55332	2.72418	0.997142
55333	2.76338	0.176704
55334	5.01623	0.97581
55335	1.89227	0.05
55336	1.89227	0.05
55337	1.89227	0.05
55338	1.89227	0.05
55339	3.78453	0.05
55340	2.39588	0.05
55341	2.14918	0.05
55342	2.14918	0.05
55343	2.37907	0.05
55344	2.20531	0.05
55345	2.4352	0.05
55346	1.89226	0.05
55347	2.64327	0.05
55348	2.36175	0.05
55349	1.89226	0.05
55350	4.25402	0.05
55351	1.89226	0.05
55352	1.89226	0.05
55353	1.89226	0.05
55354	1.89226	0.05
55355	1.89226	0.05
55356	1.89226	0.05
55357	1.05395	0.05
55358	1.05395	0.05
55359	1.03114	0.05
55360	1.03114	0.05
55361	0.936689	0.05
55362	1.07459	0.05
55363	1.10265	0.05
55364	1.01049	0.05
55365	0.93669	0.05
55366	1.01049	0.05
55367	1.07459	0.05
55368	1.10265	0.05
55369	1.18088	0.05
55370	0.776509	0.05
55371	0.946132	0.05
55372	0.946132	0.05
55373	1.04225	0.05
55374	0.830392	0.05
55375	0.931352	0.05
55376	0.931352	0.05
55377	1.13006	0.05
55378	1.04074	0.05
55379	1.0628	0.05
55380	0.851529	0.05
55381	2.40032	2.1
55382	1.89226	2.1
55383	1.89226	2.1
55384	1.89226	2.1
55385	2.38918	2.1
55386	2.38918	2.1
55387	1.89226	2.1

55388	1.89226	2.1
55389	1.89226	2.1
55390	1.89226	2.1
55391	1.89226	2.1
55392	1.89226	2.1
55393	1.6585	2.1
55394	2.37364	2.1
55395	2.39622	2.1
55396	2.39622	2.1
55397	1.6488	2.1
55398	1.6488	2.1
55399	2.44603	2.1
55400	1.89224	2.1
55401	1.89224	2.1
55402	1.89224	2.1
55403	2.40029	2.1
55404	2.40029	2.1
55405	1.89224	2.1
55406	1.89224	2.1
55407	1.89224	2.1
55408	1.89224	2.1
55409	1.89224	2.1
55410	1.89224	2.1
55411	1.01946	2.1
55412	1.01946	2.1
55413	1.02497	2.1
55414	1.02497	2.1
55415	1.21519	2.1
55416	1.19809	2.1
55417	0.835303	2.1
55418	0.82924	2.1
55419	0.835314	2.1
55420	1.2152	2.1
55421	0.829228	2.1
55422	1.19808	2.1
55423	1.91027	2.1
55424	1.05809	2.1
55425	1.00659	2.1
55426	2.06727	2.1
55427	3.11819	2.1
55428	2.37166	2.1
55429	2.06726	2.1
55430	1.66994	2.1
55431	1.67623	2.1
55432	2.40308	2.1
55433	2.40308	2.1
55434	0.960194	2.1
55435	0.939664	2.1
55436	0.531131	2.1
55437	0.503298	2.1
55438	0.729927	2.1
55439	0.729927	2.1
55440	0.733565	2.1
55441	0.733565	2.1
55442	0.960195	2.1
55443	0.531132	2.1
55444	0.503297	2.1
55445	0.939663	2.1

55446	1.20154	2.1
55447	1.21754	2.1
55448	0.960195	2.1
55449	0.939665	2.1
55450	1.07745	2.1
55451	1.07745	2.1
55452	1.07976	2.1
55453	1.07976	2.1
55454	0.960194	2.1
55455	1.21754	2.1
55456	0.939662	2.1
55457	1.20155	2.1
55458	1.89224	2.1
55459	1.89224	2.1
55460	2.08609	2.1
55461	2.08609	2.1
55462	2.09527	2.1
55463	1.90142	2.1
55464	1.65815	2.1
55465	1.71398	2.1
55466	2.2001	2.1
55467	2.23791	2.1
55468	1.4502	2.1
55469	1.4502	2.1
55470	0.793587	2.1
55471	0.793587	2.1
55472	0.792408	2.1
55473	0.792408	2.1
55474	0.952221	2.1
55475	0.858585	2.1
55476	0.725101	2.1
55477	0.633774	2.1
55478	0.633775	2.1
55479	0.858585	2.1
55480	0.952221	2.1
55481	0.725101	2.1
55482	1.5735	2.1
55483	2.56975	2.1
55484	2.63166	2.1
55485	2.63166	2.1
55486	1.54711	2.1
55487	1.54711	2.1
55488	0.946131	2.1
55489	0.946131	2.1
55490	1.28487	2.1
55491	1.28487	2.1
55492	0.946127	2.1
55493	0.946127	2.1
55494	1.28488	2.1
55495	1.31583	2.1
55496	1.31583	2.1
55497	0.94613	2.1
55498	1.28487	2.1
55499	0.946129	2.1
55500	1.0596	2.1
55501	1.0596	2.1
55502	1.06939	2.1
55503	1.06939	2.1

55504	1.34225	2.1
55505	1.31583	2.1
55506	0.793563	2.1
55507	0.786748	2.1
55508	0.793563	2.1
55509	1.34225	2.1
55510	0.786748	2.1
55511	1.31583	2.1
55512	0.946131	2.1
55513	0.946131	2.1
55514	1.31582	2.1
55515	1.31582	2.1
55516	0.946127	2.1
55517	0.946127	2.1
55518	1.34225	2.1
55519	1.31583	2.1
55520	1.34225	2.1
55521	0.946129	2.1
55522	1.31583	2.1
55523	0.946129	2.1
55524	1.89226	2.1
55525	1.89226	2.1
55526	1.89226	2.1
55527	1.89226	2.1
55528	1.89226	2.1
55529	1.89226	2.1
55530	1.89226	2.1
55531	1.89226	2.1
55532	1.89226	2.1
55533	1.89226	2.1
55534	1.89226	2.1
55535	1.89226	2.1
55536	1.26992	2.1
55537	1.75003	2.1
55538	1.79883	2.1
55539	1.26732	2.1
55540	3.06875	2.1
55541	0.875017	2.1
55542	0.899414	2.1
55543	0.811147	2.1
55544	0.85053	2.1
55545	0.851513	2.1
55546	0.851513	2.1
55547	0.859048	2.1
55548	0.859048	2.1
55549	0.899413	2.1
55550	0.85053	2.1
55551	0.811147	2.1
55552	0.875016	2.1
55553	1.89227	2.1
55554	1.89227	2.1
55555	1.89227	2.1
55556	1.89227	2.1
55557	1.89227	2.1
55558	1.89227	2.1
55559	1.89226	2.1
55560	1.89226	2.1
55561	1.89226	2.1

55562	1.89226	2.1
55563	1.89226	2.1
55564	1.89226	2.1
55565	0.633661	2.1
55566	0.642005	2.1
55567	0.93285	2.1
55568	0.899413	2.1
55569	0.764487	2.1
55570	0.764487	2.1
55571	0.776931	2.1
55572	0.776931	2.1
55573	0.642004	2.1
55574	0.93285	2.1
55575	0.899414	2.1
55576	0.633661	2.1
55577	0.932851	2.1
55578	0.899414	2.1
55579	0.905419	2.1
55580	0.85053	2.1
55581	0.886336	2.1
55582	0.886336	2.1
55583	0.897045	2.1
55584	0.897045	2.1
55585	0.93285	2.1
55586	0.905419	2.1
55587	0.899414	2.1
55588	0.850531	2.1
55589	2.39322	2.1
55590	2.74149	2.08682
55591	2.70944	2.1
55592	2.6849	2.0156
55593	2.41776	2.1
55594	2.39322	1.72126
55595	2.41776	2.1
55596	2.80992	2.1
55597	2.77256	2.1
55598	2.74149	2.09719
55599	2.44882	2.1
55600	2.41776	1.98941
55601	2.80992	2.1
55602	1.89226	1.62117
55603	2.74149	2.03875
55604	2.74149	2.09605
55605	1.89226	1.18878
55606	1.89226	2.05076
55607	1.89226	0.0536646
55608	1.89226	0.05
55609	1.89226	0.0681668
55610	1.89226	0.05
55611	1.89226	0.0705417
55612	1.89226	0.05
55613	2.38796	0.05
55614	2.0815	0.05
55615	2.0815	0.05
55616	2.49452	0.05
55617	1.55994	0.05
55618	2.14721	0.05
55619	1.89227	0.0721442

55620	1.89227	0.0524483
55621	1.89227	0.0879867
55622	1.89227	0.05
55623	1.89227	0.091573
55624	1.89227	0.0519883
55625	1.13387	0.05
55626	2.27494	0.05
55627	1.13387	0.05
55628	2.19489	0.05
55629	1.60867	0.05
55630	2.6994	0.05
55631	1.89226	0.0678751
55632	1.89226	0.05
55633	1.89226	0.05
55634	1.89226	0.0919679
55635	3.78453	0.05
55636	1.42381	2.08436
55637	1.10747	2.1
55638	1.10747	2.08878
55639	1.08911	2.1
55640	1.42866	2.07158
55641	1.4103	2.1
55642	1.89226	2.1
55643	1.88181	2.1
55644	1.88181	2.1
55645	1.76249	2.1
55646	2.0815	2.1
55647	1.97539	2.1
55648	0.946128	2.1
55649	0.946128	2.1
55650	0.946133	2.08255
55651	0.946133	1.96851
55652	0.94613	1.98395
55653	0.94613	2.1
55654	0.946131	2.1
55655	0.946131	2.06059
55656	0.946131	2.1
55657	0.946131	2.1
55658	0.946131	2.09668
55659	0.946131	2.09738
55660	1.42866	2.09181
55661	1.43213	2.08553
55662	1.10748	2.1
55663	1.10748	2.1
55664	1.13641	2.1
55665	1.43213	2.1
55666	2.0815	2.1
55667	2.10287	2.1
55668	2.10287	2.1
55669	2.0921	2.1
55670	1.89227	2.1
55671	1.83938	2.1
55672	0.810944	2.08193
55673	0.810944	2.01171
55674	0.810945	2.01943
55675	0.810945	2.1
55676	0.810933	2.08444
55677	0.810933	2.0872

55678	1.89226	2.1
55679	1.3517	2.1
55680	1.08132	2.09414
55681	1.08132	2.1
55682	1.08133	2.09842
55683	2.33097	2.1
55684	1.53822	2.1
55685	1.53822	2.1
55686	1.68437	2.1
55687	2.17996	2.1
55688	2.32612	2.1
55689	0.99189	2.1
55690	1.18948	2.08468
55691	1.19756	2.1
55692	1.19756	2.1
55693	0.978851	2.1
55694	0.926995	2.1
55695	1.26924	1.95157
55696	1.34642	2.08268
55697	1.3228	2.1
55698	0.990899	2.1
55699	1.0627	2.1
55700	1.33473	2.1
55701	1.96196	2.1
55702	1.39702	2.1
55703	1.39702	2.1
55704	1.53822	2.1
55705	2.17372	2.1
55706	2.31011	2.1
55707	1.89226	1.94276
55708	1.89226	0.477445
55709	1.89226	1.9403
55710	1.89226	0.576831
55711	3.78453	0.699185
55712	2.38475	2.1
55713	1.73957	0.588877
55714	1.88452	1.94557
55715	1.58695	0.40638
55716	3.6241	0.868692
55717	1.73958	0.589913
55718	1.3126	2.1
55719	1.58695	0.424313
55720	1.14946	2.1
55721	2.88903	1.56548
55722	1.89226	0.436265
55723	1.89226	2.1
55724	1.89226	0.526363
55725	1.89226	2.1
55726	3.78453	1.77799
55727	1.89225	1.98126
55728	1.89225	0.630118
55729	1.89225	1.75337
55730	1.89225	0.67787
55731	3.7845	0.418255
55732	1.88451	1.98149
55733	1.58694	0.241291
55734	1.74649	2.09049
55735	1.44604	0.705058

55736	3.33055	0.684333
55737	1.58694	0.259476
55738	0.995727	2.1
55739	0.995727	1.41302
55740	1.14945	2.01135
55741	1.44604	0.496023
55742	1.59976	2.05095
55743	1.89225	0.489183
55744	1.89225	2.09954
55745	1.89225	2.1
55746	1.89225	1.08952
55747	1.89225	2.1
55748	1.89225	0.54771
55749	2.01197	2.09088
55750	2.0961	1.46698
55751	2.13283	1.57472
55752	2.25399	0.463724
55753	4.22894	1.37976
55754	2.09611	0.85466
55755	2.37014	1.52689
55756	2.29031	1.77324
55757	2.10084	2.05196
55758	2.25399	0.511628
55759	2.09611	2.03848
55760	2.13283	2.0318
55761	2.25399	2.03654
55762	2.22044	2.0245
55763	2.38975	0.5156
55764	4.52258	1.36946
55765	2.38976	0.177886
55766	2.15466	1.7395
55767	2.31988	0.255678
55768	2.31988	2.05491
55769	2.25399	0.175345
55770	2.25399	2.09718
55771	2.58286	2.08392
55772	2.29193	0.45377
55773	2.40107	2.1
55774	2.13815	0.223355
55775	4.72101	1.11094
55776	2.29193	0.420504
55777	1.90104	1.98466
55778	2.13815	0.261714
55779	1.77249	1.86814
55780	4.03919	0.747588
55781	2.25197	2.1
55782	2.13815	0.439227
55783	2.00625	1.97475
55784	2.00625	0.430519
55785	2.40107	1.95742
55786	2.40107	1.10118
55787	2.13815	0.287979
55788	1.77249	1.92009
55789	2.00625	0.879144
55790	1.65815	2.04451
55791	3.7963	0.749697
55792	2.43417	0.57609
55793	2.06653	0.416402

55794	1.64587	0.470482
55795	1.65284	0.387464
55796	2.46898	0.569128
55797	2.46898	0.453882
55798	2.46895	0.47018
55799	1.89016	0.327152
55800	1.87861	0.378155
55801	1.87861	0.29762
55802	2.47161	0.470442
55803	2.47161	0.359711
55804	0.946132	1.5962
55805	0.946132	1.32211
55806	0.863311	1.7877
55807	1.34768	1.64523
55808	0.851542	1.75255
55809	0.851542	1.15405
55810	1.30451	1.3985
55811	1.45304	1.55234
55812	0.946132	1.47428
55813	1.20992	1.5343
55814	0.855162	1.67228
55815	0.946132	1.5171
55816	1.89226	1.29718
55817	1.89226	1.9753
55818	1.89226	1.9044
55819	1.89226	1.54104
55820	1.89226	1.82837
55821	1.89226	1.44682
55822	0.946132	1.08261
55823	0.946132	0.839017
55824	0.946133	1.11927
55825	0.946133	1.04084
55826	0.946132	1.12724
55827	0.946132	0.821336
55828	0.946132	0.848976
55829	0.946132	0.997053
55830	0.946132	1.06244
55831	0.946132	0.999019
55832	0.946133	1.04552
55833	0.946133	0.995054
55834	2.35574	1.99988
55835	1.89226	1.49843
55836	1.89226	2.09516
55837	1.89226	1.28282
55838	2.67456	2.08444
55839	2.67456	0.668571
55840	0.583505	0.138112
55841	0.761972	0.315058
55842	1.17787	1.76318
55843	1.33728	0.657559
55844	0.965128	0.991116
55845	0.965128	0.1717
55846	0.974714	0.743729
55847	0.974715	1.75358
55848	1.17787	0.622148
55849	0.583506	0.842084
55850	1.33728	0.517729
55851	0.761974	0.650543

55852	1.89226	0.87662
55853	1.89226	0.622261
55854	1.89226	0.584831
55855	1.89226	0.147127
55856	1.89226	0.185421
55857	1.89226	0.471507
55858	1.89226	1.4539
55859	1.89226	0.234393
55860	1.89226	0.700469
55861	1.89226	0.588457
55862	3.78453	0.527667
55863	0.946132	0.129652
55864	0.946132	0.335695
55865	0.946132	1.06961
55866	0.946132	0.584278
55867	0.946133	0.535019
55868	0.946133	0.163045
55869	0.946132	0.745293
55870	0.946132	1.03954
55871	0.946133	0.657504
55872	0.946133	0.725706
55873	0.946132	0.485645
55874	0.946132	0.791988
55875	1.01779	2.1
55876	1.50685	2.1
55877	1.12064	2.1
55878	1.65151	2.1
55879	2.6275	2.1
55880	1.35733	2.1
55881	1.53507	2.1
55882	1.50683	2.1
55883	1.73626	2.1
55884	3.09359	2.1
55885	1.73626	2.1
55886	1.52226	2.1
55887	1.75555	2.1
55888	1.75555	2.1
55889	1.53507	2.1
55890	1.53507	2.1
55891	1.65151	2.1
55892	1.73628	2.1
55893	1.69528	2.1
55894	1.50685	2.1
55895	1.92471	2.1
55896	1.73628	2.1
55897	1.73629	2.1
55898	1.75558	2.1
55899	1.9943	2.1
55900	1.9943	2.1
55901	1.92471	2.1
55902	1.73629	2.1
55903	2.5601	0.992038
55904	1.97645	1.94203
55905	2.09605	0.735593
55906	2.09605	1.82114
55907	2.45853	1.08208
55908	2.45853	1.978
55909	2.33824	1.75187

55910	1.97646	1.50605
55911	2.45853	1.1448
55912	1.94984	1.9405
55913	2.33824	1.52248
55914	1.82955	2.03046
55915	1.22926	2.00225
55916	0.946128	1.96467
55917	0.946133	2.03986
55918	1.28005	1.39055
55919	1.22927	1.42626
55920	1.22926	1.65294
55921	0.946131	1.76477
55922	0.946131	1.40582
55923	0.946131	1.45301
55924	0.946131	1.31895
55925	1.28005	1.53186
55926	1.22926	1.43105
55927	2.35349	2.01472
55928	2.61606	0.596062
55929	2.48209	2.00578
55930	2.48209	0.582784
55931	2.50057	2.00023
55932	2.50057	0.777078
55933	2.61607	0.542987
55934	2.57611	0.816279
55935	2.4821	0.760624
55936	2.44814	0.477234
55937	5.06421	0.261258
55938	2.15721	1.71088
55939	2.48209	0.7053
55940	2.31844	2.04348
55941	2.31844	0.520641
55942	2.35349	2.02458
55943	2.35349	0.595348
55944	2.44814	0.507322
55945	2.30908	0.948162
55946	2.44814	0.469382
55947	2.31844	0.584853
55948	2.4821	0.415221
55949	2.31844	0.259873
55950	0.91766	0.0720099
55951	0.91766	0.0856348
55952	0.912463	0.0849156
55953	0.912463	0.0751511
55954	1.16525	0.0725598
55955	1.26377	0.0741284
55956	0.576692	0.082915
55957	0.664871	0.0800193
55958	1.16525	0.0791598
55959	0.576692	0.0768846
55960	0.664871	0.0924605
55961	1.26377	0.0834844
55962	1.72901	2.09782
55963	1.62414	2.09411
55964	1.88971	2.09848
55965	1.8411	1.74218
55966	3.57011	2.09028
55967	1.89226	2.09967

55968	1.89226	1.85868
55969	1.89226	1.9132
55970	1.89226	1.79518
55971	3.78452	1.85719
55972	1.89226	1.68504
55973	1.89226	2.09617
55974	1.89226	2.05245
55975	1.89226	1.43659
55976	3.78452	2.05559
55977	1.62414	2.09965
55978	1.30896	2.06461
55979	1.8411	2.01192
55980	1.52776	2.09169
55981	3.15006	2.04915
55982	1.51448	1.99344
55983	1.8411	1.94568
55984	2.02311	2.0999
55985	1.99309	1.84192
55986	3.86421	1.91598
55987	1.89226	2.01505
55988	1.89226	2.01079
55989	1.89226	2.06957
55990	1.89226	1.97293
55991	3.78452	2.05139
55992	1.89226	2.09255
55993	1.89226	1.93003
55994	1.89226	2.04264
55995	1.89226	2.1
55996	3.78452	2.06088
55997	1.8411	2.06616
55998	1.52776	2.1
55999	1.99309	2.1
56000	1.72526	2.1
56001	3.56636	2.1
56002	1.89226	2.08762
56003	1.89226	2.04025
56004	1.89226	2.04725
56005	1.89226	2.09305
56006	3.78453	2.06503
56007	1.0424	2.09901
56008	1.12935	2.1
56009	0.929383	2.09707
56010	0.929383	2.0967
56011	1.24941	2.09868
56012	1.12686	2.1
56013	0.946132	2.09921
56014	0.946132	2.09404
56015	1.12944	2.09231
56016	0.851554	2.08846
56017	1.05017	2.09649
56018	0.929315	2.09756
56019	0.898029	2.09193
56020	0.973994	2.1
56021	0.946131	2.04899
56022	0.946131	2.07982
56023	1.0221	2.08119
56024	1.0221	2.1
56025	0.898028	2.03774

56026	0.898028	2.09292
56027	0.973994	2.09477
56028	0.946131	2.09847
56029	0.946131	2.09356
56030	0.898029	2.09422
56031	1.89226	2.02319
56032	1.89226	2.04131
56033	1.89226	1.99525
56034	1.89226	2.0415
56035	1.89226	1.9939
56036	1.89226	2.03937
56037	1.13537	2.03869
56038	0.879308	2.09516
56039	0.946131	2.02795
56040	0.946131	2.04083
56041	1.13537	2.0462
56042	0.85187	2.09586
56043	0.94613	2.03628
56044	0.94613	1.98177
56045	1.05777	2.05849
56046	1.05777	2.08396
56047	0.799318	2.06935
56048	0.776452	2.07561
56049	0.946116	2.1
56050	0.946116	2.1
56051	1.09531	2.1
56052	1.09531	2.1
56053	0.946118	2.1
56054	0.946118	2.1
56055	1.09135	2.1
56056	1.09531	2.1
56057	1.09134	2.1
56058	0.946105	2.1
56059	1.09532	2.1
56060	0.946131	2.1
56061	0.946129	2.1
56062	0.946129	2.1
56063	1.09137	2.1
56064	1.09137	2.1
56065	0.946131	2.1
56066	0.946131	2.1
56067	1.07713	2.1
56068	1.09137	2.1
56069	0.946131	2.1
56070	1.07713	2.1
56071	1.09137	2.1
56072	0.946131	2.1
56073	0.938916	2.1
56074	0.938916	2.1
56075	0.936995	2.1
56076	1.00447	2.1
56077	0.979819	2.1
56078	1.16821	2.1
56079	1.01263	2.1
56080	0.719167	2.1
56081	1.04536	2.1
56082	1.18901	2.1
56083	0.758057	2.1

56084	0.924111	2.1
56085	1.00532	2.1
56086	1.00532	2.1
56087	0.9925	2.1
56088	0.9925	2.1
56089	1.29322	2.1
56090	1.01985	2.1
56091	1.00365	2.1
56092	0.704601	2.1
56093	1.00365	2.1
56094	1.29322	2.1
56095	1.01985	2.1
56096	0.704602	2.1
56097	2.20011	2.1
56098	1.401	2.1
56099	1.401	2.1
56100	1.52008	2.1
56101	2.11084	2.1
56102	2.22991	2.1
56103	0.854068	2.1
56104	0.854068	2.1
56105	0.851357	2.1
56106	0.851357	2.1
56107	0.644681	2.1
56108	0.700493	2.1
56109	1.0554	2.1
56110	1.00493	2.1
56111	1.00495	2.1
56112	0.64469	2.1
56113	0.700483	2.1
56114	1.05539	2.1
56115	2.46665	0.05
56116	2.22713	0.05
56117	1.85877	0.05
56118	1.8967	0.05
56119	1.56002	0.05
56120	2.22713	0.05
56121	1.89225	2.1
56122	1.89225	2.1
56123	1.89225	2.1
56124	1.89225	2.1
56125	1.89225	2.1
56126	1.89225	2.1
56127	1.89225	2.1
56128	1.89225	2.1
56129	1.89225	2.1
56130	1.89225	2.1
56131	1.89225	2.1
56132	1.89225	2.1
56133	1.284	2.1
56134	1.86569	2.1
56135	1.95398	2.1
56136	1.31091	2.1
56137	3.23798	2.1
56138	0.932844	2.1
56139	0.976992	2.1
56140	0.960128	2.1
56141	0.905413	2.1

56142	0.938531	2.1
56143	0.938531	2.1
56144	0.943874	2.1
56145	0.943874	2.1
56146	0.976985	2.1
56147	0.960122	2.1
56148	0.93285	2.1
56149	0.905419	2.1
56150	1.89227	2.1
56151	1.89227	2.1
56152	1.89227	2.1
56153	1.89227	2.1
56154	3.78453	2.1
56155	1.89226	2.1
56156	1.89226	2.1
56157	1.89226	2.1
56158	1.89226	2.1
56159	1.89226	2.1
56160	1.89226	2.1
56161	0.65546	2.1
56162	0.665451	2.1
56163	1.00694	2.1
56164	0.976998	2.1
56165	0.816261	2.1
56166	0.816261	2.1
56167	0.826189	2.1
56168	0.826189	2.1
56169	1.00694	2.1
56170	0.665451	2.1
56171	0.976998	2.1
56172	0.65546	2.1
56173	2.01555	2.1
56174	2.01388	2.1
56175	1.92027	2.1
56176	1.92027	2.1
56177	2.01388	2.1
56178	1.954 2.1	
56179	2.75466	2.1
56180	1.89226	2.1
56181	1.89226	2.1
56182	2.54931	2.1
56183	4.44158	2.1
56184	2.47917	2.1
56185	1.92772	2.1
56186	1.80682	2.1
56187	2.54931	2.1
56188	4.47702	2.1
56189	0.946132	2.1
56190	0.946132	2.1
56191	1.17602	2.1
56192	1.27466	2.1
56193	0.946132	2.1
56194	0.946132	2.1
56195	1.17602	2.1
56196	1.17602	2.1
56197	0.946132	2.1
56198	1.17602	2.1
56199	0.946131	2.1

56200	1.27466	2.1
56201	2.35204	2.1
56202	2.54931	2.1
56203	2.40783	2.1
56204	1.50108	2.1
56205	1.64256	2.1
56206	1.64256	2.1
56207	1.89226	2.1
56208	1.89226	1.61919
56209	2.31012	2.03965
56210	2.17534	2.08262
56211	4.0676	2.06834
56212	2.17533	2.07499
56213	1.89226	1.14842
56214	2.17533	1.91635
56215	1.89226	1.61331
56216	1.66635	2.00649
56217	1.38328	1.8455
56218	1.92027	2.1
56219	2.17597	2.1
56220	2.14619	2.1
56221	2.15388	2.1
56222	1.91087	2.1
56223	1.92027	2.1
56224	1.08622	2.1
56225	1.08622	2.1
56226	0.971184	2.1
56227	0.972761	2.1
56228	1.14845	2.1
56229	1.16321	2.1
56230	0.894769	2.1
56231	0.894967	2.1
56232	0.894775	2.1
56233	1.28563	2.1
56234	1.14844	2.1
56235	1.0167	2.1
56236	1.89226	1.90155
56237	1.89226	2.1
56238	1.89226	2.1
56239	1.89226	1.93768
56240	1.89226	2.1
56241	1.89226	1.97631
56242	0.937933	2.09879
56243	0.937933	2.1
56244	1.26192	2.1
56245	1.26192	2.1
56246	0.937903	2.09068
56247	1.25372	2.1
56248	0.946132	2.1
56249	0.946132	2.1
56250	0.946133	2.1
56251	1.25372	2.1
56252	0.946132	2.1
56253	0.937902	2.1
56254	0.946132	1.79233
56255	0.946132	1.67793
56256	0.946128	2.09727
56257	0.946128	2.1

56258	0.94613	2.09035
56259	0.94613	1.66625
56260	0.94613	1.602
56261	0.94613	2.09386
56262	0.946131	1.97694
56263	0.946131	1.9997
56264	0.94613	2.02999
56265	0.94613	2.03465
56266	0.946132	1.90997
56267	0.946132	1.71416
56268	1.2778	2.09931
56269	1.00191	2.1
56270	1.00191	1.83242
56271	1.00191	2.1
56272	0.94613	2.1
56273	0.94613	1.88985
56274	0.946131	2.0772
56275	1.2778	2.07218
56276	1.00191	2.01665
56277	0.94613	2.01903
56278	1.50847	0.05
56279	2.0815	0.05
56280	1.56806	0.05
56281	2.0815	0.05
56282	1.52043	0.05
56283	1.9224	0.05
56284	1.06882	0.05
56285	0.69567	0.05
56286	0.773112	0.05
56287	1.09473	0.05
56288	0.8941	0.05
56289	0.8941	0.05
56290	0.937064	0.05
56291	0.981131	0.05
56292	0.763762	0.05
56293	0.722535	0.05
56294	1.2153	0.05
56295	1.07279	0.05
56296	1.89226	0.05
56297	1.89226	0.05
56298	1.89226	0.05
56299	1.89226	0.05
56300	1.89226	0.0502692
56301	1.89226	0.05
56302	1.89226	0.05
56303	1.89226	0.0578592
56304	1.89226	0.05
56305	1.89226	0.0562447
56306	3.78453	0.05
56307	2.51518	0.05
56308	1.89226	0.05
56309	1.89226	0.05
56310	1.89226	0.05
56311	2.37584	0.05
56312	2.37584	0.05
56313	1.25759	0.05
56314	0.946131	0.05
56315	0.946131	0.05

56316	1.24489	0.05
56317	0.946131	0.05
56318	0.946131	0.05
56319	1.25759	0.05
56320	1.25759	0.05
56321	1.25759	0.05
56322	1.24489	0.05
56323	0.946131	0.05
56324	0.946131	0.05
56325	1.08028	0.338236
56326	1.09362	0.285196
56327	0.596616	0.750923
56328	0.606432	0.740081
56329	0.844265	0.809539
56330	0.844265	0.155842
56331	0.855786	0.41332
56332	0.855786	0.700228
56333	1.08028	0.560458
56334	0.606433	0.500936
56335	0.596616	0.607852
56336	1.09362	0.572095
56337	2.837	0.338805
56338	2.16055	0.0982102
56339	2.12741	0.200693
56340	2.79312	0.419697
56341	4.95367	0.195422
56342	2.12741	0.292577
56343	1.21286	0.414089
56344	1.20859	0.575467
56345	1.21286	0.719161
56346	2.15628	0.360965
56347	2.16056	0.119283
56348	1.89226	2.1
56349	1.89226	2.1
56350	1.89226	2.1
56351	1.89226	2.1
56352	3.78453	2.1
56353	0.946102	2.1
56354	0.946102	2.1
56355	0.850039	2.1
56356	0.850039	2.1
56357	0.914891	2.1
56358	0.85001	2.1
56359	0.946131	2.1
56360	0.946131	2.1
56361	0.94613	2.1
56362	0.91489	2.1
56363	0.850011	2.1
56364	0.946131	2.1
56365	0.71107	2.1
56366	0.784813	2.1
56367	1.28453	2.1
56368	1.20328	2.1
56369	0.992128	2.1
56370	0.992128	2.1
56371	0.995965	2.1
56372	0.995965	2.1
56373	0.784813	2.1

56374	1.28453	2.1
56375	0.711071	2.1
56376	1.20328	2.1
56377	1.89226	2.1
56378	1.89226	2.1
56379	1.89226	2.1
56380	1.89226	2.1
56381	3.78453	2.1
56382	0.946132	2.1
56383	0.946132	2.1
56384	0.914889	2.1
56385	0.914889	2.1
56386	0.985255	2.1
56387	0.914891	2.1
56388	0.946131	2.1
56389	0.946131	2.1
56390	0.946131	2.1
56391	0.985255	2.1
56392	0.94613	2.1
56393	0.91489	2.1
56394	0.784813	2.1
56395	0.871551	2.1
56396	1.37791	2.1
56397	1.28453	2.1
56398	1.07644	2.1
56399	1.07644	2.1
56400	1.07964	2.1
56401	1.07964	2.1
56402	0.871551	2.1
56403	1.37791	2.1
56404	0.784813	2.1
56405	1.28453	2.1
56406	1.73075	1.8193
56407	2.10582	1.39498
56408	1.89319	1.45854
56409	1.38766	1.98536
56410	3.27777	1.63631
56411	1.08715	1.95074
56412	1.54185	1.99718
56413	1.73074	1.94314
56414	1.73074	2.03562
56415	1.18948	1.79366
56416	1.18948	1.96634
56417	1.89319	1.33133
56418	1.41689	1.94996
56419	1.11619	1.77222
56420	1.38766	1.8012
56421	1.6052	1.59066
56422	1.89319	1.75885
56423	0.647894	1.87348
56424	0.594738	1.97474
56425	0.708445	1.97264
56426	0.770926	1.85204
56427	0.738326	1.98741
56428	0.738326	2.00381
56429	0.680494	1.83425
56430	0.680494	1.69749
56431	0.708443	1.73861

56432	0.647895	1.77994
56433	0.770925	1.83593
56434	0.59474	1.62316
56435	1.89111	2.1
56436	1.06994	2.1
56437	2.06689	2.1
56438	1.37063	2.1
56439	3.13683	2.1
56440	2.06689	2.1
56441	1.39295	2.1
56442	1.56818	2.1
56443	1.37063	2.1
56444	2.26444	2.1
56445	2.06689	2.1
56446	1.49808	2.1
56447	1.50201	2.1
56448	2.57784	2.1
56449	2.58615	2.1
56450	4.08424	2.1
56451	0.946133	2.1
56452	0.946133	2.1
56453	0.946132	2.1
56454	0.946132	2.1
56455	0.946132	2.1
56456	0.946132	2.1
56457	0.946132	2.1
56458	0.946132	2.1
56459	0.946133	2.1
56460	0.946133	2.1
56461	0.946132	2.1
56462	0.946132	2.1
56463	1.89226	2.1
56464	1.89226	2.1
56465	1.89226	2.1
56466	1.89226	2.1
56467	3.78452	2.1
56468	0.977201	2.1
56469	0.977201	2.1
56470	0.980489	2.1
56471	0.980489	2.1
56472	0.657973	2.1
56473	1.20669	2.1
56474	0.751002	2.1
56475	1.29307	2.1
56476	1.29308	2.1
56477	1.20669	2.1
56478	0.751003	2.1
56479	0.657972	2.1
56480	0.946131	2.1
56481	0.946131	2.1
56482	1.29308	2.1
56483	1.29308	2.1
56484	0.946132	2.1
56485	1.29308	2.1
56486	0.946132	2.1
56487	1.28892	2.1
56488	0.946132	2.1
56489	0.946132	2.1

56490	1.28892	2.1
56491	1.29308	2.1
56492	0.946132	2.09961
56493	0.946132	2.06801
56494	0.946131	2.1
56495	0.946131	2.09432
56496	0.946132	2.1
56497	0.946132	2.09591
56498	0.946132	2.09242
56499	0.946132	2.06626
56500	0.946132	2.09222
56501	0.946132	2.1
56502	0.946132	2.09519
56503	0.946132	2.1
56504	1.89226	2.1
56505	1.89226	2.09707
56506	1.89226	2.1
56507	1.89226	2.08224
56508	1.89226	2.1
56509	1.89226	2.06648
56510	0.94613	2.1
56511	0.94613	2.1
56512	1.20669	2.1
56513	1.20669	2.1
56514	1.20669	2.1
56515	0.946132	2.1
56516	0.946129	2.1
56517	1.29307	2.1
56518	0.946131	2.1
56519	0.946131	2.1
56520	1.29308	2.1
56521	1.20669	2.1
56522	1.89226	0.166345
56523	1.89226	0.082331
56524	1.89226	0.101037
56525	1.89226	0.128123
56526	3.78453	0.131415
56527	0.946132	0.05
56528	0.946132	0.0590353
56529	0.946126	0.0716659
56530	0.946126	0.0990472
56531	0.946129	0.0752236
56532	0.946129	0.0562792
56533	0.946129	0.0683987
56534	0.946129	0.0985811
56535	0.946128	0.0636579
56536	0.946128	0.0682225
56537	0.946129	0.067073
56538	0.946129	0.0704545
56539	0.725277	0.05
56540	0.588155	0.05
56541	1.19403	0.0644961
56542	1.27681	0.05
56543	0.918903	0.05
56544	0.918903	0.05
56545	0.946068	0.0581631
56546	0.946068	0.05
56547	1.19404	0.05

56548	0.588153	0.05
56549	0.725275	0.05
56550	1.27682	0.05
56551	1.19404	0.0713009
56552	1.27682	0.0521016
56553	0.946131	0.108244
56554	0.946131	0.0815099
56555	1.19404	0.083916
56556	1.19404	0.0620782
56557	0.946129	0.0702212
56558	0.946129	0.0944514
56559	1.19404	0.0755092
56560	0.94613	0.0767287
56561	1.27682	0.0652385
56562	0.946131	0.0742331
56563	1.89226	0.0896603
56564	1.89226	0.130313
56565	1.89226	0.14809
56566	1.89226	0.0917585
56567	1.89226	0.139198
56568	1.89226	0.0752802
56569	1.4263	1.97951
56570	1.67651	0.873283
56571	1.5955	0.959939
56572	1.31022	2.08473
56573	2.98674	1.61593
56574	1.18824	2.1
56575	1.28802	2.1
56576	0.946132	2.07855
56577	0.946132	2.1
56578	0.946132	2.0943
56579	0.946132	2.1
56580	1.28802	2.08173
56581	1.28802	2.1
56582	1.18824	2.1
56583	0.946132	2.1
56584	0.946132	2.1
56585	1.28802	2.1
56586	1.89226	0.966103
56587	1.89226	2.09897
56588	1.89226	2.08758
56589	1.89226	1.11529
56590	3.78452	1.89804
56591	1.5955	1.15463
56592	1.52909	1.16984
56593	1.31022	1.63819
56594	1.31022	2.0838
56595	1.2087	2.08072
56596	1.52909	2.09191
56597	1.11825	2.1
56598	1.18824	2.1
56599	0.946132	2.0883
56600	0.946132	2.1
56601	0.946132	2.1
56602	0.946132	2.1
56603	1.18824	2.08168
56604	1.18824	2.1
56605	0.946132	2.1

56606	1.11825	2.1
56607	1.18824	2.1
56608	0.946133	2.1
56609	0.810943	2.08773
56610	0.810943	2.09802
56611	0.810945	1.25046
56612	0.810945	2.09818
56613	1.89226	1.23161
56614	1.35168	1.67071
56615	1.08132	1.24617
56616	1.0813	1.87763
56617	1.08132	2.08995
56618	0.810959	1.89385
56619	0.810959	1.86064
56620	1.89226	0.486176
56621	1.89226	0.164294
56622	1.89226	0.237151
56623	1.89226	0.371348
56624	3.78453	0.393331
56625	1.89226	0.0839143
56626	1.89226	0.12101
56627	1.89226	0.134649
56628	1.89226	0.0727165
56629	1.89226	0.124197
56630	1.89226	0.0705707
56631	0.946131	0.0515788
56632	0.946131	0.0599136
56633	0.946126	0.124185
56634	0.946126	0.0857469
56635	0.946128	0.119686
56636	0.946128	0.0730374
56637	0.946128	0.120145
56638	0.946128	0.0928292
56639	0.946128	0.0896231
56640	0.946128	0.0977516
56641	0.946128	0.111643
56642	0.946128	0.0873512
56643	1.89226	0.35924
56644	1.89226	0.108507
56645	1.89226	0.251965
56646	1.89226	0.227165
56647	1.89226	0.161318
56648	1.89226	0.149203
56649	1.89226	0.151346
56650	1.89226	0.219338
56651	1.89226	0.494969
56652	1.89226	0.121103
56653	3.78453	0.120556
56654	1.07176	0.05
56655	1.07176	0.05
56656	0.946126	0.0946134
56657	0.946126	0.0724365
56658	1.07176	0.0587328
56659	0.946129	0.05
56660	1.27474	0.0717827
56661	0.94613	0.0879478
56662	1.27474	0.0728996
56663	1.07176	0.05

56664	0.94613	0.05
56665	0.94613	0.0557289
56666	0.723486	0.05
56667	0.94613	0.05
56668	0.946126	0.0734832
56669	0.946126	0.05
56670	0.946129	0.05
56671	0.723484	0.05
56672	1.07176	0.05
56673	0.946129	0.05
56674	1.32398	0.0513353
56675	1.19835	0.05
56676	0.723471	0.05
56677	0.723471	0.0578031
56678	0.810946	0.282604
56679	0.810946	0.157584
56680	0.810946	0.259426
56681	0.810946	0.183337
56682	0.810933	0.0577929
56683	0.810933	0.182639
56684	1.89226	0.0650845
56685	1.3517	0.157562
56686	1.08132	0.0810255
56687	1.08132	0.124777
56688	1.08133	0.186908
56689	2.39923	2.1
56690	1.89226	2.1
56691	1.89226	2.1
56692	1.89226	2.1
56693	2.44606	2.1
56694	2.44606	2.1
56695	1.89226	2.1
56696	1.89226	2.1
56697	1.89226	2.1
56698	1.89226	2.1
56699	1.89226	2.1
56700	1.89226	2.1
56701	1.70302	2.1
56702	2.43041	2.1
56703	2.25275	2.1
56704	2.4747	2.1
56705	1.52079	2.1
56706	1.67063	2.1
56707	2.40644	2.1
56708	1.89226	2.1
56709	1.89226	2.1
56710	1.89226	2.1
56711	2.39923	2.1
56712	2.39923	2.1
56713	0.946131	2.1
56714	0.946131	2.1
56715	0.946131	2.1
56716	0.946131	2.1
56717	0.94613	2.1
56718	0.94613	2.1
56719	0.946131	2.1
56720	0.946131	2.1
56721	0.946131	2.1

56722	0.946131	2.1
56723	0.946131	2.1
56724	0.946131	2.1
56725	1.04938	2.1
56726	1.04938	2.1
56727	1.111 2.1	
56728	1.06978	2.1
56729	1.2748	2.1
56730	1.25472	2.1
56731	0.88782	2.1
56732	0.882987	2.1
56733	0.973779	2.1
56734	1.31997	2.1
56735	0.913905	2.1
56736	1.31312	2.1
56737	2.6536	2.1
56738	2.15287	2.1
56739	2.72238	2.1
56740	2.22887	2.1
56741	4.87525	2.1
56742	2.15288	2.1
56743	1.40719	2.1
56744	1.41839	2.1
56745	1.34239	2.1
56746	2.22887	2.1
56747	2.15288	2.1
56748	2.72237	2.1
56749	2.22887	2.1
56750	2.77314	2.1
56751	2.28748	2.1
56752	5.00985	2.1
56753	0.922802	2.1
56754	0.922802	2.1
56755	0.92107	2.1
56756	0.92107	2.1
56757	0.703593	2.1
56758	0.729436	2.1
56759	1.11444	2.1
56760	1.14374	2.1
56761	1.14374	2.1
56762	0.729436	2.1
56763	0.703593	2.1
56764	1.11444	2.1
56765	1.89226	2.1
56766	1.89226	2.1
56767	1.89226	2.1
56768	1.89226	2.1
56769	1.89226	2.1
56770	1.89226	2.1
56771	0.489927	2.1
56772	0.63008	2.1
56773	0.697385	2.1
56774	0.55524	2.1
56775	0.592141	2.1
56776	0.592141	2.1
56777	0.593179	2.1
56778	0.593179	2.1
56779	0.63008	2.1

56780	0.697385	2.1
56781	0.55524	2.1
56782	0.489927	2.1
56783	0.946133	2.1
56784	0.946133	2.1
56785	0.946132	2.08887
56786	0.946132	1.98512
56787	0.946133	2.08893
56788	0.946133	2.1
56789	0.946132	2.1
56790	0.946132	2.0239
56791	0.946132	2.1
56792	0.946132	2.1
56793	0.946132	2.1
56794	0.946132	2.1
56795	1.89226	2.1
56796	1.89226	2.1
56797	1.89226	2.1
56798	1.89226	2.1
56799	1.89226	2.1
56800	1.89226	2.1
56801	0.805746	2.1
56802	0.63008	2.1
56803	0.881598	2.1
56804	0.697385	2.1
56805	0.749417	2.1
56806	0.749417	2.1
56807	0.753714	2.1
56808	0.753714	2.1
56809	0.881598	2.1
56810	0.805746	2.1
56811	0.63008	2.1
56812	0.697385	2.1
56813	0.881599	2.1
56814	0.697385	2.1
56815	0.805746	2.1
56816	0.626731	2.09273
56817	0.755479	2.1
56818	0.755479	2.1
56819	0.752851	2.09139
56820	0.752851	2.1
56821	0.881598	2.1
56822	0.805747	2.1
56823	0.697385	2.1
56824	0.626731	2.1
56825	2.65348	2.1
56826	2.12314	2.1
56827	2.63899	2.1
56828	2.13898	2.1
56829	4.76213	2.1
56830	0.763518	2.1
56831	0.765889	2.1
56832	0.84086	2.1
56833	0.84086	2.1
56834	0.632078	2.1
56835	0.597565	2.1
56836	1.08813	2.1
56837	0.985643	2.1

56838	0.982464	2.1
56839	0.66404	2.1
56840	1.0358	2.1
56841	0.598023	2.1
56842	1.29293	2.1
56843	1.29628	2.1
56844	1.19266	2.1
56845	1.19266	2.1
56846	1.07109	2.1
56847	1.13093	2.1
56848	1.34467	2.1
56849	1.35251	2.1
56850	1.34369	2.1
56851	1.12525	2.1
56852	1.28746	2.1
56853	1.13511	2.1
56854	2.19717	2.1
56855	2.16547	2.1
56856	1.37392	2.1
56857	1.37392	2.1
56858	1.2966	2.1
56859	2.11985	2.1
56860	1.89226	2.1
56861	1.89226	2.1
56862	1.89226	2.1
56863	1.89226	2.1
56864	1.89226	2.1
56865	1.89226	2.1
56866	1.65637	2.1
56867	1.41758	2.1
56868	1.65637	2.1
56869	1.74391	2.1
56870	1.35504	2.1
56871	1.44258	2.1
56872	1.40774	2.1
56873	1.40774	2.1
56874	0.946131	2.1
56875	0.946131	2.1
56876	1.40774	2.1
56877	1.38882	2.1
56878	0.946132	2.1
56879	0.946132	2.1
56880	0.946132	2.1
56881	1.38882	2.1
56882	0.946132	2.1
56883	1.40774	2.1
56884	1.89227	2.1
56885	1.89227	2.1
56886	1.89227	2.1
56887	1.89227	2.1
56888	3.78453	2.1
56889	0.603148	2.1
56890	0.600177	2.1
56891	0.558615	2.1
56892	0.580714	2.1
56893	0.558606	2.1
56894	0.67883	2.1
56895	1.65637	2.1

56896	1.07933	2.1
56897	0.842649	2.1
56898	0.905168	2.1
56899	0.774334	2.1
56900	1.38882	2.1
56901	1.38882	2.1
56902	0.946132	2.1
56903	0.946132	2.1
56904	1.38882	2.1
56905	1.37381	2.1
56906	0.946132	2.1
56907	0.946132	2.1
56908	0.946132	2.1
56909	1.37381	2.1
56910	0.946133	2.1
56911	1.38882	2.1
56912	2.3771	2.1
56913	1.9339	2.1
56914	1.70782	2.1
56915	1.9339	2.1
56916	2.42693	2.1
56917	2.83279	2.1
56918	2.34901	0.263755
56919	1.45833	0.356107
56920	1.86695	0.295394
56921	1.03101	0.330072
56922	3.32528	0.311949
56923	1.89226	0.136961
56924	1.89226	0.11867
56925	1.89226	0.0892819
56926	1.89226	0.0731082
56927	3.78453	0.123517
56928	2.71753	0.0676465
56929	1.89226	0.10695
56930	2.76228	0.0582357
56931	2.76228	0.0648504
56932	1.89226	0.100093
56933	1.89226	0.114534
56934	0.946131	0.0730963
56935	0.946131	0.0753669
56936	0.946132	0.0808683
56937	0.946132	0.0795837
56938	0.946132	0.0956661
56939	0.946132	0.0631419
56940	0.946132	0.106424
56941	0.946132	0.063657
56942	0.946132	0.0615209
56943	0.946132	0.0695904
56944	0.946132	0.0966659
56945	0.946132	0.0867042
56946	1.89227	0.088062
56947	1.89227	0.05
56948	1.89227	0.0642687
56949	1.89227	0.0809797
56950	1.89227	0.05
56951	1.89227	0.0549622
56952	2.71753	0.05
56953	2.37907	0.05

56954	2.37907	0.0531097
56955	2.76228	0.0554676
56956	2.39588	0.05
56957	2.77909	0.05
56958	1.6571	0.401355
56959	1.11682	1.75719
56960	1.47132	0.899099
56961	1.93763	0.12155
56962	3.12842	1.36168
56963	1.93762	0.168648
56964	2.12345	1.30141
56965	1.65709	0.183006
56966	1.65709	1.95714
56967	2.12345	0.586941
56968	1.88635	1.97551
56969	1.47134	1.90305
56970	2.219	0.258446
56971	2.25734	2.1
56972	1.93765	0.565935
56973	1.79103	1.45709
56974	1.47134	0.554579
56975	1.06174	0.788114
56976	1.19349	2.08289
56977	1.1095	1.96125
56978	0.968827	0.417207
56979	1.07892	0.16847
56980	1.07892	2.1
56981	1.0834	1.03009
56982	1.0834	0.986574
56983	1.1095	0.519457
56984	1.19349	0.372689
56985	0.968825	0.799174
56986	1.06174	0.74427
56987	2.40627	2.1
56988	2.08288	1.17192
56989	2.5444	1.23055
56990	2.29285	1.72896
56991	4.62729	1.48027
56992	2.08289	0.604115
56993	1.65703	0.583335
56994	1.71105	2.1
56995	1.50108	1.6669
56996	2.29286	2.04757
56997	2.08289	1.80701
56998	2.5444	1.77686
56999	2.29285	2.1
57000	2.71429	2.1
57001	2.50288	0.777174
57002	5.04728	2.07323
57003	2.50288	0.68033
57004	2.00927	1.84548
57005	2.02116	1.69681
57006	2.02116	2.05484
57007	2.29286	1.39065
57008	2.29286	1.88171
57009	1.89226	2.1
57010	1.89226	2.1
57011	1.89226	2.1

57012	1.89226	2.1
57013	3.78453	2.1
57014	1.17418	2.1
57015	1.29741	2.1
57016	1.04921	2.1
57017	1.19352	2.1
57018	2.3677	2.1
57019	1.29741	2.1
57020	1.27575	2.1
57021	1.19352	2.1
57022	1.19473	2.1
57023	2.46927	2.1
57024	1.89226	2.1
57025	1.89226	2.1
57026	1.89226	2.1
57027	1.89226	2.1
57028	1.89226	2.1
57029	1.89226	2.1
57030	0.561332	2.1
57031	0.561332	2.1
57032	0.567597	2.1
57033	0.567597	2.1
57034	0.597365	2.1
57035	0.544699	2.1
57036	0.596761	2.1
57037	0.531564	2.1
57038	0.5447	2.1
57039	0.531563	2.1
57040	0.596759	2.1
57041	0.597366	2.1
57042	0.946131	2.1
57043	0.946131	2.1
57044	0.946131	2.1
57045	0.946131	2.1
57046	0.946129	2.1
57047	0.946129	2.1
57048	0.946133	2.1
57049	0.946133	2.1
57050	0.946131	2.1
57051	0.946131	2.1
57052	0.946131	2.1
57053	0.946131	2.1
57054	1.10938	0.817419
57055	1.17614	2.1
57056	1.02478	1.65357
57057	1.09056	2.1
57058	2.19994	2.05282
57059	1.17614	2.1
57060	1.11868	2.1
57061	1.03408	2.1
57062	1.03408	2.1
57063	1.09056	2.1
57064	1.17516	2.1
57065	1.89226	0.455216
57066	1.89226	0.35646
57067	1.89226	0.436913
57068	1.89226	0.315635
57069	1.89226	0.531976

57070	1.89226	0.38084
57071	1.89226	0.314605
57072	1.89226	0.397772
57073	1.89226	0.37813
57074	1.89226	0.263402
57075	1.89226	0.470812
57076	1.89226	0.273831
57077	0.579551	0.248727
57078	0.563873	0.216036
57079	0.611341	0.304942
57080	0.598693	0.260352
57081	0.589856	0.253431
57082	0.589856	0.220092
57083	0.588387	0.298414
57084	0.588387	0.25656
57085	0.57955	0.264395
57086	0.611341	0.257575
57087	0.563873	0.253326
57088	0.598693	0.245849
57089	0.611341	0.302229
57090	0.598693	0.259296
57091	0.586048	0.299325
57092	0.572737	0.329497
57093	0.591824	0.298072
57094	0.591824	0.263719
57095	0.592254	0.340709
57096	0.592254	0.309811
57097	0.611341	0.307614
57098	0.586048	0.294633
57099	0.598693	0.298886
57100	0.572737	0.293094
57101	0.946119	0.308634
57102	0.946119	0.44392
57103	0.946119	0.327102
57104	0.946119	0.448783
57105	0.94612	0.321708
57106	0.94612	0.287511
57107	0.946119	0.448262
57108	0.946119	0.505366
57109	0.946132	0.390186
57110	0.946132	0.374916
57111	0.946107	0.393191
57112	0.946107	0.420368
57113	1.89224	0.345686
57114	1.89224	0.282388
57115	1.89224	0.366017
57116	1.89224	0.239483
57117	1.89224	0.454122
57118	1.89224	0.297149
57119	0.562335	0.228036
57120	0.579543	0.195679
57121	0.59499	0.262298
57122	0.611333	0.226019
57123	0.586619	0.221154
57124	0.586619	0.200331
57125	0.587049	0.257406
57126	0.587049	0.23134
57127	0.594999	0.243564

57128	0.562342	0.241563
57129	0.579535	0.232032
57130	0.611325	0.225162
57131	0.59499	0.258559
57132	0.611333	0.224825
57133	0.567849	0.258485
57134	0.58604	0.287178
57135	0.590978	0.255611
57136	0.590978	0.228843
57137	0.590052	0.294743
57138	0.590052	0.265405
57139	0.567857	0.280042
57140	0.594998	0.274161
57141	0.611325	0.272356
57142	0.586032	0.261772
57143	1.69661	1.8677
57144	1.9339	0.501888
57145	1.39379	1.99626
57146	1.69107	0.238231
57147	3.32768	1.07238
57148	1.9339	0.340269
57149	1.98414	0.982945
57150	1.69107	0.280156
57151	1.72774	0.583468
57152	3.66164	0.320003
57153	1.39379	2.05545
57154	1.69107	0.259387
57155	1.14397	1.65383
57156	1.43548	0.286014
57157	2.82927	1.07147
57158	1.69107	0.157607
57159	1.72774	0.558325
57160	1.53873	0.301534
57161	1.53873	0.920407
57162	1.43548	0.232575
57163	1.62449	1.06608
57164	2.58431	0.100274
57165	1.34072	0.128521
57166	1.80841	0.112718
57167	1.34072	0.126191
57168	2.51501	0.111745
57169	2.04732	0.130487
57170	1.89226	0.123747
57171	1.89226	0.149384
57172	1.89226	0.12521
57173	1.89226	0.159742
57174	1.89226	0.132651
57175	1.89226	0.160057
57176	0.946131	0.160673
57177	0.946131	0.136463
57178	1.3639	0.114609
57179	1.1516	0.140527
57180	0.946129	0.143224
57181	0.946129	0.169165
57182	1.36391	0.12984
57183	1.36391	0.122073
57184	0.946131	0.148617
57185	1.3639	0.138606

57186	1.1516	0.13337
57187	0.946131	0.143022
57188	0.946131	0.145247
57189	0.946131	0.122173
57190	0.94613	0.152281
57191	0.94613	0.12753
57192	0.946128	0.160474
57193	0.946128	0.14424
57194	0.946133	0.121578
57195	0.946133	0.134362
57196	0.946131	0.140466
57197	0.946131	0.140037
57198	0.946131	0.138736
57199	0.946131	0.132213
57200	1.89226	1.66072
57201	2.63634	2.02575
57202	1.89226	1.81829
57203	1.83902	1.75314
57204	3.73128	1.7112
57205	1.47059	2.1
57206	2.02928	2.1
57207	1.47059	2.1
57208	1.65706	2.1
57209	1.98628	2.1
57210	2.17274	2.1
57211	2.43618	2.1
57212	2.41176	2.1
57213	1.98627	2.1
57214	2.34084	2.1
57215	4.39803	2.1
57216	1.47059	2.1
57217	1.82453	2.1
57218	1.98627	2.1
57219	1.98627	2.1
57220	1.26929	2.1
57221	1.26929	2.1
57222	1.98627	2.1
57223	2.34084	2.1
57224	1.47264	2.1
57225	2.24937	2.1
57226	4.23564	2.1
57227	1.33026	1.71951
57228	1.51171	2.06508
57229	1.51171	1.63788
57230	1.4617	2.07939
57231	1.35382	1.56031
57232	1.30382	2.09943
57233	1.89226	1.37973
57234	1.89226	1.88444
57235	1.89226	1.27411
57236	1.89226	2.04346
57237	1.89226	1.2291
57238	1.89226	2.06264
57239	1.39263	1.55029
57240	0.962704	0.670155
57241	1.0171	0.88358
57242	0.962704	1.32803
57243	1.40822	0.783812

57244	1.35382	1.0752
57245	0.755853	2.1
57246	0.789808	2.0247
57247	0.696315	1.64207
57248	0.676912	1.20573
57249	0.729751	1.24268
57250	0.729751	2.00922
57251	0.722417	2.1
57252	0.722417	1.40758
57253	0.696315	1.92543
57254	0.789808	1.90631
57255	0.755853	1.90015
57256	0.676912	1.89326
57257	0.946132	1.83425
57258	0.946132	2.00377
57259	0.946132	1.27101
57260	0.946132	1.01509
57261	0.946131	1.04881
57262	0.946131	1.82054
57263	0.946132	1.36117
57264	0.946132	2.00586
57265	0.946132	1.49118
57266	0.946132	1.50114
57267	0.946132	1.60417
57268	0.946132	1.59039
57269	0.946133	1.10918
57270	0.946133	0.912167
57271	0.946132	0.589451
57272	0.946132	0.73177
57273	0.946132	0.601452
57274	0.946132	0.89444
57275	0.946133	0.66277
57276	0.946133	1.16436
57277	0.946132	0.837068
57278	0.946132	0.835666
57279	0.946132	0.822651
57280	0.946132	0.842415
57281	1.89227	1.86686
57282	2.53014	1.89812
57283	1.89227	1.99764
57284	1.98241	1.70649
57285	1.89227	2.01178
57286	1.98241	1.76826
57287	1.89226	1.74801
57288	1.89226	2.06435
57289	1.89226	1.95242
57290	1.89226	1.949
57291	1.89226	2.03212
57292	1.89226	2.01942
57293	1.89226	1.61017
57294	1.89226	2.00515
57295	1.89226	1.80478
57296	1.89226	1.82125
57297	3.78452	1.80613
57298	2.45304	1.1921
57299	1.89226	1.88897
57300	1.89226	1.75654
57301	1.89226	1.71278

57302	1.89226	1.46085
57303	1.89226	1.44095
57304	1.89227	1.88889
57305	1.89227	1.86561
57306	1.89227	1.98416
57307	1.89227	1.68084
57308	1.89227	1.99386
57309	1.89227	1.73067
57310	1.89226	1.82293
57311	1.89226	2.05386
57312	1.89226	1.94316
57313	1.89226	1.94736
57314	1.89226	2.00572
57315	1.89226	2.00153
57316	2.09871	0.249942
57317	2.71891	0.252287
57318	1.31126	0.211703
57319	1.31126	0.374529
57320	2.55341	0.208767
57321	1.76597	0.55364
57322	1.89226	0.72047
57323	1.89226	0.875879
57324	1.89226	0.386822
57325	1.89226	1.34912
57326	1.89226	0.46115
57327	1.89226	1.57341
57328	0.946128	0.819814
57329	0.946128	0.367107
57330	0.946133	0.316839
57331	0.946133	0.242789
57332	0.946131	0.312537
57333	0.946131	0.538893
57334	0.94613	0.26807
57335	0.94613	0.380466
57336	0.946131	0.354163
57337	0.946131	0.309973
57338	0.946131	0.347837
57339	0.946131	0.393975
57340	2.71891	0.234434
57341	1.89226	0.221633
57342	2.09871	0.170669
57343	2.09871	0.384179
57344	1.89226	0.165169
57345	1.89226	0.403808
57346	1.89227	0.66838
57347	1.89227	0.673696
57348	1.89227	0.399218
57349	1.89227	1.2576
57350	1.89227	0.489652
57351	1.89227	1.40466
57352	0.946129	0.636038
57353	0.946129	0.36963
57354	0.946133	0.28369
57355	0.946133	0.218218
57356	0.946131	0.280385
57357	0.946131	0.496574
57358	0.946131	0.243
57359	0.946131	0.394389

57360	0.946131	0.332987
57361	0.946131	0.297655
57362	0.946131	0.364859
57363	0.946131	0.328767
57364	2.32462	0.05
57365	1.89226	0.05
57366	1.89226	0.05
57367	1.89226	0.05
57368	2.2291	0.05
57369	2.2291	0.05
57370	0.847669	0.05
57371	0.847669	0.05
57372	0.796966	0.05
57373	0.796966	0.05
57374	0.833552	0.05
57375	1.01049	0.05
57376	0.709709	0.05
57377	0.634147	0.05
57378	0.634148	0.05
57379	0.833549	0.05
57380	0.709711	0.05
57381	1.01049	0.05
57382	1.89226	0.05
57383	1.89226	0.05
57384	1.89226	0.05
57385	1.89226	0.05
57386	1.89226	0.05
57387	1.89226	0.05
57388	1.03611	0.05
57389	0.936655	0.05
57390	0.946132	0.05
57391	0.946132	0.05
57392	0.941918	0.05
57393	0.941918	0.05
57394	1.00308	0.05
57395	1.04117	0.05
57396	1.1406	0.05
57397	1.04074	0.05
57398	0.851529	0.05
57399	1.00311	0.05
57400	1.01814	0.05
57401	1.6671	0.05
57402	1.35561	0.05
57403	1.35561	0.05
57404	1.2683	0.05
57405	1.2683	0.05
57406	0.946128	0.05
57407	0.946128	0.05
57408	0.946129	0.05
57409	0.946129	0.05
57410	0.946132	0.05
57411	0.946132	0.05
57412	0.946126	0.05
57413	0.946126	0.05
57414	0.946129	0.05
57415	0.946129	0.05
57416	0.946129	0.05
57417	0.946129	0.05

57418	2.23338	0.05
57419	1.96	0.05
57420	2.03438	0.05
57421	1.87835	0.05
57422	4.11172	0.05
57423	1.96	0.05
57424	1.52661	0.05
57425	1.87834	0.05
57426	1.53175	0.05
57427	3.40495	0.05
57428	2.03435	0.05
57429	1.65813	0.05
57430	1.65813	0.05
57431	1.87832	0.05
57432	1.72067	0.05
57433	1.94086	0.05
57434	1.65813	0.05
57435	1.40572	0.05
57436	1.87832	0.05
57437	1.87832	0.05
57438	1.31154	0.05
57439	1.53173	0.05
57440	1.86662	2.1
57441	2.0815	2.1
57442	2.0815	2.1
57443	2.0815	2.1
57444	1.87246	2.1
57445	1.87362	2.1
57446	1.0273	2.1
57447	0.996347	2.1
57448	0.946129	2.1
57449	0.946129	2.1
57450	1.04427	2.1
57451	1.04196	2.1
57452	0.910674	2.1
57453	0.910674	2.1
57454	1.04072	2.1
57455	1.10931	2.1
57456	1.03132	2.1
57457	0.933698	2.1
57458	2.63789	0.287054
57459	1.89226	0.537264
57460	2.3033	0.310253
57461	2.3033	0.414801
57462	1.89226	0.306558
57463	1.89226	0.42157
57464	1.89226	0.7415
57465	1.89226	0.929572
57466	1.89226	0.489194
57467	1.89226	0.742874
57468	3.78452	0.768973
57469	1.51898	0.523242
57470	1.36006	0.941879
57471	2.03143	0.905416
57472	2.03143	0.962961
57473	1.04026	0.624984
57474	1.04026	0.691965
57475	0.94613	0.168754

57476	0.94613	0.258295
57477	1.31894	0.171484
57478	1.31894	0.149911
57479	0.94613	0.159532
57480	0.94613	0.318743
57481	1.31894	0.195729
57482	1.41232	0.23876
57483	0.946129	0.208817
57484	1.41232	0.211177
57485	1.31895	0.199422
57486	0.946129	0.225932
57487	1.89226	0.224806
57488	1.89226	0.701435
57489	1.89226	0.233454
57490	1.89226	0.573765
57491	1.89226	0.175837
57492	1.89226	0.498618
57493	1.89804	0.20618
57494	2.03143	0.43407
57495	2.46286	0.370157
57496	2.46287	0.410303
57497	1.51898	0.273283
57498	1.51898	0.327814
57499	1.89226	2.1
57500	1.89226	2.1
57501	1.89226	2.1
57502	1.89226	2.1
57503	3.78452	2.1
57504	1.3864	2.1
57505	1.3864	2.1
57506	0.946132	2.1
57507	0.946132	2.1
57508	1.32905	2.1
57509	1.3864	2.1
57510	0.946132	2.1
57511	0.946132	2.1
57512	0.946132	2.1
57513	1.32905	2.1
57514	0.946133	2.1
57515	1.3864	2.1
57516	2.77279	2.1
57517	1.67883	2.1
57518	1.67883	2.1
57519	1.73415	2.1
57520	2.65811	2.1
57521	2.71343	2.1
57522	0.946131	2.1
57523	0.946131	2.1
57524	0.946128	2.1
57525	0.946128	2.1
57526	0.946127	2.1
57527	0.946127	2.1
57528	0.946131	2.1
57529	0.946131	2.1
57530	0.946129	2.1
57531	0.946129	2.1
57532	0.94613	2.1
57533	0.94613	2.1

57534	1.89226	2.1
57535	1.89226	2.1
57536	1.89226	2.1
57537	1.89226	2.1
57538	3.78452	2.1
57539	1.89226	2.1
57540	2.54394	2.1
57541	2.54394	2.1
57542	2.65811	2.1
57543	1.89226	2.1
57544	2.00643	2.1
57545	2.54394	2.1
57546	2.6581	2.1
57547	1.62743	2.1
57548	1.62743	2.1
57549	1.67883	2.1
57550	2.59533	2.1
57551	1.89226	2.1
57552	1.89226	2.1
57553	1.89226	2.1
57554	1.89226	2.1
57555	1.89226	2.1
57556	1.89226	2.1
57557	1.89226	1.85787
57558	1.89226	1.80395
57559	1.89226	2.05991
57560	1.89226	1.46138
57561	1.89226	2.04032
57562	1.89226	1.43552
57563	1.89226	1.85298
57564	1.89226	1.77666
57565	1.89226	2.02838
57566	1.89226	1.61166
57567	1.89226	1.99662
57568	1.89226	1.50725
57569	1.89226	1.29675
57570	1.89226	0.794114
57571	1.89226	1.77077
57572	1.89226	0.755203
57573	1.89226	1.71142
57574	1.89226	0.75685
57575	2.68537	0.492118
57576	2.22575	2.08454
57577	2.47464	0.315086
57578	1.79093	1.94727
57579	4.70039	0.920151
57580	2.81217	2.1
57581	2.47464	0.416274
57582	2.58286	2.04604
57583	2.29193	0.313472
57584	5.0575	0.863126
57585	2.47464	0.29416
57586	2.20365	1.93838
57587	2.29193	0.503737
57588	1.90104	2.03158
57589	4.37568	0.645116
57590	1.89226	0.710043
57591	2.16957	2.05098

57592	2.52823	1.40287
57593	2.52823	2.01655
57594	1.89226	0.971045
57595	1.89226	1.61642
57596	1.36226	2.1
57597	1.55369	2.1
57598	1.33309	2.1
57599	1.62593	2.1
57600	2.98819	2.1
57601	0.802281	2.1
57602	0.802281	2.1
57603	0.835143	2.1
57604	0.835143	2.1
57605	0.824458	2.1
57606	0.92641	2.1
57607	0.776847	2.1
57608	0.812967	2.1
57609	0.812967	2.1
57610	0.926411	2.1
57611	0.776847	2.1
57612	0.824458	2.1
57613	1.33309	2.1
57614	1.62593	2.1
57615	1.32813	2.1
57616	1.7237	2.1
57617	2.95406	2.1
57618	0.814544	2.1
57619	0.875312	2.1
57620	0.910502	2.1
57621	0.910502	2.1
57622	0.886633	2.1
57623	0.975739	2.1
57624	0.792623	2.1
57625	0.840283	2.1
57626	0.796372	2.1
57627	1.06159	2.1
57628	0.81948	2.1
57629	0.933833	2.1
57630	2.63997	2.1
57631	1.29694	2.1
57632	1.28141	2.1
57633	2.62623	2.1
57634	3.92317	2.1
57635	2.63998	2.1
57636	1.89226	2.1
57637	1.89226	2.1
57638	2.62623	2.1
57639	4.5185	2.1
57640	1.89226	2.1
57641	1.89226	2.1
57642	1.89226	2.1
57643	1.89226	2.1
57644	3.78453	2.1
57645	1.89226	2.1
57646	1.89226	2.1
57647	1.89226	2.1
57648	1.89226	2.1
57649	1.89226	2.1

57650	1.89226	2.1
57651	1.25468	2.1
57652	2.62623	2.1
57653	1.28141	2.1
57654	1.28141	2.1
57655	2.60665	2.1
57656	2.60665	2.1
57657	1.89226	2.1
57658	2.62623	2.1
57659	2.60666	2.1
57660	2.62623	2.1
57661	1.87269	2.1
57662	1.89226	2.1
57663	1.89226	2.1
57664	1.89226	2.1
57665	1.89226	2.1
57666	1.89226	2.1
57667	3.78453	2.1
57668	1.89226	2.1
57669	1.89226	2.1
57670	1.89226	2.1
57671	1.89226	2.1
57672	1.89226	2.1
57673	1.89226	2.1
57674	2.30147	1.5373
57675	2.27703	1.95877
57676	1.89227	1.36784
57677	1.89227	1.86055
57678	4.1693	1.76096
57679	1.38617	0.745605
57680	1.24613	1.55026
57681	2.59886	0.809176
57682	2.6238	1.48582
57683	3.86993	0.986654
57684	0.946133	1.80514
57685	0.946133	1.28455
57686	0.946132	1.3014
57687	0.946132	1.97978
57688	0.946133	1.9812
57689	0.946133	1.88075
57690	0.946132	1.26116
57691	0.946132	1.55178
57692	0.946133	1.76396
57693	0.946133	1.73555
57694	0.946132	1.63639
57695	0.946132	1.62903
57696	2.21773	2.1
57697	2.59216	2.1
57698	1.68541	2.1
57699	1.68541	2.1
57700	1.84814	2.1
57701	2.38046	2.1
57702	1.89966	2.1
57703	1.70303	2.1
57704	1.2028	2.1
57705	1.29897	2.1
57706	2.02873	2.1
57707	2.19093	2.1

57708	2.75218	2.1
57709	1.70303	2.1
57710	3.10254	2.1
57711	2.24783	2.1
57712	2.56839	2.1
57713	1.70303	2.1
57714	2.56839	2.1
57715	1.08276	2.1
57716	2.24061	2.1
57717	1.67491	2.1
57718	1.60948	2.1
57719	1.08276	2.1
57720	1.89226	1.29788
57721	1.89226	1.36279
57722	1.89226	0.654682
57723	1.89226	1.01872
57724	3.78453	1.09949
57725	1.89226	1.45298
57726	1.89226	1.35339
57727	1.89226	0.382974
57728	1.89226	0.803366
57729	3.78452	1.10212
57730	1.89226	1.3482
57731	2.7535	0.979237
57732	1.89226	1.67359
57733	1.89226	1.67407
57734	1.89226	0.554221
57735	1.89226	0.684447
57736	1.89226	1.32697
57737	1.89226	1.19108
57738	1.89226	1.13243
57739	1.89226	1.44774
57740	1.89226	1.16392
57741	1.89226	1.39127
57742	1.89227	1.25926
57743	1.89227	0.866363
57744	1.89227	0.260296
57745	1.89227	0.479939
57746	3.78453	0.660281
57747	1.89226	1.19717
57748	2.53412	0.39535
57749	1.89226	1.63602
57750	1.89226	0.623977
57751	1.89226	1.00407
57752	1.89226	0.400133
57753	2.21126	0.0648832
57754	1.89226	0.0578978
57755	2.57464	0.0856722
57756	1.89226	0.0845332
57757	2.57464	0.05
57758	1.89226	0.05
57759	1.89226	0.190402
57760	1.89226	0.225147
57761	1.89226	0.64672
57762	1.89226	0.155255
57763	1.89226	0.579292
57764	1.89226	0.146133
57765	1.1418	0.187474

57766	0.653021	0.0844547
57767	1.37282	0.182621
57768	0.931386	0.252796
57769	1.34568	0.352748
57770	1.17669	0.121828
57771	0.666595	0.0786455
57772	0.786895	0.194178
57773	0.933982	0.266602
57774	1.05351	0.319964
57775	1.00792	0.098882
57776	1.00792	0.0810616
57777	1.89226	2.1
57778	1.89226	2.1
57779	1.89226	2.1
57780	1.89226	2.1
57781	3.78452	2.1
57782	1.89226	2.1
57783	1.89226	2.1
57784	1.89226	2.1
57785	1.89226	2.1
57786	1.89226	2.1
57787	1.89226	2.1
57788	1.65642	2.1
57789	1.30248	2.1
57790	1.38888	2.1
57791	1.25987	2.1
57792	1.70611	2.1
57793	1.65642	2.1
57794	1.65642	2.1
57795	1.81407	2.1
57796	2.19428	2.1
57797	1.92555	2.1
57798	3.8507	2.1
57799	1.89226	2.1
57800	1.89226	2.1
57801	1.89226	2.1
57802	1.89226	2.1
57803	3.78452	2.1
57804	1.89226	2.1
57805	1.89226	2.1
57806	1.89226	2.1
57807	1.89226	2.1
57808	1.89226	2.1
57809	1.89226	2.1
57810	0.705495	2.1
57811	0.809317	2.1
57812	1.1655	2.1
57813	1.09605	2.1
57814	0.983375	2.1
57815	0.797301	2.1
57816	0.906633	2.1
57817	0.906633	2.1
57818	0.85085	2.1
57819	1.16548	2.1
57820	0.705501	2.1
57821	1.00314	2.1
57822	2.19428	2.1
57823	2.31862	2.1

57824	2.27408	2.1
57825	2.45893	2.1
57826	4.46836	2.1
57827	1.89226	0.493792
57828	1.89226	0.880283
57829	1.89226	1.16744
57830	1.89226	0.442909
57831	1.89226	1.2597
57832	1.89226	0.717635
57833	2.0815	0.441603
57834	2.22136	0.598004
57835	1.5515	0.570109
57836	1.86323	0.344291
57837	2.74986	0.87235
57838	3.30238	0.435071
57839	1.89224	0.504221
57840	1.89224	0.890369
57841	1.89224	1.15204
57842	1.89224	0.422299
57843	1.89224	1.31561
57844	1.89224	0.59255
57845	1.89224	0.490271
57846	1.89224	1.18973
57847	1.89224	0.919727
57848	1.89224	0.396464
57849	1.89224	1.2591
57850	1.89224	0.701161
57851	1.17846	0.330908
57852	0.810935	0.417994
57853	0.93347	1.14492
57854	0.93347	0.391281
57855	0.810932	0.611506
57856	0.810932	0.478077
57857	2.22133	0.462662
57858	1.71919	0.468941
57859	1.32629	0.352958
57860	1.0813	0.691057
57861	1.57137	0.421296
57862	2.21149	2.1
57863	1.89226	2.1
57864	1.89226	2.1
57865	1.89226	2.1
57866	2.17159	2.1
57867	2.17159	2.1
57868	0.946131	1.04079
57869	0.946131	2.1
57870	0.946131	2.1
57871	0.946131	2.09797
57872	0.94613	1.49557
57873	0.94613	1.88267
57874	0.946132	2.1
57875	0.946132	2.1
57876	0.946131	2.04414
57877	0.946131	2.05298
57878	0.946131	2.1
57879	0.946131	2.09999
57880	1.12266	2.1
57881	1.4714	2.04554

57882	1.62322	2.1
57883	1.62322	2.03343
57884	1.02958	2.1
57885	1.02958	2.1
57886	2.2671	2.1
57887	1.89226	2.1
57888	1.89226	2.1
57889	1.89226	2.1
57890	2.21149	2.1
57891	2.21149	2.1
57892	1.89226	2.1
57893	1.89226	2.02915
57894	1.89226	2.1
57895	1.89226	1.82918
57896	1.89226	2.1
57897	1.89226	2.05972
57898	0.736914	2.08412
57899	0.736914	2.1
57900	0.822839	2.1
57901	0.822836	2.1
57902	0.791298	1.29549
57903	1.00003	2.01317
57904	0.640254	2.1
57905	0.575379	2.1
57906	0.609001	2.07889
57907	1.00005	2.09068
57908	0.575371	2.09471
57909	0.831908	2.07582
57910	1.89226	2.1
57911	1.89226	2.1
57912	1.89226	2.1
57913	1.89226	2.1
57914	1.89226	2.1
57915	1.89226	2.1
57916	1.89226	2.1
57917	1.89226	2.1
57918	1.89226	2.1
57919	1.89226	2.1
57920	1.89226	2.1
57921	1.89226	2.1
57922	0.730617	2.1
57923	0.71052	2.1
57924	0.7329	2.1
57925	0.712083	2.1
57926	0.701051	2.1
57927	0.701051	2.1
57928	0.721552	2.1
57929	0.721552	2.1
57930	0.710521	2.1
57931	0.732898	2.1
57932	0.730619	2.1
57933	0.712081	2.1
57934	0.7329	2.1
57935	0.712082	2.1
57936	0.559601	2.1
57937	0.495993	2.1
57938	0.603718	2.1
57939	0.603718	2.1

57940	0.625174	2.1
57941	0.625174	2.1
57942	0.732899	2.1
57943	0.559601	2.1
57944	0.712082	2.1
57945	0.495993	2.1
57946	0.94613	2.1
57947	0.94613	2.1
57948	0.94613	2.1
57949	0.94613	2.1
57950	0.946131	2.1
57951	0.946131	2.1
57952	0.946129	2.1
57953	0.946129	2.1
57954	0.94613	2.1
57955	0.94613	2.1
57956	0.94613	2.1
57957	0.94613	2.1
57958	1.89226	2.1
57959	1.89226	2.1
57960	1.89226	2.1
57961	1.89226	2.1
57962	1.89226	2.1
57963	1.89226	2.1
57964	1.42104	2.1
57965	1.4658	2.1
57966	1.51223	2.1
57967	1.42283	2.1
57968	2.93327	2.1
57969	0.732899	2.1
57970	0.756115	2.1
57971	0.629763	2.1
57972	0.559601	2.1
57973	0.646134	2.1
57974	0.646134	2.1
57975	0.669582	2.1
57976	0.669582	2.1
57977	0.756115	2.1
57978	0.629763	2.1
57979	0.732899	2.1
57980	0.559601	2.1
57981	2.566	2.1
57982	1.20242	2.1
57983	2.64084	2.1
57984	2.18297	2.1
57985	1.66029	2.1
57986	1.20242	2.1
57987	0.946133	1.55214
57988	0.946133	1.85331
57989	0.946129	2.1
57990	0.946129	2.1
57991	0.946131	2.1
57992	0.946131	1.47633
57993	0.946131	1.28479
57994	0.946131	2.1
57995	0.946131	2.03725
57996	0.946131	2.0581
57997	0.946131	2.07154

57998	0.946131	2.08555
57999	1.89226	0.05
58000	1.89226	0.05
58001	1.89226	0.05
58002	1.89226	0.05
58003	3.78453	0.05
58004	1.03073	0.05
58005	1.03073	0.05
58006	1.04066	0.05
58007	1.04066	0.05
58008	1.20802	0.05
58009	1.32689	0.05
58010	0.883301	0.05
58011	0.744506	0.05
58012	1.20802	0.05
58013	0.744506	0.05
58014	0.883301	0.05
58015	1.32689	0.05
58016	1.20802	0.05
58017	1.20802	0.05
58018	0.946131	0.05
58019	0.946131	0.05
58020	0.94613	0.05
58021	0.94613	0.05
58022	1.20802	0.05
58023	1.32689	0.05
58024	1.20802	0.05
58025	0.946131	0.05
58026	0.946131	0.05
58027	1.32689	0.05
58028	1.89226	0.05
58029	1.89226	0.05
58030	1.89226	0.05
58031	1.89226	0.05
58032	1.89226	0.05
58033	1.89226	0.05
58034	0.946119	0.05
58035	0.946119	0.05
58036	0.946118	0.05
58037	0.946118	0.0529094
58038	0.946118	0.0516938
58039	0.946118	0.05
58040	0.946119	0.05
58041	0.946119	0.05
58042	0.946131	0.05
58043	0.946131	0.05
58044	0.946106	0.05
58045	0.946106	0.05
58046	1.3939	2.1
58047	1.49641	2.1
58048	1.15096	2.1
58049	1.10101	2.1
58050	2.59742	2.1
58051	1.22106	2.1
58052	2.60663	2.1
58053	1.25467	2.1
58054	1.25467	2.1
58055	2.58377	2.1

58056	2.58377	2.1
58057	1.89225	2.1
58058	2.60664	2.1
58059	2.58377	2.1
58060	2.60664	2.1
58061	1.86938	2.1
58062	1.89225	2.1
58063	1.89225	2.1
58064	1.89225	2.1
58065	1.89225	2.1
58066	1.89225	2.1
58067	1.89225	2.1
58068	1.89225	2.1
58069	0.946121	2.1
58070	0.946121	2.1
58071	0.946126	2.1
58072	0.946126	2.1
58073	0.946124	2.1
58074	0.946124	2.1
58075	0.946123	2.1
58076	0.946123	2.1
58077	0.946117	2.1
58078	0.946117	2.1
58079	0.94613	2.1
58080	0.94613	2.1
58081	1.28017	2.1
58082	1.29189	2.1
58083	0.610534	2.1
58084	0.592373	2.1
58085	0.943764	2.1
58086	0.943764	2.1
58087	0.940501	2.1
58088	0.940501	2.1
58089	1.28017	2.1
58090	0.592371	2.1
58091	0.610533	2.1
58092	1.29189	2.1
58093	0.946133	2.1
58094	0.946133	2.1
58095	1.28017	2.1
58096	1.29189	2.1
58097	0.946132	2.1
58098	0.946132	2.1
58099	1.28017	2.1
58100	1.28017	2.1
58101	1.28017	2.1
58102	0.946132	2.1
58103	0.946133	2.1
58104	1.2919	2.1
58105	1.89226	2.1
58106	1.89226	2.1
58107	1.89226	2.1
58108	1.89226	2.1
58109	1.89226	2.1
58110	1.89226	2.1
58111	1.89226	2.1
58112	1.89226	2.1
58113	1.89226	2.1

58114	1.89226	2.1
58115	3.78452	2.1
58116	1.89226	2.1
58117	2.08196	2.1
58118	1.97854	2.1
58119	2.08196	2.1
58120	1.78884	2.1
58121	1.89226	2.1
58122	1.89226	2.1
58123	1.89226	2.1
58124	1.89226	2.1
58125	1.89226	2.1
58126	1.89226	2.1
58127	1.89226	2.1
58128	1.83688	2.1
58129	1.89226	2.1
58130	1.89226	2.1
58131	1.89226	2.1
58132	1.86015	2.1
58133	1.86015	2.1
58134	1.89226	2.1
58135	1.97854	2.1
58136	1.85883	2.1
58137	1.97854	2.1
58138	1.77255	2.1
58139	1.89226	2.1
58140	0.946131	2.1
58141	0.946131	2.1
58142	0.946131	2.1
58143	0.946131	2.1
58144	0.94613	2.1
58145	0.94613	2.1
58146	0.946132	2.1
58147	0.946132	2.1
58148	0.946131	2.1
58149	0.946131	2.1
58150	0.946131	2.1
58151	0.946131	2.1
58152	0.918412	2.1
58153	0.918412	2.1
58154	0.94893	2.1
58155	0.750211	2.1
58156	0.851541	2.1
58157	0.851541	2.1
58158	0.970566	2.1
58159	0.826567	2.1
58160	0.970554	2.1
58161	1.04071	2.1
58162	0.826632	2.1
58163	0.851554	2.1
58164	1.89226	0.05
58165	1.89226	0.05
58166	1.89226	0.05
58167	1.89226	0.05
58168	3.78453	0.05
58169	0.946131	0.05
58170	0.946131	0.05
58171	0.811232	0.05

58172	0.811232	0.05
58173	0.94613	0.05
58174	0.94613	0.05
58175	0.959701	0.05
58176	0.811233	0.05
58177	0.94613	0.05
58178	0.811233	0.05
58179	0.959702	0.05
58180	0.946131	0.05
58181	1.89226	0.05
58182	1.89226	0.05
58183	1.89226	0.05
58184	1.89226	0.05
58185	1.89226	0.05
58186	1.89226	0.05
58187	1.89227	0.05
58188	1.89227	0.05
58189	1.89227	0.05
58190	1.89227	0.05
58191	1.89227	0.05
58192	1.89227	0.05
58193	1.89226	0.05
58194	1.89226	0.05
58195	1.89226	0.05
58196	1.89226	0.05
58197	3.78453	0.05
58198	1.89226	0.05
58199	1.89226	0.05
58200	1.89226	0.05
58201	1.89226	0.05
58202	1.89226	0.05
58203	1.89226	0.05
58204	1.89226	2.1
58205	2.36204	2.1
58206	2.39853	2.1
58207	2.36204	2.1
58208	1.92876	2.1
58209	1.89226	2.1
58210	1.89226	2.1
58211	1.89226	2.1
58212	1.89226	2.1
58213	1.89226	2.1
58214	3.78453	2.1
58215	0.946128	2.1
58216	0.946128	2.1
58217	1.13182	2.1
58218	1.13182	2.1
58219	0.94613	2.1
58220	0.94613	2.1
58221	1.1749	2.1
58222	1.13182	2.1
58223	1.1749	2.1
58224	0.94613	2.1
58225	1.13182	2.1
58226	0.94613	2.1
58227	1.89226	2.1
58228	2.39853	2.1
58229	2.44918	2.1

58230	2.39853	2.1
58231	1.94291	2.1
58232	1.89226	2.1
58233	0.946132	2.1
58234	0.946132	2.1
58235	0.946131	2.1
58236	0.946131	2.1
58237	0.946132	2.1
58238	0.946132	2.1
58239	0.946131	2.1
58240	0.946131	2.1
58241	0.946131	2.1
58242	0.946131	2.1
58243	0.946132	2.1
58244	0.946132	2.1
58245	0.946128	2.1
58246	0.946128	2.1
58247	1.1749	2.1
58248	1.1749	2.1
58249	0.946131	2.1
58250	0.946131	2.1
58251	1.23631	2.1
58252	1.1749	2.1
58253	1.23631	2.1
58254	0.94613	2.1
58255	1.1749	2.1
58256	0.946131	2.1
58257	1.89226	2.1
58258	1.89226	2.1
58259	1.89226	2.1
58260	1.89226	2.1
58261	3.78453	2.1
58262	1.38859	2.1
58263	1.45333	2.1
58264	1.28909	2.1
58265	1.38091	2.1
58266	2.74242	2.1
58267	1.45332	2.1
58268	1.38414	2.1
58269	1.38091	2.1
58270	1.33506	2.1
58271	2.78838	2.1
58272	1.89226	2.1
58273	1.89226	2.1
58274	1.89226	2.1
58275	1.89226	2.1
58276	3.78452	2.1
58277	1.89226	2.1
58278	1.89226	2.1
58279	1.89226	2.1
58280	1.89226	2.1
58281	1.89226	2.1
58282	1.89226	2.1
58283	1.17418	2.1
58284	1.38091	2.1
58285	1.17418	2.1
58286	1.28909	2.1
58287	1.29741	2.1

58288	1.41232	2.1
58289	1.38091	2.1
58290	1.33506	2.1
58291	1.27575	2.1
58292	1.27575	2.1
58293	1.29741	2.1
58294	1.35671	2.1
58295	1.89226	2.1
58296	1.89226	2.1
58297	1.89226	2.1
58298	1.89226	2.1
58299	3.78452	2.1
58300	1.92737	0.0602644
58301	2.40223	0.0580283
58302	1.56178	0.05
58303	1.97605	0.0865
58304	3.90343	0.0547736
58305	1.89226	0.065411
58306	1.89226	0.130717
58307	1.89226	0.0959351
58308	1.89226	0.0907978
58309	3.78452	0.0977741
58310	1.89226	0.134425
58311	1.89226	0.105661
58312	1.89226	0.0982124
58313	1.89226	0.157701
58314	1.89226	0.0942312
58315	1.89226	0.153829
58316	1.13641	0.098582
58317	1.13641	0.0876341
58318	1.12763	0.0563605
58319	1.12763	0.0694489
58320	1.29348	0.0704648
58321	1.06293	0.108261
58322	1.20111	0.0579363
58323	0.988028	0.0878772
58324	0.988028	0.0801994
58325	1.06293	0.080093
58326	1.29348	0.0723772
58327	1.20111	0.0724417
58328	1.56176	0.0513004
58329	1.46387	0.0826
58330	1.97603	0.0626546
58331	1.97603	0.0619926
58332	1.08211	0.0619711
58333	1.08211	0.0624625
58334	1.89224	0.0889793
58335	1.89224	0.0866369
58336	1.89224	0.0766211
58337	1.89224	0.11232
58338	1.89224	0.0689137
58339	1.89224	0.106772
58340	1.89224	0.117505
58341	1.89224	0.10838
58342	1.89224	0.0887752
58343	1.89224	0.15465
58344	3.78448	0.112243
58345	0.820185	0.0642906

58346	0.818947	0.0887701
58347	0.911041	0.104302
58348	0.695265	0.0863379
58349	1.46388	0.0849484
58350	1.23607	0.083863
58351	0.927068	0.0827862
58352	1.02691	0.108588
58353	1.12918	0.0645114
58354	0.846843	0.0822786
58355	0.911039	0.0862776
58356	1.29129	2.1
58357	1.45547	2.1
58358	1.67327	2.1
58359	1.66698	2.1
58360	1.20149	2.1
58361	1.20149	2.1
58362	0.946133	2.1
58363	0.946133	2.1
58364	0.946129	2.1
58365	0.946129	2.1
58366	0.946131	2.1
58367	0.946131	2.09994
58368	0.946131	2.1
58369	0.946131	2.1
58370	0.946131	2.1
58371	0.946131	2.1
58372	0.946131	2.1
58373	0.946131	2.1
58374	1.89226	2.1
58375	1.89226	2.09725
58376	1.89226	2.1
58377	1.89226	1.97888
58378	3.78452	2.09822
58379	0.86875	2.09757
58380	0.86875	2.1
58381	0.899002	2.1
58382	0.966245	2.1
58383	0.849364	2.04696
58384	1.01918	2.0966
58385	0.764737	2.1
58386	0.89249	2.1
58387	0.764624	2.1
58388	0.967869	2.1
58389	0.808606	2.1
58390	0.864506	2.1
58391	0.773929	2.1
58392	0.773929	2.1
58393	0.781991	2.1
58394	0.836559	2.1
58395	0.983761	2.1
58396	0.834687	2.1
58397	0.680545	2.1
58398	0.640799	2.1
58399	0.70286	2.1
58400	0.935844	2.1
58401	0.629479	2.1
58402	0.823655	2.1
58403	0.946133	2.1

58404	0.946133	2.1
58405	0.946128	2.1
58406	0.946128	2.1
58407	0.94613	2.1
58408	0.94613	2.1
58409	0.946131	2.1
58410	0.946131	2.1
58411	0.946131	2.1
58412	0.946131	2.1
58413	0.946131	2.1
58414	0.946131	2.1
58415	1.89226	2.1
58416	1.89226	2.0475
58417	1.89226	2.1
58418	1.89226	2.1
58419	1.89226	2.07976
58420	1.89226	2.1
58421	0.931638	2.1
58422	0.931638	2.09273
58423	1.03559	2.1
58424	0.977442	2.03271
58425	0.826487	1.46846
58426	0.993902	2.0545
58427	1.04922	2.1
58428	0.894324	2.1
58429	0.912984	2.1
58430	1.01156	2.1
58431	0.944692	2.1
58432	1.15456	2.09786
58433	1.89226	0.0791137
58434	1.89226	0.112404
58435	1.89226	0.154768
58436	1.89226	0.0683674
58437	3.78453	0.0754199
58438	1.89226	0.0717513
58439	1.89226	0.0606337
58440	2.64262	0.0713525
58441	2.64262	0.05
58442	1.78052	0.05
58443	1.03015	0.0579631
58444	0.999721	0.0529101
58445	0.659011	0.05
58446	1.15564	0.0951786
58447	1.41388	0.0612749
58448	1.01583	0.0650837
58449	1.01583	0.0510865
58450	1.05706	0.0951474
58451	1.05706	0.0626175
58452	1.15564	0.0607265
58453	0.65901	0.0552952
58454	1.41388	0.0598111
58455	0.999721	0.0593666
58456	1.89226	2.1
58457	1.89226	2.1
58458	1.89226	2.1
58459	1.89226	2.1
58460	3.78453	2.1
58461	2.39022	2.1

58462	1.90734	2.1
58463	2.27634	2.1
58464	1.82666	2.1
58465	4.18368	2.1
58466	1.90734	2.1
58467	1.21564	2.1
58468	1.82666	2.1
58469	1.16938	2.1
58470	3.07672	2.1
58471	1.89226	2.1
58472	1.89226	2.1
58473	1.89226	2.1
58474	1.89226	2.1
58475	1.89226	2.1
58476	1.89226	2.1
58477	1.89227	2.1
58478	1.89227	2.1
58479	1.89227	2.1
58480	1.89227	2.1
58481	3.78453	2.1
58482	2.27634	2.1
58483	1.82666	2.1
58484	2.18042	2.1
58485	1.74738	2.1
58486	4.02372	2.1
58487	1.74738	2.1
58488	1.82666	2.1
58489	1.1181	2.1
58490	1.1181	2.1
58491	1.16938	2.1
58492	1.79865	2.1
58493	1.89226	2.1
58494	1.89226	2.1
58495	1.89226	2.1
58496	1.89226	2.1
58497	1.89226	2.1
58498	1.89226	2.1
58499	1.89226	1.20907
58500	1.89226	0.388821
58501	1.89226	0.994618
58502	1.89226	0.482783
58503	1.89226	1.26089
58504	1.89226	0.59893
58505	0.790736	0.514403
58506	1.08265	0.363471
58507	0.938909	1.07142
58508	0.638683	0.848784
58509	0.858588	1.10946
58510	0.858588	0.376811
58511	0.862743	1.19109
58512	0.862743	0.537908
58513	1.08265	1.08244
58514	0.938911	0.669518
58515	0.790735	1.07058
58516	0.638685	0.540204
58517	1.89226	0.450727
58518	1.89226	1.0479
58519	1.89226	1.09103

58520	1.89226	0.439876
58521	1.89226	1.26804
58522	1.89226	0.851757
58523	1.89226	0.447335
58524	1.89226	1.25727
58525	1.89226	1.04552
58526	1.89226	0.562863
58527	1.89226	1.19999
58528	1.89226	1.11736
58529	1.08265	0.392199
58530	1.36209	0.370557
58531	1.25233	0.591911
58532	0.938909	0.668445
58533	1.14195	0.599859
58534	1.14195	0.326187
58535	1.15904	1.21629
58536	1.15904	0.523282
58537	1.36209	0.938566
58538	1.25233	0.306731
58539	1.08265	0.782598
58540	0.938911	0.408253
58541	1.22077	0.384698
58542	0.915244	0.411356
58543	0.967835	0.685408
58544	0.742495	0.859361
58545	0.78648	0.700225
58546	0.985041	0.40695
58547	0.989098	1.16754
58548	0.989098	0.488956
58549	1.03206	0.634586
58550	0.967897	0.939356
58551	0.915404	0.95869
58552	0.706105	0.638051
58553	2.69662	0.05
58554	1.89226	0.05
58555	2.35499	0.05
58556	1.89226	0.05
58557	4.24725	0.05
58558	1.89227	0.05
58559	1.89227	0.05
58560	1.89227	0.05
58561	1.89227	0.05
58562	3.78453	0.05
58563	2.35499	0.05
58564	1.70302	0.05
58565	1.70302	0.05
58566	1.703 0.05	
58567	1.97374	0.05
58568	1.97372	0.05
58569	0.946133	0.05
58570	0.946133	0.05
58571	0.946132	0.05
58572	0.946132	0.05
58573	0.946133	0.05
58574	0.946133	0.05
58575	0.946132	0.05
58576	0.946132	0.05
58577	0.946133	0.05

58578	0.946133	0.05
58579	0.946132	0.05
58580	0.946132	0.05
58581	0.6794	2.1
58582	0.6794	2.1
58583	0.670812	2.1
58584	0.62182	2.1
58585	0.648326	2.1
58586	0.792154	2.1
58587	0.719167	2.1
58588	0.494515	2.1
58589	0.649421	2.1
58590	0.804869	2.1
58591	0.485585	2.1
58592	0.627666	2.1
58593	1.79347	2.1
58594	1.03968	2.1
58595	1.70303	2.1
58596	0.933265	2.07405
58597	2.62068	2.1
58598	1.89226	2.1
58599	1.89226	2.1
58600	1.89226	2.1
58601	1.89226	2.09956
58602	3.78452	2.1
58603	1.89226	2.1
58604	2.24604	2.1
58605	2.0554	2.1
58606	2.0554	2.1
58607	1.89226	2.1
58608	1.89226	2.1
58609	1.89226	2.1
58610	1.89226	2.06592
58611	1.89226	2.1
58612	1.89226	2.1
58613	1.89226	2.1
58614	1.89226	2.1
58615	2.0815	2.1
58616	2.64424	1.379
58617	2.80807	1.99941
58618	3.232 2.1	
58619	1.89417	2.04553
58620	2.18012	2.1
58621	0.946131	2.1
58622	0.946131	2.02722
58623	0.946131	2.1
58624	0.946131	2.1
58625	0.94613	2.1
58626	0.94613	2.05883
58627	0.946132	2.1
58628	0.946132	2.1
58629	0.946131	2.1
58630	0.946131	2.1
58631	0.946131	2.1
58632	0.946131	2.1
58633	1.89227	0.0529248
58634	1.89227	0.0966906
58635	1.89227	0.112933

58636	1.89227	0.0527974
58637	3.78453	0.0633871
58638	2.70881	0.05
58639	2.68687	0.05
58640	2.03046	0.05
58641	1.9682	0.05
58642	4.67701	0.05
58643	1.08518	0.05
58644	1.08518	0.05
58645	1.09033	0.05
58646	1.09033	0.05
58647	0.842273	0.05
58648	1.19141	0.05
58649	1.34343	0.05
58650	0.9841	0.05
58651	0.9841	0.05
58652	0.842273	0.05
58653	1.34344	0.05
58654	1.19141	0.05
58655	0.946133	0.05
58656	0.946133	0.05
58657	0.946132	0.103458
58658	0.946132	0.0787363
58659	0.946132	0.0636785
58660	0.946132	0.05
58661	0.946132	0.100291
58662	0.946132	0.0705848
58663	0.946132	0.05
58664	0.946132	0.0690674
58665	0.946133	0.0827285
58666	0.946133	0.0647615
58667	1.89227	0.0818956
58668	1.89227	0.05
58669	1.89227	0.0754949
58670	1.89227	0.0564991
58671	1.89227	0.0587585
58672	1.89227	0.05
58673	2.03046	0.05
58674	1.9682	0.05
58675	1.23886	0.05
58676	1.12971	0.05
58677	3.20706	0.05
58678	1.89226	0.260022
58679	1.89226	0.800383
58680	1.89226	0.257259
58681	1.89226	0.569799
58682	3.78452	0.385838
58683	2.45569	0.142986
58684	1.89226	0.184135
58685	2.22922	0.133109
58686	2.0815	0.258802
58687	1.85074	0.105025
58688	1.70303	0.204251
58689	2.72856	0.150316
58690	1.89226	0.333048
58691	1.89226	0.146512
58692	1.89226	0.245582
58693	3.78452	0.203747

58694	1.89226	0.34467
58695	1.89226	0.708548
58696	1.89226	0.238333
58697	1.89226	0.745828
58698	3.78452	0.485712
58699	0.946131	0.650861
58700	0.946131	0.216888
58701	0.946131	0.400751
58702	0.946131	0.179678
58703	0.94613	0.379108
58704	0.94613	0.472503
58705	0.946132	0.216308
58706	0.946132	0.234334
58707	0.946131	0.314302
58708	0.946131	0.304017
58709	0.946131	0.301176
58710	0.946131	0.316668
58711	2.0595	0.13583
58712	2.58581	0.101135
58713	1.48694	0.104961
58714	1.48694	0.118602
58715	2.60157	0.108984
58716	2.02901	0.118536
58717	1.89226	0.119983
58718	1.89226	0.17094
58719	1.89226	0.128701
58720	1.89226	0.136668
58721	1.89226	0.134466
58722	1.89226	0.151416
58723	2.04728	0.132327
58724	2.64161	0.0945131
58725	1.47606	0.107946
58726	1.47606	0.123645
58727	2.63069	0.109442
58728	2.05947	0.123164
58729	1.89224	0.100916
58730	1.89224	0.151265
58731	1.89224	0.116522
58732	1.89224	0.134052
58733	1.89224	0.111429
58734	1.89224	0.12169
58735	0.946114	0.158963
58736	0.946114	0.117224
58737	0.946118	0.16491
58738	0.946118	0.13189
58739	0.946114	0.171011
58740	0.946114	0.162809
58741	0.946118	0.13205
58742	0.946118	0.12196
58743	0.946128	0.149191
58744	0.946128	0.151509
58745	0.946103	0.141971
58746	0.946103	0.144435
58747	1.1233	2.0752
58748	1.15009	2.1
58749	2.36036	2.09347
58750	2.38225	2.1
58751	3.50554	2.1

58752	2.38224	2.1
58753	0.977775	2.1
58754	2.20473	2.1
58755	2.20473	2.1
58756	1.15009	2.1
58757	1.15009	2.1
58758	0.946119	2.1
58759	0.946119	2.0933
58760	1.19111	2.1
58761	1.19111	2.08413
58762	0.946118	2.1
58763	1.19111	2.1
58764	1.18016	2.08803
58765	0.946118	1.92377
58766	0.94613	1.76639
58767	0.94613	2.1
58768	1.18015	2.07183
58769	1.19109	2.08462
58770	0.946118	2.1
58771	0.946118	2.1
58772	1.10235	2.1
58773	1.10235	2.1
58774	0.946118	2.1
58775	1.10235	2.1
58776	0.946115	2.1
58777	1.1911	2.1
58778	0.946129	2.0999
58779	0.946129	2.1
58780	1.19109	2.1
58781	1.10234	2.1
58782	1.89226	2.1
58783	2.23932	2.1
58784	2.11568	2.1
58785	2.23932	2.1
58786	1.76862	2.1
58787	1.89226	2.1
58788	1.89226	2.1
58789	1.89226	2.1
58790	1.89226	2.1
58791	1.89226	2.1
58792	1.89226	2.1
58793	1.89226	2.1
58794	2.07838	2.1
58795	1.89226	2.1
58796	1.89226	2.1
58797	1.89226	2.1
58798	2.10264	2.1
58799	2.10264	2.1
58800	1.89226	2.1
58801	2.11568	2.1
58802	1.99753	2.1
58803	2.11568	2.1
58804	1.77411	2.1
58805	1.89226	2.1
58806	0.946128	2.1
58807	0.946128	2.1
58808	0.946133	2.1
58809	0.946133	2.1

58810	0.94613	2.1
58811	0.94613	2.1
58812	0.946131	2.1
58813	0.946131	2.1
58814	0.946131	2.1
58815	0.946131	2.1
58816	0.946131	2.1
58817	0.946131	2.1
58818	0.946132	2.1
58819	0.946132	2.1
58820	1.03919	2.1
58821	1.03919	2.1
58822	0.946131	2.1
58823	0.946131	2.1
58824	1.02744	2.1
58825	1.03919	2.1
58826	1.02744	2.1
58827	0.946132	2.1
58828	1.03919	2.1
58829	0.946132	2.1
58830	1.64834	1.85626
58831	1.82955	1.63174
58832	2.33824	1.43567
58833	2.19301	1.82861
58834	3.98658	1.67593
58835	1.40574	1.90554
58836	2.19301	1.59987
58837	1.64834	1.85801
58838	1.64834	1.71262
58839	1.99961	1.62163
58840	1.99961	1.56787
58841	2.33824	1.36952
58842	2.19301	1.83695
58843	2.68524	1.70171
58844	2.81847	1.50395
58845	5.02349	1.63549
58846	1.99961	1.85214
58847	2.19301	1.48712
58848	2.68524	1.60925
58849	2.50732	1.62731
58850	4.68485	1.63837
58851	1.89226	2.1
58852	1.89226	2.1
58853	1.89226	2.1
58854	1.89226	2.1
58855	3.78453	2.1
58856	1.75142	2.1
58857	2.47026	2.1
58858	1.46811	2.1
58859	2.26652	2.1
58860	4.01794	2.1
58861	1.89226	2.1
58862	1.89226	2.1
58863	1.89226	2.1
58864	1.89226	2.1
58865	1.89226	2.1
58866	1.89226	2.1
58867	1.89226	2.1

58868	1.89226	2.1
58869	1.89226	2.1
58870	1.89226	2.1
58871	1.89226	2.1
58872	1.89226	2.1
58873	0.812082	2.1
58874	0.878759	2.1
58875	0.882782	2.1
58876	0.882782	2.1
58877	1.05572	2.1
58878	1.04037	2.1
58879	0.73849	2.1
58880	0.626857	2.1
58881	0.638677	2.1
58882	1.05581	2.1
58883	0.715695	2.1
58884	1.10492	2.1
58885	1.17058	2.1
58886	1.07863	2.1
58887	1.21113	2.1
58888	1.21113	2.1
58889	1.2811	2.1
58890	1.37319	2.1
58891	1.01788	2.1
58892	0.999492	2.1
58893	1.04352	2.1
58894	1.32112	2.1
58895	1.10492	2.1
58896	1.34699	2.1
58897	1.89226	2.1
58898	1.89226	2.1
58899	1.89226	2.1
58900	1.89226	2.1
58901	1.89226	2.1
58902	1.89226	2.1
58903	1.19813	0.05
58904	1.19813	0.05
58905	2.43432	0.05
58906	1.21716	0.05
58907	0.946124	0.05
58908	1.21715	0.05
58909	0.946118	0.05
58910	2.56627	2.1
58911	1.89226	2.1
58912	1.89226	2.1
58913	1.89226	2.1
58914	2.43434	2.1
58915	2.43434	2.1
58916	1.89227	0.05
58917	1.89227	0.05
58918	1.89227	0.05
58919	1.89227	0.05
58920	1.89227	0.05
58921	1.89227	0.05
58922	1.89226	0.05
58923	1.89226	0.0686573
58924	1.89226	0.0715597
58925	1.89226	0.05

58926	3.78453	0.0550373
58927	1.89226	0.05
58928	2.0764	0.05
58929	1.89226	0.05
58930	2.81674	0.05
58931	1.89226	0.05
58932	2.81674	0.05
58933	0.946132	0.05
58934	0.946132	0.05
58935	0.946131	0.065577
58936	0.946131	0.05
58937	0.946132	0.05
58938	0.946132	0.05
58939	0.946132	0.0651243
58940	0.946132	0.05
58941	0.946132	0.05
58942	0.946132	0.05
58943	0.946132	0.05
58944	0.946132	0.05
58945	0.578684	1.62772
58946	0.578684	2.1
58947	0.589487	2.04708
58948	0.589487	2.1
58949	0.625461	1.94977
58950	0.5378	1.99051
58951	0.608714	2.1
58952	0.54271	1.9714
58953	0.608714	1.54151
58954	0.625461	1.15874
58955	0.54271	2.02852
58956	0.537801	1.97742
58957	0.946128	1.07257
58958	0.946128	2.0297
58959	0.946133	0.722742
58960	0.946133	2.1
58961	0.946131	0.855815
58962	0.946131	1.08334
58963	0.946131	1.0237
58964	0.946131	1.65876
58965	0.94613	0.827279
58966	0.94613	0.653683
58967	0.946131	1.18939
58968	0.946131	1.49249
58969	1.21743	2.08313
58970	1.25092	2.09942
58971	1.38847	1.9507
58972	1.46031	0.759039
58973	2.67774	1.86875
58974	0.946128	0.808785
58975	0.946128	2.09261
58976	0.946132	2.1
58977	0.946132	0.781285
58978	0.94613	1.36685
58979	0.94613	0.758045
58980	0.94613	1.72163
58981	0.94613	1.10906
58982	0.94613	0.644893
58983	0.94613	0.671029

58984	0.94613	1.47886
58985	0.94613	1.07199
58986	1.89226	0.354359
58987	1.89226	1.06952
58988	1.89226	0.574891
58989	1.89226	2.06418
58990	1.89226	2.0851
58991	1.89226	2.02956
58992	0.539566	0.955766
58993	0.534046	2.1
58994	0.577914	1.85542
58995	0.471623	1.88035
58996	1.46031	0.250148
58997	0.945865	0.742292
58998	0.770603	0.349951
58999	0.737201	2.03408
59000	0.714829	0.835123
59001	0.536085	1.74675
59002	0.471617	1.37102
59003	1.89226	1.96568
59004	1.89226	1.20913
59005	1.89226	1.17839
59006	1.89226	1.53102
59007	1.89226	1.80015
59008	1.89226	1.95181
59009	1.89225	1.41334
59010	1.89225	0.620548
59011	1.65572	0.688479
59012	1.65572	0.791752
59013	1.89225	1.12165
59014	1.89225	1.45555
59015	1.89226	1.38399
59016	1.89226	0.87254
59017	1.89226	1.29451
59018	1.89226	0.591093
59019	3.78453	1.35174
59020	1.89226	1.81243
59021	1.89226	0.788274
59022	1.89226	0.807041
59023	1.89226	1.37941
59024	1.89226	1.49566
59025	1.89226	1.84214
59026	1.89225	1.25463
59027	1.89225	0.396359
59028	1.89225	0.485949
59029	1.89225	0.6383
59030	1.89225	0.787498
59031	1.89225	1.25817
59032	0.763592	0.589885
59033	0.763592	0.983009
59034	0.987417	0.692207
59035	0.987417	0.701075
59036	0.763592	0.446594
59037	0.804881	0.391135
59038	0.946126	0.688892
59039	0.946126	0.708995
59040	0.804879	0.418377
59041	0.94613	0.676969

59042	0.946129	0.787145
59043	0.76359	0.500926
59044	1.46055	2.1
59045	2.04945	2.1
59046	2.04587	2.1
59047	2.04587	2.1
59048	1.50714	2.1
59049	1.50714	2.1
59050	2.04587	2.1
59051	2.38681	2.1
59052	2.42082	2.1
59053	2.42082	2.1
59054	2.04945	2.1
59055	2.04945	2.1
59056	0.872964	2.1
59057	0.872964	2.1
59058	0.884806	2.1
59059	0.884806	2.1
59060	1.0275	2.1
59061	1.02293	2.1
59062	0.711243	2.1
59063	0.730273	2.1
59064	0.711244	2.1
59065	1.0275	2.1
59066	0.730273	2.1
59067	1.02293	2.1
59068	2.04587	2.1
59069	2.47753	2.1
59070	2.42994	2.1
59071	2.42082	2.1
59072	2.05499	2.1
59073	2.04587	2.1
59074	0.946132	2.1
59075	0.946132	2.1
59076	1.13078	2.1
59077	1.07817	2.1
59078	0.946132	2.1
59079	0.946132	2.1
59080	1.07817	2.1
59081	1.07817	2.1
59082	0.946132	2.1
59083	1.07817	2.1
59084	1.13078	2.1
59085	0.946132	2.1
59086	1.89226	2.1
59087	1.89226	2.1
59088	1.89226	2.1
59089	1.89226	2.1
59090	1.89226	2.1
59091	1.89226	2.1
59092	1.89226	2.1
59093	1.89226	2.1
59094	1.89226	2.1
59095	1.89226	2.1
59096	1.89226	2.1
59097	1.89226	2.1
59098	1.89226	2.1
59099	1.89226	2.1

59100	1.8173	2.1
59101	2.66885	2.1
59102	2.7438	2.1
59103	2.7438	2.1
59104	1.89226	0.85451
59105	1.89226	0.0872523
59106	1.89226	0.115447
59107	1.89226	0.687254
59108	3.78453	0.616943
59109	1.89226	0.255522
59110	1.89226	0.371759
59111	1.89226	0.516589
59112	1.89226	1.1994
59113	1.89226	0.188876
59114	1.89226	0.686614
59115	1.89226	0.249945
59116	1.97385	0.510527
59117	1.7531	0.608829
59118	1.89226	1.49801
59119	1.7531	0.141039
59120	1.89226	0.328036
59121	0.946131	0.660996
59122	0.840904	0.143753
59123	0.684333	0.0879062
59124	0.94613	0.0950068
59125	0.972337	0.0995962
59126	0.946278	0.825654
59127	0.684304	0.0938484
59128	0.684304	0.361458
59129	0.999838	0.11793
59130	0.759605	0.107713
59131	0.851528	0.0869551
59132	0.851528	0.11855
59133	1.89226	0.103791
59134	2.23818	0.237921
59135	1.79771	0.136311
59136	1.89226	1.09842
59137	1.79771	0.0709288
59138	1.89226	0.182085
59139	2.79647	2.1
59140	2.01613	2.1
59141	1.46826	2.1
59142	2.54504	2.1
59143	4.56117	2.1
59144	1.89226	2.1
59145	1.89226	2.1
59146	1.89226	2.1
59147	1.89226	2.1
59148	1.89226	2.1
59149	1.89226	2.1
59150	1.89226	2.1
59151	2.66884	2.1
59152	2.56022	2.1
59153	2.66884	2.1
59154	1.78364	2.1
59155	1.89226	2.1
59156	1.14282	2.1
59157	1.24068	2.1

59158	0.880748	2.1
59159	0.834916	2.1
59160	1.05539	2.1
59161	1.05539	2.1
59162	1.04312	2.1
59163	1.04313	2.1
59164	1.2015	2.1
59165	0.83492	2.1
59166	0.789149	2.1
59167	1.24066	2.1
59168	1.89226	2.1
59169	1.89226	2.1
59170	1.89226	2.1
59171	1.89226	2.1
59172	3.78453	2.1
59173	0.946128	2.1
59174	0.946128	2.1
59175	1.28011	2.1
59176	1.22372	2.1
59177	0.946131	2.1
59178	0.946131	2.1
59179	1.22372	2.1
59180	1.22372	2.1
59181	1.22372	2.1
59182	0.94613	2.1
59183	0.946131	2.1
59184	1.28011	2.1
59185	2.28604	2.1
59186	1.34268	2.1
59187	2.43157	2.1
59188	1.50172	2.1
59189	3.77425	2.1
59190	2.6096	2.1
59191	2.43157	2.1
59192	1.66774	2.1
59193	1.66774	2.1
59194	1.50172	2.1
59195	2.43157	2.1
59196	2.16067	2.1
59197	1.08975	2.1
59198	2.19336	2.1
59199	2.06984	2.1
59200	1.21327	2.1
59201	1.08975	2.1
59202	2.28604	2.1
59203	1.21327	2.1
59204	2.29008	2.1
59205	2.16067	2.1
59206	1.34268	2.1
59207	1.21327	2.1
59208	1.89226	0.261091
59209	1.89226	0.317092
59210	1.89226	0.264141
59211	1.89226	0.135669
59212	3.78452	0.127622
59213	1.89804	0.229039
59214	2.21656	0.243835
59215	1.70537	0.114863

59216	2.50023	0.101251
59217	3.8982	0.133174
59218	1.03136	0.101437
59219	1.89486	0.118292
59220	0.949364	0.113883
59221	1.82283	0.0663981
59222	2.84422	0.088373
59223	1.89226	0.119569
59224	1.89226	0.111502
59225	1.89226	0.122934
59226	1.89226	0.135962
59227	3.78452	0.204759
59228	0.946132	0.232079
59229	0.946132	0.209726
59230	0.946129	0.219515
59231	0.946129	0.125071
59232	0.94613	0.130008
59233	0.94613	0.412075
59234	0.946131	0.12608
59235	0.946131	0.241825
59236	0.946131	0.443136
59237	0.946131	0.367517
59238	0.946131	0.281723
59239	0.946131	0.556317
59240	1.09466	0.179002
59241	1.09466	0.0854329
59242	0.866512	0.0604851
59243	1.08352	0.059903
59244	1.19189	0.05
59245	1.21883	0.123505
59246	0.971139	0.155282
59247	0.873213	0.0582863
59248	0.873202	0.0793853
59249	1.00855	0.05
59250	0.923735	0.0711983
59251	1.21885	0.0680173
59252	1.89225	0.05
59253	2.40019	0.05
59254	1.89225	0.363174
59255	1.89225	0.482067
59256	1.89225	0.352082
59257	1.89225	0.666396
59258	1.89225	0.363174
59259	1.89225	0.739902
59260	2.05088	0.352082
59261	1.89225	0.233999
59262	1.89225	0.286614
59263	1.89225	0.414875
59264	2.03956	0.352082
59265	2.03956	0.482067
59266	1.89226	2.1
59267	2.40021	2.1
59268	2.38473	2.07996
59269	2.40021	2.1
59270	1.87679	2.1
59271	1.89226	2.1
59272	1.89226	2.1
59273	1.89226	2.03925

59274	1.89226	2.1
59275	1.89226	2.00235
59276	1.89226	2.1
59277	1.89226	2.06997
59278	2.0692	2.1
59279	1.89226	2.07307
59280	1.89226	2.1
59281	1.89226	1.94782
59282	2.05089	2.1
59283	2.05089	2.0368
59284	1.89226	2.1
59285	1.89226	2.1
59286	1.89226	2.1
59287	1.89226	2.1
59288	3.78452	2.1
59289	1.89226	2.1
59290	2.50051	2.1
59291	1.89226	2.1
59292	2.30076	2.1
59293	4.39277	2.1
59294	2.50052	2.1
59295	1.70377	2.1
59296	2.30076	2.1
59297	1.54231	2.1
59298	4.04283	2.1
59299	1.89226	2.1
59300	1.89226	2.1
59301	1.89226	2.1
59302	1.89226	2.1
59303	1.89226	2.1
59304	1.89226	2.1
59305	1.89226	2.1
59306	1.89226	2.1
59307	1.89226	2.1
59308	1.89226	2.1
59309	3.78452	2.1
59310	2.07948	2.1
59311	2.30076	2.1
59312	1.3294	2.1
59313	1.3294	2.1
59314	1.54232	2.1
59315	2.29239	2.1
59316	0.810947	2.1
59317	0.810947	2.1
59318	0.810946	2.1
59319	0.810946	2.1
59320	1.89226	2.1
59321	1.35169	2.1
59322	1.08132	2.1
59323	1.08132	2.1
59324	1.08132	2.1
59325	0.810946	2.1
59326	0.810946	2.1
59327	1.34851	0.872325
59328	0.997471	2.06559
59329	1.62287	0.666509
59330	1.27096	1.799
59331	2.62034	1.46189

59332	1.89226	1.75018
59333	1.89226	2.07452
59334	1.89226	0.834867
59335	1.89226	2.1
59336	3.78452	1.94768
59337	0.946128	2.1
59338	0.946128	2.1
59339	1.29331	1.98158
59340	1.29331	2.1
59341	1.29331	2.1
59342	1.10699	2.1
59343	0.946131	1.95972
59344	0.946131	2.1
59345	0.94613	2.1
59346	1.29331	2.1
59347	1.10699	2.1
59348	0.946131	2.1
59349	1.62287	0.835026
59350	1.27096	1.94618
59351	1.86067	0.341409
59352	1.58407	1.85477
59353	3.20694	1.44505
59354	1.89226	0.975541
59355	1.89226	2.1
59356	1.89226	1.21122
59357	1.89226	1.89045
59358	3.78452	1.87001
59359	2.62761	0.117287
59360	2.55849	1.21154
59361	2.12261	0.953097
59362	2.22939	0.421847
59363	4.78788	0.594485
59364	0.772522	2.09245
59365	0.839588	1.98272
59366	1.31126	1.14023
59367	1.08787	0.589549
59368	1.01318	1.21625
59369	1.01318	2.09585
59370	0.900815	2.04314
59371	1.10799	0.687694
59372	0.772511	1.77066
59373	1.03473	1.72557
59374	1.31126	1.64277
59375	0.841464	1.70175
59376	1.89227	1.20234
59377	1.89227	2.1
59378	1.89227	1.86011
59379	1.89227	2.08909
59380	1.89227	1.72086
59381	1.89227	2.09199
59382	1.89226	0.437772
59383	1.89226	1.80303
59384	1.89226	0.655665
59385	1.89226	1.32715
59386	1.89226	0.652522
59387	1.89226	1.32275
59388	2.12261	1.34943
59389	2.22939	0.327244

59390	1.97004	0.343005
59391	1.8072	1.59908
59392	4.03658	0.712023
59393	1.5847	2.07028
59394	1.8072	1.41186
59395	2.12262	1.34673
59396	2.12262	2.09077
59397	1.23267	1.31613
59398	1.23267	2.09511
59399	1.89227	1.45723
59400	1.89227	2.1
59401	1.89227	1.85209
59402	1.89227	2.09205
59403	1.89227	1.77219
59404	1.89227	2.09383
59405	1.89226	0.348537
59406	1.89226	1.79001
59407	1.89226	1.66429
59408	1.89226	0.59224
59409	3.78452	1.03084
59410	2.49093	2.1
59411	2.67393	2.1
59412	2.47932	2.1
59413	2.47932	2.1
59414	2.67994	2.1
59415	2.67994	2.1
59416	2.49873	2.1
59417	2.67393	2.1
59418	2.67993	2.1
59419	2.69807	2.1
59420	2.49873	2.1
59421	2.51686	2.1
59422	2.49093	2.1
59423	2.67994	2.1
59424	2.69784	2.1
59425	2.51651	2.1
59426	5.18877	2.1
59427	1.24359	2.1
59428	1.24936	2.1
59429	1.33997	2.1
59430	1.34892	2.1
59431	1.28805	2.1
59432	1.28805	2.1
59433	1.2955	2.1
59434	1.2955	2.1
59435	1.34892	2.1
59436	1.24359	2.1
59437	1.24936	2.1
59438	1.33997	2.1
59439	1.15506	2.03281
59440	1.15506	2.1
59441	0.946128	2.1
59442	0.946128	2.1
59443	0.946131	2.1
59444	1.15506	2.03381
59445	1.06997	2.1
59446	0.946128	2.1
59447	1.06997	2.09633

59448	1.15506	2.09909
59449	0.946129	2.1
59450	0.946129	2.1
59451	1.70608	2.1
59452	2.13994	2.1
59453	1.33384	2.1
59454	1.43635	2.1
59455	3.14243	2.1
59456	2.13994	2.1
59457	2.31012	2.1
59458	1.43635	2.1
59459	1.23007	2.1
59460	3.74646	2.1
59461	2.46361	2.1
59462	1.89226	2.1
59463	1.89226	2.1
59464	1.89226	2.1
59465	2.39045	2.1
59466	2.39045	2.1
59467	1.89226	2.1
59468	1.89226	2.07585
59469	1.89226	2.1
59470	1.89226	1.88358
59471	3.78452	2.1
59472	1.52984	2.1
59473	2.25682	1.7778
59474	2.20831	1.68667
59475	2.20831	2.1
59476	1.61178	1.66045
59477	1.61178	2.1
59478	2.47395	2.1
59479	1.89226	2.1
59480	1.89226	2.1
59481	1.89226	2.1
59482	2.46362	2.1
59483	2.46362	2.1
59484	0.946131	1.75709
59485	0.946131	2.1
59486	0.946131	2.1
59487	0.946131	2.09012
59488	0.94613	1.95952
59489	0.94613	2.052
59490	0.946132	2.1
59491	0.946132	2.1
59492	0.946131	2.1
59493	0.946131	2.1
59494	0.946131	2.1
59495	0.946131	2.1
59496	0.982398	2.1
59497	0.982398	2.09988
59498	0.931703	2.1
59499	0.97549	2.1
59500	1.11509	2.08633
59501	1.10017	1.96796
59502	0.774576	2.1
59503	0.789859	2.1
59504	0.716502	2.1
59505	1.12793	2.1

59506	1.14398	2.1
59507	0.817011	2.1
59508	1.82499	2.1
59509	1.50524	2.1
59510	1.21304	2.1
59511	1.54191	2.1
59512	3.04715	2.1
59513	1.54191	2.1
59514	1.49823	2.1
59515	1.82499	2.1
59516	1.82499	2.1
59517	1.21147	2.1
59518	1.48977	2.1
59519	1.89226	2.1
59520	1.89226	2.1
59521	1.89226	2.1
59522	1.89226	2.1
59523	3.78452	2.1
59524	1.89226	2.1
59525	1.89226	2.1
59526	1.89226	2.1
59527	1.89226	2.1
59528	1.89226	2.1
59529	1.89226	2.1
59530	0.566674	2.1
59531	0.770957	2.1
59532	0.60652	2.1
59533	0.473557	2.1
59534	0.604441	2.1
59535	0.604441	2.1
59536	0.640073	2.1
59537	0.640073	2.1
59538	0.473557	2.1
59539	0.566674	2.1
59540	0.60652	2.1
59541	0.770956	2.1
59542	1.36203	2.1
59543	1.13335	2.1
59544	1.54191	2.1
59545	1.54191	2.1
59546	1.00965	2.1
59547	1.00965	2.1
59548	1.89226	2.1
59549	1.89226	2.1
59550	1.89226	2.1
59551	1.89226	2.1
59552	1.89226	2.1
59553	1.89226	2.1
59554	0.946131	2.1
59555	0.946131	2.1
59556	0.946131	2.1
59557	0.946131	2.1
59558	0.946132	2.1
59559	0.946132	2.1
59560	0.946131	2.1
59561	0.946131	2.1
59562	0.946132	2.1
59563	0.946132	2.1

59564	0.946131	2.1
59565	0.946131	2.1
59566	0.946132	0.05
59567	0.946132	0.05
59568	0.946133	0.05
59569	0.946133	0.05
59570	0.946132	0.05
59571	0.946132	0.05
59572	0.946132	0.05
59573	0.946132	0.05
59574	0.946132	0.05
59575	0.946132	0.05
59576	0.946133	0.05
59577	0.946133	0.05
59578	1.89226	0.05
59579	1.89226	0.05
59580	1.89226	0.05
59581	1.89226	0.05
59582	3.78453	0.05
59583	1.89226	0.05
59584	1.89226	0.05
59585	1.89226	0.05
59586	1.89226	0.05
59587	1.89226	0.05
59588	1.89226	0.05
59589	0.946132	0.05
59590	0.946132	0.05
59591	0.946131	0.05
59592	0.946131	0.05
59593	0.946132	0.05
59594	0.946132	0.05
59595	0.946132	0.05
59596	0.946132	0.05
59597	0.946132	0.05
59598	0.946132	0.05
59599	0.946132	0.05
59600	0.946132	0.05
59601	0.946129	0.05
59602	0.946129	0.05
59603	0.946128	0.05
59604	0.946128	0.05
59605	0.946132	0.05
59606	0.946132	0.05
59607	0.946126	0.05
59608	0.946126	0.05
59609	0.946129	0.05
59610	0.946129	0.05
59611	0.946129	0.05
59612	0.946129	0.05
59613	1.20674	0.05
59614	1.79877	0.05
59615	1.22586	0.05
59616	1.22586	0.05
59617	1.7919	0.05
59618	1.7919	0.05
59619	2.27622	0.05
59620	1.79877	0.05
59621	1.7919	0.05

59622	2.27102	0.05
59623	4.06979	0.05
59624	1.89226	0.05
59625	1.89226	0.0865658
59626	1.89226	0.05
59627	1.89226	0.0775359
59628	3.78452	0.0681423
59629	1.89226	0.05
59630	1.89226	0.0626956
59631	1.89226	0.05
59632	1.89226	0.0734044
59633	3.78453	0.05
59634	1.13259	0.05
59635	1.79187	0.05
59636	1.32741	0.05
59637	1.18784	0.05
59638	1.703	0.05
59639	1.49575	0.05
59640	1.46201	0.05
59641	2.27099	0.05
59642	1.79187	0.05
59643	1.79187	0.05
59644	2.12923	0.05
59645	2.12923	0.05
59646	1.89224	0.05
59647	1.89224	0.0757942
59648	1.89224	0.05
59649	1.89224	0.0795628
59650	3.78447	0.053183
59651	0.946119	0.0733507
59652	0.946119	0.05
59653	0.946118	0.0601512
59654	0.946118	0.05
59655	0.946118	0.0627449
59656	0.946118	0.0739533
59657	0.946119	0.05
59658	0.946119	0.05
59659	0.946131	0.0518339
59660	0.946131	0.05
59661	0.946106	0.0509907
59662	0.946106	0.0563772
59663	2.07526	0.373817
59664	1.89226	0.190763
59665	1.89226	0.448764
59666	2.13445	0.542081
59667	4.02671	0.269779
59668	1.89226	0.238671
59669	1.89226	0.493493
59670	1.89226	0.503545
59671	1.89226	0.187158
59672	1.89226	0.60515
59673	1.89226	0.229527
59674	2.13446	0.398853
59675	1.00013	0.63908
59676	1.1475	0.773917
59677	2.20046	0.493846
59678	3.20059	0.635644
59679	0.946133	0.278367

59680	0.946133	0.313851
59681	1.06722	0.318864
59682	1.10023	0.309499
59683	0.946131	0.355309
59684	0.946131	0.0967642
59685	1.10023	0.364138
59686	1.10023	0.227989
59687	0.946131	0.375023
59688	1.10023	0.270063
59689	0.94613	0.198977
59690	1.06723	0.206028
59691	1.89226	0.127694
59692	1.89226	0.341787
59693	1.89226	0.413066
59694	1.89226	0.267499
59695	3.78452	0.334705
59696	1.89226	0.549097
59697	1.89226	0.659281
59698	1.89226	0.708094
59699	1.89226	0.589105
59700	1.89226	0.591298
59701	1.89226	0.453776
59702	1.89226	2.1
59703	1.89226	1.05063
59704	1.89226	2.08556
59705	1.89226	1.01058
59706	1.89226	2.1
59707	0.828588	0.05
59708	0.828588	0.05
59709	0.841316	0.05
59710	0.841316	2.1
59711	0.528387	2.1
59712	0.565893	0.05
59713	1.10401	0.05
59714	1.10401	2.1
59715	0.528387	2.1
59716	1.89226	2.1
59717	1.36047	2.08556
59718	1.33973	2.1
59719	1.33973	2.1
59720	1.89226	0.05
59721	2.28951	1.05454
59722	1.89226	1.36421
59723	2.80796	1.8331
59724	2.01584	1.83608
59725	2.01584	1.45679
59726	1.89226	1.82107
59727	1.89226	1.4155
59728	1.89226	0.890174
59729	1.89226	1.61067
59730	1.89226	1.51716
59731	1.89226	1.01426
59732	1.89226	1.50621
59733	1.89226	0.996218
59734	2.15223	1.07121
59735	1.30664	1.75457
59736	1.96011	1.68558
59737	2.76516	1.20438

59738	4.11234	1.59262
59739	1.89226	0.7618
59740	1.89226	1.4125
59741	1.89226	1.38999
59742	1.89226	0.905478
59743	1.89226	1.35944
59744	1.89226	0.869208
59745	2.81143	2.1
59746	2.58517	2.1
59747	2.55318	2.1
59748	2.7542	2.1
59749	5.33937	2.1
59750	2.7777	2.1
59751	2.7542	2.1
59752	2.71672	2.1
59753	2.70401	2.1
59754	5.45821	2.1
59755	2.55318	2.1
59756	2.7542	2.1
59757	2.71672	2.1
59758	2.54198	2.1
59759	5.29618	2.1
59760	1.95416	2.1
59761	2.162 2.1	
59762	2.11563	2.1
59763	1.91813	2.1
59764	4.08013	2.1
59765	1.95417	2.1
59766	1.47712	2.1
59767	1.45182	2.1
59768	1.91813	2.1
59769	3.39525	2.1
59770	2.11562	2.1
59771	1.91813	2.1
59772	1.50979	2.1
59773	2.03974	2.1
59774	3.95787	2.1
59775	1.85605	2.1
59776	1.91813	2.1
59777	1.88885	2.1
59778	1.42255	2.1
59779	1.45182	2.1
59780	1.45182	2.1
59781	0.94613	2.1
59782	0.94613	1.09148
59783	0.946128	0.760864
59784	0.946128	2.1
59785	0.946127	2.08732
59786	0.946127	2.1
59787	0.946131	0.622074
59788	0.946131	0.640919
59789	0.946129	1.77359
59790	0.946129	1.77739
59791	0.946128	1.7963
59792	0.946128	1.84968
59793	0.543052	2.1
59794	0.54608	1.10881
59795	0.602293	0.686138

59796	0.535325	0.794155
59797	1.05446	2.1
59798	0.850496	1.97143
59799	0.699838	1.13773
59800	0.679751	2.1
59801	0.713797	1.74751
59802	0.509794	1.51245
59803	0.602304	1.31464
59804	1.40017	2.1
59805	1.40017	2.1
59806	0.94613	2.09865
59807	0.94613	2.1
59808	1.40017	2.1
59809	1.33997	2.1
59810	0.946131	2.08886
59811	0.946131	2.1
59812	1.33997	2.1
59813	0.946131	2.1
59814	0.946131	2.1
59815	1.40017	2.1
59816	1.89226	0.05
59817	1.89226	0.0773176
59818	1.89226	0.0856307
59819	1.89226	0.05
59820	3.78452	0.0573043
59821	0.946133	0.05
59822	0.946133	0.05
59823	0.946132	0.0772306
59824	0.946132	0.0528869
59825	0.946132	0.05
59826	0.946132	0.0532827
59827	0.946133	0.0765227
59828	0.946133	0.05
59829	0.946132	0.05
59830	0.946132	0.0536953
59831	0.946132	0.0506488
59832	0.946132	0.05
59833	1.89224	0.0880275
59834	1.89224	0.05
59835	1.89224	0.0628006
59836	1.89224	0.0566371
59837	1.89224	0.0737045
59838	1.89224	0.0689207
59839	0.94612	0.05
59840	0.94612	0.05
59841	0.946119	0.0767753
59842	0.946119	0.0551407
59843	0.946119	0.05
59844	0.946119	0.0568293
59845	0.94612	0.05
59846	0.94612	0.0743746
59847	0.946132	0.05
59848	0.946132	0.05
59849	0.946107	0.05
59850	0.946107	0.0524965
59851	2.63939	2.1
59852	1.2862	2.1
59853	1.29891	2.1

59854	2.64419	2.1
59855	3.93039	2.1
59856	2.6394	2.1
59857	1.89226	2.1
59858	1.89226	2.1
59859	2.64419	2.1
59860	4.53646	2.1
59861	1.89226	2.1
59862	1.89226	2.1
59863	1.89226	2.1
59864	1.89226	2.1
59865	3.78453	2.1
59866	1.89226	2.1
59867	1.89226	2.1
59868	1.89226	2.1
59869	1.89226	2.1
59870	1.89226	2.1
59871	1.89226	2.1
59872	1.31998	2.1
59873	1.32209	2.1
59874	0.649456	2.1
59875	0.648474	2.1
59876	0.984995	2.1
59877	0.984995	2.1
59878	0.985573	2.1
59879	0.985573	2.1
59880	1.31999	2.1
59881	0.648472	2.1
59882	0.649455	2.1
59883	1.32209	2.1
59884	0.946133	2.1
59885	0.946133	2.1
59886	1.31999	2.1
59887	1.3221	2.1
59888	0.946132	2.1
59889	0.946132	2.1
59890	1.31999	2.1
59891	1.31999	2.1
59892	1.31999	2.1
59893	0.946132	2.1
59894	0.946133	2.1
59895	1.3221	2.1
59896	0.946132	2.1
59897	0.946132	2.1
59898	0.946131	2.1
59899	0.946131	2.1
59900	0.946132	2.1
59901	0.946132	2.1
59902	0.946131	2.1
59903	0.946131	2.1
59904	0.946131	2.1
59905	0.946131	2.1
59906	0.946132	2.1
59907	0.946132	2.1
59908	0.946128	2.1
59909	0.946128	2.1
59910	0.946132	2.1
59911	0.946132	2.1

59912	0.946131	2.1
59913	0.946131	2.1
59914	0.94613	2.1
59915	0.94613	2.1
59916	0.94613	2.1
59917	0.94613	2.1
59918	0.946131	2.1
59919	0.946131	2.1
59920	1.89226	0.20992
59921	1.89226	0.591063
59922	1.89226	0.553827
59923	1.89226	0.547323
59924	1.89226	0.225148
59925	1.89226	0.276065
59926	1.89226	0.505927
59927	1.89226	1.07409
59928	1.89226	0.704137
59929	1.89226	0.923624
59930	3.78453	1.07273
59931	0.946133	0.384494
59932	0.946133	0.458192
59933	0.946132	0.374623
59934	0.946132	0.313531
59935	0.946133	0.542303
59936	0.946133	0.561862
59937	0.946132	0.378898
59938	0.946132	0.266692
59939	0.946132	0.45649
59940	0.946132	0.538998
59941	0.946132	0.413044
59942	0.946132	0.678871
59943	2.23638	0.18462
59944	1.89226	0.308945
59945	2.66014	0.473689
59946	2.66014	0.517472
59947	1.89226	0.563642
59948	1.89226	0.333435
59949	1.11819	0.0861695
59950	1.33007	0.106531
59951	0.660255	0.166439
59952	0.91151	0.134476
59953	0.911511	0.235054
59954	1.33007	0.132137
59955	1.11819	0.0779553
59956	0.660255	0.15128
59957	0.985327	0.112398
59958	0.985327	0.166825
59959	1.005	0.157296
59960	1.005	0.1967
59961	1.89226	2.1
59962	1.89226	2.1
59963	1.89226	2.1
59964	1.89226	2.1
59965	3.78453	2.1
59966	1.59533	2.1
59967	2.68571	2.1
59968	1.28291	2.1
59969	2.46974	2.1

59970	4.06506	2.1
59971	2.68571	2.1
59972	1.89226	2.1
59973	1.89226	2.1
59974	1.89226	2.1
59975	2.46973	2.1
59976	2.46973	2.1
59977	1.89226	2.1
59978	1.89226	2.1
59979	1.89226	2.1
59980	1.89226	2.1
59981	1.89226	2.1
59982	1.89226	2.1
59983	1.89226	2.1
59984	1.89226	2.1
59985	1.89226	2.1
59986	1.89226	2.1
59987	1.89226	2.1
59988	1.89226	2.1
59989	0.883594	2.1
59990	0.883594	2.1
59991	0.876484	2.1
59992	0.876484	2.1
59993	1.10442	2.1
59994	1.23487	2.1
59995	0.641457	2.1
59996	0.52521	2.1
59997	1.10442	2.1
59998	0.52521	2.1
59999	0.641457	2.1
60000	1.23487	2.1
60001	0.946131	2.1
60002	0.946131	2.1
60003	0.946131	2.1
60004	0.946131	2.1
60005	0.946131	2.1
60006	0.946131	2.1
60007	0.946131	2.1
60008	0.946131	2.1
60009	0.946131	2.1
60010	0.946131	2.1
60011	0.946131	2.1
60012	0.946131	2.1
60013	2.04016	0.0665448
60014	2.13042	0.0723187
60015	2.11465	0.0728435
60016	2.11465	0.0934226
60017	1.95908	0.0573533
60018	1.95908	0.0687641
60019	1.66953	0.0625825
60020	2.13041	0.0741663
60021	1.95908	0.0571431
60022	2.21952	0.0615527
60023	1.66953	0.0876622
60024	1.92997	0.0887721
60025	2.04016	0.0594255
60026	1.95908	0.05
60027	1.82014	0.05

60028	2.08757	0.05
60029	3.86029	0.05
60030	0.834764	0.05
60031	0.722102	0.05
60032	0.979537	0.05
60033	0.910065	0.05
60034	0.840053	0.05
60035	0.840052	0.0566794
60036	0.861587	0.05
60037	0.861587	0.0541457
60038	0.910067	0.05
60039	0.722101	0.05
60040	0.834763	0.0555512
60041	0.979539	0.0514362
60042	1.89226	0.0841761
60043	1.89226	0.0898419
60044	1.89226	0.0993222
60045	1.89226	0.0702764
60046	1.89226	0.106871
60047	1.89226	0.0790771
60048	1.89226	0.0936949
60049	1.89226	0.107862
60050	1.89226	0.152446
60051	1.89226	0.0693145
60052	3.78453	0.105274
60053	1.89226	0.0510584
60054	1.89226	0.0608768
60055	1.89226	0.0715367
60056	1.89226	0.05
60057	1.89226	0.072255
60058	1.89226	0.05
60059	1.20691	0.05
60060	2.27379	0.05
60061	2.58476	0.0527438
60062	1.5124	0.05
60063	3.79167	0.05
60064	1.89226	0.101113
60065	2.2738	0.0521501
60066	2.58477	0.0608643
60067	1.89226	0.0733246
60068	4.47703	0.0703302
60069	0.952225	1.53708
60070	0.952225	1.78352
60071	0.909865	1.89992
60072	0.909865	1.95839
60073	0.909895	2.00368
60074	0.946132	1.63047
60075	0.946132	1.83398
60076	0.915958	1.55183
60077	0.946132	1.81312
60078	0.946132	1.86753
60079	0.909894	1.83967
60080	0.915958	1.82735
60081	0.983493	2.1
60082	2.40055	2.1
60083	1.02986	2.1
60084	1.02986	2.1
60085	2.34217	2.1

60086	2.34217	2.1
60087	1.89226	2.1
60088	2.40056	2.1
60089	2.34217	2.1
60090	2.40056	2.1
60091	1.83388	2.1
60092	1.89226	2.1
60093	1.89226	2.1
60094	1.89226	2.1
60095	1.89226	2.1
60096	1.89226	2.1
60097	1.89226	2.1
60098	1.89226	2.1
60099	1.89226	2.1
60100	1.89226	2.1
60101	1.89226	2.1
60102	1.89226	2.1
60103	1.89226	2.1
60104	1.89226	2.1
60105	0.946133	2.1
60106	0.946133	2.1
60107	1.17109	2.1
60108	1.13078	2.1
60109	0.946132	2.1
60110	0.946132	2.1
60111	1.13078	2.1
60112	1.13078	2.1
60113	1.13078	2.1
60114	0.946133	2.1
60115	0.946132	2.1
60116	1.17109	2.1
60117	1.89226	2.1
60118	1.89226	2.1
60119	1.89226	2.1
60120	1.89226	2.1
60121	1.89226	2.1
60122	1.89226	2.1
60123	2.33963	0.955594
60124	1.89227	0.504613
60125	1.74565	0.646624
60126	1.74565	0.697238
60127	1.89227	0.437982
60128	1.89227	0.530552
60129	1.89227	0.667673
60130	1.89227	0.464265
60131	1.89227	1.1716
60132	1.89227	0.526834
60133	3.78453	0.642847
60134	1.31398	0.344729
60135	1.10392	0.433724
60136	0.796878	0.407731
60137	0.524356	0.355415
60138	0.870614	0.349193
60139	0.945739	0.298307
60140	0.89208	0.436886
60141	0.89208	0.838837
60142	1.23425	0.358038
60143	0.774658	0.592017

60144	1.03182	0.357341
60145	0.557289	0.312706
60146	2.33963	0.826006
60147	1.89226	0.357474
60148	1.89226	0.968448
60149	2.77245	0.458818
60150	4.23189	0.589802
60151	1.89226	1.03732
60152	1.89226	0.400684
60153	1.89226	1.01688
60154	1.89226	0.857318
60155	1.89226	0.528892
60156	1.89226	0.47574
60157	1.89226	0.185184
60158	1.89226	0.189492
60159	1.89226	0.152717
60160	1.89226	0.333552
60161	1.89226	0.140623
60162	1.89226	0.315559
60163	1.89226	0.142997
60164	1.89226	0.131781
60165	1.89226	0.111662
60166	1.89226	0.209195
60167	1.89226	0.0967539
60168	1.89226	0.1856
60169	0.946128	0.133113
60170	0.946128	0.0942904
60171	0.963918	0.0926857
60172	1.37543	0.0689681
60173	0.946129	0.091098
60174	0.946129	0.127417
60175	1.37542	0.0764951
60176	1.37542	0.0590538
60177	0.94613	0.0722634
60178	1.37542	0.0857773
60179	0.963916	0.0770578
60180	0.94613	0.085655
60181	0.946132	0.17587
60182	0.946132	0.123605
60183	0.946131	0.131232
60184	0.946131	0.0857357
60185	0.946131	0.133779
60186	0.946131	0.171151
60187	0.946132	0.112158
60188	0.946132	0.0826378
60189	0.946131	0.126253
60190	0.946131	0.128932
60191	0.946131	0.121612
60192	0.946131	0.121521
60193	1.89226	0.195602
60194	1.89226	0.208293
60195	1.89226	0.140755
60196	1.89226	0.326489
60197	3.78452	0.216992
60198	0.946127	0.206523
60199	0.946127	0.136321
60200	0.946131	0.137441
60201	0.946131	0.095453

60202	0.946128	0.140762
60203	0.946128	0.189772
60204	0.94613	0.102696
60205	0.94613	0.146267
60206	0.946129	0.131445
60207	0.946129	0.135544
60208	0.946129	0.141866
60209	0.946129	0.146245
60210	1.89226	1.18574
60211	1.89226	1.59219
60212	1.89226	2.06954
60213	1.89226	0.707851
60214	3.78453	0.816363
60215	1.89226	0.744165
60216	1.89226	0.143343
60217	1.89226	0.345896
60218	1.89226	0.529793
60219	3.78453	0.664308
60220	0.946131	0.396674
60221	0.946131	0.426559
60222	0.946132	1.32137
60223	0.946132	0.834811
60224	0.946132	1.16256
60225	0.946132	0.649293
60226	0.946132	1.17533
60227	0.946132	0.802537
60228	0.946132	0.842809
60229	0.946132	1.057
60230	0.946132	1.19395
60231	0.946132	0.79675
60232	0.548169	0.245876
60233	0.736603	0.161877
60234	1.18322	0.382412
60235	1.01139	0.185043
60236	1.01139	0.140942
60237	0.548168	0.164469
60238	0.736603	0.313701
60239	1.18322	0.175268
60240	0.861589	0.204606
60241	0.861589	0.368456
60242	0.869798	0.160107
60243	0.869798	0.18857
60244	1.16037	0.321068
60245	0.942012	0.368528
60246	0.942012	0.220115
60247	0.62356	0.250807
60248	0.891932	0.345349
60249	0.900255	0.306852
60250	2.02279	0.169441
60251	1.48677	0.294734
60252	1.54724	0.253893
60253	0.831456	0.23918
60254	1.19775	0.349016
60255	1.89226	1.58182
60256	2.76291	1.37789
60257	1.89226	1.56758
60258	1.89226	1.45556
60259	3.78452	1.4537

60260	1.89226	1.05803
60261	1.89226	1.13135
60262	1.89226	1.31294
60263	1.89226	1.05981
60264	1.89226	1.24363
60265	1.89226	0.978219
60266	1.89226	1.24009
60267	1.89226	1.19923
60268	1.89226	1.45224
60269	1.89226	1.05896
60270	3.78452	1.25854
60271	1.89226	0.90785
60272	1.89226	1.02268
60273	1.89226	1.23798
60274	1.89226	1.04952
60275	3.78453	1.06902
60276	0.946128	0.750789
60277	0.946128	1.06759
60278	0.946129	1.1055
60279	0.946129	0.878558
60280	0.946132	0.745518
60281	0.946132	0.982637
60282	0.946126	1.02248
60283	0.946126	1.08219
60284	0.946129	1.04505
60285	0.946129	1.08197
60286	0.946129	0.943247
60287	0.946129	0.842512
60288	2.80495	0.950529
60289	1.89225	0.929784
60290	1.89225	1.13804
60291	1.87794	1.151
60292	1.89225	1.27971
60293	1.87794	1.23633
60294	2.25195	2.1
60295	2.00624	0.981974
60296	2.13132	2.08348
60297	1.90239	0.971784
60298	4.13756	1.12154
60299	0.804661	1.90116
60300	0.804663	0.495826
60301	0.889591	0.71335
60302	0.889591	2.04169
60303	0.808324	1.73252
60304	0.761765	1.97793
60305	1.02822	0.154884
60306	0.879624	0.221159
60307	0.879617	0.959541
60308	0.800864	0.853612
60309	0.978023	1.03559
60310	0.808329	0.891562
60311	2.13133	2.08545
60312	2.00558	0.443769
60313	2.09316	0.342808
60314	2.09316	2.09662
60315	2.03253	0.950914
60316	2.03253	2.09786
60317	0.84577	1.68019

60318	0.845767	0.773301
60319	0.843917	0.624387
60320	0.843917	2.03868
60321	0.76177	1.46941
60322	0.719345	1.88881
60323	0.934435	0.181905
60324	0.884197	0.151739
60325	0.934439	0.639569
60326	0.75627	0.716493
60327	0.849424	0.562177
60328	0.76177	0.662855
60329	2.61855	0.116128
60330	1.17793	0.125149
60331	1.42965	0.12014
60332	1.17793	0.119886
60333	2.50057	0.135236
60334	2.24885	0.137772
60335	0.946133	0.115664
60336	0.946133	0.100365
60337	1.30928	0.116749
60338	1.12443	0.140299
60339	0.946132	0.148547
60340	0.946132	0.12328
60341	1.30928	0.0989213
60342	1.30928	0.12374
60343	0.946132	0.126268
60344	1.30928	0.121372
60345	1.12443	0.110839
60346	0.946133	0.117085
60347	1.89226	0.116951
60348	1.89226	0.145388
60349	1.89226	0.129094
60350	1.89226	0.139009
60351	3.78452	0.143672
60352	1.89226	0.131913
60353	1.89226	0.116873
60354	1.89226	0.117177
60355	1.89226	0.123701
60356	1.89226	0.124122
60357	1.89226	0.132566
60358	1.89226	0.13149
60359	1.89226	0.133534
60360	1.89226	0.115088
60361	1.89226	0.150821
60362	3.78452	0.121812
60363	0.94613	0.11439
60364	0.94613	0.125856
60365	0.946131	0.110774
60366	0.946131	0.119054
60367	0.946128	0.122065
60368	0.946128	0.118001
60369	0.946133	0.115646
60370	0.946133	0.122554
60371	0.946131	0.120995
60372	0.946131	0.113627
60373	0.946131	0.119365
60374	0.946131	0.130447
60375	1.89226	2.1

60376	1.89226	2.1
60377	1.89226	2.1
60378	1.89226	2.1
60379	3.78453	2.1
60380	1.0465	2.1
60381	2.73926	2.1
60382	2.03721	2.1
60383	2.03721	2.1
60384	1.44709	2.1
60385	1.44709	2.1
60386	0.946131	2.1
60387	0.946131	2.1
60388	0.946131	2.1
60389	0.946131	2.1
60390	0.946132	2.1
60391	0.946132	2.1
60392	0.94613	2.1
60393	0.94613	2.1
60394	0.946131	2.1
60395	0.946131	2.1
60396	0.946131	2.1
60397	0.946131	2.1
60398	0.946132	2.1
60399	0.946132	2.1
60400	0.946131	2.1
60401	0.946131	2.1
60402	0.946132	2.1
60403	0.946132	2.1
60404	0.946132	2.1
60405	0.946132	2.1
60406	0.946132	2.1
60407	0.946132	2.1
60408	0.946132	2.1
60409	0.946132	2.1
60410	1.89226	2.1
60411	1.89226	2.1
60412	1.89226	2.1
60413	1.89226	2.1
60414	3.78452	2.1
60415	1.61969	0.104338
60416	1.79374	0.0697593
60417	1.29382	0.07551
60418	1.29382	0.108775
60419	2.05759	0.0700968
60420	2.05759	0.10277
60421	1.89226	0.107773
60422	1.89226	0.0913894
60423	1.89226	0.102656
60424	1.89226	0.145567
60425	1.89226	0.0711688
60426	1.89226	0.0982091
60427	1.89226	0.714902
60428	1.89226	0.848262
60429	1.89226	0.723532
60430	1.89226	0.721577
60431	1.89226	0.687043
60432	1.89226	0.688541
60433	1.89226	1.79763

60434	1.89226	1.16701
60435	1.89226	1.36843
60436	1.89226	1.70405
60437	3.78453	1.7972
60438	0.946131	0.988998
60439	0.946131	1.08718
60440	0.946131	0.816291
60441	0.946131	0.989413
60442	0.946131	0.857283
60443	0.946131	0.77343
60444	0.946131	0.98163
60445	0.946131	1.20861
60446	0.946131	0.930879
60447	0.946131	0.965838
60448	0.946131	1.00501
60449	0.946131	0.915935
60450	2.45177	0.182152
60451	2.68507	0.141218
60452	2.3376	0.220976
60453	2.22086	0.130427
60454	4.78937	0.170747
60455	2.45178	0.166492
60456	2.35552	0.109146
60457	1.68062	0.151151
60458	1.81902	0.121654
60459	4.13239	0.133338
60460	2.3376	0.172622
60461	1.55612	0.110788
60462	1.69811	0.120563
60463	2.31527	0.137719
60464	1.79553	0.121048
60465	2.3376	0.141743
60466	1.55613	0.100885
60467	1.06336	0.116871
60468	1.97516	0.120477
60469	1.88966	0.0994204
60470	1.06336	0.15486
60471	1.68062	0.0983158
60472	1.89226	1.34108
60473	1.91634	1.84318
60474	2.03709	1.9933
60475	1.91634	1.57951
60476	1.83185	1.81993
60477	1.70303	0.880858
60478	1.89226	1.24325
60479	1.89226	1.75067
60480	1.89226	1.97928
60481	1.89226	1.51716
60482	1.89226	1.69527
60483	1.89226	0.910537
60484	1.89226	2.09368
60485	2.03472	2.1
60486	2.22183	2.1
60487	2.03472	2.1
60488	2.07936	2.1
60489	1.89226	1.92562
60490	1.89226	0.864301
60491	1.8519	1.27672

60492	1.92119	1.61358
60493	1.8519	1.09705
60494	1.96156	1.1405
60495	1.89226	0.632595
60496	1.89226	0.901275
60497	1.89226	1.36887
60498	1.89226	1.63896
60499	1.89226	1.14868
60500	1.89226	1.15992
60501	1.89226	0.645023
60502	1.89226	2.00142
60503	2.22183	2.09549
60504	2.39541	2.1
60505	2.22183	2.04741
60506	2.06585	2.07074
60507	1.89226	1.69251
60508	1.47001	2.1
60509	1.57962	1.99274
60510	1.65238	2.1
60511	1.60502	2.1
60512	3.18464	2.1
60513	1.02415	2.1
60514	1.60503	2.1
60515	1.47001	2.1
60516	1.47001	2.1
60517	1.28228	2.1
60518	1.28228	2.1
60519	0.946133	2.1
60520	0.946133	2.1
60521	0.946132	2.1
60522	0.946132	2.1
60523	0.946132	2.1
60524	0.946132	2.1
60525	0.946133	2.1
60526	0.946133	2.1
60527	0.946132	2.1
60528	0.946132	2.1
60529	0.946133	2.1
60530	0.946133	2.1
60531	1.89226	1.9886
60532	1.89226	2.09024
60533	1.89226	2.1
60534	1.89226	1.76905
60535	3.78452	2.03409
60536	1.76897	2.06881
60537	1.60502	2.1
60538	1.65238	1.98495
60539	1.65238	2.1
60540	1.76932	1.94893
60541	1.76932	2.1
60542	1.55998	2.1
60543	1.60503	2.1
60544	1.76932	2.1
60545	1.60503	2.1
60546	1.44657	2.1
60547	1.28228	2.1
60548	0.946133	2.1
60549	0.946133	2.1

60550	0.946132	2.1
60551	0.946132	2.07997
60552	0.946132	2.0782
60553	0.946132	2.1
60554	0.946133	2.1
60555	0.946133	2.1
60556	0.946132	2.1
60557	0.946132	2.1
60558	0.946132	2.1
60559	0.946132	2.1
60560	0.946128	2.1
60561	0.946128	2.03068
60562	0.946133	1.41377
60563	0.946133	1.8185
60564	0.94613	1.51695
60565	0.94613	2.03218
60566	0.946131	2.1
60567	0.946131	1.95452
60568	0.946131	1.96294
60569	0.946131	1.94094
60570	0.946131	2.01732
60571	0.946131	2.0201
60572	1.85779	0.15591
60573	1.00763	0.119159
60574	1.74874	0.132509
60575	1.74873	0.124157
60576	1.14467	0.163193
60577	1.14466	0.144511
60578	1.85779	0.141119
60579	1.61597	0.113202
60580	1.00053	0.14093
60581	1.03018	0.106513
60582	2.65819	0.117009
60583	1.89226	0.539474
60584	1.89226	0.328978
60585	1.89226	0.539899
60586	1.89226	0.438615
60587	3.78451	0.485055
60588	1.89226	0.49597
60589	1.89226	0.185832
60590	1.89226	0.289418
60591	1.89226	0.322057
60592	1.89226	0.257281
60593	1.89226	0.291155
60594	1.11983	0.155275
60595	0.810945	0.171675
60596	0.913906	0.145113
60597	0.913906	0.161457
60598	2.23303	0.109061
60599	1.66057	0.114015
60600	1.28724	0.127927
60601	1.08132	0.130852
60602	1.49316	0.149233
60603	0.810958	0.159939
60604	0.810958	0.151187
60605	0.827861	0.27317
60606	0.946126	0.305343
60607	0.946132	0.479098

60608	0.946132	0.255206
60609	0.827863	0.306
60610	0.827863	0.291322
60611	0.946129	0.348911
60612	0.946129	0.376401
60613	0.827863	0.290484
60614	0.946128	0.321009
60615	0.946129	0.272808
60616	0.946129	0.29389
60617	0.974718	0.05
60618	1.53671	0.0867864
60619	2.80542	0.05
60620	2.27259	0.0776791
60621	3.8093	0.0594558
60622	1.89226	0.115226
60623	1.89226	0.216032
60624	1.89226	0.106738
60625	1.89226	0.19794
60626	3.78453	0.160206
60627	0.946132	0.111553
60628	0.946132	0.169771
60629	0.946131	0.161672
60630	0.946131	0.111633
60631	0.946132	0.166876
60632	0.946132	0.186421
60633	0.946131	0.101311
60634	0.946131	0.102915
60635	0.946132	0.14114
60636	0.946132	0.157827
60637	0.946131	0.125293
60638	0.946131	0.12847
60639	2.80542	0.0688893
60640	1.89226	0.0946104
60641	2.27259	0.0695255
60642	2.27259	0.114158
60643	1.89226	0.0524918
60644	1.89226	0.0970877
60645	1.89226	0.114141
60646	1.89226	0.225901
60647	1.89226	0.124681
60648	1.89226	0.233512
60649	1.89226	0.113358
60650	1.89226	0.232395
60651	0.946132	0.215834
60652	0.946132	0.111675
60653	0.946131	0.192134
60654	0.946131	0.122348
60655	0.946132	0.199351
60656	0.946132	0.203214
60657	0.946131	0.110236
60658	0.946131	0.120829
60659	0.946131	0.148858
60660	0.946131	0.156662
60661	0.946131	0.134853
60662	0.946131	0.149686
60663	2.68213	2.1
60664	2.58234	2.1
60665	2.58234	2.1

60666	2.61243	2.1
60667	2.67035	2.1
60668	2.70044	2.1
60669	2.68213	2.1
60670	2.53921	2.1
60671	2.53303	2.1
60672	2.67035	2.1
60673	5.20956	2.1
60674	2.67033	2.1
60675	2.58232	2.1
60676	2.55004	2.1
60677	2.64567	2.1
60678	5.228	2.1
60679	2.53301	2.1
60680	2.64567	2.1
60681	2.64567	2.1
60682	2.67033	2.1
60683	2.51603	2.1
60684	2.54069	2.1
60685	0.907497	2.1
60686	0.907497	2.1
60687	0.926641	2.1
60688	0.926641	2.1
60689	1.14989	2.1
60690	1.10046	2.1
60691	0.695334	2.1
60692	0.684247	2.1
60693	0.695333	2.1
60694	1.14989	2.1
60695	0.684246	2.1
60696	1.10046	2.1
60697	0.946126	2.1
60698	0.946126	2.1
60699	0.946131	2.1
60700	0.946131	2.1
60701	0.946127	2.1
60702	0.946127	2.1
60703	0.94613	2.1
60704	0.94613	2.1
60705	0.946128	2.1
60706	0.946128	2.1
60707	0.946128	2.1
60708	0.946128	2.1
60709	1.89226	2.1
60710	1.89226	2.1
60711	1.89226	2.1
60712	1.89226	2.1
60713	1.89226	2.1
60714	1.89226	2.08021
60715	1.42174	2.1
60716	2.29978	2.1
60717	2.41233	2.1
60718	2.41233	2.1
60719	1.39067	2.1
60720	1.39067	2.1
60721	1.89226	2.1
60722	1.89226	2.1
60723	1.89226	2.1

60724	1.89226	2.1
60725	3.78452	2.1
60726	1.89226	2.1
60727	1.89226	2.1
60728	1.89226	2.1
60729	1.89226	2.1
60730	1.89226	2.1
60731	1.89226	2.1
60732	2.41234	2.1
60733	1.89226	2.1
60734	2.29979	2.1
60735	1.89226	2.1
60736	1.89226	2.1
60737	2.29979	2.1
60738	1.09506	0.05
60739	2.82921	0.05
60740	2.53251	0.05
60741	2.53251	0.05
60742	1.42901	0.05
60743	1.42901	0.05
60744	0.946132	0.05
60745	0.946132	0.05
60746	0.946128	0.0515683
60747	0.946128	0.0518607
60748	0.94613	0.05
60749	0.94613	0.05
60750	0.946131	0.0510516
60751	0.946131	0.0517569
60752	0.94613	0.05
60753	0.94613	0.0531588
60754	0.94613	0.0513306
60755	0.94613	0.05
60756	1.89226	0.0551921
60757	1.89226	0.0614645
60758	1.89226	0.0635623
60759	1.89226	0.0508874
60760	3.78452	0.0663742
60761	2.53251	0.05
60762	1.89226	0.0531599
60763	2.82921	0.05
60764	1.89226	0.0529155
60765	1.89226	0.0611659
60766	2.82921	0.0559912
60767	1.89226	0.0555616
60768	1.89226	0.05
60769	1.89226	0.05
60770	1.89226	0.05
60771	1.89226	0.05
60772	1.89226	0.05
60773	2.53251	0.05
60774	1.89226	0.05
60775	2.06158	0.05
60776	1.89226	0.05
60777	4.42477	0.05
60778	1.23296	2.1
60779	2.55352	2.1
60780	2.47476	2.1
60781	2.47476	2.1

60782	1.32368	2.1
60783	1.32368	2.1
60784	1.89226	2.1
60785	1.89226	2.1
60786	1.89226	2.1
60787	1.89226	2.1
60788	1.89226	2.1
60789	1.89226	2.1
60790	1.89226	2.1
60791	1.89226	2.1
60792	1.89226	2.1
60793	1.89226	2.1
60794	1.89226	2.1
60795	1.89226	2.1
60796	2.47476	2.1
60797	1.89226	2.1
60798	1.89226	2.1
60799	1.89226	2.1
60800	2.55353	2.1
60801	2.55353	2.1
60802	1.05983	2.1
60803	2.47476	2.1
60804	2.37664	2.1
60805	2.37664	2.1
60806	1.23296	2.1
60807	1.23296	2.1
60808	0.946127	2.1
60809	0.946127	2.1
60810	0.946131	2.1
60811	0.946131	2.1
60812	0.946127	2.1
60813	0.946127	2.1
60814	0.946131	2.1
60815	0.946131	2.1
60816	0.946129	2.1
60817	0.946129	2.1
60818	0.946128	2.1
60819	0.946128	2.1
60820	1.89226	2.1
60821	1.89226	2.1
60822	1.89226	2.1
60823	1.89226	2.1
60824	3.78452	2.1
60825	2.37665	2.1
60826	1.89226	2.1
60827	1.89226	2.1
60828	1.89226	2.1
60829	2.47476	2.1
60830	2.47476	2.1
60831	1.89226	0.541074
60832	1.92119	0.900234
60833	1.99586	1.06719
60834	1.92119	0.796069
60835	1.96693	0.649604
60836	1.89226	0.386044
60837	1.89226	0.635953
60838	1.89226	0.954718
60839	1.89226	1.16854

60840	1.89226	0.848879
60841	1.89226	0.822031
60842	1.89226	0.469147
60843	1.89226	1.62737
60844	2.39541	2.01471
60845	2.57183	2.07862
60846	2.39541	1.82978
60847	2.06868	1.79109
60848	1.89226	1.14249
60849	0.946133	0.374792
60850	0.946133	0.317462
60851	0.997928	0.620805
60852	1.03763	0.602971
60853	0.946131	0.681175
60854	0.946131	0.186476
60855	1.03763	0.342006
60856	1.03763	0.497598
60857	1.03763	0.451356
60858	0.946131	0.442812
60859	0.94613	0.416534
60860	0.99793	0.459575
60861	1.89226	0.280556
60862	1.89226	0.734909
60863	1.89226	0.765515
60864	1.89226	0.333377
60865	1.89226	0.817334
60866	1.89226	0.369861
60867	0.946131	0.906942
60868	0.946131	0.915031
60869	1.36939	1.42471
60870	1.28592	1.96726
60871	0.946129	1.49064
60872	0.946129	0.660003
60873	1.36939	0.962663
60874	1.36939	1.63149
60875	0.946131	1.13352
60876	1.36939	1.15263
60877	0.946131	1.2938
60878	1.28592	1.40239
60879	1.89226	0.180647
60880	1.89226	0.324548
60881	1.89226	0.258087
60882	1.89226	0.179548
60883	3.78453	0.212825
60884	1.89226	0.326984
60885	1.89226	0.217918
60886	1.89226	0.189816
60887	1.89226	0.317584
60888	3.78452	0.249884
60889	1.91005	0.0970328
60890	1.89226	0.228453
60891	2.60215	0.094179
60892	2.60215	0.11024
60893	1.89226	0.214817
60894	1.89226	0.203089
60895	0.946132	0.271257
60896	0.946132	0.251359
60897	0.946132	0.189783

60898	0.946132	0.17793
60899	0.946132	0.187385
60900	0.946132	0.262203
60901	0.946132	0.246215
60902	0.946132	0.172867
60903	0.946132	0.209108
60904	0.946132	0.210161
60905	0.946131	0.219623
60906	0.946131	0.217369
60907	1.89226	0.198214
60908	1.89226	0.305975
60909	1.89226	0.241459
60910	1.89226	0.298778
60911	1.89226	0.28777
60912	1.89226	0.240403
60913	0.780483	0.147737
60914	0.78928	0.239288
60915	0.818566	0.256791
60916	0.460387	0.192013
60917	1.82793	0.198082
60918	1.32039	0.201089
60919	0.613889	0.213488
60920	1.0592	0.244897
60921	1.48698	0.146523
60922	0.818555	0.206172
60923	1.11515	0.17837
60924	1.2802	0.552096
60925	2.20046	0.317843
60926	1.1475	0.528495
60927	1.1475	0.282385
60928	2.27062	0.521088
60929	2.27062	0.25721
60930	1.89226	0.498818
60931	2.20046	0.213882
60932	2.27062	0.389713
60933	2.20046	0.4042
60934	1.96242	0.140861
60935	1.89226	0.166548
60936	1.89226	0.235334
60937	1.89226	0.153121
60938	1.89226	0.311662
60939	1.89226	0.326012
60940	3.78452	0.190694
60941	1.89226	0.364518
60942	1.89226	0.478866
60943	1.89226	0.532152
60944	1.89226	0.385239
60945	3.78452	0.443819
60946	1.17403	0.301171
60947	1.13531	0.327046
60948	0.6401	0.373599
60949	0.714566	0.3625
60950	0.915987	0.409318
60951	0.915987	0.162378
60952	0.933891	0.392278
60953	0.933891	0.319698
60954	1.17403	0.360975
60955	0.714566	0.348758

60956	0.6401	0.327942
60957	1.13531	0.289819
60958	0.946133	0.246676
60959	0.946133	0.311712
60960	1.17403	0.19933
60961	1.13531	0.197046
60962	0.946131	0.259103
60963	0.946131	0.132156
60964	1.17403	0.360083
60965	1.17403	0.132827
60966	1.17403	0.234263
60967	0.94613	0.234343
60968	0.946131	0.174666
60969	1.13531	0.168485
60970	1.89226	0.137111
60971	1.89226	0.258811
60972	1.89226	0.217603
60973	1.89226	0.112963
60974	1.89226	0.28208
60975	1.89226	0.143981
60976	0.946131	0.279308
60977	0.946131	0.269021
60978	0.946131	0.365167
60979	0.946131	0.377671
60980	0.946129	0.382778
60981	0.946129	0.187234
60982	0.946133	0.472473
60983	0.946133	0.23471
60984	0.946131	0.324829
60985	0.946131	0.323247
60986	0.946131	0.26238
60987	0.946131	0.275568
60988	2.29413	2.1
60989	1.99332	2.1
60990	1.84774	2.1
60991	2.21582	2.1
60992	4.20915	2.1
60993	0.517359	2.1
60994	0.634469	2.1
60995	0.996661	2.1
60996	0.92387	2.1
60997	0.768098	2.1
60998	0.768098	2.1
60999	0.790242	2.1
61000	0.790242	2.1
61001	0.923871	2.1
61002	0.517359	2.1
61003	0.634469	2.1
61004	0.996661	2.1
61005	1.89226	2.1
61006	1.89226	2.1
61007	1.89226	2.1
61008	1.89226	2.1
61009	3.78453	2.1
61010	0.946132	2.1
61011	0.946132	2.1
61012	0.946131	2.1
61013	0.946131	2.1

61014	0.946132	2.1
61015	0.946132	2.1
61016	0.946131	2.1
61017	0.946131	2.1
61018	0.946132	2.1
61019	0.946132	2.1
61020	0.946131	2.1
61021	0.946131	2.1
61022	1.62708	2.1
61023	2.21582	2.1
61024	2.13925	2.1
61025	2.21582	2.1
61026	1.77117	2.1
61027	1.84774	2.1
61028	0.946132	2.1
61029	0.946132	2.1
61030	0.946131	2.1
61031	0.946131	2.1
61032	0.946132	2.1
61033	0.946132	2.1
61034	0.946131	2.1
61035	0.946131	2.1
61036	0.946131	2.1
61037	0.946131	2.1
61038	0.946131	2.1
61039	0.946131	2.1
61040	1.89226	1.99776
61041	1.89226	1.17681
61042	1.89226	1.20962
61043	1.89226	1.84149
61044	3.78453	1.91624
61045	1.89226	1.11122
61046	1.89226	1.80029
61047	1.89226	1.7634
61048	1.89226	2.05484
61049	1.89226	0.864991
61050	1.89226	1.74509
61051	1.89226	1.27532
61052	1.89226	2.02893
61053	1.89226	0.906983
61054	1.89226	1.9813
61055	1.89226	1.09359
61056	1.89226	2.03736
61057	0.914369	1.84541
61058	0.914369	1.93742
61059	0.810945	2.09361
61060	0.810945	1.91087
61061	2.26581	2.09897
61062	1.66199	2.08538
61063	1.49503	1.93726
61064	1.28816	2.1
61065	1.08133	2.04066
61066	0.810933	2.01871
61067	1.1212	2.01828
61068	1.89227	0.240539
61069	1.89227	0.125165
61070	1.89227	0.154327
61071	1.89227	0.250532

61072	3.78453	0.180838
61073	1.89226	0.220484
61074	1.89226	0.373372
61075	1.89226	0.365186
61076	1.89226	0.235276
61077	3.78453	0.290836
61078	1.89226	0.387402
61079	1.89226	0.216366
61080	1.89226	0.250131
61081	1.89226	0.386977
61082	3.78453	0.293637
61083	1.89226	0.134179
61084	1.89226	0.237957
61085	1.89226	0.265248
61086	2.25318	0.124169
61087	3.78453	0.182273
61088	1.89226	0.167144
61089	1.89226	0.260796
61090	1.89226	0.231567
61091	1.9637	0.114165
61092	3.78453	0.182401
61093	1.89226	0.361467
61094	1.89226	0.236461
61095	1.89226	0.222453
61096	1.89226	0.352335
61097	3.78453	0.280617
61098	1.89226	0.25341
61099	1.89226	0.398
61100	1.89226	0.361251
61101	2.53384	0.238378
61102	3.78453	0.324647
61103	1.55405	0.282406
61104	1.03818	1.36563
61105	1.03818	0.527485
61106	1.34857	0.428799
61107	1.46338	0.222296
61108	1.77377	0.997198
61109	1.86273	0.414927
61110	1.73802	0.767749
61111	1.50969	1.96331
61112	1.46337	0.584374
61113	3.20139	0.767279
61114	1.2377	0.541553
61115	1.46337	0.234141
61116	1.27408	1.27844
61117	1.32039	1.93681
61118	1.50969	1.01979
61119	1.50969	0.44425
61120	1.89226	2.1
61121	1.89226	2.1
61122	1.89226	2.1
61123	1.89226	2.1
61124	1.89226	2.1
61125	1.89226	2.1
61126	1.89169	2.1
61127	1.89226	2.1
61128	1.89226	2.1
61129	1.89226	2.1

61130	1.96624	2.1
61131	1.96624	2.1
61132	2.62871	2.1
61133	1.6201	2.1
61134	1.49149	2.1
61135	1.6201	2.1
61136	2.62559	2.1
61137	2.75419	2.1
61138	0.946129	2.0681
61139	0.946129	2.1
61140	0.946133	2.1
61141	0.946133	2.1
61142	0.946131	2.1
61143	0.946131	2.04825
61144	0.946131	2.1
61145	0.946131	2.1
61146	0.946131	2.1
61147	0.946131	2.1
61148	0.946131	2.1
61149	0.946131	2.1
61150	0.851543	2.1
61151	0.851543	2.1
61152	1.04043	2.1
61153	0.997939	2.1
61154	0.946132	2.1
61155	0.946132	2.1
61156	0.894185	2.1
61157	0.893901	2.1
61158	0.998267	2.1
61159	1.04074	2.1
61160	0.85127	2.1
61161	0.85153	2.1
61162	1.89226	2.1
61163	1.89226	2.1
61164	1.89226	2.1
61165	1.89226	2.1
61166	1.89226	2.1
61167	1.89226	2.1
61168	0.928691	2.1
61169	0.928691	2.1
61170	0.935332	2.1
61171	0.935332	2.1
61172	1.398	2.1
61173	1.14418	2.1
61174	0.479331	2.1
61175	0.719848	2.1
61176	1.398	2.1
61177	0.719848	2.1
61178	1.14417	2.1
61179	0.47933	2.1
61180	2.28835	2.1
61181	1.89226	2.1
61182	1.89226	2.1
61183	1.89226	2.1
61184	2.796	2.1
61185	2.796	2.1
61186	0.946131	2.1
61187	0.946131	2.1

61188	0.946131	2.1
61189	0.946131	2.1
61190	0.946128	2.1
61191	0.946128	2.1
61192	0.946133	2.1
61193	0.946133	2.1
61194	0.946131	2.1
61195	0.946131	2.1
61196	0.94613	2.1
61197	0.94613	2.1
61198	0.946131	2.1
61199	0.946131	2.1
61200	0.946131	2.1
61201	0.946131	2.1
61202	0.946131	2.1
61203	0.946131	2.1
61204	0.946131	2.1
61205	0.946131	2.1
61206	0.946131	2.1
61207	0.946131	2.1
61208	0.946131	2.1
61209	0.946131	2.1
61210	1.89226	2.1
61211	1.89226	2.1
61212	1.89226	2.1
61213	1.89226	2.1
61214	3.78452	2.1
61215	2.05207	0.633689
61216	1.70302	1.60693
61217	1.33794	2.07502
61218	1.98194	0.871435
61219	3.36873	1.3693
61220	2.62761	0.216567
61221	1.98194	0.706531
61222	2.28009	1.53862
61223	2.28009	0.278969
61224	2.22939	1.14474
61225	2.22939	0.37944
61226	1.89226	0.722292
61227	1.89226	0.518528
61228	1.89226	0.539829
61229	1.89226	0.330087
61230	1.89226	0.69561
61231	1.89226	0.392462
61232	1.89227	0.882444
61233	1.89227	1.38435
61234	1.89227	1.91952
61235	1.89227	1.32154
61236	1.89227	1.19951
61237	1.89227	0.45
61238	0.888355	0.47453
61239	0.990973	0.956682
61240	0.71133	1.42968
61241	0.634324	1.6925
61242	0.806209	1.43557
61243	0.806209	0.23548
61244	0.819087	1.35691
61245	0.819087	1.77301

61246	0.888355	1.4111
61247	0.634324	1.25869
61248	0.71133	1.3813
61249	0.990972	1.27893
61250	1.11469	1.10549
61251	0.985023	0.315099
61252	0.888354	0.379747
61253	0.990972	0.975877
61254	0.994754	0.570507
61255	0.994754	0.93219
61256	1.0083	0.735827
61257	1.0083	0.513888
61258	0.888354	0.304545
61259	0.985024	0.364728
61260	0.990971	0.283604
61261	1.11469	0.146869
61262	0.946132	0.599782
61263	0.946132	0.517291
61264	0.946132	0.45778
61265	0.946132	0.466492
61266	0.946132	0.46745
61267	0.946132	0.53423
61268	0.946133	1.51936
61269	0.946133	0.693191
61270	0.946133	0.415072
61271	0.946133	0.55504
61272	0.946132	0.337177
61273	0.946132	0.244621
61274	0.810944	0.662039
61275	0.810944	0.450813
61276	0.810947	1.19088
61277	0.810947	0.17175
61278	1.89227	1.74311
61279	1.35168	1.03329
61280	1.08132	1.20918
61281	1.08131	0.905339
61282	1.08132	0.570577
61283	0.810959	0.637633
61284	0.810959	0.717971
61285	1.70303	2.1
61286	2.03975	2.1
61287	1.99324	2.1
61288	2.17615	2.1
61289	1.40549	2.1
61290	1.53446	2.1
61291	0.946133	2.1
61292	0.946133	2.1
61293	0.946129	2.1
61294	0.946129	2.1
61295	0.946131	2.1
61296	0.946131	2.1
61297	0.946131	2.1
61298	0.946131	2.1
61299	0.946131	2.1
61300	0.946131	2.1
61301	0.946131	2.1
61302	0.946131	2.1
61303	1.89226	2.1

61304	1.89226	2.1
61305	1.89226	2.1
61306	1.89226	2.1
61307	1.89226	2.1
61308	1.89226	2.1
61309	2.17615	2.1
61310	2.3111	2.1
61311	2.50288	2.1
61312	2.50288	2.1
61313	2.03975	2.1
61314	2.03975	2.1
61315	1.76401	2.1
61316	1.95852	2.1
61317	2.32134	2.1
61318	2.12306	2.1
61319	1.8513	2.1
61320	1.70303	2.1
61321	1.89226	2.1
61322	1.89226	2.1
61323	1.89226	2.1
61324	1.89226	2.1
61325	3.78452	2.1
61326	1.89226	2.1
61327	1.89226	2.1
61328	1.89226	2.1
61329	1.89226	2.1
61330	3.78452	2.1
61331	1.2243	2.1
61332	1.2243	2.1
61333	1.24189	2.1
61334	1.24189	2.1
61335	1.37811	2.1
61336	1.25144	2.1
61337	1.08807	2.1
61338	1.17949	2.1
61339	1.17949	2.1
61340	1.37811	2.1
61341	1.08807	2.1
61342	1.25144	2.1
61343	1.89226	2.1
61344	1.89226	2.1
61345	1.89226	2.1
61346	1.89226	2.1
61347	1.89226	2.1
61348	1.89226	2.1
61349	1.89227	2.1
61350	1.89227	2.1
61351	1.89227	2.1
61352	1.89227	2.1
61353	1.89227	2.1
61354	1.89227	2.1
61355	1.89224	2.1
61356	1.89224	2.1
61357	1.89224	2.1
61358	1.89224	2.1
61359	3.78447	2.1
61360	1.89224	2.1
61361	1.89224	2.1

61362	1.89224	2.1
61363	1.89224	2.1
61364	1.89224	2.1
61365	1.89224	2.1
61366	1.89224	2.1
61367	1.89224	2.1
61368	1.89224	2.1
61369	1.89224	2.1
61370	3.78448	2.1
61371	1.89224	2.1
61372	1.703 2.1	
61373	1.89224	2.1
61374	1.72991	2.1
61375	1.07643	2.1
61376	1.36657	2.1
61377	1.44472	2.1
61378	2.41233	2.1
61379	2.51628	2.1
61380	2.51628	2.1
61381	1.42174	2.1
61382	1.42174	2.1
61383	1.89226	2.1
61384	1.89226	2.1
61385	1.89226	2.1
61386	1.89226	2.1
61387	1.89226	2.1
61388	1.89226	2.1
61389	1.89226	2.1
61390	1.89226	2.1
61391	1.89226	2.1
61392	1.89226	2.1
61393	3.78452	2.1
61394	2.51628	2.1
61395	1.89226	2.1
61396	1.89226	2.1
61397	1.89226	2.1
61398	2.41234	2.1
61399	2.41234	2.1
61400	0.996254	2.1
61401	0.996254	2.1
61402	1.01153	2.1
61403	1.01153	2.1
61404	1.28542	2.1
61405	1.25814	2.1
61406	0.719184	2.1
61407	0.72236	2.1
61408	0.719183	2.1
61409	1.28542	2.1
61410	0.722359	2.1
61411	1.25814	2.1
61412	0.946126	2.1
61413	0.946126	2.1
61414	0.946131	2.1
61415	0.946131	2.1
61416	0.946127	2.1
61417	0.946127	2.1
61418	0.94613	2.1
61419	0.94613	2.1

61420	0.946128	2.1
61421	0.946128	2.1
61422	0.946128	2.1
61423	0.946128	2.1
61424	1.89226	2.1
61425	1.89226	2.1
61426	1.89226	2.1
61427	1.89226	2.1
61428	1.89226	2.1
61429	1.89226	2.1
61430	0.946132	2.1
61431	0.946132	2.1
61432	1.25814	2.1
61433	1.25814	2.1
61434	0.946131	2.1
61435	0.946131	2.1
61436	1.28543	2.1
61437	1.25814	2.1
61438	1.28543	2.1
61439	0.946132	2.1
61440	1.25814	2.1
61441	0.946132	2.1
61442	1.89226	1.24927
61443	1.89226	0.569413
61444	1.89226	1.00344
61445	1.89226	0.891002
61446	1.89226	0.766078
61447	1.89226	0.676101
61448	1.89226	1.01695
61449	2.27719	0.490696
61450	1.89226	0.564553
61451	1.89226	0.648958
61452	3.78452	0.596962
61453	1.89225	1.46024
61454	1.89225	0.644859
61455	1.89225	0.928536
61456	1.89225	1.11306
61457	3.78451	1.03637
61458	1.89226	0.504279
61459	1.89226	0.53696
61460	1.89226	0.782252
61461	1.89226	0.381117
61462	3.78452	0.533943
61463	0.946132	0.326787
61464	0.946132	0.498688
61465	0.946132	0.54491
61466	0.946132	0.450564
61467	0.946131	0.395507
61468	0.946131	0.32609
61469	0.946132	0.534881
61470	0.946132	0.377536
61471	0.946132	0.399971
61472	0.946132	0.406342
61473	0.946132	0.409511
61474	0.946132	0.374729
61475	0.946126	0.582387
61476	0.946126	0.493669
61477	0.946132	1.41945

61478	0.946132	0.677595
61479	0.946128	0.499017
61480	0.946128	0.612102
61481	0.946129	0.6412
61482	0.946129	1.06263
61483	0.946129	0.765309
61484	0.946129	0.817201
61485	0.946129	0.675327
61486	0.946129	0.61304
61487	1.39858	2.1
61488	1.39858	2.1
61489	0.94613	2.1
61490	0.94613	2.1
61491	0.946132	2.1
61492	0.946132	2.1
61493	1.39858	2.1
61494	1.25002	2.1
61495	1.39858	2.1
61496	0.946131	2.1
61497	1.25002	2.1
61498	0.946131	2.1
61499	2.0815	2.1
61500	1.89226	2.1
61501	1.89226	2.1
61502	2.05298	2.1
61503	2.5593	2.1
61504	2.77667	2.1
61505	0.946133	2.1
61506	0.946133	2.1
61507	0.946132	2.1
61508	0.946132	2.1
61509	0.946132	2.1
61510	0.946132	2.1
61511	0.946133	2.1
61512	0.946133	2.1
61513	0.946132	2.1
61514	0.946132	2.1
61515	0.946132	2.1
61516	0.946132	2.1
61517	1.38908	2.1
61518	1.38908	2.1
61519	0.946132	2.1
61520	0.946132	2.1
61521	0.946132	2.1
61522	0.946132	2.1
61523	1.38908	2.1
61524	1.27965	2.1
61525	1.38908	2.1
61526	0.946132	2.1
61527	0.946133	2.1
61528	1.27965	2.1
61529	0.946133	2.1
61530	0.946133	2.1
61531	0.946132	2.1
61532	0.946132	2.1
61533	0.946132	2.1
61534	0.946132	2.1
61535	0.946133	2.1

61536	0.946133	2.1
61537	0.946132	2.1
61538	0.946132	2.1
61539	0.946133	2.1
61540	0.946133	2.1
61541	1.89227	1.33822
61542	1.89227	1.58557
61543	1.89227	0.833501
61544	1.89227	0.392696
61545	3.78453	1.07364
61546	1.33154	0.961091
61547	1.33154	0.73141
61548	0.946132	0.907783
61549	0.946132	0.787172
61550	1.19095	0.545273
61551	1.33154	0.999979
61552	0.946133	1.24083
61553	0.946133	1.36175
61554	0.946133	1.64027
61555	1.19095	0.679653
61556	0.946132	1.45012
61557	1.33154	0.888808
61558	1.0888	0.656956
61559	1.04745	0.491492
61560	0.946132	0.674466
61561	0.946132	0.551966
61562	1.04745	0.333372
61563	1.04745	1.00404
61564	0.946132	1.41487
61565	0.946132	0.366124
61566	1.04745	1.08548
61567	0.946133	0.381722
61568	0.946132	0.954953
61569	1.0888	0.637741
61570	1.89226	1.79677
61571	1.89226	0.362627
61572	1.89226	0.469099
61573	1.89226	0.90414
61574	1.89226	0.520573
61575	1.89226	0.72084
61576	1.89226	1.65546
61577	1.87592	0.331859
61578	1.87592	0.5054
61579	2.3819	0.65371
61580	1.89226	0.783924
61581	2.39825	0.853866
61582	1.89226	0.377553
61583	2.0949	1.12985
61584	1.88741	0.282841
61585	1.89226	0.281743
61586	1.89226	0.435228
61587	1.88741	0.767008
61588	1.07508	1.59955
61589	1.63419	1.94442
61590	1.63419	1.81909
61591	1.09795	1.83285
61592	1.56137	1.83893
61593	1.02513	1.94703

61594	0.967672	0.917163
61595	1.56137	1.50998
61596	1.56137	1.02841
61597	1.07508	1.26886
61598	1.41319	0.983078
61599	0.926902	1.48835
61600	1.56137	1.23145
61601	1.63419	1.81856
61602	2.29852	1.68718
61603	2.14485	1.50572
61604	3.77904	1.24014
61605	1.41319	0.504727
61606	1.92622	0.593257
61607	1.56137	0.493369
61608	1.56137	0.943484
61609	2.14485	1.21206
61610	1.92622	0.938501
61611	1.89227	2.1
61612	1.89227	2.1
61613	1.89227	2.1
61614	1.89227	2.1
61615	3.78453	2.1
61616	2.56618	2.1
61617	2.08612	2.1
61618	2.48048	2.1
61619	1.99644	2.1
61620	4.5666	2.1
61621	2.08612	2.1
61622	1.30659	2.1
61623	1.99644	2.1
61624	1.25404	2.1
61625	3.34016	2.1
61626	1.89226	2.1
61627	1.89226	2.1
61628	1.89226	2.1
61629	1.89226	2.1
61630	1.89226	2.1
61631	1.89226	2.1
61632	1.89227	2.1
61633	1.89227	2.1
61634	1.89227	2.1
61635	1.89227	2.1
61636	3.78453	2.1
61637	2.48048	2.1
61638	1.99644	2.1
61639	2.39022	2.1
61640	1.90734	2.1
61641	4.38782	2.1
61642	1.90734	2.1
61643	1.99644	2.1
61644	1.21564	2.1
61645	1.21564	2.1
61646	1.25404	2.1
61647	1.94574	2.1
61648	0.810946	2.1
61649	0.810946	2.1
61650	0.810947	2.1
61651	0.810947	2.1

61652	1.89226	2.1
61653	1.3517	2.1
61654	1.08133	2.1
61655	1.08132	2.1
61656	1.08132	2.1
61657	0.810934	2.1
61658	0.810934	2.1
61659	1.89226	2.1
61660	1.89226	1.55566
61661	1.89226	2.0544
61662	1.89226	2.1
61663	3.78453	2.1
61664	1.89226	0.715656
61665	1.89226	0.226474
61666	1.89226	1.31116
61667	1.89226	0.633952
61668	1.89226	0.876362
61669	1.89226	0.251698
61670	0.946131	0.5552
61671	0.946131	0.440871
61672	0.94613	0.990759
61673	0.94613	0.778224
61674	0.946128	0.507455
61675	0.946128	0.369648
61676	0.946133	0.294592
61677	0.946133	0.960061
61678	0.946131	0.661635
61679	0.946131	0.702368
61680	0.946131	0.875638
61681	0.946131	0.615193
61682	1.89226	2.1
61683	1.89226	1.14889
61684	1.89226	2.08717
61685	1.89226	2.0125
61686	1.89226	1.6683
61687	1.89226	1.35467
61688	2.56348	1.30184
61689	1.89226	2.1
61690	1.89226	2.1
61691	2.53234	1.58906
61692	4.45574	1.45963
61693	2.53234	0.354029
61694	1.65583	0.142419
61695	2.56348	0.331029
61696	2.56348	0.440806
61697	1.66295	0.304827
61698	1.65583	0.112321
61699	0.736602	0.219787
61700	0.827916	0.297544
61701	1.26617	0.473114
61702	1.18322	0.0793044
61703	0.7366	0.255771
61704	1.18321	0.246074
61705	1.26617	0.310273
61706	0.827918	0.309911
61707	0.99929	0.334872
61708	0.99929	0.381737
61709	1.00348	0.160387

61710	1.00348	0.173835
61711	1.26617	0.48503
61712	1.18322	0.871386
61713	0.946131	2.1
61714	0.946131	1.93491
61715	0.946129	0.870115
61716	1.18321	0.622487
61717	0.946133	2.1
61718	1.26617	2.04914
61719	1.26617	2.05362
61720	1.26617	0.352077
61721	0.946131	2.1
61722	0.946131	1.75025
61723	2.23267	0.0590005
61724	1.89226	0.0964922
61725	2.07099	0.0903888
61726	1.89226	0.117959
61727	2.07099	0.090755
61728	1.89226	0.0742467
61729	0.946132	0.155722
61730	1.35403	0.0791546
61731	1.24038	0.0856911
61732	0.946131	0.0650571
61733	0.946131	0.126471
61734	0.946131	0.144808
61735	1.24039	0.110241
61736	1.35403	0.0833601
61737	1.24038	0.13048
61738	1.24038	0.144096
61739	0.946131	0.0803802
61740	0.946131	0.186339
61741	1.89226	0.213712
61742	1.89226	0.629355
61743	1.89226	0.179801
61744	1.89226	0.188669
61745	1.89226	0.64625
61746	1.89226	0.613451
61747	1.89226	0.134593
61748	1.89226	0.453763
61749	1.89226	0.200897
61750	1.89226	0.307751
61751	3.78452	0.387466
61752	1.56971	0.0783337
61753	2.80086	0.20075
61754	2.59806	0.110551
61755	1.93489	0.10568
61756	2.59806	0.200139
61757	1.93489	0.110329
61758	0.827467	0.05
61759	1.26652	0.0740157
61760	1.13679	0.0959448
61761	0.639035	0.0964843
61762	1.11547	0.152556
61763	1.30656	0.151996
61764	0.869062	0.051916
61765	0.696608	0.0877397
61766	1.07205	0.0671241
61767	0.953656	0.165676

61768	0.970744	0.0870265
61769	0.970744	0.190004
61770	2.38681	2.1
61771	2.50438	2.09594
61772	2.42082	2.1
61773	2.59106	2.1
61774	4.92521	2.1
61775	2.50438	2.0548
61776	2.40627	2.1
61777	2.59106	2.1
61778	2.5444	1.82392
61779	5.04879	2.1
61780	2.42082	2.1
61781	2.70575	2.1
61782	2.64778	2.1
61783	2.59106	2.1
61784	2.47753	2.1
61785	2.42082	2.09778
61786	2.70575	2.1
61787	2.5444	1.98316
61788	2.76095	2.1
61789	2.59106	2.1
61790	2.71429	2.1
61791	2.5444	2.098
61792	1.89226	2.1
61793	2.38473	2.1
61794	2.36689	2.1
61795	2.38473	2.1
61796	1.87442	2.1
61797	1.89226	2.1
61798	1.89226	2.1
61799	1.89226	2.1
61800	1.89226	2.1
61801	1.89226	2.1
61802	1.89226	2.1
61803	1.89226	2.1
61804	2.14952	2.1
61805	1.89226	2.1
61806	1.89226	2.1
61807	1.89226	2.1
61808	2.0692	2.1
61809	2.0692	2.1
61810	1.89226	2.1
61811	2.36689	2.1
61812	2.36204	2.1
61813	2.36689	2.1
61814	1.88742	2.1
61815	1.89226	2.1
61816	0.946132	2.1
61817	0.946132	2.1
61818	0.946131	2.1
61819	0.946131	2.1
61820	0.946132	2.1
61821	0.946132	2.1
61822	0.946131	2.1
61823	0.946131	2.1
61824	0.946132	2.1
61825	0.946132	2.1

61826	0.946131	2.1
61827	0.946131	2.1
61828	0.946128	2.1
61829	0.946128	2.1
61830	1.07476	2.1
61831	1.07476	2.1
61832	0.946131	2.1
61833	0.946131	2.1
61834	1.13182	2.1
61835	1.07476	2.1
61836	1.13182	2.1
61837	0.946131	2.1
61838	1.07476	2.1
61839	0.94613	2.1
61840	1.89227	0.0915309
61841	1.89227	0.130258
61842	1.89227	0.18653
61843	1.89227	0.113505
61844	3.78453	0.0980884
61845	1.89226	0.0954428
61846	1.89226	0.0787542
61847	1.89226	0.0961573
61848	1.89226	0.122323
61849	3.78452	0.104471
61850	1.89226	0.0712846
61851	1.89226	0.140725
61852	1.89226	0.133309
61853	1.89226	0.108086
61854	3.78452	0.0810693
61855	1.89226	0.118949
61856	1.89226	0.0967218
61857	1.89226	0.10354
61858	1.89226	0.134981
61859	1.89226	0.139807
61860	1.89226	0.140766
61861	2.58344	0.245538
61862	2.00267	0.0912377
61863	2.14218	0.170011
61864	2.22674	0.228559
61865	2.14218	0.098305
61866	2.22674	0.158363
61867	2.83531	0.254766
61868	1.56043	0.103303
61869	2.22674	0.212365
61870	1.90912	0.262443
61871	2.22674	0.0836342
61872	1.90912	0.151193
61873	0.780217	0.159406
61874	1.11337	0.177345
61875	1.00133	0.222319
61876	0.804472	0.140584
61877	0.804472	0.138487
61878	0.780217	0.116086
61879	1.00133	0.0908742
61880	1.11337	0.205753
61881	0.856668	0.211029
61882	0.856668	0.201295
61883	0.924883	0.10438

61884	0.924883	0.137146
61885	1.93107	2.1
61886	1.68657	2.1
61887	1.7237	2.1
61888	1.89226	2.1
61889	1.53436	2.1
61890	1.68657	2.1
61891	1.89226	2.1
61892	1.89226	2.1
61893	1.89226	2.1
61894	1.89226	2.1
61895	3.78453	2.1
61896	0.946131	2.1
61897	0.946131	2.1
61898	0.855092	2.1
61899	0.83953	2.1
61900	0.946131	2.1
61901	0.946131	2.1
61902	0.839531	2.1
61903	0.839531	2.1
61904	0.946131	2.1
61905	0.855093	2.1
61906	0.839531	2.1
61907	0.946131	2.1
61908	1.89226	2.1
61909	1.70302	2.1
61910	2.21419	2.1
61911	1.93107	2.1
61912	1.97872	2.1
61913	1.70302	2.1
61914	0.946133	2.1
61915	0.946133	2.1
61916	0.946132	2.1
61917	0.946132	2.1
61918	0.946132	2.1
61919	0.946132	2.1
61920	0.946132	2.1
61921	0.946132	2.1
61922	0.946133	2.1
61923	0.946133	2.1
61924	0.946132	2.1
61925	0.946132	2.1
61926	1.90081	2.1
61927	1.89226	2.1
61928	1.51379	2.1
61929	1.36813	2.1
61930	1.71019	2.1
61931	1.89226	2.1
61932	1.89227	1.44155
61933	1.89227	0.417666
61934	1.89227	0.533519
61935	1.89227	1.40314
61936	1.89227	1.18265
61937	1.89227	1.62746
61938	1.36428	1.10099
61939	1.37869	1.16408
61940	0.946132	0.798567
61941	0.946132	1.19959

61942	1.37869	0.586688
61943	1.37869	1.90411
61944	0.946133	1.1992
61945	0.946133	1.39427
61946	1.37869	0.641733
61947	0.946132	0.885784
61948	1.36428	1.1885
61949	0.946133	0.495379
61950	0.94612	0.678605
61951	0.94612	0.874146
61952	0.946119	0.934712
61953	0.946119	0.939016
61954	0.946119	0.386693
61955	0.946119	1.89764
61956	0.94612	1.1892
61957	0.94612	1.76726
61958	0.946132	0.785089
61959	0.946132	0.474905
61960	0.946107	0.591414
61961	0.946107	1.39614
61962	1.89225	0.05
61963	1.89225	2.1
61964	1.63564	0.05
61965	1.42278	2.1
61966	1.42278	2.1
61967	1.57534	0.05
61968	1.53735	2.1
61969	1.68991	0.05
61970	1.57535	0.05
61971	1.04442	2.1
61972	1.04442	2.1
61973	1.22031	0.05
61974	1.42278	2.1
61975	1.59868	0.05
61976	0.946132	2.1
61977	0.946132	2.1
61978	0.946131	2.1
61979	0.946131	2.1
61980	0.946131	2.1
61981	0.946131	2.1
61982	0.946132	2.1
61983	0.946132	2.1
61984	0.946132	2.1
61985	0.946132	2.1
61986	0.946131	2.1
61987	0.946131	2.1
61988	1.89226	2.1
61989	1.89226	2.1
61990	1.89226	2.1
61991	1.89226	2.1
61992	1.89226	2.1
61993	1.89226	2.1
61994	2.03143	1.48381
61995	1.47111	2.03742
61996	2.28685	2.02047
61997	2.28685	2.04
61998	1.36006	1.66039
61999	1.36006	1.77487

62000	1.89226	0.877257
62001	1.89226	2.0065
62002	1.89226	1.85464
62003	1.89226	1.96616
62004	1.89226	1.05266
62005	1.89226	1.63395
62006	1.89226	1.91136
62007	1.89226	2.1
62008	1.89226	2.1
62009	1.89226	2.1
62010	1.89226	1.96714
62011	1.89226	2.0931
62012	0.902821	2.1
62013	0.902821	2.1
62014	0.889218	2.1
62015	0.889218	2.1
62016	0.675759	2.1
62017	1.05649	2.1
62018	0.735554	2.1
62019	1.14342	2.1
62020	1.14343	2.1
62021	1.05649	2.1
62022	0.735554	2.1
62023	0.675758	2.1
62024	2.46286	0.489902
62025	2.28685	1.10305
62026	2.67843	0.890939
62027	2.67843	0.996402
62028	2.03143	0.585407
62029	2.03143	0.784495
62030	1.89226	0.853947
62031	1.89226	1.13639
62032	1.89226	0.468981
62033	1.89226	0.525883
62034	3.78452	1.41879
62035	0.94613	0.716416
62036	0.94613	1.8288
62037	0.946128	2.07793
62038	0.946128	1.86749
62039	0.94613	2.1
62040	0.94613	1.80236
62041	0.946128	0.56908
62042	0.946128	1.62625
62043	0.946129	0.141534
62044	0.946129	1.92262
62045	0.946129	1.1448
62046	0.946129	2.1
62047	2.67843	1.51872
62048	2.11297	2.09074
62049	2.52453	1.99427
62050	2.52453	2.08738
62051	2.28685	1.59979
62052	2.28685	1.92585
62053	1.89226	1.41701
62054	1.89226	2.1
62055	1.89226	1.16071
62056	1.89226	2.1
62057	3.78451	2.08271

62058	2.39524	0.960094
62059	2.10188	2.1
62060	2.29839	1.14128
62061	2.02242	2.1
62062	4.40028	2.09709
62063	1.71839	2.1
62064	1.65261	2.1
62065	2.10189	2.1
62066	2.10189	2.1
62067	2.02243	2.1
62068	1.63893	2.1
62069	0.946132	2.1
62070	0.946132	2.1
62071	0.946129	2.1
62072	0.946129	2.1
62073	0.94613	2.1
62074	0.94613	2.1
62075	0.946131	2.1
62076	0.946131	2.1
62077	0.946131	2.1
62078	0.946131	2.1
62079	0.946131	2.1
62080	0.946131	2.1
62081	1.89226	1.00792
62082	1.89226	2.1
62083	1.89226	1.21981
62084	1.89226	2.1
62085	3.78452	2.0561
62086	2.2984	0.969079
62087	2.02242	2.1
62088	2.21582	1.24828
62089	1.94412	2.1
62090	4.24251	2.04625
62091	1.94412	2.1
62092	1.5854	2.1
62093	2.02243	2.1
62094	2.02243	2.1
62095	1.57431	2.1
62096	1.65261	2.1
62097	0.810943	2.1
62098	0.810943	2.1
62099	0.810945	2.1
62100	0.810945	2.1
62101	1.89226	2.1
62102	1.3517	2.1
62103	1.08133	2.1
62104	1.08132	2.1
62105	1.08132	2.1
62106	0.810933	2.1
62107	0.810933	2.1
62108	1.89226	0.05
62109	1.89226	0.0945905
62110	1.89226	0.0762506
62111	1.89226	0.0624639
62112	1.89226	0.0673259
62113	1.89226	0.0554895
62114	1.89226	0.0781315
62115	1.89226	0.160742

62116	1.89226	0.097516
62117	1.89226	0.131135
62118	3.78452	0.13515
62119	1.89226	0.0614015
62120	1.89226	0.115587
62121	1.89226	0.0911293
62122	1.89226	0.0770014
62123	1.89226	0.0864495
62124	1.89226	0.0727345
62125	0.946128	0.134217
62126	0.946128	0.0812561
62127	0.946132	0.0763386
62128	0.946132	0.106473
62129	0.94613	0.130003
62130	0.94613	0.143165
62131	0.946129	0.0889002
62132	0.946129	0.0748295
62133	0.94613	0.0786549
62134	0.94613	0.100208
62135	0.94613	0.0855428
62136	0.94613	0.100859
62137	1.89226	0.0518555
62138	1.89226	0.103442
62139	1.89226	0.0703755
62140	1.89226	0.067401
62141	1.89226	0.0900672
62142	1.89226	0.0885309
62143	2.81478	2.1
62144	1.89226	2.1
62145	2.65557	2.1
62146	1.89226	2.1
62147	4.54783	2.1
62148	1.89226	2.1
62149	1.89226	2.1
62150	1.89226	2.1
62151	1.89226	2.1
62152	3.78452	2.1
62153	2.65557	2.1
62154	1.89226	2.1
62155	1.89226	2.1
62156	1.89226	2.1
62157	2.57997	2.1
62158	2.57997	2.1
62159	0.946131	2.1
62160	0.946131	2.1
62161	0.946128	2.1
62162	0.946128	2.1
62163	0.94613	2.1
62164	0.94613	2.1
62165	0.946129	2.1
62166	0.946129	2.1
62167	0.94613	2.1
62168	0.94613	2.1
62169	0.94613	2.1
62170	0.94613	2.1
62171	1.89226	1.20793
62172	2.27703	1.92138
62173	2.35615	1.94763

62174	2.35615	1.43055
62175	1.89226	1.94873
62176	1.89226	1.41413
62177	0.946132	1.20458
62178	1.17807	2.01468
62179	1.14926	1.09636
62180	0.946132	1.82305
62181	0.946133	1.79029
62182	0.946133	1.81624
62183	1.14926	1.32541
62184	1.14926	1.29909
62185	0.946132	1.74119
62186	0.946132	1.49766
62187	1.17807	1.7833
62188	1.14926	1.44138
62189	2.16841	0.575232
62190	1.35487	1.51443
62191	1.19854	1.16095
62192	1.19854	0.63573
62193	2.46078	1.1204
62194	2.46078	0.756942
62195	0.69308	0.605606
62196	1.3119	1.31248
62197	0.677433	1.61673
62198	1.23039	0.788962
62199	0.978178	1.59105
62200	0.978178	1.51128
62201	0.945294	0.681185
62202	0.945294	0.770745
62203	1.3119	1.06228
62204	1.23039	1.01292
62205	0.693081	1.05489
62206	0.677432	1.11221
62207	1.89226	1.31728
62208	1.89226	1.96959
62209	1.89226	1.94683
62210	1.89226	1.4322
62211	1.89226	1.94666
62212	1.89226	1.45619
62213	0.946132	1.23246
62214	0.946132	1.99989
62215	0.946132	0.88432
62216	0.946132	1.8052
62217	0.946133	1.76532
62218	0.946133	1.77761
62219	0.946132	1.23887
62220	0.946132	1.07468
62221	0.946133	1.65265
62222	0.946133	1.31808
62223	0.946132	1.67198
62224	0.946132	1.38027
62225	2.60637	2.1
62226	1.89226	2.1
62227	1.89226	2.1
62228	1.89226	1.9943
62229	3.78452	2.1
62230	1.89226	1.25598
62231	1.8396	1.85007

62232	1.89226	1.974
62233	1.89226	1.28389
62234	1.60993	2.06249
62235	1.60993	1.45141
62236	0.946131	1.85247
62237	0.946131	2.02306
62238	0.946128	2.1
62239	0.946128	2.07919
62240	0.946131	1.81555
62241	0.946131	2.04463
62242	0.946127	2.1
62243	0.946127	1.98983
62244	0.946129	2.0742
62245	0.946129	2.06616
62246	0.94613	2.08759
62247	0.94613	2.08291
62248	1.89226	2.1
62249	1.89226	1.96509
62250	1.89226	2.09682
62251	1.89226	2.08119
62252	1.89226	2.09839
62253	1.89226	2.08556
62254	1.89226	1.25638
62255	1.89226	1.79975
62256	1.89226	1.96948
62257	1.89226	1.25013
62258	1.89226	2.01442
62259	1.89226	1.2713
62260	0.946131	1.79768
62261	0.946131	1.98619
62262	0.946128	2.09518
62263	0.946128	2.02195
62264	0.946131	1.77504
62265	0.946131	1.98533
62266	0.946127	1.97518
62267	0.946127	2.09612
62268	0.94613	2.03355
62269	0.94613	2.0288
62270	0.946129	2.0431
62271	0.946129	2.04545
62272	0.946133	2.1
62273	0.946133	2.1
62274	1.28153	2.1
62275	1.26781	2.1
62276	0.946132	2.1
62277	0.946132	2.1
62278	1.28153	2.1
62279	1.28153	2.1
62280	0.946132	2.1
62281	1.28153	2.1
62282	0.946133	2.1
62283	1.26781	2.1
62284	1.89226	2.1
62285	1.89226	2.1
62286	1.89226	2.1
62287	1.89226	2.1
62288	1.89226	2.1
62289	1.89226	2.1

62290	2.5844	2.1
62291	1.09563	2.1
62292	1.1468	2.1
62293	1.09563	2.1
62294	2.6142	2.1
62295	2.56304	2.1
62296	0.946126	2.1
62297	0.946126	2.1
62298	1.2922	2.1
62299	1.28152	2.1
62300	0.946126	2.1
62301	0.946126	2.1
62302	1.2922	2.1
62303	1.2922	2.1
62304	1.29221	2.1
62305	0.946132	2.1
62306	0.946119	2.1
62307	1.28151	2.1
62308	1.89225	2.1
62309	1.89225	2.1
62310	1.89225	2.1
62311	1.89225	2.1
62312	1.89225	2.1
62313	1.89225	2.1
62314	0.946121	2.1
62315	0.946121	2.1
62316	0.946126	2.1
62317	0.946126	2.1
62318	0.946124	2.1
62319	0.946124	2.1
62320	0.946123	2.1
62321	0.946123	2.1
62322	0.94613	2.1
62323	0.94613	2.1
62324	0.946117	2.1
62325	0.946117	2.1
62326	1.89226	2.08831
62327	1.89226	2.0125
62328	1.89226	1.90835
62329	1.89226	2.1
62330	1.89226	1.37212
62331	1.89226	2.1
62332	1.89226	2.09531
62333	1.89226	2.03086
62334	1.89226	1.77205
62335	1.89226	2.1
62336	1.89226	1.31079
62337	1.89226	2.1
62338	1.89226	2.1
62339	2.81281	2.1
62340	2.50369	2.02748
62341	2.50369	2.1
62342	1.89226	2.03446
62343	1.89226	2.1
62344	1.89226	2.1
62345	1.89226	2.1
62346	1.89226	2.1
62347	1.89226	2.1

62348	1.89226	2.1
62349	1.89226	2.1
62350	1.89226	2.1
62351	1.89226	2.1
62352	1.89226	2.1
62353	1.89226	2.1
62354	1.89226	2.1
62355	1.89226	2.1
62356	0.907036	2.1
62357	1.09714	2.1
62358	0.994609	2.1
62359	0.838808	2.1
62360	0.976536	2.1
62361	0.976536	2.1
62362	0.959412	2.1
62363	0.959412	2.1
62364	1.09714	2.1
62365	0.994609	2.1
62366	0.907036	2.1
62367	0.838808	2.1
62368	0.99461	2.1
62369	0.838808	2.1
62370	0.738212	2.1
62371	0.565634	2.1
62372	0.775921	2.1
62373	0.775921	2.1
62374	0.784322	2.1
62375	0.784322	2.1
62376	0.994609	2.1
62377	0.738212	2.1
62378	0.838808	2.1
62379	0.565635	2.1
62380	1.89226	2.1
62381	1.89226	2.1
62382	1.89226	2.1
62383	1.89226	2.1
62384	1.89226	2.1
62385	1.89226	2.1
62386	1.89226	2.1
62387	1.89226	2.1
62388	1.89226	2.1
62389	1.89226	2.1
62390	1.89226	2.1
62391	1.89226	2.1
62392	2.19428	2.1
62393	1.98922	2.1
62394	2.27322	2.1
62395	2.45893	2.1
62396	4.4675	2.1
62397	0.99461	2.1
62398	1.13661	2.1
62399	0.902272	2.1
62400	0.738212	2.1
62401	0.931874	2.1
62402	0.931874	2.1
62403	0.942953	2.1
62404	0.942953	2.1
62405	1.13658	2.1

62406	0.902248	2.1
62407	0.994637	2.1
62408	0.738233	2.1
62409	1.89226	2.03857
62410	1.89226	2.05649
62411	1.89226	2.0906
62412	1.89226	2.06062
62413	1.89226	2.07144
62414	1.89226	2.00533
62415	1.89227	2.03471
62416	2.58246	1.87925
62417	1.89227	2.02796
62418	1.89227	1.9555
62419	3.78453	1.94822
62420	1.89226	2.09284
62421	1.89226	2.0282
62422	1.65573	2.07964
62423	1.65573	2.08668
62424	1.89226	2.06048
62425	1.89226	2.06678
62426	1.89226	1.9122
62427	1.89226	1.83521
62428	1.89226	1.90802
62429	1.89226	1.93642
62430	1.89226	1.83795
62431	1.89226	1.85384
62432	1.89226	1.72948
62433	1.89226	1.76632
62434	1.89226	1.61507
62435	1.89226	1.84193
62436	1.89226	1.54552
62437	1.89226	1.8138
62438	0.946132	2.03894
62439	0.946132	2.00438
62440	1.01023	1.92449
62441	1.13651	2.06554
62442	1.01023	1.90842
62443	0.946133	1.99991
62444	1.0644	2.01777
62445	1.0644	2.0426
62446	0.946132	2.01785
62447	0.946132	2.02355
62448	1.13651	2.04403
62449	0.946132	2.00613
62450	1.89226	0.725559
62451	1.89226	1.55541
62452	1.89226	1.20441
62453	1.89226	0.648707
62454	3.78451	0.792034
62455	1.00934	0.439555
62456	0.741561	0.36766
62457	0.946131	0.561982
62458	0.946131	0.604315
62459	0.946131	0.733775
62460	0.741561	0.326862
62461	1.00934	0.31809
62462	0.946131	0.584551
62463	0.741586	0.475276

62464	0.741586	0.5377
62465	0.946106	0.537624
62466	0.946106	0.403833
62467	1.14895	0.693963
62468	0.957449	0.862651
62469	0.934806	1.3583
62470	0.934806	1.19432
62471	1.13536	0.615305
62472	1.02829	1.45278
62473	0.946129	1.61791
62474	0.946129	0.727731
62475	0.946127	1.1047
62476	0.946127	1.18587
62477	0.946127	0.980045
62478	1.0283	1.05474
62479	1.89226	1.65368
62480	1.89226	0.803285
62481	1.89226	0.776999
62482	1.89226	1.4784
62483	3.78452	1.28724
62484	1.89226	0.495324
62485	1.89226	0.733186
62486	1.89226	0.748948
62487	1.89226	0.484169
62488	3.78453	0.518045
62489	1.89226	0.744634
62490	2.69068	0.487747
62491	1.89226	0.496558
62492	1.89226	0.658126
62493	3.78452	0.626086
62494	1.89226	0.819275
62495	1.89226	1.55307
62496	1.89226	1.55726
62497	1.89226	0.969876
62498	1.89226	1.44508
62499	1.89226	0.825512
62500	1.89226	2.1
62501	1.89226	2.1
62502	1.89226	2.1
62503	1.89226	2.1
62504	1.89226	2.1
62505	1.89226	2.1
62506	1.89226	2.1
62507	1.89226	2.1
62508	1.89226	2.1
62509	1.89226	2.1
62510	1.89226	2.1
62511	1.89226	2.1
62512	0.985109	2.1
62513	1.31144	2.1
62514	1.45864	2.1
62515	1.10667	2.1
62516	2.44375	2.1
62517	0.655718	2.1
62518	0.729316	2.1
62519	0.684594	2.1
62520	0.782707	2.1
62521	0.700849	2.1

62522	0.700849	2.1
62523	0.71306	2.1
62524	0.71306	2.1
62525	0.729318	2.1
62526	0.782705	2.1
62527	0.684592	2.1
62528	0.655719	2.1
62529	0.946128	2.1
62530	0.946128	2.1
62531	0.946133	2.1
62532	0.946133	2.1
62533	0.946131	2.1
62534	0.946131	2.1
62535	0.946131	2.1
62536	0.946131	2.1
62537	0.94613	2.1
62538	0.94613	2.1
62539	0.946131	2.1
62540	0.946131	2.1
62541	1.89226	2.1
62542	1.89226	2.1
62543	1.89226	2.1
62544	1.89226	2.1
62545	1.89226	2.1
62546	1.89226	2.1
62547	0.553334	2.1
62548	0.629936	2.1
62549	0.828211	2.1
62550	0.729319	2.1
62551	0.674025	2.1
62552	0.674025	2.1
62553	0.68523	2.1
62554	0.68523	2.1
62555	0.828211	2.1
62556	0.629935	2.1
62557	0.72932	2.1
62558	0.553334	2.1
62559	0.828208	2.1
62560	0.729316	2.1
62561	0.907037	2.1
62562	0.782707	2.1
62563	0.799048	2.1
62564	0.799048	2.1
62565	0.811867	2.1
62566	0.811867	2.1
62567	0.828209	2.1
62568	0.907035	2.1
62569	0.729318	2.1
62570	0.782706	2.1
62571	2.32981	2.1
62572	1.89226	2.1
62573	1.89226	2.1
62574	1.89226	2.1
62575	2.2671	2.1
62576	2.2671	2.1
62577	1.89226	2.1
62578	1.89226	2.09306
62579	1.89226	2.1

62580	1.89226	2.03168
62581	1.89226	2.1
62582	1.89226	2.0976
62583	1.3991	2.1
62584	1.81793	2.04121
62585	2.07226	2.1
62586	2.07226	2.00037
62587	1.24924	2.1
62588	1.24924	2.1
62589	0.946131	2.1
62590	0.946131	2.1
62591	1.16491	2.1
62592	1.16491	2.1
62593	0.946131	2.1
62594	0.946131	2.1
62595	1.16491	2.1
62596	1.19523	2.1
62597	0.946132	2.1
62598	1.19523	2.1
62599	1.16491	2.1
62600	0.946131	2.1
62601	1.89226	2.1
62602	1.89226	2.07419
62603	1.89226	2.1
62604	1.89226	1.51391
62605	3.78452	2.0921
62606	0.901204	2.09647
62607	0.901204	2.1
62608	0.902504	2.1
62609	0.902504	2.1
62610	1.10416	2.04555
62611	1.03613	1.80951
62612	0.764922	2.1
62613	0.699552	2.1
62614	0.764921	2.1
62615	1.10416	2.1
62616	0.699551	2.1
62617	1.03613	2.09243
62618	1.89226	1.23974
62619	1.89226	1.42321
62620	1.89226	1.5111
62621	1.89226	1.26198
62622	3.78453	1.46253
62623	1.89226	1.63111
62624	1.89226	1.60281
62625	1.89226	1.55083
62626	1.89226	1.77082
62627	1.89226	1.56398
62628	1.89226	1.7762
62629	0.946131	1.38057
62630	0.946131	1.49789
62631	0.946131	1.16886
62632	0.946131	1.26831
62633	0.946131	1.17146
62634	0.946131	1.37869
62635	0.946131	1.49739
62636	0.946131	1.17253
62637	0.946131	1.14414

62638	0.946131	1.14933
62639	0.946131	1.15996
62640	0.946131	1.25779
62641	1.89226	1.59557
62642	1.89226	1.5654
62643	1.89226	1.50768
62644	1.89226	1.71076
62645	1.89226	1.49682
62646	1.89226	1.70029
62647	0.946127	1.36178
62648	0.946127	1.45501
62649	1.22788	1.44992
62650	1.35469	1.45182
62651	1.35469	1.38799
62652	1.35469	1.44088
62653	0.946128	1.45519
62654	0.946128	1.38596
62655	0.946129	1.38869
62656	1.22788	1.41382
62657	1.35469	1.41972
62658	0.946129	1.35045
62659	1.22788	1.41109
62660	1.35469	1.409
62661	1.02602	1.18603
62662	0.794861	1.2359
62663	1.15301	1.43379
62664	1.15301	1.40642
62665	1.10089	1.15829
62666	1.10089	1.35521
62667	0.794861	1.15195
62668	1.22788	1.45539
62669	1.02602	1.40078
62670	1.35469	1.37568
62671	1.89226	0.247256
62672	1.89226	1.48188
62673	1.89226	1.24276
62674	1.89226	0.472273
62675	3.78452	0.620134
62676	1.13536	2.1
62677	0.942524	1.46236
62678	0.946131	1.56998
62679	0.946131	0.554836
62680	0.946127	1.54786
62681	0.946127	2.1
62682	1.26282	0.573659
62683	0.917471	1.97416
62684	1.21023	1.81439
62685	1.00851	1.80267
62686	1.17955	1.66584
62687	0.883746	1.67726
62688	1.89226	1.53778
62689	1.89226	2.1
62690	1.89226	2.09712
62691	1.89226	2.1
62692	1.89226	2.09116
62693	1.89226	2.1
62694	1.89226	0.554651
62695	1.89226	2.0911

62696	1.89226	1.39006
62697	1.89226	1.48927
62698	1.89226	1.3675
62699	1.89226	1.82887
62700	0.94613	1.71822
62701	0.94613	0.756983
62702	0.94613	0.120904
62703	0.94613	0.683249
62704	0.946128	0.611445
62705	0.946128	1.74616
62706	0.946131	1.39095
62707	0.946131	0.0690477
62708	0.94613	0.668144
62709	0.94613	0.816976
62710	0.94613	0.669853
62711	0.94613	0.645525
62712	0.883758	2.1
62713	1.03041	1.93856
62714	0.946131	1.63813
62715	0.946131	0.792327
62716	0.851537	1.69716
62717	0.851537	2.1
62718	1.23942	0.869201
62719	1.14906	1.98286
62720	0.851484	1.66408
62721	0.985518	1.51392
62722	1.0156	1.62833
62723	0.851526	1.63629
62724	1.89226	1.6696
62725	1.89226	2.1
62726	1.89226	2.08955
62727	1.89226	2.1
62728	1.89226	2.08239
62729	1.89226	2.1
62730	1.89226	0.672759
62731	1.89226	2.07793
62732	1.89226	1.40004
62733	1.89226	1.69443
62734	1.89226	1.32128
62735	1.89226	1.8289
62736	1.89227	0.0897772
62737	1.89227	0.126655
62738	1.89227	0.123103
62739	1.89227	0.0816214
62740	3.78453	0.0815864
62741	1.89226	0.150799
62742	1.89226	0.0730862
62743	1.89226	0.136936
62744	1.89226	0.142477
62745	3.78452	0.154788
62746	0.946132	0.0960022
62747	0.946132	0.115496
62748	0.946128	0.176271
62749	0.946128	0.0792467
62750	0.94613	0.0962536
62751	0.94613	0.0685056
62752	0.94613	0.0750313
62753	0.94613	0.132529

62754	0.946131	0.103759
62755	0.946131	0.0841536
62756	0.94613	0.083459
62757	0.94613	0.0775508
62758	1.89226	0.118369
62759	1.89226	0.0652385
62760	1.89226	0.108464
62761	1.89226	0.114773
62762	1.89226	0.0612122
62763	1.89226	0.0998217
62764	1.98874	0.0885716
62765	2.33455	0.05
62766	2.01865	0.120459
62767	2.07024	0.0655207
62768	4.32329	0.0996735
62769	1.98874	0.107532
62770	2.00266	0.101099
62771	2.14218	0.196151
62772	2.07024	0.133591
62773	3.9914	0.104739
62774	1.00133	0.117503
62775	0.804471	0.077922
62776	1.03512	0.104463
62777	0.995176	0.0910747
62778	0.80447	0.168237
62779	0.995178	0.0760904
62780	1.00133	0.127614
62781	1.03512	0.181358
62782	0.880587	0.186591
62783	0.880587	0.0749512
62784	0.959002	0.0885906
62785	0.959002	0.0692372
62786	2.33455	0.0583256
62787	1.99036	0.05
62788	2.07024	0.0780359
62789	2.58645	0.05
62790	4.32491	0.05
62791	1.89226	0.552711
62792	1.89226	0.456059
62793	1.89226	0.584827
62794	1.89226	0.376686
62795	3.78453	0.398569
62796	1.89226	0.860392
62797	1.89226	0.508851
62798	1.89226	0.668758
62799	1.89226	0.581713
62800	3.78453	0.802628
62801	1.89226	0.417993
62802	1.89226	0.317258
62803	1.89226	0.321444
62804	1.89226	0.409334
62805	1.89226	0.326476
62806	1.89226	0.421947
62807	1.89226	0.380561
62808	2.1523	0.181456
62809	2.53257	0.226082
62810	2.53257	0.213843
62811	1.89226	0.360548

62812	1.89226	0.33233
62813	0.946133	0.355983
62814	0.946133	0.465553
62815	0.946132	0.266793
62816	0.946132	0.31205
62817	0.946132	0.25994
62818	0.946132	0.292263
62819	0.946132	0.483501
62820	0.946132	0.307887
62821	0.946133	0.3561
62822	0.946133	0.371599
62823	0.946132	0.448328
62824	0.946132	0.382359
62825	0.946132	0.490128
62826	0.946132	0.628514
62827	0.946132	0.403861
62828	0.946132	0.538487
62829	0.946132	0.395083
62830	0.946132	0.433162
62831	0.946132	0.640819
62832	0.946132	0.539539
62833	0.946132	0.497122
62834	0.946132	0.485115
62835	0.946131	0.556218
62836	0.946131	0.538065
62837	1.62772	0.0766371
62838	1.70303	0.0724966
62839	1.70303	0.0846329
62840	1.70303	0.0819927
62841	1.90116	0.0717609
62842	1.90116	0.0707774
62843	1.89226	0.0785111
62844	1.89226	0.0791742
62845	1.89226	0.092571
62846	1.89226	0.104468
62847	1.89226	0.0748141
62848	1.89226	0.0759883
62849	1.89226	0.096854
62850	1.89226	0.0914714
62851	1.89226	0.0865512
62852	1.89226	0.106491
62853	3.78452	0.0788101
62854	1.89226	0.0980968
62855	1.89226	0.0935387
62856	1.89226	0.0831614
62857	1.89226	0.0872626
62858	1.89226	0.0891418
62859	1.89226	0.0965022
62860	0.946132	0.0595485
62861	0.946132	0.0621597
62862	0.946129	0.0613272
62863	0.946129	0.0701899
62864	0.94613	0.0596511
62865	0.94613	0.0612504
62866	0.946131	0.0664089
62867	0.946131	0.0653295
62868	0.94613	0.083578
62869	0.94613	0.0697215

62870	0.946131	0.0706356
62871	0.946131	0.0851656
62872	1.89226	0.0670522
62873	1.89226	0.0744208
62874	1.89226	0.0904267
62875	1.89226	0.0904985
62876	1.89226	0.0620186
62877	1.89226	0.0619184
62878	1.89226	0.386272
62879	1.89226	1.0306
62880	1.89226	0.923638
62881	1.89226	0.327923
62882	3.78452	0.464866
62883	1.89226	0.769891
62884	1.89226	1.2922
62885	1.89226	0.681434
62886	1.89226	1.39073
62887	1.89226	0.6718
62888	1.89226	1.45431
62889	1.30165	0.735085
62890	0.946127	1.00586
62891	1.09659	0.352545
62892	0.946131	0.342691
62893	1.09659	0.505633
62894	1.09659	0.833281
62895	0.946128	1.70508
62896	0.946128	0.393434
62897	1.30165	0.485452
62898	1.09659	0.512057
62899	0.946129	0.905764
62900	0.946129	1.13709
62901	0.946128	0.697364
62902	0.946128	0.925678
62903	0.946133	0.329272
62904	0.946133	0.449219
62905	0.946131	0.324226
62906	0.946131	1.06794
62907	0.946131	0.881035
62908	0.946131	0.412034
62909	0.94613	0.454526
62910	0.94613	0.463031
62911	0.946131	0.529252
62912	0.946131	0.610632
62913	0.946133	1.18704
62914	0.946133	1.26457
62915	0.946132	0.664692
62916	0.946132	0.865941
62917	0.946132	0.645254
62918	0.946132	1.27301
62919	0.946132	1.28326
62920	0.946132	0.812398
62921	0.946132	0.9643
62922	0.946132	0.914928
62923	0.946133	1.11303
62924	0.946133	1.09617
62925	1.30165	0.90892
62926	0.972228	0.941774
62927	1.09659	0.363271

62928	0.656268	0.324741
62929	1.06217	0.373984
62930	1.06217	1.19542
62931	1.00665	0.4181
62932	1.00665	1.51026
62933	0.97223	0.459566
62934	0.656267	0.432444
62935	1.30165	0.78044
62936	1.09659	0.758004
62937	0.946132	2.02585
62938	0.946132	2.1
62939	1.19895	2.1
62940	1.19895	2.1
62941	0.94613	2.1
62942	0.94613	1.9347
62943	1.39356	2.1
62944	1.19895	2.1
62945	0.94613	2.1
62946	1.39356	2.1
62947	1.19895	2.1
62948	0.946131	2.1
62949	1.89226	2.0025
62950	1.89226	2.1
62951	1.89226	2.1
62952	1.89226	2.01219
62953	1.89226	2.1
62954	1.89226	1.97116
62955	0.946132	1.81434
62956	0.946132	1.96006
62957	0.946128	2.09119
62958	0.946128	2.1
62959	0.94613	2.09277
62960	0.94613	1.56146
62961	0.94613	2.00229
62962	0.94613	2.09603
62963	0.946131	2.04972
62964	0.946131	2.04859
62965	0.94613	2.09166
62966	0.94613	2.06903
62967	1.89226	0.05
62968	1.89226	0.05
62969	1.89226	0.0673353
62970	1.89226	0.05
62971	1.89226	0.0727093
62972	1.89226	0.05
62973	1.29881	0.05
62974	1.09985	0.05
62975	0.946126	0.05
62976	0.946126	0.05
62977	0.851539	0.05
62978	1.20421	0.05
62979	0.851483	0.05
62980	0.999426	0.05
62981	1.47172	0.05
62982	0.999413	0.05
62983	0.851552	0.05
62984	0.851552	0.05
62985	1.89226	0.0810396

62986	1.89226	0.0769088
62987	1.89226	0.132726
62988	1.89226	0.0612982
62989	1.89226	0.133633
62990	1.89226	0.0573082
62991	1.89226	0.0551186
62992	1.89226	0.05248
62993	1.89226	0.0843395
62994	1.89226	0.05
62995	1.89226	0.0873202
62996	1.89226	0.05
62997	1.33915	2.1
62998	1.99297	2.1
62999	2.05216	2.1
63000	2.60163	2.1
63001	4.04513	2.1
63002	1.89226	2.09064
63003	1.89226	2.1
63004	1.89226	2.1
63005	1.89226	2.1
63006	3.78452	2.1
63007	0.946132	2.1
63008	0.946132	2.1
63009	0.946133	2.1
63010	0.946133	2.1
63011	0.946132	2.1
63012	0.946132	2.08241
63013	0.946133	2.1
63014	0.946133	2.1
63015	0.946132	2.1
63016	0.946132	2.1
63017	0.946132	2.1
63018	0.946132	2.1
63019	1.89226	2.1
63020	1.89226	1.97151
63021	1.89226	2.0729
63022	1.89226	2.1
63023	3.78452	2.08867
63024	1.89227	2.00627
63025	1.89227	2.1
63026	1.89227	2.1
63027	1.89227	2.00331
63028	1.89227	2.1
63029	1.89227	2.05884
63030	2.29301	2.1
63031	1.70303	2.1
63032	2.46749	2.1
63033	2.30728	2.1
63034	4.53937	2.1
63035	1.89226	2.05975
63036	1.89226	2.1
63037	1.89226	2.1
63038	1.89226	1.9196
63039	3.78452	2.1
63040	1.89226	1.42505
63041	1.89226	2.1
63042	1.89226	2.1
63043	1.89226	2.07597

63044	1.89226	2.05625
63045	1.89226	1.57876
63046	2.30728	2.1
63047	1.00757	2.09597
63048	2.0833	2.1
63049	1.65318	2.1
63050	1.417 2.1	
63051	1.00757	2.1
63052	2.46749	2.1
63053	2.0975	2.1
63054	2.79653	2.1
63055	2.44785	2.1
63056	4.91534	2.1
63057	1.89226	2.1
63058	1.89226	2.08888
63059	1.89226	2.07656
63060	1.89226	2.1
63061	3.78452	2.1
63062	1.89226	1.98159
63063	1.89226	2.1
63064	1.89226	2.1
63065	1.89226	2.03008
63066	1.89226	2.1
63067	1.89226	1.98276
63068	0.970559	2.1
63069	0.970559	2.1
63070	0.979305	2.1
63071	0.979305	2.1
63072	0.708502	2.1
63073	0.901117	2.1
63074	1.04875	2.1
63075	1.22392	2.1
63076	1.22392	2.1
63077	0.901117	2.1
63078	1.04875	2.1
63079	0.7085	2.1
63080	1.89226	1.90831
63081	1.89226	1.15147
63082	1.89226	1.71931
63083	1.89226	1.41756
63084	3.78452	1.84669
63085	1.89226	1.8379
63086	1.89226	1.12975
63087	1.89226	1.88372
63088	1.89226	1.31608
63089	3.78452	1.43741
63090	1.89226	0.641635
63091	1.89226	0.475212
63092	1.89226	0.72516
63093	1.89226	0.526084
63094	3.78453	0.555984
63095	1.89226	0.774088
63096	1.89226	0.851541
63097	1.89226	0.652104
63098	1.89226	0.583489
63099	1.89226	0.563665
63100	1.89226	0.538337
63101	1.89226	0.951126

63102	2.35441	0.840005
63103	1.89226	0.936146
63104	2.30931	0.478201
63105	4.24667	0.727559
63106	1.11612	0.686739
63107	0.946131	0.78098
63108	1.1772	0.663712
63109	0.946131	0.607202
63110	1.17721	0.667198
63111	1.11612	0.710486
63112	0.94613	0.79829
63113	0.94613	0.972519
63114	1.11612	0.661761
63115	1.11612	0.561202
63116	0.946131	0.968466
63117	0.946131	0.703352
63118	1.11612	0.172191
63119	0.582675	0.110524
63120	1.17721	0.112945
63121	0.651969	0.227341
63122	0.651969	0.183628
63123	0.582674	0.157419
63124	1.17721	0.169583
63125	1.11612	0.159719
63126	0.877955	0.142815
63127	0.877955	0.118044
63128	0.881925	0.229683
63129	0.881925	0.187125
63130	1.17721	0.21202
63131	0.651969	0.315202
63132	1.15466	0.219966
63133	0.623642	0.175898
63134	0.623642	0.210919
63135	0.651969	0.147114
63136	1.17721	0.280831
63137	1.15466	0.185285
63138	0.904714	0.198161
63139	0.904714	0.128924
63140	0.901914	0.446125
63141	0.901914	0.222412
63142	1.89226	0.6105
63143	1.89226	0.4545
63144	1.89226	1.28668
63145	1.89226	1.18774
63146	1.89226	0.683257
63147	1.89226	1.75912
63148	1.89226	1.54368
63149	1.89226	0.448792
63150	1.89226	0.64074
63151	1.89226	1.98557
63152	1.89226	0.50119
63153	1.89226	2.04495
63154	2.33986	1.92728
63155	1.97819	0.833088
63156	2.39166	0.393376
63157	2.70141	2.03414
63158	4.73151	0.937888
63159	0.989095	0.864518

63160	1.19583	0.487373
63161	0.851194	0.250696
63162	0.581197	0.242537
63163	0.872704	0.137281
63164	0.872704	1.19339
63165	0.904322	0.362126
63166	0.904322	0.618139
63167	1.19583	0.0557981
63168	0.851193	0.174701
63169	0.989095	0.459645
63170	0.581197	0.34252
63171	1.89226	0.506634
63172	1.89226	0.758784
63173	1.89226	1.41009
63174	1.89226	1.17555
63175	1.89226	1.10976
63176	1.89226	1.70441
63177	1.89226	1.51409
63178	1.89226	0.345215
63179	1.89226	0.947411
63180	1.89226	1.92409
63181	1.89226	0.443961
63182	1.89226	2.04522
63183	2.39166	0.365164
63184	1.70239	0.989585
63185	2.13665	0.396137
63186	2.82684	0.562166
63187	4.52831	0.383224
63188	1.89226	0.05
63189	1.89226	0.127407
63190	1.89226	0.0855457
63191	1.89226	0.0769285
63192	3.78453	0.116971
63193	1.89226	0.05
63194	1.89226	0.05
63195	1.89226	0.0587984
63196	1.89226	0.05
63197	1.89226	0.0629136
63198	1.89226	0.05
63199	1.89226	0.07738
63200	1.89226	0.0918397
63201	1.89226	0.137708
63202	1.89226	0.0668767
63203	1.89226	0.146752
63204	1.89226	0.05
63205	1.89226	0.191774
63206	1.89226	0.1878
63207	1.89226	0.385257
63208	1.89226	0.122539
63209	1.89226	0.448504
63210	1.89226	0.109761
63211	1.89226	0.107331
63212	1.89226	0.121812
63213	1.89226	0.225868
63214	1.89226	0.0748377
63215	1.89226	0.245133
63216	1.89226	0.0703634
63217	1.5169	0.20586

63218	1.61969	0.127113
63219	1.17262	0.12861
63220	1.17262	0.188823
63221	1.88868	0.135684
63222	1.88868	0.195927
63223	1.79981	0.331583
63224	1.88868	0.212612
63225	1.5169	0.219897
63226	1.5169	0.303662
63227	2.12891	0.23445
63228	2.12891	0.314861
63229	1.89226	0.300178
63230	1.89226	0.191312
63231	1.89226	0.209761
63232	1.89226	0.291121
63233	3.78452	0.266762
63234	0.946132	0.174788
63235	0.946132	0.189898
63236	0.946132	0.118338
63237	0.946132	0.122775
63238	0.946132	0.120167
63239	0.946132	0.18278
63240	0.946133	0.130533
63241	0.946133	0.193654
63242	0.946133	0.141071
63243	0.946133	0.144414
63244	0.946132	0.160379
63245	0.946132	0.15841
63246	1.88868	0.175033
63247	2.05759	0.111762
63248	1.61969	0.11286
63249	1.61969	0.164053
63250	2.29747	0.121847
63251	2.29747	0.166744
63252	2.12891	0.283574
63253	2.29747	0.176266
63254	1.88868	0.181688
63255	1.88868	0.243152
63256	2.50894	0.197376
63257	2.50894	0.25629
63258	1.89226	0.171668
63259	1.89226	0.248326
63260	1.89226	0.166852
63261	1.89226	0.242131
63262	1.89226	0.168445
63263	1.89226	0.246907
63264	0.946132	0.158999
63265	0.946132	0.153516
63266	0.946132	0.123458
63267	0.946132	0.113887
63268	0.946131	0.109345
63269	0.946131	0.189027
63270	0.946132	0.114951
63271	0.946132	0.165864
63272	0.946132	0.125869
63273	0.946132	0.13559
63274	0.946132	0.14202
63275	0.946132	0.156874

63276	2.42758	2.09773
63277	2.14895	2.1
63278	2.56975	2.06408
63279	2.28142	2.1
63280	4.71869	2.1
63281	1.89226	2.08306
63282	1.89226	2.1
63283	1.89226	2.06827
63284	1.89226	2.1
63285	3.78452	2.1
63286	1.89226	2.1
63287	1.89226	2.1
63288	1.89226	2.1
63289	1.89226	2.1
63290	1.89226	2.1
63291	1.89226	2.1
63292	0.960916	2.1
63293	0.960916	2.1
63294	0.978118	2.1
63295	0.875674	2.1
63296	0.775661	2.1
63297	0.773613	2.1
63298	1.07631	2.1
63299	1.14266	2.1
63300	1.16927	2.1
63301	0.898612	2.1
63302	1.04757	2.1
63303	0.757536	2.1
63304	2.56975	2.04002
63305	2.28142	2.1
63306	2.64384	2.03986
63307	2.40621	2.1
63308	4.97596	2.1
63309	1.89226	2.04736
63310	1.89226	2.1
63311	1.89226	1.78563
63312	1.89226	2.1
63313	3.78452	2.1
63314	1.89226	2.1
63315	1.89226	2.1
63316	1.89226	2.1
63317	1.89226	2.1
63318	1.89226	2.1
63319	1.89226	2.1
63320	1.03328	2.1
63321	1.03328	2.1
63322	1.04321	2.1
63323	1.15424	2.1
63324	0.936183	2.1
63325	0.944491	2.1
63326	1.14266	2.1
63327	1.20517	2.1
63328	1.23323	2.1
63329	0.961268	2.1
63330	1.11215	2.1
63331	0.827247	2.1
63332	1.89226	2.1
63333	1.89226	2.1

63334	1.89226	2.1
63335	1.89226	2.1
63336	3.78453	2.1
63337	2.14647	2.1
63338	2.20589	2.1
63339	1.95567	2.1
63340	2.03224	2.1
63341	4.17871	2.1
63342	2.06035	2.1
63343	1.88452	1.99087
63344	1.88452	2.1
63345	2.26997	2.1
63346	2.03224	2.1
63347	2.42272	2.1
63348	1.89226	2.1
63349	1.89226	1.90489
63350	1.89226	1.46875
63351	1.89226	2.1
63352	1.89226	1.76127
63353	1.89226	2.1
63354	1.89225	2.1
63355	1.89225	2.1
63356	1.89225	2.1
63357	1.89225	2.1
63358	1.89225	2.1
63359	1.89225	2.1
63360	0.952418	2.1
63361	0.952418	2.1
63362	0.95883	2.1
63363	0.95883	2.1
63364	0.946182	2.1
63365	1.01611	2.1
63366	0.895134	2.1
63367	0.977828	2.1
63368	0.89514	2.1
63369	0.946189	2.1
63370	0.977821	2.1
63371	1.01611	2.1
63372	2.03223	2.1
63373	1.88451	2.03169
63374	1.89236	2.1
63375	1.74649	2.05904
63376	3.77687	2.1
63377	0.81094	2.1
63378	0.81094	2.05109
63379	0.810939	2.1
63380	0.810939	2.1
63381	0.810945	2.1
63382	0.810945	2.1
63383	1.89225	1.97764
63384	1.35167	2.1
63385	1.08131	2.1
63386	1.08131	1.76758
63387	1.0813	2.1
63388	1.89226	0.05
63389	1.89226	0.054568
63390	1.89226	0.0575375
63391	1.89226	0.05

63392	1.89226	0.0549633
63393	1.89226	0.05
63394	1.01842	0.05
63395	0.936656	0.05
63396	0.946131	0.05
63397	0.946131	0.05
63398	1.11157	0.05
63399	1.04281	0.05
63400	0.925803	0.05
63401	0.925803	0.05
63402	0.851553	0.05
63403	1.11158	0.05
63404	1.05017	0.05
63405	0.925798	0.05
63406	1.89226	0.05
63407	1.89226	0.0677842
63408	1.89226	0.076175
63409	1.89226	0.0504141
63410	1.89226	0.071618
63411	1.89226	0.05
63412	1.23504	0.05
63413	1.23504	0.05
63414	0.946131	0.05
63415	0.946131	0.05
63416	1.37358	0.05
63417	1.23504	0.05
63418	0.946129	0.05
63419	0.946129	0.05
63420	1.37358	0.05
63421	0.946131	0.05
63422	0.94613	0.05
63423	1.23504	0.05
63424	1.00261	0.05
63425	1.00261	0.05
63426	1.02952	0.05
63427	1.02952	0.05
63428	0.850964	0.05
63429	0.658553	0.05
63430	1.37358	0.05
63431	1.23504	0.05
63432	1.37358	0.05
63433	0.850965	0.05
63434	1.23504	0.05
63435	0.658553	0.05
63436	0.946133	0.05
63437	0.946133	0.05
63438	0.946132	0.0676367
63439	0.946132	0.05
63440	0.946132	0.05
63441	0.946132	0.05
63442	0.946133	0.0581359
63443	0.946133	0.05
63444	0.946133	0.05
63445	0.946133	0.05
63446	0.946132	0.05
63447	0.946132	0.05
63448	1.89226	0.05
63449	1.89226	0.05

63450	1.89226	0.05
63451	1.89226	0.05
63452	3.78452	0.05
63453	1.89226	0.05
63454	1.89226	0.0504659
63455	1.89226	0.05
63456	1.89226	0.05
63457	3.78452	0.05
63458	1.89227	0.05
63459	1.89227	0.05
63460	1.89227	0.05
63461	1.89227	0.05
63462	1.89227	0.05
63463	1.89227	0.05
63464	0.94613	0.05
63465	0.94613	0.05
63466	0.946127	0.05
63467	0.946127	0.05
63468	0.94613	0.05
63469	0.94613	0.05
63470	0.946127	0.05
63471	0.946127	0.05
63472	0.946128	0.05
63473	0.946128	0.05
63474	0.946128	0.05
63475	0.946128	0.05
63476	0.946132	0.05
63477	0.946132	0.05
63478	0.946128	0.05
63479	0.946128	0.05
63480	0.94613	0.05
63481	0.94613	0.05
63482	0.946131	0.05
63483	0.946131	0.05
63484	0.94613	0.05
63485	0.94613	0.05
63486	0.94613	0.05
63487	0.94613	0.05
63488	1.89226	2.1
63489	1.89226	2.09915
63490	1.89226	2.1
63491	1.89226	2.1
63492	3.78453	2.1
63493	2.72844	2.1
63494	1.89226	2.1
63495	2.26536	2.1
63496	2.26536	2.1
63497	1.89226	2.1
63498	1.89226	2.1
63499	1.89226	2.1
63500	1.89226	2.07631
63501	1.89226	2.09922
63502	1.89226	2.1
63503	1.89226	2.09843
63504	1.89226	2.1
63505	1.89226	2.1
63506	1.89226	2.1
63507	1.89226	2.1

63508	1.89226	2.1
63509	1.89226	2.1
63510	1.89226	2.1
63511	2.72844	2.1
63512	2.26536	2.1
63513	2.28367	2.1
63514	1.5791	2.1
63515	4.54903	2.1
63516	0.810945	2.1
63517	0.810945	2.09074
63518	0.810943	2.08988
63519	0.810943	2.1
63520	1.89226	2.1
63521	1.3517	2.1
63522	1.08133	2.1
63523	1.08132	2.1
63524	1.08132	2.1
63525	0.810933	2.1
63526	0.810933	2.1
63527	1.89227	2.1
63528	1.89227	2.1
63529	1.89227	2.1
63530	1.89227	2.1
63531	3.78453	2.1
63532	2.71897	2.1
63533	2.27495	2.1
63534	2.648	2.1
63535	2.20832	2.1
63536	4.92729	2.1
63537	2.27495	2.1
63538	1.38455	2.1
63539	1.38455	2.1
63540	1.46586	2.1
63541	2.20831	2.1
63542	2.28962	2.1
63543	0.946133	2.1
63544	0.946133	2.1
63545	0.946132	2.1
63546	0.946132	2.1
63547	0.946132	2.1
63548	0.946132	2.1
63549	0.946132	2.1
63550	0.946132	2.1
63551	0.946132	2.1
63552	0.946132	2.1
63553	0.946133	2.1
63554	0.946133	2.1
63555	1.89226	2.1
63556	1.89226	2.1
63557	1.89226	2.1
63558	1.89226	2.1
63559	1.89226	2.1
63560	1.89226	2.1
63561	2.648	2.1
63562	2.08612	2.1
63563	2.08612	2.1
63564	2.20831	2.1
63565	2.56618	2.1

63566	2.68837	2.1
63567	2.08612	2.1
63568	2.20831	2.1
63569	1.30659	2.1
63570	1.30659	2.1
63571	1.38455	2.1
63572	2.16408	2.1
63573	0.810946	2.1
63574	0.810946	2.1
63575	0.810946	2.1
63576	0.810946	2.1
63577	1.89226	2.1
63578	1.3517	2.1
63579	1.08133	2.1
63580	1.08132	2.1
63581	1.08132	2.1
63582	0.810934	2.1
63583	0.810934	2.1
63584	1.89226	1.96646
63585	1.89226	1.55337
63586	1.89226	1.21321
63587	1.89226	2.00253
63588	3.78452	1.67784
63589	1.89226	1.79684
63590	1.89226	2.07242
63591	1.89226	2.0913
63592	1.89226	1.75554
63593	3.78453	2.03806
63594	1.08733	2.07262
63595	0.856615	2.09871
63596	1.25964	1.76738
63597	1.12378	1.7192
63598	1.23551	1.70323
63599	0.969894	2.1
63600	0.985977	2.06416
63601	0.985977	1.7057
63602	1.16169	1.88038
63603	1.21072	1.88501
63604	0.765175	2.01589
63605	0.863419	2.00221
63606	2.39934	1.98315
63607	2.44427	1.32037
63608	2.64003	1.09706
63609	2.39077	1.96585
63610	2.39934	1.77184
63611	2.10194	2.03646
63612	1.89226	1.0959
63613	1.89226	2.03532
63614	1.89226	1.89257
63615	1.89226	0.881026
63616	3.78452	1.77886
63617	0.946132	2.04557
63618	0.946132	2.02386
63619	0.946131	1.45983
63620	0.946131	1.71135
63621	0.946132	1.53453
63622	0.946132	2.09375
63623	0.946132	2.04945

63624	0.946132	1.76732
63625	0.946132	1.89106
63626	0.946132	1.92083
63627	0.946132	1.92028
63628	0.946132	1.94613
63629	1.19967	1.99418
63630	0.946128	1.91254
63631	0.946133	1.06787
63632	1.32002	1.41363
63633	1.19967	1.91966
63634	1.19967	1.71191
63635	0.94613	1.99034
63636	0.94613	0.859486
63637	0.946131	1.44855
63638	0.946131	1.63691
63639	1.19967	1.7274
63640	1.32001	1.62071
63641	1.89226	1.89503
63642	1.89226	2.1
63643	1.89226	1.34618
63644	1.89226	2.1
63645	3.78451	2.1
63646	2.6402	1.14214
63647	2.28552	2.1
63648	2.50958	1.29213
63649	2.18948	2.1
63650	4.79509	2.09756
63651	2.28552	2.1
63652	1.86102	2.1
63653	2.18949	2.1
63654	1.78376	2.1
63655	4.06928	2.1
63656	1.89226	2.1
63657	1.89226	2.1
63658	1.89226	2.1
63659	1.89226	2.1
63660	3.78452	2.1
63661	1.89226	1.23261
63662	1.89226	2.1
63663	1.89226	1.31227
63664	1.89226	2.1
63665	3.78452	2.1
63666	2.50958	1.18155
63667	2.18948	2.1
63668	2.39524	1.07143
63669	2.10188	2.1
63670	4.61146	2.06867
63671	2.18949	2.1
63672	1.78376	2.1
63673	1.71839	2.1
63674	1.71839	2.1
63675	2.10189	2.1
63676	2.16726	2.1
63677	0.810943	2.1
63678	0.810943	2.1
63679	0.810945	2.1
63680	0.810945	2.1
63681	0.810959	2.1

63682	0.810959	2.1
63683	1.89226	2.1
63684	1.35168	2.1
63685	1.08132	2.1
63686	1.08132	2.1
63687	1.0813	2.1
63688	1.89226	0.441774
63689	1.89226	1.78454
63690	1.89226	1.32233
63691	1.89226	0.769531
63692	1.89226	1.41176
63693	1.89226	1.11088
63694	1.28421	1.17504
63695	0.992041	0.54212
63696	1.07707	2.06075
63697	0.789746	1.77336
63698	1.03815	2.00785
63699	1.03815	1.4807
63700	1.03581	1.79472
63701	1.03581	0.969332
63702	1.28421	1.71441
63703	1.07707	1.80942
63704	0.789748	1.435
63705	0.992038	1.36833
63706	1.89226	0.686426
63707	1.89226	1.71396
63708	1.89226	1.46793
63709	1.89226	0.906539
63710	1.89226	1.49041
63711	1.89226	0.945718
63712	1.89226	0.445958
63713	1.89226	1.60016
63714	1.89226	1.1599
63715	1.89226	0.818262
63716	1.89226	1.30108
63717	1.89226	1.11214
63718	0.92236	1.90199
63719	0.655971	1.77335
63720	0.916772	1.83697
63721	0.913834	1.21597
63722	1.18074	1.70257
63723	0.922346	1.84042
63724	2.15414	0.882506
63725	1.53528	1.58074
63726	1.22748	1.79195
63727	1.57442	1.07525
63728	0.874683	1.62154
63729	1.25935	2.1
63730	2.61433	2.1
63731	1.22262	2.1
63732	1.22262	2.1
63733	2.62813	2.1
63734	2.62813	2.1
63735	1.89226	2.1
63736	2.61434	2.1
63737	2.62814	2.1
63738	2.61434	2.1
63739	1.90607	2.1

63740	1.89226	2.1
63741	1.89226	2.1
63742	1.89226	2.1
63743	1.89226	2.1
63744	1.89226	2.1
63745	1.89226	2.1
63746	1.89226	2.1
63747	1.89226	2.1
63748	1.89226	2.1
63749	1.89226	2.1
63750	1.89226	2.1
63751	1.89226	2.1
63752	1.89226	2.1
63753	1.31969	2.1
63754	1.31406	2.1
63755	0.629677	2.1
63756	0.643104	2.1
63757	0.976621	2.1
63758	0.976621	2.1
63759	0.98055	2.1
63760	0.98055	2.1
63761	1.3197	2.1
63762	0.643102	2.1
63763	0.629675	2.1
63764	1.31407	2.1
63765	0.946133	2.1
63766	0.946133	2.1
63767	1.3197	2.1
63768	1.31407	2.1
63769	0.946132	2.1
63770	0.946132	2.1
63771	1.3197	2.1
63772	1.3197	2.1
63773	1.3197	2.1
63774	0.946133	2.1
63775	0.946132	2.1
63776	1.31407	2.1
63777	1.89226	2.1
63778	1.89226	2.1
63779	1.89226	2.1
63780	1.89226	2.1
63781	1.89226	2.1
63782	1.89226	2.1
63783	1.89226	2.1
63784	1.89226	2.1
63785	1.89226	2.1
63786	1.89226	2.1
63787	1.89226	2.1
63788	1.89226	2.1
63789	1.89226	0.0820935
63790	1.89226	0.060424
63791	1.89226	0.05
63792	1.89226	0.0534643
63793	3.78452	0.0555516
63794	1.89226	0.06318
63795	1.89226	0.05
63796	1.89226	0.05
63797	1.89226	0.0505595

63798	1.89226	0.0557719
63799	1.89226	0.0676779
63800	0.946131	0.05
63801	0.946131	0.05
63802	0.946128	0.05
63803	0.946128	0.05
63804	0.94613	0.05
63805	0.94613	0.05
63806	0.94613	0.05
63807	0.94613	0.05
63808	0.94613	0.05
63809	0.94613	0.05
63810	0.94613	0.05
63811	0.94613	0.05
63812	1.89226	0.05
63813	1.89226	0.05
63814	1.89226	0.05
63815	1.89226	0.05
63816	1.89226	0.05
63817	1.89226	0.05
63818	0.924614	0.05
63819	0.914275	0.05
63820	0.906003	0.05
63821	0.62479	0.05
63822	2.02733	0.05
63823	1.48332	0.05
63824	0.833106	0.05
63825	1.19364	0.05
63826	1.57482	0.05
63827	1.18103	0.05
63828	0.905988	0.05
63829	1.89226	2.1
63830	1.89226	2.1
63831	1.89226	2.1
63832	1.89226	2.1
63833	3.78452	2.1
63834	1.89226	2.1
63835	1.89226	2.1
63836	1.89226	2.1
63837	1.89226	2.1
63838	1.89226	2.1
63839	1.89226	2.1
63840	0.816805	2.1
63841	0.928516	2.1
63842	1.4045	2.1
63843	1.26268	2.1
63844	1.0881	2.1
63845	1.0881	2.1
63846	1.10309	2.1
63847	1.10309	2.1
63848	0.928518	2.1
63849	1.4045	2.1
63850	0.816807	2.1
63851	1.26267	2.1
63852	1.89226	2.1
63853	2.52535	2.1
63854	1.89226	2.1
63855	1.89226	2.1

63856	2.809	2.1
63857	2.809	2.1
63858	1.89226	2.1
63859	1.89226	2.1
63860	1.89226	2.1
63861	1.89226	2.1
63862	3.78452	2.1
63863	0.946127	2.1
63864	0.946127	2.1
63865	0.946131	2.1
63866	0.946131	2.1
63867	0.94613	2.1
63868	0.94613	2.1
63869	0.946128	2.1
63870	0.946128	2.1
63871	0.946129	2.1
63872	0.946129	2.1
63873	0.946129	2.1
63874	0.946129	2.1
63875	1.89224	2.1
63876	1.89224	2.1
63877	1.89224	2.1
63878	1.89224	2.1
63879	3.78448	2.1
63880	2.39244	2.1
63881	2.27036	2.1
63882	2.25945	2.1
63883	2.27398	2.1
63884	4.52981	2.1
63885	2.27036	2.1
63886	1.97088	2.1
63887	1.86302	2.1
63888	1.84991	2.1
63889	4.12027	2.1
63890	1.89224	2.1
63891	1.89224	2.1
63892	1.89224	2.1
63893	1.89224	2.1
63894	3.78447	2.1
63895	1.89226	2.1
63896	1.89226	2.1
63897	1.89226	2.1
63898	1.89226	2.1
63899	3.78453	2.1
63900	2.25948	2.1
63901	2.33611	2.1
63902	2.05257	2.1
63903	1.94441	2.1
63904	4.20389	2.1
63905	1.92514	2.1
63906	1.84993	2.1
63907	1.94441	2.1
63908	1.74391	2.1
63909	3.79434	2.1
63910	1.89226	2.1
63911	1.89226	2.1
63912	1.89226	2.1
63913	1.89226	2.1

63914	3.78452	2.1
63915	15.1381	1.10724
63916	15.1381	0.240704
63917	15.1381	0.206943
63918	15.1381	1.76326
63919	30.2762	0.267843
63920	15.1381	0.8915
63921	15.1381	1.7107
63922	15.1381	0.341547
63923	15.1381	0.256748
63924	30.2762	0.491399
63925	15.1381	1.93472
63926	15.1381	1.91529
63927	15.1381	1.73202
63928	15.1381	2.01846
63929	30.2762	1.99168
63930	15.1381	0.204036
63931	15.1381	1.82268
63932	15.1381	1.00999
63933	15.1381	0.183825
63934	30.2762	0.228404
63935	1.89225	2.1
63936	1.89225	2.1
63937	1.89225	2.1
63938	1.89225	2.1
63939	3.78449	2.1
63940	1.39909	2.1
63941	1.39909	2.1
63942	0.946123	2.1
63943	0.946123	2.1
63944	1.3291	2.1
63945	1.39908	2.1
63946	0.946126	2.1
63947	0.946126	2.1
63948	0.946118	2.1
63949	1.32909	2.1
63950	0.94613	2.1
63951	1.3991	2.1
63952	3.07801	2.1
63953	1.84322	2.1
63954	1.84322	2.1
63955	1.68788	2.1
63956	2.6582	2.1
63957	2.50287	2.1
63958	1.89225	2.1
63959	1.89225	2.1
63960	1.89225	2.1
63961	1.89225	2.1
63962	3.7845	2.1
63963	1.89226	2.1
63964	1.89226	2.1
63965	1.89226	2.1
63966	1.89226	2.1
63967	3.78452	2.1
63968	1.89226	2.1
63969	2.50051	2.1
63970	2.50051	2.1
63971	2.65822	2.1

63972	1.89226	2.1
63973	2.04997	2.1
63974	2.50052	2.1
63975	2.65822	2.1
63976	1.70377	2.1
63977	1.70377	2.1
63978	1.84324	2.1
63979	2.63998	2.1
63980	0.810947	2.1
63981	0.810947	2.1
63982	0.810946	2.1
63983	0.810946	2.1
63984	1.89226	2.1
63985	1.35169	2.1
63986	1.08132	2.1
63987	1.08132	2.1
63988	1.08132	2.1
63989	0.810946	2.1
63990	0.810946	2.1
63991	1.89226	2.1
63992	1.89226	2.1
63993	1.89226	2.1
63994	1.89226	2.1
63995	3.78453	2.1
63996	0.946164	2.1
63997	0.946164	2.1
63998	0.929842	2.1
63999	0.929842	2.1
64000	0.946132	2.1
64001	0.946132	2.1
64002	1.04101	2.1
64003	0.929874	2.1
64004	0.946132	2.1
64005	0.929874	2.1
64006	1.04101	2.1
64007	0.946132	2.1
64008	1.89226	2.1
64009	1.89226	2.1
64010	1.89226	2.1
64011	1.89226	2.1
64012	3.78453	2.1
64013	1.89226	2.1
64014	1.89226	2.1
64015	1.89226	2.1
64016	1.89226	2.1
64017	1.89226	2.1
64018	1.89226	2.1
64019	1.89226	2.1
64020	1.89226	2.1
64021	1.89226	2.1
64022	1.89226	2.1
64023	1.89226	2.1
64024	1.89226	2.1
64025	0.946131	2.1
64026	0.946131	2.1
64027	0.946131	2.1
64028	0.946131	2.1
64029	0.946132	2.1

64030	0.946132	2.1
64031	0.946131	2.1
64032	0.946131	2.1
64033	0.946131	2.1
64034	0.946131	2.1
64035	0.946132	2.1
64036	0.946132	2.1
64037	1.89226	2.01576
64038	1.89226	1.18923
64039	1.89226	0.95508
64040	1.89226	1.78504
64041	3.78453	0.780845
64042	2.73497	0.832845
64043	1.93823	2.1
64044	1.93823	1.30028
64045	2.20046	2.1
64046	2.42279	1.49198
64047	2.68501	2.1
64048	1.89226	1.14092
64049	1.89226	2.1
64050	1.89226	2.1
64051	1.89226	1.38354
64052	1.89226	2.1
64053	1.89226	0.927887
64054	1.89226	1.06289
64055	1.89226	0.52277
64056	1.89226	0.635163
64057	1.89226	1.87458
64058	1.89226	0.864614
64059	1.89226	0.761556
64060	2.82984	1.70158
64061	2.15276	0.656256
64062	2.15276	1.4959
64063	2.42278	0.068396
64064	2.50074	0.861452
64065	2.77077	0.558998
64066	2.42279	0.639527
64067	1.93823	2.1
64068	1.70423	0.862176
64069	1.70423	2.08508
64070	2.15276	0.988033
64071	2.38676	2.1
64072	0.810946	1.26424
64073	0.810946	2.1
64074	0.810944	0.656947
64075	0.810944	0.601087
64076	0.810946	1.76711
64077	0.810946	1.86524
64078	1.89226	2.1
64079	1.35169	1.92305
64080	1.08132	1.09389
64081	1.08132	2.1
64082	1.08132	1.96037
64083	1.89226	2.1
64084	1.89226	2.1
64085	1.89226	2.1
64086	1.89226	2.1
64087	3.78452	2.1

64088	1.89226	2.1
64089	1.89226	2.1
64090	1.89226	2.1
64091	1.89226	2.1
64092	3.78453	2.1
64093	1.04144	2.1
64094	1.04144	2.1
64095	0.938972	2.1
64096	0.938972	2.1
64097	0.969661	2.1
64098	1.03285	2.1
64099	0.946133	2.1
64100	0.946133	2.1
64101	0.946131	2.1
64102	0.969663	2.1
64103	0.946131	2.1
64104	1.03284	2.1
64105	0.946131	2.1
64106	0.946131	2.1
64107	0.946128	2.1
64108	0.946128	2.1
64109	0.946127	2.1
64110	0.946127	2.1
64111	0.946131	2.1
64112	0.946131	2.1
64113	0.94613	2.1
64114	0.94613	2.1
64115	0.946129	2.1
64116	0.946129	2.1
64117	1.89226	2.1
64118	1.89226	2.1
64119	1.89226	2.1
64120	1.89226	2.1
64121	3.78452	2.1
64122	1.89226	2.1
64123	1.89226	2.1
64124	1.89226	2.1
64125	1.89226	2.1
64126	3.78453	2.1
64127	1.89226	2.1
64128	1.89226	2.1
64129	1.85882	2.1
64130	1.85882	2.1
64131	1.93932	2.1
64132	1.97276	2.1
64133	0.94613	2.1
64134	0.94613	2.1
64135	0.946128	2.1
64136	0.946128	2.1
64137	0.946127	2.1
64138	0.946127	2.1
64139	0.946131	2.1
64140	0.946131	2.1
64141	0.946129	2.1
64142	0.946129	2.1
64143	0.946129	2.1
64144	0.946129	2.1
64145	1.89225	2.1

64146	1.89225	2.1
64147	1.89225	2.1
64148	1.89225	2.1
64149	3.7845	2.1
64150	1.0086	2.1
64151	1.09028	2.1
64152	0.946125	2.1
64153	0.946125	2.1
64154	0.916906	2.1
64155	0.916906	2.1
64156	1.13051	2.1
64157	1.1505	2.1
64158	1.11557	2.1
64159	1.04071	2.1
64160	0.851542	2.1
64161	0.991196	2.1
64162	1.89225	2.1
64163	1.89225	2.1
64164	1.89225	2.1
64165	1.89225	2.1
64166	3.7845	2.1
64167	1.89225	2.1
64168	1.89225	2.1
64169	1.89225	2.1
64170	1.89225	2.1
64171	1.89225	2.1
64172	1.89225	2.1
64173	1.89226	2.1
64174	1.89226	2.1
64175	1.89226	2.1
64176	1.89226	2.1
64177	3.78452	2.1
64178	0.890962	2.1
64179	0.952547	2.1
64180	0.946132	2.1
64181	0.946132	2.1
64182	0.851543	2.1
64183	0.851543	2.1
64184	1.11558	2.1
64185	1.06304	2.1
64186	0.994315	2.1
64187	1.04072	2.1
64188	0.900943	2.1
64189	0.851542	2.1
64190	1.89226	2.1
64191	1.89226	2.1
64192	1.89226	2.1
64193	1.89226	2.1
64194	1.89226	2.1
64195	1.89226	2.1
64196	1.89226	2.1
64197	1.89226	2.1
64198	1.89226	2.1
64199	1.89226	2.1
64200	3.78452	2.1
64201	1.89226	2.1
64202	1.89226	2.1
64203	1.89226	2.1

64204	1.89226	2.1
64205	3.78452	2.1
64206	0.782015	2.1
64207	0.929718	2.1
64208	0.946132	2.1
64209	0.946132	2.1
64210	0.782016	2.1
64211	0.782016	2.1
64212	1.06706	2.1
64213	1.11785	2.1
64214	0.782015	2.1
64215	0.946133	2.1
64216	0.878931	2.1
64217	0.946132	2.1
64218	1.89226	2.1
64219	1.89226	2.1
64220	1.89226	2.1
64221	1.89226	2.1
64222	1.89226	2.1
64223	1.89226	2.1
64224	0.946131	2.1
64225	0.946131	2.1
64226	0.946129	2.1
64227	0.946129	2.1
64228	0.946131	2.1
64229	0.946131	2.1
64230	0.94613	2.1
64231	0.94613	2.1
64232	0.946131	2.1
64233	0.946131	2.1
64234	0.94613	2.1
64235	0.94613	2.1
64236	0.827866	2.1
64237	0.946131	2.1
64238	0.946129	2.1
64239	0.946129	2.1
64240	0.94613	2.1
64241	0.94613	2.1
64242	0.94613	2.1
64243	0.94613	2.1
64244	0.827865	2.1
64245	0.94613	2.1
64246	0.94613	2.1
64247	0.94613	2.1
64248	1.89226	2.1
64249	1.89226	2.1
64250	1.89226	2.1
64251	1.89226	2.1
64252	3.78452	2.1
64253	1.89226	2.1
64254	1.89226	2.1
64255	1.89226	2.1
64256	1.89226	2.1
64257	1.89226	2.1
64258	1.89226	2.1
64259	1.89226	2.1
64260	1.89226	2.1
64261	1.89226	2.1

64262	1.89226	2.1
64263	3.78453	2.1
64264	1.89226	2.1
64265	1.89226	2.1
64266	1.89226	2.1
64267	1.89226	2.1
64268	3.78452	2.1
64269	0.744377	2.1
64270	0.541919	2.1
64271	0.652022	2.1
64272	0.544198	2.1
64273	0.764633	2.1
64274	0.673254	2.1
64275	1.89226	2.1
64276	1.31149	2.1
64277	1.05423	2.1
64278	1.02189	2.1
64279	0.870602	2.1
64280	1.89226	2.1
64281	1.89226	2.1
64282	1.89226	2.1
64283	1.89226	2.1
64284	1.89226	2.1
64285	1.89226	2.1
64286	1.89226	2.1
64287	1.89226	2.1
64288	1.89226	2.1
64289	1.89226	2.1
64290	1.89226	2.1
64291	1.89226	2.1
64292	1.89226	2.1
64293	1.89226	2.1
64294	1.89226	2.1
64295	1.89226	2.1
64296	1.89226	2.1
64297	1.89226	2.1
64298	0.815314	2.1
64299	0.815313	2.1
64300	0.714029	2.1
64301	0.782951	2.1
64302	0.810944	2.1
64303	0.782954	2.1
64304	1.89226	2.1
64305	1.30999	2.1
64306	1.08132	2.1
64307	1.03962	2.1
64308	0.95209	2.1
64309	1.89226	2.1
64310	1.89226	2.1
64311	1.89226	2.1
64312	1.89226	2.1
64313	1.89226	2.1
64314	1.89226	2.1
64315	1.89226	2.1
64316	1.89226	2.1
64317	1.89226	2.1
64318	1.89226	2.1
64319	1.89226	2.1

64320	1.89226	2.1
64321	1.89226	2.1
64322	1.89226	2.1
64323	1.89226	2.1
64324	1.89226	2.1
64325	1.89226	2.1
64326	1.89226	2.1
64327	0.871558	2.1
64328	0.713078	2.1
64329	0.756858	2.1
64330	0.703674	2.1
64331	0.838025	2.1
64332	0.862196	2.1
64333	1.89226	2.1
64334	1.33194	2.1
64335	1.10834	2.1
64336	1.06163	2.1
64337	1.01785	2.1
64338	1.89226	2.1
64339	1.89226	2.1
64340	1.89226	2.1
64341	1.89226	2.1
64342	3.78452	2.1
64343	1.89226	2.1
64344	1.89226	2.1
64345	1.89226	2.1
64346	1.89226	2.1
64347	1.89226	2.1
64348	1.89226	2.1
64349	1.89226	2.1
64350	1.89226	2.1
64351	1.89226	2.1
64352	1.89226	2.1
64353	1.89226	2.1
64354	1.89226	2.1
64355	0.958406	2.1
64356	1.1383	2.1
64357	0.865636	2.1
64358	1.10093	2.1
64359	0.838024	2.1
64360	0.975154	2.1
64361	1.89226	2.1
64362	1.38553	2.1
64363	1.10834	2.1
64364	1.11522	2.1
64365	1.15271	2.1
64366	1.89227	2.1
64367	1.89227	2.1
64368	1.89227	2.1
64369	1.89227	2.1
64370	3.78453	2.1
64371	0.946132	2.1
64372	0.946132	2.1
64373	0.946132	2.1
64374	0.946132	2.1
64375	0.946132	2.1
64376	0.946132	2.1
64377	0.946133	2.1

64378	0.946133	2.1
64379	0.946132	2.1
64380	0.946132	2.1
64381	0.946133	2.1
64382	0.946133	2.1
64383	0.719271	2.1
64384	0.774968	2.1
64385	1.28401	2.1
64386	1.17415	2.1
64387	0.960999	2.1
64388	0.960999	2.1
64389	0.988115	2.1
64390	0.988115	2.1
64391	0.774968	2.1
64392	1.28401	2.1
64393	0.719271	2.1
64394	1.17415	2.1
64395	2.77811	2.1
64396	2.34829	2.1
64397	2.56802	2.1
64398	2.52333	2.1
64399	2.56802	2.1
64400	2.52333	2.1
64401	1.89227	2.1
64402	1.89227	2.1
64403	1.89227	2.1
64404	1.89227	2.1
64405	3.78453	2.1
64406	1.89226	2.1
64407	1.89226	2.1
64408	1.89226	2.1
64409	1.89226	2.1
64410	1.89226	2.1
64411	1.89226	2.1
64412	1.89226	2.1
64413	2.77811	2.1
64414	1.89226	2.1
64415	2.56802	2.1
64416	1.89226	2.1
64417	2.56802	2.1
64418	1.89226	1.78669
64419	1.89226	2.1
64420	1.89226	1.83416
64421	1.89226	2.1
64422	3.78452	2.1
64423	2.41802	2.1
64424	1.95764	2.1
64425	1.95764	2.1
64426	2.27727	2.1
64427	2.39311	2.1
64428	2.73961	2.1
64429	0.946132	2.1
64430	0.946132	2.1
64431	0.94613	2.1
64432	0.94613	2.1
64433	0.946131	2.1
64434	0.946131	2.1
64435	0.946131	2.1

64436	0.946131	2.1
64437	0.946131	2.1
64438	0.946131	2.1
64439	0.946131	2.1
64440	0.946131	2.1
64441	1.89226	1.88247
64442	1.89226	2.1
64443	1.89226	2.1
64444	1.89226	1.92746
64445	1.89226	2.1
64446	1.89226	1.7349
64447	2.78856	1.21007
64448	2.28552	2.1
64449	2.28552	1.76489
64450	2.3931	2.1
64451	2.6402	1.69845
64452	2.74778	2.1
64453	2.39311	2.1
64454	1.95764	2.1
64455	2.28552	2.1
64456	1.86103	2.1
64457	4.24316	2.1
64458	1.89226	2.1
64459	1.89226	2.1
64460	1.89226	2.1
64461	1.89226	2.1
64462	1.89226	2.1
64463	1.89226	2.1
64464	1.47412	2.07334
64465	2.18862	1.91938
64466	2.07226	0.99393
64467	2.33645	1.78854
64468	4.26088	1.42333
64469	1.89226	1.03789
64470	1.89226	1.95902
64471	1.89226	1.1846
64472	1.89226	1.92765
64473	1.89226	1.7096
64474	1.89226	2.09314
64475	1.89226	1.46982
64476	1.89226	2.1
64477	1.89226	0.429843
64478	1.89226	2.1
64479	3.78453	1.88153
64480	2.18862	1.42485
64481	2.1128	2.1
64482	2.33645	1.08798
64483	2.28188	2.1
64484	4.47051	2.09062
64485	2.07226	2.1
64486	2.33645	2.03982
64487	2.20831	1.26491
64488	2.4646	0.847922
64489	4.53686	1.2058
64490	0.946131	1.22171
64491	0.946131	2.1
64492	0.946131	2.1
64493	0.946131	1.47855

64494	0.94613	2.1
64495	0.94613	0.92476
64496	0.946133	2.07642
64497	0.946133	2.1
64498	0.946132	0.878906
64499	0.946132	0.604836
64500	0.946131	2.015
64501	0.946131	1.87178
64502	1.89226	1.76682
64503	1.89226	2.1
64504	1.89226	0.631602
64505	1.89226	2.09987
64506	3.78453	1.94531
64507	2.33646	1.67132
64508	2.28189	2.1
64509	2.4646	0.576122
64510	2.42758	2.1
64511	4.74648	1.9105
64512	1.89226	2.1
64513	1.89226	2.1
64514	1.89226	2.1
64515	1.89226	2.1
64516	1.89226	2.1
64517	1.89226	2.1
64518	1.89226	2.1
64519	1.89226	2.1
64520	1.89226	2.1
64521	1.89226	2.1
64522	1.89226	2.1
64523	1.89226	2.1
64524	1.89226	2.1
64525	1.89226	2.1
64526	1.89226	2.1
64527	1.89226	2.1
64528	3.78453	2.1
64529	1.89226	2.1
64530	1.89226	2.1
64531	1.89226	2.1
64532	1.89226	2.1
64533	1.89226	2.1
64534	1.89226	2.1
64535	1.89226	2.1
64536	1.89226	2.1
64537	1.89226	2.1
64538	1.89226	2.1
64539	3.78452	2.1
64540	0.877238	2.1
64541	0.877238	2.1
64542	1.24746	2.1
64543	1.04764	2.1
64544	0.964969	2.1
64545	1.1521	2.1
64546	0.946132	2.1
64547	0.946132	2.1
64548	0.946132	2.1
64549	1.12659	2.1
64550	0.964969	2.1
64551	0.946131	2.1

64552	1.89226	2.1
64553	1.89226	2.1
64554	1.89226	2.1
64555	1.89226	2.1
64556	1.89226	2.1
64557	1.89226	2.1
64558	1.89226	2.1
64559	1.89226	2.1
64560	1.89226	2.1
64561	1.89226	2.1
64562	3.78453	2.1
64563	0.868144	2.1
64564	0.992762	2.1
64565	0.946131	2.1
64566	0.946131	2.1
64567	1.07072	2.1
64568	1.07072	2.1
64569	0.868171	2.1
64570	0.868171	2.1
64571	0.992762	2.1
64572	0.946131	2.1
64573	0.946131	2.1
64574	0.868144	2.1
64575	0.946132	2.1
64576	0.946132	2.1
64577	0.946131	2.1
64578	0.946131	2.1
64579	0.946132	2.1
64580	0.946132	2.1
64581	0.946132	2.1
64582	0.946132	2.1
64583	0.946132	2.1
64584	0.946132	2.1
64585	0.946132	2.1
64586	0.946132	2.1
64587	1.89227	2.1
64588	1.89227	2.1
64589	1.89227	2.1
64590	1.89227	2.1
64591	1.89227	2.1
64592	1.89227	2.1
64593	1.89226	2.1
64594	1.89226	2.1
64595	1.89226	2.1
64596	1.89226	2.1
64597	1.89226	2.1
64598	1.89226	2.1
64599	1.98552	2.1
64600	2.22298	2.1
64601	2.22298	2.1
64602	1.89226	2.1
64603	1.89226	2.1
64604	1.54525	2.1
64605	0.810946	2.1
64606	0.810946	2.1
64607	0.810947	2.1
64608	0.810947	2.1
64609	0.81096	2.1

64610	0.81096	2.1
64611	1.89226	2.1
64612	1.35168	2.1
64613	1.08132	2.1
64614	1.08132	2.1
64615	1.0813	2.1
64616	1.89226	2.1
64617	1.89226	2.1
64618	1.89226	2.1
64619	1.89226	2.1
64620	3.78452	2.1
64621	1.7753	2.1
64622	2.39703	2.1
64623	1.90577	2.1
64624	2.03445	2.1
64625	3.82335	2.1
64626	2.39703	2.1
64627	2.47949	2.09703
64628	2.26051	2.1
64629	2.36891	2.07597
64630	4.76594	2.1
64631	1.89226	2.1
64632	1.89226	2.07207
64633	1.89226	2.1
64634	1.89226	2.08385
64635	3.78452	2.1
64636	1.89226	2.1
64637	1.89226	2.1
64638	1.89226	2.1
64639	1.89226	2.1
64640	1.89226	2.1
64641	1.89226	2.1
64642	0.883745	2.1
64643	0.973143	2.1
64644	0.988173	2.1
64645	0.988173	2.1
64646	0.941927	2.1
64647	1.02971	2.1
64648	0.854065	2.1
64649	0.82702	2.1
64650	0.847835	2.1
64651	0.981126	2.1
64652	1.02996	2.1
64653	1.10704	2.1
64654	2.28031	2.1
64655	2.26369	2.08042
64656	2.26369	2.1
64657	2.36891	2.09204
64658	2.30788	2.1
64659	2.42455	2.09249
64660	1.89226	2.1
64661	1.89226	2.08139
64662	1.89226	2.04529
64663	1.89226	2.1
64664	1.89226	2.0731
64665	1.89226	2.1
64666	2.71406	2.1
64667	1.83244	2.1

64668	1.83244	2.1
64669	2.07567	2.1
64670	2.52044	2.1
64671	2.76367	2.1
64672	1.89226	2.1
64673	1.89226	2.1
64674	1.89226	2.1
64675	1.89226	2.1
64676	3.78453	2.1
64677	1.89226	2.1
64678	1.89226	2.1
64679	1.89226	2.1
64680	1.89226	2.1
64681	1.89226	2.1
64682	1.89226	2.1
64683	2.70714	2.1
64684	2.3148	2.1
64685	2.3148	2.1
64686	2.52044	2.1
64687	2.54204	2.1
64688	2.74768	2.1
64689	2.52044	2.1
64690	2.3148	2.1
64691	1.83244	2.1
64692	1.83244	2.1
64693	1.62611	2.1
64694	2.3148	2.1
64695	0.946131	2.1
64696	0.946131	2.1
64697	0.946131	2.1
64698	0.946131	2.1
64699	0.946131	2.1
64700	0.946131	2.1
64701	0.946131	2.1
64702	0.946131	2.1
64703	0.946131	2.1
64704	0.946131	2.1
64705	0.946131	2.1
64706	0.946131	2.1
64707	1.89226	2.1
64708	1.89226	2.1
64709	1.89226	2.1
64710	1.89226	2.1
64711	3.78453	2.1
64712	1.89226	2.1
64713	1.89226	2.1
64714	1.89226	2.1
64715	1.89226	2.1
64716	1.89226	2.1
64717	1.89226	2.1
64718	1.89227	2.1
64719	1.89227	2.1
64720	1.89227	2.1
64721	1.89227	2.1
64722	3.78453	2.1
64723	1.89226	2.1
64724	1.89226	2.1
64725	1.89226	2.1

64726	1.89226	2.1
64727	3.78453	2.1
64728	0.915781	2.1
64729	0.863524	2.1
64730	0.717056	2.1
64731	0.830182	2.1
64732	0.865094	2.1
64733	0.918003	2.1
64734	1.89226	2.1
64735	1.29933	2.1
64736	1.11215	2.1
64737	1.05227	2.1
64738	0.966269	2.1
64739	1.89226	2.1
64740	1.89226	2.1
64741	1.89226	2.1
64742	1.89226	2.1
64743	1.89226	2.1
64744	1.89226	2.1
64745	1.89226	2.1
64746	1.89226	2.1
64747	1.89226	2.1
64748	1.89226	2.1
64749	1.89226	2.1
64750	1.89226	2.1
64751	1.89226	2.1
64752	1.89226	2.1
64753	1.89226	2.1
64754	1.89226	2.1
64755	1.89226	2.1
64756	1.89226	2.1
64757	1.01725	2.1
64758	0.997048	2.1
64759	0.818657	2.1
64760	0.960724	2.1
64761	0.838013	2.1
64762	1.04859	2.1
64763	1.89226	2.1
64764	1.41754	2.1
64765	1.17625	2.1
64766	1.0793	2.1
64767	1.18822	2.1
64768	2.16402	2.1
64769	1.89452	2.1
64770	1.50084	2.1
64771	2.09288	2.1
64772	3.9874	2.1
64773	2.16402	2.1
64774	2.13925	2.1
64775	2.04483	2.1
64776	2.09288	2.1
64777	4.23212	2.1
64778	1.89226	2.1
64779	1.89226	2.1
64780	1.89226	2.1
64781	1.89226	2.1
64782	3.78452	2.1
64783	1.89226	2.1

64784	1.89226	2.1
64785	1.89226	2.1
64786	1.89226	2.1
64787	1.89226	2.1
64788	1.89226	2.1
64789	1.9824	2.1
64790	2.09288	2.1
64791	1.69889	2.1
64792	1.6817	2.1
64793	2.00695	2.1
64794	2.00695	2.1
64795	1.93592	2.1
64796	2.09288	2.1
64797	2.00695	2.1
64798	2.09288	2.1
64799	1.9589	2.1
64800	2.04483	2.1
64801	0.946128	2.1
64802	0.946128	2.1
64803	0.946132	2.1
64804	0.946132	2.1
64805	0.94613	2.1
64806	0.94613	2.1
64807	0.946129	2.1
64808	0.946129	2.1
64809	0.94613	2.1
64810	0.94613	2.1
64811	0.94613	2.1
64812	0.94613	2.1
64813	0.946131	2.1
64814	0.946131	2.1
64815	0.946131	2.1
64816	0.946131	2.1
64817	0.946131	2.1
64818	0.946131	2.1
64819	0.946131	2.1
64820	0.946131	2.1
64821	0.946131	2.1
64822	0.946131	2.1
64823	0.946131	2.1
64824	0.946131	2.1
64825	1.89226	2.1
64826	2.48083	2.1
64827	2.6968	2.1
64828	2.6968	2.1
64829	1.89226	2.1
64830	1.89226	2.1
64831	1.89226	2.1
64832	1.89226	2.1
64833	1.89226	2.1
64834	1.89226	2.1
64835	3.78453	2.1
64836	1.89226	2.1
64837	1.89226	2.1
64838	1.89226	2.1
64839	1.89226	2.1
64840	1.89226	2.1
64841	1.89226	2.1

64842	1.1017	2.1
64843	1.1017	2.1
64844	1.10605	2.1
64845	1.01647	2.1
64846	0.868255	2.1
64847	0.883424	2.1
64848	1.24624	2.1
64849	1.35473	2.1
64850	0.942231	2.1
64851	1.31464	2.1
64852	0.910064	2.1
64853	1.27147	2.1
64854	1.89226	2.1
64855	1.89226	2.1
64856	1.89226	2.1
64857	1.89226	2.1
64858	3.78453	2.1
64859	1.89226	2.1
64860	1.89226	2.1
64861	1.89226	2.1
64862	1.89226	2.1
64863	1.89226	2.1
64864	1.89226	2.1
64865	1.89226	2.1
64866	1.89226	2.1
64867	1.89226	2.1
64868	1.89226	2.1
64869	3.78453	2.1
64870	1.89226	2.1
64871	2.70015	2.1
64872	1.89226	2.1
64873	2.61064	2.1
64874	4.59241	2.1
64875	2.70015	2.1
64876	2.3372	2.1
64877	2.61064	2.1
64878	1.85184	2.1
64879	4.55198	2.1
64880	1.89226	2.1
64881	1.89226	2.1
64882	1.89226	2.1
64883	1.89226	2.1
64884	1.89226	2.1
64885	1.89226	2.1
64886	1.89227	2.1
64887	1.89227	2.1
64888	1.89227	2.1
64889	1.89227	2.1
64890	3.78453	2.1
64891	1.89226	2.1
64892	2.52194	2.1
64893	2.61064	2.1
64894	2.61064	2.1
64895	1.89226	2.1
64896	1.89226	2.1
64897	1.095 2.1	
64898	1.095 2.1	
64899	1.09189	2.1

64900	1.09189	2.1
64901	0.875334	2.1
64902	0.925919	2.1
64903	1.26097	2.1
64904	1.30532	2.1
64905	1.26097	2.1
64906	0.875334	2.1
64907	1.30532	2.1
64908	0.92592	2.1
64909	1.89226	2.1
64910	1.89226	2.1
64911	1.89226	2.1
64912	1.89226	2.1
64913	1.89226	2.1
64914	1.89226	2.1
64915	1.89226	2.1
64916	1.89226	2.1
64917	1.89226	2.1
64918	1.89226	2.1
64919	3.78453	2.1
64920	1.26097	2.1
64921	1.26097	2.1
64922	0.946132	2.1
64923	0.946132	2.1
64924	1.22176	2.1
64925	1.26097	2.1
64926	0.946132	2.1
64927	0.946132	2.1
64928	0.946132	2.1
64929	1.22176	2.1
64930	1.26097	2.1
64931	0.946132	2.1
64932	2.52194	2.1
64933	1.67381	2.1
64934	1.67381	2.1
64935	1.75067	2.1
64936	2.44352	2.1
64937	2.52038	2.1
64938	1.89226	2.1
64939	1.89226	2.1
64940	1.89226	2.1
64941	1.89226	2.1
64942	1.89226	2.1
64943	1.89226	2.1
64944	1.89227	2.1
64945	1.89227	2.1
64946	1.89227	2.1
64947	1.89227	2.1
64948	1.89227	2.1
64949	1.89227	2.1
64950	1.22176	2.1
64951	1.22176	2.1
64952	0.946132	2.1
64953	0.946132	2.1
64954	1.19095	2.1
64955	1.22176	2.1
64956	0.946133	2.1
64957	0.946133	2.1

64958	1.19095	2.1
64959	0.946132	2.1
64960	0.946133	2.1
64961	1.22176	2.1
64962	2.44352	2.1
64963	1.6287	2.1
64964	1.6287	2.1
64965	1.67381	2.1
64966	2.3819	2.1
64967	2.427	2.1
64968	1.89226	2.1
64969	1.89226	2.1
64970	1.89226	2.1
64971	1.89226	2.1
64972	1.89226	2.1
64973	1.89226	2.1
64974	1.89226	2.1
64975	1.89226	2.1
64976	1.89226	2.1
64977	1.89226	2.1
64978	3.78453	2.1
64979	1.89226	2.1
64980	1.89226	2.1
64981	1.89226	2.1
64982	1.89226	2.1
64983	1.89226	2.1
64984	1.89226	2.1
64985	1.89226	2.1
64986	1.89226	2.1
64987	1.89226	2.1
64988	1.89226	2.1
64989	1.89226	2.1
64990	1.89226	2.1
64991	0.851542	2.1
64992	0.851542	2.1
64993	1.1736	2.1
64994	1.29261	2.1
64995	0.852708	2.1
64996	1.18843	2.1
64997	0.946132	2.1
64998	0.946132	2.1
64999	0.946132	2.1
65000	1.07901	2.1
65001	1.04208	2.1
65002	0.936669	2.1
65003	1.89227	2.1
65004	1.89227	2.1
65005	1.89227	2.1
65006	1.89227	2.1
65007	3.78453	2.1
65008	1.89226	2.1
65009	1.89226	2.1
65010	1.89226	2.1
65011	1.89226	2.1
65012	3.78453	2.1
65013	1.89226	2.1
65014	1.89226	2.1
65015	1.89226	2.1

65016	1.89226	2.1
65017	1.89226	2.1
65018	1.89226	2.1
65019	0.946133	2.1
65020	0.946133	2.1
65021	1.28525	2.1
65022	1.28525	2.1
65023	1.07901	2.1
65024	1.28525	2.1
65025	0.946132	2.1
65026	0.946132	2.1
65027	0.946132	2.1
65028	1.28525	2.1
65029	0.946133	2.1
65030	1.07901	2.1
65031	1.89226	2.1
65032	1.89226	2.1
65033	1.89226	2.1
65034	1.89226	2.1
65035	1.89226	2.1
65036	1.89226	2.1
65037	1.89226	2.1
65038	1.89226	2.1
65039	1.89226	2.1
65040	1.89226	2.1
65041	1.89226	2.1
65042	1.89226	2.1
65043	1.24419	2.1
65044	1.09093	2.1
65045	1.22722	2.1
65046	1.18154	2.1
65047	1.27455	2.1
65048	1.08473	2.1
65049	1.15541	2.1
65050	1.15541	2.1
65051	1.29607	2.1
65052	1.23814	2.1
65053	1.13024	2.1
65054	1.12096	2.1
65055	1.18858	2.1
65056	1.13181	2.1
65057	0.988718	2.1
65058	0.802498	2.1
65059	0.994034	2.1
65060	1.15773	2.1
65061	1.0029	2.1
65062	1.0029	2.1
65063	1.23814	2.1
65064	0.997523	2.1
65065	1.11498	2.1
65066	0.899532	2.1
65067	1.89226	2.1
65068	1.89226	2.1
65069	1.89226	2.1
65070	1.89226	2.1
65071	1.89226	2.1
65072	1.89226	2.1
65073	1.89226	2.1

65074	1.89226	2.1
65075	1.89226	2.1
65076	1.89226	2.1
65077	1.89226	2.1
65078	1.89226	2.1
65079	0.993139	2.1
65080	0.834108	2.1
65081	1.01147	2.1
65082	1.01358	2.1
65083	1.20067	2.1
65084	0.993156	2.1
65085	2.4158	2.1
65086	1.69956	2.1
65087	1.3544	2.1
65088	1.60095	2.1
65089	1.11219	2.1
65090	1.89226	2.1
65091	1.89226	2.1
65092	1.89226	2.1
65093	1.89226	2.1
65094	3.78452	2.1
65095	1.89226	2.1
65096	1.89226	2.1
65097	1.89226	2.1
65098	1.89226	2.1
65099	1.89226	2.1
65100	1.89226	2.1
65101	1.89226	2.1
65102	2.83014	2.1
65103	2.32404	2.1
65104	2.32404	2.1
65105	1.89226	2.1
65106	1.89226	2.1
65107	1.37966	2.1
65108	1.13294	2.1
65109	0.881723	2.1
65110	0.582304	2.1
65111	0.977944	2.1
65112	0.977948	2.1
65113	1.0264	2.1
65114	1.0264	2.1
65115	1.37967	2.1
65116	1.03896	2.1
65117	1.19108	2.1
65118	0.582298	2.1
65119	1.89226	2.1
65120	1.89226	2.1
65121	1.89226	2.1
65122	1.89226	2.1
65123	3.78452	2.1
65124	0.946131	2.1
65125	0.946131	2.1
65126	0.946131	2.1
65127	0.946131	2.1
65128	0.946132	2.1
65129	0.946132	2.1
65130	0.946129	2.1
65131	0.946129	2.1

65132	0.946131	2.1
65133	0.946131	2.1
65134	0.94613	2.1
65135	0.94613	2.1
65136	1.89226	2.1
65137	1.89226	2.1
65138	1.89226	2.1
65139	1.89226	2.1
65140	3.78453	2.1
65141	1.89226	2.1
65142	1.89226	2.1
65143	1.89226	2.1
65144	1.89226	2.1
65145	1.89226	2.1
65146	1.89226	2.1
65147	2.65216	2.1
65148	1.93528	2.1
65149	1.78595	2.1
65150	2.48544	2.1
65151	4.42072	2.1
65152	1.89226	2.1
65153	1.89226	2.1
65154	1.89226	2.1
65155	1.89226	2.1
65156	1.89226	2.1
65157	1.89226	2.1
65158	1.89226	2.1
65159	1.89226	2.1
65160	1.89226	2.1
65161	1.89226	2.1
65162	3.78452	2.1
65163	1.66537	2.1
65164	2.48544	2.1
65165	1.78595	2.1
65166	1.78595	2.1
65167	2.36197	2.1
65168	2.36197	2.1
65169	2.80975	2.1
65170	2.48544	2.1
65171	2.36198	2.1
65172	2.65135	2.1
65173	5.13679	2.1
65174	1.89226	2.1
65175	1.89226	2.1
65176	1.89226	2.1
65177	1.89226	2.1
65178	1.89226	2.1
65179	1.89226	2.1
65180	0.946127	2.1
65181	0.946127	2.1
65182	0.946131	2.1
65183	0.946131	2.1
65184	0.946128	2.1
65185	0.946128	2.1
65186	0.94613	2.1
65187	0.94613	2.1
65188	0.946129	2.1
65189	0.946129	2.1

65190	0.946129	2.1
65191	0.946129	2.1
65192	1.11972	2.1
65193	1.18099	2.1
65194	0.832687	2.1
65195	0.764517	2.1
65196	0.974426	2.1
65197	0.974426	2.1
65198	0.971078	2.1
65199	0.971078	2.1
65200	1.11972	2.1
65201	0.764515	2.1
65202	0.832686	2.1
65203	1.18099	2.1
65204	2.36198	2.1
65205	2.48025	2.1
65206	2.48025	2.1
65207	2.65135	2.1
65208	2.23944	2.1
65209	2.41055	2.1
65210	0.946132	2.1
65211	0.946132	2.1
65212	0.946131	2.1
65213	0.946131	2.1
65214	0.946131	2.1
65215	0.946131	2.1
65216	0.946132	2.1
65217	0.946132	2.1
65218	0.946131	2.1
65219	0.946131	2.1
65220	0.946132	2.1
65221	0.946132	2.1
65222	0.810942	2.1
65223	0.810942	2.1
65224	0.810944	2.1
65225	0.810944	2.1
65226	1.89226	2.1
65227	1.3517	2.1
65228	1.08132	2.1
65229	1.08133	2.1
65230	1.08132	2.1
65231	0.810932	2.1
65232	0.810932	2.1
65233	1.89249	2.1
65234	1.9857	1.65842
65235	2.02433	2.05318
65236	2.15807	1.94606
65237	4.01003	2.02789
65238	1.89226	1.90815
65239	1.89226	1.71017
65240	1.89226	2.07046
65241	1.89226	0.724776
65242	3.78452	1.64545
65243	1.89226	1.87197
65244	1.89226	1.99565
65245	1.89226	0.377997
65246	1.89226	0.992137
65247	3.78452	0.60703

65248	1.98569	1.63521
65249	1.86067	1.64524
65250	2.15807	1.8746
65251	2.07965	0.91581
65252	4.06534	1.80286
65253	2.02433	2.1
65254	2.15807	2.09782
65255	2.12608	2.1
65256	2.3025	1.54574
65257	4.32683	2.09878
65258	1.89226	2.1
65259	1.89226	1.2735
65260	1.89226	1.81215
65261	1.89226	1.61049
65262	3.78452	1.80223
65263	1.89226	0.867617
65264	1.89226	1.59686
65265	1.89226	1.45142
65266	1.89226	1.45242
65267	3.78452	1.49829
65268	2.15807	2.09604
65269	2.07965	1.41173
65270	2.3025	1.12873
65271	2.28384	1.3744
65272	4.38215	1.20421
65273	1.89226	2.09255
65274	1.89226	0.639291
65275	1.89226	1.79622
65276	1.89226	0.733353
65277	3.78453	1.46991
65278	2.16905	0.688734
65279	2.56369	0.559269
65280	1.88394	1.52035
65281	2.41938	0.434904
65282	4.44763	0.597082
65283	2.56369	0.332283
65284	2.80663	1.50438
65285	2.41938	0.441109
65286	2.69045	0.589279
65287	5.25414	0.470985
65288	1.89226	0.377316
65289	1.89226	0.449939
65290	1.89226	0.709055
65291	1.89226	0.663487
65292	3.78453	0.147862
65293	1.89226	1.51537
65294	1.89226	0.796237
65295	1.89226	2.0433
65296	1.89226	0.856937
65297	1.89226	1.97334
65298	1.89226	0.526915
65299	1.01988	0.631364
65300	1.01988	0.576226
65301	1.03333	1.20511
65302	1.03333	0.708276
65303	1.13811	0.144534
65304	1.20969	0.355039
65305	0.941974	1.21916

65306	0.843515	0.593515
65307	0.843514	0.484157
65308	1.13811	0.478516
65309	0.941973	0.604622
65310	1.20969	0.367229
65311	2.41938	0.261334
65312	2.57414	0.590553
65313	2.57414	0.895518
65314	2.69045	1.61168
65315	2.27623	0.201088
65316	2.39253	1.42254
65317	1.89227	0.67775
65318	1.89227	0.709235
65319	1.89227	0.22248
65320	1.89227	0.252631
65321	1.89227	0.206219
65322	1.89227	0.344718
65323	2.28385	1.86825
65324	2.09114	1.90128
65325	2.46968	0.331275
65326	2.32438	0.947738
65327	4.56082	0.431684
65328	1.89226	0.789825
65329	1.89226	1.92241
65330	1.89226	0.538579
65331	1.89226	1.93152
65332	3.78452	1.28052
65333	1.89226	0.915261
65334	1.89226	2.1
65335	1.89226	1.8399
65336	1.89226	1.96984
65337	3.78452	1.94942
65338	2.09114	0.834712
65339	1.5249	1.92185
65340	2.55684	0.334275
65341	1.86899	1.97489
65342	3.96011	1.2163
65343	2.46968	0.774668
65344	2.32439	1.41625
65345	2.62768	1.65187
65346	2.506	1.41095
65347	4.97568	1.78868
65348	1.89226	1.43043
65349	1.89226	1.95283
65350	1.89226	0.389568
65351	1.89226	1.19012
65352	3.78452	0.839969
65353	1.89226	1.68565
65354	1.89226	1.86612
65355	1.89226	1.52537
65356	1.89226	2.1
65357	1.89226	0.852261
65358	1.89226	2.1
65359	2.55684	0.753259
65360	2.16506	1.85663
65361	1.86901	1.43798
65362	1.86899	1.06386
65363	2.75664	1.05396

65364	2.75662	0.750677
65365	1.89224	0.776344
65366	1.89224	1.40254
65367	1.89224	0.290756
65368	1.89224	2.09118
65369	3.78447	1.09081
65370	2.24206	0.890679
65371	2.38862	2.1
65372	2.07107	1.78715
65373	2.23097	2.08723
65374	4.47303	2.01719
65375	2.38863	2.1
65376	2.25945	2.1
65377	2.25945	2.0954
65378	2.39244	2.1
65379	2.23098	2.09179
65380	2.36397	2.1
65381	1.89224	1.36482
65382	1.89224	2.1
65383	1.89224	2.1
65384	1.89224	2.09574
65385	1.89224	2.1
65386	1.89224	2.09245
65387	1.89226	0.450796
65388	1.89226	2.07417
65389	1.89226	1.65711
65390	1.89226	0.975043
65391	1.89226	1.58498
65392	1.89226	1.3622
65393	0.920975	2.1
65394	0.920979	1.88242
65395	1.03015	2.0844
65396	1.03015	1.0552
65397	0.992407	2.06461
65398	1.08759	2.1
65399	0.854346	1.86946
65400	0.87241	1.00338
65401	0.872397	1.99219
65402	1.04334	1.99313
65403	1.00965	2.01908
65404	1.0876	2.01744
65405	2.23101	2.1
65406	2.25948	2.1
65407	2.03577	2.07897
65408	2.05257	2.1
65409	4.29524	2.1
65410	0.810947	1.64503
65411	0.810947	2.1
65412	0.810947	2.06585
65413	0.810947	1.60555
65414	0.810934	2.05409
65415	0.810934	2.06192
65416	1.89227	2.1
65417	1.3517	2.1
65418	1.08132	2.08585
65419	1.08132	2.1
65420	1.08133	2.09726
65421	1.89226	2.07174

65422	1.89226	2.07617
65423	1.89226	1.72179
65424	1.89226	2.1
65425	1.89226	1.17671
65426	1.89226	2.1
65427	1.89226	2.1
65428	1.89226	2.1
65429	1.89226	2.083
65430	1.89226	2.1
65431	1.89226	2.07813
65432	1.89226	2.1
65433	0.879432	2.1
65434	0.879432	2.08516
65435	0.900223	1.12755
65436	0.900223	2.0982
65437	0.54571	2.1
65438	0.743092	2.09804
65439	1.03656	2.09462
65440	1.19234	1.20473
65441	1.19234	2.05371
65442	0.743092	2.05777
65443	1.03656	2.08898
65444	0.545711	2.09527
65445	1.89226	0.686617
65446	2.38467	1.58845
65447	2.69293	0.316382
65448	2.69293	2.07262
65449	1.89226	0.221469
65450	1.89226	1.9771
65451	1.89226	1.89776
65452	1.89226	1.97577
65453	1.89226	0.842451
65454	1.89226	2.1
65455	3.78452	1.94385
65456	0.946129	2.1
65457	0.946129	2.1
65458	0.946133	1.78204
65459	0.946133	2.1
65460	0.946131	2.1
65461	0.946131	2.1
65462	0.946131	1.74517
65463	0.946131	2.1
65464	0.946131	2.1
65465	0.946131	2.1
65466	0.946131	2.1
65467	0.946131	2.1
65468	1.04846	1.70111
65469	1.04846	2.1
65470	1.05594	0.814323
65471	1.05594	2.04984
65472	0.743092	2.1
65473	0.912061	2.01359
65474	1.19234	0.752968
65475	1.34647	1.68602
65476	0.912061	1.62165
65477	1.34646	1.78203
65478	1.19234	1.90247
65479	0.743093	1.8918

65480	1.89226	0.931193
65481	1.89226	2.1
65482	1.89226	1.32794
65483	1.89226	1.82191
65484	3.78452	1.97093
65485	0.946128	2.1
65486	0.946128	2.1
65487	0.946133	2.1
65488	0.946133	1.81425
65489	0.94613	2.1
65490	0.94613	2.1
65491	0.946131	2.1
65492	0.946131	1.94903
65493	0.946131	2.1
65494	0.946131	2.1
65495	0.946131	2.1
65496	0.946131	2.1
65497	1.89226	1.43616
65498	1.89226	2.09844
65499	1.89226	0.364576
65500	1.89226	1.85846
65501	3.78452	1.40839
65502	1.89226	1.84309
65503	1.89226	1.82067
65504	1.89226	1.60106
65505	1.89226	2.1
65506	1.89226	0.774148
65507	1.89226	2.1
65508	1.89226	2.09266
65509	1.89226	2.09149
65510	1.89226	1.86893
65511	1.89226	2.1
65512	1.89226	1.71878
65513	1.89226	2.1
65514	0.946132	2.1
65515	0.946132	1.90092
65516	1.17758	0.736778
65517	1.17759	1.97622
65518	1.04514	2.09945
65519	1.17758	1.56876
65520	0.946133	0.775783
65521	0.946133	1.69857
65522	0.946107	1.7616
65523	1.17755	1.71447
65524	1.04517	1.77838
65525	0.946158	1.73954
65526	1.89226	2.1
65527	2.10171	2.1
65528	1.95064	2.1
65529	2.10171	2.1
65530	1.7412	2.1
65531	1.89226	2.1
65532	1.89226	2.1
65533	1.89226	2.1
65534	1.89226	2.1
65535	1.89226	2.1
65536	1.89226	2.1
65537	1.89226	2.1

65538	1.89226	2.1
65539	1.89226	2.1
65540	1.89226	2.1
65541	1.89226	2.1
65542	3.78453	2.1
65543	0.946133	2.1
65544	0.946133	2.1
65545	0.946132	2.1
65546	0.946132	2.1
65547	0.946133	2.1
65548	0.946133	2.1
65549	0.946132	2.1
65550	0.946132	2.1
65551	0.946132	2.1
65552	0.946132	2.1
65553	0.946132	2.1
65554	0.946132	2.1
65555	1.70303	2.1
65556	1.95064	2.1
65557	1.74039	2.1
65558	1.95064	2.1
65559	1.69368	2.1
65560	1.89226	2.1
65561	1.89226	2.1
65562	1.89226	2.1
65563	1.89226	2.1
65564	1.89226	2.1
65565	3.78453	2.1
65566	0.946132	2.1
65567	0.946132	2.1
65568	0.946132	2.1
65569	0.946132	2.1
65570	0.946133	2.1
65571	0.946133	2.1
65572	0.946132	2.1
65573	0.946132	2.1
65574	0.946107	2.1
65575	0.946107	2.1
65576	0.946158	2.1
65577	0.946158	2.1
65578	0.946133	2.1
65579	0.946133	2.1
65580	0.946132	2.1
65581	0.946132	2.1
65582	0.946133	2.1
65583	0.946133	2.1
65584	0.946132	2.1
65585	0.946132	2.1
65586	0.946107	2.1
65587	0.946107	2.1
65588	0.946158	2.1
65589	0.946158	2.1
65590	1.89226	2.1
65591	1.89226	2.1
65592	1.89226	2.1
65593	1.89226	2.1
65594	1.89226	2.1
65595	1.89226	2.1

65596	1.89226	2.1
65597	1.89226	2.1
65598	1.89226	2.1
65599	1.89226	2.1
65600	1.89226	2.1
65601	1.89226	2.1
65602	2.5198	2.1
65603	2.45893	2.1
65604	2.27322	2.1
65605	2.27322	2.1
65606	2.63576	2.1
65607	2.63576	2.1
65608	1.24045	2.1
65609	1.11906	2.1
65610	1.13578	2.1
65611	0.916205	2.1
65612	1.12539	2.1
65613	1.08404	2.1
65614	1.08484	2.1
65615	1.08484	2.1
65616	1.22836	2.1
65617	0.919363	2.1
65618	1.16507	2.1
65619	0.924864	2.1
65620	1.89226	2.1
65621	1.89226	2.1
65622	1.89226	2.1
65623	1.89226	2.1
65624	1.89226	2.1
65625	1.89226	2.1
65626	1.89226	2.1
65627	1.89226	2.1
65628	1.89226	2.1
65629	1.89226	2.1
65630	1.89226	2.1
65631	1.89226	2.1
65632	2.6946	2.1
65633	2.63576	2.1
65634	2.5198	2.1
65635	2.5198	2.1
65636	2.8362	2.1
65637	2.8362	2.1
65638	1.3265	2.1
65639	1.24045	2.1
65640	0.983223	2.1
65641	0.954429	2.1
65642	1.06046	2.1
65643	1.10594	2.1
65644	1.1915	2.1
65645	1.1915	2.1
65646	1.31357	2.1
65647	1.09005	2.1
65648	1.29145	2.1
65649	0.995825	2.1
65650	1.89226	2.1
65651	1.89226	2.1
65652	1.89226	2.1
65653	1.89226	2.1

65654	3.78452	2.1
65655	1.89226	2.1
65656	1.89226	2.1
65657	1.89226	2.1
65658	1.89226	2.1
65659	3.78453	2.1
65660	1.91802	2.1
65661	2.27409	2.1
65662	2.48048	2.1
65663	2.22264	2.1
65664	1.91802	2.1
65665	1.66018	2.1
65666	1.13704	2.1
65667	1.24023	2.1
65668	1.22947	2.1
65669	1.31788	2.1
65670	1.23859	2.1
65671	1.23859	2.1
65672	1.23111	2.1
65673	1.23111	2.1
65674	1.24024	2.1
65675	1.31788	2.1
65676	1.13704	2.1
65677	1.22947	2.1
65678	1.89226	2.1
65679	1.89226	2.1
65680	1.89226	2.1
65681	1.89226	2.1
65682	1.89226	2.1
65683	1.89226	2.1
65684	1.89226	2.1
65685	1.89226	2.1
65686	1.89226	2.1
65687	1.89226	2.1
65688	3.78453	2.1
65689	1.05568	2.1
65690	0.959008	2.1
65691	1.32068	2.1
65692	1.24024	2.1
65693	1.15206	2.1
65694	1.15206	2.1
65695	1.14386	2.1
65696	1.14386	2.1
65697	1.05568	2.1
65698	1.32068	2.1
65699	0.959008	2.1
65700	1.24024	2.1
65701	1.32068	2.1
65702	1.24023	2.1
65703	1.31788	2.1
65704	1.4181	2.1
65705	1.31436	2.1
65706	1.31436	2.1
65707	1.3242	2.1
65708	1.3242	2.1
65709	1.32068	2.1
65710	1.4181	2.1
65711	1.24024	2.1

65712	1.31788	2.1
65713	1.89226	2.1
65714	1.89226	0.784308
65715	1.89226	1.89934
65716	1.89226	1.92295
65717	1.89226	1.88675
65718	1.89226	2.00533
65719	1.02925	0.0881318
65720	1.16678	0.598119
65721	0.946132	0.596775
65722	0.946132	1.98447
65723	0.935677	0.64354
65724	0.935677	0.129995
65725	1.18902	1.8437
65726	1.21562	1.27454
65727	1.13433	0.497868
65728	1.04071	0.526207
65729	0.851553	0.711791
65730	1.06075	0.721542
65731	1.89226	0.807864
65732	1.89226	0.651753
65733	1.89226	0.493113
65734	1.89226	0.156428
65735	1.89226	0.294777
65736	1.89226	0.804302
65737	1.89226	1.9902
65738	1.89226	0.306052
65739	1.89226	0.775595
65740	1.89226	0.681837
65741	3.78453	0.63839
65742	0.946128	0.649046
65743	0.946128	0.645938
65744	0.946133	0.381027
65745	0.946133	0.416057
65746	0.946131	0.224069
65747	0.946131	0.576831
65748	0.94613	0.346809
65749	0.94613	0.375153
65750	0.946131	0.634945
65751	0.946131	0.20653
65752	0.946131	0.19566
65753	0.946131	0.135
65754	0.946132	0.94605
65755	0.946132	0.542836
65756	0.946132	1.72112
65757	0.946132	0.794329
65758	0.946132	0.638057
65759	0.946132	0.0990079
65760	0.946131	0.926117
65761	0.946131	1.68314
65762	0.946132	0.827555
65763	0.946132	0.873699
65764	0.946132	0.619876
65765	0.946132	0.717669
65766	0.946131	2.04068
65767	0.946131	1.64426
65768	0.946131	1.98823
65769	0.946131	0.413771

65770	0.946131	0.711901
65771	0.946131	2.02237
65772	0.946131	0.996994
65773	0.946131	0.379852
65774	0.946131	1.23921
65775	0.946131	1.28392
65776	0.946131	1.34112
65777	0.946131	1.28821
65778	1.89226	1.99979
65779	1.89226	1.849
65780	1.89226	1.52357
65781	1.89226	2.1
65782	1.89226	1.12789
65783	1.89226	2.1
65784	1.89226	1.19486
65785	1.89226	0.554208
65786	1.89226	0.947329
65787	1.89226	2.06835
65788	1.89226	0.458344
65789	1.89226	2.1
65790	1.89226	1.98722
65791	1.89226	1.44559
65792	1.89226	1.3083
65793	1.89226	2.1
65794	1.89226	0.791238
65795	1.89226	2.1
65796	1.89226	2.09369
65797	1.89226	2.05896
65798	1.89226	1.24976
65799	1.89226	2.1
65800	3.78452	2.05663
65801	0.946131	2.05711
65802	0.946131	1.38265
65803	1.17195	0.480958
65804	1.17195	1.91498
65805	0.958489	2.06496
65806	1.17195	1.26594
65807	0.946129	0.603341
65808	0.946129	1.78152
65809	0.946131	1.58971
65810	1.17195	1.58032
65811	0.958487	1.55486
65812	0.94613	1.60927
65813	2.69656	2.1
65814	2.17516	2.1
65815	2.41885	2.1
65816	2.41885	2.1
65817	2.46476	2.1
65818	2.46476	2.1
65819	2.69657	2.1
65820	2.30233	1.67903
65821	2.30233	2.1
65822	2.59476	2.09875
65823	2.46477	2.1
65824	2.7572	2.1
65825	1.89226	2.1
65826	1.89226	0.618543
65827	1.89226	1.42657

65828	1.89226	1.38788
65829	1.89226	1.27025
65830	1.89226	1.77399
65831	1.89226	2.1
65832	1.89226	2.1
65833	1.89226	2.1
65834	1.89226	1.46657
65835	3.78452	2.1
65836	2.46477	2.1
65837	2.17516	2.1
65838	1.80252	2.1
65839	2.09715	2.0917
65840	4.27231	2.1
65841	2.46477	2.1
65842	2.09715	2.05364
65843	2.30233	2.1
65844	2.30233	2.07016
65845	1.96416	1.29655
65846	2.09715	1.2064
65847	1.89227	2.02764
65848	1.89227	0.461489
65849	1.89227	1.20722
65850	1.89227	1.33784
65851	1.89227	1.24602
65852	1.89227	1.70596
65853	1.89226	2.1
65854	1.89226	1.31805
65855	1.89226	2.07747
65856	1.89226	2.0887
65857	3.78452	2.08272
65858	0.946132	2.1
65859	0.946132	2.1
65860	0.946132	2.1
65861	0.946132	2.1
65862	0.946132	2.1
65863	0.946132	2.1
65864	0.946132	2.1
65865	0.946132	2.1
65866	0.946132	2.1
65867	0.946132	2.1
65868	0.946131	2.1
65869	0.946131	2.1
65870	1.89226	2.1
65871	1.89226	2.1
65872	1.89226	2.1
65873	1.89226	2.1
65874	3.78453	2.1
65875	1.89226	2.1
65876	1.89226	2.1
65877	1.89226	2.1
65878	1.89226	2.1
65879	1.89226	2.1
65880	1.89226	2.1
65881	1.89226	2.1
65882	1.63707	2.1
65883	1.63707	2.1
65884	1.38859	2.1
65885	1.89226	2.1

65886	1.64377	2.1
65887	0.94613	2.1
65888	0.94613	2.1
65889	0.94613	2.1
65890	0.94613	2.1
65891	0.946131	2.1
65892	0.946131	2.1
65893	0.946129	2.1
65894	0.946129	2.1
65895	0.94613	2.1
65896	0.94613	2.1
65897	0.94613	2.1
65898	0.94613	2.1
65899	2.15888	2.1
65900	1.99309	2.1
65901	2.16841	2.1
65902	2.16841	2.1
65903	2.02311	2.08236
65904	2.02311	1.92266
65905	1.89226	2.07637
65906	1.89226	2.05287
65907	1.89226	2.1
65908	1.89226	2.07463
65909	1.89226	2.1
65910	1.89226	2.02553
65911	1.89226	2.0849
65912	1.89226	2.1
65913	1.89226	2.1
65914	1.89226	2.09696
65915	1.89226	2.1
65916	1.89226	2.06938
65917	1.99309	2.1
65918	1.96035	2.1
65919	1.72526	2.1
65920	1.72526	2.1
65921	2.16841	2.1
65922	2.16841	2.1
65923	2.15888	2.1
65924	2.16841	2.1
65925	2.3111	2.1
65926	2.35938	2.1
65927	4.51826	2.1
65928	1.89226	2.1
65929	1.89226	2.1
65930	1.89226	2.09766
65931	1.89226	1.93587
65932	3.78452	2.1
65933	1.89226	2.1
65934	1.89226	2.1
65935	1.89226	2.1
65936	1.89226	1.88582
65937	3.78452	2.1
65938	2.16841	2.1
65939	1.96035	2.1
65940	2.35938	2.1
65941	2.18493	2.1
65942	4.31973	2.1
65943	1.89226	2.1

65944	1.89226	2.1
65945	1.89226	2.1
65946	1.89226	2.1
65947	3.78453	2.1
65948	1.89226	2.1
65949	1.89226	2.1
65950	1.89226	2.1
65951	1.89226	2.1
65952	3.78452	2.1
65953	0.946158	2.1
65954	0.946158	2.1
65955	0.818509	2.1
65956	0.818509	2.1
65957	0.933975	2.1
65958	0.818539	2.1
65959	0.946128	2.1
65960	0.946128	2.1
65961	0.94613	2.1
65962	0.933974	2.1
65963	0.818537	2.1
65964	0.946129	2.1
65965	0.946131	2.1
65966	0.946131	2.1
65967	0.946131	2.1
65968	0.946131	2.1
65969	0.946132	2.1
65970	0.946132	2.1
65971	0.946129	2.1
65972	0.946129	2.1
65973	0.946131	2.1
65974	0.946131	2.1
65975	0.94613	2.1
65976	0.94613	2.1
65977	1.89226	2.1
65978	1.89226	2.1
65979	1.89226	2.1
65980	1.89226	2.1
65981	3.78453	2.1
65982	1.89226	2.1
65983	1.89226	2.1
65984	1.89226	2.1
65985	1.89226	2.1
65986	3.78452	2.1
65987	1.89226	2.1
65988	1.8706	2.1
65989	1.8706	2.1
65990	1.68112	2.1
65991	2.0815	2.1
65992	1.88715	2.1
65993	0.94613	2.1
65994	0.94613	2.1
65995	0.94613	2.1
65996	0.94613	2.1
65997	0.946131	2.1
65998	0.946131	2.1
65999	0.946129	2.1
66000	0.946129	2.1
66001	0.94613	2.1

66002	0.94613	2.1
66003	0.94613	2.1
66004	0.94613	2.1
66005	1.76401	2.1
66006	2.60983	2.1
66007	2.50461	2.1
66008	2.35897	2.1
66009	1.90965	2.1
66010	1.76401	2.1
66011	1.89226	2.1
66012	1.89226	2.1
66013	1.89226	2.1
66014	1.89226	2.1
66015	3.78452	2.1
66016	1.89226	2.1
66017	1.89226	2.1
66018	1.89226	2.1
66019	1.89226	2.1
66020	1.89226	2.1
66021	1.89226	2.1
66022	1.90965	2.1
66023	2.83834	2.1
66024	2.7538	2.1
66025	2.60983	2.1
66026	2.05361	2.1
66027	1.90965	2.1
66028	1.89226	2.1
66029	1.89226	2.1
66030	1.89226	2.1
66031	1.89226	2.1
66032	1.89226	2.1
66033	1.89226	2.1
66034	1.89226	2.1
66035	1.89226	2.1
66036	1.89226	2.1
66037	1.89226	2.1
66038	3.78452	2.1
66039	2.83834	2.1
66040	1.89226	2.1
66041	2.60983	2.1
66042	2.60983	2.1
66043	1.89226	2.1
66044	1.89226	2.1
66045	1.89226	2.1
66046	1.89226	2.1
66047	1.89226	2.1
66048	1.89226	2.1
66049	1.89226	2.1
66050	1.89226	2.1
66051	1.89226	2.1
66052	1.89226	2.1
66053	1.89226	2.1
66054	1.89226	2.0773
66055	1.89226	2.1
66056	1.89226	2.07082
66057	2.44785	2.1
66058	1.80223	2.1
66059	2.80894	2.1

66060	2.18296	2.1
66061	4.63081	2.1
66062	1.89226	2.1
66063	1.89226	2.1
66064	1.89226	2.1
66065	1.89226	2.1
66066	1.89226	2.1
66067	1.89226	2.1
66068	0.946131	1.95416
66069	0.946131	2.1
66070	0.946127	2.1
66071	0.946127	2.1
66072	0.946131	2.09723
66073	0.946131	2.1
66074	0.946128	2.1
66075	0.946128	2.1
66076	0.94613	2.1
66077	0.94613	2.1
66078	0.946129	2.1
66079	0.946129	2.1
66080	1.89226	2.1
66081	1.89226	2.1
66082	1.89226	2.1
66083	1.89226	2.1
66084	3.78452	2.1
66085	1.89226	2.1
66086	1.89226	2.1
66087	1.89226	2.1
66088	1.89226	2.1
66089	3.78453	2.1
66090	2.41083	2.1
66091	2.93937	2.1
66092	1.68761	2.1
66093	1.68761	2.1
66094	1.67248	2.1
66095	2.3957	2.1
66096	1.89226	2.1
66097	1.89226	2.1
66098	1.89226	2.1
66099	1.89226	2.1
66100	1.89226	2.1
66101	1.89226	2.1
66102	1.89227	2.1
66103	1.89227	2.1
66104	1.89227	2.1
66105	1.89227	2.1
66106	1.89227	2.1
66107	1.89227	2.1
66108	1.89224	1.02955
66109	1.89224	0.399355
66110	1.89224	0.690315
66111	1.89224	0.762872
66112	3.78448	0.440121
66113	1.89224	2.07179
66114	1.89224	0.979236
66115	1.89224	1.99781
66116	1.89224	1.13188
66117	1.89224	1.97014

66118	1.89224	1.41881
66119	1.11152	0.6844
66120	1.29346	1.42982
66121	0.978596	1.98175
66122	0.918324	2.05491
66123	1.06821	1.98505
66124	1.06821	0.63522
66125	0.985654	1.8993
66126	1.15278	2.0504
66127	1.07693	1.90238
66128	0.902432	1.87811
66129	0.968939	1.92298
66130	1.34644	1.80927
66131	0.946132	0.456407
66132	0.946132	0.45162
66133	0.946132	0.629488
66134	0.946132	0.599942
66135	0.946131	0.474789
66136	0.946131	0.305817
66137	0.946132	0.693532
66138	0.946132	0.483629
66139	0.946132	0.531454
66140	0.946132	0.435662
66141	0.946132	0.275238
66142	0.946132	0.325796
66143	0.946133	1.19872
66144	0.946133	0.731359
66145	0.946132	1.56531
66146	0.946132	1.88289
66147	0.946132	1.51552
66148	0.946132	0.31067
66149	0.946133	2.07861
66150	0.946133	0.968843
66151	0.946132	1.43927
66152	0.946132	1.34022
66153	0.946132	0.963121
66154	0.946132	1.03639
66155	1.89224	2.06297
66156	1.89224	2.1
66157	1.89224	2.1
66158	1.89224	2.1
66159	1.89224	2.1
66160	1.89224	2.1
66161	1.89224	2.1
66162	1.89224	2.1
66163	1.89224	2.1
66164	1.89224	2.1
66165	1.89224	2.1
66166	1.89224	2.1
66167	1.89226	2.1
66168	1.89226	2.1
66169	1.89226	2.1
66170	1.89226	2.1
66171	3.78453	2.1
66172	1.11056	2.1
66173	1.21595	2.1
66174	1.12763	2.1
66175	1.12763	2.1

66176	1.23339	2.1
66177	1.35697	2.1
66178	1.03581	2.1
66179	0.95954	2.1
66180	0.89761	2.1
66181	1.26602	2.1
66182	1.12749	2.1
66183	1.45307	2.1
66184	2.71775	2.1
66185	1.89226	2.1
66186	1.89226	2.1
66187	1.89226	2.1
66188	2.47025	2.1
66189	2.47025	2.1
66190	0.94613	2.1
66191	0.94613	2.1
66192	0.94613	2.1
66193	0.94613	2.1
66194	0.946128	2.1
66195	0.946128	2.1
66196	0.946132	2.1
66197	0.946132	2.1
66198	0.94613	2.1
66199	0.94613	2.1
66200	0.94613	2.1
66201	0.94613	2.1
66202	1.89226	1.83277
66203	1.89226	2.1
66204	1.89226	2.1
66205	1.89226	1.94144
66206	1.89226	2.1
66207	1.89226	1.94729
66208	1.89226	1.7675
66209	1.89226	2.1
66210	1.89226	2.1
66211	1.89226	1.54778
66212	1.89226	2.1
66213	1.89226	1.62632
66214	0.946132	2.1
66215	0.946132	2.1
66216	0.946133	2.1
66217	0.946133	2.1
66218	0.946133	2.1
66219	0.946133	2.1
66220	0.946132	2.1
66221	0.946132	2.1
66222	0.946133	2.1
66223	0.946133	2.1
66224	0.946132	2.1
66225	0.946132	2.1
66226	1.89226	1.89616
66227	1.89226	2.1
66228	1.89226	2.1
66229	1.89226	1.96183
66230	1.89226	2.1
66231	1.89226	1.96615
66232	0.946131	1.82912
66233	0.946131	1.76173

66234	0.946127	2.1
66235	0.946127	2.1
66236	0.946131	1.93118
66237	0.946131	2.1
66238	0.946127	2.1
66239	0.946127	1.9934
66240	0.94613	2.05727
66241	0.94613	2.05215
66242	0.946129	2.06307
66243	0.946129	2.06036
66244	1.94291	2.1
66245	2.00118	2.1
66246	2.36337	2.1
66247	2.41217	2.1
66248	4.35508	2.1
66249	1.89226	2.1
66250	1.89226	2.1
66251	1.89226	2.1
66252	1.89226	2.1
66253	3.78452	2.1
66254	1.89226	2.1
66255	1.89226	2.1
66256	1.89226	2.1
66257	1.89226	2.1
66258	3.78452	2.1
66259	1.06209	2.1
66260	1.06209	2.1
66261	1.05987	2.1
66262	1.05987	2.1
66263	0.920378	2.1
66264	1.12137	2.1
66265	1.00059	2.1
66266	1.20608	2.1
66267	1.20608	2.1
66268	1.12137	2.1
66269	1.00059	2.1
66270	0.920376	2.1
66271	2.77222	2.1
66272	2.41216	2.1
66273	2.77002	2.1
66274	2.36337	2.1
66275	2.81882	2.1
66276	2.41216	2.1
66277	1.89226	2.1
66278	1.89226	2.1
66279	1.89226	2.1
66280	1.89226	2.1
66281	1.89226	2.1
66282	1.89226	2.1
66283	1.89226	2.1
66284	1.89226	2.1
66285	1.89226	2.1
66286	1.89226	2.1
66287	3.78451	2.1
66288	2.41216	2.1
66289	2.24273	2.1
66290	2.81882	2.1
66291	2.68473	2.1

66292	5.09689	2.1
66293	1.89226	2.1
66294	1.89226	2.1
66295	1.89226	2.1
66296	1.89226	2.1
66297	3.78453	2.1
66298	2.17614	2.1
66299	2.65326	2.1
66300	1.93101	2.1
66301	2.58552	2.1
66302	4.58427	2.1
66303	2.65326	2.1
66304	1.89226	2.1
66305	1.89226	2.1
66306	1.89226	2.1
66307	2.58552	2.1
66308	2.58552	2.1
66309	1.89226	2.1
66310	1.89226	2.1
66311	1.89226	2.1
66312	1.89226	2.1
66313	3.78453	2.1
66314	1.89226	2.1
66315	1.89226	2.1
66316	1.89226	2.1
66317	1.89226	2.1
66318	3.78453	2.1
66319	1.93101	2.1
66320	2.58552	2.1
66321	1.46507	2.1
66322	2.52893	2.1
66323	4.45994	2.1
66324	1.26446	2.1
66325	1.26446	2.1
66326	0.946132	2.1
66327	0.946132	2.1
66328	0.94613	2.1
66329	0.94613	2.1
66330	1.29276	2.1
66331	1.26446	2.1
66332	1.26446	2.1
66333	0.94613	2.1
66334	0.94613	2.1
66335	1.29276	2.1
66336	1.89226	2.1
66337	1.89226	2.1
66338	1.89226	2.1
66339	1.89226	2.1
66340	3.78453	2.1
66341	1.89226	2.1
66342	1.89226	2.1
66343	1.89226	2.1
66344	1.89226	2.1
66345	3.78453	2.1
66346	1.83378	2.1
66347	2.52893	2.1
66348	1.75592	2.1
66349	2.49844	2.1

66350	4.28485	2.1
66351	1.24922	2.1
66352	1.24922	2.1
66353	0.946133	2.1
66354	0.946133	2.1
66355	0.946131	2.1
66356	0.946131	2.1
66357	1.26446	2.1
66358	1.24922	2.1
66359	1.24922	2.1
66360	0.946131	2.1
66361	1.26446	2.1
66362	0.94613	2.1
66363	0.946132	2.1
66364	0.946132	2.1
66365	0.946132	2.1
66366	0.946132	2.1
66367	0.946132	2.1
66368	0.946132	2.1
66369	0.946132	2.1
66370	0.946132	2.1
66371	0.946132	2.1
66372	0.946132	2.1
66373	0.946131	2.1
66374	0.946131	2.1
66375	1.89226	2.1
66376	1.89226	2.1
66377	1.89226	2.1
66378	1.89226	2.1
66379	1.89226	2.1
66380	1.89226	2.1
66381	1.0465	2.1
66382	1.0465	2.1
66383	1.06047	2.1
66384	1.06047	2.1
66385	1.25687	2.1
66386	1.24922	2.1
66387	0.87796	2.1
66388	0.857749	2.1
66389	0.857749	2.1
66390	1.25687	2.1
66391	0.877959	2.1
66392	1.24922	2.1
66393	2.49843	2.1
66394	1.89226	2.1
66395	2.51374	2.1
66396	1.89226	2.1
66397	4.406 2.1	
66398	1.89226	2.1
66399	1.89226	2.1
66400	1.89226	2.1
66401	1.89226	2.1
66402	1.89226	2.1
66403	1.89226	2.1
66404	1.89226	2.07744
66405	1.89226	2.1
66406	1.89226	2.1
66407	1.89226	2.08004

66408	1.89226	2.1
66409	1.89226	2.08664
66410	1.89226	2.1
66411	1.89226	2.0787
66412	1.89226	2.1
66413	1.89226	2.07819
66414	1.89226	2.1
66415	1.89226	2.0818
66416	1.03835	2.1
66417	1.03835	2.1
66418	1.15388	2.1
66419	0.94267	2.1
66420	0.736433	2.1
66421	0.849239	2.1
66422	1.15014	2.1
66423	1.39058	2.1
66424	0.838639	2.1
66425	1.39056	2.1
66426	1.09399	2.1
66427	0.736444	2.1
66428	1.89226	2.00902
66429	1.89226	2.1
66430	1.89226	2.1
66431	1.89226	1.99678
66432	1.89226	2.1
66433	1.89226	2.01396
66434	0.94613	1.99393
66435	0.94613	2.00941
66436	0.946127	2.1
66437	0.946127	2.1
66438	0.946131	2.1
66439	0.946131	1.94676
66440	0.946126	2.1
66441	0.946126	2.03921
66442	0.946128	2.1
66443	0.946128	2.1
66444	0.946128	2.09342
66445	0.946128	2.09737
66446	1.89226	2.07818
66447	1.89226	1.69138
66448	1.89226	1.46344
66449	1.89226	2.08586
66450	3.78453	1.89596
66451	0.946133	1.39214
66452	0.946133	0.911332
66453	0.946128	1.88142
66454	0.946128	1.75997
66455	0.946131	1.79595
66456	0.946131	1.49483
66457	0.946131	0.780665
66458	0.946131	1.66473
66459	0.946131	1.46409
66460	0.946131	1.48694
66461	0.94613	1.57687
66462	0.94613	1.56039
66463	1.89226	1.33975
66464	1.89226	2.04611
66465	1.89226	2.00955

66466	1.89226	1.53684
66467	1.89226	2.02707
66468	1.89226	1.6458
66469	1.89226	0.83749
66470	1.89226	1.90669
66471	1.89226	1.72421
66472	1.89226	1.07563
66473	1.89226	1.67217
66474	1.89226	1.24711
66475	1.89226	0.882929
66476	1.89226	2.00049
66477	1.89226	1.7457
66478	1.89226	1.37918
66479	1.89226	1.74929
66480	1.89226	1.46938
66481	1.89226	1.40402
66482	2.67388	2.08848
66483	2.23684	2.02441
66484	2.23684	1.7526
66485	1.89226	2.04293
66486	1.89226	1.80389
66487	1.89226	2.1
66488	1.89226	2.1
66489	1.89226	2.1
66490	1.89226	2.1
66491	1.89226	2.1
66492	1.89226	2.1
66493	1.72197	2.1
66494	2.51374	2.1
66495	2.58004	2.1
66496	2.58004	2.1
66497	1.7155	2.1
66498	1.7155	2.1
66499	2.51374	2.1
66500	1.89226	2.1
66501	1.89226	2.1
66502	1.89226	2.1
66503	2.58003	2.1
66504	2.58003	2.1
66505	0.946132	2.1
66506	0.946132	2.1
66507	0.946132	2.1
66508	0.946132	2.1
66509	0.946132	2.1
66510	0.946132	2.1
66511	0.946132	2.1
66512	0.946132	2.1
66513	0.946131	2.1
66514	0.946131	2.1
66515	0.946132	2.1
66516	0.946132	2.1
66517	1.89226	2.1
66518	1.89226	2.1
66519	1.89226	2.1
66520	1.89226	2.1
66521	3.78453	2.1
66522	1.08262	2.1
66523	1.08262	2.1

66524	1.09674	2.1
66525	1.09674	2.1
66526	1.34652	2.1
66527	1.29002	2.1
66528	0.860984	2.1
66529	0.889338	2.1
66530	1.34652	2.1
66531	0.889338	2.1
66532	0.860983	2.1
66533	1.29002	2.1
66534	1.34652	2.1
66535	1.34652	2.1
66536	0.946133	2.1
66537	0.946133	2.1
66538	0.946131	2.1
66539	0.946131	2.1
66540	1.34652	2.1
66541	1.29002	2.1
66542	1.34652	2.1
66543	0.946131	2.1
66544	0.94613	2.1
66545	1.29002	2.1
66546	1.89226	2.1
66547	1.89226	2.1
66548	1.89226	2.1
66549	1.89226	2.1
66550	1.89226	2.1
66551	1.89226	2.1
66552	1.89226	2.1
66553	1.89226	2.1
66554	1.89226	2.1
66555	1.89226	2.1
66556	1.89226	2.1
66557	1.89226	2.1
66558	0.946131	2.1
66559	0.946131	2.1
66560	0.946131	2.1
66561	0.946131	2.1
66562	0.946131	2.1
66563	0.946131	2.1
66564	0.946131	2.1
66565	0.946131	2.1
66566	0.946131	2.1
66567	0.946131	2.1
66568	0.946131	2.1
66569	0.946131	2.1
66570	1.89226	2.1
66571	1.89226	2.1
66572	1.89226	2.1
66573	1.89226	2.1
66574	1.89226	2.1
66575	1.89226	2.1
66576	1.04103	2.1
66577	1.13849	2.1
66578	0.943014	2.1
66579	0.943014	2.1
66580	0.943591	2.1
66581	0.943591	2.1

66582	1.03757	2.1
66583	1.04098	2.1
66584	1.03761	2.1
66585	0.946131	2.1
66586	0.943041	2.1
66587	0.946131	2.1
66588	0.946128	2.1
66589	0.946128	2.1
66590	0.946133	2.1
66591	0.946133	2.1
66592	0.946131	2.1
66593	0.946131	2.1
66594	0.946131	2.1
66595	0.946131	2.1
66596	0.946131	2.1
66597	0.946131	2.1
66598	0.94613	2.1
66599	0.94613	2.1
66600	0.946132	2.1
66601	0.946132	2.1
66602	0.946132	2.1
66603	0.946132	2.1
66604	0.946132	2.1
66605	0.946132	2.1
66606	0.946132	2.1
66607	0.946132	2.1
66608	0.946132	2.1
66609	0.946132	2.1
66610	0.946131	2.1
66611	0.946131	2.1
66612	1.89226	0.879518
66613	1.89226	1.93896
66614	1.89226	1.07616
66615	1.89226	0.956471
66616	3.78452	1.47578
66617	0.946132	1.2297
66618	0.946132	1.19067
66619	0.946131	0.771601
66620	0.946131	0.856908
66621	0.946132	0.914419
66622	0.946132	2.01563
66623	0.946132	1.26152
66624	0.946132	1.19607
66625	0.946132	0.976114
66626	0.946132	1.14737
66627	0.946132	1.12987
66628	0.946132	1.38991
66629	0.935482	1.67129
66630	1.08999	1.60056
66631	1.24315	1.24647
66632	1.12559	1.39067
66633	1.21343	1.0491
66634	0.889397	2.0821
66635	1.01265	0.728257
66636	1.01265	1.81445
66637	1.07332	1.08437
66638	1.34292	1.36454
66639	0.865649	1.63201

66640	0.840024	1.86819
66641	1.15433	1.51338
66642	1.02858	1.6999
66643	1.34082	1.27355
66644	1.08357	1.03452
66645	1.10938	0.593539
66646	1.33349	2.05624
66647	1.12232	1.82277
66648	1.12232	0.613286
66649	1.21357	1.27864
66650	1.3408	1.33434
66651	1.02859	1.5818
66652	1.24211	1.84161
66653	1.89226	1.28401
66654	1.89226	0.401919
66655	1.89226	1.09195
66656	1.89226	2.04195
66657	1.89226	0.422409
66658	1.89226	1.06896
66659	1.89226	0.664739
66660	1.89226	1.07445
66661	1.89226	0.877547
66662	1.89226	1.68955
66663	1.89226	1.08293
66664	1.89226	1.47856
66665	2.0815	1.58083
66666	2.79029	1.19799
66667	2.36793	1.19842
66668	2.58428	1.97172
66669	2.38161	0.645316
66670	2.59922	1.51027
66671	1.89226	2.07775
66672	1.89226	1.86994
66673	1.89226	2.02519
66674	1.89226	2.00861
66675	1.89226	2.02367
66676	1.89226	2.01761
66677	1.89226	1.8651
66678	1.89226	0.743204
66679	1.89226	1.20064
66680	1.89226	1.4568
66681	3.78452	1.55394
66682	1.89226	2.01958
66683	1.89226	1.46471
66684	1.89226	1.95833
66685	1.89226	1.84235
66686	1.89226	1.96242
66687	1.89226	1.8674
66688	0.946132	1.65752
66689	0.946132	1.98576
66690	0.94613	2.09123
66691	0.94613	2.00753
66692	0.946131	2.05882
66693	0.946131	1.63436
66694	0.946131	1.98257
66695	0.946131	2.09713
66696	0.946131	2.02833
66697	0.946131	2.02765

66698	0.946131	2.00794
66699	0.946131	1.997
66700	1.85178	1.9066
66701	1.89226	1.36752
66702	2.0815	0.985239
66703	2.27054	1.31757
66704	1.70303	0.684032
66705	1.5217	0.90507
66706	1.89226	2.05625
66707	1.89226	1.37956
66708	1.89226	1.91097
66709	1.89226	1.66795
66710	1.89226	1.97652
66711	1.89226	1.79135
66712	2.58823	0.215356
66713	2.02924	0.163327
66714	1.79398	0.146177
66715	1.79398	0.0945121
66716	2.76708	0.343733
66717	2.76708	0.14227
66718	0.946133	0.198673
66719	0.946133	0.253487
66720	1.29411	0.153972
66721	1.38353	0.10046
66722	0.946131	0.115853
66723	0.946131	0.16406
66724	1.38354	0.264381
66725	1.38354	0.115993
66726	0.946131	0.158355
66727	1.38354	0.156361
66728	0.94613	0.168584
66729	1.29411	0.169482
66730	1.89226	0.154221
66731	1.89226	0.0584982
66732	1.89226	0.1274
66733	1.89226	0.177513
66734	3.78452	0.132201
66735	0.946131	0.114469
66736	0.946131	0.12319
66737	0.946131	0.132758
66738	0.946131	0.148249
66739	0.946129	0.155861
66740	0.946129	0.0778088
66741	0.946133	0.176174
66742	0.946133	0.113841
66743	0.946131	0.141186
66744	0.946131	0.134191
66745	0.946131	0.0929449
66746	0.946131	0.102741
66747	1.89226	0.0546338
66748	1.89226	0.118158
66749	1.89226	0.160983
66750	1.89226	0.105654
66751	3.78452	0.0789247
66752	0.94613	0.114682
66753	0.94613	0.095934
66754	0.946131	0.0880139
66755	0.946131	0.100845

66756	0.946128	0.109247
66757	0.946128	0.0525015
66758	0.946133	0.136996
66759	0.946133	0.066594
66760	0.946131	0.107741
66761	0.946131	0.0895649
66762	0.946131	0.0679638
66763	0.946131	0.0678301
66764	0.946129	1.08055
66765	0.946129	0.864363
66766	1.13486	0.84566
66767	0.859753	1.07538
66768	0.851541	1.03384
66769	0.851541	0.851511
66770	1.34346	0.828293
66771	1.26212	0.837819
66772	0.851553	0.847328
66773	1.12403	0.865194
66774	0.851474	0.761007
66775	0.956163	0.900308
66776	0.946132	0.555289
66777	0.946132	0.530901
66778	0.946133	0.646649
66779	0.946133	0.515588
66780	0.946132	0.666252
66781	0.946132	0.504216
66782	0.946133	0.542085
66783	0.946133	0.527847
66784	0.946133	0.53519
66785	0.946133	0.579609
66786	0.946132	0.529814
66787	0.946132	0.552275
66788	1.89226	0.545609
66789	1.89226	0.745239
66790	1.89226	0.710057
66791	1.89226	0.681269
66792	3.78453	0.668067
66793	0.946128	1.18484
66794	0.946128	0.958592
66795	0.946132	1.40646
66796	0.946132	1.04797
66797	0.94613	1.4259
66798	0.94613	1.25321
66799	0.94613	1.1602
66800	0.94613	0.968262
66801	0.946131	1.16381
66802	0.946131	1.23144
66803	0.94613	1.25739
66804	0.94613	1.22011
66805	0.946132	0.40443
66806	0.946132	0.35752
66807	0.946133	0.422323
66808	0.946133	0.350925
66809	0.946132	0.425148
66810	0.946132	0.394055
66811	0.946133	0.351649
66812	0.946133	0.358905
66813	0.946132	0.367053

66814	0.946132	0.389758
66815	0.946132	0.373037
66816	0.946132	0.383002
66817	1.89226	0.446217
66818	1.89226	0.465487
66819	1.89226	0.575279
66820	1.89226	0.411987
66821	1.89226	0.562608
66822	1.89226	0.370159
66823	1.89226	0.848822
66824	1.89226	0.609493
66825	1.89226	0.595396
66826	1.89226	0.721337
66827	1.89226	0.635472
66828	1.89226	0.708346
66829	1.89226	2.1
66830	1.89226	2.1
66831	1.89226	2.1
66832	1.89226	2.1
66833	3.78452	2.1
66834	1.07652	2.1
66835	1.07652	2.1
66836	0.928749	2.1
66837	0.928749	2.1
66838	0.946132	2.1
66839	0.946132	2.1
66840	1.05914	2.1
66841	0.928746	2.1
66842	1.05914	2.1
66843	0.946131	2.1
66844	0.94613	2.1
66845	0.928747	2.1
66846	1.89226	2.1
66847	1.89226	2.1
66848	1.89226	2.1
66849	1.89226	2.1
66850	1.89226	2.1
66851	1.89226	2.1
66852	1.89227	2.1
66853	1.89227	2.1
66854	1.89227	2.1
66855	1.89227	2.1
66856	1.89227	2.1
66857	1.89227	2.1
66858	1.89226	2.1
66859	1.89226	2.1
66860	1.89226	2.1
66861	1.89226	2.1
66862	1.89226	2.1
66863	1.89226	2.1
66864	2.11828	2.1
66865	1.89226	2.1
66866	2.35845	2.1
66867	1.89226	2.1
66868	4.25071	2.1
66869	1.89226	2.1
66870	1.89226	2.1
66871	1.89226	2.1

66872	1.89226	2.1
66873	3.78453	2.1
66874	1.89226	2.1
66875	1.89226	2.1
66876	1.89226	2.1
66877	1.89226	2.1
66878	1.89226	2.1
66879	1.89226	2.1
66880	1.89226	0.931953
66881	1.89226	1.46203
66882	1.65573	1.42225
66883	1.65573	0.962398
66884	1.89226	1.3888
66885	1.89226	0.933095
66886	1.89226	0.662373
66887	1.89226	1.38705
66888	1.89226	1.32603
66889	1.89226	0.824221
66890	1.89226	1.30347
66891	1.89226	0.780455
66892	1.89226	0.803724
66893	1.89226	1.25816
66894	1.89226	1.3267
66895	1.89226	0.908968
66896	1.89226	1.28114
66897	1.89226	0.842094
66898	1.89226	0.586003
66899	1.89226	1.44937
66900	1.89226	1.32092
66901	1.89226	0.731534
66902	1.89226	1.28748
66903	1.89226	0.678611
66904	0.946128	0.681863
66905	0.946128	0.638963
66906	0.946125	1.12679
66907	0.946125	1.32132
66908	0.94613	0.783964
66909	0.94613	1.45901
66910	0.946124	1.39542
66911	0.946124	0.706457
66912	0.946127	1.0541
66913	0.946127	1.12492
66914	0.946127	1.05026
66915	0.946127	0.994353
66916	1.89226	0.782465
66917	1.89226	1.57456
66918	1.89226	0.796593
66919	1.89226	0.552025
66920	3.78452	0.890923
66921	1.89226	1.48356
66922	1.89226	1.46383
66923	1.89226	1.06607
66924	1.89226	1.52004
66925	3.78453	1.36474
66926	1.05926	1.79819
66927	0.891358	2.02134
66928	0.946129	1.48007
66929	0.946129	1.76404

66930	0.946101	1.35057
66931	0.946101	2.08544
66932	0.891386	1.62022
66933	0.891386	1.81551
66934	0.891357	1.76568
66935	0.946129	1.65751
66936	0.94613	1.77928
66937	1.05926	1.73519
66938	0.946126	1.00163
66939	0.946126	1.32535
66940	0.94613	0.88981
66941	0.94613	1.37116
66942	0.946131	1.16904
66943	0.946131	1.4182
66944	0.946125	1.52079
66945	0.946125	0.55326
66946	0.946127	1.10596
66947	0.946127	0.96615
66948	0.946128	1.40786
66949	0.946128	1.21873
66950	1.89226	1.24118
66951	1.89226	0.601915
66952	1.89226	0.571563
66953	1.89226	1.65164
66954	1.89226	0.641999
66955	1.89226	1.70682
66956	1.89226	1.36183
66957	1.89226	1.67798
66958	1.89226	1.47016
66959	1.89226	1.87588
66960	1.89226	1.02629
66961	1.89226	1.8512
66962	1.16109	1.89941
66963	1.23243	1.95012
66964	0.659819	1.51198
66965	0.659819	1.72873
66966	0.697965	1.11954
66967	1.00087	2.0362
66968	0.891386	1.89491
66969	0.891386	1.6774
66970	0.946129	1.7308
66971	0.65979	1.47072
66972	0.891357	1.7329
66973	0.946129	1.64263
66974	1.89225	0.648323
66975	1.89225	1.22207
66976	1.89225	1.79468
66977	1.89225	0.597014
66978	1.89225	1.9496
66979	1.89225	0.88726
66980	1.79981	0.354611
66981	2.02514	0.503419
66982	2.33247	0.637906
66983	2.12891	0.342556
66984	4.15405	0.381219
66985	2.48284	1.18662
66986	2.02514	0.559298
66987	2.33247	0.650464

66988	2.02514	1.0025
66989	2.48567	0.618957
66990	2.17834	0.959391
66991	1.89226	0.526623
66992	1.89226	0.813411
66993	1.89226	0.949692
66994	1.89226	0.519005
66995	3.78453	0.600846
66996	1.89227	0.48121
66997	1.89227	0.293652
66998	1.89227	0.307685
66999	1.89227	0.424419
67000	1.89227	0.343686
67001	1.89227	0.445909
67002	2.50894	0.281906
67003	2.70118	0.454744
67004	2.12891	0.299242
67005	2.12891	0.391099
67006	2.7125	0.293825
67007	2.33247	0.412654
67008	1.89226	0.735921
67009	1.89226	0.420879
67010	1.89226	0.42965
67011	1.89226	0.661156
67012	1.89226	0.403768
67013	1.89226	0.609569
67014	0.946133	0.401378
67015	0.946133	0.390668
67016	0.946132	0.260622
67017	0.946132	0.261771
67018	0.946132	0.262656
67019	0.946132	0.395997
67020	0.946133	0.283732
67021	0.946133	0.393671
67022	0.946132	0.309344
67023	0.946132	0.296267
67024	0.946132	0.338881
67025	0.946132	0.34636
67026	1.90098	0.131325
67027	1.36463	0.159725
67028	1.56656	0.126602
67029	1.36463	0.157466
67030	2.04386	0.120766
67031	1.89227	0.175216
67032	1.89226	0.1497
67033	1.89226	0.159972
67034	1.89226	0.131158
67035	1.89226	0.175029
67036	1.89226	0.137412
67037	1.89226	0.182245
67038	1.89226	0.184634
67039	1.89226	0.226475
67040	1.89226	0.174993
67041	1.89226	0.259474
67042	1.89226	0.17257
67043	1.89226	0.255914
67044	1.89226	0.176227
67045	1.89226	0.23471

67046	1.89226	0.178051
67047	1.89226	0.233696
67048	1.89226	0.176663
67049	1.89226	0.245266
67050	1.89226	0.107735
67051	1.70303	0.126159
67052	2.1292	0.105074
67053	1.70303	0.134338
67054	2.32302	0.102667
67055	1.90098	0.142344
67056	1.89226	0.132403
67057	1.89226	0.1381
67058	1.89226	0.111465
67059	1.89226	0.156893
67060	1.89226	0.118165
67061	1.89226	0.163908
67062	1.89226	0.157459
67063	1.89226	0.169634
67064	1.89226	0.142379
67065	1.89226	0.210437
67066	1.89226	0.13932
67067	1.89226	0.204334
67068	1.89226	0.137143
67069	1.89226	0.146601
67070	1.89226	0.119369
67071	1.89226	0.165286
67072	1.89226	0.123176
67073	1.89226	0.177111
67074	1.89226	1.3049
67075	1.89226	1.90764
67076	1.89226	1.94349
67077	1.89226	1.32402
67078	3.78453	1.30025
67079	1.89226	1.94306
67080	1.89226	2.08803
67081	1.89226	1.63217
67082	1.89226	2.09338
67083	1.89226	1.90113
67084	1.89226	2.1
67085	1.89226	1.88092
67086	1.89226	1.23806
67087	1.89226	1.2271
67088	1.89226	1.65643
67089	3.78452	1.76293
67090	1.89224	1.96707
67091	1.89224	1.06557
67092	1.89224	1.61692
67093	1.89224	1.93645
67094	1.89224	1.41283
67095	1.89224	1.76482
67096	1.89224	1.96837
67097	1.89224	2.08491
67098	1.89224	2.01318
67099	1.89224	2.1
67100	1.89224	1.66771
67101	1.89224	2.08735
67102	1.31307	2.09819
67103	1.31307	2.0872

67104	0.888948	1.93122
67105	0.888948	1.83465
67106	1.2559	1.95877
67107	0.888979	2.08526
67108	0.946114	2.08084
67109	0.946114	1.8973
67110	0.946129	1.82706
67111	0.888989	1.94407
67112	0.946104	1.76834
67113	1.25588	1.92578
67114	1.89224	0.994278
67115	1.89224	1.94757
67116	1.89224	1.03693
67117	1.89224	1.90801
67118	1.89224	1.50686
67119	1.89224	2.0179
67120	1.79375	0.0865966
67121	1.47689	0.0824146
67122	1.97732	0.0511817
67123	2.26687	0.0743707
67124	3.74375	0.0660045
67125	2.05759	0.104939
67126	2.26686	0.0849573
67127	1.79374	0.0776749
67128	1.79374	0.129559
67129	2.49889	0.0713475
67130	2.49889	0.113452
67131	1.89226	0.13152
67132	1.89226	0.128903
67133	1.89226	0.0810821
67134	1.89226	0.13621
67135	3.78452	0.116888
67136	1.89227	0.0723167
67137	1.89227	0.0706396
67138	1.89227	0.11542
67139	1.89227	0.129482
67140	1.89227	0.0849592
67141	1.89227	0.123448
67142	1.97732	0.0768659
67143	2.26687	0.101582
67144	2.6607	0.0580005
67145	2.41464	0.0698195
67146	4.68151	0.0710881
67147	1.89226	0.0968775
67148	1.89226	0.177929
67149	1.89226	0.107299
67150	1.89226	0.0885348
67151	3.78452	0.103548
67152	1.89226	0.0967403
67153	1.89226	0.0822338
67154	1.89226	0.11507
67155	1.89226	0.187104
67156	1.89226	0.0688578
67157	1.89226	0.104648
67158	1.89226	0.853058
67159	1.89226	1.85981
67160	1.89226	1.45949
67161	1.89226	0.86437

67162	3.78452	1.02255
67163	1.89226	1.01318
67164	1.89226	1.90834
67165	1.89226	0.645018
67166	1.89226	1.64136
67167	1.89226	1.05086
67168	1.89226	1.87362
67169	1.89226	1.83552
67170	1.89226	0.822855
67171	1.89226	1.00505
67172	1.89226	1.74654
67173	1.89226	0.788356
67174	1.89226	1.48191
67175	1.89226	1.22046
67176	1.89226	0.752869
67177	1.89226	1.08265
67178	1.89226	1.73527
67179	1.89226	0.619102
67180	1.89226	1.338
67181	1.89226	0.856276
67182	2.45282	1.39172
67183	1.89226	1.54706
67184	1.89226	1.80342
67185	1.89226	0.603651
67186	1.89226	1.4388
67187	1.89226	0.75504
67188	1.89226	1.73637
67189	1.89226	1.029
67190	1.89226	0.908707
67191	1.89226	1.85046
67192	1.89226	1.83949
67193	1.87308	0.0718391
67194	1.88631	0.0812841
67195	1.63908	0.0836422
67196	1.70302	0.113386
67197	2.08745	0.0643079
67198	2.16916	0.0715968
67199	2.41019	0.076066
67200	2.733	0.0539051
67201	2.08121	0.0774926
67202	2.08121	0.11265
67203	2.74362	0.0780551
67204	2.41464	0.0773276
67205	1.89226	0.0758025
67206	1.89226	0.128263
67207	1.89226	0.0678962
67208	1.89226	0.113764
67209	1.89226	0.0790361
67210	1.89226	0.145931
67211	0.946133	0.0926
67212	0.946133	0.0879546
67213	0.946132	0.0988534
67214	0.946132	0.100788
67215	0.946132	0.0968669
67216	0.946132	0.137799
67217	0.946133	0.0769709
67218	0.946133	0.101756
67219	0.946132	0.0757844

67220	0.946132	0.0915722
67221	0.946133	0.0950729
67222	0.946133	0.12656
67223	2.02912	0.0794585
67224	2.57016	0.0689537
67225	1.88631	0.0807166
67226	1.88631	0.112718
67227	2.55301	0.0816053
67228	2.4102	0.0851415
67229	1.89226	0.121552
67230	1.89226	0.133428
67231	1.89226	0.0687628
67232	1.89226	0.0786348
67233	3.78452	0.107835
67234	1.89227	0.106913
67235	1.89227	0.0900568
67236	1.89227	0.0963295
67237	1.89227	0.134912
67238	1.89227	0.0769867
67239	1.89227	0.0916748
67240	1.89226	2.09719
67241	1.89226	2.08813
67242	1.89226	2.05871
67243	1.89226	2.09837
67244	3.78452	2.09229
67245	1.2515	2.03545
67246	1.34788	2.08467
67247	0.946132	2.09703
67248	0.946132	2.08832
67249	0.973043	2.09682
67250	1.16766	2.0846
67251	0.919222	1.98904
67252	1.2159	2.09755
67253	0.946132	2.07509
67254	0.946132	2.08236
67255	1.33746	2.08855
67256	1.13537	2.08741
67257	1.89226	2.08776
67258	1.89226	2.09991
67259	1.89226	2.09982
67260	1.89226	2.09519
67261	1.89226	2.09811
67262	1.89226	2.09253
67263	1.89226	2.04461
67264	1.89226	2.08919
67265	1.89226	2.08232
67266	1.89226	2.04924
67267	3.78452	2.05916
67268	1.89227	2.07166
67269	1.89227	1.90708
67270	1.89227	2.06659
67271	1.89227	2.0156
67272	1.89227	2.08276
67273	1.89227	2.00137
67274	0.946132	2.06316
67275	0.946132	2.05586
67276	0.946129	2.08849
67277	0.946129	2.09052

67278	0.946131	2.07363
67279	0.946131	2.08991
67280	0.946131	2.08983
67281	0.946131	2.05324
67282	0.946131	2.07485
67283	0.946131	2.08021
67284	0.94613	2.07965
67285	0.94613	2.07489
67286	1.89226	1.52608
67287	1.89226	1.25007
67288	1.89226	1.2574
67289	1.89226	1.08909
67290	3.78453	1.3601
67291	0.946132	1.34568
67292	0.946132	1.14414
67293	0.946132	0.87844
67294	0.946132	1.16842
67295	0.946132	0.604722
67296	0.946132	1.85723
67297	0.946132	1.38615
67298	0.946132	1.45566
67299	0.946131	1.62243
67300	0.946131	1.03341
67301	0.946132	1.05864
67302	0.946132	1.15556
67303	1.89226	1.48741
67304	1.89226	0.609837
67305	1.89226	0.905044
67306	1.89226	1.07048
67307	1.89226	1.12772
67308	1.89226	1.03084
67309	1.89226	1.48706
67310	1.89226	1.01678
67311	1.89226	0.885116
67312	1.89226	0.871015
67313	3.78453	0.948996
67314	0.96614	1.12136
67315	0.96614	1.40971
67316	0.946131	0.767505
67317	0.946131	0.874051
67318	0.946131	0.876578
67319	0.96614	0.708916
67320	1.37736	1.415
67321	0.946131	1.39902
67322	1.37736	1.65315
67323	0.96614	0.743513
67324	0.946131	1.25175
67325	0.946131	0.722378
67326	0.946129	0.811082
67327	0.946129	0.916244
67328	0.946128	1.05893
67329	0.946128	0.912507
67330	0.946132	0.420362
67331	0.946132	1.72563
67332	0.946126	1.22693
67333	0.946126	1.11136
67334	0.946129	0.803385
67335	0.946129	0.757799

67336	0.946129	0.768189
67337	0.946129	1.03055
67338	1.89226	0.110396
67339	1.65723	0.119279
67340	1.83117	0.121932
67341	1.65723	0.115577
67342	2.0662	0.129953
67343	1.89226	0.123255
67344	1.89226	0.108796
67345	1.89226	0.135309
67346	1.89226	0.120015
67347	1.89226	0.117612
67348	1.89226	0.123716
67349	1.89226	0.118969
67350	1.89226	0.109807
67351	1.89226	0.140945
67352	1.89226	0.12719
67353	1.89226	0.13165
67354	1.89226	0.127183
67355	1.89226	0.127504
67356	1.89226	0.108496
67357	1.89226	0.11277
67358	1.89226	0.111562
67359	1.89226	0.122125
67360	1.89226	0.112512
67361	1.89226	0.119078
67362	1.89226	0.106483
67363	1.83117	0.110588
67364	1.92807	0.113845
67365	1.83117	0.111935
67366	1.98916	0.116939
67367	1.89226	0.114167
67368	1.89226	0.114766
67369	1.89226	0.126275
67370	1.89226	0.109579
67371	1.89226	0.1175
67372	3.78453	0.117839
67373	1.89226	0.113886
67374	1.89226	0.124495
67375	1.89226	0.121857
67376	1.89226	0.126874
67377	1.89226	0.118062
67378	1.89226	0.121523
67379	1.89226	0.104433
67380	1.89226	0.102644
67381	1.89226	0.105226
67382	1.89226	0.109327
67383	1.89226	0.103245
67384	1.89226	0.104526
67385	1.88037	0.0944695
67386	1.89227	0.0932568
67387	1.89227	0.0946175
67388	1.89227	0.103951
67389	1.98599	0.0954935
67390	1.98599	0.101307
67391	1.89226	0.101322
67392	1.89226	0.104275
67393	1.89226	0.102595

67394	1.89226	0.127818
67395	1.89226	0.0972379
67396	1.89226	0.110983
67397	1.89226	0.113596
67398	1.89226	0.12755
67399	1.89226	0.120308
67400	1.89226	0.162815
67401	3.78452	0.0982478
67402	1.89226	0.102337
67403	1.89226	0.133003
67404	1.89226	0.131555
67405	1.89226	0.103198
67406	1.89226	0.127523
67407	1.89226	0.110612
67408	1.90116	0.0859976
67409	1.89227	0.0818209
67410	1.89226	0.0845071
67411	1.89227	0.0932333
67412	1.88036	0.0869377
67413	1.88037	0.0846542
67414	1.89226	0.0914228
67415	1.89226	0.0909695
67416	1.89226	0.0967772
67417	1.89226	0.112656
67418	1.89226	0.0868503
67419	1.89226	0.093544
67420	0.946132	0.117806
67421	0.946132	0.104735
67422	0.946128	0.10928
67423	0.946128	0.120443
67424	0.94613	0.118876
67425	0.94613	0.11767
67426	0.94613	0.0959103
67427	0.94613	0.116365
67428	0.946131	0.188082
67429	0.946131	0.153659
67430	0.94613	0.134237
67431	0.94613	0.170411
67432	1.89226	0.0996872
67433	1.89226	0.101897
67434	1.89226	0.107943
67435	1.89226	0.103584
67436	1.89226	0.100882
67437	1.89226	0.0945286
67438	1.89226	0.556745
67439	1.89226	1.35691
67440	2.35239	1.29095
67441	2.27873	0.623728
67442	1.89226	1.28566
67443	1.83301	0.634469
67444	2.68449	0.840668
67445	1.89226	1.84247
67446	1.89226	1.08591
67447	1.89226	1.26312
67448	3.78453	1.06253
67449	0.946132	1.78728
67450	0.946132	1.17404
67451	0.946129	1.07994

67452	0.946129	1.81832
67453	0.946131	1.82623
67454	0.946131	1.62998
67455	0.94613	1.15329
67456	0.94613	1.41044
67457	0.946131	1.61625
67458	0.946131	1.47045
67459	0.946131	1.49903
67460	0.946131	1.59145
67461	1.89226	0.658239
67462	1.89226	1.17925
67463	1.89226	1.48084
67464	1.89226	0.738164
67465	1.89226	1.4748
67466	1.89226	0.74098
67467	1.70303	0.557557
67468	1.89226	1.31909
67469	1.89226	0.586733
67470	1.89226	0.824616
67471	3.78453	0.641734
67472	1.89227	1.34992
67473	1.89227	1.44474
67474	1.89227	0.845915
67475	1.89227	1.76725
67476	3.78453	1.52687
67477	1.89226	1.21462
67478	1.89226	1.88042
67479	1.89226	1.40023
67480	1.89226	1.6929
67481	3.78452	1.55755
67482	1.89226	2.03341
67483	1.89226	2.07581
67484	1.89226	2.07485
67485	1.89226	2.01516
67486	3.78452	2.02795
67487	0.946132	1.91927
67488	0.946132	2.03062
67489	0.946129	2.08574
67490	0.946129	2.06356
67491	0.94613	2.05837
67492	0.94613	1.97665
67493	0.946131	2.07084
67494	0.946131	2.00973
67495	0.946131	2.00864
67496	0.946131	2.03446
67497	0.946131	2.0264
67498	0.946131	1.96901
67499	1.89226	2.0606
67500	1.89226	1.95081
67501	1.89226	1.98193
67502	1.89226	2.04085
67503	3.78452	2.03941
67504	1.89226	1.80069
67505	1.89226	2.0149
67506	1.89226	2.04368
67507	1.89226	1.78664
67508	1.89226	2.04554
67509	1.89226	1.79854

67510	1.89227	2.00829
67511	2.48308	1.59601
67512	1.89227	1.78375
67513	1.89227	1.93403
67514	3.78453	1.9543
67515	1.89226	1.93977
67516	1.89226	2.00225
67517	1.89226	2.03862
67518	1.89226	1.89456
67519	1.89226	2.03122
67520	1.89226	1.87537
67521	1.89226	1.98466
67522	1.89226	1.95076
67523	1.89226	2.00541
67524	1.89226	1.97344
67525	3.78453	2.01983
67526	0.946131	1.94099
67527	0.946131	2.02399
67528	0.946131	2.01341
67529	0.946131	2.06744
67530	0.946131	2.00943
67531	0.946131	1.9803
67532	0.946131	2.01349
67533	0.946131	2.02852
67534	0.946131	1.99687
67535	0.946131	2.03573
67536	0.946131	2.00876
67537	0.946131	1.95429
67538	1.23719	2.02591
67539	1.28439	1.97332
67540	0.94613	1.98541
67541	0.94613	1.99829
67542	1.28439	2.02035
67543	1.6431	2.04232
67544	0.946129	1.94871
67545	0.946129	1.95122
67546	1.28439	1.88939
67547	0.946131	1.8282
67548	1.21038	1.83998
67549	1.21038	1.91887
67550	1.89226	1.96643
67551	1.89226	1.9195
67552	1.89226	1.96187
67553	1.89226	1.91166
67554	1.89226	1.94935
67555	1.89226	1.8991
67556	1.89226	1.94214
67557	1.89226	1.96873
67558	1.89226	2.01637
67559	1.89226	1.84567
67560	1.89226	2.00477
67561	1.89226	1.81558
67562	1.89226	1.27737
67563	1.89226	0.437279
67564	1.89226	0.469715
67565	1.89226	0.680852
67566	1.89226	0.882469
67567	1.89226	1.24754

67568	0.946128	0.459955
67569	0.946128	0.606144
67570	0.946125	0.201557
67571	0.946125	0.397891
67572	0.94613	0.279517
67573	0.94613	0.321709
67574	0.946124	0.570066
67575	0.946124	0.680479
67576	0.946127	0.337909
67577	0.946127	0.253224
67578	0.946127	0.437501
67579	0.946127	0.605683
67580	1.89226	0.224728
67581	2.78671	0.209556
67582	2.59302	0.217159
67583	2.59302	0.216034
67584	1.89226	0.333545
67585	1.89226	0.286905
67586	1.89226	0.398367
67587	1.89226	0.37871
67588	1.89226	0.280853
67589	1.89226	0.271188
67590	1.89226	0.561955
67591	1.89226	0.491839
67592	1.89225	0.211092
67593	1.89225	0.133479
67594	1.89225	0.293008
67595	1.89225	0.230675
67596	1.89225	0.35943
67597	1.89225	0.200278
67598	0.979536	0.146929
67599	0.816597	0.162496
67600	1.15446	0.146621
67601	1.07423	0.145255
67602	1.08452	0.147906
67603	0.603522	0.144601
67604	1.70302	0.140102
67605	1.29344	0.12589
67606	1.21806	0.143623
67607	0.723758	0.124625
67608	1.37562	0.162833
67609	1.89226	0.0974547
67610	1.92807	0.096343
67611	1.90843	0.0982384
67612	1.92807	0.101618
67613	1.87263	0.0979179
67614	1.89226	0.100136
67615	0.946132	0.107396
67616	0.946132	0.109466
67617	0.946131	0.102107
67618	0.946131	0.10281
67619	0.946132	0.10459
67620	0.946132	0.128772
67621	0.946132	0.104046
67622	0.946132	0.11086
67623	0.946132	0.0948352
67624	0.946132	0.10721
67625	0.946132	0.107772

67626	0.946132	0.11142
67627	1.89226	0.122272
67628	1.89226	0.109274
67629	1.89226	0.102257
67630	1.89226	0.109153
67631	3.78452	0.122623
67632	1.89226	0.0952239
67633	1.89226	0.0892969
67634	1.89226	0.0921796
67635	1.89226	0.09479
67636	1.89226	0.0897989
67637	1.89226	0.0914422
67638	1.70303	0.0795321
67639	1.90843	0.0796302
67640	1.77002	0.0827572
67641	1.90843	0.0959132
67642	1.75709	0.0776606
67643	1.89226	0.0856473
67644	1.89226	0.0999249
67645	1.89226	0.0968373
67646	1.89226	0.0826133
67647	1.89226	0.115089
67648	3.78453	0.0908103
67649	1.89226	0.0959174
67650	1.89226	0.114846
67651	1.89226	0.110776
67652	1.89226	0.0813365
67653	3.78452	0.08874
67654	0.946131	0.0889305
67655	0.946131	0.0713008
67656	0.946131	0.075689
67657	0.946131	0.0675708
67658	0.946129	0.0663045
67659	0.946129	0.0727987
67660	0.946133	0.0746771
67661	0.946133	0.0921882
67662	0.946131	0.0693669
67663	0.946131	0.0671419
67664	0.946131	0.0792037
67665	0.946131	0.0833616
67666	1.89226	0.137671
67667	2.10275	0.331953
67668	1.76377	0.307369
67669	1.76377	0.195877
67670	1.89226	0.273322
67671	1.89226	0.167391
67672	1.89226	0.156924
67673	1.89226	0.366468
67674	2.10275	0.333157
67675	2.55889	0.195833
67676	1.89226	0.309701
67677	2.30274	0.182609
67678	1.89226	0.35123
67679	1.89226	0.929186
67680	1.89226	0.92193
67681	1.89226	0.444693
67682	1.89226	0.859872
67683	1.89226	0.411517

67684	1.89226	0.296254
67685	1.89226	0.963877
67686	1.89226	0.844686
67687	1.89226	0.414661
67688	1.89226	0.77094
67689	1.89226	0.375934
67690	1.89226	0.157715
67691	1.89226	0.3949
67692	1.89226	0.339801
67693	1.89226	0.19836
67694	1.89226	0.31042
67695	1.89226	0.176909
67696	1.89226	0.174252
67697	1.89226	0.432151
67698	1.89226	0.395536
67699	1.89226	0.232004
67700	1.89226	0.369568
67701	1.89226	0.210931
67702	1.89226	0.391048
67703	1.89226	1.03111
67704	1.89226	0.949216
67705	1.89226	0.490986
67706	1.89226	0.91177
67707	1.89226	0.468774
67708	1.89226	0.862044
67709	1.89226	1.05037
67710	1.89226	0.33732
67711	1.89226	0.470624
67712	1.89226	0.351814
67713	1.89226	0.701325
67714	0.946131	0.141371
67715	0.976399	0.116348
67716	1.10124	0.115805
67717	0.946131	0.252292
67718	0.887633	0.244222
67719	0.887633	0.185238
67720	1.16933	0.1349
67721	1.23163	0.11307
67722	0.946131	0.177763
67723	0.946131	0.203183
67724	0.976399	0.1668
67725	1.14992	0.165456
67726	1.89226	0.244783
67727	1.89226	0.633763
67728	1.89226	0.282963
67729	1.89226	0.383364
67730	3.78452	0.25991
67731	1.89226	0.152859
67732	1.89226	0.335577
67733	1.89226	0.155883
67734	1.89226	0.244384
67735	3.78453	0.162243
67736	0.946132	0.109139
67737	0.946132	0.205421
67738	0.946131	0.184606
67739	0.946131	0.260789
67740	0.946131	0.265829
67741	0.946131	0.209124

67742	0.946132	0.101804
67743	0.946132	0.120873
67744	0.946131	0.161539
67745	0.946131	0.215788
67746	0.946132	0.16843
67747	0.946132	0.106819
67748	0.946132	0.318037
67749	0.946132	0.213025
67750	0.946131	0.291895
67751	0.946131	0.467419
67752	0.946131	0.477782
67753	0.946131	0.374751
67754	0.946132	0.254423
67755	0.946132	0.200213
67756	0.946131	0.309601
67757	0.946131	0.396883
67758	0.946132	0.330568
67759	0.946132	0.229124
67760	1.89226	0.401474
67761	1.89226	0.537786
67762	1.89226	0.268595
67763	1.89226	0.642697
67764	3.78452	0.551671
67765	1.89226	1.10045
67766	1.89226	1.01736
67767	1.89226	0.516632
67768	1.89226	0.429564
67769	3.78453	0.625897
67770	1.89226	0.386009
67771	1.89226	0.977912
67772	1.89226	0.358914
67773	1.89226	1.16864
67774	1.89226	0.336565
67775	1.89226	0.866491
67776	1.89225	0.253964
67777	1.89225	0.394134
67778	1.89225	0.288227
67779	1.89225	0.957063
67780	1.89225	0.215952
67781	1.89225	0.548252
67782	1.89226	0.395645
67783	1.89226	0.51503
67784	1.89226	0.390808
67785	1.89226	0.967097
67786	1.89226	0.247193
67787	1.89226	0.409474
67788	1.89226	1.02604
67789	1.89226	0.487695
67790	1.89226	0.256084
67791	1.89226	0.214521
67792	3.78453	0.245303
67793	1.39491	0.293859
67794	1.39491	0.362108
67795	0.924649	0.451187
67796	0.924649	0.491253
67797	0.946131	0.389861
67798	1.37343	0.166859
67799	0.924678	0.127689

67800	0.946131	1.2499
67801	0.924678	0.191798
67802	1.37343	0.189601
67803	0.946131	0.616752
67804	0.946131	0.529576
67805	1.89226	0.463169
67806	1.89226	0.15145
67807	1.89226	0.245388
67808	1.89226	0.356141
67809	1.89226	0.151541
67810	1.89226	0.223257
67811	0.946126	0.05
67812	0.975461	0.05
67813	0.946132	0.05
67814	1.12674	0.0547595
67815	0.946129	0.060327
67816	0.946129	0.05
67817	1.12673	0.05
67818	1.12673	0.05
67819	0.946129	0.05
67820	0.946129	0.0564911
67821	0.975464	0.05
67822	1.12673	0.05
67823	1.89226	0.0515386
67824	1.89226	0.0580063
67825	1.89226	0.0764452
67826	1.89226	0.050822
67827	1.89226	0.06823
67828	1.89226	0.0541803
67829	0.946126	0.0556658
67830	0.946126	0.073197
67831	0.946131	0.0629607
67832	0.946131	0.113324
67833	0.946129	0.116358
67834	0.946129	0.0986738
67835	0.946128	0.0665339
67836	0.946128	0.0530934
67837	0.946129	0.0915287
67838	0.946129	0.114782
67839	0.946128	0.0875221
67840	0.946128	0.0881001
67841	1.89226	0.05
67842	1.89226	0.0896423
67843	1.89226	0.05
67844	1.89226	0.0617122
67845	3.78451	0.05
67846	1.89226	0.0720019
67847	1.89226	0.0816072
67848	1.89226	0.0540237
67849	1.89226	0.103911
67850	3.78452	0.0932207
67851	0.946125	0.073691
67852	0.946125	0.0974516
67853	0.946131	0.0893625
67854	0.946131	0.143984
67855	0.946128	0.148377
67856	0.946128	0.130968
67857	0.946128	0.0899389

67858	0.946128	0.075306
67859	0.946128	0.102401
67860	0.946128	0.127348
67861	0.946128	0.0829811
67862	0.946128	0.108777
67863	1.89226	0.0583645
67864	1.89226	0.142126
67865	1.89226	0.0868651
67866	1.89226	0.05
67867	3.78452	0.0536168
67868	1.89226	0.08032
67869	1.89226	0.106997
67870	1.89226	0.111062
67871	1.89226	0.152372
67872	1.89226	0.0833113
67873	1.89226	0.103625
67874	1.12492	0.157053
67875	1.41201	0.170424
67876	1.12082	0.140303
67877	0.771416	0.137
67878	1.10725	0.131836
67879	1.10725	0.135888
67880	1.07618	0.218145
67881	1.07618	0.115693
67882	1.12492	0.158817
67883	0.771415	0.197549
67884	1.12082	0.142681
67885	1.41201	0.169845
67886	2.82401	0.110725
67887	1.89226	0.1164
67888	1.89226	0.0998445
67889	1.89226	0.119328
67890	2.24983	0.118006
67891	2.24983	0.129122
67892	0.946133	0.10233
67893	0.946133	0.105825
67894	0.946129	0.0889353
67895	0.946129	0.0804416
67896	0.946131	0.0854661
67897	0.946131	0.152785
67898	0.946131	0.105819
67899	0.946131	0.0743561
67900	0.946131	0.0992204
67901	0.946131	0.0927014
67902	0.946131	0.090222
67903	0.946131	0.0975132
67904	0.946132	0.129836
67905	0.946132	0.127761
67906	0.946133	0.115757
67907	0.946133	0.116185
67908	0.946132	0.106364
67909	0.946132	0.132559
67910	0.946133	0.136822
67911	0.946133	0.140963
67912	0.946132	0.125036
67913	0.946132	0.142871
67914	0.946133	0.11361
67915	0.946133	0.11644

67916	1.89227	0.344714
67917	1.89227	0.40499
67918	1.89227	0.44073
67919	1.89227	0.310874
67920	1.89227	0.448156
67921	1.89227	0.325141
67922	1.42982	0.140665
67923	1.19944	0.0936866
67924	0.936311	0.184984
67925	0.936311	0.15277
67926	0.946132	0.151916
67927	1.02993	0.138326
67928	1.41513	0.159378
67929	0.946133	0.183002
67930	0.946133	0.15359
67931	0.946133	0.164053
67932	1.02993	0.158047
67933	1.18181	0.12589
67934	1.89226	0.405406
67935	1.89226	0.50388
67936	1.89226	0.605553
67937	1.89226	0.309085
67938	3.78453	0.430963
67939	1.89226	0.198703
67940	1.89226	0.241332
67941	1.89226	0.263723
67942	1.89226	0.189495
67943	1.89226	0.262627
67944	1.89226	0.192936
67945	1.89226	0.18379
67946	1.89226	0.204207
67947	1.89226	0.225136
67948	1.89226	0.168818
67949	1.89226	0.229965
67950	1.89226	0.173383
67951	0.946131	0.301064
67952	0.946131	0.395274
67953	0.946132	0.506303
67954	0.946132	0.404918
67955	0.946132	0.300361
67956	0.946132	0.362582
67957	0.946132	0.506096
67958	0.946132	0.315354
67959	0.946132	0.367671
67960	0.946132	0.383329
67961	0.946132	0.368348
67962	0.946132	0.345677
67963	1.89226	1.14019
67964	1.89226	1.16555
67965	1.89226	1.47139
67966	1.89226	0.812214
67967	1.89226	1.5569
67968	1.89226	0.868772
67969	1.89226	1.31924
67970	2.22749	0.54704
67971	1.89226	0.671197
67972	1.89226	0.702563
67973	3.78453	0.70174

67974	1.89226	1.27101
67975	1.89226	1.41523
67976	1.89226	1.67007
67977	1.89226	0.783185
67978	3.78453	1.19223
67979	1.89226	0.754795
67980	1.89226	0.79885
67981	1.89226	1.06584
67982	1.89226	0.574859
67983	1.89226	1.21293
67984	1.89226	0.647759
67985	0.94613	0.552832
67986	0.94613	1.10498
67987	0.94613	1.50804
67988	0.94613	0.754158
67989	0.946131	0.655483
67990	0.946131	0.546756
67991	0.946129	1.40118
67992	0.946129	0.765815
67993	0.94613	0.738948
67994	0.94613	0.746058
67995	0.94613	0.72298
67996	0.94613	0.698886
67997	1.89226	0.0971767
67998	1.89226	0.106917
67999	1.89226	0.107367
68000	1.89226	0.089213
68001	1.89226	0.109814
68002	1.89226	0.0917619
68003	1.89226	0.119952
68004	1.89226	0.15909
68005	1.89226	0.175025
68006	1.89226	0.0897231
68007	3.78452	0.134016
68008	1.89225	0.0632772
68009	1.89225	0.0718329
68010	1.89225	0.0781169
68011	1.89225	0.0572771
68012	1.89225	0.0813892
68013	1.89225	0.0626349
68014	1.89226	0.05
68015	1.89226	0.0540079
68016	1.89226	0.0636758
68017	1.89226	0.05
68018	1.89226	0.062127
68019	1.89226	0.05
68020	0.946132	0.0805407
68021	0.946132	0.108563
68022	0.946132	0.148852
68023	0.946132	0.105644
68024	0.946131	0.0935676
68025	0.946131	0.0778883
68026	0.946132	0.155013
68027	0.946132	0.0849405
68028	0.946132	0.0979617
68029	0.946132	0.0997885
68030	0.946132	0.101261
68031	0.946132	0.0996766

68032	1.89226	0.105734
68033	1.89226	0.144031
68034	1.89226	0.102069
68035	1.89226	0.192931
68036	1.89226	0.0931071
68037	1.89226	0.178016
68038	1.89226	0.115244
68039	1.89226	0.0910987
68040	1.89226	0.0771973
68041	1.89226	0.138231
68042	1.89226	0.0685533
68043	1.89226	0.13311
68044	1.89226	0.0597738
68045	1.89226	0.0883294
68046	1.89226	0.061456
68047	1.89226	0.101874
68048	1.89226	0.0525713
68049	1.89226	0.0932525
68050	1.89226	0.109879
68051	1.89226	0.163271
68052	1.89226	0.0946934
68053	1.89226	0.194432
68054	3.78452	0.131208
68055	1.89226	0.0891
68056	1.89226	0.109003
68057	1.89226	0.0785548
68058	1.89226	0.159085
68059	1.89226	0.076278
68060	1.89226	0.152014
68061	1.89226	0.05
68062	1.89226	0.05
68063	1.89226	0.05
68064	1.89226	0.05
68065	3.78453	0.05
68066	1.89226	0.05
68067	2.48862	0.05
68068	1.89226	0.05
68069	2.22062	0.05
68070	1.89226	0.05
68071	2.22062	0.05
68072	1.89227	0.05
68073	1.89227	0.05
68074	1.89227	0.05
68075	1.89227	0.05
68076	1.89227	0.05
68077	1.89227	0.05
68078	0.946132	0.05
68079	0.946132	0.05
68080	0.946131	0.05
68081	0.946131	0.05
68082	0.94613	0.05
68083	0.94613	0.05
68084	0.946133	0.05
68085	0.946133	0.05
68086	0.946131	0.05
68087	0.946131	0.05
68088	0.946131	0.05
68089	0.946131	0.05

68090	1.89226	0.05
68091	1.89226	0.05
68092	1.89226	0.05
68093	1.89226	0.05
68094	3.78453	0.05
68095	1.89226	0.05
68096	1.89226	0.0610544
68097	1.89226	0.0519811
68098	1.89226	0.05
68099	3.78453	0.05
68100	1.89226	0.129788
68101	1.89226	0.0960668
68102	1.89226	0.0567988
68103	1.89226	0.124905
68104	1.89226	0.0983736
68105	1.89226	0.152658
68106	1.89226	0.132153
68107	1.89226	0.0639107
68108	1.89226	0.090931
68109	1.89226	0.145655
68110	3.78452	0.119405
68111	1.89226	0.0837174
68112	1.89226	0.109986
68113	1.89226	0.125862
68114	1.89226	0.102108
68115	3.78453	0.0804612
68116	2.57454	0.182358
68117	1.89226	0.16406
68118	1.3663	0.145789
68119	1.89226	0.174312
68120	3.25856	0.151162
68121	1.89226	0.146535
68122	1.89226	0.117746
68123	1.89226	0.113024
68124	1.89226	0.145399
68125	3.78451	0.157662
68126	1.89226	0.110079
68127	1.89226	0.157231
68128	1.89226	0.112599
68129	1.89226	0.141611
68130	1.89226	0.100833
68131	1.89226	0.128748
68132	0.810946	0.133855
68133	0.810946	0.12815
68134	0.810946	0.144332
68135	0.810946	0.167234
68136	0.810959	0.14092
68137	0.810959	0.137147
68138	1.89226	0.157005
68139	1.35168	0.16293
68140	1.08132	0.144731
68141	1.08132	0.155422
68142	1.0813	0.157586
68143	1.89226	0.10709
68144	1.89226	0.158784
68145	1.89226	0.216741
68146	1.89226	0.143114
68147	3.78453	0.132298

68148	1.89226	0.128305
68149	1.89226	0.0933393
68150	1.89226	0.12034
68151	1.89226	0.129671
68152	1.89226	0.0991748
68153	1.89226	0.107752
68154	1.89226	0.0868758
68155	1.89226	0.0988009
68156	1.89226	0.133564
68157	1.89226	0.0898917
68158	3.78452	0.0816476
68159	1.89226	0.149464
68160	1.89226	0.0968923
68161	1.89226	0.143907
68162	1.89226	0.176454
68163	3.78452	0.14991
68164	2.142	0.303761
68165	1.93949	0.134113
68166	1.39296	0.244687
68167	2.55711	0.274712
68168	1.39296	0.158531
68169	2.55711	0.214366
68170	1.89226	0.174428
68171	2.5571	0.124741
68172	2.142	0.199726
68173	1.89226	0.20572
68174	4.44936	0.20024
68175	0.946131	0.164605
68176	0.946131	0.171819
68177	1.27855	0.175057
68178	1.29171	0.151582
68179	1.29171	0.166355
68180	0.946127	0.145209
68181	1.27855	0.12204
68182	0.946131	0.199697
68183	1.29172	0.182253
68184	1.29172	0.17217
68185	0.946129	0.146774
68186	0.946129	0.133849
68187	1.27855	0.207712
68188	1.29172	0.200468
68189	0.969743	0.19568
68190	1.07109	0.187173
68191	1.29172	0.250494
68192	1.07109	0.125347
68193	0.969745	0.130328
68194	1.27855	0.258896
68195	1.10873	0.259327
68196	1.10873	0.216445
68197	1.15273	0.149664
68198	1.15273	0.133043
68199	1.89227	0.05
68200	1.89227	0.05
68201	1.89227	0.05
68202	1.89227	0.05
68203	2.1288	0.05
68204	2.1288	0.05
68205	1.89227	0.05

68206	1.89227	0.05
68207	1.89227	0.05
68208	1.89227	0.05
68209	3.78453	0.05
68210	1.89226	0.05
68211	1.89226	0.05
68212	1.89226	0.05
68213	1.89226	0.05
68214	3.78453	0.05
68215	1.89226	0.05
68216	1.89226	0.05
68217	1.89226	0.05
68218	1.89226	0.05
68219	1.89226	0.05
68220	1.89226	0.05
68221	1.94432	0.05
68222	1.89226	0.05
68223	2.07911	0.05
68224	1.89226	0.05
68225	2.07911	0.05
68226	1.89226	0.05
68227	1.89226	0.05
68228	1.89226	0.05
68229	1.89226	0.05
68230	1.89226	0.05
68231	1.89226	0.05
68232	1.89226	0.05
68233	1.89226	0.549296
68234	1.89226	0.530717
68235	1.89226	0.640203
68236	1.89226	0.416891
68237	1.89226	0.736696
68238	1.89226	0.479926
68239	1.89226	0.344765
68240	1.89226	0.309913
68241	1.89226	0.390104
68242	1.89226	0.246045
68243	1.89226	0.452695
68244	1.89226	0.30851
68245	1.86778	0.241766
68246	2.72348	0.214405
68247	1.89226	0.302453
68248	1.89226	0.144457
68249	3.76004	0.251316
68250	1.89226	0.576634
68251	1.89226	0.557632
68252	1.89226	0.91864
68253	1.89226	0.413954
68254	3.78451	0.564922
68255	1.89226	0.368868
68256	1.89226	0.361503
68257	1.89226	0.428276
68258	1.89226	0.276574
68259	1.89226	0.494759
68260	1.89226	0.330639
68261	0.94613	0.198146
68262	0.94613	0.281157
68263	0.94613	0.413058

68264	0.94613	0.252382
68265	0.946131	0.255566
68266	0.946131	0.168229
68267	0.946129	0.350351
68268	0.946129	0.26993
68269	0.94613	0.259651
68270	0.94613	0.265953
68271	0.94613	0.245824
68272	0.94613	0.243344
68273	0.946124	0.404199
68274	0.946124	0.256248
68275	0.94613	0.567411
68276	0.94613	0.366277
68277	0.946128	0.272715
68278	0.946128	0.324783
68279	0.946126	0.561889
68280	0.946126	0.408538
68281	0.946127	0.402103
68282	0.946127	0.380285
68283	0.946127	0.350352
68284	0.946127	0.367491
68285	2.09166	0.05
68286	2.1285	0.05
68287	1.78381	0.05
68288	1.78381	0.05
68289	2.47689	0.05
68290	2.47689	0.05
68291	2.09167	0.05
68292	2.26572	0.05
68293	2.73393	0.05
68294	2.4769	0.05
68295	4.74261	0.05
68296	1.89226	0.05
68297	1.89226	0.0894781
68298	1.89226	0.0825463
68299	1.89226	0.05
68300	1.89226	0.0880368
68301	1.89226	0.0574299
68302	0.946131	0.0504727
68303	0.946131	0.0896498
68304	0.946129	0.052844
68305	0.946129	0.0808094
68306	0.94613	0.0829695
68307	0.94613	0.0873128
68308	0.946131	0.05
68309	0.946131	0.05
68310	0.94613	0.0606991
68311	0.94613	0.0686875
68312	0.946131	0.0597965
68313	0.946131	0.0679885
68314	2.27622	0.05
68315	2.66786	0.05
68316	2.1285	0.05
68317	2.1285	0.0508731
68318	2.62461	0.05
68319	2.47689	0.05
68320	1.89226	0.0509617
68321	1.89226	0.0943419

68322	1.89226	0.0507026
68323	1.89226	0.0920847
68324	3.78453	0.073628
68325	1.89226	0.0503872
68326	1.89226	0.0874047
68327	1.89226	0.0619642
68328	1.89226	0.0933289
68329	1.89226	0.05
68330	1.89226	0.0807142
68331	1.89226	0.413074
68332	1.89226	0.568172
68333	1.89226	0.398726
68334	1.89226	0.570243
68335	1.89226	0.405469
68336	1.89226	0.561773
68337	1.89226	1.07719
68338	1.89226	0.592922
68339	1.89226	0.605433
68340	1.89226	0.950748
68341	3.78452	0.745737
68342	1.89227	0.613143
68343	1.89227	0.910658
68344	1.89227	0.948138
68345	1.89227	0.641084
68346	3.78453	0.812395
68347	0.946127	0.596225
68348	0.946127	0.589769
68349	1.06438	0.438157
68350	0.946131	0.428744
68351	1.06438	0.434865
68352	0.94613	0.580005
68353	0.946128	0.584242
68354	0.946128	0.407991
68355	1.06438	0.478749
68356	1.06438	0.485455
68357	0.946129	0.503886
68358	0.946129	0.499092
68359	1.89226	0.601282
68360	1.89226	0.858131
68361	1.89226	0.641651
68362	1.89226	0.939124
68363	1.89226	0.624235
68364	1.89226	0.912662
68365	0.946133	0.770938
68366	0.946133	0.829064
68367	1.39771	0.70121
68368	0.946132	0.660235
68369	1.39771	0.704744
68370	1.39772	0.805788
68371	0.946133	0.824638
68372	0.946133	0.607664
68373	0.946132	0.648251
68374	1.39772	0.680032
68375	0.946132	0.610838
68376	0.946132	0.664685
68377	1.98182	0.05
68378	2.2217	0.05
68379	2.79889	0.05

68380	2.50097	0.05
68381	4.72267	0.05
68382	2.05362	0.05
68383	1.70302	0.05
68384	2.75109	0.05
68385	2.138 0.05	
68386	2.0815	0.05
68387	1.84751	0.05
68388	0.946131	0.05
68389	0.946131	0.05
68390	0.946131	0.05
68391	0.946131	0.0526047
68392	0.946131	0.0540972
68393	0.946131	0.05
68394	0.946132	0.05
68395	0.946132	0.05
68396	0.946131	0.05
68397	0.946131	0.05
68398	0.946132	0.05
68399	0.946132	0.05
68400	1.89226	0.05
68401	1.89226	0.0582155
68402	1.89226	0.05
68403	1.89226	0.0662697
68404	3.78451	0.0529813
68405	0.946126	0.05
68406	1.25048	0.05
68407	0.946131	0.05
68408	1.39945	0.05
68409	0.946127	0.05
68410	0.946127	0.05
68411	1.39944	0.05
68412	1.39944	0.05
68413	0.946129	0.05
68414	0.946129	0.05
68415	1.39944	0.05
68416	1.25048	0.05
68417	1.89226	0.05
68418	1.89226	0.0600869
68419	1.89226	0.05
68420	1.89226	0.05
68421	1.89226	0.05
68422	1.89226	0.05
68423	0.946126	0.061875
68424	0.946126	0.05
68425	0.946131	0.05
68426	0.946131	0.0706566
68427	0.946128	0.0728351
68428	0.946128	0.0634156
68429	0.946129	0.05
68430	0.946129	0.05
68431	0.946129	0.050111
68432	0.946129	0.0540631
68433	0.946128	0.0509773
68434	0.946128	0.0554931
68435	1.89226	0.05
68436	1.89226	0.05
68437	1.89226	0.05

68438	1.89226	0.05
68439	1.89226	0.05
68440	1.89226	0.05
68441	1.01393	0.05
68442	0.775245	0.05
68443	0.946126	0.05
68444	0.946126	0.05
68445	0.78305	0.05
68446	0.78305	0.05
68447	0.946159	0.05
68448	0.929267	0.05
68449	1.14956	0.05
68450	1.04073	0.05
68451	0.851526	0.05
68452	0.704781	0.05
68453	1.89226	0.05
68454	1.89226	0.05
68455	1.89226	0.05
68456	1.89226	0.05
68457	3.78451	0.05
68458	1.89226	0.05
68459	1.89226	0.0550958
68460	1.89226	0.05
68461	1.89226	0.05
68462	1.89226	0.05
68463	1.89226	0.05
68464	1.89226	0.05
68465	1.89226	0.05
68466	1.89226	0.05
68467	1.89226	0.05
68468	1.89226	0.05
68469	1.89226	0.05
68470	1.16317	0.05
68471	1.0292	0.05
68472	0.946126	0.05
68473	0.946126	0.05
68474	0.93562	0.05
68475	0.93562	0.05
68476	1.0418	0.05
68477	1.04181	0.05
68478	1.16317	0.05
68479	0.946129	0.05
68480	1.02919	0.05
68481	0.946128	0.05
68482	0.946131	0.05
68483	0.946131	0.05
68484	0.946126	0.05
68485	0.946126	0.05
68486	0.946129	0.05
68487	0.946129	0.05
68488	0.946128	0.05
68489	0.946128	0.05
68490	0.946129	0.05
68491	0.946129	0.05
68492	0.946128	0.05
68493	0.946128	0.05
68494	1.89226	0.05
68495	1.89226	0.0591558

68496	1.89226	0.05
68497	1.89226	0.05
68498	1.89226	0.05
68499	1.89226	0.05
68500	1.89226	0.05
68501	1.89226	0.05
68502	1.89226	0.05
68503	1.89226	0.05
68504	1.89226	0.05
68505	1.89226	0.05
68506	1.89226	0.05
68507	1.89226	0.05
68508	1.89226	0.05
68509	1.89226	0.05
68510	3.78453	0.05
68511	1.89226	0.05
68512	1.89226	0.05
68513	1.89226	0.05
68514	1.89226	0.05
68515	3.78452	0.05
68516	1.89225	0.05
68517	1.89225	0.05
68518	1.89225	0.05
68519	1.89225	0.05
68520	1.89225	0.05
68521	1.89225	0.05
68522	0.946132	0.05
68523	0.946132	0.05
68524	0.946133	0.05
68525	0.946133	0.05
68526	0.946132	0.05
68527	0.946132	0.05
68528	0.946132	0.05
68529	0.946132	0.05
68530	0.946133	0.05
68531	0.946133	0.05
68532	0.946132	0.05
68533	0.946132	0.05
68534	1.89226	0.05
68535	1.89226	0.05
68536	1.89226	0.05
68537	1.89226	0.05
68538	3.78453	0.05
68539	1.13537	0.05
68540	1.09222	0.05
68541	0.946126	0.05
68542	0.946126	0.05
68543	0.946128	0.05
68544	0.946128	0.05
68545	1.16317	0.05
68546	1.13034	0.05
68547	1.30155	0.05
68548	1.08462	0.05
68549	0.992915	0.05
68550	0.807642	0.05
68551	0.946131	0.05
68552	0.946131	0.05
68553	0.946125	0.05

68554	0.946125	0.05
68555	0.946128	0.05
68556	0.946128	0.05
68557	0.946128	0.05
68558	0.946128	0.05
68559	0.946128	0.05
68560	0.946128	0.05
68561	0.946128	0.05
68562	0.946128	0.05
68563	1.89226	0.05
68564	1.89226	0.0601717
68565	1.89226	0.05
68566	1.89226	0.05
68567	1.89226	0.05
68568	1.89226	0.05
68569	1.89224	0.05
68570	1.89224	0.05
68571	1.89224	0.05
68572	1.89224	0.05
68573	1.89224	0.05
68574	1.89224	0.05
68575	0.946086	0.05
68576	1.17919	0.05
68577	0.89738	0.05
68578	0.89738	0.05
68579	0.946116	0.05
68580	0.946116	0.05
68581	1.13048	0.05
68582	1.11844	0.05
68583	0.946128	0.05
68584	0.897362	0.05
68585	1.1282	0.05
68586	0.946103	0.05
68587	1.89223	0.05
68588	1.89223	0.05
68589	1.89223	0.05
68590	1.89223	0.05
68591	3.78446	0.05
68592	0.946118	0.05
68593	0.946118	0.05
68594	0.946118	0.05
68595	0.946118	0.0605733
68596	0.946119	0.0612736
68597	0.946119	0.05
68598	0.946118	0.05
68599	0.946118	0.05
68600	0.946131	0.05
68601	0.946131	0.05
68602	0.946106	0.05
68603	0.946106	0.05
68604	1.89227	0.43336
68605	1.89227	0.624839
68606	1.89227	0.597071
68607	1.89227	0.464485
68608	3.78453	0.518196
68609	1.89226	0.623508
68610	1.89226	0.414708
68611	1.89226	0.411381

68612	1.89226	0.502438
68613	1.89226	0.492848
68614	1.89226	0.574619
68615	1.89226	0.331259
68616	2.59378	0.244247
68617	2.30348	0.247323
68618	2.30348	0.323506
68619	1.89226	0.278579
68620	1.89226	0.368905
68621	1.89226	0.551426
68622	1.89226	0.394881
68623	1.89226	0.393565
68624	1.89226	0.507148
68625	3.78453	0.498715
68626	1.89226	0.490591
68627	1.89226	0.35572
68628	1.89226	0.362356
68629	1.89226	0.495501
68630	1.89226	0.4022
68631	1.89226	0.539119
68632	0.904406	0.247932
68633	1.01667	0.264396
68634	1.11974	0.242493
68635	0.994417	0.311225
68636	1.02013	0.273641
68637	0.628203	0.301637
68638	1.70302	0.312742
68639	1.49898	0.273041
68640	1.14559	0.314183
68641	0.900571	0.266381
68642	1.58886	0.275948
68643	2.81102	0.05
68644	2.3923	0.05
68645	2.05632	0.05
68646	2.43514	0.05
68647	4.82744	0.05
68648	1.89226	0.05
68649	1.89226	0.05
68650	1.89226	0.05
68651	1.89226	0.05
68652	1.89226	0.05
68653	1.89226	0.05
68654	1.89226	0.05
68655	1.89226	0.0558813
68656	1.89226	0.05
68657	1.89226	0.061911
68658	3.78451	0.05
68659	2.50803	0.05
68660	1.903 0.05	
68661	1.903 0.05	
68662	2.19162	0.05
68663	2.09934	0.05
68664	2.39063	0.05
68665	1.00243	0.05
68666	0.731731	0.05
68667	0.876616	0.05
68668	1.00451	0.05
68669	0.986901	0.05

68670	0.986901	0.05
68671	0.887729	0.05
68672	0.887732	0.05
68673	0.876605	0.05
68674	0.769283	0.05
68675	1.00245	0.05
68676	1.18712	0.05
68677	0.946131	0.05
68678	0.946131	0.05
68679	0.946131	0.05
68680	0.946131	0.05
68681	0.946131	0.05
68682	0.946131	0.05
68683	0.946131	0.05
68684	0.946131	0.05
68685	0.946131	0.05
68686	0.946131	0.05
68687	0.946131	0.05
68688	0.946131	0.05
68689	1.89226	0.05
68690	1.89226	0.0566847
68691	1.89226	0.05
68692	1.89226	0.05
68693	1.89226	0.05
68694	1.89226	0.05
68695	1.89226	0.0672031
68696	1.89226	0.127629
68697	1.89226	0.0902554
68698	1.89226	0.0983681
68699	3.78453	0.10811
68700	1.89226	0.131189
68701	1.89226	0.137954
68702	1.89226	0.101854
68703	1.89226	0.207315
68704	3.78453	0.122006
68705	1.89226	0.0664718
68706	1.89226	0.126237
68707	1.89226	0.0905483
68708	1.89226	0.0905596
68709	1.89226	0.0984141
68710	1.89226	0.0992054
68711	0.946132	0.103106
68712	0.946132	0.0700758
68713	0.946131	0.0881929
68714	0.946131	0.0659883
68715	0.946131	0.0923035
68716	0.946131	0.109626
68717	0.946132	0.056634
68718	0.946132	0.0682983
68719	0.946131	0.077091
68720	0.946131	0.065531
68721	0.946131	0.0648381
68722	0.946131	0.0750509
68723	0.946131	0.171842
68724	0.946131	0.113444
68725	0.946131	0.122718
68726	0.946131	0.100161
68727	0.94613	0.136372

68728	0.94613	0.17567
68729	0.946132	0.0931606
68730	0.946132	0.111494
68731	0.946131	0.109498
68732	0.946131	0.128285
68733	0.946131	0.12237
68734	0.946131	0.126864
68735	1.89226	0.05
68736	1.89226	0.05
68737	1.89226	0.05
68738	1.89226	0.05
68739	1.89226	0.05
68740	1.89226	0.05
68741	1.89226	0.05
68742	1.89226	0.05
68743	1.89226	0.05
68744	1.89226	0.0625445
68745	3.78453	0.05
68746	1.89226	0.0602763
68747	1.89226	0.0679539
68748	1.89226	0.106745
68749	1.89226	0.0555042
68750	1.89226	0.102149
68751	1.89226	0.05
68752	0.946132	0.05
68753	0.946132	0.05
68754	0.946131	0.05
68755	0.946131	0.05
68756	0.946132	0.05
68757	0.946132	0.05
68758	0.946131	0.05
68759	0.946131	0.05
68760	0.946132	0.05
68761	0.946132	0.05
68762	0.946131	0.05
68763	0.946131	0.05
68764	0.946131	0.05
68765	0.946131	0.05
68766	0.946132	0.058593
68767	0.946132	0.0763293
68768	0.946132	0.0689764
68769	0.946132	0.05
68770	0.946131	0.05
68771	0.946131	0.05
68772	0.946132	0.05
68773	0.946132	0.05
68774	0.946131	0.05
68775	0.946131	0.0511978
68776	1.89226	0.409535
68777	1.89226	0.471
68778	1.89226	0.546459
68779	1.89226	0.358897
68780	3.78452	0.370101
68781	1.89226	0.923583
68782	1.89226	0.552841
68783	1.89226	0.650175
68784	1.89226	0.736279
68785	3.78452	0.862345

68786	1.89226	0.533922
68787	1.89226	0.551012
68788	1.89226	0.713582
68789	1.89226	0.40171
68790	3.78452	0.435284
68791	1.89226	0.436806
68792	2.75945	0.226537
68793	1.89226	0.32276
68794	2.35026	0.310854
68795	4.24252	0.448232
68796	0.946131	0.376718
68797	0.946131	0.461385
68798	0.946131	0.263627
68799	0.946131	0.318927
68800	0.946132	0.260678
68801	0.946132	0.305674
68802	0.946129	0.471026
68803	0.946129	0.327907
68804	0.946131	0.371605
68805	0.946131	0.370942
68806	0.94613	0.46382
68807	0.94613	0.395151
68808	1.89226	0.557215
68809	1.89226	0.616479
68810	1.89226	0.505768
68811	1.89226	0.636748
68812	1.89226	0.517951
68813	1.89226	0.694568
68814	0.946129	0.404279
68815	0.946129	0.517
68816	0.946133	0.412449
68817	0.946133	0.32741
68818	0.946131	0.308238
68819	0.946131	0.330002
68820	0.946131	0.51618
68821	0.946131	0.361658
68822	0.946131	0.44197
68823	0.946131	0.429072
68824	0.946131	0.504865
68825	0.946131	0.442148
68826	1.89226	0.227859
68827	2.35026	0.216721
68828	1.32126	0.18509
68829	1.89226	0.252434
68830	1.77925	0.189055
68831	2.35026	0.246104
68832	1.89226	0.0539423
68833	1.89226	0.115458
68834	1.89226	0.101984
68835	1.89226	0.0794932
68836	1.89226	0.0772717
68837	1.89226	0.0583825
68838	1.89226	0.097642
68839	1.89226	0.215062
68840	1.89226	0.130832
68841	1.89226	0.172942
68842	3.78453	0.188218
68843	1.89226	0.0761948

68844	1.89226	0.1677
68845	1.89226	0.134105
68846	1.89226	0.114379
68847	1.89226	0.114348
68848	1.89226	0.0943213
68849	0.946131	0.160438
68850	0.946131	0.0989833
68851	0.946131	0.0974033
68852	0.946131	0.118881
68853	0.946132	0.155515
68854	0.946132	0.171956
68855	0.946131	0.106692
68856	0.946131	0.0962227
68857	0.946132	0.0858766
68858	0.946132	0.126664
68859	0.946131	0.101154
68860	0.946131	0.120165
68861	1.89226	0.0613864
68862	1.89226	0.131252
68863	1.89226	0.079973
68864	1.89226	0.0772294
68865	1.89226	0.122066
68866	1.89226	0.126434
68867	1.89226	0.115014
68868	1.89226	0.201554
68869	1.89226	0.155464
68870	1.89226	0.166598
68871	3.78453	0.177938
68872	1.89227	0.208289
68873	1.89227	0.190634
68874	1.89227	0.170277
68875	1.89227	0.29168
68876	3.78453	0.186097
68877	1.89226	0.108526
68878	1.89226	0.167329
68879	1.89226	0.147627
68880	1.89226	0.145443
68881	3.78452	0.152213
68882	0.946132	0.149275
68883	0.946132	0.118435
68884	0.946131	0.132962
68885	0.946131	0.119194
68886	0.946131	0.146934
68887	0.946131	0.161394
68888	0.946133	0.126092
68889	0.946133	0.102593
68890	0.946132	0.109397
68891	0.946132	0.131235
68892	0.946131	0.109013
68893	0.946131	0.123438
68894	1.89227	0.148185
68895	1.89227	0.229325
68896	1.89227	0.154822
68897	1.89227	0.163663
68898	1.89227	0.196628
68899	1.89227	0.20387
68900	2.20491	0.124144
68901	1.81656	0.121663

68902	1.81656	0.121205
68903	1.89226	0.133834
68904	1.89226	0.104721
68905	1.89226	0.130683
68906	1.89226	0.201884
68907	1.89226	0.286335
68908	1.89226	0.259819
68909	1.89226	0.222101
68910	3.78452	0.226862
68911	1.89226	0.43833
68912	1.89226	0.351518
68913	1.89226	0.326458
68914	1.89226	0.509664
68915	3.78453	0.402744
68916	1.89226	0.350922
68917	1.89226	0.468986
68918	1.89226	0.372468
68919	1.89226	0.45728
68920	1.89226	0.389902
68921	1.89226	0.481101
68922	1.89226	0.212215
68923	2.11032	0.177045
68924	2.30793	0.175006
68925	1.89226	0.20032
68926	2.30793	0.187784
68927	1.89226	0.21294
68928	1.89226	0.274196
68929	1.89226	0.391577
68930	1.89226	0.294243
68931	1.89226	0.351972
68932	1.89226	0.321809
68933	1.89226	0.389361
68934	0.810943	0.375188
68935	0.810943	0.343839
68936	0.810943	0.356483
68937	0.810943	0.308527
68938	0.810933	0.335611
68939	0.810933	0.330097
68940	1.89226	0.272588
68941	1.3517	0.332854
68942	1.08132	0.300766
68943	1.08132	0.379244
68944	1.08133	0.337339
68945	1.89226	0.376322
68946	1.89226	0.17235
68947	1.89226	0.238435
68948	1.89226	0.290421
68949	3.78453	0.32219
68950	1.89226	0.160072
68951	1.89226	0.163635
68952	1.89226	0.225453
68953	1.89226	0.122202
68954	1.89226	0.214627
68955	1.89226	0.118959
68956	0.810945	0.151261
68957	0.810945	0.109175
68958	0.870829	0.14922
68959	0.870829	0.120953

68960	0.990502	0.094922
68961	0.810933	0.118779
68962	2.82777	0.0762321
68963	1.53126	0.115053
68964	1.32076	0.103722
68965	1.201	0.108265
68966	1.08133	0.116314
68967	1.89226	0.224018
68968	1.89226	0.122745
68969	1.89226	0.155911
68970	1.89226	0.154058
68971	1.89226	0.162613
68972	1.89226	0.161051
68973	0.946132	0.172679
68974	0.946132	0.212492
68975	0.946133	0.372301
68976	0.946133	0.234048
68977	0.946132	0.277659
68978	0.946132	0.202416
68979	0.946132	0.266295
68980	0.946132	0.323222
68981	0.946133	0.231054
68982	0.946133	0.190161
68983	0.946132	0.179487
68984	0.946132	0.221655
68985	0.946133	0.0990787
68986	0.946133	0.122926
68987	0.946132	0.169104
68988	0.946132	0.206453
68989	0.946132	0.159412
68990	0.946132	0.124448
68991	0.946132	0.155299
68992	0.946132	0.213391
68993	0.946133	0.108251
68994	0.946133	0.139618
68995	0.946132	0.124037
68996	0.946132	0.143776
68997	1.89226	0.119015
68998	1.89226	0.225622
68999	1.89226	0.169591
69000	1.89226	0.174969
69001	1.89226	0.140886
69002	1.89226	0.14905
69003	1.89226	0.178896
69004	1.89226	0.397795
69005	1.89226	0.247854
69006	1.89226	0.316158
69007	3.78452	0.373922
69008	1.89226	0.416406
69009	1.89226	0.43131
69010	1.89226	0.329552
69011	1.89226	0.663411
69012	3.78452	0.348983
69013	1.89226	0.17308
69014	1.89226	0.322309
69015	1.89226	0.196126
69016	1.89226	0.224712
69017	1.89226	0.287645

69018	1.89226	0.299843
69019	0.946128	0.215012
69020	0.946128	0.182873
69021	0.946132	0.189737
69022	0.946132	0.181792
69023	0.946129	0.24614
69024	0.946129	0.26388
69025	0.946131	0.173498
69026	0.946131	0.149299
69027	0.94613	0.20626
69028	0.94613	0.127355
69029	0.946131	0.145424
69030	0.946131	0.184614
69031	0.946129	0.385476
69032	0.946129	0.310468
69033	0.946133	0.300561
69034	0.946133	0.265763
69035	0.946131	0.368613
69036	0.946131	0.400425
69037	0.946131	0.236407
69038	0.946131	0.256526
69039	0.946131	0.351206
69040	0.946131	0.243682
69041	0.946131	0.289235
69042	0.946131	0.358892
69043	1.89226	0.233049
69044	1.89226	0.165488
69045	1.89226	0.143031
69046	1.89226	0.432862
69047	3.78452	0.14973
69048	1.89225	0.05
69049	1.89225	0.110674
69050	1.89225	0.095019
69051	1.89225	0.0997318
69052	1.89225	0.0533109
69053	1.89225	0.05
69054	1.89226	0.111061
69055	1.89226	0.0661865
69056	1.89226	0.0798461
69057	1.89226	0.13099
69058	3.78452	0.083414
69059	1.89226	0.166152
69060	1.89226	0.53484
69061	1.89226	0.457298
69062	1.89226	0.256769
69063	3.78453	0.371628
69064	1.89226	0.0699785
69065	1.89226	0.178649
69066	1.89226	0.146705
69067	1.89226	0.140066
69068	3.78452	0.155509
69069	1.22164	0.05
69070	1.22164	0.0692259
69071	0.806032	0.05
69072	0.806032	0.05
69073	1.08154	0.05
69074	0.946126	0.05
69075	0.946129	0.05

69076	0.806065	0.05
69077	0.806063	0.05
69078	1.08155	0.05
69079	0.946128	0.05
69080	0.946128	0.05
69081	1.28554	0.05
69082	1.28554	0.0525398
69083	0.946126	0.05
69084	0.946126	0.0678668
69085	1.28554	0.0822946
69086	0.946129	0.05
69087	1.08155	0.05
69088	0.94613	0.05
69089	1.08155	0.05
69090	1.28554	0.0544365
69091	0.94613	0.05
69092	0.94613	0.05
69093	0.946132	0.121853
69094	0.946132	0.173603
69095	0.946132	0.213319
69096	0.946132	0.338948
69097	0.946132	0.417774
69098	0.946132	0.146647
69099	0.946133	0.162961
69100	0.946133	0.141215
69101	0.946132	0.188921
69102	0.946132	0.177473
69103	0.946133	0.1568
69104	0.946133	0.183809
69105	1.89226	0.756801
69106	1.89226	0.499278
69107	1.89226	0.860318
69108	1.89226	0.536067
69109	3.78453	0.684945
69110	1.89226	0.685417
69111	1.89226	0.460933
69112	1.89226	0.49377
69113	1.89226	0.564036
69114	1.89226	0.527514
69115	1.89226	0.601212
69116	1.89226	0.304318
69117	1.89226	0.202868
69118	1.89226	0.221072
69119	1.89226	0.246541
69120	1.89226	0.24071
69121	1.89226	0.267742
69122	1.89226	0.355368
69123	1.89226	0.229817
69124	1.89226	0.267074
69125	1.89226	0.336308
69126	1.89226	0.284273
69127	1.89226	0.239228
69128	0.946132	0.471006
69129	0.946132	0.508586
69130	0.946132	0.330837
69131	0.946132	0.516303
69132	0.946132	0.367478
69133	0.946132	0.370424

69134	0.946132	0.508978
69135	0.946132	0.477186
69136	0.946131	0.297438
69137	0.946131	0.470706
69138	0.946132	0.41087
69139	0.946132	0.391825
69140	1.89226	0.48089
69141	1.89226	0.27356
69142	1.89226	0.49568
69143	1.89226	0.362917
69144	3.78453	0.393541
69145	0.851541	0.120616
69146	0.72883	0.172866
69147	0.991276	0.177107
69148	0.851485	0.139342
69149	1.00003	0.144816
69150	1.00003	0.114534
69151	0.809788	0.166368
69152	0.809788	0.155863
69153	0.890738	0.157774
69154	0.991264	0.138411
69155	0.851554	0.149489
69156	0.851554	0.157636
69157	0.8628	0.207363
69158	0.78387	0.154523
69159	0.830233	0.191412
69160	0.830233	0.21322
69161	0.808312	0.187105
69162	0.817238	0.131181
69163	1.70303	0.150415
69164	1.24116	0.17243
69165	0.995299	0.136264
69166	1.0989	0.174403
69167	1.02714	0.201015
69168	1.89226	1.01182
69169	1.89226	1.0971
69170	1.89226	0.756528
69171	1.89226	1.97316
69172	3.78451	0.796496
69173	0.946131	0.886066
69174	0.946131	0.338732
69175	0.946131	0.881098
69176	0.946131	0.737466
69177	0.94613	0.81721
69178	0.94613	0.46568
69179	0.946132	0.384513
69180	0.946132	0.532679
69181	0.946131	0.563374
69182	0.946131	0.664941
69183	0.946131	0.580738
69184	0.946131	0.480669
69185	1.89226	0.862123
69186	1.89226	2.09265
69187	1.89226	1.84904
69188	1.89226	1.85257
69189	3.78453	2.05885
69190	1.89226	2.1
69191	1.89226	2.1

69192	1.89226	2.1
69193	1.89226	1.97747
69194	1.89226	2.1
69195	1.89226	1.91357
69196	0.946126	0.510334
69197	1.17111	0.251051
69198	1.3393	0.730719
69199	0.946131	1.13593
69200	0.946127	1.0397
69201	0.946127	0.33027
69202	1.3393	0.371104
69203	1.17111	0.290002
69204	1.3393	0.531127
69205	1.3393	0.334028
69206	0.946129	0.499772
69207	0.946129	0.80243
69208	0.946126	2.07689
69209	0.946126	1.11941
69210	0.946132	2.1
69211	0.946132	2.1
69212	0.94613	2.1
69213	0.94613	1.99328
69214	0.946129	2.09413
69215	0.946129	1.99057
69216	0.946129	0.886559
69217	0.946129	2.1
69218	0.94613	1.94907
69219	0.94613	1.94446
69220	0.946131	0.753956
69221	0.946131	0.671724
69222	0.94613	0.550599
69223	0.94613	0.550821
69224	0.946128	0.771335
69225	0.946128	0.796021
69226	0.946132	0.506451
69227	0.946132	0.529668
69228	0.94613	0.735969
69229	0.94613	0.474077
69230	0.94613	0.576313
69231	0.94613	0.750883
69232	0.94613	1.9192
69233	0.94613	1.4004
69234	0.94613	1.23236
69235	0.94613	1.00346
69236	0.946128	1.78917
69237	0.946128	1.93222
69238	0.946131	1.0011
69239	0.946131	0.929877
69240	0.94613	1.71254
69241	0.94613	0.950269
69242	0.94613	1.15247
69243	0.94613	1.76994
69244	1.89226	1.97175
69245	1.89226	1.56026
69246	1.89226	1.67789
69247	1.89226	2.08406
69248	3.78453	1.73858
69249	1.89226	0.849016

69250	1.89226	0.951869
69251	1.89226	0.778392
69252	1.89226	0.876316
69253	1.89226	0.808565
69254	1.89226	0.944126
69255	1.89226	0.973544
69256	1.89226	1.41602
69257	1.89226	1.16482
69258	1.89226	1.41644
69259	1.89226	0.848425
69260	1.89226	1.05806
69261	1.89227	0.484964
69262	2.28622	0.516393
69263	2.16109	0.373776
69264	1.89227	0.393853
69265	4.17849	0.424871
69266	15.1381	0.139937
69267	15.1381	1.21614
69268	15.1381	0.25955
69269	15.1381	0.166396
69270	30.2762	0.123381
69271	15.1381	0.172556
69272	15.1381	0.613054
69273	15.1381	0.235229
69274	15.1381	0.234522
69275	30.2762	0.100676
69276	15.1381	0.872242
69277	15.1381	0.403829
69278	15.1381	0.237842
69279	15.1381	0.710945
69280	30.2762	0.502545
69281	15.1381	0.248216
69282	15.1381	1.61476
69283	15.1381	0.0679296
69284	15.1381	0.167758
69285	30.2762	0.171442
69286	15.138	1.29539
69287	15.138	1.82551
69288	15.138	1.12681
69289	15.138	1.96124
69290	30.276	1.8067
69291	15.138	1.85024
69292	15.138	2.04155
69293	15.138	1.989
69294	15.138	2.05118
69295	30.276	1.91611
69296	15.138	1.77164
69297	15.138	1.56481
69298	15.138	1.19483
69299	15.138	2.00013
69300	30.2759	1.8386
69301	15.138	1.63622
69302	15.138	1.94721
69303	15.138	2.03227
69304	15.138	1.59987
69305	30.2759	1.7532
69306	15.138	2.05732
69307	15.138	1.98046

69308	15.138	2.06966
69309	15.138	2.04881
69310	30.276	2.04432
69311	15.1381	2.0342
69312	15.1381	2.08992
69313	15.1381	2.03709
69314	15.1381	2.09734
69315	30.2762	2.0928
69316	15.1381	2.0955
69317	15.1381	2.08264
69318	15.1381	2.1
69319	15.1381	2.08975
69320	30.2762	2.09377
69321	15.138	1.94684
69322	15.138	2.03565
69323	15.138	2.01794
69324	15.138	2.02795
69325	30.276	2.02552
69326	15.138	2.05163
69327	15.138	2.01365
69328	15.138	2.05499
69329	15.138	2.05579
69330	30.276	2.02865
69331	15.1381	1.73286
69332	15.1381	1.98504
69333	15.1381	1.92211
69334	15.1381	1.90404
69335	30.2762	1.91729
69336	15.1381	1.92361
69337	15.1381	2.03365
69338	15.1381	1.95984
69339	15.1381	2.01452
69340	30.2762	1.95844
69341	15.1381	2.05318
69342	15.1381	2.01163
69343	15.1381	2.03736
69344	15.1381	2.02935
69345	30.2762	2.04922
69346	15.138	0.939554
69347	15.138	0.198977
69348	15.138	0.0986998
69349	15.138	1.15567
69350	30.276	0.18666
69351	1.89226	2.1
69352	1.89226	2.1
69353	1.89226	2.1
69354	1.89226	2.1
69355	3.78453	2.1
69356	1.89226	2.1
69357	1.89226	2.1
69358	1.89226	2.1
69359	1.89226	2.1
69360	1.89226	2.1
69361	1.89226	2.1
69362	1.89226	2.1
69363	1.89226	2.1
69364	1.89226	2.1
69365	1.89226	2.1

69366	1.89226	2.1
69367	1.89226	2.1
69368	1.89226	2.1
69369	1.89226	2.1
69370	1.89226	2.1
69371	1.89226	2.1
69372	3.78453	2.1
69373	1.89226	2.1
69374	1.89226	2.1
69375	1.89226	2.1
69376	1.89226	2.1
69377	1.89226	2.1
69378	1.89226	2.1
69379	0.946127	2.1
69380	0.946127	2.1
69381	0.946131	2.1
69382	0.946131	2.1
69383	0.946131	2.1
69384	0.946131	2.1
69385	0.946128	2.1
69386	0.946128	2.1
69387	0.94613	2.1
69388	0.94613	2.1
69389	0.946129	2.1
69390	0.946129	2.1
69391	1.89226	2.1
69392	1.89226	2.1
69393	1.89226	2.1
69394	1.89226	2.1
69395	1.89226	2.1
69396	1.89226	2.1
69397	1.89226	2.1
69398	1.89226	2.1
69399	1.89226	2.1
69400	1.89226	2.1
69401	3.78452	2.1
69402	1.89226	2.1
69403	1.89226	2.1
69404	1.89226	2.1
69405	1.89226	2.1
69406	1.89226	2.1
69407	1.89226	2.1
69408	0.946129	2.1
69409	0.946129	2.1
69410	0.946133	2.1
69411	0.946133	2.1
69412	0.946131	2.1
69413	0.946131	2.1
69414	0.946131	2.1
69415	0.946131	2.1
69416	0.946131	2.1
69417	0.946131	2.1
69418	0.946131	2.1
69419	0.946131	2.1
69420	0.946132	2.1
69421	0.946132	2.1
69422	0.946132	2.1
69423	0.946132	2.1

69424	0.946132	2.1
69425	0.946132	2.1
69426	0.946133	2.1
69427	0.946133	2.1
69428	0.946132	2.1
69429	0.946132	2.1
69430	0.946133	2.1
69431	0.946133	2.1
69432	1.89226	2.1
69433	1.89226	2.1
69434	1.89226	2.1
69435	1.89226	2.1
69436	3.78452	2.1
69437	1.89226	2.1
69438	1.89226	2.1
69439	1.89226	2.1
69440	1.89226	2.1
69441	1.89226	2.1
69442	1.89226	2.1
69443	0.946129	2.1
69444	0.946129	2.1
69445	0.946133	2.1
69446	0.946133	2.1
69447	0.946131	2.1
69448	0.946131	2.1
69449	0.946131	2.1
69450	0.946131	2.1
69451	0.946131	2.1
69452	0.946131	2.1
69453	0.946131	2.1
69454	0.946131	2.1
69455	0.946132	2.1
69456	0.946132	2.1
69457	0.946132	2.1
69458	0.946132	2.1
69459	0.946132	2.1
69460	0.946132	2.1
69461	0.946133	2.1
69462	0.946133	2.1
69463	0.946133	2.1
69464	0.946133	2.1
69465	0.946132	2.1
69466	0.946132	2.1
69467	1.89226	2.1
69468	1.89226	2.1
69469	1.89226	2.1
69470	1.89226	2.1
69471	3.78453	2.1
69472	0.946128	2.1
69473	0.946128	2.1
69474	0.946132	2.1
69475	0.946132	2.1
69476	0.94613	2.1
69477	0.94613	2.1
69478	0.94613	2.1
69479	0.94613	2.1
69480	0.94613	2.1
69481	0.94613	2.1

69482	0.94613	2.1
69483	0.94613	2.1
69484	0.946127	2.1
69485	0.946127	2.1
69486	0.946131	2.1
69487	0.946131	2.1
69488	0.94613	2.1
69489	0.94613	2.1
69490	0.946128	2.1
69491	0.946128	2.1
69492	0.946129	2.1
69493	0.946129	2.1
69494	0.946129	2.1
69495	0.946129	2.1
69496	0.94613	2.1
69497	0.94613	2.1
69498	0.94613	2.1
69499	0.94613	2.1
69500	0.946131	2.1
69501	0.946131	2.1
69502	0.946129	2.1
69503	0.946129	2.1
69504	0.94613	2.1
69505	0.94613	2.1
69506	0.94613	2.1
69507	0.94613	2.1
69508	1.89226	2.1
69509	1.89226	2.1
69510	1.89226	2.1
69511	1.89226	2.1
69512	3.78453	2.1
69513	1.89226	2.1
69514	1.89226	2.1
69515	1.89226	2.1
69516	1.89226	2.1
69517	1.89226	2.1
69518	1.89226	2.1
69519	1.89226	2.1
69520	1.89226	2.1
69521	1.89226	2.1
69522	1.89226	2.1
69523	1.89226	2.1
69524	1.89226	2.1
69525	0.946131	2.1
69526	0.946131	2.1
69527	0.946131	2.1
69528	0.946131	2.1
69529	0.946132	2.1
69530	0.946132	2.1
69531	0.946129	2.1
69532	0.946129	2.1
69533	0.946131	2.1
69534	0.946131	2.1
69535	0.94613	2.1
69536	0.94613	2.1
69537	1.89224	2.1
69538	1.89224	2.1
69539	1.89224	2.1

69540	1.89224	2.1
69541	3.78448	2.1
69542	1.89224	2.1
69543	1.89224	2.1
69544	1.89224	2.1
69545	1.89224	2.1
69546	3.78447	2.1
69547	1.89223	2.1
69548	2.38995	2.1
69549	2.25315	2.1
69550	2.25315	2.1
69551	1.89223	2.1
69552	1.89223	2.1
69553	1.89224	2.1
69554	1.89224	2.1
69555	1.89224	2.1
69556	1.89224	2.1
69557	1.89224	2.1
69558	1.89224	2.1
69559	1.89226	2.1
69560	1.89226	2.1
69561	1.89226	2.1
69562	1.89226	2.1
69563	3.78453	2.1
69564	1.89226	2.1
69565	1.89226	2.1
69566	1.89226	2.1
69567	1.89226	2.1
69568	1.89226	2.1
69569	1.89226	2.1
69570	1.89226	2.1
69571	2.60272	2.1
69572	2.38998	2.1
69573	2.38998	2.1
69574	1.89226	2.1
69575	1.89226	2.1
69576	0.94613	2.1
69577	0.94613	2.1
69578	0.94613	2.1
69579	0.94613	2.1
69580	0.946131	2.1
69581	0.946131	2.1
69582	0.946129	2.1
69583	0.946129	2.1
69584	0.94613	2.1
69585	0.94613	2.1
69586	0.94613	2.1
69587	0.94613	2.1
69588	1.89226	2.1
69589	1.89226	2.1
69590	1.89226	2.1
69591	1.89226	2.1
69592	3.78452	2.1
69593	1.89226	2.1
69594	1.89226	2.1
69595	1.89226	2.1
69596	1.89226	2.1
69597	3.78452	2.1

69598	1.89226	2.1
69599	1.89226	2.1
69600	1.89226	2.1
69601	1.89226	2.1
69602	1.89226	2.1
69603	1.89226	2.1
69604	0.946132	2.1
69605	0.946132	2.1
69606	0.946132	2.1
69607	0.946132	2.1
69608	0.946131	2.1
69609	0.946131	2.1
69610	0.946132	2.1
69611	0.946132	2.1
69612	0.946132	2.1
69613	0.946132	2.1
69614	0.946132	2.1
69615	0.946132	2.1
69616	1.89226	2.1
69617	1.89226	2.1
69618	1.89226	2.1
69619	1.89226	2.1
69620	3.78452	2.1
69621	1.89226	2.1
69622	1.89226	2.1
69623	1.89226	2.1
69624	1.89226	2.1
69625	3.78452	2.1
69626	0.946129	2.1
69627	0.946129	2.1
69628	0.946133	2.1
69629	0.946133	2.1
69630	0.946131	2.1
69631	0.946131	2.1
69632	0.946131	2.1
69633	0.946131	2.1
69634	0.946131	2.1
69635	0.946131	2.1
69636	0.946131	2.1
69637	0.946131	2.1
69638	0.946132	2.1
69639	0.946132	2.1
69640	0.946132	2.1
69641	0.946132	2.1
69642	0.946132	2.1
69643	0.946132	2.1
69644	0.946133	2.1
69645	0.946133	2.1
69646	0.946133	2.1
69647	0.946133	2.1
69648	0.946132	2.1
69649	0.946132	2.1
69650	1.89224	2.1
69651	1.89224	2.1
69652	1.89224	2.1
69653	1.89224	2.1
69654	3.78447	2.1
69655	1.89223	2.1

69656	1.89223	2.1
69657	1.89223	2.1
69658	1.89223	2.1
69659	1.89223	2.1
69660	1.89223	2.1
69661	1.89224	2.1
69662	1.89224	2.1
69663	1.89224	2.1
69664	1.89224	2.1
69665	3.78447	2.1
69666	1.89224	2.1
69667	1.89224	2.1
69668	1.89224	2.1
69669	1.89224	2.1
69670	3.78448	2.1
69671	1.89226	2.1
69672	1.89226	2.1
69673	1.89226	2.1
69674	1.89226	2.1
69675	3.78452	2.1
69676	1.89226	2.1
69677	1.89226	2.1
69678	1.89226	2.1
69679	1.89226	2.1
69680	1.89226	2.1
69681	1.89226	2.1
69682	1.89226	2.1
69683	1.89226	2.1
69684	1.89226	2.1
69685	1.89226	2.1
69686	1.89226	2.1
69687	1.89226	2.1
69688	0.810946	2.1
69689	0.810946	2.1
69690	0.810946	2.1
69691	0.810946	2.1
69692	0.810933	2.1
69693	0.810933	2.1
69694	1.89226	2.1
69695	1.3517	2.1
69696	1.08132	2.1
69697	1.08132	2.1
69698	1.08133	2.1
69699	1.89226	2.1
69700	1.89226	2.1
69701	1.89226	2.1
69702	1.89226	2.1
69703	3.78453	2.1
69704	1.89226	2.1
69705	1.89226	2.1
69706	1.89226	2.1
69707	1.89226	2.1
69708	3.78452	2.1
69709	1.89226	2.1
69710	1.89226	2.1
69711	1.89226	2.1
69712	1.89226	2.1
69713	1.89226	2.1

69714	1.89226	2.1
69715	0.94613	2.1
69716	0.94613	2.1
69717	0.94613	2.1
69718	0.94613	2.1
69719	0.946131	2.1
69720	0.946131	2.1
69721	0.946129	2.1
69722	0.946129	2.1
69723	0.94613	2.1
69724	0.94613	2.1
69725	0.94613	2.1
69726	0.94613	2.1
69727	1.89226	2.1
69728	1.89226	2.1
69729	1.89226	2.1
69730	1.89226	2.1
69731	3.78453	2.1
69732	1.89226	2.1
69733	1.89226	2.1
69734	1.89226	2.1
69735	1.89226	2.1
69736	3.78452	2.1
69737	1.89226	2.1
69738	1.89226	2.1
69739	1.89226	2.1
69740	1.89226	2.1
69741	3.78452	2.1
69742	1.89226	2.1
69743	1.89226	2.1
69744	1.89226	2.1
69745	1.89226	2.1
69746	1.89226	2.1
69747	1.89226	2.1
69748	1.89226	2.1
69749	1.89226	2.1
69750	1.89226	2.1
69751	1.89226	2.1
69752	3.78452	2.1
69753	1.89226	2.1
69754	1.89226	2.1
69755	1.89226	2.1
69756	1.89226	2.1
69757	3.78452	2.1
69758	0.946128	2.1
69759	0.946128	2.1
69760	0.946133	2.1
69761	0.946133	2.1
69762	0.94613	2.1
69763	0.94613	2.1
69764	0.946131	2.1
69765	0.946131	2.1
69766	0.946131	2.1
69767	0.946131	2.1
69768	0.946131	2.1
69769	0.946131	2.1
69770	0.946132	2.1
69771	0.946132	2.1

69772	0.946132	2.1
69773	0.946132	2.1
69774	0.946131	2.1
69775	0.946131	2.1
69776	0.946132	2.1
69777	0.946132	2.1
69778	0.946132	2.1
69779	0.946132	2.1
69780	0.946132	2.1
69781	0.946132	2.1
69782	1.89226	2.1
69783	1.89226	2.1
69784	1.89226	2.1
69785	1.89226	2.1
69786	3.78452	2.1
69787	1.89226	2.1
69788	1.89226	2.1
69789	1.89226	2.1
69790	1.89226	2.1
69791	3.78452	2.1
69792	0.946129	2.1
69793	0.946129	2.1
69794	0.946133	2.1
69795	0.946133	2.1
69796	0.946131	2.1
69797	0.946131	2.1
69798	0.946131	2.1
69799	0.946131	2.1
69800	0.946131	2.1
69801	0.946131	2.1
69802	0.946131	2.1
69803	0.946131	2.1
69804	0.946132	2.1
69805	0.946132	2.1
69806	0.946132	2.1
69807	0.946132	2.1
69808	0.946132	2.1
69809	0.946132	2.1
69810	0.946133	2.1
69811	0.946133	2.1
69812	0.946132	2.1
69813	0.946132	2.1
69814	0.946133	2.1
69815	0.946133	2.1
69816	1.89226	2.1
69817	1.89226	2.1
69818	1.89226	2.1
69819	1.89226	2.1
69820	1.89226	2.1
69821	1.89226	2.1
69822	0.946128	2.1
69823	0.946128	2.1
69824	0.946133	2.1
69825	0.946133	2.1
69826	0.946131	2.1
69827	0.946131	2.1
69828	0.94613	2.1
69829	0.94613	2.1

69830	0.946131	2.1
69831	0.946131	2.1
69832	0.946131	2.1
69833	0.946131	2.1
69834	1.89226	2.1
69835	1.89226	2.1
69836	1.89226	2.1
69837	1.89226	2.1
69838	1.89226	2.1
69839	1.89226	2.1
69840	1.89226	2.1
69841	1.89226	2.1
69842	1.89226	2.1
69843	1.89226	2.1
69844	3.78453	2.1
69845	1.89226	2.1
69846	1.89226	2.1
69847	1.89226	2.1
69848	1.89226	2.1
69849	1.89226	2.1
69850	1.89226	2.1
69851	0.946127	2.1
69852	0.946127	2.1
69853	0.946131	2.1
69854	0.946131	2.1
69855	0.946131	2.1
69856	0.946131	2.1
69857	0.946127	2.1
69858	0.946127	2.1
69859	0.946129	2.1
69860	0.946129	2.1
69861	0.94613	2.1
69862	0.94613	2.1
69863	0.946131	2.1
69864	0.946131	2.1
69865	0.946131	2.1
69866	0.946131	2.1
69867	0.946133	2.1
69868	0.946133	2.1
69869	0.946128	2.1
69870	0.946128	2.1
69871	0.94613	2.1
69872	0.94613	2.1
69873	0.946131	2.1
69874	0.946131	2.1
69875	1.89226	2.1
69876	1.89226	2.1
69877	1.89226	2.1
69878	1.89226	2.1
69879	1.89226	2.1
69880	1.89226	2.1
69881	1.89226	2.1
69882	1.89226	2.1
69883	1.89226	2.1
69884	1.89226	2.1
69885	3.78452	2.1
69886	1.89226	2.1
69887	1.89226	2.1

69888	1.89226	2.1
69889	1.89226	2.1
69890	3.78452	2.1
69891	1.89226	2.1
69892	1.89226	2.1
69893	1.89226	2.1
69894	1.89226	2.1
69895	1.89226	2.1
69896	1.89226	2.1
[truncated: 397,460 more chars]
