# Supplementary material for: On the Use of Bone Remodelling Models to Estimate the Density Distribution of Bones. Uniqueness of the Solution
Source: PLoS One. 2016 Feb 9;11(2):e0148603. doi: 10.1371/journal.pone.0148603 (PMC4747586; doi:10.1371/journal.pone.0148603)
Supplement: S6 File — (PDF) [file pone.0148603.s006.pdf]

N° Elem	Volume (mm3)	Density (g/cm3)
1	1.32415	2.1
2	1.75823	2.1
3	0.984099	2.1
4	1.46442	2.1
5	1.53082	2.1
6	1.32068	2.1
7	1.91404	0.0698591
8	1.17036	0.0698591
9	0.929209	0.0698591
10	1.27254	0.0698591
11	2.0926	0.0698591
12	2.20407	0.0698591
13	1.21488	0.0698591
14	1.26024	0.0698591
15	0.794454	0.0698591
16	1.95087	0.0698591
17	0.953925	2.1
18	0.98949	2.1
19	1.27875	2.1
20	1.04598	2.1
21	0.594005	2.1
22	0.578861	2.1
23	1.09506	2.1
24	1.45327	2.1
25	2.58396	2.1
26	1.58581	2.1
27	1.5858	2.1
28	0.636946	2.1
29	0.94202	2.1
30	0.946119	2.09788
31	0.636953	2.1
32	0.946107	2.1
33	1.09505	2.1
34	1.27389	2.09249
35	1.00232	2.1
36	1.00231	2.1
37	1.83583	2.1
38	1.00231	2.1
39	1.28828	0.0698591
40	1.81696	0.0698591
41	1.28828	0.0698591
42	1.28828	0.0698591
43	1.0044	0.0698591
44	0.85148	0.0698591
45	0.851539	0.0698591
46	1.57767	0.0698591
47	1.52227	0.0698591
48	0.730657	0.0698591
49	0.94613	0.0698591
50	1.28228	2.1
51	1.64268	2.1
52	1.10101	2.1
53	1.3939	2.1
54	0.959471	2.1
55	1.10101	2.1

56	1.2873	2.1
57	1.59659	2.1
58	1.80841	0.668814
59	1.80841	1.99959
60	0.946129	0.812644
61	1.36991	0.333122
62	1.32262	2.08655
63	1.32261	1.20018
64	1.38464	1.23047
65	0.962877	0.700156
66	0.962878	0.214169
67	1.82129	0.259092
68	1.82129	0.842869
69	0.94613	0.701859
70	1.39751	0.767248
71	0.946131	0.471509
72	1.34493	0.153013
73	1.39751	0.206513
74	0.837577	0.212198
75	0.823478	0.199332
76	1.10889	0.285595
77	1.01686	0.226458
78	1.1942	0.287012
79	0.836129	0.168469
80	0.937165	0.169275
81	0.969438	0.178263
82	0.895386	0.117815
83	1.30554	0.220379
84	1.15798	0.144628
85	1.22578	0.251743
86	1.15255	0.133899
87	0.9765	0.146015
88	2.02178	0.297676
89	1.63755	0.258454
90	0.727713	0.173082
91	0.810945	0.212241
92	1.00559	0.138807
93	0.879098	0.152043
94	1.07852	0.227919
95	0.657764	0.0902021
96	0.829406	0.183949
97	0.720201	0.165958
98	0.946131	0.0704108
99	1.11827	0.0755611
100	1.50754	0.0720822
101	1.64835	0.164998
102	1.71903	0.208402
103	0.851554	0.0706573
104	1.75626	0.203298
105	2.1646	0.257525
106	1.5563	0.215457
107	1.5563	0.291443
108	2.10876	0.350244
109	1.05438	0.317076
110	0.946133	0.155802
111	1.05438	0.559814
112	1.20256	0.205606
113	1.47879	1.17316

114	1.47879	1.33715
115	0.77815	1.43549
116	0.947114	2.1
117	1.31257	2.1
118	1.09235	2.1
119	0.946132	2.1
120	1.54246	2.1
121	1.54246	2.1
122	1.09235	2.1
123	0.946131	2.1
124	1.29507	2.1
125	1.0353	2.1
126	1.03529	2.1
127	1.28667	2.1
128	1.44777	2.1
129	1.61121	2.1
130	1.22176	0.0777863
131	0.946129	0.119609
132	1.08848	0.0698591
133	0.946131	0.115628
134	1.61201	0.0719673
135	1.61201	0.0698591
136	1.19993	0.0892789
137	1.35822	0.0796966
138	1.1539	0.0702897
139	1.00765	0.0698958
140	1.28228	2.1
141	2.65339	2.1
142	1.28228	2.1
143	1.55998	2.1
144	1.00509	2.1
145	0.93845	2.1
146	1.27674	2.1
147	0.810958	2.1
148	1.91381	2.1
149	1.1861	2.1
150	1.24886	2.1
151	2.02842	2.1
152	0.991436	0.0698591
153	1.34588	0.0698591
154	2.02729	0.0698591
155	0.72904	0.0698591
156	1.06139	0.0698591
157	0.835368	0.0698591
158	1.63819	0.0698591
159	1.10745	0.0698591
160	1.79925	0.0698591
161	1.00085	0.0698591
162	0.946133	2.1
163	1.41123	2.1
164	1.8327	2.1
165	1.8327	2.1
166	0.946132	2.07929
167	1.41123	2.1
168	1.3676	2.1
169	1.11048	2.1
170	1.11048	2.1
171	1.65815	2.1

172	1.9004	2.1
173	1.88691	2.1
174	1.82232	2.1
175	1.46591	2.1
176	1.09115	2.1
177	1.02051	1.68796
178	1.03373	2.03308
179	1.10307	2.1
180	1.05556	2.1
181	1.82533	0.728705
182	1.19496	0.539621
183	1.28597	2.1
184	1.20421	2.1
185	2.23644	2.1
186	1.08378	2.1
187	0.983724	2.1
188	0.602075	2.1
189	1.45876	2.1
190	1.45875	2.1
191	0.602103	2.1
192	1.11822	2.1
193	0.946133	2.1
194	0.602103	2.1
195	1.20421	2.1
196	1.26371	0.33741
197	1.55179	0.157365
198	1.14042	0.112066
199	1.56876	0.127172
200	2.27496	0.255666
201	1.13748	0.196213
202	1.02884	0.388202
203	1.25124	0.323085
204	0.82703	0.201499
205	0.607775	0.342882
206	1.49242	0.304997
207	1.35674	0.201534
208	1.6697	2.1
209	1.81655	2.1
210	0.900108	1.88392
211	0.851537	1.32383
212	0.703076	2.00823
213	0.639143	1.75808
214	1.42358	1.91744
215	1.4976	2.02571
216	0.86284	1.8391
217	1.2965	1.88015
218	1.27835	2.1
219	1.00012	1.10729
220	1.74332	2.05488
221	1.08576	2.1
222	1.19 2.1	
223	1.08576	2.1
224	1.20854	2.1
225	0.946131	2.1
226	0.946131	2.1
227	1.75985	2.1
228	1.75985	2.1
229	1.41232	2.1

230	1.29365	2.1
231	0.973559	2.1
232	1.23698	0.0698591
233	1.45055	0.0698591
234	1.26947	0.0698591
235	0.856218	0.0698591
236	1.75303	0.0698591
237	1.14938	0.0698591
238	0.984711	0.0698591
239	2.05771	0.0698591
240	0.625171	0.0698591
241	0.965675	0.0698591
242	1.17631	0.0698591
243	1.4677	0.0698591
244	1.35403	0.0698591
245	0.975149	0.0698591
246	0.946131	0.0698591
247	1.80893	0.0698591
248	1.80893	0.0698591
249	0.975149	0.0698591
250	0.946132	0.0698591
251	1.9503	0.0698591
252	2.80207	0.0698591
253	1.76411	0.0698591
254	2.77469	0.0698591
255	1.9503	0.0698591
256	1.26784	0.0698591
257	2.31093	0.0706476
258	0.851541	0.0816488
259	1.39521	0.0698591
260	1.45613	0.0715522
261	0.851541	0.0838151
262	0.996013	0.0698591
263	1.07989	0.0758002
264	0.946131	0.0757609
265	1.08213	0.0705173
266	1.74289	0.0700014
267	1.75838	0.0708019
268	2.11163	0.0790866
269	1.23773	2.1
270	1.25398	2.1
271	1.0304	2.1
272	1.32368	2.1
273	1.11712	0.80043
274	1.07999	0.490299
275	1.1766	2.1
276	1.35735	2.1
277	1.089 0.0698591	
278	1.81193	0.0698591
279	1.11081	0.0698591
280	1.78069	0.0698591
281	0.717512	0.0698591
282	0.810946	0.0698591
283	0.703901	0.0698591
284	1.09028	0.0698591
285	0.958031	0.0698591
286	0.892585	0.0698591
287	0.873431	0.0698591

288	0.997755	0.0698591
289	1.85479	0.0698591
290	0.983029	0.114592
291	1.27922	0.124219
292	1.64346	0.431335
293	1.27922	0.178964
294	1.09795	2.06652
295	1.07508	1.56194
296	1.02975	2.03857
297	1.01121	2.1
298	1.72369	0.0698591
299	1.12035	0.0698591
300	2.13511	0.0698591
301	0.9705	0.0698591
302	0.946131	0.0698591
303	0.86182	0.0698591
304	1.51379	0.0698591
305	1.51379	0.0698591
306	0.946132	0.0698591
307	0.861849	0.0698591
308	1.34591	0.358597
309	1.14721	0.223636
310	1.15539	2.1
311	1.90938	2.1
312	2.19436	2.1
313	1.41563	2.1
314	0.785641	2.1
315	0.736384	2.1
316	1.46489	2.1
317	1.46488	2.1
318	0.736372	2.1
319	0.946132	2.1
320	1.90941	2.1
321	1.18907	2.1
322	1.20409	2.1
323	0.973559	2.1
324	1.29461	2.1
325	0.946129	2.1
326	0.94613	2.1
327	1.78751	2.1
328	1.78751	2.1
329	1.38885	2.1
330	1.34479	2.1
331	1.07283	2.1
332	1.6869	2.1
333	1.52544	2.1
334	1.07797	2.1
335	1.13819	2.09926
336	0.776179	2.1
337	1.61092	2.1
338	1.36155	2.1
339	0.77618	2.1
340	1.2933	2.1
341	1.15946	2.1
342	0.69857	2.1
343	1.87607	2.1
344	1.07612	2.1
345	1.31976	1.26096

346	1.01243	1.97546
347	1.41279	1.94097
348	1.35146	0.0789061
349	1.90664	0.0749779
350	1.35145	0.0806781
351	1.22811	0.0999903
352	1.93147	0.0865031
353	1.42486	2.1
354	1.56558	2.1
355	1.0476	0.0698591
356	2.31605	0.0698591
357	1.89367	0.0698591
358	0.804706	0.0698591
359	0.783919	0.0698591
360	1.3116	0.0698591
361	1.03891	0.0698591
362	0.960473	0.0698591
363	1.08462	0.0698591
364	0.785994	0.0698591
365	1.1318	0.0698591
366	1.29576	0.0698591
367	1.3946	0.0698591
368	1.11924	0.0698591
369	0.834322	2.1
370	1.0256	2.1
371	1.74321	0.857023
372	1.41798	0.77837
373	0.871608	0.745805
374	1.47253	0.720146
375	1.47252	0.762073
376	0.817064	0.751937
377	0.817064	0.761647
378	0.946129	0.766194
379	1.63413	0.685957
380	1.9194	0.0698591
381	1.93562	0.0698591
382	2.06307	0.0698591
383	1.46021	0.0698591
384	0.955002	1.16436
385	0.833409	1.54526
386	0.838039	1.12032
387	1.48702	1.04484
388	1.1697	1.02883
389	1.23549	1.09569
390	1.18373	1.1318
391	2.02254	0.927529
392	1.68867	1.09382
393	0.875481	0.759075
394	0.821635	1.28839
395	0.858878	0.675242
396	1.30245	0.681044
397	1.07476	0.682904
398	1.11907	0.71243
399	1.10453	0.752152
400	1.70233	0.719337
401	1.70233	0.769287
402	2.6558	1.06484
403	1.7818	2.1

404	1.87059	2.1
405	1.34412	0.0698591
406	1.98068	0.0698591
407	2.56369	0.0698591
408	1.49512	0.0698591
409	1.35977	0.204023
410	1.34254	0.262911
411	1.75618	0.219174
412	1.75618	0.186981
413	0.946129	0.356647
414	0.946129	0.268191
415	1.17665	0.260425
416	1.01976	0.221219
417	1.27376	0.153377
418	1.35978	0.192421
419	1.68741	0.163604
420	1.68741	0.140346
421	0.946132	0.262758
422	0.946133	0.200408
423	1.14241	0.19303
424	0.996831	0.164883
425	0.996828	0.195114
426	1.12774	0.244337
427	1.13664	2.1
428	1.09627	2.1
429	2.8814	2.1
430	3.49751	2.1
431	1.79109	0.3181
432	1.54197	0.251804
433	2.75153	1.65673
434	1.93019	1.34239
435	1.51569	0.718725
436	1.55145	0.954551
437	1.78595	2.1
438	3.74452	2.1
439	1.66537	2.1
440	3.55161	2.1
441	1.35002	0.699397
442	1.19901	0.696355
443	1.61028	1.95865
444	1.35002	0.715828
445	1.65712	0.150949
446	1.10585	0.195613
447	0.961347	0.15906
448	0.810944	0.128508
449	1.16148	0.251322
450	1.08066	0.368755
451	0.934577	0.176284
452	1.05164	0.170308
453	0.79216	0.172993
454	2.99194	2.1
455	1.2832	2.1
456	2.97842	2.1
457	1.39517	2.1
458	1.03391	2.1
459	1.74416	2.1
460	1.05071	2.1
461	0.923782	2.1

462	1.55369	2.1
463	1.86444	2.1
464	1.01617	2.1
465	1.27174	2.1
466	1.20573	2.1
467	1.01616	2.1
468	1.13621	2.09206
469	1.75366	2.09025
470	2.27009	1.92961
471	1.6813	2.0224
472	1.47904	0.0698591
473	2.62714	0.0704428
474	1.80085	0.0835297
475	1.80085	0.0871522
476	2.74104	0.0811412
477	1.37052	0.0762963
478	0.946131	0.0698591
479	1.37052	0.0748925
480	1.31357	0.0698591
481	1.7837	0.179847
482	1.7837	0.158885
483	0.900397	0.0839743
484	1.74487	0.241639
485	2.88672	2.1
486	2.88249	2.1
487	1.0961	0.172849
488	1.20143	0.0715148
489	1.66739	0.117365
490	1.85795	0.0698591
491	1.45789	0.0698591
492	2.11407	0.0698591
493	0.713524	0.0698591
494	1.58391	0.0698591
495	1.58391	0.0698591
496	1.04765	0.0698591
497	1.24979	0.0698591
498	0.713551	0.0698591
499	0.946127	0.0698591
500	1.36768	0.0698591
501	1.14433	0.0698591
502	1.66923	0.0698591
503	0.699479	0.0698591
504	0.70351	0.0698591
505	0.758416	0.0698591
506	1.07678	0.0698591
507	0.909874	0.0698591
508	0.940218	0.0698591
509	0.876604	0.0698591
510	1.27148	0.0698591
511	2.42348	0.0698591
512	1.19837	0.0698591
513	0.764764	0.0698591
514	0.829259	0.0698591
515	0.740584	0.0698591
516	1.2772	0.0698591
517	0.964465	0.0698591
518	1.06231	0.0698591
519	1.06924	0.0698591

520	1.17566	0.0698591
521	1.3063	0.0699979
522	2.51253	0.0713287
523	2.42267	0.0709789
524	1.89226	0.0707732
525	1.46492	0.0698591
526	0.946132	0.0729316
527	0.653151	0.0706053
528	0.653124	0.0741044
529	1.53995	0.0698591
530	1.53995	0.0698591
531	0.936806	0.0699431
532	1.25626	0.0698591
533	1.1893	0.077514
534	1.62206	0.0698591
535	1.04921	2.1
536	1.22927	2.1
537	0.801765	2.1
538	0.774917	2.1
539	0.844623	2.1
540	0.737481	2.1
541	3.24379	2.1
542	2.48573	2.1
543	1.40967	1.12091
544	1.47203	1.35246
545	2.07659	0.816795
546	1.40967	1.23286
547	2.06638	0.852617
548	1.17418	2.1
549	1.04921	2.1
550	1.74593	2.1
551	1.74593	2.1
552	0.946132	2.1
553	1.38651	2.1
554	1.20761	1.95985
555	1.20761	2.1
556	1.30555	2.1
557	1.46812	0.441327
558	2.41602	0.684313
559	1.46812	0.606336
560	1.46812	0.529478
561	0.734059	0.559626
562	0.946132	1.44366
563	1.21261	0.937614
564	1.34225	0.742728
565	0.734084	1.04617
566	1.82077	0.837497
567	1.82077	0.6535
568	1.13751	0.291229
569	0.946131	0.642767
570	1.13751	0.471919
571	1.13748	0.315077
572	0.784392	0.564644
573	1.4906	0.409657
574	1.4906	0.209205
575	1.17879	1.52079
576	1.28291	1.35424
577	0.626495	2.1

578	1.45334	2.1
579	1.45334	2.1
580	0.626494	2.1
581	1.3852	2.1
582	0.946116	2.1
583	0.626499	2.1
584	1.47203	0.937013
585	1.42639	2.1
586	1.42639	2.1
587	0.795469	2.1
588	0.795469	2.1
589	0.946132	2.1
590	0.850877	2.1
591	1.37098	2.1
592	1.87194	2.1
593	1.76988	2.1
594	3.16295	0.0698591
595	3.26725	0.0698591
596	1.13322	2.1
597	1.20761	2.1
598	0.686933	0.2393
599	0.493248	0.0698591
600	1.2053	0.651122
601	0.947149	0.123321
602	1.27591	0.105515
603	1.75732	0.170299
604	0.493261	0.166539
605	1.34337	0.388488
606	2.73207	0.154695
607	1.19857	0.124792
608	0.671688	0.0794059
609	0.946134	0.326528
610	1.36603	0.111499
611	2.07817	0.116327
612	1.82573	0.181561
613	0.671699	0.117384
614	1.47772	0.0836804
615	0.975349	0.311471
616	1.22615	2.1
617	1.47868	2.1
618	2.29351	2.1
619	1.47642	2.1
620	2.94443	2.09911
621	1.80454	2.1
622	0.949135	2.1
623	0.949134	2.1
624	1.10476	0.116747
625	0.982857	0.188126
626	1.10938	0.124709
627	1.19052	1.22387
628	1.01012	0.0698591
629	1.35562	0.0698591
630	0.691742	0.0698591
631	2.39596	0.0698591
632	2.35604	0.0698591
633	0.974081	0.0698591
634	3.22444	0.0698591
635	1.3294	0.272873

636	1.37265	0.59874
637	1.3294	0.901919
638	1.51113	2.1
639	1.43643	2.1
640	1.72726	2.1
641	1.634 2.1	
642	0.755566	2.1
643	1.29319	2.1
644	0.755567	2.1
645	0.946131	2.1
646	1.29319	2.1
647	0.973563	2.1
648	0.904398	2.1
649	0.904406	2.1
650	1.1538	2.1
651	1.10437	2.1
652	0.935418	2.1
653	1.16649	2.1
654	0.975667	2.1
655	1.17952	2.1
656	1.95053	2.1
657	0.948676	2.1
658	1.44287	2.1
659	0.746095	2.1
660	1.51224	2.1
661	1.27354	2.1
662	0.746066	2.1
663	1.20573	2.1
664	1.12442	2.1
665	0.671487	2.1
666	1.04355	2.1
667	0.948675	2.1
668	0.967673	1.01603
669	1.12931	0.0698591
670	1.38212	0.0698591
671	2.40113	0.0698591
672	1.08054	0.0698591
673	1.04072	0.0698591
674	0.691062	0.0698591
675	1.58052	0.0698591
676	1.33502	0.0698591
677	0.946131	0.0698591
678	0.691062	0.0698591
679	1.02663	0.0698591
680	1.80569	0.0698591
681	1.26062	0.0698591
682	1.18203	0.0698591
683	0.946131	0.0698591
684	0.795065	0.0698591
685	1.58753	0.0698591
686	1.58753	0.0698591
687	0.946131	0.0698591
688	0.795093	0.0698591
689	0.805057	2.1
690	0.812415	2.1
691	1.23868	0.074175
692	1.34448	0.100371
693	1.67201	2.1

694	1.61202	2.1
695	1.41103	2.1
696	1.41103	2.1
697	0.841088	2.1
698	1.38302	2.1
699	0.841089	2.1
700	0.869097	2.1
701	1.38302	2.1
702	0.949941	2.1
703	0.94994	2.1
704	1.88608	2.1
705	0.854942	2.1
706	1.68893	2.1
707	1.70002	2.1
708	2.02107	0.117655
709	2.04982	0.117375
710	3.11063	0.155024
711	2.03805	0.113593
712	1.67638	2.1
713	0.967492	2.1
714	2.00209	2.1
715	0.967491	2.1
716	0.870742	2.1
717	1.30266	0.0698591
718	0.651334	0.0698591
719	1.69413	0.0698591
720	1.69413	0.0698591
721	0.946132	0.0698591
722	1.03079	0.0698591
723	1.31468	0.0698591
724	0.946132	0.0698591
725	0.919055	0.0698591
726	1.16815	0.0698591
727	2.79004	0.0698591
728	1.2136	0.0698591
729	2.62935	0.0698591
730	0.947692	0.202823
731	1.51835	0.177727
732	0.947693	0.128371
733	0.947692	0.14542
734	0.980841	2.1
735	1.43577	2.1
736	0.980841	2.1
737	1.67858	2.1
738	1.34124	0.336933
739	1.39818	0.250209
740	1.2065	2.1
741	0.946133	2.1
742	1.2079	2.1
743	1.65229	2.1
744	1.65229	2.1
745	0.946132	2.1
746	1.2079	2.1
747	1.39053	2.1
748	1.20651	2.1
749	1.36919	2.1
750	1.12717	2.1
751	1.09483	2.1

752	0.994294	2.1
753	0.97356	2.1
754	1.06827	2.1
755	1.40571	2.1
756	1.29259	2.1
757	0.946131	2.1
758	1.7787	2.1
759	1.7787	2.1
760	1.29259	2.1
761	0.946132	2.1
762	1.09408	0.763026
763	1.03824	0.336177
764	0.851541	0.353949
765	1.23608	0.352689
766	0.851539	0.343223
767	1.5021	0.334366
768	1.54839	0.351873
769	1.12373	0.335714
770	1.22617	0.331091
771	1.42762	0.473244
772	2.24746	0.439292
773	1.05475	0.097572
774	1.46845	0.0895814
775	1.05475	0.0698591
776	0.929726	0.0762258
777	1.52072	0.0829494
778	1.82488	0.0807475
779	1.02271	0.0781146
780	1.19225	0.0749097
781	1.43071	0.071956
782	0.94613	0.0781071
783	1.05475	0.0698591
784	1.39223	2.1
785	1.71255	2.1
786	0.946132	0.480424
787	1.10531	0.41967
788	1.46355	0.37819
789	1.46355	0.359043
790	0.78086	0.813197
791	1.10531	0.478705
792	1.1391	0.409147
793	1.56166	0.537397
794	2.21062	0.462056
795	1.0361	2.1
796	1.73577	2.1
797	1.73577	2.1
798	0.946116	2.1
799	1.28174	2.1
800	0.946118	2.1
801	1.22883	2.1
802	1.28172	2.1
803	1.03609	2.1
804	0.553567	0.0698591
805	1.60581	0.0698591
806	1.60581	0.0698591
807	0.553594	0.0698591
808	0.966748	0.0698591
809	0.966748	0.0698591

810	0.946133	0.0698591
811	1.05602	0.0698591
812	2.38525	0.0698591
813	1.14503	0.0698591
814	1.24678	0.0698591
815	0.56262	0.0698591
816	1.24678	0.0698591
817	0.978467	0.0698591
818	1.66262	0.0698591
819	1.66262	0.0698591
820	0.562633	0.0698591
821	1.09522	0.0698591
822	2.49356	0.0698591
823	0.592547	0.0698591
824	1.04321	1.62265
825	1.25133	1.64798
826	1.70508	1.30847
827	1.81043	1.24809
828	0.494934	1.11343
829	0.601047	0.854175
830	0.993428	1.28644
831	0.946573	1.15238
832	1.35039	0.923441
833	0.816895	1.19997
834	1.5063	1.09297
835	0.816895	0.904418
836	0.946132	0.984804
837	1.9067	1.40869
838	2.14504	1.42551
839	0.816895	1.11529
840	2.29624	0.99641
841	2.76933	0.463115
842	1.254 0.968162	
843	0.946128	2.1
844	1.38813	2.1
845	1.79998	2.1
846	1.79998	2.1
847	0.94613	2.1
848	1.38813	2.1
849	1.35798	2.1
850	1.27809	2.1
851	1.35798	2.1
852	1.68994	2.1
853	1.68994	2.1
854	0.946132	2.1
855	0.946132	2.1
856	1.18067	2.1
857	0.958256	2.1
858	0.958255	2.1
859	0.958257	2.1
860	0.958255	2.1
861	1.06457	2.1
862	1.14195	2.1
863	1.70423	2.1
864	1.14195	2.1
865	1.64285	2.1
866	2.02057	2.1
867	0.946132	2.1

868	1.44186	2.1
869	0.946133	2.1
870	1.16366	2.1
871	1.17233	2.1
872	1.31259	2.1
873	1.2016	2.1
874	1.17129	2.1
875	1.05724	0.546142
876	1.32697	2.09835
877	1.05724	1.02132
878	1.04292	2.1
879	3.39037	2.1
880	2.57275	2.1
881	0.629212	2.1
882	2.32525	2.1
883	1.76949	0.308098
884	1.65809	0.307429
885	1.27665	2.1
886	1.19861	2.1
887	1.20045	2.1
888	1.1635	2.1
889	0.953633	0.171016
890	0.953634	0.0940629
891	1.21408	2.1
892	1.25716	2.1
893	2.01791	2.1
894	1.89318	2.1
895	0.947921	2.1
896	0.939049	2.1
897	0.947921	2.1
898	0.740753	2.1
899	0.916104	2.1
900	1.16941	2.1
901	1.72038	2.1
902	2.04441	2.1
903	0.973182	2.1
904	1.11751	2.1
905	0.611089	2.1
906	1.53584	0.0760939
907	0.767923	0.170197
908	1.38925	0.127064
909	1.38926	0.117441
910	0.946131	0.205979
911	0.961347	0.164637
912	1.19583	0.105541
913	0.946131	0.163322
914	1.3663	0.233607
915	1.21067	0.0730506
916	1.00166	0.0754708
917	1.40707	0.0779138
918	1.50435	0.0786564
919	1.47954	1.08917
920	1.1233	2.1
921	2.36036	1.97432
922	1.04024	0.826318
923	0.739729	1.0255
924	1.45533	0.656247
925	1.45532	0.569718

926	0.946118	0.789445
927	1.15163	0.433773
928	1.15165	0.876803
929	0.739748	0.69397
930	1.04025	1.67079
931	1.47608	0.155268
932	0.946131	0.239624
933	1.02028	0.20506
934	0.946129	0.226546
935	1.78223	0.156792
936	1.78223	0.150558
937	1.34153	0.198755
938	1.38683	0.196222
939	1.3407	0.134109
940	1.3407	0.125247
941	2.04729	0.142227
942	0.851529	0.201459
943	1.26671	0.156265
944	0.946116	0.192978
945	1.4869	0.132947
946	1.29553	0.12877
947	1.03645	0.186917
948	1.20731	0.170401
949	0.73665	2.1
950	0.693596	2.1
951	2.11152	2.1
952	1.73627	2.1
953	1.06191	2.1
954	1.52226	2.1
955	1.76869	2.1
956	1.1881	2.1
957	1.32349	2.1
958	1.51041	2.1
959	1.61591	2.1
960	0.946132	2.1
961	0.946132	2.1
962	1.13537	2.1
963	3.0048	2.1
964	0.985109	2.1
965	1.68899	2.1
966	3.23261	2.1
967	1.08831	2.1
968	2.20623	2.1
969	0.91749	2.1
970	1.15515	2.1
971	0.550438	2.1
972	1.66294	2.1
973	1.08257	2.1
974	0.87851	2.1
975	2.09046	2.1
976	0.738711	2.1
977	1.31242	0.0698591
978	1.26311	0.0698591
979	1.67652	0.0698591
980	0.968594	2.1
981	0.968594	2.1
982	0.981099	0.0698591
983	1.32418	0.0698591

984	2.67175	0.0698591
985	2.09678	0.0698591
986	1.2511	0.0698591
987	0.912698	0.0698591
988	0.810946	0.0698591
989	0.810959	0.0698591
990	1.67479	0.0698591
991	1.65677	0.0698591
992	1.28469	0.0698591
993	1.48817	0.0698591
994	1.08131	0.0698591
995	1.04349	1.29665
996	1.42492	0.554763
997	1.05724	0.393689
998	1.65723	0.115347
999	1.95313	0.110514
1000	1.9293	0.105155
1001	2.02729	0.101195
1002	0.963393	1.61967
1003	0.963392	1.10193
1004	2.90171	2.1
1005	2.65447	2.1
1006	1.64158	2.1
1007	1.6488	2.1
1008	1.15991	0.161789
1009	0.946132	0.230397
1010	1.58199	0.174874
1011	1.58199	0.202002
1012	1.03333	0.239992
1013	1.25436	0.178948
1014	1.25436	0.213272
1015	0.946132	0.211751
1016	1.3771	0.192412
1017	0.687745	0.174061
1018	1.37709	0.158333
1019	0.812386	0.202253
1020	1.50172	0.190697
1021	1.50172	0.127545
1022	0.687758	0.205015
1023	1.17506	0.122734
1024	2.00539	0.14265
1025	2.2441	0.203948
1026	1.09667	0.126811
1027	1.43577	2.1
1028	2.24741	2.1
1029	1.26103	2.1
1030	0.851544	2.1
1031	1.5543	2.1
1032	1.59756	2.1
1033	0.851543	2.1
1034	1.44673	2.1
1035	1.31947	2.1
1036	1.1237	2.1
1037	1.26103	2.1
1038	1.089	0.0698591
1039	0.946128	0.071105
1040	0.965824	0.0701993
1041	0.94613	0.0715531

1042	1.51344	0.0698591
1043	1.51344	0.0698591
1044	1.13508	0.0698591
1045	1.32449	0.0698591
1046	1.089	0.0698591
1047	3.33706	0.129217
1048	2.7542	0.157315
1049	1.27256	0.254237
1050	0.954172	0.19787
1051	1.55527	0.188358
1052	1.05765	0.307477
1053	1.57326	0.250678
1054	1.38251	0.123152
1055	0.94613	0.239288
1056	2.38829	0.141454
1057	2.00539	0.149718
1058	1.53798	0.124551
1059	1.20969	2.1
1060	1.15864	2.1
1061	1.7986	2.1
1062	1.74533	2.1
1063	0.944827	1.4461
1064	0.946131	1.14658
1065	0.851543	1.33716
1066	1.52816	1.13254
1067	1.5314	1.54027
1068	1.39266	1.06125
1069	0.851528	1.08428
1070	1.39589	1.24907
1071	1.7348	1.08463
1072	1.85439	1.02794
1073	1.86367	1.18363
1074	1.90905	0.799759
1075	1.22176	0.0854138
1076	1.59492	0.0880617
1077	2.97929	2.1
1078	2.1905	2.1
1079	1.24128	2.1
1080	0.994293	2.1
1081	2.49767	0.0698591
1082	2.37105	0.0698591
1083	1.10834	2.03384
1084	1.53214	0.373202
1085	1.417	2.1
1086	2.13599	0.520868
1087	1.61727	2.1
1088	1.75004	2.1
1089	1.96301	2.1
1090	0.966631	2.1
1091	1.37791	2.1
1092	1.47302	2.1
1093	1.47302	2.1
1094	0.871519	2.1
1095	0.94613	2.1
1096	0.87155	2.1
1097	2.38731	2.1
1098	2.12399	0.877711
1099	1.74865	0.845904

1100	1.46724	0.392147
1101	2.38056	0.452474
1102	1.46724	0.399047
1103	0.818887	0.561606
1104	0.810944	1.19297
1105	0.805227	0.562348
1106	1.06558	0.583848
1107	0.841487	0.520845
1108	1.08456	0.624767
1109	0.903083	0.705622
1110	1.08929	0.0698591
1111	1.68454	0.0698591
1112	1.08929	0.0698591
1113	1.07658	0.0698591
1114	0.904439	0.0698591
1115	1.58501	0.0698591
1116	1.19729	0.0698591
1117	1.05427	0.0698591
1118	1.47764	0.0698591
1119	0.810959	0.0698591
1120	1.60946	0.792464
1121	2.28887	0.756067
1122	0.946133	0.817056
1123	1.14444	0.54949
1124	1.67964	0.465761
1125	1.67964	0.517166
1126	0.755334	0.820896
1127	1.14444	0.675406
1128	1.29054	0.59924
1129	1.37762	0.566257
1130	0.946132	0.724026
1131	0.533645	0.563094
1132	1.48361	0.462981
1133	1.48361	0.557418
1134	0.533645	0.665495
1135	0.872823	0.708962
1136	1.14444	0.555526
1137	1.37762	0.505023
1138	2.10101	0.481568
1139	1.02311	0.628085
1140	1.04083	2.1
1141	1.04084	2.1
1142	1.04084	2.1
1143	1.30116	2.1
1144	1.23477	0.0698591
1145	1.81893	0.0698591
1146	1.23477	0.0698591
1147	1.23477	0.0698591
1148	1.11967	0.0698591
1149	0.946131	0.0698591
1150	1.11966	0.0698591
1151	1.12628	0.0698591
1152	0.629641	0.0698591
1153	1.6163	0.0698591
1154	1.6163	0.0698591
1155	1.11117	2.1
1156	1.13006	2.1
1157	1.15149	2.1

1158	1.13179	2.1
1159	0.585368	1.10758
1160	0.986036	1.10036
1161	1.94788	0.275994
1162	1.34857	0.692408
1163	2.31806	2.1
1164	2.06363	2.1
1165	1.00423	2.1
1166	0.829065	2.1
1167	0.551816	2.1
1168	0.99406	2.1
1169	1.98339	2.1
1170	1.68073	2.1
1171	2.558 2.1	
1172	0.711814	2.1
1173	2.97316	2.1
1174	2.41579	2.1
1175	1.15248	0.0700508
1176	1.14305	0.0698591
1177	1.15483	0.0698591
1178	1.90981	0.0698591
1179	0.998957	0.0698591
1180	1.83742	0.0698591
1181	1.27581	2.1
1182	0.94613	2.1
1183	1.6315	2.1
1184	1.6315	2.1
1185	0.946132	2.1
1186	1.28441	2.1
1187	1.28441	2.1
1188	1.00365	2.1
1189	0.968536	2.1
1190	1.57575	2.1
1191	1.27581	2.1
1192	1.99297	2.1
1193	1.32784	2.1
1194	1.4259	2.1
1195	2.27952	2.1
1196	1.5685	2.1
1197	1.54231	2.1
1198	3.54638	2.1
1199	1.3294	2.1
1200	2.98237	2.1
1201	2.01633	0.430325
1202	2.0985	0.448537
1203	2.26047	0.243007
1204	1.7913	0.1842
1205	1.09504	2.1
1206	1.74126	2.1
1207	1.05983	2.1
1208	2.37664	2.1
1209	2.28084	2.1
1210	2.19948	2.1
1211	1.33593	0.0698591
1212	1.31508	0.0698591
1213	1.30647	2.1
1214	1.28846	2.1
1215	1.2955	2.1

1216	1.09044	2.1
1217	1.4099	2.1
1218	1.14984	2.1
1219	0.946132	2.09647
1220	1.70727	2.1
1221	1.70727	2.1
1222	1.14984	2.1
1223	0.946132	2.1
1224	0.911818	2.1
1225	2.12209	2.1
1226	2.4158	2.1
1227	1.20867	2.1
1228	2.84085	2.1
1229	1.42284	2.1
1230	2.45689	2.1
1231	1.41562	2.1
1232	0.918358	2.1
1233	1.11222	2.1
1234	0.999083	2.1
1235	0.696781	2.1
1236	1.30869	2.1
1237	1.66132	2.1
1238	0.696807	2.1
1239	0.745315	2.1
1240	1.23296	2.1
1241	0.780548	2.1
1242	1.93242	1.08742
1243	1.27362	1.94526
1244	2.77828	1.90096
1245	1.02653	0.64357
1246	0.808576	0.674254
1247	0.744012	1.2195
1248	0.605174	0.487008
1249	1.16501	0.386477
1250	0.803432	0.442692
1251	0.961016	0.391567
1252	1.10425	0.508393
1253	1.3756	0.895876
1254	2.17765	0.373054
1255	1.84578	0.323725
1256	1.32801	0.191843
1257	1.76377	0.203908
1258	0.946131	0.213443
1259	1.3022	0.348802
1260	0.694814	0.448641
1261	1.53028	0.216573
1262	1.53028	0.189543
1263	1.3022	0.315593
1264	0.922889	0.31893
1265	1.57766	2.1
1266	2.06911	2.1
1267	1.57766	2.1
1268	1.37721	0.0698591
1269	1.72174	0.0698591
1270	1.4468	0.0698591
1271	1.37721	0.0698591
1272	1.73972	0.1151
1273	1.62324	0.12095

1274	0.946125	0.0880103
1275	1.34259	0.076922
1276	1.04187	0.0705343
1277	1.05019	0.0777234
1278	1.2361	0.0928647
1279	1.69055	0.0698591
1280	2.25056	0.114136
1281	1.10574	1.84283
1282	1.21632	2.06177
1283	1.10574	2.08784
1284	1.46181	2.1
1285	0.946131	1.79725
1286	1.74111	1.93941
1287	1.74111	1.64305
1288	0.94613	1.52693
1289	1.26891	0.617697
1290	1.26891	0.675442
1291	1.23452	1.68619
1292	1.00255	1.95913
1293	1.72286	0.161115
1294	1.12233	0.11116
1295	1.30682	0.208697
1296	1.97397	0.239189
1297	1.67425	2.1
1298	1.77775	2.1
1299	1.73571	1.98962
1300	0.426265	2.05625
1301	1.31817	1.93135
1302	1.81042	2.08257
1303	1.85456	2.07878
1304	0.466532	1.90092
1305	1.28025	1.90598
1306	0.964467	1.83
1307	1.27921	2.09031
1308	2.11851	1.87386
1309	2.37121	1.96682
1310	1.49357	0.266436
1311	1.42486	2.1
1312	1.00278	2.1
1313	1.00278	2.1
1314	1.12503	2.1
1315	0.568697	2.1
1316	0.555211	2.1
1317	0.982272	2.1
1318	1.57558	2.1
1319	0.851539	2.1
1320	1.07307	2.1
1321	1.58292	2.1
1322	1.50731	2.1
1323	0.851541	2.1
1324	1.00304	2.1
1325	1.36003	2.1
1326	1.13127	2.1
1327	0.672234	2.1
1328	0.763044	2.1
1329	0.966979	2.1
1330	1.31502	2.1
1331	1.67969	2.1

1332	1.62408	2.1
1333	0.661955	2.1
1334	1.35048	2.1
1335	1.40447	2.1
1336	1.40447	2.1
1337	0.607961	2.1
1338	0.946131	2.1
1339	0.607963	2.1
1340	1.01531	2.1
1341	1.01531	2.1
1342	1.91802	2.1
1343	0.777749	2.1
1344	1.22585	0.0698591
1345	1.20672	0.0698591
1346	1.03166	0.171561
1347	0.553805	2.1
1348	2.00551	2.1
1349	2.07203	2.1
1350	0.589479	2.1
1351	1.22352	2.1
1352	0.984227	2.1
1353	0.802455	2.1
1354	1.40942	2.1
1355	1.6662	2.1
1356	0.802485	2.1
1357	0.936669	2.1
1358	1.31127	0.280139
1359	0.946132	0.367166
1360	1.09729	0.220504
1361	0.946133	0.401966
1362	1.68703	0.244481
1363	1.68703	0.189453
1364	1.3689	0.290255
1365	1.26426	0.300722
1366	1.26923	2.1
1367	1.23614	2.1
1368	0.828371	2.1
1369	2.04556	2.1
1370	0.772648	2.1
1371	0.791059	2.1
1372	0.809958	2.1
1373	1.70012	2.1
1374	0.267032	2.1
1375	1.67604	2.1
1376	0.803823	2.1
1377	0.267305	2.1
1378	0.80246	2.1
1379	0.874582	2.1
1380	1.00188	2.1
1381	0.709588	2.1
1382	1.50679	2.1
1383	1.22456	2.1
1384	0.870622	2.1
1385	1.05754	2.1
1386	1.1689	2.1
1387	1.54556	2.09584
1388	1.89226	2.08022
1389	0.995311	2.1

1390	1.26195	2.1
1391	1.9	2.1
1392	0.845055	2.1
1393	0.737836	2.1
1394	0.823707	2.1
1395	0.626587	2.1
1396	1.16109	2.1
1397	0.85517	2.1
1398	0.932036	2.1
1399	1.08321	2.1
1400	1.43863	2.1
1401	2.72297	2.1
1402	2.90798	2.1
1403	1.47818	1.20864
1404	1.47819	1.56123
1405	0.694357	1.12652
1406	0.694382	1.29946
1407	0.946132	0.593927
1408	0.694382	1.04739
1409	1.22641	0.745685
1410	1.31155	1.50063
1411	2.45282	1.32524
1412	0.787843	0.709387
1413	0.808984	0.443147
1414	1.14232	1.41074
1415	0.795037	1.26537
1416	0.887382	1.12416
1417	1.14493	0.903351
1418	0.78393	0.588322
1419	1.09129	1.12076
1420	0.791482	1.02985
1421	1.1137	1.52805
1422	1.11819	1.09378
1423	0.960173	1.29994
1424	1.09447	1.56716
1425	0.874365	1.02664
1426	1.2474	0.197978
1427	1.75038	0.384352
1428	1.75038	0.443252
1429	0.94613	0.859557
1430	1.30076	0.420767
1431	1.12817	1.22561
1432	0.94613	0.6453
1433	1.30076	0.53067
1434	1.51841	0.511458
1435	1.51841	0.617091
1436	0.946132	1.36362
1437	1.39575	0.58162
1438	0.970358	1.66032
1439	0.946133	0.997014
1440	1.39575	0.834798
1441	1.06486	0.294307
1442	1.06486	0.284945
1443	1.38622	0.341051
1444	1.04521	0.0698591
1445	1.00809	0.0698591
1446	1.28952	0.069861
1447	0.849316	0.0698591

1448	0.946132	0.0698591
1449	0.849344	0.0737838
1450	1.53631	0.0698591
1451	1.53631	0.0702389
1452	1.77843	0.0698591
1453	1.69863	0.0698591
1454	1.91646	2.1
1455	1.8582	2.1
1456	2.8349	0.0698591
1457	1.36307	0.0698591
1458	1.23886	0.0698591
1459	1.12971	0.0698591
1460	1.59662	0.0698591
1461	1.59662	0.0698591
1462	0.640304	0.0698591
1463	0.640333	0.0698591
1464	0.886276	0.0698591
1465	0.886276	0.0698591
1466	1.35065	0.0698591
1467	2.09002	0.0698591
1468	1.35543	0.0698591
1469	1.94688	2.1
1470	1.97221	2.1
1471	0.719563	2.1
1472	1.20358	2.1
1473	0.874264	2.1
1474	1.27676	2.1
1475	1.46557	2.1
1476	1.46557	2.1
1477	0.68546	2.1
1478	0.94613	2.1
1479	0.685489	2.1
1480	1.39615	0.0978212
1481	1.05404	0.0713733
1482	1.06474	0.0963364
1483	1.00713	0.0797044
1484	0.714535	0.0850276
1485	0.714563	0.116913
1486	1.41646	0.0708729
1487	1.52582	0.0698591
1488	0.721694	0.0804164
1489	0.93665	0.0762606
1490	1.21067	0.0716921
1491	1.21067	0.0717341
1492	1.73577	0.0896357
1493	1.17799	0.0707183
1494	3.21432	0.238802
1495	2.41452	0.224884
1496	1.36208	0.194029
1497	2.53256	0.23088
1498	0.834593	0.174394
1499	2.5853	0.202533
1500	1.77259	0.176316
1501	0.615724	0.362974
1502	1.11374	0.469665
1503	0.615726	0.296438
1504	0.946129	0.644233
1505	0.615714	0.528084

1506	1.75489	0.476725
1507	1.75489	0.379173
1508	1.23146	0.228511
1509	2.2772	0.276733
1510	0.74463	0.235099
1511	1.3546	0.293373
1512	1.80252	0.192053
1513	2.38304	0.225942
1514	1.19151	0.324237
1515	1.1231	0.358588
1516	1.82345	0.279048
1517	1.79805	0.247124
1518	0.761337	0.471172
1519	0.946127	0.355863
1520	0.557726	0.302703
1521	1.05353	0.117996
1522	1.05353	0.129896
1523	1.68599	0.184526
1524	1.05353	0.13647
1525	1.40588	2.1
1526	1.09373	2.1
1527	1.18382	0.919668
1528	1.46903	0.729274
1529	1.25103	2.1
1530	1.10378	2.1
1531	1.58941	2.1
1532	1.29639	2.1
1533	1.14874	0.0698591
1534	0.899817	0.0698591
1535	0.666898	0.0698591
1536	1.76519	0.0698591
1537	1.17289	0.0698591
1538	1.03526	0.0698591
1539	1.97708	0.0698591
1540	1.31711	0.0698591
1541	0.992517	0.0698591
1542	1.70193	0.0698591
1543	1.13852	0.0698591
1544	1.23943	1.57644
1545	1.40014	2.1
1546	1.04598	2.1
1547	1.1483	2.1
1548	1.11433	1.21622
1549	1.32037	0.422136
1550	1.00381	1.42182
1551	1.11435	0.239823
1552	0.973502	0.779314
1553	0.946128	1.93516
1554	1.83697	0.869166
1555	1.83697	2.1
1556	1.31721	1.84231
1557	1.38614	1.08096
1558	1.39696	0.927358
1559	1.31721	1.58953
1560	1.0756	1.86978
1561	1.08542	0.910292
1562	0.946127	2.09996
1563	1.81052	0.584746

1564	1.81052	2.08721
1565	1.24315	2.0988
1566	1.39696	0.403383
1567	1.35969	1.84799
1568	1.24315	1.7177
1569	1.0756	0.230868
1570	1.20669	0.126007
1571	1.18508	0.156109
1572	1.71059	2.08136
1573	1.66762	2.1
1574	2.50853	2.1
1575	1.8236	2.1
1576	1.78872	2.1
1577	1.41721	2.1
1578	1.01335	1.9242
1579	1.42439	0.619143
1580	0.946129	2.03124
1581	1.33266	1.55808
1582	1.79776	1.84325
1583	1.79776	2.09651
1584	0.946131	0.911917
1585	1.33266	1.96338
1586	1.41123	2.04964
1587	1.25346	2.1
1588	0.946132	0.703624
1589	0.827287	1.11323
1590	1.57878	0.692038
1591	1.57878	1.0731
1592	0.827287	0.520301
1593	1.07341	0.771861
1594	1.33266	0.900717
1595	1.25346	1.79545
1596	1.61149	2.1
1597	1.15644	1.97172
1598	1.27558	1.04491
1599	1.3764	0.583308
1600	1.89526	1.4175
1601	1.37641	0.505909
1602	1.2064	2.1
1603	1.38426	2.1
1604	1.01595	2.1
1605	1.15067	2.1
1606	1.82276	2.1
1607	1.82276	2.1
1608	0.946131	2.09755
1609	1.40609	2.09718
1610	0.946132	2.1
1611	1.29143	2.1
1612	1.40609	2.1
1613	1.30272	2.1
1614	1.19548	2.1
1615	2.95451	2.1
1616	1.46124	2.1
1617	2.93215	2.1
1618	1.42105	2.1
1619	1.75477	0.452611
1620	2.31301	0.452609
1621	2.10275	0.212567

1622	1.80099	0.222353
1623	0.97541	2.1
1624	1.49733	2.1
1625	0.97541	2.1
1626	1.30751	2.1
1627	1.28614	0.0698591
1628	1.74759	0.0848909
1629	1.19756	0.0698591
1630	1.71053	0.0755898
1631	1.19757	0.0854784
1632	1.28954	0.0700611
1633	2.02703	0.070121
1634	2.29453	0.0698591
1635	1.28265	0.0698591
1636	0.793267	0.0699054
1637	1.19974	0.0959867
1638	0.509551	0.0698591
1639	1.04072	0.0718194
1640	1.35302	0.0713044
1641	1.53133	0.122853
1642	0.566153	0.0715438
1643	1.09634	0.0930863
1644	1.12792	0.0928726
1645	1.68908	2.1
1646	1.68908	2.1
1647	0.768212	2.1
1648	0.768213	2.1
1649	0.946132	2.1
1650	0.768212	2.1
1651	1.32461	2.1
1652	1.87291	2.1
1653	2.64921	2.1
1654	1.53642	2.1
1655	2.26537	2.1
1656	1.53642	2.1
1657	1.79874	2.1
1658	1.79874	2.1
1659	0.726635	2.08381
1660	0.726636	2.1
1661	0.946128	2.09078
1662	0.726634	2.09076
1663	1.23339	2.07029
1664	0.992674	2.1
1665	1.8023	2.1
1666	1.12324	0.0698591
1667	1.29163	0.0698591
1668	0.996831	0.171147
1669	1.0019	0.162336
1670	0.993831	0.0856811
1671	1.07283	2.1
1672	1.04788	2.1
1673	1.44489	2.1
1674	1.04788	2.1
1675	1.07652	2.1
1676	0.991406	2.1
1677	1.37	2.1
1678	1.14195	2.1
1679	1.47904	0.0753563

1680	1.26593	2.0206
1681	1.75366	1.93887
1682	1.57621	0.111015
1683	1.24538	0.0811723
1684	0.969118	2.1
1685	0.946133	2.09299
1686	1.69387	2.1
1687	1.69387	2.1
1688	0.94613	2.1
1689	1.28713	2.1
1690	1.28713	2.1
1691	1.23877	2.1
1692	0.969118	2.1
1693	1.22615	2.1
1694	0.98084	2.1
1695	1.01843	2.1
1696	1.50876	2.1
1697	1.85391	2.1
1698	1.84322	2.1
1699	1.70377	2.1
1700	1.78919	2.1
1701	2.66787	2.1
1702	1.41549	2.1
1703	1.41549	2.1
1704	0.86707	2.1
1705	0.896172	2.1
1706	0.946126	2.1
1707	0.867074	2.1
1708	0.946118	2.1
1709	1.54468	0.0706248
1710	1.54468	0.0715353
1711	2.27259	0.0763341
1712	0.946131	0.118381
1713	1.25837	0.0931901
1714	0.663171	0.136891
1715	1.68931	0.0752651
1716	1.68931	0.0721901
1717	1.25837	0.0975136
1718	1.09411	0.0914918
1719	1.73571	0.070387
1720	2.34894	0.0739698
1721	0.946128	0.0853641
1722	1.30497	0.0759687
1723	0.772312	0.094257
1724	1.66895	0.0703339
1725	1.66895	0.0698591
1726	1.30497	0.0757471
1727	1.13629	0.0723187
1728	1.22852	0.441423
1729	1.578	0.415078
1730	2.07781	2.09745
1731	1.5478	2.08989
1732	1.70048	2.1
1733	1.42329	2.1
1734	1.05481	2.09467
1735	1.1032	1.97792
1736	1.19267	2.1
1737	1.77324	2.1

1738	1.01121	0.0796783
1739	0.991867	0.129412
1740	2.0744	2.09857
1741	1.75142	2.1
1742	1.35418	2.09862
1743	1.40041	2.1
1744	1.50045	2.1
1745	1.37704	2.1
1746	1.50045	2.1
1747	1.87875	2.1
1748	1.35308	0.301489
1749	1.29983	0.273293
1750	1.62784	2.1
1751	1.44807	2.1
1752	1.00965	2.1
1753	2.69264	2.1
1754	1.04075	2.1
1755	0.946131	2.1
1756	0.946132	2.1
1757	1.52704	2.1
1758	1.83246	2.1
1759	1.56757	2.1
1760	1.16687	2.1
1761	2.04857	2.1
1762	1.32707	2.1
1763	1.25519	0.46189
1764	1.32101	1.08062
1765	1.25055	2.06768
1766	0.827853	1.75237
1767	1.11835	0.981792
1768	1.07281	2.1
1769	1.54302	1.7252
1770	0.724064	2.03072
1771	1.32709	2.1
1772	3.44123	2.1
1773	2.46889	0.918301
1774	0.730076	0.0704531
1775	1.25809	0.148662
1776	0.730077	0.0817554
1777	0.946129	0.104554
1778	1.72777	0.0699086
1779	1.72777	0.144386
1780	0.73005	0.0991134
1781	1.7522	0.0723552
1782	2.51618	0.0698591
1783	0.628177	0.0701275
1784	1.1068	0.180231
1785	0.747435	0.0827934
1786	0.946133	0.139513
1787	1.84668	0.0699305
1788	1.8563	0.143826
1789	0.628177	0.135159
1790	1.45501	0.150409
1791	2.19809	0.129341
1792	1.45501	0.0720958
1793	1.48974	0.0699012
1794	1.7709	2.1
1795	2.63288	2.1

1796	1.5362	2.1
1797	1.56327	2.1
1798	3.03945	0.0698591
1799	0.899979	0.0698591
1800	0.869386	0.0698591
1801	1.85864	0.0698591
1802	1.78028	0.0698591
1803	1.28719	0.0698591
1804	1.09532	0.0698591
1805	0.881898	0.0698591
1806	1.28911	0.0698591
1807	1.39644	0.0698591
1808	0.688829	0.0698591
1809	2.14611	0.0698591
1810	1.80313	0.0698591
1811	0.68883	0.0698591
1812	0.946131	0.0698591
1813	1.25334	0.0698591
1814	1.88987	0.0698591
1815	1.94432	0.0698591
1816	1.37592	0.0698591
1817	1.11691	2.1
1818	1.02923	2.1
1819	0.45824	0.0698591
1820	0.646512	0.0698591
1821	2.46292	0.0698591
1822	0.493451	0.0698591
1823	2.00826	2.1
1824	2.49784	2.1
1825	1.17054	2.1
1826	1.01485	2.1
1827	1.26045	2.1
1828	1.17054	2.1
1829	1.44247	2.1
1830	2.02687	0.0698591
1831	1.66365	0.0698591
1832	1.64656	0.0698591
1833	1.56597	0.0698591
1834	1.21419	2.1
1835	1.5008	2.1
1836	1.10711	2.1
1837	1.44287	2.1
1838	1.44287	2.1
1839	0.750368	2.1
1840	1.25876	2.1
1841	0.82542	2.1
1842	1.04074	2.1
1843	1.25874	2.1
1844	1.04159	0.0698591
1845	1.2489	0.0698591
1846	0.980547	0.123472
1847	1.62235	0.0984621
1848	1.15991	0.126331
1849	0.980547	0.100952
1850	0.94613	0.152658
1851	1.54662	0.114323
1852	1.54661	0.132975
1853	1.07912	0.163323

1854	1.1032	0.116879
1855	1.1032	0.134371
1856	0.94613	0.131901
1857	1.12468	2.1
1858	1.2724	2.1
1859	1.06521	2.1
1860	1.0752	2.1
1861	1.94936	0.0921841
1862	1.62772	0.0904378
1863	1.94733	0.0801963
1864	1.42901	0.0813596
1865	1.33022	2.1
1866	1.70839	2.1
1867	1.75048	2.1
1868	1.41201	2.1
1869	1.52314	2.1
1870	1.44039	2.1
1871	1.51113	2.1
1872	2.58638	2.1
1873	0.987247	0.12097
1874	1.38622	0.325474
1875	0.858359	0.134629
1876	1.19967	0.187423
1877	1.06414	0.132252
1878	0.946132	0.320513
1879	0.94613	0.12811
1880	0.687079	0.538421
1881	0.946132	0.280874
1882	0.822611	0.313336
1883	1.70242	0.151488
1884	1.70242	0.280796
1885	0.946132	0.543432
1886	1.11155	0.181083
1887	0.946132	0.682389
1888	1.11155	0.404213
1889	1.37981	0.470783
1890	1.18348	0.172286
1891	2.44179	0.249359
1892	2.00403	0.431248
1893	1.10961	2.1
1894	1.85415	2.1
1895	1.44825	2.1
1896	0.932272	2.1
1897	0.810942	2.1
1898	1.00189	2.1
1899	1.11779	2.1
1900	1.3359	2.1
1901	0.965279	2.1
1902	0.833779	2.1
1903	1.05337	2.1
1904	0.526656	2.1
1905	1.44599	2.1
1906	1.44599	2.1
1907	1.24853	2.1
1908	0.946132	2.1
1909	1.24853	2.1
1910	0.724122	2.1
1911	1.91547	2.1

1912	2.49705	2.1
1913	1.05931	2.1
1914	1.1729	2.1
1915	1.34526	2.1
1916	1.5379	2.1
1917	1.90855	2.1
1918	2.08597	2.1
1919	1.68165	2.1
1920	1.45887	2.1
1921	1.50022	2.1
1922	1.4748	1.94915
1923	2.63645	1.81189
1924	1.74431	1.86831
1925	1.25288	1.65915
1926	1.25289	1.44528
1927	0.970025	1.58107
1928	0.747542	2.00495
1929	0.810959	1.49478
1930	1.15369	1.51624
1931	0.977472	1.51883
1932	1.12695	1.52884
1933	0.996762	1.57694
1934	1.28249	0.503104
1935	0.946132	0.555061
1936	0.528621	0.597527
1937	1.59186	0.362925
1938	1.44072	0.371812
1939	0.633488	0.491026
1940	0.598506	0.582463
1941	1.51618	0.520651
1942	1.19999	0.349568
1943	1.46511	0.309618
1944	1.44056	0.274727
1945	0.912754	0.264868
1946	1.4725	0.194142
1947	1.4725	0.185103
1948	0.912753	0.269343
1949	1.23351	0.236173
1950	1.23351	0.263236
1951	0.946131	0.307849
1952	1.17129	0.192037
1953	1.82551	0.173678
1954	1.27575	1.90226
1955	0.946129	1.9943
1956	0.946129	2.04025
1957	0.946128	1.90464
1958	0.792754	2.04052
1959	0.94613	1.86637
1960	0.792752	1.89425
1961	2.08054	2.0019
1962	1.20091	2.04203
1963	2.1669	1.95366
1964	1.24383	1.71
1965	0.904669	1.90815
1966	0.83804	1.57295
1967	1.90486	1.56446
1968	1.29162	1.53828
1969	1.37383	1.69629

1970	1.86533	1.62718
1971	1.76472	1.71522
1972	1.60428	1.66214
1973	2.43993	1.89046
1974	1.37833	2.1
1975	1.18632	2.1
1976	0.946132	2.1
1977	1.27544	2.1
1978	1.05085	2.1
1979	0.961178	2.1
1980	0.859844	2.1
1981	1.0499	2.1
1982	1.75592	2.1
1983	2.08668	2.1
1984	1.75592	2.1
1985	1.4402	2.1
1986	1.72285	2.1
1987	0.83312	0.0698591
1988	1.07295	0.0698591
1989	1.37607	0.0698591
1990	1.32097	0.0698591
1991	1.11332	0.0698591
1992	1.18568	0.0698591
1993	0.877482	0.0698591
1994	1.4458	0.0698591
1995	2.34112	0.0698591
1996	2.68021	0.0698591
1997	0.681677	0.0698591
1998	0.946131	0.0698591
1999	0.681649	0.0698591
2000	1.83115	0.0698591
2001	1.68029	0.0698591
2002	1.05482	0.0698591
2003	1.31485	0.0698591
2004	2.08247	0.70969
2005	1.33153	0.573174
2006	0.935062	0.925613
2007	0.806958	1.41039
2008	0.827683	0.849747
2009	1.01041	0.874936
2010	0.741832	0.753157
2011	1.15668	0.934187
2012	0.908649	0.914544
2013	0.59561	0.367834
2014	1.19028	0.509411
2015	0.59561	0.448682
2016	0.946132	0.613109
2017	0.595623	0.700732
2018	1.58164	0.520861
2019	1.58164	0.495435
2020	0.946131	2.1
2021	1.55201	2.1
2022	1.55201	2.1
2023	0.946127	2.1
2024	1.13312	2.1
2025	1.13312	2.1
2026	1.08108	2.1
2027	1.04973	2.1

2028	1.20149	2.1
2029	1.86902	1.71807
2030	0.613447	2.1
2031	1.22618	1.84428
2032	1.38663	1.98854
2033	1.38662	2.1
2034	0.613447	1.97599
2035	1.22617	2.04952
2036	0.773899	2.08177
2037	1.23515	1.95516
2038	1.87823	1.70412
2039	1.70303	0.644781
2040	1.4714	2.1
2041	1.74067	2.1
2042	0.735699	2.1
2043	1.73461	2.1
2044	1.47224	2.1
2045	1.22827	2.1
2046	1.1634	2.1
2047	1.36471	2.1
2048	0.735701	2.1
2049	2.8386	1.51969
2050	2.39056	2.1
2051	2.44128	2.06955
2052	1.54102	2.1
2053	1.54102	2.1
2054	0.936141	2.1
2055	1.40594	2.1
2056	1.07121	2.06973
2057	0.936142	2.1
2058	1.07121	2.1
2059	1.22691	0.0716521
2060	1.42907	0.0698591
2061	2.18048	0.0698591
2062	0.947662	0.0698591
2063	2.18394	2.1
2064	1.21292	2.1
2065	2.18394	2.1
2066	2.19064	2.1
2067	1.27229	2.1
2068	1.20859	0.474511
2069	1.21073	0.325238
2070	1.73899	2.1
2071	1.3164	2.1
2072	1.61245	2.1
2073	1.41444	2.1
2074	2.55116	2.1
2075	1.26193	2.1
2076	1.89553	2.1
2077	0.916438	2.1
2078	1.26113	2.1
2079	0.810934	2.1
2080	2.00419	2.1
2081	1.27872	2.1
2082	1.35306	2.1
2083	1.96749	2.1
2084	1.07955	2.1
2085	1.31854	2.1

2086	1.29505	0.324069
2087	1.29504	0.372614
2088	0.612469	0.530397
2089	0.990053	0.387113
2090	1.04072	0.498646
2091	0.985266	0.353146
2092	0.990039	0.338451
2093	1.06892	0.500201
2094	1.57158	0.250408
2095	1.895	0.317235
2096	1.05698	0.260284
2097	0.925053	2.1
2098	0.624712	2.1
2099	1.42465	2.1
2100	1.49014	2.1
2101	0.687198	2.1
2102	1.22058	2.1
2103	0.851488	2.1
2104	0.588128	2.1
2105	1.12452	2.1
2106	1.02783	2.1
2107	1.7083	2.1
2108	1.06977	0.21306
2109	0.818569	0.133617
2110	1.14386	0.174651
2111	0.983791	0.134038
2112	1.12214	0.230705
2113	1.0915	0.15921
2114	0.810934	0.182825
2115	0.764114	0.379752
2116	1.87956	0.287711
2117	1.87956	0.324658
2118	0.764141	0.424149
2119	1.37675	0.261455
2120	0.764141	0.392474
2121	0.946132	0.323481
2122	1.9637	0.279444
2123	2.10107	0.252545
2124	1.67814	0.408036
2125	3.02886	0.303931
2126	0.908982	0.0775747
2127	0.621727	0.163827
2128	0.525963	0.107332
2129	0.946128	0.174848
2130	1.60979	0.0799584
2131	1.45596	0.116715
2132	0.556172	0.16245
2133	2.06779	0.0777437
2134	2.17911	0.0843816
2135	1.00205	0.0698591
2136	1.30685	0.0783577
2137	1.16534	0.0852698
2138	1.65532	2.1
2139	2.20346	2.1
2140	0.993889	2.1
2141	1.09328	2.1
2142	2.09372	2.1
2143	2.10036	2.1

2144	1.33727	0.472661
2145	2.53383	0.349611
2146	0.893379	0.517012
2147	1.52828	0.43068
2148	1.09599	0.0698591
2149	1.09599	0.0698591
2150	1.50524	2.1
2151	1.54711	2.1
2152	1.41469	2.1
2153	1.99459	2.03525
2154	1.79805	2.1
2155	1.79805	2.1
2156	0.707344	2.1
2157	1.30318	2.1
2158	0.707344	2.1
2159	0.946129	2.1
2160	1.30318	2.1
2161	2.40442	2.1
2162	1.89226	2.1
2163	1.40794	2.09456
2164	0.898721	2.09235
2165	0.791921	1.78884
2166	0.810933	2.09443
2167	1.10904	2.06172
2168	0.991102	2.09101
2169	0.808502	1.71027
2170	1.1398	2.06987
2171	1.31346	2.1
2172	1.11833	2.1
2173	1.669	2.1
2174	1.25715	2.1
2175	1.3199	2.1
2176	1.63092	2.1
2177	1.63092	2.1
2178	0.946131	2.1
2179	0.946131	2.1
2180	1.13534	2.1
2181	0.979853	2.1
2182	2.19994	2.1
2183	0.725844	2.1
2184	2.19994	2.1
2185	2.35055	2.1
2186	1.52204	2.1
2187	1.40118	2.1
2188	3.42285	2.1
2189	2.05217	2.1
2190	1.50319	2.1
2191	0.947516	2.03584
2192	0.903572	2.04215
2193	0.76996	2.07157
2194	1.90959	2.1
2195	1.18897	2.1
2196	1.24166	2.1
2197	2.26785	2.09239
2198	2.05217	2.1
2199	2.73306	2.1
2200	1.42729	0.0699602
2201	1.387	0.0727744

2202	1.59458	0.348186
2203	1.91051	0.396968
2204	1.68108	0.202334
2205	2.03357	0.288254
2206	0.928982	0.0803276
2207	1.55687	0.091638
2208	1.55687	0.0698591
2209	0.92898	0.0754754
2210	1.3495	0.103098
2211	1.3495	0.0726914
2212	0.946133	0.0946624
2213	1.85795	0.0698591
2214	2.2727	0.0850141
2215	1.20324	2.06221
2216	1.07784	0.193547
2217	0.957792	0.13104
2218	1.2745	0.126406
2219	1.4659	0.145599
2220	0.946129	0.209039
2221	0.95778	0.198002
2222	0.851553	0.259286
2223	1.74103	0.14753
2224	2.28994	0.158206
2225	2.08213	0.118731
2226	2.1556	0.131195
2227	2.20372	0.108287
2228	0.838027	0.0698591
2229	1.11373	0.0698591
2230	1.2909	0.0698591
2231	0.998424	0.0698591
2232	1.40621	0.0698591
2233	1.19478	0.0698591
2234	1.11783	0.0698591
2235	1.6695	0.0698591
2236	1.7498	0.0698591
2237	0.477652	0.0698591
2238	1.77291	0.0698591
2239	0.590448	0.0698591
2240	1.07668	0.0698591
2241	0.810946	0.0698591
2242	1.1946	0.0698591
2243	2.03914	0.0698591
2244	1.25585	0.0698591
2245	2.02622	0.0698591
2246	1.39091	0.0698591
2247	1.31871	0.0698591
2248	1.15789	2.1
2249	1.15755	2.1
2250	1.82268	2.1
2251	1.87119	2.1
2252	3.10636	2.1
2253	2.96285	2.1
2254	1.73443	0.702742
2255	2.33812	0.696328
2256	1.59457	0.376869
2257	1.73443	0.93003
2258	2.00665	2.1
2259	1.99841	2.1

2260	1.19901	2.1
2261	1.16661	2.1
2262	2.15883	0.109468
2263	1.20951	0.110681
2264	2.49192	0.147767
2265	0.65112	0.100187
2266	2.45317	0.0698591
2267	1.73091	0.0698591
2268	2.0795	0.0698591
2269	1.45891	0.0698591
2270	1.6531	0.0698591
2271	1.78304	0.0698591
2272	0.775132	0.0698591
2273	1.35051	0.0698591
2274	1.0461	0.0698591
2275	1.37837	0.0698591
2276	1.00259	0.0698591
2277	1.25796	0.0727124
2278	1.10719	0.080507
2279	1.51067	2.1
2280	2.65632	2.07504
2281	1.30521	2.1
2282	2.456 2.1	
2283	3.19218	2.1
2284	2.67387	2.06442
2285	0.988862	2.1
2286	1.917 2.1	
2287	0.946132	2.1
2288	1.16202	2.1
2289	1.53871	2.1
2290	1.53871	2.1
2291	0.946132	2.1
2292	1.16202	2.1
2293	1.32282	2.1
2294	2.46356	2.1
2295	2.32404	2.1
2296	1.37562	0.0704845
2297	0.985546	0.072125
2298	1.28983	0.0698591
2299	1.05566	0.0698591
2300	0.895312	0.0698591
2301	1.53325	0.0698591
2302	1.04342	0.0698591
2303	1.80881	0.0698591
2304	0.876865	0.0698591
2305	0.5689	0.0698591
2306	1.09506	0.0698591
2307	1.51789	2.1
2308	1.49872	2.1
2309	0.949364	0.105347
2310	2.01233	0.0927069
2311	1.55063	0.100293
2312	1.57783	0.218375
2313	1.57783	0.220423
2314	0.674605	0.241556
2315	0.98537	0.121431
2316	0.674633	0.0906957
2317	0.98537	0.0959046

2318	1.26706	0.248123
2319	0.980664	0.427733
2320	1.03298	0.108725
2321	1.84936	0.377761
2322	1.51696	0.0839475
2323	1.97074	0.0950625
2324	1.03298	0.168197
2325	1.51324	0.0819935
2326	1.51324	0.0730899
2327	0.758454	0.070758
2328	1.28632	0.0726391
2329	0.758482	0.0698591
2330	0.758482	0.0698591
2331	0.985372	0.0773298
2332	1.14105	0.0698591
2333	1.59787	0.24649
2334	2.46244	0.317937
2335	1.70367	0.147285
2336	2.13757	1.62479
2337	1.98142	1.60943
2338	1.35878	0.0698591
2339	2.39947	0.0698591
2340	1.24554	0.0698591
2341	1.7522	0.0698591
2342	1.49693	1.65627
2343	1.49652	1.70923
2344	1.6211	1.74796
2345	1.45938	1.78997
2346	0.737431	1.96085
2347	1.18643	1.80055
2348	1.04072	1.99962
2349	0.9459	1.8779
2350	0.66364	1.94934
2351	0.985109	1.52056
2352	0.638368	1.89408
2353	0.83804	1.4351
2354	0.998024	1.36106
2355	0.914471	1.17856
2356	1.14281	1.43144
2357	0.833972	1.50437
2358	0.991865	0.0698591
2359	1.42208	0.0698591
2360	1.70718	2.1
2361	1.79888	2.1
2362	1.16851	0.348506
2363	1.16851	0.309822
2364	1.51021	0.450313
2365	0.946132	0.246818
2366	1.14897	0.148375
2367	0.946132	0.278625
2368	1.77177	0.258645
2369	1.77177	0.155801
2370	1.33038	0.218741
2371	1.38752	0.229033
2372	1.1726	0.157751
2373	1.51688	0.242954
2374	1.06455	2.1
2375	0.98511	2.1

2376	1.31144	2.1
2377	1.79825	2.1
2378	1.79825	2.1
2379	1.73044	2.1
2380	1.59589	0.0698591
2381	1.59589	0.0698591
2382	2.78009	2.1
2383	1.68761	2.1
2384	3.05511	2.1
2385	2.25318	2.1
2386	0.578998	2.1
2387	1.03512	2.1
2388	2.26156	2.1
2389	1.44859	0.246031
2390	1.35897	0.59146
2391	1.18301	2.1
2392	1.25173	2.1
2393	1.98851	2.1
2394	2.70685	2.1
2395	0.682342	2.1
2396	1.52491	2.1
2397	1.37119	2.1
2398	0.682313	2.1
2399	1.35342	2.1
2400	0.947555	2.1
2401	0.614107	2.1
2402	1.23007	2.1
2403	1.5735	2.1
2404	1.58713	2.1
2405	1.57979	0.0698591
2406	1.30723	0.0698591
2407	0.935658	0.546462
2408	0.8952	0.410952
2409	1.27352	1.36534
2410	1.0985	0.915209
2411	1.07163	0.728811
2412	1.15823	0.690811
2413	0.836451	0.639275
2414	2.08051	0.328415
2415	1.92601	1.56487
2416	2.0644	0.312391
2417	1.37259	2.1
2418	1.37259	2.1
2419	0.752217	2.1
2420	1.28569	2.1
2421	0.752186	2.1
2422	0.839124	2.1
2423	1.28568	2.1
2424	1.04199	2.1
2425	1.04199	2.1
2426	2.59268	2.1
2427	1.04199	2.1
2428	1.5098	2.1
2429	1.5098	2.1
2430	0.520997	2.1
2431	1.29634	2.1
2432	0.520996	2.1
2433	0.73446	2.1

2434	1.29634	2.1
2435	0.983724	2.1
2436	1.23914	2.1
2437	2.65095	2.1
2438	1.52851	2.1
2439	2.399	2.1
2440	1.68137	2.1
2441	1.11285	2.1
2442	1.04921	2.1
2443	1.30711	2.1
2444	1.04921	2.1
2445	1.07496	0.618629
2446	1.55831	0.2814
2447	1.07497	0.337874
2448	1.07497	0.826849
2449	1.27391	2.1
2450	2.5615	2.1
2451	1.2739	2.1
2452	1.75253	2.09613
2453	1.12907	0.0713923
2454	1.9194	0.0699192
2455	1.96617	0.0698591
2456	1.01225	2.1
2457	0.947979	2.1
2458	1.01225	2.09835
2459	1.06561	2.1
2460	1.01225	2.1
2461	3.56876	2.1
2462	3.68767	2.1
2463	1.52301	2.1
2464	1.61013	2.1
2465	1.43726	2.1
2466	2.04275	2.1
2467	0.995734	2.1
2468	1.16199	2.1
2469	1.73704	0.0698591
2470	1.04019	0.0698591
2471	0.956294	2.1
2472	1.81488	2.1
2473	1.63719	2.1
2474	1.50103	0.0698591
2475	1.75289	0.0698591
2476	1.31491	0.0698591
2477	1.03567	0.0698591
2478	0.672257	0.0698591
2479	1.04072	0.0698591
2480	0.746903	0.0698591
2481	1.43317	0.0698591
2482	1.39986	0.0698591
2483	1.06385	0.0698591
2484	1.1624	0.0698591
2485	1.61667	0.0698591
2486	1.27258	0.0698591
2487	2.559	0.0698591
2488	1.66093	0.0698591
2489	1.10719	0.0877251
2490	2.03235	0.086755
2491	1.17105	0.156049

2492	1.10719	0.0957643
2493	1.3028	2.1
2494	0.946131	2.1
2495	0.946127	2.09704
2496	1.52912	2.1
2497	1.52912	2.1
2498	0.946128	2.05963
2499	0.949997	2.08941
2500	1.31565	2.1
2501	1.46643	2.1
2502	1.5482	2.1
2503	1.47102	2.1
2504	1.47102	2.1
2505	0.774101	2.1
2506	1.30583	2.1
2507	0.774103	2.1
2508	0.939287	2.1
2509	1.30584	2.1
2510	0.958661	2.1
2511	2.28835	2.1
2512	1.43213	0.0698591
2513	2.13255	0.0700646
2514	1.43213	0.0698591
2515	1.10476	0.0698591
2516	1.93165	0.0698591
2517	1.03235	0.927682
2518	1.31	0.994834
2519	1.22082	2.1
2520	1.35827	2.1
2521	0.790936	2.1
2522	0.716644	2.1
2523	1.43257	2.1
2524	1.43257	2.1
2525	0.716615	2.1
2526	0.946131	2.1
2527	0.730157	2.1
2528	1.47212	2.1
2529	1.47212	2.1
2530	0.946131	2.1
2531	0.790792	2.1
2532	1.41149	2.1
2533	0.790791	2.1
2534	1.01006	2.1
2535	1.53045	0.481845
2536	1.53045	0.651528
2537	0.946132	1.33953
2538	1.40779	0.548699
2539	1.06879	1.58156
2540	0.990713	0.942648
2541	1.06879	1.07144
2542	1.06486	0.306258
2543	1.18347	0.285832
2544	1.38622	0.323744
2545	1.18347	0.187885
2546	1.7304	0.297127
2547	1.7304	0.401004
2548	0.946131	0.755611
2549	1.26874	0.385407

2550	1.13337	1.39123
2551	1.13337	0.615751
2552	1.40779	0.638253
2553	1.695 2.1	
2554	1.77071	2.1
2555	0.649313	2.1
2556	0.692829	2.1
2557	1.40395	2.1
2558	1.34243	2.1
2559	0.813292	0.130872
2560	0.632691	0.414946
2561	1.75766	0.339808
2562	2.25727	1.84077
2563	1.74206	0.114855
2564	0.826073	0.127757
2565	1.30022	0.129287
2566	1.45641	0.0913141
2567	0.946131	0.134272
2568	0.752245	0.0906966
2569	0.960952	0.169317
2570	0.743477	0.118585
2571	1.04355	0.0783355
2572	1.6253	0.115718
2573	0.960577	0.107864
2574	1.34852	0.554123
2575	1.03235	0.588339
2576	1.78168	0.175062
2577	1.2966	0.0698591
2578	2.21116	0.0698591
2579	1.6395	0.0698591
2580	1.6395	0.0698591
2581	0.94613	0.0698591
2582	1.22187	0.0698591
2583	0.962737	0.0698591
2584	0.962735	0.0698591
2585	1.36376	0.0698591
2586	1.2966	0.0698591
2587	1.2966	0.0698591
2588	1.19669	0.524457
2589	2.37636	0.666426
2590	1.08894	0.98712
2591	1.62551	0.792118
2592	2.51472	1.02017
2593	1.89226	0.65029
2594	0.980038	0.961265
2595	1.10187	2.1
2596	1.01479	2.1
2597	1.81663	2.1
2598	1.83247	2.1
2599	0.921851	0.252874
2600	0.983148	2.1
2601	1.82816	2.1
2602	1.26103	2.1
2603	2.34818	2.1
2604	1.23152	2.1
2605	2.15738	2.1
2606	1.23152	2.1
2607	1.51042	2.1

2608	0.842995	2.1
2609	1.22257	2.1
2610	0.810959	2.1
2611	1.68242	2.1
2612	0.95701	2.1
2613	1.12389	2.1
2614	1.95255	2.1
2615	1.57974	2.1
2616	1.94632	2.1
2617	0.81534	2.1
2618	1.13996	2.1
2619	1.18958	0.0698591
2620	1.25929	0.0698591
2621	3.02933	1.1744
2622	2.37434	0.638067
2623	1.10609	2.05673
2624	1.64132	1.35724
2625	1.64132	0.990149
2626	0.946132	0.441577
2627	1.40029	1.33553
2628	1.08946	0.344786
2629	1.08946	0.439948
2630	1.18717	0.138986
2631	2.71421	0.093708
2632	1.08189	0.0706206
2633	0.94613	0.0698591
2634	1.37109	0.0721675
2635	1.17925	0.0707547
2636	0.851541	0.0700204
2637	0.942512	0.0753812
2638	1.18541	0.0698591
2639	0.851526	0.074919
2640	1.0819	0.0698591
2641	1.86009	0.0755611
2642	0.946132	0.0698591
2643	1.67409	0.0698591
2644	1.67409	0.0698591
2645	0.946131	0.0698591
2646	1.31716	0.0698591
2647	1.30307	0.0698591
2648	0.946131	0.0698591
2649	1.18601	0.0698591
2650	3.15646	2.1
2651	0.985109	2.1
2652	3.37048	2.1
2653	1.10667	2.1
2654	1.77065	1.20485
2655	1.45837	1.04126
2656	1.77063	0.811555
2657	2.58104	0.76966
2658	1.37652	2.1
2659	1.54168	2.1
2660	1.00224	0.0698591
2661	1.05851	0.0698591
2662	1.39093	0.0698591
2663	1.43358	0.0698591
2664	0.575741	0.0698591
2665	0.518161	0.0698591

2666	1.04071	0.0698591
2667	1.13092	1.77646
2668	1.17042	2.02371
2669	1.53347	1.67504
2670	1.52938	1.76551
2671	1.00613	0.858023
2672	1.81618	2.1
2673	1.81618	1.0394
2674	0.946127	2.1
2675	1.36479	2.1
2676	0.94613	0.577946
2677	1.31724	1.70995
2678	1.3648	1.67176
2679	1.00613	0.9658
2680	0.781375	0.135453
2681	1.13431	0.167648
2682	1.03547	0.160956
2683	1.40668	0.174913
2684	0.94613	0.160716
2685	0.713285	0.210617
2686	1.72885	0.19421
2687	1.72885	0.211756
2688	0.713298	0.129065
2689	3.30075	0.112486
2690	2.81337	0.155548
2691	1.39296	0.235736
2692	0.830164	2.1
2693	1.50493	2.1
2694	1.50494	2.1
2695	0.830136	2.1
2696	1.28298	2.1
2697	1.0521	2.1
2698	0.830147	2.1
2699	2.0799	2.1
2700	1.52115	2.09992
2701	2.5142	2.1
2702	1.31253	0.428565
2703	1.94446	0.922169
2704	1.18817	0.642466
2705	0.598348	0.480234
2706	1.69315	0.53152
2707	1.69315	0.730195
2708	0.598377	0.488366
2709	0.598347	0.494363
2710	0.94613	0.470705
2711	1.28418	0.821532
2712	2.05205	1.08193
2713	1.28418	0.870902
2714	1.0737	2.1
2715	1.81024	2.1
2716	2.73834	2.1
2717	1.71369	2.1
2718	1.71368	2.1
2719	0.946118	2.1
2720	1.29063	2.1
2721	1.36917	2.1
2722	0.94613	2.1
2723	1.29062	2.1

2724	0.947921	2.1
2725	1.58661	1.82196
2726	1.37658	1.58855
2727	1.2364	1.46933
2728	1.66685	0.372973
2729	1.66685	1.92336
2730	0.946133	0.25428
2731	0.946133	0.881042
2732	1.11978	1.54929
2733	1.09194	0.358858
2734	1.21858	1.71118
2735	1.37658	1.20253
2736	1.64903	0.393886
2737	1.64903	2.08083
2738	0.946132	0.424408
2739	0.946132	1.05088
2740	1.05496	1.13199
2741	1.22851	0.296612
2742	1.22852	1.91879
2743	1.58482	0.199181
2744	1.60578	0.0909601
2745	1.06336	0.0861974
2746	1.68062	0.0933532
2747	1.14721	0.0754637
2748	1.11924	0.0698591
2749	1.18421	0.0698591
2750	2.01276	0.0698591
2751	1.23502	1.8147
2752	1.34926	1.93845
2753	0.546751	1.54223
2754	0.790446	1.75696
2755	1.04072	1.68233
2756	0.555868	1.44172
2757	0.930305	1.50337
2758	1.09344	1.51217
2759	1.18351	1.50314
2760	1.97215	1.58528
2761	1.07416	1.64544
2762	1.33204	1.3734
2763	1.22038	0.908087
2764	0.597449	0.832693
2765	0.821422	1.22596
2766	1.04071	1.19451
2767	0.608026	0.642088
2768	0.929427	1.054
2769	1.64929	0.0698591
2770	1.68541	0.0698591
2771	0.496093	0.0698591
2772	1.3385	0.0698591
2773	0.790798	0.0698591
2774	1.22971	0.0698591
2775	0.898853	0.0698591
2776	2.14361	0.0698591
2777	2.10813	0.0698591
2778	1.05417	0.0698591
2779	1.80768	0.0698591
2780	1.09725	2.1
2781	1.41131	2.1

2782	1.1656	2.1
2783	0.946129	2.1
2784	1.68365	2.1
2785	1.68365	2.1
2786	1.1656	2.1
2787	0.94613	2.1
2788	1.21269	0.0698591
2789	1.48867	0.0698591
2790	1.27806	0.0698591
2791	1.68264	0.0698591
2792	1.37813	1.70794
2793	1.50743	1.15699
2794	1.04121	1.3578
2795	1.12434	0.4794
2796	1.39553	0.525401
2797	1.44721	1.78848
2798	0.946132	0.676998
2799	1.23943	0.81742
2800	1.05292	1.07405
2801	1.28607	0.440168
2802	1.16915	0.339791
2803	1.76306	2.04139
2804	1.42266	1.90195
2805	1.36844	2.1
2806	1.36844	2.1
2807	1.67352	2.1
2808	1.11256	1.18937
2809	1.03217	0.949998
2810	1.18422	0.0698591
2811	1.04159	0.0698591
2812	1.60139	0.0698591
2813	1.04159	0.0698591
2814	0.988805	2.1
2815	1.05481	2.1
2816	1.52938	2.00949
2817	1.94724	1.76529
2818	0.896336	0.0756495
2819	0.911484	0.0755558
2820	1.18315	0.0698591
2821	1.10515	0.0699612
2822	1.04067	0.0698591
2823	1.12758	0.0717298
2824	0.904106	0.0721655
2825	1.6901	0.0698591
2826	1.68671	0.0698591
2827	1.99908	0.0698591
2828	0.772426	0.484223
2829	0.810945	1.80521
2830	0.667562	1.18813
2831	0.496617	0.26798
2832	0.847689	0.867932
2833	0.914378	1.34367
2834	0.934003	0.749766
2835	1.07459	0.727588
2836	1.40225	2.1
2837	1.07043	2.1
2838	0.94613	2.1
2839	1.63072	2.1

2840	1.63072	2.1
2841	1.07043	2.1
2842	0.946131	2.1
2843	1.53082	2.1
2844	1.69916	2.1
2845	1.75211	1.46233
2846	1.96831	2.1
2847	1.75211	2.07813
2848	0.946127	0.625809
2849	0.817065	0.583996
2850	1.41798	0.573236
2851	1.41798	0.469028
2852	0.791656	0.614724
2853	0.791656	0.639681
2854	1.41798	0.587
2855	1.58331	0.553263
2856	1.41776	0.422773
2857	0.791647	0.416375
2858	1.4512	0.381576
2859	1.45119	0.394134
2860	0.75821	0.488797
2861	0.758248	0.456854
2862	0.946104	0.469679
2863	1.36482	0.440948
2864	1.29389	1.98436
2865	1.04164	1.90127
2866	1.40574	1.75181
2867	1.64833	1.80475
2868	1.71183	0.151791
2869	1.71183	0.266726
2870	0.946131	0.59223
2871	1.35721	0.260101
2872	0.946131	0.971603
2873	0.946131	0.634414
2874	1.30076	0.572346
2875	1.2474	0.193163
2876	2.85225	0.357176
2877	2.71441	0.156782
2878	1.2474	0.131652
2879	1.38622	0.339434
2880	0.858359	0.123325
2881	1.3682	0.160909
2882	1.57364	0.124483
2883	0.729695	0.32
2884	0.729723	0.111491
2885	0.822611	0.664294
2886	0.946133	0.329448
2887	0.729723	0.187651
2888	0.974704	0.339664
2889	0.974702	0.33522
2890	1.81603	2.1
2891	1.96348	2.1
2892	1.43612	0.52669
2893	1.43612	0.856938
2894	0.634295	0.867858
2895	0.93796	0.906527
2896	0.946133	1.13139
2897	0.634326	0.455158

2898	0.946133	0.662325
2899	1.2654	0.345864
2900	1.26865	0.562205
2901	2.2649	0.530752
2902	1.00864	0.503295
2903	1.09689	0.358315
2904	0.832021	0.368659
2905	1.53229	0.321238
2906	1.1145	0.373073
2907	0.929583	0.363826
2908	1.613 0.287618	
2909	0.644729	0.346643
2910	1.03471	2.1
2911	2.7739	2.1
2912	1.36133	2.08641
2913	2.50215	2.1
2914	2.52195	2.1
2915	1.0465	2.1
2916	1.42673	2.1
2917	1.42673	2.1
2918	0.946132	2.1
2919	1.31904	2.1
2920	0.986499	2.1
2921	0.946132	2.1
2922	1.31904	2.1
2923	0.966978	2.1
2924	1.42237	0.0702325
2925	2.60925	0.0741703
2926	0.753659	0.0875542
2927	1.77241	0.071641
2928	1.77241	0.0770797
2929	0.946129	0.0931641
2930	0.75366	0.0711454
2931	1.22144	0.0851232
2932	0.94613	0.0850015
2933	1.50732	0.0714731
2934	1.18514	0.0763743
2935	0.902187	0.0839532
2936	1.89858	0.0698995
2937	1.24294	0.0724036
2938	1.67211	0.0752528
2939	1.41786	0.0703416
2940	0.810959	0.079915
2941	1.50732	0.0730125
2942	2.44288	0.0820197
2943	1.40969	0.0698591
2944	2.06654	0.0698591
2945	1.40969	0.0698591
2946	1.40882	0.0700633
2947	1.94063	0.0731407
2948	0.867921	0.0698591
2949	1.29289	0.0698591
2950	0.867918	0.0698591
2951	0.946128	0.0698591
2952	0.867944	0.0698591
2953	1.66589	0.0698591
2954	1.66589	0.0698591
2955	0.968595	2.1

2956	1.5534	2.1
2957	1.27793	2.1
2958	1.13433	2.1
2959	1.3439	0.0698591
2960	2.53709	0.0698591
2961	0.571998	0.0698591
2962	0.64837	0.0698591
2963	1.99755	0.0698591
2964	1.99854	0.0698591
2965	0.764229	2.1
2966	1.61842	2.1
2967	1.61842	2.1
2968	1.18315	2.1
2969	1.1995	2.1
2970	1.18315	2.1
2971	0.764256	2.1
2972	1.52364	2.1
2973	2.36631	2.1
2974	0.641912	2.04256
2975	1.7181	2.1
2976	1.459 2.1	
2977	0.634845	2.00391
2978	1.18315	2.1
2979	1.17615	2.1
2980	0.571367	2.09949
2981	1.3324	0.143739
2982	0.798214	0.14915
2983	0.946128	0.156205
2984	0.627252	0.138928
2985	1.50337	0.134215
2986	1.50337	0.101895
2987	0.62725	0.170547
2988	1.07763	0.0951558
2989	1.07763	0.127179
2990	2.16109	0.133186
2991	1.07763	0.124026
2992	1.12486	2.1
2993	1.11827	2.1
2994	1.0491	2.1
2995	1.09034	2.1
2996	1.09431	2.1
2997	0.946131	2.1
2998	0.748607	2.1
2999	1.44002	2.1
3000	1.44002	2.1
3001	0.748635	2.1
3002	0.782505	2.1
3003	1.40615	2.1
3004	1.19539	2.1
3005	1.29389	1.91521
3006	1.4867	1.32426
3007	1.50103	0.0698591
3008	1.63025	0.0698591
3009	2.72109	0.0698591
3010	0.915755	0.0698591
3011	0.946132	0.0698591
3012	0.915755	0.0698591
3013	1.70236	0.0698591

3014	1.70236	0.0698591
3015	1.26155	0.0698591
3016	1.35657	0.0698591
3017	1.83151	0.0698591
3018	1.83151	0.0698591
3019	2.52309	0.0698591
3020	0.983607	2.1
3021	1.06838	0.820518
3022	0.983609	2.1
3023	0.983596	2.06694
3024	2.74384	0.0878847
3025	1.6261	0.0712678
3026	1.15477	2.1
3027	1.02136	2.1
3028	1.83561	2.1
3029	1.83561	2.1
3030	0.946131	2.1
3031	1.39523	2.1
3032	1.29313	2.1
3033	1.29313	2.1
3034	1.38651	2.1
3035	1.06313	2.1
3036	0.974888	2.1
3037	1.0894	2.1
3038	0.974888	2.1
3039	1.82282	2.1
3040	1.82282	2.1
3041	0.946131	2.1
3042	1.37372	2.1
3043	1.32374	2.1
3044	1.32374	2.1
3045	1.39523	2.1
3046	1.40882	0.0717991
3047	0.861998	0.0725918
3048	1.24448	0.0773799
3049	0.861999	0.0737511
3050	0.946129	0.0835879
3051	0.862011	0.0812216
3052	1.67537	0.0778879
3053	1.67537	0.0707395
3054	1.72399	0.0698591
3055	2.58579	0.0698591
3056	1.72399	0.0698591
3057	1.50951	0.0698591
3058	2.48897	0.0711904
3059	1.26755	2.1
3060	1.03824	2.1
3061	1.27875	2.1
3062	1.48901	2.1
3063	1.18902	2.1
3064	1.11764	2.1
3065	1.87436	2.1
3066	0.95345	2.00722
3067	0.724191	1.7759
3068	0.86668	1.81265
3069	0.811523	1.88786
3070	1.04074	1.50536
3071	0.843097	1.68059

3072	0.833434	1.85723
3073	0.965727	1.79709
3074	0.85346	1.77085
3075	1.00185	0.899521
3076	1.00184	1.74268
3077	0.980651	1.88015
3078	0.980664	1.6783
3079	0.475737	1.43403
3080	0.719696	1.44109
3081	0.946109	1.07007
3082	0.946116	1.22723
3083	0.719693	1.34784
3084	0.946117	1.22692
3085	1.14879	1.79794
3086	0.719683	1.69603
3087	0.900977	1.34862
3088	0.972364	1.45418
3089	1.22961	1.41712
3090	0.405384	1.30794
3091	1.34406	2.1
3092	1.2187	2.1
3093	0.963571	0.502472
3094	0.946131	1.2656
3095	1.57246	1.17627
3096	1.57246	2.1
3097	0.946131	1.22318
3098	1.1235	2.1
3099	1.1235	2.08158
3100	1.07439	2.09657
3101	1.19612	1.39157
3102	1.24844	1.4753
3103	1.00007	2.01288
3104	1.02429	2.1
3105	1.10459	2.1
3106	1.06724	0.204996
3107	0.67519	0.50493
3108	0.49762	0.237567
3109	1.43435	0.170735
3110	0.833315	0.374592
3111	0.58135	0.271722
3112	1.14749	0.70092
3113	1.85091	1.71984
3114	1.2802	0.529641
3115	1.27737	1.29055
3116	2.3655	0.948005
3117	1.30584	2.1
3118	2.06424	2.1
3119	0.946131	0.0773291
3120	1.04257	0.0910252
3121	0.94613	0.0991332
3122	1.67201	0.0881068
3123	1.67201	0.0725413
3124	1.27696	0.0768397
3125	1.34118	0.0699052
3126	1.3413	0.0765657
3127	2.36238	0.0725999
3128	0.6036	0.100373
3129	1.11745	0.112261

3130	1.46126	0.0855272
3131	1.39612	0.0736268
3132	0.670678	0.0990366
3133	1.03402	0.109879
3134	1.11271	0.102718
3135	0.838028	0.0710221
3136	0.807019	0.082962
3137	1.27787	0.0698591
3138	0.994705	0.0701711
3139	1.20014	0.0698591
3140	0.964793	0.0784931
3141	0.863868	0.0720981
3142	1.3413	0.0698628
3143	1.79549	0.0712426
3144	1.35877	2.1
3145	1.63648	2.1
3146	1.08171	2.1
3147	1.27839	2.1
3148	1.52601	2.1
3149	1.27841	2.1
3150	1.71255	2.1
3151	2.16307	2.1
3152	1.91592	2.1
3153	1.78508	2.1
3154	0.946128	2.1
3155	1.41199	2.1
3156	0.946128	2.1
3157	0.901088	2.1
3158	1.55244	2.1
3159	1.59475	2.1
3160	1.40921	2.1
3161	0.960106	2.1
3162	1.40921	2.1
3163	2.0073	2.1
3164	2.2973	0.113514
3165	1.90705	0.119747
3166	1.53671	0.0837652
3167	1.86035	0.0809772
3168	1.33747	2.1
3169	1.14007	2.1
3170	1.3212	2.1
3171	1.66867	0.0698591
3172	1.30203	0.0698591
3173	1.27696	2.1
3174	1.05025	2.1
3175	1.05025	2.1
3176	1.3704	2.1
3177	1.15677	2.1
3178	0.946129	2.1
3179	1.65879	2.1
3180	1.65879	2.1
3181	1.15676	2.1
3182	0.94613	2.1
3183	0.867217	1.52852
3184	1.28684	1.39062
3185	1.58866	1.19466
3186	1.58866	0.807846
3187	0.867246	1.80738

3188	1.28684	1.23972
3189	1.16906	1.44478
3190	1.13438	1.18014
3191	1.37224	1.41326
3192	1.61519	1.52604
3193	0.883939	1.84181
3194	1.40092	1.58021
3195	1.80383	1.7865
3196	1.80383	1.58733
3197	0.883935	1.87515
3198	1.40092	1.50894
3199	1.28684	1.75109
3200	1.76788	1.16445
3201	2.57368	1.32714
3202	1.37224	1.84354
3203	1.65411	0.837087
3204	1.77042	2.1
3205	1.77042	2.1
3206	1.90446	2.1
3207	1.28667	2.1
3208	1.55635	2.1
3209	1.30646	0.248514
3210	2.31836	0.0965864
3211	1.30645	0.09881
3212	1.86429	0.176325
3213	1.48676	0.1094
3214	0.743379	0.117539
3215	1.41848	0.114841
3216	1.41848	0.100235
3217	0.946131	0.113469
3218	0.812649	0.105765
3219	1.34921	0.111432
3220	0.812649	0.114319
3221	0.949366	0.105129
3222	1.32974	0.100436
3223	0.794204	0.0698591
3224	0.634352	2.1
3225	0.838025	2.08838
3226	1.08802	2.04044
3227	0.838038	2.01347
3228	1.18314	2.04126
3229	1.12495	2.05391
3230	1.22774	2.0316
3231	1.04343	2.04801
3232	0.921111	0.0698591
3233	2.1609	0.0698591
3234	1.09987	0.0698591
3235	2.5043	0.0698591
3236	1.05492	0.0698591
3237	0.590344	0.0698591
3238	1.05492	0.0698591
3239	1.25215	0.0698591
3240	1.71674	0.0698591
3241	1.71674	0.0698591
3242	0.590331	0.0698591
3243	1.17078	0.0698591
3244	0.625542	0.0698591
3245	0.724673	2.1

3246	1.08011	2.1
3247	1.87598	2.1
3248	1.50738	2.1
3249	1.44951	0.103791
3250	1.50102	0.0987452
3251	1.8042	0.389794
3252	0.687769	0.738704
3253	1.66483	0.472717
3254	1.66483	0.543419
3255	0.946132	0.696993
3256	0.687798	0.474974
3257	1.20885	0.720048
3258	0.687799	0.617235
3259	0.886063	0.232459
3260	0.798751	0.253373
3261	0.584813	0.207178
3262	1.49483	0.148226
3263	0.888005	0.190322
3264	1.03589	0.169857
3265	1.70435	0.209383
3266	0.710414	2.1
3267	0.917609	2.06433
3268	0.810947	2.07792
3269	0.81096	2.05307
3270	1.48658	1.90198
3271	1.53653	2.00293
3272	1.19329	2.07114
3273	0.946153	2.00629
3274	1.50798	2.03937
3275	1.85017	2.1
3276	1.19768	2.1
3277	0.864304	2.1
3278	0.792906	2.09186
3279	0.989025	1.99118
3280	1.19768	1.83852
3281	1.54548	1.9822
3282	1.18798	2.06789
3283	1.31875	1.8513
3284	1.05725	1.99825
3285	2.17365	0.0698591
3286	2.48106	0.0698591
3287	1.87451	0.0698591
3288	1.27189	1.44092
3289	2.79399	1.86633
3290	0.859565	1.42468
3291	0.926174	1.28141
3292	0.604744	1.20769
3293	1.41832	0.603682
3294	1.05494	0.983997
3295	0.874749	0.972872
3296	1.50067	0.797266
3297	0.917817	1.64004
3298	0.63591	1.66126
3299	1.72849	1.38199
3300	1.72849	1.01609
3301	0.635943	1.26048
3302	1.39699	1.59723
3303	0.967411	1.9549

3304	0.635943	1.41238
3305	1.39908	1.06546
3306	1.26971	1.31322
3307	1.02969	1.70146
3308	1.15326	1.91466
3309	2.30741	1.78706
3310	0.93545	1.28448
3311	0.802747	0.948318
3312	1.15466	1.56284
3313	0.859164	1.65369
3314	1.02616	1.73167
3315	1.23149	1.47364
3316	0.83804	1.09125
3317	1.13266	1.92858
3318	1.64142	1.89629
3319	1.21223	2.1
3320	1.43051	2.1
3321	1.32418	1.14749
3322	0.621319	2.03948
3323	1.47134	2.08825
3324	1.79103	0.875653
3325	3.25951	0.0698591
3326	3.16764	0.0698591
3327	1.65583	0.18137
3328	1.4732	0.099513
3329	1.76476	2.1
3330	1.80114	2.09265
3331	0.754121	2.1
3332	0.94613	1.72544
3333	0.754119	2.1
3334	1.45636	2.06065
3335	1.45636	1.08964
3336	1.30029	1.9786
3337	0.910183	1.95272
3338	1.29099	2.1
3339	1.08039	2.1
3340	1.05498	2.1
3341	1.24445	2.1
3342	1.2873	2.1
3343	0.987703	2.1
3344	0.964782	2.1
3345	1.73645	0.0698591
3346	0.871279	0.0734193
3347	0.884744	0.0788632
3348	1.85193	0.0698591
3349	1.68681	0.0701617
3350	1.33689	0.0722251
3351	1.23493	0.0698591
3352	0.892482	0.0760292
3353	2.95185	0.0699777
3354	2.10496	0.0698591
3355	1.87334	2.1
3356	0.94411	2.1
3357	0.754196	2.1
3358	1.43122	2.1
3359	1.58684	2.1
3360	0.838027	2.1
3361	1.1536	2.1

3362	1.24878	2.1
3363	1.23125	2.1
3364	2.86862	2.1
3365	1.57636	2.1
3366	0.998451	0.353338
3367	1.5176	0.278885
3368	1.13271	0.0698591
3369	1.60248	0.0698591
3370	1.22083	2.1
3371	1.79996	2.1
3372	1.19087	2.1
3373	2.00342	2.1
3374	1.3715	2.1
3375	2.79227	2.1
3376	2.7873	2.1
3377	0.987265	0.0698591
3378	1.62715	2.1
3379	1.70117	2.1
3380	1.27806	0.0698591
3381	1.45246	2.1
3382	1.45244	2.1
3383	1.87214	2.1
3384	0.995796	2.1
3385	1.68189	0.879799
3386	1.49155	2.1
3387	1.58054	2.1
3388	1.59169	2.1
3389	0.736364	0.0698591
3390	0.70236	0.0698591
3391	2.24367	0.0698591
3392	2.57085	0.0698591
3393	1.70952	0.0698591
3394	2.60516	0.0698591
3395	1.45158	2.1
3396	1.00371	2.1
3397	1.65748	2.1
3398	1.23706	0.162856
3399	1.55834	1.1655
3400	1.72671	0.673114
3401	0.597656	0.480601
3402	1.39137	1.18235
3403	0.855578	0.122343
3404	1.48398	0.56222
3405	0.745117	0.143814
3406	1.23706	0.363608
3407	1.57916	0.772018
3408	0.893025	0.228885
3409	3.22433	0.277277
3410	2.47324	1.7747
3411	1.63852	2.1
3412	1.63852	2.1
3413	0.946132	2.1
3414	1.19095	2.1
3415	0.946133	2.1
3416	1.16684	2.1
3417	1.19095	2.1
3418	1.06698	0.16024
3419	1.48692	0.135745

3420	1.08334	2.00983
3421	1.07283	2.1
3422	1.28517	1.4963
3423	1.2746	0.0698591
3424	1.30723	0.0698591
3425	2.02069	0.0698591
3426	1.05579	0.0698591
3427	1.59475	2.1
3428	2.40113	2.09877
3429	1.58875	2.1
3430	1.6881	2.1
3431	1.6881	2.1
3432	0.819953	2.1
3433	0.819954	2.1
3434	0.946131	2.1
3435	0.946131	2.09571
3436	1.34782	2.1
3437	1.65619	2.04923
3438	1.65619	2.03087
3439	0.79437	2.0727
3440	1.25	2.04965
3441	0.94613	2.08091
3442	0.946131	2.04089
3443	0.794371	2.08381
3444	1.58875	2.08023
3445	2.5	2.05031
3446	1.04147	2.1
3447	0.946131	0.776917
3448	1.79686	2.1
3449	1.79686	2.1
3450	1.25144	0.750994
3451	1.35714	2.06162
3452	1.38585	1.89616
3453	1.25144	2.0847
3454	1.15564	2.1
3455	1.34389	2.1
3456	0.946133	0.678112
3457	1.74353	2.04821
3458	1.74353	2.1
3459	1.14553	0.640558
3460	1.38585	1.54879
3461	1.30382	1.13638
3462	1.14553	1.97242
3463	1.15564	2.1
3464	1.90618	2.1
3465	2.11056	2.1
3466	1.032	2.1
3467	0.953636	2.1
3468	1.23131	1.33829
3469	1.39763	1.08148
3470	2.01024	0.90639
3471	1.9186	0.983089
3472	1.83658	0.0852818
3473	1.81656	0.0887598
3474	2.2049	0.107942
3475	2.16081	0.0895639
3476	1.12219	0.109649
3477	0.599771	0.129326

3478	1.61401	0.0833994
3479	1.05912	0.106655
3480	0.951648	0.106482
3481	1.89328	0.0868708
3482	0.913682	0.124876
3483	1.00763	0.0934499
3484	1.29129	2.1
3485	1.37138	2.1
3486	1.41986	0.0698591
3487	1.41986	0.0715875
3488	0.944166	0.0725187
3489	1.41789	0.0698591
3490	0.944167	0.0698591
3491	0.944166	0.0698591
3492	0.946132	0.0698591
3493	1.98713	0.0698591
3494	1.23626	0.0698591
3495	0.932791	0.0698591
3496	0.810251	0.0698591
3497	0.986748	0.0703743
3498	0.898155	0.0709168
3499	0.92624	0.0698591
3500	1.1349	0.0698591
3501	0.934572	0.0698591
3502	1.23104	0.661573
3503	1.23104	0.816233
3504	0.799994	0.407892
3505	0.94613	0.675339
3506	0.802822	0.40267
3507	1.40944	0.620945
3508	0.800035	0.695034
3509	1.41223	0.608518
3510	1.41223	0.449125
3511	1.6	0.362629
3512	1.60565	0.41291
3513	1.23042	2.1
3514	1.0645	2.1
3515	1.00319	2.1
3516	1.29435	2.1
3517	1.2243	2.1
3518	1.2243	2.1
3519	1.05655	1.5141
3520	1.29764	1.73649
3521	2.55252	2.1
3522	1.15676	2.08647
3523	0.855295	2.07899
3524	0.810959	2.09135
3525	1.65857	2.08854
3526	1.00849	2.05175
3527	1.16021	2.08652
3528	1.75869	2.08842
3529	1.13775	2.08017
3530	1.20091	2.09576
3531	1.61582	2.1
3532	1.88031	2.1
3533	0.968536	2.1
3534	0.946131	2.1
3535	1.61481	2.1

3536	1.61481	2.1
3537	1.09547	2.1
3538	1.18486	2.1
3539	1.37609	2.1
3540	1.09546	2.1
3541	1.02461	2.1
3542	1.83223	2.1
3543	1.74002	2.1
3544	1.70939	2.1
3545	1.7094	2.1
3546	0.726287	2.1
3547	1.01712	2.0957
3548	1.30318	2.1
3549	0.946131	2.1
3550	0.87155	2.1
3551	1.77093	2.00736
3552	2.02356	1.97656
3553	1.32449	2.1
3554	1.10003	2.1
3555	1.5161	2.1
3556	1.5161	2.1
3557	0.946132	2.1
3558	1.09206	2.1
3559	0.946132	2.1
3560	1.04404	2.1
3561	1.09206	2.1
3562	0.635628	1.2101
3563	1.14623	0.354972
3564	1.73197	0.500443
3565	1.73197	1.09539
3566	0.529655	0.626782
3567	1.14623	0.584515
3568	0.992085	0.717172
3569	1.16915	1.30876
3570	2.29245	0.408206
3571	0.584573	0.557927
3572	0.956756	0.227429
3573	1.52471	0.40655
3574	1.51409	1.73893
3575	0.584575	0.392154
3576	0.94613	0.227422
3577	1.14622	0.307596
3578	1.28096	1.17998
3579	1.34695	1.55306
3580	1.04193	2.1
3581	1.05378	2.1
3582	0.798209	2.1
3583	1.32007	2.04117
3584	1.64327	0.0698591
3585	2.64674	0.373949
3586	2.38936	0.0698591
3587	2.88475	0.0698591
3588	1.07745	2.1
3589	1.08911	2.1
3590	1.49127	2.1
3591	1.49127	2.1
3592	0.709714	2.1
3593	1.37199	2.1

3594	0.82899	2.1
3595	0.709714	2.1
3596	0.946132	2.1
3597	1.00133	2.1
3598	1.40182	2.1
3599	1.53278	2.1
3600	1.40182	2.1
3601	1.31764	0.0698591
3602	1.00691	0.0698591
3603	1.24094	0.0698591
3604	0.64868	0.0698591
3605	0.946133	0.0698591
3606	0.64868	0.0698591
3607	1.71762	0.0698591
3608	1.71762	0.0698591
3609	1.41662	0.0698591
3610	0.975477	0.0698591
3611	1.73584	0.0698591
3612	2.48188	0.0698591
3613	2.2045	0.300493
3614	2.08134	0.261062
3615	0.993366	1.43054
3616	0.993368	1.41622
3617	1.14187	0.238109
3618	0.946132	0.607838
3619	0.946133	0.744194
3620	1.68951	1.06296
3621	1.68951	0.905552
3622	1.34612	0.63765
3623	1.28953	0.469636
3624	1.34611	1.76887
3625	1.31892	1.73098
3626	0.946131	1.07078
3627	1.80988	1.72181
3628	1.80988	2.1
3629	1.31892	1.50846
3630	0.946131	1.60866
3631	2.13214	2.1
3632	1.94691	2.1
3633	1.52661	0.0698591
3634	1.53175	0.0698591
3635	0.956005	0.0698591
3636	0.955991	0.0698591
3637	1.40572	0.0698591
3638	0.955992	0.0698591
3639	1.51084	2.1
3640	1.67115	2.1
3641	1.73143	2.1
3642	1.55234	2.1
3643	1.69377	2.1
3644	2.51483	2.1
3645	1.25987	2.1
3646	1.4472	2.1
3647	1.83151	0.331848
3648	2.01001	0.642121
3649	0.993998	0.308974
3650	1.83151	0.223418
3651	1.40915	1.29399

3652	1.24653	1.209
3653	1.74472	2.1
3654	1.74472	2.1
3655	1.74244	2.1
3656	1.04788	2.1
3657	1.28522	2.1
3658	1.03785	2.1
3659	0.896519	2.1
3660	1.60428	2.1
3661	1.73305	2.1
3662	1.39072	0.135228
3663	2.27501	0.285841
3664	1.39073	0.191691
3665	1.39073	0.238791
3666	1.06838	2.0972
3667	0.968462	1.10526
3668	1.06837	1.8699
3669	1.13455	1.13078
3670	1.23527	1.19066
3671	2.71686	0.564971
3672	1.86881	0.882386
3673	1.10586	2.09545
3674	1.03852	2.1
3675	1.03899	2.1
3676	0.963973	2.1
3677	1.00319	2.1
3678	1.00319	2.1
3679	0.987132	2.1
3680	0.974889	2.1
3681	0.987131	2.1
3682	1.7677	2.1
3683	1.7677	2.1
3684	0.946131	2.1
3685	1.31185	2.1
3686	0.946132	2.1
3687	1.30203	2.1
3688	1.31185	2.1
3689	1.82958	2.1
3690	1.82958	2.1
3691	0.946132	2.1
3692	1.40199	2.1
3693	0.946133	2.1
3694	1.36994	2.1
3695	1.40199	2.1
3696	0.97489	2.1
3697	1.05939	1.88114
3698	1.01922	1.96392
3699	0.810941	1.73549
3700	1.05372	1.85044
3701	0.754065	1.99766
3702	0.926131	1.9587
3703	1.35905	1.81228
3704	0.810931	1.82037
3705	1.75647	1.0497
3706	1.68265	2.1
3707	1.41995	2.1
3708	1.36457	1.55994
3709	0.933392	1.25373

3710	1.42255	2.1
3711	1.3868	2.1
3712	1.08074	2.1
3713	1.07196	2.1
3714	1.13537	0.409566
3715	1.01179	0.308587
3716	1.60196	0.279857
3717	1.72798	0.291183
3718	1.3715	0.298164
3719	1.56409	1.0291
3720	1.56409	0.183386
3721	1.97819	0.966509
3722	1.47954	0.688603
3723	0.798655	0.247243
3724	1.47952	0.445771
3725	2.30327	0.345428
3726	1.49111	2.1
3727	1.96013	2.02992
3728	1.54599	2.1
3729	2.06632	2.1
3730	1.54601	2.1
3731	1.10557	0.103777
3732	1.17624	0.210578
3733	1.60497	2.1
3734	1.61587	2.1
3735	1.65977	0.0698591
3736	1.69044	0.0698591
3737	1.00659	2.1
3738	1.06226	2.1
3739	1.15171	2.1
3740	1.50853	2.1
3741	1.80252	2.1
3742	1.41432	2.1
3743	1.04687	2.1
3744	0.940878	2.1
3745	1.52031	2.1
3746	1.52031	2.1
3747	0.940877	2.1
3748	0.946132	2.1
3749	1.50853	2.1
3750	0.963973	2.1
3751	1.70562	0.0698591
3752	2.43203	0.0698591
3753	1.36004	0.0698591
3754	1.06648	0.0698591
3755	1.66009	0.0698591
3756	1.66009	0.0698591
3757	0.766427	0.0698591
3758	0.766452	0.0698591
3759	0.946132	0.0698591
3760	1.98012	0.0698591
3761	1.20357	2.1
3762	1.20356	2.1
3763	1.67324	2.1
3764	1.52008	2.1
3765	1.19964	2.1
3766	1.36861	2.1
3767	1.19964	2.1

3768	2.85415	2.05504
3769	1.43687	2.08666
3770	0.935852	2.1
3771	1.09972	2.08332
3772	0.810933	2.04718
3773	2.25974	2.06252
3774	1.74149	2.05735
3775	1.36984	2.07737
3776	1.87602	2.07413
3777	0.890748	2.05538
3778	1.20587	1.9159
3779	0.810956	1.81478
3780	2.03174	1.82034
3781	1.34403	1.86959
3782	1.58825	1.87022
3783	1.63096	1.8939
3784	2.53735	1.90077
3785	1.98241	1.91048
3786	2.53014	2.0351
3787	1.1775	2.04198
3788	1.06538	1.74014
3789	0.946132	0.981619
3790	1.4101	2.02708
3791	1.75162	2.1
3792	1.75162	1.0076
3793	0.946132	1.47405
3794	1.4101	1.23314
3795	1.28765	1.03855
3796	1.1427	0.503399
3797	1.1427	0.502345
3798	1.3109	2.1
3799	1.02721	1.981
3800	0.846447	1.86961
3801	0.862076	2.1
3802	0.644098	1.83228
3803	0.581308	2.00583
3804	1.02306	1.99721
3805	0.322641	0.952127
3806	0.589346	1.17062
3807	2.25572	1.8608
3808	1.31372	2.1
3809	1.18873	2.1
3810	1.78225	2.1
3811	1.764 2.1	
3812	1.17503	2.05711
3813	1.06821	2.07663
3814	1.06821	1.87341
3815	1.7777	1.79633
3816	1.9096	0.240112
3817	1.70239	0.24217
3818	1.16182	0.0698591
3819	1.20132	0.0698591
3820	1.23475	2.1
3821	1.56859	2.1
3822	2.46263	2.1
3823	0.528744	2.1
3824	2.93337	2.1
3825	2.42504	2.1

3826	1.38646	0.302666
3827	1.61963	0.56908
3828	1.88016	0.251937
3829	0.622283	0.148001
3830	1.03243	0.500633
3831	0.710307	1.53344
3832	0.798124	0.247546
3833	0.931073	1.16184
3834	0.892862	0.976517
3835	1.06791	0.311264
3836	0.552787	0.235936
3837	0.946132	0.0903391
3838	1.36665	1.55209
3839	1.36665	0.538338
3840	0.506966	0.182025
3841	0.506964	0.13116
3842	1.36665	0.336486
3843	0.653469	0.881096
3844	1.36665	0.109551
3845	1.21339	0.13427
3846	1.21339	0.414581
3847	0.552757	0.599842
3848	0.552786	0.172878
3849	1.21336	0.195205
3850	1.70744	1.78825
3851	1.0775	0.0994817
3852	1.59527	0.523962
3853	1.48312	0.247816
3854	2.01868	0.219204
3855	1.18947	0.0958993
3856	1.48312	0.253442
3857	0.785626	0.114216
3858	1.19601	0.130921
3859	0.785626	0.127022
3860	0.946132	0.13141
3861	0.7856	0.187898
3862	1.59087	0.13363
3863	1.51158	0.123746
3864	1.74098	0.119333
3865	2.39202	0.119133
3866	0.870489	0.226034
3867	1.34534	0.280127
3868	0.870488	0.198705
3869	0.946133	0.318277
3870	0.870489	0.422808
3871	1.67086	0.285586
3872	1.67086	0.266055
3873	2.11311	2.1
3874	1.98765	1.94054
3875	0.946133	1.09948
3876	1.30881	2.06558
3877	1.72211	2.09843
3878	1.72211	1.66029
3879	0.94613	1.99192
3880	1.30881	1.76282
3881	1.35943	1.58913
3882	1.01294	0.436395
3883	1.01294	0.404196

3884	1.38963	2.08671
3885	1.01294	0.818365
3886	1.60056	0.612539
3887	1.60056	2.01254
3888	0.946132	2.09496
3889	1.204	1.9274
3890	0.946132	0.29596
3891	1.11288	1.31712
3892	1.204	0.840107
3893	1.14599	2.07681
3894	1.00065	2.04529
3895	1.74685	2.09887
3896	1.74685	2.1
3897	0.946128	2.09626
3898	1.38463	2.00418
3899	1.2275	2.1
3900	1.2275	2.1
3901	1.30835	2.1
3902	2.04407	2.1
3903	2.50425	2.1
3904	1.19647	0.279961
3905	1.19647	0.34471
3906	1.19645	0.420906
3907	1.75446	0.263523
3908	1.1315	1.39096
3909	1.1315	1.02107
3910	1.45569	1.02456
3911	1.1315	0.790458
3912	1.14357	2.08205
3913	1.25793	2.0934
3914	1.14357	2.06938
3915	2.08181	1.99489
3916	1.4714	2.1
3917	0.71295	2.1
3918	1.68285	2.1
3919	1.68285	2.1
3920	1.25603	2.1
3921	1.13976	2.1
3922	1.25603	2.1
3923	0.71295	2.1
3924	1.61095	2.1
3925	2.51207	2.1
3926	1.63762	2.1
3927	2.74451	2.1
3928	0.671255	2.02562
3929	1.87601	2.1
3930	1.87601	2.1
3931	0.671226	2.01597
3932	1.25603	2.1
3933	1.29123	2.09406
3934	0.671226	2.09328
3935	1.63723	2.1
3936	0.872928	0.0701547
3937	1.47904	0.0698591
3938	1.18793	0.0698591
3939	2.57464	0.0698591
3940	1.14986	2.09984
3941	1.19611	2.08643

3942	0.96029	1.19568
3943	1.7961	2.1
3944	0.928721	2.1
3945	1.34352	2.1
3946	1.37935	2.1
3947	1.37935	2.1
3948	0.892888	2.1
3949	0.946131	2.1
3950	0.892888	2.1
3951	1.75422	2.1
3952	1.05556	2.1
3953	1.27362	0.229155
3954	1.27421	0.169828
3955	1.3003	1.23655
3956	1.31415	1.30297
3957	1.01431	2.1
3958	0.673951	2.1
3959	1.63384	2.1
3960	1.63661	2.1
3961	1.52245	2.1
3962	1.50714	2.1
3963	0.996778	0.0703028
3964	1.56091	0.0698591
3965	1.06543	2.1
3966	2.66689	2.1
3967	1.01474	2.1
3968	2.76422	2.1
3969	1.45321	0.111369
3970	0.974716	0.0806394
3971	0.810943	0.0865801
3972	0.909982	0.150685
3973	0.937793	0.0982616
3974	1.05138	0.0778701
3975	1.25044	0.0798281
3976	0.92095	0.0871151
3977	0.830414	0.107317
3978	2.1402	0.0958936
3979	1.75938	2.1
3980	1.05556	2.1
3981	1.78558	2.1
3982	1.379 2.1	
3983	0.970214	0.293562
3984	0.9702	0.286844
3985	1.08894	1.00855
3986	2.17251	1.04671
3987	1.51569	0.62878
3988	1.06012	0.560591
3989	0.98966	0.496219
3990	1.55926	0.378652
3991	1.92645	0.467053
3992	1.37427	0.0698591
3993	2.16338	0.0698591
3994	1.5456	0.0698591
3995	1.5456	0.0698591
3996	1.94002	0.0698591
3997	1.33135	0.0698591
3998	1.31948	0.0698591
3999	2.23255	0.0698591

4000	2.34061	0.0698591
4001	1.52903	2.1
4002	3.38327	2.1
4003	1.35977	2.1
4004	2.22414	2.1
4005	1.6812	0.0801543
4006	2.33397	0.0890796
4007	1.16699	0.121443
4008	0.946131	0.119636
4009	1.58115	0.118119
4010	1.58115	0.105517
4011	0.519726	0.18306
4012	0.946131	0.144732
4013	0.519697	0.120302
4014	1.26868	0.0856682
4015	1.16699	0.0945401
4016	1.5951	0.0823194
4017	1.5951	0.0768871
4018	0.840568	0.114789
4019	0.946131	0.0941597
4020	0.840598	0.0893556
4021	1.30047	2.1
4022	1.5586	2.1
4023	1.31825	1.52204
4024	1.31924	1.43829
4025	1.15818	1.86252
4026	1.52782	0.56332
4027	1.15818	1.10946
4028	1.15818	1.36296
4029	0.99518	0.0698591
4030	0.94613	0.0698591
4031	0.922045	0.0698591
4032	1.29322	0.0700038
4033	1.29321	0.0698591
4034	1.29321	0.142967
4035	0.922056	0.0698591
4036	1.06848	0.0799113
4037	1.84409	0.0700581
4038	1.9186	1.00463
4039	2.72244	0.752848
4040	0.946132	0.873807
4041	1.36122	0.632404
4042	1.79658	0.602769
4043	1.79658	0.570396
4044	0.946133	1.06855
4045	1.36122	0.786249
4046	1.38149	0.628108
4047	1.59106	0.579788
4048	0.946116	0.79679
4049	1.29052	0.567616
4050	1.76641	0.512973
4051	1.76641	0.542213
4052	0.885309	0.949058
4053	1.29054	0.707531
4054	1.36118	0.599111
4055	1.59103	0.621167
4056	2.27816	0.461763
4057	1.56164	0.566967

4058	1.41114	0.0698591
4059	2.04128	0.0698591
4060	0.986723	2.1
4061	1.03645	0.134306
4062	1.79224	0.120867
4063	1.2627	2.1
4064	1.23036	2.1
4065	1.45257	2.1
4066	1.41469	2.1
4067	1.41469	2.1
4068	2.60636	2.1
4069	1.45789	0.0698591
4070	0.851541	0.0698591
4071	1.57877	0.0698591
4072	1.53134	0.0698591
4073	0.851479	0.0698591
4074	1.08982	0.0698591
4075	0.716284	0.0698591
4076	0.946131	0.0698591
4077	1.73193	0.0698591
4078	2.57309	0.0698591
4079	1.36307	0.0698591
4080	2.42175	0.0698591
4081	1.72948	0.0698591
4082	0.978698	2.1
4083	0.978699	2.1
4084	2.08398	2.1
4085	0.978698	2.1
4086	1.44096	2.1
4087	1.44096	2.1
4088	0.638164	2.1
4089	0.681588	2.1
4090	0.946133	2.1
4091	0.638165	2.1
4092	0.94613	2.1
4093	1.55432	2.1
4094	0.901147	2.1
4095	0.85777	2.1
4096	1.36073	2.1
4097	1.44004	2.1
4098	0.946133	2.1
4099	1.09982	2.1
4100	1.27562	2.1
4101	1.55432	2.1
4102	1.62229	2.1
4103	1.0101	2.1
4104	2.28459	2.09323
4105	2.50554	1.97722
4106	0.946131	1.28333
4107	1.25276	1.9865
4108	1.73721	2.01423
4109	1.73721	1.56511
4110	0.93406	1.9007
4111	1.25277	1.78351
4112	1.4185	1.71308
4113	1.53064	1.56165
4114	0.946133	1.66737
4115	0.609462	2.01238

4116	1.65123	2.00439
4117	1.65123	1.78754
4118	0.609466	2.05737
4119	1.00792	1.94625
4120	1.25277	1.8593
4121	1.53064	1.86937
4122	2.15223	1.36948
4123	1.30664	1.81913
4124	1.10674	2.1
4125	1.22862	2.1
4126	1.49358	0.131595
4127	2.0207	0.098291
4128	2.09374	2.1
4129	1.88175	2.1
4130	1.10938	0.739849
4131	1.02478	1.79856
4132	1.13272	0.0698591
4133	1.13272	0.0698591
4134	1.03935	1.06533
4135	0.810945	1.32851
4136	0.527573	0.690046
4137	0.925011	1.09778
4138	1.03193	0.63593
4139	1.38587	0.757796
4140	0.703469	0.710671
4141	0.912282	1.02601
4142	1.75505	2.1
4143	1.78529	2.1
4144	1.60017	0.509517
4145	1.35608	0.182724
4146	1.01479	2.1
4147	1.47561	1.87944
4148	1.01479	2.1
4149	1.28418	1.93917
4150	2.62605	2.1
4151	1.71233	2.1
4152	3.22222	2.1
4153	1.97526	2.1
4154	0.949712	0.104172
4155	1.78986	0.100123
4156	2.72879	0.0911999
4157	2.66428	0.0948587
4158	1.68108	0.168306
4159	1.67265	0.141815
4160	2.24877	0.190346
4161	1.15558	0.225698
4162	1.39708	2.1
4163	1.39327	2.1
4164	1.69788	0.0698591
4165	1.1273	0.0698591
4166	1.17302	1.58907
4167	1.04349	1.27963
4168	0.979853	2.1
4169	1.19094	2.1
4170	1.39408	2.1
4171	1.61857	2.1
4172	1.66523	2.1
4173	0.946132	2.1

4174	0.838554	2.1
4175	1.05151	2.1
4176	1.18901	2.1
4177	1.18901	2.1
4178	1.61149	2.1
4179	1.22857	0.341279
4180	1.25848	0.368356
4181	1.09154	2.1
4182	2.20358	2.1
4183	1.23013	2.1
4184	1.55698	2.1
4185	1.55828	2.09051
4186	1.52391	2.1
4187	0.60346	1.97127
4188	1.06336	2.09806
4189	0.870714	1.87533
4190	0.543109	1.98287
4191	1.10367	1.92829
4192	1.20686	1.98638
4193	1.20684	2.04955
4194	2.5118	1.95123
4195	1.20684	2.08187
4196	1.52468	1.99087
4197	1.52469	1.99102
4198	0.532308	1.7606
4199	0.903301	1.99186
4200	0.946119	1.76944
4201	0.532315	1.71232
4202	0.946107	1.81536
4203	1.46129	2.1
4204	0.804766	2.1
4205	1.4809	2.1
4206	1.4809	2.1
4207	1.11822	2.1
4208	1.16744	2.1
4209	1.11822	2.1
4210	0.804737	2.1
4211	1.04193	2.1
4212	0.983723	2.1
4213	1.8014	2.1
4214	1.1729	2.1
4215	1.03951	2.1
4216	1.03951	2.1
4217	1.17583	2.1
4218	1.42866	2.1
4219	1.78503	2.1
4220	1.17822	0.788966
4221	1.89121	2.1
4222	1.44472	2.1
4223	1.62143	2.1
4224	1.43837	2.1
4225	1.01121	0.0731191
4226	0.962703	1.00179
4227	0.946129	0.605207
4228	1.10941	0.366699
4229	0.946131	0.677288
4230	1.60326	0.746555
4231	1.60326	0.419273

4232	1.14215	0.548405
4233	1.40724	0.555029
4234	0.962703	0.703844
4235	1.53336	0.0698591
4236	1.40272	0.0698591
4237	1.64984	0.0698591
4238	1.67345	0.0698591
4239	1.08212	2.1
4240	1.40905	2.1
4241	0.946132	0.0698591
4242	0.946132	0.0698591
4243	0.81268	0.0698591
4244	0.946131	0.0698591
4245	0.812681	0.0698591
4246	0.946131	0.0698591
4247	0.946132	0.0698591
4248	0.997755	0.0698591
4249	1.94585	0.0698591
4250	1.2925	0.0698591
4251	2.52454	0.0698591
4252	1.69182	0.0698591
4253	1.69182	0.0698591
4254	0.92361	0.0698591
4255	1.35316	0.0698591
4256	1.26227	0.0698591
4257	0.946131	0.0698591
4258	1.35316	0.0698591
4259	1.48676	0.0974443
4260	1.33917	0.0844215
4261	0.757292	2.1
4262	1.02415	2.1
4263	1.48495	2.1
4264	0.97587	2.1
4265	0.946132	2.1
4266	0.747365	2.1
4267	1.55432	2.1
4268	1.55432	2.1
4269	0.946132	2.1
4270	0.747394	2.1
4271	1.17464	0.0698591
4272	1.0372	0.0698591
4273	2.16553	0.0698591
4274	2.00616	0.0698591
4275	1.12184	0.0698591
4276	1.33177	0.0698591
4277	0.936674	0.0698591
4278	0.946129	0.0698591
4279	0.851552	0.0698591
4280	1.60205	0.0698591
4281	1.77391	0.0698591
4282	1.03327	0.0779452
4283	1.41512	0.0848627
4284	1.03327	0.0722161
4285	0.946133	0.0862474
4286	0.946131	0.0927666
4287	1.80077	0.0866964
4288	1.80077	0.0780943
4289	1.54848	2.1

4290	1.29931	2.1
4291	1.04482	0.115547
4292	2.08069	0.130255
4293	0.946127	0.116099
4294	0.515539	0.155674
4295	0.515566	0.210638
4296	1.38423	0.117071
4297	1.38423	0.106644
4298	0.972058	0.163552
4299	0.927737	0.163086
4300	0.94971	0.0911002
4301	2.19682	0.115848
4302	0.946132	0.168876
4303	1.09841	0.121606
4304	0.522382	0.213979
4305	1.5962	0.0993882
4306	1.72791	0.0850376
4307	1.07428	0.140462
4308	1.05215	0.126861
4309	1.54632	1.7783
4310	1.15693	1.81254
4311	1.41238	2.1
4312	1.41238	2.1
4313	1.77966	2.1
4314	1.77966	2.1
4315	0.946129	2.1
4316	1.40525	2.1
4317	0.946131	2.1
4318	0.94613	2.1
4319	1.32054	2.1
4320	1.41238	2.1
4321	1.94026	2.1
4322	1.0171	0.623735
4323	1.1211	0.543666
4324	0.993022	2.1
4325	0.773813	2.1
4326	1.4073	2.1
4327	1.4073	2.1
4328	0.94613	2.1
4329	0.787615	2.1
4330	0.94613	2.1
4331	0.787615	2.1
4332	1.764	2.1
4333	1.90964	2.1
4334	1.05787	2.1
4335	1.403	0.959341
4336	0.744189	1.2991
4337	2.32834	2.1
4338	2.34986	2.1
4339	1.17106	0.345984
4340	1.94826	0.0699391
4341	2.40606	0.0698591
4342	1.26387	0.0707573
4343	0.954013	0.0710864
4344	0.946131	0.0742931
4345	1.71	0.0698591
4346	1.51926	0.0698591
4347	0.953997	0.0698591

4348	0.851554	0.0713333
4349	1.96502	0.0698591
4350	1.72954	0.0702364
4351	2.89117	0.0698591
4352	0.971958	0.0723686
4353	0.985575	0.0785099
4354	1.41943	0.0698591
4355	1.20021	0.0700808
4356	1.29599	0.0714353
4357	1.15687	0.0698591
4358	0.810958	0.0751331
4359	1.12919	0.0698591
4360	1.98859	2.1
4361	1.82044	2.1
4362	1.90488	2.1
4363	0.851542	2.1
4364	1.02973	2.1
4365	1.39286	2.1
4366	1.44873	2.1
4367	0.946132	2.1
4368	1.11826	2.1
4369	1.26842	2.1
4370	2.09461	2.1
4371	1.76952	0.0698591
4372	1.6473	0.0698591
4373	0.946131	0.0698591
4374	1.36486	0.0698591
4375	1.04243	0.0698591
4376	1.05017	0.0698591
4377	1.24059	0.0698591
4378	2.11646	0.0698591
4379	1.50368	0.0698591
4380	2.38796	0.0698591
4381	1.50368	0.0698591
4382	1.71844	0.0698591
4383	1.71844	0.0698591
4384	0.914836	0.0698591
4385	1.24416	0.0698591
4386	0.914805	0.0698591
4387	1.24416	0.0698591
4388	1.38911	0.0698591
4389	1.46859	0.0698591
4390	1.35348	0.0698591
4391	0.946131	0.0698591
4392	1.1226	0.0698591
4393	0.857078	0.0698591
4394	0.9746	0.0698591
4395	1.37624	0.0698591
4396	1.15131	0.0698591
4397	1.139	0.0698591
4398	1.90456	0.0698591
4399	2.09692	0.0698591
4400	1.16655	2.1
4401	1.23017	2.1
4402	2.14592	2.1
4403	1.23017	2.1
4404	1.0752	2.1
4405	1.52482	1.3771

4406	1.09252	2.1
4407	1.10725	2.1
4408	1.14305	0.0698591
4409	0.851541	0.0733711
4410	1.48073	0.0698591
4411	1.45607	0.0698591
4412	0.946131	0.0751537
4413	0.932823	0.0698591
4414	1.45828	0.0698591
4415	0.851528	0.0698834
4416	1.14305	0.0698591
4417	1.92737	0.0713664
4418	1.08211	0.0730363
4419	1.69831	0.0698591
4420	1.69831	0.0698591
4421	0.946131	0.0698591
4422	1.26868	0.0698591
4423	1.23377	0.0698591
4424	1.23377	0.0698591
4425	0.94723	0.0698591
4426	1.64322	0.0698591
4427	1.64322	0.0698591
4428	0.946131	1.51805
4429	1.32068	0.0698591
4430	0.946129	0.0698591
4431	1.18789	1.83437
4432	1.32068	0.0698591
4433	1.32813	2.1
4434	1.32461	2.1
4435	1.42729	0.0698591
4436	2.47846	0.0698591
4437	1.00024	0.0865161
4438	1.23923	0.0698591
4439	0.946131	0.0923227
4440	0.712304	0.0698591
4441	1.77501	0.0906627
4442	1.77501	0.0698591
4443	0.593593	0.119939
4444	1.23923	0.107279
4445	1.23175	0.0785071
4446	0.94613	0.130339
4447	0.713646	0.0823153
4448	1.75735	0.115325
4449	1.75735	0.0698591
4450	0.713633	0.176468
4451	1.37157	0.0698591
4452	1.47169	0.0698591
4453	2.21808	0.075184
4454	1.47169	0.0698591
4455	1.04759	2.1
4456	0.987132	2.1
4457	1.11181	2.04856
4458	2.61682	1.95282
4459	1.11181	2.07237
4460	1.06461	1.58917
4461	1.69733	2.1
4462	2.79705	2.1
4463	1.08245	2.1

4464	1.40583	2.1
4465	1.39852	2.1
4466	2.0239	2.1
4467	2.0239	2.1
4468	0.780455	2.1
4469	0.946132	2.1
4470	0.780455	2.1
4471	1.01885	2.1
4472	0.564217	2.1
4473	0.733461	2.1
4474	0.537126	2.1
4475	0.683838	2.1
4476	0.779662	2.1
4477	0.634141	2.1
4478	0.685337	2.1
4479	1.58848	2.09754
4480	1.17129	0.133474
4481	0.663168	0.197975
4482	1.55587	0.159556
4483	1.31342	0.145817
4484	0.596868	0.201983
4485	1.1666	0.207922
4486	0.867261	0.218134
4487	0.946132	0.241271
4488	1.28843	0.146229
4489	1.31248	0.181224
4490	2.59921	0.163544
4491	1.32634	0.120997
4492	1.35564	0.0698591
4493	1.52661	0.0698591
4494	1.12323	0.0698591
4495	1.17701	1.18746
4496	1.36405	1.10718
4497	1.19837	1.12356
4498	2.25636	1.05446
4499	1.0823	0.0758311
4500	1.31774	0.0785461
4501	1.0823	0.0698591
4502	1.01121	0.0897141
4503	1.27408	0.0698591
4504	0.946131	0.192098
4505	0.566074	0.170536
4506	0.79576	0.171248
4507	1.24596	0.180914
4508	1.47567	0.182949
4509	1.47567	0.0983885
4510	0.56605	0.178828
4511	1.6567	0.12809
4512	0.967792	0.124006
4513	1.00366	0.124042
4514	1.00366	0.132836
4515	1.56409	0.0698591
4516	1.56409	0.0698591
4517	0.774911	0.0698591
4518	1.09483	0.0698591
4519	0.774912	0.0698591
4520	1.09483	0.0698591
4521	1.24417	0.0698591

4522	1.13387	0.0698591
4523	1.13387	0.0698591
4524	1.30572	0.0698591
4525	2.18966	0.0698591
4526	0.98575	0.0698591
4527	1.54982	0.0698591
4528	2.48833	0.0698591
4529	1.54982	0.0698591
4530	1.28954	0.0755735
4531	1.64002	0.0698591
4532	1.64002	0.0698591
4533	1.85594	0.0698591
4534	1.12968	2.1
4535	1.23536	2.1
4536	1.4882	2.1
4537	1.4882	2.1
4538	0.876836	2.1
4539	0.946131	2.1
4540	0.876835	2.1
4541	1.43166	2.1
4542	2.25936	2.1
4543	1.0305	2.1
4544	0.992095	2.1
4545	1.12968	2.1
4546	1.53813	2.1
4547	1.53812	2.1
4548	0.583653	2.1
4549	0.94613	2.1
4550	0.583651	2.1
4551	0.964033	2.1
4552	0.964034	2.1
4553	1.54457	2.1
4554	3.54109	0.0725643
4555	0.99974	0.0847776
4556	0.78814	0.096986
4557	1.65729	0.0727255
4558	1.1283	0.0806216
4559	1.09407	0.0840783
4560	1.55091	0.0734685
4561	0.870773	0.0906343
4562	1.19967	0.0763619
4563	1.56521	0.0766863
4564	1.30896	1.9422
4565	1.17989	2.1
4566	1.72526	2.1
4567	0.851537	1.07329
4568	1.58944	2.08379
4569	1.56915	2.0856
4570	0.891039	1.03813
4571	1.4172	1.80265
4572	0.963818	1.55386
4573	0.851474	1.86968
4574	1.17989	2.1
4575	1.56261	2.1
4576	1.56261	2.1
4577	0.761574	2.1
4578	1.31323	2.1
4579	0.761573	2.1

4580	0.946133	2.1
4581	1.31323	2.1
4582	1.16759	2.1
4583	1.16759	2.1
4584	2.11829	2.1
4585	1.16759	2.1
4586	1.10748	0.0698591
4587	1.47688	0.0698591
4588	0.946133	0.0698591
4589	0.953319	0.080826
4590	0.946132	0.0715507
4591	1.51168	0.0698591
4592	1.51168	0.072881
4593	1.25797	0.0703384
4594	1.19984	0.0699251
4595	1.95729	2.1
4596	2.34853	2.1
4597	1.94343	2.1
4598	2.00551	2.1
4599	1.20067	1.00959
4600	1.87244	1.33298
4601	1.4178	2.1
4602	1.33538	2.1
4603	0.946131	2.1
4604	1.22895	2.1
4605	1.12433	2.1
4606	0.956757	2.1
4607	0.91826	2.1
4608	1.44221	2.1
4609	1.14206	2.1
4610	1.80514	1.07503
4611	0.890304	1.85994
4612	1.1362	1.0523
4613	1.32819	1.3067
4614	1.46311	2.03611
4615	0.8654	1.35281
4616	1.1699	1.63472
4617	1.00267	1.6811
4618	1.02882	1.14135
4619	1.00832	2.1
4620	0.974137	2.1
4621	1.02368	2.1
4622	0.974138	2.1
4623	0.995734	2.02198
4624	1.44605	0.541342
4625	1.18549	0.586326
4626	1.33621	0.277454
4627	1.5176	0.283486
4628	1.91762	0.273469
4629	1.33621	0.258088
4630	1.6638	0.26341
4631	1.66774	2.1
4632	1.11833	2.1
4633	0.926499	2.1
4634	1.51229	2.1
4635	1.51229	2.1
4636	0.926528	2.1
4637	1.40335	2.1

4638	1.03544	2.1
4639	0.926527	2.1
4640	1.85305	2.1
4641	1.5812	2.1
4642	1.15529	2.1
4643	1.31086	2.1
4644	0.999206	2.1
4645	1.46714	2.1
4646	1.73087	2.1
4647	0.772299	2.1
4648	1.24341	2.1
4649	0.997867	2.1
4650	0.772311	2.1
4651	0.936657	2.1
4652	1.35986	2.1
4653	0.959414	2.1
4654	1.36541	2.1
4655	1.49268	2.1
4656	1.49268	2.1
4657	0.832137	2.1
4658	0.946132	2.1
4659	0.832138	2.1
4660	1.64523	2.1
4661	1.11764	2.1
4662	1.05776	2.1
4663	0.972465	2.1
4664	0.972464	2.1
4665	1.98425	2.1
4666	0.969822	2.1
4667	1.30566	2.1
4668	1.52724	2.1
4669	2.32237	0.110202
4670	2.30274	0.119667
4671	2.27761	0.0730002
4672	3.33012	0.07432
4673	1.24001	0.38561
4674	1.41821	0.380316
4675	0.978584	2.1
4676	1.18076	2.1
4677	1.07645	2.1
4678	0.877351	1.51732
4679	0.946132	2.1
4680	0.877353	1.89903
4681	1.58444	2.1
4682	1.49614	2.1
4683	1.28355	2.1
4684	1.13491	2.1
4685	1.56875	0.175402
4686	0.499592	0.0698591
4687	0.946131	0.0698591
4688	1.20538	0.0698591
4689	1.09913	0.0698591
4690	1.8049	0.0698591
4691	1.8049	0.0698591
4692	0.499606	0.0698591
4693	2.41076	0.0698591
4694	0.860202	0.0698591
4695	0.906798	0.0698591

4696	1.8626	0.0698591
4697	1.33986	0.0698591
4698	1.2992	0.0698591
4699	1.58702	0.0698591
4700	1.05939	0.0698591
4701	1.02074	2.08999
4702	1.07866	2.09975
4703	1.5082	0.0891614
4704	1.5522	0.074662
4705	1.65903	0.125937
4706	1.86766	2.1
4707	2.11684	2.1
4708	0.946132	2.1
4709	1.6585	2.1
4710	1.6585	2.1
4711	0.94613	2.1
4712	1.1872	2.1
4713	1.1872	2.1
4714	1.05842	2.1
4715	1.20455	2.1
4716	1.34141	2.1
4717	1.34141	2.1
4718	1.42416	2.1
4719	1.75818	2.1
4720	2.63715	2.0271
4721	1.80454	2.1
4722	2.26198	2.1
4723	0.696255	2.1
4724	0.624075	2.1
4725	1.50905	1.07103
4726	1.50905	2.06578
4727	0.624075	2.1
4728	1.31857	1.0094
4729	0.946131	2.06231
4730	0.624075	2.01416
4731	0.914628	2.1
4732	1.14721	2.1
4733	1.16153	2.1
4734	1.47664	2.1
4735	1.54791	2.1
4736	1.19098	2.1
4737	1.13341	2.1
4738	1.00503	0.174735
4739	1.00503	1.03453
4740	1.22655	2.1
4741	0.999489	2.1
4742	1.3268	2.1
4743	0.946132	2.1
4744	1.60061	2.1
4745	1.60061	2.1
4746	1.08034	2.1
4747	1.40372	2.1
4748	1.14302	2.1
4749	1.08034	2.1
4750	0.999491	2.1
4751	1.17152	0.213805
4752	1.17152	0.136964
4753	1.17152	0.151472

4754	2.05423	0.168587
4755	1.40984	2.03492
4756	1.2489	2.1
4757	0.885089	2.1
4758	0.810931	2.1
4759	1.84128	2.1
4760	1.11206	2.1
4761	1.21926	2.1
4762	1.99084	2.1
4763	0.976334	0.707442
4764	0.599363	0.637033
4765	1.02621	0.334244
4766	0.462959	0.565777
4767	1.42612	0.25367
4768	0.299682	0.375747
4769	1.51379	2.1
4770	0.719531	2.1
4771	1.3702	2.1
4772	1.33293	2.1
4773	0.851539	2.1
4774	0.62998	2.1
4775	0.851543	2.1
4776	0.629966	2.1
4777	1.43132	2.1
4778	1.13328	0.615102
4779	1.93212	0.317967
4780	1.62345	1.00322
4781	1.38511	1.16467
4782	1.40013	0.567891
4783	1.04132	0.51652
4784	0.83473	1.08929
4785	0.868732	0.951271
4786	0.810595	1.28008
4787	0.729919	0.915101
4788	0.992428	0.917054
4789	0.577107	0.766148
4790	1.18464	0.935315
4791	0.791199	0.957253
4792	0.810943	0.0698591
4793	0.860723	0.0698591
4794	1.33348	0.0698591
4795	1.57467	0.0698591
4796	0.981032	0.0698591
4797	0.731569	0.0698591
4798	0.770312	0.0698591
4799	1.46055	2.1
4800	1.42249	2.1
4801	1.75999	0.216991
4802	1.6868	0.251993
4803	1.17042	1.18844
4804	1.01485	2.1
4805	1.19952	0.0698591
4806	1.40592	0.0698591
4807	1.19952	0.0698591
4808	2.20145	0.0698591
4809	1.33649	0.22836
4810	1.11612	0.638101
4811	0.957588	0.457927

4812	0.946126	0.665056
4813	1.50648	0.217374
4814	1.50648	0.345952
4815	0.946128	0.457338
4816	2.10671	0.0706162
4817	1.91518	0.128139
4818	2.23223	0.188802
4819	1.53556	2.1
4820	1.56253	2.1
4821	1.27806	0.0698591
4822	1.44593	0.0698591
4823	1.03818	0.831156
4824	1.03818	0.739266
4825	1.38969	0.285121
4826	1.85548	0.170304
4827	2.6044	0.287823
4828	1.85548	0.172766
4829	1.49346	1.67394
4830	1.83192	1.69582
4831	0.834043	1.72495
4832	0.810943	1.08775
4833	0.887113	1.26299
4834	0.893827	1.05516
4835	0.991061	1.0868
4836	0.91939	1.18728
4837	0.793307	1.31941
4838	1.75291	1.21993
4839	1.35435	1.24781
4840	1.16989	0.206279
4841	1.36717	0.172676
4842	2.12276	0.0989829
4843	2.30493	0.1477
4844	2.47857	0.160449
4845	1.92269	0.0911918
4846	1.66426	0.140831
4847	0.956698	2.08478
4848	0.956698	2.1
4849	0.857957	2.1
4850	1.4706	2.1
4851	1.65706	2.1
4852	1.47059	2.1
4853	1.04713	0.101912
4854	0.946131	0.119328
4855	1.47374	0.148389
4856	1.47374	0.0995318
4857	0.951675	0.10856
4858	1.2582	0.160428
4859	1.2582	0.113758
4860	0.94613	0.144842
4861	1.4468	0.0699353
4862	1.90335	0.103439
4863	1.14886	2.1
4864	2.59689	2.1
4865	1.02747	2.1
4866	0.946131	2.1
4867	0.934061	2.1
4868	1.63979	2.1
4869	1.65206	2.1

4870	1.48	2.1
4871	1.27467	2.1
4872	2.20449	2.1
4873	1.1333	0.0698591
4874	1.95334	2.1
4875	2.20984	2.1
4876	1.46389	0.0726444
4877	1.4639	0.0771748
4878	1.4639	0.0931469
4879	0.946132	0.122108
4880	1.5144	0.0831821
4881	1.5144	0.103404
4882	0.946132	0.125092
4883	1.35732	0.0837758
4884	1.10321	0.111162
4885	0.946132	0.107383
4886	0.980549	0.0965028
4887	1.32819	2.1
4888	1.30171	1.52925
4889	1.68377	1.5156
4890	1.68377	2.1
4891	0.946132	1.36161
4892	0.946132	1.95584
4893	1.1952	2.00643
4894	0.990019	1.66456
4895	1.08195	1.95342
4896	1.2269	2.08918
4897	1.19219	2.05834
4898	1.01485	2.1
4899	1.57125	0.0722387
4900	2.10696	0.0714084
4901	1.29666	0.0698591
4902	1.57125	0.074384
4903	1.47712	2.1
4904	1.45182	2.1
4905	1.17816	0.0698591
4906	1.4516	0.0698591
4907	1.17816	0.0698591
4908	1.17816	0.0698591
4909	0.596954	0.746185
4910	1.65133	0.737273
4911	0.583859	0.524835
4912	1.04072	1.44277
4913	1.28795	0.580526
4914	1.59423	0.695318
4915	0.483559	0.708535
4916	1.74088	0.556163
4917	1.09545	1.21378
4918	1.02519	1.72821
4919	2.47579	0.728844
4920	1.54732	0.859888
4921	2.17663	0.822763
4922	1.75006	1.02491
4923	1.32354	1.1798
4924	1.09962	2.1
4925	1.85057	2.1
4926	1.91046	2.1
4927	2.17037	2.1

4928	1.99528	0.0701891
4929	1.97969	0.0698591
4930	3.82765	2.1
4931	1.85767	2.1
4932	2.77042	2.1
4933	1.37236	0.0698591
4934	2.23254	0.0698591
4935	1.10089	0.0698591
4936	0.994294	2.1
4937	1.81921	2.1
4938	1.00073	2.1
4939	0.681043	2.1
4940	0.946131	2.1
4941	0.681073	2.1
4942	1.51804	2.1
4943	1.60653	2.1
4944	1.30188	2.1
4945	0.919622	2.1
4946	1.00965	2.1
4947	1.93821	2.1
4948	2.60377	2.1
4949	1.36416	0.0698591
4950	1.36416	0.0698591
4951	1.36416	0.0698591
4952	2.44132	0.0698591
4953	2.15871	0.254986
4954	2.22171	0.1966
4955	1.70747	0.15327
4956	1.49515	0.123488
4957	0.773007	0.163337
4958	0.946125	0.184366
4959	0.946131	0.224929
4960	0.773034	0.118002
4961	1.15844	0.15884
4962	1.98104	0.124503
4963	2.44283	0.135895
4964	1.05417	0.0698591
4965	1.1596	0.0698591
4966	0.829921	0.0698591
4967	0.946131	0.0698591
4968	0.951911	0.0698591
4969	1.39027	0.0698591
4970	0.829946	0.0698591
4971	1.51223	0.0698591
4972	1.51223	0.0698591
4973	1.65984	0.0698591
4974	1.90382	0.0698591
4975	1.93228	0.82603
4976	2.47924	1.02685
4977	1.73904	0.839446
4978	1.40547	0.578521
4979	2.37589	0.668624
4980	0.662605	1.05389
4981	2.33293	0.50109
4982	1.72249	2.1
4983	1.68478	2.1
4984	1.00514	0.449059
4985	1.4537	2.1

4986	1.92698	2.1
4987	1.46041	0.0698591
4988	2.04817	0.0698591
4989	1.65954	2.1
4990	1.42342	2.1
4991	1.04405	0.0698591
4992	1.17056	0.0698591
4993	1.11339	0.0698591
4994	0.946133	0.0698591
4995	0.929724	0.0698591
4996	1.35424	0.0698591
4997	1.35424	0.0698591
4998	0.929713	0.0698591
4999	1.85945	0.0698591
5000	1.38905	2.1
5001	1.39056	2.1
5002	1.52852	2.1
5003	1.06472	2.1
5004	0.749096	2.1
5005	1.46076	2.1
5006	1.29263	2.1
5007	0.94613	2.1
5008	0.69097	2.1
5009	0.982284	2.1
5010	0.67419	2.1
5011	1.52852	2.1
5012	1.91121	2.1
5013	1.00775	2.046
5014	1.61201	2.00613
5015	0.529987	2.1
5016	0.506125	2.1
5017	1.44696	2.1
5018	2.32981	2.1
5019	1.32784	2.1
5020	1.94158	2.1
5021	1.31595	2.1
5022	0.663922	2.1
5023	1.44663	2.1
5024	1.44663	2.1
5025	1.13976	2.1
5026	0.970788	2.1
5027	1.13976	2.1
5028	0.663921	2.1
5029	0.966114	2.1
5030	1.05498	2.02129
5031	1.38086	2.1
5032	0.946127	2.1
5033	0.922513	2.1
5034	1.49903	2.1
5035	1.49903	2.1
5036	0.92251	2.1
5037	1.06105	2.1
5038	1.36049	2.1
5039	1.38086	2.1
5040	1.52534	2.1
5041	1.04101	2.1
5042	1.30861	0.0698591
5043	0.949112	0.0698591

5044	0.894275	0.0702512
5045	1.10232	0.0698591
5046	0.830889	0.0698591
5047	1.26554	0.0698591
5048	0.948347	0.0698591
5049	0.81096	0.0698591
5050	1.18338	0.0698591
5051	0.883764	0.0708266
5052	1.54793	0.0698591
5053	1.02867	0.0698591
5054	1.8573	0.0698591
5055	1.06065	0.0698591
5056	0.64842	0.0698591
5057	1.99319	0.0698591
5058	1.9476	0.0702545
5059	2.51436	0.0698591
5060	1.68337	0.0698591
5061	1.92321	0.0698591
5062	0.810942	0.0698591
5063	0.921001	0.0698591
5064	0.99758	0.0698591
5065	0.960785	0.0698591
5066	1.01565	0.0698591
5067	0.908624	0.0698591
5068	0.912835	0.0698591
5069	1.18351	0.0698591
5070	1.40239	2.1
5071	1.26091	2.1
5072	0.947979	2.1
5073	0.946131	2.1
5074	1.81959	2.1
5075	1.81959	2.1
5076	0.94613	2.1
5077	1.38415	2.05812
5078	1.38414	2.1
5079	1.33266	2.1
5080	0.946132	2.1
5081	1.76225	2.1
5082	1.76225	2.1
5083	1.27599	2.1
5084	1.38158	2.1
5085	1.3268	2.1
5086	1.27599	2.1
5087	0.947979	2.1
5088	1.0142	0.453206
5089	1.01422	0.341468
5090	1.11435	0.306572
5091	1.42186	0.820201
5092	1.24001	0.913361
5093	1.77342	1.11225
5094	1.42186	1.37888
5095	1.99817	0.74893
5096	1.27361	2.1
5097	1.68914	2.1
5098	1.30116	2.1
5099	1.32819	2.01892
5100	1.68322	2.04298
5101	1.68322	2.1

5102	0.94613	1.88468
5103	0.94613	2.09347
5104	1.14666	2.1
5105	1.07612	2.02528
5106	1.31151	2.1
5107	1.22095	2.1
5108	1.57256	2.1
5109	1.6679	2.1
5110	0.946132	2.09727
5111	0.946133	2.1
5112	1.13537	2.1
5113	1.07855	2.1
5114	1.07855	2.1
5115	1.43895	1.80402
5116	1.38481	0.0698591
5117	1.72385	0.0698591
5118	0.550441	0.0698591
5119	0.79807	0.0698591
5120	1.11627	0.0698591
5121	1.30349	0.0698591
5122	0.94613	0.0698591
5123	0.686176	0.0698591
5124	1.11627	0.0698591
5125	1.73363	0.0698591
5126	1.73363	0.0698591
5127	0.686139	0.0698591
5128	1.24932	0.0698591
5129	0.899582	0.0698591
5130	2.00472	0.0698591
5131	1.17121	0.0698591
5132	1.2874	0.0698591
5133	2.0194	0.0698591
5134	0.88601	0.0698591
5135	1.77409	0.0698591
5136	1.73293	0.0698591
5137	2.74045	0.0698591
5138	1.83787	0.0698591
5139	1.45448	0.0802506
5140	1.27778	0.0743353
5141	1.1192	2.1
5142	1.25953	2.1
5143	1.93611	2.1
5144	1.92104	2.1
5145	1.15506	0.0698591
5146	1.15506	0.0698591
5147	1.23943	1.94437
5148	0.851543	2.1
5149	1.6061	2.1
5150	1.80058	2.1
5151	0.869471	2.1
5152	1.3931	2.1
5153	1.06454	2.1
5154	0.93667	2.1
5155	2.16916	2.1
5156	3.10246	2.1
5157	1.91539	2.09149
5158	1.91539	2.06956
5159	0.620263	2.04493

5160	0.620265	2.08799
5161	0.946132	2.03183
5162	0.620265	2.03516
5163	1.30226	1.97959
5164	1.87292	2.1
5165	1.18769	2.0962
5166	2.60452	2.0881
5167	1.26574	2.1
5168	2.46679	2.09482
5169	1.36459	2.1
5170	1.26865	1.63715
5171	1.1744	1.0666
5172	1.15841	0.341873
5173	1.02247	0.201783
5174	1.93649	2.1
5175	1.64718	2.1
5176	1.59654	2.1
5177	1.57823	2.1
5178	1.00665	0.0917132
5179	1.28239	0.10289
5180	0.887895	2.1
5181	1.22083	2.1
5182	1.42127	2.1
5183	1.07374	2.1
5184	1.00503	1.13226
5185	1.23082	0.651995
5186	2.78663	2.1
5187	2.20883	2.1
5188	2.54588	2.1
5189	0.946128	2.1
5190	1.13716	2.1
5191	1.58724	2.1
5192	1.58724	2.1
5193	0.946131	2.1
5194	1.13716	2.1
5195	1.39621	2.1
5196	1.02438	2.1
5197	1.49543	0.0698591
5198	2.78336	0.0698591
5199	1.27148	0.0698591
5200	1.0906	0.0698591
5201	1.04072	0.0698591
5202	0.63574	0.0698591
5203	1.45506	0.0698591
5204	1.5863	0.0698591
5205	0.928352	0.0698591
5206	0.647687	0.0698591
5207	2.2375	1.47754
5208	1.50998	1.58693
5209	2.75021	1.31672
5210	1.91912	0.948762
5211	1.99872	0.931922
5212	0.918127	1.69955
5213	1.03031	1.19442
5214	0.951169	1.06619
5215	1.27975	0.914314
5216	1.12735	1.01288
5217	1.15741	1.07108

5218	1.24998	1.10663
5219	1.65953	0.276631
5220	0.967555	0.502767
5221	0.967542	0.307306
5222	1.03824	0.217256
5223	1.48693	0.188246
5224	1.48693	0.154368
5225	2.02901	0.159814
5226	0.946127	0.267351
5227	1.07139	0.246323
5228	0.946126	0.248828
5229	1.84644	0.187853
5230	1.84644	0.185
5231	1.38683	0.217427
5232	1.40574	0.218242
5233	1.36866	0.0964128
5234	0.649325	0.178823
5235	1.93631	2.1
5236	1.50145	2.1
5237	1.6287	2.1
5238	1.31302	2.1
5239	1.18041	2.1
5240	1.18041	2.1
5241	0.974196	2.1
5242	0.946131	2.1
5243	1.74169	2.1
5244	1.74169	2.1
5245	1.23163	2.1
5246	1.27451	2.1
5247	1.41331	2.1
5248	1.23163	2.1
5249	1.28037	2.1
5250	1.28158	2.1
5251	1.28037	2.1
5252	2.28015	0.0698591
5253	2.06561	0.0698591
5254	2.61343	0.0698591
5255	1.11712	0.0698591
5256	1.63689	0.238567
5257	1.01393	0.204344
5258	0.996775	2.1
5259	1.54277	2.1
5260	1.26397	2.1
5261	1.46129	2.1
5262	1.62391	2.1
5263	1.08848	0.0698591
5264	1.11833	2.1
5265	1.24041	2.1
5266	2.31884	2.1
5267	1.335	2.1
5268	1.48589	0.0698591
5269	1.77131	0.0698591
5270	1.8422	1.16078
5271	2.47453	1.59339
5272	1.23726	1.52106
5273	1.21996	1.48794
5274	1.65512	1.45261
5275	1.65512	1.37031

5276	0.802107	1.80514
5277	0.946129	1.40918
5278	0.802137	1.37302
5279	1.3279	1.08934
5280	1.23726	1.07164
5281	1.79759	0.979048
5282	1.79759	0.882225
5283	0.767577	1.56276
5284	0.946131	1.02072
5285	0.767577	0.981422
5286	1.03534	0.433862
5287	1.09962	2.01261
5288	0.929217	2.09131
5289	1.70126	2.09591
5290	0.991407	2.1
5291	1.41077	2.1
5292	0.991406	2.1
5293	1.62966	2.1
5294	1.62966	2.1
5295	0.946128	2.1
5296	1.18364	2.1
5297	0.946131	2.1
5298	1.14186	2.1
5299	1.18364	2.1
5300	1.16858	2.1
5301	1.66137	0.797982
5302	1.90165	0.509728
5303	1.66139	0.133744
5304	1.26893	0.139253
5305	1.15918	0.0972183
5306	0.653228	0.130883
5307	0.946133	0.073713
5308	1.77488	0.259789
5309	1.77489	0.194736
5310	0.653228	0.109377
5311	1.56043	0.163315
5312	1.60894	0.180112
5313	1.56043	0.0851984
5314	1.06848	0.119154
5315	1.04307	2.1
5316	1.14188	2.1
5317	1.09195	2.1
5318	0.615121	2.1
5319	1.50867	2.1
5320	1.27152	2.1
5321	0.615094	2.1
5322	1.07297	2.1
5323	1.1372	2.1
5324	0.553608	2.1
5325	1.23019	2.1
5326	0.992675	2.1
5327	1.40984	2.04246
5328	2.55682	2.1
5329	3.66069	2.1
5330	3.58726	2.1
5331	1.4375	0.0793187
5332	1.88424	0.117711
5333	1.56714	2.1

5334	1.6721	2.1
5335	1.18474	2.1
5336	1.2295	2.1
5337	1.28754	2.1
5338	1.36838	2.1
5339	2.78661	2.1
5340	2.64055	2.1
5341	1.35065	0.932227
5342	2.26675	0.557224
5343	1.35065	0.842884
5344	1.21558	0.582229
5345	1.49709	0.0698591
5346	1.4971	0.0698591
5347	0.85472	0.0698591
5348	0.854718	0.0698591
5349	0.946129	0.0698591
5350	0.854717	0.0698591
5351	1.1604	0.0698591
5352	1.25265	0.0698591
5353	2.32081	0.0698591
5354	1.15371	0.0698591
5355	1.14945	2.1
5356	0.995725	2.1
5357	1.00307	0.215152
5358	1.10907	0.160035
5359	2.30315	0.162598
5360	1.10908	0.177933
5361	1.27558	1.08678
5362	1.71613	1.72215
5363	1.32977	2.1
5364	0.815276	2.1
5365	1.41697	2.1
5366	1.41697	2.1
5367	0.815276	2.1
5368	1.38897	2.1
5369	0.946131	2.1
5370	0.815275	2.1
5371	1.44684	2.1
5372	1.21489	1.6809
5373	1.22476	1.59241
5374	0.870366	1.03314
5375	0.862572	0.824721
5376	0.835435	0.667032
5377	1.6993	0.519776
5378	1.23618	0.584364
5379	1.18624	0.534725
5380	1.53058	0.663998
5381	2.16108	0.748003
5382	2.21589	0.642812
5383	1.04037	2.08935
5384	1.03933	2.09569
5385	1.57753	0.0937126
5386	1.87644	0.12172
5387	1.24728	0.119161
5388	1.30394	0.0867519
5389	1.3538	2.1
5390	1.69858	2.1
5391	1.1726	0.192438

5392	0.94612	0.132467
5393	1.09805	0.0824231
5394	0.946118	0.178325
5395	1.72576	0.136502
5396	1.72575	0.0759047
5397	1.34152	0.119343
5398	1.33034	0.1192
5399	0.784151	2.1
5400	0.946131	2.1
5401	0.784151	2.1
5402	1.47963	2.1
5403	1.47963	2.1
5404	1.3335	2.1
5405	0.930281	2.1
5406	1.29435	2.1
5407	0.883308	2.1
5408	1.31589	2.1
5409	1.67846	2.1
5410	0.993598	0.0698591
5411	2.23193	0.0698591
5412	0.9936	0.0698591
5413	0.615629	0.0698591
5414	1.65925	2.1
5415	2.05992	2.1
5416	1.65925	2.1
5417	4.08735	1.6262
5418	2.72856	1.25311
5419	0.869602	2.04685
5420	3.42833	0.804204
5421	1.14397	1.60851
5422	1.34563	0.0851574
5423	1.08189	0.0792537
5424	0.782128	1.97924
5425	1.0022	2.0741
5426	0.900427	1.99015
5427	1.51499	2.00362
5428	1.05588	1.95779
5429	1.13739	2.07614
5430	1.37004	1.99971
5431	1.14603	1.98964
5432	0.851541	1.51383
5433	0.946799	1.90977
5434	1.14854	1.82039
5435	1.33793	1.63255
5436	0.94613	1.81786
5437	1.24336	1.8349
5438	0.851473	1.85227
5439	2.08792	1.69857
5440	1.01869	1.80248
5441	0.986953	0.0709633
5442	0.896238	0.0907361
5443	0.906574	0.0891749
5444	0.852145	0.0758249
5445	2.14191	0.0710286
5446	1.15979	0.0740772
5447	1.18327	0.0713531
5448	2.32742	0.0753149
5449	2.35961	0.0963851

5450	2.38126	0.0713458
5451	1.13632	0.0715873
5452	1.17897	0.0698591
5453	1.76199	0.0698591
5454	1.00573	2.1
5455	0.946127	1.77522
5456	1.7077	2.1
5457	1.7077	2.1
5458	0.946127	2.08307
5459	1.24887	2.1
5460	1.24887	2.1
5461	1.22441	2.1
5462	1.08542	2.1
5463	1.21743	2.03727
5464	1.12572	2.1
5465	1.05499	0.867546
5466	1.00521	0.850567
5467	2.86191	0.0698591
5468	2.27972	0.0698591
5469	1.01169	2.1
5470	1.03373	2.1
5471	1.39319	2.1
5472	0.636837	2.1
5473	1.63908	2.09567
5474	1.45713	1.95485
5475	1.04072	2.1
5476	1.25025	1.98001
5477	1.38914	2.0823
5478	0.636807	2.09671
5479	1.50211	2.1
5480	1.36555	2.1
5481	2.77545	1.91214
5482	0.682805	2.1
5483	1.50267	1.95982
5484	1.50267	2.1
5485	0.797749	2.1
5486	1.38773	1.95837
5487	1.38773	2.1
5488	0.682776	2.1
5489	2.01245	0.0698591
5490	0.925918	0.0698591
5491	0.75689	0.0698591
5492	1.25823	0.0698591
5493	1.16505	0.0698591
5494	0.838779	0.0698591
5495	1.14722	0.0698591
5496	0.756849	0.0698591
5497	1.52298	0.0698591
5498	2.11034	0.0698591
5499	1.92174	0.0698591
5500	1.92174	0.0698591
5501	0.576083	0.0698591
5502	1.23146	0.0698591
5503	1.26637	0.0698591
5504	1.23146	0.0698591
5505	1.26637	0.0698591
5506	0.946133	0.589786
5507	1.37034	0.453619

5508	1.84145	0.482947
5509	1.84145	0.379466
5510	0.946132	0.733714
5511	1.37034	0.494681
5512	1.41724	0.485633
5513	1.0685	0.360034
5514	1.14547	0.379084
5515	1.24976	2.1
5516	1.30682	2.1
5517	2.12095	2.1
5518	1.46392	2.1
5519	1.46392	2.1
5520	0.935738	2.1
5521	1.32949	2.1
5522	1.07017	2.1
5523	0.935766	2.1
5524	1.07017	2.1
5525	1.78095	2.1
5526	1.05619	0.294039
5527	1.05793	0.226182
5528	1.77677	2.1
5529	2.04766	2.1
5530	1.02524	2.1
5531	1.18803	2.1
5532	1.70047	2.1
5533	1.556 2.1	
5534	1.24201	2.1
5535	1.3592	2.1
5536	2.11824	0.0698591
5537	2.20248	0.0884602
5538	3.43878	0.0699547
5539	3.12466	0.0698591
5540	2.09351	2.1
5541	0.946128	2.1
5542	1.51269	2.1
5543	1.51269	2.1
5544	0.946131	2.1
5545	1.01306	2.1
5546	1.04676	2.1
5547	1.01306	2.1
5548	1.07444	2.1
5549	2.17465	2.1
5550	1.14808	2.1
5551	1.39067	2.0695
5552	1.20958	1.98981
5553	0.662832	0.075815
5554	1.93512	0.0755666
5555	1.15471	2.1
5556	1.75119	2.1
5557	1.75118	2.1
5558	0.946131	2.1
5559	1.28606	2.1
5560	1.27087	2.1
5561	0.946131	2.1
5562	1.28606	2.1
5563	1.58916	0.0702419
5564	0.927822	0.0822548
5565	2.24367	0.0698591

5566	1.8734	0.0698591
5567	3.51668	0.0698591
5568	2.68986	0.0698591
5569	2.32395	2.1
5570	1.39564	2.1
5571	2.10862	2.1
5572	1.36763	2.1
5573	0.970963	0.0698591
5574	0.97096	0.0698591
5575	1.11897	0.135398
5576	1.26885	0.194357
5577	1.11605	0.146003
5578	2.06612	0.119311
5579	1.717	0.132826
5580	0.59502	0.187988
5581	0.825755	0.108467
5582	0.709624	0.21369
5583	1.65741	0.117268
5584	0.307486	0.109622
5585	1.4785	0.125727
5586	0.983143	2.1
5587	1.08247	2.1
5588	1.42353	2.1
5589	1.42353	2.1
5590	0.781667	2.1
5591	0.797634	2.1
5592	0.946131	2.1
5593	0.781638	2.1
5594	0.946131	2.1
5595	0.98315	2.1
5596	1.083	2.1
5597	1.61975	0.0698591
5598	1.11946	0.0698591
5599	1.18977	0.0700768
5600	0.996484	0.0711217
5601	2.24343	0.0698591
5602	1.4787	0.0698591
5603	1.66127	0.0698591
5604	1.86028	0.0698591
5605	1.09128	0.0698591
5606	1.03115	0.0701186
5607	0.838025	0.0698591
5608	1.3736	0.0698591
5609	1.44009	0.0698591
5610	0.92917	0.0698591
5611	1.03889	0.0699578
5612	0.956974	0.0698591
5613	3.23468	0.0698591
5614	0.953922	0.0762156
5615	0.957104	0.0743013
5616	1.66231	0.0698591
5617	1.26778	0.0698591
5618	1.47615	0.0698591
5619	1.28621	0.0719238
5620	1.08043	0.0701867
5621	2.35026	2.1
5622	2.35025	2.1
5623	2.60659	2.1

5624	1.57627	2.1
5625	1.5493	2.1
5626	1.24682	0.399853
5627	2.14277	0.396977
5628	1.24682	0.5477
5629	1.00307	0.33379
5630	2.04056	0.31686
5631	1.45408	2.1
5632	1.54249	2.1
5633	1.24474	1.59234
5634	1.37813	1.71197
5635	0.924158	0.0698591
5636	0.916623	0.0698591
5637	1.35344	0.0698591
5638	1.00859	0.0698591
5639	1.34786	0.0698591
5640	0.952457	0.0698591
5641	0.85404	0.0698591
5642	1.06341	2.1
5643	1.09194	2.1
5644	1.93707	2.1
5645	2.14087	2.1
5646	1.93707	2.1
5647	1.07519	2.1
5648	1.07519	2.1
5649	1.62518	2.1
5650	1.3592	2.1
5651	1.13183	2.1
5652	1.20357	2.1
5653	1.4858	2.1
5654	1.23694	2.1
5655	1.44011	2.1
5656	1.23989	2.1
5657	2.21604	2.1
5658	1.85574	1.09961
5659	1.55649	2.1
5660	1.48861	0.704736
5661	1.15968	0.0698591
5662	0.946132	0.0698591
5663	0.663056	0.0698591
5664	1.70837	0.0698591
5665	1.70837	0.0698591
5666	0.946131	0.0698591
5667	0.663026	0.0698591
5668	2.09604	0.0698591
5669	0.81094	0.0698591
5670	1.06559	0.0698591
5671	0.810956	0.0698591
5672	1.15474	0.0698591
5673	0.990051	0.0698591
5674	0.894971	0.0698591
5675	1.32536	0.0698591
5676	0.946133	2.1
5677	1.33125	2.1
5678	1.75823	2.1
5679	1.75823	2.1
5680	0.946133	2.1
5681	1.33125	2.1

5682	1.37312	2.1
5683	1.27985	2.1
5684	1.37312	2.1
5685	1.70684	2.1
5686	1.70684	2.1
5687	0.946132	2.1
5688	0.946132	2.1
5689	1.25081	2.1
5690	0.956293	2.1
5691	0.956294	2.1
5692	1.36035	2.1
5693	1.41944	2.1
5694	0.830638	2.1
5695	0.747568	2.1
5696	0.888498	2.1
5697	1.04071	2.1
5698	1.17128	2.1
5699	1.92339	2.1
5700	2.91208	2.1
5701	1.82208	0.652663
5702	1.89653	0.23364
5703	1.82588	1.37062
5704	1.26703	0.240312
5705	1.26703	0.443811
5706	0.6157	0.719408
5707	1.26706	1.6261
5708	0.61567	0.11725
5709	0.615671	0.131714
5710	1.26706	0.263183
5711	1.89158	0.197071
5712	1.23134	0.23405
5713	2.49497	0.0698591
5714	1.68103	0.0698591
5715	2.0554	2.1
5716	1.72647	2.1
5717	1.67375	2.1
5718	1.65037	2.1
5719	1.04349	1.36042
5720	1.69055	0.0698591
5721	1.53463	0.0698591
5722	1.19022	1.78973
5723	1.89226	1.2216
5724	1.15009	1.24294
5725	1.7054	1.12059
5726	1.31612	0.356945
5727	1.31611	0.957148
5728	1.67345	0.909264
5729	1.06577	0.207576
5730	1.40193	0.127953
5731	1.72411	0.111412
5732	1.46123	0.131489
5733	0.808808	0.193031
5734	0.897714	0.133876
5735	0.851554	0.232198
5736	1.3499	0.0698591
5737	1.29001	0.0698591
5738	1.22083	2.1
5739	1.06191	2.1

5740	1.05758	0.922221
5741	1.348	1.81063
5742	1.43485	0.905981
5743	1.34799	1.71849
5744	1.36307	0.0698591
5745	2.28188	0.0698591
5746	1.7926	0.0698591
5747	1.38957	2.1
5748	1.47446	2.1
5749	0.953638	2.1
5750	1.11868	2.1
5751	0.953636	2.1
5752	1.03408	2.1
5753	1.52443	0.0698591
5754	1.38539	0.0698591
5755	0.946132	0.0698591
5756	1.12747	0.0698591
5757	1.04393	0.0698976
5758	1.05017	0.0698591
5759	1.20529	0.0698591
5760	1.3663	0.247878
5761	1.89226	0.217843
5762	1.17885	0.188507
5763	0.867881	0.135339
5764	1.32917	0.100514
5765	1.32917	0.0780117
5766	0.946127	0.22535
5767	1.19583	0.143868
5768	1.00122	0.0754725
5769	0.946128	0.188183
5770	1.71405	0.239244
5771	0.871815	2.06701
5772	1.16435	2.1
5773	0.686619	2.09693
5774	1.63102	2.1
5775	1.06589	2.09305
5776	1.05375	2.1
5777	1.9023	2.1
5778	1.27394	0.500396
5779	1.88345	0.436074
5780	2.05636	0.423156
5781	0.672977	0.49545
5782	1.41274	0.462079
5783	1.65606	0.450969
5784	0.869329	0.493917
5785	1.2207	0.479831
5786	1.35631	0.47865
5787	0.908776	0.516596
5788	0.955081	0.391431
5789	1.2243	2.1
5790	1.23169	2.1
5791	1.52228	2.1
5792	1.23169	2.1
5793	0.990892	0.223048
5794	1.08376	0.161903
5795	1.79348	0.160301
5796	1.50043	0.126589
5797	1.32599	0.325981

5798	1.37924	0.181631
5799	0.838014	0.179235
5800	0.87737	0.447742
5801	0.947086	0.286908
5802	1.27199	0.325581
5803	1.04918	0.223048
5804	1.04851	0.491017
5805	1.1699	0.358039
5806	0.810934	0.428854
5807	1.83602	0.517135
5808	2.21909	0.352717
5809	0.619441	0.431931
5810	2.03755	0.293994
5811	1.83472	0.389453
5812	1.20416	0.474723
5813	1.26691	0.264932
5814	0.655795	0.473728
5815	0.94613	0.380133
5816	2.35169	0.467009
5817	3.54705	2.0841
5818	2.60741	2.08583
5819	1.59019	0.0698591
5820	1.24526	0.0698591
5821	1.59019	0.0698591
5822	2.36406	0.0698591
5823	1.16776	2.09413
5824	1.67311	2.1
5825	1.67311	2.01601
5826	0.94613	1.72297
5827	1.38645	2.1
5828	1.13563	0.669559
5829	1.13563	1.71484
5830	1.23279	1.46407
5831	1.05042	2.1
5832	1.17418	2.1
5833	1.28909	2.1
5834	0.973448	2.1
5835	1.02958	2.1
5836	3.3005	2.1
5837	2.86325	2.1
5838	1.35879	2.1
5839	1.69332	2.1
5840	1.69332	2.1
5841	0.946128	2.1
5842	1.39659	2.1
5843	1.12035	2.1
5844	1.12035	2.1
5845	1.24286	2.1
5846	1.10407	2.1
5847	1.14599	2.1
5848	1.32259	2.06518
5849	0.788592	0.0698591
5850	2.4588	2.1
5851	0.909581	1.9776
5852	1.35656	1.8536
5853	0.986895	1.90596
5854	2.30602	1.77003
5855	1.46873	1.85109

5856	1.55584	1.81405
5857	2.1547	1.83347
5858	1.87794	1.8951
5859	0.808286	1.99898
5860	0.928464	1.94442
5861	0.810959	1.91385
5862	1.50962	1.66313
5863	1.1053	1.8144
5864	1.0712	1.8577
5865	1.40504	1.86375
5866	1.87794	1.39222
5867	2.80496	1.48717
5868	1.41479	1.87781
5869	1.38634	2.1
5870	1.38634	2.1
5871	1.67762	2.1
5872	0.946131	2.1
5873	1.188 2.1	
5874	1.62585	2.1
5875	1.62585	2.1
5876	0.946129	2.1
5877	1.18801	2.1
5878	1.38397	2.1
5879	1.04546	2.1
5880	1.16817	2.1
5881	1.06007	2.1
5882	1.19785	2.1
5883	1.43652	2.1
5884	1.06994	2.1
5885	1.89111	2.1
5886	1.07144	2.1
5887	1.51754	2.1
5888	2.0873	2.1
5889	0.758799	2.1
5890	1.59518	2.1
5891	1.59518	2.1
5892	0.946132	2.1
5893	0.758771	2.1
5894	1.06957	2.1
5895	0.758771	2.1
5896	1.33916	2.1
5897	2.03045	1.62148
5898	2.80184	1.7851
5899	1.76788	1.77919
5900	1.27442	2.05621
5901	1.06756	2.1
5902	0.966979	2.1
5903	1.7054	2.1
5904	1.07156	2.1
5905	0.986096	2.1
5906	1.32813	2.1
5907	1.3331	2.1
5908	2.08369	0.105609
5909	2.23818	0.763704
5910	1.10335	0.366739
5911	0.622314	0.178862
5912	1.72451	0.187935
5913	1.72451	0.502117

5914	0.622283	0.257378
5915	0.622314	0.264211
5916	0.946131	0.363629
5917	1.31254	0.987687
5918	1.15704	0.848559
5919	1.24463	0.247184
5920	2.2067	0.526644
5921	1.30001	0.984593
5922	1.29175	1.15657
5923	1.1233	0.0698591
5924	1.8689	0.0698591
5925	1.29494	0.0698591
5926	1.11895	0.0698591
5927	0.851539	0.0698591
5928	0.851474	0.0698591
5929	1.58352	0.0698591
5930	1.58228	0.0698591
5931	0.851527	0.0698591
5932	2.6389	0.0698591
5933	1.77548	0.0698591
5934	1.24555	2.1
5935	2.0512	2.1
5936	1.46101	2.1
5937	2.60635	2.1
5938	1.10476	0.0698591
5939	1.44103	0.0698591
5940	0.652391	2.1
5941	0.618322	2.1
5942	2.22915	2.1
5943	1.39067	2.1
5944	1.99737	2.1
5945	1.42174	2.1
5946	0.946132	0.381734
5947	1.29735	1.77094
5948	1.61765	2.1
5949	1.61765	0.65695
5950	0.946132	1.85525
5951	1.29735	1.11588
5952	1.26643	1.18797
5953	1.01335	0.803797
5954	1.01335	0.167794
5955	0.947669	2.1
5956	2.17167	2.0969
5957	2.59733	1.96939
5958	1.50954	2.03386
5959	1.02638	1.79282
5960	0.555785	1.97791
5961	1.91101	1.95597
5962	2.09809	1.83066
5963	0.641974	2.02953
5964	1.2022	1.95779
5965	1.36359	1.89897
5966	1.8128	1.92206
5967	2.34348	0.192874
5968	1.66343	0.133715
5969	0.974717	0.385917
5970	1.53908	0.300067
5971	1.27737	1.40719

5972	1.09408	0.875785
5973	2.07382	0.751113
5974	1.10466	1.77517
5975	1.05654	1.53069
5976	1.23498	1.05243
5977	1.48104	1.40082
5978	1.23498	1.00862
5979	1.17261	2.1
5980	1.18059	2.1
5981	2.43005	2.1
5982	1.04546	2.1
5983	2.81222	2.1
5984	1.16817	2.1
5985	1.75766	0.869614
5986	1.47021	1.23392
5987	1.07236	0.259069
5988	1.81759	0.724129
5989	1.42913	0.385181
5990	1.00636	0.473627
5991	1.00636	0.311112
5992	1.79397	0.309051
5993	0.946131	0.456604
5994	1.03691	0.508935
5995	0.851539	0.454213
5996	1.46001	0.472928
5997	1.37878	0.521207
5998	0.933258	0.460418
5999	1.32423	0.468553
6000	1.32614	0.0698591
6001	1.11356	0.0698591
6002	1.32614	0.0698591
6003	1.28895	0.0698591
6004	1.66895	0.0698591
6005	1.66895	0.0698591
6006	0.946132	0.0698591
6007	1.29576	0.0698591
6008	1.1318	0.0698591
6009	1.37621	1.74662
6010	1.94071	1.68971
6011	1.10517	2.1
6012	1.97098	2.1
6013	0.811 2.1	
6014	0.956127	2.1
6015	1.18647	1.99682
6016	0.988455	1.58359
6017	1.12457	0.0831873
6018	1.7453	0.102238
6019	0.92492	2.1
6020	1.24353	2.1
6021	1.84536	0.554673
6022	1.67662	0.458557
6023	1.38247	0.0698591
6024	1.02663	0.0698591
6025	0.946132	0.493569
6026	1.41723	0.396118
6027	1.72976	0.438015
6028	1.72976	0.329301
6029	0.946131	0.658817

6030	1.41723	0.462802
6031	1.25866	0.464517
6032	1.34254	0.282303
6033	1.25866	0.363525
6034	1.65507	0.293263
6035	1.65507	0.24799
6036	0.946132	0.495823
6037	0.946132	0.378794
6038	1.11929	0.366106
6039	0.961779	0.293695
6040	0.961779	0.300417
6041	0.946132	2.1
6042	1.69361	2.1
6043	1.69361	2.1
6044	0.94613	2.1
6045	1.36523	2.1
6046	1.36523	2.1
6047	1.19802	2.1
6048	0.974196	2.1
6049	1.09518	2.1
6050	0.946131	2.1
6051	1.7161	2.1
6052	1.7161	2.1
6053	0.94613	2.1
6054	1.297	2.1
6055	1.297	2.1
6056	1.14599	2.1
6057	0.974197	2.1
6058	0.974197	2.1
6059	1.80778	2.1
6060	2.20473	2.1
6061	1.80777	2.1
6062	1.3515	2.1
6063	0.583237	2.1
6064	1.39359	2.1
6065	1.39359	2.1
6066	1.07296	2.1
6067	0.946114	2.1
6068	1.07297	2.1
6069	0.903862	2.1
6070	0.956572	2.1
6071	1.03437	2.1
6072	0.975409	2.1
6073	1.31167	0.0698699
6074	2.70246	0.0698591
6075	1.12018	0.0698592
6076	0.655836	0.0699726
6077	0.655837	0.0698692
6078	1.81557	0.0698591
6079	1.81557	0.0698591
6080	0.655836	0.0699006
6081	0.946131	0.0713622
6082	1.01847	0.0698591
6083	1.61334	0.0698591
6084	0.810946	0.070842
6085	0.8311	0.0738674
6086	1.2466	0.0698591
6087	1.20286	0.0698591

6088	0.97508	0.0711269
6089	0.870805	0.0698591
6090	0.760087	0.0725892
6091	0.853389	2.1
6092	0.820894	2.1
6093	0.928291	2.1
6094	1.06922	2.1
6095	1.32829	2.1
6096	1.12173	2.1
6097	1.16615	2.1
6098	0.957748	2.1
6099	0.612491	2.00544
6100	0.548141	2.1
6101	0.979614	2.1
6102	0.658184	2.09442
6103	0.678456	2.00307
6104	0.529544	1.99796
6105	0.739013	2.0992
6106	0.694037	2.08681
6107	0.475716	2.03665
6108	1.58794	1.99797
6109	1.58794	1.8674
6110	0.475743	1.94265
6111	1.08301	2.07132
6112	0.980664	2.09407
6113	0.475737	2.05674
6114	1.32709	2.1
6115	1.59396	2.1
6116	1.19719	0.17602
6117	1.34921	0.182957
6118	1.69061	0.170239
6119	1.69061	0.124328
6120	0.946132	0.222865
6121	1.34921	0.182085
6122	1.28753	0.178521
6123	0.95895	0.136369
6124	1.01051	0.152908
6125	1.12468	0.170142
6126	1.22588	0.217671
6127	1.34254	0.194612
6128	1.74561	0.181819
6129	1.74561	0.146882
6130	0.946128	0.24598
6131	1.34253	0.194829
6132	1.3492	0.198887
6133	1.01051	0.142516
6134	1.922 2.1	
6135	1.922 2.1	
6136	2.12556	2.1
6137	0.946131	2.1
6138	1.06278	2.1
6139	1.47631	2.1
6140	1.47631	2.1
6141	0.946129	2.1
6142	1.06278	2.1
6143	1.35966	2.1
6144	0.991985	2.1
6145	0.738507	1.09748

6146	0.536935	1.77455
6147	0.799004	1.15981
6148	1.04281	0.907396
6149	0.879197	0.867193
6150	1.06538	0.926126
6151	0.715942	1.09754
6152	1.00636	0.325067
6153	1.03824	0.234895
6154	1.70147	0.238926
6155	1.21223	2.1
6156	1.01243	2.1
6157	1.01242	2.1
6158	1.06935	2.1
6159	1.54241	2.1
6160	0.998971	0.0698591
6161	1.60312	0.0698591
6162	1.44836	0.0698591
6163	2.05632	0.0698591
6164	1.44836	0.0698591
6165	0.973765	0.0698591
6166	0.946131	0.0698591
6167	0.851541	0.0698591
6168	1.73854	0.0698591
6169	1.91752	0.0698591
6170	1.49622	0.0698591
6171	1.23351	0.0698591
6172	3.33289	2.1
6173	1.86606	2.1
6174	2.71233	2.1
6175	0.788816	2.1
6176	0.963972	2.1
6177	1.25866	2.1
6178	1.10817	2.1
6179	1.41908	2.1
6180	1.48734	2.1
6181	1.40822	0.153895
6182	1.40822	0.153426
6183	2.09871	0.239977
6184	0.946133	0.196151
6185	1.14926	0.107807
6186	0.946133	0.205369
6187	1.78098	0.130651
6188	1.78098	0.0946813
6189	1.35822	0.160623
6190	1.3689	0.161291
6191	1.22176	0.0755501
6192	2.03426	0.138664
6193	0.946131	0.207245
6194	1.01713	0.143447
6195	0.704112	0.218126
6196	1.36237	0.136956
6197	1.36237	0.113514
6198	1.01713	0.187909
6199	1.04936	0.182081
6200	1.87377	2.1
6201	1.60501	2.1
6202	0.981584	0.0698591
6203	1.69435	0.0698591

6204	0.85154	2.1
6205	1.47841	2.1
6206	1.4952	2.1
6207	1.31941	2.1
6208	1.04023	2.1
6209	1.1995	2.1
6210	0.914791	2.1
6211	1.1427	1.83131
6212	1.64787	0.75605
6213	1.07526	1.61759
6214	1.07527	0.889784
6215	1.07527	1.16317
6216	1.58551	1.52696
6217	2.77362	1.19767
6218	2.17974	0.818904
6219	2.71723	1.19435
6220	1.29868	0.0748702
6221	1.3594	0.0730006
6222	1.04786	0.0709853
6223	0.527876	0.0706622
6224	1.4085	0.0747012
6225	1.48538	0.0698591
6226	0.76829	0.0698591
6227	1.18514	0.0735133
6228	0.617954	0.0698591
6229	0.864161	0.070224
6230	1.18835	0.0698591
6231	1.16817	2.1
6232	1.04263	2.1
6233	1.50524	2.1
6234	1.85355	2.1
6235	1.23963	2.1
6236	1.06279	2.1
6237	0.94613	2.1
6238	1.35229	2.1
6239	1.15947	2.1
6240	0.965593	2.1
6241	0.851553	2.1
6242	2.21296	2.1
6243	1.11764	2.1
6244	2.46399	2.1
6245	1.39292	2.1
6246	1.34701	2.1
6247	1.34701	2.1
6248	1.47001	2.1
6249	0.963166	0.0698591
6250	0.893445	0.0698591
6251	1.21056	0.0698591
6252	1.67375	2.1
6253	2.0977	2.1
6254	1.09154	2.1
6255	1.09154	2.1
6256	1.57122	0.72386
6257	1.60976	0.182924
6258	1.69533	0.140897
6259	1.6951	0.107251
6260	1.5831	0.0953819
6261	1.20969	2.1

6262	1.12076	2.1
6263	1.49808	2.1
6264	1.50201	2.1
6265	1.00278	2.1
6266	1.22811	0.0963102
6267	1.11545	2.1
6268	1.09646	2.1
6269	1.03107	0.0698591
6270	1.44293	0.0698591
6271	1.66752	2.1
6272	1.11023	2.1
6273	2.57831	1.97588
6274	2.20191	0.888946
6275	2.18051	1.98291
6276	1.48035	0.2814
6277	1.0961	0.153685
6278	1.63241	0.223036
6279	1.0961	0.387805
6280	1.20959	2.1
6281	2.86136	2.1
6282	1.64933	2.1
6283	1.47217	2.1
6284	0.915178	2.1
6285	1.19074	2.1
6286	0.897276	2.1
6287	0.915177	2.1
6288	0.987037	2.1
6289	1.04288	2.1
6290	2.39323	2.1
6291	0.976218	0.0698591
6292	2.23933	0.0698591
6293	0.976215	0.0698591
6294	1.07384	0.0698591
6295	1.07652	2.1
6296	1.55678	2.1
6297	1.54168	2.1
6298	1.69136	2.1
6299	1.64391	1.27662
6300	1.76477	1.54752
6301	0.611486	1.50257
6302	0.683421	0.838477
6303	0.946131	1.51946
6304	1.1019	1.25828
6305	1.12946	1.05092
6306	1.54226	1.79444
6307	1.19496	0.992258
6308	1.96199	0.99819
6309	2.0775	1.48908
6310	1.22297	1.07258
6311	1.3469	1.1154
6312	1.25366	1.389
6313	1.65443	1.3877
6314	1.65443	1.48644
6315	0.946132	1.68676
6316	0.946132	1.76132
6317	1.12873	1.63644
6318	1.07468	1.34248
6319	1.2854	0.667363

6320	1.3469	1.23364
6321	1.68616	1.15878
6322	1.68616	0.943147
6323	0.946132	1.70302
6324	0.946132	1.60088
6325	1.16109	1.44637
6326	1.17879	1.07962
6327	1.17879	1.13448
6328	1.45304	1.51148
6329	2.21058	2.1
6330	2.81552	2.1
6331	1.45535	0.129944
6332	1.49906	0.0956401
6333	1.04413	2.1
6334	1.74586	2.1
6335	1.34526	2.1
6336	1.17402	1.05105
6337	1.74564	0.742458
6338	1.52724	2.1
6339	1.68142	2.1
6340	1.09651	2.1
6341	1.49149	2.1
6342	1.36814	2.1
6343	1.46783	2.1
6344	2.18116	2.09933
6345	1.23602	2.09065
6346	1.74538	1.98923
6347	0.851539	2.1
6348	1.45775	2.1
6349	1.28919	2.1
6350	0.851483	2.1
6351	1.26102	2.1
6352	0.869889	2.1
6353	0.85151	2.1
6354	1.23451	2.09861
6355	1.23602	2.1
6356	1.11955	2.1
6357	1.08022	2.1
6358	0.977776	2.1
6359	1.27896	2.1
6360	1.64268	2.1
6361	1.97548	2.1
6362	1.72527	2.06209
6363	0.946132	2.1
6364	1.69702	2.1
6365	1.69702	2.1
6366	1.13635	2.1
6367	1.40274	2.1
6368	1.24041	2.1
6369	1.13635	2.1
6370	1.78427	0.0698591
6371	1.68338	0.0698591
6372	1.00681	0.0698591
6373	1.12988	0.0698591
6374	0.946132	0.0698591
6375	0.957543	0.0698591
6376	1.40515	0.0698591
6377	1.40515	0.0698591

6378	0.797932	0.0698591
6379	1.30187	0.0698591
6380	1.14846	0.0698591
6381	0.665137	0.173964
6382	1.09575	0.214512
6383	0.596894	0.178475
6384	1.04072	0.271158
6385	0.663171	0.27426
6386	1.47218	0.220105
6387	1.32889	0.180065
6388	1.32639	0.0928357
6389	1.94887	0.110947
6390	1.32639	0.100771
6391	1.19323	0.146334
6392	2.19794	0.180862
6393	0.754756	0.0923443
6394	1.11015	0.10871
6395	0.754755	0.0937216
6396	0.946133	0.127979
6397	0.754768	0.124382
6398	1.59986	0.109952
6399	1.59986	0.0842156
6400	1.84082	2.1
6401	1.79044	2.1
6402	1.75922	2.1
6403	1.86766	2.1
6404	1.58995	2.1
6405	1.58995	2.1
6406	1.74351	2.1
6407	1.04442	2.1
6408	1.42278	2.1
6409	1.08576	2.1
6410	0.994294	2.1
6411	1.44884	2.1
6412	1.71036	2.08451
6413	0.714101	1.6517
6414	1.17541	2.1
6415	1.00872	1.00529
6416	0.714072	1.91921
6417	0.936677	1.82563
6418	0.966979	2.1
6419	1.4908	2.1
6420	1.03108	0.152736
6421	0.949712	0.0912537
6422	0.810946	0.146325
6423	0.88209	0.148092
6424	0.646702	0.0998296
6425	0.972663	0.0732206
6426	0.862301	0.0849095
6427	0.868143	0.0894974
6428	1.11094	0.0922485
6429	1.35809	0.0866213
6430	1.32455	2.1
6431	1.32328	2.1
6432	1.69586	2.1
6433	1.78301	2.1
6434	1.67906	2.1
6435	1.60635	2.1

6436	1.57234	2.1
6437	1.47012	2.1
6438	1.22811	0.109635
6439	1.08732	0.13033
6440	2.08849	0.109677
6441	1.08732	0.120426
6442	1.30635	0.0698591
6443	0.77315	0.0698591
6444	1.25732	0.0698591
6445	2.43845	2.1
6446	2.38577	2.08815
6447	0.946132	1.31952
6448	1.19288	2.1
6449	1.54508	2.1
6450	1.54508	1.8446
6451	0.94515	2.1
6452	1.19288	2.03059
6453	1.29735	1.98056
6454	1.12087	1.25438
6455	1.54699	1.59597
6456	1.43635	2.1
6457	1.33385	2.1
6458	1.43635	2.1
6459	1.02368	2.1
6460	1.035 2.1	
6461	1.00128	2.1
6462	1.04009	2.1
6463	1.14052	2.1
6464	0.443552	2.1
6465	0.984351	2.1
6466	0.591443	2.1
6467	0.929561	2.1
6468	1.52079	2.1
6469	1.035 2.1	
6470	3.5282	2.1
6471	1.88288	2.1
6472	2.73877	2.02163
6473	0.906059	2.1
6474	1.23107	0.0698591
6475	0.906712	0.070102
6476	0.841322	0.0727383
6477	1.53005	0.0698591
6478	1.10007	0.0700043
6479	1.38033	0.06997
6480	1.03442	0.0698591
6481	0.787223	0.0698591
6482	1.42589	0.0698606
6483	2.35665	0.0698837
6484	1.17793	0.125415
6485	1.81356	0.128763
6486	1.42964	0.125154
6487	1.95641	0.118339
6488	1.39139	0.106735
6489	1.39139	0.175085
6490	0.564533	0.0982064
6491	1.20016	0.139357
6492	0.755765	0.0779697
6493	0.755766	0.089901

6494	1.20016	0.097952
6495	1.91011	0.0795153
6496	1.4695	0.0814589
6497	1.70048	2.1
6498	1.93765	2.1
6499	0.741307	2.1
6500	0.780371	2.1
6501	0.946133	0.388906
6502	1.28918	1.01509
6503	1.5111	1.87516
6504	1.5111	0.567262
6505	0.946131	1.39456
6506	1.28918	0.742349
6507	1.16805	0.433594
6508	1.01179	0.417877
6509	0.946132	0.531161
6510	1.35328	1.12297
6511	1.69633	1.524
6512	1.69633	0.682134
6513	0.946133	1.38118
6514	1.35328	0.863069
6515	1.28918	0.523
6516	1.01179	0.743805
6517	1.58147	1.07307
6518	1.27737	0.53716
6519	2.17223	0.0698591
6520	0.697168	0.0698591
6521	1.62495	0.0980338
6522	1.62495	0.133737
6523	1.93948	0.235377
6524	0.601592	2.1
6525	1.93528	2.1
6526	2.13757	2.1
6527	1.67561	2.1
6528	2.70875	2.1
6529	1.93528	2.1
6530	3.98475	2.1
6531	1.12376	2.1
6532	1.19016	2.1
6533	1.67323	0.14421
6534	1.74019	0.125542
6535	1.84719	0.146049
6536	1.00764	0.113191
6537	1.23475	2.1
6538	1.06757	2.1
6539	1.57916	2.1
6540	1.06758	2.1
6541	1.28014	2.1
6542	1.33339	2.1
6543	1.2679	2.1
6544	1.23504	2.1
6545	1.50108	2.1
6546	1.3715	2.1
6547	0.58831	0.596015
6548	0.865971	0.505558
6549	0.813704	0.697753
6550	0.658856	0.538235
6551	0.495869	0.69414

6552	1.13845	0.651043
6553	0.780804	0.63055
6554	0.642023	0.692815
6555	0.707748	0.564198
6556	1.64476	0.465058
6557	1.64476	0.54152
6558	0.99611	0.573368
6559	1.35639	0.443312
6560	1.3564	0.512336
6561	0.946129	0.511321
6562	1.99222	0.544165
6563	1.4777	2.1
6564	1.03968	2.1
6565	1.82516	2.1
6566	0.851539	2.1
6567	1.63693	2.1
6568	1.59416	2.1
6569	0.878566	2.1
6570	1.40796	2.1
6571	1.13172	2.1
6572	0.851527	2.1
6573	1.14366	2.1
6574	0.823822	0.195346
6575	0.882001	0.201788
6576	1.09756	0.0792219
6577	1.11857	0.120385
6578	1.0934	0.118257
6579	0.960104	0.180582
6580	0.982001	0.282368
6581	1.74319	0.132272
6582	1.03043	0.143848
6583	0.967194	0.104314
6584	1.16938	2.1
6585	2.48377	2.1
6586	1.1181	2.1
6587	2.57349	2.1
6588	1.0465	2.1
6589	1.44709	2.1
6590	0.946132	2.1
6591	1.29302	2.1
6592	1.73502	2.1
6593	1.73502	2.1
6594	0.946132	2.1
6595	1.29302	2.1
6596	1.38813	2.1
6597	1.03933	2.1
6598	1.01976	0.263931
6599	1.11764	0.31542
6600	0.961779	0.390738
6601	1.0685	0.320815
6602	1.08426	1.70533
6603	0.99695	1.91241
6604	2.25593	1.57439
6605	1.47992	1.70473
6606	1.88989	1.46172
6607	1.65813	1.59324
6608	1.20278	1.69871
6609	0.981842	1.96455

6610	0.802655	2.09608
6611	0.953489	2.01097
6612	0.961719	2.00163
6613	0.975614	1.9786
6614	0.991617	1.96719
6615	0.940748	1.95097
6616	2.25019	2.0245
6617	2.30712	1.81475
6618	1.98823	1.9962
6619	0.522844	1.93216
6620	0.946122	1.94801
6621	0.946132	1.95739
6622	0.593846	1.89567
6623	1.26501	1.84513
6624	1.21171	0.0698591
6625	0.851542	0.0736028
6626	0.940327	0.0747707
6627	0.940356	0.0752221
6628	1.52117	0.0698591
6629	1.58072	0.0698591
6630	1.35169	0.0699003
6631	1.13889	0.0698619
6632	1.35007	0.0698591
6633	1.35007	0.0698591
6634	1.78381	0.0698591
6635	0.946132	0.0733169
6636	1.06627	0.0707869
6637	0.946131	0.0747171
6638	1.73005	0.0698591
6639	1.73005	0.0698591
6640	1.32449	0.0698591
6641	1.35169	0.0698591
6642	1.42446	0.304766
6643	1.86697	0.261162
6644	1.13413	0.275272
6645	1.42446	0.232821
6646	1.33575	0.107982
6647	1.20473	0.123869
6648	1.87517	0.0698591
6649	1.87517	0.0698591
6650	0.488712	0.0698591
6651	1.13242	0.0698591
6652	0.488713	0.0698591
6653	0.488713	0.0698591
6654	1.23146	0.0698591
6655	1.57932	0.0698591
6656	1.01698	0.0698591
6657	1.42085	0.0698591
6658	0.246725	0.0698591
6659	0.996283	0.0698591
6660	1.36965	0.0698591
6661	0.86361	0.0698591
6662	0.810945	0.0698591
6663	1.72938	0.0728578
6664	1.21649	0.0715956
6665	1.1865	0.0698591
6666	1.62387	0.0698591
6667	1.05302	0.0698591

6668	1.10049	0.855403
6669	0.946129	2.1
6670	1.70184	1.45214
6671	1.70184	2.1
6672	1.12538	2.1
6673	1.35969	1.25087
6674	1.28828	2.09947
6675	1.12538	2.08635
6676	0.99747	1.93685
6677	1.34851	0.942688
6678	0.923724	2.1
6679	1.47154	2.09494
6680	1.47154	2.1
6681	1.10698	2.1
6682	1.28828	2.04957
6683	1.10698	2.1
6684	0.923695	2.1
6685	0.99747	1.93893
6686	1.65677	2.1
6687	1.77677	2.1
6688	1.05689	0.176501
6689	2.11031	0.159736
6690	1.07367	0.122264
6691	0.656239	0.179321
6692	1.4563	0.151489
6693	1.4563	0.137057
6694	0.656238	0.178996
6695	0.946133	0.162912
6696	0.812937	0.193498
6697	0.946131	0.202446
6698	1.05689	0.132412
6699	1.34009	2.1
6700	1.398 2.1	
6701	3.29047	2.1
6702	2.53897	2.1
6703	1.24509	2.1
6704	3.06162	2.1
6705	1.01595	2.1
6706	1.52661	2.1
6707	1.5266	2.1
6708	0.946131	2.1
6709	1.31049	2.1
6710	1.16225	2.1
6711	1.05978	2.1
6712	1.16225	2.1
6713	1.06561	2.1
6714	0.953927	2.1
6715	1.15114	2.1
6716	1.1807	0.332678
6717	0.507259	0.282778
6718	1.47709	0.264056
6719	1.36192	0.23784
6720	0.487293	0.266287
6721	1.65569	0.31533
6722	0.679725	0.218977
6723	0.721928	0.275791
6724	1.03931	0.261979
6725	1.0775	0.136379

6726	1.0775	0.394989
6727	2.30207	0.665126
6728	1.01814	0.630586
6729	2.33963	0.558925
6730	2.42589	0.558985
6731	1.33962	2.1
6732	1.27956	2.1
6733	2.14624	0.0698591
6734	1.6812	0.0698591
6735	1.25698	0.0698591
6736	1.35725	0.0698591
6737	1.86387	0.0698591
6738	1.86387	0.0698591
6739	0.750365	0.0698591
6740	0.946128	0.0698591
6741	0.750365	0.0698591
6742	1.72271	0.0698591
6743	1.72272	0.0702025
6744	2.53736	0.0734566
6745	1.07327	2.1
6746	1.05376	2.1
6747	1.00387	1.15081
6748	2.0554	1.45476
6749	1.00387	0.832714
6750	1.00387	0.483588
6751	1.27119	2.1
6752	1.51748	2.1
6753	3.15271	2.1
6754	3.45801	2.1
6755	1.99871	2.1
6756	1.41186	2.1
6757	1.07624	2.1
6758	1.5954	2.1
6759	1.19323	1.20443
6760	1.21286	0.743984
6761	0.946114	2.1
6762	1.38395	2.1
6763	1.73389	2.1
6764	1.7339	2.1
6765	0.946115	2.1
6766	1.38397	2.1
6767	1.29604	2.1
6768	1.04544	2.1
6769	1.60578	0.0840216
6770	1.93407	0.0883553
6771	1.0525	0.780103
6772	1.0525	1.03606
6773	1.0525	2.00857
6774	1.38506	1.58753
6775	2.34737	0.0698591
6776	1.3531	0.0698591
6777	1.2925	0.0698591
6778	0.871466	0.0698591
6779	1.26623	0.0698591
6780	0.700631	0.0698591
6781	1.88774	0.0698591
6782	1.14167	0.0698591
6783	1.18606	0.0698591

6784	2.1238	0.0698591
6785	1.42762	0.767816
6786	1.67791	0.0698591
6787	1.47118	0.0698591
6788	0.946128	2.1
6789	1.31641	2.1
6790	1.75933	2.1
6791	1.75933	2.1
6792	0.946131	2.1
6793	1.31641	2.1
6794	1.38905	2.1
6795	1.38437	2.1
6796	1.51829	2.1
6797	0.947114	2.1
6798	1.13335	2.1
6799	1.47521	2.1
6800	1.14911	2.1
6801	1.53822	2.1
6802	1.39702	2.1
6803	0.739166	2.1
6804	0.706056	2.1
6805	0.964033	2.1
6806	1.58993	2.09745
6807	2.51892	2.08075
6808	0.595905	0.0698591
6809	1.18155	0.0698591
6810	1.73364	0.0698591
6811	0.952735	0.0698591
6812	2.10381	0.0698591
6813	1.12115	0.0698591
6814	1.27826	0.0698591
6815	2.74021	0.0698591
6816	1.78416	2.1
6817	1.78416	2.1
6818	0.946132	2.1
6819	1.41126	2.1
6820	1.21275	2.1
6821	0.946133	2.1
6822	1.41126	2.1
6823	1.33125	2.1
6824	1.33125	2.1
6825	1.78918	2.1
6826	1.0465	2.1
6827	1.99715	2.1
6828	1.17683	2.1
6829	1.25889	2.1
6830	0.860834	0.0698591
6831	0.946131	0.0698591
6832	1.05008	0.0698591
6833	0.970444	0.0698591
6834	1.19401	0.0698591
6835	1.25989	0.0698591
6836	0.702942	0.0698591
6837	1.40591	0.0698591
6838	1.72168	0.0698591
6839	0.446352	0.0698591
6840	1.04072	0.0698591
6841	0.711038	0.0698591

6842	0.980737	0.0698591
6843	1.05099	0.0698591
6844	1.26003	0.0698591
6845	0.495934	0.0698591
6846	1.03114	0.0698591
6847	1.09911	0.0698591
6848	1.24825	0.0698591
6849	2.19827	0.0698591
6850	2.0953	0.0698591
6851	2.30437	0.0698591
6852	0.775923	0.0698591
6853	2.2283	0.0698591
6854	1.19583	2.1
6855	1.14496	2.1
6856	1.62183	2.1
6857	1.62316	2.1
6858	1.02305	0.236599
6859	1.21136	0.123736
6860	1.44426	0.192055
6861	1.21137	0.0891539
6862	1.20143	0.193147
6863	1.15009	0.352242
6864	1.24965	2.1
6865	1.41077	2.1
6866	1.00052	0.107427
6867	1.79553	0.107903
6868	1.06336	0.0827517
6869	1.00053	0.0864298
6870	1.18041	2.1
6871	1.80818	2.1
6872	1.73571	1.60227
6873	1.94429	1.73303
6874	1.4375	0.0705826
6875	1.44421	0.0698591
6876	1.66953	0.0745381
6877	1.09529	0.0698591
6878	0.952395	2.1
6879	0.717337	2.1
6880	1.03609	2.1
6881	1.33949	2.09474
6882	1.2774	0.217059
6883	2.24983	0.197749
6884	1.28727	0.166247
6885	0.583592	0.141216
6886	0.583591	0.176608
6887	1.82859	0.213688
6888	1.82859	0.225224
6889	0.583593	0.232456
6890	0.946128	0.135822
6891	0.692233	0.31142
6892	1.12226	0.255463
6893	2.57454	0.242765
6894	0.810944	0.205853
6895	0.810945	0.174026
6896	1.12226	0.241018
6897	1.45867	0.240074
6898	1.22403	0.202209
6899	1.15262	0.222855

6900	1.08132	0.216231
6901	0.846671	0.174254
6902	0.480954	0.265478
6903	0.917975	0.251881
6904	0.917975	0.304172
6905	0.720957	0.297232
6906	0.795998	0.2347
6907	0.69316	0.291648
6908	0.641306	0.30639
6909	0.690817	0.198364
6910	0.961899	2.1
6911	2.49579	2.1
6912	2.31106	2.1
6913	1.70482	0.0883642
6914	1.93156	0.118025
6915	1.23868	0.14942
6916	1.73878	0.133036
6917	1.26267	2.1
6918	1.26441	2.1
6919	1.58095	2.1
6920	1.58095	2.1
6921	0.946132	2.1
6922	0.946132	2.1
6923	1.11077	2.1
6924	1.62905	2.1
6925	1.49692	2.1
6926	3.49966	2.1
6927	2.39213	2.1
6928	2.12603	2.1
6929	0.946129	2.1
6930	0.461661	2.1
6931	1.52841	2.1
6932	1.39091	2.1
6933	0.550679	2.1
6934	0.509894	2.1
6935	1.49508	2.1
6936	2.05862	2.1
6937	2.47851	2.1
6938	1.07213	2.1
6939	1.09148	1.8024
6940	1.99776	2.03647
6941	1.38333	2.01396
6942	1.04412	2.1
6943	1.04412	2.1
6944	1.1893	0.0712139
6945	0.946132	0.0761938
6946	0.965737	0.101729
6947	0.946132	0.0794191
6948	1.54061	0.0755987
6949	1.54061	0.0989443
6950	1.22878	0.0817871
6951	1.25797	0.0824794
6952	1.00665	0.0815375
6953	1.00665	0.095051
6954	1.80369	0.0740657
6955	0.946128	0.0941997
6956	1.04424	0.120717
6957	0.85154	0.0922733

6958	1.39322	0.0877464
6959	1.35464	0.121536
6960	0.939857	0.101582
6961	1.28272	0.104418
6962	1.19323	0.154576
6963	1.03498	0.0776748
6964	2.22029	0.0867428
6965	0.540005	0.0777883
6966	0.970382	0.108784
6967	2.14514	0.0814476
6968	1.31542	0.0795393
6969	1.05575	0.120045
6970	0.810943	0.144094
6971	1.31542	0.0728449
6972	1.70493	0.082862
6973	1.22582	0.106962
6974	1.31681	0.116385
6975	1.40775	0.0859636
6976	0.928633	0.168585
6977	1.2545	0.0951
6978	2.66481	0.104938
6979	1.32051	0.0730816
6980	1.32051	0.118572
6981	2.66305	2.1
6982	1.3551	2.1
6983	2.45325	2.1
6984	1.3685	2.1
6985	1.51748	2.1
6986	1.78941	2.1
6987	1.02963	0.0698591
6988	1.28643	0.0698591
6989	0.86131	0.0698591
6990	0.851539	0.0698591
6991	1.46585	0.0698591
6992	1.38822	0.0698591
6993	0.996402	0.0698591
6994	0.851553	0.0698591
6995	1.73156	0.0698591
6996	1.91695	0.0698591
6997	1.92535	0.0698591
6998	2.57287	0.0698591
6999	1.77743	0.0698591
7000	1.02836	0.264465
7001	1.36362	0.279673
7002	1.36936	0.217866
7003	1.78684	0.195413
7004	1.78685	0.215255
7005	0.946132	0.341607
7006	1.27581	0.309605
7007	0.946132	0.390612
7008	1.02836	0.183944
7009	1.55398	2.1
7010	1.07167	2.1
7011	1.87557	2.1
7012	0.751639	2.1
7013	1.50884	2.1
7014	1.33793	2.1
7015	0.751664	2.1

7016	1.37277	2.1
7017	0.979515	2.1
7018	0.676508	2.1
7019	1.09578	2.1
7020	0.953925	2.1
7021	0.94613	2.1
7022	1.64707	2.1
7023	1.64707	2.1
7024	1.16658	2.1
7025	1.40638	2.1
7026	1.18682	2.1
7027	1.16658	2.1
7028	0.543765	0.162209
7029	1.57462	0.150015
7030	1.57462	0.122632
7031	1.00083	0.151271
7032	1.16834	0.187036
7033	1.16834	0.169693
7034	0.889626	0.17635
7035	1.25944	0.173793
7036	0.824061	0.184437
7037	1.9397	0.108603
7038	1.21893	0.13417
7039	1.91364	0.126435
7040	1.33672	0.165522
7041	0.928069	0.227482
7042	1.41484	0.172402
7043	0.538379	0.14329
7044	1.41484	0.150326
7045	1.35594	0.182203
7046	2.11414	0.159427
7047	2.11414	0.104591
7048	0.538378	0.161621
7049	1.38532	0.113324
7050	0.962877	1.40676
7051	1.07497	0.558096
7052	1.00613	0.815238
7053	0.962878	0.279972
7054	0.711447	2.1
7055	1.40008	0.0787763
7056	2.51674	0.0886429
7057	1.40008	0.0747312
7058	1.7263	0.0698591
7059	1.40008	0.0812085
7060	1.9216	0.0698591
7061	2.7013	0.0698591
7062	2.58584	0.0698591
7063	0.979296	0.0698591
7064	1.46459	1.22665
7065	0.899254	0.894625
7066	1.15038	2.1
7067	1.24687	2.1
7068	1.16738	2.1
7069	0.942323	2.1
7070	1.65573	2.01005
7071	0.844791	2.1
7072	0.54674	2.09791
7073	0.795811	2.1

7074	1.08131	2.1
7075	1.03553	2.1
7076	0.758334	2.1
7077	0.708665	2.04864
7078	1.22626	2.01658
7079	0.835439	2.01168
7080	0.861131	1.95791
7081	0.68296	2.06468
7082	1.65224	2.03159
7083	2.82624	2.07776
7084	1.0151	1.80308
7085	1.15905	1.93928
7086	1.71076	1.80604
7087	1.4529	1.50388
7088	0.494895	2.07017
7089	0.946132	1.98051
7090	0.613132	1.91281
7091	0.863631	1.95737
7092	1.78391	2.1
7093	1.1706	0.0698591
7094	2.02734	0.0698591
7095	1.1706	0.0698591
7096	0.681532	0.0698591
7097	1.50316	0.0698591
7098	1.50316	0.0698591
7099	0.681534	0.0698591
7100	0.973819	0.0698591
7101	0.97382	0.0698591
7102	0.94613	0.0698591
7103	1.1706	0.0698591
7104	1.67727	0.0698591
7105	1.60329	0.0698591
7106	1.3227	2.1
7107	1.40846	2.1
7108	1.07889	2.1
7109	1.07721	2.1
7110	0.953523	0.571357
7111	1.79675	0.622752
7112	2.09859	0.886983
7113	1.21309	2.1
7114	1.54567	0.815963
7115	1.19722	0.237052
7116	1.82793	0.176561
7117	1.1599	0.205222
7118	1.73621	0.154018
7119	0.933474	0.291041
7120	1.51477	0.218512
7121	1.51477	0.257457
7122	0.93347	0.305689
7123	1.27374	0.216432
7124	1.27376	0.261062
7125	0.946105	0.254821
7126	1.1972	0.201769
7127	0.851541	0.0698591
7128	1.36971	0.0698591
7129	1.27832	0.0698591
7130	0.853489	0.0698591
7131	0.973847	0.0698591

7132	0.976308	0.0698591
7133	0.851528	0.0698591
7134	2.72654	0.0698591
7135	1.77867	0.0698591
7136	3.23032	2.1
7137	2.78208	2.1
7138	1.0227	0.0698591
7139	1.02269	0.0698591
7140	1.02269	0.0698591
7141	1.24293	0.0698591
7142	0.831695	2.1
7143	0.67513	2.1
7144	0.956294	2.1
7145	1.08623	2.1
7146	1.05556	2.1
7147	1.38859	2.1
7148	1.13179	2.1
7149	1.02438	2.1
7150	1.06659	2.1
7151	1.36595	2.1
7152	1.06659	2.1
7153	1.06659	2.1
7154	0.946131	2.02017
7155	0.875912	2.1
7156	1.65971	2.1
7157	1.65971	2.08851
7158	0.875912	2.1
7159	1.1229	2.1
7160	1.41273	2.1
7161	1.54699	2.05435
7162	1.93655	1.73294
7163	1.42307	2.0078
7164	1.97907	2.03417
7165	0.996774	2.1
7166	1.15505	0.0698591
7167	1.28463	0.0698591
7168	1.86353	0.0834303
7169	2.71398	0.0759804
7170	1.56787	0.304601
7171	1.66295	0.231155
7172	1.73432	1.12091
7173	1.05019	0.0698591
7174	1.42117	0.0698591
7175	0.888299	0.0698591
7176	1.38127	0.0698591
7177	1.72993	0.0698591
7178	0.664129	0.0698591
7179	0.99302	2.1
7180	0.993023	2.1
7181	1.13491	2.1
7182	0.876316	2.1
7183	1.2588	2.1
7184	0.895613	2.1
7185	2.07526	2.1
7186	1.23369	2.1
7187	1.25146	2.1
7188	2.15282	2.1
7189	1.22239	2.09999

7190	1.10962	2.1
7191	1.77402	2.09427
7192	1.0305	2.1
7193	1.50108	2.04772
7194	1.20761	2.1
7195	1.3626	2.1
7196	1.14825	2.1
7197	1.78711	2.1
7198	1.17933	2.1
7199	1.14824	2.1
7200	1.09651	0.116151
7201	1.23851	0.111056
7202	1.83284	0.135384
7203	1.23851	0.0812409
7204	1.53521	2.1
7205	1.46279	2.1
7206	1.9233	0.0698591
7207	1.2966	0.0698591
7208	2.11443	0.0698591
7209	1.30265	0.0698679
7210	1.13528	0.135418
7211	0.946131	0.231763
7212	1.41169	0.175633
7213	1.33879	0.112529
7214	0.946128	0.173372
7215	1.80435	0.154428
7216	1.80435	0.0750858
7217	1.234	2.1
7218	1.45633	2.1
7219	1.234	2.1
7220	1.98448	2.1
7221	2.02612	2.1
7222	1.23527	1.33961
7223	1.44472	1.86411
7224	0.993366	2.1
7225	1.22025	2.0645
7226	0.993368	1.52982
7227	2.43669	2.1
7228	2.56707	2.1
7229	1.80348	0.737666
7230	1.74406	0.412647
7231	1.90937	0.760556
7232	1.49357	0.120561
7233	2.5832	0.264002
7234	1.82274	0.347853
7235	1.6957	0.298307
7236	2.22495	0.292797
7237	2.49959	0.300397
7238	2.01183	0.0731011
7239	1.37427	0.0698591
7240	1.15069	2.1
7241	1.39379	2.1
7242	1.69661	1.89081
7243	1.39379	1.99534
7244	1.39654	2.1
7245	1.93505	2.1
7246	1.5525	2.1
7247	1.5525	2.1

7248	0.615065	2.1
7249	1.38908	2.1
7250	0.615065	2.1
7251	0.77849	2.1
7252	1.38908	2.1
7253	1.56839	2.098
7254	1.09142	2.1
7255	2.07313	2.08217
7256	0.998288	0.0698591
7257	1.31481	0.0745768
7258	1.88175	0.0698591
7259	2.23267	0.0698591
7260	1.06935	2.1
7261	1.06936	2.1
7262	1.17989	2.09762
7263	1.00757	2.07333
7264	1.65318	2.1
7265	1.4185	0.224322
7266	2.22495	0.260564
7267	1.02011	0.157519
7268	0.59676	0.21025
7269	1.43606	0.180357
7270	1.43606	0.161442
7271	0.89771	0.189445
7272	1.13511	0.218192
7273	0.59676	0.14684
7274	0.897709	0.200933
7275	1.79542	0.18426
7276	2.38566	2.0845
7277	1.63838	1.4056
7278	1.63837	1.75251
7279	0.916275	2.05026
7280	1.36183	1.58652
7281	1.19282	2.07036
7282	0.916275	1.92154
7283	1.19283	2.00615
7284	2.01582	2.1
7285	1.00215	0.0698591
7286	2.60643	2.1
7287	2.15971	2.1
7288	1.28267	2.1
7289	1.29954	2.1
7290	3.40327	2.1
7291	3.49128	2.1
7292	1.3864	2.1
7293	1.3201	2.1
7294	1.45666	0.0698591
7295	1.55798	0.0698591
7296	0.857202	0.0698591
7297	1.24374	0.0698591
7298	1.04157	0.0698591
7299	0.85723	0.0698591
7300	1.02722	0.0698591
7301	1.01361	0.0698591
7302	0.974077	0.0698591
7303	1.01814	0.0698591
7304	0.974077	0.0698591
7305	1.43987	0.0698591

7306	1.43986	0.0698591
7307	0.927881	0.0698591
7308	0.946132	0.0698591
7309	0.927849	0.0698591
7310	0.927852	0.0698591
7311	1.2493	0.0698591
7312	1.22482	0.445255
7313	1.41319	0.705793
7314	1.22482	0.287791
7315	0.999492	2.1
7316	1.14657	2.1
7317	1.3617	0.0698591
7318	0.983269	0.0698591
7319	1.33174	0.0698591
7320	1.62816	0.0698591
7321	1.62817	0.0698591
7322	0.81938	0.0698591
7323	1.33174	0.0698591
7324	1.01332	0.0698591
7325	0.874788	0.0698591
7326	1.16725	2.1
7327	1.63991	2.1
7328	1.08848	0.0698591
7329	1.15354	0.0698591
7330	1.12665	2.1
7331	1.58742	2.1
7332	1.57523	2.1
7333	1.32201	1.48018
7334	1.32097	1.44788
7335	0.851542	0.142017
7336	0.877188	0.0866878
7337	0.797489	0.143654
7338	1.44721	0.0804931
7339	1.61761	0.0709848
7340	0.943799	0.130841
7341	1.36079	0.0986908
7342	1.16361	2.1
7343	1.15852	2.09725
7344	1.07624	2.1
7345	1.07624	2.1
7346	0.946132	0.616058
7347	1.37938	0.980012
7348	1.78653	1.22121
7349	1.78653	0.650557
7350	0.946131	1.27043
7351	1.37938	0.851075
7352	1.35328	0.589534
7353	1.44847	0.570881
7354	0.946133	0.756369
7355	1.38149	0.799857
7356	1.81474	0.898415
7357	1.81474	0.612054
7358	0.946133	1.20128
7359	1.38149	0.820348
7360	1.37938	0.628492
7361	1.44847	0.77909
7362	1.93665	0.544311
7363	1.5515	0.573541

7364	1.05025	2.1
7365	1.05025	2.1
7366	1.71468	2.1
7367	1.04759	2.1
7368	1.04646	0.0698591
7369	1.00739	0.0698591
7370	1.37086	0.0698591
7371	1.12547	0.0698591
7372	1.03853	0.0698591
7373	1.34824	0.0698591
7374	0.799786	0.0698591
7375	2.10105	0.0698591
7376	2.72007	0.0698591
7377	0.589918	0.0698591
7378	0.589918	0.0698591
7379	1.1509	0.0698591
7380	0.619832	0.0698591
7381	1.1509	0.0698591
7382	0.705903	0.0698591
7383	1.53703	0.0698591
7384	1.53703	0.0698591
7385	0.705904	0.0698591
7386	0.946133	0.0698591
7387	0.946132	0.0698591
7388	0.946132	0.0698591
7389	0.588467	0.0698591
7390	1.1078	0.0698591
7391	0.408061	0.0698591
7392	1.38703	0.0698591
7393	1.68328	0.0698591
7394	1.87031	0.0698591
7395	1.86268	0.0938852
7396	1.27472	0.078395
7397	0.947862	0.606233
7398	1.19854	1.01598
7399	0.946129	0.801285
7400	0.963111	0.47256
7401	0.946131	1.02735
7402	1.39055	0.461787
7403	1.39055	0.344167
7404	1.26426	0.53627
7405	1.07242	0.564691
7406	1.31126	0.2759
7407	1.76597	0.52546
7408	0.94613	1.38779
7409	1.27214	0.854013
7410	1.90751	0.913157
7411	1.90751	0.773085
7412	0.767114	0.987069
7413	1.27214	1.13044
7414	1.40248	1.01794
7415	1.41479	1.31336
7416	2.22625	1.35363
7417	0.913373	1.4145
7418	1.0158	0.815714
7419	1.61259	0.853284
7420	1.69051	0.666503
7421	0.560949	1.04807

7422	0.898559	1.23216
7423	1.28011	1.0736
7424	1.51026	0.0755419
7425	1.98252	0.0901798
7426	1.05129	0.0844005
7427	1.2951	0.0935697
7428	1.46041	0.0698591
7429	1.7155	2.1
7430	1.72196	2.1
7431	0.933885	2.1
7432	0.986723	2.1
7433	1.28072	1.73849
7434	1.40765	2.1
7435	1.28072	2.09676
7436	1.61687	2.1
7437	1.60965	2.1
7438	1.21424	2.1
7439	2.07297	2.1
7440	1.19943	2.1
7441	2.38784	2.1
7442	1.10278	0.100785
7443	1.43678	0.11708
7444	1.1142	0.1386
7445	1.18034	0.12118
7446	0.94613	0.147357
7447	0.752356	0.121838
7448	0.752329	0.141441
7449	1.46634	0.108525
7450	1.31789	0.1136
7451	0.958159	0.164255
7452	1.22752	0.154222
7453	1.50472	0.124157
7454	1.98974	2.1
7455	2.54186	2.1
7456	0.76512	2.1
7457	2.08777	2.1
7458	1.7753	2.1
7459	3.07372	2.1
7460	1.9756	2.1
7461	1.48162	2.1
7462	1.23204	2.1
7463	0.946131	1.07553
7464	1.13505	2.06511
7465	1.47432	2.09949
7466	1.47432	1.53035
7467	0.876831	1.90763
7468	1.13505	1.78644
7469	1.2161	1.65782
7470	1.05267	1.56161
7471	1.05267	1.34235
7472	1.5795	1.31796
7473	0.98186	2.1
7474	1.56735	2.1
7475	0.859099	2.1
7476	1.69858	2.1
7477	1.93586	2.1
7478	1.09233	2.1
7479	1.08732	0.114721

7480	1.3357	0.112433
7481	1.84826	2.1
7482	0.924132	2.1
7483	1.39188	2.1
7484	1.43685	2.1
7485	0.851541	2.1
7486	1.16251	2.1
7487	0.946131	2.1
7488	1.05685	2.1
7489	2.13781	2.1
7490	1.98859	2.1
7491	1.99731	2.1
7492	1.51379	2.1
7493	1.48796	2.1
7494	0.851542	2.1
7495	0.802558	2.1
7496	1.09244	2.1
7497	1.33891	2.1
7498	0.844958	2.1
7499	0.710864	2.1
7500	1.26087	2.1
7501	1.03679	2.1
7502	1.51026	0.0735027
7503	1.05248	0.462704
7504	0.827258	0.434333
7505	1.05248	0.451338
7506	1.28435	0.449548
7507	1.50953	0.451645
7508	1.50953	0.140192
7509	0.827298	0.473365
7510	1.00366	0.120288
7511	2.10495	0.222106
7512	1.17885	2.1
7513	1.25185	2.1
7514	1.02784	2.1
7515	1.76764	2.1
7516	1.23746	0.104931
7517	1.24082	0.116928
7518	1.46506	0.0762509
7519	1.33061	0.0698591
7520	0.768407	0.267553
7521	0.972058	0.158537
7522	1.16536	0.15615
7523	1.13325	0.110942
7524	0.978906	0.662428
7525	1.02291	0.180203
7526	0.838015	0.16347
7527	1.01052	0.176105
7528	1.08166	0.198278
7529	1.42607	0.0698591
7530	1.47354	0.0712576
7531	1.21703	2.1
7532	1.29897	2.1
7533	1.83293	2.1
7534	1.29897	2.1
7535	1.24688	0.121595
7536	0.782563	2.1
7537	0.946129	2.1

7538	0.782591	2.1
7539	1.49487	2.1
7540	1.49487	2.1
7541	1.37713	2.1
7542	0.900337	2.1
7543	1.31284	2.1
7544	0.924468	2.1
7545	1.40602	2.1
7546	1.25669	2.1
7547	0.986901	2.1
7548	0.958565	2.1
7549	1.37266	2.1
7550	1.64146	2.1
7551	0.49345	2.1
7552	1.08276	2.1
7553	1.08276	2.1
7554	1.26929	2.1
7555	1.09847	2.1
7556	0.827081	0.215054
7557	1.07236	0.366077
7558	1.25063	1.17275
7559	1.43548	0.197178
7560	1.25063	0.21173
7561	1.3659	0.0698591
7562	1.72441	0.0698591
7563	1.3659	0.0698591
7564	1.4677	0.0698591
7565	1.86114	0.0698591
7566	1.40104	0.0698591
7567	0.962675	0.0698591
7568	0.946131	0.0699369
7569	1.74134	0.0698591
7570	1.74134	0.0698591
7571	0.962676	0.0698591
7572	0.946131	0.0698591
7573	1.05548	0.0789415
7574	0.902187	0.0918592
7575	2.14178	0.0700391
7576	1.74998	0.0745616
7577	1.38541	0.0781155
7578	1.64468	0.0703139
7579	0.949317	0.0860423
7580	2.06654	0.0713086
7581	0.839626	0.0796836
7582	0.810945	0.0879052
7583	1.29203	0.0698591
7584	1.18091	0.0716305
7585	1.06872	0.078055
7586	1.11956	0.069867
7587	0.926911	0.0905711
7588	0.901716	0.0865226
7589	0.909146	0.0967117
7590	1.33776	0.0713757
7591	1.02048	0.0804632
7592	1.08365	0.0861207
7593	1.21042	0.072045
7594	0.865634	0.0981502
7595	0.590354	2.1

7596	0.61173	2.1
7597	3.46825	2.1
7598	3.28404	2.1
7599	1.56004	0.10096
7600	1.69344	0.317309
7601	1.7151	0.887987
7602	0.946131	0.335236
7603	1.40265	0.225246
7604	1.13537	1.31869
7605	0.965708	0.445646
7606	1.13537	0.580525
7607	1.29681	0.523071
7608	1.40915	0.310351
7609	1.61652	1.82062
7610	1.33171	2.09731
7611	1.37681	2.1
7612	0.81094	0.0698591
7613	0.598606	0.0698591
7614	0.723111	0.0698591
7615	1.04562	0.0698591
7616	0.979052	0.0698591
7617	0.798185	0.0698591
7618	0.880117	0.0698591
7619	0.979341	0.208095
7620	0.800229	0.158761
7621	1.39126	0.199257
7622	1.13005	0.218027
7623	0.913877	0.227086
7624	1.26959	0.184266
7625	0.699741	0.219712
7626	1.19496	0.994957
7627	1.13392	0.0698591
7628	1.11913	0.0698591
7629	1.06866	0.0698591
7630	0.615546	0.0698591
7631	0.831006	0.0698591
7632	1.53286	0.0698591
7633	0.690977	0.0698591
7634	1.01956	0.0698591
7635	2.33818	0.0698591
7636	1.6585	2.1
7637	1.67061	2.1
7638	1.41414	2.1
7639	1.16756	2.1
7640	1.00898	0.0698591
7641	0.966964	2.1
7642	2.85443	2.1
7643	0.966977	2.1
7644	2.08427	2.1
7645	2.94901	2.1
7646	1.10735	0.353492
7647	1.65815	2.1
7648	1.4502	2.1
7649	1.45026	2.1
7650	1.53446	2.1
7651	0.946129	0.768402
7652	1.81075	2.1
7653	1.81075	1.07663

7654	0.946131	1.65665
7655	1.37075	2.1
7656	1.37075	1.15582
7657	1.34245	1.81815
7658	0.973502	1.11508
7659	1.08542	2.08537
7660	1.5814	0.0698591
7661	1.43836	0.0698591
7662	0.946132	0.0698591
7663	1.13747	0.0698591
7664	1.04429	0.0698591
7665	1.05017	0.0698591
7666	1.24732	0.0698591
7667	1.13387	0.0698591
7668	2.19489	0.0698591
7669	1.10748	2.1
7670	1.25006	2.1
7671	1.58834	2.1
7672	1.58834	2.1
7673	0.94613	2.1
7674	1.16548	2.1
7675	0.946132	2.1
7676	1.08998	2.1
7677	1.16548	2.1
7678	0.995732	2.07645
7679	0.657014	2.1
7680	0.957173	2.1
7681	1.01485	2.1
7682	1.12468	2.1
7683	1.43871	2.1
7684	1.23716	2.1
7685	1.89277	0.589722
7686	2.06653	0.413196
7687	0.967544	0.305306
7688	1.89016	0.346486
7689	2.22165	2.1
7690	0.83514	2.1
7691	1.76565	2.1
7692	1.76565	2.1
7693	0.94613	2.1
7694	0.835112	2.1
7695	1.23426	2.1
7696	1.36653	2.1
7697	0.895892	1.77567
7698	0.859278	1.82292
7699	0.791461	1.78929
7700	1.86249	1.76916
7701	1.14607	1.85234
7702	1.19904	1.76822
7703	1.84898	1.79508
7704	1.03452	1.81111
7705	0.946131	1.51109
7706	0.940472	0.7127
7707	1.53478	2.1
7708	1.84175	1.85558
7709	1.55687	1.55159
7710	1.17787	1.23437
7711	1.60589	0.332638

7712	2.35574	0.411283
7713	1.3305	0.686767
7714	1.15008	1.12174
7715	1.12457	0.0714046
7716	1.12457	0.0762563
7717	1.01778	2.1
7718	1.35733	2.1
7719	1.50683	2.1
7720	1.12064	2.1
7721	1.42716	0.858554
7722	1.42717	1.01736
7723	1.45927	1.42904
7724	1.04321	1.60046
7725	1.32634	0.179254
7726	2.27022	0.156929
7727	1.19352	0.132103
7728	1.32634	0.122926
7729	1.40924	1.72515
7730	1.22927	1.12416
7731	1.69237	1.6205
7732	1.69237	2.08553
7733	0.946132	0.982466
7734	0.946133	1.59427
7735	1.16912	1.50988
7736	1.0417	1.27218
7737	1.14605	1.67966
7738	1.1233	2.1
7739	1.96882	1.53141
7740	1.03804	0.0929488
7741	1.15338	0.0837977
7742	1.15338	0.0820396
7743	2.33051	0.0796544
7744	1.81507	2.1
7745	2.42949	2.1
7746	0.725879	2.1
7747	1.88394	2.09713
7748	2.2699	1.03456
7749	2.78952	2.1
7750	1.88394	1.24318
7751	1.79606	2.1
7752	1.20091	2.0777
7753	1.9614	2.07875
7754	1.19901	2.07793
7755	3.07915	2.1
7756	1.32592	2.1
7757	2.86461	2.1
7758	1.34817	2.1
7759	1.44859	2.1
7760	2.03969	2.1
7761	0.946132	2.1
7762	1.50709	2.1
7763	1.50709	2.1
7764	0.946132	2.1
7765	1.29322	2.1
7766	1.29322	2.1
7767	1.01985	2.1
7768	0.968537	2.1
7769	1.401	2.1

7770	1.28936	2.1
7771	1.56002	0.0698591
7772	1.92548	0.0698591
7773	1.46041	0.0698591
7774	2.22712	0.0698591
7775	1.56001	0.0698591
7776	1.50524	2.1
7777	1.21304	2.1
7778	1.60665	1.9315
7779	1.85178	1.33951
7780	2.17534	2.09407
7781	2.31012	2.1
7782	2.17533	1.98493
7783	0.986724	2.1
7784	2.01895	2.1
7785	2.00433	2.1
7786	1.45752	0.125121
7787	1.12695	0.123679
7788	0.652605	2.1
7789	1.5133	2.1
7790	1.5133	2.1
7791	0.652603	2.1
7792	1.228	2.1
7793	0.946132	2.1
7794	0.652604	2.1
7795	0.946132	1.95064
7796	0.716815	2.1
7797	1.62203	2.04929
7798	1.62203	2.1
7799	0.716814	2.1
7800	1.00191	2.09421
7801	1.33694	2.06451
7802	1.38216	2.1
7803	2.53338	2.09954
7804	1.46932	0.0698591
7805	1.50848	0.0698591
7806	1.50108	1.4155
7807	1.18382	0.445963
7808	2.02115	0.230719
7809	0.921823	0.501417
7810	2.08356	0.0698591
7811	1.543	0.0698591
7812	1.00763	0.218461
7813	1.4185	0.233861
7814	1.08028	0.201804
7815	0.946131	0.390844
7816	1.56599	0.183512
7817	1.56599	0.38925
7818	1.08028	0.188006
7819	0.946131	0.0957179
7820	1.37518	2.1
7821	1.63453	2.1
7822	1.04627	1.42787
7823	1.04626	1.49219
7824	1.35111	1.53679
7825	1.22297	1.41509
7826	1.64499	2.1
7827	1.87598	2.1

7828	1.06994	2.1
7829	1.37063	2.1
7830	1.99943	0.0698591
7831	2.38807	0.0698591
7832	0.99972	0.0771381
7833	1.41388	0.0854285
7834	1.66179	0.0785204
7835	1.66179	0.0705985
7836	0.946129	0.0920666
7837	1.41388	0.0833334
7838	1.19404	0.0814833
7839	1.31023	2.08282
7840	1.20871	2.08191
7841	1.69731	2.1
7842	1.67971	2.1
7843	0.998971	0.0698591
7844	1.32988	0.0698591
7845	0.701079	0.0698591
7846	0.946131	0.0698591
7847	0.582785	0.0698591
7848	1.48592	0.0698591
7849	1.48592	0.0698591
7850	1.23351	0.0698591
7851	0.835199	0.0698591
7852	0.92427	0.0698591
7853	1.68262	0.0698591
7854	2.46702	0.0698591
7855	1.08715	2.03189
7856	1.01606	1.87078
7857	1.29579	1.94091
7858	1.01606	1.93093
7859	1.63823	2.1
7860	1.67687	2.1
7861	1.11048	2.1
7862	1.39477	2.1
7863	0.991114	2.1
7864	2.90978	0.340137
7865	0.906811	2.06346
7866	0.872849	0.4356
7867	1.17091	1.34533
7868	0.685763	0.846586
7869	1.6386	1.44121
7870	1.06907	0.460169
7871	1.05516	1.7383
7872	1.9189	0.911686
7873	1.04615	0.11106
7874	1.2966	2.1
7875	1.37392	2.1
7876	1.35504	2.1
7877	1.30347	2.1
7878	1.50877	2.1
7879	1.11193	2.1
7880	1.40984	2.08223
7881	1.67682	2.08806
7882	1.00371	2.1
7883	1.15412	2.1
7884	1.41638	2.1
7885	1.03796	2.1

7886	0.946117	2.1
7887	1.50823	2.1
7888	1.50822	2.1
7889	0.966923	2.1
7890	0.946131	2.1
7891	2.07593	2.1
7892	1.9339	2.1
7893	1.1817	0.265484
7894	0.729178	0.357878
7895	1.5079	0.266188
7896	1.26991	0.341993
7897	0.994049	0.367367
7898	1.17452	0.25308
7899	1.05711	0.316001
7900	1.04071	0.307124
7901	1.80299	0.354158
7902	1.60417	0.335453
7903	0.943405	0.0698591
7904	1.01129	0.0698591
7905	1.42972	0.0698591
7906	1.15904	0.0698591
7907	0.680409	0.0698591
7908	1.2816	0.0698591
7909	0.806176	0.0698591
7910	1.18954	0.0698591
7911	1.26055	0.0698591
7912	1.06313	2.1
7913	1.19352	2.1
7914	1.55766	2.1
7915	1.53729	2.1
7916	0.993021	1.73106
7917	1.09056	2.1
7918	1.03408	2.1
7919	0.993023	2.1
7920	0.659901	2.1
7921	0.632905	2.1
7922	1.34072	0.133244
7923	1.17793	0.119489
7924	2.24885	0.130114
7925	0.946132	0.120857
7926	1.12443	0.132051
7927	0.904207	0.12105
7928	1.51237	0.0972183
7929	1.51237	0.117704
7930	1.12443	0.13156
7931	1.29215	0.128978
7932	1.6943	2.06394
7933	2.43858	2.07452
7934	2.0589	1.85612
7935	1.67204	2.09041
7936	1.67204	2.0464
7937	0.555875	1.96679
7938	0.91951	2.09863
7939	0.946131	1.8582
7940	0.555904	1.93989
7941	0.946131	1.97037
7942	1.6943	2.1
7943	1.04744	1.82649

7944	1.50938	2.1
7945	1.60861	2.1
7946	1.92406	0.0701313
7947	1.18716	0.0698591
7948	0.962705	0.831844
7949	1.35382	1.51602
7950	1.43632	0.0698591
7951	1.09386	0.0698591
7952	1.92547	0.0698591
7953	0.994164	0.0698591
7954	0.903974	1.94629
7955	0.928829	1.7398
7956	0.810958	1.62827
7957	1.92853	1.441
7958	1.598	1.49147
7959	1.28967	1.61874
7960	1.44993	1.64216
7961	0.946132	0.762868
7962	0.882984	0.676427
7963	0.882955	0.89031
7964	1.56071	0.294778
7965	1.56071	0.263847
7966	1.35946	0.542399
7967	1.08421	0.604149
7968	1.41943	0.0698591
7969	1.26265	0.0698591
7970	1.03235	0.50608
7971	1.50778	2.1
7972	1.70387	2.1
7973	1.65608	2.1
7974	1.39076	2.1
7975	1.6104	2.1
7976	1.37456	2.1
7977	1.51898	0.405272
7978	1.9726	0.202493
7979	2.13757	1.00429
7980	1.74856	1.08011
7981	0.635949	0.619938
7982	2.46448	1.01857
7983	1.23224	1.06198
7984	1.12786	1.59595
7985	1.82721	1.11974
7986	1.82721	0.759133
7987	0.532892	1.94959
7988	0.946131	1.66891
7989	0.53292	1.57718
7990	1.41394	2.1
7991	1.45506	2.1
7992	1.39523	2.1
7993	1.09006	2.1
7994	1.80264	0.73336
7995	1.80264	2.07241
7996	0.946131	0.674482
7997	1.34268	0.126073
7998	0.946132	1.93341
7999	1.23732	1.14622
8000	1.34268	1.10219
8001	1.2064	2.1

8002	1.58164	2.1
8003	1.73853	2.1
8004	1.01243	1.75767
8005	0.910672	1.51056
8006	1.47793	1.74954
8007	1.47793	2.09869
8008	0.910702	1.54001
8009	1.30382	1.12272
8010	1.08478	0.898399
8011	0.910702	1.99787
8012	2.60709	1.17936
8013	1.01243	1.64724
8014	1.85516	0.731054
8015	2.52823	0.419719
8016	1.24001	0.529581
8017	1.89793	0.638967
8018	1.73584	0.0698591
8019	1.24613	1.60988
8020	1.37074	1.36412
8021	1.23763	1.39348
8022	1.04072	1.96425
8023	0.75499	1.61603
8024	1.72329	1.36645
8025	1.46195	1.22176
8026	0.946132	1.57664
8027	0.747438	1.74001
8028	1.24613	1.42162
8029	2.59886	0.847205
8030	1.87279	2.1
8031	1.07977	2.1
8032	1.29606	2.1
8033	1.45178	2.1
8034	1.45177	2.1
8035	0.92406	2.1
8036	0.946131	2.1
8037	0.924047	2.1
8038	2.04529	2.1
8039	1.04544	2.1
8040	1.08276	2.1
8041	1.67491	2.1
8042	0.961826	1.48691
8043	0.810942	0.438778
8044	0.826622	1.27459
8045	0.914378	1.38419
8046	0.89022	1.10378
8047	0.922383	1.09325
8048	0.988165	0.830979
8049	1.31357	0.0734873
8050	0.946131	0.0698591
8051	1.31357	0.0717704
8052	1.10564	0.0698591
8053	1.6797	0.104814
8054	1.6797	0.0946453
8055	0.739506	0.0698591
8056	1.33957	0.102307
8057	1.33957	0.164109
8058	1.33957	0.135246
8059	2.34223	0.162948

8060	1.5515	0.674137
8061	2.22137	0.498609
8062	0.94612	0.44187
8063	1.13908	0.463441
8064	1.45423	0.383348
8065	1.45422	0.495039
8066	0.795519	0.903824
8067	1.1391	0.501354
8068	1.11065	0.44926
8069	1.12266	2.1
8070	1.24925	2.1
8071	1.20242	2.1
8072	2.18297	2.1
8073	3.00107	2.05277
8074	2.58297	0.682892
8075	0.884783	2.1
8076	0.851538	1.06199
8077	0.713587	2.07275
8078	1.47949	1.13881
8079	1.66673	2.09309
8080	0.696199	2.1
8081	0.9383	1.93144
8082	1.29149	1.5253
8083	1.32266	2.1
8084	2.11428	0.0698591
8085	2.17439	0.0698591
8086	1.40489	0.0698591
8087	1.21071	0.0698591
8088	0.946118	0.0698591
8089	1.0746	0.0698591
8090	0.973119	0.0698591
8091	1.31333	0.0698591
8092	1.08723	0.0698591
8093	0.994292	2.1
8094	1.4964	2.1
8095	1.15096	2.1
8096	1.10101	2.1
8097	1.50438	2.1
8098	1.67825	2.1
8099	2.02103	2.1
8100	2.01978	2.1
8101	1.50931	0.0698591
8102	1.50931	0.0698591
8103	0.65023	0.0698591
8104	1.34831	0.0698591
8105	0.811233	0.0698591
8106	1.34831	0.0698591
8107	0.94613	0.0698591
8108	1.00754	1.58136
8109	0.714132	2.02685
8110	1.28877	1.36043
8111	1.39905	1.40815
8112	1.03282	1.42955
8113	0.997769	1.5314
8114	1.11743	1.55389
8115	1.86863	1.11417
8116	3.30723	1.50228
8117	1.66635	1.98987

8118	1.35609	0.0698591
8119	1.38212	0.0698591
8120	1.95597	2.1
8121	1.64257	2.1
8122	2.93316	2.1
8123	3.13417	2.1
8124	0.733947	2.1
8125	1.15259	2.1
8126	1.07976	2.1
8127	1.30237	2.1
8128	1.99943	0.0699877
8129	0.907431	0.0702417
8130	0.878161	0.0711513
8131	1.07544	0.0698591
8132	1.03401	0.0698591
8133	0.991975	0.0698591
8134	1.03635	0.0698591
8135	0.788549	0.0698591
8136	1.44984	0.0698591
8137	1.84007	0.0708432
8138	2.31127	0.0703264
8139	1.27362	2.1
8140	1.27737	0.868343
8141	1.87783	0.541174
8142	2.35499	0.0698591
8143	2.69662	0.0698591
8144	2.24065	0.0698591
8145	0.996082	2.1
8146	0.996083	2.1
8147	1.44859	2.1
8148	1.03968	2.08961
8149	1.60018	0.940634
8150	1.73082	1.54876
8151	0.946132	1.66423
8152	1.49742	1.77735
8153	1.49742	0.730047
8154	1.00501	1.15533
8155	1.41585	1.9482
8156	1.41585	1.2217
8157	0.946131	1.90142
8158	1.03697	1.21176
8159	2.95786	2.1
8160	2.32185	2.1
8161	1.65783	2.02575
8162	1.772 2.1	
8163	0.946131	2.06935
8164	1.40404	1.769
8165	1.13537	2.1
8166	1.04559	2.1
8167	1.16091	2.1
8168	2.63751	2.1
8169	1.68455	0.0698591
8170	1.9682	0.0698591
8171	2.16834	0.117826
8172	2.45569	0.187175
8173	0.946128	0.131683
8174	1.36428	0.20076
8175	0.663978	0.27125

8176	1.58218	0.139749
8177	1.58219	0.124258
8178	1.36428	0.18981
8179	0.881886	0.188657
8180	1.73849	2.1
8181	1.35668	2.1
8182	1.1233	2.1
8183	1.15009	2.1
8184	1.16482	0.254581
8185	1.29313	0.278062
8186	1.47608	0.14022
8187	2.05949	0.152692
8188	0.946119	0.0972713
8189	1.02974	0.117889
8190	0.946119	0.104162
8191	1.66758	0.0789098
8192	1.66757	0.0839208
8193	1.32082	0.09039
8194	1.29287	0.0858672
8195	1.36005	2.1
8196	1.47109	2.1
8197	2.45958	2.1
8198	2.2975	2.1
8199	0.953521	0.808005
8200	0.953521	0.556054
8201	1.67054	2.1
8202	1.67054	2.1
8203	0.946131	2.1
8204	1.38154	2.1
8205	1.13326	2.1
8206	1.13326	2.1
8207	1.23513	2.1
8208	1.50998	1.85036
8209	2.47982	0.0905177
8210	1.45091	0.0893985
8211	0.958953	0.0698591
8212	0.79239	0.107508
8213	2.11929	0.189699
8214	1.26511	0.177736
8215	1.27733	0.0921651
8216	2.18127	0.121824
8217	1.30169	0.0812989
8218	1.19259	0.238052
8219	0.963395	1.7825
8220	0.999947	2.09837
8221	1.62241	2.1
8222	1.62027	2.1
8223	0.981586	0.0698591
8224	0.981585	0.0698591
8225	0.721961	2.1
8226	0.891321	2.1
8227	1.04442	2.1
8228	1.66554	2.1
8229	2.43434	2.1
8230	1.73944	0.0698591
8231	1.88032	0.0698591
8232	0.711391	0.0698591
8233	1.40837	0.0698591

8234	0.946133	0.0698591
8235	0.92252	0.0698591
8236	1.13964	0.0698591
8237	1.83394	0.0698591
8238	2.81674	0.0698591
8239	1.10049	1.57487
8240	1.25092	0.250628
8241	0.760299	0.781479
8242	1.48017	0.954828
8243	1.48017	0.938472
8244	0.760271	0.645687
8245	1.35861	0.949245
8246	0.946129	0.804186
8247	0.760269	0.712185
8248	2.26155	2.1
8249	2.15634	2.1
8250	1.39824	2.1
8251	1.07817	2.1
8252	0.946132	2.1
8253	1.53028	2.1
8254	1.53028	2.1
8255	1.00807	2.1
8256	0.946133	2.1
8257	2.06126	0.200522
8258	1.97385	0.942282
8259	1.75754	0.103704
8260	2.01613	2.1
8261	1.97995	2.1
8262	1.47696	2.1
8263	1.50172	2.1
8264	1.5177	0.0698591
8265	1.44146	0.0698591
8266	1.8807	0.0698591
8267	2.07068	0.0699695
8268	1.84267	0.0698591
8269	1.08975	2.1
8270	1.21327	2.1
8271	1.10106	0.0698591
8272	1.34496	0.0698591
8273	2.08452	0.188841
8274	1.70537	0.0742909
8275	2.62464	2.1
8276	1.5726	2.1
8277	1.5726	2.1
8278	0.94613	2.1
8279	1.15038	2.1
8280	0.946132	2.1
8281	1.03974	2.1
8282	1.15038	2.1
8283	0.99747	1.98013
8284	1.03472	2.09371
8285	1.58407	1.69963
8286	1.21148	2.1
8287	1.35025	2.1
8288	1.3428	0.217762
8289	1.00775	2.1
8290	1.00775	2.1
8291	1.3879	2.1

8292	1.39958	2.1
8293	0.853042	2.1
8294	1.06997	2.1
8295	0.853042	2.1
8296	0.853042	2.1
8297	1.12491	2.1
8298	1.17879	2.1
8299	1.07162	2.08784
8300	1.61179	2.1
8301	1.76016	2.1
8302	0.946132	0.545507
8303	1.23449	0.39821
8304	1.70392	0.405345
8305	1.70392	0.359581
8306	0.94613	0.601359
8307	1.23449	0.43516
8308	1.41556	0.431299
8309	1.14547	0.388496
8310	1.1721	0.299767
8311	0.96478	0.352377
8312	1.41843	0.368118
8313	1.2358	0.314769
8314	1.70678	0.307708
8315	1.70678	0.311512
8316	0.946118	0.500892
8317	1.23582	0.423041
8318	0.946105	0.508539
8319	0.964769	0.262831
8320	1.1357	0.273293
8321	0.964767	0.263988
8322	0.619856	0.0698591
8323	1.72366	0.0698591
8324	1.72367	0.0698591
8325	0.619831	0.0698591
8326	1.19262	0.0698591
8327	0.619829	0.0698591
8328	0.946129	0.0698591
8329	1.57459	2.1
8330	1.86512	2.1
8331	0.93841	2.1
8332	1.57459	2.1
8333	1.10476	0.0698591
8334	1.00013	1.01835
8335	2.13446	0.686732
8336	0.759781	2.1
8337	1.96011	1.51275
8338	0.946129	0.8777
8339	1.32056	1.53941
8340	1.75702	1.05519
8341	1.75702	1.67141
8342	0.946124	1.45457
8343	1.32056	1.26349
8344	1.38258	1.03204
8345	1.96011	1.50627
8346	2.31099	1.29867
8347	0.97356	2.1
8348	1.38885	2.1
8349	1.3771	2.1

8350	0.946131	2.1
8351	1.84843	2.1
8352	1.84843	2.1
8353	1.3771	2.1
8354	0.946131	2.1
8355	1.00278	2.1
8356	1.05447	2.1
8357	1.05446	2.1
8358	1.24913	0.736504
8359	1.05447	2.1
8360	1.80882	2.1
8361	1.80882	2.1
8362	0.946129	2.1
8363	1.33997	2.1
8364	0.946131	2.08328
8365	1.28831	2.1
8366	1.33997	2.1
8367	1.44391	0.0698591
8368	1.44391	0.0698591
8369	0.850964	0.0698591
8370	0.850963	0.0698591
8371	0.946132	0.0698591
8372	0.921299	0.0698591
8373	1.37358	0.0698591
8374	1.46297	0.0698591
8375	1.46298	0.0698591
8376	0.823988	0.0698591
8377	0.921287	0.0698591
8378	0.94612	0.0698591
8379	0.823995	0.0698591
8380	0.946107	0.0698591
8381	1.44437	2.1
8382	1.06203	2.1
8383	2.21397	2.1
8384	0.637677	2.1
8385	1.8892	2.1
8386	2.075 2.1	
8387	1.32051	0.0702695
8388	2.23637	0.0936744
8389	1.10904	0.20089
8390	1.11819	0.125684
8391	0.94613	0.221925
8392	0.735845	0.116388
8393	1.4914	0.197584
8394	1.4914	0.0818492
8395	0.735831	0.315118
8396	1.61683	2.1
8397	1.61683	2.1
8398	0.946131	2.1
8399	1.3281	2.1
8400	1.10441	2.1
8401	1.10442	2.1
8402	1.23487	2.1
8403	1.09914	2.1
8404	1.20691	0.0698591
8405	2.27379	0.0698591
8406	1.1369	0.0715044
8407	1.26868	0.0751041

8408	1.54422	0.0720641
8409	1.54422	0.0703061
8410	0.861357	0.0814914
8411	0.94613	0.0740104
8412	0.861359	0.0726501
8413	1.2858	0.289978
8414	1.31011	0.508482
8415	1.294	0.228126
8416	1.03079	0.352715
8417	1.81979	1.89901
8418	1.57829	2.01969
8419	1.19023	1.59166
8420	1.19022	1.75853
8421	1.46458	1.78036
8422	1.46458	1.9262
8423	0.789173	2.01156
8424	1.34386	1.92505
8425	0.909896	2.02609
8426	0.946131	1.87326
8427	0.909893	1.9068
8428	1.27725	2.1
8429	1.68451	2.1
8430	2.26155	2.1
8431	2.34217	2.1
8432	1.55485	0.494417
8433	0.90854	0.400407
8434	0.858256	0.327472
8435	1.47042	0.263889
8436	1.62771	0.272696
8437	0.777398	0.517738
8438	0.982525	0.340347
8439	1.23865	0.311062
8440	2.09928	0.3588
8441	0.946128	0.135406
8442	0.474856	0.111344
8443	0.474855	0.150535
8444	1.58747	0.0771175
8445	1.58747	0.0725739
8446	0.963917	0.117483
8447	1.09841	0.105724
8448	2.23904	2.1
8449	2.60055	1.83155
8450	1.02599	2.1
8451	2.17837	2.1
8452	2.07122	2.1
8453	2.7309	2.02346
8454	2.08482	2.1
8455	1.18322	0.262291
8456	1.35381	0.994915
8457	1.01139	0.547389
8458	0.946133	1.14524
8459	1.59089	0.88526
8460	1.59089	0.352242
8461	0.946133	0.751312
8462	1.09634	0.11164
8463	2.02279	0.124682
8464	1.03108	0.142631
8465	0.808978	1.50783

8466	1.07317	1.42722
8467	0.831695	1.36482
8468	1.22583	1.18495
8469	1.10215	1.27139
8470	1.07508	1.30534
8471	1.22204	1.24589
8472	1.04679	1.98756
8473	1.92535	0.0698591
8474	1.11081	0.0698591
8475	0.946131	0.124624
8476	0.714824	0.12914
8477	0.714795	0.114935
8478	1.4231	0.115758
8479	1.4231	0.147728
8480	0.828617	0.12514
8481	1.30928	0.141971
8482	1.49894	2.1
8483	1.55702	2.1
8484	0.946131	2.1
8485	1.10689	2.1
8486	0.946227	2.1
8487	0.946131	2.1
8488	1.21754	2.1
8489	1.504 2.1	
8490	1.504 2.1	
8491	0.723573	2.1
8492	1.24512	2.1
8493	0.723544	2.1
8494	0.946131	2.1
8495	1.24512	2.1
8496	1.29383	0.115001
8497	1.29383	0.0807279
8498	1.61969	0.126136
8499	0.946132	0.0764152
8500	1.12818	0.0715184
8501	0.946132	0.180291
8502	1.80922	0.076267
8503	1.80922	0.0698591
8504	1.41383	0.0762403
8505	1.34153	0.0699403
8506	1.47689	0.0715603
8507	1.79375	0.0763291
8508	1.24333	0.341679
8509	0.768989	0.287109
8510	1.24333	0.257804
8511	1.0027	0.401151
8512	1.47705	0.34394
8513	1.47705	0.146419
8514	0.768977	0.3205
8515	1.28436	0.529231
8516	0.928583	0.602823
8517	1.28436	0.230864
8518	1.24333	0.586739
8519	1.5991	0.552361
8520	1.5991	0.110735
8521	0.928583	0.578348
8522	1.85717	0.154466
8523	2.48665	0.22444

8524	1.18423	0.0698591
8525	1.9493	2.08974
8526	1.8519	1.98243
8527	1.68802	2.08115
8528	1.0302	0.111337
8529	0.900473	0.0993299
8530	1.99663	0.0978609
8531	2.61301	0.0869797
8532	0.745772	0.158477
8533	0.946131	0.110535
8534	1.48881	0.112703
8535	1.48881	0.0897006
8536	0.745745	0.13819
8537	0.809786	0.141496
8538	1.3065	0.134262
8539	0.946132	0.0862608
8540	0.768357	0.0924547
8541	0.768387	0.100331
8542	1.76897	0.0706865
8543	1.76896	0.0698591
8544	1.40271	0.0761024
8545	1.00777	0.0772413
8546	1.37427	0.0698591
8547	1.1967	0.660423
8548	0.946132	2.1
8549	1.59397	2.1
8550	1.59397	2.1
8551	0.946131	2.1
8552	1.14989	2.1
8553	1.14989	2.1
8554	1.10046	2.1
8555	1.09506	0.0698591
8556	2.53251	0.0698591
8557	1.9212	1.53116
8558	1.99586	0.981089
8559	0.913967	0.188292
8560	1.55028	0.100066
8561	1.55028	0.130379
8562	0.913966	0.203684
8563	1.16318	0.103191
8564	1.16318	0.139659
8565	0.946132	0.13462
8566	1.20478	2.1
8567	1.09662	2.1
8568	1.03472	2.1
8569	1.84774	2.1
8570	1.7984	2.1
8571	2.1221	2.1
8572	1.68223	2.09198
8573	1.68224	2.05314
8574	0.514872	1.93023
8575	0.888991	2.03096
8576	0.946127	1.77581
8577	0.514845	1.95631
8578	0.946129	1.95743
8579	1.10127	1.85923
8580	2.61623	1.86267
8581	0.983581	0.215124

8582	0.869663	0.152187
8583	1.1818	0.166207
8584	1.10406	0.137401
8585	1.07133	0.22628
8586	1.09405	0.166764
8587	0.838014	0.195825
8588	0.842998	1.09488
8589	1.03818	0.850196
8590	1.46338	0.155194
8591	1.903 2.1	
8592	1.23942	2.1
8593	0.965933	2.1
8594	0.810075	2.1
8595	1.40571	2.1
8596	1.66219	2.1
8597	0.810047	2.1
8598	0.936678	2.1
8599	1.45808	2.1
8600	1.6384	2.1
8601	1.54699	2.1
8602	0.719877	2.1
8603	1.33256	2.1
8604	0.753547	2.1
8605	1.04073	2.1
8606	1.25822	2.1
8607	0.851542	2.1
8608	0.714102	2.1
8609	1.45886	2.1
8610	1.80171	2.1
8611	0.597337	2.1
8612	0.957713	2.1
8613	1.3033	2.1
8614	0.596613	0.248706
8615	1.1386	0.351435
8616	0.596612	0.263536
8617	0.946133	0.460259
8618	0.596652	0.430674
8619	1.64092	0.358559
8620	1.64092	0.282537
8621	1.991 2.1	
8622	1.23013	2.1
8623	2.77816	2.1
8624	2.74107	0.474718
8625	2.07681	0.37181
8626	1.47764	0.0699812
8627	1.72677	0.0701981
8628	1.52161	2.1
8629	1.28526	2.1
8630	1.90502	2.1
8631	1.87359	0.119151
8632	1.93489	0.127917
8633	2.70895	2.1
8634	2.83141	2.1
8635	1.41766	0.167355
8636	1.11337	0.153398
8637	1.41765	0.26457
8638	1.29172	0.181701
8639	1.76324	0.190321

8640	1.76324	0.239672
8641	0.946131	0.133848
8642	1.52428	2.1
8643	1.71459	2.1
8644	1.93852	0.34294
8645	1.07119	1.01384
8646	1.29698	0.953375
8647	1.42203	0.33408
8648	1.42203	1.44009
8649	0.946132	0.369233
8650	0.946132	0.655689
8651	0.96926	0.864879
8652	0.769913	1.75975
8653	0.946119	1.39367
8654	1.37867	0.891983
8655	1.94028	0.82217
8656	0.94612	0.549766
8657	0.946132	1.22947
8658	1.37865	0.769835
8659	2.14234	0.895935
8660	3.06459	0.902566
8661	1.44421	0.0698591
8662	1.33924	0.0698591
8663	1.33671	0.0698749
8664	1.10733	0.0698591
8665	0.68396	0.0719812
8666	1.76008	0.0698591
8667	1.76008	0.0698591
8668	0.68393	0.0698591
8669	0.946131	0.070142
8670	3.79242	2.1
8671	2.46293	2.1
8672	3.56947	2.1
8673	1.35487	1.32677
8674	1.38616	1.53511
8675	0.954048	0.115964
8676	1.36663	0.0944361
8677	0.909487	0.131479
8678	0.887288	2.03404
8679	0.810947	1.40638
8680	0.810934	1.81108
8681	1.07046	2.00086
8682	1.13979	2.02623
8683	0.927838	1.61205
8684	0.919995	1.88701
8685	1.61447	2.1
8686	2.56305	2.1
8687	0.943414	2.1
8688	1.17016	2.1
8689	1.7238	2.1
8690	1.89334	2.1
8691	0.946128	2.02622
8692	1.25184	2.1
8693	1.55639	2.1
8694	3.12098	2.05453
8695	3.57307	1.18412
8696	2.81281	2.1
8697	0.851542	2.1

8698	1.27689	2.1
8699	1.52568	2.1
8700	0.839689	2.1
8701	1.36471	2.1
8702	0.827865	2.1
8703	0.93667	2.1
8704	0.900861	2.1
8705	0.943152	2.1
8706	1.00667	2.1
8707	1.18141	2.1
8708	1.15298	2.1
8709	1.20335	2.1
8710	1.00713	2.1
8711	0.597537	2.1
8712	1.0577	2.1
8713	0.967342	0.556377
8714	0.838027	0.828882
8715	0.893587	0.502141
8716	1.06436	0.563969
8717	0.850048	0.473879
8718	1.22026	0.618098
8719	0.988077	0.548212
8720	1.18769	0.210972
8721	1.3991	2.1
8722	1.52984	2.1
8723	1.35469	1.58292
8724	1.25083	1.43828
8725	1.6594	1.72738
8726	1.6594	1.60714
8727	0.946129	1.22342
8728	1.02602	1.12286
8729	0.94613	0.910786
8730	1.29724	0.0851686
8731	1.14501	0.202781
8732	1.13078	0.161786
8733	1.48381	0.157702
8734	1.45921	0.142843
8735	0.851539	0.218114
8736	0.890836	0.220338
8737	0.946131	0.268431
8738	1.60428	2.1
8739	1.50097	2.1
8740	0.714507	0.074928
8741	1.51396	0.0862779
8742	1.51396	0.0921091
8743	0.946132	0.0753304
8744	0.813862	0.0912474
8745	1.41461	0.0792857
8746	0.813862	0.085937
8747	0.921311	2.1
8748	1.25084	1.30428
8749	1.30165	0.874436
8750	1.60636	1.44589
8751	1.60636	1.04887
8752	0.94613	0.903976
8753	0.972232	0.839921
8754	0.946131	0.772121
8755	2.35003	2.1

8756	0.92546	2.1
8757	1.00713	2.1
8758	0.810947	2.1
8759	0.810934	2.1
8760	1.056	2.1
8761	0.926811	2.1
8762	0.766408	2.1
8763	1.34292	2.1
8764	1.57932	0.0698591
8765	2.67595	0.0698591
8766	1.77737	0.0698591
8767	1.77738	0.0698591
8768	0.789633	0.0698591
8769	1.22903	0.0698591
8770	0.789663	0.0698591
8771	0.789662	0.0698591
8772	1.33797	0.0698591
8773	0.946128	2.1
8774	1.47351	2.1
8775	1.47351	2.1
8776	0.94613	2.1
8777	1.30082	2.1
8778	1.30082	2.1
8779	0.996486	2.1
8780	0.946127	2.1
8781	1.72122	2.1
8782	1.72122	2.1
8783	0.94613	2.1
8784	1.36653	2.1
8785	1.36653	2.1
8786	1.02608	2.1
8787	0.709123	1.69742
8788	0.946127	1.4092
8789	1.41342	0.554828
8790	1.818	1.72718
8791	1.818	0.970909
8792	0.946127	0.332961
8793	1.41342	1.17495
8794	1.35071	1.28926
8795	1.8993	0.309002
8796	1.4748	2.03655
8797	1.18711	0.0698591
8798	0.946131	0.0698591
8799	1.18711	0.0698591
8800	1.20416	0.0698591
8801	1.91358	0.0702261
8802	1.91358	0.0706179
8803	0.477691	0.0698591
8804	2.16798	0.0698591
8805	2.22307	0.0698591
8806	0.758971	0.0698591
8807	1.80694	0.0698591
8808	1.80694	0.0698591
8809	0.946132	0.0698591
8810	0.75897	0.0698591
8811	1.25124	0.0698591
8812	0.946131	0.0698591
8813	1.76837	2.1

8814	1.76837	2.1
8815	0.946131	2.1
8816	1.36422	2.1
8817	0.946131	2.1
8818	1.14184	2.1
8819	1.36422	2.1
8820	0.793303	0.0698591
8821	1.79584	0.0698591
8822	1.35333	1.96549
8823	1.19967	1.51345
8824	1.55088	1.72985
8825	1.64246	2.00276
8826	0.946132	1.37683
8827	0.886293	1.91318
8828	1.25018	1.98441
8829	1.5795	1.80355
8830	2.15414	1.38369
8831	0.94613	0.551943
8832	1.37758	1.69213
8833	1.71567	0.975038
8834	1.71567	2.04207
8835	0.946125	1.42258
8836	1.37759	1.19908
8837	1.28421	0.751093
8838	0.933959	1.13931
8839	0.946133	0.0698591
8840	1.7813	0.0698591
8841	1.7813	0.0698591
8842	1.05704	0.0698591
8843	1.37794	0.0698591
8844	1.37794	0.0698591
8845	0.946132	0.0698591
8846	0.946132	0.0698591
8847	1.68197	0.0698591
8848	1.68197	0.0698591
8849	1.01367	0.0698591
8850	1.25017	0.0698591
8851	1.25017	0.0698591
8852	0.946132	0.0698591
8853	0.838953	2.1
8854	1.4045	2.1
8855	1.56388	2.1
8856	1.3182	2.1
8857	0.928518	2.1
8858	1.04073	2.1
8859	0.835676	2.1
8860	1.47507	2.1
8861	1.47506	2.1
8862	0.797664	2.1
8863	1.34285	2.1
8864	0.929875	2.1
8865	0.797663	2.1
8866	0.946133	2.1
8867	1.83628	1.25977
8868	1.83627	1.72106
8869	0.946131	1.65691
8870	1.36748	0.65235
8871	0.946133	1.22187

8872	1.21139	0.742929
8873	1.36748	0.502345
8874	1.06795	2.1
8875	1.08192	2.1
8876	0.827866	2.1
8877	1.02548	2.1
8878	0.95006	2.1
8879	1.53933	2.1
8880	0.586511	2.1
8881	0.868145	2.1
8882	1.28401	2.1
8883	1.37719	2.1
8884	1.37719	2.1
8885	0.77497	2.1
8886	0.946132	2.1
8887	0.774968	2.1
8888	1.7053	1.80545
8889	1.7053	2.1
8890	0.94613	2.1
8891	1.25715	2.1
8892	0.946132	1.41234
8893	1.19655	2.09886
8894	1.25716	2.1
8895	0.851542	2.1
8896	0.80043	2.1
8897	1.27239	2.1
8898	1.02719	2.1
8899	0.946131	2.1
8900	0.981318	2.1
8901	1.06628	2.1
8902	1.26022	2.1
8903	0.946132	2.1
8904	0.946132	2.1
8905	1.76447	2.1
8906	1.76447	2.1
8907	1.35357	2.1
8908	1.35703	2.1
8909	0.946131	2.1
8910	1.50538	2.1
8911	1.50538	2.1
8912	1.10311	2.1
8913	1.3484	2.1
8914	1.10312	2.1
8915	0.966428	2.1
8916	0.946133	2.1
8917	1.4008	2.1
8918	1.71908	2.1
8919	1.71908	2.1
8920	0.946133	2.1
8921	1.40081	2.1
8922	1.26441	2.1
8923	0.851543	2.1
8924	0.961732	2.1
8925	1.54576	2.1
8926	1.35364	2.1
8927	0.946133	2.1
8928	1.02913	2.1
8929	1.2735	2.1

8930	1.32608	2.1
8931	0.967643	2.1
8932	0.94613	2.1
8933	1.44873	2.1
8934	1.44873	2.1
8935	0.967641	2.1
8936	0.946131	2.1
8937	1.40488	2.1
8938	1.24272	2.1
8939	0.946131	2.1
8940	1.78483	2.1
8941	1.78483	2.1
8942	1.24272	2.1
8943	0.946131	2.1
8944	0.94613	2.0925
8945	1.5627	0.428474
8946	1.5627	1.86871
8947	1.03656	2.09512
8948	1.31649	0.273337
8949	1.19233	1.64218
8950	1.03656	1.41706
8951	0.912059	2.1
8952	1.47954	0.852005
8953	1.47954	2.04209
8954	0.912061	2.1
8955	1.34647	0.732109
8956	1.04514	2.04909
8957	0.912061	1.89563
8958	0.761972	0.0840071
8959	0.946132	0.608222
8960	0.761974	0.324109
8961	1.70225	1.44519
8962	1.70225	0.786829
8963	1.33728	0.617751
8964	1.01188	0.522767
8965	0.94613	1.8489
8966	0.746361	1.57777
8967	1.6369	2.05751
8968	1.62454	1.79835
8969	0.736738	0.985355
8970	0.958487	1.62943
8971	1.40641	1.89487
8972	1.2284	2.1
8973	0.92609	2.1
8974	0.595102	2.1
8975	1.80871	2.1
8976	1.1516	2.1
8977	0.962474	2.1
8978	2.24024	2.1
8979	0.946131	2.1
8980	1.73689	2.1
8981	1.73689	2.1
8982	0.946132	2.1
8983	1.30492	2.1
8984	1.30492	2.1
8985	1.17949	2.1
8986	0.946131	2.1
8987	1.8566	2.1

8988	1.8566	2.1
8989	1.22392	2.1
8990	1.39826	2.1
8991	1.40447	2.1
8992	1.22392	2.1
8993	0.946131	2.1
8994	1.74134	2.1
8995	1.74134	2.1
8996	1.09148	2.1
8997	1.40447	2.1
8998	1.283 2.1	
8999	1.09148	2.1
9000	1.33607	2.1
9001	1.41022	2.1
9002	1.80016	2.1
9003	1.80015	2.1
9004	0.946127	2.1
9005	0.946129	2.1
9006	1.20541	2.1
9007	1.13078	2.1
9008	1.33607	2.1
9009	1.54624	2.1
9010	1.85553	2.1
9011	0.920606	2.1
9012	0.946131	2.1
9013	1.01271	2.1
9014	1.32664	1.90989
9015	0.954692	2.07143
9016	0.946116	1.71653
9017	1.47771	1.90965
9018	1.4777	2.1
9019	0.95468	1.95573
9020	0.94613	1.98097
9021	1.31381	1.11824
9022	1.14005	0.755094
9023	0.946128	0.754235
9024	1.69433	0.494416
9025	1.69433	1.55535
9026	1.14005	0.684305
9027	0.94613	0.558764
9028	1.521 2.1	
9029	1.411 2.1	
9030	0.94612	2.1
9031	1.35115	2.1
9032	1.04127	2.1
9033	1.05017	2.1
9034	1.03564	2.1
9035	0.607156	2.1
9036	1.54549	2.1
9037	1.36715	2.1
9038	0.607127	2.1
9039	1.2571	2.1
9040	0.998701	2.1
9041	0.546436	2.1
9042	0.946133	2.1
9043	1.75286	2.1
9044	1.75286	2.1
9045	1.12206	2.1

9046	1.34237	2.1
9047	1.35663	2.1
9048	1.12206	2.1
9049	0.919455	2.1
9050	1.66517	2.1
9051	1.99822	2.1
9052	1.228 2.1	
9053	1.35662	2.1
9054	1.228 2.1	
9055	1.01143	2.1
9056	0.851543	1.53847
9057	0.986856	2.09799
9058	1.42762	1.878
9059	1.23095	2.1
9060	0.946132	2.09352
9061	1.00659	1.99039
9062	1.1779	1.98624
9063	1.40025	2.1
9064	1.40025	2.1
9065	0.889308	2.1
9066	1.34652	2.1
9067	0.943042	2.1
9068	0.943041	2.1
9069	1.34652	2.1
9070	1.39514	1.20276
9071	1.30809	1.0302
9072	1.75711	0.86856
9073	1.7571	2.07253
9074	0.946125	0.377667
9075	0.946127	1.21671
9076	1.18396	1.21636
9077	1.4844	1.24188
9078	1.4844	1.53077
9079	0.803322	1.92774
9080	0.946133	0.997663
9081	0.803324	2.06908
9082	0.803324	1.76784
9083	1.36183	1.72522
9084	0.946131	0.131524
9085	1.01462	0.11846
9086	0.946132	0.350768
9087	1.59749	0.190902
9088	1.59749	0.0925737
9089	1.16009	0.12912
9090	1.38354	0.0851349
9091	0.851539	1.03062
9092	1.36005	1.40172
9093	1.5532	1.09949
9094	1.80402	1.47244
9095	0.851537	1.53058
9096	0.902632	1.37146
9097	1.44437	1.16565
9098	0.633098	2.1
9099	1.00307	0.393918
9100	0.94613	0.706333
9101	1.16623	0.457582
9102	0.946128	0.80296
9103	1.64588	0.68346

9104	1.64588	0.454401
9105	1.35059	0.628779
9106	1.24142	0.719661
9107	0.946131	0.0943851
9108	1.13343	0.0843806
9109	0.946127	0.111921
9110	1.63367	0.0809931
9111	1.63367	0.0779294
9112	1.33035	0.0886814
9113	1.24945	0.100294
9114	1.78733	1.68938
9115	1.70856	1.73951
9116	0.637995	1.27003
9117	0.712454	1.29289
9118	0.946132	1.36062
9119	0.637964	1.16993
9120	1.13021	1.26949
9121	0.94613	0.0780591
9122	1.20509	0.0828222
9123	0.946127	0.0762444
9124	1.70545	0.0736318
9125	1.70545	0.0840993
9126	1.28508	0.0738023
9127	1.3665	0.0842616
9128	1.58612	2.08578
9129	1.51845	2.09884
9130	0.797374	2.1
9131	1.20057	2.09875
9132	0.946132	2.09505
9133	0.946132	2.08952
9134	0.899819	2.09949
9135	1.50471	0.973615
9136	1.50472	0.748983
9137	0.59387	0.864934
9138	0.593844	0.506744
9139	0.946126	1.05734
9140	0.966137	0.618551
9141	1.13245	0.632191
9142	1.20801	0.593008
9143	0.946131	1.09102
9144	1.47644	0.75168
9145	0.733223	0.569277
9146	0.563609	0.738218
9147	1.69218	0.709706
9148	1.6173	0.67984
9149	1.8126	1.902
9150	1.8126	1.92891
9151	0.678938	1.94232
9152	0.678935	1.92098
9153	0.946133	2.03916
9154	0.946132	1.93627
9155	0.678936	2.0382
9156	1.21997	1.83226
9157	1.28439	1.83522
9158	1.65282	1.88416
9159	1.86735	1.85802
9160	0.851539	1.9324
9161	0.94613	1.67256

9162	0.87688	1.64566
9163	0.804877	0.356249
9164	1.40747	0.419701
9165	1.41312	0.162776
9166	0.885349	0.234474
9167	1.25405	0.424799
9168	1.34463	0.252674
9169	0.804878	0.257121
9170	0.810299	0.0836706
9171	1.04072	0.151997
9172	1.25601	0.123672
9173	1.04943	0.0837432
9174	0.900294	0.159347
9175	1.38777	0.118586
9176	1.33131	0.0711666
9177	1.39168	0.0698591
9178	0.946132	0.0698591
9179	0.747712	0.0698591
9180	1.61944	0.0698591
9181	1.61944	0.0698591
9182	0.946132	0.0698591
9183	0.747712	0.0698591
9184	1.41201	0.114975
9185	1.12082	0.0893683
9186	0.946128	0.104331
9187	1.79415	0.132579
9188	1.79415	0.114277
9189	1.12082	0.0844299
9190	0.94613	0.096199
9191	0.711721	0.110256
9192	1.12316	0.130873
9193	0.707038	0.111152
9194	1.04072	0.148806
9195	0.704409	0.158409
9196	1.49036	0.13522
9197	1.27689	0.104161
9198	0.793044	0.478668
9199	0.810886	0.72495
9200	0.885957	0.509449
9201	1.38254	0.461487
9202	1.35991	0.44912
9203	1.01375	0.499248
9204	1.03169	0.577088
9205	0.946131	0.578749
9206	1.3279	0.737188
9207	1.42284	0.615217
9208	1.42284	0.5472
9209	0.851192	1.12454
9210	0.946131	0.827942
9211	0.851165	0.703194
9212	0.748062	0.0698591
9213	1.27511	0.0698591
9214	0.74806	0.0698591
9215	0.94613	0.0698591
9216	0.748059	0.0698591
9217	1.86293	0.0698591
9218	1.86293	0.0698591
9219	0.851542	0.0820616

9220	1.38186	0.0735883
9221	0.851542	0.0873371
9222	1.67869	0.0698591
9223	1.66579	0.0698591
9224	1.25626	0.0727059
9225	1.23046	0.0708975
9226	1.46023	0.0698591
9227	1.27083	0.0698591
9228	0.946131	0.0698591
9229	0.999304	0.0698591
9230	0.946803	0.0698591
9231	0.851554	0.0698591
9232	0.999227	0.0698591
9233	0.750097	0.0698591
9234	1.91815	0.0698591
9235	0.946132	0.0698591
9236	0.768117	0.0698591
9237	1.78003	0.0698591
9238	1.99572	0.0698591
9239	0.650453	0.0698591
9240	0.946132	0.0698591
9241	1.23845	0.0698591
9242	0.946131	0.0698591
9243	1.75477	0.0698591
9244	1.75477	0.0698591
9245	1.33393	0.0698591
9246	1.36697	0.0698591
9247	0.749018	0.675637
9248	1.02366	0.58337
9249	1.43924	0.688711
9250	1.00018	0.479416
9251	1.06438	0.480712
9252	0.946127	0.51369
9253	0.74906	0.5488
9254	0.946131	0.528007
9255	1.26488	0.0698591
9256	0.946131	0.0698591
9257	0.94613	0.0698591
9258	1.49051	0.0698591
9259	1.59837	0.0698591
9260	0.907797	0.0698591
9261	1.04455	0.0698591
9262	1.25214	0.0698591
9263	0.68452	0.0698591
9264	1.25215	0.0698591
9265	1.24677	0.0698591
9266	1.8144	0.0698591
9267	1.81441	0.0698591
9268	0.684518	0.0698591
9269	0.851542	0.367305
9270	1.52275	0.304514
9271	1.45223	0.317628
9272	1.0269	0.345418
9273	1.29689	0.334409
9274	1.32822	0.370884
9275	0.851553	0.409849
9276	1.21756	0.0698591
9277	0.946131	0.0698591

9278	0.946127	0.0698591
9279	1.78014	0.0698591
9280	1.78014	0.0698591
9281	1.32076	0.0698591
9282	1.40551	0.0698591
9283	0.711214	0.0758296
9284	1.82057	0.0704477
9285	1.82057	0.0698591
9286	0.946129	0.0788378
9287	1.22716	0.0698591
9288	1.30463	0.0746959
9289	0.94613	0.0725268
9290	0.568927	0.0698591
9291	0.946132	0.0698591
9292	0.568927	0.0698591
9293	1.99266	0.0698591
9294	1.99266	0.0698591
9295	1.26201	0.0698591
9296	1.29957	0.0698591
9297	1.21614	0.071644
9298	0.851484	0.0711608
9299	0.851543	0.0742259
9300	1.85815	0.0698591
9301	1.81937	0.0698591
9302	0.697705	0.0698591
9303	0.946132	0.0720533
9304	0.593295	0.105237
9305	1.53642	0.0917163
9306	1.53642	0.0795682
9307	0.908276	0.116996
9308	0.593296	0.0779917
9309	0.593295	0.0880563
9310	0.94613	0.100344
9311	0.867593	0.227143
9312	1.42107	0.160443
9313	1.42107	0.162579
9314	0.867621	0.218965
9315	1.05516	0.196207
9316	0.867624	0.166203
9317	0.946129	0.220493
9318	0.766681	0.082131
9319	1.18119	0.0990672
9320	1.57018	0.0808151
9321	1.57018	0.0723097
9322	0.766652	0.102203
9323	1.18119	0.0900379
9324	1.15564	0.0890169
9325	0.946131	0.128294
9326	1.239 0.100812	
9327	0.851541	0.143477
9328	0.851487	0.0983396
9329	1.40021	0.128576
9330	1.38373	0.0811142
9331	0.851554	0.152419
9332	1.12266	0.290512
9333	0.946131	0.193473
9334	1.08234	0.250851
9335	1.2882	0.128718

9336	1.49963	0.413872
9337	1.46188	0.355582
9338	0.851555	0.253415
9339	1.33007	0.292042
9340	1.08054	0.257349
9341	0.946129	0.417179
9342	0.91151	0.224542
9343	1.49914	0.262084
9344	1.49914	0.140534
9345	0.911471	0.537686
9346	0.946132	0.709139
9347	1.35943	2.05179
9348	1.68117	1.50043
9349	1.68117	2.1
9350	0.946132	1.86843
9351	1.35943	1.49454
9352	1.26787	1.0242
9353	1.81612	2.1
9354	1.81612	1.61009
9355	0.946131	1.56009
9356	1.41492	1.20963
9357	0.946132	2.1
9358	1.25037	2.1
9359	1.41492	2.1
9360	1.30541	0.0698591
9361	1.23646	0.0698591
9362	0.94613	0.0698591
9363	1.77304	0.0698591
9364	1.77304	0.0698591
9365	1.23646	0.0698591
9366	0.946132	0.0698591
9367	0.946131	2.1
9368	1.26689	2.1
9369	1.66806	2.1
9370	1.66806	2.1
9371	0.94613	2.1
9372	1.26689	2.1
9373	1.3473	2.1
9374	1.39384	2.1
9375	1.4181	2.1
9376	1.86581	2.1
9377	1.86581	2.1
9378	0.946128	2.1
9379	0.946129	2.1
9380	1.32068	2.1
9381	1.20328	2.1
9382	1.39384	2.1
9383	1.651	2.1
9384	1.651	2.1
9385	0.946129	2.1
9386	0.946131	2.1
9387	1.12913	2.1
9388	1.21474	2.1
9389	0.946132	2.1
9390	0.94613	2.1
9391	1.79724	2.1
9392	1.79724	2.1
9393	1.40987	2.1

9394	1.3335	2.1
9395	1.65642	2.1
9396	1.65642	2.1
9397	0.946132	2.1
9398	1.19063	2.1
9399	0.946133	2.1
9400	1.15203	2.1
9401	1.19063	2.1
9402	0.946131	1.86422
9403	1.77815	2.1
9404	1.77815	2.1
9405	0.946131	2.09322
9406	1.31745	2.1
9407	1.31745	2.1
9408	1.2597	2.1
9409	0.946131	0.760093
9410	1.85184	1.97053
9411	1.85184	2.1
9412	0.946128	1.56518
9413	1.40684	2.1
9414	1.40684	1.96002
9415	1.34049	2.00352
9416	0.946132	1.78738
9417	1.71333	0.532477
9418	1.71333	2.0998
9419	1.1848	1.71134
9420	1.34297	0.421464
9421	1.31649	0.677497
9422	1.1848	0.918156
9423	1.36604	0.131842
9424	0.954559	0.133852
9425	1.36604	0.133897
9426	1.41766	0.123865
9427	1.83756	0.147365
9428	1.83756	0.248206
9429	0.946131	0.117292
9430	0.946127	0.213456
9431	1.14874	0.156426
9432	0.946127	0.238822
9433	1.66393	0.202278
9434	1.66393	0.136295
9435	1.35559	0.203286
9436	1.25447	0.213915
9437	0.94613	0.075428
9438	1.20732	0.0792152
9439	0.946129	0.07534
9440	1.75073	0.0699277
9441	1.75073	0.0790617
9442	1.3665	0.070573
9443	1.33035	0.0813475
9444	1.73256	2.1
9445	1.73256	2.1
9446	0.946131	2.1
9447	1.34921	2.1
9448	1.19462	2.1
9449	1.19462	2.1
9450	1.32949	2.1
9451	1.75754	0.356951

9452	1.75754	1.2065
9453	0.946132	1.35268
9454	1.3201	1.04629
9455	0.946132	0.337782
9456	1.25479	0.468702
9457	1.3201	0.358957
9458	1.8087	2.07834
9459	1.8087	0.53449
9460	0.946131	0.755118
9461	1.38357	0.371296
9462	0.946133	2.06861
9463	1.31034	1.34847
9464	1.38357	1.32827
9465	1.12702	0.130605
9466	0.946131	0.222505
9467	1.23923	0.183041
9468	1.4117	0.125223
9469	0.946131	0.204838
9470	1.70479	0.160251
9471	1.70479	0.0792985
9472	0.946119	2.1
9473	1.55309	2.1
9474	1.55309	2.1
9475	0.946119	2.1
9476	1.08006	2.1
9477	1.08005	2.1
9478	1.02681	2.1
9479	1.36581	2.1
9480	1.93075	2.1
9481	0.946133	2.1
9482	0.772538	2.1
9483	0.946131	2.1
9484	0.946131	2.1
9485	1.33748	2.1
9486	1.44991	2.1
9487	1.31183	2.1
9488	0.946132	2.1
9489	0.944104	2.1
9490	0.915236	2.1
9491	1.05017	2.1
9492	0.944148	2.1
9493	0.946132	0.0924722
9494	1.60171	0.0701376
9495	1.60171	0.0698591
9496	0.946131	0.113615
9497	1.16234	0.0715308
9498	1.3855	0.0714754
9499	0.946132	0.0772722
9500	0.946133	0.102319
9501	1.73285	0.0707551
9502	1.73285	0.0698591
9503	0.946133	0.10924
9504	1.3855	0.0711173
9505	1.29348	0.0808049
9506	0.946132	0.0820301
9507	1.34828	2.1
9508	1.20943	2.1
9509	0.946129	2.1

9510	1.71013	2.1
9511	1.71013	2.1
9512	1.20943	2.1
9513	0.94613	2.1
9514	1.19613	0.0698591
9515	0.946118	0.0698591
9516	0.946117	0.0698591
9517	1.70868	0.0698591
9518	1.70869	0.0698591
9519	1.40551	0.0698591
9520	1.2493	0.0698591
9521	0.946131	2.1
9522	1.73123	2.1
9523	1.73123	2.1
9524	0.946132	2.1
9525	1.37811	2.1
9526	1.37811	2.1
9527	1.25144	2.1
9528	0.946132	2.1
9529	1.75139	2.1
9530	1.75139	2.1
9531	1.23374	2.1
9532	1.29926	2.1
9533	1.39827	2.1
9534	1.23374	2.1
9535	1.41375	0.0698591
9536	1.25697	0.0698591
9537	0.946118	0.0698591
9538	1.80377	0.0698591
9539	1.80377	0.0698591
9540	1.25699	0.0698591
9541	0.946106	0.0698591
9542	0.946133	2.03423
9543	1.81806	1.31899
9544	1.81806	2.1
9545	0.946133	2.07319
9546	1.37089	2.1
9547	1.37089	1.21815
9548	1.34999	2.00763
9549	0.946131	2.1
9550	1.80855	1.83029
9551	1.80855	2.1
9552	1.31184	2.1
9553	1.3933	1.71504
9554	1.36139	2.08993
9555	1.31184	2.0967
9556	1.01175	2.00513
9557	0.94613	1.04913
9558	0.919796	2.01959
9559	1.50484	0.700847
9560	1.80584	1.84494
9561	1.53512	1.38573
9562	1.14539	1.29824
9563	0.946133	2.1
9564	1.72797	2.1
9565	1.72797	2.1
9566	1.2111	2.1
9567	1.39731	2.09848

9568	1.27679	2.1
9569	1.21111	2.1
9570	0.946119	1.02378
9571	1.67673	1.70911
9572	1.67673	2.1
9573	0.946118	1.60436
9574	1.21519	2.1
9575	1.2152	2.07128
9576	1.19808	2.1
9577	0.946127	2.1
9578	1.54442	2.1
9579	1.54442	2.1
9580	1.04806	2.1
9581	1.13485	2.1
9582	1.3557	2.1
9583	1.04805	2.1
9584	0.946129	2.1
9585	1.32282	2.1
9586	1.77749	2.1
9587	1.77749	2.1
9588	0.946131	2.1
9589	1.32282	2.1
9590	1.4008	2.1
9591	0.946128	0.109101
9592	1.02366	0.129671
9593	0.94613	0.114678
9594	1.66684	0.0865242
9595	1.66684	0.100384
9596	1.29215	0.110349
9597	1.32082	0.107707
9598	0.946133	0.0795638
9599	1.01451	0.107413
9600	0.946132	0.0843281
9601	1.62374	0.078032
9602	1.62374	0.0772095
9603	1.2929	0.0786462
9604	1.27697	0.0730988
9605	1.37869	2.1
9606	1.17215	2.1
9607	0.946132	2.1
9608	1.70508	2.1
9609	1.70508	2.1
9610	1.17215	2.1
9611	0.946132	2.1
9612	1.03784	2.1
9613	0.946132	2.1
9614	0.946132	2.1
9615	1.56718	2.1
9616	1.56717	2.1
9617	1.35703	2.1
9618	1.15628	2.1
9619	1.35356	2.1
9620	1.28976	2.1
9621	0.946131	2.1
9622	1.78612	2.1
9623	1.78612	2.1
9624	1.28976	2.1
9625	0.94613	2.1

9626	0.946132	2.1
9627	1.36373	2.1
9628	1.92382	2.1
9629	0.946131	2.1
9630	1.92382	2.1
9631	1.33471	2.1
9632	0.946132	2.1
9633	0.94613	0.217782
9634	1.29149	0.760432
9635	1.75878	0.411828
9636	1.75878	2.1
9637	0.946128	1.12146
9638	1.29149	0.354509
9639	1.41342	0.386589
9640	1.41022	2.1
9641	1.37034	2.1
9642	1.83443	2.1
9643	1.83443	2.1
9644	0.946128	2.1
9645	0.946131	2.1
9646	1.26928	2.1
9647	0.946128	0.723494
9648	1.38259	1.7181
9649	1.81405	1.08883
9650	1.81405	1.90676
9651	0.946126	1.55151
9652	1.38259	1.24341
9653	1.37759	0.854077
9654	1.68131	0.317448
9655	1.68131	1.15566
9656	0.946131	0.608632
9657	1.38896	0.250854
9658	1.17438	2.04591
9659	1.17438	0.802246
9660	1.23848	0.981915
9661	1.80088	1.14458
9662	1.80088	0.652979
9663	0.946132	0.647904
9664	1.40332	1.23194
9665	1.28185	0.586174
9666	1.28185	0.389059
9667	1.34369	0.218991
9668	1.71939	2.1
9669	1.71939	2.1
9670	0.946131	2.1
9671	1.28308	2.1
9672	0.946133	2.1
9673	1.24024	2.1
9674	1.28309	2.1
9675	1.8525	2.1
9676	1.8525	2.1
9677	0.946132	2.1
9678	1.38244	2.1
9679	0.946132	2.1
9680	1.33469	2.1
9681	1.38244	2.1
9682	1.26654	1.13258
9683	1.39514	1.06492

9684	1.71556	1.00009
9685	1.71556	2.02115
9686	0.946126	0.602459
9687	0.946129	1.41395
9688	1.1908	1.267
9689	1.49995	2.1
9690	1.63875	2.1
9691	0.93608	2.1
9692	0.937208	2.1
9693	0.943708	2.1
9694	0.936134	2.1
9695	1.14341	2.1
9696	1.17802	0.0698591
9697	1.3542	0.0698591
9698	1.73981	0.0698591
9699	1.73981	0.0698591
9700	0.94613	0.0698591
9701	1.3542	0.0698591
9702	1.33174	0.0698591
9703	1.59452	2.1
9704	1.59452	2.1
9705	0.946132	2.1
9706	1.25	2.1
9707	0.975266	2.1
9708	0.946132	2.1
9709	1.25	2.1
9710	0.946131	2.1
9711	1.48685	2.1
9712	1.48685	2.1
9713	0.946132	2.1
9714	1.17273	2.1
9715	1.17273	2.1
9716	0.955602	2.1
9717	1.32827	0.10212
9718	1.11166	0.0915217
9719	0.946127	0.0951959
9720	1.6136	0.118829
9721	1.6136	0.0863937
9722	1.11166	0.083918
9723	0.946129	0.0989004
9724	0.851541	0.429845
9725	1.22107	0.371706
9726	1.64594	0.296061
9727	1.5271	0.312251
9728	0.94613	0.620219
9729	1.22112	0.400401
9730	1.25002	0.342337
9731	1.65795	1.26772
9732	1.65795	2.1
9733	0.946132	2.1
9734	1.2366	2.1
9735	0.946132	0.933726
9736	1.10023	2.05201
9737	1.2366	1.9793
9738	1.35856	0.0698591
9739	0.946132	0.0698591
9740	1.35856	0.0698591
9741	1.08019	0.0698591

9742	1.49261	0.0698591
9743	1.49261	0.0698591
9744	0.94613	0.0698591
9745	1.29322	0.0698591
9746	0.946132	0.0698591
9747	1.29322	0.0698591
9748	1.35856	0.0736185
9749	1.70565	0.0698591
9750	1.70565	0.0753598
9751	0.946132	0.0698591
9752	1.66635	2.1
9753	1.66635	2.1
9754	0.946133	2.1
9755	1.39558	2.1
9756	1.14793	2.1
9757	1.14793	2.1
9758	1.21691	2.1
9759	1.35381	0.113017
9760	1.3365	1.16329
9761	1.09905	0.741849
9762	0.946132	1.05266
9763	1.74418	0.818726
9764	1.74418	0.336152
9765	0.946132	0.713715
9766	1.84759	2.1
9767	1.84759	1.72503
9768	0.94613	2.01615
9769	1.3904	2.1
9770	1.34522	0.654934
9771	1.34522	1.78063
9772	1.40331	1.51695
9773	1.86047	2.1
9774	1.86047	2.1
9775	0.946132	2.1
9776	1.41619	2.1
9777	0.946132	2.00559
9778	1.3659	2.1
9779	1.41619	2.1
9780	1.28464	2.06885
9781	1.14496	1.92296
9782	1.57806	1.94951
9783	1.7753	2.01976
9784	0.851541	2.0199
9785	0.946131	2.02067
9786	0.872786	2.01509
9787	0.85062	0.386798
9788	1.52635	0.566068
9789	1.63785	0.389249
9790	1.69972	0.994636
9791	0.946132	1.2074
9792	1.37493	0.494366
9793	1.23854	0.447805
9794	1.41134	2.09695
9795	1.27079	1.89601
9796	1.736	1.17093
9797	1.736	2.1
9798	0.946132	0.445281
9799	0.946133	1.44032

9800	1.21177	1.78166
9801	0.946131	0.0699799
9802	0.946129	0.0698591
9803	0.94613	0.0698675
9804	0.946131	0.0698591
9805	0.946131	0.0698591
9806	1.35403	0.0698591
9807	0.946131	0.0699322
9808	1.37052	0.153286
9809	0.946132	0.505843
9810	0.94613	0.23088
9811	0.946133	0.105885
9812	0.946131	0.121945
9813	0.946131	0.314279
9814	0.946131	0.123807
9815	1.24249	0.181843
9816	0.946132	0.197512
9817	0.946133	0.140214
9818	0.946132	0.0797475
9819	0.946132	0.128602
9820	0.946132	0.215112
9821	0.946132	0.111258
9822	0.946132	2.1
9823	0.946132	2.1
9824	0.946131	2.1
9825	0.946132	2.1
9826	0.946132	2.1
9827	0.946132	2.1
9828	1.20608	2.1
9829	1.41232	2.1
9830	0.946128	2.1
9831	0.946129	2.1
9832	0.94613	2.1
9833	0.94613	2.1
9834	0.94613	2.1
9835	0.94613	2.1
9836	0.946132	0.0737806
9837	0.946131	0.0723215
9838	0.946131	0.0756233
9839	0.946132	0.0698591
9840	0.946132	0.0698591
9841	0.946131	0.0723731
9842	0.946132	0.0701394
9843	0.946116	1.83202
9844	0.94612	2.1
9845	0.946118	2.09169
9846	0.946124	2.1
9847	0.946118	2.09475
9848	1.41561	2.1
9849	0.946131	2.09927
9850	0.945846	2.1
9851	1.03508	2.1
9852	0.77579	2.1
9853	0.658908	2.1
9854	1.17474	2.1
9855	0.94303	2.1
9856	0.849189	2.1
9857	0.94298	2.1

9858	1.03963	2.1
9859	0.946131	1.99656
9860	0.946131	1.41326
9861	0.946131	2.1
9862	0.946131	1.29976
9863	0.946131	0.875607
9864	0.946131	1.77304
9865	1.32819	1.89325
9866	0.946133	0.775471
9867	0.946129	2.05476
9868	0.946128	2.01686
9869	0.94613	1.57788
9870	0.946131	2.1
9871	0.946131	1.27729
9872	0.946131	1.71074
9873	0.954072	0.0698591
9874	1.28895	0.0698591
9875	0.946133	0.0698591
9876	1.15803	0.0698591
9877	1.29689	0.0698591
9878	1.29689	0.0698591
9879	0.946132	0.0698591
9880	0.946132	0.966568
9881	0.946132	0.935995
9882	0.946132	0.751188
9883	0.946132	1.00905
9884	0.946132	1.56835
9885	0.946131	1.31354
9886	1.0264	1.28877
9887	0.946126	0.275455
9888	0.946132	0.250815
9889	0.946131	0.196006
9890	0.94613	0.252312
9891	0.946129	0.345618
9892	0.94613	0.270978
9893	1.35977	0.253098
9894	0.946132	0.177328
9895	0.946133	0.122118
9896	0.876632	0.32554
9897	0.946132	0.0956674
9898	1.01566	0.160396
9899	1.01566	0.120896
9900	0.876606	0.150911
9901	0.946131	0.0698591
9902	0.946128	0.0698591
9903	0.946128	0.0698591
9904	0.94613	0.0698591
9905	0.94613	0.0698591
9906	1.24979	0.0698591
9907	0.94613	0.0698591
9908	0.946131	0.0698591
9909	0.946131	0.0698591
9910	0.921762	0.0698591
9911	0.9705	0.0698591
9912	0.9705	0.0698591
9913	0.946131	0.0698591
9914	0.921791	0.0698591
9915	0.810944	0.101247

9916	0.810945	0.108287
9917	0.810959	0.0869746
9918	2.42267	0.0711331
9919	1.35168	0.0747944
9920	1.08132	0.0868062
9921	1.08132	0.0736769
9922	1.08131	0.0813995
9923	0.946133	0.0736418
9924	0.946132	0.0907199
9925	0.946132	0.0739105
9926	0.946133	0.0859868
9927	0.946133	0.070821
9928	0.946132	0.0812091
9929	0.946133	0.0960705
9930	0.946131	0.524718
9931	0.946131	0.943143
9932	0.94613	0.542859
9933	0.851514	0.453014
9934	0.946131	0.767375
9935	0.965268	0.522183
9936	0.851513	0.533914
9937	0.946122	2.1
9938	0.946123	2.1
9939	0.946126	2.1
9940	0.946124	2.1
9941	0.946124	2.1
9942	0.946118	2.1
9943	0.94613	2.1
9944	0.946131	0.0762856
9945	1.30448	0.0702421
9946	0.946132	0.12508
9947	0.946129	0.077852
9948	0.946131	0.13558
9949	0.946131	0.0760389
9950	0.94613	0.117405
9951	0.946133	2.0771
9952	0.946128	2.1
9953	0.946128	2.1
9954	0.94613	2.1
9955	1.36503	2.1
9956	0.946131	2.1
9957	0.946131	2.1
9958	0.810947	0.0698591
9959	0.810947	0.0698591
9960	2.48233	0.0698591
9961	1.3517	0.0698591
9962	1.08132	0.0698591
9963	1.08132	0.0698591
9964	1.08133	0.0698591
9965	0.810934	0.0698591
9966	1.48874	0.0698591
9967	2.48233	0.0698591
9968	2.2369	0.0698591
9969	0.946133	0.790271
9970	0.946132	0.157503
9971	0.946132	0.895759
9972	0.946133	0.407471
9973	0.946133	0.882981

9974	1.41851	0.436774
9975	0.946132	0.481251
9976	1.03925	2.1
9977	0.946131	2.1
9978	0.94613	2.1
9979	0.946128	2.1
9980	0.946133	2.1
9981	0.944743	2.1
9982	1.13703	2.1
9983	0.946132	2.1
9984	0.946128	2.1
9985	0.946128	2.1
9986	0.946131	2.1
9987	0.94613	2.1
9988	0.94613	2.1
9989	0.94613	2.1
9990	0.946131	0.0698591
9991	0.946132	0.0698591
9992	0.946131	0.0698591
9993	0.946132	0.0698591
9994	0.946132	0.0698591
9995	0.946131	0.0698591
9996	1.20057	0.0698591
9997	0.946129	2.1
9998	0.94613	2.1
9999	0.94613	2.1
10000	0.946128	2.1
10001	0.946132	2.1
10002	0.94613	2.1
10003	1.38302	2.1
10004	0.856567	0.0698591
10005	1.14319	0.0698591
10006	0.716526	0.0698591
10007	1.20631	0.0698591
10008	0.626936	0.0698591
10009	0.946131	0.0698591
10010	0.94613	0.0698591
10011	1.39502	0.0698591
10012	0.881445	0.0698591
10013	0.946128	0.0698591
10014	0.94613	0.0698591
10015	0.881447	0.0698591
10016	0.946117	0.0698591
10017	0.946116	0.0698591
10018	0.88146	0.0698591
10019	0.946132	2.1
10020	1.39053	2.1
10021	0.946132	2.1
10022	0.946132	2.1
10023	0.946132	2.1
10024	0.946132	2.1
10025	0.946131	2.1
10026	0.946131	2.1
10027	0.946131	2.1
10028	0.946131	2.1
10029	0.946132	2.1
10030	0.946132	2.1
10031	1.40571	2.1

10032	0.946132	2.1
10033	0.94613	0.11571
10034	0.946127	0.170821
10035	0.946128	0.137791
10036	0.946131	0.124852
10037	0.946127	0.123322
10038	1.25819	0.132402
10039	0.946129	0.121674
10040	0.946129	0.523196
10041	1.29054	0.563329
10042	0.94613	0.45387
10043	0.94613	0.548876
10044	0.946131	1.18897
10045	0.946131	0.677744
10046	0.94613	0.547441
10047	0.810944	0.0698591
10048	0.810941	0.0698591
10049	1.61558	0.0698591
10050	1.3517	0.0698591
10051	1.08131	0.0698591
10052	1.08132	0.0698591
10053	1.08133	0.0698591
10054	0.810931	0.0698591
10055	1.02036	1.54353
10056	0.946132	1.12545
10057	1.02036	1.10974
10058	0.946132	0.598174
10059	1.1576	0.919538
10060	0.964656	1.10731
10061	0.927608	0.698471
10062	0.946128	2.1
10063	1.38813	2.1
10064	0.946129	2.1
10065	0.946131	2.1
10066	0.946131	2.1
10067	0.94613	2.1
10068	0.946131	2.1
10069	0.946131	2.1
10070	0.94613	2.1
10071	0.94613	2.1
10072	1.41665	2.1
10073	0.946132	2.1
10074	0.946131	2.1
10075	0.94613	2.1
10076	0.946118	0.186641
10077	0.946118	0.169327
10078	0.946116	0.185687
10079	0.946126	0.133586
10080	0.94612	0.146642
10081	0.946131	0.1607
10082	0.946105	0.150594
10083	0.946132	2.1
10084	0.946132	2.1
10085	0.946132	2.1
10086	0.946131	2.1
10087	0.946132	2.1
10088	0.946132	2.1
10089	0.946132	2.1

10090	0.864997	0.0698591
10091	0.786573	0.0698591
10092	0.978561	0.0698591
10093	1.47385	0.0698591
10094	1.68652	0.0698591
10095	1.27803	0.0698591
10096	1.19771	0.0698591
10097	1.27595	0.0698591
10098	0.946127	0.457424
10099	1.3771	0.336548
10100	0.946127	0.33552
10101	0.946131	0.535554
10102	0.946129	0.308795
10103	0.946129	0.464418
10104	0.946129	0.440941
10105	0.95697	0.0698591
10106	1.48133	0.0698591
10107	1.24499	0.0698591
10108	1.5295	0.0698591
10109	0.938161	0.0698591
10110	0.808717	0.0698591
10111	1.3542	0.0698591
10112	0.946133	0.549819
10113	1.14444	0.53129
10114	0.946132	0.449677
10115	0.946132	0.539899
10116	0.946133	1.07198
10117	0.946132	0.639778
10118	0.946132	0.538613
10119	0.946131	0.0698591
10120	0.946132	0.0712001
10121	0.946131	0.0698591
10122	0.946132	0.0698591
10123	0.946131	0.0700414
10124	0.946132	0.0698591
10125	0.946132	0.0698591
10126	0.946132	2.1
10127	0.946133	2.1
10128	0.946133	2.1
10129	0.946132	2.1
10130	1.29322	2.1
10131	0.946132	2.1
10132	0.946132	2.1
10133	0.946131	2.1
10134	0.946132	2.1
10135	0.946131	2.1
10136	0.946132	2.1
10137	1.28441	2.1
10138	0.946131	2.1
10139	0.946131	2.1
10140	0.946113	2.1
10141	0.946118	2.1
10142	0.946118	2.09296
10143	1.28073	2.1
10144	0.946117	2.1
10145	0.946131	2.1
10146	0.946105	2.1
10147	0.810945	2.1

10148	0.810942	2.1
10149	2.75904	2.1
10150	1.3517	2.1
10151	1.08131	2.1
10152	1.08133	2.1
10153	1.08132	2.1
10154	0.81093	2.1
10155	0.946127	2.1
10156	1.37952	2.1
10157	1.86965	2.1
10158	0.946127	2.1
10159	1.86965	2.1
10160	1.28228	2.1
10161	1.37952	2.1
10162	0.946128	2.1
10163	0.946132	2.1
10164	1.14042	2.1
10165	0.946132	2.1
10166	1.14042	2.1
10167	1.09974	2.1
10168	1.09974	2.1
10169	0.946132	2.1
10170	0.946132	0.0833116
10171	0.946132	0.0721797
10172	0.946133	0.0722173
10173	0.946132	0.0700909
10174	0.946132	0.0733812
10175	0.946132	0.0698591
10176	0.946133	0.0788145
10177	1.31817	1.86387
10178	0.946131	1.51936
10179	0.946131	1.81221
10180	0.946131	1.76921
10181	0.946131	1.28696
10182	0.946131	1.61277
10183	0.946131	1.62464
10184	0.946132	2.1
10185	0.946132	2.1
10186	0.946132	2.1
10187	0.946132	2.1
10188	0.946132	2.1
10189	0.946132	2.1
10190	1.35048	2.1
10191	0.946131	2.1
10192	0.946131	2.1
10193	0.94613	2.1
10194	0.946133	2.1
10195	0.946133	2.1
10196	1.35943	2.1
10197	0.946131	2.1
10198	0.946133	2.1
10199	0.946132	2.1
10200	0.946132	2.1
10201	0.946133	2.1
10202	0.946133	2.1
10203	0.946133	2.1
10204	0.990712	2.1
10205	0.965725	1.99271

10206	0.946129	1.26458
10207	0.946133	1.36682
10208	1.21721	1.3002
10209	0.877931	0.788592
10210	0.946131	1.58522
10211	0.946131	1.10496
10212	0.946132	0.498034
10213	0.946131	0.402217
10214	0.946131	0.933861
10215	0.946133	0.480706
10216	0.946128	1.42235
10217	0.946131	0.828555
10218	1.39575	0.746018
10219	0.810943	0.347717
10220	0.810942	0.361003
10221	2.58794	0.242853
10222	1.21652	0.229391
10223	0.946154	0.300723
10224	0.979952	0.294174
10225	1.0813	0.303529
10226	0.810959	0.328338
10227	0.924071	0.251708
10228	1.16453	0.179323
10229	1.28586	0.180141
10230	0.946131	0.277991
10231	1.1227	0.173464
10232	1.16462	0.218367
10233	0.851553	0.307706
10234	0.709578	0.238179
10235	0.810943	0.388765
10236	0.810959	0.26515
10237	1.82615	0.20066
10238	1.21651	0.212594
10239	1.08132	0.255996
10240	0.946155	0.244088
10241	0.979937	0.312406
10242	0.946128	1.76115
10243	0.946132	2.1
10244	0.946132	0.782275
10245	0.94613	2.02625
10246	0.94613	2.07311
10247	0.94613	0.461505
10248	0.94613	1.25337
10249	0.946131	1.75472
10250	1.29735	2.04798
10251	0.946131	2.1
10252	0.946131	0.87264
10253	0.946131	1.85191
10254	0.946131	0.969842
10255	0.946131	1.7839
10256	0.946128	2.09978
10257	1.33266	1.2559
10258	0.94613	1.36267
10259	0.946131	2.09229
10260	0.94613	0.861835
10261	0.946131	1.75131
10262	0.946131	1.51964
10263	0.946131	2.1

10264	0.946131	2.1
10265	0.946131	2.05667
10266	1.40608	2.1
10267	0.946131	2.1
10268	0.946131	2.1
10269	0.946131	2.1
10270	0.946131	1.87757
10271	0.946131	2.08718
10272	0.946131	1.97869
10273	1.2334	2.00028
10274	0.946129	2.02666
10275	0.946131	2.01185
10276	0.946131	2.07074
10277	0.946133	2.1
10278	0.946133	2.1
10279	0.946132	2.0716
10280	1.29198	2.1
10281	0.946132	2.09433
10282	0.946132	2.1
10283	0.946132	2.1
10284	0.946131	2.1
10285	0.946128	2.1
10286	0.946128	2.1
10287	0.946131	2.1
10288	0.946127	2.1
10289	0.94613	2.1
10290	0.946129	2.1
10291	0.946123	2.1
10292	0.946126	2.1
10293	0.946125	2.1
10294	1.38639	2.1
10295	0.946126	2.1
10296	0.946132	2.1
10297	0.946119	2.1
10298	0.946128	0.144301
10299	0.946132	0.126223
10300	0.946131	0.159729
10301	0.94613	0.0798304
10302	0.94613	0.0920075
10303	0.946131	0.121155
10304	0.94613	0.101332
10305	0.946131	0.130438
10306	0.946131	0.104771
10307	0.946131	0.146151
10308	0.946131	0.0728496
10309	0.946131	0.0794754
10310	0.946131	0.105202
10311	0.946131	0.085831
10312	0.810945	2.1
10313	0.810943	2.1
10314	0.810933	2.1
10315	2.24688	2.1
10316	1.3517	2.1
10317	1.08132	2.1
10318	1.08132	2.1
10319	1.08133	2.1
10320	1.25809	0.14703
10321	0.946131	0.516339

10322	1.25809	0.155255
10323	0.946129	0.447509
10324	0.94613	0.150523
10325	0.946131	0.433411
10326	0.946131	0.242513
10327	0.946131	0.0698591
10328	0.946131	0.0698591
10329	0.946129	0.0698591
10330	1.28911	0.0698591
10331	0.946131	0.0698591
10332	0.946131	0.0698591
10333	0.946131	0.0698591
10334	0.94613	0.171521
10335	0.946131	0.14097
10336	0.94613	0.116417
10337	0.946128	0.170022
10338	0.946132	0.114904
10339	1.38954	0.145108
10340	0.946131	0.152365
10341	0.946131	0.167608
10342	0.946131	0.373382
10343	0.946131	0.584539
10344	1.37981	0.267334
10345	0.946131	0.997969
10346	0.946131	0.445951
10347	0.946131	0.510295
10348	0.946131	2.1
10349	0.946127	2.1
10350	0.946127	2.1
10351	0.946131	2.1
10352	0.946127	2.1
10353	0.946129	2.1
10354	0.946128	2.1
10355	0.94613	2.1
10356	0.946126	2.1
10357	0.946127	2.1
10358	0.946131	2.1
10359	0.946126	2.1
10360	0.946128	2.1
10361	0.946128	2.1
10362	0.946133	0.296539
10363	0.946132	0.213709
10364	0.946132	0.237229
10365	0.946132	0.254645
10366	0.946132	0.279743
10367	1.13511	0.192664
10368	0.946132	0.234259
10369	0.946132	1.87607
10370	0.946129	1.83502
10371	0.94613	1.57557
10372	0.946131	1.87876
10373	0.946131	1.97843
10374	0.94613	2.03761
10375	1.27575	1.98206
10376	0.946129	2.1
10377	0.946131	2.1
10378	0.946131	2.1
10379	0.946128	2.1

10380	0.946133	2.1
10381	0.946131	2.1
10382	0.94613	2.1
10383	1.15581	0.0698591
10384	0.946132	0.0698591
10385	0.946131	0.0698591
10386	0.94613	0.0698591
10387	1.15581	0.0698591
10388	0.94613	0.0698591
10389	0.946131	0.0698591
10390	0.611407	1.26414
10391	0.946131	0.804034
10392	1.37335	1.07371
10393	1.15086	1.46109
10394	0.617549	1.29764
10395	0.851553	1.42435
10396	1.14592	1.43577
10397	1.22618	1.94001
10398	0.946132	1.00718
10399	0.946132	1.9597
10400	0.946132	1.2341
10401	0.946131	1.4385
10402	0.946132	1.75498
10403	0.946132	1.68521
10404	0.946131	2.1
10405	0.94613	2.1
10406	0.946131	2.1
10407	0.946133	2.1
10408	0.946129	2.1
10409	0.946131	2.1
10410	0.946131	2.1
10411	0.810945	0.930846
10412	0.810943	2.04831
10413	2.50715	1.71091
10414	1.3517	1.41846
10415	1.08133	1.3247
10416	1.08132	2.02785
10417	1.08132	0.319684
10418	0.810932	1.1789
10419	1.07811	2.05265
10420	0.94613	1.24338
10421	0.946128	2.05945
10422	1.25358	0.679975
10423	0.946131	1.50765
10424	1.25358	1.23131
10425	0.94613	1.27234
10426	0.946129	2.1
10427	1.09197	2.1
10428	0.946131	2.1
10429	1.09197	2.1
10430	1.09532	2.1
10431	1.09532	2.1
10432	0.946131	2.1
10433	1.58788	2.1
10434	1.34255	2.1
10435	0.594912	2.1
10436	0.58894	2.1
10437	0.946129	2.1

10438	1.16679	2.1
10439	1.04071	2.1
10440	0.535418	2.1
10441	0.946131	2.1
10442	0.946128	2.1
10443	0.946131	2.09566
10444	1.20221	2.1
10445	0.946128	2.1
10446	0.94613	2.1
10447	1.20221	2.1
10448	0.946132	2.1
10449	0.946133	2.1
10450	0.946132	2.1
10451	0.946132	2.1
10452	0.946133	2.1
10453	0.946133	2.1
10454	0.946132	2.1
10455	0.946132	1.96079
10456	0.946132	2.08793
10457	0.946133	2.1
10458	0.946132	2.06812
10459	1.411 2.09217	
10460	0.946132	2.0887
10461	0.946133	2.08959
10462	1.05575	0.0756591
10463	0.563734	0.0799527
10464	1.05575	0.081348
10465	0.773833	0.0698591
10466	0.751686	0.0709963
10467	0.75884	0.073346
10468	0.815612	0.0704309
10469	0.793465	0.0751317
10470	0.768563	0.0982715
10471	0.946129	0.0753543
10472	0.946125	0.0699321
10473	0.946126	0.0839745
10474	0.94613	0.072516
10475	0.946124	0.0871171
10476	1.31678	0.0698921
10477	0.946128	0.0743453
10478	0.946132	0.282537
10479	1.29914	0.199781
10480	0.946132	0.202884
10481	0.946132	0.214494
10482	0.946132	0.302568
10483	1.29914	0.156864
10484	0.946132	0.228328
10485	0.810946	0.268755
10486	0.810943	0.262476
10487	2.21082	0.150537
10488	1.35168	0.16507
10489	1.08132	0.199136
10490	1.08132	0.208178
10491	1.0813	0.253156
10492	0.81096	0.24382
10493	0.625813	0.0698591
10494	0.946132	0.0698591
10495	0.946131	0.0698591

10496	0.946132	0.0698591
10497	0.946131	0.0698591
10498	1.39301	0.0698591
10499	0.625812	0.0698591
10500	0.810946	2.02656
10501	0.810945	2.1
10502	0.810931	2.1
10503	2.70043	2.1
10504	1.3517	2.1
10505	1.08131	2.05378
10506	1.08131	2.1
10507	1.08133	2.08987
10508	0.810943	2.1
10509	0.810944	2.1
10510	0.810958	2.1
10511	2.44802	2.1
10512	1.35168	2.1
10513	1.08132	2.1
10514	1.08132	2.1
10515	1.0813	2.1
10516	0.946131	0.13379
10517	0.946129	0.116941
10518	0.946128	0.0958624
10519	0.946131	0.168983
10520	1.3492	0.136106
10521	1.3492	0.135622
10522	0.94613	0.1076
10523	0.946132	0.275565
10524	0.946132	0.14172
10525	0.946133	0.291721
10526	0.946133	0.122348
10527	0.946132	0.421436
10528	0.946132	0.117909
10529	0.946132	0.393683
10530	0.946129	0.359894
10531	0.946133	0.252666
10532	0.946131	0.36489
10533	0.94613	0.243923
10534	0.946131	0.401568
10535	0.946131	0.317957
10536	0.946131	0.303363
10537	0.946119	0.19959
10538	0.946119	0.134558
10539	0.946119	0.208836
10540	0.946125	0.123054
10541	0.94612	0.226379
10542	0.946132	0.164404
10543	0.946107	0.156611
10544	0.946133	2.1
10545	0.946132	2.1
10546	0.946132	2.1
10547	0.946132	2.1
10548	0.946132	2.1
10549	0.946133	2.1
10550	1.3868	2.1
10551	0.810947	2.1
10552	0.810946	2.1
10553	0.81096	2.1

10554	2.66094	2.1
10555	1.35168	2.1
10556	1.08132	2.1
10557	1.08132	2.1
10558	1.0813	2.1
10559	0.810946	2.1
10560	0.810946	2.1
10561	0.810933	2.1
10562	2.75737	2.1
10563	1.3517	2.1
10564	1.08132	2.1
10565	1.08132	2.1
10566	1.08133	2.1
10567	0.946133	2.1
10568	0.946132	2.1
10569	0.946132	2.1
10570	0.946132	2.1
10571	0.946133	2.1
10572	0.946132	2.1
10573	0.946133	2.1
10574	0.946133	2.1
10575	0.946133	2.1
10576	0.946132	2.1
10577	0.946132	2.1
10578	0.946133	2.1
10579	0.946132	2.1
10580	1.28569	2.1
10581	0.946132	2.04548
10582	0.946133	2.1
10583	0.946133	2.1
10584	0.946132	2.09042
10585	0.946132	2.1
10586	0.946132	2.1
10587	0.946133	2.1
10588	0.946132	0.0698591
10589	0.946131	0.0698591
10590	0.94613	0.0698591
10591	0.946133	0.0698591
10592	0.946128	0.0698591
10593	0.946131	0.0698591
10594	0.946131	0.0698591
10595	0.946131	2.1
10596	0.94613	2.1
10597	0.946131	2.1
10598	0.94613	2.1
10599	1.41833	2.006
10600	0.946131	2.1
10601	0.946131	2.1
10602	1.26155	0.0698591
10603	0.946131	0.0698591
10604	0.946131	0.0698591
10605	0.946132	0.0698591
10606	0.946132	0.0698591
10607	0.946131	0.0698591
10608	0.946132	0.0698591
10609	0.810946	2.1
10610	0.810946	2.1
10611	0.810959	2.1

10612	2.47938	2.1
10613	1.35168	2.1
10614	1.08132	2.1
10615	1.08132	2.1
10616	1.08131	2.1
10617	0.946131	0.47297
10618	0.94613	0.364444
10619	0.94613	0.851572
10620	0.946132	0.340062
10621	0.946128	1.28111
10622	0.94613	0.716886
10623	0.94613	0.796691
10624	0.94613	0.0698591
10625	0.946131	0.0698591
10626	0.94613	0.084197
10627	0.946129	0.0729623
10628	0.946132	0.0720537
10629	0.94613	0.072757
10630	0.946131	0.0704143
10631	0.946132	2.1
10632	0.946131	2.1
10633	0.946128	2.1
10634	1.40756	2.1
10635	0.946131	2.1
10636	0.946129	2.1
10637	0.94613	2.1
10638	0.946131	2.09838
10639	0.946131	2.00458
10640	0.946131	2.1
10641	0.946132	2.1
10642	1.31947	2.08955
10643	0.946131	2.1
10644	0.946131	2.1
10645	0.946131	2.1
10646	1.25768	2.1
10647	0.946132	2.1
10648	0.946132	2.1
10649	0.946129	2.1
10650	0.946131	2.1
10651	0.94613	2.1
10652	0.946132	0.0698591
10653	0.946129	0.0698591
10654	0.946129	0.0929564
10655	0.94613	0.0760783
10656	1.31716	0.0762954
10657	1.31716	0.0705546
10658	0.946131	0.0935877
10659	0.810943	1.26457
10660	0.810942	2.1
10661	0.81093	2.06373
10662	2.37052	2.1
10663	1.3517	2.07522
10664	1.08132	1.45504
10665	1.08132	2.1
10666	1.08133	1.91874
10667	0.946128	0.722283
10668	1.10335	0.360426
10669	0.94613	0.340622

10670	0.946131	0.797744
10671	0.946131	0.416416
10672	1.10335	0.697259
10673	0.94613	0.779579
10674	0.946132	1.04167
10675	1.18818	0.672
10676	0.946132	0.621907
10677	0.946132	1.05515
10678	0.946132	0.702784
10679	1.18818	1.12488
10680	0.946132	1.17683
10681	0.946125	2.1
10682	0.94612	2.1
10683	0.946119	2.1
10684	0.946119	2.1
10685	0.946119	2.1
10686	0.946132	2.1
10687	1.31522	2.1
10688	1.28803	2.1
10689	0.946132	2.00779
10690	0.946132	2.1
10691	0.946133	2.1
10692	0.946133	1.8471
10693	0.946133	2.1
10694	0.946132	2.1
10695	0.946133	1.04282
10696	0.946132	1.60781
10697	0.946132	1.25576
10698	0.946132	0.340619
10699	0.946133	0.232173
10700	0.946132	0.257786
10701	1.37658	0.551207
10702	1.02239	1.60926
10703	0.946128	1.24891
10704	0.946132	1.48103
10705	1.02239	1.07565
10706	0.94613	1.0675
10707	0.96283	1.55642
10708	0.92943	1.08333
10709	0.946132	0.0698591
10710	0.946131	0.0698591
10711	0.946126	0.0698591
10712	0.946129	0.0698591
10713	0.946128	0.0698591
10714	0.946128	0.0698591
10715	0.946129	0.0698591
10716	0.810946	0.0698591
10717	0.810944	0.0698591
10718	2.22493	0.0698591
10719	1.21651	0.0698591
10720	1.0813	0.0698591
10721	0.946154	0.0698591
10722	0.979951	0.0698591
10723	0.70959	0.0698591
10724	0.946132	0.0698591
10725	0.946131	0.0801566
10726	0.946131	0.0698634
10727	0.946133	0.0698591

10728	0.946131	0.0750914
10729	0.946132	0.0698591
10730	0.946132	0.0746116
10731	0.946132	1.91481
10732	0.946131	1.63943
10733	0.946132	2.06523
10734	0.946132	1.13781
10735	0.946132	0.798682
10736	0.946132	1.20688
10737	1.40924	1.72396
10738	0.946131	2.1
10739	0.946128	2.1
10740	0.946127	2.1
10741	0.94613	2.1
10742	1.41743	2.1
10743	0.946128	2.1
10744	0.946129	2.1
10745	1.37675	1.24228
10746	0.946128	1.29351
10747	0.94613	0.620549
10748	0.94613	1.46223
10749	0.94613	0.873752
10750	0.946129	0.94462
10751	0.94613	1.15714
10752	0.946132	0.194534
10753	0.946132	0.371783
10754	0.946132	0.582166
10755	1.35721	0.217827
10756	0.946132	0.952637
10757	0.946132	0.526988
10758	1.35721	0.455925
10759	0.946133	0.578912
10760	0.946133	0.397706
10761	0.946132	0.506538
10762	1.10003	0.292909
10763	0.946133	0.307215
10764	0.946132	0.430196
10765	0.946132	0.38952
10766	0.946132	0.0859445
10767	0.946131	0.0717101
10768	0.946131	0.0760796
10769	0.946131	0.0938369
10770	1.0616	0.0714005
10771	1.0616	0.0736926
10772	0.946132	0.083136
10773	0.946132	0.106566
10774	0.946131	0.0792681
10775	0.946131	0.0885946
10776	0.946131	0.119202
10777	1.09402	0.0789236
10778	1.09402	0.0837236
10779	0.946131	0.11087
10780	0.946132	0.0698591
10781	0.946126	0.0698724
10782	1.24093	0.0698591
10783	0.946129	0.0723173
10784	0.946129	0.0700945
10785	0.946128	0.0703852

10786	0.946129	0.0703994
10787	0.946131	0.0825547
10788	0.946131	0.0842601
10789	1.2929	0.079765
10790	0.946131	0.109407
10791	0.946131	0.0900283
10792	0.946131	0.0926699
10793	0.946131	0.0896781
10794	0.810946	0.0698591
10795	0.810945	0.0698591
10796	0.810959	0.0698591
10797	2.41233	0.0698591
10798	1.35168	0.0698591
10799	1.08132	0.0698591
10800	1.08132	0.0698591
10801	1.0813	0.0698591
10802	0.810946	1.903
10803	0.810943	2.1
10804	0.750103	2.07088
10805	2.22778	2.08884
10806	1.27059	2.07013
10807	1.00021	1.99244
10808	1.02049	2.09914
10809	1.08133	2.04086
10810	0.946128	2.08385
10811	0.946132	2.1
10812	0.946132	2.1
10813	0.94613	2.1
10814	0.94613	2.1
10815	0.94613	2.1
10816	0.946131	2.1
10817	0.946126	0.314604
10818	0.946131	0.265562
10819	0.946127	0.393892
10820	1.3324	0.222999
10821	0.946127	0.228679
10822	0.946128	0.324246
10823	0.946128	0.446104
10824	0.94613	2.1
10825	0.946131	2.1
10826	0.94613	2.1
10827	0.946128	2.1
10828	0.946133	2.1
10829	0.946131	2.1
10830	0.946131	2.1
10831	0.946133	2.1
10832	0.946132	2.1
10833	0.946133	2.1
10834	0.946133	2.1
10835	0.946132	2.1
10836	0.946132	2.1
10837	1.36541	2.1
10838	0.946131	1.06549
10839	0.94613	2.1
10840	0.946131	1.63293
10841	0.94613	0.939071
10842	1.39509	1.87252
10843	0.946131	0.334989

10844	0.946131	1.93552
10845	0.946133	2.1
10846	0.946133	2.1
10847	0.946132	2.1
10848	0.946132	2.1
10849	0.946133	2.1
10850	0.946132	2.1
10851	1.29319	2.1
10852	1.01196	0.0739383
10853	0.946131	0.12643
10854	0.946132	0.0746716
10855	0.946132	0.0875943
10856	0.946132	0.119916
10857	0.946132	0.105057
10858	0.946132	0.0975341
10859	0.946112	2.1
10860	0.946118	2.1
10861	0.946116	2.1
10862	0.946117	2.1
10863	0.946118	2.1
10864	0.94613	2.1
10865	0.946105	2.1
10866	1.28684	1.29333
10867	0.946131	1.45321
10868	0.946132	1.34048
10869	0.946132	1.63524
10870	0.946132	1.81974
10871	0.946132	1.46711
10872	0.946132	1.69334
10873	0.94613	2.00534
10874	0.94613	2.05625
10875	0.946128	1.97119
10876	0.946131	1.76586
10877	0.946131	1.91742
10878	0.94613	1.96913
10879	1.10185	2.00289
10880	0.810947	0.0698591
10881	0.810945	0.0698591
10882	1.57348	0.0698591
10883	1.3517	0.0698591
10884	1.08131	0.0698591
10885	1.08132	0.0698591
10886	1.08133	0.0698591
10887	0.810932	0.0698591
10888	0.946119	0.237902
10889	0.946116	0.199926
10890	0.946117	0.24401
10891	0.946121	0.169747
10892	0.946118	0.192366
10893	0.94613	0.200071
10894	0.946105	0.193232
10895	0.94612	0.397192
10896	0.946122	0.245711
10897	0.946119	0.34124
10898	0.946119	0.422966
10899	1.20884	0.270772
10900	0.946132	0.3502
10901	0.946107	0.319818

10902	2.67849	1.67063
10903	1.17709	1.53154
10904	0.605418	1.72087
10905	0.286984	1.58618
10906	0.172654	1.62006
10907	0.810946	1.08868
10908	0.940878	0.925659
10909	0.941235	0.814155
10910	1.26696	0.69511
10911	1.56837	0.754221
10912	1.13737	0.833417
10913	1.34115	0.740108
10914	1.24889	0.802134
10915	0.946132	1.84018
10916	0.946129	1.01784
10917	0.946128	1.05362
10918	0.94613	1.35628
10919	0.94613	1.06439
10920	0.946131	1.28034
10921	0.94613	1.24192
10922	0.946116	2.1
10923	0.94612	2.1
10924	0.946118	2.1
10925	0.946123	2.1
10926	0.946118	2.1
10927	0.946131	2.1
10928	1.23836	2.1
10929	0.810946	0.0698591
10930	0.810946	0.0698591
10931	0.810959	0.0698591
10932	2.80677	0.0698591
10933	1.35168	0.0698591
10934	1.08132	0.0698591
10935	1.08132	0.0698591
10936	1.08131	0.0698591
10937	0.810947	0.146477
10938	0.810947	0.699085
10939	2.41574	0.38198
10940	1.21653	0.393901
10941	1.08133	0.26332
10942	0.946153	1.01522
10943	0.979949	0.665401
10944	0.709567	0.159385
10945	0.94613	2.08977
10946	0.946131	2.09437
10947	0.946128	2.1
10948	1.34782	2.09771
10949	0.946128	2.09914
10950	0.946129	2.1
10951	1.34782	2.1
10952	0.946132	2.01137
10953	0.946132	2.05837
10954	0.94613	2.07712
10955	1.16023	2.0552
10956	0.946131	2.07934
10957	0.946131	2.1
10958	1.16023	2.09482
10959	0.946131	0.961538

10960	0.946127	2.1
10961	0.946127	2.1
10962	0.946131	1.74552
10963	0.946128	2.1
10964	0.94613	1.82215
10965	0.946129	1.981
10966	0.946133	0.160306
10967	0.946132	0.122826
10968	0.946132	0.137655
10969	0.946133	0.179405
10970	1.20045	0.123328
10971	1.20045	0.130163
10972	0.946132	0.170543
10973	0.918871	0.0698591
10974	0.971961	0.0698591
10975	1.37324	0.0698591
10976	1.78255	0.0698591
10977	1.25155	0.0698591
10978	1.42172	0.0698591
10979	1.40554	0.0698591
10980	1.08862	0.0698591
10981	1.03803	0.0698591
10982	0.946132	0.0698591
10983	0.946126	0.0703739
10984	0.943647	0.0698601
10985	0.948611	0.0698591
10986	0.946129	0.0702657
10987	0.946129	0.0698591
10988	1.1699	1.00157
10989	0.946132	0.480433
10990	0.946133	1.11488
10991	0.946133	0.52381
10992	0.946132	0.7082
10993	0.946133	0.863321
10994	0.946132	1.486
10995	0.946132	2.1
10996	0.946131	2.1
10997	0.946131	2.1
10998	0.946132	2.1
10999	0.946131	2.1
11000	0.946131	2.1
11001	0.946132	2.1
11002	0.946132	0.608659
11003	1.38623	0.496541
11004	0.946132	0.345939
11005	0.946132	0.404682
11006	0.946133	0.621933
11007	0.946132	0.404815
11008	0.946133	0.638986
11009	0.946132	2.1
11010	1.40098	2.1
11011	0.946131	2.1
11012	0.946131	2.1
11013	0.946132	2.1
11014	0.946132	2.1
11015	0.946132	2.1
11016	1.01777	0.548115
11017	0.946132	0.278316

11018	0.946133	1.24331
11019	1.68068	0.453476
11020	1.68068	1.20193
11021	1.41894	0.290679
11022	1.20787	0.0810895
11023	0.946132	0.190989
11024	0.946133	2.1
11025	0.946133	2.1
11026	0.946132	2.1
11027	1.40199	2.1
11028	0.946133	2.1
11029	0.946132	2.1
11030	0.946132	2.1
11031	0.810946	1.56358
11032	0.810947	0.925697
11033	2.37827	1.73275
11034	1.3517	1.4959
11035	1.08132	1.48609
11036	1.08132	1.77694
11037	1.08133	1.53052
11038	0.810934	1.48763
11039	0.946124	2.01312
11040	0.94613	1.61369
11041	1.40248	1.3782
11042	0.946131	1.30048
11043	0.946125	0.770652
11044	0.946127	1.64199
11045	0.946128	1.0247
11046	1.25703	1.74761
11047	0.87821	1.29297
11048	1.18912	1.74145
11049	0.946119	1.28791
11050	0.87825	1.45007
11051	1.1891	1.65549
11052	0.946107	1.87544
11053	0.946132	1.97039
11054	1.28543	1.26609
11055	0.946132	0.940889
11056	0.946132	1.30264
11057	0.946131	1.17468
11058	0.946132	0.319649
11059	0.946132	0.293249
11060	0.946115	2.1
11061	0.946119	2.1
11062	0.946116	2.1
11063	0.946123	2.1
11064	0.946118	2.1
11065	1.41636	2.1
11066	0.946129	2.1
11067	0.946132	2.08415
11068	0.946132	2.1
11069	0.946132	2.1
11070	0.946131	2.1
11071	0.946131	2.1
11072	1.41432	2.1
11073	0.946132	2.1
11074	0.810947	0.0698591
11075	0.810947	0.0698591

11076	2.44433	0.0698591
11077	1.21654	0.0698591
11078	0.946154	0.0698591
11079	0.97995	0.0698591
11080	1.08133	0.0698591
11081	0.810934	0.0698591
11082	0.851542	0.0698591
11083	0.835698	0.0698591
11084	1.22216	0.0698591
11085	0.946133	0.0698591
11086	1.01217	0.0698591
11087	1.09996	0.0698591
11088	0.851529	0.0698591
11089	0.946132	1.77681
11090	0.946132	1.99773
11091	0.946129	2.07684
11092	0.94613	1.92995
11093	0.946131	2.02284
11094	0.946131	2.02673
11095	1.2254	2.04169
11096	0.800897	1.83267
11097	0.838029	2.09666
11098	0.838013	1.86999
11099	0.93679	1.36659
11100	1.20217	1.48508
11101	1.16923	1.68858
11102	0.94948	1.83026
11103	0.958118	1.95985
11104	0.36751	1.43402
11105	0.800535	1.72371
11106	0.272087	0.993901
11107	0.428084	1.10925
11108	0.986335	1.73066
11109	0.946132	0.264919
11110	0.946133	0.327159
11111	1.19601	0.259298
11112	0.946132	0.322341
11113	0.946132	0.349363
11114	0.946133	0.341036
11115	0.946133	0.270868
11116	0.946132	1.99397
11117	1.36956	2.1
11118	0.946132	2.1
11119	0.946132	2.00881
11120	0.946132	2.1
11121	0.946133	2.06799
11122	0.946132	2.0885
11123	0.946132	0.834908
11124	0.946131	2.07424
11125	0.946131	1.03324
11126	1.34268	0.240372
11127	0.946133	2.0256
11128	0.946132	0.823328
11129	0.946131	1.22844
11130	0.946132	2.08867
11131	0.946131	2.1
11132	0.946131	2.1
11133	0.946132	2.1

11134	0.946132	2.1
11135	0.946132	2.1
11136	0.946132	2.1
11137	0.810946	1.17274
11138	0.810943	0.910153
11139	2.51472	1.09076
11140	1.3517	1.03007
11141	1.08132	0.861705
11142	1.08132	1.12693
11143	1.08133	1.11111
11144	0.810933	1.15623
11145	0.902316	0.509234
11146	1.04517	0.545481
11147	0.974924	0.378614
11148	0.530063	0.679687
11149	1.1272	0.535708
11150	0.68207	0.558681
11151	0.477061	0.596944
11152	0.810946	0.0880004
11153	0.810943	0.135799
11154	0.810959	0.103797
11155	1.99211	0.0858708
11156	1.35168	0.0849027
11157	1.08132	0.0893305
11158	1.08132	0.0918754
11159	1.08131	0.11585
11160	0.94613	0.142093
11161	0.946131	0.138267
11162	0.946132	0.115194
11163	0.946132	0.138773
11164	0.946129	0.214499
11165	0.946131	0.142973
11166	1.16699	0.132334
11167	0.810943	1.8947
11168	0.810942	1.78009
11169	1.6943	2.02148
11170	1.27059	2.03559
11171	1.00021	2.01838
11172	1.02049	1.97414
11173	1.08133	1.91111
11174	0.750103	1.8569
11175	0.957813	1.99266
11176	0.946128	2.03008
11177	0.946132	1.78625
11178	1.04832	2.0563
11179	0.870714	1.70324
11180	0.94613	1.89175
11181	0.94613	1.94896
11182	1.86374	2.09912
11183	2.0815	1.84613
11184	1.15918	0.117848
11185	0.946129	0.0785906
11186	1.15918	0.127226
11187	0.946131	0.0907581
11188	0.946131	0.134457
11189	0.946131	0.0999063
11190	0.94613	0.0917584
11191	0.946119	0.502799

11192	1.3612	0.655302
11193	0.946125	0.511898
11194	0.946119	0.588565
11195	0.94612	1.18175
11196	0.946132	0.774951
11197	0.946107	0.581946
11198	0.810947	0.142215
11199	0.810947	0.205584
11200	2.50398	0.135932
11201	1.3517	0.135989
11202	1.08132	0.11716
11203	1.08133	0.152545
11204	1.08132	0.143332
11205	0.810934	0.134203
11206	0.946132	2.1
11207	0.946132	2.1
11208	0.946132	2.1
11209	1.39754	2.1
11210	0.946132	2.1
11211	0.946132	2.1
11212	0.946131	2.1
11213	0.946129	2.1
11214	1.41732	2.1
11215	0.946129	2.1
11216	0.946131	2.1
11217	0.946131	2.1
11218	0.946131	2.1
11219	0.946131	2.1
11220	0.946132	1.19698
11221	1.25276	1.70956
11222	0.946131	1.82773
11223	0.94613	1.29474
11224	0.94613	1.72799
11225	0.94613	1.47219
11226	0.946131	1.4961
11227	1.21261	0.836262
11228	0.94613	1.07625
11229	0.94613	1.68959
11230	0.946128	0.915661
11231	0.946131	1.35096
11232	0.94613	1.52251
11233	0.946129	1.34414
11234	0.946128	2.1
11235	0.946133	2.1
11236	0.946132	2.1
11237	0.946131	2.1
11238	0.94613	2.1
11239	0.946131	2.1
11240	1.39407	2.1
11241	0.946116	1.83707
11242	0.946119	1.93465
11243	0.946119	1.47731
11244	1.15369	1.83596
11245	0.946119	1.35214
11246	0.946131	1.76336
11247	0.946106	1.73698
11248	0.946131	2.1
11249	0.946131	2.1

11250	0.946131	2.1
11251	0.946133	2.1
11252	0.946129	2.1
11253	0.946131	2.1
11254	0.946131	2.1
11255	1.19564	0.0698591
11256	0.498881	0.0698591
11257	1.33964	0.0698591
11258	0.498878	0.0698591
11259	0.923583	0.0698591
11260	0.831259	0.0698591
11261	0.553735	0.0698591
11262	0.946132	0.0698591
11263	0.946132	0.0698591
11264	0.946131	0.0698591
11265	0.946131	0.0698591
11266	0.946132	0.0698591
11267	0.946131	0.0698591
11268	1.28673	0.0698591
11269	0.946132	0.0845823
11270	0.946132	0.0880609
11271	1.33178	0.0838198
11272	0.946132	0.0906692
11273	0.946132	0.0905334
11274	0.946131	0.10036
11275	0.946131	0.0846995
11276	0.946128	0.215443
11277	0.946132	0.14785
11278	0.946129	0.236906
11279	0.946131	0.0931837
11280	0.946131	0.117379
11281	0.946129	0.138788
11282	0.94613	0.118721
11283	0.946131	2.1
11284	0.946131	2.1
11285	0.946129	2.1
11286	1.40525	2.1
11287	0.94613	2.1
11288	0.94613	2.1
11289	1.40525	2.1
11290	0.946131	2.1
11291	0.946126	2.1
11292	0.946127	2.1
11293	0.946127	2.1
11294	1.4073	2.1
11295	0.946129	2.1
11296	0.946129	2.1
11297	0.946131	0.0804468
11298	0.946131	0.072547
11299	0.946132	0.0832158
11300	0.946131	0.0698591
11301	0.946131	0.0704361
11302	1.05016	0.0708423
11303	0.946132	0.079092
11304	0.946128	0.0837029
11305	0.946132	0.0749681
11306	0.946131	0.087211
11307	0.946129	0.0700333

11308	0.94613	0.0711961
11309	1.13398	0.0715633
11310	0.94613	0.0892345
11311	0.946132	0.0698591
11312	0.946132	0.0698591
11313	0.946131	0.0698591
11314	0.946131	0.0698591
11315	0.946132	0.0698591
11316	0.946131	0.0698591
11317	0.946132	0.0698591
11318	0.946131	0.0845309
11319	0.94613	0.0698591
11320	0.946131	0.0718787
11321	0.94613	0.0898897
11322	1.38645	0.0698826
11323	1.38645	0.0720205
11324	0.946131	0.075766
11325	0.946131	0.195204
11326	0.946131	0.119934
11327	0.94613	0.232081
11328	1.23923	0.104517
11329	0.94613	0.081283
11330	0.946131	0.203148
11331	0.946131	0.295821
11332	0.946132	0.0698591
11333	0.946131	0.0698591
11334	0.946131	0.0698591
11335	0.946132	0.0698591
11336	0.946132	0.0698591
11337	0.946132	0.0698591
11338	1.40511	0.0698591
11339	0.946132	2.1
11340	0.946132	2.1
11341	0.946132	2.1
11342	0.946132	2.1
11343	0.946132	2.1
11344	0.946131	2.1
11345	1.12968	2.1
11346	0.946132	1.54762
11347	0.946128	2.1
11348	0.946128	2.08503
11349	0.94613	1.95909
11350	0.94613	2.1
11351	0.94613	1.93812
11352	0.94613	1.9762
11353	0.946133	2.1
11354	0.946133	2.1
11355	0.946132	2.1
11356	0.946132	2.1
11357	0.946132	2.1
11358	0.946133	2.1
11359	1.31323	2.1
11360	0.94613	2.1
11361	0.946131	2.0933
11362	0.946131	2.09396
11363	0.94613	2.02476
11364	0.946131	2.1
11365	0.946131	2.1

11366	0.946131	2.1
11367	0.946132	2.08475
11368	0.946132	0.859456
11369	0.946132	2.1
11370	1.36899	2.00509
11371	0.946132	0.188345
11372	0.946132	1.67701
11373	0.946133	1.18941
11374	1.03543	0.0698591
11375	0.946131	0.0698591
11376	0.946131	0.0698591
11377	0.946132	0.0698591
11378	0.94613	0.0698591
11379	0.950987	0.0698591
11380	0.941275	0.0698591
11381	0.946131	0.0972509
11382	0.94613	0.107161
11383	1.28952	0.113738
11384	0.946132	0.0884755
11385	0.94613	0.142555
11386	0.946131	0.132397
11387	0.946131	0.100845
11388	0.94613	2.1
11389	1.35966	2.1
11390	0.946131	2.1
11391	0.946131	2.1
11392	0.946129	2.1
11393	0.94613	2.1
11394	0.94613	2.1
11395	0.810946	2.1
11396	0.810942	2.04418
11397	0.810932	2.1
11398	2.6655	2.1
11399	1.3517	2.1
11400	1.08132	2.1
11401	1.08131	2.04502
11402	1.08133	2.1
11403	0.94613	2.1
11404	0.946131	2.1
11405	0.94613	2.1
11406	0.946128	2.1
11407	0.946131	2.1
11408	0.94613	2.1
11409	0.94613	2.1
11410	0.946128	2.1
11411	0.946128	2.1
11412	0.946133	2.1
11413	1.25375	2.1
11414	0.94613	2.1
11415	0.946131	2.1
11416	1.25375	2.1
11417	0.94613	2.1
11418	0.94613	2.1
11419	0.946132	2.1
11420	0.946132	2.1
11421	0.946128	2.1
11422	0.94613	2.1
11423	1.31453	2.1

11424	0.946131	0.0718743
11425	0.946129	0.0698591
11426	0.94613	0.0719586
11427	0.94613	0.0698591
11428	0.94613	0.0698591
11429	1.12018	0.0698591
11430	0.94613	0.0715118
11431	0.946131	0.0924011
11432	0.946129	0.0698635
11433	0.946131	0.0978777
11434	1.16373	0.0698591
11435	0.94613	0.0698591
11436	0.94613	0.0916632
11437	0.946131	0.109152
11438	1.36811	0.314053
11439	0.946132	0.50515
11440	1.36811	0.268884
11441	0.946129	0.4563
11442	0.94613	0.34125
11443	0.946129	0.46651
11444	0.946129	0.407314
11445	0.946132	0.135573
11446	0.946133	0.0838021
11447	0.946132	0.105231
11448	0.946133	0.136726
11449	1.35732	0.0904942
11450	1.35732	0.1024
11451	0.946133	0.113635
11452	1.36399	0.623057
11453	0.946129	1.23945
11454	0.94613	0.671535
11455	0.946131	1.19758
11456	0.94613	1.78183
11457	0.946131	1.04319
11458	0.946131	1.35702
11459	0.946133	2.1
11460	0.946132	2.1
11461	0.946132	2.1
11462	1.38302	2.1
11463	0.946133	2.1
11464	0.946132	2.1
11465	0.946133	2.1
11466	0.810943	2.1
11467	0.810944	2.1
11468	0.810945	2.1
11469	2.80542	2.1
11470	1.35169	2.1
11471	1.08132	2.1
11472	1.08132	2.1
11473	1.08132	2.1
11474	1.30189	2.1
11475	0.946131	2.1
11476	0.946132	2.1
11477	0.946131	2.1
11478	0.946131	2.1
11479	0.946131	2.1
11480	0.946132	2.1
11481	0.810946	1.03741

11482	0.810947	0.766058
11483	2.33293	0.648989
11484	1.24357	0.630782
11485	1.08132	0.809264
11486	0.973183	1.04845
11487	1.00023	0.865911
11488	0.810934	1.02353
11489	1.06584	0.529918
11490	0.946129	0.601282
11491	0.84731	0.377877
11492	1.16646	0.571327
11493	0.946126	1.07914
11494	1.2831	0.427649
11495	0.851527	0.678697
11496	0.946131	2.1
11497	0.946128	2.1
11498	0.946128	2.1
11499	0.94613	2.1
11500	1.31961	2.1
11501	0.94613	2.1
11502	0.94613	2.1
11503	0.946131	2.1
11504	1.3605	2.1
11505	0.946133	2.1
11506	0.946132	2.1
11507	0.94613	2.1
11508	0.946131	2.1
11509	0.946131	2.1
11510	0.946128	2.1
11511	0.946133	1.87558
11512	0.946132	2.1
11513	0.946131	1.8715
11514	0.946131	1.55644
11515	0.946131	2.02841
11516	1.30116	2.0929
11517	0.946132	0.0698591
11518	0.946129	0.0698591
11519	0.94613	0.0698591
11520	1.3035	0.0698591
11521	0.946131	0.0698591
11522	0.94613	0.0698591
11523	0.946131	0.0698591
11524	0.946132	2.1
11525	1.2051	2.1
11526	1.2051	2.1
11527	0.946132	2.1
11528	1.37869	2.1
11529	0.946132	2.1
11530	0.946131	2.1
11531	0.810946	2.1
11532	0.810945	2.1
11533	2.69247	2.1
11534	1.35169	2.1
11535	1.08132	2.1
11536	1.08132	2.1
11537	1.08132	2.1
11538	0.810946	2.1
11539	1.22993	2.1

11540	0.946131	2.1
11541	0.946131	2.1
11542	1.34623	2.1
11543	0.946131	2.1
11544	1.34623	2.1
11545	0.946131	2.1
11546	0.946131	0.0698591
11547	0.946132	0.0698591
11548	0.946132	0.0698591
11549	0.946132	0.0698591
11550	0.946131	0.0698591
11551	0.946132	0.0698591
11552	1.32299	0.0698591
11553	0.94613	0.181144
11554	0.946131	0.203851
11555	0.946128	0.182362
11556	0.946132	0.163035
11557	0.946133	0.165256
11558	0.946131	0.190611
11559	0.946131	0.17394
11560	0.946132	2.1
11561	0.946129	2.1
11562	0.946128	2.1
11563	0.94613	2.1
11564	0.94613	2.1
11565	0.946131	2.1
11566	0.94613	2.1
11567	0.94613	2.1
11568	0.94613	2.1
11569	0.946131	2.1
11570	0.946132	2.1
11571	0.946129	2.1
11572	0.946131	2.1
11573	0.946131	2.1
11574	0.946132	1.51491
11575	0.946131	1.47234
11576	1.13731	1.1593
11577	0.94775	1.55629
11578	0.944514	1.85967
11579	0.946132	1.83885
11580	1.039	1.77309
11581	0.946131	1.50789
11582	0.946128	1.30062
11583	0.94613	0.957099
11584	0.946131	1.38972
11585	0.946128	1.74755
11586	0.946129	1.73766
11587	1.23726	1.65571
11588	0.946132	2.1
11589	0.946132	2.1
11590	0.946129	2.1
11591	1.39214	2.1
11592	0.946131	2.1
11593	0.946131	2.1
11594	0.94613	2.1
11595	0.946125	1.17975
11596	0.946119	0.465479
11597	0.946118	0.598154

11598	0.946118	0.310571
11599	0.94612	1.52757
11600	0.946106	0.665789
11601	0.946131	0.714804
11602	1.26893	0.108883
11603	0.946131	0.0818583
11604	1.26893	0.136235
11605	0.94613	0.109333
11606	0.946131	0.113744
11607	0.946131	0.106038
11608	0.946131	0.152292
11609	0.946133	0.0698591
11610	0.946133	0.0698591
11611	0.946132	0.0704084
11612	1.28924	0.0698591
11613	0.946133	0.0706089
11614	0.946132	0.0698591
11615	0.946132	0.0698591
11616	0.946133	0.538786
11617	0.946132	0.547311
11618	0.946132	0.554127
11619	0.946133	0.413792
11620	0.946133	0.452133
11621	0.946132	0.510305
11622	0.946133	0.462411
11623	0.946132	1.42341
11624	0.946133	0.553079
11625	0.946133	1.0887
11626	0.946132	1.45101
11627	1.15388	0.800255
11628	0.946132	1.28066
11629	0.946133	0.943881
11630	0.946132	0.446394
11631	0.946133	0.415073
11632	0.946132	0.461168
11633	0.946133	0.337476
11634	0.946133	0.374228
11635	0.946132	0.424356
11636	0.946132	0.378515
11637	1.0644	1.21554
11638	1.12486	1.71495
11639	1.08674	1.74143
11640	0.946132	1.37863
11641	0.946132	1.79025
11642	0.946133	1.60352
11643	0.946132	1.56721
11644	0.808978	1.2747
11645	0.911537	1.28348
11646	0.932832	1.30532
11647	1.14604	1.35827
11648	1.56953	1.24162
11649	1.25866	1.206
11650	1.18488	1.33368
11651	1.341	1.15758
11652	0.946131	0.128372
11653	0.946131	0.075158
11654	0.946131	0.0919892
11655	0.94613	0.131381

11656	1.33922	0.0803815
11657	0.946131	0.102093
11658	0.946131	0.0900805
11659	0.946132	0.0715445
11660	0.946133	0.0700934
11661	0.946132	0.0764707
11662	0.946132	0.0712347
11663	0.946132	0.0768434
11664	0.946133	0.0722788
11665	0.946132	0.0712414
11666	0.946129	0.677106
11667	0.946133	2.1
11668	0.946133	1.4821
11669	0.946131	1.44632
11670	1.40496	2.1
11671	0.946131	1.20204
11672	0.946131	2.09569
11673	0.94613	1.8642
11674	0.946127	0.759605
11675	0.946127	1.6609
11676	0.94613	1.93263
11677	1.38913	1.21645
11678	0.946128	1.84539
11679	0.946128	1.4502
11680	1.98863	0.0698591
11681	2.59915	0.0698591
11682	1.40338	0.0698591
11683	0.946131	0.0698591
11684	1.22266	0.0698591
11685	1.40338	0.0698591
11686	0.946132	0.0698591
11687	0.946132	0.0698591
11688	1.22266	0.0698591
11689	0.94613	0.0698591
11690	0.94613	0.0698591
11691	0.946124	0.0846782
11692	1.21428	0.0698591
11693	0.946126	0.0833204
11694	0.946127	0.0781306
11695	0.946127	0.0701566
11696	1.03411	0.0698591
11697	0.946131	0.12156
11698	1.03411	0.069867
11699	0.946129	0.113168
11700	1.14258	0.0698591
11701	0.952143	0.108157
11702	0.940118	0.0790538
11703	0.810943	2.1
11704	0.810945	2.1
11705	0.810958	2.1
11706	2.82603	2.1
11707	1.35168	2.1
11708	1.08132	2.1
11709	1.08132	2.1
11710	1.0813	2.1
11711	0.946133	0.0698591
11712	0.946132	0.0698591
11713	0.946132	0.0698591

11714	0.946132	0.0698591
11715	0.946132	0.0698591
11716	1.11539	0.0698591
11717	0.946133	0.0698591
11718	0.946133	2.1
11719	0.946132	2.1
11720	0.946133	2.1
11721	0.946133	2.1
11722	0.946132	2.1
11723	1.41195	2.1
11724	0.946133	2.1
11725	0.946131	0.0698591
11726	0.946131	0.0698591
11727	0.946131	0.0698591
11728	0.946131	0.0698591
11729	0.946131	0.0698591
11730	0.946131	0.0698591
11731	1.15968	0.0698591
11732	0.946131	1.47518
11733	0.94613	1.63844
11734	0.94613	2.00272
11735	1.34386	1.71928
11736	0.946132	1.93695
11737	0.94613	2.01305
11738	1.34386	1.8804
11739	0.946131	1.24677
11740	0.935202	1.0067
11741	0.946131	1.8137
11742	1.25054	1.29586
11743	0.850155	1.76296
11744	0.946131	1.7602
11745	0.935205	1.60154
11746	0.946131	0.281462
11747	1.40193	0.205644
11748	0.946132	0.209064
11749	0.946132	0.219168
11750	0.946129	0.324042
11751	1.40193	0.17377
11752	0.946131	0.244286
11753	0.808779	0.185994
11754	0.561937	0.130205
11755	1.40193	0.127843
11756	1.40193	0.122511
11757	0.561936	0.161392
11758	0.561937	0.10434
11759	1.40193	0.127272
11760	0.946132	0.0698591
11761	0.946132	0.0698591
11762	0.946132	0.0702085
11763	0.946132	0.0698591
11764	0.946133	0.0698616
11765	0.946132	0.0698591
11766	0.946133	0.0698591
11767	0.946127	0.203518
11768	0.946131	0.207565
11769	0.946131	0.105846
11770	0.946127	0.132003
11771	1.19583	0.0797188

11772	1.19583	0.096658
11773	0.946129	0.155586
11774	0.589428	0.215543
11775	1.08737	0.188796
11776	1.10961	0.246875
11777	0.872901	0.149688
11778	0.541327	0.166935
11779	0.541312	0.130648
11780	0.85153	0.195002
11781	0.946132	2.05299
11782	0.946131	2.1
11783	0.946131	2.1
11784	0.946132	2.08838
11785	0.946132	2.1
11786	0.946132	2.1
11787	0.946132	2.1
11788	0.946132	1.71951
11789	1.2778	2.06974
11790	0.946131	2.1
11791	0.946131	1.78489
11792	0.94613	2.08433
11793	0.94613	1.90594
11794	0.94613	1.9615
11795	0.946118	0.598669
11796	0.946112	0.500157
11797	0.946118	0.472191
11798	0.946119	0.558024
11799	0.946117	0.495843
11800	1.35631	0.455235
11801	0.946105	0.511954
11802	0.946133	2.1
11803	0.946133	2.1
11804	0.946132	2.1
11805	0.946132	2.1
11806	0.946133	1.76292
11807	0.946132	2.1
11808	0.946132	2.1
11809	0.94613	2.1
11810	0.946131	2.09939
11811	0.946128	2.09897
11812	0.946128	2.06505
11813	0.94613	2.1
11814	0.946129	2.1
11815	0.946129	2.1
11816	0.946132	1.54895
11817	1.15139	1.33977
11818	0.946132	1.27059
11819	0.946132	1.60866
11820	0.946132	1.52152
11821	0.946132	1.66481
11822	0.946132	1.51099
11823	0.946133	2.1
11824	1.36151	2.1
11825	0.946132	2.1
11826	0.946132	2.1
11827	0.946132	2.1
11828	0.946132	2.1
11829	0.946133	2.1

11830	0.946127	2.1
11831	1.38397	2.1
11832	0.94613	2.1
11833	0.946131	2.1
11834	0.946128	2.1
11835	0.94613	2.1
11836	0.946129	2.1
11837	0.946131	0.278659
11838	0.946127	0.324379
11839	0.946127	0.28417
11840	0.946132	0.305874
11841	0.946131	0.290225
11842	0.946128	0.295591
11843	0.946129	0.290792
11844	1.0268	0.0698591
11845	1.23823	0.0698591
11846	1.2171	0.0698591
11847	0.883446	0.0698591
11848	1.23827	0.0698591
11849	1.32614	0.0698591
11850	0.94613	0.0698591
11851	0.946132	2.1
11852	0.946129	2.1
11853	0.946129	2.1
11854	0.94613	2.1
11855	1.36523	2.1
11856	0.946131	2.1
11857	0.94613	2.1
11858	0.946132	0.0877677
11859	0.946131	0.0725308
11860	0.946132	0.0948006
11861	0.946131	0.0698591
11862	0.946131	0.0704036
11863	1.01653	0.0698963
11864	0.946131	0.0961896
11865	0.94612	2.1
11866	0.946121	2.07015
11867	0.946119	2.1
11868	0.946119	2.08914
11869	0.946119	2.1
11870	0.946132	2.1
11871	0.946107	2.1
11872	0.810942	2.1
11873	0.810944	2.00663
11874	2.60324	2.1
11875	1.21651	2.1
11876	1.0813	2.0994
11877	0.946153	2.00692
11878	0.979948	2.1
11879	0.709588	2.1
11880	1.3492	0.1988
11881	0.946132	0.217576
11882	0.946131	0.179471
11883	0.94613	0.214755
11884	0.946129	0.297527
11885	0.946129	0.222889
11886	0.94613	0.238635
11887	0.946131	0.223044

11888	0.94613	0.262065
11889	0.946128	0.227785
11890	0.946133	0.238735
11891	0.946133	0.234552
11892	0.946131	0.244865
11893	0.946131	0.234599
11894	1.23351	0.0698591
11895	0.946131	0.0698591
11896	0.946131	0.0698591
11897	0.946132	0.0698591
11898	0.946132	0.0698591
11899	0.946131	0.0698591
11900	0.946132	0.0698591
11901	1.35657	0.0698591
11902	0.946132	0.0698591
11903	0.946131	0.0698591
11904	0.946132	0.0698591
11905	0.946132	0.0698591
11906	0.946131	0.0698591
11907	0.946131	0.0698591
11908	0.946132	0.289483
11909	0.946132	0.212757
11910	0.946132	0.293794
11911	0.946131	0.155321
11912	0.946131	0.219747
11913	0.946132	0.190596
11914	0.946132	0.180863
11915	0.946133	0.171158
11916	0.946132	0.11076
11917	0.946133	0.171971
11918	0.946132	0.0797209
11919	0.946132	0.127906
11920	0.946132	0.107126
11921	0.946133	0.0968031
11922	0.946131	0.785676
11923	1.4101	1.78837
11924	0.946131	2.1
11925	0.946131	0.477807
11926	0.946131	1.46611
11927	0.946131	0.543027
11928	0.946131	1.61137
11929	0.810944	1.30319
11930	0.810939	1.36185
11931	0.709586	0.865342
11932	2.71261	0.877545
11933	1.21651	1.12989
11934	0.946151	0.772369
11935	0.979943	1.21861
11936	1.0813	1.39094
11937	0.946132	2.1
11938	0.946131	2.1
11939	0.946132	2.1
11940	0.946131	2.1
11941	0.946131	2.1
11942	1.37733	2.1
11943	0.946131	2.1
11944	0.946133	2.1
11945	0.946132	2.1

11946	0.946132	2.1
11947	0.946133	2.1
11948	0.946133	2.1
11949	0.946132	2.1
11950	1.32582	2.1
11951	0.946132	2.1
11952	0.946132	2.1
11953	0.946132	2.1
11954	0.946132	2.1
11955	0.946132	2.1
11956	0.946132	2.1
11957	0.946131	2.1
11958	0.946133	1.80684
11959	0.946132	1.52126
11960	0.946132	1.52914
11961	0.946132	1.40224
11962	0.946132	1.53272
11963	0.946133	1.43861
11964	1.3469	1.46823
11965	0.946126	2.09794
11966	0.94613	2.1
11967	0.946131	2.1
11968	0.946127	2.1
11969	1.41206	2.1
11970	0.946128	2.1
11971	0.946128	2.1
11972	0.946127	2.1
11973	0.94613	2.1
11974	0.946131	2.0314
11975	0.94613	2.1
11976	0.946128	2.02208
11977	0.946129	2.1
11978	0.946129	2.1
11979	0.946131	0.134904
11980	0.94613	0.124517
11981	1.24448	0.111633
11982	0.946129	0.192168
11983	0.946131	0.144877
11984	0.94613	0.151141
11985	0.946131	0.137783
11986	0.946132	0.197366
11987	0.946133	0.183103
11988	1.11015	0.160251
11989	0.946133	0.314573
11990	0.946132	0.22063
11991	0.946132	0.212217
11992	0.946132	0.208743
11993	1.38775	2.1
11994	0.946131	2.1
11995	0.946131	2.1
11996	0.946132	2.1
11997	0.946132	2.1
11998	0.946131	2.1
11999	0.946131	2.1
12000	0.946129	2.1
12001	0.946129	2.1
12002	0.946133	2.0998
12003	0.946131	2.1

12004	0.946131	1.71633
12005	0.946131	2.1
12006	0.946131	2.1
12007	0.946132	2.1
12008	0.946133	2.1
12009	0.946132	2.1
12010	1.40774	2.1
12011	0.946132	2.1
12012	0.946133	2.1
12013	0.946132	2.1
12014	0.610968	0.0698591
12015	0.688233	0.0698591
12016	0.715226	0.0698591
12017	0.792409	0.0698591
12018	1.13418	0.0698591
12019	0.956667	0.0698591
12020	0.757321	0.0698591
12021	0.968845	0.0698591
12022	0.595679	0.0698591
12023	0.946133	1.06042
12024	1.28917	1.24782
12025	0.946131	1.59479
12026	0.94613	0.930193
12027	0.946131	1.31807
12028	0.946131	1.21149
12029	0.946131	1.20603
12030	0.946132	0.153677
12031	0.946128	0.182777
12032	0.94613	0.205189
12033	1.40668	0.193107
12034	0.946131	0.240773
12035	0.946131	0.1566
12036	0.94613	0.181436
12037	0.810946	2.1
12038	0.810945	2.1
12039	2.78163	2.1
12040	1.3517	2.1
12041	1.08132	2.1
12042	1.08133	2.1
12043	1.08132	2.1
12044	0.810933	2.1
12045	0.946131	2.1
12046	1.39081	2.1
12047	0.94613	2.1
12048	1.30339	2.1
12049	1.30339	2.1
12050	1.39081	2.1
12051	0.946131	2.1
12052	0.946127	0.557131
12053	0.821631	0.466788
12054	0.946131	0.425251
12055	0.946131	0.557367
12056	0.946128	0.421296
12057	0.996109	0.545059
12058	0.827876	0.540375
12059	0.946131	2.1
12060	0.946129	2.1
12061	0.946129	2.1

12062	0.94613	2.1
12063	0.94613	2.1
12064	0.94613	2.1
12065	0.94613	2.1
12066	0.946132	0.377238
12067	1.41723	0.412818
12068	0.946132	0.37381
12069	0.946133	0.319635
12070	0.946132	0.616518
12071	0.946132	0.451341
12072	0.946133	0.471765
12073	0.946133	0.366491
12074	0.946132	0.316018
12075	0.946133	0.261732
12076	0.946133	0.325181
12077	0.946132	0.48173
12078	0.946132	0.370984
12079	1.34254	0.345023
12080	1.26501	1.37113
12081	0.946132	1.68739
12082	0.946133	1.56375
12083	0.946133	1.85997
12084	0.946132	1.86567
12085	0.946133	1.65137
12086	0.946132	1.89265
12087	0.946131	0.0752158
12088	0.946131	0.0744648
12089	0.946131	0.0777975
12090	0.946131	0.0698591
12091	0.946131	0.0698591
12092	0.946131	0.0729548
12093	0.946131	0.0706963
12094	0.878843	0.0698591
12095	0.82561	0.0698591
12096	0.586974	0.0698591
12097	1.05343	0.0698591
12098	0.80631	0.0698591
12099	0.782663	0.0698591
12100	0.789405	0.0698591
12101	0.902491	0.0698591
12102	1.05344	0.0698591
12103	0.94613	0.0698591
12104	0.946131	0.0698591
12105	0.946131	0.0764732
12106	0.94613	0.0699539
12107	0.946131	0.0698591
12108	0.946131	0.0710124
12109	0.946131	0.0698591
12110	0.946128	2.1
12111	0.946133	2.09804
12112	0.946133	1.07881
12113	0.94613	2.1
12114	0.946131	1.24933
12115	0.946131	1.95118
12116	0.946131	2.03177
12117	0.946131	0.297473
12118	0.946131	0.241058
12119	0.94613	0.229089

12120	0.946128	0.26333
12121	0.946133	0.258744
12122	1.2996	0.213001
12123	0.946131	0.252372
12124	0.946132	2.1
12125	0.946132	2.1
12126	0.946132	2.1
12127	0.946132	2.1
12128	0.946131	2.1
12129	0.946132	2.1
12130	0.946132	2.1
12131	1.99211	0.0724774
12132	2.55024	0.0698634
12133	0.946131	0.0735231
12134	0.946131	0.0744792
12135	0.946131	0.0735997
12136	0.946131	0.0746389
12137	0.946131	0.0850728
12138	0.946131	0.0764115
12139	1.35726	0.0744623
12140	0.946128	2.1
12141	0.946132	2.0684
12142	0.946131	2.1
12143	0.94613	2.0892
12144	0.94613	2.1
12145	0.94613	2.1
12146	1.39852	2.1
12147	1.31666	0.0698591
12148	0.946131	0.0698591
12149	0.946128	0.0698591
12150	0.94613	0.0698591
12151	0.946128	0.0698591
12152	0.946128	0.0698591
12153	0.946128	0.0698591
12154	1.32275	0.0698591
12155	0.946131	0.0734304
12156	0.946129	0.0698591
12157	0.946128	0.0698591
12158	0.946129	0.0698591
12159	0.946128	0.0698591
12160	0.946129	0.0698591
12161	0.946132	2.1
12162	0.946131	2.1
12163	0.946131	2.1
12164	0.946132	2.1
12165	0.94613	2.1
12166	0.946131	2.1
12167	1.41125	2.1
12168	0.946132	2.1
12169	0.946132	2.1
12170	0.946131	2.1
12171	0.946132	2.1
12172	0.946132	2.1
12173	0.946132	2.1
12174	1.31904	2.1
12175	1.02425	0.0698591
12176	0.946132	0.0698591
12177	0.94613	0.0698591

12178	0.946131	0.0698591
12179	1.1534	0.0698591
12180	0.961161	0.0698591
12181	0.9311	0.0698591
12182	1.40092	1.38699
12183	0.946133	1.5219
12184	0.946132	1.46919
12185	0.946131	1.79408
12186	0.946131	1.53429
12187	0.94613	1.5168
12188	0.946131	1.78844
12189	0.946113	2.1
12190	0.946119	2.09984
12191	0.946117	2.1
12192	1.40015	2.1
12193	0.946118	2.0996
12194	0.946131	2.1
12195	0.946105	2.1
12196	0.946132	0.177263
12197	0.946133	0.178227
12198	0.946132	0.224341
12199	1.03547	0.209349
12200	0.946133	0.182699
12201	0.946133	0.224898
12202	0.946132	0.148395
12203	0.946133	2.1
12204	1.36694	2.1
12205	0.946133	2.1
12206	0.946132	2.1
12207	0.946133	2.1
12208	0.946133	2.1
12209	0.946132	2.1
12210	0.946132	0.0841411
12211	0.946133	0.110078
12212	0.946132	0.0843916
12213	0.946133	0.107688
12214	0.946133	0.0807024
12215	0.946132	0.0951612
12216	0.946132	0.107366
12217	0.491552	0.123145
12218	0.913078	0.155545
12219	0.946132	0.207717
12220	0.491552	0.108127
12221	0.946132	0.187308
12222	0.49154	0.171726
12223	1.53168	0.15861
12224	1.53168	0.116402
12225	0.946127	2.1
12226	1.38752	2.1
12227	0.946129	2.1
12228	0.94613	2.1
12229	0.946128	2.1
12230	0.946129	2.1
12231	0.946129	2.1
12232	0.946132	2.1
12233	0.946132	2.1
12234	0.946132	2.1
12235	0.946132	2.1

12236	0.946132	2.1
12237	0.946131	2.1
12238	0.946132	2.1
12239	0.94613	0.289626
12240	0.94613	0.202841
12241	0.94613	0.219258
12242	0.946131	0.229339
12243	0.946129	0.280536
12244	1.16834	0.161602
12245	0.94613	0.231624
12246	0.946127	0.320496
12247	1.35594	0.206377
12248	0.946131	0.235704
12249	0.946128	0.332909
12250	0.946127	0.163616
12251	0.946129	0.287943
12252	0.94613	0.256166
12253	0.94613	2.0665
12254	0.946131	0.653053
12255	0.946128	0.879182
12256	0.946128	0.296162
12257	0.946131	2.05558
12258	0.94613	1.3692
12259	0.94613	1.27914
12260	0.946132	0.774975
12261	0.946132	0.347909
12262	0.946129	0.699056
12263	1.39751	0.550363
12264	0.94613	0.599588
12265	0.946131	0.313058
12266	0.946131	0.344409
12267	0.946132	0.101267
12268	0.700041	0.0878334
12269	0.700013	0.110635
12270	1.7697	0.0708732
12271	1.7697	0.0698914
12272	1.21134	0.0811294
12273	0.946131	0.0816748
12274	1.25837	0.0790566
12275	0.946133	2.1
12276	0.946132	2.06199
12277	0.946132	2.06241
12278	0.946132	2.081
12279	0.946132	2.1
12280	0.946133	2.1
12281	1.40583	2.1
12282	0.946131	1.84973
12283	0.946131	2.03518
12284	0.946131	1.83485
12285	0.946131	2.1
12286	0.946131	2.03999
12287	0.827865	2.04968
12288	0.946131	2.06858
12289	0.946132	0.0698591
12290	0.946129	0.0698591
12291	0.946129	0.0698591
12292	0.946131	0.0698591
12293	0.94613	0.0698591

12294	1.21087	0.0698591
12295	0.946131	0.0698591
12296	0.94613	0.301114
12297	0.946131	0.237781
12298	0.94613	0.21177
12299	0.946128	0.294528
12300	0.946132	0.211227
12301	1.17452	0.254775
12302	0.94613	0.26482
12303	0.946119	0.250952
12304	0.946114	0.209703
12305	0.946119	0.179188
12306	0.946119	0.244604
12307	0.946119	0.180321
12308	1.27376	0.210633
12309	0.946106	0.217038
12310	0.946133	2.1
12311	1.39621	2.1
12312	0.946132	2.1
12313	0.946132	2.1
12314	0.946133	2.1
12315	0.946132	2.1
12316	0.946132	2.1
12317	0.946132	2.1
12318	0.946132	2.1
12319	0.946131	2.1
12320	0.946131	2.1
12321	0.946132	2.1
12322	0.946132	2.1
12323	1.34352	2.1
12324	0.946132	1.93638
12325	1.41272	2.09269
12326	0.946131	2.1
12327	0.94613	1.82146
12328	0.94613	2.08865
12329	0.94613	1.92347
12330	0.94613	2.02623
12331	0.946132	2.1
12332	0.946131	2.05143
12333	0.946132	2.1
12334	0.946132	2.06578
12335	0.946132	2.07622
12336	0.946132	2.1
12337	1.23536	2.1
12338	0.946132	2.1
12339	0.946131	2.1
12340	0.946131	2.1
12341	0.946132	2.1
12342	0.946132	2.1
12343	1.41131	2.1
12344	0.946131	2.1
12345	1.41894	1.01402
12346	0.946132	0.896828
12347	0.946133	0.529832
12348	0.946132	1.01404
12349	0.946132	1.67581
12350	0.946133	0.765144
12351	0.946132	0.801254

12352	0.946128	0.650805
12353	0.946133	1.98626
12354	0.946131	1.30917
12355	0.946129	2.1
12356	0.94613	1.80251
12357	1.4099	1.99806
12358	0.946131	1.78162
12359	0.810947	2.1
12360	0.810945	2.08721
12361	0.810933	2.1
12362	2.62883	2.1
12363	1.3517	2.1
12364	1.08132	2.1
12365	1.08132	2.09263
12366	1.08133	2.1
12367	0.946131	0.759026
12368	0.946126	2.02541
12369	0.946127	2.06342
12370	0.94613	1.43938
12371	0.946128	2.1
12372	0.946129	1.5419
12373	0.946129	1.40803
12374	1.21347	2.07766
12375	0.946129	2.1
12376	0.94613	2.0795
12377	1.21347	2.1
12378	1.33064	2.1
12379	0.94613	2.1
12380	1.33064	2.1
12381	0.94613	0.0698591
12382	0.946129	0.0698591
12383	0.94613	0.0698591
12384	0.946132	0.0698591
12385	0.946126	0.0698591
12386	0.946129	0.0698591
12387	0.94613	0.0698591
12388	0.946132	0.572798
12389	1.37938	0.950056
12390	0.946131	1.01917
12391	0.94613	0.649387
12392	0.946131	1.46723
12393	0.94613	1.02151
12394	0.94613	0.804492
12395	0.946133	0.0698591
12396	0.946133	0.0698591
12397	1.2968	0.0698591
12398	0.946132	0.0698591
12399	0.946133	0.0698591
12400	1.2968	0.0698591
12401	0.946133	0.0698591
12402	1.43643	0.0698591
12403	0.946129	0.679043
12404	0.946133	0.444591
12405	0.946131	0.740602
12406	0.94613	0.275951
12407	0.946131	0.398667
12408	0.946131	0.366832
12409	0.946131	0.345284

12410	0.872447	1.61026
12411	0.946132	1.24525
12412	0.872447	1.16073
12413	0.827867	1.48597
12414	0.946132	1.46528
12415	0.827867	1.71167
12416	0.872447	1.66665
12417	0.946117	0.89226
12418	1.27213	0.698675
12419	0.946124	0.639419
12420	0.946119	0.858087
12421	0.946113	0.933978
12422	0.946129	0.928539
12423	0.946104	0.82046
12424	0.94613	1.65904
12425	0.946131	2.1
12426	0.94613	1.88696
12427	0.946129	2.1
12428	0.946132	0.479686
12429	0.946131	1.63408
12430	0.94613	1.62735
12431	0.810942	2.1
12432	0.810943	2.1
12433	2.80212	2.1
12434	1.3517	2.1
12435	1.08133	2.1
12436	1.08132	2.1
12437	1.08132	2.1
12438	0.810932	2.1
12439	1.29521	2.1
12440	0.946129	2.1
12441	0.946131	2.1
12442	1.40106	2.1
12443	0.94613	2.1
12444	1.40106	2.1
12445	0.946131	2.1
12446	0.946132	0.481777
12447	1.24596	0.298998
12448	0.94613	0.369897
12449	0.946131	0.453457
12450	0.946131	0.195385
12451	0.946131	0.443631
12452	0.946131	0.397428
12453	0.946131	2.1
12454	0.946131	2.1
12455	0.946131	2.1
12456	0.946128	2.1
12457	0.946133	2.1
12458	0.94613	2.1
12459	0.946131	2.1
12460	0.879548	0.174223
12461	1.21527	0.0807098
12462	0.967506	0.0837912
12463	0.946132	0.200201
12464	0.946133	0.143315
12465	0.967511	0.140606
12466	0.946132	0.267979
12467	0.946132	0.389646

12468	0.946132	0.204329
12469	0.974253	0.204617
12470	0.974253	0.437123
12471	0.91801	0.257823
12472	0.918009	0.280865
12473	0.946132	0.38013
12474	0.946132	0.0707927
12475	0.946131	0.0698591
12476	0.946131	0.0705739
12477	0.946133	0.0698591
12478	0.946132	0.0698591
12479	1.28644	0.0698591
12480	0.946132	0.0701365
12481	0.946131	0.0720283
12482	0.946131	0.0698591
12483	0.946131	0.0718921
12484	0.946131	0.0698591
12485	0.946131	0.0698591
12486	1.40103	0.0698591
12487	0.946131	0.0700795
12488	0.946128	0.135107
12489	0.946132	0.0998942
12490	0.946129	0.159131
12491	1.03327	0.0816633
12492	0.946129	0.0811812
12493	0.946129	0.132016
12494	0.94613	0.168658
12495	0.946134	0.608637
12496	0.946133	1.04665
12497	0.946132	0.331716
12498	0.946133	1.01723
12499	0.946132	0.155097
12500	0.946132	0.311713
12501	0.946133	0.247673
12502	0.856654	0.0698591
12503	0.946131	0.0698591
12504	0.946131	0.0698591
12505	0.946132	0.0698591
12506	0.856616	0.0698591
12507	1.03565	0.0698591
12508	1.03565	0.0698591
12509	1.15844	0.206083
12510	0.946132	0.277768
12511	0.946132	0.144149
12512	0.946132	0.195293
12513	0.946132	0.149539
12514	0.946132	0.207478
12515	0.946131	0.195881
12516	1.4582	2.1
12517	1.35067	2.1
12518	0.946131	2.1
12519	1.04106	2.1
12520	0.938748	2.1
12521	0.943015	2.1
12522	1.05017	2.1
12523	0.938793	2.1
12524	0.946131	0.0698591
12525	0.946132	0.0698591

12526	0.946131	0.0710117
12527	0.94613	0.0698591
12528	0.946133	0.0698591
12529	0.946131	0.0698591
12530	0.946131	0.0698591
12531	0.946132	0.795999
12532	0.946133	1.00381
12533	0.946132	0.845664
12534	0.946133	0.890699
12535	0.946133	0.820661
12536	0.946132	0.88079
12537	0.946133	0.898523
12538	0.946128	1.92173
12539	0.946132	2.07287
12540	0.946132	1.7307
12541	0.94613	1.99054
12542	1.23426	1.93288
12543	0.94613	2.01493
12544	0.946131	1.96717
12545	0.810943	0.253039
12546	0.810946	0.868698
12547	2.60388	0.378005
12548	1.3517	0.338462
12549	1.08133	0.267056
12550	1.08132	0.838239
12551	1.08132	0.248039
12552	0.810933	0.0860855
12553	0.810945	2.09322
12554	0.810945	1.84348
12555	2.72377	2.1
12556	1.3517	2.1
12557	1.08133	2.09781
12558	1.08132	1.9448
12559	1.08132	2.1
12560	0.810932	2.1
12561	1.25104	1.32876
12562	0.946131	2.04432
12563	0.946131	1.58828
12564	1.36188	2.1
12565	0.946131	2.07661
12566	1.36188	2.1
12567	0.946132	2.1
12568	0.946132	2.05693
12569	0.946132	2.0594
12570	0.946132	2.05799
12571	0.946132	2.06524
12572	0.946132	2.0546
12573	0.946132	2.09427
12574	1.25488	2.09018
12575	1.32132	0.0698591
12576	0.946126	0.0768991
12577	0.946127	0.0698591
12578	0.946128	0.0740191
12579	0.946128	0.0773467
12580	0.946128	0.0715198
12581	0.946128	0.0734337
12582	0.946133	2.1
12583	1.41123	2.1

12584	0.946134	2.1
12585	0.946133	2.1
12586	0.946132	2.0386
12587	0.946133	2.1
12588	0.946132	2.1
12589	1.32163	0.0698591
12590	0.946133	0.0699361
12591	0.946133	0.0698591
12592	0.946132	0.0702195
12593	0.946132	0.0698591
12594	0.946132	0.0698591
12595	0.946133	0.0708928
12596	0.946133	2.1
12597	0.946133	2.1
12598	0.946132	2.1
12599	0.946132	2.1
12600	0.946132	2.1
12601	0.946133	2.1
12602	0.946132	2.1
12603	0.94613	0.149509
12604	0.946131	0.141202
12605	0.946128	0.148263
12606	0.946133	0.124066
12607	0.946133	0.121122
12608	0.946131	0.119661
12609	0.946131	0.122936
12610	0.946133	1.13437
12611	0.946132	0.604265
12612	0.946132	1.20752
12613	0.946134	0.668781
12614	0.946133	1.51028
12615	0.946133	0.91994
12616	0.946132	0.879571
12617	0.94613	1.85175
12618	0.946128	1.98738
12619	0.946127	1.72399
12620	0.94613	1.3888
12621	0.946131	1.79096
12622	0.946128	1.86767
12623	1.21099	1.90575
12624	0.946132	0.674636
12625	0.946131	0.593271
12626	0.946131	0.745952
12627	0.946133	0.230273
12628	0.946133	0.279171
12629	0.946131	0.353292
12630	0.946132	0.302096
12631	1.34794	0.852818
12632	0.517994	0.953537
12633	0.517965	0.631482
12634	0.946132	1.03504
12635	0.9198	1.79625
12636	0.946132	1.31911
12637	0.9198	1.29165
12638	0.946132	1.40221
12639	0.94613	1.47139
12640	0.946131	0.726737
12641	0.946131	1.42517

12642	0.946131	1.8693
12643	0.946131	1.38413
12644	1.23224	1.01289
12645	0.946132	1.80597
12646	0.946132	1.90973
12647	0.946133	1.73833
12648	0.946132	1.14793
12649	0.946132	1.49662
12650	0.946133	1.7492
12651	1.37511	1.67936
12652	0.946133	0.680956
12653	0.946129	0.806171
12654	0.851104	1.48072
12655	0.946131	0.209403
12656	1.0412	0.3736
12657	1.0412	0.248158
12658	0.851066	0.862762
12659	1.31357	0.106791
12660	0.946131	0.224965
12661	0.94613	0.128775
12662	0.946131	0.103651
12663	0.94613	0.0866488
12664	0.946131	0.166567
12665	0.946131	0.0788041
12666	0.946133	0.476841
12667	1.38149	0.784118
12668	0.946132	0.741287
12669	0.946132	0.566616
12670	0.946133	1.37562
12671	0.946132	0.916174
12672	0.946133	0.661629
12673	0.810944	1.54156
12674	0.810945	2.1
12675	0.810958	2.09322
12676	2.65782	2.1
12677	1.35168	2.09784
12678	1.08132	1.84531
12679	1.08132	2.1
12680	1.0813	1.97801
12681	0.946113	0.0698591
12682	0.946119	0.0698591
12683	0.946118	0.0698591
12684	0.946118	0.0698591
12685	0.946119	0.0698591
12686	0.946131	0.0698591
12687	0.946106	0.0698591
12688	0.946132	0.0698591
12689	0.946132	0.0698591
12690	0.946128	0.0698591
12691	0.946131	0.0698591
12692	0.94613	0.0698591
12693	0.94613	0.0698591
12694	0.94613	0.0698591
12695	1.00099	0.0704023
12696	0.946132	0.0986874
12697	1.17876	0.0704977
12698	0.982282	0.0769786
12699	0.909981	0.105267

12700	0.946132	0.0861384
12701	0.946131	0.0839724
12702	0.946132	0.657363
12703	1.35328	1.06745
12704	0.946132	1.45752
12705	0.946132	0.598592
12706	0.946133	1.41108
12707	0.946132	1.02177
12708	0.946133	1.06262
12709	0.946133	0.0698591
12710	0.946133	0.0698591
12711	0.946132	0.0698591
12712	0.946132	0.0698591
12713	0.946132	0.0698591
12714	0.946132	0.0698591
12715	0.946133	0.0698591
12716	0.900195	0.0698591
12717	0.946132	0.0698591
12718	0.900167	0.0698591
12719	1.41963	0.0698591
12720	0.900195	0.0698591
12721	0.946133	0.0698591
12722	1.35385	0.0698591
12723	0.946132	1.86375
12724	0.946129	1.91031
12725	0.946129	2.02538
12726	0.946131	1.87545
12727	0.946131	2.04371
12728	1.41585	1.5929
12729	0.94613	2.00982
12730	0.810946	2.1
12731	0.810946	2.1
12732	2.68895	2.1
12733	1.3517	2.1
12734	1.08133	2.1
12735	1.08132	2.1
12736	1.08132	2.1
12737	0.810933	2.1
12738	1.19886	2.1
12739	0.946131	2.1
12740	0.946131	2.1
12741	1.34447	2.1
12742	0.946131	2.1
12743	1.34447	2.1
12744	0.946131	2.1
12745	0.946133	0.0698591
12746	0.946133	0.0698591
12747	0.946132	0.0724353
12748	1.35065	0.0698591
12749	0.946132	0.0749366
12750	0.946133	0.0708929
12751	0.946132	0.0698591
12752	0.946131	0.449388
12753	0.946131	0.382452
12754	0.94613	0.537615
12755	0.946131	0.181899
12756	0.946131	0.2127
12757	0.946131	0.286621

12758	0.946131	0.233498
12759	0.946127	0.193749
12760	0.946131	0.189807
12761	0.946127	0.192101
12762	0.94613	0.150229
12763	0.946131	0.158377
12764	0.946128	0.181298
12765	0.946129	0.164937
12766	0.810946	0.0698591
12767	0.810947	0.0698591
12768	0.810959	0.0698591
12769	2.4391	0.0698591
12770	1.35168	0.0698591
12771	1.08132	0.0698591
12772	1.08132	0.0698591
12773	1.08131	0.0698591
12774	0.946132	0.0698591
12775	0.946132	0.0698591
12776	0.946132	0.0698591
12777	1.40837	0.0698591
12778	0.946132	0.0698591
12779	0.946132	0.0698591
12780	1.40837	0.0698591
12781	0.946127	2.0768
12782	0.946132	2.1
12783	0.946131	0.341979
12784	0.946127	2.09302
12785	0.946131	1.31452
12786	0.946129	1.2305
12787	0.94613	1.61593
12788	0.946133	2.00034
12789	0.946132	1.74599
12790	0.946132	1.80293
12791	0.946132	1.43039
12792	0.946132	1.87139
12793	0.946132	1.36128
12794	0.946133	2.01426
12795	0.946132	0.43883
12796	1.24344	0.152251
12797	0.946132	0.133733
12798	0.946132	0.808417
12799	0.946132	0.234477
12800	1.24344	0.236833
12801	0.946132	0.761699
12802	0.946131	0.182893
12803	0.946128	0.151061
12804	0.946128	0.0939277
12805	0.946131	0.200938
12806	1.41232	0.102205
12807	0.946129	0.100746
12808	0.94613	0.127762
12809	0.946128	2.1
12810	0.946133	2.1
12811	0.946133	1.82399
12812	0.946131	2.1
12813	0.946131	1.8686
12814	0.946131	2.1
12815	0.94613	2.1

12816	0.946133	0.397583
12817	1.41556	0.391152
12818	0.946133	0.347257
12819	0.946133	0.332019
12820	0.946132	0.611403
12821	0.946132	0.419941
12822	0.946132	0.498606
12823	0.94612	0.354034
12824	1.41843	0.343856
12825	0.946114	0.338367
12826	0.94612	0.307967
12827	0.946119	0.548665
12828	1.41845	0.344861
12829	0.946107	0.463682
12830	0.946129	0.0698591
12831	0.946129	0.0698591
12832	0.94613	0.0698591
12833	0.946132	0.0698591
12834	0.946126	0.0698591
12835	1.1509	0.0698591
12836	0.94613	0.0698591
12837	0.946131	0.0698591
12838	0.946132	0.0698591
12839	0.946132	0.0698591
12840	0.946132	0.0698591
12841	0.946132	0.0698591
12842	1.19263	0.0698591
12843	0.946132	0.0698591
12844	0.94613	0.617155
12845	0.946131	0.920502
12846	0.946128	0.660095
12847	0.946133	1.03881
12848	0.946133	0.702251
12849	0.946131	0.736659
12850	0.946131	0.873516
12851	0.946133	1.1177
12852	1.22848	1.38648
12853	1.00336	1.36709
12854	0.827866	1.20685
12855	0.946132	1.62989
12856	0.827865	1.43889
12857	0.946133	1.34018
12858	0.946131	2.1
12859	0.94613	0.829786
12860	0.946127	2.0886
12861	1.41498	2.09556
12862	0.94613	0.644426
12863	0.946128	1.84378
12864	0.946128	1.40082
12865	0.94613	0.0698591
12866	0.94613	0.0698591
12867	0.94613	0.0698591
12868	1.37358	0.0698591
12869	0.946129	0.0698591
12870	0.94613	0.0698591
12871	1.37357	0.0698591
12872	0.946111	0.0698591
12873	0.946118	0.0698591

12874	0.946118	0.0698591
12875	1.36568	0.0698591
12876	0.946116	0.0698591
12877	0.94613	0.0698591
12878	0.946105	0.0698591
12879	0.946132	0.222673
12880	0.946131	0.157228
12881	0.94613	0.29228
12882	1.23175	0.127751
12883	0.946131	0.108516
12884	0.946131	0.238868
12885	0.946131	0.336071
12886	0.946132	0.0698591
12887	0.946126	0.0698591
12888	0.946128	0.0698591
12889	0.946129	0.0698591
12890	0.946129	0.0707341
12891	0.946128	0.0698591
12892	1.25698	0.0698591
12893	0.810943	2.1
12894	0.810944	2.1
12895	2.81171	2.1
12896	1.3517	2.1
12897	1.08132	2.1
12898	1.08133	2.1
12899	1.08132	2.1
12900	0.810932	2.1
12901	0.946132	0.170882
12902	0.946131	0.139732
12903	0.946131	0.181956
12904	0.946132	0.0836876
12905	0.946132	0.10018
12906	0.946132	0.127375
12907	0.946131	0.109049
12908	0.810936	2.04987
12909	0.810935	2.1
12910	2.80914	2.1
12911	1.35167	2.1
12912	1.0813	2.1
12913	1.0813	2.1
12914	1.0813	1.83924
12915	0.810934	2.09837
12916	1.23851	2.1
12917	0.946132	2.09788
12918	0.946131	2.1
12919	1.40459	2.0342
12920	0.946132	2.1
12921	1.40459	2.1
12922	0.946132	2.1
12923	1.19974	0.115156
12924	0.946131	0.335695
12925	1.19974	0.11965
12926	0.946132	0.266296
12927	0.946132	0.0848782
12928	0.946132	0.316502
12929	0.946132	0.148897
12930	0.939141	0.848044
12931	1.14375	1.00792

12932	1.03306	0.952173
12933	0.946132	1.04085
12934	0.946126	1.16835
12935	0.946129	1.15877
12936	0.94613	0.992913
12937	0.946132	0.117459
12938	0.946131	0.134766
12939	0.946132	0.118127
12940	0.946132	0.127826
12941	0.946132	0.1061
12942	0.946132	0.116118
12943	0.946132	0.119519
12944	0.946131	2.1
12945	0.94613	2.1
12946	0.94613	2.1
12947	0.946131	2.1
12948	0.946129	2.1
12949	0.94613	2.1
12950	1.24512	2.1
12951	0.946133	0.110538
12952	0.946129	0.0788008
12953	0.946131	0.149043
12954	0.946132	0.0716967
12955	0.946131	0.122304
12956	0.946131	0.0960619
12957	0.946131	0.0865259
12958	0.946132	0.0811033
12959	0.946133	0.0812354
12960	0.946132	0.0842052
12961	0.946132	0.0707231
12962	0.946133	0.0904425
12963	0.946132	0.0739902
12964	0.946132	0.0871695
12965	0.946132	0.559073
12966	1.41392	0.39103
12967	0.946132	0.403283
12968	0.946132	0.632713
12969	0.946132	0.288403
12970	0.946132	0.551074
12971	0.946132	0.504939
12972	0.946132	0.872295
12973	1.24333	0.674398
12974	0.946132	0.641638
12975	0.946133	0.996611
12976	0.946132	0.441598
12977	0.946132	0.93397
12978	0.946133	0.762645
12979	1.30649	0.165893
12980	0.94613	0.197688
12981	0.94613	0.156227
12982	0.946128	0.174072
12983	0.946126	0.218562
12984	0.946127	0.191949
12985	0.946127	0.194155
12986	0.709578	0.26983
12987	0.810943	0.245214
12988	2.53303	0.13345
12989	1.21651	0.133447

12990	0.979951	0.176326
12991	1.08132	0.149696
12992	0.946141	0.255062
12993	0.81096	0.221105
12994	0.946131	0.0949169
12995	0.946131	0.102983
12996	0.946131	0.103273
12997	0.946131	0.07272
12998	0.946131	0.0767487
12999	0.946131	0.0992068
13000	0.946131	0.083978
13001	0.946132	0.0698591
13002	0.946129	0.0698591
13003	0.946129	0.0698591
13004	0.94613	0.0698591
13005	1.20634	0.0698591
13006	1.20634	0.0698591
13007	0.946131	0.0698591
13008	0.946133	0.188938
13009	0.946132	0.0926083
13010	0.946132	0.0873045
13011	0.946132	0.203197
13012	0.946132	0.0831848
13013	1.30107	0.141455
13014	0.946133	0.175828
13015	0.810946	2.1
13016	0.810944	2.1
13017	2.73936	2.1
13018	1.35168	2.1
13019	1.0813	2.1
13020	1.08132	2.1
13021	1.08132	2.1
13022	0.810958	2.1
13023	0.946129	2.08174
13024	0.946129	2.1
13025	0.946133	1.77609
13026	1.30812	2.07949
13027	0.946131	1.97022
13028	0.946131	2.05028
13029	0.946131	2.04746
13030	1.00901	1.56619
13031	0.94613	1.95874
13032	0.94613	0.844057
13033	1.17001	1.69917
13034	0.917259	1.05587
13035	0.94613	1.38533
13036	0.94613	1.45487
13037	0.946126	2.1
13038	0.946127	2.1
13039	0.946131	2.1
13040	0.946127	2.1
13041	0.946131	2.1
13042	0.946129	2.1
13043	1.398	2.1
13044	0.946133	2.1
13045	1.3033	2.1
13046	0.946132	2.1
13047	0.946132	2.1

13048	0.946132	2.1
13049	0.946132	2.1
13050	0.946133	2.1
13051	0.946131	0.44625
13052	0.946131	0.473224
13053	1.09897	0.350035
13054	0.946129	0.647045
13055	0.946131	0.491186
13056	0.94613	0.475831
13057	0.94613	0.449954
13058	0.946132	2.1
13059	0.946131	2.1
13060	0.946131	2.1
13061	0.946132	2.1
13062	0.94613	2.1
13063	0.946131	2.1
13064	1.39858	2.1
13065	1.07117	0.476297
13066	1.08878	0.551266
13067	0.946119	0.972973
13068	1.08878	1.03553
13069	0.946119	0.285758
13070	0.946132	0.793224
13071	1.07116	0.538586
13072	0.946132	0.0730326
13073	0.946132	0.0702522
13074	0.946132	0.0738585
13075	0.946132	0.0698591
13076	0.946132	0.0698591
13077	1.10733	0.0698591
13078	0.946132	0.0703952
13079	0.946128	0.0778979
13080	0.946132	0.0711031
13081	0.94613	0.0793619
13082	0.946129	0.0698591
13083	0.94613	0.0698591
13084	1.3367	0.0699049
13085	0.94613	0.0739986
13086	0.810943	2.1
13087	0.810944	2.1
13088	2.70234	2.1
13089	1.21653	2.1
13090	1.08132	2.1
13091	0.946155	2.1
13092	0.979951	2.1
13093	0.709578	2.1
13094	1.27174	2.1
13095	0.946129	2.1
13096	0.94613	2.1
13097	1.35117	2.1
13098	0.94613	2.1
13099	1.35117	2.1
13100	0.94613	2.1
13101	0.946133	1.50567
13102	0.946133	1.659
13103	0.946132	1.98319
13104	0.946133	1.44692
13105	0.946132	2.08507

13106	0.946133	1.96939
13107	0.997296	2.02962
13108	0.810943	2.1
13109	0.810944	2.1
13110	0.810945	2.1
13111	2.78833	2.1
13112	1.35169	2.1
13113	1.08132	2.1
13114	1.08132	2.1
13115	1.08132	2.1
13116	0.810946	2.1
13117	0.810943	0.761341
13118	0.810933	1.68009
13119	2.70049	1.86003
13120	1.3517	1.30846
13121	1.08132	2.08488
13122	1.08132	0.291499
13123	1.08133	1.23392
13124	0.946127	1.49095
13125	1.35469	1.68518
13126	0.94613	1.67841
13127	0.946131	1.52042
13128	0.946128	1.50727
13129	1.35469	1.71267
13130	0.946129	1.71098
13131	0.946131	0.251938
13132	1.2722	0.201671
13133	0.946132	0.200702
13134	0.946132	0.208125
13135	0.946129	0.285028
13136	1.2722	0.175024
13137	0.94613	0.222413
13138	0.946133	0.0744109
13139	0.946132	0.0869701
13140	0.946132	0.0856421
13141	0.946132	0.0821422
13142	1.41461	0.093962
13143	1.41461	0.0810188
13144	0.946133	0.0832283
13145	0.946131	1.3235
13146	1.30165	0.581771
13147	0.946132	0.520936
13148	0.946132	1.47513
13149	0.946129	0.747249
13150	1.30165	1.21879
13151	0.946131	1.55934
13152	0.946132	1.94438
13153	0.946129	2.1
13154	0.94613	1.99999
13155	1.39356	2.1
13156	0.946131	2.03809
13157	0.946131	2.04863
13158	0.94613	2.09349
13159	0.810946	2.1
13160	0.810947	2.1
13161	0.810934	2.1
13162	2.35003	2.1
13163	1.3517	2.1

13164	1.08132	2.1
13165	1.08132	2.1
13166	1.08133	2.1
13167	0.946131	0.0698591
13168	0.946131	0.0698591
13169	0.946125	0.0702305
13170	0.946127	0.0698591
13171	0.946129	0.0698591
13172	0.946128	0.0698591
13173	0.946128	0.0698591
13174	0.946131	0.0698591
13175	0.946131	0.0698591
13176	0.946131	0.0698591
13177	0.946131	0.0698591
13178	0.946132	0.0698591
13179	0.946132	0.0698591
13180	0.946131	0.0698591
13181	0.946129	2.1
13182	0.946132	2.1
13183	0.946132	2.1
13184	0.94613	2.1
13185	1.30082	2.1
13186	0.94613	2.1
13187	0.946131	2.1
13188	1.20416	0.0698591
13189	0.946132	0.0839146
13190	0.946131	0.0698591
13191	0.946132	0.0698591
13192	0.946131	0.0698591
13193	0.946131	0.0733234
13194	0.946132	0.0698591
13195	1.18711	0.0698591
13196	0.946131	0.0849414
13197	0.946131	0.0710782
13198	0.946132	0.0698591
13199	0.946131	0.0698591
13200	0.946131	0.085745
13201	0.946132	0.0698591
13202	0.946133	0.0698591
13203	0.946133	0.0698591
13204	0.946132	0.0698591
13205	1.30212	0.0698591
13206	0.946133	0.0698591
13207	0.946132	0.0698591
13208	1.30212	0.0698591
13209	0.946131	0.0698591
13210	0.946131	0.0698591
13211	0.946131	0.0698591
13212	0.946132	0.0698591
13213	1.31467	0.0698591
13214	1.31467	0.0698591
13215	0.946131	0.0698591
13216	0.946133	0.0698591
13217	0.946132	0.0698591
13218	0.946132	0.0698591
13219	0.946132	0.0698591
13220	1.25124	0.0698591
13221	1.25124	0.0698591

13222	0.946132	0.0698591
13223	0.946132	2.1
13224	0.946132	2.1
13225	0.946132	2.1
13226	1.36422	2.1
13227	0.946132	2.1
13228	0.946131	2.1
13229	0.946132	2.1
13230	0.946131	1.10421
13231	1.41851	1.72307
13232	0.946132	1.97713
13233	0.946132	1.12517
13234	0.946132	1.67953
13235	0.946132	1.32349
13236	0.946132	1.45564
13237	0.946133	0.0698591
13238	0.946132	0.0698591
13239	0.946132	0.0698591
13240	0.946132	0.0698591
13241	0.946132	0.0698591
13242	1.37794	0.0698591
13243	0.946132	0.0698591
13244	0.946132	2.1
13245	0.946132	2.1
13246	0.946131	2.1
13247	0.946131	2.1
13248	0.946132	2.1
13249	0.946132	2.1
13250	1.4045	2.1
13251	0.946133	2.1
13252	0.946132	2.1
13253	0.946131	2.1
13254	0.946132	2.1
13255	0.946131	2.1
13256	0.946131	2.1
13257	0.946132	2.1
13258	0.946132	2.1
13259	0.946132	2.1
13260	0.946131	2.1
13261	0.946132	2.1
13262	0.946131	2.1
13263	0.946131	2.1
13264	0.946131	2.1
13265	0.946128	2.1
13266	1.31641	2.1
13267	0.946129	2.1
13268	0.94613	2.1
13269	0.94613	2.1
13270	0.94613	2.1
13271	0.94613	2.1
13272	0.946132	2.1
13273	0.946131	2.1
13274	0.946131	2.1
13275	0.946132	2.1
13276	0.946132	2.1
13277	0.946132	2.1
13278	0.946132	2.1
13279	0.946132	2.1

13280	1.41507	2.1
13281	0.946132	2.1
13282	0.946132	2.1
13283	0.946133	2.1
13284	0.946133	2.1
13285	0.946132	2.1
13286	0.946132	2.1
13287	0.946131	2.1
13288	0.946131	2.1
13289	0.946133	2.1
13290	0.946132	2.1
13291	1.32608	2.1
13292	0.946132	2.1
13293	0.94613	2.03598
13294	0.94613	2.1
13295	0.94613	0.313021
13296	0.946131	2.06323
13297	0.946129	1.34025
13298	0.94613	0.701604
13299	0.94613	0.972125
13300	1.33728	2.00001
13301	0.946131	1.9423
13302	0.94613	1.00777
13303	0.946132	2.02718
13304	0.946132	2.1
13305	0.946131	1.71984
13306	0.946131	1.79718
13307	0.946127	2.1
13308	1.40641	2.09117
13309	0.946129	2.097
13310	0.94613	2.1
13311	0.946128	1.9016
13312	0.946129	2.1
13313	0.946129	2.1
13314	0.946129	2.1
13315	0.946128	2.1
13316	0.946133	2.1
13317	1.37467	2.1
13318	0.946131	2.1
13319	0.946131	2.1
13320	1.37467	2.1
13321	0.946131	2.1
13322	0.946131	2.1
13323	0.946132	2.1
13324	0.946132	2.1
13325	0.946129	2.1
13326	0.946131	2.1
13327	1.34912	2.1
13328	0.946132	2.1
13329	0.946133	2.1
13330	0.946133	2.1
13331	0.946132	2.1
13332	0.946132	2.1
13333	0.946133	2.1
13334	0.946132	2.1
13335	0.946127	2.1
13336	0.946131	2.1
13337	0.946132	2.1

13338	0.946131	2.1
13339	0.946127	2.1
13340	0.94613	2.1
13341	1.33607	2.1
13342	0.946117	0.211355
13343	0.946117	1.06654
13344	0.946115	1.02095
13345	0.946124	2.07422
13346	0.946119	0.598875
13347	1.32662	0.896427
13348	0.94613	0.55762
13349	0.946119	2.1
13350	0.946114	2.1
13351	0.946118	2.1
13352	0.946114	2.1
13353	0.946118	2.1
13354	0.946104	2.1
13355	0.946129	2.1
13356	0.946132	2.1
13357	0.946133	2.1
13358	0.946133	2.1
13359	0.946133	2.1
13360	0.946132	2.1
13361	0.946132	2.1
13362	0.946133	2.1
13363	0.946133	2.1
13364	0.946132	2.1
13365	0.946132	2.1
13366	0.946133	2.1
13367	0.946132	2.1
13368	0.946132	2.1
13369	0.946132	2.1
13370	0.946133	1.67328
13371	1.30881	2.0158
13372	0.946132	2.1
13373	0.946131	1.51668
13374	0.946131	1.85736
13375	0.946131	1.57525
13376	0.946131	1.82967
13377	0.946128	2.1
13378	0.946128	2.1
13379	0.946132	2.1
13380	0.94613	2.1
13381	0.94613	2.1
13382	0.94613	2.1
13383	1.34652	2.1
13384	0.946133	1.40326
13385	0.946133	1.27502
13386	0.946132	1.9119
13387	0.946133	1.18615
13388	0.946132	2.01755
13389	0.946132	1.68111
13390	0.946132	1.90046
13391	0.946132	0.162558
13392	0.946128	0.0874732
13393	0.94613	0.18369
13394	0.946131	0.0739928
13395	0.94613	0.217421

13396	0.94613	0.136968
13397	0.94613	0.122769
13398	0.946128	0.569008
13399	0.946132	0.623849
13400	0.94613	0.580026
13401	0.94613	0.483319
13402	0.946131	0.494933
13403	0.94613	0.561457
13404	0.94613	0.527678
13405	0.946129	0.755536
13406	1.32056	1.26251
13407	0.946131	1.46614
13408	0.94613	0.874611
13409	0.946124	1.39152
13410	0.946128	1.10963
13411	0.946127	1.06213
13412	0.946129	0.78883
13413	1.20351	1.11916
13414	0.946132	1.11958
13415	0.946132	0.956931
13416	0.946126	1.28654
13417	0.946129	1.14487
13418	0.946129	0.993025
13419	0.946127	2.03188
13420	0.946127	2.08942
13421	0.946131	1.76191
13422	1.25592	2.03712
13423	0.946127	1.86364
13424	0.946129	1.97761
13425	0.94613	1.99872
13426	1.02332	1.97591
13427	0.946133	1.7407
13428	0.946132	1.44968
13429	1.02332	1.72345
13430	0.946132	1.06803
13431	0.96196	1.72223
13432	0.930306	1.44556
13433	0.946133	1.97972
13434	0.946133	2.0135
13435	0.946132	2.08313
13436	1.20057	2.03345
13437	0.946132	2.06808
13438	0.946133	2.09052
13439	1.20057	2.07579
13440	0.946128	1.492
13441	0.946129	0.887447
13442	0.946129	1.18072
13443	1.13245	0.626887
13444	0.946126	0.528345
13445	0.946128	1.0581
13446	0.946129	0.848511
13447	0.946131	1.71398
13448	0.946131	1.94882
13449	0.94613	2.04093
13450	1.25	1.90242
13451	0.946129	2.04411
13452	0.946131	2.07374
13453	1.25	2.03368

13454	0.540425	1.96468
13455	0.748493	1.93939
13456	1.46266	2.05273
13457	1.20046	2.01101
13458	0.600454	1.99317
13459	1.41283	2.02683
13460	0.582567	1.95784
13461	0.946131	1.91282
13462	0.946131	1.88257
13463	0.946132	1.74067
13464	0.946132	1.9093
13465	0.94613	1.885
13466	0.946131	2.05049
13467	1.28439	2.01429
13468	0.946132	0.578363
13469	0.946131	0.374542
13470	0.946131	0.596836
13471	0.946132	0.406295
13472	0.946132	0.746256
13473	1.39335	0.351673
13474	0.946132	0.435749
13475	0.946131	0.199752
13476	0.946131	0.432146
13477	0.94613	0.212595
13478	0.946132	0.173949
13479	0.946131	0.380717
13480	0.946131	0.202001
13481	0.946131	0.239352
13482	0.946131	0.183582
13483	0.94613	0.14814
13484	0.946126	0.238014
13485	0.946131	0.113808
13486	0.946125	0.501997
13487	0.946127	0.122394
13488	0.946128	0.266567
13489	0.946131	0.0698591
13490	0.946131	0.0702893
13491	0.946131	0.0698591
13492	0.946131	0.0698591
13493	0.946131	0.0698591
13494	0.946131	0.0698591
13495	1.39168	0.0698591
13496	0.946131	0.0963593
13497	0.946126	0.0717851
13498	0.946127	0.0825258
13499	0.946131	0.0849708
13500	0.94613	0.099599
13501	1.412	0.0829554
13502	0.946128	0.0810948
13503	0.948901	0.258907
13504	0.943364	0.262777
13505	1.03768	0.223481
13506	0.946132	0.390603
13507	0.946133	0.320181
13508	1.03767	0.303254
13509	0.946132	0.27978
13510	1.00902	0.495832
13511	0.88324	0.451003

13512	0.971584	0.375267
13513	0.946132	0.466357
13514	0.946129	0.784569
13515	0.946131	0.549637
13516	0.971572	0.534555
13517	0.946131	0.0698591
13518	0.946125	0.0698591
13519	1.33587	0.0698591
13520	0.946126	0.0723369
13521	0.946127	0.0698591
13522	0.946126	0.0699064
13523	0.946128	0.069972
13524	0.946132	0.0740278
13525	0.946132	0.0751835
13526	1.27512	0.0702896
13527	0.946133	0.0951233
13528	0.946132	0.0761122
13529	0.946133	0.0770742
13530	0.946133	0.0821102
13531	0.946128	0.0905593
13532	0.946133	0.0857224
13533	0.946131	0.0948827
13534	0.94613	0.0698882
13535	0.946131	0.0723669
13536	0.94613	0.0815062
13537	0.946131	0.0749584
13538	0.946131	0.0801395
13539	0.946131	0.0752104
13540	0.946131	0.0821463
13541	0.946131	0.0698591
13542	0.946131	0.0700574
13543	0.946131	0.0743697
13544	0.946131	0.0709375
13545	0.94613	0.0698591
13546	0.946131	0.0698591
13547	0.946131	0.0698591
13548	0.94613	0.0698591
13549	0.946132	0.0698591
13550	0.946131	0.0698591
13551	1.35316	0.0698591
13552	0.94613	0.148454
13553	0.94613	0.118162
13554	0.946128	0.127832
13555	0.946132	0.116089
13556	0.946132	0.13491
13557	1.28727	0.122522
13558	0.94613	0.0958615
13559	0.946131	0.0698591
13560	0.946132	0.0698591
13561	0.946132	0.0698591
13562	1.39502	0.0698591
13563	0.946132	0.0698591
13564	0.946132	0.0698591
13565	0.946132	0.0698591
13566	0.946125	0.358632
13567	0.946131	0.317585
13568	0.94613	0.250972
13569	0.946129	0.331249

13570	0.946126	0.521129
13571	0.946127	0.3877
13572	1.36173	0.351734
13573	0.946129	0.603223
13574	1.02363	0.442109
13575	0.94613	0.438602
13576	0.946131	0.608368
13577	0.946131	0.446244
13578	1.02363	0.607084
13579	0.946131	0.595442
13580	0.946128	0.0698591
13581	1.24678	0.0698591
13582	0.94613	0.0698591
13583	0.94613	0.0698591
13584	0.94613	0.0698591
13585	0.946131	0.0698591
13586	0.94613	0.0698591
13587	0.946129	0.0698591
13588	1.25215	0.0698591
13589	0.946132	0.0698591
13590	0.946126	0.0698591
13591	0.946129	0.0698591
13592	0.94613	0.0698591
13593	0.946129	0.0698591
13594	0.946129	0.400808
13595	0.946133	0.35301
13596	0.946133	0.288664
13597	0.946131	0.362536
13598	0.946131	0.319443
13599	1.29689	0.283108
13600	0.946131	0.316892
13601	0.946128	0.0791043
13602	0.946132	0.0698591
13603	0.946132	0.0714419
13604	0.94613	0.0839397
13605	1.22716	0.0698591
13606	1.22716	0.0707098
13607	0.946131	0.0747106
13608	0.946127	0.0975187
13609	0.946131	0.0739943
13610	0.946131	0.0827904
13611	0.946128	0.109275
13612	1.30463	0.0747378
13613	1.30463	0.0799363
13614	0.946129	0.088134
13615	1.29958	0.0698591
13616	0.946132	0.0698591
13617	0.946132	0.0698591
13618	0.946132	0.0698591
13619	0.946132	0.0698591
13620	0.946132	0.0698591
13621	0.946133	0.0698591
13622	1.26201	0.0698591
13623	0.946133	0.0698591
13624	0.946132	0.0698591
13625	0.946132	0.0698591
13626	0.946132	0.0698591
13627	0.946133	0.0698591

13628	0.946132	0.0698591
13629	0.946133	0.0821369
13630	0.946132	0.0711013
13631	0.946132	0.0829266
13632	0.946133	0.0698591
13633	0.946132	0.0698702
13634	1.40427	0.0698676
13635	0.946133	0.0776992
13636	0.946132	0.0830195
13637	0.946132	0.070323
13638	0.946132	0.0830503
13639	0.946132	0.0698591
13640	0.946132	0.0698591
13641	1.35123	0.0698591
13642	0.946131	0.0779698
13643	0.946128	0.12493
13644	0.946132	0.0845938
13645	0.946132	0.100727
13646	0.946129	0.142111
13647	1.22144	0.0860977
13648	1.22144	0.0953043
13649	0.946131	0.115184
13650	0.946127	0.289387
13651	0.946132	0.235455
13652	0.946131	0.215009
13653	0.946127	0.263667
13654	0.946131	0.237866
13655	1.23351	0.208275
13656	0.94613	0.238755
13657	1.18119	0.103517
13658	0.946131	0.149856
13659	0.946132	0.0997433
13660	0.946132	0.12489
13661	0.94613	0.165618
13662	0.946131	0.13434
13663	0.946131	0.1385
13664	0.946132	0.190751
13665	0.946132	0.160645
13666	0.946133	0.233329
13667	1.30563	0.13547
13668	0.946132	0.147398
13669	0.946132	0.187821
13670	0.946133	0.237131
13671	1.20256	0.426585
13672	0.946132	2.00456
13673	0.946133	1.32707
13674	0.946132	0.681792
13675	0.946132	0.415655
13676	0.946132	1.67925
13677	0.946133	1.06373
13678	0.94613	0.878439
13679	0.94613	0.659229
13680	0.946128	1.0084
13681	1.33007	0.522958
13682	0.946129	0.437052
13683	0.94613	0.922757
13684	0.946131	1.34169
13685	0.946127	2.1

13686	0.946127	2.1
13687	0.946131	2.1
13688	1.30136	2.1
13689	0.946128	2.1
13690	0.946129	2.1
13691	1.30136	2.1
13692	0.810942	2.1
13693	0.810942	2.1
13694	0.81093	2.1
13695	2.3208	2.1
13696	1.3517	2.1
13697	1.08132	2.1
13698	1.08132	2.1
13699	1.08133	2.1
13700	0.946132	2.1
13701	0.946132	2.1
13702	0.946132	2.1
13703	0.946132	2.1
13704	0.946132	2.1
13705	1.39823	2.1
13706	0.946132	2.1
13707	1.37713	2.1
13708	0.946131	2.1
13709	0.946131	2.1
13710	0.946131	2.1
13711	0.946131	2.1
13712	0.946131	2.1
13713	0.946131	2.1
13714	0.946128	2.1
13715	0.946128	2.1
13716	0.946133	2.1
13717	0.946131	2.1
13718	0.946131	2.1
13719	0.946131	2.1
13720	0.94613	2.1
13721	0.946129	2.1
13722	0.946129	2.1
13723	0.946133	2.1
13724	0.946131	2.1
13725	0.946131	2.1
13726	0.946131	2.1
13727	0.946131	2.1
13728	0.946132	1.40665
13729	1.33694	2.02471
13730	0.946133	2.1
13731	0.946133	1.42326
13732	0.946132	2.02369
13733	0.946132	1.6666
13734	0.946133	1.82187
13735	0.946131	2.1
13736	0.94613	2.1
13737	0.946131	2.1
13738	0.946133	2.1
13739	0.946129	2.1
13740	0.946131	2.1
13741	0.946131	2.1
13742	0.946132	1.88884
13743	1.35943	2.00848

13744	0.946132	2.1
13745	0.946132	1.19511
13746	0.946131	1.72458
13747	0.946132	1.25641
13748	0.946132	1.88554
13749	0.946132	0.0698591
13750	0.946132	0.0698591
13751	0.946133	0.0698591
13752	0.946133	0.0698591
13753	0.946132	0.0698591
13754	0.946132	0.0698591
13755	0.946132	0.0698591
13756	1.29322	0.0698591
13757	0.946131	0.0698591
13758	0.946132	0.0698591
13759	0.946129	0.0698591
13760	0.946131	0.0698591
13761	0.946131	0.0698591
13762	0.946131	0.0698591
13763	0.946132	1.82342
13764	0.946132	2.1
13765	0.946131	1.72528
13766	1.41492	1.22696
13767	0.946132	2.1
13768	0.946132	2.05928
13769	0.946131	2.1
13770	0.946132	2.1
13771	0.946132	2.1
13772	0.946132	2.1
13773	1.35028	2.1
13774	0.946132	2.1
13775	0.946132	2.1
13776	0.946132	2.1
13777	0.946132	1.96229
13778	0.946132	2.09975
13779	0.946132	2.08556
13780	1.32461	2.05016
13781	0.946131	2.1
13782	0.946132	2.08011
13783	0.946132	2.1
13784	0.946131	2.1
13785	0.946131	2.1
13786	0.946131	2.1
13787	0.946132	2.1
13788	0.946129	2.1
13789	0.94613	2.1
13790	1.40369	2.1
13791	0.946132	0.108701
13792	0.946131	0.0785509
13793	0.946131	0.123782
13794	0.979598	0.0718032
13795	0.946131	0.0706548
13796	0.946131	0.105562
13797	0.946132	0.132659
13798	0.946131	2.1
13799	1.41897	2.1
13800	0.946132	2.1
13801	0.946132	2.1

13802	0.94613	2.1
13803	0.946131	2.1
13804	0.946131	2.1
13805	0.946131	2.1
13806	0.94613	2.1
13807	0.946132	2.1
13808	0.946132	2.1
13809	0.946129	2.1
13810	0.946131	2.1
13811	1.37791	2.1
13812	0.946127	2.1
13813	0.946131	2.1
13814	0.946131	2.1
13815	0.94613	2.1
13816	0.946128	2.1
13817	0.946129	2.1
13818	1.39384	2.1
13819	0.810945	2.05556
13820	0.810944	1.04804
13821	0.810958	1.73647
13822	2.70852	1.56519
13823	1.35168	1.12932
13824	1.08132	1.91663
13825	1.08132	0.364545
13826	1.0813	1.32206
13827	1.3335	2.1
13828	0.946132	2.1
13829	0.94613	2.1
13830	0.946132	2.1
13831	0.946131	2.1
13832	0.94613	2.1
13833	0.946131	2.1
13834	0.946132	2.1
13835	0.946131	1.97023
13836	0.94613	2.1
13837	1.41192	2.1
13838	0.946132	2.00059
13839	0.946131	2.1
13840	0.94613	2.09898
13841	0.946133	1.58832
13842	0.946133	0.737121
13843	0.946132	1.50142
13844	1.39428	1.11547
13845	0.946132	0.215851
13846	0.946132	0.784015
13847	0.946133	0.564592
13848	0.94613	0.923384
13849	0.946129	2.1
13850	0.94613	1.97167
13851	0.946128	1.63038
13852	1.40684	2.1
13853	0.94613	1.82906
13854	0.94613	2.1
13855	0.946131	2.08266
13856	0.946131	2.1
13857	0.946131	2.1
13858	0.946131	2.1
13859	1.39021	2.1

13860	0.946131	2.1
13861	0.946131	2.1
13862	0.946131	2.05204
13863	0.946131	2.07871
13864	0.946131	0.782599
13865	0.946131	2.07445
13866	0.946131	2.1
13867	0.946131	0.592056
13868	0.946131	0.914707
13869	0.810946	2.1
13870	0.810946	2.1
13871	2.69264	2.1
13872	1.35168	2.1
13873	1.0813	2.1
13874	1.08132	2.1
13875	1.08132	2.1
13876	0.810958	2.1
13877	1.16687	2.1
13878	0.946131	2.1
13879	0.946131	2.1
13880	1.34632	2.1
13881	0.946131	2.1
13882	1.34632	2.1
13883	0.946131	2.1
13884	0.946128	0.514033
13885	0.946133	0.419849
13886	0.946133	0.358304
13887	0.946131	0.499227
13888	0.946131	0.358196
13889	1.3564	0.44206
13890	0.94613	0.469184
13891	0.946128	2.1
13892	0.946132	2.1
13893	0.94613	2.1
13894	0.94613	2.1
13895	0.946131	2.1
13896	1.35827	2.1
13897	0.94613	2.1
13898	0.946133	0.928443
13899	0.946132	1.31938
13900	0.946132	0.995008
13901	0.946133	1.30036
13902	0.946133	1.02159
13903	0.946132	1.06402
13904	0.946133	1.19307
13905	0.946132	2.1
13906	0.946132	1.90868
13907	0.946132	0.374486
13908	0.946131	2.09759
13909	0.946132	0.858949
13910	0.946132	1.52786
13911	0.946132	1.37485
13912	0.94613	0.56441
13913	0.94613	0.390158
13914	0.946128	0.60638
13915	0.946132	0.344395
13916	0.946132	0.60095
13917	0.94613	0.425108

13918	0.946131	0.425414
13919	0.946126	0.177623
13920	0.946131	0.128613
13921	0.946127	0.188909
13922	0.946129	0.114966
13923	0.94613	0.175919
13924	0.946128	0.136555
13925	0.946128	0.13516
13926	0.946131	0.083694
13927	0.946127	0.0773244
13928	0.946127	0.0833775
13929	0.946131	0.0756076
13930	0.946131	0.0761803
13931	0.946128	0.0724764
13932	0.946129	0.0858377
13933	0.94613	0.0761661
13934	0.946131	0.0790145
13935	0.946129	0.0746264
13936	0.946132	0.085592
13937	0.946132	0.0717604
13938	0.946131	0.0723181
13939	0.94613	0.0944251
13940	0.94613	0.098666
13941	0.946131	0.0760167
13942	0.946129	0.0909192
13943	0.946132	0.0735849
13944	0.946132	0.0963364
13945	1.32827	0.0799419
13946	0.946131	0.0946139
13947	0.946132	2.1
13948	0.946132	2.1
13949	0.946131	2.1
13950	0.946131	2.1
13951	0.946132	2.1
13952	0.946131	2.1
13953	0.946131	2.1
13954	0.946132	0.726941
13955	0.946132	2.09279
13956	0.946131	0.677652
13957	1.38356	0.347518
13958	0.946133	2.07207
13959	0.946131	1.11694
13960	0.946131	1.48575
13961	0.94613	2.1
13962	0.946131	2.1
13963	0.946131	2.1
13964	0.946128	2.1
13965	0.946133	2.1
13966	0.946131	2.1
13967	1.36918	2.1
13968	0.946131	1.91392
13969	0.94613	2.1
13970	0.946129	2.09629
13971	0.946133	2.1
13972	0.946132	2.1
13973	1.3771	2.1
13974	0.946131	2.1
13975	1.30029	1.28463

13976	0.94613	0.616783
13977	0.946129	1.93173
13978	0.946132	1.68316
13979	0.946132	0.135579
13980	0.946131	1.05638
13981	0.94613	0.786448
13982	0.946133	0.0698591
13983	0.946132	0.0698591
13984	0.946132	0.0698591
13985	0.946132	0.0698591
13986	0.946132	0.0698591
13987	0.946132	0.0698591
13988	1.21174	0.0698591
13989	0.810946	2.1
13990	0.810945	2.1
13991	2.63045	2.1
13992	1.35169	2.1
13993	1.08132	2.1
13994	1.08132	2.1
13995	1.08132	2.1
13996	0.810946	2.1
13997	1.1796	2.1
13998	0.946131	2.1
13999	0.946132	2.1
14000	1.31522	2.1
14001	0.946132	2.1
14002	1.31522	2.1
14003	0.946132	2.1
14004	0.946131	2.1
14005	1.40615	2.1
14006	0.946129	2.1
14007	0.946128	2.1
14008	0.946133	2.1
14009	0.946131	2.1
14010	0.94613	2.1
14011	0.810945	2.1
14012	0.810944	2.1
14013	2.80479	2.1
14014	1.35169	2.1
14015	1.08132	2.1
14016	1.08132	2.1
14017	1.08132	2.1
14018	0.810945	2.1
14019	1.35562	2.1
14020	0.94613	2.1
14021	1.35561	2.1
14022	0.946133	2.1
14023	0.946131	2.1
14024	1.40239	2.1
14025	0.946131	2.1
14026	0.946125	0.229414
14027	0.946131	0.408323
14028	0.946127	0.215327
14029	0.946129	0.220413
14030	0.946128	0.43438
14031	0.946129	0.276105
14032	0.946128	0.270256
14033	0.946119	2.1

14034	0.946113	2.1
14035	0.94612	2.1
14036	0.946119	2.1
14037	1.41915	2.1
14038	0.946107	2.1
14039	0.946132	2.1
14040	0.810946	1.84646
14041	0.810943	2.1
14042	0.810934	2.04168
14043	2.31411	2.09044
14044	1.35171	2.06589
14045	1.08132	1.93749
14046	1.08132	2.0962
14047	1.08133	2.00521
14048	0.946127	2.1
14049	0.94613	2.1
14050	0.94613	2.1
14051	0.946131	2.1
14052	0.946126	2.1
14053	0.946128	2.1
14054	0.946128	2.1
14055	0.810944	2.1
14056	0.810947	2.1
14057	0.81096	2.1
14058	2.78019	2.1
14059	1.35168	2.1
14060	1.08132	2.1
14061	1.08132	2.1
14062	1.08131	2.1
14063	0.946133	0.0794664
14064	0.946132	0.0899951
14065	0.946132	0.0984047
14066	0.946133	0.0763538
14067	0.946132	0.103995
14068	1.3495	0.0763435
14069	0.946133	0.0950883
14070	0.946133	2.1
14071	0.946132	2.1
14072	0.946132	2.1
14073	1.39529	2.1
14074	0.946133	2.1
14075	0.946132	2.1
14076	0.946133	2.1
14077	0.946133	2.1
14078	0.946132	2.1
14079	0.946132	2.1
14080	0.946132	2.1
14081	0.946132	2.1
14082	0.946133	2.1
14083	0.946132	2.1
14084	0.810946	2.1
14085	0.810945	2.1
14086	2.67861	2.1
14087	1.35169	2.1
14088	1.08132	2.1
14089	1.08132	2.1
14090	1.08132	2.1
14091	0.810946	2.1

14092	1.24616	2.1
14093	0.946132	2.1
14094	0.946132	2.1
14095	1.33931	2.1
14096	0.946132	2.1
14097	1.33931	2.1
14098	0.946133	2.1
14099	0.946133	0.111047
14100	0.946133	0.0703917
14101	0.946132	0.073789
14102	0.946133	0.101949
14103	1.3855	0.0698775
14104	1.3855	0.0753741
14105	0.946133	0.0940621
14106	0.946128	2.1
14107	0.946132	2.1
14108	0.946128	2.1
14109	0.94613	2.1
14110	0.946131	2.1
14111	1.40225	2.1
14112	0.94613	2.1
14113	1.40548	0.0698591
14114	0.946118	0.0698591
14115	0.946114	0.0698591
14116	0.946112	0.0698591
14117	0.946117	0.0698591
14118	0.946128	0.0698591
14119	0.946103	0.0698591
14120	0.94613	2.1
14121	0.946132	2.1
14122	0.946131	2.1
14123	0.946132	2.1
14124	1.37811	2.1
14125	0.946131	2.1
14126	0.946131	2.1
14127	0.946132	2.1
14128	0.946133	2.1
14129	0.946133	2.1
14130	0.946132	2.1
14131	0.946133	2.1
14132	0.946132	2.1
14133	0.946132	2.1
14134	0.946118	2.1
14135	0.946113	2.1
14136	0.946118	2.1
14137	0.946119	2.1
14138	0.946117	2.1
14139	0.946105	2.1
14140	0.946131	2.1
14141	0.946118	0.0698591
14142	0.946118	0.0698591
14143	0.946117	0.0698591
14144	0.946113	0.0698591
14145	0.94612	0.0698591
14146	1.41377	0.0698591
14147	0.946106	0.0698591
14148	0.946132	1.09912
14149	1.28436	0.776676

14150	0.946131	0.810674
14151	0.946131	1.11289
14152	0.946131	0.332162
14153	0.94613	1.12575
14154	0.946131	0.901056
14155	0.810947	0.327363
14156	0.810946	0.355693
14157	2.68675	0.208008
14158	1.35168	0.224775
14159	1.08132	0.274947
14160	1.08132	0.267433
14161	1.0813	0.338309
14162	0.81096	0.30126
14163	0.946132	0.211814
14164	1.34338	0.177495
14165	0.946132	0.189744
14166	1.34338	0.229884
14167	0.946132	0.246751
14168	1.34337	0.202098
14169	0.946132	0.207455
14170	0.946127	2.08604
14171	0.946131	2.07828
14172	0.946127	2.06901
14173	0.946131	1.99286
14174	0.946131	2.05625
14175	0.946128	2.05351
14176	1.30281	2.0865
14177	0.946132	0.471374
14178	0.946132	0.351817
14179	0.946132	0.389084
14180	0.946131	0.402307
14181	0.946132	0.459108
14182	1.37973	0.29577
14183	0.946132	0.37069
14184	0.946131	0.655983
14185	1.41798	0.510435
14186	0.946132	0.458277
14187	0.946132	0.525905
14188	0.946129	0.857447
14189	0.946131	0.552047
14190	0.946131	0.798468
14191	0.946118	0.548831
14192	1.41776	0.430698
14193	0.946113	0.425162
14194	0.946119	0.432417
14195	0.946117	0.700625
14196	1.41778	0.43539
14197	0.946105	0.688682
14198	0.946131	0.763561
14199	1.41798	0.853684
14200	0.946132	0.936546
14201	0.946132	0.674609
14202	0.94613	1.01266
14203	1.41799	0.626704
14204	0.946131	0.708535
14205	0.946133	2.1
14206	0.946132	2.1
14207	0.946132	1.65459

14208	0.946132	2.1
14209	0.946133	1.86494
14210	0.946132	2.1
14211	0.946132	2.1
14212	0.946131	2.1
14213	0.946131	2.1
14214	0.94613	1.00132
14215	0.946129	2.1
14216	0.946132	1.341
14217	0.94613	1.92431
14218	0.946131	2.07771
14219	0.94612	1.32474
14220	0.946126	2.1
14221	0.946119	1.37881
14222	0.94612	1.86644
14223	1.40766	2.06834
14224	0.946132	0.534714
14225	0.946107	2.09815
14226	0.946128	2.1
14227	1.31213	2.1
14228	0.946129	2.1
14229	0.94613	2.1
14230	0.94613	2.1
14231	0.94613	2.1
14232	0.94613	2.1
14233	0.946127	2.1
14234	0.946131	2.1
14235	0.94613	2.1
14236	0.94613	2.1
14237	1.36653	2.1
14238	0.946128	2.1
14239	0.946128	2.1
14240	0.946127	2.1
14241	0.94613	2.1
14242	0.946131	2.1
14243	0.94613	2.1
14244	0.946128	2.1
14245	0.946128	2.1
14246	0.946129	2.1
14247	0.946133	2.1
14248	1.40081	2.1
14249	0.946132	2.1
14250	0.946132	2.1
14251	0.946133	2.1
14252	0.946132	2.1
14253	0.946133	2.1
14254	0.946128	2.1
14255	1.32282	2.1
14256	0.946129	2.1
14257	0.94613	2.1
14258	0.946131	2.1
14259	0.946131	2.1
14260	0.946131	2.1
14261	0.810945	2.1
14262	0.810943	2.1
14263	0.810933	2.1
14264	2.44097	2.1
14265	1.3517	2.1

14266	1.08132	2.1
14267	1.08132	2.1
14268	1.08133	2.1
14269	0.946131	2.1
14270	0.94613	2.1
14271	0.946133	2.1
14272	0.946133	2.1
14273	0.946128	2.1
14274	0.946131	2.1
14275	1.27676	2.1
14276	0.946126	1.33202
14277	0.946126	2.03872
14278	0.946131	1.40666
14279	0.946127	2.0345
14280	0.94613	0.315141
14281	0.946128	1.2938
14282	0.946128	1.21871
14283	0.946132	0.0873137
14284	0.946133	0.113458
14285	0.946132	0.089197
14286	0.946132	0.0877643
14287	0.946132	0.0773508
14288	0.946133	0.0956739
14289	0.946132	0.0910789
14290	0.946128	0.108207
14291	0.946131	0.129392
14292	0.94613	0.10989
14293	0.946129	0.106419
14294	0.94613	0.0900354
14295	0.94613	0.109936
14296	0.94613	0.107789
14297	0.946132	0.0698591
14298	0.946128	0.0705762
14299	0.94613	0.0698591
14300	0.946131	0.0698591
14301	0.94613	0.0706796
14302	0.94613	0.0702739
14303	0.946131	0.0698591
14304	0.94612	0.0707489
14305	0.946119	0.088399
14306	0.946119	0.0726617
14307	0.946124	0.0724912
14308	0.946119	0.0702258
14309	0.946132	0.0766013
14310	0.946107	0.0733225
14311	0.946128	2.1
14312	0.946133	2.1
14313	0.946131	2.1
14314	0.94613	2.1
14315	0.946131	2.1
14316	1.37869	2.1
14317	0.946131	2.1
14318	1.35703	2.1
14319	0.946132	2.1
14320	0.946132	2.1
14321	0.946132	2.1
14322	0.946132	2.1
14323	0.946132	2.1

14324	0.946133	2.1
14325	0.946133	2.1
14326	0.946133	2.1
14327	0.946132	2.1
14328	1.37017	2.1
14329	0.946133	2.1
14330	0.946132	2.1
14331	0.946133	2.1
14332	0.946114	0.0698591
14333	0.946119	0.0698591
14334	0.946119	0.0698591
14335	0.94612	0.0698591
14336	0.946119	0.0698591
14337	0.946132	0.0698591
14338	0.946107	0.0698591
14339	0.946131	0.0698591
14340	0.946131	0.0698591
14341	0.946126	0.0717533
14342	0.946127	0.0703167
14343	0.94613	0.0698591
14344	0.946129	0.07005
14345	0.946128	0.0698591
14346	0.946131	1.10025
14347	0.946131	0.893485
14348	0.946132	0.678735
14349	0.946132	0.965114
14350	0.94613	1.54981
14351	0.946131	1.34882
14352	1.3279	1.27455
14353	0.946132	2.1
14354	1.40932	2.1
14355	0.946132	2.1
14356	0.946132	2.1
14357	0.946132	2.1
14358	0.946131	2.1
14359	0.946132	2.1
14360	0.946131	0.0998181
14361	0.946131	0.104349
14362	0.946132	0.0889174
14363	0.946132	0.10608
14364	0.94613	0.142908
14365	0.946131	0.11337
14366	1.26868	0.103161
14367	0.810943	2.1
14368	0.810945	2.1
14369	0.810932	2.1
14370	2.82321	2.1
14371	1.3517	2.1
14372	1.08132	2.1
14373	1.08132	2.1
14374	1.08133	2.1
14375	0.810943	1.08456
14376	0.810944	0.545659
14377	2.63079	1.19886
14378	1.35168	0.86986
14379	1.0813	0.85367
14380	1.08132	0.45259
14381	1.08132	2.0058

14382	0.810957	1.05125
14383	1.17856	0.108078
14384	0.946132	0.769034
14385	0.946128	0.467619
14386	1.3154	2.00996
14387	0.94613	0.816847
14388	1.31539	1.07158
14389	0.94613	1.4083
14390	0.94613	1.76839
14391	0.94613	1.65804
14392	0.946131	1.3421
14393	0.946131	1.71969
14394	0.946129	1.8608
14395	0.94613	1.96359
14396	1.21997	1.90415
14397	0.946131	1.55163
14398	1.25083	1.39901
14399	0.946132	1.31403
14400	0.946132	1.60424
14401	0.946129	1.23582
14402	1.25083	1.72442
14403	0.946131	1.75086
14404	0.946127	1.68503
14405	1.41342	0.405584
14406	0.94613	1.32829
14407	0.946131	1.41944
14408	0.946127	0.445846
14409	0.946129	0.817696
14410	0.946129	0.639166
14411	0.946131	1.93519
14412	1.29149	1.58795
14413	0.946133	2.1
14414	0.946133	0.778928
14415	0.946128	2.00127
14416	0.946131	0.484205
14417	0.94613	0.961193
14418	0.94613	2.1
14419	0.94613	2.09848
14420	0.946132	2.1
14421	0.946132	2.09948
14422	0.946128	2.04348
14423	0.94613	2.1
14424	1.41022	2.1
14425	0.946132	0.0936257
14426	0.946132	0.0855127
14427	0.946128	0.0884462
14428	0.94613	0.0793164
14429	0.94613	0.11128
14430	0.94613	0.0742957
14431	0.946131	0.14079
14432	0.946129	0.699597
14433	1.37758	1.63573
14434	0.946131	2.08834
14435	0.94613	0.597976
14436	0.946124	1.52475
14437	0.946127	0.768161
14438	0.946127	1.36038
14439	0.946129	0.716206

14440	1.38259	1.41265
14441	0.946132	1.853
14442	0.946132	0.743141
14443	0.946126	1.50156
14444	0.946129	0.988596
14445	0.94613	1.17976
14446	0.946131	0.0738352
14447	0.946132	0.0698591
14448	0.946132	0.0851066
14449	0.946132	0.0698591
14450	0.946132	0.0911485
14451	0.946132	0.0738652
14452	0.946132	0.0698591
14453	0.946131	0.0698591
14454	0.94613	0.0698591
14455	0.94613	0.0698591
14456	0.946133	0.0698591
14457	0.946128	0.0698591
14458	1.25017	0.0698591
14459	0.946131	0.0698591
14460	0.946131	2.1
14461	0.946131	2.1
14462	0.946131	2.1
14463	1.3937	2.1
14464	0.946133	2.1
14465	0.946131	2.1
14466	0.94613	2.1
14467	0.946131	1.95939
14468	0.946131	0.717149
14469	0.946131	0.894116
14470	0.94613	0.426492
14471	0.946133	2.00115
14472	0.946132	1.16234
14473	0.946131	1.01462
14474	0.946132	1.04908
14475	0.946132	1.04591
14476	0.946132	0.714893
14477	0.946132	0.942791
14478	0.946131	0.347236
14479	0.946132	0.260317
14480	0.946132	0.28961
14481	0.946129	1.41461
14482	0.94613	2.1
14483	0.94613	2.01403
14484	0.946128	2.1
14485	0.946132	0.490023
14486	0.946131	1.67087
14487	0.94613	1.81331
14488	0.946132	2.1
14489	0.946132	2.1
14490	0.946132	2.1
14491	1.41619	2.1
14492	0.946133	1.95582
14493	0.946133	2.1
14494	0.946132	2.1
14495	0.946132	2.1
14496	0.946132	2.1
14497	0.946132	2.1

14498	1.38244	2.1
14499	0.946132	2.1
14500	0.946131	2.1
14501	0.946132	2.1
14502	0.810946	0.741721
14503	0.810943	1.36581
14504	0.810934	0.698384
14505	2.42741	0.363653
14506	1.35171	0.504646
14507	1.08132	0.615789
14508	1.08132	0.710484
14509	1.08133	0.742292
14510	0.810947	0.65231
14511	0.810943	1.35127
14512	2.44179	0.370319
14513	1.35171	0.537044
14514	1.08132	0.56296
14515	1.08132	0.564041
14516	1.08133	0.638513
14517	0.810934	0.599032
14518	0.946132	1.87658
14519	0.946132	1.79693
14520	0.946133	1.31815
14521	0.946133	1.77429
14522	0.946132	1.98262
14523	0.946132	1.80214
14524	1.12786	1.72129
14525	1.27806	0.907612
14526	0.946132	1.56538
14527	0.946133	1.10636
14528	0.946133	1.63437
14529	0.946132	1.88953
14530	0.946132	1.46192
14531	0.946133	1.78452
14532	0.946132	2.1
14533	0.946132	2.1
14534	0.946132	2.1
14535	1.37098	2.1
14536	0.946133	2.1
14537	0.946132	2.1
14538	1.37098	2.1
14539	0.946125	1.19684
14540	0.946129	0.785479
14541	0.946131	1.99115
14542	0.94613	0.4524
14543	0.946124	0.400188
14544	0.946127	0.591471
14545	1.39514	1.18696
14546	0.946132	2.1
14547	0.946131	2.1
14548	0.94613	2.1
14549	1.39909	2.1
14550	0.946132	2.1
14551	0.946131	2.1
14552	0.94613	2.1
14553	0.946133	2.1
14554	0.946133	1.76321
14555	0.946132	2.1

14556	1.36835	2.1
14557	0.946132	1.89047
14558	0.946133	2.1
14559	0.946132	2.05442
14560	0.946132	2.1
14561	0.946133	2.1
14562	0.946133	2.1
14563	0.946132	2.1
14564	1.39047	2.1
14565	0.946132	2.1
14566	0.946132	2.1
14567	1.33174	0.0698591
14568	0.946128	0.0698591
14569	0.94613	0.0698591
14570	0.94613	0.0698591
14571	0.94613	0.0698591
14572	0.94613	0.0698591
14573	0.946131	0.0698591
14574	0.946128	0.0698591
14575	1.36004	0.0698591
14576	0.946129	0.0698591
14577	0.94613	0.0698591
14578	0.94613	0.0698591
14579	1.36003	0.0698591
14580	0.94613	0.0698591
14581	0.946124	2.1
14582	0.946119	2.1
14583	0.946119	2.1
14584	0.946119	2.1
14585	0.94612	2.1
14586	0.946132	2.1
14587	1.29062	2.1
14588	0.946128	2.1
14589	0.946128	2.1
14590	0.946133	2.1
14591	0.94613	2.1
14592	0.946131	2.1
14593	0.946131	2.1
14594	1.30584	2.1
14595	0.946131	2.1
14596	0.946131	2.1
14597	0.946131	2.1
14598	0.946132	2.1
14599	1.39549	2.1
14600	0.946131	2.1
14601	0.946131	2.1
14602	0.913386	0.942199
14603	1.17466	0.741152
14604	1.00473	0.678144
14605	0.946132	0.82489
14606	0.946126	1.13417
14607	0.94613	0.953173
14608	0.946129	0.915333
14609	0.946132	0.370674
14610	1.38894	0.372607
14611	0.946132	0.279311
14612	0.946132	0.310849
14613	0.946132	1.02991

14614	0.946132	0.400379
14615	0.946133	0.397811
14616	0.946128	0.335252
14617	1.23865	0.328011
14618	0.946129	0.255344
14619	0.94613	0.27888
14620	0.94613	0.47912
14621	0.94613	0.320096
14622	0.94613	0.35551
14623	0.946126	0.782402
14624	1.40248	0.748764
14625	0.946129	0.688281
14626	0.94613	0.837833
14627	0.946124	1.05032
14628	0.946127	0.956469
14629	0.946128	0.809967
14630	0.946133	0.364162
14631	1.1391	0.463169
14632	0.946132	0.39586
14633	0.946132	0.335525
14634	0.946132	1.11717
14635	0.946133	0.451119
14636	0.946132	0.433449
14637	1.17156	1.19945
14638	0.946133	1.17401
14639	1.33893	1.04059
14640	0.827864	1.02385
14641	0.995232	1.08798
14642	0.827864	1.1609
14643	0.94613	1.08071
14644	1.21387	0.435792
14645	0.946132	0.416659
14646	0.946131	0.212635
14647	0.94613	0.304741
14648	0.94613	0.276404
14649	0.94613	0.321574
14650	0.94613	0.309892
14651	0.810943	0.132489
14652	0.810945	0.0830712
14653	2.42463	0.0862631
14654	1.3517	0.0889898
14655	1.08132	0.113195
14656	1.08132	0.121077
14657	1.08133	0.0922311
14658	0.810932	0.126115
14659	0.946133	1.88898
14660	0.946132	1.413
14661	0.946131	1.35953
14662	1.36748	1.22881
14663	0.946133	1.61612
14664	0.946131	1.06851
14665	0.946132	0.318783
14666	1.35856	0.0698591
14667	0.946131	0.0698591
14668	0.94613	0.0698591
14669	0.94613	0.0698591
14670	0.946131	0.0698591
14671	0.94613	0.0698591

14672	0.94613	0.0698591
14673	0.946132	2.1
14674	0.946131	2.1
14675	0.946131	2.1
14676	0.946131	2.1
14677	1.41149	2.1
14678	1.41149	2.1
14679	0.946131	2.1
14680	0.946128	2.1
14681	0.946128	2.1
14682	0.946133	2.1
14683	0.946131	2.1
14684	0.946131	2.1
14685	0.946131	2.1
14686	0.94613	2.1
14687	1.41388	0.0910231
14688	0.94613	0.130847
14689	0.946132	0.0889683
14690	0.946132	0.117485
14691	0.946129	0.151109
14692	0.946131	0.114991
14693	0.946131	0.126211
14694	1.02798	0.0730838
14695	0.946132	0.16188
14696	1.02798	0.0750135
14697	0.946133	0.141938
14698	1.14931	0.0714504
14699	0.957746	0.135255
14700	0.93452	0.106119
14701	0.946129	2.1
14702	0.946134	2.1
14703	0.946133	2.1
14704	0.946131	2.1
14705	0.946131	2.1
14706	0.946131	2.1
14707	0.946131	2.1
14708	0.946133	2.07133
14709	0.946132	1.62459
14710	0.946132	1.90429
14711	0.946132	2.05417
14712	1.38773	1.74687
14713	0.946132	1.98343
14714	0.946133	1.8461
14715	0.946131	0.168694
14716	0.946131	0.168915
14717	1.41512	0.152908
14718	0.946133	0.233496
14719	0.946131	0.223498
14720	0.946131	0.203685
14721	0.946131	0.1741
14722	1.35381	1.11037
14723	0.946132	2.1
14724	1.35381	1.13261
14725	0.946133	2.1
14726	0.946132	1.17792
14727	0.946132	2.1
14728	0.946133	2.08918
14729	1.3365	1.16733

14730	0.946132	1.96756
14731	1.3365	1.05055
14732	0.946129	1.80224
14733	0.94613	1.23596
14734	0.946128	1.85372
14735	0.946129	1.61294
14736	0.939895	1.9556
14737	1.14285	2.09296
14738	0.952366	2.09391
14739	0.946131	2.03484
14740	0.94613	2.09887
14741	0.946131	2.06576
14742	0.94613	2.06854
14743	0.946131	2.1
14744	0.946131	2.1
14745	0.946131	2.1
14746	0.946129	2.1
14747	0.946133	2.1
14748	0.946131	2.1
14749	0.946131	2.1
14750	0.946133	2.1
14751	0.946132	2.1
14752	0.946132	2.1
14753	0.946133	2.1
14754	0.946132	2.1
14755	0.946132	2.1
14756	0.946133	2.1
14757	0.946133	2.09784
14758	0.946132	1.72626
14759	0.946132	2.08539
14760	0.946133	2.02392
14761	0.946132	2.1
14762	0.946132	2.1
14763	0.946132	1.75748
14764	0.946132	2.1
14765	0.946131	2.1
14766	0.946131	2.1
14767	0.946131	2.1
14768	1.38969	2.1
14769	0.946131	2.1
14770	0.946132	2.1
14771	0.946128	2.05469
14772	0.946133	1.83888
14773	0.946132	1.89585
14774	0.946131	1.85215
14775	0.94613	1.9451
14776	0.946131	1.96772
14777	1.28464	1.96732
14778	0.946119	0.448356
14779	1.37492	0.595544
14780	0.946125	0.959578
14781	0.946119	0.304939
14782	0.946119	1.2048
14783	0.946132	0.421294
14784	0.946107	0.642776
14785	0.810942	2.1
14786	0.810943	2.1
14787	0.709587	2.1

14788	2.64488	2.1
14789	1.21651	2.1
14790	0.946151	2.1
14791	0.979948	2.1
14792	1.0813	2.1
14793	0.946133	1.76113
14794	0.946132	2.1
14795	0.946132	2.1
14796	0.946133	0.585686
14797	0.946132	0.37643
14798	0.946132	0.651965
14799	1.41134	1.46233
14800	0.946133	1.984
14801	0.946132	1.61432
14802	0.946133	2.0303
14803	0.946133	1.44719
14804	0.946132	1.13921
14805	0.946133	1.70153
14806	1.31338	1.81788
14807	2.07612	0.380754
14808	0.946102	0.0698591
14809	0.752448	0.0698591
14810	1.20617	0.0698591
14811	0.94613	0.0698591
14812	0.75242	0.0698591
14813	0.946131	0.0698591
14814	0.752419	0.0698591
14815	0.946132	1.03162
14816	1.25736	0.911495
14817	1.25736	0.915418
14818	0.946132	0.868716
14819	1.08054	0.787392
14820	1.25736	0.897108
14821	0.946132	0.961413
14822	0.946128	2.1
14823	1.1896	2.1
14824	1.18959	2.1
14825	0.946131	2.0921
14826	1.33271	2.1
14827	0.946105	2.1
14828	0.946156	2.1
14829	0.741946	0.0698591
14830	1.33587	0.0698591
14831	0.741947	0.0698591
14832	1.21954	0.0698591
14833	0.94613	0.0698591
14834	0.741946	0.0698591
14835	1.81347	0.0698591
14836	1.81347	0.0698591
14837	0.653322	0.0698591
14838	1.36935	0.0698591
14839	0.661882	0.0698591
14840	1.04753	0.0698591
14841	0.507464	0.0698591
14842	0.945844	1.02772
14843	1.59507	2.1
14844	1.04043	1.07204
14845	0.946131	2.1

14846	1.32891	2.04964
14847	0.946131	1.92667
14848	0.946131	1.65899
14849	1.28866	1.79827
14850	1.12682	0.0955201
14851	0.851543	0.128825
14852	1.04072	0.0945237
14853	0.946132	0.1715
14854	1.13437	0.0861028
14855	1.04167	0.0897037
14856	0.936652	0.0943526
14857	1.258	2.1
14858	0.946131	2.1
14859	0.946132	2.1
14860	1.36968	2.1
14861	0.946132	2.1
14862	1.36968	2.1
14863	0.946131	2.1
14864	2.30046	0.169704
14865	2.82969	0.19179
14866	0.946132	2.08224
14867	1.35022	2.1
14868	1.12356	2.06735
14869	0.94613	2.1
14870	1.35022	2.1
14871	0.946131	2.1
14872	0.94613	2.1
14873	0.946131	2.1
14874	1.22401	2.1
14875	0.952456	2.1
14876	0.94613	2.1
14877	1.22401	2.1
14878	0.946131	2.1
14879	0.946131	2.1
14880	1.33047	2.1
14881	0.946132	2.1
14882	1.33047	2.1
14883	0.946131	2.1
14884	1.33047	2.1
14885	0.946132	2.1
14886	1.2107	2.1
14887	0.61957	2.05758
14888	1.80666	2.1
14889	1.53743	2.1
14890	0.619572	2.00155
14891	1.32547	2.1
14892	1.16166	2.0975
14893	1.04074	2.09911
14894	0.557621	2.09781
14895	0.946128	2.1
14896	1.36607	2.1
14897	0.94613	2.1
14898	1.36607	2.1
14899	1.39416	2.1
14900	0.94613	2.1
14901	0.94613	2.1
14902	1.23969	2.1
14903	0.946131	2.1

14904	0.946131	2.1
14905	1.13537	2.1
14906	1.05719	2.1
14907	0.806848	2.1
14908	1.30251	2.1
14909	0.851526	1.57086
14910	1.14176	2.1
14911	0.93666	1.49947
14912	0.946114	2.1
14913	1.15832	2.07865
14914	0.924624	2.07369
14915	0.967609	1.94117
14916	0.771764	0.119026
14917	1.26652	0.139229
14918	0.946133	0.141715
14919	1.17957	0.0803051
14920	0.771735	0.108701
14921	0.946122	0.144319
14922	0.946133	0.121561
14923	0.924325	1.47371
14924	0.656739	1.81141
14925	0.637876	1.88935
14926	0.530092	1.64058
14927	0.914415	1.53221
14928	0.821311	1.91775
14929	0.630589	1.86471
14930	0.738823	1.80924
14931	0.839109	1.85936
14932	1.55029	0.0698591
14933	1.13537	1.80056
14934	0.946131	2.09238
14935	0.946128	1.98183
14936	1.30162	2.1
14937	0.952476	2.1
14938	1.29289	2.1
14939	0.939783	2.1
14940	0.94612	2.1
14941	0.946131	2.1
14942	1.07998	2.1
14943	0.836985	2.1
14944	1.1604	2.1
14945	0.94613	2.1
14946	0.837016	2.1
14947	0.712461	0.584611
14948	1.36399	0.619066
14949	1.8724	0.426044
14950	1.8724	0.299668
14951	0.623426	1.18351
14952	1.36399	0.542094
14953	1.22089	0.563114
14954	0.946132	0.562402
14955	0.946131	2.1
14956	1.32241	2.1
14957	1.32241	2.1
14958	0.946133	2.1
14959	1.40271	2.1
14960	0.946131	2.1
14961	0.946131	2.1

14962	1.1989	1.2857
14963	0.954106	1.68261
14964	0.820068	1.28461
14965	0.563139	1.58329
14966	1.06448	1.55914
14967	0.892472	1.69136
14968	0.862598	1.32346
14969	0.745851	1.74198
14970	1.13336	1.57457
14971	0.946129	2.1
14972	1.28254	2.1
14973	1.28254	2.1
14974	0.946131	2.1
14975	1.41302	2.1
14976	0.946131	2.1
14977	0.946131	2.1
14978	0.851543	2.02445
14979	1.21961	2.1
14980	0.851484	1.9804
14981	0.851543	2.1
14982	1.15705	2.1
14983	0.946133	2.1
14984	0.909417	2.07727
14985	1.13808	0.918141
14986	0.946133	0.203916
14987	0.946131	1.02324
14988	1.30194	0.186354
14989	0.946131	0.331316
14990	1.30194	0.126744
14991	0.946131	0.183321
14992	0.930941	1.09631
14993	1.20185	0.860886
14994	1.02404	0.824825
14995	0.946124	1.31166
14996	1.3563	0.937803
14997	0.946127	1.33136
14998	0.946128	1.09101
14999	0.946116	2.1
15000	1.3144	2.1
15001	1.13088	2.1
15002	0.946118	2.09303
15003	1.31438	2.1
15004	0.946105	2.1
15005	0.946131	2.1
15006	1.76972	0.0801789
15007	1.42091	0.0699749
15008	0.946128	0.080319
15009	0.946101	0.12158
15010	0.863552	0.112227
15011	0.94613	0.071985
15012	1.21232	0.0886643
15013	0.94613	0.128912
15014	1.2137	0.419654
15015	1.23224	0.842861
15016	1.86512	0.560298
15017	1.86512	0.321074
15018	0.580825	1.507
15019	1.2137	0.931374

15020	0.946133	0.910263
15021	0.580822	0.807155
15022	0.946128	2.1
15023	1.40586	2.1
15024	0.94613	2.1
15025	1.3719	2.1
15026	1.3719	2.1
15027	1.40586	2.1
15028	0.94613	2.1
15029	0.946132	2.09382
15030	1.35025	1.86146
15031	1.1794	2.07251
15032	0.946132	0.582423
15033	1.35025	1.53184
15034	0.946131	1.53677
15035	0.946132	1.44243
15036	0.946133	2.1
15037	1.60149	2.1
15038	1.67058	2.1
15039	0.851542	2.1
15040	1.20296	2.1
15041	1.17501	2.1
15042	0.946132	2.1
15043	1.04993	2.1
15044	0.851541	2.1
15045	1.48219	2.1
15046	0.874565	2.09993
15047	0.946129	2.1
15048	1.22049	2.1
15049	0.946131	2.1
15050	0.946131	2.1
15051	0.94613	2.07641
15052	0.848719	1.99882
15053	1.17195	2.02759
15054	0.946129	0.751158
15055	1.35426	1.49266
15056	0.94613	1.70815
15057	0.94613	1.75259
15058	0.946133	2.1
15059	1.14342	2.1
15060	1.14342	2.1
15061	0.946133	2.1
15062	1.39009	2.1
15063	0.946132	2.1
15064	0.946132	2.1
15065	1.38819	2.1
15066	0.946133	2.1
15067	1.38819	2.1
15068	0.946131	2.1
15069	1.4116	2.1
15070	0.946131	2.1
15071	1.38819	2.1
15072	0.946127	2.1
15073	1.32244	2.1
15074	1.2176	2.1
15075	0.946131	2.1
15076	1.32244	2.1
15077	0.94613	2.1

15078	0.946129	2.1
15079	1.32415	2.1
15080	1.11239	2.1
15081	0.984099	2.1
15082	1.65532	2.1
15083	1.53082	2.1
15084	1.32068	2.1
15085	1.32068	2.1
15086	1.08172	2.1
15087	3.76293	0.0698591
15088	1.36335	0.0698591
15089	0.953925	2.1
15090	0.989497	2.1
15091	0.98949	2.1
15092	1.06341	2.1
15093	1.27875	2.1
15094	1.04598	2.1
15095	0.603154	2.1
15096	0.582457	2.1
15097	0.582457	2.1
15098	0.568994	2.1
15099	1.5791	2.1
15100	1.09506	2.1
15101	1.45327	2.1
15102	1.88406	2.1
15103	1.88404	2.1
15104	1.27389	2.08769
15105	1.14206	2.1
15106	1.00232	2.1
15107	1.00231	2.1
15108	1.68436	2.1
15109	1.4677	0.0698591
15110	1.28828	0.0698591
15111	1.28828	0.0698591
15112	1.46932	0.0698591
15113	1.28228	2.1
15114	1.64268	2.09735
15115	0.959473	2.1
15116	1.10101	2.1
15117	1.07976	2.1
15118	1.2873	2.1
15119	1.70303	0.168749
15120	1.40066	0.352149
15121	1.5563	0.125651
15122	1.13727	0.352242
15123	1.03529	2.1
15124	1.11285	2.1
15125	1.03529	2.1
15126	1.28667	2.1
15127	1.32119	2.1
15128	1.44777	2.1
15129	1.44778	2.1
15130	1.61121	2.1
15131	1.09372	0.0698591
15132	1.17664	0.0698591
15133	1.17664	0.0702289
15134	0.872931	0.0699448
15135	1.02415	2.1

15136	1.28228	2.1
15137	2.14692	2.1
15138	3.90469	2.1
15139	1.00509	2.1
15140	1.28228	2.1
15141	3.32936	0.0698591
15142	1.1333	0.0698591
15143	1.11048	2.1
15144	0.979854	2.1
15145	1.65815	2.1
15146	1.00185	2.1
15147	1.84887	2.1
15148	1.59597	2.1
15149	1.78468	2.1
15150	1.87508	2.1
15151	1.82716	2.1
15152	1.33429	2.1
15153	1.34626	2.1
15154	0.797142	2.1
15155	1.00007	1.85988
15156	1.02051	2.1
15157	1.02051	2.04442
15158	1.03373	2.1
15159	1.05786	2.1
15160	0.887631	2.1
15161	0.992773	2.1
15162	1.28462	2.1
15163	2.35274	1.60067
15164	1.82533	0.656164
15165	1.82533	1.3024
15166	1.19496	0.516208
15167	1.28597	0.0698591
15168	1.09725	2.1
15169	1.60948	2.1
15170	1.20421	2.1
15171	1.16566	2.1
15172	1.88522	2.1
15173	1.81948	0.154285
15174	1.14042	0.130247
15175	1.21558	0.253163
15176	1.56876	0.188116
15177	1.6697	2.1
15178	1.81655	2.1
15179	1.81655	2.1
15180	1.5956	2.1
15181	0.906059	2.1
15182	1.63045	2.1
15183	0.9533	1.56562
15184	1.1475	1.11334
15185	1.72231	2.09944
15186	1.94936	2.07218
15187	1.06826	2.1
15188	1.08576	2.1
15189	1.06826	2.1
15190	0.973559	2.1
15191	1.62737	0.0698591
15192	0.965676	0.0698591
15193	1.45055	0.0698591

15194	1.17631	0.0698591
15195	1.92535	0.0698591
15196	1.9503	0.0698591
15197	1.99828	0.0698591
15198	1.70302	0.0698591
15199	1.65139	0.0698591
15200	1.26784	0.0705009
15201	1.35923	0.0744432
15202	1.70302	0.0785837
15203	1.33033	2.1
15204	1.12481	2.1
15205	1.29452	2.1
15206	1.25399	2.1
15207	1.12481	2.1
15208	0.918358	2.1
15209	1.39332	2.1
15210	1.32368	2.1
15211	1.11712	1.44121
15212	1.07999	0.808263
15213	1.07999	0.936629
15214	1.05619	0.53171
15215	1.1766	2.1
15216	1.35735	2.1
15217	1.42456	0.0698591
15218	1.089	0.0698591
15219	1.11081	0.0698591
15220	2.88521	0.0698591
15221	1.09754	0.0698591
15222	1.85479	0.0698591
15223	1.27922	0.642748
15224	1.03556	0.372174
15225	1.07508	2.02451
15226	1.09795	2.08615
15227	0.967672	1.3622
15228	1.07508	1.89057
15229	1.03933	2.0789
15230	1.02975	2.1
15231	1.02975	2.1
15232	1.01121	2.1
15233	1.24526	0.0698591
15234	1.72368	0.0698591
15235	1.89607	0.0698591
15236	2.13512	0.0698591
15237	1.34591	0.305118
15238	1.14721	0.344875
15239	1.15539	2.1
15240	1.90938	2.06123
15241	1.90941	2.1
15242	1.89242	2.1
15243	1.18907	2.1
15244	1.20408	2.1
15245	1.20409	2.1
15246	1.22655	2.1
15247	0.701447	2.1
15248	1.57567	2.1
15249	1.38806	2.1
15250	2.09407	2.1
15251	1.07612	1.36263

15252	0.990019	1.25124
15253	1.15564	2.08741
15254	1.01243	1.97431
15255	1.28956	0.0721046
15256	1.35146	0.0756419
15257	1.35145	0.0792453
15258	1.22811	0.0885664
15259	1.09662	2.1
15260	1.42486	2.1
15261	1.10106	0.0698591
15262	1.90814	0.0698591
15263	1.3946	0.0698591
15264	1.11926	0.0698591
15265	0.725839	2.1
15266	0.925068	2.1
15267	0.925074	2.1
15268	1.43282	2.1
15269	1.91756	0.894558
15270	1.74321	0.988345
15271	1.74321	0.687114
15272	1.63413	0.763156
15273	1.9194	0.0698591
15274	1.62246	0.0698591
15275	1.90456	0.0698591
15276	1.06578	0.0698591
15277	1.46019	0.0698591
15278	1.17036	0.0698591
15279	1.70233	1.01215
15280	1.53515	0.801817
15281	2.03494	2.1
15282	1.78441	2.1
15283	1.75992	2.1
15284	1.94351	2.1
15285	1.51057	0.0698591
15286	0.991435	0.0698591
15287	1.98068	0.0698591
15288	1.64283	0.0698591
15289	2.96126	0.0698591
15290	2.20692	0.0698591
15291	1.95054	0.0698591
15292	1.21325	0.0698591
15293	0.996828	0.247772
15294	1.01976	0.21362
15295	1.17129	2.1
15296	1.13664	2.1
15297	1.13664	2.1
15298	1.09627	2.1
15299	2.65123	2.1
15300	3.23062	2.1
15301	3.23062	2.1
15302	3.92038	2.1
15303	1.89019	0.246443
15304	1.79109	0.284007
15305	1.79109	0.209144
15306	1.54197	0.219349
15307	3.41824	2.1
15308	1.93553	0.930698
15309	2.26215	2.1

15310	2.0103	0.780297
15311	1.51569	0.970568
15312	1.01683	0.829176
15313	1.78595	2.1
15314	1.66537	2.1
15315	3.73426	2.1
15316	3.53905	2.1
15317	1.66537	2.1
15318	1.52903	2.1
15319	3.53905	2.1
15320	3.3837	2.1
15321	1.19901	1.35437
15322	1.35002	1.92009
15323	1.35002	0.124849
15324	1.11683	0.217058
15325	1.75326	1.19574
15326	0.712404	0.173942
15327	2.97768	2.1
15328	2.98949	2.1
15329	1.2832	2.1
15330	1.39518	2.1
15331	2.98949	2.1
15332	2.99339	2.1
15333	1.39517	2.1
15334	1.46124	2.1
15335	1.28534	2.1
15336	1.03391	2.1
15337	1.75366	1.6675
15338	1.26247	1.92633
15339	1.13621	2.03793
15340	2.43994	2.1
15341	1.47904	0.0698591
15342	1.80085	0.0862346
15343	1.80085	0.0698606
15344	1.64656	0.134495
15345	2.87331	2.1
15346	2.82863	2.1
15347	2.82859	2.1
15348	2.84673	2.1
15349	1.0961	0.0776803
15350	1.39448	0.180068
15351	1.21366	0.205216
15352	1.20143	0.105416
15353	1.85795	0.0698591
15354	1.36768	0.0698591
15355	1.36769	0.0698591
15356	1.45789	0.0698591
15357	1.32605	0.0698591
15358	1.27148	0.0698591
15359	1.70218	0.0698591
15360	1.3063	0.0728727
15361	1.45603	0.0699906
15362	1.89226	0.0886359
15363	1.2742	0.0698591
15364	2.10112	0.0698591
15365	1.10748	0.0698591
15366	1.1893	0.0701582
15367	1.04921	2.1

15368	1.22927	2.1
15369	1.22927	2.1
15370	1.46568	2.1
15371	0.811186	2.1
15372	0.792162	2.1
15373	0.792162	2.1
15374	0.758749	2.1
15375	1.03581	2.1
15376	0.737481	2.1
15377	2.24071	2.1
15378	1.34868	2.1
15379	1.47203	0.796622
15380	1.40967	0.814889
15381	1.40967	1.0001
15382	1.27916	0.880518
15383	1.17418	2.1
15384	1.04921	2.1
15385	1.28291	0.698295
15386	1.3213	0.474476
15387	1.46812	0.510886
15388	2.42522	0.801907
15389	1.17879	0.918082
15390	1.28291	0.961812
15391	1.28291	1.0494
15392	1.13438	0.918593
15393	1.75004	2.1
15394	1.78247	2.1
15395	1.78247	2.1
15396	1.76421	2.1
15397	2.25975	0.0698591
15398	3.66111	0.0698591
15399	3.6611	0.0698591
15400	3.44043	0.0698591
15401	1.13339	2.1
15402	1.13322	2.1
15403	1.13323	2.1
15404	1.20761	2.1
15405	1.34337	0.132228
15406	1.90912	0.347182
15407	1.04399	0.138661
15408	2.00578	0.408655
15409	1.04973	2.1
15410	1.22615	2.1
15411	1.93384	2.1
15412	2.62816	1.85971
15413	1.13127	2.1
15414	1.47642	2.1
15415	2.62816	2.1
15416	3.31755	1.58269
15417	1.47642	2.1
15418	1.80454	2.1
15419	0.964782	2.1
15420	0.949135	2.1
15421	0.949134	2.1
15422	0.949134	2.1
15423	1.10476	1.55225
15424	0.982858	0.230571
15425	1.10938	1.11924

15426	0.982858	1.14354
15427	1.01012	0.0698591
15428	0.974081	0.0698591
15429	1.82643	0.0698591
15430	1.29585	0.0698591
15431	1.00763	0.590514
15432	1.3294	0.758673
15433	1.3294	0.288112
15434	1.30001	1.01113
15435	1.59151	2.1
15436	1.99443	2.1
15437	1.07369	2.1
15438	1.30584	2.1
15439	1.05071	2.1
15440	0.948676	2.1
15441	1.13842	2.1
15442	1.42884	2.1
15443	1.59019	0.0698591
15444	1.38212	0.0698591
15445	1.34102	0.0698591
15446	1.02663	0.0698591
15447	0.797011	2.1
15448	0.81301	2.1
15449	0.81301	2.1
15450	0.811187	2.1
15451	0.991869	0.0955593
15452	1.23868	0.0968405
15453	1.67201	2.1
15454	1.61201	2.1
15455	1.61202	2.1
15456	1.57627	2.1
15457	0.94994	2.1
15458	1.77867	2.1
15459	0.854945	2.1
15460	0.973905	2.1
15461	1.64193	2.1
15462	1.68893	2.1
15463	1.68893	2.1
15464	1.70002	2.1
15465	2.08149	0.220391
15466	1.69456	0.149259
15467	1.67399	0.0698591
15468	1.83922	2.1
15469	1.91703	2.1
15470	0.967492	2.1
15471	1.13317	2.1
15472	0.980766	2.1
15473	2.06158	0.0698591
15474	0.986498	0.0698591
15475	1.76289	0.0698591
15476	1.17248	0.0698591
15477	1.51794	0.0698591
15478	1.30266	0.0698591
15479	1.03165	0.170065
15480	0.947692	0.197646
15481	1.20473	0.136254
15482	0.947693	0.138219
15483	1.54357	2.1

15484	0.980841	2.1
15485	1.34124	0.140364
15486	1.39818	0.380459
15487	1.39818	0.130982
15488	1.48035	0.520257
15489	1.03932	2.1
15490	1.2065	2.1
15491	1.20651	2.1
15492	1.12717	2.1
15493	1.09483	2.1
15494	0.994293	2.1
15495	0.994293	2.1
15496	0.97356	2.1
15497	0.995252	0.751484
15498	1.66294	0.78865
15499	1.82775	0.458099
15500	1.42762	0.483218
15501	1.04713	0.0872752
15502	1.05475	0.0971522
15503	1.29002	0.0698591
15504	1.05475	0.0698591
15505	1.17585	2.1
15506	1.39223	2.1
15507	1.39223	2.1
15508	1.71255	2.1
15509	1.56166	0.596242
15510	1.37762	0.424524
15511	1.16858	2.1
15512	1.0361	2.1
15513	1.23966	0.0698591
15514	1.10718	0.0698591
15515	1.99957	0.0698591
15516	0.658392	0.0698591
15517	1.36904	0.0698591
15518	1.12524	0.0698591
15519	1.04321	1.67916
15520	1.04627	1.66183
15521	1.91351	1.34142
15522	1.63379	1.07906
15523	1.79718	1.24242
15524	0.59634	1.07344
15525	0.958257	2.1
15526	1.06457	2.1
15527	0.958255	2.1
15528	1.03933	2.1
15529	1.14195	2.1
15530	1.49438	2.09842
15531	1.49438	2.1
15532	1.31259	2.1
15533	1.23036	2.1
15534	1.2016	2.1
15535	1.2016	2.1
15536	1.17129	2.1
15537	1.05724	2.09691
15538	1.04291	2.1
15539	1.12341	2.1
15540	2.32525	2.1
15541	3.39038	2.1

15542	2.09374	2.1
15543	1.76185	0.291979
15544	1.7934	0.298718
15545	1.65809	0.296522
15546	1.54852	0.303918
15547	1.38766	2.1
15548	1.28765	2.1
15549	1.26599	2.1
15550	1.21397	2.1
15551	1.28765	2.1
15552	1.20045	2.1
15553	1.21398	2.1
15554	1.15234	2.1
15555	0.953634	0.322149
15556	1.39132	0.110725
15557	1.17285	2.1
15558	1.21408	2.1
15559	1.21408	2.1
15560	0.982848	2.1
15561	2.00101	2.1
15562	2.00973	2.1
15563	2.20314	2.1
15564	1.89318	2.1
15565	1.23267	2.1
15566	0.947921	2.1
15567	1.18872	2.1
15568	0.740752	2.1
15569	1.92269	0.139238
15570	1.53584	0.0856692
15571	1.53584	0.0954592
15572	1.21067	0.0707391
15573	1.40707	0.0720891
15574	1.50437	0.0739991
15575	1.50435	0.0711506
15576	1.45446	0.0735028
15577	1.47954	1.00228
15578	2.08687	1.69223
15579	2.08687	2.07545
15580	1.1233	2.08759
15581	1.04025	1.34975
15582	1.36005	2.00872
15583	1.47606	0.126849
15584	1.3407	0.144594
15585	0.758743	2.1
15586	0.716917	2.1
15587	0.716922	2.1
15588	0.672964	2.1
15589	2.40014	2.1
15590	1.96848	2.1
15591	1.96848	2.1
15592	1.53555	2.1
15593	1.06191	2.1
15594	1.29579	2.1
15595	1.29579	2.1
15596	1.71289	2.1
15597	1.78584	2.1
15598	2.15211	2.1
15599	2.00427	2.1

15600	3.41615	2.1
15601	1.66408	2.1
15602	1.73954	2.1
15603	2.91663	2.1
15604	3.65818	2.1
15605	1.93286	2.1
15606	1.08831	2.1
15607	1.31242	0.0698591
15608	1.11712	0.0698591
15609	1.26311	0.0698591
15610	1.31242	0.0698591
15611	1.10378	2.1
15612	0.968594	2.1
15613	1.48389	0.0698591
15614	1.49613	0.0698591
15615	1.04349	0.89882
15616	1.26409	0.572469
15617	1.26409	0.494669
15618	1.05724	1.42801
15619	1.65723	0.135167
15620	1.83116	0.115137
15621	2.06284	0.124954
15622	2.00512	0.111954
15623	1.83116	0.132977
15624	2.03139	0.104362
15625	2.20564	0.121831
15626	1.90048	0.101353
15627	0.963392	2.1
15628	1.01346	0.954932
15629	2.98092	2.1
15630	2.70295	2.1
15631	2.70295	2.1
15632	2.48828	2.1
15633	1.63661	2.1
15634	1.64158	2.1
15635	1.64158	2.1
15636	1.6488	2.1
15637	1.17506	0.127124
15638	1.09667	0.108306
15639	2.00539	0.342695
15640	1.09667	0.152572
15641	1.54946	2.1
15642	1.43577	2.1
15643	1.26103	2.1
15644	1.49808	2.1
15645	1.089	0.0698591
15646	1.22586	0.0698591
15647	2.35027	0.234017
15648	1.37549	0.14607
15649	3.33706	0.139678
15650	1.69894	0.118047
15651	2.00539	0.157379
15652	1.53798	0.116163
15653	1.28938	2.1
15654	1.20969	2.1
15655	1.20969	2.1
15656	1.15864	2.1
15657	1.7986	2.1

15658	1.75869	2.1
15659	1.58281	2.1
15660	1.7392	2.1
15661	1.30733	1.22132
15662	1.5419	1.54524
15663	1.50883	1.02292
15664	1.85439	0.991412
15665	1.73531	0.989764
15666	1.70225	0.910047
15667	1.22176	0.0698591
15668	1.59492	0.0929481
15669	1.59492	0.0780888
15670	2.15542	0.10506
15671	3.24379	2.1
15672	2.64775	2.1
15673	3.16707	2.1
15674	1.93032	2.1
15675	1.50738	2.1
15676	1.24128	2.1
15677	1.24128	2.1
15678	0.994293	2.1
15679	2.90808	0.0698591
15680	2.45797	0.0698591
15681	1.85501	0.0698591
15682	2.90809	0.0698591
15683	0.892835	1.99098
15684	1.55871	2.1
15685	1.04615	0.439808
15686	1.66643	1.26333
15687	1.70044	2.1
15688	1.80224	2.1
15689	1.70186	0.595635
15690	3.30123	1.78725
15691	1.74789	2.1
15692	1.77446	2.1
15693	1.77901	2.1
15694	1.57995	2.1
15695	1.81518	2.1
15696	2.17716	2.1
15697	2.17716	2.1
15698	2.65123	2.1
15699	2.01899	0.939216
15700	1.77479	0.854222
15701	1.19122	0.45274
15702	1.46724	0.381383
15703	1.70944	0.0698591
15704	1.08929	0.0698591
15705	1.68454	0.0698591
15706	1.08929	0.0698591
15707	1.51067	1.03735
15708	1.06729	0.677265
15709	1.37762	0.578086
15710	1.13992	0.418328
15711	1.26397	2.1
15712	1.04083	2.1
15713	1.00278	2.1
15714	1.04084	2.1
15715	1.1016	0.0698591

15716	1.23477	0.0698591
15717	1.23477	0.0698591
15718	1.64985	0.0698591
15719	1.11117	2.1
15720	1.13006	2.1
15721	1.13006	2.1
15722	1.14721	2.1
15723	1.21946	2.1
15724	1.0186	2.1
15725	1.13178	2.1
15726	1.00133	2.1
15727	1.67046	0.0724934
15728	0.986036	0.291263
15729	1.67046	0.251324
15730	1.34857	1.777
15731	2.46016	2.1
15732	2.07161	2.1
15733	2.10732	2.1
15734	2.08371	2.1
15735	1.35602	2.1
15736	0.829065	2.1
15737	1.81667	2.1
15738	0.99406	2.1
15739	1.81667	2.1
15740	1.68073	2.1
15741	2.05736	2.1
15742	2.97311	2.1
15743	2.14011	2.1
15744	1.23411	2.1
15745	1.13632	0.0698591
15746	1.40862	0.0698591
15747	1.28055	0.0698591
15748	1.14305	0.0698591
15749	1.12035	0.0698591
15750	1.15483	0.0698591
15751	1.94099	0.0698591
15752	1.82126	0.0698591
15753	1.15481	0.0698591
15754	0.998957	0.0698591
15755	2.10078	0.0698591
15756	1.81617	0.0698591
15757	1.27581	2.1
15758	0.968536	2.1
15759	1.33916	2.1
15760	1.27581	2.1
15761	1.32784	2.1
15762	1.4259	2.1
15763	1.70377	2.1
15764	1.54231	2.1
15765	3.77569	2.1
15766	3.42809	2.1
15767	1.54231	2.1
15768	1.51298	2.1
15769	3.55866	2.1
15770	2.86136	2.1
15771	1.83301	0.425794
15772	1.26371	0.362114
15773	1.98301	0.465701

15774	2.0609	0.40345
15775	2.26047	0.239853
15776	2.00537	0.20336
15777	1.7913	0.180757
15778	1.48864	0.160975
15779	1.09504	2.1
15780	1.39827	2.1
15781	2.28084	2.1
15782	1.05983	2.1
15783	1.33593	0.0698591
15784	1.31508	0.0698591
15785	1.31508	0.0698591
15786	1.09599	0.0698591
15787	1.30647	2.1
15788	1.28846	2.1
15789	1.28846	2.1
15790	1.26923	2.1
15791	1.2955	2.1
15792	1.09044	2.1
15793	2.12209	2.1
15794	1.26438	2.1
15795	0.911819	2.1
15796	1.38752	2.1
15797	2.57704	2.1
15798	2.71736	2.1
15799	1.42284	2.1
15800	1.41562	2.1
15801	2.69131	2.1
15802	2.36178	2.1
15803	1.41562	2.1
15804	1.39564	2.1
15805	1.32368	2.1
15806	0.908053	2.1
15807	0.908053	2.1
15808	0.629213	2.1
15809	1.8758	1.96812
15810	1.27362	2.04372
15811	1.3756	0.793073
15812	2.28749	1.45252
15813	1.84577	0.361124
15814	2.17765	0.358515
15815	1.38969	0.319569
15816	1.84578	0.325879
15817	1.32801	0.207469
15818	1.76377	0.191817
15819	1.22845	2.1
15820	1.57766	2.06187
15821	1.57766	2.1
15822	1.39067	2.1
15823	1.15743	0.0698591
15824	1.37721	0.0698591
15825	1.69055	0.0698591
15826	1.14858	0.0743833
15827	1.38333	2.1
15828	1.10575	1.33146
15829	1.22418	2.1
15830	1.10574	2.1
15831	1.91509	0.128586

15832	1.02001	0.112597
15833	1.03074	0.24872
15834	1.19122	0.203138
15835	1.67887	2.1
15836	1.67597	2.1
15837	1.84984	2.1
15838	1.77775	2.1
15839	1.73571	2.0637
15840	1.27921	2.06917
15841	2.11851	1.86122
15842	1.27921	2.04858
15843	1.42486	2.1
15844	1.12503	2.1
15845	1.12503	2.1
15846	1.30116	2.1
15847	0.568995	2.1
15848	0.557348	2.1
15849	0.557348	2.1
15850	0.53907	2.1
15851	0.633575	2.1
15852	1.33756	2.1
15853	1.51074	2.1
15854	1.93384	2.03367
15855	0.655202	2.1
15856	0.731934	2.1
15857	0.731934	2.1
15858	1.09061	2.1
15859	0.966979	2.1
15860	1.31502	2.1
15861	1.31502	2.1
15862	1.72116	2.1
15863	1.70002	2.1
15864	1.67969	2.1
15865	1.67969	2.1
15866	1.62408	2.1
15867	1.01531	2.1
15868	1.66018	2.1
15869	1.01531	2.1
15870	0.886968	2.1
15871	1.22585	0.0698591
15872	1.20674	0.0698591
15873	1.20672	0.0698591
15874	1.02962	0.0698591
15875	0.956467	2.1
15876	2.00551	2.1
15877	0.553801	2.1
15878	0.617006	2.1
15879	1.26923	2.1
15880	1.23614	2.1
15881	1.23614	2.1
15882	1.16817	2.1
15883	2.14482	2.1
15884	1.72637	2.07436
15885	1.09368	2.1
15886	1.26195	2.1
15887	0.760546	2.1
15888	1.84094	2.1
15889	2.62868	2.1

15890	2.79784	2.1
15891	2.79784	2.1
15892	3.00129	2.1
15893	1.27593	0.929033
15894	1.38877	1.79359
15895	1.06486	0.346405
15896	1.2474	0.179381
15897	1.76234	0.0698591
15898	1.8685	0.0698591
15899	1.69863	0.0698591
15900	1.29666	0.0698591
15901	1.91646	2.1
15902	1.8582	2.1
15903	1.8582	2.1
15904	1.7986	2.1
15905	3.44043	0.0698591
15906	1.94764	0.0698591
15907	1.94764	0.0698591
15908	1.36307	0.0698591
15909	1.32075	0.0698591
15910	1.12971	0.0698591
15911	2.06319	0.0698591
15912	1.94982	0.0698591
15913	1.77255	0.0698591
15914	0.979297	0.0698591
15915	1.59997	2.1
15916	1.93506	2.1
15917	1.94409	2.1
15918	1.9597	2.1
15919	0.57899	2.1
15920	0.953036	2.1
15921	0.953049	2.1
15922	1.53503	2.1
15923	1.39615	0.078004
15924	1.06474	0.084076
15925	0.947564	0.0896859
15926	1.45506	0.0939645
15927	1.17799	0.0698591
15928	1.21067	0.0767083
15929	1.35945	0.209819
15930	2.05648	0.266587
15931	1.36208	0.239403
15932	2.15229	0.182648
15933	2.89288	0.168177
15934	1.95719	0.177051
15935	2.25169	0.140892
15936	1.52756	0.157824
15937	1.19322	0.280773
15938	1.23146	0.235417
15939	2.45024	0.414002
15940	0.794122	0.250654
15941	1.6343	0.240837
15942	1.34161	0.170851
15943	1.05353	0.624001
15944	1.50795	0.152732
15945	1.05353	0.130469
15946	1.20669	0.178982
15947	1.59289	2.1

15948	0.989543	2.1
15949	1.40588	2.1
15950	0.991427	2.1
15951	0.921822	1.76425
15952	1.46903	0.342335
15953	1.46903	2.07166
15954	1.8214	0.195877
15955	1.39702	2.1
15956	1.25103	2.1
15957	1.25103	2.1
15958	1.10378	2.1
15959	1.71813	2.1
15960	1.39595	2.1
15961	1.386 2.1	
15962	1.13008	2.1
15963	1.31711	0.0698591
15964	1.70191	0.0698591
15965	0.893249	0.0698591
15966	1.42669	0.0698591
15967	1.23943	2.1
15968	1.1483	2.1
15969	1.04598	2.1
15970	1.1483	2.1
15971	1.00381	0.502939
15972	1.11433	0.184073
15973	0.973501	1.042
15974	1.0756	0.92522
15975	1.20669	0.133454
15976	1.18508	0.170626
15977	1.18508	0.137428
15978	0.983027	0.141915
15979	1.43687	2.08395
15980	1.71059	2.1
15981	1.71059	2.1
15982	1.66762	2.1
15983	2.8415	2.1
15984	2.40555	2.1
15985	1.91546	2.1
15986	1.61385	2.1
15987	2.01569	2.1
15988	1.33183	2.1
15989	1.23218	2.1
15990	1.23999	2.1
15991	1.01335	0.453665
15992	1.12088	0.647795
15993	1.25346	2.09023
15994	1.15644	2.1
15995	1.3764	1.36418
15996	1.27558	0.937755
15997	1.2064	2.1
15998	1.15067	2.1
15999	1.01595	2.1
16000	1.15067	2.1
16001	1.41902	2.1
16002	1.30272	2.1
16003	1.30272	2.1
16004	1.19548	2.1
16005	2.99339	2.1

16006	2.98041	2.1
16007	1.46124	2.1
16008	1.42105	2.1
16009	2.98041	2.1
16010	2.86339	2.1
16011	1.42105	2.1
16012	1.42284	2.1
16013	2.31301	0.417353
16014	1.80273	0.420471
16015	2.17765	0.418955
16016	2.31301	0.372745
16017	1.76378	0.21416
16018	2.10275	0.192309
16019	2.10275	0.215207
16020	2.26047	0.215297
16021	0.97541	2.1
16022	1.40863	2.1
16023	1.40863	2.1
16024	1.30751	2.1
16025	1.20143	0.0835337
16026	1.19756	0.0718409
16027	1.43707	0.0698806
16028	1.28954	0.069918
16029	3.34254	0.0864789
16030	1.92916	0.0698591
16031	1.80089	0.0707398
16032	1.1323	0.0698591
16033	1.47321	0.234007
16034	1.23756	0.184716
16035	1.87291	2.1
16036	1.53642	2.1
16037	1.53642	2.1
16038	1.5791	2.1
16039	1.0446	2.1
16040	0.992674	2.1
16041	1.12324	0.0698591
16042	1.29163	0.0698591
16043	1.29163	0.0698591
16044	1.15371	0.0698591
16045	0.996831	0.132379
16046	1.00192	0.156887
16047	1.0019	0.130448
16048	0.958937	0.151947
16049	1.07283	2.1
16050	1.25294	2.1
16051	1.25294	2.1
16052	1.04788	2.1
16053	1.14496	2.1
16054	1.07652	2.1
16055	1.07652	2.1
16056	0.991406	2.1
16057	1.60976	2.1
16058	1.37 2.1	
16059	1.37 2.1	
16060	1.14195	2.1
16061	1.26593	1.98239
16062	1.75366	2.1
16063	1.57621	0.103816

16064	1.24538	0.115492
16065	0.969118	2.1
16066	1.04147	2.1
16067	0.969118	2.1
16068	1.22615	2.1
16069	0.98084	2.1
16070	1.01843	2.1
16071	1.09435	2.1
16072	1.50876	2.1
16073	1.5976	2.1
16074	1.84322	2.1
16075	1.84324	2.1
16076	1.70377	2.1
16077	1.20709	2.1
16078	2.54729	2.1
16079	0.974716	0.0702653
16080	1.54468	0.0798348
16081	1.39021	0.0698601
16082	1.70303	0.0757606
16083	1.22852	1.62713
16084	1.578	0.597418
16085	1.578	1.70305
16086	1.93852	0.397931
16087	2.2506	2.1
16088	1.39302	2.07481
16089	1.5478	2.1
16090	1.2269	2.1
16091	1.70048	2.1
16092	1.45477	2.1
16093	1.30929	2.1
16094	1.15069	2.1
16095	1.00255	2.1
16096	1.05481	0.639864
16097	1.05481	2.1
16098	1.1032	0.705365
16099	0.993888	2.1
16100	1.19294	2.1
16101	1.01121	0.0820893
16102	0.991866	0.0813233
16103	2.20052	1.97222
16104	1.7409	2.1
16105	1.75142	2.1
16106	1.46811	2.1
16107	1.7409	2.08196
16108	1.23267	2.1
16109	1.46811	2.1
16110	1.18872	2.1
16111	1.12665	2.1
16112	1.50045	2.1
16113	1.50045	2.1
16114	1.37704	2.1
16115	1.35308	0.7983
16116	1.29983	0.182288
16117	1.29983	0.938673
16118	0.970212	0.211373
16119	1.82272	2.1
16120	1.62784	2.1
16121	1.62784	2.1

16122	1.87691	2.1
16123	3.65294	2.1
16124	2.33374	2.1
16125	2.12888	2.1
16126	1.62229	2.1
16127	1.41496	2.1
16128	1.32707	2.05542
16129	2.18871	1.87309
16130	1.4235	0.968311
16131	1.46015	0.0771728
16132	1.25635	0.0745609
16133	1.45501	0.179448
16134	1.3543	0.205251
16135	1.38139	2.1
16136	1.98159	2.1
16137	2.15868	2.1
16138	4.04308	2.1
16139	1.5362	2.1
16140	1.56328	2.1
16141	1.56327	2.1
16142	1.58163	2.1
16143	1.88987	0.0698591
16144	1.08761	0.0698591
16145	0.862154	0.0698591
16146	1.77688	0.0698591
16147	1.14238	2.1
16148	1.11691	2.1
16149	1.01537	2.1
16150	1.02923	2.1
16151	0.977425	0.0698591
16152	1.15211	0.0698591
16153	2.0815	2.1
16154	1.18621	2.1
16155	1.01485	2.1
16156	1.17054	2.1
16157	1.17054	2.1
16158	1.12665	2.1
16159	1.87213	0.0698591
16160	1.82244	0.0698591
16161	1.45039	0.0698591
16162	1.7226	0.0698591
16163	1.841	0.0698591
16164	1.79011	0.0698591
16165	1.8792	0.0698591
16166	1.45053	0.0698591
16167	1.08947	2.1
16168	1.5008	2.1
16169	1.5008	2.1
16170	1.83186	2.1
16171	1.04159	0.0698591
16172	1.2489	0.0698591
16173	1.2489	0.0698591
16174	1.33592	0.0698591
16175	1.51973	0.097461
16176	0.980547	0.107885
16177	1.51974	0.119841
16178	1.15991	0.155737
16179	1.34965	2.1

16180	1.2724	2.1
16181	1.2724	2.1
16182	1.38438	2.1
16183	1.02923	2.1
16184	1.06521	2.1
16185	1.06521	2.1
16186	1.0752	2.1
16187	2.03139	0.110238
16188	1.92951	0.089618
16189	1.74852	0.0958949
16190	1.62772	0.0930822
16191	1.73655	0.0958544
16192	1.76809	0.0795465
16193	1.62772	0.0908601
16194	1.1432	0.0785756
16195	1.09235	2.1
16196	1.48081	2.1
16197	1.46712	2.1
16198	1.89321	2.1
16199	1.94548	2.1
16200	1.5636	2.1
16201	1.5636	2.1
16202	1.21124	2.1
16203	1.52314	2.1
16204	2.0219	2.1
16205	1.51113	2.1
16206	2.0219	2.1
16207	0.987247	0.359034
16208	1.18347	0.176208
16209	1.89226	0.490341
16210	1.61441	0.156651
16211	1.42884	2.1
16212	1.10961	2.1
16213	1.56991	2.1
16214	1.05337	2.1
16215	1.05337	2.1
16216	1.91547	2.1
16217	1.05931	2.1
16218	1.1729	2.1
16219	1.34526	2.1
16220	1.0593	2.1
16221	1.0856	2.1
16222	1.90854	2.1
16223	1.91674	2.1
16224	1.11345	2.1
16225	1.75067	2.1
16226	1.50022	2.1
16227	1.50022	2.1
16228	1.53619	2.1
16229	1.4748	2.02802
16230	1.74431	1.7763
16231	2.02581	1.42655
16232	1.25288	1.46394
16233	1.01814	0.516375
16234	1.2881	0.391326
16235	1.2881	0.322583
16236	1.54433	0.263581
16237	1.19999	0.285836

16238	1.44056	0.193482
16239	1.02011	0.165579
16240	1.17129	0.212151
16241	1.13775	1.91757
16242	3.34815	1.95354
16243	1.60428	1.86915
16244	1.85928	1.82353
16245	1.75592	2.1
16246	1.25989	2.1
16247	1.75592	2.1
16248	1.71549	2.1
16249	1.37634	2.1
16250	1.64693	2.1
16251	1.64693	2.1
16252	1.64193	2.1
16253	1.18351	0.0698591
16254	2.19398	0.0698591
16255	2.16272	0.61612
16256	1.19837	0.573151
16257	1.04973	2.1
16258	1.20149	2.1
16259	1.60574	2.1
16260	1.11164	1.06686
16261	1.87823	0.983317
16262	1.11164	1.71793
16263	0.772882	2.1
16264	1.4714	2.1
16265	2.14242	2.03109
16266	1.12336	2.1
16267	2.8386	1.62759
16268	2.13346	2.1
16269	1.06788	0.0789475
16270	0.947662	0.0704872
16271	1.05297	0.0698591
16272	1.42907	0.0698591
16273	2.17465	2.1
16274	2.18394	2.1
16275	1.14808	2.1
16276	1.21292	2.1
16277	1.21292	2.1
16278	1.27229	2.1
16279	1.20859	0.717109
16280	1.21073	0.459973
16281	1.21073	0.485374
16282	1.22857	0.386528
16283	1.79158	2.1
16284	1.73899	2.1
16285	1.34012	2.1
16286	1.39991	2.1
16287	1.73898	2.1
16288	1.61245	2.1
16289	1.40136	2.1
16290	1.57234	2.1
16291	1.82162	2.1
16292	3.09875	2.1
16293	1.10264	2.1
16294	1.02339	2.1
16295	1.02339	2.1

16296	1.23042	2.1
16297	2.82266	0.348711
16298	1.05698	0.209513
16299	1.96384	0.329697
16300	1.10244	0.286848
16301	0.929061	2.1
16302	1.47007	2.1
16303	0.985303	2.1
16304	1.24554	2.1
16305	1.05993	2.1
16306	2.02074	2.1
16307	2.10106	0.300237
16308	1.18171	0.185376
16309	1.68111	0.319743
16310	1.84226	0.350208
16311	1.82131	0.129008
16312	1.32371	0.079882
16313	1.41902	0.0775139
16314	1.00205	0.0699713
16315	1.65532	2.1
16316	1.18977	2.1
16317	1.15471	2.1
16318	0.993889	2.1
16319	2.08521	2.1
16320	2.09885	2.1
16321	2.09497	2.1
16322	2.10604	2.1
16323	0.881028	0.358956
16324	1.52828	0.422798
16325	1.40574	0.0698591
16326	1.09599	0.0698591
16327	1.45506	2.1
16328	1.50524	2.1
16329	1.50524	2.1
16330	1.54711	2.1
16331	1.41469	2.1
16332	2.40442	2.1
16333	1.40794	2.1
16334	1.89226	2.1
16335	1.31346	2.1
16336	1.51067	2.1
16337	1.31363	2.1
16338	1.11833	2.1
16339	2.3373	2.1
16340	2.19994	2.1
16341	2.19994	2.1
16342	1.09616	2.1
16343	1.63719	2.1
16344	1.52204	2.1
16345	1.52205	2.1
16346	1.40118	2.1
16347	2.5946	2.1
16348	4.07065	2.1
16349	1.33916	2.1
16350	2.05217	2.1
16351	1.67023	2.1
16352	2.05217	2.1
16353	1.59458	0.560864

16354	1.02247	0.404828
16355	1.68108	0.283635
16356	1.03079	0.200507
16357	1.1643	0.0933902
16358	1.85795	0.0806195
16359	1.70302	0.170423
16360	1.42076	0.13262
16361	1.91351	0.142482
16362	1.01148	0.126735
16363	2.52402	0.0698591
16364	0.752625	0.0698591
16365	0.886968	2.1
16366	1.28845	2.1
16367	1.28845	2.1
16368	1.27766	2.1
16369	1.82268	2.1
16370	1.87118	2.1
16371	1.87119	2.1
16372	1.91704	2.1
16373	3.13938	2.1
16374	2.97839	2.1
16375	2.97839	2.1
16376	2.87331	2.1
16377	1.73443	1.22315
16378	1.59457	0.690141
16379	2.01827	2.1
16380	2.04586	2.1
16381	2.04586	2.1
16382	2.00865	2.1
16383	1.19901	2.07806
16384	1.16661	2.1
16385	1.13215	0.193184
16386	1.96256	0.130958
16387	2.34043	0.0698591
16388	2.04154	0.0698591
16389	1.58991	0.0698591
16390	1.45893	0.0698591
16391	2.56818	0.0698591
16392	1.70532	0.0698591
16393	1.17798	0.0712665
16394	1.25796	0.0773028
16395	1.25797	0.0785271
16396	1.10719	0.0843613
16397	1.51067	2.1
16398	1.60707	2.1
16399	2.06946	2.09174
16400	3.19218	2.1
16401	2.19965	2.1
16402	1.30521	2.1
16403	2.23684	1.99268
16404	1.43363	2.06503
16405	1.51007	2.1
16406	2.46356	2.1
16407	1.90428	2.1
16408	1.13618	2.1
16409	2.13276	0.0724307
16410	0.998345	0.0698632
16411	1.2861	0.0734109

16412	1.20457	0.0721862
16413	1.51789	2.1
16414	1.49872	2.1
16415	1.49872	2.1
16416	1.47712	2.1
16417	1.87129	0.0713694
16418	0.949364	0.0857853
16419	1.83142	0.0735727
16420	1.55063	0.101607
16421	1.03298	0.089849
16422	0.980664	0.216381
16423	1.51696	0.0744973
16424	1.34927	0.173868
16425	1.70302	0.330231
16426	1.65952	0.230848
16427	1.76658	0.18981
16428	0.986848	0.138914
16429	2.13757	1.67016
16430	1.98142	0.727373
16431	1.98142	1.45099
16432	1.65411	0.726301
16433	1.1323	0.0753904
16434	1.46015	0.0736084
16435	2.15376	1.58914
16436	1.64622	1.71158
16437	0.991867	0.0698591
16438	1.58074	0.0698591
16439	1.69368	2.1
16440	1.76403	2.1
16441	1.76403	2.1
16442	1.83318	2.1
16443	1.28418	0.565643
16444	1.16851	0.411233
16445	1.1685	0.266674
16446	1.1726	0.230397
16447	1.06455	2.1
16448	1.18773	2.1
16449	0.98511	2.1
16450	1.18774	2.1
16451	1.76476	2.1
16452	1.79825	2.1
16453	1.79825	2.1
16454	0.961895	2.1
16455	1.59589	0.0698591
16456	2.25975	0.0698591
16457	2.76339	2.1
16458	3.0551	2.1
16459	1.68761	2.1
16460	1.59561	2.1
16461	2.17257	2.1
16462	1.05845	2.1
16463	1.03512	2.1
16464	2.19011	2.1
16465	1.44859	2.1
16466	1.35897	0.139407
16467	1.35897	2.00558
16468	1.17822	0.290145
16469	1.13641	2.1

16470	1.18301	2.1
16471	1.18301	2.1
16472	1.25173	2.1
16473	2.41297	2.1
16474	1.36463	2.1
16475	1.54711	2.1
16476	1.5735	2.1
16477	1.5735	2.1
16478	1.58713	2.1
16479	1.69044	0.0698591
16480	1.57979	0.0698591
16481	1.57979	0.0698591
16482	1.14387	0.0698591
16483	1.04199	2.1
16484	1.96491	2.1
16485	1.04199	2.1
16486	1.46892	2.1
16487	0.983724	2.1
16488	1.70579	2.1
16489	1.88522	2.1
16490	1.23914	2.1
16491	1.79424	2.1
16492	1.52851	2.1
16493	1.04921	2.1
16494	1.11285	2.1
16495	1.25334	0.4948
16496	1.07496	0.184263
16497	1.07497	2.04336
16498	1.34852	0.403486
16499	1.27391	2.1
16500	2.20307	2.1
16501	1.81311	2.1
16502	2.10305	2.1
16503	1.21059	0.0805356
16504	1.44253	0.0882471
16505	1.9194	0.0698591
16506	1.21159	0.0748774
16507	0.947979	2.1
16508	1.01225	2.01613
16509	0.947979	2.1
16510	1.01225	2.1
16511	1.00255	2.1
16512	1.01225	2.1
16513	1.01225	2.1
16514	1.06561	2.1
16515	3.50562	2.1
16516	3.61336	2.1
16517	3.61336	2.1
16518	3.73635	2.1
16519	1.61245	2.1
16520	1.52301	2.1
16521	1.57612	2.1
16522	1.83035	2.1
16523	1.52301	2.1
16524	1.24789	2.1
16525	1.85156	2.1
16526	1.89072	2.1
16527	0.995735	2.1

16528	1.57738	2.1
16529	1.87141	0.0698591
16530	1.73388	0.0698591
16531	1.61344	0.0698591
16532	0.922676	0.0698591
16533	1.56614	2.1
16534	0.956294	2.1
16535	1.82589	2.1
16536	1.63719	2.1
16537	1.12399	0.0698591
16538	1.50103	0.0698591
16539	1.56976	0.0698591
16540	1.03567	0.0698591
16541	1.21776	0.0698591
16542	1.89708	0.0698591
16543	1.16323	0.0698591
16544	1.63533	0.0698591
16545	2.29397	0.0698591
16546	3.01596	0.0698591
16547	1.80145	0.0698591
16548	1.7666	0.0698591
16549	1.91621	0.0772004
16550	1.10719	0.0792307
16551	1.57828	0.353284
16552	1.17105	0.110961
16553	1.15806	2.1
16554	1.31565	2.1
16555	1.31565	2.1
16556	1.46643	2.1
16557	1.47507	2.1
16558	1.87858	2.1
16559	0.958661	2.1
16560	1.87858	2.1
16561	1.44293	0.0698591
16562	1.43213	0.0705469
16563	1.43213	0.0698591
16564	1.10476	0.0698645
16565	1.03235	1.93114
16566	1.31	0.265449
16567	1.31	1.97535
16568	1.50108	0.282722
16569	1.32215	2.1
16570	1.10611	2.1
16571	1.10611	2.1
16572	0.904978	2.1
16573	1.06486	0.329441
16574	1.18347	0.181182
16575	1.70535	2.1
16576	1.94779	2.1
16577	1.77071	2.1
16578	1.82268	2.1
16579	0.61701	2.1
16580	0.672495	2.1
16581	0.672494	2.1
16582	0.713174	2.1
16583	1.40395	2.1
16584	1.34245	2.1
16585	1.34243	2.1

16586	1.30646	2.1
16587	1.06204	0.247184
16588	0.632691	0.590896
16589	1.75766	0.801235
16590	1.14287	1.79736
16591	1.52101	0.10441
16592	1.71414	0.108564
16593	1.86411	0.121379
16594	1.6253	0.110226
16595	1.04355	0.108961
16596	1.0443	0.109953
16597	1.34852	2.09868
16598	1.56016	0.164376
16599	1.56016	0.28803
16600	1.03235	0.165795
16601	1.2966	0.0698591
16602	1.82354	0.0698591
16603	1.2966	0.0698591
16604	1.67687	0.0698591
16605	1.19669	0.723577
16606	1.82979	0.785183
16607	1.58972	1.06912
16608	1.09219	0.898295
16609	1.42077	0.894884
16610	1.89226	0.786992
16611	1.19547	2.1
16612	1.10187	2.1
16613	1.10188	2.1
16614	1.01479	2.1
16615	1.81663	2.1
16616	1.81533	2.1
16617	1.83247	2.1
16618	1.51511	2.1
16619	0.983148	2.1
16620	1.7513	2.1
16621	1.85017	2.1
16622	1.26103	2.1
16623	1.23152	2.1
16624	1.65508	2.1
16625	1.23152	2.1
16626	2.15082	2.1
16627	1.57974	2.1
16628	1.07289	2.1
16629	0.81534	2.1
16630	1.51007	2.1
16631	1.00898	0.0698591
16632	1.18959	0.0698591
16633	1.32178	0.0698591
16634	1.25929	0.0698591
16635	2.17892	0.388803
16636	1.22938	0.497535
16637	3.02933	0.984894
16638	2.34619	0.527261
16639	1.47679	0.0714657
16640	1.0819	0.0887654
16641	3.10558	2.1
16642	3.31457	2.1
16643	0.985109	2.1

16644	1.10667	2.1
16645	3.31457	2.1
16646	3.54443	2.1
16647	1.10667	2.1
16648	1.25987	2.1
16649	1.77063	1.11264
16650	1.51065	0.698281
16651	1.24688	2.1
16652	1.37652	2.1
16653	1.37652	2.1
16654	1.54168	2.1
16655	1.17042	1.10213
16656	1.13092	1.64859
16657	1.13092	1.81827
16658	1.52938	2.08593
16659	1.11787	0.93424
16660	1.00613	2.1
16661	0.781373	0.206489
16662	1.6885	0.202957
16663	2.49439	0.084893
16664	1.42657	0.145256
16665	1.77903	2.1
16666	2.63606	2.1
16667	2.10425	2.1
16668	1.21425	2.1
16669	1.31253	1.13997
16670	1.28418	0.830384
16671	1.28418	1.20931
16672	1.58972	1.13682
16673	0.858948	2.1
16674	1.90196	2.1
16675	1.81024	2.1
16676	1.90194	2.1
16677	1.4263	1.69111
16678	0.947921	1.86573
16679	1.22852	0.388891
16680	1.09194	0.240111
16681	1.60578	0.0788157
16682	1.14721	0.0824851
16683	1.06336	0.112836
16684	1.14721	0.0941873
16685	1.11924	0.0698591
16686	1.53814	0.0698591
16687	1.18421	0.0698591
16688	1.8458	0.0698591
16689	1.7565	1.93553
16690	1.09344	1.06274
16691	1.09344	1.1653
16692	1.16971	1.94443
16693	1.71465	0.0698591
16694	1.19412	0.0698591
16695	1.64985	0.0698591
16696	1.05417	0.0698885
16697	1.2127	0.0698591
16698	1.48867	0.0698591
16699	1.27806	0.0698591
16700	1.21269	0.0698591
16701	1.37813	1.03582

16702	1.0417	1.05519
16703	1.88626	0.242095
16704	1.14977	0.748506
16705	1.78803	2.1
16706	1.33793	2.07703
16707	1.42266	2.09979
16708	1.26865	2.01989
16709	1.09518	2.1
16710	1.36844	2.1
16711	1.20455	2.1
16712	1.36844	2.1
16713	1.22297	1.11763
16714	1.11256	1.41823
16715	1.11256	0.929658
16716	1.03217	1.16189
16717	1.18422	0.0698591
16718	1.4282	0.0698591
16719	1.04159	0.0698591
16720	1.4282	0.0698591
16721	0.988805	2.1
16722	1.05481	2.1
16723	1.52938	1.21728
16724	1.26593	1.77631
16725	1.53082	2.1
16726	1.09847	2.1
16727	1.75211	2.02997
16728	1.22222	2.1
16729	1.75211	1.37337
16730	1.51592	2.1
16731	1.63413	0.541928
16732	1.26662	0.563477
16733	1.42495	0.382016
16734	1.4356	0.426849
16735	1.29389	1.63433
16736	1.04164	1.98809
16737	1.40574	1.79663
16738	1.04164	1.65631
16739	2.22344	0.635492
16740	1.45945	0.147261
16741	0.987248	0.369615
16742	1.2474	0.186741
16743	0.974702	0.460429
16744	1.43777	0.289232
16745	1.71289	2.1
16746	1.91529	2.1
16747	1.91529	2.1
16748	2.01827	2.1
16749	1.87111	0.693937
16750	1.00864	0.298672
16751	1.26865	0.432514
16752	1.18769	0.692029
16753	1.24167	2.1
16754	1.36133	1.97548
16755	2.28168	2.1
16756	3.98524	2.1
16757	1.36133	2.1
16758	1.69436	1.7535
16759	2.21926	2.1

16760	1.70302	2.1
16761	1.50732	0.0710882
16762	1.42237	0.0734075
16763	1.18659	0.0777506
16764	1.50732	0.0803292
16765	1.19785	0.0698591
16766	1.40969	0.0698591
16767	1.28339	0.0734751
16768	1.40882	0.0737069
16769	0.968595	2.1
16770	1.41903	2.1
16771	1.12567	2.1
16772	1.15898	2.1
16773	1.27241	2.1
16774	0.995796	2.1
16775	1.89983	0.0698591
16776	1.05417	0.0698591
16777	0.571999	0.0698591
16778	0.743597	0.0698591
16779	1.89353	0.0698591
16780	0.924068	0.0698591
16781	1.52851	2.1
16782	1.26969	2.1
16783	1.07763	0.203974
16784	1.82301	0.129527
16785	1.07763	0.109413
16786	1.17506	0.109161
16787	1.20045	2.1
16788	1.12486	2.1
16789	1.15234	2.1
16790	1.11598	2.1
16791	1.12486	2.1
16792	1.0491	2.1
16793	1.11598	2.1
16794	1.1047	2.1
16795	1.05902	2.1
16796	1.14361	2.1
16797	1.14361	2.1
16798	1.26851	2.1
16799	1.29389	1.33083
16800	0.988456	1.21984
16801	1.83688	0.0698591
16802	1.70302	0.0698591
16803	1.49386	0.0698591
16804	1.83151	0.0698591
16805	1.01346	0.930206
16806	0.983607	0.964399
16807	0.983596	2.1
16808	1.08332	2.0085
16809	2.36848	0.121762
16810	2.97066	0.102525
16811	1.12352	0.0756245
16812	2.5311	0.0698721
16813	1.09062	2.1
16814	0.962182	2.1
16815	0.962181	2.1
16816	1.2258	2.1
16817	0.974888	2.1

16818	1.06312	2.1
16819	1.73583	0.0698591
16820	1.72399	0.0698591
16821	1.724	0.0719238
16822	1.50951	0.0714469
16823	1.26755	2.1
16824	1.03824	2.1
16825	1.27875	2.1
16826	1.03824	2.1
16827	1.18902	2.1
16828	1.534	2.1
16829	1.11764	2.1
16830	1.534	2.1
16831	1.52008	2.1
16832	1.34406	2.1
16833	1.34406	2.1
16834	1.2187	2.1
16835	0.963571	1.26228
16836	1.19612	0.91243
16837	0.963571	2.05405
16838	1.00007	2.1
16839	0.903333	2.1
16840	1.12724	2.1
16841	1.23771	2.1
16842	1.17683	2.1
16843	1.20059	0.556285
16844	0.772023	0.272284
16845	1.14749	0.831782
16846	1.2802	0.714823
16847	1.85091	1.96882
16848	2.11193	1.74975
16849	1.2802	0.623038
16850	1.42913	0.529794
16851	2.11193	1.16574
16852	1.27737	1.00008
16853	1.30584	2.1
16854	1.60936	2.1
16855	1.3413	0.0917969
16856	1.53336	0.0731118
16857	2.60679	0.0729715
16858	1.3413	0.0698591
16859	1.35877	2.1
16860	1.11024	2.1
16861	1.27839	2.1
16862	1.08171	2.1
16863	1.27841	2.1
16864	1.0577	2.1
16865	1.71255	2.1
16866	1.10611	2.1
16867	0.960106	2.1
16868	1.40921	2.1
16869	1.90705	0.113722
16870	2.79564	0.105776
16871	1.45321	0.120245
16872	1.90705	0.109043
16873	0.974718	0.0840429
16874	1.53671	0.0762094
16875	1.69039	0.078919

16876	1.78229	0.071868
16877	1.0101	2.1
16878	1.3167	2.1
16879	1.19699	2.1
16880	1.3212	2.1
16881	1.60811	0.0792205
16882	1.43224	0.0698591
16883	1.35401	0.0698591
16884	1.17464	0.0698591
16885	1.27696	2.1
16886	1.05025	2.1
16887	1.05025	2.1
16888	1.18076	2.1
16889	1.37224	1.6652
16890	1.13438	1.61532
16891	1.76788	1.6528
16892	1.73444	1.38336
16893	1.37704	2.1
16894	1.77042	2.1
16895	1.77042	2.1
16896	1.09778	2.1
16897	1.28667	2.1
16898	1.28228	2.1
16899	1.30646	0.149822
16900	1.68867	0.117845
16901	1.30645	0.625101
16902	0.986494	0.33238
16903	1.6253	0.108619
16904	1.48676	0.111547
16905	1.48676	0.105174
16906	1.32974	0.103799
16907	0.634347	0.0698591
16908	1.15142	0.0698591
16909	1.11395	0.0698591
16910	1.18068	0.0698591
16911	1.36904	0.0698591
16912	0.764563	0.0698591
16913	2.09213	0.0698591
16914	1.56631	2.1
16915	1.08011	2.1
16916	1.80283	2.1
16917	1.50738	2.1
16918	1.44951	0.105193
16919	1.50102	0.0926428
16920	1.50102	0.0922842
16921	1.39615	0.087973
16922	1.02653	0.34181
16923	2.98258	0.343217
16924	2.0815	0.0698591
16925	1.39525	0.0698591
16926	1.82176	0.0698591
16927	0.977607	0.0698591
16928	1.96133	1.81744
16929	1.27189	1.071
16930	0.826032	1.11038
16931	2.12831	0.959685
16932	1.77798	2.0558
16933	1.02969	1.79272

16934	1.06463	1.65963
16935	1.49219	2.01503
16936	1.10003	2.1
16937	1.21223	2.1
16938	1.00513	1.11302
16939	1.43788	2.09961
16940	1.47134	0.887808
16941	0.968249	0.93897
16942	3.22585	0.118053
16943	3.36933	0.0708119
16944	3.36932	0.115092
16945	3.03865	0.0698591
16946	1.66295	0.333534
16947	1.65583	0.225304
16948	1.65583	0.340318
16949	1.4732	0.230285
16950	1.73704	0.0698591
16951	1.76476	2.1
16952	2.13345	1.95297
16953	1.55816	2.1
16954	1.55816	2.06611
16955	1.02601	2.1
16956	1.08039	2.1
16957	1.05498	1.93361
16958	1.08039	2.1
16959	1.2873	2.1
16960	1.01121	2.1
16961	0.987703	2.1
16962	0.98771	2.1
16963	0.964782	2.1
16964	1.35719	2.1
16965	2.61092	2.1
16966	2.6607	2.1
16967	3.41693	2.1
16968	1.23125	2.1
16969	1.73772	2.1
16970	0.99845	0.25654
16971	1.5176	0.347289
16972	1.13271	0.0698591
16973	1.332	0.0698591
16974	1.63159	2.1
16975	1.61342	2.1
16976	1.03679	2.1
16977	1.19087	2.1
16978	1.97172	2.1
16979	2.68634	2.1
16980	1.19086	2.1
16981	1.50091	2.1
16982	2.84677	2.1
16983	2.77894	2.1
16984	2.77894	2.1
16985	2.74206	2.1
16986	1.3284	2.1
16987	1.83524	2.1
16988	1.65853	2.1
16989	1.56741	2.1
16990	1.00509	2.1
16991	1.45246	2.1

16992	1.26193	2.1
16993	1.45244	2.1
16994	1.61963	0.440577
16995	1.68189	0.365117
16996	1.68189	1.62553
16997	1.49155	1.14918
16998	1.58054	2.1
16999	1.59169	2.1
17000	1.59169	2.1
17001	1.60497	2.1
17002	0.743595	0.0698591
17003	0.702359	0.0698591
17004	1.15317	0.0698591
17005	2.24367	0.0698591
17006	1.15252	0.0698591
17007	2.60354	0.0698591
17008	1.45158	2.1
17009	1.56004	2.1
17010	1.64527	2.1
17011	1.00371	2.1
17012	1.38287	0.438402
17013	0.893024	0.658804
17014	1.23527	1.47971
17015	1.23706	0.688729
17016	2.20993	0.465061
17017	1.54858	1.69982
17018	0.798666	0.15443
17019	1.22487	0.153719
17020	1.33158	0.126808
17021	1.85902	0.131153
17022	1.08334	2.1
17023	1.17885	1.94494
17024	1.17885	1.45676
17025	1.07283	1.69565
17026	1.30723	0.0698591
17027	1.62507	0.0698591
17028	1.62507	0.0698591
17029	1.08854	0.0698591
17030	1.59475	2.1
17031	1.58874	2.09783
17032	1.58875	2.0893
17033	1.35787	2.04412
17034	1.04147	2.1
17035	1.15564	2.1
17036	1.78301	2.1
17037	1.90618	2.1
17038	1.71555	2.1
17039	2.13143	2.1
17040	1.10817	2.1
17041	1.032	2.1
17042	1.032	2.1
17043	0.953636	2.1
17044	1.27916	1.1511
17045	1.11938	0.922724
17046	1.11937	0.895015
17047	1.9186	0.92652
17048	1.19967	0.0805578
17049	1.63489	0.0854975

17050	1.00763	0.102309
17051	1.81656	0.108744
17052	1.31814	0.0797205
17053	2.98183	0.0833053
17054	1.20149	2.1
17055	1.29129	2.1
17056	1.29129	2.1
17057	1.37138	2.1
17058	1.23104	0.612141
17059	1.75236	0.954984
17060	1.6	0.243327
17061	1.60565	0.389818
17062	1.60565	0.356789
17063	1.40013	0.454015
17064	1.23042	2.1
17065	1.18278	2.1
17066	1.10853	2.1
17067	1.04724	2.1
17068	1.1766	2.1
17069	1.00319	2.1
17070	1.29435	2.1
17071	1.00319	2.1
17072	1.05655	1.70983
17073	1.01606	1.72828
17074	1.80391	2.1
17075	2.7699	2.1
17076	1.52073	2.1
17077	2.06835	2.1
17078	2.00202	2.1
17079	2.04906	2.1
17080	0.968536	2.1
17081	1.02461	2.1
17082	1.59784	2.1
17083	1.78249	2.1
17084	1.81409	2.1
17085	1.79844	2.1
17086	1.77093	2.01582
17087	2.02356	1.79651
17088	1.32449	2.1
17089	1.10003	2.1
17090	1.16915	0.481153
17091	1.05937	0.408036
17092	1.28096	2.09255
17093	1.34696	1.94353
17094	1.34695	2.09998
17095	1.36457	1.87294
17096	1.06478	2.1
17097	1.06239	2.1
17098	1.06239	2.1
17099	1.09629	2.1
17100	0.798209	2.02928
17101	1.8532	2.1
17102	2.10112	0.0698591
17103	2.8704	0.0698591
17104	2.8704	0.0698591
17105	2.92457	0.0698591
17106	1.0752	2.1
17107	1.07745	2.1

17108	1.07745	2.1
17109	1.08911	2.1
17110	1.40182	2.1
17111	1.00133	2.1
17112	1.40182	2.1
17113	1.24509	2.1
17114	2.22243	0.0698591
17115	1.07303	0.0698591
17116	1.29736	0.0698591
17117	1.73584	0.0698591
17118	2.0815	0.323893
17119	1.07999	0.205302
17120	2.1692	2.1
17121	1.99992	2.1
17122	1.99992	2.1
17123	1.78327	2.1
17124	1.52661	0.0698591
17125	0.956003	0.0698591
17126	1.53172	0.0698591
17127	0.955991	0.0698591
17128	1.35025	2.1
17129	1.51084	2.1
17130	1.51084	2.1
17131	1.67115	2.1
17132	2.00977	2.1
17133	1.63632	2.1
17134	1.63632	2.1
17135	2.66128	2.1
17136	1.13525	2.1
17137	2.37566	2.1
17138	1.25987	2.1
17139	1.4472	2.1
17140	1.4472	2.1
17141	1.66018	2.1
17142	0.994001	0.647227
17143	1.83151	0.593478
17144	1.83151	0.20356
17145	1.57621	0.210933
17146	1.40915	0.392289
17147	1.24653	1.93914
17148	1.24653	0.250937
17149	1.10609	2.07498
17150	1.61121	2.1
17151	1.74472	2.1
17152	1.74472	2.1
17153	1.0368	2.1
17154	1.04788	2.1
17155	1.28522	2.1
17156	1.28522	2.1
17157	1.54196	2.1
17158	1.23387	2.1
17159	0.896519	2.1
17160	1.60428	2.1
17161	1.08467	2.1
17162	1.56875	0.27978
17163	1.39072	0.199387
17164	1.39073	0.159723
17165	1.6	0.27908

17166	1.01346	2.09242
17167	1.06838	0.974848
17168	0.968463	1.1785
17169	1.06837	1.07186
17170	3.22433	0.0836555
17171	2.05993	1.96568
17172	2.01836	0.219677
17173	1.66154	1.96175
17174	1.20871	2.09967
17175	1.10586	2.1
17176	1.10586	2.09878
17177	1.03853	2.1
17178	1.12076	2.1
17179	1.03899	2.1
17180	1.03899	2.1
17181	0.963973	2.1
17182	0.987132	2.1
17183	1.0894	2.1
17184	2.37823	1.89672
17185	1.75647	1.25684
17186	1.64625	2.1
17187	1.56195	2.1
17188	1.41995	2.1
17189	1.15171	2.1
17190	1.36457	2.1
17191	1.33847	1.5645
17192	1.33847	2.06536
17193	0.869601	1.23428
17194	1.42255	2.1
17195	1.3868	2.1
17196	1.3868	2.1
17197	1.33962	2.1
17198	1.05481	2.1
17199	1.08074	2.1
17200	1.08074	2.1
17201	0.857557	2.1
17202	1.13537	1.5811
17203	1.09194	0.162275
17204	1.01179	1.90132
17205	1.13537	0.239427
17206	1.15645	1.95065
17207	1.56409	0.196139
17208	1.56409	0.730501
17209	1.16239	1.00586
17210	1.10746	0.372973
17211	1.47952	0.483155
17212	1.14733	2.01433
17213	3.77068	2.1
17214	1.38497	2.1
17215	1.54599	2.1
17216	1.54601	2.1
17217	1.27697	2.1
17218	1.01393	0.292833
17219	1.10558	0.74295
17220	0.995012	0.1712
17221	1.17624	0.683947
17222	1.60497	2.1
17223	1.61588	2.1

17224	1.61587	2.1
17225	1.62182	2.1
17226	1.48866	0.0698591
17227	1.65977	0.0698591
17228	1.65977	0.0698591
17229	1.69044	0.0698591
17230	0.961892	2.1
17231	1.00659	2.1
17232	1.0066	2.1
17233	1.06226	2.1
17234	1.15171	2.1
17235	1.50853	2.1
17236	1.50853	2.1
17237	1.2955	2.1
17238	1.3204	0.0698591
17239	2.13295	0.0698591
17240	2.75524	0.0698591
17241	1.18156	0.0698591
17242	1.73729	2.1
17243	1.20356	2.1
17244	1.62483	2.1
17245	1.52008	2.1
17246	1.19964	2.1
17247	1.0577	2.1
17248	1.19964	2.1
17249	1.07156	2.1
17250	1.98241	2.03633
17251	1.43687	1.98676
17252	1.27863	2.1
17253	1.19915	2.06417
17254	0.97269	2.05138
17255	1.0739	1.81471
17256	1.1427	1.98856
17257	1.22476	0.368041
17258	1.06584	1.85949
17259	1.22626	1.57898
17260	1.31388	2.1
17261	1.16932	2.1
17262	1.28819	2.1
17263	1.0499	2.1
17264	1.55894	2.1
17265	1.78709	2.1
17266	1.75986	2.1
17267	1.764 2.1	
17268	1.44801	2.05891
17269	1.28186	2.05022
17270	1.20797	1.79916
17271	1.06821	1.97145
17272	1.87287	0.225466
17273	2.08014	0.247164
17274	1.63225	0.248104
17275	1.39501	0.254303
17276	1.20132	0.0698591
17277	1.16182	0.0698591
17278	1.08848	0.0698591
17279	1.20132	0.0698591
17280	1.23475	2.1
17281	1.16661	2.1

17282	2.31317	2.1
17283	2.50145	2.1
17284	1.04367	2.1
17285	2.23875	2.1
17286	0.953632	0.215018
17287	1.38646	0.318355
17288	1.38646	0.352997
17289	1.61963	0.354844
17290	1.62934	1.6077
17291	1.88626	1.35024
17292	1.0775	0.834262
17293	1.119	0.434355
17294	1.08133	0.231936
17295	1.48312	0.196412
17296	1.57125	0.124228
17297	1.74098	0.121007
17298	1.94372	1.90874
17299	1.22237	1.93773
17300	1.18549	0.838511
17301	1.01294	2.01305
17302	1.01294	2.06983
17303	1.2064	2.1
17304	1.14599	2.1
17305	1.00065	2.03181
17306	1.76357	2.1
17307	2.23468	2.1
17308	2.21735	2.1
17309	3.32769	2.1
17310	1.19647	0.711335
17311	1.35307	0.235189
17312	1.19645	0.281072
17313	1.43778	0.211554
17314	1.34412	1.20291
17315	1.1315	2.1
17316	1.1315	1.44383
17317	1.23943	1.05094
17318	1.57829	2.06412
17319	1.22537	2.09525
17320	1.14357	2.06717
17321	1.32772	1.98989
17322	1.4259	2.1
17323	1.34245	2.1
17324	1.47953	2.1
17325	1.70303	2.1
17326	2.21127	0.0775167
17327	1.47904	0.0855963
17328	1.18793	0.0698591
17329	2.21126	0.0737359
17330	1.1032	2.08088
17331	1.14986	1.05373
17332	1.14986	2.1
17333	1.19611	0.727399
17334	1.91883	2.1
17335	1.83019	2.1
17336	1.83019	2.1
17337	1.82462	2.1
17338	1.27362	0.161007
17339	1.27421	0.304735

17340	1.27421	0.172615
17341	1.10736	0.352085
17342	1.3003	0.636922
17343	1.31415	1.4187
17344	1.31415	0.788096
17345	1.32201	1.57924
17346	1.21124	2.1
17347	0.81568	2.1
17348	0.81568	2.1
17349	0.551816	2.1
17350	1.67119	2.1
17351	1.63384	2.1
17352	1.63384	2.1
17353	1.63661	2.1
17354	1.48734	2.1
17355	1.52245	2.1
17356	1.52245	2.1
17357	1.50714	2.1
17358	0.996778	0.0710706
17359	1.56091	0.0698591
17360	1.56091	0.0700475
17361	1.90457	0.0698591
17362	1.1181	2.1
17363	1.06543	2.1
17364	2.66828	2.1
17365	2.76932	2.1
17366	1.06543	2.1
17367	1.01474	2.1
17368	2.76932	2.1
17369	2.86704	2.1
17370	1.29724	0.0900743
17371	1.96938	0.0787344
17372	1.82462	2.1
17373	1.84553	2.1
17374	1.05556	2.1
17375	1.25363	2.1
17376	1.84552	2.1
17377	1.53208	2.1
17378	1.12826	2.1
17379	1.39075	2.1
17380	0.9702	0.796543
17381	1.16481	0.294057
17382	1.08894	1.06599
17383	1.91756	1.05961
17384	1.50865	0.472028
17385	1.06012	0.380257
17386	1.5456	0.0698591
17387	1.37427	0.0698591
17388	1.1211	0.0698591
17389	1.5456	0.0698591
17390	1.33135	0.0698591
17391	1.07956	0.0698591
17392	2.23255	0.0698591
17393	1.21031	0.0698591
17394	1.52903	2.1
17395	1.35977	2.1
17396	3.3837	2.1
17397	3.20581	2.1

17398	1.35977	2.1
17399	1.15414	2.1
17400	3.20582	2.1
17401	2.07596	2.1
17402	1.6812	0.0862633
17403	1.03939	0.078847
17404	1.30047	2.1
17405	1.5586	2.1
17406	1.5586	2.1
17407	1.84214	2.1
17408	1.31825	0.843612
17409	1.31923	1.88819
17410	1.31924	0.84271
17411	1.33026	1.79327
17412	1.15818	0.444169
17413	1.17822	0.602336
17414	1.15818	2.00573
17415	1.28096	1.32883
17416	1.06848	0.0718598
17417	1.89639	0.075655
17418	1.9186	1.0545
17419	1.77065	0.773312
17420	1.59103	0.638595
17421	1.56164	0.430849
17422	1.09697	0.0698591
17423	2.38719	0.0698591
17424	2.22123	0.0698591
17425	2.46059	0.0698591
17426	1.45506	0.134879
17427	1.03645	0.141306
17428	1.30347	2.1
17429	1.2627	2.1
17430	1.2627	2.1
17431	1.23036	2.1
17432	1.41469	2.1
17433	1.45257	2.1
17434	1.46907	0.0698591
17435	1.70302	0.0698591
17436	1.49196	0.0698591
17437	1.36307	0.0698591
17438	1.05677	2.1
17439	0.978698	2.1
17440	0.978699	2.1
17441	2.01822	2.1
17442	1.47446	2.1
17443	1.55432	2.1
17444	1.55432	2.1
17445	1.06053	2.1
17446	1.86813	1.89476
17447	1.21892	1.97464
17448	1.53064	1.28198
17449	1.30664	1.37958
17450	1.09629	2.1
17451	1.19244	2.1
17452	1.19244	2.1
17453	1.37635	2.1
17454	1.49358	0.181645
17455	1.57702	0.0921172

17456	2.09374	2.1
17457	1.88175	2.1
17458	1.88175	2.1
17459	1.51085	2.1
17460	1.10938	0.176647
17461	1.02478	1.60804
17462	1.33061	0.0698591
17463	1.13272	0.0698591
17464	1.75505	2.1
17465	1.78529	2.1
17466	1.6353	2.1
17467	1.59307	2.1
17468	1.60017	0.376207
17469	1.35608	0.43196
17470	1.35608	0.186979
17471	1.04713	0.188381
17472	1.01479	1.91316
17473	1.3868	1.4576
17474	1.3868	1.48865
17475	1.28418	2.1
17476	2.15949	2.1
17477	2.90429	2.1
17478	1.51902	2.1
17479	1.54755	2.1
17480	2.90429	2.1
17481	3.49966	2.1
17482	1.70842	2.1
17483	1.97526	2.1
17484	0.949712	0.0784533
17485	1.92784	0.0802829
17486	1.34093	0.078667
17487	2.53426	0.0987044
17488	1.92783	0.0782562
17489	3.89373	0.0752076
17490	2.53426	0.0839855
17491	2.35961	0.0856774
17492	1.68108	0.216256
17493	1.15558	0.182036
17494	1.67265	0.190587
17495	1.27608	0.162061
17496	1.42249	2.1
17497	1.39708	2.1
17498	1.39708	2.1
17499	1.39327	2.1
17500	1.97046	0.0699214
17501	1.11688	0.0698591
17502	1.28418	1.17196
17503	1.17302	2.1
17504	1.17302	1.36983
17505	1.04349	2.1
17506	0.979853	2.1
17507	1.26016	2.1
17508	1.18901	2.1
17509	1.26016	2.1
17510	1.22857	0.323786
17511	1.25848	0.374492
17512	1.25848	0.244596
17513	1.2858	0.503609

17514	1.09154	2.1
17515	2.01915	2.1
17516	1.23013	2.1
17517	1.55698	2.1
17518	1.55698	2.1
17519	1.8575	2.1
17520	2.01854	2.1
17521	1.20686	2.05214
17522	1.20684	2.02802
17523	1.77796	2.09674
17524	1.46129	2.1
17525	1.04193	2.1
17526	1.10051	2.1
17527	0.983723	2.1
17528	1.1729	2.1
17529	0.98505	2.1
17530	0.985052	2.1
17531	1.03951	2.1
17532	1.03949	2.1
17533	1.17583	2.1
17534	1.42866	2.1
17535	1.97261	2.1
17536	1.73274	2.1
17537	1.53629	2.1
17538	1.98182	2.1
17539	1.72511	2.1
17540	1.42174	2.1
17541	1.44472	2.1
17542	1.72511	2.1
17543	1.45411	2.1
17544	1.44472	2.1
17545	1.43836	2.1
17546	0.962703	0.465242
17547	1.0171	0.89329
17548	1.53336	0.0698591
17549	1.40274	0.0698591
17550	1.40272	0.0698591
17551	1.00213	0.0698591
17552	1.50892	0.0698591
17553	1.64984	0.0698591
17554	1.67464	0.0698591
17555	1.67481	0.0698591
17556	1.08212	2.1
17557	1.28095	2.1
17558	1.46956	2.1
17559	1.70303	2.1
17560	1.2925	0.0698591
17561	1.84716	0.0698591
17562	1.48676	0.115816
17563	1.48797	0.0960723
17564	1.33917	0.0933009
17565	1.22691	0.079897
17566	0.528744	2.1
17567	1.13728	2.1
17568	1.13728	2.1
17569	1.99843	2.1
17570	1.17464	0.0698591
17571	1.0372	0.0698591

17572	2.0815	0.0698591
17573	0.933462	0.0698591
17574	1.54931	2.1
17575	1.5485	2.1
17576	1.4077	2.1
17577	1.29931	2.1
17578	1.14931	0.125061
17579	2.02223	0.116246
17580	1.04482	0.0933181
17581	0.94971	0.115917
17582	1.54632	1.77506
17583	1.15693	1.76558
17584	1.66763	2.1
17585	1.41238	2.1
17586	1.41238	2.1
17587	1.48068	2.1
17588	1.0171	0.370813
17589	1.1211	0.772453
17590	1.1211	0.333743
17591	1.28418	0.603935
17592	0.993021	2.1
17593	1.764	2.1
17594	0.993023	2.1
17595	1.05787	2.1
17596	1.90914	0.471622
17597	1.12229	2.05237
17598	1.02025	0.937711
17599	0.585368	2.06711
17600	1.99323	2.1
17601	2.34988	2.1
17602	2.34988	2.1
17603	2.36987	2.1
17604	1.23107	0.0698591
17605	1.90664	0.0705895
17606	1.53496	0.0698591
17607	1.70302	0.0713937
17608	1.98859	2.1
17609	1.61057	2.1
17610	1.79844	2.1
17611	2.10811	2.1
17612	1.4618	2.1
17613	1.96722	2.1
17614	1.95191	2.1
17615	2.13418	2.1
17616	1.50368	0.0698591
17617	1.61417	0.0698591
17618	1.15008	0.0698591
17619	1.19685	0.0698591
17620	1.90456	0.0698673
17621	1.90899	0.0698591
17622	2.08864	0.0698591
17623	2.10447	0.0698591
17624	1.16653	2.1
17625	1.23017	2.1
17626	1.0752	1.63615
17627	1.34412	1.13201
17628	1.06226	2.1
17629	1.09252	2.1

17630	1.09252	2.1
17631	0.88579	2.1
17632	1.56178	0.0698643
17633	1.14305	0.071291
17634	1.56175	0.0700879
17635	1.08211	0.0698591
17636	1.32813	2.1
17637	1.32461	2.1
17638	1.32461	2.1
17639	1.32455	2.1
17640	1.42729	0.0777859
17641	1.18716	0.0699015
17642	1.47169	0.127848
17643	1.24687	0.0718273
17644	1.09627	2.1
17645	1.04759	2.1
17646	1.04759	2.1
17647	0.987132	2.1
17648	1.11181	1.87729
17649	1.80662	2.08664
17650	1.8066	2.056
17651	1.06461	1.71647
17652	1.56091	2.1
17653	1.9842	2.1
17654	1.73525	0.136477
17655	1.40557	0.136825
17656	2.26906	0.146019
17657	1.31248	0.193447
17658	1.66832	0.129487
17659	1.32634	0.115381
17660	1.03314	0.0698591
17661	1.24682	0.0698591
17662	1.38536	0.0698591
17663	1.52661	0.0698591
17664	1.177	1.17478
17665	1.36405	0.81665
17666	1.19837	1.27656
17667	1.17701	0.52173
17668	0.993831	0.0739639
17669	1.0823	0.076562
17670	1.0823	0.0698591
17671	1.01121	0.0723767
17672	1.49653	0.094183
17673	1.89583	0.11723
17674	0.67242	0.0910926
17675	1.36047	0.112399
17676	1.00366	0.099323
17677	1.24728	0.113108
17678	1.13387	0.0698591
17679	1.60867	0.0698591
17680	1.30572	0.0698591
17681	1.54982	0.0698591
17682	0.788592	0.0698591
17683	1.82961	0.0698591
17684	1.54982	0.0698591
17685	1.82961	0.0698591
17686	1.26062	0.0698591
17687	1.64002	0.0698591

17688	1.12035	0.0698591
17689	1.64002	0.0698591
17690	1.1673	2.1
17691	1.75367	2.1
17692	0.964034	2.1
17693	1.0305	2.0977
17694	1.30896	2.01934
17695	1.52776	2.02989
17696	1.52776	2.1
17697	1.17989	2.09037
17698	1.16759	2.1
17699	1.8575	2.1
17700	1.16759	2.1
17701	1.60936	2.1
17702	1.10747	0.0698591
17703	1.47688	0.0703084
17704	1.40794	2.1
17705	1.95729	1.97685
17706	1.95729	2.1
17707	3.18156	1.89656
17708	1.87598	2.1
17709	1.94343	2.1
17710	1.94343	2.1
17711	2.00551	2.1
17712	1.20067	1.4533
17713	1.70744	1.26588
17714	1.87244	1.51801
17715	1.32075	1.2785
17716	1.64955	2.1
17717	1.44221	2.1
17718	1.31109	2.1
17719	1.14206	2.1
17720	1.60452	1.93931
17721	1.12111	0.822224
17722	1.00832	2.1
17723	0.974137	2.1
17724	0.974137	2.1
17725	0.98505	2.1
17726	1.02368	2.1
17727	0.985051	2.1
17728	0.995734	0.787461
17729	1.31993	0.650167
17730	1.31993	0.493218
17731	1.18549	2.00405
17732	1.5176	0.308592
17733	1.33622	0.225152
17734	1.33621	0.271347
17735	1.02782	0.221661
17736	1.66774	2.1
17737	1.85305	2.1
17738	1.85305	2.1
17739	2.07088	2.1
17740	1.78873	2.1
17741	1.47533	2.1
17742	1.23152	2.1
17743	0.99922	2.1
17744	1.48493	2.1
17745	1.1394	2.1

17746	1.19179	2.1
17747	1.64289	2.1
17748	1.47858	2.1
17749	1.89687	2.1
17750	0.972464	2.1
17751	1.79473	2.1
17752	1.06971	2.1
17753	1.21089	2.1
17754	1.12572	2.1
17755	1.30566	2.1
17756	1.30566	2.1
17757	1.52724	2.1
17758	2.32237	0.107735
17759	2.20248	0.0926801
17760	2.79564	0.117789
17761	2.32237	0.105032
17762	1.59354	0.0737448
17763	3.098	0.0718963
17764	3.09801	0.0737732
17765	3.45132	0.0701929
17766	1.24001	1.90176
17767	1.41822	0.301505
17768	1.41821	2.1
17769	1.44859	0.162084
17770	1.34539	2.1
17771	1.06991	2.1
17772	1.18076	2.1
17773	0.978586	2.1
17774	0.999189	0.0698591
17775	2.41076	0.0698591
17776	0.968475	2.09503
17777	1.02074	2.1
17778	1.02074	2.1
17779	1.07866	2.1
17780	0.817702	0.0776977
17781	1.5082	0.0754125
17782	1.48376	0.0944043
17783	1.18947	0.094187
17784	1.10638	2.1
17785	1.86766	2.1
17786	1.19785	2.1
17787	1.34141	2.1
17788	1.34141	2.1
17789	0.991985	2.1
17790	2.35516	1.01982
17791	1.24815	2.03086
17792	1.80454	2.1
17793	1.22633	2.1
17794	0.696255	2.1
17795	1.19379	2.05322
17796	1.14721	2.1
17797	1.16153	2.1
17798	1.16153	2.1
17799	1.17261	2.1
17800	1.20814	2.1
17801	1.54791	2.1
17802	1.54791	2.1
17803	1.60477	2.1

17804	1.29931	2.1
17805	1.19098	2.1
17806	1.19098	2.1
17807	1.13341	2.1
17808	1.00503	1.86933
17809	1.25334	0.277063
17810	1.22655	2.1
17811	1.26282	2.1
17812	1.26282	2.1
17813	0.999489	2.1
17814	1.54197	0.169843
17815	1.17152	0.177905
17816	1.17152	0.174728
17817	1.49627	0.15491
17818	1.36405	0.744597
17819	0.976334	0.643397
17820	1.12495	2.1
17821	1.54096	2.1
17822	1.75263	2.1
17823	1.49114	2.1
17824	0.842998	1.71204
17825	1.60244	0.265047
17826	1.48911	1.09569
17827	2.18685	0.798076
17828	1.75236	0.888231
17829	1.54143	1.25504
17830	1.40013	0.430799
17831	0.946646	0.522608
17832	1.50714	2.1
17833	1.46055	2.1
17834	1.46055	2.1
17835	1.42249	2.1
17836	2.12839	0.158065
17837	1.75211	0.160017
17838	1.76297	0.246313
17839	1.70736	0.153002
17840	1.19952	0.0698591
17841	1.56475	0.0698591
17842	1.74312	0.0738637
17843	1.48973	0.100465
17844	1.91518	0.159163
17845	1.16534	0.134458
17846	1.53556	2.1
17847	1.56253	2.1
17848	1.56253	2.1
17849	1.58054	2.1
17850	1.27806	0.0698591
17851	1.44593	0.0698591
17852	1.44593	0.0698591
17853	1.3946	0.0698591
17854	1.34857	0.478395
17855	1.03818	1.94076
17856	1.38969	0.171859
17857	1.85548	0.257715
17858	1.03108	0.16334
17859	1.85548	0.172007
17860	1.49346	1.59096
17861	1.83192	1.92704

17862	1.75291	1.10512
17863	1.35435	1.56195
17864	0.921851	0.20415
17865	1.09646	0.199123
17866	1.2183	0.183478
17867	1.36717	0.16165
17868	2.14179	0.100464
17869	1.79805	0.129845
17870	1.3428	0.155997
17871	2.30493	0.156056
17872	1.81545	0.15152
17873	1.92269	0.11102
17874	2.30492	0.207746
17875	1.3663	0.196776
17876	1.22418	2.1
17877	0.956698	2.03157
17878	1.4706	2.1
17879	0.953295	2.1
17880	1.47059	2.1
17881	1.26929	2.1
17882	1.24538	0.100655
17883	1.4468	0.0807292
17884	2.86762	2.1
17885	2.32911	2.1
17886	2.10864	2.1
17887	1.94548	2.1
17888	1.83134	2.1
17889	2.19519	2.1
17890	2.19853	2.1
17891	2.63855	2.1
17892	1.08213	0.0711708
17893	1.46389	0.0773549
17894	1.62235	0.0953003
17895	0.980549	0.10484
17896	1.08195	1.76927
17897	0.99002	2.06612
17898	1.2269	2.1
17899	1.0838	2.1
17900	0.889491	2.09805
17901	1.19587	2.08501
17902	1.18801	0.0724957
17903	1.57125	0.0719703
17904	1.47712	2.1
17905	1.45182	2.1
17906	1.45182	2.1
17907	1.42255	2.1
17908	1.17816	0.0698591
17909	1.00982	0.0698591
17910	1.21056	0.0698591
17911	1.17816	0.0698591
17912	3.80902	0.592665
17913	0.976991	0.526074
17914	1.09962	2.1
17915	1.60976	2.1
17916	1.87029	2.1
17917	2.33806	2.1
17918	2.12549	2.1
17919	2.21472	2.1

17920	2.16221	0.0698591
17921	1.99528	0.0698591
17922	1.99528	0.0698591
17923	1.97969	0.0698591
17924	3.60854	2.1
17925	4.00847	2.1
17926	2.70372	2.1
17927	1.253	2.1
17928	1.65739	2.1
17929	0.994294	2.1
17930	1.36203	2.1
17931	1.00965	2.1
17932	2.33374	2.1
17933	1.36209	2.1
17934	1.64983	0.0698591
17935	1.36416	0.0698591
17936	2.19766	0.0698591
17937	1.36416	0.0698591
17938	2.15871	0.325274
17939	1.81454	0.204288
17940	1.54607	0.110421
17941	1.57702	0.19958
17942	0.948748	0.0698591
17943	1.04522	0.0698591
17944	1.60994	0.0698591
17945	1.90382	0.0698591
17946	1.90382	0.0698591
17947	1.57999	0.0698591
17948	1.73905	0.543257
17949	1.26535	1.28454
17950	2.12552	0.79268
17951	1.06891	0.578946
17952	1.13999	1.28137
17953	2.29812	0.779458
17954	2.83329	0.592702
17955	2.16272	0.431031
17956	1.71277	2.1
17957	1.68401	2.1
17958	1.68401	2.1
17959	1.69368	2.1
17960	1.06472	2.1
17961	1.56017	2.1
17962	1.2765	2.1
17963	1.99541	2.1
17964	1.46041	0.0698591
17965	2.04817	0.0698591
17966	2.04817	0.0698591
17967	2.33181	0.0698591
17968	2.06353	2.1
17969	1.35449	2.1
17970	1.52045	2.1
17971	1.45437	2.1
17972	1.04405	0.0698591
17973	1.85945	0.0698591
17974	1.85945	0.0698591
17975	2.22679	0.0698591
17976	1.33339	2.1
17977	1.19376	2.1

17978	1.33266	2.1
17979	1.44244	2.1
17980	1.20455	2.1
17981	1.52852	2.1
17982	1.03882	2.1
17983	1.52852	2.1
17984	1.44801	2.01884
17985	1.00775	2.07303
17986	0.53907	2.1
17987	0.506124	2.1
17988	2.39045	2.1
17989	0.973794	2.1
17990	1.11193	2.1
17991	1.32784	2.1
17992	1.31595	2.1
17993	0.966115	2.1
17994	1.25953	2.1
17995	1.38086	2.1
17996	1.38086	2.1
17997	1.04101	2.1
17998	1.99319	0.0698591
17999	1.23107	0.0698591
18000	3.3606	0.0698591
18001	1.23112	0.0698591
18002	1.40239	2.1
18003	1.26091	2.1
18004	1.26091	2.1
18005	1.05776	2.1
18006	1.23768	0.231938
18007	1.0142	1.26977
18008	1.11435	1.24464
18009	1.01422	0.332662
18010	1.24001	0.447434
18011	1.42186	1.01621
18012	1.42186	0.630434
18013	1.47204	0.72763
18014	1.27361	2.1
18015	1.56642	2.1
18016	1.71676	2.1
18017	2.16815	2.1
18018	1.07855	1.85506
18019	1.07612	1.75169
18020	1.703	0.0698591
18021	1.46853	0.0698591
18022	1.45448	0.0721299
18023	1.27778	0.0753961
18024	0.991986	2.1
18025	1.1192	2.1
18026	1.1192	2.1
18027	1.25953	2.1
18028	2.08506	2.1
18029	1.75162	2.1
18030	1.94523	2.1
18031	1.88405	1.54745
18032	1.15506	0.0698591
18033	1.1233	0.0698591
18034	2.09736	2.1
18035	2.98303	2.1

18036	2.71184	2.1
18037	3.51614	2.1
18038	1.18769	2.09231
18039	1.24053	2.09661
18040	1.24053	2.1
18041	1.45327	2.1
18042	1.26865	2.07358
18043	1.1744	1.72875
18044	1.1744	1.89569
18045	1.11712	1.24106
18046	1.15841	0.158799
18047	1.02247	0.204322
18048	1.93649	2.1
18049	1.81191	2.1
18050	1.64718	2.1
18051	1.47521	2.1
18052	1.59654	2.1
18053	1.57823	2.1
18054	1.57823	2.1
18055	1.55768	2.1
18056	1.00665	0.111849
18057	1.28239	0.088635
18058	1.28239	0.122337
18059	1.44951	0.0938438
18060	1.22083	2.1
18061	0.957622	2.1
18062	0.887896	2.1
18063	1.27387	2.1
18064	1.00521	1.07233
18065	1.00503	0.768288
18066	1.95844	2.1
18067	1.05042	2.1
18068	2.78662	2.1
18069	2.22399	2.1
18070	2.3356	0.0698591
18071	1.49543	0.0698591
18072	1.96854	0.0698591
18073	1.21681	0.0698591
18074	2.17803	1.40214
18075	1.50998	1.87401
18076	2.35239	0.924579
18077	2.26987	0.811063
18078	1.03824	0.212669
18079	1.51396	0.218995
18080	1.51396	0.148733
18081	1.48693	0.164972
18082	1.08784	1.06009
18083	1.36865	0.724246
18084	0.712405	1.64221
18085	1.08784	1.0262
18086	2.02522	2.1
18087	1.6738	2.1
18088	1.35175	2.1
18089	1.50094	2.1
18090	1.6738	2.1
18091	1.62871	2.1
18092	1.35084	2.1
18093	1.34276	2.1

18094	1.18041	2.1
18095	1.43652	2.1
18096	0.974196	2.1
18097	1.28037	2.1
18098	1.28037	2.1
18099	1.05071	2.1
18100	2.44737	0.0698591
18101	1.4672	0.0698591
18102	1.22883	0.0698591
18103	2.08149	0.0698591
18104	1.82208	1.45843
18105	1.59826	0.205141
18106	1.59826	0.658577
18107	1.01393	0.370514
18108	1.42757	2.1
18109	0.996775	2.1
18110	1.42757	2.1
18111	1.26397	2.1
18112	1.02461	2.1
18113	1.46129	2.1
18114	1.11833	2.1
18115	1.335 2.1	
18116	2.07087	2.1
18117	1.24041	2.1
18118	1.335 2.1	
18119	1.55398	2.1
18120	1.21171	0.0698591
18121	1.48589	0.0698591
18122	1.32886	0.0698591
18123	1.8027	0.0698591
18124	1.53515	1.54875
18125	1.60428	1.33046
18126	1.00035	0.0698591
18127	1.03534	0.356872
18128	1.03535	2.1
18129	1.09962	2.1
18130	0.62132	2.06827
18131	1.21033	2.1
18132	1.30326	2.1
18133	2.6823	2.1
18134	0.991407	2.1
18135	1.29729	2.1
18136	1.29729	2.1
18137	1.16858	2.1
18138	1.66137	0.39418
18139	1.23012	0.371642
18140	1.66139	0.84634
18141	1.41821	0.647363
18142	1.56043	0.149884
18143	1.06848	0.19785
18144	1.56043	0.270118
18145	1.90911	0.249894
18146	0.982847	2.1
18147	1.05863	2.1
18148	1.05863	2.1
18149	1.22352	2.1
18150	1.23019	2.07254
18151	1.60509	2.0873

18152	1.19122	2.1
18153	1.47769	2.1
18154	2.18868	2.1
18155	1.40984	2.09736
18156	3.55197	2.1
18157	3.57227	2.1
18158	3.57227	2.1
18159	3.48926	2.1
18160	1.4375	0.108569
18161	1.11314	0.100249
18162	1.62408	2.1
18163	1.74438	2.1
18164	1.72387	2.1
18165	1.50018	2.1
18166	1.1486	2.1
18167	1.18474	2.1
18168	1.18474	2.1
18169	1.2295	2.1
18170	1.28754	2.1
18171	1.36838	2.1
18172	1.36838	2.1
18173	1.21703	2.1
18174	2.74206	2.1
18175	2.70795	2.1
18176	2.70795	2.1
18177	2.55918	2.1
18178	1.35065	0.986729
18179	1.65411	0.504984
18180	1.48572	0.825528
18181	1.28393	0.547828
18182	1.25265	0.0698591
18183	1.70944	0.0698591
18184	1.31259	2.1
18185	1.14945	2.1
18186	1.14945	2.06699
18187	0.995725	2.1
18188	1.00307	0.19819
18189	2.05786	0.215085
18190	1.56357	0.140191
18191	1.10907	0.181627
18192	1.27558	1.71814
18193	1.05175	1.33074
18194	1.2685	2.1
18195	1.41369	2.1
18196	1.4137	2.1
18197	1.49654	2.1
18198	1.21489	2.05401
18199	1.14605	1.33806
18200	1.22476	2.1
18201	1.21489	0.530653
18202	2.51472	0.871066
18203	2.52091	0.813712
18204	2.52091	0.726642
18205	1.79171	0.622858
18206	1.03373	2.0992
18207	1.04037	2.1
18208	1.04038	2.1
18209	1.03933	2.1

18210	1.74123	0.0883891
18211	1.41902	0.0773513
18212	1.89777	0.095326
18213	1.89899	0.100909
18214	1.24728	0.0871092
18215	1.30394	0.0897484
18216	1.30394	0.0737592
18217	1.16535	0.101204
18218	1.09914	2.1
18219	1.54259	2.1
18220	1.40233	2.1
18221	1.69858	2.1
18222	1.53555	2.1
18223	1.09859	2.1
18224	1.09859	2.1
18225	0.725879	2.1
18226	1.07213	2.1
18227	1.31589	2.1
18228	1.36904	2.1
18229	1.69711	2.1
18230	1.51028	0.0698591
18231	0.993598	0.0698591
18232	0.61563	0.0698591
18233	1.26311	0.0698591
18234	1.19496	2.1
18235	1.65925	2.1
18236	1.65925	2.1
18237	1.48162	2.1
18238	2.75738	1.43043
18239	1.24699	1.01506
18240	4.08729	1.64568
18241	2.35271	1.04803
18242	1.55063	0.0776128
18243	1.34563	0.0852462
18244	1.34563	0.0724822
18245	1.08189	0.0771286
18246	1.09662	0.0717928
18247	2.35961	0.108679
18248	0.986952	0.0707086
18249	0.813251	0.0698668
18250	1.17897	0.0698591
18251	1.67685	0.0698591
18252	1.00573	2.0399
18253	1.08542	1.28415
18254	1.00573	2.1
18255	1.12572	2.1
18256	1.05499	2.1
18257	1.00521	1.02239
18258	2.22679	0.0698591
18259	2.86746	0.0698591
18260	2.86746	0.0698591
18261	1.99958	0.0698591
18262	0.996517	2.1
18263	1.01169	2.1
18264	1.01169	2.1
18265	1.26346	2.1
18266	1.68274	2.1
18267	1.75505	2.1

18268	2.28748	2.03263
18269	1.36555	2.06357
18270	3.47888	0.0698591
18271	0.692788	0.0698591
18272	1.0685	0.348941
18273	1.14547	0.40786
18274	1.19017	2.1
18275	1.24976	2.1
18276	1.24976	2.1
18277	1.30682	2.1
18278	2.2153	2.1
18279	1.85457	2.1
18280	2.03267	2.1
18281	1.51042	2.1
18282	1.05619	0.571927
18283	1.05793	0.317005
18284	1.05793	0.395275
18285	1.00763	0.255535
18286	1.77677	2.1
18287	1.02524	2.1
18288	1.02524	2.1
18289	1.18803	2.1
18290	1.68669	2.1
18291	1.71162	2.1
18292	1.556	2.1
18293	1.39523	2.1
18294	0.960288	2.0395
18295	1.24201	2.1
18296	1.24202	2.1
18297	1.3592	2.1
18298	2.14045	0.0698591
18299	1.62054	0.0698591
18300	2.20248	0.0853971
18301	2.34699	0.0757097
18302	3.45132	0.0698708
18303	3.38241	0.0698591
18304	3.38241	0.0698591
18305	2.96127	0.0698591
18306	2.02612	2.1
18307	2.09351	2.1
18308	2.09351	2.1
18309	2.17465	2.1
18310	1.07445	2.1
18311	1.14808	2.1
18312	1.39067	2.1
18313	1.20958	2.0995
18314	1.20958	2.1
18315	1.00382	2.01423
18316	0.986496	0.163213
18317	1.27142	0.643176
18318	1.39857	0.26853
18319	2.26482	0.513971
18320	2.24367	0.0698591
18321	1.9234	0.0698591
18322	1.59076	0.0698591
18323	1.185	0.0698591
18324	3.86179	0.0698591
18325	3.0638	0.0698591

18326	2.7574	0.0698591
18327	1.83266	0.0698591
18328	2.59418	2.1
18329	2.23048	2.1
18330	1.39564	2.1
18331	1.36763	2.1
18332	2.23048	2.1
18333	2.02522	2.1
18334	1.36763	2.1
18335	1.35175	2.1
18336	1.16181	0.0698591
18337	0.97096	0.0698591
18338	2.05665	0.130424
18339	0.483583	0.120823
18340	0.601928	0.0988414
18341	3.88545	0.126862
18342	1.04909	2.1
18343	0.983143	2.1
18344	1.10469	2.1
18345	1.10516	2.1
18346	1.10517	2.1
18347	1.09951	2.1
18348	2.33985	2.1
18349	2.35026	2.1
18350	2.35025	2.1
18351	1.19462	2.1
18352	1.57627	2.1
18353	1.5493	2.1
18354	1.5493	2.1
18355	1.53556	2.1
18356	1.42763	0.391381
18357	1.24682	0.441488
18358	1.24682	0.303117
18359	1.00307	0.370886
18360	1.37392	2.1
18361	1.45408	2.1
18362	1.45408	2.1
18363	1.54249	2.1
18364	1.24474	1.5143
18365	1.07467	1.52979
18366	1.37813	1.5735
18367	1.24474	1.62351
18368	1.06341	2.1
18369	1.09194	2.1
18370	1.09194	2.1
18371	1.11117	2.1
18372	1.1658	2.1
18373	1.93707	2.1
18374	1.93707	2.1
18375	1.47729	2.1
18376	1.25669	2.1
18377	1.07519	2.1
18378	1.65417	2.1
18379	1.50956	2.1
18380	1.37231	2.1
18381	1.27285	2.1
18382	1.23612	2.1
18383	1.1899	2.1

18384	1.08172	2.1
18385	1.20357	2.1
18386	1.5664	2.1
18387	1.7472	2.1
18388	1.48434	2.1
18389	1.23989	2.1
18390	1.58836	2.1
18391	1.38766	2.1
18392	1.23989	2.1
18393	1.26599	2.1
18394	2.56248	2.1
18395	2.08673	2.08673
18396	1.87216	2.09568
18397	1.60476	0.694813
18398	1.84355	2.1
18399	1.25971	2.0793
18400	1.44141	2.05048
18401	1.25521	0.443219
18402	1.19837	0.0698591
18403	2.31936	0.0698591
18404	1.38153	2.1
18405	2.41734	2.1
18406	2.70856	2.1
18407	3.48385	2.1
18408	1.89226	0.857031
18409	1.64617	0.375382
18410	1.89112	0.212687
18411	1.23135	0.167407
18412	1.23134	0.214498
18413	1.34927	0.189956
18414	3.60096	0.0698591
18415	1.68103	0.0698591
18416	2.25644	2.1
18417	1.91955	2.1
18418	1.91955	2.1
18419	1.47688	2.1
18420	1.67375	2.1
18421	1.50033	2.1
18422	1.5098	2.1
18423	1.46908	2.1
18424	1.69055	0.0698591
18425	1.53463	0.0698591
18426	1.53463	0.0698591
18427	1.41943	0.0698591
18428	1.19022	1.817
18429	1.95981	1.23778
18430	1.15009	1.91497
18431	1.31612	1.5544
18432	1.31611	0.861636
18433	1.10735	0.501851
18434	1.18601	0.0698591
18435	1.3499	0.0698591
18436	1.3499	0.0698591
18437	1.29001	0.0698591
18438	1.22083	2.1
18439	1.06191	2.1
18440	1.348 0.642517	
18441	1.05758	1.35888

18442	1.34799	1.75123
18443	1.18647	1.72493
18444	2.92457	0.0698591
18445	1.99182	0.0698591
18446	2.25313	0.0698591
18447	1.30859	0.0698591
18448	1.30682	2.1
18449	1.38957	2.1
18450	1.38957	2.1
18451	1.47446	2.1
18452	0.953638	2.1
18453	1.03408	2.1
18454	1.17885	0.23204
18455	1.3663	0.168525
18456	1.08265	0.185954
18457	1.71948	0.23198
18458	1.29501	0.574346
18459	0.955093	0.499707
18460	0.955081	0.410643
18461	1.88345	0.436541
18462	1.2243	2.1
18463	1.23169	2.1
18464	2.04726	0.566848
18465	0.842869	0.372307
18466	3.18156	2.1
18467	3.60786	1.79462
18468	3.60786	2.0919
18469	2.38566	1.64426
18470	1.24526	0.0698591
18471	1.59019	0.0698591
18472	1.16776	1.84042
18473	1.05042	2.1
18474	1.17418	2.1
18475	1.05042	2.1
18476	0.949135	2.1
18477	0.973448	2.1
18478	0.973447	2.1
18479	1.02958	2.1
18480	3.57768	2.1
18481	3.04246	2.1
18482	3.04246	2.1
18483	2.47824	2.1
18484	1.35879	2.1
18485	1.10407	2.1
18486	1.14599	2.0812
18487	1.10407	2.0932
18488	1.87794	1.69509
18489	1.53429	1.46457
18490	1.12717	2.1
18491	1.38634	2.1
18492	1.38634	2.1
18493	1.13127	2.1
18494	1.04546	2.1
18495	1.06007	2.1
18496	1.06007	2.1
18497	1.19785	2.1
18498	1.43652	2.1
18499	1.71741	2.1

18500	1.70921	2.1
18501	1.06994	2.1
18502	0.960106	2.1
18503	1.48077	2.1
18504	1.48077	2.1
18505	2.5946	2.1
18506	2.03045	1.86376
18507	1.76788	1.90735
18508	2.03045	2.05169
18509	0.633098	2.09576
18510	1.06756	2.1
18511	1.38667	2.1
18512	0.966979	2.1
18513	1.38669	2.1
18514	1.07156	2.1
18515	0.986095	2.1
18516	1.32813	2.1
18517	0.986095	2.1
18518	1.98697	0.348623
18519	1.12016	0.689089
18520	1.24463	0.585658
18521	1.1967	0.555166
18522	1.30001	0.531839
18523	1.29175	1.17171
18524	1.29175	0.570657
18525	1.3003	1.27069
18526	1.5606	0.0698591
18527	2.32098	0.0698591
18528	2.57888	0.0698591
18529	2.97463	0.0698591
18530	1.24555	2.1
18531	1.46101	2.1
18532	1.90723	2.1
18533	2.29126	2.1
18534	1.46101	2.1
18535	1.84089	2.1
18536	2.5519	2.1
18537	2.91663	2.1
18538	1.10476	0.0698591
18539	1.44103	0.0698591
18540	1.44103	0.0698591
18541	1.53336	0.0698591
18542	0.672964	2.1
18543	0.633884	2.1
18544	0.633884	2.1
18545	0.603154	2.1
18546	2.29663	2.1
18547	2.06892	2.1
18548	1.3685	2.1
18549	1.39067	2.1
18550	2.06892	2.1
18551	1.78208	2.1
18552	1.39067	2.1
18553	1.42174	2.1
18554	1.01335	1.66393
18555	1.08195	0.698873
18556	1.86463	1.98914
18557	1.39827	1.96243

18558	0.861515	2.04088
18559	2.10074	2.1
18560	2.09511	1.15286
18561	2.04853	0.546998
18562	2.21488	0.837887
18563	1.38287	1.1209
18564	0.974717	0.333839
18565	0.998451	0.271457
18566	1.42281	1.56349
18567	1.82595	1.43022
18568	1.50458	0.841694
18569	1.09408	0.80704
18570	1.23498	1.38817
18571	1.05654	1.2073
18572	1.17261	2.1
18573	1.18058	2.1
18574	1.18059	2.1
18575	1.18907	2.1
18576	2.38998	2.1
18577	2.77437	2.1
18578	1.04546	2.1
18579	1.16817	2.1
18580	2.77437	2.1
18581	2.97768	2.1
18582	1.16817	2.1
18583	1.2832	2.1
18584	1.75766	0.134543
18585	1.4934	1.92956
18586	1.4934	0.277077
18587	1.20324	2.0387
18588	1.07236	0.727174
18589	1.37641	0.511379
18590	1.42913	0.415904
18591	1.93373	0.387228
18592	1.42629	0.321152
18593	1.00636	0.299659
18594	0.933959	1.88254
18595	2.22873	0.957088
18596	2.22873	1.6968
18597	2.13757	0.719884
18598	1.10517	2.1
18599	1.86737	2.1
18600	0.811 2.1	
18601	1.19179	2.1
18602	1.18647	1.8354
18603	0.988453	2.05705
18604	1.12457	0.101489
18605	1.23851	0.0891428
18606	0.92492	2.1
18607	1.45656	2.1
18608	1.61251	0.585342
18609	1.56696	0.582091
18610	1.90245	0.439452
18611	1.46046	0.427292
18612	1.60329	0.0698591
18613	1.38247	0.0698591
18614	1.38247	0.0698591
18615	1.23198	0.0698591

18616	1.09518	2.1
18617	1.28159	2.1
18618	0.977777	2.1
18619	1.80778	2.1
18620	1.80777	2.1
18621	1.16655	2.1
18622	1.3515	2.1
18623	0.956573	2.1
18624	1.08022	2.1
18625	1.03437	2.1
18626	1.03437	2.1
18627	0.975409	2.1
18628	1.47369	0.0698591
18629	1.31167	0.0704513
18630	1.01847	0.0698591
18631	2.75562	0.0698591
18632	1.13443	2.1
18633	1.32709	2.1
18634	1.01051	0.202631
18635	0.958947	0.17052
18636	1.7211	2.1
18637	1.922 2.1	
18638	1.922 2.1	
18639	0.991113	2.1
18640	1.00636	0.333072
18641	1.31658	0.330209
18642	1.44627	0.245271
18643	1.03824	0.234466
18644	1.21223	2.1
18645	1.01242	2.1
18646	1.01243	2.1
18647	0.956698	2.1
18648	1.23167	2.1
18649	1.06935	2.1
18650	1.29215	0.0698591
18651	0.998971	0.0698591
18652	1.50103	0.0698591
18653	1.44836	0.0698591
18654	3.02513	2.1
18655	3.7335	2.1
18656	2.49988	2.1
18657	1.24948	2.1
18658	0.963972	2.1
18659	1.1822	2.1
18660	1.1822	2.1
18661	1.10817	2.1
18662	1.30237	2.1
18663	1.41908	2.1
18664	1.41908	2.1
18665	1.48734	2.1
18666	1.31127	0.191538
18667	1.40822	0.222109
18668	1.40822	0.110919
18669	1.22176	0.127224
18670	1.82525	2.1
18671	1.59394	2.1
18672	1.22731	0.0698591
18673	0.981584	0.0698591

18674	1.1427	0.359129
18675	1.44091	0.621129
18676	1.49154	1.30236
18677	1.07526	1.03706
18678	1.07527	0.649989
18679	0.96029	1.31827
18680	2.40568	1.08124
18681	3.46486	1.0815
18682	2.417	1.14609
18683	1.52054	0.8552
18684	1.51638	0.0851858
18685	1.04786	0.0718704
18686	1.51404	0.0834266
18687	1.3461	0.0710378
18688	1.27696	0.0698591
18689	0.736457	0.0698591
18690	1.16817	2.1
18691	1.04263	2.1
18692	1.13449	2.1
18693	1.50524	2.1
18694	2.1182	2.1
18695	2.49001	2.1
18696	1.11764	2.1
18697	1.39292	2.1
18698	2.49001	2.1
18699	2.76339	2.1
18700	1.39292	2.1
18701	1.68761	2.1
18702	1.25173	2.1
18703	1.34701	2.1
18704	1.02415	2.1
18705	1.34701	2.1
18706	1.332	0.0698591
18707	0.989764	0.0698591
18708	0.963167	0.0698591
18709	1.21056	0.0698591
18710	1.67375	2.1
18711	1.17965	2.1
18712	1.09154	2.1
18713	1.52314	2.1
18714	1.54433	0.209991
18715	1.81969	0.167524
18716	1.3598	0.151448
18717	1.71041	0.123076
18718	1.70934	0.118605
18719	1.33871	0.0973252
18720	1.79485	0.104474
18721	1.51404	0.081603
18722	1.30751	2.1
18723	1.20969	2.1
18724	1.20969	2.1
18725	1.12076	2.1
18726	1.49808	2.1
18727	1.50201	2.1
18728	1.50201	2.1
18729	1.31594	2.1
18730	1.2187	2.1
18731	1.11545	2.1

18732	1.227	2.1
18733	1.09645	2.1
18734	1.05902	0.0698591
18735	1.41595	1.60606
18736	1.35696	2.1
18737	1.29446	1.95848
18738	0.857957	2.1
18739	1.21125	0.0698591
18740	2.43115	2.1
18741	2.1151	0.284491
18742	2.0815	1.50758
18743	1.1723	1.96774
18744	1.48035	0.126131
18745	1.59971	0.461752
18746	1.33668	0.280513
18747	1.0961	0.242985
18748	1.46235	2.1
18749	0.882195	2.1
18750	2.46624	2.1
18751	2.72161	2.1
18752	2.96094	2.1
18753	2.25644	2.1
18754	1.25923	0.0698591
18755	0.976218	0.0698591
18756	1.10319	0.0698591
18757	1.82583	0.0698591
18758	0.888955	2.1
18759	1.30817	2.1
18760	1.30817	2.1
18761	1.60787	2.1
18762	0.988805	2.1
18763	1.54168	2.1
18764	1.19496	0.675679
18765	1.12224	1.46443
18766	1.93597	1.40478
18767	1.31113	1.15057
18768	1.17879	1.29753
18769	1.07468	1.30989
18770	2.0523	2.1
18771	2.41385	2.1
18772	2.41385	2.1
18773	3.0355	2.1
18774	1.36717	0.145047
18775	1.45535	0.119083
18776	1.45535	0.109369
18777	1.49906	0.0871016
18778	1.37785	2.1
18779	1.04413	2.1
18780	1.06729	0.671052
18781	1.54305	0.591216
18782	1.07444	2.1
18783	1.52724	2.1
18784	1.49151	2.1
18785	1.36814	2.1
18786	1.36814	2.1
18787	1.15541	2.1
18788	1.19379	2.1
18789	1.78657	1.8824

18790	1.6079	2.1
18791	2.59469	1.53622
18792	1.35545	0.951391
18793	1.23602	1.98277
18794	1.23452	2.1
18795	1.56842	2.05673
18796	1.15864	2.1
18797	1.11955	2.1
18798	1.11955	2.1
18799	1.08022	2.1
18800	1.64268	2.1
18801	1.26247	2.1
18802	1.16912	2.1
18803	2.1095	1.19718
18804	1.75549	0.0698591
18805	1.7491	0.0698591
18806	1.59833	0.0698591
18807	1.68338	0.0698591
18808	1.14846	0.0698591
18809	1.06515	0.0698591
18810	1.2721	0.114445
18811	1.32639	0.101705
18812	1.32639	0.184078
18813	1.19323	0.159765
18814	1.81034	2.1
18815	1.9034	2.1
18816	2.08245	2.1
18817	1.84887	2.1
18818	1.41448	2.1
18819	1.77326	2.1
18820	1.76872	2.1
18821	1.86766	2.1
18822	1.46643	2.1
18823	1.58995	2.1
18824	1.58995	2.1
18825	1.13854	2.1
18826	1.23654	2.1
18827	1.04442	2.1
18828	1.23654	2.1
18829	1.08576	2.1
18830	0.966979	2.1
18831	1.41758	2.1
18832	1.03108	0.0954136
18833	1.94412	0.109169
18834	1.32455	2.1
18835	1.32328	2.1
18836	1.32328	2.1
18837	1.34008	2.1
18838	1.60861	2.1
18839	1.69586	2.1
18840	1.69586	2.1
18841	1.78301	2.1
18842	1.7513	2.1
18843	1.67906	2.1
18844	1.67906	2.1
18845	1.60635	2.1
18846	1.68073	2.1
18847	1.57234	2.1

18848	1.57233	2.1
18849	1.47012	2.1
18850	1.22811	0.132027
18851	1.79683	0.0950757
18852	1.53417	0.101711
18853	1.08732	0.106548
18854	1.8903	2.09146
18855	1.20247	2.08972
18856	1.12087	1.69721
18857	1.54699	2.08827
18858	1.43635	2.1
18859	1.02368	2.1
18860	1.43635	2.1
18861	1.23007	2.1
18862	1.035 2.1	
18863	1.07889	2.1
18864	3.0355	2.1
18865	3.78805	2.1
18866	2.57183	2.1
18867	1.27835	2.05792
18868	1.29813	0.135905
18869	1.42965	0.119969
18870	1.69832	0.132192
18871	2.18359	0.118256
18872	1.42964	0.137043
18873	1.65723	0.126398
18874	1.98508	0.129163
18875	2.06284	0.115483
18876	1.50705	0.0715507
18877	1.51153	0.100563
18878	1.36037	0.0847412
18879	1.09767	0.0946045
18880	1.70048	2.1
18881	1.15177	2.1
18882	0.713174	2.1
18883	0.763575	2.1
18884	0.763575	2.1
18885	0.797011	2.1
18886	1.01179	0.425695
18887	1.27737	0.536767
18888	3.16531	0.0698591
18889	1.0141	0.0698591
18890	1.47354	0.165164
18891	1.62495	0.134032
18892	1.62495	0.112276
18893	1.39296	0.224675
18894	0.998682	2.1
18895	1.93528	2.1
18896	2.71383	2.1
18897	1.13987	2.1
18898	1.93528	2.1
18899	1.78595	2.1
18900	3.98933	2.1
18901	3.73426	2.1
18902	1.02438	2.1
18903	1.12376	2.1
18904	1.12375	2.1
18905	1.19016	2.1

18906	1.37157	0.12083
18907	1.60814	0.118119
18908	1.70303	0.153662
18909	1.01219	0.131891
18910	1.06757	2.1
18911	1.23475	2.1
18912	1.2295	2.1
18913	1.28014	2.1
18914	1.28014	2.1
18915	1.33339	2.1
18916	1.3201	2.1
18917	1.2679	2.1
18918	1.2679	2.1
18919	1.23504	2.1
18920	1.50108	2.1
18921	1.3715	2.1
18922	1.3715	2.1
18923	1.25075	2.1
18924	1.99222	0.681589
18925	1.4155	0.531684
18926	1.4777	2.1
18927	1.52613	2.1
18928	1.91031	2.1
18929	1.03968	2.1
18930	2.04289	0.144732
18931	0.967192	0.1168
18932	1.03043	0.0947407
18933	1.65452	0.143635
18934	1.21564	2.1
18935	1.16938	2.1
18936	2.47056	2.1
18937	2.56853	2.1
18938	1.16938	2.1
18939	1.1181	2.1
18940	2.56853	2.1
18941	2.66828	2.1
18942	1.0465	2.1
18943	1.44709	2.1
18944	1.44709	2.1
18945	1.96491	2.1
18946	1.01976	0.323272
18947	0.961779	0.289336
18948	1.11764	0.318302
18949	0.961779	0.290072
18950	1.21171	0.0698591
18951	1.35007	0.0698591
18952	1.03102	0.254573
18953	1.42446	0.269069
18954	1.42446	0.241613
18955	1.03166	0.215487
18956	1.2951	0.104686
18957	1.33575	0.117856
18958	1.33575	0.122723
18959	1.20473	0.132687
18960	1.10049	0.587033
18961	0.99747	0.943205
18962	1.54249	2.1
18963	1.65677	2.1

18964	1.65677	2.1
18965	1.77677	2.1
18966	1.73525	0.147139
18967	1.05689	0.185209
18968	1.26051	0.125642
18969	1.16259	0.118383
18970	1.34009	2.1
18971	1.398	2.1
18972	1.398	2.1
18973	1.50437	2.1
18974	2.3245	2.1
18975	1.18237	2.1
18976	1.24509	2.1
18977	1.01595	2.1
18978	3.29047	2.1
18979	2.79168	2.1
18980	1.06561	2.1
18981	1.11077	2.1
18982	1.11077	2.1
18983	0.953927	2.1
18984	1.30509	0.288952
18985	1.04685	0.303182
18986	1.04685	0.221677
18987	0.834593	0.23316
18988	1.0775	0.139134
18989	1.27362	0.128872
18990	1.71762	0.590786
18991	2.5736	0.532497
18992	2.04717	0.683751
18993	1.45521	0.427389
18994	1.33962	2.1
18995	1.27956	2.1
18996	1.27956	2.1
18997	1.20478	2.1
18998	3.65841	0.0698591
18999	1.18656	0.0698591
19000	1.72272	0.0723741
19001	1.6812	0.0699202
19002	1.09951	2.1
19003	1.08231	2.1
19004	1.08231	2.1
19005	1.06478	2.1
19006	2.16196	1.70336
19007	1.00387	1.29144
19008	1.00387	0.500445
19009	0.993999	0.455143
19010	1.04101	2.1
19011	1.27119	2.1
19012	1.27119	2.1
19013	1.51748	2.1
19014	3.00129	2.1
19015	3.27303	2.1
19016	3.27303	2.1
19017	3.60855	2.1
19018	2.22399	2.1
19019	1.71995	2.1
19020	1.54793	2.1
19021	1.227	2.1

19022	1.07624	2.1
19023	1.264 2.1	
19024	1.19323	1.86768
19025	1.21286	1.23631
19026	1.21286	1.19814
19027	1.20859	0.703238
19028	1.60578	0.114275
19029	1.27778	0.090056
19030	1.0525	2.1
19031	1.05758	0.75344
19032	1.0525	0.71312
19033	1.17042	1.46696
19034	1.62536	0.0698591
19035	3.71662	0.0698591
19036	1.78169	0.0698591
19037	1.91434	0.0698591
19038	2.12331	0.0698591
19039	1.33406	0.0698591
19040	1.38437	2.1
19041	1.51829	2.1
19042	1.51829	2.1
19043	1.6697	2.1
19044	0.947114	2.1
19045	1.13335	2.1
19046	1.13335	2.1
19047	1.00965	2.1
19048	1.47521	2.1
19049	1.27679	2.1
19050	1.14911	2.1
19051	0.93841	2.1
19052	1.68437	2.1
19053	1.53822	2.1
19054	1.53822	2.1
19055	1.39702	2.1
19056	0.726453	2.1
19057	0.754101	2.1
19058	0.754101	2.1
19059	0.69776	2.1
19060	0.964033	2.1
19061	1.62397	2.1
19062	1.70302	2.08107
19063	1.78637	2.06483
19064	3.68158	0.0698591
19065	2.11685	0.0698591
19066	1.33125	2.1
19067	1.15471	2.1
19068	1.0465	2.1
19069	1.33125	2.1
19070	1.17683	2.1
19071	1.25889	2.1
19072	1.25889	2.1
19073	1.34239	2.1
19074	1.40591	0.0698591
19075	0.79348	0.0698591
19076	1.04019	0.0698591
19077	0.999187	0.0698591
19078	1.30218	0.0698591
19079	1.83339	0.0698591

19080	1.88574	0.0698591
19081	1.5177	0.0698591
19082	1.23504	2.1
19083	1.19583	2.1
19084	1.19583	2.1
19085	1.14496	2.1
19086	1.62183	2.1
19087	1.62316	2.1
19088	1.62316	2.1
19089	1.62241	2.1
19090	1.02305	0.195474
19091	1.21136	0.178691
19092	0.993832	0.0851916
19093	1.21137	0.117036
19094	1.12064	2.1
19095	1.24965	2.1
19096	1.24965	2.1
19097	1.41077	2.1
19098	1.00052	0.137167
19099	1.49842	0.106618
19100	1.49843	0.0879975
19101	1.10801	0.0776367
19102	1.54196	2.1
19103	1.18041	2.1
19104	1.73571	1.85197
19105	1.37224	1.93189
19106	1.4375	0.0789927
19107	1.09529	0.0720297
19108	1.44421	0.0698675
19109	1.0953	0.0714838
19110	1.0753	2.1
19111	0.815365	2.1
19112	0.815365	2.1
19113	0.597537	2.1
19114	1.03609	2.1
19115	1.18952	2.1
19116	1.54283	0.216758
19117	1.16719	0.190698
19118	1.16719	0.266698
19119	1.89226	0.233394
19120	2.55919	2.1
19121	2.37492	2.1
19122	2.37492	2.1
19123	2.07435	2.1
19124	1.36016	0.112713
19125	1.70302	0.103343
19126	1.85252	0.120645
19127	1.2111	0.205583
19128	1.62905	2.1
19129	1.49692	2.1
19130	1.49692	2.1
19131	1.40395	2.1
19132	2.19083	2.1
19133	1.30542	2.1
19134	1.58736	2.1
19135	2.16579	2.1
19136	2.0815	2.1
19137	1.17935	2.1

19138	1.53803	2.1
19139	1.09148	2.03319
19140	1.81301	2.1
19141	1.38333	1.67234
19142	1.04412	2.1
19143	1.44437	2.1
19144	1.4675	0.0733235
19145	1.00665	0.0789056
19146	1.50951	0.089657
19147	0.983105	0.0826173
19148	1.80252	0.128428
19149	0.540005	0.0895439
19150	1.2545	0.142909
19151	2.14514	0.099339
19152	1.32051	0.0790295
19153	1.82302	0.0741336
19154	2.71057	2.1
19155	2.51107	2.1
19156	1.34817	2.1
19157	1.3551	2.1
19158	2.51107	2.1
19159	2.29663	2.1
19160	1.3551	2.1
19161	1.3685	2.1
19162	1.51748	2.1
19163	1.23389	2.1
19164	0.901928	0.0698591
19165	1.70302	0.0698591
19166	1.66787	0.0698591
19167	1.92535	0.0698591
19168	1.13571	0.226621
19169	1.02836	0.250825
19170	1.55398	2.1
19171	1.54179	2.1
19172	1.89608	2.1
19173	1.07167	2.1
19174	3.3488	0.119824
19175	0.550044	0.105325
19176	0.962877	0.397777
19177	1.00065	0.78982
19178	1.00613	0.417206
19179	1.07497	0.467781
19180	1.00486	0.0698591
19181	1.00486	0.0698591
19182	0.726453	2.1
19183	1.32634	0.0743668
19184	1.40008	0.0935118
19185	1.40008	0.0703877
19186	2.42267	0.0702616
19187	1.9216	0.0698591
19188	1.28066	0.0698591
19189	1.79102	2.09761
19190	1.18219	0.737323
19191	1.18219	2.03837
19192	0.709123	0.655728
19193	1.07866	2.1
19194	1.15038	2.1
19195	1.15038	2.1

19196	1.24687	2.1
19197	1.83613	2.07599
19198	1.99157	2.05395
19199	1.45789	0.0698591
19200	1.1706	0.0698591
19201	1.74746	0.0698591
19202	1.1706	0.0698591
19203	1.96383	0.0698591
19204	1.67727	0.0698591
19205	1.67727	0.0698591
19206	1.60329	0.0698591
19207	1.18803	2.1
19208	1.3227	2.1
19209	1.3227	2.1
19210	1.40844	2.1
19211	1.07889	2.1
19212	1.07721	2.1
19213	1.07721	2.1
19214	1.00832	2.1
19215	0.953523	0.624389
19216	1.62875	0.521874
19217	1.07427	0.186024
19218	1.19722	0.211358
19219	1.1972	0.154945
19220	1.1599	0.177196
19221	2.97463	0.0698591
19222	1.9477	0.0698591
19223	1.95222	0.0698591
19224	1.75083	0.0698591
19225	3.46787	2.1
19226	3.02896	2.1
19227	3.02896	2.1
19228	2.46145	2.1
19229	0.970962	0.0698591
19230	1.0227	0.0698591
19231	1.12328	0.0698591
19232	1.02269	0.0698591
19233	0.904978	2.1
19234	0.725887	2.1
19235	0.725887	2.1
19236	0.601592	2.1
19237	0.956294	2.1
19238	1.13179	2.1
19239	0.956294	2.1
19240	1.13179	2.1
19241	1.05556	2.1
19242	1.13179	2.1
19243	1.38859	2.1
19244	1.13179	2.1
19245	1.06659	2.1
19246	1.05776	2.1
19247	1.06659	2.1
19248	1.04291	2.1
19249	1.54699	1.42037
19250	1.42307	1.6761
19251	1.5654	2.04585
19252	1.97906	2.1
19253	1.15354	0.0698591

19254	1.15505	0.0698591
19255	1.26455	0.127455
19256	2.50042	0.0774415
19257	2.50042	0.123443
19258	3.22585	0.0736993
19259	1.19258	0.302302
19260	1.34562	0.250078
19261	1.49515	0.352242
19262	1.66295	0.22545
19263	1.02395	2.1
19264	1.51067	2.1
19265	1.35821	2.0961
19266	2.10671	2.1
19267	1.0305	2.05593
19268	1.07855	2.03155
19269	1.20761	2.1
19270	0.98488	2.1
19271	1.4162	2.1
19272	1.14825	2.1
19273	1.29415	2.1
19274	1.6067	2.1
19275	1.23851	0.136035
19276	1.09651	0.113679
19277	1.60636	2.1
19278	1.53521	2.1
19279	1.53521	2.1
19280	1.46279	2.1
19281	1.28044	0.0698591
19282	1.64147	0.070577
19283	1.18422	0.0698591
19284	1.68208	0.0736775
19285	1.09726	2.1
19286	1.234 2.1	
19287	1.234 2.1	
19288	1.09483	2.1
19289	1.89318	2.1
19290	1.98448	2.1
19291	1.98448	2.1
19292	2.02612	2.1
19293	1.22026	2.08376
19294	1.23527	1.88386
19295	1.09044	2.06935
19296	0.993366	1.49601
19297	1.22026	2.02846
19298	0.993368	1.14026
19299	2.61357	2.1
19300	2.48208	2.1
19301	2.48208	2.1
19302	2.62868	2.1
19303	1.85439	0.806716
19304	1.81776	0.472974
19305	1.81776	0.359385
19306	2.1587	0.397093
19307	1.8914	0.699017
19308	2.23961	0.407043
19309	1.49357	0.111048
19310	2.23961	0.391177
19311	1.78014	0.357089

19312	1.67635	0.317123
19313	1.66219	0.287165
19314	1.56036	0.264351
19315	1.14721	0.279116
19316	2.22495	0.350013
19317	1.88509	0.0723961
19318	1.95249	0.0735217
19319	2.11959	0.0698591
19320	1.37427	0.0698591
19321	1.39379	2.06082
19322	1.17614	1.92798
19323	1.39379	2.1
19324	1.14397	1.52598
19325	1.09651	2.1
19326	1.42843	2.1
19327	1.7069	2.1
19328	2.31293	2.1
19329	1.56839	2.1
19330	1.78252	1.82374
19331	1.78254	1.37217
19332	1.09142	2.08363
19333	0.99829	0.0698591
19334	1.09372	0.0698591
19335	1.88175	0.070491
19336	0.898452	0.0698591
19337	1.06936	2.1
19338	1.4162	2.1
19339	1.17989	2.1
19340	1.47411	2.1
19341	1.25376	2.1
19342	1.00757	2.09248
19343	1.4185	0.192622
19344	1.02011	0.204596
19345	2.22495	0.222415
19346	1.79542	0.203724
19347	1.79542	0.172034
19348	1.19352	0.163393
19349	2.38566	2.1
19350	1.83255	1.80554
19351	1.62667	2.1
19352	1.17879	1.91941
19353	2.79168	2.1
19354	2.28162	2.1
19355	2.28162	2.1
19356	1.90905	2.1
19357	1.40117	2.1
19358	1.28268	2.1
19359	1.41096	2.1
19360	1.22978	2.1
19361	3.20034	2.1
19362	3.42052	2.1
19363	3.42052	2.1
19364	3.50562	2.1
19365	1.46279	2.1
19366	1.3864	2.1
19367	1.3864	2.1
19368	1.3201	2.1
19369	1.35561	0.0698591

19370	0.974077	0.0698591
19371	0.967673	0.765525
19372	1.22482	0.640621
19373	1.02305	0.26698
19374	1.22482	0.403837
19375	0.999492	2.1
19376	1.14657	2.1
19377	1.14657	2.1
19378	1.31346	2.1
19379	1.84417	0.0698591
19380	1.31777	0.0698591
19381	1.19796	0.0698591
19382	0.691742	0.0698591
19383	1.16725	2.1
19384	1.9679	2.1
19385	1.08848	0.0698591
19386	1.15356	0.0698591
19387	1.58713	2.1
19388	1.58742	2.1
19389	1.58742	2.1
19390	1.49662	2.1
19391	1.32201	0.809572
19392	1.32097	1.66082
19393	1.32097	0.837904
19394	1.31825	1.73305
19395	1.12028	2.1
19396	1.15852	2.1
19397	1.15852	2.1
19398	1.16239	2.1
19399	1.07624	2.1
19400	1.30047	2.1
19401	1.44847	0.57439
19402	1.55292	0.519281
19403	1.21125	0.0698591
19404	1.15262	2.1
19405	1.05025	2.1
19406	1.55351	2.1
19407	1.5329	0.0698591
19408	2.26758	0.0698591
19409	1.94968	0.0698591
19410	1.65747	0.0698591
19411	1.42549	0.0698591
19412	1.57656	0.0698591
19413	2.11157	0.690412
19414	1.56531	0.63714
19415	1.69279	0.54044
19416	1.06204	0.713859
19417	0.947863	0.927343
19418	1.19854	1.07319
19419	0.947862	0.37639
19420	1.31126	0.404585
19421	1.3425	1.66098
19422	1.29976	1.34594
19423	1.05129	0.0834305
19424	1.51026	0.0908562
19425	1.05129	0.0886251
19426	1.2951	0.100452
19427	1.7155	2.1

19428	1.72196	2.1
19429	1.72196	2.1
19430	1.77867	2.1
19431	0.964755	2.1
19432	1.14142	2.1
19433	1.03766	2.1
19434	0.986723	2.1
19435	1.28072	2.1
19436	1.10609	2.1
19437	1.28072	1.77017
19438	1.16776	2.1
19439	1.61687	2.1
19440	1.60965	2.1
19441	1.60965	2.1
19442	1.59654	2.1
19443	1.26512	2.1
19444	1.23964	2.1
19445	2.07997	2.1
19446	2.60048	2.1
19447	1.23732	2.1
19448	1.3372	2.1
19449	2.62253	2.1
19450	2.47056	2.1
19451	0.92782	0.0898085
19452	1.40335	0.0974331
19453	1.26301	0.110161
19454	1.52668	0.107258
19455	0.99818	0.113024
19456	1.51165	0.136
19457	1.66213	0.109458
19458	1.55246	0.112119
19459	2.31255	2.1
19460	1.34366	2.1
19461	1.90577	2.1
19462	0.969739	2.1
19463	3.4245	2.1
19464	2.8415	2.1
19465	1.97559	2.1
19466	1.82362	2.1
19467	1.48162	2.1
19468	1.23204	2.1
19469	1.23204	2.1
19470	0.95947	2.1
19471	1.05267	2.07893
19472	1.44091	1.1546
19473	1.05267	1.43787
19474	1.5795	1.97145
19475	0.738711	2.1
19476	1.28549	2.1
19477	1.15694	2.1
19478	1.72061	2.1
19479	1.69858	2.1
19480	0.954563	2.1
19481	0.859087	2.1
19482	0.788886	2.1
19483	1.08732	0.137407
19484	1.3357	0.118974
19485	1.3357	0.131347

19486	1.48676	0.102923
19487	2.15079	2.1
19488	2.12414	2.1
19489	2.12414	2.1
19490	2.04664	2.1
19491	1.98859	2.1
19492	0.992159	2.1
19493	1.48064	2.1
19494	1.2163	2.1
19495	1.26337	2.1
19496	1.63159	2.1
19497	1.00366	0.327143
19498	1.65452	0.187867
19499	1.10408	2.1
19500	1.37704	2.1
19501	1.50224	2.1
19502	1.42866	2.1
19503	1.43939	2.1
19504	1.02784	2.1
19505	1.00763	0.100923
19506	1.23746	0.102198
19507	1.23747	0.110572
19508	1.13642	0.116565
19509	1.49906	0.0837648
19510	1.46506	0.0729897
19511	1.46506	0.0699511
19512	1.33061	0.0698591
19513	1.08166	0.163745
19514	1.01052	0.149683
19515	1.02836	0.178871
19516	1.08166	0.163908
19517	1.25334	0.0698591
19518	1.23749	0.0698591
19519	1.37501	0.0798389
19520	1.47354	0.0930545
19521	1.30167	2.1
19522	1.29897	2.1
19523	1.51042	2.1
19524	1.07684	2.1
19525	1.07684	2.1
19526	0.765121	2.1
19527	1.5535	2.1
19528	1.40602	2.1
19529	1.40603	2.1
19530	1.25669	2.1
19531	1.08276	2.1
19532	1.60948	2.1
19533	1.26929	2.1
19534	1.09847	2.1
19535	0.827082	0.176595
19536	1.15841	0.191404
19537	1.25063	0.459988
19538	1.14397	0.451264
19539	1.25063	1.77853
19540	1.10476	0.25517
19541	1.14872	0.0698591
19542	1.3659	0.0698591
19543	1.36589	0.0698591

19544	1.4677	0.0698591
19545	0.590354	2.1
19546	0.655202	2.1
19547	3.48926	2.1
19548	3.32709	2.1
19549	3.32709	2.1
19550	3.13938	2.1
19551	1.24458	0.879852
19552	1.29681	0.623941
19553	1.40915	1.0429
19554	1.17891	1.79579
19555	1.33171	0.0698591
19556	1.37681	1.68603
19557	1.37681	2.1
19558	1.4516	2.1
19559	2.68241	0.0698591
19560	1.61031	0.0698591
19561	1.6488	2.1
19562	1.6585	2.1
19563	1.65848	2.1
19564	1.67061	2.1
19565	1.15152	2.1
19566	1.32548	2.1
19567	1.43677	2.1
19568	1.10264	2.1
19569	1.01472	2.1
19570	0.966964	2.1
19571	2.867 2.1	
19572	2.94897	2.1
19573	1.65815	2.1
19574	1.4502	2.1
19575	1.4502	2.1
19576	1.26755	2.1
19577	1.37138	2.1
19578	1.45026	2.1
19579	1.45026	2.1
19580	1.53446	2.1
19581	0.973502	2.07867
19582	1.08542	2.1
19583	1.13387	0.0698591
19584	1.71031	0.0698591
19585	1.08911	2.1
19586	1.10748	2.1
19587	1.21823	2.1
19588	1.25005	2.1
19589	0.657014	2.1
19590	1.38139	2.1
19591	1.01485	2.1
19592	1.26045	2.1
19593	1.12468	2.1
19594	1.26045	2.1
19595	1.89278	0.500113
19596	1.06433	0.393101
19597	1.87861	0.405637
19598	0.967544	0.317867
19599	1.50319	2.1
19600	3.47245	2.1
19601	2.2354	1.13147

19602	1.16701	0.337337
19603	1.3592	2.1
19604	1.3305	1.53427
19605	1.3305	2.1
19606	1.15008	0.822627
19607	1.12457	0.0708185
19608	1.40707	0.0734516
19609	1.01778	2.1
19610	1.35733	2.1
19611	1.01779	2.1
19612	1.12064	2.1
19613	1.30694	0.485899
19614	1.42716	1.37686
19615	1.42717	1.61886
19616	1.04321	1.56856
19617	1.19352	0.147252
19618	1.32634	0.195311
19619	1.14605	1.9347
19620	1.0417	1.67538
19621	1.19676	0.0974026
19622	1.03804	0.106131
19623	2.09455	0.0783099
19624	1.15338	0.0822705
19625	2.21119	2.1
19626	1.26097	2.1
19627	1.88394	1.74043
19628	1.24106	0.95115
19629	2.98143	2.1
19630	2.56248	2.1
19631	1.88394	2.1
19632	1.68703	0.762158
19633	1.5846	2.1
19634	1.94799	2.1
19635	1.20091	2.06091
19636	1.40393	2.0981
19637	3.09031	2.1
19638	2.90243	2.1
19639	1.27227	2.1
19640	1.32592	2.1
19641	2.90247	2.1
19642	2.71057	2.1
19643	1.32594	2.1
19644	1.34817	2.1
19645	1.4092	2.1
19646	1.44859	2.1
19647	1.52008	2.1
19648	1.401	2.1
19649	1.40099	2.1
19650	1.28936	2.1
19651	1.56002	0.0698591
19652	1.08811	0.0698591
19653	1.46041	0.0698591
19654	1.56002	0.0698591
19655	1.50524	2.1
19656	1.21304	2.1
19657	1.21304	2.1
19658	0.947115	2.1
19659	1.60665	2.0335

19660	1.85179	1.77662
19661	1.85178	1.79266
19662	2.06277	1.15302
19663	2.17534	2.1
19664	1.23007	2.1
19665	2.17533	2.09644
19666	1.66635	1.98674
19667	2.00869	2.1
19668	0.986724	2.1
19669	2.00475	2.1
19670	1.99394	2.1
19671	1.67244	0.12571
19672	1.39604	0.112818
19673	1.39604	0.126946
19674	0.954046	0.100177
19675	0.988863	2.1
19676	1.9864	2.1
19677	1.9864	2.1
19678	3.38645	2.1
19679	1.46932	0.0698591
19680	1.50847	0.0698591
19681	1.50848	0.0698591
19682	1.42705	0.0698591
19683	1.50108	2.1
19684	1.51621	0.290929
19685	1.94317	0.413648
19686	1.18382	0.167015
19687	2.05091	0.0698591
19688	1.88593	0.0698591
19689	1.54299	0.0698591
19690	0.987265	0.0698591
19691	1.27766	2.1
19692	1.5178	2.1
19693	1.5178	2.1
19694	1.81518	2.1
19695	1.04626	1.464
19696	1.31057	1.50251
19697	1.31057	1.32418
19698	1.22297	1.60986
19699	1.67844	2.1
19700	1.62862	2.1
19701	1.62112	2.1
19702	1.87598	2.1
19703	1.17844	2.1
19704	1.37063	2.1
19705	1.37063	2.1
19706	1.68723	2.1
19707	1.99943	0.0755042
19708	1.17631	0.0698591
19709	1.4263	1.96832
19710	1.31023	2.1
19711	1.31023	2.07897
19712	1.20871	2.1
19713	1.50356	2.1
19714	1.69311	2.1
19715	1.69599	2.1
19716	1.6801	2.1
19717	1.5843	0.0698591

19718	0.840233	0.0698591
19719	1.95358	0.0698591
19720	1.40216	0.0698591
19721	1.39078	2.05138
19722	1.18948	2.04172
19723	1.01606	1.88929
19724	1.18948	2.00769
19725	1.63823	2.1
19726	1.67687	2.1
19727	1.67687	2.1
19728	1.52855	2.1
19729	1.11048	2.1
19730	1.25346	2.1
19731	2.42984	1.2234
19732	3.41731	1.50891
19733	1.2258	2.1
19734	1.2966	2.1
19735	1.2966	2.1
19736	1.37392	2.1
19737	1.41758	2.1
19738	1.35504	2.1
19739	1.35504	2.1
19740	1.30347	2.1
19741	1.50877	2.1
19742	1.11193	2.1
19743	1.40984	2.05699
19744	1.54537	2.07467
19745	1.71638	2.08264
19746	1.77795	2.00459
19747	2.07593	2.1
19748	1.93387	2.1
19749	1.9339	2.1
19750	1.75669	2.1
19751	1.80299	0.430975
19752	1.31251	0.341797
19753	1.31249	0.421005
19754	1.13411	0.330675
19755	1.06313	2.1
19756	1.04921	2.1
19757	1.55766	2.1
19758	1.53728	2.1
19759	1.53729	2.1
19760	1.51789	2.1
19761	0.993021	2.1
19762	1.02478	2.1
19763	1.03408	2.1
19764	1.09056	2.1
19765	0.697755	2.1
19766	0.632901	2.1
19767	1.34072	0.116659
19768	1.80841	0.120646
19769	1.80841	0.113291
19770	1.17793	0.12017
19771	1.75032	2.04465
19772	1.13076	2.0982
19773	2.11851	2.1
19774	1.73255	2.01144
19775	1.73255	2.1

19776	1.11181	1.92739
19777	1.43772	2.1
19778	1.67381	2.1
19779	1.52164	2.1
19780	1.60861	2.1
19781	1.30588	0.0698591
19782	1.75241	0.0698591
19783	1.33026	1.83389
19784	0.962705	1.51575
19785	0.99678	0.0698591
19786	1.30572	0.0698591
19787	1.41943	0.0698591
19788	1.2683	0.0698591
19789	1.14145	0.0698591
19790	1.01361	0.0698591
19791	1.29943	2.1
19792	1.3483	2.1
19793	1.34832	2.1
19794	1.83488	2.1
19795	1.71065	2.1
19796	1.66178	2.1
19797	1.39076	2.1
19798	1.37456	2.1
19799	1.66126	2.1
19800	1.562	2.1
19801	1.37456	2.1
19802	1.36065	2.1
19803	1.04026	0.414769
19804	1.51898	0.393976
19805	1.67088	0.233605
19806	1.9726	0.169185
19807	1.74852	0.916122
19808	1.95512	1.10466
19809	1.16165	0.956054
19810	1.06584	0.734432
19811	1.39327	2.1
19812	1.41394	2.1
19813	1.41394	2.1
19814	1.45506	2.1
19815	1.39523	2.1
19816	1.21119	2.1
19817	1.09005	2.1
19818	0.921311	2.1
19819	1.58164	2.1
19820	1.77574	2.1
19821	1.73184	2.1
19822	1.59423	2.1
19823	1.8214	2.1
19824	3.10544	0.565653
19825	1.01243	1.81969
19826	1.30896	1.80568
19827	2.16956	0.874185
19828	1.29608	0.166211
19829	1.38617	1.51074
19830	1.49536	1.34021
19831	2.35239	0.845102
19832	1.24613	1.62272
19833	1.94351	2.1

19834	2.05361	2.1
19835	2.05358	2.1
19836	2.38994	2.1
19837	1.13533	2.1
19838	1.08276	2.1
19839	1.55611	0.238918
19840	1.33957	0.168256
19841	1.33957	0.135271
19842	1.69011	0.305209
19843	1.5515	0.559289
19844	1.59106	0.471253
19845	1.02958	2.1
19846	1.12266	2.1
19847	1.12266	2.1
19848	1.24925	2.1
19849	1.80223	2.1
19850	1.20242	2.1
19851	2.13665	0.892807
19852	1.3924	0.783744
19853	0.904894	2.06584
19854	1.82633	2.1
19855	1.94138	0.0698591
19856	1.34009	0.0698591
19857	1.15096	2.1
19858	0.994292	2.1
19859	1.10101	2.1
19860	1.15096	2.1
19861	1.50438	2.1
19862	1.67825	2.1
19863	1.67825	2.1
19864	1.91894	2.1
19865	2.04664	2.1
19866	1.92063	2.1
19867	1.72857	2.1
19868	1.89757	2.1
19869	1.61843	0.0698591
19870	1.49431	0.0698591
19871	2.33925	2.1
19872	1.80683	2.1
19873	1.64257	2.1
19874	1.50108	2.1
19875	2.82561	2.1
19876	2.97847	2.1
19877	2.97848	2.1
19878	3.20034	2.1
19879	0.66055	2.1
19880	1.95919	2.1
19881	1.07976	2.1
19882	1.30237	2.1
19883	1.99943	0.0698591
19884	1.31802	0.0698591
19885	1.53336	0.0781292
19886	1.31802	0.0708859
19887	1.27737	0.473936
19888	1.44848	0.474606
19889	2.35499	0.0698591
19890	1.30041	0.0698591
19891	2.35499	0.0698591

19892	1.65381	0.0698591
19893	0.996083	2.1
19894	1.28754	2.1
19895	0.996081	2.1
19896	1.44859	2.1
19897	1.24763	2.05189
19898	1.03697	2.09101
19899	0.951273	1.30553
19900	1.60018	1.68416
19901	2.0815	2.1
19902	1.20488	2.1
19903	2.95785	2.1
19904	2.52245	2.1
19905	1.68455	0.0698591
19906	1.12971	0.0698591
19907	1.1952	0.120063
19908	2.0815	0.174935
19909	1.3963	2.1
19910	1.4934	2.1
19911	1.47973	2.1
19912	1.25559	2.1
19913	1.1233	2.1
19914	1.15009	2.1
19915	1.15009	2.1
19916	0.977775	2.1
19917	1.16482	0.681887
19918	1.29313	0.249588
19919	1.29313	0.421188
19920	1.294	0.251278
19921	1.48693	0.139369
19922	1.47608	0.156813
19923	1.36005	2.04491
19924	1.47109	2.1
19925	1.47109	2.1
19926	1.3515	2.1
19927	2.48828	2.1
19928	2.32378	2.1
19929	2.32378	2.1
19930	2.1692	2.1
19931	1.03217	0.732546
19932	0.953521	0.920106
19933	1.4515	0.108143
19934	3.72406	0.086274
19935	0.963395	2.06072
19936	1.01346	1.13812
19937	1.62241	2.1
19938	1.62027	2.1
19939	1.62027	2.1
19940	1.61687	2.1
19941	1.2593	0.0698591
19942	0.981586	0.0698591
19943	0.721961	2.1
19944	0.888955	2.1
19945	1.22031	0.0698591
19946	1.27566	2.1
19947	2.50066	2.1
19948	1.19814	2.1
19949	1.42278	0.0698591

19950	2.02657	0.0698591
19951	1.0756	0.230894
19952	1.10049	0.258029
19953	2.26155	2.1
19954	2.15634	2.1
19955	2.15634	2.1
19956	2.01613	2.1
19957	1.94233	1.01926
19958	1.18127	0.752443
19959	1.36865	0.129235
19960	1.75755	0.391273
19961	2.01613	2.1
19962	1.78958	2.1
19963	1.97995	2.1
19964	1.79198	2.1
19965	1.20841	2.1
19966	1.6519	2.1
19967	1.80207	2.1
19968	1.66774	2.1
19969	1.17935	0.0698591
19970	1.88071	0.0712466
19971	1.58723	0.0698591
19972	1.7361	0.0698591
19973	0.794165	2.1
19974	1.21327	2.1
19975	1.21327	2.1
19976	1.34268	2.1
19977	1.34497	0.0698591
19978	0.793303	0.0698591
19979	1.12323	0.0698591
19980	1.34496	0.0698591
19981	0.928226	0.0716859
19982	1.89804	0.118964
19983	2.60141	0.0698591
19984	2.60142	2.1
19985	2.60772	2.09675
19986	0.99747	2.1
19987	1.27096	1.64839
19988	1.27096	1.08642
19989	1.03472	1.70577
19990	1.09962	2.08317
19991	1.21148	2.1
19992	1.21148	2.1
19993	1.35025	2.1
19994	1.09795	2.09809
19995	1.00775	2.1
19996	1.07162	2.1
19997	1.60665	2.02946
19998	1.52984	2.1
19999	1.61179	2.1
20000	1.70057	2.1
20001	1.76063	2.1
20002	1.14547	0.329158
20003	0.964782	0.294831
20004	1.17208	0.294502
20005	0.964769	0.259539
20006	1.04268	2.1
20007	1.57459	2.1

20008	1.57459	2.1
20009	1.32449	2.1
20010	2.07527	0.687916
20011	1.00013	0.739323
20012	0.813711	0.0698591
20013	1.05677	2.1
20014	1.30664	1.49728
20015	1.96011	1.82849
20016	1.7151	1.24903
20017	1.61309	1.31257
20018	1.18954	2.1
20019	1.05447	2.1
20020	1.05446	0.954428
20021	1.11787	0.876778
20022	1.44437	2.1
20023	1.84739	2.1
20024	1.84739	2.1
20025	1.06203	2.1
20026	1.78391	2.1
20027	1.89267	2.1
20028	1.9212	2.1
20029	2.02997	2.1
20030	1.32051	0.167657
20031	1.47169	0.0892505
20032	1.20691	0.0698591
20033	1.72271	0.0698591
20034	1.2858	0.169564
20035	1.31011	0.851823
20036	1.31011	0.130215
20037	1.34124	0.625686
20038	1.294	0.454194
20039	1.03079	0.305859
20040	1.83192	1.88679
20041	1.81979	2.00328
20042	1.81979	1.98692
20043	1.57829	2.07187
20044	1.35435	1.49521
20045	1.19023	1.79757
20046	0.991115	2.1
20047	1.27725	2.1
20048	1.38204	2.1
20049	1.48013	2.1
20050	0.983493	2.1
20051	2.26155	2.1
20052	1.02311	0.432197
20053	1.55485	0.423571
20054	1.71033	0.318108
20055	2.07319	0.299136
20056	2.29074	1.10776
20057	1.50822	1.85304
20058	2.08147	2.1
20059	1.1585	2.1
20060	3.74092	1.9125
20061	2.40799	2.1
20062	2.05349	2.1
20063	1.92656	2.1
20064	1.09634	0.163409
20065	1.45502	0.27758

20066	1.17261	0.132866
20067	1.29383	0.125796
20068	1.29383	0.077552
20069	1.47689	0.089272
20070	1.85717	0.445938
20071	1.53798	0.178971
20072	1.9493	2.1
20073	2.0371	2.09074
20074	1.8519	2.1
20075	1.92119	2.02125
20076	1.00764	0.105377
20077	1.0302	0.0938882
20078	1.0302	0.0932708
20079	0.90047	0.0897399
20080	1.49155	0.151024
20081	2.26403	0.0870025
20082	2.06158	0.0698591
20083	1.09506	0.0698591
20084	1.9212	1.95672
20085	1.99586	1.56979
20086	1.99586	1.6603
20087	2.07527	1.01215
20088	1.20478	2.1
20089	1.09662	2.1
20090	1.55961	2.1
20091	1.03472	2.1
20092	1.54983	2.1
20093	2.02953	2.1
20094	1.84502	2.1
20095	2.1221	2.1
20096	1.02969	1.99923
20097	2.23523	2.08436
20098	1.2377	0.069962
20099	1.03818	0.0960379
20100	1.978	2.1
20101	1.78159	2.1
20102	1.45762	2.1
20103	1.34234	2.1
20104	2.01624	2.1
20105	2.52072	2.1
20106	1.23013	2.1
20107	2.5593	2.1
20108	3.06464	0.941575
20109	2.63697	0.406282
20110	2.63697	0.777197
20111	1.87111	0.338952
20112	1.04574	0.0699332
20113	2.13428	0.0698591
20114	1.61715	2.1
20115	1.44137	2.1
20116	1.44867	2.1
20117	1.18283	2.1
20118	1.50584	0.0765764
20119	1.45466	0.0964509
20120	1.4959	0.0950781
20121	1.93489	0.0865141
20122	2.60772	2.1
20123	2.68868	2.1

20124	2.68868	2.1
20125	2.82561	2.1
20126	1.49654	2.1
20127	1.38672	2.1
20128	1.36813	2.1
20129	1.98396	2.1
20130	1.93852	1.34214
20131	2.14237	0.5306
20132	1.44421	0.0698591
20133	1.21749	0.0698591
20134	1.09572	0.0698591
20135	1.11081	0.0698591
20136	2.32363	2.1
20137	1.24375	2.1
20138	3.79242	2.1
20139	3.46787	2.1
20140	1.19854	1.25313
20141	1.35487	1.2914
20142	1.35486	1.47494
20143	1.38616	1.36717
20144	1.67265	0.141652
20145	0.909487	0.102922
20146	2.53562	2.1
20147	1.09564	2.1
20148	2.9068	2.1
20149	3.57307	0.944932
20150	2.50369	2.03753
20151	1.47348	2.1
20152	1.24925	2.1
20153	1.3991	2.1
20154	1.3991	2.1
20155	1.52984	2.1
20156	1.60428	2.1
20157	1.50097	2.1
20158	1.50097	2.1
20159	1.40239	2.1
20160	2.19621	2.1
20161	2.35003	2.1
20162	1.57932	0.0698591
20163	1.61194	0.0698591
20164	2.0815	0.0698591
20165	1.18539	0.0698591
20166	1.5795	0.880585
20167	1.53064	1.33718
20168	1.89227	0.0698591
20169	3.01037	0.0698591
20170	2.08149	2.082
20171	2.06678	2.02922
20172	1.89226	0.0698591
20173	3.47888	0.0698591
20174	1.89226	0.0862488
20175	3.65842	0.0698591
20176	3.88546	0.124553
20177	1.89226	0.195924
20178	1.11239	2.1
20179	1.29133	0.0698591
20180	0.889309	0.0698591
20181	0.881934	0.0698591

20182	1.88406	2.1
20183	0.636946	2.1
20184	0.636945	2.08453
20185	1.28073	2.1
20186	1.28075	2.1
20187	0.942007	2.1
20188	1.68436	2.1
20189	1.4677	0.0698591
20190	0.851542	0.0698591
20191	1.39063	0.0698591
20192	0.851539	0.0698591
20193	1.11597	0.0698591
20194	1.35403	0.0698591
20195	0.959473	2.1
20196	1.07976	2.1
20197	0.946129	2.1
20198	0.946129	0.469779
20199	1.38464	1.98426
20200	1.36991	1.02462
20201	0.946131	1.16782
20202	0.94613	1.20114
20203	1.34493	0.552957
20204	1.36991	0.379604
20205	1.36991	0.135084
20206	0.946131	0.215156
20207	0.78392	0.225086
20208	0.80282	0.175654
20209	1.10351	0.22262
20210	0.80846	0.194596
20211	0.838026	0.22353
20212	1.08558	0.179132
20213	0.718203	0.188164
20214	0.501866	0.0957664
20215	0.85426	0.168728
20216	1.24249	0.0706203
20217	1.37052	0.0847539
20218	0.851541	0.0703671
20219	1.0092	0.117395
20220	0.851554	0.0770131
20221	1.49826	0.346827
20222	1.13727	0.311821
20223	0.778149	0.208481
20224	1.20257	0.268922
20225	0.946133	0.213465
20226	0.778149	0.348691
20227	0.77815	0.313911
20228	0.946132	2.1
20229	1.17602	2.1
20230	0.946132	2.1
20231	1.31257	2.1
20232	1.17602	2.1
20233	1.35821	0.0899442
20234	1.19993	0.0698591
20235	0.946131	0.0698591
20236	0.946131	0.0927229
20237	1.08848	0.0822544
20238	1.00509	2.1
20239	1.19629	2.1

20240	0.867593	2.1
20241	0.757921	2.1
20242	0.729116	0.0698591
20243	0.89162	0.0698591
20244	1.16811	0.0698591
20245	1.3676	2.1
20246	1.2638	2.0773
20247	0.946132	2.1
20248	0.946132	2.1
20249	1.2638	2.1
20250	1.00185	2.1
20251	1.60948	2.1
20252	0.602075	2.1
20253	0.942611	2.1
20254	0.946132	2.1
20255	0.942611	2.1
20256	1.11822	2.1
20257	1.21558	0.11374
20258	0.607788	0.166333
20259	0.827029	0.198701
20260	1.00691	0.408809
20261	0.607789	0.161564
20262	0.607775	0.310091
20263	1.23248	1.75553
20264	0.902653	2.04203
20265	0.639143	2.03628
20266	0.851545	1.61243
20267	0.605153	1.94847
20268	1.06826	2.1
20269	1.29365	2.1
20270	1.41232	2.1
20271	0.946131	2.1
20272	1.20854	2.1
20273	0.946131	2.1
20274	0.909005	0.0698591
20275	1.16672	0.0698591
20276	0.810959	0.0698591
20277	0.946131	0.0698611
20278	1.40104	0.0698591
20279	0.946131	0.0698619
20280	1.35403	0.0698591
20281	1.40104	0.0698591
20282	1.92535	0.0698591
20283	2.14534	0.0698591
20284	1.77532	0.0698591
20285	0.851541	0.0714797
20286	1.05585	0.0767303
20287	1.27098	0.0711422
20288	0.870925	0.0757226
20289	1.15547	0.0754653
20290	1.47583	0.0703272
20291	1.35007	0.0698591
20292	0.740095	0.0698591
20293	0.644912	0.0698591
20294	0.819489	0.0698591
20295	0.983028	0.0980132
20296	0.861849	0.0698591
20297	1.40511	0.0698591

20298	0.86182	0.0698591
20299	0.9705	0.0698591
20300	1.40511	0.0698591
20301	1.15539	2.1
20302	0.946119	2.1
20303	0.736382	2.1
20304	0.736384	2.1
20305	1.41561	2.1
20306	0.785651	2.1
20307	1.34479	2.1
20308	1.38885	2.1
20309	0.94613	2.1
20310	1.29461	2.1
20311	0.94613	2.1
20312	0.776179	2.1
20313	0.858611	2.1
20314	0.946128	2.1
20315	1.04073	2.1
20316	1.16399	2.1
20317	0.990019	2.1
20318	1.15564	2.07469
20319	1.28956	0.0787549
20320	1.35145	0.10129
20321	1.09662	2.1
20322	1.8155	0.0698591
20323	0.811459	0.0698591
20324	0.731982	0.0698591
20325	0.900554	0.0698591
20326	0.946127	0.7883
20327	0.817066	0.747478
20328	0.817064	0.808145
20329	1.41798	0.742559
20330	0.871606	0.789993
20331	1.30265	0.0698591
20332	0.849402	1.70512
20333	0.868582	1.1085
20334	0.909137	1.1976
20335	0.807016	1.13776
20336	0.83393	0.568717
20337	0.9034	0.738237
20338	1.53515	0.716715
20339	1.17665	0.277947
20340	0.946132	0.395507
20341	0.946129	0.273181
20342	1.35977	0.271993
20343	1.34254	0.252522
20344	1.14241	0.194527
20345	0.946132	0.29524
20346	0.946132	0.194764
20347	1.27376	0.200313
20348	1.35978	0.186903
20349	1.01976	0.184393
20350	1.23498	0.681086
20351	1.35002	0.752666
20352	1.11683	1.50517
20353	0.88359	0.128436
20354	0.751374	0.831172
20355	0.791714	0.156054

20356	1.6067	2.1
20357	0.923782	2.1
20358	1.44689	2.1
20359	0.94613	2.1
20360	0.946131	2.1
20361	1.5261	2.1
20362	1.26247	2.09325
20363	1.80085	0.108126
20364	1.7693	0.140494
20365	0.900422	0.0699915
20366	1.31357	0.0818913
20367	0.94613	0.0698624
20368	0.900424	0.107815
20369	0.900397	0.0702468
20370	1.39448	0.0704258
20371	1.36769	0.0698591
20372	0.713524	0.0698591
20373	0.713554	0.0698591
20374	0.946124	0.0698591
20375	1.24979	0.0698591
20376	1.04765	0.0698591
20377	0.70054	0.0698591
20378	0.776991	0.0698591
20379	0.738905	0.0698591
20380	1.32605	0.0698591
20381	0.784957	0.0698591
20382	0.803955	0.0698591
20383	0.819486	0.0698591
20384	1.79306	0.0698591
20385	1.18275	0.0698591
20386	1.25626	0.0698591
20387	0.936806	0.0698591
20388	0.653124	0.0702685
20389	0.946132	0.0698591
20390	0.653151	0.0699699
20391	1.10748	0.0779103
20392	1.95244	2.1
20393	1.40967	1.49615
20394	1.27916	1.23031
20395	0.946132	1.97152
20396	0.946132	2.1
20397	1.30555	2.1
20398	1.38651	2.1
20399	0.946132	2.1
20400	1.23571	0.390484
20401	1.21261	0.826295
20402	1.34225	0.88198
20403	0.946132	1.41676
20404	0.734059	0.539151
20405	0.734084	0.892152
20406	0.78438	0.246738
20407	1.13748	0.434773
20408	0.946131	0.634901
20409	0.78438	0.238332
20410	0.784392	0.433943
20411	0.626495	2.1
20412	0.694635	2.1
20413	0.946125	2.1

20414	0.694632	2.1
20415	1.38521	2.1
20416	0.795469	2.1
20417	0.850877	2.1
20418	1.37098	2.1
20419	0.946131	2.1
20420	0.79547	2.1
20421	0.946132	0.171246
20422	1.30448	0.0812325
20423	0.628361	0.0730038
20424	0.686964	0.163426
20425	0.493261	0.0773256
20426	1.90912	0.259015
20427	0.946127	0.168885
20428	1.36604	0.086951
20429	0.671687	0.0814831
20430	0.946128	0.271346
20431	0.671699	0.0978703
20432	1.04973	2.1
20433	0.982858	0.235648
20434	0.974081	0.0698591
20435	1.69993	0.0698591
20436	1.00763	0.265224
20437	0.755566	2.1
20438	0.946129	2.1
20439	1.04184	2.1
20440	1.13509	2.1
20441	0.755566	2.1
20442	1.114 2.1	
20443	0.810946	2.1
20444	0.786259	2.1
20445	1.43455	2.1
20446	1.05071	2.1
20447	0.746095	2.1
20448	0.82737	2.1
20449	0.946126	2.1
20450	1.04071	2.1
20451	1.0852	2.1
20452	1.59019	0.0698591
20453	0.621972	0.0698591
20454	1.14475	0.0698591
20455	0.691062	0.0698591
20456	1.20057	0.0698591
20457	0.846868	0.0698591
20458	1.26062	0.0698591
20459	0.795093	0.0698591
20460	1.20056	0.0698591
20461	0.795065	0.0698591
20462	1.18203	0.0698591
20463	1.20057	0.0698591
20464	0.991869	0.0770841
20465	0.841088	2.1
20466	0.946131	2.1
20467	0.869097	2.1
20468	0.946131	2.1
20469	0.841089	2.1
20470	1.77867	2.1
20471	1.99296	0.0973378

20472	1.91703	2.1
20473	0.651334	0.0698591
20474	1.31467	0.0698591
20475	0.651332	0.0698591
20476	0.651332	0.0698591
20477	1.03079	0.0698591
20478	0.808519	0.0698591
20479	1.41417	0.0698591
20480	1.03165	0.169807
20481	1.43577	2.1
20482	1.39053	2.1
20483	1.07006	2.1
20484	0.946132	2.1
20485	0.946133	2.1
20486	1.07006	2.1
20487	1.12717	2.1
20488	0.994293	2.1
20489	0.946132	2.1
20490	1.31912	2.1
20491	0.946131	2.1
20492	1.40571	2.1
20493	1.31912	2.1
20494	1.12292	0.319962
20495	1.07755	0.376084
20496	0.851539	0.348926
20497	0.946131	0.326278
20498	0.929834	0.337621
20499	1.83919	0.436494
20500	1.04713	0.0950502
20501	0.929726	0.0717103
20502	1.50985	0.0799569
20503	0.946127	0.0710687
20504	1.0227	0.0744697
20505	1.2582	0.0738342
20506	1.1391	0.34967
20507	0.780831	0.570543
20508	0.78086	0.397675
20509	0.946133	0.544355
20510	0.780831	0.379467
20511	1.37762	0.468976
20512	0.946116	2.1
20513	1.22882	2.1
20514	1.40015	2.1
20515	1.40017	2.1
20516	0.946105	2.1
20517	0.553567	0.0698591
20518	1.19263	0.0698591
20519	0.946132	0.0698591
20520	0.553594	0.0698591
20521	1.19263	0.0698591
20522	1.18237	0.0698591
20523	0.946133	0.0698591
20524	0.978467	0.0698591
20525	0.56262	0.0698591
20526	0.946132	0.0698591
20527	0.562633	0.0698591
20528	0.900175	0.0698591
20529	1.04627	1.82203

20530	0.549928	0.9884
20531	1.17765	0.714456
20532	1.11479	1.52809
20533	0.398261	1.13108
20534	0.927608	1.14423
20535	1.38467	1.01748
20536	1.04824	0.669043
20537	1.33893	0.710894
20538	1.55775	1.07016
20539	0.816895	1.23172
20540	1.96057	1.22799
20541	1.35798	2.1
20542	1.29262	2.1
20543	0.94613	2.1
20544	0.94613	2.1
20545	1.29261	2.1
20546	1.18067	2.1
20547	0.946131	2.1
20548	0.946132	2.1
20549	1.27809	2.1
20550	1.35799	2.1
20551	1.03933	2.1
20552	1.49438	2.1
20553	0.946132	2.1
20554	1.13537	2.1
20555	1.41666	2.1
20556	1.74237	2.1
20557	0.946132	2.1
20558	1.04291	0.862727
20559	1.63802	2.1
20560	1.24714	2.1
20561	0.842013	2.1
20562	0.810959	2.1
20563	0.767923	0.1417
20564	1.19583	0.122967
20565	0.767921	0.0997952
20566	0.767921	0.0758671
20567	0.961348	0.128681
20568	2.08687	1.98826
20569	0.739729	0.699547
20570	1.04342	0.625014
20571	0.739757	0.606501
20572	0.946129	0.730903
20573	1.04341	1.04914
20574	1.38683	0.190993
20575	1.34153	0.165849
20576	0.946129	0.20203
20577	0.946131	0.202727
20578	1.02028	0.192332
20579	1.47606	0.11812
20580	1.20739	0.158551
20581	0.957684	0.128371
20582	0.946116	0.166626
20583	0.851528	0.191495
20584	0.957685	0.18362
20585	1.04504	2.1
20586	0.902938	2.1
20587	0.946132	2.1

20588	1.07566	2.1
20589	1.4558	2.1
20590	1.93286	2.1
20591	1.27241	2.1
20592	0.828671	2.1
20593	0.810933	2.1
20594	1.31335	0.0698591
20595	1.40305	0.0698591
20596	0.912698	0.0698591
20597	0.810946	0.0698591
20598	1.11608	0.0698591
20599	1.26409	1.14724
20600	0.946132	0.204081
20601	1.27376	0.208817
20602	0.946132	0.201504
20603	1.03333	0.212851
20604	1.27376	0.206763
20605	0.94613	0.233797
20606	0.812384	0.203112
20607	0.687744	0.149562
20608	0.946132	0.229367
20609	0.687758	0.202914
20610	1.09667	0.133236
20611	1.56977	2.1
20612	0.851544	2.1
20613	1.01136	2.1
20614	0.851481	2.1
20615	0.946131	2.1
20616	0.705409	2.1
20617	1.32449	0.0698591
20618	1.13508	0.0698591
20619	0.94613	0.0701327
20620	0.94613	0.0698591
20621	0.965822	0.0698591
20622	1.95301	0.115413
20623	0.851538	0.302913
20624	1.29262	0.260815
20625	0.95417	0.173983
20626	0.851541	0.311467
20627	0.94613	0.272749
20628	1.02727	0.97216
20629	1.37675	1.28655
20630	0.955646	1.05858
20631	0.95114	1.61775
20632	0.851528	1.13821
20633	1.76513	1.41083
20634	0.87155	2.1
20635	0.94613	2.1
20636	0.871519	2.1
20637	0.966631	2.1
20638	1.37791	2.1
20639	1.19122	0.347573
20640	0.818971	0.783668
20641	0.628799	0.446222
20642	0.861027	0.590478
20643	0.800034	0.0698591
20644	0.91846	0.0698591
20645	0.765645	0.0698591

20646	1.13709	0.643981
20647	1.29054	0.609944
20648	0.755336	0.711264
20649	0.755334	0.642145
20650	0.946133	0.792621
20651	0.755336	0.509727
20652	1.14444	0.621787
20653	0.872823	0.654726
20654	0.533645	0.626867
20655	0.946132	0.550892
20656	0.533645	0.423753
20657	1.02311	0.433989
20658	1.00278	2.1
20659	1.1016	0.0698591
20660	0.629615	0.0698591
20661	1.12628	0.0698591
20662	0.946131	0.0698591
20663	0.629615	0.0698591
20664	0.629641	0.0698591
20665	1.67046	1.06609
20666	1.81667	2.1
20667	1.7145	2.1
20668	0.94613	2.1
20669	1.29322	2.1
20670	1.00365	2.1
20671	0.946131	2.1
20672	1.29322	2.1
20673	1.27581	2.1
20674	1.33916	2.1
20675	1.32784	2.1
20676	1.74787	2.1
20677	2.28084	2.1
20678	2.98092	2.1
20679	0.946132	2.06519
20680	1.2435	2.1
20681	0.946132	2.1
20682	1.4099	2.1
20683	1.2435	2.1
20684	1.26438	2.1
20685	0.745287	2.1
20686	0.696827	2.1
20687	0.696781	2.1
20688	1.41195	2.1
20689	0.946131	2.1
20690	1.8758	1.53616
20691	0.832979	0.51197
20692	0.75054	0.440507
20693	0.810958	0.435495
20694	0.922889	0.555605
20695	0.694844	0.205841
20696	0.694814	0.38271
20697	0.946131	0.312327
20698	0.694844	0.290023
20699	1.02369	2.1
20700	1.4468	0.0698591
20701	0.946125	0.102559
20702	0.93487	0.0814419
20703	1.23608	0.15252

20704	1.34257	0.0736379
20705	0.851525	0.0899717
20706	1.03298	0.0746123
20707	1.22418	2.09919
20708	0.946131	2.08705
20709	1.41833	1.92814
20710	1.23452	1.16337
20711	0.946131	0.582384
20712	1.41833	1.57681
20713	1.13077	0.186529
20714	0.942874	1.91399
20715	0.946132	1.86967
20716	0.504366	1.8641
20717	0.593647	2.0927
20718	0.898829	1.87767
20719	1.27921	2.04903
20720	1.12503	2.1
20721	1.22539	2.1
20722	1.10332	2.1
20723	0.851541	2.1
20724	0.94613	2.1
20725	0.945109	2.1
20726	0.607962	2.1
20727	0.946133	2.1
20728	0.607961	2.1
20729	0.661956	2.1
20730	1.35048	2.1
20731	1.66018	2.1
20732	0.956467	2.1
20733	0.851542	2.1
20734	0.890734	2.1
20735	0.802455	2.1
20736	1.35943	2.1
20737	1.04798	2.1
20738	1.26426	0.273385
20739	1.3689	0.176965
20740	0.946133	0.226329
20741	0.946133	0.290052
20742	1.09729	0.266317
20743	0.765259	2.1
20744	0.810945	2.1
20745	0.810957	2.1
20746	1.10284	2.07657
20747	1.09368	2.1
20748	0.772601	2.1
20749	0.788011	2.1
20750	0.810934	2.1
20751	0.694357	1.14923
20752	1.22641	1.13705
20753	0.946132	1.95196
20754	0.946132	0.433563
20755	0.946133	0.612565
20756	1.42754	1.67899
20757	0.776414	0.645723
20758	0.806525	1.33329
20759	0.575339	1.0242
20760	0.97154	0.689629
20761	0.8217	1.42052

20762	0.838039	1.3839
20763	0.94613	1.20478
20764	0.946132	0.802895
20765	1.39575	0.462274
20766	1.39575	0.610054
20767	1.12818	0.686245
20768	0.946132	1.63922
20769	0.946133	1.30098
20770	1.06879	0.658658
20771	1.06879	0.972206
20772	0.970358	0.956198
20773	1.2474	0.282136
20774	1.01919	0.0698591
20775	0.94613	0.0716369
20776	1.28952	0.0698803
20777	1.00809	0.0698591
20778	0.849344	0.0698591
20779	0.640304	0.0701227
20780	1.35065	0.0698591
20781	0.946132	0.0698591
20782	0.640333	0.0698591
20783	0.946132	0.0698591
20784	0.685489	2.1
20785	0.94613	2.1
20786	0.68546	2.1
20787	0.874264	2.1
20788	1.27676	2.1
20789	0.946129	0.0957276
20790	1.09024	0.0732723
20791	0.714563	0.0725332
20792	1.12335	0.07749
20793	1.07931	0.0735436
20794	1.17799	0.0698591
20795	1.8795	0.181508
20796	2.15229	0.177999
20797	1.13859	0.395709
20798	0.946132	0.747656
20799	1.11374	0.419758
20800	1.1386	0.492195
20801	0.615713	0.518073
20802	1.19322	0.198984
20803	1.3377	0.161308
20804	0.670801	0.213068
20805	0.94613	0.524158
20806	0.66617	0.385859
20807	1.36173	0.362537
20808	0.980473	0.34125
20809	1.39132	0.328554
20810	0.864653	0.0698591
20811	1.23837	0.0698591
20812	0.810959	0.0698591
20813	1.1483	1.7645
20814	1.00381	1.92595
20815	0.946128	1.79937
20816	1.39696	1.0763
20817	0.946131	0.77325
20818	0.946131	1.35424
20819	1.38613	1.64457

20820	0.973501	1.95459
20821	0.946127	1.36242
20822	1.35968	1.60688
20823	0.946131	0.924316
20824	0.946129	1.8587
20825	1.39696	1.80674
20826	1.12088	2.1
20827	1.41122	2.1
20828	1.14464	0.856411
20829	0.946131	2.06802
20830	0.946131	1.99455
20831	1.14464	2.0536
20832	1.33266	1.59103
20833	1.07341	0.228829
20834	0.827287	1.94913
20835	0.946132	1.78491
20836	0.827287	2.03992
20837	1.15644	1.851
20838	1.27558	0.591123
20839	1.15067	2.1
20840	0.946131	2.1
20841	1.29143	2.05549
20842	1.36281	2.1
20843	1.36281	2.1
20844	0.946131	2.1
20845	1.40863	2.1
20846	1.20143	0.0698591
20847	1.19755	0.0701454
20848	0.566153	0.0698591
20849	0.946131	0.0801969
20850	1.07979	0.0698591
20851	1.00463	0.0887361
20852	0.566153	0.123908
20853	0.768212	2.1
20854	1.32461	2.1
20855	1.13268	2.1
20856	1.13268	2.1
20857	0.946133	2.09899
20858	1.53642	2.1
20859	1.5791	2.1
20860	0.726635	2.1
20861	1.2334	2.09511
20862	1.29198	2.1
20863	1.29198	2.0698
20864	0.946131	2.02015
20865	0.956585	2.1
20866	1.25294	2.1
20867	0.946133	2.1
20868	1.35287	2.1
20869	1.23877	2.1
20870	0.946131	2.1
20871	1.35287	2.1
20872	1.01843	2.1
20873	3.77569	2.1
20874	0.86707	2.1
20875	0.867066	2.1
20876	1.38639	2.1
20877	1.38639	2.1

20878	0.896168	2.1
20879	0.974716	0.0699772
20880	1.09411	0.0893684
20881	0.663171	0.0718148
20882	0.663171	0.102008
20883	0.946132	0.0994025
20884	0.66317	0.0798565
20885	1.47104	0.0698591
20886	1.13629	0.0745663
20887	0.772342	0.0698591
20888	0.772312	0.0781674
20889	0.94613	0.0769465
20890	0.77234	0.0743976
20891	1.43154	2.1
20892	1.37704	2.1
20893	1.40025	2.1
20894	1.3063	2.1
20895	0.946132	2.1
20896	1.04075	2.1
20897	0.946131	2.1
20898	0.946118	2.1
20899	0.750011	1.72813
20900	1.0971	2.04068
20901	0.889667	1.87513
20902	0.709598	2.03099
20903	2.39737	2.1
20904	1.19974	0.0710721
20905	0.94613	0.133221
20906	1.25809	0.0698591
20907	1.19974	0.11116
20908	0.73005	0.196168
20909	1.50764	0.0698591
20910	1.25809	0.0751689
20911	0.946133	0.16792
20912	1.10357	0.0976663
20913	1.25809	0.133616
20914	0.628177	0.208451
20915	1.3543	0.107076
20916	0.793711	0.0698591
20917	1.18455	0.0698591
20918	1.03966	0.0698591
20919	0.946131	0.0698591
20920	0.767453	0.0698591
20921	0.612292	0.0698591
20922	0.946131	0.0698591
20923	1.24128	0.0698591
20924	1.08761	0.0698591
20925	0.762059	0.0698591
20926	1.005 2.1	
20927	1.17054	2.1
20928	1.12665	2.1
20929	0.750368	2.1
20930	0.851542	2.1
20931	0.975436	2.1
20932	0.975442	2.1
20933	0.675367	2.1
20934	1.51973	0.116301
20935	0.94613	0.13113

20936	1.38954	0.136057
20937	0.946131	0.128402
20938	1.07912	0.137667
20939	1.38954	0.12866
20940	2.0219	2.1
20941	1.18347	0.127413
20942	0.946132	0.638279
20943	0.94613	0.319399
20944	1.00202	0.232606
20945	1.00201	0.261786
20946	0.946132	0.334505
20947	0.946132	0.794956
20948	1.37981	0.475624
20949	1.26874	0.373201
20950	1.26874	0.397005
20951	0.946132	0.477137
20952	2.8687	0.156602
20953	1.42884	2.1
20954	1.05188	2.1
20955	0.625307	2.1
20956	0.810956	2.1
20957	0.526656	2.1
20958	0.526684	2.1
20959	0.724122	2.1
20960	0.946132	2.1
20961	0.526684	2.1
20962	1.05337	2.1
20963	1.0593	2.1
20964	1.47981	2.1
20965	1.74431	1.87968
20966	0.885612	1.95418
20967	0.810946	1.34977
20968	0.845185	1.56816
20969	1.38622	0.453585
20970	0.723194	0.478513
20971	0.554302	0.556853
20972	0.709597	0.409891
20973	0.909507	0.398047
20974	0.912754	0.266236
20975	1.15174	0.194886
20976	0.946131	0.268691
20977	0.912753	0.257025
20978	1.15174	0.256967
20979	1.02011	0.154491
20980	0.792754	1.96209
20981	0.946129	2.05894
20982	0.792754	1.9481
20983	1.27575	1.90014
20984	0.94613	1.95781
20985	1.60132	1.98243
20986	0.922455	1.98257
20987	1.18713	1.59042
20988	0.926957	1.69447
20989	1.66525	1.71648
20990	0.946132	2.1
20991	0.851541	2.1
20992	0.961191	2.1
20993	1.27534	2.1

20994	0.851554	2.1
20995	1.0499	2.1
20996	1.01776	0.0698591
20997	0.97622	0.0698591
20998	0.810934	0.0698591
20999	2.6802	0.0698591
21000	1.31485	0.0698591
21001	1.09699	0.0698591
21002	0.681649	0.0698591
21003	0.790369	0.0698591
21004	0.946131	0.0698591
21005	0.838026	1.20779
21006	0.567833	0.644626
21007	0.891492	0.899807
21008	0.986984	0.426394
21009	0.946132	0.910002
21010	1.19028	0.451615
21011	0.986984	0.477537
21012	0.595623	0.562775
21013	0.946131	2.1
21014	1.36502	2.1
21015	1.08109	2.1
21016	0.946129	2.1
21017	1.36503	2.1
21018	0.773897	2.08747
21019	0.946133	1.94368
21020	0.613447	2.09009
21021	0.613449	2.04848
21022	0.946131	2.08888
21023	1.11163	2.04346
21024	0.966114	2.1
21025	0.735699	2.1
21026	0.662147	2.1
21027	1.04072	2.1
21028	0.946131	2.1
21029	0.862978	2.1
21030	1.48839	2.02943
21031	0.936141	2.05245
21032	0.946131	2.1
21033	0.936142	2.1
21034	1.40595	2.1
21035	0.946131	2.09989
21036	1.05297	0.0698591
21037	3.09035	2.1
21038	1.19952	2.1
21039	0.907198	2.1
21040	0.849144	2.1
21041	0.612469	0.298052
21042	0.551237	0.274969
21043	0.985256	0.43492
21044	0.551209	0.301935
21045	0.85153	0.602624
21046	0.624712	2.1
21047	0.900018	2.1
21048	0.851542	2.1
21049	0.864787	2.1
21050	1.27669	2.1
21051	0.946711	0.227587

21052	0.810947	0.273001
21053	0.841547	0.185906
21054	0.764114	0.373817
21055	1.26692	0.353996
21056	0.946132	0.385737
21057	1.37675	0.32136
21058	1.26692	0.337021
21059	1.37545	0.406822
21060	1.49637	0.104759
21061	0.709598	0.17126
21062	0.751252	0.162217
21063	1.36811	0.152707
21064	0.635625	0.173066
21065	1.31713	0.0715629
21066	1.18977	2.1
21067	0.893377	0.477205
21068	0.707344	2.1
21069	0.946131	2.1
21070	1.20221	2.1
21071	1.20221	2.1
21072	0.707345	2.1
21073	1.26786	2.1
21074	0.988048	2.09028
21075	0.751293	2.08206
21076	0.632008	2.08874
21077	1.51067	2.1
21078	1.13534	2.1
21079	0.946133	2.1
21080	0.946131	2.1
21081	1.25716	2.1
21082	1.3199	2.1
21083	1.09616	2.1
21084	1.36677	2.07299
21085	1.34752	2.09329
21086	0.838041	2.04569
21087	1.67023	2.1
21088	1.02247	0.305697
21089	1.03079	0.176484
21090	0.928982	0.0913714
21091	1.13635	0.0783617
21092	0.946132	0.0921838
21093	0.92898	0.0805626
21094	1.13635	0.085727
21095	1.1643	0.0698591
21096	0.851541	0.220364
21097	1.39939	0.172016
21098	0.946129	0.229384
21099	1.07775	0.189177
21100	1.14503	0.204515
21101	1.78573	0.122144
21102	1.29827	0.110897
21103	0.872373	0.0698591
21104	1.01162	0.0698591
21105	0.83804	0.0698591
21106	1.48851	0.0698591
21107	1.47385	0.0698591
21108	0.843858	0.0698591
21109	0.938626	0.0698591

21110	0.89567	0.0698591
21111	1.59457	0.581379
21112	2.09666	0.0956256
21113	0.775132	0.0698591
21114	0.927005	0.0698591
21115	0.854224	0.0698591
21116	0.775142	0.0698591
21117	0.946106	0.0698591
21118	1.80784	2.1
21119	2.04593	2.1
21120	1.32282	2.1
21121	0.952138	2.1
21122	0.946132	2.1
21123	0.946132	2.1
21124	0.952138	2.1
21125	1.46988	2.1
21126	0.80465	0.0698591
21127	1.14155	0.0698591
21128	0.810931	0.0698591
21129	1.66203	0.112556
21130	0.674605	0.246859
21131	0.946132	0.477855
21132	1.26706	0.411372
21133	0.674633	0.134326
21134	0.946132	1.13564
21135	0.980664	0.265266
21136	1.34927	0.166151
21137	0.758454	0.0831226
21138	0.946133	0.0768464
21139	0.985371	0.0759059
21140	1.28632	0.0698591
21141	0.946133	0.111726
21142	1.46868	0.200649
21143	1.7522	0.0698591
21144	0.737431	2.0171
21145	0.663679	2.02166
21146	1.16541	1.79687
21147	1.3179	1.9063
21148	1.04081	1.9352
21149	0.887866	1.6968
21150	0.806961	1.09576
21151	0.929061	1.47461
21152	1.28418	0.313603
21153	1.38752	0.266508
21154	1.33038	0.18038
21155	0.946132	0.155757
21156	0.946132	0.215774
21157	1.14897	0.212135
21158	1.1685	0.159763
21159	1.18774	2.1
21160	0.961899	2.1
21161	1.48841	2.1
21162	1.79105	2.1
21163	3.51614	2.1
21164	0.682342	2.1
21165	0.783184	2.1
21166	0.946132	2.1
21167	0.868396	2.1

21168	1.21813	2.1
21169	0.848305	0.463625
21170	0.94559	0.498303
21171	0.805691	0.36176
21172	0.752217	2.1
21173	0.946132	2.1
21174	0.839123	2.1
21175	0.946131	2.1
21176	0.752187	2.1
21177	1.96491	2.1
21178	0.520997	2.1
21179	0.946132	2.1
21180	0.73446	2.1
21181	0.946133	2.1
21182	0.520995	2.1
21183	1.88522	2.1
21184	1.8361	2.1
21185	1.11285	2.1
21186	1.25334	0.97325
21187	2.00756	2.1
21188	1.44252	0.0728598
21189	1.00255	2.0837
21190	1.64516	2.1
21191	1.12399	0.0698591
21192	1.13542	0.0698591
21193	1.09038	0.0698591
21194	0.746903	0.0698591
21195	0.672241	0.0698591
21196	0.871774	0.0698591
21197	1.73513	0.107481
21198	0.949995	2.1
21199	1.17244	2.09413
21200	0.946127	2.1
21201	1.3028	2.09144
21202	1.17244	2.09181
21203	0.774101	2.1
21204	0.946127	2.1
21205	0.939289	2.1
21206	0.946129	2.1
21207	0.774101	2.1
21208	1.87858	2.1
21209	1.44293	0.0698591
21210	1.43213	0.0698591
21211	0.946131	2.1
21212	0.716614	2.1
21213	0.716644	2.1
21214	1.35828	2.1
21215	0.790935	2.1
21216	0.730157	2.1
21217	1.41149	2.1
21218	0.730186	2.1
21219	0.730186	2.1
21220	0.946131	2.1
21221	0.946132	1.61315
21222	0.946132	1.33593
21223	0.990713	0.667532
21224	1.40779	0.919791
21225	0.946132	1.0929

21226	1.06486	0.327093
21227	0.946131	1.31449
21228	0.946133	0.69639
21229	1.40779	0.508787
21230	1.26874	0.567329
21231	0.946131	0.668132
21232	1.02588	0.706131
21233	0.826073	0.110125
21234	1.41233	0.106029
21235	0.861269	0.108117
21236	1.04073	0.159608
21237	1.27111	0.170568
21238	1.56016	1.61536
21239	1.60866	0.0698591
21240	0.94613	0.0698591
21241	0.946129	0.0698591
21242	1.36376	0.0698591
21243	1.22187	0.0698591
21244	0.94613	0.0698591
21245	1.82581	0.608825
21246	1.19705	1.00395
21247	1.7513	2.1
21248	1.85017	2.1
21249	1.72116	2.1
21250	1.12058	2.1
21251	0.899991	2.1
21252	0.616288	2.1
21253	1.07289	2.1
21254	1.7092	0.745138
21255	0.946132	0.498861
21256	0.946133	0.47927
21257	1.18717	0.347948
21258	1.40029	0.849944
21259	0.946132	0.177348
21260	0.94613	0.0816041
21261	1.18547	0.0698591
21262	0.960326	0.0785268
21263	0.960345	0.0698649
21264	0.942557	0.0772628
21265	1.18601	0.0698591
21266	0.946132	0.0699276
21267	1.30307	0.0698591
21268	1.08169	0.0698591
21269	1.08169	0.0698591
21270	1.31716	0.069907
21271	1.51065	0.653729
21272	0.877642	0.0698591
21273	0.617384	0.0698591
21274	0.575741	0.0698591
21275	0.972448	0.0698591
21276	1.11334	0.0698591
21277	1.13092	2.1
21278	0.946127	1.70266
21279	1.31724	2.1
21280	1.39751	0.601711
21281	1.39751	1.48608
21282	0.946128	1.56769
21283	0.946132	0.181008

21284	0.713284	0.147946
21285	1.40668	0.187381
21286	1.03547	0.170641
21287	0.713298	0.167145
21288	1.88774	0.145215
21289	0.830164	2.1
21290	1.05211	2.1
21291	0.946119	2.1
21292	0.946107	2.1
21293	1.283 2.1	
21294	2.63609	2.1
21295	1.28418	0.459922
21296	0.946128	0.683438
21297	1.10335	0.448962
21298	0.598377	0.82241
21299	1.18818	0.471965
21300	1.10335	0.464645
21301	1.58972	1.079
21302	1.90194	2.1
21303	0.946118	2.1
21304	0.946115	2.1
21305	0.950969	2.1
21306	1.36918	2.1
21307	0.950955	2.1
21308	1.4263	2.1
21309	1.11978	0.259983
21310	0.946132	1.02428
21311	0.946133	1.478
21312	1.37658	0.258567
21313	1.2364	0.440222
21314	1.05496	0.508443
21315	0.946132	1.36484
21316	0.946132	1.15777
21317	1.21858	0.396678
21318	1.37658	0.820892
21319	1.09194	1.10943
21320	1.14721	0.0770479
21321	1.53814	0.0698591
21322	0.546751	0.93807
21323	0.492063	1.20392
21324	1.16889	1.97569
21325	1.00349	1.77036
21326	0.863559	1.0588
21327	1.38102	1.51756
21328	0.597449	1.28192
21329	0.537748	0.53591
21330	1.16297	1.49615
21331	0.999366	0.899723
21332	0.896554	0.770029
21333	0.566962	0.0698591
21334	0.946129	0.0698591
21335	0.452729	0.0698591
21336	0.696552	0.0698591
21337	0.852524	0.0698591
21338	1.57992	0.0698591
21339	1.64985	0.0698591
21340	0.946128	2.1
21341	1.21847	2.1

21342	0.946129	2.1
21343	1.41131	2.1
21344	1.21847	2.1
21345	1.21269	0.0698591
21346	1.0417	1.95615
21347	1.15821	1.38183
21348	0.946132	0.635664
21349	0.946132	1.92203
21350	1.37967	0.871688
21351	0.851529	1.17936
21352	1.20455	2.1
21353	1.4282	0.0698591
21354	1.26593	2.08928
21355	0.838026	0.0780435
21356	0.985096	0.0726871
21357	0.83804	0.0766679
21358	0.822395	1.26223
21359	0.420272	0.247788
21360	0.946131	2.1
21361	1.17461	2.1
21362	0.94613	2.1
21363	1.40225	2.1
21364	1.17461	2.1
21365	1.09847	2.1
21366	1.22222	2.07381
21367	1.41798	0.470768
21368	0.791658	0.607581
21369	0.791656	0.662059
21370	0.946129	0.610511
21371	0.817064	0.59692
21372	0.946114	0.528944
21373	0.75824	0.49454
21374	0.75821	0.542184
21375	1.41778	0.435093
21376	0.791635	0.499122
21377	1.04164	1.78698
21378	0.946131	0.956599
21379	1.11172	0.692365
21380	1.30076	0.494365
21381	1.35721	0.600027
21382	1.11172	0.562682
21383	1.87218	0.165061
21384	0.987248	0.16678
21385	0.729695	0.701708
21386	0.974389	0.302471
21387	1.18002	0.286386
21388	0.822612	0.307312
21389	1.35721	0.299577
21390	0.634295	0.551141
21391	0.634326	0.376937
21392	1.13245	1.15913
21393	1.13245	0.580303
21394	0.937961	0.397948
21395	1.18769	0.471792
21396	0.854949	0.294972
21397	0.967357	0.353183
21398	0.810934	0.286367
21399	3.91082	2.1

21400	0.946132	2.1
21401	0.946132	2.1
21402	1.05383	2.1
21403	1.05383	2.1
21404	0.986498	2.1
21405	1.50732	0.0699279
21406	0.753659	0.0762558
21407	1.22144	0.0871639
21408	1.30463	0.0756319
21409	0.753659	0.083355
21410	1.30463	0.0826705
21411	0.886189	0.0832857
21412	1.04321	0.0799757
21413	0.909928	0.0757416
21414	1.18659	0.0726251
21415	1.19785	0.0698591
21416	1.40969	0.0712313
21417	1.24094	0.0698591
21418	0.946126	0.0709117
21419	1.2929	0.0698591
21420	1.24093	0.0698591
21421	0.867944	0.0698591
21422	1.41903	2.1
21423	2.47881	0.0698591
21424	1.12943	0.0698591
21425	0.764229	2.1
21426	0.764256	2.1
21427	0.94613	2.1
21428	0.94613	2.1
21429	1.1995	2.1
21430	1.83423	2.1
21431	0.634816	2.08977
21432	0.877837	2.1
21433	0.94613	2.0982
21434	1.04073	2.1
21435	1.06485	2.1
21436	0.94613	0.167434
21437	0.62725	0.14624
21438	0.798212	0.167397
21439	1.33241	0.111335
21440	0.62725	0.147063
21441	1.82301	0.0802945
21442	1.40615	2.1
21443	0.782504	2.1
21444	0.748635	2.1
21445	0.946131	2.1
21446	0.748607	2.1
21447	0.988456	1.97631
21448	1.64833	0.0698591
21449	1.35657	0.0698591
21450	1.26155	0.0698591
21451	0.915755	0.0698591
21452	0.915755	0.0698591
21453	0.946132	0.0698591
21454	1.49386	0.0698591
21455	1.01346	2.1
21456	0.946131	2.1
21457	0.946132	2.1

21458	1.38651	2.1
21459	1.39523	2.1
21460	0.946132	2.1
21461	1.06312	2.1
21462	0.946131	2.1
21463	0.946129	2.1
21464	1.39523	2.1
21465	1.37372	2.1
21466	0.946131	2.1
21467	1.2929	0.0731805
21468	0.94613	0.10088
21469	1.24448	0.0718203
21470	1.2929	0.0797095
21471	0.862011	0.0735659
21472	1.73583	0.0698591
21473	1.724	0.0698591
21474	1.03824	2.1
21475	1.534	2.1
21476	0.725492	1.8962
21477	0.771967	2.01831
21478	0.61731	1.4602
21479	0.475743	1.94602
21480	0.719694	1.71599
21481	1.14878	1.30479
21482	0.946117	1.92798
21483	0.94613	1.89137
21484	0.946105	1.93723
21485	0.482666	1.42993
21486	0.609172	1.445
21487	0.946131	2.1
21488	1.39509	0.986333
21489	1.07439	2.1
21490	0.946131	2.085
21491	1.39509	2.09949
21492	0.963571	1.76198
21493	0.591011	0.425602
21494	1.1357	0.226433
21495	2.11193	1.32593
21496	1.60936	2.1
21497	1.34118	0.0773138
21498	1.27696	0.0779758
21499	0.94613	0.0874662
21500	0.94613	0.0746242
21501	1.04257	0.0698591
21502	1.53336	0.0698654
21503	1.0631	0.0761884
21504	1.04072	0.119473
21505	0.670678	0.0911827
21506	0.709531	0.0978582
21507	0.891275	0.109276
21508	0.683583	0.105226
21509	0.902478	0.0760372
21510	0.807671	0.0787113
21511	1.11024	2.1
21512	1.08171	2.1
21513	1.16725	2.1
21514	0.946128	2.1
21515	1.13537	2.1

21516	1.40525	2.1
21517	1.28365	2.1
21518	1.04523	2.1
21519	0.960106	2.1
21520	0.946128	2.1
21521	1.23452	2.1
21522	0.946129	2.1
21523	1.3704	2.1
21524	1.23452	2.1
21525	1.16906	1.00632
21526	0.946131	1.78542
21527	0.867246	1.28137
21528	0.867217	1.56896
21529	0.946131	1.43135
21530	1.13438	1.47631
21531	1.28684	1.5985
21532	0.946132	1.87514
21533	0.883935	1.56299
21534	0.883938	1.9999
21535	0.946133	1.61401
21536	1.73444	1.1532
21537	1.21835	2.1
21538	1.28228	2.1
21539	1.8643	0.417057
21540	0.743379	0.112597
21541	1.34921	0.0994261
21542	0.743381	0.112918
21543	0.743381	0.116159
21544	0.946131	0.118776
21545	0.915528	2.05631
21546	0.982742	2.08297
21547	0.975965	2.05031
21548	0.891132	0.0698591
21549	1.27533	0.0698591
21550	0.946132	0.0698591
21551	1.25215	0.0698591
21552	0.590343	0.0698591
21553	0.946133	0.0698591
21554	0.590331	0.0698591
21555	1.62375	2.1
21556	0.687769	0.625713
21557	1.14374	0.60635
21558	1.20885	0.501951
21559	0.946132	0.682464
21560	1.14374	0.832666
21561	1.09308	0.214923
21562	1.02055	0.214002
21563	0.810934	0.160722
21564	0.917609	2.03871
21565	1.13094	2.08053
21566	0.70959	1.94761
21567	1.11223	2.1
21568	0.83051	1.95498
21569	0.810946	2.06285
21570	0.810959	1.97807
21571	1.5773	0.0698591
21572	1.96133	1.1791
21573	1.0214	1.22338

21574	0.762783	1.72213
21575	0.838036	0.928953
21576	0.63591	1.80846
21577	0.967411	1.18903
21578	0.946132	2.0485
21579	0.946132	2.03283
21580	1.39699	1.88439
21581	1.61642	1.94187
21582	0.860954	1.15352
21583	0.957411	1.81283
21584	0.660856	1.55363
21585	1.10003	2.1
21586	0.968249	2.09706
21587	0.910184	2.0543
21588	1.30029	0.964222
21589	0.754119	1.8201
21590	0.754121	1.97632
21591	0.94613	1.93527
21592	1.08039	2.1
21593	0.810942	0.0771907
21594	1.15153	0.0755918
21595	1.06968	0.0716376
21596	1.38456	2.1
21597	1.04118	2.1
21598	0.838027	2.1
21599	1.04073	2.1
21600	0.754209	2.1
21601	1.332	0.0698591
21602	1.26193	2.1
21603	1.15317	0.0698591
21604	3.86179	0.0698591
21605	1.56005	2.1
21606	0.683035	0.212128
21607	0.931463	0.305276
21608	0.556019	0.609174
21609	0.929172	0.567435
21610	0.756874	0.138707
21611	1.23527	0.541668
21612	2.05961	0.608636
21613	0.946132	2.1
21614	1.16684	2.1
21615	1.3937	2.1
21616	1.3937	2.1
21617	0.946132	2.1
21618	1.17885	2.1
21619	1.62507	0.0698591
21620	1.58874	2.1
21621	0.819953	2.1
21622	1.16023	2.1
21623	1.34782	2.09685
21624	1.16023	2.09762
21625	0.819955	2.1
21626	0.79437	2.08734
21627	0.794373	2.0887
21628	1.20056	2.02425
21629	1.25	2.05041
21630	1.20056	2.06648
21631	1.35787	2.08075

21632	0.946131	2.06685
21633	1.38585	2.01889
21634	0.946128	1.32872
21635	0.946129	1.91565
21636	1.35714	2.04127
21637	1.04147	2.1
21638	0.946133	1.55226
21639	1.30382	1.32648
21640	0.946131	0.614428
21641	0.946131	1.32896
21642	1.38585	1.64331
21643	1.22305	0.957785
21644	1.00763	0.0880639
21645	0.813199	0.130696
21646	0.810948	0.117137
21647	1.15928	0.111761
21648	0.944166	0.0698591
21649	0.94613	0.0698591
21650	0.946133	0.0698591
21651	1.4179	0.0708765
21652	0.946132	0.0698591
21653	1.36965	0.0706642
21654	1.05444	0.0698591
21655	0.690302	0.0704357
21656	0.838038	0.0698591
21657	0.802821	0.420353
21658	1.40944	0.628351
21659	0.946126	0.759358
21660	0.799998	0.336748
21661	0.800035	0.523701
21662	1.1766	2.1
21663	1.00319	2.1
21664	1.01606	1.59665
21665	0.872158	2.09078
21666	1.04884	2.05094
21667	0.679494	2.08634
21668	0.946131	2.1
21669	1.37609	2.1
21670	0.94613	2.1
21671	0.946131	2.1
21672	1.18486	2.1
21673	0.726287	2.1
21674	0.946131	2.1
21675	0.726286	2.1
21676	1.30318	2.1
21677	1.01712	2.1
21678	0.946132	2.1
21679	1.04404	2.1
21680	1.37017	2.1
21681	1.37017	2.1
21682	0.946133	2.1
21683	0.992084	1.27695
21684	0.946131	0.806074
21685	0.529655	1.9765
21686	0.529686	1.25943
21687	0.946131	0.942492
21688	1.05937	1.65245
21689	1.14622	0.452405

21690	0.946131	0.495104
21691	0.584575	1.94432
21692	0.584575	0.283528
21693	0.946129	0.284851
21694	0.709714	2.1
21695	0.946132	2.1
21696	0.709714	2.1
21697	1.37199	2.1
21698	0.82899	2.1
21699	1.00133	2.1
21700	0.778424	0.0698591
21701	0.946132	0.0698591
21702	1.24094	0.0698591
21703	1.00691	0.0698591
21704	0.64868	0.0698591
21705	1.29736	0.0698591
21706	1.24767	0.266435
21707	1.28953	0.395793
21708	1.34612	1.04858
21709	0.946133	0.691368
21710	1.14187	1.07418
21711	0.946132	0.519327
21712	0.946129	0.971028
21713	1.4099	2.1
21714	0.946131	1.85404
21715	1.34612	1.31007
21716	1.4099	1.69722
21717	0.956003	0.0698591
21718	1.40572	0.0698591
21719	3.54443	2.1
21720	0.994001	0.206197
21721	1.03679	2.1
21722	1.08467	2.1
21723	1.56875	0.149404
21724	0.968463	2.1
21725	0.974889	2.1
21726	0.946131	2.1
21727	1.30203	2.1
21728	1.40199	2.1
21729	1.40199	2.1
21730	0.946132	2.1
21731	0.946132	2.1
21732	1.36994	2.1
21733	1.37372	2.1
21734	1.37372	2.1
21735	0.946132	2.1
21736	0.928979	1.28959
21737	0.565509	1.51344
21738	1.60196	0.286803
21739	1.16239	0.22717
21740	1.10746	0.329146
21741	1.15412	2.1
21742	1.15171	2.1
21743	0.946133	2.1
21744	0.940877	2.1
21745	0.940878	2.1
21746	1.41432	2.1
21747	1.04687	2.1

21748	2.75524	0.0698591
21749	0.946132	0.0698591
21750	0.766452	0.0698591
21751	0.766427	0.0698591
21752	1.36004	0.0698591
21753	1.06648	0.0698591
21754	1.62483	2.1
21755	1.0577	2.1
21756	1.39158	2.07162
21757	0.94255	2.09901
21758	1.05029	2.07628
21759	1.03735	1.92047
21760	0.931055	2.04035
21761	1.03797	1.93093
21762	1.43687	1.88629
21763	1.28765	1.35629
21764	1.1811	1.17671
21765	0.946132	1.27422
21766	0.946132	1.5647
21767	1.1811	1.11167
21768	0.67805	1.0601
21769	1.50998	1.74601
21770	1.16661	2.1
21771	1.36749	2.1
21772	0.810945	0.954768
21773	1.14745	0.455766
21774	0.865588	0.255622
21775	1.36665	0.170269
21776	0.506964	0.128049
21777	0.506966	2.02527
21778	0.552786	0.113431
21779	0.946133	0.138715
21780	1.21336	0.401449
21781	0.552786	0.139872
21782	0.552757	2.1
21783	0.653468	0.257389
21784	1.36665	1.98736
21785	1.27922	0.133751
21786	1.08133	0.210242
21787	1.05348	0.115661
21788	0.946132	0.194452
21789	1.19601	0.119481
21790	1.13276	0.127994
21791	0.7856	0.128502
21792	1.57125	0.11328
21793	1.19601	0.221858
21794	0.946131	0.518579
21795	1.34534	0.296535
21796	1.19601	0.254459
21797	0.870489	0.285304
21798	1.49404	2.1
21799	1.35943	1.48113
21800	1.10427	1.96377
21801	0.94613	1.44699
21802	0.946131	1.76467
21803	1.10427	1.71897
21804	1.2064	1.16646
21805	0.946132	1.00053

21806	1.11288	2.09438
21807	1.34268	0.178557
21808	1.34268	0.773958
21809	0.946132	0.557276
21810	0.946128	2.1
21811	0.946128	2.06706
21812	1.30835	2.1
21813	1.38463	2.1
21814	0.946129	2.1
21815	1.43778	0.506975
21816	1.23943	1.63678
21817	1.4748	2.0424
21818	0.71295	2.1
21819	0.712951	2.1
21820	0.946132	2.1
21821	0.946133	2.1
21822	1.13976	2.1
21823	1.71109	2.1
21824	1.32426	2.1
21825	0.671255	2.09347
21826	1.29123	2.09963
21827	0.946132	2.09856
21828	0.946132	2.08757
21829	1.25603	2.09707
21830	2.21126	0.076319
21831	0.892889	2.1
21832	0.946129	2.1
21833	0.892888	2.1
21834	0.92872	2.1
21835	1.34352	2.1
21836	0.622784	0.175993
21837	1.02947	0.107289
21838	0.810957	0.101921
21839	1.91756	1.14461
21840	1.06012	0.64008
21841	1.09569	0.334051
21842	1.5456	0.0698591
21843	1.1211	0.0698591
21844	1.21031	0.0698591
21845	1.03939	0.07634
21846	0.519697	0.0934542
21847	0.933889	0.182691
21848	0.519726	0.155178
21849	1.16699	0.142105
21850	0.933889	0.153361
21851	0.840599	0.0723736
21852	0.94613	0.131003
21853	0.840568	0.096605
21854	1.26868	0.0952669
21855	1.16699	0.0922637
21856	1.17822	1.93311
21857	0.922044	0.0698591
21858	1.29322	0.0698591
21859	0.946132	0.0698591
21860	0.995178	0.0698591
21861	0.922056	0.0698591
21862	1.76232	0.0930134
21863	1.77065	0.732409

21864	1.38149	0.54714
21865	0.959301	0.943191
21866	0.946133	0.686275
21867	0.946132	0.755528
21868	0.959301	0.63396
21869	1.3612	0.568881
21870	0.885314	0.804979
21871	0.885309	0.650501
21872	0.94613	0.78215
21873	0.8853	0.561621
21874	1.56164	0.521414
21875	1.45506	0.129545
21876	1.45257	2.1
21877	0.851541	0.0698591
21878	1.29018	0.0698591
21879	0.851541	0.0698591
21880	1.21087	0.0698591
21881	1.24979	0.0698591
21882	1.56983	0.0698591
21883	1.60151	0.0698591
21884	1.78952	2.1
21885	0.638164	2.1
21886	0.638163	2.1
21887	1.39754	2.1
21888	1.39754	2.1
21889	0.681589	2.1
21890	1.34993	2.1
21891	1.04525	2.1
21892	0.946133	2.1
21893	1.04072	2.1
21894	0.857805	2.1
21895	1.06053	2.1
21896	1.49066	2.09468
21897	1.4185	1.31189
21898	0.934059	1.8508
21899	0.93406	1.37364
21900	0.94613	1.76395
21901	0.93406	1.6451
21902	1.25277	1.64428
21903	1.00792	2.02217
21904	0.609466	1.77868
21905	0.946132	1.94325
21906	0.609463	1.84935
21907	1.30664	1.89016
21908	0.810943	1.39903
21909	1.05741	0.977415
21910	1.3868	2.1
21911	1.15558	0.133133
21912	1.13537	2.1
21913	0.946132	2.1
21914	0.946132	2.1
21915	1.08266	2.1
21916	1.54934	2.1
21917	1.26016	2.1
21918	2.01915	2.1
21919	0.60346	1.88196
21920	0.651529	1.91062
21921	1.15581	2.05305

21922	1.13037	1.8955
21923	1.04071	1.87026
21924	1.77796	2.03068
21925	0.532308	1.78694
21926	0.532308	1.33159
21927	1.15369	1.99819
21928	1.1537	1.82095
21929	0.903288	1.81867
21930	0.804766	2.1
21931	0.804739	2.1
21932	0.946128	2.1
21933	0.946131	2.1
21934	1.16745	2.1
21935	1.04193	2.1
21936	0.98505	2.1
21937	1.40724	0.763015
21938	1.14216	0.475582
21939	0.946131	0.377431
21940	0.946131	0.53517
21941	1.10941	0.54446
21942	0.81268	0.0698591
21943	0.946131	0.0698591
21944	1.28673	0.0698591
21945	1.28673	0.0698591
21946	0.812681	0.0698591
21947	1.84716	0.0698591
21948	1.84716	0.0698591
21949	0.92361	0.0698591
21950	0.94613	0.0698591
21951	0.923582	0.0698591
21952	1.26227	0.0698591
21953	0.923581	0.0698591
21954	0.747394	2.1
21955	1.32582	2.1
21956	0.747365	2.1
21957	0.97587	2.1
21958	1.32582	2.1
21959	1.04182	0.0698591
21960	0.961743	0.0698591
21961	0.946126	0.0700171
21962	1.47463	0.0698591
21963	1.12183	0.0698591
21964	0.851552	0.0698591
21965	1.33177	0.0761464
21966	0.946131	0.116289
21967	1.41512	0.088647
21968	1.33178	0.0837472
21969	0.946131	0.0783979
21970	0.927735	0.218672
21971	0.97206	0.109347
21972	0.515566	0.16736
21973	0.946129	0.153986
21974	0.515538	0.128347
21975	1.04482	0.0821305
21976	0.975535	0.125565
21977	0.522411	0.0846929
21978	0.522382	0.137026
21979	0.925346	0.140228

21980	0.575817	0.102197
21981	0.946129	2.1
21982	1.14256	2.1
21983	1.32054	2.1
21984	1.40525	2.1
21985	1.14256	2.1
21986	1.59475	2.1
21987	0.773813	2.1
21988	1.4073	2.1
21989	0.773813	2.1
21990	0.773813	2.1
21991	1.4073	2.1
21992	0.993021	2.1
21993	1.50466	0.0698591
21994	0.851539	0.0742588
21995	1.20299	0.0698591
21996	0.946131	0.0709309
21997	1.54464	0.0706138
21998	1.20309	0.0699664
21999	2.00701	0.0698591
22000	0.810946	0.0780132
22001	1.07678	0.0755347
22002	0.900128	0.0719129
22003	1.26842	2.1
22004	1.11206	2.1
22005	0.946132	2.1
22006	1.04074	2.1
22007	0.948809	2.1
22008	0.946131	0.0698591
22009	0.929617	0.0698591
22010	1.24057	0.0698591
22011	1.36485	0.0698591
22012	0.851554	0.0698591
22013	1.96823	0.0698591
22014	0.914836	0.0698591
22015	0.94613	0.0698591
22016	1.38911	0.0698591
22017	0.914805	0.0698591
22018	0.946131	0.0698591
22019	0.946131	0.0698642
22020	1.04072	0.0698591
22021	1.26837	0.0698591
22022	0.970054	0.0698591
22023	0.923958	0.0698591
22024	1.16653	2.1
22025	1.34412	2.1
22026	0.851541	0.0698591
22027	1.38645	0.0700676
22028	0.891637	0.0698591
22029	0.851487	0.0698707
22030	0.890871	0.0698969
22031	1.56178	0.0698591
22032	0.946128	0.0698591
22033	1.26868	0.0698591
22034	0.946131	0.0698591
22035	1.18789	0.327175
22036	1.26868	0.0698591
22037	1.26868	0.0698591

22038	0.946128	0.0698591
22039	1.18716	0.0698591
22040	0.946131	0.0814133
22041	0.59358	0.0704788
22042	1.23923	0.0910836
22043	1.00024	0.0698591
22044	0.593593	0.0720939
22045	0.946131	0.107474
22046	0.713645	0.092346
22047	1.23175	0.13311
22048	1.23923	0.0704364
22049	0.713633	0.099433
22050	1.24687	0.0698591
22051	1.80662	2.04303
22052	2.15762	2.1
22053	0.780455	2.1
22054	0.946131	2.1
22055	0.780455	2.1
22056	1.40583	2.1
22057	1.39852	2.1
22058	0.565619	2.1
22059	1.1428	2.1
22060	0.663168	0.225419
22061	1.02163	0.162389
22062	1.04072	0.227008
22063	0.663169	0.196726
22064	1.13511	0.201883
22065	1.85277	0.121055
22066	1.17701	1.00568
22067	0.993831	0.0731948
22068	1.0823	0.0758694
22069	0.795761	0.185831
22070	1.24596	0.125822
22071	0.566075	0.186673
22072	0.94613	0.209201
22073	0.56605	0.191445
22074	0.774911	0.0698591
22075	0.946131	0.0698591
22076	1.24417	0.0698591
22077	0.774911	0.0698591
22078	0.946132	0.0698591
22079	1.54982	0.0698591
22080	1.82961	0.0698591
22081	1.12035	0.0698591
22082	0.876835	2.1
22083	0.946131	2.1
22084	0.876836	2.1
22085	1.12968	2.1
22086	1.23536	2.1
22087	2.15082	2.1
22088	0.58365	2.1
22089	0.946133	2.1
22090	0.583653	2.1
22091	0.992097	2.1
22092	1.12968	2.1
22093	1.0305	2.1
22094	0.82613	0.0945546
22095	0.879644	0.0852802

22096	1.00688	0.0838254
22097	1.52776	2.1
22098	0.851537	1.69102
22099	1.00851	1.11675
22100	0.946128	0.92886
22101	0.851552	1.24532
22102	1.4895	1.76946
22103	0.761574	2.1
22104	0.946132	2.1
22105	1.01095	2.1
22106	1.01095	2.1
22107	0.761572	2.1
22108	1.8575	2.1
22109	1.19984	0.0698591
22110	1.25797	0.0755134
22111	0.946132	0.0871226
22112	0.946132	0.0727616
22113	0.95332	0.0802241
22114	0.946131	2.1
22115	0.851542	2.1
22116	1.03172	2.1
22117	1.22889	2.1
22118	1.0313	2.1
22119	1.15977	1.9924
22120	0.918875	1.41326
22121	0.8654	1.95428
22122	0.865374	1.54846
22123	0.946132	1.67301
22124	0.985051	2.1
22125	1.31993	2.01207
22126	1.33622	0.269302
22127	0.959992	0.23884
22128	0.926499	2.1
22129	1.03544	2.1
22130	0.946132	2.1
22131	0.946132	2.1
22132	1.40335	2.1
22133	0.772299	2.1
22134	0.851529	2.1
22135	0.857243	2.1
22136	1.38155	2.1
22137	1.05462	2.1
22138	0.832138	2.1
22139	0.946132	2.1
22140	0.832137	2.1
22141	0.959414	2.1
22142	1.36541	2.1
22143	1.91894	2.1
22144	1.04661	2.1
22145	1.28355	2.1
22146	0.877353	2.1
22147	0.877353	2.1
22148	0.94613	2.1
22149	1.20537	0.0698591
22150	1.09914	0.0698591
22151	0.94613	0.0698591
22152	0.499593	0.0698591
22153	0.499606	0.0698591

22154	1.0959	0.0698591
22155	0.838027	0.0698591
22156	0.883644	0.0698591
22157	0.95019	0.0830901
22158	1.10638	2.1
22159	0.946132	2.1
22160	1.41744	2.1
22161	1.05842	2.1
22162	0.94613	2.1
22163	1.41744	2.1
22164	0.991985	2.1
22165	3.31755	2.0974
22166	1.05556	2.1
22167	0.624075	2.04386
22168	0.814554	2.03187
22169	0.946131	2.03442
22170	0.814554	2.07187
22171	1.31857	2.05671
22172	1.26282	2.1
22173	0.946132	2.1
22174	1.14302	2.1
22175	0.946132	2.1
22176	0.946132	2.1
22177	1.40372	2.1
22178	1.24688	0.142583
22179	0.89889	2.1
22180	1.17286	2.1
22181	0.71741	2.1
22182	0.491096	0.572256
22183	1.05432	0.297999
22184	0.719531	2.1
22185	1.25145	2.1
22186	0.864686	2.1
22187	0.867974	2.1
22188	1.21734	2.1
22189	0.405517	0.68122
22190	0.818878	0.925208
22191	0.502457	0.0698591
22192	1.05868	0.0698591
22193	0.912018	0.0698591
22194	1.40591	0.0698591
22195	0.957584	0.447978
22196	0.946131	0.658888
22197	1.11612	0.440193
22198	1.3365	0.458982
22199	0.946128	0.708126
22200	1.16534	0.120047
22201	1.38969	0.173898
22202	0.891597	1.35647
22203	0.640032	1.8001
22204	0.956758	1.36301
22205	1.3428	0.179559
22206	0.953295	2.1
22207	0.946131	0.117602
22208	1.16167	0.113891
22209	0.94613	0.122446
22210	0.951675	0.134831
22211	1.16167	0.120234

22212	1.24538	0.0698591
22213	1.13482	2.1
22214	1.38775	2.1
22215	0.934061	2.1
22216	0.91408	2.1
22217	1.06099	2.1
22218	0.980549	0.08308
22219	0.946132	0.0970367
22220	1.10321	0.114067
22221	0.99126	0.100787
22222	0.991261	0.110999
22223	1.35732	0.104765
22224	1.1952	2.1
22225	0.946132	1.63974
22226	0.946132	2.1
22227	1.32819	1.92552
22228	1.30171	1.96007
22229	1.29667	0.0722846
22230	1.00982	0.0698591
22231	0.407589	0.546743
22232	1.18266	1.56644
22233	1.04037	0.553291
22234	0.956694	0.882476
22235	0.552639	0.810739
22236	0.810943	1.9052
22237	1.43144	1.04765
22238	0.918074	1.06601
22239	1.60976	2.1
22240	4.00847	2.1
22241	2.3995	0.0698591
22242	1.36203	2.1
22243	0.919619	2.1
22244	1.30188	2.1
22245	0.681073	2.1
22246	0.681043	2.1
22247	1.04687	2.1
22248	3.32085	2.1
22249	1.80396	0.0698591
22250	0.773007	0.157462
22251	0.773034	0.108311
22252	1.22141	0.170394
22253	1.22141	0.149774
22254	0.946131	0.234076
22255	2.02071	0.0964476
22256	0.951912	0.0698591
22257	1.39027	0.0698591
22258	0.94613	0.0698591
22259	0.829922	0.0698591
22260	0.829946	0.0698591
22261	1.24902	1.30965
22262	2.8333	0.898259
22263	0.946131	0.0698591
22264	0.929725	0.0698591
22265	1.1134	0.0698591
22266	1.17056	0.0698591
22267	0.929713	0.0698591
22268	0.749096	2.1
22269	1.31961	2.1

22270	0.870191	2.1
22271	1.04071	2.1
22272	1.1877	2.1
22273	1.03882	2.1
22274	1.44801	2.03194
22275	3.55198	2.1
22276	1.11193	2.1
22277	0.663922	2.1
22278	0.663922	2.1
22279	0.946129	2.1
22280	0.946131	2.1
22281	0.97079	2.1
22282	1.36049	2.1
22283	1.06105	2.1
22284	0.92251	2.1
22285	0.94613	2.1
22286	0.922511	2.1
22287	1.04101	2.1
22288	0.810944	0.0700178
22289	0.994109	0.0698591
22290	0.623143	0.0698591
22291	0.864244	0.0706949
22292	1.14627	0.0698591
22293	0.64586	0.0698591
22294	0.721422	0.0698591
22295	0.870885	0.0698591
22296	0.855175	0.0698591
22297	0.946131	2.08148
22298	1.38157	2.1
22299	1.33266	1.89876
22300	0.946131	2.1
22301	1.38158	2.1
22302	0.946132	2.1
22303	1.3268	2.1
22304	0.946132	2.1
22305	0.946132	2.1
22306	1.38158	2.1
22307	1.42186	1.89599
22308	1.47204	1.68843
22309	1.14665	2.1
22310	0.946132	1.98786
22311	0.94613	2.1
22312	1.30116	2.09355
22313	1.32819	2.1
22314	1.25388	2.1
22315	0.887808	2.1
22316	0.946132	2.1
22317	1.23536	2.1
22318	1.34309	2.1
22319	1.07612	2.1
22320	0.728982	0.0698591
22321	1.3157	0.0698591
22322	0.946131	0.0698591
22323	0.686176	0.0698591
22324	1.3035	0.0698591
22325	0.686139	0.0698591
22326	0.906806	0.0698591
22327	0.794122	0.0698591

22328	1.17173	0.0698591
22329	1.8194	0.0698591
22330	0.851543	2.1
22331	1.19345	2.1
22332	0.946133	2.1
22333	0.946132	2.1
22334	1.56179	2.1
22335	0.620263	2.08219
22336	1.30226	2.07867
22337	1.2334	2.0077
22338	1.2334	1.9482
22339	0.946133	1.8983
22340	1.24053	2.09787
22341	1.4828	2.1
22342	0.957622	2.1
22343	1.00521	1.77707
22344	1.49463	2.1
22345	1.39621	2.1
22346	1.09861	2.1
22347	0.946131	2.1
22348	0.946131	2.1
22349	1.09861	2.1
22350	2.16987	0.0698591
22351	0.572182	0.0698591
22352	1.15802	0.0698591
22353	0.63574	0.0698591
22354	1.18897	0.0698591
22355	0.996733	0.0698591
22356	1.82402	1.41874
22357	0.969602	1.16944
22358	0.794777	1.66918
22359	1.02626	1.3235
22360	1.51396	0.141721
22361	1.40574	0.213535
22362	1.38683	0.197376
22363	0.946126	0.237147
22364	0.946128	0.213926
22365	1.07138	0.20547
22366	0.946131	2.1
22367	1.41331	2.1
22368	0.94613	2.1
22369	0.94613	2.1
22370	1.27451	2.1
22371	0.974196	2.1
22372	1.2049	0.0698591
22373	1.42757	2.1
22374	1.02461	2.1
22375	2.07087	2.1
22376	1.92515	1.15168
22377	0.802138	1.21944
22378	0.946128	1.90278
22379	0.802107	1.5393
22380	1.23726	1.49734
22381	1.21997	1.42192
22382	0.767577	0.762607
22383	0.946131	1.73218
22384	0.767577	1.16647
22385	1.3279	1.10537

22386	1.23726	1.00597
22387	1.29729	2.1
22388	0.946128	2.1
22389	1.14186	2.1
22390	1.39214	2.1
22391	1.39214	2.1
22392	0.946129	2.1
22393	1.23012	0.455368
22394	0.653228	0.0784898
22395	0.946133	0.0949399
22396	1.15918	0.0901649
22397	1.26893	0.095192
22398	0.653228	0.080691
22399	1.06848	0.191116
22400	0.615121	2.1
22401	0.841488	2.09648
22402	0.946131	2.1
22403	1.04071	2.1
22404	0.965717	2.1
22405	1.77893	2.03489
22406	1.11314	0.0773713
22407	1.65411	0.823929
22408	0.85472	0.0698591
22409	1.1604	0.0698591
22410	1.19141	0.0698591
22411	1.19141	0.0698591
22412	0.94613	0.0698591
22413	1.70944	0.0698591
22414	1.89082	0.127123
22415	1.05175	1.06573
22416	0.815276	2.1
22417	0.843283	2.1
22418	0.946131	2.1
22419	0.843284	2.1
22420	1.38897	2.1
22421	1.01019	0.681764
22422	1.05197	0.665328
22423	0.810959	0.636897
22424	1.33037	0.137922
22425	1.3415	0.0964096
22426	0.946118	0.0901448
22427	0.946131	0.121148
22428	1.09803	0.116194
22429	0.930281	2.1
22430	1.3335	2.1
22431	0.784151	2.1
22432	0.78415	2.1
22433	0.946132	2.1
22434	1.6299	0.0698591
22435	0.995795	2.1
22436	1.84846	1.21348
22437	0.929873	1.95513
22438	0.889245	2.00006
22439	0.808072	1.98269
22440	0.851541	1.81695
22441	1.51956	1.82675
22442	0.94613	1.82252
22443	0.851553	1.82912

22444	0.946787	1.86796
22445	1.15544	1.74103
22446	1.30289	0.0785948
22447	1.28323	0.0761376
22448	0.85024	0.0712897
22449	1.09662	0.0801639
22450	1.67687	0.0698591
22451	0.946127	2.1
22452	1.40496	2.1
22453	1.22442	2.1
22454	0.946128	2.1
22455	1.40496	2.1
22456	1.00573	2.1
22457	0.636837	2.09831
22458	0.988642	2.09869
22459	0.573141	2.08182
22460	0.946128	2.08189
22461	0.786538	2.08035
22462	2.28748	1.86993
22463	0.682805	2.1
22464	0.946132	2.1
22465	0.682776	2.08278
22466	0.946132	2.09999
22467	0.79775	2.1
22468	1.05403	0.0698591
22469	0.888273	0.0698591
22470	0.876373	0.0698591
22471	0.576083	0.0698591
22472	0.946132	0.0698591
22473	0.576057	0.0698591
22474	0.576058	0.0698591
22475	0.946131	0.0698591
22476	1.41724	0.385341
22477	1.31678	0.654318
22478	0.946132	0.644477
22479	0.946132	0.542803
22480	1.31678	0.5045
22481	0.935738	2.1
22482	0.946132	2.1
22483	0.935765	2.1
22484	1.32949	2.1
22485	0.946131	2.1
22486	1.02525	2.1
22487	0.946128	2.1
22488	1.41207	2.1
22489	1.04675	2.1
22490	0.946131	2.1
22491	1.41207	2.1
22492	0.946131	2.1
22493	0.946132	2.1
22494	1.41125	2.1
22495	1.41125	2.1
22496	1.27087	2.1
22497	0.924071	0.20375
22498	0.608893	0.152042
22499	0.528907	0.203972
22500	1.78249	0.181377
22501	0.720094	0.207446

22502	0.781667	2.1
22503	0.781638	2.1
22504	1.40756	2.1
22505	1.40756	2.1
22506	0.797635	2.1
22507	1.11946	0.0698591
22508	1.32068	0.0698591
22509	0.88757	0.071614
22510	1.27891	0.0698629
22511	0.810957	0.07024
22512	0.914656	0.0698591
22513	0.640819	0.0698591
22514	0.745643	0.0698676
22515	0.865108	0.0772642
22516	1.06883	0.0729058
22517	0.806001	0.0786447
22518	1.19462	2.1
22519	1.42763	0.361114
22520	1.24682	0.279562
22521	0.78392	0.0698591
22522	1.02874	0.0698591
22523	0.884512	0.0698591
22524	1.1658	2.1
22525	0.663026	0.0698591
22526	1.21174	0.0698591
22527	0.663056	0.0698591
22528	1.15968	0.0698591
22529	1.21174	0.0698591
22530	0.993976	0.0698591
22531	0.945495	0.0698591
22532	0.671202	0.0698591
22533	1.37312	2.1
22534	1.28499	2.1
22535	0.946133	2.1
22536	0.946132	2.1
22537	1.28499	2.1
22538	1.25081	2.1
22539	0.946132	2.1
22540	0.946132	2.1
22541	1.27985	2.1
22542	1.37312	2.1
22543	0.830638	2.1
22544	1.05358	2.1
22545	1.22209	2.1
22546	1.09874	2.1
22547	0.74758	2.1
22548	2.50011	1.19059
22549	0.6157	0.190311
22550	0.674634	0.138948
22551	0.946128	1.82407
22552	0.674633	0.163955
22553	0.946131	0.419245
22554	1.96192	1.59679
22555	1.31612	0.62699
22556	1.10735	0.412338
22557	0.936676	0.168283
22558	0.808779	0.183575
22559	0.808808	0.209622

22560	1.00245	0.188469
22561	1.26179	0.161463
22562	1.05758	2.04782
22563	1.03408	2.1
22564	0.946132	0.0699919
22565	0.914368	0.0698591
22566	1.20524	0.0698591
22567	1.12743	0.0698591
22568	0.851555	0.0698591
22569	1.17885	0.179328
22570	0.867881	0.130481
22571	1.00122	0.106866
22572	0.867883	0.0889331
22573	0.867881	0.071361
22574	1.19583	0.129163
22575	1.17743	2.07802
22576	0.878684	2.05623
22577	0.687087	2.09792
22578	0.955079	0.393657
22579	0.672977	0.526698
22580	0.735937	0.467696
22581	0.851531	0.534288
22582	0.672957	0.485998
22583	0.923974	0.493572
22584	1.2243	2.1
22585	0.808463	0.25139
22586	0.971078	0.316912
22587	1.06161	0.182491
22588	0.810947	0.439631
22589	0.886074	0.484825
22590	0.786829	0.466427
22591	0.55062	0.420084
22592	0.574458	0.360704
22593	0.946129	0.429227
22594	1.42526	0.356826
22595	1.15962	0.364248
22596	1.24526	0.0698591
22597	0.94613	0.621879
22598	0.946128	1.71802
22599	1.23279	1.88859
22600	1.38645	1.82899
22601	0.94613	1.43414
22602	1.05042	2.1
22603	0.946128	2.1
22604	0.946129	2.1
22605	1.24286	2.1
22606	1.39659	2.1
22607	0.94613	2.1
22608	1.10407	2.1
22609	1.32809	1.88455
22610	0.993999	1.97687
22611	0.810959	1.86033
22612	0.983643	1.92459
22613	0.869552	2.01542
22614	0.747801	1.86343
22615	1.53429	1.26184
22616	1.13127	2.1
22617	1.38397	2.1

22618	1.08366	2.1
22619	0.946129	2.1
22620	0.946131	2.1
22621	1.08366	2.1
22622	1.06007	2.1
22623	1.79641	2.1
22624	0.758799	2.1
22625	1.28442	2.1
22626	1.06957	2.1
22627	0.946133	2.1
22628	1.28442	2.1
22629	1.76788	1.85682
22630	1.38669	2.1
22631	0.986095	2.1
22632	1.38478	0.118794
22633	0.946132	0.414502
22634	1.24344	0.182412
22635	0.622283	0.89423
22636	1.10335	0.257206
22637	1.24344	0.369531
22638	1.1967	0.256794
22639	0.946131	0.0698591
22640	0.901005	0.0698591
22641	1.05751	0.0698591
22642	1.37771	0.0698591
22643	0.851527	0.0698591
22644	1.26643	0.539417
22645	1.08992	1.79378
22646	0.946132	0.917794
22647	0.946132	1.10753
22648	1.08992	1.1931
22649	1.53534	2.09388
22650	1.58825	1.77507
22651	1.0447	1.99425
22652	0.593818	1.83626
22653	0.89883	1.97192
22654	0.734088	1.85578
22655	0.998451	0.377526
22656	1.64156	0.855142
22657	1.05654	1.034
22658	1.37641	0.25704
22659	1.74553	0.318988
22660	1.31112	0.442145
22661	0.74518	0.561809
22662	0.851539	0.503777
22663	0.851554	0.446131
22664	0.851475	0.463016
22665	0.946132	0.0698591
22666	1.28895	0.0698591
22667	1.11356	0.0698591
22668	0.946132	0.0698591
22669	0.946132	0.0698591
22670	1.84214	2.1
22671	1.23851	0.0799764
22672	1.25866	0.339658
22673	1.28655	0.596103
22674	0.946131	0.546817
22675	0.946132	0.497382

22676	1.28655	0.451717
22677	1.11929	0.390249
22678	0.946132	0.552636
22679	0.946132	0.384924
22680	1.34254	0.379223
22681	1.25866	0.351472
22682	0.946132	2.1
22683	1.27451	2.1
22684	1.19803	2.1
22685	0.94613	2.1
22686	1.27451	2.1
22687	0.946131	2.1
22688	1.36523	2.1
22689	1.14599	2.1
22690	0.946129	2.1
22691	1.36523	2.1
22692	1.09518	2.1
22693	0.977777	2.1
22694	0.583237	2.1
22695	0.583267	2.1
22696	0.903872	2.1
22697	0.946129	2.1
22698	0.583258	2.1
22699	1.58179	0.0698591
22700	0.946131	0.06987
22701	1.35123	0.0698591
22702	0.655837	0.0701367
22703	1.12018	0.0698591
22704	1.35123	0.0698591
22705	0.62967	0.0698613
22706	0.860497	0.0725855
22707	0.816639	0.0698591
22708	0.746716	2.1
22709	0.810947	2.1
22710	0.810959	2.1
22711	0.653422	2.07565
22712	0.289127	1.93233
22713	0.475716	2.05673
22714	0.980651	1.86218
22715	0.946119	2.08881
22716	0.946132	2.09632
22717	1.08299	2.1
22718	1.34539	2.1
22719	1.28753	0.121321
22720	0.946132	0.222631
22721	0.946132	0.175892
22722	1.19719	0.182696
22723	0.946133	0.177406
22724	0.958947	0.1398
22725	1.3492	0.145983
22726	0.946132	0.240061
22727	0.946128	0.213073
22728	1.22588	0.20397
22729	0.946129	0.199815
22730	0.991113	2.1
22731	1.35966	2.1
22732	0.961 2.1	
22733	0.946129	2.1

22734	0.946131	2.1
22735	0.961001	2.1
22736	0.727096	1.66562
22737	0.810946	0.834904
22738	0.810959	1.06004
22739	1.31658	0.237202
22740	1.23167	2.1
22741	1.44836	0.0698591
22742	1.50103	0.0698591
22743	1.23351	0.0698591
22744	1.35657	0.0698591
22745	0.851541	0.0698591
22746	0.93668	0.0698591
22747	0.946131	0.0698591
22748	3.7335	2.1
22749	1.1822	2.1
22750	1.31127	0.168457
22751	1.3689	0.153847
22752	1.35822	0.0844161
22753	0.946133	0.11606
22754	0.946132	0.162297
22755	1.14926	0.146158
22756	1.40822	0.0819414
22757	1.04935	0.157494
22758	0.704112	0.112506
22759	0.704112	0.153364
22760	0.946131	0.182249
22761	0.704112	0.16242
22762	1.37721	0.0698591
22763	0.85154	2.1
22764	1.06949	2.1
22765	0.851541	2.1
22766	0.946128	2.1
22767	1.1175	2.1
22768	1.44091	2.1
22769	0.96029	1.68643
22770	3.46486	0.978401
22771	0.603286	0.0798357
22772	0.432283	0.0698591
22773	0.851541	0.0761523
22774	1.09355	0.0698591
22775	0.851534	0.0831463
22776	1.04263	2.1
22777	0.851541	2.1
22778	0.965604	2.1
22779	0.94613	2.1
22780	1.23953	2.1
22781	0.869615	2.1
22782	1.02415	2.1
22783	0.983032	2.1
22784	1.35385	1.32656
22785	1.59971	0.124648
22786	0.915178	2.1
22787	0.962435	2.1
22788	1.3116	2.1
22789	1.38302	2.1
22790	1.13227	2.1
22791	1.25923	0.0698591

22792	0.988805	2.1
22793	0.611486	1.27351
22794	1.1019	0.804424
22795	1.11124	1.78085
22796	0.716713	0.877821
22797	0.946131	1.42298
22798	1.22297	0.849257
22799	1.12873	1.66861
22800	0.946131	1.68735
22801	0.946132	1.69334
22802	1.3469	1.74872
22803	1.25366	1.87151
22804	1.1611	1.30433
22805	0.946132	1.64065
22806	0.946132	1.40867
22807	1.2854	1.59838
22808	1.3469	1.73202
22809	1.07468	1.37394
22810	1.79317	2.1
22811	1.07444	2.1
22812	1.69436	2.09078
22813	0.851539	2.1
22814	0.870925	2.1
22815	0.851541	2.1
22816	0.870951	2.1
22817	1.26097	2.1
22818	1.26247	2.1
22819	0.946132	2.1
22820	1.24041	2.1
22821	0.946132	2.1
22822	0.946132	2.1
22823	1.40274	2.1
22824	0.946131	0.0698591
22825	0.797945	0.0698591
22826	1.12988	0.0698591
22827	1.00681	0.0698591
22828	0.797932	0.0698591
22829	0.977483	0.188199
22830	0.943362	0.358622
22831	0.9891	0.177523
22832	1.1703	0.226881
22833	0.663171	0.230778
22834	1.40882	0.091336
22835	1.32639	0.139691
22836	1.24448	0.0931374
22837	0.946132	0.153073
22838	1.11015	0.0897697
22839	1.24448	0.114308
22840	0.754768	0.106424
22841	1.13854	2.1
22842	1.23654	2.1
22843	0.714101	0.847301
22844	0.851538	1.71912
22845	0.79261	2.05498
22846	1.30598	1.98885
22847	1.05338	1.72719
22848	1.41758	2.1
22849	0.83317	0.107932

22850	1.00058	0.107736
22851	0.810958	0.107016
22852	1.63791	0.136805
22853	1.55116	2.1
22854	1.29735	1.57569
22855	0.945147	2.09918
22856	0.94515	1.79759
22857	0.94613	2.03474
22858	0.945149	1.97646
22859	1.02368	2.1
22860	1.12787	2.1
22861	1.04007	2.1
22862	3.78805	2.1
22863	0.816891	0.07217
22864	0.943996	0.0713689
22865	0.870002	0.0699169
22866	0.564533	0.0771387
22867	0.564536	0.082042
22868	0.946128	0.13462
22869	0.564535	0.107221
22870	0.946128	0.0982178
22871	1.15177	2.1
22872	1.16806	0.499408
22873	0.999083	1.08037
22874	0.946131	0.675321
22875	0.946131	0.830326
22876	0.999084	0.708294
22877	1.28918	0.510263
22878	1.0383	1.13897
22879	0.946133	0.731117
22880	0.946132	0.84257
22881	1.0383	0.673958
22882	1.27737	1.42644
22883	1.39296	0.24013
22884	0.998682	2.1
22885	3.98933	2.1
22886	1.05328	0.117681
22887	1.23475	2.1
22888	0.900856	0.69632
22889	0.94001	0.587691
22890	0.707748	0.520421
22891	0.707751	0.555694
22892	0.946128	0.512409
22893	0.70775	0.519578
22894	0.996109	0.517192
22895	1.68551	2.1
22896	0.851539	2.1
22897	0.962688	2.1
22898	0.94613	2.1
22899	1.04073	2.1
22900	1.44824	2.1
22901	0.794313	0.20696
22902	0.838031	0.217599
22903	1.11076	0.188111
22904	1.38813	2.1
22905	1.17204	2.1
22906	0.946132	2.1
22907	0.946131	2.1

22908	1.17204	2.1
22909	0.961779	0.247863
22910	1.0685	0.380823
22911	0.810947	2.00799
22912	1.2899	1.50139
22913	0.890404	1.49909
22914	0.909981	2.08648
22915	0.810946	1.86192
22916	0.890402	1.83089
22917	0.522844	2.03245
22918	0.593846	2.01386
22919	1.30226	1.74915
22920	1.30226	1.86978
22921	0.946132	1.82956
22922	1.03537	0.0707874
22923	1.50035	0.0698591
22924	0.940356	0.0728754
22925	0.946131	0.0702687
22926	1.03441	0.0707
22927	1.21171	0.0698591
22928	1.35169	0.0698858
22929	1.32449	0.0698591
22930	0.946131	0.0707014
22931	0.946131	0.0699064
22932	1.06627	0.0700378
22933	1.03102	0.276186
22934	0.488712	0.0698591
22935	0.946131	0.0698591
22936	1.23146	0.0698591
22937	1.13242	0.0698591
22938	0.946131	0.0698591
22939	0.409199	0.0698591
22940	0.429959	0.0698591
22941	1.08357	0.0698591
22942	0.80926	0.0698591
22943	0.879791	0.0698591
22944	0.946129	2.06129
22945	1.28828	2.1
22946	0.946131	2.03397
22947	0.946131	2.09956
22948	1.35969	2.08668
22949	1.10049	1.96128
22950	0.923724	2.1
22951	0.923695	2.1
22952	0.94613	2.1
22953	0.94613	2.1
22954	1.28828	2.1
22955	1.73525	0.134199
22956	0.656239	0.191332
22957	1.2996	0.146225
22958	0.812939	0.207321
22959	0.65624	0.179478
22960	1.2996	0.17326
22961	1.67372	2.1
22962	0.946131	2.1
22963	0.946132	2.1
22964	1.05978	2.1
22965	1.31049	2.1

22966	0.946132	2.1
22967	1.11077	2.1
22968	0.579725	0.360311
22969	0.373532	0.237165
22970	1.13537	0.368261
22971	1.20726	0.256222
22972	0.946131	0.264945
22973	1.45694	0.476663
22974	0.750367	0.0698591
22975	0.946126	0.0699673
22976	0.750365	0.0698591
22977	1.25698	0.0698591
22978	1.35726	0.0698591
22979	1.6812	0.0698891
22980	1.78665	1.78227
22981	1.46568	2.1
22982	1.29606	2.1
22983	1.25298	2.1
22984	0.946115	2.1
22985	0.946129	2.1
22986	1.25296	2.1
22987	1.27778	0.0757747
22988	1.17042	2.1
22989	1.25858	0.0698591
22990	0.93106	0.0698591
22991	0.810959	0.0698591
22992	1.38905	2.1
22993	1.19216	2.1
22994	0.946131	2.1
22995	0.946131	2.1
22996	1.19216	2.1
22997	1.58339	2.09397
22998	0.81094	0.0698591
22999	0.845129	0.0698591
23000	0.918535	0.0698591
23001	0.946132	2.1
23002	0.946132	2.1
23003	1.31904	2.1
23004	1.31904	2.1
23005	1.21275	2.1
23006	1.15471	2.1
23007	1.33125	2.1
23008	0.702954	0.0698591
23009	0.942973	0.0698591
23010	0.94613	0.0698591
23011	0.641772	0.0698591
23012	0.702942	0.0698591
23013	0.991865	0.0698591
23014	0.639952	0.0698591
23015	1.17333	0.0698591
23016	0.946132	0.0698591
23017	0.490975	0.0698591
23018	0.495934	0.0698591
23019	1.19904	0.0698591
23020	1.30488	0.0698591
23021	1.02305	0.127838
23022	1.49842	0.0873971
23023	1.54196	2.1

23024	1.37224	1.88951
23025	1.0953	0.0698591
23026	1.18952	2.1
23027	1.68851	0.253879
23028	0.946131	0.143137
23029	1.12491	0.141518
23030	0.583591	0.137075
23031	1.28727	0.17986
23032	1.12491	0.144074
23033	0.884641	0.291278
23034	0.810944	0.217793
23035	0.917973	0.195451
23036	0.846671	0.185589
23037	0.596968	0.178141
23038	0.716347	0.228963
23039	1.36255	0.0986893
23040	1.11077	2.1
23041	0.946132	2.1
23042	0.946132	2.1
23043	1.26267	2.1
23044	1.26441	2.1
23045	1.85238	2.1
23046	1.36693	2.1
23047	0.616123	2.1
23048	0.481844	2.1
23049	0.709599	2.1
23050	0.815889	2.1
23051	1.1502	2.1
23052	1.63937	1.98047
23053	1.25797	0.0743696
23054	1.22878	0.0913028
23055	0.946132	0.108529
23056	0.946132	0.0856013
23057	0.965737	0.0930745
23058	1.1893	0.0922865
23059	1.22877	0.0882002
23060	0.735217	0.116979
23061	0.85154	0.123942
23062	0.851553	0.106893
23063	0.851473	0.110098
23064	1.58916	0.0769498
23065	1.25545	0.073967
23066	0.810942	0.125394
23067	0.919314	0.138949
23068	0.928633	0.116832
23069	2.14514	0.0803999
23070	1.23389	2.1
23071	0.946128	0.0698608
23072	0.969504	0.0698591
23073	0.851539	0.0698591
23074	1.41503	0.0698591
23075	0.958517	0.0698591
23076	0.990615	0.0698591
23077	1.7943	0.0698591
23078	0.946133	0.346585
23079	1.27581	0.360848
23080	0.946132	0.357041
23081	1.36362	0.315047

23082	1.36936	0.318366
23083	1.71903	2.1
23084	0.751639	2.1
23085	0.77464	2.1
23086	0.946133	2.1
23087	1.04073	2.1
23088	1.23551	2.1
23089	0.94613	2.1
23090	1.18682	2.1
23091	0.946131	2.1
23092	0.94613	2.1
23093	1.40638	2.1
23094	0.483346	0.194328
23095	0.432527	0.119581
23096	0.946132	0.205967
23097	0.483312	0.156369
23098	0.946132	0.160522
23099	0.878798	0.195034
23100	0.810944	0.191005
23101	1.14604	0.168063
23102	0.946129	0.184202
23103	1.35594	0.148872
23104	0.538378	0.149985
23105	0.946131	0.193458
23106	0.538378	0.16918
23107	1.00065	1.95608
23108	0.962878	0.905771
23109	1.32634	0.0726708
23110	1.28066	0.0698591
23111	0.785651	2.1
23112	1.20327	2.1
23113	1.47058	2.02234
23114	0.551834	1.47836
23115	1.04072	2.05127
23116	0.506719	1.91554
23117	1.12786	1.97823
23118	1.45789	0.0698591
23119	0.681532	0.0698591
23120	1.21087	0.0698591
23121	0.94613	0.0698591
23122	0.681534	0.0698591
23123	1.21087	0.0698591
23124	1.98618	0.566644
23125	1.71742	1.46655
23126	1.07427	0.252752
23127	1.1972	0.194438
23128	0.933474	0.254101
23129	1.1745	0.267479
23130	0.94612	0.251805
23131	0.933485	0.263454
23132	1.17449	0.256435
23133	0.851541	0.0698591
23134	1.12774	0.0698591
23135	0.946129	0.0698591
23136	0.851486	0.0698591
23137	1.12517	0.0698591
23138	1.12328	0.0698591
23139	1.13179	2.1

23140	1.02438	2.1
23141	1.05776	2.1
23142	1.41273	2.04653
23143	1.1229	2.1
23144	0.875912	2.07415
23145	0.946132	2.1
23146	0.875912	2.1
23147	1.42307	2.1
23148	1.15354	0.0698591
23149	0.848382	0.0698591
23150	1.2685	2.1
23151	0.938104	2.1
23152	0.793166	2.1
23153	1.07855	2.1
23154	0.98488	2.1
23155	1.4162	2.1
23156	1.09651	0.102187
23157	1.98502	0.0728384
23158	1.41168	0.122316
23159	1.33879	0.15974
23160	0.946127	0.230963
23161	1.13528	0.126123
23162	0.946128	0.165708
23163	1.09726	2.1
23164	1.22026	1.63161
23165	1.09044	2.1
23166	2.23961	0.363879
23167	1.14721	0.227608
23168	1.17614	2.1
23169	0.615065	2.1
23170	0.946131	2.1
23171	0.778488	2.1
23172	0.94613	2.1
23173	0.615066	2.1
23174	1.78254	2.1
23175	0.898452	0.0698591
23176	1.47411	2.1
23177	0.59676	0.247483
23178	0.59676	0.162988
23179	0.94613	0.253321
23180	1.13511	0.196193
23181	0.946131	0.187175
23182	0.916275	2.07582
23183	0.946132	2.0259
23184	0.916274	1.69096
23185	1.36182	1.91918
23186	0.946131	2.00822
23187	0.857202	0.0698591
23188	0.941918	0.0698591
23189	0.943349	0.0698591
23190	1.2493	0.0698591
23191	0.946133	0.0698591
23192	1.01814	0.0698591
23193	0.927881	0.0698591
23194	1.11845	0.0698591
23195	1.2493	0.0698591
23196	1.11845	0.0698591
23197	0.946129	0.0698591

23198	0.967673	0.466793
23199	1.01332	0.0698591
23200	0.946133	0.0698591
23201	0.81938	0.0698591
23202	0.819377	0.0698591
23203	0.946133	0.0698591
23204	1.22474	0.115606
23205	1.15321	0.0906803
23206	0.797489	0.0908784
23207	0.946131	0.122004
23208	0.797461	0.103727
23209	1.35328	0.519711
23210	1.03318	1.14718
23211	0.946131	0.711372
23212	0.94613	0.770418
23213	1.03319	0.684094
23214	1.37938	0.537549
23215	1.00512	1.05402
23216	0.946133	0.688332
23217	0.946132	0.763948
23218	1.00512	0.669679
23219	1.5515	0.870884
23220	1.55351	2.1
23221	0.94261	0.0698591
23222	0.828156	0.0698591
23223	0.916495	0.0698591
23224	3.06309	0.0698591
23225	0.619832	0.0698591
23226	0.705903	0.0698591
23227	1.2968	0.0698591
23228	0.946133	0.0698591
23229	0.705904	0.0698591
23230	1.2968	0.0698591
23231	1.01971	0.0698591
23232	0.60302	0.0698591
23233	1.07242	0.505626
23234	1.26426	0.333958
23235	0.946131	0.475516
23236	0.946131	0.524997
23237	0.963109	0.523351
23238	0.947862	0.297088
23239	1.40248	1.15503
23240	0.767143	1.09256
23241	0.767114	1.30469
23242	0.946129	1.19501
23243	0.767143	0.981495
23244	1.22637	1.16053
23245	1.2281	1.20004
23246	0.590909	1.1917
23247	0.490832	1.26081
23248	0.804174	1.27959
23249	0.747368	1.11509
23250	1.05129	0.0750055
23251	1.10609	2.06335
23252	1.3639	0.12225
23253	0.776117	0.110367
23254	0.752329	0.126686
23255	1.04073	0.162753

23256	0.67713	0.14426
23257	3.4245	2.1
23258	1.18526	2.1
23259	1.2161	1.2639
23260	0.876834	1.83906
23261	0.876831	1.20207
23262	0.946132	1.77118
23263	0.876834	1.57153
23264	0.954563	2.1
23265	0.924132	2.1
23266	1.25075	2.1
23267	1.01652	2.1
23268	0.92413	2.1
23269	1.37579	2.1
23270	0.992159	2.1
23271	1.26092	2.1
23272	0.929435	2.1
23273	0.844958	2.1
23274	0.79598	2.1
23275	0.852559	2.1
23276	0.946131	0.483046
23277	1.28435	0.225003
23278	0.827261	0.400523
23279	0.946128	0.514129
23280	0.827298	0.504131
23281	1.65452	0.095713
23282	1.79929	2.1
23283	0.791947	0.244347
23284	0.902072	0.388195
23285	0.882672	0.189852
23286	1.21703	2.1
23287	0.900338	2.1
23288	1.37712	2.1
23289	0.782591	2.1
23290	0.782562	2.1
23291	0.94613	2.1
23292	1.34749	2.1
23293	0.61359	2.1
23294	1.14397	1.42997
23295	1.02963	0.0698591
23296	1.36589	0.0698591
23297	0.946132	0.0699866
23298	1.28644	0.0698591
23299	0.946131	0.0698591
23300	1.40104	0.0698591
23301	1.28644	0.0698591
23302	0.810946	0.0914029
23303	1.09178	0.0867499
23304	1.26956	0.078181
23305	0.810943	0.0830121
23306	0.885633	0.0853358
23307	1.00844	0.076818
23308	0.825671	0.0943099
23309	0.792093	0.088971
23310	0.925037	0.085344
23311	0.946131	1.89332
23312	0.946131	0.283718
23313	1.18052	0.703656

23314	1.40265	0.573141
23315	0.946132	1.06638
23316	1.17891	0.580346
23317	0.734248	0.0698591
23318	0.810943	0.0698591
23319	0.731621	0.0698591
23320	0.799857	0.190819
23321	0.834507	0.273117
23322	0.810932	0.193688
23323	1.01316	0.0698591
23324	1.23884	0.0698591
23325	1.88438	2.1
23326	0.946129	2.1
23327	1.38614	2.08509
23328	1.34245	1.85887
23329	0.946131	1.24777
23330	1.38614	1.77317
23331	0.946132	0.0698591
23332	0.910733	0.0698591
23333	1.24726	0.0698591
23334	1.13742	0.0698591
23335	0.851555	0.0698591
23336	1.50368	0.0698591
23337	0.94613	2.1
23338	1.08998	2.1
23339	1.36899	2.00246
23340	1.36898	2.1
23341	0.946131	2.1
23342	1.26045	2.1
23343	1.06432	0.561318
23344	1.89016	0.370179
23345	0.83514	2.1
23346	1.36653	2.1
23347	1.23426	2.1
23348	0.94613	2.1
23349	0.835112	2.1
23350	1.14567	1.79775
23351	1.12096	1.8646
23352	0.881106	1.70957
23353	1.17787	0.651062
23354	1.29738	2.09374
23355	0.940472	1.37398
23356	1.03452	1.4247
23357	0.946131	1.25192
23358	2.7489	0.558775
23359	1.01778	2.1
23360	1.04321	1.06923
23361	1.19352	0.130263
23362	1.16912	1.6269
23363	0.946132	1.19335
23364	0.946132	1.72272
23365	1.40924	1.74557
23366	1.22927	1.93075
23367	2.09455	0.084328
23368	3.00575	2.1
23369	1.08928	2.1
23370	1.94799	2.0987
23371	1.4092	2.1

23372	0.946132	2.1
23373	1.16	2.1
23374	1.01985	2.1
23375	0.946133	2.1
23376	1.16	2.1
23377	1.15371	0.0698591
23378	1.56002	0.0698591
23379	1.23007	2.08569
23380	1.9992	2.1
23381	0.652605	2.1
23382	0.937903	2.1
23383	0.946132	2.1
23384	0.937902	2.1
23385	1.228	2.1
23386	1.33694	1.98161
23387	1.00191	2.1
23388	0.716814	2.01301
23389	0.94613	2.09452
23390	0.716816	2.07038
23391	1.70388	1.34106
23392	0.946129	0.188645
23393	1.09362	0.172498
23394	0.946131	0.296248
23395	1.4185	0.21362
23396	1.09362	0.114501
23397	1.31057	1.64212
23398	1.17631	0.0698591
23399	1.19404	0.0699887
23400	0.94613	0.100489
23401	0.946129	0.0753984
23402	0.99972	0.0797279
23403	0.94613	0.080846
23404	0.835199	0.0698591
23405	1.23351	0.0698591
23406	0.582785	0.0698591
23407	0.701079	0.0698591
23408	0.946132	0.0698591
23409	2.17385	0.0698591
23410	1.18948	1.98773
23411	1.25346	2.1
23412	1.18586	0.356838
23413	0.880876	0.41292
23414	0.68632	0.792484
23415	0.946118	2.1
23416	0.966936	2.1
23417	0.946117	2.1
23418	1.41636	2.1
23419	1.03798	2.1
23420	0.729178	0.311684
23421	0.721888	0.346099
23422	0.946132	0.310906
23423	0.656286	0.320002
23424	1.13898	0.314204
23425	1.02351	0.0698591
23426	1.48803	0.0698967
23427	0.946131	0.0702877
23428	1.04921	2.1
23429	1.02478	1.7749

23430	0.993023	2.1
23431	1.80841	0.127653
23432	1.29215	0.100176
23433	0.904206	0.115605
23434	0.904207	0.131843
23435	0.946132	0.137682
23436	0.904207	0.119406
23437	1.07347	2.1
23438	0.555875	1.83911
23439	0.555904	1.7585
23440	1.30841	2.02114
23441	1.30841	1.99721
23442	0.91951	1.99293
23443	1.33026	0.820648
23444	1.43289	0.0698591
23445	1.15703	1.67297
23446	0.886594	1.92232
23447	1.05129	1.72995
23448	1.08421	0.360471
23449	1.35946	0.2426
23450	0.882955	0.63776
23451	0.946131	0.543098
23452	0.882985	0.456412
23453	0.583501	0.58898
23454	0.532921	0.700369
23455	0.94613	1.9003
23456	0.532892	1.61255
23457	1.23224	1.67944
23458	1.12786	1.6345
23459	0.946131	2.08966
23460	1.23732	0.306022
23461	1.40609	2.1
23462	1.40609	1.2993
23463	0.946131	1.0131
23464	0.910672	1.53233
23465	1.08478	0.75063
23466	0.94613	0.505248
23467	0.94613	1.31752
23468	1.30382	1.11819
23469	3.10544	1.63315
23470	0.679509	1.92515
23471	1.15966	1.61421
23472	0.75499	1.96535
23473	1.37511	1.67633
23474	1.01776	1.67297
23475	2.35239	0.805088
23476	0.92406	2.1
23477	0.946118	2.1
23478	0.92406	2.1
23479	1.07979	2.1
23480	1.29604	2.1
23481	1.29897	2.1
23482	0.902645	0.409669
23483	0.729493	1.8072
23484	0.906844	0.864239
23485	0.739518	0.0698591
23486	1.10564	0.078521
23487	0.946131	0.0698591

23488	0.739519	0.0698591
23489	0.739506	0.0698591
23490	1.79907	0.271135
23491	1.59106	0.613836
23492	1.11067	0.341822
23493	0.795516	0.605447
23494	0.795519	0.419166
23495	0.946132	0.555296
23496	0.795506	0.436191
23497	1.80223	2.1
23498	1.95572	2.0708
23499	1.16237	0.894476
23500	1.07518	2.1
23501	0.696199	1.59259
23502	0.94613	1.91395
23503	0.696199	1.6789
23504	1.6379	0.0698591
23505	0.946118	0.0698591
23506	0.851529	0.0698591
23507	1.08716	0.0698591
23508	0.973118	0.0698591
23509	0.851528	0.0698591
23510	1.15096	2.1
23511	0.65023	0.0698591
23512	0.94613	0.0698591
23513	0.650205	0.0698591
23514	0.650205	0.0698591
23515	0.811233	0.0698591
23516	0.778721	1.979
23517	0.810945	1.23676
23518	0.997518	1.45882
23519	0.838028	0.072677
23520	0.952893	0.0698591
23521	0.838037	0.0702913
23522	1.58166	0.0698591
23523	1.44848	1.13064
23524	1.30041	0.0698591
23525	1.03697	0.698083
23526	0.946132	1.93804
23527	1.0277	1.12005
23528	0.946131	1.98966
23529	1.00501	1.4165
23530	1.0277	1.70593
23531	1.64988	2.1
23532	0.946131	2.1
23533	0.94613	2.03456
23534	1.27702	2.1
23535	1.55163	2.1
23536	0.897905	2.1
23537	1.12971	0.0698591
23538	1.35715	0.111318
23539	0.881884	0.321678
23540	0.664007	0.134058
23541	0.663978	0.207664
23542	0.94613	0.191498
23543	0.664006	0.190707
23544	1.48693	0.13097
23545	1.29289	0.0794501

23546	1.32081	0.088978
23547	0.946119	0.111951
23548	0.946132	0.0929837
23549	1.02972	0.0896763
23550	0.946131	2.1
23551	0.946127	2.1
23552	1.23513	2.1
23553	1.38154	2.1
23554	0.94613	2.1
23555	1.25933	0.0929427
23556	0.917954	0.100489
23557	0.868589	0.0698591
23558	3.0957	2.1
23559	0.711391	0.0698591
23560	0.711391	0.0698591
23561	0.991303	0.0698591
23562	1.37322	0.0698591
23563	0.729145	0.0698591
23564	2.55648	0.0698591
23565	1.0756	1.9086
23566	0.760299	1.10991
23567	0.763592	0.570591
23568	0.946126	0.81892
23569	0.76359	0.560483
23570	1.35862	1.02474
23571	0.946133	2.1
23572	1.00807	2.1
23573	0.946132	2.1
23574	1.39823	2.1
23575	1.07817	2.1
23576	1.44379	0.202348
23577	1.17934	0.0698591
23578	1.13451	0.145137
23579	0.94613	2.1
23580	1.03974	2.1
23581	1.36835	2.1
23582	1.36835	2.1
23583	0.94613	2.1
23584	1.27096	2.09518
23585	0.853042	2.1
23586	0.946131	2.1
23587	1.13658	2.1
23588	1.06997	2.1
23589	0.94613	2.1
23590	1.41556	0.364917
23591	1.21708	0.546897
23592	0.94613	0.570861
23593	0.946131	0.473452
23594	1.21708	0.465408
23595	0.964782	0.365286
23596	0.946119	0.514046
23597	1.23447	0.458639
23598	0.946118	0.52088
23599	1.41844	0.419739
23600	1.23446	0.472313
23601	1.1357	0.29809
23602	0.619856	0.0698591
23603	1.1509	0.0698591

23604	0.946126	0.0698591
23605	1.19262	0.0698591
23606	1.1509	0.0698591
23607	1.04268	2.1
23608	2.07527	0.950428
23609	1.38259	0.833791
23610	0.980054	1.42009
23611	0.946124	0.975869
23612	0.946127	1.1802
23613	0.980056	1.03446
23614	1.62916	1.60303
23615	0.946131	2.1
23616	1.40571	2.1
23617	0.946131	2.1
23618	1.38885	2.1
23619	1.40571	2.1
23620	1.11787	2.1
23621	0.946129	2.1
23622	1.28831	2.1
23623	1.41498	2.09791
23624	1.41498	2.1
23625	0.946131	2.1
23626	0.850964	0.0698591
23627	0.921299	0.0698591
23628	1.37358	0.0698591
23629	0.946132	0.0698591
23630	0.850964	0.0698591
23631	0.823988	0.0698591
23632	0.823984	0.0698591
23633	1.36568	0.0698591
23634	1.36569	0.0698591
23635	0.921275	0.0698591
23636	1.84739	2.1
23637	1.47169	0.0704595
23638	0.946131	0.158705
23639	0.735844	0.130961
23640	1.11819	0.202958
23641	1.10904	0.0889331
23642	0.735831	0.134393
23643	0.946131	2.1
23644	0.946132	2.1
23645	1.23487	2.1
23646	1.3281	2.1
23647	0.946131	2.1
23648	1.72271	0.0698591
23649	0.861359	0.0698591
23650	0.946131	0.0911916
23651	0.861357	0.0736368
23652	1.1369	0.0746792
23653	1.26868	0.0737859
23654	0.789173	1.99139
23655	0.789143	2.03844
23656	0.946133	1.69145
23657	1.34386	1.8956
23658	0.789144	1.93993
23659	0.983493	2.1
23660	1.18944	0.33671
23661	1.05121	0.379644

23662	0.777398	0.394755
23663	0.946132	0.531029
23664	0.777423	0.53135
23665	1.09841	0.10471
23666	0.963919	0.086873
23667	0.474855	0.109323
23668	0.946131	0.114168
23669	0.474855	0.105814
23670	3.40074	2.09926
23671	1.29453	2.1
23672	1.01139	0.402886
23673	0.946133	1.15605
23674	1.35381	0.182642
23675	1.18322	0.820077
23676	0.946133	1.23597
23677	1.45502	0.13857
23678	0.970088	1.31278
23679	0.960333	1.54765
23680	0.838036	1.41971
23681	1.30927	0.113739
23682	0.828617	0.130312
23683	0.714795	0.127978
23684	0.946132	0.131804
23685	0.714824	0.12223
23686	0.946131	2.1
23687	0.851542	2.1
23688	1.38204	2.1
23689	1.24512	2.1
23690	1.04086	2.1
23691	0.723573	2.1
23692	0.946131	2.1
23693	0.982454	2.1
23694	0.982454	2.1
23695	0.723544	2.1
23696	1.17261	0.0801867
23697	1.34153	0.0765256
23698	1.41383	0.0703969
23699	0.946132	0.085725
23700	0.946133	0.0834846
23701	1.12818	0.114144
23702	1.29383	0.0699939
23703	0.946133	0.391457
23704	1.0027	0.365759
23705	0.768989	0.22223
23706	0.946133	0.419403
23707	0.768977	0.396359
23708	0.946132	0.563237
23709	1.24333	0.350553
23710	0.928583	0.551006
23711	0.946132	0.588242
23712	0.928583	0.599246
23713	1.53798	0.114547
23714	2.95436	0.0736184
23715	1.30649	0.0928681
23716	0.809789	0.162888
23717	0.745744	0.156846
23718	0.745774	0.139503
23719	0.946128	0.144018

23720	1.00777	0.0755075
23721	1.40271	0.0700883
23722	0.768387	0.0834898
23723	0.946132	0.0777381
23724	0.922045	0.0765627
23725	0.946132	2.1
23726	1.39021	2.1
23727	1.10046	2.1
23728	0.946132	2.1
23729	1.39021	2.1
23730	2.06158	0.0698591
23731	0.913967	0.176492
23732	1.30107	0.149117
23733	0.946132	0.186544
23734	0.913966	0.140522
23735	1.30107	0.143955
23736	1.64117	2.1
23737	0.514872	1.95569
23738	0.514846	1.58661
23739	1.30811	2.06973
23740	1.30812	1.75709
23741	0.888989	1.92156
23742	1.99279	1.93919
23743	0.910271	0.254209
23744	0.838027	0.318171
23745	0.928444	0.194447
23746	1.2377	0.91309
23747	0.851537	2.1
23748	0.899142	2.1
23749	0.810075	2.1
23750	1.3771	2.1
23751	1.03281	2.1
23752	0.719877	2.1
23753	0.901838	2.1
23754	1.01748	2.1
23755	1.12315	2.1
23756	0.64787	2.1
23757	1.173 2.1	
23758	0.876903	2.1
23759	0.597337	2.1
23760	1.00291	2.1
23761	0.597309	2.1
23762	1.09897	0.287035
23763	0.946132	0.550023
23764	1.1386	0.275445
23765	1.09897	0.358234
23766	0.596652	0.387256
23767	2.5593	2.1
23768	0.946133	0.173436
23769	1.29172	0.17158
23770	1.11337	0.115205
23771	0.946131	0.161221
23772	0.946131	0.120658
23773	0.96926	0.533264
23774	0.946132	0.891724
23775	0.946132	1.09937
23776	1.07118	0.330483
23777	1.29698	0.433933

23778	1.37867	0.872629
23779	0.946119	1.72252
23780	0.94612	1.8454
23781	1.33154	0.698797
23782	0.946107	1.06381
23783	2.17757	0.978056
23784	0.946131	0.0720426
23785	0.68393	0.0698591
23786	0.68396	0.0698614
23787	1.33671	0.0698591
23788	1.10733	0.0699511
23789	2.31367	2.1
23790	0.989326	1.91856
23791	0.689952	2.0779
23792	0.854787	1.83225
23793	3.73635	2.1
23794	1.41489	2.1
23795	1.25544	2.03293
23796	0.946128	2.1
23797	0.946129	2.1
23798	1.13536	2.1
23799	2.10284	1.67755
23800	0.851542	2.1
23801	0.828523	2.1
23802	0.946132	2.1
23803	0.946132	2.1
23804	1.50873	2.1
23805	0.989048	2.09978
23806	0.925055	2.1
23807	0.838041	2.09806
23808	0.807714	0.652149
23809	0.656831	0.378995
23810	0.997603	0.546168
23811	0.946131	1.41977
23812	1.02602	1.26101
23813	0.946129	1.42299
23814	1.35469	1.10891
23815	1.25083	1.12015
23816	0.851541	0.236086
23817	0.851483	0.215483
23818	0.851539	0.241948
23819	1.19785	0.208437
23820	1.07615	0.219647
23821	0.714507	0.0854661
23822	1.41461	0.0799304
23823	0.714507	0.0830145
23824	0.714507	0.0804491
23825	0.946133	0.0869424
23826	0.946131	1.21524
23827	0.972232	0.882524
23828	0.94613	1.38706
23829	1.25083	0.786929
23830	1.30165	0.936217
23831	1.05332	2.1
23832	0.574768	2.1
23833	1.94541	0.0698591
23834	0.789633	0.0698591
23835	0.946127	0.0698591

23836	1.33798	0.0698591
23837	1.22903	0.0698591
23838	0.946128	0.0698591
23839	0.946128	2.1
23840	1.11882	2.1
23841	0.996487	2.1
23842	0.94613	2.1
23843	1.11883	2.1
23844	0.946127	2.1
23845	1.30082	2.1
23846	1.02608	2.1
23847	0.946128	2.1
23848	1.30081	2.1
23849	1.3507	1.85079
23850	1.19583	0.194855
23851	0.946127	1.72828
23852	0.94613	1.16415
23853	1.19583	1.43316
23854	0.477652	0.0698591
23855	1.20416	0.0698591
23856	0.946131	0.0698591
23857	0.477652	0.0698591
23858	0.477691	0.0698591
23859	1.34536	0.0698591
23860	0.758971	0.0698591
23861	1.25124	0.0698591
23862	1.31467	0.0698591
23863	0.758971	0.0698591
23864	1.31467	0.0698591
23865	0.946131	2.1
23866	1.14184	2.1
23867	1.35028	2.1
23868	1.35028	2.1
23869	0.946131	2.1
23870	1.13537	2.04683
23871	0.946132	1.53434
23872	0.946132	2.04888
23873	1.23031	1.92078
23874	1.27555	1.93905
23875	1.53064	1.98595
23876	1.28421	0.758983
23877	1.07707	1.22792
23878	0.946125	0.845358
23879	0.946127	1.19584
23880	1.07707	0.971634
23881	0.946133	0.0698591
23882	1.3495	0.0698591
23883	0.946132	0.0698591
23884	1.05704	0.0698591
23885	1.3495	0.0698591
23886	0.946132	0.0698591
23887	1.37794	0.0698591
23888	0.946132	0.0698591
23889	1.01367	0.0698591
23890	1.37794	0.0698591
23891	0.928516	2.1
23892	0.946133	2.1
23893	0.928518	2.1

23894	1.14105	2.1
23895	1.26406	2.1
23896	0.797664	2.1
23897	0.946132	2.1
23898	0.797663	2.1
23899	1.34285	2.1
23900	0.929875	2.1
23901	0.946131	2.08411
23902	1.21139	0.927574
23903	1.41492	1.22279
23904	1.41492	0.63861
23905	0.946131	0.426326
23906	0.946131	2.1
23907	0.689388	2.1
23908	0.94365	2.1
23909	0.777345	2.1
23910	0.709599	2.1
23911	0.774968	2.1
23912	0.946132	2.1
23913	0.77497	2.1
23914	0.868145	2.1
23915	1.28401	2.1
23916	0.94613	2.0688
23917	1.19655	2.1
23918	1.39428	1.1438
23919	1.39428	2.08176
23920	0.946131	2.0978
23921	1.1848	2.1
23922	1.07591	2.1
23923	0.946131	2.1
23924	0.946131	2.1
23925	0.874788	2.1
23926	1.35703	2.1
23927	1.35357	2.1
23928	0.946132	2.1
23929	1.26022	2.1
23930	0.946132	2.1
23931	0.946131	2.1
23932	0.966426	2.1
23933	0.946133	2.1
23934	0.946131	2.1
23935	1.3484	2.1
23936	1.26441	2.1
23937	1.2079	2.1
23938	0.946133	2.1
23939	0.946132	2.1
23940	1.2079	2.1
23941	1.2736	2.1
23942	1.25774	2.1
23943	0.946133	2.1
23944	0.851555	2.1
23945	0.96172	2.1
23946	0.946128	2.1
23947	1.06879	2.1
23948	0.94613	2.1
23949	1.32608	2.1
23950	1.06879	2.1
23951	0.946132	2.1

23952	1.32608	2.1
23953	0.946131	2.1
23954	1.40488	2.1
23955	1.32608	2.1
23956	0.94613	1.50697
23957	1.19234	0.947399
23958	0.946129	1.15362
23959	0.94613	1.64214
23960	1.31649	1.75272
23961	0.912059	1.83647
23962	1.04514	1.77202
23963	0.946132	1.78331
23964	0.946132	2.05941
23965	1.34646	2.02122
23966	1.01187	0.4186
23967	1.33728	1.51794
23968	0.761974	0.664825
23969	0.761974	0.544191
23970	0.94613	0.535044
23971	1.40641	2.08812
23972	0.955752	0.796116
23973	0.736738	2.00799
23974	0.94613	1.54872
23975	0.736739	1.77326
23976	0.861471	2.1
23977	1.34556	2.1
23978	0.810957	2.1
23979	0.946131	2.1
23980	1.37811	2.1
23981	1.17949	2.1
23982	0.946132	2.1
23983	1.37811	2.1
23984	0.946131	2.1
23985	1.40447	2.1
23986	0.946132	2.1
23987	0.946132	2.1
23988	1.39826	2.1
23989	0.946131	2.1
23990	1.283	2.1
23991	0.94613	2.1
23992	0.946131	2.1
23993	1.40447	2.1
23994	1.20541	2.1
23995	0.946131	2.1
23996	0.946127	2.1
23997	1.33607	2.1
23998	1.41021	2.1
23999	1.01271	2.1
24000	0.946131	2.1
24001	0.920606	2.1
24002	1.13078	2.1
24003	1.60331	2.1
24004	0.946118	1.43704
24005	1.09718	2.1
24006	0.946116	2.07536
24007	1.32662	1.87374
24008	1.0972	2.00821
24009	0.94613	0.65642

24010	1.32666	1.5691
24011	0.946128	0.838325
24012	1.31381	0.552722
24013	1.32666	0.607595
24014	0.94612	2.1
24015	0.940748	2.1
24016	1.03561	2.1
24017	1.35111	2.1
24018	0.851527	2.1
24019	0.607156	2.1
24020	0.789158	2.1
24021	0.946129	2.1
24022	1.04071	2.1
24023	1.13144	2.1
24024	0.946133	2.1
24025	1.35663	2.1
24026	0.946132	2.1
24027	0.946133	2.1
24028	1.34237	2.1
24029	0.919455	2.1
24030	1.01143	2.1
24031	0.946128	2.1
24032	0.946131	2.1
24033	1.62796	2.1
24034	1.17796	1.76473
24035	1.23024	2.07948
24036	0.946132	1.75676
24037	0.85153	1.99439
24038	0.986867	1.96756
24039	0.889308	2.1
24040	0.94613	2.1
24041	0.889338	2.1
24042	0.946131	2.1
24043	0.889337	2.1
24044	1.18396	1.03707
24045	0.94613	0.455602
24046	0.946125	1.83343
24047	1.39514	0.831445
24048	1.30809	1.29494
24049	0.803322	2.03743
24050	0.925893	1.89515
24051	1.36183	1.62593
24052	0.925892	1.62108
24053	0.946133	1.68496
24054	1.38354	0.1414
24055	1.16009	0.128376
24056	0.946132	0.119901
24057	0.946132	0.114425
24058	1.01462	0.0824655
24059	1.18854	0.935819
24060	0.888265	1.5522
24061	0.745095	1.10455
24062	0.709597	1.35281
24063	0.936541	1.2548
24064	1.24142	0.699622
24065	1.35059	0.469147
24066	0.946128	0.451188
24067	0.94613	0.61062

24068	1.16623	0.621896
24069	1.24945	0.0867598
24070	1.33035	0.0829169
24071	0.946127	0.099681
24072	0.946128	0.0940466
24073	1.13343	0.127515
24074	0.637995	1.17355
24075	1.10455	1.07356
24076	1.22641	1.68958
24077	1.22641	1.71362
24078	0.946132	1.36352
24079	1.3665	0.0809902
24080	1.28507	0.0859917
24081	0.946127	0.105194
24082	0.946129	0.0782729
24083	1.2051	0.124345
24084	0.797374	2.09894
24085	0.797376	2.1
24086	1.13113	2.08089
24087	1.20057	2.09169
24088	1.12049	2.09695
24089	0.59387	0.967152
24090	1.13245	0.512644
24091	0.966134	1.11323
24092	0.593842	0.536164
24093	0.946128	0.862745
24094	0.641455	0.520646
24095	0.916507	0.480451
24096	1.04072	1.11619
24097	0.415585	0.429496
24098	0.507244	0.829239
24099	0.678938	2.03831
24100	1.24154	2.0053
24101	1.25	1.88202
24102	1.24154	1.94095
24103	1.25	1.96649
24104	0.851009	1.71635
24105	0.946131	1.99112
24106	0.851539	1.80848
24107	1.21997	1.75108
24108	1.45109	1.74975
24109	0.804877	0.585066
24110	1.00062	0.20902
24111	0.851536	0.478426
24112	0.909673	0.224566
24113	0.946129	0.475707
24114	1.20494	0.0969042
24115	1.09088	0.131303
24116	0.887634	0.155688
24117	0.81027	0.0797148
24118	0.900294	0.100173
24119	0.747713	0.0698591
24120	0.975467	0.0698591
24121	0.747712	0.0698591
24122	1.39168	0.0698591
24123	0.975467	0.0698591
24124	0.94613	0.0948066
24125	1.32827	0.0957248

24126	0.946128	0.0861058
24127	1.41201	0.105575
24128	1.32827	0.093403
24129	0.981121	0.112254
24130	0.936311	0.190413
24131	1.00715	0.108928
24132	1.15914	0.139507
24133	0.704409	0.131411
24134	0.713444	0.688356
24135	0.954538	0.563679
24136	0.944508	0.524762
24137	0.851165	0.921401
24138	0.946131	1.4359
24139	0.851192	0.811519
24140	0.946131	0.778814
24141	1.3279	0.707235
24142	1.33587	0.0698591
24143	0.946126	0.0698981
24144	1.27512	0.0698591
24145	1.33587	0.0698591
24146	0.748059	0.0698591
24147	1.17451	0.0711004
24148	1.04299	0.0698591
24149	0.851542	0.0753482
24150	0.946131	0.0734318
24151	0.892109	0.0719149
24152	0.946131	0.0698591
24153	0.851541	0.0698591
24154	1.2178	0.0698591
24155	1.2179	0.0698591
24156	0.946793	0.0698591
24157	0.709599	0.0698591
24158	0.987426	0.0698591
24159	1.39502	0.0698591
24160	1.13624	0.0698591
24161	0.73176	0.0698591
24162	1.36697	0.0698591
24163	1.33393	0.0698591
24164	0.946131	0.0698853
24165	0.946132	0.0698591
24166	1.23845	0.0698591
24167	0.50702	0.628951
24168	1.06442	0.460258
24169	0.946131	0.634574
24170	1.02363	0.511966
24171	1.13537	0.0698591
24172	1.25049	0.0698591
24173	0.94613	0.0698591
24174	1.14993	0.0698591
24175	1.38056	0.0698591
24176	0.946126	0.0698591
24177	1.24678	0.0698591
24178	0.684521	0.0698591
24179	0.946124	0.0698591
24180	0.684518	0.0698591
24181	0.851542	0.358561
24182	0.925459	0.315584
24183	0.946131	0.358526

24184	0.851474	0.373918
24185	0.923305	0.374447
24186	1.4055	0.0698591
24187	1.32077	0.0698591
24188	0.946127	0.0698591
24189	1.21757	0.0698591
24190	0.946129	0.0698591
24191	0.711214	0.0700492
24192	1.30463	0.0753216
24193	0.711185	0.0706096
24194	0.711185	0.0740092
24195	1.22716	0.0720623
24196	1.29958	0.0698591
24197	1.26201	0.0698591
24198	0.568927	0.0698591
24199	0.568927	0.0698591
24200	0.946132	0.0698591
24201	0.851542	0.0746196
24202	1.44968	0.0698591
24203	0.851543	0.0711348
24204	1.35123	0.0711748
24205	1.40427	0.0698979
24206	0.593295	0.0908392
24207	0.946129	0.0988962
24208	1.22144	0.0841539
24209	0.908278	0.104969
24210	1.22144	0.0965605
24211	0.867593	0.221863
24212	1.23351	0.169617
24213	0.946131	0.218021
24214	1.05516	0.206385
24215	1.23351	0.19008
24216	1.15564	0.072056
24217	0.946131	0.112718
24218	0.766652	0.0800526
24219	0.766681	0.0765969
24220	0.946131	0.0900775
24221	0.946131	0.1218
24222	0.897851	0.109818
24223	1.1751	0.141439
24224	1.00273	0.105194
24225	0.851554	0.1166
24226	0.883272	0.153266
24227	1.17111	0.349459
24228	0.851541	0.187342
24229	1.01665	0.334174
24230	0.851555	0.153808
24231	0.946131	0.349768
24232	0.911508	0.218777
24233	1.08054	0.38656
24234	1.33007	0.145265
24235	0.911471	0.274705
24236	1.26787	1.80599
24237	1.16641	1.86456
24238	0.946132	1.37533
24239	0.946133	1.56119
24240	1.16641	1.7349
24241	0.946131	2.1

24242	1.25037	0.856203
24243	1.34733	2.1
24244	1.34733	2.1
24245	0.946131	2.1
24246	0.946131	0.0698591
24247	1.41377	0.0698591
24248	0.94613	0.0698591
24249	1.30541	0.0698591
24250	1.41377	0.0698591
24251	1.3473	2.1
24252	1.11804	2.1
24253	0.94613	2.1
24254	0.946131	2.1
24255	1.11805	2.1
24256	1.32068	2.1
24257	0.946131	2.1
24258	0.946128	2.1
24259	1.39384	2.1
24260	1.4181	2.1
24261	1.12913	2.1
24262	0.94613	2.1
24263	0.946129	2.1
24264	1.20328	2.1
24265	1.39385	2.1
24266	1.3335	2.1
24267	1.40987	2.1
24268	0.94613	2.1
24269	1.21474	2.1
24270	0.94613	2.1
24271	0.946132	2.1
24272	1.15203	2.1
24273	1.41192	2.1
24274	1.41192	2.1
24275	0.946132	2.1
24276	0.946131	2.1
24277	1.40684	2.1
24278	1.2597	2.1
24279	0.946131	2.1
24280	1.40684	2.1
24281	0.946131	2.09035
24282	1.39113	2.0839
24283	1.34049	2.06043
24284	0.946131	1.91801
24285	1.39113	1.99272
24286	0.946132	1.31122
24287	1.31649	0.367019
24288	0.946132	0.407789
24289	0.946132	0.94118
24290	1.34297	1.15818
24291	0.946131	0.140843
24292	1.41766	0.149522
24293	0.954559	0.238154
24294	0.946131	0.155356
24295	0.946131	0.103814
24296	1.25447	0.208243
24297	1.35559	0.140668
24298	0.946127	0.159882
24299	0.946128	0.205019

24300	1.14874	0.197813
24301	1.33035	0.0752555
24302	1.3665	0.0821049
24303	0.946129	0.101288
24304	0.94613	0.0743912
24305	1.20732	0.118793
24306	0.946131	2.1
24307	0.946131	2.1
24308	1.32949	2.1
24309	1.34921	2.1
24310	0.946131	2.1
24311	0.946132	0.787519
24312	1.25479	1.27642
24313	1.38357	0.4403
24314	1.38357	0.326153
24315	0.946132	0.19249
24316	0.946131	2.1
24317	1.31034	0.380233
24318	1.37127	2.09988
24319	1.37127	1.25713
24320	0.946131	1.39387
24321	1.23923	0.108235
24322	1.41169	0.156988
24323	0.94613	0.221771
24324	1.12703	0.106048
24325	0.946131	0.145755
24326	0.946119	2.1
24327	1.41915	2.1
24328	1.02679	2.1
24329	0.946107	2.1
24330	1.41917	2.1
24331	0.946133	2.1
24332	1.36581	2.1
24333	0.946131	2.1
24334	1.36581	2.1
24335	0.946131	2.1
24336	0.946132	2.1
24337	1.04384	2.1
24338	1.34973	2.1
24339	1.34967	2.1
24340	0.851555	2.1
24341	0.946132	0.0803463
24342	1.3855	0.0717025
24343	1.08603	0.0805291
24344	1.08602	0.0717878
24345	1.16234	0.0773958
24346	0.946133	0.0815514
24347	1.29348	0.0739827
24348	1.20111	0.0823892
24349	1.20112	0.078948
24350	1.3855	0.0818228
24351	0.946128	2.1
24352	1.30798	2.1
24353	0.946129	2.1
24354	1.34828	2.1
24355	1.30797	2.1
24356	1.24932	0.0698591
24357	1.40549	0.0698591

24358	0.946117	0.0698591
24359	1.19615	0.0698591
24360	0.946106	0.0698591
24361	0.946131	2.1
24362	1.29926	2.1
24363	1.25144	2.1
24364	0.946131	2.1
24365	1.29925	2.1
24366	0.946132	2.1
24367	1.39826	2.1
24368	0.946133	2.1
24369	0.946133	2.1
24370	1.29926	2.1
24371	0.946119	0.0698591
24372	1.33614	0.0698591
24373	0.946118	0.0698591
24374	1.41377	0.0698591
24375	1.33612	0.0698591
24376	0.946133	1.68576
24377	1.3933	2.0908
24378	1.34999	0.750472
24379	0.946133	1.67799
24380	1.3933	1.63668
24381	0.946131	2.05824
24382	1.36139	2.1
24383	0.946132	1.7511
24384	0.946131	2.09424
24385	1.39329	2.08382
24386	1.37449	1.84781
24387	1.27925	0.524443
24388	0.919796	0.987727
24389	1.01176	1.47075
24390	0.94613	1.24191
24391	0.946133	2.1
24392	1.27679	2.1
24393	0.946133	2.1
24394	0.946132	2.1
24395	1.39731	2.1
24396	0.946119	2.1
24397	1.40765	1.92945
24398	1.1981	2.1
24399	0.946131	2.05714
24400	1.40764	2.1
24401	0.946127	2.1
24402	1.3557	2.1
24403	0.946127	2.1
24404	0.946128	2.1
24405	1.13485	2.1
24406	1.4008	2.1
24407	1.1587	2.1
24408	0.946131	2.1
24409	0.946131	2.1
24410	1.1587	2.1
24411	1.32082	0.0890197
24412	1.29215	0.102329
24413	0.94613	0.123949
24414	0.946131	0.116071
24415	1.02366	0.106382

24416	1.27697	0.0745856
24417	1.2929	0.0819934
24418	0.946132	0.100217
24419	0.946132	0.0764935
24420	1.01451	0.0743233
24421	0.946132	2.1
24422	1.27252	2.1
24423	0.946132	2.1
24424	1.37869	2.1
24425	1.27252	2.1
24426	1.15627	2.1
24427	1.35703	2.1
24428	0.946132	2.1
24429	1.03783	2.1
24430	0.946132	2.1
24431	0.946128	2.1
24432	1.37869	2.1
24433	0.946131	2.1
24434	1.35357	2.1
24435	1.37869	2.1
24436	0.946132	2.1
24437	0.946131	2.1
24438	1.33471	2.1
24439	0.946131	2.1
24440	1.36373	2.1
24441	1.41342	0.445217
24442	1.06832	0.927773
24443	0.946128	0.506279
24444	0.94613	0.2452
24445	1.06832	0.577762
24446	1.26929	2.1
24447	0.946131	2.1
24448	0.946128	2.1
24449	1.41022	2.1
24450	1.37034	2.1
24451	1.37759	0.796066
24452	1.07612	1.46159
24453	0.946126	0.95108
24454	0.946129	1.18818
24455	1.07612	1.02599
24456	0.946131	2.0664
24457	0.94613	0.499282
24458	1.23848	1.22268
24459	1.38897	0.939239
24460	0.946131	1.06769
24461	0.946132	0.763216
24462	0.946133	0.532657
24463	1.34369	0.156182
24464	1.40332	0.57698
24465	0.946132	0.193327
24466	0.946131	2.1
24467	1.24023	2.1
24468	1.38244	2.1
24469	1.38244	2.1
24470	0.946131	2.1
24471	0.946132	2.1
24472	1.33468	2.1
24473	1.41619	2.1

24474	1.41619	2.1
24475	0.946131	2.1
24476	1.1908	1.19763
24477	0.946128	0.797121
24478	0.946126	1.54243
24479	1.26654	1.49874
24480	1.39514	1.85342
24481	0.93608	2.1
24482	1.04105	2.1
24483	1.39551	2.1
24484	1.52471	2.1
24485	1.04072	2.1
24486	1.33174	0.0698591
24487	0.946128	0.0698591
24488	0.94613	0.0698591
24489	1.17801	0.0698591
24490	0.94613	0.0698591
24491	0.946132	2.1
24492	0.946132	2.1
24493	1.29065	2.1
24494	1.29065	2.1
24495	0.975266	2.1
24496	0.946131	2.1
24497	1.26025	2.1
24498	0.955601	2.1
24499	0.946131	2.1
24500	1.26025	2.1
24501	0.94613	0.0981901
24502	1.23146	0.091009
24503	0.946127	0.0946481
24504	1.32827	0.0881496
24505	1.23146	0.0956392
24506	1.25008	0.342696
24507	1.05018	0.41958
24508	0.94613	0.371865
24509	0.932744	0.546976
24510	1.04207	0.425485
24511	0.946132	1.47091
24512	1.10023	2.1
24513	1.36748	0.832301
24514	1.36748	1.95768
24515	0.946133	1.97883
24516	1.06023	0.0698591
24517	1.08019	0.0698591
24518	0.94613	0.0698591
24519	1.06023	0.0698591
24520	0.94613	0.0698591
24521	1.16727	0.0698591
24522	1.35856	0.0698591
24523	0.946132	0.0698591
24524	1.16727	0.0698591
24525	0.946132	0.0698591
24526	0.946133	2.1
24527	0.946131	2.1
24528	1.21691	2.1
24529	1.39558	2.1
24530	0.946131	2.1
24531	1.09905	0.442933

24532	0.946132	1.11375
24533	1.3365	0.447647
24534	1.35381	0.706614
24535	0.946132	1.27773
24536	0.94613	0.78749
24537	0.946128	2.04047
24538	1.40332	1.47268
24539	1.39041	1.86369
24540	0.94613	1.58658
24541	0.946132	2.08067
24542	1.3659	2.1
24543	1.39041	2.1
24544	1.39041	2.1
24545	0.946132	2.1
24546	0.840292	2.03733
24547	0.946133	2.01868
24548	0.851541	2.06287
24549	1.28464	2.02242
24550	1.28806	2.01791
24551	1.12591	0.36077
24552	1.05033	0.663573
24553	0.946132	0.459539
24554	0.946132	0.753932
24555	1.13537	0.505016
24556	1.21177	1.25129
24557	0.946132	1.25059
24558	0.946132	2.08863
24559	1.41134	0.864636
24560	1.27079	1.59299
24561	0.946131	0.0698591
24562	1.35403	0.0698591
24563	0.94613	0.0703629
24564	0.94613	0.0698591
24565	0.946131	0.0698591
24566	0.946131	0.107261
24567	0.946132	0.117338
24568	0.94613	0.3268
24569	1.37052	0.125274
24570	0.946131	0.116346
24571	0.946133	0.0944722
24572	0.946132	0.141397
24573	0.946133	0.110639
24574	1.24249	0.0891899
24575	0.946132	0.120664
24576	1.20608	2.1
24577	0.946132	2.1
24578	0.946131	2.1
24579	0.946132	2.1
24580	0.946132	2.1
24581	0.946131	2.1
24582	0.946128	2.1
24583	0.946129	2.1
24584	1.41232	2.1
24585	0.94613	2.1
24586	0.946132	0.069863
24587	1.32449	0.0698591
24588	0.946131	0.0725622
24589	0.946131	0.0710897

24590	1.32449	0.0699657
24591	0.946116	2.09562
24592	1.41563	2.1
24593	0.946118	2.1
24594	0.946105	2.1
24595	0.946131	2.1
24596	1.26563	2.1
24597	1.32819	2.04114
24598	0.946131	0.896142
24599	0.946131	2.05645
24600	0.946131	1.60748
24601	0.946131	1.94768
24602	0.946133	1.56449
24603	1.30382	2.1
24604	0.946131	1.54654
24605	0.946131	1.49554
24606	1.30382	1.55195
24607	0.946133	0.0698591
24608	1.15803	0.0698591
24609	1.28895	0.0698591
24610	0.954071	0.0698591
24611	0.946132	0.0698591
24612	1.0264	0.673637
24613	0.946132	1.53865
24614	0.946132	1.10478
24615	0.946131	1.22458
24616	0.946132	1.02628
24617	1.35977	0.19015
24618	0.946132	0.326335
24619	0.946129	0.291412
24620	0.94613	0.281187
24621	0.946129	0.250361
24622	0.876631	0.11675
24623	0.946133	0.116624
24624	0.946132	0.109758
24625	0.946132	0.211113
24626	0.876606	0.114659
24627	0.946131	0.0698591
24628	1.24979	0.0698591
24629	0.94613	0.0698591
24630	0.94613	0.0698591
24631	0.94613	0.0698591
24632	0.921791	0.0698591
24633	0.946131	0.0698591
24634	0.921762	0.0698591
24635	0.946131	0.0698591
24636	0.946131	0.0698591
24637	0.810944	0.0790365
24638	0.810945	0.0899936
24639	0.810959	0.0815672
24640	0.946133	0.0726485
24641	1.25797	0.0896476
24642	0.946132	0.103816
24643	0.946132	0.073286
24644	1.25797	0.0809124
24645	1.13748	0.336506
24646	0.851512	0.613513
24647	0.94613	0.965076

24648	1.02373	0.33079
24649	0.946131	0.635039
24650	0.946122	2.1
24651	1.3852	2.1
24652	0.946124	2.1
24653	0.946118	2.1
24654	1.38521	2.1
24655	0.946131	0.0871713
24656	0.946131	0.0732429
24657	1.30448	0.084351
24658	0.946129	0.116656
24659	0.94613	0.0704134
24660	0.946133	2.1
24661	0.94613	2.1
24662	0.946131	2.1
24663	0.946131	2.1
24664	1.36503	2.1
24665	0.810947	0.0698591
24666	0.810947	0.0698591
24667	0.810934	0.0698591
24668	3.34066	0.0698591
24669	0.946133	0.790563
24670	1.4185	0.185094
24671	0.946132	0.342222
24672	0.946133	0.481086
24673	0.946132	0.459689
24674	0.94613	2.1
24675	0.946128	2.1
24676	1.03925	2.1
24677	0.944743	2.1
24678	0.947518	2.1
24679	0.946132	2.1
24680	1.20573	2.1
24681	0.94613	2.1
24682	0.94613	2.1
24683	1.20573	2.1
24684	1.20056	0.0698591
24685	0.946132	0.0698591
24686	0.946131	0.0698591
24687	0.946131	0.0698591
24688	0.946131	0.0698591
24689	0.94613	2.1
24690	0.946128	2.1
24691	1.38302	2.1
24692	0.94613	2.1
24693	0.94613	2.1
24694	0.827866	0.0698591
24695	0.913552	0.0698591
24696	0.716526	0.0698591
24697	0.82784	0.0698591
24698	1.20634	0.0698591
24699	0.946131	0.0698591
24700	0.94613	0.0698591
24701	1.39502	0.0698591
24702	0.88146	0.0698591
24703	0.946132	2.1
24704	0.946132	2.1
24705	0.946132	2.1

24706	0.946132	2.1
24707	1.39053	2.1
24708	0.946131	2.1
24709	1.40571	2.1
24710	0.946131	2.1
24711	0.946131	2.1
24712	0.946132	2.1
24713	0.94613	0.102123
24714	1.2582	0.173116
24715	0.946127	0.102894
24716	0.946129	0.138089
24717	0.946129	0.114203
24718	0.946129	0.904611
24719	0.946132	0.813155
24720	0.946131	0.556759
24721	0.946131	0.845072
24722	1.29054	0.528029
24723	0.810944	0.0698591
24724	0.810941	0.0698591
24725	0.810931	0.0698591
24726	0.946132	0.70538
24727	0.946132	0.692228
24728	0.946132	1.51514
24729	0.946132	0.925449
24730	0.927608	0.512759
24731	0.946128	2.1
24732	0.946133	2.1
24733	0.946131	2.1
24734	0.94613	2.1
24735	1.38813	2.1
24736	0.94613	2.1
24737	0.946128	2.1
24738	0.946132	2.1
24739	1.41666	2.1
24740	0.94613	2.1
24741	0.946118	0.157379
24742	1.34151	0.128733
24743	0.946116	0.175714
24744	0.946131	0.155444
24745	1.34149	0.145493
24746	0.946132	2.1
24747	1.31889	2.1
24748	0.946132	2.1
24749	0.946132	2.1
24750	1.31889	2.1
24751	0.864997	0.0698591
24752	0.928981	0.0698591
24753	0.872219	0.0698591
24754	0.946127	0.556934
24755	0.946131	0.44564
24756	1.37709	0.340708
24757	0.946131	0.542467
24758	0.946129	0.446983
24759	1.21882	0.0698591
24760	0.938168	0.0698591
24761	0.938161	0.0698591
24762	1.06327	0.0698591
24763	1.33457	0.0698591

24764	0.946133	0.80896
24765	0.946132	0.658775
24766	0.946133	0.568024
24767	0.946132	0.86649
24768	1.14444	0.505561
24769	1.12628	0.0698591
24770	0.946132	0.0698782
24771	0.946131	0.072007
24772	1.12628	0.0698591
24773	0.946131	0.0698591
24774	0.946132	2.1
24775	0.946132	2.1
24776	0.946133	2.1
24777	0.946132	2.1
24778	1.29322	2.1
24779	0.946131	2.1
24780	0.946132	2.1
24781	0.94613	2.1
24782	0.946131	2.1
24783	1.28441	2.1
24784	0.946118	2.1
24785	0.946119	2.1
24786	0.946117	2.1
24787	1.28075	2.1
24788	0.946105	2.1
24789	0.810945	2.1
24790	0.810942	2.1
24791	0.81093	2.1
24792	0.946127	2.1
24793	0.946127	2.1
24794	1.28228	2.1
24795	0.946129	2.1
24796	0.946132	2.1
24797	1.14042	2.1
24798	0.946132	2.1
24799	0.946133	2.1
24800	1.14042	2.1
24801	0.946133	0.0793817
24802	0.946132	0.0750172
24803	1.28632	0.0714204
24804	1.28632	0.0698591
24805	0.946133	0.0750848
24806	0.946131	1.69813
24807	0.946131	1.50819
24808	0.946131	1.54574
24809	1.31817	1.70387
24810	0.946131	1.39466
24811	1.35048	2.1
24812	0.946132	2.1
24813	0.946132	2.1
24814	0.946132	2.1
24815	0.946131	2.1
24816	0.946131	2.1
24817	1.35943	2.1
24818	0.94613	2.1
24819	0.946132	2.1
24820	0.946131	2.1
24821	0.990713	2.1

24822	0.946132	2.1
24823	0.946132	2.1
24824	0.946133	2.1
24825	0.946132	2.1
24826	0.946133	1.5447
24827	1.01433	1.35221
24828	0.877931	1.78969
24829	0.96573	0.914471
24830	0.946131	0.994268
24831	0.946131	1.44074
24832	0.946133	1.02687
24833	1.39575	0.638642
24834	0.946131	0.799796
24835	0.94613	0.774573
24836	0.810943	0.43191
24837	0.810942	0.443679
24838	0.709589	0.322394
24839	0.924071	0.290493
24840	1.04293	0.218761
24841	1.15747	0.163519
24842	0.946131	0.294855
24843	1.04071	0.239092
24844	0.810946	0.371285
24845	0.810943	0.326688
24846	0.810959	0.285898
24847	0.946128	1.17519
24848	1.39696	1.8428
24849	0.94613	0.691433
24850	0.94613	0.596094
24851	1.39696	1.3727
24852	0.946131	0.648373
24853	0.946131	1.87559
24854	0.946131	1.26446
24855	0.946131	0.964498
24856	1.29735	1.73174
24857	0.946128	1.99859
24858	0.946133	0.700408
24859	0.94613	2.04978
24860	0.946131	1.78751
24861	1.33266	1.42533
24862	0.946131	2.1
24863	0.94613	1.90513
24864	0.946131	2.1
24865	1.40609	2.1
24866	0.946131	2.1
24867	0.946131	2.03825
24868	0.946133	2.04936
24869	0.946129	2.07574
24870	1.23339	2.084
24871	0.946131	2.03606
24872	0.946132	2.0991
24873	0.946133	2.09925
24874	0.946132	2.1
24875	1.29198	2.1
24876	0.946132	2.09105
24877	0.946131	2.1
24878	1.07297	2.1
24879	0.946127	2.1

24880	0.94613	2.1
24881	1.07297	2.1
24882	0.946125	2.1
24883	0.946126	2.1
24884	0.946126	2.1
24885	1.3864	2.1
24886	0.946119	2.1
24887	0.946128	0.103136
24888	1.09411	0.0874777
24889	0.946131	0.132568
24890	0.946131	0.116004
24891	1.09411	0.0972187
24892	0.946131	0.0867806
24893	1.25837	0.0768767
24894	0.946131	0.110303
24895	0.946131	0.0975044
24896	1.25837	0.0828166
24897	0.810945	2.1
24898	0.810943	2.1
24899	0.810933	2.1
24900	0.94613	0.150838
24901	0.946131	0.555967
24902	0.946132	0.201092
24903	0.946129	0.217067
24904	0.946131	0.488964
24905	0.946131	0.0698591
24906	1.28911	0.0698591
24907	0.946129	0.0698591
24908	0.946133	0.0698591
24909	0.946131	0.0698591
24910	0.94613	0.146269
24911	1.38954	0.145246
24912	0.946132	0.142018
24913	0.94613	0.14781
24914	0.946131	0.147052
24915	0.946131	0.931154
24916	0.946131	0.576884
24917	0.946131	0.422608
24918	1.37981	0.438754
24919	0.946131	0.515158
24920	0.946131	2.1
24921	1.26958	2.1
24922	0.946127	2.1
24923	0.946129	2.1
24924	1.26957	2.1
24925	0.94613	2.1
24926	1.16889	2.1
24927	0.946126	2.1
24928	0.946128	2.1
24929	1.16889	2.1
24930	0.946133	0.312533
24931	1.13511	0.210295
24932	0.946132	0.318479
24933	0.946133	0.240537
24934	0.946132	0.23957
24935	1.27575	1.48198
24936	0.946129	1.98557
24937	0.946131	1.87045

24938	0.94613	1.98746
24939	0.946131	1.90524
24940	0.946131	2.1
24941	0.946128	2.1
24942	1.41712	2.1
24943	1.41712	2.1
24944	0.94613	2.1
24945	0.946129	0.0698591
24946	0.946132	0.0698591
24947	0.946131	0.0698591
24948	0.946131	0.0698591
24949	0.946131	0.0698591
24950	0.979045	1.9606
24951	0.946131	1.39081
24952	0.617549	2.01275
24953	0.555844	1.99441
24954	1.04071	1.43889
24955	0.946132	1.9852
24956	0.946132	1.56201
24957	0.946131	2.03646
24958	1.22618	1.74998
24959	0.946132	1.50386
24960	0.946131	2.1
24961	1.13976	2.1
24962	0.946129	2.1
24963	0.946131	2.1
24964	1.13976	2.1
24965	0.810945	0.446076
24966	0.810943	0.907588
24967	0.810932	1.19074
24968	1.25358	1.61569
24969	0.94613	0.715608
24970	0.946128	1.27774
24971	1.25358	1.76056
24972	1.07811	1.49127
24973	0.946129	2.1
24974	1.09197	2.1
24975	0.946131	2.1
24976	0.946131	2.1
24977	1.09197	2.1
24978	0.594912	2.1
24979	0.993703	2.1
24980	1.10173	2.1
24981	0.9916	2.1
24982	0.946131	2.1
24983	0.946128	2.1
24984	0.946131	2.1
24985	0.94613	2.1
24986	0.946129	2.1
24987	0.946132	2.1
24988	1.40335	2.1
24989	0.946133	2.1
24990	0.946133	2.1
24991	1.40335	2.1
24992	0.946132	2.0351
24993	0.946132	2.1
24994	0.946132	2.0512
24995	0.946132	2.05436

24996	1.411	2.09428
24997	0.611675	0.0882334
24998	0.946129	0.0929531
24999	1.31678	0.0699672
25000	0.946124	0.100102
25001	0.946127	0.0702672
25002	0.946128	0.0997049
25003	0.946132	0.35495
25004	0.946131	0.231112
25005	0.946132	0.370364
25006	0.946132	0.243678
25007	0.946132	0.2473
25008	0.810946	0.287097
25009	0.810943	0.308314
25010	0.81096	0.260465
25011	1.39301	0.0698591
25012	0.946132	0.0698591
25013	0.625812	0.0698591
25014	0.946132	0.0698591
25015	0.625812	0.0698591
25016	0.810946	1.98326
25017	0.810945	2.06403
25018	0.810931	2.1
25019	0.810943	2.1
25020	0.810944	2.1
25021	0.810958	2.1
25022	0.946131	0.143851
25023	0.946131	0.11513
25024	0.946128	0.163785
25025	0.946129	0.128595
25026	0.94613	0.10693
25027	0.946133	0.220385
25028	0.946133	0.475958
25029	0.98537	0.130248
25030	0.985371	0.083419
25031	0.946132	0.375943
25032	0.946129	0.395822
25033	1.38752	0.274747
25034	0.946131	0.255837
25035	0.946131	0.32375
25036	1.38752	0.299018
25037	0.946119	0.219249
25038	1.33036	0.139947
25039	0.946119	0.140359
25040	0.946132	0.170358
25041	1.33035	0.153322
25042	1.3868	2.1
25043	0.946132	2.1
25044	0.946132	2.1
25045	0.946133	2.1
25046	0.946132	2.1
25047	0.810947	2.1
25048	0.810946	2.1
25049	0.81096	2.1
25050	0.810946	2.1
25051	0.810946	2.1
25052	0.810933	2.1
25053	0.946133	2.1

25054	1.35342	2.1
25055	0.946133	2.1
25056	0.946132	2.1
25057	1.35342	2.1
25058	0.946132	2.1
25059	0.946132	2.1
25060	1.28569	2.1
25061	0.946132	2.1
25062	0.946133	2.1
25063	0.946132	2.07436
25064	1.32547	2.1
25065	0.946132	2.07999
25066	0.946132	2.1
25067	1.32547	2.1
25068	0.94613	0.0698591
25069	0.946133	0.0698591
25070	1.39664	0.0698591
25071	1.39664	0.0698591
25072	0.946131	0.0698591
25073	0.946131	2.03343
25074	0.94613	2.1
25075	0.946132	1.71743
25076	0.946131	2.1
25077	1.41833	2.1
25078	0.946131	0.0698591
25079	0.946131	0.0698591
25080	0.946131	0.0698591
25081	1.26155	0.0698591
25082	0.946132	0.0698591
25083	0.810946	2.1
25084	0.810946	2.1
25085	0.810959	2.1
25086	0.94613	1.43986
25087	0.946132	0.83379
25088	1.40779	0.523723
25089	1.40779	0.685045
25090	0.94613	0.844839
25091	0.94613	0.0744523
25092	0.946129	0.0839854
25093	1.36376	0.0698762
25094	1.36376	0.0698591
25095	0.946131	0.0715544
25096	0.946128	2.1
25097	0.946127	2.1
25098	0.946131	2.1
25099	1.40756	2.1
25100	0.94613	2.1
25101	0.946131	2.08515
25102	0.946132	2.1
25103	0.94613	2.08803
25104	0.946131	2.1
25105	1.31947	2.1
25106	0.946131	2.1
25107	0.946131	2.1
25108	0.946129	2.1
25109	0.946131	2.1
25110	1.25768	2.1
25111	0.946132	0.100254

25112	0.94613	0.0767962
25113	0.946131	0.0807261
25114	0.946131	0.0700625
25115	0.946131	0.0976923
25116	0.810943	1.23691
25117	0.810942	1.89807
25118	0.81093	2.06118
25119	0.946128	0.817736
25120	0.946133	0.40008
25121	0.946131	0.971116
25122	0.946131	0.512698
25123	0.94613	0.839857
25124	0.946132	1.1267
25125	0.946131	0.729428
25126	0.946132	1.16916
25127	0.946132	0.975726
25128	0.946132	1.22611
25129	0.946119	2.1
25130	0.946119	2.1
25131	1.31523	2.1
25132	0.946132	2.1
25133	0.946107	2.1
25134	0.946133	2.1
25135	0.946132	1.79781
25136	0.946132	2.02174
25137	1.28803	2.1
25138	0.946132	2.1
25139	1.37658	1.12725
25140	0.946132	0.399962
25141	0.946133	1.47886
25142	0.946132	0.424654
25143	0.946132	1.07495
25144	0.946132	1.49163
25145	0.94613	1.11026
25146	0.94613	1.87993
25147	1.15541	0.908955
25148	0.92943	1.54364
25149	0.946126	0.0698591
25150	0.946129	0.0698591
25151	1.33849	0.0698591
25152	1.33849	0.0698591
25153	0.946129	0.0698591
25154	0.810946	0.0698591
25155	0.810944	0.0698591
25156	0.810959	0.0698591
25157	1.39027	0.0698591
25158	0.946131	0.0742686
25159	0.946131	0.0887793
25160	1.39027	0.0698591
25161	0.946131	0.0698591
25162	1.40924	1.85309
25163	0.946131	1.31546
25164	0.946132	1.82044
25165	0.946132	0.97723
25166	0.946132	1.51448
25167	0.946131	2.1
25168	0.94613	2.1
25169	0.946128	2.1

25170	0.946128	2.1
25171	1.41744	2.1
25172	0.946132	0.696695
25173	0.946128	1.14522
25174	0.94613	1.17141
25175	1.37675	0.864459
25176	0.94613	1.28979
25177	0.946132	1.10172
25178	0.946132	0.572996
25179	0.946132	0.371487
25180	0.946132	0.518131
25181	0.946131	0.511586
25182	0.946132	0.717033
25183	0.946132	0.330065
25184	0.946133	1.15541
25185	1.10003	0.283566
25186	0.946132	0.685976
25187	0.946132	0.074694
25188	0.946131	0.079795
25189	0.946132	0.0731329
25190	0.946131	0.0795755
25191	0.946132	0.077096
25192	0.946132	0.0934463
25193	0.946131	0.0983803
25194	0.946132	0.084192
25195	0.946132	0.0984984
25196	0.946131	0.0935754
25197	1.24094	0.0698591
25198	0.946126	0.0715474
25199	0.946128	0.0698591
25200	0.946129	0.0714167
25201	0.946129	0.0698591
25202	1.2929	0.0733296
25203	0.946131	0.106654
25204	0.946131	0.0837317
25205	0.946131	0.0963762
25206	0.946131	0.0855869
25207	0.810946	0.0698591
25208	0.810945	0.0698591
25209	0.810959	0.0698591
25210	0.810946	1.98842
25211	0.810943	2.00006
25212	0.810933	2.07117
25213	0.946128	2.1
25214	1.18315	2.1
25215	0.94613	2.09987
25216	0.94613	2.1
25217	1.18316	2.1
25218	0.946126	0.325682
25219	1.3324	0.216245
25220	0.946127	0.34002
25221	0.94613	0.222189
25222	0.946128	0.272373
25223	0.94613	2.1
25224	0.946128	2.1
25225	1.39523	2.1
25226	1.39523	2.1
25227	0.946131	2.1

25228	1.36541	2.1
25229	0.946132	2.1
25230	0.946132	2.1
25231	0.946132	2.1
25232	0.946132	2.1
25233	0.946131	2.09615
25234	0.94613	1.47022
25235	0.946132	1.91249
25236	0.946131	0.296628
25237	1.39509	1.58869
25238	0.946132	2.1
25239	0.946132	2.1
25240	1.29319	2.1
25241	0.946132	2.1
25242	0.946133	2.1
25243	0.946132	0.0792777
25244	0.946131	0.140295
25245	0.946132	0.0865637
25246	1.01196	0.0763647
25247	0.946132	0.125883
25248	0.946116	2.1
25249	0.946117	2.1
25250	1.38153	2.1
25251	1.38155	2.1
25252	0.946105	2.1
25253	0.946132	1.76143
25254	0.946131	1.89041
25255	0.946132	1.68224
25256	1.28684	1.67941
25257	0.946132	1.53269
25258	1.10185	1.77183
25259	0.94613	1.92176
25260	0.946128	2.05721
25261	0.94613	2.00711
25262	0.94613	1.92302
25263	0.810947	0.0698591
25264	0.810945	0.0698591
25265	0.810932	0.0698591
25266	0.946119	0.218333
25267	1.16717	0.159179
25268	0.946117	0.214467
25269	0.94613	0.168721
25270	1.16715	0.166709
25271	0.94612	0.347606
25272	0.946119	0.362707
25273	0.946119	0.308591
25274	0.946132	0.235377
25275	1.20882	0.272806
25276	1.07043	0.807377
25277	0.823268	0.843096
25278	0.810959	0.789405
25279	0.946132	1.62793
25280	1.14879	0.881114
25281	0.94613	1.56886
25282	0.946131	1.55867
25283	1.14879	0.753197
25284	1.23837	2.1
25285	0.94612	2.1

25286	0.946118	2.1
25287	0.946131	2.1
25288	0.946105	2.1
25289	0.810946	0.0698591
25290	0.810946	0.0698591
25291	0.810959	0.0698591
25292	0.810947	1.48791
25293	0.810947	0.240985
25294	0.810934	0.25348
25295	0.946128	2.1
25296	0.946131	2.1
25297	0.946128	2.09331
25298	0.946129	2.1
25299	0.94613	2.1
25300	0.94613	2.08844
25301	0.946131	2.08229
25302	0.946131	2.05335
25303	0.946131	2.09122
25304	0.946131	2.08639
25305	0.946131	1.89978
25306	1.38585	2.1
25307	0.946128	1.93818
25308	0.94613	1.93374
25309	1.38585	1.96604
25310	0.946133	0.151892
25311	0.946133	0.148204
25312	0.946132	0.131125
25313	0.946132	0.153722
25314	0.946132	0.144388
25315	0.918871	0.0698591
25316	0.810248	0.0698591
25317	0.838037	0.0698591
25318	0.946126	0.0699158
25319	0.943647	0.069979
25320	1.13836	0.0698591
25321	1.03802	0.0698591
25322	0.946129	0.0698591
25323	0.946133	1.50549
25324	0.946132	1.0383
25325	0.946132	2.07417
25326	1.1699	0.846132
25327	0.946132	0.859487
25328	0.946131	2.1
25329	0.946132	2.1
25330	1.37199	2.1
25331	1.37199	2.1
25332	0.946132	2.1
25333	0.946132	0.784017
25334	0.946133	0.515222
25335	0.946133	0.828771
25336	0.946132	0.595417
25337	1.38623	0.458515
25338	0.946132	2.1
25339	0.946132	2.1
25340	0.946132	2.1
25341	0.946132	2.1
25342	1.40098	2.1
25343	1.20787	0.173694

25344	1.41894	0.506345
25345	0.946133	0.301567
25346	1.01777	0.369843
25347	0.946132	2.1
25348	0.946132	2.1
25349	0.946133	2.1
25350	1.40199	2.1
25351	0.946132	2.1
25352	0.810946	1.46104
25353	0.810947	1.96063
25354	0.810934	1.60222
25355	0.946126	1.14266
25356	0.94613	1.63357
25357	0.946125	1.7398
25358	1.40247	1.0349
25359	0.946128	1.21494
25360	1.25703	1.72234
25361	0.87821	1.37929
25362	0.878238	0.920158
25363	0.946119	1.93295
25364	0.946132	2.0301
25365	0.946132	0.363889
25366	0.946132	0.317303
25367	0.946131	0.833366
25368	0.946132	0.2006
25369	1.28543	0.262308
25370	0.946115	2.1
25371	1.41638	2.1
25372	0.946116	2.1
25373	0.946104	2.1
25374	0.946129	2.1
25375	0.946132	2.1
25376	1.41432	2.1
25377	0.946132	2.1
25378	0.946132	2.1
25379	0.946132	2.1
25380	0.810947	0.0698591
25381	0.810947	0.0698591
25382	0.709567	0.0698591
25383	0.851542	0.0698591
25384	1.01217	0.0698591
25385	0.697993	0.0698591
25386	0.946133	0.0698591
25387	0.851488	0.0698591
25388	0.946129	2.03308
25389	0.94613	2.08466
25390	1.2254	1.86728
25391	0.946131	2.04262
25392	0.946131	1.98401
25393	0.700785	2.09886
25394	0.915893	2.0097
25395	0.838013	1.87262
25396	0.760188	1.29035
25397	1.19601	0.189918
25398	0.946133	0.332627
25399	0.946132	0.326793
25400	0.946132	0.264438
25401	0.946132	0.324156

25402	0.946132	1.87237
25403	0.946133	2.1
25404	0.946132	2.01025
25405	0.946133	2.07435
25406	1.36956	2.08739
25407	0.946131	2.09922
25408	0.94613	0.217954
25409	0.946133	2.08233
25410	1.34268	0.454854
25411	0.946131	1.03028
25412	0.946132	2.1
25413	1.25603	2.1
25414	0.946132	2.1
25415	0.946132	2.1
25416	1.25603	2.1
25417	0.810946	1.25051
25418	0.810943	1.24568
25419	0.810933	1.14267
25420	0.496545	0.393984
25421	0.805767	0.683883
25422	0.936671	0.578382
25423	0.530063	0.565367
25424	1.05954	0.551773
25425	0.810946	0.131512
25426	0.810943	0.115775
25427	0.810959	0.0972302
25428	1.16699	0.10682
25429	0.946131	0.197826
25430	0.946129	0.169167
25431	0.946131	0.149299
25432	0.946131	0.14286
25433	0.810943	1.85285
25434	0.810942	1.92073
25435	0.810933	1.82586
25436	0.946132	1.84913
25437	1.04832	1.80972
25438	0.870714	2.00781
25439	0.957819	2.02193
25440	0.94613	1.84947
25441	1.82931	2.1
25442	0.946132	0.105507
25443	0.946129	0.0749491
25444	0.94613	0.112476
25445	0.946131	0.132644
25446	0.94613	0.0769431
25447	0.946119	0.88334
25448	0.94612	0.930906
25449	0.94612	0.550442
25450	0.946132	0.757369
25451	1.36118	0.587658
25452	0.810947	0.134226
25453	0.810947	0.101801
25454	0.810934	0.126368
25455	0.946132	2.1
25456	0.946132	2.1
25457	0.946132	2.1
25458	1.39754	2.1
25459	0.946131	2.1

25460	0.946129	2.1
25461	0.946133	2.1
25462	0.946131	2.1
25463	0.946131	2.1
25464	1.41732	2.1
25465	0.946132	1.06662
25466	0.946128	1.68904
25467	0.94613	1.22579
25468	0.94613	1.4403
25469	1.25277	1.51713
25470	0.94613	1.53595
25471	0.946128	1.77952
25472	1.21261	0.869726
25473	0.94613	1.46175
25474	0.94613	1.16748
25475	1.39407	2.1
25476	0.946133	2.1
25477	0.94613	2.1
25478	0.946131	2.1
25479	0.946131	2.1
25480	0.946119	1.66117
25481	0.946119	1.80307
25482	0.946119	1.96858
25483	1.1537	1.81374
25484	0.946106	1.58343
25485	0.946131	2.1
25486	1.11822	2.1
25487	0.946129	2.1
25488	0.946131	2.1
25489	1.11822	2.1
25490	1.46806	0.0698591
25491	0.498881	0.0698591
25492	0.996658	0.0698591
25493	1.2179	0.0698591
25494	0.991519	0.0698591
25495	0.946131	0.0698591
25496	0.946131	0.0698591
25497	1.28673	0.0698591
25498	0.946131	0.0698591
25499	0.946131	0.0698591
25500	1.33178	0.0737934
25501	0.946132	0.0895254
25502	0.946132	0.0858188
25503	0.946132	0.0873342
25504	0.946132	0.0871307
25505	0.946128	0.13642
25506	0.972061	0.09577
25507	0.946129	0.156238
25508	0.946129	0.143814
25509	0.972059	0.10463
25510	0.946129	2.1
25511	0.94613	2.1
25512	0.94613	2.1
25513	0.94613	2.1
25514	0.94613	2.1
25515	0.946131	2.1
25516	0.946127	2.1
25517	0.946131	2.1

25518	0.946129	2.1
25519	1.4073	2.1
25520	0.946131	0.071531
25521	1.05016	0.0700442
25522	0.946132	0.0767175
25523	0.946131	0.0749531
25524	0.946132	0.0729801
25525	0.946128	0.0737329
25526	1.13398	0.070786
25527	0.946131	0.0860581
25528	0.946131	0.0791885
25529	0.94613	0.0755164
25530	0.946131	0.0698591
25531	0.946131	0.0698591
25532	1.38911	0.0698591
25533	1.38911	0.0698591
25534	0.946132	0.0698591
25535	0.946131	0.0757929
25536	0.94613	0.0714866
25537	0.946131	0.0754694
25538	0.946131	0.0724085
25539	0.946131	0.0757154
25540	0.946131	0.15937
25541	1.23923	0.111669
25542	0.94613	0.200806
25543	0.946131	0.113423
25544	0.946131	0.12925
25545	1.40511	0.0698591
25546	0.946131	0.0698591
25547	0.946131	0.0698591
25548	0.946132	0.0698591
25549	0.946131	0.0698591
25550	1.12968	2.1
25551	0.946132	2.1
25552	0.946132	2.1
25553	0.946131	2.1
25554	0.946132	2.1
25555	0.946132	1.76604
25556	1.4172	2.1
25557	0.94613	1.84265
25558	0.94613	1.86689
25559	1.4172	1.99359
25560	0.946132	2.1
25561	0.946132	2.1
25562	1.31323	2.1
25563	0.946133	2.1
25564	0.946132	2.1
25565	0.946131	2.1
25566	0.94613	2.06344
25567	1.36546	2.1
25568	1.36546	2.1
25569	0.946131	2.1
25570	0.946132	1.9018
25571	0.946133	2.1
25572	0.946132	0.255966
25573	1.36899	1.47874
25574	0.946133	1.01576
25575	0.946131	0.0698591

25576	0.946132	0.0698591
25577	1.03543	0.0698591
25578	1.1412	0.0698591
25579	0.941275	0.0698591
25580	1.28952	0.0845111
25581	0.94613	0.104376
25582	0.946129	0.115867
25583	0.946132	0.0875312
25584	0.94613	0.102479
25585	0.94613	2.1
25586	0.94613	2.1
25587	0.946129	2.1
25588	0.94613	2.1
25589	1.35966	2.1
25590	0.810946	2.1
25591	0.810942	2.1
25592	0.810932	2.1
25593	0.94613	2.1
25594	1.40372	2.1
25595	0.946131	2.1
25596	0.94613	2.1
25597	1.40372	2.1
25598	0.946133	2.1
25599	0.946131	2.1
25600	0.94613	2.1
25601	0.946131	2.1
25602	0.946131	2.1
25603	1.31453	2.1
25604	0.94613	2.1
25605	0.946128	2.1
25606	0.94613	2.1
25607	0.94613	2.1
25608	0.946131	0.0698591
25609	1.12018	0.0698591
25610	0.94613	0.0704073
25611	0.94613	0.0706756
25612	0.94613	0.0698591
25613	0.946131	0.072928
25614	1.16373	0.0698591
25615	0.946131	0.0925594
25616	0.94613	0.0701211
25617	0.946131	0.0702641
25618	0.946126	0.327289
25619	0.946132	0.505802
25620	0.946128	0.381836
25621	0.946129	0.502051
25622	0.946129	0.580001
25623	0.946132	0.106504
25624	0.946133	0.111203
25625	0.946132	0.105934
25626	0.946132	0.105997
25627	0.946133	0.112852
25628	0.946132	1.20229
25629	0.946129	1.70864
25630	0.94613	1.09705
25631	1.36399	1.19572
25632	0.946131	1.25804
25633	0.946132	2.1

25634	0.946132	2.1
25635	0.946133	2.1
25636	1.38302	2.1
25637	0.946133	2.1
25638	0.810943	2.1
25639	0.810944	2.1
25640	0.810945	2.1
25641	0.946132	2.1
25642	0.946131	2.1
25643	0.946132	2.1
25644	1.30188	2.1
25645	0.946132	2.1
25646	0.810946	1.03568
25647	0.72985	1.63423
25648	0.810934	1.13802
25649	0.856243	0.509693
25650	0.946129	0.710964
25651	0.946132	0.504123
25652	1.19686	0.377229
25653	0.851527	0.649027
25654	0.946131	2.1
25655	0.94613	2.1
25656	0.94613	2.1
25657	0.94613	2.1
25658	1.31961	2.1
25659	0.946131	2.1
25660	0.946131	2.1
25661	0.94613	2.1
25662	0.946131	2.1
25663	1.3605	2.1
25664	1.30116	2.1
25665	0.946133	1.60225
25666	0.946131	2.1
25667	0.946131	2.01807
25668	0.94613	2.09335
25669	0.946132	0.0698591
25670	1.3035	0.0698591
25671	0.94613	0.0698591
25672	0.946131	0.0698591
25673	0.946131	0.0698591
25674	0.946132	2.1
25675	1.37869	2.1
25676	1.2051	2.1
25677	0.946132	2.1
25678	1.2051	2.1
25679	0.810946	2.1
25680	0.810945	2.1
25681	0.810946	2.1
25682	1.34623	2.1
25683	0.946131	2.1
25684	0.946131	2.1
25685	1.34623	2.1
25686	1.22993	2.1
25687	1.32299	0.0698591
25688	0.946132	0.0698591
25689	0.946132	0.0698591
25690	0.946132	0.0698591
25691	0.946131	0.0698591

25692	0.94613	0.160898
25693	1.40574	0.151455
25694	0.946128	0.202336
25695	0.946131	0.192977
25696	1.40574	0.189318
25697	0.946132	2.1
25698	1.41331	2.1
25699	0.94613	2.1
25700	0.946131	2.1
25701	1.41331	2.1
25702	0.94613	2.1
25703	1.37609	2.1
25704	0.946129	2.1
25705	0.946131	2.1
25706	1.37609	2.1
25707	1.039 0.987721	
25708	0.946131	1.86015
25709	0.944514	1.57693
25710	0.946132	1.74098
25711	0.946132	1.58183
25712	1.23726	0.925172
25713	0.946128	1.72786
25714	0.946128	1.53899
25715	0.946129	1.65216
25716	0.94613	1.46813
25717	0.946129	2.1
25718	0.946131	2.1
25719	0.946131	2.1
25720	1.39214	2.1
25721	0.94613	2.1
25722	0.946118	1.99616
25723	0.946118	0.643852
25724	1.41078	0.957592
25725	1.41076	0.5649
25726	0.946131	0.894475
25727	0.946131	0.134268
25728	0.946131	0.089857
25729	0.946132	0.0984932
25730	0.94613	0.151129
25731	0.946131	0.0951197
25732	0.946132	0.0733479
25733	0.946132	0.0698591
25734	0.946133	0.0698591
25735	1.28924	0.0698591
25736	0.946132	0.0698924
25737	0.946133	0.475187
25738	1.09812	0.350392
25739	0.946132	0.551331
25740	0.946132	0.460122
25741	1.09812	0.505311
25742	0.946132	1.25928
25743	0.946132	1.12167
25744	0.946132	1.11283
25745	0.946132	0.999317
25746	1.15388	1.07389
25747	0.946132	0.408697
25748	0.974688	0.26706
25749	0.946132	0.433723

25750	0.946132	0.372527
25751	0.974687	0.400132
25752	1.0644	1.54316
25753	0.946133	1.75237
25754	0.946132	1.53598
25755	0.946133	1.54473
25756	1.26547	1.54385
25757	1.02905	1.11479
25758	0.911537	1.24258
25759	0.838038	1.25235
25760	0.946131	0.100175
25761	0.94613	0.096729
25762	0.946132	0.0921994
25763	0.946131	0.088219
25764	1.33922	0.0869764
25765	0.946132	0.0829171
25766	0.946132	0.0749526
25767	1.40926	0.0716954
25768	1.40927	0.070187
25769	0.946132	0.0721211
25770	0.946129	2.09562
25771	0.946131	2.014
25772	0.946131	2.00481
25773	0.946131	1.2604
25774	1.40496	2.08763
25775	0.94613	1.81823
25776	0.94613	1.68658
25777	0.946127	1.73832
25778	0.946128	1.45087
25779	1.38914	1.47227
25780	2.675	0.0698591
25781	1.22266	0.0698591
25782	0.946131	0.0698591
25783	0.946132	0.0698591
25784	0.946132	0.0698591
25785	1.22266	0.0698591
25786	0.946124	0.0982211
25787	0.946128	0.0698591
25788	0.946126	0.0698591
25789	1.21428	0.0698591
25790	0.946127	0.0827839
25791	0.946131	0.0706438
25792	0.946131	0.136035
25793	0.946132	0.0712747
25794	0.946129	0.0782731
25795	0.940118	0.128682
25796	0.810943	2.1
25797	0.810945	2.1
25798	0.810958	2.1
25799	0.946133	0.0698591
25800	1.11539	0.0698591
25801	0.946132	0.0698591
25802	0.946132	0.0698591
25803	0.946133	0.0698591
25804	0.946133	2.1
25805	1.41195	2.1
25806	0.946133	2.1
25807	0.946132	2.1

25808	0.946133	2.1
25809	1.15968	0.0698591
25810	0.946131	0.0698591
25811	0.946131	0.0698591
25812	0.946131	0.0698591
25813	0.946131	0.0698591
25814	0.94613	1.98124
25815	0.946128	2.004
25816	0.946132	1.60438
25817	0.94613	1.97843
25818	0.94613	1.81386
25819	0.946131	1.81962
25820	1.04211	1.80396
25821	0.850155	1.25524
25822	0.946131	1.71977
25823	0.946131	1.45502
25824	0.946131	0.345802
25825	0.94613	0.254662
25826	0.946129	0.365602
25827	0.946131	0.261515
25828	0.946131	0.259366
25829	1.40193	0.131888
25830	1.19756	0.142027
25831	0.561936	0.181417
25832	0.808778	0.162035
25833	1.19756	0.154676
25834	0.946132	0.0700005
25835	0.946132	0.069912
25836	1.33916	0.0698591
25837	1.33916	0.0698591
25838	0.946133	0.0698591
25839	0.946127	0.196363
25840	0.946127	0.144316
25841	0.946131	0.177665
25842	0.946128	0.12491
25843	0.946129	0.1299
25844	0.589428	0.21105
25845	0.851501	0.196918
25846	0.868924	0.201942
25847	0.877703	0.167415
25848	0.85153	0.192164
25849	0.946132	2.04728
25850	1.25372	2.1
25851	0.946132	2.07364
25852	0.946132	2.1
25853	1.25372	2.1
25854	0.946132	1.47549
25855	0.946128	2.05254
25856	0.94613	1.70637
25857	0.94613	1.91901
25858	1.2778	1.95278
25859	0.946118	0.590784
25860	1.35629	0.489133
25861	0.946117	0.580824
25862	0.946131	0.494178
25863	0.946105	0.495362
25864	0.946132	1.88406
25865	0.946132	2.1

25866	1.38646	2.1
25867	1.38646	2.1
25868	0.946132	2.1
25869	0.946128	2.1
25870	0.946128	2.07626
25871	1.39659	2.1
25872	1.39659	2.1
25873	0.946129	2.1
25874	0.946132	1.71317
25875	0.946131	1.56927
25876	0.946132	1.71771
25877	0.946132	1.67533
25878	1.15139	1.60386
25879	0.946133	2.1
25880	0.946132	2.1
25881	0.946132	2.1
25882	0.946132	2.1
25883	1.36151	2.1
25884	0.946127	2.1
25885	0.946131	2.1
25886	0.946128	2.1
25887	0.94613	2.1
25888	1.38397	2.1
25889	0.946131	0.276825
25890	1.31112	0.309619
25891	0.946127	0.321979
25892	0.946128	0.308546
25893	1.31112	0.296503
25894	1.29778	0.0698591
25895	0.883417	0.0698591
25896	1.32613	0.0698591
25897	0.94613	0.0698591
25898	0.94613	0.0698591
25899	0.946132	2.1
25900	0.94613	2.1
25901	0.946131	2.1
25902	0.946131	2.1
25903	1.36523	2.1
25904	0.946132	0.0725685
25905	1.01653	0.0703712
25906	0.946132	0.089108
25907	0.946131	0.0829853
25908	0.946131	0.0735151
25909	0.94612	2.1
25910	1.08301	2.05689
25911	0.946119	2.1
25912	0.946132	2.1
25913	1.08299	2.0893
25914	0.810942	2.1
25915	0.810944	2.1
25916	0.810957	2.1
25917	0.946126	0.245717
25918	0.946132	0.290701
25919	0.946129	0.247326
25920	1.3492	0.222285
25921	0.94613	0.242186
25922	0.946131	0.223168
25923	1.24766	0.222875

25924	0.946128	0.258617
25925	0.946131	0.253998
25926	1.24766	0.253027
25927	0.946132	0.0698591
25928	0.946131	0.0698591
25929	0.946131	0.0698591
25930	1.23351	0.0698591
25931	0.946132	0.0698591
25932	0.946131	0.0698591
25933	0.946132	0.0698591
25934	0.946131	0.0698591
25935	1.35657	0.0698591
25936	0.946131	0.0698591
25937	0.946132	0.229523
25938	1.3689	0.162565
25939	0.946132	0.214791
25940	0.946132	0.211215
25941	1.3689	0.17063
25942	0.946133	0.13848
25943	1.35822	0.0822685
25944	0.946133	0.116409
25945	0.946132	0.117861
25946	1.35822	0.0864614
25947	0.946131	0.335298
25948	0.946131	1.17107
25949	0.946131	0.792999
25950	0.946131	0.642252
25951	1.4101	1.17974
25952	0.810944	1.5885
25953	0.810939	1.48707
25954	0.810955	1.2451
25955	0.946132	2.1
25956	1.37733	2.1
25957	0.946132	2.1
25958	0.946131	2.1
25959	0.946131	2.1
25960	1.32582	2.1
25961	0.946132	2.1
25962	0.946132	2.1
25963	0.946132	2.1
25964	0.946132	2.1
25965	0.946132	2.1
25966	0.946132	2.1
25967	1.31401	2.1
25968	1.31401	2.1
25969	0.946131	2.1
25970	1.3469	1.54776
25971	0.946132	1.78714
25972	0.946132	1.77889
25973	0.946133	1.28908
25974	0.946132	1.44641
25975	0.946126	2.1
25976	0.946127	2.1
25977	0.94613	2.1
25978	0.946128	2.1
25979	1.41206	2.1
25980	0.946127	2.1
25981	1.40109	2.1

25982	0.946128	2.1
25983	0.946129	2.1
25984	1.40109	2.1
25985	1.24448	0.0939775
25986	0.94613	0.178399
25987	0.946132	0.127878
25988	0.946129	0.159999
25989	0.946131	0.148143
25990	1.11015	0.140078
25991	0.946133	0.296075
25992	0.946132	0.18904
25993	0.946133	0.241539
25994	0.946132	0.226896
25995	0.946132	2.1
25996	0.946131	2.1
25997	0.946131	2.1
25998	1.38775	2.1
25999	0.946131	2.1
26000	0.946133	1.80988
26001	0.946131	2.1
26002	1.30598	2.1
26003	1.30598	2.1
26004	0.946131	2.1
26005	0.946132	2.1
26006	0.946132	2.1
26007	0.946132	2.1
26008	1.40774	2.1
26009	0.946132	2.1
26010	0.798396	0.0698591
26011	0.946133	0.397683
26012	0.946128	1.3561
26013	0.946131	0.778398
26014	0.946131	1.10336
26015	1.28918	1.05338
26016	0.946132	0.15661
26017	1.40668	0.126383
26018	0.94613	0.122062
26019	0.94613	0.164533
26020	0.94613	0.169365
26021	0.810946	2.1
26022	0.810945	2.1
26023	0.810933	2.1
26024	0.946131	2.1
26025	1.30338	2.1
26026	0.94613	2.1
26027	1.30339	2.1
26028	0.946131	2.1
26029	0.946127	0.532049
26030	0.87161	0.474573
26031	0.946128	0.547045
26032	0.946129	0.527535
26033	0.827876	0.553576
26034	0.946131	2.1
26035	1.40796	2.1
26036	0.94613	2.1
26037	0.94613	2.1
26038	1.40796	2.1
26039	0.946132	0.542535

26040	0.946132	0.662022
26041	0.946132	0.523036
26042	0.946132	0.482412
26043	1.41724	0.425832
26044	1.34254	0.252809
26045	0.946132	0.453124
26046	0.946132	0.399799
26047	0.946132	0.376178
26048	0.946133	0.346584
26049	0.946133	1.90566
26050	0.946132	1.8528
26051	0.946132	1.79169
26052	1.26501	1.93741
26053	0.946132	1.76406
26054	0.946131	0.0700461
26055	1.35169	0.0699088
26056	0.946131	0.0751711
26057	0.946131	0.0713347
26058	1.35169	0.0701209
26059	0.676844	0.0698591
26060	0.946131	0.0698591
26061	0.94613	0.0718389
26062	1.13242	0.0698591
26063	1.13242	0.0698591
26064	0.946131	0.0698591
26065	0.946128	1.85144
26066	1.28828	2.08634
26067	0.946131	1.79307
26068	0.946131	1.9616
26069	1.28828	2.02659
26070	0.946131	0.307984
26071	1.2996	0.237657
26072	0.946133	0.292486
26073	0.946131	0.265096
26074	0.946131	0.247033
26075	0.946132	2.1
26076	1.40638	2.1
26077	0.946131	2.1
26078	0.946132	2.1
26079	1.40638	2.1
26080	2.84909	0.069867
26081	1.35726	0.0701828
26082	0.946131	0.0841869
26083	0.946131	0.0776774
26084	0.946131	0.0737169
26085	0.946131	0.0750633
26086	1.39852	2.1
26087	0.946132	2.1
26088	0.94613	2.1
26089	0.94613	2.1
26090	0.94613	2.1
26091	0.946126	0.0698591
26092	0.946131	0.0698591
26093	0.946128	0.0698591
26094	1.31666	0.0698591
26095	0.946128	0.0698591
26096	0.946126	0.0698591
26097	0.946131	0.0698591

26098	0.946129	0.0706078
26099	1.32275	0.0698591
26100	0.946129	0.0698591
26101	0.946131	2.1
26102	0.946132	2.1
26103	1.41125	2.1
26104	0.946131	2.1
26105	0.946131	2.1
26106	0.946131	2.1
26107	0.946132	2.1
26108	1.31904	2.1
26109	0.946132	2.1
26110	0.946132	2.1
26111	0.946129	0.0698591
26112	0.946132	0.0698591
26113	0.94613	0.0698591
26114	1.02426	0.0698591
26115	0.9311	0.0698591
26116	0.946128	1.7933
26117	0.946133	1.6132
26118	0.946131	1.63833
26119	1.40092	1.81063
26120	0.946131	1.61024
26121	0.946117	2.1
26122	0.946118	2.1
26123	0.946118	2.09345
26124	1.40017	2.1
26125	0.946105	2.1
26126	0.946132	0.177165
26127	1.03547	0.157161
26128	0.946132	0.127052
26129	0.946133	0.184434
26130	0.946132	0.165087
26131	0.946133	2.1
26132	0.946132	2.1
26133	0.946133	2.1
26134	0.946133	2.1
26135	1.36694	2.1
26136	0.946132	0.0838233
26137	1.22878	0.105261
26138	0.946132	0.120256
26139	0.946132	0.0850642
26140	1.22878	0.0977004
26141	1.11014	0.127141
26142	0.913078	0.127933
26143	1.11015	0.165323
26144	0.49154	0.163802
26145	0.946127	2.1
26146	0.946131	2.1
26147	0.946128	2.1
26148	0.946129	2.1
26149	1.38752	2.1
26150	0.946132	2.1
26151	1.37277	2.1
26152	0.946132	2.1
26153	0.946131	2.1
26154	1.37277	2.1
26155	0.94613	0.348817

26156	1.16834	0.190909
26157	0.946129	0.342505
26158	0.94613	0.238241
26159	0.94613	0.237178
26160	0.946127	0.377914
26161	0.946131	0.249951
26162	1.35594	0.203292
26163	0.946128	0.380068
26164	0.94613	0.272868
26165	0.946128	2.09975
26166	0.946128	0.53908
26167	1.38464	1.97863
26168	1.38463	1.38844
26169	0.94613	1.38385
26170	0.946129	1.09877
26171	0.946131	0.604074
26172	0.94613	0.367703
26173	1.39751	0.286101
26174	0.946131	0.369035
26175	1.25837	0.079206
26176	1.21134	0.0730992
26177	0.700013	0.0846862
26178	0.700041	0.0823998
26179	1.40583	2.09684
26180	0.946132	2.1
26181	0.946132	2.1
26182	0.946133	2.1
26183	0.946132	2.1
26184	0.827865	2.1
26185	0.946131	2.1
26186	0.946131	2.03492
26187	0.946131	2.09803
26188	0.946131	2.03401
26189	0.946132	0.0698591
26190	1.21087	0.0698591
26191	0.94613	0.0698591
26192	0.94613	0.0698591
26193	0.946131	0.0698591
26194	0.94613	0.273192
26195	1.17452	0.240487
26196	0.946132	0.273446
26197	0.94613	0.253141
26198	0.94613	0.262598
26199	0.946119	0.222908
26200	1.27374	0.211551
26201	0.946119	0.221285
26202	0.946131	0.215273
26203	0.946106	0.213787
26204	0.946133	2.1
26205	0.946132	2.1
26206	0.946133	2.1
26207	0.946132	2.1
26208	1.39621	2.1
26209	1.34352	2.1
26210	0.946132	2.1
26211	0.946132	2.1
26212	0.946132	2.1
26213	0.946132	2.1

26214	0.946132	1.32611
26215	0.946128	2.07431
26216	0.94613	1.71613
26217	0.94613	1.9515
26218	1.41273	2.02677
26219	1.23536	2.1
26220	0.946131	2.08055
26221	0.946132	2.1
26222	0.946132	2.1
26223	0.946132	2.1
26224	0.946132	2.1
26225	1.41131	2.1
26226	0.946131	2.1
26227	0.946131	2.1
26228	0.946131	2.1
26229	0.946133	0.548331
26230	0.946132	2.06048
26231	0.946133	0.832542
26232	1.41894	0.610011
26233	0.946132	1.01325
26234	0.946128	1.79276
26235	1.4099	2.1
26236	0.946131	1.98882
26237	0.946131	2.01297
26238	0.946131	1.8068
26239	0.810947	2.1
26240	0.810945	2.1
26241	0.810933	2.1
26242	0.946131	0.75252
26243	1.25765	2.06399
26244	0.946128	1.16826
26245	0.946129	1.29145
26246	1.25765	1.62725
26247	1.21347	2.1
26248	0.946129	2.1
26249	0.94613	2.1
26250	1.21347	2.1
26251	0.94613	2.1
26252	0.94613	0.0698591
26253	0.946132	0.0698591
26254	1.2493	0.0698591
26255	1.2493	0.0698591
26256	0.94613	0.0698591
26257	0.946132	0.742685
26258	0.946128	1.22879
26259	0.946131	0.567802
26260	0.94613	0.967715
26261	1.37938	0.787963
26262	0.946133	0.0698591
26263	0.946132	0.0698591
26264	0.946133	0.0698591
26265	0.946132	0.0698591
26266	0.946133	0.0698591
26267	0.946129	0.428222
26268	1.26426	0.296535
26269	0.946131	0.449767
26270	0.946131	0.421116
26271	1.26426	0.3278

26272	0.872447	1.87417
26273	0.946132	1.59602
26274	0.946132	1.93774
26275	0.946132	1.87035
26276	0.946133	1.75322
26277	0.946117	1.25487
26278	0.946116	0.832607
26279	0.946113	1.11659
26280	0.946129	1.07201
26281	1.27211	0.755794
26282	0.94613	0.825527
26283	0.946129	1.86067
26284	1.40029	1.40007
26285	1.40028	1.5799
26286	0.94613	1.58099
26287	0.810942	2.1
26288	0.810943	2.1
26289	0.810932	2.1
26290	1.29521	2.1
26291	0.946129	2.1
26292	0.946131	2.1
26293	1.29521	2.1
26294	1.29521	2.1
26295	0.946132	0.511789
26296	0.946129	0.314178
26297	1.24596	0.286813
26298	0.946131	0.525878
26299	0.946131	0.398169
26300	0.946131	2.1
26301	1.35616	2.1
26302	0.946133	2.1
26303	0.94613	2.1
26304	1.35616	2.1
26305	0.879548	0.235557
26306	1.01272	0.105171
26307	0.946133	0.392699
26308	0.946133	0.13353
26309	0.946132	0.161003
26310	0.946132	0.415765
26311	0.918009	0.220401
26312	0.91801	0.486998
26313	0.946132	0.264612
26314	0.946132	0.358587
26315	0.946132	0.0698591
26316	1.28644	0.0698591
26317	0.946131	0.0704721
26318	0.946131	0.0698591
26319	0.946132	0.0698591
26320	0.946131	0.0698591
26321	1.40103	0.0698591
26322	0.946131	0.0707832
26323	0.946131	0.0698639
26324	0.946131	0.0698591
26325	0.946128	0.114538
26326	1.03327	0.0872177
26327	0.946129	0.134949
26328	0.946131	0.0876571
26329	0.94613	0.107264

26330	0.946132	0.60633
26331	0.946133	0.448258
26332	1.40265	0.311851
26333	1.40265	0.296239
26334	0.946133	0.213486
26335	0.946131	0.0698591
26336	0.946131	0.0698591
26337	0.946131	0.0698591
26338	0.856655	0.0698591
26339	0.856616	0.0698591
26340	0.946132	0.178649
26341	0.946132	0.227778
26342	0.946132	0.414115
26343	1.15844	0.140244
26344	0.946131	0.350563
26345	0.946131	2.1
26346	1.4031	2.1
26347	1.40304	2.1
26348	0.851554	2.1
26349	0.946131	0.0698591
26350	0.94613	0.07161
26351	1.24417	0.0698591
26352	1.24416	0.0698591
26353	0.946131	0.0698591
26354	0.946132	0.808006
26355	1.20992	0.847693
26356	0.946132	0.988226
26357	0.946132	0.862809
26358	1.20992	0.934801
26359	0.946128	1.88348
26360	0.94613	2.06748
26361	0.94613	1.89951
26362	0.94613	1.99453
26363	1.23426	2.01475
26364	0.810943	0.526199
26365	0.810946	0.137196
26366	0.810933	0.0878291
26367	0.810945	2.1
26368	0.810945	2.07418
26369	0.810932	2.1
26370	1.25104	2.1
26371	0.946131	2.1
26372	0.946131	2.01744
26373	1.25104	2.1
26374	1.25104	2.06804
26375	1.25488	2.05639
26376	0.946132	2.06754
26377	0.946132	2.0514
26378	0.946132	2.08062
26379	0.946131	2.08474
26380	0.946131	0.069998
26381	0.946126	0.0826394
26382	0.946128	0.0699649
26383	1.32131	0.0708539
26384	0.946128	0.0745482
26385	0.946133	2.1
26386	0.946132	2.03309
26387	0.946132	2.1

26388	0.946133	2.1
26389	1.41123	2.1
26390	0.946132	0.0698591
26391	0.946133	0.0698591
26392	0.946132	0.0739004
26393	1.32163	0.0698591
26394	0.946133	0.0724827
26395	0.946132	2.1
26396	0.946132	2.1
26397	1.38651	2.1
26398	1.38651	2.1
26399	0.946132	2.1
26400	0.94613	0.127711
26401	1.3639	0.117874
26402	0.946128	0.147739
26403	0.946131	0.121511
26404	1.3639	0.113094
26405	0.946133	1.43132
26406	1.40724	0.760551
26407	0.946132	0.622817
26408	0.946133	0.946117
26409	1.40724	0.870949
26410	1.21099	1.40901
26411	0.946128	1.80292
26412	0.946127	2.00009
26413	0.946128	1.92224
26414	0.946129	1.71714
26415	0.946132	0.312948
26416	1.35946	0.252308
26417	0.946131	0.606491
26418	0.946131	0.390749
26419	1.35946	0.280781
26420	1.34794	0.673692
26421	0.517994	1.64857
26422	0.946132	0.848167
26423	0.946132	0.945614
26424	0.517965	1.2039
26425	1.23224	0.85805
26426	0.94613	1.92811
26427	0.946131	1.62644
26428	0.946131	1.29065
26429	0.946131	1.22443
26430	1.37511	1.25384
26431	0.946132	1.51534
26432	0.946133	1.9122
26433	0.946133	1.81589
26434	0.946132	1.51557
26435	0.851106	0.371957
26436	0.946129	0.555407
26437	0.946131	0.286718
26438	0.946131	1.64922
26439	0.851066	0.548202
26440	0.946131	0.114614
26441	0.946131	0.144412
26442	0.94613	0.150235
26443	1.31357	0.111329
26444	0.946131	0.110489
26445	0.946133	0.851129

26446	0.946132	1.14479
26447	0.946133	0.54753
26448	0.946132	0.868616
26449	1.38149	0.66564
26450	0.810944	1.49264
26451	0.810945	1.95498
26452	0.810958	2.09242
26453	0.946118	0.0698591
26454	0.946118	0.0698591
26455	1.37835	0.0698591
26456	1.37837	0.0698591
26457	0.946106	0.0698591
26458	0.946128	0.0698591
26459	0.946131	0.0698591
26460	1.34831	0.0698591
26461	1.34831	0.0698591
26462	0.94613	0.0698591
26463	0.946132	0.074106
26464	0.946132	0.119316
26465	0.909981	0.0766785
26466	1.00099	0.0727645
26467	0.946131	0.0948551
26468	0.946132	0.436599
26469	0.946133	1.2813
26470	0.946133	0.57497
26471	0.946132	0.932658
26472	1.35328	0.888064
26473	0.946132	0.0698591
26474	0.946132	0.0698591
26475	1.35386	0.0698591
26476	1.35385	0.0698591
26477	0.946133	0.0698591
26478	0.946121	0.0698591
26479	0.946132	0.0698591
26480	0.900167	0.0698591
26481	1.01189	0.0698591
26482	0.946132	0.0698591
26483	0.946132	2.07357
26484	1.41585	1.69986
26485	0.946131	2.08443
26486	0.946131	1.75509
26487	0.94613	2.07447
26488	0.810946	2.1
26489	0.810946	2.1
26490	0.810933	2.1
26491	1.34447	2.1
26492	0.946131	2.1
26493	0.946131	2.1
26494	1.34447	2.1
26495	1.19886	2.1
26496	0.946132	0.0797785
26497	0.946132	0.0698591
26498	0.946132	0.0698591
26499	1.35065	0.0698591
26500	0.946132	0.073512
26501	0.946131	0.236033
26502	1.3022	0.199767
26503	0.94613	0.398126

26504	0.946131	0.303621
26505	1.3022	0.220577
26506	0.946127	0.162103
26507	1.38683	0.143454
26508	0.946127	0.192133
26509	0.946128	0.179339
26510	1.38683	0.171012
26511	0.810946	0.0698591
26512	0.810947	0.0698591
26513	0.810959	0.0698591
26514	0.946132	0.0698591
26515	0.946131	0.0698591
26516	0.946132	0.0698591
26517	0.946132	0.0698591
26518	0.946132	0.0698591
26519	0.946127	1.29586
26520	1.35968	1.96691
26521	0.946131	1.09958
26522	0.946129	1.10729
26523	1.35969	1.76085
26524	0.946133	2.03609
26525	1.35862	1.44611
26526	0.946132	2.05501
26527	0.946132	1.66227
26528	1.35862	1.72545
26529	0.946132	0.704053
26530	0.946132	0.203637
26531	0.946132	0.88193
26532	0.946131	0.232539
26533	0.946132	0.345051
26534	0.946131	0.173805
26535	0.946131	0.119168
26536	0.946128	0.195702
26537	0.946129	0.0880665
26538	1.41232	0.0947557
26539	0.946128	2.0958
26540	1.29331	2.1
26541	0.946131	2.09649
26542	0.946131	2.1
26543	1.29331	2.1
26544	0.946133	0.54194
26545	0.946132	0.602429
26546	0.946132	0.541685
26547	0.946132	0.483594
26548	1.41556	0.422921
26549	0.94612	0.49563
26550	0.946119	0.460845
26551	0.946119	0.501747
26552	0.946132	0.403775
26553	0.946107	0.399344
26554	0.946129	0.0698591
26555	1.1509	0.0698591
26556	0.946126	0.0698591
26557	0.946129	0.0698591
26558	0.94613	0.0698591
26559	0.946131	0.0698591
26560	1.19263	0.0698591
26561	0.946132	0.0698591

26562	0.946132	0.0698591
26563	0.946132	0.0698591
26564	0.94613	0.652954
26565	1.36939	1.07964
26566	0.946128	0.914775
26567	0.946131	0.788051
26568	1.36939	0.890137
26569	0.946133	1.1618
26570	0.827865	1.55048
26571	0.946132	1.20182
26572	0.946132	1.458
26573	1.40398	1.3702
26574	0.946127	0.925246
26575	0.946127	2.08527
26576	0.94613	0.687683
26577	1.41498	1.86456
26578	0.946128	1.30232
26579	0.94613	0.0698591
26580	0.946131	0.0698591
26581	0.946129	0.0698591
26582	0.94613	0.0698591
26583	0.94613	0.0698591
26584	0.946118	0.0698591
26585	0.946119	0.0698591
26586	0.946116	0.0698591
26587	1.36569	0.0698591
26588	0.946105	0.0698591
26589	0.946132	0.211137
26590	1.23175	0.145657
26591	0.94613	0.224743
26592	0.946133	0.134322
26593	0.946131	0.160142
26594	1.25698	0.0698591
26595	0.946126	0.0710077
26596	0.946129	0.0698591
26597	0.946128	0.0698591
26598	0.946129	0.0698591
26599	0.810943	2.1
26600	0.810944	2.1
26601	0.810932	2.1
26602	0.946132	0.11589
26603	1.09841	0.0901696
26604	0.946131	0.146131
26605	0.946132	0.125983
26606	1.09841	0.102363
26607	0.810936	1.79517
26608	0.810935	2.05985
26609	0.810934	2.1
26610	1.23851	2.1
26611	0.946132	1.9994
26612	0.946131	2.1
26613	1.23851	2.1
26614	1.23851	2.1
26615	0.946132	0.10468
26616	0.946131	0.329771
26617	0.946132	0.11957
26618	0.946132	0.198693
26619	0.946132	0.400735

26620	0.939141	1.16534
26621	0.953118	1.17752
26622	0.946126	1.05386
26623	0.946129	1.06252
26624	1.03306	1.0833
26625	0.946132	0.107426
26626	1.30927	0.123916
26627	0.946132	0.137446
26628	0.946132	0.112709
26629	1.30928	0.115695
26630	0.94613	2.1
26631	0.946131	2.1
26632	1.24511	2.1
26633	0.94613	2.1
26634	0.94613	2.1
26635	0.946133	0.116134
26636	1.34152	0.0792407
26637	0.946131	0.0852086
26638	0.946131	0.0889335
26639	1.34152	0.0799059
26640	0.946132	0.0872536
26641	1.41383	0.0849503
26642	0.946132	0.0914464
26643	0.946132	0.0716295
26644	1.41383	0.0717655
26645	0.946132	0.654039
26646	0.946131	0.496574
26647	1.41392	0.379676
26648	0.946132	0.661574
26649	0.946132	0.511329
26650	0.946132	0.926403
26651	0.946132	0.762822
26652	1.24333	0.635406
26653	0.946133	1.00501
26654	0.946133	0.826722
26655	0.946124	0.188153
26656	0.94613	0.222702
26657	0.946126	0.214697
26658	1.3065	0.169637
26659	0.946127	0.215922
26660	0.810946	0.277007
26661	0.810943	0.199435
26662	0.81096	0.264252
26663	0.946131	0.0785323
26664	1.40271	0.079462
26665	0.946131	0.104787
26666	0.946131	0.0905512
26667	1.40271	0.0836792
26668	0.946132	0.0698591
26669	0.94613	0.0698591
26670	0.946131	0.0698591
26671	0.94613	0.0698591
26672	0.946131	0.0698591
26673	0.946133	0.171283
26674	1.30107	0.0852643
26675	0.946132	0.219393
26676	0.946132	0.13218
26677	0.946133	0.196692

26678	0.810946	2.1
26679	0.810944	2.1
26680	0.810958	2.1
26681	0.946133	1.96247
26682	0.946131	1.95157
26683	0.946131	2.1
26684	1.30812	2.06383
26685	0.946131	2.02637
26686	0.94613	1.14241
26687	0.975002	1.46025
26688	0.917259	2.03185
26689	1.00902	1.79226
26690	0.94613	1.14742
26691	0.946131	2.1
26692	0.946127	2.1
26693	1.398 2.1	
26694	0.946129	2.1
26695	0.946129	2.1
26696	0.946133	2.1
26697	0.946132	2.1
26698	0.946132	2.1
26699	0.946132	2.1
26700	1.3033	2.1
26701	1.09897	0.28886
26702	0.946131	0.593319
26703	0.946132	0.464904
26704	0.946129	0.502376
26705	0.946131	0.509966
26706	0.946131	2.1
26707	0.946132	2.1
26708	1.39858	2.1
26709	0.946131	2.1
26710	0.946131	2.1
26711	0.946119	0.383526
26712	1.08878	0.412627
26713	0.946119	1.22993
26714	1.0888	0.417031
26715	0.946107	1.03039
26716	0.946132	0.0698591
26717	1.10733	0.0698591
26718	0.946132	0.0711394
26719	0.946131	0.0700959
26720	0.946132	0.0699212
26721	0.946128	0.069884
26722	1.33671	0.0698591
26723	0.94613	0.0738584
26724	0.94613	0.0717953
26725	0.94613	0.0710456
26726	0.810943	2.1
26727	0.810944	2.1
26728	0.810945	2.1
26729	1.35117	2.1
26730	0.946129	2.1
26731	0.94613	2.1
26732	1.35117	2.1
26733	1.27174	2.1
26734	0.946132	2.0769
26735	0.946133	2.03966

26736	0.997296	1.81643
26737	0.946133	2.00663
26738	0.946132	1.95839
26739	0.810943	2.1
26740	0.810944	2.1
26741	0.810945	2.1
26742	0.810946	1.5447
26743	0.810943	1.97395
26744	0.810933	1.54079
26745	0.946127	1.60701
26746	0.946131	1.55147
26747	0.946128	1.57827
26748	0.94613	1.68921
26749	0.946129	1.6845
26750	0.946131	0.306867
26751	0.946131	0.239644
26752	0.946129	0.315955
26753	0.946131	0.228819
26754	0.94613	0.236979
26755	0.946133	0.0855635
26756	0.946132	0.0818641
26757	0.946132	0.0864992
26758	0.946132	0.0803376
26759	0.946133	0.0872498
26760	0.946131	1.39959
26761	0.94613	0.769293
26762	0.946129	1.4477
26763	0.946131	1.08373
26764	0.946131	1.42277
26765	0.946132	2.01463
26766	0.946129	2.1
26767	0.94613	2.09954
26768	0.946131	2.05252
26769	1.39356	2.09351
26770	0.810946	2.1
26771	0.810947	2.1
26772	0.810934	2.1
26773	0.946125	0.0698591
26774	0.946127	0.070253
26775	1.33798	0.0698591
26776	1.33797	0.0698591
26777	0.946128	0.0698591
26778	0.946131	0.0698591
26779	0.946131	0.0698591
26780	1.23146	0.0698591
26781	1.23146	0.0698591
26782	0.946131	0.0698591
26783	0.946129	2.1
26784	0.94613	2.1
26785	0.946131	2.1
26786	0.94613	2.1
26787	1.30082	2.1
26788	0.946131	0.0698591
26789	0.946132	0.0698591
26790	0.946131	0.0713906
26791	1.20416	0.0698591
26792	0.946132	0.0698591
26793	0.946132	0.0698591

26794	0.946131	0.0698591
26795	0.946131	0.0726522
26796	1.18711	0.0698591
26797	0.946132	0.0698591
26798	0.946132	0.0698591
26799	0.946132	0.0698591
26800	0.946133	0.0698591
26801	0.946132	0.0698591
26802	0.946133	0.0698591
26803	0.946131	0.0698591
26804	0.946132	0.0698591
26805	0.94613	0.0698591
26806	0.946131	0.0698591
26807	0.946131	0.0698591
26808	0.946133	0.0698591
26809	0.946132	0.0698591
26810	0.946133	0.0698591
26811	0.946132	0.0698591
26812	0.946132	0.0698591
26813	0.946132	2.1
26814	0.946132	2.1
26815	0.946132	2.1
26816	1.36422	2.1
26817	0.946132	2.1
26818	0.946131	0.770844
26819	0.946132	1.64446
26820	0.946132	1.1187
26821	0.946132	1.29334
26822	1.4185	1.47454
26823	0.946133	0.0698591
26824	1.37794	0.0698591
26825	0.946132	0.0698591
26826	0.946133	0.0698591
26827	0.946132	0.0698591
26828	1.4045	2.1
26829	0.946132	2.1
26830	0.946132	2.1
26831	0.946132	2.1
26832	0.946132	2.1
26833	0.946131	2.1
26834	0.946132	2.1
26835	1.34285	2.1
26836	1.34285	2.1
26837	0.946132	2.1
26838	0.946131	2.1
26839	0.946132	2.1
26840	1.40739	2.1
26841	1.40739	2.1
26842	0.946131	2.1
26843	0.946128	2.1
26844	0.946132	2.1
26845	0.94613	2.1
26846	0.94613	2.1
26847	1.3164	2.1
26848	0.946132	2.1
26849	1.3484	2.1
26850	0.946132	2.1
26851	0.946132	2.1

26852	1.3484	2.1
26853	0.946132	2.1
26854	0.946132	2.1
26855	0.946133	2.1
26856	0.946133	2.1
26857	1.41507	2.1
26858	0.946132	2.1
26859	1.32608	2.1
26860	0.946131	2.1
26861	0.946131	2.1
26862	0.946132	2.1
26863	0.94613	0.852256
26864	1.34646	1.87056
26865	0.946129	0.930563
26866	0.94613	0.743457
26867	1.34646	1.15761
26868	0.946131	0.992415
26869	0.946131	2.1
26870	0.94613	1.94554
26871	1.33728	1.46067
26872	0.946131	1.85228
26873	0.946127	2.1
26874	0.946131	1.87255
26875	0.946128	2.1
26876	0.946129	2.1
26877	1.4064	2.09984
26878	0.946133	2.1
26879	0.946131	2.1
26880	0.946131	2.1
26881	0.946131	2.1
26882	0.94613	2.1
26883	1.34912	2.1
26884	0.946131	2.1
26885	0.946129	2.1
26886	0.946131	2.1
26887	0.94613	2.1
26888	0.946132	2.1
26889	1.40447	2.1
26890	0.946132	2.1
26891	0.946133	2.1
26892	1.40447	2.1
26893	1.33607	2.1
26894	0.946131	2.1
26895	0.946127	2.1
26896	0.94613	2.1
26897	0.946129	2.1
26898	0.946117	0.493231
26899	1.32664	1.50739
26900	0.946115	1.1356
26901	0.946105	1.11517
26902	0.94613	0.673946
26903	0.946118	2.1
26904	0.946114	2.1
26905	1.35886	2.1
26906	1.35884	2.1
26907	0.946129	2.1
26908	0.946132	2.1
26909	1.2571	2.1

26910	0.946132	2.1
26911	0.946132	2.1
26912	1.2571	2.1
26913	0.946133	2.1
26914	1.35663	2.1
26915	0.946132	2.1
26916	0.946132	2.1
26917	1.35663	2.1
26918	0.946133	0.962813
26919	0.946129	1.86088
26920	0.946131	1.51106
26921	0.946131	1.57264
26922	1.30881	1.81907
26923	0.946132	2.1
26924	0.94613	2.1
26925	1.34652	2.1
26926	0.94613	2.1
26927	0.94613	2.1
26928	0.946132	2.03287
26929	0.946133	1.85533
26930	1.36183	1.56131
26931	1.36183	1.73089
26932	0.946132	1.8993
26933	0.946132	0.202085
26934	1.38353	0.0953567
26935	0.94613	0.101428
26936	0.94613	0.146269
26937	1.38354	0.122008
26938	0.946128	0.507394
26939	1.24887	0.436154
26940	0.94613	0.62125
26941	0.94613	0.538157
26942	1.24887	0.562329
26943	0.946129	0.828114
26944	0.946126	1.36683
26945	0.946124	0.85527
26946	0.946128	1.01232
26947	1.32056	1.1029
26948	0.946129	0.990592
26949	0.946128	1.25887
26950	0.946126	0.915325
26951	0.946129	1.06169
26952	1.20351	1.06594
26953	0.946131	1.96033
26954	0.946131	1.96856
26955	0.946127	2.08845
26956	1.25591	2.04078
26957	0.94613	1.87447
26958	0.946132	1.69752
26959	0.946133	1.52057
26960	0.946132	1.96171
26961	1.15436	1.42109
26962	0.930306	1.38871
26963	0.946132	2.08231
26964	0.946132	2.08468
26965	0.946132	2.0074
26966	0.946133	2.07799
26967	0.946132	2.06343

26968	0.946129	1.40528
26969	0.946132	0.899612
26970	0.946126	1.70085
26971	1.13245	0.633214
26972	0.946129	0.992792
26973	0.94613	2.05588
26974	0.946132	2.04651
26975	0.946129	1.94204
26976	0.946131	2.05503
26977	0.946131	1.99711
26978	1.41279	2.017
26979	1.76254	2.0557
26980	0.600454	1.96276
26981	0.858991	1.97135
26982	0.940722	1.99224
26983	1.28439	1.71682
26984	0.946131	1.91786
26985	0.94613	1.88777
26986	0.946131	2.00494
26987	0.946131	1.97195
26988	0.946132	0.865144
26989	1.39335	0.329214
26990	0.946132	0.910009
26991	0.946132	0.475331
26992	0.946132	0.76185
26993	1.33879	0.141331
26994	0.946131	0.275515
26995	0.94613	0.468137
26996	1.33879	0.140729
26997	0.946131	0.270685
26998	0.946126	0.271408
26999	0.946131	0.19676
27000	1.37342	0.15202
27001	1.37343	0.120478
27002	0.946128	0.412994
27003	1.39168	0.0698591
27004	0.946131	0.0698591
27005	0.946131	0.070042
27006	0.946131	0.0698591
27007	0.946131	0.0698591
27008	0.946131	0.0953613
27009	1.412	0.0790967
27010	0.946127	0.0732388
27011	0.946128	0.0766567
27012	0.946128	0.0730626
27013	1.1387	0.181058
27014	0.943364	0.388055
27015	0.946132	0.273908
27016	0.946132	0.302241
27017	0.946133	0.306641
27018	1.21085	0.348755
27019	0.88324	0.70824
27020	0.946129	0.570791
27021	0.946131	0.561471
27022	0.946131	0.498254
27023	1.33587	0.0698591
27024	0.946125	0.0717739
27025	0.946129	0.0698591

27026	0.946126	0.070772
27027	0.946127	0.0698591
27028	1.27512	0.0707469
27029	0.946132	0.0965565
27030	0.946132	0.0926541
27031	0.946133	0.0848509
27032	0.946132	0.0737127
27033	0.946128	0.0747591
27034	1.30498	0.0713889
27035	0.946131	0.0879376
27036	0.94613	0.0789319
27037	1.30497	0.0741577
27038	0.946131	0.0708803
27039	1.25626	0.0698591
27040	0.946131	0.0762095
27041	0.946131	0.0725435
27042	1.25626	0.0705849
27043	0.946131	0.0698591
27044	0.94613	0.0698591
27045	1.35316	0.0698591
27046	0.946131	0.0698591
27047	0.946131	0.0698591
27048	0.94613	0.146873
27049	1.28727	0.156382
27050	0.946128	0.0926936
27051	0.946131	0.128658
27052	0.94613	0.102576
27053	0.946131	0.0698591
27054	1.39502	0.0698591
27055	0.946132	0.0698591
27056	0.946132	0.0698591
27057	0.946132	0.0698591
27058	1.36173	0.234897
27059	0.946131	0.487591
27060	0.946126	0.408423
27061	0.946127	0.404323
27062	0.946128	0.342016
27063	0.946129	0.599243
27064	0.946133	0.44814
27065	0.946131	0.629376
27066	0.946131	0.558201
27067	0.946131	0.621683
27068	0.946128	0.0698591
27069	0.946132	0.0698591
27070	1.24678	0.0698591
27071	0.94613	0.0698591
27072	0.94613	0.0698591
27073	0.946129	0.0698591
27074	0.94613	0.0698591
27075	1.25215	0.0698591
27076	0.946126	0.0698591
27077	0.946129	0.0698591
27078	0.946129	0.402826
27079	1.29689	0.336639
27080	0.946131	0.398267
27081	0.946131	0.335208
27082	0.946131	0.308576
27083	0.946128	0.0715359

27084	0.94613	0.0722577
27085	0.946131	0.0709298
27086	0.94613	0.072418
27087	0.946131	0.0728742
27088	0.946127	0.0798403
27089	0.946128	0.0841138
27090	0.94613	0.0788838
27091	0.946129	0.0846314
27092	0.946129	0.0849067
27093	0.946133	0.0698591
27094	0.946132	0.0698591
27095	0.946132	0.0698591
27096	1.29957	0.0698591
27097	0.946133	0.0698591
27098	0.946132	0.0698591
27099	0.946133	0.0698591
27100	0.946132	0.0698591
27101	1.26201	0.0698591
27102	0.946132	0.0698591
27103	0.946133	0.0702739
27104	1.40427	0.0698591
27105	0.946132	0.0764249
27106	0.946132	0.0741426
27107	0.946133	0.0712868
27108	0.946132	0.0701067
27109	1.35123	0.0698591
27110	0.946132	0.0773036
27111	0.946132	0.0753328
27112	0.946131	0.0704233
27113	0.946128	0.0999608
27114	0.946129	0.106086
27115	0.946131	0.0934652
27116	0.94613	0.109527
27117	0.946131	0.104996
27118	0.946127	0.287292
27119	1.23351	0.230477
27120	0.946131	0.281741
27121	0.946129	0.245169
27122	0.94613	0.234028
27123	0.946131	0.121901
27124	0.946131	0.175053
27125	0.94613	0.129122
27126	1.18119	0.11568
27127	0.946131	0.14877
27128	0.946132	0.169396
27129	1.30563	0.13364
27130	0.946133	0.192796
27131	0.946132	0.140593
27132	0.946133	0.167405
27133	0.946132	0.371741
27134	0.946132	0.731078
27135	0.946133	1.74065
27136	1.20256	0.459832
27137	0.946133	0.589576
27138	0.94613	1.01962
27139	1.33007	0.596573
27140	0.946128	1.02083
27141	0.946132	0.587002

27142	0.946131	0.718878
27143	0.946131	2.1
27144	0.946131	2.1
27145	0.946128	2.1
27146	0.946129	2.1
27147	0.94613	2.1
27148	0.810942	2.1
27149	0.810942	2.1
27150	0.81093	2.1
27151	0.946132	2.1
27152	1.39823	2.1
27153	0.946132	2.1
27154	0.946131	2.1
27155	0.946132	2.1
27156	0.946131	2.1
27157	0.946131	2.1
27158	0.946131	2.1
27159	1.37713	2.1
27160	0.946131	2.1
27161	0.946133	2.1
27162	0.946131	2.1
27163	1.40594	2.1
27164	1.40594	2.1
27165	0.94613	2.1
27166	0.946133	2.1
27167	0.946131	2.1
27168	1.38155	2.1
27169	1.38155	2.1
27170	0.946131	2.1
27171	0.946132	1.23964
27172	0.946133	1.98497
27173	0.946132	1.51039
27174	0.946132	1.66422
27175	1.33694	1.82138
27176	0.946131	2.1
27177	1.228 2.1	
27178	0.946129	2.1
27179	0.946131	2.1
27180	1.228 2.1	
27181	0.946132	0.981461
27182	0.946132	1.74191
27183	0.946131	1.8585
27184	0.946132	1.43917
27185	1.35943	1.83224
27186	0.946133	0.0698591
27187	0.946133	0.0698591
27188	1.02377	0.0698591
27189	1.02377	0.0698591
27190	0.946132	0.0698591
27191	0.94613	0.0698591
27192	0.946131	0.0698591
27193	0.946132	0.0698591
27194	1.29322	0.0698591
27195	0.946131	0.0698591
27196	0.946131	2.1
27197	0.946131	1.24378
27198	0.946132	2.1
27199	1.41492	2.00758

27200	0.946131	2.1
27201	0.946132	2.1
27202	0.946131	2.1
27203	0.946132	2.1
27204	1.35028	2.1
27205	0.946132	2.1
27206	0.946132	2.1
27207	0.946132	2.09608
27208	0.946131	2.09676
27209	1.32461	2.09992
27210	0.946132	2.09276
27211	0.946131	2.1
27212	0.946132	2.1
27213	1.40369	2.1
27214	0.94613	2.1
27215	0.946131	2.1
27216	0.946132	0.0858823
27217	0.979597	0.0748138
27218	0.946131	0.106189
27219	0.946132	0.0749931
27220	0.946132	0.0826746
27221	0.946131	2.1
27222	0.946131	2.1
27223	0.94613	2.1
27224	0.946131	2.1
27225	1.41897	2.1
27226	1.37791	2.1
27227	0.94613	2.1
27228	0.946129	2.1
27229	0.946131	2.1
27230	0.946131	2.1
27231	1.39384	2.1
27232	0.946131	2.1
27233	0.946128	2.1
27234	0.946129	2.1
27235	0.946129	2.1
27236	0.810945	1.6516
27237	0.810944	2.1
27238	0.810958	1.35159
27239	0.946128	2.1
27240	0.946132	2.1
27241	0.94613	2.1
27242	1.3335	2.1
27243	0.946131	2.1
27244	0.94613	2.05401
27245	0.946129	2.1
27246	0.946132	1.79714
27247	1.41192	2.1
27248	0.94613	2.08688
27249	0.946132	0.826425
27250	0.946133	1.44855
27251	0.946132	0.355963
27252	1.39428	0.643898
27253	0.946133	0.303601
27254	0.94613	2.05988
27255	0.946128	2.06592
27256	0.946132	2.00689
27257	0.94613	1.99658

27258	1.40684	2.1
27259	0.946131	2.1
27260	0.946131	2.1
27261	0.946131	2.1
27262	0.946131	2.1
27263	1.39021	2.1
27264	0.946131	0.75695
27265	1.31649	1.84186
27266	0.946131	0.577766
27267	0.946131	0.511506
27268	1.31649	0.920358
27269	0.810946	2.1
27270	0.810946	2.1
27271	0.810958	2.1
27272	1.16687	2.1
27273	0.946131	2.1
27274	0.946131	2.1
27275	1.16687	2.1
27276	1.16687	2.1
27277	0.946128	0.494965
27278	1.3564	0.430444
27279	0.946131	0.495843
27280	0.946131	0.448189
27281	0.94613	0.461051
27282	0.946128	2.1
27283	1.35828	2.1
27284	0.94613	2.1
27285	0.946131	2.1
27286	0.94613	2.1
27287	0.946133	0.98811
27288	1.36477	1.32674
27289	0.946132	1.29379
27290	0.946132	1.04567
27291	1.36477	1.21727
27292	0.946132	1.25466
27293	1.31857	1.90555
27294	0.946132	1.4646
27295	0.946132	1.69884
27296	1.31857	1.43959
27297	0.94613	0.572772
27298	1.35059	0.385108
27299	0.946128	0.386509
27300	0.94613	0.457773
27301	1.35059	0.421559
27302	0.946126	0.174645
27303	1.35559	0.125882
27304	0.946127	0.132804
27305	0.946128	0.145777
27306	1.35559	0.128663
27307	0.946131	0.0813224
27308	1.33035	0.0763919
27309	0.946127	0.0846592
27310	0.946128	0.0702603
27311	1.33035	0.0765164
27312	0.94613	0.0780693
27313	1.3665	0.081797
27314	0.946129	0.0904487
27315	0.946131	0.0698591

27316	1.3665	0.0818849
27317	0.94613	0.0936523
27318	1.32828	0.0783024
27319	0.946129	0.0883519
27320	0.94613	0.0815546
27321	0.946131	0.0796734
27322	0.946131	2.1
27323	0.946131	2.1
27324	1.32949	2.1
27325	1.32949	2.1
27326	0.946131	2.1
27327	0.946131	2.1
27328	0.94613	0.219102
27329	0.946133	2.08246
27330	1.38357	0.987589
27331	0.946131	1.43317
27332	0.946131	2.1
27333	0.946128	2.1
27334	1.36919	2.1
27335	0.946131	2.1
27336	0.94613	2.1
27337	0.946131	2.1
27338	1.3771	2.1
27339	0.946129	2.1
27340	0.94613	2.1
27341	0.946131	2.1
27342	0.946131	1.88817
27343	0.94613	0.513456
27344	0.946129	0.537559
27345	1.30029	1.04125
27346	0.94613	0.79643
27347	1.21174	0.0698591
27348	0.946132	0.0698591
27349	0.946132	0.0698591
27350	0.946132	0.0698591
27351	0.946133	0.0698591
27352	0.810946	2.1
27353	0.810945	2.1
27354	0.810946	2.1
27355	1.1796	2.1
27356	0.946131	2.1
27357	0.946132	2.1
27358	1.1796	2.1
27359	1.1796	2.1
27360	0.946131	2.1
27361	0.946131	2.1
27362	0.946133	2.1
27363	0.946131	2.1
27364	1.40615	2.1
27365	0.810945	2.1
27366	0.810944	2.1
27367	0.810945	2.1
27368	1.40239	2.1
27369	0.94613	2.1
27370	1.35561	2.1
27371	0.946133	2.1
27372	1.35562	2.1
27373	1.41169	0.161168

27374	0.946131	0.327975
27375	0.946127	0.452714
27376	1.41169	0.167982
27377	0.946128	0.324336
27378	0.946119	2.1
27379	0.946119	2.1
27380	0.946119	2.1
27381	0.946107	2.1
27382	1.41917	2.1
27383	0.810946	1.85852
27384	0.810943	1.92722
27385	0.810934	2.04096
27386	0.946127	2.1
27387	1.1995	2.1
27388	0.946126	2.1
27389	0.946128	2.1
27390	1.1995	2.1
27391	0.810944	2.1
27392	0.810947	2.1
27393	0.81096	2.1
27394	0.946133	0.121457
27395	1.3495	0.0797948
27396	0.946132	0.0936645
27397	0.946132	0.0808496
27398	0.946133	0.0867557
27399	0.946132	2.1
27400	0.946132	2.1
27401	0.946133	2.1
27402	1.39529	2.1
27403	0.946133	2.1
27404	0.946133	2.1
27405	1.28525	2.1
27406	0.946132	2.1
27407	0.946133	2.1
27408	1.28525	2.1
27409	0.810946	2.1
27410	0.810945	2.1
27411	0.810946	2.1
27412	1.33931	2.1
27413	0.946132	2.1
27414	0.946132	2.1
27415	1.33931	2.1
27416	1.24616	2.1
27417	0.946133	0.096988
27418	0.946133	0.0775097
27419	0.946132	0.101849
27420	0.946132	0.0755964
27421	0.946133	0.0907539
27422	0.946128	2.1
27423	1.40225	2.1
27424	0.946128	2.1
27425	0.94613	2.1
27426	0.94613	2.1
27427	0.946113	0.0698591
27428	0.946118	0.0698591
27429	0.946114	0.0698591
27430	1.40551	0.0698591
27431	0.946103	0.0698591

27432	0.94613	2.1
27433	0.946132	2.1
27434	0.946129	2.1
27435	0.946131	2.1
27436	1.37811	2.1
27437	0.946132	2.1
27438	1.39826	2.1
27439	0.946133	2.1
27440	0.946132	2.1
27441	1.39826	2.1
27442	0.946118	2.1
27443	1.28298	2.1
27444	0.946117	2.1
27445	0.946105	2.1
27446	1.283 2.1	
27447	0.946118	0.0698591
27448	1.41375	0.0698591
27449	0.946117	0.0698591
27450	0.946131	0.0698591
27451	0.946106	0.0698591
27452	0.946132	1.13455
27453	0.946129	0.811471
27454	1.28436	0.763483
27455	0.946131	1.16277
27456	0.946131	0.916081
27457	0.810947	0.363531
27458	0.810946	0.366065
27459	0.81096	0.313455
27460	0.946132	0.243251
27461	1.34338	0.206755
27462	0.961435	0.180447
27463	0.961435	0.204455
27464	0.946132	0.216877
27465	1.3028	2.00907
27466	0.946131	2.02688
27467	0.946127	2.07697
27468	0.946128	2.08162
27469	0.946129	2.04245
27470	0.946132	0.540535
27471	1.37973	0.333356
27472	0.946132	0.54676
27473	0.946132	0.366313
27474	0.946132	0.378537
27475	0.946131	1.02362
27476	0.94613	0.753374
27477	0.946129	1.01454
27478	0.946131	0.673946
27479	1.41798	0.624473
27480	0.946118	0.747889
27481	0.946118	0.547494
27482	0.946117	0.755618
27483	0.946131	0.546157
27484	0.946105	0.548823
27485	0.946131	1.13113
27486	0.946131	0.875711
27487	0.94613	1.11994
27488	0.946131	0.761958
27489	0.946131	0.726762

27490	0.946133	1.93894
27491	1.40274	2.1
27492	0.946133	1.71016
27493	0.946132	2.1
27494	1.40274	2.1
27495	0.946131	1.78034
27496	1.39731	2.1
27497	0.946132	1.05511
27498	0.94613	1.93258
27499	1.39731	2.07546
27500	0.94612	2.09958
27501	0.94612	1.81835
27502	0.946119	1.91439
27503	0.946132	1.19666
27504	1.40764	2.04993
27505	0.946128	2.1
27506	0.946132	2.1
27507	0.94613	2.1
27508	0.94613	2.1
27509	1.31213	2.1
27510	0.946127	2.1
27511	0.94613	2.1
27512	0.946127	2.1
27513	0.946128	2.1
27514	1.36653	2.1
27515	0.946127	2.1
27516	1.3557	2.1
27517	0.946128	2.1
27518	0.946128	2.1
27519	1.3557	2.1
27520	0.946133	2.1
27521	0.946132	2.1
27522	0.946133	2.1
27523	0.946132	2.1
27524	1.40081	2.1
27525	0.946128	2.1
27526	0.946133	2.1
27527	0.946131	2.1
27528	0.946131	2.1
27529	1.32282	2.1
27530	0.810945	2.1
27531	0.810943	2.1
27532	0.810933	2.1
27533	1.27676	2.1
27534	0.94613	2.1
27535	0.946128	2.1
27536	0.946131	2.1
27537	0.946131	2.1
27538	0.946131	0.659375
27539	0.946127	1.32614
27540	1.41328	1.21054
27541	1.41327	1.27141
27542	0.946128	1.25445
27543	0.946132	0.0770652
27544	1.32083	0.0938664
27545	0.946132	0.113786
27546	0.946133	0.0919403
27547	1.32082	0.0875988

27548	0.946128	0.0914978
27549	1.29215	0.110747
27550	0.94613	0.131072
27551	0.94613	0.105915
27552	1.29215	0.102273
27553	0.946132	0.0698591
27554	1.27696	0.0698591
27555	0.94613	0.0710642
27556	0.94613	0.0708443
27557	1.27696	0.0698591
27558	0.94612	0.0698591
27559	1.29289	0.0753536
27560	0.946119	0.0861035
27561	0.946132	0.0759913
27562	1.29287	0.0739819
27563	0.946128	2.1
27564	1.37869	2.1
27565	0.946131	2.1
27566	0.94613	2.1
27567	0.946131	2.1
27568	0.946133	2.1
27569	0.946132	2.1
27570	0.946132	2.1
27571	1.35703	2.1
27572	0.946133	2.1
27573	0.946132	2.1
27574	0.946132	2.1
27575	0.946133	2.1
27576	1.37017	2.1
27577	0.946133	2.1
27578	0.946119	0.0698591
27579	0.94612	0.0698591
27580	1.38583	0.0698591
27581	1.38585	0.0698591
27582	0.946107	0.0698591
27583	0.946126	0.0698591
27584	0.946127	0.0725976
27585	1.22904	0.0698591
27586	1.22904	0.0698591
27587	0.946128	0.0698591
27588	1.3279	0.665224
27589	0.946131	1.50097
27590	0.94613	1.18999
27591	0.946131	1.27065
27592	0.946131	1.02317
27593	0.946132	2.1
27594	0.946132	2.1
27595	0.946132	2.1
27596	0.946131	2.1
27597	1.40932	2.1
27598	1.26868	0.0793967
27599	0.946131	0.138213
27600	0.94613	0.111247
27601	0.946131	0.108716
27602	0.946131	0.101895
27603	0.810943	2.1
27604	0.810945	2.1
27605	0.810932	2.1

27606	0.810943	2.07769
27607	0.810944	0.96711
27608	0.810957	1.10371
27609	1.31539	0.987309
27610	0.946132	2.1
27611	0.946128	0.834559
27612	1.3154	1.65195
27613	1.17856	0.817957
27614	1.21997	1.27358
27615	0.94613	1.86995
27616	0.946129	1.75526
27617	0.94613	1.89124
27618	0.94613	1.79488
27619	0.946131	1.53992
27620	0.946131	1.33142
27621	0.946129	1.54935
27622	0.94613	1.61758
27623	0.946131	1.75008
27624	0.946127	0.996131
27625	0.946131	0.159656
27626	0.946127	1.80917
27627	0.946129	0.893794
27628	1.41342	0.554655
27629	0.946131	0.341311
27630	0.946131	1.49722
27631	0.946128	1.44614
27632	0.946131	0.507749
27633	1.29149	0.826408
27634	1.41022	2.1
27635	0.94613	2.05285
27636	0.946128	2.1
27637	0.94613	2.1
27638	0.94613	2.1
27639	0.946128	0.0844816
27640	0.94613	0.124552
27641	1.27864	0.0775509
27642	1.27864	0.0722071
27643	0.946131	0.101585
27644	0.946129	0.415923
27645	0.946125	1.5058
27646	0.946124	0.781547
27647	0.946127	0.741621
27648	1.37759	1.19982
27649	0.946129	0.626175
27650	0.94613	1.47751
27651	0.946126	0.844155
27652	0.946129	0.921444
27653	1.38259	1.14425
27654	0.946131	0.0819609
27655	1.34118	0.0698591
27656	0.946132	0.0698591
27657	0.946132	0.0748739
27658	1.34118	0.0698591
27659	0.946131	0.0698591
27660	1.25017	0.0698591
27661	0.946128	0.0698591
27662	0.946131	0.0698591
27663	0.946131	0.0698591

27664	0.946131	2.1
27665	0.946128	2.1
27666	0.946133	2.1
27667	1.3937	2.1
27668	0.94613	2.1
27669	0.946131	2.09514
27670	0.94613	0.686828
27671	1.40404	1.71978
27672	1.40404	1.05701
27673	0.946131	1.16498
27674	0.946132	1.63255
27675	0.946132	0.489682
27676	1.38897	0.298567
27677	1.38897	0.214181
27678	0.946132	0.363169
27679	0.94613	0.868436
27680	0.946128	1.99139
27681	1.40332	1.27533
27682	1.40331	1.61486
27683	0.94613	1.7548
27684	0.946132	2.00785
27685	0.946132	2.1
27686	0.946133	2.1
27687	1.41619	2.1
27688	0.946132	2.1
27689	0.946132	2.1
27690	0.946132	2.1
27691	0.946132	2.1
27692	1.38244	2.1
27693	0.946132	2.1
27694	0.810946	1.48979
27695	0.810943	0.830309
27696	0.810934	0.69455
27697	0.810947	1.33111
27698	0.810943	0.593405
27699	0.810934	0.658832
27700	1.12786	1.5966
27701	0.946132	2.02149
27702	0.946132	1.93196
27703	0.946132	1.76194
27704	0.946133	1.78238
27705	0.946132	1.7518
27706	0.946132	1.85254
27707	0.946132	1.64875
27708	1.27806	1.75634
27709	0.946133	1.70059
27710	0.946132	2.1
27711	0.946132	2.1
27712	0.946133	2.1
27713	0.946132	2.1
27714	0.946133	2.1
27715	1.39514	1.47058
27716	0.946129	1.2041
27717	0.946124	1.5065
27718	0.946127	0.50129
27719	0.946127	1.43949
27720	0.94613	2.1
27721	0.946129	2.1

27722	0.946132	2.1
27723	1.3991	2.1
27724	0.94613	2.1
27725	0.946132	1.56189
27726	0.946133	2.1
27727	0.946132	2.08293
27728	1.36835	2.1
27729	0.946132	2.08347
27730	0.946132	2.1
27731	0.946132	2.1
27732	0.946133	2.1
27733	0.946132	2.1
27734	1.39047	2.1
27735	0.946132	0.0698591
27736	0.946128	0.0698591
27737	0.94613	0.0698591
27738	1.33174	0.0698591
27739	0.946131	0.0698591
27740	0.946128	0.0698591
27741	0.946131	0.0698591
27742	0.94613	0.0698591
27743	0.94613	0.0698591
27744	0.94613	0.0698591
27745	0.946119	2.1
27746	0.946119	2.1
27747	1.29064	2.1
27748	0.946132	2.1
27749	0.946107	2.1
27750	0.946133	2.1
27751	0.94613	2.1
27752	1.30584	2.1
27753	0.946131	2.1
27754	0.946131	2.1
27755	0.946131	2.1
27756	0.946132	2.1
27757	0.94613	2.1
27758	0.946131	2.1
27759	1.39549	2.1
27760	0.913386	1.25385
27761	0.978873	0.934754
27762	0.946126	1.07451
27763	0.94613	1.1869
27764	1.00473	0.739884
27765	0.946132	0.533606
27766	0.946133	0.464159
27767	0.946132	0.364694
27768	0.946132	0.56089
27769	1.38894	0.312951
27770	0.946128	0.419364
27771	0.946131	0.369387
27772	0.94613	0.353221
27773	0.94613	0.40159
27774	1.23865	0.289239
27775	0.946126	1.21235
27776	0.946129	0.913438
27777	0.946124	1.02015
27778	0.946127	1.03719
27779	1.40248	0.771549

27780	0.946133	0.625538
27781	0.946132	0.66962
27782	0.946132	0.382744
27783	0.946133	0.574927
27784	1.1391	0.393457
27785	0.827862	0.976197
27786	0.946133	1.13988
27787	0.946131	1.296
27788	0.946131	1.34939
27789	0.94613	1.11292
27790	0.946128	0.283169
27791	0.946132	0.558618
27792	0.94613	0.604652
27793	1.21388	0.211051
27794	0.94613	0.470976
27795	0.810943	0.128876
27796	0.810945	0.185686
27797	0.810932	0.159206
27798	0.946131	1.8305
27799	0.946131	1.23023
27800	0.946133	1.54547
27801	1.36748	0.571267
27802	0.946132	0.370532
27803	0.946128	0.0698591
27804	0.946131	0.0698591
27805	0.94613	0.0698591
27806	1.35856	0.0698591
27807	0.94613	0.0698591
27808	0.946132	2.1
27809	0.946131	2.1
27810	0.946133	2.1
27811	0.946132	2.1
27812	0.946131	2.1
27813	0.946133	2.1
27814	0.946131	2.1
27815	1.39558	2.1
27816	1.39558	2.1
27817	0.94613	2.1
27818	0.946131	0.109591
27819	0.94613	0.158229
27820	0.946129	0.111779
27821	1.41388	0.104614
27822	0.946131	0.130742
27823	0.946133	0.0794959
27824	0.946132	0.169933
27825	0.946132	0.0873953
27826	0.946133	0.0996968
27827	0.93452	0.166882
27828	0.946129	2.1
27829	1.38897	2.1
27830	0.946131	2.1
27831	0.946131	2.1
27832	1.38897	2.1
27833	0.946133	2.00327
27834	0.946132	1.91703
27835	0.946133	1.96467
27836	0.946132	1.69282
27837	1.38773	1.79909

27838	1.41512	0.123146
27839	0.946131	0.233443
27840	0.946129	0.177792
27841	0.946133	0.185352
27842	0.946131	0.198506
27843	0.946132	1.57902
27844	0.946132	2.1
27845	0.946132	1.7323
27846	0.946133	2.1
27847	0.946133	2.1
27848	0.946126	1.18534
27849	0.946132	1.958
27850	0.946129	1.36511
27851	0.946129	1.95679
27852	0.946129	2.06566
27853	0.939895	2.03869
27854	1.03386	2.09334
27855	0.94613	2.03645
27856	0.946131	2.04542
27857	1.03386	2.06258
27858	0.946131	2.1
27859	1.39309	2.1
27860	0.946133	2.1
27861	0.946131	2.1
27862	1.39309	2.1
27863	0.946133	2.1
27864	1.24853	2.1
27865	0.946132	2.1
27866	0.946132	2.1
27867	1.24853	2.1
27868	0.946133	2.02418
27869	1.39699	1.19961
27870	0.946132	1.95757
27871	0.946132	2.1
27872	1.39699	1.30802
27873	0.946132	2.1
27874	0.946131	2.1
27875	0.946133	2.1
27876	0.946131	2.1
27877	1.38969	2.1
27878	1.28464	1.97719
27879	0.946133	2.00752
27880	0.94613	2.04565
27881	0.946131	1.94262
27882	0.946131	1.96387
27883	0.946119	0.585467
27884	0.94612	0.860071
27885	0.946119	0.435361
27886	0.946132	0.537458
27887	1.3749	0.424916
27888	0.810942	2.1
27889	0.810943	2.1
27890	0.810957	2.1
27891	1.41134	2.00935
27892	0.946132	0.545653
27893	0.946132	2.01645
27894	0.946132	0.799873
27895	0.946132	1.78434

27896	1.31338	1.96689
27897	0.946132	1.39198
27898	0.946132	1.95298
27899	0.946133	1.59841
27900	0.946132	1.8145
27901	0.946103	0.0698591
27902	0.752448	0.0698591
27903	0.946132	0.0698591
27904	0.946131	0.0698591
27905	1.20617	0.0698591
27906	0.946132	1.08835
27907	1.08054	0.75506
27908	1.25736	0.916093
27909	0.946132	1.09034
27910	0.946131	0.89159
27911	0.946128	2.1
27912	1.33275	2.0955
27913	1.1896	2.1
27914	0.946131	2.1
27915	1.18962	2.1
27916	1.21955	0.0698591
27917	0.946131	0.0698591
27918	1.33587	0.0698591
27919	0.741946	0.0698591
27920	1.07387	0.0698591
27921	0.945844	1.15092
27922	1.3292	2.1
27923	1.59472	2.05479
27924	0.946131	1.59322
27925	1.04043	1.84665
27926	1.13529	0.077648
27927	0.946132	0.0808773
27928	1.24893	0.0925052
27929	1.12675	0.101242
27930	0.936652	0.100335
27931	1.36968	2.1
27932	0.946131	2.1
27933	0.946132	2.1
27934	1.36968	2.1
27935	1.258 2.1	
27936	3.17458	0.105916
27937	0.946132	2.0787
27938	1.35022	2.1
27939	1.35022	2.1
27940	0.94613	2.08883
27941	1.12356	2.09954
27942	0.946131	2.1
27943	1.22401	2.1
27944	1.22401	2.1
27945	0.94613	2.1
27946	0.952455	2.1
27947	1.2107	2.1
27948	0.946132	2.1
27949	1.33047	2.1
27950	0.946131	2.1
27951	0.946132	2.1
27952	0.61957	2.0637
27953	0.8636	2.1

27954	0.946132	2.0715
27955	1.19294	2.09799
27956	0.946128	2.1
27957	1.36607	2.1
27958	0.94613	2.1
27959	1.39416	2.1
27960	1.36607	2.1
27961	1.21474	2.1
27962	0.946131	2.1
27963	0.946131	2.1
27964	1.16288	2.1
27965	1.08542	2.1
27966	0.851526	1.23847
27967	1.30374	2.1
27968	1.01708	2.1
27969	0.946114	1.78252
27970	0.96976	1.97379
27971	0.946132	0.0784369
27972	1.49997	0.0996437
27973	0.771735	0.0987138
27974	0.771764	0.107328
27975	0.946133	0.15003
27976	0.700863	1.89289
27977	1.42222	2.1
27978	0.946131	2.1
27979	0.946128	2.09555
27980	1.42509	2.1
27981	1.14298	2.1
27982	0.837014	2.1
27983	0.946131	2.1
27984	1.29426	2.1
27985	0.836985	2.1
27986	0.946129	2.1
27987	1.22089	0.384955
27988	0.946132	1.11823
27989	0.712486	0.522657
27990	0.6234	0.518353
27991	0.946131	2.1
27992	1.40271	2.1
27993	1.32241	2.1
27994	0.946133	2.1
27995	1.32241	2.1
27996	0.769373	1.35093
27997	0.946129	2.1
27998	1.41302	2.1
27999	1.28254	2.1
28000	0.946131	2.1
28001	1.28254	2.1
28002	0.851543	2.00152
28003	1.23056	2.1
28004	1.24356	2.1
28005	0.851543	2.02624
28006	0.909417	2.07513
28007	1.13808	0.194964
28008	0.946133	0.506683
28009	0.946131	0.300689
28010	1.13808	1.00384
28011	1.13808	0.31

28012	0.930941	1.39907
28013	1.37808	1.00278
28014	1.17573	0.775653
28015	0.946124	1.23204
28016	1.02404	0.87941
28017	0.946116	2.1
28018	1.3144	2.09645
28019	1.3144	2.1
28020	0.946118	2.1
28021	1.13089	2.1
28022	0.946128	0.0778861
28023	0.946101	0.0723686
28024	1.2606	0.0817024
28025	1.26055	0.070186
28026	0.580823	0.648127
28027	0.946132	1.39911
28028	0.580825	0.763839
28029	1.23224	0.881579
28030	0.946128	2.1
28031	1.3719	2.1
28032	0.94613	2.1
28033	1.3719	2.1
28034	0.94613	2.1
28035	0.946132	1.72189
28036	1.35025	0.404689
28037	1.35025	0.983844
28038	0.946132	1.82267
28039	1.1794	1.53556
28040	0.946133	2.1
28041	1.25598	2.1
28042	1.29249	2.1
28043	1.38154	2.1
28044	0.851541	2.1
28045	1.31508	2.1
28046	1.37558	2.1
28047	0.946129	2.1
28048	0.867663	2.1
28049	0.94613	1.57616
28050	1.35426	0.54762
28051	0.84872	0.617544
28052	0.946129	1.52353
28053	1.17195	1.7291
28054	0.946133	2.1
28055	1.39009	2.1
28056	1.14342	2.1
28057	0.946133	2.1
28058	1.14343	2.1
28059	1.38819	2.1
28060	0.946133	2.1
28061	1.4116	2.1
28062	0.946131	2.1
28063	0.946131	2.1
28064	0.946127	2.1
28065	1.32244	2.1
28066	1.32244	2.1
28067	0.946131	2.1
28068	1.2176	2.1
28069	1.75823	2.1

28070	1.11239	2.1
28071	2.17979	2.1
28072	1.65533	2.1
28073	3.41356	2.1
28074	1.94841	2.1
28075	1.91767	2.1
28076	1.76726	2.1
28077	1.70302	2.1
28078	3.76137	2.1
28079	1.94841	2.1
28080	1.33962	2.1
28081	1.31365	2.1
28082	1.70302	2.1
28083	3.2123	2.1
28084	1.76726	2.1
28085	1.70302	2.1
28086	1.47581	2.1
28087	1.7457	2.1
28088	3.22764	2.1
28089	1.20478	2.1
28090	1.70302	2.1
28091	1.27956	2.1
28092	1.18569	2.1
28093	1.63979	2.1
28094	1.51949	2.1
28095	1.53082	2.1
28096	1.92911	2.1
28097	1.32068	2.1
28098	1.74454	2.1
28099	3.27536	2.1
28100	1.08172	2.1
28101	1.74454	2.1
28102	1.52603	2.1
28103	1.52603	2.1
28104	1.32068	2.1
28105	1.32068	2.1
28106	1.89226	2.1
28107	2.37364	2.1
28108	1.89226	2.1
28109	2.42769	2.1
28110	4.31995	2.1
28111	2.37365	2.1
28112	1.5362	2.1
28113	2.4277	2.1
28114	1.56328	2.1
28115	3.9639	2.1
28116	1.89225	2.1
28117	2.42768	2.1
28118	1.89213	2.1
28119	1.89225	2.1
28120	2.42756	2.1
28121	2.42768	2.1
28122	2.42768	2.1
28123	1.58163	2.1
28124	1.56315	2.1
28125	1.56327	2.1
28126	2.42757	2.1
28127	2.42768	2.1

28128	1.01641	2.1
28129	2.15005	2.1
28130	1.00099	2.1
28131	2.15114	2.1
28132	3.15104	2.1
28133	0.994813	2.1
28134	2.15114	2.1
28135	2.17159	2.1
28136	2.17159	2.1
28137	1.00099	2.1
28138	1.00099	2.1
28139	1.29608	2.1
28140	1.29608	2.1
28141	0.942035	2.1
28142	0.942035	2.08999
28143	1.29198	2.1
28144	0.942033	2.1
28145	0.946132	2.1
28146	0.946132	2.1
28147	0.946132	2.1
28148	0.942033	2.1
28149	0.946133	2.08329
28150	1.29198	2.1
28151	1.99985	2.1
28152	1.14207	2.1
28153	1.83584	2.1
28154	1.00232	2.1
28155	3.00217	2.1
28156	1.99867	0.0698591
28157	2.0815	0.0698591
28158	1.56806	0.0698591
28159	1.28828	0.0698591
28160	3.58519	0.0698591
28161	1.89226	0.0698591
28162	1.89226	0.0698645
28163	1.89226	0.0698591
28164	1.89226	0.0701811
28165	1.89226	0.0698591
28166	1.89226	0.0700947
28167	1.89226	0.0698591
28168	1.89226	0.0698591
28169	2.23193	0.0698591
28170	2.48978	0.0698591
28171	4.38204	0.0698591
28172	1.85293	2.08734
28173	1.64279	0.431681
28174	1.76268	2.09928
28175	1.54214	0.411904
28176	3.39507	1.14349
28177	1.6428	0.409178
28178	1.17302	1.38569
28179	1.17302	1.97796
28180	1.28419	1.01758
28181	1.54214	0.357195
28182	1.65331	1.06219
28183	1.76268	2.1
28184	1.54214	0.64313
28185	1.65324	2.07034

28186	1.42491	0.763582
28187	3.19538	1.06455
28188	0.651043	1.84334
28189	0.651043	1.0664
28190	0.647925	0.57759
28191	0.647925	1.78275
28192	0.521747	1.17917
28193	0.58651	1.31816
28194	0.77107	0.238388
28195	0.712457	0.428956
28196	0.712457	0.578631
28197	0.521747	0.452451
28198	0.58651	0.500715
28199	0.77107	0.564623
28200	1.2873	2.1
28201	1.48751	2.1
28202	1.59659	2.1
28203	1.77596	2.08762
28204	3.06326	2.1
28205	1.07976	2.1
28206	1.59659	2.1
28207	1.30237	2.1
28208	1.77723	2.1
28209	2.89897	2.1
28210	1.77723	2.1
28211	1.77596	2.1
28212	1.82614	2.1
28213	1.59659	2.1
28214	2.0055	2.1
28215	1.77596	2.1
28216	2.64523	2.07524
28217	2.73982	0.55588
28218	2.50057	2.06144
28219	2.61606	0.477502
28220	5.26129	1.27788
28221	2.73982	0.273382
28222	2.68987	0.649953
28223	2.61607	0.528773
28224	2.57611	0.791952
28225	5.30593	0.0740605
28226	1.89226	0.138889
28227	1.89226	0.180769
28228	1.89226	0.168992
28229	2.02178	0.101363
28230	3.78452	0.148685
28231	1.89226	0.610368
28232	1.89226	0.56347
28233	1.89226	1.56116
28234	1.89226	0.343921
28235	1.89226	1.38675
28236	1.89226	0.255148
28237	2.35204	2.1
28238	1.50108	2.1
28239	1.3715	2.1
28240	2.18469	2.1
28241	3.68577	2.1
28242	2.62514	2.1
28243	2.1847	2.1

28244	2.0487	2.1
28245	2.45213	2.1
28246	4.63682	2.1
28247	1.25075	2.1
28248	2.18469	2.1
28249	1.3715	2.1
28250	1.3715	2.1
28251	2.0487	2.1
28252	2.0487	2.1
28253	1.28667	2.1
28254	1.11285	2.1
28255	1.03529	2.1
28256	1.03529	2.1
28257	1.30711	2.1
28258	1.30711	2.1
28259	2.35053	2.1
28260	1.89225	2.1
28261	1.89225	2.1
28262	2.51216	2.1
28263	4.24278	2.1
28264	1.89225	2.1
28265	1.89225	2.1
28266	1.89225	2.1
28267	1.89225	2.1
28268	3.7845	2.1
28269	1.92025	2.1
28270	1.32119	2.1
28271	1.44777	2.1
28272	1.33834	2.1
28273	1.92025	2.1
28274	1.81082	2.1
28275	1.89226	2.1
28276	2.67272	2.1
28277	2.51218	2.1
28278	2.51218	2.1
28279	1.89226	2.1
28280	1.89226	2.1
28281	1.89226	2.1
28282	1.89226	2.1
28283	1.89226	2.1
28284	1.89226	2.1
28285	3.78452	2.1
28286	1.92027	2.1
28287	1.44778	2.1
28288	1.61121	2.1
28289	2.01555	2.1
28290	3.53147	2.1
28291	1.90727	0.0699286
28292	2.29851	0.111294
28293	2.71643	0.0841012
28294	2.17695	0.0710117
28295	4.47547	0.0703096
28296	1.90728	0.0723314
28297	1.56339	0.0698591
28298	1.58143	0.0698591
28299	2.17696	0.0735366
28300	3.74035	0.0698773
28301	1.91368	0.0698591

28302	2.17696	0.0748105
28303	2.39986	0.0698591
28304	1.75904	0.0698591
28305	3.936	0.0698591
28306	1.18793	0.0698591
28307	2.74744	0.0698591
28308	1.38716	0.0698591
28309	2.57465	0.0799238
28310	3.93537	0.0698591
28311	1.3148	0.0698591
28312	2.74744	0.0699778
28313	2.70805	0.0698591
28314	1.38716	0.0698591
28315	2.70805	0.0716989
28316	1.38716	0.0698591
28317	1.20608	2.1
28318	0.975871	2.1
28319	0.946132	2.1
28320	0.946132	2.1
28321	0.946132	2.1
28322	0.946132	2.1
28323	0.975871	2.1
28324	0.975871	2.1
28325	1.20608	2.1
28326	0.946132	2.1
28327	0.946133	2.1
28328	0.975871	2.1
28329	2.8198	2.1
28330	2.29967	2.1
28331	2.09542	2.1
28332	2.63784	2.02048
28333	4.93751	2.1
28334	2.09542	2.1
28335	1.39523	2.1
28336	1.21119	2.1
28337	1.39523	2.1
28338	2.11563	2.1
28339	2.29967	2.1
28340	1.88584	2.1
28341	2.63784	1.84027
28342	2.11262	2.1
28343	1.91132	2.1
28344	4.02153	2.1
28345	1.88584	2.1
28346	1.21119	2.1
28347	1.12606	2.1
28348	1.91132	2.1
28349	3.1225	2.1
28350	2.18077	2.09082
28351	1.78167	1.98931
28352	2.2947	0.807591
28353	1.93139	1.60285
28354	4.07637	1.04769
28355	1.5709	0.990558
28356	1.03235	1.80648
28357	2.05933	1.62703
28358	1.78168	0.755693
28359	1.31	0.298924

28360	1.03235	0.791517
28361	2.2947	1.4457
28362	2.31785	2.00174
28363	2.40627	2.1
28364	2.08288	0.703999
28365	4.37758	2.02586
28366	1.95737	1.88463
28367	1.31	1.84885
28368	2.08289	0.257956
28369	1.50108	0.262746
28370	3.39289	0.403762
28371	2.49676	2.1
28372	2.24781	2.1
28373	2.5276	2.09976
28374	2.7352	2.1
28375	5.02436	2.1
28376	2.49676	2.1
28377	2.43549	2.1
28378	2.6398	2.1
28379	2.7352	2.1
28380	5.13656	2.1
28381	1.89226	2.1
28382	2.7352	2.1
28383	1.89226	2.1
28384	2.6398	2.1
28385	1.89226	2.1
28386	2.6398	2.1
28387	2.16843	0.0698591
28388	1.00185	0.0698591
28389	1.90039	2.1
28390	3.1547	0.0698591
28391	2.54064	0.0698591
28392	2.46752	2.1
28393	1.90039	2.1
28394	2.26044	2.1
28395	4.36791	2.00735
28396	1.65815	2.1
28397	2.48652	2.1
28398	1.65815	2.1
28399	1.9004	2.1
28400	2.21711	2.1
28401	2.5034	2.1
28402	2.37167	2.1
28403	2.59965	2.1
28404	2.62812	2.1
28405	2.40308	2.1
28406	4.99979	2.1
28407	2.50137	2.1
28408	2.59965	2.1
28409	2.62812	2.1
28410	2.5214	2.1
28411	5.12948	2.1
28412	2.62814	2.1
28413	2.4031	2.1
28414	2.43509	2.1
28415	2.65066	2.1
28416	5.06323	2.1
28417	2.62814	2.1

28418	2.52142	2.1
28419	2.53008	2.1
28420	2.65066	2.1
28421	5.15822	2.1
28422	2.0815	2.1
28423	1.92067	2.1
28424	1.59611	2.1
28425	1.89226	2.1
28426	1.62453	2.1
28427	1.92067	2.1
28428	1.89226	2.1
28429	1.89226	2.1
28430	1.89226	2.1
28431	1.89226	2.1
28432	3.78453	2.1
28433	0.946131	2.1
28434	0.946131	2.1
28435	0.929501	2.1
28436	0.910761	2.1
28437	0.9461	2.1
28438	0.9461	2.1
28439	0.910792	2.1
28440	0.910792	2.1
28441	0.946131	2.1
28442	0.929501	2.1
28443	0.910761	2.1
28444	0.946131	2.1
28445	2.0815	2.1
28446	1.81661	2.1
28447	2.1341	2.1
28448	2.0815	2.1
28449	1.86921	2.1
28450	1.81661	2.1
28451	1.89226	2.1
28452	1.89226	2.1
28453	1.89226	2.1
28454	1.89226	2.1
28455	1.89226	2.1
28456	1.89226	2.1
28457	0.946131	2.1
28458	0.946131	2.1
28459	0.949104	2.1
28460	0.9295	2.1
28461	0.946132	2.1
28462	0.946132	2.1
28463	0.929499	2.1
28464	0.929499	2.1
28465	0.946131	2.1
28466	0.949105	2.1
28467	0.946131	2.1
28468	0.929501	2.1
28469	1.66104	2.1
28470	2.34044	2.1
28471	2.08889	2.1
28472	1.33264	2.1
28473	3.67308	2.1
28474	2.0889	2.1
28475	1.08011	2.1

28476	1.08011	2.1
28477	1.33264	2.1
28478	1.87598	2.1
28479	2.12851	2.1
28480	2.27472	2.1
28481	1.89226	1.94303
28482	2.27703	2.1
28483	1.89226	1.81986
28484	4.16699	2.07676
28485	1.89226	2.03043
28486	1.89226	1.43159
28487	1.89226	1.95265
28488	1.89226	1.36987
28489	3.78452	1.53984
28490	1.00007	2.01197
28491	1.32316	0.965033
28492	1.29417	0.996744
28493	1.29417	1.98902
28494	1.02051	1.59861
28495	1.02051	1.89618
28496	2.2846	2.1
28497	1.89226	2.07067
28498	2.27703	2.07165
28499	2.27703	2.1
28500	1.89226	1.79783
28501	1.89226	2.1
28502	1.89226	2.1
28503	1.89226	2.037
28504	1.89226	1.71103
28505	1.89226	0.495604
28506	3.78452	1.02507
28507	1.02051	2.1
28508	1.32316	1.99473
28509	1.03373	1.6701
28510	1.34066	1.51783
28511	2.35689	2.06262
28512	1.703	2.1
28513	1.89224	2.1
28514	1.52266	2.1
28515	1.89224	2.1
28516	1.23344	2.1
28517	1.53282	2.1
28518	1.89223	2.1
28519	1.89223	2.1
28520	1.89223	2.1
28521	1.89223	2.1
28522	3.78447	2.1
28523	0.796845	2.1
28524	0.796845	2.1
28525	0.812146	2.1
28526	0.812146	2.1
28527	1.08006	2.1
28528	1.02679	2.1
28529	0.551538	2.1
28530	0.528929	2.1
28531	0.551529	2.1
28532	1.08005	2.1
28533	0.528935	2.1

28534	1.0268	2.1
28535	1.5001	2.1
28536	1.70302	2.1
28537	2.0726	2.1
28538	1.70302	2.1
28539	1.99601	2.1
28540	1.70303	2.1
28541	1.89226	2.1
28542	1.89226	2.1
28543	1.89226	2.1
28544	1.89226	2.1
28545	1.89226	2.1
28546	1.89226	2.1
28547	1.17285	2.1
28548	2.16015	2.1
28549	2.23563	2.1
28550	2.23563	2.1
28551	1.10309	2.1
28552	1.10309	2.1
28553	0.946128	2.1
28554	0.946128	2.1
28555	1.21847	2.1
28556	1.2884	2.1
28557	0.94613	2.1
28558	0.94613	2.1
28559	1.21847	2.1
28560	1.21847	2.1
28561	0.946131	2.1
28562	1.21847	2.1
28563	0.94613	2.1
28564	1.2884	2.1
28565	1.83922	2.1
28566	2.5768	2.1
28567	1.83488	2.1
28568	1.82846	0.0698591
28569	2.43693	2.1
28570	2.43693	2.1
28571	2.36134	2.1
28572	1.81656	2.1
28573	1.86454	2.1
28574	1.6697	2.1
28575	2.55618	2.1
28576	2.36134	2.1
28577	2.55618	2.1
28578	1.9323	2.1
28579	1.9302	2.1
28580	1.81656	2.1
28581	2.66983	2.1
28582	2.55618	2.1
28583	1.89226	1.06209
28584	1.70302	1.41146
28585	1.94935	1.72537
28586	1.70302	1.28889
28587	1.95569	1.25248
28588	1.70302	0.821192
28589	2.82465	2.1
28590	2.41707	2.1
28591	2.24481	2.1

28592	2.68958	2.1
28593	5.10665	2.1
28594	1.41232	2.1
28595	1.34479	2.1
28596	0.946129	2.1
28597	0.946129	2.1
28598	0.94613	2.1
28599	0.94613	2.1
28600	1.41232	2.1
28601	1.41232	2.1
28602	1.34479	2.1
28603	0.94613	2.1
28604	0.946131	2.1
28605	1.41232	2.1
28606	1.86859	0.0698591
28607	1.89226	0.0698591
28608	1.89226	0.0702629
28609	1.89226	0.0698591
28610	1.45875	0.0698591
28611	1.45875	0.0698591
28612	2.1769	0.122751
28613	2.50259	0.184803
28614	1.94955	0.0991723
28615	2.25959	0.0891375
28616	4.45214	0.132607
28617	1.89226	0.257033
28618	1.89226	0.310843
28619	1.89226	0.14843
28620	1.89226	0.294105
28621	3.78452	0.173377
28622	1.89226	0.386652
28623	1.89226	0.68301
28624	1.89226	0.301142
28625	1.89226	0.451872
28626	3.78452	0.823933
28627	2.50259	0.257928
28628	2.49445	0.187166
28629	2.25959	0.0911805
28630	2.20843	0.289165
28631	4.71102	0.174163
28632	1.94955	0.117762
28633	2.25959	0.0850949
28634	1.24689	0.0698591
28635	1.79906	0.0979815
28636	3.74861	0.0815321
28637	1.89226	0.18766
28638	1.89226	0.18173
28639	1.89226	0.0918405
28640	1.89226	0.12264
28641	3.78452	0.274913
28642	1.89226	0.195767
28643	1.89226	0.283844
28644	1.89226	0.119593
28645	1.89226	0.164535
28646	3.78452	0.12379
28647	2.25959	0.0873122
28648	2.20843	0.174125
28649	1.79906	0.0944989

28650	1.98391	0.116788
28651	4.00748	0.124964
28652	1.89226	2.1
28653	1.89226	2.1
28654	1.89226	2.1
28655	1.89226	2.1
28656	3.78452	2.1
28657	1.89226	2.1
28658	1.89226	2.1
28659	1.89226	2.1
28660	1.89226	2.1
28661	3.78452	2.1
28662	1.64718	2.1
28663	1.23085	2.1
28664	1.46028	2.1
28665	1.39564	2.1
28666	1.68372	2.1
28667	1.64718	2.1
28668	1.64718	2.1
28669	1.64981	2.1
28670	1.70303	2.1
28671	1.6991	2.1
28672	3.3199	2.1
28673	1.89226	2.1
28674	1.89226	2.1
28675	1.89226	2.1
28676	1.89226	2.1
28677	3.78452	2.1
28678	1.89226	2.1
28679	1.89226	2.1
28680	1.89226	2.1
28681	1.89226	2.1
28682	3.78452	2.1
28683	0.666701	2.1
28684	0.65896	2.1
28685	0.920797	2.1
28686	0.895441	2.1
28687	0.775871	2.1
28688	0.687493	2.1
28689	0.777393	2.1
28690	0.777393	2.1
28691	0.677856	2.1
28692	0.90095	2.1
28693	0.621435	2.1
28694	0.858124	2.1
28695	1.70303	2.1
28696	1.70731	2.1
28697	1.79663	2.1
28698	1.68783	2.1
28699	3.17337	2.1
28700	1.22724	2.1
28701	2.0021	2.1
28702	1.90427	2.1
28703	1.08698	2.1
28704	3.08907	2.1
28705	2.41689	2.1
28706	2.00209	2.1
28707	1.90427	2.1

28708	2.35245	2.1
28709	4.35454	2.1
28710	1.90427	2.1
28711	0.956294	2.1
28712	0.956294	2.1
28713	1.08698	2.1
28714	1.81488	2.1
28715	1.94556	2.1
28716	1.90427	2.1
28717	2.35245	2.1
28718	2.28979	2.1
28719	1.81488	2.1
28720	4.16733	2.1
28721	1.6272	2.1
28722	1.70302	2.1
28723	1.70303	2.1
28724	1.70302	2.1
28725	1.91167	2.1
28726	1.91167	2.1
28727	1.49063	2.1
28728	1.89226	2.1
28729	1.89226	2.1
28730	1.89226	2.1
28731	1.6272	2.1
28732	1.6272	2.1
28733	2.25988	1.65037
28734	1.89226	0.414168
28735	1.89226	0.858607
28736	2.23152	1.18799
28737	4.12379	0.831678
28738	2.25988	1.85269
28739	1.16361	2.09429
28740	1.15853	2.1
28741	2.23153	1.52794
28742	3.39513	2.08102
28743	1.89226	0.586384
28744	2.23153	0.839237
28745	2.21022	1.31885
28746	2.23153	0.719376
28747	1.87095	0.786186
28748	1.89226	0.268446
28749	1.16239	2.0882
28750	2.23153	0.963988
28751	1.15853	2.03485
28752	1.15853	1.41901
28753	2.21022	2.03463
28754	2.21022	1.28379
28755	1.78381	0.0698591
28756	1.70302	0.0698591
28757	1.15453	0.0698591
28758	1.35007	0.0698591
28759	1.82021	0.0698591
28760	2.12851	0.0698591
28761	1.89226	0.0698591
28762	1.89226	0.075895
28763	1.89226	0.0701834
28764	1.89226	0.0805216
28765	1.89226	0.0698591

28766	1.89226	0.0758293
28767	1.89258	0.0698591
28768	1.089	0.0698591
28769	1.22586	0.0698591
28770	1.79877	0.0698591
28771	2.88777	0.0698591
28772	1.91564	0.0698591
28773	1.79877	0.0698591
28774	1.96356	0.0698591
28775	1.70302	0.0698601
28776	2.37828	0.0698591
28777	2.04859	0.06987
28778	1.89226	0.0698591
28779	1.89226	0.0757981
28780	1.89226	0.0698591
28781	1.89226	0.0795874
28782	3.78453	0.0707322
28783	1.89226	0.0698591
28784	1.89226	0.0736121
28785	1.89226	0.0698591
28786	1.89226	0.0750999
28787	1.89226	0.0698591
28788	1.89226	0.073297
28789	2.28593	2.1
28790	1.89226	2.08292
28791	2.2731	2.1
28792	1.89226	1.95356
28793	4.17819	2.1
28794	1.89226	2.1
28795	1.89226	2.07233
28796	1.89226	2.01852
28797	1.89226	1.39945
28798	3.78452	1.76038
28799	1.03933	2.1
28800	1.34355	1.02273
28801	1.35048	0.916017
28802	1.35048	2.09479
28803	1.02975	1.1079
28804	1.02975	2.06905
28805	2.25105	2.1
28806	1.89226	2.1
28807	2.27311	2.1
28808	2.27311	2.1
28809	1.89226	2.08063
28810	1.89226	2.1
28811	1.89226	2.1
28812	1.89226	2.03219
28813	1.89226	2.06441
28814	1.89226	0.313813
28815	3.78452	1.20534
28816	1.02975	2.1
28817	1.34355	2.0094
28818	1.01121	2.0311
28819	1.33079	1.19906
28820	2.35476	2.0997
28821	1.24526	0.0698591
28822	2.36406	0.0698591
28823	2.81021	0.0698591

28824	1.7237	0.0698591
28825	4.08776	0.0698591
28826	1.89226	2.1
28827	1.89226	2.1
28828	1.89226	2.1
28829	1.89226	2.1
28830	3.78453	2.1
28831	1.79028	2.1
28832	1.89238	2.1
28833	1.65533	2.1
28834	1.77821	2.1
28835	3.5477	2.1
28836	1.89238	2.1
28837	1.7465	2.0988
28838	1.77821	2.1
28839	1.63557	2.05009
28840	3.52795	2.1
28841	1.89226	2.1
28842	1.89226	1.91177
28843	1.89226	2.1
28844	1.89226	2.04037
28845	3.78452	2.1
28846	1.89226	2.1
28847	1.89226	2.1
28848	1.89226	2.1
28849	1.89226	2.1
28850	3.78452	2.1
28851	1.65532	2.1
28852	1.77821	2.1
28853	1.53278	2.1
28854	1.67571	2.1
28855	3.33103	2.1
28856	1.77821	2.1
28857	1.63557	2.09407
28858	1.67571	2.1
28859	1.52615	2.0881
28860	3.31128	2.1
28861	1.89226	2.1
28862	1.89226	2.09034
28863	1.89226	2.1
28864	1.89226	1.91887
28865	3.78452	2.1
28866	2.83129	2.1
28867	1.89226	1.53996
28868	1.89226	2.07684
28869	2.6491	2.1
28870	4.54136	2.07956
28871	2.83129	2.1
28872	1.47278	2.1
28873	1.3909	2.1
28874	2.6491	2.1
28875	4.12188	2.1
28876	1.89225	2.1
28877	2.65393	2.1
28878	1.89225	2.1
28879	2.69591	2.1
28880	4.58816	2.1
28881	1.91703	2.1

28882	2.00209	2.1
28883	2.65393	2.1
28884	2.65393	2.1
28885	2.69591	2.1
28886	2.00209	2.1
28887	1.89226	2.1
28888	2.69593	2.1
28889	1.93967	2.1
28890	1.89226	2.1
28891	2.74334	2.1
28892	2.69593	2.1
28893	2.74335	2.1
28894	2.0021	2.1
28895	2.76367	2.1
28896	2.69593	2.1
28897	2.06984	2.1
28898	2.0021	2.1
28899	2.24481	2.1
28900	2.58921	2.1
28901	2.68958	2.1
28902	2.68958	2.1
28903	2.11984	2.1
28904	2.11984	2.1
28905	2.11984	2.1
28906	2.58921	2.1
28907	2.46488	2.1
28908	1.98339	2.1
28909	4.5726	2.1
28910	2.7777	2.1
28911	2.58921	2.1
28912	2.46488	2.1
28913	2.70401	2.1
28914	5.29322	2.1
28915	2.17741	2.1
28916	2.54197	2.1
28917	2.54018	2.1
28918	2.18608	2.1
28919	4.72805	2.1
28920	2.17742	2.1
28921	1.58054	2.1
28922	1.59169	2.1
28923	2.18608	2.1
28924	3.76662	2.1
28925	2.54018	2.1
28926	2.18608	2.1
28927	2.19104	2.1
28928	2.5392	2.1
28929	4.72528	2.1
28930	1.60497	2.1
28931	2.18608	2.1
28932	1.59169	2.1
28933	1.59169	2.1
28934	2.19105	2.1
28935	2.19105	2.1
28936	2.58661	2.1
28937	1.06203	2.1
28938	2.7043	2.1
28939	2.21397	2.1

28940	1.55236	2.1
28941	1.06203	2.1
28942	1.89226	1.61227
28943	1.89226	1.60571
28944	1.89226	0.354429
28945	1.89226	2.00591
28946	3.78453	1.30048
28947	1.08195	1.36314
28948	0.990019	2.1
28949	1.31976	1.98062
28950	1.4539	0.637451
28951	2.40171	1.99648
28952	1.89226	0.702034
28953	1.89226	2.08939
28954	1.89226	1.58412
28955	1.89226	0.249971
28956	3.78453	1.30458
28957	1.89226	1.92558
28958	1.89226	1.39808
28959	1.89226	0.931909
28960	1.89226	2.04844
28961	3.78453	1.64357
28962	1.43895	2.02925
28963	1.31976	1.82671
28964	1.70066	0.980025
28965	1.33783	2.09497
28966	1.43895	1.94101
28967	1.07612	2.1
28968	0.65988	1.91244
28969	0.850329	1.58853
28970	0.919598	0.500281
28971	0.726952	1.73853
28972	0.788106	0.340234
28973	0.788106	2.1
28974	0.789175	2.05787
28975	0.789175	0.243461
28976	0.850329	0.706411
28977	0.919598	0.76421
28978	0.659879	1.58111
28979	0.726952	1.49737
28980	1.34389	2.1
28981	1.41279	2.02726
28982	1.15564	2.1
28983	1.15564	2.1
28984	1.55998	2.09292
28985	1.55998	2.1
28986	1.89226	1.93356
28987	1.89226	2.03073
28988	1.89226	2.1
28989	1.89226	1.47489
28990	1.89226	2.1
28991	1.89226	1.38579
28992	1.55998	2.1
28993	1.41279	2.06371
28994	1.72901	2.09283
28995	1.62414	2.09316
28996	3.1418	2.09686
28997	1.89226	2.0321

28998	1.89226	2.08079
28999	1.89226	2.09293
29000	1.89226	1.35985
29001	3.78452	2.03782
29002	1.89226	1.14639
29003	1.89226	2.03234
29004	1.89226	1.91312
29005	1.89226	1.93073
29006	1.89226	1.50449
29007	1.89226	1.36894
29008	1.62414	2.09946
29009	1.01243	2.01632
29010	1.70932	2.09113
29011	1.41279	1.99527
29012	1.30896	2.02254
29013	1.01243	1.92532
29014	1.9719	2.1
29015	1.10725	2.1
29016	1.12411	2.1
29017	1.98691	2.1
29018	3.09601	2.1
29019	1.97189	2.1
29020	2.47932	2.1
29021	2.49092	2.1
29022	1.98691	2.1
29023	4.46282	2.1
29024	1.1486	2.1
29025	1.98691	2.1
29026	1.12411	2.1
29027	1.12411	2.1
29028	2.01602	2.1
29029	2.01602	2.1
29030	2.49093	2.1
29031	1.98691	2.1
29032	2.01601	2.1
29033	2.51651	2.1
29034	4.50694	2.1
29035	1.93147	0.098155
29036	2.51593	0.0710218
29037	1.35146	0.091628
29038	1.35146	0.0773997
29039	2.48665	0.0851933
29040	1.90664	0.0747652
29041	1.63979	2.1
29042	1.7457	2.1
29043	1.66046	2.1
29044	1.56558	2.1
29045	3.31128	2.1
29046	0.819895	2.1
29047	0.782792	2.1
29048	0.60239	2.1
29049	0.54831	2.1
29050	0.688347	2.1
29051	0.688347	2.1
29052	0.679857	2.1
29053	0.679857	2.1
29054	0.782792	2.1
29055	0.54831	2.1

29056	0.60239	2.1
29057	0.819895	2.1
29058	1.66046	2.1
29059	1.42485	2.1
29060	1.56558	2.1
29061	1.56558	2.1
29062	1.52995	2.1
29063	1.52995	2.1
29064	2.58101	2.07753
29065	2.09803	2.1
29066	2.408	2.02275
29067	1.86893	2.1
29068	4.44994	2.1
29069	2.36062	2.1
29070	1.25104	2.1
29071	1.25104	2.1
29072	1.39703	2.1
29073	1.86893	2.1
29074	2.01492	2.1
29075	2.408	1.97988
29076	1.86893	2.1
29077	2.22575	2.09678
29078	1.70677	2.1
29079	4.09468	2.1
29080	0.737605	2.1
29081	0.737605	2.1
29082	0.741302	2.1
29083	0.741302	2.1
29084	0.55189	2.1
29085	0.625517	2.1
29086	0.934468	2.1
29087	0.853389	2.1
29088	0.853389	2.1
29089	0.55189	2.1
29090	0.625517	2.1
29091	0.934467	2.1
29092	2.35053	2.1
29093	1.09615	2.1
29094	1.25998	2.1
29095	2.51216	2.1
29096	3.61051	2.1
29097	1.33636	2.1
29098	1.25609	2.1
29099	0.698455	2.1
29100	0.629995	2.1
29101	0.986092	2.1
29102	0.986092	2.1
29103	0.980263	2.1
29104	0.980263	2.1
29105	1.33636	2.1
29106	0.698455	2.1
29107	0.629995	2.1
29108	1.25609	2.1
29109	1.89226	1.17261
29110	1.89226	1.1987
29111	1.89226	1.41597
29112	1.89226	1.18038
29113	3.78452	1.28247

29114	1.74321	0.935289
29115	1.89226	0.890392
29116	1.91755	0.99088
29117	1.91755	1.01922
29118	1.89226	0.94718
29119	1.89226	1.00347
29120	0.946132	0.849647
29121	0.946132	0.96261
29122	0.949798	0.658944
29123	1.08054	0.752768
29124	1.08054	0.72664
29125	1.08054	0.751843
29126	0.946132	0.964574
29127	0.946132	0.736103
29128	0.946132	0.763593
29129	0.949798	0.645329
29130	1.08054	0.743142
29131	0.946131	0.848081
29132	1.89226	1.34585
29133	1.89226	0.958831
29134	1.89226	1.05044
29135	1.89226	1.02862
29136	1.89226	1.13959
29137	1.89226	1.11982
29138	2.39378	0.0721588
29139	2.29234	0.0698591
29140	2.29234	0.0698591
29141	2.7933	0.0698591
29142	1.18422	0.0698591
29143	1.68518	0.0698591
29144	2.79329	0.0698591
29145	1.89226	0.0698591
29146	2.29233	0.0698591
29147	1.89226	0.0698591
29148	4.18459	0.0698591
29149	0.857046	0.0698591
29150	0.857037	0.0698591
29151	0.94613	0.0698591
29152	0.94613	0.0698591
29153	0.851539	0.0698591
29154	0.851539	0.0698591
29155	1.0315	0.0698591
29156	0.952867	0.0698591
29157	0.851486	0.0698591
29158	0.952869	0.0698591
29159	1.03156	0.0698591
29160	0.851528	0.0698591
29161	1.61981	2.1
29162	1.93096	2.1
29163	2.00776	2.1
29164	1.62218	2.1
29165	3.62757	2.1
29166	2.00776	2.1
29167	1.68541	2.1
29168	1.83215	2.1
29169	1.62218	2.1
29170	2.21773	2.1
29171	2.00776	2.1

29172	1.89188	0.0698591
29173	1.70303	0.0698591
29174	1.89226	0.0698591
29175	1.72465	0.0698591
29176	1.89226	0.0698591
29177	1.70552	0.0698591
29178	2.48659	0.0698591
29179	2.0815	0.0704835
29180	1.89226	0.0755604
29181	2.08111	0.0702794
29182	1.89226	0.0725669
29183	2.08111	0.0703802
29184	2.35332	0.262459
29185	1.83284	0.140876
29186	2.28483	0.175277
29187	2.71955	0.160015
29188	4.63815	0.156599
29189	1.89227	2.1
29190	1.89227	2.1
29191	1.89227	2.1
29192	1.89227	2.1
29193	3.78453	2.1
29194	1.33506	2.1
29195	1.17129	2.1
29196	1.33506	2.1
29197	1.38415	2.1
29198	1.13664	2.1
29199	1.18573	2.1
29200	1.89226	2.1
29201	2.68785	2.1
29202	1.89226	2.1
29203	2.6692	2.1
29204	4.58011	2.1
29205	1.89226	2.1
29206	1.89226	2.1
29207	1.89226	2.1
29208	1.89226	2.1
29209	3.78453	2.1
29210	1.33506	2.1
29211	1.13664	2.1
29212	1.27575	2.1
29213	1.09627	2.1
29214	2.43133	2.1
29215	1.89226	2.1
29216	2.65349	2.1
29217	2.66919	2.1
29218	2.66919	2.1
29219	1.89226	2.1
29220	1.89226	2.1
29221	1.89226	2.1
29222	1.89226	2.1
29223	1.89226	2.1
29224	1.89226	2.1
29225	3.78453	2.1
29226	1.16834	2.1
29227	1.16834	2.1
29228	0.946132	2.1
29229	0.946132	2.1

29230	1.26978	2.1
29231	1.16834	2.1
29232	0.94613	2.1
29233	0.94613	2.1
29234	0.94613	2.1
29235	1.26978	2.1
29236	0.94613	2.1
29237	1.16834	2.1
29238	2.14698	2.1
29239	1.89226	2.1
29240	1.89226	2.1
29241	1.89226	2.1
29242	2.40719	2.1
29243	2.40719	2.1
29244	1.89226	2.1
29245	1.89226	2.1
29246	1.89226	2.1
29247	1.89226	2.1
29248	3.78453	2.1
29249	1.26977	2.1
29250	1.26977	2.1
29251	0.946133	2.1
29252	0.946133	2.1
29253	1.37467	2.1
29254	1.26978	2.1
29255	0.946131	2.1
29256	0.946131	2.1
29257	1.37467	2.1
29258	0.94613	2.1
29259	0.946131	2.1
29260	1.26978	2.1
29261	1.2036	2.1
29262	1.34912	2.1
29263	0.946131	2.1
29264	0.946131	2.1
29265	1.34912	2.1
29266	1.34912	2.1
29267	0.946129	2.1
29268	0.946129	2.1
29269	1.34912	2.1
29270	0.94613	2.1
29271	0.946131	2.1
29272	1.2036	2.1
29273	2.47164	0.393887
29274	1.89019	0.246127
29275	1.79109	0.367752
29276	2.41632	0.246144
29277	4.26273	0.369284
29278	1.54197	0.305413
29279	2.41632	0.197824
29280	1.79109	0.251605
29281	1.79109	0.309175
29282	2.20929	0.2176
29283	2.20929	0.289419
29284	1.70302	0.911124
29285	1.48104	1.11342
29286	1.99365	1.27174
29287	1.32897	0.812131

29288	1.70302	1.31099
29289	1.14034	0.859128
29290	1.70302	0.92853
29291	1.99365	0.918143
29292	1.99297	1.18741
29293	2.36337	0.656388
29294	4.04804	0.855507
29295	1.51569	0.729773
29296	2.49093	0.662907
29297	1.6026	0.794785
29298	1.43229	0.564803
29299	2.0815	0.880914
29300	1.83402	0.616802
29301	2.54665	2.1
29302	2.34044	2.1
29303	2.08889	2.1
29304	2.37653	2.1
29305	4.71697	2.1
29306	2.54665	2.1
29307	2.45212	2.1
29308	2.31012	2.1
29309	2.37653	2.1
29310	4.82865	2.1
29311	2.08889	2.1
29312	2.37653	2.1
29313	2.23366	2.1
29314	1.87598	2.1
29315	4.25251	2.1
29316	2.31012	2.1
29317	2.37653	2.1
29318	2.23367	2.1
29319	2.17384	2.1
29320	4.55037	2.1
29321	0.946131	2.1
29322	0.946131	2.1
29323	1.30338	2.1
29324	1.23186	2.1
29325	0.94613	2.1
29326	0.94613	2.1
29327	1.23187	2.1
29328	1.23187	2.1
29329	0.946131	2.1
29330	1.23186	2.1
29331	1.30338	2.1
29332	0.946131	2.1
29333	1.89226	2.1
29334	2.46373	2.1
29335	2.34725	2.1
29336	2.46373	2.1
29337	1.77579	2.1
29338	1.89226	2.1
29339	2.61901	2.1
29340	2.45899	2.1
29341	2.64067	2.1
29342	2.52708	2.1
29343	5.14609	2.1
29344	2.41795	2.1
29345	2.0815	2.1

29346	2.52708	2.1
29347	1.92277	2.1
29348	4.31368	2.1
29349	2.64067	2.1
29350	2.52708	2.1
29351	2.58638	2.1
29352	2.51039	2.1
29353	5.11346	2.1
29354	2.52708	2.1
29355	2.13642	2.1
29356	2.51039	2.1
29357	2.16633	2.1
29358	4.69341	2.1
29359	1.61028	1.65036
29360	1.65712	0.222307
29361	1.35002	0.174039
29362	1.35002	1.8208
29363	1.88637	0.201997
29364	1.88637	2.02104
29365	2.57663	2.1
29366	2.24227	2.1
29367	2.48354	2.1
29368	2.14317	2.1
29369	4.7198	2.1
29370	2.24227	2.1
29371	1.77353	2.1
29372	2.14317	2.1
29373	1.69956	2.1
29374	3.9167	2.1
29375	2.48353	2.1
29376	2.14317	2.1
29377	2.39282	2.1
29378	1.79824	2.1
29379	4.53599	2.1
29380	2.14317	2.1
29381	1.69956	2.1
29382	1.91427	2.1
29383	1.6347	2.1
29384	3.77787	2.1
29385	1.89226	0.222631
29386	1.89226	0.177016
29387	1.89226	0.100241
29388	1.89226	0.125081
29389	3.78453	0.125729
29390	1.89226	0.152153
29391	1.89226	0.159931
29392	1.89226	0.147846
29393	1.89226	0.130566
29394	3.78452	0.16101
29395	1.89226	0.167725
29396	1.89652	0.221322
29397	1.89226	0.198708
29398	1.89226	0.920383
29399	1.19352	0.130577
29400	1.19352	0.256089
29401	1.89226	0.187805
29402	1.89653	0.164991
29403	1.89226	0.468153

29404	1.89226	1.28046
29405	1.89226	0.122516
29406	1.89226	0.131887
29407	1.89226	0.246567
29408	1.75326	0.212814
29409	1.89226	0.591521
29410	1.12948	0.212726
29411	1.89226	0.0966128
29412	1.12948	0.0997495
29413	2.61064	2.1
29414	1.89226	2.1
29415	1.89226	2.1
29416	1.89226	2.1
29417	2.6085	2.1
29418	2.6085	2.1
29419	2.6085	2.1
29420	1.89226	2.1
29421	1.89226	2.1
29422	1.89226	2.1
29423	2.62161	2.1
29424	2.62161	2.1
29425	2.16535	2.1
29426	1.44603	2.1
29427	2.79784	2.1
29428	1.84756	2.1
29429	4.01292	2.1
29430	2.65262	2.1
29431	1.03391	2.1
29432	2.20239	2.1
29433	1.74416	2.1
29434	1.49213	2.1
29435	1.03391	2.1
29436	2.29066	2.1
29437	2.49174	2.1
29438	2.56502	2.1
29439	2.33802	2.1
29440	4.85568	2.1
29441	2.39663	2.1
29442	2.49174	2.1
29443	2.56502	2.1
29444	2.45929	2.1
29445	4.96165	2.1
29446	2.56502	2.1
29447	2.33802	2.1
29448	2.37168	2.1
29449	2.59967	2.1
29450	4.9367	2.1
29451	2.56502	2.1
29452	2.45929	2.1
29453	2.50138	2.1
29454	2.59967	2.1
29455	5.0664	2.1
29456	2.27009	1.52443
29457	1.26247	2.06136
29458	1.86812	2.02287
29459	2.837	1.7747
29460	4.13822	1.95238
29461	1.89226	2.1

29462	1.89226	2.1
29463	1.89226	2.1
29464	1.89226	2.1
29465	3.78453	2.1
29466	1.75835	2.1
29467	1.72866	2.1
29468	1.65313	2.1
29469	1.64822	2.1
29470	3.3818	2.1
29471	1.72866	2.1
29472	1.58401	2.1
29473	1.64822	2.1
29474	1.52441	2.1
29475	3.25307	2.1
29476	1.89226	2.1
29477	1.89226	2.1
29478	1.89226	2.1
29479	1.89226	2.1
29480	3.78452	2.1
29481	1.89226	2.1
29482	1.89226	2.1
29483	1.89226	2.1
29484	1.89226	2.1
29485	3.78453	2.1
29486	1.65313	2.1
29487	1.64822	2.1
29488	1.55779	2.1
29489	1.57968	2.1
29490	3.23281	2.1
29491	1.64822	2.1
29492	1.52441	2.1
29493	1.57967	2.1
29494	1.474 2.1	
29495	3.10408	2.1
29496	1.89226	2.1
29497	1.89226	2.1
29498	1.89226	2.1
29499	1.89226	2.1
29500	3.78452	2.1
29501	1.86067	1.3289
29502	1.58407	2.09241
29503	2.07965	0.321702
29504	1.83123	1.58977
29505	3.66372	0.952773
29506	1.89226	1.07772
29507	1.89226	2.09327
29508	1.89226	0.470724
29509	1.89226	2.04899
29510	3.78452	1.5332
29511	1.89226	2.03201
29512	1.89226	2.09947
29513	1.89226	1.66984
29514	1.89226	2.1
29515	1.89226	1.61892
29516	1.89226	2.1
29517	1.83123	0.689686
29518	1.03472	2.09981
29519	1.91068	1.95612

29520	1.58406	1.74799
29521	1.36133	1.89963
29522	1.03472	1.76614
29523	2.07965	0.609787
29524	1.83123	1.83381
29525	2.28385	0.775262
29526	2.09114	1.66373
29527	4.1708	1.20957
29528	1.89226	0.989743
29529	1.89226	2.05321
29530	1.89226	0.697501
29531	1.89226	1.65263
29532	3.78452	1.33903
29533	1.89226	1.95613
29534	1.89226	2.09719
29535	1.89226	1.08601
29536	1.89226	2.1
29537	3.78452	2.04121
29538	1.83123	0.653055
29539	1.36133	2.09642
29540	2.09114	0.96277
29541	1.69436	1.74437
29542	3.45248	1.63589
29543	1.89226	2.1
29544	1.89226	2.1
29545	1.89226	2.1
29546	1.89226	2.1
29547	3.78453	2.1
29548	1.53278	2.1
29549	1.67571	2.1
29550	1.40182	2.1
29551	1.55686	2.1
29552	3.07752	2.1
29553	1.67571	2.1
29554	1.52615	2.08166
29555	1.55686	2.1
29556	1.38963	1.9017
29557	3.06534	2.1
29558	1.89226	2.1
29559	1.89226	2.01415
29560	1.89226	2.1
29561	1.89226	1.91414
29562	3.78452	2.1
29563	1.89226	2.1
29564	1.89226	2.1
29565	1.89226	2.1
29566	1.89226	2.1
29567	3.78453	2.1
29568	1.40182	2.1
29569	1.55686	2.1
29570	1.24509	2.1
29571	1.38426	2.1
29572	2.78608	2.1
29573	1.55686	2.1
29574	1.38963	2.08378
29575	1.2064	2.1
29576	1.2064	2.1
29577	1.38426	2.1

29578	1.56749	2.1
29579	0.810946	2.1
29580	0.810946	2.0796
29581	0.810945	2.1
29582	0.810945	2.1
29583	0.810945	2.1
29584	0.810945	2.1
29585	1.89226	2.08892
29586	1.35169	2.1
29587	1.08132	2.1
29588	1.08132	2.04257
29589	1.08132	2.1
29590	1.28418	0.508796
29591	1.59382	0.719555
29592	1.78289	0.924964
29593	1.51021	0.49054
29594	3.10403	0.596737
29595	1.76897	1.96756
29596	1.59382	0.729702
29597	1.78289	1.09414
29598	1.94302	1.51495
29599	3.53684	1.51749
29600	1.89226	0.716995
29601	1.89226	1.15948
29602	1.89226	1.42897
29603	1.89226	0.680614
29604	3.78453	0.877807
29605	1.89227	0.713726
29606	1.89227	0.391052
29607	1.89227	0.411616
29608	1.89227	0.557636
29609	1.89227	0.474673
29610	1.89227	0.61112
29611	1.78286	0.747643
29612	1.51019	0.399327
29613	1.79979	0.445865
29614	2.02511	0.578045
29615	3.5353	0.539725
29616	1.78286	0.870871
29617	1.94299	1.24307
29618	2.17831	1.74566
29619	2.02511	0.661417
29620	3.9681	0.823786
29621	1.89224	1.17103
29622	1.89224	0.533608
29623	1.89224	0.63013
29624	1.89224	0.994659
29625	3.78448	0.870245
29626	1.89224	0.557912
29627	1.89224	0.350808
29628	1.89224	0.391956
29629	1.89224	0.550606
29630	1.89224	0.38061
29631	1.89224	0.54772
29632	1.83198	0.234099
29633	1.0961	0.0908084
29634	1.39448	0.148844
29635	1.99568	0.198198

29636	3.09178	0.171165
29637	1.25503	0.093251
29638	1.99568	0.167995
29639	2.3661	0.238747
29640	1.70302	0.114951
29641	3.55833	0.119568
29642	2.69899	0.0698591
29643	1.85796	0.0698591
29644	2.11407	0.0698591
29645	1.36769	0.0698591
29646	3.97203	0.0698591
29647	1.89226	0.0706299
29648	1.89226	0.0698591
29649	1.89226	0.0698591
29650	1.89226	0.0698591
29651	3.78452	0.0712469
29652	1.89225	0.0698591
29653	2.63355	0.0698591
29654	2.09529	0.0698591
29655	2.09529	0.0698591
29656	1.89225	0.0698591
29657	1.89225	0.0698591
29658	1.89226	0.0740592
29659	1.89226	0.0826427
29660	1.89226	0.0712618
29661	1.89226	0.101126
29662	1.89226	0.0710388
29663	1.89226	0.0931751
29664	2.12204	0.0698617
29665	1.89226	0.0793611
29666	2.05798	0.0698675
29667	2.01954	0.0698591
29668	1.70302	0.0707124
29669	1.3865	0.0731567
29670	1.28326	0.869911
29671	1.33326	2.1
29672	1.19052	1.18247
29673	1.25369	2.0982
29674	2.53694	1.99209
29675	1.33327	2.1
29676	1.27339	2.1
29677	1.25369	2.09974
29678	1.19733	2.1
29679	2.52708	2.1
29680	1.19052	1.09279
29681	1.25369	2.1
29682	1.10938	0.934414
29683	1.17614	2.1
29684	2.36307	1.93824
29685	1.25369	2.1
29686	1.19733	2.1
29687	1.17614	2.1
29688	1.11868	2.1
29689	2.37237	2.1
29690	1.10747	0.0747989
29691	1.47688	0.0815627
29692	1.97732	0.0698591
29693	1.62206	0.0720893

29694	3.09894	0.0753314
29695	1.89226	0.083355
29696	1.89226	0.0869927
29697	1.89226	0.0935737
29698	1.89226	0.104208
29699	1.89226	0.071561
29700	1.89226	0.0703901
29701	1.62206	0.0780282
29702	1.70302	0.079626
29703	1.22962	0.0887407
29704	1.1893	0.092917
29705	2.15177	0.0755177
29706	2.08121	0.0750822
29707	1.97732	0.0722877
29708	2.08121	0.0746493
29709	1.62206	0.0788035
29710	1.62206	0.100097
29711	2.41464	0.0702651
29712	2.41464	0.0754121
29713	1.89226	0.0956082
29714	1.89226	0.136554
29715	1.89226	0.0743781
29716	1.89226	0.0773118
29717	3.78452	0.0794304
29718	0.946133	0.0865855
29719	0.946133	0.0892316
29720	0.946132	0.104487
29721	0.946132	0.106482
29722	0.946132	0.0987186
29723	0.946132	0.107473
29724	0.946133	0.0778195
29725	0.946133	0.0854071
29726	0.946132	0.0763611
29727	0.946132	0.089224
29728	0.946132	0.115293
29729	0.946132	0.0861514
29730	1.83157	1.70008
29731	2.19989	1.5187
29732	2.20664	0.645463
29733	1.81487	0.667657
29734	4.03146	1.298
29735	2.41055	2.1
29736	2.19989	2.1
29737	2.20665	2.07568
29738	2.38023	1.98013
29739	4.61044	2.08396
29740	2.19989	2.1
29741	2.22558	2.1
29742	2.38023	2.07322
29743	2.07065	0.973247
29744	4.42547	2.0998
29745	2.19988	1.85915
29746	1.70303	1.08905
29747	1.89939	0.770021
29748	1.86357	0.525336
29749	3.78536	0.877896
29750	1.04921	2.1
29751	1.30711	2.1

29752	1.53076	2.1
29753	1.22927	2.1
29754	2.57997	2.1
29755	1.53076	2.1
29756	1.46568	2.1
29757	1.56056	2.1
29758	1.22927	2.1
29759	1.86206	2.1
29760	1.53076	2.1
29761	1.18353	2.1
29762	2.28593	2.1
29763	1.16979	2.1
29764	2.2731	2.1
29765	3.45571	2.1
29766	0.854776	2.1
29767	0.854776	2.1
29768	0.855641	2.1
29769	0.855641	2.1
29770	1.12552	2.1
29771	1.13655	2.1
29772	0.57216	2.1
29773	0.584893	2.1
29774	0.57216	2.1
29775	1.12552	2.1
29776	0.584893	2.1
29777	1.13655	2.1
29778	1.54462	2.1
29779	2.76309	2.1
29780	1.34869	2.1
29781	2.48573	2.1
29782	4.03035	2.1
29783	1.35328	0.803652
29784	1.0383	0.769202
29785	1.03318	1.22106
29786	0.704834	1.32774
29787	1.0257	1.24374
29788	1.0257	0.737584
29789	1.03241	0.658732
29790	1.03241	1.31271
29791	1.35328	1.13894
29792	1.03319	1.26768
29793	1.0383	1.16807
29794	0.704835	1.18297
29795	2.27126	0.654592
29796	2.77291	2.1
29797	1.81771	1.83869
29798	2.6111	1.90414
29799	4.88236	1.92983
29800	2.77291	2.1
29801	1.89227	2.1
29802	1.89227	2.01306
29803	1.89227	2.1
29804	2.6111	1.95661
29805	2.6111	2.1
29806	2.22061	1.79986
29807	2.41522	1.96227
29808	2.41522	1.17568
29809	2.6111	2.1

29810	1.75765	1.12719
29811	1.95353	2.1
29812	2.27502	0.497588
29813	1.89227	1.39061
29814	1.89227	0.650178
29815	2.8189	0.477791
29816	4.71116	0.48579
29817	1.89226	0.564231
29818	1.89226	1.55043
29819	1.89226	0.983071
29820	1.89226	0.781177
29821	1.89226	1.1001
29822	1.89226	0.85375
29823	1.89226	1.77013
29824	1.89226	1.64062
29825	1.89226	1.63792
29826	1.89226	1.23155
29827	3.78453	1.69404
29828	1.89226	1.76327
29829	2.57079	0.839632
29830	2.5932	1.48201
29831	1.89226	1.26401
29832	4.48546	1.33697
29833	0.589396	1.3426
29834	0.641454	0.972187
29835	0.814062	1.57578
29836	0.726518	1.33964
29837	0.675126	1.50046
29838	0.675125	1.4056
29839	0.692847	1.57029
29840	0.692847	1.37378
29841	0.641454	1.12573
29842	0.814062	1.46328
29843	0.726518	1.60554
29844	0.589396	1.3986
29845	1.89226	1.72832
29846	1.89226	1.23799
29847	1.89226	1.65367
29848	1.89226	1.75654
29849	1.89226	1.51026
29850	1.89226	1.7422
29851	1.16906	0.61705
29852	1.2966	1.38016
29853	0.946132	1.24757
29854	0.946132	1.8165
29855	1.16906	1.21271
29856	1.16906	0.708207
29857	0.946132	1.45961
29858	0.946132	1.64411
29859	1.16906	1.41605
29860	0.946131	1.38857
29861	1.2966	1.3887
29862	0.946132	1.63008
29863	0.641455	1.32685
29864	0.567191	0.972868
29865	0.807594	1.658
29866	0.814062	1.38082
29867	0.673735	1.61086

29868	0.673734	1.22157
29869	0.707519	1.3581
29870	0.707519	1.68119
29871	0.807594	1.50264
29872	0.56719	1.64985
29873	0.641455	1.55524
29874	0.814063	1.77723
29875	1.89225	2.1
29876	1.89225	2.1
29877	1.89225	2.1
29878	1.89225	2.1
29879	3.7845	2.1
29880	1.89225	2.1
29881	1.89225	2.1
29882	1.89225	2.1
29883	1.89225	2.1
29884	1.89225	2.1
29885	1.89225	2.1
29886	1.89226	2.1
29887	1.89226	2.1
29888	1.89226	2.1
29889	1.89226	2.1
29890	3.78452	2.1
29891	1.89226	2.1
29892	1.89226	2.1
29893	1.89226	2.1
29894	1.89226	2.1
29895	1.89226	2.1
29896	1.89226	2.1
29897	1.89226	2.1
29898	1.89226	2.1
29899	1.89226	2.1
29900	1.89226	2.1
29901	3.78452	2.1
29902	1.89226	2.1
29903	1.38928	2.1
29904	1.89226	2.1
29905	1.51378	2.1
29906	1.89061	2.1
29907	1.89226	2.1
29908	1.89226	2.1
29909	1.89226	2.08823
29910	1.89226	2.08738
29911	1.89226	2.1
29912	3.78453	2.1
29913	1.04572	2.1
29914	0.994213	2.1
29915	0.912466	2.09356
29916	0.912466	2.0993
29917	0.947743	2.09527
29918	1.00388	2.1
29919	0.900913	2.08776
29920	0.900913	2.1
29921	0.900915	2.1
29922	0.946131	2.1
29923	0.946131	2.1
29924	0.999196	2.1
29925	1.89226	2.06358

29926	1.89226	2.1
29927	1.89226	2.1
29928	1.89226	2.0787
29929	3.78453	2.09602
29930	0.946161	2.1
29931	1.1354	2.1
29932	0.850847	2.1
29933	0.850847	2.1
29934	1.01445	2.1
29935	1.02106	2.1
29936	0.946131	2.1
29937	0.946131	2.1
29938	1.13537	2.1
29939	1.01444	2.1
29940	0.850877	2.1
29941	0.946131	2.1
29942	1.80183	2.1
29943	1.89226	2.08416
29944	1.89226	2.08008
29945	1.89226	2.1
29946	1.79606	2.0813
29947	1.79606	2.1
29948	1.24001	2.02557
29949	1.41822	0.703803
29950	1.95665	0.25268
29951	1.77342	1.60534
29952	3.19666	0.661497
29953	1.95665	1.2329
29954	1.44859	1.77982
29955	1.41821	1.89133
29956	1.41821	1.90535
29957	1.95543	1.42456
29958	1.95543	0.953692
29959	1.95665	0.268913
29960	1.77342	1.47483
29961	2.33611	1.43349
29962	2.49694	0.426403
29963	4.29276	1.68496
29964	1.95665	1.60463
29965	1.95544	0.508324
29966	2.4728	0.127898
29967	2.49693	1.55007
29968	4.42945	0.29844
29969	2.24937	2.1
29970	2.49053	2.1
29971	2.16323	2.1
29972	2.36377	2.1
29973	4.61314	2.1
29974	2.49053	2.1
29975	2.58639	2.1
29976	2.36377	2.1
29977	2.47395	2.1
29978	4.95016	2.1
29979	2.45054	2.1
29980	2.36377	2.1
29981	1.92911	2.1
29982	2.181 2.1	
29983	4.29288	2.1

29984	2.36377	2.1
29985	2.47395	2.1
29986	2.181 2.1	
29987	2.29881	2.1
29988	4.66258	2.1
29989	1.57885	2.1
29990	1.13339	2.1
29991	1.48594	2.1
29992	1.13322	2.1
29993	2.71207	2.1
29994	1.48596	2.1
29995	1.20761	2.1
29996	1.20761	2.1
29997	1.13323	2.1
29998	1.46568	2.1
29999	1.3913	2.1
30000	1.89226	0.0698591
30001	1.89226	0.147965
30002	1.89226	0.0926268
30003	1.89226	0.392614
30004	1.89226	0.0707662
30005	1.89226	0.114447
30006	1.11712	0.104023
30007	0.946129	0.382622
30008	0.686934	0.0698591
30009	0.946133	0.0698591
30010	0.9461	0.0698821
30011	1.10729	0.58354
30012	0.686963	0.147817
30013	0.686963	0.0698591
30014	1.10945	0.0948908
30015	0.80397	0.415277
30016	0.946131	0.0698591
30017	0.946131	0.0805353
30018	1.89226	2.1
30019	1.89226	1.99689
30020	1.89226	2.07971
30021	1.89226	2.09572
30022	3.78452	2.09079
30023	1.22615	2.1
30024	1.3439	2.1
30025	1.47868	2.1
30026	1.55998	2.09809
30027	2.78613	2.1
30028	1.04973	2.1
30029	1.47868	2.1
30030	1.20149	2.1
30031	1.61721	2.1
30032	2.68017	2.1
30033	1.89226	2.1
30034	1.89226	2.1
30035	1.89226	2.1
30036	1.89226	2.06633
30037	3.78452	2.1
30038	0.946133	1.74197
30039	0.946133	2.09987
30040	0.946128	2.1
30041	0.946128	2.1

30042	0.946131	1.62839
30043	0.946131	2.09121
30044	0.946131	2.1
30045	0.946131	2.05422
30046	0.94613	2.1
30047	0.94613	2.09421
30048	0.946131	2.1
30049	0.946131	2.09892
30050	0.798126	2.1
30051	0.798126	2.1
30052	0.805719	2.1
30053	0.805719	2.1
30054	0.779992	2.1
30055	0.864507	2.1
30056	0.739338	2.1
30057	0.808607	2.1
30058	0.808606	2.1
30059	0.864506	2.1
30060	0.739339	2.1
30061	0.779993	2.1
30062	0.946128	2.1
30063	0.946128	2.1
30064	1.18959	2.1
30065	1.00304	2.1
30066	1.00304	2.1
30067	1.00304	2.1
30068	0.94613	2.1
30069	0.94613	2.1
30070	0.94613	2.1
30071	1.18959	2.1
30072	1.00304	2.1
30073	0.94613	2.1
30074	2.19163	2.1
30075	1.89226	2.1
30076	2.16541	2.1
30077	1.89226	2.1
30078	4.08389	2.1
30079	1.89226	2.1
30080	1.89226	1.9592
30081	1.89226	2.1
30082	1.89226	1.24972
30083	3.78452	2.08834
30084	0.964782	2.1
30085	1.30151	1.05355
30086	1.30318	0.964416
30087	1.30318	2.1
30088	0.949134	1.32808
30089	0.949134	2.1
30090	2.15005	2.1
30091	1.89226	2.1
30092	2.16541	2.1
30093	2.16541	2.1
30094	1.89226	2.1
30095	1.89226	2.1
30096	1.89226	2.1
30097	1.89226	2.04849
30098	1.89226	2.1
30099	1.89226	0.576603

30100	3.78452	1.91914
30101	0.949134	2.1
30102	1.30151	2.03841
30103	0.949134	2.1
30104	1.32143	1.1071
30105	2.25064	2.1
30106	0.982859	0.233384
30107	1.28326	0.840012
30108	1.19052	0.255703
30109	1.19052	1.12278
30110	1.10476	0.239569
30111	1.10476	1.14859
30112	1.37265	0.687193
30113	1.38247	1.61788
30114	1.36636	1.73551
30115	1.3294	0.735463
30116	2.71187	1.22552
30117	1.89226	1.70831
30118	1.89226	0.787473
30119	1.89226	0.812218
30120	1.89226	1.62787
30121	1.89226	0.805252
30122	1.89226	1.61913
30123	1.36636	1.8915
30124	1.3294	0.943513
30125	1.30001	1.01116
30126	1.36416	1.82516
30127	2.69356	1.52192
30128	1.89226	1.86967
30129	1.89226	0.862836
30130	1.89226	1.01516
30131	1.89226	1.90499
30132	1.89226	0.941676
30133	1.89226	1.80523
30134	2.83701	0.313371
30135	1.89226	0.840114
30136	1.89226	1.01807
30137	2.79312	0.303632
30138	4.68539	0.659027
30139	1.44288	2.1
30140	0.948677	2.1
30141	1.84076	2.1
30142	1.42884	2.1
30143	2.78943	2.1
30144	1.89226	2.1
30145	1.89226	2.1
30146	1.89226	2.1
30147	1.89226	2.1
30148	3.78452	2.1
30149	0.909402	2.1
30150	0.909402	2.1
30151	1.3063	2.1
30152	1.56757	2.1
30153	1.00038	2.1
30154	1.5235	2.1
30155	0.946127	2.1
30156	0.946127	2.1
30157	0.946128	2.1

30158	1.26957	2.1
30159	1.00038	2.1
30160	0.946129	2.1
30161	1.17256	2.1
30162	2.2846	2.1
30163	1.1844	2.1
30164	2.28904	2.1
30165	3.469 2.1	
30166	0.867886	2.1
30167	0.867886	2.1
30168	0.867277	2.1
30169	0.867277	2.1
30170	1.14296	2.1
30171	1.14452	2.1
30172	0.591766	2.1
30173	0.592199	2.1
30174	0.591766	2.1
30175	1.14296	2.1
30176	0.5922	2.1
30177	1.14452	2.1
30178	1.27408	0.0698591
30179	1.56339	0.0954268
30180	1.52522	0.0698591
30181	1.34447	0.0698591
30182	2.90786	0.0698591
30183	1.27408	0.0875091
30184	1.01121	0.0777274
30185	0.991868	0.0698591
30186	1.34447	0.0715968
30187	2.35569	0.0734268
30188	0.944818	0.0773175
30189	0.883008	0.0740475
30190	0.66957	0.0698591
30191	0.616883	0.0698591
30192	0.755679	0.0698591
30193	0.755679	0.109943
30194	0.791912	0.0762345
30195	0.701943	0.0698591
30196	0.901541	0.0703415
30197	0.634847	0.072084
30198	0.82781	0.0722726
30199	0.655405	0.0782717
30200	1.73819	2.1
30201	1.70303	2.1
30202	1.70303	2.1
30203	1.70303	2.1
30204	1.94516	2.1
30205	1.94517	2.1
30206	0.94994	2.1
30207	2.07469	2.1
30208	1.08506	2.1
30209	2.0815	2.1
30210	3.12902	2.1
30211	0.946131	2.1
30212	0.946131	2.1
30213	0.946131	2.1
30214	0.946131	2.1
30215	0.946128	2.1

30216	0.946128	2.1
30217	0.946133	2.1
30218	0.946133	2.1
30219	0.946131	2.1
30220	0.946131	2.1
30221	0.94613	2.1
30222	0.94613	2.1
30223	2.51796	2.1
30224	1.64193	2.1
30225	2.51172	2.1
30226	1.68893	2.1
30227	4.20689	2.1
30228	2.51172	2.1
30229	1.70002	2.1
30230	1.70002	2.1
30231	1.68893	2.1
30232	2.48689	2.1
30233	2.4758	2.1
30234	2.27963	0.0698591
30235	2.21582	0.0698591
30236	2.60486	0.0698591
30237	2.5105	0.0698591
30238	4.79013	0.0698591
30239	2.21582	0.0698591
30240	2.02069	0.0698591
30241	2.5105	0.0698591
30242	2.28615	0.0698591
30243	4.53119	0.0698591
30244	2.60486	0.0698591
30245	2.5105	0.0698591
30246	2.55791	0.0698591
30247	2.47141	0.0698591
30248	5.06842	0.0698591
30249	2.5105	0.0698591
30250	2.28615	0.0698591
30251	2.47141	0.0698591
30252	2.23337	0.0698591
30253	4.74387	0.0698591
30254	2.06984	2.1
30255	0.967499	2.1
30256	2.12435	2.1
30257	2.0021	2.1
30258	1.08975	2.1
30259	0.967499	2.1
30260	1.89226	0.0698591
30261	1.89226	0.0698591
30262	1.89226	0.0698591
30263	1.89226	0.0698591
30264	1.89226	0.0698591
30265	1.89226	0.0698591
30266	1.89226	2.1
30267	1.89226	2.1
30268	1.89226	2.1
30269	1.89226	2.1
30270	3.78452	2.1
30271	1.89227	2.1
30272	1.89227	2.1
30273	1.89227	2.1

30274	1.89227	2.1
30275	3.78453	2.1
30276	0.55882	2.1
30277	0.696462	2.1
30278	1.07003	2.1
30279	0.937179	2.1
30280	0.818077	2.1
30281	0.818077	2.1
30282	0.815565	2.1
30283	0.815565	2.1
30284	0.696462	2.1
30285	1.07003	2.1
30286	0.93718	2.1
30287	0.55882	2.1
30288	2.53857	2.1
30289	1.87436	2.1
30290	2.27541	2.1
30291	2.27541	2.1
30292	2.14005	2.1
30293	2.14005	2.1
30294	1.89226	2.1
30295	1.89226	2.1
30296	1.89226	2.1
30297	1.89226	2.1
30298	3.78452	2.1
30299	1.89227	2.1
30300	1.89227	2.1
30301	1.89227	2.1
30302	1.89227	2.1
30303	3.78453	2.1
30304	1.39292	2.1
30305	2.14006	2.1
30306	2.41083	2.1
30307	1.68761	2.1
30308	3.80376	2.1
30309	2.53857	2.1
30310	2.14005	2.1
30311	2.41083	2.1
30312	2.82043	2.1
30313	4.9494	2.1
30314	1.90439	0.172593
30315	1.51835	0.168027
30316	1.20473	0.145154
30317	0.947693	0.193539
30318	2.85208	0.17281
30319	2.586	2.1
30320	1.46262	2.1
30321	2.14009	2.1
30322	2.78101	2.1
30323	4.72609	2.1
30324	1.89224	2.1
30325	2.78101	2.1
30326	2.586	2.1
30327	2.586	2.1
30328	1.89224	2.1
30329	1.89224	2.1
30330	0.994293	2.1
30331	1.19	2.1

30332	1.09483	2.1
30333	1.09483	2.1
30334	1.06827	2.1
30335	1.06827	2.1
30336	1.89226	2.1
30337	1.89226	2.1
30338	1.89226	2.1
30339	1.89226	2.1
30340	3.78452	2.1
30341	0.946132	2.1
30342	0.946132	2.1
30343	1.31912	2.1
30344	1.3572	2.1
30345	0.946131	2.1
30346	0.946131	2.1
30347	1.31912	2.1
30348	1.31912	2.1
30349	0.946131	2.1
30350	1.31912	2.1
30351	1.35721	2.1
30352	0.946131	2.1
30353	2.24577	0.43971
30354	1.09408	0.519952
30355	1.60239	0.510237
30356	1.09408	0.517738
30357	2.39404	0.447582
30358	2.07382	0.477745
30359	1.70303	0.0792382
30360	1.3216	0.117678
30361	1.23104	0.0698914
30362	1.05475	0.106861
30363	2.60408	0.0807275
30364	1.89226	0.129233
30365	1.89226	0.150923
30366	1.89226	0.087255
30367	1.89226	0.141206
30368	3.78452	0.160052
30369	1.89226	0.0725925
30370	2.3845	0.0827308
30371	2.6875	0.0848605
30372	1.89226	0.0836376
30373	2.6875	0.0698591
30374	1.89226	0.0707731
30375	1.46759	2.1
30376	1.17585	2.1
30377	1.85189	2.1
30378	1.39223	2.1
30379	2.85981	2.1
30380	1.85189	2.1
30381	1.71255	2.1
30382	1.71255	2.1
30383	1.39223	2.1
30384	2.28511	2.1
30385	1.96479	2.1
30386	1.89227	0.532127
30387	1.89227	0.809594
30388	1.89227	1.09269
30389	1.89227	0.416873

30390	1.89227	1.28636
30391	1.89227	0.508116
30392	1.89226	0.619408
30393	1.89226	0.721363
30394	1.89226	1.00208
30395	1.89226	0.404439
30396	1.89226	1.22841
30397	1.89226	0.485006
30398	1.89226	0.535159
30399	1.89226	0.984755
30400	1.89226	1.01351
30401	1.89226	0.376789
30402	1.89226	1.38918
30403	1.89226	0.498047
30404	1.89226	0.379846
30405	2.77788	0.408257
30406	2.21062	0.558113
30407	2.21062	0.306604
30408	1.89226	0.996497
30409	1.89226	0.359665
30410	0.868548	0.362451
30411	0.610104	0.480879
30412	0.796678	0.361861
30413	0.865021	0.30148
30414	1.0883	0.367052
30415	0.904938	0.453306
30416	2.21062	0.402858
30417	1.49548	0.381
30418	1.2068	0.698641
30419	1.54764	0.350346
30420	0.790005	0.330437
30421	1.89226	2.1
30422	2.67457	2.1
30423	1.89226	2.1
30424	2.56352	2.1
30425	4.56683	2.1
30426	2.67456	2.1
30427	2.20011	2.1
30428	2.56351	2.1
30429	2.11083	2.1
30430	4.78539	2.1
30431	2.56348	2.1
30432	2.1108	2.1
30433	2.45763	2.1
30434	2.00986	2.1
30435	4.56843	2.1
30436	0.946133	0.0698591
30437	0.946133	0.0698591
30438	0.946132	0.0698591
30439	0.946132	0.0698591
30440	0.946132	0.0698591
30441	0.946132	0.0698591
30442	0.946132	0.0698591
30443	0.946132	0.0698591
30444	0.946132	0.0698591
30445	0.946132	0.0698591
30446	0.946133	0.0698591
30447	0.946133	0.0698591

30448	1.45927	1.42679
30449	1.25133	1.80968
30450	1.00082	1.82391
30451	1.41689	1.72416
30452	1.0432	1.56656
30453	1.45927	1.69707
30454	1.41689	1.70755
30455	1.29579	1.81671
30456	1.34874	1.62186
30457	1.25133	1.88419
30458	2.76563	1.94095
30459	0.675555	1.68134
30460	0.674371	1.63981
30461	0.625664	1.71519
30462	0.523133	1.72083
30463	0.676509	1.78822
30464	0.676509	1.77124
30465	0.62471	1.60485
30466	0.62471	1.60636
30467	0.675556	1.49237
30468	0.523132	1.31303
30469	0.674373	1.32182
30470	0.625663	1.50148
30471	1.89226	1.38927
30472	1.89226	1.16912
30473	1.89226	0.599612
30474	1.89226	1.05627
30475	3.78453	1.17255
30476	0.851517	1.18226
30477	0.946133	1.01638
30478	0.946132	1.21749
30479	0.946132	1.31809
30480	0.946133	0.77735
30481	0.851517	1.78941
30482	0.946132	1.37893
30483	0.946132	1.12578
30484	0.851516	1.11737
30485	0.851516	0.891537
30486	0.946133	1.51501
30487	0.946133	1.16175
30488	0.569995	0.777523
30489	0.816895	1.34554
30490	1.33893	1.29775
30491	1.18795	0.845265
30492	1.18795	0.827186
30493	0.569995	1.50565
30494	0.816895	1.57874
30495	1.33893	1.23823
30496	0.859572	1.70248
30497	0.859572	0.875369
30498	1.04936	1.08147
30499	1.04936	0.894252
30500	1.89226	2.1
30501	1.89226	2.1
30502	2.55618	2.1
30503	2.55618	2.1
30504	2.66983	2.1
30505	2.00591	2.1

30506	1.89226	2.1
30507	2.64586	2.1
30508	2.83331	2.1
30509	2.83331	2.1
30510	1.89226	2.1
30511	1.89226	2.1
30512	2.64586	2.1
30513	2.0815	2.1
30514	1.99985	2.1
30515	1.94005	2.1
30516	2.83332	2.1
30517	2.74859	2.1
30518	1.98061	2.1
30519	1.68356	2.1
30520	1.83879	2.1
30521	1.55987	2.1
30522	3.54048	2.1
30523	1.68356	2.1
30524	1.20969	2.1
30525	1.20969	2.1
30526	1.30751	2.1
30527	1.55987	2.1
30528	1.65769	2.1
30529	1.83879	2.1
30530	1.55987	2.1
30531	1.68443	2.1
30532	1.4449	2.1
30533	3.2443	2.1
30534	0.660368	2.1
30535	0.660368	2.1
30536	0.666922	2.1
30537	0.666922	2.1
30538	0.560377	2.1
30539	0.604842	2.1
30540	0.779935	2.1
30541	0.722448	2.1
30542	0.722446	2.1
30543	0.560377	2.1
30544	0.604844	2.1
30545	0.779934	2.1
30546	1.89227	2.1
30547	1.89227	2.1
30548	1.89227	2.1
30549	1.89227	2.1
30550	3.78453	2.1
30551	1.42896	2.1
30552	1.23037	2.1
30553	1.42896	2.1
30554	1.474 2.1	
30555	1.2016	2.1
30556	1.24665	2.1
30557	1.89226	2.1
30558	2.7188	2.1
30559	1.89226	2.1
30560	2.70702	2.1
30561	4.61106	2.1
30562	1.89226	2.1
30563	1.89226	2.1

30564	1.89226	2.1
30565	1.89226	2.1
30566	3.78453	2.1
30567	0.644603	2.1
30568	0.644603	2.1
30569	0.648272	2.1
30570	0.648272	2.1
30571	0.585644	2.1
30572	0.600802	2.1
30573	0.692073	2.1
30574	0.714478	2.1
30575	0.585644	2.1
30576	0.692073	2.1
30577	0.714478	2.1
30578	0.600802	2.1
30579	1.35351	2.1
30580	1.35351	2.1
30581	0.946131	2.1
30582	0.946131	2.1
30583	1.34392	2.1
30584	1.35351	2.1
30585	0.946132	2.1
30586	0.946132	2.1
30587	0.946132	2.1
30588	1.34392	2.1
30589	0.946132	2.1
30590	1.35351	2.1
30591	1.87609	2.1
30592	1.79155	2.1
30593	1.79155	2.1
30594	1.89226	2.1
30595	1.89226	2.1
30596	1.99211	2.1
30597	1.89226	2.1
30598	1.71706	2.1
30599	1.71706	2.1
30600	1.79155	2.1
30601	1.89226	2.1
30602	1.96675	2.1
30603	1.46869	2.1
30604	1.89226	2.1
30605	1.89226	2.1
30606	1.89226	2.1
30607	1.5001	2.1
30608	1.5001	2.1
30609	1.89226	2.1
30610	1.89226	2.1
30611	1.89226	2.1
30612	1.89226	2.1
30613	1.89226	2.1
30614	1.89226	2.1
30615	1.21408	2.1
30616	2.23563	2.1
30617	2.34021	2.1
30618	2.34021	2.1
30619	1.17285	2.1
30620	1.17285	2.1
30621	1.89226	2.1

30622	1.89226	2.1
30623	1.47937	2.1
30624	1.89226	2.1
30625	1.14822	2.1
30626	1.46869	2.1
30627	1.89226	2.1
30628	1.89226	2.1
30629	1.89226	2.1
30630	1.89226	2.1
30631	1.89226	2.1
30632	1.89226	2.1
30633	1.25716	2.1
30634	2.34021	2.1
30635	2.4542	2.1
30636	2.4542	2.1
30637	1.21408	2.1
30638	1.21408	2.1
30639	1.66824	2.1
30640	2.44883	2.1
30641	1.93003	2.1
30642	2.24795	2.1
30643	3.88907	2.1
30644	1.89319	2.1
30645	2.24795	2.1
30646	2.82724	2.1
30647	2.31318	2.1
30648	2.30868	2.1
30649	1.93003	2.1
30650	1.89226	2.1
30651	1.42589	2.1
30652	1.42589	2.1
30653	1.80501	2.1
30654	1.89226	2.1
30655	2.27137	2.1
30656	1.21067	0.0717382
30657	2.39166	0.139307
30658	1.73577	0.0729125
30659	1.73577	0.0738012
30660	1.53584	0.10985
30661	1.53584	0.0901663
30662	1.35025	2.1
30663	2.0599	1.64093
30664	1.51084	2.1
30665	2.27218	0.645895
30666	3.57074	2.03136
30667	2.0599	1.41698
30668	2.51818	2.1
30669	2.27218	0.52857
30670	2.66532	2.06215
30671	4.72522	1.69674
30672	1.67115	2.1
30673	2.27218	1.30286
30674	2.39605	2.1
30675	2.39605	0.99917
30676	1.51084	2.1
30677	1.51084	1.96466
30678	2.27218	0.989907
30679	2.66532	2.1

30680	2.39605	0.631748
30681	2.76829	2.06151
30682	5.06137	1.11169
30683	2.08684	1.49894
30684	2.36033	2.00303
30685	1.89224	1.19665
30686	1.89224	1.93275
30687	4.25257	1.41905
30688	2.41473	0.164943
30689	1.00307	0.212414
30690	2.01365	0.178826
30691	1.00307	0.226091
30692	3.05113	0.164617
30693	2.04056	0.244363
30694	1.11099	2.1
30695	2.25103	2.1
30696	2.22227	2.1
30697	2.22227	2.1
30698	1.14431	2.1
30699	1.14431	2.1
30700	1.07485	2.1
30701	2.22229	2.1
30702	2.19163	2.1
30703	2.19163	2.1
30704	1.111 2.1	
30705	1.111 2.1	
30706	1.0547	2.1
30707	1.19886	2.1
30708	0.946131	2.1
30709	0.946131	2.1
30710	0.946131	2.1
30711	0.946131	2.1
30712	1.19886	2.1
30713	1.19886	2.1
30714	1.0547	2.1
30715	0.946131	2.1
30716	0.946132	2.1
30717	1.19886	2.1
30718	1.89417	2.1
30719	2.64424	1.35351
30720	1.76585	2.1
30721	2.47695	2.05555
30722	4.37112	2.1
30723	1.89226	2.1
30724	1.89226	2.1
30725	1.89226	2.1
30726	1.89226	2.0749
30727	3.78453	2.1
30728	0.930281	2.1
30729	1.0547	2.1
30730	0.946131	2.1
30731	0.946131	2.1
30732	0.930309	2.1
30733	0.930309	2.1
30734	1.07052	2.1
30735	1.07052	2.1
30736	0.93028	2.1
30737	0.946131	2.1

30738	1.0547	2.1
30739	0.946131	2.1
30740	1.00015	1.06463
30741	1.00015	2.1
30742	1.01918	2.1
30743	1.01918	1.97376
30744	1.17437	2.01737
30745	1.23847	0.915737
30746	0.882926	2.1
30747	0.780859	2.1
30748	0.780859	2.06252
30749	1.17438	2.08195
30750	0.882925	2.09788
30751	1.23848	2.0965
30752	1.89226	2.1
30753	1.89226	2.02726
30754	1.89226	2.1
30755	1.89226	2.1
30756	1.89226	2.1
30757	1.89226	2.1
30758	1.22083	2.1
30759	1.29579	2.1
30760	1.26236	2.1
30761	1.06191	2.1
30762	1.42128	2.1
30763	1.22083	2.1
30764	1.42128	2.1
30765	1.45806	2.1
30766	1.43502	2.1
30767	1.29579	2.1
30768	1.5605	2.1
30769	1.42128	2.1
30770	1.02813	2.1
30771	1.12121	2.1
30772	1.40557	2.1
30773	1.35298	2.1
30774	1.1314	2.1
30775	1.28679	2.1
30776	1.1715	2.1
30777	1.1715	2.1
30778	0.975656	2.1
30779	1.24908	2.1
30780	0.98956	2.1
30781	1.2681	2.1
30782	0.946132	2.1
30783	0.946132	2.1
30784	1.31889	2.1
30785	1.31889	2.1
30786	1.16546	2.1
30787	1.31889	2.1
30788	0.946133	2.1
30789	0.946133	2.1
30790	0.946133	2.1
30791	1.31889	2.1
30792	0.946132	2.1
30793	1.16546	2.1
30794	2.51518	0.0698591
30795	2.37584	0.0698591

30796	1.11712	0.0698591
30797	1.31242	0.0698591
30798	3.68826	0.0698591
30799	2.51518	0.0698591
30800	1.26311	0.0698591
30801	2.48977	0.0698591
30802	2.48977	0.0698591
30803	1.31242	0.0698591
30804	1.31242	0.0698591
30805	1.89226	1.0804
30806	1.89226	2.1
30807	1.89226	0.91862
30808	1.89226	2.1
30809	3.78452	1.97412
30810	2.04737	0.663873
30811	1.77122	2.1
30812	1.94498	0.893244
30813	1.65174	2.1
30814	3.71621	2.03227
30815	1.41077	2.1
30816	1.29729	2.1
30817	1.77123	2.1
30818	1.77123	2.1
30819	1.65175	2.1
30820	1.29129	2.1
30821	1.89226	2.1
30822	1.89226	2.1
30823	1.89226	2.1
30824	1.89226	2.1
30825	1.89226	2.1
30826	1.89226	2.1
30827	1.89226	0.75147
30828	1.89226	2.1
30829	1.89226	0.884199
30830	1.89226	2.1
30831	3.78451	1.83415
30832	1.94498	0.737862
30833	1.65174	2.1
30834	1.7968	1.16207
30835	1.49831	2.1
30836	3.44329	1.93924
30837	1.49831	2.1
30838	1.29729	2.1
30839	1.49831	2.1
30840	1.65175	2.1
30841	1.16858	2.1
30842	1.32201	2.1
30843	1.89226	2.1
30844	1.89226	2.1
30845	1.89226	2.1
30846	1.89226	2.1
30847	3.78452	2.1
30848	1.89226	0.0698591
30849	1.89226	0.0698591
30850	1.89226	0.0698591
30851	1.89226	0.0698591
30852	1.89226	0.0698591
30853	1.89226	0.0698591

30854	1.65324	2.0607
30855	1.26409	0.389203
30856	1.42491	0.382952
30857	1.42491	2.0672
30858	1.52268	0.933523
30859	1.52268	2.0445
30860	1.52268	2.1
30861	1.26409	0.713552
30862	1.32697	2.06498
30863	1.05724	0.67513
30864	2.57991	1.52631
30865	1.89226	0.107448
30866	1.96548	0.113381
30867	2.0178	0.116635
30868	1.96548	0.109833
30869	1.94458	0.125402
30870	1.89226	0.115367
30871	1.99366	0.0994835
30872	2.0178	0.10607
30873	1.89226	0.108356
30874	2.0178	0.100653
30875	1.75999	0.120453
30876	1.89226	0.102952
30877	0.946126	2.1
30878	0.946126	2.1
30879	1.28228	2.1
30880	1.19444	2.1
30881	0.946127	2.1
30882	0.946127	2.1
30883	1.19444	2.1
30884	1.19444	2.1
30885	0.946128	2.1
30886	1.19444	2.1
30887	1.28228	2.1
30888	0.946128	2.1
30889	1.89226	2.1
30890	1.89226	2.1
30891	1.89226	2.1
30892	1.89226	2.1
30893	3.78452	2.1
30894	2.14104	2.1
30895	1.89226	2.1
30896	2.19948	2.1
30897	1.89226	2.1
30898	1.89226	2.1
30899	2.19948	2.1
30900	1.89226	2.1
30901	2.38888	2.1
30902	2.23932	2.1
30903	2.38888	2.1
30904	1.74269	2.1
30905	1.89226	2.1
30906	1.89226	2.1
30907	1.89226	2.1
30908	1.89226	2.1
30909	1.89226	2.1
30910	3.78452	2.1
30911	0.946132	2.1

30912	0.946132	2.1
30913	1.07052	2.1
30914	1.07052	2.1
30915	0.946132	2.1
30916	0.946132	2.1
30917	1.05132	2.1
30918	1.07052	2.1
30919	1.05132	2.1
30920	0.946132	2.1
30921	1.07052	2.1
30922	0.946133	2.1
30923	2.40171	2.1
30924	1.89226	2.1
30925	1.89226	2.1
30926	1.89226	2.1
30927	2.42723	2.1
30928	2.42723	2.1
30929	1.89226	2.1
30930	1.89226	2.1
30931	1.89226	2.1
30932	1.89226	2.09877
30933	3.78452	2.1
30934	1.63661	2.1
30935	2.3532	2.08938
30936	2.33287	2.09719
30937	2.33287	2.1
30938	1.64158	2.1
30939	1.64158	2.1
30940	2.38918	2.1
30941	1.89226	2.1
30942	1.89226	2.1
30943	1.89226	2.1
30944	2.40171	2.1
30945	2.40171	2.1
30946	1.89226	2.1
30947	1.89226	2.1
30948	1.89226	2.1
30949	1.89226	2.1
30950	1.89226	2.1
30951	1.89226	2.1
30952	1.64158	2.1
30953	2.3532	2.1
30954	1.6488	2.1
30955	2.37364	2.1
30956	4.002 2.1	
30957	2.54752	0.205533
30958	1.42445	0.268025
30959	1.86697	0.260379
30960	1.86697	0.230105
30961	2.06666	0.254385
30962	2.06666	0.231306
30963	2.50872	0.161602
30964	2.06666	0.223246
30965	1.90439	0.187297
30966	1.51835	0.186724
30967	3.97105	0.184409
30968	0.755161	0.239861
30969	0.755161	0.2135

30970	0.793994	0.187943
30971	0.793994	0.209731
30972	0.712225	0.230609
30973	0.515826	0.213711
30974	1.03333	0.21806
30975	0.759175	0.193367
30976	0.759173	0.208417
30977	0.515827	0.190283
30978	0.712226	0.201638
30979	1.03333	0.204931
30980	1.69633	0.206619
30981	1.89226	0.207862
30982	1.62477	0.187893
30983	1.89226	0.214696
30984	1.62477	0.199485
30985	1.89226	0.219225
30986	1.89226	0.418225
30987	1.89226	0.537715
30988	1.89226	0.528257
30989	1.89226	0.467717
30990	3.78452	0.48425
30991	2.28622	0.215624
30992	1.09667	0.134008
30993	1.17506	0.138107
30994	2.24411	0.18887
30995	1.17506	0.166182
30996	2.24411	0.235404
30997	2.24741	2.1
30998	1.70303	2.1
30999	2.49168	2.1
31000	1.57936	2.1
31001	2.53729	2.1
31002	1.70303	2.1
31003	2.24741	2.1
31004	2.47557	2.1
31005	1.89227	2.1
31006	1.89227	2.1
31007	4.36784	2.1
31008	2.27016	0.0698591
31009	1.10476	0.0698591
31010	1.44103	0.0698591
31011	1.10476	0.0700802
31012	2.26791	0.0698591
31013	1.93165	0.0698702
31014	2.27016	0.0698591
31015	1.89226	0.0719904
31016	1.89226	0.0698591
31017	2.4065	0.0698591
31018	4.29876	0.0698591
31019	2.27016	0.0698591
31020	1.44103	0.0698591
31021	1.53336	0.0698591
31022	2.4065	0.0698591
31023	3.84753	0.0698591
31024	2.7542	0.302944
31025	1.89226	0.385742
31026	2.35027	0.355513
31027	1.89226	0.353189

31028	2.35027	0.325045
31029	1.89226	0.326202
31030	2.3301	2.1
31031	2.36071	2.1
31032	2.07629	2.1
31033	2.01105	2.1
31034	4.37176	2.1
31035	1.86512	2.1
31036	2.01105	2.1
31037	2.01105	2.1
31038	2.3301	2.1
31039	1.57459	2.1
31040	1.89364	2.1
31041	2.52671	2.1
31042	2.01105	2.1
31043	1.78251	2.1
31044	1.70302	2.1
31045	3.98362	2.1
31046	1.227 2.1	
31047	2.01105	2.1
31048	1.45869	2.1
31049	1.41713	2.1
31050	1.70302	2.1
31051	1.64994	2.1
31052	2.00989	2.1
31053	1.28938	2.1
31054	1.8769	2.1
31055	1.20969	2.1
31056	3.21958	2.1
31057	1.8769	2.1
31058	1.15864	2.1
31059	1.15864	2.1
31060	1.20969	2.1
31061	1.77353	2.1
31062	1.82458	2.1
31063	1.7986	2.1
31064	2.48717	2.1
31065	2.50161	2.1
31066	1.75869	2.1
31067	4.30021	2.1
31068	2.50161	2.1
31069	1.75251	2.1
31070	1.81678	2.1
31071	1.75869	2.1
31072	2.5597	2.1
31073	2.50161	2.1
31074	1.91334	0.647104
31075	1.85439	1.19359
31076	1.91334	0.45805
31077	1.70303	1.16682
31078	1.70303	0.433263
31079	1.49692	1.06492
31080	2.39986	0.0698591
31081	1.89226	0.112286
31082	1.89226	0.0949236
31083	2.50756	0.0699053
31084	4.39982	0.0698591
31085	2.39986	0.0784201

31086	1.70303	0.0698591
31087	1.75564	0.0698591
31088	1.75904	0.083907
31089	2.50272	0.077258
31090	2.50756	0.093514
31091	1.89226	0.102314
31092	2.50756	0.0701731
31093	2.47857	0.0896711
31094	2.50756	0.110609
31095	1.86327	0.0958069
31096	1.89226	0.0960838
31097	1.22241	0.0916507
31098	1.20827	0.121183
31099	0.772416	0.0874485
31100	0.555208	0.13845
31101	1.004	0.0888137
31102	1.004	0.0967912
31103	0.93504	0.13889
31104	0.744364	0.11438
31105	1.2089	0.127467
31106	0.654935	0.128442
31107	0.773927	0.0846408
31108	1.03618	0.100708
31109	1.89227	0.0698591
31110	1.89227	0.0698591
31111	1.89227	0.0698591
31112	1.89227	0.0698591
31113	3.78453	0.0698591
31114	1.89226	0.0698591
31115	1.89226	0.0698591
31116	1.89226	0.0698591
31117	1.89226	0.0698591
31118	3.78452	0.0698591
31119	1.82643	0.0698591
31120	2.7084	0.0698591
31121	2.12654	0.0698591
31122	2.65614	0.0698591
31123	1.82643	0.0698591
31124	2.35603	0.0698591
31125	1.29385	0.0698591
31126	0.946133	0.0698591
31127	1.05492	0.0698591
31128	0.946132	0.0698591
31129	1.05492	0.0698591
31130	1.29385	0.0698591
31131	0.946133	0.0698591
31132	0.946133	0.0698591
31133	1.29385	0.0698591
31134	1.29385	0.0698591
31135	0.946132	0.0698591
31136	0.946132	0.0698591
31137	0.946132	0.0698591
31138	1.27329	0.0698591
31139	1.29385	0.0698591
31140	0.946129	0.0698591
31141	1.29385	0.0698591
31142	1.27328	0.0698591
31143	0.946131	0.0698591

31144	0.946131	0.0698591
31145	1.27328	0.0698591
31146	1.27328	0.0698591
31147	0.94613	0.0698591
31148	0.94613	0.0698591
31149	1.89226	1.92854
31150	1.89226	2.00051
31151	1.89226	0.606698
31152	1.89226	1.15171
31153	3.78453	1.56662
31154	1.89226	1.62319
31155	1.89226	1.85088
31156	1.89226	0.460803
31157	1.89226	1.60795
31158	3.78453	0.891896
31159	1.21488	1.78476
31160	1.14605	2.05655
31161	1.62974	1.31143
31162	1.52399	0.659863
31163	2.84463	1.7284
31164	0.577078	0.938477
31165	0.81251	2.07397
31166	0.821003	1.25913
31167	0.543413	1.88442
31168	0.683929	1.05564
31169	0.722433	2.1
31170	0.730674	2.00327
31171	0.730674	0.94125
31172	0.857766	1.19216
31173	0.862119	0.951469
31174	0.600938	1.92772
31175	0.596094	1.30769
31176	1.89226	1.19032
31177	1.89226	1.71008
31178	1.89226	0.338344
31179	1.89226	1.34986
31180	3.78453	1.05721
31181	1.89226	0.783172
31182	1.89226	2.07728
31183	1.89226	0.770953
31184	1.89226	1.23061
31185	3.78453	1.59379
31186	1.62974	2.01789
31187	1.56958	2.02497
31188	2.2362	0.462541
31189	2.11424	1.20017
31190	3.86595	1.20171
31191	2.2362	0.715663
31192	1.85414	2.07513
31193	2.28211	0.270645
31194	1.78367	0.324558
31195	2.2362	1.65676
31196	1.73256	1.93544
31197	0.851539	0.548143
31198	0.851539	0.587168
31199	1.14454	2.01551
31200	0.938988	1.89756
31201	0.851485	0.547199

31202	0.920593	1.39863
31203	0.851541	0.435877
31204	0.851541	1.74592
31205	1.04073	0.671862
31206	1.16342	0.942931
31207	0.825315	1.20177
31208	0.825316	0.676944
31209	1.89226	2.1
31210	1.89226	2.1
31211	1.89226	2.1
31212	1.89226	2.1
31213	3.78453	2.1
31214	2.57016	2.1
31215	1.74789	2.1
31216	2.57016	2.1
31217	2.522	2.1
31218	1.61727	2.1
31219	1.56911	2.1
31220	1.28075	2.1
31221	1.406	2.1
31222	0.946131	2.1
31223	0.946131	2.1
31224	1.28075	2.1
31225	1.00333	2.1
31226	0.94613	2.1
31227	0.94613	2.1
31228	1.041	2.1
31229	1.041	2.1
31230	0.943484	2.1
31231	1.27717	2.1
31232	1.89226	2.1
31233	1.89226	2.09383
31234	1.89226	2.09687
31235	1.89226	2.1
31236	1.89226	2.09899
31237	1.89226	2.1
31238	2.57016	2.1
31239	1.61727	2.1
31240	2.74196	2.1
31241	1.59094	2.1
31242	4.16109	2.1
31243	1.89226	2.09523
31244	2.0815	2.1
31245	2.2034	2.1
31246	1.82493	2.09218
31247	1.87847	2.1
31248	2.16142	2.1
31249	1.89226	2.1
31250	1.89226	2.1
31251	1.89226	2.1
31252	1.89226	2.1
31253	3.78453	2.1
31254	0.985252	2.1
31255	0.985252	2.1
31256	0.946132	2.1
31257	0.946132	2.1
31258	1.0655	2.1
31259	0.985255	2.1

31260	0.94613	2.1
31261	0.94613	2.1
31262	0.94613	2.1
31263	1.0655	2.1
31264	0.94613	2.1
31265	0.985255	2.1
31266	1.89226	2.1
31267	1.89226	2.1
31268	1.89226	2.1
31269	1.89226	2.1
31270	3.78453	2.1
31271	1.06549	2.1
31272	1.06549	2.1
31273	0.946133	2.1
31274	0.946133	2.1
31275	1.16834	2.1
31276	1.0655	2.1
31277	0.946131	2.1
31278	0.946131	2.1
31279	0.946131	2.1
31280	1.16834	2.1
31281	0.94613	2.1
31282	1.0655	2.1
31283	0.966631	2.1
31284	1.07349	2.1
31285	0.946131	2.1
31286	0.946131	2.1
31287	1.07349	2.1
31288	1.07349	2.1
31289	0.946129	2.1
31290	0.946129	2.1
31291	1.07349	2.1
31292	0.946131	2.1
31293	0.94613	2.1
31294	0.966631	2.1
31295	2.38055	0.668171
31296	1.89226	1.15393
31297	1.89226	1.09733
31298	1.46724	0.43903
31299	3.35949	0.738115
31300	1.42249	2.1
31301	2.05499	2.1
31302	1.39708	2.1
31303	2.09033	2.1
31304	3.45207	2.1
31305	2.05499	2.1
31306	2.57425	2.1
31307	2.51287	2.1
31308	2.47753	2.1
31309	2.09033	2.1
31310	2.05499	2.1
31311	1.39327	2.1
31312	2.09033	2.1
31313	2.16217	2.1
31314	2.16217	2.1
31315	1.39708	2.1
31316	1.39708	2.1
31317	2.16217	2.1

31318	2.57426	2.1
31319	2.73006	2.1
31320	2.73006	2.1
31321	2.09033	2.1
31322	2.09033	2.1
31323	1.89226	0.0759555
31324	1.89226	0.0698591
31325	1.89226	0.0698591
31326	1.89226	0.0723683
31327	3.78452	0.072545
31328	0.838749	0.0698591
31329	0.838749	0.0698591
31330	0.85824	0.0698591
31331	0.85824	0.0698591
31332	0.544644	0.0698591
31333	0.854718	0.0698591
31334	0.842271	0.0698591
31335	1.19141	0.0698591
31336	0.842272	0.0698591
31337	0.544643	0.0698591
31338	0.854718	0.0698591
31339	1.19141	0.0698591
31340	1.34214	0.0698591
31341	1.34214	0.0698591
31342	0.893236	0.0699397
31343	0.893236	0.0698591
31344	0.893265	0.0698591
31345	1.28924	0.0698591
31346	0.946133	0.0698591
31347	0.946133	0.0698591
31348	0.946132	0.0698591
31349	0.893265	0.0699147
31350	0.946133	0.0698591
31351	1.28924	0.0698591
31352	2.7013	0.0698591
31353	1.89226	0.0700126
31354	1.9216	0.0704851
31355	1.9216	0.0698591
31356	1.89226	0.0701492
31357	1.89226	0.0698591
31358	1.26397	2.1
31359	1.52995	2.1
31360	1.30116	2.1
31361	1.04084	2.1
31362	2.57079	2.1
31363	1.47013	2.1
31364	2.17459	2.1
31365	2.10668	2.1
31366	1.38328	2.1
31367	3.55787	2.1
31368	2.55006	2.1
31369	2.17459	2.1
31370	2.10668	2.1
31371	2.49168	2.1
31372	4.66626	2.1
31373	0.613622	2.1
31374	0.691639	2.1
31375	1.00105	2.1

31376	1.05334	2.1
31377	0.839874	2.1
31378	0.839874	2.1
31379	0.852814	2.1
31380	0.852814	2.1
31381	0.613622	2.1
31382	1.00105	2.1
31383	1.05334	2.1
31384	0.691639	2.1
31385	1.00105	2.1
31386	1.05334	2.1
31387	1.20844	2.1
31388	1.24584	2.1
31389	1.12717	2.1
31390	1.12717	2.1
31391	1.13462	2.1
31392	1.13462	2.1
31393	1.20845	2.1
31394	1.00105	2.1
31395	1.05334	2.1
31396	1.24584	2.1
31397	2.22558	2.1
31398	1.85981	2.1
31399	2.18943	2.00188
31400	1.89142	0.794034
31401	4.0854	2.07969
31402	2.02641	2.1
31403	1.85981	2.1
31404	2.18943	1.94419
31405	1.67346	2.01282
31406	3.88622	1.99373
31407	1.85981	0.776507
31408	1.10736	0.393345
31409	1.27421	0.654844
31410	1.67346	0.684563
31411	1.27421	0.487067
31412	1.67346	1.89856
31413	1.85981	1.21098
31414	1.27362	0.773111
31415	1.89142	0.457501
31416	1.27421	0.420698
31417	3.13343	0.572785
31418	1.81893	0.0698591
31419	1.64985	0.0705977
31420	1.23477	0.0698591
31421	2.25405	0.073666
31422	3.48882	0.0706973
31423	2.23933	0.0698591
31424	1.89226	0.0698591
31425	1.89226	0.0698591
31426	2.78053	0.0698591
31427	1.89226	0.0698591
31428	2.78053	0.0698591
31429	0.946131	0.0699581
31430	0.946131	0.0698591
31431	0.946132	0.0767062
31432	0.946132	0.0936186
31433	0.946131	0.100491

31434	0.946131	0.089469
31435	0.946132	0.0699127
31436	0.946132	0.0849846
31437	0.946131	0.0715607
31438	0.946131	0.0876811
31439	0.946132	0.0698591
31440	0.946132	0.085492
31441	1.89226	2.1
31442	2.48596	2.1
31443	1.89226	2.1
31444	2.51506	2.1
31445	4.40732	2.1
31446	2.48596	2.1
31447	1.63823	2.1
31448	2.51507	2.1
31449	1.67687	2.1
31450	4.1533	2.1
31451	1.89226	2.1
31452	2.51506	2.1
31453	1.92044	2.1
31454	1.89226	2.1
31455	2.54325	2.1
31456	2.51506	2.1
31457	2.51507	2.1
31458	1.5704	2.1
31459	1.70505	2.1
31460	1.67687	2.1
31461	2.54325	2.1
31462	2.51507	2.1
31463	2.0815	2.1
31464	1.70303	2.1
31465	1.70303	2.1
31466	1.80354	2.1
31467	1.98172	2.1
31468	2.09868	2.1
31469	1.27944	2.1
31470	1.79028	2.1
31471	1.13179	2.1
31472	1.65533	2.1
31473	2.93477	2.1
31474	1.89226	2.1
31475	1.89226	2.1
31476	1.89226	2.1
31477	1.89226	2.1
31478	1.89226	2.1
31479	1.89226	2.1
31480	1.98171	2.1
31481	1.89226	2.1
31482	1.89226	2.1
31483	1.89226	2.1
31484	1.65798	2.1
31485	1.65797	2.1
31486	1.00133	2.1
31487	1.65532	2.1
31488	1.53278	2.1
31489	1.53278	2.1
31490	1.13179	2.1
31491	1.13179	2.1

31492	0.810946	2.1
31493	0.810946	2.1
31494	0.810944	2.1
31495	0.810944	2.1
31496	0.810946	2.1
31497	0.810946	2.1
31498	1.89226	2.1
31499	1.35169	2.1
31500	1.08132	2.1
31501	1.08132	2.1
31502	1.08132	2.1
31503	1.99528	0.0702503
31504	1.70303	0.072434
31505	2.08152	0.0698591
31506	1.70303	0.078348
31507	2.33877	0.0700615
31508	1.93967	0.0781416
31509	1.89226	0.0726601
31510	1.89226	0.1157
31511	1.89226	0.0763558
31512	1.89226	0.0788571
31513	3.78452	0.0827256
31514	1.26259	0.0698591
31515	2.17205	0.0761433
31516	2.32468	0.0698591
31517	2.32468	0.070673
31518	1.28055	0.0698591
31519	1.28055	0.070942
31520	0.946131	0.0744763
31521	0.946131	0.0698779
31522	0.997642	0.0698591
31523	0.997642	0.0698591
31524	0.946131	0.0698591
31525	0.946131	0.0767424
31526	0.989846	0.0698622
31527	0.997642	0.0698591
31528	0.946132	0.0698591
31529	0.989846	0.0698591
31530	0.997641	0.0699277
31531	0.946131	0.0702488
31532	1.89226	0.0769588
31533	1.89226	0.0759425
31534	1.89226	0.0698618
31535	1.89226	0.0985348
31536	3.78452	0.0737898
31537	1.28055	0.0698591
31538	2.17205	0.0737658
31539	1.14305	0.0698591
31540	1.92737	0.0698591
31541	3.3151	0.0699696
31542	0.954908	0.0698591
31543	0.9705	0.0698591
31544	0.946131	0.0698591
31545	0.946131	0.0698591
31546	0.946131	0.0698591
31547	0.946131	0.0698591
31548	0.9705	0.0698591
31549	0.9705	0.0698591

31550	0.954908	0.0698591
31551	0.946131	0.0698591
31552	0.946131	0.0698591
31553	0.9705	0.0698591
31554	0.835187	0.0698591
31555	0.954896	0.0698591
31556	0.946118	0.0698591
31557	0.946118	0.0698591
31558	0.835186	0.0698591
31559	0.835186	0.0698591
31560	1.06583	0.0698591
31561	1.06583	0.0698591
31562	0.835198	0.0698591
31563	0.946131	0.0698591
31564	0.954883	0.0698591
31565	0.946106	0.0698591
31566	1.89227	2.1
31567	1.89227	2.1
31568	1.89227	2.1
31569	1.89227	2.1
31570	1.89227	2.1
31571	1.89227	2.1
31572	0.885265	2.1
31573	0.885265	2.1
31574	0.871467	2.1
31575	0.871467	2.1
31576	1.11883	2.1
31577	0.787875	2.1
31578	0.996487	2.1
31579	0.637907	2.1
31580	0.996487	2.1
31581	1.11883	2.1
31582	0.787875	2.1
31583	0.637906	2.1
31584	1.89226	2.1
31585	2.39258	2.1
31586	2.39258	2.1
31587	2.54729	2.1
31588	1.89226	2.1
31589	2.04697	2.1
31590	1.89226	2.1
31591	2.0815	2.1
31592	2.0815	2.1
31593	2.48371	2.1
31594	2.0815	2.1
31595	2.51469	2.1
31596	1.00035	0.0698591
31597	1.55755	0.884549
31598	1.03534	1.15797
31599	1.61333	1.68169
31600	2.59289	0.466114
31601	1.55755	0.884549
31602	1.85217	2.1
31603	1.61333	2.09261
31604	1.92875	2.1
31605	3.48629	2.1
31606	1.09962	1.11797
31607	1.61334	0.985817

31608	1.70868	2.09325
31609	1.70868	1.89935
31610	1.03535	2.1
31611	1.03535	1.1979
31612	1.61334	1.08708
31613	1.92876	2.1
31614	1.70868	1.41017
31615	2.05274	2.1
31616	3.63744	1.94941
31617	1.82233	0.262163
31618	2.05768	0.46732
31619	1.89226	0.534359
31620	1.65406	0.303849
31621	1.89226	0.517397
31622	1.65406	0.313999
31623	2.26047	0.310078
31624	2.0815	0.636501
31625	1.89226	0.681206
31626	2.01156	0.373725
31627	1.89226	0.621149
31628	2.01156	0.366122
31629	1.89226	2.1
31630	1.89226	2.1
31631	1.89226	2.1
31632	1.89226	2.1
31633	3.78453	2.1
31634	2.70055	2.1
31635	2.28367	2.1
31636	2.56456	2.1
31637	2.33565	2.1
31638	4.84823	2.1
31639	2.28367	2.1
31640	1.5791	2.1
31641	1.96966	2.1
31642	1.09506	2.1
31643	3.37873	2.1
31644	1.89226	2.1
31645	1.89226	2.1
31646	1.89226	2.1
31647	1.89226	2.1
31648	3.78452	2.1
31649	0.94612	2.1
31650	0.94612	2.1
31651	0.946119	2.1
31652	0.946119	2.1
31653	0.946119	2.1
31654	0.946119	2.1
31655	0.946119	2.1
31656	0.946119	2.1
31657	0.946132	2.1
31658	0.946132	2.1
31659	0.946107	2.1
31660	0.946107	2.1
31661	2.56452	2.1
31662	1.57307	2.1
31663	2.10299	2.1
31664	2.1115	2.1
31665	2.52196	2.1

31666	2.52196	2.1
31667	1.89226	2.1
31668	1.89226	2.1
31669	1.89226	2.1
31670	1.89226	2.1
31671	3.78452	2.1
31672	2.37665	2.1
31673	1.89226	2.1
31674	2.28084	2.1
31675	1.89226	2.1
31676	4.26891	2.1
31677	0.946129	2.1
31678	0.946129	2.1
31679	0.946133	2.1
31680	0.946133	2.1
31681	0.946131	2.1
31682	0.946131	2.1
31683	0.946131	2.1
31684	0.946131	2.1
31685	0.946131	2.1
31686	0.946131	2.1
31687	0.946131	2.1
31688	0.946131	2.1
31689	1.7207	0.0698591
31690	1.65815	0.0698591
31691	1.33593	0.0698591
31692	1.31508	0.0698591
31693	2.99408	0.0698591
31694	0.676047	0.0698591
31695	0.676047	0.0698591
31696	0.684362	0.0698591
31697	0.684362	0.0698591
31698	0.547994	0.0698591
31699	0.702871	0.0698591
31700	0.829076	0.0698591
31701	0.657538	0.0698591
31702	0.657538	0.0698591
31703	0.547994	0.0698591
31704	0.829077	0.0698591
31705	0.702871	0.0698591
31706	1.9518	2.1
31707	1.30648	2.1
31708	1.28846	2.1
31709	1.92033	2.1
31710	3.22681	2.1
31711	1.89226	2.1
31712	1.89226	2.1
31713	1.89226	2.1
31714	1.89226	2.1
31715	3.78453	2.1
31716	0.946128	2.1
31717	0.946128	2.1
31718	1.10113	2.1
31719	1.09587	2.1
31720	0.946131	2.1
31721	0.946131	2.1
31722	1.09587	2.1
31723	1.09587	2.1

31724	0.946131	2.1
31725	1.09587	2.1
31726	1.10112	2.1
31727	0.94613	2.1
31728	1.26923	2.1
31729	1.92033	2.1
31730	1.28846	2.1
31731	1.28846	2.1
31732	1.89452	2.1
31733	1.89452	2.1
31734	1.89226	2.1
31735	1.89226	2.1
31736	1.89226	2.1
31737	1.89226	2.1
31738	3.78453	2.1
31739	1.89226	2.1
31740	2.19174	2.1
31741	2.15616	2.1
31742	2.19174	2.1
31743	1.85669	2.1
31744	1.89226	2.1
31745	1.89226	2.1
31746	1.89226	2.1
31747	1.89226	2.1
31748	1.89226	2.1
31749	3.78452	2.1
31750	1.89226	2.1
31751	1.89226	2.1
31752	1.89226	2.1
31753	1.89226	2.1
31754	3.78452	2.1
31755	1.51223	2.1
31756	1.41562	2.1
31757	1.5139	2.1
31758	1.42284	2.1
31759	1.6033	2.1
31760	1.51223	2.1
31761	1.51223	2.1
31762	1.25953	2.1
31763	1.38085	2.1
31764	1.6033	2.1
31765	2.89309	2.1
31766	0.946131	2.1
31767	0.946131	2.1
31768	0.946131	2.1
31769	0.946131	2.1
31770	0.946132	2.1
31771	0.946132	2.1
31772	0.946129	2.1
31773	0.946129	2.1
31774	0.946131	2.1
31775	0.946131	2.1
31776	0.94613	2.1
31777	0.94613	2.1
31778	1.89226	2.1
31779	1.89226	2.1
31780	1.89226	2.1
31781	1.89226	2.1

31782	3.78452	2.1
31783	0.707806	2.1
31784	0.697816	2.1
31785	0.823593	2.1
31786	0.801649	2.1
31787	0.74182	2.1
31788	0.74182	2.1
31789	0.757645	2.1
31790	0.757645	2.1
31791	0.823592	2.1
31792	0.697818	2.1
31793	0.707807	2.1
31794	0.801647	2.1
31795	1.52534	2.1
31796	1.6033	2.1
31797	1.64719	2.1
31798	1.38085	2.1
31799	1.64719	2.1
31800	1.38085	2.1
31801	2.67041	2.1
31802	1.89226	2.07758
31803	1.89226	2.1
31804	1.89226	2.1
31805	2.487	2.1
31806	2.487	2.1
31807	2.70097	2.1
31808	1.70303	2.1
31809	1.71163	2.1
31810	2.487	2.1
31811	4.46723	2.1
31812	1.14984	2.1
31813	1.2435	2.1
31814	0.778	2.1
31815	0.697616	2.1
31816	0.967211	2.1
31817	0.967211	2.1
31818	0.973907	2.1
31819	0.973907	2.1
31820	0.697616	2.1
31821	1.14984	2.1
31822	0.778	2.1
31823	1.2435	2.1
31824	2.4158	2.1
31825	1.26438	2.1
31826	1.676	2.1
31827	2.77505	2.1
31828	4.0918	2.1
31829	2.58258	2.1
31830	1.89226	2.1
31831	1.89226	2.1
31832	1.89226	2.1
31833	2.47032	2.1
31834	2.47032	2.1
31835	1.23516	2.1
31836	1.18734	2.1
31837	0.946131	2.1
31838	0.946131	2.1
31839	1.18734	2.1

31840	1.18734	2.1
31841	0.94613	2.1
31842	0.94613	2.1
31843	1.18734	2.1
31844	0.946131	2.1
31845	0.946131	2.1
31846	1.23516	2.1
31847	2.8239	2.1
31848	1.39361	2.1
31849	1.12342	2.1
31850	2.57275	2.1
31851	3.96637	2.1
31852	0.937929	1.53869
31853	0.937929	0.963231
31854	1.39734	0.734864
31855	1.39734	0.656017
31856	1.38914	0.877324
31857	0.94613	1.29129
31858	0.937902	1.13619
31859	0.94613	0.407144
31860	0.94613	0.644632
31861	0.94613	0.932376
31862	1.38914	0.816025
31863	0.937902	0.688819
31864	1.89226	0.218702
31865	2.17765	0.658186
31866	1.84577	0.555311
31867	1.84577	0.323081
31868	1.89226	0.492542
31869	1.89226	0.273347
31870	2.11262	2.05741
31871	1.57766	2.08275
31872	2.22689	2.1
31873	1.91132	1.51762
31874	2.19624	2.1
31875	1.79689	1.13534
31876	1.99656	1.28405
31877	1.57766	2.07882
31878	1.39067	2.1
31879	1.73802	1.25479
31880	3.31568	2.03937
31881	1.89226	0.289353
31882	1.89226	0.0933506
31883	1.89226	0.150393
31884	1.89226	0.544564
31885	1.89226	0.0728089
31886	1.89226	0.154605
31887	1.84936	0.30205
31888	2.0815	0.0714578
31889	2.0815	0.102867
31890	2.47217	0.20564
31891	1.04528	0.0706505
31892	1.62774	0.153496
31893	1.89226	0.160558
31894	1.89226	0.0804193
31895	1.89226	0.1051
31896	1.89226	0.24957
31897	1.89226	0.0729723

31898	1.89226	0.123694
31899	1.69055	0.0698591
31900	2.68518	0.122532
31901	1.69055	0.0698591
31902	2.25055	0.113175
31903	2.39587	0.0698591
31904	3.01801	0.0901672
31905	1.89226	0.0754668
31906	1.89226	0.0937781
31907	1.89226	0.0698591
31908	1.89226	0.0698591
31909	3.78452	0.0734975
31910	1.24499	2.1
31911	1.70302	2.1
31912	1.41207	2.1
31913	1.10574	1.9645
31914	2.66056	2.1
31915	1.70303	2.1
31916	2.39605	1.97607
31917	2.72674	2.1
31918	2.46904	1.34412
31919	1.84557	2.1
31920	1.67115	2.00671
31921	2.46904	0.482406
31922	2.76829	2.1
31923	2.83666	0.318254
31924	2.83666	1.89813
31925	2.39605	0.560164
31926	2.39605	2.08565
31927	1.07323	0.720435
31928	1.07323	2.1
31929	1.06715	2.1
31930	1.18052	1.84611
31931	1.30067	0.621004
31932	1.26542	0.914669
31933	0.914516	2.1
31934	0.839633	2.1
31935	0.936958	2.00143
31936	1.26751	2.01889
31937	0.847764	2.06995
31938	1.35395	2.06213
31939	2.69069	0.30162
31940	1.03074	0.243852
31941	1.74098	0.196589
31942	1.97397	0.237191
31943	3.71495	0.235934
31944	2.44554	2.1
31945	1.89226	2.1
31946	1.89226	2.1
31947	1.89226	2.1
31948	2.40645	2.1
31949	2.40645	2.1
31950	1.89226	2.1
31951	1.89226	2.1
31952	1.89226	2.1
31953	1.89226	2.1
31954	3.78452	2.1
31955	1.82117	2.1

31956	2.55786	2.1
31957	2.51431	2.1
31958	2.51431	2.1
31959	1.85054	2.1
31960	1.84375	2.1
31961	2.38467	2.1
31962	1.89226	2.1
31963	1.89226	2.1
31964	1.89226	2.1
31965	2.44554	2.1
31966	2.44554	2.1
31967	1.89226	2.1
31968	1.89226	2.1
31969	1.89226	2.1
31970	1.89226	2.1
31971	1.89226	2.1
31972	1.89226	2.1
31973	2.02397	2.1
31974	2.55786	2.1
31975	1.77775	2.1
31976	2.61404	2.1
31977	4.33561	2.1
31978	1.89226	1.63939
31979	1.89226	1.6168
31980	1.89226	1.29054
31981	1.89226	1.30636
31982	3.78453	1.64097
31983	1.89226	1.85273
31984	2.80184	1.77111
31985	1.89226	1.57096
31986	1.89226	1.7832
31987	2.03045	1.88466
31988	2.03045	1.94157
31989	2.52263	1.67595
31990	1.27921	2.01247
31991	1.58429	2.05714
31992	1.73571	1.94537
31993	2.37121	1.81528
31994	2.52263	1.67792
31995	0.764974	2.1
31996	0.650577	2.1
31997	0.712428	2.1
31998	0.562516	2.1
31999	0.67264	2.1
32000	0.67264	2.1
32001	0.654851	2.1
32002	0.654851	2.1
32003	0.562515	2.1
32004	0.650579	2.1
32005	0.712427	2.1
32006	0.764976	2.1
32007	1.89227	2.1
32008	1.89227	2.1
32009	1.89227	2.1
32010	1.89227	2.1
32011	3.78453	2.1
32012	1.89226	2.1
32013	1.89226	2.1

32014	1.89226	2.1
32015	1.89226	2.1
32016	3.78453	2.1
32017	1.49248	2.1
32018	1.24959	2.1
32019	1.28211	2.1
32020	1.26432	2.1
32021	1.51027	2.1
32022	1.49248	2.1
32023	1.49248	2.1
32024	1.19017	2.1
32025	1.24977	2.1
32026	1.51027	2.1
32027	2.74225	2.1
32028	1.89226	2.1
32029	1.89226	2.1
32030	1.89226	2.1
32031	1.89226	2.1
32032	3.78453	2.1
32033	1.89226	2.1
32034	1.89226	2.1
32035	1.89226	2.1
32036	1.89226	2.1
32037	3.78453	2.1
32038	0.624796	2.1
32039	0.618472	2.1
32040	0.770147	2.1
32041	0.755132	2.1
32042	0.681445	2.1
32043	0.681445	2.1
32044	0.69216	2.1
32045	0.69216	2.1
32046	0.618472	2.1
32047	0.770147	2.1
32048	0.624796	2.1
32049	0.755132	2.1
32050	1.54029	2.1
32051	1.51027	2.1
32052	1.54029	2.1
32053	1.30682	2.1
32054	1.24977	2.1
32055	1.24977	2.1
32056	0.994293	2.1
32057	2.17159	2.1
32058	2.21149	2.1
32059	2.21149	2.1
32060	0.994814	2.1
32061	0.994814	2.1
32062	0.990481	2.1
32063	2.21149	2.1
32064	2.2671	2.1
32065	2.2671	2.1
32066	0.994293	2.1
32067	0.994293	2.1
32068	2.41579	2.1
32069	1.09847	2.1
32070	1.70302	2.1
32071	2.45069	2.1

32072	3.61169	2.1
32073	2.48689	2.1
32074	1.67969	2.1
32075	1.67969	2.1
32076	1.70002	2.1
32077	2.43826	2.1
32078	2.45859	2.1
32079	2.43827	2.1
32080	1.62408	2.1
32081	1.62408	2.1
32082	1.67969	2.1
32083	2.36729	2.1
32084	2.4229	2.1
32085	2.47199	2.1
32086	1.0823	2.1
32087	1.0607	2.1
32088	2.44276	2.1
32089	3.52506	2.1
32090	2.47199	2.1
32091	1.89226	2.1
32092	1.89226	2.1
32093	2.44277	2.1
32094	4.33503	2.1
32095	1.89226	2.1
32096	1.89226	2.1
32097	1.89226	2.1
32098	1.89226	2.1
32099	3.78453	2.1
32100	1.89226	2.1
32101	1.89226	2.1
32102	1.89226	2.1
32103	1.89226	2.1
32104	1.89226	2.1
32105	1.89226	2.1
32106	1.02986	2.1
32107	2.44276	2.1
32108	1.0607	2.1
32109	1.0607	2.1
32110	2.40055	2.1
32111	2.40055	2.1
32112	1.89226	2.1
32113	2.44277	2.1
32114	2.40056	2.1
32115	2.44277	2.1
32116	1.85005	2.1
32117	1.89226	2.1
32118	1.89226	2.1
32119	1.89226	2.1
32120	1.89226	2.1
32121	1.89226	2.1
32122	3.78453	2.1
32123	1.89226	2.1
32124	1.89226	2.1
32125	1.89226	2.1
32126	1.89226	2.1
32127	3.78452	2.1
32128	2.79502	0.74822
32129	2.63448	2.09678

32130	2.68986	0.609903
32131	2.53868	2.1
32132	5.32434	1.65506
32133	2.63449	2.09294
32134	2.39282	2.1
32135	2.53869	2.1
32136	2.3085	2.1
32137	4.94299	2.1
32138	2.68986	0.536011
32139	2.53868	2.1
32140	2.5761	0.858577
32141	2.43404	2.0831
32142	5.1239	1.55481
32143	2.53869	2.1
32144	2.3085	2.1
32145	2.43405	2.08999
32146	2.22575	2.1
32147	4.74255	2.1
32148	1.89226	2.1
32149	1.89226	2.1
32150	1.89226	2.1
32151	1.89226	2.1
32152	3.78453	2.1
32153	1.32391	2.1
32154	1.44951	2.1
32155	1.44951	2.1
32156	1.89226	2.1
32157	1.89226	2.1
32158	2.20203	2.1
32159	0.507656	2.1
32160	0.578945	2.1
32161	1.05568	2.1
32162	0.959007	2.1
32163	0.76264	2.1
32164	0.76264	2.1
32165	0.775313	2.1
32166	0.775313	2.1
32167	0.578945	2.1
32168	1.05568	2.1
32169	0.959007	2.1
32170	0.507656	2.1
32171	1.89226	0.0698591
32172	2.4065	0.0701127
32173	2.36561	0.0698591
32174	2.4065	0.070624
32175	1.85138	0.0698591
32176	1.89226	0.070225
32177	1.40274	0.0698591
32178	2.4065	0.0698591
32179	1.53336	0.0698591
32180	1.53336	0.0698591
32181	2.36562	0.0698591
32182	2.36562	0.0698591
32183	0.946115	0.0702685
32184	0.946115	0.0698591
32185	1.06496	0.0698591
32186	1.18279	0.0698591
32187	0.946117	0.0698591

32188	0.946117	0.0708855
32189	1.06496	0.0698591
32190	1.06496	0.0698591
32191	0.94613	0.0698591
32192	1.06497	0.0698591
32193	0.946105	0.0698591
32194	1.18278	0.0698591
32195	1.00213	0.0698591
32196	2.36559	0.0698591
32197	1.40272	0.0698591
32198	1.40272	0.0698591
32199	2.12992	0.0698591
32200	2.12992	0.0698591
32201	0.95646	2.1
32202	2.07201	2.1
32203	1.01808	2.1
32204	2.14876	2.1
32205	3.09009	2.1
32206	1.89226	2.1
32207	1.89226	2.1
32208	1.89226	2.1
32209	1.89226	2.1
32210	3.78453	2.1
32211	1.89227	2.1
32212	1.89227	2.1
32213	1.89227	2.1
32214	1.89227	2.1
32215	1.89227	2.1
32216	1.89227	2.1
32217	2.71886	2.1
32218	1.60497	2.1
32219	1.43935	2.1
32220	2.55151	2.1
32221	4.15648	2.1
32222	2.71886	2.1
32223	1.89226	2.1
32224	1.89226	2.1
32225	2.55151	2.1
32226	4.44377	2.1
32227	1.89226	2.1
32228	1.89226	2.1
32229	1.89226	2.1
32230	1.89226	2.1
32231	3.78453	2.1
32232	1.89226	2.1
32233	1.89226	2.1
32234	1.89226	2.1
32235	1.89226	2.1
32236	3.78453	2.1
32237	1.58942	0.264184
32238	1.92622	0.408458
32239	2.52852	0.335645
32240	2.19457	0.261037
32241	4.12079	0.258066
32242	1.92649	0.214693
32243	1.44426	0.15569
32244	1.87529	0.166887
32245	2.19457	0.237584

32246	3.63883	0.208749
32247	1.87529	0.12649
32248	2.19457	0.204378
32249	2.7378	0.178557
32250	2.29852	0.134584
32251	4.49309	0.130943
32252	1.35976	2.1
32253	1.89452	2.1
32254	1.39616	2.1
32255	1.1423	2.1
32256	1.8996	2.1
32257	1.52068	2.1
32258	1.89226	2.1
32259	1.89226	2.1
32260	1.89226	2.1
32261	1.89226	2.1
32262	1.89226	2.1
32263	1.89226	2.1
32264	1.89226	2.1
32265	2.15617	2.1
32266	2.0953	2.1
32267	2.15617	2.1
32268	1.83139	2.1
32269	1.89226	2.1
32270	1.16817	2.1
32271	1.8996	2.1
32272	1.23614	2.1
32273	1.48338	2.1
32274	1.80218	2.1
32275	2.16263	2.1
32276	1.89226	2.1
32277	1.89226	2.1
32278	1.89226	2.1
32279	1.89226	2.1
32280	1.89226	2.1
32281	1.89226	2.1
32282	1.89226	2.1
32283	2.0953	2.1
32284	2.03074	2.1
32285	2.0953	2.1
32286	1.8277	2.1
32287	1.89226	2.1
32288	1.74455	2.1
32289	2.181	2.1
32290	1.74455	2.1
32291	1.92911	2.1
32292	1.98195	2.1
32293	2.16651	2.1
32294	2.181	2.1
32295	2.29881	2.1
32296	1.98195	2.1
32297	1.80729	2.1
32298	4.28075	2.1
32299	1.74455	2.1
32300	1.98195	2.1
32301	1.52603	2.1
32302	1.76603	2.1
32303	3.51058	2.1

32304	1.76603	2.1
32305	1.94506	2.1
32306	1.76603	2.1
32307	1.98195	2.1
32308	1.86795	2.1
32309	2.08387	2.1
32310	0.984305	2.1
32311	1.9	2.1
32312	2.86619	2.1
32313	1.86189	2.09958
32314	3.76189	2.1
32315	1.4621	2.1
32316	1.89226	2.1
32317	2.81374	2.1
32318	1.89226	2.1
32319	3.35437	2.1
32320	0.946133	2.09698
32321	0.946133	2.1
32322	0.946132	2.09577
32323	0.946132	2.1
32324	0.946132	2.1
32325	0.946132	2.1
32326	0.946133	2.1
32327	0.946133	2.09773
32328	0.946132	2.1
32329	0.946132	2.09555
32330	0.946132	2.1
32331	0.946132	2.1
32332	1.64268	2.1
32333	1.93673	2.1
32334	2.25284	2.1
32335	1.97548	2.1
32336	3.89552	2.1
32337	1.86206	2.1
32338	1.93673	2.1
32339	2.25284	2.1
32340	2.20075	2.1
32341	4.1149	2.1
32342	2.25284	2.1
32343	2.24781	2.1
32344	1.97548	2.1
32345	1.97548	2.1
32346	2.49676	2.1
32347	2.49676	2.1
32348	2.25284	2.1
32349	2.43549	2.1
32350	2.44467	2.1
32351	2.20075	2.1
32352	2.49676	2.1
32353	2.25284	2.1
32354	2.57928	2.1
32355	2.32606	2.1
32356	2.59522	2.1
32357	2.39022	2.1
32358	4.9695	2.1
32359	2.32606	2.1
32360	1.77677	2.1
32361	2.39022	2.1

32362	2.28669	2.1
32363	4.16699	2.1
32364	2.59522	2.1
32365	2.39022	2.1
32366	2.61901	2.1
32367	2.45899	2.1
32368	5.00923	2.1
32369	2.39022	2.1
32370	2.11575	2.1
32371	2.41795	2.1
32372	2.0815	2.1
32373	4.67085	2.1
32374	1.89226	2.1
32375	2.24723	2.1
32376	1.95405	2.1
32377	1.89226	2.1
32378	2.30902	2.1
32379	2.24723	2.1
32380	1.89226	2.1
32381	1.89226	2.1
32382	1.89226	2.1
32383	1.89226	2.1
32384	3.78453	2.1
32385	0.946131	2.1
32386	0.946131	2.1
32387	1.14643	2.1
32388	1.10673	2.1
32389	1.10673	2.1
32390	1.10673	2.1
32391	0.946133	2.1
32392	0.946133	2.1
32393	0.946131	2.1
32394	1.14643	2.1
32395	1.10673	2.1
32396	0.946131	2.1
32397	1.89226	2.1
32398	2.30902	2.1
32399	1.96635	2.1
32400	1.89226	2.1
32401	2.38311	2.1
32402	2.30902	2.1
32403	1.89226	2.1
32404	1.89226	2.1
32405	1.89226	2.1
32406	1.89226	2.1
32407	1.89226	2.1
32408	1.89226	2.1
32409	0.946131	2.1
32410	0.946131	2.1
32411	1.19156	2.1
32412	1.14643	2.1
32413	1.14643	2.1
32414	1.14643	2.1
32415	0.946133	2.1
32416	0.946133	2.1
32417	0.946131	2.1
32418	1.19156	2.1
32419	0.946131	2.1

32420	1.14643	2.1
32421	1.89226	1.70029
32422	1.89226	0.739737
32423	1.89226	1.05328
32424	1.89226	1.74496
32425	1.89226	0.700308
32426	1.89226	1.44646
32427	1.89226	0.948746
32428	1.89226	0.800925
32429	1.89226	0.41012
32430	1.9645	0.909514
32431	3.78452	1.3371
32432	2.39489	2.1
32433	2.15715	2.1
32434	2.07821	2.1
32435	2.33541	2.1
32436	4.49256	2.1
32437	2.39489	2.1
32438	2.34898	2.1
32439	2.30729	2.1
32440	2.33541	2.1
32441	4.6844	2.1
32442	2.07821	2.1
32443	2.33541	2.1
32444	2.26415	2.1
32445	1.99632	2.1
32446	4.33174	2.1
32447	2.30729	2.1
32448	2.33542	2.1
32449	2.26416	2.1
32450	2.26618	2.1
32451	4.60159	2.1
32452	1.19837	1.75376
32453	2.79151	0.464622
32454	2.25636	1.24112
32455	2.25636	0.852696
32456	1.94072	1.20281
32457	1.94072	0.896083
32458	2.53725	0.0701365
32459	1.16699	0.0714062
32460	1.69864	0.0698591
32461	2.0815	0.0714142
32462	3.58273	0.0705007
32463	1.91646	2.1
32464	2.51686	2.1
32465	2.49872	2.1
32466	1.85819	2.1
32467	4.41518	2.1
32468	0.929098	2.1
32469	0.8993	2.1
32470	1.24359	2.1
32471	1.24936	2.1
32472	1.06832	2.1
32473	1.06832	2.1
32474	1.08034	2.1
32475	1.08034	2.1
32476	0.899298	2.1
32477	1.24359	2.1

32478	1.24936	2.1
32479	0.929097	2.1
32480	2.0815	0.0699225
32481	1.89226	0.0698591
32482	2.0815	0.0698591
32483	1.82811	0.0698591
32484	1.94982	0.0698591
32485	1.71246	0.0698591
32486	2.3256	2.1
32487	1.89226	2.1
32488	1.89226	2.1
32489	1.89226	2.1
32490	2.38467	2.1
32491	2.38467	2.1
32492	1.89226	2.1
32493	1.89226	2.1
32494	1.89226	2.1
32495	1.89226	2.1
32496	1.89226	2.1
32497	1.89226	2.1
32498	1.89763	2.1
32499	2.61405	2.1
32500	2.15035	2.1
32501	2.68796	2.1
32502	1.42219	2.1
32503	1.77775	2.1
32504	2.26306	2.1
32505	1.89226	2.1
32506	1.89226	2.1
32507	1.89226	2.1
32508	2.3256	2.1
32509	2.3256	2.1
32510	1.89226	2.1
32511	1.89226	2.1
32512	1.89226	2.1
32513	1.89226	2.1
32514	1.89226	2.1
32515	1.89226	2.1
32516	1.92887	2.1
32517	2.41915	2.1
32518	2.50142	2.1
32519	2.50142	2.1
32520	1.90388	2.1
32521	1.89763	2.1
32522	1.89224	2.1
32523	1.89224	2.1
32524	1.89224	2.1
32525	1.89224	2.1
32526	3.78448	2.1
32527	2.25315	2.1
32528	1.05843	2.1
32529	2.38995	2.1
32530	1.31541	2.1
32531	3.56856	2.1
32532	0.517552	2.1
32533	0.68548	2.1
32534	1.27675	2.1
32535	1.13076	2.1

32536	0.913611	2.1
32537	0.913612	2.1
32538	0.902633	2.1
32539	0.902633	2.1
32540	0.68547	2.1
32541	1.27673	2.1
32542	1.13078	2.1
32543	0.517559	2.1
32544	1.89226	2.1
32545	1.89226	2.1
32546	1.89226	2.1
32547	1.89226	2.1
32548	3.78453	2.1
32549	0.962936	2.1
32550	0.962936	2.1
32551	0.996141	2.1
32552	0.996141	2.1
32553	0.830609	2.1
32554	0.657714	2.1
32555	1.30136	2.1
32556	1.19499	2.1
32557	1.30136	2.1
32558	0.830609	2.1
32559	1.19499	2.1
32560	0.657713	2.1
32561	1.60057	0.102152
32562	1.39615	0.0927095
32563	1.06474	0.0876231
32564	1.39615	0.134598
32565	1.64655	0.0827012
32566	2.04424	0.115974
32567	1.89226	0.0903198
32568	1.89226	0.134968
32569	1.89226	0.101042
32570	1.89226	0.122446
32571	1.89226	0.107319
32572	1.89226	0.134872
32573	2.56322	0.0793736
32574	1.89226	0.122265
32575	1.89226	0.0917607
32576	1.89226	0.105617
32577	2.18047	0.0868331
32578	2.18047	0.0992398
32579	1.89226	0.494176
32580	1.89226	0.47111
32581	1.89226	0.548879
32582	1.65573	0.384282
32583	1.89226	0.603883
32584	1.65573	0.438863
32585	1.05353	0.529891
32586	1.83158	0.679021
32587	1.50282	0.344898
32588	1.20669	0.240358
32589	2.88511	0.259597
32590	1.44807	2.1
32591	1.93592	2.1
32592	1.80703	2.1
32593	1.27806	2.1

32594	3.21398	2.1
32595	2.0815	2.1
32596	1.87465	2.1
32597	1.87465	2.1
32598	1.50566	2.1
32599	2.0815	2.1
32600	1.69263	2.1
32601	1.89226	2.1
32602	1.89226	2.1
32603	1.89226	2.1
32604	1.89226	2.1
32605	1.89226	2.1
32606	1.89226	2.1
32607	1.62632	2.1
32608	0.994294	2.1
32609	0.994294	2.1
32610	1.27806	2.1
32611	1.81921	2.1
32612	2.10776	2.1
32613	2.0815	2.1
32614	1.70303	2.1
32615	1.70303	2.1
32616	1.85749	2.1
32617	1.90451	2.1
32618	2.07724	2.1
32619	1.89226	2.1
32620	1.89226	2.1
32621	1.89226	2.1
32622	1.89226	2.1
32623	1.89226	2.1
32624	1.89226	2.1
32625	2.69773	2.1
32626	1.88129	2.1
32627	2.55196	2.1
32628	2.26469	2.1
32629	4.94367	2.1
32630	1.88129	2.1
32631	1.25889	2.1
32632	1.26614	2.1
32633	1.05914	2.1
32634	2.26469	2.1
32635	1.88129	2.1
32636	2.55196	2.1
32637	2.0588	2.1
32638	2.6536	2.1
32639	2.15287	2.1
32640	4.70484	2.1
32641	0.8516	2.1
32642	0.8516	2.1
32643	0.849002	2.1
32644	0.849002	2.1
32645	0.629445	2.1
32646	0.671197	2.1
32647	1.0294	2.1
32648	1.07644	2.1
32649	1.07644	2.1
32650	0.671197	2.1
32651	0.629446	2.1

32652	1.0294	2.1
32653	2.02115	2.04695
32654	1.18382	1.82999
32655	2.29107	0.224816
32656	1.46903	0.565796
32657	3.47488	0.45878
32658	2.60763	1.55889
32659	2.29107	1.48051
32660	1.82141	1.02422
32661	1.82141	1.6173
32662	1.46903	1.66894
32663	2.29107	1.82108
32664	1.64007	2.1
32665	2.2024	2.1
32666	1.44044	2.1
32667	2.02634	2.1
32668	3.6664	2.1
32669	1.25565	2.1
32670	2.02635	2.1
32671	1.87228	2.1
32672	1.87228	2.1
32673	1.44045	2.1
32674	1.44045	2.1
32675	1.89226	0.0698591
32676	1.78503	0.0698591
32677	1.78503	0.0698591
32678	1.40048	0.0698591
32679	2.0815	0.0698591
32680	1.62972	0.0698591
32681	1.27875	2.1
32682	1.1483	2.1
32683	1.40014	2.1
32684	1.40014	2.1
32685	1.04598	2.1
32686	1.04598	2.1
32687	2.63443	1.75112
32688	2.26444	2.07076
32689	2.79392	0.633057
32690	2.4863	2.1
32691	5.12073	2.00591
32692	1.83158	0.803803
32693	1.18508	0.235209
32694	1.20669	0.188592
32695	1.81488	0.483303
32696	1.20669	0.328898
32697	1.81488	0.474266
32698	1.08134	0.220587
32699	1.70303	0.548222
32700	1.24027	0.369718
32701	1.30359	0.223195
32702	1.89939	0.577957
32703	1.70303	0.587212
32704	1.89226	2.09578
32705	2.69027	2.1
32706	1.89226	2.1
32707	2.2078	2.08049
32708	1.89226	2.1
32709	2.2078	2.08481

32710	1.89226	2.1
32711	2.78977	2.1
32712	1.89226	2.1
32713	2.69027	2.09987
32714	1.89226	2.1
32715	2.69027	2.1
32716	1.89226	2.09184
32717	1.89226	2.0757
32718	1.89226	1.94254
32719	1.89226	2.09509
32720	3.78452	2.07244
32721	1.43687	1.99808
32722	1.71059	2.09574
32723	2.53014	2.02897
32724	2.6521	2.06317
32725	4.24073	2.04779
32726	2.6521	2.0637
32727	1.66762	2.1
32728	2.50299	2.08218
32729	2.50299	2.09745
32730	1.71059	2.09565
32731	1.71059	2.09828
32732	1.89226	2.09518
32733	1.89226	2.09769
32734	1.89226	2.1
32735	1.89226	2.07072
32736	1.89226	2.1
32737	1.89226	2.07545
32738	2.10662	0.278267
32739	2.77417	0.567043
32740	2.78873	0.717505
32741	2.11661	0.256658
32742	4.89078	0.42767
32743	2.10662	0.356449
32744	1.22857	0.10458
32745	1.25849	0.304109
32746	2.11661	0.340765
32747	3.34518	0.256658
32748	2.78873	0.82269
32749	2.11661	0.270232
32750	2.12475	0.382532
32751	2.79029	0.634487
32752	4.9069	0.440292
32753	1.2858	0.258565
32754	2.11661	0.325207
32755	1.25849	0.245174
32756	1.25849	0.435371
32757	2.12475	0.234211
32758	2.12475	0.398844
32759	1.13537	2.1
32760	1.20714	2.1
32761	0.946129	2.1
32762	0.946129	2.1
32763	0.946131	2.1
32764	0.946131	2.1
32765	1.29521	2.1
32766	1.20713	2.1
32767	1.13537	2.1

32768	0.946131	2.1
32769	0.94613	2.1
32770	1.29521	2.1
32771	1.00533	2.1
32772	0.876055	2.1
32773	0.946128	2.1
32774	0.946128	2.1
32775	0.913908	2.1
32776	0.913908	2.1
32777	1.17081	2.1
32778	0.896413	2.1
32779	0.955905	2.1
32780	0.893715	2.1
32781	1.20327	2.1
32782	0.998544	2.1
32783	1.89226	1.88138
32784	1.89226	1.99675
32785	1.89226	0.278725
32786	1.89226	0.968358
32787	3.78452	0.452987
32788	0.540975	1.34834
32789	0.726949	2.1
32790	0.712198	2.06455
32791	0.506675	0.817691
32792	0.611916	1.01185
32793	0.611916	2.1
32794	0.621708	1.8834
32795	0.621708	0.874146
32796	0.726951	1.01308
32797	0.712196	0.665732
32798	0.506674	1.55011
32799	0.540976	1.67008
32800	1.89226	0.873083
32801	1.89226	2.1
32802	1.89226	1.66995
32803	1.89226	0.96181
32804	3.78453	1.70389
32805	1.89226	0.62096
32806	1.89226	1.2568
32807	1.89226	0.507812
32808	1.89226	2.05981
32809	1.89226	0.503638
32810	1.89226	2.1
32811	0.919594	0.796115
32812	0.726949	1.79323
32813	0.898318	1.10548
32814	0.712198	1.67772
32815	0.817559	0.668026
32816	0.817559	2.1
32817	0.814234	0.72843
32818	0.814234	1.92494
32819	0.919597	0.420915
32820	0.898316	0.311474
32821	0.726951	1.74959
32822	0.712196	1.20563
32823	1.79663	0.542356
32824	1.12088	2.07476
32825	1.54699	1.74264

32826	1.17475	1.52088
32827	1.79663	2.05687
32828	1.42439	1.97481
32829	2.28928	1.19634
32830	1.23077	0.992256
32831	1.65458	0.241772
32832	2.66533	1.87846
32833	3.94385	0.89999
32834	1.89226	2.1
32835	1.89226	2.1
32836	1.89226	2.1
32837	1.89226	2.1
32838	3.78452	2.1
32839	1.38426	2.1
32840	1.15067	2.1
32841	1.38426	2.1
32842	1.01595	2.1
32843	1.24509	2.1
32844	1.01595	2.1
32845	2.62097	2.1
32846	1.89226	2.1
32847	2.72561	2.1
32848	2.72561	2.1
32849	1.89226	2.1
32850	1.89226	2.1
32851	1.90103	1.95252
32852	1.41902	2.1
32853	1.77249	1.97678
32854	1.30272	2.1
32855	3.20375	2.1
32856	1.77249	1.90453
32857	1.19548	2.1
32858	1.19548	2.1
32859	1.30272	2.1
32860	1.65815	2.05014
32861	1.76539	2.1
32862	2.62161	2.1
32863	1.89226	2.1
32864	1.89226	2.1
32865	1.89226	2.1
32866	2.6446	2.1
32867	2.6446	2.1
32868	2.64459	2.1
32869	1.89226	2.1
32870	1.89226	2.1
32871	1.89226	2.1
32872	2.58257	2.1
32873	2.58257	2.1
32874	1.89226	0.298508
32875	1.89226	0.833001
32876	2.31301	0.789082
32877	2.76309	0.378045
32878	1.89226	0.740191
32879	2.26047	0.362102
32880	1.89226	0.272025
32881	2.31301	0.794305
32882	2.17765	0.698782
32883	2.17765	0.380231

32884	1.89226	0.653314
32885	1.89226	0.333483
32886	2.18516	2.1
32887	1.85196	2.1
32888	1.78595	2.1
32889	2.14606	2.1
32890	3.99802	2.1
32891	2.18516	2.1
32892	2.21189	2.1
32893	2.18937	2.1
32894	2.14606	2.1
32895	4.35796	2.1
32896	1.60735	2.1
32897	2.14606	2.1
32898	2.15714	2.1
32899	1.70302	2.1
32900	3.70055	2.1
32901	2.18937	2.1
32902	2.14606	2.1
32903	2.0927	2.1
32904	2.162 2.1	
32905	4.30806	2.1
32906	2.17326	2.1
32907	2.58517	2.1
32908	2.55318	2.1
32909	2.1665	2.1
32910	4.75167	2.1
32911	2.17326	2.1
32912	1.53556	2.1
32913	1.56253	2.1
32914	2.1665	2.1
32915	3.70206	2.1
32916	2.55317	2.1
32917	2.1665	2.1
32918	2.17741	2.1
32919	2.54197	2.1
32920	4.70847	2.1
32921	2.17742	2.1
32922	2.1665	2.1
32923	2.18452	2.1
32924	1.58054	2.1
32925	1.56253	2.1
32926	1.56253	2.1
32927	1.71051	0.0698591
32928	1.89807	0.0705171
32929	1.19755	0.0699088
32930	1.19755	0.0698591
32931	2.25632	0.0734372
32932	2.25632	0.0698591
32933	0.876046	0.0733954
32934	1.27293	0.0718303
32935	0.946132	0.176389
32936	0.946132	0.0978123
32937	0.946131	0.0785001
32938	0.869312	0.0786132
32939	1.12767	0.137308
32940	0.946132	0.117136
32941	1.22225	0.153562

32942	1.37968	0.074022
32943	0.851555	0.164912
32944	0.851555	0.0780234
32945	0.946133	2.06264
32946	0.946133	2.1
32947	0.946132	2.07243
32948	0.946132	1.95964
32949	0.946133	2.03506
32950	0.946133	2.07358
32951	0.946132	2.1
32952	0.946132	2.07146
32953	0.946132	2.09946
32954	0.946132	2.07998
32955	0.946133	2.06075
32956	0.946133	2.08493
32957	1.35152	2.1
32958	0.948936	2.1
32959	1.90166	2.1
32960	1.70303	2.1
32961	2.90858	2.1
32962	1.89226	2.1
32963	1.89226	2.1
32964	1.89226	2.1
32965	1.89226	2.1
32966	3.78452	2.1
32967	1.90166	2.1
32968	1.70303	2.1
32969	2.52454	2.1
32970	2.13919	2.1
32971	3.99529	2.1
32972	1.89226	2.09336
32973	1.89226	2.1
32974	1.89226	2.1
32975	1.89226	2.1
32976	3.78452	2.1
32977	1.89226	2.1
32978	1.89226	2.1
32979	1.89226	2.1
32980	1.89226	2.1
32981	1.89226	2.09349
32982	1.89226	2.1
32983	2.13918	2.1
32984	0.992674	2.1
32985	2.3397	2.1
32986	1.8023	2.1
32987	1.47769	2.1
32988	0.992674	2.1
32989	1.89226	0.273155
32990	1.89226	0.258317
32991	1.89226	0.368861
32992	1.89226	0.196606
32993	3.78452	0.277938
32994	1.89226	0.264232
32995	2.54752	0.14177
32996	2.63974	0.201053
32997	1.89226	0.190177
32998	4.53201	0.187194
32999	0.498412	0.195795

33000	0.500955	0.176709
33001	0.57955	0.245088
33002	0.563873	0.21284
33003	0.529107	0.208103
33004	0.529107	0.178331
33005	0.53572	0.25913
33006	0.53572	0.190974
33007	0.500956	0.201412
33008	0.579548	0.197965
33009	0.563871	0.201989
33010	0.498414	0.204757
33011	1.89223	0.319198
33012	1.89223	0.190911
33013	1.89223	0.244037
33014	1.89223	0.273973
33015	1.89223	0.244591
33016	1.89223	0.274675
33017	1.28752	0.143278
33018	1.31985	0.183191
33019	0.946119	0.184299
33020	0.946119	0.248958
33021	1.28752	0.185361
33022	1.28752	0.145612
33023	0.946119	0.180152
33024	0.946119	0.306
33025	1.28753	0.185243
33026	0.946132	0.184062
33027	1.31984	0.197134
33028	0.946107	0.19455
33029	0.500948	0.180054
33030	0.479465	0.16706
33031	0.562335	0.227721
33032	0.579542	0.195125
33033	0.528436	0.191855
33034	0.528436	0.169105
33035	0.530571	0.179685
33036	0.530571	0.236159
33037	0.56234	0.19053
33038	0.479473	0.191885
33039	0.500943	0.195573
33040	0.579533	0.191208
33041	1.89226	2.1
33042	1.89226	2.1
33043	1.89226	2.1
33044	1.89226	2.1
33045	3.78452	2.1
33046	1.50767	2.1
33047	1.14496	2.1
33048	1.50767	2.1
33049	1.58539	2.1
33050	1.07653	2.1
33051	1.15425	2.1
33052	1.89226	2.1
33053	2.43826	2.1
33054	2.43826	2.1
33055	2.48688	2.1
33056	1.89226	2.1
33057	1.94088	2.1

33058	1.89226	2.1
33059	1.89226	2.1
33060	1.89226	2.1
33061	1.89226	2.1
33062	3.78452	2.1
33063	1.50767	2.1
33064	1.07653	2.1
33065	1.41077	2.1
33066	0.991407	2.1
33067	2.49907	2.1
33068	1.21913	2.1
33069	1.21913	2.1
33070	0.946128	2.1
33071	0.946128	2.1
33072	1.21913	2.1
33073	1.18364	2.1
33074	0.946131	2.1
33075	0.946131	2.1
33076	1.18364	2.1
33077	0.946129	2.1
33078	1.21913	2.1
33079	0.94613	2.1
33080	1.89226	2.1
33081	1.89226	2.1
33082	1.89226	2.1
33083	1.89226	2.1
33084	3.78452	2.1
33085	1.93823	2.1
33086	1.60976	2.1
33087	1.93823	2.1
33088	2.20045	2.1
33089	1.36999	2.1
33090	1.63222	2.1
33091	1.38302	2.1
33092	1.38302	2.1
33093	0.946132	2.1
33094	0.946132	2.1
33095	1.27258	2.1
33096	1.38302	2.1
33097	0.946133	2.1
33098	0.946133	2.1
33099	0.946133	2.1
33100	1.27258	2.1
33101	0.946132	2.1
33102	1.38302	2.1
33103	1.89226	2.1
33104	1.89226	2.1
33105	1.89226	2.1
33106	1.89226	2.1
33107	3.78452	2.1
33108	1.93823	2.1
33109	1.36999	2.1
33110	1.70423	2.1
33111	1.14195	2.1
33112	3.08018	2.1
33113	1.27258	2.1
33114	1.27258	2.1
33115	0.946132	2.1

33116	0.946132	2.1
33117	1.17233	2.1
33118	1.27258	2.1
33119	0.946132	2.1
33120	0.946132	2.1
33121	1.17233	2.1
33122	0.946132	2.1
33123	0.946132	2.1
33124	1.27258	2.1
33125	2.70575	2.1
33126	1.89226	1.65906
33127	2.71429	2.1
33128	2.71429	2.1
33129	1.89226	2.1
33130	1.89226	2.07814
33131	0.946133	2.08149
33132	0.946133	2.1
33133	1.28713	2.1
33134	1.28713	2.1
33135	0.946131	2.1
33136	0.946131	2.06799
33137	1.28713	2.1
33138	1.36503	2.1
33139	0.946131	2.1
33140	1.36503	2.1
33141	1.28713	2.1
33142	0.946131	2.1
33143	1.89226	2.1
33144	1.89226	1.95906
33145	1.89226	2.1
33146	1.89226	2.1
33147	1.89226	2.1
33148	1.89226	2.09385
33149	1.22615	2.1
33150	1.04147	2.1
33151	1.3439	2.1
33152	1.3439	2.1
33153	0.969118	2.1
33154	0.969118	2.1
33155	0.980841	2.1
33156	0.992505	2.1
33157	1.34289	2.1
33158	1.70303	2.1
33159	2.56081	2.1
33160	1.89225	2.1
33161	2.66785	2.1
33162	2.79506	2.1
33163	2.79506	2.1
33164	1.89225	2.1
33165	1.89225	2.1
33166	0.978778	2.1
33167	0.978778	2.1
33168	0.993303	2.1
33169	0.993303	2.1
33170	0.603538	2.1
33171	0.638158	2.1
33172	1.33393	2.1
33173	1.39753	2.1

33174	1.33391	2.1
33175	0.603535	2.1
33176	0.638164	2.1
33177	1.39754	2.1
33178	1.33393	2.1
33179	1.33393	2.1
33180	0.946132	2.1
33181	0.946132	2.1
33182	1.33393	2.1
33183	1.27365	2.1
33184	0.946132	2.1
33185	0.946132	2.1
33186	0.946132	2.1
33187	1.27365	2.1
33188	1.33393	2.1
33189	0.946131	2.1
33190	1.89226	2.1
33191	1.89226	2.1
33192	1.89226	2.1
33193	1.89226	2.1
33194	3.78452	2.1
33195	1.89226	2.1
33196	1.89226	2.1
33197	1.89226	2.1
33198	1.89226	2.1
33199	3.78453	2.1
33200	0.979364	2.1
33201	0.979364	2.1
33202	0.89618	2.1
33203	0.89618	2.1
33204	0.896178	2.1
33205	0.929411	2.1
33206	0.946133	2.1
33207	0.946133	2.1
33208	0.946131	2.1
33209	0.89618	2.1
33210	0.946131	2.1
33211	0.929413	2.1
33212	1.89226	2.1
33213	1.89226	2.1
33214	1.89226	2.1
33215	1.89226	2.1
33216	1.89226	2.1
33217	1.89226	2.1
33218	1.89225	2.1
33219	1.89225	2.1
33220	1.89225	2.1
33221	1.89225	2.1
33222	3.7845	2.1
33223	1.89225	2.1
33224	1.89225	2.1
33225	1.89225	2.1
33226	1.89225	2.1
33227	1.89225	2.1
33228	1.89225	2.1
33229	0.946128	0.169289
33230	0.946128	0.108847
33231	0.946132	0.140415

33232	0.946132	0.0879539
33233	0.94613	0.144496
33234	0.94613	0.173623
33235	0.946129	0.0968035
33236	0.946129	0.0801394
33237	0.94613	0.122063
33238	0.94613	0.125056
33239	0.94613	0.112684
33240	0.94613	0.108156
33241	1.89227	1.24609
33242	1.89227	1.30886
33243	1.89227	0.571717
33244	1.89227	0.456986
33245	3.78453	0.798347
33246	1.89226	1.16438
33247	2.43717	1.74497
33248	2.63259	0.906635
33249	1.89226	0.48335
33250	4.52485	1.29549
33251	0.616808	0.777204
33252	0.792277	0.943254
33253	1.04986	0.530982
33254	0.72303	1.15252
33255	0.702675	0.200637
33256	0.76862	2.06045
33257	0.809154	1.61877
33258	0.809154	0.508244
33259	0.808757	0.942629
33260	1.07789	0.275994
33261	0.772568	0.635823
33262	0.598878	1.18404
33263	1.89226	1.15183
33264	1.89226	1.3585
33265	1.89226	0.757132
33266	1.89226	0.608935
33267	3.78453	1.19552
33268	1.36428	1.39981
33269	1.31629	1.10847
33270	0.946132	0.907387
33271	0.946132	0.908426
33272	1.36428	0.598768
33273	1.36428	1.89391
33274	0.946132	1.31083
33275	0.946132	1.69313
33276	1.36428	1.54284
33277	0.946132	0.77539
33278	1.31629	1.47322
33279	0.946132	0.816726
33280	1.578	1.36771
33281	2.85336	0.331763
33282	2.11368	0.273013
33283	2.14375	1.14153
33284	1.93852	0.65599
33285	1.93852	1.1986
33286	2.34806	0.219328
33287	1.42913	0.239839
33288	1.61539	0.305048
33289	2.45322	0.259644

33290	3.88235	0.274306
33291	2.34806	0.13764
33292	1.89226	0.185304
33293	1.89226	0.414982
33294	2.45322	0.298657
33295	4.34548	0.167737
33296	1.89226	0.175454
33297	1.89226	0.114184
33298	1.89226	0.197141
33299	1.89226	0.254607
33300	3.78452	0.224494
33301	1.89226	0.240834
33302	1.89226	0.166542
33303	1.89226	0.256626
33304	1.89226	0.315033
33305	3.78452	0.220215
33306	1.25972	0.165919
33307	2.45322	0.165384
33308	2.58823	0.316474
33309	1.79398	0.247882
33310	4.2472	0.175477
33311	1.89226	0.282405
33312	2.45322	0.0984989
33313	2.58823	0.201985
33314	2.45322	0.249417
33315	2.02727	0.113647
33316	1.89226	0.161887
33317	1.89226	0.091931
33318	1.89226	0.139083
33319	1.89226	0.236128
33320	1.89226	0.185576
33321	3.78452	0.105106
33322	1.89226	0.165721
33323	1.89226	0.214595
33324	1.89226	0.200602
33325	1.89226	0.106188
33326	1.89226	0.316858
33327	1.89226	0.156956
33328	1.70048	2.1
33329	2.1572	1.65167
33330	1.45477	2.1
33331	1.93973	2.02094
33332	3.64021	2.1
33333	1.17614	2.1
33334	1.93973	1.95099
33335	1.69661	2.1
33336	1.69661	1.88337
33337	1.45477	2.1
33338	1.45477	1.9514
33339	1.94343	2.1
33340	2.53782	1.98459
33341	2.59903	2.1
33342	2.59903	1.35056
33343	1.87598	2.1
33344	1.87598	1.85068
33345	0.946131	1.2335
33346	0.946131	1.97543
33347	1.26891	2.1

33348	1.26891	0.647683
33349	0.94613	2.03509
33350	0.94613	1.69888
33351	1.26891	1.83874
33352	1.29952	1.0824
33353	1.29952	0.200242
33354	0.946131	0.34327
33355	1.26891	1.09118
33356	0.946131	1.19902
33357	2.00551	2.1
33358	2.59903	2.04265
33359	2.65811	2.1
33360	2.65811	1.85044
33361	1.94343	2.1
33362	1.94343	2.0721
33363	2.65811	1.26223
33364	1.89226	2.08096
33365	1.89226	0.604634
33366	1.89226	1.69267
33367	2.59903	0.907269
33368	2.59903	2.06181
33369	1.89227	2.1
33370	1.89227	2.1
33371	1.89227	2.1
33372	1.89227	2.1
33373	3.78453	2.1
33374	1.15471	2.1
33375	0.993889	2.1
33376	1.78919	2.1
33377	1.77325	2.1
33378	2.78308	2.1
33379	1.89226	2.1
33380	1.89226	2.1
33381	1.89226	2.1
33382	1.89226	2.1
33383	3.78453	2.1
33384	1.78919	2.1
33385	1.77325	2.1
33386	2.45969	2.1
33387	2.70835	2.1
33388	4.49753	2.1
33389	0.939314	2.1
33390	0.939315	2.1
33391	1.01133	2.1
33392	1.01133	2.1
33393	0.596471	2.1
33394	1.20813	2.1
33395	0.886619	2.1
33396	1.35417	2.1
33397	1.35417	2.1
33398	1.20813	2.1
33399	0.886621	2.1
33400	0.59647	2.1
33401	0.946131	2.1
33402	0.946131	2.1
33403	0.94613	2.1
33404	0.94613	2.1
33405	0.946133	2.1

33406	0.946133	2.1
33407	0.946128	2.1
33408	0.946128	2.1
33409	0.946131	2.1
33410	0.946131	2.1
33411	0.946131	2.1
33412	0.946131	2.1
33413	1.03939	2.1
33414	1.03939	2.1
33415	0.946132	2.08766
33416	0.946132	2.1
33417	0.946132	2.1
33418	0.946132	2.1
33419	1.23851	2.09967
33420	1.03939	2.1
33421	1.03939	2.1
33422	0.946132	2.1
33423	0.946133	2.1
33424	1.23851	2.1
33425	0.902503	2.1
33426	0.946129	2.1
33427	0.946132	2.09064
33428	0.946132	2.1
33429	0.902472	2.1
33430	0.902472	2.1
33431	1.08736	2.09984
33432	0.994093	2.1
33433	0.946133	2.1
33434	0.902504	2.1
33435	1.03939	2.1
33436	0.946132	2.1
33437	1.35306	1.138
33438	1.29982	0.370696
33439	1.7216	0.258507
33440	1.89183	0.298369
33441	3.07466	0.321381
33442	0.485098	0.343938
33443	0.582401	0.354323
33444	0.649908	0.543364
33445	0.8608	0.418945
33446	0.587739	0.212236
33447	0.58774	0.590884
33448	0.644567	0.332647
33449	0.644567	1.25018
33450	0.58241	0.400234
33451	0.86081	0.225412
33452	0.649898	0.707455
33453	0.485093	0.379396
33454	2.25936	2.1
33455	2.71441	2.1
33456	2.63824	2.1
33457	2.20652	2.1
33458	4.92092	2.1
33459	2.25936	2.1
33460	1.57627	2.1
33461	1.5493	2.1
33462	2.20652	2.1
33463	3.78279	2.1

33464	2.63824	2.1
33465	2.20652	2.1
33466	2.17326	2.1
33467	2.58517	2.1
33468	4.79169	2.1
33469	2.17326	2.1
33470	2.20652	2.1
33471	2.19278	2.1
33472	1.53556	2.1
33473	1.5493	2.1
33474	1.5493	2.1
33475	1.97465	2.1
33476	2.13925	2.1
33477	2.04483	2.1
33478	1.62784	2.1
33479	3.76709	2.1
33480	1.89226	2.1
33481	1.89226	2.1
33482	1.89226	2.1
33483	1.89226	2.1
33484	1.89226	2.1
33485	1.89226	2.1
33486	0.724037	2.1
33487	0.81392	2.1
33488	0.96796	2.1
33489	1.02242	2.1
33490	0.882091	2.1
33491	0.882091	2.1
33492	0.899789	2.1
33493	0.899789	2.1
33494	0.724036	2.1
33495	0.96796	2.1
33496	1.02242	2.1
33497	0.81392	2.1
33498	1.13537	2.1
33499	1.63054	2.1
33500	0.946131	2.1
33501	0.946131	2.1
33502	0.946131	2.1
33503	0.946131	2.1
33504	1.258	2.1
33505	1.63054	2.1
33506	1.22632	2.1
33507	1.22632	2.1
33508	0.946131	2.1
33509	1.258	2.1
33510	1.89226	2.1
33511	1.89226	2.1
33512	1.89226	2.1
33513	1.89226	2.1
33514	1.89226	2.1
33515	1.89226	2.1
33516	0.809739	2.1
33517	0.862674	2.1
33518	1.1995	2.1
33519	1.33899	2.1
33520	1.06324	2.1
33521	1.06324	2.1

33522	0.981932	2.1
33523	1.19563	2.1
33524	0.809727	2.1
33525	1.14092	2.1
33526	1.33902	2.1
33527	0.863494	2.1
33528	2.23742	2.1
33529	1.84902	2.1
33530	1.88088	2.1
33531	2.20392	2.1
33532	4.08644	2.1
33533	1.3592	2.1
33534	1.84902	2.1
33535	1.88088	2.1
33536	1.3305	2.1
33537	1.88088	2.1
33538	1.3305	2.1
33539	1.84902	2.1
33540	1.15008	1.00438
33541	1.3305	2.1
33542	1.70539	2.1
33543	2.9991	2.1
33544	1.84901	2.1
33545	2.0264	2.1
33546	2.20391	2.1
33547	1.70539	2.1
33548	3.87541	2.1
33549	1.03852	2.1
33550	1.38247	1.64885
33551	1.36635	1.73917
33552	1.01537	2.09942
33553	2.39784	2.04607
33554	2.0815	2.1
33555	1.91744	2.1
33556	1.91744	2.08227
33557	2.06496	2.1
33558	1.89227	2.08804
33559	2.03474	2.1
33560	1.89226	1.74714
33561	1.89226	2.08688
33562	1.89226	1.76393
33563	1.89226	2.1
33564	1.89226	1.53351
33565	1.89226	2.09974
33566	1.36635	1.89161
33567	1.02923	2.09938
33568	1.02923	2.00806
33569	1.01537	2.1
33570	1.36416	1.84259
33571	1.3503	2.1
33572	1.89226	2.1
33573	1.89359	2.1
33574	1.89359	2.09834
33575	1.91744	2.1
33576	1.89226	2.09446
33577	1.91611	2.1
33578	0.946128	2.09973
33579	0.946128	2.09199

33580	0.946133	1.86784
33581	0.946133	1.73532
33582	0.94613	1.71527
33583	0.94613	2.09293
33584	0.946131	2.1
33585	0.946131	1.84545
33586	0.946131	2.07098
33587	0.946131	2.0781
33588	0.946131	2.06708
33589	0.946131	2.07759
33590	2.689 2.1	
33591	2.0815	2.1
33592	2.72786	2.1
33593	2.48295	2.1
33594	5.02397	2.1
33595	2.72786	2.1
33596	1.86424	2.1
33597	2.7188	2.1
33598	2.08981	2.1
33599	4.81768	2.1
33600	2.08982	2.1
33601	0.986987	2.1
33602	2.08982	2.1
33603	1.87249	2.1
33604	1.01964	2.1
33605	0.906115	2.1
33606	1.44247	2.1
33607	1.43872	2.1
33608	1.17054	2.1
33609	1.17054	2.1
33610	1.43872	2.1
33611	1.26045	2.1
33612	2.0815	0.0698591
33613	2.0815	0.0698591
33614	2.0815	0.0698591
33615	1.81468	0.0698591
33616	1.89065	0.0698591
33617	1.55625	0.0698591
33618	1.89062	0.0698591
33619	1.89224	0.0698591
33620	1.89224	0.0698591
33621	2.27073	0.0698591
33622	1.86856	0.0698591
33623	2.24232	0.0698591
33624	1.21419	2.1
33625	2.79716	2.1
33626	2.50005	2.1
33627	2.50005	2.1
33628	1.5008	2.1
33629	1.5008	2.1
33630	1.60139	0.0698591
33631	1.7103	0.0698591
33632	1.04159	0.0698591
33633	1.2489	0.0698591
33634	2.75189	0.0698591
33635	0.732812	0.0698591
33636	0.732812	0.0698591
33637	0.751986	0.0698591

33638	0.751986	0.0698591
33639	0.860346	0.0698591
33640	0.667961	0.0698591
33641	0.85515	0.0698591
33642	0.624452	0.0698591
33643	0.62445	0.0698591
33644	0.667962	0.0698591
33645	0.855148	0.0698591
33646	0.860348	0.0698591
33647	1.89226	0.178438
33648	2.50872	0.171379
33649	2.77909	0.159549
33650	1.89226	0.198761
33651	2.77909	0.150956
33652	1.89226	0.194658
33653	2.15825	0.167425
33654	2.50872	0.158753
33655	1.90439	0.133674
33656	1.90439	0.185039
33657	2.77909	0.136172
33658	2.77909	0.186022
33659	1.12468	2.1
33660	1.43872	2.1
33661	1.64926	2.1
33662	1.2724	2.1
33663	2.77395	2.1
33664	1.64926	2.1
33665	1.38438	2.1
33666	1.47244	2.1
33667	1.2724	2.1
33668	1.8493	2.1
33669	1.64926	2.1
33670	1.36416	1.96995
33671	1.0652	2.1
33672	1.0652	2.03174
33673	1.02923	2.1
33674	1.37496	1.93433
33675	1.33898	2.1
33676	1.89227	2.1
33677	1.90433	2.1
33678	1.90433	2.1
33679	1.89359	2.1
33680	1.89227	2.09997
33681	1.88153	2.1
33682	0.946129	2.1
33683	0.946129	2.09905
33684	0.946133	1.95558
33685	0.946133	1.91751
33686	0.946131	1.90861
33687	0.946131	2.09955
33688	0.946131	2.1
33689	0.946131	1.91868
33690	0.946131	2.07097
33691	0.946131	2.07758
33692	0.946131	2.04607
33693	0.946131	2.05214
33694	1.37496	2.00152
33695	1.07519	2.1

33696	1.07519	2.0564
33697	1.0652	2.1
33698	1.39725	1.98751
33699	1.38726	2.1
33700	1.89227	2.1
33701	1.9052	2.1
33702	1.9052	2.1
33703	1.90433	2.1
33704	1.89227	2.1
33705	1.8914	2.1
33706	0.946129	2.1
33707	0.946129	2.1
33708	0.946133	2.01022
33709	0.946133	1.94524
33710	0.946131	1.94185
33711	0.946131	2.1
33712	0.946131	2.1
33713	0.946131	1.97412
33714	0.946131	2.08767
33715	0.946131	2.09233
33716	0.946131	2.06744
33717	0.946131	2.07142
33718	1.91316	0.0884328
33719	1.89226	0.0964753
33720	1.89226	0.0985287
33721	1.89226	0.0899737
33722	1.99366	0.103317
33723	1.99366	0.0912387
33724	1.93083	0.0783954
33725	1.89226	0.0854571
33726	1.51379	0.0808878
33727	1.89226	0.0778019
33728	1.53051	0.0887
33729	1.91316	0.0797638
33730	2.16731	2.1
33731	1.21373	2.1
33732	1.47804	2.1
33733	2.37601	2.1
33734	3.64535	2.1
33735	1.7211	2.1
33736	2.37601	2.1
33737	1.47804	2.1
33738	1.47804	2.1
33739	2.56843	2.1
33740	2.56843	2.1
33741	2.58731	2.1
33742	1.59094	2.1
33743	1.59094	2.1
33744	1.76816	2.1
33745	2.41707	2.1
33746	2.5943	2.1
33747	0.718346	2.1
33748	0.795468	2.1
33749	1.20854	2.1
33750	1.1224	2.1
33751	0.961203	2.1
33752	0.961203	2.1
33753	0.95667	2.1

33754	0.95667	2.1
33755	0.718346	2.1
33756	1.1224	2.1
33757	0.795469	2.1
33758	1.20854	2.1
33759	1.84076	2.1
33760	1.42884	2.1
33761	2.24274	2.1
33762	1.85415	2.1
33763	3.69491	2.1
33764	1.89226	2.1
33765	1.89226	2.1
33766	1.89226	2.1
33767	1.89226	2.1
33768	3.78452	2.1
33769	2.24273	2.1
33770	2.24412	2.1
33771	1.85415	2.1
33772	1.85415	2.1
33773	2.68473	2.1
33774	2.68473	2.1
33775	1.89226	2.1
33776	1.89226	2.1
33777	1.89226	2.1
33778	1.89226	2.09099
33779	1.89226	2.1
33780	1.89226	2.08829
33781	1.89226	2.08388
33782	1.89226	2.1
33783	1.89226	2.1
33784	1.89226	2.09698
33785	3.78451	2.1
33786	1.85415	2.1
33787	1.10961	2.1
33788	2.24411	2.1
33789	1.51067	2.1
33790	3.35373	2.1
33791	1.70302	2.1
33792	1.89226	2.1
33793	1.76399	2.1
33794	1.43577	2.1
33795	2.08149	2.1
33796	1.70302	2.1
33797	1.89226	2.1
33798	1.89226	2.1
33799	1.89226	2.1
33800	1.89226	2.1
33801	1.89226	2.1
33802	1.89226	2.1
33803	1.0593	2.1
33804	1.17289	2.1
33805	1.5379	2.1
33806	1.46757	2.1
33807	2.71079	2.1
33808	0.931235	2.1
33809	1.1151	2.1
33810	0.975236	2.1
33811	0.975236	2.1

33812	1.31147	2.1
33813	0.762882	2.1
33814	1.13152	2.1
33815	0.505799	2.1
33816	1.31149	2.1
33817	1.07629	2.1
33818	0.884817	2.1
33819	0.505793	2.1
33820	2.69027	2.1
33821	1.60441	2.1
33822	2.69027	2.1
33823	1.64771	2.1
33824	2.78977	2.1
33825	1.72731	2.1
33826	0.77878	0.0698591
33827	0.843226	0.0698591
33828	0.845997	0.0698591
33829	0.926091	0.0698591
33830	0.77878	0.0698591
33831	0.926093	0.0698591
33832	1.3922	1.64594
33833	1.3922	1.44499
33834	0.872156	1.96602
33835	0.872156	1.90359
33836	0.872157	1.98408
33837	0.946132	1.32428
33838	0.946129	1.38828
33839	1.31822	2.02235
33840	0.94613	1.85563
33841	0.94613	1.86857
33842	1.31823	1.8072
33843	0.872157	1.90611
33844	1.81534	0.414253
33845	1.89226	0.294488
33846	1.59602	0.456781
33847	1.59602	0.540547
33848	1.89226	0.211473
33849	1.89226	0.273833
33850	1.89227	0.33591
33851	1.89227	0.455346
33852	1.89227	0.477698
33853	1.89227	0.334643
33854	3.78453	0.348026
33855	1.89226	0.432702
33856	1.89226	0.255636
33857	1.89226	0.322386
33858	1.89226	0.389928
33859	3.78453	0.416625
33860	2.30348	0.183454
33861	1.4185	0.227753
33862	1.82551	0.263903
33863	1.02011	0.194413
33864	3.24401	0.195412
33865	2.46702	0.168324
33866	1.17129	0.214119
33867	1.82551	0.177486
33868	1.82551	0.19573
33869	1.73525	0.171264

33870	1.73525	0.20957
33871	1.89227	0.384967
33872	1.89227	0.268366
33873	1.89227	0.268626
33874	1.89227	0.311126
33875	1.89227	0.338339
33876	1.89227	0.380929
33877	2.80034	2.1
33878	2.45767	2.1
33879	2.67994	2.1
33880	2.35395	2.1
33881	5.1376	2.1
33882	2.45766	2.1
33883	1.8769	2.1
33884	2.00989	2.1
33885	2.00989	2.1
33886	2.35395	2.1
33887	2.35395	2.1
33888	2.57662	2.1
33889	2.35395	2.1
33890	2.57662	2.1
33891	2.67993	2.1
33892	2.24227	2.1
33893	2.34558	2.1
33894	1.02874	2.1
33895	1.02874	2.1
33896	1.03084	2.1
33897	1.03084	2.1
33898	0.938448	2.1
33899	0.886762	2.1
33900	1.17698	2.1
33901	1.12114	2.1
33902	1.12113	2.1
33903	0.886764	2.1
33904	0.93845	2.1
33905	1.17697	2.1
33906	1.91969	2.1
33907	2.5508	2.1
33908	1.88273	1.90115
33909	2.76609	2.1
33910	4.68578	2.1
33911	2.83423	2.1
33912	1.89226	2.1
33913	2.76609	2.1
33914	1.89226	2.1
33915	4.65835	2.1
33916	1.56685	0.204036
33917	1.11712	0.355099
33918	1.07999	1.18443
33919	1.51448	0.395864
33920	2.6316	0.451139
33921	1.56685	0.107479
33922	1.67652	0.929846
33923	1.5955	1.07247
33924	1.51448	0.299875
33925	3.19099	0.490464
33926	1.89226	1.03385
33927	1.89226	0.310921

33928	1.89226	0.335128
33929	1.89226	1.25546
33930	3.78453	0.639042
33931	1.89226	0.420663
33932	1.89226	0.351588
33933	1.89226	0.673403
33934	1.89226	1.36
33935	1.89226	0.245017
33936	1.89226	0.320841
33937	1.0562	0.45937
33938	1.51448	0.268887
33939	1.07999	0.413541
33940	1.07999	0.24064
33941	1.46441	0.463419
33942	1.46441	0.236551
33943	1.5955	1.1259
33944	1.51448	0.283814
33945	1.46441	0.422437
33946	1.52909	1.22687
33947	3.04357	0.738787
33948	1.89226	0.329025
33949	1.89226	1.34857
33950	1.89226	1.2903
33951	1.89226	0.439958
33952	3.78453	0.742558
33953	1.89227	0.323864
33954	1.89227	0.246169
33955	1.89227	0.466264
33956	1.89227	0.514184
33957	3.78453	0.358714
33958	1.89994	0.740261
33959	1.70303	1.11509
33960	1.68576	0.366947
33961	1.89608	0.391539
33962	3.8237	0.71635
33963	1.89994	1.28466
33964	2.31116	2.09554
33965	1.9386	0.869792
33966	1.88653	1.56836
33967	3.74885	1.77874
33968	2.31117	2.1
33969	2.20665	1.98323
33970	2.09617	2.04539
33971	2.41055	2.1
33972	2.09617	1.90132
33973	2.41055	2.1
33974	1.10777	0.751511
33975	1.0523	1.29233
33976	0.912098	0.884408
33977	0.757533	0.766591
33978	0.89287	1.55827
33979	1.07571	0.300621
33980	0.77485	1.09808
33981	0.98645	0.473907
33982	0.989171	0.356482
33983	0.875089	0.400641
33984	0.979686	1.11774
33985	0.979686	1.72859

33986	2.52442	2.1
33987	1.49998	2.1
33988	2.51692	2.1
33989	1.56957	2.1
33990	4.09399	2.1
33991	1.01298	2.1
33992	1.01298	2.1
33993	1.03079	2.1
33994	1.03079	2.1
33995	0.820964	2.1
33996	0.784786	2.1
33997	1.25898	2.1
33998	1.25846	2.1
33999	1.25898	2.1
34000	0.820964	2.1
34001	1.25846	2.1
34002	0.784786	2.1
34003	1.89226	0.0698591
34004	1.89226	0.0698591
34005	2.6297	0.0698591
34006	1.89226	0.0698591
34007	3.78452	0.0698591
34008	1.89226	0.0698591
34009	1.89226	0.0698591
34010	1.89226	0.0698591
34011	1.89226	0.0698591
34012	1.89226	0.0698591
34013	1.89226	0.0698591
34014	2.19397	0.0698591
34015	1.89226	0.0698591
34016	2.34112	0.0698591
34017	2.34112	0.0698591
34018	1.89226	0.0698591
34019	1.89226	0.0698591
34020	2.23366	2.1
34021	1.87598	2.1
34022	1.64716	2.1
34023	2.06048	2.1
34024	3.93645	2.1
34025	2.23367	2.1
34026	2.17384	2.1
34027	2.04818	2.1
34028	2.06048	2.1
34029	4.23432	2.1
34030	1.9855	2.1
34031	2.06048	2.1
34032	1.93163	2.1
34033	1.50737	2.1
34034	3.56785	2.1
34035	2.04818	2.1
34036	2.06048	2.1
34037	2.27465	2.1
34038	1.92264	2.1
34039	3.98312	2.1
34040	2.08149	0.616219
34041	1.89226	1.36435
34042	1.89226	1.21826
34043	1.33153	0.574723

34044	1.68038	1.59583
34045	1.18244	0.698465
34046	1.89226	0.904342
34047	1.89226	1.63502
34048	1.89226	1.55675
34049	1.89226	0.708194
34050	3.78452	1.00709
34051	2.69069	0.489313
34052	1.89226	0.625568
34053	1.89226	0.63764
34054	1.97397	0.485314
34055	1.89226	0.631596
34056	1.97397	0.472792
34057	1.89226	1.6297
34058	2.38056	0.669524
34059	1.89226	0.72788
34060	1.89226	1.10308
34061	3.78452	0.949286
34062	0.904435	2.1
34063	0.904435	2.1
34064	0.925315	2.1
34065	0.925315	2.1
34066	1.13312	2.1
34067	1.08109	2.1
34068	0.70697	2.1
34069	0.696633	2.1
34070	0.70697	2.1
34071	1.13312	2.1
34072	0.696633	2.1
34073	1.08109	2.1
34074	1.45506	2.1
34075	2.26623	2.1
34076	2.38363	2.1
34077	2.38363	2.1
34078	1.41394	2.1
34079	1.41394	2.1
34080	0.946131	2.1
34081	0.946131	2.1
34082	1.13311	2.1
34083	1.13311	2.1
34084	0.946127	2.1
34085	0.946127	2.1
34086	1.13312	2.1
34087	1.19182	2.1
34088	1.19181	2.1
34089	0.946129	2.1
34090	1.13312	2.1
34091	0.94613	2.1
34092	1.89226	1.11285
34093	1.89226	2.00202
34094	1.89226	1.21499
34095	1.89226	2.00637
34096	1.89226	1.37696
34097	1.89226	2.01773
34098	1.05168	2.09976
34099	0.696527	2.0875
34100	0.907442	1.79512
34101	0.907442	1.95135

34102	0.953145	1.71116
34103	0.782285	2.1
34104	0.773928	2.09757
34105	0.773928	1.8898
34106	0.696538	1.932
34107	0.851554	1.92652
34108	1.04071	1.98718
34109	1.12335	1.99077
34110	1.89226	0.605529
34111	1.89226	0.220637
34112	1.89226	0.309814
34113	1.89226	0.17357
34114	3.78453	0.195522
34115	1.89226	0.509059
34116	1.89226	0.224131
34117	1.89226	0.357045
34118	1.89226	0.208286
34119	3.78453	0.348845
34120	2.50912	0.201386
34121	1.89226	0.156979
34122	1.89226	0.200347
34123	2.63195	0.151117
34124	4.40139	0.154529
34125	2.50912	0.197579
34126	1.3598	0.149462
34127	1.70303	0.157501
34128	2.36874	0.126048
34129	3.82055	0.166685
34130	1.89227	0.24347
34131	1.89227	0.167053
34132	1.89227	0.15465
34133	1.89227	0.113702
34134	3.78453	0.203952
34135	1.89227	0.280031
34136	1.89227	0.179522
34137	1.89227	0.194694
34138	1.89227	0.123992
34139	3.78453	0.14707
34140	1.89226	0.169184
34141	2.67088	0.108818
34142	2.63195	0.0923613
34143	2.63195	0.13552
34144	1.89226	0.107807
34145	1.89226	0.162254
34146	1.30202	0.124695
34147	1.28304	0.122001
34148	0.809902	0.110685
34149	0.888801	0.117682
34150	1.13262	0.0851914
34151	0.968276	0.111929
34152	1.0733	0.124653
34153	1.0733	0.112708
34154	1.35419	0.106633
34155	0.801222	0.0876753
34156	0.899732	0.145463
34157	1.23967	0.104568
34158	1.31595	2.1
34159	0.869501	2.1

34160	2.65473	2.1
34161	1.88782	2.1
34162	3.20377	2.1
34163	1.89227	2.1
34164	1.89227	2.1
34165	1.89227	2.1
34166	1.89227	2.1
34167	3.78453	2.1
34168	2.65473	2.1
34169	1.89226	2.1
34170	1.88781	2.1
34171	1.88782	2.1
34172	2.0815	2.1
34173	2.0815	2.1
34174	1.89226	2.09759
34175	1.89226	2.1
34176	1.89226	2.1
34177	1.89226	2.1
34178	1.89226	2.1
34179	1.89226	2.1
34180	1.89226	2.09871
34181	1.89226	2.1
34182	1.89226	2.1
34183	1.89226	2.1
34184	1.89226	2.1
34185	1.89226	2.09729
34186	2.39057	2.1
34187	1.89226	2.1
34188	1.89226	2.05051
34189	1.89226	2.1
34190	2.14243	2.05187
34191	2.14243	2.1
34192	1.89226	0.934778
34193	1.89226	2.1
34194	1.89226	1.90298
34195	1.89226	2.0205
34196	1.89226	1.87687
34197	1.89226	2.05528
34198	1.89226	1.25639
34199	1.89226	2.1
34200	1.89226	2.00458
34201	1.89226	2.04608
34202	1.89226	1.93707
34203	1.89226	2.07562
34204	2.56323	0.0778261
34205	1.36324	0.0811711
34206	1.05296	0.073786
34207	2.18048	0.0809699
34208	3.54372	0.075018
34209	0.946128	2.1
34210	0.946128	2.1
34211	1.08733	2.1
34212	1.08733	2.1
34213	0.946131	2.1
34214	0.946131	2.1
34215	1.09197	2.1
34216	1.08732	2.1
34217	0.946131	2.1

34218	1.09197	2.1
34219	1.08732	2.1
34220	0.946131	2.1
34221	1.35111	1.53122
34222	1.51327	1.18817
34223	1.64493	1.22364
34224	1.31058	1.51274
34225	2.99605	1.54709
34226	1.8124	1.07572
34227	1.51327	1.28919
34228	1.64493	1.45765
34229	1.31358	0.970071
34230	1.8124	1.44424
34231	1.48105	0.979989
34232	1.64493	1.20935
34233	1.31058	1.3925
34234	1.22297	1.36224
34235	1.70101	1.0702
34236	2.8679	1.10541
34237	1.70101	1.18113
34238	1.99297	1.07072
34239	1.64493	1.29101
34240	1.64493	0.877949
34241	1.99297	1.37519
34242	1.8124	0.81944
34243	2.0815	2.1
34244	1.80258	2.1
34245	1.80258	2.1
34246	1.83506	2.1
34247	2.0815	2.1
34248	2.1212	2.1
34249	2.0815	2.1
34250	1.82129	2.1
34251	1.82129	2.1
34252	1.80445	2.1
34253	2.0815	2.1
34254	2.06092	2.1
34255	1.89224	2.1
34256	1.703 2.1	
34257	1.97839	2.1
34258	1.703 2.1	
34259	1.97402	2.1
34260	1.703 2.1	
34261	1.89226	0.23228
34262	1.89226	0.362356
34263	1.89226	0.327312
34264	2.75351	0.239266
34265	3.78453	0.291825
34266	1.89226	0.314383
34267	3.02887	0.304493
34268	2.03149	0.259851
34269	2.03149	0.361972
34270	1.89226	0.231113
34271	1.89226	0.324675
34272	2.17979	2.1
34273	1.65533	2.1
34274	2.57212	2.1
34275	2.20346	2.1

34276	4.22745	2.1
34277	1.89227	2.1
34278	2.20347	2.1
34279	1.89227	2.1
34280	2.57212	2.1
34281	1.89227	2.1
34282	2.57212	2.1
34283	2.05274	2.1
34284	2.16783	2.1
34285	2.24429	2.1
34286	2.38404	2.1
34287	4.41212	2.1
34288	2.16783	2.1
34289	2.05967	2.1
34290	2.38404	2.1
34291	2.24695	2.1
34292	4.41478	2.1
34293	2.24429	2.1
34294	2.38404	2.1
34295	2.51819	2.1
34296	2.60723	2.1
34297	4.85152	2.1
34298	2.38404	2.1
34299	2.24695	2.1
34300	2.60723	2.1
34301	2.45247	2.1
34302	4.85418	2.1
34303	1.89227	2.1
34304	1.89227	2.1
34305	1.89227	2.1
34306	1.89227	2.1
34307	3.78453	2.1
34308	1.89226	2.1
34309	1.89226	2.1
34310	1.89226	2.1
34311	1.89226	2.1
34312	3.78453	2.1
34313	0.618473	2.1
34314	0.619945	2.1
34315	0.797692	2.1
34316	0.770147	2.1
34317	0.68852	2.1
34318	0.68852	2.1
34319	0.701573	2.1
34320	0.701573	2.1
34321	0.619945	2.1
34322	0.797691	2.1
34323	0.618473	2.1
34324	0.770147	2.1
34325	0.797692	2.1
34326	0.770148	2.1
34327	0.694787	2.1
34328	0.653411	2.1
34329	0.722102	2.1
34330	0.722102	2.1
34331	0.729001	2.1
34332	0.729001	2.1
34333	0.797692	2.1

34334	0.694787	2.1
34335	0.770148	2.1
34336	0.653412	2.1
34337	1.89227	2.1
34338	1.89227	2.1
34339	1.89227	2.1
34340	1.89227	2.1
34341	3.78453	2.1
34342	1.89226	2.1
34343	1.89226	2.1
34344	1.89226	2.1
34345	1.89226	2.1
34346	3.78453	2.1
34347	1.23989	2.1
34348	1.59538	2.1
34349	1.66647	2.1
34350	1.26599	2.1
34351	2.90636	2.1
34352	1.66647	2.1
34353	1.38957	2.1
34354	1.47446	2.1
34355	1.40337	2.1
34356	1.66647	2.1
34357	1.59538	2.1
34358	2.80619	2.1
34359	2.0815	2.1
34360	2.0815	2.1
34361	2.0815	2.1
34362	2.73721	2.1
34363	2.74192	2.1
34364	2.73721	2.1
34365	2.0815	2.1
34366	2.0815	2.1
34367	2.0815	2.1
34368	2.689	2.1
34369	2.69166	2.1
34370	2.62369	2.1
34371	2.19717	2.1
34372	2.60405	2.1
34373	2.20566	2.1
34374	4.80122	2.1
34375	2.19717	2.1
34376	1.37392	2.1
34377	2.20566	2.1
34378	1.45408	2.1
34379	3.65125	2.1
34380	2.60405	2.1
34381	2.20566	2.1
34382	2.57466	2.1
34383	2.21715	2.1
34384	4.8212	2.1
34385	2.20566	2.1
34386	1.54249	2.1
34387	1.54249	2.1
34388	1.45408	2.1
34389	2.21715	2.1
34390	2.12874	2.1
34391	1.60647	2.1

34392	1.31346	2.1
34393	1.82649	2.1
34394	1.51067	2.1
34395	3.13995	2.1
34396	1.89226	2.1
34397	1.89226	2.1
34398	1.89226	2.1
34399	1.89226	2.1
34400	3.78452	2.1
34401	1.89226	2.1
34402	2.6096	2.1
34403	2.8067	2.1
34404	2.8067	2.1
34405	1.89226	2.1
34406	1.89226	2.1
34407	1.82649	2.1
34408	1.3596	2.1
34409	2.17708	2.1
34410	1.70302	2.1
34411	3.40243	2.1
34412	1.89226	2.1
34413	1.89226	2.1
34414	1.89226	2.1
34415	1.89226	2.1
34416	3.78452	2.1
34417	2.27067	2.1
34418	2.43549	2.1
34419	2.63979	2.1
34420	2.41828	2.1
34421	2.27067	2.1
34422	2.04916	2.1
34423	1.30047	2.1
34424	2.04916	2.1
34425	2.27068	2.1
34426	1.5586	2.1
34427	3.57115	2.1
34428	2.27068	2.1
34429	1.84214	2.1
34430	1.80224	2.1
34431	1.5586	2.1
34432	2.51432	2.1
34433	2.27068	2.1
34434	1.63719	2.1
34435	2.15714	2.1
34436	2.07821	2.1
34437	1.52204	2.1
34438	3.67919	2.1
34439	2.07821	2.1
34440	1.40118	2.1
34441	1.40118	2.1
34442	1.52204	2.1
34443	1.99632	2.1
34444	2.11718	2.1
34445	0.946131	2.1
34446	0.946131	2.1
34447	1.06957	2.1
34448	1.06957	2.1
34449	0.946132	2.1

34450	0.946132	2.06394
34451	1.411	2.1
34452	1.06957	2.1
34453	0.946132	2.1
34454	1.411	2.1
34455	1.06957	2.1
34456	0.946132	2.1
34457	1.99875	0.877475
34458	2.57079	0.675254
34459	2.59319	1.49048
34460	1.9105	0.495525
34461	4.59194	0.799392
34462	1.99875	0.803849
34463	1.02247	0.266526
34464	1.07017	0.373285
34465	1.15841	0.389345
34466	1.91051	0.359165
34467	1.99875	0.402714
34468	2.59319	1.3851
34469	1.9105	0.520979
34470	2.33812	0.757215
34471	2.59319	0.809964
34472	1.59457	0.787636
34473	1.59457	0.894491
34474	2.50906	0.436879
34475	2.0467	0.202308
34476	2.03357	0.228849
34477	2.53176	0.187332
34478	4.54263	0.221743
34479	1.294	0.398925
34480	1.66636	0.285564
34481	2.03357	0.214632
34482	1.03079	0.197602
34483	3.32757	0.264332
34484	1.68108	0.216859
34485	2.53176	0.202649
34486	2.36417	0.161673
34487	2.71666	0.161197
34488	1.68108	0.536146
34489	2.03357	0.32644
34490	1.89226	0.115266
34491	2.59302	0.138147
34492	2.2727	0.116448
34493	2.2727	0.111813
34494	1.89226	0.151589
34495	1.89226	0.12162
34496	1.89226	0.224048
34497	1.89226	0.188529
34498	1.89226	0.141749
34499	1.89226	0.0930905
34500	3.78452	0.202797
34501	1.89225	0.265873
34502	1.89225	0.0768202
34503	1.89225	0.106119
34504	1.89225	0.123797
34505	1.89225	0.106401
34506	1.89225	0.12598
34507	0.934114	0.128216

34508	0.934114	0.0847093
34509	0.944544	0.101696
34510	0.944544	0.109406
34511	0.721493	0.0824828
34512	0.58215	0.0787028
34513	1.2965	0.103125
34514	1.13634	0.0842149
34515	1.13635	0.0774641
34516	0.582148	0.0772474
34517	1.29651	0.102771
34518	0.721491	0.0924079
34519	1.89226	0.135264
34520	1.89226	0.0925217
34521	1.89226	0.077614
34522	1.89226	0.0750834
34523	3.78452	0.0736802
34524	0.946131	0.377079
34525	0.946131	0.421095
34526	0.946131	0.309844
34527	0.946131	0.400474
34528	0.946131	0.299545
34529	0.946131	0.337476
34530	0.946131	0.427961
34531	0.946131	0.459391
34532	0.946131	0.348662
34533	0.946131	0.362428
34534	0.946131	0.388525
34535	0.946131	0.362819
34536	1.89226	0.0698591
34537	1.89226	0.0698591
34538	2.78602	0.0698591
34539	1.89226	0.0698591
34540	3.78453	0.0698591
34541	1.52601	2.1
34542	1.76601	2.1
34543	1.27839	2.1
34544	1.56095	2.1
34545	3.0444	2.1
34546	1.76601	2.1
34547	1.86793	2.1
34548	1.56095	2.1
34549	1.68152	2.1
34550	3.44753	2.1
34551	1.27841	2.1
34552	1.56097	2.1
34553	1.0577	2.1
34554	1.36861	2.1
34555	2.64702	2.1
34556	1.56097	2.1
34557	1.68154	2.1
34558	1.36861	2.1
34559	1.53296	2.1
34560	3.05015	2.1
34561	1.89226	2.1
34562	1.89226	2.1
34563	1.89226	2.1
34564	1.89226	2.1
34565	3.78453	2.1

34566	0.983075	2.1
34567	0.788361	2.1
34568	0.724726	2.1
34569	0.724726	2.1
34570	0.983027	2.1
34571	0.724734	2.1
34572	0.788364	2.1
34573	0.788364	2.1
34574	0.788335	2.1
34575	0.94613	2.1
34576	0.724757	2.1
34577	0.946131	2.1
34578	1.15789	2.1
34579	2.11135	2.1
34580	2.25826	2.1
34581	1.28845	2.1
34582	3.41615	2.1
34583	1.89226	2.1
34584	1.89226	2.1
34585	1.89226	2.1
34586	1.89226	2.1
34587	3.78453	2.1
34588	1.57667	2.1
34589	1.70002	2.1
34590	1.70002	2.1
34591	1.89226	2.1
34592	1.89226	2.1
34593	2.05244	2.1
34594	2.25826	2.1
34595	1.42214	2.1
34596	1.43675	2.1
34597	1.28845	2.1
34598	2.40656	2.1
34599	2.25826	2.1
34600	1.58066	2.1
34601	1.74247	2.1
34602	1.85097	2.1
34603	1.61155	2.1
34604	3.43163	2.1
34605	0.785612	2.1
34606	0.789638	2.1
34607	0.882561	2.1
34608	0.763529	2.1
34609	0.686921	2.1
34610	0.86048	2.1
34611	0.871812	2.1
34612	0.871812	2.1
34613	0.830183	2.1
34614	0.882572	2.1
34615	0.902315	2.1
34616	0.785602	2.1
34617	1.01972	0.0698591
34618	0.936657	0.0698591
34619	0.946131	0.0698591
34620	0.946131	0.0698591
34621	0.927018	0.0698591
34622	0.927018	0.0698591
34623	1.1376	0.0698591

34624	0.953663	0.0698591
34625	1.1257	0.0698591
34626	1.04073	0.0698591
34627	1.00358	0.0698591
34628	0.851528	0.0698591
34629	1.73576	0.0776257
34630	2.00244	0.134661
34631	1.17798	0.0698591
34632	1.25796	0.0723681
34633	2.99373	0.0698591
34634	2.00244	0.0751156
34635	1.10719	0.0735646
34636	2.03236	0.0829232
34637	2.03236	0.15024
34638	1.25797	0.0698591
34639	1.25797	0.0909511
34640	1.89226	2.1
34641	1.89226	2.1
34642	1.89226	2.1
34643	1.89226	2.1
34644	3.78452	2.1
34645	2.3174	2.1
34646	1.50005	2.1
34647	1.90428	2.1
34648	2.64564	2.1
34649	4.22168	2.1
34650	1.89226	2.1
34651	1.89226	2.1
34652	1.89226	2.1
34653	1.89226	2.1
34654	1.89226	2.1
34655	1.89226	2.1
34656	1.32749	0.0699043
34657	1.70302	0.0743878
34658	2.08149	0.0722666
34659	1.70302	0.0708748
34660	2.19634	0.0722182
34661	1.93083	0.071713
34662	1.89226	1.82775
34663	1.89226	1.84198
34664	1.89226	0.486148
34665	1.89226	2.07219
34666	3.78453	1.02842
34667	1.89226	1.38
34668	1.89226	2.01358
34669	1.89226	0.66883
34670	1.89226	1.00614
34671	3.78452	1.70458
34672	2.17499	0.388426
34673	1.79663	1.98662
34674	2.1538	0.487901
34675	1.818	0.82388
34676	2.17499	1.81548
34677	1.83919	2.1
34678	0.898316	1.77066
34679	1.0769	0.79027
34680	0.968276	2.06309
34681	0.773495	1.88194

34682	0.921126	1.97466
34683	0.921126	1.99685
34684	0.92927	1.56688
34685	0.92927	1.93175
34686	1.0769	1.79091
34687	0.968276	1.58564
34688	0.898316	1.89717
34689	0.773495	1.67275
34690	1.89226	0.713993
34691	1.89226	2.09671
34692	1.89226	1.68624
34693	1.89226	0.985121
34694	3.78453	1.87945
34695	1.89226	1.57533
34696	1.89226	2.06618
34697	1.89226	0.37334
34698	1.89226	1.80672
34699	3.78452	0.861757
34700	2.17499	1.71031
34701	2.1538	2.1
34702	2.53574	0.933111
34703	2.54157	0.426712
34704	4.71073	1.8314
34705	1.0769	1.53151
34706	1.26787	0.915676
34707	1.16641	1.77827
34708	0.968276	2.01787
34709	1.11625	1.83549
34710	1.11625	1.8478
34711	1.11989	1.98839
34712	1.11989	0.569777
34713	1.26787	1.48077
34714	1.16641	1.9875
34715	1.0769	2.0259
34716	0.968277	1.86893
34717	1.89226	0.207741
34718	1.89226	0.242175
34719	1.89226	0.208237
34720	1.89226	0.198303
34721	3.78452	0.166608
34722	1.91739	0.172362
34723	2.20045	0.0698591
34724	1.79005	0.0773627
34725	2.33802	0.0983737
34726	4.25539	0.116852
34727	2.52754	0.116973
34728	1.89226	0.122701
34729	2.69841	0.105751
34730	2.69841	0.141174
34731	1.89226	0.0918688
34732	1.89226	0.120617
34733	1.89226	0.186452
34734	1.89226	0.212516
34735	1.89226	0.159613
34736	1.89226	0.191645
34737	3.78452	0.207795
34738	1.93544	0.100007
34739	2.33802	0.132566

34740	1.55063	0.125932
34741	2.1769	0.0698591
34742	3.88865	0.0874888
34743	2.20929	0.245916
34744	2.55162	0.174083
34745	2.27807	0.273326
34746	1.48831	0.177653
34747	4.48736	0.189306
34748	2.27807	0.281014
34749	2.72723	0.17834
34750	2.55162	0.20844
34751	2.55162	0.266357
34752	2.43157	0.184318
34753	2.43157	0.253288
34754	2.27807	0.213634
34755	1.51612	0.151784
34756	1.96048	0.161339
34757	1.73022	0.154317
34758	1.70302	0.21678
34759	1.61016	0.189024
34760	2.27807	0.217939
34761	2.08048	0.142848
34762	1.98522	0.159607
34763	2.43157	0.191304
34764	1.83798	0.162959
34765	2.27807	0.196768
34766	1.70302	0.272338
34767	2.68507	0.217929
34768	2.23856	0.275835
34769	2.52174	0.195566
34770	2.35331	0.317483
34771	2.65099	0.241521
34772	1.70303	0.256732
34773	0.986848	0.148559
34774	2.41566	0.171674
34775	1.70303	0.163249
34776	1.88139	0.165445
34777	1.64955	0.161166
34778	1.89226	2.00232
34779	2.34978	1.79993
34780	1.89226	1.98422
34781	2.68773	1.71197
34782	1.89226	2.00081
34783	2.68773	1.80323
34784	1.89226	1.99763
34785	1.89226	1.68825
34786	1.89226	1.74214
34787	1.89226	1.94736
34788	3.78453	1.92199
34789	1.00825	1.07847
34790	1.09847	1.15432
34791	0.939987	1.2088
34792	0.783516	1.75777
34793	0.838041	1.46759
34794	0.838041	1.7598
34795	1.49693	1.85956
34796	1.26602	1.40894
34797	1.10875	1.06017

34798	1.05187	1.93146
34799	1.28743	1.46152
34800	0.946132	1.4613
34801	0.946132	1.69476
34802	0.946132	1.99666
34803	0.946132	1.93463
34804	0.946132	1.29978
34805	1.31823	2.0015
34806	0.946132	1.68021
34807	0.946132	1.92889
34808	0.946131	1.95925
34809	0.946131	2.02989
34810	0.946132	1.86431
34811	1.31823	1.92544
34812	1.51688	0.248715
34813	1.79978	0.390477
34814	1.1685	0.261415
34815	1.1685	0.35503
34816	1.85857	0.25649
34817	1.51018	0.38026
34818	0.946116	0.334415
34819	0.946116	0.359235
34820	0.94612	0.208966
34821	0.94612	0.229913
34822	0.946118	0.218519
34823	0.946118	0.330506
34824	0.946118	0.248847
34825	0.946118	0.358851
34826	0.946131	0.267748
34827	0.946131	0.278051
34828	0.946105	0.297609
34829	0.946105	0.299553
34830	1.89224	2.1
34831	1.89224	2.1
34832	1.89224	2.1
34833	1.89224	2.1
34834	1.89224	2.1
34835	1.89224	2.1
34836	1.38678	2.1
34837	1.3349	2.1
34838	0.946119	2.1
34839	0.946119	2.1
34840	1.38678	2.1
34841	1.38678	2.1
34842	0.946119	2.1
34843	0.946119	2.1
34844	1.38676	2.1
34845	0.946107	2.1
34846	0.946132	2.1
34847	1.33491	2.1
34848	1.06455	2.1
34849	1.03932	2.1
34850	1.20649	2.1
34851	1.18773	2.1
34852	2.27105	2.1
34853	1.89226	2.1
34854	1.89226	2.1
34855	1.89226	2.1

34856	1.89226	2.1
34857	1.89226	2.1
34858	1.89226	2.1
34859	1.36918	2.1
34860	1.18774	2.1
34861	1.31144	2.1
34862	1.20651	2.1
34863	1.31144	2.1
34864	1.20651	2.1
34865	2.29066	2.1
34866	1.76476	2.1
34867	1.79825	2.1
34868	2.33801	2.1
34869	4.08891	2.1
34870	1.79825	2.1
34871	2.33801	2.1
34872	2.37168	2.1
34873	1.49325	2.1
34874	4.16993	2.1
34875	1.13641	2.1
34876	1.43213	2.03913
34877	1.44085	2.1
34878	1.18301	2.1
34879	2.61513	2.1
34880	1.89226	2.1
34881	2.23875	2.1
34882	2.23875	2.1
34883	2.10287	2.1
34884	1.89226	2.1
34885	1.75639	2.1
34886	1.89226	2.0525
34887	1.89226	2.1
34888	1.89226	2.07278
34889	1.89226	2.1
34890	1.89226	2.05193
34891	1.89226	2.1
34892	1.44085	2.1
34893	1.25173	2.1
34894	1.25173	2.09557
34895	1.18301	2.1
34896	1.4617	2.08724
34897	1.39298	2.1
34898	1.89227	2.1
34899	2.23875	2.1
34900	2.42504	2.1
34901	1.89227	2.1
34902	4.13101	2.1
34903	1.89226	2.06887
34904	1.89226	2.1
34905	1.89226	2.04595
34906	1.89226	2.1
34907	1.89226	2.03805
34908	1.89226	2.1
34909	1.89226	2.1
34910	2.41297	2.1
34911	2.70685	2.1
34912	2.70685	2.1
34913	1.89226	2.1

34914	1.89226	2.1
34915	1.89226	2.1
34916	1.89226	2.1
34917	1.89226	2.1
34918	1.89226	2.1
34919	1.89226	2.1
34920	1.89226	2.1
34921	1.89226	2.1
34922	1.89226	2.1
34923	1.89226	2.1
34924	1.89226	2.1
34925	3.78453	2.1
34926	1.89226	2.1
34927	1.89226	2.1
34928	1.89226	2.1
34929	1.89226	2.1
34930	1.89226	2.1
34931	1.89226	2.1
34932	1.69044	0.0698591
34933	1.57979	0.0698591
34934	2.27963	0.0698591
34935	2.21582	0.0698591
34936	3.85942	0.0698591
34937	2.21582	0.0698591
34938	1.30722	0.0698591
34939	2.02069	0.0698591
34940	2.02069	0.0698591
34941	1.57979	0.0698591
34942	1.57979	0.0698591
34943	1.67825	2.1
34944	1.89226	2.1
34945	1.89226	2.1
34946	1.89226	2.1
34947	1.91894	2.1
34948	1.91894	2.1
34949	0.946133	2.1
34950	0.946133	2.1
34951	0.946132	2.1
34952	0.946132	2.1
34953	0.946132	2.1
34954	0.946132	2.1
34955	0.946133	2.1
34956	0.946133	2.1
34957	0.946133	2.1
34958	0.946133	2.1
34959	0.946132	2.1
34960	0.946132	2.1
34961	0.942609	2.1
34962	0.942609	2.1
34963	1.329	2.1
34964	1.329	2.1
34965	1.32547	2.1
34966	0.94261	2.1
34967	0.946132	2.1
34968	0.946132	2.1
34969	0.946132	2.1
34970	1.32547	2.1
34971	0.946132	2.1

34972	0.94261	2.1
34973	1.55831	2.1
34974	1.07496	2.09489
34975	1.80327	0.280064
34976	1.34851	0.344891
34977	2.87824	0.679731
34978	2.14346	0.484303
34979	2.77937	0.667563
34980	2.79173	0.94172
34981	2.17129	0.409054
34982	4.95066	0.527219
34983	2.14346	0.582832
34984	1.34124	0.193401
34985	1.39818	0.300549
34986	2.1713	0.427292
34987	3.51253	0.348769
34988	2.79173	0.847357
34989	2.21882	0.414502
34990	2.17129	0.466569
34991	2.17129	0.628309
34992	2.81448	0.509995
34993	2.81448	0.643571
34994	1.10941	0.404365
34995	1.08565	0.491241
34996	0.699089	0.205326
34997	0.740176	0.288404
34998	0.908594	0.215212
34999	0.908594	0.378729
35000	0.91723	0.624467
35001	0.91723	0.230615
35002	1.10941	0.331812
35003	0.740176	0.32014
35004	0.699089	0.312397
35005	1.08565	0.324744
35006	1.89227	0.077634
35007	1.89227	0.0870373
35008	1.89227	0.0747859
35009	1.89227	0.0698591
35010	3.78453	0.0774605
35011	2.40032	0.160448
35012	2.19526	0.0770335
35013	1.12907	0.0719232
35014	1.44253	0.131768
35015	3.32433	0.0823089
35016	2.19526	0.0727063
35017	2.71093	0.109071
35018	1.29826	0.0928453
35019	2.0815	0.0698591
35020	4.18911	0.0896861
35021	1.89226	0.0789001
35022	1.89226	0.071541
35023	1.89226	0.0698591
35024	1.89226	0.0698591
35025	3.78452	0.0698591
35026	2.30822	0.0732174
35027	1.89226	0.0698591
35028	2.79329	0.0698591
35029	2.79329	0.0698591

35030	1.89226	0.0830817
35031	1.89226	0.0753113
35032	0.946131	2.1
35033	0.946131	2.00598
35034	1.38414	2.1
35035	1.38414	1.97755
35036	0.94613	2.1
35037	0.94613	2.1
35038	1.38415	2.02143
35039	1.41833	1.3698
35040	0.946132	2.1
35041	1.41833	2.1
35042	1.38414	2.1
35043	0.946131	2.1
35044	1.89226	2.1
35045	2.6536	2.1
35046	1.89226	2.1
35047	2.72238	2.1
35048	4.61465	2.1
35049	0.946132	2.1
35050	0.946132	2.1
35051	1.38657	2.1
35052	1.38657	2.1
35053	1.38657	2.1
35054	1.36119	2.1
35055	0.946131	2.1
35056	0.946131	2.1
35057	0.946132	2.1
35058	1.38657	2.1
35059	0.946132	2.1
35060	1.36119	2.1
35061	1.89226	2.1
35062	2.4717	2.1
35063	2.5026	2.1
35064	2.4717	2.1
35065	1.92316	2.1
35066	1.89226	2.1
35067	1.89226	2.1
35068	1.89226	2.1
35069	1.89226	2.1
35070	1.89226	2.1
35071	3.78453	2.1
35072	0.946128	2.1
35073	0.946128	2.1
35074	1.3419	2.1
35075	1.3419	2.1
35076	0.946131	2.1
35077	0.946131	2.1
35078	1.36607	2.1
35079	1.3419	2.1
35080	0.946131	2.1
35081	1.36607	2.1
35082	1.3419	2.1
35083	0.94613	2.1
35084	1.89226	2.1
35085	2.50259	2.1
35086	2.53563	2.1
35087	2.50259	2.1

35088	1.92529	2.1
35089	1.89226	2.1
35090	0.946132	2.1
35091	0.946132	2.1
35092	0.946131	2.1
35093	0.946131	2.1
35094	0.946132	2.1
35095	0.946132	2.1
35096	0.946131	2.1
35097	0.946131	2.1
35098	0.946131	2.1
35099	0.946131	2.1
35100	0.946131	2.1
35101	0.946131	2.1
35102	2.0815	2.1
35103	1.86506	2.1
35104	1.86506	2.1
35105	1.82567	2.1
35106	2.0815	2.1
35107	2.03335	2.1
35108	2.0815	2.1
35109	2.0815	2.1
35110	2.0815	2.1
35111	1.88668	2.1
35112	1.83221	2.1
35113	1.57275	2.1
35114	2.34061	0.0698591
35115	1.89226	0.0698591
35116	2.23254	0.0698591
35117	1.89226	0.0698591
35118	2.23254	0.0698591
35119	1.89226	0.0698591
35120	1.8619	0.0698591
35121	1.89226	0.0698591
35122	2.34061	0.0698591
35123	1.89226	0.0698591
35124	2.34061	0.0698591
35125	1.89226	0.0698591
35126	1.89227	0.0698591
35127	1.89227	0.0698591
35128	1.89227	0.0698591
35129	1.89227	0.0698591
35130	1.89227	0.0698591
35131	1.89227	0.0698591
35132	1.89227	0.0698591
35133	1.89227	0.0698591
35134	1.89227	0.0698591
35135	1.89227	0.0698591
35136	1.89227	0.0698591
35137	1.89227	0.0698591
35138	1.06365	0.0698591
35139	0.503305	0.0698591
35140	0.794912	0.0698591
35141	1.42752	0.0698591
35142	1.34826	0.0698591
35143	1.07163	0.0698591
35144	0.533113	0.0698591
35145	0.761277	0.0698591

35146	0.848403	0.0698591
35147	0.876191	0.0698591
35148	0.963372	0.0698591
35149	0.963372	0.0698591
35150	1.55571	0.0698591
35151	2.76574	0.0698591
35152	2.92453	0.0698591
35153	1.70301	0.0698591
35154	2.33089	0.0698591
35155	1.06905	0.0698591
35156	2.28979	2.1
35157	1.81488	2.1
35158	1.63722	2.1
35159	2.22627	2.1
35160	4.04115	2.1
35161	1.98246	2.1
35162	2.15715	2.1
35163	2.22627	2.1
35164	2.22627	2.1
35165	1.63719	2.1
35166	1.63719	2.1
35167	2.1644	0.0698591
35168	1.903	0.0698591
35169	1.23639	0.0698591
35170	1.42704	0.0698591
35171	3.61546	0.0698591
35172	1.89226	0.0698591
35173	1.89226	0.0698591
35174	1.89226	0.0698591
35175	1.89226	0.0698591
35176	3.78451	0.0698591
35177	2.61343	0.0698591
35178	1.89226	0.0698591
35179	2.08149	0.0698591
35180	1.74357	0.0698591
35181	2.08149	0.0698591
35182	1.74357	0.0698591
35183	1.63483	0.0698591
35184	1.8631	0.0698591
35185	1.8631	0.0698591
35186	1.70301	0.0698591
35187	2.0815	0.0698591
35188	1.94319	0.0698591
35189	2.0815	0.0698591
35190	1.89226	0.0698591
35191	1.89226	0.0698591
35192	2.05236	0.0698591
35193	2.23014	0.0698591
35194	2.41882	0.0698591
35195	2.14993	2.1
35196	1.87217	2.1
35197	2.34488	2.1
35198	2.5254	2.09647
35199	4.39757	2.09865
35200	0.949995	2.1
35201	0.630972	2.1
35202	0.936084	2.1
35203	1.17244	2.1

35204	0.922394	2.1
35205	0.922394	2.1
35206	0.88102	2.1
35207	0.88102	2.1
35208	0.949998	2.1
35209	1.17244	2.1
35210	0.936081	2.1
35211	0.630973	2.1
35212	1.89226	2.1
35213	1.89226	2.1
35214	1.89226	2.1
35215	1.89226	2.1
35216	3.78452	2.1
35217	1.89226	2.09139
35218	1.89226	2.1
35219	1.89226	2.09262
35220	1.89226	2.1
35221	1.89226	2.08616
35222	1.89226	2.1
35223	2.34488	2.08956
35224	2.5254	2.06886
35225	1.89226	2.05312
35226	1.89226	2.09325
35227	4.41766	2.09948
35228	1.89226	2.09685
35229	1.89226	2.1
35230	1.89226	2.09353
35231	1.89226	2.09764
35232	3.78452	2.08132
35233	1.89226	2.05369
35234	1.89226	2.09105
35235	1.89226	2.05584
35236	1.89226	2.07442
35237	1.89226	2.07241
35238	1.89226	2.08353
35239	1.5605	2.1
35240	1.15806	2.1
35241	1.31565	2.1
35242	1.65642	2.1
35243	2.87615	2.1
35244	1.46643	2.1
35245	1.65642	2.1
35246	1.31565	2.1
35247	1.31565	2.1
35248	1.74246	2.1
35249	1.74246	2.1
35250	1.89226	2.1
35251	1.89226	2.1
35252	1.89226	2.1
35253	1.89226	2.1
35254	1.89226	2.1
35255	1.89226	2.1
35256	1.15099	2.1
35257	1.15099	2.1
35258	0.939316	2.1
35259	0.939316	2.1
35260	0.946127	2.1
35261	0.946127	2.1

35262	1.14418	2.1
35263	0.939289	2.1
35264	1.14417	2.1
35265	0.946129	2.1
35266	0.939287	2.1
35267	0.94613	2.1
35268	1.89226	2.1
35269	1.89226	2.1
35270	1.89226	2.1
35271	1.89226	2.1
35272	3.78452	2.1
35273	1.93164	0.0700597
35274	2.64898	0.0698591
35275	1.43213	0.0698591
35276	1.43213	0.070944
35277	2.63206	0.0698591
35278	2.13254	0.0702881
35279	1.89226	2.1
35280	1.89226	2.1
35281	1.89226	2.1
35282	1.89226	2.1
35283	3.78452	2.1
35284	2.71655	2.1
35285	1.43323	2.1
35286	1.28413	2.1
35287	2.53614	2.1
35288	3.96937	2.1
35289	1.89226	2.1
35290	1.89226	2.1
35291	1.89226	2.1
35292	1.89226	2.1
35293	1.89226	2.1
35294	1.89226	2.1
35295	1.33356	2.1
35296	2.82297	2.1
35297	2.771	2.1
35298	2.771	2.1
35299	1.46037	2.1
35300	1.46037	2.1
35301	2.56034	2.1
35302	1.18474	2.1
35303	1.15113	2.1
35304	2.53724	2.1
35305	3.72198	2.1
35306	2.56034	2.1
35307	1.89226	2.1
35308	1.89226	2.1
35309	2.53725	2.1
35310	4.42951	2.1
35311	1.89226	2.1
35312	1.89226	2.1
35313	1.89226	2.1
35314	1.89226	2.1
35315	3.78453	2.1
35316	1.89226	2.1
35317	1.89226	2.1
35318	1.89226	2.1
35319	1.89226	2.1

35320	3.78452	2.1
35321	1.1229	2.1
35322	2.53724	2.1
35323	1.15113	2.1
35324	1.15113	2.1
35325	2.51559	2.1
35326	2.51559	2.1
35327	1.89226	2.1
35328	2.53724	2.1
35329	2.51559	2.1
35330	2.53724	2.1
35331	1.87061	2.1
35332	1.89226	2.1
35333	1.89226	2.1
35334	1.89226	2.1
35335	1.89226	2.1
35336	1.89226	2.1
35337	3.78453	2.1
35338	1.89226	2.1
35339	1.89226	2.1
35340	1.89226	2.1
35341	1.89226	2.1
35342	3.78452	2.1
35343	1.98143	1.57205
35344	2.81559	0.445579
35345	1.65411	0.660162
35346	2.26675	1.32147
35347	4.24818	0.901912
35348	0.810656	2.1
35349	0.810656	2.1
35350	0.821888	2.1
35351	0.821888	2.1
35352	1.1235	2.1
35353	1.07439	2.1
35354	0.535693	2.1
35355	0.509042	2.1
35356	0.535693	2.1
35357	1.1235	2.1
35358	0.509043	2.1
35359	1.07439	2.1
35360	1.10378	2.1
35361	2.247 2.1	
35362	2.27472	2.1
35363	2.27472	2.1
35364	1.07139	2.1
35365	1.07139	2.1
35366	2.08081	2.1
35367	1.40395	2.1
35368	1.34245	2.1
35369	2.00239	2.1
35370	3.40634	2.1
35371	1.89226	2.1
35372	1.89226	2.1
35373	1.89226	2.1
35374	1.89226	2.1
35375	3.78453	2.1
35376	0.946128	2.1
35377	0.946128	2.1

35378	1.13785	2.1
35379	1.11496	2.1
35380	0.946131	2.1
35381	0.946131	2.1
35382	1.11495	2.1
35383	1.11495	2.1
35384	0.94613	2.1
35385	1.11495	2.1
35386	1.13785	2.1
35387	0.946131	2.1
35388	0.975888	2.1
35389	1.00118	2.1
35390	0.671218	2.1
35391	0.653229	2.1
35392	0.825372	2.1
35393	0.825372	2.1
35394	0.829038	2.1
35395	0.829038	2.1
35396	0.975901	2.1
35397	0.653238	2.1
35398	0.671209	2.1
35399	1.00117	2.1
35400	1.89224	2.1
35401	1.89224	2.1
35402	1.89224	2.1
35403	1.89224	2.1
35404	3.78448	2.1
35405	0.946115	2.1
35406	0.946115	2.1
35407	1.11494	2.1
35408	1.10111	2.1
35409	0.946118	2.1
35410	0.946118	2.1
35411	1.10111	2.1
35412	1.10111	2.1
35413	1.10112	2.1
35414	0.94613	2.1
35415	0.946105	2.1
35416	1.11492	2.1
35417	1.04028	0.639585
35418	2.27126	0.596226
35419	2.0815	0.343199
35420	1.82249	1.72698
35421	1.33723	0.437965
35422	1.18684	1.55049
35423	0.812616	0.114823
35424	0.812616	0.113196
35425	0.997777	0.107537
35426	1.08122	0.110329
35427	0.94613	0.108724
35428	0.94613	0.116102
35429	0.812648	0.114591
35430	0.932779	0.10894
35431	0.812648	0.0978208
35432	0.94613	0.0935564
35433	0.849335	0.082393
35434	0.94613	0.0943765
35435	1.80327	0.690077

35436	1.56016	0.482554
35437	1.57731	1.96006
35438	1.34851	1.71724
35439	2.03207	1.38255
35440	1.80327	1.96968
35441	2.03207	1.80027
35442	1.56016	1.59475
35443	2.18076	1.80262
35444	1.78167	0.558872
35445	3.81374	1.55481
35446	1.37597	2.09387
35447	1.60058	2.1
35448	1.70302	0.67695
35449	1.85448	1.13768
35450	3.15028	1.14625
35451	1.60058	1.17358
35452	1.4054	2.07925
35453	1.66202	0.538456
35454	1.70302	2.0794
35455	3.09979	1.7595
35456	1.70302	1.92198
35457	1.85448	1.69319
35458	2.21317	1.76689
35459	2.0961	0.763761
35460	4.01757	1.41532
35461	1.66202	1.01814
35462	1.70302	2.09737
35463	2.09611	0.434457
35464	2.00892	2.05313
35465	3.6468	1.1957
35466	1.2966	0.0698591
35467	2.25495	0.0698591
35468	1.2966	0.0698591
35469	2.21117	0.0698591
35470	1.67687	0.0698591
35471	2.6167	0.0698591
35472	1.89227	0.0871448
35473	1.89227	0.0698602
35474	1.89227	0.0713319
35475	1.89227	0.106541
35476	3.78453	0.0746631
35477	2.44375	0.0698591
35478	1.89227	0.076313
35479	2.81853	0.0703423
35480	2.81853	0.0714841
35481	1.89227	0.0698591
35482	1.89227	0.0698591
35483	1.89226	0.935768
35484	1.75853	0.803443
35485	2.37636	0.764497
35486	2.37636	1.10621
35487	1.89226	0.622484
35488	1.89226	0.967183
35489	1.65814	1.89843
35490	1.10187	2.1
35491	1.10187	2.1
35492	1.19547	2.1
35493	1.56263	2.02506

35494	1.65624	2.1
35495	1.56264	1.76066
35496	1.01479	2.1
35497	1.01479	2.1
35498	1.10187	2.1
35499	1.47561	1.92578
35500	1.5627	2.1
35501	1.89225	2.1
35502	1.89225	2.1
35503	1.89225	2.1
35504	1.89225	2.1
35505	3.7845	2.1
35506	2.54386	2.1
35507	1.91546	2.1
35508	2.46008	2.1
35509	1.82815	2.1
35510	4.37554	2.1
35511	1.91546	2.1
35512	1.04909	2.1
35513	1.82815	2.1
35514	0.983141	2.1
35515	2.8986	2.1
35516	1.89225	2.1
35517	1.89225	2.1
35518	1.89225	2.1
35519	1.89225	2.1
35520	3.78449	2.1
35521	1.89226	2.1
35522	1.89226	2.1
35523	1.89226	2.1
35524	1.89226	2.1
35525	1.89226	2.1
35526	1.89226	2.1
35527	2.46009	2.1
35528	1.7513	2.1
35529	1.82817	2.1
35530	1.82817	2.1
35531	2.38127	2.1
35532	2.38127	2.1
35533	2.55006	2.1
35534	2.64569	2.1
35535	2.60128	2.1
35536	2.49168	2.1
35537	5.13737	2.1
35538	2.51605	2.1
35539	2.64569	2.1
35540	2.60128	2.1
35541	2.48891	2.1
35542	5.1346	2.1
35543	2.60128	2.1
35544	2.49168	2.1
35545	2.41689	2.1
35546	2.55248	2.1
35547	5.04416	2.1
35548	2.60128	2.1
35549	2.48891	2.1
35550	2.45922	2.1
35551	2.55249	2.1

35552	5.04139	2.1
35553	1.89227	2.1
35554	1.89227	2.1
35555	1.89227	2.1
35556	1.89227	2.1
35557	1.89227	2.1
35558	1.89227	2.1
35559	0.96063	2.1
35560	0.96063	2.1
35561	0.958806	2.1
35562	0.958806	2.1
35563	1.28892	2.1
35564	0.749042	2.1
35565	1.17409	2.1
35566	0.630516	2.1
35567	1.17409	2.1
35568	1.28892	2.1
35569	0.630515	2.1
35570	0.749042	2.1
35571	0.92511	2.1
35572	0.92511	2.1
35573	1.19511	2.1
35574	1.19511	2.06147
35575	0.946132	2.1
35576	1.17409	2.1
35577	0.925081	2.1
35578	0.946129	2.07939
35579	0.946131	2.1
35580	0.946131	2.1
35581	1.17409	2.1
35582	0.925083	2.1
35583	1.89226	1.75931
35584	1.89226	2.03046
35585	1.89226	1.49653
35586	1.89226	1.94876
35587	1.89226	1.59211
35588	1.89226	1.9693
35589	1.89226	1.97132
35590	1.89226	2.08028
35591	1.89226	2.0212
35592	1.89226	2.09362
35593	3.78453	2.04725
35594	0.946132	2.1
35595	0.946132	2.1
35596	1.28892	2.1
35597	1.28892	2.1
35598	0.946132	2.1
35599	1.28892	2.1
35600	0.946133	2.1
35601	1.17409	2.1
35602	0.946132	2.1
35603	0.946132	2.1
35604	1.17409	2.1
35605	1.28892	2.1
35606	0.625229	2.1
35607	0.890321	2.1
35608	1.21636	2.1
35609	1.13022	2.1

35610	1.07094	2.1
35611	1.03541	2.1
35612	0.942207	2.1
35613	0.942207	2.1
35614	0.898766	2.1
35615	1.26626	2.1
35616	1.0853	2.1
35617	0.600358	2.1
35618	0.719012	2.1
35619	0.719012	2.1
35620	1.17957	2.1
35621	1.17957	2.1
35622	0.719012	2.1
35623	0.952456	2.1
35624	0.946129	2.1
35625	0.946129	2.1
35626	0.946131	2.1
35627	0.952454	2.1
35628	0.946131	2.1
35629	0.719011	2.1
35630	0.94613	2.1
35631	0.94613	2.1
35632	0.94613	2.1
35633	0.94613	2.1
35634	0.946131	2.1
35635	0.946131	2.1
35636	0.946129	2.1
35637	0.946129	2.1
35638	0.94613	2.1
35639	0.94613	2.1
35640	0.94613	2.1
35641	0.94613	2.1
35642	1.1587	2.1
35643	0.973158	2.1
35644	0.750023	2.1
35645	0.536443	2.1
35646	0.84054	2.1
35647	0.84054	2.1
35648	0.854602	2.1
35649	0.854602	2.1
35650	1.1587	2.1
35651	0.750023	2.1
35652	0.973158	2.1
35653	0.536443	2.1
35654	1.89227	1.6724
35655	1.89227	2.1
35656	1.89227	2.1
35657	1.89227	1.97414
35658	3.78453	2.09909
35659	1.89226	2.1
35660	1.89226	2.1
35661	1.89226	2.1
35662	1.89226	2.1
35663	3.78453	2.1
35664	1.7632	2.1
35665	1.61149	2.1
35666	1.98749	2.1
35667	2.11615	2.1

35668	3.75068	2.1
35669	1.7632	2.1
35670	1.61149	2.1
35671	1.97733	2.01949
35672	2.11615	2.1
35673	3.74052	2.1
35674	1.89226	2.1
35675	1.89226	1.89147
35676	1.89226	1.56477
35677	1.89226	2.1
35678	3.78453	2.04808
35679	1.89226	2.1
35680	1.89226	2.1
35681	1.89226	2.1
35682	1.89226	2.1
35683	3.78453	2.1
35684	1.98749	2.1
35685	2.11615	2.1
35686	2.43151	2.1
35687	2.27541	2.1
35688	4.41899	2.1
35689	1.97733	1.92361
35690	2.11615	2.1
35691	2.43151	2.1
35692	2.33986	2.01275
35693	4.40883	2.1
35694	2.17892	0.231611
35695	2.80057	1.86687
35696	1.88349	0.666773
35697	2.63062	0.63088
35698	4.80954	0.669879
35699	1.89226	2.09275
35700	1.89226	0.67087
35701	1.89226	2.06956
35702	1.89226	0.384728
35703	3.78452	1.39337
35704	2.0155	2.09875
35705	1.97652	0.670647
35706	1.8247	2.05461
35707	1.80645	0.403868
35708	3.80122	1.18152
35709	1.97652	0.447209
35710	1.79681	1.013
35711	1.80645	0.399243
35712	1.59244	0.782516
35713	3.56896	0.267669
35714	1.89226	0.512646
35715	1.89226	1.3899
35716	1.89226	0.337262
35717	1.89226	0.79664
35718	3.78452	0.174981
35719	1.89223	2.06989
35720	1.89223	0.605422
35721	1.89223	2.07049
35722	1.89223	0.399347
35723	3.78447	1.15486
35724	1.82467	2.05742
35725	1.80643	0.465867

35726	1.55159	2.03462
35727	1.53969	0.334529
35728	3.36437	1.17647
35729	1.80643	0.424795
35730	1.59242	0.948929
35731	1.5397	0.376989
35732	1.40538	0.971442
35733	3.13211	0.302618
35734	1.89224	0.450353
35735	1.89224	0.983953
35736	1.89224	0.419869
35737	1.89224	0.903981
35738	3.78447	0.333608
35739	1.37427	0.0698591
35740	2.37086	0.0698591
35741	2.37972	0.0698591
35742	1.94703	0.0698591
35743	2.19299	0.0698591
35744	1.91813	0.0698591
35745	1.07566	2.1
35746	1.12954	2.1
35747	0.946131	2.1
35748	0.946131	2.1
35749	1.12954	2.1
35750	1.12954	2.1
35751	0.946131	2.1
35752	0.946131	2.1
35753	1.12954	2.1
35754	0.946131	2.1
35755	0.946131	2.1
35756	1.07566	2.1
35757	2.25908	2.1
35758	1.89226	2.1
35759	1.89226	2.1
35760	1.89226	2.1
35761	2.37567	2.1
35762	2.37567	2.1
35763	1.37597	2.02768
35764	1.70302	0.936955
35765	1.24162	2.1
35766	1.12219	2.07087
35767	1.37072	2.08823
35768	1.70302	2.1
35769	1.37072	2.1
35770	1.70302	1.65915
35771	1.54168	2.1
35772	2.21318	1.67563
35773	3.39238	2.07505
35774	1.89226	0.0698591
35775	1.89226	0.0698591
35776	1.89226	0.0698591
35777	1.89226	0.0698591
35778	3.78452	0.0698591
35779	1.89227	0.0698591
35780	1.89227	0.0698591
35781	1.89227	0.0698591
35782	1.89227	0.0698591
35783	3.78453	0.0698591

35784	1.89226	0.0698591
35785	1.89226	0.0698591
35786	1.89226	0.0698591
35787	1.89226	0.0698591
35788	3.78453	0.0698591
35789	1.89226	0.0698591
35790	2.21654	0.0698591
35791	2.72007	0.0698591
35792	2.72007	0.0698591
35793	1.89226	0.0698591
35794	1.89226	0.0698591
35795	0.946128	0.0698591
35796	0.946128	0.0698591
35797	0.946132	0.0698591
35798	0.946132	0.0698591
35799	0.94613	0.0698591
35800	0.94613	0.0698591
35801	0.946131	0.0698591
35802	0.946131	0.0698591
35803	0.94613	0.0698591
35804	0.94613	0.0698591
35805	0.94613	0.0698591
35806	0.94613	0.0698591
35807	1.89226	0.0698591
35808	1.89226	0.0698591
35809	1.89226	0.0698591
35810	1.89226	0.0698591
35811	3.78453	0.0698591
35812	1.33457	0.0698591
35813	0.946131	0.0698591
35814	1.08045	0.0698591
35815	0.946132	0.0698591
35816	1.08045	0.0698591
35817	1.08045	0.0698591
35818	0.946132	0.0698591
35819	0.946132	0.0698591
35820	1.33457	0.0698591
35821	1.08045	0.0698591
35822	0.946132	0.0698591
35823	0.946132	0.0698591
35824	1.53347	1.19889
35825	1.13092	2.09543
35826	1.52938	1.93775
35827	1.93568	1.55705
35828	3.06285	1.94828
35829	1.89226	0.785928
35830	2.72959	2.1
35831	2.82996	2.09986
35832	2.82996	1.07229
35833	1.89226	2.1
35834	1.89226	0.826016
35835	2.7296	2.1
35836	2.57663	2.1
35837	2.48354	2.1
35838	2.48354	2.1
35839	2.82996	2.1
35840	2.82996	2.1
35841	2.63449	2.1

35842	2.7296	2.1
35843	2.39282	2.1
35844	2.39282	2.1
35845	2.48354	2.1
35846	2.72521	2.1
35847	1.89224	2.1
35848	1.89224	1.93997
35849	1.89224	1.85519
35850	1.89224	2.1
35851	3.78447	2.0345
35852	1.89226	2.1
35853	2.10425	2.1
35854	2.5142	2.1
35855	2.5142	2.1
35856	1.89226	2.1
35857	1.89226	2.1
35858	0.946133	1.42617
35859	0.946133	1.91028
35860	0.946128	2.1
35861	0.946128	2.1
35862	0.946131	2.1
35863	0.946131	1.48217
35864	0.94613	1.49054
35865	0.94613	2.1
35866	0.946131	2.07675
35867	0.946131	2.0895
35868	0.946131	2.09199
35869	0.946131	2.0924
35870	1.89226	0.748209
35871	1.89226	1.13575
35872	1.89226	0.709188
35873	1.89226	1.21587
35874	1.89226	0.700446
35875	1.89226	1.19878
35876	1.94446	0.858066
35877	2.50167	1.37193
35878	1.28418	0.776998
35879	1.94446	1.21673
35880	2.05205	1.07068
35881	2.05205	1.19347
35882	1.27835	2.1
35883	1.08341	2.1
35884	0.946132	2.1
35885	0.946132	2.1
35886	0.851542	2.1
35887	0.851542	2.1
35888	0.977356	2.1
35889	1.18368	2.1
35890	1.18378	2.1
35891	0.851555	2.1
35892	0.977346	2.1
35893	0.851476	2.1
35894	1.07369	2.1
35895	2.5813	2.1
35896	1.95053	2.1
35897	1.95053	2.1
35898	1.90196	2.1
35899	1.90196	2.1

35900	1.89226	2.1
35901	1.89226	2.1
35902	1.89226	2.1
35903	1.89226	2.1
35904	3.78452	2.1
35905	1.89224	2.1
35906	1.89224	2.1
35907	1.89224	2.1
35908	1.89224	2.1
35909	1.89224	2.1
35910	1.89224	2.1
35911	1.97004	0.240028
35912	1.8072	1.73755
35913	1.5866	1.47099
35914	1.79478	0.588271
35915	3.60198	1.02762
35916	1.23267	2.1
35917	1.8072	1.73965
35918	1.58661	1.2618
35919	0.947921	2.1
35920	2.75512	2.04757
35921	1.89226	1.78578
35922	1.89226	2.1
35923	1.89226	2.02539
35924	1.89226	2.1
35925	1.89226	1.93854
35926	1.89226	2.1
35927	1.89226	1.78282
35928	1.89226	0.812341
35929	1.89226	0.65876
35930	1.89226	1.99755
35931	3.78452	1.28498
35932	1.4263	1.7792
35933	1.79478	0.753071
35934	1.67651	0.72872
35935	1.79478	1.87663
35936	1.46834	0.763791
35937	1.5866	1.71168
35938	1.89226	0.847726
35939	1.89226	2.01978
35940	1.89226	1.2209
35941	1.89226	1.77035
35942	1.89226	0.929071
35943	1.89226	1.82911
35944	2.23956	1.52384
35945	1.81847	1.60103
35946	2.10991	0.672743
35947	2.75317	0.464234
35948	4.34947	1.19987
35949	1.60578	0.102142
35950	1.94974	0.103504
35951	1.68062	0.140924
35952	1.14721	0.0917191
35953	3.2864	0.10035
35954	1.3946	0.0698591
35955	1.692	0.0698591
35956	2.0154	0.0698591
35957	2.0154	0.0698591

35958	1.11926	0.0698591
35959	1.11926	0.0698591
35960	1.90347	0.0698591
35961	1.83626	0.0698591
35962	1.84583	0.0698591
35963	1.89434	0.0698591
35964	3.79781	0.0698591
35965	1.89226	1.81816
35966	1.89226	1.32091
35967	1.89226	0.872778
35968	1.89226	1.53938
35969	3.78452	1.20291
35970	1.20986	1.60908
35971	1.20986	1.07183
35972	0.912635	1.2372
35973	0.912635	1.41679
35974	0.912667	0.753536
35975	1.17637	1.54562
35976	0.946125	1.7285
35977	0.946125	1.83851
35978	0.946128	1.61019
35979	0.912664	1.28264
35980	0.946127	1.82504
35981	1.17637	1.34457
35982	1.89226	0.0698591
35983	1.65573	0.0698591
35984	1.65573	0.0698591
35985	1.89226	0.0698591
35986	1.89226	0.0698591
35987	2.12879	0.0698591
35988	1.89226	0.0698591
35989	1.89226	0.0698591
35990	1.89226	0.0700534
35991	1.89226	0.0698591
35992	1.89226	0.0698868
35993	1.89226	0.0698591
35994	2.40928	0.0698591
35995	1.89226	0.0698591
35996	1.95959	0.0698591
35997	1.70302	0.0698591
35998	3.47338	0.0698591
35999	1.89226	0.0698591
36000	1.89226	0.0698591
36001	1.89226	0.0698591
36002	1.89226	0.0698591
36003	3.78452	0.0698591
36004	1.64985	0.0717132
36005	2.47846	0.101118
36006	2.25405	0.0764192
36007	1.80768	0.0782512
36008	4.06173	0.0736072
36009	1.98846	0.0698591
36010	0.912729	0.0780472
36011	1.05417	0.0698591
36012	1.70303	0.0762006
36013	2.82107	0.0761254
36014	2.47846	0.103221
36015	1.70303	0.114637

36016	2.86918	0.133331
36017	2.05973	0.125152
36018	2.51808	0.0957858
36019	1.80768	0.0915636
36020	0.946131	0.0960705
36021	0.946131	0.0852433
36022	0.946132	0.0955493
36023	0.946132	0.156741
36024	0.946131	0.172906
36025	0.946131	0.139669
36026	0.946133	0.0906022
36027	0.946133	0.0827551
36028	0.946131	0.124072
36029	0.946131	0.166083
36030	0.946132	0.124039
36031	0.946132	0.133666
36032	0.946133	0.0737973
36033	0.946133	0.0728699
36034	0.946132	0.0937613
36035	0.946132	0.0904542
36036	0.946133	0.0945765
36037	0.946133	0.0933182
36038	0.946132	0.0707058
36039	0.946132	0.100422
36040	0.946132	0.0754635
36041	0.946132	0.0985857
36042	0.946133	0.0716385
36043	0.946133	0.0901219
36044	1.89227	0.172809
36045	1.89227	0.184058
36046	1.89227	0.190835
36047	1.89227	0.14354
36048	1.89227	0.243026
36049	1.89227	0.183212
36050	1.1656	2.1
36051	1.21847	2.1
36052	0.836006	2.1
36053	0.806007	2.1
36054	1.00653	2.1
36055	1.00653	2.1
36056	1.01794	2.1
36057	1.01794	2.1
36058	1.1656	2.1
36059	0.806007	2.1
36060	0.836006	2.1
36061	1.21847	2.1
36062	2.82263	2.1
36063	2.3312	2.1
36064	2.25936	2.1
36065	2.71441	2.1
36066	5.0456	2.1
36067	2.25936	2.1
36068	1.61201	2.1
36069	1.57627	2.1
36070	1.61201	2.1
36071	2.29545	2.1
36072	2.3312	2.1
36073	1.21269	0.0698591

36074	1.48867	0.0698591
36075	1.68264	0.0698591
36076	1.9898	0.0698591
36077	3.17131	0.0698591
36078	2.0487	2.1
36079	2.45212	2.1
36080	2.31012	2.1
36081	1.94095	2.1
36082	4.39307	2.1
36083	1.02435	2.1
36084	0.970469	2.1
36085	0.625375	2.1
36086	0.567166	2.1
36087	0.796854	2.1
36088	0.796854	2.1
36089	0.79466	2.1
36090	0.79466	2.1
36091	0.970471	2.1
36092	0.567165	2.1
36093	0.625374	2.1
36094	1.02435	2.1
36095	2.31012	2.1
36096	1.94095	2.1
36097	1.4825	2.1
36098	2.17384	2.1
36099	4.11479	2.1
36100	0.946187	2.1
36101	0.803763	2.1
36102	0.581357	2.1
36103	0.510356	2.1
36104	0.745632	2.1
36105	0.745632	2.1
36106	0.734566	2.1
36107	0.734563	2.1
36108	0.896142	2.1
36109	0.510366	2.1
36110	0.946173	2.1
36111	0.552965	2.1
36112	1.89226	0.849147
36113	1.89226	1.47766
36114	1.89226	1.64755
36115	1.89226	1.80569
36116	1.89226	0.88298
36117	1.89226	1.7132
36118	1.50743	1.37874
36119	1.0417	2.02675
36120	1.50743	1.81078
36121	1.62974	0.875246
36122	1.14605	0.804856
36123	1.14605	1.99819
36124	1.89226	0.641142
36125	1.89226	1.78874
36126	1.89226	0.462673
36127	1.89226	1.64828
36128	1.89226	0.535598
36129	1.89226	1.86113
36130	1.89226	1.7119
36131	1.89226	1.35773

36132	1.89226	0.659464
36133	1.89226	1.66003
36134	3.78453	1.1063
36135	1.77186	1.57127
36136	1.35668	1.83841
36137	1.70254	0.929408
36138	1.38756	1.81758
36139	1.77186	1.62694
36140	1.37814	2.026
36141	0.734857	1.94908
36142	1.00549	1.68155
36143	0.835261	0.808505
36144	1.08357	1.91705
36145	0.97665	1.18495
36146	0.795559	2.09725
36147	0.891385	1.59314
36148	0.891385	0.506777
36149	0.874231	0.836932
36150	1.08356	1.08321
36151	0.734863	1.30389
36152	0.79449	1.64948
36153	1.89226	1.63436
36154	1.89226	0.21334
36155	2.42671	0.331847
36156	1.98783	1.25474
36157	4.31897	1.05477
36158	0.579854	0.594917
36159	0.965582	0.800877
36160	0.634403	0.888869
36161	1.17796	0.333129
36162	1.00592	0.284193
36163	0.797259	2.08791
36164	0.905246	1.41558
36165	0.905246	0.355288
36166	0.695715	0.38727
36167	0.637106	0.372479
36168	1.24374	1.18421
36169	0.95617	0.593142
36170	2.0815	0.736613
36171	1.60521	1.57697
36172	1.29556	0.73494
36173	2.32205	1.77061
36174	1.28445	0.589416
36175	2.0815	1.45656
36176	2.3085	2.1
36177	2.21147	2.1
36178	2.3085	2.1
36179	2.39282	2.1
36180	1.98899	2.1
36181	2.0733	2.1
36182	2.3275	2.1
36183	1.63471	2.1
36184	1.98899	2.1
36185	1.56801	2.1
36186	3.62369	2.1
36187	2.3085	2.1
36188	1.98899	2.1
36189	2.22575	2.1

36190	1.90502	2.1
36191	4.21353	2.1
36192	1.90502	2.1
36193	1.56801	2.1
36194	1.90502	2.1
36195	1.98899	2.1
36196	1.49733	2.1
36197	1.58129	2.1
36198	1.89226	2.03543
36199	2.6491	2.1
36200	2.49266	2.1
36201	2.6491	2.05785
36202	1.73582	2.1
36203	1.89226	1.5104
36204	1.31854	2.1
36205	2.6491	2.1
36206	1.3909	2.1
36207	1.3909	2.1
36208	2.49266	2.1
36209	2.49266	2.1
36210	1.89226	1.89332
36211	2.49266	2.06852
36212	2.38634	2.1
36213	2.49266	1.92883
36214	1.78595	2.06651
36215	1.89226	1.28193
36216	1.23042	2.1
36217	2.49266	2.08679
36218	1.31854	2.1
36219	1.31854	2.1
36220	2.38635	2.1
36221	2.38635	2.1
36222	2.83487	2.1
36223	1.89226	2.1
36224	2.594	2.1
36225	2.594	2.1
36226	1.89226	2.1
36227	1.89226	2.1
36228	1.89226	2.1
36229	1.89226	2.1
36230	1.89226	2.1
36231	1.89226	2.1
36232	3.78452	2.1
36233	1.09518	2.1
36234	1.28158	2.1
36235	1.36844	2.1
36236	1.59972	2.1
36237	2.65003	2.1
36238	1.89226	2.1
36239	1.89226	2.1
36240	1.89226	2.1
36241	1.89226	2.1
36242	3.78452	2.1
36243	1.36844	2.1
36244	1.59972	2.1
36245	1.67352	2.1
36246	1.94291	2.1
36247	3.31135	2.1

36248	1.52938	1.93514
36249	1.93568	1.74012
36250	2.36221	0.814095
36251	1.94723	2.07822
36252	3.89158	1.77958
36253	1.94724	1.28764
36254	1.26593	2.07998
36255	1.75367	1.98415
36256	2.4322	1.63746
36257	3.7009	1.93281
36258	2.36221	0.78029
36259	2.43219	1.97703
36260	1.94723	1.69282
36261	1.94723	1.49387
36262	2.8202	1.73434
36263	2.8202	1.55645
36264	2.51604	2.1
36265	2.18533	2.1
36266	2.17228	2.1
36267	2.4889	2.1
36268	4.67423	2.1
36269	2.17228	2.1
36270	1.62183	2.1
36271	1.62316	2.1
36272	1.62183	2.1
36273	2.18666	2.1
36274	2.18533	2.1
36275	2.17227	2.1
36276	2.4889	2.1
36277	2.45922	2.1
36278	2.15686	2.1
36279	4.64576	2.1
36280	2.17228	2.1
36281	1.62316	2.1
36282	1.62241	2.1
36283	2.15686	2.1
36284	3.78002	2.1
36285	1.89226	1.41187
36286	1.89226	1.36604
36287	1.89226	1.192
36288	1.89226	1.18711
36289	3.78453	1.30136
36290	1.89226	1.15455
36291	1.89226	1.1638
36292	1.89226	0.996163
36293	1.89226	0.37713
36294	3.78452	0.956247
36295	1.89226	1.36954
36296	1.89226	0.554349
36297	1.89226	1.15934
36298	1.89226	1.05549
36299	1.89226	0.916336
36300	1.89226	1.02871
36301	1.89226	1.17747
36302	1.89226	1.25903
36303	2.53413	0.317253
36304	1.89226	0.546435
36305	3.78453	1.10816

36306	1.38048	0.46033
36307	1.38048	0.761893
36308	0.490332	0.914619
36309	0.490332	0.563942
36310	0.924679	0.140766
36311	0.946131	1.65541
36312	0.946131	1.44005
36313	0.490332	0.1238
36314	0.924678	0.16525
36315	0.490332	0.144282
36316	0.946131	0.716961
36317	0.946131	0.492987
36318	1.89227	0.351727
36319	1.89227	0.950856
36320	1.89227	0.325805
36321	1.89227	1.1915
36322	1.89227	0.400965
36323	1.89227	0.804726
36324	1.30803	2.1
36325	2.55151	2.1
36326	1.43935	2.1
36327	1.43935	2.1
36328	2.34922	2.1
36329	2.34922	2.1
36330	0.946131	2.1
36331	0.946131	2.1
36332	1.27575	2.1
36333	1.17461	2.1
36334	0.94613	2.1
36335	0.94613	2.1
36336	1.17461	2.1
36337	1.17461	2.1
36338	0.946131	2.1
36339	1.17461	2.1
36340	0.946132	2.1
36341	1.27575	2.1
36342	1.89226	2.1
36343	1.89226	2.1
36344	1.89226	2.1
36345	1.89226	2.1
36346	1.89226	2.1
36347	1.89226	2.1
36348	0.946133	2.1
36349	0.946133	2.1
36350	0.946132	2.1
36351	0.946132	2.1
36352	0.946133	2.1
36353	0.946133	2.1
36354	0.946132	2.1
36355	0.946132	2.1
36356	0.946132	2.1
36357	0.946132	2.1
36358	0.946133	2.1
36359	0.946133	2.1
36360	1.07043	2.1
36361	1.17461	2.1
36362	0.654016	2.1
36363	0.5829	2.1

36364	0.870481	2.1
36365	0.870481	2.1
36366	0.887027	2.1
36367	0.887027	2.1
36368	1.07043	2.1
36369	0.5829	2.1
36370	0.654016	2.1
36371	1.17461	2.1
36372	1.89226	2.1
36373	1.89226	2.1
36374	1.89226	2.1
36375	1.89226	2.1
36376	3.78453	2.1
36377	1.89226	2.1
36378	1.89226	2.1
36379	1.89226	2.1
36380	1.89226	2.1
36381	3.78453	2.1
36382	1.26929	2.1
36383	2.19313	2.1
36384	1.09847	2.1
36385	1.69916	2.1
36386	2.96844	2.1
36387	1.84124	2.1
36388	2.24937	2.1
36389	1.69915	2.1
36390	1.75691	2.1
36391	3.94852	2.1
36392	1.53082	2.1
36393	2.04422	2.1
36394	1.53082	2.1
36395	1.69915	2.1
36396	1.92911	2.1
36397	2.09744	2.1
36398	1.39263	1.34461
36399	1.0171	0.560825
36400	1.1211	0.786261
36401	1.46482	1.08187
36402	2.48192	0.928659
36403	1.39263	1.43143
36404	1.57962	1.95036
36405	1.65238	2.1
36406	1.46482	1.21997
36407	3.04443	1.68157
36408	1.89226	1.94675
36409	1.89226	0.839504
36410	1.89226	1.18168
36411	1.89226	1.63929
36412	3.78453	1.50503
36413	1.89227	0.558532
36414	1.89227	0.802491
36415	1.89227	1.0598
36416	1.89227	0.538764
36417	3.78453	0.659901
36418	1.28418	0.646005
36419	1.46482	0.951236
36420	1.1211	0.614481
36421	1.1211	1.1682

36422	1.59382	0.594181
36423	1.59382	1.11439
36424	1.76897	2.0284
36425	1.46482	1.04431
36426	1.59382	1.43453
36427	1.46482	1.82899
36428	1.78138	1.24586
36429	1.65238	1.71008
36430	1.89226	1.01286
36431	1.89226	1.33067
36432	1.89226	0.844794
36433	1.89226	1.67231
36434	1.89226	0.867792
36435	1.89226	1.78653
36436	1.89226	0.935129
36437	1.89226	0.46742
36438	1.89226	0.515728
36439	1.89226	0.726174
36440	1.89226	0.588722
36441	1.89226	0.762808
36442	1.96832	2.1
36443	2.40858	2.1
36444	1.75211	2.04812
36445	2.20797	2.1
36446	4.1607	2.1
36447	1.75211	2.08029
36448	2.20797	2.1
36449	1.51592	2.1
36450	2.02064	2.1
36451	3.77275	2.1
36452	2.71793	0.289093
36453	2.67099	0.158452
36454	2.55105	0.201244
36455	2.71297	0.146221
36456	5.26898	0.18092
36457	2.36417	0.293173
36458	2.67098	0.180026
36459	2.55104	0.220792
36460	1.83418	0.129299
36461	4.91522	0.184925
36462	2.55105	0.20425
36463	2.71297	0.150356
36464	2.58032	0.177492
36465	2.28922	0.112138
36466	5.13137	0.161775
36467	2.55104	0.224984
36468	2.23787	0.13528
36469	1.67265	0.142098
36470	2.28922	0.126721
36471	4.22369	0.145715
36472	0.851542	0.678126
36473	0.851542	0.73938
36474	0.981419	0.558651
36475	0.854774	0.585998
36476	0.949431	0.576338
36477	0.753763	0.587606
36478	0.946131	0.739131
36479	0.946131	0.626215

36480	1.04073	0.633208
36481	0.981433	0.513024
36482	0.851528	0.701209
36483	0.854828	0.626952
36484	1.89226	1.19891
36485	1.89226	0.712224
36486	1.89226	0.771228
36487	1.89226	0.778721
36488	1.89226	0.944523
36489	1.89226	0.968288
36490	0.814227	0.521962
36491	0.814227	0.589206
36492	0.946117	0.471876
36493	1.07285	0.444983
36494	0.879132	0.452529
36495	0.923293	0.434103
36496	0.946119	0.587843
36497	0.946119	0.508462
36498	0.946131	0.501793
36499	0.814236	0.401659
36500	0.946106	0.553003
36501	0.870405	0.503844
36502	1.89224	0.91754
36503	1.89224	0.537391
36504	1.89224	0.592969
36505	1.89224	0.611924
36506	1.89224	0.758342
36507	1.89224	0.798549
36508	1.29388	1.50895
36509	1.04164	1.90575
36510	1.64834	1.87286
36511	1.82955	1.45886
36512	2.94222	1.81942
36513	1.4934	1.95976
36514	2.41522	1.84527
36515	2.20387	1.12983
36516	2.20387	2.1
36517	1.75765	1.36706
36518	1.75765	2.1
36519	2.41522	1.86653
36520	2.77303	2.1
36521	2.20387	2.1
36522	2.58627	2.1
36523	5.00149	2.1
36524	1.22222	2.00231
36525	2.20387	2.08346
36526	1.96831	1.28712
36527	1.96831	2.1
36528	1.4934	1.4703
36529	1.4934	2.1
36530	1.96832	2.1
36531	2.58627	2.1
36532	2.40858	2.08658
36533	2.40858	2.1
36534	2.20387	2.1
36535	2.20387	2.1
36536	1.5605	2.1
36537	1.53775	2.1

36538	1.55398	2.1
36539	1.45806	2.1
36540	1.65642	2.1
36541	1.5605	2.1
36542	0.768876	2.1
36543	0.79033	2.1
36544	0.828211	2.1
36545	0.871233	2.1
36546	0.803843	2.1
36547	0.803843	2.1
36548	0.814698	2.1
36549	0.814698	2.1
36550	0.79033	2.1
36551	0.871233	2.1
36552	0.768876	2.1
36553	0.828212	2.1
36554	1.80252	2.1
36555	2.09715	2.08176
36556	1.9394	2.1
36557	1.50853	2.1
36558	3.60567	2.1
36559	1.96416	1.88443
36560	2.09715	2.04757
36561	1.88484	2.1
36562	1.57916	1.15303
36563	3.67631	2.03638
36564	1.89226	2.00427
36565	1.89226	0.384936
36566	1.89226	1.15149
36567	1.89226	1.37699
36568	3.78453	1.08829
36569	1.89226	2.05286
36570	1.89226	2.09515
36571	1.89226	2.1
36572	1.89226	1.51462
36573	1.89226	2.1
36574	1.89226	1.10489
36575	1.5852	2.1
36576	1.50853	2.1
36577	1.2955	2.1
36578	1.44472	2.05322
36579	2.95325	2.1
36580	1.53065	2.1
36581	1.57916	0.90493
36582	1.44472	2.00495
36583	1.78861	1.97181
36584	1.23527	2.00024
36585	1.23527	1.86683
36586	1.89227	0.876355
36587	1.89227	1.11495
36588	1.89227	2.01204
36589	1.89227	0.526165
36590	3.78453	1.10907
36591	1.89226	2.06751
36592	1.89226	2.08063
36593	1.89226	2.1
36594	1.89226	2.0025
36595	1.89226	2.1

36596	1.89226	1.2798
36597	1.89227	1.28577
36598	1.89227	0.735684
36599	1.89227	0.650316
36600	1.89227	0.429643
36601	3.78453	0.440531
36602	0.943708	0.443133
36603	0.752219	0.331919
36604	0.946132	0.427982
36605	0.946132	0.43757
36606	0.946133	0.258409
36607	0.946133	0.930064
36608	0.752218	1.23259
36609	0.752218	0.539336
36610	0.752218	0.392857
36611	0.946132	0.318542
36612	0.946133	0.687896
36613	0.943708	0.483615
36614	0.943992	2.1
36615	0.943992	2.1
36616	1.23507	2.1
36617	1.48209	2.1
36618	1.0384	2.1
36619	1.47953	2.1
36620	0.946128	2.09923
36621	0.946128	2.1
36622	0.946129	2.1
36623	1.23293	2.1
36624	1.0384	2.1
36625	0.946129	2.1
36626	1.89226	2.1
36627	2.46586	2.1
36628	2.80218	2.08501
36629	2.80218	2.1
36630	1.89226	2.09132
36631	1.89226	2.1
36632	1.32455	2.1
36633	1.89128	2.1
36634	1.32328	2.1
36635	1.97299	2.1
36636	3.29754	2.1
36637	1.89128	2.1
36638	2.4255	2.1
36639	1.973	2.1
36640	2.63807	2.1
36641	4.3985	2.1
36642	1.34008	2.1
36643	1.97299	2.1
36644	2.10766	2.1
36645	2.10766	2.1
36646	1.32328	2.1
36647	1.32328	2.1
36648	2.41055	2.1
36649	2.37491	2.1
36650	2.40754	2.1
36651	2.38023	2.1
36652	4.78546	2.1
36653	2.23743	2.1

36654	2.37491	2.1
36655	2.40754	2.1
36656	2.20392	2.1
36657	4.61234	2.1
36658	2.37491	2.1
36659	2.02641	2.1
36660	2.20392	2.1
36661	2.18943	2.1
36662	4.40132	2.1
36663	2.37491	2.1
36664	2.22558	2.1
36665	2.38023	2.1
36666	2.18943	2.1
36667	4.60049	2.1
36668	1.89226	0.0821189
36669	1.89226	0.152573
36670	1.89226	0.109295
36671	1.89226	0.105729
36672	1.89226	0.103721
36673	1.89226	0.101065
36674	1.03599	0.0788155
36675	0.706698	0.0758822
36676	1.41141	0.0926238
36677	1.17573	0.0842771
36678	1.11957	0.0737849
36679	0.765034	0.0754605
36680	1.05913	0.0730468
36681	1.45054	0.0889722
36682	1.04847	0.0734947
36683	1.04847	0.0809344
36684	1.15323	0.0822987
36685	1.05825	0.0726658
36686	1.89226	0.0997505
36687	1.89226	0.114597
36688	1.89226	0.115608
36689	1.89226	0.0971386
36690	1.89226	0.116513
36691	1.89226	0.0991024
36692	2.22575	2.06762
36693	1.70677	2.1
36694	1.90157	2.04616
36695	1.5534	2.1
36696	3.77914	2.1
36697	1.70678	2.1
36698	0.968594	2.1
36699	0.968594	2.1
36700	1.10378	2.1
36701	1.5534	2.1
36702	1.68859	2.1
36703	2.31429	1.91042
36704	1.5534	2.1
36705	1.90103	2.05585
36706	1.41902	2.1
36707	3.45443	2.1
36708	2.53709	0.0698591
36709	1.209	0.0698591
36710	1.05417	0.0698591
36711	2.6513	0.0698591

36712	3.70547	0.0698591
36713	1.02675	0.0698591
36714	1.16384	0.0698591
36715	2.22062	0.0698591
36716	2.48862	0.0698591
36717	3.38446	0.0698591
36718	1.89226	0.0698591
36719	1.89226	0.0698591
36720	1.89226	0.0698591
36721	1.89226	0.0698591
36722	1.89226	0.0698591
36723	1.89226	0.0698591
36724	1.89226	1.87897
36725	1.89226	2.1
36726	1.89226	2.1
36727	1.89226	1.94122
36728	1.89226	2.1
36729	1.89226	1.92576
36730	1.89226	1.75081
36731	1.89226	2.02224
36732	1.89226	2.07099
36733	1.89226	1.79565
36734	1.89226	2.06264
36735	1.89226	1.72809
36736	1.89227	1.75518
36737	1.89227	1.98822
36738	1.75032	2.04243
36739	1.75032	1.73908
36740	1.89227	2.02762
36741	1.89227	1.68036
36742	1.89226	0.38603
36743	1.89226	0.497329
36744	1.89226	0.340307
36745	1.89226	0.39355
36746	1.89226	0.399931
36747	1.89226	0.459842
36748	0.946132	0.201383
36749	0.946132	0.183489
36750	0.798215	0.177671
36751	0.848163	0.169666
36752	0.848163	0.196301
36753	0.946131	0.199564
36754	0.946132	0.164632
36755	0.798216	0.170124
36756	0.996105	0.183924
36757	0.996105	0.152057
36758	0.79819	0.177606
36759	0.79819	0.211271
36760	1.07763	0.202151
36761	2.28622	0.19506
36762	2.16109	0.133382
36763	1.17506	0.144051
36764	3.36385	0.158625
36765	1.89226	2.1
36766	1.6663	2.1
36767	1.6663	2.1
36768	1.71706	2.1
36769	1.89226	2.1

36770	1.94302	2.1
36771	1.89226	2.1
36772	1.62801	2.1
36773	1.62801	2.1
36774	1.6663	2.1
36775	1.89226	2.1
36776	1.93055	2.1
36777	1.48734	2.1
36778	1.99609	2.1
36779	1.52245	2.1
36780	2.04137	2.1
36781	3.51854	2.1
36782	1.99609	2.1
36783	2.28563	2.1
36784	2.04136	2.1
36785	2.35019	2.1
36786	4.34628	2.1
36787	1.50714	2.1
36788	2.04137	2.1
36789	2.04945	2.1
36790	2.04945	2.1
36791	1.52245	2.1
36792	1.52245	2.1
36793	2.04136	2.1
36794	2.35019	2.1
36795	2.04945	2.1
36796	2.38681	2.1
36797	4.39964	2.1
36798	2.50924	0.0698591
36799	1.38305	2.1
36800	2.56918	2.1
36801	1.44535	2.1
36802	3.95222	2.1
36803	1.89226	2.1
36804	1.89226	2.1
36805	1.89226	2.1
36806	1.89226	2.1
36807	3.78453	2.1
36808	1.40615	2.1
36809	1.38851	2.1
36810	0.748607	2.1
36811	0.724468	2.1
36812	1.06362	2.1
36813	1.06362	2.1
36814	1.067 2.1	
36815	1.067 2.1	
36816	1.40615	2.1
36817	0.748607	2.1
36818	0.724468	2.1
36819	1.38851	2.1
36820	2.56918	2.1
36821	1.53377	2.1
36822	1.53706	2.1
36823	1.44535	2.1
36824	2.66088	2.1
36825	2.56918	2.1
36826	1.89226	2.1
36827	1.89226	2.1

36828	1.89226	2.1
36829	1.89226	2.1
36830	3.78453	2.1
36831	1.40239	2.1
36832	1.68466	2.1
36833	1.26091	2.1
36834	1.54641	2.1
36835	2.94881	2.1
36836	1.68466	2.1
36837	1.65324	2.08228
36838	1.54641	2.1
36839	1.52268	2.07925
36840	3.19965	2.1
36841	1.05776	2.1
36842	1.54641	2.1
36843	1.36595	2.1
36844	1.36595	2.1
36845	1.26091	2.1
36846	1.26091	2.1
36847	1.54641	2.1
36848	1.32697	2.06929
36849	1.52268	2.08215
36850	1.52268	2.1
36851	1.36595	2.08648
36852	1.36595	2.1
36853	1.18647	1.40063
36854	0.988454	2.09988
36855	1.4867	1.9195
36856	1.63892	1.30201
36857	2.67317	2.02643
36858	1.4867	2.05343
36859	1.63892	1.5443
36860	2.09605	0.701861
36861	1.97645	1.90847
36862	3.58275	1.3436
36863	1.29389	1.91546
36864	1.97645	1.47702
36865	1.4867	1.65827
36866	1.4867	1.98113
36867	1.82955	1.20014
36868	1.82955	1.92357
36869	1.13454	1.06909
36870	0.983594	2.1
36871	1.16809	2.1
36872	1.06836	2.06095
36873	1.08332	2.09738
36874	0.983594	1.90755
36875	1.48751	2.1
36876	1.48047	1.84821
36877	1.77596	2.07347
36878	1.75506	2.01859
36879	3.25643	2.06878
36880	1.48047	1.92497
36881	1.25334	2.05552
36882	1.75505	1.97958
36883	1.55831	0.50317
36884	3.03878	1.7866
36885	1.77596	2.0961

36886	1.75506	2.08867
36887	2.00551	2.1
36888	2.00083	1.39396
36889	3.77679	2.09339
36890	1.75506	2.08241
36891	1.80328	0.536522
36892	1.55831	1.94121
36893	1.55831	2.07776
36894	2.00083	0.984937
36895	2.00083	1.63416
36896	2.58627	2.1
36897	2.79047	2.1
36898	2.40858	2.1
36899	2.64748	2.1
36900	5.23375	2.1
36901	1.26755	2.1
36902	1.71717	2.1
36903	1.03824	2.1
36904	1.48901	2.1
36905	2.75656	2.1
36906	1.89226	2.1
36907	1.89226	2.1
36908	1.89226	2.1
36909	1.89226	2.1
36910	1.89226	2.1
36911	1.89226	2.1
36912	1.36541	2.1
36913	1.22156	2.1
36914	0.946132	2.1
36915	0.946132	2.1
36916	1.36541	2.1
36917	1.36541	2.1
36918	0.946132	2.1
36919	0.946132	2.1
36920	1.36541	2.1
36921	0.946133	2.1
36922	0.946132	2.1
36923	1.22156	2.1
36924	1.18901	2.1
36925	1.61149	2.1
36926	1.98748	2.1
36927	1.534 2.1	
36928	3.1765	2.1
36929	1.89226	2.1
36930	1.89226	2.1
36931	1.89226	2.1
36932	1.89226	2.1
36933	3.78452	2.1
36934	1.98748	2.1
36935	1.534 2.1	
36936	2.27541	2.1
36937	1.98748	2.1
36938	1.87436	2.1
36939	1.87436	2.1
36940	2.2988	2.1
36941	2.2604	2.1
36942	2.20831	2.1
36943	2.02802	2.1

36944	4.32683	2.1
36945	2.26041	2.1
36946	1.98957	2.1
36947	2.02803	2.1
36948	1.75021	2.1
36949	4.0176	2.1
36950	2.34607	2.1
36951	2.02803	2.1
36952	1.86795	2.1
36953	1.81448	2.1
36954	3.89597	2.1
36955	2.02803	2.1
36956	1.75021	2.1
36957	1.81448	2.1
36958	1.57887	2.1
36959	3.6069	2.1
36960	0.946131	1.66082
36961	0.946131	2.1
36962	1.1235	2.05692
36963	1.1235	2.1
36964	0.946131	1.81106
36965	0.946131	1.45674
36966	1.1235	2.1
36967	1.13736	2.1
36968	1.13736	2.08195
36969	0.946131	2.09642
36970	1.1235	2.1
36971	0.946131	2.1
36972	1.89226	2.08876
36973	1.89226	2.1
36974	1.89226	1.07028
36975	1.89226	0.59028
36976	3.78452	0.599874
36977	0.563266	2.1
36978	0.563266	1.74445
36979	0.565606	0.769765
36980	0.565606	2.1
36981	0.647086	2.0789
36982	0.624219	2.05821
36983	0.500035	1.46448
36984	0.481785	1.17639
36985	0.500034	0.424745
36986	0.647087	0.392479
36987	0.624219	1.93438
36988	0.481785	1.5075
36989	0.915788	0.2126
36990	0.915788	0.302934
36991	1.02732	0.713717
36992	1.02732	0.486902
36993	0.660831	0.512008
36994	1.11155	0.224213
36995	0.713295	0.269073
36996	1.26874	0.648019
36997	0.713295	0.391259
36998	0.660831	0.341043
36999	1.11155	0.377482
37000	1.26874	0.360864
37001	1.89226	0.727171

37002	1.85091	0.950618
37003	2.11193	1.01888
37004	1.85091	0.875265
37005	2.15328	0.750999
37006	1.89226	0.641325
37007	0.946131	0.465756
37008	0.946131	0.518769
37009	1.18275	0.612785
37010	1.05596	0.725672
37011	0.946127	0.639327
37012	0.946127	0.395225
37013	1.18275	0.54602
37014	1.18275	0.635221
37015	0.946128	0.564269
37016	1.18275	0.563185
37017	0.946129	0.574227
37018	1.05596	0.586834
37019	1.60936	2.1
37020	2.35846	2.1
37021	2.06424	2.1
37022	2.57651	2.1
37023	4.42269	2.1
37024	1.89226	2.1
37025	1.89226	2.1
37026	1.89226	2.1
37027	1.89226	2.1
37028	3.78453	2.1
37029	1.89226	2.1
37030	1.89226	2.1
37031	1.89226	2.1
37032	1.89226	2.1
37033	1.89226	2.1
37034	1.89226	2.1
37035	1.95053	2.1
37036	2.06424	2.1
37037	1.95053	2.1
37038	1.30584	2.1
37039	2.5	2.1
37040	1.8553	2.1
37041	2.49999	2.1
37042	2.57651	2.1
37043	2.49999	2.1
37044	2.06423	2.1
37045	2.80738	2.1
37046	2.37162	2.1
37047	1.89226	2.1
37048	1.89226	2.1
37049	1.89226	2.1
37050	1.89226	2.1
37051	1.89226	2.1
37052	1.89226	2.1
37053	1.49295	0.148126
37054	1.03824	0.16023
37055	1.51396	0.168481
37056	2.08514	0.159586
37057	3.12338	0.156366
37058	1.49798	0.101073
37059	2.32017	0.0749955

37060	2.68236	0.145167
37061	2.08514	0.123205
37062	4.4053	0.100464
37063	1.89226	0.0751403
37064	1.89226	0.120124
37065	1.89226	0.119067
37066	1.89226	0.104076
37067	3.78452	0.101681
37068	1.89226	0.16142
37069	1.89226	0.195297
37070	1.89226	0.168665
37071	1.89226	0.178378
37072	1.89226	0.164425
37073	1.89226	0.168198
37074	1.51396	0.108108
37075	2.08514	0.101118
37076	2.55393	0.117683
37077	2.02901	0.133642
37078	4.11415	0.113009
37079	1.89226	0.0745758
37080	1.89226	0.0698591
37081	1.89226	0.0859009
37082	1.89226	0.112038
37083	3.78451	0.0731583
37084	1.89226	0.101488
37085	1.89226	0.156515
37086	1.89226	0.120987
37087	1.89226	0.101827
37088	1.89226	0.132047
37089	1.89226	0.118307
37090	0.946131	0.123728
37091	0.946131	0.142783
37092	0.946132	0.272096
37093	0.946132	0.168737
37094	0.946132	0.18094
37095	0.946132	0.151285
37096	0.946132	0.194981
37097	0.946132	0.262085
37098	0.946132	0.137027
37099	0.946132	0.186786
37100	0.946132	0.20424
37101	0.946132	0.168885
37102	1.89226	2.1
37103	1.89226	2.1
37104	1.89226	2.1
37105	1.89226	2.1
37106	3.78452	2.1
37107	1.28367	2.1
37108	1.82707	2.1
37109	1.11025	2.1
37110	1.6365	2.1
37111	2.92016	2.1
37112	1.82707	2.1
37113	1.8247	2.05943
37114	1.8247	2.1
37115	2.01549	2.09866
37116	1.6365	2.1
37117	1.82729	2.09937

37118	1.89226	2.1
37119	1.89226	2.06615
37120	1.89226	2.02771
37121	1.89226	2.1
37122	1.89226	2.06211
37123	1.89226	2.1
37124	1.63648	2.1
37125	1.82467	2.06962
37126	1.35877	2.1
37127	1.55159	2.02802
37128	3.18345	2.1
37129	1.89223	2.1
37130	1.89223	2.07367
37131	1.89223	2.1
37132	1.89223	2.07306
37133	3.78447	2.1
37134	1.59972	2.1
37135	1.28037	2.1
37136	1.63702	2.1
37137	1.28158	2.1
37138	1.63581	2.1
37139	1.28037	2.1
37140	1.89226	2.1
37141	1.89226	2.1
37142	1.89226	2.1
37143	1.89226	2.1
37144	1.89226	2.1
37145	1.89226	2.1
37146	1.89226	2.1
37147	1.89226	2.1
37148	1.89226	2.1
37149	1.89226	2.1
37150	3.78451	2.1
37151	1.28037	2.1
37152	1.05071	2.1
37153	1.63581	2.1
37154	1.44287	2.1
37155	2.72324	2.1
37156	1.59972	2.1
37157	1.63581	2.1
37158	1.94291	2.1
37159	2.00118	2.1
37160	3.57872	2.1
37161	1.89226	2.1
37162	1.89226	2.1
37163	1.89226	2.1
37164	1.89226	2.1
37165	3.78452	2.1
37166	0.946131	2.1
37167	0.946131	2.1
37168	0.946127	2.1
37169	0.946127	2.1
37170	0.94613	2.1
37171	0.94613	2.1
37172	0.946128	2.1
37173	0.946128	2.1
37174	0.946128	2.1
37175	0.946128	2.1

37176	0.946129	2.1
37177	0.946129	2.1
37178	0.865056	2.1
37179	0.865056	2.1
37180	0.873224	2.1
37181	0.873224	2.1
37182	0.721437	2.1
37183	0.920378	2.1
37184	0.817903	2.1
37185	1.00059	2.1
37186	1.00059	2.1
37187	0.920376	2.1
37188	0.817904	2.1
37189	0.721437	2.1
37190	3.09157	2.1
37191	1.71255	2.1
37192	2.0815	2.1
37193	1.9918	2.1
37194	2.51364	2.1
37195	2.39396	2.1
37196	2.0815	2.1
37197	1.05051	2.1
37198	2.56724	2.1
37199	1.63991	2.1
37200	3.47929	2.1
37201	1.89226	0.0923901
37202	2.2973	0.176516
37203	1.90705	0.166043
37204	1.90705	0.113446
37205	1.89226	0.146431
37206	1.89226	0.0903093
37207	1.89226	0.0892307
37208	1.90705	0.163749
37209	1.45321	0.168225
37210	1.45321	0.115687
37211	1.89226	0.146158
37212	1.89226	0.0933143
37213	0.901146	2.1
37214	0.901146	2.1
37215	1.15227	2.1
37216	1.21279	2.1
37217	0.991265	2.1
37218	1.15003	2.1
37219	0.946132	2.1
37220	0.946132	2.1
37221	0.946132	2.1
37222	1.09997	2.1
37223	0.991266	2.1
37224	0.946132	2.1
37225	1.89226	2.1
37226	1.89226	2.1
37227	1.89226	2.1
37228	1.89226	2.1
37229	1.89226	2.1
37230	1.89226	2.1
37231	1.62229	2.1
37232	1.06053	2.1
37233	1.19698	2.1

37234	1.70106	2.1
37235	2.81928	2.1
37236	0.946132	2.1
37237	0.946132	2.1
37238	1.17527	2.1
37239	1.09997	2.1
37240	1.09997	2.1
37241	1.09997	2.1
37242	0.946133	2.1
37243	0.946133	2.1
37244	1.17527	2.1
37245	0.946132	2.1
37246	0.946133	2.1
37247	1.09997	2.1
37248	1.89226	2.1
37249	1.89226	2.1
37250	1.89226	2.1
37251	1.89226	2.1
37252	1.89226	2.1
37253	1.89226	2.1
37254	0.905416	2.1
37255	0.850527	2.1
37256	0.660601	2.1
37257	0.598494	2.1
37258	0.750137	2.1
37259	0.750137	2.1
37260	0.753774	2.1
37261	0.753774	2.1
37262	0.660599	2.1
37263	0.905418	2.1
37264	0.598493	2.1
37265	0.850529	2.1
37266	2.43509	2.1
37267	2.65066	2.1
37268	2.66602	2.1
37269	2.46339	2.1
37270	5.10111	2.1
37271	2.53008	2.1
37272	2.65066	2.1
37273	2.66602	2.1
37274	2.52852	2.1
37275	5.1961	2.1
37276	2.66602	2.1
37277	2.46339	2.1
37278	2.47932	2.1
37279	2.67393	2.1
37280	5.14535	2.1
37281	2.66602	2.1
37282	2.52853	2.1
37283	2.51686	2.1
37284	2.67393	2.1
37285	5.18288	2.1
37286	1.89226	2.09119
37287	1.89226	0.602119
37288	1.89226	2.07043
37289	1.89226	0.513545
37290	3.78452	1.40338
37291	1.55161	2.08466

37292	1.53972	0.548467
37293	1.32259	2.05264
37294	1.35082	0.415547
37295	2.8623	1.19081
37296	1.53972	0.474535
37297	1.4054	0.961688
37298	1.35083	0.421064
37299	1.24913	0.651345
37300	2.78885	0.377474
37301	1.89226	0.507315
37302	1.89226	1.31568
37303	1.89226	0.531406
37304	1.89226	0.635783
37305	3.78452	0.193769
37306	1.89226	2.06147
37307	1.89226	0.720875
37308	1.89226	2.07318
37309	1.89226	0.760198
37310	1.89226	2.07779
37311	1.89226	1.10596
37312	1.14599	2.05786
37313	1.35082	0.580278
37314	1.14599	1.2136
37315	1.32259	2.05954
37316	1.19993	0.554969
37317	1.37652	2.05867
37318	1.35083	0.4581
37319	1.24913	1.18126
37320	1.19993	0.743995
37321	1.11787	0.962414
37322	2.44906	0.343276
37323	1.89226	0.671982
37324	1.89226	1.16283
37325	1.89226	0.825975
37326	1.89226	0.543907
37327	3.78452	0.356557
37328	1.51697	0.0698591
37329	2.57264	0.0698591
37330	1.30203	0.0698591
37331	2.41701	0.0698591
37332	3.93398	0.0698591
37333	1.17464	0.0698591
37334	2.41701	0.0698591
37335	2.32462	0.0698591
37336	2.32462	0.0698591
37337	1.30203	0.0698591
37338	1.30203	0.0698591
37339	2.08149	2.1
37340	1.9839	2.1
37341	1.71398	2.1
37342	1.99182	2.09805
37343	3.71121	2.1
37344	2.16061	2.1
37345	2.01399	0.718523
37346	1.99182	2.1
37347	1.83895	2.07257
37348	4.00581	2.08024
37349	1.90444	2.1

37350	1.99182	2.1
37351	1.71717	2.1
37352	1.805	2.1
37353	3.70899	2.1
37354	1.99182	2.1
37355	1.83896	1.09974
37356	1.805	2.1
37357	1.65243	1.15988
37358	3.64425	2.05878
37359	1.18076	2.1
37360	1.27697	2.1
37361	1.05025	2.1
37362	1.27697	2.1
37363	1.25	2.1
37364	1.47671	2.1
37365	1.89226	2.1
37366	1.89226	2.1
37367	1.89226	2.1
37368	1.89226	2.1
37369	3.78452	2.1
37370	2.64124	2.1
37371	1.89226	2.1
37372	1.89226	2.1
37373	1.89226	2.1
37374	2.46904	2.1
37375	2.46904	2.1
37376	1.89226	2.1
37377	1.89226	2.1
37378	2.7408	2.1
37379	2.82858	2.1
37380	4.72084	2.1
37381	1.87875	2.1
37382	1.37704	2.1
37383	1.77042	2.1
37384	2.27801	2.1
37385	3.64916	2.1
37386	1.77042	2.1
37387	2.27801	2.1
37388	2.58523	2.1
37389	1.90178	2.1
37390	4.35565	2.1
37391	1.28667	2.1
37392	1.30711	2.1
37393	1.53077	2.1
37394	1.55635	2.1
37395	2.81743	2.1
37396	1.55635	2.1
37397	1.28228	2.1
37398	1.64268	2.1
37399	1.93673	2.1
37400	3.19903	2.1
37401	1.53077	2.1
37402	1.93673	2.1
37403	1.55635	2.1
37404	1.55635	2.1
37405	1.86206	2.1
37406	1.86206	2.1
37407	1.64892	2.1

37408	1.60532	2.1
37409	1.85282	2.1
37410	1.96716	2.1
37411	3.61608	2.1
37412	1.60531	2.1
37413	1.32414	2.1
37414	1.96716	2.1
37415	1.75823	2.1
37416	3.2913	2.1
37417	1.91219	2.1
37418	1.96716	2.1
37419	2.0815	2.1
37420	2.51396	2.1
37421	4.30843	2.1
37422	1.96716	2.1
37423	2.17979	2.1
37424	1.75823	2.1
37425	1.75823	2.1
37426	2.28541	2.1
37427	2.28541	2.1
37428	1.89226	1.3372
37429	1.89226	1.69999
37430	1.89226	1.36745
37431	1.89226	1.13758
37432	3.78453	1.34181
37433	1.89226	1.59851
37434	1.89226	1.52562
37435	1.89226	1.48951
37436	1.89226	1.56711
37437	1.89226	1.48192
37438	1.89226	1.56151
37439	1.61016	1.47527
37440	1.45303	1.68424
37441	1.62812	1.48359
37442	1.86379	1.48117
37443	3.23828	1.47005
37444	1.61016	1.6555
37445	1.78716	1.78316
37446	2.0815	1.08257
37447	1.6774	1.54244
37448	3.54454	1.5796
37449	1.89226	1.5416
37450	1.89226	1.41888
37451	1.89226	1.04433
37452	1.89226	1.39131
37453	3.78453	1.41059
37454	1.89226	1.6863
37455	1.89226	1.58752
37456	1.89226	1.65358
37457	1.89226	1.81589
37458	1.89226	1.17963
37459	1.89226	1.26462
37460	0.807591	1.7333
37461	0.814059	1.48151
37462	1.00217	1.79804
37463	0.931896	1.61488
37464	0.850586	1.66519
37465	0.850586	1.7815

37466	0.8889	1.4691
37467	0.8889	1.69996
37468	0.807592	1.14198
37469	1.00217	1.71364
37470	0.814061	1.62815
37471	0.931894	1.8898
37472	2.0815	1.3499
37473	1.6774	1.8801
37474	2.00435	1.38502
37475	2.36104	1.35462
37476	3.86809	1.39169
37477	1.00532	0.097141
37478	1.00532	0.110138
37479	1.00183	0.119778
37480	1.00183	0.0945284
37481	1.26377	0.0948694
37482	1.34921	0.0925721
37483	0.664872	0.107056
37484	0.743381	0.110016
37485	0.664872	0.104817
37486	1.26377	0.109754
37487	1.34921	0.103796
37488	0.743381	0.115214
37489	1.26826	0.0698591
37490	0.946131	2.06935
37491	1.02404	2.01152
37492	0.94613	2.09869
37493	1.30281	2.04868
37494	0.930941	2.09568
37495	0.930941	2.07323
37496	1.318	2.04061
37497	1.58161	2.02178
37498	0.94613	2.054
37499	0.94613	2.01438
37500	1.56338	2.03553
37501	1.02404	2.0265
37502	2.81374	2.04596
37503	1.89226	2.09959
37504	1.89226	2.06667
37505	1.89226	2.08015
37506	3.78453	2.08463
37507	0.946133	2.09878
37508	0.946133	2.05949
37509	0.946132	2.07137
37510	0.946132	2.09435
37511	0.946132	2.09406
37512	0.946132	2.0846
37513	0.946133	2.0887
37514	0.946133	2.0603
37515	0.946132	2.08565
37516	0.946132	2.06424
37517	0.946133	2.1
37518	0.946133	2.08749
37519	1.89226	1.90325
37520	1.89226	2.07334
37521	1.89226	1.95811
37522	1.89226	2.01174
37523	3.78452	2.01587

37524	1.89226	2.06117
37525	1.89226	2.04878
37526	1.89226	2.08526
37527	1.89226	1.99143
37528	1.89226	2.08506
37529	1.89226	1.99187
37530	0.946133	2.01163
37531	0.946133	2.0692
37532	0.946132	2.01538
37533	0.946132	2.07536
37534	0.946132	2.0759
37535	0.946132	2.05999
37536	0.946133	2.0705
37537	0.946133	2.01363
37538	0.946132	2.06094
37539	0.946132	2.03132
37540	0.946132	2.08079
37541	0.946132	2.05387
37542	2.56953	2.1
37543	2.64148	2.1
37544	2.48631	2.1
37545	2.62213	2.1
37546	5.19166	2.1
37547	2.64148	2.1
37548	2.57929	2.1
37549	2.62213	2.1
37550	2.59522	2.1
37551	5.20142	2.1
37552	2.48631	2.1
37553	2.62213	2.1
37554	2.41176	2.1
37555	2.59262	2.1
37556	5.03389	2.1
37557	2.62213	2.1
37558	2.59522	2.1
37559	2.59262	2.1
37560	2.61901	2.1
37561	5.24114	2.1
37562	0.946132	0.0698591
37563	0.946132	0.0698591
37564	0.946133	0.0698591
37565	0.946133	0.0698591
37566	0.946132	0.0698591
37567	0.946132	0.0698591
37568	0.946133	0.0698591
37569	0.946133	0.0698591
37570	0.946132	0.0698591
37571	0.946132	0.0698591
37572	0.946133	0.0698591
37573	0.946133	0.0698591
37574	1.5681	0.0698591
37575	1.01248	0.0698591
37576	1.10749	0.0698591
37577	0.570913	0.0698591
37578	1.15589	0.0698591
37579	1.15588	0.0698591
37580	0.977549	0.0698591
37581	0.977549	0.0698591

37582	1.01249	0.0698591
37583	0.654445	0.0698591
37584	1.36795	0.0698591
37585	1.10748	0.0698591
37586	1.73883	0.0698591
37587	1.87638	0.0698591
37588	1.85219	0.0698591
37589	1.93113	2.1
37590	3.7407	0.0698591
37591	1.87638	0.0698591
37592	1.73883	2.1
37593	1.93113	2.1
37594	1.80426	2.1
37595	3.66996	2.1
37596	2.02912	0.119777
37597	1.44951	0.113032
37598	1.50103	0.087589
37599	2.02248	0.107004
37600	3.47199	0.0957717
37601	1.89226	0.108708
37602	1.89226	0.143634
37603	1.89226	0.105722
37604	1.89226	0.105051
37605	3.78453	0.113481
37606	2.57881	0.103464
37607	1.89226	0.112289
37608	1.89226	0.116324
37609	1.89226	0.120293
37610	2.6428	0.100948
37611	2.6428	0.106683
37612	1.01379	0.107197
37613	0.985937	0.102596
37614	0.769295	0.0964461
37615	0.715547	0.0889083
37616	0.853042	0.0988627
37617	0.853042	0.134806
37618	0.864117	0.106121
37619	0.864114	0.0891515
37620	0.715556	0.0997058
37621	1.03047	0.094725
37622	0.731721	0.107514
37623	0.985921	0.108798
37624	1.89227	0.128001
37625	1.89227	0.116929
37626	1.89227	0.0932233
37627	1.89227	0.138013
37628	3.78453	0.110246
37629	0.946129	0.121945
37630	0.946129	0.109904
37631	1.28161	0.0888088
37632	1.3214	0.0951095
37633	0.94613	0.0993532
37634	0.94613	0.132917
37635	1.28161	0.0970698
37636	1.28161	0.0908078
37637	1.28161	0.0905186
37638	0.94613	0.0987359
37639	0.946131	0.107411

37640	1.3214	0.102338
37641	0.946132	0.379494
37642	0.946132	0.333117
37643	0.706045	0.296292
37644	1.16718	0.261203
37645	0.706017	0.28579
37646	0.706017	0.476582
37647	1.4073	0.257812
37648	1.4073	0.239823
37649	0.94613	0.313229
37650	1.16719	0.309028
37651	0.706046	0.346764
37652	0.946131	0.330127
37653	1.89226	0.482228
37654	1.89226	0.47487
37655	1.89226	0.297837
37656	1.89226	0.696204
37657	1.89226	0.294573
37658	1.89226	0.601604
37659	0.946119	0.337904
37660	0.946119	0.307581
37661	0.946115	0.313911
37662	0.946115	0.247251
37663	0.946117	0.31838
37664	0.946117	0.527365
37665	0.946117	0.278569
37666	0.946117	0.242167
37667	0.94613	0.24178
37668	0.94613	0.322223
37669	0.946105	0.384143
37670	0.946105	0.290478
37671	0.946132	1.53515
37672	0.946132	1.13492
37673	0.946131	1.47938
37674	0.946131	0.817835
37675	0.946132	1.73703
37676	0.946132	1.52608
37677	0.946132	1.04523
37678	0.827865	0.951388
37679	0.946132	0.986375
37680	0.946132	1.26559
37681	0.946132	1.17007
37682	0.827865	1.12798
37683	1.89226	1.56203
37684	1.89226	1.23674
37685	1.89226	1.60807
37686	1.89226	1.22836
37687	1.89226	1.81274
37688	1.89226	1.6519
37689	0.93094	1.01002
37690	0.93094	1.11322
37691	1.04649	0.923091
37692	0.614468	0.798699
37693	0.950358	0.809682
37694	0.799012	0.715318
37695	0.682742	0.936174
37696	0.682742	1.01521
37697	1.04073	1.37228

37698	1.15596	1.11147
37699	0.614478	1.36431
37700	0.851524	1.02328
37701	2.22575	2.1
37702	1.90502	2.1
37703	2.10893	2.1
37704	1.80258	2.1
37705	4.01395	2.1
37706	1.90502	2.1
37707	1.40863	2.1
37708	1.49733	2.1
37709	1.49733	2.1
37710	1.80258	2.1
37711	1.80258	2.1
37712	2.10893	2.1
37713	1.68356	2.1
37714	1.80258	2.1
37715	1.80258	2.1
37716	1.98061	2.1
37717	1.98061	2.1
37718	1.80258	2.1
37719	1.40863	2.1
37720	1.68356	2.1
37721	1.30751	2.1
37722	3.11009	2.1
37723	1.35374	1.90147
37724	1.35374	1.83623
37725	0.746096	1.53551
37726	0.746096	1.47217
37727	1.1537	1.56684
37728	0.746095	1.8128
37729	0.946132	1.79269
37730	0.946132	1.37887
37731	0.946132	1.07725
37732	0.746095	1.50525
37733	0.946131	1.16416
37734	1.1537	1.40119
37735	2.15247	2.1
37736	1.70303	2.1
37737	1.41207	2.1
37738	1.43052	2.1
37739	3.22793	2.1
37740	0.940246	2.1
37741	0.728442	2.1
37742	0.650037	2.1
37743	0.539874	2.1
37744	0.694756	2.1
37745	0.694756	2.1
37746	0.776346	2.1
37747	0.656772	2.1
37748	0.733146	2.1
37749	0.563769	2.1
37750	0.66316	2.1
37751	0.899692	2.1
37752	1.21223	2.1
37753	1.22418	2.1
37754	1.43052	2.1
37755	1.43052	2.1

37756	1.24353	2.1
37757	1.46181	2.1
37758	1.89226	0.179482
37759	2.39894	0.0932022
37760	1.89226	0.149649
37761	2.37417	0.0813963
37762	1.89226	0.201856
37763	2.37417	0.125885
37764	1.12767	0.0810836
37765	1.19947	0.113512
37766	0.94613	0.224791
37767	0.94613	0.0945268
37768	1.12766	0.0821268
37769	0.946128	0.0802184
37770	1.19947	0.112727
37771	0.946132	0.147133
37772	1.19947	0.179272
37773	1.19947	0.0915836
37774	0.94613	0.160414
37775	0.94613	0.0756972
37776	2.22621	0.0698591
37777	1.14533	2.1
37778	1.1131	2.1
37779	0.88238	2.1
37780	0.868521	2.1
37781	1.01153	2.1
37782	1.01153	2.1
37783	1.00232	2.1
37784	1.00232	2.1
37785	1.14533	2.1
37786	0.882378	2.1
37787	0.868519	2.1
37788	1.11311	2.1
37789	0.910183	2.08808
37790	1.07811	1.90916
37791	0.94613	0.772774
37792	0.94613	1.47141
37793	0.910212	1.44537
37794	0.910212	2.09277
37795	1.11403	0.864794
37796	1.11403	1.94448
37797	0.910183	1.78563
37798	0.94613	1.82036
37799	0.94613	1.86645
37800	1.07811	1.84864
37801	1.87228	2.08941
37802	2.81189	2.1
37803	1.6197	2.1
37804	2.56716	2.1
37805	4.43944	2.1
37806	0.946132	2.08172
37807	0.946132	2.1
37808	0.946132	1.40432
37809	0.946132	2.00532
37810	0.946131	2.03794
37811	0.946131	2.1
37812	0.946132	1.51167
37813	0.946132	2.08221

37814	0.946132	2.097
37815	0.946132	2.09702
37816	0.946132	2.09724
37817	0.946132	2.0976
37818	1.28724	2.1
37819	2.56716	2.1
37820	2.3014	2.1
37821	2.3014	2.1
37822	1.6197	2.1
37823	1.6197	2.1
37824	0.946132	2.1
37825	0.946132	2.1
37826	0.946132	1.66459
37827	0.946132	2.07318
37828	0.946132	2.05712
37829	0.946132	2.1
37830	0.946133	2.1
37831	0.946133	1.78931
37832	0.946133	2.0664
37833	0.946133	2.04941
37834	0.946132	2.07901
37835	0.946132	2.07243
37836	1.08039	2.1
37837	1.24445	2.09804
37838	1.2873	2.1
37839	1.48751	2.1
37840	2.53175	2.1
37841	0.946125	1.9658
37842	0.946125	2.1
37843	1.12552	2.1
37844	1.12552	2.1
37845	0.946124	2.08009
37846	0.946124	2.09468
37847	1.12552	2.1
37848	1.11114	2.1
37849	1.11113	2.1
37850	0.946118	2.1
37851	1.12552	2.1
37852	0.946131	2.1
37853	1.89225	1.90988
37854	1.89225	1.53537
37855	1.89225	2.08753
37856	1.89225	2.1
37857	1.89225	1.10761
37858	1.89225	2.1
37859	0.987703	1.83014
37860	1.33078	2.02945
37861	1.31565	1.87971
37862	1.31565	1.96631
37863	1.01121	1.91847
37864	1.01121	2.09176
37865	2.19163	2.1
37866	1.89226	2.1
37867	1.89226	2.1
37868	1.89226	2.1
37869	2.22229	2.1
37870	2.22229	2.1
37871	1.89226	2.1

37872	1.89226	0.536864
37873	1.89226	0.471877
37874	1.89226	2.1
37875	1.89226	1.04876
37876	1.89226	2.1
37877	0.57141	2.1
37878	0.57141	1.48947
37879	0.574036	2.03051
37880	0.574036	2.1
37881	0.651591	2.04081
37882	0.657827	1.5852
37883	0.482391	2.06908
37884	0.493855	2.09026
37885	0.482391	1.04479
37886	0.651591	1.00319
37887	0.493855	2.03039
37888	0.657828	2.00921
37889	1.88428	2.1
37890	2.03975	2.1
37891	1.97316	2.1
37892	1.8266	2.1
37893	3.86635	2.1
37894	2.23053	2.1
37895	1.42255	2.1
37896	1.3868	2.1
37897	1.8266	2.1
37898	3.24915	2.1
37899	1.97316	2.1
37900	1.8266	2.1
37901	1.77128	2.1
37902	1.91767	2.1
37903	3.74427	2.1
37904	1.77128	2.1
37905	1.8266	2.1
37906	1.77942	2.1
37907	1.33962	2.1
37908	1.3868	2.1
37909	1.3868	2.1
37910	0.944111	2.1
37911	0.944111	2.1
37912	1.26036	2.1
37913	1.16968	2.1
37914	1.03853	2.1
37915	1.16646	2.1
37916	0.946129	2.1
37917	0.946129	2.1
37918	0.94613	2.1
37919	1.25768	2.1
37920	1.03853	2.1
37921	0.946131	2.1
37922	1.82441	0.0698591
37923	1.33061	0.0698591
37924	1.13272	0.0698591
37925	1.60249	0.0698591
37926	2.95712	0.0698591
37927	1.82441	0.0698591
37928	2.08757	0.0698591
37929	1.81292	0.0698591

37930	1.60249	0.0698591
37931	3.63733	0.0698591
37932	1.81292	0.0698591
37933	1.332	0.0698591
37934	1.60249	0.0698591
37935	1.60249	0.0698591
37936	1.5344	0.0698591
37937	1.5344	0.0698591
37938	1.10107	2.1
37939	2.51559	2.1
37940	1.1229	2.1
37941	1.1229	2.1
37942	2.49456	2.1
37943	2.49456	2.1
37944	1.89226	2.1
37945	2.51559	2.1
37946	2.49457	2.1
37947	2.51559	2.1
37948	1.87124	2.1
37949	1.89226	2.1
37950	1.89226	2.1
37951	1.89226	2.1
37952	1.89226	2.1
37953	1.89226	2.1
37954	1.89226	2.1
37955	1.89226	2.1
37956	1.89226	2.1
37957	1.89226	2.1
37958	1.89226	2.1
37959	1.89226	2.1
37960	1.89226	2.1
37961	1.89226	2.1
37962	1.23599	2.1
37963	1.24728	2.1
37964	0.550534	2.1
37965	0.541151	2.1
37966	0.893754	2.1
37967	0.893754	2.1
37968	0.894678	2.1
37969	0.894678	2.1
37970	1.23599	2.1
37971	0.541149	2.1
37972	0.550533	2.1
37973	1.24728	2.1
37974	0.946132	2.1
37975	0.946132	2.1
37976	1.23599	2.1
37977	1.24728	2.1
37978	0.946132	2.1
37979	0.946132	2.1
37980	1.23599	2.1
37981	1.23599	2.1
37982	1.23599	2.1
37983	0.946132	2.1
37984	0.946132	2.1
37985	1.24728	2.1
37986	1.89226	2.1
37987	1.89226	2.1

37988	1.89226	2.1
37989	1.89226	2.1
37990	1.89226	2.1
37991	1.89226	2.1
37992	0.946128	2.1
37993	0.946128	2.1
37994	0.946132	2.1
37995	0.946132	2.1
37996	0.94613	2.1
37997	0.94613	2.1
37998	0.946129	2.1
37999	0.946129	2.1
38000	0.94613	2.1
38001	0.94613	2.1
38002	0.94613	2.1
38003	0.94613	2.1
38004	2.58696	0.080355
38005	2.57073	0.130051
38006	2.12585	0.10451
38007	2.07607	0.0956396
38008	4.69658	0.101069
38009	1.89226	0.160797
38010	1.89226	0.13067
38011	1.89226	0.103774
38012	1.89226	0.201427
38013	3.78452	0.140788
38014	1.89226	0.133034
38015	1.89226	0.235408
38016	1.89226	0.205359
38017	1.89226	0.159869
38018	3.78453	0.17909
38019	2.57074	0.132717
38020	2.32635	0.13416
38021	2.07608	0.0997211
38022	1.73623	0.160086
38023	4.30697	0.121267
38024	2.12582	0.108995
38025	1.51971	0.107548
38026	1.57255	0.0951312
38027	2.07604	0.128272
38028	1.62233	0.0927243
38029	2.12582	0.123348
38030	1.89223	0.108599
38031	1.89223	0.20098
38032	1.89223	0.132344
38033	1.89223	0.15105
38034	1.89223	0.134219
38035	1.89223	0.154721
38036	0.946119	0.21317
38037	0.946119	0.195469
38038	0.946119	0.131156
38039	0.946119	0.167262
38040	0.946119	0.172092
38041	0.946119	0.208698
38042	0.94612	0.201424
38043	0.94612	0.13104
38044	0.946132	0.177105
38045	0.946132	0.173231

38046	0.946107	0.175732
38047	0.946107	0.176142
38048	0.811456	0.139151
38049	0.811456	0.171918
38050	0.806521	0.111136
38051	0.806521	0.153695
38052	0.868104	0.149378
38053	0.579949	0.172114
38054	0.759859	0.112319
38055	1.03802	0.131675
38056	0.579957	0.139693
38057	0.759868	0.138317
38058	1.03801	0.145117
38059	0.868093	0.145105
38060	2.7408	2.1
38061	2.82858	2.1
38062	2.57368	2.1
38063	2.52633	2.1
38064	5.3549	2.1
38065	2.7408	2.1
38066	2.31353	2.1
38067	2.14063	2.1
38068	2.52632	2.1
38069	4.83985	2.1
38070	2.57368	2.1
38071	2.52633	2.1
38072	2.3301	2.1
38073	2.36071	2.1
38074	4.88704	2.1
38075	1.86512	2.1
38076	2.52632	2.1
38077	2.14063	2.1
38078	2.14063	2.1
38079	2.3301	2.1
38080	2.3301	2.1
38081	1.86431	2.1
38082	1.89227	2.1
38083	1.68992	2.1
38084	1.68992	2.1
38085	1.89227	2.1
38086	1.89227	2.1
38087	2.0815	2.1
38088	1.89226	2.1
38089	2.04022	2.1
38090	1.86431	2.1
38091	2.07081	2.1
38092	1.89226	2.1
38093	1.91686	2.05768
38094	1.65683	2.1
38095	1.86871	2.1
38096	1.65683	2.09329
38097	2.13075	2.1
38098	1.89226	1.73106
38099	1.89226	2.1
38100	1.89226	1.68164
38101	1.89226	1.99061
38102	1.89226	2.1
38103	3.78452	2.0566

38104	0.851541	2.1
38105	0.851541	2.1
38106	0.952803	2.1
38107	0.851573	2.1
38108	0.946129	2.1
38109	0.946129	2.1
38110	0.929184	2.1
38111	0.757482	2.1
38112	0.836274	2.1
38113	0.851528	2.1
38114	0.952816	2.1
38115	1.04073	2.1
38116	1.70303	1.8511
38117	1.70302	2.05486
38118	1.5544	2.09976
38119	1.70302	1.95768
38120	1.74957	2.02598
38121	1.91686	1.30901
38122	1.89226	1.71366
38123	1.89226	2.07311
38124	1.89226	2.09546
38125	1.89226	1.92848
38126	1.89226	1.99881
38127	1.89226	1.18222
38128	1.89226	2.1
38129	1.89226	2.1
38130	2.03472	2.1
38131	1.89226	2.1
38132	1.99827	2.1
38133	1.85836	2.09727
38134	1.00509	2.1
38135	1.55998	2.1
38136	1.83347	2.1
38137	1.45246	2.1
38138	3.01244	2.1
38139	1.23839	2.1
38140	0.884251	2.1
38141	0.946133	2.1
38142	0.946133	2.1
38143	0.884226	2.1
38144	0.884226	2.1
38145	0.946158	2.1
38146	0.946159	2.1
38147	1.23839	2.1
38148	0.946131	2.1
38149	0.946131	2.1
38150	0.884254	2.1
38151	1.89226	2.1
38152	1.89226	2.1
38153	1.89226	2.1
38154	1.89226	2.1
38155	1.89226	2.1
38156	1.89226	2.1
38157	2.1499	2.1
38158	1.45244	2.1
38159	1.83345	2.1
38160	1.83345	2.1
38161	1.87214	2.1

38162	1.87214	2.1
38163	1.89224	2.1
38164	1.89224	2.1
38165	1.89224	2.1
38166	1.89224	2.1
38167	3.78448	2.1
38168	1.49155	1.99743
38169	2.0815	2.1
38170	1.77515	1.99393
38171	1.72377	2.1
38172	3.50659	1.9893
38173	1.69876	0.423233
38174	2.0815	2.09757
38175	1.9133	1.41179
38176	1.68189	0.710602
38177	2.17628	2.06554
38178	1.94335	2.1
38179	2.65129	0.0698591
38180	1.15317	0.0698591
38181	2.57085	0.0698591
38182	1.209	0.0698591
38183	2.57085	0.0698591
38184	1.209	0.0698591
38185	1.89226	0.0698591
38186	1.89226	0.0698591
38187	1.89226	0.0698591
38188	1.89226	0.0698591
38189	3.78452	0.0698591
38190	2.48861	0.0698591
38191	1.15252	0.0698591
38192	2.60515	0.0698591
38193	2.60515	0.0698591
38194	1.16384	0.0698591
38195	1.16384	0.0698591
38196	1.59903	0.512709
38197	1.26865	0.958992
38198	1.30486	1.59204
38199	1.70302	0.615986
38200	2.98573	0.912301
38201	1.59903	0.278028
38202	1.79478	0.832252
38203	1.79478	0.760958
38204	1.97005	0.298123
38205	1.50365	0.42841
38206	1.64192	0.992669
38207	1.89226	0.264379
38208	1.89226	0.832165
38209	1.89226	0.900859
38210	1.89226	0.375027
38211	3.78453	0.308792
38212	1.89226	1.13976
38213	1.89226	0.49138
38214	1.89226	1.15568
38215	1.89226	0.382982
38216	1.89226	0.928127
38217	1.89226	0.690039
38218	0.761672	0.239462
38219	0.896406	0.331835

38220	0.603509	0.680266
38221	0.574071	0.784076
38222	0.695159	0.874452
38223	0.695159	0.193628
38224	0.762564	1.26328
38225	0.770159	0.755735
38226	0.803021	0.639593
38227	0.572529	0.791801
38228	0.696466	0.463266
38229	0.899733	0.446772
38230	1.66034	0.165303
38231	1.79478	0.838755
38232	1.56685	0.316582
38233	1.68512	1.46895
38234	1.67652	0.380568
38235	1.67652	0.718324
38236	1.89226	0.918052
38237	1.89226	0.408711
38238	1.89226	0.521217
38239	1.89226	0.996303
38240	1.89226	0.546882
38241	1.89226	1.00433
38242	0.946133	0.255929
38243	0.946133	0.332317
38244	0.946132	0.729088
38245	0.946132	0.631451
38246	0.946132	0.625193
38247	0.946132	0.141075
38248	0.946133	1.44636
38249	0.946133	0.47248
38250	0.946133	0.516411
38251	0.946133	0.63137
38252	0.946132	0.250428
38253	0.946132	0.296029
38254	1.80424	2.1
38255	1.45158	2.1
38256	1.9098	2.1
38257	1.56003	2.1
38258	3.36138	2.1
38259	1.90982	2.1
38260	1.71606	2.1
38261	2.15314	2.1
38262	1.70302	2.1
38263	3.89051	2.1
38264	1.96416	1.8208
38265	1.23706	0.62725
38266	1.00053	1.02051
38267	1.38287	0.476224
38268	1.57916	0.822037
38269	1.9615	0.769122
38270	0.827865	0.364538
38271	0.946132	0.202023
38272	0.946132	0.463487
38273	0.946132	0.663715
38274	0.946132	0.45932
38275	0.946132	0.378319
38276	0.946131	0.491187
38277	0.946131	0.715769

38278	0.827865	0.743933
38279	0.946132	0.300747
38280	0.946132	0.255881
38281	0.946132	0.218224
38282	1.89227	2.1
38283	1.89227	2.1
38284	1.89227	2.1
38285	1.89227	2.1
38286	3.78453	2.1
38287	2.3819	2.1
38288	1.58089	2.1
38289	1.58089	2.1
38290	1.6287	2.1
38291	2.33368	2.1
38292	2.3815	2.1
38293	0.946133	2.1
38294	0.946133	2.1
38295	0.946132	2.1
38296	0.946132	2.1
38297	0.946132	2.1
38298	0.946132	2.1
38299	0.946132	2.1
38300	0.946132	2.1
38301	0.946133	2.1
38302	0.946133	2.1
38303	0.946132	2.1
38304	0.946132	2.1
38305	1.89226	2.1
38306	1.89226	2.1
38307	1.89226	2.1
38308	1.89226	2.1
38309	3.78453	2.1
38310	2.7874	2.1
38311	2.33368	2.1
38312	2.71897	2.1
38313	2.27495	2.1
38314	5.05266	2.1
38315	0.970991	2.1
38316	0.970991	2.1
38317	0.956929	2.1
38318	0.956929	2.1
38319	0.732928	2.1
38320	0.790444	2.1
38321	1.16684	2.1
38322	1.13748	2.1
38323	1.13748	2.1
38324	0.732928	2.1
38325	0.790444	2.1
38326	1.16684	2.1
38327	0.946132	2.1
38328	0.946132	2.1
38329	0.946131	2.1
38330	0.946131	2.1
38331	0.946132	2.1
38332	0.946132	2.1
38333	0.946132	2.1
38334	0.946132	2.1
38335	0.946132	2.1

38336	0.946132	2.1
38337	0.946132	2.1
38338	0.946132	2.1
38339	1.10747	0.207658
38340	2.30331	0.281382
38341	1.47954	0.169886
38342	2.63789	0.189855
38343	3.78285	0.206163
38344	1.65215	0.122063
38345	2.63789	0.154138
38346	2.82465	0.147578
38347	2.82465	0.168971
38348	1.47954	0.121192
38349	1.47954	0.144399
38350	1.13456	2.0754
38351	1.08334	2.1
38352	1.20674	0.483092
38353	1.17885	2.08704
38354	2.29008	2.03244
38355	1.20674	1.77227
38356	1.17885	2.1
38357	1.28649	0.7397
38358	1.28516	2.00672
38359	2.4919	1.94803
38360	1.89226	2.06635
38361	1.89226	2.08469
38362	1.89226	2.09003
38363	1.89226	2.05942
38364	1.89226	2.08623
38365	1.89226	2.05269
38366	2.50288	1.6986
38367	2.02115	1.87259
38368	2.7717	2.014
38369	2.29107	0.551681
38370	4.79395	1.8846
38371	1.27339	2.1
38372	1.10817	2.1
38373	1.19733	2.1
38374	1.032 2.1	
38375	2.30539	2.1
38376	1.19733	2.1
38377	0.953637	2.1
38378	0.953637	2.1
38379	1.032 2.1	
38380	1.11868	2.1
38381	1.19704	2.1
38382	2.01024	0.832213
38383	1.11938	0.729675
38384	1.9186	0.985066
38385	2.76298	0.838927
38386	3.92884	0.786923
38387	0.851541	0.122899
38388	0.851541	0.10714
38389	0.957577	0.100899
38390	0.848952	0.104079
38391	1.08043	0.12592
38392	0.851541	0.131805
38393	0.851484	0.103953

38394	0.957578	0.0908737
38395	1.20045	0.118554
38396	0.726289	0.088771
38397	0.946131	0.107812
38398	0.946131	0.122997
38399	1.50524	2.1
38400	2.38363	2.1
38401	2.49018	2.1
38402	2.49018	2.1
38403	1.45506	2.1
38404	1.45506	2.1
38405	2.49017	2.1
38406	1.89226	2.1
38407	1.89226	2.1
38408	1.89226	2.1
38409	2.38363	2.1
38410	2.38363	2.1
38411	1.54711	2.1
38412	2.49018	2.1
38413	2.56975	2.1
38414	2.56975	2.1
38415	1.50524	2.1
38416	1.50524	2.1
38417	2.56975	2.1
38418	1.89226	2.1
38419	1.89226	2.1
38420	1.89226	2.1
38421	2.49017	2.1
38422	2.49017	2.1
38423	1.89226	0.0781069
38424	1.89226	0.0878558
38425	1.89226	0.139233
38426	1.89226	0.0723589
38427	1.89226	0.141762
38428	1.89226	0.0698591
38429	1.89226	0.131702
38430	1.89226	0.138336
38431	1.89226	0.274624
38432	1.89226	0.089445
38433	1.89226	0.271275
38434	1.89226	0.0862722
38435	1.89226	0.0700341
38436	1.89226	0.0745526
38437	2.8358	0.0698591
38438	1.89226	0.166417
38439	3.78453	0.0699374
38440	1.60565	0.393226
38441	1.89226	1.01691
38442	1.89226	0.888608
38443	1.40013	0.587532
38444	1.89226	0.783469
38445	1.40013	0.561213
38446	1.1766	2.1
38447	1.29435	2.1
38448	1.35735	2.1
38449	1.53509	2.1
38450	2.65169	2.1
38451	0.69701	2.1

38452	0.69701	2.1
38453	0.711303	2.1
38454	0.711303	2.1
38455	0.761138	2.1
38456	0.612149	2.1
38457	0.647174	2.1
38458	0.767548	2.1
38459	0.767548	2.1
38460	0.761139	2.1
38461	0.612149	2.1
38462	0.647174	2.1
38463	1.29764	1.59838
38464	1.29579	1.88473
38465	1.34875	1.82954
38466	1.06717	1.7518
38467	1.29764	1.77263
38468	1.01606	1.64352
38469	0.756635	1.46107
38470	0.648821	1.58225
38471	0.675557	1.79259
38472	0.674374	1.75505
38473	0.74214	1.80134
38474	0.74214	1.58888
38475	0.688868	1.46843
38476	0.688868	1.7418
38477	0.675557	1.42725
38478	0.756634	1.53041
38479	0.648821	1.31696
38480	0.674374	1.49464
38481	1.51327	1.51361
38482	1.48105	1.24922
38483	1.29764	1.58777
38484	1.29764	1.28374
38485	1.48105	1.66654
38486	1.05655	1.17649
38487	1.13537	2.1
38488	1.07326	2.1
38489	0.94613	2.1
38490	0.94613	2.1
38491	0.946132	2.1
38492	0.946132	2.1
38493	1.13537	2.1
38494	1.08927	2.1
38495	1.13537	2.1
38496	0.946131	2.1
38497	0.946131	2.1
38498	0.95947	2.1
38499	1.46892	2.1
38500	1.89226	2.1
38501	1.89226	2.1
38502	1.89226	2.1
38503	1.88031	2.1
38504	1.88031	2.1
38505	1.89226	2.1
38506	1.89226	2.1
38507	1.89226	2.1
38508	1.89226	2.1
38509	3.78453	2.1

38510	2.18254	2.1
38511	1.89226	2.1
38512	2.50005	2.1
38513	1.89226	2.1
38514	4.39231	2.1
38515	1.88031	2.1
38516	2.0815	2.1
38517	2.0815	2.1
38518	1.92078	2.1
38519	2.0815	2.1
38520	1.9218	2.1
38521	1.89227	2.1
38522	1.89227	2.1
38523	1.89227	2.1
38524	1.89227	2.1
38525	1.89227	2.1
38526	1.89227	2.1
38527	1.98693	2.1
38528	1.82857	2.1
38529	2.32	2.1
38530	2.36972	2.1
38531	4.14857	2.1
38532	1.82857	2.1
38533	1.58688	2.1
38534	2.36971	2.1
38535	2.19093	2.1
38536	4.0195	2.1
38537	0.946132	2.1
38538	0.946132	2.1
38539	1.18486	2.1
38540	1.18486	2.1
38541	1.18486	2.1
38542	0.946132	2.1
38543	0.946132	2.1
38544	1.16	2.1
38545	0.946133	2.1
38546	0.946133	2.1
38547	1.16	2.1
38548	1.18486	2.1
38549	1.07798	2.05628
38550	1.08101	1.94566
38551	0.910786	2.1
38552	0.910786	2.1
38553	0.851537	2.09304
38554	0.901377	1.93031
38555	1.14652	2.09876
38556	1.04072	2.1
38557	0.946129	2.09759
38558	0.946129	2.09684
38559	1.0019	2.09037
38560	1.04263	2.09864
38561	1.89226	2.1
38562	1.89226	2.1
38563	1.89226	2.1
38564	1.89226	2.1
38565	3.78452	2.1
38566	1.89226	2.1
38567	2.30146	2.1

38568	1.89226	2.1
38569	2.18412	2.1
38570	4.19372	2.1
38571	2.30146	2.1
38572	1.39962	2.1
38573	2.18411	2.1
38574	1.28765	2.1
38575	3.5891	2.1
38576	1.89226	2.1
38577	1.89226	2.1
38578	1.89226	2.1
38579	1.89226	2.1
38580	1.89226	2.1
38581	1.89226	2.1
38582	1.89226	2.1
38583	1.89226	2.1
38584	1.89226	2.1
38585	1.89226	2.1
38586	3.78452	2.1
38587	2.18412	2.1
38588	1.20045	2.1
38589	1.20045	2.1
38590	1.28765	2.1
38591	2.08808	2.1
38592	2.17528	2.1
38593	0.946131	2.1
38594	0.946131	2.1
38595	0.946128	2.1
38596	0.946128	2.1
38597	0.946127	2.1
38598	0.946127	2.1
38599	0.946131	2.1
38600	0.946131	2.1
38601	0.946129	2.1
38602	0.946129	2.1
38603	0.94613	2.1
38604	0.94613	2.1
38605	1.89226	0.386539
38606	1.89226	1.70897
38607	1.89226	1.76154
38608	1.89226	0.311252
38609	3.78452	0.360752
38610	1.89227	0.630704
38611	1.89227	1.94233
38612	1.89227	0.511413
38613	1.89227	1.63495
38614	1.89227	0.54964
38615	1.89227	1.8299
38616	1.89226	1.71601
38617	1.89226	0.521715
38618	1.89226	0.307946
38619	2.29245	0.646339
38620	3.78452	1.77153
38621	1.89226	1.74986
38622	1.89226	0.237517
38623	1.89226	0.330112
38624	1.89226	1.7808
38625	1.89226	0.341266

38626	1.89226	0.452793
38627	1.28095	1.75586
38628	1.81846	1.77219
38629	2.10991	0.759086
38630	1.34695	1.48774
38631	3.39086	1.50529
38632	2.10991	1.13865
38633	1.36457	1.50591
38634	1.67421	1.80757
38635	1.34695	1.74707
38636	2.43717	1.08141
38637	2.10991	1.58132
38638	2.69541	2.1
38639	1.48572	2.1
38640	2.63553	2.1
38641	1.45385	2.1
38642	4.14926	2.1
38643	2.63553	2.1
38644	1.44207	2.1
38645	1.44207	2.1
38646	1.45385	2.1
38647	2.588 2.1	
38648	2.59978	2.1
38649	1.89227	2.1
38650	1.89227	2.1
38651	1.89227	2.1
38652	1.89227	2.1
38653	3.78453	2.1
38654	1.89226	2.1
38655	1.89226	2.1
38656	1.89226	2.1
38657	1.89226	2.1
38658	3.78453	2.1
38659	2.15929	2.1
38660	1.38276	2.1
38661	1.43617	2.1
38662	1.35614	2.1
38663	2.23932	2.1
38664	2.15929	2.1
38665	2.15929	2.1
38666	2.25168	2.1
38667	2.3558	2.1
38668	2.23932	2.1
38669	4.51509	2.1
38670	1.89226	2.1
38671	1.89226	2.1
38672	1.89226	2.1
38673	1.89226	2.1
38674	3.78453	2.1
38675	1.89226	2.1
38676	1.89226	2.1
38677	1.89226	2.1
38678	1.89226	2.1
38679	1.89226	2.1
38680	1.89226	2.1
38681	2.23932	2.1
38682	1.43854	2.1
38683	1.49173	2.1

38684	1.38276	2.1
38685	2.34829	2.1
38686	2.23932	2.1
38687	2.3558	2.1
38688	2.34829	2.1
38689	2.40685	2.1
38690	2.23932	2.1
38691	2.52332	2.1
38692	2.3558	2.1
38693	1.89226	0.0698591
38694	1.87361	0.0699989
38695	2.25082	0.0698591
38696	1.87361	0.0711765
38697	2.26947	0.0698591
38698	1.89226	0.0706424
38699	1.89226	0.0710678
38700	1.89226	0.0824055
38701	1.89226	0.070571
38702	1.89226	0.0982666
38703	1.89226	0.0702366
38704	1.89226	0.0923383
38705	2.41314	0.0698591
38706	1.89226	0.0720953
38707	1.89226	0.0698591
38708	1.89226	0.078762
38709	2.12204	0.0698591
38710	2.12204	0.0767854
38711	1.89226	0.0698591
38712	2.25082	0.0698591
38713	2.28189	0.0698591
38714	2.25082	0.0716647
38715	1.92333	0.0698591
38716	1.89226	0.0709344
38717	1.89226	0.0712805
38718	1.89226	0.0756893
38719	1.89226	0.0699446
38720	1.89226	0.0940311
38721	1.89226	0.0700221
38722	1.89226	0.0867231
38723	2.42521	0.0698591
38724	1.89226	0.0703
38725	1.89226	0.0698591
38726	1.89226	0.073386
38727	2.41314	0.0698591
38728	2.41314	0.0723938
38729	1.39725	2.03783
38730	1.07745	2.1
38731	1.07745	2.07354
38732	1.07519	2.1
38733	1.41336	2.02002
38734	1.41111	2.1
38735	1.89226	2.1
38736	1.92039	2.1
38737	1.92039	2.1
38738	1.9052	2.1
38739	1.89226	2.1
38740	1.87708	2.1
38741	0.946128	2.1

38742	0.946128	2.1
38743	0.946133	2.03427
38744	0.946133	1.97791
38745	0.94613	1.97707
38746	0.94613	2.1
38747	0.946131	2.1
38748	0.946131	2.00379
38749	0.946131	2.09159
38750	0.946131	2.09422
38751	0.946131	2.07808
38752	0.946131	2.081
38753	1.41336	2.06485
38754	1.08911	2.1
38755	1.08911	2.08659
38756	1.07745	2.1
38757	1.42381	2.05238
38758	1.41215	2.1
38759	1.89227	2.1
38760	1.95833	2.1
38761	1.95833	2.1
38762	1.92039	2.1
38763	1.89227	2.1
38764	1.85433	2.1
38765	0.946129	2.1
38766	0.946129	2.1
38767	0.946133	2.06476
38768	0.946133	2.01318
38769	0.946131	2.01212
38770	0.946131	2.1
38771	0.946131	2.1
38772	0.946131	2.03816
38773	0.946131	2.09986
38774	0.946131	2.1
38775	0.946131	2.09131
38776	0.946131	2.09288
38777	1.41943	2.1
38778	2.74398	2.1
38779	1.18238	2.1
38780	2.53897	2.1
38781	3.9584	2.1
38782	2.74398	2.1
38783	1.89226	2.1
38784	1.89226	2.1
38785	1.89226	2.1
38786	2.53897	2.1
38787	2.53897	2.1
38788	1.89227	0.816171
38789	1.89227	0.384196
38790	1.89227	1.07973
38791	1.89227	0.403549
38792	3.78453	0.516444
38793	1.89226	0.379549
38794	2.08134	0.24522
38795	2.00632	0.271173
38796	2.00632	0.251106
38797	1.89226	0.385442
38798	1.89226	0.321843
38799	1.89227	0.561274

38800	1.89227	0.310618
38801	1.89227	0.600931
38802	1.89227	0.277982
38803	3.78453	0.423463
38804	1.89227	0.868502
38805	1.89227	0.272201
38806	1.89227	0.305034
38807	1.89227	0.336007
38808	1.89227	0.699367
38809	1.89227	0.662922
38810	2.50912	0.249869
38811	1.89226	0.298649
38812	1.89226	0.183051
38813	2.31261	0.236812
38814	2.31261	0.222108
38815	1.89226	0.29266
38816	2.50912	0.255117
38817	1.19999	0.190804
38818	1.44056	0.178466
38819	1.24405	0.179567
38820	2.50912	0.226616
38821	2.31261	0.238603
38822	2.69224	1.16445
38823	2.28373	0.256777
38824	1.94788	0.927805
38825	2.4106	0.444811
38826	4.69434	0.473924
38827	2.69223	1.0306
38828	2.63783	1.55598
38829	2.34737	2.08137
38830	2.4106	0.636801
38831	5.04843	1.50666
38832	1.89226	2.1
38833	1.99753	2.1
38834	1.87366	2.1
38835	1.99753	2.1
38836	1.76839	2.1
38837	1.89226	2.1
38838	1.89226	2.1
38839	1.89226	2.1
38840	1.89226	2.1
38841	1.89226	2.1
38842	3.78452	2.1
38843	0.946132	2.1
38844	0.946132	2.1
38845	1.02744	2.1
38846	1.02744	2.1
38847	0.946132	2.1
38848	0.946132	2.1
38849	1.00989	2.1
38850	1.02744	2.1
38851	1.00989	2.1
38852	0.946133	2.1
38853	1.02744	2.1
38854	0.946132	2.1
38855	1.70302	2.1
38856	1.87366	2.1
38857	1.71618	2.1

38858	1.87366	2.1
38859	1.73105	2.1
38860	1.89226	2.1
38861	1.89226	2.1
38862	1.89226	2.1
38863	1.89226	2.1
38864	1.89226	2.1
38865	1.89226	2.1
38866	1.89226	2.1
38867	1.96624	2.1
38868	1.89226	2.1
38869	1.89226	2.1
38870	1.89226	2.1
38871	2.01977	2.1
38872	2.01977	2.1
38873	1.1032	2.09607
38874	1.15114	2.1
38875	1.14986	1.20482
38876	1.19242	2.1
38877	2.301 2.1	
38878	1.89226	1.84846
38879	1.89226	2.1
38880	1.89226	1.21193
38881	1.89226	2.1
38882	3.78453	2.09927
38883	0.946132	2.1
38884	0.946132	2.1
38885	0.946132	2.1
38886	0.946132	2.1
38887	0.946133	2.1
38888	0.946133	2.1
38889	0.946132	2.1
38890	0.946132	2.1
38891	0.946132	2.1
38892	0.946132	2.1
38893	0.946133	2.1
38894	0.946133	2.1
38895	1.19243	2.1
38896	0.953928	2.1
38897	1.18671	2.1
38898	1.15114	2.1
38899	0.9895	2.1
38900	0.953928	2.1
38901	1.19611	1.13298
38902	1.19242	2.1
38903	1.24693	1.82009
38904	1.24693	2.1
38905	1.14986	1.51562
38906	1.14986	2.1
38907	1.89225	1.1609
38908	1.89225	2.1
38909	1.89225	2.1
38910	1.89225	1.91344
38911	1.89225	2.1
38912	1.89225	2.0833
38913	1.89225	2.1
38914	1.89225	2.1
38915	1.89225	2.1

38916	1.89225	2.1
38917	1.89225	2.1
38918	1.89225	2.1
38919	0.561532	2.1
38920	0.561532	2.1
38921	0.566382	2.1
38922	0.566382	2.1
38923	0.494747	2.1
38924	0.531705	2.1
38925	0.596208	2.1
38926	0.623464	2.1
38927	0.623469	2.1
38928	0.531708	2.1
38929	0.596205	2.1
38930	0.494743	2.1
38931	1.25742	2.1
38932	1.18783	2.1
38933	0.946131	2.1
38934	0.946131	2.1
38935	1.25742	2.1
38936	1.25742	2.1
38937	0.946131	2.1
38938	0.946131	2.1
38939	1.25742	2.1
38940	0.946131	2.1
38941	0.946131	2.1
38942	1.18783	2.1
38943	1.35048	2.1
38944	1.25742	2.1
38945	0.946132	2.1
38946	0.946132	2.1
38947	1.35048	2.1
38948	1.35048	2.1
38949	0.946132	2.1
38950	0.946132	2.1
38951	1.35044	2.1
38952	0.946106	2.1
38953	1.25745	2.1
38954	0.946158	2.1
38955	1.13525	2.1
38956	2.70095	2.1
38957	2.51483	2.1
38958	2.51483	2.1
38959	1.21593	2.1
38960	1.21593	2.1
38961	1.93142	1.39149
38962	2.8053	0.403462
38963	1.559	0.681689
38964	2.60359	0.21294
38965	4.53501	0.447735
38966	2.80531	0.337615
38967	1.89226	0.395008
38968	2.60359	0.25886
38969	1.89226	1.35951
38970	4.49586	0.391337
38971	1.22938	0.582422
38972	2.60359	0.212248
38973	2.37434	1.2413

38974	2.37434	0.60146
38975	1.55901	1.12413
38976	1.55901	0.231597
38977	1.18717	1.2737
38978	1.18717	0.189156
38979	0.946132	0.125764
38980	0.946132	0.580548
38981	0.946133	0.426602
38982	0.946133	1.27539
38983	1.3018	0.14283
38984	1.18717	0.808975
38985	1.18714	0.392784
38986	0.946107	0.444587
38987	1.30183	0.451901
38988	0.946158	0.481795
38989	1.89226	2.1
38990	2.80196	2.1
38991	2.67272	2.1
38992	2.67272	2.1
38993	1.89226	2.1
38994	1.89226	2.1
38995	1.89226	2.1
38996	1.89226	2.1
38997	1.89226	2.1
38998	1.89226	2.1
38999	1.89226	2.1
39000	1.89226	2.1
39001	1.74471	2.1
39002	2.01555	2.1
39003	1.61121	2.1
39004	1.61121	2.1
39005	2.13878	2.1
39006	2.13878	2.1
39007	1.89226	2.1
39008	1.89226	2.1
39009	1.89226	2.1
39010	1.89226	2.1
39011	1.89226	2.1
39012	1.89226	2.1
39013	1.09766	2.1
39014	1.04263	2.1
39015	0.881794	2.1
39016	0.894188	2.1
39017	0.795402	2.1
39018	0.99524	2.1
39019	1.00647	2.1
39020	1.00647	2.1
39021	1.15401	2.1
39022	0.881789	2.1
39023	0.850526	2.1
39024	1.04263	2.1
39025	1.4054	2.01833
39026	1.04788	2.1
39027	1.70302	2.067
39028	1.41375	2.1
39029	2.82706	2.1
39030	1.70302	2.01588
39031	1.38776	2.1

39032	1.59457	2.09576
39033	1.41375	2.1
39034	1.77261	1.89384
39035	1.70302	2.1
39036	1.89226	2.1
39037	1.89226	2.1
39038	1.89226	2.1
39039	1.89226	2.1
39040	3.78453	2.1
39041	2.18042	2.1
39042	1.74738	2.1
39043	2.08433	2.1
39044	1.66347	2.1
39045	3.83171	2.1
39046	1.74738	2.1
39047	1.1181	2.1
39048	1.66347	2.1
39049	1.06543	2.1
39050	2.81281	2.1
39051	1.89226	2.1
39052	1.89226	2.1
39053	1.89226	2.1
39054	1.89226	2.1
39055	3.78453	2.1
39056	1.89227	2.1
39057	1.89227	2.1
39058	1.89227	2.1
39059	1.89227	2.1
39060	3.78453	2.1
39061	2.08433	2.1
39062	1.66347	2.1
39063	1.97092	2.1
39064	1.57431	2.1
39065	3.65864	2.1
39066	1.66347	2.1
39067	1.01474	2.1
39068	1.01474	2.1
39069	1.06544	2.1
39070	1.57431	2.1
39071	1.625 2.1	
39072	1.89226	2.1
39073	1.89226	2.1
39074	1.89226	2.1
39075	1.89226	2.1
39076	1.89226	2.1
39077	1.89226	2.1
39078	1.08467	2.1
39079	1.87228	2.1
39080	1.73305	2.1
39081	1.73305	2.1
39082	1.25565	2.1
39083	1.25565	2.1
39084	0.695361	0.270044
39085	0.799996	0.294053
39086	1.13751	0.450112
39087	1.40945	0.470389
39088	1.40944	0.527999
39089	0.799999	0.267483

39090	1.13751	0.302968
39091	0.695363	0.220097
39092	1.01059	0.306852
39093	1.01059	0.496266
39094	0.926908	0.50491
39095	0.926909	0.216849
39096	1.05481	2.1
39097	1.78069	2.1
39098	1.08074	2.1
39099	1.83284	2.1
39100	2.86143	2.1
39101	1.78069	2.1
39102	2.22044	2.1
39103	1.83284	2.1
39104	2.28696	1.98278
39105	4.06766	2.1
39106	1.07196	2.1
39107	1.64955	2.1
39108	2.02665	2.1
39109	2.0284	2.1
39110	1.08074	2.1
39111	1.08074	2.1
39112	1.64955	2.1
39113	2.28696	2.09554
39114	1.92033	2.1
39115	2.1004	2.1
39116	3.74988	2.1
39117	2.83787	0.607598
39118	1.70303	1.10703
39119	1.70303	0.397052
39120	1.83195	0.146951
39121	2.64075	0.242981
39122	2.70231	0.767458
39123	1.2087	2.09983
39124	1.52909	1.30179
39125	1.43063	1.14207
39126	1.10586	2.09508
39127	2.63494	1.92882
39128	1.89227	2.09792
39129	2.10732	2.1
39130	2.10732	2.1
39131	2.2365	2.1
39132	1.89227	2.1
39133	2.02145	2.1
39134	1.89226	1.36892
39135	1.89226	2.1
39136	1.89226	1.91243
39137	1.89226	2.09601
39138	1.89226	1.64858
39139	1.89226	2.09701
39140	0.51926	2.1
39141	0.552927	2.09822
39142	0.691235	1.22923
39143	0.715314	1.38471
39144	0.619684	1.36969
39145	0.619684	2.1
39146	0.624477	2.1
39147	0.624477	1.32115

39148	0.519262	2.01731
39149	0.691234	2.00844
39150	0.715313	2.01784
39151	0.552928	2.01823
39152	1.89226	2.1
39153	1.87321	2.1
39154	1.87321	2.1
39155	1.89658	2.1
39156	2.0815	2.1
39157	2.14789	2.1
39158	0.946128	2.1
39159	0.946128	2.1
39160	0.946133	1.43636
39161	0.946133	1.75128
39162	0.94613	1.52978
39163	0.94613	2.1
39164	0.946131	1.62133
39165	0.946131	2.1
39166	0.946131	1.95401
39167	0.946131	1.94568
39168	0.946131	2.0639
39169	0.946131	2.07457
39170	1.99297	1.14825
39171	2.19514	0.570313
39172	2.46153	0.82757
39173	2.187	0.740765
39174	4.45451	0.753104
39175	2.0815	0.671562
39176	2.33402	0.572516
39177	2.46154	0.861812
39178	2.83046	0.457565
39179	4.94496	0.634506
39180	2.46153	0.7617
39181	2.187	0.634003
39182	2.27351	0.955469
39183	2.63356	0.499355
39184	4.73504	0.705422
39185	2.46154	0.796495
39186	2.83447	0.415655
39187	2.74513	0.541017
39188	2.5731	0.697982
39189	2.63357	0.515294
39190	2.46154	0.63999
39191	2.80398	2.1
39192	2.60406	2.1
39193	2.73988	2.1
39194	2.57466	2.1
39195	5.34394	2.1
39196	1.89226	1.80544
39197	1.89226	0.761487
39198	1.89226	0.577173
39199	1.89226	1.76841
39200	1.89226	0.664556
39201	1.89226	1.92654
39202	1.89224	1.9204
39203	1.89224	0.811029
39204	1.89224	0.793718
39205	1.89224	1.93086

39206	1.89224	0.828766
39207	1.89224	1.81908
39208	1.40245	1.37833
39209	1.40246	1.47457
39210	0.946117	1.41749
39211	0.946117	1.66158
39212	1.17635	0.821176
39213	1.40246	1.69065
39214	0.946112	0.920378
39215	0.946112	1.93756
39216	0.946127	1.48439
39217	1.17637	0.977596
39218	0.946102	1.85817
39219	1.40244	1.3101
39220	2.53269	2.1
39221	1.70302	2.1
39222	1.56194	2.1
39223	2.17516	2.1
39224	4.19807	2.1
39225	1.89226	2.1
39226	1.89226	2.1
39227	1.89226	2.1
39228	1.89226	2.1
39229	3.78453	2.1
39230	2.57276	2.1
39231	1.89226	2.1
39232	1.89226	2.1
39233	1.89226	2.1
39234	2.32525	2.1
39235	2.32525	2.1
39236	1.15171	2.1
39237	2.17517	2.1
39238	1.41995	2.1
39239	1.41995	2.1
39240	1.80252	2.1
39241	1.80252	2.1
39242	1.89226	2.1
39243	1.89226	2.1
39244	1.89226	2.1
39245	1.89226	2.09914
39246	3.78453	2.1
39247	0.946133	2.1
39248	0.946133	2.1
39249	1.16263	2.1
39250	1.04687	2.1
39251	0.946133	2.1
39252	0.946133	2.1
39253	1.04687	2.1
39254	1.04687	2.1
39255	0.946133	2.1
39256	1.04687	2.1
39257	0.946132	2.1
39258	1.16263	2.1
39259	0.682281	1.63812
39260	0.669233	1.70085
39261	1.21859	1.72052
39262	1.31629	1.27035
39263	0.916158	0.736576

39264	0.916158	1.98329
39265	0.971658	1.73644
39266	0.971658	1.77191
39267	0.669234	1.7597
39268	1.31629	1.4011
39269	0.682283	1.67258
39270	1.21858	1.80498
39271	0.669232	1.51104
39272	0.623495	1.51713
39273	1.36428	1.42039
39274	1.31629	1.12667
39275	0.946475	0.64845
39276	0.946476	1.90571
39277	0.99331	1.67597
39278	0.99331	1.60382
39279	1.36428	1.30618
39280	0.623497	0.992552
39281	0.669234	1.45007
39282	1.31629	1.18718
39283	2.02069	0.0698591
39284	1.46253	0.0698591
39285	2.51479	0.0698591
39286	1.87136	0.0698591
39287	3.89203	0.0698591
39288	1.46253	0.0698591
39289	1.38536	0.0698591
39290	1.38536	0.0698591
39291	1.05579	0.0698591
39292	1.73281	0.0698591
39293	1.46916	0.0698591
39294	2.51479	0.0698591
39295	1.87136	0.0698591
39296	2.23338	0.0698591
39297	2.15603	0.0698591
39298	4.55146	0.0698591
39299	0.822553	0.0698591
39300	0.822563	0.0698591
39301	0.842935	0.0698591
39302	0.842935	0.0698591
39303	0.744203	0.0698591
39304	0.675346	0.0698591
39305	0.874795	0.0698591
39306	1.00453	0.0698591
39307	1.00454	0.0698591
39308	0.782417	0.0698591
39309	0.675337	0.0698591
39310	0.858007	0.0698591
39311	0.946132	1.2058
39312	1.24847	0.250146
39313	0.946132	0.992901
39314	1.16806	1.10145
39315	1.24847	0.310788
39316	1.24847	1.99116
39317	0.946133	0.196449
39318	0.946133	1.56377
39319	0.946133	0.485536
39320	0.946133	0.336656
39321	1.16805	0.9145

39322	1.24847	0.343006
39323	1.2364	1.56973
39324	0.946129	0.336077
39325	0.946133	1.06616
39326	1.24847	0.565829
39327	1.2364	0.278182
39328	1.2364	1.68086
39329	0.946131	0.146037
39330	0.946131	2.09535
39331	0.946131	1.21161
39332	0.946131	0.292798
39333	1.2364	1.62971
39334	1.24847	0.490361
39335	1.89226	0.747191
39336	1.89226	1.45884
39337	1.89226	0.674531
39338	1.89226	0.411079
39339	1.89226	1.37986
39340	1.89226	1.4521
39341	1.89226	1.16992
39342	1.89226	0.699501
39343	1.89226	0.263988
39344	1.89226	1.20011
39345	1.89226	0.578276
39346	1.89226	1.55444
39347	1.69014	1.10927
39348	1.09194	1.14013
39349	1.13537	1.39844
39350	1.13537	1.20875
39351	1.58481	0.746488
39352	1.58481	0.438457
39353	1.58147	1.0138
39354	1.13537	0.422215
39355	1.69015	1.14892
39356	1.01179	0.647008
39357	1.69015	0.447469
39358	1.01179	0.602982
39359	0.946132	1.30717
39360	0.946132	0.620863
39361	1.28543	0.217994
39362	1.07341	1.32941
39363	1.07341	1.10856
39364	1.07341	0.0751059
39365	0.946132	0.556179
39366	0.946132	0.237793
39367	0.946133	0.236636
39368	1.28543	0.336644
39369	1.07341	0.338373
39370	0.946132	0.3802
39371	1.89226	1.66492
39372	1.89226	0.771963
39373	1.89226	0.822886
39374	1.89226	1.9998
39375	1.89226	0.599547
39376	1.89226	2.07604
39377	0.805744	2.1
39378	0.988659	2.02596
39379	0.782044	0.840114

39380	0.578223	1.36366
39381	0.77823	1.28732
39382	0.778231	2.1
39383	0.788653	0.839174
39384	0.788653	1.98885
39385	0.988662	1.61353
39386	0.782042	1.70303
39387	0.805745	1.94977
39388	0.578222	1.91247
39389	1.89226	0.771428
39390	1.89226	1.51843
39391	1.89226	1.30366
39392	1.89226	1.95048
39393	1.89226	0.57054
39394	1.89226	1.99957
39395	1.97732	1.92856
39396	1.97819	0.738242
39397	1.56408	0.839117
39398	1.56408	2.03617
39399	2.33986	0.877039
39400	2.33986	2.06134
39401	1.84516	2.1
39402	2.21152	2.1
39403	1.90702	2.1
39404	1.54599	2.1
39405	3.75751	2.1
39406	1.89224	2.1
39407	1.89224	2.1
39408	1.89224	2.1
39409	1.89224	2.1
39410	1.89224	2.1
39411	1.89224	2.1
39412	1.47672	2.1
39413	1.54601	2.1
39414	1.27697	2.1
39415	1.54601	2.1
39416	1.638	2.1
39417	1.90705	2.1
39418	0.946132	2.1
39419	0.946132	2.1
39420	0.946131	2.1
39421	0.946131	2.1
39422	0.946131	2.1
39423	0.946131	2.1
39424	0.946132	2.1
39425	0.946132	2.1
39426	0.946132	2.1
39427	0.946132	2.1
39428	0.946131	2.1
39429	0.946131	2.1
39430	0.946128	2.1
39431	0.946128	2.1
39432	1.32062	2.1
39433	1.4164	2.1
39434	0.946129	2.1
39435	0.946129	2.1
39436	1.32062	2.1
39437	1.32062	2.1

39438	1.32062	2.1
39439	0.94613	2.1
39440	1.41639	2.1
39441	0.946129	2.1
39442	1.98979	0.0698591
39443	1.65977	0.0698591
39444	2.21772	0.0698591
39445	2.21772	0.0698591
39446	1.48866	0.0698591
39447	1.48866	0.0698591
39448	0.973129	0.0698591
39449	0.973129	0.0698591
39450	0.980954	0.0698591
39451	0.980954	0.0698591
39452	0.845218	0.0698591
39453	1.13981	0.0698591
39454	0.829886	0.0698591
39455	1.10886	0.0698591
39456	1.10886	0.0698591
39457	1.13982	0.0698591
39458	0.845219	0.0698591
39459	0.829886	0.0698591
39460	0.946132	2.1
39461	0.946132	2.1
39462	1.3352	2.1
39463	1.41432	2.1
39464	0.946133	2.1
39465	0.946133	2.06566
39466	1.3352	2.1
39467	1.3352	2.1
39468	1.3352	2.1
39469	0.946132	2.1
39470	1.41432	2.1
39471	0.946133	2.1
39472	2.70097	2.1
39473	1.88175	2.1
39474	1.51482	2.1
39475	1.88175	2.1
39476	2.37784	2.1
39477	2.82864	2.1
39478	1.89227	0.0698591
39479	1.89227	0.0698591
39480	1.89227	0.0698591
39481	1.89227	0.0698591
39482	3.78453	0.0698591
39483	1.89226	0.0698591
39484	2.13295	0.0698591
39485	2.43203	0.0698591
39486	2.43203	0.0698591
39487	1.89226	0.0698591
39488	1.89226	0.0698591
39489	0.946133	0.0698591
39490	0.946133	0.0698591
39491	0.946132	0.0698591
39492	0.946132	0.0698591
39493	0.946132	0.0698591
39494	0.946132	0.0698591
39495	0.946133	0.0698591

39496	0.946133	0.0698591
39497	0.946132	0.0698591
39498	0.946132	0.0698591
39499	0.946132	0.0698591
39500	0.946132	0.0698591
39501	1.54987	2.1
39502	1.24128	2.1
39503	1.24128	2.1
39504	1.50738	2.1
39505	1.75702	2.1
39506	2.02312	2.1
39507	1.89289	2.1
39508	1.92264	2.1
39509	1.72994	2.1
39510	1.75702	2.1
39511	3.67966	2.1
39512	1.75702	2.1
39513	0.994291	2.1
39514	0.994291	2.1
39515	1.24128	2.1
39516	1.4964	2.1
39517	1.74339	2.1
39518	1.72993	2.1
39519	1.49641	2.1
39520	1.49641	2.1
39521	1.75702	2.1
39522	1.3939	2.1
39523	1.65452	2.1
39524	1.19964	2.1
39525	1.53296	2.1
39526	1.19964	2.1
39527	1.36861	2.1
39528	1.42624	2.1
39529	1.59521	2.1
39530	1.19964	2.1
39531	1.42624	2.1
39532	1.07156	2.1
39533	1.36226	2.1
39534	2.5619	2.1
39535	2.14058	0.903194
39536	1.93569	2.07364
39537	2.36221	1.69913
39538	2.5753	1.40725
39539	4.50279	1.93612
39540	2.14057	1.90668
39541	2.20831	2.04075
39542	2.64725	0.63022
39543	2.57529	1.85257
39544	4.78782	1.73937
39545	2.64725	1.05029
39546	2.57529	1.9426
39547	1.89226	1.15163
39548	1.89226	0.378626
39549	4.53951	1.63357
39550	2.0815	2.1
39551	1.86137	2.1
39552	1.86137	2.1
39553	1.86159	2.1

39554	2.0815	2.1
39555	2.10235	2.1
39556	1.33552	2.1
39557	2.32417	2.1
39558	1.18872	2.1
39559	1.93101	2.1
39560	3.26653	2.1
39561	1.89226	2.1
39562	1.89226	2.1
39563	1.89226	2.1
39564	1.89226	2.1
39565	1.89226	2.1
39566	1.89226	2.1
39567	0.955191	2.1
39568	1.03372	2.1
39569	0.946131	2.1
39570	0.946131	2.1
39571	0.930654	2.1
39572	0.930654	2.1
39573	1.04233	2.1
39574	1.05247	2.1
39575	0.955341	2.1
39576	0.946131	2.1
39577	1.02375	2.1
39578	1.13537	2.1
39579	1.1549	2.1
39580	1.93101	2.1
39581	1.91108	2.1
39582	1.52487	2.1
39583	1.3076	2.1
39584	1.06985	2.1
39585	1.89226	2.1
39586	1.89226	2.1
39587	1.89226	2.1
39588	1.89226	2.1
39589	1.89226	2.1
39590	1.89226	2.1
39591	1.58713	2.1
39592	2.6845	2.1
39593	1.58742	2.1
39594	2.74201	2.1
39595	4.27191	2.1
39596	2.68449	2.1
39597	1.89226	2.1
39598	2.74201	2.1
39599	1.89226	2.1
39600	4.57675	2.1
39601	1.57523	2.1
39602	2.74201	2.1
39603	2.8146	2.1
39604	2.8146	2.1
39605	1.58742	2.1
39606	1.58742	2.1
39607	0.946131	2.1
39608	0.946131	2.1
39609	1.371	2.1
39610	1.371	2.1
39611	0.946127	2.1

39612	0.946127	2.1
39613	1.37101	2.1
39614	1.4073	2.1
39615	0.94613	2.1
39616	1.4073	2.1
39617	1.371 2.1	
39618	0.946129	2.1
39619	1.44801	1.82591
39620	1.06821	1.96204
39621	2.00542	1.80371
39622	2.27703	1.9858
39623	3.34524	1.83454
39624	1.70301	0.197476
39625	1.89226	0.171298
39626	1.65809	0.18735
39627	1.89226	0.198653
39628	1.62758	0.156705
39629	1.85744	0.165231
39630	1.89226	0.13638
39631	1.89226	0.188121
39632	1.89226	0.147046
39633	1.89226	0.128594
39634	3.78452	0.129053
39635	1.89226	0.189925
39636	1.89226	0.138002
39637	1.89226	0.11948
39638	1.89226	0.161657
39639	3.78452	0.157204
39640	0.851541	0.184979
39641	0.851541	0.184894
39642	0.91193	0.169799
39643	0.824837	0.173196
39644	0.854559	0.191395
39645	0.851534	0.159747
39646	0.81851	0.168342
39647	0.717219	0.188376
39648	0.871743	0.162221
39649	0.837985	0.159738
39650	0.82902	0.174364
39651	0.82902	0.183972
39652	1.89226	0.184876
39653	1.92659	0.0759536
39654	2.03235	0.0822724
39655	1.89226	0.244127
39656	2.03235	0.0712709
39657	1.89226	0.189804
39658	0.585557	0.188413
39659	0.585557	0.167541
39660	1.22612	0.138794
39661	1.09436	0.145512
39662	0.851537	0.207045
39663	0.526987	0.137147
39664	0.983879	0.0901351
39665	1.04072	0.264071
39666	0.585526	0.0726156
39667	0.792325	0.0723238
39668	0.946129	0.195398
39669	0.946129	0.178342

39670	1.68152	2.1
39671	1.81445	2.1
39672	1.68152	2.1
39673	1.86792	2.1
39674	1.65888	2.1
39675	1.84529	2.1
39676	1.81445	2.1
39677	1.57885	2.1
39678	1.65889	2.1
39679	1.48594	2.1
39680	3.23773	2.1
39681	1.68154	2.1
39682	1.65891	2.1
39683	1.53296	2.1
39684	1.56299	2.1
39685	3.24453	2.1
39686	1.56299	2.1
39687	1.48596	2.1
39688	1.56299	2.1
39689	1.65891	2.1
39690	1.46568	2.1
39691	1.5616	2.1
39692	2.68785	2.1
39693	2.08831	2.1
39694	2.66919	2.1
39695	2.1024	2.1
39696	4.79025	2.1
39697	2.08832	2.1
39698	1.15478	2.1
39699	1.15478	2.1
39700	1.09062	2.1
39701	2.10241	2.1
39702	2.03825	2.1
39703	2.65348	2.1
39704	2.1024	2.1
39705	2.12313	2.1
39706	2.12313	2.1
39707	2.66919	2.1
39708	2.66919	2.1
39709	0.813169	2.1
39710	0.813169	2.1
39711	0.825788	2.1
39712	0.825788	2.1
39713	0.612902	2.1
39714	0.577387	2.1
39715	1.0512	2.1
39716	1.06157	2.1
39717	1.06157	2.1
39718	0.612902	2.1
39719	0.577387	2.1
39720	1.0512	2.1
39721	1.27227	2.1
39722	1.91724	2.1
39723	1.32591	2.1
39724	1.99837	2.1
39725	3.24315	2.1
39726	1.89223	2.1
39727	1.89223	2.1

39728	1.89223	2.1
39729	1.89223	2.1
39730	3.78446	2.1
39731	1.89224	2.1
39732	1.89224	2.08053
39733	1.89224	2.1
39734	1.89224	2.1
39735	3.78447	2.1
39736	1.91724	2.1
39737	2.321	2.1
39738	1.99838	2.1
39739	2.4149	2.1
39740	4.33214	2.1
39741	1.34817	2.1
39742	1.9984	2.1
39743	2.06238	2.1
39744	2.06238	2.1
39745	1.32593	2.1
39746	1.32593	2.1
39747	1.89226	2.1
39748	1.89226	2.1
39749	1.89226	2.1
39750	1.89226	2.1
39751	1.89226	2.1
39752	1.89226	2.1
39753	1.89226	2.1
39754	1.89226	2.1
39755	1.89226	2.1
39756	1.89226	2.08305
39757	3.78452	2.1
39758	1.9984	2.1
39759	2.41494	2.1
39760	2.06239	2.1
39761	2.5194	2.1
39762	4.47732	2.1
39763	1.19901	1.62971
39764	1.16661	2.1
39765	1.56859	2.1
39766	1.61029	1.73661
39767	2.7676	2.09779
39768	1.56859	2.1
39769	1.88637	0.95833
39770	1.61029	0.96427
39771	1.61029	2.08159
39772	1.856	1.01941
39773	1.856	2.08505
39774	1.856	2.04272
39775	1.57916	2.1
39776	1.56859	2.08984
39777	1.56859	2.1
39778	1.57916	2.1
39779	1.23475	2.1
39780	0.890924	0.471254
39781	1.07608	0.412689
39782	0.72877	0.844125
39783	0.910106	0.715395
39784	1.00196	1.02763
39785	1.09519	0.933749

39786	0.839967	0.51922
39787	0.719043	0.375551
39788	0.893859	0.530083
39789	0.942408	1.19628
39790	0.873497	0.702259
39791	0.873497	1.59046
39792	1.53046	0.348093
39793	1.89994	0.54314
39794	1.049	0.379593
39795	1.049	0.297781
39796	1.52512	0.253073
39797	1.89994	0.738471
39798	1.65573	1.67541
39799	1.89226	0.335223
39800	1.89226	0.168661
39801	2.73329	1.06498
39802	3.548	1.57259
39803	1.65573	0.201222
39804	2.42671	0.693305
39805	2.73329	0.205774
39806	1.89226	0.195732
39807	2.73329	0.181274
39808	1.89226	0.23669
39809	0.941078	0.395148
39810	1.01599	0.723426
39811	0.818936	0.447442
39812	0.651554	0.78272
39813	0.904729	0.726131
39814	0.665756	0.661487
39815	0.843423	1.18334
39816	0.918649	0.742232
39817	0.855806	0.772565
39818	0.814384	0.231964
39819	0.844437	1.01396
39820	0.844437	0.93148
39821	2.22558	2.04243
39822	1.95785	0.453118
39823	2.07065	1.00371
39824	1.89142	1.16403
39825	2.07065	1.81875
39826	1.89142	1.85935
39827	0.640813	0.869474
39828	0.951654	0.744109
39829	0.797442	0.261562
39830	0.535364	0.728767
39831	0.525269	0.821871
39832	0.620874	0.401913
39833	0.916953	0.482522
39834	0.969374	0.615358
39835	0.788925	0.860717
39836	0.69596	0.304586
39837	0.743154	0.852747
39838	0.743154	0.914915
39839	1.89226	2.01335
39840	1.89226	1.7024
39841	1.89226	1.09769
39842	1.89226	2.06713
39843	3.78452	1.66794

39844	1.32541	1.71112
39845	1.13722	1.29489
39846	0.91103	2.1
39847	0.881123	2.03187
39848	1.03363	2.08137
39849	1.03364	1.90458
39850	1.1505	1.73407
39851	1.1505	2.03678
39852	1.32539	2.01455
39853	1.07662	2.06152
39854	0.881121	1.90945
39855	0.962303	1.91032
39856	1.89226	1.77675
39857	1.89226	2.1
39858	1.89226	2.09375
39859	1.89226	1.88242
39860	3.78453	2.06532
39861	1.89226	1.01633
39862	1.89226	2.02119
39863	1.89226	1.87258
39864	1.89226	1.45112
39865	1.89226	1.86792
39866	1.89226	1.55658
39867	1.27606	1.94077
39868	1.07664	1.80374
39869	0.905895	2.1
39870	0.696099	2.09987
39871	0.984902	2.09627
39872	0.984905	1.98564
39873	1.01878	2.0946
39874	1.01878	1.60422
39875	1.27604	2.04972
39876	1.06044	2.03655
39877	1.13192	2.04901
39878	0.696111	2.04746
39879	1.89226	1.97169
39880	1.89226	0.669486
39881	1.89226	0.251864
39882	1.89226	2.00142
39883	1.89226	0.684576
39884	1.89226	1.87949
39885	1.52615	2.08359
39886	1.01294	0.93327
39887	1.01294	2.09467
39888	1.18549	0.353714
39889	1.38963	1.85034
39890	1.56218	0.714077
39891	1.89226	1.01812
39892	2.40801	1.88164
39893	2.58101	2.06315
39894	2.58101	1.08638
39895	1.89226	2.07994
39896	1.89226	0.414268
39897	2.24071	2.1
39898	2.79317	2.1
39899	1.99136	2.1
39900	2.6167	2.1
39901	4.85741	2.1

39902	2.79317	2.1
39903	1.89226	2.0826
39904	1.89226	2.1
39905	1.89226	2.09783
39906	2.6167	2.1
39907	2.6167	2.0824
39908	1.08354	2.1
39909	1.08354	2.1
39910	1.10222	2.1
39911	1.10222	2.1
39912	1.2275	2.1
39913	1.30835	2.1
39914	0.995678	2.1
39915	0.877412	2.1
39916	0.877411	2.1
39917	1.2275	2.1
39918	0.995678	2.1
39919	1.30835	2.1
39920	1.05793	0.348571
39921	1.46441	0.37043
39922	1.0562	0.265646
39923	1.0562	0.359133
39924	1.41787	0.327827
39925	1.41787	0.345712
39926	1.41787	0.436401
39927	1.52909	1.30648
39928	1.46441	0.430498
39929	1.46441	1.12327
39930	1.43063	0.491358
39931	1.43063	1.0861
39932	1.89226	1.3607
39933	1.89226	0.508635
39934	1.89226	0.487922
39935	1.89226	1.77479
39936	3.78453	0.990174
39937	1.89227	0.343161
39938	1.89227	0.49722
39939	1.89227	0.276132
39940	1.89227	0.529702
39941	1.89227	0.297655
39942	1.89227	0.41143
39943	1.00763	0.181189
39944	1.41787	0.522803
39945	1.05793	0.179199
39946	1.05793	0.522732
39947	1.37265	0.193669
39948	1.37265	0.468371
39949	1.37265	0.463007
39950	1.43063	1.38424
39951	1.41787	0.496996
39952	1.41787	1.24997
39953	1.38247	0.568819
39954	1.38247	1.22521
39955	0.946132	1.39858
39956	0.946132	1.62152
39957	0.946132	0.502375
39958	0.946132	0.637685
39959	0.946131	0.622618

39960	0.946131	1.62169
39961	0.946132	1.56763
39962	0.946132	0.700783
39963	0.946132	1.12242
39964	0.946132	1.14472
39965	0.946132	1.20725
39966	0.946132	1.1807
39967	1.89226	0.216425
39968	1.89226	0.711107
39969	1.89226	0.219836
39970	1.89226	0.673024
39971	1.89226	0.211144
39972	1.89226	0.550445
39973	1.19645	0.940411
39974	1.89183	0.235127
39975	1.91106	0.506234
39976	1.35306	0.277646
39977	1.75446	0.431482
39978	1.19645	0.252582
39979	2.37576	2.03277
39980	1.90139	2.1
39981	2.1845	2.1
39982	1.86568	2.1
39983	4.24145	2.1
39984	2.29435	2.1
39985	1.40602	2.1
39986	1.40602	2.09608
39987	1.55351	2.1
39988	1.86568	2.1
39989	2.01317	2.1
39990	2.18452	2.1
39991	1.8657	1.46526
39992	2.01399	1.95995
39993	1.70141	2.1
39994	3.87969	2.0791
39995	0.774999	2.1
39996	0.774999	2.08826
39997	0.778721	2.1
39998	0.778721	2.1
39999	0.628348	2.1
40000	0.703016	2.1
40001	0.932848	2.1
40002	0.850704	2.1
40003	0.850704	2.1
40004	0.628348	2.1
40005	0.703015	2.1
40006	0.932848	2.1
40007	1.65243	1.01271
40008	1.34412	0.735993
40009	1.45569	1.88842
40010	1.1315	1.8692
40011	2.78393	0.867225
40012	1.48901	2.1
40013	1.805	2.1
40014	1.48901	2.1
40015	1.71717	2.1
40016	1.60635	2.1
40017	1.83451	2.1

40018	1.805	2.1
40019	1.65243	0.960733
40020	1.60635	2.1
40021	1.45569	1.65602
40022	3.25879	2.0288
40023	1.27875	2.1
40024	1.60635	2.1
40025	1.27875	2.1
40026	1.48901	2.1
40027	1.40014	2.1
40028	1.6104	2.1
40029	0.715199	2.03834
40030	0.715199	2.1
40031	0.712714	2.1
40032	0.712714	1.27495
40033	0.727843	1.07886
40034	0.619713	1.27965
40035	0.803177	2.1
40036	0.70007	2.1
40037	0.700069	2.1
40038	0.619713	2.09458
40039	0.803177	2.04765
40040	0.727844	2.06299
40041	1.14357	2.06977
40042	2.3506	1.98895
40043	1.14357	2.04995
40044	2.68773	1.94242
40045	1.57829	2.04085
40046	2.68773	1.93205
40047	2.00551	2.1
40048	2.65811	2.06024
40049	2.07203	2.1
40050	2.72117	1.03479
40051	4.72667	2.1
40052	0.946131	1.43556
40053	0.946131	2.1
40054	1.32905	2.1
40055	1.32905	0.843317
40056	0.94613	1.1637
40057	0.94613	1.98545
40058	1.32905	1.88331
40059	1.36058	1.69554
40060	1.36058	0.273938
40061	0.946131	0.174828
40062	1.32905	1.63826
40063	0.946131	1.78789
40064	1.21649	1.19708
40065	1.21649	2.1
40066	1.21459	2.1
40067	1.21459	2.1
40068	1.39508	1.74522
40069	1.36057	1.60929
40070	1.03601	2.1
40071	1.07438	2.1
40072	1.07439	2.08112
40073	1.39509	2.09192
40074	1.036	2.09434
40075	1.36056	2.09355

40076	2.79015	0.72167
40077	2.72114	2.1
40078	1.89225	0.997241
40079	1.89225	2.1
40080	1.89225	2.0179
40081	2.72114	1.86841
40082	0.876258	2.1
40083	0.892889	2.1
40084	1.34352	2.1
40085	1.27985	2.1
40086	1.07462	2.1
40087	1.07462	2.1
40088	1.09812	2.1
40089	1.09812	2.1
40090	0.892888	2.1
40091	1.34352	2.1
40092	0.876256	2.1
40093	1.27985	2.1
40094	1.39725	2.03752
40095	1.30029	1.20544
40096	1.31415	1.42712
40097	1.41336	2.01356
40098	2.71366	1.84178
40099	1.89226	1.27298
40100	1.89226	1.97602
40101	1.89226	2.02994
40102	1.89226	1.24212
40103	3.78453	1.80745
40104	0.946133	1.31616
40105	0.946133	1.21348
40106	1.38709	0.563996
40107	1.39437	0.662957
40108	0.946132	0.577694
40109	0.946132	1.20127
40110	1.39437	1.3156
40111	1.39437	0.63156
40112	0.946132	0.91863
40113	1.39437	0.906004
40114	1.38709	0.912979
40115	0.946132	0.918371
40116	0.706681	2.06782
40117	0.711908	2.04174
40118	0.657077	1.31415
40119	0.661003	1.56073
40120	0.684188	1.33915
40121	0.684188	2.04138
40122	0.683496	2.07842
40123	0.683496	1.46021
40124	0.711908	1.90355
40125	0.661003	1.89311
40126	0.657076	1.85623
40127	0.706681	1.86464
40128	0.946132	2.05491
40129	0.946132	2.0055
40130	0.946132	1.45324
40131	0.946132	1.16916
40132	0.946132	1.18422
40133	0.946132	2.01492

40134	0.946133	1.53501
40135	0.946133	2.06138
40136	0.946133	1.80097
40137	0.946133	1.85729
40138	0.946132	1.80574
40139	0.946132	1.83738
40140	2.79029	0.616816
40141	1.89227	1.462
40142	2.78873	0.836482
40143	2.78873	1.16214
40144	1.89383	0.774933
40145	1.89227	1.14506
40146	1.43669	2.1
40147	2.11984	2.1
40148	2.24481	2.1
40149	2.24481	2.1
40150	1.20311	2.1
40151	1.20311	2.1
40152	0.49703	2.1
40153	0.601553	2.1
40154	1.05992	2.1
40155	0.991694	2.1
40156	0.787558	2.1
40157	0.787558	2.1
40158	0.805689	2.1
40159	0.805689	2.1
40160	0.991694	2.1
40161	0.49703	2.1
40162	1.05992	2.1
40163	0.601553	2.1
40164	2.45905	2.1
40165	1.89226	2.1
40166	1.89226	2.1
40167	1.89226	2.1
40168	2.47395	2.1
40169	2.47395	2.1
40170	1.89226	2.1
40171	1.89226	2.09758
40172	1.89226	2.1
40173	1.89226	2.03768
40174	3.78452	2.1
40175	1.38427	2.1
40176	2.31203	1.99444
40177	2.28743	2.01474
40178	2.28743	2.1
40179	1.63384	2.00925
40180	1.63384	2.1
40181	2.42723	2.1
40182	1.89226	2.1
40183	1.89226	2.1
40184	1.89226	2.1
40185	2.45905	2.1
40186	2.45905	2.1
40187	0.946131	1.88126
40188	0.946131	2.1
40189	0.946131	2.1
40190	0.946131	2.1
40191	0.94613	2.06828

40192	0.94613	2.07206
40193	0.946132	2.1
40194	0.946132	2.1
40195	0.946131	2.1
40196	0.946131	2.1
40197	0.946131	2.1
40198	0.946131	2.1
40199	0.989441	2.1
40200	0.989441	2.1
40201	0.993918	2.1
40202	0.993918	2.1
40203	1.16644	2.1
40204	1.15601	2.07823
40205	0.818308	2.1
40206	0.816922	2.1
40207	0.818307	2.1
40208	1.16644	2.1
40209	1.15601	2.1
40210	0.816922	2.1
40211	0.996778	0.0698591
40212	2.44375	0.0698591
40213	0.996778	0.0698591
40214	1.92547	0.0698591
40215	1.56091	0.0698591
40216	2.4896	0.0698591
40217	1.56091	0.0698591
40218	2.44375	0.0698591
40219	1.90456	0.0698591
40220	1.90456	0.0698591
40221	2.81853	0.0698591
40222	2.47487	0.0698591
40223	1.32143	2.0624
40224	1.43294	2.1
40225	1.37444	0.605048
40226	1.51691	2.09665
40227	2.80738	2.02141
40228	1.89226	1.7335
40229	1.89226	2.1
40230	1.89226	0.81813
40231	1.89226	1.90076
40232	3.78453	1.26817
40233	1.89226	2.08191
40234	1.89226	2.1
40235	1.89226	1.56073
40236	1.89226	2.1
40237	3.78453	2.1
40238	1.43294	2.07621
40239	1.3268	2.1
40240	1.51691	2.02698
40241	1.44091	2.1
40242	2.87385	2.1
40243	1.37444	2.04808
40244	1.51691	2.1
40245	1.4714	0.658014
40246	1.65434	1.95226
40247	3.02878	1.78453
40248	1.89226	2.04616
40249	1.89226	2.09478

40250	1.89226	0.892253
40251	1.89226	1.98413
40252	3.78452	1.75374
40253	1.89226	1.90227
40254	1.89226	2.1
40255	1.89226	1.64571
40256	1.89226	2.1
40257	3.78453	2.1
40258	1.51691	2.09571
40259	1.44091	2.1
40260	1.65434	1.65938
40261	1.60647	2.1
40262	3.09526	2.1
40263	1.05013	2.1
40264	1.05013	2.1
40265	0.94613	2.1
40266	0.94613	2.1
40267	1.04959	2.1
40268	1.05014	2.1
40269	0.946129	2.1
40270	0.946129	2.1
40271	0.946131	2.1
40272	1.04958	2.1
40273	0.946131	2.1
40274	1.05013	2.1
40275	1.04959	2.1
40276	1.04959	2.1
40277	0.946131	2.1
40278	0.946131	2.1
40279	1.04765	2.1
40280	1.04959	2.1
40281	0.94613	2.1
40282	0.94613	2.1
40283	0.946131	2.1
40284	1.04765	2.1
40285	0.946131	2.1
40286	1.04959	2.1
40287	1.85744	2.1
40288	1.89226	2.1
40289	1.89226	2.1
40290	1.89226	2.1
40291	1.94839	2.1
40292	1.94839	2.1
40293	0.97419	2.1
40294	0.822054	2.1
40295	0.946123	2.1
40296	0.946123	2.1
40297	1.04202	2.1
40298	0.84898	2.1
40299	0.933307	2.1
40300	0.933307	2.1
40301	0.933285	2.1
40302	1.05018	2.1
40303	0.851528	2.1
40304	0.876787	2.1
40305	1.87528	0.138579
40306	1.21137	0.0969088
40307	1.57893	0.0979531

40308	1.21137	0.161008
40309	1.81182	0.10168
40310	1.44426	0.155039
40311	0.993833	0.0761604
40312	1.21137	0.116596
40313	1.57893	0.10243
40314	1.31774	0.0748703
40315	2.5291	0.0795328
40316	1.87528	0.112656
40317	1.57893	0.0830647
40318	1.90728	0.0792376
40319	2.29851	0.124599
40320	3.87744	0.109872
40321	1.90728	0.0735186
40322	1.31774	0.0739332
40323	1.56339	0.0698591
40324	1.31774	0.0874568
40325	1.82458	0.0698619
40326	1.57893	0.0954676
40327	1.89226	1.07001
40328	1.89226	1.24175
40329	1.89226	1.12638
40330	1.89226	1.35855
40331	1.89226	1.1241
40332	1.89226	1.35505
40333	1.89226	1.44783
40334	1.89226	1.4391
40335	1.89226	1.66246
40336	1.89226	1.40839
40337	3.78452	1.60451
40338	1.89226	1.3271
40339	2.17251	1.28593
40340	2.45575	1.23282
40341	2.45575	1.21287
40342	1.89226	1.23789
40343	1.89226	1.22584
40344	1.08625	1.24944
40345	1.22788	1.17976
40346	0.54447	1.08336
40347	0.794861	1.17823
40348	0.967729	1.3493
40349	0.967729	1.24544
40350	0.913386	1.15346
40351	0.913386	1.03692
40352	1.08625	1.33638
40353	0.544469	1.04168
40354	0.794862	1.30662
40355	1.22788	1.22792
40356	0.946128	1.34376
40357	0.946128	1.57834
40358	0.946132	1.30553
40359	0.946132	1.40307
40360	0.94613	1.3022
40361	0.94613	1.36261
40362	0.94613	1.57311
40363	0.94613	1.29247
40364	0.94613	1.24211
40365	0.94613	1.24984

40366	0.94613	1.22664
40367	0.94613	1.35997
40368	0.946127	1.0431
40369	0.946127	1.17172
40370	1.08625	1.1135
40371	0.958779	1.1278
40372	1.08625	1.02621
40373	1.08625	1.08867
40374	0.946128	1.1238
40375	0.946128	1.17677
40376	0.946129	1.13233
40377	0.958777	1.14459
40378	1.08625	1.15778
40379	0.946129	1.16496
40380	2.16338	0.0698591
40381	1.94002	0.0698591
40382	1.98836	0.0698591
40383	2.60614	0.0698591
40384	1.5456	0.0698591
40385	2.16338	0.0698591
40386	1.19952	0.0698591
40387	2.34061	0.0698591
40388	1.21031	0.0698591
40389	2.1711	0.0698591
40390	3.54013	0.0698591
40391	1.13624	2.1
40392	1.70302	2.1
40393	1.8267	2.1
40394	1.11545	2.1
40395	2.85455	2.1
40396	1.82671	2.1
40397	1.91767	2.1
40398	1.81563	2.1
40399	1.91767	2.1
40400	1.71362	2.1
40401	1.83277	2.1
40402	1.66064	2.1
40403	0.996776	2.1
40404	0.996776	2.1
40405	1.11545	2.1
40406	1.54278	2.1
40407	1.66145	2.1
40408	1.66064	2.1
40409	1.7457	2.1
40410	1.81563	2.1
40411	1.81563	2.1
40412	1.54278	2.1
40413	1.54278	2.1
40414	2.71672	2.1
40415	2.70402	2.1
40416	2.64996	2.1
40417	2.69443	2.1
40418	5.39845	2.1
40419	2.71672	2.1
40420	2.54198	2.1
40421	2.54019	2.1
40422	2.69443	2.1
40423	5.23641	2.1

40424	2.64996	2.1
40425	2.69443	2.1
40426	2.68213	2.1
40427	2.61243	2.1
40428	5.30686	2.1
40429	2.54019	2.1
40430	2.69443	2.1
40431	2.68213	2.1
40432	2.5392	2.1
40433	5.23363	2.1
40434	1.89226	2.1
40435	2.34725	2.1
40436	2.22414	2.1
40437	2.34725	2.1
40438	1.76914	2.1
40439	1.89226	2.1
40440	0.946131	2.1
40441	0.946131	2.1
40442	1.11207	2.1
40443	1.03798	2.1
40444	0.94613	2.1
40445	0.94613	2.1
40446	1.03798	2.1
40447	1.03798	2.1
40448	0.946131	2.1
40449	1.03798	2.1
40450	0.946131	2.1
40451	1.11207	2.1
40452	1.89226	0.196388
40453	1.89226	0.17865
40454	1.89226	0.211059
40455	1.89226	0.150306
40456	1.89226	0.238244
40457	1.89226	0.175083
40458	1.89226	0.218757
40459	1.89226	0.22284
40460	1.89226	0.263616
40461	1.89226	0.173593
40462	1.89226	0.28919
40463	1.89226	0.197552
40464	1.89226	0.122683
40465	1.89226	0.122717
40466	1.89226	0.147827
40467	1.89226	0.105926
40468	1.89226	0.155438
40469	1.89226	0.115147
40470	1.44085	2.09858
40471	1.31825	1.46383
40472	1.31923	1.8845
40473	1.31825	2.07017
40474	1.43311	1.77691
40475	1.43213	2.03264
40476	1.89226	1.51361
40477	1.89226	2.03786
40478	1.89226	1.49142
40479	1.89226	2.04875
40480	1.89226	1.30948
40481	1.89226	2.05975

40482	0.946133	1.5707
40483	0.946133	1.24486
40484	1.38969	0.692649
40485	1.39587	0.920421
40486	0.946132	0.706564
40487	0.946132	1.20521
40488	1.39587	1.58998
40489	1.39587	0.82949
40490	0.946132	0.968978
40491	1.39587	0.962206
40492	1.38969	0.977196
40493	0.946132	1.00901
40494	1.44085	2.1
40495	1.31923	1.37158
40496	1.33026	1.8616
40497	1.4617	2.07881
40498	2.78093	2.02672
40499	1.89226	1.41259
40500	1.89226	2.02204
40501	1.89226	1.48024
40502	1.89226	2.06535
40503	1.89226	1.32044
40504	1.89226	2.08145
40505	0.946133	1.46576
40506	0.946133	1.21832
40507	1.39587	0.862297
40508	1.40724	0.63493
40509	0.946132	0.663054
40510	0.946132	1.19603
40511	1.40724	1.49414
40512	1.40724	0.837673
40513	0.946132	0.988353
40514	1.40724	0.984528
40515	0.946133	1.14212
40516	1.39587	1.04151
40517	1.15818	1.81733
40518	1.81846	1.47784
40519	1.52782	0.775161
40520	1.52782	2.06198
40521	1.28095	0.506056
40522	1.28095	1.81798
40523	2.56709	2.1
40524	1.4349	1.61709
40525	1.4349	2.09425
40526	1.75471	2.1
40527	2.29909	2.09048
40528	2.61889	2.1
40529	2.5671	2.1
40530	2.82858	2.1
40531	2.57368	2.1
40532	2.29909	2.1
40533	5.12767	2.1
40534	2.29909	2.1
40535	1.09148	1.19504
40536	1.09148	2.09291
40537	1.4349	2.0268
40538	1.99776	2.06113
40539	2.34119	2.08283

40540	2.57368	2.1
40541	1.99777	2.08733
40542	1.99777	2.1
40543	2.29909	2.1
40544	2.36071	2.1
40545	2.66203	2.1
40546	1.89226	1.16033
40547	1.89226	1.54218
40548	1.89226	1.52998
40549	1.89226	1.12139
40550	3.78453	1.15304
40551	0.946128	1.7882
40552	0.946128	1.95136
40553	0.946133	1.53623
40554	0.946133	1.75022
40555	0.946131	1.73902
40556	0.946131	1.87604
40557	0.946131	1.89888
40558	0.946131	1.77115
40559	0.94613	1.92564
40560	0.94613	1.78527
40561	0.946131	1.67791
40562	0.946131	1.84471
40563	1.89226	1.57259
40564	1.89226	1.61593
40565	1.89226	1.11015
40566	1.89226	1.73416
40567	3.78453	1.69529
40568	1.89226	1.7112
40569	1.89226	1.48212
40570	1.89226	1.23648
40571	1.89226	1.63278
40572	3.78453	1.65202
40573	1.89226	1.48837
40574	2.61682	1.90714
40575	1.89226	1.84604
40576	1.89226	1.89444
40577	1.89226	1.37054
40578	1.89226	1.86027
40579	1.89226	0.0897623
40580	1.89226	0.0698591
40581	1.89226	0.0714167
40582	1.89226	0.0754742
40583	3.78452	0.0935018
40584	1.89226	0.111879
40585	1.89226	0.0841641
40586	1.89226	0.0759191
40587	1.89226	0.0950383
40588	1.89226	0.105858
40589	1.89226	0.103255
40590	1.89227	0.0699251
40591	1.89227	0.0713208
40592	1.89227	0.0785674
40593	1.89227	0.0791168
40594	1.89227	0.0698591
40595	1.89227	0.0698591
40596	0.80447	0.105594
40597	0.534241	0.117032

40598	0.995175	0.153933
40599	0.92204	0.0763488
40600	0.922042	0.0769439
40601	0.534241	0.0779771
40602	0.804468	0.129082
40603	0.995176	0.0699733
40604	0.71543	0.152147
40605	0.71543	0.0705574
40606	0.813988	0.074035
40607	0.813988	0.0783961
40608	0.922045	0.0698591
40609	0.942899	0.0712146
40610	0.946132	0.0698591
40611	0.946132	0.0698591
40612	1.05169	0.0698591
40613	0.936661	0.0698831
40614	0.922045	0.0698591
40615	0.946133	0.0698591
40616	1.01665	0.0698591
40617	1.13008	0.0698591
40618	0.851529	0.0698591
40619	0.851529	0.0698591
40620	1.41908	2.1
40621	1.77723	2.1
40622	1.90485	2.1
40623	1.90485	2.1
40624	1.30237	2.1
40625	1.30237	2.1
40626	1.77723	2.1
40627	2.0055	2.1
40628	1.90485	2.1
40629	2.17158	2.1
40630	3.91035	2.1
40631	0.850906	2.1
40632	0.850906	2.1
40633	0.85668	2.1
40634	0.85668	2.1
40635	0.998045	2.1
40636	0.952425	2.1
40637	0.74367	2.1
40638	0.709541	2.1
40639	0.74367	2.1
40640	0.998045	2.1
40641	0.709541	2.1
40642	0.952425	2.1
40643	1.90485	2.1
40644	2.28563	2.1
40645	2.26282	2.1
40646	2.17158	2.1
40647	1.99609	2.1
40648	1.90485	2.1
40649	2.00098	0.133226
40650	1.90462	0.0928387
40651	1.60057	0.105016
40652	1.79224	0.142309
40653	3.69686	0.110185
40654	2.65654	0.140014
40655	1.79224	0.0959997

40656	2.22332	0.0983749
40657	2.22332	0.122342
40658	2.24163	0.115852
40659	2.24163	0.123007
40660	1.89226	0.12911
40661	1.89226	0.115851
40662	1.89226	0.096045
40663	1.89226	0.13294
40664	3.78452	0.126353
40665	1.89226	0.0916988
40666	1.89226	0.175466
40667	1.89226	0.108677
40668	1.89226	0.147011
40669	1.89226	0.109038
40670	1.89226	0.146217
40671	2.24163	0.167311
40672	1.03645	0.142231
40673	1.79224	0.151979
40674	1.79224	0.150166
40675	1.54283	0.172337
40676	1.54283	0.162459
40677	0.946132	0.133917
40678	0.946132	0.131035
40679	0.946129	0.13849
40680	0.946129	0.131906
40681	0.94613	0.115448
40682	0.94613	0.133398
40683	0.946131	0.149015
40684	0.946131	0.152694
40685	0.94613	0.131868
40686	0.94613	0.164085
40687	0.946131	0.131077
40688	0.946131	0.11023
40689	1.89226	2.1
40690	1.89226	2.1
40691	1.89226	2.1
40692	1.89226	2.1
40693	3.78453	2.1
40694	1.52441	2.1
40695	1.30347	2.1
40696	1.52441	2.1
40697	1.58401	2.1
40698	1.2627	2.1
40699	1.3223	2.1
40700	1.89226	2.1
40701	2.72787	2.1
40702	2.72787	2.1
40703	2.74762	2.1
40704	1.89226	2.1
40705	1.91202	2.1
40706	1.89227	2.1
40707	1.89227	2.1
40708	1.89227	2.1
40709	1.89227	2.1
40710	3.78453	2.1
40711	0.56414	2.1
40712	0.561557	2.1
40713	0.527284	2.1

40714	0.541143	2.1
40715	0.527276	2.1
40716	0.631685	2.1
40717	1.52441	2.1
40718	1.00232	2.1
40719	0.784495	2.1
40720	0.842304	2.1
40721	0.721569	2.1
40722	1.36393	2.1
40723	1.36393	2.1
40724	0.946132	2.1
40725	0.946132	2.1
40726	1.36393	2.1
40727	1.3594	2.1
40728	0.946132	2.1
40729	0.946132	2.1
40730	0.946107	2.1
40731	1.35936	2.1
40732	1.36397	2.1
40733	0.946158	2.1
40734	1.89226	2.1
40735	1.89226	2.1
40736	1.89226	2.1
40737	1.89226	2.1
40738	3.78453	2.1
40739	2.20802	2.1
40740	1.05677	2.1
40741	2.30479	2.1
40742	0.978699	2.1
40743	3.18672	2.1
40744	0.94613	2.1
40745	0.94613	2.1
40746	0.669866	2.1
40747	0.669866	2.1
40748	0.681588	2.1
40749	0.669865	2.1
40750	0.946133	2.1
40751	0.946133	2.1
40752	0.94613	2.1
40753	0.681589	2.1
40754	0.946131	2.1
40755	0.669867	2.1
40756	2.73514	2.1
40757	1.78301	2.1
40758	1.90618	2.1
40759	2.83464	2.1
40760	4.64132	2.1
40761	2.73514	2.1
40762	1.89226	2.1
40763	1.89226	2.1
40764	2.83464	2.1
40765	4.6274	2.1
40766	2.03915	2.1
40767	2.26616	2.1
40768	2.23549	2.1
40769	2.01704	2.1
40770	4.2832	2.1
40771	2.03916	2.1

40772	1.55766	2.1
40773	1.53728	2.1
40774	2.01704	2.1
40775	3.5747	2.1
40776	2.23551	2.1
40777	2.01705	2.1
40778	1.99823	2.1
40779	2.21189	2.1
40780	4.22894	2.1
40781	1.51788	2.1
40782	2.01705	2.1
40783	1.53729	2.1
40784	1.53729	2.1
40785	1.99823	2.1
40786	1.99823	2.1
40787	2.588	2.1
40788	1.45581	2.1
40789	1.45581	2.1
40790	1.44207	2.1
40791	2.54949	2.1
40792	2.53574	2.1
40793	0.98641	2.1
40794	0.98641	2.1
40795	1.00371	2.1
40796	1.00371	2.1
40797	0.749992	2.1
40798	0.727904	2.1
40799	1.26221	2.1
40800	1.27474	2.1
40801	1.26221	2.1
40802	0.749993	2.1
40803	0.727904	2.1
40804	1.27474	2.1
40805	1.23105	0.704919
40806	1.35435	1.68118
40807	1.89226	1.36064
40808	1.89226	0.929435
40809	1.89226	1.41924
40810	1.7043	1.00125
40811	1.89226	1.85321
40812	1.89226	1.38261
40813	1.89226	0.933784
40814	1.89226	1.84665
40815	3.78453	1.69797
40816	1.89226	0.757103
40817	1.89226	1.83696
40818	1.89226	1.31459
40819	1.89226	1.29682
40820	1.89226	1.49803
40821	1.89226	1.46705
40822	0.946131	0.712813
40823	1.40945	0.644044
40824	0.94613	0.926153
40825	0.94613	1.52548
40826	0.946129	1.54007
40827	0.946129	0.999887
40828	0.946132	1.48136
40829	1.40945	0.59846

40830	0.94613	0.957687
40831	0.94613	1.04547
40832	0.946131	0.949633
40833	0.94613	0.716201
40834	0.946133	0.906849
40835	0.946133	0.874492
40836	0.946132	1.51701
40837	0.946132	1.76073
40838	0.946132	1.74981
40839	0.946132	1.13565
40840	0.946133	1.42354
40841	0.946133	0.884117
40842	0.946132	1.3517
40843	0.946132	1.40742
40844	0.946133	0.954899
40845	0.946133	1.1822
40846	1.89226	1.25389
40847	1.89226	1.90575
40848	1.89226	1.70444
40849	1.89226	1.55116
40850	1.89226	1.82061
40851	1.89226	1.69258
40852	1.89226	2.1
40853	1.89226	2.1
40854	1.89226	2.1
40855	1.89226	2.1
40856	1.89226	2.1
40857	1.89226	2.1
40858	1.89226	2.1
40859	1.89226	2.1
40860	1.89226	2.1
40861	1.89226	2.1
40862	3.78453	2.1
40863	1.66647	2.1
40864	1.15399	2.1
40865	1.25443	2.1
40866	1.26599	2.1
40867	1.58697	2.1
40868	1.66647	2.1
40869	1.66647	2.1
40870	1.47446	2.1
40871	1.55433	2.1
40872	1.74241	2.1
40873	3.2208	2.1
40874	1.89227	2.1
40875	1.89227	2.1
40876	1.89227	2.1
40877	1.89227	2.1
40878	3.78453	2.1
40879	0.946132	2.1
40880	0.946132	2.1
40881	0.946132	2.1
40882	0.946132	2.1
40883	0.946132	2.1
40884	0.946132	2.1
40885	0.946133	2.1
40886	0.946133	2.1
40887	0.946132	2.1

40888	0.946132	2.1
40889	0.946133	2.1
40890	0.946133	2.1
40891	0.625067	2.1
40892	0.61907	2.1
40893	0.896913	2.1
40894	0.946199	2.1
40895	0.806287	2.1
40896	0.677685	2.1
40897	0.750657	2.1
40898	0.750657	2.1
40899	0.646004	2.1
40900	0.890236	2.1
40901	0.61049	2.1
40902	0.888979	2.1
40903	1.6223	2.1
40904	1.98034	2.1
40905	1.75003	2.1
40906	1.55433	2.1
40907	1.75003	2.1
40908	1.55433	2.1
40909	1.88602	2.1
40910	2.08149	2.1
40911	2.07991	2.1
40912	1.88473	2.1
40913	2.08149	2.1
40914	1.88602	2.1
40915	1.88458	2.1
40916	2.08149	2.1
40917	1.7069	2.1
40918	1.56284	2.1
40919	2.08149	2.1
40920	1.88458	2.1
40921	1.5955	2.1
40922	1.89226	2.1
40923	1.91975	2.09869
40924	1.62299	2.1
40925	1.89226	2.1
40926	1.5955	2.1
40927	1.89226	2.1
40928	1.89226	2.1
40929	1.89226	2.02166
40930	1.89226	2.1
40931	1.89226	2.01284
40932	1.89226	2.1
40933	1.89226	2.1
40934	1.89226	2.1
40935	1.89226	2.09852
40936	1.89226	2.1
40937	1.89226	2.08209
40938	1.89226	2.1
40939	1.62299	2.1
40940	1.89226	2.1
40941	1.71752	2.1
40942	1.44824	2.1
40943	1.89226	2.1
40944	1.62299	2.1
40945	1.89226	1.00336

40946	2.8317	0.628976
40947	1.89226	0.874694
40948	2.64055	0.559869
40949	4.53281	0.86459
40950	2.8317	0.724898
40951	1.83151	0.42876
40952	2.01001	0.502188
40953	2.01001	0.879796
40954	2.64055	0.340316
40955	2.64055	0.6714
40956	0.946132	0.228987
40957	0.946132	0.296665
40958	1.16167	0.266455
40959	1.16167	0.304474
40960	1.16167	0.253449
40961	1.32027	0.204413
40962	0.946131	0.247781
40963	0.946131	0.321797
40964	0.94613	0.229122
40965	1.16167	0.183906
40966	0.946131	0.236878
40967	1.32027	0.219232
40968	2.32335	0.264806
40969	1.83151	0.240063
40970	2.38525	0.204375
40971	2.64055	0.256896
40972	1.57621	0.180269
40973	1.83151	0.201773
40974	2.25677	2.1
40975	1.88158	2.1
40976	1.86856	2.1
40977	2.0815	2.1
40978	1.70303	2.1
40979	1.88158	2.1
40980	2.50381	2.1
40981	1.89227	2.1
40982	2.25677	2.1
40983	2.25677	2.1
40984	1.89227	2.1
40985	1.89227	2.1
40986	1.89226	0.101
40987	1.50899	0.102275
40988	2.13659	0.101399
40989	1.50899	0.144206
40990	2.51986	0.083486
40991	1.89226	0.128696
40992	0.946131	0.150034
40993	0.946131	0.111369
40994	1.06829	0.0962236
40995	0.895375	0.0884107
40996	0.94613	0.0928475
40997	0.94613	0.150107
40998	1.33922	0.0967168
40999	0.895376	0.0854523
41000	0.946131	0.110327
41001	1.33922	0.10745
41002	0.946131	0.112563
41003	1.06829	0.123066

41004	0.841467	0.189794
41005	0.578428	0.226372
41006	1.18079	0.181309
41007	0.920237	0.233919
41008	0.772317	0.146903
41009	0.856555	0.317913
41010	0.910271	0.187577
41011	0.910271	0.308749
41012	0.975752	0.186647
41013	0.536929	0.157363
41014	0.81952	0.264419
41015	1.15252	0.186514
41016	1.89226	2.1
41017	2.51431	2.1
41018	1.89226	2.1
41019	1.89226	2.1
41020	2.78815	2.1
41021	2.78815	2.1
41022	2.0815	2.1
41023	2.0516	2.1
41024	2.0815	2.1
41025	3.06699	2.1
41026	2.76576	2.1
41027	2.8002	2.1
41028	1.94112	2.1
41029	1.89226	2.1
41030	2.20358	2.1
41031	1.89226	2.1
41032	4.09584	2.1
41033	1.09154	2.1
41034	2.62647	2.1
41035	2.20358	2.1
41036	2.20358	2.1
41037	1.52314	2.1
41038	1.52314	2.1
41039	1.31323	2.1
41040	1.31323	2.1
41041	0.946132	2.1
41042	0.946132	2.1
41043	0.946132	2.1
41044	0.946132	2.1
41045	1.31323	2.1
41046	1.10179	2.1
41047	1.31323	2.1
41048	0.946132	2.1
41049	1.10179	2.1
41050	0.946133	2.1
41051	1.89226	1.17492
41052	1.89226	1.82975
41053	1.89226	1.75747
41054	1.89226	1.93312
41055	1.89226	1.05406
41056	1.89226	1.72901
41057	1.35126	1.97673
41058	1.35126	2.04411
41059	0.903285	1.71844
41060	0.903285	1.86179
41061	1.30841	1.84348

41062	0.903313	2.02159
41063	0.946132	1.91759
41064	0.946132	1.67698
41065	0.946132	1.82279
41066	0.903312	1.86463
41067	1.30841	1.79572
41068	0.946133	1.78158
41069	2.62097	2.1
41070	2.72561	2.1
41071	2.39016	2.1
41072	2.51062	2.1
41073	5.11577	2.1
41074	2.51062	2.1
41075	2.58286	2.08328
41076	2.40107	2.1
41077	2.40107	2.1
41078	2.72561	2.1
41079	2.72561	2.1
41080	2.39016	2.1
41081	2.51062	2.1
41082	2.20242	2.1
41083	2.34107	2.1
41084	4.73124	2.1
41085	2.51062	2.1
41086	2.40107	2.08108
41087	2.34107	2.1
41088	2.25197	2.1
41089	4.74215	2.1
41090	2.75218	2.1
41091	1.89226	2.1
41092	2.3349	2.1
41093	2.3349	2.1
41094	1.89226	2.1
41095	1.89226	2.1
41096	1.46129	2.1
41097	0.996308	2.1
41098	1.88649	2.1
41099	1.70302	2.1
41100	2.95419	2.1
41101	1.89227	2.1
41102	1.89227	2.1
41103	1.89227	2.1
41104	1.89227	2.1
41105	3.78453	2.1
41106	0.646558	2.1
41107	0.646557	2.1
41108	0.60697	2.1
41109	0.60697	2.1
41110	0.587917	2.1
41111	0.519747	2.1
41112	0.586443	2.1
41113	0.733782	2.1
41114	0.733793	2.1
41115	0.587924	2.1
41116	0.519739	2.1
41117	0.586436	2.1
41118	2.1642	2.1
41119	1.42867	2.1

41120	2.28847	2.1
41121	1.68299	2.1
41122	3.71714	2.1
41123	1.89226	2.1
41124	1.89226	2.1
41125	1.89226	2.1
41126	1.89226	2.1
41127	3.78453	2.1
41128	0.946131	2.1
41129	0.946131	2.1
41130	1.24994	2.1
41131	1.24994	2.1
41132	1.11582	2.1
41133	1.24994	2.1
41134	0.946133	2.1
41135	0.946133	2.1
41136	0.946131	2.1
41137	1.24994	2.1
41138	0.94613	2.1
41139	1.11582	2.1
41140	2.28847	2.1
41141	1.53629	2.1
41142	1.57498	2.1
41143	1.47417	2.1
41144	2.42221	2.1
41145	2.28847	2.1
41146	1.89226	2.1
41147	1.89226	2.1
41148	1.89226	2.1
41149	1.89226	2.1
41150	3.78453	2.1
41151	1.89226	2.1
41152	2.49988	2.1
41153	2.71233	2.1
41154	2.71233	2.1
41155	1.89226	2.1
41156	1.89226	2.1
41157	0.924534	2.1
41158	0.924534	2.1
41159	0.990395	2.1
41160	0.990395	2.1
41161	0.946131	2.1
41162	0.946131	2.1
41163	0.924505	2.1
41164	0.968797	2.1
41165	0.924505	2.1
41166	0.946131	2.1
41167	0.968797	2.1
41168	0.946131	2.1
41169	0.924533	2.1
41170	0.924533	2.1
41171	0.948264	2.1
41172	0.945345	2.1
41173	0.851541	2.1
41174	0.851541	2.1
41175	0.958934	2.1
41176	1.02617	2.1
41177	0.936668	2.1

41178	1.04829	2.1
41179	0.924505	2.1
41180	0.946131	2.1
41181	2.28431	0.477139
41182	1.48035	0.203275
41183	1.59971	0.31051
41184	1.48035	0.393433
41185	2.33818	0.269364
41186	2.21882	0.342453
41187	2.28431	0.439242
41188	1.89226	0.548917
41189	1.89226	0.733061
41190	2.3968	0.374253
41191	4.28906	0.456775
41192	2.28431	0.445415
41193	1.70303	0.209723
41194	1.5428	0.227546
41195	1.59971	0.319401
41196	2.31152	0.284747
41197	2.3968	0.357223
41198	1.99823	2.1
41199	2.21189	2.1
41200	2.18937	2.1
41201	1.979 2.1	
41202	4.19089	2.1
41203	0.999114	2.1
41204	0.9895	2.1
41205	0.758942	2.1
41206	0.749358	2.1
41207	0.874236	2.1
41208	0.874236	2.1
41209	0.874236	2.1
41210	0.874236	2.1
41211	0.9895	2.1
41212	0.749358	2.1
41213	0.758942	2.1
41214	0.999114	2.1
41215	2.18937	2.1
41216	1.979 2.1	
41217	1.95416	2.1
41218	2.162 2.1	
41219	4.14099	2.1
41220	1.95416	2.1
41221	1.49871	2.1
41222	1.47712	2.1
41223	1.49871	2.1
41224	1.9574	2.1
41225	1.979 2.1	
41226	1.68508	0.0698591
41227	1.64984	0.0698591
41228	2.67711	0.0698591
41229	2.76407	0.0698591
41230	4.41391	0.0698591
41231	1.84227	0.0698591
41232	2.76407	0.0698591
41233	2.76407	0.0698591
41234	1.85007	0.0698591
41235	2.72769	0.0698591

41236	1.80812	0.0698591
41237	1.94516	2.1
41238	1.89226	2.1
41239	1.89226	2.1
41240	1.89226	2.1
41241	1.98853	2.1
41242	1.98852	2.1
41243	1.28095	2.1
41244	2.0815	2.1
41245	2.29123	2.1
41246	2.06833	2.1
41247	1.08212	2.1
41248	0.976848	2.1
41249	1.89226	2.1
41250	1.89226	2.1
41251	1.89226	2.1
41252	1.89226	2.1
41253	1.89226	2.1
41254	1.89226	2.1
41255	1.13537	2.1
41256	1.18233	2.1
41257	0.946132	2.1
41258	0.946132	2.1
41259	0.946132	2.1
41260	0.946132	2.1
41261	0.994264	2.1
41262	1.18233	2.1
41263	1.13537	2.1
41264	0.946131	2.1
41265	0.946132	2.1
41266	0.994264	2.1
41267	0.953204	2.1
41268	0.953204	2.1
41269	0.966515	2.1
41270	0.966515	2.1
41271	1.30583	2.1
41272	1.14561	2.1
41273	0.640475	2.1
41274	0.774103	2.1
41275	1.30584	2.1
41276	0.774101	2.1
41277	0.640474	2.1
41278	1.14562	2.1
41279	0.946131	2.1
41280	0.946131	2.1
41281	0.946131	2.1
41282	0.946131	2.1
41283	0.946128	2.1
41284	0.946128	2.1
41285	0.946133	2.1
41286	0.946133	2.1
41287	0.94613	2.1
41288	0.94613	2.1
41289	0.946131	2.1
41290	0.946131	2.1
41291	0.946132	0.0698591
41292	0.946132	0.0698591
41293	0.946131	0.0698591

41294	0.946131	0.0698591
41295	0.946131	0.0698591
41296	0.946131	0.0698591
41297	0.946132	0.0698591
41298	0.946132	0.0698591
41299	0.946131	0.0698591
41300	0.946131	0.0698591
41301	0.946132	0.0698591
41302	0.946132	0.0698591
41303	0.946131	0.0698591
41304	0.946131	0.0698591
41305	0.946131	0.0698591
41306	0.946131	0.0698591
41307	0.94613	0.0698591
41308	0.94613	0.0698591
41309	0.946132	0.0698591
41310	0.946132	0.0698591
41311	0.946131	0.0698591
41312	0.946131	0.0698591
41313	0.946131	0.0698591
41314	0.946131	0.0698591
41315	2.57882	0.100325
41316	1.48676	0.116108
41317	1.48797	0.0989348
41318	2.64281	0.102477
41319	4.12957	0.100897
41320	2.56323	0.0841011
41321	2.64281	0.0980107
41322	2.51807	0.0891145
41323	1.36324	0.0869325
41324	1.48797	0.0933086
41325	1.48797	0.0970054
41326	1.04367	2.1
41327	2.42504	2.1
41328	2.65164	2.1
41329	1.49479	2.1
41330	3.91983	2.1
41331	2.15622	2.1
41332	1.70539	2.1
41333	2.02369	2.1
41334	2.39039	2.1
41335	4.17992	2.1
41336	0.966979	2.1
41337	1.7054	2.1
41338	2.0237	2.1
41339	1.31502	2.1
41340	2.99067	2.1
41341	2.02369	2.1
41342	2.65637	2.09904
41343	2.39039	2.09398
41344	2.39039	2.1
41345	2.29332	2.09417
41346	2.29332	2.1
41347	2.0237	2.1
41348	1.72116	2.1
41349	1.58465	2.1
41350	1.31502	2.1
41351	2.29332	2.1

41352	2.0237	2.1
41353	1.0372	0.0698591
41354	2.32462	0.0698591
41355	2.2291	0.0698591
41356	2.2291	0.0698591
41357	1.17464	0.0698591
41358	1.17464	0.0698591
41359	2.68289	0.0698591
41360	1.07805	0.0698591
41361	1.70302	0.0698591
41362	2.06653	0.0698591
41363	3.5779	0.0698591
41364	1.89226	0.105844
41365	1.89226	0.187341
41366	1.89226	0.156152
41367	1.89226	0.0842628
41368	3.78453	0.116351
41369	1.89226	0.0702934
41370	1.89226	0.0763119
41371	1.89226	0.0749245
41372	1.89226	0.0726645
41373	1.89226	0.0727174
41374	1.89226	0.0708903
41375	1.89225	0.0710781
41376	1.89225	0.0736936
41377	1.89225	0.0742003
41378	1.89225	0.0718418
41379	1.89225	0.0738118
41380	1.89225	0.0717852
41381	0.94613	0.0914087
41382	0.94613	0.113689
41383	0.94613	0.179785
41384	0.94613	0.137936
41385	0.946128	0.107678
41386	0.946128	0.125059
41387	0.946132	0.193383
41388	0.946132	0.0870392
41389	0.94613	0.107202
41390	0.94613	0.132639
41391	0.94613	0.122914
41392	0.94613	0.0979303
41393	1.29417	2.1
41394	1.32732	2.1
41395	1.32316	1.02737
41396	1.34973	2.1
41397	2.65048	2.1
41398	1.89226	1.51449
41399	1.89226	2.1
41400	1.89226	1.37231
41401	1.89226	2.1
41402	3.78453	2.0775
41403	1.89226	2.1
41404	1.89226	2.1
41405	1.89226	2.1
41406	1.89226	2.1
41407	3.78453	2.1
41408	1.32732	2.1
41409	1.13006	2.1

41410	1.13358	2.1
41411	1.11117	2.1
41412	1.34973	2.1
41413	1.32732	2.1
41414	1.32316	2.01349
41415	1.34973	2.1
41416	1.34066	1.65378
41417	1.36601	2.1
41418	2.68918	2.1
41419	1.89226	2.02995
41420	1.89226	2.1
41421	1.89226	0.511441
41422	1.89226	2.1
41423	3.78453	2.09249
41424	1.89226	2.1
41425	1.89226	2.1
41426	1.89226	2.1
41427	1.89226	2.1
41428	1.89226	2.1
41429	1.89226	2.1
41430	0.624134	2.1
41431	0.624134	2.1
41432	0.624334	2.1
41433	0.624334	2.1
41434	0.56503	2.1
41435	0.573605	2.1
41436	0.674864	2.1
41437	0.683007	2.1
41438	0.683007	2.1
41439	0.573605	2.1
41440	0.565029	2.1
41441	0.674864	2.1
41442	2.16633	2.1
41443	1.40846	2.1
41444	2.13977	2.1
41445	1.40771	2.1
41446	3.57404	2.1
41447	2.13977	2.1
41448	1.29932	2.1
41449	1.29932	2.1
41450	1.40771	2.1
41451	1.98957	2.1
41452	2.09796	2.1
41453	2.6044	0.182598
41454	1.85548	0.222951
41455	1.89226	0.123656
41456	1.89226	0.314684
41457	3.74774	0.216279
41458	1.89226	0.134054
41459	1.89226	0.362926
41460	1.89226	0.177141
41461	1.89226	0.23066
41462	3.78453	0.178099
41463	1.89226	2.1
41464	2.64107	2.1
41465	1.89226	2.1
41466	2.78977	2.1
41467	1.89226	2.1

41468	2.78977	2.1
41469	1.89226	2.1
41470	1.89226	2.1
41471	1.89226	2.1
41472	1.89226	2.09657
41473	1.89226	2.1
41474	1.89226	2.09782
41475	2.43173	2.0964
41476	1.41238	2.1
41477	2.0815	2.1
41478	1.98048	2.1
41479	1.62014	2.1
41480	1.50086	2.1
41481	0.946129	0.577865
41482	0.946129	0.496693
41483	1.27636	0.402579
41484	1.1984	0.318895
41485	0.946131	0.325912
41486	0.946131	0.491558
41487	1.27636	0.60399
41488	1.27636	0.362989
41489	0.946131	0.422886
41490	1.27636	0.420281
41491	0.946131	0.442598
41492	1.1984	0.430257
41493	1.99569	0.206259
41494	2.55272	0.415297
41495	1.77403	0.233848
41496	1.70303	0.292123
41497	3.05772	0.241704
41498	2.55121	0.311656
41499	0.946128	0.430484
41500	0.946128	0.498276
41501	1.38752	0.367
41502	1.27636	0.278795
41503	0.94613	0.284661
41504	0.94613	0.427542
41505	1.38752	0.516716
41506	1.38752	0.31425
41507	0.946131	0.381457
41508	1.38752	0.376602
41509	0.946131	0.401295
41510	1.27636	0.382501
41511	2.77504	0.347622
41512	1.99569	0.176574
41513	2.29794	0.21541
41514	1.99569	0.275699
41515	2.85497	0.200995
41516	2.55272	0.25574
41517	1.53284	2.1
41518	1.89226	2.1
41519	1.89226	2.1
41520	1.89226	2.1
41521	1.54763	2.1
41522	1.54763	2.1
41523	0.946131	2.1
41524	0.946131	2.1
41525	0.946127	2.1

41526	0.946127	2.1
41527	0.946127	2.1
41528	0.946127	2.1
41529	0.946131	2.1
41530	0.946131	2.1
41531	0.94613	2.1
41532	0.94613	2.1
41533	0.946129	2.1
41534	0.946129	2.1
41535	0.751754	2.1
41536	0.751754	2.1
41537	0.771562	2.1
41538	0.771561	2.1
41539	1.0268	2.1
41540	0.95482	2.1
41541	0.528937	2.1
41542	0.496512	2.1
41543	0.528936	2.1
41544	1.0268	2.1
41545	0.954821	2.1
41546	0.49651	2.1
41547	2.64075	0.264676
41548	1.35181	1.06536
41549	1.35181	0.317443
41550	1.83823	0.387456
41551	2.28373	0.415904
41552	2.88566	1.15696
41553	0.493018	1.18755
41554	0.675903	0.904678
41555	1.14187	0.299416
41556	0.973937	0.0752338
41557	0.821166	0.368514
41558	0.821166	1.26968
41559	0.828674	1.65825
41560	0.828674	0.256518
41561	0.973937	0.5507
41562	0.493018	0.579404
41563	0.675903	0.50143
41564	1.14187	0.545087
41565	1.89226	2.1
41566	2.11667	2.1
41567	1.91184	2.1
41568	1.89226	2.1
41569	2.13625	2.1
41570	2.11667	2.1
41571	1.89226	2.1
41572	1.89226	2.1
41573	1.89226	2.1
41574	1.89226	2.1
41575	3.78453	2.1
41576	0.907729	2.1
41577	0.907729	2.1
41578	1.04459	2.1
41579	0.890955	2.1
41580	0.988181	2.1
41581	0.841856	2.1
41582	0.946133	2.1
41583	0.946133	2.1

41584	1.04072	2.1
41585	1.1064	2.1
41586	0.89099	2.1
41587	0.851541	2.1
41588	1.89225	2.1
41589	2.13623	2.1
41590	1.91044	2.1
41591	1.89225	2.1
41592	2.15442	2.1
41593	2.13623	2.1
41594	1.89225	2.1
41595	1.89225	2.1
41596	1.89225	2.1
41597	1.89225	2.1
41598	1.89225	2.1
41599	1.89225	2.1
41600	0.907723	2.1
41601	0.907723	2.1
41602	1.07536	2.1
41603	1.07814	2.1
41604	0.998499	2.1
41605	1.03575	2.1
41606	0.946126	2.1
41607	0.946126	2.1
41608	1.04072	2.1
41609	1.14025	2.1
41610	0.851528	2.1
41611	0.898284	2.1
41612	1.89226	0.0713948
41613	1.89226	0.13285
41614	1.89226	0.0890264
41615	1.89226	0.0832738
41616	1.89226	0.0812051
41617	1.89226	0.0769465
41618	2.53008	2.1
41619	1.94534	2.1
41620	1.98702	2.1
41621	1.98858	2.1
41622	2.52852	2.1
41623	2.53008	2.1
41624	1.03267	2.1
41625	1.12591	2.1
41626	1.2959	2.1
41627	1.28992	2.1
41628	1.10851	2.1
41629	1.33078	2.1
41630	1.10661	2.1
41631	1.10661	2.1
41632	0.982208	2.1
41633	1.28992	2.1
41634	1.03267	2.1
41635	1.23262	2.1
41636	2.2712	2.1
41637	1.89226	2.1
41638	1.95954	2.1
41639	1.95954	2.1
41640	1.89226	2.1
41641	1.89226	2.1

41642	1.89226	2.1
41643	1.89226	2.1
41644	1.89226	2.1
41645	1.89226	2.1
41646	3.78453	2.1
41647	1.33887	2.1
41648	1.25395	2.1
41649	0.88327	2.1
41650	0.676665	2.1
41651	0.84881	2.1
41652	0.987503	2.1
41653	1.13243	2.1
41654	1.13243	2.1
41655	1.15338	2.1
41656	0.849966	2.1
41657	0.793074	2.1
41658	1.11957	2.1
41659	0.946129	2.1
41660	0.946129	2.1
41661	1.28254	2.1
41662	1.1356	2.1
41663	1.1356	2.1
41664	1.1356	2.1
41665	0.946131	2.1
41666	0.946131	2.1
41667	0.946131	2.1
41668	1.28254	2.1
41669	1.1356	2.1
41670	0.946131	2.1
41671	1.89227	2.1
41672	1.89227	2.1
41673	1.89227	2.1
41674	1.89227	2.1
41675	3.78453	2.1
41676	0.936287	0.0698591
41677	1.01399	0.0698591
41678	0.959415	0.0698591
41679	0.959415	0.0698591
41680	0.910786	0.0698591
41681	1.38301	0.0698591
41682	1.0286	0.0698591
41683	0.535742	0.0698591
41684	0.505202	0.0698591
41685	0.937685	0.0698591
41686	1.11066	0.0698591
41687	1.46907	0.0698591
41688	1.00546	0.0698591
41689	1.01524	0.0698591
41690	0.94613	0.0698591
41691	0.94613	0.0698591
41692	1.05018	0.0698591
41693	0.914025	0.0698591
41694	0.913512	0.0698591
41695	0.851542	0.0699159
41696	1.05385	0.0698591
41697	1.05494	0.0698591
41698	0.91404	0.0698591
41699	0.91404	0.0698591

41700	2.03253	2.0746
41701	1.82325	0.475249
41702	1.93938	2.1
41703	1.73756	0.364143
41704	3.77009	1.40143
41705	1.82325	0.365442
41706	1.47562	1.94947
41707	1.73756	0.418273
41708	1.3868	1.55642
41709	3.21317	0.563536
41710	1.93938	2.1
41711	1.73756	0.486823
41712	1.85293	2.07841
41713	1.64279	0.419343
41714	3.59049	1.12188
41715	1.73756	0.337119
41716	1.3868	1.51523
41717	1.64279	0.489292
41718	1.28419	1.85737
41719	3.02174	0.629783
41720	1.35048	2.09968
41721	1.37999	2.1
41722	1.34355	0.989233
41723	1.37854	2.1
41724	2.72354	2.1
41725	1.89226	1.62566
41726	1.89226	2.1
41727	1.89226	0.892712
41728	1.89226	2.07963
41729	3.78452	1.91488
41730	1.89226	2.1
41731	1.89226	2.1
41732	1.89226	2.06634
41733	1.89226	2.1
41734	3.78453	2.1
41735	1.37999	2.1
41736	1.18059	2.1
41737	1.17117	2.1
41738	1.17262	2.1
41739	1.37855	2.1
41740	1.37999	2.1
41741	1.34355	2.01334
41742	1.37854	2.1
41743	1.33079	1.5641
41744	1.3741	2.1
41745	2.71765	2.08426
41746	1.89226	1.95412
41747	1.89226	2.1
41748	1.89226	0.47445
41749	1.89226	2.1
41750	3.78453	2.02287
41751	1.89226	2.1
41752	1.89226	2.1
41753	1.89226	2.1
41754	1.89226	2.1
41755	3.78453	2.1
41756	0.640305	2.1
41757	0.640305	2.1

41758	0.643507	2.1
41759	0.643507	2.1
41760	0.590294	2.1
41761	0.594539	2.1
41762	0.689273	2.1
41763	0.687048	2.1
41764	0.687048	2.1
41765	0.594538	2.1
41766	0.590294	2.1
41767	0.689273	2.1
41768	2.01399	0.965021
41769	1.70141	1.65116
41770	1.83896	2.09929
41771	1.52482	2.08508
41772	3.53881	1.85213
41773	1.70141	2.06602
41774	1.0752	2.1
41775	1.0752	2.02403
41776	1.25669	2.1
41777	1.52482	2.1
41778	1.70632	2.1
41779	1.83896	1.85588
41780	1.52482	1.1083
41781	1.65244	1.36582
41782	1.34412	2.05401
41783	3.17725	1.50945
41784	1.26782	0.0698591
41785	2.90353	0.0698591
41786	2.54203	0.0698591
41787	2.41979	0.0717998
41788	1.77007	0.0698591
41789	1.703 0.0708169	
41790	0.946118	0.0829829
41791	0.946118	0.0714665
41792	1.38643	0.0727178
41793	1.38644	0.0698591
41794	0.946117	0.0752439
41795	0.946117	0.0852671
41796	1.38644	0.0698591
41797	1.15545	0.0718084
41798	0.946131	0.0731132
41799	1.15546	0.0717067
41800	1.38642	0.0727367
41801	0.946105	0.07292
41802	2.42256	0.175565
41803	2.63056	0.131023
41804	2.43174	0.13483
41805	2.25035	0.126537
41806	4.8543	0.12584
41807	2.58031	0.168954
41808	2.63056	0.138347
41809	2.43174	0.133304
41810	2.32127	0.106131
41811	5.01205	0.145434
41812	2.43174	0.129382
41813	2.25035	0.121397
41814	2.11465	0.0998888
41815	2.13042	0.0954629

41816	4.54639	0.115953
41817	2.43174	0.129235
41818	2.32127	0.105454
41819	1.92997	0.0873057
41820	2.13041	0.0984191
41821	4.36171	0.097784
41822	1.89227	2.1
41823	1.89227	2.1
41824	1.89227	2.1
41825	1.89227	2.1
41826	3.78453	2.1
41827	0.568724	2.1
41828	0.568724	2.1
41829	0.57678	2.1
41830	0.57678	2.1
41831	0.523796	2.1
41832	0.548137	2.1
41833	0.597368	2.1
41834	0.637877	2.1
41835	0.597367	2.1
41836	0.523796	2.1
41837	0.637877	2.1
41838	0.548137	2.1
41839	1.32674	2.1
41840	1.32674	2.1
41841	0.946132	2.1
41842	0.946132	2.1
41843	1.3195	2.1
41844	1.32674	2.1
41845	0.946132	2.1
41846	0.946132	2.1
41847	0.946132	2.1
41848	1.3195	2.1
41849	1.32674	2.1
41850	0.946133	2.1
41851	1.89227	2.1
41852	1.89227	2.1
41853	1.89227	2.1
41854	1.89227	2.1
41855	3.78453	2.1
41856	1.19473	2.1
41857	0.987132	2.1
41858	0.987132	2.1
41859	1.04759	2.1
41860	1.0894	2.1
41861	1.14986	2.1
41862	1.3195	2.1
41863	1.3195	2.1
41864	0.946132	2.1
41865	0.946132	2.1
41866	1.31185	2.1
41867	1.3195	2.1
41868	0.946132	2.1
41869	0.946132	2.1
41870	1.31185	2.1
41871	0.946133	2.1
41872	0.946132	2.1
41873	1.3195	2.1

41874	1.11256	1.17874
41875	1.70101	0.891305
41876	1.22297	1.11878
41877	1.22297	1.09983
41878	1.7252	1.14334
41879	1.7252	1.12316
41880	1.99298	1.02061
41881	1.70101	0.970308
41882	1.7252	1.20353
41883	2.18701	0.714914
41884	3.71818	0.927802
41885	1.03218	0.987112
41886	1.7252	0.778573
41887	1.11256	0.965631
41888	1.11256	0.942464
41889	1.7457	0.962943
41890	1.7457	0.940907
41891	1.7457	1.03062
41892	2.187	0.61496
41893	1.7252	0.8253
41894	1.7252	0.908174
41895	2.27351	0.773838
41896	2.27351	0.830213
41897	1.95544	0.304606
41898	1.35897	2.03158
41899	1.44859	0.855005
41900	1.44859	1.87872
41901	1.80709	0.29615
41902	1.80709	1.54297
41903	1.80709	0.22037
41904	1.17822	1.68624
41905	1.35897	0.363126
41906	1.35897	1.97605
41907	1.52782	0.208036
41908	1.52782	1.43
41909	1.11978	1.62481
41910	0.903546	0.332574
41911	0.977719	1.83121
41912	1.2364	0.484305
41913	1.03812	0.221963
41914	1.03812	2.03674
41915	1.05938	0.18267
41916	1.05938	2.08919
41917	1.11978	0.317237
41918	1.2364	0.14944
41919	0.97772	1.19657
41920	0.903546	0.415819
41921	1.80709	2.0128
41922	1.81847	0.861785
41923	1.96029	1.77261
41924	1.52782	1.37449
41925	2.23956	0.495901
41926	1.80709	1.26758
41927	1.89226	0.299289
41928	2.32962	0.184306
41929	2.5992	0.188156
41930	1.89226	0.207814
41931	2.5992	0.265253

41932	1.89226	0.287045
41933	2.55791	0.0698591
41934	2.47141	0.0698591
41935	2.13515	0.0698591
41936	2.10939	0.0698591
41937	4.66731	0.0698591
41938	2.47141	0.0698591
41939	2.23337	0.0698591
41940	2.10939	0.0698591
41941	2.03438	0.0698591
41942	4.34276	0.0698591
41943	2.13512	0.0698591
41944	1.71027	0.0698591
41945	1.71027	0.0698591
41946	2.10936	0.0698591
41947	1.60137	0.0698591
41948	2.00046	0.0698591
41949	1.71027	0.0698591
41950	2.03435	0.0698591
41951	1.71027	0.0698591
41952	2.10936	0.0698591
41953	1.72067	0.0698591
41954	2.11976	0.0698591
41955	1.177	0.505675
41956	1.36405	0.929956
41957	2.25635	1.10456
41958	2.60151	0.358736
41959	3.6204	0.822855
41960	0.658869	0.0742708
41961	0.781697	0.0755204
41962	0.637037	0.0698591
41963	0.541148	0.0698591
41964	0.654682	0.0698591
41965	0.654682	0.109567
41966	0.641224	0.0698591
41967	0.641224	0.0716307
41968	0.637039	0.0698591
41969	0.781696	0.0698591
41970	0.658867	0.0759986
41971	0.541149	0.0751726
41972	1.89226	0.246055
41973	1.59152	0.165243
41974	1.89226	0.154934
41975	1.82125	0.155432
41976	1.89226	0.279992
41977	1.82125	0.253938
41978	2.18967	0.0698591
41979	1.89227	0.0720679
41980	2.72752	0.0698782
41981	2.72752	0.0706387
41982	1.89227	0.0698591
41983	1.89227	0.0698591
41984	2.18967	0.0698591
41985	1.92547	0.0698591
41986	1.92547	0.0698591
41987	2.72752	0.0698591
41988	1.30572	0.0698591
41989	2.10777	0.0698591

41990	2.39663	2.1
41991	1.92293	2.1
41992	1.96889	2.1
41993	2.45928	2.1
41994	4.36552	2.1
41995	1.02605	2.1
41996	1.92293	2.1
41997	1.9689	2.1
41998	1.04679	2.1
41999	2.99495	2.1
42000	1.96889	2.1
42001	2.45928	2.1
42002	2.50138	2.1
42003	1.99782	2.1
42004	4.47027	2.1
42005	1.9689	2.1
42006	1.03449	2.1
42007	1.07572	2.1
42008	1.04679	2.1
42009	1.99783	2.1
42010	1.9689	2.1
42011	1.72162	0.666027
42012	1.89186	0.321663
42013	2.29771	0.370797
42014	1.80149	0.168121
42015	4.01933	0.352255
42016	1.16482	0.670668
42017	1.97003	0.21351
42018	1.72162	0.185014
42019	1.72162	0.347231
42020	1.29313	0.28833
42021	1.29313	0.46065
42022	2.29771	0.521909
42023	2.84159	0.142967
42024	2.19622	0.25083
42025	1.97366	0.23427
42026	2.50906	0.255886
42027	2.29771	0.277777
42028	1.29313	0.612692
42029	2.4483	0.224286
42030	2.14223	0.205278
42031	2.14228	0.341105
42032	1.294	0.2646
42033	1.294	0.382184
42034	1.21495	0.0698591
42035	1.70303	0.0698591
42036	1.7924	0.0698591
42037	1.64002	0.0698591
42038	3.11963	0.0698591
42039	2.81021	0.0698591
42040	1.89226	0.0698591
42041	1.89226	0.0698591
42042	1.89226	0.0698591
42043	2.36406	0.0698591
42044	2.36406	0.0698591
42045	1.89226	0.0698591
42046	1.89226	0.0698591
42047	1.89226	0.0698591

42048	1.89226	0.0698591
42049	1.89226	0.0698591
42050	1.89226	0.0698591
42051	2.29976	0.0698591
42052	1.64002	0.0698591
42053	1.8959	0.0698591
42054	1.90873	0.0698591
42055	1.85594	0.0698591
42056	1.85594	0.0698591
42057	1.89226	0.0698591
42058	1.89226	0.0698591
42059	1.89226	0.0698591
42060	1.89226	0.0698591
42061	3.78453	0.0698591
42062	1.89226	0.991201
42063	2.52823	2.1
42064	2.83441	1.83512
42065	2.83441	2.07259
42066	1.89226	1.40163
42067	1.89226	1.88459
42068	1.04522	1.52123
42069	1.04522	1.33644
42070	1.12831	1.875
42071	1.18364	2.1
42072	0.640525	1.57941
42073	0.924882	1.1163
42074	1.27877	1.81821
42075	1.43363	0.790088
42076	0.93474	0.912008
42077	1.58926	1.3812
42078	1.29573	1.74884
42079	0.664247	1.93861
42080	2.02191	2.1
42081	1.89226	2.1
42082	1.89226	2.1
42083	1.89226	2.1
42084	2.58639	2.1
42085	2.58639	2.1
42086	1.16759	2.1
42087	2.11828	2.1
42088	1.60936	2.1
42089	2.35846	2.1
42090	3.52605	2.1
42091	1.89226	2.1
42092	1.89226	2.1
42093	1.89226	2.1
42094	1.89226	2.1
42095	3.78453	2.1
42096	2.82765	0.0698591
42097	1.89809	0.0700733
42098	2.15971	0.0698591
42099	1.89809	0.0700998
42100	2.29233	0.0698591
42101	2.03071	0.0698591
42102	1.90664	0.0826792
42103	2.15971	0.0698591
42104	1.28956	0.0759071
42105	1.28956	0.0698591

42106	2.64414	0.07103
42107	2.02706	0.0698591
42108	2.82765	0.06994
42109	1.89226	0.0767091
42110	2.39968	0.0867907
42111	2.39968	0.0868615
42112	1.89226	0.0698591
42113	1.89226	0.0698591
42114	0.978643	2.1
42115	0.703968	2.1
42116	0.946129	2.1
42117	0.946129	2.1
42118	0.703939	2.1
42119	0.703939	2.1
42120	0.946159	2.1
42121	0.946159	2.1
42122	0.978642	2.1
42123	0.94613	2.1
42124	0.94613	2.1
42125	0.703968	2.1
42126	1.89226	2.09943
42127	2.17309	1.63782
42128	2.17309	2.07732
42129	1.47475	1.9189
42130	1.89226	2.07507
42131	1.19393	1.84514
42132	1.89226	2.1
42133	1.89226	2.1
42134	1.89226	2.1
42135	1.89226	2.0602
42136	3.78452	2.1
42137	1.17426	2.1
42138	0.978644	2.1
42139	0.946129	2.1
42140	0.946129	2.1
42141	0.946131	2.1
42142	0.946131	2.1
42143	0.978643	2.1
42144	0.978642	2.1
42145	1.17426	2.1
42146	0.946131	2.1
42147	0.978642	2.1
42148	0.94613	2.1
42149	1.89226	2.09983
42150	1.89226	1.60195
42151	1.89226	2.05075
42152	2.17309	1.68607
42153	2.56346	2.03705
42154	2.94389	1.81451
42155	0.946131	2.09701
42156	0.946131	2.0447
42157	0.946128	2.1
42158	0.946128	2.09661
42159	0.946131	2.09256
42160	0.946131	2.04385
42161	0.946128	2.1
42162	0.946128	2.1
42163	0.94613	2.08576

42164	0.94613	2.08109
42165	0.946129	2.08737
42166	0.946129	2.07349
42167	1.89226	1.21255
42168	1.89226	0.827068
42169	1.89226	0.661897
42170	1.89226	0.517888
42171	3.78453	0.819428
42172	1.89226	0.769979
42173	1.89226	0.590865
42174	1.89226	0.756496
42175	1.89226	1.318
42176	1.89226	0.755405
42177	1.89226	1.07182
42178	1.89226	0.638278
42179	1.30733	1.13754
42180	1.89226	1.36926
42181	1.92403	1.07184
42182	3.38635	0.856504
42183	1.89226	0.777503
42184	3.02887	1.1318
42185	2.0815	1.09652
42186	1.88422	1.2121
42187	2.0815	1.07916
42188	1.88422	1.1158
42189	1.89226	1.0693
42190	1.70221	0.772532
42191	1.89226	0.937789
42192	1.20068	1.21961
42193	1.89226	0.674412
42194	1.20068	0.268298
42195	1.01393	1.05025
42196	1.20067	0.396063
42197	1.89226	0.425337
42198	2.44958	1.47766
42199	1.01393	0.192068
42200	1.89226	0.197673
42201	1.89226	2.1
42202	1.89226	2.1
42203	1.89226	2.1
42204	1.89226	2.1
42205	1.89226	2.1
42206	1.89226	2.1
42207	1.89226	2.1
42208	1.89226	2.09216
42209	1.89226	2.1
42210	1.89226	2.1
42211	1.89226	2.1
42212	1.89226	2.1
42213	1.89226	2.1
42214	1.89226	2.1
42215	1.89226	2.1
42216	1.89226	2.1
42217	1.89226	2.1
42218	1.89226	2.1
42219	1.91161	2.1
42220	2.45782	2.1
42221	1.39653	2.1

42222	2.44652	2.1
42223	4.35812	2.1
42224	2.73092	2.1
42225	1.89226	2.1
42226	1.89226	2.1
42227	1.89226	2.05893
42228	2.44652	2.1
42229	2.44652	2.00891
42230	1.89226	2.1
42231	1.89226	2.1
42232	1.89226	2.1
42233	1.89226	2.1
42234	1.89226	2.1
42235	1.89226	2.1
42236	2.64924	2.1
42237	1.31109	2.1
42238	1.31109	2.1
42239	1.49958	2.1
42240	1.95039	2.1
42241	2.14451	2.1
42242	0.825157	2.1
42243	0.634856	2.1
42244	0.816836	2.1
42245	0.816836	2.1
42246	0.556743	2.1
42247	0.639142	2.1
42248	0.985106	2.1
42249	0.795045	2.1
42250	0.967461	2.1
42251	0.552494	2.1
42252	0.676829	2.1
42253	0.98803	2.1
42254	2.28564	2.1
42255	2.34084	2.0497
42256	2.3502	2.1
42257	2.42908	2.1
42258	4.69104	2.1
42259	2.34084	2.07215
42260	2.18077	2.09792
42261	2.42908	2.1
42262	2.2947	1.15158
42263	4.63554	2.08331
42264	2.3502	2.1
42265	2.42908	2.1
42266	2.38681	2.1
42267	2.50438	2.06492
42268	4.85458	2.1
42269	2.42908	2.1
42270	2.2947	1.5835
42271	2.50438	1.97653
42272	2.40627	2.1
42273	4.79908	2.00455
42274	1.89226	1.20509
42275	2.20711	1.89146
42276	1.89226	1.15042
42277	1.89226	1.94479
42278	2.02693	1.14588
42279	2.02693	1.9272

42280	1.2269	2.08058
42281	2.20711	1.89398
42282	2.0815	1.9134
42283	1.11506	2.09973
42284	3.22109	2.06717
42285	0.545802	2.0157
42286	0.547489	2.06532
42287	0.85914	1.9163
42288	1.11641	2.00886
42289	0.899974	2.02928
42290	0.849893	2.07188
42291	0.747885	2.01568
42292	0.747885	1.85303
42293	0.557184	2.05882
42294	0.887039	2.05873
42295	1.08948	2.05557
42296	0.59815	2.06764
42297	0.974139	2.1
42298	1.33385	2.1
42299	1.02368	2.1
42300	1.02368	2.1
42301	1.00833	2.1
42302	1.00833	2.1
42303	1.89226	1.85939
42304	1.89226	0.505934
42305	1.89226	2.06184
42306	1.89226	0.373363
42307	3.78453	0.711842
42308	1.74651	2.09632
42309	1.44605	0.300523
42310	1.63557	2.05171
42311	1.31993	0.453676
42312	3.08162	0.796665
42313	1.89226	2.08592
42314	1.89226	0.765943
42315	1.89226	1.92751
42316	1.89226	0.760155
42317	3.78452	0.650469
42318	1.63557	2.08175
42319	1.31992	0.37627
42320	1.52615	2.09816
42321	1.18549	0.675699
42322	2.82106	1.17098
42323	1.89226	0.383194
42324	2.58101	1.95862
42325	2.73797	2.08363
42326	2.73797	0.866048
42327	1.89226	2.09861
42328	1.89226	0.426596
42329	1.91762	0.238804
42330	1.44354	0.213477
42331	1.33622	0.222135
42332	1.33622	0.192456
42333	2.20248	0.310276
42334	2.20248	0.237789
42335	1.11712	2.1
42336	1.11712	2.1
42337	1.11421	2.1

42338	1.11421	2.1
42339	0.833871	2.1
42340	0.926528	2.1
42341	1.3048	2.1
42342	1.40335	2.1
42343	0.926527	2.1
42344	1.40335	2.1
42345	1.3048	2.1
42346	0.833871	2.1
42347	0.879657	2.1
42348	0.937695	2.1
42349	0.946129	2.1
42350	0.946129	2.1
42351	0.851541	2.1
42352	0.851541	2.1
42353	1.1126	2.1
42354	1.04519	2.1
42355	0.851486	2.1
42356	0.980501	2.1
42357	0.898251	2.1
42358	0.851528	2.1
42359	1.37756	2.1
42360	2.73081	2.1
42361	2.44311	2.1
42362	2.44311	2.1
42363	1.66428	2.1
42364	1.66428	2.1
42365	1.89226	2.1
42366	1.89226	2.1
42367	1.89226	2.1
42368	1.89226	2.1
42369	3.78452	2.1
42370	1.65781	2.1
42371	1.89837	2.1
42372	1.46713	2.1
42373	1.98027	2.1
42374	3.63808	2.1
42375	2.29054	2.1
42376	2.26369	2.08716
42377	1.98027	2.1
42378	2.15617	2.03162
42379	4.24396	2.1
42380	1.89226	2.1
42381	1.89226	2.08769
42382	1.89226	2.1
42383	1.89226	2.07338
42384	3.78452	2.1
42385	0.946131	2.1
42386	0.946131	2.1
42387	0.946128	2.1
42388	0.946128	2.1
42389	0.946129	2.1
42390	0.946129	2.1
42391	0.94613	2.1
42392	0.94613	2.1
42393	0.94613	2.1
42394	0.94613	2.1
42395	0.94613	2.1

42396	0.94613	2.1
42397	0.812186	2.1
42398	0.812186	2.1
42399	0.819781	2.1
42400	0.819781	2.1
42401	0.913532	2.1
42402	0.990134	2.1
42403	0.733564	2.1
42404	0.641833	2.1
42405	0.641832	2.1
42406	0.913533	2.1
42407	0.733564	2.1
42408	0.990134	2.1
42409	0.994262	2.08628
42410	0.994262	2.1
42411	0.997354	2.1
42412	0.997354	2.06927
42413	1.00775	1.95801
42414	1.07808	2.00745
42415	0.913533	2.1
42416	0.990135	2.1
42417	0.913532	2.1
42418	1.00775	2.1
42419	0.990134	2.1
42420	1.07808	2.1
42421	0.946131	2.1
42422	0.946131	2.08693
42423	0.946128	2.1
42424	0.946128	2.0907
42425	0.946128	2.06842
42426	0.946128	1.97054
42427	0.94613	2.1
42428	0.94613	2.1
42429	0.946129	2.1
42430	0.946129	2.1
42431	0.946129	2.1
42432	0.946129	2.1
42433	0.816232	2.1
42434	0.760038	2.1
42435	0.845999	2.1
42436	0.845999	2.1
42437	1.0631	2.1
42438	1.28131	2.1
42439	0.474065	2.1
42440	0.591905	2.1
42441	0.601756	2.1
42442	1.23903	2.1
42443	0.988592	2.1
42444	0.49051	2.1
42445	2.57021	2.1
42446	1.89226	2.1
42447	2.68172	2.1
42448	1.89226	2.1
42449	4.46247	2.1
42450	1.89226	2.1
42451	1.89226	1.82795
42452	1.89226	2.08892
42453	1.89226	2.01682

42454	3.78452	2.07381
42455	1.12573	2.1
42456	1.65504	1.99931
42457	1.38848	2.1
42458	1.38848	2.1
42459	1.30567	2.08798
42460	1.30567	2.1
42461	2.82413	2.1
42462	1.89226	2.1
42463	2.68172	2.1
42464	2.68172	2.1
42465	1.89226	2.1
42466	1.89226	2.1
42467	1.89226	2.1
42468	1.89226	2.09338
42469	1.89226	2.1
42470	1.89226	1.60846
42471	3.78452	2.09656
42472	1.30567	2.1
42473	1.65504	2.08914
42474	1.52724	2.1
42475	1.89249	2.1
42476	3.18228	2.1
42477	2.57386	0.0847709
42478	1.89226	0.163201
42479	1.89226	0.16368
42480	2.66753	0.0956285
42481	1.89226	0.138914
42482	2.66753	0.0898351
42483	1.89226	0.084545
42484	1.89226	0.18002
42485	2.2973	0.170202
42486	3.12479	0.105148
42487	1.89226	0.153552
42488	2.57385	0.0939342
42489	0.489293	2.1
42490	0.672698	2.1
42491	0.59038	2.1
42492	0.738357	2.1
42493	0.62269	2.1
42494	0.62269	2.1
42495	0.640388	2.1
42496	0.640388	2.1
42497	0.59038	2.1
42498	0.489293	2.1
42499	0.738357	2.1
42500	0.672698	2.1
42501	1.89226	2.1
42502	1.89226	2.1
42503	1.89226	2.1
42504	1.89226	2.09836
42505	3.78452	2.1
42506	2.82858	2.1
42507	1.89226	2.1
42508	2.5671	2.1
42509	1.89226	2.1
42510	2.5671	2.1
42511	1.89226	2.1

42512	2.06581	0.0698591
42513	2.47308	0.0698591
42514	1.89433	0.0698591
42515	1.67571	0.0698591
42516	3.87274	0.0698591
42517	2.55791	0.0698591
42518	2.10276	0.0698591
42519	2.47308	0.0698591
42520	2.47308	0.0698591
42521	1.83415	0.0698591
42522	1.90922	0.0698591
42523	1.18421	0.0698591
42524	2.03277	0.0698591
42525	1.18421	0.0698591
42526	2.01275	0.0698591
42527	1.42818	0.0698591
42528	2.25673	0.0698591
42529	0.882904	0.0698591
42530	0.958492	0.0698591
42531	0.905756	0.0698591
42532	0.905756	0.0698591
42533	1.06352	0.0698591
42534	0.797654	0.0698591
42535	1.11319	0.0698591
42536	0.716795	0.0698591
42537	0.820721	0.0698591
42538	0.731963	0.0698591
42539	1.13143	0.0698591
42540	1.10528	0.0698591
42541	1.89226	0.0698591
42542	1.89226	0.0698591
42543	1.89226	0.0698591
42544	1.89226	0.0698591
42545	1.89226	0.0698591
42546	1.89226	0.0698591
42547	1.01241	0.0698591
42548	0.703929	0.0698591
42549	0.946133	0.0698591
42550	0.946133	0.0698591
42551	1.04072	0.0698591
42552	1.163	0.0698591
42553	0.639952	0.0698591
42554	0.851543	0.0698591
42555	0.946132	0.0698591
42556	0.920761	0.0698591
42557	0.711038	0.0698591
42558	0.711038	0.0698591
42559	1.89226	0.0698591
42560	1.89226	0.0698591
42561	1.89226	0.0698591
42562	1.89226	0.0698591
42563	1.89226	0.0698591
42564	1.89226	0.0698591
42565	1.89226	0.0698591
42566	1.89226	0.0698591
42567	2.41075	0.0698591
42568	1.89226	0.0698591
42569	3.78451	0.0698591

42570	0.968476	2.06394
42571	1.20674	0.660408
42572	1.13456	0.633227
42573	1.13456	2.1
42574	1.02074	0.945084
42575	1.02074	2.0904
42576	1.02074	2.1
42577	1.20674	1.87239
42578	1.07866	1.79074
42579	1.28649	0.891287
42580	2.2854	2.04337
42581	1.94221	0.149189
42582	1.36717	0.13815
42583	1.45535	0.147042
42584	1.93771	0.125806
42585	3.39756	0.14597
42586	1.94222	0.174215
42587	2.25035	0.127622
42588	2.41806	0.145175
42589	2.42256	0.131631
42590	1.93772	0.155382
42591	1.94222	0.136919
42592	1.49906	0.0992091
42593	1.93771	0.117936
42594	1.45535	0.120734
42595	1.45535	0.129206
42596	1.91484	0.0936129
42597	1.91484	0.0967692
42598	1.91484	0.0991044
42599	2.25035	0.122628
42600	1.93772	0.124678
42601	1.93772	0.127048
42602	2.11465	0.1008
42603	2.11465	0.0977111
42604	2.61735	0.147065
42605	1.5082	0.14239
42606	2.36481	0.182366
42607	1.58597	0.171375
42608	2.36481	0.141897
42609	1.46653	0.126831
42610	2.36482	0.189008
42611	1.08133	0.22648
42612	1.5082	0.136708
42613	2.01868	0.233003
42614	3.52688	0.228733
42615	0.94613	0.191234
42616	0.94613	0.179675
42617	0.946131	0.28709
42618	0.946131	0.302615
42619	0.946129	0.302138
42620	0.946129	0.229093
42621	0.946132	0.181178
42622	0.946132	0.245777
42623	0.94613	0.253944
42624	0.94613	0.279077
42625	0.946131	0.234215
42626	0.946131	0.229588
42627	2.53725	0.103818

42628	1.89227	0.136315
42629	1.89227	0.131685
42630	2.27073	0.128287
42631	1.89227	0.110798
42632	2.27073	0.111037
42633	1.89227	0.208264
42634	1.89227	0.344428
42635	1.89227	0.356299
42636	1.89227	0.273681
42637	1.89227	0.276847
42638	1.89227	0.212733
42639	1.86766	2.1
42640	2.83487	2.1
42641	2.84318	2.1
42642	2.59401	2.1
42643	2.11684	2.1
42644	1.86766	2.1
42645	1.10638	2.1
42646	2.11684	2.1
42647	1.29111	2.1
42648	2.37439	2.1
42649	3.40796	2.1
42650	2.56843	2.1
42651	1.89226	2.1
42652	1.89226	2.1
42653	2.71932	2.1
42654	4.46069	2.1
42655	1.89226	2.1
42656	1.89226	2.1
42657	1.89226	2.1
42658	1.89226	2.1
42659	3.78452	2.1
42660	0.6416	2.1
42661	0.697585	2.1
42662	0.670706	2.1
42663	0.598927	2.1
42664	0.644313	2.1
42665	0.644313	2.1
42666	0.652199	2.1
42667	0.652199	2.1
42668	0.697587	2.1
42669	0.670705	2.1
42670	0.598926	2.1
42671	0.641601	2.1
42672	1.89226	2.1
42673	1.89226	2.1
42674	1.89226	2.1
42675	1.89226	2.1
42676	1.89226	2.1
42677	1.89226	2.1
42678	0.697585	2.1
42679	0.730617	2.1
42680	0.712082	2.1
42681	0.670706	2.1
42682	0.69854	2.1
42683	0.69854	2.1
42684	0.702783	2.1
42685	0.702783	2.1

42686	0.730618	2.1
42687	0.712081	2.1
42688	0.697586	2.1
42689	0.670705	2.1
42690	1.89226	2.1
42691	1.89226	2.1
42692	1.89226	2.05458
42693	1.89226	2.1
42694	1.89226	2.03768
42695	1.89226	2.1
42696	2.0815	2.1
42697	1.12076	2.1
42698	1.38662	2.1
42699	2.01244	2.1
42700	3.41508	2.1
42701	1.89226	2.1
42702	2.54324	2.1
42703	1.91981	2.1
42704	1.89226	2.1
42705	2.57079	2.1
42706	2.54324	2.1
42707	2.54325	2.1
42708	1.94779	2.1
42709	1.76879	2.1
42710	1.73811	2.1
42711	2.5708	2.1
42712	2.54325	2.1
42713	1.89226	2.1
42714	2.57079	2.1
42715	1.91894	2.1
42716	1.89226	2.1
42717	2.59747	2.1
42718	2.57079	2.1
42719	2.5708	2.1
42720	1.82268	2.1
42721	1.79738	2.1
42722	1.77071	2.1
42723	2.59747	2.1
42724	2.5708	2.1
42725	1.89226	1.30586
42726	1.89226	2.1
42727	1.89226	1.02831
42728	1.89226	2.1
42729	3.78451	1.93959
42730	2.21582	0.99097
42731	1.94412	2.1
42732	2.13428	0.990641
42733	1.86292	2.1
42734	4.07839	1.92356
42735	1.94412	2.1
42736	1.5854	2.1
42737	1.86293	2.1
42738	1.50767	2.1
42739	3.45179	2.1
42740	1.89226	2.1
42741	1.89226	2.1
42742	1.89226	2.1
42743	1.89226	2.1

42744	3.78452	2.1
42745	1.89226	0.916473
42746	1.89226	2.1
42747	1.89226	1.03277
42748	1.89226	2.1
42749	3.78452	2.0417
42750	2.13428	0.871211
42751	1.86293	2.1
42752	2.04737	0.942174
42753	1.77122	2.1
42754	3.9055	1.89803
42755	1.86293	2.1
42756	1.50767	2.1
42757	1.41077	2.1
42758	1.41077	2.1
42759	1.77123	2.1
42760	1.86813	2.1
42761	0.810943	2.1
42762	0.810943	2.1
42763	0.810945	2.1
42764	0.810945	2.1
42765	0.810958	2.1
42766	0.810958	2.1
42767	1.89226	2.1
42768	1.35168	2.1
42769	1.08132	2.1
42770	1.08132	2.1
42771	1.0813	2.1
42772	0.745979	2.1
42773	0.745979	2.1
42774	0.778781	2.1
42775	0.778781	2.1
42776	0.59549	2.1
42777	0.649658	2.1
42778	0.994784	2.1
42779	0.875102	2.1
42780	0.875102	2.1
42781	0.59549	2.1
42782	0.649658	2.1
42783	0.994784	2.1
42784	1.57887	2.1
42785	1.75021	2.1
42786	1.13341	2.1
42787	1.13341	2.1
42788	1.19098	2.1
42789	1.63644	2.1
42790	2.5761	0.786242
42791	2.43404	2.07943
42792	2.44813	0.570643
42793	2.31751	2.09622
42794	4.88218	1.58779
42795	2.43405	2.07729
42796	2.22575	2.1
42797	2.31751	2.09995
42798	2.10893	2.1
42799	4.54298	2.1
42800	2.44813	0.461992
42801	2.31751	2.09523

42802	2.30908	0.754747
42803	2.19815	2.0677
42804	4.64628	1.4508
42805	2.31751	2.09485
42806	2.10893	2.1
42807	2.19815	2.07986
42808	1.98061	2.1
42809	4.30708	2.1
42810	0.94613	2.1
42811	0.94613	2.1
42812	1.40372	2.1
42813	1.40372	2.1
42814	1.40372	2.1
42815	1.37167	2.1
42816	0.946132	2.1
42817	0.946132	2.1
42818	0.946131	2.1
42819	1.40372	2.1
42820	0.94613	2.1
42821	1.37167	2.1
42822	2.80744	2.1
42823	2.06984	2.1
42824	2.83418	2.1
42825	2.74335	2.1
42826	2.16067	2.1
42827	2.06984	2.1
42828	1.077	0.231986
42829	0.809116	0.186206
42830	0.600419	0.26208
42831	0.790277	0.191475
42832	0.927647	0.175669
42833	0.758598	0.177959
42834	0.85749	0.238905
42835	0.85749	0.241538
42836	1.02417	0.19756
42837	0.600411	0.211913
42838	0.751701	0.203579
42839	1.07702	0.194531
42840	1.89226	2.1
42841	1.89226	2.1
42842	1.89226	2.1
42843	1.89226	2.1
42844	1.89226	2.1
42845	1.89226	2.1
42846	1.89226	2.1
42847	1.89226	2.1
42848	1.89226	2.1
42849	1.89226	2.1
42850	3.78453	2.1
42851	1.89226	2.1
42852	1.89226	2.1
42853	1.89226	2.1
42854	1.89226	2.1
42855	1.89226	2.1
42856	1.89226	2.1
42857	0.946131	2.1
42858	0.946131	2.1
42859	0.946131	2.1

42860	0.946131	2.1
42861	0.946133	2.1
42862	0.946133	2.1
42863	0.946128	2.1
42864	0.946128	2.1
42865	0.946131	2.1
42866	0.946131	2.1
42867	0.94613	2.1
42868	0.94613	2.1
42869	2.17159	2.1
42870	2.00083	2.00209
42871	2.21305	2.1
42872	2.00551	2.1
42873	2.20837	2.1
42874	2.00083	2.09933
42875	2.20837	2.0925
42876	1.80328	0.929074
42877	2.22962	2.03091
42878	2.00083	1.92583
42879	2.03207	1.08677
42880	1.80328	1.50161
42881	2.28564	2.1
42882	2.20837	1.96414
42883	2.30406	2.1
42884	2.17159	2.1
42885	2.34084	2.1
42886	2.20837	2.1
42887	1.09523	0.846394
42888	1.09523	1.98183
42889	1.09934	2.1
42890	1.09934	2.1
42891	1.01603	0.731419
42892	1.09038	1.95575
42893	1.10419	1.83119
42894	1.17042	2.1
42895	1.17042	2.03057
42896	1.09038	2.00825
42897	1.10419	1.89615
42898	1.01603	2.03175
42899	2.60152	0.366222
42900	0.976334	0.607856
42901	2.22344	0.708754
42902	2.22344	0.387506
42903	1.36405	0.826404
42904	1.36405	0.23602
42905	1.89226	2.1
42906	1.70302	2.1
42907	1.69019	2.1
42908	1.70302	2.1
42909	1.73933	2.1
42910	1.70303	2.08578
42911	1.89226	2.1
42912	1.89226	2.02855
42913	1.89226	2.1
42914	1.89226	2.1
42915	3.78452	2.1
42916	1.89226	2.09822
42917	1.59036	2.1

42918	1.65683	2.1
42919	1.59036	2.1
42920	1.95873	2.1
42921	1.89226	1.9948
42922	0.946131	1.85416
42923	0.946131	2.03748
42924	0.946127	2.1
42925	0.946127	2.1
42926	0.946127	2.1
42927	0.946127	1.70864
42928	0.946131	2.1
42929	0.946131	2.05617
42930	0.946129	2.1
42931	0.946129	2.1
42932	0.94613	2.09891
42933	0.94613	2.1
42934	0.851541	2.1
42935	0.851541	2.1
42936	0.851483	2.1
42937	1.15713	2.1
42938	0.851539	2.1
42939	0.851539	2.1
42940	0.942127	2.1
42941	1.14455	2.1
42942	0.838683	2.1
42943	0.946131	2.1
42944	0.957503	2.1
42945	0.957504	2.1
42946	1.89226	1.41415
42947	1.89226	0.879045
42948	1.89226	0.750513
42949	1.89226	2.1
42950	3.78452	0.771067
42951	1.89226	0.130873
42952	1.89226	0.48991
42953	1.89226	0.470896
42954	1.89226	0.491496
42955	1.89226	0.17
42956	1.89226	0.0841375
42957	1.89226	0.518278
42958	1.89226	0.384813
42959	1.89226	0.33172
42960	1.89226	0.773487
42961	3.78453	0.300043
42962	1.89226	0.979505
42963	1.89226	2.1
42964	1.89226	2.1
42965	1.89226	2.06478
42966	3.78453	2.1
42967	1.22575	0.404643
42968	1.05438	1.26826
42969	0.946131	0.741192
42970	0.946131	0.952706
42971	0.946129	1.92509
42972	1.22575	0.474529
42973	1.05438	0.531913
42974	0.946132	0.337221
42975	1.05438	0.766758

42976	1.05438	1.14285
42977	0.946131	0.587156
42978	0.946131	1.12895
42979	2.4515	0.364967
42980	1.13726	0.334765
42981	2.10875	0.22564
42982	1.49515	0.216015
42983	2.10875	0.350446
42984	1.49515	0.173847
42985	0.831477	0.122272
42986	0.747575	0.34885
42987	1.22575	0.352242
42988	1.28174	0.183614
42989	1.28174	0.287779
42990	0.831476	0.245355
42991	1.22575	0.352245
42992	0.747575	0.295212
42993	1.03561	0.352257
42994	1.03561	0.357647
42995	1.02162	0.182967
42996	1.02162	0.103653
42997	1.89227	2.09605
42998	2.56348	1.30328
42999	1.89227	2.1
43000	2.45151	2.0932
43001	1.89227	2.1
43002	2.45151	1.02983
43003	1.40013	0.588063
43004	1.89226	1.25043
43005	1.89226	0.926447
43006	0.946643	0.574438
43007	1.89226	0.981381
43008	0.946643	0.665364
43009	1.89226	0.0698591
43010	1.89226	0.0724643
43011	1.89226	0.0698591
43012	1.89226	0.0707718
43013	3.78453	0.0705961
43014	1.89226	0.0922185
43015	1.89226	0.14
43016	1.89226	0.0935505
43017	1.89226	0.193006
43018	3.78453	0.0974907
43019	0.946131	0.155861
43020	0.946131	0.129597
43021	0.946128	0.0816082
43022	0.946128	0.0896211
43023	0.94613	0.140526
43024	0.94613	0.164334
43025	0.946129	0.0868986
43026	0.946129	0.085492
43027	0.94613	0.122362
43028	0.94613	0.0740969
43029	0.94613	0.100942
43030	0.94613	0.163958
43031	1.89226	0.0698591
43032	1.89226	0.0698591
43033	1.17244	0.0698591

43034	1.89226	0.0698591
43035	0.975291	0.0698591
43036	1.57408	0.0698591
43037	0.946132	0.0911539
43038	0.946132	0.0714083
43039	0.946131	0.0911612
43040	0.946131	0.0709716
43041	0.946132	0.0836736
43042	0.946132	0.0984278
43043	0.946131	0.0715853
43044	0.946131	0.0721192
43045	0.946131	0.0862255
43046	0.946131	0.0827566
43047	0.946132	0.0731237
43048	0.946132	0.0898521
43049	2.74744	0.0853707
43050	1.89226	0.207649
43051	2.70805	0.113396
43052	1.89226	0.126687
43053	2.70805	0.147087
43054	1.89226	0.171526
43055	2.74744	0.0759501
43056	1.89226	0.0844751
43057	2.57465	0.107716
43058	1.89226	0.178484
43059	4.6397	0.0850172
43060	1.89226	0.223139
43061	1.89226	0.55562
43062	1.89226	0.393979
43063	1.89226	0.668709
43064	3.78452	0.452179
43065	1.89226	0.287991
43066	1.89226	0.749741
43067	1.89226	0.284551
43068	1.89226	0.209098
43069	1.89226	0.740408
43070	1.89226	0.678604
43071	2.16206	0.19578
43072	2.67861	0.16741
43073	3.11996	0.206307
43074	1.70303	0.111606
43075	2.89015	0.33352
43076	1.486	0.198959
43077	2.12839	0.116504
43078	2.83631	0.214733
43079	3.08096	0.158004
43080	1.84157	0.114546
43081	3.08096	0.298826
43082	1.84157	0.167201
43083	1.89225	1.67538
43084	1.89225	0.827096
43085	1.89225	1.01666
43086	1.89225	1.47445
43087	1.89225	0.881179
43088	1.89225	1.21799
43089	1.89226	0.610514
43090	1.89226	0.765827
43091	1.89226	1.15501

43092	1.89226	0.489103
43093	3.78452	0.511237
43094	1.89226	0.307415
43095	2.49041	0.286637
43096	1.89226	0.346253
43097	1.89226	0.168128
43098	2.27073	0.169593
43099	2.27073	0.28616
43100	1.09904	0.209382
43101	0.677147	0.141712
43102	1.3365	0.0987089
43103	0.95759	0.434632
43104	0.957591	0.197619
43105	0.677148	0.23288
43106	1.09904	0.171614
43107	1.3365	0.254828
43108	0.996015	0.230792
43109	0.996015	0.173378
43110	1.01764	0.462196
43111	1.01764	0.197112
43112	2.77349	0.27192
43113	2.72723	0.170028
43114	2.43156	0.293381
43115	2.4564	0.166016
43116	5.20505	0.247609
43117	2.77349	0.25793
43118	2.68508	0.152825
43119	2.33761	0.232248
43120	2.4564	0.161149
43121	5.1111	0.182495
43122	2.43156	0.200367
43123	2.70208	0.144127
43124	1.85779	0.22284
43125	2.10695	0.138974
43126	4.77572	0.15319
43127	2.33761	0.182991
43128	2.70208	0.140118
43129	1.85779	0.200003
43130	1.61597	0.132274
43131	4.19542	0.164572
43132	0.946132	1.20567
43133	0.946132	1.54978
43134	0.746729	1.60906
43135	0.746729	1.84874
43136	0.946132	1.87267
43137	0.915958	1.63987
43138	0.946133	1.18781
43139	0.74673	1.90647
43140	0.946133	1.57682
43141	0.946133	1.57565
43142	0.915958	1.63521
43143	0.746729	1.48743
43144	1.50743	2.1
43145	2.45501	2.1
43146	2.29118	2.1
43147	2.29118	2.1
43148	1.75482	2.1
43149	1.75482	2.1

43150	2.45501	2.1
43151	2.76927	2.05146
43152	2.29118	2.1
43153	2.64523	2.08012
43154	5.10024	2.1
43155	1.25559	2.1
43156	2.29118	2.1
43157	2.0815	2.1
43158	2.26387	2.1
43159	1.35668	2.1
43160	1.47667	2.1
43161	2.28481	2.1
43162	2.64523	2.08407
43163	2.50057	2.1
43164	2.50057	2.07492
43165	2.29118	2.1
43166	2.29118	2.08541
43167	0.946129	0.126079
43168	0.946129	0.111042
43169	1.23929	0.0814563
43170	1.15246	0.0991305
43171	0.946131	0.112018
43172	0.946131	0.141828
43173	1.15246	0.0992806
43174	1.15246	0.106072
43175	0.946131	0.0966523
43176	1.15246	0.120367
43177	0.946131	0.0953481
43178	1.23928	0.107974
43179	0.946128	0.167025
43180	0.946128	0.153057
43181	1.15246	0.12215
43182	0.942814	0.198189
43183	0.946131	0.120126
43184	0.946131	0.164379
43185	0.961347	0.111781
43186	0.942811	0.167312
43187	0.946131	0.131666
43188	0.961347	0.116424
43189	0.946131	0.112661
43190	1.15246	0.151239
43191	2.32334	0.152447
43192	1.57621	0.108635
43193	1.90335	0.118616
43194	1.24538	0.0962924
43195	3.47956	0.0972915
43196	0.965762	0.0736879
43197	0.965762	0.0861498
43198	1.00245	0.074331
43199	1.00245	0.0755688
43200	0.92786	0.0789663
43201	0.705297	0.0720827
43202	0.856414	0.0804794
43203	1.28968	0.0753402
43204	1.00306	0.0749312
43205	0.705303	0.0731396
43206	1.28967	0.0761053
43207	0.975478	0.0860904

43208	3.05308	2.1
43209	1.76816	2.1
43210	1.76816	2.1
43211	1.69129	2.1
43212	2.58731	2.1
43213	2.51043	2.1
43214	1.89226	0.0853282
43215	2.71464	0.0882204
43216	2.34627	0.0828515
43217	1.89226	0.104181
43218	2.34627	0.0768887
43219	1.89226	0.0964983
43220	2.34627	0.0811284
43221	2.71464	0.0825142
43222	1.98252	0.0758974
43223	1.98252	0.100915
43224	1.51026	0.0763554
43225	2.34627	0.0894849
43226	2.38698	0.815121
43227	2.35142	2.09731
43228	2.60343	1.79246
43229	2.6636	0.858651
43230	4.99041	1.61771
43231	1.07811	2.1
43232	1.1952	2.1
43233	1.17571	1.98469
43234	1.30171	1.69342
43235	1.18322	1.8045
43236	1.18322	2.1
43237	1.18769	2.1
43238	1.18769	1.84487
43239	1.1952	2.08356
43240	1.30171	2.0737
43241	1.17571	2.08658
43242	1.07811	2.08576
43243	1.89226	1.75217
43244	2.6636	1.71095
43245	1.89226	0.471236
43246	2.60343	1.61551
43247	1.89226	1.01587
43248	2.60343	2.1
43249	1.5344	0.0698591
43250	1.4516	0.0698591
43251	1.17816	0.0698591
43252	1.21056	0.0698591
43253	2.71257	0.0698591
43254	1.89226	1.95899
43255	1.89226	0.94591
43256	1.89226	1.54073
43257	1.89226	1.63367
43258	3.78452	1.61951
43259	1.89226	1.52993
43260	1.89226	0.514355
43261	1.89226	1.03887
43262	1.89226	0.753955
43263	1.65573	1.12445
43264	1.65573	0.829484
43265	1.89226	1.47804

43266	1.89226	1.66861
43267	1.89226	1.9839
43268	2.64212	0.956151
43269	3.78452	1.60511
43270	1.89226	2.1
43271	1.89226	2.1
43272	1.89226	2.1
43273	1.89226	2.1
43274	3.78452	2.1
43275	2.7367	2.1
43276	2.07947	2.1
43277	2.47319	2.1
43278	1.85057	2.1
43279	4.55267	2.1
43280	2.07948	2.1
43281	1.3294	2.1
43282	1.85057	2.1
43283	1.09962	2.1
43284	3.1791	2.1
43285	1.89226	2.1
43286	1.89226	2.1
43287	1.89226	2.1
43288	1.89226	2.1
43289	3.78453	2.1
43290	0.94613	2.1
43291	0.94613	2.1
43292	0.94613	2.1
43293	0.94613	2.1
43294	0.946128	2.1
43295	0.946128	2.1
43296	0.946132	2.1
43297	0.946132	2.1
43298	0.94613	2.1
43299	0.94613	2.1
43300	0.946131	2.1
43301	0.946131	2.1
43302	1.00875	2.1
43303	1.00875	2.1
43304	1.01676	2.1
43305	1.01676	2.1
43306	0.925282	2.1
43307	0.804877	2.1
43308	1.2366	2.1
43309	1.10023	2.1
43310	0.804878	2.1
43311	1.10023	2.1
43312	1.2366	2.1
43313	0.925284	2.1
43314	1.89226	2.1
43315	1.89226	2.1
43316	1.89226	2.1
43317	1.89226	2.1
43318	3.78453	2.1
43319	1.89226	2.1
43320	1.89226	2.1
43321	1.89226	2.1
43322	1.89226	2.1
43323	1.89226	2.1

43324	1.89226	2.1
43325	1.09766	2.1
43326	1.14842	2.1
43327	1.02909	2.1
43328	1.07659	2.1
43329	1.16718	2.1
43330	0.954189	2.1
43331	1.06653	2.1
43332	1.06653	2.1
43333	1.20738	2.1
43334	1.02909	2.1
43335	1.09767	2.1
43336	0.815951	2.1
43337	2.00793	2.1
43338	1.14048	2.1
43339	1.19086	2.1
43340	1.13723	2.1
43341	2.00793	2.1
43342	2.11772	2.1
43343	1.89226	2.1
43344	1.89226	2.1
43345	1.89226	2.1
43346	1.89226	2.1
43347	1.89226	2.1
43348	1.89226	2.1
43349	1.89226	2.1
43350	1.89226	2.1
43351	1.89226	2.1
43352	1.89226	2.1
43353	1.89226	2.1
43354	1.89226	2.1
43355	1.1779	2.1
43356	1.26166	2.1
43357	1.0875	2.1
43358	1.00397	2.1
43359	1.13286	2.1
43360	1.13286	2.1
43361	1.13277	2.1
43362	1.13277	2.1
43363	1.26166	2.1
43364	1.08749	2.1
43365	1.1779	2.1
43366	1.00396	2.1
43367	1.0875	2.1
43368	1.00397	2.1
43369	0.685749	2.1
43370	0.595432	2.1
43371	0.83976	2.1
43372	0.83976	2.1
43373	0.843169	2.1
43374	0.843169	2.1
43375	1.0875	2.1
43376	0.685749	2.1
43377	1.00397	2.1
43378	0.595432	2.1
43379	1.89226	2.1
43380	1.86443	2.1
43381	2.4988	2.1

43382	2.0815	2.1
43383	2.28173	2.1
43384	1.86443	2.1
43385	1.89226	2.1
43386	1.89226	2.1
43387	1.89226	2.1
43388	1.89226	2.1
43389	1.89226	2.1
43390	1.89226	2.1
43391	0.946131	2.1
43392	0.946131	2.1
43393	0.969491	2.1
43394	0.949105	2.1
43395	0.949103	2.1
43396	0.949103	2.1
43397	0.946133	2.1
43398	0.946133	2.1
43399	0.946131	2.1
43400	0.969492	2.1
43401	0.946131	2.1
43402	0.949105	2.1
43403	1.89226	2.1
43404	2.07428	2.1
43405	1.93464	2.1
43406	1.89226	2.1
43407	2.11667	2.1
43408	2.07428	2.1
43409	0.946132	2.1
43410	0.946132	2.1
43411	0.946131	2.1
43412	0.946131	2.1
43413	0.946132	2.1
43414	0.946132	2.1
43415	0.946132	2.1
43416	0.946132	2.1
43417	0.946132	2.1
43418	0.946132	2.1
43419	0.946132	2.1
43420	0.946132	2.1
43421	1.97992	2.1
43422	1.89226	2.1
43423	1.89226	2.1
43424	1.93898	2.1
43425	1.93898	2.1
43426	1.89226	2.1
43427	2.12475	0.385831
43428	2.79029	0.665678
43429	2.7803	0.965232
43430	2.13052	0.369059
43431	4.92081	0.510476
43432	1.06238	0.504036
43433	1.06526	0.372364
43434	0.642901	0.15461
43435	0.655055	0.266308
43436	0.856391	0.202019
43437	0.856391	0.325946
43438	0.86104	1.42018
43439	0.86104	0.152607

43440	1.06526	0.299023
43441	0.655055	0.324351
43442	0.642901	0.280967
43443	1.06238	0.273375
43444	2.7803	0.886495
43445	2.13053	0.329787
43446	2.14346	0.45687
43447	2.77937	0.664836
43448	4.9099	0.548487
43449	2.14346	0.500229
43450	1.31011	0.153774
43451	1.34124	0.349624
43452	1.31011	0.509552
43453	2.16166	0.231807
43454	2.13053	0.352271
43455	1.89226	2.1
43456	2.58176	2.1
43457	2.01422	2.1
43458	1.89226	2.1
43459	2.70373	2.1
43460	2.58176	2.1
43461	1.89226	2.1
43462	1.89226	2.1
43463	1.89226	2.1
43464	1.89226	2.1
43465	1.89226	2.1
43466	1.89226	2.1
43467	0.946128	2.1
43468	0.946128	2.1
43469	1.38521	2.1
43470	1.38521	2.1
43471	1.38521	2.1
43472	1.35186	2.1
43473	0.946131	2.1
43474	0.946131	2.1
43475	0.94613	2.1
43476	1.38521	2.1
43477	0.946131	2.1
43478	1.35186	2.1
43479	0.946133	2.1
43480	0.946133	2.1
43481	0.946132	2.1
43482	0.946132	2.1
43483	0.946132	2.1
43484	0.946132	2.1
43485	0.946132	2.1
43486	0.946132	2.1
43487	0.946132	2.1
43488	0.946132	2.1
43489	0.946133	2.1
43490	0.946133	2.1
43491	2.33374	2.1
43492	1.89226	2.1
43493	1.89226	2.1
43494	1.89226	2.1
43495	2.60377	2.1
43496	2.60377	2.1
43497	2.6771	0.0698591

43498	1.36416	0.0698591
43499	1.64983	0.0698591
43500	1.64983	0.0698591
43501	2.44131	0.0698591
43502	2.44131	0.0698591
43503	2.58321	0.175357
43504	1.89226	0.210727
43505	2.44283	0.113277
43506	2.44283	0.140457
43507	1.89226	0.381418
43508	1.89226	0.624193
43509	2.58321	0.248124
43510	2.44283	0.131712
43511	1.49358	0.0936294
43512	1.57702	0.139747
43513	3.93641	0.116334
43514	0.951913	0.0698591
43515	0.81556	0.0698591
43516	0.946132	0.0698591
43517	0.946132	0.0698591
43518	1.04072	0.0698591
43519	1.12182	0.0698591
43520	0.856745	0.0698591
43521	0.851542	0.0698591
43522	0.923163	0.0698591
43523	0.923163	0.0700093
43524	1.04304	0.0698591
43525	0.85098	0.0698591
43526	2.70125	1.11826
43527	2.37734	2.02979
43528	2.63495	1.18084
43529	2.30539	1.69484
43530	5.01229	1.81217
43531	1.89226	1.97771
43532	1.89226	2.1
43533	1.89226	1.48586
43534	1.89226	2.09893
43535	3.78452	1.7541
43536	1.89226	2.1
43537	1.89226	2.1
43538	1.89226	2.1
43539	1.89226	2.1
43540	3.78452	2.1
43541	2.37734	2.1
43542	1.79346	2.1
43543	2.53594	1.58687
43544	1.70302	2.1
43545	4.27682	2.1
43546	2.63495	0.915924
43547	2.30539	1.10263
43548	2.49445	0.258258
43549	2.12089	0.988549
43550	4.75584	0.679127
43551	1.89226	1.22417
43552	1.89226	1.9678
43553	1.89226	1.04162
43554	1.89226	1.77952
43555	3.78452	1.98147

43556	1.89226	2.0144
43557	1.89226	2.04742
43558	1.89226	2.08844
43559	1.89226	2.1
43560	1.89226	1.54572
43561	1.89226	1.73961
43562	2.53594	0.776743
43563	1.60017	1.61049
43564	1.57079	1.01419
43565	1.70302	1.33689
43566	2.15772	1.07159
43567	2.33299	1.12405
43568	2.09114	2.1
43569	2.34898	2.1
43570	2.30729	2.1
43571	2.06556	2.1
43572	4.41454	2.1
43573	2.09114	2.1
43574	1.59654	2.1
43575	1.57823	2.1
43576	2.06556	2.1
43577	3.6621	2.1
43578	2.30729	2.1
43579	2.06556	2.1
43580	2.03917	2.1
43581	2.26617	2.1
43582	4.33173	2.1
43583	2.03917	2.1
43584	2.06556	2.1
43585	2.045	2.1
43586	1.55767	2.1
43587	1.57823	2.1
43588	1.57823	2.1
43589	1.89225	2.1
43590	1.09726	2.1
43591	1.61196	2.1
43592	1.28597	2.1
43593	1.28597	0.0698591
43594	1.45633	2.1
43595	1.45633	2.1
43596	1.9544	0.0698591
43597	1.61196	2.1
43598	1.45633	2.1
43599	1.53735	2.1
43600	3.14931	2.1
43601	1.89226	2.1
43602	1.89226	2.1
43603	1.89226	2.1
43604	1.89226	2.1
43605	3.78453	2.1
43606	0.810946	2.1
43607	0.810946	2.1
43608	0.810947	2.1
43609	0.810947	2.1
43610	0.810933	2.1
43611	0.810933	2.1
43612	1.89226	2.1
43613	1.3517	2.1

43614	1.08132	2.1
43615	1.08132	2.1
43616	1.08133	2.1
43617	0.851539	2.1
43618	0.851539	2.1
43619	0.972053	2.1
43620	1.05632	2.1
43621	0.94613	2.1
43622	0.94613	2.1
43623	1.0279	2.1
43624	0.94991	2.1
43625	1.0279	2.1
43626	0.946131	2.1
43627	0.865643	2.1
43628	0.94613	2.1
43629	1.89226	2.1
43630	1.89226	2.1
43631	1.89226	2.1
43632	1.89226	2.1
43633	3.78452	2.1
43634	1.03882	2.1
43635	1.91121	2.1
43636	1.41833	2.1
43637	2.34547	2.1
43638	3.32954	2.1
43639	2.3979	2.1
43640	1.89226	2.1
43641	1.89226	2.1
43642	1.89226	2.1
43643	2.0558	2.1
43644	2.0558	2.1
43645	1.89226	2.1
43646	1.89226	2.07797
43647	1.89226	2.1
43648	1.89226	2.1
43649	3.78452	2.1
43650	1.05816	2.1
43651	1.05816	2.1
43652	0.842045	2.1
43653	1.05252	2.1
43654	1.36057	2.1
43655	1.14339	2.1
43656	0.873397	2.1
43657	0.585073	2.1
43658	0.873388	2.1
43659	1.15129	2.1
43660	0.69143	2.1
43661	1.1434	2.1
43662	1.93338	2.1
43663	1.85883	2.1
43664	1.51714	2.1
43665	1.85883	2.1
43666	1.54795	2.1
43667	1.89226	2.1
43668	1.89226	2.1
43669	1.89226	2.1
43670	1.89226	2.1
43671	1.89226	2.1

43672	3.78452	2.1
43673	0.844726	2.1
43674	0.844726	2.1
43675	0.960524	2.1
43676	1.16288	2.1
43677	0.851541	2.1
43678	0.851541	2.1
43679	0.929147	2.1
43680	1.15533	2.1
43681	0.844697	2.1
43682	0.946131	2.1
43683	0.851632	2.1
43684	0.946131	2.1
43685	1.89226	2.1
43686	1.70303	2.1
43687	1.74006	2.1
43688	1.70303	2.1
43689	1.99471	2.1
43690	1.93338	2.1
43691	0.946131	2.1
43692	0.946131	2.1
43693	0.946131	2.1
43694	0.946131	2.1
43695	0.94613	2.1
43696	0.94613	2.1
43697	0.946132	2.1
43698	0.946132	2.1
43699	0.946131	2.1
43700	0.946131	2.1
43701	0.946131	2.1
43702	0.946131	2.1
43703	1.58158	2.1
43704	1.89226	2.1
43705	1.89226	2.1
43706	1.89226	2.1
43707	1.68939	2.1
43708	1.68939	2.1
43709	0.946132	0.0698591
43710	0.946132	0.0698591
43711	1.12988	0.0698591
43712	1.30783	0.0698591
43713	1.30783	0.0698591
43714	0.946133	0.0698591
43715	0.946132	0.0698591
43716	1.12988	0.0698591
43717	1.30783	0.0698591
43718	1.30783	0.0698591
43719	0.946133	0.0698591
43720	0.946133	0.0698591
43721	1.89227	0.0698591
43722	1.89227	0.0698591
43723	1.89227	0.0698591
43724	1.89227	0.0698591
43725	1.89227	0.0698591
43726	1.89227	0.0698591
43727	1.89226	0.0698591
43728	1.89226	0.0698591
43729	1.89226	0.0698591

43730	1.89226	0.0698591
43731	1.89226	0.0698591
43732	1.89226	0.0698591
43733	0.946131	0.0698591
43734	0.946131	0.0698591
43735	1.30783	0.0698591
43736	1.37715	0.0698591
43737	1.37715	0.0698591
43738	0.946133	0.0698591
43739	1.30783	0.0698591
43740	0.946129	0.0698591
43741	1.37715	0.0698591
43742	1.37715	0.0698591
43743	0.946131	0.0698591
43744	0.946131	0.0698591
43745	1.17056	0.0698591
43746	0.591757	0.0698591
43747	0.522025	0.0698591
43748	0.929727	0.0698591
43749	0.929728	0.0698591
43750	1.17056	0.0698591
43751	0.591756	0.0698591
43752	0.522025	0.0698591
43753	0.889054	0.0698591
43754	0.889054	0.0698591
43755	0.803535	0.0698591
43756	0.803535	0.0698591
43757	1.49814	2.1
43758	2.37439	2.1
43759	2.63923	2.1
43760	2.63923	2.1
43761	1.29111	2.1
43762	1.29111	2.1
43763	0.946132	2.1
43764	0.946132	2.1
43765	1.18719	2.1
43766	1.18719	2.1
43767	0.94613	2.1
43768	0.94613	2.1
43769	1.1872	2.1
43770	1.31961	2.1
43771	0.946131	2.1
43772	1.31961	2.1
43773	1.1872	2.1
43774	0.94613	2.1
43775	2.49445	0.151123
43776	2.12089	0.586088
43777	2.20843	0.243538
43778	1.82805	0.260941
43779	4.32932	0.319148
43780	1.89226	0.664229
43781	1.89226	1.1386
43782	1.89226	0.445556
43783	1.89226	0.692596
43784	3.78452	0.437581
43785	0.94613	0.793496
43786	0.94613	0.868546
43787	0.946128	0.594282

43788	0.946128	1.06228
43789	0.946131	1.27796
43790	0.946131	1.05055
43791	0.946127	0.844172
43792	0.946127	0.767823
43793	0.946129	0.767189
43794	0.946129	0.621657
43795	0.946129	1.20877
43796	0.946129	0.89517
43797	1.82804	0.232572
43798	1.60017	0.694097
43799	1.87679	0.360578
43800	2.12088	0.388223
43801	1.35608	0.41027
43802	1.60017	0.439398
43803	2.20843	0.133087
43804	1.3216	0.128228
43805	1.44426	0.123838
43806	1.82805	0.172942
43807	1.80399	0.0978815
43808	2.20843	0.157151
43809	1.89226	0.213568
43810	1.89226	0.244122
43811	1.89226	0.394604
43812	1.89226	0.29383
43813	1.89226	0.270738
43814	1.89226	0.237892
43815	1.89226	0.402745
43816	1.89226	0.28855
43817	1.89226	0.230846
43818	1.89226	0.293961
43819	1.89226	0.505963
43820	1.89226	0.23577
43821	0.627705	0.112422
43822	0.634941	0.175505
43823	0.629317	0.115433
43824	0.783422	0.140391
43825	0.629328	0.156329
43826	0.448764	0.162215
43827	1.35608	0.201707
43828	1.00805	0.190074
43829	0.814295	0.149588
43830	0.598375	0.189072
43831	1.04461	0.160931
43832	1.09795	1.92507
43833	1.00775	2.05817
43834	1.61202	2.00966
43835	1.63419	1.98366
43836	2.64194	1.94025
43837	1.44801	1.71051
43838	1.61201	2.00774
43839	2.35614	1.75968
43840	2.27703	1.96428
43841	3.88904	1.91406
43842	2.35615	1.66616
43843	1.63419	1.89928
43844	2.29852	1.66168
43845	1.63419	1.90164

43846	2.27634	1.71892
43847	1.61202	2.00987
43848	0.973793	2.1
43849	2.2671	2.1
43850	2.32981	2.1
43851	2.32981	2.1
43852	0.990481	2.1
43853	0.990481	2.1
43854	1.50877	2.1
43855	1.11193	2.1
43856	2.29667	2.1
43857	1.94158	2.1
43858	3.45035	2.1
43859	2.29667	2.1
43860	1.89227	2.1
43861	1.94158	2.1
43862	1.94158	2.1
43863	1.89227	2.1
43864	1.89227	2.1
43865	2.51379	2.1
43866	1.30026	2.1
43867	1.84502	2.1
43868	2.72099	2.1
43869	4.35882	2.1
43870	1.89226	2.1
43871	2.721	2.1
43872	2.5138	2.1
43873	2.5138	2.1
43874	1.89226	2.1
43875	1.89226	2.1
43876	1.89226	0.0702423
43877	1.89226	0.072312
43878	2.59101	0.0698591
43879	1.89226	0.0847115
43880	3.78453	0.069907
43881	0.851541	0.0729512
43882	0.851541	0.0698591
43883	1.42502	0.0701
43884	0.948679	0.0698591
43885	0.946132	0.0720782
43886	0.946132	0.075328
43887	0.853617	0.069886
43888	0.853618	0.0698591
43889	0.851475	0.0719021
43890	0.948666	0.0735081
43891	1.166	0.0706988
43892	0.851554	0.0714263
43893	2.51818	2.09843
43894	2.60723	2.1
43895	2.66532	2.05979
43896	2.76315	2.1
43897	5.27255	2.1
43898	2.60723	2.1
43899	2.45247	2.1
43900	2.76315	2.1
43901	2.55197	2.1
43902	5.1592	2.1
43903	1.23768	0.236164

43904	1.11433	0.418386
43905	1.32037	1.70803
43906	1.32037	0.894889
43907	1.0142	0.887294
43908	1.0142	0.569348
43909	0.886711	0.568369
43910	1.16806	0.502674
43911	0.999083	1.83429
43912	0.710926	1.50103
43913	0.937757	1.57522
43914	0.937757	0.783627
43915	0.941224	0.392186
43916	0.941224	1.76986
43917	1.16805	1.27913
43918	0.999084	1.93575
43919	0.886709	1.44314
43920	0.710928	1.33598
43921	2.29332	2.1
43922	1.54904	2.1
43923	2.0815	2.1
43924	2.44199	2.1
43925	4.00418	2.1
43926	1.53873	0.805712
43927	1.53422	2.1
43928	1.39661	1.20829
43929	1.42387	2.09551
43930	2.9626	1.90317
43931	1.53423	2.1
43932	1.44091	2.1
43933	1.42387	2.09931
43934	1.35215	2.1
43935	2.86479	2.1
43936	1.39661	1.2639
43937	1.42387	2.09861
43938	1.28325	0.830435
43939	1.33326	2.1
43940	2.70713	1.88146
43941	1.42387	2.09666
43942	1.35215	2.1
43943	1.33327	2.1
43944	1.27339	2.1
43945	2.69726	2.1
43946	2.58442	2.1
43947	1.1468	2.1
43948	1.18775	2.1
43949	2.60047	2.1
43950	3.74728	2.1
43951	2.58442	2.1
43952	1.89226	2.1
43953	1.89226	2.1
43954	2.60048	2.1
43955	4.49274	2.1
43956	1.89226	2.1
43957	1.89226	2.1
43958	1.89226	2.1
43959	1.89226	2.1
43960	3.78453	2.1
43961	1.89226	2.1

43962	1.89226	2.1
43963	1.89226	2.1
43964	1.89226	2.1
43965	3.78452	2.1
43966	1.22262	2.1
43967	2.60047	2.1
43968	1.18775	2.1
43969	1.18775	2.1
43970	2.61433	2.1
43971	2.61433	2.1
43972	1.89226	2.1
43973	2.60048	2.1
43974	2.61434	2.1
43975	2.60048	2.1
43976	1.90612	2.1
43977	1.89226	2.1
43978	1.89226	2.1
43979	1.89226	2.1
43980	1.89226	2.1
43981	1.89226	2.1
43982	3.78453	2.1
43983	1.89226	2.1
43984	1.89226	2.1
43985	1.89226	2.1
43986	1.89226	2.1
43987	3.78452	2.1
43988	2.12556	2.1
43989	0.991113	2.1
43990	1.27725	2.1
43991	2.31081	2.1
43992	3.40281	2.1
43993	1.89226	2.1
43994	2.31082	2.1
43995	2.12556	2.1
43996	2.12556	2.1
43997	1.89226	2.1
43998	1.89226	2.1
43999	1.22544	2.1
44000	1.12649	2.1
44001	0.775254	2.1
44002	0.643133	2.1
44003	0.917924	2.1
44004	0.934955	2.1
44005	0.975579	2.1
44006	0.975579	2.1
44007	1.23113	2.1
44008	0.685272	2.1
44009	0.651719	2.1
44010	1.1791	2.1
44011	1.89226	2.1
44012	2.5138	2.1
44013	2.31082	2.1
44014	2.31082	2.1
44015	1.89226	2.1
44016	1.89226	2.1
44017	1.89226	2.1
44018	1.89226	2.1
44019	1.89226	2.1

44020	1.89226	2.1
44021	3.78453	2.1
44022	0.922224	2.1
44023	0.922224	2.1
44024	1.23555	2.1
44025	1.19715	2.1
44026	1.01445	2.1
44027	1.16061	2.1
44028	0.946132	2.1
44029	0.946132	2.1
44030	0.946131	2.1
44031	1.2051	2.1
44032	1.13537	2.1
44033	1.01445	2.1
44034	0.946133	2.1
44035	0.946133	2.1
44036	1.20648	2.1
44037	1.20648	2.1
44038	1.06454	2.1
44039	1.20648	2.1
44040	0.946133	2.1
44041	0.946133	2.1
44042	1.20649	2.1
44043	0.946133	2.1
44044	1.06454	2.1
44045	0.946132	2.1
44046	0.946132	2.1
44047	0.946132	2.1
44048	0.946132	2.1
44049	0.946132	2.1
44050	0.946132	2.1
44051	0.946132	2.1
44052	0.946133	2.1
44053	0.946133	2.1
44054	0.946133	2.1
44055	0.946133	2.1
44056	0.946132	2.1
44057	0.946132	2.1
44058	1.33078	2.0726
44059	1.37409	2.1
44060	1.31565	0.447806
44061	1.37114	2.1
44062	2.68973	2.09557
44063	1.89225	1.88802
44064	1.89225	2.1
44065	1.89225	0.466115
44066	1.89225	2.0802
44067	3.7845	1.54314
44068	1.89225	2.1
44069	1.89225	2.1
44070	1.89225	2.05071
44071	1.89225	2.1
44072	3.7845	2.1
44073	1.37409	2.1
44074	1.20408	2.1
44075	1.18612	2.1
44076	1.18907	2.1
44077	1.37114	2.1

44078	1.37409	2.1
44079	1.31566	2.05326
44080	1.37115	2.1
44081	1.30318	0.656691
44082	1.37433	2.1
44083	2.68998	2.04021
44084	1.89226	2.06275
44085	1.89226	2.1
44086	1.89226	0.489866
44087	1.89226	2.1
44088	3.78453	1.81661
44089	1.89226	2.09304
44090	1.89226	2.1
44091	1.89226	2.1
44092	1.89226	2.1
44093	1.89226	2.1
44094	1.89226	2.1
44095	0.647001	2.1
44096	0.647001	2.1
44097	0.651851	2.1
44098	0.651851	2.1
44099	0.602046	2.1
44100	0.613277	2.1
44101	0.685576	2.1
44102	0.687164	2.1
44103	0.687164	2.1
44104	0.613276	2.1
44105	0.602046	2.1
44106	0.685576	2.1
44107	0.946128	1.46459
44108	0.946128	0.751868
44109	1.15307	2.08361
44110	1.19317	2.03786
44111	0.94613	2.0301
44112	0.94613	0.660638
44113	1.15307	1.68394
44114	1.15307	2.07317
44115	0.946131	1.80297
44116	1.15307	1.8424
44117	0.946131	1.77379
44118	1.19317	1.76035
44119	1.18278	2.1
44120	2.38635	2.05479
44121	1.23042	2.1
44122	1.23042	2.1
44123	2.30614	2.1
44124	2.30614	2.1
44125	0.946129	0.913086
44126	0.946129	0.785323
44127	1.12994	1.92823
44128	1.15307	1.80254
44129	0.946131	1.76187
44130	0.946131	0.47981
44131	1.12994	1.04635
44132	1.12994	1.86439
44133	1.12994	1.35236
44134	0.946131	1.34
44135	0.946131	1.337

44136	1.15307	1.37686
44137	2.25988	2.0967
44138	2.30614	1.84946
44139	2.28696	2.1
44140	1.16361	2.1
44141	1.18278	2.1
44142	1.18278	2.09224
44143	2.3771	2.1
44144	1.90121	2.1
44145	1.81191	2.1
44146	2.46904	2.1
44147	4.37025	2.1
44148	1.15676	2.1
44149	1.23452	2.1
44150	0.823592	2.1
44151	0.737606	2.1
44152	0.988113	2.1
44153	0.988113	2.1
44154	0.984014	2.1
44155	0.984014	2.1
44156	1.15676	2.1
44157	0.737605	2.1
44158	0.823592	2.1
44159	1.23452	2.1
44160	1.30318	2.1
44161	1.37433	2.1
44162	1.30151	0.670078
44163	1.39067	2.1
44164	2.67584	2.05352
44165	1.89226	1.92492
44166	1.89226	2.1
44167	1.89226	0.656815
44168	1.89226	1.97677
44169	3.78453	1.5696
44170	1.89226	1.82802
44171	1.89226	2.1
44172	1.89226	2.06942
44173	1.89226	2.1
44174	1.89226	2.06404
44175	1.89226	2.1
44176	0.656819	2.1
44177	0.656819	2.09895
44178	0.661755	2.1
44179	0.661755	2.1
44180	0.613277	2.1
44181	0.63141	2.1
44182	0.687164	2.08296
44183	0.695336	2.1
44184	0.695337	2.1
44185	0.63141	2.1
44186	0.687164	2.1
44187	0.613276	2.1
44188	1.30151	2.02341
44189	1.39067	2.1
44190	1.32143	0.839889
44191	1.43294	2.09571
44192	2.73445	2.06302
44193	1.89226	1.96576

44194	1.89226	2.1
44195	1.89226	0.450943
44196	1.89226	2.1
44197	3.78452	1.76025
44198	1.89226	2.03045
44199	1.89226	2.06111
44200	1.89226	2.08059
44201	1.89226	2.1
44202	1.89226	2.1
44203	1.89226	2.1
44204	1.43294	2.02071
44205	1.26282	2.1
44206	1.3268	2.09938
44207	1.3268	2.1
44208	1.39067	2.09903
44209	1.39067	2.1
44210	1.5511	0.0946823
44211	1.00665	0.115097
44212	1.28239	0.0847953
44213	1.88631	0.0849717
44214	2.89296	0.0894042
44215	1.89227	0.0841224
44216	1.89227	0.133171
44217	1.89227	0.102708
44218	1.89227	0.0926408
44219	1.89227	0.113805
44220	1.89227	0.0995087
44221	2.08848	0.114936
44222	1.89226	0.11368
44223	1.89226	0.0867989
44224	2.37947	0.118952
44225	4.27173	0.113992
44226	1.01456	0.0936111
44227	0.943157	0.0918562
44228	0.641198	0.100134
44229	0.724757	0.0952837
44230	0.830923	0.100637
44231	0.830923	0.117051
44232	0.836991	0.0842957
44233	0.836991	0.100364
44234	1.01456	0.0890257
44235	0.724757	0.0984722
44236	0.641197	0.0979955
44237	0.943157	0.107467
44238	1.89227	0.110826
44239	1.89227	0.140152
44240	1.89227	0.0952679
44241	1.89227	0.0986803
44242	3.78453	0.100669
44243	0.946129	0.113612
44244	0.946129	0.110033
44245	1.28941	0.107789
44246	1.18974	0.111352
44247	0.94613	0.112311
44248	0.94613	0.121968
44249	1.28941	0.0994153
44250	1.28941	0.118294
44251	1.28941	0.104996

44252	0.946131	0.111383
44253	0.94613	0.110892
44254	1.18974	0.12029
44255	0.710636	2.1
44256	0.780249	2.1
44257	0.579033	2.1
44258	0.478812	2.1
44259	0.621822	2.1
44260	0.621822	2.1
44261	0.63724	2.1
44262	0.63724	2.1
44263	0.780251	2.1
44264	0.579032	2.1
44265	0.710637	2.1
44266	0.478811	2.1
44267	1.05499	2.099
44268	1.00521	2.05077
44269	1.24445	2.1
44270	1.23082	1.02417
44271	2.28581	2.06003
44272	1.48751	2.1
44273	1.23083	1.64683
44274	1.4941	2.08462
44275	1.24445	2.06335
44276	1.48047	1.8544
44277	1.23083	2.05989
44278	0.621185	1.96414
44279	0.621185	2.04858
44280	0.620899	1.85902
44281	0.620899	2.1
44282	0.502515	1.13175
44283	0.626672	0.752678
44284	0.615412	0.93683
44285	0.740234	2.1
44286	0.740233	0.807987
44287	0.626672	0.812521
44288	0.615413	1.04836
44289	0.502516	1.6064
44290	1.95844	2.1
44291	2.6562	2.1
44292	1.95844	2.1
44293	2.20883	2.1
44294	2.38225	2.1
44295	2.63264	2.1
44296	1.89226	2.1
44297	1.89226	2.1
44298	1.89226	2.1
44299	1.89226	2.1
44300	3.78453	2.1
44301	1.95844	2.1
44302	2.38225	2.1
44303	1.71082	2.1
44304	2.14647	2.1
44305	4.1049	2.1
44306	0.946131	2.1
44307	0.946131	2.1
44308	0.946131	2.1
44309	0.946131	2.1

44310	0.946131	2.1
44311	0.946131	2.1
44312	0.946131	2.1
44313	0.946131	2.1
44314	0.946131	2.1
44315	0.946131	2.1
44316	0.946131	2.1
44317	0.946131	2.1
44318	2.12597	2.1
44319	1.2295	2.1
44320	1.28014	2.1
44321	2.19723	2.1
44322	3.40611	2.1
44323	2.12597	2.1
44324	2.79242	2.1
44325	2.73375	2.1
44326	2.66249	2.1
44327	2.19723	2.1
44328	2.12597	2.1
44329	1.13716	2.1
44330	1.09861	2.1
44331	0.666692	2.1
44332	0.640071	2.1
44333	0.891617	2.1
44334	0.891617	2.1
44335	0.885614	2.1
44336	0.885614	2.1
44337	1.13716	2.1
44338	0.666692	2.1
44339	0.640071	2.1
44340	1.09861	2.1
44341	1.89226	0.0698591
44342	1.70303	0.0698591
44343	1.89226	0.0698591
44344	2.64598	0.0698591
44345	1.81091	0.0698591
44346	2.53222	0.0698591
44347	2.32422	0.0698591
44348	1.89226	0.0698591
44349	2.78336	0.0698591
44350	2.78336	0.0698591
44351	1.89226	0.0698591
44352	1.89226	0.0698591
44353	0.946132	0.0698591
44354	0.946132	0.0698591
44355	0.946131	0.0698591
44356	0.946131	0.0698591
44357	0.946132	0.0698591
44358	0.946132	0.0698591
44359	0.946131	0.0698591
44360	0.946131	0.0698591
44361	0.946131	0.0698591
44362	0.946131	0.0698591
44363	0.946131	0.0698591
44364	0.946131	0.0698591
44365	0.946131	0.0698591
44366	0.946131	0.0698591
44367	0.946132	0.0698591

44368	0.946132	0.0711569
44369	0.946132	0.0717211
44370	0.946132	0.0700363
44371	0.946131	0.0698591
44372	0.946131	0.0698591
44373	0.946131	0.0698591
44374	0.946131	0.06992
44375	0.946131	0.0698591
44376	0.946131	0.0698591
44377	2.24323	1.09545
44378	1.89226	1.82699
44379	2.75021	1.59611
44380	2.75021	1.66402
44381	1.89226	0.904492
44382	1.89226	1.24686
44383	2.30908	0.83638
44384	2.19815	2.06905
44385	2.1609	0.665124
44386	1.83796	2.1
44387	4.35904	1.64134
44388	2.19815	2.0667
44389	2.02268	2.1
44390	2.17869	2.1
44391	1.9559	2.1
44392	2.0815	2.1
44393	1.83424	2.1
44394	2.1609	0.644199
44395	1.88877	2.1
44396	1.98413	0.993389
44397	1.87355	2.09048
44398	4.03445	1.77661
44399	1.87355	2.09315
44400	2.02268	2.1
44401	1.87355	2.1
44402	2.10488	2.1
44403	1.68443	2.1
44404	1.89448	2.1
44405	0.946131	0.243263
44406	0.946131	0.203538
44407	1.40574	0.22769
44408	1.12373	0.281923
44409	0.946129	0.2641
44410	0.946129	0.245059
44411	1.40574	0.22509
44412	1.40574	0.223051
44413	0.946131	0.225238
44414	1.40574	0.236779
44415	1.12373	0.265482
44416	0.946131	0.242297
44417	2.14277	0.329606
44418	2.81148	0.281308
44419	1.42762	0.285157
44420	1.42762	0.355224
44421	2.96261	0.289858
44422	2.24746	0.333736
44423	1.89226	0.276278
44424	1.89226	0.146969
44425	1.89226	0.0817486

44426	1.89226	0.136044
44427	3.78453	0.180825
44428	1.89226	0.0902341
44429	1.89226	0.317935
44430	1.89226	0.204455
44431	1.89226	0.0710012
44432	3.78452	0.0881561
44433	0.68433	0.0846088
44434	0.946127	0.136487
44435	0.543921	0.123127
44436	0.946131	0.0739809
44437	0.946131	0.206448
44438	0.946131	0.145035
44439	0.543918	1.52575
44440	0.543918	0.111683
44441	0.684332	0.0879393
44442	0.54392	0.657171
44443	0.946129	0.102642
44444	0.946129	1.18938
44445	1.89226	0.153187
44446	1.08784	0.669971
44447	1.19352	0.184733
44448	1.89226	0.208484
44449	1.19352	0.937129
44450	1.89226	0.189368
44451	0.635711	0.0779264
44452	0.946132	0.204541
44453	0.946132	0.166968
44454	0.56474	0.075431
44455	0.750045	0.112396
44456	1.25657	0.677184
44457	0.635682	0.0937646
44458	0.635682	0.0959496
44459	0.891954	0.0815587
44460	1.32751	0.355967
44461	0.564766	0.0870582
44462	0.564766	0.473828
44463	0.858345	0.0911516
44464	0.946128	0.575414
44465	0.635712	0.102902
44466	0.946133	0.0887455
44467	0.977198	0.0876546
44468	0.944521	0.664851
44469	0.635682	0.584344
44470	0.635682	0.0750682
44471	1.02924	0.118467
44472	0.705625	0.903725
44473	0.851528	0.0820412
44474	0.851528	1.54135
44475	2.18903	2.1
44476	2.10182	2.1
44477	2.10182	2.1
44478	1.89226	2.1
44479	1.89226	2.1
44480	1.64983	2.1
44481	1.89226	2.1
44482	1.83867	2.1
44483	1.83867	2.1

44484	1.89164	2.1
44485	2.0815	2.1
44486	2.17638	2.1
44487	2.57957	0.837572
44488	2.3506	2.07396
44489	2.69056	0.384597
44490	2.49439	1.83391
44491	5.04116	1.11515
44492	2.3506	2.0131
44493	1.8911	2.1
44494	2.49439	1.48298
44495	2.06689	2.1
44496	4.41748	2.1
44497	2.69056	0.785713
44498	2.49439	2.04438
44499	2.77227	0.335164
44500	2.63443	1.89667
44501	5.32499	1.43002
44502	2.49439	1.34046
44503	2.06689	2.1
44504	2.63443	1.54391
44505	2.26444	2.1
44506	4.70131	2.07895
44507	2.46327	2.1
44508	2.54347	2.1
44509	2.52869	2.1
44510	2.16534	2.1
44511	2.82661	2.1
44512	2.46327	2.1
44513	0.946131	2.1
44514	0.946131	2.1
44515	1.27174	2.1
44516	1.27174	2.1
44517	1.27174	2.1
44518	0.94613	2.1
44519	0.946128	2.1
44520	1.4133	2.1
44521	0.946129	2.1
44522	0.946129	2.1
44523	1.27174	2.1
44524	1.4133	2.1
44525	1.42757	2.1
44526	1.7457	2.1
44527	1.66047	2.1
44528	1.7457	2.1
44529	1.45754	2.1
44530	1.54278	2.1
44531	1.42757	2.1
44532	1.52996	2.1
44533	1.66047	2.1
44534	1.66047	2.1
44535	1.26397	2.1
44536	1.26397	2.1
44537	0.968536	2.1
44538	1.02461	2.1
44539	1.57575	2.1
44540	1.62391	2.1
44541	2.59245	2.1

44542	0.946131	2.1
44543	0.946131	2.1
44544	0.946132	2.1
44545	0.946132	2.1
44546	0.946132	2.1
44547	0.946132	2.1
44548	0.946132	2.1
44549	0.946132	2.1
44550	0.946132	2.1
44551	0.946132	2.1
44552	0.946132	2.1
44553	0.946132	2.1
44554	2.23765	2.1
44555	1.62391	2.1
44556	2.22154	2.1
44557	1.57575	2.1
44558	2.2697	2.1
44559	1.62391	2.1
44560	1.89226	2.1
44561	1.89226	2.1
44562	1.89226	2.1
44563	1.89226	2.1
44564	1.89226	2.1
44565	1.89226	2.1
44566	1.89226	2.1
44567	1.89226	2.1
44568	1.89226	2.1
44569	1.89226	2.1
44570	1.89226	2.1
44571	1.89226	2.1
44572	2.2697	2.1
44573	1.46129	2.1
44574	2.25873	2.1
44575	1.62391	2.1
44576	2.09611	2.1
44577	1.46129	2.1
44578	1.89226	2.1
44579	2.07088	2.1
44580	1.89226	2.1
44581	2.31884	2.1
44582	4.21111	2.1
44583	1.89226	2.1
44584	2.31884	2.1
44585	2.11713	2.1
44586	1.89226	2.1
44587	2.54371	2.1
44588	2.31884	2.1
44589	2.54371	2.1
44590	1.24041	2.1
44591	2.55208	2.1
44592	2.31884	2.1
44593	1.23712	2.1
44594	1.00388	2.1
44595	2.72769	0.0698591
44596	2.57507	0.0743897
44597	1.89226	0.0698591
44598	1.89226	0.0734398
44599	4.46733	0.0699912

44600	1.89226	0.0744226
44601	2.57506	0.0739596
44602	1.89226	0.0698591
44603	2.30076	0.0706369
44604	1.89226	0.0698591
44605	2.30076	0.0747778
44606	1.21171	0.0698591
44607	1.48589	0.0698591
44608	2.09166	0.0698591
44609	1.78381	0.0698591
44610	3.2697	0.0698591
44611	2.09166	0.0698591
44612	1.70302	0.0698591
44613	1.48589	0.0698591
44614	1.18027	0.0698591
44615	2.26571	0.0698591
44616	1.79969	0.0698591
44617	1.89226	0.0698591
44618	1.89226	0.0761791
44619	1.89226	0.0797805
44620	1.89226	0.0699592
44621	1.89226	0.0779996
44622	1.89226	0.0700046
44623	0.946131	0.0698591
44624	0.946131	0.0822273
44625	0.946128	0.0774913
44626	0.946128	0.070262
44627	0.946129	0.078498
44628	0.946129	0.0793901
44629	0.94613	0.0698591
44630	0.94613	0.0698591
44631	0.94613	0.0709511
44632	0.94613	0.0726058
44633	0.94613	0.072981
44634	0.94613	0.0713904
44635	1.89226	1.82179
44636	1.89226	1.85399
44637	1.89226	1.95519
44638	1.89226	1.5733
44639	1.89226	1.9542
44640	1.89226	1.5599
44641	1.89226	1.8173
44642	1.89226	1.81434
44643	1.89226	1.92523
44644	1.89226	1.5907
44645	1.89226	1.93339
44646	1.89226	1.60246
44647	1.89226	1.59097
44648	1.89226	1.61429
44649	1.89226	1.82585
44650	1.89226	1.17524
44651	1.89226	1.85442
44652	1.89226	1.23618
44653	1.79103	1.33536
44654	0.968249	2.1
44655	1.44807	2.08801
44656	2.17984	0.873326
44657	3.2391	2.04505

44658	1.29735	1.54053
44659	1.08992	1.56786
44660	0.945147	2.09993
44661	0.724033	2.1
44662	1.00723	2.1
44663	1.00723	1.87442
44664	1.01416	1.20912
44665	1.01416	2.09166
44666	1.29735	2.0155
44667	0.94515	2.00459
44668	0.724034	2.06992
44669	1.08992	2.0825
44670	1.39214	2.1
44671	1.39214	2.1
44672	0.946129	2.1
44673	0.946129	2.1
44674	1.33728	2.1
44675	1.39214	2.1
44676	0.94613	2.1
44677	0.94613	2.1
44678	0.94613	2.1
44679	1.33728	2.1
44680	0.94613	2.1
44681	1.39214	2.1
44682	2.78428	2.1
44683	2.28373	2.1
44684	2.67456	2.1
44685	2.20011	2.1
44686	4.95829	2.1
44687	1.90165	0.277119
44688	2.24206	0.810041
44689	1.66137	0.196815
44690	2.07108	1.80621
44691	3.90343	0.404426
44692	1.89224	0.643723
44693	1.89224	0.847045
44694	1.89224	0.32068
44695	1.89224	0.270006
44696	3.78448	0.312794
44697	2.82159	0.79519
44698	1.89226	0.661029
44699	2.50138	1.77761
44700	1.89226	0.339306
44701	4.39365	0.648696
44702	1.66139	0.177478
44703	1.86399	1.88597
44704	1.56005	0.490343
44705	1.90324	0.963101
44706	3.56463	0.609271
44707	0.946133	0.103666
44708	0.946133	1.01204
44709	0.946132	0.309583
44710	0.946132	0.794289
44711	0.946133	0.364016
44712	0.946133	0.992957
44713	0.946132	0.298205
44714	0.946132	0.367157
44715	0.946133	0.245684

44716	0.946133	0.305119
44717	0.946132	0.170357
44718	0.946132	0.259443
44719	1.5176	0.362886
44720	1.97964	0.20374
44721	2.32889	0.285754
44722	1.91762	0.262044
44723	3.84649	0.23675
44724	2.3376	0.265054
44725	1.97963	0.234094
44726	2.32889	0.348662
44727	2.58303	0.200814
44728	4.66649	0.30448
44729	2.32889	0.250591
44730	2.20248	0.212589
44731	1.91762	0.209957
44732	1.91762	0.191689
44733	2.5615	0.32287
44734	2.5615	0.256622
44735	2.32889	0.347356
44736	2.58303	0.187251
44737	2.71793	0.264629
44738	2.56149	0.208006
44739	5.04681	0.208315
44740	1.89226	0.1502
44741	2.53785	0.184769
44742	1.89226	0.104838
44743	2.60895	0.135596
44744	1.89226	0.0928768
44745	2.60895	0.095581
44746	0.493248	0.102807
44747	1.30448	0.197175
44748	1.26893	0.306947
44749	0.653226	0.0899118
44750	0.653227	0.146556
44751	0.493247	0.728135
44752	1.26893	0.309978
44753	1.30448	0.198453
44754	0.832232	0.302634
44755	0.832233	0.401994
44756	0.929944	0.0917361
44757	0.929944	0.291655
44758	1.89226	2.1
44759	1.47937	2.1
44760	1.50143	2.1
44761	1.47937	2.1
44762	1.91432	2.1
44763	1.89226	2.1
44764	1.89226	2.1
44765	1.89226	2.1
44766	1.89226	2.1
44767	1.89226	2.1
44768	1.89226	2.1
44769	1.89226	2.1
44770	1.33419	2.1
44771	2.4542	2.1
44772	2.59834	2.1
44773	2.59834	2.1

44774	1.25716	2.1
44775	1.25716	2.1
44776	1.70303	2.1
44777	1.50143	2.1
44778	1.60878	2.1
44779	1.50143	2.1
44780	1.93599	2.1
44781	1.89226	2.1
44782	0.946131	2.09395
44783	0.946131	2.1
44784	0.946127	2.1
44785	0.946127	2.1
44786	0.946127	2.1
44787	0.946127	2.07063
44788	0.946131	2.1
44789	0.946131	2.1
44790	0.94613	2.1
44791	0.94613	2.1
44792	0.946129	2.1
44793	0.946129	2.1
44794	1.43912	2.1
44795	2.59834	2.1
44796	2.78093	2.1
44797	2.78093	2.1
44798	1.33419	2.1
44799	1.33419	2.1
44800	1.89226	2.1
44801	2.26322	2.1
44802	2.55682	2.1
44803	2.55682	2.1
44804	1.89226	2.1
44805	1.89226	2.1
44806	2.58032	0.185102
44807	2.28922	0.117556
44808	1.88424	0.119375
44809	2.32127	0.111721
44810	4.46456	0.120484
44811	1.67265	0.179361
44812	2.28922	0.135202
44813	1.88424	0.100216
44814	1.11314	0.100338
44815	3.55689	0.117222
44816	1.43751	0.0861282
44817	2.32127	0.111283
44818	1.92998	0.0809148
44819	2.37671	0.0878716
44820	1.43751	0.148242
44821	1.88424	0.143556
44822	2.36729	2.1
44823	1.56714	2.1
44824	1.56714	2.1
44825	1.62408	2.1
44826	2.28373	2.1
44827	2.34067	2.1
44828	0.937268	2.1
44829	0.937268	2.1
44830	0.94636	2.1
44831	0.94636	2.1

44832	0.76004	2.1
44833	0.783572	2.1
44834	1.14187	2.1
44835	1.10006	2.1
44836	1.10006	2.1
44837	0.76004	2.1
44838	0.783572	2.1
44839	1.14187	2.1
44840	1.28753	2.1
44841	1.36838	2.1
44842	1.62617	2.1
44843	1.82857	2.1
44844	3.1161	2.1
44845	1.82856	2.1
44846	1.17996	2.1
44847	1.53417	2.1
44848	1.56618	2.1
44849	1.36838	2.1
44850	1.36838	2.1
44851	1.35065	0.98828
44852	1.42659	0.283969
44853	2.26675	0.690759
44854	2.26675	0.522292
44855	1.62137	0.521732
44856	2.53747	0.655537
44857	1.89226	0.0826167
44858	1.89226	0.0698591
44859	1.89226	0.0698591
44860	1.89226	0.0764764
44861	3.78452	0.0740296
44862	1.36569	0.0698591
44863	1.36569	0.0698591
44864	0.946131	0.0698591
44865	0.946131	0.0698591
44866	1.16041	0.0698591
44867	1.36569	0.0698591
44868	0.946129	0.0698591
44869	0.946129	0.0698591
44870	1.1604	0.0698591
44871	0.94613	0.0698591
44872	0.946131	0.0698591
44873	1.36569	0.0698591
44874	2.73139	0.0698591
44875	1.25265	0.0698591
44876	1.25265	0.0698591
44877	1.64799	0.0698591
44878	2.32081	0.0698591
44879	2.71615	0.0698591
44880	0.946133	0.0698591
44881	0.946133	0.0698591
44882	0.946132	0.074786
44883	0.946132	0.0708006
44884	0.946132	0.0698591
44885	0.946132	0.0708229
44886	0.946133	0.0698591
44887	0.946133	0.0713525
44888	0.946132	0.0698591
44889	0.946132	0.0703098

44890	0.946133	0.0698591
44891	0.946133	0.0698591
44892	0.946131	0.0698591
44893	0.946131	0.0698591
44894	0.94613	0.0897055
44895	0.94613	0.0730864
44896	0.946132	0.0767611
44897	0.946132	0.0698677
44898	0.946129	0.0714842
44899	0.946129	0.0872731
44900	0.946131	0.0718149
44901	0.946131	0.0698591
44902	0.946131	0.0698591
44903	0.946131	0.0719476
44904	1.89226	2.1
44905	2.48503	2.1
44906	2.64586	2.1
44907	2.64586	2.1
44908	1.89226	2.1
44909	1.89226	2.1
44910	2.48504	2.1
44911	1.99985	2.1
44912	1.83584	2.1
44913	1.83584	2.1
44914	2.64586	2.1
44915	2.64586	2.1
44916	1.89225	2.1
44917	2.33095	2.1
44918	2.48501	2.1
44919	2.48501	2.1
44920	1.89225	2.1
44921	1.89225	2.1
44922	2.33096	2.1
44923	1.83583	2.1
44924	1.68436	2.1
44925	1.68436	2.1
44926	2.48502	2.1
44927	2.48502	2.1
44928	1.14807	2.1
44929	1.76545	2.1
44930	1.21292	2.1
44931	1.83936	2.1
44932	2.97837	2.1
44933	1.89226	2.1
44934	1.89226	2.1
44935	1.89226	2.1
44936	1.89226	2.1
44937	3.78451	2.1
44938	1.89226	2.1
44939	1.89226	2.03508
44940	1.89226	2.1
44941	1.89226	2.1
44942	3.78452	2.1
44943	1.76545	2.1
44944	2.12608	2.1
44945	1.83937	2.1
44946	2.22529	2.1
44947	3.99074	2.1

44948	0.780222	2.1
44949	0.780222	2.1
44950	0.784871	2.1
44951	0.784871	2.1
44952	0.95863	2.1
44953	0.919681	2.1
44954	0.636145	2.1
44955	0.606461	2.1
44956	0.636144	2.1
44957	0.958631	2.1
44958	0.60646	2.1
44959	0.919683	2.1
44960	1.89226	2.1
44961	1.89226	2.1
44962	1.89226	2.1
44963	1.89226	2.1
44964	3.78452	2.1
44965	1.89226	2.1
44966	1.89226	2.1
44967	1.89226	2.1
44968	1.89226	2.04747
44969	3.78452	2.1
44970	1.03789	2.1
44971	1.03789	2.1
44972	1.04231	2.1
44973	1.04231	2.1
44974	1.16052	2.1
44975	1.11265	2.1
44976	0.958634	2.1
44977	0.919685	2.1
44978	0.958634	2.1
44979	1.16052	2.1
44980	0.919685	2.1
44981	1.11265	2.1
44982	1.43837	2.1
44983	2.57085	2.1
44984	1.39332	2.1
44985	2.58228	2.1
44986	3.96417	2.1
44987	1.89226	2.1
44988	1.89226	2.1
44989	1.89226	2.1
44990	1.89226	2.1
44991	1.89226	2.1
44992	1.89226	2.1
44993	1.89226	2.1
44994	1.89226	2.1
44995	1.89226	2.1
44996	1.89226	2.1
44997	3.78452	2.1
44998	2.57085	2.1
44999	1.89226	2.1
45000	2.58229	2.1
45001	1.89226	2.1
45002	4.46312	2.1
45003	1.32368	2.1
45004	2.58228	2.1
45005	2.55352	2.1

45006	2.55352	2.1
45007	1.39332	2.1
45008	1.39332	2.1
45009	1.89226	2.1
45010	1.89226	2.1
45011	1.89226	2.1
45012	1.89226	2.1
45013	1.89226	2.1
45014	1.89226	2.1
45015	1.89226	2.1
45016	1.89226	2.1
45017	1.89226	2.1
45018	1.89226	2.1
45019	1.89226	2.1
45020	1.89226	2.1
45021	2.55353	2.1
45022	1.89226	2.1
45023	2.58228	2.1
45024	1.89226	2.1
45025	1.89226	2.1
45026	2.58228	2.1
45027	1.40574	1.83192
45028	1.05175	1.42424
45029	1.71613	1.74497
45030	1.99961	1.57769
45031	3.12187	1.62252
45032	1.71613	1.71985
45033	2.50733	1.4801
45034	1.99961	1.87257
45035	1.99961	1.6199
45036	2.25746	1.55747
45037	2.25746	1.35157
45038	2.25746	1.39018
45039	1.89526	1.46119
45040	1.71613	1.75541
45041	1.71613	1.03535
45042	1.89526	1.53517
45043	1.27558	1.02747
45044	1.07536	2.1
45045	1.07536	2.1
45046	1.07034	2.1
45047	1.07034	2.1
45048	0.766879	2.1
45049	0.81527	2.1
45050	1.38896	2.1
45051	1.33043	2.1
45052	0.815264	2.1
45053	1.38895	2.1
45054	0.766884	2.1
45055	1.33044	2.1
45056	1.89225	2.1
45057	1.89225	2.1
45058	1.89225	2.1
45059	1.89225	2.1
45060	1.89225	2.1
45061	1.89225	2.1
45062	1.64059	2.1
45063	1.89225	2.1

45064	1.565	2.1
45065	1.565	2.1
45066	1.89225	2.1
45067	1.89225	2.1
45068	1.89226	2.1
45069	1.89226	2.1
45070	1.89226	2.1
45071	1.89226	2.1
45072	1.89226	2.1
45073	1.89226	2.1
45074	1.67906	2.1
45075	1.89226	2.1
45076	1.6406	2.1
45077	1.6406	2.1
45078	1.89226	2.1
45079	1.89226	2.1
45080	2.18106	1.7461
45081	2.20831	2.09232
45082	2.64725	0.734217
45083	2.63102	1.12286
45084	4.82831	1.48173
45085	2.18106	1.2925
45086	2.09605	2.1
45087	2.5601	1.54554
45088	2.63102	1.17327
45089	4.74116	2.03364
45090	2.64725	1.00725
45091	1.89226	1.37803
45092	1.87603	2.1
45093	2.63102	1.20971
45094	1.89226	1.92921
45095	2.64725	0.656842
45096	0.946132	1.35725
45097	1.28005	1.22758
45098	0.946132	2.04393
45099	1.31551	0.898427
45100	1.28005	1.60346
45101	1.28005	1.95404
45102	0.946132	1.12426
45103	0.946132	1.42919
45104	0.946132	1.12011
45105	0.946132	1.30652
45106	1.28005	1.59207
45107	1.31551	1.69937
45108	2.0815	2.1
45109	1.59318	2.1
45110	2.0815	2.1
45111	1.40161	2.1
45112	2.49782	2.1
45113	1.68553	2.1
45114	0.819885	2.1
45115	0.935082	2.1
45116	0.867363	2.1
45117	0.867363	2.1
45118	0.715431	2.1
45119	0.902777	2.1
45120	0.738933	2.1
45121	0.980053	2.1

45122	0.936716	2.1
45123	0.917651	2.1
45124	0.775853	2.1
45125	0.74037	2.1
45126	2.78977	2.1
45127	2.38239	2.1
45128	2.78977	2.1
45129	1.96468	2.1
45130	2.64107	2.1
45131	1.80733	2.1
45132	1.80141	2.1
45133	2.28512	2.1
45134	2.28512	2.1
45135	1.8519	2.1
45136	2.64107	2.1
45137	2.20785	2.1
45138	0.946133	0.832411
45139	0.946133	0.617458
45140	0.946132	0.765441
45141	0.946132	0.563492
45142	0.946132	0.768638
45143	0.946132	0.987426
45144	0.946132	0.577996
45145	0.946132	0.573259
45146	0.946132	0.638998
45147	0.946132	0.714775
45148	0.946133	0.66037
45149	0.946133	0.786347
45150	1.89226	0.326604
45151	1.89226	0.343575
45152	1.89226	0.258704
45153	1.89226	0.394732
45154	1.89226	0.280533
45155	1.89226	0.391826
45156	1.89226	0.418445
45157	1.89226	0.468992
45158	1.89226	0.36115
45159	1.89226	0.554082
45160	3.78453	0.471282
45161	1.89226	0.66909
45162	1.89226	0.497304
45163	1.89226	0.423468
45164	1.89226	0.80891
45165	1.89226	0.489991
45166	1.89226	0.666905
45167	2.12925	0.0698591
45168	2.51397	0.0698591
45169	2.22488	0.0698591
45170	1.86113	0.0698591
45171	4.3751	0.0698591
45172	2.82754	0.0698591
45173	2.51397	0.0698591
45174	2.22489	0.0698591
45175	2.47291	0.0698591
45176	4.98688	0.0698591
45177	1.89226	0.0698591
45178	1.89226	0.078816
45179	1.89226	0.0698591

45180	1.89226	0.0774084
45181	3.78453	0.0731712
45182	1.89226	0.0698591
45183	1.89226	0.0759166
45184	1.89226	0.0698591
45185	1.89226	0.0779284
45186	3.78452	0.0699819
45187	0.90848	0.0698591
45188	1.11244	0.0698591
45189	0.930566	0.0698591
45190	0.73385	0.0698591
45191	0.921298	0.0698591
45192	0.921298	0.0698591
45193	0.924994	0.0698591
45194	0.924994	0.0698591
45195	0.908478	0.0698591
45196	0.733851	0.0698591
45197	1.11244	0.0698591
45198	0.930567	0.0698591
45199	2.22489	0.0698591
45200	2.0815	0.0698591
45201	2.0815	0.0698591
45202	2.30674	0.0698591
45203	1.99867	0.0698591
45204	2.33358	0.0698591
45205	1.89226	0.0698591
45206	1.89226	0.0746983
45207	1.89226	0.0698591
45208	1.89226	0.0707225
45209	1.89226	0.0698591
45210	1.89226	0.0702282
45211	0.946131	0.0752124
45212	0.946131	0.0702271
45213	0.946128	0.0719758
45214	0.946128	0.0698591
45215	0.946129	0.0727959
45216	0.946129	0.0771666
45217	0.94613	0.0698591
45218	0.94613	0.0698591
45219	0.94613	0.0703694
45220	0.94613	0.0698591
45221	0.94613	0.0698591
45222	0.94613	0.0712523
45223	2.28904	2.1
45224	1.89226	2.09096
45225	2.2846	2.09719
45226	2.2846	2.1
45227	1.89226	1.89953
45228	1.89226	2.1
45229	1.89226	1.90475
45230	1.89226	1.40374
45231	1.89226	2.1
45232	1.89226	2.09981
45233	1.89226	0.77499
45234	1.89226	2.09914
45235	1.04037	1.63605
45236	1.34066	2.0773
45237	1.34955	1.46477

45238	1.34955	2.06204
45239	1.03373	1.59753
45240	1.03373	2.1
45241	0.946131	1.82469
45242	0.946131	2.1
45243	1.14452	2.1
45244	1.14452	2.1
45245	0.946131	1.97606
45246	0.946131	2.01413
45247	1.14296	2.1
45248	1.14452	2.1
45249	1.14296	2.09963
45250	0.946131	2.1
45251	1.14452	2.1
45252	0.946132	2.1
45253	1.89226	1.62691
45254	1.89226	2.04908
45255	1.89226	2.00352
45256	1.89226	2.1
45257	1.89226	0.742549
45258	1.89226	2.1
45259	0.597454	1.60814
45260	0.597454	2.00681
45261	0.597972	1.74921
45262	0.597972	2.1
45263	0.675238	2.06893
45264	0.674776	1.61329
45265	0.519667	1.72551
45266	0.520188	1.85726
45267	0.519667	0.822286
45268	0.675238	0.867907
45269	0.520188	1.77982
45270	0.674777	1.79117
45271	1.89226	0.239062
45272	1.82125	0.18557
45273	1.89226	0.171668
45274	1.84314	0.151693
45275	1.89226	0.332116
45276	1.84314	0.285604
45277	1.89226	0.190992
45278	1.84314	0.200604
45279	1.89226	0.164549
45280	1.65794	0.155368
45281	1.89226	0.203071
45282	1.65794	0.189249
45283	1.348	2.06241
45284	1.43485	2.1
45285	1.79929	0.529537
45286	1.75309	1.75898
45287	3.14728	1.89678
45288	1.348	1.17139
45289	1.63892	1.85041
45290	1.59157	1.98556
45291	1.18647	1.92298
45292	1.75309	1.20257
45293	1.348	1.87534
45294	1.79929	1.5332
45295	1.75309	2.1

45296	2.18106	1.21706
45297	2.20831	1.1324
45298	3.98034	1.91914
45299	2.18106	0.887088
45300	1.63892	1.95196
45301	1.75309	0.914793
45302	1.75309	2.06976
45303	2.09605	1.01625
45304	2.09605	2.06566
45305	2.66076	0.182697
45306	1.70302	0.0888704
45307	2.41575	0.113192
45308	1.70302	0.159934
45309	3.08598	0.098611
45310	2.3661	0.140153
45311	1.71053	0.0716333
45312	2.41575	0.138218
45313	1.20143	0.0698591
45314	1.20143	0.0768785
45315	2.2123	0.0698591
45316	1.66739	0.0801321
45317	2.25633	0.0747411
45318	2.68302	0.0953777
45319	1.71051	0.070676
45320	1.71051	0.0774531
45321	2.74192	0.0698591
45322	2.1961	0.0731971
45323	1.32553	2.1
45324	2.34875	1.8662
45325	2.19139	2.1
45326	2.19139	1.42282
45327	1.56172	2.1
45328	1.56172	1.19843
45329	1.89226	2.1
45330	1.89226	1.92036
45331	1.89226	2.1
45332	1.89226	2.0993
45333	3.78453	2.1
45334	2.667 2.1	
45335	1.26097	2.1
45336	1.26097	2.1
45337	1.5683	2.1
45338	2.42949	2.1
45339	2.73682	2.1
45340	1.13213	2.1
45341	2.19139	1.72179
45342	2.0815	2.1
45343	1.77615	1.181
45344	1.37767	2.1
45345	1.19297	1.08186
45346	0.946132	1.97822
45347	0.946132	2.1
45348	0.946131	2.1
45349	0.946131	2.0971
45350	0.946131	2.09324
45351	0.946131	1.70741
45352	0.946132	2.1
45353	0.946132	2.1

45354	0.946132	2.1
45355	0.946132	2.1
45356	0.946131	2.1
45357	0.946131	2.1
45358	2.02064	2.1
45359	2.3606	2.1
45360	1.47478	2.1
45361	2.22997	2.1
45362	4.25061	2.1
45363	2.36059	2.1
45364	2.56952	2.1
45365	2.22997	2.1
45366	2.48631	2.1
45367	4.79949	2.1
45368	1.84261	2.1
45369	2.22997	2.1
45370	1.65706	2.1
45371	1.78837	2.1
45372	3.88703	2.1
45373	2.22997	2.1
45374	2.48631	2.1
45375	2.19528	2.1
45376	2.41175	2.1
45377	4.64172	2.1
45378	1.6466	2.1
45379	1.89226	2.1
45380	1.35972	2.1
45381	1.35972	2.1
45382	1.89226	2.1
45383	1.89226	2.1
45384	1.89226	2.1
45385	1.89226	2.1
45386	1.89226	2.1
45387	1.89226	2.1
45388	3.78453	2.1
45389	2.44797	2.1
45390	1.07214	2.1
45391	1.31589	2.1
45392	1.1211	2.1
45393	2.44797	2.1
45394	2.25318	2.1
45395	1.95954	2.1
45396	1.89226	2.1
45397	1.6466	2.1
45398	1.6466	2.1
45399	1.89226	2.1
45400	1.89226	2.1
45401	0.946133	2.1
45402	0.946133	2.1
45403	0.946132	2.1
45404	0.946132	2.1
45405	0.946132	2.1
45406	0.946132	2.1
45407	0.946133	2.1
45408	0.946133	2.1
45409	0.946132	2.1
45410	0.946132	2.1
45411	0.946133	2.1

45412	0.946133	2.1
45413	2.44797	2.1
45414	1.36904	2.1
45415	1.70303	2.1
45416	2.37586	2.1
45417	4.00507	2.1
45418	0.631553	0.0698591
45419	1.24489	0.0698591
45420	1.11597	0.0698591
45421	0.496799	0.0698591
45422	0.872272	0.0698591
45423	0.872272	0.0698591
45424	0.869415	0.0698591
45425	0.869415	0.0698591
45426	0.4968	0.0698591
45427	0.631553	0.0698591
45428	1.11597	0.0698591
45429	1.24489	0.0698591
45430	1.84253	2.1
45431	2.17384	2.1
45432	2.04818	2.1
45433	1.65925	2.1
45434	3.83309	2.1
45435	2.04818	2.1
45436	1.65925	2.1
45437	1.48162	2.1
45438	1.92264	2.1
45439	3.58189	2.1
45440	2.3305	0.096638
45441	1.89226	0.102235
45442	2.52753	0.088727
45443	2.52753	0.139792
45444	1.89226	0.0792754
45445	1.89226	0.110748
45446	1.89226	0.107234
45447	1.89226	0.173862
45448	1.89226	0.154818
45449	1.89226	0.249709
45450	1.89226	0.096549
45451	1.89226	0.163987
45452	1.34563	0.0904761
45453	2.1769	0.0758664
45454	1.94955	0.0797446
45455	1.94955	0.149599
45456	1.55063	0.0802552
45457	1.55063	0.144221
45458	2.09455	0.0812491
45459	1.89226	0.0865541
45460	2.3305	0.0794376
45461	2.3305	0.107857
45462	1.89226	0.0725182
45463	1.89226	0.0879375
45464	1.89226	0.0847195
45465	1.89226	0.090471
45466	1.89226	0.122304
45467	1.89226	0.0992707
45468	1.89226	0.117907
45469	1.89226	0.105072

45470	0.773392	0.0729738
45471	0.612765	0.081283
45472	0.79504	0.077109
45473	0.575323	0.0727718
45474	1.0819	0.0772426
45475	1.06106	0.0758398
45476	1.04675	0.0700568
45477	0.86858	0.103764
45478	0.796873	0.0770118
45479	0.557469	0.0889552
45480	0.807663	0.0825723
45481	2.2024	2.1
45482	2.34106	2.1
45483	2.02634	2.1
45484	2.20125	2.1
45485	4.36739	2.1
45486	2.34106	2.1
45487	2.25195	2.1
45488	2.20125	2.1
45489	2.13132	2.08823
45490	4.47237	2.1
45491	2.02635	2.1
45492	2.22465	2.1
45493	1.68504	2.1
45494	2.0815	2.1
45495	3.92673	2.1
45496	2.22465	2.1
45497	2.13133	2.09199
45498	2.0815	2.1
45499	2.23579	2.1
45500	4.44862	2.1
45501	1.89226	1.23776
45502	1.89226	1.22387
45503	1.89226	1.52723
45504	1.89226	1.25337
45505	1.89226	1.44781
45506	1.89226	1.10338
45507	1.93967	0.0703555
45508	1.70302	0.0774769
45509	1.21883	0.0708158
45510	1.70302	0.0812006
45511	1.19889	0.0707714
45512	1.70302	0.0805866
45513	0.946131	0.114173
45514	0.946131	0.0887034
45515	0.946131	0.0759056
45516	0.946131	0.0828946
45517	0.94613	0.0817489
45518	0.94613	0.132523
45519	0.946131	0.0788924
45520	0.946131	0.0843116
45521	0.946131	0.08235
45522	0.946131	0.0824089
45523	0.946131	0.0990972
45524	0.946131	0.0866486
45525	0.883128	0.0732709
45526	0.883128	0.0797177
45527	0.827519	0.0698591

45528	0.82752	0.07178
45529	1.16234	0.0714289
45530	1.19063	0.0872193
45531	0.631294	0.0734526
45532	0.548311	0.0726713
45533	0.631293	0.0699989
45534	1.16234	0.0699804
45535	1.19063	0.0751527
45536	0.54831	0.0747095
45537	1.89226	0.0699769
45538	1.89226	0.109034
45539	1.89226	0.11395
45540	1.89226	0.0735459
45541	3.78453	0.0783801
45542	2.33328	0.0698591
45543	1.87991	0.0698591
45544	1.87991	0.0698644
45545	2.82938	0.0698591
45546	1.84457	0.0698591
45547	2.79403	0.0698591
45548	2.82937	0.0698591
45549	2.70881	0.0698591
45550	1.8799	0.0698591
45551	1.82739	0.0698591
45552	4.58874	0.0698591
45553	1.89226	0.116588
45554	1.89226	0.0698612
45555	1.89226	0.0751752
45556	1.89226	0.104355
45557	3.78452	0.090565
45558	1.17897	0.0698591
45559	1.82739	0.0698591
45560	1.17897	0.0698591
45561	1.76199	0.0698591
45562	1.23885	0.0698591
45563	1.80989	0.0698591
45564	0.946131	0.0698636
45565	0.946131	0.0699256
45566	0.946131	0.082796
45567	0.946131	0.0783501
45568	0.946129	0.0837419
45569	0.946129	0.0698591
45570	0.946133	0.0758199
45571	0.946133	0.0829916
45572	0.946131	0.0754887
45573	0.946131	0.0698614
45574	0.946131	0.0701291
45575	0.946131	0.0718909
45576	0.946126	1.96734
45577	0.946126	2.1
45578	1.24887	2.1
45579	1.24887	2.1
45580	0.946127	2.1
45581	0.946127	2.03419
45582	1.24887	2.1
45583	1.28511	2.1
45584	1.2851	2.1
45585	0.946128	2.1

45586	1.24887	2.1
45587	0.946128	2.1
45588	1.89226	2.1
45589	1.89226	2.05391
45590	1.89226	2.1
45591	1.89226	0.888802
45592	3.78452	2.0223
45593	0.592142	2.08742
45594	0.592142	2.1
45595	0.60496	2.1
45596	0.60496	2.1
45597	0.694237	2.1
45598	0.608714	1.80329
45599	0.562863	2.1
45600	0.502864	2.1
45601	0.562863	2.1
45602	0.694237	2.1
45603	0.608714	2.1
45604	0.502864	2.1
45605	1.89226	2.1
45606	1.89226	2.1
45607	1.89226	2.1
45608	1.89226	2.1
45609	3.78453	2.1
45610	1.55779	2.1
45611	1.57968	2.1
45612	1.47186	2.1
45613	1.51725	2.1
45614	3.05154	2.1
45615	1.57967	2.1
45616	1.474	2.1
45617	1.51725	2.1
45618	1.42895	2.1
45619	3.00863	2.1
45620	1.89226	2.1
45621	1.89226	2.1
45622	1.89226	2.1
45623	1.89226	2.1
45624	3.78452	2.1
45625	1.89226	2.1
45626	1.89226	2.1
45627	1.89226	2.1
45628	1.89226	2.1
45629	3.78453	2.1
45630	1.47186	2.1
45631	1.51725	2.1
45632	1.38859	2.1
45633	1.45332	2.1
45634	2.92519	2.1
45635	1.51725	2.1
45636	1.42895	2.1
45637	1.45332	2.1
45638	1.38414	2.1
45639	2.88228	2.1
45640	1.89226	2.1
45641	1.89226	2.1
45642	1.89226	2.1
45643	1.89226	2.1

45644	3.78452	2.1
45645	1.34497	0.0698591
45646	1.36747	0.0698591
45647	1.18919	0.0698591
45648	2.08149	0.0698591
45649	1.23556	0.0698591
45650	2.13702	0.0698591
45651	1.10106	0.0698591
45652	2.08001	0.0698591
45653	1.34497	0.0698591
45654	2.31605	0.0698591
45655	1.34497	0.0698591
45656	2.31605	0.0698591
45657	2.31605	0.0698591
45658	2.0815	0.0698591
45659	1.9419	0.0698591
45660	2.46665	0.0698591
45661	4.52922	0.0698591
45662	1.20349	0.0698591
45663	2.08149	0.0698591
45664	1.36948	0.0698591
45665	1.34737	0.0698591
45666	2.08149	0.0698591
45667	2.08149	0.0698591
45668	0.946132	0.0698591
45669	0.946132	0.0698591
45670	1.37715	0.0698591
45671	0.823199	0.0698591
45672	1.21598	0.0698591
45673	0.946132	0.0698591
45674	1.37715	0.0698591
45675	0.946132	0.0698591
45676	1.21598	0.0698591
45677	0.823198	0.0698591
45678	0.946133	0.0698591
45679	0.946133	0.0698591
45680	1.89226	0.0698591
45681	1.89226	0.0698591
45682	1.89226	0.0698591
45683	1.89226	0.0698591
45684	3.78453	0.0698591
45685	1.89227	0.0698591
45686	1.89227	0.0698591
45687	1.89227	0.0698591
45688	1.89227	0.0698591
45689	3.78453	0.0698591
45690	0.946132	0.0698591
45691	0.946132	0.0698591
45692	1.21599	0.0698591
45693	1.21599	0.0698591
45694	0.966749	0.0698591
45695	0.946133	0.0698591
45696	1.21598	0.0698591
45697	0.946132	0.0698591
45698	0.966748	0.0698591
45699	1.21598	0.0698591
45700	0.946133	0.0698591
45701	0.946133	0.0698591

45702	0.946133	0.0698591
45703	0.946133	0.0698591
45704	1.1134	0.0698591
45705	1.11555	0.0698591
45706	1.11555	0.0698591
45707	0.946132	0.0698591
45708	0.946132	0.0698591
45709	1.1134	0.0698591
45710	1.11555	0.0698591
45711	1.11555	0.0698591
45712	0.946132	0.0698591
45713	0.946132	0.0698591
45714	0.946133	0.0698591
45715	0.946133	0.0698591
45716	1.11555	0.0698591
45717	0.978467	0.0698591
45718	0.978468	0.0698591
45719	0.946133	0.0698591
45720	1.11555	0.0698591
45721	0.946132	0.0698591
45722	0.978468	0.0698591
45723	0.978468	0.0698591
45724	0.946132	0.0698591
45725	0.946132	0.0698591
45726	0.851542	2.1
45727	0.851542	2.1
45728	1.14168	2.1
45729	1.27897	2.08739
45730	1.02817	2.1
45731	1.13536	2.1
45732	0.946131	2.1
45733	0.861207	2.1
45734	0.94613	2.1
45735	0.94613	2.1
45736	0.868751	2.1
45737	1.15181	2.1
45738	0.946132	2.1
45739	0.946132	2.02289
45740	0.946128	2.07254
45741	0.946128	1.49536
45742	0.94613	2.1
45743	0.94613	2.07356
45744	0.946131	1.97265
45745	0.946131	1.76032
45746	0.94613	1.86947
45747	0.94613	2.07058
45748	0.94613	1.96998
45749	0.94613	2.02949
45750	2.53273	0.0698591
45751	1.89226	0.0698591
45752	1.89226	0.0698591
45753	1.89226	0.0698591
45754	1.89226	0.0698591
45755	1.89226	0.0698591
45756	1.89226	2.1
45757	1.89226	2.1
45758	1.89226	2.1
45759	1.89226	2.1

45760	3.78452	2.1
45761	1.89226	2.1
45762	2.54394	2.1
45763	1.89226	2.1
45764	2.42221	2.1
45765	4.43621	2.1
45766	2.54394	2.1
45767	1.62743	2.1
45768	2.42221	2.1
45769	1.5437	2.1
45770	4.08764	2.1
45771	1.89226	2.1
45772	1.89226	2.1
45773	1.89226	2.1
45774	1.89226	2.1
45775	3.78452	2.1
45776	1.89226	2.1
45777	1.89226	2.1
45778	1.89226	2.1
45779	1.89226	2.1
45780	3.78452	2.1
45781	1.89226	2.1
45782	2.30146	2.1
45783	2.42221	2.1
45784	1.89226	2.1
45785	1.89226	2.1
45786	2.42221	2.1
45787	2.30146	2.1
45788	2.42221	2.1
45789	1.39962	2.1
45790	1.39962	2.1
45791	1.5437	2.1
45792	2.44553	2.1
45793	1.89226	2.1
45794	1.89226	2.1
45795	1.89226	2.1
45796	1.89226	2.1
45797	3.78452	2.1
45798	1.62196	2.1
45799	2.00695	2.1
45800	1.95288	2.1
45801	1.70303	2.1
45802	3.53316	2.1
45803	1.80703	2.1
45804	2.00695	2.1
45805	1.92991	2.1
45806	2.00695	2.1
45807	1.85888	2.1
45808	1.93592	2.1
45809	1.89226	2.1
45810	1.89226	2.1
45811	1.89226	2.1
45812	1.89226	2.1
45813	1.89226	2.1
45814	1.89226	2.1
45815	1.89226	2.1
45816	1.89226	2.1
45817	1.89226	2.1

45818	1.89226	2.1
45819	3.78453	2.1
45820	1.95288	2.1
45821	1.50524	2.1
45822	1.83564	2.1
45823	1.70303	2.1
45824	2.00749	2.1
45825	1.84671	2.1
45826	1.62632	2.1
45827	1.82498	2.1
45828	1.92991	2.1
45829	1.92991	2.1
45830	1.47928	2.1
45831	1.47751	2.1
45832	0.946128	2.1
45833	0.946128	2.1
45834	0.946132	2.1
45835	0.946132	2.1
45836	0.94613	2.1
45837	0.94613	2.1
45838	0.946129	2.1
45839	0.946129	2.1
45840	0.94613	2.1
45841	0.94613	2.1
45842	0.94613	2.1
45843	0.94613	2.1
45844	1.89226	2.1
45845	1.89226	2.1
45846	1.89226	2.1
45847	1.89226	2.1
45848	1.89226	2.1
45849	1.89226	2.1
45850	2.27351	0.835952
45851	2.74068	0.462624
45852	2.72278	0.555974
45853	2.63356	0.702305
45854	2.36273	0.544351
45855	2.27351	0.682545
45856	2.36273	0.69255
45857	2.83112	0.412504
45858	2.81212	0.493166
45859	2.74068	0.587055
45860	2.43416	0.48691
45861	2.36273	0.57532
45862	1.89227	0.574679
45863	2.83112	0.410306
45864	2.74069	0.512304
45865	2.74069	0.388495
45866	1.89227	0.675887
45867	1.89227	0.473864
45868	2.40887	2.1
45869	1.4494	2.1
45870	1.52164	2.1
45871	2.47534	2.1
45872	3.93051	2.1
45873	1.89226	2.1
45874	2.47534	2.1
45875	2.40887	2.1

45876	2.40887	2.1
45877	1.89226	2.1
45878	1.89226	2.1
45879	1.60861	2.1
45880	2.47534	2.1
45881	1.52164	2.1
45882	1.52164	2.1
45883	2.55301	2.1
45884	2.55301	2.1
45885	0.946128	2.1
45886	0.946128	2.1
45887	1.23767	2.1
45888	1.2765	2.1
45889	1.23767	2.1
45890	1.23767	2.1
45891	0.946131	2.1
45892	0.946131	2.1
45893	0.946131	2.1
45894	1.2765	2.1
45895	1.23767	2.1
45896	0.946131	2.1
45897	1.89226	2.1
45898	1.87511	2.1
45899	1.87511	2.1
45900	1.95852	2.1
45901	2.0815	2.1
45902	2.22169	2.1
45903	1.89226	2.1
45904	1.89226	2.1
45905	1.89226	2.1
45906	1.89226	2.1
45907	1.89226	2.1
45908	1.89226	2.1
45909	2.0815	2.1
45910	1.80067	2.1
45911	1.80067	2.1
45912	2.05518	2.1
45913	1.89226	2.1
45914	2.16509	2.1
45915	1.87153	2.1
45916	2.65897	2.1
45917	1.58724	2.1
45918	2.38924	2.1
45919	4.26077	2.1
45920	1.89226	2.1
45921	1.89226	2.1
45922	1.89226	2.1
45923	1.89226	2.1
45924	1.89226	2.1
45925	1.89226	2.1
45926	1.89226	0.400993
45927	2.21022	0.593907
45928	2.19769	0.862466
45929	2.21022	0.474302
45930	1.87973	0.47495
45931	1.89226	0.160161
45932	1.18376	1.83425
45933	2.21022	0.701073

45934	1.16239	1.6902
45935	1.16239	0.937233
45936	2.19769	1.68498
45937	2.19769	0.822208
45938	0.946128	0.168184
45939	0.946128	0.202422
45940	1.09362	0.483057
45941	1.09884	0.395099
45942	0.94613	0.400384
45943	0.94613	0.118237
45944	1.09362	0.226242
45945	1.09362	0.370656
45946	0.946131	0.241439
45947	1.09362	0.242559
45948	0.946131	0.151249
45949	1.09884	0.138144
45950	1.19323	1.18705
45951	2.19769	0.442389
45952	1.18376	1.07305
45953	1.18376	0.648002
45954	2.18724	1.08649
45955	2.18724	0.617202
45956	2.0815	2.1
45957	0.922714	2.1
45958	2.0815	2.1
45959	1.84882	2.1
45960	1.20787	2.1
45961	1.08321	2.1
45962	2.6238	0.0710618
45963	1.89226	0.0911303
45964	1.89226	0.0987765
45965	2.4866	0.0730897
45966	1.89226	0.0856552
45967	2.4866	0.0732896
45968	2.66753	0.07747
45969	1.89226	0.134278
45970	1.89226	0.1354
45971	2.6238	0.0820136
45972	1.89226	0.110541
45973	2.6238	0.0813502
45974	0.946129	2.1
45975	0.946129	2.1
45976	1.04676	2.1
45977	1.04676	2.1
45978	0.946131	2.1
45979	0.946131	2.1
45980	1.08732	2.1
45981	1.04676	2.1
45982	1.08732	2.1
45983	0.946131	2.1
45984	1.04676	2.1
45985	0.946131	2.1
45986	1.20958	2.09664
45987	1.73802	1.13181
45988	1.39067	1.97908
45989	1.39067	1.45073
45990	1.50969	1.94092
45991	1.50969	1.60184

45992	1.00382	1.93907
45993	1.50969	0.522588
45994	1.20958	1.89269
45995	1.20958	0.816574
45996	1.32039	1.92107
45997	1.32039	0.899463
45998	2.0815	2.1
45999	2.51396	2.1
46000	2.19826	2.1
46001	2.54175	2.1
46002	4.79599	2.1
46003	2.54174	2.1
46004	2.17978	2.1
46005	2.54174	2.1
46006	2.2854	2.1
46007	2.57212	2.1
46008	2.31578	2.1
46009	2.27103	2.1
46010	2.54175	2.1
46011	2.4255	2.1
46012	2.82251	2.1
46013	4.96725	2.1
46014	2.60614	0.0698591
46015	2.75374	0.0698591
46016	1.94002	0.0698591
46017	2.08392	0.0698591
46018	4.69006	0.0698591
46019	2.75373	0.0698591
46020	2.6875	0.0698591
46021	2.08392	0.0698591
46022	1.99944	0.0698591
46023	4.77142	0.0698591
46024	1.1211	0.0698591
46025	2.08392	0.0698591
46026	1.17787	0.0698591
46027	1.94002	0.0698591
46028	1.32178	0.0698591
46029	2.08392	0.0698591
46030	2.08392	0.0698591
46031	1.99944	0.0698591
46032	1.32178	0.0698591
46033	1.25929	0.0698591
46034	3.34321	0.0698591
46035	1.22266	0.0698591
46036	0.779473	0.0698591
46037	0.94613	0.0698591
46038	0.94613	0.0698591
46039	1.04072	0.0698591
46040	1.1407	0.0698591
46041	1.10042	0.0698591
46042	0.851542	0.0698591
46043	0.942046	0.0698591
46044	0.942046	0.0698591
46045	1.04114	0.0698591
46046	0.826602	0.0698591
46047	2.37468	2.1
46048	1.89226	2.1
46049	1.89226	2.1

46050	1.89226	2.1
46051	2.27917	2.1
46052	2.27917	2.1
46053	1.13959	2.1
46054	1.09451	2.1
46055	0.946131	2.1
46056	0.946131	2.1
46057	1.09451	2.1
46058	1.09451	2.1
46059	0.94613	2.1
46060	0.94613	2.1
46061	1.09451	2.1
46062	0.946131	2.1
46063	0.946131	2.1
46064	1.13959	2.1
46065	2.52454	2.01612
46066	2.12221	2.1
46067	2.67624	1.60588
46068	2.33865	2.1
46069	4.86319	2.1
46070	1.89226	2.09984
46071	1.89226	2.1
46072	1.89226	2.0846
46073	1.89226	2.1
46074	3.78452	2.1
46075	1.89226	2.1
46076	1.89226	2.1
46077	1.89226	2.1
46078	1.89226	2.1
46079	3.78452	2.1
46080	2.12221	2.1
46081	1.47769	2.1
46082	2.33865	2.1
46083	1.62898	2.1
46084	3.81634	2.1
46085	2.67624	2.1
46086	2.37734	2.1
46087	2.33865	2.1
46088	2.33865	2.1
46089	2.70125	2.1
46090	2.70125	2.1
46091	1.89226	2.09494
46092	1.89226	2.1
46093	1.89226	2.1
46094	1.89226	2.1
46095	1.89226	1.68702
46096	1.89226	1.82646
46097	1.89226	2.1
46098	1.89226	2.1
46099	1.89226	2.1
46100	1.89226	2.1
46101	1.89226	2.1
46102	1.89226	2.1
46103	2.37733	2.1
46104	1.95927	2.1
46105	1.82516	2.08604
46106	1.82516	2.1
46107	2.33865	2.1

46108	2.33865	2.1
46109	2.57466	2.1
46110	2.21715	2.1
46111	2.55653	2.1
46112	2.24775	2.1
46113	4.77367	2.1
46114	0.937095	2.1
46115	0.937095	2.1
46116	0.958028	2.1
46117	0.958028	2.1
46118	0.828386	2.1
46119	0.771244	2.1
46120	1.10858	2.1
46121	1.12388	2.1
46122	1.12388	2.1
46123	0.828386	2.1
46124	0.771244	2.1
46125	1.10858	2.1
46126	2.55653	2.1
46127	2.32606	2.1
46128	2.24775	2.1
46129	2.24775	2.1
46130	2.57928	2.1
46131	2.57928	2.1
46132	0.990481	2.1
46133	0.990481	2.1
46134	1.00094	2.1
46135	1.00094	2.1
46136	0.888385	2.1
46137	0.828386	2.1
46138	1.16303	2.1
46139	1.12388	2.1
46140	1.16303	2.1
46141	0.888385	2.1
46142	1.12388	2.1
46143	0.828387	2.1
46144	0.962524	2.1
46145	0.962524	2.1
46146	0.7976	2.1
46147	0.7976	2.1
46148	0.797628	2.1
46149	0.814 2.1	
46150	0.946125	2.1
46151	0.946125	2.1
46152	0.946118	2.1
46153	0.797623	2.1
46154	0.814006	2.1
46155	0.946131	2.1
46156	1.8978	0.0698591
46157	2.23892	0.0698591
46158	1.71469	0.0698591
46159	4.13672	0.0698591
46160	2.64136	0.0698591
46161	2.23892	1.59774
46162	2.37578	1.99713
46163	1.77489	2.1
46164	4.6147	2.08925
46165	1.02284	2.1

46166	0.898796	2.06768
46167	0.948554	2.1
46168	0.948554	2.1
46169	0.878797	2.1
46170	0.796197	2.1
46171	1.09145	2.1
46172	0.963109	2.1
46173	1.12114	2.1
46174	0.782739	2.1
46175	1.11084	2.1
46176	0.850742	2.1
46177	2.48024	2.1
46178	2.405	2.1
46179	2.24585	2.1
46180	2.34795	2.1
46181	4.75295	2.1
46182	1.93794	2.1
46183	2.405	2.1
46184	2.24585	2.1
46185	1.67594	2.1
46186	4.08094	2.1
46187	1.89226	2.1
46188	1.89226	2.03439
46189	1.89226	2.1
46190	1.89226	2.1
46191	3.78452	2.1
46192	1.89226	2.1
46193	1.89226	2.1
46194	1.89226	2.1
46195	1.89226	2.1
46196	3.78452	2.1
46197	2.24585	2.1
46198	2.34795	2.1
46199	2.21155	2.1
46200	2.06626	2.1
46201	4.4142	2.1
46202	2.24585	2.1
46203	1.41498	2.1
46204	1.41498	2.1
46205	1.67594	2.1
46206	2.06625	2.1
46207	2.32721	2.1
46208	1.89226	2.1
46209	1.89226	2.1
46210	1.89226	2.1
46211	1.89226	2.09539
46212	1.89226	2.1
46213	1.89226	2.0529
46214	1.89226	2.1
46215	1.89226	2.1
46216	1.89226	2.1
46217	1.89226	2.1
46218	3.78452	2.1
46219	1.89226	0.11347
46220	1.89226	0.176679
46221	1.89226	0.225323
46222	1.89226	0.0870855
46223	3.78452	0.0917232

46224	1.89226	0.131586
46225	2.53655	0.0703383
46226	1.89226	0.0706142
46227	1.89226	0.0744852
46228	1.89226	0.123465
46229	1.89226	0.129752
46230	1.89227	2.1
46231	1.89227	2.1
46232	1.89227	2.1
46233	1.89227	2.1
46234	1.89227	2.1
46235	1.89227	2.1
46236	0.91339	2.1
46237	0.91339	2.1
46238	1.2158	2.1
46239	1.20629	2.1
46240	1.00473	2.1
46241	1.15749	2.1
46242	0.946132	2.1
46243	0.946132	2.1
46244	0.946133	2.1
46245	1.17513	2.1
46246	1.00473	2.1
46247	0.946132	2.1
46248	1.89226	2.1
46249	1.89226	2.1
46250	1.89226	2.1
46251	1.89226	2.1
46252	1.89226	2.1
46253	1.89226	2.1
46254	1.89226	2.1
46255	2.6066	2.1
46256	2.35026	2.1
46257	2.35026	2.1
46258	1.89226	2.1
46259	1.89226	2.1
46260	2.04056	0.271824
46261	2.77366	0.22841
46262	1.24682	0.215984
46263	1.24682	0.314408
46264	2.93651	0.233119
46265	2.14277	0.293781
46266	2.68524	1.70607
46267	2.81847	1.56547
46268	1.89226	1.13205
46269	1.89226	1.51041
46270	4.57751	1.36514
46271	2.68524	1.58343
46272	1.89226	1.77444
46273	1.89226	1.74893
46274	2.50732	1.51745
46275	1.89226	1.62161
46276	2.50732	1.67258
46277	1.89226	1.22561
46278	1.89226	1.58877
46279	1.89226	1.46154
46280	1.89226	1.75521
46281	1.89226	1.19284

46282	1.89226	1.72173
46283	1.89226	1.79117
46284	1.89226	1.43711
46285	1.89226	1.69519
46286	1.89226	1.85151
46287	1.89226	1.14375
46288	1.89226	1.27088
46289	1.61016	1.38048
46290	1.07467	1.57448
46291	1.24474	1.27157
46292	1.24474	1.81061
46293	1.45303	1.40771
46294	1.45303	1.81685
46295	1.61016	1.53557
46296	1.24474	1.89935
46297	1.61016	1.55213
46298	1.77186	1.39154
46299	1.37813	1.31599
46300	1.37813	1.52119
46301	1.73305	2.1
46302	2.0815	2.1
46303	1.97658	2.1
46304	1.75791	2.1
46305	1.87228	2.1
46306	1.66515	2.1
46307	2.0815	2.1
46308	1.93938	2.1
46309	2.03253	2.08664
46310	2.2142	2.1
46311	1.97658	2.1
46312	2.15324	2.1
46313	1.73305	2.1
46314	1.97658	2.1
46315	1.60428	2.1
46316	1.8589	2.1
46317	3.58085	2.1
46318	1.97657	2.1
46319	1.93938	2.1
46320	1.51316	2.1
46321	1.85293	2.07993
46322	3.82951	2.1
46323	1.70303	0.0705609
46324	1.96087	0.0755121
46325	1.42308	0.0703559
46326	1.96087	0.0702363
46327	1.25023	0.0747502
46328	1.70303	0.0730383
46329	0.946132	0.0784989
46330	0.946132	0.0794051
46331	0.946131	0.0761162
46332	0.946131	0.0773582
46333	0.946132	0.0782116
46334	0.946132	0.0893138
46335	0.946132	0.0779914
46336	0.946132	0.080901
46337	0.946132	0.0784319
46338	0.946132	0.0768926
46339	0.946132	0.0812745

46340	0.946132	0.0820935
46341	1.89226	0.0854755
46342	1.89226	0.0818397
46343	1.89226	0.0759207
46344	1.89226	0.0748443
46345	3.78452	0.0821342
46346	1.89226	0.0698905
46347	1.89226	0.0747629
46348	1.89226	0.0708251
46349	1.89226	0.0710049
46350	1.89226	0.0738128
46351	1.89226	0.0733377
46352	1.89226	0.0698591
46353	1.89226	0.0698591
46354	1.89226	0.0698591
46355	1.89226	0.0698591
46356	1.89226	0.0698591
46357	1.89226	0.0698591
46358	1.89226	0.0698591
46359	1.89226	0.0698591
46360	2.48255	0.0698591
46361	1.89226	0.0698591
46362	3.78452	0.0698591
46363	1.89226	2.1
46364	2.42758	2.1
46365	1.9209	2.1
46366	1.89226	2.1
46367	2.45622	2.1
46368	2.42758	2.1
46369	2.42758	2.1
46370	1.49987	2.1
46371	1.61028	2.1
46372	1.58164	2.1
46373	2.45622	2.1
46374	2.42758	2.1
46375	0.94613	2.1
46376	0.94613	2.1
46377	1.24298	2.1
46378	1.24298	2.1
46379	1.24298	2.1
46380	1.22811	2.1
46381	0.946131	2.1
46382	0.946131	2.1
46383	0.94613	2.1
46384	1.24298	2.1
46385	0.94613	2.1
46386	1.22811	2.1
46387	2.48596	2.1
46388	2.45622	2.1
46389	1.63823	2.1
46390	1.4304	2.1
46391	1.45739	2.1
46392	2.45622	2.1
46393	2.4169	2.1
46394	2.55249	2.1
46395	2.51125	2.1
46396	2.35245	2.1
46397	4.90494	2.1

46398	2.45922	2.1
46399	2.55249	2.1
46400	2.51126	2.1
46401	2.43226	2.1
46402	4.98474	2.1
46403	2.51125	2.1
46404	2.35245	2.1
46405	2.28979	2.1
46406	2.47228	2.1
46407	4.82473	2.1
46408	2.51126	2.1
46409	2.43226	2.1
46410	2.40668	2.1
46411	2.47228	2.1
46412	4.90453	2.1
46413	2.8045	2.1
46414	1.93707	2.1
46415	2.14087	2.1
46416	2.14087	2.1
46417	2.61595	2.1
46418	2.61595	2.1
46419	1.89226	2.1
46420	1.89226	2.1
46421	1.89226	2.1
46422	1.89226	2.1
46423	1.89226	2.1
46424	1.89226	2.1
46425	1.22368	2.1
46426	1.32339	2.1
46427	0.957125	2.1
46428	0.750074	2.1
46429	1.08438	2.1
46430	1.08438	2.1
46431	1.13094	2.1
46432	0.970797	2.1
46433	1.17568	2.1
46434	0.764392	2.1
46435	0.944286	2.1
46436	1.27523	2.1
46437	1.89226	2.1
46438	1.89226	2.1
46439	1.89226	2.1
46440	1.89226	2.1
46441	3.78453	2.1
46442	2.8239	2.1
46443	1.89227	2.1
46444	2.57276	2.1
46445	2.57276	2.1
46446	1.89227	2.1
46447	1.89227	2.1
46448	1.18139	2.1
46449	1.85195	2.1
46450	1.78595	2.1
46451	1.08172	2.1
46452	2.93367	2.1
46453	1.60735	2.1
46454	1.20356	2.1
46455	0.967034	2.1

46456	1.08172	2.1
46457	1.67324	2.1
46458	1.77293	2.1
46459	1.89226	2.1
46460	1.89226	2.1
46461	1.8213	2.1
46462	1.82129	2.1
46463	2.14171	2.1
46464	2.40192	2.1
46465	0.810943	2.1
46466	0.810943	2.1
46467	0.798469	2.1
46468	0.788336	2.1
46469	0.804015	2.1
46470	0.810945	2.1
46471	1.947	2.1
46472	1.36506	2.1
46473	1.08686	2.1
46474	1.07208	2.1
46475	1.08132	2.1
46476	1.13537	1.17389
46477	1.14754	2.09842
46478	0.946131	2.00317
46479	0.946131	2.1
46480	0.94613	1.97907
46481	0.94613	1.17179
46482	1.25104	2.1
46483	1.14754	2.08275
46484	1.13537	2.04913
46485	0.946131	2.05568
46486	0.946131	2.08855
46487	1.25104	2.08052
46488	0.96542	0.829753
46489	0.922284	2.04344
46490	0.946131	2.1
46491	0.946131	2.02376
46492	0.946131	1.99404
46493	0.946131	0.845649
46494	1.13537	2.1
46495	0.924824	2.07839
46496	0.859476	2.04412
46497	0.842305	2.04367
46498	1.25996	2.03497
46499	1.04996	2.04309
46500	1.71007	0.0698591
46501	2.73283	0.0698591
46502	2.73283	0.0698591
46503	1.89226	0.0698591
46504	1.89226	0.0698591
46505	1.13262	0.0698591
46506	0.946131	0.0698591
46507	0.946131	0.0698591
46508	0.946131	0.0698591
46509	0.946131	0.0698591
46510	0.946132	0.0698591
46511	0.946132	0.0698591
46512	0.946131	0.0698591
46513	0.946131	0.0698591

46514	0.946131	0.0698591
46515	0.946131	0.0698591
46516	0.946132	0.0698591
46517	0.946132	0.0698591
46518	0.946131	0.0698591
46519	1.36642	0.0698591
46520	0.946125	0.0698591
46521	0.946125	0.0698591
46522	0.946128	0.0698591
46523	0.946128	0.0698591
46524	0.946126	0.0698591
46525	0.946128	0.0698591
46526	0.946128	0.0698591
46527	0.946128	0.0698591
46528	1.36641	0.0698591
46529	0.946128	0.0698591
46530	1.89226	0.0698591
46531	1.89226	0.0698591
46532	1.89226	0.0698591
46533	1.89226	0.0698591
46534	1.89226	0.0698591
46535	1.89226	0.0698591
46536	2.69784	2.1
46537	2.51651	2.1
46538	2.56999	2.1
46539	2.74623	2.1
46540	5.26783	2.1
46541	2.69784	2.1
46542	2.48718	2.1
46543	2.50161	2.1
46544	2.74623	2.1
46545	5.19945	2.1
46546	1.89226	2.1
46547	1.89226	2.1
46548	1.89226	2.1
46549	1.89226	2.1
46550	3.78453	2.1
46551	0.874264	2.1
46552	1.09879	2.1
46553	0.946131	2.1
46554	0.946131	2.1
46555	1.17062	2.1
46556	1.17062	2.1
46557	0.874293	2.1
46558	0.874293	2.1
46559	1.09879	2.1
46560	0.94613	2.1
46561	0.946131	2.1
46562	0.874265	2.1
46563	1.89226	2.1
46564	1.89226	2.1
46565	1.89226	2.1
46566	1.89226	2.1
46567	3.78453	2.1
46568	0.977382	2.1
46569	1.16424	2.1
46570	0.946133	2.1
46571	0.946133	2.1

46572	1.33012	2.1
46573	1.25589	2.1
46574	0.888498	2.1
46575	0.888498	2.1
46576	1.25375	2.1
46577	0.946131	2.1
46578	0.946131	2.1
46579	0.977384	2.1
46580	1.89226	2.1
46581	2.19757	2.1
46582	2.62906	2.1
46583	1.89226	2.1
46584	1.89226	2.1
46585	2.62906	2.1
46586	1.27944	0.259387
46587	1.27731	1.43412
46588	0.615701	1.26806
46589	0.615701	0.343954
46590	0.615673	0.12013
46591	0.946133	1.94625
46592	0.948264	0.171975
46593	0.946132	0.323203
46594	0.94752	0.164423
46595	0.61706	0.158487
46596	0.946132	0.277084
46597	0.946132	0.442116
46598	2.76604	2.1
46599	1.66728	2.1
46600	1.66728	2.1
46601	1.83036	2.1
46602	2.54515	2.1
46603	2.70823	2.1
46604	2.54515	2.1
46605	1.49958	2.1
46606	1.49958	2.1
46607	1.66728	2.1
46608	2.34465	2.1
46609	2.51235	2.1
46610	1.21128	2.1
46611	1.21128	2.1
46612	0.946131	2.1
46613	0.946131	2.1
46614	0.946131	2.1
46615	0.946131	2.1
46616	1.31401	2.1
46617	1.21128	2.1
46618	1.21128	2.1
46619	0.946131	2.1
46620	0.946131	2.1
46621	1.31401	2.1
46622	1.67374	2.1
46623	2.29586	2.1
46624	1.50033	2.1
46625	2.16598	2.1
46626	3.83972	2.1
46627	1.89226	2.1
46628	1.89226	2.1
46629	1.89226	2.1

46630	1.89226	2.1
46631	3.78452	2.1
46632	1.13537	2.1
46633	1.02188	2.1
46634	0.946132	2.1
46635	0.946132	2.1
46636	0.946132	2.1
46637	0.946132	2.1
46638	1.21128	2.1
46639	1.02188	2.1
46640	1.04672	2.1
46641	1.04672	2.1
46642	1.21128	2.1
46643	0.946132	2.1
46644	0.815411	2.1
46645	0.870464	2.1
46646	0.885437	2.1
46647	0.885437	2.1
46648	1.06395	2.1
46649	1.00483	2.1
46650	0.757125	2.1
46651	0.673956	2.1
46652	0.684461	2.1
46653	1.06979	2.1
46654	0.731403	2.1
46655	1.0559	2.1
46656	1.89226	2.1
46657	1.89226	2.1
46658	1.89226	2.1
46659	1.89226	2.1
46660	1.89226	2.1
46661	1.89226	2.1
46662	1.76897	2.02193
46663	1.76932	2.06777
46664	1.97455	2.1
46665	1.94302	1.78535
46666	3.71233	2.04725
46667	1.83347	2.1
46668	1.76932	2.08069
46669	1.97455	2.1
46670	1.76932	2.1
46671	1.76522	2.1
46672	1.55999	2.1
46673	1.89227	2.08103
46674	1.89227	2.1
46675	1.89227	2.1
46676	1.89227	2.03899
46677	3.78453	2.08776
46678	1.89226	2.0689
46679	1.89226	1.31958
46680	1.89226	1.69866
46681	1.89226	1.99956
46682	3.78452	2.00226
46683	1.97452	2.09915
46684	1.94299	1.52789
46685	2.17831	1.88952
46686	2.23195	2.02501
46687	4.17495	2.0306

46688	2.23196	2.06205
46689	2.1499	2.1
46690	1.97452	2.1
46691	1.97452	2.1
46692	2.09088	2.1
46693	1.83345	2.1
46694	0.94612	2.1
46695	0.94612	2.1
46696	0.946119	2.00281
46697	0.946119	1.99219
46698	0.946119	2.00117
46699	0.946119	2.09797
46700	0.94612	2.05798
46701	0.94612	2.1
46702	0.946132	2.07644
46703	0.946132	2.04488
46704	0.946107	2.09389
46705	0.946107	2.08958
46706	1.89224	1.40936
46707	1.89224	1.86628
46708	1.89224	2.00226
46709	1.89224	1.08573
46710	3.78447	1.33318
46711	1.70302	0.216752
46712	1.056	0.193447
46713	1.21829	0.275825
46714	1.89906	0.192615
46715	3.07892	0.203366
46716	2.20248	0.216169
46717	1.56498	0.187119
46718	1.46539	0.188449
46719	1.48787	0.175248
46720	2.37625	0.26306
46721	2.37625	0.212055
46722	1.14104	0.173271
46723	1.00675	0.186239
46724	0.700689	0.17875
46725	0.624389	0.186108
46726	0.782149	0.157273
46727	0.940197	0.164932
46728	0.803437	0.179407
46729	0.803437	0.192849
46730	0.995423	0.175512
46731	0.700699	0.153544
46732	0.593895	0.22207
46733	1.00673	0.172673
46734	1.18097	0.170697
46735	1.15839	0.173696
46736	0.995407	0.175185
46737	0.865932	0.176781
46738	1.17299	0.15648
46739	0.957054	0.162108
46740	1.08016	0.184486
46741	1.08016	0.22424
46742	0.995425	0.196514
46743	1.24161	0.150966
46744	0.796771	0.160507
46745	1.15837	0.205141

46746	2.57264	0.0698591
46747	1.89226	0.0698591
46748	2.41701	0.0698591
46749	1.89226	0.0698591
46750	4.30928	0.0698591
46751	1.69055	0.0698591
46752	2.39587	0.0698591
46753	1.53463	0.0698591
46754	2.2053	0.0698591
46755	3.89585	0.0698591
46756	0.946129	0.0698591
46757	0.946129	0.0698591
46758	0.94613	0.0698591
46759	0.94613	0.0698591
46760	0.946132	0.0698591
46761	0.946132	0.0698591
46762	0.946126	0.0698591
46763	0.946126	0.0698591
46764	0.94613	0.0698591
46765	0.94613	0.0698591
46766	0.946129	0.0698591
46767	0.946129	0.0698591
46768	2.41701	0.0698591
46769	1.89226	0.0698591
46770	1.89226	0.0698591
46771	1.89226	0.0698591
46772	2.32462	0.0698591
46773	2.32462	0.0698591
46774	1.41942	0.0698591
46775	2.2053	0.0698591
46776	2.02097	0.0698591
46777	2.02097	0.0698591
46778	1.53463	0.0698591
46779	1.53463	0.0698591
46780	1.89226	0.0698591
46781	1.89226	0.0698591
46782	1.89226	0.0698591
46783	1.89226	0.0698591
46784	1.89226	0.0698591
46785	1.89226	0.0698591
46786	1.89226	1.37595
46787	1.89226	1.95019
46788	1.89226	1.92576
46789	1.89226	1.65643
46790	1.89226	1.82231
46791	1.89226	1.45186
46792	1.02816	1.0391
46793	1.02816	1.3059
46794	0.595141	1.59478
46795	0.595141	1.68929
46796	0.946132	1.20774
46797	0.595113	1.80496
46798	0.677174	1.68692
46799	0.946132	1.01825
46800	0.946132	1.46771
46801	0.946132	1.52418
46802	0.595113	1.54121
46803	0.677174	1.3987

46804	1.89226	1.5463
46805	1.89226	1.9842
46806	1.89226	1.87177
46807	1.89226	1.64484
46808	1.89226	1.90472
46809	1.89226	1.7001
46810	0.946133	1.11674
46811	0.946133	0.97459
46812	0.946132	1.80736
46813	0.946132	1.82297
46814	0.946132	1.80727
46815	0.946132	1.33895
46816	0.946132	1.63482
46817	0.946132	0.989162
46818	0.946132	1.55046
46819	0.946132	1.69625
46820	0.946133	1.22586
46821	0.946133	1.40786
46822	0.946131	1.7283
46823	0.946131	1.72564
46824	0.946131	1.98088
46825	0.946131	1.9736
46826	0.946128	1.99141
46827	0.946128	1.73692
46828	0.946133	1.9789
46829	0.946133	1.6784
46830	0.94613	1.86383
46831	0.94613	1.93443
46832	0.946131	1.89984
46833	0.946131	1.81717
46834	2.0264	2.1
46835	1.67345	2.03042
46836	2.0264	2.05372
46837	1.70539	1.27103
46838	1.31611	1.65328
46839	1.31611	1.86337
46840	1.89226	0.502836
46841	1.89226	0.434546
46842	1.89226	0.646995
46843	1.89226	0.373774
46844	3.78452	0.396544
46845	1.89226	0.653145
46846	1.89226	0.511107
46847	1.89226	0.540038
46848	1.89226	0.712912
46849	1.89226	0.561104
46850	1.89226	0.760795
46851	1.02645	0.192513
46852	0.810942	0.233324
46853	0.882781	0.277996
46854	0.882781	0.247592
46855	2.80386	0.200633
46856	1.56719	0.236133
46857	1.22499	0.197093
46858	1.08132	0.26735
46859	1.36865	0.223854
46860	0.810957	0.254882
46861	0.810957	0.224974

46862	0.946132	0.370346
46863	0.946132	0.432334
46864	0.946129	0.264743
46865	0.946129	0.310326
46866	0.946131	0.250372
46867	0.946131	0.28283
46868	0.946131	0.43887
46869	0.946131	0.374776
46870	0.94613	0.353238
46871	0.94613	0.372889
46872	0.946131	0.48942
46873	0.946131	0.356793
46874	1.89226	0.481652
46875	1.89226	0.322131
46876	1.89226	0.551076
46877	1.89226	0.311115
46878	3.78453	0.329351
46879	1.30462	0.0698591
46880	1.79906	0.0828257
46881	1.01299	0.0956635
46882	1.44185	0.0758417
46883	1.3499	0.0698591
46884	1.79906	0.0698715
46885	1.89226	0.0716794
46886	1.89226	0.100252
46887	1.89226	0.0698591
46888	1.89226	0.0902693
46889	1.89226	0.0749071
46890	1.89226	0.1399
46891	1.89226	0.0962435
46892	1.89226	0.0977458
46893	1.89226	0.0980073
46894	1.89226	0.0979614
46895	1.89226	0.0954437
46896	1.89226	0.106291
46897	1.3499	0.0698591
46898	1.79905	0.0800296
46899	1.29001	0.0698591
46900	1.36005	0.0834372
46901	1.88294	0.0888767
46902	1.79905	0.0749447
46903	1.89226	0.0710509
46904	2.75374	0.0698591
46905	2.60614	0.0703562
46906	1.89226	0.0730399
46907	2.60614	0.0698591
46908	1.89226	0.0698591
46909	0.946131	0.0698591
46910	0.946131	0.0702719
46911	1.37687	0.0731053
46912	1.37687	0.0770463
46913	0.946131	0.0736179
46914	1.34375	0.0830279
46915	1.37686	0.0698591
46916	0.946128	0.0698591
46917	1.37687	0.0698591
46918	1.34375	0.0698591
46919	0.94613	0.0751092

46920	0.94613	0.0748687
46921	1.44247	2.1
46922	1.12665	2.1
46923	1.50044	2.1
46924	1.73651	2.1
46925	2.94291	2.1
46926	1.73652	2.1
46927	1.64926	2.1
46928	1.44247	2.1
46929	1.44247	2.1
46930	1.64926	2.1
46931	1.43872	2.1
46932	1.50044	2.1
46933	1.73651	2.1
46934	2.00955	2.1
46935	1.87875	2.1
46936	3.50999	2.1
46937	1.64926	2.1
46938	1.73652	2.1
46939	2.00955	2.1
46940	1.8493	2.1
46941	3.65881	2.1
46942	1.89226	0.0698591
46943	2.28188	0.0698591
46944	1.99182	0.0698591
46945	2.28188	0.0713257
46946	1.6022	0.0698591
46947	1.89226	0.070509
46948	1.89226	0.0713539
46949	1.89226	0.0756617
46950	1.89226	0.069958
46951	1.89226	0.09408
46952	1.89226	0.0699967
46953	1.89226	0.0857205
46954	1.89226	0.0698591
46955	1.89226	0.0698739
46956	2.19578	0.0698591
46957	1.89226	0.0751333
46958	2.81422	0.0698591
46959	2.42521	0.072623
46960	1.89224	0.0698591
46961	1.99179	0.0698591
46962	1.454	0.0698591
46963	1.99179	0.0698591
46964	1.35444	0.0698591
46965	1.89224	0.0698591
46966	1.89224	0.069895
46967	1.89224	0.0726934
46968	1.89224	0.0698725
46969	1.89224	0.0862955
46970	1.89224	0.0698613
46971	1.89224	0.0799101
46972	1.703	0.0698591
46973	2.19575	0.0698591
46974	1.9911	0.0698591
46975	2.19575	0.0718206
46976	1.67811	0.0698591
46977	1.89223	0.0706046

46978	1.69586	2.1
46979	2.55301	2.1
46980	1.60861	2.1
46981	1.60861	2.1
46982	2.65233	2.1
46983	2.65233	2.1
46984	0.946129	2.1
46985	0.946129	2.1
46986	1.2765	2.1
46987	1.32617	2.1
46988	1.2765	2.1
46989	1.2765	2.1
46990	0.946131	2.1
46991	0.946131	2.1
46992	0.946131	2.1
46993	1.32616	2.1
46994	0.946131	2.1
46995	1.2765	2.1
46996	1.36757	2.1
46997	1.32617	2.1
46998	0.891503	2.1
46999	0.847932	2.1
47000	1.10722	2.1
47001	1.10722	2.1
47002	1.10829	2.1
47003	1.10829	2.1
47004	1.36757	2.1
47005	0.891504	2.1
47006	0.847932	2.1
47007	1.32617	2.1
47008	0.946129	2.1
47009	0.946129	2.1
47010	1.36757	2.1
47011	1.32617	2.1
47012	1.32616	2.1
47013	1.32616	2.1
47014	0.946131	2.1
47015	0.946131	2.1
47016	1.36757	2.1
47017	0.946131	2.1
47018	0.946131	2.1
47019	1.32616	2.1
47020	1.89226	0.0856766
47021	1.89226	0.0700025
47022	1.89226	0.0703811
47023	1.89226	0.100573
47024	3.78452	0.0742555
47025	2.36336	0.0698591
47026	1.89226	0.0698591
47027	2.67831	0.0698591
47028	2.67831	0.0698591
47029	1.89226	0.0698591
47030	1.89226	0.0698591
47031	1.89227	0.0702454
47032	1.89227	0.115204
47033	1.89227	0.103902
47034	1.89227	0.0739296
47035	3.78453	0.0833593

47036	1.89226	0.0698591
47037	1.89226	0.0966518
47038	1.89226	0.0944436
47039	1.89226	0.0723632
47040	3.78452	0.0790548
47041	2.36336	0.0698591
47042	2.0815	0.0698681
47043	2.0815	0.0698591
47044	2.41048	0.0698591
47045	1.66267	0.0698591
47046	2.13452	0.0698591
47047	1.89226	0.100429
47048	1.89226	0.0732924
47049	1.89226	0.0930915
47050	1.89226	0.0704964
47051	1.89226	0.110754
47052	1.89226	0.0797617
47053	1.89226	0.144404
47054	1.89226	0.151767
47055	1.89226	0.144628
47056	1.89226	0.184593
47057	1.89226	0.10485
47058	1.89226	0.15218
47059	0.946128	0.1638
47060	0.946128	0.160793
47061	1.00122	0.0929537
47062	1.00122	0.0912734
47063	0.94613	0.213305
47064	1.01618	0.0867595
47065	0.946131	0.192683
47066	1.00122	0.0709743
47067	1.00122	0.070953
47068	1.01618	0.0730222
47069	0.946131	0.188415
47070	0.946131	0.172025
47071	1.89226	2.00299
47072	1.89226	1.4687
47073	1.89226	1.56585
47074	1.89226	2.04404
47075	3.78452	1.82209
47076	1.89226	1.92335
47077	1.89226	2.1
47078	1.89226	2.1
47079	1.89226	1.97184
47080	1.89226	2.1
47081	1.89226	1.98392
47082	1.89226	1.2658
47083	1.89226	1.97044
47084	1.89226	1.91116
47085	1.89226	1.34332
47086	1.89226	1.93107
47087	1.89226	1.37819
47088	1.89226	1.36096
47089	1.89226	2.01902
47090	1.89226	1.98856
47091	1.89226	1.482
47092	1.89226	1.99286
47093	1.89226	1.55848

47094	2.71279	0.431009
47095	1.4155	0.533118
47096	2.28488	0.47389
47097	1.06122	0.500169
47098	3.70038	0.477201
47099	1.89226	0.666029
47100	1.89226	0.554824
47101	1.89226	0.497404
47102	1.89226	0.671703
47103	3.78452	0.626526
47104	0.851542	0.602802
47105	0.851542	0.587036
47106	0.851484	0.515457
47107	0.865567	0.588377
47108	0.981616	0.599525
47109	0.864767	0.58917
47110	0.851542	0.520875
47111	0.851542	0.581999
47112	0.851477	0.602451
47113	0.851477	0.595991
47114	0.865635	0.583526
47115	0.816472	0.591781
47116	1.68443	2.1
47117	1.44489	2.1
47118	1.54924	2.1
47119	1.34389	2.1
47120	3.02832	2.1
47121	1.44489	2.1
47122	1.03899	2.1
47123	1.03899	2.1
47124	1.12075	2.1
47125	1.34389	2.1
47126	1.42566	2.1
47127	1.54924	2.1
47128	1.34389	2.1
47129	1.44091	2.1
47130	1.25866	2.1
47131	2.7848	2.1
47132	0.573141	2.1
47133	0.573141	2.1
47134	0.575681	2.1
47135	0.575681	2.1
47136	0.481985	2.1
47137	0.519492	2.1
47138	0.671948	2.1
47139	0.629331	2.1
47140	0.629329	2.1
47141	0.481986	2.1
47142	0.519494	2.1
47143	0.671947	2.1
47144	1.89226	0.247113
47145	1.89226	0.394699
47146	1.89226	0.406715
47147	2.31925	0.238257
47148	3.78452	0.352208
47149	1.30916	2.08492
47150	1.17426	2.1
47151	0.946129	2.1

47152	0.946129	2.1
47153	0.94613	2.1
47154	0.94613	2.08246
47155	1.17426	2.1
47156	1.17426	2.1
47157	1.30916	2.1
47158	0.94613	2.1
47159	1.17426	2.1
47160	0.94613	2.1
47161	2.56346	2.07725
47162	1.89226	1.59503
47163	1.89226	2.02911
47164	1.89226	1.66228
47165	2.6074	2.02079
47166	2.6074	1.66384
47167	1.89226	2.08144
47168	1.89226	2.08781
47169	1.89226	2.1
47170	1.89226	2.0214
47171	3.78452	2.09827
47172	1.33064	2.07764
47173	1.84121	2.08587
47174	0.946129	2.1
47175	0.946129	2.09964
47176	0.946131	2.09984
47177	0.946131	2.07882
47178	1.30916	2.1
47179	1.8412	2.1
47180	1.33064	2.1
47181	0.94613	2.1
47182	1.30916	2.1
47183	0.946131	2.1
47184	1.19283	1.60565
47185	1.19283	1.38701
47186	0.946131	2.0769
47187	0.946131	2.01416
47188	0.946132	2.02808
47189	0.946132	1.55548
47190	1.3037	2.07571
47191	1.19283	1.46133
47192	1.19283	1.91775
47193	0.946131	1.91993
47194	0.946131	1.87922
47195	1.3037	1.94128
47196	1.89226	2.09976
47197	1.89226	2.01607
47198	1.89226	2.07408
47199	1.89226	2.07963
47200	1.89226	2.07941
47201	1.89226	2.08189
47202	2.17708	2.1
47203	1.70302	2.1
47204	2.28188	2.1
47205	2.13621	2.1
47206	4.28253	2.1
47207	1.89226	2.1
47208	1.89226	2.1
47209	1.89226	2.1

47210	1.89226	2.1
47211	3.78452	2.1
47212	0.946132	2.1
47213	0.946132	2.1
47214	0.946132	2.1
47215	0.946132	2.1
47216	0.946131	2.1
47217	0.946131	2.1
47218	0.946132	2.1
47219	0.946132	2.1
47220	0.946132	2.1
47221	0.946132	2.1
47222	0.946132	2.1
47223	0.946132	2.1
47224	1.74933	2.1
47225	1.11833	2.1
47226	1.90074	2.1
47227	1.669	2.1
47228	1.335	2.1
47229	1.11833	2.1
47230	2.28188	2.1
47231	2.31804	2.1
47232	2.42758	2.1
47233	2.14895	2.1
47234	4.43083	2.1
47235	1.89226	2.1
47236	1.89226	2.1
47237	1.89226	2.08871
47238	1.89226	2.1
47239	3.78452	2.1
47240	1.89226	2.1
47241	1.89226	2.1
47242	1.89226	2.1
47243	1.89226	2.1
47244	1.89226	2.1
47245	1.89226	2.1
47246	0.864133	2.1
47247	0.864133	2.1
47248	0.967939	2.1
47249	0.793739	2.1
47250	0.650797	2.1
47251	0.757546	2.1
47252	1.08941	2.1
47253	1.10136	2.1
47254	1.10138	2.1
47255	0.796445	2.1
47256	0.866907	2.1
47257	0.650788	2.1
47258	2.63062	1.84102
47259	1.89226	1.62328
47260	1.89226	1.03238
47261	1.89226	2.09145
47262	2.46557	0.850723
47263	2.46557	2.09422
47264	1.16777	2.09416
47265	1.38859	2.1
47266	1.05042	2.1
47267	1.28909	2.1

47268	2.45686	2.1
47269	1.89227	2.09007
47270	1.89227	2.1
47271	1.89227	1.71614
47272	1.89227	2.1
47273	3.78453	2.09952
47274	2.15114	2.1
47275	1.89226	2.1
47276	2.15005	2.1
47277	2.15005	2.1
47278	1.89226	2.1
47279	1.89226	2.1
47280	1.89226	2.1
47281	1.89226	1.55732
47282	1.89226	2.1
47283	1.89226	2.1
47284	1.89226	1.30329
47285	1.89226	2.1
47286	0.973447	2.1
47287	1.32143	2.0655
47288	1.37444	2.1
47289	1.37444	2.07495
47290	0.949134	2.1
47291	0.949134	2.1
47292	0.946131	2.1
47293	0.946131	2.1
47294	1.07557	2.1
47295	1.07557	2.1
47296	0.946131	2.1
47297	0.946131	2.1
47298	1.07557	2.1
47299	1.08579	2.1
47300	1.08579	2.1
47301	0.946131	2.1
47302	1.07557	2.1
47303	0.946131	2.1
47304	0.946131	1.1656
47305	0.946131	2.1
47306	0.946131	2.1
47307	0.946131	2.07452
47308	0.94613	1.19201
47309	0.94613	1.57327
47310	0.946131	2.1
47311	0.946131	2.1
47312	0.946131	2.06716
47313	0.946131	2.02906
47314	0.946131	2.07962
47315	0.946131	2.09242
47316	0.606115	2.1
47317	0.606115	2.08169
47318	0.616308	2.1
47319	0.616308	2.1
47320	0.68722	1.40356
47321	0.735699	2.00069
47322	0.514789	2.1
47323	0.486724	2.1
47324	0.514788	2.04761
47325	0.735699	2.08095

47326	0.486723	2.09397
47327	0.687221	2.07976
47328	2.33761	0.581638
47329	2.51749	0.196586
47330	2.68092	0.256331
47331	2.58304	0.222499
47332	5.01853	0.214072
47333	2.50906	0.320915
47334	2.51749	0.234998
47335	2.68091	0.302952
47336	2.53176	0.158894
47337	5.18997	0.294387
47338	2.68092	0.297438
47339	2.58304	0.213448
47340	2.71793	0.287838
47341	2.67098	0.157348
47342	5.39885	0.250324
47343	2.68091	0.366525
47344	2.53175	0.171059
47345	2.36417	0.227706
47346	2.67098	0.177017
47347	5.04508	0.205674
47348	2.76309	2.1
47349	1.89226	2.1
47350	1.89226	2.1
47351	1.89226	2.1
47352	2.48573	2.1
47353	2.48573	2.1
47354	1.35879	2.1
47355	1.55161	2.09027
47356	1.10407	2.1
47357	1.32259	2.0536
47358	2.68138	2.1
47359	1.89226	2.1
47360	1.89226	2.08944
47361	1.89226	2.1
47362	1.89226	2.06813
47363	3.78452	2.1
47364	1.19612	2.053
47365	1.24694	2.1
47366	1.24844	0.998911
47367	1.29747	2.1
47368	2.49358	2.1
47369	1.89226	2.06885
47370	1.89226	2.1
47371	1.89226	0.756158
47372	1.89226	2.1
47373	3.78453	2.09625
47374	1.89226	2.1
47375	1.89226	2.1
47376	1.89226	2.1
47377	1.89226	2.1
47378	1.89226	2.1
47379	1.89226	2.1
47380	0.587463	2.1
47381	0.587463	2.1
47382	0.581977	2.1
47383	0.581977	2.1

47384	0.531709	2.1
47385	0.545972	2.1
47386	0.623469	2.1
47387	0.648733	2.1
47388	0.648733	2.1
47389	0.545971	2.1
47390	0.623469	2.1
47391	0.531709	2.1
47392	1.29417	1.5864
47393	1.29747	2.1
47394	1.27829	2.1
47395	1.24844	2.00883
47396	1.32732	2.1
47397	1.29747	2.06024
47398	1.89226	0.612529
47399	1.89226	2.1
47400	1.89226	1.59338
47401	1.89226	2.1
47402	1.89226	1.624
47403	1.89226	2.1
47404	1.89226	2.1
47405	1.89226	2.1
47406	1.89226	2.1
47407	1.89226	2.1
47408	1.89226	2.1
47409	1.89226	2.1
47410	0.60346	2.1
47411	0.60346	2.1
47412	0.60086	2.1
47413	0.60086	2.1
47414	0.545971	2.1
47415	0.555587	2.1
47416	0.648733	2.1
47417	0.663661	2.1
47418	0.663661	2.1
47419	0.555587	2.1
47420	0.648733	2.1
47421	0.545971	2.1
47422	1.34066	2.08079
47423	1.36601	2.1
47424	1.34955	0.585505
47425	1.37623	2.1
47426	2.71556	2.1
47427	1.89226	1.5595
47428	1.89226	2.1
47429	1.89226	0.581938
47430	1.89226	2.1
47431	3.78452	1.79729
47432	0.946132	2.1
47433	0.946132	2.1
47434	0.946132	2.1
47435	0.946132	2.1
47436	0.946132	2.1
47437	0.946132	2.1
47438	0.946131	2.1
47439	0.946131	2.1
47440	0.946132	2.1
47441	0.946132	2.1

47442	0.946132	2.1
47443	0.946132	2.1
47444	0.631351	2.1
47445	0.631351	2.1
47446	0.632421	2.1
47447	0.632421	2.1
47448	0.573604	2.1
47449	0.580765	2.1
47450	0.683006	2.1
47451	0.688118	2.1
47452	0.688118	2.1
47453	0.580765	2.1
47454	0.683007	2.1
47455	0.573604	2.1
47456	1.34955	1.9712
47457	1.37624	2.1
47458	1.35048	0.918563
47459	1.37999	2.1
47460	2.72954	2.06526
47461	0.946131	2.1
47462	0.946131	1.69489
47463	0.946131	2.09526
47464	0.946131	2.1
47465	0.94613	2.1
47466	0.94613	2.1
47467	0.946133	0.94966
47468	0.946133	1.16109
47469	0.946131	2.07157
47470	0.946131	2.07023
47471	0.946132	2.03047
47472	0.946132	1.9695
47473	1.89226	2.1
47474	1.89226	2.1
47475	1.89226	2.1
47476	1.89226	2.1
47477	1.89226	2.1
47478	1.89226	2.1
47479	0.532189	2.1
47480	0.531141	2.1
47481	0.497784	2.1
47482	0.502536	2.1
47483	1.37999	2.1
47484	0.92433	2.1
47485	0.670081	2.1
47486	0.727155	2.1
47487	0.786438	2.1
47488	0.589798	2.1
47489	0.497784	2.1
47490	1.89226	2.1
47491	2.72301	2.1
47492	2.4158	2.1
47493	2.4158	2.1
47494	1.89226	2.1
47495	1.89226	2.1
47496	1.89226	2.1
47497	1.89226	2.1
47498	1.89226	2.1
47499	1.89226	2.1

47500	1.89226	2.1
47501	1.89226	2.1
47502	1.36919	2.1
47503	1.12717	2.1
47504	1.38634	2.1
47505	1.56541	2.1
47506	2.75552	2.1
47507	1.89226	2.1
47508	1.89226	2.1
47509	1.89226	2.1
47510	1.89226	2.1
47511	1.89226	2.1
47512	1.89226	2.1
47513	0.907036	2.1
47514	0.782706	2.1
47515	0.838808	2.1
47516	0.693168	2.1
47517	0.794747	2.1
47518	0.794747	2.1
47519	0.805458	2.1
47520	0.805458	2.1
47521	0.907036	2.1
47522	0.838808	2.1
47523	0.693169	2.1
47524	0.782707	2.1
47525	0.94613	2.1
47526	0.94613	2.1
47527	1.188 2.1	
47528	1.28421	2.1
47529	1.18801	2.1
47530	1.18801	2.1
47531	0.946129	2.1
47532	0.946129	2.1
47533	1.28421	2.1
47534	0.94613	2.1
47535	1.188 2.1	
47536	0.94613	2.1
47537	1.89226	2.1
47538	1.89226	2.1
47539	1.89226	2.1
47540	1.89226	2.1
47541	1.89226	2.1
47542	1.89226	2.1
47543	1.16817	2.1
47544	1.06007	2.1
47545	1.19785	2.1
47546	1.2832	2.1
47547	2.36602	2.1
47548	0.960106	2.1
47549	2.0073	2.1
47550	1.51754	2.1
47551	2.56883	2.1
47552	3.52484	2.1
47553	1.06756	2.1
47554	1.57913	2.1
47555	1.87532	2.1
47556	1.38666	2.1
47557	2.94287	2.1

47558	2.15622	2.1
47559	1.38668	2.1
47560	1.87534	2.1
47561	1.87534	2.1
47562	1.70539	2.1
47563	1.70539	2.1
47564	0.986095	2.1
47565	1.36226	2.1
47566	1.3331	2.1
47567	1.3331	2.1
47568	1.07156	2.1
47569	1.07156	2.1
47570	0.682081	1.96627
47571	0.68748	1.91703
47572	0.650003	0.98078
47573	0.645873	1.13892
47574	0.666344	1.01042
47575	0.666344	1.93061
47576	0.66161	1.98046
47577	0.66161	1.14222
47578	0.68748	1.57641
47579	0.645873	1.54567
47580	0.650004	1.49947
47581	0.682082	1.52367
47582	0.946132	1.95016
47583	0.946132	1.92429
47584	0.946132	1.05282
47585	0.946132	0.925192
47586	0.946132	0.908209
47587	0.946132	1.93512
47588	0.946133	1.13709
47589	0.946133	1.9891
47590	0.946132	1.53279
47591	0.946132	1.6182
47592	0.946133	1.54376
47593	0.946133	1.58603
47594	1.89227	1.08269
47595	2.79312	0.361527
47596	2.77591	0.566222
47597	2.79312	1.05388
47598	1.87505	0.510869
47599	1.89227	0.945856
47600	0.68748	2.00178
47601	0.698626	1.97418
47602	0.645873	1.14485
47603	0.650148	1.22325
47604	0.670561	1.14991
47605	0.670561	1.98336
47606	0.667067	2.01902
47607	0.667067	1.18783
47608	0.698627	1.72758
47609	0.650148	1.69541
47610	0.68748	1.69292
47611	0.645873	1.68408
47612	0.946132	2.00149
47613	0.946132	1.94268
47614	0.946132	1.24349
47615	0.946132	1.15507

47616	0.946132	1.12271
47617	0.946132	1.94578
47618	0.946133	1.34796
47619	0.946133	2.02693
47620	0.946133	1.50051
47621	0.946133	1.5023
47622	0.946132	1.54591
47623	0.946132	1.58223
47624	0.946133	1.25119
47625	0.946133	1.1551
47626	1.38709	0.580129
47627	1.38795	0.470786
47628	0.946132	0.485916
47629	0.946132	1.16093
47630	1.38709	1.23044
47631	1.38709	0.553599
47632	1.38709	0.829984
47633	0.946133	0.839658
47634	0.946132	0.798466
47635	1.38795	0.828395
47636	1.70301	0.0698591
47637	2.07659	0.0698591
47638	1.89226	0.0698591
47639	1.89226	0.0698591
47640	1.76332	0.0698591
47641	1.76331	0.0698591
47642	1.89226	0.0698591
47643	1.89226	0.0698591
47644	1.89226	0.0698591
47645	1.89226	0.0698591
47646	1.89226	0.0698591
47647	1.89226	0.0698591
47648	1.89226	0.0698591
47649	1.89226	0.0698591
47650	1.89226	0.0698591
47651	1.89226	0.0698591
47652	3.78452	0.0698591
47653	1.89226	0.0698591
47654	2.12492	0.0698591
47655	1.89226	0.0698591
47656	1.89226	0.0698591
47657	2.07659	0.0698591
47658	2.07659	0.0698591
47659	0.94613	0.0698591
47660	0.94613	0.0698591
47661	1.17498	0.0698591
47662	1.32446	0.0698591
47663	1.32446	0.0698591
47664	0.94613	0.0698591
47665	0.946127	0.0698591
47666	1.17498	0.0698591
47667	1.32446	0.0698591
47668	1.32446	0.0698591
47669	0.946128	0.0698591
47670	0.946128	0.0698591
47671	1.89226	2.1
47672	1.86472	2.1
47673	2.11049	2.1

47674	1.89226	2.1
47675	2.08295	2.1
47676	1.86472	2.1
47677	1.89226	2.1
47678	2.08295	2.1
47679	2.14023	2.1
47680	1.89226	2.1
47681	2.33091	2.1
47682	2.08295	2.1
47683	0.809215	2.1
47684	0.809215	2.1
47685	0.810915	2.1
47686	0.810915	2.1
47687	1.09581	2.1
47688	1.08271	2.1
47689	0.520996	2.1
47690	0.537426	2.1
47691	0.520997	2.1
47692	1.08271	2.1
47693	0.537426	2.1
47694	1.09581	2.1
47695	0.79674	2.1
47696	0.79674	2.1
47697	0.799279	2.1
47698	0.799279	2.1
47699	1.07502	2.1
47700	1.0827	2.1
47701	0.508206	2.1
47702	0.520996	2.1
47703	0.508206	2.1
47704	1.07502	2.1
47705	0.520996	2.1
47706	1.0827	2.1
47707	2.15714	2.1
47708	1.70302	2.1
47709	1.78732	2.1
47710	2.02219	2.1
47711	3.98971	2.1
47712	2.0927	2.1
47713	2.162 2.1	
47714	2.11563	2.1
47715	2.02219	2.1
47716	4.18419	2.1
47717	1.62483	2.1
47718	2.02219	2.1
47719	1.93617	2.1
47720	1.52008	2.1
47721	3.54227	2.1
47722	2.11563	2.1
47723	2.02219	2.1
47724	2.29218	2.1
47725	2.03975	2.1
47726	4.06194	2.1
47727	2.007 2.1	
47728	1.89226	2.1
47729	1.89226	2.1
47730	1.89226	2.1
47731	2.06776	2.1

47732	2.06776	2.1
47733	0.946129	2.1
47734	0.946129	2.1
47735	1.0035	2.1
47736	1.0035	2.1
47737	0.946131	2.1
47738	0.946131	2.1
47739	0.968797	2.1
47740	1.0035	2.1
47741	0.968771	2.1
47742	0.946105	2.1
47743	1.00353	2.1
47744	0.946157	2.1
47745	1.79103	2.03085
47746	2.53286	0.279935
47747	2.60781	2.1
47748	2.219	0.775564
47749	2.17985	1.24023
47750	1.79103	0.201734
47751	2.219	1.42648
47752	2.66359	1.75005
47753	2.70084	2.08905
47754	2.38698	1.21715
47755	2.53285	0.334833
47756	2.219	0.565434
47757	1.33179	1.17763
47758	0.946128	0.543635
47759	0.946133	1.07013
47760	1.26643	1.94064
47761	1.3318	0.513258
47762	1.3318	1.88714
47763	0.946131	0.617108
47764	0.946131	1.33808
47765	0.946131	0.244632
47766	0.946131	0.190902
47767	1.33179	0.84998
47768	1.26643	2.01074
47769	2.28979	2.1
47770	2.47227	2.1
47771	2.43451	2.1
47772	2.22627	2.1
47773	4.69855	2.1
47774	2.40667	2.1
47775	2.47228	2.1
47776	2.43451	2.1
47777	2.38069	2.1
47778	4.85296	2.1
47779	2.43451	2.1
47780	2.15715	2.1
47781	2.15715	2.1
47782	2.22627	2.1
47783	2.39489	2.1
47784	2.46402	2.1
47785	2.43451	2.1
47786	2.34899	2.1
47787	2.38069	2.1
47788	2.38069	2.1
47789	2.3949	2.1

47790	2.3949	2.1
47791	2.85425	2.09867
47792	1.70302	0.522529
47793	1.70302	1.24418
47794	1.52254	1.01619
47795	2.34181	1.28935
47796	2.08499	1.37578
47797	1.89226	1.22934
47798	1.89226	0.482491
47799	1.89226	0.596884
47800	1.89226	0.366383
47801	1.89226	0.419825
47802	1.89226	0.693918
47803	2.34181	1.93508
47804	1.38287	0.567764
47805	1.38287	1.3517
47806	1.84132	0.7176
47807	1.96416	1.12553
47808	2.4907	1.12714
47809	0.931432	1.19128
47810	0.946135	0.203322
47811	0.946133	0.110649
47812	0.946133	0.255154
47813	0.931464	0.402583
47814	0.931464	1.20081
47815	1.15573	0.142814
47816	0.96378	1.00094
47817	0.931434	0.505672
47818	0.946131	0.236074
47819	0.946131	0.207312
47820	1.13808	0.172548
47821	0.946132	0.582027
47822	0.946132	0.433404
47823	0.946132	0.936664
47824	0.946132	0.452765
47825	0.946133	0.557747
47826	0.946133	0.194591
47827	0.946132	0.634449
47828	0.946132	0.900767
47829	0.946133	0.499634
47830	0.946133	0.590828
47831	0.946132	0.224474
47832	0.946132	0.268508
47833	1.68074	2.1
47834	2.29459	2.1
47835	2.23361	2.1
47836	1.57234	2.1
47837	3.86693	2.1
47838	2.61243	2.1
47839	2.29458	2.1
47840	2.2336	2.1
47841	2.58234	2.1
47842	4.87692	2.1
47843	0.73506	2.1
47844	0.786166	2.1
47845	1.08729	2.1
47846	1.11679	2.1
47847	0.93134	2.1

47848	0.93134	2.1
47849	0.942113	2.1
47850	0.942113	2.1
47851	0.735065	2.1
47852	1.08729	2.1
47853	1.11679	2.1
47854	0.78616	2.1
47855	1.08729	2.1
47856	1.11679	2.1
47857	1.27502	2.1
47858	1.29116	2.1
47859	1.19259	2.1
47860	1.19259	2.1
47861	1.19923	2.1
47862	1.19923	2.1
47863	1.27503	2.1
47864	1.08729	2.1
47865	1.11679	2.1
47866	1.29115	2.1
47867	0.974715	0.559789
47868	1.43779	0.254821
47869	1.93915	0.311602
47870	1.53908	0.347374
47871	2.91387	0.290302
47872	1.53908	0.277192
47873	0.99845	0.284727
47874	1.5176	0.53868
47875	1.97963	0.241527
47876	3.05668	0.323734
47877	1.93915	0.289092
47878	1.97963	0.255599
47879	1.53908	0.276665
47880	1.53908	0.201221
47881	2.3376	0.573804
47882	2.3376	0.288227
47883	1.89226	2.1
47884	2.59747	2.1
47885	1.91802	2.1
47886	1.89226	2.1
47887	2.62322	2.1
47888	2.59747	2.1
47889	2.59747	2.1
47890	1.87119	2.1
47891	1.84844	2.1
47892	1.82268	2.1
47893	2.62323	2.1
47894	2.59747	2.1
47895	0.94613	2.1
47896	0.94613	2.1
47897	1.32697	2.1
47898	1.32697	2.1
47899	1.32697	2.1
47900	1.31161	2.1
47901	0.946132	2.1
47902	0.946132	2.1
47903	0.94613	2.1
47904	1.32697	2.1
47905	0.946131	2.1

47906	1.31161	2.1
47907	2.62323	2.1
47908	1.91704	2.1
47909	1.90191	2.1
47910	1.87119	2.1
47911	2.65395	2.1
47912	2.62323	2.1
47913	1.89226	2.1
47914	1.89226	2.1
47915	2.15958	2.1
47916	2.15958	2.1
47917	2.46558	2.1
47918	2.19826	2.1
47919	2.46558	2.1
47920	1.89226	2.1
47921	1.89226	2.1
47922	1.89226	2.1
47923	2.61064	2.1
47924	2.61064	2.1
47925	1.81759	0.792666
47926	1.89526	0.894342
47927	2.32219	1.38969
47928	1.881	0.61025
47929	1.81759	1.16726
47930	1.3764	0.586738
47931	1.81759	0.879735
47932	2.57079	0.7008
47933	2.32219	1.50675
47934	2.32219	0.698125
47935	1.99875	1.14947
47936	1.99875	0.497009
47937	0.536182	0.409868
47938	0.579206	0.227268
47939	0.908796	0.816534
47940	0.999375	0.508653
47941	0.732091	0.533037
47942	0.732091	0.248481
47943	0.755911	0.371136
47944	0.755911	0.692615
47945	0.999376	0.478276
47946	0.579206	0.495357
47947	0.908796	0.519004
47948	0.536182	0.50722
47949	0.94613	0.373755
47950	0.94613	0.389701
47951	1.31112	0.440024
47952	1.18275	0.451095
47953	0.946126	0.470982
47954	0.946126	0.306393
47955	1.31112	0.379865
47956	1.31112	0.40609
47957	0.946128	0.399764
47958	1.31112	0.406603
47959	0.946128	0.391609
47960	1.18275	0.396023
47961	2.64617	0.653264
47962	1.27737	0.755929
47963	1.83076	0.783774

47964	1.27737	0.738502
47965	3.03764	0.676842
47966	2.3655	0.603932
47967	1.12991	0.0698591
47968	1.22375	0.0698591
47969	1.35931	0.0698591
47970	1.04971	0.0698591
47971	1.0419	0.0698591
47972	1.15237	0.0698591
47973	1.19593	0.0698591
47974	1.30306	0.0698591
47975	1.12937	0.0698591
47976	1.31934	0.0698591
47977	1.15203	0.0698591
47978	1.15203	0.0698591
47979	2.65227	0.0698591
47980	1.46041	0.0698591
47981	2.22712	0.0698591
47982	2.04816	0.0698591
47983	2.22712	0.0698591
47984	2.04816	0.0698591
47985	1.89226	0.416924
47986	1.89226	2.09663
47987	1.89226	1.37153
47988	1.89226	1.3732
47989	3.78452	1.45732
47990	1.24653	1.96319
47991	1.66554	2.09745
47992	1.53538	1.38186
47993	1.53538	2.1
47994	1.40915	1.52712
47995	1.40915	2.1
47996	1.66555	2.09863
47997	1.75835	2.1
47998	1.53538	2.1
47999	1.65313	2.1
48000	3.31868	2.1
48001	1.89226	2.0967
48002	1.89226	2.1
48003	1.89226	1.4951
48004	1.89226	2.1
48005	3.78453	2.1
48006	1.89226	1.06561
48007	1.89226	1.6712
48008	1.89226	0.504392
48009	1.89226	2.1
48010	3.78452	1.18864
48011	0.658778	2.1
48012	0.658778	2.00732
48013	0.661956	1.3238
48014	0.661956	2.1
48015	0.703822	2.1
48016	0.767689	2.1
48017	0.623266	1.20421
48018	0.553046	2.05522
48019	0.703804	2.09964
48020	0.55303	2.09975
48021	0.623282	2.00211

48022	0.767711	2.0171
48023	1.53538	2.1
48024	1.65313	2.1
48025	1.40765	2.1
48026	1.55779	2.1
48027	3.06078	2.1
48028	1.89227	1.64945
48029	1.89227	2.1
48030	1.89227	2.1
48031	1.89227	2.1
48032	3.78453	2.1
48033	1.10517	2.1
48034	2.16532	2.1
48035	1.94374	2.1
48036	1.94001	2.1
48037	1.37756	2.1
48038	1.37756	2.1
48039	1.7453	0.111622
48040	1.83284	0.125131
48041	2.28483	0.154164
48042	1.77804	0.0995631
48043	1.7453	0.176707
48044	1.23851	0.111122
48045	1.7453	0.135929
48046	2.54752	0.124796
48047	2.28483	0.148513
48048	2.28483	0.109707
48049	2.0189	0.160039
48050	2.0189	0.120537
48051	1.12457	0.0911171
48052	2.0189	0.0905783
48053	1.7453	0.109711
48054	1.7453	0.0833493
48055	1.40707	0.113367
48056	1.40707	0.0878726
48057	2.41176	2.1
48058	2.59262	2.1
48059	2.34085	2.1
48060	2.55827	2.1
48061	4.97003	2.1
48062	2.59262	2.1
48063	2.61901	2.1
48064	2.55827	2.1
48065	2.64067	2.1
48066	5.17728	2.1
48067	2.34084	2.1
48068	2.55827	2.1
48069	2.24937	2.1
48070	2.49053	2.1
48071	4.80764	2.1
48072	2.55826	2.1
48073	2.64067	2.1
48074	2.49053	2.1
48075	2.58639	2.1
48076	5.14465	2.1
48077	2.27918	0.0698591
48078	2.44132	0.0698591
48079	1.89226	0.0698591

48080	1.89226	0.0698591
48081	4.33359	0.0698591
48082	1.89226	0.0698591
48083	2.44132	0.0698591
48084	2.6771	0.0698591
48085	2.6771	0.0698591
48086	1.89226	0.0698591
48087	1.89226	0.0698591
48088	1.60329	0.0698591
48089	1.38246	0.0698591
48090	1.98182	0.0698591
48091	2.2217	0.0698591
48092	3.60417	0.0698591
48093	2.05362	0.0698591
48094	1.02663	0.0698591
48095	1.43255	0.0698591
48096	1.52072	0.0698591
48097	1.70302	0.0698591
48098	1.7685	0.0698591
48099	0.946131	0.0698591
48100	0.946131	0.0698591
48101	0.946131	0.0698591
48102	0.946131	0.0698591
48103	0.946131	0.0698591
48104	0.946131	0.0698591
48105	0.946131	0.0698591
48106	0.946131	0.0698591
48107	0.946131	0.0698591
48108	0.946131	0.0698591
48109	0.946131	0.0698591
48110	0.946131	0.0698591
48111	1.89226	0.0698591
48112	1.89226	0.0698976
48113	1.89226	0.070144
48114	1.89226	0.0698591
48115	1.89226	0.0703245
48116	1.89226	0.0698591
48117	1.92645	0.503751
48118	2.83045	0.448834
48119	1.92225	0.534385
48120	2.01873	0.395321
48121	2.51732	0.61274
48122	2.51732	0.436888
48123	2.51732	0.34754
48124	2.23857	0.351176
48125	1.92645	0.409493
48126	1.92645	0.294861
48127	2.23857	0.489568
48128	1.50865	0.295113
48129	2.02687	2.1
48130	2.39604	2.1
48131	2.29198	2.1
48132	2.73046	2.1
48133	4.68803	2.1
48134	1.21148	1.72667
48135	1.87957	1.90933
48136	2.03555	1.95666
48137	2.03555	1.11034

48138	1.09962	1.7908
48139	1.09962	1.87388
48140	1.92558	0.81135
48141	2.05274	2.1
48142	2.2443	1.57195
48143	2.24429	2.1
48144	1.70869	0.796145
48145	1.70868	2.1
48146	0.818397	0.389397
48147	0.818397	2.1
48148	0.827911	2.09262
48149	0.827908	1.96474
48150	1.00418	0.88852
48151	1.02054	0.958527
48152	0.692018	1.99849
48153	0.620899	1.89624
48154	0.65823	1.14769
48155	1.00418	0.571044
48156	0.620899	1.03407
48157	1.0262	0.762247
48158	1.92558	1.81076
48159	2.51818	2.1
48160	1.94816	0.890233
48161	2.01985	2.1
48162	1.85389	0.908644
48163	1.92558	2.1
48164	0.903844	2.1
48165	0.903844	2.1
48166	0.946148	2.1
48167	0.946151	2.1
48168	0.946118	2.1
48169	0.903876	2.1
48170	0.946118	2.1
48171	1.10235	2.1
48172	0.946131	2.1
48173	0.946131	2.1
48174	1.10234	2.1
48175	0.903863	2.1
48176	1.63471	2.1
48177	1.08022	2.1
48178	1.56801	2.1
48179	1.03437	2.1
48180	2.66908	2.1
48181	1.56801	2.1
48182	0.97541	2.1
48183	0.97541	2.1
48184	1.03437	2.1
48185	1.49733	2.1
48186	1.55629	2.1
48187	1.89226	0.0713333
48188	1.89226	0.121729
48189	1.89226	0.0777314
48190	1.89226	0.106695
48191	3.78452	0.11516
48192	0.946131	0.0703031
48193	1.16373	0.0704431
48194	0.946131	0.0727049
48195	0.734637	0.0706063

48196	0.946132	0.072074
48197	0.946132	0.0758877
48198	1.16373	0.0698591
48199	0.734636	0.0698591
48200	0.946093	0.0717169
48201	0.946093	0.0699312
48202	0.734675	0.0698591
48203	0.734675	0.0723045
48204	1.98413	0.949146
48205	1.87355	2.0954
48206	1.72774	0.72386
48207	1.67865	2.1
48208	3.60128	1.71732
48209	1.87355	2.09481
48210	1.54924	2.1
48211	1.68443	2.1
48212	1.68443	2.0998
48213	1.67866	2.1
48214	1.67866	2.1
48215	1.72774	0.546381
48216	1.53422	2.1
48217	1.67865	2.0472
48218	1.67865	0.937014
48219	1.53873	2.03478
48220	1.53873	0.902604
48221	1.67866	2.05901
48222	1.54924	2.1
48223	1.53423	2.1
48224	1.44091	2.1
48225	3.11957	2.1
48226	1.89226	2.1
48227	1.89226	1.99542
48228	1.65573	2.07301
48229	1.65573	2.09526
48230	1.89226	2.065
48231	1.89226	2.0928
48232	1.89226	2.08019
48233	1.89614	1.78508
48234	1.7508	2.00492
48235	1.72666	2.04061
48236	1.89226	1.98541
48237	1.89226	2.02136
48238	1.89223	2.08196
48239	1.89223	1.8084
48240	1.89223	1.86352
48241	1.89223	2.04192
48242	1.89223	1.89895
48243	1.89223	2.0808
48244	1.89224	2.01019
48245	1.89224	1.27832
48246	1.89224	1.70448
48247	1.89224	1.92858
48248	1.89224	1.76415
48249	1.89224	2.01861
48250	2.09773	2.1
48251	2.06623	2.1
48252	2.43268	2.1
48253	2.33713	2.1

48254	1.703	2.1
48255	1.61372	2.1
48256	0.646929	2.1
48257	0.689776	2.1
48258	1.05897	2.1
48259	0.956364	2.1
48260	0.905594	2.1
48261	0.904315	2.1
48262	0.727014	2.1
48263	0.850757	2.1
48264	0.798445	2.1
48265	0.654101	2.1
48266	0.717539	2.1
48267	1.0478	2.1
48268	1.89223	2.1
48269	1.89223	2.1
48270	1.89223	2.1
48271	1.6557	1.94069
48272	3.54794	2.1
48273	0.946118	2.1
48274	0.946118	2.1
48275	0.946118	2.1
48276	0.946118	2.1
48277	0.946117	2.1
48278	0.946117	2.1
48279	0.946119	2.1
48280	0.946119	2.1
48281	0.946105	2.1
48282	0.946105	2.1
48283	0.946131	2.1
48284	0.946131	2.1
48285	1.47671	2.1
48286	1.70303	2.1
48287	1.90705	2.1
48288	1.98945	2.1
48289	1.3454	2.1
48290	1.40353	2.1
48291	1.89226	2.1
48292	1.89226	2.1
48293	1.89226	2.1
48294	1.89226	2.1
48295	1.89226	2.1
48296	1.89226	2.09924
48297	2.39438	0.158177
48298	2.69842	0.138387
48299	2.45177	0.204804
48300	2.28563	0.121228
48301	4.84615	0.151667
48302	1.28421	2.1
48303	1.35966	2.1
48304	0.961001	2.1
48305	0.860549	2.1
48306	1.1039	2.1
48307	1.1039	2.1
48308	1.11631	2.1
48309	1.11631	2.1
48310	1.35966	2.1
48311	0.961001	2.1

48312	0.860549	2.1
48313	1.28421	2.1
48314	1.89226	1.66252
48315	1.89226	1.90433
48316	1.89226	1.98582
48317	1.89226	1.32848
48318	3.78452	1.71735
48319	1.48702	0.911271
48320	1.40656	0.866017
48321	0.626442	1.58321
48322	0.626442	1.44753
48323	0.626442	1.75116
48324	0.946132	0.842175
48325	1.05489	0.805283
48326	1.05012	1.77183
48327	0.946131	1.32364
48328	0.946131	1.36185
48329	0.626442	1.40807
48330	1.05012	1.19103
48331	1.89226	1.89667
48332	1.97212	1.10707
48333	1.89226	1.30915
48334	1.89226	1.57763
48335	3.78453	1.60482
48336	1.89226	1.96344
48337	1.89226	1.40181
48338	1.89226	1.52499
48339	1.89226	1.86456
48340	3.78452	1.8269
48341	1.89226	1.16165
48342	1.89226	1.51037
48343	1.89226	1.76151
48344	1.89226	0.806719
48345	1.89226	1.81613
48346	1.89226	0.92892
48347	0.946161	0.686456
48348	0.946161	1.1437
48349	0.932156	1.47726
48350	0.932156	1.06789
48351	0.932184	1.26706
48352	0.946132	0.583404
48353	0.946133	1.52328
48354	0.946133	0.819832
48355	0.946132	0.909121
48356	0.946132	0.938022
48357	0.932185	0.925702
48358	0.946133	0.840234
48359	0.946132	1.26564
48360	0.946132	0.900043
48361	0.946132	1.97404
48362	0.946132	1.69397
48363	0.946132	1.61445
48364	0.946132	1.14344
48365	0.946133	1.38332
48366	0.946133	1.8248
48367	0.946132	1.58811
48368	0.946132	1.71184
48369	0.946133	1.30562

48370	0.946133	1.44969
48371	1.79397	0.192396
48372	1.00636	0.208916
48373	1.31658	0.261705
48374	2.02924	0.21237
48375	3.0356	0.218886
48376	1.89226	0.211414
48377	1.89226	0.236353
48378	1.89226	0.233597
48379	1.89226	0.226049
48380	1.89226	0.204342
48381	1.89226	0.181869
48382	0.946131	0.317983
48383	0.946131	0.286776
48384	1.24766	0.338537
48385	1.03691	0.381391
48386	0.946129	0.370135
48387	0.946129	0.299159
48388	1.24766	0.316736
48389	1.24766	0.323482
48390	0.946131	0.316087
48391	1.24766	0.330738
48392	1.03691	0.350192
48393	0.946131	0.327754
48394	1.44824	0.157456
48395	2.02924	0.14099
48396	1.7065	0.179508
48397	1.38604	0.235053
48398	2.32017	0.172926
48399	2.32017	0.1425
48400	1.89226	0.140218
48401	1.89226	0.171967
48402	1.89226	0.152498
48403	1.89226	0.107357
48404	1.89226	0.190377
48405	1.89226	0.145554
48406	0.478349	2.07767
48407	0.612092	2.1
48408	0.606115	2.1
48409	0.506213	2.1
48410	0.550677	2.1
48411	0.550677	2.07849
48412	0.567628	2.1
48413	0.567628	2.1
48414	0.506213	2.1
48415	0.478349	2.1
48416	0.606116	2.1
48417	0.612092	2.1
48418	1.52226	2.1
48419	1.54241	2.1
48420	1.23167	2.1
48421	1.23167	2.1
48422	1.75555	2.1
48423	1.75555	2.1
48424	1.75557	2.1
48425	1.54243	2.1
48426	1.9943	2.1
48427	1.85871	2.1

48428	3.53673	2.1
48429	1.85871	2.1
48430	1.06936	2.1
48431	1.88926	2.1
48432	1.54243	2.1
48433	1.41619	2.1
48434	1.06936	2.1
48435	1.28649	1.96991
48436	1.28516	2.1
48437	1.38986	0.300745
48438	1.42399	1.89993
48439	2.67502	1.82339
48440	1.42399	1.20227
48441	1.07283	2.1
48442	1.46528	2.1
48443	1.28517	1.81864
48444	1.25294	2.1
48445	1.07283	1.95633
48446	1.38986	2.05156
48447	1.42399	2.1
48448	1.52885	1.07819
48449	1.60058	1.19789
48450	2.99043	1.55333
48451	0.715316	2.1
48452	0.715316	1.74442
48453	0.719126	0.865612
48454	0.719126	2.1
48455	0.722447	2.1
48456	0.626469	2.1
48457	0.711995	0.972488
48458	0.800291	0.955302
48459	0.80029	1.95352
48460	0.722447	1.91278
48461	0.711995	2.02754
48462	0.62647	2.02999
48463	2.05632	0.0698591
48464	1.70303	0.0698591
48465	2.3923	0.0698591
48466	2.61277	0.0698591
48467	1.44836	0.0698591
48468	1.58184	0.0698591
48469	1.89226	0.0698591
48470	1.89226	0.0698591
48471	1.89226	0.0698591
48472	1.89226	0.0698591
48473	3.78453	0.0698591
48474	1.44091	2.1
48475	1.1822	2.1
48476	1.25866	2.1
48477	1.25866	2.1
48478	1.35215	2.1
48479	1.35215	2.1
48480	1.27338	2.1
48481	1.1822	2.1
48482	1.27338	2.1
48483	1.35215	2.1
48484	1.10817	2.1
48485	1.18694	2.1

48486	0.946132	0.265202
48487	0.946132	0.210573
48488	0.946132	0.174342
48489	0.946132	0.137302
48490	0.946133	0.172147
48491	0.946133	0.249857
48492	0.946132	0.201634
48493	0.946132	0.14016
48494	0.946132	0.199847
48495	0.946132	0.179583
48496	0.946133	0.18839
48497	0.946133	0.201234
48498	2.6875	0.0698591
48499	2.40558	0.0698591
48500	2.1994	0.0698591
48501	1.70303	0.0698591
48502	4.68305	0.0698591
48503	0.915523	0.0698591
48504	0.915523	0.0698591
48505	0.861057	0.0698591
48506	0.946796	0.0698591
48507	0.838652	0.0698591
48508	0.654182	0.0698591
48509	1.14044	0.0698591
48510	0.80252	0.0698591
48511	0.836435	0.0698591
48512	0.608659	0.0698591
48513	1.22198	0.0698591
48514	0.863472	0.0698591
48515	2.1994	0.0698591
48516	0.981586	0.0698591
48517	1.59619	0.0698591
48518	1.70303	0.0698591
48519	1.24816	0.0698591
48520	1.38523	0.0698591
48521	2.71141	2.1
48522	1.70302	2.1
48523	2.56527	2.1
48524	1.88649	2.1
48525	2.41279	2.1
48526	1.70302	2.1
48527	1.89226	2.09908
48528	1.89226	2.1
48529	1.89226	2.1
48530	1.89226	2.1
48531	3.78453	2.1
48532	1.89226	2.1
48533	1.89226	2.1
48534	1.89226	2.1
48535	1.89226	2.1
48536	1.89226	2.1
48537	1.89226	2.09868
48538	2.18867	2.1
48539	0.983723	2.1
48540	2.42263	2.1
48541	1.8014	2.1
48542	1.62992	2.1
48543	0.983723	2.1

48544	2.71141	2.1
48545	1.89226	2.1
48546	2.31157	2.1
48547	2.31157	2.1
48548	1.89226	2.1
48549	1.89226	2.1
48550	1.89227	2.1
48551	1.89227	2.05663
48552	1.89227	2.08605
48553	1.89227	2.1
48554	3.78453	2.1
48555	1.89226	2.07482
48556	1.89226	2.1
48557	1.89226	2.1
48558	1.89226	2.05006
48559	1.89226	2.1
48560	1.89226	2.04927
48561	1.89226	1.7668
48562	1.89226	1.00018
48563	1.89226	2.07657
48564	1.89226	0.46444
48565	1.89226	1.60982
48566	1.89226	0.230218
48567	1.1427	1.99266
48568	1.14956	2.08465
48569	1.70302	0.689409
48570	1.81267	2.0526
48571	2.88983	1.90716
48572	1.89226	0.358337
48573	1.89226	2.00631
48574	1.89226	1.04317
48575	1.89226	1.31275
48576	3.78453	1.20002
48577	1.89226	1.62843
48578	1.89226	1.37035
48579	1.89226	0.213332
48580	1.89226	1.88141
48581	3.78452	0.874308
48582	0.884197	2.07515
48583	1.1125	1.17481
48584	1.13273	1.21873
48585	0.844557	1.67282
48586	0.981137	1.07933
48587	1.13971	1.23353
48588	0.980432	1.57028
48589	0.980432	1.24172
48590	1.15859	0.888761
48591	1.12206	0.359582
48592	0.898619	2.0417
48593	0.94426	1.03965
48594	2.21016	0.913524
48595	1.44091	2.1
48596	1.98408	1.31878
48597	1.4218	0.761045
48598	2.21016	2.00206
48599	1.64788	1.94877
48600	1.07526	0.74434
48601	1.91531	2.1

48602	1.49155	2.1
48603	1.58551	1.99168
48604	1.49155	1.63344
48605	1.58551	2.1
48606	1.98979	0.0698591
48607	2.21773	0.0698591
48608	2.30533	0.0698591
48609	2.55895	0.0698591
48610	4.52305	0.0698591
48611	2.55896	0.0698591
48612	2.27963	0.0698591
48613	2.60486	0.0698591
48614	2.60486	0.0698591
48615	2.21773	0.0698591
48616	2.21773	0.0698591
48617	2.30533	0.0698591
48618	2.55895	0.0698591
48619	2.29539	0.0698591
48620	2.47308	0.0698591
48621	4.7784	0.0698591
48622	2.55896	0.0698591
48623	2.55792	0.0698591
48624	2.55792	0.0698591
48625	2.60486	0.0698591
48626	2.47308	0.0698591
48627	2.52003	0.0698591
48628	0.946133	1.94692
48629	0.946133	2.02661
48630	1.20851	1.85015
48631	1.20851	1.70666
48632	0.967411	1.60119
48633	1.20851	1.46599
48634	0.946132	2.07767
48635	0.946132	1.98583
48636	0.946132	2.01427
48637	1.20851	1.74557
48638	0.967412	1.74696
48639	0.946133	1.96203
48640	1.89226	1.87454
48641	1.89226	1.43007
48642	1.89226	1.58903
48643	1.89226	1.44659
48644	1.89226	1.92874
48645	1.89226	1.90595
48646	1.89226	2.0375
48647	2.41701	1.79234
48648	2.71724	1.7854
48649	2.71724	1.84895
48650	1.89226	2.05985
48651	1.89226	2.05515
48652	0.946131	1.96292
48653	0.946131	2.03286
48654	0.946128	1.87063
48655	0.946128	1.80754
48656	0.94613	1.40479
48657	0.94613	1.53313
48658	0.94613	1.97686
48659	0.94613	1.97434

48660	0.94613	1.92163
48661	0.94613	1.59066
48662	0.94613	1.7193
48663	0.94613	1.96693
48664	0.851541	0.0910418
48665	0.851541	0.103054
48666	0.851481	0.0928171
48667	0.997219	0.111009
48668	0.997112	0.0821302
48669	0.992853	0.075353
48670	0.85154	0.0796631
48671	0.85154	0.0970497
48672	0.894824	0.0739634
48673	0.894824	0.0926733
48674	0.94613	0.116371
48675	0.906485	0.0784985
48676	0.802947	2.1
48677	0.845556	2.1
48678	0.582339	2.1
48679	0.519756	2.1
48680	0.714508	2.1
48681	0.714508	2.1
48682	0.628932	2.1
48683	0.708518	2.1
48684	0.801358	2.1
48685	0.476016	2.1
48686	0.569477	2.1
48687	0.878553	2.1
48688	1.89226	2.1
48689	1.89226	2.1
48690	1.89226	2.1
48691	1.89226	2.1
48692	1.89226	2.1
48693	1.89226	2.1
48694	0.946128	2.1
48695	0.946128	2.1
48696	1.01537	2.1
48697	0.9662	2.1
48698	0.946131	2.1
48699	0.946131	2.1
48700	0.966198	2.1
48701	0.966198	2.1
48702	0.94613	2.1
48703	0.966198	2.1
48704	0.946131	2.1
48705	1.01537	2.1
48706	1.89226	2.1
48707	1.89226	2.1
48708	1.91883	2.1
48709	1.91883	2.1
48710	2.15905	2.1
48711	2.13249	2.1
48712	1.07953	2.1
48713	1.2107	2.1
48714	0.946132	2.1
48715	0.946132	2.1
48716	1.2107	2.1
48717	1.2107	2.1

48718	0.946132	2.1
48719	0.946132	2.1
48720	1.2107	2.1
48721	0.946133	2.1
48722	0.946132	2.1
48723	1.07953	2.1
48724	2.46752	2.1
48725	2.53733	2.1
48726	2.26044	2.1
48727	2.33222	2.1
48728	4.79974	2.1
48729	2.53733	2.1
48730	2.37576	2.05424
48731	2.33222	2.1
48732	2.1845	2.1
48733	4.70798	2.1
48734	2.48652	2.1
48735	2.33224	2.1
48736	2.08149	2.1
48737	1.9839	2.1
48738	4.45623	2.1
48739	2.33223	2.1
48740	2.18452	2.09203
48741	2.16061	2.1
48742	2.01399	1.9021
48743	4.34623	2.1
48744	1.4617	2.08486
48745	1.34701	2.1
48746	1.34701	2.09582
48747	1.25173	2.1
48748	1.5117	2.08403
48749	1.41642	2.1
48750	2.65164	2.1
48751	1.89227	2.1
48752	1.89227	2.1
48753	1.89227	2.1
48754	2.42504	2.1
48755	2.42504	2.1
48756	1.89226	2.05117
48757	1.89226	2.1
48758	1.89226	2.1
48759	1.89226	1.96308
48760	3.78452	2.09628
48761	1.5117	2.1
48762	1.34701	2.1
48763	1.47001	2.1
48764	1.57961	2.07056
48765	2.92663	2.1
48766	1.89226	2.1
48767	1.89226	1.89914
48768	1.89226	2.0699
48769	1.89226	2.1
48770	3.78452	2.09987
48771	0.412773	0.0698591
48772	0.570839	0.0698591
48773	0.50721	0.0698591
48774	0.513638	0.0698591
48775	0.518805	0.0698591

48776	0.41278	0.0698591
48777	1.5344	0.0698591
48778	0.939282	0.0698591
48779	0.720051	0.0698591
48780	0.691767	0.0698591
48781	0.761152	0.0698591
48782	1.89226	2.1
48783	1.89226	2.1
48784	1.89226	2.1
48785	1.89226	2.1
48786	1.89226	2.1
48787	1.89226	2.1
48788	1.24618	2.1
48789	2.10585	2.1
48790	1.08134	2.1
48791	1.93032	2.1
48792	3.1765	2.1
48793	1.98474	2.1
48794	2.57777	2.1
48795	1.93031	2.1
48796	2.19041	2.1
48797	3.99251	2.1
48798	1.89226	2.1
48799	1.89226	2.1
48800	1.89226	2.1
48801	1.89226	2.1
48802	3.78452	2.1
48803	1.67374	2.1
48804	2.19042	2.1
48805	1.67374	2.1
48806	2.09769	2.1
48807	2.29586	2.1
48808	2.7198	2.1
48809	1.89226	2.1
48810	1.89226	2.1
48811	1.89226	2.1
48812	1.89226	2.1
48813	1.89226	2.1
48814	1.89226	2.1
48815	0.946132	0.271986
48816	0.946132	0.227761
48817	0.947763	0.220149
48818	0.909304	0.242424
48819	0.946127	0.185401
48820	0.947831	0.16248
48821	0.907673	0.195119
48822	0.907673	0.252915
48823	0.94613	0.190158
48824	0.947834	0.16865
48825	0.907671	0.31195
48826	0.94613	0.17384
48827	0.906519	0.136746
48828	0.906519	0.113205
48829	0.946097	0.128752
48830	0.946097	0.147445
48831	0.947834	0.127314
48832	0.906485	0.109123
48833	0.946131	0.11485

48834	0.946131	0.14185
48835	0.94613	0.170948
48836	0.906485	0.0977662
48837	0.946131	0.153025
48838	0.947835	0.142317
48839	2.58638	2.1
48840	2.51039	2.1
48841	2.47395	2.1
48842	2.41539	2.1
48843	5.00178	2.1
48844	2.51039	2.1
48845	2.16633	2.1
48846	2.4154	2.1
48847	2.13977	2.1
48848	4.58173	2.1
48849	2.47395	2.1
48850	2.41539	2.1
48851	2.2988	2.1
48852	2.2604	2.1
48853	4.7142	2.1
48854	2.4154	2.1
48855	2.13977	2.1
48856	2.26041	2.1
48857	1.98957	2.1
48858	4.40497	2.1
48859	2.15721	1.7197
48860	2.31843	0.643422
48861	1.93973	2.02346
48862	2.13243	0.144117
48863	4.25816	0.7286
48864	2.31844	0.481627
48865	2.30908	1.01418
48866	2.13243	0.313603
48867	2.1609	0.561544
48868	4.47934	0.331932
48869	1.93973	1.9809
48870	2.13243	0.270847
48871	1.69661	1.85338
48872	1.9339	0.332787
48873	3.87363	0.937079
48874	2.13243	0.157195
48875	2.1609	0.642641
48876	1.9339	0.375467
48877	1.98414	0.936695
48878	4.0948	0.170666
48879	1.88857	0.0698591
48880	2.34107	0.0698591
48881	0.502431	0.0698591
48882	0.71416	0.0698591
48883	0.50243	0.0698591
48884	0.944284	0.0698591
48885	1.88856	2.1
48886	2.34107	2.1
48887	2.39662	2.1
48888	1.92293	2.1
48889	4.28519	2.1
48890	1.00486	2.1
48891	1.92293	2.1

48892	1.88857	2.1
48893	1.88857	2.1
48894	1.02605	2.1
48895	1.02605	2.1
48896	2.53728	2.1
48897	1.70303	2.1
48898	2.03734	2.1
48899	1.3624	2.1
48900	2.54706	2.1
48901	1.70303	2.1
48902	2.54706	2.1
48903	1.50876	2.1
48904	2.74067	2.1
48905	1.85391	2.1
48906	2.29667	2.1
48907	1.50876	2.1
48908	0.946133	2.1
48909	0.946133	2.1
48910	1.24191	2.1
48911	1.24191	2.1
48912	0.946132	2.1
48913	1.24191	2.1
48914	1.23779	2.1
48915	0.946132	2.1
48916	0.946132	2.1
48917	0.946132	2.1
48918	1.23779	2.1
48919	1.24191	2.1
48920	0.946131	2.1
48921	0.946131	2.1
48922	1.14833	2.1
48923	1.14833	2.1
48924	1.14834	2.1
48925	0.946132	2.1
48926	0.94613	2.1
48927	1.24191	2.1
48928	0.946131	2.1
48929	0.946131	2.1
48930	1.24191	2.1
48931	1.14833	2.1
48932	2.08149	2.1
48933	2.31116	2.1
48934	1.9701	1.9917
48935	2.18328	2.1
48936	4.49563	2.1
48937	2.08149	2.1
48938	2.14059	2.1
48939	1.72377	2.1
48940	2.18328	2.1
48941	3.88931	2.1
48942	2.14059	2.1
48943	2.40755	2.1
48944	2.31145	2.1
48945	2.23743	2.1
48946	2.31145	2.1
48947	2.23743	2.1
48948	2.31117	2.1
48949	2.40754	2.1

48950	2.41055	2.1
48951	2.31145	2.1
48952	2.41055	2.1
48953	2.31145	2.1
48954	2.5615	0.210268
48955	2.37625	0.17283
48956	2.20248	0.175594
48957	2.20248	0.166523
48958	2.65967	0.264053
48959	2.65967	0.22024
48960	2.71793	0.250494
48961	2.65966	0.16769
48962	2.56149	0.158959
48963	2.56149	0.161421
48964	2.71296	0.229375
48965	2.71296	0.221816
48966	1.31528	0.163086
48967	1.32983	0.156575
48968	1.21128	0.165656
48969	1.18812	0.166815
48970	1.24232	0.146591
48971	1.24232	0.148801
48972	1.26109	0.170749
48973	1.26109	0.23228
48974	1.21128	0.190995
48975	1.31528	0.140837
48976	1.18812	0.15014
48977	1.32983	0.206339
48978	2.63056	0.141758
48979	2.58032	0.16601
48980	2.65966	0.123108
48981	2.65966	0.147597
48982	2.58032	0.219584
48983	2.71296	0.206164
48984	1.92039	2.1
48985	1.06226	2.1
48986	1.09252	2.1
48987	1.96052	2.1
48988	3.01291	2.1
48989	1.92039	2.1
48990	2.46338	2.1
48991	2.47522	2.1
48992	2.43508	2.1
48993	1.96052	2.1
48994	1.92039	2.1
48995	0.985949	2.1
48996	0.980261	2.1
48997	0.553625	2.1
48998	0.546262	2.1
48999	0.765671	2.1
49000	0.765671	2.1
49001	0.766539	2.1
49002	0.766539	2.1
49003	0.985949	2.1
49004	0.553625	2.1
49005	0.546261	2.1
49006	0.980261	2.1
49007	1.96052	2.1

49008	2.46338	2.1
49009	1.97189	2.1
49010	1.96052	2.1
49011	2.47932	2.1
49012	2.47932	2.1
49013	0.964608	0.0698591
49014	0.691524	0.0698591
49015	1.25462	0.0698591
49016	0.691524	0.0698591
49017	1.89226	0.0698591
49018	1.89226	0.0698591
49019	1.44894	0.0698591
49020	2.77655	0.0698591
49021	1.4237	0.0698591
49022	2.77701	0.0698591
49023	2.77701	0.0698591
49024	1.38847	2.1
49025	1.46031	1.74089
49026	1.65504	1.87035
49027	1.75396	1.44207
49028	3.11535	1.87768
49029	1.89226	1.94338
49030	1.89226	1.74239
49031	1.89226	1.91657
49032	1.89226	0.726462
49033	3.78452	1.54239
49034	1.89226	1.50882
49035	1.89226	2.07222
49036	1.89226	0.365148
49037	1.89226	1.6806
49038	3.78452	0.725141
49039	1.46031	1.03421
49040	1.34851	2.04943
49041	1.75395	1.03646
49042	1.62287	1.56382
49043	3.08318	1.61147
49044	1.65504	2.06721
49045	1.75396	1.98706
49046	1.89249	2.1
49047	1.9857	1.04704
49048	3.64073	1.97424
49049	1.89226	2.07778
49050	1.89226	1.37564
49051	1.89226	1.41647
49052	1.89226	1.11205
49053	3.78452	1.44016
49054	1.89226	0.74741
49055	1.89226	2.02327
49056	1.89226	0.847454
49057	1.89226	1.84483
49058	3.78452	1.6037
49059	1.75395	2.03579
49060	1.62287	1.95657
49061	1.98569	0.611314
49062	1.86067	0.801054
49063	3.60856	1.00391
49064	1.35697	2.1
49065	2.02064	2.1

49066	1.89989	2.1
49067	1.49571	2.1
49068	1.66753	2.1
49069	1.36431	2.1
49070	0.744004	2.1
49071	0.743997	2.1
49072	0.717972	2.1
49073	0.717972	2.1
49074	0.997982	2.1
49075	0.807796	2.1
49076	0.632235	2.1
49077	0.488575	2.1
49078	0.849275	2.1
49079	0.488581	2.1
49080	0.724729	2.1
49081	0.997967	2.1
49082	2.5276	2.09978
49083	1.70791	2.07822
49084	1.82872	1.6945
49085	1.56362	2.1
49086	2.5276	1.8852
49087	2.24781	2.1
49088	1.37591	2.1
49089	1.23217	2.08349
49090	1.00442	1.86925
49091	0.945282	1.30768
49092	1.15446	1.72863
49093	1.09123	2.1
49094	1.21506	2.09035
49095	1.21506	1.59055
49096	1.39452	1.99998
49097	1.13108	2.01107
49098	1.27876	2.02201
49099	0.929006	2.01215
49100	1.46505	0.0762742
49101	1.91484	0.0899125
49102	1.49906	0.0863942
49103	1.49906	0.0914319
49104	1.90196	0.0746393
49105	1.90196	0.0767718
49106	1.90196	0.0757391
49107	2.11465	0.0940488
49108	1.91484	0.0911989
49109	1.91484	0.0918911
49110	2.04016	0.0774219
49111	2.04016	0.0771063
49112	0.912201	0.0705795
49113	0.950978	0.0698591
49114	0.665307	0.0698591
49115	0.732528	0.070213
49116	0.829512	0.0698591
49117	0.829512	0.0716417
49118	0.815217	0.0698591
49119	0.815217	0.0701125
49120	0.912202	0.0698591
49121	0.665305	0.0698591
49122	0.732527	0.074364
49123	0.95098	0.0703127

49124	1.90196	0.0713035
49125	2.08757	0.0698591
49126	1.9626	0.0715949
49127	2.04016	0.0698611
49128	1.82441	0.0698591
49129	1.90196	0.0698591
49130	1.13048	2.1
49131	0.821162	2.1
49132	0.946131	2.1
49133	0.946131	2.1
49134	1.04567	2.1
49135	0.882347	2.1
49136	0.897276	2.1
49137	0.897276	2.1
49138	0.863109	2.1
49139	1.01055	2.1
49140	0.940305	2.1
49141	0.942166	2.1
49142	2.23933	0.0698591
49143	0.976217	0.0698591
49144	2.78053	0.0698591
49145	2.78053	0.0698591
49146	0.976217	0.0698591
49147	1.65984	0.0698591
49148	1.54168	2.1
49149	2.01197	2.04597
49150	1.69136	2.1
49151	2.13284	1.69629
49152	3.67452	2.1
49153	0.988805	2.1
49154	1.78069	2.1
49155	1.69136	2.1
49156	1.69136	2.1
49157	1.05481	2.1
49158	1.05481	2.1
49159	1.69136	2.1
49160	2.13284	2.00263
49161	1.78069	2.1
49162	2.22044	2.05124
49163	3.91353	2.1
49164	2.69379	1.16414
49165	2.32219	1.39992
49166	2.25746	1.81773
49167	2.25746	1.11011
49168	2.32219	1.48117
49169	1.89526	1.03175
49170	1.4556	2.1
49171	1.85028	2.1
49172	2.4909	1.96112
49173	2.03799	2.1
49174	2.73495	2.1
49175	1.85028	2.1
49176	2.71938	1.59099
49177	1.85028	2.1
49178	3.36051	2.1
49179	2.73495	2.098
49180	2.47584	2.1
49181	1.85028	2.09868

49182	2.47584	2.1
49183	1.04413	2.1
49184	2.1461	2.1
49185	1.74586	2.1
49186	1.44437	2.1
49187	1.04413	2.1
49188	1.89226	2.1
49189	1.89226	1.88541
49190	1.89226	2.1
49191	1.89226	2.0859
49192	3.78452	2.1
49193	1.52724	2.1
49194	1.89249	2.1
49195	1.68143	2.1
49196	2.02433	2.08149
49197	3.55157	2.1
49198	1.07444	2.1
49199	1.76545	2.1
49200	1.68142	2.1
49201	1.68142	2.1
49202	1.14807	2.1
49203	1.14807	2.1
49204	1.89226	2.1
49205	1.89226	2.1
49206	1.89226	2.1
49207	1.89226	2.1
49208	1.89226	2.1
49209	1.89226	2.1
49210	1.89226	2.1
49211	1.89226	2.1
49212	1.89226	2.1
49213	1.89226	1.897
49214	3.78452	2.1
49215	1.68143	2.1
49216	2.02433	2.1
49217	1.76545	2.1
49218	2.12608	2.1
49219	3.78978	2.1
49220	2.62871	2.1
49221	1.49149	2.1
49222	1.36814	2.1
49223	2.44761	2.1
49224	3.9391	2.1
49225	1.89226	2.1
49226	1.89226	2.04085
49227	1.89226	2.1
49228	1.89226	2.1
49229	3.78452	2.1
49230	0.851542	2.1
49231	0.851542	2.1
49232	1.00897	2.1
49233	0.931224	2.1
49234	0.838952	2.1
49235	0.838952	2.1
49236	0.956848	2.1
49237	0.942474	2.1
49238	0.946132	2.1
49239	0.838952	2.1

49240	0.878743	2.1
49241	0.946132	2.1
49242	1.15541	2.1
49243	2.44761	2.09783
49244	1.36814	2.1
49245	1.36814	2.1
49246	2.19439	2.1
49247	2.19439	2.1
49248	1.89226	2.09734
49249	1.89226	2.1
49250	1.89226	2.1
49251	1.89226	2.07798
49252	3.78452	2.1
49253	1.89226	2.1
49254	1.67791	2.1
49255	1.5713	2.1
49256	1.67791	2.1
49257	1.78566	2.1
49258	1.89226	2.1
49259	1.89226	0.746921
49260	1.62911	2.1
49261	2.24602	1.87571
49262	1.89226	2.03727
49263	1.98287	1.73262
49264	1.62911	1.92125
49265	1.89226	2.1
49266	1.89226	2.1
49267	1.89226	1.91974
49268	1.89226	2.1
49269	3.78452	2.1
49270	1.26688	2.1
49271	1.11804	2.1
49272	0.852467	2.1
49273	0.693314	2.1
49274	0.977459	2.1
49275	0.977459	2.1
49276	0.982741	2.1
49277	0.982741	2.1
49278	1.26689	2.1
49279	0.852464	2.1
49280	0.693312	2.1
49281	1.11804	2.1
49282	0.946131	2.1
49283	0.946131	1.39544
49284	1.1794	0.68278
49285	1.1794	1.84469
49286	0.991434	2.09836
49287	1.1794	1.37795
49288	0.946131	1.43916
49289	0.946131	0.72317
49290	0.946131	2.06759
49291	1.1794	1.99272
49292	0.946131	2.07066
49293	0.991434	1.92268
49294	1.89226	1.46943
49295	1.89226	2.1
49296	1.89226	2.09679
49297	1.89226	2.08366

49298	3.78452	2.0997
49299	2.53377	2.1
49300	1.53443	2.1
49301	2.0815	2.09176
49302	2.82979	2.1
49303	4.35462	2.1
49304	1.96192	0.882638
49305	1.23602	2.1
49306	2.16506	1.72441
49307	1.56841	2.04051
49308	3.40108	2.02401
49309	1.89226	2.1
49310	1.89226	2.1
49311	1.89226	1.76008
49312	1.89226	2.1
49313	3.78452	2.1
49314	0.946129	2.1
49315	0.946129	2.1
49316	1.13067	2.1
49317	1.12952	2.1
49318	0.946129	2.1
49319	1.13089	2.1
49320	0.944978	2.1
49321	0.944978	2.1
49322	0.946131	2.1
49323	1.13089	2.1
49324	0.944977	2.1
49325	0.946131	2.1
49326	0.710208	2.1
49327	0.710208	2.1
49328	0.718894	2.1
49329	0.718894	2.1
49330	0.559773	2.1
49331	0.579321	2.1
49332	0.886767	2.1
49333	0.849781	2.1
49334	0.849781	2.1
49335	0.559774	2.1
49336	0.579321	2.1
49337	0.886766	2.1
49338	1.63471	2.1
49339	1.69956	2.1
49340	1.08022	2.1
49341	1.08022	2.1
49342	1.11955	2.1
49343	1.67403	2.1
49344	1.03766	2.1
49345	2.02825	2.1
49346	2.02794	2.1
49347	2.0348	2.1
49348	1.07196	2.1
49349	1.07196	2.1
49350	1.9276	2.1
49351	2.1004	2.1
49352	2.1306	2.1
49353	2.1306	2.1
49354	1.92178	2.1
49355	1.92032	2.1

49356	0.986724	2.1
49357	2.03064	2.1
49358	2.01385	2.1
49359	2.02404	2.1
49360	1.03766	2.1
49361	1.03766	2.1
49362	1.92027	2.1
49363	2.1306	2.1
49364	2.15388	2.1
49365	2.15388	2.1
49366	1.92614	2.1
49367	1.9276	2.1
49368	1.09578	2.1
49369	0.963018	2.1
49370	0.782956	2.01643
49371	0.529693	2.1
49372	0.6994	2.09007
49373	0.862532	2.1
49374	0.898605	2.1
49375	0.898605	2.05735
49376	0.776756	2.1
49377	1.14805	2.1
49378	0.966521	2.1
49379	0.617676	2.1
49380	2.67185	2.1
49381	2.099 2.1	
49382	2.80548	2.1
49383	2.2727	2.1
49384	4.94455	2.1
49385	1.89226	2.1
49386	1.89226	2.1
49387	1.89226	2.1
49388	1.89226	2.1
49389	3.78453	2.1
49390	1.89226	2.1
49391	1.89226	2.1
49392	1.89226	2.1
49393	1.89226	2.1
49394	1.89226	2.1
49395	1.89226	2.1
49396	0.884801	2.1
49397	0.884801	2.1
49398	0.895199	2.1
49399	0.895199	2.1
49400	0.622774	2.1
49401	0.730501	2.1
49402	1.0495	2.1
49403	1.13635	2.1
49404	1.13635	2.1
49405	0.730503	2.1
49406	1.0495	2.1
49407	0.622775	2.1
49408	1.89226	2.1
49409	1.89226	2.1
49410	1.89226	2.1
49411	1.89226	2.1
49412	1.89226	2.1
49413	1.89226	2.1

49414	1.89226	2.1
49415	1.89226	2.1
49416	1.89226	2.1
49417	1.89226	2.1
49418	1.89226	2.1
49419	1.89226	2.1
49420	2.2727	2.1
49421	1.89586	2.1
49422	1.66913	2.1
49423	1.46101	2.1
49424	2.48082	2.1
49425	2.2727	2.1
49426	1.89226	2.1
49427	1.89226	2.1
49428	1.84641	2.1
49429	1.89226	2.1
49430	1.30868	2.1
49431	1.34118	2.0852
49432	1.89226	1.71711
49433	1.89226	2.00684
49434	1.89226	2.06092
49435	1.89226	1.89076
49436	1.89226	1.90911
49437	1.89226	1.38635
49438	1.89226	2.02841
49439	1.89226	1.24724
49440	1.89226	1.7415
49441	1.89226	1.96793
49442	3.78452	1.82903
49443	0.946132	2.1
49444	0.946132	2.07262
49445	0.946131	2.1
49446	0.946131	2.1
49447	0.946132	2.1
49448	0.946132	2.03582
49449	0.946132	2.1
49450	0.946132	2.1
49451	0.946132	2.1
49452	0.946132	2.1
49453	0.946132	2.1
49454	0.946132	2.1
49455	1.89227	2.06925
49456	1.84641	2.1
49457	2.29517	2.1
49458	1.84641	2.09297
49459	2.34102	2.08098
49460	1.89227	1.92445
49461	1.89226	1.16476
49462	1.89226	1.50634
49463	1.89226	1.7464
49464	1.89226	1.3003
49465	1.89226	1.29928
49466	1.89226	0.889401
49467	1.89226	1.12636
49468	1.89226	1.56773
49469	1.89226	1.74762
49470	1.89226	1.30811
49471	1.89226	1.34621

49472	1.89226	0.965715
49473	1.89226	2.1
49474	1.89226	2.0539
49475	1.89226	2.1
49476	1.89226	2.02258
49477	1.89226	2.09072
49478	1.89226	1.94505
49479	0.851544	0.0698591
49480	0.757542	0.0698591
49481	1.13421	0.0698591
49482	1.04072	0.0698591
49483	0.946132	0.0698591
49484	0.946132	0.0698591
49485	0.841686	0.0698591
49486	0.834428	0.0698591
49487	1.13649	0.0698591
49488	0.937687	0.0698591
49489	0.841713	0.0698591
49490	0.841713	0.0698591
49491	1.89226	0.290053
49492	1.89226	0.32353
49493	1.89226	0.338826
49494	1.89226	0.244879
49495	1.89226	0.352749
49496	1.89226	0.25758
49497	2.31988	1.43886
49498	2.00082	2.1
49499	2.4594	0.922804
49500	2.17638	2.08021
49501	4.46022	1.9062
49502	2.00082	2.1
49503	1.41829	2.1
49504	1.61207	2.09873
49505	1.43652	2.1
49506	2.17637	2.08108
49507	2.00082	2.1
49508	2.4594	0.928248
49509	2.17638	2.09434
49510	2.57957	0.419827
49511	2.3506	2.08082
49512	4.81	1.68627
49513	2.3506	2.06416
49514	1.57368	2.1
49515	1.89111	1.86296
49516	1.89111	2.1
49517	2.17638	1.83144
49518	2.17638	2.1
49519	1.32813	2.1
49520	1.7237	2.1
49521	1.32461	2.1
49522	1.81656	2.1
49523	3.14468	2.1
49524	1.55132	2.1
49525	2.0815	2.1
49526	1.81656	2.1
49527	2.19826	2.1
49528	3.65818	2.1
49529	0.775951	2.1

49530	0.775951	2.1
49531	0.794601	2.1
49532	0.794601	2.1
49533	0.945641	2.1
49534	0.90828	2.1
49535	0.662302	2.1
49536	0.662273	2.1
49537	0.662274	2.1
49538	0.945639	2.1
49539	0.662304	2.1
49540	0.908279	2.1
49541	1.03062	2.1
49542	1.03062	2.1
49543	1.05054	2.1
49544	1.05054	2.1
49545	1.13551	2.1
49546	1.21275	2.1
49547	0.945641	2.1
49548	0.90828	2.1
49549	0.945641	2.1
49550	1.21275	2.1
49551	0.90828	2.1
49552	1.13552	2.1
49553	1.89226	2.1
49554	1.79883	2.1
49555	1.95341	2.1
49556	1.89226	2.1
49557	1.85998	2.1
49558	1.79883	2.1
49559	1.89226	2.1
49560	1.89226	2.1
49561	1.89226	2.1
49562	1.89226	2.1
49563	3.78453	2.1
49564	0.851541	2.1
49565	0.851541	2.1
49566	0.970398	2.1
49567	0.954365	2.1
49568	0.855356	2.1
49569	0.855357	2.1
49570	0.946133	2.1
49571	0.946133	2.1
49572	1.04072	2.1
49573	0.970397	2.1
49574	0.950003	2.1
49575	0.851538	2.1
49576	1.89226	2.1
49577	1.85998	2.1
49578	1.95296	2.1
49579	1.89226	2.1
49580	1.92067	2.1
49581	1.85998	2.1
49582	0.946133	2.1
49583	0.946133	2.1
49584	0.946132	2.1
49585	0.946132	2.1
49586	0.946132	2.1
49587	0.946132	2.1

49588	0.946132	2.1
49589	0.946132	2.1
49590	0.946132	2.1
49591	0.946132	2.1
49592	0.946133	2.1
49593	0.946133	2.1
49594	1.82152	2.1
49595	1.89226	2.1
49596	1.89226	2.1
49597	2.11152	2.1
49598	1.7472	2.1
49599	1.89226	2.1
49600	2.02687	2.1
49601	2.29199	2.1
49602	1.2734	2.1
49603	1.41077	2.1
49604	1.47381	2.1
49605	2.29199	2.1
49606	1.46889	2.1
49607	2.29199	2.1
49608	1.86766	2.1
49609	2.59401	2.1
49610	4.15965	2.1
49611	1.58995	2.1
49612	1.74246	2.1
49613	1.46643	2.1
49614	1.46643	2.1
49615	1.85097	2.1
49616	1.85097	2.1
49617	1.58995	2.1
49618	1.66584	2.1
49619	1.89214	2.1
49620	1.91787	2.1
49621	3.48208	2.1
49622	1.18474	2.1
49623	1.8144	2.1
49624	1.1486	2.1
49625	1.1486	2.1
49626	2.13071	2.1
49627	2.1257	2.1
49628	2.0815	2.1
49629	2.51651	2.1
49630	1.73681	2.1
49631	2.01601	2.1
49632	2.21406	2.1
49633	2.56998	2.1
49634	1.19995	2.1
49635	1.06256	2.1
49636	0.620677	2.1
49637	0.598084	2.1
49638	0.831426	2.1
49639	0.946056	2.1
49640	0.827692	2.1
49641	0.827692	2.1
49642	1.08958	2.1
49643	0.630132	2.1
49644	0.577547	2.1
49645	1.06104	2.1

49646	2.0815	2.1
49647	2.66249	2.1
49648	2.78966	2.1
49649	2.47108	2.1
49650	2.33858	2.1
49651	2.0815	2.1
49652	1.53735	2.1
49653	1.42278	2.1
49654	1.23654	2.1
49655	1.34678	2.1
49656	2.76955	2.1
49657	1.89226	2.1
49658	1.89226	2.1
49659	1.89226	2.1
49660	1.89226	2.1
49661	3.78452	2.1
49662	1.23654	2.1
49663	1.34678	2.1
49664	1.19	2.1
49665	1.08576	2.1
49666	2.43254	2.1
49667	1.38776	2.1
49668	1.29366	2.1
49669	0.946131	2.1
49670	0.946131	2.1
49671	0.946131	2.1
49672	0.946131	2.1
49673	1.38775	2.1
49674	1.38775	2.1
49675	1.29366	2.1
49676	0.946132	2.1
49677	0.946131	2.1
49678	1.38775	2.1
49679	0.946131	2.1
49680	0.946131	2.1
49681	0.946129	2.1
49682	0.946129	2.1
49683	0.94613	2.1
49684	0.94613	2.1
49685	0.946131	2.1
49686	0.946131	2.1
49687	0.946131	2.1
49688	0.946131	2.1
49689	0.94613	2.1
49690	0.94613	2.1
49691	1.89226	0.195302
49692	1.89226	1.81176
49693	1.89226	0.866865
49694	1.89226	0.830386
49695	1.89226	0.882208
49696	1.89226	1.18181
49697	1.89226	0.751589
49698	1.89226	2.1
49699	1.89226	1.50069
49700	1.89226	1.68117
49701	1.89226	1.38201
49702	1.89226	1.85978
49703	1.89226	0.360298

49704	1.89226	2.02172
49705	1.89226	0.956657
49706	1.89226	1.148
49707	1.89226	0.837773
49708	1.89226	1.58922
49709	0.92338	2.1
49710	0.92338	2.01842
49711	0.94428	2.03997
49712	0.94428	1.24335
49713	1.19528	2.05415
49714	1.30598	2.1
49715	0.714073	1.39522
49716	0.561679	2.07464
49717	1.19528	2.08474
49718	0.561678	2.09359
49719	0.714072	2.09216
49720	1.30598	2.07785
49721	2.61197	2.1
49722	1.89226	2.1
49723	1.89226	2.08169
49724	1.89226	2.1
49725	2.39056	2.06101
49726	2.39056	2.1
49727	0.946132	2.1
49728	0.946132	2.02675
49729	0.946132	0.758727
49730	0.946132	1.7319
49731	0.946131	1.658
49732	0.946131	2.1
49733	0.946132	2.02388
49734	0.946132	1.06324
49735	0.946132	1.81236
49736	0.946132	1.85533
49737	0.946132	1.95621
49738	0.946132	1.95311
49739	1.89224	2.1
49740	1.89224	2.1
49741	1.89224	2.1
49742	1.89224	2.1
49743	3.78448	2.1
49744	1.97089	2.1
49745	1.57428	2.1
49746	1.84991	2.1
49747	1.49078	2.1
49748	3.42419	2.1
49749	0.621927	2.1
49750	0.621927	2.1
49751	0.630824	2.1
49752	0.630824	2.1
49753	0.483483	2.1
49754	0.507361	2.1
49755	0.787142	2.1
49756	0.745391	2.1
49757	0.745381	2.1
49758	0.483476	2.1
49759	0.507368	2.1
49760	0.787152	2.1
49761	1.89224	2.1

49762	1.89224	2.1
49763	1.89224	2.1
49764	1.89224	2.1
49765	1.89224	2.1
49766	1.89224	2.1
49767	1.89227	2.1
49768	1.89227	2.1
49769	1.89227	2.1
49770	1.89227	2.1
49771	1.89227	2.1
49772	1.89227	2.1
49773	1.84994	2.1
49774	1.41758	2.1
49775	1.4908	2.1
49776	1.4908	2.1
49777	1.74391	2.1
49778	1.74391	2.1
49779	1.89226	2.1
49780	1.89226	2.1
49781	1.89226	2.1
49782	1.89226	2.1
49783	3.78452	2.1
49784	1.01323	2.1
49785	1.01323	2.1
49786	1.01445	2.1
49787	1.01445	2.1
49788	0.839531	2.1
49789	0.875652	2.1
49790	1.19063	2.1
49791	1.15203	2.1
49792	1.15203	2.1
49793	0.839531	2.1
49794	0.875652	2.1
49795	1.19063	2.1
49796	1.89226	2.1
49797	2.75587	2.1
49798	2.81512	2.1
49799	2.81512	2.1
49800	1.89226	2.1
49801	1.89226	2.1
49802	1.89226	2.1
49803	1.89226	2.1
49804	1.89226	2.1
49805	1.89226	2.1
49806	3.78452	2.1
49807	0.974831	2.1
49808	0.974831	2.1
49809	0.976681	2.1
49810	0.976681	2.1
49811	0.803179	2.1
49812	0.83953	2.1
49813	1.11198	2.1
49814	1.15203	2.1
49815	1.11198	2.1
49816	0.803179	2.1
49817	1.15203	2.1
49818	0.83953	2.1
49819	1.37794	2.1

49820	1.37794	2.1
49821	0.946128	2.1
49822	0.946128	2.1
49823	1.3477	2.1
49824	1.37793	2.1
49825	0.946131	2.1
49826	0.946131	2.1
49827	0.946129	2.1
49828	1.3477	2.1
49829	1.37793	2.1
49830	0.946129	2.1
49831	2.38577	2.06935
49832	1.20247	2.1
49833	1.75182	2.1
49834	2.82546	2.07457
49835	4.1376	2.1
49836	2.82546	2.09536
49837	1.89226	1.3228
49838	2.38577	2.07244
49839	2.38577	1.79428
49840	1.89226	2.04467
49841	1.89226	1.78092
49842	1.035 2.1	
49843	2.15429	2.1
49844	0.970998	2.1
49845	2.0815	2.1
49846	2.89672	2.1
49847	0.946131	0.131058
49848	0.946131	0.118259
49849	0.853249	0.128406
49850	1.02715	0.11806
49851	0.851537	0.133172
49852	0.851537	0.13244
49853	1.02663	0.116884
49854	1.13139	0.126922
49855	0.946128	0.110914
49856	0.932039	0.10646
49857	0.843985	0.0985989
49858	0.946129	0.110368
49859	1.89226	0.115634
49860	1.86408	0.119898
49861	1.96548	0.118076
49862	1.86408	0.116592
49863	1.99366	0.128396
49864	1.89226	0.124271
49865	2.70702	2.1
49866	2.08981	2.1
49867	2.08693	2.1
49868	2.08693	2.1
49869	2.7188	2.1
49870	2.7188	2.1
49871	2.08982	2.1
49872	1.01964	2.1
49873	2.08693	2.1
49874	1.04615	2.1
49875	3.13597	2.1
49876	2.70702	2.1
49877	2.08693	2.1

49878	2.68784	2.1
49879	2.08831	2.1
49880	4.77477	2.1
49881	0.778233	2.1
49882	0.778233	2.1
49883	0.789001	2.1
49884	0.789001	2.1
49885	0.545307	2.1
49886	0.523075	2.1
49887	1.04347	2.1
49888	1.04416	2.1
49889	1.04416	2.1
49890	0.545308	2.1
49891	1.04347	2.1
49892	0.523075	2.1
49893	1.89226	0.0715502
49894	1.89226	0.087745
49895	1.89226	0.0799375
49896	1.89226	0.0698591
49897	3.78453	0.071266
49898	1.39237	0.0951293
49899	1.17933	0.159273
49900	0.755796	0.0881199
49901	0.755796	0.100326
49902	0.755767	0.0748611
49903	0.94613	0.138784
49904	1.15918	0.0795702
49905	0.94613	0.0800239
49906	1.11632	0.0894695
49907	0.92595	0.109292
49908	0.94613	0.081709
49909	0.94613	0.0911156
49910	1.89226	0.0915747
49911	1.89226	0.0792969
49912	1.89226	0.0698591
49913	1.89226	0.0806025
49914	3.78452	0.0747465
49915	0.950067	0.0720287
49916	0.75071	0.0710274
49917	0.906516	0.0698591
49918	0.906516	0.0698591
49919	0.81586	0.0698591
49920	0.851541	0.0748499
49921	0.962101	0.0698591
49922	0.851476	0.0702423
49923	0.962105	0.0698591
49924	0.815808	0.0709022
49925	0.851529	0.0747436
49926	0.851529	0.0698591
49927	1.05032	0.0752439
49928	0.972326	0.0761767
49929	0.906515	0.0698591
49930	0.906515	0.0698591
49931	1.12245	0.0698591
49932	1.04072	0.0698591
49933	0.815857	0.0698591
49934	0.851539	0.076349
49935	1.04451	0.0698591

49936	0.815853	0.0772099
49937	0.919795	0.0718225
49938	0.919795	0.0768586
49939	1.5855	2.1
49940	2.14058	2.1
49941	1.9153	2.1
49942	1.79922	2.1
49943	3.72608	2.1
49944	1.58551	2.1
49945	1.24201	2.1
49946	0.960287	2.1
49947	1.79922	2.1
49948	2.82752	2.1
49949	0.679602	2.1
49950	0.621009	2.1
49951	0.899614	2.1
49952	0.940441	2.1
49953	0.679602	2.1
49954	0.94044	2.1
49955	0.621009	2.1
49956	0.899615	2.1
49957	0.794027	2.1
49958	0.794027	2.1
49959	0.785189	2.1
49960	0.785189	2.1
49961	2.14058	2.1
49962	1.79922	2.1
49963	2.23742	2.1
49964	2.23742	2.1
49965	1.78404	2.1
49966	1.88088	2.1
49967	1.15177	2.1
49968	2.0815	2.1
49969	1.93765	2.1
49970	1.78342	2.1
49971	1.39511	2.1
49972	1.28406	2.1
49973	1.97719	2.1
49974	2.50057	2.05148
49975	2.35349	2.1
49976	2.35349	2.02866
49977	2.31686	2.1
49978	2.32857	2.06312
49979	1.93765	2.1
49980	2.35349	2.06439
49981	1.70048	2.1
49982	2.15721	1.77891
49983	4.05397	2.1
49984	0.850388	2.1
49985	0.850388	2.1
49986	0.840019	2.1
49987	0.840019	2.1
49988	1.13736	2.1
49989	1.13852	2.1
49990	0.573661	2.1
49991	0.551891	2.1
49992	0.573661	2.1
49993	1.13852	2.1

49994	0.551892	2.1
49995	1.13736	2.1
49996	0.860203	2.1
49997	0.860203	2.1
49998	0.855758	2.1
49999	0.855758	2.1
50000	1.1423	2.1
50001	1.13852	2.1
50002	0.58628	2.1
50003	0.573661	2.1
50004	0.586281	2.1
50005	1.1423	2.1
50006	0.573661	2.1
50007	1.13852	2.1
50008	1.28918	0.745151
50009	0.999086	0.682745
50010	1.0383	1.53449
50011	0.736019	1.42532
50012	1.00954	1.37171
50013	1.00954	0.732719
50014	1.01566	0.567808
50015	1.01566	1.51542
50016	1.28918	1.18078
50017	1.0383	1.6386
50018	0.999086	1.32092
50019	0.736019	1.29132
50020	2.20797	2.1
50021	2.64748	2.1
50022	2.50375	2.1
50023	2.50375	2.1
50024	2.40858	2.1
50025	2.40858	2.1
50026	2.64748	2.1
50027	2.74744	2.1
50028	2.50375	2.1
50029	2.6521	2.1
50030	5.29958	2.1
50031	2.20797	2.1
50032	2.50375	2.1
50033	2.02064	2.1
50034	2.3606	2.1
50035	4.52439	2.1
50036	2.36059	2.1
50037	2.6521	2.1
50038	2.56952	2.1
50039	2.56952	2.1
50040	2.50375	2.1
50041	2.50375	2.1
50042	2.49439	0.0847679
50043	1.89226	0.113852
50044	2.81337	0.154065
50045	1.89226	0.113696
50046	4.38665	0.111793
50047	1.89226	0.0940927
50048	1.89226	0.156836
50049	1.89226	0.192196
50050	1.89226	0.13719
50051	3.78452	0.115468

50052	1.89226	0.112897
50053	1.89226	0.081614
50054	1.89226	0.119031
50055	1.89226	0.113746
50056	3.78452	0.118546
50057	1.93948	0.240113
50058	1.98874	0.0956991
50059	1.62494	0.162532
50060	2.14218	0.205311
50061	1.62494	0.106737
50062	2.14218	0.148629
50063	1.47354	0.159534
50064	1.98874	0.0812407
50065	2.01865	0.14071
50066	1.62495	0.0998193
50067	2.01865	0.130706
50068	1.62495	0.0821982
50069	1.89226	2.1
50070	1.89226	2.1
50071	1.89226	2.1
50072	1.89226	2.1
50073	3.78453	2.1
50074	2.05257	2.1
50075	1.94441	2.1
50076	1.88487	2.1
50077	1.82441	2.1
50078	3.82928	2.1
50079	1.94441	2.1
50080	1.74391	2.1
50081	1.82441	2.1
50082	1.65636	2.1
50083	3.60077	2.1
50084	1.89226	2.1
50085	1.89226	2.1
50086	1.89226	2.1
50087	1.89226	2.1
50088	3.78452	2.1
50089	1.89226	2.1
50090	1.89226	2.1
50091	1.89226	2.1
50092	1.89226	2.1
50093	3.78453	2.1
50094	1.88487	2.1
50095	1.82441	2.1
50096	1.75835	2.1
50097	1.72866	2.1
50098	3.61354	2.1
50099	1.82441	2.1
50100	1.65636	2.1
50101	1.72866	2.1
50102	1.58401	2.1
50103	3.38503	2.1
50104	1.89226	2.1
50105	1.89226	2.1
50106	1.89226	2.1
50107	1.89226	2.1
50108	3.78452	2.1
50109	2.27432	2.1

50110	1.33339	2.1
50111	1.38905	2.1
50112	2.34471	2.1
50113	3.66337	2.1
50114	1.89226	2.1
50115	2.34471	2.1
50116	2.27432	2.1
50117	2.27432	2.1
50118	1.89226	2.1
50119	1.89226	2.1
50120	1.20443	2.1
50121	1.17235	2.1
50122	0.724695	2.1
50123	0.694522	2.1
50124	0.949994	2.1
50125	0.949993	2.1
50126	0.948955	2.1
50127	0.948955	2.1
50128	1.20443	2.1
50129	0.7247	2.1
50130	0.694517	2.1
50131	1.17234	2.1
50132	1.89225	2.1
50133	2.40885	2.1
50134	2.3447	2.1
50135	2.3447	2.1
50136	1.89225	2.1
50137	1.89225	2.1
50138	1.37138	2.1
50139	1.82597	2.1
50140	1.45027	2.1
50141	1.92772	2.1
50142	3.27624	2.1
50143	1.89226	2.1
50144	1.89226	2.1
50145	1.89226	2.1
50146	1.89226	2.1
50147	3.78452	2.1
50148	1.89226	2.1
50149	1.89226	2.09459
50150	1.89226	2.1
50151	1.89226	2.07911
50152	3.78452	2.1
50153	1.82597	2.1
50154	2.02312	1.53743
50155	1.92772	2.1
50156	2.15888	2.1
50157	3.98485	2.1
50158	1.53446	2.1
50159	1.92772	2.1
50160	2.03975	2.1
50161	2.03975	2.1
50162	1.45027	2.1
50163	1.45027	2.1
50164	1.89226	2.1
50165	1.89226	2.1
50166	1.89226	2.1
50167	1.89226	2.1

50168	1.89226	2.1
50169	1.89226	2.1
50170	1.89226	2.1
50171	1.89226	2.1
50172	1.89226	2.1
50173	1.89226	2.1
50174	3.78452	2.1
50175	1.92772	2.1
50176	2.15888	2.1
50177	2.03975	2.1
50178	2.3111	2.1
50179	4.19863	2.1
50180	1.89226	2.1
50181	1.89226	2.1
50182	1.89226	2.1
50183	1.89226	2.1
50184	3.78452	2.1
50185	1.78376	2.1
50186	1.3201	2.1
50187	1.78376	2.1
50188	1.86102	2.1
50189	1.2679	2.1
50190	1.34517	2.1
50191	1.89226	2.1
50192	2.52442	2.1
50193	1.89226	2.1
50194	2.51691	2.1
50195	4.41667	2.1
50196	1.89226	2.1
50197	1.89226	2.1
50198	1.89226	2.1
50199	1.89226	2.1
50200	3.78452	2.1
50201	1.78376	2.1
50202	1.2679	2.1
50203	1.71839	2.1
50204	1.23504	2.1
50205	3.0188	2.1
50206	1.89226	2.1
50207	2.51795	2.1
50208	2.51691	2.1
50209	2.51691	2.1
50210	1.89226	2.1
50211	1.89226	2.1
50212	1.89226	0.543579
50213	1.89226	0.768599
50214	1.89226	0.79846
50215	1.89226	0.591488
50216	3.78452	0.66278
50217	1.89227	0.578746
50218	1.89227	0.775497
50219	3.26088	0.670386
50220	2.79543	0.786084
50221	2.20733	0.717009
50222	1.89227	0.816979
50223	1.89226	0.767067
50224	1.89226	0.594816
50225	1.89226	0.570529

50226	1.89226	0.711538
50227	1.89226	0.556571
50228	1.89226	0.709915
50229	1.89227	0.565981
50230	2.20733	0.706783
50231	1.46973	0.666544
50232	1.89227	0.682413
50233	1.7848	0.670281
50234	2.20733	0.701015
50235	1.89226	2.1
50236	2.55682	2.1
50237	2.81593	2.1
50238	2.81593	2.1
50239	1.89226	2.1
50240	1.89226	2.1
50241	1.0607	2.1
50242	1.0607	2.1
50243	1.12617	2.1
50244	0.986957	2.1
50245	0.699328	2.1
50246	0.785567	2.1
50247	1.28855	2.1
50248	1.41913	2.1
50249	0.766737	2.1
50250	1.34711	2.1
50251	1.24643	2.1
50252	0.687289	2.1
50253	3.11063	0.243454
50254	1.89226	0.343469
50255	1.96536	0.295986
50256	1.89226	0.303839
50257	1.96536	0.258553
50258	1.89226	0.262364
50259	1.89226	0.702749
50260	1.89226	0.451656
50261	1.89226	0.566939
50262	1.89226	0.506047
50263	3.78452	0.700601
50264	1.89226	0.322479
50265	1.96256	0.152736
50266	1.89226	0.199409
50267	1.89226	0.312703
50268	2.49192	0.299268
50269	2.49192	0.183393
50270	1.85716	0.307645
50271	2.56871	0.166788
50272	1.03043	0.216096
50273	1.03043	0.240934
50274	2.48125	0.137754
50275	1.65452	0.24248
50276	1.89226	2.1
50277	2.09622	2.1
50278	2.09622	2.1
50279	2.08508	2.1
50280	1.89226	2.1
50281	1.88112	2.1
50282	1.04811	2.1
50283	1.04811	2.1

50284	0.946131	2.1
50285	0.946131	2.1
50286	1.05014	2.1
50287	1.04811	2.1
50288	0.94613	2.1
50289	0.94613	2.1
50290	0.946131	2.1
50291	1.05013	2.1
50292	1.04811	2.1
50293	0.946131	2.1
50294	1.398	2.1
50295	2.10766	2.1
50296	2.3018	2.1
50297	2.3018	2.1
50298	1.34008	2.1
50299	1.34008	2.1
50300	1.1509	2.1
50301	1.1509	2.1
50302	0.946132	2.1
50303	0.946132	2.1
50304	0.946132	2.1
50305	0.946132	2.1
50306	1.05383	2.1
50307	1.1509	2.1
50308	1.1509	2.1
50309	0.946133	2.1
50310	0.946132	2.1
50311	1.05383	2.1
50312	0.931141	2.1
50313	0.931141	2.1
50314	0.971946	2.1
50315	0.971946	2.1
50316	1.28569	2.1
50317	1.1509	2.1
50318	0.699	2.1
50319	0.752185	2.1
50320	1.28568	2.1
50321	0.752186	2.1
50322	0.699001	2.1
50323	1.1509	2.1
50324	2.30181	2.1
50325	1.89226	2.1
50326	1.89226	2.1
50327	1.89226	2.1
50328	2.57137	2.1
50329	2.57137	2.1
50330	2.0815	2.1
50331	1.21962	2.1
50332	1.55173	2.1
50333	2.57849	2.1
50334	3.79001	2.1
50335	1.38779	2.1
50336	1.2923	2.1
50337	1.03974	2.1
50338	1.28832	2.1
50339	1.33566	2.1
50340	1.21116	2.1
50341	1.22343	2.1

50342	1.22343	2.1
50343	1.42288	2.1
50344	1.20138	2.1
50345	1.26025	2.1
50346	1.00644	2.1
50347	1.26066	2.1
50348	1.14271	2.1
50349	0.886635	2.1
50350	0.795277	2.1
50351	1.02966	2.1
50352	1.02967	2.1
50353	1.05216	2.1
50354	1.05216	2.1
50355	1.26068	2.1
50356	0.790042	2.1
50357	0.795269	2.1
50358	1.20135	2.1
50359	1.89226	0.49165
50360	1.89226	0.387474
50361	1.89226	0.483224
50362	1.89226	0.341714
50363	1.89226	0.576864
50364	1.89226	0.402946
50365	1.89227	0.373899
50366	1.89227	0.332283
50367	1.89227	0.403458
50368	1.89227	0.277638
50369	1.89227	0.462208
50370	1.89227	0.314236
50371	0.563873	0.262204
50372	0.50988	0.228875
50373	0.598693	0.328232
50374	0.558819	0.27888
50375	0.564855	0.269408
50376	0.564855	0.233688
50377	0.557838	0.322145
50378	0.557838	0.274601
50379	0.563873	0.284451
50380	0.598694	0.277934
50381	0.558819	0.262711
50382	0.50988	0.270624
50383	0.598693	0.324959
50384	0.558819	0.276955
50385	0.572737	0.387538
50386	0.534248	0.345612
50387	0.566788	0.337341
50388	0.566788	0.287908
50389	0.566153	0.379171
50390	0.566153	0.336832
50391	0.598694	0.343342
50392	0.572737	0.33799
50393	0.534248	0.31788
50394	0.558819	0.318718
50395	1.85217	2.1
50396	1.93111	2.1
50397	1.92875	2.1
50398	2.02391	2.1
50399	3.85986	2.1

50400	1.93112	2.1
50401	1.80425	2.1
50402	2.02392	2.1
50403	1.90981	2.1
50404	3.84092	2.1
50405	1.92876	2.1
50406	2.02393	2.1
50407	2.05274	2.1
50408	2.16783	2.1
50409	4.09659	2.1
50410	2.02393	2.1
50411	1.90982	2.1
50412	2.16784	2.1
50413	2.05967	2.1
50414	4.07766	2.1
50415	1.89226	1.92136
50416	1.89226	1.6895
50417	1.89226	1.83543
50418	1.89226	1.83425
50419	3.78453	1.66201
50420	1.89226	1.60666
50421	1.89226	1.88351
50422	1.89226	2.02576
50423	2.44829	1.41678
50424	3.78453	1.89609
50425	1.89226	1.92984
50426	1.89226	1.94191
50427	1.89226	1.94129
50428	2.60452	1.69375
50429	3.78453	1.99801
50430	1.58723	0.0698591
50431	1.88071	0.0737718
50432	2.97376	0.0698591
50433	2.13255	0.0706825
50434	4.43982	0.0698591
50435	2.20641	0.106424
50436	1.33575	0.152238
50437	2.15824	0.124767
50438	2.15824	0.122578
50439	1.2951	0.123573
50440	1.2951	0.127256
50441	0.825383	0.171383
50442	0.825383	0.144366
50443	0.856104	0.127397
50444	0.856104	0.136661
50445	0.952196	0.164398
50446	0.602363	0.14056
50447	0.667877	0.155446
50448	1.07913	0.127753
50449	0.602363	0.155303
50450	0.667876	0.152415
50451	1.07913	0.152385
50452	0.952197	0.144194
50453	2.57657	2.1
50454	1.44437	2.1
50455	2.65378	2.1
50456	2.25076	2.1
50457	1.84739	2.1

50458	1.44437	2.1
50459	2.0189	0.130632
50460	2.54752	0.131602
50461	2.63975	0.183176
50462	2.31049	0.099581
50463	4.65865	0.132534
50464	1.40707	0.0899437
50465	1.89605	0.0933111
50466	2.0189	0.107592
50467	2.0189	0.0825338
50468	1.50438	0.110992
50469	1.50438	0.0851903
50470	2.63971	0.170497
50471	2.38246	0.102511
50472	2.13402	0.127914
50473	2.57503	0.121197
50474	4.77373	0.125809
50475	1.50436	0.089063
50476	1.96803	0.0938138
50477	2.13402	0.121089
50478	1.45446	0.0774242
50479	3.63838	0.0909573
50480	2.30793	0.163107
50481	2.11032	0.141282
50482	1.24082	0.139873
50483	1.05689	0.17316
50484	3.36482	0.158794
50485	1.89226	0.206109
50486	1.89226	0.291589
50487	1.89226	0.301653
50488	1.89226	0.233943
50489	3.78453	0.248398
50490	0.812967	0.183663
50491	0.812967	0.193307
50492	1.00181	0.168225
50493	1.08141	0.162264
50494	0.946132	0.157806
50495	0.812938	0.202987
50496	0.932815	0.204478
50497	1.05577	0.162803
50498	0.812938	0.183315
50499	0.932812	0.185066
50500	0.946133	0.191742
50501	0.946133	0.173759
50502	2.53897	2.1
50503	1.89226	2.1
50504	1.89226	2.1
50505	1.89226	2.1
50506	2.3245	2.1
50507	2.3245	2.1
50508	1.00255	2.09576
50509	1.06561	2.1
50510	1.05481	1.32038
50511	1.11077	2.1
50512	2.12042	2.1
50513	1.89226	2.01102
50514	1.89226	2.1
50515	1.89226	1.43067

50516	1.89226	2.1
50517	3.78453	2.1
50518	1.89226	2.1
50519	2.77314	2.1
50520	2.81276	2.1
50521	2.81276	2.1
50522	1.89226	2.1
50523	1.89226	2.1
50524	1.11077	2.1
50525	1.15114	2.1
50526	1.10274	2.08761
50527	1.1032	2.1
50528	1.05481	2.09696
50529	1.1032	1.94723
50530	1.89226	1.97944
50531	1.89226	2.1
50532	1.89226	0.739372
50533	1.89226	2.1
50534	3.78452	2.09176
50535	0.721957	0.381377
50536	0.721957	0.424955
50537	0.946103	0.388695
50538	0.946103	0.314815
50539	0.814237	0.342466
50540	0.721929	0.309557
50541	0.946132	0.434917
50542	0.946132	0.395449
50543	0.721928	0.430864
50544	0.946131	0.460174
50545	0.946132	0.492089
50546	0.814237	0.457808
50547	1.89226	0.717044
50548	1.89226	0.412397
50549	1.89226	0.451642
50550	1.89226	0.451296
50551	1.89226	0.59922
50552	1.89226	0.612588
50553	1.37196	0.375081
50554	2.83556	0.281089
50555	1.5165	0.331161
50556	1.5165	0.405854
50557	2.74923	0.303725
50558	2.74923	0.385561
50559	0.946128	0.415178
50560	0.946128	0.514442
50561	0.827866	0.316534
50562	0.946132	0.402756
50563	0.94613	0.308234
50564	0.94613	0.34431
50565	0.94613	0.518982
50566	0.94613	0.470785
50567	0.94613	0.457856
50568	0.827864	0.500161
50569	0.94613	0.478542
50570	0.94613	0.601257
50571	1.36208	0.293647
50572	2.74923	0.218388
50573	1.37196	0.2523

50574	1.37196	0.301735
50575	2.53256	0.231484
50576	2.53256	0.29063
50577	1.07705	2.1
50578	1.07705	2.1
50579	1.08253	2.1
50580	1.08253	2.1
50581	0.762985	2.1
50582	0.781638	2.1
50583	1.37794	2.1
50584	1.40756	2.1
50585	1.37794	2.1
50586	0.762986	2.1
50587	0.781638	2.1
50588	1.40756	2.1
50589	2.69541	2.1
50590	2.75587	2.1
50591	1.48572	2.1
50592	1.48572	2.1
50593	1.52597	2.1
50594	2.73566	2.1
50595	2.0554	1.10507
50596	1.00387	0.641865
50597	2.01001	0.88007
50598	0.994002	0.55761
50599	3.01389	0.586483
50600	1.89226	2.1
50601	2.41579	2.1
50602	2.12209	2.1
50603	2.12209	2.1
50604	1.89226	2.1
50605	1.89226	2.1
50606	1.89226	2.1
50607	1.89226	2.1
50608	1.89226	2.1
50609	1.89226	2.1
50610	1.89226	2.1
50611	1.89226	2.1
50612	0.88209	2.1
50613	0.899471	2.1
50614	0.651501	2.1
50615	0.533531	2.1
50616	0.724105	2.1
50617	0.711243	2.1
50618	0.692622	2.1
50619	0.692622	2.1
50620	0.890668	2.1
50621	0.738066	2.1
50622	0.778223	2.1
50623	0.531121	2.1
50624	1.89226	2.1
50625	2.77504	2.1
50626	2.4158	2.1
50627	2.4158	2.1
50628	1.89226	2.1
50629	1.89226	2.1
50630	1.89226	2.1
50631	1.89226	2.1

50632	1.89226	2.1
50633	1.89226	2.1
50634	1.89226	2.1
50635	1.89226	2.1
50636	1.70303	2.1
50637	1.39832	2.1
50638	1.51749	2.1
50639	1.68783	2.1
50640	3.24844	2.1
50641	1.89226	2.1
50642	2.38311	2.1
50643	1.98321	2.1
50644	1.89226	2.1
50645	2.47406	2.1
50646	2.38311	2.1
50647	1.89226	2.1
50648	1.89226	2.1
50649	1.89226	2.1
50650	1.89226	2.1
50651	1.89226	2.1
50652	1.89226	2.1
50653	0.946131	2.1
50654	0.946131	2.1
50655	1.25167	2.1
50656	1.19156	2.1
50657	1.19156	2.1
50658	1.19156	2.1
50659	0.946133	2.1
50660	0.946133	2.1
50661	0.946131	2.1
50662	1.25167	2.1
50663	0.946131	2.1
50664	1.19156	2.1
50665	1.89226	2.1
50666	2.47406	2.1
50667	1.99996	2.1
50668	1.89226	2.1
50669	2.58176	2.1
50670	2.47406	2.1
50671	1.89226	2.1
50672	1.89226	2.1
50673	1.89226	2.1
50674	1.89226	2.1
50675	1.89226	2.1
50676	1.89226	2.1
50677	0.946131	2.1
50678	0.946131	2.1
50679	1.32241	2.1
50680	1.25167	2.1
50681	1.25167	2.1
50682	1.25167	2.1
50683	0.946133	2.1
50684	0.946133	2.1
50685	0.946131	2.1
50686	1.32241	2.1
50687	0.946131	2.1
50688	1.25167	2.1
50689	1.65151	2.1

50690	1.92471	2.1
50691	1.81834	2.1
50692	2.13272	2.1
50693	3.74306	2.1
50694	1.92472	2.1
50695	1.9943	2.1
50696	2.13273	2.1
50697	2.24475	2.1
50698	4.16947	2.1
50699	1.81834	2.1
50700	2.13272	2.1
50701	2.02687	2.1
50702	2.39605	2.1
50703	4.21439	2.1
50704	2.13273	2.1
50705	2.24475	2.1
50706	2.39605	2.1
50707	2.54903	2.1
50708	4.6408	2.1
50709	1.10534	2.1
50710	1.22993	2.1
50711	0.946132	2.1
50712	0.946132	2.1
50713	0.946132	2.1
50714	0.946132	2.1
50715	1.22993	2.1
50716	1.22993	2.1
50717	1.10534	2.1
50718	0.946131	2.1
50719	0.946132	2.1
50720	1.22993	2.1
50721	1.47221	2.1
50722	2.14647	2.1
50723	1.95567	2.1
50724	1.95567	2.1
50725	1.71082	2.1
50726	1.71082	2.1
50727	0.946131	2.1
50728	0.946131	2.1
50729	0.946131	2.1
50730	0.946131	2.1
50731	0.946132	2.1
50732	0.946132	2.1
50733	0.946131	2.1
50734	0.946131	2.1
50735	0.946131	2.1
50736	0.946131	2.1
50737	0.946132	2.1
50738	0.946132	2.1
50739	0.998086	2.1
50740	1.09429	2.1
50741	0.946125	2.1
50742	0.946125	2.1
50743	0.90732	2.1
50744	0.90732	2.1
50745	1.14414	2.1
50746	1.16553	2.1
50747	1.05018	2.1

50748	0.907332	2.1
50749	0.99482	2.1
50750	0.851529	2.1
50751	0.80534	2.1
50752	0.80534	2.1
50753	0.812206	2.1
50754	0.812206	2.1
50755	0.895134	2.1
50756	0.977828	2.1
50757	0.736099	2.1
50758	0.639718	2.1
50759	0.639722	2.1
50760	0.89514	2.1
50761	0.736095	2.1
50762	0.977821	2.1
50763	0.946125	2.1
50764	0.946125	2.1
50765	0.946125	2.1
50766	0.946125	2.1
50767	0.946125	2.1
50768	0.946125	2.1
50769	0.946124	2.1
50770	0.946124	2.1
50771	0.946131	2.1
50772	0.946131	2.1
50773	0.946118	2.1
50774	0.946118	2.1
50775	2.22622	0.0698591
50776	2.43347	0.0698591
50777	2.34108	0.0698591
50778	1.24587	2.1
50779	1.21674	2.1
50780	1.14533	2.1
50781	1.11311	2.1
50782	1.17874	2.1
50783	1.17874	2.1
50784	1.18024	2.1
50785	1.18024	2.1
50786	1.14533	2.1
50787	1.24587	2.1
50788	1.21674	2.1
50789	1.11311	2.1
50790	2.43347	2.1
50791	2.34107	2.1
50792	2.39663	2.1
50793	2.49174	2.1
50794	4.8301	2.1
50795	1.86206	2.1
50796	1.41058	2.1
50797	1.70302	2.1
50798	2.42083	2.1
50799	3.68129	2.1
50800	1.70302	2.1
50801	2.43549	2.1
50802	2.20075	2.1
50803	2.50358	2.1
50804	2.04916	2.1
50805	2.33113	2.1

50806	1.07624	2.1
50807	2.12621	2.1
50808	1.57297	2.1
50809	1.60383	2.1
50810	1.30047	2.1
50811	1.30047	2.1
50812	2.33379	2.1
50813	2.56149	1.43051
50814	2.36734	2.07827
50815	2.62796	1.86944
50816	4.92883	2.05945
50817	2.5615	1.61373
50818	2.57957	1.49151
50819	2.62796	1.88769
50820	2.69056	0.943709
50821	5.25206	1.45664
50822	2.39322	2.1
50823	2.62796	2.08469
50824	2.6849	2.1
50825	2.6849	2.07148
50826	2.36734	2.1
50827	2.36734	2.06885
50828	2.62796	2.09383
50829	2.69056	1.85975
50830	2.6849	1.09018
50831	2.77227	0.681205
50832	5.37546	1.19724
50833	1.89226	0.0698591
50834	1.89226	0.0698591
50835	1.89226	0.0698591
50836	1.89226	0.0698591
50837	1.89226	0.0698591
50838	1.89226	0.0698591
50839	1.06974	0.0698591
50840	1.06974	0.0698591
50841	1.02299	0.0698591
50842	1.02299	0.0698591
50843	1.35299	0.0698591
50844	1.26227	0.0698591
50845	0.646252	0.0698591
50846	0.830464	0.0698591
50847	0.830463	0.0698591
50848	1.35299	0.0698591
50849	1.26227	0.0698591
50850	0.646252	0.0698591
50851	2.52454	0.0698591
50852	1.89226	0.0698591
50853	2.70598	0.0698591
50854	1.89226	0.0698591
50855	4.59824	0.0698591
50856	1.89226	0.0698591
50857	1.89226	0.0698591
50858	1.89226	0.0698591
50859	1.89226	0.0698591
50860	1.89226	0.0698591
50861	1.89226	0.0698591
50862	1.89226	0.0698591
50863	1.89226	0.0698591

50864	1.89226	0.0698591
50865	1.89226	0.0698591
50866	1.89226	0.0698591
50867	1.89226	0.0698591
50868	1.7666	0.0698591
50869	2.70598	0.0698591
50870	2.65378	0.0698591
50871	2.65378	0.0698591
50872	1.66093	0.0698591
50873	1.66093	0.0698591
50874	2.70598	0.0698591
50875	1.89226	0.0698591
50876	1.89226	0.0698591
50877	1.89226	0.0698591
50878	2.65377	0.0698591
50879	2.65377	0.0698591
50880	0.946132	0.0698591
50881	0.946132	0.0698591
50882	0.946131	0.0698591
50883	0.946131	0.0698591
50884	0.946131	0.0698591
50885	0.946131	0.0698591
50886	0.946132	0.0698591
50887	0.946132	0.0698591
50888	0.946132	0.0698591
50889	0.946132	0.0698591
50890	0.946131	0.0698591
50891	0.946131	0.0698591
50892	1.69858	2.1
50893	2.18238	2.1
50894	2.506	2.1
50895	1.93586	2.1
50896	4.20458	2.1
50897	2.21772	2.1
50898	2.18238	2.1
50899	2.506	2.1
50900	2.59215	2.1
50901	4.72372	2.1
50902	2.50596	2.1
50903	1.93584	2.1
50904	2.16728	2.1
50905	2.7679	2.1
50906	4.67325	2.1
50907	1.53296	2.1
50908	1.56299	2.1
50909	1.42624	2.1
50910	1.52961	2.1
50911	2.98923	2.1
50912	1.56299	2.1
50913	1.46568	2.1
50914	1.52961	2.1
50915	1.51916	2.1
50916	3.08215	2.1
50917	1.36226	2.1
50918	1.52961	2.1
50919	1.36226	2.1
50920	1.42624	2.1
50921	1.55369	2.1

50922	1.61768	2.1
50923	1.52961	2.1
50924	1.51916	2.1
50925	1.55369	2.1
50926	1.64892	2.1
50927	3.07285	2.1
50928	2.13405	0.121219
50929	2.57507	0.117962
50930	2.39438	0.167086
50931	1.93408	0.0982958
50932	4.52843	0.122249
50933	2.13405	0.114403
50934	1.27778	0.080503
50935	1.2545	0.0930664
50936	1.45448	0.0896089
50937	1.93408	0.0891675
50938	2.13405	0.0884278
50939	2.39438	0.148218
50940	1.93407	0.0978034
50941	1.60578	0.116255
50942	1.87985	0.113221
50943	4.00016	0.117257
50944	1.0525	2.1
50945	1.05758	2.09685
50946	1.43484	0.560336
50947	1.38506	1.92043
50948	2.48734	2.00168
50949	1.20455	2.1
50950	1.67352	2.1
50951	1.52852	2.1
50952	2.05164	2.1
50953	3.20203	2.1
50954	1.89226	2.1
50955	1.89226	2.1
50956	1.89226	2.1
50957	1.89226	2.1
50958	3.78452	2.1
50959	1.89226	2.1
50960	1.89226	2.1
50961	1.89226	2.1
50962	1.89226	2.1
50963	1.89226	2.1
50964	1.89226	2.1
50965	2.05165	2.1
50966	1.94291	2.1
50967	2.09398	2.1
50968	1.67352	2.1
50969	2.36337	2.1
50970	1.94291	2.1
50971	1.52851	2.1
50972	2.52049	2.1
50973	2.43433	2.1
50974	2.05164	2.1
50975	1.9112	2.1
50976	1.52851	2.1
50977	1.89226	2.1
50978	1.89226	2.1
50979	1.89226	2.1

50980	1.89226	2.1
50981	3.78451	2.1
50982	1.89226	2.1
50983	1.89226	2.1
50984	1.89226	2.1
50985	1.89226	2.1
50986	3.78452	2.1
50987	2.5205	2.1
50988	2.36337	2.1
50989	2.4605	2.1
50990	2.05165	2.1
50991	2.77222	2.1
50992	2.36337	2.1
50993	1.89226	2.1
50994	1.89226	2.1
50995	1.89226	2.1
50996	1.89226	2.1
50997	1.89226	2.1
50998	1.89226	2.1
50999	1.89226	2.1
51000	1.89226	2.1
51001	1.89226	2.1
51002	1.89226	2.1
51003	3.78452	2.1
51004	1.38905	2.1
51005	1.26166	2.1
51006	1.19216	2.1
51007	1.0875	2.1
51008	1.24393	2.1
51009	1.24393	2.1
51010	1.23261	2.1
51011	1.23261	2.1
51012	1.38905	2.1
51013	1.19216	2.1
51014	1.26166	2.1
51015	1.0875	2.1
51016	1.16233	2.1
51017	1.06028	2.1
51018	0.777395	2.1
51019	0.705508	2.1
51020	0.940018	2.1
51021	0.936465	2.1
51022	0.954216	2.1
51023	0.954216	2.1
51024	1.16125	2.1
51025	0.685505	2.1
51026	1.1157	2.1
51027	0.703534	2.1
51028	1.89226	2.1
51029	1.89226	2.1
51030	1.89226	2.1
51031	1.89226	2.1
51032	1.89226	2.1
51033	1.89226	2.1
51034	1.89226	2.1
51035	1.89226	2.1
51036	1.89226	2.1
51037	1.89226	2.1

51038	1.89226	2.1
51039	1.89226	2.1
51040	2.38433	2.1
51041	1.43456	2.1
51042	1.93881	2.1
51043	2.36952	2.1
51044	4.32313	2.1
51045	1.8493	2.1
51046	1.5927	2.1
51047	1.45535	2.1
51048	1.38438	2.1
51049	1.89657	2.1
51050	1.8493	2.1
51051	2.0815	2.1
51052	1.6697	2.1
51053	2.0657	2.1
51054	1.5927	2.1
51055	2.59749	2.1
51056	2.0815	2.1
51057	2.76597	2.1
51058	1.89226	2.1
51059	1.89226	2.1
51060	1.89226	2.1
51061	2.6126	2.1
51062	2.6126	2.1
51063	2.62513	2.1
51064	1.89226	2.1
51065	1.89226	2.1
51066	1.89226	2.1
51067	2.76598	2.1
51068	2.76598	2.1
51069	2.54665	2.1
51070	2.6126	2.1
51071	2.76598	2.1
51072	2.76598	2.1
51073	2.34044	2.1
51074	2.34044	2.1
51075	2.62514	2.1
51076	2.76598	2.1
51077	2.54666	2.1
51078	2.45212	2.1
51079	5.2181	2.1
51080	2.14063	2.1
51081	1.47521	2.1
51082	1.27679	2.1
51083	1.47521	2.1
51084	2.11511	2.1
51085	2.31353	2.1
51086	0.93256	2.1
51087	1.07032	2.1
51088	0.638396	2.1
51089	0.521342	2.1
51090	0.790674	2.1
51091	0.790674	2.1
51092	0.80098	2.1
51093	0.800981	2.1
51094	0.932535	2.1
51095	0.521328	2.1

51096	1.07034	2.1
51097	0.638414	2.1
51098	1.89226	2.1
51099	1.89226	2.04119
51100	1.89226	2.08883
51101	1.89226	2.1
51102	3.78453	2.096
51103	1.89226	2.1
51104	1.9842	2.1
51105	1.89226	2.1
51106	1.89226	2.1
51107	2.79705	2.1
51108	2.79705	2.1
51109	0.469955	2.1
51110	0.791665	2.1
51111	0.989115	2.06224
51112	0.763235	2.07575
51113	0.776356	2.06538
51114	0.805461	2.1
51115	0.776759	2.08086
51116	0.776759	2.1
51117	0.799151	2.09076
51118	0.940869	2.09462
51119	0.78449	2.09368
51120	0.489635	2.0954
51121	1.89226	2.02657
51122	1.89226	2.06797
51123	1.89226	1.95284
51124	1.89226	2.1
51125	1.89226	1.94016
51126	1.89226	2.1
51127	2.46058	1.91761
51128	2.28991	2.0761
51129	1.8525	2.03946
51130	1.87697	2.07359
51131	2.28991	2.01764
51132	1.62397	2.06911
51133	0.946131	0.0698591
51134	0.974033	0.0698591
51135	1.31667	0.0698591
51136	0.946131	0.0698591
51137	0.885497	0.0698591
51138	0.885497	0.0698591
51139	1.0468	0.0698591
51140	1.04678	0.0698591
51141	1.31667	0.0698591
51142	0.974019	0.0698591
51143	0.946131	0.0698591
51144	0.946131	0.0698591
51145	1.89226	0.0698591
51146	1.89226	0.0807899
51147	1.89226	0.0743644
51148	1.89226	0.0698591
51149	3.78452	0.0761246
51150	0.946125	0.0838867
51151	0.946125	0.076734
51152	0.946131	0.0900026
51153	0.946131	0.0975768

51154	0.946128	0.0912575
51155	0.946128	0.0863293
51156	0.946128	0.0715283
51157	0.946128	0.0794358
51158	0.946128	0.0819583
51159	0.946128	0.082727
51160	0.946128	0.081731
51161	0.946128	0.0783677
51162	0.946131	0.0698591
51163	0.98453	0.0698591
51164	1.32276	0.0698591
51165	0.946131	0.0698591
51166	0.946132	0.0698591
51167	0.946132	0.0698591
51168	0.98453	0.0698591
51169	1.32276	0.0698591
51170	1.32276	0.0698591
51171	1.32276	0.0698591
51172	0.946132	0.0698591
51173	0.946132	0.0698591
51174	1.89227	2.1
51175	1.89227	2.1
51176	1.89227	2.1
51177	1.89227	2.1
51178	1.89227	2.1
51179	1.89227	2.1
51180	1.89226	2.1
51181	1.89226	2.1
51182	1.89226	2.1
51183	1.89226	2.1
51184	1.89226	2.1
51185	1.89226	2.1
51186	1.05556	2.1
51187	1.38859	2.1
51188	1.46996	2.1
51189	1.25364	2.1
51190	2.52552	2.1
51191	0.694296	2.1
51192	0.73498	2.1
51193	0.561881	2.1
51194	0.512189	2.1
51195	0.621304	2.1
51196	0.621304	2.1
51197	0.625865	2.1
51198	0.625865	2.1
51199	0.734979	2.1
51200	0.561881	2.1
51201	0.51219	2.1
51202	0.694296	2.1
51203	0.946126	2.1
51204	0.946126	2.1
51205	0.946126	2.1
51206	0.946126	2.1
51207	0.946126	2.1
51208	0.946126	2.1
51209	0.946126	2.1
51210	0.946126	2.1
51211	0.946132	2.1

51212	0.946132	2.1
51213	0.94612	2.1
51214	0.94612	2.1
51215	1.89225	2.1
51216	1.89225	2.1
51217	1.89225	2.1
51218	1.89225	2.1
51219	1.89225	2.1
51220	1.89225	2.1
51221	0.626814	2.1
51222	0.632155	2.1
51223	0.746234	2.1
51224	0.734974	2.1
51225	0.68202	2.1
51226	0.68202	2.1
51227	0.685108	2.1
51228	0.685108	2.1
51229	0.746239	2.1
51230	0.632159	2.1
51231	0.734969	2.1
51232	0.62681	2.1
51233	0.746235	2.1
51234	0.734975	2.1
51235	0.595081	2.1
51236	0.561877	2.1
51237	0.648584	2.1
51238	0.648584	2.1
51239	0.659528	2.1
51240	0.659528	2.1
51241	0.746239	2.1
51242	0.595085	2.1
51243	0.734969	2.1
51244	0.561874	2.1
51245	1.89249	2.1
51246	2.2137	2.1
51247	1.33125	2.1
51248	1.06499	2.1
51249	2.12571	2.1
51250	1.78918	2.1
51251	0.946131	2.1
51252	0.946131	2.1
51253	0.94613	2.1
51254	0.94613	2.1
51255	0.946132	2.1
51256	0.946132	2.1
51257	0.946129	2.1
51258	0.946129	2.1
51259	0.946131	2.1
51260	0.946131	2.1
51261	0.946131	2.1
51262	0.946131	2.1
51263	1.89227	2.1
51264	1.89227	2.1
51265	1.89227	2.1
51266	1.89227	2.1
51267	3.78453	2.1
51268	1.89226	2.1
51269	1.89226	2.1

51270	1.89226	2.1
51271	1.89226	2.1
51272	1.89226	2.1
51273	1.89226	2.1
51274	2.01388	2.1
51275	1.38757	2.1
51276	1.24229	2.1
51277	1.3309	2.1
51278	1.88011	2.1
51279	2.01388	2.1
51280	2.01388	2.1
51281	2.01555	2.1
51282	2.13879	2.1
51283	1.80766	2.1
51284	4.15267	2.1
51285	0.946133	2.1
51286	0.946133	2.1
51287	0.946132	2.1
51288	0.946132	2.1
51289	0.946132	2.1
51290	0.946132	2.1
51291	0.946133	2.1
51292	0.946133	2.1
51293	0.946132	2.1
51294	0.946132	2.1
51295	0.946132	2.1
51296	0.946132	2.1
51297	1.89226	2.1
51298	1.89226	2.1
51299	1.89226	2.1
51300	1.89226	2.1
51301	1.89226	2.1
51302	1.89226	2.1
51303	0.676745	2.1
51304	0.68382	2.1
51305	1.07048	2.1
51306	1.1327	2.1
51307	0.929324	2.1
51308	0.871438	2.1
51309	0.867362	2.1
51310	0.867362	2.1
51311	1.10666	2.1
51312	0.695036	2.1
51313	0.744098	2.1
51314	1.10144	2.1
51315	2.25168	2.1
51316	2.22499	2.1
51317	2.15929	2.1
51318	2.13879	2.1
51319	2.15929	2.1
51320	2.13879	2.1
51321	2.15314	2.1
51322	1.70302	2.1
51323	2.24694	2.1
51324	1.65245	2.1
51325	3.82722	2.1
51326	1.65245	2.1
51327	1.00371	2.1

51328	1.7437	2.1
51329	1.65748	2.1
51330	1.10459	2.1
51331	1.00371	2.1
51332	2.24694	2.1
51333	1.65246	2.1
51334	2.69773	2.1
51335	1.88129	2.1
51336	4.12824	2.1
51337	1.65246	2.1
51338	1.10459	2.1
51339	1.88129	2.1
51340	1.05914	2.1
51341	2.76496	2.1
51342	1.89226	2.1
51343	1.89226	2.1
51344	1.89226	2.1
51345	1.89226	2.1
51346	3.78452	2.1
51347	0.718577	2.1
51348	0.718577	2.1
51349	0.725249	2.1
51350	0.725249	2.1
51351	0.597916	2.1
51352	0.61752	2.1
51353	0.826306	2.1
51354	0.859194	2.1
51355	0.826306	2.1
51356	0.597916	2.1
51357	0.859194	2.1
51358	0.61752	2.1
51359	1.25898	2.1
51360	1.25898	2.1
51361	0.946128	2.1
51362	0.946128	2.1
51363	1.25897	2.1
51364	1.25585	2.1
51365	0.946131	2.1
51366	0.946131	2.1
51367	0.946129	2.1
51368	1.25586	2.1
51369	1.25898	2.1
51370	0.946129	2.1
51371	1.89226	2.1
51372	1.89226	2.1
51373	1.89226	2.1
51374	1.89226	2.1
51375	3.78452	2.1
51376	0.693258	2.1
51377	0.693258	2.1
51378	0.697356	2.1
51379	0.697356	2.1
51380	0.572479	2.1
51381	0.597916	2.1
51382	0.792698	2.1
51383	0.826306	2.1
51384	0.572478	2.1
51385	0.792697	2.1

51386	0.826307	2.1
51387	0.597917	2.1
51388	1.25586	2.1
51389	1.25586	2.1
51390	0.946128	2.1
51391	0.946128	2.1
51392	1.25585	2.1
51393	1.24344	2.1
51394	0.946131	2.1
51395	0.946131	2.1
51396	0.946129	2.1
51397	1.24344	2.1
51398	0.94613	2.1
51399	1.25586	2.1
51400	1.65151	2.1
51401	1.81835	2.1
51402	1.12064	2.1
51403	1.12064	2.1
51404	1.24965	2.1
51405	1.81835	2.1
51406	1.24965	2.1
51407	1.81835	2.1
51408	1.41077	2.1
51409	2.02687	2.1
51410	3.22911	2.1
51411	1.89122	1.92168
51412	1.38776	2.1
51413	2.37014	1.36294
51414	1.80818	2.1
51415	3.6994	2.0963
51416	2.15466	1.11651
51417	2.00083	2.1
51418	1.80818	2.0598
51419	1.80818	1.68384
51420	2.31988	2.0707
51421	2.31988	1.46742
51422	1.80818	2.05284
51423	1.18041	2.1
51424	2.00082	2.1
51425	1.43652	2.1
51426	3.18123	2.1
51427	1.89226	1.1284
51428	1.89226	1.54021
51429	1.89226	1.23692
51430	1.89226	1.09943
51431	3.78453	1.13908
51432	1.89226	1.65997
51433	1.89226	1.68774
51434	1.89226	1.45287
51435	1.89226	1.25053
51436	3.78452	1.76039
51437	0.80759	1.47915
51438	0.686119	1.66399
51439	0.972147	1.67524
51440	1.00217	1.69454
51441	0.821334	1.89652
51442	0.821334	1.66165
51443	0.866957	1.63356

51444	0.866957	1.46788
51445	0.972145	1.75599
51446	0.686121	2.00775
51447	0.807592	1.83046
51448	1.00217	2.01925
51449	0.972146	1.585
51450	1.00217	1.55223
51451	1.26654	1.76252
51452	1.1908	1.70268
51453	1.05501	1.62667
51454	1.05501	1.94552
51455	1.10794	1.51398
51456	1.10794	1.03455
51457	0.972146	0.937332
51458	1.26654	1.6053
51459	1.00217	1.55062
51460	1.1908	1.88745
51461	1.89226	1.43781
51462	1.89226	1.05701
51463	1.89226	1.32996
51464	1.89226	1.78296
51465	1.89226	0.913366
51466	1.89226	1.23506
51467	0.972147	1.58269
51468	0.867854	1.79307
51469	1.26132	1.40992
51470	1.26654	1.52979
51471	1.0424	1.75554
51472	1.0424	1.86538
51473	1.092	1.03736
51474	1.092	1.67841
51475	1.26132	1.70084
51476	0.867854	2.07749
51477	0.972147	1.86131
51478	1.26654	2.0333
51479	1.4375	0.103523
51480	1.92998	0.0966038
51481	1.66953	0.0707517
51482	1.0953	0.0744101
51483	3.10703	0.0765837
51484	1.89226	2.1
51485	1.89226	2.1
51486	1.89226	2.1
51487	1.89226	2.1
51488	3.78452	2.1
51489	2.74034	2.1
51490	2.08808	2.1
51491	2.63859	2.1
51492	2.00343	2.1
51493	4.72667	2.1
51494	0.799825	2.1
51495	0.799825	2.1
51496	0.80211	2.1
51497	0.80211	2.1
51498	0.562428	2.1
51499	0.600223	2.1
51500	1.04404	2.1
51501	1.00171	2.1

51502	1.00171	2.1
51503	0.56243	2.1
51504	0.600225	2.1
51505	1.04404	2.1
51506	0.94613	2.1
51507	0.94613	2.1
51508	0.946128	2.1
51509	0.946128	2.1
51510	0.946127	2.1
51511	0.946127	2.1
51512	0.946131	2.1
51513	0.946131	2.1
51514	0.946129	2.1
51515	0.946129	2.1
51516	0.946129	2.1
51517	0.946129	2.1
51518	1.89226	2.1
51519	1.89226	2.1
51520	1.89226	2.1
51521	1.89226	2.1
51522	3.78452	2.1
51523	2.63859	2.1
51524	2.00343	2.1
51525	2.54388	2.1
51526	1.91548	2.1
51527	4.55407	2.1
51528	1.91548	2.1
51529	2.00342	2.1
51530	1.04909	2.1
51531	1.04909	2.1
51532	1.12486	2.1
51533	1.99124	2.1
51534	0.810942	2.1
51535	0.810942	2.1
51536	0.810942	2.1
51537	0.810942	2.1
51538	1.89226	2.1
51539	1.35169	2.1
51540	1.08131	2.1
51541	1.08132	2.1
51542	1.08132	2.1
51543	0.810944	2.1
51544	0.810944	2.1
51545	1.89226	0.863191
51546	1.89226	2.1
51547	1.89226	0.944748
51548	1.89226	2.1
51549	3.78452	1.82259
51550	1.7968	0.821779
51551	1.49831	2.1
51552	1.59244	0.866659
51553	1.3395	2.09193
51554	3.09074	1.86804
51555	1.49831	2.1
51556	1.16858	2.1
51557	1.33951	2.09247
51558	1.0361	2.1
51559	2.53442	2.1

51560	1.89226	2.1
51561	1.89226	2.1
51562	1.89226	2.1
51563	1.89226	2.1
51564	1.89226	2.1
51565	1.89226	2.1
51566	1.89223	0.800206
51567	1.89223	2.1
51568	1.89223	1.57255
51569	1.89223	2.09309
51570	1.89223	1.00329
51571	1.89223	2.09551
51572	1.59241	0.724995
51573	1.18952	2.1
51574	1.33949	2.09205
51575	1.33949	1.18305
51576	1.40538	2.09242
51577	1.40538	0.891298
51578	1.89226	0.113285
51579	1.89226	0.132229
51580	1.89226	0.122027
51581	1.89226	0.135523
51582	1.89226	0.15412
51583	1.89226	0.163749
51584	1.89226	2.1
51585	1.89226	2.1
51586	1.89226	2.1
51587	1.89226	2.1
51588	3.78452	2.1
51589	1.89226	2.1
51590	1.89226	2.1
51591	1.89226	2.1
51592	1.89226	2.1
51593	3.78452	2.1
51594	2.01867	2.1
51595	1.46955	2.1
51596	1.58476	2.1
51597	1.38189	2.1
51598	2.22153	2.1
51599	2.01867	2.1
51600	2.01867	2.1
51601	2.52881	2.1
51602	2.21732	2.1
51603	2.21732	2.1
51604	2.22154	2.1
51605	2.22154	2.1
51606	1.89226	2.1
51607	1.89226	2.1
51608	1.89226	2.1
51609	1.89226	2.1
51610	1.89226	2.1
51611	1.89226	2.1
51612	1.89226	2.1
51613	1.89226	2.1
51614	1.89226	2.1
51615	1.89226	2.1
51616	1.89226	2.1
51617	1.89226	2.1

51618	2.22153	2.1
51619	1.63361	2.1
51620	1.77336	2.1
51621	1.46955	2.1
51622	2.52534	2.1
51623	2.22153	2.1
51624	2.34431	2.1
51625	1.62905	2.1
51626	1.49693	2.1
51627	2.19197	2.1
51628	3.82103	2.1
51629	1.89226	2.1
51630	1.89226	2.1
51631	1.89226	2.1
51632	1.89226	2.1
51633	3.78453	2.1
51634	0.946128	2.1
51635	0.946128	2.1
51636	1.22372	2.1
51637	1.17331	2.1
51638	0.946131	2.1
51639	0.946131	2.1
51640	1.17331	2.1
51641	1.17331	2.1
51642	0.946131	2.1
51643	1.17331	2.1
51644	1.22372	2.1
51645	0.94613	2.1
51646	1.04041	2.1
51647	1.09599	2.1
51648	0.748462	2.1
51649	0.701976	2.1
51650	0.896701	2.1
51651	0.896701	2.1
51652	0.901262	2.1
51653	0.901262	2.1
51654	1.04041	2.1
51655	0.701977	2.1
51656	0.748463	2.1
51657	1.09599	2.1
51658	1.89226	2.1
51659	1.89226	2.1
51660	1.89226	2.1
51661	1.89226	2.1
51662	3.78453	2.1
51663	0.946128	2.1
51664	0.946128	2.1
51665	1.17331	2.1
51666	1.13785	2.1
51667	0.94613	2.1
51668	0.94613	2.1
51669	1.13785	2.1
51670	1.13785	2.1
51671	1.13785	2.1
51672	0.94613	2.1
51673	0.94613	2.1
51674	1.17331	2.1
51675	2.50381	2.1

51676	1.89226	2.1
51677	1.89226	2.1
51678	2.73387	2.1
51679	4.39607	2.1
51680	1.80045	2.1
51681	1.99777	2.06471
51682	2.45134	2.1
51683	2.12463	2.1
51684	1.70302	2.1
51685	1.42655	2.1
51686	2.20231	2.1
51687	1.70302	2.1
51688	1.93128	2.1
51689	1.66768	2.1
51690	1.24499	2.04871
51691	1.70302	2.04771
51692	2.55572	0.0849337
51693	1.22811	0.12629
51694	1.82584	0.106489
51695	1.22811	0.089969
51696	2.62764	0.113146
51697	1.93147	0.0951448
51698	2.14513	0.126452
51699	1.89226	0.235637
51700	2.66481	0.134972
51701	1.89226	0.146941
51702	2.66481	0.243994
51703	1.89226	0.254359
51704	2.11649	2.1
51705	1.89226	2.1
51706	1.89226	2.1
51707	1.89226	2.1
51708	2.15426	2.1
51709	2.15426	2.1
51710	2.06776	2.1
51711	1.89226	2.1
51712	1.89226	2.1
51713	1.89226	2.1
51714	2.11649	2.1
51715	2.11649	2.1
51716	1.89226	2.1
51717	1.89226	2.1
51718	1.89226	2.1
51719	1.89226	2.1
51720	1.89226	2.1
51721	1.89226	2.1
51722	0.998134	2.1
51723	0.918904	2.1
51724	0.894708	2.1
51725	0.758742	2.1
51726	0.864276	2.1
51727	0.864276	2.1
51728	0.8926	2.1
51729	0.8926	2.1
51730	0.998134	2.1
51731	0.894709	2.1
51732	0.758742	2.1
51733	0.918904	2.1

51734	1.89226	2.1
51735	1.89226	2.1
51736	1.89226	2.1
51737	1.89226	2.1
51738	1.89226	2.1
51739	1.89226	2.1
51740	1.89226	2.1
51741	1.89226	2.1
51742	1.89226	2.1
51743	1.89226	2.1
51744	1.89226	2.1
51745	1.89226	2.1
51746	1.99627	2.1
51747	1.61047	2.1
51748	2.0815	2.1
51749	2.07569	2.1
51750	3.75063	2.1
51751	0.848077	2.1
51752	1.07748	2.1
51753	0.802498	2.1
51754	0.626808	2.1
51755	0.875552	2.1
51756	0.737527	2.1
51757	0.835602	2.1
51758	0.835602	2.1
51759	0.983245	2.1
51760	0.781108	2.1
51761	0.872306	2.1
51762	0.601497	2.1
51763	2.74745	2.1
51764	2.73988	2.1
51765	2.6521	2.1
51766	2.67792	2.1
51767	5.39198	2.1
51768	2.73988	2.1
51769	2.57466	2.1
51770	2.67791	2.1
51771	2.55653	2.1
51772	5.29641	2.1
51773	2.6521	2.1
51774	2.64148	2.1
51775	2.67792	2.1
51776	2.67792	2.1
51777	2.56953	2.1
51778	2.56953	2.1
51779	2.64148	2.1
51780	2.55653	2.1
51781	2.57929	2.1
51782	2.57929	2.1
51783	2.67792	2.1
51784	2.67792	2.1
51785	1.89226	1.38512
51786	1.89226	0.877799
51787	1.89226	1.99566
51788	1.89226	0.297523
51789	3.78453	0.900345
51790	1.68703	1.36502
51791	2.50385	0.300306

51792	1.45958	0.628413
51793	2.0815	0.169014
51794	3.93806	0.430597
51795	2.50385	0.262969
51796	2.57414	0.673006
51797	2.0815	0.243274
51798	2.39921	1.60694
51799	4.91181	0.443915
51800	1.89226	0.855683
51801	1.89226	0.734416
51802	1.89226	0.268299
51803	1.89226	0.235701
51804	3.78453	0.373392
51805	1.89226	1.99653
51806	1.89226	0.202026
51807	1.89226	1.03051
51808	1.89226	1.03388
51809	1.89226	0.605464
51810	1.89226	1.33184
51811	1.23013	0.438245
51812	2.0815	0.130464
51813	1.90167	0.894463
51814	1.71243	0.440445
51815	1.45887	0.757928
51816	1.31369	0.236259
51817	2.0815	0.208416
51818	2.39921	1.73523
51819	1.7115	0.332094
51820	2.2421	0.698475
51821	4.09829	0.51792
51822	1.89227	0.155321
51823	1.89227	0.332358
51824	1.89227	0.62138
51825	1.89227	0.842258
51826	3.78453	0.120357
51827	2.39697	0.0698591
51828	1.85594	0.0698591
51829	1.91161	0.0698591
51830	2.49867	0.0698591
51831	4.35461	0.0698591
51832	1.12035	0.0698591
51833	1.85594	0.0698591
51834	1.91161	0.0698591
51835	1.15483	0.0698591
51836	3.01077	0.0698591
51837	1.89226	0.0698591
51838	1.89226	0.0698591
51839	1.89226	0.0698591
51840	1.89226	0.0698591
51841	1.89226	0.0698591
51842	1.89226	0.0698591
51843	1.89226	0.0698591
51844	1.89226	0.0698591
51845	1.89226	0.0698591
51846	1.89226	0.0698591
51847	1.89226	0.0698591
51848	1.89226	0.0698591
51849	0.936516	0.0698591

51850	1.03496	0.0698591
51851	1.22607	0.0698591
51852	1.28058	0.0698591
51853	1.06317	0.0698591
51854	1.06317	0.0698591
51855	1.19735	0.0698591
51856	1.0813	0.0698591
51857	0.858168	0.0698591
51858	1.32278	0.0698591
51859	0.957674	0.0698591
51860	1.25178	0.0698591
51861	1.92659	0.0698591
51862	0.998957	0.0698591
51863	0.998957	0.0698591
51864	1.15481	0.0698591
51865	1.6031	0.0698591
51866	1.74459	0.0698591
51867	0.946119	0.0698591
51868	0.946119	0.0698591
51869	0.946118	0.0698591
51870	0.946118	0.0698591
51871	0.946118	0.0698591
51872	0.946118	0.0698591
51873	0.946119	0.0698591
51874	0.946119	0.0698591
51875	0.946131	0.0698591
51876	0.946131	0.0698591
51877	0.946106	0.0698591
51878	0.946106	0.0698591
51879	0.946118	0.0698591
51880	0.946118	0.0698591
51881	0.946119	0.0698591
51882	0.946119	0.0698591
51883	0.946118	0.0698591
51884	0.946118	0.0698591
51885	0.946119	0.0698591
51886	0.946119	0.0698591
51887	0.946131	0.0698591
51888	0.946131	0.0698591
51889	0.946106	0.0698591
51890	0.946106	0.0698591
51891	2.47164	0.411236
51892	2.83689	0.247298
51893	2.73872	0.442838
51894	2.41632	0.251653
51895	5.21036	0.271683
51896	0.946133	0.33548
51897	0.946133	0.449149
51898	1.41845	0.272569
51899	1.36936	0.415049
51900	1.41845	0.266505
51901	1.41845	0.25156
51902	0.946132	0.455947
51903	0.946132	0.453404
51904	0.946132	0.36951
51905	1.36936	0.306787
51906	1.41845	0.336067
51907	0.946133	0.397493

51908	1.36936	0.188734
51909	1.27581	0.308812
51910	1.20816	0.216749
51911	1.10464	0.357387
51912	1.24454	0.199415
51913	1.24454	0.194522
51914	1.23943	0.323133
51915	1.23943	0.339072
51916	1.10465	0.29223
51917	1.27581	0.272914
51918	1.36936	0.285061
51919	1.20816	0.259098
51920	0.946131	2.1
51921	0.946131	2.1
51922	1.37277	2.1
51923	1.37277	2.1
51924	1.37277	2.1
51925	1.27186	2.1
51926	0.946131	2.1
51927	0.946131	2.1
51928	0.946131	2.1
51929	1.37277	2.1
51930	0.946131	2.1
51931	1.27186	2.1
51932	2.74554	2.1
51933	1.23712	2.1
51934	2.80992	2.1
51935	2.54371	2.1
51936	1.50333	2.1
51937	1.23712	2.1
51938	2.77314	2.1
51939	2.33316	2.1
51940	2.28748	2.1
51941	2.28748	2.1
51942	2.81275	2.1
51943	2.81275	2.1
51944	2.28749	2.1
51945	1.45887	2.1
51946	2.33317	2.1
51947	1.50022	2.1
51948	3.79204	2.1
51949	2.37364	2.1
51950	2.33317	2.1
51951	1.5362	2.1
51952	1.5362	2.1
51953	1.50022	2.1
51954	2.33317	2.1
51955	1.89226	0.465544
51956	1.89226	0.501105
51957	1.89226	0.400522
51958	1.89226	0.520572
51959	1.89226	0.412209
51960	1.89226	0.545538
51961	0.946128	0.276665
51962	0.946128	0.319785
51963	1.0644	0.212792
51964	0.946133	0.272678
51965	1.0644	0.20388

51966	0.94613	0.240312
51967	0.946131	0.339118
51968	0.946131	0.27474
51969	1.0644	0.25397
51970	1.0644	0.264104
51971	0.946131	0.27939
51972	0.946131	0.255514
51973	1.89226	2.1
51974	1.89226	0.927219
51975	1.89226	2.06665
51976	1.89226	0.309148
51977	3.78452	1.15889
51978	1.14599	2.09961
51979	1.19993	0.602864
51980	1.00065	2.04056
51981	1.07497	0.297272
51982	2.22096	1.66326
51983	0.546551	1.20385
51984	0.546551	0.659896
51985	0.54987	0.568971
51986	0.54987	1.6966
51987	0.503067	0.749044
51988	0.558937	0.79205
51989	0.599966	0.215472
51990	0.537484	0.296826
51991	0.503067	0.230581
51992	0.537485	0.213382
51993	0.599966	0.252994
51994	0.558936	0.285546
51995	0.946132	0.530015
51996	0.946132	1.22537
51997	0.94613	0.329271
51998	0.94613	1.02322
51999	0.946131	0.670752
52000	0.946131	0.787453
52001	0.946131	0.334467
52002	0.946131	0.274448
52003	0.946131	0.28038
52004	0.946131	0.0970636
52005	0.946131	0.448373
52006	0.946131	0.538816
52007	2.19104	2.1
52008	2.5392	2.1
52009	2.53302	2.1
52010	2.19226	2.1
52011	4.73146	2.1
52012	1.09552	2.1
52013	1.09613	2.1
52014	0.802484	2.1
52015	0.807941	2.1
52016	0.95052	2.1
52017	0.95052	2.1
52018	0.952944	2.1
52019	0.952944	2.1
52020	1.09613	2.1
52021	0.807941	2.1
52022	0.802484	2.1
52023	1.09552	2.1

52024	2.533	2.1
52025	2.18531	2.1
52026	2.19224	2.1
52027	2.19224	2.1
52028	2.51603	2.1
52029	2.51603	2.1
52030	1.09266	2.1
52031	1.09612	2.1
52032	0.807935	2.1
52033	0.81091	2.1
52034	0.951897	2.1
52035	0.951897	2.1
52036	0.955134	2.1
52037	0.955134	2.1
52038	0.810915	2.1
52039	1.09266	2.1
52040	1.09611	2.1
52041	0.80793	2.1
52042	2.12741	0.125517
52043	2.79312	0.371393
52044	2.77591	0.638548
52045	2.10714	0.20742
52046	4.90026	0.230231
52047	2.12741	0.187345
52048	1.20859	0.242025
52049	1.21073	0.556689
52050	2.10714	0.277478
52051	3.31573	0.317268
52052	1.38708	0.596812
52053	1.38795	0.479651
52054	1.05331	0.229415
52055	1.05357	0.192938
52056	1.22049	0.240443
52057	1.22049	0.534782
52058	1.22078	0.720091
52059	1.22078	0.180334
52060	1.05331	0.363218
52061	1.38709	0.38167
52062	1.38795	0.312812
52063	1.05357	0.312223
52064	2.10662	0.40502
52065	2.10714	0.163853
52066	2.12499	0.131423
52067	1.22857	0.364461
52068	1.21073	0.276586
52069	1.21073	0.13315
52070	1.82014	0.0698591
52071	2.08757	0.0698591
52072	1.81292	0.0698591
52073	1.67485	0.0698591
52074	3.63306	0.0698591
52075	1.82013	0.0698591
52076	1.21748	0.0698591
52077	1.29891	0.0698591
52078	1.4442	0.0698591
52079	1.67484	0.0698591
52080	1.82013	0.0698591
52081	1.81292	0.0698591

52082	1.4516	0.0698591
52083	1.39633	0.0698591
52084	1.67485	0.0698591
52085	1.5344	0.0698591
52086	1.81292	0.0698591
52087	1.67484	0.0698591
52088	1.00982	0.0698591
52089	0.994235	0.0698591
52090	1.21748	0.0698591
52091	1.45159	0.0698591
52092	1.67484	0.0698591
52093	1.55216	2.1
52094	1.34407	2.1
52095	1.34407	2.1
52096	1.52008	2.1
52097	1.81783	2.1
52098	1.99384	2.1
52099	1.90817	2.1
52100	2.03975	2.1
52101	1.97317	2.1
52102	1.81783	2.1
52103	3.85758	2.1
52104	0.599049	2.1
52105	0.660673	2.1
52106	0.785063	2.1
52107	0.924278	2.1
52108	0.760416	2.1
52109	0.760416	2.1
52110	0.719088	2.1
52111	0.694532	2.1
52112	0.522441	2.1
52113	0.796789	2.1
52114	0.655207	2.1
52115	0.886155	2.1
52116	1.97317	2.1
52117	1.81783	2.1
52118	1.65098	2.1
52119	1.91767	2.1
52120	3.7355	2.1
52121	1.15038	2.02877
52122	1.28649	1.9646
52123	1.38986	2.05524
52124	1.38986	2.02829
52125	1.07866	2.05821
52126	1.07866	2.07444
52127	0.664498	0.822482
52128	0.664498	2.1
52129	0.675122	2.1
52130	0.675122	2.1
52131	0.69493	1.04253
52132	0.764429	1.91851
52133	0.623438	2.08306
52134	0.575191	2.1
52135	0.764429	1.84545
52136	0.623438	1.50432
52137	0.575191	2.0767
52138	0.69493	2.06156
52139	1.89226	1.47533

52140	1.89226	0.530558
52141	1.89226	1.79875
52142	1.89226	0.830188
52143	3.78453	0.402033
52144	2.50075	1.91073
52145	2.15276	0.723847
52146	2.24902	1.76719
52147	2.30422	0.678564
52148	4.40178	0.279326
52149	1.70424	2.1
52150	1.34494	2.1
52151	2.15276	0.984448
52152	2.15276	2.09415
52153	2.0793	0.896703
52154	1.63078	1.578
52155	1.89226	0.976021
52156	1.89226	2.1
52157	1.89226	2.1
52158	1.89226	0.722748
52159	1.89226	2.1
52160	1.89226	1.64748
52161	1.89226	1.82884
52162	1.89226	0.934409
52163	1.89226	1.3462
52164	1.89226	0.363369
52165	3.78452	0.37993
52166	2.24902	1.81648
52167	1.91497	0.649
52168	1.97135	2.09384
52169	1.73957	0.45209
52170	3.98859	0.68242
52171	1.73957	0.455131
52172	1.34494	2.1
52173	1.73957	2.1
52174	1.69006	0.546406
52175	1.3126	2.1
52176	1.34848	1.31939
52177	0.946132	0.558107
52178	0.946132	2.1
52179	0.946132	1.30626
52180	0.946132	2.1
52181	0.946132	2.1
52182	0.946132	2.1
52183	0.946131	0.473612
52184	0.946131	0.131591
52185	0.946132	1.05739
52186	0.946132	1.61888
52187	0.946132	1.48926
52188	0.946132	1.89324
52189	1.89226	2.1
52190	1.89226	2.04576
52191	1.89226	2.0979
52192	1.89226	2.026
52193	3.78453	2.08071
52194	1.89226	2.0392
52195	2.82624	1.93003
52196	1.89226	2.04494
52197	2.02536	1.91448

52198	1.89226	2.03995
52199	2.02536	1.86225
52200	2.25027	0.0698591
52201	1.1706	0.0698591
52202	2.02733	0.0698591
52203	2.02733	0.0698591
52204	1.61462	0.0698591
52205	1.69832	0.0698591
52206	1.89226	0.0698591
52207	1.89226	0.0698591
52208	1.89226	0.0698591
52209	1.89226	0.0698591
52210	3.78452	0.0698591
52211	0.946131	0.0698591
52212	0.946131	0.0698591
52213	1.18182	0.0698591
52214	1.18182	0.0698591
52215	0.973819	0.0698591
52216	0.94613	0.0698591
52217	1.18182	0.0698591
52218	0.94613	0.0698591
52219	0.973819	0.0698591
52220	1.18182	0.0698591
52221	0.94613	0.0698591
52222	0.94613	0.0698591
52223	2.23944	2.1
52224	1.52903	2.1
52225	1.35977	2.1
52226	2.04847	2.1
52227	3.5775	2.1
52228	2.23944	2.1
52229	2.34795	2.1
52230	2.34795	2.1
52231	2.48025	2.1
52232	2.04847	2.1
52233	2.18077	2.1
52234	1.89226	2.1
52235	1.89226	2.1
52236	1.89226	2.1
52237	1.89226	2.1
52238	3.78452	2.1
52239	0.946127	2.1
52240	0.946127	2.1
52241	0.946131	2.1
52242	0.946131	2.1
52243	0.946128	2.1
52244	0.946128	2.1
52245	0.94613	2.1
52246	0.94613	2.1
52247	0.946129	2.1
52248	0.946129	2.1
52249	0.946129	2.1
52250	0.946129	2.1
52251	1.26956	2.1
52252	2.04847	2.1
52253	1.49576	2.1
52254	1.22377	2.1
52255	1.91172	2.1

52256	1.50198	2.1
52257	1.48568	2.1
52258	2.34795	2.1
52259	2.04847	2.1
52260	2.04847	2.1
52261	2.21155	2.1
52262	2.21155	2.1
52263	1.89226	2.1
52264	1.89226	2.1
52265	1.89226	2.1
52266	1.89226	2.1
52267	1.89226	2.1
52268	1.89226	2.1
52269	0.810943	2.1
52270	0.810943	2.1
52271	0.810944	2.1
52272	0.810944	2.1
52273	0.810957	2.1
52274	0.810957	2.1
52275	1.89226	2.1
52276	1.35168	2.1
52277	1.08132	2.1
52278	1.08132	2.1
52279	1.0813	2.1
52280	0.946132	0.0698591
52281	1.33855	0.0698591
52282	0.946131	0.0698591
52283	1.38204	0.0715003
52284	0.946131	0.0717527
52285	0.946131	0.070286
52286	1.38204	0.0698591
52287	1.38204	0.0698591
52288	0.946131	0.0698591
52289	0.946131	0.0698591
52290	1.33855	0.0698591
52291	1.38204	0.0699312
52292	1.89226	0.072971
52293	2.76407	0.0717023
52294	1.89226	0.0698591
52295	2.72769	0.0704405
52296	1.89226	0.0698591
52297	2.72769	0.0725685
52298	2.03914	0.0698591
52299	1.67727	0.0698591
52300	1.94831	0.0698591
52301	1.81591	0.0698591
52302	2.32098	0.0698591
52303	2.32098	0.0698591
52304	0.801647	0.0698591
52305	1.11086	0.0698591
52306	0.838634	0.0698591
52307	1.16049	0.0698591
52308	0.977891	0.0698591
52309	0.977891	0.0698591
52310	0.984246	0.0698591
52311	0.984246	0.0698591
52312	1.11086	0.0698591
52313	1.16049	0.0698591

52314	0.801647	0.0698591
52315	0.838634	0.0698591
52316	0.946132	0.0698591
52317	0.946132	0.0710016
52318	0.946131	0.0698591
52319	0.946131	0.0732452
52320	0.946131	0.0735919
52321	0.946131	0.0716866
52322	0.946132	0.0698591
52323	0.946132	0.0698591
52324	0.946131	0.0698591
52325	0.946131	0.0698824
52326	0.946131	0.0698591
52327	0.946131	0.0702592
52328	0.946131	0.0698591
52329	0.946131	0.0744294
52330	0.946131	0.0744208
52331	0.946131	0.0698591
52332	0.946131	0.0746673
52333	0.946131	0.0740558
52334	0.946131	0.0698591
52335	0.946131	0.0698591
52336	0.946131	0.069901
52337	0.946131	0.0706803
52338	0.946131	0.0716429
52339	0.946131	0.0702978
52340	2.0815	2.1
52341	1.20787	2.1
52342	1.92277	2.1
52343	1.3227	2.1
52344	3.29401	2.1
52345	2.16633	2.1
52346	2.13642	2.1
52347	1.40846	2.1
52348	1.40846	2.1
52349	1.3227	2.1
52350	2.08058	2.1
52351	0.938106	2.1
52352	0.790258	2.1
52353	0.742975	2.1
52354	0.742975	2.1
52355	0.874389	2.1
52356	1.17364	2.1
52357	0.525948	2.1
52358	0.52513	2.1
52359	0.553732	2.1
52360	0.876996	2.1
52361	0.530954	2.1
52362	1.095 2.1	
52363	1.00833	2.1
52364	1.70608	2.1
52365	1.33385	2.1
52366	1.33385	2.1
52367	1.07722	2.1
52368	1.07722	2.1
52369	0.953523	0.860846
52370	1.7457	0.66919
52371	1.03218	0.771855

52372	1.03218	0.780147
52373	1.79675	0.760341
52374	1.79675	0.769747
52375	1.13676	0.791186
52376	1.18136	0.545672
52377	0.87285	0.684805
52378	0.898375	0.835597
52379	1.03191	0.651923
52380	1.03191	0.545851
52381	1.0223	0.74716
52382	1.0223	0.85784
52383	1.18136	0.643258
52384	0.898374	0.584781
52385	0.87285	0.651173
52386	1.13676	0.643686
52387	2.36273	0.643128
52388	1.46965	0.500803
52389	1.79675	0.565891
52390	1.79675	0.462225
52391	2.43417	0.670303
52392	2.43417	0.514432
52393	1.4714	2.07871
52394	1.65434	2.09589
52395	1.62322	0.717386
52396	1.86068	2.02442
52397	3.27756	1.76293
52398	1.89226	2.1
52399	1.89226	2.09857
52400	1.89226	0.822238
52401	1.89226	1.61783
52402	3.78453	1.26329
52403	1.89226	1.92437
52404	1.89226	2.1
52405	1.89226	1.09371
52406	1.89226	2.1
52407	3.78453	2.08465
52408	1.65434	1.92534
52409	1.60647	2.1
52410	1.86068	1.62757
52411	1.82649	2.1
52412	3.48083	2.1
52413	1.62322	2.09134
52414	1.86068	2.097
52415	1.84368	0.986939
52416	2.18862	1.59528
52417	3.81184	1.51806
52418	1.89226	2.09843
52419	1.89226	2.01965
52420	1.89226	0.848446
52421	1.89226	1.73717
52422	3.78453	1.51535
52423	1.89226	1.7716
52424	1.89226	2.1
52425	1.89226	1.16686
52426	1.89226	2.1
52427	3.78453	2.09877
52428	1.86068	1.81418
52429	1.82649	2.1

52430	2.18862	1.0707
52431	2.1128	2.1
52432	4.01511	2.06281
52433	1.74351	2.1
52434	1.13854	2.1
52435	1.40233	2.1
52436	1.922 2.1	
52437	3.14584	2.1
52438	1.922 2.1	
52439	2.00775	2.1
52440	1.7435	2.1
52441	2.09224	2.1
52442	2.00775	2.1
52443	2.09295	2.1
52444	1.69858	2.1
52445	1.922 2.1	
52446	1.40233	2.1
52447	1.40233	2.1
52448	2.18238	2.1
52449	2.18238	2.1
52450	1.00387	2.1
52451	1.10886	2.1
52452	0.961001	2.1
52453	1.09119	2.1
52454	1.02868	2.1
52455	1.02868	2.1
52456	1.04118	2.1
52457	1.04118	2.1
52458	1.10886	2.1
52459	1.09119	2.1
52460	0.960999	2.1
52461	1.00388	2.1
52462	0.885958	0.193642
52463	0.885958	0.167077
52464	0.875826	0.132478
52465	0.875826	0.175555
52466	0.913965	0.171077
52467	0.598609	0.207315
52468	1.16317	0.132662
52469	0.868115	0.165495
52470	0.868115	0.174005
52471	0.598609	0.178184
52472	1.16317	0.167538
52473	0.913965	0.167422
52474	0.946131	0.264289
52475	0.946131	0.246071
52476	0.946131	0.196829
52477	0.946131	0.170932
52478	0.946131	0.198619
52479	0.946131	0.255016
52480	0.946131	0.246932
52481	0.946131	0.172972
52482	0.946131	0.211743
52483	0.946131	0.20797
52484	0.946131	0.222845
52485	0.946131	0.223909
52486	0.946133	0.0698591
52487	0.946133	0.0698591

52488	1.06246	0.0698591
52489	1.06246	0.0698591
52490	0.973851	0.0698591
52491	0.946133	0.0698591
52492	1.06246	0.0698591
52493	0.946132	0.0698591
52494	0.97385	0.0698591
52495	1.06246	0.0698591
52496	0.946133	0.0698591
52497	0.946133	0.0698591
52498	1.89226	0.0698591
52499	1.89226	0.0698591
52500	1.89226	0.0698591
52501	1.89226	0.0698591
52502	1.89226	0.0698591
52503	1.89226	0.0698591
52504	0.946131	0.0698591
52505	0.946131	0.0698591
52506	0.946127	0.0698591
52507	0.946127	0.0698591
52508	0.946131	0.0698591
52509	0.946131	0.0698591
52510	0.946127	0.0698591
52511	0.946127	0.0698591
52512	0.946128	0.0698591
52513	0.946128	0.0698591
52514	0.946129	0.0698591
52515	0.946129	0.0698591
52516	0.946132	0.0698591
52517	0.946132	0.0698591
52518	1.32446	0.0698591
52519	0.794912	0.0698591
52520	1.32429	0.0698591
52521	0.946131	0.0698591
52522	1.32446	0.0698591
52523	0.94613	0.0698591
52524	1.32429	0.0698591
52525	0.794915	0.0698591
52526	0.94613	0.0698591
52527	0.94613	0.0698591
52528	1.89226	0.0698591
52529	2.36365	0.0698591
52530	2.64857	0.0698591
52531	1.89226	0.0698591
52532	2.64857	0.0698591
52533	1.89226	0.0698591
52534	1.18463	2.1
52535	1.27174	2.1
52536	0.946129	2.1
52537	0.946129	2.1
52538	0.946131	2.1
52539	0.946131	2.1
52540	1.27174	2.1
52541	1.27174	2.1
52542	1.18463	2.1
52543	0.94613	2.1
52544	0.946131	2.1
52545	1.27174	2.1

52546	2.57997	2.1
52547	1.89226	2.1
52548	1.89226	2.1
52549	1.89226	2.1
52550	2.41915	2.1
52551	2.41915	2.1
52552	1.89226	2.1
52553	1.89226	2.1
52554	1.89226	2.1
52555	1.89226	2.1
52556	3.78452	2.1
52557	1.08807	2.1
52558	1.18463	2.1
52559	0.946129	2.1
52560	0.946129	2.1
52561	0.946131	2.1
52562	0.946131	2.1
52563	1.18463	2.1
52564	1.18463	2.1
52565	0.946131	2.1
52566	1.08807	2.1
52567	1.18463	2.1
52568	0.94613	2.1
52569	1.07017	2.1
52570	1.07017	2.1
52571	0.946131	2.1
52572	0.946131	2.1
52573	0.946132	2.1
52574	0.946132	2.1
52575	1.20957	2.1
52576	1.07017	2.1
52577	1.07017	2.1
52578	0.946132	2.1
52579	0.946131	2.1
52580	1.20957	2.1
52581	1.89226	2.1
52582	1.89226	2.1
52583	1.89226	2.1
52584	1.89226	2.1
52585	1.89226	2.1
52586	1.89226	2.1
52587	1.16181	0.0698591
52588	1.0227	0.0698591
52589	1.0227	0.0698591
52590	0.970959	0.0698591
52591	1.24295	0.0698591
52592	1.19121	0.0698591
52593	1.12076	2.1
52594	2.53614	2.1
52595	1.28413	2.1
52596	1.28413	2.1
52597	2.36423	2.1
52598	2.36423	2.1
52599	1.89226	2.1
52600	1.89226	2.1
52601	1.89226	2.1
52602	1.89226	2.1
52603	3.78452	2.1

52604	1.22557	2.1
52605	2.771 2.1	
52606	2.73012	2.1
52607	2.73012	2.1
52608	1.33356	2.1
52609	1.33356	2.1
52610	0.998682	2.1
52611	2.36423	2.1
52612	1.12076	2.1
52613	1.12076	2.1
52614	2.13757	2.1
52615	2.13757	2.1
52616	1.89226	2.1
52617	1.89226	2.1
52618	1.89226	2.1
52619	1.89226	2.1
52620	1.89226	2.1
52621	1.89226	2.1
52622	1.13987	2.1
52623	2.73011	2.1
52624	2.70875	2.1
52625	2.70875	2.1
52626	1.22557	2.1
52627	1.22557	2.1
52628	1.89227	2.1
52629	2.79243	2.1
52630	2.6625	2.1
52631	2.6625	2.1
52632	1.89227	2.1
52633	1.89227	2.1
52634	1.89226	2.1
52635	2.55971	2.1
52636	1.89226	2.1
52637	1.89226	2.1
52638	2.68704	2.1
52639	2.68704	2.1
52640	2.22043	2.1
52641	2.38975	1.47124
52642	2.28696	1.90131
52643	2.48682	1.18791
52644	4.67671	1.7036
52645	2.38976	0.820787
52646	2.31988	2.01617
52647	2.48683	0.913947
52648	2.4594	1.73138
52649	4.84915	0.842643
52650	2.28696	2.09121
52651	2.48682	1.91932
52652	2.33379	2.1
52653	2.56149	0.851343
52654	4.84845	1.92694
52655	2.48683	2.01428
52656	2.4594	1.93177
52657	2.5615	0.544121
52658	2.57957	1.14358
52659	5.0209	0.778366
52660	1.06659	2.1
52661	1.04291	2.1

52662	1.36595	2.09842
52663	1.36595	2.1
52664	1.02761	2.1
52665	1.32697	2.09672
52666	0.946132	2.04684
52667	0.946132	2.02964
52668	1.36956	2.1
52669	1.1229	2.1
52670	1.1229	2.02717
52671	1.1229	2.1
52672	0.946132	1.9496
52673	0.946132	2.1
52674	0.946132	2.09772
52675	1.36956	2.09762
52676	1.1229	2.09956
52677	0.946133	2.1
52678	0.946133	1.41275
52679	0.946133	1.39374
52680	0.946128	2.09353
52681	0.946128	2.06755
52682	0.946131	2.03404
52683	0.946131	1.64127
52684	0.946131	1.11889
52685	0.946131	2.0634
52686	0.94613	1.9277
52687	0.94613	1.90214
52688	0.946131	1.905
52689	0.946131	1.92435
52690	0.966427	1.44291
52691	0.802265	1.8366
52692	0.803714	2.05199
52693	0.808476	1.87719
52694	1.94371	2.04642
52695	1.36851	2.02852
52696	0.853864	2.08073
52697	1.26635	1.87903
52698	1.09085	1.67433
52699	0.857653	1.96599
52700	0.630243	1.93867
52701	1.20132	0.0698591
52702	1.24295	0.0698591
52703	1.24295	0.0698591
52704	1.16182	0.0698591
52705	1.28465	0.0698591
52706	1.20351	0.0698591
52707	0.600658	0.0698591
52708	0.642323	0.0698591
52709	0.54424	0.0698591
52710	0.576779	0.0698591
52711	0.590985	0.0698591
52712	0.590985	0.0698591
52713	0.586452	0.0698591
52714	0.586452	0.0698591
52715	0.642323	0.0698591
52716	0.576779	0.0698591
52717	0.600658	0.0698591
52718	0.54424	0.0698591
52719	1.28463	0.0698591

52720	1.24294	0.0698591
52721	1.12328	0.0698591
52722	1.15505	0.0698591
52723	2.39798	0.0698591
52724	1.98431	0.0986568
52725	2.18735	0.0715397
52726	1.98431	0.071669
52727	1.70303	0.0754407
52728	1.70302	0.172261
52729	1.37788	0.161709
52730	1.89226	0.15402
52731	2.37417	0.0814666
52732	1.89226	0.113521
52733	2.18735	0.0702309
52734	1.89226	0.206981
52735	2.18735	0.156589
52736	1.89226	0.405914
52737	1.89226	2.07493
52738	1.89226	0.980127
52739	1.89226	1.50601
52740	3.78452	1.32622
52741	1.8613	1.85475
52742	2.03576	2.0829
52743	1.61652	0.986062
52744	1.83082	2.1
52745	3.65228	1.96466
52746	2.03576	2.08207
52747	2.05257	2.1
52748	1.83082	2.1
52749	1.88487	2.1
52750	3.92064	2.1
52751	1.89226	2.0696
52752	1.89226	2.1
52753	1.89226	1.60653
52754	1.89226	2.1
52755	3.78453	2.1
52756	0.946131	2.09643
52757	0.946131	0.959839
52758	0.94613	0.324613
52759	0.94613	1.8326
52760	0.946129	1.57123
52761	0.946129	2.09149
52762	0.946132	1.24441
52763	0.946132	0.322932
52764	0.94613	1.23207
52765	0.94613	1.43118
52766	0.946131	1.12777
52767	0.946131	1.17249
52768	0.804708	2.1
52769	0.804708	1.81759
52770	0.815274	2.09195
52771	0.815274	1.0771
52772	0.83277	2.09111
52773	0.915407	2.1
52774	0.80826	1.7839
52775	0.704574	1.03304
52776	0.832771	2.07999
52777	0.704573	2.08486

52778	0.808259	2.03472
52779	0.915409	2.04226
52780	1.83082	2.1
52781	1.88487	2.1
52782	1.66555	2.09601
52783	1.75835	2.1
52784	3.55042	2.1
52785	0.810947	1.64863
52786	0.810947	2.1
52787	0.810947	1.59561
52788	0.810947	2.09005
52789	0.810934	2.08771
52790	0.810934	2.08896
52791	1.89227	2.1
52792	1.3517	2.1
52793	1.08132	2.09702
52794	1.08132	2.1
52795	1.08133	2.1
52796	1.89226	2.09518
52797	1.70303	2.09876
52798	1.89226	2.1
52799	2.24712	2.1
52800	2.07363	2.1
52801	1.74617	2.09867
52802	1.89226	2.09936
52803	1.89226	1.77409
52804	1.89226	1.67445
52805	1.89226	2.08082
52806	3.78452	1.9685
52807	1.50108	2.07994
52808	1.43894	1.97382
52809	1.8322	1.59567
52810	1.40967	2.09518
52811	1.50108	2.00449
52812	1.07854	2.1
52813	1.89226	1.39344
52814	1.89226	2.07221
52815	1.89226	2.03983
52816	1.89226	1.06804
52817	3.78452	1.92791
52818	1.89226	2.1
52819	1.89226	1.96836
52820	1.89226	1.99433
52821	1.89226	2.1
52822	3.78453	2.05744
52823	1.54457	2.1
52824	1.35097	1.9965
52825	1.70754	1.93286
52826	1.29629	2.1
52827	1.54457	2.06971
52828	1.0305	2.1
52829	0.731763	2.08048
52830	1.00043	2.04693
52831	1.13883	1.35436
52832	0.939026	1.62642
52833	1.04003	1.30277
52834	0.853868	2.1
52835	0.927188	2.05986

52836	0.927188	1.05394
52837	0.861565	1.75161
52838	1.13882	1.87522
52839	0.731769	1.97641
52840	0.89319	1.99914
52841	1.46568	2.1
52842	1.20761	2.1
52843	1.51916	2.1
52844	1.36261	2.1
52845	2.72677	2.1
52846	1.51916	2.1
52847	1.60532	2.1
52848	1.36261	2.1
52849	1.36261	2.1
52850	1.64892	2.1
52851	1.64892	2.1
52852	1.3626	2.1
52853	0.98488	2.1
52854	1.60532	2.1
52855	1.32414	2.1
52856	2.68675	2.1
52857	1.78711	2.1
52858	1.14825	2.1
52859	2.16535	2.1
52860	1.6067	2.1
52861	3.3136	2.1
52862	1.89226	2.1
52863	1.89226	2.1
52864	1.89226	2.1
52865	1.89226	2.1
52866	3.78452	2.1
52867	2.34552	2.1
52868	1.53521	2.1
52869	1.53521	2.1
52870	1.60636	2.1
52871	1.92797	2.1
52872	1.99589	2.1
52873	1.34771	2.1
52874	1.34771	2.1
52875	0.946128	2.1
52876	0.946128	2.1
52877	1.3477	2.1
52878	1.31776	2.1
52879	0.946131	2.1
52880	0.946131	2.1
52881	0.94613	2.1
52882	1.31776	2.1
52883	0.946129	2.1
52884	1.3477	2.1
52885	1.89226	2.1
52886	1.89226	2.1
52887	1.89226	2.1
52888	1.89226	2.1
52889	3.78452	2.1
52890	0.92843	2.1
52891	0.672114	2.1
52892	0.89514	2.1
52893	0.89514	2.1

52894	0.720104	2.1
52895	0.755753	2.1
52896	0.789158	2.1
52897	0.967302	2.1
52898	1.06565	2.1
52899	0.7397	2.1
52900	0.758893	2.1
52901	0.94672	2.1
52902	1.89226	2.1
52903	2.588	2.1
52904	2.588	2.1
52905	2.63552	2.1
52906	1.89226	2.1
52907	1.93978	2.1
52908	1.49347	0.0698591
52909	2.67757	0.092252
52910	1.80061	0.0717383
52911	2.27056	0.0751007
52912	4.07117	0.0698591
52913	1.1016	0.0698591
52914	2.27057	0.0704541
52915	1.49348	0.0734169
52916	1.81893	0.0777953
52917	1.49348	0.0698591
52918	1.81893	0.0698591
52919	1.81893	0.0705096
52920	2.82339	0.109813
52921	2.27057	0.0759824
52922	2.25405	0.077272
52923	4.52462	0.0707851
52924	1.89226	0.0787042
52925	1.89226	0.123365
52926	1.89226	0.13838
52927	1.89226	0.0881787
52928	1.89226	0.140208
52929	1.89226	0.102441
52930	1.89226	0.0698591
52931	1.89226	0.0995102
52932	1.89226	0.0706655
52933	1.89226	0.0728817
52934	3.78453	0.0698591
52935	1.89226	0.119822
52936	1.89226	0.157377
52937	1.89226	0.0759167
52938	1.89226	0.195996
52939	3.78452	0.1841
52940	1.89226	0.143266
52941	1.89226	0.359566
52942	1.89226	0.166784
52943	1.89226	0.211836
52944	3.78451	0.140638
52945	1.45633	2.1
52946	1.34678	2.1
52947	1.34678	2.1
52948	1.53735	2.1
52949	1.234	2.1
52950	1.42457	2.1
52951	1.89226	2.1

52952	1.89226	2.1
52953	1.89226	2.1
52954	1.89226	2.1
52955	1.89226	2.1
52956	1.89226	2.1
52957	1.09483	2.1
52958	1.34678	2.1
52959	1.234	2.1
52960	1.234	2.1
52961	1.19	2.1
52962	1.19	2.1
52963	0.946131	2.1
52964	0.946131	2.1
52965	0.946131	2.1
52966	0.946131	2.1
52967	0.946131	2.1
52968	0.946131	2.1
52969	0.946132	2.1
52970	0.946132	2.1
52971	0.946132	2.1
52972	0.946132	2.1
52973	0.946131	2.1
52974	0.946131	2.1
52975	2.82263	2.1
52976	1.89226	2.1
52977	2.71441	2.1
52978	2.71441	2.1
52979	1.89226	2.1
52980	1.89226	2.1
52981	1.1412	2.1
52982	1.1412	2.1
52983	1.14626	2.1
52984	1.14626	2.1
52985	1.28511	2.1
52986	1.34086	2.1
52987	0.992242	2.1
52988	0.946593	2.1
52989	0.992242	2.1
52990	1.34086	2.1
52991	0.946594	2.1
52992	1.28511	2.1
52993	1.18954	2.1
52994	1.18954	2.1
52995	1.21477	2.1
52996	1.21477	2.1
52997	1.41207	2.1
52998	1.34086	2.1
52999	1.01306	2.1
53000	0.992242	2.1
53001	1.01306	2.1
53002	1.41207	2.1
53003	0.992242	2.1
53004	1.34086	2.1
53005	1.09044	2.1
53006	1.44472	1.94021
53007	1.2955	2.1
53008	1.2955	2.1
53009	1.22025	2.1

53010	1.22025	2.1
53011	0.810944	2.04742
53012	0.810944	2.1
53013	0.810946	2.04455
53014	0.810946	0.749581
53015	0.810959	1.98241
53016	0.810959	1.99258
53017	1.89226	1.98239
53018	1.35167	2.02622
53019	1.08132	2.1
53020	1.08132	0.805868
53021	1.0813	2.01753
53022	1.41894	0.523936
53023	1.28953	0.132897
53024	0.946132	0.598425
53025	0.946132	1.97966
53026	0.946133	0.778696
53027	0.946133	0.18684
53028	1.41894	1.48338
53029	1.41894	1.10385
53030	1.28953	0.474143
53031	0.946132	0.524788
53032	0.946132	0.992348
53033	1.41894	0.595115
53034	1.89226	2.1
53035	2.15444	2.1
53036	1.93239	2.1
53037	1.89226	2.1
53038	2.19457	2.1
53039	2.15444	2.1
53040	1.89226	2.1
53041	1.89226	2.1
53042	1.89226	2.1
53043	1.89226	2.1
53044	1.89226	2.1
53045	1.89226	2.1
53046	0.938507	2.1
53047	0.938507	2.1
53048	1.07871	2.1
53049	1.15801	2.1
53050	1.03236	2.1
53051	1.14766	2.1
53052	0.946133	2.1
53053	0.946133	2.1
53054	0.946131	2.1
53055	1.07009	2.1
53056	0.946131	2.1
53057	1.03237	2.1
53058	1.89226	2.1
53059	2.19457	2.1
53060	1.94492	2.1
53061	1.89226	2.1
53062	2.24723	2.1
53063	2.19457	2.1
53064	0.946132	2.1
53065	0.946132	2.1
53066	0.946131	2.1
53067	0.946131	2.1

53068	0.946132	2.1
53069	0.946132	2.1
53070	0.946132	2.1
53071	0.946132	2.1
53072	0.946132	2.1
53073	0.946132	2.1
53074	0.946132	2.1
53075	0.946132	2.1
53076	2.21345	2.1
53077	1.89226	2.1
53078	1.89226	2.1
53079	2.14017	2.1
53080	2.14017	2.1
53081	1.89226	2.1
53082	1.89226	0.281315
53083	1.89226	1.22503
53084	1.89226	0.268081
53085	1.85439	1.13722
53086	2.23961	0.197244
53087	2.19479	0.596481
53088	1.2916	0.241838
53089	1.2916	0.231302
53090	0.946128	0.265035
53091	0.946128	0.239587
53092	1.2916	0.177617
53093	0.946131	0.252488
53094	0.946126	1.0647
53095	1.1198	0.164373
53096	1.2916	0.183132
53097	1.1198	0.385481
53098	0.946128	0.385756
53099	0.946128	0.34019
53100	1.89226	0.50878
53101	2.71262	0.366371
53102	1.89226	0.384119
53103	2.49959	0.465607
53104	4.39185	0.509726
53105	2.71262	0.356318
53106	1.34592	0.382903
53107	2.49959	0.435022
53108	1.14721	0.34258
53109	3.84551	0.329588
53110	0.946131	0.394924
53111	0.946131	0.433245
53112	1.11247	0.328882
53113	1.11247	0.297499
53114	1.24979	0.31498
53115	1.11247	0.367328
53116	0.946129	0.448277
53117	0.946129	0.341758
53118	0.946131	0.318932
53119	1.11247	0.262515
53120	0.946131	0.274795
53121	1.24979	0.31692
53122	0.946131	2.1
53123	0.946131	2.1
53124	0.94613	2.1
53125	0.94613	2.1

53126	0.946132	2.1
53127	0.946132	2.1
53128	0.946129	2.1
53129	0.946129	2.1
53130	0.946131	2.1
53131	0.946131	2.1
53132	0.946131	2.1
53133	0.946131	2.1
53134	2.77816	2.1
53135	1.89226	2.1
53136	1.89226	2.1
53137	1.89226	2.1
53138	3.78453	2.1
53139	1.89226	2.1
53140	1.89226	2.1
53141	1.89226	2.1
53142	1.89226	2.1
53143	1.89226	2.1
53144	1.89226	2.1
53145	1.89226	2.1
53146	1.89226	2.1
53147	1.89226	2.1
53148	1.89226	2.1
53149	3.78452	2.1
53150	1.55698	2.1
53151	1.89226	2.1
53152	1.89226	2.1
53153	1.89226	2.1
53154	1.8575	2.1
53155	1.8575	2.1
53156	1.89226	2.1
53157	1.89226	2.1
53158	1.89226	2.1
53159	1.89226	2.1
53160	1.89226	2.1
53161	1.89226	2.1
53162	1.89227	2.1
53163	1.89227	2.1
53164	1.89227	2.1
53165	1.89227	2.1
53166	1.89227	2.1
53167	1.89227	2.1
53168	1.35509	2.1
53169	2.06238	2.1
53170	2.12244	2.1
53171	2.12244	2.1
53172	1.34817	2.1
53173	1.34817	2.1
53174	0.946126	2.1
53175	0.946126	2.1
53176	0.946131	2.1
53177	0.946131	2.1
53178	0.946127	2.1
53179	0.946127	2.1
53180	0.94613	2.1
53181	0.94613	2.1
53182	0.946128	2.1
53183	0.946128	2.1

53184	0.946128	2.1
53185	0.946128	2.1
53186	1.89226	2.1
53187	1.89226	2.1
53188	1.89226	2.1
53189	1.89226	2.1
53190	1.89226	2.1
53191	1.89226	2.1
53192	1.16742	2.1
53193	1.16742	2.1
53194	1.18122	2.1
53195	1.18122	2.1
53196	1.31745	2.1
53197	1.2597	2.1
53198	1.03119	2.1
53199	1.06122	2.1
53200	1.06122	2.1
53201	1.31745	2.1
53202	1.03119	2.1
53203	1.2597	2.1
53204	1.36849	2.1
53205	2.12244	2.1
53206	2.20092	2.1
53207	2.20092	2.1
53208	1.35509	2.1
53209	1.35509	2.1
53210	1.89226	2.1
53211	1.89226	2.1
53212	1.89226	2.1
53213	1.89226	2.1
53214	1.89226	2.1
53215	1.89226	2.1
53216	1.89226	2.1
53217	1.89226	2.1
53218	1.89226	2.1
53219	1.89226	2.1
53220	3.78452	2.1
53221	1.21731	2.1
53222	1.21731	2.1
53223	1.23412	2.1
53224	1.23412	2.1
53225	1.39021	2.1
53226	1.31745	2.1
53227	1.06122	2.1
53228	1.10046	2.1
53229	1.10046	2.1
53230	1.39021	2.1
53231	1.06122	2.1
53232	1.31745	2.1
53233	2.16503	1.39536
53234	1.78252	1.7314
53235	1.77293	1.26746
53236	1.56839	2.09976
53237	2.36957	0.66264
53238	2.16503	2.1
53239	1.89223	1.43357
53240	1.89223	2.1
53241	1.89223	2.05923

53242	1.89223	2.08676
53243	3.78447	2.0954
53244	2.3696	1.66154
53245	1.78254	1.80667
53246	2.63298	0.311091
53247	2.07312	2.08192
53248	4.44272	1.5308
53249	1.89226	2.09325
53250	1.89226	2.07982
53251	1.89226	1.98462
53252	1.89226	2.1
53253	1.89226	1.26253
53254	1.89226	2.1
53255	1.89226	2.08811
53256	1.89226	0.703648
53257	1.89226	2.07266
53258	1.89226	0.36609
53259	3.78452	1.13518
53260	2.26369	2.08676
53261	2.2168	0.561892
53262	2.15617	2.02332
53263	2.10864	0.289755
53264	4.37234	1.19705
53265	2.2168	0.408741
53266	1.94499	0.861571
53267	1.94499	0.712885
53268	2.04737	0.764009
53269	2.10864	0.207766
53270	2.21103	0.977648
53271	1.89226	0.774286
53272	1.89226	0.730403
53273	1.89226	0.846174
53274	1.89226	0.449363
53275	1.89226	1.00901
53276	1.89226	0.315174
53277	1.89226	2.0643
53278	1.89226	0.768414
53279	1.89226	1.12242
53280	1.89226	2.0322
53281	1.89226	0.507676
53282	1.89226	2.06786
53283	1.02995	0.618648
53284	1.02995	2.06308
53285	1.03212	0.710028
53286	1.03212	1.96142
53287	0.988256	0.427361
53288	1.05432	0.332846
53289	1.00775	1.95838
53290	1.07809	2.00829
53291	1.00775	1.12392
53292	0.988257	1.13456
53293	1.07808	1.29916
53294	1.05432	1.18828
53295	2.10864	0.663485
53296	1.94499	0.963661
53297	1.97652	0.463314
53298	1.79681	0.932163
53299	3.9215	0.286751

53300	0.810943	0.763766
53301	0.810943	1.16003
53302	0.810943	0.490614
53303	0.810943	0.53302
53304	0.810958	0.201757
53305	0.810958	0.318926
53306	1.89226	0.811183
53307	1.35168	0.330282
53308	1.08132	0.748719
53309	1.08132	0.680948
53310	1.0813	0.263304
53311	0.657403	0.0698591
53312	1.35403	0.0698591
53313	1.24038	0.0698591
53314	0.499144	0.0706292
53315	1.24039	0.070062
53316	1.35403	0.0708236
53317	0.657402	0.0698591
53318	0.499144	0.0698591
53319	0.915415	0.0698591
53320	0.915415	0.0733281
53321	0.937755	0.0698591
53322	0.937755	0.0701334
53323	1.72526	2.1
53324	1.17989	2.1
53325	1.96035	2.1
53326	1.47412	2.1
53327	3.14024	2.1
53328	1.89226	1.88598
53329	1.89226	2.1
53330	1.89226	2.1
53331	1.89226	1.21198
53332	3.78452	2.1
53333	0.946131	0.717782
53334	0.946131	1.4418
53335	1.25764	2.05764
53336	1.25764	2.0749
53337	1.07087	0.717313
53338	1.25765	1.4773
53339	0.946128	1.91186
53340	0.946128	0.682828
53341	0.94613	1.02209
53342	1.25765	1.44088
53343	1.07086	1.55183
53344	0.946129	1.402
53345	1.96035	2.1
53346	1.32668	2.1
53347	2.29301	2.1
53348	1.70303	2.1
53349	3.56428	2.1
53350	1.89226	2.1
53351	1.89226	1.41292
53352	1.89226	1.74136
53353	1.89226	2.1
53354	3.78452	1.37601
53355	0.897738	0.298371
53356	0.897738	0.325715
53357	0.946102	0.258537

53358	0.946102	0.196797
53359	1.11247	0.251935
53360	0.89771	0.197547
53361	0.94613	0.253023
53362	0.94613	0.340233
53363	0.897709	0.309138
53364	0.946131	0.317293
53365	0.946131	0.37216
53366	1.11247	0.297715
53367	1.89226	2.1
53368	1.89226	2.02254
53369	1.89226	2.08627
53370	1.89226	2.06549
53371	1.89226	2.08784
53372	1.89226	2.07023
53373	2.15429	2.1
53374	1.89226	2.1
53375	1.89226	2.1
53376	1.89226	2.09667
53377	2.27074	2.1
53378	2.27074	2.0954
53379	0.993311	1.64213
53380	0.993311	1.94997
53381	0.904322	2.1
53382	0.904322	2.03095
53383	0.803324	2.06493
53384	1.36183	1.57366
53385	0.916274	2.09836
53386	0.535808	1.84878
53387	0.535808	1.96002
53388	0.803322	1.97386
53389	0.916276	1.95757
53390	1.36183	1.95334
53391	1.89226	2.1
53392	1.89226	1.97491
53393	1.89226	2.08967
53394	1.89226	2.09495
53395	1.89226	2.09827
53396	1.89226	2.09956
53397	2.11955	2.1
53398	2.62097	2.1
53399	1.85542	2.1
53400	2.39016	2.1
53401	4.50971	2.1
53402	2.39016	2.1
53403	2.20242	2.1
53404	2.39016	2.1
53405	1.64008	2.1
53406	1.85542	2.1
53407	1.64008	2.1
53408	1.79667	2.1
53409	1.28267	2.1
53410	1.28267	2.1
53411	1.40117	2.1
53412	1.74514	2.1
53413	1.83994	2.1
53414	0.575914	2.1
53415	0.625292	2.1

53416	0.949144	2.1
53417	0.882782	2.1
53418	0.773697	2.1
53419	0.773697	2.1
53420	0.786497	2.1
53421	0.786494	2.1
53422	0.605477	2.1
53423	0.949148	2.1
53424	0.625291	2.1
53425	0.862374	2.1
53426	1.89226	2.1
53427	2.44918	2.1
53428	2.4616	2.1
53429	2.44918	2.1
53430	1.90468	2.1
53431	1.89226	2.1
53432	1.89226	2.1
53433	1.89226	2.1
53434	1.89226	2.1
53435	1.89226	2.1
53436	3.78453	2.1
53437	0.946128	2.1
53438	0.946128	2.1
53439	1.23631	2.1
53440	1.23631	2.1
53441	0.946131	2.1
53442	0.946131	2.1
53443	1.3147	2.1
53444	1.23631	2.1
53445	1.3147	2.1
53446	0.946131	2.1
53447	1.23631	2.1
53448	0.94613	2.1
53449	1.89226	2.1
53450	2.4616	2.1
53451	2.4717	2.1
53452	2.4616	2.1
53453	1.90237	2.1
53454	1.89226	2.1
53455	0.946132	2.1
53456	0.946132	2.1
53457	0.946131	2.1
53458	0.946131	2.1
53459	0.946132	2.1
53460	0.946132	2.1
53461	0.946131	2.1
53462	0.946131	2.1
53463	0.946131	2.1
53464	0.946131	2.1
53465	0.946131	2.1
53466	0.946131	2.1
53467	0.946128	2.1
53468	0.946128	2.1
53469	1.3147	2.1
53470	1.3147	2.1
53471	0.94613	2.1
53472	0.94613	2.1
53473	1.3419	2.1

53474	1.3147	2.1
53475	1.3419	2.1
53476	0.94613	2.1
53477	1.3147	2.1
53478	0.94613	2.1
53479	1.89226	2.1
53480	1.89226	2.1
53481	1.89226	2.1
53482	1.89226	2.1
53483	3.78452	2.1
53484	2.3333	2.1
53485	1.3864	2.1
53486	1.3864	2.1
53487	1.4628	2.1
53488	1.95763	2.1
53489	2.03403	2.1
53490	1.294 2.1	
53491	1.294 2.1	
53492	0.946128	2.1
53493	0.946128	2.1
53494	1.294 2.1	
53495	1.27474	2.1
53496	0.946131	2.1
53497	0.946131	2.1
53498	0.946129	2.1
53499	1.27474	2.1
53500	1.294 2.1	
53501	0.94613	2.1
53502	1.89226	2.1
53503	1.89226	2.1
53504	1.89226	2.1
53505	1.89226	2.1
53506	1.89226	2.1
53507	1.89226	2.1
53508	0.808088	2.1
53509	0.808088	2.1
53510	0.815623	2.1
53511	0.815623	2.1
53512	0.660053	2.1
53513	0.693199	2.1
53514	0.930512	2.1
53515	0.978817	2.1
53516	0.660053	2.1
53517	0.930513	2.1
53518	0.693198	2.1
53519	0.978817	2.1
53520	1.27474	2.1
53521	1.27474	2.1
53522	0.946128	2.1
53523	0.946128	2.1
53524	1.26221	2.1
53525	1.27474	2.1
53526	0.946131	2.1
53527	0.946131	2.1
53528	0.94613	2.1
53529	1.26221	2.1
53530	0.946129	2.1
53531	1.27474	2.1

53532	0.90207	0.0698591
53533	0.90207	0.0698591
53534	0.895719	0.0698591
53535	0.895719	0.0698591
53536	0.927855	0.0698591
53537	1.2493	0.0698591
53538	0.85723	0.0698591
53539	0.548484	0.0698591
53540	0.548485	0.0698591
53541	0.927855	0.0698591
53542	0.85723	0.0698591
53543	1.2493	0.0698591
53544	1.41319	0.461168
53545	1.22482	0.290603
53546	1.46629	0.327473
53547	1.92622	0.508036
53548	3.15104	0.423647
53549	1.02305	0.203269
53550	1.22482	0.290281
53551	1.80336	0.292419
53552	1.44426	0.20426
53553	2.66908	0.216393
53554	1.3268	2.1
53555	0.999489	2.1
53556	1.44091	2.1
53557	1.14657	2.1
53558	2.4404	2.1
53559	1.89226	2.1
53560	1.89226	2.1
53561	1.89226	2.1
53562	1.89226	2.1
53563	3.78452	2.1
53564	1.89226	2.1
53565	2.28605	2.1
53566	2.43157	2.1
53567	2.43157	2.1
53568	1.89226	2.1
53569	1.89226	2.1
53570	1.60647	2.1
53571	1.44091	2.1
53572	1.31346	2.1
53573	1.31346	2.1
53574	1.14657	2.1
53575	1.44091	2.1
53576	1.89226	2.1
53577	1.89226	2.1
53578	1.89226	2.1
53579	1.89226	2.1
53580	3.78452	2.1
53581	0.946132	2.1
53582	0.946132	2.1
53583	1.3048	2.1
53584	1.3048	2.1
53585	1.3048	2.1
53586	1.21578	2.1
53587	0.946132	2.1
53588	0.946132	2.1
53589	1.3048	2.1

53590	0.946132	2.1
53591	0.946132	2.1
53592	1.21578	2.1
53593	1.89227	0.0698591
53594	1.89227	0.0698591
53595	1.89227	0.0698591
53596	1.89227	0.0698591
53597	3.78453	0.0698591
53598	1.6671	0.0698591
53599	1.87338	0.0698591
53600	1.6671	0.0698591
53601	2.02098	0.0698591
53602	1.38328	0.0698591
53603	1.73715	0.0698591
53604	1.89226	0.0698591
53605	1.89226	0.0698591
53606	1.89226	0.0698591
53607	1.89226	0.0698591
53608	3.78453	0.0698591
53609	1.6671	0.0698591
53610	1.38328	0.0698591
53611	1.35562	0.0698591
53612	1.01012	0.0698591
53613	2.67722	0.0698591
53614	0.819377	0.0698591
53615	0.647925	0.0698591
53616	1.17802	0.0698591
53617	1.33174	0.0698591
53618	0.98543	0.0698591
53619	0.98543	0.0698591
53620	0.994237	0.0698591
53621	0.994237	0.0698591
53622	1.17802	0.0698591
53623	0.647926	0.0698591
53624	1.33174	0.0698591
53625	0.819377	0.0698591
53626	1.27806	0.0698591
53627	1.68264	0.0698591
53628	1.44593	0.0698591
53629	1.878	0.0698591
53630	3.15606	0.0698591
53631	1.878	0.0698591
53632	1.9898	0.0698591
53633	1.878	0.0698591
53634	1.68264	0.0698591
53635	2.30533	0.0698591
53636	2.10997	0.0698591
53637	0.814587	0.0698591
53638	0.814596	0.0698591
53639	0.82775	0.0698591
53640	0.82775	0.0698591
53641	1.0107	0.0698591
53642	0.91549	0.0698591
53643	0.741058	0.0698591
53644	0.714748	0.0698591
53645	0.714758	0.0698591
53646	1.03225	0.0698591
53647	0.704864	0.0698591

53648	0.915488	0.0698591
53649	1.90347	0.0698591
53650	2.06584	0.0698591
53651	1.878 0.0698591	
53652	1.50236	0.0698591
53653	2.05749	0.0698591
53654	2.30533	0.0698591
53655	2.24475	2.1
53656	1.85871	2.1
53657	2.13711	2.1
53658	2.13711	2.1
53659	1.9943	2.1
53660	1.9943	2.1
53661	1.85871	2.1
53662	1.7871	2.1
53663	1.41619	2.1
53664	1.41619	2.1
53665	2.13711	2.1
53666	2.13711	2.1
53667	2.24475	2.1
53668	2.13711	2.1
53669	2.54902	2.1
53670	2.46327	2.1
53671	4.70802	2.1
53672	1.06913	2.1
53673	1.06913	2.1
53674	1.0821	2.1
53675	1.0821	2.1
53676	0.893552	2.1
53677	1.08267	2.1
53678	1.06855	2.1
53679	1.23163	2.1
53680	1.23163	2.1
53681	1.08267	2.1
53682	0.893551	2.1
53683	1.06855	2.1
53684	1.67046	0.415087
53685	2.4106	0.189468
53686	2.05835	1.43156
53687	2.4106	1.2934
53688	1.59562	0.493366
53689	1.94788	0.301125
53690	2.34737	1.98593
53691	2.4106	0.379006
53692	2.05834	1.01551
53693	1.99655	1.79238
53694	4.40715	1.00143
53695	1.67046	0.255895
53696	2.05835	0.183755
53697	1.588 1.11673	
53698	1.34857	1.71801
53699	3.40692	0.375489
53700	1.73802	0.882613
53701	1.89668	0.987436
53702	1.99655	2.06887
53703	1.99655	0.405899
53704	1.79981	1.3509
53705	2.05834	0.330554

53706	1.89226	1.48646
53707	1.89226	0.458194
53708	1.89226	0.260148
53709	1.89226	1.96191
53710	1.89226	0.286742
53711	1.89226	2.1
53712	1.89226	0.870913
53713	1.89226	2.07831
53714	1.89226	1.93136
53715	1.89226	0.630131
53716	3.78453	1.7046
53717	0.719473	2.06618
53718	0.916103	1.94428
53719	1.02744	0.84305
53720	0.850328	1.72759
53721	0.888094	0.806264
53722	0.888094	2.1
53723	0.878337	2.04813
53724	0.878337	0.61569
53725	0.916103	1.42153
53726	1.02744	1.66988
53727	0.719473	1.8296
53728	0.850328	1.90624
53729	1.02744	1.45246
53730	0.850329	1.98565
53731	1.0875	0.62787
53732	0.919597	1.18385
53733	0.975841	0.31212
53734	0.975841	2.1
53735	0.971196	0.313245
53736	0.971196	1.99652
53737	1.02744	1.75952
53738	1.0875	0.655577
53739	0.850328	1.79687
53740	0.919598	1.74651
53741	1.89226	2.02561
53742	1.89226	1.55397
53743	1.89226	0.326216
53744	1.89226	1.76383
53745	3.78453	1.24687
53746	1.89226	1.86131
53747	1.89226	0.677964
53748	1.89226	0.660308
53749	1.89226	1.96759
53750	1.89226	0.633024
53751	1.89226	2.09789
53752	1.11103	1.84231
53753	0.916103	2.04019
53754	1.21177	0.868771
53755	1.02744	0.896989
53756	1.07188	0.637183
53757	1.07188	2.1
53758	1.06659	0.652411
53759	1.06659	1.98562
53760	1.11103	1.90644
53761	1.21177	1.68106
53762	0.916104	1.80617
53763	1.02744	1.95558

53764	2.54158	0.597319
53765	2.42354	1.52196
53766	2.17499	0.403286
53767	2.17499	2.1
53768	2.42354	1.80336
53769	2.05488	2.1
53770	1.89226	0.0698591
53771	1.89226	0.0706447
53772	1.89226	0.0698591
53773	1.89226	0.0706733
53774	3.78453	0.0698591
53775	1.89226	0.0698591
53776	1.89226	0.0698591
53777	1.89226	0.0698591
53778	1.89226	0.0698591
53779	1.89226	0.0698591
53780	1.89226	0.0698591
53781	1.89226	0.0969465
53782	1.89226	0.120942
53783	1.89226	0.106495
53784	1.89226	0.074886
53785	1.89226	0.169755
53786	1.89226	0.114079
53787	1.89226	0.0883702
53788	1.89226	0.157219
53789	1.89226	0.104019
53790	1.89226	0.168882
53791	3.78452	0.13615
53792	0.802544	0.0698591
53793	0.946132	0.0698591
53794	0.946132	0.0698591
53795	0.787038	0.0698591
53796	0.787039	0.0698591
53797	0.787039	0.0698591
53798	0.961637	0.0698591
53799	0.961637	0.0698591
53800	0.787038	0.0698591
53801	0.802544	0.0698591
53802	0.946132	0.0698591
53803	0.946132	0.0698591
53804	1.89226	0.0698591
53805	1.60509	0.0698591
53806	1.89226	0.0698591
53807	1.89226	0.0698591
53808	1.3905	0.0698591
53809	1.3905	0.0698591
53810	0.946132	0.0741615
53811	0.946132	0.0698591
53812	0.946131	0.075028
53813	0.946131	0.0733598
53814	0.946132	0.073308
53815	0.946132	0.074137
53816	0.946131	0.071334
53817	0.946131	0.0713727
53818	0.946131	0.0711423
53819	0.946131	0.0726577
53820	0.946132	0.0702746
53821	0.946132	0.070116

53822	0.946131	0.0851622
53823	0.946131	0.0909817
53824	0.946131	0.0929533
53825	0.946131	0.0707709
53826	0.946132	0.0825026
53827	0.946132	0.095178
53828	0.946131	0.0757857
53829	0.946131	0.0733522
53830	0.946131	0.0843849
53831	0.946131	0.0701117
53832	0.946132	0.0727481
53833	0.946132	0.0756316
53834	1.42382	2.085
53835	1.32201	1.41632
53836	1.32096	1.6943
53837	1.42866	2.07083
53838	2.75067	1.96786
53839	1.89226	2.07993
53840	1.89226	1.18508
53841	1.89226	1.70421
53842	1.89226	2.03031
53843	1.89226	1.41374
53844	1.89226	1.94478
53845	1.89226	1.70795
53846	2.79029	0.657149
53847	2.7803	0.882486
53848	2.79029	1.2936
53849	1.88227	0.793371
53850	1.89226	1.16775
53851	0.716065	2.07386
53852	0.714333	2.09654
53853	0.660483	1.49478
53854	0.659126	1.75062
53855	0.687538	1.52148
53856	0.687538	2.07261
53857	0.685921	2.1
53858	0.685921	1.62908
53859	0.659126	1.95857
53860	0.716065	1.968
53861	0.660483	1.94373
53862	0.714333	1.94683
53863	1.89226	2.07392
53864	1.89226	1.26973
53865	1.89226	1.6647
53866	1.89226	2.01452
53867	3.78453	1.89362
53868	0.946133	1.26712
53869	0.946133	1.56145
53870	1.39015	0.854824
53871	1.38969	0.631217
53872	0.946132	0.647067
53873	0.946132	1.25186
53874	1.38969	1.52849
53875	1.38969	0.835423
53876	1.38969	0.999825
53877	0.946132	1.02507
53878	0.946133	1.09361
53879	1.39015	1.02284

53880	2.72157	0.100222
53881	1.22176	0.0818025
53882	1.59492	0.0822753
53883	1.22176	0.142769
53884	2.40743	0.0733953
53885	2.03426	0.135023
53886	0.946131	0.195203
53887	0.946131	0.146244
53888	1.36079	0.101763
53889	1.01713	0.140316
53890	0.94613	0.137389
53891	0.94613	0.184188
53892	1.36079	0.126656
53893	1.36079	0.0918091
53894	0.946131	0.122199
53895	1.36079	0.122764
53896	1.01713	0.111748
53897	0.946131	0.122672
53898	1.89226	0.197972
53899	1.89226	0.149158
53900	1.89226	0.137235
53901	1.89226	0.261516
53902	3.78453	0.220041
53903	1.89226	0.135949
53904	1.89226	0.0905115
53905	1.89226	0.085342
53906	1.89226	0.152431
53907	1.89226	0.0814179
53908	1.89226	0.150814
53909	0.946131	0.193801
53910	0.946131	0.152527
53911	0.946131	0.131378
53912	1.36079	0.0921683
53913	0.94613	0.132279
53914	0.94613	0.175485
53915	0.946133	0.142391
53916	0.946133	0.0932788
53917	0.946131	0.150964
53918	0.946131	0.140012
53919	0.946132	0.138353
53920	1.36079	0.121389
53921	1.89226	0.136486
53922	1.89226	0.246052
53923	1.89226	0.16909
53924	1.89226	0.179316
53925	1.89226	0.162536
53926	1.89226	0.182064
53927	1.89227	0.139317
53928	1.89227	0.0856901
53929	1.89227	0.0922178
53930	1.89227	0.163823
53931	1.89227	0.0845979
53932	1.89227	0.15659
53933	1.60428	2.1
53934	2.2367	2.1
53935	1.50097	2.1
53936	1.80125	2.1
53937	3.40553	2.1

53938	1.89095	2.1
53939	1.85293	2.07982
53940	1.80125	2.1
53941	1.76268	2.09868
53942	3.65418	2.1
53943	0.803177	2.1
53944	0.803177	2.1
53945	0.798644	2.1
53946	0.798644	2.1
53947	0.84233	2.1
53948	0.900624	2.1
53949	0.750483	2.1
53950	0.701197	2.1
53951	0.701197	2.1
53952	0.84233	2.1
53953	0.750483	2.1
53954	0.900625	2.1
53955	0.860925	2.1
53956	0.860925	2.1
53957	0.862743	2.1
53958	0.862743	2.1
53959	0.881338	2.09843
53960	0.826621	2.07764
53961	0.84233	2.1
53962	0.900625	2.1
53963	0.84233	2.1
53964	0.826621	2.1
53965	0.900624	2.1
53966	0.881339	2.1
53967	1.37938	0.77855
53968	1.03319	0.912082
53969	1.00512	0.928772
53970	0.639582	1.17375
53971	1.00463	1.00996
53972	1.00463	0.711906
53973	1.01434	0.868681
53974	1.01434	1.10808
53975	1.37938	0.991227
53976	1.00512	1.04436
53977	1.03319	0.973337
53978	0.639582	1.03717
53979	0.728713	1.1426
53980	0.728713	1.11969
53981	0.73425	1.26386
53982	0.73425	2.08353
53983	0.525122	2.1
53984	0.605622	0.244893
53985	0.948901	0.244893
53986	0.857342	0.244893
53987	0.85734	0.270614
53988	0.525123	2.1
53989	0.948899	0.270614
53990	0.605624	0.270614
53991	1.75449	0.301727
53992	1.4378	0.351822
53993	1.93916	0.497506
53994	2.22512	0.205065
53995	3.69364	0.422633

53996	1.75448	0.832156
53997	1.89186	0.25472
53998	2.29771	0.277564
53999	2.22512	0.266253
54000	4.05219	0.282592
54001	1.93916	0.59475
54002	2.51749	0.182778
54003	2.62357	0.292211
54004	2.22512	0.23322
54005	2.33761	0.396671
54006	1.93916	0.270891
54007	2.29771	0.391997
54008	2.51749	0.213747
54009	2.22512	0.21195
54010	2.22512	0.189563
54011	2.50905	0.478191
54012	2.50905	0.349464
54013	0.87382	0.0698591
54014	1.33662	0.0698591
54015	1.20756	0.0698591
54016	0.890467	0.0727296
54017	1.08859	0.0720156
54018	1.08859	0.0700247
54019	1.13084	0.0698591
54020	1.08793	0.0698591
54021	1.34722	0.0698591
54022	1.27184	0.0700876
54023	0.917678	0.0698664
54024	0.97821	0.0698591
54025	1.28396	0.0698591
54026	1.70302	0.0723715
54027	2.30076	0.0703314
54028	1.20417	0.0698591
54029	2.57506	0.070905
54030	2.2656	0.0698591
54031	1.03642	1.9871
54032	1.03642	0.46767
54033	1.07029	0.125042
54034	1.07029	0.706598
54035	1.31531	1.94026
54036	1.23279	0.706471
54037	0.941743	0.232776
54038	0.791399	0.950774
54039	0.791399	0.692499
54040	1.23279	0.660714
54041	0.941744	0.609689
54042	1.31531	0.630281
54043	0.937354	1.99641
54044	0.937354	0.40795
54045	0.954794	0.150477
54046	0.954794	0.686566
54047	1.13563	0.651796
54048	1.23279	1.92923
54049	0.791398	0.209331
54050	0.659359	0.747916
54051	1.13563	0.575619
54052	0.659359	0.647273
54053	0.791399	0.512952

54054	1.23279	0.53379
54055	2.29852	1.04572
54056	1.89227	1.15513
54057	2.14485	0.652446
54058	2.14485	1.58263
54059	1.89227	0.687625
54060	1.89227	1.59808
54061	0.947863	0.426395
54062	1.19854	1.09582
54063	2.16841	0.484028
54064	1.76597	0.678421
54065	2.96451	0.498745
54066	1.89227	0.777441
54067	1.89227	0.969492
54068	1.89227	0.550265
54069	1.89227	1.50219
54070	1.89227	0.604371
54071	1.89227	1.5638
54072	2.46489	2.1
54073	1.98339	2.1
54074	1.81667	2.1
54075	2.36914	2.1
54076	4.35253	2.1
54077	2.46488	2.1
54078	2.70401	2.1
54079	2.64996	2.1
54080	2.36914	2.1
54081	5.07315	2.1
54082	1.81667	2.1
54083	2.29459	2.1
54084	2.36914	2.1
54085	2.36914	2.1
54086	1.68073	2.1
54087	1.68073	2.1
54088	2.64996	2.1
54089	2.29458	2.1
54090	2.29458	2.1
54091	2.36914	2.1
54092	2.61243	2.1
54093	2.68698	2.1
54094	1.89226	1.61658
54095	1.89226	0.992754
54096	1.89226	1.39642
54097	1.89226	1.40715
54098	1.89226	1.35458
54099	1.89226	1.29667
54100	1.89224	1.82108
54101	1.89224	0.981741
54102	1.65571	1.11915
54103	1.65571	1.2392
54104	1.89224	1.21532
54105	1.89224	1.57365
54106	1.89224	1.42544
54107	1.89224	0.918118
54108	1.89224	1.26902
54109	1.89224	1.16465
54110	1.89224	1.37201
54111	1.89224	1.22199

54112	1.98252	0.0945593
54113	2.20641	0.101332
54114	1.05129	0.0860317
54115	1.2951	0.124918
54116	3.27762	0.0937941
54117	1.85598	2.06498
54118	1.88635	1.07062
54119	2.12346	0.805327
54120	2.10838	2.0861
54121	3.97943	1.62084
54122	2.10838	2.06521
54123	1.87532	2.1
54124	1.85597	1.99898
54125	1.85597	2.1
54126	1.87532	2.08181
54127	1.57914	2.1
54128	2.12348	0.843281
54129	2.35141	2.05755
54130	2.37191	1.6112
54131	2.10841	1.89597
54132	2.38698	1.18219
54133	2.12348	1.94527
54134	1.07811	2.1
54135	0.937672	2.1
54136	1.17571	2.02993
54137	1.05421	1.67097
54138	1.07087	1.99192
54139	1.07087	2.1
54140	1.06145	2.1
54141	1.06145	1.91573
54142	1.17571	2.09601
54143	1.07811	2.09634
54144	1.05421	2.1
54145	0.937672	2.1
54146	1.67334	2.1
54147	2.76609	2.1
54148	2.74144	2.1
54149	2.74144	2.1
54150	1.71157	2.1
54151	1.71157	2.1
54152	2.76609	2.1
54153	1.89226	2.1
54154	1.89226	2.1
54155	1.89226	2.1
54156	2.74143	2.1
54157	2.74143	2.1
54158	1.10393	2.1
54159	1.10393	2.1
54160	1.10788	2.1
54161	1.10788	2.1
54162	1.38302	2.1
54163	1.37072	2.1
54164	0.836672	2.1
54165	0.841089	2.1
54166	1.38302	2.1
54167	0.84109	2.1
54168	0.836671	2.1
54169	1.37072	2.1

54170	1.38302	2.1
54171	1.38302	2.1
54172	0.946131	2.1
54173	0.946131	2.1
54174	0.946128	2.1
54175	0.946128	2.1
54176	1.38302	2.1
54177	1.37072	2.1
54178	1.38302	2.1
54179	0.946131	2.1
54180	0.94613	2.1
54181	1.37072	2.1
54182	1.28072	2.1
54183	1.55779	2.1
54184	1.28072	2.1
54185	1.40765	2.1
54186	1.47186	2.1
54187	1.59879	2.1
54188	1.89227	2.1
54189	1.89227	2.1
54190	1.89227	1.89538
54191	1.89227	2.1
54192	1.89227	1.93707
54193	1.89227	2.1
54194	2.80057	1.80912
54195	1.89226	1.87308
54196	1.89226	1.21275
54197	1.89226	2.09455
54198	2.63061	0.809106
54199	2.63061	2.09576
54200	1.28072	2.0966
54201	1.38859	2.1
54202	1.47186	2.1
54203	1.47186	2.1
54204	1.16777	2.1
54205	1.16777	2.1
54206	1.89226	2.09325
54207	1.89226	2.1
54208	1.89226	1.99888
54209	1.89226	2.1
54210	1.89226	1.91183
54211	1.89226	2.1
54212	1.83221	2.1
54213	2.0815	2.1
54214	2.0815	2.1
54215	2.08153	2.1
54216	1.86703	2.1
54217	1.87402	2.1
54218	1.86703	2.1
54219	2.08508	2.1
54220	2.08508	2.1
54221	2.29001	2.1
54222	1.89226	2.1
54223	2.08048	2.1
54224	2.7278	0.120445
54225	1.10909	0.135335
54226	1.50472	0.117702
54227	1.10909	0.151775

54228	2.69881	0.111071
54229	2.30319	0.164966
54230	1.23204	2.1
54231	1.92264	2.1
54232	1.48162	2.1
54233	1.48162	2.1
54234	1.72994	2.1
54235	1.72994	2.1
54236	0.864968	2.1
54237	0.696952	2.1
54238	0.61602	2.1
54239	0.479736	2.1
54240	0.66444	2.1
54241	0.66444	2.1
54242	0.680264	2.1
54243	0.680264	2.1
54244	0.696952	2.1
54245	0.479736	2.1
54246	0.61602	2.1
54247	0.864968	2.1
54248	1.89226	0.567385
54249	2.43219	1.9645
54250	1.89226	1.52336
54251	2.82019	1.44397
54252	1.89226	1.60186
54253	2.82019	1.14482
54254	1.89226	0.833279
54255	2.83701	1.96194
54256	2.2701	1.71684
54257	2.2701	1.17706
54258	1.89226	1.75962
54259	1.89226	1.32838
54260	1.89226	1.80832
54261	1.89226	0.515793
54262	1.89226	1.0257
54263	1.89226	0.79444
54264	1.89226	1.20323
54265	1.89226	0.710086
54266	1.5795	1.7776
54267	1.44091	1.2605
54268	1.05267	1.86274
54269	1.05267	0.942814
54270	1.98408	1.89886
54271	1.98408	0.988861
54272	2.20624	2.1
54273	1.08831	2.1
54274	2.49053	2.1
54275	1.42486	2.1
54276	3.57883	2.1
54277	1.89226	2.1
54278	1.89226	2.1
54279	1.89226	2.1
54280	1.89226	2.1
54281	3.78453	2.1
54282	0.946132	2.1
54283	0.946132	2.1
54284	0.853591	2.1
54285	0.642207	2.1

54286	0.94613	2.1
54287	0.94613	2.1
54288	0.642209	2.1
54289	0.642209	2.1
54290	0.946131	2.1
54291	0.853591	2.1
54292	0.642207	2.1
54293	0.946132	2.1
54294	1.0506	2.1
54295	1.0506	2.1
54296	1.0879	2.1
54297	0.960424	2.1
54298	0.708687	2.1
54299	0.782927	2.1
54300	1.40041	2.1
54301	1.2518	2.1
54302	0.767748	2.1
54303	1.31801	2.1
54304	1.21412	2.1
54305	0.69461	2.1
54306	1.89226	2.1
54307	1.89226	2.1
54308	1.89226	2.1
54309	1.89226	2.1
54310	1.89226	2.1
54311	1.89226	2.1
54312	0.922223	2.1
54313	0.922223	2.1
54314	1.04548	2.1
54315	0.768229	2.1
54316	0.948441	2.1
54317	0.806483	2.1
54318	0.85362	2.1
54319	0.85362	2.1
54320	1.05942	2.1
54321	0.922187	2.1
54322	0.851554	2.1
54323	0.768264	2.1
54324	2.16728	2.1
54325	0.95455	2.1
54326	1.21372	2.1
54327	0.98227	2.1
54328	2.16728	2.1
54329	1.93584	2.1
54330	2.08849	0.108402
54331	1.08732	0.130503
54332	1.3357	0.124546
54333	2.37948	0.111962
54334	3.4668	0.12138
54335	2.57882	0.0991902
54336	2.37948	0.116656
54337	2.53055	0.118112
54338	1.48676	0.111584
54339	1.3357	0.12227
54340	1.3357	0.113923
54341	1.89226	2.1
54342	1.89226	2.1
54343	2.17471	2.1

54344	1.89226	2.1
54345	2.60085	2.1
54346	2.26306	2.1
54347	0.946131	2.1
54348	0.946131	2.1
54349	0.946131	2.1
54350	0.946131	2.1
54351	0.94613	2.1
54352	0.94613	2.1
54353	0.946132	2.1
54354	0.946132	2.1
54355	0.946131	2.1
54356	0.946131	2.1
54357	0.946131	2.1
54358	0.946131	2.1
54359	1.89226	2.1
54360	2.17471	2.1
54361	2.08196	2.1
54362	2.17471	2.1
54363	1.79952	2.1
54364	1.89226	2.1
54365	1.89226	2.1
54366	1.89226	2.1
54367	1.89226	2.1
54368	1.89226	2.1
54369	1.89226	2.1
54370	1.89226	2.1
54371	1.86016	2.1
54372	1.89226	2.1
54373	1.89226	2.1
54374	1.89226	2.1
54375	1.84826	2.1
54376	1.84826	2.1
54377	2.50136	2.1
54378	1.99781	2.1
54379	1.99729	2.1
54380	2.5214	2.1
54381	4.49865	2.1
54382	0.51724	2.1
54383	0.496076	2.1
54384	0.998906	2.1
54385	0.998645	2.1
54386	0.742249	2.1
54387	0.742249	2.1
54388	0.752734	2.1
54389	0.752734	2.1
54390	0.998639	2.1
54391	0.496073	2.1
54392	0.517243	2.1
54393	0.998913	2.1
54394	0.998653	2.1
54395	0.994293	2.1
54396	1.26071	2.1
54397	1.26504	2.1
54398	1.12529	2.1
54399	1.12529	2.1
54400	1.12971	2.1
54401	1.12971	2.1

54402	1.26504	2.1
54403	0.994292	2.1
54404	0.998651	2.1
54405	1.26071	2.1
54406	1.89226	2.1
54407	1.89226	2.1
54408	1.89226	2.1
54409	1.89226	2.1
54410	3.78452	2.1
54411	1.89226	2.1
54412	1.89226	2.1
54413	1.89226	2.1
54414	1.89226	2.1
54415	3.78452	2.1
54416	0.675872	2.1
54417	0.675641	2.1
54418	0.943072	2.1
54419	0.898317	2.1
54420	0.775717	2.1
54421	0.775717	2.1
54422	0.79824	2.1
54423	0.79824	2.1
54424	0.675642	2.1
54425	0.94307	2.1
54426	0.675874	2.1
54427	0.898315	2.1
54428	0.943071	2.1
54429	0.898316	2.1
54430	0.998133	2.1
54431	0.918903	2.1
54432	0.922369	2.1
54433	0.922369	2.1
54434	0.939606	2.1
54435	0.939606	2.1
54436	0.943071	2.1
54437	0.998133	2.1
54438	0.898316	2.1
54439	0.918903	2.1
54440	0.946131	2.1
54441	0.946131	2.1
54442	0.946131	2.1
54443	0.946131	2.1
54444	0.946132	2.1
54445	0.946132	2.1
54446	0.946129	2.1
54447	0.946129	2.1
54448	0.94613	2.1
54449	0.94613	2.1
54450	0.946131	2.1
54451	0.946131	2.1
54452	1.89226	2.1
54453	1.89226	2.1
54454	1.89226	2.1
54455	1.89226	2.1
54456	1.89226	2.1
54457	1.89226	2.1
54458	1.35129	2.1
54459	1.88614	2.1

54460	2.01867	2.1
54461	1.38189	2.1
54462	3.36995	2.1
54463	0.943071	2.1
54464	1.00934	2.1
54465	0.998134	2.1
54466	1.10866	2.1
54467	0.992617	2.1
54468	0.992617	2.1
54469	1.01485	2.1
54470	1.01485	2.1
54471	1.00934	2.1
54472	1.10866	2.1
54473	0.943072	2.1
54474	0.998134	2.1
54475	2.67272	2.1
54476	1.61859	2.1
54477	1.70303	2.1
54478	2.52175	2.1
54479	4.5326	2.1
54480	2.00954	2.1
54481	2.27801	2.1
54482	1.87875	2.1
54483	1.87875	2.1
54484	2.37837	2.1
54485	2.37837	2.1
54486	2.00955	2.1
54487	1.78	2.1
54488	2.21813	2.1
54489	1.8493	2.1
54490	2.37838	2.1
54491	2.00955	2.1
54492	2.37838	2.1
54493	2.27801	2.1
54494	2.58523	2.1
54495	2.71597	2.1
54496	4.9636	2.1
54497	2.37838	2.1
54498	2.71597	2.1
54499	1.72709	2.1
54500	2.0815	2.1
54501	2.59749	2.1
54502	2.78034	2.1
54503	0.94613	0.990368
54504	0.94613	1.09717
54505	0.946128	0.817442
54506	0.946128	0.728522
54507	0.94613	0.71173
54508	0.94613	0.829673
54509	0.946128	1.11209
54510	0.946128	0.745027
54511	0.946129	0.894665
54512	0.946129	0.90565
54513	0.946129	0.91012
54514	0.946129	0.872012
54515	1.89226	1.59161
54516	1.89226	1.18421
54517	1.89226	1.64522

54518	1.89226	1.01329
54519	3.78452	1.09536
54520	0.946133	0.670473
54521	0.946133	0.701331
54522	0.946132	0.476705
54523	0.946132	0.512886
54524	0.946132	0.491867
54525	0.946132	0.53243
54526	0.946133	0.717793
54527	0.946133	0.658648
54528	0.946132	0.622294
54529	0.946132	0.272491
54530	0.946132	0.572157
54531	0.946132	0.536151
54532	1.89226	0.937894
54533	2.10495	0.314265
54534	1.89226	0.448522
54535	2.30932	0.50993
54536	1.89226	0.78159
54537	2.30932	0.880451
54538	2.30932	0.44193
54539	1.00366	0.194635
54540	2.10496	0.144777
54541	1.24728	0.180487
54542	3.31298	0.201391
54543	1.87557	2.1
54544	1.07167	2.1
54545	2.03045	2.1
54546	1.25185	2.1
54547	3.10212	2.1
54548	1.89226	2.1
54549	1.89226	2.1
54550	1.89226	2.1
54551	1.89226	2.1
54552	3.78453	2.1
54553	0.851541	2.1
54554	0.851541	2.1
54555	1.12236	2.1
54556	1.05783	2.1
54557	0.870663	2.1
54558	0.951856	2.1
54559	0.946132	2.1
54560	0.946132	2.1
54561	1.04073	2.1
54562	1.1296	2.1
54563	0.776062	2.1
54564	0.851527	2.1
54565	0.859395	2.1
54566	0.859395	2.1
54567	0.870165	2.1
54568	0.870165	2.1
54569	0.625924	2.1
54570	0.714333	2.1
54571	1.0821	2.1
54572	1.01523	2.1
54573	1.0821	2.1
54574	0.714333	2.1
54575	1.01523	2.1

54576	0.625924	2.1
54577	1.89226	2.1
54578	1.89226	2.1
54579	1.89226	2.1
54580	1.89226	2.1
54581	3.78453	2.1
54582	1.89226	2.1
54583	1.89226	2.1
54584	2.23164	2.1
54585	2.67802	2.1
54586	2.03663	2.1
54587	1.89226	2.1
54588	2.42221	2.1
54589	1.61933	2.1
54590	2.80896	2.1
54591	1.87043	2.1
54592	4.29262	2.1
54593	1.89226	2.1
54594	1.89226	2.1
54595	1.89226	2.1
54596	1.89226	2.1
54597	1.89226	2.1
54598	1.89226	2.1
54599	2.80892	2.1
54600	1.87041	2.1
54601	2.67182	2.1
54602	2.30891	2.1
54603	5.07665	2.1
54604	1.89224	2.1
54605	1.89224	2.1
54606	1.89224	2.1
54607	1.89224	2.1
54608	3.78448	2.1
54609	0.946118	2.1
54610	0.946118	2.1
54611	0.946118	2.1
54612	0.946118	2.1
54613	0.946115	2.1
54614	0.946115	2.1
54615	0.94612	2.1
54616	0.94612	2.1
54617	0.94613	2.1
54618	0.94613	2.1
54619	0.946105	2.1
54620	0.946105	2.1
54621	2.3089	2.1
54622	1.02783	2.1
54623	1.98533	2.1
54624	1.76762	2.1
54625	1.24553	2.1
54626	1.02783	2.1
54627	2.2049	0.118144
54628	2.34673	0.112049
54629	1.00763	0.107051
54630	1.23746	0.144025
54631	3.44236	0.113828
54632	1.89226	0.155476
54633	1.89226	0.220271

54634	1.89226	0.186939
54635	1.89226	0.197536
54636	3.78452	0.207309
54637	1.89226	0.230179
54638	1.89226	0.197554
54639	1.89226	0.202997
54640	1.89226	0.285954
54641	3.78453	0.215166
54642	0.891618	0.158168
54643	0.891618	0.146284
54644	0.902158	0.117772
54645	0.902158	0.130696
54646	1.15396	0.150112
54647	0.620408	0.136745
54648	1.17337	0.119398
54649	0.618733	0.14881
54650	0.620408	0.141036
54651	0.618733	0.133495
54652	1.17337	0.138062
54653	1.15396	0.145198
54654	0.69978	0.140763
54655	0.69978	0.135571
54656	0.946102	0.135658
54657	0.946102	0.136976
54658	0.831702	0.151805
54659	0.946129	0.15267
54660	0.699752	0.138672
54661	0.946132	0.124678
54662	0.831703	0.143766
54663	0.699751	0.122619
54664	0.946131	0.128062
54665	0.946131	0.135563
54666	0.831675	0.169332
54667	0.831675	0.173926
54668	0.946163	0.155231
54669	0.864397	0.157975
54670	0.853471	0.192537
54671	0.962623	0.156277
54672	0.748555	0.153336
54673	0.851543	0.157657
54674	0.864714	0.169305
54675	0.760411	0.158544
54676	0.946133	0.159756
54677	0.946133	0.174918
54678	1.89226	0.104931
54679	1.89226	0.308564
54680	1.89226	0.248081
54681	1.89226	0.111299
54682	3.78452	0.143835
54683	1.89226	0.41513
54684	1.89226	0.214692
54685	1.89226	0.239541
54686	1.89226	0.410357
54687	3.78452	0.314361
54688	0.946132	0.363058
54689	0.946132	0.475245
54690	0.946132	0.16788
54691	0.946132	0.225613

54692	0.946132	0.167539
54693	0.946132	0.496688
54694	0.946133	0.212393
54695	0.946133	0.426936
54696	0.946133	0.259527
54697	0.946133	0.27149
54698	0.946132	0.342622
54699	0.946132	0.41818
54700	1.89226	0.265464
54701	2.39715	0.117403
54702	1.89226	0.142784
54703	1.89226	0.196376
54704	1.89226	0.154942
54705	1.89226	0.197646
54706	0.94613	0.376238
54707	0.94613	0.401006
54708	0.94613	0.245895
54709	0.94613	0.19777
54710	0.946131	0.198688
54711	0.946131	0.357711
54712	0.946129	0.401128
54713	0.946129	0.230414
54714	0.94613	0.279966
54715	0.94613	0.269554
54716	0.94613	0.29504
54717	0.94613	0.289016
54718	2.54204	2.1
54719	2.3148	2.1
54720	2.55328	2.1
54721	2.39646	2.1
54722	4.71126	2.1
54723	1.62611	2.1
54724	2.3148	2.1
54725	2.16986	2.1
54726	1.43745	2.1
54727	3.75225	2.1
54728	1.89226	2.1
54729	1.89226	2.1
54730	1.89226	2.1
54731	1.89226	2.1
54732	3.78453	2.1
54733	1.89226	2.1
54734	1.89226	2.1
54735	1.89226	2.1
54736	1.89226	2.1
54737	3.78453	2.1
54738	2.12125	2.1
54739	2.39642	2.1
54740	2.2941	2.1
54741	1.9933	2.1
54742	4.38972	2.1
54743	1.73784	2.1
54744	1.26892	2.1
54745	1.26892	2.1
54746	1.43743	2.1
54747	1.9933	2.1
54748	2.1618	2.1
54749	0.946118	2.1

54750	0.946118	2.1
54751	0.946118	2.1
54752	0.946118	2.1
54753	0.946119	2.1
54754	0.946119	2.1
54755	0.946118	2.1
54756	0.946118	2.1
54757	0.946131	2.1
54758	0.946131	2.1
54759	0.946106	2.1
54760	0.946106	2.1
54761	1.89224	2.1
54762	1.89224	2.1
54763	1.89224	2.1
54764	1.89224	2.1
54765	3.78448	2.1
54766	2.45922	2.1
54767	2.15686	2.1
54768	2.14259	2.1
54769	2.43225	2.1
54770	4.58911	2.1
54771	1.62027	2.1
54772	2.15686	2.1
54773	1.62241	2.1
54774	1.62241	2.1
54775	2.1426	2.1
54776	2.1426	2.1
54777	2.14259	2.1
54778	2.43225	2.1
54779	2.40667	2.1
54780	2.1281	2.1
54781	4.56035	2.1
54782	1.0713	2.1
54783	1.06405	2.1
54784	0.810136	2.1
54785	0.808433	2.1
54786	0.938508	2.1
54787	0.938508	2.1
54788	0.941223	2.1
54789	0.941223	2.1
54790	1.06405	2.1
54791	0.808433	2.1
54792	0.810135	2.1
54793	1.0713	2.1
54794	1.89226	0.44389
54795	1.89226	0.267122
54796	1.89226	0.395474
54797	1.89226	0.299417
54798	3.78453	0.310237
54799	1.89227	0.321115
54800	1.89227	0.264719
54801	1.89227	0.38253
54802	1.89227	0.226921
54803	3.78453	0.304222
54804	1.19	0.242051
54805	1.12469	0.178262
54806	1.01052	0.208759
54807	1.08166	0.196048

54808	2.20051	0.194912
54809	0.594999	0.242773
54810	0.540832	0.208735
54811	0.514182	0.272874
54812	0.567857	0.225256
54813	0.554259	0.218922
54814	0.554259	0.201887
54815	0.55443	0.2445
54816	0.55443	0.264536
54817	0.540832	0.22723
54818	0.514182	0.214243
54819	0.567857	0.219237
54820	0.594999	0.215082
54821	1.38674	0.255178
54822	0.946132	0.326272
54823	1.36361	0.261956
54824	0.946132	0.323459
54825	1.36362	0.25157
54826	1.36362	0.200742
54827	0.946131	0.332538
54828	0.946131	0.369499
54829	1.38674	0.281797
54830	1.36361	0.231993
54831	0.946132	0.318265
54832	0.946132	0.291877
54833	1.89226	0.316238
54834	2.68508	0.197065
54835	2.77349	0.226765
54836	1.89226	0.256545
54837	2.77349	0.239342
54838	1.89226	0.265516
54839	2.15315	0.0700552
54840	1.96254	0.0698591
54841	1.51377	0.0698591
54842	1.89226	0.0698591
54843	1.47799	0.072901
54844	1.89226	0.0698591
54845	2.49439	0.0866795
54846	1.89226	0.092844
54847	2.15315	0.0916762
54848	1.89226	0.0862274
54849	2.15315	0.119771
54850	1.89226	0.0842057
54851	1.89226	0.0928966
54852	1.89226	0.0827002
54853	1.89226	0.0907312
54854	1.89226	0.0861149
54855	1.89226	0.0803708
54856	1.89226	0.0806983
54857	1.89226	0.0698591
54858	1.89226	0.0698591
54859	1.89226	0.0698591
54860	1.89226	0.0698591
54861	1.89226	0.0698591
54862	1.89226	0.0698591
54863	1.375	0.0791178
54864	2.01865	0.0698591
54865	2.12047	0.0714409

54866	1.47354	0.0712607
54867	2.12047	0.0757163
54868	1.47354	0.0708722
54869	0.687499	0.0698591
54870	1.06023	0.0698591
54871	1.08019	0.0698591
54872	0.604229	0.0698591
54873	1.08018	0.0698591
54874	1.06023	0.0698591
54875	0.687502	0.0698591
54876	0.60423	0.0698591
54877	0.80646	0.0698591
54878	0.806461	0.0698591
54879	0.858001	0.0698591
54880	0.858001	0.0698591
54881	2.26414	2.1
54882	1.87342	2.1
54883	1.87342	2.1
54884	1.79667	2.1
54885	2.44045	2.1
54886	2.31924	2.1
54887	2.26414	2.1
54888	2.26616	2.1
54889	2.2355	2.1
54890	2.21858	2.1
54891	4.48474	2.1
54892	2.18516	2.1
54893	1.87344	2.1
54894	2.21859	2.1
54895	2.66233	2.1
54896	1.85196	2.1
54897	2.22236	2.1
54898	2.23551	2.1
54899	2.21859	2.1
54900	2.18516	2.1
54901	2.21189	2.1
54902	4.43049	2.1
54903	1.17171	2.1
54904	2.38924	2.1
54905	2.0815	2.1
54906	2.33348	2.1
54907	1.42851	2.1
54908	1.5959	2.1
54909	1.89226	2.1
54910	1.89226	2.1
54911	1.89226	2.1
54912	1.89226	2.1
54913	3.78452	2.1
54914	2.75425	2.1
54915	1.34366	2.1
54916	1.34366	2.1
54917	1.56512	2.1
54918	2.54186	2.1
54919	2.76332	2.1
54920	1.07751	2.1
54921	2.0815	2.1
54922	1.97259	2.1
54923	1.76455	2.1

54924	1.30191	2.1
54925	1.16461	2.1
54926	0.946131	2.1
54927	0.946131	2.1
54928	0.946129	2.1
54929	0.946129	2.1
54930	0.946131	2.1
54931	0.946131	2.1
54932	0.94613	2.1
54933	0.94613	2.1
54934	0.94613	2.1
54935	0.94613	2.1
54936	0.946131	2.1
54937	0.946131	2.1
54938	1.89226	0.169862
54939	1.89226	0.108299
54940	1.89226	0.0836798
54941	1.89226	0.0816649
54942	3.78453	0.0804848
54943	1.89226	0.168029
54944	1.89226	0.121477
54945	1.89226	0.091536
54946	1.89226	0.0776266
54947	3.78453	0.124709
54948	0.946132	0.100155
54949	0.946132	0.105107
54950	1.30327	0.0817149
54951	1.33544	0.105933
54952	1.33544	0.0879928
54953	1.33544	0.0796963
54954	0.946132	0.0891489
54955	0.946132	0.0754319
54956	0.946132	0.0764547
54957	1.30327	0.0731944
54958	1.33544	0.0941589
54959	0.946132	0.101792
54960	1.30327	0.077224
54961	1.33544	0.0877426
54962	0.709426	0.0764774
54963	0.790129	0.081864
54964	1.05886	0.0717854
54965	1.05886	0.0832754
54966	1.03454	0.092455
54967	1.03454	0.0775522
54968	1.30327	0.0746595
54969	0.709426	0.0714763
54970	1.33544	0.0975795
54971	0.790128	0.0812945
54972	0.946132	0.0715442
54973	0.946132	0.0759424
54974	0.827866	0.0698591
54975	0.946133	0.0734393
54976	0.946132	0.0698591
54977	0.946132	0.0707873
54978	0.946133	0.0698591
54979	0.946133	0.0822731
54980	0.946132	0.0698591
54981	0.827866	0.0702262

54982	0.946133	0.0871982
54983	0.946133	0.0790038
54984	1.89227	0.0907611
54985	1.89227	0.0698591
54986	1.89227	0.0752907
54987	1.89227	0.0723624
54988	1.89227	0.0704993
54989	1.89227	0.0698591
54990	0.946133	0.0716407
54991	0.946133	0.0727059
54992	1.21602	0.0698591
54993	1.30327	0.0754431
54994	1.30327	0.0699186
54995	1.30327	0.0698591
54996	0.946132	0.0698591
54997	0.946132	0.0706001
54998	0.946132	0.0698591
54999	1.21602	0.0698591
55000	0.946133	0.0746446
55001	1.30327	0.0715275
55002	1.04227	0.0698591
55003	1.11706	0.0699775
55004	0.968413	0.0698591
55005	0.945482	0.0699971
55006	1.3204	0.0698591
55007	1.23215	0.0698591
55008	0.810791	0.0698591
55009	1.38978	0.0698591
55010	1.07728	0.0699802
55011	1.11704	0.0713517
55012	0.608052	0.0698751
55013	1.89226	0.145666
55014	1.89226	0.0780119
55015	1.89226	0.0876158
55016	1.89226	0.109132
55017	3.78453	0.10106
55018	1.89226	0.070237
55019	1.89226	0.0742839
55020	1.89226	0.0829998
55021	1.89226	0.0698591
55022	3.78451	0.0710339
55023	2.58476	0.0717945
55024	1.56598	0.0698591
55025	1.55045	0.070082
55026	1.5124	0.0698591
55027	2.62281	0.0698591
55028	2.58476	0.0698591
55029	2.58477	0.0746263
55030	1.89226	0.084783
55031	1.89226	0.0958372
55032	1.89226	0.0749226
55033	2.62282	0.0900871
55034	2.62282	0.0732506
55035	1.89224	0.0933015
55036	1.89224	0.119021
55037	1.89224	0.1597
55038	1.89224	0.081097
55039	3.78448	0.0987357

55040	1.89223	0.0874333
55041	1.89223	0.0698805
55042	1.89223	0.073979
55043	1.89223	0.0711631
55044	1.89223	0.0759277
55045	1.89223	0.0730088
55046	2.62278	0.0734486
55047	1.45053	0.0698591
55048	1.49678	0.0698591
55049	1.56596	0.0698591
55050	2.5536	0.0698591
55051	2.62278	0.0698591
55052	1.31139	0.0756291
55053	1.2768	0.0704081
55054	0.946118	0.117596
55055	0.946118	0.0864917
55056	1.2768	0.08717
55057	1.2768	0.0748118
55058	0.946116	0.108468
55059	0.946116	0.0754591
55060	1.27682	0.0762047
55061	0.94613	0.0896618
55062	0.946105	0.085175
55063	1.31138	0.0787933
55064	1.38506	1.51579
55065	1.53348	2.05598
55066	1.17042	1.92079
55067	1.17042	1.9096
55068	1.73669	1.91082
55069	1.73669	1.84348
55070	1.73669	1.62694
55071	1.79928	0.602059
55072	1.38506	1.41054
55073	1.38506	1.92474
55074	1.79928	1.98114
55075	1.43484	2.01777
55076	1.73669	1.19945
55077	1.93568	1.98654
55078	1.53347	1.55548
55079	1.53347	1.87914
55080	2.14058	1.57742
55081	2.14058	1.93145
55082	0.89964	0.536223
55083	1.10415	2.09501
55084	0.868346	1.62892
55085	1.07029	2.00515
55086	0.98687	1.7581
55087	0.98687	2.1
55088	0.985627	0.306444
55089	0.985627	2.06502
55090	1.07029	1.92262
55091	1.10415	1.63062
55092	0.868344	1.90392
55093	0.899642	2.00096
55094	2.40667	2.1
55095	2.1281	2.1
55096	2.11176	2.1
55097	2.38069	2.1

55098	4.50878	2.1
55099	2.11176	2.1
55100	1.61687	2.1
55101	1.60965	2.1
55102	1.61687	2.1
55103	2.12088	2.1
55104	2.1281	2.1
55105	2.11176	2.1
55106	2.34898	2.1
55107	2.34898	2.1
55108	2.38069	2.1
55109	2.09114	2.1
55110	2.12285	2.1
55111	1.05588	2.1
55112	1.04557	2.1
55113	0.804824	2.1
55114	0.798269	2.1
55115	0.92615	2.1
55116	0.92615	2.1
55117	0.927999	2.1
55118	0.927999	2.1
55119	1.04557	2.1
55120	0.798269	2.1
55121	1.05588	2.1
55122	0.804824	2.1
55123	1.43548	0.325783
55124	1.53873	0.949853
55125	1.25063	0.395089
55126	1.39662	1.25448
55127	2.78937	0.347613
55128	1.25063	0.386909
55129	1.39662	1.29922
55130	1.10476	0.287436
55131	1.28326	0.803601
55132	2.53389	0.503586
55133	1.51147	0.0698591
55134	1.86114	0.0698591
55135	1.86114	0.0698591
55136	2.12926	0.0698591
55137	1.3659	0.0698591
55138	1.63401	0.0698591
55139	1.89226	0.0698591
55140	1.89226	0.0748646
55141	1.89226	0.0703289
55142	1.89226	0.0698591
55143	1.89226	0.0727186
55144	1.89226	0.0707043
55145	1.89226	0.082127
55146	1.89226	0.114758
55147	2.46286	0.076345
55148	1.89226	0.139954
55149	3.78452	0.0770202
55150	1.89226	0.121896
55151	1.89226	0.234816
55152	1.89226	0.18193
55153	1.89226	0.178829
55154	1.89226	0.133235
55155	1.89226	0.139855

55156	1.89226	0.0945666
55157	1.89226	0.117035
55158	2.28992	0.0873011
55159	1.89226	0.145329
55160	3.78452	0.0869634
55161	1.13537	0.123053
55162	0.886595	0.687975
55163	0.946131	1.63555
55164	0.946131	0.700766
55165	0.94613	0.504426
55166	0.94613	0.0950375
55167	1.25069	1.63293
55168	0.841855	1.00981
55169	1.27436	0.457946
55170	1.06196	0.551868
55171	1.09758	0.754962
55172	0.830306	0.460922
55173	0.826931	2.02064
55174	0.826933	0.475396
55175	0.764631	0.147973
55176	0.764631	0.805576
55177	1.00427	1.94816
55178	0.788034	0.865943
55179	0.726852	0.0989446
55180	0.604206	0.658323
55181	0.828475	0.711693
55182	0.604198	0.716434
55183	0.8332	0.647063
55184	1.00428	0.642006
55185	1.89226	0.581764
55186	1.89226	0.488227
55187	1.89226	0.170528
55188	1.89226	0.76507
55189	1.89226	0.677019
55190	1.89226	0.884489
55191	0.768789	2.1
55192	0.849132	0.0698591
55193	0.666423	2.1
55194	0.688993	1.95252
55195	0.868671	0.0698591
55196	0.89117	2.1
55197	0.705632	2.1
55198	1.80425	2.1
55199	1.73883	2.1
55200	1.45159	2.1
55201	1.45159	2.1
55202	1.37681	2.1
55203	1.73883	2.1
55204	1.39679	0.0698591
55205	2.08149	0.0698591
55206	1.89226	0.0698591
55207	2.32763	0.0698591
55208	1.89226	0.0698591
55209	2.31274	0.0698591
55210	1.89226	0.0698591
55211	1.89226	0.0698591
55212	1.89226	0.0698591
55213	1.89226	0.0698591

55214	1.89226	0.0698591
55215	1.89226	0.0698591
55216	1.89226	0.0698591
55217	1.89226	0.0698591
55218	1.89226	0.0698591
55219	1.89226	0.0698591
55220	1.89226	0.0698591
55221	1.89226	0.0698591
55222	1.95181	0.0698591
55223	1.89226	0.0698591
55224	1.89226	0.0698591
55225	1.89226	0.0698591
55226	3.78451	0.0698591
55227	0.946131	0.0698591
55228	0.946131	0.0698591
55229	0.946125	0.0698591
55230	0.946125	0.0698591
55231	0.946127	0.0698591
55232	0.946127	0.0698591
55233	0.946128	0.0698591
55234	0.946128	0.0698591
55235	0.946128	0.0698591
55236	0.946128	0.0698591
55237	0.946128	0.0698591
55238	0.946128	0.0698591
55239	1.89226	0.0698591
55240	1.89226	0.0711254
55241	1.89226	0.0698591
55242	1.89226	0.0698591
55243	1.89226	0.0698591
55244	1.89226	0.0698591
55245	1.89226	0.893864
55246	1.89226	0.515744
55247	1.89226	0.314285
55248	1.89226	0.260341
55249	3.78453	0.562524
55250	1.89226	0.917784
55251	1.89226	0.379471
55252	1.89226	0.389327
55253	1.89226	0.286046
55254	3.78452	0.311649
55255	1.21387	0.28403
55256	0.8164	0.208284
55257	0.946133	0.265009
55258	0.946133	0.342312
55259	0.946102	0.183235
55260	0.946102	0.393749
55261	0.816431	0.788809
55262	0.816431	0.315255
55263	0.816401	0.25004
55264	0.94613	0.210767
55265	0.946131	0.73425
55266	1.21388	0.30183
55267	0.946132	0.197167
55268	0.946132	0.22901
55269	0.946132	0.307234
55270	0.946132	0.272593
55271	0.946132	0.170145

55272	0.946132	0.276548
55273	0.946132	0.455634
55274	0.946132	0.236573
55275	0.946131	0.16411
55276	0.946131	0.247483
55277	0.946132	0.392612
55278	0.946132	0.278156
55279	1.89226	0.404649
55280	1.89226	0.162756
55281	1.89226	0.206552
55282	1.89226	0.458988
55283	1.89226	0.215872
55284	1.89226	0.382116
55285	1.89226	0.485798
55286	1.89226	0.332978
55287	2.15871	0.19838
55288	2.15871	0.383191
55289	1.81454	0.174814
55290	1.54809	0.475982
55291	1.89226	0.355204
55292	1.89226	1.56449
55293	1.89226	0.975084
55294	1.89226	0.408276
55295	3.78453	1.18414
55296	1.89226	1.19993
55297	1.89226	0.260265
55298	1.89226	0.235352
55299	1.89226	0.910527
55300	1.89226	1.55105
55301	1.89226	0.934889
55302	2.29205	1.15152
55303	1.42633	0.72207
55304	1.69015	1.1458
55305	1.69015	1.15883
55306	2.10364	0.448438
55307	2.05344	0.2942
55308	2.29205	0.344541
55309	1.58147	0.892482
55310	2.16529	0.474649
55311	1.56339	0.279825
55312	2.29205	1.26672
55313	1.69015	1.02315
55314	1.89226	1.05459
55315	1.89226	1.00552
55316	1.89226	0.292817
55317	1.89226	1.04846
55318	3.78453	0.4299
55319	1.89226	1.0255
55320	1.89226	1.21211
55321	1.89226	1.18063
55322	1.89226	0.376746
55323	3.78452	1.41287
55324	2.29205	1.12134
55325	2.85335	0.211807
55326	2.55409	1.1096
55327	2.04345	0.392621
55328	2.76338	0.614139
55329	2.29205	0.367752

55330	2.29205	0.795547
55331	2.16529	1.00115
55332	2.72418	0.996241
55333	2.76338	0.176779
55334	5.01623	0.979068
55335	1.89227	0.0698591
55336	1.89227	0.0698591
55337	1.89227	0.0698591
55338	1.89227	0.0698591
55339	3.78453	0.0698591
55340	2.39588	0.0698591
55341	2.14918	0.0698591
55342	2.14918	0.0698591
55343	2.37907	0.0698591
55344	2.20531	0.0698591
55345	2.4352	0.0698591
55346	1.89226	0.0698591
55347	2.64327	0.0698591
55348	2.36175	0.0698591
55349	1.89226	0.0698591
55350	4.25402	0.0698591
55351	1.89226	0.0698591
55352	1.89226	0.0698591
55353	1.89226	0.0698591
55354	1.89226	0.0698591
55355	1.89226	0.0698591
55356	1.89226	0.0698591
55357	1.05395	0.0698591
55358	1.05395	0.0698591
55359	1.03114	0.0698591
55360	1.03114	0.0698591
55361	0.936689	0.0698591
55362	1.07459	0.0698591
55363	1.10265	0.0698591
55364	1.01049	0.0698591
55365	0.93669	0.0698591
55366	1.01049	0.0698591
55367	1.07459	0.0698591
55368	1.10265	0.0698591
55369	1.18088	0.0698591
55370	0.776509	0.0698591
55371	0.946132	0.0698591
55372	0.946132	0.0698591
55373	1.04225	0.0698591
55374	0.830392	0.0698591
55375	0.931352	0.0698591
55376	0.931352	0.0698591
55377	1.13006	0.0698591
55378	1.04074	0.0698591
55379	1.0628	0.0698591
55380	0.851529	0.0698591
55381	2.40032	2.1
55382	1.89226	2.1
55383	1.89226	2.1
55384	1.89226	2.1
55385	2.38918	2.1
55386	2.38918	2.1
55387	1.89226	2.1

55388	1.89226	2.1
55389	1.89226	2.1
55390	1.89226	2.1
55391	1.89226	2.1
55392	1.89226	2.1
55393	1.6585	2.1
55394	2.37364	2.1
55395	2.39622	2.1
55396	2.39622	2.1
55397	1.6488	2.1
55398	1.6488	2.1
55399	2.44603	2.1
55400	1.89224	2.1
55401	1.89224	2.1
55402	1.89224	2.1
55403	2.40029	2.1
55404	2.40029	2.1
55405	1.89224	2.1
55406	1.89224	2.1
55407	1.89224	2.1
55408	1.89224	2.1
55409	1.89224	2.1
55410	1.89224	2.1
55411	1.01946	2.1
55412	1.01946	2.1
55413	1.02497	2.1
55414	1.02497	2.1
55415	1.21519	2.1
55416	1.19809	2.1
55417	0.835303	2.1
55418	0.82924	2.1
55419	0.835314	2.1
55420	1.2152	2.1
55421	0.829228	2.1
55422	1.19808	2.1
55423	1.91027	2.1
55424	1.05809	2.1
55425	1.00659	2.1
55426	2.06727	2.1
55427	3.11819	2.1
55428	2.37166	2.1
55429	2.06726	2.1
55430	1.66994	2.1
55431	1.67623	2.1
55432	2.40308	2.1
55433	2.40308	2.1
55434	0.960194	2.1
55435	0.939664	2.1
55436	0.531131	2.1
55437	0.503298	2.1
55438	0.729927	2.1
55439	0.729927	2.1
55440	0.733565	2.1
55441	0.733565	2.1
55442	0.960195	2.1
55443	0.531132	2.1
55444	0.503297	2.1
55445	0.939663	2.1

55446	1.20154	2.1
55447	1.21754	2.1
55448	0.960195	2.1
55449	0.939665	2.1
55450	1.07745	2.1
55451	1.07745	2.1
55452	1.07976	2.1
55453	1.07976	2.1
55454	0.960194	2.1
55455	1.21754	2.1
55456	0.939662	2.1
55457	1.20155	2.1
55458	1.89224	2.1
55459	1.89224	2.1
55460	2.08609	2.1
55461	2.08609	2.1
55462	2.09527	2.1
55463	1.90142	2.1
55464	1.65815	2.1
55465	1.71398	2.1
55466	2.2001	2.1
55467	2.23791	2.1
55468	1.4502	2.1
55469	1.4502	2.1
55470	0.793587	2.1
55471	0.793587	2.1
55472	0.792408	2.1
55473	0.792408	2.1
55474	0.952221	2.1
55475	0.858585	2.1
55476	0.725101	2.1
55477	0.633774	2.1
55478	0.633775	2.1
55479	0.858585	2.1
55480	0.952221	2.1
55481	0.725101	2.1
55482	1.5735	2.1
55483	2.56975	2.1
55484	2.63166	2.1
55485	2.63166	2.1
55486	1.54711	2.1
55487	1.54711	2.1
55488	0.946131	2.1
55489	0.946131	2.1
55490	1.28487	2.1
55491	1.28487	2.1
55492	0.946127	2.1
55493	0.946127	2.1
55494	1.28488	2.1
55495	1.31583	2.1
55496	1.31583	2.1
55497	0.94613	2.1
55498	1.28487	2.1
55499	0.946129	2.1
55500	1.0596	2.1
55501	1.0596	2.1
55502	1.06939	2.1
55503	1.06939	2.1

55504	1.34225	2.1
55505	1.31583	2.1
55506	0.793563	2.1
55507	0.786748	2.1
55508	0.793563	2.1
55509	1.34225	2.1
55510	0.786748	2.1
55511	1.31583	2.1
55512	0.946131	2.1
55513	0.946131	2.1
55514	1.31582	2.1
55515	1.31582	2.1
55516	0.946127	2.1
55517	0.946127	2.1
55518	1.34225	2.1
55519	1.31583	2.1
55520	1.34225	2.1
55521	0.946129	2.1
55522	1.31583	2.1
55523	0.946129	2.1
55524	1.89226	2.1
55525	1.89226	2.1
55526	1.89226	2.1
55527	1.89226	2.1
55528	1.89226	2.1
55529	1.89226	2.1
55530	1.89226	2.1
55531	1.89226	2.1
55532	1.89226	2.1
55533	1.89226	2.1
55534	1.89226	2.1
55535	1.89226	2.1
55536	1.26992	2.1
55537	1.75003	2.1
55538	1.79883	2.1
55539	1.26732	2.1
55540	3.06875	2.1
55541	0.875017	2.1
55542	0.899414	2.1
55543	0.811147	2.1
55544	0.85053	2.1
55545	0.851513	2.1
55546	0.851513	2.1
55547	0.859048	2.1
55548	0.859048	2.1
55549	0.899413	2.1
55550	0.85053	2.1
55551	0.811147	2.1
55552	0.875016	2.1
55553	1.89227	2.1
55554	1.89227	2.1
55555	1.89227	2.1
55556	1.89227	2.1
55557	1.89227	2.1
55558	1.89227	2.1
55559	1.89226	2.1
55560	1.89226	2.1
55561	1.89226	2.1

55562	1.89226	2.1
55563	1.89226	2.1
55564	1.89226	2.1
55565	0.633661	2.1
55566	0.642005	2.1
55567	0.93285	2.1
55568	0.899413	2.1
55569	0.764487	2.1
55570	0.764487	2.1
55571	0.776931	2.1
55572	0.776931	2.1
55573	0.642004	2.1
55574	0.93285	2.1
55575	0.899414	2.1
55576	0.633661	2.1
55577	0.932851	2.1
55578	0.899414	2.1
55579	0.905419	2.1
55580	0.85053	2.1
55581	0.886336	2.1
55582	0.886336	2.1
55583	0.897045	2.1
55584	0.897045	2.1
55585	0.93285	2.1
55586	0.905419	2.1
55587	0.899414	2.1
55588	0.850531	2.1
55589	2.39322	2.1
55590	2.74149	2.08691
55591	2.70944	2.1
55592	2.6849	2.01563
55593	2.41776	2.1
55594	2.39322	1.72104
55595	2.41776	2.1
55596	2.80992	2.1
55597	2.77256	2.1
55598	2.74149	2.09729
55599	2.44882	2.1
55600	2.41776	1.98941
55601	2.80992	2.1
55602	1.89226	1.62107
55603	2.74149	2.0388
55604	2.74149	2.09615
55605	1.89226	1.18844
55606	1.89226	2.05081
55607	1.89226	0.0698591
55608	1.89226	0.0698591
55609	1.89226	0.0743527
55610	1.89226	0.0698591
55611	1.89226	0.0738935
55612	1.89226	0.0698591
55613	2.38796	0.0698591
55614	2.0815	0.0698591
55615	2.0815	0.0698591
55616	2.49452	0.0698591
55617	1.55994	0.0698591
55618	2.14721	0.0698591
55619	1.89227	0.0738773

55620	1.89227	0.0699484
55621	1.89227	0.0835595
55622	1.89227	0.0698591
55623	1.89227	0.0857808
55624	1.89227	0.0698591
55625	1.13387	0.0698591
55626	2.27494	0.0698591
55627	1.13387	0.0698591
55628	2.19489	0.0698591
55629	1.60867	0.0698591
55630	2.6994	0.0698591
55631	1.89226	0.0725296
55632	1.89226	0.0698591
55633	1.89226	0.0698591
55634	1.89226	0.0910718
55635	3.78453	0.0698591
55636	1.42381	2.08449
55637	1.10747	2.1
55638	1.10747	2.08891
55639	1.08911	2.1
55640	1.42866	2.07171
55641	1.4103	2.1
55642	1.89226	2.1
55643	1.88181	2.1
55644	1.88181	2.1
55645	1.76249	2.1
55646	2.0815	2.1
55647	1.97539	2.1
55648	0.946128	2.1
55649	0.946128	2.1
55650	0.946133	2.0827
55651	0.946133	1.9687
55652	0.94613	1.98412
55653	0.94613	2.1
55654	0.946131	2.1
55655	0.946131	2.06073
55656	0.946131	2.1
55657	0.946131	2.1
55658	0.946131	2.0968
55659	0.946131	2.09751
55660	1.42866	2.09194
55661	1.43213	2.08569
55662	1.10748	2.1
55663	1.10748	2.1
55664	1.13641	2.1
55665	1.43213	2.1
55666	2.0815	2.1
55667	2.10287	2.1
55668	2.10287	2.1
55669	2.0921	2.1
55670	1.89227	2.1
55671	1.83938	2.1
55672	0.810944	2.08211
55673	0.810944	2.01196
55674	0.810945	2.01966
55675	0.810945	2.1
55676	0.810933	2.08459
55677	0.810933	2.08734

55678	1.89226	2.1
55679	1.3517	2.1
55680	1.08132	2.09429
55681	1.08132	2.1
55682	1.08133	2.09854
55683	2.33097	2.1
55684	1.53822	2.1
55685	1.53822	2.1
55686	1.68437	2.1
55687	2.17996	2.1
55688	2.32612	2.1
55689	0.99189	2.1
55690	1.18948	2.08475
55691	1.19756	2.1
55692	1.19756	2.1
55693	0.978851	2.1
55694	0.926995	2.1
55695	1.26924	1.95152
55696	1.34642	2.08275
55697	1.3228	2.1
55698	0.990899	2.1
55699	1.0627	2.1
55700	1.33473	2.1
55701	1.96196	2.1
55702	1.39702	2.1
55703	1.39702	2.1
55704	1.53822	2.1
55705	2.17372	2.1
55706	2.31011	2.1
55707	1.89226	1.94272
55708	1.89226	0.477169
55709	1.89226	1.94024
55710	1.89226	0.576579
55711	3.78453	0.698918
55712	2.38475	2.1
55713	1.73957	0.588635
55714	1.88452	1.94551
55715	1.58695	0.406197
55716	3.6241	0.868389
55717	1.73958	0.589686
55718	1.3126	2.1
55719	1.58695	0.42405
55720	1.14946	2.1
55721	2.88903	1.56512
55722	1.89226	0.435905
55723	1.89226	2.1
55724	1.89226	0.526128
55725	1.89226	2.1
55726	3.78453	1.77781
55727	1.89225	1.98123
55728	1.89225	0.629862
55729	1.89225	1.75312
55730	1.89225	0.677634
55731	3.7845	0.417905
55732	1.88451	1.98146
55733	1.58694	0.241156
55734	1.74649	2.09058
55735	1.44604	0.704839

55736	3.33055	0.684062
55737	1.58694	0.259321
55738	0.995727	2.1
55739	0.995727	1.41268
55740	1.14945	2.01135
55741	1.44604	0.495836
55742	1.59976	2.05099
55743	1.89225	0.488971
55744	1.89225	2.09965
55745	1.89225	2.1
55746	1.89225	1.08917
55747	1.89225	2.1
55748	1.89225	0.547468
55749	2.01197	2.09098
55750	2.0961	1.46683
55751	2.13283	1.57457
55752	2.25399	0.463356
55753	4.22894	1.37957
55754	2.09611	0.854379
55755	2.37014	1.52674
55756	2.29031	1.77327
55757	2.10084	2.05204
55758	2.25399	0.511328
55759	2.09611	2.03856
55760	2.13283	2.03187
55761	2.25399	2.03666
55762	2.22044	2.02455
55763	2.38975	0.515459
55764	4.52258	1.36917
55765	2.38976	0.177798
55766	2.15466	1.73939
55767	2.31988	0.255408
55768	2.31988	2.05499
55769	2.25399	0.175259
55770	2.25399	2.09729
55771	2.58286	2.084
55772	2.29193	0.453483
55773	2.40107	2.1
55774	2.13815	0.223245
55775	4.72101	1.11058
55776	2.29193	0.420218
55777	1.90104	1.98464
55778	2.13815	0.261603
55779	1.77249	1.86801
55780	4.03919	0.747325
55781	2.25197	2.1
55782	2.13815	0.438927
55783	2.00625	1.97467
55784	2.00625	0.430253
55785	2.40107	1.95739
55786	2.40107	1.1008
55787	2.13815	0.287799
55788	1.77249	1.92001
55789	2.00625	0.878845
55790	1.65815	2.04454
55791	3.7963	0.749424
55792	2.43417	0.575733
55793	2.06653	0.417693

55794	1.64587	0.470416
55795	1.65284	0.388738
55796	2.46898	0.569572
55797	2.46898	0.454571
55798	2.46895	0.47047
55799	1.89016	0.328466
55800	1.87861	0.379035
55801	1.87861	0.299107
55802	2.47161	0.47068
55803	2.47161	0.360732
55804	0.946132	1.59583
55805	0.946132	1.3208
55806	0.863311	1.78747
55807	1.34768	1.64493
55808	0.851542	1.75217
55809	0.851542	1.15247
55810	1.30451	1.39752
55811	1.45304	1.55189
55812	0.946132	1.47321
55813	1.20992	1.53335
55814	0.855162	1.67172
55815	0.946132	1.51614
55816	1.89226	1.29613
55817	1.89226	1.97559
55818	1.89226	1.90417
55819	1.89226	1.54082
55820	1.89226	1.82834
55821	1.89226	1.44696
55822	0.946132	1.08248
55823	0.946132	0.837714
55824	0.946133	1.11821
55825	0.946133	1.03937
55826	0.946132	1.12617
55827	0.946132	0.819953
55828	0.946132	0.847694
55829	0.946132	0.995788
55830	0.946132	1.06181
55831	0.946132	0.997973
55832	0.946133	1.04409
55833	0.946133	0.993745
55834	2.35574	1.99989
55835	1.89226	1.4981
55836	1.89226	2.09525
55837	1.89226	1.28249
55838	2.67456	2.08452
55839	2.67456	0.668303
55840	0.583505	0.138159
55841	0.761972	0.314919
55842	1.17787	1.76299
55843	1.33728	0.657282
55844	0.965128	0.990969
55845	0.965128	0.171563
55846	0.974714	0.743489
55847	0.974715	1.75338
55848	1.17787	0.621892
55849	0.583506	0.842082
55850	1.33728	0.517561
55851	0.761974	0.6504

55852	1.89226	0.876285
55853	1.89226	0.622021
55854	1.89226	0.58456
55855	1.89226	0.146979
55856	1.89226	0.185312
55857	1.89226	0.471281
55858	1.89226	1.45356
55859	1.89226	0.234265
55860	1.89226	0.700218
55861	1.89226	0.588199
55862	3.78453	0.52745
55863	0.946132	0.12958
55864	0.946132	0.335516
55865	0.946132	1.06927
55866	0.946132	0.584038
55867	0.946133	0.534776
55868	0.946133	0.162958
55869	0.946132	0.745002
55870	0.946132	1.03922
55871	0.946133	0.657251
55872	0.946133	0.725405
55873	0.946132	0.485393
55874	0.946132	0.79169
55875	1.01779	2.1
55876	1.50685	2.1
55877	1.12064	2.1
55878	1.65151	2.1
55879	2.6275	2.1
55880	1.35733	2.1
55881	1.53507	2.1
55882	1.50683	2.1
55883	1.73626	2.1
55884	3.09359	2.1
55885	1.73626	2.1
55886	1.52226	2.1
55887	1.75555	2.1
55888	1.75555	2.1
55889	1.53507	2.1
55890	1.53507	2.1
55891	1.65151	2.1
55892	1.73628	2.1
55893	1.69528	2.1
55894	1.50685	2.1
55895	1.92471	2.1
55896	1.73628	2.1
55897	1.73629	2.1
55898	1.75558	2.1
55899	1.9943	2.1
55900	1.9943	2.1
55901	1.92471	2.1
55902	1.73629	2.1
55903	2.5601	0.991768
55904	1.97645	1.94229
55905	2.09605	0.735185
55906	2.09605	1.82132
55907	2.45853	1.08236
55908	2.45853	1.97821
55909	2.33824	1.75198

55910	1.97646	1.50625
55911	2.45853	1.14469
55912	1.94984	1.94079
55913	2.33824	1.52256
55914	1.82955	2.03067
55915	1.22926	2.0025
55916	0.946128	1.96491
55917	0.946133	2.0401
55918	1.28005	1.39063
55919	1.22927	1.42623
55920	1.22926	1.65318
55921	0.946131	1.76493
55922	0.946131	1.40588
55923	0.946131	1.45295
55924	0.946131	1.31882
55925	1.28005	1.532
55926	1.22926	1.43114
55927	2.35349	2.01472
55928	2.61606	0.595797
55929	2.48209	2.00578
55930	2.48209	0.582493
55931	2.50057	2.00023
55932	2.50057	0.776818
55933	2.61607	0.542716
55934	2.57611	0.815946
55935	2.4821	0.760324
55936	2.44814	0.477087
55937	5.06421	0.261281
55938	2.15721	1.71062
55939	2.48209	0.705084
55940	2.31844	2.04351
55941	2.31844	0.52037
55942	2.35349	2.0246
55943	2.35349	0.594951
55944	2.44814	0.507123
55945	2.30908	0.947882
55946	2.44814	0.469207
55947	2.31844	0.584624
55948	2.4821	0.414945
55949	2.31844	0.25965
55950	0.91766	0.0827596
55951	0.91766	0.10555
55952	0.912463	0.106838
55953	0.912463	0.0887947
55954	1.16525	0.0860157
55955	1.26377	0.0839823
55956	0.576692	0.0993468
55957	0.664871	0.104225
55958	1.16525	0.0938637
55959	0.576692	0.0937469
55960	0.664871	0.108771
55961	1.26377	0.0991321
55962	1.72901	2.09792
55963	1.62414	2.0942
55964	1.88971	2.09858
55965	1.8411	1.74209
55966	3.57011	2.09037
55967	1.89226	2.09978

55968	1.89226	1.85865
55969	1.89226	1.91316
55970	1.89226	1.79512
55971	3.78452	1.85712
55972	1.89226	1.68485
55973	1.89226	2.09628
55974	1.89226	2.05252
55975	1.89226	1.43631
55976	3.78452	2.05568
55977	1.62414	2.09976
55978	1.30896	2.06469
55979	1.8411	2.01196
55980	1.52776	2.09178
55981	3.15006	2.04922
55982	1.51448	1.99348
55983	1.8411	1.94569
55984	2.02311	2.1
55985	1.99309	1.84183
55986	3.86421	1.91596
55987	1.89226	2.01509
55988	1.89226	2.01084
55989	1.89226	2.06965
55990	1.89226	1.97293
55991	3.78452	2.05146
55992	1.89226	2.09265
55993	1.89226	1.93001
55994	1.89226	2.04269
55995	1.89226	2.1
55996	3.78452	2.06095
55997	1.8411	2.06623
55998	1.52776	2.1
55999	1.99309	2.1
56000	1.72526	2.1
56001	3.56636	2.1
56002	1.89226	2.08757
56003	1.89226	2.04021
56004	1.89226	2.04728
56005	1.89226	2.09302
56006	3.78453	2.06494
56007	1.0424	2.09905
56008	1.12935	2.1
56009	0.929383	2.09709
56010	0.929383	2.0967
56011	1.24941	2.09871
56012	1.12686	2.1
56013	0.946132	2.09924
56014	0.946132	2.09404
56015	1.12944	2.09226
56016	0.851554	2.08838
56017	1.05017	2.09649
56018	0.929315	2.09757
56019	0.898029	2.09189
56020	0.973994	2.1
56021	0.946131	2.04901
56022	0.946131	2.07975
56023	1.0221	2.08112
56024	1.0221	2.1
56025	0.898028	2.03772

56026	0.898028	2.09289
56027	0.973994	2.09476
56028	0.946131	2.0985
56029	0.946131	2.09355
56030	0.898029	2.09421
56031	1.89226	2.02332
56032	1.89226	2.04122
56033	1.89226	1.99535
56034	1.89226	2.0415
56035	1.89226	1.99397
56036	1.89226	2.03935
56037	1.13537	2.03864
56038	0.879308	2.09517
56039	0.946131	2.02809
56040	0.946131	2.04088
56041	1.13537	2.04626
56042	0.85187	2.09588
56043	0.94613	2.0362
56044	0.94613	1.98192
56045	1.05777	2.05847
56046	1.05777	2.08399
56047	0.799318	2.06929
56048	0.776452	2.07559
56049	0.946116	2.1
56050	0.946116	2.1
56051	1.09531	2.1
56052	1.09531	2.1
56053	0.946118	2.1
56054	0.946118	2.1
56055	1.09135	2.1
56056	1.09531	2.1
56057	1.09134	2.1
56058	0.946105	2.1
56059	1.09532	2.1
56060	0.946131	2.1
56061	0.946129	2.1
56062	0.946129	2.1
56063	1.09137	2.1
56064	1.09137	2.1
56065	0.946131	2.1
56066	0.946131	2.1
56067	1.07713	2.1
56068	1.09137	2.1
56069	0.946131	2.1
56070	1.07713	2.1
56071	1.09137	2.1
56072	0.946131	2.1
56073	0.938916	2.1
56074	0.938916	2.1
56075	0.936995	2.1
56076	1.00447	2.1
56077	0.979819	2.1
56078	1.16821	2.1
56079	1.01263	2.1
56080	0.719167	2.1
56081	1.04536	2.1
56082	1.18901	2.1
56083	0.758057	2.1

56084	0.924111	2.1
56085	1.00532	2.1
56086	1.00532	2.1
56087	0.9925	2.1
56088	0.9925	2.1
56089	1.29322	2.1
56090	1.01985	2.1
56091	1.00365	2.1
56092	0.704601	2.1
56093	1.00365	2.1
56094	1.29322	2.1
56095	1.01985	2.1
56096	0.704602	2.1
56097	2.20011	2.1
56098	1.401	2.1
56099	1.401	2.1
56100	1.52008	2.1
56101	2.11084	2.1
56102	2.22991	2.1
56103	0.854068	2.1
56104	0.854068	2.1
56105	0.851357	2.1
56106	0.851357	2.1
56107	0.644681	2.1
56108	0.700493	2.1
56109	1.0554	2.1
56110	1.00493	2.1
56111	1.00495	2.1
56112	0.64469	2.1
56113	0.700483	2.1
56114	1.05539	2.1
56115	2.46665	0.0698591
56116	2.22713	0.0698591
56117	1.85877	0.0698591
56118	1.8967	0.0698591
56119	1.56002	0.0698591
56120	2.22713	0.0698591
56121	1.89225	2.1
56122	1.89225	2.1
56123	1.89225	2.1
56124	1.89225	2.1
56125	1.89225	2.1
56126	1.89225	2.1
56127	1.89225	2.1
56128	1.89225	2.1
56129	1.89225	2.1
56130	1.89225	2.1
56131	1.89225	2.1
56132	1.89225	2.1
56133	1.284	2.1
56134	1.86569	2.1
56135	1.95398	2.1
56136	1.31091	2.1
56137	3.23798	2.1
56138	0.932844	2.1
56139	0.976992	2.1
56140	0.960128	2.1
56141	0.905413	2.1

56142	0.938531	2.1
56143	0.938531	2.1
56144	0.943874	2.1
56145	0.943874	2.1
56146	0.976985	2.1
56147	0.960122	2.1
56148	0.93285	2.1
56149	0.905419	2.1
56150	1.89227	2.1
56151	1.89227	2.1
56152	1.89227	2.1
56153	1.89227	2.1
56154	3.78453	2.1
56155	1.89226	2.1
56156	1.89226	2.1
56157	1.89226	2.1
56158	1.89226	2.1
56159	1.89226	2.1
56160	1.89226	2.1
56161	0.65546	2.1
56162	0.665451	2.1
56163	1.00694	2.1
56164	0.976998	2.1
56165	0.816261	2.1
56166	0.816261	2.1
56167	0.826189	2.1
56168	0.826189	2.1
56169	1.00694	2.1
56170	0.665451	2.1
56171	0.976998	2.1
56172	0.65546	2.1
56173	2.01555	2.1
56174	2.01388	2.1
56175	1.92027	2.1
56176	1.92027	2.1
56177	2.01388	2.1
56178	1.954 2.1	
56179	2.75466	2.1
56180	1.89226	2.1
56181	1.89226	2.1
56182	2.54931	2.1
56183	4.44158	2.1
56184	2.47917	2.1
56185	1.92772	2.1
56186	1.80682	2.1
56187	2.54931	2.1
56188	4.47702	2.1
56189	0.946132	2.1
56190	0.946132	2.1
56191	1.17602	2.1
56192	1.27466	2.1
56193	0.946132	2.1
56194	0.946132	2.1
56195	1.17602	2.1
56196	1.17602	2.1
56197	0.946132	2.1
56198	1.17602	2.1
56199	0.946131	2.1

56200	1.27466	2.1
56201	2.35204	2.1
56202	2.54931	2.1
56203	2.40783	2.1
56204	1.50108	2.1
56205	1.64256	2.1
56206	1.64256	2.1
56207	1.89226	2.1
56208	1.89226	1.61915
56209	2.31012	2.03968
56210	2.17534	2.08272
56211	4.0676	2.06838
56212	2.17533	2.07509
56213	1.89226	1.14824
56214	2.17533	1.91654
56215	1.89226	1.61329
56216	1.66635	2.00665
56217	1.38328	1.84555
56218	1.92027	2.1
56219	2.17597	2.1
56220	2.14619	2.1
56221	2.15388	2.1
56222	1.91087	2.1
56223	1.92027	2.1
56224	1.08622	2.1
56225	1.08622	2.1
56226	0.971184	2.1
56227	0.972761	2.1
56228	1.14845	2.1
56229	1.16321	2.1
56230	0.894769	2.1
56231	0.894967	2.1
56232	0.894775	2.1
56233	1.28563	2.1
56234	1.14844	2.1
56235	1.0167	2.1
56236	1.89226	1.90143
56237	1.89226	2.1
56238	1.89226	2.1
56239	1.89226	1.93752
56240	1.89226	2.1
56241	1.89226	1.97618
56242	0.937933	2.09887
56243	0.937933	2.1
56244	1.26192	2.1
56245	1.26192	2.1
56246	0.937903	2.09071
56247	1.25372	2.1
56248	0.946132	2.1
56249	0.946132	2.1
56250	0.946133	2.1
56251	1.25372	2.1
56252	0.946132	2.1
56253	0.937902	2.1
56254	0.946132	1.79206
56255	0.946132	1.67764
56256	0.946128	2.09733
56257	0.946128	2.1

56258	0.94613	2.09039
56259	0.94613	1.66586
56260	0.94613	1.60167
56261	0.94613	2.09392
56262	0.946131	1.97678
56263	0.946131	1.9996
56264	0.94613	2.02993
56265	0.94613	2.0346
56266	0.946132	1.9097
56267	0.946132	1.71381
56268	1.2778	2.09939
56269	1.00191	2.1
56270	1.00191	1.83206
56271	1.00191	2.1
56272	0.94613	2.1
56273	0.94613	1.88962
56274	0.946131	2.07718
56275	1.2778	2.07215
56276	1.00191	2.01655
56277	0.94613	2.01893
56278	1.50847	0.0698591
56279	2.0815	0.0698591
56280	1.56806	0.0698591
56281	2.0815	0.0698591
56282	1.52043	0.0698591
56283	1.9224	0.0698591
56284	1.06882	0.0698591
56285	0.69567	0.0698591
56286	0.773112	0.0698591
56287	1.09473	0.0698591
56288	0.8941	0.0698591
56289	0.8941	0.0698591
56290	0.937064	0.0698591
56291	0.981131	0.0698591
56292	0.763762	0.0698591
56293	0.722535	0.0698591
56294	1.2153	0.0698591
56295	1.07279	0.0698591
56296	1.89226	0.0698591
56297	1.89226	0.0698591
56298	1.89226	0.0698591
56299	1.89226	0.0698591
56300	1.89226	0.0698591
56301	1.89226	0.0698591
56302	1.89226	0.0698591
56303	1.89226	0.0700735
56304	1.89226	0.0698591
56305	1.89226	0.0701119
56306	3.78453	0.0698591
56307	2.51518	0.0698591
56308	1.89226	0.0698591
56309	1.89226	0.0698591
56310	1.89226	0.0698591
56311	2.37584	0.0698591
56312	2.37584	0.0698591
56313	1.25759	0.0698591
56314	0.946131	0.0698591
56315	0.946131	0.0698591

56316	1.24489	0.0698591
56317	0.946131	0.0698591
56318	0.946131	0.0698591
56319	1.25759	0.0698591
56320	1.25759	0.0698591
56321	1.25759	0.0698591
56322	1.24489	0.0698591
56323	0.946131	0.0698591
56324	0.946131	0.0698591
56325	1.08028	0.33814
56326	1.09362	0.285123
56327	0.596616	0.750431
56328	0.606432	0.739542
56329	0.844265	0.808945
56330	0.844265	0.155817
56331	0.855786	0.41326
56332	0.855786	0.699735
56333	1.08028	0.560109
56334	0.606433	0.500698
56335	0.596616	0.607509
56336	1.09362	0.571782
56337	2.837	0.338663
56338	2.16055	0.0981992
56339	2.12741	0.200652
56340	2.79312	0.419429
56341	4.95367	0.195356
56342	2.12741	0.292528
56343	1.21286	0.413932
56344	1.20859	0.575056
56345	1.21286	0.718877
56346	2.15628	0.360893
56347	2.16056	0.119281
56348	1.89226	2.1
56349	1.89226	2.1
56350	1.89226	2.1
56351	1.89226	2.1
56352	3.78453	2.1
56353	0.946102	2.1
56354	0.946102	2.1
56355	0.850039	2.1
56356	0.850039	2.1
56357	0.914891	2.1
56358	0.85001	2.1
56359	0.946131	2.1
56360	0.946131	2.1
56361	0.94613	2.1
56362	0.91489	2.1
56363	0.850011	2.1
56364	0.946131	2.1
56365	0.71107	2.1
56366	0.784813	2.1
56367	1.28453	2.1
56368	1.20328	2.1
56369	0.992128	2.1
56370	0.992128	2.1
56371	0.995965	2.1
56372	0.995965	2.1
56373	0.784813	2.1

56374	1.28453	2.1
56375	0.711071	2.1
56376	1.20328	2.1
56377	1.89226	2.1
56378	1.89226	2.1
56379	1.89226	2.1
56380	1.89226	2.1
56381	3.78453	2.1
56382	0.946132	2.1
56383	0.946132	2.1
56384	0.914889	2.1
56385	0.914889	2.1
56386	0.985255	2.1
56387	0.914891	2.1
56388	0.946131	2.1
56389	0.946131	2.1
56390	0.946131	2.1
56391	0.985255	2.1
56392	0.94613	2.1
56393	0.91489	2.1
56394	0.784813	2.1
56395	0.871551	2.1
56396	1.37791	2.1
56397	1.28453	2.1
56398	1.07644	2.1
56399	1.07644	2.1
56400	1.07964	2.1
56401	1.07964	2.1
56402	0.871551	2.1
56403	1.37791	2.1
56404	0.784813	2.1
56405	1.28453	2.1
56406	1.73075	1.82011
56407	2.10582	1.39603
56408	1.89319	1.46075
56409	1.38766	1.98584
56410	3.27777	1.63758
56411	1.08715	1.95091
56412	1.54185	1.99752
56413	1.73074	1.94393
56414	1.73074	2.0359
56415	1.18948	1.7944
56416	1.18948	1.96654
56417	1.89319	1.33294
56418	1.41689	1.95112
56419	1.11619	1.77492
56420	1.38766	1.80282
56421	1.6052	1.59222
56422	1.89319	1.76019
56423	0.647894	1.87424
56424	0.594738	1.97531
56425	0.708445	1.97372
56426	0.770926	1.85338
56427	0.738326	1.98846
56428	0.738326	2.00451
56429	0.680494	1.83488
56430	0.680494	1.69887
56431	0.708443	1.73966

56432	0.647895	1.78105
56433	0.770925	1.83698
56434	0.59474	1.62376
56435	1.89111	2.1
56436	1.06994	2.1
56437	2.06689	2.1
56438	1.37063	2.1
56439	3.13683	2.1
56440	2.06689	2.1
56441	1.39295	2.1
56442	1.56818	2.1
56443	1.37063	2.1
56444	2.26444	2.1
56445	2.06689	2.1
56446	1.49808	2.1
56447	1.50201	2.1
56448	2.57784	2.1
56449	2.58615	2.1
56450	4.08424	2.1
56451	0.946133	2.1
56452	0.946133	2.1
56453	0.946132	2.1
56454	0.946132	2.1
56455	0.946132	2.1
56456	0.946132	2.1
56457	0.946132	2.1
56458	0.946132	2.1
56459	0.946133	2.1
56460	0.946133	2.1
56461	0.946132	2.1
56462	0.946132	2.1
56463	1.89226	2.1
56464	1.89226	2.1
56465	1.89226	2.1
56466	1.89226	2.1
56467	3.78452	2.1
56468	0.977201	2.1
56469	0.977201	2.1
56470	0.980489	2.1
56471	0.980489	2.1
56472	0.657973	2.1
56473	1.20669	2.1
56474	0.751002	2.1
56475	1.29307	2.1
56476	1.29308	2.1
56477	1.20669	2.1
56478	0.751003	2.1
56479	0.657972	2.1
56480	0.946131	2.1
56481	0.946131	2.1
56482	1.29308	2.1
56483	1.29308	2.1
56484	0.946132	2.1
56485	1.29308	2.1
56486	0.946132	2.1
56487	1.28892	2.1
56488	0.946132	2.1
56489	0.946132	2.1

56490	1.28892	2.1
56491	1.29308	2.1
56492	0.946132	2.09974
56493	0.946132	2.06836
56494	0.946131	2.1
56495	0.946131	2.09436
56496	0.946132	2.1
56497	0.946132	2.09621
56498	0.946132	2.09251
56499	0.946132	2.06659
56500	0.946132	2.09231
56501	0.946132	2.1
56502	0.946132	2.09534
56503	0.946132	2.1
56504	1.89226	2.1
56505	1.89226	2.09719
56506	1.89226	2.1
56507	1.89226	2.08249
56508	1.89226	2.1
56509	1.89226	2.06681
56510	0.94613	2.1
56511	0.94613	2.1
56512	1.20669	2.1
56513	1.20669	2.1
56514	1.20669	2.1
56515	0.946132	2.1
56516	0.946129	2.1
56517	1.29307	2.1
56518	0.946131	2.1
56519	0.946131	2.1
56520	1.29308	2.1
56521	1.20669	2.1
56522	1.89226	0.162773
56523	1.89226	0.0825565
56524	1.89226	0.0925529
56525	1.89226	0.125414
56526	3.78453	0.12623
56527	0.946132	0.0698591
56528	0.946132	0.071157
56529	0.946126	0.0761525
56530	0.946126	0.0920324
56531	0.946129	0.0780132
56532	0.946129	0.0711216
56533	0.946129	0.0736981
56534	0.946129	0.0913895
56535	0.946128	0.0725998
56536	0.946128	0.0739704
56537	0.946129	0.0742162
56538	0.946129	0.075304
56539	0.725277	0.0698591
56540	0.588155	0.0698591
56541	1.19403	0.0743063
56542	1.27681	0.06992
56543	0.918903	0.0699121
56544	0.918903	0.0698591
56545	0.946068	0.0715961
56546	0.946068	0.0698591
56547	1.19404	0.069971

56548	0.588153	0.0699056
56549	0.725275	0.0698591
56550	1.27682	0.0698591
56551	1.19404	0.0770433
56552	1.27682	0.0707724
56553	0.946131	0.106152
56554	0.946131	0.084705
56555	1.19404	0.0868197
56556	1.19404	0.0751405
56557	0.946129	0.0763628
56558	0.946129	0.0894799
56559	1.19404	0.0802503
56560	0.94613	0.0798631
56561	1.27682	0.0736466
56562	0.946131	0.0824077
56563	1.89226	0.0845239
56564	1.89226	0.129975
56565	1.89226	0.147852
56566	1.89226	0.0917294
56567	1.89226	0.128973
56568	1.89226	0.0770138
56569	1.4263	1.97948
56570	1.67651	0.872868
56571	1.5955	0.959508
56572	1.31022	2.0848
56573	2.98674	1.6155
56574	1.18824	2.1
56575	1.28802	2.1
56576	0.946132	2.07862
56577	0.946132	2.1
56578	0.946132	2.09439
56579	0.946132	2.1
56580	1.28802	2.0818
56581	1.28802	2.1
56582	1.18824	2.1
56583	0.946132	2.1
56584	0.946132	2.1
56585	1.28802	2.1
56586	1.89226	0.965664
56587	1.89226	2.09908
56588	1.89226	2.08767
56589	1.89226	1.11487
56590	3.78452	1.89796
56591	1.5955	1.15413
56592	1.52909	1.16936
56593	1.31022	1.63782
56594	1.31022	2.08388
56595	1.2087	2.0808
56596	1.52909	2.092
56597	1.11825	2.1
56598	1.18824	2.1
56599	0.946132	2.08839
56600	0.946132	2.1
56601	0.946132	2.1
56602	0.946132	2.1
56603	1.18824	2.08176
56604	1.18824	2.1
56605	0.946132	2.1

56606	1.11825	2.1
56607	1.18824	2.1
56608	0.946133	2.1
56609	0.810943	2.08782
56610	0.810943	2.09813
56611	0.810945	1.25
56612	0.810945	2.09829
56613	1.89226	1.23112
56614	1.35168	1.67042
56615	1.08132	1.24571
56616	1.0813	1.87753
56617	1.08132	2.09004
56618	0.810959	1.89374
56619	0.810959	1.8605
56620	1.89226	0.485331
56621	1.89226	0.162715
56622	1.89226	0.233376
56623	1.89226	0.371094
56624	3.78453	0.391173
56625	1.89226	0.0895698
56626	1.89226	0.12006
56627	1.89226	0.136657
56628	1.89226	0.0765926
56629	1.89226	0.130964
56630	1.89226	0.0748483
56631	0.946131	0.0702501
56632	0.946131	0.0714156
56633	0.946126	0.123564
56634	0.946126	0.086586
56635	0.946128	0.127212
56636	0.946128	0.0769781
56637	0.946128	0.120372
56638	0.946128	0.0929271
56639	0.946128	0.093215
56640	0.946128	0.098152
56641	0.946128	0.10702
56642	0.946128	0.0841803
56643	1.89226	0.357686
56644	1.89226	0.105496
56645	1.89226	0.252676
56646	1.89226	0.223714
56647	1.89226	0.16326
56648	1.89226	0.148043
56649	1.89226	0.15097
56650	1.89226	0.219678
56651	1.89226	0.503699
56652	1.89226	0.122782
56653	3.78453	0.121648
56654	1.07176	0.0698591
56655	1.07176	0.0698896
56656	0.946126	0.0936711
56657	0.946126	0.0788366
56658	1.07176	0.0703787
56659	0.946129	0.0698591
56660	1.27474	0.0821671
56661	0.94613	0.0933233
56662	1.27474	0.0796089
56663	1.07176	0.0698591

56664	0.94613	0.0698591
56665	0.94613	0.0704219
56666	0.723486	0.0698591
56667	0.94613	0.0698591
56668	0.946126	0.0785004
56669	0.946126	0.0698591
56670	0.946129	0.0698591
56671	0.723484	0.0698591
56672	1.07176	0.0698591
56673	0.946129	0.0698591
56674	1.32398	0.0703575
56675	1.19835	0.0698591
56676	0.723471	0.0698591
56677	0.723471	0.0713412
56678	0.810946	0.284591
56679	0.810946	0.156988
56680	0.810946	0.264742
56681	0.810946	0.186984
56682	0.810933	0.0720281
56683	0.810933	0.187347
56684	1.89226	0.0721951
56685	1.3517	0.161596
56686	1.08132	0.0858546
56687	1.08132	0.132568
56688	1.08133	0.182297
56689	2.39923	2.1
56690	1.89226	2.1
56691	1.89226	2.1
56692	1.89226	2.1
56693	2.44606	2.1
56694	2.44606	2.1
56695	1.89226	2.1
56696	1.89226	2.1
56697	1.89226	2.1
56698	1.89226	2.1
56699	1.89226	2.1
56700	1.89226	2.1
56701	1.70302	2.1
56702	2.43041	2.1
56703	2.25275	2.1
56704	2.4747	2.1
56705	1.52079	2.1
56706	1.67063	2.1
56707	2.40644	2.1
56708	1.89226	2.1
56709	1.89226	2.1
56710	1.89226	2.1
56711	2.39923	2.1
56712	2.39923	2.1
56713	0.946131	2.1
56714	0.946131	2.1
56715	0.946131	2.1
56716	0.946131	2.1
56717	0.94613	2.1
56718	0.94613	2.1
56719	0.946131	2.1
56720	0.946131	2.1
56721	0.946131	2.1

56722	0.946131	2.1
56723	0.946131	2.1
56724	0.946131	2.1
56725	1.04938	2.1
56726	1.04938	2.1
56727	1.111 2.1	
56728	1.06978	2.1
56729	1.2748	2.1
56730	1.25472	2.1
56731	0.88782	2.1
56732	0.882987	2.1
56733	0.973779	2.1
56734	1.31997	2.1
56735	0.913905	2.1
56736	1.31312	2.1
56737	2.6536	2.1
56738	2.15287	2.1
56739	2.72238	2.1
56740	2.22887	2.1
56741	4.87525	2.1
56742	2.15288	2.1
56743	1.40719	2.1
56744	1.41839	2.1
56745	1.34239	2.1
56746	2.22887	2.1
56747	2.15288	2.1
56748	2.72237	2.1
56749	2.22887	2.1
56750	2.77314	2.1
56751	2.28748	2.1
56752	5.00985	2.1
56753	0.922802	2.1
56754	0.922802	2.1
56755	0.92107	2.1
56756	0.92107	2.1
56757	0.703593	2.1
56758	0.729436	2.1
56759	1.11444	2.1
56760	1.14374	2.1
56761	1.14374	2.1
56762	0.729436	2.1
56763	0.703593	2.1
56764	1.11444	2.1
56765	1.89226	2.1
56766	1.89226	2.1
56767	1.89226	2.1
56768	1.89226	2.1
56769	1.89226	2.1
56770	1.89226	2.1
56771	0.489927	2.1
56772	0.63008	2.1
56773	0.697385	2.1
56774	0.55524	2.1
56775	0.592141	2.1
56776	0.592141	2.1
56777	0.593179	2.1
56778	0.593179	2.1
56779	0.63008	2.1

56780	0.697385	2.1
56781	0.55524	2.1
56782	0.489927	2.1
56783	0.946133	2.1
56784	0.946133	2.1
56785	0.946132	2.08896
56786	0.946132	1.98511
56787	0.946133	2.08901
56788	0.946133	2.1
56789	0.946132	2.1
56790	0.946132	2.02391
56791	0.946132	2.1
56792	0.946132	2.1
56793	0.946132	2.1
56794	0.946132	2.1
56795	1.89226	2.1
56796	1.89226	2.1
56797	1.89226	2.1
56798	1.89226	2.1
56799	1.89226	2.1
56800	1.89226	2.1
56801	0.805746	2.1
56802	0.63008	2.1
56803	0.881598	2.1
56804	0.697385	2.1
56805	0.749417	2.1
56806	0.749417	2.1
56807	0.753714	2.1
56808	0.753714	2.1
56809	0.881598	2.1
56810	0.805746	2.1
56811	0.63008	2.1
56812	0.697385	2.1
56813	0.881599	2.1
56814	0.697385	2.1
56815	0.805746	2.1
56816	0.626731	2.09282
56817	0.755479	2.1
56818	0.755479	2.1
56819	0.752851	2.09147
56820	0.752851	2.1
56821	0.881598	2.1
56822	0.805747	2.1
56823	0.697385	2.1
56824	0.626731	2.1
56825	2.65348	2.1
56826	2.12314	2.1
56827	2.63899	2.1
56828	2.13898	2.1
56829	4.76213	2.1
56830	0.763518	2.1
56831	0.765889	2.1
56832	0.84086	2.1
56833	0.84086	2.1
56834	0.632078	2.1
56835	0.597565	2.1
56836	1.08813	2.1
56837	0.985643	2.1

56838	0.982464	2.1
56839	0.66404	2.1
56840	1.0358	2.1
56841	0.598023	2.1
56842	1.29293	2.1
56843	1.29628	2.1
56844	1.19266	2.1
56845	1.19266	2.1
56846	1.07109	2.1
56847	1.13093	2.1
56848	1.34467	2.1
56849	1.35251	2.1
56850	1.34369	2.1
56851	1.12525	2.1
56852	1.28746	2.1
56853	1.13511	2.1
56854	2.19717	2.1
56855	2.16547	2.1
56856	1.37392	2.1
56857	1.37392	2.1
56858	1.2966	2.1
56859	2.11985	2.1
56860	1.89226	2.1
56861	1.89226	2.1
56862	1.89226	2.1
56863	1.89226	2.1
56864	1.89226	2.1
56865	1.89226	2.1
56866	1.65637	2.1
56867	1.41758	2.1
56868	1.65637	2.1
56869	1.74391	2.1
56870	1.35504	2.1
56871	1.44258	2.1
56872	1.40774	2.1
56873	1.40774	2.1
56874	0.946131	2.1
56875	0.946131	2.1
56876	1.40774	2.1
56877	1.38882	2.1
56878	0.946132	2.1
56879	0.946132	2.1
56880	0.946132	2.1
56881	1.38882	2.1
56882	0.946132	2.1
56883	1.40774	2.1
56884	1.89227	2.1
56885	1.89227	2.1
56886	1.89227	2.1
56887	1.89227	2.1
56888	3.78453	2.1
56889	0.603148	2.1
56890	0.600177	2.1
56891	0.558615	2.1
56892	0.580714	2.1
56893	0.558606	2.1
56894	0.67883	2.1
56895	1.65637	2.1

56896	1.07933	2.1
56897	0.842649	2.1
56898	0.905168	2.1
56899	0.774334	2.1
56900	1.38882	2.1
56901	1.38882	2.1
56902	0.946132	2.1
56903	0.946132	2.1
56904	1.38882	2.1
56905	1.37381	2.1
56906	0.946132	2.1
56907	0.946132	2.1
56908	0.946132	2.1
56909	1.37381	2.1
56910	0.946133	2.1
56911	1.38882	2.1
56912	2.3771	2.1
56913	1.9339	2.1
56914	1.70782	2.1
56915	1.9339	2.1
56916	2.42693	2.1
56917	2.83279	2.1
56918	2.34901	0.264595
56919	1.45833	0.356593
56920	1.86695	0.295907
56921	1.03101	0.330922
56922	3.32528	0.312795
56923	1.89226	0.117666
56924	1.89226	0.119799
56925	1.89226	0.0789798
56926	1.89226	0.0722868
56927	3.78453	0.10103
56928	2.71753	0.0746762
56929	1.89226	0.108685
56930	2.76228	0.0698836
56931	2.76228	0.0730197
56932	1.89226	0.106344
56933	1.89226	0.24048
56934	0.946131	0.0806178
56935	0.946131	0.0791531
56936	0.946132	0.0769827
56937	0.946132	0.0849276
56938	0.946132	0.111236
56939	0.946132	0.0743342
56940	0.946132	0.0985623
56941	0.946132	0.070971
56942	0.946132	0.0717975
56943	0.946132	0.0731878
56944	0.946132	0.0951055
56945	0.946132	0.0895018
56946	1.89227	0.0895173
56947	1.89227	0.0698591
56948	1.89227	0.0698591
56949	1.89227	0.0812161
56950	1.89227	0.0698591
56951	1.89227	0.0699337
56952	2.71753	0.0786711
56953	2.37907	0.0698591

56954	2.37907	0.0698591
56955	2.76228	0.0977784
56956	2.39588	0.0698591
56957	2.77909	0.0698591
56958	1.6571	0.401086
56959	1.11682	1.75736
56960	1.47132	0.898815
56961	1.93763	0.121483
56962	3.12842	1.3616
56963	1.93762	0.168563
56964	2.12345	1.30133
56965	1.65709	0.182867
56966	1.65709	1.95723
56967	2.12345	0.586865
56968	1.88635	1.97559
56969	1.47134	1.90314
56970	2.219	0.258188
56971	2.25734	2.1
56972	1.93765	0.565584
56973	1.79103	1.45694
56974	1.47134	0.554087
56975	1.06174	0.787742
56976	1.19349	2.08302
56977	1.1095	1.96144
56978	0.968827	0.416896
56979	1.07892	0.168392
56980	1.07892	2.1
56981	1.0834	1.0297
56982	1.0834	0.986341
56983	1.1095	0.519146
56984	1.19349	0.372409
56985	0.968825	0.799095
56986	1.06174	0.743871
56987	2.40627	2.1
56988	2.08288	1.17172
56989	2.5444	1.23021
56990	2.29285	1.72881
56991	4.62729	1.4801
56992	2.08289	0.603977
56993	1.65703	0.58318
56994	1.71105	2.1
56995	1.50108	1.66693
56996	2.29286	2.04767
56997	2.08289	1.80712
56998	2.5444	1.77679
56999	2.29285	2.1
57000	2.71429	2.1
57001	2.50288	0.776908
57002	5.04728	2.07331
57003	2.50288	0.680088
57004	2.00927	1.84552
57005	2.02116	1.69664
57006	2.02116	2.05495
57007	2.29286	1.39029
57008	2.29286	1.88178
57009	1.89226	2.1
57010	1.89226	2.1
57011	1.89226	2.1

57012	1.89226	2.1
57013	3.78453	2.1
57014	1.17418	2.1
57015	1.29741	2.1
57016	1.04921	2.1
57017	1.19352	2.1
57018	2.3677	2.1
57019	1.29741	2.1
57020	1.27575	2.1
57021	1.19352	2.1
57022	1.19473	2.1
57023	2.46927	2.1
57024	1.89226	2.1
57025	1.89226	2.1
57026	1.89226	2.1
57027	1.89226	2.1
57028	1.89226	2.1
57029	1.89226	2.1
57030	0.561332	2.1
57031	0.561332	2.1
57032	0.567597	2.1
57033	0.567597	2.1
57034	0.597365	2.1
57035	0.544699	2.1
57036	0.596761	2.1
57037	0.531564	2.1
57038	0.5447	2.1
57039	0.531563	2.1
57040	0.596759	2.1
57041	0.597366	2.1
57042	0.946131	2.1
57043	0.946131	2.1
57044	0.946131	2.1
57045	0.946131	2.1
57046	0.946129	2.1
57047	0.946129	2.1
57048	0.946133	2.1
57049	0.946133	2.1
57050	0.946131	2.1
57051	0.946131	2.1
57052	0.946131	2.1
57053	0.946131	2.1
57054	1.10938	0.81713
57055	1.17614	2.1
57056	1.02478	1.65324
57057	1.09056	2.1
57058	2.19994	2.05287
57059	1.17614	2.1
57060	1.11868	2.1
57061	1.03408	2.1
57062	1.03408	2.1
57063	1.09056	2.1
57064	1.17516	2.1
57065	1.89226	0.454929
57066	1.89226	0.357228
57067	1.89226	0.437356
57068	1.89226	0.31642
57069	1.89226	0.531386

57070	1.89226	0.380786
57071	1.89226	0.315455
57072	1.89226	0.397373
57073	1.89226	0.378728
57074	1.89226	0.264286
57075	1.89226	0.470296
57076	1.89226	0.274132
57077	0.579551	0.249791
57078	0.563873	0.217673
57079	0.611341	0.30603
57080	0.598693	0.262031
57081	0.589856	0.255225
57082	0.589856	0.221783
57083	0.588387	0.299435
57084	0.588387	0.257506
57085	0.57955	0.265721
57086	0.611341	0.259237
57087	0.563873	0.254526
57088	0.598693	0.247346
57089	0.611341	0.303117
57090	0.598693	0.260761
57091	0.586048	0.300831
57092	0.572737	0.330638
57093	0.591824	0.299583
57094	0.591824	0.265166
57095	0.592254	0.341624
57096	0.592254	0.310624
57097	0.611341	0.308789
57098	0.586048	0.296046
57099	0.598693	0.299921
57100	0.572737	0.294269
57101	0.946119	0.309288
57102	0.946119	0.44363
57103	0.946119	0.327949
57104	0.946119	0.448845
57105	0.94612	0.322609
57106	0.94612	0.28852
57107	0.946119	0.447909
57108	0.946119	0.505204
57109	0.946132	0.390287
57110	0.946132	0.37508
57111	0.946107	0.393223
57112	0.946107	0.42007
57113	1.89224	0.345403
57114	1.89224	0.28341
57115	1.89224	0.366349
57116	1.89224	0.240698
57117	1.89224	0.453511
57118	1.89224	0.297243
57119	0.562335	0.229336
57120	0.579543	0.197831
57121	0.59499	0.263585
57122	0.611333	0.227993
57123	0.586619	0.223247
57124	0.586619	0.202431
57125	0.587049	0.258691
57126	0.587049	0.23257
57127	0.594999	0.244908

57128	0.562342	0.243005
57129	0.579535	0.233332
57130	0.611325	0.226822
57131	0.59499	0.259876
57132	0.611333	0.226707
57133	0.567849	0.26021
57134	0.58604	0.288523
57135	0.590978	0.257369
57136	0.590978	0.230669
57137	0.590052	0.295915
57138	0.590052	0.266648
57139	0.567857	0.281307
57140	0.594998	0.2755
57141	0.611325	0.273529
57142	0.586032	0.263159
57143	1.69661	1.86757
57144	1.9339	0.501736
57145	1.39379	1.99624
57146	1.69107	0.238077
57147	3.32768	1.07203
57148	1.9339	0.340042
57149	1.98414	0.982623
57150	1.69107	0.280027
57151	1.72774	0.583233
57152	3.66164	0.319901
57153	1.39379	2.05549
57154	1.69107	0.259218
57155	1.14397	1.65353
57156	1.43548	0.285872
57157	2.82927	1.07114
57158	1.69107	0.157424
57159	1.72774	0.558059
57160	1.53873	0.301331
57161	1.53873	0.920114
57162	1.43548	0.232455
57163	1.62449	1.0657
57164	2.58431	0.102827
57165	1.34072	0.129714
57166	1.80841	0.114889
57167	1.34072	0.127144
57168	2.51501	0.11317
57169	2.04732	0.130615
57170	1.89226	0.124449
57171	1.89226	0.150563
57172	1.89226	0.126873
57173	1.89226	0.159223
57174	1.89226	0.132672
57175	1.89226	0.15884
57176	0.946131	0.162026
57177	0.946131	0.137597
57178	1.3639	0.118939
57179	1.1516	0.14553
57180	0.946129	0.148462
57181	0.946129	0.169378
57182	1.36391	0.131344
57183	1.36391	0.12466
57184	0.946131	0.151535
57185	1.3639	0.141519

57186	1.1516	0.136468
57187	0.946131	0.145905
57188	0.946131	0.143991
57189	0.946131	0.122081
57190	0.94613	0.151558
57191	0.94613	0.128142
57192	0.946128	0.159079
57193	0.946128	0.142671
57194	0.946133	0.121578
57195	0.946133	0.13412
57196	0.946131	0.139625
57197	0.946131	0.139063
57198	0.946131	0.138126
57199	0.946131	0.132117
57200	1.89226	1.66015
57201	2.63634	2.02555
57202	1.89226	1.81806
57203	1.83902	1.75227
57204	3.73128	1.71056
57205	1.47059	2.1
57206	2.02928	2.1
57207	1.47059	2.1
57208	1.65706	2.1
57209	1.98628	2.1
57210	2.17274	2.1
57211	2.43618	2.1
57212	2.41176	2.1
57213	1.98627	2.1
57214	2.34084	2.1
57215	4.39803	2.1
57216	1.47059	2.1
57217	1.82453	2.1
57218	1.98627	2.1
57219	1.98627	2.1
57220	1.26929	2.1
57221	1.26929	2.1
57222	1.98627	2.1
57223	2.34084	2.1
57224	1.47264	2.1
57225	2.24937	2.1
57226	4.23564	2.1
57227	1.33026	1.71956
57228	1.51171	2.06538
57229	1.51171	1.63822
57230	1.4617	2.07964
57231	1.35382	1.56051
57232	1.30382	2.09958
57233	1.89226	1.37912
57234	1.89226	1.88489
57235	1.89226	1.27358
57236	1.89226	2.04381
57237	1.89226	1.22841
57238	1.89226	2.06293
57239	1.39263	1.5508
57240	0.962704	0.669027
57241	1.0171	0.882299
57242	0.962704	1.32792
57243	1.40822	0.782413

57244	1.35382	1.07401
57245	0.755853	2.1
57246	0.789808	2.02518
57247	0.696315	1.64288
57248	0.676912	1.20502
57249	0.729751	1.24202
57250	0.729751	2.00975
57251	0.722417	2.1
57252	0.722417	1.40692
57253	0.696315	1.92619
57254	0.789808	1.90702
57255	0.755853	1.90082
57256	0.676912	1.89393
57257	0.946132	1.83472
57258	0.946132	2.00429
57259	0.946132	1.27064
57260	0.946132	1.01412
57261	0.946131	1.0479
57262	0.946131	1.82098
57263	0.946132	1.3613
57264	0.946132	2.00637
57265	0.946132	1.49129
57266	0.946132	1.50118
57267	0.946132	1.60453
57268	0.946132	1.59073
57269	0.946133	1.10806
57270	0.946133	0.91094
57271	0.946132	0.588596
57272	0.946132	0.730672
57273	0.946132	0.600541
57274	0.946132	0.893193
57275	0.946133	0.661659
57276	0.946133	1.16356
57277	0.946132	0.835879
57278	0.946132	0.834468
57279	0.946132	0.821384
57280	0.946132	0.841222
57281	1.89227	1.86657
57282	2.53014	1.89775
57283	1.89227	1.99742
57284	1.98241	1.70603
57285	1.89227	2.01157
57286	1.98241	1.76778
57287	1.89226	1.74786
57288	1.89226	2.0643
57289	1.89226	1.95234
57290	1.89226	1.94879
57291	1.89226	2.03205
57292	1.89226	2.01927
57293	1.89226	1.61031
57294	1.89226	2.00511
57295	1.89226	1.8049
57296	1.89226	1.82119
57297	3.78452	1.80618
57298	2.45304	1.19177
57299	1.89226	1.8888
57300	1.89226	1.75641
57301	1.89226	1.71258

57302	1.89226	1.46063
57303	1.89226	1.44067
57304	1.89227	1.88869
57305	1.89227	1.8654
57306	1.89227	1.98399
57307	1.89227	1.68065
57308	1.89227	1.99368
57309	1.89227	1.73043
57310	1.89226	1.82285
57311	1.89226	2.05382
57312	1.89226	1.9431
57313	1.89226	1.9472
57314	1.89226	2.00567
57315	1.89226	2.00141
57316	2.09871	0.249928
57317	2.71891	0.252629
57318	1.31126	0.212401
57319	1.31126	0.373903
57320	2.55341	0.209582
57321	1.76597	0.551535
57322	1.89226	0.718506
57323	1.89226	0.874166
57324	1.89226	0.385221
57325	1.89226	1.35051
57326	1.89226	0.458055
57327	1.89226	1.57403
57328	0.946128	0.816529
57329	0.946128	0.365624
57330	0.946133	0.31623
57331	0.946133	0.242889
57332	0.946131	0.311776
57333	0.946131	0.537732
57334	0.94613	0.267671
57335	0.94613	0.379249
57336	0.946131	0.353047
57337	0.946131	0.310031
57338	0.946131	0.347164
57339	0.946131	0.392608
57340	2.71891	0.233988
57341	1.89226	0.221456
57342	2.09871	0.171515
57343	2.09871	0.383865
57344	1.89226	0.16605
57345	1.89226	0.401327
57346	1.89227	0.666497
57347	1.89227	0.67248
57348	1.89227	0.396813
57349	1.89227	1.25937
57350	1.89227	0.484775
57351	1.89227	1.40505
57352	0.946129	0.63265
57353	0.946129	0.367248
57354	0.946133	0.283105
57355	0.946133	0.218148
57356	0.946131	0.27972
57357	0.946131	0.49548
57358	0.946131	0.242388
57359	0.946131	0.392299

57360	0.946131	0.331729
57361	0.946131	0.297492
57362	0.946131	0.362883
57363	0.946131	0.327784
57364	2.32462	0.0698591
57365	1.89226	0.0698591
57366	1.89226	0.0698591
57367	1.89226	0.0698591
57368	2.2291	0.0698591
57369	2.2291	0.0698591
57370	0.847669	0.0698591
57371	0.847669	0.0698591
57372	0.796966	0.0698591
57373	0.796966	0.0698591
57374	0.833552	0.0698591
57375	1.01049	0.0698591
57376	0.709709	0.0698591
57377	0.634147	0.0698591
57378	0.634148	0.0698591
57379	0.833549	0.0698591
57380	0.709711	0.0698591
57381	1.01049	0.0698591
57382	1.89226	0.0698591
57383	1.89226	0.0698591
57384	1.89226	0.0698591
57385	1.89226	0.0698591
57386	1.89226	0.0698591
57387	1.89226	0.0698591
57388	1.03611	0.0698591
57389	0.936655	0.0698591
57390	0.946132	0.0698591
57391	0.946132	0.0698591
57392	0.941918	0.0698591
57393	0.941918	0.0698591
57394	1.00308	0.0698591
57395	1.04117	0.0698591
57396	1.1406	0.0698591
57397	1.04074	0.0698591
57398	0.851529	0.0698591
57399	1.00311	0.0698591
57400	1.01814	0.0698591
57401	1.6671	0.0698591
57402	1.35561	0.0698591
57403	1.35561	0.0698591
57404	1.2683	0.0698591
57405	1.2683	0.0698591
57406	0.946128	0.0698591
57407	0.946128	0.0698591
57408	0.946129	0.0698591
57409	0.946129	0.0698591
57410	0.946132	0.0698591
57411	0.946132	0.0698591
57412	0.946126	0.0698591
57413	0.946126	0.0698591
57414	0.946129	0.0698591
57415	0.946129	0.0698591
57416	0.946129	0.0698591
57417	0.946129	0.0698591

57418	2.23338	0.0698591
57419	1.96	0.0698591
57420	2.03438	0.0698591
57421	1.87835	0.0698591
57422	4.11172	0.0698591
57423	1.96	0.0698591
57424	1.52661	0.0698591
57425	1.87834	0.0698591
57426	1.53175	0.0698591
57427	3.40495	0.0698591
57428	2.03435	0.0698591
57429	1.65813	0.0698591
57430	1.65813	0.0698591
57431	1.87832	0.0698591
57432	1.72067	0.0698591
57433	1.94086	0.0698591
57434	1.65813	0.0698591
57435	1.40572	0.0698591
57436	1.87832	0.0698591
57437	1.87832	0.0698591
57438	1.31154	0.0698591
57439	1.53173	0.0698591
57440	1.86662	2.1
57441	2.0815	2.1
57442	2.0815	2.1
57443	2.0815	2.1
57444	1.87246	2.1
57445	1.87362	2.1
57446	1.0273	2.1
57447	0.996347	2.1
57448	0.946129	2.1
57449	0.946129	2.1
57450	1.04427	2.1
57451	1.04196	2.1
57452	0.910674	2.1
57453	0.910674	2.1
57454	1.04072	2.1
57455	1.10931	2.1
57456	1.03132	2.1
57457	0.933698	2.1
57458	2.63789	0.284064
57459	1.89226	0.532619
57460	2.3033	0.30593
57461	2.3033	0.412962
57462	1.89226	0.301915
57463	1.89226	0.420272
57464	1.89226	0.739626
57465	1.89226	0.929379
57466	1.89226	0.488582
57467	1.89226	0.741212
57468	3.78452	0.767542
57469	1.51898	0.522761
57470	1.36006	0.94118
57471	2.03143	0.904589
57472	2.03143	0.962147
57473	1.04026	0.624048
57474	1.04026	0.691352
57475	0.94613	0.162199

57476	0.94613	0.257703
57477	1.31894	0.167251
57478	1.31894	0.144771
57479	0.94613	0.155402
57480	0.94613	0.313602
57481	1.31894	0.191623
57482	1.41232	0.236348
57483	0.946129	0.20651
57484	1.41232	0.207499
57485	1.31895	0.195623
57486	0.946129	0.224348
57487	1.89226	0.21823
57488	1.89226	0.699947
57489	1.89226	0.228558
57490	1.89226	0.570313
57491	1.89226	0.169319
57492	1.89226	0.496426
57493	1.89804	0.198853
57494	2.03143	0.433519
57495	2.46286	0.369
57496	2.46287	0.40907
57497	1.51898	0.269717
57498	1.51898	0.325556
57499	1.89226	2.1
57500	1.89226	2.1
57501	1.89226	2.1
57502	1.89226	2.1
57503	3.78452	2.1
57504	1.3864	2.1
57505	1.3864	2.1
57506	0.946132	2.1
57507	0.946132	2.1
57508	1.32905	2.1
57509	1.3864	2.1
57510	0.946132	2.1
57511	0.946132	2.1
57512	0.946132	2.1
57513	1.32905	2.1
57514	0.946133	2.1
57515	1.3864	2.1
57516	2.77279	2.1
57517	1.67883	2.1
57518	1.67883	2.1
57519	1.73415	2.1
57520	2.65811	2.1
57521	2.71343	2.1
57522	0.946131	2.1
57523	0.946131	2.1
57524	0.946128	2.1
57525	0.946128	2.1
57526	0.946127	2.1
57527	0.946127	2.1
57528	0.946131	2.1
57529	0.946131	2.1
57530	0.946129	2.1
57531	0.946129	2.1
57532	0.94613	2.1
57533	0.94613	2.1

57534	1.89226	2.1
57535	1.89226	2.1
57536	1.89226	2.1
57537	1.89226	2.1
57538	3.78452	2.1
57539	1.89226	2.1
57540	2.54394	2.1
57541	2.54394	2.1
57542	2.65811	2.1
57543	1.89226	2.1
57544	2.00643	2.1
57545	2.54394	2.1
57546	2.6581	2.1
57547	1.62743	2.1
57548	1.62743	2.1
57549	1.67883	2.1
57550	2.59533	2.1
57551	1.89226	2.1
57552	1.89226	2.1
57553	1.89226	2.1
57554	1.89226	2.1
57555	1.89226	2.1
57556	1.89226	2.1
57557	1.89226	1.8578
57558	1.89226	1.80385
57559	1.89226	2.05996
57560	1.89226	1.46117
57561	1.89226	2.04034
57562	1.89226	1.4352
57563	1.89226	1.85295
57564	1.89226	1.77656
57565	1.89226	2.02843
57566	1.89226	1.61154
57567	1.89226	1.99663
57568	1.89226	1.50705
57569	1.89226	1.2965
57570	1.89226	0.793894
57571	1.89226	1.77072
57572	1.89226	0.754995
57573	1.89226	1.71126
57574	1.89226	0.756647
57575	2.68537	0.491802
57576	2.22575	2.08462
57577	2.47464	0.314954
57578	1.79093	1.94719
57579	4.70039	0.919844
57580	2.81217	2.1
57581	2.47464	0.415976
57582	2.58286	2.04607
57583	2.29193	0.313289
57584	5.0575	0.862836
57585	2.47464	0.293964
57586	2.20365	1.93832
57587	2.29193	0.50363
57588	1.90104	2.03159
57589	4.37568	0.644846
57590	1.89226	0.709787
57591	2.16957	2.05109

57592	2.52823	1.40258
57593	2.52823	2.01666
57594	1.89226	0.970744
57595	1.89226	1.61637
57596	1.36226	2.1
57597	1.55369	2.1
57598	1.33309	2.1
57599	1.62593	2.1
57600	2.98819	2.1
57601	0.802281	2.1
57602	0.802281	2.1
57603	0.835143	2.1
57604	0.835143	2.1
57605	0.824458	2.1
57606	0.92641	2.1
57607	0.776847	2.1
57608	0.812967	2.1
57609	0.812967	2.1
57610	0.926411	2.1
57611	0.776847	2.1
57612	0.824458	2.1
57613	1.33309	2.1
57614	1.62593	2.1
57615	1.32813	2.1
57616	1.7237	2.1
57617	2.95406	2.1
57618	0.814544	2.1
57619	0.875312	2.1
57620	0.910502	2.1
57621	0.910502	2.1
57622	0.886633	2.1
57623	0.975739	2.1
57624	0.792623	2.1
57625	0.840283	2.1
57626	0.796372	2.1
57627	1.06159	2.1
57628	0.81948	2.1
57629	0.933833	2.1
57630	2.63997	2.1
57631	1.29694	2.1
57632	1.28141	2.1
57633	2.62623	2.1
57634	3.92317	2.1
57635	2.63998	2.1
57636	1.89226	2.1
57637	1.89226	2.1
57638	2.62623	2.1
57639	4.5185	2.1
57640	1.89226	2.1
57641	1.89226	2.1
57642	1.89226	2.1
57643	1.89226	2.1
57644	3.78453	2.1
57645	1.89226	2.1
57646	1.89226	2.1
57647	1.89226	2.1
57648	1.89226	2.1
57649	1.89226	2.1

57650	1.89226	2.1
57651	1.25468	2.1
57652	2.62623	2.1
57653	1.28141	2.1
57654	1.28141	2.1
57655	2.60665	2.1
57656	2.60665	2.1
57657	1.89226	2.1
57658	2.62623	2.1
57659	2.60666	2.1
57660	2.62623	2.1
57661	1.87269	2.1
57662	1.89226	2.1
57663	1.89226	2.1
57664	1.89226	2.1
57665	1.89226	2.1
57666	1.89226	2.1
57667	3.78453	2.1
57668	1.89226	2.1
57669	1.89226	2.1
57670	1.89226	2.1
57671	1.89226	2.1
57672	1.89226	2.1
57673	1.89226	2.1
57674	2.30147	1.5377
57675	2.27703	1.95918
57676	1.89227	1.36828
57677	1.89227	1.86084
57678	4.1693	1.7617
57679	1.38617	0.744236
57680	1.24613	1.55072
57681	2.59886	0.808353
57682	2.6238	1.48626
57683	3.86993	0.985766
57684	0.946133	1.80574
57685	0.946133	1.28574
57686	0.946132	1.3026
57687	0.946132	1.98028
57688	0.946133	1.98166
57689	0.946133	1.88114
57690	0.946132	1.26211
57691	0.946132	1.55302
57692	0.946133	1.76477
57693	0.946133	1.73629
57694	0.946132	1.63727
57695	0.946132	1.62995
57696	2.21773	2.1
57697	2.59216	2.1
57698	1.68541	2.1
57699	1.68541	2.1
57700	1.84814	2.1
57701	2.38046	2.1
57702	1.89966	2.1
57703	1.70303	2.1
57704	1.2028	2.1
57705	1.29897	2.1
57706	2.02873	2.1
57707	2.19093	2.1

57708	2.75218	2.1
57709	1.70303	2.1
57710	3.10254	2.1
57711	2.24783	2.1
57712	2.56839	2.1
57713	1.70303	2.1
57714	2.56839	2.1
57715	1.08276	2.1
57716	2.24061	2.1
57717	1.67491	2.1
57718	1.60948	2.1
57719	1.08276	2.1
57720	1.89226	1.28798
57721	1.89226	1.34827
57722	1.89226	0.671545
57723	1.89226	1.04533
57724	3.78453	1.1024
57725	1.89226	1.43746
57726	1.89226	1.34396
57727	1.89226	0.372487
57728	1.89226	0.830882
57729	3.78452	1.11356
57730	1.89226	1.33878
57731	2.7535	1.04924
57732	1.89226	1.67994
57733	1.89226	1.64531
57734	1.89226	0.583559
57735	1.89226	0.708992
57736	1.89226	1.31451
57737	1.89226	1.19993
57738	1.89226	1.1611
57739	1.89226	1.43842
57740	1.89226	1.19748
57741	1.89226	1.38695
57742	1.89227	1.24214
57743	1.89227	0.831362
57744	1.89227	0.242528
57745	1.89227	0.486988
57746	3.78453	0.648429
57747	1.89226	1.19457
57748	2.53412	0.402157
57749	1.89226	1.59909
57750	1.89226	0.637253
57751	1.89226	0.975157
57752	1.89226	0.402759
57753	2.21126	0.0759106
57754	1.89226	0.0728595
57755	2.57464	0.0910117
57756	1.89226	0.0879924
57757	2.57464	0.0699093
57758	1.89226	0.0698591
57759	1.89226	0.195413
57760	1.89226	0.229849
57761	1.89226	0.658019
57762	1.89226	0.158821
57763	1.89226	0.578701
57764	1.89226	0.153582
57765	1.1418	0.19084

57766	0.653021	0.0913268
57767	1.37282	0.185344
57768	0.931386	0.257714
57769	1.34568	0.352797
57770	1.17669	0.119831
57771	0.666595	0.0835482
57772	0.786895	0.196246
57773	0.933982	0.269845
57774	1.05351	0.319331
57775	1.00792	0.0965131
57776	1.00792	0.0850276
57777	1.89226	2.1
57778	1.89226	2.1
57779	1.89226	2.1
57780	1.89226	2.1
57781	3.78452	2.1
57782	1.89226	2.1
57783	1.89226	2.1
57784	1.89226	2.1
57785	1.89226	2.1
57786	1.89226	2.1
57787	1.89226	2.1
57788	1.65642	2.1
57789	1.30248	2.1
57790	1.38888	2.1
57791	1.25987	2.1
57792	1.70611	2.1
57793	1.65642	2.1
57794	1.65642	2.1
57795	1.81407	2.1
57796	2.19428	2.1
57797	1.92555	2.1
57798	3.8507	2.1
57799	1.89226	2.1
57800	1.89226	2.1
57801	1.89226	2.1
57802	1.89226	2.1
57803	3.78452	2.1
57804	1.89226	2.1
57805	1.89226	2.1
57806	1.89226	2.1
57807	1.89226	2.1
57808	1.89226	2.1
57809	1.89226	2.1
57810	0.705495	2.1
57811	0.809317	2.1
57812	1.1655	2.1
57813	1.09605	2.1
57814	0.983375	2.1
57815	0.797301	2.1
57816	0.906633	2.1
57817	0.906633	2.1
57818	0.85085	2.1
57819	1.16548	2.1
57820	0.705501	2.1
57821	1.00314	2.1
57822	2.19428	2.1
57823	2.31862	2.1

57824	2.27408	2.1
57825	2.45893	2.1
57826	4.46836	2.1
57827	1.89226	0.493434
57828	1.89226	0.880749
57829	1.89226	1.16347
57830	1.89226	0.4436
57831	1.89226	1.25707
57832	1.89226	0.722153
57833	2.0815	0.441439
57834	2.22136	0.599232
57835	1.5515	0.571704
57836	1.86323	0.346217
57837	2.74986	0.874858
57838	3.30238	0.437309
57839	1.89224	0.504585
57840	1.89224	0.886547
57841	1.89224	1.14731
57842	1.89224	0.423574
57843	1.89224	1.31393
57844	1.89224	0.596923
57845	1.89224	0.489273
57846	1.89224	1.18872
57847	1.89224	0.917557
57848	1.89224	0.397207
57849	1.89224	1.2526
57850	1.89224	0.706047
57851	1.17846	0.332963
57852	0.810935	0.416653
57853	0.93347	1.1455
57854	0.93347	0.392022
57855	0.810932	0.612784
57856	0.810932	0.47836
57857	2.22133	0.466425
57858	1.71919	0.471741
57859	1.32629	0.355677
57860	1.0813	0.68991
57861	1.57137	0.423127
57862	2.21149	2.1
57863	1.89226	2.1
57864	1.89226	2.1
57865	1.89226	2.1
57866	2.17159	2.1
57867	2.17159	2.1
57868	0.946131	1.04047
57869	0.946131	2.1
57870	0.946131	2.1
57871	0.946131	2.09808
57872	0.94613	1.49524
57873	0.94613	1.88263
57874	0.946132	2.1
57875	0.946132	2.1
57876	0.946131	2.04419
57877	0.946131	2.05305
57878	0.946131	2.1
57879	0.946131	2.1
57880	1.12266	2.1
57881	1.4714	2.04559

57882	1.62322	2.1
57883	1.62322	2.03349
57884	1.02958	2.1
57885	1.02958	2.1
57886	2.2671	2.1
57887	1.89226	2.1
57888	1.89226	2.1
57889	1.89226	2.1
57890	2.21149	2.1
57891	2.21149	2.1
57892	1.89226	2.1
57893	1.89226	2.02918
57894	1.89226	2.1
57895	1.89226	1.82902
57896	1.89226	2.1
57897	1.89226	2.05978
57898	0.736914	2.0842
57899	0.736914	2.1
57900	0.822839	2.1
57901	0.822836	2.1
57902	0.791298	1.29515
57903	1.00003	2.01322
57904	0.640254	2.1
57905	0.575379	2.1
57906	0.609001	2.07895
57907	1.00005	2.09077
57908	0.575371	2.09481
57909	0.831908	2.07589
57910	1.89226	2.1
57911	1.89226	2.1
57912	1.89226	2.1
57913	1.89226	2.1
57914	1.89226	2.1
57915	1.89226	2.1
57916	1.89226	2.1
57917	1.89226	2.1
57918	1.89226	2.1
57919	1.89226	2.1
57920	1.89226	2.1
57921	1.89226	2.1
57922	0.730617	2.1
57923	0.71052	2.1
57924	0.7329	2.1
57925	0.712083	2.1
57926	0.701051	2.1
57927	0.701051	2.1
57928	0.721552	2.1
57929	0.721552	2.1
57930	0.710521	2.1
57931	0.732898	2.1
57932	0.730619	2.1
57933	0.712081	2.1
57934	0.7329	2.1
57935	0.712082	2.1
57936	0.559601	2.1
57937	0.495993	2.1
57938	0.603718	2.1
57939	0.603718	2.1

57940	0.625174	2.1
57941	0.625174	2.1
57942	0.732899	2.1
57943	0.559601	2.1
57944	0.712082	2.1
57945	0.495993	2.1
57946	0.94613	2.1
57947	0.94613	2.1
57948	0.94613	2.1
57949	0.94613	2.1
57950	0.946131	2.1
57951	0.946131	2.1
57952	0.946129	2.1
57953	0.946129	2.1
57954	0.94613	2.1
57955	0.94613	2.1
57956	0.94613	2.1
57957	0.94613	2.1
57958	1.89226	2.1
57959	1.89226	2.1
57960	1.89226	2.1
57961	1.89226	2.1
57962	1.89226	2.1
57963	1.89226	2.1
57964	1.42104	2.1
57965	1.4658	2.1
57966	1.51223	2.1
57967	1.42283	2.1
57968	2.93327	2.1
57969	0.732899	2.1
57970	0.756115	2.1
57971	0.629763	2.1
57972	0.559601	2.1
57973	0.646134	2.1
57974	0.646134	2.1
57975	0.669582	2.1
57976	0.669582	2.1
57977	0.756115	2.1
57978	0.629763	2.1
57979	0.732899	2.1
57980	0.559601	2.1
57981	2.566 2.1	
57982	1.20242	2.1
57983	2.64084	2.1
57984	2.18297	2.1
57985	1.66029	2.1
57986	1.20242	2.1
57987	0.946133	1.55178
57988	0.946133	1.85323
57989	0.946129	2.1
57990	0.946129	2.1
57991	0.946131	2.1
57992	0.946131	1.47597
57993	0.946131	1.28446
57994	0.946131	2.1
57995	0.946131	2.03728
57996	0.946131	2.05816
57997	0.946131	2.0716

57998	0.946131	2.08562
57999	1.89226	0.0698591
58000	1.89226	0.0698591
58001	1.89226	0.0698591
58002	1.89226	0.0698591
58003	3.78453	0.0698591
58004	1.03073	0.0698591
58005	1.03073	0.0698591
58006	1.04066	0.0698591
58007	1.04066	0.0698591
58008	1.20802	0.0698591
58009	1.32689	0.0698591
58010	0.883301	0.0698591
58011	0.744506	0.0698591
58012	1.20802	0.0698591
58013	0.744506	0.0698591
58014	0.883301	0.0698591
58015	1.32689	0.0698591
58016	1.20802	0.0698591
58017	1.20802	0.0698591
58018	0.946131	0.0698591
58019	0.946131	0.0698591
58020	0.94613	0.0698591
58021	0.94613	0.0698591
58022	1.20802	0.0698591
58023	1.32689	0.0698591
58024	1.20802	0.0698591
58025	0.946131	0.0698591
58026	0.946131	0.0698591
58027	1.32689	0.0698591
58028	1.89226	0.0698591
58029	1.89226	0.0698591
58030	1.89226	0.0698591
58031	1.89226	0.0698591
58032	1.89226	0.0698591
58033	1.89226	0.0698591
58034	0.946119	0.0698591
58035	0.946119	0.0698591
58036	0.946118	0.0698591
58037	0.946118	0.0698591
58038	0.946118	0.0698591
58039	0.946118	0.0698591
58040	0.946119	0.0698591
58041	0.946119	0.0698591
58042	0.946131	0.0698591
58043	0.946131	0.0698591
58044	0.946106	0.0698591
58045	0.946106	0.0698591
58046	1.3939	2.1
58047	1.49641	2.1
58048	1.15096	2.1
58049	1.10101	2.1
58050	2.59742	2.1
58051	1.22106	2.1
58052	2.60663	2.1
58053	1.25467	2.1
58054	1.25467	2.1
58055	2.58377	2.1

58056	2.58377	2.1
58057	1.89225	2.1
58058	2.60664	2.1
58059	2.58377	2.1
58060	2.60664	2.1
58061	1.86938	2.1
58062	1.89225	2.1
58063	1.89225	2.1
58064	1.89225	2.1
58065	1.89225	2.1
58066	1.89225	2.1
58067	1.89225	2.1
58068	1.89225	2.1
58069	0.946121	2.1
58070	0.946121	2.1
58071	0.946126	2.1
58072	0.946126	2.1
58073	0.946124	2.1
58074	0.946124	2.1
58075	0.946123	2.1
58076	0.946123	2.1
58077	0.946117	2.1
58078	0.946117	2.1
58079	0.94613	2.1
58080	0.94613	2.1
58081	1.28017	2.1
58082	1.29189	2.1
58083	0.610534	2.1
58084	0.592373	2.1
58085	0.943764	2.1
58086	0.943764	2.1
58087	0.940501	2.1
58088	0.940501	2.1
58089	1.28017	2.1
58090	0.592371	2.1
58091	0.610533	2.1
58092	1.29189	2.1
58093	0.946133	2.1
58094	0.946133	2.1
58095	1.28017	2.1
58096	1.29189	2.1
58097	0.946132	2.1
58098	0.946132	2.1
58099	1.28017	2.1
58100	1.28017	2.1
58101	1.28017	2.1
58102	0.946132	2.1
58103	0.946133	2.1
58104	1.2919	2.1
58105	1.89226	2.1
58106	1.89226	2.1
58107	1.89226	2.1
58108	1.89226	2.1
58109	1.89226	2.1
58110	1.89226	2.1
58111	1.89226	2.1
58112	1.89226	2.1
58113	1.89226	2.1

58114	1.89226	2.1
58115	3.78452	2.1
58116	1.89226	2.1
58117	2.08196	2.1
58118	1.97854	2.1
58119	2.08196	2.1
58120	1.78884	2.1
58121	1.89226	2.1
58122	1.89226	2.1
58123	1.89226	2.1
58124	1.89226	2.1
58125	1.89226	2.1
58126	1.89226	2.1
58127	1.89226	2.1
58128	1.83688	2.1
58129	1.89226	2.1
58130	1.89226	2.1
58131	1.89226	2.1
58132	1.86015	2.1
58133	1.86015	2.1
58134	1.89226	2.1
58135	1.97854	2.1
58136	1.85883	2.1
58137	1.97854	2.1
58138	1.77255	2.1
58139	1.89226	2.1
58140	0.946131	2.1
58141	0.946131	2.1
58142	0.946131	2.1
58143	0.946131	2.1
58144	0.94613	2.1
58145	0.94613	2.1
58146	0.946132	2.1
58147	0.946132	2.1
58148	0.946131	2.1
58149	0.946131	2.1
58150	0.946131	2.1
58151	0.946131	2.1
58152	0.918412	2.1
58153	0.918412	2.1
58154	0.94893	2.1
58155	0.750211	2.1
58156	0.851541	2.1
58157	0.851541	2.1
58158	0.970566	2.1
58159	0.826567	2.1
58160	0.970554	2.1
58161	1.04071	2.1
58162	0.826632	2.1
58163	0.851554	2.1
58164	1.89226	0.0698591
58165	1.89226	0.0698591
58166	1.89226	0.0698591
58167	1.89226	0.0698591
58168	3.78453	0.0698591
58169	0.946131	0.0698591
58170	0.946131	0.0698591
58171	0.811232	0.0698591

58172	0.811232	0.0698591
58173	0.94613	0.0698591
58174	0.94613	0.0698591
58175	0.959701	0.0698591
58176	0.811233	0.0698591
58177	0.94613	0.0698591
58178	0.811233	0.0698591
58179	0.959702	0.0698591
58180	0.946131	0.0698591
58181	1.89226	0.0698591
58182	1.89226	0.0698591
58183	1.89226	0.0698591
58184	1.89226	0.0698591
58185	1.89226	0.0698591
58186	1.89226	0.0698591
58187	1.89227	0.0698591
58188	1.89227	0.0698591
58189	1.89227	0.0698591
58190	1.89227	0.0698591
58191	1.89227	0.0698591
58192	1.89227	0.0698591
58193	1.89226	0.0698591
58194	1.89226	0.0698591
58195	1.89226	0.0698591
58196	1.89226	0.0698591
58197	3.78453	0.0698591
58198	1.89226	0.0698591
58199	1.89226	0.0698591
58200	1.89226	0.0698591
58201	1.89226	0.0698591
58202	1.89226	0.0698591
58203	1.89226	0.0698591
58204	1.89226	2.1
58205	2.36204	2.1
58206	2.39853	2.1
58207	2.36204	2.1
58208	1.92876	2.1
58209	1.89226	2.1
58210	1.89226	2.1
58211	1.89226	2.1
58212	1.89226	2.1
58213	1.89226	2.1
58214	3.78453	2.1
58215	0.946128	2.1
58216	0.946128	2.1
58217	1.13182	2.1
58218	1.13182	2.1
58219	0.94613	2.1
58220	0.94613	2.1
58221	1.1749	2.1
58222	1.13182	2.1
58223	1.1749	2.1
58224	0.94613	2.1
58225	1.13182	2.1
58226	0.94613	2.1
58227	1.89226	2.1
58228	2.39853	2.1
58229	2.44918	2.1

58230	2.39853	2.1
58231	1.94291	2.1
58232	1.89226	2.1
58233	0.946132	2.1
58234	0.946132	2.1
58235	0.946131	2.1
58236	0.946131	2.1
58237	0.946132	2.1
58238	0.946132	2.1
58239	0.946131	2.1
58240	0.946131	2.1
58241	0.946131	2.1
58242	0.946131	2.1
58243	0.946132	2.1
58244	0.946132	2.1
58245	0.946128	2.1
58246	0.946128	2.1
58247	1.1749	2.1
58248	1.1749	2.1
58249	0.946131	2.1
58250	0.946131	2.1
58251	1.23631	2.1
58252	1.1749	2.1
58253	1.23631	2.1
58254	0.94613	2.1
58255	1.1749	2.1
58256	0.946131	2.1
58257	1.89226	2.1
58258	1.89226	2.1
58259	1.89226	2.1
58260	1.89226	2.1
58261	3.78453	2.1
58262	1.38859	2.1
58263	1.45333	2.1
58264	1.28909	2.1
58265	1.38091	2.1
58266	2.74242	2.1
58267	1.45332	2.1
58268	1.38414	2.1
58269	1.38091	2.1
58270	1.33506	2.1
58271	2.78838	2.1
58272	1.89226	2.1
58273	1.89226	2.1
58274	1.89226	2.1
58275	1.89226	2.1
58276	3.78452	2.1
58277	1.89226	2.1
58278	1.89226	2.1
58279	1.89226	2.1
58280	1.89226	2.1
58281	1.89226	2.1
58282	1.89226	2.1
58283	1.17418	2.1
58284	1.38091	2.1
58285	1.17418	2.1
58286	1.28909	2.1
58287	1.29741	2.1

58288	1.41232	2.1
58289	1.38091	2.1
58290	1.33506	2.1
58291	1.27575	2.1
58292	1.27575	2.1
58293	1.29741	2.1
58294	1.35671	2.1
58295	1.89226	2.1
58296	1.89226	2.1
58297	1.89226	2.1
58298	1.89226	2.1
58299	3.78452	2.1
58300	1.92737	0.0716394
58301	2.40223	0.0720724
58302	1.56178	0.0698591
58303	1.97605	0.0863743
58304	3.90343	0.0708496
58305	1.89226	0.0726653
58306	1.89226	0.127225
58307	1.89226	0.0903575
58308	1.89226	0.0883331
58309	3.78452	0.0916625
58310	1.89226	0.130542
58311	1.89226	0.103146
58312	1.89226	0.0936821
58313	1.89226	0.156957
58314	1.89226	0.0903694
58315	1.89226	0.153135
58316	1.13641	0.0980701
58317	1.13641	0.0866835
58318	1.12763	0.0716432
58319	1.12763	0.0793552
58320	1.29348	0.0794917
58321	1.06293	0.106941
58322	1.20111	0.0720809
58323	0.988028	0.0873173
58324	0.988028	0.0834656
58325	1.06293	0.0831947
58326	1.29348	0.0776186
58327	1.20111	0.0776858
58328	1.56176	0.0704327
58329	1.46387	0.0843683
58330	1.97603	0.0743862
58331	1.97603	0.0731878
58332	1.08211	0.0742083
58333	1.08211	0.0734351
58334	1.89224	0.0862085
58335	1.89224	0.0868211
58336	1.89224	0.0793704
58337	1.89224	0.109398
58338	1.89224	0.075844
58339	1.89224	0.102995
58340	1.89224	0.114392
58341	1.89224	0.107406
58342	1.89224	0.088237
58343	1.89224	0.153884
58344	3.78448	0.110989
58345	0.820185	0.0752045

58346	0.818947	0.0931196
58347	0.911041	0.105883
58348	0.695265	0.0917246
58349	1.46388	0.0859449
58350	1.23607	0.0866566
58351	0.927068	0.0859887
58352	1.02691	0.109946
58353	1.12918	0.0752974
58354	0.846843	0.0867413
58355	0.911039	0.0893791
58356	1.29129	2.1
58357	1.45547	2.1
58358	1.67327	2.1
58359	1.66698	2.1
58360	1.20149	2.1
58361	1.20149	2.1
58362	0.946133	2.1
58363	0.946133	2.1
58364	0.946129	2.1
58365	0.946129	2.1
58366	0.946131	2.1
58367	0.946131	2.1
58368	0.946131	2.1
58369	0.946131	2.1
58370	0.946131	2.1
58371	0.946131	2.1
58372	0.946131	2.1
58373	0.946131	2.1
58374	1.89226	2.1
58375	1.89226	2.09735
58376	1.89226	2.1
58377	1.89226	1.9789
58378	3.78452	2.09832
58379	0.86875	2.09767
58380	0.86875	2.1
58381	0.899002	2.1
58382	0.966245	2.1
58383	0.849364	2.04701
58384	1.01918	2.09669
58385	0.764737	2.1
58386	0.89249	2.1
58387	0.764624	2.1
58388	0.967869	2.1
58389	0.808606	2.1
58390	0.864506	2.1
58391	0.773929	2.1
58392	0.773929	2.1
58393	0.781991	2.1
58394	0.836559	2.1
58395	0.983761	2.1
58396	0.834687	2.1
58397	0.680545	2.1
58398	0.640799	2.1
58399	0.70286	2.1
58400	0.935844	2.1
58401	0.629479	2.1
58402	0.823655	2.1
58403	0.946133	2.1

58404	0.946133	2.1
58405	0.946128	2.1
58406	0.946128	2.1
58407	0.94613	2.1
58408	0.94613	2.1
58409	0.946131	2.1
58410	0.946131	2.1
58411	0.946131	2.1
58412	0.946131	2.1
58413	0.946131	2.1
58414	0.946131	2.1
58415	1.89226	2.1
58416	1.89226	2.04755
58417	1.89226	2.1
58418	1.89226	2.1
58419	1.89226	2.07984
58420	1.89226	2.1
58421	0.931638	2.1
58422	0.931638	2.09282
58423	1.03559	2.1
58424	0.977442	2.03275
58425	0.826487	1.46811
58426	0.993902	2.05456
58427	1.04922	2.1
58428	0.894324	2.1
58429	0.912984	2.1
58430	1.01156	2.1
58431	0.944692	2.1
58432	1.15456	2.09796
58433	1.89226	0.0778117
58434	1.89226	0.113047
58435	1.89226	0.151777
58436	1.89226	0.0747544
58437	3.78453	0.0771883
58438	1.89226	0.0754239
58439	1.89226	0.0723751
58440	2.64262	0.0773736
58441	2.64262	0.0698591
58442	1.78052	0.0698591
58443	1.03015	0.075222
58444	0.999721	0.0708666
58445	0.659011	0.0698591
58446	1.15564	0.0947466
58447	1.41388	0.0744696
58448	1.01583	0.0759942
58449	1.01583	0.0713921
58450	1.05706	0.0939005
58451	1.05706	0.0732692
58452	1.15564	0.073127
58453	0.65901	0.0722671
58454	1.41388	0.0741223
58455	0.999721	0.0723187
58456	1.89226	2.1
58457	1.89226	2.1
58458	1.89226	2.1
58459	1.89226	2.1
58460	3.78453	2.1
58461	2.39022	2.1

58462	1.90734	2.1
58463	2.27634	2.1
58464	1.82666	2.1
58465	4.18368	2.1
58466	1.90734	2.1
58467	1.21564	2.1
58468	1.82666	2.1
58469	1.16938	2.1
58470	3.07672	2.1
58471	1.89226	2.1
58472	1.89226	2.1
58473	1.89226	2.1
58474	1.89226	2.1
58475	1.89226	2.1
58476	1.89226	2.1
58477	1.89227	2.1
58478	1.89227	2.1
58479	1.89227	2.1
58480	1.89227	2.1
58481	3.78453	2.1
58482	2.27634	2.1
58483	1.82666	2.1
58484	2.18042	2.1
58485	1.74738	2.1
58486	4.02372	2.1
58487	1.74738	2.1
58488	1.82666	2.1
58489	1.1181	2.1
58490	1.1181	2.1
58491	1.16938	2.1
58492	1.79865	2.1
58493	1.89226	2.1
58494	1.89226	2.1
58495	1.89226	2.1
58496	1.89226	2.1
58497	1.89226	2.1
58498	1.89226	2.1
58499	1.89226	1.21163
58500	1.89226	0.389289
58501	1.89226	0.992518
58502	1.89226	0.483167
58503	1.89226	1.25917
58504	1.89226	0.599173
58505	0.790736	0.517378
58506	1.08265	0.36387
58507	0.938909	1.07414
58508	0.638683	0.849179
58509	0.858588	1.11087
58510	0.858588	0.377453
58511	0.862743	1.19167
58512	0.862743	0.538403
58513	1.08265	1.08414
58514	0.938911	0.670949
58515	0.790735	1.07289
58516	0.638685	0.540763
58517	1.89226	0.450383
58518	1.89226	1.04947
58519	1.89226	1.08753

58520	1.89226	0.440298
58521	1.89226	1.26517
58522	1.89226	0.854171
58523	1.89226	0.446782
58524	1.89226	1.25802
58525	1.89226	1.04097
58526	1.89226	0.562771
58527	1.89226	1.19999
58528	1.89226	1.11716
58529	1.08265	0.392723
58530	1.36209	0.370561
58531	1.25233	0.592001
58532	0.938909	0.669136
58533	1.14195	0.602566
58534	1.14195	0.326856
58535	1.15904	1.21624
58536	1.15904	0.523432
58537	1.36209	0.939973
58538	1.25233	0.307296
58539	1.08265	0.785285
58540	0.938911	0.408817
58541	1.22077	0.384738
58542	0.915244	0.412358
58543	0.967835	0.688374
58544	0.742495	0.859576
58545	0.78648	0.704062
58546	0.985041	0.408141
58547	0.989098	1.17004
58548	0.989098	0.489284
58549	1.03206	0.636637
58550	0.967897	0.942838
58551	0.915404	0.961977
58552	0.706105	0.64008
58553	2.69662	0.0698591
58554	1.89226	0.0698591
58555	2.35499	0.0698591
58556	1.89226	0.0698591
58557	4.24725	0.0698591
58558	1.89227	0.0698591
58559	1.89227	0.0698591
58560	1.89227	0.0698591
58561	1.89227	0.0698591
58562	3.78453	0.0698591
58563	2.35499	0.0698591
58564	1.70302	0.0698591
58565	1.70302	0.0698591
58566	1.703	0.0698591
58567	1.97374	0.0698591
58568	1.97372	0.0698591
58569	0.946133	0.0698591
58570	0.946133	0.0698591
58571	0.946132	0.0698591
58572	0.946132	0.0698591
58573	0.946133	0.0698591
58574	0.946133	0.0698591
58575	0.946132	0.0698591
58576	0.946132	0.0698591
58577	0.946133	0.0698591

58578	0.946133	0.0698591
58579	0.946132	0.0698591
58580	0.946132	0.0698591
58581	0.6794	2.1
58582	0.6794	2.1
58583	0.670812	2.1
58584	0.62182	2.1
58585	0.648326	2.1
58586	0.792154	2.1
58587	0.719167	2.1
58588	0.494515	2.1
58589	0.649421	2.1
58590	0.804869	2.1
58591	0.485585	2.1
58592	0.627666	2.1
58593	1.79347	2.1
58594	1.03968	2.1
58595	1.70303	2.1
58596	0.933265	2.07523
58597	2.62068	2.1
58598	1.89226	2.1
58599	1.89226	2.1
58600	1.89226	2.1
58601	1.89226	2.09951
58602	3.78452	2.1
58603	1.89226	2.1
58604	2.24604	2.1
58605	2.0554	2.1
58606	2.0554	2.1
58607	1.89226	2.1
58608	1.89226	2.1
58609	1.89226	2.1
58610	1.89226	2.06598
58611	1.89226	2.1
58612	1.89226	2.1
58613	1.89226	2.1
58614	1.89226	2.1
58615	2.0815	2.1
58616	2.64424	1.37866
58617	2.80807	1.99941
58618	3.232 2.1	
58619	1.89417	2.04558
58620	2.18012	2.1
58621	0.946131	2.1
58622	0.946131	2.02725
58623	0.946131	2.1
58624	0.946131	2.1
58625	0.94613	2.1
58626	0.94613	2.05889
58627	0.946132	2.1
58628	0.946132	2.1
58629	0.946131	2.1
58630	0.946131	2.1
58631	0.946131	2.1
58632	0.946131	2.1
58633	1.89227	0.0698905
58634	1.89227	0.0889988
58635	1.89227	0.110367

58636	1.89227	0.0700022
58637	3.78453	0.0712609
58638	2.70881	0.0698591
58639	2.68687	0.0698591
58640	2.03046	0.0698591
58641	1.9682	0.0698591
58642	4.67701	0.0698591
58643	1.08518	0.0698591
58644	1.08518	0.0698591
58645	1.09033	0.0698591
58646	1.09033	0.0698591
58647	0.842273	0.0698591
58648	1.19141	0.0698591
58649	1.34343	0.0698591
58650	0.9841	0.0698591
58651	0.9841	0.0698591
58652	0.842273	0.0698591
58653	1.34344	0.0698591
58654	1.19141	0.0698591
58655	0.946133	0.0698591
58656	0.946133	0.0698591
58657	0.946132	0.0960337
58658	0.946132	0.0772454
58659	0.946132	0.0716013
58660	0.946132	0.0698591
58661	0.946132	0.0951202
58662	0.946132	0.0756915
58663	0.946132	0.0698591
58664	0.946132	0.0749923
58665	0.946133	0.0790352
58666	0.946133	0.0715457
58667	1.89227	0.0846931
58668	1.89227	0.0698591
58669	1.89227	0.0801672
58670	1.89227	0.0703261
58671	1.89227	0.0719787
58672	1.89227	0.0698591
58673	2.03046	0.0698591
58674	1.9682	0.0698591
58675	1.23886	0.0698591
58676	1.12971	0.0698591
58677	3.20706	0.0698591
58678	1.89226	0.257004
58679	1.89226	0.800658
58680	1.89226	0.253191
58681	1.89226	0.566315
58682	3.78452	0.382858
58683	2.45569	0.142022
58684	1.89226	0.183743
58685	2.22922	0.133427
58686	2.0815	0.262021
58687	1.85074	0.105641
58688	1.70303	0.204015
58689	2.72856	0.148268
58690	1.89226	0.334012
58691	1.89226	0.142005
58692	1.89226	0.244041
58693	3.78452	0.201992

58694	1.89226	0.340581
58695	1.89226	0.708291
58696	1.89226	0.233535
58697	1.89226	0.746566
58698	3.78452	0.482512
58699	0.946131	0.649858
58700	0.946131	0.2125
58701	0.946131	0.398621
58702	0.946131	0.177607
58703	0.94613	0.375844
58704	0.94613	0.471507
58705	0.946132	0.21268
58706	0.946132	0.230866
58707	0.946131	0.309968
58708	0.946131	0.301659
58709	0.946131	0.297937
58710	0.946131	0.312021
58711	2.0595	0.137626
58712	2.58581	0.105336
58713	1.48694	0.108675
58714	1.48694	0.119481
58715	2.60157	0.111589
58716	2.02901	0.119397
58717	1.89226	0.12048
58718	1.89226	0.171078
58719	1.89226	0.130287
58720	1.89226	0.136168
58721	1.89226	0.136041
58722	1.89226	0.149262
58723	2.04728	0.134076
58724	2.64161	0.0984783
58725	1.47606	0.110889
58726	1.47606	0.124057
58727	2.63069	0.111467
58728	2.05947	0.123628
58729	1.89224	0.101167
58730	1.89224	0.151436
58731	1.89224	0.117815
58732	1.89224	0.13231
58733	1.89224	0.112518
58734	1.89224	0.120596
58735	0.946114	0.157944
58736	0.946114	0.117911
58737	0.946118	0.16481
58738	0.946118	0.133257
58739	0.946114	0.170741
58740	0.946114	0.161301
58741	0.946118	0.133591
58742	0.946118	0.12226
58743	0.946128	0.149056
58744	0.946128	0.151515
58745	0.946103	0.142298
58746	0.946103	0.144128
58747	1.1233	2.07527
58748	1.15009	2.1
58749	2.36036	2.09364
58750	2.38225	2.1
58751	3.50554	2.1

58752	2.38224	2.1
58753	0.977775	2.1
58754	2.20473	2.1
58755	2.20473	2.1
58756	1.15009	2.1
58757	1.15009	2.1
58758	0.946119	2.1
58759	0.946119	2.0934
58760	1.19111	2.1
58761	1.19111	2.0842
58762	0.946118	2.1
58763	1.19111	2.1
58764	1.18016	2.08811
58765	0.946118	1.92401
58766	0.94613	1.76681
58767	0.94613	2.1
58768	1.18015	2.0719
58769	1.19109	2.08468
58770	0.946118	2.1
58771	0.946118	2.1
58772	1.10235	2.1
58773	1.10235	2.1
58774	0.946118	2.1
58775	1.10235	2.1
58776	0.946115	2.1
58777	1.1911	2.1
58778	0.946129	2.1
58779	0.946129	2.1
58780	1.19109	2.1
58781	1.10234	2.1
58782	1.89226	2.1
58783	2.23932	2.1
58784	2.11568	2.1
58785	2.23932	2.1
58786	1.76862	2.1
58787	1.89226	2.1
58788	1.89226	2.1
58789	1.89226	2.1
58790	1.89226	2.1
58791	1.89226	2.1
58792	1.89226	2.1
58793	1.89226	2.1
58794	2.07838	2.1
58795	1.89226	2.1
58796	1.89226	2.1
58797	1.89226	2.1
58798	2.10264	2.1
58799	2.10264	2.1
58800	1.89226	2.1
58801	2.11568	2.1
58802	1.99753	2.1
58803	2.11568	2.1
58804	1.77411	2.1
58805	1.89226	2.1
58806	0.946128	2.1
58807	0.946128	2.1
58808	0.946133	2.1
58809	0.946133	2.1

58810	0.94613	2.1
58811	0.94613	2.1
58812	0.946131	2.1
58813	0.946131	2.1
58814	0.946131	2.1
58815	0.946131	2.1
58816	0.946131	2.1
58817	0.946131	2.1
58818	0.946132	2.1
58819	0.946132	2.1
58820	1.03919	2.1
58821	1.03919	2.1
58822	0.946131	2.1
58823	0.946131	2.1
58824	1.02744	2.1
58825	1.03919	2.1
58826	1.02744	2.1
58827	0.946132	2.1
58828	1.03919	2.1
58829	0.946132	2.1
58830	1.64834	1.85635
58831	1.82955	1.63193
58832	2.33824	1.43562
58833	2.19301	1.82884
58834	3.98658	1.67604
58835	1.40574	1.90561
58836	2.19301	1.6
58837	1.64834	1.85811
58838	1.64834	1.71273
58839	1.99961	1.62156
58840	1.99961	1.56787
58841	2.33824	1.36947
58842	2.19301	1.83722
58843	2.68524	1.70177
58844	2.81847	1.50416
58845	5.02349	1.63567
58846	1.99961	1.85221
58847	2.19301	1.48724
58848	2.68524	1.60924
58849	2.50732	1.62744
58850	4.68485	1.63846
58851	1.89226	2.1
58852	1.89226	2.1
58853	1.89226	2.1
58854	1.89226	2.1
58855	3.78453	2.1
58856	1.75142	2.1
58857	2.47026	2.1
58858	1.46811	2.1
58859	2.26652	2.1
58860	4.01794	2.1
58861	1.89226	2.1
58862	1.89226	2.1
58863	1.89226	2.1
58864	1.89226	2.1
58865	1.89226	2.1
58866	1.89226	2.1
58867	1.89226	2.1

58868	1.89226	2.1
58869	1.89226	2.1
58870	1.89226	2.1
58871	1.89226	2.1
58872	1.89226	2.1
58873	0.812082	2.1
58874	0.878759	2.1
58875	0.882782	2.1
58876	0.882782	2.1
58877	1.05572	2.1
58878	1.04037	2.1
58879	0.73849	2.1
58880	0.626857	2.1
58881	0.638677	2.1
58882	1.05581	2.1
58883	0.715695	2.1
58884	1.10492	2.1
58885	1.17058	2.1
58886	1.07863	2.1
58887	1.21113	2.1
58888	1.21113	2.1
58889	1.2811	2.1
58890	1.37319	2.1
58891	1.01788	2.1
58892	0.999492	2.1
58893	1.04352	2.1
58894	1.32112	2.1
58895	1.10492	2.1
58896	1.34699	2.1
58897	1.89226	2.1
58898	1.89226	2.1
58899	1.89226	2.1
58900	1.89226	2.1
58901	1.89226	2.1
58902	1.89226	2.1
58903	1.19813	0.0698591
58904	1.19813	0.0698591
58905	2.43432	0.0698591
58906	1.21716	0.0698591
58907	0.946124	0.0698591
58908	1.21715	0.0698591
58909	0.946118	0.0698591
58910	2.56627	2.1
58911	1.89226	2.1
58912	1.89226	2.1
58913	1.89226	2.1
58914	2.43434	2.1
58915	2.43434	2.1
58916	1.89227	0.0698591
58917	1.89227	0.0698591
58918	1.89227	0.0698591
58919	1.89227	0.0698591
58920	1.89227	0.0698591
58921	1.89227	0.0698591
58922	1.89226	0.0698591
58923	1.89226	0.0717962
58924	1.89226	0.0718282
58925	1.89226	0.0698591

58926	3.78453	0.0698591
58927	1.89226	0.0698591
58928	2.0764	0.0698591
58929	1.89226	0.0698591
58930	2.81674	0.0698591
58931	1.89226	0.0698591
58932	2.81674	0.0698591
58933	0.946132	0.0698591
58934	0.946132	0.0698591
58935	0.946131	0.0705309
58936	0.946131	0.0698591
58937	0.946132	0.0698591
58938	0.946132	0.0698591
58939	0.946132	0.0708247
58940	0.946132	0.0698591
58941	0.946132	0.0698591
58942	0.946132	0.0698591
58943	0.946132	0.0698591
58944	0.946132	0.0698591
58945	0.578684	1.62776
58946	0.578684	2.1
58947	0.589487	2.04717
58948	0.589487	2.1
58949	0.625461	1.94994
58950	0.5378	1.99064
58951	0.608714	2.1
58952	0.54271	1.97149
58953	0.608714	1.54142
58954	0.625461	1.15839
58955	0.54271	2.02862
58956	0.537801	1.97752
58957	0.946128	1.07226
58958	0.946128	2.02982
58959	0.946133	0.722485
58960	0.946133	2.1
58961	0.946131	0.855508
58962	0.946131	1.08319
58963	0.946131	1.02339
58964	0.946131	1.65868
58965	0.94613	0.826964
58966	0.94613	0.653436
58967	0.946131	1.18912
58968	0.946131	1.49236
58969	1.21743	2.08322
58970	1.25092	2.09954
58971	1.38847	1.95067
58972	1.46031	0.758862
58973	2.67774	1.86875
58974	0.946128	0.808492
58975	0.946128	2.09273
58976	0.946132	2.1
58977	0.946132	0.781027
58978	0.94613	1.36681
58979	0.94613	0.757779
58980	0.94613	1.72151
58981	0.94613	1.10885
58982	0.94613	0.644745
58983	0.94613	0.670778

58984	0.94613	1.4787
58985	0.94613	1.07168
58986	1.89226	0.3541
58987	1.89226	1.0692
58988	1.89226	0.574449
58989	1.89226	2.06429
58990	1.89226	2.08521
58991	1.89226	2.02963
58992	0.539566	0.955453
58993	0.534046	2.1
58994	0.577914	1.85538
58995	0.471623	1.88038
58996	1.46031	0.249997
58997	0.945865	0.742009
58998	0.770603	0.349717
58999	0.737201	2.03418
59000	0.714829	0.834848
59001	0.536085	1.74676
59002	0.471617	1.3707
59003	1.89226	1.96556
59004	1.89226	1.20321
59005	1.89226	1.1726
59006	1.89226	1.52896
59007	1.89226	1.79674
59008	1.89226	1.95108
59009	1.89225	1.41168
59010	1.89225	0.61688
59011	1.65572	0.684641
59012	1.65572	0.787134
59013	1.89225	1.1166
59014	1.89225	1.45534
59015	1.89226	1.38687
59016	1.89226	0.866929
59017	1.89226	1.29836
59018	1.89226	0.582828
59019	3.78453	1.35376
59020	1.89226	1.81356
59021	1.89226	0.786311
59022	1.89226	0.801467
59023	1.89226	1.38015
59024	1.89226	1.49438
59025	1.89226	1.84282
59026	1.89225	1.25534
59027	1.89225	0.39088
59028	1.89225	0.481865
59029	1.89225	0.632254
59030	1.89225	0.785648
59031	1.89225	1.25715
59032	0.763592	0.58715
59033	0.763592	0.980753
59034	0.987417	0.689508
59035	0.987417	0.699443
59036	0.763592	0.445677
59037	0.804881	0.390263
59038	0.946126	0.686374
59039	0.946126	0.704188
59040	0.804879	0.413526
59041	0.94613	0.672124

59042	0.946129	0.783804
59043	0.76359	0.498676
59044	1.46055	2.1
59045	2.04945	2.1
59046	2.04587	2.1
59047	2.04587	2.1
59048	1.50714	2.1
59049	1.50714	2.1
59050	2.04587	2.1
59051	2.38681	2.1
59052	2.42082	2.1
59053	2.42082	2.1
59054	2.04945	2.1
59055	2.04945	2.1
59056	0.872964	2.1
59057	0.872964	2.1
59058	0.884806	2.1
59059	0.884806	2.1
59060	1.0275	2.1
59061	1.02293	2.1
59062	0.711243	2.1
59063	0.730273	2.1
59064	0.711244	2.1
59065	1.0275	2.1
59066	0.730273	2.1
59067	1.02293	2.1
59068	2.04587	2.1
59069	2.47753	2.1
59070	2.42994	2.1
59071	2.42082	2.1
59072	2.05499	2.1
59073	2.04587	2.1
59074	0.946132	2.1
59075	0.946132	2.1
59076	1.13078	2.1
59077	1.07817	2.1
59078	0.946132	2.1
59079	0.946132	2.1
59080	1.07817	2.1
59081	1.07817	2.1
59082	0.946132	2.1
59083	1.07817	2.1
59084	1.13078	2.1
59085	0.946132	2.1
59086	1.89226	2.1
59087	1.89226	2.1
59088	1.89226	2.1
59089	1.89226	2.1
59090	1.89226	2.1
59091	1.89226	2.1
59092	1.89226	2.1
59093	1.89226	2.1
59094	1.89226	2.1
59095	1.89226	2.1
59096	1.89226	2.1
59097	1.89226	2.1
59098	1.89226	2.1
59099	1.89226	2.1

59100	1.8173	2.1
59101	2.66885	2.1
59102	2.7438	2.1
59103	2.7438	2.1
59104	1.89226	0.853036
59105	1.89226	0.0890277
59106	1.89226	0.116416
59107	1.89226	0.675523
59108	3.78453	0.617591
59109	1.89226	0.251796
59110	1.89226	0.373333
59111	1.89226	0.518656
59112	1.89226	1.19159
59113	1.89226	0.189266
59114	1.89226	0.678205
59115	1.89226	0.252314
59116	1.97385	0.513361
59117	1.7531	0.622607
59118	1.89226	1.48367
59119	1.7531	0.1424
59120	1.89226	0.329288
59121	0.946131	0.648939
59122	0.840904	0.14573
59123	0.684333	0.0902302
59124	0.94613	0.0971143
59125	0.972337	0.100618
59126	0.946278	0.817782
59127	0.684304	0.0956948
59128	0.684304	0.3535
59129	0.999838	0.119774
59130	0.759605	0.116783
59131	0.851528	0.0892381
59132	0.851528	0.119885
59133	1.89226	0.106695
59134	2.23818	0.236354
59135	1.79771	0.13732
59136	1.89226	1.09579
59137	1.79771	0.077004
59138	1.89226	0.182334
59139	2.79647	2.1
59140	2.01613	2.1
59141	1.46826	2.1
59142	2.54504	2.1
59143	4.56117	2.1
59144	1.89226	2.1
59145	1.89226	2.1
59146	1.89226	2.1
59147	1.89226	2.1
59148	1.89226	2.1
59149	1.89226	2.1
59150	1.89226	2.1
59151	2.66884	2.1
59152	2.56022	2.1
59153	2.66884	2.1
59154	1.78364	2.1
59155	1.89226	2.1
59156	1.14282	2.1
59157	1.24068	2.1

59158	0.880748	2.1
59159	0.834916	2.1
59160	1.05539	2.1
59161	1.05539	2.1
59162	1.04312	2.1
59163	1.04313	2.1
59164	1.2015	2.1
59165	0.83492	2.1
59166	0.789149	2.1
59167	1.24066	2.1
59168	1.89226	2.1
59169	1.89226	2.1
59170	1.89226	2.1
59171	1.89226	2.1
59172	3.78453	2.1
59173	0.946128	2.1
59174	0.946128	2.1
59175	1.28011	2.1
59176	1.22372	2.1
59177	0.946131	2.1
59178	0.946131	2.1
59179	1.22372	2.1
59180	1.22372	2.1
59181	1.22372	2.1
59182	0.94613	2.1
59183	0.946131	2.1
59184	1.28011	2.1
59185	2.28604	2.1
59186	1.34268	2.1
59187	2.43157	2.1
59188	1.50172	2.1
59189	3.77425	2.1
59190	2.6096	2.1
59191	2.43157	2.1
59192	1.66774	2.1
59193	1.66774	2.1
59194	1.50172	2.1
59195	2.43157	2.1
59196	2.16067	2.1
59197	1.08975	2.1
59198	2.19336	2.1
59199	2.06984	2.1
59200	1.21327	2.1
59201	1.08975	2.1
59202	2.28604	2.1
59203	1.21327	2.1
59204	2.29008	2.1
59205	2.16067	2.1
59206	1.34268	2.1
59207	1.21327	2.1
59208	1.89226	0.255449
59209	1.89226	0.312901
59210	1.89226	0.25867
59211	1.89226	0.125325
59212	3.78452	0.123699
59213	1.89804	0.221879
59214	2.21656	0.245184
59215	1.70537	0.106561

59216	2.50023	0.096256
59217	3.8982	0.125421
59218	1.03136	0.103937
59219	1.89486	0.114706
59220	0.949364	0.115323
59221	1.82283	0.0751269
59222	2.84422	0.090209
59223	1.89226	0.122904
59224	1.89226	0.108414
59225	1.89226	0.123633
59226	1.89226	0.133996
59227	3.78452	0.201047
59228	0.946132	0.227398
59229	0.946132	0.205902
59230	0.946129	0.214665
59231	0.946129	0.109918
59232	0.94613	0.1159
59233	0.94613	0.407821
59234	0.946131	0.118258
59235	0.946131	0.235876
59236	0.946131	0.444633
59237	0.946131	0.363559
59238	0.946131	0.2756
59239	0.946131	0.55665
59240	1.09466	0.171936
59241	1.09466	0.081242
59242	0.866512	0.070574
59243	1.08352	0.0703909
59244	1.19189	0.0698591
59245	1.21883	0.106993
59246	0.971139	0.14251
59247	0.873213	0.0707326
59248	0.873202	0.0772302
59249	1.00855	0.0698591
59250	0.923735	0.076898
59251	1.21885	0.0751748
59252	1.89225	0.0698591
59253	2.40019	0.0698591
59254	1.89225	0.363273
59255	1.89225	0.481951
59256	1.89225	0.352242
59257	1.89225	0.666057
59258	1.89225	0.363273
59259	1.89225	0.739596
59260	2.05088	0.352242
59261	1.89225	0.233884
59262	1.89225	0.286534
59263	1.89225	0.414727
59264	2.03956	0.352242
59265	2.03956	0.481951
59266	1.89226	2.1
59267	2.40021	2.1
59268	2.38473	2.08003
59269	2.40021	2.1
59270	1.87679	2.1
59271	1.89226	2.1
59272	1.89226	2.1
59273	1.89226	2.03927

59274	1.89226	2.1
59275	1.89226	2.00233
59276	1.89226	2.1
59277	1.89226	2.07003
59278	2.0692	2.1
59279	1.89226	2.07313
59280	1.89226	2.1
59281	1.89226	1.94775
59282	2.05089	2.1
59283	2.05089	2.03683
59284	1.89226	2.1
59285	1.89226	2.1
59286	1.89226	2.1
59287	1.89226	2.1
59288	3.78452	2.1
59289	1.89226	2.1
59290	2.50051	2.1
59291	1.89226	2.1
59292	2.30076	2.1
59293	4.39277	2.1
59294	2.50052	2.1
59295	1.70377	2.1
59296	2.30076	2.1
59297	1.54231	2.1
59298	4.04283	2.1
59299	1.89226	2.1
59300	1.89226	2.1
59301	1.89226	2.1
59302	1.89226	2.1
59303	1.89226	2.1
59304	1.89226	2.1
59305	1.89226	2.1
59306	1.89226	2.1
59307	1.89226	2.1
59308	1.89226	2.1
59309	3.78452	2.1
59310	2.07948	2.1
59311	2.30076	2.1
59312	1.3294	2.1
59313	1.3294	2.1
59314	1.54232	2.1
59315	2.29239	2.1
59316	0.810947	2.1
59317	0.810947	2.1
59318	0.810946	2.1
59319	0.810946	2.1
59320	1.89226	2.1
59321	1.35169	2.1
59322	1.08132	2.1
59323	1.08132	2.1
59324	1.08132	2.1
59325	0.810946	2.1
59326	0.810946	2.1
59327	1.34851	0.872025
59328	0.997471	2.06566
59329	1.62287	0.666255
59330	1.27096	1.79897
59331	2.62034	1.46165

59332	1.89226	1.74998
59333	1.89226	2.07459
59334	1.89226	0.834575
59335	1.89226	2.1
59336	3.78452	1.94763
59337	0.946128	2.1
59338	0.946128	2.1
59339	1.29331	1.98155
59340	1.29331	2.1
59341	1.29331	2.1
59342	1.10699	2.1
59343	0.946131	1.95967
59344	0.946131	2.1
59345	0.94613	2.1
59346	1.29331	2.1
59347	1.10699	2.1
59348	0.946131	2.1
59349	1.62287	0.834705
59350	1.27096	1.94627
59351	1.86067	0.341103
59352	1.58407	1.85473
59353	3.20694	1.4448
59354	1.89226	0.975209
59355	1.89226	2.1
59356	1.89226	1.21088
59357	1.89226	1.89044
59358	3.78452	1.87
59359	2.62761	0.117237
59360	2.55849	1.21115
59361	2.12261	0.95276
59362	2.22939	0.42162
59363	4.78788	0.594241
59364	0.772522	2.09253
59365	0.839588	1.98269
59366	1.31126	1.13987
59367	1.08787	0.58929
59368	1.01318	1.21585
59369	1.01318	2.09595
59370	0.900815	2.04317
59371	1.10799	0.687414
59372	0.772511	1.77038
59373	1.03473	1.72524
59374	1.31126	1.64243
59375	0.841464	1.70146
59376	1.89227	1.20194
59377	1.89227	2.1
59378	1.89227	1.86003
59379	1.89227	2.08917
59380	1.89227	1.72063
59381	1.89227	2.09208
59382	1.89226	0.437527
59383	1.89226	1.8029
59384	1.89226	0.655422
59385	1.89226	1.32679
59386	1.89226	0.652237
59387	1.89226	1.32234
59388	2.12261	1.34917
59389	2.22939	0.327124

59390	1.97004	0.342798
59391	1.8072	1.59875
59392	4.03658	0.711741
59393	1.5847	2.07033
59394	1.8072	1.41147
59395	2.12262	1.3463
59396	2.12262	2.09085
59397	1.23267	1.31572
59398	1.23267	2.0952
59399	1.89227	1.45685
59400	1.89227	2.1
59401	1.89227	1.85198
59402	1.89227	2.09213
59403	1.89227	1.77198
59404	1.89227	2.09392
59405	1.89226	0.348405
59406	1.89226	1.78988
59407	1.89226	1.66404
59408	1.89226	0.591983
59409	3.78452	1.03048
59410	2.49093	2.1
59411	2.67393	2.1
59412	2.47932	2.1
59413	2.47932	2.1
59414	2.67994	2.1
59415	2.67994	2.1
59416	2.49873	2.1
59417	2.67393	2.1
59418	2.67993	2.1
59419	2.69807	2.1
59420	2.49873	2.1
59421	2.51686	2.1
59422	2.49093	2.1
59423	2.67994	2.1
59424	2.69784	2.1
59425	2.51651	2.1
59426	5.18877	2.1
59427	1.24359	2.1
59428	1.24936	2.1
59429	1.33997	2.1
59430	1.34892	2.1
59431	1.28805	2.1
59432	1.28805	2.1
59433	1.2955	2.1
59434	1.2955	2.1
59435	1.34892	2.1
59436	1.24359	2.1
59437	1.24936	2.1
59438	1.33997	2.1
59439	1.15506	2.03281
59440	1.15506	2.1
59441	0.946128	2.1
59442	0.946128	2.1
59443	0.946131	2.1
59444	1.15506	2.03383
59445	1.06997	2.1
59446	0.946128	2.1
59447	1.06997	2.09639

59448	1.15506	2.09917
59449	0.946129	2.1
59450	0.946129	2.1
59451	1.70608	2.1
59452	2.13994	2.1
59453	1.33384	2.1
59454	1.43635	2.1
59455	3.14243	2.1
59456	2.13994	2.1
59457	2.31012	2.1
59458	1.43635	2.1
59459	1.23007	2.1
59460	3.74646	2.1
59461	2.46361	2.1
59462	1.89226	2.1
59463	1.89226	2.1
59464	1.89226	2.1
59465	2.39045	2.1
59466	2.39045	2.1
59467	1.89226	2.1
59468	1.89226	2.07591
59469	1.89226	2.1
59470	1.89226	1.88355
59471	3.78452	2.1
59472	1.52984	2.1
59473	2.25682	1.77767
59474	2.20831	1.68641
59475	2.20831	2.1
59476	1.61178	1.66018
59477	1.61178	2.1
59478	2.47395	2.1
59479	1.89226	2.1
59480	1.89226	2.1
59481	1.89226	2.1
59482	2.46362	2.1
59483	2.46362	2.1
59484	0.946131	1.75689
59485	0.946131	2.1
59486	0.946131	2.1
59487	0.946131	2.0902
59488	0.94613	1.95948
59489	0.94613	2.05206
59490	0.946132	2.1
59491	0.946132	2.1
59492	0.946131	2.1
59493	0.946131	2.1
59494	0.946131	2.1
59495	0.946131	2.1
59496	0.982398	2.1
59497	0.982398	2.09999
59498	0.931703	2.1
59499	0.97549	2.1
59500	1.11509	2.08641
59501	1.10017	1.96791
59502	0.774576	2.1
59503	0.789859	2.1
59504	0.716502	2.1
59505	1.12793	2.1

59506	1.14398	2.1
59507	0.817011	2.1
59508	1.82499	2.1
59509	1.50524	2.1
59510	1.21304	2.1
59511	1.54191	2.1
59512	3.04715	2.1
59513	1.54191	2.1
59514	1.49823	2.1
59515	1.82499	2.1
59516	1.82499	2.1
59517	1.21147	2.1
59518	1.48977	2.1
59519	1.89226	2.1
59520	1.89226	2.1
59521	1.89226	2.1
59522	1.89226	2.1
59523	3.78452	2.1
59524	1.89226	2.1
59525	1.89226	2.1
59526	1.89226	2.1
59527	1.89226	2.1
59528	1.89226	2.1
59529	1.89226	2.1
59530	0.566674	2.1
59531	0.770957	2.1
59532	0.60652	2.1
59533	0.473557	2.1
59534	0.604441	2.1
59535	0.604441	2.1
59536	0.640073	2.1
59537	0.640073	2.1
59538	0.473557	2.1
59539	0.566674	2.1
59540	0.60652	2.1
59541	0.770956	2.1
59542	1.36203	2.1
59543	1.13335	2.1
59544	1.54191	2.1
59545	1.54191	2.1
59546	1.00965	2.1
59547	1.00965	2.1
59548	1.89226	2.1
59549	1.89226	2.1
59550	1.89226	2.1
59551	1.89226	2.1
59552	1.89226	2.1
59553	1.89226	2.1
59554	0.946131	2.1
59555	0.946131	2.1
59556	0.946131	2.1
59557	0.946131	2.1
59558	0.946132	2.1
59559	0.946132	2.1
59560	0.946131	2.1
59561	0.946131	2.1
59562	0.946132	2.1
59563	0.946132	2.1

59564	0.946131	2.1
59565	0.946131	2.1
59566	0.946132	0.0698591
59567	0.946132	0.0698591
59568	0.946133	0.0698591
59569	0.946133	0.0698591
59570	0.946132	0.0698591
59571	0.946132	0.0698591
59572	0.946132	0.0698591
59573	0.946132	0.0698591
59574	0.946132	0.0698591
59575	0.946132	0.0698591
59576	0.946133	0.0698591
59577	0.946133	0.0698591
59578	1.89226	0.0698591
59579	1.89226	0.0698591
59580	1.89226	0.0698591
59581	1.89226	0.0698591
59582	3.78453	0.0698591
59583	1.89226	0.0698591
59584	1.89226	0.0698591
59585	1.89226	0.0698591
59586	1.89226	0.0698591
59587	1.89226	0.0698591
59588	1.89226	0.0698591
59589	0.946132	0.0698591
59590	0.946132	0.0698591
59591	0.946131	0.0698591
59592	0.946131	0.0698591
59593	0.946132	0.0698591
59594	0.946132	0.0698591
59595	0.946132	0.0698591
59596	0.946132	0.0698591
59597	0.946132	0.0698591
59598	0.946132	0.0698591
59599	0.946132	0.0698591
59600	0.946132	0.0698591
59601	0.946129	0.0698591
59602	0.946129	0.0698591
59603	0.946128	0.0698591
59604	0.946128	0.0698591
59605	0.946132	0.0698591
59606	0.946132	0.0698591
59607	0.946126	0.0698591
59608	0.946126	0.0698591
59609	0.946129	0.0698591
59610	0.946129	0.0698591
59611	0.946129	0.0698591
59612	0.946129	0.0698591
59613	1.20674	0.0698591
59614	1.79877	0.0698591
59615	1.22586	0.0698591
59616	1.22586	0.0698591
59617	1.7919	0.0698591
59618	1.7919	0.0698591
59619	2.27622	0.0698591
59620	1.79877	0.0698591
59621	1.7919	0.0698591

59622	2.27102	0.0698591
59623	4.06979	0.0698591
59624	1.89226	0.0698591
59625	1.89226	0.0808516
59626	1.89226	0.0698591
59627	1.89226	0.0762604
59628	3.78452	0.0732065
59629	1.89226	0.0698591
59630	1.89226	0.0715985
59631	1.89226	0.0698591
59632	1.89226	0.0746025
59633	3.78453	0.0698591
59634	1.13259	0.0698591
59635	1.79187	0.0698591
59636	1.32741	0.0698591
59637	1.18784	0.0698591
59638	1.703 0.0698591	
59639	1.49575	0.0698591
59640	1.46201	0.0698591
59641	2.27099	0.0698591
59642	1.79187	0.0698591
59643	1.79187	0.0698591
59644	2.12923	0.0698591
59645	2.12923	0.0698591
59646	1.89224	0.0698591
59647	1.89224	0.0754901
59648	1.89224	0.0698591
59649	1.89224	0.0764964
59650	3.78447	0.069919
59651	0.946119	0.0746837
59652	0.946119	0.0698591
59653	0.946118	0.0711827
59654	0.946118	0.0698591
59655	0.946118	0.0716504
59656	0.946118	0.074726
59657	0.946119	0.0698591
59658	0.946119	0.0698591
59659	0.946131	0.0699428
59660	0.946131	0.0698591
59661	0.946106	0.0699568
59662	0.946106	0.0703249
59663	2.07526	0.373816
59664	1.89226	0.190805
59665	1.89226	0.448414
59666	2.13445	0.541668
59667	4.02671	0.269835
59668	1.89226	0.238629
59669	1.89226	0.493142
59670	1.89226	0.503273
59671	1.89226	0.187104
59672	1.89226	0.604391
59673	1.89226	0.229447
59674	2.13446	0.398753
59675	1.00013	0.638459
59676	1.1475	0.772883
59677	2.20046	0.493598
59678	3.20059	0.634995
59679	0.946133	0.278411

59680	0.946133	0.313726
59681	1.06722	0.318899
59682	1.10023	0.3096
59683	0.946131	0.355382
59684	0.946131	0.0968087
59685	1.10023	0.363894
59686	1.10023	0.228249
59687	0.946131	0.375699
59688	1.10023	0.270166
59689	0.94613	0.199156
59690	1.06723	0.206109
59691	1.89226	0.127764
59692	1.89226	0.341788
59693	1.89226	0.412998
59694	1.89226	0.26751
59695	3.78452	0.334603
59696	1.89226	0.548597
59697	1.89226	0.658611
59698	1.89226	0.707319
59699	1.89226	0.58858
59700	1.89226	0.590792
59701	1.89226	0.453556
59702	1.89226	2.1
59703	1.89226	1.0503
59704	1.89226	2.08565
59705	1.89226	1.01026
59706	1.89226	2.1
59707	0.828588	0.0698591
59708	0.828588	0.0698591
59709	0.841316	0.0698591
59710	0.841316	2.1
59711	0.528387	2.1
59712	0.565893	0.0698591
59713	1.10401	0.0698591
59714	1.10401	2.1
59715	0.528387	2.1
59716	1.89226	2.1
59717	1.36047	2.08565
59718	1.33973	2.1
59719	1.33973	2.1
59720	1.89226	0.0698591
59721	2.28951	1.05421
59722	1.89226	1.3638
59723	2.80796	1.83296
59724	2.01584	1.83578
59725	2.01584	1.45642
59726	1.89226	1.82082
59727	1.89226	1.41529
59728	1.89226	0.890198
59729	1.89226	1.61048
59730	1.89226	1.51679
59731	1.89226	1.01402
59732	1.89226	1.50584
59733	1.89226	0.996067
59734	2.15223	1.07072
59735	1.30664	1.75475
59736	1.96011	1.68546
59737	2.76516	1.20407

59738	4.11234	1.59249
59739	1.89226	0.761852
59740	1.89226	1.41227
59741	1.89226	1.38939
59742	1.89226	0.904876
59743	1.89226	1.35888
59744	1.89226	0.868746
59745	2.81143	2.1
59746	2.58517	2.1
59747	2.55318	2.1
59748	2.7542	2.1
59749	5.33937	2.1
59750	2.7777	2.1
59751	2.7542	2.1
59752	2.71672	2.1
59753	2.70401	2.1
59754	5.45821	2.1
59755	2.55318	2.1
59756	2.7542	2.1
59757	2.71672	2.1
59758	2.54198	2.1
59759	5.29618	2.1
59760	1.95416	2.1
59761	2.162 2.1	
59762	2.11563	2.1
59763	1.91813	2.1
59764	4.08013	2.1
59765	1.95417	2.1
59766	1.47712	2.1
59767	1.45182	2.1
59768	1.91813	2.1
59769	3.39525	2.1
59770	2.11562	2.1
59771	1.91813	2.1
59772	1.50979	2.1
59773	2.03974	2.1
59774	3.95787	2.1
59775	1.85605	2.1
59776	1.91813	2.1
59777	1.88885	2.1
59778	1.42255	2.1
59779	1.45182	2.1
59780	1.45182	2.1
59781	0.94613	2.1
59782	0.94613	1.09113
59783	0.946128	0.760588
59784	0.946128	2.1
59785	0.946127	2.0874
59786	0.946127	2.1
59787	0.946131	0.621829
59788	0.946131	0.640672
59789	0.946129	1.77336
59790	0.946129	1.77716
59791	0.946128	1.79607
59792	0.946128	1.84952
59793	0.543052	2.1
59794	0.54608	1.10849
59795	0.602293	0.68588

59796	0.535325	0.793892
59797	1.05446	2.1
59798	0.850496	1.97139
59799	0.699838	1.13741
59800	0.679751	2.1
59801	0.713797	1.74721
59802	0.509794	1.51208
59803	0.602304	1.31428
59804	1.40017	2.1
59805	1.40017	2.1
59806	0.94613	2.09875
59807	0.94613	2.1
59808	1.40017	2.1
59809	1.33997	2.1
59810	0.946131	2.08894
59811	0.946131	2.1
59812	1.33997	2.1
59813	0.946131	2.1
59814	0.946131	2.1
59815	1.40017	2.1
59816	1.89226	0.0698591
59817	1.89226	0.0758321
59818	1.89226	0.0784324
59819	1.89226	0.0698591
59820	3.78452	0.0698943
59821	0.946133	0.0698591
59822	0.946133	0.0698591
59823	0.946132	0.075414
59824	0.946132	0.069895
59825	0.946132	0.0698591
59826	0.946132	0.070017
59827	0.946133	0.0744514
59828	0.946133	0.0698591
59829	0.946132	0.0698591
59830	0.946132	0.0699207
59831	0.946132	0.0698591
59832	0.946132	0.0698591
59833	1.89224	0.0798101
59834	1.89224	0.0698591
59835	1.89224	0.07081
59836	1.89224	0.0699951
59837	1.89224	0.0744557
59838	1.89224	0.0728228
59839	0.94612	0.0698591
59840	0.94612	0.0698591
59841	0.946119	0.0746904
59842	0.946119	0.0700555
59843	0.946119	0.0698591
59844	0.946119	0.070404
59845	0.94612	0.0698591
59846	0.94612	0.0733391
59847	0.946132	0.0698591
59848	0.946132	0.0698591
59849	0.946107	0.0698591
59850	0.946107	0.0699003
59851	2.63939	2.1
59852	1.2862	2.1
59853	1.29891	2.1

59854	2.64419	2.1
59855	3.93039	2.1
59856	2.6394	2.1
59857	1.89226	2.1
59858	1.89226	2.1
59859	2.64419	2.1
59860	4.53646	2.1
59861	1.89226	2.1
59862	1.89226	2.1
59863	1.89226	2.1
59864	1.89226	2.1
59865	3.78453	2.1
59866	1.89226	2.1
59867	1.89226	2.1
59868	1.89226	2.1
59869	1.89226	2.1
59870	1.89226	2.1
59871	1.89226	2.1
59872	1.31998	2.1
59873	1.32209	2.1
59874	0.649456	2.1
59875	0.648474	2.1
59876	0.984995	2.1
59877	0.984995	2.1
59878	0.985573	2.1
59879	0.985573	2.1
59880	1.31999	2.1
59881	0.648472	2.1
59882	0.649455	2.1
59883	1.32209	2.1
59884	0.946133	2.1
59885	0.946133	2.1
59886	1.31999	2.1
59887	1.3221	2.1
59888	0.946132	2.1
59889	0.946132	2.1
59890	1.31999	2.1
59891	1.31999	2.1
59892	1.31999	2.1
59893	0.946132	2.1
59894	0.946133	2.1
59895	1.3221	2.1
59896	0.946132	2.1
59897	0.946132	2.1
59898	0.946131	2.1
59899	0.946131	2.1
59900	0.946132	2.1
59901	0.946132	2.1
59902	0.946131	2.1
59903	0.946131	2.1
59904	0.946131	2.1
59905	0.946131	2.1
59906	0.946132	2.1
59907	0.946132	2.1
59908	0.946128	2.1
59909	0.946128	2.1
59910	0.946132	2.1
59911	0.946132	2.1

59912	0.946131	2.1
59913	0.946131	2.1
59914	0.94613	2.1
59915	0.94613	2.1
59916	0.94613	2.1
59917	0.94613	2.1
59918	0.946131	2.1
59919	0.946131	2.1
59920	1.89226	0.209188
59921	1.89226	0.590802
59922	1.89226	0.553923
59923	1.89226	0.544819
59924	1.89226	0.227003
59925	1.89226	0.276559
59926	1.89226	0.504501
59927	1.89226	1.07338
59928	1.89226	0.702974
59929	1.89226	0.923489
59930	3.78453	1.07223
59931	0.946133	0.383726
59932	0.946133	0.45809
59933	0.946132	0.375113
59934	0.946132	0.314102
59935	0.946133	0.54279
59936	0.946133	0.562319
59937	0.946132	0.379204
59938	0.946132	0.267246
59939	0.946132	0.456142
59940	0.946132	0.53918
59941	0.946132	0.412647
59942	0.946132	0.677905
59943	2.23638	0.1858
59944	1.89226	0.309911
59945	2.66014	0.474172
59946	2.66014	0.518131
59947	1.89226	0.564055
59948	1.89226	0.334476
59949	1.11819	0.0882969
59950	1.33007	0.107803
59951	0.660255	0.167509
59952	0.91151	0.135252
59953	0.911511	0.236201
59954	1.33007	0.134606
59955	1.11819	0.0828341
59956	0.660255	0.152808
59957	0.985327	0.114672
59958	0.985327	0.167929
59959	1.005	0.158202
59960	1.005	0.198171
59961	1.89226	2.1
59962	1.89226	2.1
59963	1.89226	2.1
59964	1.89226	2.1
59965	3.78453	2.1
59966	1.59533	2.1
59967	2.68571	2.1
59968	1.28291	2.1
59969	2.46974	2.1

59970	4.06506	2.1
59971	2.68571	2.1
59972	1.89226	2.1
59973	1.89226	2.1
59974	1.89226	2.1
59975	2.46973	2.1
59976	2.46973	2.1
59977	1.89226	2.1
59978	1.89226	2.1
59979	1.89226	2.1
59980	1.89226	2.1
59981	1.89226	2.1
59982	1.89226	2.1
59983	1.89226	2.1
59984	1.89226	2.1
59985	1.89226	2.1
59986	1.89226	2.1
59987	1.89226	2.1
59988	1.89226	2.1
59989	0.883594	2.1
59990	0.883594	2.1
59991	0.876484	2.1
59992	0.876484	2.1
59993	1.10442	2.1
59994	1.23487	2.1
59995	0.641457	2.1
59996	0.52521	2.1
59997	1.10442	2.1
59998	0.52521	2.1
59999	0.641457	2.1
60000	1.23487	2.1
60001	0.946131	2.1
60002	0.946131	2.1
60003	0.946131	2.1
60004	0.946131	2.1
60005	0.946131	2.1
60006	0.946131	2.1
60007	0.946131	2.1
60008	0.946131	2.1
60009	0.946131	2.1
60010	0.946131	2.1
60011	0.946131	2.1
60012	0.946131	2.1
60013	2.04016	0.0751881
60014	2.13042	0.0916464
60015	2.11465	0.0923918
60016	2.11465	0.0969421
60017	1.95908	0.0794753
60018	1.95908	0.0798721
60019	1.66953	0.0727537
60020	2.13041	0.0945039
60021	1.95908	0.0726694
60022	2.21952	0.0784507
60023	1.66953	0.0958091
60024	1.92997	0.108728
60025	2.04016	0.0718439
60026	1.95908	0.0738277
60027	1.82014	0.0698591

60028	2.08757	0.0698591
60029	3.86029	0.0698591
60030	0.834764	0.0719066
60031	0.722102	0.0698591
60032	0.979537	0.0703675
60033	0.910065	0.0698591
60034	0.840053	0.0698591
60035	0.840052	0.0781504
60036	0.861587	0.0698591
60037	0.861587	0.0700476
60038	0.910067	0.0698591
60039	0.722101	0.0698591
60040	0.834763	0.0755677
60041	0.979539	0.0736997
60042	1.89226	0.0806856
60043	1.89226	0.08821
60044	1.89226	0.0957616
60045	1.89226	0.0776502
60046	1.89226	0.0956993
60047	1.89226	0.07865
60048	1.89226	0.0860824
60049	1.89226	0.10666
60050	1.89226	0.146411
60051	1.89226	0.0740797
60052	3.78453	0.0954273
60053	1.89226	0.0698742
60054	1.89226	0.0716647
60055	1.89226	0.0752443
60056	1.89226	0.0698798
60057	1.89226	0.0738036
60058	1.89226	0.0698591
60059	1.20691	0.0698591
60060	2.27379	0.0698591
60061	2.58476	0.070179
60062	1.5124	0.0698591
60063	3.79167	0.0698591
60064	1.89226	0.0986354
60065	2.2738	0.0714609
60066	2.58477	0.0716819
60067	1.89226	0.0811759
60068	4.47703	0.0780027
60069	0.952225	1.53644
60070	0.952225	1.78294
60071	0.909865	1.89961
60072	0.909865	1.95808
60073	0.909895	2.00342
60074	0.946132	1.62981
60075	0.946132	1.83359
60076	0.915958	1.5512
60077	0.946132	1.8127
60078	0.946132	1.86717
60079	0.909894	1.83921
60080	0.915958	1.82683
60081	0.983493	2.1
60082	2.40055	2.1
60083	1.02986	2.1
60084	1.02986	2.1
60085	2.34217	2.1

60086	2.34217	2.1
60087	1.89226	2.1
60088	2.40056	2.1
60089	2.34217	2.1
60090	2.40056	2.1
60091	1.83388	2.1
60092	1.89226	2.1
60093	1.89226	2.1
60094	1.89226	2.1
60095	1.89226	2.1
60096	1.89226	2.1
60097	1.89226	2.1
60098	1.89226	2.1
60099	1.89226	2.1
60100	1.89226	2.1
60101	1.89226	2.1
60102	1.89226	2.1
60103	1.89226	2.1
60104	1.89226	2.1
60105	0.946133	2.1
60106	0.946133	2.1
60107	1.17109	2.1
60108	1.13078	2.1
60109	0.946132	2.1
60110	0.946132	2.1
60111	1.13078	2.1
60112	1.13078	2.1
60113	1.13078	2.1
60114	0.946133	2.1
60115	0.946132	2.1
60116	1.17109	2.1
60117	1.89226	2.1
60118	1.89226	2.1
60119	1.89226	2.1
60120	1.89226	2.1
60121	1.89226	2.1
60122	1.89226	2.1
60123	2.33963	0.959645
60124	1.89227	0.503799
60125	1.74565	0.646267
60126	1.74565	0.697857
60127	1.89227	0.437557
60128	1.89227	0.530879
60129	1.89227	0.669338
60130	1.89227	0.46565
60131	1.89227	1.17437
60132	1.89227	0.526535
60133	3.78453	0.635177
60134	1.31398	0.35109
60135	1.10392	0.433789
60136	0.796878	0.409768
60137	0.524356	0.359897
60138	0.870614	0.353445
60139	0.945739	0.304562
60140	0.89208	0.439823
60141	0.89208	0.836923
60142	1.23425	0.361028
60143	0.774658	0.600746

60144	1.03182	0.360772
60145	0.557289	0.315426
60146	2.33963	0.827022
60147	1.89226	0.358034
60148	1.89226	0.971015
60149	2.77245	0.46139
60150	4.23189	0.591713
60151	1.89226	1.03955
60152	1.89226	0.401201
60153	1.89226	1.01538
60154	1.89226	0.860214
60155	1.89226	0.522665
60156	1.89226	0.472174
60157	1.89226	0.180753
60158	1.89226	0.188053
60159	1.89226	0.151424
60160	1.89226	0.334337
60161	1.89226	0.139001
60162	1.89226	0.311578
60163	1.89226	0.141368
60164	1.89226	0.132266
60165	1.89226	0.113199
60166	1.89226	0.209445
60167	1.89226	0.099162
60168	1.89226	0.183515
60169	0.946128	0.131752
60170	0.946128	0.0941699
60171	0.963918	0.0946505
60172	1.37543	0.0778127
60173	0.946129	0.0917328
60174	0.946129	0.127024
60175	1.37542	0.0804407
60176	1.37542	0.0731289
60177	0.94613	0.0797291
60178	1.37542	0.0895237
60179	0.963916	0.0821352
60180	0.94613	0.0884667
60181	0.946132	0.172818
60182	0.946132	0.120534
60183	0.946131	0.128376
60184	0.946131	0.0861063
60185	0.946131	0.130668
60186	0.946131	0.17022
60187	0.946132	0.107436
60188	0.946132	0.0843958
60189	0.946131	0.123237
60190	0.946131	0.128657
60191	0.946131	0.119569
60192	0.946131	0.117367
60193	1.89226	0.191859
60194	1.89226	0.206317
60195	1.89226	0.136803
60196	1.89226	0.325088
60197	3.78452	0.212175
60198	0.946127	0.202753
60199	0.946127	0.132838
60200	0.946131	0.136118
60201	0.946131	0.0961884

60202	0.946128	0.139231
60203	0.946128	0.188521
60204	0.94613	0.101544
60205	0.94613	0.143931
60206	0.946129	0.13034
60207	0.946129	0.137117
60208	0.946129	0.139941
60209	0.946129	0.145643
60210	1.89226	1.18438
60211	1.89226	1.59169
60212	1.89226	2.0696
60213	1.89226	0.706753
60214	3.78453	0.815328
60215	1.89226	0.743904
60216	1.89226	0.145427
60217	1.89226	0.349949
60218	1.89226	0.530077
60219	3.78453	0.664303
60220	0.946131	0.396657
60221	0.946131	0.425872
60222	0.946132	1.32126
60223	0.946132	0.834318
60224	0.946132	1.16361
60225	0.946132	0.648548
60226	0.946132	1.17511
60227	0.946132	0.801961
60228	0.946132	0.842181
60229	0.946132	1.0564
60230	0.946132	1.19315
60231	0.946132	0.79581
60232	0.548169	0.24779
60233	0.736603	0.163967
60234	1.18322	0.382223
60235	1.01139	0.18589
60236	1.01139	0.141891
60237	0.548168	0.165974
60238	0.736603	0.316708
60239	1.18322	0.175485
60240	0.861589	0.205778
60241	0.861589	0.368661
60242	0.869798	0.162919
60243	0.869798	0.189589
60244	1.16037	0.320926
60245	0.942012	0.368796
60246	0.942012	0.220442
60247	0.62356	0.250821
60248	0.891932	0.345528
60249	0.900255	0.306723
60250	2.02279	0.170557
60251	1.48677	0.295065
60252	1.54724	0.254152
60253	0.831456	0.239395
60254	1.19775	0.349706
60255	1.89226	1.58186
60256	2.76291	1.37611
60257	1.89226	1.56684
60258	1.89226	1.45452
60259	3.78452	1.4523

60260	1.89226	1.0584
60261	1.89226	1.13122
60262	1.89226	1.31302
60263	1.89226	1.05908
60264	1.89226	1.24391
60265	1.89226	0.977954
60266	1.89226	1.23963
60267	1.89226	1.19826
60268	1.89226	1.45265
60269	1.89226	1.05814
60270	3.78452	1.25869
60271	1.89226	0.908466
60272	1.89226	1.02259
60273	1.89226	1.23882
60274	1.89226	1.04829
60275	3.78453	1.06955
60276	0.946128	0.751681
60277	0.946128	1.06724
60278	0.946129	1.10578
60279	0.946129	0.87947
60280	0.946132	0.745694
60281	0.946132	0.981886
60282	0.946126	1.02302
60283	0.946126	1.08186
60284	0.946129	1.04444
60285	0.946129	1.08032
60286	0.946129	0.942289
60287	0.946129	0.842815
60288	2.80495	0.950643
60289	1.89225	0.929865
60290	1.89225	1.13814
60291	1.87794	1.15093
60292	1.89225	1.27952
60293	1.87794	1.23697
60294	2.25195	2.1
60295	2.00624	0.981638
60296	2.13132	2.08355
60297	1.90239	0.971478
60298	4.13756	1.12122
60299	0.804661	1.90104
60300	0.804663	0.495482
60301	0.889591	0.71307
60302	0.889591	2.04173
60303	0.808324	1.73224
60304	0.761765	1.97791
60305	1.02822	0.154806
60306	0.879624	0.221052
60307	0.879617	0.959189
60308	0.800864	0.853314
60309	0.978023	1.0353
60310	0.808329	0.891263
60311	2.13133	2.08552
60312	2.00558	0.44355
60313	2.09316	0.342626
60314	2.09316	2.09672
60315	2.03253	0.950613
60316	2.03253	2.09796
60317	0.84577	1.67985

60318	0.845767	0.772987
60319	0.843917	0.624158
60320	0.843917	2.03871
60321	0.76177	1.46911
60322	0.719345	1.88871
60323	0.934435	0.181815
60324	0.884197	0.151664
60325	0.934439	0.639316
60326	0.75627	0.716241
60327	0.849424	0.561924
60328	0.76177	0.662597
60329	2.61855	0.116337
60330	1.17793	0.127686
60331	1.42965	0.122259
60332	1.17793	0.119618
60333	2.50057	0.136362
60334	2.24885	0.13693
60335	0.946133	0.115061
60336	0.946133	0.1009
60337	1.30928	0.117889
60338	1.12443	0.140367
60339	0.946132	0.147902
60340	0.946132	0.12224
60341	1.30928	0.0997791
60342	1.30928	0.12446
60343	0.946132	0.126219
60344	1.30928	0.121859
60345	1.12443	0.111951
60346	0.946133	0.117327
60347	1.89226	0.116517
60348	1.89226	0.144632
60349	1.89226	0.129197
60350	1.89226	0.137372
60351	3.78452	0.143122
60352	1.89226	0.135955
60353	1.89226	0.118095
60354	1.89226	0.121173
60355	1.89226	0.125045
60356	1.89226	0.127831
60357	1.89226	0.132765
60358	1.89226	0.130688
60359	1.89226	0.130886
60360	1.89226	0.11308
60361	1.89226	0.149823
60362	3.78452	0.120396
60363	0.94613	0.114096
60364	0.94613	0.125297
60365	0.946131	0.116161
60366	0.946131	0.125277
60367	0.946128	0.128116
60368	0.946128	0.119417
60369	0.946133	0.114988
60370	0.946133	0.128233
60371	0.946131	0.123216
60372	0.946131	0.11741
60373	0.946131	0.122288
60374	0.946131	0.132455
60375	1.89226	2.1

60376	1.89226	2.1
60377	1.89226	2.1
60378	1.89226	2.1
60379	3.78453	2.1
60380	1.0465	2.1
60381	2.73926	2.1
60382	2.03721	2.1
60383	2.03721	2.1
60384	1.44709	2.1
60385	1.44709	2.1
60386	0.946131	2.1
60387	0.946131	2.1
60388	0.946131	2.1
60389	0.946131	2.1
60390	0.946132	2.1
60391	0.946132	2.1
60392	0.94613	2.1
60393	0.94613	2.1
60394	0.946131	2.1
60395	0.946131	2.1
60396	0.946131	2.1
60397	0.946131	2.1
60398	0.946132	2.1
60399	0.946132	2.1
60400	0.946131	2.1
60401	0.946131	2.1
60402	0.946132	2.1
60403	0.946132	2.1
60404	0.946132	2.1
60405	0.946132	2.1
60406	0.946132	2.1
60407	0.946132	2.1
60408	0.946132	2.1
60409	0.946132	2.1
60410	1.89226	2.1
60411	1.89226	2.1
60412	1.89226	2.1
60413	1.89226	2.1
60414	3.78452	2.1
60415	1.61969	0.108435
60416	1.79374	0.0766769
60417	1.29382	0.0807951
60418	1.29382	0.111095
60419	2.05759	0.0771278
60420	2.05759	0.105097
60421	1.89226	0.109562
60422	1.89226	0.0892336
60423	1.89226	0.0888086
60424	1.89226	0.132487
60425	1.89226	0.0773499
60426	1.89226	0.100711
60427	1.89226	0.714664
60428	1.89226	0.847887
60429	1.89226	0.723389
60430	1.89226	0.721423
60431	1.89226	0.686793
60432	1.89226	0.688304
60433	1.89226	1.79745

60434	1.89226	1.1665
60435	1.89226	1.36792
60436	1.89226	1.70383
60437	3.78453	1.79705
60438	0.946131	0.988647
60439	0.946131	1.08677
60440	0.946131	0.815938
60441	0.946131	0.989016
60442	0.946131	0.856961
60443	0.946131	0.7732
60444	0.946131	0.981199
60445	0.946131	1.20823
60446	0.946131	0.930586
60447	0.946131	0.965486
60448	0.946131	1.00463
60449	0.946131	0.915585
60450	2.45177	0.182965
60451	2.68507	0.144685
60452	2.3376	0.221177
60453	2.22086	0.133781
60454	4.78937	0.172142
60455	2.45178	0.167578
60456	2.35552	0.114276
60457	1.68062	0.151356
60458	1.81902	0.125558
60459	4.13239	0.136181
60460	2.3376	0.173599
60461	1.55612	0.115248
60462	1.69811	0.124552
60463	2.31527	0.1405
60464	1.79553	0.124253
60465	2.3376	0.143528
60466	1.55613	0.105875
60467	1.06336	0.118349
60468	1.97516	0.12377
60469	1.88966	0.105124
60470	1.06336	0.156068
60471	1.68062	0.102667
60472	1.89226	1.34008
60473	1.91634	1.84287
60474	2.03709	1.99327
60475	1.91634	1.57882
60476	1.83185	1.81949
60477	1.70303	0.88024
60478	1.89226	1.24241
60479	1.89226	1.75029
60480	1.89226	1.97924
60481	1.89226	1.51653
60482	1.89226	1.69465
60483	1.89226	0.909967
60484	1.89226	2.09383
60485	2.03472	2.1
60486	2.22183	2.1
60487	2.03472	2.1
60488	2.07936	2.1
60489	1.89226	1.92557
60490	1.89226	0.863477
60491	1.8519	1.27567

60492	1.92119	1.61274
60493	1.8519	1.09618
60494	1.96156	1.13937
60495	1.89226	0.632195
60496	1.89226	0.90055
60497	1.89226	1.36798
60498	1.89226	1.63822
60499	1.89226	1.14789
60500	1.89226	1.15896
60501	1.89226	0.644628
60502	1.89226	2.00147
60503	2.22183	2.09562
60504	2.39541	2.1
60505	2.22183	2.04752
60506	2.06585	2.07085
60507	1.89226	1.69203
60508	1.47001	2.1
60509	1.57962	1.99333
60510	1.65238	2.1
60511	1.60502	2.1
60512	3.18464	2.1
60513	1.02415	2.1
60514	1.60503	2.1
60515	1.47001	2.1
60516	1.47001	2.1
60517	1.28228	2.1
60518	1.28228	2.1
60519	0.946133	2.1
60520	0.946133	2.1
60521	0.946132	2.1
60522	0.946132	2.1
60523	0.946132	2.1
60524	0.946132	2.1
60525	0.946133	2.1
60526	0.946133	2.1
60527	0.946132	2.1
60528	0.946132	2.1
60529	0.946133	2.1
60530	0.946133	2.1
60531	1.89226	1.98923
60532	1.89226	2.0905
60533	1.89226	2.1
60534	1.89226	1.76968
60535	3.78452	2.03462
60536	1.76897	2.06933
60537	1.60502	2.1
60538	1.65238	1.98568
60539	1.65238	2.1
60540	1.76932	1.94972
60541	1.76932	2.1
60542	1.55998	2.1
60543	1.60503	2.1
60544	1.76932	2.1
60545	1.60503	2.1
60546	1.44657	2.1
60547	1.28228	2.1
60548	0.946133	2.1
60549	0.946133	2.1

60550	0.946132	2.1
60551	0.946132	2.0803
60552	0.946132	2.07853
60553	0.946132	2.1
60554	0.946133	2.1
60555	0.946133	2.1
60556	0.946132	2.1
60557	0.946132	2.1
60558	0.946132	2.1
60559	0.946132	2.1
60560	0.946128	2.1
60561	0.946128	2.03121
60562	0.946133	1.41374
60563	0.946133	1.81934
60564	0.94613	1.51733
60565	0.94613	2.03273
60566	0.946131	2.1
60567	0.946131	1.9553
60568	0.946131	1.96369
60569	0.946131	1.94171
60570	0.946131	2.01789
60571	0.946131	2.0207
60572	1.85779	0.15761
60573	1.00763	0.122372
60574	1.74874	0.135026
60575	1.74873	0.127463
60576	1.14467	0.164349
60577	1.14466	0.146681
60578	1.85779	0.143185
60579	1.61597	0.116787
60580	1.00053	0.142698
60581	1.03018	0.110287
60582	2.65819	0.120235
60583	1.89226	0.539566
60584	1.89226	0.329072
60585	1.89226	0.540187
60586	1.89226	0.438677
60587	3.78451	0.484742
60588	1.89226	0.496258
60589	1.89226	0.185973
60590	1.89226	0.288851
60591	1.89226	0.321674
60592	1.89226	0.257761
60593	1.89226	0.291753
60594	1.11983	0.156228
60595	0.810945	0.172049
60596	0.913906	0.145766
60597	0.913906	0.161065
60598	2.23303	0.11216
60599	1.66057	0.115836
60600	1.28724	0.130002
60601	1.08132	0.131762
60602	1.49316	0.149326
60603	0.810958	0.160358
60604	0.810958	0.152097
60605	0.827861	0.273651
60606	0.946126	0.30553
60607	0.946132	0.479869

60608	0.946132	0.255118
60609	0.827863	0.306952
60610	0.827863	0.291617
60611	0.946129	0.349087
60612	0.946129	0.376754
60613	0.827863	0.292091
60614	0.946128	0.32152
60615	0.946129	0.273268
60616	0.946129	0.294189
60617	0.974718	0.0698759
60618	1.53671	0.0893055
60619	2.80542	0.0698617
60620	2.27259	0.0806805
60621	3.8093	0.0721654
60622	1.89226	0.108978
60623	1.89226	0.218163
60624	1.89226	0.0976812
60625	1.89226	0.19487
60626	3.78453	0.154173
60627	0.946132	0.106037
60628	0.946132	0.165625
60629	0.946131	0.158557
60630	0.946131	0.107944
60631	0.946132	0.163229
60632	0.946132	0.185549
60633	0.946131	0.0951397
60634	0.946131	0.0980916
60635	0.946132	0.137973
60636	0.946132	0.159106
60637	0.946131	0.122064
60638	0.946131	0.124347
60639	2.80542	0.0759339
60640	1.89226	0.0908317
60641	2.27259	0.0757637
60642	2.27259	0.119998
60643	1.89226	0.0701622
60644	1.89226	0.0967255
60645	1.89226	0.102853
60646	1.89226	0.22444
60647	1.89226	0.114551
60648	1.89226	0.232037
60649	1.89226	0.104553
60650	1.89226	0.23086
60651	0.946132	0.212812
60652	0.946132	0.102294
60653	0.946131	0.189504
60654	0.946131	0.115859
60655	0.946132	0.197453
60656	0.946132	0.201381
60657	0.946131	0.101812
60658	0.946131	0.114203
60659	0.946131	0.142219
60660	0.946131	0.152719
60661	0.946131	0.126044
60662	0.946131	0.143215
60663	2.68213	2.1
60664	2.58234	2.1
60665	2.58234	2.1

60666	2.61243	2.1
60667	2.67035	2.1
60668	2.70044	2.1
60669	2.68213	2.1
60670	2.53921	2.1
60671	2.53303	2.1
60672	2.67035	2.1
60673	5.20956	2.1
60674	2.67033	2.1
60675	2.58232	2.1
60676	2.55004	2.1
60677	2.64567	2.1
60678	5.228 2.1	
60679	2.53301	2.1
60680	2.64567	2.1
60681	2.64567	2.1
60682	2.67033	2.1
60683	2.51603	2.1
60684	2.54069	2.1
60685	0.907497	2.1
60686	0.907497	2.1
60687	0.926641	2.1
60688	0.926641	2.1
60689	1.14989	2.1
60690	1.10046	2.1
60691	0.695334	2.1
60692	0.684247	2.1
60693	0.695333	2.1
60694	1.14989	2.1
60695	0.684246	2.1
60696	1.10046	2.1
60697	0.946126	2.1
60698	0.946126	2.1
60699	0.946131	2.1
60700	0.946131	2.1
60701	0.946127	2.1
60702	0.946127	2.1
60703	0.94613	2.1
60704	0.94613	2.1
60705	0.946128	2.1
60706	0.946128	2.1
60707	0.946128	2.1
60708	0.946128	2.1
60709	1.89226	2.1
60710	1.89226	2.1
60711	1.89226	2.1
60712	1.89226	2.1
60713	1.89226	2.1
60714	1.89226	2.08028
60715	1.42174	2.1
60716	2.29978	2.1
60717	2.41233	2.1
60718	2.41233	2.1
60719	1.39067	2.1
60720	1.39067	2.1
60721	1.89226	2.1
60722	1.89226	2.1
60723	1.89226	2.1

60724	1.89226	2.1
60725	3.78452	2.1
60726	1.89226	2.1
60727	1.89226	2.1
60728	1.89226	2.1
60729	1.89226	2.1
60730	1.89226	2.1
60731	1.89226	2.1
60732	2.41234	2.1
60733	1.89226	2.1
60734	2.29979	2.1
60735	1.89226	2.1
60736	1.89226	2.1
60737	2.29979	2.1
60738	1.09506	0.0719594
60739	2.82921	0.0729553
60740	2.53251	0.073062
60741	2.53251	0.071728
60742	1.42901	0.0731864
60743	1.42901	0.072068
60744	0.946132	0.0698591
60745	0.946132	0.0711557
60746	0.946128	0.0734697
60747	0.946128	0.0724941
60748	0.94613	0.0710501
60749	0.94613	0.0705708
60750	0.946131	0.0751677
60751	0.946131	0.0709354
60752	0.94613	0.070099
60753	0.94613	0.070229
60754	0.94613	0.0733674
60755	0.94613	0.0724022
60756	1.89226	0.074532
60757	1.89226	0.0755845
60758	1.89226	0.0747447
60759	1.89226	0.0699407
60760	3.78452	0.0755607
60761	2.53251	0.0716144
60762	1.89226	0.0733887
60763	2.82921	0.0726432
60764	1.89226	0.0731843
60765	1.89226	0.0744994
60766	2.82921	0.0745832
60767	1.89226	0.0700123
60768	1.89226	0.0698591
60769	1.89226	0.0698591
60770	1.89226	0.0698591
60771	1.89226	0.0698591
60772	1.89226	0.0698591
60773	2.53251	0.0698591
60774	1.89226	0.0698591
60775	2.06158	0.0698591
60776	1.89226	0.0698591
60777	4.42477	0.0698591
60778	1.23296	2.1
60779	2.55352	2.1
60780	2.47476	2.1
60781	2.47476	2.1

60782	1.32368	2.1
60783	1.32368	2.1
60784	1.89226	2.1
60785	1.89226	2.1
60786	1.89226	2.1
60787	1.89226	2.1
60788	1.89226	2.1
60789	1.89226	2.1
60790	1.89226	2.1
60791	1.89226	2.1
60792	1.89226	2.1
60793	1.89226	2.1
60794	1.89226	2.1
60795	1.89226	2.1
60796	2.47476	2.1
60797	1.89226	2.1
60798	1.89226	2.1
60799	1.89226	2.1
60800	2.55353	2.1
60801	2.55353	2.1
60802	1.05983	2.1
60803	2.47476	2.1
60804	2.37664	2.1
60805	2.37664	2.1
60806	1.23296	2.1
60807	1.23296	2.1
60808	0.946127	2.1
60809	0.946127	2.1
60810	0.946131	2.1
60811	0.946131	2.1
60812	0.946127	2.1
60813	0.946127	2.1
60814	0.946131	2.1
60815	0.946131	2.1
60816	0.946129	2.1
60817	0.946129	2.1
60818	0.946128	2.1
60819	0.946128	2.1
60820	1.89226	2.1
60821	1.89226	2.1
60822	1.89226	2.1
60823	1.89226	2.1
60824	3.78452	2.1
60825	2.37665	2.1
60826	1.89226	2.1
60827	1.89226	2.1
60828	1.89226	2.1
60829	2.47476	2.1
60830	2.47476	2.1
60831	1.89226	0.540705
60832	1.92119	0.899342
60833	1.99586	1.06599
60834	1.92119	0.795345
60835	1.96693	0.649117
60836	1.89226	0.385897
60837	1.89226	0.635446
60838	1.89226	0.953871
60839	1.89226	1.16745

60840	1.89226	0.848156
60841	1.89226	0.821278
60842	1.89226	0.468881
60843	1.89226	1.62676
60844	2.39541	2.01475
60845	2.57183	2.07874
60846	2.39541	1.82952
60847	2.06868	1.79071
60848	1.89226	1.14155
60849	0.946133	0.374685
60850	0.946133	0.317406
60851	0.997928	0.620311
60852	1.03763	0.60248
60853	0.946131	0.680516
60854	0.946131	0.186507
60855	1.03763	0.34194
60856	1.03763	0.49739
60857	1.03763	0.451176
60858	0.946131	0.442669
60859	0.94613	0.41639
60860	0.99793	0.459067
60861	1.89226	0.280467
60862	1.89226	0.734183
60863	1.89226	0.764785
60864	1.89226	0.333156
60865	1.89226	0.81638
60866	1.89226	0.369675
60867	0.946131	0.906188
60868	0.946131	0.914172
60869	1.36939	1.42363
60870	1.28592	1.96715
60871	0.946129	1.48958
60872	0.946129	0.659485
60873	1.36939	0.961727
60874	1.36939	1.63071
60875	0.946131	1.13248
60876	1.36939	1.1516
60877	0.946131	1.29271
60878	1.28592	1.40132
60879	1.89226	0.180121
60880	1.89226	0.324612
60881	1.89226	0.25835
60882	1.89226	0.18018
60883	3.78453	0.213641
60884	1.89226	0.327055
60885	1.89226	0.218321
60886	1.89226	0.190486
60887	1.89226	0.317719
60888	3.78452	0.249809
60889	1.91005	0.0976285
60890	1.89226	0.228856
60891	2.60215	0.094771
60892	2.60215	0.110339
60893	1.89226	0.215537
60894	1.89226	0.203468
60895	0.946132	0.271459
60896	0.946132	0.251459
60897	0.946132	0.190222

60898	0.946132	0.178807
60899	0.946132	0.18797
60900	0.946132	0.26246
60901	0.946132	0.24645
60902	0.946132	0.174215
60903	0.946132	0.209357
60904	0.946132	0.210508
60905	0.946131	0.219908
60906	0.946131	0.21752
60907	1.89226	0.19866
60908	1.89226	0.306229
60909	1.89226	0.241782
60910	1.89226	0.298873
60911	1.89226	0.287905
60912	1.89226	0.240711
60913	0.780483	0.1478
60914	0.78928	0.239919
60915	0.818566	0.257402
60916	0.460387	0.192645
60917	1.82793	0.198606
60918	1.32039	0.201199
60919	0.613889	0.21357
60920	1.0592	0.245105
60921	1.48698	0.146364
60922	0.818555	0.206103
60923	1.11515	0.178223
60924	1.2802	0.5517
60925	2.20046	0.318012
60926	1.1475	0.528168
60927	1.1475	0.282414
60928	2.27062	0.520831
60929	2.27062	0.25721
60930	1.89226	0.499316
60931	2.20046	0.21427
60932	2.27062	0.390148
60933	2.20046	0.403696
60934	1.96242	0.141273
60935	1.89226	0.166242
60936	1.89226	0.235625
60937	1.89226	0.153066
60938	1.89226	0.311445
60939	1.89226	0.325965
60940	3.78452	0.190913
60941	1.89226	0.364506
60942	1.89226	0.478704
60943	1.89226	0.531763
60944	1.89226	0.385122
60945	3.78452	0.443537
60946	1.17403	0.301527
60947	1.13531	0.327083
60948	0.6401	0.373819
60949	0.714566	0.36277
60950	0.915987	0.409412
60951	0.915987	0.162644
60952	0.933891	0.393204
60953	0.933891	0.32015
60954	1.17403	0.361296
60955	0.714566	0.348797

60956	0.6401	0.328325
60957	1.13531	0.290182
60958	0.946133	0.246807
60959	0.946133	0.311713
60960	1.17403	0.199903
60961	1.13531	0.197374
60962	0.946131	0.260623
60963	0.946131	0.131994
60964	1.17403	0.359931
60965	1.17403	0.133267
60966	1.17403	0.23468
60967	0.94613	0.234722
60968	0.946131	0.174927
60969	1.13531	0.168606
60970	1.89226	0.136911
60971	1.89226	0.258638
60972	1.89226	0.21796
60973	1.89226	0.112562
60974	1.89226	0.282624
60975	1.89226	0.144024
60976	0.946131	0.279358
60977	0.946131	0.269081
60978	0.946131	0.365168
60979	0.946131	0.377874
60980	0.946129	0.382797
60981	0.946129	0.187412
60982	0.946133	0.472355
60983	0.946133	0.23469
60984	0.946131	0.324898
60985	0.946131	0.323307
60986	0.946131	0.262552
60987	0.946131	0.275787
60988	2.29413	2.1
60989	1.99332	2.1
60990	1.84774	2.1
60991	2.21582	2.1
60992	4.20915	2.1
60993	0.517359	2.1
60994	0.634469	2.1
60995	0.996661	2.1
60996	0.92387	2.1
60997	0.768098	2.1
60998	0.768098	2.1
60999	0.790242	2.1
61000	0.790242	2.1
61001	0.923871	2.1
61002	0.517359	2.1
61003	0.634469	2.1
61004	0.996661	2.1
61005	1.89226	2.1
61006	1.89226	2.1
61007	1.89226	2.1
61008	1.89226	2.1
61009	3.78453	2.1
61010	0.946132	2.1
61011	0.946132	2.1
61012	0.946131	2.1
61013	0.946131	2.1

61014	0.946132	2.1
61015	0.946132	2.1
61016	0.946131	2.1
61017	0.946131	2.1
61018	0.946132	2.1
61019	0.946132	2.1
61020	0.946131	2.1
61021	0.946131	2.1
61022	1.62708	2.1
61023	2.21582	2.1
61024	2.13925	2.1
61025	2.21582	2.1
61026	1.77117	2.1
61027	1.84774	2.1
61028	0.946132	2.1
61029	0.946132	2.1
61030	0.946131	2.1
61031	0.946131	2.1
61032	0.946132	2.1
61033	0.946132	2.1
61034	0.946131	2.1
61035	0.946131	2.1
61036	0.946131	2.1
61037	0.946131	2.1
61038	0.946131	2.1
61039	0.946131	2.1
61040	1.89226	1.99816
61041	1.89226	1.17651
61042	1.89226	1.20929
61043	1.89226	1.84259
61044	3.78453	1.91737
61045	1.89226	1.11049
61046	1.89226	1.80165
61047	1.89226	1.76508
61048	1.89226	2.0552
61049	1.89226	0.863577
61050	1.89226	1.74601
61051	1.89226	1.27546
61052	1.89226	2.02951
61053	1.89226	0.9058
61054	1.89226	1.9818
61055	1.89226	1.09283
61056	1.89226	2.03772
61057	0.914369	1.84575
61058	0.914369	1.93779
61059	0.810945	2.0937
61060	0.810945	1.91117
61061	2.26581	2.09907
61062	1.66199	2.08545
61063	1.49503	1.9375
61064	1.28816	2.1
61065	1.08133	2.04077
61066	0.810933	2.01895
61067	1.1212	2.01849
61068	1.89227	0.241373
61069	1.89227	0.125607
61070	1.89227	0.15376
61071	1.89227	0.251036

61072	3.78453	0.181088
61073	1.89226	0.220547
61074	1.89226	0.373148
61075	1.89226	0.365214
61076	1.89226	0.235478
61077	3.78453	0.290889
61078	1.89226	0.387197
61079	1.89226	0.216414
61080	1.89226	0.250421
61081	1.89226	0.386863
61082	3.78453	0.293553
61083	1.89226	0.134481
61084	1.89226	0.238084
61085	1.89226	0.265828
61086	2.25318	0.12428
61087	3.78453	0.182063
61088	1.89226	0.16746
61089	1.89226	0.261367
61090	1.89226	0.231794
61091	1.9637	0.114266
61092	3.78453	0.182673
61093	1.89226	0.361507
61094	1.89226	0.236713
61095	1.89226	0.222597
61096	1.89226	0.352396
61097	3.78453	0.280717
61098	1.89226	0.25368
61099	1.89226	0.397904
61100	1.89226	0.361353
61101	2.53384	0.238618
61102	3.78453	0.324836
61103	1.55405	0.282401
61104	1.03818	1.36531
61105	1.03818	0.527352
61106	1.34857	0.428633
61107	1.46338	0.222197
61108	1.77377	0.997116
61109	1.86273	0.414415
61110	1.73802	0.767501
61111	1.50969	1.96336
61112	1.46337	0.584109
61113	3.20139	0.767044
61114	1.2377	0.541479
61115	1.46337	0.233958
61116	1.27408	1.2783
61117	1.32039	1.93689
61118	1.50969	1.01997
61119	1.50969	0.443791
61120	1.89226	2.1
61121	1.89226	2.1
61122	1.89226	2.1
61123	1.89226	2.1
61124	1.89226	2.1
61125	1.89226	2.1
61126	1.89169	2.1
61127	1.89226	2.1
61128	1.89226	2.1
61129	1.89226	2.1

61130	1.96624	2.1
61131	1.96624	2.1
61132	2.62871	2.1
61133	1.6201	2.1
61134	1.49149	2.1
61135	1.6201	2.1
61136	2.62559	2.1
61137	2.75419	2.1
61138	0.946129	2.06816
61139	0.946129	2.1
61140	0.946133	2.1
61141	0.946133	2.1
61142	0.946131	2.1
61143	0.946131	2.04829
61144	0.946131	2.1
61145	0.946131	2.1
61146	0.946131	2.1
61147	0.946131	2.1
61148	0.946131	2.1
61149	0.946131	2.1
61150	0.851543	2.1
61151	0.851543	2.1
61152	1.04043	2.1
61153	0.997939	2.1
61154	0.946132	2.1
61155	0.946132	2.1
61156	0.894185	2.1
61157	0.893901	2.1
61158	0.998267	2.1
61159	1.04074	2.1
61160	0.85127	2.1
61161	0.85153	2.1
61162	1.89226	2.1
61163	1.89226	2.1
61164	1.89226	2.1
61165	1.89226	2.1
61166	1.89226	2.1
61167	1.89226	2.1
61168	0.928691	2.1
61169	0.928691	2.1
61170	0.935332	2.1
61171	0.935332	2.1
61172	1.398	2.1
61173	1.14418	2.1
61174	0.479331	2.1
61175	0.719848	2.1
61176	1.398	2.1
61177	0.719848	2.1
61178	1.14417	2.1
61179	0.47933	2.1
61180	2.28835	2.1
61181	1.89226	2.1
61182	1.89226	2.1
61183	1.89226	2.1
61184	2.796	2.1
61185	2.796	2.1
61186	0.946131	2.1
61187	0.946131	2.1

61188	0.946131	2.1
61189	0.946131	2.1
61190	0.946128	2.1
61191	0.946128	2.1
61192	0.946133	2.1
61193	0.946133	2.1
61194	0.946131	2.1
61195	0.946131	2.1
61196	0.94613	2.1
61197	0.94613	2.1
61198	0.946131	2.1
61199	0.946131	2.1
61200	0.946131	2.1
61201	0.946131	2.1
61202	0.946131	2.1
61203	0.946131	2.1
61204	0.946131	2.1
61205	0.946131	2.1
61206	0.946131	2.1
61207	0.946131	2.1
61208	0.946131	2.1
61209	0.946131	2.1
61210	1.89226	2.1
61211	1.89226	2.1
61212	1.89226	2.1
61213	1.89226	2.1
61214	3.78452	2.1
61215	2.05207	0.633426
61216	1.70302	1.60652
61217	1.33794	2.07509
61218	1.98194	0.871111
61219	3.36873	1.36888
61220	2.62761	0.216454
61221	1.98194	0.706233
61222	2.28009	1.53825
61223	2.28009	0.278753
61224	2.22939	1.14469
61225	2.22939	0.37909
61226	1.89226	0.721976
61227	1.89226	0.518336
61228	1.89226	0.539545
61229	1.89226	0.32989
61230	1.89226	0.695245
61231	1.89226	0.392208
61232	1.89227	0.882114
61233	1.89227	1.38393
61234	1.89227	1.91944
61235	1.89227	1.32112
61236	1.89227	1.19911
61237	1.89227	0.449975
61238	0.888355	0.474237
61239	0.990973	0.956302
61240	0.71133	1.42921
61241	0.634324	1.69215
61242	0.806209	1.43508
61243	0.806209	0.235309
61244	0.819087	1.35636
61245	0.819087	1.77274

61246	0.888355	1.41059
61247	0.634324	1.25821
61248	0.71133	1.3808
61249	0.990972	1.27846
61250	1.11469	1.10613
61251	0.985023	0.314992
61252	0.888354	0.379544
61253	0.990972	0.975812
61254	0.994754	0.570205
61255	0.994754	0.930185
61256	1.0083	0.735623
61257	1.0083	0.513655
61258	0.888354	0.304413
61259	0.985024	0.364588
61260	0.990971	0.283412
61261	1.11469	0.146797
61262	0.946132	0.599826
61263	0.946132	0.51709
61264	0.946132	0.457792
61265	0.946132	0.46619
61266	0.946132	0.46713
61267	0.946132	0.534066
61268	0.946133	1.51946
61269	0.946133	0.693212
61270	0.946133	0.414832
61271	0.946133	0.554579
61272	0.946132	0.337075
61273	0.946132	0.244519
61274	0.810944	0.661727
61275	0.810944	0.450565
61276	0.810947	1.19041
61277	0.810947	0.171666
61278	1.89227	1.74284
61279	1.35168	1.03292
61280	1.08132	1.20876
61281	1.08131	0.904987
61282	1.08132	0.57033
61283	0.810959	0.637369
61284	0.810959	0.717703
61285	1.70303	2.1
61286	2.03975	2.1
61287	1.99324	2.1
61288	2.17615	2.1
61289	1.40549	2.1
61290	1.53446	2.1
61291	0.946133	2.1
61292	0.946133	2.1
61293	0.946129	2.1
61294	0.946129	2.1
61295	0.946131	2.1
61296	0.946131	2.1
61297	0.946131	2.1
61298	0.946131	2.1
61299	0.946131	2.1
61300	0.946131	2.1
61301	0.946131	2.1
61302	0.946131	2.1
61303	1.89226	2.1

61304	1.89226	2.1
61305	1.89226	2.1
61306	1.89226	2.1
61307	1.89226	2.1
61308	1.89226	2.1
61309	2.17615	2.1
61310	2.3111	2.1
61311	2.50288	2.1
61312	2.50288	2.1
61313	2.03975	2.1
61314	2.03975	2.1
61315	1.76401	2.1
61316	1.95852	2.1
61317	2.32134	2.1
61318	2.12306	2.1
61319	1.8513	2.1
61320	1.70303	2.1
61321	1.89226	2.1
61322	1.89226	2.1
61323	1.89226	2.1
61324	1.89226	2.1
61325	3.78452	2.1
61326	1.89226	2.1
61327	1.89226	2.1
61328	1.89226	2.1
61329	1.89226	2.1
61330	3.78452	2.1
61331	1.2243	2.1
61332	1.2243	2.1
61333	1.24189	2.1
61334	1.24189	2.1
61335	1.37811	2.1
61336	1.25144	2.1
61337	1.08807	2.1
61338	1.17949	2.1
61339	1.17949	2.1
61340	1.37811	2.1
61341	1.08807	2.1
61342	1.25144	2.1
61343	1.89226	2.1
61344	1.89226	2.1
61345	1.89226	2.1
61346	1.89226	2.1
61347	1.89226	2.1
61348	1.89226	2.1
61349	1.89227	2.1
61350	1.89227	2.1
61351	1.89227	2.1
61352	1.89227	2.1
61353	1.89227	2.1
61354	1.89227	2.1
61355	1.89224	2.1
61356	1.89224	2.1
61357	1.89224	2.1
61358	1.89224	2.1
61359	3.78447	2.1
61360	1.89224	2.1
61361	1.89224	2.1

61362	1.89224	2.1
61363	1.89224	2.1
61364	1.89224	2.1
61365	1.89224	2.1
61366	1.89224	2.1
61367	1.89224	2.1
61368	1.89224	2.1
61369	1.89224	2.1
61370	3.78448	2.1
61371	1.89224	2.1
61372	1.703 2.1	
61373	1.89224	2.1
61374	1.72991	2.1
61375	1.07643	2.1
61376	1.36657	2.1
61377	1.44472	2.1
61378	2.41233	2.1
61379	2.51628	2.1
61380	2.51628	2.1
61381	1.42174	2.1
61382	1.42174	2.1
61383	1.89226	2.1
61384	1.89226	2.1
61385	1.89226	2.1
61386	1.89226	2.1
61387	1.89226	2.1
61388	1.89226	2.1
61389	1.89226	2.1
61390	1.89226	2.1
61391	1.89226	2.1
61392	1.89226	2.1
61393	3.78452	2.1
61394	2.51628	2.1
61395	1.89226	2.1
61396	1.89226	2.1
61397	1.89226	2.1
61398	2.41234	2.1
61399	2.41234	2.1
61400	0.996254	2.1
61401	0.996254	2.1
61402	1.01153	2.1
61403	1.01153	2.1
61404	1.28542	2.1
61405	1.25814	2.1
61406	0.719184	2.1
61407	0.72236	2.1
61408	0.719183	2.1
61409	1.28542	2.1
61410	0.722359	2.1
61411	1.25814	2.1
61412	0.946126	2.1
61413	0.946126	2.1
61414	0.946131	2.1
61415	0.946131	2.1
61416	0.946127	2.1
61417	0.946127	2.1
61418	0.94613	2.1
61419	0.94613	2.1

61420	0.946128	2.1
61421	0.946128	2.1
61422	0.946128	2.1
61423	0.946128	2.1
61424	1.89226	2.1
61425	1.89226	2.1
61426	1.89226	2.1
61427	1.89226	2.1
61428	1.89226	2.1
61429	1.89226	2.1
61430	0.946132	2.1
61431	0.946132	2.1
61432	1.25814	2.1
61433	1.25814	2.1
61434	0.946131	2.1
61435	0.946131	2.1
61436	1.28543	2.1
61437	1.25814	2.1
61438	1.28543	2.1
61439	0.946132	2.1
61440	1.25814	2.1
61441	0.946132	2.1
61442	1.89226	1.2489
61443	1.89226	0.568387
61444	1.89226	1.00262
61445	1.89226	0.891353
61446	1.89226	0.766019
61447	1.89226	0.676212
61448	1.89226	1.01588
61449	2.27719	0.489756
61450	1.89226	0.563693
61451	1.89226	0.648109
61452	3.78452	0.596018
61453	1.89225	1.46026
61454	1.89225	0.645072
61455	1.89225	0.928332
61456	1.89225	1.11335
61457	3.78451	1.03664
61458	1.89226	0.504547
61459	1.89226	0.536294
61460	1.89226	0.782543
61461	1.89226	0.37909
61462	3.78452	0.534318
61463	0.946132	0.323199
61464	0.946132	0.500035
61465	0.946132	0.545158
61466	0.946132	0.449162
61467	0.946131	0.393538
61468	0.946131	0.322998
61469	0.946132	0.534212
61470	0.946132	0.37531
61471	0.946132	0.397625
61472	0.946132	0.404364
61473	0.946132	0.407667
61474	0.946132	0.371732
61475	0.946126	0.582247
61476	0.946126	0.492727
61477	0.946132	1.41928

61478	0.946132	0.677128
61479	0.946128	0.498415
61480	0.946128	0.61137
61481	0.946129	0.641385
61482	0.946129	1.06314
61483	0.946129	0.765258
61484	0.946129	0.816826
61485	0.946129	0.675309
61486	0.946129	0.612563
61487	1.39858	2.1
61488	1.39858	2.1
61489	0.94613	2.1
61490	0.94613	2.1
61491	0.946132	2.1
61492	0.946132	2.1
61493	1.39858	2.1
61494	1.25002	2.1
61495	1.39858	2.1
61496	0.946131	2.1
61497	1.25002	2.1
61498	0.946131	2.1
61499	2.0815	2.1
61500	1.89226	2.1
61501	1.89226	2.1
61502	2.05298	2.1
61503	2.5593	2.1
61504	2.77667	2.1
61505	0.946133	2.1
61506	0.946133	2.1
61507	0.946132	2.1
61508	0.946132	2.1
61509	0.946132	2.1
61510	0.946132	2.1
61511	0.946133	2.1
61512	0.946133	2.1
61513	0.946132	2.1
61514	0.946132	2.1
61515	0.946132	2.1
61516	0.946132	2.1
61517	1.38908	2.1
61518	1.38908	2.1
61519	0.946132	2.1
61520	0.946132	2.1
61521	0.946132	2.1
61522	0.946132	2.1
61523	1.38908	2.1
61524	1.27965	2.1
61525	1.38908	2.1
61526	0.946132	2.1
61527	0.946133	2.1
61528	1.27965	2.1
61529	0.946133	2.1
61530	0.946133	2.1
61531	0.946132	2.1
61532	0.946132	2.1
61533	0.946132	2.1
61534	0.946132	2.1
61535	0.946133	2.1

61536	0.946133	2.1
61537	0.946132	2.1
61538	0.946132	2.1
61539	0.946133	2.1
61540	0.946133	2.1
61541	1.89227	1.33313
61542	1.89227	1.58286
61543	1.89227	0.830837
61544	1.89227	0.392792
61545	3.78453	1.07151
61546	1.33154	0.959595
61547	1.33154	0.732471
61548	0.946132	0.905705
61549	0.946132	0.78391
61550	1.19095	0.545869
61551	1.33154	1.00086
61552	0.946133	1.23826
61553	0.946133	1.35866
61554	0.946133	1.63932
61555	1.19095	0.677647
61556	0.946132	1.4472
61557	1.33154	0.887221
61558	1.0888	0.656969
61559	1.04745	0.494621
61560	0.946132	0.673926
61561	0.946132	0.555076
61562	1.04745	0.33476
61563	1.04745	1.01381
61564	0.946132	1.41542
61565	0.946132	0.366106
61566	1.04745	1.08292
61567	0.946133	0.382621
61568	0.946132	0.956486
61569	1.0888	0.638343
61570	1.89226	1.79637
61571	1.89226	0.361722
61572	1.89226	0.466328
61573	1.89226	0.901594
61574	1.89226	0.517017
61575	1.89226	0.718205
61576	1.89226	1.65373
61577	1.87592	0.332165
61578	1.87592	0.504465
61579	2.3819	0.653443
61580	1.89226	0.780645
61581	2.39825	0.856655
61582	1.89226	0.376783
61583	2.0949	1.12828
61584	1.88741	0.28327
61585	1.89226	0.28231
61586	1.89226	0.437797
61587	1.88741	0.772566
61588	1.07508	1.59955
61589	1.63419	1.94517
61590	1.63419	1.81983
61591	1.09795	1.83315
61592	1.56137	1.8397
61593	1.02513	1.94743

61594	0.967672	0.915751
61595	1.56137	1.5093
61596	1.56137	1.0269
61597	1.07508	1.26755
61598	1.41319	0.980727
61599	0.926902	1.48673
61600	1.56137	1.22912
61601	1.63419	1.81938
61602	2.29852	1.68692
61603	2.14485	1.50597
61604	3.77904	1.23933
61605	1.41319	0.503515
61606	1.92622	0.591343
61607	1.56137	0.492723
61608	1.56137	0.940852
61609	2.14485	1.2108
61610	1.92622	0.937048
61611	1.89227	2.1
61612	1.89227	2.1
61613	1.89227	2.1
61614	1.89227	2.1
61615	3.78453	2.1
61616	2.56618	2.1
61617	2.08612	2.1
61618	2.48048	2.1
61619	1.99644	2.1
61620	4.5666	2.1
61621	2.08612	2.1
61622	1.30659	2.1
61623	1.99644	2.1
61624	1.25404	2.1
61625	3.34016	2.1
61626	1.89226	2.1
61627	1.89226	2.1
61628	1.89226	2.1
61629	1.89226	2.1
61630	1.89226	2.1
61631	1.89226	2.1
61632	1.89227	2.1
61633	1.89227	2.1
61634	1.89227	2.1
61635	1.89227	2.1
61636	3.78453	2.1
61637	2.48048	2.1
61638	1.99644	2.1
61639	2.39022	2.1
61640	1.90734	2.1
61641	4.38782	2.1
61642	1.90734	2.1
61643	1.99644	2.1
61644	1.21564	2.1
61645	1.21564	2.1
61646	1.25404	2.1
61647	1.94574	2.1
61648	0.810946	2.1
61649	0.810946	2.1
61650	0.810947	2.1
61651	0.810947	2.1

61652	1.89226	2.1
61653	1.3517	2.1
61654	1.08133	2.1
61655	1.08132	2.1
61656	1.08132	2.1
61657	0.810934	2.1
61658	0.810934	2.1
61659	1.89226	2.1
61660	1.89226	1.55345
61661	1.89226	2.05442
61662	1.89226	2.1
61663	3.78453	2.1
61664	1.89226	0.71668
61665	1.89226	0.233994
61666	1.89226	1.31402
61667	1.89226	0.635237
61668	1.89226	0.87666
61669	1.89226	0.257825
61670	0.946131	0.55514
61671	0.946131	0.440724
61672	0.94613	0.989333
61673	0.94613	0.77746
61674	0.946128	0.508773
61675	0.946128	0.375111
61676	0.946133	0.303288
61677	0.946133	0.9598
61678	0.946131	0.662594
61679	0.946131	0.702557
61680	0.946131	0.875313
61681	0.946131	0.615638
61682	1.89226	2.1
61683	1.89226	1.14773
61684	1.89226	2.08726
61685	1.89226	2.01235
61686	1.89226	1.66763
61687	1.89226	1.35303
61688	2.56348	1.30188
61689	1.89226	2.1
61690	1.89226	2.1
61691	2.53234	1.58919
61692	4.45574	1.45978
61693	2.53234	0.354351
61694	1.65583	0.144797
61695	2.56348	0.33168
61696	2.56348	0.440581
61697	1.66295	0.305436
61698	1.65583	0.116182
61699	0.736602	0.222644
61700	0.827916	0.298067
61701	1.26617	0.472555
61702	1.18322	0.0826011
61703	0.7366	0.260078
61704	1.18321	0.248099
61705	1.26617	0.311848
61706	0.827918	0.311614
61707	0.99929	0.335738
61708	0.99929	0.382058
61709	1.00348	0.162629

61710	1.00348	0.17436
61711	1.26617	0.48614
61712	1.18322	0.871347
61713	0.946131	2.1
61714	0.946131	1.93476
61715	0.946129	0.870051
61716	1.18321	0.623419
61717	0.946133	2.1
61718	1.26617	2.04916
61719	1.26617	2.05363
61720	1.26617	0.352229
61721	0.946131	2.1
61722	0.946131	1.74976
61723	2.23267	0.0725029
61724	1.89226	0.099915
61725	2.07099	0.0946837
61726	1.89226	0.120867
61727	2.07099	0.0956265
61728	1.89226	0.0815903
61729	0.946132	0.15576
61730	1.35403	0.0897822
61731	1.24038	0.0896095
61732	0.946131	0.0771274
61733	0.946131	0.129865
61734	0.946131	0.145518
61735	1.24039	0.116064
61736	1.35403	0.0896103
61737	1.24038	0.133561
61738	1.24038	0.151088
61739	0.946131	0.0855451
61740	0.946131	0.190868
61741	1.89226	0.216354
61742	1.89226	0.627407
61743	1.89226	0.181566
61744	1.89226	0.188008
61745	1.89226	0.646305
61746	1.89226	0.613741
61747	1.89226	0.134881
61748	1.89226	0.453814
61749	1.89226	0.204251
61750	1.89226	0.311863
61751	3.78452	0.387491
61752	1.56971	0.0828005
61753	2.80086	0.201667
61754	2.59806	0.115073
61755	1.93489	0.108044
61756	2.59806	0.201991
61757	1.93489	0.111984
61758	0.827467	0.0698591
61759	1.26652	0.0798723
61760	1.13679	0.0997082
61761	0.639035	0.102742
61762	1.11547	0.150857
61763	1.30656	0.154576
61764	0.869062	0.0706558
61765	0.696608	0.0911998
61766	1.07205	0.0772847
61767	0.953656	0.172839

61768	0.970744	0.0902949
61769	0.970744	0.185899
61770	2.38681	2.1
61771	2.50438	2.09604
61772	2.42082	2.1
61773	2.59106	2.1
61774	4.92521	2.1
61775	2.50438	2.05486
61776	2.40627	2.1
61777	2.59106	2.1
61778	2.5444	1.82387
61779	5.04879	2.1
61780	2.42082	2.1
61781	2.70575	2.1
61782	2.64778	2.1
61783	2.59106	2.1
61784	2.47753	2.1
61785	2.42082	2.09788
61786	2.70575	2.1
61787	2.5444	1.98317
61788	2.76095	2.1
61789	2.59106	2.1
61790	2.71429	2.1
61791	2.5444	2.0981
61792	1.89226	2.1
61793	2.38473	2.1
61794	2.36689	2.1
61795	2.38473	2.1
61796	1.87442	2.1
61797	1.89226	2.1
61798	1.89226	2.1
61799	1.89226	2.1
61800	1.89226	2.1
61801	1.89226	2.1
61802	1.89226	2.1
61803	1.89226	2.1
61804	2.14952	2.1
61805	1.89226	2.1
61806	1.89226	2.1
61807	1.89226	2.1
61808	2.0692	2.1
61809	2.0692	2.1
61810	1.89226	2.1
61811	2.36689	2.1
61812	2.36204	2.1
61813	2.36689	2.1
61814	1.88742	2.1
61815	1.89226	2.1
61816	0.946132	2.1
61817	0.946132	2.1
61818	0.946131	2.1
61819	0.946131	2.1
61820	0.946132	2.1
61821	0.946132	2.1
61822	0.946131	2.1
61823	0.946131	2.1
61824	0.946132	2.1
61825	0.946132	2.1

61826	0.946131	2.1
61827	0.946131	2.1
61828	0.946128	2.1
61829	0.946128	2.1
61830	1.07476	2.1
61831	1.07476	2.1
61832	0.946131	2.1
61833	0.946131	2.1
61834	1.13182	2.1
61835	1.07476	2.1
61836	1.13182	2.1
61837	0.946131	2.1
61838	1.07476	2.1
61839	0.94613	2.1
61840	1.89227	0.0890291
61841	1.89227	0.130279
61842	1.89227	0.1881
61843	1.89227	0.116792
61844	3.78453	0.0958297
61845	1.89226	0.0953255
61846	1.89226	0.0792081
61847	1.89226	0.0941175
61848	1.89226	0.123076
61849	3.78452	0.100271
61850	1.89226	0.0764946
61851	1.89226	0.142282
61852	1.89226	0.133668
61853	1.89226	0.105217
61854	3.78452	0.0830359
61855	1.89226	0.122134
61856	1.89226	0.0941923
61857	1.89226	0.0994505
61858	1.89226	0.137086
61859	1.89226	0.138554
61860	1.89226	0.139678
61861	2.58344	0.254557
61862	2.00267	0.0909283
61863	2.14218	0.182264
61864	2.22674	0.248237
61865	2.14218	0.0955777
61866	2.22674	0.153792
61867	2.83531	0.26008
61868	1.56043	0.0988476
61869	2.22674	0.208834
61870	1.90912	0.266651
61871	2.22674	0.0833559
61872	1.90912	0.148996
61873	0.780217	0.166659
61874	1.11337	0.17526
61875	1.00133	0.218434
61876	0.804472	0.139968
61877	0.804472	0.141681
61878	0.780217	0.113661
61879	1.00133	0.0875084
61880	1.11337	0.20935
61881	0.856668	0.216503
61882	0.856668	0.209292
61883	0.924883	0.0998152

61884	0.924883	0.132019
61885	1.93107	2.1
61886	1.68657	2.1
61887	1.7237	2.1
61888	1.89226	2.1
61889	1.53436	2.1
61890	1.68657	2.1
61891	1.89226	2.1
61892	1.89226	2.1
61893	1.89226	2.1
61894	1.89226	2.1
61895	3.78453	2.1
61896	0.946131	2.1
61897	0.946131	2.1
61898	0.855092	2.1
61899	0.83953	2.1
61900	0.946131	2.1
61901	0.946131	2.1
61902	0.839531	2.1
61903	0.839531	2.1
61904	0.946131	2.1
61905	0.855093	2.1
61906	0.839531	2.1
61907	0.946131	2.1
61908	1.89226	2.1
61909	1.70302	2.1
61910	2.21419	2.1
61911	1.93107	2.1
61912	1.97872	2.1
61913	1.70302	2.1
61914	0.946133	2.1
61915	0.946133	2.1
61916	0.946132	2.1
61917	0.946132	2.1
61918	0.946132	2.1
61919	0.946132	2.1
61920	0.946132	2.1
61921	0.946132	2.1
61922	0.946133	2.1
61923	0.946133	2.1
61924	0.946132	2.1
61925	0.946132	2.1
61926	1.90081	2.1
61927	1.89226	2.1
61928	1.51379	2.1
61929	1.36813	2.1
61930	1.71019	2.1
61931	1.89226	2.1
61932	1.89227	1.44015
61933	1.89227	0.41782
61934	1.89227	0.531463
61935	1.89227	1.40215
61936	1.89227	1.1822
61937	1.89227	1.62727
61938	1.36428	1.10073
61939	1.37869	1.16293
61940	0.946132	0.796064
61941	0.946132	1.19866

61942	1.37869	0.587124
61943	1.37869	1.90437
61944	0.946133	1.19836
61945	0.946133	1.39243
61946	1.37869	0.64211
61947	0.946132	0.88429
61948	1.36428	1.18831
61949	0.946133	0.496425
61950	0.94612	0.677343
61951	0.94612	0.871721
61952	0.946119	0.931109
61953	0.946119	0.936932
61954	0.946119	0.386945
61955	0.946119	1.89588
61956	0.94612	1.18832
61957	0.94612	1.76563
61958	0.946132	0.784601
61959	0.946132	0.474769
61960	0.946107	0.590932
61961	0.946107	1.39499
61962	1.89225	0.0698591
61963	1.89225	2.1
61964	1.63564	0.0698591
61965	1.42278	2.1
61966	1.42278	2.1
61967	1.57534	0.0698591
61968	1.53735	2.1
61969	1.68991	0.0698591
61970	1.57535	0.0698591
61971	1.04442	2.1
61972	1.04442	2.1
61973	1.22031	0.0698591
61974	1.42278	2.1
61975	1.59868	0.0698591
61976	0.946132	2.1
61977	0.946132	2.1
61978	0.946131	2.1
61979	0.946131	2.1
61980	0.946131	2.1
61981	0.946131	2.1
61982	0.946132	2.1
61983	0.946132	2.1
61984	0.946132	2.1
61985	0.946132	2.1
61986	0.946131	2.1
61987	0.946131	2.1
61988	1.89226	2.1
61989	1.89226	2.1
61990	1.89226	2.1
61991	1.89226	2.1
61992	1.89226	2.1
61993	1.89226	2.1
61994	2.03143	1.48258
61995	1.47111	2.0372
61996	2.28685	2.02017
61997	2.28685	2.03977
61998	1.36006	1.6598
61999	1.36006	1.77424

62000	1.89226	0.87622
62001	1.89226	2.00617
62002	1.89226	1.8542
62003	1.89226	1.96566
62004	1.89226	1.05297
62005	1.89226	1.63333
62006	1.89226	1.91095
62007	1.89226	2.1
62008	1.89226	2.1
62009	1.89226	2.1
62010	1.89226	1.96691
62011	1.89226	2.0931
62012	0.902821	2.1
62013	0.902821	2.1
62014	0.889218	2.1
62015	0.889218	2.1
62016	0.675759	2.1
62017	1.05649	2.1
62018	0.735554	2.1
62019	1.14342	2.1
62020	1.14343	2.1
62021	1.05649	2.1
62022	0.735554	2.1
62023	0.675758	2.1
62024	2.46286	0.48947
62025	2.28685	1.101
62026	2.67843	0.891679
62027	2.67843	0.995154
62028	2.03143	0.58514
62029	2.03143	0.784067
62030	1.89226	0.851974
62031	1.89226	1.13441
62032	1.89226	0.468525
62033	1.89226	0.525475
62034	3.78452	1.41648
62035	0.94613	0.714996
62036	0.94613	1.82701
62037	0.946128	2.07775
62038	0.946128	1.86587
62039	0.94613	2.1
62040	0.94613	1.80102
62041	0.946128	0.568143
62042	0.946128	1.62458
62043	0.946129	0.136112
62044	0.946129	1.92165
62045	0.946129	1.14371
62046	0.946129	2.1
62047	2.67843	1.51743
62048	2.11297	2.09071
62049	2.52453	1.99332
62050	2.52453	2.08729
62051	2.28685	1.5981
62052	2.28685	1.92504
62053	1.89226	1.41667
62054	1.89226	2.1
62055	1.89226	1.16037
62056	1.89226	2.1
62057	3.78451	2.08279

62058	2.39524	0.959781
62059	2.10188	2.1
62060	2.29839	1.14095
62061	2.02242	2.1
62062	4.40028	2.09719
62063	1.71839	2.1
62064	1.65261	2.1
62065	2.10189	2.1
62066	2.10189	2.1
62067	2.02243	2.1
62068	1.63893	2.1
62069	0.946132	2.1
62070	0.946132	2.1
62071	0.946129	2.1
62072	0.946129	2.1
62073	0.94613	2.1
62074	0.94613	2.1
62075	0.946131	2.1
62076	0.946131	2.1
62077	0.946131	2.1
62078	0.946131	2.1
62079	0.946131	2.1
62080	0.946131	2.1
62081	1.89226	1.00759
62082	1.89226	2.1
62083	1.89226	1.21947
62084	1.89226	2.1
62085	3.78452	2.05614
62086	2.2984	0.968754
62087	2.02242	2.1
62088	2.21582	1.24796
62089	1.94412	2.1
62090	4.24251	2.04628
62091	1.94412	2.1
62092	1.5854	2.1
62093	2.02243	2.1
62094	2.02243	2.1
62095	1.57431	2.1
62096	1.65261	2.1
62097	0.810943	2.1
62098	0.810943	2.1
62099	0.810945	2.1
62100	0.810945	2.1
62101	1.89226	2.1
62102	1.3517	2.1
62103	1.08133	2.1
62104	1.08132	2.1
62105	1.08132	2.1
62106	0.810933	2.1
62107	0.810933	2.1
62108	1.89226	0.0698591
62109	1.89226	0.0878037
62110	1.89226	0.0768152
62111	1.89226	0.0710955
62112	1.89226	0.0736968
62113	1.89226	0.0701358
62114	1.89226	0.0770217
62115	1.89226	0.154395

62116	1.89226	0.0892799
62117	1.89226	0.122194
62118	3.78452	0.122873
62119	1.89226	0.0716341
62120	1.89226	0.106095
62121	1.89226	0.085455
62122	1.89226	0.0766263
62123	1.89226	0.0838631
62124	1.89226	0.0755321
62125	0.946128	0.126107
62126	0.946128	0.0790901
62127	0.946132	0.0784364
62128	0.946132	0.097466
62129	0.94613	0.122627
62130	0.94613	0.136998
62131	0.946129	0.0844154
62132	0.946129	0.0778629
62133	0.94613	0.0791577
62134	0.94613	0.0931775
62135	0.94613	0.0838839
62136	0.94613	0.0939114
62137	1.89226	0.0701886
62138	1.89226	0.0955772
62139	1.89226	0.0762423
62140	1.89226	0.0744712
62141	1.89226	0.086704
62142	1.89226	0.0855377
62143	2.81478	2.1
62144	1.89226	2.1
62145	2.65557	2.1
62146	1.89226	2.1
62147	4.54783	2.1
62148	1.89226	2.1
62149	1.89226	2.1
62150	1.89226	2.1
62151	1.89226	2.1
62152	3.78452	2.1
62153	2.65557	2.1
62154	1.89226	2.1
62155	1.89226	2.1
62156	1.89226	2.1
62157	2.57997	2.1
62158	2.57997	2.1
62159	0.946131	2.1
62160	0.946131	2.1
62161	0.946128	2.1
62162	0.946128	2.1
62163	0.94613	2.1
62164	0.94613	2.1
62165	0.946129	2.1
62166	0.946129	2.1
62167	0.94613	2.1
62168	0.94613	2.1
62169	0.94613	2.1
62170	0.94613	2.1
62171	1.89226	1.20801
62172	2.27703	1.92194
62173	2.35615	1.94846

62174	2.35615	1.431
62175	1.89226	1.94949
62176	1.89226	1.4146
62177	0.946132	1.20462
62178	1.17807	2.01538
62179	1.14926	1.09483
62180	0.946132	1.82403
62181	0.946133	1.79111
62182	0.946133	1.81718
62183	1.14926	1.3251
62184	1.14926	1.2997
62185	0.946132	1.74222
62186	0.946132	1.49774
62187	1.17807	1.78435
62188	1.14926	1.44153
62189	2.16841	0.574042
62190	1.35487	1.51508
62191	1.19854	1.15946
62192	1.19854	0.634095
62193	2.46078	1.11865
62194	2.46078	0.755719
62195	0.69308	0.604821
62196	1.3119	1.31294
62197	0.677433	1.61755
62198	1.23039	0.787811
62199	0.978178	1.59164
62200	0.978178	1.51207
62201	0.945294	0.680004
62202	0.945294	0.769351
62203	1.3119	1.06168
62204	1.23039	1.01228
62205	0.693081	1.05486
62206	0.677432	1.11216
62207	1.89226	1.3178
62208	1.89226	1.97013
62209	1.89226	1.94784
62210	1.89226	1.43326
62211	1.89226	1.94758
62212	1.89226	1.45715
62213	0.946132	1.23279
62214	0.946132	2.00068
62215	0.946132	0.882383
62216	0.946132	1.80607
62217	0.946133	1.76597
62218	0.946133	1.77868
62219	0.946132	1.23952
62220	0.946132	1.07354
62221	0.946133	1.6538
62222	0.946133	1.3181
62223	0.946132	1.67336
62224	0.946132	1.38071
62225	2.60637	2.1
62226	1.89226	2.1
62227	1.89226	2.1
62228	1.89226	1.99426
62229	3.78452	2.1
62230	1.89226	1.25562
62231	1.8396	1.84996

62232	1.89226	1.97397
62233	1.89226	1.28355
62234	1.60993	2.0625
62235	1.60993	1.45097
62236	0.946131	1.85237
62237	0.946131	2.02306
62238	0.946128	2.1
62239	0.946128	2.07924
62240	0.946131	1.81544
62241	0.946131	2.04465
62242	0.946127	2.1
62243	0.946127	1.98979
62244	0.946129	2.07425
62245	0.946129	2.06621
62246	0.94613	2.08764
62247	0.94613	2.08296
62248	1.89226	2.1
62249	1.89226	1.96505
62250	1.89226	2.09691
62251	1.89226	2.08124
62252	1.89226	2.09848
62253	1.89226	2.08561
62254	1.89226	1.256
62255	1.89226	1.79964
62256	1.89226	1.96946
62257	1.89226	1.24989
62258	1.89226	2.01441
62259	1.89226	1.27098
62260	0.946131	1.79758
62261	0.946131	1.98618
62262	0.946128	2.09526
62263	0.946128	2.02196
62264	0.946131	1.77494
62265	0.946131	1.98533
62266	0.946127	1.97516
62267	0.946127	2.0962
62268	0.94613	2.03357
62269	0.94613	2.02883
62270	0.946129	2.04312
62271	0.946129	2.04548
62272	0.946133	2.1
62273	0.946133	2.1
62274	1.28153	2.1
62275	1.26781	2.1
62276	0.946132	2.1
62277	0.946132	2.1
62278	1.28153	2.1
62279	1.28153	2.1
62280	0.946132	2.1
62281	1.28153	2.1
62282	0.946133	2.1
62283	1.26781	2.1
62284	1.89226	2.1
62285	1.89226	2.1
62286	1.89226	2.1
62287	1.89226	2.1
62288	1.89226	2.1
62289	1.89226	2.1

62290	2.5844	2.1
62291	1.09563	2.1
62292	1.1468	2.1
62293	1.09563	2.1
62294	2.6142	2.1
62295	2.56304	2.1
62296	0.946126	2.1
62297	0.946126	2.1
62298	1.2922	2.1
62299	1.28152	2.1
62300	0.946126	2.1
62301	0.946126	2.1
62302	1.2922	2.1
62303	1.2922	2.1
62304	1.29221	2.1
62305	0.946132	2.1
62306	0.946119	2.1
62307	1.28151	2.1
62308	1.89225	2.1
62309	1.89225	2.1
62310	1.89225	2.1
62311	1.89225	2.1
62312	1.89225	2.1
62313	1.89225	2.1
62314	0.946121	2.1
62315	0.946121	2.1
62316	0.946126	2.1
62317	0.946126	2.1
62318	0.946124	2.1
62319	0.946124	2.1
62320	0.946123	2.1
62321	0.946123	2.1
62322	0.94613	2.1
62323	0.94613	2.1
62324	0.946117	2.1
62325	0.946117	2.1
62326	1.89226	2.0884
62327	1.89226	2.01252
62328	1.89226	1.90832
62329	1.89226	2.1
62330	1.89226	1.37177
62331	1.89226	2.1
62332	1.89226	2.09541
62333	1.89226	2.0309
62334	1.89226	1.77188
62335	1.89226	2.1
62336	1.89226	1.31045
62337	1.89226	2.1
62338	1.89226	2.1
62339	2.81281	2.1
62340	2.50369	2.02751
62341	2.50369	2.1
62342	1.89226	2.03449
62343	1.89226	2.1
62344	1.89226	2.1
62345	1.89226	2.1
62346	1.89226	2.1
62347	1.89226	2.1

62348	1.89226	2.1
62349	1.89226	2.1
62350	1.89226	2.1
62351	1.89226	2.1
62352	1.89226	2.1
62353	1.89226	2.1
62354	1.89226	2.1
62355	1.89226	2.1
62356	0.907036	2.1
62357	1.09714	2.1
62358	0.994609	2.1
62359	0.838808	2.1
62360	0.976536	2.1
62361	0.976536	2.1
62362	0.959412	2.1
62363	0.959412	2.1
62364	1.09714	2.1
62365	0.994609	2.1
62366	0.907036	2.1
62367	0.838808	2.1
62368	0.99461	2.1
62369	0.838808	2.1
62370	0.738212	2.1
62371	0.565634	2.1
62372	0.775921	2.1
62373	0.775921	2.1
62374	0.784322	2.1
62375	0.784322	2.1
62376	0.994609	2.1
62377	0.738212	2.1
62378	0.838808	2.1
62379	0.565635	2.1
62380	1.89226	2.1
62381	1.89226	2.1
62382	1.89226	2.1
62383	1.89226	2.1
62384	1.89226	2.1
62385	1.89226	2.1
62386	1.89226	2.1
62387	1.89226	2.1
62388	1.89226	2.1
62389	1.89226	2.1
62390	1.89226	2.1
62391	1.89226	2.1
62392	2.19428	2.1
62393	1.98922	2.1
62394	2.27322	2.1
62395	2.45893	2.1
62396	4.4675	2.1
62397	0.99461	2.1
62398	1.13661	2.1
62399	0.902272	2.1
62400	0.738212	2.1
62401	0.931874	2.1
62402	0.931874	2.1
62403	0.942953	2.1
62404	0.942953	2.1
62405	1.13658	2.1

62406	0.902248	2.1
62407	0.994637	2.1
62408	0.738233	2.1
62409	1.89226	2.03867
62410	1.89226	2.05625
62411	1.89226	2.09059
62412	1.89226	2.06064
62413	1.89226	2.07129
62414	1.89226	2.00516
62415	1.89227	2.0347
62416	2.58246	1.87789
62417	1.89227	2.02756
62418	1.89227	1.95505
62419	3.78453	1.94746
62420	1.89226	2.09292
62421	1.89226	2.02774
62422	1.65573	2.07953
62423	1.65573	2.08673
62424	1.89226	2.06022
62425	1.89226	2.06673
62426	1.89226	1.91247
62427	1.89226	1.83423
62428	1.89226	1.90754
62429	1.89226	1.93643
62430	1.89226	1.83725
62431	1.89226	1.85368
62432	1.89226	1.72822
62433	1.89226	1.76582
62434	1.89226	1.61354
62435	1.89226	1.84183
62436	1.89226	1.54374
62437	1.89226	1.81363
62438	0.946132	2.03874
62439	0.946132	2.00408
62440	1.01023	1.92333
62441	1.13651	2.06534
62442	1.01023	1.90714
62443	0.946133	1.99952
62444	1.0644	2.01724
62445	1.0644	2.04247
62446	0.946132	2.01762
62447	0.946132	2.02327
62448	1.13651	2.04373
62449	0.946132	2.00565
62450	1.89226	0.725926
62451	1.89226	1.55459
62452	1.89226	1.20468
62453	1.89226	0.648445
62454	3.78451	0.792424
62455	1.00934	0.436173
62456	0.741561	0.364456
62457	0.946131	0.560633
62458	0.946131	0.602619
62459	0.946131	0.733263
62460	0.741561	0.322442
62461	1.00934	0.313084
62462	0.946131	0.584451
62463	0.741586	0.474736

62464	0.741586	0.536156
62465	0.946106	0.536098
62466	0.946106	0.401487
62467	1.14895	0.693029
62468	0.957449	0.862808
62469	0.934806	1.35801
62470	0.934806	1.19379
62471	1.13536	0.61442
62472	1.02829	1.452
62473	0.946129	1.61697
62474	0.946129	0.727593
62475	0.946127	1.10493
62476	0.946127	1.18605
62477	0.946127	0.979908
62478	1.0283	1.05478
62479	1.89226	1.65305
62480	1.89226	0.804425
62481	1.89226	0.777817
62482	1.89226	1.47825
62483	3.78452	1.28774
62484	1.89226	0.495519
62485	1.89226	0.732756
62486	1.89226	0.749153
62487	1.89226	0.484062
62488	3.78453	0.518142
62489	1.89226	0.744025
62490	2.69068	0.485867
62491	1.89226	0.497156
62492	1.89226	0.657712
62493	3.78452	0.62586
62494	1.89226	0.820497
62495	1.89226	1.55274
62496	1.89226	1.55682
62497	1.89226	0.970649
62498	1.89226	1.44504
62499	1.89226	0.82644
62500	1.89226	2.1
62501	1.89226	2.1
62502	1.89226	2.1
62503	1.89226	2.1
62504	1.89226	2.1
62505	1.89226	2.1
62506	1.89226	2.1
62507	1.89226	2.1
62508	1.89226	2.1
62509	1.89226	2.1
62510	1.89226	2.1
62511	1.89226	2.1
62512	0.985109	2.1
62513	1.31144	2.1
62514	1.45864	2.1
62515	1.10667	2.1
62516	2.44375	2.1
62517	0.655718	2.1
62518	0.729316	2.1
62519	0.684594	2.1
62520	0.782707	2.1
62521	0.700849	2.1

62522	0.700849	2.1
62523	0.71306	2.1
62524	0.71306	2.1
62525	0.729318	2.1
62526	0.782705	2.1
62527	0.684592	2.1
62528	0.655719	2.1
62529	0.946128	2.1
62530	0.946128	2.1
62531	0.946133	2.1
62532	0.946133	2.1
62533	0.946131	2.1
62534	0.946131	2.1
62535	0.946131	2.1
62536	0.946131	2.1
62537	0.94613	2.1
62538	0.94613	2.1
62539	0.946131	2.1
62540	0.946131	2.1
62541	1.89226	2.1
62542	1.89226	2.1
62543	1.89226	2.1
62544	1.89226	2.1
62545	1.89226	2.1
62546	1.89226	2.1
62547	0.553334	2.1
62548	0.629936	2.1
62549	0.828211	2.1
62550	0.729319	2.1
62551	0.674025	2.1
62552	0.674025	2.1
62553	0.68523	2.1
62554	0.68523	2.1
62555	0.828211	2.1
62556	0.629935	2.1
62557	0.72932	2.1
62558	0.553334	2.1
62559	0.828208	2.1
62560	0.729316	2.1
62561	0.907037	2.1
62562	0.782707	2.1
62563	0.799048	2.1
62564	0.799048	2.1
62565	0.811867	2.1
62566	0.811867	2.1
62567	0.828209	2.1
62568	0.907035	2.1
62569	0.729318	2.1
62570	0.782706	2.1
62571	2.32981	2.1
62572	1.89226	2.1
62573	1.89226	2.1
62574	1.89226	2.1
62575	2.2671	2.1
62576	2.2671	2.1
62577	1.89226	2.1
62578	1.89226	2.09315
62579	1.89226	2.1

62580	1.89226	2.03172
62581	1.89226	2.1
62582	1.89226	2.0977
62583	1.3991	2.1
62584	1.81793	2.04125
62585	2.07226	2.1
62586	2.07226	2.00038
62587	1.24924	2.1
62588	1.24924	2.1
62589	0.946131	2.1
62590	0.946131	2.1
62591	1.16491	2.1
62592	1.16491	2.1
62593	0.946131	2.1
62594	0.946131	2.1
62595	1.16491	2.1
62596	1.19523	2.1
62597	0.946132	2.1
62598	1.19523	2.1
62599	1.16491	2.1
62600	0.946131	2.1
62601	1.89226	2.1
62602	1.89226	2.07425
62603	1.89226	2.1
62604	1.89226	1.51357
62605	3.78452	2.09219
62606	0.901204	2.09656
62607	0.901204	2.1
62608	0.902504	2.1
62609	0.902504	2.1
62610	1.10416	2.0456
62611	1.03613	1.80933
62612	0.764922	2.1
62613	0.699552	2.1
62614	0.764921	2.1
62615	1.10416	2.1
62616	0.699551	2.1
62617	1.03613	2.09252
62618	1.89226	1.23939
62619	1.89226	1.4234
62620	1.89226	1.51105
62621	1.89226	1.26171
62622	3.78453	1.46266
62623	1.89226	1.63138
62624	1.89226	1.60305
62625	1.89226	1.55123
62626	1.89226	1.77112
62627	1.89226	1.56439
62628	1.89226	1.77649
62629	0.946131	1.38033
62630	0.946131	1.49752
62631	0.946131	1.16817
62632	0.946131	1.26754
62633	0.946131	1.17076
62634	0.946131	1.37847
62635	0.946131	1.49703
62636	0.946131	1.17194
62637	0.946131	1.14333

62638	0.946131	1.1485
62639	0.946131	1.15956
62640	0.946131	1.25714
62641	1.89226	1.59566
62642	1.89226	1.5655
62643	1.89226	1.5079
62644	1.89226	1.71088
62645	1.89226	1.49702
62646	1.89226	1.7004
62647	0.946127	1.36227
62648	0.946127	1.45508
62649	1.22788	1.45027
62650	1.35469	1.45287
62651	1.35469	1.38878
62652	1.35469	1.44199
62653	0.946128	1.45529
62654	0.946128	1.38623
62655	0.946129	1.3891
62656	1.22788	1.41444
62657	1.35469	1.42032
62658	0.946129	1.35083
62659	1.22788	1.41118
62660	1.35469	1.40986
62661	1.02602	1.18584
62662	0.794861	1.23599
62663	1.15301	1.43441
62664	1.15301	1.4072
62665	1.10089	1.15792
62666	1.10089	1.35531
62667	0.794861	1.15149
62668	1.22788	1.45576
62669	1.02602	1.40138
62670	1.35469	1.37603
62671	1.89226	0.247233
62672	1.89226	1.48154
62673	1.89226	1.24258
62674	1.89226	0.472013
62675	3.78452	0.619885
62676	1.13536	2.1
62677	0.942524	1.46202
62678	0.946131	1.56964
62679	0.946131	0.554595
62680	0.946127	1.54753
62681	0.946127	2.1
62682	1.26282	0.573387
62683	0.917471	1.97413
62684	1.21023	1.81419
62685	1.00851	1.80246
62686	1.17955	1.66555
62687	0.883746	1.67697
62688	1.89226	1.53744
62689	1.89226	2.1
62690	1.89226	2.09722
62691	1.89226	2.1
62692	1.89226	2.09125
62693	1.89226	2.1
62694	1.89226	0.554334
62695	1.89226	2.09119

62696	1.89226	1.38973
62697	1.89226	1.48894
62698	1.89226	1.36717
62699	1.89226	1.82877
62700	0.94613	1.71803
62701	0.94613	0.756701
62702	0.94613	0.120347
62703	0.94613	0.683131
62704	0.946128	0.611211
62705	0.946128	1.74599
62706	0.946131	1.39081
62707	0.946131	0.0755952
62708	0.94613	0.66794
62709	0.94613	0.816708
62710	0.94613	0.66957
62711	0.94613	0.645362
62712	0.883758	2.1
62713	1.03041	1.9385
62714	0.946131	1.63786
62715	0.946131	0.792045
62716	0.851537	1.69692
62717	0.851537	2.1
62718	1.23942	0.86892
62719	1.14906	1.98286
62720	0.851484	1.66383
62721	0.985518	1.51359
62722	1.0156	1.62806
62723	0.851526	1.63603
62724	1.89226	1.66934
62725	1.89226	2.1
62726	1.89226	2.08964
62727	1.89226	2.1
62728	1.89226	2.08247
62729	1.89226	2.1
62730	1.89226	0.672506
62731	1.89226	2.07801
62732	1.89226	1.39969
62733	1.89226	1.69422
62734	1.89226	1.32095
62735	1.89226	1.82879
62736	1.89227	0.0811188
62737	1.89227	0.107871
62738	1.89227	0.113522
62739	1.89227	0.0878319
62740	3.78453	0.0791801
62741	1.89226	0.143792
62742	1.89226	0.0772754
62743	1.89226	0.134535
62744	1.89226	0.149555
62745	3.78452	0.156752
62746	0.946132	0.0950883
62747	0.946132	0.110129
62748	0.946128	0.158413
62749	0.946128	0.0831779
62750	0.94613	0.0996143
62751	0.94613	0.077314
62752	0.94613	0.0755038
62753	0.94613	0.13087

62754	0.946131	0.101858
62755	0.946131	0.0958119
62756	0.94613	0.0839551
62757	0.94613	0.0837384
62758	1.89226	0.0933432
62759	1.89226	0.076728
62760	1.89226	0.0914347
62761	1.89226	0.100985
62762	1.89226	0.0740428
62763	1.89226	0.0964519
62764	1.98874	0.0822228
62765	2.33455	0.0698591
62766	2.01865	0.152444
62767	2.07024	0.106589
62768	4.32329	0.127084
62769	1.98874	0.0965952
62770	2.00266	0.101885
62771	2.14218	0.207799
62772	2.07024	0.17602
62773	3.9914	0.106181
62774	1.00133	0.123966
62775	0.804471	0.0832574
62776	1.03512	0.0988034
62777	0.995176	0.0959163
62778	0.80447	0.231335
62779	0.995178	0.0809104
62780	1.00133	0.12768
62781	1.03512	0.209943
62782	0.880587	0.218893
62783	0.880587	0.0861531
62784	0.959002	0.0841717
62785	0.959002	0.0757223
62786	2.33455	0.0698591
62787	1.99036	0.0698591
62788	2.07024	0.113332
62789	2.58645	0.0723222
62790	4.32491	0.0698591
62791	1.89226	0.55254
62792	1.89226	0.456058
62793	1.89226	0.584557
62794	1.89226	0.37696
62795	3.78453	0.398649
62796	1.89226	0.860438
62797	1.89226	0.508843
62798	1.89226	0.668667
62799	1.89226	0.581154
62800	3.78453	0.80252
62801	1.89226	0.417947
62802	1.89226	0.317381
62803	1.89226	0.322143
62804	1.89226	0.409241
62805	1.89226	0.327134
62806	1.89226	0.421793
62807	1.89226	0.380562
62808	2.1523	0.183367
62809	2.53257	0.227216
62810	2.53257	0.215161
62811	1.89226	0.360568

62812	1.89226	0.332718
62813	0.946133	0.355962
62814	0.946133	0.465343
62815	0.946132	0.267628
62816	0.946132	0.312383
62817	0.946132	0.260757
62818	0.946132	0.292614
62819	0.946132	0.483176
62820	0.946132	0.308308
62821	0.946133	0.356522
62822	0.946133	0.372031
62823	0.946132	0.448333
62824	0.946132	0.382312
62825	0.946132	0.48979
62826	0.946132	0.628646
62827	0.946132	0.403891
62828	0.946132	0.538126
62829	0.946132	0.395052
62830	0.946132	0.433137
62831	0.946132	0.640153
62832	0.946132	0.539192
62833	0.946132	0.497082
62834	0.946132	0.484893
62835	0.946131	0.555988
62836	0.946131	0.537651
62837	1.62772	0.0928103
62838	1.70303	0.0927852
62839	1.70303	0.108959
62840	1.70303	0.0957447
62841	1.90116	0.0978469
62842	1.90116	0.0883717
62843	1.89226	0.0876806
62844	1.89226	0.0947455
62845	1.89226	0.101789
62846	1.89226	0.102414
62847	1.89226	0.0938839
62848	1.89226	0.0885916
62849	1.89226	0.110459
62850	1.89226	0.096368
62851	1.89226	0.0921712
62852	1.89226	0.100454
62853	3.78452	0.0889744
62854	1.89226	0.115519
62855	1.89226	0.0915372
62856	1.89226	0.0889958
62857	1.89226	0.101981
62858	1.89226	0.0904991
62859	1.89226	0.108326
62860	0.946132	0.0759802
62861	0.946132	0.0798997
62862	0.946129	0.0775979
62863	0.946129	0.0908764
62864	0.94613	0.0774586
62865	0.94613	0.0788093
62866	0.946131	0.0880051
62867	0.946131	0.0798703
62868	0.94613	0.0827572
62869	0.94613	0.0803818

62870	0.946131	0.0864081
62871	0.946131	0.094015
62872	1.89226	0.0843948
62873	1.89226	0.0789144
62874	1.89226	0.104343
62875	1.89226	0.0920788
62876	1.89226	0.0785306
62877	1.89226	0.0747305
62878	1.89226	0.386038
62879	1.89226	1.02826
62880	1.89226	0.920697
62881	1.89226	0.328
62882	3.78452	0.464305
62883	1.89226	0.768741
62884	1.89226	1.29235
62885	1.89226	0.680012
62886	1.89226	1.39122
62887	1.89226	0.670488
62888	1.89226	1.45383
62889	1.30165	0.731734
62890	0.946127	1.00424
62891	1.09659	0.3543
62892	0.946131	0.342854
62893	1.09659	0.510026
62894	1.09659	0.832614
62895	0.946128	1.70108
62896	0.946128	0.393524
62897	1.30165	0.488254
62898	1.09659	0.51975
62899	0.946129	0.910039
62900	0.946129	1.13421
62901	0.946128	0.694191
62902	0.946128	0.921483
62903	0.946133	0.329237
62904	0.946133	0.447317
62905	0.946131	0.324094
62906	0.946131	1.05933
62907	0.946131	0.877223
62908	0.946131	0.410627
62909	0.94613	0.453921
62910	0.94613	0.462505
62911	0.946131	0.527745
62912	0.946131	0.60638
62913	0.946133	1.18632
62914	0.946133	1.2638
62915	0.946132	0.663547
62916	0.946132	0.864542
62917	0.946132	0.64338
62918	0.946132	1.2723
62919	0.946132	1.28268
62920	0.946132	0.811004
62921	0.946132	0.963827
62922	0.946132	0.913795
62923	0.946133	1.11216
62924	0.946133	1.09491
62925	1.30165	0.907529
62926	0.972228	0.938308
62927	1.09659	0.363147

62928	0.656268	0.326045
62929	1.06217	0.373862
62930	1.06217	1.19491
62931	1.00665	0.41685
62932	1.00665	1.50644
62933	0.97223	0.458883
62934	0.656267	0.431971
62935	1.30165	0.774172
62936	1.09659	0.754487
62937	0.946132	2.02598
62938	0.946132	2.1
62939	1.19895	2.1
62940	1.19895	2.1
62941	0.94613	2.1
62942	0.94613	1.93469
62943	1.39356	2.1
62944	1.19895	2.1
62945	0.94613	2.1
62946	1.39356	2.1
62947	1.19895	2.1
62948	0.946131	2.1
62949	1.89226	2.00259
62950	1.89226	2.1
62951	1.89226	2.1
62952	1.89226	2.0123
62953	1.89226	2.1
62954	1.89226	1.97124
62955	0.946132	1.8144
62956	0.946132	1.96011
62957	0.946128	2.09135
62958	0.946128	2.1
62959	0.94613	2.09293
62960	0.94613	1.56109
62961	0.94613	2.00238
62962	0.94613	2.09618
62963	0.946131	2.04988
62964	0.946131	2.04875
62965	0.94613	2.09182
62966	0.94613	2.06919
62967	1.89226	0.0698591
62968	1.89226	0.0698591
62969	1.89226	0.0765384
62970	1.89226	0.0698591
62971	1.89226	0.0780218
62972	1.89226	0.0698591
62973	1.29881	0.0698591
62974	1.09985	0.0698591
62975	0.946126	0.0698591
62976	0.946126	0.0698591
62977	0.851539	0.0698591
62978	1.20421	0.0698591
62979	0.851483	0.0698591
62980	0.999426	0.0698591
62981	1.47172	0.0698591
62982	0.999413	0.0698591
62983	0.851552	0.0698591
62984	0.851552	0.0698591
62985	1.89226	0.0783872

62986	1.89226	0.0793297
62987	1.89226	0.139287
62988	1.89226	0.0707834
62989	1.89226	0.132715
62990	1.89226	0.0703623
62991	1.89226	0.0701266
62992	1.89226	0.0702208
62993	1.89226	0.0886112
62994	1.89226	0.0698591
62995	1.89226	0.087789
62996	1.89226	0.0698591
62997	1.33915	2.1
62998	1.99297	2.1
62999	2.05216	2.1
63000	2.60163	2.1
63001	4.04513	2.1
63002	1.89226	2.09083
63003	1.89226	2.1
63004	1.89226	2.1
63005	1.89226	2.1
63006	3.78452	2.1
63007	0.946132	2.1
63008	0.946132	2.1
63009	0.946133	2.1
63010	0.946133	2.1
63011	0.946132	2.1
63012	0.946132	2.08265
63013	0.946133	2.1
63014	0.946133	2.1
63015	0.946132	2.1
63016	0.946132	2.1
63017	0.946132	2.1
63018	0.946132	2.1
63019	1.89226	2.1
63020	1.89226	1.97192
63021	1.89226	2.07316
63022	1.89226	2.1
63023	3.78452	2.08892
63024	1.89227	2.0066
63025	1.89227	2.1
63026	1.89227	2.1
63027	1.89227	2.00372
63028	1.89227	2.1
63029	1.89227	2.05931
63030	2.29301	2.1
63031	1.70303	2.1
63032	2.46749	2.1
63033	2.30728	2.1
63034	4.53937	2.1
63035	1.89226	2.05981
63036	1.89226	2.1
63037	1.89226	2.1
63038	1.89226	1.91955
63039	3.78452	2.1
63040	1.89226	1.42472
63041	1.89226	2.1
63042	1.89226	2.1
63043	1.89226	2.07605

63044	1.89226	2.05631
63045	1.89226	1.57846
63046	2.30728	2.1
63047	1.00757	2.09607
63048	2.0833	2.1
63049	1.65318	2.1
63050	1.417	2.1
63051	1.00757	2.1
63052	2.46749	2.1
63053	2.0975	2.1
63054	2.79653	2.1
63055	2.44785	2.1
63056	4.91534	2.1
63057	1.89226	2.1
63058	1.89226	2.08897
63059	1.89226	2.07663
63060	1.89226	2.1
63061	3.78452	2.1
63062	1.89226	1.98158
63063	1.89226	2.1
63064	1.89226	2.1
63065	1.89226	2.03012
63066	1.89226	2.1
63067	1.89226	1.98277
63068	0.970559	2.1
63069	0.970559	2.1
63070	0.979305	2.1
63071	0.979305	2.1
63072	0.708502	2.1
63073	0.901117	2.1
63074	1.04875	2.1
63075	1.22392	2.1
63076	1.22392	2.1
63077	0.901117	2.1
63078	1.04875	2.1
63079	0.7085	2.1
63080	1.89226	1.90829
63081	1.89226	1.15095
63082	1.89226	1.71915
63083	1.89226	1.41726
63084	3.78452	1.84667
63085	1.89226	1.83782
63086	1.89226	1.12925
63087	1.89226	1.88368
63088	1.89226	1.3157
63089	3.78452	1.4369
63090	1.89226	0.641768
63091	1.89226	0.475074
63092	1.89226	0.725238
63093	1.89226	0.52643
63094	3.78453	0.556295
63095	1.89226	0.773904
63096	1.89226	0.851715
63097	1.89226	0.652046
63098	1.89226	0.583283
63099	1.89226	0.563914
63100	1.89226	0.538179
63101	1.89226	0.950802

63102	2.35441	0.839957
63103	1.89226	0.936345
63104	2.30931	0.47859
63105	4.24667	0.727638
63106	1.11612	0.686941
63107	0.946131	0.780998
63108	1.1772	0.663941
63109	0.946131	0.60755
63110	1.17721	0.667571
63111	1.11612	0.710604
63112	0.94613	0.798288
63113	0.94613	0.972196
63114	1.11612	0.662059
63115	1.11612	0.561667
63116	0.946131	0.968109
63117	0.946131	0.703454
63118	1.11612	0.173245
63119	0.582675	0.111795
63120	1.17721	0.114065
63121	0.651969	0.228276
63122	0.651969	0.18491
63123	0.582674	0.15914
63124	1.17721	0.170427
63125	1.11612	0.161703
63126	0.877955	0.143773
63127	0.877955	0.11924
63128	0.881925	0.230802
63129	0.881925	0.188414
63130	1.17721	0.213386
63131	0.651969	0.317011
63132	1.15466	0.220848
63133	0.623642	0.176796
63134	0.623642	0.211942
63135	0.651969	0.148729
63136	1.17721	0.282333
63137	1.15466	0.18618
63138	0.904714	0.198927
63139	0.904714	0.130147
63140	0.901914	0.446513
63141	0.901914	0.223431
63142	1.89226	0.610235
63143	1.89226	0.454284
63144	1.89226	1.28639
63145	1.89226	1.18761
63146	1.89226	0.683006
63147	1.89226	1.75933
63148	1.89226	1.54342
63149	1.89226	0.448539
63150	1.89226	0.640546
63151	1.89226	1.98562
63152	1.89226	0.500765
63153	1.89226	2.04503
63154	2.33986	1.92727
63155	1.97819	0.832755
63156	2.39166	0.393088
63157	2.70141	2.03418
63158	4.73151	0.937555
63159	0.989095	0.864213

63160	1.19583	0.486799
63161	0.851194	0.250663
63162	0.581197	0.242324
63163	0.872704	0.136545
63164	0.872704	1.19292
63165	0.904322	0.361793
63166	0.904322	0.617876
63167	1.19583	0.0709567
63168	0.851193	0.173944
63169	0.989095	0.459326
63170	0.581197	0.342397
63171	1.89226	0.506409
63172	1.89226	0.758472
63173	1.89226	1.40983
63174	1.89226	1.17546
63175	1.89226	1.1094
63176	1.89226	1.70456
63177	1.89226	1.51392
63178	1.89226	0.345
63179	1.89226	0.947107
63180	1.89226	1.92417
63181	1.89226	0.443575
63182	1.89226	2.04534
63183	2.39166	0.36499
63184	1.70239	0.989363
63185	2.13665	0.396048
63186	2.82684	0.561793
63187	4.52831	0.383072
63188	1.89226	0.0698591
63189	1.89226	0.125826
63190	1.89226	0.0922256
63191	1.89226	0.0850311
63192	3.78453	0.116909
63193	1.89226	0.0698591
63194	1.89226	0.0698591
63195	1.89226	0.071462
63196	1.89226	0.0698591
63197	1.89226	0.0769407
63198	1.89226	0.0698591
63199	1.89226	0.0814257
63200	1.89226	0.0996825
63201	1.89226	0.152277
63202	1.89226	0.072457
63203	1.89226	0.153281
63204	1.89226	0.069886
63205	1.89226	0.18777
63206	1.89226	0.187692
63207	1.89226	0.408846
63208	1.89226	0.116747
63209	1.89226	0.449513
63210	1.89226	0.106579
63211	1.89226	0.112175
63212	1.89226	0.128461
63213	1.89226	0.234907
63214	1.89226	0.081417
63215	1.89226	0.257019
63216	1.89226	0.083663
63217	1.5169	0.207719

63218	1.61969	0.128731
63219	1.17262	0.131187
63220	1.17262	0.189861
63221	1.88868	0.138137
63222	1.88868	0.19707
63223	1.79981	0.332564
63224	1.88868	0.213425
63225	1.5169	0.221114
63226	1.5169	0.304119
63227	2.12891	0.235818
63228	2.12891	0.31556
63229	1.89226	0.300588
63230	1.89226	0.191567
63231	1.89226	0.210269
63232	1.89226	0.289911
63233	3.78452	0.265816
63234	0.946132	0.175636
63235	0.946132	0.190551
63236	0.946132	0.119705
63237	0.946132	0.12444
63238	0.946132	0.120118
63239	0.946132	0.182719
63240	0.946133	0.13227
63241	0.946133	0.194641
63242	0.946133	0.142963
63243	0.946133	0.142988
63244	0.946132	0.160183
63245	0.946132	0.158909
63246	1.88868	0.177759
63247	2.05759	0.112949
63248	1.61969	0.115525
63249	1.61969	0.165184
63250	2.29747	0.124468
63251	2.29747	0.16846
63252	2.12891	0.284989
63253	2.29747	0.176891
63254	1.88868	0.183084
63255	1.88868	0.243447
63256	2.50894	0.198926
63257	2.50894	0.257008
63258	1.89226	0.172205
63259	1.89226	0.247341
63260	1.89226	0.167903
63261	1.89226	0.242082
63262	1.89226	0.168674
63263	1.89226	0.245934
63264	0.946132	0.158716
63265	0.946132	0.153771
63266	0.946132	0.121342
63267	0.946132	0.113375
63268	0.946131	0.108019
63269	0.946131	0.183825
63270	0.946132	0.116155
63271	0.946132	0.166901
63272	0.946132	0.127085
63273	0.946132	0.132413
63274	0.946132	0.142941
63275	0.946132	0.153197

63276	2.42758	2.09783
63277	2.14895	2.1
63278	2.56975	2.06414
63279	2.28142	2.1
63280	4.71869	2.1
63281	1.89226	2.08314
63282	1.89226	2.1
63283	1.89226	2.06834
63284	1.89226	2.1
63285	3.78452	2.1
63286	1.89226	2.1
63287	1.89226	2.1
63288	1.89226	2.1
63289	1.89226	2.1
63290	1.89226	2.1
63291	1.89226	2.1
63292	0.960916	2.1
63293	0.960916	2.1
63294	0.978118	2.1
63295	0.875674	2.1
63296	0.775661	2.1
63297	0.773613	2.1
63298	1.07631	2.1
63299	1.14266	2.1
63300	1.16927	2.1
63301	0.898612	2.1
63302	1.04757	2.1
63303	0.757536	2.1
63304	2.56975	2.04005
63305	2.28142	2.1
63306	2.64384	2.03991
63307	2.40621	2.1
63308	4.97596	2.1
63309	1.89226	2.04739
63310	1.89226	2.1
63311	1.89226	1.78547
63312	1.89226	2.1
63313	3.78452	2.1
63314	1.89226	2.1
63315	1.89226	2.1
63316	1.89226	2.1
63317	1.89226	2.1
63318	1.89226	2.1
63319	1.89226	2.1
63320	1.03328	2.1
63321	1.03328	2.1
63322	1.04321	2.1
63323	1.15424	2.1
63324	0.936183	2.1
63325	0.944491	2.1
63326	1.14266	2.1
63327	1.20517	2.1
63328	1.23323	2.1
63329	0.961268	2.1
63330	1.11215	2.1
63331	0.827247	2.1
63332	1.89226	2.1
63333	1.89226	2.1

63334	1.89226	2.1
63335	1.89226	2.1
63336	3.78453	2.1
63337	2.14647	2.1
63338	2.20589	2.1
63339	1.95567	2.1
63340	2.03224	2.1
63341	4.17871	2.1
63342	2.06035	2.1
63343	1.88452	1.99086
63344	1.88452	2.1
63345	2.26997	2.1
63346	2.03224	2.1
63347	2.42272	2.1
63348	1.89226	2.1
63349	1.89226	1.90479
63350	1.89226	1.46843
63351	1.89226	2.1
63352	1.89226	1.76105
63353	1.89226	2.1
63354	1.89225	2.1
63355	1.89225	2.1
63356	1.89225	2.1
63357	1.89225	2.1
63358	1.89225	2.1
63359	1.89225	2.1
63360	0.952418	2.1
63361	0.952418	2.1
63362	0.95883	2.1
63363	0.95883	2.1
63364	0.946182	2.1
63365	1.01611	2.1
63366	0.895134	2.1
63367	0.977828	2.1
63368	0.89514	2.1
63369	0.946189	2.1
63370	0.977821	2.1
63371	1.01611	2.1
63372	2.03223	2.1
63373	1.88451	2.03171
63374	1.89236	2.1
63375	1.74649	2.05908
63376	3.77687	2.1
63377	0.81094	2.1
63378	0.81094	2.05113
63379	0.810939	2.1
63380	0.810939	2.1
63381	0.810945	2.1
63382	0.810945	2.1
63383	1.89225	1.97761
63384	1.35167	2.1
63385	1.08131	2.1
63386	1.08131	1.76735
63387	1.0813	2.1
63388	1.89226	0.0698591
63389	1.89226	0.0698591
63390	1.89226	0.0699519
63391	1.89226	0.0698591

63392	1.89226	0.0698591
63393	1.89226	0.0698591
63394	1.01842	0.0698591
63395	0.936656	0.0698591
63396	0.946131	0.0698591
63397	0.946131	0.0698591
63398	1.11157	0.0698591
63399	1.04281	0.0698591
63400	0.925803	0.0698591
63401	0.925803	0.0698591
63402	0.851553	0.0698591
63403	1.11158	0.0698591
63404	1.05017	0.0698591
63405	0.925798	0.0698591
63406	1.89226	0.0698591
63407	1.89226	0.0724894
63408	1.89226	0.0747858
63409	1.89226	0.0698591
63410	1.89226	0.0717648
63411	1.89226	0.0698591
63412	1.23504	0.0698591
63413	1.23504	0.0698591
63414	0.946131	0.0698591
63415	0.946131	0.0698591
63416	1.37358	0.0698591
63417	1.23504	0.0698591
63418	0.946129	0.0698591
63419	0.946129	0.0698591
63420	1.37358	0.0698591
63421	0.946131	0.0698591
63422	0.94613	0.0698591
63423	1.23504	0.0698591
63424	1.00261	0.0698591
63425	1.00261	0.0698591
63426	1.02952	0.0698591
63427	1.02952	0.0698591
63428	0.850964	0.0698591
63429	0.658553	0.0698591
63430	1.37358	0.0698591
63431	1.23504	0.0698591
63432	1.37358	0.0698591
63433	0.850965	0.0698591
63434	1.23504	0.0698591
63435	0.658553	0.0698591
63436	0.946133	0.0698591
63437	0.946133	0.0698591
63438	0.946132	0.0720109
63439	0.946132	0.0698591
63440	0.946132	0.0698591
63441	0.946132	0.0698591
63442	0.946133	0.0701023
63443	0.946133	0.0698591
63444	0.946133	0.0698591
63445	0.946133	0.0698591
63446	0.946132	0.0698591
63447	0.946132	0.0698591
63448	1.89226	0.0698591
63449	1.89226	0.0698591

63450	1.89226	0.0698591
63451	1.89226	0.0698591
63452	3.78452	0.0698591
63453	1.89226	0.0698591
63454	1.89226	0.0698591
63455	1.89226	0.0698591
63456	1.89226	0.0698591
63457	3.78452	0.0698591
63458	1.89227	0.0698591
63459	1.89227	0.0698591
63460	1.89227	0.0698591
63461	1.89227	0.0698591
63462	1.89227	0.0698591
63463	1.89227	0.0698591
63464	0.94613	0.0698591
63465	0.94613	0.0698591
63466	0.946127	0.0698591
63467	0.946127	0.0698591
63468	0.94613	0.0698591
63469	0.94613	0.0698591
63470	0.946127	0.0698591
63471	0.946127	0.0698591
63472	0.946128	0.0698591
63473	0.946128	0.0698591
63474	0.946128	0.0698591
63475	0.946128	0.0698591
63476	0.946132	0.0698591
63477	0.946132	0.0698591
63478	0.946128	0.0698591
63479	0.946128	0.0698591
63480	0.94613	0.0698591
63481	0.94613	0.0698591
63482	0.946131	0.0698591
63483	0.946131	0.0698591
63484	0.94613	0.0698591
63485	0.94613	0.0698591
63486	0.94613	0.0698591
63487	0.94613	0.0698591
63488	1.89226	2.1
63489	1.89226	2.09925
63490	1.89226	2.1
63491	1.89226	2.1
63492	3.78453	2.1
63493	2.72844	2.1
63494	1.89226	2.1
63495	2.26536	2.1
63496	2.26536	2.1
63497	1.89226	2.1
63498	1.89226	2.1
63499	1.89226	2.1
63500	1.89226	2.07645
63501	1.89226	2.09933
63502	1.89226	2.1
63503	1.89226	2.09853
63504	1.89226	2.1
63505	1.89226	2.1
63506	1.89226	2.1
63507	1.89226	2.1

63508	1.89226	2.1
63509	1.89226	2.1
63510	1.89226	2.1
63511	2.72844	2.1
63512	2.26536	2.1
63513	2.28367	2.1
63514	1.5791	2.1
63515	4.54903	2.1
63516	0.810945	2.1
63517	0.810945	2.09086
63518	0.810943	2.09001
63519	0.810943	2.1
63520	1.89226	2.1
63521	1.3517	2.1
63522	1.08133	2.1
63523	1.08132	2.1
63524	1.08132	2.1
63525	0.810933	2.1
63526	0.810933	2.1
63527	1.89227	2.1
63528	1.89227	2.1
63529	1.89227	2.1
63530	1.89227	2.1
63531	3.78453	2.1
63532	2.71897	2.1
63533	2.27495	2.1
63534	2.648	2.1
63535	2.20832	2.1
63536	4.92729	2.1
63537	2.27495	2.1
63538	1.38455	2.1
63539	1.38455	2.1
63540	1.46586	2.1
63541	2.20831	2.1
63542	2.28962	2.1
63543	0.946133	2.1
63544	0.946133	2.1
63545	0.946132	2.1
63546	0.946132	2.1
63547	0.946132	2.1
63548	0.946132	2.1
63549	0.946132	2.1
63550	0.946132	2.1
63551	0.946132	2.1
63552	0.946132	2.1
63553	0.946133	2.1
63554	0.946133	2.1
63555	1.89226	2.1
63556	1.89226	2.1
63557	1.89226	2.1
63558	1.89226	2.1
63559	1.89226	2.1
63560	1.89226	2.1
63561	2.648	2.1
63562	2.08612	2.1
63563	2.08612	2.1
63564	2.20831	2.1
63565	2.56618	2.1

63566	2.68837	2.1
63567	2.08612	2.1
63568	2.20831	2.1
63569	1.30659	2.1
63570	1.30659	2.1
63571	1.38455	2.1
63572	2.16408	2.1
63573	0.810946	2.1
63574	0.810946	2.1
63575	0.810946	2.1
63576	0.810946	2.1
63577	1.89226	2.1
63578	1.3517	2.1
63579	1.08133	2.1
63580	1.08132	2.1
63581	1.08132	2.1
63582	0.810934	2.1
63583	0.810934	2.1
63584	1.89226	1.96653
63585	1.89226	1.55332
63586	1.89226	1.21301
63587	1.89226	2.00266
63588	3.78452	1.6778
63589	1.89226	1.79677
63590	1.89226	2.0725
63591	1.89226	2.09139
63592	1.89226	1.75552
63593	3.78453	2.03813
63594	1.08733	2.07271
63595	0.856615	2.09882
63596	1.25964	1.76738
63597	1.12378	1.7192
63598	1.23551	1.70322
63599	0.969894	2.1
63600	0.985977	2.06424
63601	0.985977	1.70568
63602	1.16169	1.88044
63603	1.21072	1.88507
63604	0.765175	2.01596
63605	0.863419	2.00228
63606	2.39934	1.98328
63607	2.44427	1.32016
63608	2.64003	1.09679
63609	2.39077	1.96602
63610	2.39934	1.77194
63611	2.10194	2.03661
63612	1.89226	1.09563
63613	1.89226	2.03545
63614	1.89226	1.89265
63615	1.89226	0.88072
63616	3.78452	1.77896
63617	0.946132	2.04562
63618	0.946132	2.02392
63619	0.946131	1.4596
63620	0.946131	1.71126
63621	0.946132	1.53437
63622	0.946132	2.09385
63623	0.946132	2.04951

63624	0.946132	1.76725
63625	0.946132	1.89108
63626	0.946132	1.92089
63627	0.946132	1.92029
63628	0.946132	1.94616
63629	1.19967	1.99433
63630	0.946128	1.91265
63631	0.946133	1.06751
63632	1.32002	1.41352
63633	1.19967	1.91984
63634	1.19967	1.71199
63635	0.94613	1.99048
63636	0.94613	0.859192
63637	0.946131	1.44857
63638	0.946131	1.63698
63639	1.19967	1.72746
63640	1.32001	1.62067
63641	1.89226	1.89491
63642	1.89226	2.1
63643	1.89226	1.34584
63644	1.89226	2.1
63645	3.78451	2.1
63646	2.6402	1.14181
63647	2.28552	2.1
63648	2.50958	1.29179
63649	2.18948	2.1
63650	4.79509	2.09765
63651	2.28552	2.1
63652	1.86102	2.1
63653	2.18949	2.1
63654	1.78376	2.1
63655	4.06928	2.1
63656	1.89226	2.1
63657	1.89226	2.1
63658	1.89226	2.1
63659	1.89226	2.1
63660	3.78452	2.1
63661	1.89226	1.23227
63662	1.89226	2.1
63663	1.89226	1.31192
63664	1.89226	2.1
63665	3.78452	2.1
63666	2.50958	1.18122
63667	2.18948	2.1
63668	2.39524	1.07109
63669	2.10188	2.1
63670	4.61146	2.06874
63671	2.18949	2.1
63672	1.78376	2.1
63673	1.71839	2.1
63674	1.71839	2.1
63675	2.10189	2.1
63676	2.16726	2.1
63677	0.810943	2.1
63678	0.810943	2.1
63679	0.810945	2.1
63680	0.810945	2.1
63681	0.810959	2.1

63682	0.810959	2.1
63683	1.89226	2.1
63684	1.35168	2.1
63685	1.08132	2.1
63686	1.08132	2.1
63687	1.0813	2.1
63688	1.89226	0.441745
63689	1.89226	1.78457
63690	1.89226	1.3221
63691	1.89226	0.769414
63692	1.89226	1.41164
63693	1.89226	1.11114
63694	1.28421	1.17503
63695	0.992041	0.542121
63696	1.07707	2.06075
63697	0.789746	1.77323
63698	1.03815	2.00784
63699	1.03815	1.48065
63700	1.03581	1.79462
63701	1.03581	0.969146
63702	1.28421	1.71433
63703	1.07707	1.80931
63704	0.789748	1.43496
63705	0.992038	1.36818
63706	1.89226	0.686444
63707	1.89226	1.71389
63708	1.89226	1.4676
63709	1.89226	0.906316
63710	1.89226	1.49014
63711	1.89226	0.94571
63712	1.89226	0.445744
63713	1.89226	1.60019
63714	1.89226	1.15976
63715	1.89226	0.817995
63716	1.89226	1.30074
63717	1.89226	1.11231
63718	0.92236	1.90199
63719	0.655971	1.77319
63720	0.916772	1.83694
63721	0.913834	1.21581
63722	1.18074	1.70244
63723	0.922346	1.84025
63724	2.15414	0.88224
63725	1.53528	1.58041
63726	1.22748	1.79184
63727	1.57442	1.07508
63728	0.874683	1.62123
63729	1.25935	2.1
63730	2.61433	2.1
63731	1.22262	2.1
63732	1.22262	2.1
63733	2.62813	2.1
63734	2.62813	2.1
63735	1.89226	2.1
63736	2.61434	2.1
63737	2.62814	2.1
63738	2.61434	2.1
63739	1.90607	2.1

63740	1.89226	2.1
63741	1.89226	2.1
63742	1.89226	2.1
63743	1.89226	2.1
63744	1.89226	2.1
63745	1.89226	2.1
63746	1.89226	2.1
63747	1.89226	2.1
63748	1.89226	2.1
63749	1.89226	2.1
63750	1.89226	2.1
63751	1.89226	2.1
63752	1.89226	2.1
63753	1.31969	2.1
63754	1.31406	2.1
63755	0.629677	2.1
63756	0.643104	2.1
63757	0.976621	2.1
63758	0.976621	2.1
63759	0.98055	2.1
63760	0.98055	2.1
63761	1.3197	2.1
63762	0.643102	2.1
63763	0.629675	2.1
63764	1.31407	2.1
63765	0.946133	2.1
63766	0.946133	2.1
63767	1.3197	2.1
63768	1.31407	2.1
63769	0.946132	2.1
63770	0.946132	2.1
63771	1.3197	2.1
63772	1.3197	2.1
63773	1.3197	2.1
63774	0.946133	2.1
63775	0.946132	2.1
63776	1.31407	2.1
63777	1.89226	2.1
63778	1.89226	2.1
63779	1.89226	2.1
63780	1.89226	2.1
63781	1.89226	2.1
63782	1.89226	2.1
63783	1.89226	2.1
63784	1.89226	2.1
63785	1.89226	2.1
63786	1.89226	2.1
63787	1.89226	2.1
63788	1.89226	2.1
63789	1.89226	0.0873855
63790	1.89226	0.0698591
63791	1.89226	0.0698591
63792	1.89226	0.0698591
63793	3.78452	0.0698591
63794	1.89226	0.0710653
63795	1.89226	0.0698591
63796	1.89226	0.0698591
63797	1.89226	0.0698591

63798	1.89226	0.0698591
63799	1.89226	0.0703497
63800	0.946131	0.0698591
63801	0.946131	0.0698591
63802	0.946128	0.0698591
63803	0.946128	0.0698591
63804	0.94613	0.0698591
63805	0.94613	0.0698591
63806	0.94613	0.0698591
63807	0.94613	0.0698591
63808	0.94613	0.0698591
63809	0.94613	0.0698591
63810	0.94613	0.0698591
63811	0.94613	0.0698591
63812	1.89226	0.0698591
63813	1.89226	0.0698591
63814	1.89226	0.0698591
63815	1.89226	0.0698591
63816	1.89226	0.0698591
63817	1.89226	0.0698591
63818	0.924614	0.0698591
63819	0.914275	0.0698591
63820	0.906003	0.0698591
63821	0.62479	0.0698591
63822	2.02733	0.0698591
63823	1.48332	0.0698591
63824	0.833106	0.0698591
63825	1.19364	0.0698591
63826	1.57482	0.0698591
63827	1.18103	0.0698591
63828	0.905988	0.0698591
63829	1.89226	2.1
63830	1.89226	2.1
63831	1.89226	2.1
63832	1.89226	2.1
63833	3.78452	2.1
63834	1.89226	2.1
63835	1.89226	2.1
63836	1.89226	2.1
63837	1.89226	2.1
63838	1.89226	2.1
63839	1.89226	2.1
63840	0.816805	2.1
63841	0.928516	2.1
63842	1.4045	2.1
63843	1.26268	2.1
63844	1.0881	2.1
63845	1.0881	2.1
63846	1.10309	2.1
63847	1.10309	2.1
63848	0.928518	2.1
63849	1.4045	2.1
63850	0.816807	2.1
63851	1.26267	2.1
63852	1.89226	2.1
63853	2.52535	2.1
63854	1.89226	2.1
63855	1.89226	2.1

63856	2.809	2.1
63857	2.809	2.1
63858	1.89226	2.1
63859	1.89226	2.1
63860	1.89226	2.1
63861	1.89226	2.1
63862	3.78452	2.1
63863	0.946127	2.1
63864	0.946127	2.1
63865	0.946131	2.1
63866	0.946131	2.1
63867	0.94613	2.1
63868	0.94613	2.1
63869	0.946128	2.1
63870	0.946128	2.1
63871	0.946129	2.1
63872	0.946129	2.1
63873	0.946129	2.1
63874	0.946129	2.1
63875	1.89224	2.1
63876	1.89224	2.1
63877	1.89224	2.1
63878	1.89224	2.1
63879	3.78448	2.1
63880	2.39244	2.1
63881	2.27036	2.1
63882	2.25945	2.1
63883	2.27398	2.1
63884	4.52981	2.1
63885	2.27036	2.1
63886	1.97088	2.1
63887	1.86302	2.1
63888	1.84991	2.1
63889	4.12027	2.1
63890	1.89224	2.1
63891	1.89224	2.1
63892	1.89224	2.1
63893	1.89224	2.1
63894	3.78447	2.1
63895	1.89226	2.1
63896	1.89226	2.1
63897	1.89226	2.1
63898	1.89226	2.1
63899	3.78453	2.1
63900	2.25948	2.1
63901	2.33611	2.1
63902	2.05257	2.1
63903	1.94441	2.1
63904	4.20389	2.1
63905	1.92514	2.1
63906	1.84993	2.1
63907	1.94441	2.1
63908	1.74391	2.1
63909	3.79434	2.1
63910	1.89226	2.1
63911	1.89226	2.1
63912	1.89226	2.1
63913	1.89226	2.1

63914	3.78452	2.1
63915	15.1381	1.11879
63916	15.1381	0.240201
63917	15.1381	0.206676
63918	15.1381	1.76562
63919	30.2762	0.267652
63920	15.1381	0.887407
63921	15.1381	1.7115
63922	15.1381	0.340532
63923	15.1381	0.257471
63924	30.2762	0.491479
63925	15.1381	1.93465
63926	15.1381	1.91512
63927	15.1381	1.7315
63928	15.1381	2.01853
63929	30.2762	1.99157
63930	15.1381	0.203516
63931	15.1381	1.82261
63932	15.1381	1.00589
63933	15.1381	0.183985
63934	30.2762	0.229048
63935	1.89225	2.1
63936	1.89225	2.1
63937	1.89225	2.1
63938	1.89225	2.1
63939	3.78449	2.1
63940	1.39909	2.1
63941	1.39909	2.1
63942	0.946123	2.1
63943	0.946123	2.1
63944	1.3291	2.1
63945	1.39908	2.1
63946	0.946126	2.1
63947	0.946126	2.1
63948	0.946118	2.1
63949	1.32909	2.1
63950	0.94613	2.1
63951	1.3991	2.1
63952	3.07801	2.1
63953	1.84322	2.1
63954	1.84322	2.1
63955	1.68788	2.1
63956	2.6582	2.1
63957	2.50287	2.1
63958	1.89225	2.1
63959	1.89225	2.1
63960	1.89225	2.1
63961	1.89225	2.1
63962	3.7845	2.1
63963	1.89226	2.1
63964	1.89226	2.1
63965	1.89226	2.1
63966	1.89226	2.1
63967	3.78452	2.1
63968	1.89226	2.1
63969	2.50051	2.1
63970	2.50051	2.1
63971	2.65822	2.1

63972	1.89226	2.1
63973	2.04997	2.1
63974	2.50052	2.1
63975	2.65822	2.1
63976	1.70377	2.1
63977	1.70377	2.1
63978	1.84324	2.1
63979	2.63998	2.1
63980	0.810947	2.1
63981	0.810947	2.1
63982	0.810946	2.1
63983	0.810946	2.1
63984	1.89226	2.1
63985	1.35169	2.1
63986	1.08132	2.1
63987	1.08132	2.1
63988	1.08132	2.1
63989	0.810946	2.1
63990	0.810946	2.1
63991	1.89226	2.1
63992	1.89226	2.1
63993	1.89226	2.1
63994	1.89226	2.1
63995	3.78453	2.1
63996	0.946164	2.1
63997	0.946164	2.1
63998	0.929842	2.1
63999	0.929842	2.1
64000	0.946132	2.1
64001	0.946132	2.1
64002	1.04101	2.1
64003	0.929874	2.1
64004	0.946132	2.1
64005	0.929874	2.1
64006	1.04101	2.1
64007	0.946132	2.1
64008	1.89226	2.1
64009	1.89226	2.1
64010	1.89226	2.1
64011	1.89226	2.1
64012	3.78453	2.1
64013	1.89226	2.1
64014	1.89226	2.1
64015	1.89226	2.1
64016	1.89226	2.1
64017	1.89226	2.1
64018	1.89226	2.1
64019	1.89226	2.1
64020	1.89226	2.1
64021	1.89226	2.1
64022	1.89226	2.1
64023	1.89226	2.1
64024	1.89226	2.1
64025	0.946131	2.1
64026	0.946131	2.1
64027	0.946131	2.1
64028	0.946131	2.1
64029	0.946132	2.1

64030	0.946132	2.1
64031	0.946131	2.1
64032	0.946131	2.1
64033	0.946131	2.1
64034	0.946131	2.1
64035	0.946132	2.1
64036	0.946132	2.1
64037	1.89226	2.01581
64038	1.89226	1.18891
64039	1.89226	0.954756
64040	1.89226	1.78488
64041	3.78453	0.780573
64042	2.73497	0.832623
64043	1.93823	2.1
64044	1.93823	1.29997
64045	2.20046	2.1
64046	2.42279	1.49166
64047	2.68501	2.1
64048	1.89226	1.14058
64049	1.89226	2.1
64050	1.89226	2.1
64051	1.89226	1.3832
64052	1.89226	2.1
64053	1.89226	0.927592
64054	1.89226	1.06256
64055	1.89226	0.522488
64056	1.89226	0.634926
64057	1.89226	1.87456
64058	1.89226	0.864307
64059	1.89226	0.761289
64060	2.82984	1.70139
64061	2.15276	0.656017
64062	2.15276	1.49559
64063	2.42278	0.0753472
64064	2.50074	0.861165
64065	2.77077	0.558661
64066	2.42279	0.63901
64067	1.93823	2.1
64068	1.70423	0.861876
64069	1.70423	2.08515
64070	2.15276	0.987762
64071	2.38676	2.1
64072	0.810946	1.2639
64073	0.810946	2.1
64074	0.810944	0.656671
64075	0.810944	0.60085
64076	0.810946	1.76693
64077	0.810946	1.86515
64078	1.89226	2.1
64079	1.35169	1.92295
64080	1.08132	1.09352
64081	1.08132	2.1
64082	1.08132	1.9603
64083	1.89226	2.1
64084	1.89226	2.1
64085	1.89226	2.1
64086	1.89226	2.1
64087	3.78452	2.1

64088	1.89226	2.1
64089	1.89226	2.1
64090	1.89226	2.1
64091	1.89226	2.1
64092	3.78453	2.1
64093	1.04144	2.1
64094	1.04144	2.1
64095	0.938972	2.1
64096	0.938972	2.1
64097	0.969661	2.1
64098	1.03285	2.1
64099	0.946133	2.1
64100	0.946133	2.1
64101	0.946131	2.1
64102	0.969663	2.1
64103	0.946131	2.1
64104	1.03284	2.1
64105	0.946131	2.1
64106	0.946131	2.1
64107	0.946128	2.1
64108	0.946128	2.1
64109	0.946127	2.1
64110	0.946127	2.1
64111	0.946131	2.1
64112	0.946131	2.1
64113	0.94613	2.1
64114	0.94613	2.1
64115	0.946129	2.1
64116	0.946129	2.1
64117	1.89226	2.1
64118	1.89226	2.1
64119	1.89226	2.1
64120	1.89226	2.1
64121	3.78452	2.1
64122	1.89226	2.1
64123	1.89226	2.1
64124	1.89226	2.1
64125	1.89226	2.1
64126	3.78453	2.1
64127	1.89226	2.1
64128	1.89226	2.1
64129	1.85882	2.1
64130	1.85882	2.1
64131	1.93932	2.1
64132	1.97276	2.1
64133	0.94613	2.1
64134	0.94613	2.1
64135	0.946128	2.1
64136	0.946128	2.1
64137	0.946127	2.1
64138	0.946127	2.1
64139	0.946131	2.1
64140	0.946131	2.1
64141	0.946129	2.1
64142	0.946129	2.1
64143	0.946129	2.1
64144	0.946129	2.1
64145	1.89225	2.1

64146	1.89225	2.1
64147	1.89225	2.1
64148	1.89225	2.1
64149	3.7845	2.1
64150	1.0086	2.1
64151	1.09028	2.1
64152	0.946125	2.1
64153	0.946125	2.1
64154	0.916906	2.1
64155	0.916906	2.1
64156	1.13051	2.1
64157	1.1505	2.1
64158	1.11557	2.1
64159	1.04071	2.1
64160	0.851542	2.1
64161	0.991196	2.1
64162	1.89225	2.1
64163	1.89225	2.1
64164	1.89225	2.1
64165	1.89225	2.1
64166	3.7845	2.1
64167	1.89225	2.1
64168	1.89225	2.1
64169	1.89225	2.1
64170	1.89225	2.1
64171	1.89225	2.1
64172	1.89225	2.1
64173	1.89226	2.1
64174	1.89226	2.1
64175	1.89226	2.1
64176	1.89226	2.1
64177	3.78452	2.1
64178	0.890962	2.1
64179	0.952547	2.1
64180	0.946132	2.1
64181	0.946132	2.1
64182	0.851543	2.1
64183	0.851543	2.1
64184	1.11558	2.1
64185	1.06304	2.1
64186	0.994315	2.1
64187	1.04072	2.1
64188	0.900943	2.1
64189	0.851542	2.1
64190	1.89226	2.1
64191	1.89226	2.1
64192	1.89226	2.1
64193	1.89226	2.1
64194	1.89226	2.1
64195	1.89226	2.1
64196	1.89226	2.1
64197	1.89226	2.1
64198	1.89226	2.1
64199	1.89226	2.1
64200	3.78452	2.1
64201	1.89226	2.1
64202	1.89226	2.1
64203	1.89226	2.1

64204	1.89226	2.1
64205	3.78452	2.1
64206	0.782015	2.1
64207	0.929718	2.1
64208	0.946132	2.1
64209	0.946132	2.1
64210	0.782016	2.1
64211	0.782016	2.1
64212	1.06706	2.1
64213	1.11785	2.1
64214	0.782015	2.1
64215	0.946133	2.1
64216	0.878931	2.1
64217	0.946132	2.1
64218	1.89226	2.1
64219	1.89226	2.1
64220	1.89226	2.1
64221	1.89226	2.1
64222	1.89226	2.1
64223	1.89226	2.1
64224	0.946131	2.1
64225	0.946131	2.1
64226	0.946129	2.1
64227	0.946129	2.1
64228	0.946131	2.1
64229	0.946131	2.1
64230	0.94613	2.1
64231	0.94613	2.1
64232	0.946131	2.1
64233	0.946131	2.1
64234	0.94613	2.1
64235	0.94613	2.1
64236	0.827866	2.1
64237	0.946131	2.1
64238	0.946129	2.1
64239	0.946129	2.1
64240	0.94613	2.1
64241	0.94613	2.1
64242	0.94613	2.1
64243	0.94613	2.1
64244	0.827865	2.1
64245	0.94613	2.1
64246	0.94613	2.1
64247	0.94613	2.1
64248	1.89226	2.1
64249	1.89226	2.1
64250	1.89226	2.1
64251	1.89226	2.1
64252	3.78452	2.1
64253	1.89226	2.1
64254	1.89226	2.1
64255	1.89226	2.1
64256	1.89226	2.1
64257	1.89226	2.1
64258	1.89226	2.1
64259	1.89226	2.1
64260	1.89226	2.1
64261	1.89226	2.1

64262	1.89226	2.1
64263	3.78453	2.1
64264	1.89226	2.1
64265	1.89226	2.1
64266	1.89226	2.1
64267	1.89226	2.1
64268	3.78452	2.1
64269	0.744377	2.1
64270	0.541919	2.1
64271	0.652022	2.1
64272	0.544198	2.1
64273	0.764633	2.1
64274	0.673254	2.1
64275	1.89226	2.1
64276	1.31149	2.1
64277	1.05423	2.1
64278	1.02189	2.1
64279	0.870602	2.1
64280	1.89226	2.1
64281	1.89226	2.1
64282	1.89226	2.1
64283	1.89226	2.1
64284	1.89226	2.1
64285	1.89226	2.1
64286	1.89226	2.1
64287	1.89226	2.1
64288	1.89226	2.1
64289	1.89226	2.1
64290	1.89226	2.1
64291	1.89226	2.1
64292	1.89226	2.1
64293	1.89226	2.1
64294	1.89226	2.1
64295	1.89226	2.1
64296	1.89226	2.1
64297	1.89226	2.1
64298	0.815314	2.1
64299	0.815313	2.1
64300	0.714029	2.1
64301	0.782951	2.1
64302	0.810944	2.1
64303	0.782954	2.1
64304	1.89226	2.1
64305	1.30999	2.1
64306	1.08132	2.1
64307	1.03962	2.1
64308	0.95209	2.1
64309	1.89226	2.1
64310	1.89226	2.1
64311	1.89226	2.1
64312	1.89226	2.1
64313	1.89226	2.1
64314	1.89226	2.1
64315	1.89226	2.1
64316	1.89226	2.1
64317	1.89226	2.1
64318	1.89226	2.1
64319	1.89226	2.1

64320	1.89226	2.1
64321	1.89226	2.1
64322	1.89226	2.1
64323	1.89226	2.1
64324	1.89226	2.1
64325	1.89226	2.1
64326	1.89226	2.1
64327	0.871558	2.1
64328	0.713078	2.1
64329	0.756858	2.1
64330	0.703674	2.1
64331	0.838025	2.1
64332	0.862196	2.1
64333	1.89226	2.1
64334	1.33194	2.1
64335	1.10834	2.1
64336	1.06163	2.1
64337	1.01785	2.1
64338	1.89226	2.1
64339	1.89226	2.1
64340	1.89226	2.1
64341	1.89226	2.1
64342	3.78452	2.1
64343	1.89226	2.1
64344	1.89226	2.1
64345	1.89226	2.1
64346	1.89226	2.1
64347	1.89226	2.1
64348	1.89226	2.1
64349	1.89226	2.1
64350	1.89226	2.1
64351	1.89226	2.1
64352	1.89226	2.1
64353	1.89226	2.1
64354	1.89226	2.1
64355	0.958406	2.1
64356	1.1383	2.1
64357	0.865636	2.1
64358	1.10093	2.1
64359	0.838024	2.1
64360	0.975154	2.1
64361	1.89226	2.1
64362	1.38553	2.1
64363	1.10834	2.1
64364	1.11522	2.1
64365	1.15271	2.1
64366	1.89227	2.1
64367	1.89227	2.1
64368	1.89227	2.1
64369	1.89227	2.1
64370	3.78453	2.1
64371	0.946132	2.1
64372	0.946132	2.1
64373	0.946132	2.1
64374	0.946132	2.1
64375	0.946132	2.1
64376	0.946132	2.1
64377	0.946133	2.1

64378	0.946133	2.1
64379	0.946132	2.1
64380	0.946132	2.1
64381	0.946133	2.1
64382	0.946133	2.1
64383	0.719271	2.1
64384	0.774968	2.1
64385	1.28401	2.1
64386	1.17415	2.1
64387	0.960999	2.1
64388	0.960999	2.1
64389	0.988115	2.1
64390	0.988115	2.1
64391	0.774968	2.1
64392	1.28401	2.1
64393	0.719271	2.1
64394	1.17415	2.1
64395	2.77811	2.1
64396	2.34829	2.1
64397	2.56802	2.1
64398	2.52333	2.1
64399	2.56802	2.1
64400	2.52333	2.1
64401	1.89227	2.1
64402	1.89227	2.1
64403	1.89227	2.1
64404	1.89227	2.1
64405	3.78453	2.1
64406	1.89226	2.1
64407	1.89226	2.1
64408	1.89226	2.1
64409	1.89226	2.1
64410	1.89226	2.1
64411	1.89226	2.1
64412	1.89226	2.1
64413	2.77811	2.1
64414	1.89226	2.1
64415	2.56802	2.1
64416	1.89226	2.1
64417	2.56802	2.1
64418	1.89226	1.78649
64419	1.89226	2.1
64420	1.89226	1.83402
64421	1.89226	2.1
64422	3.78452	2.1
64423	2.41802	2.1
64424	1.95764	2.1
64425	1.95764	2.1
64426	2.27727	2.1
64427	2.39311	2.1
64428	2.73961	2.1
64429	0.946132	2.1
64430	0.946132	2.1
64431	0.94613	2.1
64432	0.94613	2.1
64433	0.946131	2.1
64434	0.946131	2.1
64435	0.946131	2.1

64436	0.946131	2.1
64437	0.946131	2.1
64438	0.946131	2.1
64439	0.946131	2.1
64440	0.946131	2.1
64441	1.89226	1.88235
64442	1.89226	2.1
64443	1.89226	2.1
64444	1.89226	1.9274
64445	1.89226	2.1
64446	1.89226	1.73466
64447	2.78856	1.20972
64448	2.28552	2.1
64449	2.28552	1.76468
64450	2.3931	2.1
64451	2.6402	1.69814
64452	2.74778	2.1
64453	2.39311	2.1
64454	1.95764	2.1
64455	2.28552	2.1
64456	1.86103	2.1
64457	4.24316	2.1
64458	1.89226	2.1
64459	1.89226	2.1
64460	1.89226	2.1
64461	1.89226	2.1
64462	1.89226	2.1
64463	1.89226	2.1
64464	1.47412	2.07345
64465	2.18862	1.91941
64466	2.07226	0.993615
64467	2.33645	1.7886
64468	4.26088	1.42316
64469	1.89226	1.03757
64470	1.89226	1.95906
64471	1.89226	1.18427
64472	1.89226	1.92774
64473	1.89226	1.70954
64474	1.89226	2.09325
64475	1.89226	1.46948
64476	1.89226	2.1
64477	1.89226	0.4296
64478	1.89226	2.1
64479	3.78453	1.88145
64480	2.18862	1.42451
64481	2.1128	2.1
64482	2.33645	1.08764
64483	2.28188	2.1
64484	4.47051	2.09071
64485	2.07226	2.1
64486	2.33645	2.0399
64487	2.20831	1.26457
64488	2.4646	0.847649
64489	4.53686	1.20552
64490	0.946131	1.22137
64491	0.946131	2.1
64492	0.946131	2.1
64493	0.946131	1.47838

64494	0.94613	2.1
64495	0.94613	0.924457
64496	0.946133	2.07652
64497	0.946133	2.1
64498	0.946132	0.878641
64499	0.946132	0.604442
64500	0.946131	2.01507
64501	0.946131	1.87178
64502	1.89226	1.76669
64503	1.89226	2.1
64504	1.89226	0.631338
64505	1.89226	2.09998
64506	3.78453	1.94529
64507	2.33646	1.67113
64508	2.28189	2.1
64509	2.4646	0.575848
64510	2.42758	2.1
64511	4.74648	1.91046
64512	1.89226	2.1
64513	1.89226	2.1
64514	1.89226	2.1
64515	1.89226	2.1
64516	1.89226	2.1
64517	1.89226	2.1
64518	1.89226	2.1
64519	1.89226	2.1
64520	1.89226	2.1
64521	1.89226	2.1
64522	1.89226	2.1
64523	1.89226	2.1
64524	1.89226	2.1
64525	1.89226	2.1
64526	1.89226	2.1
64527	1.89226	2.1
64528	3.78453	2.1
64529	1.89226	2.1
64530	1.89226	2.1
64531	1.89226	2.1
64532	1.89226	2.1
64533	1.89226	2.1
64534	1.89226	2.1
64535	1.89226	2.1
64536	1.89226	2.1
64537	1.89226	2.1
64538	1.89226	2.1
64539	3.78452	2.1
64540	0.877238	2.1
64541	0.877238	2.1
64542	1.24746	2.1
64543	1.04764	2.1
64544	0.964969	2.1
64545	1.1521	2.1
64546	0.946132	2.1
64547	0.946132	2.1
64548	0.946132	2.1
64549	1.12659	2.1
64550	0.964969	2.1
64551	0.946131	2.1

64552	1.89226	2.1
64553	1.89226	2.1
64554	1.89226	2.1
64555	1.89226	2.1
64556	1.89226	2.1
64557	1.89226	2.1
64558	1.89226	2.1
64559	1.89226	2.1
64560	1.89226	2.1
64561	1.89226	2.1
64562	3.78453	2.1
64563	0.868144	2.1
64564	0.992762	2.1
64565	0.946131	2.1
64566	0.946131	2.1
64567	1.07072	2.1
64568	1.07072	2.1
64569	0.868171	2.1
64570	0.868171	2.1
64571	0.992762	2.1
64572	0.946131	2.1
64573	0.946131	2.1
64574	0.868144	2.1
64575	0.946132	2.1
64576	0.946132	2.1
64577	0.946131	2.1
64578	0.946131	2.1
64579	0.946132	2.1
64580	0.946132	2.1
64581	0.946132	2.1
64582	0.946132	2.1
64583	0.946132	2.1
64584	0.946132	2.1
64585	0.946132	2.1
64586	0.946132	2.1
64587	1.89227	2.1
64588	1.89227	2.1
64589	1.89227	2.1
64590	1.89227	2.1
64591	1.89227	2.1
64592	1.89227	2.1
64593	1.89226	2.1
64594	1.89226	2.1
64595	1.89226	2.1
64596	1.89226	2.1
64597	1.89226	2.1
64598	1.89226	2.1
64599	1.98552	2.1
64600	2.22298	2.1
64601	2.22298	2.1
64602	1.89226	2.1
64603	1.89226	2.1
64604	1.54525	2.1
64605	0.810946	2.1
64606	0.810946	2.1
64607	0.810947	2.1
64608	0.810947	2.1
64609	0.81096	2.1

64610	0.81096	2.1
64611	1.89226	2.1
64612	1.35168	2.1
64613	1.08132	2.1
64614	1.08132	2.1
64615	1.0813	2.1
64616	1.89226	2.1
64617	1.89226	2.1
64618	1.89226	2.1
64619	1.89226	2.1
64620	3.78452	2.1
64621	1.7753	2.1
64622	2.39703	2.1
64623	1.90577	2.1
64624	2.03445	2.1
64625	3.82335	2.1
64626	2.39703	2.1
64627	2.47949	2.09712
64628	2.26051	2.1
64629	2.36891	2.07603
64630	4.76594	2.1
64631	1.89226	2.1
64632	1.89226	2.07212
64633	1.89226	2.1
64634	1.89226	2.08393
64635	3.78452	2.1
64636	1.89226	2.1
64637	1.89226	2.1
64638	1.89226	2.1
64639	1.89226	2.1
64640	1.89226	2.1
64641	1.89226	2.1
64642	0.883745	2.1
64643	0.973143	2.1
64644	0.988173	2.1
64645	0.988173	2.1
64646	0.941927	2.1
64647	1.02971	2.1
64648	0.854065	2.1
64649	0.82702	2.1
64650	0.847835	2.1
64651	0.981126	2.1
64652	1.02996	2.1
64653	1.10704	2.1
64654	2.28031	2.1
64655	2.26369	2.08049
64656	2.26369	2.1
64657	2.36891	2.09212
64658	2.30788	2.1
64659	2.42455	2.09257
64660	1.89226	2.1
64661	1.89226	2.08146
64662	1.89226	2.04533
64663	1.89226	2.1
64664	1.89226	2.07317
64665	1.89226	2.1
64666	2.71406	2.1
64667	1.83244	2.1

64668	1.83244	2.1
64669	2.07567	2.1
64670	2.52044	2.1
64671	2.76367	2.1
64672	1.89226	2.1
64673	1.89226	2.1
64674	1.89226	2.1
64675	1.89226	2.1
64676	3.78453	2.1
64677	1.89226	2.1
64678	1.89226	2.1
64679	1.89226	2.1
64680	1.89226	2.1
64681	1.89226	2.1
64682	1.89226	2.1
64683	2.70714	2.1
64684	2.3148	2.1
64685	2.3148	2.1
64686	2.52044	2.1
64687	2.54204	2.1
64688	2.74768	2.1
64689	2.52044	2.1
64690	2.3148	2.1
64691	1.83244	2.1
64692	1.83244	2.1
64693	1.62611	2.1
64694	2.3148	2.1
64695	0.946131	2.1
64696	0.946131	2.1
64697	0.946131	2.1
64698	0.946131	2.1
64699	0.946131	2.1
64700	0.946131	2.1
64701	0.946131	2.1
64702	0.946131	2.1
64703	0.946131	2.1
64704	0.946131	2.1
64705	0.946131	2.1
64706	0.946131	2.1
64707	1.89226	2.1
64708	1.89226	2.1
64709	1.89226	2.1
64710	1.89226	2.1
64711	3.78453	2.1
64712	1.89226	2.1
64713	1.89226	2.1
64714	1.89226	2.1
64715	1.89226	2.1
64716	1.89226	2.1
64717	1.89226	2.1
64718	1.89227	2.1
64719	1.89227	2.1
64720	1.89227	2.1
64721	1.89227	2.1
64722	3.78453	2.1
64723	1.89226	2.1
64724	1.89226	2.1
64725	1.89226	2.1

64726	1.89226	2.1
64727	3.78453	2.1
64728	0.915781	2.1
64729	0.863524	2.1
64730	0.717056	2.1
64731	0.830182	2.1
64732	0.865094	2.1
64733	0.918003	2.1
64734	1.89226	2.1
64735	1.29933	2.1
64736	1.11215	2.1
64737	1.05227	2.1
64738	0.966269	2.1
64739	1.89226	2.1
64740	1.89226	2.1
64741	1.89226	2.1
64742	1.89226	2.1
64743	1.89226	2.1
64744	1.89226	2.1
64745	1.89226	2.1
64746	1.89226	2.1
64747	1.89226	2.1
64748	1.89226	2.1
64749	1.89226	2.1
64750	1.89226	2.1
64751	1.89226	2.1
64752	1.89226	2.1
64753	1.89226	2.1
64754	1.89226	2.1
64755	1.89226	2.1
64756	1.89226	2.1
64757	1.01725	2.1
64758	0.997048	2.1
64759	0.818657	2.1
64760	0.960724	2.1
64761	0.838013	2.1
64762	1.04859	2.1
64763	1.89226	2.1
64764	1.41754	2.1
64765	1.17625	2.1
64766	1.0793	2.1
64767	1.18822	2.1
64768	2.16402	2.1
64769	1.89452	2.1
64770	1.50084	2.1
64771	2.09288	2.1
64772	3.9874	2.1
64773	2.16402	2.1
64774	2.13925	2.1
64775	2.04483	2.1
64776	2.09288	2.1
64777	4.23212	2.1
64778	1.89226	2.1
64779	1.89226	2.1
64780	1.89226	2.1
64781	1.89226	2.1
64782	3.78452	2.1
64783	1.89226	2.1

64784	1.89226	2.1
64785	1.89226	2.1
64786	1.89226	2.1
64787	1.89226	2.1
64788	1.89226	2.1
64789	1.9824	2.1
64790	2.09288	2.1
64791	1.69889	2.1
64792	1.6817	2.1
64793	2.00695	2.1
64794	2.00695	2.1
64795	1.93592	2.1
64796	2.09288	2.1
64797	2.00695	2.1
64798	2.09288	2.1
64799	1.9589	2.1
64800	2.04483	2.1
64801	0.946128	2.1
64802	0.946128	2.1
64803	0.946132	2.1
64804	0.946132	2.1
64805	0.94613	2.1
64806	0.94613	2.1
64807	0.946129	2.1
64808	0.946129	2.1
64809	0.94613	2.1
64810	0.94613	2.1
64811	0.94613	2.1
64812	0.94613	2.1
64813	0.946131	2.1
64814	0.946131	2.1
64815	0.946131	2.1
64816	0.946131	2.1
64817	0.946131	2.1
64818	0.946131	2.1
64819	0.946131	2.1
64820	0.946131	2.1
64821	0.946131	2.1
64822	0.946131	2.1
64823	0.946131	2.1
64824	0.946131	2.1
64825	1.89226	2.1
64826	2.48083	2.1
64827	2.6968	2.1
64828	2.6968	2.1
64829	1.89226	2.1
64830	1.89226	2.1
64831	1.89226	2.1
64832	1.89226	2.1
64833	1.89226	2.1
64834	1.89226	2.1
64835	3.78453	2.1
64836	1.89226	2.1
64837	1.89226	2.1
64838	1.89226	2.1
64839	1.89226	2.1
64840	1.89226	2.1
64841	1.89226	2.1

64842	1.1017	2.1
64843	1.1017	2.1
64844	1.10605	2.1
64845	1.01647	2.1
64846	0.868255	2.1
64847	0.883424	2.1
64848	1.24624	2.1
64849	1.35473	2.1
64850	0.942231	2.1
64851	1.31464	2.1
64852	0.910064	2.1
64853	1.27147	2.1
64854	1.89226	2.1
64855	1.89226	2.1
64856	1.89226	2.1
64857	1.89226	2.1
64858	3.78453	2.1
64859	1.89226	2.1
64860	1.89226	2.1
64861	1.89226	2.1
64862	1.89226	2.1
64863	1.89226	2.1
64864	1.89226	2.1
64865	1.89226	2.1
64866	1.89226	2.1
64867	1.89226	2.1
64868	1.89226	2.1
64869	3.78453	2.1
64870	1.89226	2.1
64871	2.70015	2.1
64872	1.89226	2.1
64873	2.61064	2.1
64874	4.59241	2.1
64875	2.70015	2.1
64876	2.3372	2.1
64877	2.61064	2.1
64878	1.85184	2.1
64879	4.55198	2.1
64880	1.89226	2.1
64881	1.89226	2.1
64882	1.89226	2.1
64883	1.89226	2.1
64884	1.89226	2.1
64885	1.89226	2.1
64886	1.89227	2.1
64887	1.89227	2.1
64888	1.89227	2.1
64889	1.89227	2.1
64890	3.78453	2.1
64891	1.89226	2.1
64892	2.52194	2.1
64893	2.61064	2.1
64894	2.61064	2.1
64895	1.89226	2.1
64896	1.89226	2.1
64897	1.095 2.1	
64898	1.095 2.1	
64899	1.09189	2.1

64900	1.09189	2.1
64901	0.875334	2.1
64902	0.925919	2.1
64903	1.26097	2.1
64904	1.30532	2.1
64905	1.26097	2.1
64906	0.875334	2.1
64907	1.30532	2.1
64908	0.92592	2.1
64909	1.89226	2.1
64910	1.89226	2.1
64911	1.89226	2.1
64912	1.89226	2.1
64913	1.89226	2.1
64914	1.89226	2.1
64915	1.89226	2.1
64916	1.89226	2.1
64917	1.89226	2.1
64918	1.89226	2.1
64919	3.78453	2.1
64920	1.26097	2.1
64921	1.26097	2.1
64922	0.946132	2.1
64923	0.946132	2.1
64924	1.22176	2.1
64925	1.26097	2.1
64926	0.946132	2.1
64927	0.946132	2.1
64928	0.946132	2.1
64929	1.22176	2.1
64930	1.26097	2.1
64931	0.946132	2.1
64932	2.52194	2.1
64933	1.67381	2.1
64934	1.67381	2.1
64935	1.75067	2.1
64936	2.44352	2.1
64937	2.52038	2.1
64938	1.89226	2.1
64939	1.89226	2.1
64940	1.89226	2.1
64941	1.89226	2.1
64942	1.89226	2.1
64943	1.89226	2.1
64944	1.89227	2.1
64945	1.89227	2.1
64946	1.89227	2.1
64947	1.89227	2.1
64948	1.89227	2.1
64949	1.89227	2.1
64950	1.22176	2.1
64951	1.22176	2.1
64952	0.946132	2.1
64953	0.946132	2.1
64954	1.19095	2.1
64955	1.22176	2.1
64956	0.946133	2.1
64957	0.946133	2.1

64958	1.19095	2.1
64959	0.946132	2.1
64960	0.946133	2.1
64961	1.22176	2.1
64962	2.44352	2.1
64963	1.6287	2.1
64964	1.6287	2.1
64965	1.67381	2.1
64966	2.3819	2.1
64967	2.427	2.1
64968	1.89226	2.1
64969	1.89226	2.1
64970	1.89226	2.1
64971	1.89226	2.1
64972	1.89226	2.1
64973	1.89226	2.1
64974	1.89226	2.1
64975	1.89226	2.1
64976	1.89226	2.1
64977	1.89226	2.1
64978	3.78453	2.1
64979	1.89226	2.1
64980	1.89226	2.1
64981	1.89226	2.1
64982	1.89226	2.1
64983	1.89226	2.1
64984	1.89226	2.1
64985	1.89226	2.1
64986	1.89226	2.1
64987	1.89226	2.1
64988	1.89226	2.1
64989	1.89226	2.1
64990	1.89226	2.1
64991	0.851542	2.1
64992	0.851542	2.1
64993	1.1736	2.1
64994	1.29261	2.1
64995	0.852708	2.1
64996	1.18843	2.1
64997	0.946132	2.1
64998	0.946132	2.1
64999	0.946132	2.1
65000	1.07901	2.1
65001	1.04208	2.1
65002	0.936669	2.1
65003	1.89227	2.1
65004	1.89227	2.1
65005	1.89227	2.1
65006	1.89227	2.1
65007	3.78453	2.1
65008	1.89226	2.1
65009	1.89226	2.1
65010	1.89226	2.1
65011	1.89226	2.1
65012	3.78453	2.1
65013	1.89226	2.1
65014	1.89226	2.1
65015	1.89226	2.1

65016	1.89226	2.1
65017	1.89226	2.1
65018	1.89226	2.1
65019	0.946133	2.1
65020	0.946133	2.1
65021	1.28525	2.1
65022	1.28525	2.1
65023	1.07901	2.1
65024	1.28525	2.1
65025	0.946132	2.1
65026	0.946132	2.1
65027	0.946132	2.1
65028	1.28525	2.1
65029	0.946133	2.1
65030	1.07901	2.1
65031	1.89226	2.1
65032	1.89226	2.1
65033	1.89226	2.1
65034	1.89226	2.1
65035	1.89226	2.1
65036	1.89226	2.1
65037	1.89226	2.1
65038	1.89226	2.1
65039	1.89226	2.1
65040	1.89226	2.1
65041	1.89226	2.1
65042	1.89226	2.1
65043	1.24419	2.1
65044	1.09093	2.1
65045	1.22722	2.1
65046	1.18154	2.1
65047	1.27455	2.1
65048	1.08473	2.1
65049	1.15541	2.1
65050	1.15541	2.1
65051	1.29607	2.1
65052	1.23814	2.1
65053	1.13024	2.1
65054	1.12096	2.1
65055	1.18858	2.1
65056	1.13181	2.1
65057	0.988718	2.1
65058	0.802498	2.1
65059	0.994034	2.1
65060	1.15773	2.1
65061	1.0029	2.1
65062	1.0029	2.1
65063	1.23814	2.1
65064	0.997523	2.1
65065	1.11498	2.1
65066	0.899532	2.1
65067	1.89226	2.1
65068	1.89226	2.1
65069	1.89226	2.1
65070	1.89226	2.1
65071	1.89226	2.1
65072	1.89226	2.1
65073	1.89226	2.1

65074	1.89226	2.1
65075	1.89226	2.1
65076	1.89226	2.1
65077	1.89226	2.1
65078	1.89226	2.1
65079	0.993139	2.1
65080	0.834108	2.1
65081	1.01147	2.1
65082	1.01358	2.1
65083	1.20067	2.1
65084	0.993156	2.1
65085	2.4158	2.1
65086	1.69956	2.1
65087	1.3544	2.1
65088	1.60095	2.1
65089	1.11219	2.1
65090	1.89226	2.1
65091	1.89226	2.1
65092	1.89226	2.1
65093	1.89226	2.1
65094	3.78452	2.1
65095	1.89226	2.1
65096	1.89226	2.1
65097	1.89226	2.1
65098	1.89226	2.1
65099	1.89226	2.1
65100	1.89226	2.1
65101	1.89226	2.1
65102	2.83014	2.1
65103	2.32404	2.1
65104	2.32404	2.1
65105	1.89226	2.1
65106	1.89226	2.1
65107	1.37966	2.1
65108	1.13294	2.1
65109	0.881723	2.1
65110	0.582304	2.1
65111	0.977944	2.1
65112	0.977948	2.1
65113	1.0264	2.1
65114	1.0264	2.1
65115	1.37967	2.1
65116	1.03896	2.1
65117	1.19108	2.1
65118	0.582298	2.1
65119	1.89226	2.1
65120	1.89226	2.1
65121	1.89226	2.1
65122	1.89226	2.1
65123	3.78452	2.1
65124	0.946131	2.1
65125	0.946131	2.1
65126	0.946131	2.1
65127	0.946131	2.1
65128	0.946132	2.1
65129	0.946132	2.1
65130	0.946129	2.1
65131	0.946129	2.1

65132	0.946131	2.1
65133	0.946131	2.1
65134	0.94613	2.1
65135	0.94613	2.1
65136	1.89226	2.1
65137	1.89226	2.1
65138	1.89226	2.1
65139	1.89226	2.1
65140	3.78453	2.1
65141	1.89226	2.1
65142	1.89226	2.1
65143	1.89226	2.1
65144	1.89226	2.1
65145	1.89226	2.1
65146	1.89226	2.1
65147	2.65216	2.1
65148	1.93528	2.1
65149	1.78595	2.1
65150	2.48544	2.1
65151	4.42072	2.1
65152	1.89226	2.1
65153	1.89226	2.1
65154	1.89226	2.1
65155	1.89226	2.1
65156	1.89226	2.1
65157	1.89226	2.1
65158	1.89226	2.1
65159	1.89226	2.1
65160	1.89226	2.1
65161	1.89226	2.1
65162	3.78452	2.1
65163	1.66537	2.1
65164	2.48544	2.1
65165	1.78595	2.1
65166	1.78595	2.1
65167	2.36197	2.1
65168	2.36197	2.1
65169	2.80975	2.1
65170	2.48544	2.1
65171	2.36198	2.1
65172	2.65135	2.1
65173	5.13679	2.1
65174	1.89226	2.1
65175	1.89226	2.1
65176	1.89226	2.1
65177	1.89226	2.1
65178	1.89226	2.1
65179	1.89226	2.1
65180	0.946127	2.1
65181	0.946127	2.1
65182	0.946131	2.1
65183	0.946131	2.1
65184	0.946128	2.1
65185	0.946128	2.1
65186	0.94613	2.1
65187	0.94613	2.1
65188	0.946129	2.1
65189	0.946129	2.1

65190	0.946129	2.1
65191	0.946129	2.1
65192	1.11972	2.1
65193	1.18099	2.1
65194	0.832687	2.1
65195	0.764517	2.1
65196	0.974426	2.1
65197	0.974426	2.1
65198	0.971078	2.1
65199	0.971078	2.1
65200	1.11972	2.1
65201	0.764515	2.1
65202	0.832686	2.1
65203	1.18099	2.1
65204	2.36198	2.1
65205	2.48025	2.1
65206	2.48025	2.1
65207	2.65135	2.1
65208	2.23944	2.1
65209	2.41055	2.1
65210	0.946132	2.1
65211	0.946132	2.1
65212	0.946131	2.1
65213	0.946131	2.1
65214	0.946131	2.1
65215	0.946131	2.1
65216	0.946132	2.1
65217	0.946132	2.1
65218	0.946131	2.1
65219	0.946131	2.1
65220	0.946132	2.1
65221	0.946132	2.1
65222	0.810942	2.1
65223	0.810942	2.1
65224	0.810944	2.1
65225	0.810944	2.1
65226	1.89226	2.1
65227	1.3517	2.1
65228	1.08132	2.1
65229	1.08133	2.1
65230	1.08132	2.1
65231	0.810932	2.1
65232	0.810932	2.1
65233	1.89249	2.1
65234	1.9857	1.65823
65235	2.02433	2.05324
65236	2.15807	1.94606
65237	4.01003	2.02794
65238	1.89226	1.90814
65239	1.89226	1.71002
65240	1.89226	2.07054
65241	1.89226	0.724528
65242	3.78452	1.64527
65243	1.89226	1.87205
65244	1.89226	1.99573
65245	1.89226	0.377754
65246	1.89226	0.992053
65247	3.78452	0.606774

65248	1.98569	1.63514
65249	1.86067	1.64509
65250	2.15807	1.87456
65251	2.07965	0.915601
65252	4.06534	1.803
65253	2.02433	2.1
65254	2.15807	2.09792
65255	2.12608	2.1
65256	2.3025	1.54552
65257	4.32683	2.09889
65258	1.89226	2.1
65259	1.89226	1.27315
65260	1.89226	1.81205
65261	1.89226	1.6103
65262	3.78452	1.80213
65263	1.89226	0.867287
65264	1.89226	1.59688
65265	1.89226	1.45124
65266	1.89226	1.45244
65267	3.78452	1.49841
65268	2.15807	2.09614
65269	2.07965	1.41156
65270	2.3025	1.12866
65271	2.28384	1.37425
65272	4.38215	1.20392
65273	1.89226	2.09263
65274	1.89226	0.639064
65275	1.89226	1.79603
65276	1.89226	0.733076
65277	3.78453	1.46956
65278	2.16905	0.68845
65279	2.56369	0.559046
65280	1.88394	1.51999
65281	2.41938	0.434671
65282	4.44763	0.596844
65283	2.56369	0.332035
65284	2.80663	1.50404
65285	2.41938	0.440865
65286	2.69045	0.589033
65287	5.25414	0.470897
65288	1.89226	0.377026
65289	1.89226	0.44974
65290	1.89226	0.708787
65291	1.89226	0.663251
65292	3.78453	0.147788
65293	1.89226	1.51503
65294	1.89226	0.795961
65295	1.89226	2.04333
65296	1.89226	0.85665
65297	1.89226	1.97334
65298	1.89226	0.526491
65299	1.01988	0.631138
65300	1.01988	0.575985
65301	1.03333	1.20476
65302	1.03333	0.707926
65303	1.13811	0.144461
65304	1.20969	0.354863
65305	0.941974	1.21881

65306	0.843515	0.593256
65307	0.843514	0.483827
65308	1.13811	0.478259
65309	0.941973	0.604182
65310	1.20969	0.366972
65311	2.41938	0.261221
65312	2.57414	0.590316
65313	2.57414	0.895161
65314	2.69045	1.61142
65315	2.27623	0.200988
65316	2.39253	1.42218
65317	1.89227	0.677494
65318	1.89227	0.708955
65319	1.89227	0.222344
65320	1.89227	0.252461
65321	1.89227	0.206117
65322	1.89227	0.344469
65323	2.28385	1.86834
65324	2.09114	1.90133
65325	2.46968	0.331054
65326	2.32438	0.947723
65327	4.56082	0.431406
65328	1.89226	0.78954
65329	1.89226	1.9225
65330	1.89226	0.538215
65331	1.89226	1.9315
65332	3.78452	1.28025
65333	1.89226	0.914958
65334	1.89226	2.1
65335	1.89226	1.83979
65336	1.89226	1.96983
65337	3.78452	1.94945
65338	2.09114	0.834426
65339	1.5249	1.92186
65340	2.55684	0.333989
65341	1.86899	1.97489
65342	3.96011	1.21596
65343	2.46968	0.774376
65344	2.32439	1.41608
65345	2.62768	1.6518
65346	2.506	1.41097
65347	4.97568	1.7889
65348	1.89226	1.43049
65349	1.89226	1.95284
65350	1.89226	0.389353
65351	1.89226	1.19
65352	3.78452	0.839677
65353	1.89226	1.68539
65354	1.89226	1.86609
65355	1.89226	1.52516
65356	1.89226	2.1
65357	1.89226	0.851969
65358	1.89226	2.1
65359	2.55684	0.752968
65360	2.16506	1.8566
65361	1.86901	1.43779
65362	1.86899	1.06353
65363	2.75664	1.05366

65364	2.75662	0.7504
65365	1.89224	0.776079
65366	1.89224	1.40219
65367	1.89224	0.290536
65368	1.89224	2.09127
65369	3.78447	1.09047
65370	2.24206	0.890389
65371	2.38862	2.1
65372	2.07107	1.78693
65373	2.23097	2.08731
65374	4.47303	2.0172
65375	2.38863	2.1
65376	2.25945	2.1
65377	2.25945	2.0955
65378	2.39244	2.1
65379	2.23098	2.09188
65380	2.36397	2.1
65381	1.89224	1.36446
65382	1.89224	2.1
65383	1.89224	2.1
65384	1.89224	2.09584
65385	1.89224	2.1
65386	1.89224	2.09254
65387	1.89226	0.450688
65388	1.89226	2.07424
65389	1.89226	1.65678
65390	1.89226	0.974743
65391	1.89226	1.58465
65392	1.89226	1.36186
65393	0.920975	2.1
65394	0.920979	1.88229
65395	1.03015	2.08448
65396	1.03015	1.05489
65397	0.992407	2.06467
65398	1.08759	2.1
65399	0.854346	1.86931
65400	0.87241	1.00307
65401	0.872397	1.99218
65402	1.04334	1.99312
65403	1.00965	2.01909
65404	1.0876	2.01745
65405	2.23101	2.1
65406	2.25948	2.1
65407	2.03577	2.07904
65408	2.05257	2.1
65409	4.29524	2.1
65410	0.810947	1.64472
65411	0.810947	2.1
65412	0.810947	2.06592
65413	0.810947	1.60521
65414	0.810934	2.05415
65415	0.810934	2.06198
65416	1.89227	2.1
65417	1.3517	2.1
65418	1.08132	2.08593
65419	1.08132	2.1
65420	1.08133	2.09736
65421	1.89226	2.07181

65422	1.89226	2.07624
65423	1.89226	1.72159
65424	1.89226	2.1
65425	1.89226	1.17638
65426	1.89226	2.1
65427	1.89226	2.1
65428	1.89226	2.1
65429	1.89226	2.08307
65430	1.89226	2.1
65431	1.89226	2.0782
65432	1.89226	2.1
65433	0.879432	2.1
65434	0.879432	2.08524
65435	0.900223	1.12721
65436	0.900223	2.0983
65437	0.54571	2.1
65438	0.743092	2.09814
65439	1.03656	2.09472
65440	1.19234	1.20438
65441	1.19234	2.05376
65442	0.743092	2.05783
65443	1.03656	2.08906
65444	0.545711	2.09536
65445	1.89226	0.686363
65446	2.38467	1.58836
65447	2.69293	0.316088
65448	2.69293	2.07272
65449	1.89226	0.221328
65450	1.89226	1.97714
65451	1.89226	1.89769
65452	1.89226	1.97579
65453	1.89226	0.842162
65454	1.89226	2.1
65455	3.78452	1.9438
65456	0.946129	2.1
65457	0.946129	2.1
65458	0.946133	1.78185
65459	0.946133	2.1
65460	0.946131	2.1
65461	0.946131	2.1
65462	0.946131	1.74495
65463	0.946131	2.1
65464	0.946131	2.1
65465	0.946131	2.1
65466	0.946131	2.1
65467	0.946131	2.1
65468	1.04846	1.70086
65469	1.04846	2.1
65470	1.05594	0.814026
65471	1.05594	2.04989
65472	0.743092	2.1
65473	0.912061	2.01361
65474	1.19234	0.752676
65475	1.34647	1.68578
65476	0.912061	1.62135
65477	1.34646	1.78191
65478	1.19234	1.90236
65479	0.743093	1.89168

65480	1.89226	0.930869
65481	1.89226	2.1
65482	1.89226	1.32761
65483	1.89226	1.82179
65484	3.78452	1.97097
65485	0.946128	2.1
65486	0.946128	2.1
65487	0.946133	2.1
65488	0.946133	1.81408
65489	0.94613	2.1
65490	0.94613	2.1
65491	0.946131	2.1
65492	0.946131	1.94899
65493	0.946131	2.1
65494	0.946131	2.1
65495	0.946131	2.1
65496	0.946131	2.1
65497	1.89226	1.43601
65498	1.89226	2.09855
65499	1.89226	0.364307
65500	1.89226	1.85845
65501	3.78452	1.40821
65502	1.89226	1.84298
65503	1.89226	1.82061
65504	1.89226	1.60085
65505	1.89226	2.1
65506	1.89226	0.773881
65507	1.89226	2.1
65508	1.89226	2.09275
65509	1.89226	2.09157
65510	1.89226	1.86885
65511	1.89226	2.1
65512	1.89226	1.71858
65513	1.89226	2.1
65514	0.946132	2.1
65515	0.946132	1.90088
65516	1.17758	0.736506
65517	1.17759	1.97627
65518	1.04514	2.09955
65519	1.17758	1.56848
65520	0.946133	0.775495
65521	0.946133	1.69837
65522	0.946107	1.7615
65523	1.17755	1.71433
65524	1.04517	1.77823
65525	0.946158	1.73934
65526	1.89226	2.1
65527	2.10171	2.1
65528	1.95064	2.1
65529	2.10171	2.1
65530	1.7412	2.1
65531	1.89226	2.1
65532	1.89226	2.1
65533	1.89226	2.1
65534	1.89226	2.1
65535	1.89226	2.1
65536	1.89226	2.1
65537	1.89226	2.1

65538	1.89226	2.1
65539	1.89226	2.1
65540	1.89226	2.1
65541	1.89226	2.1
65542	3.78453	2.1
65543	0.946133	2.1
65544	0.946133	2.1
65545	0.946132	2.1
65546	0.946132	2.1
65547	0.946133	2.1
65548	0.946133	2.1
65549	0.946132	2.1
65550	0.946132	2.1
65551	0.946132	2.1
65552	0.946132	2.1
65553	0.946132	2.1
65554	0.946132	2.1
65555	1.70303	2.1
65556	1.95064	2.1
65557	1.74039	2.1
65558	1.95064	2.1
65559	1.69368	2.1
65560	1.89226	2.1
65561	1.89226	2.1
65562	1.89226	2.1
65563	1.89226	2.1
65564	1.89226	2.1
65565	3.78453	2.1
65566	0.946132	2.1
65567	0.946132	2.1
65568	0.946132	2.1
65569	0.946132	2.1
65570	0.946133	2.1
65571	0.946133	2.1
65572	0.946132	2.1
65573	0.946132	2.1
65574	0.946107	2.1
65575	0.946107	2.1
65576	0.946158	2.1
65577	0.946158	2.1
65578	0.946133	2.1
65579	0.946133	2.1
65580	0.946132	2.1
65581	0.946132	2.1
65582	0.946133	2.1
65583	0.946133	2.1
65584	0.946132	2.1
65585	0.946132	2.1
65586	0.946107	2.1
65587	0.946107	2.1
65588	0.946158	2.1
65589	0.946158	2.1
65590	1.89226	2.1
65591	1.89226	2.1
65592	1.89226	2.1
65593	1.89226	2.1
65594	1.89226	2.1
65595	1.89226	2.1

65596	1.89226	2.1
65597	1.89226	2.1
65598	1.89226	2.1
65599	1.89226	2.1
65600	1.89226	2.1
65601	1.89226	2.1
65602	2.5198	2.1
65603	2.45893	2.1
65604	2.27322	2.1
65605	2.27322	2.1
65606	2.63576	2.1
65607	2.63576	2.1
65608	1.24045	2.1
65609	1.11906	2.1
65610	1.13578	2.1
65611	0.916205	2.1
65612	1.12539	2.1
65613	1.08404	2.1
65614	1.08484	2.1
65615	1.08484	2.1
65616	1.22836	2.1
65617	0.919363	2.1
65618	1.16507	2.1
65619	0.924864	2.1
65620	1.89226	2.1
65621	1.89226	2.1
65622	1.89226	2.1
65623	1.89226	2.1
65624	1.89226	2.1
65625	1.89226	2.1
65626	1.89226	2.1
65627	1.89226	2.1
65628	1.89226	2.1
65629	1.89226	2.1
65630	1.89226	2.1
65631	1.89226	2.1
65632	2.6946	2.1
65633	2.63576	2.1
65634	2.5198	2.1
65635	2.5198	2.1
65636	2.8362	2.1
65637	2.8362	2.1
65638	1.3265	2.1
65639	1.24045	2.1
65640	0.983223	2.1
65641	0.954429	2.1
65642	1.06046	2.1
65643	1.10594	2.1
65644	1.1915	2.1
65645	1.1915	2.1
65646	1.31357	2.1
65647	1.09005	2.1
65648	1.29145	2.1
65649	0.995825	2.1
65650	1.89226	2.1
65651	1.89226	2.1
65652	1.89226	2.1
65653	1.89226	2.1

65654	3.78452	2.1
65655	1.89226	2.1
65656	1.89226	2.1
65657	1.89226	2.1
65658	1.89226	2.1
65659	3.78453	2.1
65660	1.91802	2.1
65661	2.27409	2.1
65662	2.48048	2.1
65663	2.22264	2.1
65664	1.91802	2.1
65665	1.66018	2.1
65666	1.13704	2.1
65667	1.24023	2.1
65668	1.22947	2.1
65669	1.31788	2.1
65670	1.23859	2.1
65671	1.23859	2.1
65672	1.23111	2.1
65673	1.23111	2.1
65674	1.24024	2.1
65675	1.31788	2.1
65676	1.13704	2.1
65677	1.22947	2.1
65678	1.89226	2.1
65679	1.89226	2.1
65680	1.89226	2.1
65681	1.89226	2.1
65682	1.89226	2.1
65683	1.89226	2.1
65684	1.89226	2.1
65685	1.89226	2.1
65686	1.89226	2.1
65687	1.89226	2.1
65688	3.78453	2.1
65689	1.05568	2.1
65690	0.959008	2.1
65691	1.32068	2.1
65692	1.24024	2.1
65693	1.15206	2.1
65694	1.15206	2.1
65695	1.14386	2.1
65696	1.14386	2.1
65697	1.05568	2.1
65698	1.32068	2.1
65699	0.959008	2.1
65700	1.24024	2.1
65701	1.32068	2.1
65702	1.24023	2.1
65703	1.31788	2.1
65704	1.4181	2.1
65705	1.31436	2.1
65706	1.31436	2.1
65707	1.3242	2.1
65708	1.3242	2.1
65709	1.32068	2.1
65710	1.4181	2.1
65711	1.24024	2.1

65712	1.31788	2.1
65713	1.89226	2.1
65714	1.89226	0.784045
65715	1.89226	1.89926
65716	1.89226	1.92288
65717	1.89226	1.88667
65718	1.89226	2.00535
65719	1.02925	0.0884643
65720	1.16678	0.597894
65721	0.946132	0.596541
65722	0.946132	1.98444
65723	0.935677	0.64327
65724	0.935677	0.129839
65725	1.18902	1.84356
65726	1.21562	1.27419
65727	1.13433	0.497706
65728	1.04071	0.525983
65729	0.851553	0.711517
65730	1.06075	0.721299
65731	1.89226	0.807561
65732	1.89226	0.651506
65733	1.89226	0.492627
65734	1.89226	0.156221
65735	1.89226	0.294636
65736	1.89226	0.803999
65737	1.89226	1.99019
65738	1.89226	0.305803
65739	1.89226	0.775345
65740	1.89226	0.681595
65741	3.78453	0.638175
65742	0.946128	0.648727
65743	0.946128	0.645719
65744	0.946133	0.380907
65745	0.946133	0.415687
65746	0.946131	0.223953
65747	0.946131	0.576595
65748	0.94613	0.346592
65749	0.94613	0.37499
65750	0.946131	0.634636
65751	0.946131	0.206417
65752	0.946131	0.195554
65753	0.946131	0.134924
65754	0.946132	0.945736
65755	0.946132	0.542581
65756	0.946132	1.72091
65757	0.946132	0.794063
65758	0.946132	0.637819
65759	0.946132	0.0989283
65760	0.946131	0.925815
65761	0.946131	1.68287
65762	0.946132	0.827275
65763	0.946132	0.873412
65764	0.946132	0.619654
65765	0.946132	0.717431
65766	0.946131	2.04076
65767	0.946131	1.64415
65768	0.946131	1.9884
65769	0.946131	0.413515

65770	0.946131	0.7117
65771	0.946131	2.02244
65772	0.946131	0.996692
65773	0.946131	0.379586
65774	0.946131	1.23901
65775	0.946131	1.28375
65776	0.946131	1.3409
65777	0.946131	1.28791
65778	1.89226	1.9998
65779	1.89226	1.84893
65780	1.89226	1.52333
65781	1.89226	2.1
65782	1.89226	1.12756
65783	1.89226	2.1
65784	1.89226	1.19454
65785	1.89226	0.554102
65786	1.89226	0.947001
65787	1.89226	2.06847
65788	1.89226	0.457845
65789	1.89226	2.1
65790	1.89226	1.98724
65791	1.89226	1.44529
65792	1.89226	1.30794
65793	1.89226	2.1
65794	1.89226	0.790952
65795	1.89226	2.1
65796	1.89226	2.09378
65797	1.89226	2.05902
65798	1.89226	1.24943
65799	1.89226	2.1
65800	3.78452	2.05667
65801	0.946131	2.05718
65802	0.946131	1.38231
65803	1.17195	0.480902
65804	1.17195	1.91505
65805	0.958489	2.06504
65806	1.17195	1.26559
65807	0.946129	0.603042
65808	0.946129	1.78147
65809	0.946131	1.5896
65810	1.17195	1.5802
65811	0.958487	1.55461
65812	0.94613	1.60907
65813	2.69656	2.1
65814	2.17516	2.1
65815	2.41885	2.1
65816	2.41885	2.1
65817	2.46476	2.1
65818	2.46476	2.1
65819	2.69657	2.1
65820	2.30233	1.67877
65821	2.30233	2.1
65822	2.59476	2.09886
65823	2.46477	2.1
65824	2.7572	2.1
65825	1.89226	2.1
65826	1.89226	0.618231
65827	1.89226	1.42623

65828	1.89226	1.38752
65829	1.89226	1.26991
65830	1.89226	1.7738
65831	1.89226	2.1
65832	1.89226	2.1
65833	1.89226	2.1
65834	1.89226	1.46622
65835	3.78452	2.1
65836	2.46477	2.1
65837	2.17516	2.1
65838	1.80252	2.1
65839	2.09715	2.09179
65840	4.27231	2.1
65841	2.46477	2.1
65842	2.09715	2.0537
65843	2.30233	2.1
65844	2.30233	2.07023
65845	1.96416	1.29623
65846	2.09715	1.20605
65847	1.89227	2.02768
65848	1.89227	0.461428
65849	1.89227	1.20687
65850	1.89227	1.33753
65851	1.89227	1.24567
65852	1.89227	1.70588
65853	1.89226	2.1
65854	1.89226	1.3177
65855	1.89226	2.07755
65856	1.89226	2.08878
65857	3.78452	2.08281
65858	0.946132	2.1
65859	0.946132	2.1
65860	0.946132	2.1
65861	0.946132	2.1
65862	0.946132	2.1
65863	0.946132	2.1
65864	0.946132	2.1
65865	0.946132	2.1
65866	0.946132	2.1
65867	0.946132	2.1
65868	0.946131	2.1
65869	0.946131	2.1
65870	1.89226	2.1
65871	1.89226	2.1
65872	1.89226	2.1
65873	1.89226	2.1
65874	3.78453	2.1
65875	1.89226	2.1
65876	1.89226	2.1
65877	1.89226	2.1
65878	1.89226	2.1
65879	1.89226	2.1
65880	1.89226	2.1
65881	1.89226	2.1
65882	1.63707	2.1
65883	1.63707	2.1
65884	1.38859	2.1
65885	1.89226	2.1

65886	1.64377	2.1
65887	0.94613	2.1
65888	0.94613	2.1
65889	0.94613	2.1
65890	0.94613	2.1
65891	0.946131	2.1
65892	0.946131	2.1
65893	0.946129	2.1
65894	0.946129	2.1
65895	0.94613	2.1
65896	0.94613	2.1
65897	0.94613	2.1
65898	0.94613	2.1
65899	2.15888	2.1
65900	1.99309	2.1
65901	2.16841	2.1
65902	2.16841	2.1
65903	2.02311	2.08243
65904	2.02311	1.92262
65905	1.89226	2.07644
65906	1.89226	2.05293
65907	1.89226	2.1
65908	1.89226	2.07471
65909	1.89226	2.1
65910	1.89226	2.02557
65911	1.89226	2.08498
65912	1.89226	2.1
65913	1.89226	2.1
65914	1.89226	2.09706
65915	1.89226	2.1
65916	1.89226	2.06945
65917	1.99309	2.1
65918	1.96035	2.1
65919	1.72526	2.1
65920	1.72526	2.1
65921	2.16841	2.1
65922	2.16841	2.1
65923	2.15888	2.1
65924	2.16841	2.1
65925	2.3111	2.1
65926	2.35938	2.1
65927	4.51826	2.1
65928	1.89226	2.1
65929	1.89226	2.1
65930	1.89226	2.09776
65931	1.89226	1.93578
65932	3.78452	2.1
65933	1.89226	2.1
65934	1.89226	2.1
65935	1.89226	2.1
65936	1.89226	1.88581
65937	3.78452	2.1
65938	2.16841	2.1
65939	1.96035	2.1
65940	2.35938	2.1
65941	2.18493	2.1
65942	4.31973	2.1
65943	1.89226	2.1

65944	1.89226	2.1
65945	1.89226	2.1
65946	1.89226	2.1
65947	3.78453	2.1
65948	1.89226	2.1
65949	1.89226	2.1
65950	1.89226	2.1
65951	1.89226	2.1
65952	3.78452	2.1
65953	0.946158	2.1
65954	0.946158	2.1
65955	0.818509	2.1
65956	0.818509	2.1
65957	0.933975	2.1
65958	0.818539	2.1
65959	0.946128	2.1
65960	0.946128	2.1
65961	0.94613	2.1
65962	0.933974	2.1
65963	0.818537	2.1
65964	0.946129	2.1
65965	0.946131	2.1
65966	0.946131	2.1
65967	0.946131	2.1
65968	0.946131	2.1
65969	0.946132	2.1
65970	0.946132	2.1
65971	0.946129	2.1
65972	0.946129	2.1
65973	0.946131	2.1
65974	0.946131	2.1
65975	0.94613	2.1
65976	0.94613	2.1
65977	1.89226	2.1
65978	1.89226	2.1
65979	1.89226	2.1
65980	1.89226	2.1
65981	3.78453	2.1
65982	1.89226	2.1
65983	1.89226	2.1
65984	1.89226	2.1
65985	1.89226	2.1
65986	3.78452	2.1
65987	1.89226	2.1
65988	1.8706	2.1
65989	1.8706	2.1
65990	1.68112	2.1
65991	2.0815	2.1
65992	1.88715	2.1
65993	0.94613	2.1
65994	0.94613	2.1
65995	0.94613	2.1
65996	0.94613	2.1
65997	0.946131	2.1
65998	0.946131	2.1
65999	0.946129	2.1
66000	0.946129	2.1
66001	0.94613	2.1

66002	0.94613	2.1
66003	0.94613	2.1
66004	0.94613	2.1
66005	1.76401	2.1
66006	2.60983	2.1
66007	2.50461	2.1
66008	2.35897	2.1
66009	1.90965	2.1
66010	1.76401	2.1
66011	1.89226	2.1
66012	1.89226	2.1
66013	1.89226	2.1
66014	1.89226	2.1
66015	3.78452	2.1
66016	1.89226	2.1
66017	1.89226	2.1
66018	1.89226	2.1
66019	1.89226	2.1
66020	1.89226	2.1
66021	1.89226	2.1
66022	1.90965	2.1
66023	2.83834	2.1
66024	2.7538	2.1
66025	2.60983	2.1
66026	2.05361	2.1
66027	1.90965	2.1
66028	1.89226	2.1
66029	1.89226	2.1
66030	1.89226	2.1
66031	1.89226	2.1
66032	1.89226	2.1
66033	1.89226	2.1
66034	1.89226	2.1
66035	1.89226	2.1
66036	1.89226	2.1
66037	1.89226	2.1
66038	3.78452	2.1
66039	2.83834	2.1
66040	1.89226	2.1
66041	2.60983	2.1
66042	2.60983	2.1
66043	1.89226	2.1
66044	1.89226	2.1
66045	1.89226	2.1
66046	1.89226	2.1
66047	1.89226	2.1
66048	1.89226	2.1
66049	1.89226	2.1
66050	1.89226	2.1
66051	1.89226	2.1
66052	1.89226	2.1
66053	1.89226	2.1
66054	1.89226	2.07737
66055	1.89226	2.1
66056	1.89226	2.07089
66057	2.44785	2.1
66058	1.80223	2.1
66059	2.80894	2.1

66060	2.18296	2.1
66061	4.63081	2.1
66062	1.89226	2.1
66063	1.89226	2.1
66064	1.89226	2.1
66065	1.89226	2.1
66066	1.89226	2.1
66067	1.89226	2.1
66068	0.946131	1.95415
66069	0.946131	2.1
66070	0.946127	2.1
66071	0.946127	2.1
66072	0.946131	2.09733
66073	0.946131	2.1
66074	0.946128	2.1
66075	0.946128	2.1
66076	0.94613	2.1
66077	0.94613	2.1
66078	0.946129	2.1
66079	0.946129	2.1
66080	1.89226	2.1
66081	1.89226	2.1
66082	1.89226	2.1
66083	1.89226	2.1
66084	3.78452	2.1
66085	1.89226	2.1
66086	1.89226	2.1
66087	1.89226	2.1
66088	1.89226	2.1
66089	3.78453	2.1
66090	2.41083	2.1
66091	2.93937	2.1
66092	1.68761	2.1
66093	1.68761	2.1
66094	1.67248	2.1
66095	2.3957	2.1
66096	1.89226	2.1
66097	1.89226	2.1
66098	1.89226	2.1
66099	1.89226	2.1
66100	1.89226	2.1
66101	1.89226	2.1
66102	1.89227	2.1
66103	1.89227	2.1
66104	1.89227	2.1
66105	1.89227	2.1
66106	1.89227	2.1
66107	1.89227	2.1
66108	1.89224	1.02921
66109	1.89224	0.399076
66110	1.89224	0.690042
66111	1.89224	0.762589
66112	3.78448	0.439881
66113	1.89224	2.07185
66114	1.89224	0.978888
66115	1.89224	1.99779
66116	1.89224	1.13153
66117	1.89224	1.9701

66118	1.89224	1.41844
66119	1.11152	0.684146
66120	1.29346	1.42945
66121	0.978596	1.98171
66122	0.918324	2.05495
66123	1.06821	1.98501
66124	1.06821	0.634963
66125	0.985654	1.89921
66126	1.15278	2.05045
66127	1.07693	1.90226
66128	0.902432	1.87796
66129	0.968939	1.9229
66130	1.34644	1.80911
66131	0.946132	0.456269
66132	0.946132	0.451369
66133	0.946132	0.629239
66134	0.946132	0.599699
66135	0.946131	0.474552
66136	0.946131	0.305604
66137	0.946132	0.693155
66138	0.946132	0.483477
66139	0.946132	0.531163
66140	0.946132	0.435566
66141	0.946132	0.275041
66142	0.946132	0.325572
66143	0.946133	1.19833
66144	0.946133	0.731072
66145	0.946132	1.56494
66146	0.946132	1.88274
66147	0.946132	1.51512
66148	0.946132	0.310454
66149	0.946133	2.07867
66150	0.946133	0.968489
66151	0.946132	1.43884
66152	0.946132	1.33981
66153	0.946132	0.962768
66154	0.946132	1.03603
66155	1.89224	2.06303
66156	1.89224	2.1
66157	1.89224	2.1
66158	1.89224	2.1
66159	1.89224	2.1
66160	1.89224	2.1
66161	1.89224	2.1
66162	1.89224	2.1
66163	1.89224	2.1
66164	1.89224	2.1
66165	1.89224	2.1
66166	1.89224	2.1
66167	1.89226	2.1
66168	1.89226	2.1
66169	1.89226	2.1
66170	1.89226	2.1
66171	3.78453	2.1
66172	1.11056	2.1
66173	1.21595	2.1
66174	1.12763	2.1
66175	1.12763	2.1

66176	1.23339	2.1
66177	1.35697	2.1
66178	1.03581	2.1
66179	0.95954	2.1
66180	0.89761	2.1
66181	1.26602	2.1
66182	1.12749	2.1
66183	1.45307	2.1
66184	2.71775	2.1
66185	1.89226	2.1
66186	1.89226	2.1
66187	1.89226	2.1
66188	2.47025	2.1
66189	2.47025	2.1
66190	0.94613	2.1
66191	0.94613	2.1
66192	0.94613	2.1
66193	0.94613	2.1
66194	0.946128	2.1
66195	0.946128	2.1
66196	0.946132	2.1
66197	0.946132	2.1
66198	0.94613	2.1
66199	0.94613	2.1
66200	0.94613	2.1
66201	0.94613	2.1
66202	1.89226	1.8326
66203	1.89226	2.1
66204	1.89226	2.1
66205	1.89226	1.94138
66206	1.89226	2.1
66207	1.89226	1.94724
66208	1.89226	1.76731
66209	1.89226	2.1
66210	1.89226	2.1
66211	1.89226	1.54739
66212	1.89226	2.1
66213	1.89226	1.62598
66214	0.946132	2.1
66215	0.946132	2.1
66216	0.946133	2.1
66217	0.946133	2.1
66218	0.946133	2.1
66219	0.946133	2.1
66220	0.946132	2.1
66221	0.946132	2.1
66222	0.946133	2.1
66223	0.946133	2.1
66224	0.946132	2.1
66225	0.946132	2.1
66226	1.89226	1.89607
66227	1.89226	2.1
66228	1.89226	2.1
66229	1.89226	1.96179
66230	1.89226	2.1
66231	1.89226	1.9661
66232	0.946131	1.82896
66233	0.946131	1.76151

66234	0.946127	2.1
66235	0.946127	2.1
66236	0.946131	1.93111
66237	0.946131	2.1
66238	0.946127	2.1
66239	0.946127	1.99338
66240	0.94613	2.05732
66241	0.94613	2.05219
66242	0.946129	2.06311
66243	0.946129	2.0604
66244	1.94291	2.1
66245	2.00118	2.1
66246	2.36337	2.1
66247	2.41217	2.1
66248	4.35508	2.1
66249	1.89226	2.1
66250	1.89226	2.1
66251	1.89226	2.1
66252	1.89226	2.1
66253	3.78452	2.1
66254	1.89226	2.1
66255	1.89226	2.1
66256	1.89226	2.1
66257	1.89226	2.1
66258	3.78452	2.1
66259	1.06209	2.1
66260	1.06209	2.1
66261	1.05987	2.1
66262	1.05987	2.1
66263	0.920378	2.1
66264	1.12137	2.1
66265	1.00059	2.1
66266	1.20608	2.1
66267	1.20608	2.1
66268	1.12137	2.1
66269	1.00059	2.1
66270	0.920376	2.1
66271	2.77222	2.1
66272	2.41216	2.1
66273	2.77002	2.1
66274	2.36337	2.1
66275	2.81882	2.1
66276	2.41216	2.1
66277	1.89226	2.1
66278	1.89226	2.1
66279	1.89226	2.1
66280	1.89226	2.1
66281	1.89226	2.1
66282	1.89226	2.1
66283	1.89226	2.1
66284	1.89226	2.1
66285	1.89226	2.1
66286	1.89226	2.1
66287	3.78451	2.1
66288	2.41216	2.1
66289	2.24273	2.1
66290	2.81882	2.1
66291	2.68473	2.1

66292	5.09689	2.1
66293	1.89226	2.1
66294	1.89226	2.1
66295	1.89226	2.1
66296	1.89226	2.1
66297	3.78453	2.1
66298	2.17614	2.1
66299	2.65326	2.1
66300	1.93101	2.1
66301	2.58552	2.1
66302	4.58427	2.1
66303	2.65326	2.1
66304	1.89226	2.1
66305	1.89226	2.1
66306	1.89226	2.1
66307	2.58552	2.1
66308	2.58552	2.1
66309	1.89226	2.1
66310	1.89226	2.1
66311	1.89226	2.1
66312	1.89226	2.1
66313	3.78453	2.1
66314	1.89226	2.1
66315	1.89226	2.1
66316	1.89226	2.1
66317	1.89226	2.1
66318	3.78453	2.1
66319	1.93101	2.1
66320	2.58552	2.1
66321	1.46507	2.1
66322	2.52893	2.1
66323	4.45994	2.1
66324	1.26446	2.1
66325	1.26446	2.1
66326	0.946132	2.1
66327	0.946132	2.1
66328	0.94613	2.1
66329	0.94613	2.1
66330	1.29276	2.1
66331	1.26446	2.1
66332	1.26446	2.1
66333	0.94613	2.1
66334	0.94613	2.1
66335	1.29276	2.1
66336	1.89226	2.1
66337	1.89226	2.1
66338	1.89226	2.1
66339	1.89226	2.1
66340	3.78453	2.1
66341	1.89226	2.1
66342	1.89226	2.1
66343	1.89226	2.1
66344	1.89226	2.1
66345	3.78453	2.1
66346	1.83378	2.1
66347	2.52893	2.1
66348	1.75592	2.1
66349	2.49844	2.1

66350	4.28485	2.1
66351	1.24922	2.1
66352	1.24922	2.1
66353	0.946133	2.1
66354	0.946133	2.1
66355	0.946131	2.1
66356	0.946131	2.1
66357	1.26446	2.1
66358	1.24922	2.1
66359	1.24922	2.1
66360	0.946131	2.1
66361	1.26446	2.1
66362	0.94613	2.1
66363	0.946132	2.1
66364	0.946132	2.1
66365	0.946132	2.1
66366	0.946132	2.1
66367	0.946132	2.1
66368	0.946132	2.1
66369	0.946132	2.1
66370	0.946132	2.1
66371	0.946132	2.1
66372	0.946132	2.1
66373	0.946131	2.1
66374	0.946131	2.1
66375	1.89226	2.1
66376	1.89226	2.1
66377	1.89226	2.1
66378	1.89226	2.1
66379	1.89226	2.1
66380	1.89226	2.1
66381	1.0465	2.1
66382	1.0465	2.1
66383	1.06047	2.1
66384	1.06047	2.1
66385	1.25687	2.1
66386	1.24922	2.1
66387	0.87796	2.1
66388	0.857749	2.1
66389	0.857749	2.1
66390	1.25687	2.1
66391	0.877959	2.1
66392	1.24922	2.1
66393	2.49843	2.1
66394	1.89226	2.1
66395	2.51374	2.1
66396	1.89226	2.1
66397	4.406	2.1
66398	1.89226	2.1
66399	1.89226	2.1
66400	1.89226	2.1
66401	1.89226	2.1
66402	1.89226	2.1
66403	1.89226	2.1
66404	1.89226	2.07752
66405	1.89226	2.1
66406	1.89226	2.1
66407	1.89226	2.08011

66408	1.89226	2.1
66409	1.89226	2.08671
66410	1.89226	2.1
66411	1.89226	2.07874
66412	1.89226	2.1
66413	1.89226	2.07825
66414	1.89226	2.1
66415	1.89226	2.08186
66416	1.03835	2.1
66417	1.03835	2.1
66418	1.15388	2.1
66419	0.94267	2.1
66420	0.736433	2.1
66421	0.849239	2.1
66422	1.15014	2.1
66423	1.39058	2.1
66424	0.838639	2.1
66425	1.39056	2.1
66426	1.09399	2.1
66427	0.736444	2.1
66428	1.89226	2.00905
66429	1.89226	2.1
66430	1.89226	2.1
66431	1.89226	1.99679
66432	1.89226	2.1
66433	1.89226	2.01397
66434	0.94613	1.99386
66435	0.94613	2.00941
66436	0.946127	2.1
66437	0.946127	2.1
66438	0.946131	2.1
66439	0.946131	1.94665
66440	0.946126	2.1
66441	0.946126	2.03923
66442	0.946128	2.1
66443	0.946128	2.1
66444	0.946128	2.09349
66445	0.946128	2.09746
66446	1.89226	2.07821
66447	1.89226	1.69103
66448	1.89226	1.46313
66449	1.89226	2.0859
66450	3.78453	1.89583
66451	0.946133	1.39183
66452	0.946133	0.911053
66453	0.946128	1.88132
66454	0.946128	1.75979
66455	0.946131	1.79589
66456	0.946131	1.49452
66457	0.946131	0.780437
66458	0.946131	1.66453
66459	0.946131	1.46386
66460	0.946131	1.48672
66461	0.94613	1.57659
66462	0.94613	1.56008
66463	1.89226	1.33942
66464	1.89226	2.04609
66465	1.89226	2.00953

66466	1.89226	1.53645
66467	1.89226	2.02707
66468	1.89226	1.64546
66469	1.89226	0.837237
66470	1.89226	1.90671
66471	1.89226	1.72414
66472	1.89226	1.0752
66473	1.89226	1.6721
66474	1.89226	1.24679
66475	1.89226	0.882649
66476	1.89226	2.00051
66477	1.89226	1.74561
66478	1.89226	1.37878
66479	1.89226	1.7492
66480	1.89226	1.46904
66481	1.89226	1.40362
66482	2.67388	2.0885
66483	2.23684	2.02437
66484	2.23684	1.75228
66485	1.89226	2.0429
66486	1.89226	1.8036
66487	1.89226	2.1
66488	1.89226	2.1
66489	1.89226	2.1
66490	1.89226	2.1
66491	1.89226	2.1
66492	1.89226	2.1
66493	1.72197	2.1
66494	2.51374	2.1
66495	2.58004	2.1
66496	2.58004	2.1
66497	1.7155	2.1
66498	1.7155	2.1
66499	2.51374	2.1
66500	1.89226	2.1
66501	1.89226	2.1
66502	1.89226	2.1
66503	2.58003	2.1
66504	2.58003	2.1
66505	0.946132	2.1
66506	0.946132	2.1
66507	0.946132	2.1
66508	0.946132	2.1
66509	0.946132	2.1
66510	0.946132	2.1
66511	0.946132	2.1
66512	0.946132	2.1
66513	0.946131	2.1
66514	0.946131	2.1
66515	0.946132	2.1
66516	0.946132	2.1
66517	1.89226	2.1
66518	1.89226	2.1
66519	1.89226	2.1
66520	1.89226	2.1
66521	3.78453	2.1
66522	1.08262	2.1
66523	1.08262	2.1

66524	1.09674	2.1
66525	1.09674	2.1
66526	1.34652	2.1
66527	1.29002	2.1
66528	0.860984	2.1
66529	0.889338	2.1
66530	1.34652	2.1
66531	0.889338	2.1
66532	0.860983	2.1
66533	1.29002	2.1
66534	1.34652	2.1
66535	1.34652	2.1
66536	0.946133	2.1
66537	0.946133	2.1
66538	0.946131	2.1
66539	0.946131	2.1
66540	1.34652	2.1
66541	1.29002	2.1
66542	1.34652	2.1
66543	0.946131	2.1
66544	0.94613	2.1
66545	1.29002	2.1
66546	1.89226	2.1
66547	1.89226	2.1
66548	1.89226	2.1
66549	1.89226	2.1
66550	1.89226	2.1
66551	1.89226	2.1
66552	1.89226	2.1
66553	1.89226	2.1
66554	1.89226	2.1
66555	1.89226	2.1
66556	1.89226	2.1
66557	1.89226	2.1
66558	0.946131	2.1
66559	0.946131	2.1
66560	0.946131	2.1
66561	0.946131	2.1
66562	0.946131	2.1
66563	0.946131	2.1
66564	0.946131	2.1
66565	0.946131	2.1
66566	0.946131	2.1
66567	0.946131	2.1
66568	0.946131	2.1
66569	0.946131	2.1
66570	1.89226	2.1
66571	1.89226	2.1
66572	1.89226	2.1
66573	1.89226	2.1
66574	1.89226	2.1
66575	1.89226	2.1
66576	1.04103	2.1
66577	1.13849	2.1
66578	0.943014	2.1
66579	0.943014	2.1
66580	0.943591	2.1
66581	0.943591	2.1

66582	1.03757	2.1
66583	1.04098	2.1
66584	1.03761	2.1
66585	0.946131	2.1
66586	0.943041	2.1
66587	0.946131	2.1
66588	0.946128	2.1
66589	0.946128	2.1
66590	0.946133	2.1
66591	0.946133	2.1
66592	0.946131	2.1
66593	0.946131	2.1
66594	0.946131	2.1
66595	0.946131	2.1
66596	0.946131	2.1
66597	0.946131	2.1
66598	0.94613	2.1
66599	0.94613	2.1
66600	0.946132	2.1
66601	0.946132	2.1
66602	0.946132	2.1
66603	0.946132	2.1
66604	0.946132	2.1
66605	0.946132	2.1
66606	0.946132	2.1
66607	0.946132	2.1
66608	0.946132	2.1
66609	0.946132	2.1
66610	0.946131	2.1
66611	0.946131	2.1
66612	1.89226	0.879059
66613	1.89226	1.93921
66614	1.89226	1.07602
66615	1.89226	0.957869
66616	3.78452	1.4759
66617	0.946132	1.22955
66618	0.946132	1.19064
66619	0.946131	0.771457
66620	0.946131	0.856683
66621	0.946132	0.914291
66622	0.946132	2.01584
66623	0.946132	1.26144
66624	0.946132	1.19593
66625	0.946132	0.975861
66626	0.946132	1.14737
66627	0.946132	1.12973
66628	0.946132	1.38996
66629	0.935482	1.67156
66630	1.08999	1.60072
66631	1.24315	1.24659
66632	1.12559	1.39098
66633	1.21343	1.04911
66634	0.889397	2.08227
66635	1.01265	0.728052
66636	1.01265	1.81468
66637	1.07332	1.08438
66638	1.34292	1.3648
66639	0.865649	1.63219

66640	0.840024	1.86852
66641	1.15433	1.51333
66642	1.02858	1.70009
66643	1.34082	1.27371
66644	1.08357	1.03383
66645	1.10938	0.592848
66646	1.33349	2.05647
66647	1.12232	1.82299
66648	1.12232	0.611832
66649	1.21357	1.27867
66650	1.3408	1.33476
66651	1.02859	1.58182
66652	1.24211	1.84201
66653	1.89226	1.28382
66654	1.89226	0.401603
66655	1.89226	1.09151
66656	1.89226	2.04213
66657	1.89226	0.422105
66658	1.89226	1.06896
66659	1.89226	0.664533
66660	1.89226	1.07454
66661	1.89226	0.877129
66662	1.89226	1.68979
66663	1.89226	1.08319
66664	1.89226	1.47862
66665	2.0815	1.58096
66666	2.79029	1.19821
66667	2.36793	1.19833
66668	2.58428	1.97199
66669	2.38161	0.645385
66670	2.59922	1.5106
66671	1.89226	2.0778
66672	1.89226	1.86992
66673	1.89226	2.02525
66674	1.89226	2.00861
66675	1.89226	2.02371
66676	1.89226	2.01761
66677	1.89226	1.86509
66678	1.89226	0.742993
66679	1.89226	1.20053
66680	1.89226	1.45675
66681	3.78452	1.55393
66682	1.89226	2.01963
66683	1.89226	1.46466
66684	1.89226	1.95846
66685	1.89226	1.84233
66686	1.89226	1.96252
66687	1.89226	1.86739
66688	0.946132	1.65753
66689	0.946132	1.98576
66690	0.94613	2.09129
66691	0.94613	2.00761
66692	0.946131	2.05889
66693	0.946131	1.63431
66694	0.946131	1.98256
66695	0.946131	2.09722
66696	0.946131	2.02836
66697	0.946131	2.02768

66698	0.946131	2.00796
66699	0.946131	1.99702
66700	1.85178	1.90664
66701	1.89226	1.36746
66702	2.0815	0.984995
66703	2.27054	1.31736
66704	1.70303	0.684073
66705	1.5217	0.904828
66706	1.89226	2.05633
66707	1.89226	1.3795
66708	1.89226	1.91111
66709	1.89226	1.66795
66710	1.89226	1.97664
66711	1.89226	1.7914
66712	2.58823	0.215004
66713	2.02924	0.163003
66714	1.79398	0.146689
66715	1.79398	0.0939778
66716	2.76708	0.350615
66717	2.76708	0.142602
66718	0.946133	0.198589
66719	0.946133	0.253583
66720	1.29411	0.154252
66721	1.38353	0.100619
66722	0.946131	0.116931
66723	0.946131	0.163505
66724	1.38354	0.264619
66725	1.38354	0.116289
66726	0.946131	0.158229
66727	1.38354	0.156416
66728	0.94613	0.168255
66729	1.29411	0.168668
66730	1.89226	0.153999
66731	1.89226	0.0713695
66732	1.89226	0.128105
66733	1.89226	0.176892
66734	3.78452	0.131554
66735	0.946131	0.112057
66736	0.946131	0.12298
66737	0.946131	0.132487
66738	0.946131	0.147879
66739	0.946129	0.155851
66740	0.946129	0.0809243
66741	0.946133	0.17974
66742	0.946133	0.113076
66743	0.946131	0.141541
66744	0.946131	0.13306
66745	0.946131	0.0926799
66746	0.946131	0.101512
66747	1.89226	0.0704937
66748	1.89226	0.116147
66749	1.89226	0.161385
66750	1.89226	0.0925889
66751	3.78452	0.0802665
66752	0.94613	0.110889
66753	0.94613	0.0920258
66754	0.946131	0.0884383
66755	0.946131	0.0996132

66756	0.946128	0.108651
66757	0.946128	0.0702344
66758	0.946133	0.132976
66759	0.946133	0.0748384
66760	0.946131	0.107999
66761	0.946131	0.0933621
66762	0.946131	0.0748527
66763	0.946131	0.0741145
66764	0.946129	1.07847
66765	0.946129	0.863328
66766	1.13486	0.845428
66767	0.859753	1.07389
66768	0.851541	1.03265
66769	0.851541	0.849744
66770	1.34346	0.827806
66771	1.26212	0.837652
66772	0.851553	0.846535
66773	1.12403	0.864351
66774	0.851474	0.76049
66775	0.956163	0.899143
66776	0.946132	0.553458
66777	0.946132	0.530381
66778	0.946133	0.645498
66779	0.946133	0.515857
66780	0.946132	0.665224
66781	0.946132	0.503151
66782	0.946133	0.541739
66783	0.946133	0.527477
66784	0.946133	0.534252
66785	0.946133	0.578855
66786	0.946132	0.529535
66787	0.946132	0.55157
66788	1.89226	0.544591
66789	1.89226	0.743941
66790	1.89226	0.70924
66791	1.89226	0.680333
66792	3.78453	0.666701
66793	0.946128	1.18453
66794	0.946128	0.957325
66795	0.946132	1.40574
66796	0.946132	1.04655
66797	0.94613	1.42536
66798	0.94613	1.25265
66799	0.94613	1.15986
66800	0.94613	0.967012
66801	0.946131	1.16385
66802	0.946131	1.23026
66803	0.94613	1.25607
66804	0.94613	1.22004
66805	0.946132	0.402238
66806	0.946132	0.357354
66807	0.946133	0.421261
66808	0.946133	0.351408
66809	0.946132	0.424194
66810	0.946132	0.392753
66811	0.946133	0.351578
66812	0.946133	0.358803
66813	0.946132	0.366003

66814	0.946132	0.389072
66815	0.946132	0.372575
66816	0.946132	0.382936
66817	1.89226	0.445249
66818	1.89226	0.464127
66819	1.89226	0.574237
66820	1.89226	0.410784
66821	1.89226	0.56149
66822	1.89226	0.369086
66823	1.89226	0.847539
66824	1.89226	0.608542
66825	1.89226	0.595102
66826	1.89226	0.719314
66827	1.89226	0.634713
66828	1.89226	0.706841
66829	1.89226	2.1
66830	1.89226	2.1
66831	1.89226	2.1
66832	1.89226	2.1
66833	3.78452	2.1
66834	1.07652	2.1
66835	1.07652	2.1
66836	0.928749	2.1
66837	0.928749	2.1
66838	0.946132	2.1
66839	0.946132	2.1
66840	1.05914	2.1
66841	0.928746	2.1
66842	1.05914	2.1
66843	0.946131	2.1
66844	0.94613	2.1
66845	0.928747	2.1
66846	1.89226	2.1
66847	1.89226	2.1
66848	1.89226	2.1
66849	1.89226	2.1
66850	1.89226	2.1
66851	1.89226	2.1
66852	1.89227	2.1
66853	1.89227	2.1
66854	1.89227	2.1
66855	1.89227	2.1
66856	1.89227	2.1
66857	1.89227	2.1
66858	1.89226	2.1
66859	1.89226	2.1
66860	1.89226	2.1
66861	1.89226	2.1
66862	1.89226	2.1
66863	1.89226	2.1
66864	2.11828	2.1
66865	1.89226	2.1
66866	2.35845	2.1
66867	1.89226	2.1
66868	4.25071	2.1
66869	1.89226	2.1
66870	1.89226	2.1
66871	1.89226	2.1

66872	1.89226	2.1
66873	3.78453	2.1
66874	1.89226	2.1
66875	1.89226	2.1
66876	1.89226	2.1
66877	1.89226	2.1
66878	1.89226	2.1
66879	1.89226	2.1
66880	1.89226	0.931736
66881	1.89226	1.46175
66882	1.65573	1.42199
66883	1.65573	0.961808
66884	1.89226	1.38853
66885	1.89226	0.933123
66886	1.89226	0.662393
66887	1.89226	1.38608
66888	1.89226	1.32522
66889	1.89226	0.823518
66890	1.89226	1.3026
66891	1.89226	0.77999
66892	1.89226	0.803515
66893	1.89226	1.25771
66894	1.89226	1.32638
66895	1.89226	0.907857
66896	1.89226	1.28079
66897	1.89226	0.841281
66898	1.89226	0.586024
66899	1.89226	1.44831
66900	1.89226	1.31981
66901	1.89226	0.730566
66902	1.89226	1.28625
66903	1.89226	0.678256
66904	0.946128	0.681859
66905	0.946128	0.638709
66906	0.946125	1.12703
66907	0.946125	1.32
66908	0.94613	0.783832
66909	0.94613	1.45894
66910	0.946124	1.39469
66911	0.946124	0.705984
66912	0.946127	1.05317
66913	0.946127	1.12406
66914	0.946127	1.0496
66915	0.946127	0.993766
66916	1.89226	0.78167
66917	1.89226	1.57318
66918	1.89226	0.795618
66919	1.89226	0.551932
66920	3.78452	0.889859
66921	1.89226	1.48245
66922	1.89226	1.46253
66923	1.89226	1.06471
66924	1.89226	1.5186
66925	3.78453	1.36326
66926	1.05926	1.7977
66927	0.891358	2.02103
66928	0.946129	1.47946
66929	0.946129	1.76354

66930	0.946101	1.35042
66931	0.946101	2.08545
66932	0.891386	1.61925
66933	0.891386	1.81494
66934	0.891357	1.76399
66935	0.946129	1.65674
66936	0.94613	1.77838
66937	1.05926	1.73455
66938	0.946126	1.00097
66939	0.946126	1.32433
66940	0.94613	0.889215
66941	0.94613	1.37093
66942	0.946131	1.16821
66943	0.946131	1.41723
66944	0.946125	1.51962
66945	0.946125	0.553185
66946	0.946127	1.10473
66947	0.946127	0.965193
66948	0.946128	1.40731
66949	0.946128	1.21781
66950	1.89226	1.23958
66951	1.89226	0.601689
66952	1.89226	0.571214
66953	1.89226	1.64992
66954	1.89226	0.641726
66955	1.89226	1.70519
66956	1.89226	1.36031
66957	1.89226	1.67655
66958	1.89226	1.46825
66959	1.89226	1.87556
66960	1.89226	1.02478
66961	1.89226	1.85081
66962	1.16109	1.89883
66963	1.23243	1.94959
66964	0.659819	1.51118
66965	0.659819	1.72758
66966	0.697965	1.1188
66967	1.00087	2.03604
66968	0.891386	1.89436
66969	0.891386	1.67621
66970	0.946129	1.72961
66971	0.65979	1.46988
66972	0.891357	1.73191
66973	0.946129	1.64218
66974	1.89225	0.648233
66975	1.89225	1.22068
66976	1.89225	1.7936
66977	1.89225	0.597287
66978	1.89225	1.94853
66979	1.89225	0.88647
66980	1.79981	0.355441
66981	2.02514	0.502651
66982	2.33247	0.637119
66983	2.12891	0.3426
66984	4.15405	0.381419
66985	2.48284	1.18498
66986	2.02514	0.55827
66987	2.33247	0.649455

66988	2.02514	1.00046
66989	2.48567	0.618432
66990	2.17834	0.957581
66991	1.89226	0.525834
66992	1.89226	0.811481
66993	1.89226	0.947791
66994	1.89226	0.517862
66995	3.78453	0.599832
66996	1.89227	0.480648
66997	1.89227	0.293602
66998	1.89227	0.307535
66999	1.89227	0.423371
67000	1.89227	0.34377
67001	1.89227	0.445602
67002	2.50894	0.282118
67003	2.70118	0.454763
67004	2.12891	0.299636
67005	2.12891	0.390525
67006	2.7125	0.294552
67007	2.33247	0.412671
67008	1.89226	0.734241
67009	1.89226	0.42007
67010	1.89226	0.429166
67011	1.89226	0.659432
67012	1.89226	0.403596
67013	1.89226	0.608409
67014	0.946133	0.400657
67015	0.946133	0.390158
67016	0.946132	0.260459
67017	0.946132	0.261654
67018	0.946132	0.262366
67019	0.946132	0.39444
67020	0.946133	0.283959
67021	0.946133	0.393272
67022	0.946132	0.309278
67023	0.946132	0.295736
67024	0.946132	0.337985
67025	0.946132	0.34615
67026	1.90098	0.138149
67027	1.36463	0.163385
67028	1.56656	0.131421
67029	1.36463	0.163097
67030	2.04386	0.126101
67031	1.89227	0.180032
67032	1.89226	0.155724
67033	1.89226	0.166013
67034	1.89226	0.138798
67035	1.89226	0.180734
67036	1.89226	0.144465
67037	1.89226	0.186478
67038	1.89226	0.189441
67039	1.89226	0.226884
67040	1.89226	0.179694
67041	1.89226	0.259754
67042	1.89226	0.177754
67043	1.89226	0.256072
67044	1.89226	0.18017
67045	1.89226	0.232759

67046	1.89226	0.179368
67047	1.89226	0.233085
67048	1.89226	0.178374
67049	1.89226	0.243598
67050	1.89226	0.116412
67051	1.70303	0.134291
67052	2.1292	0.114318
67053	1.70303	0.142798
67054	2.32302	0.111813
67055	1.90098	0.149748
67056	1.89226	0.137962
67057	1.89226	0.146447
67058	1.89226	0.120745
67059	1.89226	0.162742
67060	1.89226	0.126927
67061	1.89226	0.168404
67062	1.89226	0.162632
67063	1.89226	0.172599
67064	1.89226	0.149651
67065	1.89226	0.210549
67066	1.89226	0.147067
67067	1.89226	0.205565
67068	1.89226	0.144552
67069	1.89226	0.148719
67070	1.89226	0.126062
67071	1.89226	0.167936
67072	1.89226	0.130461
67073	1.89226	0.178592
67074	1.89226	1.30523
67075	1.89226	1.90816
67076	1.89226	1.94399
67077	1.89226	1.3243
67078	3.78453	1.30029
67079	1.89226	1.94351
67080	1.89226	2.08823
67081	1.89226	1.63264
67082	1.89226	2.09353
67083	1.89226	1.90165
67084	1.89226	2.1
67085	1.89226	1.88143
67086	1.89226	1.23793
67087	1.89226	1.22703
67088	1.89226	1.65724
67089	3.78452	1.76358
67090	1.89224	1.96755
67091	1.89224	1.06514
67092	1.89224	1.61783
67093	1.89224	1.93699
67094	1.89224	1.41366
67095	1.89224	1.76556
67096	1.89224	1.96879
67097	1.89224	2.08512
67098	1.89224	2.01364
67099	1.89224	2.1
67100	1.89224	1.66824
67101	1.89224	2.08751
67102	1.31307	2.09835
67103	1.31307	2.0875

67104	0.888948	1.93172
67105	0.888948	1.83554
67106	1.2559	1.95946
67107	0.888979	2.0856
67108	0.946114	2.0811
67109	0.946114	1.8979
67110	0.946129	1.82792
67111	0.888989	1.94477
67112	0.946104	1.76925
67113	1.25588	1.92659
67114	1.89224	0.993437
67115	1.89224	1.94829
67116	1.89224	1.03674
67117	1.89224	1.90856
67118	1.89224	1.50792
67119	1.89224	2.01823
67120	1.79375	0.0864224
67121	1.47689	0.0849873
67122	1.97732	0.0698599
67123	2.26687	0.0774802
67124	3.74375	0.0745024
67125	2.05759	0.107747
67126	2.26686	0.0837423
67127	1.79374	0.0828124
67128	1.79374	0.126415
67129	2.49889	0.0782035
67130	2.49889	0.112894
67131	1.89226	0.128014
67132	1.89226	0.116336
67133	1.89226	0.0819443
67134	1.89226	0.1347
67135	3.78452	0.114668
67136	1.89227	0.0784638
67137	1.89227	0.0761616
67138	1.89227	0.1028
67139	1.89227	0.112181
67140	1.89227	0.0896945
67141	1.89227	0.101473
67142	1.97732	0.0773538
67143	2.26687	0.0939241
67144	2.6607	0.0705791
67145	2.41464	0.0772478
67146	4.68151	0.0754663
67147	1.89226	0.094458
67148	1.89226	0.174555
67149	1.89226	0.105059
67150	1.89226	0.0880619
67151	3.78452	0.098182
67152	1.89226	0.0922266
67153	1.89226	0.0846601
67154	1.89226	0.104927
67155	1.89226	0.184103
67156	1.89226	0.0766562
67157	1.89226	0.0976998
67158	1.89226	0.853794
67159	1.89226	1.86014
67160	1.89226	1.4608
67161	1.89226	0.863945

67162	3.78452	1.02313
67163	1.89226	1.01351
67164	1.89226	1.90921
67165	1.89226	0.644159
67166	1.89226	1.64212
67167	1.89226	1.05117
67168	1.89226	1.8739
67169	1.89226	1.83578
67170	1.89226	0.820977
67171	1.89226	1.00291
67172	1.89226	1.74535
67173	1.89226	0.788203
67174	1.89226	1.48179
67175	1.89226	1.22034
67176	1.89226	0.753097
67177	1.89226	1.08195
67178	1.89226	1.73513
67179	1.89226	0.620401
67180	1.89226	1.34084
67181	1.89226	0.855574
67182	2.45282	1.39347
67183	1.89226	1.54901
67184	1.89226	1.80332
67185	1.89226	0.602423
67186	1.89226	1.44032
67187	1.89226	0.755458
67188	1.89226	1.73683
67189	1.89226	1.02532
67190	1.89226	0.90752
67191	1.89226	1.8499
67192	1.89226	1.8409
67193	1.87308	0.077817
67194	1.88631	0.0847223
67195	1.63908	0.087309
67196	1.70302	0.105279
67197	2.08745	0.0746729
67198	2.16916	0.07789
67199	2.41019	0.0802368
67200	2.733	0.0704649
67201	2.08121	0.0806545
67202	2.08121	0.101153
67203	2.74362	0.0790852
67204	2.41464	0.0778365
67205	1.89226	0.0799588
67206	1.89226	0.117719
67207	1.89226	0.0753306
67208	1.89226	0.101011
67209	1.89226	0.0820281
67210	1.89226	0.1336
67211	0.946133	0.0912324
67212	0.946133	0.0867839
67213	0.946132	0.0981112
67214	0.946132	0.0988356
67215	0.946132	0.096614
67216	0.946132	0.130664
67217	0.946133	0.0815673
67218	0.946133	0.0962139
67219	0.946132	0.0805971

67220	0.946132	0.0918247
67221	0.946133	0.0918012
67222	0.946133	0.125953
67223	2.02912	0.0834567
67224	2.57016	0.0768841
67225	1.88631	0.085124
67226	1.88631	0.110025
67227	2.55301	0.0854025
67228	2.4102	0.0870237
67229	1.89226	0.111341
67230	1.89226	0.128121
67231	1.89226	0.0763886
67232	1.89226	0.08256
67233	3.78452	0.100522
67234	1.89227	0.104019
67235	1.89227	0.0913555
67236	1.89227	0.0977859
67237	1.89227	0.131945
67238	1.89227	0.0816923
67239	1.89227	0.0919447
67240	1.89226	2.09721
67241	1.89226	2.08805
67242	1.89226	2.05853
67243	1.89226	2.09841
67244	3.78452	2.09226
67245	1.2515	2.03517
67246	1.34788	2.08455
67247	0.946132	2.09702
67248	0.946132	2.08826
67249	0.973043	2.09681
67250	1.16766	2.08448
67251	0.919222	1.98862
67252	1.2159	2.09757
67253	0.946132	2.07495
67254	0.946132	2.08225
67255	1.33746	2.08846
67256	1.13537	2.08732
67257	1.89226	2.08768
67258	1.89226	2.09997
67259	1.89226	2.09989
67260	1.89226	2.09515
67261	1.89226	2.09814
67262	1.89226	2.09247
67263	1.89226	2.04449
67264	1.89226	2.08918
67265	1.89226	2.0823
67266	1.89226	2.0491
67267	3.78452	2.05908
67268	1.89227	2.07156
67269	1.89227	1.90672
67270	1.89227	2.06648
67271	1.89227	2.01536
67272	1.89227	2.08269
67273	1.89227	2.0011
67274	0.946132	2.06302
67275	0.946132	2.05575
67276	0.946129	2.08848
67277	0.946129	2.0905

67278	0.946131	2.07352
67279	0.946131	2.08989
67280	0.946131	2.08983
67281	0.946131	2.05311
67282	0.946131	2.07478
67283	0.946131	2.08015
67284	0.94613	2.07959
67285	0.94613	2.07481
67286	1.89226	1.52651
67287	1.89226	1.24487
67288	1.89226	1.25856
67289	1.89226	1.08372
67290	3.78453	1.35971
67291	0.946132	1.35005
67292	0.946132	1.14191
67293	0.946132	0.875873
67294	0.946132	1.16575
67295	0.946132	0.601965
67296	0.946132	1.85747
67297	0.946132	1.38669
67298	0.946132	1.45522
67299	0.946131	1.63071
67300	0.946131	1.02979
67301	0.946132	1.05981
67302	0.946132	1.15349
67303	1.89226	1.4858
67304	1.89226	0.602261
67305	1.89226	0.88765
67306	1.89226	1.07025
67307	1.89226	1.12713
67308	1.89226	1.02546
67309	1.89226	1.48966
67310	1.89226	1.01617
67311	1.89226	0.879805
67312	1.89226	0.870748
67313	3.78453	0.949874
67314	0.96614	1.12873
67315	0.96614	1.40463
67316	0.946131	0.760267
67317	0.946131	0.855973
67318	0.946131	0.862757
67319	0.96614	0.700579
67320	1.37736	1.41988
67321	0.946131	1.40226
67322	1.37736	1.66701
67323	0.96614	0.73051
67324	0.946131	1.25798
67325	0.946131	0.718512
67326	0.946129	0.813144
67327	0.946129	0.906701
67328	0.946128	1.05155
67329	0.946128	0.906295
67330	0.946132	0.416645
67331	0.946132	1.73652
67332	0.946126	1.21991
67333	0.946126	1.11263
67334	0.946129	0.780782
67335	0.946129	0.753097

67336	0.946129	0.749013
67337	0.946129	1.01965
67338	1.89226	0.106288
67339	1.65723	0.123374
67340	1.83117	0.122846
67341	1.65723	0.110999
67342	2.0662	0.133705
67343	1.89226	0.121447
67344	1.89226	0.106052
67345	1.89226	0.135491
67346	1.89226	0.118805
67347	1.89226	0.114038
67348	1.89226	0.124492
67349	1.89226	0.116844
67350	1.89226	0.106529
67351	1.89226	0.142066
67352	1.89226	0.125713
67353	1.89226	0.127483
67354	1.89226	0.127964
67355	1.89226	0.124335
67356	1.89226	0.106094
67357	1.89226	0.119917
67358	1.89226	0.115784
67359	1.89226	0.120914
67360	1.89226	0.118413
67361	1.89226	0.11926
67362	1.89226	0.101434
67363	1.83117	0.117009
67364	1.92807	0.114359
67365	1.83117	0.103636
67366	1.98916	0.12355
67367	1.89226	0.111638
67368	1.89226	0.110637
67369	1.89226	0.128263
67370	1.89226	0.110116
67371	1.89226	0.111807
67372	3.78453	0.116811
67373	1.89226	0.107962
67374	1.89226	0.127959
67375	1.89226	0.11869
67376	1.89226	0.119816
67377	1.89226	0.119893
67378	1.89226	0.118642
67379	1.89226	0.0983884
67380	1.89226	0.111711
67381	1.89226	0.109536
67382	1.89226	0.107258
67383	1.89226	0.111182
67384	1.89226	0.105574
67385	1.88037	0.108607
67386	1.89227	0.108937
67387	1.89227	0.114538
67388	1.89227	0.115074
67389	1.98599	0.115989
67390	1.98599	0.113381
67391	1.89226	0.108646
67392	1.89226	0.116555
67393	1.89226	0.116047

67394	1.89226	0.131648
67395	1.89226	0.111825
67396	1.89226	0.118103
67397	1.89226	0.123591
67398	1.89226	0.130353
67399	1.89226	0.125073
67400	1.89226	0.159817
67401	3.78452	0.107925
67402	1.89226	0.116067
67403	1.89226	0.134188
67404	1.89226	0.13742
67405	1.89226	0.115179
67406	1.89226	0.12978
67407	1.89226	0.1187
67408	1.90116	0.101977
67409	1.89227	0.100083
67410	1.89226	0.107022
67411	1.89227	0.105762
67412	1.88036	0.111969
67413	1.88037	0.0996767
67414	1.89226	0.0990208
67415	1.89226	0.105466
67416	1.89226	0.110562
67417	1.89226	0.115274
67418	1.89226	0.104048
67419	1.89226	0.102591
67420	0.946132	0.117648
67421	0.946132	0.112973
67422	0.946128	0.119385
67423	0.946128	0.119735
67424	0.94613	0.11951
67425	0.94613	0.120744
67426	0.94613	0.109327
67427	0.94613	0.117364
67428	0.946131	0.158265
67429	0.946131	0.14747
67430	0.94613	0.132239
67431	0.94613	0.16003
67432	1.89226	0.115263
67433	1.89226	0.106693
67434	1.89226	0.108849
67435	1.89226	0.11364
67436	1.89226	0.105432
67437	1.89226	0.107371
67438	1.89226	0.555286
67439	1.89226	1.35785
67440	2.35239	1.29099
67441	2.27873	0.622083
67442	1.89226	1.28543
67443	1.83301	0.632784
67444	2.68449	0.839933
67445	1.89226	1.84224
67446	1.89226	1.08583
67447	1.89226	1.26295
67448	3.78453	1.06201
67449	0.946132	1.78696
67450	0.946132	1.17423
67451	0.946129	1.08135

67452	0.946129	1.81852
67453	0.946131	1.82636
67454	0.946131	1.63038
67455	0.94613	1.15362
67456	0.94613	1.41128
67457	0.946131	1.61687
67458	0.946131	1.47171
67459	0.946131	1.49982
67460	0.946131	1.59175
67461	1.89226	0.657572
67462	1.89226	1.17971
67463	1.89226	1.48161
67464	1.89226	0.738589
67465	1.89226	1.47544
67466	1.89226	0.741325
67467	1.70303	0.556236
67468	1.89226	1.31915
67469	1.89226	0.585297
67470	1.89226	0.825079
67471	3.78453	0.640439
67472	1.89227	1.35099
67473	1.89227	1.44488
67474	1.89227	0.847291
67475	1.89227	1.76723
67476	3.78453	1.52772
67477	1.89226	1.21614
67478	1.89226	1.88045
67479	1.89226	1.40138
67480	1.89226	1.69296
67481	3.78452	1.55829
67482	1.89226	2.03333
67483	1.89226	2.0758
67484	1.89226	2.07485
67485	1.89226	2.01504
67486	3.78452	2.02789
67487	0.946132	1.91912
67488	0.946132	2.03053
67489	0.946129	2.08574
67490	0.946129	2.06351
67491	0.94613	2.05832
67492	0.94613	1.97653
67493	0.946131	2.07084
67494	0.946131	2.00962
67495	0.946131	2.00856
67496	0.946131	2.03439
67497	0.946131	2.02632
67498	0.946131	1.96888
67499	1.89226	2.06062
67500	1.89226	1.95076
67501	1.89226	1.98191
67502	1.89226	2.04086
67503	3.78452	2.0394
67504	1.89226	1.80057
67505	1.89226	2.01482
67506	1.89226	2.04364
67507	1.89226	1.78647
67508	1.89226	2.04552
67509	1.89226	1.79843

67510	1.89227	2.00822
67511	2.48308	1.59572
67512	1.89227	1.78361
67513	1.89227	1.93392
67514	3.78453	1.95417
67515	1.89226	1.93973
67516	1.89226	2.00224
67517	1.89226	2.03862
67518	1.89226	1.89451
67519	1.89226	2.03123
67520	1.89226	1.87535
67521	1.89226	1.9849
67522	1.89226	1.95079
67523	1.89226	2.00547
67524	1.89226	1.97365
67525	3.78453	2.01992
67526	0.946131	1.9409
67527	0.946131	2.02395
67528	0.946131	2.01342
67529	0.946131	2.06749
67530	0.946131	2.00942
67531	0.946131	1.98025
67532	0.946131	2.01344
67533	0.946131	2.02857
67534	0.946131	1.99684
67535	0.946131	2.03574
67536	0.946131	2.00875
67537	0.946131	1.95424
67538	1.23719	2.02591
67539	1.28439	1.97339
67540	0.94613	1.98568
67541	0.94613	1.99838
67542	1.28439	2.02046
67543	1.6431	2.04237
67544	0.946129	1.94876
67545	0.946129	1.95145
67546	1.28439	1.88958
67547	0.946131	1.82837
67548	1.21038	1.84009
67549	1.21038	1.91905
67550	1.89226	1.96661
67551	1.89226	1.91988
67552	1.89226	1.96223
67553	1.89226	1.91185
67554	1.89226	1.94976
67555	1.89226	1.89932
67556	1.89226	1.9422
67557	1.89226	1.96885
67558	1.89226	2.01647
67559	1.89226	1.84574
67560	1.89226	2.00489
67561	1.89226	1.81569
67562	1.89226	1.28818
67563	1.89226	0.42368
67564	1.89226	0.459146
67565	1.89226	0.676738
67566	1.89226	0.90531
67567	1.89226	1.2618

67568	0.946128	0.442021
67569	0.946128	0.609356
67570	0.946125	0.197811
67571	0.946125	0.386448
67572	0.94613	0.266832
67573	0.94613	0.326025
67574	0.946124	0.555998
67575	0.946124	0.679644
67576	0.946127	0.320824
67577	0.946127	0.239324
67578	0.946127	0.424836
67579	0.946127	0.60163
67580	1.89226	0.184849
67581	2.78671	0.221849
67582	2.59302	0.208474
67583	2.59302	0.210066
67584	1.89226	0.319445
67585	1.89226	0.270817
67586	1.89226	0.373321
67587	1.89226	0.385578
67588	1.89226	0.247219
67589	1.89226	0.246588
67590	1.89226	0.564923
67591	1.89226	0.494808
67592	1.89225	0.220522
67593	1.89225	0.117989
67594	1.89225	0.281528
67595	1.89225	0.203182
67596	1.89225	0.363368
67597	1.89225	0.174533
67598	0.979536	0.147328
67599	0.816597	0.167057
67600	1.15446	0.146121
67601	1.07423	0.129168
67602	1.08452	0.141796
67603	0.603522	0.139791
67604	1.70302	0.142538
67605	1.29344	0.121576
67606	1.21806	0.134749
67607	0.723758	0.121221
67608	1.37562	0.163879
67609	1.89226	0.0901509
67610	1.92807	0.105541
67611	1.90843	0.0982095
67612	1.92807	0.0935179
67613	1.87263	0.107864
67614	1.89226	0.100627
67615	0.946132	0.100144
67616	0.946132	0.102856
67617	0.946131	0.100033
67618	0.946131	0.105221
67619	0.946132	0.10646
67620	0.946132	0.118687
67621	0.946132	0.107781
67622	0.946132	0.101318
67623	0.946132	0.0933675
67624	0.946132	0.100165
67625	0.946132	0.103101

67626	0.946132	0.106522
67627	1.89226	0.112482
67628	1.89226	0.115927
67629	1.89226	0.102182
67630	1.89226	0.0999571
67631	3.78452	0.118954
67632	1.89226	0.0898794
67633	1.89226	0.101375
67634	1.89226	0.0961297
67635	1.89226	0.0922998
67636	1.89226	0.100057
67637	1.89226	0.0942048
67638	1.70303	0.0789938
67639	1.90843	0.0919512
67640	1.77002	0.0845949
67641	1.90843	0.081876
67642	1.75709	0.0890003
67643	1.89226	0.0862711
67644	1.89226	0.0929714
67645	1.89226	0.099727
67646	1.89226	0.0864756
67647	1.89226	0.095601
67648	3.78453	0.0912789
67649	1.89226	0.0984124
67650	1.89226	0.101386
67651	1.89226	0.0932285
67652	1.89226	0.0852746
67653	3.78452	0.0867667
67654	0.946131	0.0819163
67655	0.946131	0.0781005
67656	0.946131	0.0828562
67657	0.946131	0.0819452
67658	0.946129	0.0809227
67659	0.946129	0.0822987
67660	0.946133	0.0891966
67661	0.946133	0.0814738
67662	0.946131	0.0784812
67663	0.946131	0.0777957
67664	0.946131	0.0878512
67665	0.946131	0.089657
67666	1.89226	0.137343
67667	2.10275	0.332456
67668	1.76377	0.308001
67669	1.76377	0.194396
67670	1.89226	0.270936
67671	1.89226	0.164986
67672	1.89226	0.153496
67673	1.89226	0.367466
67674	2.10275	0.333122
67675	2.55889	0.193894
67676	1.89226	0.307855
67677	2.30274	0.179841
67678	1.89226	0.346311
67679	1.89226	0.929339
67680	1.89226	0.922137
67681	1.89226	0.441858
67682	1.89226	0.86022
67683	1.89226	0.40805

67684	1.89226	0.291432
67685	1.89226	0.964811
67686	1.89226	0.844586
67687	1.89226	0.411426
67688	1.89226	0.76994
67689	1.89226	0.372376
67690	1.89226	0.152379
67691	1.89226	0.395081
67692	1.89226	0.338689
67693	1.89226	0.192947
67694	1.89226	0.306059
67695	1.89226	0.170337
67696	1.89226	0.166971
67697	1.89226	0.433267
67698	1.89226	0.394652
67699	1.89226	0.225573
67700	1.89226	0.366993
67701	1.89226	0.203022
67702	1.89226	0.387516
67703	1.89226	1.0315
67704	1.89226	0.952441
67705	1.89226	0.48923
67706	1.89226	0.914169
67707	1.89226	0.466632
67708	1.89226	0.863401
67709	1.89226	1.05132
67710	1.89226	0.332617
67711	1.89226	0.468322
67712	1.89226	0.34734
67713	1.89226	0.698513
67714	0.946131	0.139622
67715	0.976399	0.11657
67716	1.10124	0.111107
67717	0.946131	0.256796
67718	0.887633	0.245936
67719	0.887633	0.183146
67720	1.16933	0.136177
67721	1.23163	0.112496
67722	0.946131	0.176327
67723	0.946131	0.193127
67724	0.976399	0.164235
67725	1.14992	0.163108
67726	1.89226	0.239399
67727	1.89226	0.633792
67728	1.89226	0.275711
67729	1.89226	0.378481
67730	3.78452	0.252862
67731	1.89226	0.144468
67732	1.89226	0.335287
67733	1.89226	0.142221
67734	1.89226	0.239421
67735	3.78453	0.152957
67736	0.946132	0.0941334
67737	0.946132	0.246724
67738	0.946131	0.172318
67739	0.946131	0.26072
67740	0.946131	0.264799
67741	0.946131	0.200531

67742	0.946132	0.0899629
67743	0.946132	0.106134
67744	0.946131	0.148895
67745	0.946131	0.201404
67746	0.946132	0.157095
67747	0.946132	0.100829
67748	0.946132	0.319115
67749	0.946132	0.201062
67750	0.946131	0.286234
67751	0.946131	0.466821
67752	0.946131	0.477563
67753	0.946131	0.372061
67754	0.946132	0.246999
67755	0.946132	0.187761
67756	0.946131	0.305507
67757	0.946131	0.396985
67758	0.946132	0.325966
67759	0.946132	0.223193
67760	1.89226	0.397578
67761	1.89226	0.537844
67762	1.89226	0.262754
67763	1.89226	0.643907
67764	3.78452	0.553079
67765	1.89226	1.11641
67766	1.89226	1.02269
67767	1.89226	0.586039
67768	1.89226	0.443485
67769	3.78453	0.650095
67770	1.89226	0.373697
67771	1.89226	0.958006
67772	1.89226	0.380176
67773	1.89226	1.15949
67774	1.89226	0.343094
67775	1.89226	0.938911
67776	1.89225	0.237662
67777	1.89225	0.379326
67778	1.89225	0.282685
67779	1.89225	0.936002
67780	1.89225	0.179745
67781	1.89225	0.517469
67782	1.89226	0.397769
67783	1.89226	0.508383
67784	1.89226	0.40026
67785	1.89226	0.959205
67786	1.89226	0.241206
67787	1.89226	0.417189
67788	1.89226	1.04347
67789	1.89226	0.466394
67790	1.89226	0.231375
67791	1.89226	0.181552
67792	3.78453	0.229698
67793	1.39491	0.244298
67794	1.39491	0.25514
67795	0.924649	0.37534
67796	0.924649	0.380584
67797	0.946131	0.359438
67798	1.37343	0.143108
67799	0.924678	0.110051

67800	0.946131	1.38324
67801	0.924678	0.188266
67802	1.37343	0.145169
67803	0.946131	0.559691
67804	0.946131	0.535058
67805	1.89226	0.43331
67806	1.89226	0.127209
67807	1.89226	0.225281
67808	1.89226	0.345324
67809	1.89226	0.123731
67810	1.89226	0.198138
67811	0.946126	0.0698591
67812	0.975461	0.0698591
67813	0.946132	0.0698591
67814	1.12674	0.0700327
67815	0.946129	0.0701714
67816	0.946129	0.0698591
67817	1.12673	0.0698591
67818	1.12673	0.0698591
67819	0.946129	0.0698591
67820	0.946129	0.0700042
67821	0.975464	0.0698591
67822	1.12673	0.0698591
67823	1.89226	0.0698591
67824	1.89226	0.0699165
67825	1.89226	0.0744318
67826	1.89226	0.0698591
67827	1.89226	0.0723258
67828	1.89226	0.0701115
67829	0.946126	0.0699612
67830	0.946126	0.0730981
67831	0.946131	0.0708769
67832	0.946131	0.104425
67833	0.946129	0.105068
67834	0.946129	0.0875471
67835	0.946128	0.0718378
67836	0.946128	0.0698804
67837	0.946129	0.0801829
67838	0.946129	0.0954583
67839	0.946128	0.0827018
67840	0.946128	0.0824233
67841	1.89226	0.0698591
67842	1.89226	0.0846191
67843	1.89226	0.0698591
67844	1.89226	0.0701866
67845	3.78451	0.0698591
67846	1.89226	0.0735995
67847	1.89226	0.0772086
67848	1.89226	0.0698591
67849	1.89226	0.0879971
67850	3.78452	0.0813095
67851	0.946125	0.0732614
67852	0.946125	0.0842964
67853	0.946131	0.0794455
67854	0.946131	0.13311
67855	0.946128	0.136058
67856	0.946128	0.114218
67857	0.946128	0.0823347

67858	0.946128	0.0740233
67859	0.946128	0.0878337
67860	0.946128	0.108294
67861	0.946128	0.0778926
67862	0.946128	0.0937007
67863	1.89226	0.0708688
67864	1.89226	0.128021
67865	1.89226	0.0820752
67866	1.89226	0.0698591
67867	3.78452	0.0701189
67868	1.89226	0.0849735
67869	1.89226	0.0992748
67870	1.89226	0.102757
67871	1.89226	0.145773
67872	1.89226	0.0833318
67873	1.89226	0.10331
67874	1.12492	0.157179
67875	1.41201	0.172258
67876	1.12082	0.140471
67877	0.771416	0.136248
67878	1.10725	0.131081
67879	1.10725	0.13677
67880	1.07618	0.219654
67881	1.07618	0.115658
67882	1.12492	0.158703
67883	0.771415	0.197933
67884	1.12082	0.144918
67885	1.41201	0.171182
67886	2.82401	0.11001
67887	1.89226	0.115697
67888	1.89226	0.0997173
67889	1.89226	0.120094
67890	2.24983	0.117977
67891	2.24983	0.129663
67892	0.946133	0.0993254
67893	0.946133	0.104071
67894	0.946129	0.0895479
67895	0.946129	0.081319
67896	0.946131	0.0837941
67897	0.946131	0.125965
67898	0.946131	0.104929
67899	0.946131	0.0781655
67900	0.946131	0.0983434
67901	0.946131	0.0911612
67902	0.946131	0.0901737
67903	0.946131	0.0924844
67904	0.946132	0.13077
67905	0.946132	0.123341
67906	0.946133	0.112381
67907	0.946133	0.114122
67908	0.946132	0.104607
67909	0.946132	0.13495
67910	0.946133	0.135989
67911	0.946133	0.141378
67912	0.946132	0.124656
67913	0.946132	0.141629
67914	0.946133	0.115692
67915	0.946133	0.115134

67916	1.89227	0.343376
67917	1.89227	0.402758
67918	1.89227	0.439631
67919	1.89227	0.307187
67920	1.89227	0.448269
67921	1.89227	0.322531
67922	1.42982	0.132974
67923	1.19944	0.0935304
67924	0.936311	0.181194
67925	0.936311	0.14671
67926	0.946132	0.147178
67927	1.02993	0.1365
67928	1.41513	0.153247
67929	0.946133	0.178002
67930	0.946133	0.146872
67931	0.946133	0.16093
67932	1.02993	0.155636
67933	1.18181	0.125046
67934	1.89226	0.405971
67935	1.89226	0.503302
67936	1.89226	0.606209
67937	1.89226	0.308345
67938	3.78453	0.431915
67939	1.89226	0.193937
67940	1.89226	0.238473
67941	1.89226	0.262437
67942	1.89226	0.184094
67943	1.89226	0.260053
67944	1.89226	0.187046
67945	1.89226	0.178411
67946	1.89226	0.198857
67947	1.89226	0.221007
67948	1.89226	0.162168
67949	1.89226	0.226003
67950	1.89226	0.166735
67951	0.946131	0.298851
67952	0.946131	0.397151
67953	0.946132	0.506506
67954	0.946132	0.404187
67955	0.946132	0.297512
67956	0.946132	0.361328
67957	0.946132	0.505372
67958	0.946132	0.314309
67959	0.946132	0.367278
67960	0.946132	0.382416
67961	0.946132	0.367335
67962	0.946132	0.344079
67963	1.89226	1.14003
67964	1.89226	1.16509
67965	1.89226	1.47124
67966	1.89226	0.811821
67967	1.89226	1.55696
67968	1.89226	0.868126
67969	1.89226	1.31885
67970	2.22749	0.546579
67971	1.89226	0.670679
67972	1.89226	0.701959
67973	3.78453	0.701226

67974	1.89226	1.27096
67975	1.89226	1.41517
67976	1.89226	1.67026
67977	1.89226	0.78253
67978	3.78453	1.19206
67979	1.89226	0.754521
67980	1.89226	0.798053
67981	1.89226	1.06556
67982	1.89226	0.574316
67983	1.89226	1.21291
67984	1.89226	0.646945
67985	0.94613	0.552231
67986	0.94613	1.10495
67987	0.94613	1.50837
67988	0.94613	0.753946
67989	0.946131	0.655263
67990	0.946131	0.546441
67991	0.946129	1.40103
67992	0.946129	0.765146
67993	0.94613	0.738318
67994	0.94613	0.745748
67995	0.94613	0.722644
67996	0.94613	0.698163
67997	1.89226	0.0890165
67998	1.89226	0.0991231
67999	1.89226	0.100033
68000	1.89226	0.0869307
68001	1.89226	0.0971791
68002	1.89226	0.0851046
68003	1.89226	0.103936
68004	1.89226	0.160812
68005	1.89226	0.170098
68006	1.89226	0.0803037
68007	3.78452	0.12032
68008	1.89225	0.0707433
68009	1.89225	0.0734193
68010	1.89225	0.0753295
68011	1.89225	0.0703396
68012	1.89225	0.0757849
68013	1.89225	0.0708994
68014	1.89226	0.0698591
68015	1.89226	0.0699676
68016	1.89226	0.0716346
68017	1.89226	0.0698591
68018	1.89226	0.0705861
68019	1.89226	0.0698591
68020	0.946132	0.0782779
68021	0.946132	0.0939782
68022	0.946132	0.146105
68023	0.946132	0.0982107
68024	0.946131	0.0877181
68025	0.946131	0.0794909
68026	0.946132	0.15383
68027	0.946132	0.0787737
68028	0.946132	0.0867223
68029	0.946132	0.0944533
68030	0.946132	0.0959638
68031	0.946132	0.0911542

68032	1.89226	0.0954798
68033	1.89226	0.139768
68034	1.89226	0.0952801
68035	1.89226	0.193813
68036	1.89226	0.0881239
68037	1.89226	0.170376
68038	1.89226	0.109717
68039	1.89226	0.0864642
68040	1.89226	0.0794308
68041	1.89226	0.137712
68042	1.89226	0.0748959
68043	1.89226	0.125652
68044	1.89226	0.071673
68045	1.89226	0.0842713
68046	1.89226	0.0717805
68047	1.89226	0.0953605
68048	1.89226	0.0701196
68049	1.89226	0.08786
68050	1.89226	0.0988958
68051	1.89226	0.158856
68052	1.89226	0.0872301
68053	1.89226	0.190499
68054	3.78452	0.117441
68055	1.89226	0.0832361
68056	1.89226	0.103194
68057	1.89226	0.0789789
68058	1.89226	0.161907
68059	1.89226	0.077262
68060	1.89226	0.142178
68061	1.89226	0.0698591
68062	1.89226	0.0698591
68063	1.89226	0.0698591
68064	1.89226	0.0698591
68065	3.78453	0.0698591
68066	1.89226	0.0698591
68067	2.48862	0.0698591
68068	1.89226	0.0698591
68069	2.22062	0.0698591
68070	1.89226	0.0698591
68071	2.22062	0.0698591
68072	1.89227	0.0698591
68073	1.89227	0.0698591
68074	1.89227	0.0698591
68075	1.89227	0.0698591
68076	1.89227	0.0698591
68077	1.89227	0.0698591
68078	0.946132	0.0698591
68079	0.946132	0.0698591
68080	0.946131	0.0698591
68081	0.946131	0.0698591
68082	0.94613	0.0698591
68083	0.94613	0.0698591
68084	0.946133	0.0698591
68085	0.946133	0.0698591
68086	0.946131	0.0698591
68087	0.946131	0.0698591
68088	0.946131	0.0698591
68089	0.946131	0.0698591

68090	1.89226	0.0698591
68091	1.89226	0.0698591
68092	1.89226	0.0698591
68093	1.89226	0.0698591
68094	3.78453	0.0698591
68095	1.89226	0.0698591
68096	1.89226	0.0699712
68097	1.89226	0.0698591
68098	1.89226	0.0698591
68099	3.78453	0.0698591
68100	1.89226	0.127548
68101	1.89226	0.0969949
68102	1.89226	0.0713025
68103	1.89226	0.121497
68104	1.89226	0.0990184
68105	1.89226	0.150821
68106	1.89226	0.128367
68107	1.89226	0.0737619
68108	1.89226	0.0910772
68109	1.89226	0.143837
68110	3.78452	0.118115
68111	1.89226	0.081981
68112	1.89226	0.100671
68113	1.89226	0.125184
68114	1.89226	0.102032
68115	3.78453	0.0806578
68116	2.57454	0.181818
68117	1.89226	0.162472
68118	1.3663	0.146421
68119	1.89226	0.173842
68120	3.25856	0.150514
68121	1.89226	0.144464
68122	1.89226	0.117318
68123	1.89226	0.113695
68124	1.89226	0.144561
68125	3.78451	0.156354
68126	1.89226	0.108863
68127	1.89226	0.156657
68128	1.89226	0.11236
68129	1.89226	0.140009
68130	1.89226	0.101726
68131	1.89226	0.128028
68132	0.810946	0.13408
68133	0.810946	0.12689
68134	0.810946	0.144541
68135	0.810946	0.164643
68136	0.810959	0.141059
68137	0.810959	0.137137
68138	1.89226	0.154999
68139	1.35168	0.161447
68140	1.08132	0.143872
68141	1.08132	0.154162
68142	1.0813	0.155518
68143	1.89226	0.103572
68144	1.89226	0.154999
68145	1.89226	0.215353
68146	1.89226	0.140019
68147	3.78453	0.128302

68148	1.89226	0.127691
68149	1.89226	0.0897643
68150	1.89226	0.119504
68151	1.89226	0.12775
68152	1.89226	0.0960599
68153	1.89226	0.103465
68154	1.89226	0.0857387
68155	1.89226	0.0955683
68156	1.89226	0.131715
68157	1.89226	0.0883792
68158	3.78452	0.0818151
68159	1.89226	0.146108
68160	1.89226	0.0932497
68161	1.89226	0.14227
68162	1.89226	0.177615
68163	3.78452	0.148866
68164	2.142	0.30218
68165	1.93949	0.12842
68166	1.39296	0.246371
68167	2.55711	0.274996
68168	1.39296	0.155846
68169	2.55711	0.210027
68170	1.89226	0.172297
68171	2.5571	0.122902
68172	2.142	0.199366
68173	1.89226	0.206789
68174	4.44936	0.200144
68175	0.946131	0.164523
68176	0.946131	0.171388
68177	1.27855	0.177854
68178	1.29171	0.150342
68179	1.29171	0.165944
68180	0.946127	0.142818
68181	1.27855	0.119991
68182	0.946131	0.199555
68183	1.29172	0.184801
68184	1.29172	0.172759
68185	0.946129	0.144047
68186	0.946129	0.131163
68187	1.27855	0.210578
68188	1.29172	0.199983
68189	0.969743	0.203046
68190	1.07109	0.186135
68191	1.29172	0.261743
68192	1.07109	0.125678
68193	0.969745	0.126522
68194	1.27855	0.259561
68195	1.10873	0.261859
68196	1.10873	0.230287
68197	1.15273	0.142966
68198	1.15273	0.130498
68199	1.89227	0.0698591
68200	1.89227	0.0698591
68201	1.89227	0.0698591
68202	1.89227	0.0698591
68203	2.1288	0.0698591
68204	2.1288	0.0698591
68205	1.89227	0.0698591

68206	1.89227	0.0698591
68207	1.89227	0.0698591
68208	1.89227	0.0698591
68209	3.78453	0.0698591
68210	1.89226	0.0698591
68211	1.89226	0.0698591
68212	1.89226	0.0698591
68213	1.89226	0.0698591
68214	3.78453	0.0698591
68215	1.89226	0.0698591
68216	1.89226	0.0698591
68217	1.89226	0.0698591
68218	1.89226	0.0698591
68219	1.89226	0.0698591
68220	1.89226	0.0698591
68221	1.94432	0.0698591
68222	1.89226	0.0698591
68223	2.07911	0.0698591
68224	1.89226	0.0698591
68225	2.07911	0.0698591
68226	1.89226	0.0698591
68227	1.89226	0.0698591
68228	1.89226	0.0698591
68229	1.89226	0.0698591
68230	1.89226	0.0698591
68231	1.89226	0.0698591
68232	1.89226	0.0698591
68233	1.89226	0.548892
68234	1.89226	0.530127
68235	1.89226	0.640053
68236	1.89226	0.415807
68237	1.89226	0.736367
68238	1.89226	0.479095
68239	1.89226	0.343217
68240	1.89226	0.309244
68241	1.89226	0.390564
68242	1.89226	0.244954
68243	1.89226	0.451656
68244	1.89226	0.30598
68245	1.86778	0.2412
68246	2.72348	0.216175
68247	1.89226	0.30206
68248	1.89226	0.14494
68249	3.76004	0.250492
68250	1.89226	0.57618
68251	1.89226	0.557257
68252	1.89226	0.918878
68253	1.89226	0.412782
68254	3.78451	0.564478
68255	1.89226	0.367778
68256	1.89226	0.360816
68257	1.89226	0.428241
68258	1.89226	0.274667
68259	1.89226	0.494629
68260	1.89226	0.328363
68261	0.94613	0.196671
68262	0.94613	0.280492
68263	0.94613	0.412996

68264	0.94613	0.252175
68265	0.946131	0.2568
68266	0.946131	0.168328
68267	0.946129	0.349605
68268	0.946129	0.268124
68269	0.94613	0.259496
68270	0.94613	0.267901
68271	0.94613	0.245709
68272	0.94613	0.244841
68273	0.946124	0.403037
68274	0.946124	0.255356
68275	0.94613	0.567667
68276	0.94613	0.366215
68277	0.946128	0.272292
68278	0.946128	0.324846
68279	0.946126	0.561215
68280	0.946126	0.407219
68281	0.946127	0.401041
68282	0.946127	0.380476
68283	0.946127	0.34987
68284	0.946127	0.366009
68285	2.09166	0.0698591
68286	2.1285	0.0698591
68287	1.78381	0.0698591
68288	1.78381	0.0698672
68289	2.47689	0.0698591
68290	2.47689	0.0698591
68291	2.09167	0.0698591
68292	2.26572	0.0698591
68293	2.73393	0.0698591
68294	2.4769	0.0698591
68295	4.74261	0.0698591
68296	1.89226	0.0698591
68297	1.89226	0.0823598
68298	1.89226	0.0793488
68299	1.89226	0.0698721
68300	1.89226	0.0802164
68301	1.89226	0.070359
68302	0.946131	0.0698653
68303	0.946131	0.0819107
68304	0.946129	0.0701008
68305	0.946129	0.0784136
68306	0.94613	0.0788133
68307	0.94613	0.0809287
68308	0.946131	0.0698591
68309	0.946131	0.0698654
68310	0.94613	0.0712263
68311	0.94613	0.0729587
68312	0.946131	0.0711637
68313	0.946131	0.0731679
68314	2.27622	0.0698779
68315	2.66786	0.0698591
68316	2.1285	0.0698591
68317	2.1285	0.0699611
68318	2.62461	0.0698591
68319	2.47689	0.0698591
68320	1.89226	0.0699035
68321	1.89226	0.0844829

68322	1.89226	0.0698591
68323	1.89226	0.0826789
68324	3.78453	0.0744089
68325	1.89226	0.0699178
68326	1.89226	0.0801289
68327	1.89226	0.071322
68328	1.89226	0.0841969
68329	1.89226	0.0698708
68330	1.89226	0.0790264
68331	1.89226	0.412972
68332	1.89226	0.567438
68333	1.89226	0.39845
68334	1.89226	0.570184
68335	1.89226	0.405204
68336	1.89226	0.561448
68337	1.89226	1.07612
68338	1.89226	0.592278
68339	1.89226	0.604808
68340	1.89226	0.949686
68341	3.78452	0.744688
68342	1.89227	0.612564
68343	1.89227	0.909419
68344	1.89227	0.94714
68345	1.89227	0.64056
68346	3.78453	0.811216
68347	0.946127	0.59607
68348	0.946127	0.589574
68349	1.06438	0.438181
68350	0.946131	0.428709
68351	1.06438	0.434852
68352	0.94613	0.579894
68353	0.946128	0.584173
68354	0.946128	0.407951
68355	1.06438	0.478671
68356	1.06438	0.485448
68357	0.946129	0.503819
68358	0.946129	0.49892
68359	1.89226	0.600883
68360	1.89226	0.85738
68361	1.89226	0.641178
68362	1.89226	0.937814
68363	1.89226	0.623883
68364	1.89226	0.911644
68365	0.946133	0.770503
68366	0.946133	0.82849
68367	1.39771	0.700804
68368	0.946132	0.659922
68369	1.39771	0.704251
68370	1.39772	0.805362
68371	0.946133	0.824056
68372	0.946133	0.60758
68373	0.946132	0.647813
68374	1.39772	0.679664
68375	0.946132	0.610212
68376	0.946132	0.664162
68377	1.98182	0.0698591
68378	2.2217	0.0698591
68379	2.79889	0.0698591

68380	2.50097	0.0698591
68381	4.72267	0.0698591
68382	2.05362	0.0698591
68383	1.70302	0.0698591
68384	2.75109	0.0698591
68385	2.138	0.0698591
68386	2.0815	0.0698591
68387	1.84751	0.0698591
68388	0.946131	0.0698591
68389	0.946131	0.0698591
68390	0.946131	0.0698591
68391	0.946131	0.0698591
68392	0.946131	0.0698591
68393	0.946131	0.0698591
68394	0.946132	0.0698591
68395	0.946132	0.0698591
68396	0.946131	0.0698591
68397	0.946131	0.0698591
68398	0.946132	0.0698591
68399	0.946132	0.0698591
68400	1.89226	0.0698591
68401	1.89226	0.0701705
68402	1.89226	0.0698591
68403	1.89226	0.0709888
68404	3.78451	0.0698591
68405	0.946126	0.0698591
68406	1.25048	0.0698591
68407	0.946131	0.0698591
68408	1.39945	0.0698591
68409	0.946127	0.0698591
68410	0.946127	0.0698591
68411	1.39944	0.0698591
68412	1.39944	0.0698591
68413	0.946129	0.0698591
68414	0.946129	0.0698591
68415	1.39944	0.0698591
68416	1.25048	0.0698591
68417	1.89226	0.0698591
68418	1.89226	0.0702232
68419	1.89226	0.0698591
68420	1.89226	0.0698591
68421	1.89226	0.0698591
68422	1.89226	0.0698591
68423	0.946126	0.0705831
68424	0.946126	0.0698591
68425	0.946131	0.0698591
68426	0.946131	0.0717817
68427	0.946128	0.0727822
68428	0.946128	0.0707134
68429	0.946129	0.0698591
68430	0.946129	0.0698591
68431	0.946129	0.0698591
68432	0.946129	0.0698591
68433	0.946128	0.0698591
68434	0.946128	0.0698835
68435	1.89226	0.0698591
68436	1.89226	0.0698591
68437	1.89226	0.0698591

68438	1.89226	0.0698591
68439	1.89226	0.0698591
68440	1.89226	0.0698591
68441	1.01393	0.0698591
68442	0.775245	0.0698591
68443	0.946126	0.0698591
68444	0.946126	0.0698591
68445	0.78305	0.0698591
68446	0.78305	0.0698591
68447	0.946159	0.0698591
68448	0.929267	0.0698591
68449	1.14956	0.0698591
68450	1.04073	0.0698591
68451	0.851526	0.0698591
68452	0.704781	0.0698591
68453	1.89226	0.0698591
68454	1.89226	0.0698591
68455	1.89226	0.0698591
68456	1.89226	0.0698591
68457	3.78451	0.0698591
68458	1.89226	0.0698591
68459	1.89226	0.0698591
68460	1.89226	0.0698591
68461	1.89226	0.0698591
68462	1.89226	0.0698591
68463	1.89226	0.0698591
68464	1.89226	0.0698591
68465	1.89226	0.0698591
68466	1.89226	0.0698591
68467	1.89226	0.0698591
68468	1.89226	0.0698591
68469	1.89226	0.0698591
68470	1.16317	0.0698591
68471	1.0292	0.0698591
68472	0.946126	0.0698591
68473	0.946126	0.0698591
68474	0.93562	0.0698591
68475	0.93562	0.0698591
68476	1.0418	0.0698591
68477	1.04181	0.0698591
68478	1.16317	0.0698591
68479	0.946129	0.0698591
68480	1.02919	0.0698591
68481	0.946128	0.0698591
68482	0.946131	0.0698591
68483	0.946131	0.0698591
68484	0.946126	0.0698591
68485	0.946126	0.0698591
68486	0.946129	0.0698591
68487	0.946129	0.0698591
68488	0.946128	0.0698591
68489	0.946128	0.0698591
68490	0.946129	0.0698591
68491	0.946129	0.0698591
68492	0.946128	0.0698591
68493	0.946128	0.0698591
68494	1.89226	0.0698591
68495	1.89226	0.069893

68496	1.89226	0.0698591
68497	1.89226	0.0698591
68498	1.89226	0.0698591
68499	1.89226	0.0698591
68500	1.89226	0.0698591
68501	1.89226	0.0698591
68502	1.89226	0.0698591
68503	1.89226	0.0698591
68504	1.89226	0.0698591
68505	1.89226	0.0698591
68506	1.89226	0.0698591
68507	1.89226	0.0698591
68508	1.89226	0.0698591
68509	1.89226	0.0698591
68510	3.78453	0.0698591
68511	1.89226	0.0698591
68512	1.89226	0.0698591
68513	1.89226	0.0698591
68514	1.89226	0.0698591
68515	3.78452	0.0698591
68516	1.89225	0.0698591
68517	1.89225	0.0698591
68518	1.89225	0.0698591
68519	1.89225	0.0698591
68520	1.89225	0.0698591
68521	1.89225	0.0698591
68522	0.946132	0.0698591
68523	0.946132	0.0698591
68524	0.946133	0.0698591
68525	0.946133	0.0698591
68526	0.946132	0.0698591
68527	0.946132	0.0698591
68528	0.946132	0.0698591
68529	0.946132	0.0698591
68530	0.946133	0.0698591
68531	0.946133	0.0698591
68532	0.946132	0.0698591
68533	0.946132	0.0698591
68534	1.89226	0.0698591
68535	1.89226	0.0698591
68536	1.89226	0.0698591
68537	1.89226	0.0698591
68538	3.78453	0.0698591
68539	1.13537	0.0698591
68540	1.09222	0.0698591
68541	0.946126	0.0698591
68542	0.946126	0.0698591
68543	0.946128	0.0698591
68544	0.946128	0.0698591
68545	1.16317	0.0698591
68546	1.13034	0.0698591
68547	1.30155	0.0698591
68548	1.08462	0.0698591
68549	0.992915	0.0698591
68550	0.807642	0.0698591
68551	0.946131	0.0698591
68552	0.946131	0.0698591
68553	0.946125	0.0698591

68554	0.946125	0.0698591
68555	0.946128	0.0698591
68556	0.946128	0.0698591
68557	0.946128	0.0698591
68558	0.946128	0.0698591
68559	0.946128	0.0698591
68560	0.946128	0.0698591
68561	0.946128	0.0698591
68562	0.946128	0.0698591
68563	1.89226	0.0698591
68564	1.89226	0.0699398
68565	1.89226	0.0698591
68566	1.89226	0.0698591
68567	1.89226	0.0698591
68568	1.89226	0.0698591
68569	1.89224	0.0698591
68570	1.89224	0.0698591
68571	1.89224	0.0698591
68572	1.89224	0.0698591
68573	1.89224	0.0698591
68574	1.89224	0.0698591
68575	0.946086	0.0698591
68576	1.17919	0.0698591
68577	0.89738	0.0698591
68578	0.89738	0.0698591
68579	0.946116	0.0698591
68580	0.946116	0.0698591
68581	1.13048	0.0698591
68582	1.11844	0.0698591
68583	0.946128	0.0698591
68584	0.897362	0.0698591
68585	1.1282	0.0698591
68586	0.946103	0.0698591
68587	1.89223	0.0698591
68588	1.89223	0.0698591
68589	1.89223	0.0698591
68590	1.89223	0.0698591
68591	3.78446	0.0698591
68592	0.946118	0.0698591
68593	0.946118	0.0698591
68594	0.946118	0.0698591
68595	0.946118	0.0701201
68596	0.946119	0.0700692
68597	0.946119	0.0698591
68598	0.946118	0.0698591
68599	0.946118	0.0698591
[truncated: 438,132 more chars]
