# Supplementary material for: On the Use of Bone Remodelling Models to Estimate the Density Distribution of Bones. Uniqueness of the Solution
Source: PLoS One. 2016 Feb 9;11(2):e0148603. doi: 10.1371/journal.pone.0148603 (PMC4747586; doi:10.1371/journal.pone.0148603)
Supplement: S7 File — (PDF) [file pone.0148603.s007.pdf]

N° Elem	Volume (mm3)	Density (g/cm3)
1	1.32415	2.1
2	1.75823	2.1
3	0.984099	2.1
4	1.46442	2.1
5	1.53082	2.1
6	1.32068	2.1
7	1.91404	0.149715
8	1.17036	0.150392
9	0.929209	0.0938281
10	1.27254	0.0792892
11	2.0926	0.0897192
12	2.20407	0.0815229
13	1.21488	0.09259
14	1.26024	0.063869
15	0.794454	0.0680543
16	1.95087	0.05
17	0.953925	2.1
18	0.98949	2.1
19	1.27875	2.1
20	1.04598	2.1
21	0.594005	2.1
22	0.578861	2.1
23	1.09506	2.1
24	1.45327	2.06722
25	2.58396	1.5291
26	1.58581	2.1
27	1.5858	2.1
28	0.636946	1.94946
29	0.94202	2.1
30	0.946119	1.30841
31	0.636953	2.1
32	0.946107	2.1
33	1.09505	2.1
34	1.27389	1.28115
35	1.00232	2.1
36	1.00231	2.1
37	1.83583	2.1
38	1.00231	2.1
39	1.28828	0.0714327
40	1.81696	0.0642887
41	1.28828	0.0607977
42	1.28828	0.0547404
43	1.0044	0.102378
44	0.85148	0.102477
45	0.851539	0.108187
46	1.57767	0.0691169
47	1.52227	0.0655251
48	0.730657	0.06307
49	0.94613	0.0980686
50	1.28228	2.1
51	1.64268	2.06695
52	1.10101	2.1
53	1.3939	2.1
54	0.959471	2.1
55	1.10101	2.1

56	1.2873	2.1
57	1.59659	2.1
58	1.80841	0.404005
59	1.80841	0.573231
60	0.946129	0.413563
61	1.36991	0.366048
62	1.32262	0.639427
63	1.32261	0.46855
64	1.38464	0.48314
65	0.962877	0.399593
66	0.962878	0.360909
67	1.82129	0.37584
68	1.82129	0.414161
69	0.94613	0.424767
70	1.39751	0.415434
71	0.946131	0.43029
72	1.34493	0.349395
73	1.39751	0.357237
74	0.837577	0.780453
75	0.823478	0.550395
76	1.10889	1.59683
77	1.01686	0.809745
78	1.1942	1.62929
79	0.836129	0.746098
80	0.937165	0.550009
81	0.969438	0.909239
82	0.895386	0.664225
83	1.30554	1.88298
84	1.15798	0.724837
85	1.22578	2.06821
86	1.15255	0.876149
87	0.9765	0.561673
88	2.02178	1.79747
89	1.63755	1.62308
90	0.727713	0.94458
91	0.810945	0.950959
92	1.00559	1.75046
93	0.879098	0.999142
94	1.07852	1.84309
95	0.657764	0.817217
96	0.829406	0.710238
97	0.720201	0.174001
98	0.946131	0.298609
99	1.11827	0.223698
100	1.50754	0.150646
101	1.64835	0.0848323
102	1.71903	0.267763
103	0.851554	0.219889
104	1.75626	0.0867236
105	2.1646	0.116642
106	1.5563	0.155045
107	1.5563	0.146923
108	2.10876	0.233804
109	1.05438	0.427337
110	0.946133	0.520987
111	1.05438	0.576022
112	1.20256	0.203206
113	1.47879	0.443577

114	1.47879	0.714951
115	0.77815	0.555303
116	0.947114	2.1
117	1.31257	2.1
118	1.09235	2.1
119	0.946132	2.1
120	1.54246	2.1
121	1.54246	2.1
122	1.09235	2.1
123	0.946131	2.1
124	1.29507	1.40209
125	1.0353	2.1
126	1.03529	2.1
127	1.28667	2.1
128	1.44777	2.1
129	1.61121	2.1
130	1.22176	0.452776
131	0.946129	0.430584
132	1.08848	0.359718
133	0.946131	0.444571
134	1.61201	0.38999
135	1.61201	0.345187
136	1.19993	0.401111
137	1.35822	0.413091
138	1.1539	0.0982212
139	1.00765	0.06248
140	1.28228	2.1
141	2.65339	2.1
142	1.28228	2.1
143	1.55998	2.1
144	1.00509	2.1
145	0.93845	2.1
146	1.27674	2.1
147	0.810958	2.1
148	1.91381	2.1
149	1.1861	2.1
150	1.24886	2.1
151	2.02842	2.1
152	0.991436	0.0550754
153	1.34588	0.0519095
154	2.02729	0.050193
155	0.72904	0.0675463
156	1.06139	0.0709584
157	0.835368	0.0709275
158	1.63819	0.05
159	1.10745	0.0532666
160	1.79925	0.0527322
161	1.00085	0.0694794
162	0.946133	1.97469
163	1.41123	1.79569
164	1.8327	1.94849
165	1.8327	2.1
166	0.946132	0.864726
167	1.41123	2.1
168	1.3676	2.1
169	1.11048	2.1
170	1.11048	2.1
171	1.65815	2.1

172	1.9004	2.1
173	1.88691	2.1
174	1.82232	2.1
175	1.46591	2.1
176	1.09115	2.1
177	1.02051	0.824923
178	1.03373	0.872136
179	1.10307	2.1
180	1.05556	2.1
181	1.82533	0.876958
182	1.19496	0.734418
183	1.28597	2.1
184	1.20421	2.1
185	2.23644	2.1
186	1.08378	2.1
187	0.983724	2.1
188	0.602075	2.1
189	1.45876	2.1
190	1.45875	2.1
191	0.602103	1.55601
192	1.11822	2.1
193	0.946133	2.1
194	0.602103	2.1
195	1.20421	2.1
196	1.26371	0.72492
197	1.55179	0.505784
198	1.14042	0.274942
199	1.56876	0.326947
200	2.27496	0.579203
201	1.13748	0.491487
202	1.02884	0.663391
203	1.25124	0.598715
204	0.82703	0.522685
205	0.607775	0.486453
206	1.49242	0.588236
207	1.35674	0.604347
208	1.6697	2.1
209	1.81655	2.1
210	0.900108	0.998234
211	0.851537	0.742977
212	0.703076	0.938425
213	0.639143	0.793402
214	1.42358	1.01018
215	1.4976	1.18043
216	0.86284	0.816699
217	1.2965	0.935443
218	1.27835	1.97835
219	1.00012	0.850482
220	1.74332	1.11758
221	1.08576	2.1
222	1.19 2.1	
223	1.08576	2.1
224	1.20854	2.1
225	0.946131	2.1
226	0.946131	2.1
227	1.75985	2.1
228	1.75985	2.1
229	1.41232	2.1

230	1.29365	2.1
231	0.973559	2.1
232	1.23698	0.05
233	1.45055	0.05
234	1.26947	0.0548932
235	0.856218	0.054903
236	1.75303	0.05
237	1.14938	0.05
238	0.984711	0.05
239	2.05771	0.05
240	0.625171	0.05
241	0.965675	0.05
242	1.17631	0.05
243	1.4677	0.0645014
244	1.35403	0.109769
245	0.975149	0.116616
246	0.946131	0.125088
247	1.80893	0.0719169
248	1.80893	0.067254
249	0.975149	0.0697676
250	0.946132	0.116677
251	1.9503	0.1121
252	2.80207	0.0916918
253	1.76411	0.111491
254	2.77469	0.0913402
255	1.9503	0.0838631
256	1.26784	0.093791
257	2.31093	0.150066
258	0.851541	0.228605
259	1.39521	0.115077
260	1.45613	0.180182
261	0.851541	0.256675
262	0.996013	0.130905
263	1.07989	0.223845
264	0.946131	0.243669
265	1.08213	0.138206
266	1.74289	0.135766
267	1.75838	0.140865
268	2.11163	0.214289
269	1.23773	2.1
270	1.25398	2.1
271	1.0304	2.1
272	1.32368	2.1
273	1.11712	0.89962
274	1.07999	0.815805
275	1.1766	2.1
276	1.35735	2.1
277	1.089	0.073672
278	1.81193	0.093295
279	1.11081	0.05
280	1.78069	0.05
281	0.717512	0.05
282	0.810946	0.05
283	0.703901	0.05
284	1.09028	0.05
285	0.958031	0.05
286	0.892585	0.05
287	0.873431	0.05

288	0.997755	0.05
289	1.85479	0.05
290	0.983029	0.442657
291	1.27922	0.415314
292	1.64346	0.635343
293	1.27922	0.54552
294	1.09795	1.56595
295	1.07508	0.99746
296	1.02975	1.07643
297	1.01121	1.20885
298	1.72369	0.05
299	1.12035	0.05
300	2.13511	0.05
301	0.9705	0.05
302	0.946131	0.05
303	0.86182	0.05
304	1.51379	0.05
305	1.51379	0.05
306	0.946132	0.05
307	0.861849	0.05
308	1.34591	0.551936
309	1.14721	0.373049
310	1.15539	2.1
311	1.90938	2.1
312	2.19436	0.962263
313	1.41563	2.1
314	0.785641	2.1
315	0.736384	2.04472
316	1.46489	2.1
317	1.46488	2.1
318	0.736372	2.1
319	0.946132	2.1
320	1.90941	0.978404
321	1.18907	2.1
322	1.20409	2.1
323	0.973559	2.1
324	1.29461	2.1
325	0.946129	2.1
326	0.94613	2.1
327	1.78751	2.1
328	1.78751	2.1
329	1.38885	2.1
330	1.34479	2.1
331	1.07283	0.944962
332	1.6869	2.1
333	1.52544	2.1
334	1.07797	2.1
335	1.13819	0.786693
336	0.776179	2.1
337	1.61092	0.892163
338	1.36155	2.1
339	0.77618	2.1
340	1.2933	0.840797
341	1.15946	2.1
342	0.69857	2.08529
343	1.87607	2.1
344	1.07612	2.1
345	1.31976	1.05628

346	1.01243	2.06366
347	1.41279	1.08457
348	1.35146	0.3237
349	1.90664	0.322522
350	1.35145	0.329806
351	1.22811	0.32959
352	1.93147	0.326032
353	1.42486	2.1
354	1.56558	2.1
355	1.0476	0.05
356	2.31605	0.05
357	1.89367	0.05
358	0.804706	0.05
359	0.783919	0.05
360	1.3116	0.05
361	1.03891	0.05
362	0.960473	0.05
363	1.08462	0.05
364	0.785994	0.05
365	1.1318	0.05
366	1.29576	0.05
367	1.3946	0.05
368	1.11924	0.05
369	0.834322	2.1
370	1.0256	2.1
371	1.74321	0.834355
372	1.41798	0.801705
373	0.871608	0.809143
374	1.47253	0.804665
375	1.47252	0.809092
376	0.817064	0.769888
377	0.817064	0.790388
378	0.946129	0.761984
379	1.63413	0.760226
380	1.9194	0.119373
381	1.93562	0.124699
382	2.06307	0.146686
383	1.46021	0.1033
384	0.955002	1.22798
385	0.833409	1.47885
386	0.838039	1.15009
387	1.48702	1.27006
388	1.1697	1.18288
389	1.23549	1.32145
390	1.18373	1.19011
391	2.02254	1.22607
392	1.68867	1.33517
393	0.875481	0.925243
394	0.821635	1.34072
395	0.858878	0.817609
396	1.30245	0.986735
397	1.07476	0.932518
398	1.11907	1.00197
399	1.10453	0.916102
400	1.70233	1.01186
401	1.70233	0.896502
402	2.6558	1.22511
403	1.7818	2.1

404	1.87059	2.1
405	1.34412	0.0822654
406	1.98068	0.109423
407	2.56369	0.0982209
408	1.49512	0.0661813
409	1.35977	0.262887
410	1.34254	0.352451
411	1.75618	0.260193
412	1.75618	0.209664
413	0.946129	0.470874
414	0.946129	0.308189
415	1.17665	0.302522
416	1.01976	0.24324
417	1.27376	0.177494
418	1.35978	0.216407
419	1.68741	0.168544
420	1.68741	0.144472
421	0.946132	0.28945
422	0.946133	0.20723
423	1.14241	0.199302
424	0.996831	0.157617
425	0.996828	0.217967
426	1.12774	0.277976
427	1.13664	2.1
428	1.09627	2.1
429	2.8814	2.1
430	3.49751	2.1
431	1.79109	0.488884
432	1.54197	0.3658
433	2.75153	0.268292
434	1.93019	0.233256
435	1.51569	0.836424
436	1.55145	1.04305
437	1.78595	2.1
438	3.74452	2.1
439	1.66537	2.1
440	3.55161	2.1
441	1.35002	0.802492
442	1.19901	0.703943
443	1.61028	2.06496
444	1.35002	0.835593
445	1.65712	0.765324
446	1.10585	0.825121
447	0.961347	0.887767
448	0.810944	1.18392
449	1.16148	1.88866
450	1.08066	1.51311
451	0.934577	0.721269
452	1.05164	1.26443
453	0.79216	0.776573
454	2.99194	2.1
455	1.2832	2.1
456	2.97842	2.1
457	1.39517	2.1
458	1.03391	2.1
459	1.74416	2.1
460	1.05071	2.1
461	0.923782	2.1

462	1.55369	2.1
463	1.86444	2.1
464	1.01617	2.00389
465	1.27174	2.1
466	1.20573	2.1
467	1.01616	2.1
468	1.13621	2.01965
469	1.75366	2.09703
470	2.27009	1.21713
471	1.6813	1.4112
472	1.47904	0.067772
473	2.62714	0.0791291
474	1.80085	0.0863487
475	1.80085	0.0580263
476	2.74104	0.0923908
477	1.37052	0.0919295
478	0.946131	0.157862
479	1.37052	0.0958416
480	1.31357	0.0943701
481	1.7837	0.0730303
482	1.7837	0.126005
483	0.900397	0.0867408
484	1.74487	0.100989
485	2.88672	2.1
486	2.88249	2.1
487	1.0961	0.394937
488	1.20143	0.343336
489	1.66739	0.360944
490	1.85795	0.152751
491	1.45789	0.0849631
492	2.11407	0.112522
493	0.713524	0.100441
494	1.58391	0.115566
495	1.58391	0.0698991
496	1.04765	0.0922754
497	1.24979	0.127763
498	0.713551	0.0730181
499	0.946127	0.127915
500	1.36768	0.10715
501	1.14433	0.05
502	1.66923	0.05
503	0.699479	0.05
504	0.70351	0.05
505	0.758416	0.05
506	1.07678	0.05
507	0.909874	0.05
508	0.940218	0.05
509	0.876604	0.05
510	1.27148	0.05
511	2.42348	0.05
512	1.19837	0.05
513	0.764764	0.05
514	0.829259	0.05
515	0.740584	0.05
516	1.2772	0.05
517	0.964465	0.05
518	1.06231	0.05
519	1.06924	0.05

520	1.17566	0.125099
521	1.3063	0.115953
522	2.51253	0.171631
523	2.42267	0.161842
524	1.89226	0.141441
525	1.46492	0.190591
526	0.946132	0.155428
527	0.653151	0.16938
528	0.653124	0.228562
529	1.53995	0.102918
530	1.53995	0.0950513
531	0.936806	0.154505
532	1.25626	0.127194
533	1.1893	0.318278
534	1.62206	0.315805
535	1.04921	2.1
536	1.22927	2.1
537	0.801765	2.1
538	0.774917	2.1
539	0.844623	2.1
540	0.737481	2.1
541	3.24379	2.1
542	2.48573	2.1
543	1.40967	0.972335
544	1.47203	1.37358
545	2.07659	0.923961
546	1.40967	1.34229
547	2.06638	0.938399
548	1.17418	1.7624
549	1.04921	2.1
550	1.74593	2.06128
551	1.74593	1.00517
552	0.946132	0.787755
553	1.38651	1.95302
554	1.20761	0.527728
555	1.20761	0.707334
556	1.30555	0.67053
557	1.46812	0.794621
558	2.41602	1.01925
559	1.46812	1.10085
560	1.46812	0.503488
561	0.734059	0.701556
562	0.946132	1.45766
563	1.21261	1.01118
564	1.34225	1.05477
565	0.734084	0.939499
566	1.82077	0.943769
567	1.82077	0.927358
568	1.13751	0.549312
569	0.946131	0.867479
570	1.13751	0.717372
571	1.13748	0.692337
572	0.784392	0.683639
573	1.4906	0.67083
574	1.4906	0.472488
575	1.17879	1.08527
576	1.28291	1.04522
577	0.626495	2.1

578	1.45334	2.1
579	1.45334	2.1
580	0.626494	2.1
581	1.3852	2.1
582	0.946116	2.1
583	0.626499	2.1
584	1.47203	0.856182
585	1.42639	2.1
586	1.42639	2.1
587	0.795469	2.1
588	0.795469	2.1
589	0.946132	2.1
590	0.850877	2.1
591	1.37098	2.1
592	1.87194	2.1
593	1.76988	2.1
594	3.16295	0.05
595	3.26725	0.05
596	1.13322	2.1
597	1.20761	2.1
598	0.686933	1.03847
599	0.493248	0.784977
600	1.2053	2.1
601	0.947149	0.914923
602	1.27591	0.992313
603	1.75732	2.1
604	0.493261	1.10958
605	1.34337	2.1
606	2.73207	0.752891
607	1.19857	0.964314
608	0.671688	0.655308
609	0.946134	2.1
610	1.36603	0.731675
611	2.07817	0.964608
612	1.82573	2.09253
613	0.671699	1.12739
614	1.47772	0.735395
615	0.975349	2.1
616	1.22615	2.1
617	1.47868	2.1
618	2.29351	1.11313
619	1.47642	2.1
620	2.94443	1.03046
621	1.80454	2.1
622	0.949135	1.63858
623	0.949134	1.78534
624	1.10476	0.31074
625	0.982857	0.347662
626	1.10938	0.334581
627	1.19052	0.438906
628	1.01012	0.05
629	1.35562	0.05
630	0.691742	0.05
631	2.39596	0.05
632	2.35604	0.05
633	0.974081	0.05
634	3.22444	0.05
635	1.3294	0.599877

636	1.37265	0.933591
637	1.3294	0.815109
638	1.51113	2.1
639	1.43643	2.1
640	1.72726	2.1
641	1.634	2.1
642	0.755566	2.1
643	1.29319	2.1
644	0.755567	2.1
645	0.946131	2.1
646	1.29319	2.1
647	0.973563	2.1
648	0.904398	2.1
649	0.904406	2.1
650	1.1538	2.1
651	1.10437	2.1
652	0.935418	2.1
653	1.16649	2.1
654	0.975667	2.1
655	1.17952	2.1
656	1.95053	2.1
657	0.948676	2.1
658	1.44287	2.1
659	0.746095	2.04049
660	1.51224	2.1
661	1.27354	2.1
662	0.746066	1.78254
663	1.20573	2.1
664	1.12442	2.1
665	0.671487	2.1
666	1.04355	2.1
667	0.948675	2.1
668	0.967673	0.870613
669	1.12931	0.05
670	1.38212	0.0500912
671	2.40113	0.05
672	1.08054	0.0544522
673	1.04072	0.0655581
674	0.691062	0.05
675	1.58052	0.05
676	1.33502	0.05
677	0.946131	0.0546831
678	0.691062	0.0618079
679	1.02663	0.05
680	1.80569	0.05
681	1.26062	0.05
682	1.18203	0.05
683	0.946131	0.0563067
684	0.795065	0.05
685	1.58753	0.05
686	1.58753	0.05
687	0.946131	0.05
688	0.795093	0.05
689	0.805057	2.1
690	0.812415	2.1
691	1.23868	0.345277
692	1.34448	0.352053
693	1.67201	2.1

694	1.61202	2.1
695	1.41103	2.1
696	1.41103	2.1
697	0.841088	2.1
698	1.38302	2.1
699	0.841089	2.1
700	0.869097	2.1
701	1.38302	2.1
702	0.949941	2.1
703	0.94994	2.1
704	1.88608	2.1
705	0.854942	2.1
706	1.68893	2.1
707	1.70002	2.1
708	2.02107	0.257131
709	2.04982	0.244351
710	3.11063	0.371384
711	2.03805	0.258963
712	1.67638	2.1
713	0.967492	2.1
714	2.00209	2.1
715	0.967491	2.1
716	0.870742	2.1
717	1.30266	0.252555
718	0.651334	0.283937
719	1.69413	0.180631
720	1.69413	0.294145
721	0.946132	0.291324
722	1.03079	0.26721
723	1.31468	0.24248
724	0.946132	0.305609
725	0.919055	0.156029
726	1.16815	0.190956
727	2.79004	0.171806
728	1.2136	0.105039
729	2.62935	0.159064
730	0.947692	0.446499
731	1.51835	0.434833
732	0.947693	0.220809
733	0.947692	0.29733
734	0.980841	2.1
735	1.43577	2.1
736	0.980841	2.1
737	1.67858	2.1
738	1.34124	0.538769
739	1.39818	0.462319
740	1.2065	2.1
741	0.946133	2.1
742	1.2079	2.1
743	1.65229	2.1
744	1.65229	2.1
745	0.946132	2.1
746	1.2079	2.1
747	1.39053	2.1
748	1.20651	2.1
749	1.36919	2.1
750	1.12717	2.1
751	1.09483	2.1

752	0.994294	2.1
753	0.97356	2.1
754	1.06827	2.1
755	1.40571	2.1
756	1.29259	2.1
757	0.946131	2.1
758	1.7787	2.1
759	1.7787	2.1
760	1.29259	2.1
761	0.946132	2.1
762	1.09408	0.697013
763	1.03824	0.513146
764	0.851541	0.37147
765	1.23608	0.340641
766	0.851539	0.438547
767	1.5021	0.49974
768	1.54839	0.422768
769	1.12373	0.302263
770	1.22617	0.253404
771	1.42762	0.526333
772	2.24746	0.531088
773	1.05475	0.333171
774	1.46845	0.38354
775	1.05475	0.25412
776	0.929726	0.31532
777	1.52072	0.326359
778	1.82488	0.285964
779	1.02271	0.304043
780	1.19225	0.306179
781	1.43071	0.272987
782	0.94613	0.324967
783	1.05475	0.212165
784	1.39223	2.1
785	1.71255	2.1
786	0.946132	0.744891
787	1.10531	0.776063
788	1.46355	0.761266
789	1.46355	0.693887
790	0.78086	0.941877
791	1.10531	0.779309
792	1.1391	0.747955
793	1.56166	0.845469
794	2.21062	0.756063
795	1.0361	0.95617
796	1.73577	2.1
797	1.73577	1.8605
798	0.946116	2.1
799	1.28174	2.1
800	0.946118	1.19943
801	1.22883	2.1
802	1.28172	2.1
803	1.03609	1.13644
804	0.553567	0.05
805	1.60581	0.05
806	1.60581	0.05
807	0.553594	0.05
808	0.966748	0.05
809	0.966748	0.05

810	0.946133	0.05
811	1.05602	0.05
812	2.38525	0.05
813	1.14503	0.05
814	1.24678	0.05
815	0.56262	0.05
816	1.24678	0.05
817	0.978467	0.05
818	1.66262	0.05
819	1.66262	0.05
820	0.562633	0.05
821	1.09522	0.05
822	2.49356	0.05
823	0.592547	0.05
824	1.04321	0.893477
825	1.25133	1.09184
826	1.70508	0.997361
827	1.81043	0.764551
828	0.494934	0.852332
829	0.601047	0.793692
830	0.993428	0.813281
831	0.946573	0.90678
832	1.35039	0.745045
833	0.816895	1.28448
834	1.5063	0.750695
835	0.816895	0.659013
836	0.946132	0.713683
837	1.9067	0.991166
838	2.14504	1.28164
839	0.816895	0.635482
840	2.29624	0.648486
841	2.76933	0.696878
842	1.254	1.07149
843	0.946128	2.1
844	1.38813	2.1
845	1.79998	2.1
846	1.79998	2.1
847	0.94613	2.1
848	1.38813	2.1
849	1.35798	2.1
850	1.27809	2.1
851	1.35798	2.1
852	1.68994	2.1
853	1.68994	2.1
854	0.946132	2.1
855	0.946132	2.1
856	1.18067	2.1
857	0.958256	2.1
858	0.958255	2.1
859	0.958257	2.1
860	0.958255	2.1
861	1.06457	2.1
862	1.14195	1.21572
863	1.70423	0.556951
864	1.14195	1.13989
865	1.64285	2.1
866	2.02057	2.1
867	0.946132	2.1

868	1.44186	2.1
869	0.946133	2.1
870	1.16366	2.1
871	1.17233	2.1
872	1.31259	0.574456
873	1.2016	2.1
874	1.17129	2.1
875	1.05724	0.398185
876	1.32697	0.710502
877	1.05724	0.531294
878	1.04292	0.825633
879	3.39037	2.1
880	2.57275	2.1
881	0.629212	2.1
882	2.32525	2.1
883	1.76949	0.782196
884	1.65809	1.11818
885	1.27665	2.1
886	1.19861	2.1
887	1.20045	2.1
888	1.1635	2.1
889	0.953633	0.141268
890	0.953634	0.186497
891	1.21408	2.1
892	1.25716	2.1
893	2.01791	2.1
894	1.89318	2.1
895	0.947921	0.944694
896	0.939049	2.1
897	0.947921	1.21383
898	0.740753	2.1
899	0.916104	1.5488
900	1.16941	2.1
901	1.72038	2.1
902	2.04441	2.1
903	0.973182	2.1
904	1.11751	1.73398
905	0.611089	2.1
906	1.53584	0.407927
907	0.767923	0.445073
908	1.38925	0.441206
909	1.38926	0.416784
910	0.946131	0.471449
911	0.961347	0.4494
912	1.19583	0.487263
913	0.946131	0.476015
914	1.3663	0.552892
915	1.21067	0.409371
916	1.00166	0.0573804
917	1.40707	0.0629454
918	1.50435	0.0603447
919	1.47954	0.5556
920	1.1233	2.1
921	2.36036	1.06331
922	1.04024	0.575625
923	0.739729	0.631237
924	1.45533	0.53795
925	1.45532	0.519936

926	0.946118	0.592361
927	1.15163	0.441334
928	1.15165	0.644802
929	0.739748	0.54155
930	1.04025	0.783287
931	1.47608	0.374615
932	0.946131	0.406008
933	1.02028	0.395515
934	0.946129	0.395564
935	1.78223	0.369772
936	1.78223	0.366558
937	1.34153	0.381681
938	1.38683	0.357938
939	1.3407	0.355968
940	1.3407	0.350605
941	2.04729	0.355985
942	0.851529	0.38945
943	1.26671	0.371601
944	0.946116	0.386967
945	1.4869	0.353809
946	1.29553	0.35114
947	1.03645	0.378048
948	1.20731	0.368006
949	0.73665	2.1
950	0.693596	2.1
951	2.11152	1.23938
952	1.73627	0.967981
953	1.06191	2.1
954	1.52226	2.1
955	1.76869	2.1
956	1.1881	2.1
957	1.32349	2.1
958	1.51041	2.1
959	1.61591	2.1
960	0.946132	2.1
961	0.946132	2.1
962	1.13537	2.1
963	3.0048	2.1
964	0.985109	2.1
965	1.68899	2.1
966	3.23261	2.1
967	1.08831	2.1
968	2.20623	2.1
969	0.91749	2.1
970	1.15515	2.1
971	0.550438	2.1
972	1.66294	2.1
973	1.08257	2.1
974	0.87851	2.1
975	2.09046	2.1
976	0.738711	2.1
977	1.31242	0.0640336
978	1.26311	0.0682964
979	1.67652	0.05
980	0.968594	2.1
981	0.968594	2.1
982	0.981099	0.05
983	1.32418	0.05

984	2.67175	0.05
985	2.09678	0.05
986	1.2511	0.05
987	0.912698	0.05
988	0.810946	0.05
989	0.810959	0.05
990	1.67479	0.05
991	1.65677	0.05
992	1.28469	0.05
993	1.48817	0.05
994	1.08131	0.05
995	1.04349	0.522075
996	1.42492	0.445612
997	1.05724	0.424717
998	1.65723	0.343685
999	1.95313	0.352392
1000	1.9293	0.341156
1001	2.02729	0.351359
1002	0.963393	0.809574
1003	0.963392	0.723145
1004	2.90171	2.1
1005	2.65447	2.1
1006	1.64158	2.1
1007	1.6488	2.1
1008	1.15991	0.460745
1009	0.946132	0.512239
1010	1.58199	0.485095
1011	1.58199	0.505
1012	1.03333	0.5117
1013	1.25436	0.491813
1014	1.25436	0.519035
1015	0.946132	0.535342
1016	1.3771	0.403522
1017	0.687745	0.396103
1018	1.37709	0.362971
1019	0.812386	0.419431
1020	1.50172	0.410341
1021	1.50172	0.290073
1022	0.687758	0.404838
1023	1.17506	0.289979
1024	2.00539	0.373167
1025	2.2441	0.441907
1026	1.09667	0.306056
1027	1.43577	2.1
1028	2.24741	2.1
1029	1.26103	2.1
1030	0.851544	2.1
1031	1.5543	2.1
1032	1.59756	2.1
1033	0.851543	2.1
1034	1.44673	2.1
1035	1.31947	2.1
1036	1.1237	2.1
1037	1.26103	2.1
1038	1.089	0.0806852
1039	0.946128	0.123988
1040	0.965824	0.125751
1041	0.94613	0.159128

1042	1.51344	0.0932145
1043	1.51344	0.0897156
1044	1.13508	0.109647
1045	1.32449	0.107827
1046	1.089	0.116039
1047	3.33706	0.260383
1048	2.7542	0.373229
1049	1.27256	0.446286
1050	0.954172	0.423148
1051	1.55527	0.410111
1052	1.05765	0.479645
1053	1.57326	0.45383
1054	1.38251	0.295086
1055	0.94613	0.43302
1056	2.38829	0.311025
1057	2.00539	0.361714
1058	1.53798	0.354139
1059	1.20969	2.1
1060	1.15864	2.1
1061	1.7986	2.1
1062	1.74533	2.1
1063	0.944827	1.67385
1064	0.946131	0.934289
1065	0.851543	0.848663
1066	1.52816	0.899233
1067	1.5314	1.16325
1068	1.39266	0.666806
1069	0.851528	0.655722
1070	1.39589	0.63156
1071	1.7348	0.883176
1072	1.85439	1.20818
1073	1.86367	1.0045
1074	1.90905	0.828406
1075	1.22176	0.448845
1076	1.59492	0.443067
1077	2.97929	2.1
1078	2.1905	2.1
1079	1.24128	2.1
1080	0.994293	2.1
1081	2.49767	0.05
1082	2.37105	0.05
1083	1.10834	0.895788
1084	1.53214	0.940206
1085	1.417	1.91738
1086	2.13599	0.689628
1087	1.61727	2.1
1088	1.75004	2.1
1089	1.96301	2.1
1090	0.966631	2.1
1091	1.37791	2.1
1092	1.47302	2.1
1093	1.47302	2.1
1094	0.871519	2.1
1095	0.94613	2.1
1096	0.87155	2.1
1097	2.38731	2.1
1098	2.12399	1.24362
1099	1.74865	1.18709

1100	1.46724	0.762249
1101	2.38056	0.666301
1102	1.46724	0.774093
1103	0.818887	0.723651
1104	0.810944	1.372
1105	0.805227	0.721957
1106	1.06558	0.93781
1107	0.841487	0.786685
1108	1.08456	0.998305
1109	0.903083	0.936806
1110	1.08929	0.05
1111	1.68454	0.05
1112	1.08929	0.05
1113	1.07658	0.0571873
1114	0.904439	0.0599395
1115	1.58501	0.05
1116	1.19729	0.05
1117	1.05427	0.05
1118	1.47764	0.055166
1119	0.810959	0.05
1120	1.60946	0.847488
1121	2.28887	0.822598
1122	0.946133	0.88625
1123	1.14444	0.782234
1124	1.67964	0.697872
1125	1.67964	0.724048
1126	0.755334	0.954778
1127	1.14444	0.864668
1128	1.29054	0.802548
1129	1.37762	0.76508
1130	0.946132	0.867444
1131	0.533645	0.711004
1132	1.48361	0.647423
1133	1.48361	0.7373
1134	0.533645	0.846502
1135	0.872823	0.886324
1136	1.14444	0.71562
1137	1.37762	0.764709
1138	2.10101	0.764548
1139	1.02311	0.784107
1140	1.04083	2.1
1141	1.04084	2.1
1142	1.04084	2.1
1143	1.30116	2.1
1144	1.23477	0.0609308
1145	1.81893	0.0782362
1146	1.23477	0.0660098
1147	1.23477	0.0624869
1148	1.11967	0.0585924
1149	0.946131	0.0707387
1150	1.11966	0.0743383
1151	1.12628	0.0582409
1152	0.629641	0.070242
1153	1.6163	0.071485
1154	1.6163	0.05
1155	1.11117	2.1
1156	1.13006	2.1
1157	1.15149	2.1

1158	1.13179	2.1
1159	0.585368	0.539127
1160	0.986036	1.1973
1161	1.94788	0.525479
1162	1.34857	0.563317
1163	2.31806	2.1
1164	2.06363	2.1
1165	1.00423	2.1
1166	0.829065	2.1
1167	0.551816	2.1
1168	0.99406	2.1
1169	1.98339	2.1
1170	1.68073	2.1
1171	2.558	2.1
1172	0.711814	0.855366
1173	2.97316	1.27635
1174	2.41579	2.1
1175	1.15248	0.184035
1176	1.14305	0.199506
1177	1.15483	0.05
1178	1.90981	0.05
1179	0.998957	0.05
1180	1.83742	0.05
1181	1.27581	2.1
1182	0.94613	2.1
1183	1.6315	2.1
1184	1.6315	2.1
1185	0.946132	2.1
1186	1.28441	2.1
1187	1.28441	2.1
1188	1.00365	2.1
1189	0.968536	2.1
1190	1.57575	2.1
1191	1.27581	2.1
1192	1.99297	2.1
1193	1.32784	2.1
1194	1.4259	2.1
1195	2.27952	2.1
1196	1.5685	2.1
1197	1.54231	2.1
1198	3.54638	2.1
1199	1.3294	2.1
1200	2.98237	2.1
1201	2.01633	0.890044
1202	2.0985	0.943807
1203	2.26047	0.65546
1204	1.7913	0.592488
1205	1.09504	2.1
1206	1.74126	2.1
1207	1.05983	2.1
1208	2.37664	2.1
1209	2.28084	2.1
1210	2.19948	2.1
1211	1.33593	0.05
1212	1.31508	0.05
1213	1.30647	2.1
1214	1.28846	2.1
1215	1.2955	2.1

1216	1.09044	1.34051
1217	1.4099	1.31256
1218	1.14984	1.60728
1219	0.946132	0.775876
1220	1.70727	1.14572
1221	1.70727	2.1
1222	1.14984	0.942005
1223	0.946132	1.32577
1224	0.911818	2.1
1225	2.12209	2.1
1226	2.4158	2.1
1227	1.20867	2.1
1228	2.84085	2.1
1229	1.42284	2.1
1230	2.45689	2.1
1231	1.41562	2.1
1232	0.918358	2.1
1233	1.11222	2.1
1234	0.999083	2.1
1235	0.696781	2.1
1236	1.30869	2.1
1237	1.66132	2.1
1238	0.696807	2.1
1239	0.745315	2.1
1240	1.23296	2.1
1241	0.780548	2.1
1242	1.93242	0.832546
1243	1.27362	0.830172
1244	2.77828	1.23642
1245	1.02653	0.599356
1246	0.808576	0.62143
1247	0.744012	0.753395
1248	0.605174	0.532242
1249	1.16501	0.49428
1250	0.803432	0.509889
1251	0.961016	0.499252
1252	1.10425	0.56365
1253	1.3756	0.645764
1254	2.17765	0.847992
1255	1.84578	0.754457
1256	1.32801	0.619759
1257	1.76377	0.617262
1258	0.946131	0.557249
1259	1.3022	0.695645
1260	0.694814	0.729369
1261	1.53028	0.57278
1262	1.53028	0.579253
1263	1.3022	0.64688
1264	0.922889	0.664532
1265	1.57766	2.04816
1266	2.06911	0.95622
1267	1.57766	1.13317
1268	1.37721	0.109007
1269	1.72174	0.166926
1270	1.4468	0.172771
1271	1.37721	0.0752993
1272	1.73972	0.419073
1273	1.62324	0.43478

1274	0.946125	0.379904
1275	1.34259	0.32923
1276	1.04187	0.225859
1277	1.05019	0.345896
1278	1.2361	0.382076
1279	1.69055	0.320419
1280	2.25056	0.370038
1281	1.10574	0.598573
1282	1.21632	0.739138
1283	1.10574	0.743005
1284	1.46181	1.00395
1285	0.946131	0.811837
1286	1.74111	1.04355
1287	1.74111	0.862244
1288	0.94613	0.814284
1289	1.26891	0.699075
1290	1.26891	0.646159
1291	1.23452	0.972045
1292	1.00255	0.832811
1293	1.72286	0.292857
1294	1.12233	0.132678
1295	1.30682	0.325727
1296	1.97397	0.40584
1297	1.67425	2.1
1298	1.77775	2.1
1299	1.73571	1.90419
1300	0.426265	2.1
1301	1.31817	1.2911
1302	1.81042	2.1
1303	1.85456	2.1
1304	0.466532	1.49979
1305	1.28025	1.19531
1306	0.964467	1.25787
1307	1.27921	2.1
1308	2.11851	1.52738
1309	2.37121	2.06096
1310	1.49357	0.505829
1311	1.42486	2.1
1312	1.00278	2.1
1313	1.00278	2.1
1314	1.12503	2.1
1315	0.568697	2.1
1316	0.555211	2.1
1317	0.982272	1.04387
1318	1.57558	0.946463
1319	0.851539	1.80344
1320	1.07307	2.1
1321	1.58292	2.1
1322	1.50731	2.1
1323	0.851541	0.841202
1324	1.00304	2.1
1325	1.36003	2.1
1326	1.13127	2.1
1327	0.672234	2.1
1328	0.763044	2.1
1329	0.966979	2.1
1330	1.31502	2.1
1331	1.67969	2.1

1332	1.62408	2.1
1333	0.661955	2.1
1334	1.35048	2.1
1335	1.40447	2.1
1336	1.40447	2.1
1337	0.607961	2.1
1338	0.946131	2.1
1339	0.607963	2.1
1340	1.01531	2.1
1341	1.01531	2.1
1342	1.91802	2.1
1343	0.777749	2.1
1344	1.22585	0.0968716
1345	1.20672	0.0863161
1346	1.03166	0.382597
1347	0.553805	2.1
1348	2.00551	2.1
1349	2.07203	1.01834
1350	0.589479	2.1
1351	1.22352	2.1
1352	0.984227	2.1
1353	0.802455	2.1
1354	1.40942	2.1
1355	1.6662	2.1
1356	0.802485	2.1
1357	0.936669	2.1
1358	1.31127	0.704105
1359	0.946132	0.640639
1360	1.09729	0.476229
1361	0.946133	0.659677
1362	1.68703	0.53278
1363	1.68703	0.490754
1364	1.3689	0.594898
1365	1.26426	0.622384
1366	1.26923	2.1
1367	1.23614	2.1
1368	0.828371	2.1
1369	2.04556	2.1
1370	0.772648	2.1
1371	0.791059	2.1
1372	0.809958	2.1
1373	1.70012	2.1
1374	0.267032	2.1
1375	1.67604	2.1
1376	0.803823	2.1
1377	0.267305	2.1
1378	0.80246	2.1
1379	0.874582	2.1
1380	1.00188	2.1
1381	0.709588	2.1
1382	1.50679	1.97333
1383	1.22456	1.94492
1384	0.870622	2.1
1385	1.05754	1.8968
1386	1.1689	2.1
1387	1.54556	2.1
1388	1.89226	2.1
1389	0.995311	2.1

1390	1.26195	2.1
1391	1.9	2.1
1392	0.845055	2.1
1393	0.737836	2.1
1394	0.823707	2.1
1395	0.626587	2.1
1396	1.16109	2.1
1397	0.85517	2.1
1398	0.932036	2.1
1399	1.08321	2.1
1400	1.43863	2.1
1401	2.72297	2.1
1402	2.90798	2.1
1403	1.47818	1.03579
1404	1.47819	0.974036
1405	0.694357	0.909166
1406	0.694382	0.914547
1407	0.946132	0.697817
1408	0.694382	0.82664
1409	1.22641	0.746825
1410	1.31155	1.17143
1411	2.45282	0.968579
1412	0.787843	0.793478
1413	0.808984	0.629943
1414	1.14232	0.969181
1415	0.795037	0.924841
1416	0.887382	0.893349
1417	1.14493	0.834694
1418	0.78393	0.717269
1419	1.09129	0.914158
1420	0.791482	0.841291
1421	1.1137	1.16463
1422	1.11819	0.931532
1423	0.960173	0.851315
1424	1.09447	1.23676
1425	0.874365	0.812865
1426	1.2474	0.247983
1427	1.75038	0.391522
1428	1.75038	0.536789
1429	0.94613	0.697534
1430	1.30076	0.399476
1431	1.12817	0.871179
1432	0.94613	0.612806
1433	1.30076	0.581198
1434	1.51841	0.460137
1435	1.51841	0.590056
1436	0.946132	0.863517
1437	1.39575	0.487729
1438	0.970358	1.09946
1439	0.946133	0.744765
1440	1.39575	0.690338
1441	1.06486	0.317215
1442	1.06486	0.337739
1443	1.38622	0.375696
1444	1.04521	0.05
1445	1.00809	0.0628103
1446	1.28952	0.0683998
1447	0.849316	0.0571185

1448	0.946132	0.0631062
1449	0.849344	0.0758557
1450	1.53631	0.072041
1451	1.53631	0.0761844
1452	1.77843	0.05
1453	1.69863	0.0502943
1454	1.91646	2.1
1455	1.8582	2.1
1456	2.8349	0.05
1457	1.36307	0.05
1458	1.23886	0.05
1459	1.12971	0.05
1460	1.59662	0.05
1461	1.59662	0.05
1462	0.640304	0.0824521
1463	0.640333	0.05
1464	0.886276	0.088058
1465	0.886276	0.0835156
1466	1.35065	0.0614792
1467	2.09002	0.052867
1468	1.35543	0.0627646
1469	1.94688	2.1
1470	1.97221	2.1
1471	0.719563	2.1
1472	1.20358	2.1
1473	0.874264	2.1
1474	1.27676	2.1
1475	1.46557	2.1
1476	1.46557	2.1
1477	0.68546	2.1
1478	0.94613	2.1
1479	0.685489	2.1
1480	1.39615	0.312868
1481	1.05404	0.173835
1482	1.06474	0.296337
1483	1.00713	0.277938
1484	0.714535	0.309731
1485	0.714563	0.319996
1486	1.41646	0.203062
1487	1.52582	0.218058
1488	0.721694	0.318418
1489	0.93665	0.310674
1490	1.21067	0.41686
1491	1.21067	0.415742
1492	1.73577	0.476764
1493	1.17799	0.442108
1494	3.21432	0.449175
1495	2.41452	0.453049
1496	1.36208	0.322979
1497	2.53256	0.357152
1498	0.834593	0.210759
1499	2.5853	0.362321
1500	1.77259	0.263118
1501	0.615724	0.385325
1502	1.11374	0.715337
1503	0.615726	0.334825
1504	0.946129	0.869939
1505	0.615714	0.814597

1506	1.75489	0.717453
1507	1.75489	0.582918
1508	1.23146	0.411645
1509	2.2772	0.387681
1510	0.74463	0.407698
1511	1.3546	0.31874
1512	1.80252	0.247881
1513	2.38304	0.338209
1514	1.19151	0.497338
1515	1.1231	0.513226
1516	1.82345	0.413555
1517	1.79805	0.358628
1518	0.761337	0.699783
1519	0.946127	0.472595
1520	0.557726	0.528902
1521	1.05353	0.179808
1522	1.05353	0.145069
1523	1.68599	0.206883
1524	1.05353	0.158687
1525	1.40588	2.1
1526	1.09373	2.1
1527	1.18382	0.928003
1528	1.46903	0.858544
1529	1.25103	2.1
1530	1.10378	2.1
1531	1.58941	2.1
1532	1.29639	2.1
1533	1.14874	0.05
1534	0.899817	0.05
1535	0.666898	0.05
1536	1.76519	0.05
1537	1.17289	0.05
1538	1.03526	0.05
1539	1.97708	0.05
1540	1.31711	0.05
1541	0.992517	0.05
1542	1.70193	0.05
1543	1.13852	0.05
1544	1.23943	0.16629
1545	1.40014	0.692082
1546	1.04598	0.883825
1547	1.1483	0.763741
1548	1.11433	0.930744
1549	1.32037	0.579311
1550	1.00381	0.679491
1551	1.11435	0.508226
1552	0.973502	0.816011
1553	0.946128	1.69015
1554	1.83697	1.02784
1555	1.83697	2.1
1556	1.31721	1.60563
1557	1.38614	1.13972
1558	1.39696	1.00945
1559	1.31721	1.36627
1560	1.0756	1.66943
1561	1.08542	0.877268
1562	0.946127	2.09838
1563	1.81052	0.760345

1564	1.81052	2.1
1565	1.24315	1.90993
1566	1.39696	0.689324
1567	1.35969	1.18718
1568	1.24315	1.05678
1569	1.0756	0.60217
1570	1.20669	0.242045
1571	1.18508	0.295291
1572	1.71059	2.1
1573	1.66762	2.1
1574	2.50853	2.1
1575	1.8236	2.1
1576	1.78872	2.1
1577	1.41721	2.1
1578	1.01335	1.37716
1579	1.42439	0.952626
1580	0.946129	1.00679
1581	1.33266	1.20114
1582	1.79776	1.25065
1583	1.79776	1.87578
1584	0.946131	0.754639
1585	1.33266	1.29913
1586	1.41123	1.11566
1587	1.25346	2.1
1588	0.946132	0.803447
1589	0.827287	1.91468
1590	1.57878	1.04138
1591	1.57878	1.22239
1592	0.827287	1.02221
1593	1.07341	1.07936
1594	1.33266	1.02605
1595	1.25346	1.03593
1596	1.61149	2.1
1597	1.15644	1.93816
1598	1.27558	0.759837
1599	1.3764	0.549272
1600	1.89526	0.935559
1601	1.37641	0.53823
1602	1.2064	0.485636
1603	1.38426	1.95539
1604	1.01595	2.1
1605	1.15067	2.1
1606	1.82276	1.47972
1607	1.82276	0.313381
1608	0.946131	0.551913
1609	1.40609	0.458483
1610	0.946132	0.889212
1611	1.29143	0.699694
1612	1.40609	0.700174
1613	1.30272	0.866372
1614	1.19548	0.7222
1615	2.95451	2.1
1616	1.46124	2.1
1617	2.93215	2.1
1618	1.42105	2.1
1619	1.75477	0.937984
1620	2.31301	0.995746
1621	2.10275	0.648511

1622	1.80099	0.704077
1623	0.97541	2.1
1624	1.49733	2.1
1625	0.97541	2.1
1626	1.30751	2.1
1627	1.28614	0.172163
1628	1.74759	0.0583938
1629	1.19756	0.326651
1630	1.71053	0.335161
1631	1.19757	0.360726
1632	1.28954	0.319905
1633	2.02703	0.323724
1634	2.29453	0.0845902
1635	1.28265	0.05
1636	0.793267	0.0857329
1637	1.19974	0.172956
1638	0.509551	0.064955
1639	1.04072	0.157969
1640	1.35302	0.0553692
1641	1.53133	0.171882
1642	0.566153	0.0899134
1643	1.09634	0.0702818
1644	1.12792	0.115747
1645	1.68908	2.1
1646	1.68908	2.1
1647	0.768212	1.89136
1648	0.768213	2.1
1649	0.946132	2.1
1650	0.768212	1.87307
1651	1.32461	1.87972
1652	1.87291	1.83981
1653	2.64921	1.99831
1654	1.53642	2.1
1655	2.26537	2.1
1656	1.53642	2.1
1657	1.79874	2.1
1658	1.79874	2.1
1659	0.726635	1.18737
1660	0.726636	2.1
1661	0.946128	1.2096
1662	0.726634	1.21441
1663	1.23339	0.978177
1664	0.992674	2.1
1665	1.8023	2.1
1666	1.12324	0.05
1667	1.29163	0.05
1668	0.996831	0.143991
1669	1.0019	0.133519
1670	0.993831	0.347575
1671	1.07283	0.972012
1672	1.04788	2.1
1673	1.44489	0.932853
1674	1.04788	1.78145
1675	1.07652	2.1
1676	0.991406	2.1
1677	1.37	2.1
1678	1.14195	2.1
1679	1.47904	0.0700497

1680	1.26593	1.34409
1681	1.75366	1.17668
1682	1.57621	0.39852
1683	1.24538	0.279579
1684	0.969118	2.1
1685	0.946133	0.801296
1686	1.69387	2.1
1687	1.69387	2.1
1688	0.94613	1.3412
1689	1.28713	2.1
1690	1.28713	2.06423
1691	1.23877	2.09618
1692	0.969118	2.1
1693	1.22615	2.1
1694	0.98084	2.1
1695	1.01843	2.1
1696	1.50876	2.1
1697	1.85391	2.1
1698	1.84322	2.1
1699	1.70377	2.1
1700	1.78919	2.1
1701	2.66787	2.1
1702	1.41549	2.1
1703	1.41549	2.1
1704	0.86707	2.1
1705	0.896172	2.1
1706	0.946126	2.1
1707	0.867074	2.1
1708	0.946118	2.1
1709	1.54468	0.185753
1710	1.54468	0.187178
1711	2.27259	0.196841
1712	0.946131	0.336628
1713	1.25837	0.308685
1714	0.663171	0.405657
1715	1.68931	0.212629
1716	1.68931	0.212091
1717	1.25837	0.292855
1718	1.09411	0.298211
1719	1.73571	0.156131
1720	2.34894	0.187818
1721	0.946128	0.213934
1722	1.30497	0.208428
1723	0.772312	0.289303
1724	1.66895	0.150942
1725	1.66895	0.125746
1726	1.30497	0.182266
1727	1.13629	0.156045
1728	1.22852	0.798771
1729	1.578	0.74534
1730	2.07781	1.93816
1731	1.5478	1.8904
1732	1.70048	1.05046
1733	1.42329	1.01701
1734	1.05481	1.54908
1735	1.1032	0.918709
1736	1.19267	2.1
1737	1.77324	2.1

1738	1.01121	0.337099
1739	0.991867	0.33932
1740	2.0744	0.942435
1741	1.75142	2.1
1742	1.35418	0.925668
1743	1.40041	2.1
1744	1.50045	2.1
1745	1.37704	2.1
1746	1.50045	2.1
1747	1.87875	2.1
1748	1.35308	0.64757
1749	1.29983	0.655241
1750	1.62784	2.1
1751	1.44807	2.1
1752	1.00965	2.1
1753	2.69264	2.1
1754	1.04075	2.1
1755	0.946131	2.1
1756	0.946132	2.1
1757	1.52704	2.1
1758	1.83246	2.1
1759	1.56757	2.1
1760	1.16687	2.1
1761	2.04857	2.1
1762	1.32707	0.945852
1763	1.25519	0.240278
1764	1.32101	0.241478
1765	1.25055	0.689109
1766	0.827853	0.351908
1767	1.11835	0.202135
1768	1.07281	0.946699
1769	1.54302	0.443168
1770	0.724064	0.679416
1771	1.32709	2.07073
1772	3.44123	0.782464
1773	2.46889	0.213337
1774	0.730076	0.0911858
1775	1.25809	0.284804
1776	0.730077	0.131758
1777	0.946129	0.260168
1778	1.72777	0.0717403
1779	1.72777	0.252527
1780	0.73005	0.161112
1781	1.7522	0.0732736
1782	2.51618	0.103106
1783	0.628177	0.0985587
1784	1.1068	0.281644
1785	0.747435	0.113151
1786	0.946133	0.25516
1787	1.84668	0.10163
1788	1.8563	0.233516
1789	0.628177	0.187785
1790	1.45501	0.124449
1791	2.19809	0.259778
1792	1.45501	0.131061
1793	1.48974	0.101154
1794	1.7709	2.01219
1795	2.63288	1.84605

1796	1.5362	2.1
1797	1.56327	2.1
1798	3.03945	0.192586
1799	0.899979	0.266936
1800	0.869386	0.27024
1801	1.85864	0.217723
1802	1.78028	0.248278
1803	1.28719	0.255858
1804	1.09532	0.216592
1805	0.881898	0.272177
1806	1.28911	0.250737
1807	1.39644	0.29844
1808	0.688829	0.264752
1809	2.14611	0.215462
1810	1.80313	0.209329
1811	0.68883	0.289274
1812	0.946131	0.3057
1813	1.25334	0.10641
1814	1.88987	0.198154
1815	1.94432	0.189772
1816	1.37592	0.152806
1817	1.11691	0.996988
1818	1.02923	1.09754
1819	0.45824	0.05
1820	0.646512	0.0973312
1821	2.46292	0.0659006
1822	0.493451	0.0724401
1823	2.00826	2.1
1824	2.49784	2.1
1825	1.17054	2.1
1826	1.01485	2.1
1827	1.26045	2.1
1828	1.17054	2.1
1829	1.44247	2.1
1830	2.02687	0.05
1831	1.66365	0.05
1832	1.64656	0.05
1833	1.56597	0.05
1834	1.21419	2.1
1835	1.5008	2.1
1836	1.10711	2.1
1837	1.44287	2.1
1838	1.44287	2.1
1839	0.750368	2.1
1840	1.25876	2.1
1841	0.82542	2.1
1842	1.04074	2.1
1843	1.25874	2.1
1844	1.04159	0.05
1845	1.2489	0.05
1846	0.980547	0.416064
1847	1.62235	0.357961
1848	1.15991	0.416176
1849	0.980547	0.279858
1850	0.94613	0.426506
1851	1.54662	0.389366
1852	1.54661	0.405571
1853	1.07912	0.435878

1854	1.1032	0.38464
1855	1.1032	0.406078
1856	0.94613	0.417783
1857	1.12468	2.1
1858	1.2724	2.1
1859	1.06521	1.24151
1860	1.0752	1.52051
1861	1.94936	0.336248
1862	1.62772	0.346508
1863	1.94733	0.328419
1864	1.42901	0.34221
1865	1.33022	2.1
1866	1.70839	2.1
1867	1.75048	2.1
1868	1.41201	2.1
1869	1.52314	2.1
1870	1.44039	2.1
1871	1.51113	2.1
1872	2.58638	2.1
1873	0.987247	0.234899
1874	1.38622	0.426988
1875	0.858359	0.340259
1876	1.19967	0.390557
1877	1.06414	0.248344
1878	0.946132	0.46014
1879	0.94613	0.33889
1880	0.687079	0.522079
1881	0.946132	0.44985
1882	0.822611	0.429572
1883	1.70242	0.290026
1884	1.70242	0.438433
1885	0.946132	0.541634
1886	1.11155	0.409724
1887	0.946132	0.608168
1888	1.11155	0.481519
1889	1.37981	0.503651
1890	1.18348	0.290098
1891	2.44179	0.437355
1892	2.00403	0.511806
1893	1.10961	2.1
1894	1.85415	2.1
1895	1.44825	2.1
1896	0.932272	2.1
1897	0.810942	2.1
1898	1.00189	2.1
1899	1.11779	2.1
1900	1.3359	2.1
1901	0.965279	2.1
1902	0.833779	2.1
1903	1.05337	2.1
1904	0.526656	2.1
1905	1.44599	2.1
1906	1.44599	2.1
1907	1.24853	2.1
1908	0.946132	2.1
1909	1.24853	2.1
1910	0.724122	2.1
1911	1.91547	2.1

1912	2.49705	2.1
1913	1.05931	2.1
1914	1.1729	2.1
1915	1.34526	2.1
1916	1.5379	2.1
1917	1.90855	2.1
1918	2.08597	2.1
1919	1.68165	2.1
1920	1.45887	2.1
1921	1.50022	2.1
1922	1.4748	2.01403
1923	2.63645	1.86799
1924	1.74431	2.00999
1925	1.25288	1.92141
1926	1.25289	1.68325
1927	0.970025	1.54417
1928	0.747542	2.1
1929	0.810959	1.44333
1930	1.15369	1.68757
1931	0.977472	1.55515
1932	1.12695	1.708
1933	0.996762	1.5326
1934	1.28249	0.666183
1935	0.946132	0.698018
1936	0.528621	0.777765
1937	1.59186	0.611484
1938	1.44072	0.626247
1939	0.633488	0.632344
1940	0.598506	0.710332
1941	1.51618	0.744997
1942	1.19999	0.589402
1943	1.46511	0.568157
1944	1.44056	0.550607
1945	0.912754	0.474866
1946	1.4725	0.443272
1947	1.4725	0.428238
1948	0.912753	0.472554
1949	1.23351	0.477568
1950	1.23351	0.490492
1951	0.946131	0.527376
1952	1.17129	0.425631
1953	1.82551	0.386919
1954	1.27575	1.71923
1955	0.946129	1.99151
1956	0.946129	2.1
1957	0.946128	1.87115
1958	0.792754	2.1
1959	0.94613	1.49277
1960	0.792752	1.39956
1961	2.08054	2.1
1962	1.20091	2.1
1963	2.1669	1.93361
1964	1.24383	1.55123
1965	0.904669	1.8724
1966	0.83804	1.30917
1967	1.90486	1.65758
1968	1.29162	1.45344
1969	1.37383	1.78086

1970	1.86533	1.44131
1971	1.76472	1.83048
1972	1.60428	1.43019
1973	2.43993	1.87427
1974	1.37833	2.1
1975	1.18632	2.1
1976	0.946132	2.1
1977	1.27544	2.1
1978	1.05085	2.1
1979	0.961178	2.1
1980	0.859844	2.1
1981	1.0499	2.1
1982	1.75592	2.1
1983	2.08668	2.1
1984	1.75592	2.1
1985	1.4402	2.1
1986	1.72285	2.1
1987	0.83312	0.102722
1988	1.07295	0.0878466
1989	1.37607	0.114991
1990	1.32097	0.0847162
1991	1.11332	0.130247
1992	1.18568	0.0753042
1993	0.877482	0.0808481
1994	1.4458	0.0579511
1995	2.34112	0.05
1996	2.68021	0.0883191
1997	0.681677	0.05
1998	0.946131	0.057307
1999	0.681649	0.05
2000	1.83115	0.05
2001	1.68029	0.0520489
2002	1.05482	0.05
2003	1.31485	0.05
2004	2.08247	1.06576
2005	1.33153	1.00197
2006	0.935062	1.11369
2007	0.806958	1.61923
2008	0.827683	0.9999
2009	1.01041	1.23507
2010	0.741832	0.959453
2011	1.15668	1.32626
2012	0.908649	1.09669
2013	0.59561	0.270509
2014	1.19028	0.784245
2015	0.59561	0.751691
2016	0.946132	0.858688
2017	0.595623	1.04134
2018	1.58164	0.813054
2019	1.58164	0.867811
2020	0.946131	1.06853
2021	1.55201	2.1
2022	1.55201	2.1
2023	0.946127	2.1
2024	1.13312	2.1
2025	1.13312	2.1
2026	1.08108	2.1
2027	1.04973	2.1

2028	1.20149	2.1
2029	1.86902	0.853514
2030	0.613447	2.1
2031	1.22618	0.972767
2032	1.38663	1.0932
2033	1.38662	2.1
2034	0.613447	1.0119
2035	1.22617	1.82589
2036	0.773899	1.4471
2037	1.23515	1.17256
2038	1.87823	1.04157
2039	1.70303	0.591805
2040	1.4714	2.1
2041	1.74067	2.1
2042	0.735699	2.1
2043	1.73461	2.1
2044	1.47224	2.1
2045	1.22827	2.1
2046	1.1634	2.1
2047	1.36471	2.1
2048	0.735701	2.1
2049	2.8386	0.625977
2050	2.39056	2.1
2051	2.44128	0.775671
2052	1.54102	2.1
2053	1.54102	1.7779
2054	0.936141	0.96926
2055	1.40594	2.1
2056	1.07121	0.689291
2057	0.936142	1.25966
2058	1.07121	0.946083
2059	1.22691	0.284719
2060	1.42907	0.24985
2061	2.18048	0.210092
2062	0.947662	0.229007
2063	2.18394	2.1
2064	1.21292	2.1
2065	2.18394	2.1
2066	2.19064	2.1
2067	1.27229	2.1
2068	1.20859	0.730263
2069	1.21073	0.673491
2070	1.73899	2.1
2071	1.3164	2.1
2072	1.61245	2.1
2073	1.41444	2.1
2074	2.55116	2.1
2075	1.26193	2.1
2076	1.89553	2.1
2077	0.916438	2.1
2078	1.26113	2.1
2079	0.810934	2.1
2080	2.00419	2.1
2081	1.27872	2.1
2082	1.35306	2.1
2083	1.96749	2.1
2084	1.07955	2.1
2085	1.31854	2.1

2086	1.29505	0.65039
2087	1.29504	0.644448
2088	0.612469	0.729084
2089	0.990053	0.705227
2090	1.04072	0.671278
2091	0.985266	0.6523
2092	0.990039	0.617272
2093	1.06892	0.76651
2094	1.57158	0.556171
2095	1.895	0.642092
2096	1.05698	0.575731
2097	0.925053	2.1
2098	0.624712	2.1
2099	1.42465	2.1
2100	1.49014	2.1
2101	0.687198	2.1
2102	1.22058	2.1
2103	0.851488	2.1
2104	0.588128	2.1
2105	1.12452	2.1
2106	1.02783	2.1
2107	1.7083	2.1
2108	1.06977	1.10694
2109	0.818569	0.795271
2110	1.14386	1.3786
2111	0.983791	0.908363
2112	1.12214	1.59411
2113	1.0915	0.953563
2114	0.810934	0.893264
2115	0.764114	1.21283
2116	1.87956	0.911516
2117	1.87956	1.4684
2118	0.764141	0.865817
2119	1.37675	0.811849
2120	0.764141	0.851401
2121	0.946132	1.05495
2122	1.9637	1.80802
2123	2.10107	0.713661
2124	1.67814	1.24746
2125	3.02886	1.14945
2126	0.908982	0.175286
2127	0.621727	0.241248
2128	0.525963	0.217161
2129	0.946128	0.271065
2130	1.60979	0.189936
2131	1.45596	0.23036
2132	0.556172	0.218838
2133	2.06779	0.117077
2134	2.17911	0.178673
2135	1.00205	0.0851796
2136	1.30685	0.112145
2137	1.16534	0.123068
2138	1.65532	2.1
2139	2.20346	2.1
2140	0.993889	2.1
2141	1.09328	2.1
2142	2.09372	2.1
2143	2.10036	2.1

2144	1.33727	1.70578
2145	2.53383	1.72036
2146	0.893379	1.62364
2147	1.52828	1.41876
2148	1.09599	0.05
2149	1.09599	0.05
2150	1.50524	2.1
2151	1.54711	2.1
2152	1.41469	2.1
2153	1.99459	1.74273
2154	1.79805	1.97192
2155	1.79805	2.1
2156	0.707344	2.1
2157	1.30318	1.72539
2158	0.707344	2.1
2159	0.946129	2.1
2160	1.30318	2.1
2161	2.40442	2.1
2162	1.89226	2.1
2163	1.40794	2.1
2164	0.898721	2.06803
2165	0.791921	1.02016
2166	0.810933	1.82153
2167	1.10904	1.33932
2168	0.991102	2.04995
2169	0.808502	0.952107
2170	1.1398	1.58791
2171	1.31346	2.1
2172	1.11833	2.1
2173	1.669	2.1
2174	1.25715	2.1
2175	1.3199	2.1
2176	1.63092	2.1
2177	1.63092	2.1
2178	0.946131	2.1
2179	0.946131	2.1
2180	1.13534	2.1
2181	0.979853	2.1
2182	2.19994	2.1
2183	0.725844	2.1
2184	2.19994	2.1
2185	2.35055	2.1
2186	1.52204	2.1
2187	1.40118	2.1
2188	3.42285	1.96786
2189	2.05217	2.1
2190	1.50319	2.1
2191	0.947516	0.986868
2192	0.903572	0.960504
2193	0.76996	1.03714
2194	1.90959	2.1
2195	1.18897	1.74961
2196	1.24166	1.57697
2197	2.26785	1.2145
2198	2.05217	2.1
2199	2.73306	2.1
2200	1.42729	0.173509
2201	1.387	0.171659

2202	1.59458	0.482732
2203	1.91051	0.506567
2204	1.68108	0.491187
2205	2.03357	0.568896
2206	0.928982	0.284052
2207	1.55687	0.303349
2208	1.55687	0.148229
2209	0.92898	0.250342
2210	1.3495	0.33293
2211	1.3495	0.251895
2212	0.946133	0.337047
2213	1.85795	0.215733
2214	2.2727	0.310771
2215	1.20324	1.01971
2216	1.07784	0.333592
2217	0.957792	0.169061
2218	1.2745	0.173565
2219	1.4659	0.197255
2220	0.946129	0.366131
2221	0.95778	0.335638
2222	0.851553	0.438963
2223	1.74103	0.232145
2224	2.28994	0.238148
2225	2.08213	0.198319
2226	2.1556	0.213616
2227	2.20372	0.154406
2228	0.838027	0.0696943
2229	1.11373	0.0614241
2230	1.2909	0.05
2231	0.998424	0.0629202
2232	1.40621	0.05
2233	1.19478	0.0537533
2234	1.11783	0.0574571
2235	1.6695	0.05
2236	1.7498	0.056432
2237	0.477652	0.0612136
2238	1.77291	0.0681869
2239	0.590448	0.0551923
2240	1.07668	0.0630833
2241	0.810946	0.0897465
2242	1.1946	0.0775232
2243	2.03914	0.0650423
2244	1.25585	0.0840026
2245	2.02622	0.066476
2246	1.39091	0.0692383
2247	1.31871	0.0781814
2248	1.15789	2.1
2249	1.15755	2.1
2250	1.82268	2.1
2251	1.87119	2.1
2252	3.10636	2.1
2253	2.96285	2.1
2254	1.73443	0.627058
2255	2.33812	0.657896
2256	1.59457	0.523983
2257	1.73443	0.940137
2258	2.00665	2.1
2259	1.99841	2.1

2260	1.19901	2.1
2261	1.16661	2.1
2262	2.15883	0.209722
2263	1.20951	0.193942
2264	2.49192	0.331208
2265	0.65112	0.168891
2266	2.45317	0.05
2267	1.73091	0.05
2268	2.0795	0.05
2269	1.45891	0.05
2270	1.6531	0.05
2271	1.78304	0.05
2272	0.775132	0.05
2273	1.35051	0.05
2274	1.0461	0.05
2275	1.37837	0.05
2276	1.00259	0.05
2277	1.25796	0.512464
2278	1.10719	0.639047
2279	1.51067	2.1
2280	2.65632	1.21451
2281	1.30521	2.1
2282	2.456 2.1	
2283	3.19218	2.1
2284	2.67387	1.61612
2285	0.988862	2.1
2286	1.917 2.1	
2287	0.946132	2.1
2288	1.16202	2.1
2289	1.53871	2.1
2290	1.53871	2.1
2291	0.946132	2.1
2292	1.16202	2.1
2293	1.32282	2.1
2294	2.46356	2.1
2295	2.32404	2.1
2296	1.37562	0.320466
2297	0.985546	0.330728
2298	1.28983	0.260157
2299	1.05566	0.297924
2300	0.895312	0.319801
2301	1.53325	0.26612
2302	1.04342	0.320999
2303	1.80881	0.29512
2304	0.876865	0.26778
2305	0.5689	0.293532
2306	1.09506	0.316163
2307	1.51789	2.1
2308	1.49872	2.1
2309	0.949364	0.395778
2310	2.01233	0.402437
2311	1.55063	0.386532
2312	1.57783	0.565781
2313	1.57783	0.605528
2314	0.674605	0.512867
2315	0.98537	0.389116
2316	0.674633	0.272467
2317	0.98537	0.374996

2318	1.26706	0.574757
2319	0.980664	0.660951
2320	1.03298	0.410914
2321	1.84936	0.593115
2322	1.51696	0.430883
2323	1.97074	0.383525
2324	1.03298	0.38544
2325	1.51324	0.288589
2326	1.51324	0.356771
2327	0.758454	0.301721
2328	1.28632	0.246024
2329	0.758482	0.162394
2330	0.758482	0.240845
2331	0.985372	0.336828
2332	1.14105	0.0936397
2333	1.59787	0.348588
2334	2.46244	0.485309
2335	1.70367	0.137696
2336	2.13757	1.07127
2337	1.98142	1.08559
2338	1.35878	0.0663873
2339	2.39947	0.0988672
2340	1.24554	0.0719882
2341	1.7522	0.0807417
2342	1.49693	1.73222
2343	1.49652	1.89189
2344	1.6211	1.69247
2345	1.45938	1.83921
2346	0.737431	1.94761
2347	1.18643	1.85774
2348	1.04072	2.1
2349	0.9459	1.77604
2350	0.66364	1.94327
2351	0.985109	1.47824
2352	0.638368	1.89899
2353	0.83804	1.38513
2354	0.998024	1.63241
2355	0.914471	1.29776
2356	1.14281	1.71727
2357	0.833972	1.46241
2358	0.991865	0.464724
2359	1.42208	0.470097
2360	1.70718	2.1
2361	1.79888	2.1
2362	1.16851	0.467259
2363	1.16851	0.445668
2364	1.51021	0.480755
2365	0.946132	0.370496
2366	1.14897	0.294969
2367	0.946132	0.404151
2368	1.77177	0.381214
2369	1.77177	0.308837
2370	1.33038	0.338677
2371	1.38752	0.337691
2372	1.1726	0.375831
2373	1.51688	0.362434
2374	1.06455	2.1
2375	0.98511	2.1

2376	1.31144	2.1
2377	1.79825	2.1
2378	1.79825	2.1
2379	1.73044	2.1
2380	1.59589	0.0554818
2381	1.59589	0.0582457
2382	2.78009	2.1
2383	1.68761	2.1
2384	3.05511	2.1
2385	2.25318	2.1
2386	0.578998	2.1
2387	1.03512	2.1
2388	2.26156	2.1
2389	1.44859	1.04824
2390	1.35897	1.47879
2391	1.18301	1.84462
2392	1.25173	1.79781
2393	1.98851	2.1
2394	2.70685	2.1
2395	0.682342	2.1
2396	1.52491	2.1
2397	1.37119	2.1
2398	0.682313	2.1
2399	1.35342	2.1
2400	0.947555	2.1
2401	0.614107	2.1
2402	1.23007	2.1
2403	1.5735	2.1
2404	1.58713	2.1
2405	1.57979	0.05
2406	1.30723	0.05
2407	0.935658	0.715226
2408	0.8952	0.68451
2409	1.27352	0.890827
2410	1.0985	0.798239
2411	1.07163	0.669415
2412	1.15823	0.783421
2413	0.836451	0.64646
2414	2.08051	0.52929
2415	1.92601	0.927453
2416	2.0644	0.621731
2417	1.37259	2.1
2418	1.37259	2.1
2419	0.752217	2.1
2420	1.28569	2.1
2421	0.752186	2.1
2422	0.839124	2.1
2423	1.28568	2.1
2424	1.04199	2.1
2425	1.04199	2.1
2426	2.59268	2.1
2427	1.04199	2.1
2428	1.5098	2.1
2429	1.5098	2.1
2430	0.520997	2.1
2431	1.29634	2.1
2432	0.520996	2.1
2433	0.73446	2.1

2434	1.29634	2.1
2435	0.983724	2.1
2436	1.23914	2.1
2437	2.65095	2.1
2438	1.52851	2.1
2439	2.399 2.1	
2440	1.68137	2.1
2441	1.11285	2.1
2442	1.04921	2.1
2443	1.30711	2.1
2444	1.04921	2.1
2445	1.07496	0.962147
2446	1.55831	0.683198
2447	1.07497	0.559833
2448	1.07497	0.786504
2449	1.27391	2.1
2450	2.5615	1.90631
2451	1.2739	2.1
2452	1.75253	1.68938
2453	1.12907	0.299907
2454	1.9194	0.341197
2455	1.96617	0.264458
2456	1.01225	2.07283
2457	0.947979	1.5154
2458	1.01225	0.819556
2459	1.06561	2.1
2460	1.01225	0.900828
2461	3.56876	2.1
2462	3.68767	2.1
2463	1.52301	2.1
2464	1.61013	2.1
2465	1.43726	2.1
2466	2.04275	2.1
2467	0.995734	2.1
2468	1.16199	2.1
2469	1.73704	0.120061
2470	1.04019	0.168547
2471	0.956294	2.1
2472	1.81488	2.1
2473	1.63719	2.1
2474	1.50103	0.05
2475	1.75289	0.05
2476	1.31491	0.05
2477	1.03567	0.05
2478	0.672257	0.05
2479	1.04072	0.0640595
2480	0.746903	0.0570101
2481	1.43317	0.0509025
2482	1.39986	0.05
2483	1.06385	0.0572618
2484	1.1624	0.0565996
2485	1.61667	0.05
2486	1.27258	0.05
2487	2.559 0.05	
2488	1.66093	0.05
2489	1.10719	0.595417
2490	2.03235	0.721113
2491	1.17105	0.998523

2492	1.10719	0.739853
2493	1.3028	2.1
2494	0.946131	2.1
2495	0.946127	2.0437
2496	1.52912	2.1
2497	1.52912	2.1
2498	0.946128	1.31595
2499	0.949997	1.75365
2500	1.31565	2.1
2501	1.46643	2.1
2502	1.5482	2.1
2503	1.47102	2.1
2504	1.47102	2.1
2505	0.774101	2.1
2506	1.30583	2.1
2507	0.774103	2.1
2508	0.939287	2.1
2509	1.30584	2.1
2510	0.958661	2.1
2511	2.28835	2.1
2512	1.43213	0.0965036
2513	2.13255	0.12906
2514	1.43213	0.0963082
2515	1.10476	0.0848153
2516	1.93165	0.123969
2517	1.03235	0.867898
2518	1.31	0.907643
2519	1.22082	2.1
2520	1.35827	2.1
2521	0.790936	2.1
2522	0.716644	2.1
2523	1.43257	2.1
2524	1.43257	2.1
2525	0.716615	2.1
2526	0.946131	2.1
2527	0.730157	2.1
2528	1.47212	2.1
2529	1.47212	2.1
2530	0.946131	2.1
2531	0.790792	2.1
2532	1.41149	2.1
2533	0.790791	2.1
2534	1.01006	2.1
2535	1.53045	0.42716
2536	1.53045	0.59455
2537	0.946132	0.853544
2538	1.40779	0.511719
2539	1.06879	1.03881
2540	0.990713	0.701744
2541	1.06879	0.75546
2542	1.06486	0.331085
2543	1.18347	0.345479
2544	1.38622	0.373669
2545	1.18347	0.264177
2546	1.7304	0.329799
2547	1.7304	0.444691
2548	0.946131	0.639409
2549	1.26874	0.478132

2550	1.13337	0.885318
2551	1.13337	0.592213
2552	1.40779	0.597772
2553	1.695 2.1	
2554	1.77071	2.1
2555	0.649313	2.1
2556	0.692829	2.1
2557	1.40395	2.1
2558	1.34243	2.1
2559	0.813292	0.378225
2560	0.632691	0.421988
2561	1.75766	0.44154
2562	2.25727	0.703611
2563	1.74206	0.357007
2564	0.826073	0.388027
2565	1.30022	0.387768
2566	1.45641	0.370574
2567	0.946131	0.386615
2568	0.752245	0.363982
2569	0.960952	0.401436
2570	0.743477	0.366705
2571	1.04355	0.358909
2572	1.6253	0.360222
2573	0.960577	0.366075
2574	1.34852	0.85539
2575	1.03235	0.915007
2576	1.78168	0.673355
2577	1.2966	0.0543478
2578	2.21116	0.05
2579	1.6395	0.0582203
2580	1.6395	0.0549663
2581	0.94613	0.0765479
2582	1.22187	0.0722209
2583	0.962737	0.0889236
2584	0.962735	0.0707052
2585	1.36376	0.0692081
2586	1.2966	0.05
2587	1.2966	0.05
2588	1.19669	1.01365
2589	2.37636	1.07423
2590	1.08894	1.03087
2591	1.62551	1.45534
2592	2.51472	1.2328
2593	1.89226	0.965361
2594	0.980038	1.05185
2595	1.10187	0.697982
2596	1.01479	0.708367
2597	1.81663	2.1
2598	1.83247	2.1
2599	0.921851	0.576437
2600	0.983148	2.1
2601	1.82816	2.1
2602	1.26103	2.1
2603	2.34818	2.1
2604	1.23152	2.1
2605	2.15738	2.1
2606	1.23152	2.1
2607	1.51042	2.1

2608	0.842995	2.1
2609	1.22257	2.1
2610	0.810959	2.1
2611	1.68242	2.1
2612	0.95701	2.1
2613	1.12389	2.1
2614	1.95255	2.1
2615	1.57974	2.1
2616	1.94632	2.1
2617	0.81534	2.1
2618	1.13996	2.1
2619	1.18958	0.0700482
2620	1.25929	0.0562441
2621	3.02933	0.567256
2622	2.37434	0.351574
2623	1.10609	0.560101
2624	1.64132	0.521319
2625	1.64132	0.527587
2626	0.946132	0.423317
2627	1.40029	0.481793
2628	1.08946	0.414606
2629	1.08946	0.432918
2630	1.18717	0.291495
2631	2.71421	0.26586
2632	1.08189	0.345604
2633	0.94613	0.340766
2634	1.37109	0.352753
2635	1.17925	0.330413
2636	0.851541	0.347633
2637	0.942512	0.352035
2638	1.18541	0.333154
2639	0.851526	0.355206
2640	1.0819	0.330845
2641	1.86009	0.362728
2642	0.946132	0.323261
2643	1.67409	0.312941
2644	1.67409	0.333561
2645	0.946131	0.331264
2646	1.31716	0.328624
2647	1.30307	0.319563
2648	0.946131	0.336394
2649	1.18601	0.32197
2650	3.15646	2.1
2651	0.985109	2.1
2652	3.37048	2.1
2653	1.10667	2.1
2654	1.77065	1.01629
2655	1.45837	0.875508
2656	1.77063	0.935829
2657	2.58104	0.867533
2658	1.37652	1.65438
2659	1.54168	1.4842
2660	1.00224	0.05
2661	1.05851	0.0517663
2662	1.39093	0.05
2663	1.43358	0.0515653
2664	0.575741	0.05
2665	0.518161	0.05

2666	1.04071	0.0547196
2667	1.13092	1.08468
2668	1.17042	1.83306
2669	1.53347	1.53999
2670	1.52938	1.15294
2671	1.00613	0.405625
2672	1.81618	0.582694
2673	1.81618	0.41473
2674	0.946127	0.585118
2675	1.36479	0.562841
2676	0.94613	0.385907
2677	1.31724	0.465668
2678	1.3648	0.469422
2679	1.00613	0.406766
2680	0.781375	0.334796
2681	1.13431	0.36447
2682	1.03547	0.376103
2683	1.40668	0.385212
2684	0.94613	0.382865
2685	0.713285	0.409905
2686	1.72885	0.429817
2687	1.72885	0.405186
2688	0.713298	0.377472
2689	3.30075	0.329319
2690	2.81337	0.370138
2691	1.39296	0.476412
2692	0.830164	1.00679
2693	1.50493	2.1
2694	1.50494	2.1
2695	0.830136	0.943084
2696	1.28298	2.1
2697	1.0521	2.1
2698	0.830147	2.1
2699	2.0799	1.19262
2700	1.52115	0.981897
2701	2.5142	0.943449
2702	1.31253	0.79859
2703	1.94446	1.1654
2704	1.18817	1.19646
2705	0.598348	1.86231
2706	1.69315	1.26465
2707	1.69315	1.68178
2708	0.598377	0.81117
2709	0.598347	0.942416
2710	0.94613	0.814002
2711	1.28418	0.85697
2712	2.05205	0.965678
2713	1.28418	1.62328
2714	1.0737	2.1
2715	1.81024	2.1
2716	2.73834	2.1
2717	1.71369	2.1
2718	1.71368	2.1
2719	0.946118	2.1
2720	1.29063	2.1
2721	1.36917	2.1
2722	0.94613	2.1
2723	1.29062	2.1

2724	0.947921	2.08011
2725	1.58661	0.697718
2726	1.37658	1.63112
2727	1.2364	1.55035
2728	1.66685	0.874225
2729	1.66685	1.95992
2730	0.946133	0.812568
2731	0.946133	1.14533
2732	1.11978	1.38397
2733	1.09194	1.00169
2734	1.21858	1.9045
2735	1.37658	1.36083
2736	1.64903	0.739632
2737	1.64903	2.1
2738	0.946132	0.808217
2739	0.946132	1.19293
2740	1.05496	0.983297
2741	1.22851	0.749992
2742	1.22852	2.08766
2743	1.58482	0.69363
2744	1.60578	0.0808348
2745	1.06336	0.0772783
2746	1.68062	0.0858544
2747	1.14721	0.0650138
2748	1.11924	0.05
2749	1.18421	0.05
2750	2.01276	0.05
2751	1.23502	1.85979
2752	1.34926	1.6661
2753	0.546751	1.20393
2754	0.790446	1.70642
2755	1.04072	1.1811
2756	0.555868	1.14407
2757	0.930305	1.11603
2758	1.09344	1.3394
2759	1.18351	1.61958
2760	1.97215	1.17149
2761	1.07416	1.80174
2762	1.33204	1.15725
2763	1.22038	1.08212
2764	0.597449	0.880527
2765	0.821422	1.27945
2766	1.04071	1.04276
2767	0.608026	0.790199
2768	0.929427	0.973063
2769	1.64929	0.05
2770	1.68541	0.05
2771	0.496093	0.0530448
2772	1.3385	0.05
2773	0.790798	0.05
2774	1.22971	0.05
2775	0.898853	0.05
2776	2.14361	0.05
2777	2.10813	0.05
2778	1.05417	0.0542814
2779	1.80768	0.0880122
2780	1.09725	2.1
2781	1.41131	2.1

2782	1.1656	2.1
2783	0.946129	2.1
2784	1.68365	2.1
2785	1.68365	2.1
2786	1.1656	2.1
2787	0.94613	2.1
2788	1.21269	0.05
2789	1.48867	0.05
2790	1.27806	0.05
2791	1.68264	0.05
2792	1.37813	1.79465
2793	1.50743	1.13242
2794	1.04121	0.911889
2795	1.12434	0.615378
2796	1.39553	0.558461
2797	1.44721	1.21192
2798	0.946132	0.636741
2799	1.23943	0.655226
2800	1.05292	0.742635
2801	1.28607	0.612198
2802	1.16915	0.683164
2803	1.76306	0.885436
2804	1.42266	0.979968
2805	1.36844	2.1
2806	1.36844	2.1
2807	1.67352	2.1
2808	1.11256	0.953456
2809	1.03217	0.884798
2810	1.18422	0.05
2811	1.04159	0.05
2812	1.60139	0.05
2813	1.04159	0.05
2814	0.988805	2.1
2815	1.05481	2.1
2816	1.52938	1.58539
2817	1.94724	1.39144
2818	0.896336	0.103721
2819	0.911484	0.10955
2820	1.18315	0.0576108
2821	1.10515	0.0745899
2822	1.04067	0.0561829
2823	1.12758	0.0930711
2824	0.904106	0.0839116
2825	1.6901	0.0538507
2826	1.68671	0.0522687
2827	1.99908	0.0721072
2828	0.772426	0.769932
2829	0.810945	1.6663
2830	0.667562	1.03122
2831	0.496617	0.540941
2832	0.847689	0.910142
2833	0.914378	1.24758
2834	0.934003	0.693148
2835	1.07459	0.928808
2836	1.40225	2.1
2837	1.07043	2.1
2838	0.94613	2.1
2839	1.63072	2.1

2840	1.63072	2.1
2841	1.07043	2.1
2842	0.946131	2.1
2843	1.53082	2.1
2844	1.69916	2.1
2845	1.75211	0.54819
2846	1.96831	1.58341
2847	1.75211	0.692504
2848	0.946127	0.698238
2849	0.817065	0.722831
2850	1.41798	0.719372
2851	1.41798	0.640872
2852	0.791656	0.709871
2853	0.791656	0.736383
2854	1.41798	0.693774
2855	1.58331	0.686445
2856	1.41776	0.607411
2857	0.791647	0.603448
2858	1.4512	0.587904
2859	1.45119	0.594642
2860	0.75821	0.630257
2861	0.758248	0.620428
2862	0.946104	0.611046
2863	1.36482	0.60931
2864	1.29389	2.1
2865	1.04164	1.51244
2866	1.40574	1.66353
2867	1.64833	1.29645
2868	1.71183	0.28173
2869	1.71183	0.351186
2870	0.946131	0.581754
2871	1.35721	0.444389
2872	0.946131	0.75501
2873	0.946131	0.600443
2874	1.30076	0.576952
2875	1.2474	0.31105
2876	2.85225	0.488285
2877	2.71441	0.405883
2878	1.2474	0.22268
2879	1.38622	0.427371
2880	0.858359	0.279978
2881	1.3682	0.33195
2882	1.57364	0.29901
2883	0.729695	0.465028
2884	0.729723	0.297273
2885	0.822611	0.580804
2886	0.946133	0.466022
2887	0.729723	0.379821
2888	0.974704	0.659068
2889	0.974702	0.668817
2890	1.81603	2.1
2891	1.96348	2.1
2892	1.43612	0.835227
2893	1.43612	0.943627
2894	0.634295	0.85622
2895	0.93796	1.15473
2896	0.946133	0.962737
2897	0.634326	0.72465

2898	0.946133	0.827206
2899	1.2654	0.638679
2900	1.26865	0.863263
2901	2.2649	0.77669
2902	1.00864	0.811105
2903	1.09689	0.636662
2904	0.832021	0.639943
2905	1.53229	0.609778
2906	1.1145	0.642555
2907	0.929583	0.641468
2908	1.613	0.58372
2909	0.644729	0.626489
2910	1.03471	0.950085
2911	2.7739	2.1
2912	1.36133	0.95874
2913	2.50215	2.1
2914	2.52195	2.1
2915	1.0465	2.1
2916	1.42673	2.1
2917	1.42673	2.1
2918	0.946132	2.1
2919	1.31904	2.1
2920	0.986499	2.1
2921	0.946132	2.1
2922	1.31904	2.1
2923	0.966978	2.1
2924	1.42237	0.11305
2925	2.60925	0.163146
2926	0.753659	0.219765
2927	1.77241	0.136228
2928	1.77241	0.176867
2929	0.946129	0.251018
2930	0.75366	0.138181
2931	1.22144	0.214115
2932	0.94613	0.241525
2933	1.50732	0.137104
2934	1.18514	0.166001
2935	0.902187	0.196743
2936	1.89858	0.110244
2937	1.24294	0.136354
2938	1.67211	0.162588
2939	1.41786	0.118377
2940	0.810959	0.196139
2941	1.50732	0.141867
2942	2.44288	0.191324
2943	1.40969	0.0526672
2944	2.06654	0.0628231
2945	1.40969	0.0535914
2946	1.40882	0.0742999
2947	1.94063	0.0797231
2948	0.867921	0.0547432
2949	1.29289	0.0577779
2950	0.867918	0.0562255
2951	0.946128	0.058134
2952	0.867944	0.0616048
2953	1.66589	0.058511
2954	1.66589	0.0527252
2955	0.968595	2.1

2956	1.5534	0.824944
2957	1.27793	2.1
2958	1.13433	2.1
2959	1.3439	0.05
2960	2.53709	0.05
2961	0.571998	0.05
2962	0.64837	0.05
2963	1.99755	0.05
2964	1.99854	0.05
2965	0.764229	2.1
2966	1.61842	2.1
2967	1.61842	2.1
2968	1.18315	2.1
2969	1.1995	2.1
2970	1.18315	2.1
2971	0.764256	2.1
2972	1.52364	2.1
2973	2.36631	2.1
2974	0.641912	1.34644
2975	1.7181	2.1
2976	1.459 2.1	
2977	0.634845	1.2525
2978	1.18315	2.1
2979	1.17615	2.1
2980	0.571367	2.1
2981	1.3324	0.344883
2982	0.798214	0.352603
2983	0.946128	0.379833
2984	0.627252	0.314996
2985	1.50337	0.337575
2986	1.50337	0.250167
2987	0.62725	0.38462
2988	1.07763	0.246146
2989	1.07763	0.302152
2990	2.16109	0.386565
2991	1.07763	0.28245
2992	1.12486	2.1
2993	1.11827	2.1
2994	1.0491	2.1
2995	1.09034	2.1
2996	1.09431	2.1
2997	0.946131	2.1
2998	0.748607	2.1
2999	1.44002	2.1
3000	1.44002	2.1
3001	0.748635	2.1
3002	0.782505	2.1
3003	1.40615	2.1
3004	1.19539	2.1
3005	1.29389	2.08151
3006	1.4867	1.08137
3007	1.50103	0.05
3008	1.63025	0.05
3009	2.72109	0.05
3010	0.915755	0.05
3011	0.946132	0.0548634
3012	0.915755	0.0567883
3013	1.70236	0.05

3014	1.70236	0.05
3015	1.26155	0.0507069
3016	1.35657	0.0515165
3017	1.83151	0.05
3018	1.83151	0.05
3019	2.52309	0.05
3020	0.983607	2.1
3021	1.06838	0.673319
3022	0.983609	0.84651
3023	0.983596	0.833852
3024	2.74384	0.321521
3025	1.6261	0.296555
3026	1.15477	2.1
3027	1.02136	2.1
3028	1.83561	2.1
3029	1.83561	2.1
3030	0.946131	2.1
3031	1.39523	2.1
3032	1.29313	0.691379
3033	1.29313	2.042
3034	1.38651	1.89044
3035	1.06313	2.1
3036	0.974888	2.1
3037	1.0894	2.1
3038	0.974888	2.1
3039	1.82282	2.1
3040	1.82282	2.1
3041	0.946131	2.1
3042	1.37372	2.1
3043	1.32374	2.1
3044	1.32374	2.1
3045	1.39523	2.1
3046	1.40882	0.0762292
3047	0.861998	0.0780275
3048	1.24448	0.0849084
3049	0.861999	0.0791995
3050	0.946129	0.0908171
3051	0.862011	0.0984006
3052	1.67537	0.0870069
3053	1.67537	0.0747469
3054	1.72399	0.05
3055	2.58579	0.0523654
3056	1.72399	0.05
3057	1.50951	0.0605578
3058	2.48897	0.0724384
3059	1.26755	2.1
3060	1.03824	2.1
3061	1.27875	2.1
3062	1.48901	2.1
3063	1.18902	2.1
3064	1.11764	2.1
3065	1.87436	2.1
3066	0.95345	1.98415
3067	0.724191	1.33406
3068	0.86668	1.28773
3069	0.811523	1.50779
3070	1.04074	0.931618
3071	0.843097	1.15828

3072	0.833434	1.38686
3073	0.965727	1.16077
3074	0.85346	1.42313
3075	1.00185	0.788743
3076	1.00184	1.17337
3077	0.980651	1.33201
3078	0.980664	1.17638
3079	0.475737	0.902708
3080	0.719696	1.00024
3081	0.946109	0.916751
3082	0.946116	1.03446
3083	0.719693	0.931362
3084	0.946117	1.04026
3085	1.14879	1.38535
3086	0.719683	1.31758
3087	0.900977	0.972742
3088	0.972364	1.09589
3089	1.22961	1.08248
3090	0.405384	0.972829
3091	1.34406	2.1
3092	1.2187	2.1
3093	0.963571	0.503006
3094	0.946131	0.518189
3095	1.57246	0.665025
3096	1.57246	2.1
3097	0.946131	0.68235
3098	1.1235	0.9321
3099	1.1235	0.950366
3100	1.07439	0.990201
3101	1.19612	0.968589
3102	1.24844	0.994766
3103	1.00007	1.14662
3104	1.02429	2.1
3105	1.10459	2.1
3106	1.06724	0.428646
3107	0.67519	0.576411
3108	0.49762	0.446989
3109	1.43435	0.403681
3110	0.833315	0.485551
3111	0.58135	0.465348
3112	1.14749	0.733645
3113	1.85091	0.958749
3114	1.2802	0.665555
3115	1.27737	0.728569
3116	2.3655	0.727056
3117	1.30584	2.1
3118	2.06424	2.1
3119	0.946131	0.327057
3120	1.04257	0.32473
3121	0.94613	0.333595
3122	1.67201	0.349293
3123	1.67201	0.310907
3124	1.27696	0.32391
3125	1.34118	0.217734
3126	1.3413	0.0922811
3127	2.36238	0.0705212
3128	0.6036	0.125212
3129	1.11745	0.12967

3130	1.46126	0.0907369
3131	1.39612	0.0777474
3132	0.670678	0.108901
3133	1.03402	0.139375
3134	1.11271	0.107841
3135	0.838028	0.0831699
3136	0.807019	0.128132
3137	1.27787	0.0671301
3138	0.994705	0.0841121
3139	1.20014	0.0693503
3140	0.964793	0.111299
3141	0.863868	0.0949361
3142	1.3413	0.0684579
3143	1.79549	0.0732856
3144	1.35877	2.1
3145	1.63648	1.62446
3146	1.08171	2.1
3147	1.27839	2.1
3148	1.52601	2.1
3149	1.27841	2.1
3150	1.71255	2.1
3151	2.16307	2.1
3152	1.91592	2.1
3153	1.78508	2.1
3154	0.946128	2.1
3155	1.41199	2.1
3156	0.946128	2.1
3157	0.901088	2.1
3158	1.55244	2.1
3159	1.59475	2.1
3160	1.40921	2.1
3161	0.960106	2.1
3162	1.40921	2.1
3163	2.0073	2.1
3164	2.2973	0.44077
3165	1.90705	0.438878
3166	1.53671	0.284247
3167	1.86035	0.27502
3168	1.33747	2.1
3169	1.14007	2.1
3170	1.3212	2.1
3171	1.66867	0.301005
3172	1.30203	0.266862
3173	1.27696	2.1
3174	1.05025	2.1
3175	1.05025	2.1
3176	1.3704	2.1
3177	1.15677	2.1
3178	0.946129	2.1
3179	1.65879	2.1
3180	1.65879	2.1
3181	1.15676	2.1
3182	0.94613	2.1
3183	0.867217	1.0664
3184	1.28684	0.930744
3185	1.58866	1.03469
3186	1.58866	0.757624
3187	0.867246	1.70186

3188	1.28684	0.829662
3189	1.16906	1.09199
3190	1.13438	1.03234
3191	1.37224	1.11595
3192	1.61519	1.17871
3193	0.883939	1.79106
3194	1.40092	1.02362
3195	1.80383	1.83643
3196	1.80383	1.48447
3197	0.883935	1.59553
3198	1.40092	0.947855
3199	1.28684	1.67842
3200	1.76788	0.811335
3201	2.57368	1.00055
3202	1.37224	1.92554
3203	1.65411	0.761273
3204	1.77042	2.1
3205	1.77042	2.1
3206	1.90446	2.1
3207	1.28667	2.1
3208	1.55635	2.1
3209	1.30646	1.10396
3210	2.31836	1.37583
3211	1.30645	0.895183
3212	1.86429	1.10189
3213	1.48676	0.360041
3214	0.743379	0.384728
3215	1.41848	0.382323
3216	1.41848	0.375894
3217	0.946131	0.392837
3218	0.812649	0.381693
3219	1.34921	0.3785
3220	0.812649	0.374757
3221	0.949366	0.367093
3222	1.32974	0.359337
3223	0.794204	0.05
3224	0.634352	2.1
3225	0.838025	2.1
3226	1.08802	1.87015
3227	0.838038	1.80913
3228	1.18314	2.1
3229	1.12495	2.1
3230	1.22774	2.05569
3231	1.04343	2.04296
3232	0.921111	0.05
3233	2.1609	0.05
3234	1.09987	0.05
3235	2.5043	0.05
3236	1.05492	0.05
3237	0.590344	0.05
3238	1.05492	0.05
3239	1.25215	0.05
3240	1.71674	0.05
3241	1.71674	0.05
3242	0.590331	0.05
3243	1.17078	0.05
3244	0.625542	0.05
3245	0.724673	2.1

3246	1.08011	2.1
3247	1.87598	2.1
3248	1.50738	2.1
3249	1.44951	0.321201
3250	1.50102	0.315797
3251	1.8042	0.47668
3252	0.687769	0.583581
3253	1.66483	0.494593
3254	1.66483	0.51743
3255	0.946132	0.577395
3256	0.687798	0.493917
3257	1.20885	0.59414
3258	0.687799	0.549962
3259	0.886063	0.428835
3260	0.798751	0.430948
3261	0.584813	0.407596
3262	1.49483	0.365276
3263	0.888005	0.380557
3264	1.03589	0.382128
3265	1.70435	0.39866
3266	0.710414	2.1
3267	0.917609	0.91637
3268	0.810947	0.928455
3269	0.81096	0.940836
3270	1.48658	1.0288
3271	1.53653	1.02676
3272	1.19329	0.996591
3273	0.946153	1.01375
3274	1.50798	1.00378
3275	1.85017	2.1
3276	1.19768	2.1
3277	0.864304	1.52745
3278	0.792906	1.00045
3279	0.989025	1.01876
3280	1.19768	1.29875
3281	1.54548	1.09271
3282	1.18798	1.32371
3283	1.31875	0.948428
3284	1.05725	1.05007
3285	2.17365	0.05
3286	2.48106	0.05
3287	1.87451	0.05
3288	1.27189	1.0157
3289	2.79399	1.56176
3290	0.859565	1.1004
3291	0.926174	0.990375
3292	0.604744	0.950989
3293	1.41832	0.673751
3294	1.05494	0.84467
3295	0.874749	0.874887
3296	1.50067	0.773234
3297	0.917817	1.14895
3298	0.63591	1.17779
3299	1.72849	1.08727
3300	1.72849	0.839716
3301	0.635943	0.934183
3302	1.39699	1.15774
3303	0.967411	1.55441

3304	0.635943	1.0544
3305	1.39908	0.975559
3306	1.26971	1.07331
3307	1.02969	0.945003
3308	1.15326	1.91368
3309	2.30741	1.31902
3310	0.93545	0.991971
3311	0.802747	0.771566
3312	1.15466	1.07253
3313	0.859164	1.2798
3314	1.02616	1.31812
3315	1.23149	1.00259
3316	0.83804	0.830416
3317	1.13266	1.90299
3318	1.64142	1.82438
3319	1.21223	2.1
3320	1.43051	2.1
3321	1.32418	0.89274
3322	0.621319	0.791982
3323	1.47134	1.61484
3324	1.79103	0.659159
3325	3.25951	0.0798714
3326	3.16764	0.0514026
3327	1.65583	0.0843772
3328	1.4732	0.0804581
3329	1.76476	2.1
3330	1.80114	0.913626
3331	0.754121	1.77487
3332	0.94613	0.74191
3333	0.754119	1.82338
3334	1.45636	1.89939
3335	1.45636	0.648881
3336	1.30029	0.931899
3337	0.910183	0.945242
3338	1.29099	1.13128
3339	1.08039	2.1
3340	1.05498	2.1
3341	1.24445	1.97963
3342	1.2873	2.1
3343	0.987703	1.6292
3344	0.964782	1.24714
3345	1.73645	0.10799
3346	0.871279	0.161199
3347	0.884744	0.180772
3348	1.85193	0.106242
3349	1.68681	0.134179
3350	1.33689	0.15882
3351	1.23493	0.112556
3352	0.892482	0.174752
3353	2.95185	0.12343
3354	2.10496	0.132062
3355	1.87334	2.1
3356	0.94411	2.1
3357	0.754196	2.1
3358	1.43122	2.1
3359	1.58684	2.1
3360	0.838027	2.1
3361	1.1536	2.1

3362	1.24878	2.1
3363	1.23125	2.1
3364	2.86862	2.1
3365	1.57636	2.1
3366	0.998451	0.681623
3367	1.5176	0.639271
3368	1.13271	0.079212
3369	1.60248	0.0700018
3370	1.22083	2.1
3371	1.79996	2.1
3372	1.19087	2.1
3373	2.00342	2.1
3374	1.3715	2.1
3375	2.79227	2.1
3376	2.7873	2.1
3377	0.987265	0.05
3378	1.62715	2.1
3379	1.70117	1.66859
3380	1.27806	0.05
3381	1.45246	2.1
3382	1.45244	2.1
3383	1.87214	2.1
3384	0.995796	2.1
3385	1.68189	0.449529
3386	1.49155	1.68385
3387	1.58054	2.1
3388	1.59169	2.1
3389	0.736364	0.05
3390	0.70236	0.05
3391	2.24367	0.05
3392	2.57085	0.05
3393	1.70952	0.05
3394	2.60516	0.05
3395	1.45158	2.1
3396	1.00371	2.1
3397	1.65748	2.1
3398	1.23706	0.431736
3399	1.55834	0.673951
3400	1.72671	0.610784
3401	0.597656	0.523407
3402	1.39137	0.615065
3403	0.855578	0.418565
3404	1.48398	0.524658
3405	0.745117	0.456256
3406	1.23706	0.683236
3407	1.57916	0.495394
3408	0.893025	0.449274
3409	3.22433	0.490041
3410	2.47324	1.03189
3411	1.63852	2.1
3412	1.63852	2.1
3413	0.946132	2.1
3414	1.19095	2.1
3415	0.946133	2.1
3416	1.16684	2.1
3417	1.19095	2.1
3418	1.06698	0.375967
3419	1.48692	0.365925

3420	1.08334	0.814823
3421	1.07283	2.1
3422	1.28517	0.818964
3423	1.2746	0.05
3424	1.30723	0.05
3425	2.02069	0.05
3426	1.05579	0.05
3427	1.59475	2.1
3428	2.40113	2.1
3429	1.58875	2.1
3430	1.6881	2.1
3431	1.6881	2.1
3432	0.819953	2.1
3433	0.819954	2.1
3434	0.946131	2.1
3435	0.946131	2.1
3436	1.34782	2.1
3437	1.65619	2.1
3438	1.65619	2.02682
3439	0.79437	2.1
3440	1.25	2.1
3441	0.94613	2.1
3442	0.946131	2.05837
3443	0.794371	2.1
3444	1.58875	2.1
3445	2.5	2.1
3446	1.04147	2.1
3447	0.946131	0.733948
3448	1.79686	2.1
3449	1.79686	2.1
3450	1.25144	0.747876
3451	1.35714	1.29339
3452	1.38585	1.32505
3453	1.25144	2.09149
3454	1.15564	2.06057
3455	1.34389	1.36379
3456	0.946133	1.02319
3457	1.74353	1.97611
3458	1.74353	2.1
3459	1.14553	0.964542
3460	1.38585	0.931334
3461	1.30382	1.07888
3462	1.14553	1.96504
3463	1.15564	1.69687
3464	1.90618	2.1
3465	2.11056	2.1
3466	1.032	2.1
3467	0.953636	2.1
3468	1.23131	1.14682
3469	1.39763	1.34572
3470	2.01024	0.945839
3471	1.9186	0.961238
3472	1.83658	0.206547
3473	1.81656	0.195997
3474	2.2049	0.273633
3475	2.16081	0.220811
3476	1.12219	0.272251
3477	0.599771	0.32938

3478	1.61401	0.193702
3479	1.05912	0.256338
3480	0.951648	0.266168
3481	1.89328	0.19874
3482	0.913682	0.327865
3483	1.00763	0.210015
3484	1.29129	2.1
3485	1.37138	2.1
3486	1.41986	0.0779784
3487	1.41986	0.078161
3488	0.944166	0.132319
3489	1.41789	0.10298
3490	0.944167	0.170721
3491	0.944166	0.10074
3492	0.946132	0.0907436
3493	1.98713	0.0660632
3494	1.23626	0.0559221
3495	0.932791	0.0885663
3496	0.810251	0.0564704
3497	0.986748	0.0633088
3498	0.898155	0.0846063
3499	0.92624	0.0713271
3500	1.1349	0.0599674
3501	0.934572	0.0751254
3502	1.23104	1.09411
3503	1.23104	1.12003
3504	0.799994	0.518664
3505	0.94613	0.90484
3506	0.802822	0.27242
3507	1.40944	1.05456
3508	0.800035	0.748094
3509	1.41223	0.824634
3510	1.41223	0.834345
3511	1.6	0.632832
3512	1.60565	0.536161
3513	1.23042	2.1
3514	1.0645	2.1
3515	1.00319	2.1
3516	1.29435	2.1
3517	1.2243	2.1
3518	1.2243	2.1
3519	1.05655	1.2664
3520	1.29764	1.41037
3521	2.55252	2.1
3522	1.15676	2.1
3523	0.855295	2.1
3524	0.810959	2.1
3525	1.65857	2.1
3526	1.00849	2.03616
3527	1.16021	2.1
3528	1.75869	2.1
3529	1.13775	2.1
3530	1.20091	2.1
3531	1.61582	2.1
3532	1.88031	2.1
3533	0.968536	2.1
3534	0.946131	2.1
3535	1.61481	2.1

3536	1.61481	2.1
3537	1.09547	2.1
3538	1.18486	2.1
3539	1.37609	2.1
3540	1.09546	2.1
3541	1.02461	2.1
3542	1.83223	2.1
3543	1.74002	2.1
3544	1.70939	1.46734
3545	1.7094	2.1
3546	0.726287	2.1
3547	1.01712	1.42008
3548	1.30318	2.1
3549	0.946131	2.1
3550	0.87155	2.1
3551	1.77093	1.6541
3552	2.02356	1.6846
3553	1.32449	2.1
3554	1.10003	2.1
3555	1.5161	2.1
3556	1.5161	2.1
3557	0.946132	2.1
3558	1.09206	2.1
3559	0.946132	2.1
3560	1.04404	2.1
3561	1.09206	2.1
3562	0.635628	0.978908
3563	1.14623	0.604852
3564	1.73197	0.639431
3565	1.73197	0.873374
3566	0.529655	0.640864
3567	1.14623	0.77513
3568	0.992085	0.735807
3569	1.16915	1.19264
3570	2.29245	0.540945
3571	0.584573	0.836831
3572	0.956756	0.622435
3573	1.52471	0.61027
3574	1.51409	1.10442
3575	0.584575	0.679261
3576	0.94613	0.733167
3577	1.14622	0.569493
3578	1.28096	1.01381
3579	1.34695	1.29962
3580	1.04193	2.1
3581	1.05378	2.1
3582	0.798209	2.1
3583	1.32007	1.83801
3584	1.64327	0.05
3585	2.64674	2.1
3586	2.38936	0.147113
3587	2.88475	0.133655
3588	1.07745	1.73826
3589	1.08911	1.92077
3590	1.49127	2.1
3591	1.49127	2.1
3592	0.709714	2.1
3593	1.37199	2.1

3594	0.82899	2.1
3595	0.709714	2.1
3596	0.946132	2.1
3597	1.00133	2.1
3598	1.40182	2.1
3599	1.53278	2.1
3600	1.40182	2.1
3601	1.31764	0.05
3602	1.00691	0.05
3603	1.24094	0.05
3604	0.64868	0.05
3605	0.946133	0.05
3606	0.64868	0.05
3607	1.71762	0.05
3608	1.71762	0.05
3609	1.41662	0.05
3610	0.975477	0.05
3611	1.73584	0.05
3612	2.48188	0.05
3613	2.2045	0.577805
3614	2.08134	0.565418
3615	0.993366	0.320951
3616	0.993368	0.692565
3617	1.14187	0.59709
3618	0.946132	0.547602
3619	0.946133	0.927563
3620	1.68951	0.687601
3621	1.68951	1.31177
3622	1.34612	0.594532
3623	1.28953	0.550472
3624	1.34611	0.867339
3625	1.31892	0.573835
3626	0.946131	0.618675
3627	1.80988	0.557209
3628	1.80988	1.77642
3629	1.31892	0.71798
3630	0.946131	0.650073
3631	2.13214	2.1
3632	1.94691	2.1
3633	1.52661	0.05
3634	1.53175	0.05
3635	0.956005	0.05
3636	0.955991	0.05
3637	1.40572	0.05
3638	0.955992	0.05
3639	1.51084	0.754151
3640	1.67115	0.843983
3641	1.73143	2.1
3642	1.55234	2.1
3643	1.69377	2.1
3644	2.51483	2.1
3645	1.25987	2.1
3646	1.4472	2.1
3647	1.83151	0.601506
3648	2.01001	0.947276
3649	0.993998	0.612551
3650	1.83151	0.539504
3651	1.40915	0.193168

3652	1.24653	0.430356
3653	1.74472	2.1
3654	1.74472	2.1
3655	1.74244	2.1
3656	1.04788	1.70584
3657	1.28522	1.13874
3658	1.03785	2.1
3659	0.896519	2.1
3660	1.60428	2.1
3661	1.73305	2.1
3662	1.39072	0.315803
3663	2.27501	0.54875
3664	1.39073	0.494107
3665	1.39073	0.376604
3666	1.06838	1.94217
3667	0.968462	0.762826
3668	1.06837	1.21196
3669	1.13455	1.15614
3670	1.23527	0.617087
3671	2.71686	0.506191
3672	1.86881	0.521896
3673	1.10586	0.839782
3674	1.03852	0.945976
3675	1.03899	2.1
3676	0.963973	2.1
3677	1.00319	2.1
3678	1.00319	2.1
3679	0.987132	2.1
3680	0.974889	2.1
3681	0.987131	2.1
3682	1.7677	2.1
3683	1.7677	2.1
3684	0.946131	2.1
3685	1.31185	2.1
3686	0.946132	2.1
3687	1.30203	2.1
3688	1.31185	2.1
3689	1.82958	2.1
3690	1.82958	2.1
3691	0.946132	2.1
3692	1.40199	2.1
3693	0.946133	2.1
3694	1.36994	2.1
3695	1.40199	2.1
3696	0.97489	2.1
3697	1.05939	1.99421
3698	1.01922	1.96914
3699	0.810941	1.41192
3700	1.05372	2.1
3701	0.754065	2.1
3702	0.926131	2.01789
3703	1.35905	1.93672
3704	0.810931	1.81348
3705	1.75647	0.937076
3706	1.68265	2.1
3707	1.41995	2.1
3708	1.36457	1.31106
3709	0.933392	1.09544

3710	1.42255	2.1
3711	1.3868	2.1
3712	1.08074	2.1
3713	1.07196	2.1
3714	1.13537	1.01907
3715	1.01179	0.756982
3716	1.60196	0.630467
3717	1.72798	0.730426
3718	1.3715	0.780382
3719	1.56409	0.980439
3720	1.56409	0.700934
3721	1.97819	1.1539
3722	1.47954	0.57214
3723	0.798655	0.401535
3724	1.47952	0.521833
3725	2.30327	0.422091
3726	1.49111	2.1
3727	1.96013	1.89745
3728	1.54599	2.1
3729	2.06632	2.1
3730	1.54601	2.1
3731	1.10557	0.858281
3732	1.17624	0.685064
3733	1.60497	2.1
3734	1.61587	2.1
3735	1.65977	0.05
3736	1.69044	0.05
3737	1.00659	2.1
3738	1.06226	2.1
3739	1.15171	2.1
3740	1.50853	2.1
3741	1.80252	2.1
3742	1.41432	2.1
3743	1.04687	2.1
3744	0.940878	2.1
3745	1.52031	2.1
3746	1.52031	2.1
3747	0.940877	2.1
3748	0.946132	2.1
3749	1.50853	2.1
3750	0.963973	2.1
3751	1.70562	0.152065
3752	2.43203	0.125009
3753	1.36004	0.0821129
3754	1.06648	0.0978962
3755	1.66009	0.0621253
3756	1.66009	0.0768232
3757	0.766427	0.0938501
3758	0.766452	0.0821789
3759	0.946132	0.105358
3760	1.98012	0.0593225
3761	1.20357	2.1
3762	1.20356	2.1
3763	1.67324	2.1
3764	1.52008	2.1
3765	1.19964	2.1
3766	1.36861	2.1
3767	1.19964	2.1

3768	2.85415	2.1
3769	1.43687	2.1
3770	0.935852	2.1
3771	1.09972	2.1
3772	0.810933	1.89778
3773	2.25974	2.1
3774	1.74149	2.1
3775	1.36984	2.1
3776	1.87602	2.1
3777	0.890748	2.1
3778	1.20587	1.73287
3779	0.810956	1.52817
3780	2.03174	1.90301
3781	1.34403	1.99208
3782	1.58825	1.84314
3783	1.63096	1.76467
3784	2.53735	2.03982
3785	1.98241	1.90606
3786	2.53014	2.1
3787	1.1775	1.05135
3788	1.06538	0.934145
3789	0.946132	1.04328
3790	1.4101	1.98639
3791	1.75162	2.1
3792	1.75162	1.00296
3793	0.946132	1.0912
3794	1.4101	0.962453
3795	1.28765	1.01573
3796	1.1427	0.917671
3797	1.1427	0.737501
3798	1.3109	2.07075
3799	1.02721	1.14185
3800	0.846447	1.40293
3801	0.862076	2.1
3802	0.644098	1.47653
3803	0.581308	1.40847
3804	1.02306	1.23272
3805	0.322641	0.774907
3806	0.589346	0.786897
3807	2.25572	1.27338
3808	1.31372	2.1
3809	1.18873	2.1
3810	1.78225	2.1
3811	1.764	2.1
3812	1.17503	2.1
3813	1.06821	2.1
3814	1.06821	1.63315
3815	1.7777	1.87717
3816	1.9096	1.03245
3817	1.70239	1.23096
3818	1.16182	0.05
3819	1.20132	0.0557063
3820	1.23475	2.1
3821	1.56859	1.08077
3822	2.46263	2.1
3823	0.528744	2.1
3824	2.93337	2.1
3825	2.42504	2.1

3826	1.38646	0.170269
3827	1.61963	0.351206
3828	1.88016	0.632485
3829	0.622283	0.810859
3830	1.03243	0.674456
3831	0.710307	1.05547
3832	0.798124	0.735624
3833	0.931073	0.892942
3834	0.892862	1.32336
3835	1.06791	0.643811
3836	0.552787	1.16319
3837	0.946132	0.865736
3838	1.36665	0.902145
3839	1.36665	1.46882
3840	0.506966	0.902755
3841	0.506964	1.64886
3842	1.36665	0.831298
3843	0.653469	0.87061
3844	1.36665	0.654981
3845	1.21339	0.701846
3846	1.21339	1.2898
3847	0.552757	0.769753
3848	0.552786	1.50217
3849	1.21336	0.563246
3850	1.70744	1.37117
3851	1.0775	0.451745
3852	1.59527	0.635144
3853	1.48312	0.535495
3854	2.01868	0.389277
3855	1.18947	0.120079
3856	1.48312	0.535636
3857	0.785626	0.147346
3858	1.19601	0.187648
3859	0.785626	0.212188
3860	0.946132	0.173596
3861	0.7856	0.27136
3862	1.59087	0.211094
3863	1.51158	0.210456
3864	1.74098	0.222077
3865	2.39202	0.170611
3866	0.870489	0.277286
3867	1.34534	0.41306
3868	0.870488	0.182661
3869	0.946133	0.463234
3870	0.870489	0.640766
3871	1.67086	0.467636
3872	1.67086	0.451978
3873	2.11311	2.09654
3874	1.98765	1.10022
3875	0.946133	0.82486
3876	1.30881	1.90834
3877	1.72211	2.1
3878	1.72211	1.14437
3879	0.94613	1.56937
3880	1.30881	1.08951
3881	1.35943	0.89065
3882	1.01294	0.363058
3883	1.01294	0.126854

3884	1.38963	0.563415
3885	1.01294	0.395514
3886	1.60056	0.379323
3887	1.60056	0.52605
3888	0.946132	0.5518
3889	1.204	0.492544
3890	0.946132	0.345338
3891	1.11288	0.350769
3892	1.204	0.398675
3893	1.14599	0.599084
3894	1.00065	0.571881
3895	1.74685	0.611189
3896	1.74685	1.32808
3897	0.946128	0.550018
3898	1.38463	0.382119
3899	1.2275	1.82367
3900	1.2275	0.729868
3901	1.30835	0.762513
3902	2.04407	2.1
3903	2.50425	2.1
3904	1.19647	0.59914
3905	1.19647	0.673429
3906	1.19645	0.739139
3907	1.75446	0.641685
3908	1.1315	0.43515
3909	1.1315	0.462451
3910	1.45569	0.418575
3911	1.1315	0.399898
3912	1.14357	2.1
3913	1.25793	2.1
3914	1.14357	2.1
3915	2.08181	2.05601
3916	1.4714	2.1
3917	0.71295	2.1
3918	1.68285	2.1
3919	1.68285	2.1
3920	1.25603	2.1
3921	1.13976	2.1
3922	1.25603	2.1
3923	0.71295	2.1
3924	1.61095	2.1
3925	2.51207	2.1
3926	1.63762	2.1
3927	2.74451	2.1
3928	0.671255	1.38326
3929	1.87601	2.01488
3930	1.87601	2.09484
3931	0.671226	1.43056
3932	1.25603	2.02998
3933	1.29123	2.1
3934	0.671226	2.0662
3935	1.63723	2.1
3936	0.872928	0.0577525
3937	1.47904	0.0803998
3938	1.18793	0.0694464
3939	2.57464	0.084033
3940	1.14986	1.7223
3941	1.19611	1.82632

3942	0.96029	0.643548
3943	1.7961	2.1
3944	0.928721	2.1
3945	1.34352	2.1
3946	1.37935	2.1
3947	1.37935	2.1
3948	0.892888	2.1
3949	0.946131	2.1
3950	0.892888	2.1
3951	1.75422	2.1
3952	1.05556	2.1
3953	1.27362	0.576027
3954	1.27421	0.138398
3955	1.3003	0.861614
3956	1.31415	0.720492
3957	1.01431	2.1
3958	0.673951	2.1
3959	1.63384	2.08785
3960	1.63661	2.1
3961	1.52245	2.1
3962	1.50714	2.1
3963	0.996778	0.05
3964	1.56091	0.0604021
3965	1.06543	2.1
3966	2.66689	2.1
3967	1.01474	2.1
3968	2.76422	2.1
3969	1.45321	0.433652
3970	0.974716	0.291374
3971	0.810943	0.287336
3972	0.909982	0.459894
3973	0.937793	0.324664
3974	1.05138	0.301259
3975	1.25044	0.295862
3976	0.92095	0.290109
3977	0.830414	0.379075
3978	2.1402	0.354261
3979	1.75938	2.1
3980	1.05556	2.1
3981	1.78558	2.1
3982	1.379 2.1	
3983	0.970214	0.675475
3984	0.9702	0.627709
3985	1.08894	0.938066
3986	2.17251	0.881968
3987	1.51569	0.837786
3988	1.06012	0.776306
3989	0.98966	0.790032
3990	1.55926	0.54322
3991	1.92645	0.699949
3992	1.37427	0.206278
3993	2.16338	0.303679
3994	1.5456	0.153109
3995	1.5456	0.0997012
3996	1.94002	0.131416
3997	1.33135	0.131426
3998	1.31948	0.159786
3999	2.23255	0.157607

4000	2.34061	0.123971
4001	1.52903	2.1
4002	3.38327	2.1
4003	1.35977	2.1
4004	2.22414	2.1
4005	1.6812	0.101525
4006	2.33397	0.108087
4007	1.16699	0.153919
4008	0.946131	0.114185
4009	1.58115	0.144099
4010	1.58115	0.132332
4011	0.519726	0.229373
4012	0.946131	0.164562
4013	0.519697	0.178077
4014	1.26868	0.106447
4015	1.16699	0.106497
4016	1.5951	0.0890454
4017	1.5951	0.0866114
4018	0.840568	0.123276
4019	0.946131	0.10159
4020	0.840598	0.0967119
4021	1.30047	2.1
4022	1.5586	2.1
4023	1.31825	0.723894
4024	1.31924	0.682037
4025	1.15818	2.1
4026	1.52782	0.974755
4027	1.15818	1.21072
4028	1.15818	1.11203
4029	0.99518	0.450591
4030	0.94613	0.432633
4031	0.922045	0.467169
4032	1.29322	0.568593
4033	1.29321	0.401935
4034	1.29321	0.564056
4035	0.922056	0.463841
4036	1.06848	0.51309
4037	1.84409	0.537789
4038	1.9186	1.19508
4039	2.72244	0.894428
4040	0.946132	0.827713
4041	1.36122	0.93637
4042	1.79658	0.99892
4043	1.79658	0.801875
4044	0.946133	1.21223
4045	1.36122	0.906607
4046	1.38149	0.845676
4047	1.59106	0.77615
4048	0.946116	0.838544
4049	1.29052	0.844714
4050	1.76641	0.81443
4051	1.76641	0.766841
4052	0.885309	1.12523
4053	1.29054	0.882729
4054	1.36118	0.830337
4055	1.59103	0.916392
4056	2.27816	0.75963
4057	1.56164	0.772851

4058	1.41114	0.05
4059	2.04128	0.05
4060	0.986723	2.1
4061	1.03645	0.348777
4062	1.79224	0.333353
4063	1.2627	2.1
4064	1.23036	2.1
4065	1.45257	2.1
4066	1.41469	2.1
4067	1.41469	2.1
4068	2.60636	2.1
4069	1.45789	0.0772052
4070	0.851541	0.0677514
4071	1.57877	0.0737028
4072	1.53134	0.05
4073	0.851479	0.0683352
4074	1.08982	0.0828702
4075	0.716284	0.0533937
4076	0.946131	0.083207
4077	1.73193	0.0607602
4078	2.57309	0.0751986
4079	1.36307	0.0511141
4080	2.42175	0.0532322
4081	1.72948	0.0574311
4082	0.978698	2.1
4083	0.978699	2.1
4084	2.08398	2.1
4085	0.978698	2.1
4086	1.44096	2.1
4087	1.44096	2.1
4088	0.638164	2.1
4089	0.681588	2.1
4090	0.946133	2.1
4091	0.638165	2.1
4092	0.94613	2.1
4093	1.55432	2.1
4094	0.901147	2.1
4095	0.85777	2.1
4096	1.36073	2.1
4097	1.44004	2.1
4098	0.946133	2.1
4099	1.09982	2.1
4100	1.27562	2.1
4101	1.55432	2.1
4102	1.62229	2.1
4103	1.0101	2.1
4104	2.28459	1.99899
4105	2.50554	1.60567
4106	0.946131	0.852787
4107	1.25276	2.1
4108	1.73721	2.1
4109	1.73721	1.16437
4110	0.93406	1.84012
4111	1.25277	1.46153
4112	1.4185	1.00375
4113	1.53064	0.997742
4114	0.946133	1.00545
4115	0.609462	2.1

4116	1.65123	2.1
4117	1.65123	1.39839
4118	0.609466	1.92066
4119	1.00792	1.57111
4120	1.25277	1.17066
4121	1.53064	1.90041
4122	2.15223	1.1098
4123	1.30664	1.38515
4124	1.10674	2.1
4125	1.22862	2.1
4126	1.49358	0.424907
4127	2.0207	0.363236
4128	2.09374	2.1
4129	1.88175	2.1
4130	1.10938	0.3895
4131	1.02478	0.473904
4132	1.13272	0.0614264
4133	1.13272	0.0662429
4134	1.03935	1.24918
4135	0.810945	1.48159
4136	0.527573	0.839851
4137	0.925011	1.17276
4138	1.03193	0.944413
4139	1.38587	1.07879
4140	0.703469	0.998373
4141	0.912282	1.22125
4142	1.75505	2.1
4143	1.78529	2.1
4144	1.60017	0.727071
4145	1.35608	0.503821
4146	1.01479	0.728227
4147	1.47561	0.49268
4148	1.01479	0.714454
4149	1.28418	0.488872
4150	2.62605	2.1
4151	1.71233	2.1
4152	3.22222	2.1
4153	1.97526	2.1
4154	0.949712	0.456565
4155	1.78986	0.40872
4156	2.72879	0.343903
4157	2.66428	0.385991
4158	1.68108	0.458264
4159	1.67265	0.417481
4160	2.24877	0.476542
4161	1.15558	0.523123
4162	1.39708	2.1
4163	1.39327	2.1
4164	1.69788	0.0967318
4165	1.1273	0.0791609
4166	1.17302	0.461157
4167	1.04349	0.43847
4168	0.979853	2.1
4169	1.19094	2.1
4170	1.39408	2.1
4171	1.61857	2.1
4172	1.66523	2.1
4173	0.946132	2.1

4174	0.838554	2.1
4175	1.05151	2.1
4176	1.18901	2.1
4177	1.18901	2.1
4178	1.61149	2.1
4179	1.22857	0.679282
4180	1.25848	0.660663
4181	1.09154	2.1
4182	2.20358	2.1
4183	1.23013	2.1
4184	1.55698	2.1
4185	1.55828	1.75086
4186	1.52391	2.1
4187	0.60346	1.25818
4188	1.06336	2.1
4189	0.870714	0.96215
4190	0.543109	1.23309
4191	1.10367	0.957458
4192	1.20686	1.34529
4193	1.20684	2.06528
4194	2.5118	1.09345
4195	1.20684	2.1
4196	1.52468	1.68931
4197	1.52469	1.94293
4198	0.532308	1.25594
4199	0.903301	1.93156
4200	0.946119	1.12134
4201	0.532315	1.16242
4202	0.946107	1.20177
4203	1.46129	2.1
4204	0.804766	2.1
4205	1.4809	2.1
4206	1.4809	2.1
4207	1.11822	2.1
4208	1.16744	2.1
4209	1.11822	2.1
4210	0.804737	2.1
4211	1.04193	2.1
4212	0.983723	2.1
4213	1.8014	2.1
4214	1.1729	2.1
4215	1.03951	2.1
4216	1.03951	2.1
4217	1.17583	2.1
4218	1.42866	2.1
4219	1.78503	2.1
4220	1.17822	1.24473
4221	1.89121	2.1
4222	1.44472	2.1
4223	1.62143	2.1
4224	1.43837	2.1
4225	1.01121	0.330595
4226	0.962703	0.638922
4227	0.946129	0.497735
4228	1.10941	0.434982
4229	0.946131	0.511794
4230	1.60326	0.563393
4231	1.60326	0.458964

4232	1.14215	0.483998
4233	1.40724	0.489086
4234	0.962703	0.529979
4235	1.53336	0.100882
4236	1.40272	0.0899062
4237	1.64984	0.0872917
4238	1.67345	0.0983047
4239	1.08212	2.1
4240	1.40905	2.1
4241	0.946132	0.05
4242	0.946132	0.05
4243	0.81268	0.05
4244	0.946131	0.05
4245	0.812681	0.05
4246	0.946131	0.05
4247	0.946132	0.05
4248	0.997755	0.05
4249	1.94585	0.05
4250	1.2925	0.05
4251	2.52454	0.05
4252	1.69182	0.05
4253	1.69182	0.05
4254	0.92361	0.05
4255	1.35316	0.05
4256	1.26227	0.05
4257	0.946131	0.05
4258	1.35316	0.05
4259	1.48676	0.325104
4260	1.33917	0.319785
4261	0.757292	2.1
4262	1.02415	2.1
4263	1.48495	2.1
4264	0.97587	2.1
4265	0.946132	2.1
4266	0.747365	2.1
4267	1.55432	2.1
4268	1.55432	2.1
4269	0.946132	2.1
4270	0.747394	2.1
4271	1.17464	0.146811
4272	1.0372	0.165548
4273	2.16553	0.0627978
4274	2.00616	0.115938
4275	1.12184	0.0552137
4276	1.33177	0.0567835
4277	0.936674	0.0517036
4278	0.946129	0.0566538
4279	0.851552	0.0694259
4280	1.60205	0.0556802
4281	1.77391	0.0555186
4282	1.03327	0.0890002
4283	1.41512	0.0999505
4284	1.03327	0.0767074
4285	0.946133	0.0966503
4286	0.946131	0.124282
4287	1.80077	0.110884
4288	1.80077	0.102594
4289	1.54848	2.1

4290	1.29931	2.1
4291	1.04482	0.487523
4292	2.08069	0.494359
4293	0.946127	0.405474
4294	0.515539	0.523575
4295	0.515566	0.560561
4296	1.38423	0.412494
4297	1.38423	0.44278
4298	0.972058	0.478164
4299	0.927737	0.51966
4300	0.94971	0.368633
4301	2.19682	0.412541
4302	0.946132	0.48947
4303	1.09841	0.416457
4304	0.522382	0.534917
4305	1.5962	0.33087
4306	1.72791	0.324714
4307	1.07428	0.418882
4308	1.05215	0.428002
4309	1.54632	1.26512
4310	1.15693	1.36554
4311	1.41238	2.1
4312	1.41238	2.1
4313	1.77966	2.1
4314	1.77966	2.1
4315	0.946129	2.1
4316	1.40525	2.1
4317	0.946131	2.1
4318	0.94613	2.1
4319	1.32054	2.1
4320	1.41238	2.1
4321	1.94026	2.1
4322	1.0171	0.508274
4323	1.1211	0.427601
4324	0.993022	2.1
4325	0.773813	2.1
4326	1.4073	2.1
4327	1.4073	2.1
4328	0.94613	2.1
4329	0.787615	2.1
4330	0.94613	2.1
4331	0.787615	2.1
4332	1.764	2.1
4333	1.90964	2.1
4334	1.05787	2.1
4335	1.403	0.531238
4336	0.744189	0.622255
4337	2.32834	2.1
4338	2.34986	2.1
4339	1.17106	1.45415
4340	1.94826	0.127869
4341	2.40606	0.103367
4342	1.26387	0.139904
4343	0.954013	0.147729
4344	0.946131	0.168556
4345	1.71	0.0898666
4346	1.51926	0.0844658
4347	0.953997	0.0857667

4348	0.851554	0.143405
4349	1.96502	0.104321
4350	1.72954	0.140193
4351	2.89117	0.114034
4352	0.971958	0.147527
4353	0.985575	0.172942
4354	1.41943	0.0990953
4355	1.20021	0.122329
4356	1.29599	0.14219
4357	1.15687	0.104738
4358	0.810958	0.171913
4359	1.12919	0.05
4360	1.98859	2.1
4361	1.82044	2.1
4362	1.90488	2.1
4363	0.851542	2.1
4364	1.02973	2.1
4365	1.39286	2.1
4366	1.44873	2.1
4367	0.946132	2.1
4368	1.11826	2.1
4369	1.26842	2.1
4370	2.09461	2.1
4371	1.76952	0.05
4372	1.6473	0.05
4373	0.946131	0.0563943
4374	1.36486	0.05
4375	1.04243	0.050203
4376	1.05017	0.05
4377	1.24059	0.05
4378	2.11646	0.05
4379	1.50368	0.05
4380	2.38796	0.05
4381	1.50368	0.05
4382	1.71844	0.05
4383	1.71844	0.05
4384	0.914836	0.0666998
4385	1.24416	0.057751
4386	0.914805	0.0633002
4387	1.24416	0.0559868
4388	1.38911	0.0502778
4389	1.46859	0.0627537
4390	1.35348	0.0610434
4391	0.946131	0.0752277
4392	1.1226	0.0661239
4393	0.857078	0.0854706
4394	0.9746	0.0739554
4395	1.37624	0.0693325
4396	1.15131	0.05
4397	1.139 0.05	
4398	1.90456	0.051847
4399	2.09692	0.0651532
4400	1.16655	2.1
4401	1.23017	2.1
4402	2.14592	2.1
4403	1.23017	2.1
4404	1.0752	0.731201
4405	1.52482	0.438953

4406	1.09252	2.1
4407	1.10725	2.1
4408	1.14305	0.148862
4409	0.851541	0.165539
4410	1.48073	0.148599
4411	1.45607	0.100964
4412	0.946131	0.205707
4413	0.932823	0.096089
4414	1.45828	0.178595
4415	0.851528	0.182685
4416	1.14305	0.138087
4417	1.92737	0.237378
4418	1.08211	0.196413
4419	1.69831	0.05
4420	1.69831	0.05
4421	0.946131	0.05
4422	1.26868	0.05
4423	1.23377	0.05
4424	1.23377	0.05
4425	0.94723	0.05
4426	1.64322	0.05
4427	1.64322	0.05
4428	0.946131	0.151431
4429	1.32068	0.05
4430	0.946129	0.05
4431	1.18789	0.22773
4432	1.32068	0.05
4433	1.32813	2.1
4434	1.32461	2.1
4435	1.42729	0.123831
4436	2.47846	0.178589
4437	1.00024	0.282625
4438	1.23923	0.178949
4439	0.946131	0.295132
4440	0.712304	0.151187
4441	1.77501	0.296936
4442	1.77501	0.123898
4443	0.593593	0.403066
4444	1.23923	0.318627
4445	1.23175	0.226531
4446	0.94613	0.366913
4447	0.713646	0.200934
4448	1.75735	0.345129
4449	1.75735	0.135655
4450	0.713633	0.449359
4451	1.37157	0.18022
4452	1.47169	0.157246
4453	2.21808	0.269258
4454	1.47169	0.23268
4455	1.04759	2.1
4456	0.987132	2.1
4457	1.11181	2.1
4458	2.61682	1.6291
4459	1.11181	2.1
4460	1.06461	1.08553
4461	1.69733	2.1
4462	2.79705	2.1
4463	1.08245	2.1

4464	1.40583	2.04588
4465	1.39852	1.91848
4466	2.0239	1.70498
4467	2.0239	1.91034
4468	0.780455	2.1
4469	0.946132	2.1
4470	0.780455	2.1
4471	1.01885	2.1
4472	0.564217	2.1
4473	0.733461	2.1
4474	0.537126	2.1
4475	0.683838	2.1
4476	0.779662	2.1
4477	0.634141	2.1
4478	0.685337	2.1
4479	1.58848	1.65378
4480	1.17129	0.210148
4481	0.663168	0.415015
4482	1.55587	0.338923
4483	1.31342	0.295579
4484	0.596868	0.412606
4485	1.1666	0.430511
4486	0.867261	0.435694
4487	0.946132	0.451074
4488	1.28843	0.26526
4489	1.31248	0.403344
4490	2.59921	0.382509
4491	1.32634	0.186447
4492	1.35564	0.05
4493	1.52661	0.05
4494	1.12323	0.05
4495	1.17701	0.877897
4496	1.36405	0.867363
4497	1.19837	0.885665
4498	2.25636	0.821908
4499	1.0823	0.31976
4500	1.31774	0.35154
4501	1.0823	0.339366
4502	1.01121	0.320651
4503	1.27408	0.337507
4504	0.946131	0.398208
4505	0.566074	0.331482
4506	0.79576	0.386566
4507	1.24596	0.38805
4508	1.47567	0.399277
4509	1.47567	0.207626
4510	0.56605	0.37799
4511	1.6567	0.230499
4512	0.967792	0.191859
4513	1.00366	0.359995
4514	1.00366	0.303482
4515	1.56409	0.0504403
4516	1.56409	0.05
4517	0.774911	0.0736912
4518	1.09483	0.0700132
4519	0.774912	0.0650663
4520	1.09483	0.0671043
4521	1.24417	0.0568879

4522	1.13387	0.05
4523	1.13387	0.05
4524	1.30572	0.0722929
4525	2.18966	0.0630336
4526	0.98575	0.05
4527	1.54982	0.0613544
4528	2.48833	0.05294
4529	1.54982	0.0546599
4530	1.28954	0.336971
4531	1.64002	0.05
4532	1.64002	0.05
4533	1.85594	0.05
4534	1.12968	2.1
4535	1.23536	1.55486
4536	1.4882	2.0005
4537	1.4882	2.1
4538	0.876836	1.56917
4539	0.946131	2.1
4540	0.876835	2.1
4541	1.43166	2.1
4542	2.25936	2.1
4543	1.0305	1.82917
4544	0.992095	2.1
4545	1.12968	2.09043
4546	1.53813	2.1
4547	1.53812	2.1
4548	0.583653	2.1
4549	0.94613	2.1
4550	0.583651	2.1
4551	0.964033	2.1
4552	0.964034	2.1
4553	1.54457	1.7309
4554	3.54109	0.146276
4555	0.99974	0.208588
4556	0.78814	0.261042
4557	1.65729	0.140612
4558	1.1283	0.179021
4559	1.09407	0.20829
4560	1.55091	0.144196
4561	0.870773	0.237606
4562	1.19967	0.161919
4563	1.56521	0.168594
4564	1.30896	1.07433
4565	1.17989	2.1
4566	1.72526	2.1
4567	0.851537	1.65481
4568	1.58944	2.1
4569	1.56915	2.1
4570	0.891039	1.4692
4571	1.4172	1.46465
4572	0.963818	1.24384
4573	0.851474	1.89313
4574	1.17989	2.1
4575	1.56261	2.1
4576	1.56261	2.1
4577	0.761574	2.1
4578	1.31323	2.1
4579	0.761573	2.1

4580	0.946133	2.1
4581	1.31323	2.1
4582	1.16759	2.1
4583	1.16759	2.1
4584	2.11829	2.1
4585	1.16759	2.1
4586	1.10748	0.319584
4587	1.47688	0.312496
4588	0.946133	0.313159
4589	0.953319	0.320294
4590	0.946132	0.317325
4591	1.51168	0.315125
4592	1.51168	0.317449
4593	1.25797	0.314374
4594	1.19984	0.284688
4595	1.95729	2.1
4596	2.34853	2.1
4597	1.94343	1.08133
4598	2.00551	1.23899
4599	1.20067	0.899838
4600	1.87244	0.933474
4601	1.4178	1.69891
4602	1.33538	2.1
4603	0.946131	1.84079
4604	1.22895	0.994677
4605	1.12433	2.1
4606	0.956757	2.1
4607	0.91826	2.1
4608	1.44221	2.1
4609	1.14206	2.1
4610	1.80514	0.788625
4611	0.890304	1.07972
4612	1.1362	0.675629
4613	1.32819	0.70239
4614	1.46311	1.6962
4615	0.8654	0.730582
4616	1.1699	1.05165
4617	1.00267	1.02708
4618	1.02882	0.810427
4619	1.00832	2.1
4620	0.974137	2.1
4621	1.02368	2.1
4622	0.974138	2.1
4623	0.995734	0.472798
4624	1.44605	0.376577
4625	1.18549	0.374595
4626	1.33621	0.628654
4627	1.5176	0.642881
4628	1.91762	0.652497
4629	1.33621	0.606667
4630	1.6638	0.61302
4631	1.66774	2.1
4632	1.11833	2.1
4633	0.926499	2.1
4634	1.51229	2.1
4635	1.51229	2.1
4636	0.926528	2.1
4637	1.40335	2.1

4638	1.03544	2.1
4639	0.926527	2.1
4640	1.85305	2.1
4641	1.5812	2.1
4642	1.15529	2.1
4643	1.31086	2.1
4644	0.999206	2.1
4645	1.46714	2.1
4646	1.73087	2.1
4647	0.772299	2.1
4648	1.24341	2.1
4649	0.997867	2.1
4650	0.772311	2.1
4651	0.936657	2.1
4652	1.35986	2.1
4653	0.959414	2.1
4654	1.36541	2.1
4655	1.49268	2.1
4656	1.49268	2.1
4657	0.832137	2.1
4658	0.946132	2.1
4659	0.832138	2.1
4660	1.64523	2.1
4661	1.11764	2.1
4662	1.05776	2.1
4663	0.972465	2.1
4664	0.972464	2.1
4665	1.98425	2.1
4666	0.969822	2.1
4667	1.30566	2.1
4668	1.52724	2.1
4669	2.32237	0.376734
4670	2.30274	0.43804
4671	2.27761	0.242949
4672	3.33012	0.222579
4673	1.24001	0.820753
4674	1.41821	1.10014
4675	0.978584	2.1
4676	1.18076	2.1
4677	1.07645	2.1
4678	0.877351	0.286458
4679	0.946132	0.752032
4680	0.877353	0.575532
4681	1.58444	2.1
4682	1.49614	1.27021
4683	1.28355	0.827179
4684	1.13491	0.784945
4685	1.56875	0.418323
4686	0.499592	0.204475
4687	0.946131	0.137443
4688	1.20538	0.144873
4689	1.09913	0.150178
4690	1.8049	0.159929
4691	1.8049	0.128508
4692	0.499606	0.213766
4693	2.41076	0.176076
4694	0.860202	0.133706
4695	0.906798	0.155461

4696	1.8626	0.19739
4697	1.33986	0.13067
4698	1.2992	0.235007
4699	1.58702	0.119496
4700	1.05939	0.142934
4701	1.02074	1.89536
4702	1.07866	1.71889
4703	1.5082	0.155835
4704	1.5522	0.105173
4705	1.65903	0.227693
4706	1.86766	2.1
4707	2.11684	2.1
4708	0.946132	2.1
4709	1.6585	2.1
4710	1.6585	2.1
4711	0.94613	2.1
4712	1.1872	2.1
4713	1.1872	2.1
4714	1.05842	2.1
4715	1.20455	2.1
4716	1.34141	2.1
4717	1.34141	2.1
4718	1.42416	2.1
4719	1.75818	1.70997
4720	2.63715	0.96897
4721	1.80454	2.1
4722	2.26198	2.1
4723	0.696255	2.1
4724	0.624075	1.99468
4725	1.50905	0.787087
4726	1.50905	2.1
4727	0.624075	2.1
4728	1.31857	0.723987
4729	0.946131	1.7174
4730	0.624075	0.942145
4731	0.914628	1.83001
4732	1.14721	2.1
4733	1.16153	2.1
4734	1.47664	2.1
4735	1.54791	2.1
4736	1.19098	2.1
4737	1.13341	2.1
4738	1.00503	0.611121
4739	1.00503	1.00178
4740	1.22655	1.93315
4741	0.999489	2.1
4742	1.3268	1.43007
4743	0.946132	2.1
4744	1.60061	2.1
4745	1.60061	2.1
4746	1.08034	2.1
4747	1.40372	2.1
4748	1.14302	2.1
4749	1.08034	2.1
4750	0.999491	2.1
4751	1.17152	0.290103
4752	1.17152	0.139358
4753	1.17152	0.197891

4754	2.05423	0.215346
4755	1.40984	1.70731
4756	1.2489	2.1
4757	0.885089	2.1
4758	0.810931	2.1
4759	1.84128	2.1
4760	1.11206	2.1
4761	1.21926	2.1
4762	1.99084	2.1
4763	0.976334	0.759035
4764	0.599363	0.629371
4765	1.02621	0.502266
4766	0.462959	0.62142
4767	1.42612	0.476505
4768	0.299682	0.514365
4769	1.51379	2.1
4770	0.719531	1.93909
4771	1.3702	2.1
4772	1.33293	2.1
4773	0.851539	2.1
4774	0.62998	2.1
4775	0.851543	2.1
4776	0.629966	2.1
4777	1.43132	2.1
4778	1.13328	1.13822
4779	1.93212	0.862542
4780	1.62345	1.28917
4781	1.38511	1.43911
4782	1.40013	0.937031
4783	1.04132	0.863731
4784	0.83473	1.24979
4785	0.868732	1.20917
4786	0.810595	1.55451
4787	0.729919	1.15192
4788	0.992428	1.28972
4789	0.577107	0.940305
4790	1.18464	1.32989
4791	0.791199	1.23352
4792	0.810943	0.117683
4793	0.860723	0.124035
4794	1.33348	0.0789449
4795	1.57467	0.0877203
4796	0.981032	0.118505
4797	0.731569	0.0841973
4798	0.770312	0.125006
4799	1.46055	2.1
4800	1.42249	2.1
4801	1.75999	0.132512
4802	1.6868	0.128647
4803	1.17042	0.775889
4804	1.01485	2.1
4805	1.19952	0.263489
4806	1.40592	0.380033
4807	1.19952	0.327829
4808	2.20145	0.299144
4809	1.33649	0.323457
4810	1.11612	0.55782
4811	0.957588	0.397929

4812	0.946126	0.566652
4813	1.50648	0.248279
4814	1.50648	0.410223
4815	0.946128	0.43013
4816	2.10671	0.0960508
4817	1.91518	0.233986
4818	2.23223	0.265299
4819	1.53556	2.1
4820	1.56253	2.1
4821	1.27806	0.05
4822	1.44593	0.05
4823	1.03818	0.575386
4824	1.03818	0.56041
4825	1.38969	0.737676
4826	1.85548	0.601073
4827	2.6044	0.679984
4828	1.85548	0.577128
4829	1.49346	1.95178
4830	1.83192	1.70315
4831	0.834043	1.84484
4832	0.810943	1.15874
4833	0.887113	1.20703
4834	0.893827	1.31892
4835	0.991061	1.3335
4836	0.91939	1.40363
4837	0.793307	1.34645
4838	1.75291	1.61228
4839	1.35435	1.39247
4840	1.16989	0.536953
4841	1.36717	0.481827
4842	2.12276	0.430746
4843	2.30493	0.414938
4844	2.47857	0.446519
4845	1.92269	0.435617
4846	1.66426	0.450086
4847	0.956698	0.707265
4848	0.956698	1.76074
4849	0.857957	2.1
4850	1.4706	1.10153
4851	1.65706	2.1
4852	1.47059	1.9627
4853	1.04713	0.361413
4854	0.946131	0.401564
4855	1.47374	0.451704
4856	1.47374	0.381836
4857	0.951675	0.378031
4858	1.2582	0.447204
4859	1.2582	0.400023
4860	0.94613	0.432503
4861	1.4468	0.209355
4862	1.90335	0.31849
4863	1.14886	2.1
4864	2.59689	2.1
4865	1.02747	2.1
4866	0.946131	2.1
4867	0.934061	2.1
4868	1.63979	2.1
4869	1.65206	2.1

4870	1.48	2.1
4871	1.27467	2.1
4872	2.20449	2.1
4873	1.1333	0.05
4874	1.95334	2.1
4875	2.20984	2.1
4876	1.46389	0.192353
4877	1.4639	0.220705
4878	1.4639	0.338645
4879	0.946132	0.365365
4880	1.5144	0.226037
4881	1.5144	0.326467
4882	0.946132	0.395838
4883	1.35732	0.230647
4884	1.10321	0.349125
4885	0.946132	0.367584
4886	0.980549	0.246761
4887	1.32819	2.1
4888	1.30171	1.10403
4889	1.68377	0.922889
4890	1.68377	2.1
4891	0.946132	0.794278
4892	0.946132	1.87232
4893	1.1952	1.0936
4894	0.990019	1.2002
4895	1.08195	1.73933
4896	1.2269	1.90065
4897	1.19219	1.70595
4898	1.01485	2.1
4899	1.57125	0.0877277
4900	2.10696	0.0776185
4901	1.29666	0.05
4902	1.57125	0.0911813
4903	1.47712	2.1
4904	1.45182	2.1
4905	1.17816	0.056054
4906	1.4516	0.0632886
4907	1.17816	0.05
4908	1.17816	0.05
4909	0.596954	0.857857
4910	1.65133	0.623545
4911	0.583859	0.572589
4912	1.04072	0.888806
4913	1.28795	0.637447
4914	1.59423	0.619762
4915	0.483559	0.664938
4916	1.74088	0.582499
4917	1.09545	0.796141
4918	1.02519	1.15644
4919	2.47579	0.771487
4920	1.54732	0.887837
4921	2.17663	0.715569
4922	1.75006	0.737395
4923	1.32354	0.842968
4924	1.09962	2.1
4925	1.85057	2.1
4926	1.91046	2.1
4927	2.17037	2.1

4928	1.99528	0.183557
4929	1.97969	0.156862
4930	3.82765	2.1
4931	1.85767	2.1
4932	2.77042	2.1
4933	1.37236	0.151464
4934	2.23254	0.123927
4935	1.10089	0.109054
4936	0.994294	2.1
4937	1.81921	2.1
4938	1.00073	2.1
4939	0.681043	2.1
4940	0.946131	2.1
4941	0.681073	2.1
4942	1.51804	2.1
4943	1.60653	2.1
4944	1.30188	2.1
4945	0.919622	2.1
4946	1.00965	2.1
4947	1.93821	2.1
4948	2.60377	2.1
4949	1.36416	0.0566995
4950	1.36416	0.0701536
4951	1.36416	0.0706354
4952	2.44132	0.0602099
4953	2.15871	0.616331
4954	2.22171	0.51667
4955	1.70747	0.443101
4956	1.49515	0.419169
4957	0.773007	0.451241
4958	0.946125	0.48006
4959	0.946131	0.494328
4960	0.773034	0.401119
4961	1.15844	0.449484
4962	1.98104	0.403843
4963	2.44283	0.443267
4964	1.05417	0.05
4965	1.1596	0.0545187
4966	0.829921	0.0505772
4967	0.946131	0.0608966
4968	0.951911	0.0601062
4969	1.39027	0.05
4970	0.829946	0.0576481
4971	1.51223	0.0562335
4972	1.51223	0.05
4973	1.65984	0.05
4974	1.90382	0.05
4975	1.93228	0.904181
4976	2.47924	0.864176
4977	1.73904	0.996191
4978	1.40547	0.652245
4979	2.37589	0.731034
4980	0.662605	0.95762
4981	2.33293	0.795746
4982	1.72249	2.1
4983	1.68478	2.1
4984	1.00514	0.546581
4985	1.4537	2.1

4986	1.92698	2.1
4987	1.46041	0.05
4988	2.04817	0.05
4989	1.65954	2.1
4990	1.42342	2.1
4991	1.04405	0.0535727
4992	1.17056	0.05
4993	1.11339	0.05
4994	0.946133	0.05
4995	0.929724	0.05
4996	1.35424	0.05
4997	1.35424	0.05
4998	0.929713	0.05
4999	1.85945	0.05
5000	1.38905	2.1
5001	1.39056	2.1
5002	1.52852	2.1
5003	1.06472	2.1
5004	0.749096	2.1
5005	1.46076	2.1
5006	1.29263	2.1
5007	0.94613	2.1
5008	0.69097	2.1
5009	0.982284	2.1
5010	0.67419	2.1
5011	1.52852	2.1
5012	1.91121	2.1
5013	1.00775	1.88555
5014	1.61201	2.07508
5015	0.529987	2.1
5016	0.506125	2.1
5017	1.44696	2.1
5018	2.32981	2.1
5019	1.32784	2.1
5020	1.94158	2.1
5021	1.31595	2.1
5022	0.663922	2.1
5023	1.44663	2.1
5024	1.44663	2.1
5025	1.13976	2.1
5026	0.970788	2.1
5027	1.13976	2.1
5028	0.663921	2.1
5029	0.966114	2.1
5030	1.05498	0.888725
5031	1.38086	2.1
5032	0.946127	2.1
5033	0.922513	2.1
5034	1.49903	2.1
5035	1.49903	2.1
5036	0.92251	2.1
5037	1.06105	2.1
5038	1.36049	2.1
5039	1.38086	2.1
5040	1.52534	2.1
5041	1.04101	2.1
5042	1.30861	0.0810705
5043	0.949112	0.10082

5044	0.894275	0.129491
5045	1.10232	0.0788469
5046	0.830889	0.0938181
5047	1.26554	0.0971776
5048	0.948347	0.085933
5049	0.81096	0.0970166
5050	1.18338	0.108272
5051	0.883764	0.141651
5052	1.54793	0.0807288
5053	1.02867	0.107294
5054	1.8573	0.106161
5055	1.06065	0.0869213
5056	0.64842	0.0977779
5057	1.99319	0.12709
5058	1.9476	0.139632
5059	2.51436	0.05
5060	1.68337	0.11837
5061	1.92321	0.133028
5062	0.810942	0.05
5063	0.921001	0.0822963
5064	0.99758	0.12003
5065	0.960785	0.0880865
5066	1.01565	0.120085
5067	0.908624	0.0771485
5068	0.912835	0.0764041
5069	1.18351	0.0937707
5070	1.40239	2.1
5071	1.26091	2.1
5072	0.947979	2.1
5073	0.946131	2.04385
5074	1.81959	1.66053
5075	1.81959	0.704924
5076	0.94613	1.93074
5077	1.38415	0.767648
5078	1.38414	1.32402
5079	1.33266	1.82672
5080	0.946132	2.1
5081	1.76225	2.1
5082	1.76225	2.1
5083	1.27599	2.1
5084	1.38158	1.15235
5085	1.3268	2.1
5086	1.27599	2.1
5087	0.947979	2.04495
5088	1.0142	0.559875
5089	1.01422	0.567665
5090	1.11435	0.797522
5091	1.42186	0.783662
5092	1.24001	1.04224
5093	1.77342	1.16963
5094	1.42186	1.25276
5095	1.99817	0.900665
5096	1.27361	2.1
5097	1.68914	2.1
5098	1.30116	2.1
5099	1.32819	1.1803
5100	1.68322	1.11964
5101	1.68322	2.1

5102	0.94613	0.776938
5103	0.94613	1.90097
5104	1.14666	1.08145
5105	1.07612	1.04866
5106	1.31151	2.1
5107	1.22095	1.2301
5108	1.57256	1.55545
5109	1.6679	2.1
5110	0.946132	0.885733
5111	0.946133	2.03169
5112	1.13537	1.87494
5113	1.07855	1.22311
5114	1.07855	2.1
5115	1.43895	1.05441
5116	1.38481	0.082543
5117	1.72385	0.0794356
5118	0.550441	0.0741607
5119	0.79807	0.0934836
5120	1.11627	0.098529
5121	1.30349	0.113594
5122	0.94613	0.12631
5123	0.686176	0.157104
5124	1.11627	0.0945441
5125	1.73363	0.0788758
5126	1.73363	0.0666806
5127	0.686139	0.157096
5128	1.24932	0.0793126
5129	0.899582	0.0727136
5130	2.00472	0.0611226
5131	1.17121	0.102476
5132	1.2874	0.0663373
5133	2.0194	0.0762193
5134	0.88601	0.1301
5135	1.77409	0.100365
5136	1.73293	0.0754775
5137	2.74045	0.0926327
5138	1.83787	0.0750456
5139	1.45448	0.0636637
5140	1.27778	0.0600981
5141	1.1192	2.1
5142	1.25953	2.1
5143	1.93611	2.1
5144	1.92104	2.1
5145	1.15506	0.05
5146	1.15506	0.0511934
5147	1.23943	0.501456
5148	0.851543	2.1
5149	1.6061	2.1
5150	1.80058	2.1
5151	0.869471	2.1
5152	1.3931	2.1
5153	1.06454	2.1
5154	0.93667	2.1
5155	2.16916	2.1
5156	3.10246	2.1
5157	1.91539	2.03963
5158	1.91539	1.72724
5159	0.620263	1.12988

5160	0.620265	1.93875
5161	0.946132	1.21817
5162	0.620265	1.0844
5163	1.30226	0.994112
5164	1.87292	2.01242
5165	1.18769	2.1
5166	2.60452	1.94712
5167	1.26574	2.1
5168	2.46679	1.32422
5169	1.36459	2.1
5170	1.26865	1.0649
5171	1.1744	0.802489
5172	1.15841	0.469819
5173	1.02247	0.418346
5174	1.93649	2.1
5175	1.64718	2.1
5176	1.59654	2.1
5177	1.57823	2.1
5178	1.00665	0.322071
5179	1.28239	0.322079
5180	0.887895	2.1
5181	1.22083	2.1
5182	1.42127	2.1
5183	1.07374	2.1
5184	1.00503	1.32247
5185	1.23082	0.875249
5186	2.78663	2.1
5187	2.20883	2.1
5188	2.54588	2.1
5189	0.946128	2.1
5190	1.13716	2.1
5191	1.58724	2.1
5192	1.58724	2.1
5193	0.946131	2.1
5194	1.13716	2.1
5195	1.39621	2.1
5196	1.02438	2.1
5197	1.49543	0.0669278
5198	2.78336	0.0591193
5199	1.27148	0.05
5200	1.0906	0.0510697
5201	1.04072	0.0585198
5202	0.63574	0.05
5203	1.45506	0.05
5204	1.5863	0.05
5205	0.928352	0.0533047
5206	0.647687	0.0569578
5207	2.2375	1.82666
5208	1.50998	1.46044
5209	2.75021	1.46647
5210	1.91912	1.38071
5211	1.99872	1.34572
5212	0.918127	1.77035
5213	1.03031	1.18925
5214	0.951169	1.04073
5215	1.27975	1.19462
5216	1.12735	1.32076
5217	1.15741	1.2156

5218	1.24998	1.17539
5219	1.65953	0.397507
5220	0.967555	0.685346
5221	0.967542	0.423277
5222	1.03824	0.364871
5223	1.48693	0.402056
5224	1.48693	0.368809
5225	2.02901	0.373817
5226	0.946127	0.416193
5227	1.07139	0.391132
5228	0.946126	0.400999
5229	1.84644	0.38967
5230	1.84644	0.350476
5231	1.38683	0.380603
5232	1.40574	0.260858
5233	1.36866	1.03189
5234	0.649325	0.890004
5235	1.93631	2.1
5236	1.50145	2.1
5237	1.6287	2.1
5238	1.31302	2.1
5239	1.18041	2.1
5240	1.18041	2.1
5241	0.974196	2.1
5242	0.946131	2.1
5243	1.74169	2.1
5244	1.74169	2.1
5245	1.23163	2.1
5246	1.27451	2.1
5247	1.41331	2.1
5248	1.23163	2.1
5249	1.28037	2.1
5250	1.28158	2.1
5251	1.28037	2.1
5252	2.28015	0.05
5253	2.06561	0.05
5254	2.61343	0.0592656
5255	1.11712	0.0573612
5256	1.63689	1.17519
5257	1.01393	0.855741
5258	0.996775	2.1
5259	1.54277	2.1
5260	1.26397	2.1
5261	1.46129	2.1
5262	1.62391	2.1
5263	1.08848	0.0579405
5264	1.11833	2.1
5265	1.24041	2.1
5266	2.31884	2.1
5267	1.335 2.1	
5268	1.48589	0.0956927
5269	1.77131	0.0798616
5270	1.8422	1.14179
5271	2.47453	1.58601
5272	1.23726	1.29793
5273	1.21996	1.26851
5274	1.65512	1.41764
5275	1.65512	1.39412

5276	0.802107	1.50704
5277	0.946129	1.09757
5278	0.802137	1.10619
5279	1.3279	1.07743
5280	1.23726	1.08955
5281	1.79759	1.13558
5282	1.79759	1.05427
5283	0.767577	1.34901
5284	0.946131	0.973467
5285	0.767577	0.953894
5286	1.03534	0.0809944
5287	1.09962	0.531971
5288	0.929217	0.992959
5289	1.70126	1.01812
5290	0.991407	2.1
5291	1.41077	1.67132
5292	0.991406	2.1
5293	1.62966	2.1
5294	1.62966	2.1
5295	0.946128	2.1
5296	1.18364	2.1
5297	0.946131	2.1
5298	1.14186	2.1
5299	1.18364	2.1
5300	1.16858	1.18054
5301	1.66137	0.349264
5302	1.90165	0.219637
5303	1.66139	0.091912
5304	1.26893	0.519654
5305	1.15918	0.571502
5306	0.653228	0.69405
5307	0.946133	0.620935
5308	1.77488	2.04715
5309	1.77489	1.03292
5310	0.653228	1.01619
5311	1.56043	0.673252
5312	1.60894	1.02626
5313	1.56043	0.566949
5314	1.06848	1.31551
5315	1.04307	2.1
5316	1.14188	2.1
5317	1.09195	2.1
5318	0.615121	2.1
5319	1.50867	2.1
5320	1.27152	1.97133
5321	0.615094	1.59882
5322	1.07297	2.1
5323	1.1372	2.1
5324	0.553608	2.1
5325	1.23019	2.09584
5326	0.992675	2.1
5327	1.40984	1.03221
5328	2.55682	2.1
5329	3.66069	2.1
5330	3.58726	2.1
5331	1.4375	0.347173
5332	1.88424	0.407027
5333	1.56714	2.1

5334	1.6721	2.1
5335	1.18474	2.1
5336	1.2295	2.1
5337	1.28754	2.1
5338	1.36838	2.1
5339	2.78661	2.1
5340	2.64055	2.1
5341	1.35065	0.978458
5342	2.26675	0.583492
5343	1.35065	0.737652
5344	1.21558	0.606876
5345	1.49709	0.05
5346	1.4971	0.05
5347	0.85472	0.0520784
5348	0.854718	0.05
5349	0.946129	0.055034
5350	0.854717	0.0500679
5351	1.1604	0.05
5352	1.25265	0.05
5353	2.32081	0.05
5354	1.15371	0.05
5355	1.14945	0.591765
5356	0.995725	0.562919
5357	1.00307	0.407445
5358	1.10907	0.381538
5359	2.30315	0.378754
5360	1.10908	0.3863
5361	1.27558	0.777648
5362	1.71613	1.22157
5363	1.32977	2.1
5364	0.815276	2.1
5365	1.41697	2.1
5366	1.41697	2.1
5367	0.815276	2.1
5368	1.38897	2.1
5369	0.946131	2.1
5370	0.815275	2.1
5371	1.44684	2.1
5372	1.21489	1.77545
5373	1.22476	1.69607
5374	0.870366	0.709893
5375	0.862572	0.64006
5376	0.835435	0.598881
5377	1.6993	0.592983
5378	1.23618	0.583328
5379	1.18624	0.597827
5380	1.53058	0.616765
5381	2.16108	0.852374
5382	2.21589	0.762412
5383	1.04037	1.24021
5384	1.03933	1.23853
5385	1.57753	0.13527
5386	1.87644	0.211265
5387	1.24728	0.294076
5388	1.30394	0.143794
5389	1.3538	2.1
5390	1.69858	2.1
5391	1.1726	0.400094

5392	0.94612	0.328784
5393	1.09805	0.284835
5394	0.946118	0.364092
5395	1.72576	0.331879
5396	1.72575	0.292007
5397	1.34152	0.307596
5398	1.33034	0.27941
5399	0.784151	0.91881
5400	0.946131	1.97178
5401	0.784151	1.0366
5402	1.47963	2.1
5403	1.47963	2.1
5404	1.3335	2.1
5405	0.930281	2.1
5406	1.29435	0.895942
5407	0.883308	0.823632
5408	1.31589	2.1
5409	1.67846	2.1
5410	0.993598	0.0935637
5411	2.23193	0.0826167
5412	0.9936	0.0719298
5413	0.615629	0.0745504
5414	1.65925	2.1
5415	2.05992	2.1
5416	1.65925	2.1
5417	4.08735	1.48968
5418	2.72856	1.65342
5419	0.869602	2.1
5420	3.42833	0.900913
5421	1.14397	0.52069
5422	1.34563	0.384034
5423	1.08189	0.362168
5424	0.782128	1.75012
5425	1.0022	1.96476
5426	0.900427	1.52233
5427	1.51499	1.58359
5428	1.05588	1.58694
5429	1.13739	1.97123
5430	1.37004	1.5838
5431	1.14603	1.96552
5432	0.851541	1.11464
5433	0.946799	1.59616
5434	1.14854	1.53385
5435	1.33793	1.20584
5436	0.94613	1.227
5437	1.24336	1.38478
5438	0.851473	1.38826
5439	2.08792	1.57151
5440	1.01869	1.6273
5441	0.986953	0.199308
5442	0.896238	0.264975
5443	0.906574	0.323617
5444	0.852145	0.257521
5445	2.14191	0.200526
5446	1.15979	0.208289
5447	1.18327	0.194486
5448	2.32742	0.222343
5449	2.35961	0.270459

5450	2.38126	0.289828
5451	1.13632	0.214176
5452	1.17897	0.05
5453	1.76199	0.05
5454	1.00573	1.22145
5455	0.946127	0.622362
5456	1.7077	1.12495
5457	1.7077	2.1
5458	0.946127	1.07666
5459	1.24887	2.1
5460	1.24887	1.45653
5461	1.22441	1.70409
5462	1.08542	1.54585
5463	1.21743	1.63842
5464	1.12572	2.1
5465	1.05499	0.861014
5466	1.00521	0.933007
5467	2.86191	0.05
5468	2.27972	0.05
5469	1.01169	2.1
5470	1.03373	2.1
5471	1.39319	2.1
5472	0.636837	2.1
5473	1.63908	1.80187
5474	1.45713	1.81615
5475	1.04072	1.91203
5476	1.25025	1.88344
5477	1.38914	1.68235
5478	0.636807	1.36866
5479	1.50211	2.1
5480	1.36555	1.98946
5481	2.77545	1.18376
5482	0.682805	2.1
5483	1.50267	1.94797
5484	1.50267	2.1
5485	0.797749	2.1
5486	1.38773	1.83027
5487	1.38773	2.1
5488	0.682776	2.1
5489	2.01245	0.05
5490	0.925918	0.0606767
5491	0.75689	0.05
5492	1.25823	0.05
5493	1.16505	0.0566328
5494	0.838779	0.05
5495	1.14722	0.05
5496	0.756849	0.05
5497	1.52298	0.0605647
5498	2.11034	0.0538627
5499	1.92174	0.0516996
5500	1.92174	0.0520939
5501	0.576083	0.0799405
5502	1.23146	0.070566
5503	1.26637	0.0611589
5504	1.23146	0.0685289
5505	1.26637	0.0613561
5506	0.946133	0.711394
5507	1.37034	0.608875

5508	1.84145	0.66045
5509	1.84145	0.510508
5510	0.946132	0.76879
5511	1.37034	0.668611
5512	1.41724	0.672576
5513	1.0685	0.507751
5514	1.14547	0.533879
5515	1.24976	2.1
5516	1.30682	2.1
5517	2.12095	2.1
5518	1.46392	2.1
5519	1.46392	2.1
5520	0.935738	2.1
5521	1.32949	2.1
5522	1.07017	2.1
5523	0.935766	2.1
5524	1.07017	2.1
5525	1.78095	2.1
5526	1.05619	0.749174
5527	1.05793	0.743482
5528	1.77677	2.1
5529	2.04766	2.1
5530	1.02524	2.1
5531	1.18803	2.1
5532	1.70047	2.1
5533	1.556 2.1	
5534	1.24201	1.40796
5535	1.3592	2.1
5536	2.11824	0.149177
5537	2.20248	0.299533
5538	3.43878	0.168443
5539	3.12466	0.131994
5540	2.09351	2.1
5541	0.946128	1.56946
5542	1.51269	2.1
5543	1.51269	2.1
5544	0.946131	2.1
5545	1.01306	2.1
5546	1.04676	2.1
5547	1.01306	2.1
5548	1.07444	2.1
5549	2.17465	2.1
5550	1.14808	2.1
5551	1.39067	0.993729
5552	1.20958	1.02954
5553	0.662832	0.647267
5554	1.93512	0.90951
5555	1.15471	2.1
5556	1.75119	2.1
5557	1.75118	2.1
5558	0.946131	2.1
5559	1.28606	2.1
5560	1.27087	2.1
5561	0.946131	2.1
5562	1.28606	2.1
5563	1.58916	0.0640418
5564	0.927822	0.181517
5565	2.24367	0.05

5566	1.8734	0.05
5567	3.51668	0.05
5568	2.68986	0.05
5569	2.32395	2.1
5570	1.39564	2.1
5571	2.10862	2.1
5572	1.36763	2.1
5573	0.970963	0.05
5574	0.97096	0.05
5575	1.11897	0.19748
5576	1.26885	0.377861
5577	1.11605	0.230884
5578	2.06612	0.146964
5579	1.717	0.162611
5580	0.59502	0.352935
5581	0.825755	0.107268
5582	0.709624	0.432425
5583	1.65741	0.129757
5584	0.307486	0.167681
5585	1.4785	0.209848
5586	0.983143	2.1
5587	1.08247	2.1
5588	1.42353	2.1
5589	1.42353	2.1
5590	0.781667	2.1
5591	0.797634	2.1
5592	0.946131	2.1
5593	0.781638	2.1
5594	0.946131	2.1
5595	0.98315	2.1
5596	1.083	2.1
5597	1.61975	0.05
5598	1.11946	0.05
5599	1.18977	0.0798939
5600	0.996484	0.0875344
5601	2.24343	0.05
5602	1.4787	0.0559719
5603	1.66127	0.0538869
5604	1.86028	0.0727357
5605	1.09128	0.0660346
5606	1.03115	0.106791
5607	0.838025	0.0998212
5608	1.3736	0.0526796
5609	1.44009	0.0606025
5610	0.92917	0.0572293
5611	1.03889	0.103905
5612	0.956974	0.0716668
5613	3.23468	0.0631451
5614	0.953922	0.111562
5615	0.957104	0.116644
5616	1.66231	0.0600344
5617	1.26778	0.0703194
5618	1.47615	0.0693408
5619	1.28621	0.102285
5620	1.08043	0.0900886
5621	2.35026	2.1
5622	2.35025	2.1
5623	2.60659	2.1

5624	1.57627	2.1
5625	1.5493	2.1
5626	1.24682	0.506888
5627	2.14277	0.511028
5628	1.24682	0.605462
5629	1.00307	0.469345
5630	2.04056	0.46832
5631	1.45408	2.1
5632	1.54249	2.1
5633	1.24474	1.48788
5634	1.37813	1.7753
5635	0.924158	0.267444
5636	0.916623	0.307242
5637	1.35344	0.254193
5638	1.00859	0.300095
5639	1.34786	0.276395
5640	0.952457	0.241582
5641	0.85404	0.265835
5642	1.06341	2.1
5643	1.09194	2.1
5644	1.93707	2.1
5645	2.14087	2.1
5646	1.93707	2.1
5647	1.07519	0.697257
5648	1.07519	0.64654
5649	1.62518	2.1
5650	1.3592	2.1
5651	1.13183	2.1
5652	1.20357	2.1
5653	1.4858	2.1
5654	1.23694	2.1
5655	1.44011	2.1
5656	1.23989	2.1
5657	2.21604	0.983784
5658	1.85574	0.240407
5659	1.55649	0.967503
5660	1.48861	0.188724
5661	1.15968	0.05
5662	0.946132	0.0517358
5663	0.663056	0.05
5664	1.70837	0.05
5665	1.70837	0.05
5666	0.946131	0.05
5667	0.663026	0.05
5668	2.09604	0.05
5669	0.81094	0.05
5670	1.06559	0.05
5671	0.810956	0.05
5672	1.15474	0.05
5673	0.990051	0.05
5674	0.894971	0.05
5675	1.32536	0.05
5676	0.946133	2.1
5677	1.33125	2.1
5678	1.75823	2.1
5679	1.75823	2.1
5680	0.946133	2.1
5681	1.33125	2.1

5682	1.37312	2.1
5683	1.27985	2.1
5684	1.37312	2.1
5685	1.70684	2.1
5686	1.70684	2.1
5687	0.946132	2.1
5688	0.946132	2.1
5689	1.25081	2.1
5690	0.956293	2.1
5691	0.956294	2.1
5692	1.36035	2.1
5693	1.41944	2.1
5694	0.830638	2.1
5695	0.747568	2.1
5696	0.888498	2.1
5697	1.04071	2.1
5698	1.17128	2.1
5699	1.92339	2.1
5700	2.91208	2.1
5701	1.82208	1.01284
5702	1.89653	2.1
5703	1.82588	1.17451
5704	1.26703	0.578926
5705	1.26703	0.895779
5706	0.6157	0.532042
5707	1.26706	1.12608
5708	0.61567	0.373195
5709	0.615671	0.435456
5710	1.26706	0.603512
5711	1.89158	0.897315
5712	1.23134	0.513448
5713	2.49497	0.0599154
5714	1.68103	0.05
5715	2.0554	2.1
5716	1.72647	2.1
5717	1.67375	2.1
5718	1.65037	2.1
5719	1.04349	0.440326
5720	1.69055	0.175005
5721	1.53463	0.111514
5722	1.19022	1.97588
5723	1.89226	1.32241
5724	1.15009	0.731138
5725	1.7054	0.464094
5726	1.31612	0.100621
5727	1.31611	0.561422
5728	1.67345	0.406431
5729	1.06577	0.375598
5730	1.40193	0.229565
5731	1.72411	0.187983
5732	1.46123	0.211431
5733	0.808808	0.356353
5734	0.897714	0.222232
5735	0.851554	0.409799
5736	1.3499	0.321451
5737	1.29001	0.321153
5738	1.22083	2.1
5739	1.06191	2.1

5740	1.05758	1.27444
5741	1.348	1.8726
5742	1.43485	1.21103
5743	1.34799	1.81947
5744	1.36307	0.05
5745	2.28188	0.125814
5746	1.7926	0.111147
5747	1.38957	2.1
5748	1.47446	2.1
5749	0.953638	2.1
5750	1.11868	1.25077
5751	0.953636	2.1
5752	1.03408	2.06393
5753	1.52443	0.05
5754	1.38539	0.05
5755	0.946132	0.0575697
5756	1.12747	0.05
5757	1.04393	0.0653628
5758	1.05017	0.0543128
5759	1.20529	0.0518965
5760	1.3663	0.569271
5761	1.89226	0.530757
5762	1.17885	0.693479
5763	0.867881	0.479512
5764	1.32917	0.451868
5765	1.32917	0.477946
5766	0.946127	0.513775
5767	1.19583	0.499695
5768	1.00122	0.533189
5769	0.946128	0.523109
5770	1.71405	0.803577
5771	0.871815	1.2451
5772	1.16435	2.1
5773	0.686619	2.06366
5774	1.63102	2.1
5775	1.06589	1.96399
5776	1.05375	2.1
5777	1.9023	2.1
5778	1.27394	0.726028
5779	1.88345	0.692248
5780	2.05636	0.85156
5781	0.672977	0.64607
5782	1.41274	0.662714
5783	1.65606	0.639564
5784	0.869329	0.645841
5785	1.2207	0.683629
5786	1.35631	0.667983
5787	0.908776	0.643594
5788	0.955081	0.598206
5789	1.2243	2.1
5790	1.23169	2.1
5791	1.52228	2.1
5792	1.23169	2.1
5793	0.990892	1.15892
5794	1.08376	0.742968
5795	1.79348	2.09733
5796	1.50043	0.925539
5797	1.32599	2.1

5798	1.37924	1.02816
5799	0.838014	0.746629
5800	0.87737	1.61803
5801	0.947086	0.859038
5802	1.27199	1.91801
5803	1.04918	0.943375
5804	1.04851	2.1
5805	1.1699	1.35241
5806	0.810934	1.17883
5807	1.83602	2.1
5808	2.21909	2.09325
5809	0.619441	1.43522
5810	2.03755	0.910481
5811	1.83472	1.96874
5812	1.20416	1.60119
5813	1.26691	0.784006
5814	0.655795	0.920015
5815	0.94613	1.15139
5816	2.35169	1.53975
5817	3.54705	2.1
5818	2.60741	2.1
5819	1.59019	0.05
5820	1.24526	0.05
5821	1.59019	0.05
5822	2.36406	0.05
5823	1.16776	0.63062
5824	1.67311	0.744256
5825	1.67311	0.661715
5826	0.94613	0.567289
5827	1.38645	0.671898
5828	1.13563	0.421594
5829	1.13563	0.556628
5830	1.23279	0.496355
5831	1.05042	1.10796
5832	1.17418	0.699648
5833	1.28909	2.1
5834	0.973448	2.09302
5835	1.02958	1.93634
5836	3.3005	2.1
5837	2.86325	2.1
5838	1.35879	1.7472
5839	1.69332	2.1
5840	1.69332	2.1
5841	0.946128	1.52538
5842	1.39659	0.957359
5843	1.12035	2.1
5844	1.12035	2.1
5845	1.24286	2.1
5846	1.10407	1.20971
5847	1.14599	0.892552
5848	1.32259	0.590269
5849	0.788592	0.05
5850	2.4588	2.1
5851	0.909581	1.88399
5852	1.35656	1.15506
5853	0.986895	1.46022
5854	2.30602	1.1182
5855	1.46873	1.41821

5856	1.55584	1.09367
5857	2.1547	1.18479
5858	1.87794	1.80585
5859	0.808286	1.92897
5860	0.928464	1.40885
5861	0.810959	1.39048
5862	1.50962	0.997561
5863	1.1053	1.26667
5864	1.0712	1.20773
5865	1.40504	1.18249
5866	1.87794	1.13439
5867	2.80496	1.27973
5868	1.41479	1.61759
5869	1.38634	2.1
5870	1.38634	2.1
5871	1.67762	2.1
5872	0.946131	2.1
5873	1.188 2.1	
5874	1.62585	2.1
5875	1.62585	2.1
5876	0.946129	2.1
5877	1.18801	2.1
5878	1.38397	2.1
5879	1.04546	2.1
5880	1.16817	2.1
5881	1.06007	2.1
5882	1.19785	2.1
5883	1.43652	2.09633
5884	1.06994	2.1
5885	1.89111	1.10413
5886	1.07144	2.1
5887	1.51754	2.1
5888	2.0873	2.1
5889	0.758799	2.1
5890	1.59518	2.1
5891	1.59518	2.1
5892	0.946132	2.1
5893	0.758771	2.1
5894	1.06957	2.1
5895	0.758771	2.1
5896	1.33916	2.1
5897	2.03045	1.06717
5898	2.80184	1.35443
5899	1.76788	1.88563
5900	1.27442	1.98083
5901	1.06756	2.1
5902	0.966979	2.1
5903	1.7054	2.1
5904	1.07156	2.1
5905	0.986096	2.1
5906	1.32813	2.1
5907	1.3331	2.1
5908	2.08369	0.868264
5909	2.23818	2.007
5910	1.10335	0.979613
5911	0.622314	1.71083
5912	1.72451	1.13493
5913	1.72451	1.9886

5914	0.622283	0.760823
5915	0.622314	0.812454
5916	0.946131	0.774377
5917	1.31254	2.1
5918	1.15704	2.1
5919	1.24463	0.914877
5920	2.2067	1.50658
5921	1.30001	0.743988
5922	1.29175	0.791299
5923	1.1233	0.05
5924	1.8689	0.0675984
5925	1.29494	0.0579906
5926	1.11895	0.0554319
5927	0.851539	0.0725183
5928	0.851474	0.070383
5929	1.58352	0.05
5930	1.58228	0.05
5931	0.851527	0.083839
5932	2.6389	0.0533235
5933	1.77548	0.0781692
5934	1.24555	2.1
5935	2.0512	2.1
5936	1.46101	2.1
5937	2.60635	2.1
5938	1.10476	0.139137
5939	1.44103	0.119662
5940	0.652391	2.1
5941	0.618322	2.1
5942	2.22915	2.1
5943	1.39067	2.1
5944	1.99737	2.1
5945	1.42174	2.1
5946	0.946132	0.751392
5947	1.29735	1.2842
5948	1.61765	2.1
5949	1.61765	0.958475
5950	0.946132	1.73124
5951	1.29735	0.926444
5952	1.26643	0.88075
5953	1.01335	1.19975
5954	1.01335	0.616428
5955	0.947669	2.1
5956	2.17167	2.1
5957	2.59733	1.60951
5958	1.50954	1.76651
5959	1.02638	1.17601
5960	0.555785	1.99003
5961	1.91101	1.97634
5962	2.09809	1.63063
5963	0.641974	1.80133
5964	1.2022	1.62698
5965	1.36359	1.39671
5966	1.8128	1.9188
5967	2.34348	0.401403
5968	1.66343	0.403787
5969	0.974717	0.749827
5970	1.53908	0.666926
5971	1.27737	0.882028

5972	1.09408	0.681406
5973	2.07382	0.674812
5974	1.10466	1.02284
5975	1.05654	1.48509
5976	1.23498	1.05163
5977	1.48104	1.25286
5978	1.23498	1.16311
5979	1.17261	2.1
5980	1.18059	2.1
5981	2.43005	2.1
5982	1.04546	2.1
5983	2.81222	2.1
5984	1.16817	2.1
5985	1.75766	0.486539
5986	1.47021	0.561461
5987	1.07236	0.434059
5988	1.81759	0.644086
5989	1.42913	0.585546
5990	1.00636	0.627403
5991	1.00636	0.478179
5992	1.79397	0.520286
5993	0.946131	0.388278
5994	1.03691	0.38205
5995	0.851539	0.456284
5996	1.46001	0.602315
5997	1.37878	0.56837
5998	0.933258	0.342765
5999	1.32423	0.388472
6000	1.32614	0.05
6001	1.11356	0.05
6002	1.32614	0.05
6003	1.28895	0.05
6004	1.66895	0.05
6005	1.66895	0.05
6006	0.946132	0.05
6007	1.29576	0.05
6008	1.1318	0.05
6009	1.37621	1.23154
6010	1.94071	1.12725
6011	1.10517	2.1
6012	1.97098	2.1
6013	0.811	2.1
6014	0.956127	2.1
6015	1.18647	2.1
6016	0.988455	1.35105
6017	1.12457	0.100268
6018	1.7453	0.102043
6019	0.92492	2.1
6020	1.24353	2.1
6021	1.84536	0.699021
6022	1.67662	0.619482
6023	1.38247	0.0606183
6024	1.02663	0.0514696
6025	0.946132	0.65491
6026	1.41723	0.511903
6027	1.72976	0.624531
6028	1.72976	0.418414
6029	0.946131	0.76564

6030	1.41723	0.646037
6031	1.25866	0.627664
6032	1.34254	0.389765
6033	1.25866	0.539171
6034	1.65507	0.40215
6035	1.65507	0.318862
6036	0.946132	0.671879
6037	0.946132	0.488791
6038	1.11929	0.476453
6039	0.961779	0.383441
6040	0.961779	0.42486
6041	0.946132	2.1
6042	1.69361	2.1
6043	1.69361	2.1
6044	0.94613	2.1
6045	1.36523	2.1
6046	1.36523	2.1
6047	1.19802	2.1
6048	0.974196	2.1
6049	1.09518	2.1
6050	0.946131	2.1
6051	1.7161	2.1
6052	1.7161	2.1
6053	0.94613	2.1
6054	1.297	2.1
6055	1.297	2.1
6056	1.14599	2.1
6057	0.974197	2.1
6058	0.974197	2.1
6059	1.80778	2.1
6060	2.20473	2.1
6061	1.80777	2.1
6062	1.3515	2.1
6063	0.583237	2.1
6064	1.39359	2.1
6065	1.39359	2.1
6066	1.07296	2.1
6067	0.946114	2.1
6068	1.07297	2.1
6069	0.903862	2.1
6070	0.956572	2.1
6071	1.03437	2.1
6072	0.975409	2.1
6073	1.31167	0.146353
6074	2.70246	0.120668
6075	1.12018	0.133441
6076	0.655836	0.147824
6077	0.655837	0.135254
6078	1.81557	0.0847617
6079	1.81557	0.0820705
6080	0.655836	0.131269
6081	0.946131	0.153893
6082	1.01847	0.110744
6083	1.61334	0.0931557
6084	0.810946	0.164465
6085	0.8311	0.179275
6086	1.2466	0.106224
6087	1.20286	0.121711

6088	0.97508	0.175215
6089	0.870805	0.114983
6090	0.760087	0.180972
6091	0.853389	2.1
6092	0.820894	2.1
6093	0.928291	2.1
6094	1.06922	2.07828
6095	1.32829	2.1
6096	1.12173	2.1
6097	1.16615	2.1
6098	0.957748	2.1
6099	0.612491	1.67439
6100	0.548141	2.1
6101	0.979614	2.1
6102	0.658184	2.1
6103	0.678456	1.31265
6104	0.529544	1.47403
6105	0.739013	2.1
6106	0.694037	2.02175
6107	0.475716	1.58783
6108	1.58794	1.3386
6109	1.58794	1.05419
6110	0.475743	1.18735
6111	1.08301	1.94067
6112	0.980664	2.1
6113	0.475737	1.73256
6114	1.32709	2.1
6115	1.59396	2.1
6116	1.19719	0.176409
6117	1.34921	0.181418
6118	1.69061	0.174136
6119	1.69061	0.114541
6120	0.946132	0.21052
6121	1.34921	0.201765
6122	1.28753	0.191079
6123	0.95895	0.111257
6124	1.01051	0.143003
6125	1.12468	0.150741
6126	1.22588	0.256357
6127	1.34254	0.222373
6128	1.74561	0.208918
6129	1.74561	0.157944
6130	0.946128	0.2736
6131	1.34253	0.253229
6132	1.3492	0.241693
6133	1.01051	0.112226
6134	1.922 2.1	
6135	1.922 2.1	
6136	2.12556	2.1
6137	0.946131	2.1
6138	1.06278	2.1
6139	1.47631	2.1
6140	1.47631	2.1
6141	0.946129	2.1
6142	1.06278	2.1
6143	1.35966	2.1
6144	0.991985	2.1
6145	0.738507	1.31038

6146	0.536935	1.7381
6147	0.799004	1.44897
6148	1.04281	1.21538
6149	0.879197	1.09574
6150	1.06538	1.18374
6151	0.715942	1.3304
6152	1.00636	0.498516
6153	1.03824	0.421909
6154	1.70147	0.453681
6155	1.21223	2.1
6156	1.01243	1.07095
6157	1.01242	2.1
6158	1.06935	2.1
6159	1.54241	2.1
6160	0.998971	0.05
6161	1.60312	0.05
6162	1.44836	0.05
6163	2.05632	0.05
6164	1.44836	0.05
6165	0.973765	0.05
6166	0.946131	0.05
6167	0.851541	0.05
6168	1.73854	0.05
6169	1.91752	0.05
6170	1.49622	0.05
6171	1.23351	0.05
6172	3.33289	2.1
6173	1.86606	2.1
6174	2.71233	2.1
6175	0.788816	2.1
6176	0.963972	2.1
6177	1.25866	2.1
6178	1.10817	2.1
6179	1.41908	2.1
6180	1.48734	2.1
6181	1.40822	0.55075
6182	1.40822	0.51957
6183	2.09871	0.609056
6184	0.946133	0.497231
6185	1.14926	0.356695
6186	0.946133	0.534091
6187	1.78098	0.410592
6188	1.78098	0.359759
6189	1.35822	0.440671
6190	1.3689	0.458855
6191	1.22176	0.414003
6192	2.03426	0.493409
6193	0.946131	0.548832
6194	1.01713	0.438349
6195	0.704112	0.56577
6196	1.36237	0.441519
6197	1.36237	0.439315
6198	1.01713	0.513847
6199	1.04936	0.541554
6200	1.87377	2.1
6201	1.60501	2.1
6202	0.981584	0.0657367
6203	1.69435	0.0667123

6204	0.85154	2.1
6205	1.47841	2.1
6206	1.4952	2.1
6207	1.31941	2.1
6208	1.04023	2.1
6209	1.1995	2.1
6210	0.914791	2.1
6211	1.1427	1.45085
6212	1.64787	1.10673
6213	1.07526	0.518254
6214	1.07527	0.643787
6215	1.07527	0.62335
6216	1.58551	0.60522
6217	2.77362	1.06198
6218	2.17974	0.771998
6219	2.71723	0.891161
6220	1.29868	0.294556
6221	1.3594	0.23155
6222	1.04786	0.232488
6223	0.527876	0.204738
6224	1.4085	0.281147
6225	1.48538	0.136536
6226	0.76829	0.171252
6227	1.18514	0.277469
6228	0.617954	0.134874
6229	0.864161	0.185396
6230	1.18835	0.13184
6231	1.16817	2.1
6232	1.04263	2.1
6233	1.50524	2.1
6234	1.85355	2.1
6235	1.23963	2.1
6236	1.06279	2.1
6237	0.94613	2.1
6238	1.35229	2.1
6239	1.15947	2.1
6240	0.965593	2.1
6241	0.851553	2.1
6242	2.21296	2.1
6243	1.11764	2.1
6244	2.46399	2.1
6245	1.39292	2.1
6246	1.34701	1.88363
6247	1.34701	2.1
6248	1.47001	2.1
6249	0.963166	0.05
6250	0.893445	0.05
6251	1.21056	0.05
6252	1.67375	2.1
6253	2.0977	2.1
6254	1.09154	2.1
6255	1.09154	2.1
6256	1.57122	0.842566
6257	1.60976	0.481162
6258	1.69533	0.419554
6259	1.6951	0.385647
6260	1.5831	0.361787
6261	1.20969	2.1

6262	1.12076	2.1
6263	1.49808	2.1
6264	1.50201	2.1
6265	1.00278	2.1
6266	1.22811	0.329294
6267	1.11545	2.1
6268	1.09646	2.1
6269	1.03107	0.05
6270	1.44293	0.122423
6271	1.66752	0.875112
6272	1.11023	0.907449
6273	2.57831	1.82189
6274	2.20191	1.10032
6275	2.18051	2.0705
6276	1.48035	0.462969
6277	1.0961	0.391294
6278	1.63241	0.414351
6279	1.0961	0.472666
6280	1.20959	2.1
6281	2.86136	2.1
6282	1.64933	2.1
6283	1.47217	2.1
6284	0.915178	2.1
6285	1.19074	2.1
6286	0.897276	2.1
6287	0.915177	2.1
6288	0.987037	2.1
6289	1.04288	2.1
6290	2.39323	2.1
6291	0.976218	0.05
6292	2.23933	0.05
6293	0.976215	0.05
6294	1.07384	0.05
6295	1.07652	2.1
6296	1.55678	2.1
6297	1.54168	2.1
6298	1.69136	1.67332
6299	1.64391	1.26847
6300	1.76477	1.10929
6301	0.611486	1.4253
6302	0.683421	0.907666
6303	0.946131	1.05842
6304	1.1019	1.0597
6305	1.12946	0.913841
6306	1.54226	1.69275
6307	1.19496	1.11933
6308	1.96199	0.992841
6309	2.0775	1.52568
6310	1.22297	1.02656
6311	1.3469	0.885493
6312	1.25366	1.09752
6313	1.65443	1.14266
6314	1.65443	1.21458
6315	0.946132	1.28207
6316	0.946132	1.66117
6317	1.12873	1.25733
6318	1.07468	0.997925
6319	1.2854	0.63619

6320	1.3469	0.918386
6321	1.68616	0.882129
6322	1.68616	0.739226
6323	0.946132	1.23055
6324	0.946132	1.14914
6325	1.16109	0.963371
6326	1.17879	0.82492
6327	1.17879	0.859296
6328	1.45304	1.1199
6329	2.21058	2.1
6330	2.81552	2.1
6331	1.45535	0.327448
6332	1.49906	0.213878
6333	1.04413	2.1
6334	1.74586	2.08828
6335	1.34526	2.1
6336	1.17402	0.883719
6337	1.74564	0.795836
6338	1.52724	2.1
6339	1.68142	2.1
6340	1.09651	2.1
6341	1.49149	2.1
6342	1.36814	2.1
6343	1.46783	1.01604
6344	2.18116	1.36111
6345	1.23602	1.02549
6346	1.74538	0.906737
6347	0.851539	2.1
6348	1.45775	0.998863
6349	1.28919	2.1
6350	0.851483	2.1
6351	1.26102	0.97656
6352	0.869889	2.1
6353	0.85151	2.03009
6354	1.23451	0.779069
6355	1.23602	0.952992
6356	1.11955	2.1
6357	1.08022	2.1
6358	0.977776	2.1
6359	1.27896	2.1
6360	1.64268	2.1
6361	1.97548	2.1
6362	1.72527	1.75581
6363	0.946132	2.1
6364	1.69702	1.12812
6365	1.69702	2.1
6366	1.13635	2.1
6367	1.40274	0.987773
6368	1.24041	2.1
6369	1.13635	2.1
6370	1.78427	0.0735261
6371	1.68338	0.106563
6372	1.00681	0.05
6373	1.12988	0.05
6374	0.946132	0.0545793
6375	0.957543	0.05
6376	1.40515	0.05
6377	1.40515	0.05

6378	0.797932	0.0646335
6379	1.30187	0.0710869
6380	1.14846	0.0624661
6381	0.665137	0.200133
6382	1.09575	0.352423
6383	0.596894	0.296
6384	1.04072	0.391383
6385	0.663171	0.4988
6386	1.47218	0.383816
6387	1.32889	0.334899
6388	1.32639	0.131194
6389	1.94887	0.145386
6390	1.32639	0.135842
6391	1.19323	0.205374
6392	2.19794	0.277871
6393	0.754756	0.114697
6394	1.11015	0.144919
6395	0.754755	0.124979
6396	0.946133	0.15037
6397	0.754768	0.187026
6398	1.59986	0.147914
6399	1.59986	0.117568
6400	1.84082	2.1
6401	1.79044	2.1
6402	1.75922	2.1
6403	1.86766	2.1
6404	1.58995	2.1
6405	1.58995	2.1
6406	1.74351	2.1
6407	1.04442	2.1
6408	1.42278	2.1
6409	1.08576	2.1
6410	0.994294	2.1
6411	1.44884	1.26007
6412	1.71036	0.927425
6413	0.714101	0.653243
6414	1.17541	0.999515
6415	1.00872	0.51028
6416	0.714072	0.68826
6417	0.936677	0.641392
6418	0.966979	2.1
6419	1.4908	2.1
6420	1.03108	0.533755
6421	0.949712	0.380952
6422	0.810946	0.456627
6423	0.88209	0.4844
6424	0.646702	0.429021
6425	0.972663	0.376653
6426	0.862301	0.374055
6427	0.868143	0.313545
6428	1.11094	0.408748
6429	1.35809	0.421997
6430	1.32455	2.1
6431	1.32328	2.1
6432	1.69586	2.1
6433	1.78301	2.1
6434	1.67906	2.1
6435	1.60635	2.1

6436	1.57234	2.1
6437	1.47012	2.1
6438	1.22811	0.330573
6439	1.08732	0.338209
6440	2.08849	0.329374
6441	1.08732	0.335544
6442	1.30635	0.05
6443	0.77315	0.05
6444	1.25732	0.05
6445	2.43845	2.1
6446	2.38577	0.991214
6447	0.946132	0.865756
6448	1.19288	2.1
6449	1.54508	2.1
6450	1.54508	1.53719
6451	0.94515	2.08919
6452	1.19288	1.67861
6453	1.29735	1.06236
6454	1.12087	0.785166
6455	1.54699	1.06723
6456	1.43635	2.1
6457	1.33385	2.1
6458	1.43635	2.1
6459	1.02368	2.1
6460	1.035 2.1	
6461	1.00128	2.1
6462	1.04009	2.1
6463	1.14052	2.1
6464	0.443552	2.1
6465	0.984351	2.1
6466	0.591443	2.1
6467	0.929561	2.1
6468	1.52079	2.1
6469	1.035 2.1	
6470	3.5282	1.90523
6471	1.88288	1.87219
6472	2.73877	1.00311
6473	0.906059	2.1
6474	1.23107	0.101504
6475	0.906712	0.122698
6476	0.841322	0.148551
6477	1.53005	0.100331
6478	1.10007	0.122479
6479	1.38033	0.123828
6480	1.03442	0.100767
6481	0.787223	0.111647
6482	1.42589	0.108757
6483	2.35665	0.115593
6484	1.17793	0.345949
6485	1.81356	0.354741
6486	1.42964	0.344511
6487	1.95641	0.353514
6488	1.39139	0.411267
6489	1.39139	0.603622
6490	0.564533	0.439253
6491	1.20016	0.615739
6492	0.755765	0.238412
6493	0.755766	0.4077

6494	1.20016	0.422637
6495	1.91011	0.715933
6496	1.4695	0.413467
6497	1.70048	2.1
6498	1.93765	1.14708
6499	0.741307	2.1
6500	0.780371	2.1
6501	0.946133	0.76929
6502	1.28918	1.10646
6503	1.5111	1.96596
6504	1.5111	0.788285
6505	0.946131	1.37001
6506	1.28918	0.827191
6507	1.16805	0.727713
6508	1.01179	0.638343
6509	0.946132	0.771793
6510	1.35328	1.12834
6511	1.69633	1.80928
6512	1.69633	0.807031
6513	0.946133	1.4751
6514	1.35328	0.840039
6515	1.28918	0.725269
6516	1.01179	0.99246
6517	1.58147	1.1268
6518	1.27737	0.693475
6519	2.17223	0.214275
6520	0.697168	0.165888
6521	1.62495	0.361145
6522	1.62495	0.359668
6523	1.93948	0.572788
6524	0.601592	2.1
6525	1.93528	2.1
6526	2.13757	2.1
6527	1.67561	2.1
6528	2.70875	2.1
6529	1.93528	2.1
6530	3.98475	2.1
6531	1.12376	2.1
6532	1.19016	2.1
6533	1.67323	0.173536
6534	1.74019	0.165973
6535	1.84719	0.18112
6536	1.00764	0.161155
6537	1.23475	2.1
6538	1.06757	2.1
6539	1.57916	1.4992
6540	1.06758	2.1
6541	1.28014	2.1
6542	1.33339	2.1
6543	1.2679	2.1
6544	1.23504	2.1
6545	1.50108	2.1
6546	1.3715	2.1
6547	0.58831	0.904077
6548	0.865971	1.05466
6549	0.813704	1.36152
6550	0.658856	1.3465
6551	0.495869	1.29584

6552	1.13845	1.01592
6553	0.780804	1.3784
6554	0.642023	1.0401
6555	0.707748	0.898247
6556	1.64476	0.846243
6557	1.64476	1.15319
6558	0.99611	0.860409
6559	1.35639	0.875366
6560	1.3564	1.39979
6561	0.946129	1.47066
6562	1.99222	0.811043
6563	1.4777	2.1
6564	1.03968	2.1
6565	1.82516	2.1
6566	0.851539	2.1
6567	1.63693	2.1
6568	1.59416	2.1
6569	0.878566	2.1
6570	1.40796	2.1
6571	1.13172	1.65529
6572	0.851527	0.893649
6573	1.14366	2.1
6574	0.823822	0.425252
6575	0.882001	0.433067
6576	1.09756	0.134419
6577	1.11857	0.245948
6578	1.0934	0.228878
6579	0.960104	0.41791
6580	0.982001	0.467472
6581	1.74319	0.336555
6582	1.03043	0.29994
6583	0.967194	0.262311
6584	1.16938	2.1
6585	2.48377	2.1
6586	1.1181	2.1
6587	2.57349	2.1
6588	1.0465	2.1
6589	1.44709	2.1
6590	0.946132	2.1
6591	1.29302	2.1
6592	1.73502	2.1
6593	1.73502	2.1
6594	0.946132	2.1
6595	1.29302	2.1
6596	1.38813	2.1
6597	1.03933	2.1
6598	1.01976	0.336652
6599	1.11764	0.407714
6600	0.961779	0.566036
6601	1.0685	0.451173
6602	1.08426	1.00274
6603	0.99695	1.47784
6604	2.25593	1.48062
6605	1.47992	1.69646
6606	1.88989	1.01684
6607	1.65813	0.943221
6608	1.20278	1.23272
6609	0.981842	0.989861

6610	0.802655	2.01161
6611	0.953489	1.90699
6612	0.961719	1.70757
6613	0.975614	1.71873
6614	0.991617	1.01049
6615	0.940748	1.17703
6616	2.25019	2.03664
6617	2.30712	1.23939
6618	1.98823	1.90227
6619	0.522844	1.12115
6620	0.946122	1.7762
6621	0.946132	1.46457
6622	0.593846	1.07125
6623	1.26501	1.12781
6624	1.21171	0.0926338
6625	0.851542	0.137588
6626	0.940327	0.169895
6627	0.940356	0.178257
6628	1.52117	0.112944
6629	1.58072	0.0928349
6630	1.35169	0.122888
6631	1.13889	0.112191
6632	1.35007	0.0777301
6633	1.35007	0.0755551
6634	1.78381	0.0909207
6635	0.946132	0.138085
6636	1.06627	0.136049
6637	0.946131	0.174486
6638	1.73005	0.100647
6639	1.73005	0.101577
6640	1.32449	0.119471
6641	1.35169	0.125268
6642	1.42446	0.577854
6643	1.86697	0.561473
6644	1.13413	0.533196
6645	1.42446	0.474978
6646	1.33575	0.245524
6647	1.20473	0.288905
6648	1.87517	0.0603698
6649	1.87517	0.0611963
6650	0.488712	0.107328
6651	1.13242	0.095302
6652	0.488713	0.122377
6653	0.488713	0.0957384
6654	1.23146	0.0817999
6655	1.57932	0.0613697
6656	1.01698	0.0628368
6657	1.42085	0.06248
6658	0.246725	0.0544296
6659	0.996283	0.055929
6660	1.36965	0.0675732
6661	0.86361	0.0911658
6662	0.810945	0.05
6663	1.72938	0.063049
6664	1.21649	0.0887668
6665	1.1865	0.0626318
6666	1.62387	0.0662826
6667	1.05302	0.073337

6668	1.10049	0.64145
6669	0.946129	2.1
6670	1.70184	0.857095
6671	1.70184	2.1
6672	1.12538	2.1
6673	1.35969	0.783002
6674	1.28828	1.72962
6675	1.12538	1.1077
6676	0.99747	1.29111
6677	1.34851	0.838093
6678	0.923724	2.1
6679	1.47154	0.863467
6680	1.47154	2.1
6681	1.10698	2.1
6682	1.28828	0.788875
6683	1.10698	2.01131
6684	0.923695	1.31502
6685	0.99747	0.555315
6686	1.65677	2.1
6687	1.77677	2.1
6688	1.05689	0.403272
6689	2.11031	0.383033
6690	1.07367	0.205388
6691	0.656239	0.375521
6692	1.4563	0.339216
6693	1.4563	0.299079
6694	0.656238	0.351105
6695	0.946133	0.384627
6696	0.812937	0.419391
6697	0.946131	0.42471
6698	1.05689	0.231634
6699	1.34009	2.1
6700	1.398 2.1	
6701	3.29047	2.1
6702	2.53897	2.1
6703	1.24509	2.1
6704	3.06162	2.1
6705	1.01595	2.1
6706	1.52661	2.1
6707	1.5266	2.1
6708	0.946131	2.1
6709	1.31049	2.1
6710	1.16225	2.1
6711	1.05978	2.1
6712	1.16225	2.1
6713	1.06561	0.820194
6714	0.953927	2.1
6715	1.15114	0.945837
6716	1.1807	0.530359
6717	0.507259	0.486194
6718	1.47709	0.47931
6719	1.36192	0.413746
6720	0.487293	0.422063
6721	1.65569	0.513609
6722	0.679725	0.292936
6723	0.721928	0.425184
6724	1.03931	0.402197
6725	1.0775	0.5219

6726	1.0775	0.556578
6727	2.30207	0.761452
6728	1.01814	0.711824
6729	2.33963	0.666476
6730	2.42589	0.749794
6731	1.33962	2.1
6732	1.27956	2.1
6733	2.14624	0.05
6734	1.6812	0.0552806
6735	1.25698	0.051568
6736	1.35725	0.0563014
6737	1.86387	0.05
6738	1.86387	0.05
6739	0.750365	0.057198
6740	0.946128	0.0523676
6741	0.750365	0.05
6742	1.72271	0.05
6743	1.72272	0.0697486
6744	2.53736	0.0756498
6745	1.07327	2.1
6746	1.05376	2.1
6747	1.00387	0.871842
6748	2.0554	0.791263
6749	1.00387	0.846867
6750	1.00387	0.817105
6751	1.27119	2.1
6752	1.51748	2.1
6753	3.15271	2.1
6754	3.45801	2.1
6755	1.99871	2.1
6756	1.41186	2.1
6757	1.07624	2.1
6758	1.5954	2.1
6759	1.19323	0.868254
6760	1.21286	0.78583
6761	0.946114	2.1
6762	1.38395	2.1
6763	1.73389	2.1
6764	1.7339	2.1
6765	0.946115	2.1
6766	1.38397	2.1
6767	1.29604	2.1
6768	1.04544	2.1
6769	1.60578	0.0801057
6770	1.93407	0.0741331
6771	1.0525	0.843573
6772	1.0525	1.15673
6773	1.0525	1.51561
6774	1.38506	1.67271
6775	2.34737	0.05
6776	1.3531	0.05
6777	1.2925	0.05
6778	0.871466	0.05
6779	1.26623	0.05
6780	0.700631	0.05
6781	1.88774	0.05
6782	1.14167	0.05
6783	1.18606	0.05

6784	2.1238	0.05
6785	1.42762	0.69572
6786	1.67791	0.143253
6787	1.47118	0.132946
6788	0.946128	2.1
6789	1.31641	2.1
6790	1.75933	2.1
6791	1.75933	2.1
6792	0.946131	2.1
6793	1.31641	2.1
6794	1.38905	2.1
6795	1.38437	2.1
6796	1.51829	2.1
6797	0.947114	2.1
6798	1.13335	2.1
6799	1.47521	2.1
6800	1.14911	2.1
6801	1.53822	2.1
6802	1.39702	2.1
6803	0.739166	2.1
6804	0.706056	2.1
6805	0.964033	2.1
6806	1.58993	2.1
6807	2.51892	1.71091
6808	0.595905	0.10057
6809	1.18155	0.0684579
6810	1.73364	0.0641909
6811	0.952735	0.0732605
6812	2.10381	0.0625331
6813	1.12115	0.0668784
6814	1.27826	0.0672442
6815	2.74021	0.0614023
6816	1.78416	2.1
6817	1.78416	2.1
6818	0.946132	2.1
6819	1.41126	2.1
6820	1.21275	2.1
6821	0.946133	2.1
6822	1.41126	2.1
6823	1.33125	2.1
6824	1.33125	2.1
6825	1.78918	2.1
6826	1.0465	2.1
6827	1.99715	2.1
6828	1.17683	2.1
6829	1.25889	2.1
6830	0.860834	0.308701
6831	0.946131	0.28081
6832	1.05008	0.138337
6833	0.970444	0.316992
6834	1.19401	0.272212
6835	1.25989	0.12865
6836	0.702942	0.369803
6837	1.40591	0.418971
6838	1.72168	0.387033
6839	0.446352	0.403677
6840	1.04072	0.309658
6841	0.711038	0.389124

6842	0.980737	0.391266
6843	1.05099	0.313858
6844	1.26003	0.2025
6845	0.495934	0.426231
6846	1.03114	0.173324
6847	1.09911	0.239194
6848	1.24825	0.200508
6849	2.19827	0.160575
6850	2.0953	0.101769
6851	2.30437	0.141049
6852	0.775923	0.116464
6853	2.2283	0.0790406
6854	1.19583	2.1
6855	1.14496	2.1
6856	1.62183	2.1
6857	1.62316	2.1
6858	1.02305	0.470449
6859	1.21136	0.377093
6860	1.44426	0.449858
6861	1.21137	0.31373
6862	1.20143	0.390161
6863	1.15009	0.648689
6864	1.24965	2.1
6865	1.41077	2.1
6866	1.00052	0.102267
6867	1.79553	0.10763
6868	1.06336	0.0609708
6869	1.00053	0.0816417
6870	1.18041	2.1
6871	1.80818	1.01532
6872	1.73571	1.17569
6873	1.94429	1.40962
6874	1.4375	0.209365
6875	1.44421	0.16671
6876	1.66953	0.256827
6877	1.09529	0.225032
6878	0.952395	2.1
6879	0.717337	2.1
6880	1.03609	1.12613
6881	1.33949	0.54207
6882	1.2774	0.451928
6883	2.24983	0.407911
6884	1.28727	0.437264
6885	0.583592	0.397212
6886	0.583591	0.489978
6887	1.82859	0.453067
6888	1.82859	0.440756
6889	0.583593	0.485207
6890	0.946128	0.402566
6891	0.692233	0.534901
6892	1.12226	0.500707
6893	2.57454	0.527389
6894	0.810944	0.485601
6895	0.810945	0.468108
6896	1.12226	0.483301
6897	1.45867	0.483767
6898	1.22403	0.443795
6899	1.15262	0.525543

6900	1.08132	0.481463
6901	0.846671	0.466577
6902	0.480954	0.503574
6903	0.917975	0.478463
6904	0.917975	0.635046
6905	0.720957	0.490289
6906	0.795998	0.453221
6907	0.69316	0.589877
6908	0.641306	0.524238
6909	0.690817	0.438625
6910	0.961899	2.1
6911	2.49579	2.1
6912	2.31106	2.1
6913	1.70482	0.376252
6914	1.93156	0.389246
6915	1.23868	0.371135
6916	1.73878	0.41379
6917	1.26267	2.1
6918	1.26441	2.1
6919	1.58095	2.1
6920	1.58095	2.1
6921	0.946132	2.1
6922	0.946132	2.1
6923	1.11077	2.1
6924	1.62905	2.1
6925	1.49692	2.1
6926	3.49966	2.1
6927	2.39213	2.1
6928	2.12603	2.1
6929	0.946129	2.1
6930	0.461661	2.1
6931	1.52841	2.1
6932	1.39091	2.1
6933	0.550679	2.1
6934	0.509894	2.1
6935	1.49508	2.1
6936	2.05862	2.1
6937	2.47851	2.1
6938	1.07213	2.1
6939	1.09148	0.617743
6940	1.99776	0.648497
6941	1.38333	0.639652
6942	1.04412	2.1
6943	1.04412	2.1
6944	1.1893	0.317939
6945	0.946132	0.319359
6946	0.965737	0.32678
6947	0.946132	0.323348
6948	1.54061	0.318836
6949	1.54061	0.323496
6950	1.22878	0.320977
6951	1.25797	0.312289
6952	1.00665	0.320451
6953	1.00665	0.323296
6954	1.80369	0.318209
6955	0.946128	0.324193
6956	1.04424	0.330638
6957	0.85154	0.327471

6958	1.39322	0.322463
6959	1.35464	0.328087
6960	0.939857	0.325494
6961	1.28272	0.326642
6962	1.19323	0.227276
6963	1.03498	0.101747
6964	2.22029	0.114308
6965	0.540005	0.0976089
6966	0.970382	0.105947
6967	2.14514	0.224687
6968	1.31542	0.219461
6969	1.05575	0.322851
6970	0.810943	0.382169
6971	1.31542	0.170031
6972	1.70493	0.224256
6973	1.22582	0.284205
6974	1.31681	0.334608
6975	1.40775	0.220702
6976	0.928633	0.428767
6977	1.2545	0.21287
6978	2.66481	0.284955
6979	1.32051	0.242161
6980	1.32051	0.316092
6981	2.66305	2.1
6982	1.3551	2.1
6983	2.45325	2.1
6984	1.3685	2.1
6985	1.51748	2.1
6986	1.78941	2.1
6987	1.02963	0.0695448
6988	1.28643	0.102503
6989	0.86131	0.108266
6990	0.851539	0.122616
6991	1.46585	0.0720193
6992	1.38822	0.0661676
6993	0.996402	0.0759305
6994	0.851553	0.104404
6995	1.73156	0.0992422
6996	1.91695	0.0822371
6997	1.92535	0.106109
6998	2.57287	0.0869794
6999	1.77743	0.0827577
7000	1.02836	0.363068
7001	1.36362	0.441319
7002	1.36936	0.33871
7003	1.78684	0.238058
7004	1.78685	0.271785
7005	0.946132	0.500685
7006	1.27581	0.465321
7007	0.946132	0.531847
7008	1.02836	0.191826
7009	1.55398	2.1
7010	1.07167	2.1
7011	1.87557	2.1
7012	0.751639	2.1
7013	1.50884	2.1
7014	1.33793	2.1
7015	0.751664	2.1

7016	1.37277	2.1
7017	0.979515	2.1
7018	0.676508	2.1
7019	1.09578	2.1
7020	0.953925	2.1
7021	0.94613	2.1
7022	1.64707	2.1
7023	1.64707	2.1
7024	1.16658	2.1
7025	1.40638	2.1
7026	1.18682	2.1
7027	1.16658	2.1
7028	0.543765	0.250129
7029	1.57462	0.278855
7030	1.57462	0.214198
7031	1.00083	0.275363
7032	1.16834	0.394354
7033	1.16834	0.351788
7034	0.889626	0.33009
7035	1.25944	0.377363
7036	0.824061	0.399439
7037	1.9397	0.202224
7038	1.21893	0.261645
7039	1.91364	0.244857
7040	1.33672	0.369524
7041	0.928069	0.436739
7042	1.41484	0.351122
7043	0.538379	0.319912
7044	1.41484	0.312979
7045	1.35594	0.379906
7046	2.11414	0.354291
7047	2.11414	0.173811
7048	0.538378	0.35815
7049	1.38532	0.175521
7050	0.962877	0.526394
7051	1.07497	0.399185
7052	1.00613	0.403964
7053	0.962878	0.362651
7054	0.711447	2.1
7055	1.40008	0.238468
7056	2.51674	0.301619
7057	1.40008	0.238549
7058	1.7263	0.152373
7059	1.40008	0.3408
7060	1.9216	0.0534004
7061	2.7013	0.0534928
7062	2.58584	0.05
7063	0.979296	0.056846
7064	1.46459	0.288591
7065	0.899254	0.324213
7066	1.15038	1.90618
7067	1.24687	1.2589
7068	1.16738	1.87324
7069	0.942323	2.1
7070	1.65573	1.29765
7071	0.844791	2.1
7072	0.54674	1.42072
7073	0.795811	1.72565

7074	1.08131	2.05886
7075	1.03553	1.87171
7076	0.758334	2.1
7077	0.708665	1.54619
7078	1.22626	1.57154
7079	0.835439	1.5625
7080	0.861131	1.36963
7081	0.68296	1.69636
7082	1.65224	1.77857
7083	2.82624	1.92377
7084	1.0151	1.13604
7085	1.15905	1.47538
7086	1.71076	1.29088
7087	1.4529	1.0023
7088	0.494895	2.1
7089	0.946132	1.40939
7090	0.613132	1.2388
7091	0.863631	1.28272
7092	1.78391	2.1
7093	1.1706	0.0566198
7094	2.02734	0.0678303
7095	1.1706	0.05
7096	0.681532	0.05
7097	1.50316	0.05
7098	1.50316	0.05
7099	0.681534	0.05
7100	0.973819	0.050286
7101	0.97382	0.0522842
7102	0.94613	0.0525899
7103	1.1706	0.05
7104	1.67727	0.0778998
7105	1.60329	0.0667773
7106	1.3227	2.1
7107	1.40846	2.1
7108	1.07889	2.1
7109	1.07721	2.1
7110	0.953523	0.715602
7111	1.79675	0.719237
7112	2.09859	0.730061
7113	1.21309	2.1
7114	1.54567	0.623333
7115	1.19722	0.716082
7116	1.82793	0.755689
7117	1.1599	0.550427
7118	1.73621	0.516649
7119	0.933474	0.597013
7120	1.51477	0.579419
7121	1.51477	0.618044
7122	0.93347	0.580037
7123	1.27374	0.586972
7124	1.27376	0.631925
7125	0.946105	0.67317
7126	1.1972	0.557271
7127	0.851541	0.0522747
7128	1.36971	0.05
7129	1.27832	0.05
7130	0.853489	0.05
7131	0.973847	0.0526204

7132	0.976308	0.0574257
7133	0.851528	0.0589588
7134	2.72654	0.05
7135	1.77867	0.05
7136	3.23032	2.1
7137	2.78208	2.1
7138	1.0227	0.05
7139	1.02269	0.05
7140	1.02269	0.05
7141	1.24293	0.05
7142	0.831695	2.1
7143	0.67513	2.1
7144	0.956294	2.1
7145	1.08623	2.1
7146	1.05556	2.1
7147	1.38859	2.1
7148	1.13179	2.1
7149	1.02438	2.1
7150	1.06659	2.1
7151	1.36595	2.05983
7152	1.06659	2.1
7153	1.06659	0.68898
7154	0.946131	0.973849
7155	0.875912	2.1
7156	1.65971	2.1
7157	1.65971	1.52457
7158	0.875912	2.1
7159	1.1229	2.06604
7160	1.41273	2.00598
7161	1.54699	1.2904
7162	1.93655	1.42879
7163	1.42307	0.932133
7164	1.97907	1.18717
7165	0.996774	2.1
7166	1.15505	0.0571571
7167	1.28463	0.0510836
7168	1.86353	0.0575188
7169	2.71398	0.0757089
7170	1.56787	0.0978796
7171	1.66295	0.144741
7172	1.73432	1.5426
7173	1.05019	0.05
7174	1.42117	0.05
7175	0.888299	0.05
7176	1.38127	0.05
7177	1.72993	0.05
7178	0.664129	0.05
7179	0.99302	0.625592
7180	0.993023	0.616066
7181	1.13491	2.1
7182	0.876316	1.33191
7183	1.2588	2.1
7184	0.895613	2.1
7185	2.07526	2.1
7186	1.23369	2.01979
7187	1.25146	2.1
7188	2.15282	2.1
7189	1.22239	1.43551

7190	1.10962	2.1
7191	1.77402	1.41733
7192	1.0305	2.1
7193	1.50108	1.1642
7194	1.20761	2.1
7195	1.3626	2.1
7196	1.14825	2.1
7197	1.78711	2.1
7198	1.17933	2.1
7199	1.14824	2.1
7200	1.09651	0.136555
7201	1.23851	0.129318
7202	1.83284	0.150959
7203	1.23851	0.0834771
7204	1.53521	2.1
7205	1.46279	2.1
7206	1.9233	0.336929
7207	1.2966	0.244489
7208	2.11443	0.243555
7209	1.30265	0.252351
7210	1.13528	0.302335
7211	0.946131	0.459226
7212	1.41169	0.342135
7213	1.33879	0.264152
7214	0.946128	0.226904
7215	1.80435	0.302441
7216	1.80435	0.154235
7217	1.234 2.1	
7218	1.45633	2.1
7219	1.234 2.1	
7220	1.98448	2.1
7221	2.02612	2.1
7222	1.23527	0.717458
7223	1.44472	0.621821
7224	0.993366	0.76963
7225	1.22025	0.707312
7226	0.993368	0.656358
7227	2.43669	2.1
7228	2.56707	2.1
7229	1.80348	1.08438
7230	1.74406	0.769958
7231	1.90937	0.952245
7232	1.49357	0.377704
7233	2.5832	0.557415
7234	1.82274	0.554701
7235	1.6957	0.510804
7236	2.22495	0.511423
7237	2.49959	0.52237
7238	2.01183	0.342781
7239	1.37427	0.326064
7240	1.15069	0.944068
7241	1.39379	0.817582
7242	1.69661	0.411406
7243	1.39379	0.563589
7244	1.39654	2.1
7245	1.93505	2.1
7246	1.5525	2.1
7247	1.5525	2.1

7248	0.615065	2.1
7249	1.38908	2.1
7250	0.615065	2.1
7251	0.77849	2.1
7252	1.38908	2.1
7253	1.56839	0.944678
7254	1.09142	1.15186
7255	2.07313	0.963353
7256	0.998288	0.0879874
7257	1.31481	0.0918763
7258	1.88175	0.100922
7259	2.23267	0.123568
7260	1.06935	2.1
7261	1.06936	2.1
7262	1.17989	1.61832
7263	1.00757	0.954946
7264	1.65318	0.775009
7265	1.4185	0.449695
7266	2.22495	0.474708
7267	1.02011	0.258663
7268	0.59676	0.430222
7269	1.43606	0.393351
7270	1.43606	0.32726
7271	0.89771	0.383075
7272	1.13511	0.443172
7273	0.59676	0.245346
7274	0.897709	0.411581
7275	1.79542	0.352337
7276	2.38566	2.1
7277	1.63838	0.831991
7278	1.63837	1.2077
7279	0.916275	2.083
7280	1.36183	0.912309
7281	1.19282	2.1
7282	0.916275	1.53282
7283	1.19283	1.84197
7284	2.01582	2.1
7285	1.00215	0.0799028
7286	2.60643	2.1
7287	2.15971	2.1
7288	1.28267	2.1
7289	1.29954	2.1
7290	3.40327	2.1
7291	3.49128	2.1
7292	1.3864	2.1
7293	1.3201	2.1
7294	1.45666	0.05
7295	1.55798	0.05
7296	0.857202	0.05
7297	1.24374	0.05
7298	1.04157	0.05
7299	0.85723	0.05
7300	1.02722	0.05
7301	1.01361	0.05
7302	0.974077	0.05
7303	1.01814	0.05
7304	0.974077	0.05
7305	1.43987	0.05

7306	1.43986	0.05
7307	0.927881	0.05
7308	0.946132	0.05
7309	0.927849	0.05
7310	0.927852	0.05
7311	1.2493	0.05
7312	1.22482	0.657231
7313	1.41319	0.777054
7314	1.22482	0.503216
7315	0.999492	2.1
7316	1.14657	2.1
7317	1.3617	0.0528699
7318	0.983269	0.0605293
7319	1.33174	0.0653874
7320	1.62816	0.0661435
7321	1.62817	0.056688
7322	0.81938	0.0507565
7323	1.33174	0.0587019
7324	1.01332	0.0657443
7325	0.874788	0.05
7326	1.16725	2.1
7327	1.63991	2.1
7328	1.08848	0.0700932
7329	1.15354	0.0547505
7330	1.12665	2.1
7331	1.58742	2.1
7332	1.57523	2.1
7333	1.32201	0.785709
7334	1.32097	0.697281
7335	0.851542	0.454993
7336	0.877188	0.388667
7337	0.797489	0.474934
7338	1.44721	0.364752
7339	1.61761	0.356314
7340	0.943799	0.405584
7341	1.36079	0.399189
7342	1.16361	1.94934
7343	1.15852	1.14143
7344	1.07624	2.1
7345	1.07624	2.1
7346	0.946132	0.772104
7347	1.37938	1.0911
7348	1.78653	1.63797
7349	1.78653	0.825522
7350	0.946131	1.52533
7351	1.37938	0.879626
7352	1.35328	0.75752
7353	1.44847	0.726666
7354	0.946133	0.801821
7355	1.38149	1.0279
7356	1.81474	1.29202
7357	1.81474	0.832257
7358	0.946133	1.55573
7359	1.38149	0.90637
7360	1.37938	0.799256
7361	1.44847	0.964854
7362	1.93665	0.804231
7363	1.5515	0.747734

7364	1.05025	2.1
7365	1.05025	2.1
7366	1.71468	1.30458
7367	1.04759	2.1
7368	1.04646	0.06558
7369	1.00739	0.081473
7370	1.37086	0.0564699
7371	1.12547	0.078935
7372	1.03853	0.0621402
7373	1.34824	0.0553947
7374	0.799786	0.0582187
7375	2.10105	0.0757221
7376	2.72007	0.0601125
7377	0.589918	0.05
7378	0.589918	0.058576
7379	1.1509	0.050194
7380	0.619832	0.05
7381	1.1509	0.0558428
7382	0.705903	0.0508256
7383	1.53703	0.05
7384	1.53703	0.0542962
7385	0.705904	0.0516594
7386	0.946133	0.0577303
7387	0.946132	0.0541801
7388	0.946132	0.0605281
7389	0.588467	0.05
7390	1.1078	0.05
7391	0.408061	0.05
7392	1.38703	0.0515935
7393	1.68328	0.111311
7394	1.87031	0.129296
7395	1.86268	0.290306
7396	1.27472	0.357216
7397	0.947862	0.963178
7398	1.19854	1.12527
7399	0.946129	0.802876
7400	0.963111	0.669603
7401	0.946131	0.833181
7402	1.39055	0.705931
7403	1.39055	0.682518
7404	1.26426	0.751983
7405	1.07242	0.771675
7406	1.31126	0.6831
7407	1.76597	0.835665
7408	0.94613	1.14542
7409	1.27214	0.91859
7410	1.90751	0.900138
7411	1.90751	0.847747
7412	0.767114	1.08195
7413	1.27214	1.04439
7414	1.40248	0.985864
7415	1.41479	1.07244
7416	2.22625	1.08528
7417	0.913373	1.16454
7418	1.0158	0.850677
7419	1.61259	0.794888
7420	1.69051	0.718899
7421	0.560949	1.07092

7422	0.898559	1.09013
7423	1.28011	0.983893
7424	1.51026	0.168084
7425	1.98252	0.233211
7426	1.05129	0.185428
7427	1.2951	0.196833
7428	1.46041	0.05
7429	1.7155	2.1
7430	1.72196	2.1
7431	0.933885	2.1
7432	0.986723	2.1
7433	1.28072	0.393952
7434	1.40765	0.757037
7435	1.28072	0.649804
7436	1.61687	2.1
7437	1.60965	2.1
7438	1.21424	2.1
7439	2.07297	2.1
7440	1.19943	2.1
7441	2.38784	2.1
7442	1.10278	0.217745
7443	1.43678	0.247178
7444	1.1142	0.364082
7445	1.18034	0.343402
7446	0.94613	0.363027
7447	0.752356	0.354546
7448	0.752329	0.363464
7449	1.46634	0.341179
7450	1.31789	0.339665
7451	0.958159	0.364599
7452	1.22752	0.353478
7453	1.50472	0.352229
7454	1.98974	2.1
7455	2.54186	2.1
7456	0.76512	2.1
7457	2.08777	2.1
7458	1.7753	2.1
7459	3.07372	2.1
7460	1.9756	2.1
7461	1.48162	2.1
7462	1.23204	2.1
7463	0.946131	0.897091
7464	1.13505	2.1
7465	1.47432	2.1
7466	1.47432	1.24963
7467	0.876831	1.83194
7468	1.13505	1.59638
7469	1.2161	1.062
7470	1.05267	1.50199
7471	1.05267	0.93533
7472	1.5795	1.08446
7473	0.98186	2.1
7474	1.56735	2.1
7475	0.859099	2.1
7476	1.69858	2.1
7477	1.93586	2.1
7478	1.09233	2.1
7479	1.08732	0.332937

7480	1.3357	0.330494
7481	1.84826	2.1
7482	0.924132	1.03865
7483	1.39188	2.1
7484	1.43685	2.1
7485	0.851541	2.1
7486	1.16251	2.1
7487	0.946131	2.1
7488	1.05685	2.1
7489	2.13781	2.1
7490	1.98859	2.1
7491	1.99731	2.1
7492	1.51379	2.1
7493	1.48796	2.1
7494	0.851542	2.1
7495	0.802558	2.1
7496	1.09244	2.1
7497	1.33891	2.1
7498	0.844958	2.1
7499	0.710864	2.1
7500	1.26087	2.1
7501	1.03679	2.1
7502	1.51026	0.163977
7503	1.05248	0.551653
7504	0.827258	0.516149
7505	1.05248	0.526528
7506	1.28435	0.550375
7507	1.50953	0.57247
7508	1.50953	0.398054
7509	0.827298	0.536578
7510	1.00366	0.371974
7511	2.10495	0.46281
7512	1.17885	2.1
7513	1.25185	2.1
7514	1.02784	2.1
7515	1.76764	2.1
7516	1.23746	0.207432
7517	1.24082	0.255402
7518	1.46506	0.16869
7519	1.33061	0.111312
7520	0.768407	1.18789
7521	0.972058	0.795063
7522	1.16536	2.09261
7523	1.13325	1.08902
7524	0.978906	2.1
7525	1.02291	0.982029
7526	0.838015	0.793239
7527	1.01052	0.167405
7528	1.08166	0.211393
7529	1.42607	0.219902
7530	1.47354	0.315754
7531	1.21703	2.1
7532	1.29897	2.1
7533	1.83293	2.1
7534	1.29897	2.1
7535	1.24688	0.145882
7536	0.782563	2.1
7537	0.946129	2.1

7538	0.782591	2.1
7539	1.49487	2.1
7540	1.49487	2.1
7541	1.37713	2.1
7542	0.900337	2.1
7543	1.31284	2.1
7544	0.924468	2.1
7545	1.40602	1.1899
7546	1.25669	1.58278
7547	0.986901	2.1
7548	0.958565	2.1
7549	1.37266	2.1
7550	1.64146	2.1
7551	0.49345	2.1
7552	1.08276	2.1
7553	1.08276	2.1
7554	1.26929	2.1
7555	1.09847	2.1
7556	0.827081	0.425446
7557	1.07236	0.485333
7558	1.25063	0.491071
7559	1.43548	0.351226
7560	1.25063	0.33585
7561	1.3659	0.0805136
7562	1.72441	0.0757361
7563	1.3659	0.0673345
7564	1.4677	0.0810115
7565	1.86114	0.0750636
7566	1.40104	0.109135
7567	0.962675	0.118243
7568	0.946131	0.132168
7569	1.74134	0.07388
7570	1.74134	0.0686662
7571	0.962676	0.0738029
7572	0.946131	0.109912
7573	1.05548	0.188675
7574	0.902187	0.233408
7575	2.14178	0.128216
7576	1.74998	0.168734
7577	1.38541	0.194024
7578	1.64468	0.125383
7579	0.949317	0.218357
7580	2.06654	0.158347
7581	0.839626	0.204862
7582	0.810945	0.232287
7583	1.29203	0.117055
7584	1.18091	0.158988
7585	1.06872	0.205687
7586	1.11956	0.134728
7587	0.926911	0.240953
7588	0.901716	0.223311
7589	0.909146	0.264174
7590	1.33776	0.145742
7591	1.02048	0.191607
7592	1.08365	0.224713
7593	1.21042	0.153312
7594	0.865634	0.251378
7595	0.590354	2.1

7596	0.61173	2.1
7597	3.46825	2.1
7598	3.28404	2.1
7599	1.56004	0.0875003
7600	1.69344	0.182146
7601	1.7151	0.46173
7602	0.946131	0.10874
7603	1.40265	0.152028
7604	1.13537	0.277337
7605	0.965708	0.213522
7606	1.13537	0.233452
7607	1.29681	0.159904
7608	1.40915	0.230546
7609	1.61652	0.426707
7610	1.33171	0.519986
7611	1.37681	2.1
7612	0.81094	0.05
7613	0.598606	0.05
7614	0.723111	0.05
7615	1.04562	0.05
7616	0.979052	0.05
7617	0.798185	0.05
7618	0.880117	0.05
7619	0.979341	0.506141
7620	0.800229	0.433298
7621	1.39126	0.499083
7622	1.13005	0.516335
7623	0.913877	0.527706
7624	1.26959	0.482168
7625	0.699741	0.523189
7626	1.19496	1.20261
7627	1.13392	0.117082
7628	1.11913	0.0998177
7629	1.06866	0.0944078
7630	0.615546	0.128829
7631	0.831006	0.166667
7632	1.53286	0.111898
7633	0.690977	0.115438
7634	1.01956	0.125153
7635	2.33818	0.0801367
7636	1.6585	2.1
7637	1.67061	2.1
7638	1.41414	2.1
7639	1.16756	2.1
7640	1.00898	0.0736016
7641	0.966964	2.1
7642	2.85443	2.1
7643	0.966977	2.1
7644	2.08427	2.1
7645	2.94901	2.1
7646	1.10735	0.134587
7647	1.65815	2.1
7648	1.4502	2.1
7649	1.45026	2.1
7650	1.53446	2.1
7651	0.946129	0.848812
7652	1.81075	2.1
7653	1.81075	0.811058

7654	0.946131	1.30647
7655	1.37075	2.1
7656	1.37075	0.839349
7657	1.34245	1.55512
7658	0.973502	0.850151
7659	1.08542	2.015
7660	1.5814	0.05
7661	1.43836	0.05
7662	0.946132	0.054305
7663	1.13747	0.05
7664	1.04429	0.0570368
7665	1.05017	0.05
7666	1.24732	0.05
7667	1.13387	0.05
7668	2.19489	0.05
7669	1.10748	2.0531
7670	1.25006	2.1
7671	1.58834	1.38571
7672	1.58834	0.509114
7673	0.94613	1.81163
7674	1.16548	0.980005
7675	0.946132	0.528172
7676	1.08998	0.676435
7677	1.16548	0.657088
7678	0.995732	0.52519
7679	0.657014	2.1
7680	0.957173	2.09506
7681	1.01485	2.1
7682	1.12468	2.1
7683	1.43871	2.1
7684	1.23716	2.1
7685	1.89277	0.709914
7686	2.06653	0.605134
7687	0.967544	0.436072
7688	1.89016	0.554381
7689	2.22165	1.67741
7690	0.83514	1.26168
7691	1.76565	2.1
7692	1.76565	2.1
7693	0.94613	1.1904
7694	0.835112	2.1
7695	1.23426	1.2635
7696	1.36653	1.82705
7697	0.895892	0.882358
7698	0.859278	0.834171
7699	0.791461	0.893311
7700	1.86249	1.05762
7701	1.14607	0.909274
7702	1.19904	1.02124
7703	1.84898	0.951816
7704	1.03452	0.778693
7705	0.946131	0.530938
7706	0.940472	0.461756
7707	1.53478	0.740858
7708	1.84175	0.618984
7709	1.55687	0.595631
7710	1.17787	0.485385
7711	1.60589	0.473796

7712	2.35574	0.408504
7713	1.3305	0.167373
7714	1.15008	0.791301
7715	1.12457	0.0629866
7716	1.12457	0.0625821
7717	1.01778	2.1
7718	1.35733	2.1
7719	1.50683	2.1
7720	1.12064	2.1
7721	1.42716	0.680184
7722	1.42717	0.744089
7723	1.45927	1.03021
7724	1.04321	1.57388
7725	1.32634	0.389175
7726	2.27022	0.335324
7727	1.19352	0.19665
7728	1.32634	0.196357
7729	1.40924	1.95874
7730	1.22927	1.17699
7731	1.69237	1.63949
7732	1.69237	2.1
7733	0.946132	0.907198
7734	0.946133	1.56815
7735	1.16912	1.2877
7736	1.0417	1.2423
7737	1.14605	1.90964
7738	1.1233	2.1
7739	1.96882	1.7434
7740	1.03804	0.357441
7741	1.15338	0.350649
7742	1.15338	0.348604
7743	2.33051	0.360064
7744	1.81507	0.980105
7745	2.42949	2.02899
7746	0.725879	0.734484
7747	1.88394	0.77771
7748	2.2699	0.179034
7749	2.78952	1.97454
7750	1.88394	0.234566
7751	1.79606	2.1
7752	1.20091	2.1
7753	1.9614	2.1
7754	1.19901	1.76955
7755	3.07915	2.1
7756	1.32592	2.1
7757	2.86461	2.1
7758	1.34817	2.1
7759	1.44859	2.1
7760	2.03969	2.1
7761	0.946132	2.1
7762	1.50709	2.1
7763	1.50709	2.1
7764	0.946132	2.1
7765	1.29322	2.1
7766	1.29322	2.1
7767	1.01985	2.1
7768	0.968537	2.1
7769	1.401 2.1	

7770	1.28936	2.1
7771	1.56002	0.05
7772	1.92548	0.05
7773	1.46041	0.05
7774	2.22712	0.05
7775	1.56001	0.05
7776	1.50524	2.1
7777	1.21304	2.1
7778	1.60665	1.36114
7779	1.85178	0.930713
7780	2.17534	2.1
7781	2.31012	2.1
7782	2.17533	1.19063
7783	0.986724	2.1
7784	2.01895	2.1
7785	2.00433	2.1
7786	1.45752	0.253017
7787	1.12695	0.21588
7788	0.652605	2.01303
7789	1.5133	2.1
7790	1.5133	2.1
7791	0.652603	1.50383
7792	1.228 2.1	
7793	0.946132	2.1
7794	0.652604	2.1
7795	0.946132	0.976219
7796	0.716815	2.1
7797	1.62203	1.62361
7798	1.62203	2.1
7799	0.716814	2.1
7800	1.00191	1.95071
7801	1.33694	1.39278
7802	1.38216	2.1
7803	2.53338	1.96342
7804	1.46932	0.0536274
7805	1.50848	0.0515181
7806	1.50108	1.08954
7807	1.18382	0.793695
7808	2.02115	0.671185
7809	0.921823	0.55499
7810	2.08356	0.0503878
7811	1.543 0.05	
7812	1.00763	0.675131
7813	1.4185	0.668724
7814	1.08028	0.594822
7815	0.946131	0.749505
7816	1.56599	0.658106
7817	1.56599	0.809868
7818	1.08028	0.675377
7819	0.946131	0.560072
7820	1.37518	2.1
7821	1.63453	2.1
7822	1.04627	1.04287
7823	1.04626	0.968471
7824	1.35111	1.08464
7825	1.22297	1.0293
7826	1.64499	0.914293
7827	1.87598	0.830881

7828	1.06994	2.1
7829	1.37063	2.1
7830	1.99943	0.0518986
7831	2.38807	0.05
7832	0.99972	0.0726692
7833	1.41388	0.0780482
7834	1.66179	0.0701126
7835	1.66179	0.0547907
7836	0.946129	0.0811667
7837	1.41388	0.0782612
7838	1.19404	0.0736019
7839	1.31023	0.824831
7840	1.20871	0.858401
7841	1.69731	2.1
7842	1.67971	2.1
7843	0.998971	0.05
7844	1.32988	0.05
7845	0.701079	0.05
7846	0.946131	0.05
7847	0.582785	0.05
7848	1.48592	0.05
7849	1.48592	0.05
7850	1.23351	0.05
7851	0.835199	0.05
7852	0.92427	0.05
7853	1.68262	0.05
7854	2.46702	0.05
7855	1.08715	1.73572
7856	1.01606	1.47026
7857	1.29579	1.37995
7858	1.01606	1.87833
7859	1.63823	2.1
7860	1.67687	2.1
7861	1.11048	2.04505
7862	1.39477	2.1
7863	0.991114	2.1
7864	2.90978	0.77411
7865	0.906811	0.863958
7866	0.872849	0.595845
7867	1.17091	0.781345
7868	0.685763	0.769358
7869	1.6386	1.71843
7870	1.06907	0.849773
7871	1.05516	0.946357
7872	1.9189	0.970604
7873	1.04615	0.711838
7874	1.2966	2.1
7875	1.37392	2.1
7876	1.35504	2.1
7877	1.30347	2.1
7878	1.50877	2.1
7879	1.11193	2.1
7880	1.40984	2.04938
7881	1.67682	2.09685
7882	1.00371	2.1
7883	1.15412	2.1
7884	1.41638	2.1
7885	1.03796	2.1

7886	0.946117	2.1
7887	1.50823	2.1
7888	1.50822	2.1
7889	0.966923	2.1
7890	0.946131	2.1
7891	2.07593	2.1
7892	1.9339	2.1
7893	1.1817	0.726533
7894	0.729178	0.728295
7895	1.5079	0.774188
7896	1.26991	0.926462
7897	0.994049	0.714582
7898	1.17452	0.765434
7899	1.05711	0.859215
7900	1.04071	0.854868
7901	1.80299	0.70125
7902	1.60417	0.630882
7903	0.943405	0.257029
7904	1.01129	0.18886
7905	1.42972	0.210186
7906	1.15904	0.212105
7907	0.680409	0.175426
7908	1.2816	0.111195
7909	0.806176	0.188668
7910	1.18954	0.195418
7911	1.26055	0.207619
7912	1.06313	2.1
7913	1.19352	2.1
7914	1.55766	2.1
7915	1.53729	2.1
7916	0.993021	0.203802
7917	1.09056	0.559805
7918	1.03408	2.1
7919	0.993023	0.518781
7920	0.659901	2.1
7921	0.632905	2.1
7922	1.34072	0.345824
7923	1.17793	0.346324
7924	2.24885	0.343774
7925	0.946132	0.339896
7926	1.12443	0.341555
7927	0.904207	0.343094
7928	1.51237	0.329685
7929	1.51237	0.333742
7930	1.12443	0.339022
7931	1.29215	0.336731
7932	1.6943	1.99744
7933	2.43858	2.1
7934	2.0589	1.40212
7935	1.67204	2.1
7936	1.67204	1.93233
7937	0.555875	1.77776
7938	0.91951	2.1
7939	0.946131	1.17817
7940	0.555904	1.63046
7941	0.946131	1.53734
7942	1.6943	2.1
7943	1.04744	1.39796

7944	1.50938	2.1
7945	1.60861	2.1
7946	1.92406	0.126583
7947	1.18716	0.132636
7948	0.962705	0.563863
7949	1.35382	0.693758
7950	1.43632	0.0541908
7951	1.09386	0.059777
7952	1.92547	0.0601433
7953	0.994164	0.0548393
7954	0.903974	2.09814
7955	0.928829	1.6306
7956	0.810958	1.34252
7957	1.92853	1.62361
7958	1.598	1.57393
7959	1.28967	1.73551
7960	1.44993	1.52343
7961	0.946132	0.844218
7962	0.882984	0.783978
7963	0.882955	0.895756
7964	1.56071	0.619788
7965	1.56071	0.669888
7966	1.35946	0.77173
7967	1.08421	0.798633
7968	1.41943	0.0746634
7969	1.26265	0.0511182
7970	1.03235	0.600717
7971	1.50778	2.1
7972	1.70387	2.1
7973	1.65608	2.1
7974	1.39076	2.1
7975	1.6104	2.1
7976	1.37456	2.1
7977	1.51898	0.453062
7978	1.9726	0.405457
7979	2.13757	1.31271
7980	1.74856	1.38626
7981	0.635949	0.660525
7982	2.46448	0.882119
7983	1.23224	0.844465
7984	1.12786	0.982196
7985	1.82721	0.809814
7986	1.82721	0.656643
7987	0.532892	1.60626
7988	0.946131	1.03232
7989	0.53292	0.986786
7990	1.41394	2.1
7991	1.45506	2.1
7992	1.39523	2.1
7993	1.09006	2.1
7994	1.80264	0.192294
7995	1.80264	0.55397
7996	0.946131	0.395053
7997	1.34268	0.329954
7998	0.946132	0.516598
7999	1.23732	0.418711
8000	1.34268	0.445518
8001	1.2064	0.637299

8002	1.58164	2.1
8003	1.73853	2.1
8004	1.01243	0.93826
8005	0.910672	1.50676
8006	1.47793	1.69344
8007	1.47793	2.1
8008	0.910702	1.43393
8009	1.30382	0.9541
8010	1.08478	0.975704
8011	0.910702	1.88357
8012	2.60709	1.13622
8013	1.01243	0.940552
8014	1.85516	1.44034
8015	2.52823	0.750648
8016	1.24001	0.692972
8017	1.89793	1.0428
8018	1.73584	0.05
8019	1.24613	2.05429
8020	1.37074	1.64084
8021	1.23763	1.38129
8022	1.04072	2.03947
8023	0.75499	1.20137
8024	1.72329	1.51633
8025	1.46195	1.3748
8026	0.946132	1.25856
8027	0.747438	1.83876
8028	1.24613	1.35495
8029	2.59886	1.17628
8030	1.87279	2.1
8031	1.07977	2.1
8032	1.29606	2.1
8033	1.45178	2.1
8034	1.45177	2.1
8035	0.92406	2.1
8036	0.946131	2.1
8037	0.924047	2.1
8038	2.04529	2.1
8039	1.04544	2.1
8040	1.08276	2.1
8041	1.67491	2.1
8042	0.961826	0.847745
8043	0.810942	0.88769
8044	0.826622	1.60359
8045	0.914378	1.67971
8046	0.89022	0.790724
8047	0.922383	0.994455
8048	0.988165	0.697715
8049	1.31357	0.0834117
8050	0.946131	0.163763
8051	1.31357	0.118691
8052	1.10564	0.104613
8053	1.6797	0.0790755
8054	1.6797	0.154926
8055	0.739506	0.158195
8056	1.33957	0.0949946
8057	1.33957	0.079171
8058	1.33957	0.0662641
8059	2.34223	0.100294

8060	1.5515	0.891358
8061	2.22137	0.783271
8062	0.94612	0.727958
8063	1.13908	0.812774
8064	1.45423	0.70479
8065	1.45422	0.868409
8066	0.795519	1.02853
8067	1.1391	0.785896
8068	1.11065	0.780744
8069	1.12266	1.96143
8070	1.24925	2.04511
8071	1.20242	1.09125
8072	2.18297	1.05564
8073	3.00107	1.27063
8074	2.58297	0.63804
8075	0.884783	2.06442
8076	0.851538	0.78858
8077	0.713587	1.88119
8078	1.47949	0.839041
8079	1.66673	2.09975
8080	0.696199	1.72605
8081	0.9383	0.956311
8082	1.29149	0.999682
8083	1.32266	0.948389
8084	2.11428	0.05
8085	2.17439	0.05
8086	1.40489	0.05
8087	1.21071	0.05
8088	0.946118	0.05
8089	1.0746	0.05
8090	0.973119	0.05
8091	1.31333	0.05
8092	1.08723	0.05
8093	0.994292	2.1
8094	1.4964	2.1
8095	1.15096	2.1
8096	1.10101	2.1
8097	1.50438	2.1
8098	1.67825	2.1
8099	2.02103	2.1
8100	2.01978	2.1
8101	1.50931	0.0842346
8102	1.50931	0.0806849
8103	0.65023	0.0609483
8104	1.34831	0.0754249
8105	0.811233	0.0785115
8106	1.34831	0.0864502
8107	0.94613	0.0782184
8108	1.00754	0.908867
8109	0.714132	1.99198
8110	1.28877	1.22661
8111	1.39905	1.1231
8112	1.03282	1.13337
8113	0.997769	0.911002
8114	1.11743	0.995112
8115	1.86863	0.904732
8116	3.30723	1.36011
8117	1.66635	1.96331

8118	1.35609	0.05
8119	1.38212	0.0521903
8120	1.95597	2.1
8121	1.64257	2.1
8122	2.93316	2.1
8123	3.13417	2.1
8124	0.733947	2.1
8125	1.15259	2.1
8126	1.07976	2.1
8127	1.30237	2.1
8128	1.99943	0.0568458
8129	0.907431	0.0737204
8130	0.878161	0.0790841
8131	1.07544	0.05
8132	1.03401	0.060347
8133	0.991975	0.05
8134	1.03635	0.0641367
8135	0.788549	0.0576249
8136	1.44984	0.05
8137	1.84007	0.0687844
8138	2.31127	0.0566186
8139	1.27362	2.1
8140	1.27737	1.02364
8141	1.87783	0.828625
8142	2.35499	0.0554512
8143	2.69662	0.0848332
8144	2.24065	0.05
8145	0.996082	2.1
8146	0.996083	2.1
8147	1.44859	2.1
8148	1.03968	1.86606
8149	1.60018	0.883681
8150	1.73082	1.45522
8151	0.946132	1.05746
8152	1.49742	1.39343
8153	1.49742	0.607681
8154	1.00501	0.832191
8155	1.41585	1.81646
8156	1.41585	0.896852
8157	0.946131	1.28869
8158	1.03697	1.01576
8159	2.95786	2.1
8160	2.32185	1.71497
8161	1.65783	0.547987
8162	1.772	1.31433
8163	0.946131	0.449035
8164	1.40404	0.219095
8165	1.13537	1.19864
8166	1.04559	0.711278
8167	1.16091	0.748088
8168	2.63751	1.67428
8169	1.68455	0.05
8170	1.9682	0.05
8171	2.16834	0.481006
8172	2.45569	0.548016
8173	0.946128	0.428896
8174	1.36428	0.552708
8175	0.663978	0.594252

8176	1.58218	0.447682
8177	1.58219	0.444489
8178	1.36428	0.518358
8179	0.881886	0.535344
8180	1.73849	2.1
8181	1.35668	2.1
8182	1.1233	2.1
8183	1.15009	2.1
8184	1.16482	0.581657
8185	1.29313	0.602398
8186	1.47608	0.359799
8187	2.05949	0.365813
8188	0.946119	0.333897
8189	1.02974	0.339327
8190	0.946119	0.331485
8191	1.66758	0.326944
8192	1.66757	0.327942
8193	1.32082	0.326568
8194	1.29287	0.290726
8195	1.36005	1.91001
8196	1.47109	2.1
8197	2.45958	2.1
8198	2.2975	2.1
8199	0.953521	0.824948
8200	0.953521	0.707367
8201	1.67054	2.1
8202	1.67054	2.1
8203	0.946131	2.1
8204	1.38154	2.1
8205	1.13326	2.1
8206	1.13326	2.1
8207	1.23513	2.1
8208	1.50998	2.09875
8209	2.47982	0.0653938
8210	1.45091	0.0560683
8211	0.958953	0.0728036
8212	0.79239	0.0720838
8213	2.11929	0.0681399
8214	1.26511	0.0855433
8215	1.27733	0.0598824
8216	2.18127	0.0574025
8217	1.30169	0.0609505
8218	1.19259	0.0732062
8219	0.963395	1.01756
8220	0.999947	1.80983
8221	1.62241	2.1
8222	1.62027	2.1
8223	0.981586	0.05
8224	0.981585	0.05
8225	0.721961	2.1
8226	0.891321	2.1
8227	1.04442	2.1
8228	1.66554	2.1
8229	2.43434	2.1
8230	1.73944	0.05
8231	1.88032	0.05
8232	0.711391	0.05
8233	1.40837	0.05

8234	0.946133	0.05
8235	0.92252	0.05
8236	1.13964	0.05
8237	1.83394	0.05
8238	2.81674	0.05
8239	1.10049	1.37306
8240	1.25092	0.717404
8241	0.760299	0.890602
8242	1.48017	0.980606
8243	1.48017	1.08898
8244	0.760271	0.792386
8245	1.35861	0.921016
8246	0.946129	0.820894
8247	0.760269	0.813052
8248	2.26155	2.1
8249	2.15634	2.1
8250	1.39824	2.1
8251	1.07817	2.1
8252	0.946132	2.1
8253	1.53028	2.1
8254	1.53028	2.1
8255	1.00807	2.1
8256	0.946133	2.1
8257	2.06126	0.731783
8258	1.97385	1.70179
8259	1.75754	0.692576
8260	2.01613	2.1
8261	1.97995	2.1
8262	1.47696	2.1
8263	1.50172	2.1
8264	1.5177	0.0774235
8265	1.44146	0.126575
8266	1.8807	0.106328
8267	2.07068	0.119621
8268	1.84267	0.130604
8269	1.08975	2.1
8270	1.21327	2.1
8271	1.10106	0.05
8272	1.34496	0.05
8273	2.08452	0.430933
8274	1.70537	0.38052
8275	2.62464	0.854262
8276	1.5726	0.711063
8277	1.5726	2.1
8278	0.94613	2.1
8279	1.15038	2.1
8280	0.946132	0.698139
8281	1.03974	0.921271
8282	1.15038	1.06777
8283	0.99747	0.812505
8284	1.03472	1.25796
8285	1.58407	0.835953
8286	1.21148	0.585731
8287	1.35025	0.722207
8288	1.3428	0.455441
8289	1.00775	2.1
8290	1.00775	2.1
8291	1.3879	2.1

8292	1.39958	2.1
8293	0.853042	2.1
8294	1.06997	2.1
8295	0.853042	2.1
8296	0.853042	2.1
8297	1.12491	2.1
8298	1.17879	2.1
8299	1.07162	2.08918
8300	1.61179	1.95519
8301	1.76016	2.1
8302	0.946132	0.661541
8303	1.23449	0.574501
8304	1.70392	0.599001
8305	1.70392	0.524354
8306	0.94613	0.674479
8307	1.23449	0.606682
8308	1.41556	0.609424
8309	1.14547	0.565164
8310	1.1721	0.381423
8311	0.96478	0.516486
8312	1.41843	0.556775
8313	1.2358	0.472429
8314	1.70678	0.454037
8315	1.70678	0.461521
8316	0.946118	0.615865
8317	1.23582	0.586784
8318	0.946105	0.635105
8319	0.964769	0.331989
8320	1.1357	0.374984
8321	0.964767	0.354255
8322	0.619856	0.0516742
8323	1.72366	0.0578163
8324	1.72367	0.05
8325	0.619831	0.0523381
8326	1.19262	0.0608984
8327	0.619829	0.05
8328	0.946129	0.0627753
8329	1.57459	2.1
8330	1.86512	2.1
8331	0.93841	2.1
8332	1.57459	2.1
8333	1.10476	0.0925434
8334	1.00013	0.740895
8335	2.13446	0.744962
8336	0.759781	2.1
8337	1.96011	1.10185
8338	0.946129	0.826882
8339	1.32056	1.56691
8340	1.75702	0.999502
8341	1.75702	2.1
8342	0.946124	1.61511
8343	1.32056	1.10236
8344	1.38258	0.867446
8345	1.96011	1.21063
8346	2.31099	1.13186
8347	0.97356	2.1
8348	1.38885	2.1
8349	1.3771	2.1

8350	0.946131	2.1
8351	1.84843	2.1
8352	1.84843	2.1
8353	1.3771	2.1
8354	0.946131	2.1
8355	1.00278	2.1
8356	1.05447	0.56758
8357	1.05446	0.571826
8358	1.24913	0.396978
8359	1.05447	0.558396
8360	1.80882	0.570593
8361	1.80882	1.95262
8362	0.946129	1.30692
8363	1.33997	1.90665
8364	0.946131	0.469799
8365	1.28831	0.674525
8366	1.33997	0.629268
8367	1.44391	0.05
8368	1.44391	0.05
8369	0.850964	0.05
8370	0.850963	0.05
8371	0.946132	0.05
8372	0.921299	0.05
8373	1.37358	0.05
8374	1.46297	0.05
8375	1.46298	0.05
8376	0.823988	0.05
8377	0.921287	0.05
8378	0.94612	0.05
8379	0.823995	0.05
8380	0.946107	0.05
8381	1.44437	2.1
8382	1.06203	2.1
8383	2.21397	2.1
8384	0.637677	2.1
8385	1.8892	2.1
8386	2.075 2.1	
8387	1.32051	0.203675
8388	2.23637	0.329077
8389	1.10904	0.437677
8390	1.11819	0.376252
8391	0.94613	0.465699
8392	0.735845	0.339072
8393	1.4914	0.47312
8394	1.4914	0.19166
8395	0.735831	0.506
8396	1.61683	2.1
8397	1.61683	2.1
8398	0.946131	2.1
8399	1.3281	2.1
8400	1.10441	2.1
8401	1.10442	2.1
8402	1.23487	2.1
8403	1.09914	2.1
8404	1.20691	0.0516123
8405	2.27379	0.0552171
8406	1.1369	0.0710309
8407	1.26868	0.0738534

8408	1.54422	0.0663778
8409	1.54422	0.0643151
8410	0.861357	0.0795373
8411	0.94613	0.0701549
8412	0.861359	0.068031
8413	1.2858	0.583812
8414	1.31011	0.583088
8415	1.294	0.530826
8416	1.03079	0.648534
8417	1.81979	1.86099
8418	1.57829	2.1
8419	1.19023	1.66367
8420	1.19022	1.91206
8421	1.46458	1.64735
8422	1.46458	2.01986
8423	0.789173	2.0989
8424	1.34386	2.01829
8425	0.909896	2.1
8426	0.946131	1.79573
8427	0.909893	1.80476
8428	1.27725	2.1
8429	1.68451	2.1
8430	2.26155	2.1
8431	2.34217	2.1
8432	1.55485	0.731191
8433	0.90854	0.681785
8434	0.858256	0.652171
8435	1.47042	0.596577
8436	1.62771	0.601085
8437	0.777398	0.724375
8438	0.982525	0.652774
8439	1.23865	0.642757
8440	2.09928	0.634071
8441	0.946128	0.407929
8442	0.474856	0.43526
8443	0.474855	0.464502
8444	1.58747	0.229944
8445	1.58747	0.251613
8446	0.963917	0.349211
8447	1.09841	0.366844
8448	2.23904	1.3158
8449	2.60055	0.785023
8450	1.02599	2.1
8451	2.17837	2.03043
8452	2.07122	2.1
8453	2.7309	0.769526
8454	2.08482	2.1
8455	1.18322	0.345221
8456	1.35381	0.81855
8457	1.01139	0.620149
8458	0.946133	0.809164
8459	1.59089	0.821804
8460	1.59089	0.0992677
8461	0.946133	0.667955
8462	1.09634	0.0952323
8463	2.02279	0.211295
8464	1.03108	0.552272
8465	0.808978	1.24644

8466	1.07317	1.2775
8467	0.831695	1.25262
8468	1.22583	1.26663
8469	1.10215	1.12263
8470	1.07508	1.34394
8471	1.22204	1.1871
8472	1.04679	2.06712
8473	1.92535	0.084373
8474	1.11081	0.0618034
8475	0.946131	0.341423
8476	0.714824	0.347682
8477	0.714795	0.344968
8478	1.4231	0.335516
8479	1.4231	0.34136
8480	0.828617	0.350215
8481	1.30928	0.341498
8482	1.49894	2.1
8483	1.55702	2.1
8484	0.946131	2.1
8485	1.10689	2.1
8486	0.946227	2.1
8487	0.946131	2.1
8488	1.21754	2.1
8489	1.504 2.1	
8490	1.504 2.1	
8491	0.723573	2.1
8492	1.24512	2.1
8493	0.723544	2.1
8494	0.946131	2.1
8495	1.24512	2.1
8496	1.29383	0.340477
8497	1.29383	0.329339
8498	1.61969	0.299601
8499	0.946132	0.292497
8500	1.12818	0.289115
8501	0.946132	0.321418
8502	1.80922	0.298676
8503	1.80922	0.286251
8504	1.41383	0.281013
8505	1.34153	0.224077
8506	1.47689	0.316497
8507	1.79375	0.309481
8508	1.24333	0.50381
8509	0.768989	0.484478
8510	1.24333	0.452244
8511	1.0027	0.543624
8512	1.47705	0.532372
8513	1.47705	0.375067
8514	0.768977	0.478425
8515	1.28436	0.55862
8516	0.928583	0.565112
8517	1.28436	0.473161
8518	1.24333	0.590486
8519	1.5991	0.599415
8520	1.5991	0.301466
8521	0.928583	0.561507
8522	1.85717	0.407337
8523	2.48665	0.467087

8524	1.18423	0.05
8525	1.9493	1.13273
8526	1.8519	0.978213
8527	1.68802	2.1
8528	1.0302	0.122023
8529	0.900473	0.100704
8530	1.99663	0.140525
8531	2.61301	0.113145
8532	0.745772	0.236654
8533	0.946131	0.15817
8534	1.48881	0.156934
8535	1.48881	0.134316
8536	0.745745	0.198842
8537	0.809786	0.193207
8538	1.3065	0.181068
8539	0.946132	0.234291
8540	0.768357	0.27646
8541	0.768387	0.296509
8542	1.76897	0.166095
8543	1.76896	0.169818
8544	1.40271	0.204129
8545	1.00777	0.217265
8546	1.37427	0.280595
8547	1.1967	2.1
8548	0.946132	1.02535
8549	1.59397	2.1
8550	1.59397	2.1
8551	0.946131	2.1
8552	1.14989	2.1
8553	1.14989	2.1
8554	1.10046	2.1
8555	1.09506	0.297629
8556	2.53251	0.326627
8557	1.9212	0.871905
8558	1.99586	0.80357
8559	0.913967	0.613782
8560	1.55028	0.643917
8561	1.55028	0.756353
8562	0.913966	0.594459
8563	1.16318	0.575349
8564	1.16318	0.586033
8565	0.946132	0.566832
8566	1.20478	2.1
8567	1.09662	2.1
8568	1.03472	2.1
8569	1.84774	2.1
8570	1.7984	2.1
8571	2.1221	2.1
8572	1.68223	1.96925
8573	1.68224	1.60609
8574	0.514872	1.57287
8575	0.888991	1.76769
8576	0.946127	0.839252
8577	0.514845	1.49994
8578	0.946129	1.0151
8579	1.10127	1.3921
8580	2.61623	0.922184
8581	0.983581	1.01831

8582	0.869663	0.72425
8583	1.1818	1.87786
8584	1.10406	0.857437
8585	1.07133	2.03616
8586	1.09405	0.917913
8587	0.838014	0.791749
8588	0.842998	1.44899
8589	1.03818	0.957111
8590	1.46338	0.528262
8591	1.903 2.1	
8592	1.23942	2.1
8593	0.965933	2.1
8594	0.810075	2.02407
8595	1.40571	2.1
8596	1.66219	2.1
8597	0.810047	2.1
8598	0.936678	2.1
8599	1.45808	2.1
8600	1.6384	2.1
8601	1.54699	2.1
8602	0.719877	2.1
8603	1.33256	2.1
8604	0.753547	2.1
8605	1.04073	2.1
8606	1.25822	2.1
8607	0.851542	2.1
8608	0.714102	2.1
8609	1.45886	2.1
8610	1.80171	2.1
8611	0.597337	2.1
8612	0.957713	2.1
8613	1.3033	2.1
8614	0.596613	0.210207
8615	1.1386	0.560376
8616	0.596612	0.408061
8617	0.946133	0.695276
8618	0.596652	0.749679
8619	1.64092	0.571886
8620	1.64092	0.519477
8621	1.991 2.1	
8622	1.23013	2.1
8623	2.77816	2.1
8624	2.74107	0.743188
8625	2.07681	0.647134
8626	1.47764	0.148863
8627	1.72677	0.16566
8628	1.52161	2.1
8629	1.28526	2.1
8630	1.90502	2.1
8631	1.87359	0.167303
8632	1.93489	0.228262
8633	2.70895	2.1
8634	2.83141	2.1
8635	1.41766	0.569031
8636	1.11337	0.557983
8637	1.41765	1.32827
8638	1.29172	0.5694
8639	1.76324	0.632093

8640	1.76324	1.0206
8641	0.946131	0.578832
8642	1.52428	2.1
8643	1.71459	2.1
8644	1.93852	0.626363
8645	1.07119	1.32194
8646	1.29698	1.08002
8647	1.42203	0.647329
8648	1.42203	1.33786
8649	0.946132	0.729326
8650	0.946132	0.860822
8651	0.96926	0.917105
8652	0.769913	1.72611
8653	0.946119	1.55756
8654	1.37867	0.927228
8655	1.94028	0.883381
8656	0.94612	0.692023
8657	0.946132	1.32172
8658	1.37865	0.81288
8659	2.14234	0.986211
8660	3.06459	1.19191
8661	1.44421	0.104644
8662	1.33924	0.0709018
8663	1.33671	0.120973
8664	1.10733	0.109651
8665	0.68396	0.146388
8666	1.76008	0.0876583
8667	1.76008	0.0834285
8668	0.68393	0.0929129
8669	0.946131	0.122635
8670	3.79242	2.1
8671	2.46293	2.1
8672	3.56947	2.1
8673	1.35487	1.38589
8674	1.38616	1.71391
8675	0.954048	0.211865
8676	1.36663	0.379421
8677	0.909487	0.409911
8678	0.887288	1.51156
8679	0.810947	0.863433
8680	0.810934	1.33306
8681	1.07046	1.51428
8682	1.13979	1.79781
8683	0.927838	0.903905
8684	0.919995	1.30881
8685	1.61447	2.1
8686	2.56305	2.1
8687	0.943414	1.12608
8688	1.17016	2.1
8689	1.7238	2.1
8690	1.89334	1.80961
8691	0.946128	0.804275
8692	1.25184	1.47821
8693	1.55639	1.65962
8694	3.12098	1.01713
8695	3.57307	0.789252
8696	2.81281	2.1
8697	0.851542	2.1

8698	1.27689	1.50754
8699	1.52568	2.08407
8700	0.839689	2.1
8701	1.36471	1.96792
8702	0.827865	2.1
8703	0.93667	2.1
8704	0.900861	2.1
8705	0.943152	2.1
8706	1.00667	2.1
8707	1.18141	2.1
8708	1.15298	2.1
8709	1.20335	2.06978
8710	1.00713	2.1
8711	0.597537	2.1
8712	1.0577	2.1
8713	0.967342	0.815438
8714	0.838027	1.23021
8715	0.893587	0.652448
8716	1.06436	0.923278
8717	0.850048	0.643261
8718	1.22026	1.04919
8719	0.988077	0.815278
8720	1.18769	0.327472
8721	1.3991	2.08833
8722	1.52984	2.1
8723	1.35469	1.02097
8724	1.25083	0.891642
8725	1.6594	1.20043
8726	1.6594	0.993232
8727	0.946129	0.807042
8728	1.02602	0.804831
8729	0.94613	0.746913
8730	1.29724	0.337799
8731	1.14501	0.345644
8732	1.13078	0.247198
8733	1.48381	0.203635
8734	1.45921	0.183915
8735	0.851539	0.377011
8736	0.890836	0.37747
8737	0.946131	0.464413
8738	1.60428	2.1
8739	1.50097	2.1
8740	0.714507	0.345603
8741	1.51396	0.355088
8742	1.51396	0.348921
8743	0.946132	0.349713
8744	0.813862	0.352353
8745	1.41461	0.346439
8746	0.813862	0.349806
8747	0.921311	2.1
8748	1.25084	0.898774
8749	1.30165	0.792989
8750	1.60636	1.31514
8751	1.60636	0.901759
8752	0.94613	0.721333
8753	0.972232	0.719399
8754	0.946131	0.670776
8755	2.35003	2.1

8756	0.92546	1.83023
8757	1.00713	2.1
8758	0.810947	2.1
8759	0.810934	2.1
8760	1.056	2.1
8761	0.926811	2.1
8762	0.766408	2.1
8763	1.34292	2.1
8764	1.57932	0.0711674
8765	2.67595	0.0612073
8766	1.77737	0.0570741
8767	1.77738	0.0564307
8768	0.789633	0.0810608
8769	1.22903	0.0755428
8770	0.789663	0.0879548
8771	0.789662	0.0735966
8772	1.33797	0.0698002
8773	0.946128	2.1
8774	1.47351	2.1
8775	1.47351	2.1
8776	0.94613	2.1
8777	1.30082	2.1
8778	1.30082	2.1
8779	0.996486	2.1
8780	0.946127	2.1
8781	1.72122	2.1
8782	1.72122	2.1
8783	0.94613	2.1
8784	1.36653	2.1
8785	1.36653	2.1
8786	1.02608	2.1
8787	0.709123	0.624104
8788	0.946127	0.914476
8789	1.41342	0.973574
8790	1.818	1.13739
8791	1.818	0.928363
8792	0.946127	0.634133
8793	1.41342	1.01983
8794	1.35071	0.859666
8795	1.8993	1.17973
8796	1.4748	2.1
8797	1.18711	0.0660714
8798	0.946131	0.110841
8799	1.18711	0.0950499
8800	1.20416	0.0765305
8801	1.91358	0.112917
8802	1.91358	0.0553542
8803	0.477691	0.0924473
8804	2.16798	0.05
8805	2.22307	0.05
8806	0.758971	0.172827
8807	1.80694	0.180506
8808	1.80694	0.0947616
8809	0.946132	0.185758
8810	0.75897	0.160495
8811	1.25124	0.142369
8812	0.946131	0.216536
8813	1.76837	2.1

8814	1.76837	2.1
8815	0.946131	2.1
8816	1.36422	2.1
8817	0.946131	2.1
8818	1.14184	2.1
8819	1.36422	2.1
8820	0.793303	0.05
8821	1.79584	0.05
8822	1.35333	2.1
8823	1.19967	1.32365
8824	1.55088	1.55036
8825	1.64246	2.1
8826	0.946132	0.866594
8827	0.886293	1.93739
8828	1.25018	1.99162
8829	1.5795	1.46685
8830	2.15414	1.26771
8831	0.94613	0.860649
8832	1.37758	1.48886
8833	1.71567	0.921634
8834	1.71567	2.1
8835	0.946125	1.10826
8836	1.37759	0.891203
8837	1.28421	0.810516
8838	0.933959	0.876312
8839	0.946133	0.171702
8840	1.7813	0.175224
8841	1.7813	0.104224
8842	1.05704	0.150892
8843	1.37794	0.197356
8844	1.37794	0.167826
8845	0.946132	0.205787
8846	0.946132	0.0941421
8847	1.68197	0.0902934
8848	1.68197	0.0659826
8849	1.01367	0.0826069
8850	1.25017	0.100264
8851	1.25017	0.0906064
8852	0.946132	0.112668
8853	0.838953	2.1
8854	1.4045	2.1
8855	1.56388	2.1
8856	1.3182	2.1
8857	0.928518	2.1
8858	1.04073	2.1
8859	0.835676	2.1
8860	1.47507	2.1
8861	1.47506	2.1
8862	0.797664	2.1
8863	1.34285	2.1
8864	0.929875	2.1
8865	0.797663	2.1
8866	0.946133	2.1
8867	1.83628	0.438401
8868	1.83627	0.455689
8869	0.946131	0.520557
8870	1.36748	0.400183
8871	0.946133	0.44417

8872	1.21139	0.433597
8873	1.36748	0.377199
8874	1.06795	2.1
8875	1.08192	2.1
8876	0.827866	2.1
8877	1.02548	2.1
8878	0.95006	2.1
8879	1.53933	2.1
8880	0.586511	2.1
8881	0.868145	2.1
8882	1.28401	2.1
8883	1.37719	2.1
8884	1.37719	2.1
8885	0.77497	2.1
8886	0.946132	2.1
8887	0.774968	2.1
8888	1.7053	0.470065
8889	1.7053	0.678661
8890	0.94613	0.7853
8891	1.25715	0.653812
8892	0.946132	0.463358
8893	1.19655	0.553065
8894	1.25716	0.558898
8895	0.851542	2.1
8896	0.80043	2.1
8897	1.27239	2.1
8898	1.02719	2.1
8899	0.946131	2.1
8900	0.981318	2.1
8901	1.06628	2.1
8902	1.26022	2.1
8903	0.946132	2.1
8904	0.946132	2.1
8905	1.76447	2.1
8906	1.76447	2.1
8907	1.35357	2.1
8908	1.35703	2.1
8909	0.946131	2.1
8910	1.50538	2.1
8911	1.50538	2.1
8912	1.10311	2.1
8913	1.3484	1.8329
8914	1.10312	2.1
8915	0.966428	2.1
8916	0.946133	2.1
8917	1.4008	2.1
8918	1.71908	2.1
8919	1.71908	2.1
8920	0.946133	2.1
8921	1.40081	2.1
8922	1.26441	2.1
8923	0.851543	2.1
8924	0.961732	2.1
8925	1.54576	2.1
8926	1.35364	2.1
8927	0.946133	2.1
8928	1.02913	2.1
8929	1.2735	2.1

8930	1.32608	2.1
8931	0.967643	2.1
8932	0.94613	2.1
8933	1.44873	2.1
8934	1.44873	2.1
8935	0.967641	2.1
8936	0.946131	2.1
8937	1.40488	2.1
8938	1.24272	2.1
8939	0.946131	2.1
8940	1.78483	2.1
8941	1.78483	2.1
8942	1.24272	2.1
8943	0.946131	2.1
8944	0.94613	1.93319
8945	1.5627	0.711172
8946	1.5627	2.06697
8947	1.03656	1.88032
8948	1.31649	0.6077
8949	1.19233	1.15386
8950	1.03656	0.925007
8951	0.912059	2.08079
8952	1.47954	0.747763
8953	1.47954	2.1
8954	0.912061	2.04097
8955	1.34647	0.681787
8956	1.04514	1.43066
8957	0.912061	0.937789
8958	0.761972	0.387193
8959	0.946132	0.432016
8960	0.761974	0.438243
8961	1.70225	0.572164
8962	1.70225	0.494466
8963	1.33728	0.463672
8964	1.01188	0.459849
8965	0.94613	0.95428
8966	0.746361	1.31846
8967	1.6369	1.64879
8968	1.62454	1.27904
8969	0.736738	0.866497
8970	0.958487	0.946694
8971	1.40641	1.05644
8972	1.2284	2.1
8973	0.92609	2.1
8974	0.595102	2.1
8975	1.80871	2.1
8976	1.1516	2.1
8977	0.962474	2.1
8978	2.24024	2.1
8979	0.946131	2.1
8980	1.73689	2.1
8981	1.73689	2.1
8982	0.946132	2.1
8983	1.30492	2.1
8984	1.30492	2.1
8985	1.17949	2.1
8986	0.946131	2.00765
8987	1.8566	2.1

8988	1.8566	2.1
8989	1.22392	2.07734
8990	1.39826	2.1
8991	1.40447	2.1
8992	1.22392	2.1
8993	0.946131	1.12485
8994	1.74134	2.1
8995	1.74134	2.1
8996	1.09148	0.993642
8997	1.40447	2.1
8998	1.283 2.1	
8999	1.09148	2.1
9000	1.33607	2.1
9001	1.41022	1.92323
9002	1.80016	2.1
9003	1.80015	2.1
9004	0.946127	1.09721
9005	0.946129	2.1
9006	1.20541	2.1
9007	1.13078	2.1
9008	1.33607	2.1
9009	1.54624	2.1
9010	1.85553	2.1
9011	0.920606	2.1
9012	0.946131	2.1
9013	1.01271	2.1
9014	1.32664	1.39907
9015	0.954692	0.952267
9016	0.946116	0.84873
9017	1.47771	0.811611
9018	1.4777	2.1
9019	0.95468	1.00102
9020	0.94613	0.835509
9021	1.31381	1.11752
9022	1.14005	0.684724
9023	0.946128	0.91162
9024	1.69433	0.594184
9025	1.69433	1.34539
9026	1.14005	0.888052
9027	0.94613	0.716042
9028	1.521 2.1	
9029	1.411 2.1	
9030	0.94612	2.1
9031	1.35115	2.1
9032	1.04127	1.28433
9033	1.05017	2.1
9034	1.03564	2.1
9035	0.607156	1.39926
9036	1.54549	2.1
9037	1.36715	2.1
9038	0.607127	1.27054
9039	1.2571	2.1
9040	0.998701	1.95316
9041	0.546436	2.1
9042	0.946133	2.1
9043	1.75286	2.1
9044	1.75286	2.1
9045	1.12206	2.07399

9046	1.34237	2.1
9047	1.35663	2.1
9048	1.12206	2.1
9049	0.919455	2.1
9050	1.66517	2.1
9051	1.99822	2.1
9052	1.228	2.1
9053	1.35662	2.1
9054	1.228	2.1
9055	1.01143	2.1
9056	0.851543	0.893163
9057	0.986856	2.1
9058	1.42762	1.56246
9059	1.23095	2.1
9060	0.946132	2.1
9061	1.00659	1.58508
9062	1.1779	1.0414
9063	1.40025	2.1
9064	1.40025	2.1
9065	0.889308	2.1
9066	1.34652	2.1
9067	0.943042	2.1
9068	0.943041	2.1
9069	1.34652	2.1
9070	1.39514	1.4448
9071	1.30809	1.10703
9072	1.75711	1.046
9073	1.7571	2.1
9074	0.946125	0.677096
9075	0.946127	1.1527
9076	1.18396	1.11467
9077	1.4844	0.788525
9078	1.4844	1.07528
9079	0.803322	1.40362
9080	0.946133	0.713683
9081	0.803324	2.1
9082	0.803324	1.23874
9083	1.36183	1.27852
9084	0.946131	0.370976
9085	1.01462	0.363588
9086	0.946132	0.375963
9087	1.59749	0.414741
9088	1.59749	0.384841
9089	1.16009	0.378547
9090	1.38354	0.365099
9091	0.851539	0.892416
9092	1.36005	1.49808
9093	1.5532	1.07374
9094	1.80402	2.01574
9095	0.851537	1.70375
9096	0.902632	1.32681
9097	1.44437	1.12564
9098	0.633098	2.1
9099	1.00307	0.501856
9100	0.94613	0.643989
9101	1.16623	0.397372
9102	0.946128	0.666461
9103	1.64588	0.644646

9104	1.64588	0.562138
9105	1.35059	0.621985
9106	1.24142	0.670057
9107	0.946131	0.324545
9108	1.13343	0.301119
9109	0.946127	0.352842
9110	1.63367	0.334819
9111	1.63367	0.293609
9112	1.33035	0.305689
9113	1.24945	0.289923
9114	1.78733	1.22855
9115	1.70856	1.66961
9116	0.637995	0.967428
9117	0.712454	0.968227
9118	0.946132	0.981014
9119	0.637964	0.895218
9120	1.13021	0.968315
9121	0.94613	0.328201
9122	1.20509	0.323248
9123	0.946127	0.337234
9124	1.70545	0.329004
9125	1.70545	0.319785
9126	1.28508	0.321932
9127	1.3665	0.31927
9128	1.58612	2.1
9129	1.51845	2.1
9130	0.797374	2.1
9131	1.20057	2.1
9132	0.946132	2.1
9133	0.946132	2.1
9134	0.899819	2.1
9135	1.50471	0.835513
9136	1.50472	0.906626
9137	0.59387	0.935598
9138	0.593844	0.748851
9139	0.946126	0.854502
9140	0.966137	0.82025
9141	1.13245	0.766836
9142	1.20801	0.838643
9143	0.946131	1.13146
9144	1.47644	0.898504
9145	0.733223	0.875976
9146	0.563609	0.804938
9147	1.69218	0.872999
9148	1.6173	1.06086
9149	1.8126	1.79709
9150	1.8126	1.97503
9151	0.678938	1.84808
9152	0.678935	1.96012
9153	0.946133	2.1
9154	0.946132	1.83432
9155	0.678936	2.1
9156	1.21997	1.48843
9157	1.28439	1.4393
9158	1.65282	1.75455
9159	1.86735	1.7467
9160	0.851539	1.5778
9161	0.94613	1.12988

9162	0.87688	1.1292
9163	0.804877	0.574907
9164	1.40747	0.687534
9165	1.41312	0.440267
9166	0.885349	0.512496
9167	1.25405	0.671795
9168	1.34463	0.50593
9169	0.804878	0.529663
9170	0.810299	0.203901
9171	1.04072	0.331689
9172	1.25601	0.279127
9173	1.04943	0.186871
9174	0.900294	0.287734
9175	1.38777	0.270282
9176	1.33131	0.15992
9177	1.39168	0.0684632
9178	0.946132	0.0845378
9179	0.747712	0.0668735
9180	1.61944	0.0588554
9181	1.61944	0.056956
9182	0.946132	0.0838506
9183	0.747712	0.0822974
9184	1.41201	0.37008
9185	1.12082	0.34684
9186	0.946128	0.375396
9187	1.79415	0.364895
9188	1.79415	0.360137
9189	1.12082	0.354943
9190	0.94613	0.351612
9191	0.711721	0.126891
9192	1.12316	0.168941
9193	0.707038	0.149028
9194	1.04072	0.16828
9195	0.704409	0.237378
9196	1.49036	0.179622
9197	1.27689	0.158018
9198	0.793044	0.677505
9199	0.810886	0.931028
9200	0.885957	0.718731
9201	1.38254	0.720193
9202	1.35991	0.671663
9203	1.01375	0.789999
9204	1.03169	0.783199
9205	0.946131	0.729344
9206	1.3279	0.895512
9207	1.42284	0.798162
9208	1.42284	0.725671
9209	0.851192	1.10442
9210	0.946131	0.988065
9211	0.851165	0.821427
9212	0.748062	0.0566631
9213	1.27511	0.0566214
9214	0.74806	0.0503761
9215	0.94613	0.0549456
9216	0.748059	0.0567201
9217	1.86293	0.0583803
9218	1.86293	0.0515026
9219	0.851542	0.195239

9220	1.38186	0.185867
9221	0.851542	0.275117
9222	1.67869	0.125507
9223	1.66579	0.112034
9224	1.25626	0.160186
9225	1.23046	0.143684
9226	1.46023	0.05
9227	1.27083	0.05
9228	0.946131	0.05
9229	0.999304	0.05
9230	0.946803	0.05
9231	0.851554	0.05
9232	0.999227	0.05
9233	0.750097	0.204575
9234	1.91815	0.227226
9235	0.946132	0.228996
9236	0.768117	0.176297
9237	1.78003	0.175135
9238	1.99572	0.148001
9239	0.650453	0.237701
9240	0.946132	0.0859057
9241	1.23845	0.0848201
9242	0.946131	0.0990721
9243	1.75477	0.0645968
9244	1.75477	0.0680556
9245	1.33393	0.0746576
9246	1.36697	0.0794699
9247	0.749018	1.19488
9248	1.02366	1.63809
9249	1.43924	1.31031
9250	1.00018	1.29793
9251	1.06438	0.901328
9252	0.946127	1.18715
9253	0.74906	0.980389
9254	0.946131	1.18486
9255	1.26488	0.05
9256	0.946131	0.061869
9257	0.94613	0.0512361
9258	1.49051	0.05
9259	1.59837	0.05
9260	0.907797	0.05
9261	1.04455	0.0526939
9262	1.25214	0.05
9263	0.68452	0.05
9264	1.25215	0.05
9265	1.24677	0.05
9266	1.8144	0.05
9267	1.81441	0.05
9268	0.684518	0.05
9269	0.851542	0.574942
9270	1.52275	0.566027
9271	1.45223	0.560779
9272	1.0269	0.552503
9273	1.29689	0.603092
9274	1.32822	0.634009
9275	0.851553	0.684502
9276	1.21756	0.0580726
9277	0.946131	0.0584528

9278	0.946127	0.0577153
9279	1.78014	0.05
9280	1.78014	0.05
9281	1.32076	0.0523736
9282	1.40551	0.0548541
9283	0.711214	0.15681
9284	1.82057	0.134537
9285	1.82057	0.108751
9286	0.946129	0.191019
9287	1.22716	0.103573
9288	1.30463	0.175943
9289	0.94613	0.177194
9290	0.568927	0.0606363
9291	0.946132	0.0598192
9292	0.568927	0.0592319
9293	1.99266	0.05
9294	1.99266	0.05
9295	1.26201	0.0606093
9296	1.29957	0.0529054
9297	1.21614	0.151586
9298	0.851484	0.153963
9299	0.851543	0.171704
9300	1.85815	0.0909165
9301	1.81937	0.0852723
9302	0.697705	0.0775431
9303	0.946132	0.157004
9304	0.593295	0.275528
9305	1.53642	0.217144
9306	1.53642	0.174402
9307	0.908276	0.322617
9308	0.593296	0.170716
9309	0.593295	0.20705
9310	0.94613	0.289102
9311	0.867593	0.446003
9312	1.42107	0.396901
9313	1.42107	0.384001
9314	0.867621	0.438026
9315	1.05516	0.437485
9316	0.867624	0.386456
9317	0.946129	0.454335
9318	0.766681	0.0896466
9319	1.18119	0.103336
9320	1.57018	0.0754621
9321	1.57018	0.0658805
9322	0.766652	0.103078
9323	1.18119	0.100924
9324	1.15564	0.0860326
9325	0.946131	0.335434
9326	1.239 0.234846	
9327	0.851541	0.369394
9328	0.851487	0.210051
9329	1.40021	0.333384
9330	1.38373	0.184803
9331	0.851554	0.39284
9332	1.12266	0.125173
9333	0.946131	0.233547
9334	1.08234	0.225754
9335	1.2882	0.157178

9336	1.49963	0.486138
9337	1.46188	0.206251
9338	0.851555	0.288923
9339	1.33007	0.498012
9340	1.08054	0.487194
9341	0.946129	0.537835
9342	0.91151	0.431544
9343	1.49914	0.520689
9344	1.49914	0.359448
9345	0.911471	0.553249
9346	0.946132	0.910894
9347	1.35943	1.87341
9348	1.68117	1.34063
9349	1.68117	2.1
9350	0.946132	1.47153
9351	1.35943	1.05631
9352	1.26787	0.878087
9353	1.81612	0.679835
9354	1.81612	0.396465
9355	0.946131	0.224507
9356	1.41492	0.148353
9357	0.946132	0.700733
9358	1.25037	0.503698
9359	1.41492	0.323253
9360	1.30541	0.0672022
9361	1.23646	0.0844002
9362	0.94613	0.0757193
9363	1.77304	0.0533212
9364	1.77304	0.0518592
9365	1.23646	0.069124
9366	0.946132	0.0716156
9367	0.946131	2.1
9368	1.26689	2.1
9369	1.66806	2.1
9370	1.66806	2.1
9371	0.94613	1.73615
9372	1.26689	2.1
9373	1.3473	2.1
9374	1.39384	2.1
9375	1.4181	2.1
9376	1.86581	2.1
9377	1.86581	2.1
9378	0.946128	2.1
9379	0.946129	2.1
9380	1.32068	2.1
9381	1.20328	2.1
9382	1.39384	2.1
9383	1.651	2.1
9384	1.651	2.1
9385	0.946129	2.1
9386	0.946131	2.1
9387	1.12913	2.1
9388	1.21474	1.9821
9389	0.946132	2.1
9390	0.94613	2.1
9391	1.79724	2.1
9392	1.79724	2.1
9393	1.40987	2.1

9394	1.3335	2.1
9395	1.65642	0.742153
9396	1.65642	2.1
9397	0.946132	2.1
9398	1.19063	2.1
9399	0.946133	0.703445
9400	1.15203	2.1
9401	1.19063	2.1
9402	0.946131	0.682806
9403	1.77815	1.53465
9404	1.77815	2.1
9405	0.946131	1.10334
9406	1.31745	2.1
9407	1.31745	1.07776
9408	1.2597	1.82568
9409	0.946131	0.655186
9410	1.85184	0.94363
9411	1.85184	2.1
9412	0.946128	1.138
9413	1.40684	2.1
9414	1.40684	0.887146
9415	1.34049	1.11463
9416	0.946132	1.59651
9417	1.71333	1.12996
9418	1.71333	2.1
9419	1.1848	1.5955
9420	1.34297	1.00744
9421	1.31649	1.06625
9422	1.1848	1.58648
9423	1.36604	0.659562
9424	0.954559	0.703885
9425	1.36604	0.707383
9426	1.41766	0.632329
9427	1.83756	0.792764
9428	1.83756	1.9125
9429	0.946131	0.759834
9430	0.946127	0.350376
9431	1.14874	0.265711
9432	0.946127	0.401215
9433	1.66393	0.331666
9434	1.66393	0.265927
9435	1.35559	0.330506
9436	1.25447	0.350431
9437	0.94613	0.328826
9438	1.20732	0.318231
9439	0.946129	0.339377
9440	1.75073	0.329734
9441	1.75073	0.316363
9442	1.3665	0.320517
9443	1.33035	0.300731
9444	1.73256	2.1
9445	1.73256	2.1
9446	0.946131	1.67539
9447	1.34921	1.883
9448	1.19462	2.1
9449	1.19462	2.1
9450	1.32949	2.1
9451	1.75754	0.431146

9452	1.75754	0.510454
9453	0.946132	0.593057
9454	1.3201	0.51412
9455	0.946132	0.486713
9456	1.25479	0.418573
9457	1.3201	0.425948
9458	1.8087	0.641895
9459	1.8087	0.38259
9460	0.946131	0.430617
9461	1.38357	0.408278
9462	0.946133	0.624875
9463	1.31034	0.491692
9464	1.38357	0.511181
9465	1.12702	0.242823
9466	0.946131	0.420982
9467	1.23923	0.346692
9468	1.4117	0.276368
9469	0.946131	0.283059
9470	1.70479	0.300955
9471	1.70479	0.130709
9472	0.946119	2.1
9473	1.55309	2.1
9474	1.55309	2.1
9475	0.946119	2.1
9476	1.08006	2.1
9477	1.08005	2.1
9478	1.02681	2.1
9479	1.36581	2.1
9480	1.93075	2.1
9481	0.946133	2.1
9482	0.772538	2.1
9483	0.946131	2.1
9484	0.946131	2.1
9485	1.33748	2.1
9486	1.44991	2.1
9487	1.31183	2.1
9488	0.946132	2.1
9489	0.944104	2.1
9490	0.915236	2.1
9491	1.05017	2.1
9492	0.944148	2.1
9493	0.946132	0.367783
9494	1.60171	0.353866
9495	1.60171	0.259363
9496	0.946131	0.423116
9497	1.16234	0.240688
9498	1.3855	0.408777
9499	0.946132	0.411845
9500	0.946133	0.431345
9501	1.73285	0.422388
9502	1.73285	0.362613
9503	0.946133	0.446652
9504	1.3855	0.359963
9505	1.29348	0.452465
9506	0.946132	0.449544
9507	1.34828	2.1
9508	1.20943	2.1
9509	0.946129	0.859109

9510	1.71013	2.1
9511	1.71013	2.1
9512	1.20943	2.1
9513	0.94613	2.08521
9514	1.19613	0.05
9515	0.946118	0.051034
9516	0.946117	0.05
9517	1.70868	0.05
9518	1.70869	0.05
9519	1.40551	0.05
9520	1.2493	0.05
9521	0.946131	2.1
9522	1.73123	2.1
9523	1.73123	2.1
9524	0.946132	2.1
9525	1.37811	2.1
9526	1.37811	2.1
9527	1.25144	2.1
9528	0.946132	2.1
9529	1.75139	2.1
9530	1.75139	2.1
9531	1.23374	2.1
9532	1.29926	2.1
9533	1.39827	2.1
9534	1.23374	2.1
9535	1.41375	0.0777449
9536	1.25697	0.100518
9537	0.946118	0.0968565
9538	1.80377	0.0561783
9539	1.80377	0.0519859
9540	1.25699	0.0832503
9541	0.946106	0.0865525
9542	0.946133	1.70022
9543	1.81806	0.992511
9544	1.81806	2.00105
9545	0.946133	1.92735
9546	1.37089	2.1
9547	1.37089	1.0441
9548	1.34999	1.84793
9549	0.946131	2.1
9550	1.80855	0.912304
9551	1.80855	2.1
9552	1.31184	1.94081
9553	1.3933	1.04035
9554	1.36139	1.16034
9555	1.31184	1.26941
9556	1.01175	1.0812
9557	0.94613	0.777016
9558	0.919796	1.36598
9559	1.50484	0.667379
9560	1.80584	1.72879
9561	1.53512	0.763377
9562	1.14539	0.852571
9563	0.946133	2.1
9564	1.72797	1.02636
9565	1.72797	2.1
9566	1.2111	2.1
9567	1.39731	1.00447

9568	1.27679	1.96201
9569	1.21111	1.81174
9570	0.946119	0.598567
9571	1.67673	0.942504
9572	1.67673	2.1
9573	0.946118	0.941799
9574	1.21519	1.88311
9575	1.2152	1.61491
9576	1.19808	1.82097
9577	0.946127	2.1
9578	1.54442	2.1
9579	1.54442	2.1
9580	1.04806	2.1
9581	1.13485	2.1
9582	1.3557	2.1
9583	1.04805	2.1
9584	0.946129	2.1
9585	1.32282	2.1
9586	1.77749	2.1
9587	1.77749	2.1
9588	0.946131	2.1
9589	1.32282	2.1
9590	1.4008	2.1
9591	0.946128	0.3374
9592	1.02366	0.341696
9593	0.94613	0.339027
9594	1.66684	0.326656
9595	1.66684	0.329667
9596	1.29215	0.332419
9597	1.32082	0.320741
9598	0.946133	0.323824
9599	1.01451	0.329231
9600	0.946132	0.313146
9601	1.62374	0.332439
9602	1.62374	0.331682
9603	1.2929	0.317082
9604	1.27697	0.249032
9605	1.37869	2.1
9606	1.17215	2.1
9607	0.946132	2.1
9608	1.70508	2.1
9609	1.70508	2.1
9610	1.17215	2.1
9611	0.946132	2.1
9612	1.03784	2.1
9613	0.946132	2.1
9614	0.946132	2.1
9615	1.56718	2.1
9616	1.56717	2.1
9617	1.35703	2.1
9618	1.15628	2.1
9619	1.35356	2.1
9620	1.28976	2.1
9621	0.946131	2.1
9622	1.78612	2.1
9623	1.78612	2.1
9624	1.28976	2.1
9625	0.94613	2.1

9626	0.946132	2.1
9627	1.36373	2.1
9628	1.92382	2.1
9629	0.946131	2.1
9630	1.92382	2.1
9631	1.33471	2.1
9632	0.946132	2.1
9633	0.94613	0.701385
9634	1.29149	1.02595
9635	1.75878	0.812991
9636	1.75878	1.86372
9637	0.946128	1.00717
9638	1.29149	0.740375
9639	1.41342	0.702286
9640	1.41022	2.1
9641	1.37034	1.02088
9642	1.83443	1.12262
9643	1.83443	2.1
9644	0.946128	0.843822
9645	0.946131	1.47737
9646	1.26928	0.973272
9647	0.946128	0.82231
9648	1.38259	1.62708
9649	1.81405	0.93776
9650	1.81405	2.1
9651	0.946126	1.28614
9652	1.38259	0.949845
9653	1.37759	0.795248
9654	1.68131	0.362766
9655	1.68131	0.479999
9656	0.946131	0.380195
9657	1.38896	0.34918
9658	1.17438	0.611325
9659	1.17438	0.415724
9660	1.23848	0.463641
9661	1.80088	0.440725
9662	1.80088	0.306311
9663	0.946132	0.395049
9664	1.40332	0.441889
9665	1.28185	0.395478
9666	1.28185	0.371304
9667	1.34369	0.344602
9668	1.71939	2.1
9669	1.71939	2.1
9670	0.946131	2.1
9671	1.28308	2.1
9672	0.946133	2.1
9673	1.24024	2.1
9674	1.28309	2.1
9675	1.8525	1.70331
9676	1.8525	2.1
9677	0.946132	2.1
9678	1.38244	2.1
9679	0.946132	0.674434
9680	1.33469	2.01884
9681	1.38244	2.04929
9682	1.26654	1.24134
9683	1.39514	1.07823

9684	1.71556	1.03436
9685	1.71556	2.1
9686	0.946126	0.706305
9687	0.946129	1.46319
9688	1.1908	1.10467
9689	1.49995	2.1
9690	1.63875	2.1
9691	0.93608	2.1
9692	0.937208	2.1
9693	0.943708	2.1
9694	0.936134	2.1
9695	1.14341	2.1
9696	1.17802	0.05
9697	1.3542	0.05
9698	1.73981	0.05
9699	1.73981	0.05
9700	0.94613	0.05
9701	1.3542	0.05
9702	1.33174	0.05
9703	1.59452	2.1
9704	1.59452	2.1
9705	0.946132	2.1
9706	1.25 2.1	
9707	0.975266	2.1
9708	0.946132	2.1
9709	1.25 2.1	
9710	0.946131	2.1
9711	1.48685	2.1
9712	1.48685	2.1
9713	0.946132	2.1
9714	1.17273	2.1
9715	1.17273	2.1
9716	0.955602	2.1
9717	1.32827	0.343888
9718	1.11166	0.335656
9719	0.946127	0.351612
9720	1.6136	0.335474
9721	1.6136	0.332323
9722	1.11166	0.341327
9723	0.946129	0.339255
9724	0.851541	0.718729
9725	1.22107	0.723893
9726	1.64594	0.653084
9727	1.5271	0.668093
9728	0.94613	0.797371
9729	1.22112	0.742022
9730	1.25002	0.684095
9731	1.65795	0.434653
9732	1.65795	1.1005
9733	0.946132	1.03278
9734	1.2366	0.63075
9735	0.946132	0.415202
9736	1.10023	0.550772
9737	1.2366	0.48615
9738	1.35856	0.318415
9739	0.946132	0.327559
9740	1.35856	0.254852
9741	1.08019	0.299356

9742	1.49261	0.243987
9743	1.49261	0.286463
9744	0.94613	0.343338
9745	1.29322	0.411501
9746	0.946132	0.380626
9747	1.29322	0.361839
9748	1.35856	0.406088
9749	1.70565	0.341814
9750	1.70565	0.39247
9751	0.946132	0.394311
9752	1.66635	2.1
9753	1.66635	2.1
9754	0.946133	2.1
9755	1.39558	2.1
9756	1.14793	2.1
9757	1.14793	2.1
9758	1.21691	2.1
9759	1.35381	0.319905
9760	1.3365	0.730603
9761	1.09905	0.531153
9762	0.946132	0.667372
9763	1.74418	0.616792
9764	1.74418	0.13127
9765	0.946132	0.498841
9766	1.84759	0.554393
9767	1.84759	0.174792
9768	0.94613	0.497232
9769	1.3904	0.550331
9770	1.34522	0.386928
9771	1.34522	0.466875
9772	1.40331	0.199552
9773	1.86047	0.570154
9774	1.86047	2.01813
9775	0.946132	0.735676
9776	1.41619	1.95712
9777	0.946132	0.219192
9778	1.3659	0.576397
9779	1.41619	0.640125
9780	1.28464	2.07931
9781	1.14496	1.30433
9782	1.57806	1.47626
9783	1.7753	2.01394
9784	0.851541	1.27092
9785	0.946131	1.70023
9786	0.872786	1.57522
9787	0.85062	0.701359
9788	1.52635	0.839337
9789	1.63785	0.672556
9790	1.69972	1.04241
9791	0.946132	1.2451
9792	1.37493	0.721211
9793	1.23854	0.727417
9794	1.41134	2.1
9795	1.27079	1.82179
9796	1.736	1.19505
9797	1.736	2.1
9798	0.946132	0.811759
9799	0.946133	1.60608

9800	1.21177	1.85232
9801	0.946131	0.110959
9802	0.946129	0.0909274
9803	0.94613	0.112568
9804	0.946131	0.0774869
9805	0.946131	0.0830282
9806	1.35403	0.0858188
9807	0.946131	0.117513
9808	1.37052	0.219952
9809	0.946132	0.657726
9810	0.94613	0.369887
9811	0.946133	0.232627
9812	0.946131	0.356237
9813	0.946131	0.343974
9814	0.946131	0.322541
9815	1.24249	0.169014
9816	0.946132	0.14482
9817	0.946133	0.101685
9818	0.946132	0.202554
9819	0.946132	0.145499
9820	0.946132	0.121032
9821	0.946132	0.176378
9822	0.946132	2.1
9823	0.946132	1.89277
9824	0.946131	2.1
9825	0.946132	2.1
9826	0.946132	2.1
9827	0.946132	2.1
9828	1.20608	2.1
9829	1.41232	2.1
9830	0.946128	2.1
9831	0.946129	2.1
9832	0.94613	2.1
9833	0.94613	2.1
9834	0.94613	2.1
9835	0.94613	2.1
9836	0.946132	0.140228
9837	0.946131	0.157794
9838	0.946131	0.172936
9839	0.946132	0.0787786
9840	0.946132	0.10395
9841	0.946131	0.150661
9842	0.946132	0.126204
9843	0.946116	0.598277
9844	0.94612	1.07641
9845	0.946118	0.837334
9846	0.946124	2.1
9847	0.946118	0.780616
9848	1.41561	1.30342
9849	0.946131	0.765327
9850	0.945846	2.04817
9851	1.03508	2.1
9852	0.77579	1.10674
9853	0.658908	2.1
9854	1.17474	2.1
9855	0.94303	2.1
9856	0.849189	2.1
9857	0.94298	2.1

9858	1.03963	2.1
9859	0.946131	1.29206
9860	0.946131	1.20091
9861	0.946131	2.1
9862	0.946131	0.978513
9863	0.946131	0.619474
9864	0.946131	1.05331
9865	1.32819	1.26781
9866	0.946133	0.966421
9867	0.946129	2.1
9868	0.946128	1.90634
9869	0.94613	1.8339
9870	0.946131	2.1
9871	0.946131	0.9191
9872	0.946131	1.63958
9873	0.954072	0.05
9874	1.28895	0.05
9875	0.946133	0.05
9876	1.15803	0.05
9877	1.29689	0.05
9878	1.29689	0.05
9879	0.946132	0.05
9880	0.946132	1.00016
9881	0.946132	1.01725
9882	0.946132	0.840112
9883	0.946132	1.09816
9884	0.946132	1.36437
9885	0.946131	1.38175
9886	1.0264	1.36231
9887	0.946126	0.334706
9888	0.946132	0.289646
9889	0.946131	0.253675
9890	0.94613	0.302808
9891	0.946129	0.443637
9892	0.94613	0.375646
9893	1.35977	0.351353
9894	0.946132	0.651344
9895	0.946133	0.628348
9896	0.876632	0.965618
9897	0.946132	0.884308
9898	1.01566	1.41142
9899	1.01566	0.823539
9900	0.876606	1.01495
9901	0.946131	0.0959043
9902	0.946128	0.0992638
9903	0.946128	0.11088
9904	0.94613	0.0900211
9905	0.94613	0.120678
9906	1.24979	0.0781636
9907	0.94613	0.119396
9908	0.946131	0.05
9909	0.946131	0.0580511
9910	0.921762	0.05
9911	0.9705	0.05
9912	0.9705	0.05
9913	0.946131	0.0532534
9914	0.921791	0.0531183
9915	0.810944	0.260648

9916	0.810945	0.326833
9917	0.810959	0.272602
9918	2.42267	0.147108
9919	1.35168	0.226843
9920	1.08132	0.263574
9921	1.08132	0.174819
9922	1.08131	0.245432
9923	0.946133	0.315799
9924	0.946132	0.321857
9925	0.946132	0.31855
9926	0.946133	0.317648
9927	0.946133	0.316215
9928	0.946132	0.326081
9929	0.946133	0.320992
9930	0.946131	0.722513
9931	0.946131	0.951259
9932	0.94613	0.58608
9933	0.851514	0.658033
9934	0.946131	0.784497
9935	0.965268	0.824502
9936	0.851513	0.650994
9937	0.946122	2.1
9938	0.946123	2.1
9939	0.946126	2.1
9940	0.946124	2.1
9941	0.946124	2.1
9942	0.946118	2.1
9943	0.94613	2.1
9944	0.946131	0.561433
9945	1.30448	0.674914
9946	0.946132	0.7393
9947	0.946129	0.629321
9948	0.946131	1.43835
9949	0.946131	0.58218
9950	0.94613	1.18867
9951	0.946133	0.730732
9952	0.946128	2.1
9953	0.946128	1.49313
9954	0.94613	0.860199
9955	1.36503	2.1
9956	0.946131	1.37623
9957	0.946131	2.1
9958	0.810947	0.05
9959	0.810947	0.05
9960	2.48233	0.05
9961	1.3517	0.05
9962	1.08132	0.05
9963	1.08132	0.05
9964	1.08133	0.05
9965	0.810934	0.05
9966	1.48874	0.05
9967	2.48233	0.05
9968	2.2369	0.05
9969	0.946133	0.655358
9970	0.946132	0.538631
9971	0.946132	0.719268
9972	0.946133	0.746703
9973	0.946133	0.833915

9974	1.41851	0.64177
9975	0.946132	0.624907
9976	1.03925	2.1
9977	0.946131	2.1
9978	0.94613	2.1
9979	0.946128	2.1
9980	0.946133	2.1
9981	0.944743	2.1
9982	1.13703	2.1
9983	0.946132	2.06207
9984	0.946128	2.1
9985	0.946128	2.1
9986	0.946131	2.1
9987	0.94613	2.1
9988	0.94613	2.1
9989	0.94613	2.1
9990	0.946131	0.0538204
9991	0.946132	0.0657576
9992	0.946131	0.0531408
9993	0.946132	0.0500955
9994	0.946132	0.05
9995	0.946131	0.0547597
9996	1.20057	0.053475
9997	0.946129	2.1
9998	0.94613	2.1
9999	0.94613	2.1
10000	0.946128	2.1
10001	0.946132	2.1
10002	0.94613	2.1
10003	1.38302	2.1
10004	0.856567	0.288323
10005	1.14319	0.302064
10006	0.716526	0.2634
10007	1.20631	0.257516
10008	0.626936	0.222141
10009	0.946131	0.276433
10010	0.94613	0.316186
10011	1.39502	0.171237
10012	0.881445	0.133842
10013	0.946128	0.256621
10014	0.94613	0.168188
10015	0.881447	0.20974
10016	0.946117	0.168356
10017	0.946116	0.154248
10018	0.88146	0.292608
10019	0.946132	2.1
10020	1.39053	2.1
10021	0.946132	2.1
10022	0.946132	2.1
10023	0.946132	2.1
10024	0.946132	2.1
10025	0.946131	2.1
10026	0.946131	2.1
10027	0.946131	2.1
10028	0.946131	2.1
10029	0.946132	2.1
10030	0.946132	2.1
10031	1.40571	2.1

10032	0.946132	2.1
10033	0.94613	0.409437
10034	0.946127	0.490551
10035	0.946128	0.431002
10036	0.946131	0.418543
10037	0.946127	0.418827
10038	1.25819	0.419084
10039	0.946129	0.40467
10040	0.946129	0.798208
10041	1.29054	0.819449
10042	0.94613	0.767166
10043	0.94613	0.775978
10044	0.946131	1.34684
10045	0.946131	0.877682
10046	0.94613	0.823182
10047	0.810944	0.05
10048	0.810941	0.05
10049	1.61558	0.05
10050	1.3517	0.05
10051	1.08131	0.05
10052	1.08132	0.05
10053	1.08133	0.05
10054	0.810931	0.05
10055	1.02036	1.2995
10056	0.946132	0.904409
10057	1.02036	0.837167
10058	0.946132	0.67106
10059	1.1576	0.789722
10060	0.964656	0.91155
10061	0.927608	0.640514
10062	0.946128	2.1
10063	1.38813	2.1
10064	0.946129	2.1
10065	0.946131	2.1
10066	0.946131	2.1
10067	0.94613	2.1
10068	0.946131	2.1
10069	0.946131	2.03282
10070	0.94613	0.586586
10071	0.94613	2.1
10072	1.41665	1.40041
10073	0.946132	0.580797
10074	0.946131	1.13739
10075	0.94613	0.671604
10076	0.946118	0.376221
10077	0.946118	0.370032
10078	0.946116	0.375024
10079	0.946126	0.349974
10080	0.94612	0.35599
10081	0.946131	0.368527
10082	0.946105	0.359765
10083	0.946132	2.1
10084	0.946132	2.1
10085	0.946132	2.1
10086	0.946131	2.1
10087	0.946132	2.1
10088	0.946132	2.1
10089	0.946132	2.1

10090	0.864997	0.05
10091	0.786573	0.05
10092	0.978561	0.05
10093	1.47385	0.05
10094	1.68652	0.05
10095	1.27803	0.05
10096	1.19771	0.05
10097	1.27595	0.05
10098	0.946127	0.516709
10099	1.3771	0.478642
10100	0.946127	0.467741
10101	0.946131	0.56583
10102	0.946129	0.452845
10103	0.946129	0.529106
10104	0.946129	0.486622
10105	0.95697	0.05
10106	1.48133	0.05
10107	1.24499	0.05
10108	1.5295	0.05
10109	0.938161	0.05
10110	0.808717	0.05
10111	1.3542	0.05
10112	0.946133	0.805534
10113	1.14444	0.741749
10114	0.946132	0.721126
10115	0.946132	0.77243
10116	0.946133	1.20627
10117	0.946132	0.893563
10118	0.946132	0.792704
10119	0.946131	0.0792153
10120	0.946132	0.118892
10121	0.946131	0.0672316
10122	0.946132	0.0760143
10123	0.946131	0.0961625
10124	0.946132	0.0754962
10125	0.946132	0.099521
10126	0.946132	2.1
10127	0.946133	2.1
10128	0.946133	2.1
10129	0.946132	2.1
10130	1.29322	2.1
10131	0.946132	2.1
10132	0.946132	2.1
10133	0.946131	2.1
10134	0.946132	2.1
10135	0.946131	2.1
10136	0.946132	2.1
10137	1.28441	2.1
10138	0.946131	2.1
10139	0.946131	2.1
10140	0.946113	2.1
10141	0.946118	2.1
10142	0.946118	1.13469
10143	1.28073	2.1
10144	0.946117	1.69038
10145	0.946131	2.1
10146	0.946105	2.1
10147	0.810945	2.1

10148	0.810942	2.1
10149	2.75904	2.1
10150	1.3517	2.1
10151	1.08131	2.1
10152	1.08133	2.1
10153	1.08132	2.1
10154	0.81093	2.1
10155	0.946127	2.1
10156	1.37952	2.1
10157	1.86965	2.1
10158	0.946127	2.1
10159	1.86965	2.1
10160	1.28228	2.1
10161	1.37952	2.1
10162	0.946128	2.1
10163	0.946132	2.1
10164	1.14042	2.1
10165	0.946132	2.1
10166	1.14042	2.1
10167	1.09974	2.1
10168	1.09974	2.1
10169	0.946132	2.1
10170	0.946132	0.337228
10171	0.946132	0.278644
10172	0.946133	0.332002
10173	0.946132	0.258512
10174	0.946132	0.341286
10175	0.946132	0.256319
10176	0.946133	0.343113
10177	1.31817	1.33923
10178	0.946131	1.02243
10179	0.946131	1.25297
10180	0.946131	1.19198
10181	0.946131	0.860907
10182	0.946131	1.06932
10183	0.946131	1.07398
10184	0.946132	2.1
10185	0.946132	2.1
10186	0.946132	2.1
10187	0.946132	2.1
10188	0.946132	2.1
10189	0.946132	2.1
10190	1.35048	2.1
10191	0.946131	2.1
10192	0.946131	2.1
10193	0.94613	2.1
10194	0.946133	2.1
10195	0.946133	2.1
10196	1.35943	2.1
10197	0.946131	2.1
10198	0.946133	2.1
10199	0.946132	2.1
10200	0.946132	2.1
10201	0.946133	2.1
10202	0.946133	2.1
10203	0.946133	2.1
10204	0.990712	2.1
10205	0.965725	1.95118

10206	0.946129	0.804253
10207	0.946133	1.41416
10208	1.21721	0.939336
10209	0.877931	0.707327
10210	0.946131	1.33693
10211	0.946131	0.771887
10212	0.946132	0.515998
10213	0.946131	0.409239
10214	0.946131	0.700261
10215	0.946133	0.510484
10216	0.946128	0.89812
10217	0.946131	0.639131
10218	1.39575	0.617606
10219	0.810943	0.513881
10220	0.810942	0.521976
10221	2.58794	0.440868
10222	1.21652	0.448235
10223	0.946154	0.492243
10224	0.979952	0.488802
10225	1.0813	0.487724
10226	0.810959	0.492843
10227	0.924071	0.456091
10228	1.16453	0.302592
10229	1.28586	0.261353
10230	0.946131	0.469621
10231	1.1227	0.245394
10232	1.16462	0.354714
10233	0.851553	0.489434
10234	0.709578	0.283781
10235	0.810943	0.563344
10236	0.810959	0.458285
10237	1.82615	0.281138
10238	1.21651	0.305401
10239	1.08132	0.324987
10240	0.946155	0.336226
10241	0.979937	0.47653
10242	0.946128	1.32745
10243	0.946132	2.1
10244	0.946132	0.907059
10245	0.94613	2.04385
10246	0.94613	1.8485
10247	0.94613	0.814816
10248	0.94613	1.39641
10249	0.946131	1.85048
10250	1.29735	2.1
10251	0.946131	2.1
10252	0.946131	0.926192
10253	0.946131	1.48412
10254	0.946131	0.867333
10255	0.946131	1.99329
10256	0.946128	2.1
10257	1.33266	1.09144
10258	0.94613	1.11216
10259	0.946131	1.8879
10260	0.94613	0.692999
10261	0.946131	1.07918
10262	0.946131	0.954068
10263	0.946131	0.333242

10264	0.946131	1.66862
10265	0.946131	0.332093
10266	1.40608	0.336645
10267	0.946131	1.0848
10268	0.946131	0.557119
10269	0.946131	0.711882
10270	0.946131	0.840194
10271	0.946131	1.42988
10272	0.946131	0.974843
10273	1.2334	0.988091
10274	0.946129	1.069
10275	0.946131	1.00044
10276	0.946131	1.34222
10277	0.946133	1.54554
10278	0.946133	2.1
10279	0.946132	1.01643
10280	1.29198	1.79125
10281	0.946132	1.38899
10282	0.946132	1.31026
10283	0.946132	1.89565
10284	0.946131	2.1
10285	0.946128	2.1
10286	0.946128	2.1
10287	0.946131	2.1
10288	0.946127	2.1
10289	0.94613	2.1
10290	0.946129	2.1
10291	0.946123	2.1
10292	0.946126	2.1
10293	0.946125	2.1
10294	1.38639	2.1
10295	0.946126	2.1
10296	0.946132	2.1
10297	0.946119	2.1
10298	0.946128	0.362673
10299	0.946132	0.367703
10300	0.946131	0.416087
10301	0.94613	0.194439
10302	0.94613	0.256953
10303	0.946131	0.350225
10304	0.94613	0.304973
10305	0.946131	0.314653
10306	0.946131	0.318772
10307	0.946131	0.397424
10308	0.946131	0.134103
10309	0.946131	0.203538
10310	0.946131	0.329264
10311	0.946131	0.257048
10312	0.810945	2.1
10313	0.810943	2.1
10314	0.810933	2.1
10315	2.24688	2.1
10316	1.3517	2.1
10317	1.08132	2.1
10318	1.08132	2.1
10319	1.08133	2.1
10320	1.25809	0.270201
10321	0.946131	0.568849

10322	1.25809	0.287282
10323	0.946129	0.547757
10324	0.94613	0.309792
10325	0.946131	0.537051
10326	0.946131	0.41042
10327	0.946131	0.319981
10328	0.946131	0.318106
10329	0.946129	0.32209
10330	1.28911	0.297756
10331	0.946131	0.295797
10332	0.946131	0.320556
10333	0.946131	0.323069
10334	0.94613	0.459745
10335	0.946131	0.470153
10336	0.94613	0.423561
10337	0.946128	0.46344
10338	0.946132	0.42036
10339	1.38954	0.447755
10340	0.946131	0.450608
10341	0.946131	0.268239
10342	0.946131	0.481131
10343	0.946131	0.545848
10344	1.37981	0.442607
10345	0.946131	0.713917
10346	0.946131	0.499877
10347	0.946131	0.519068
10348	0.946131	1.35658
10349	0.946127	2.1
10350	0.946127	2.1
10351	0.946131	1.89591
10352	0.946127	2.1
10353	0.946129	2.1
10354	0.946128	2.1
10355	0.94613	2.1
10356	0.946126	2.1
10357	0.946127	2.1
10358	0.946131	2.1
10359	0.946126	2.01265
10360	0.946128	2.1
10361	0.946128	2.1
10362	0.946133	0.476344
10363	0.946132	0.444465
10364	0.946132	0.454628
10365	0.946132	0.453649
10366	0.946132	0.479508
10367	1.13511	0.404328
10368	0.946132	0.440252
10369	0.946132	1.57549
10370	0.946129	1.59515
10371	0.94613	1.22456
10372	0.946131	1.70732
10373	0.946131	1.94148
10374	0.94613	2.1
10375	1.27575	2.09001
10376	0.946129	2.1
10377	0.946131	2.1
10378	0.946131	2.1
10379	0.946128	2.1

10380	0.946133	2.1
10381	0.946131	2.1
10382	0.94613	2.1
10383	1.15581	0.05
10384	0.946132	0.05
10385	0.946131	0.0627134
10386	0.94613	0.05
10387	1.15581	0.05
10388	0.94613	0.0513593
10389	0.946131	0.0664853
10390	0.611407	0.876875
10391	0.946131	0.689902
10392	1.37335	0.779269
10393	1.15086	0.886395
10394	0.617549	0.759208
10395	0.851553	0.851268
10396	1.14592	0.895511
10397	1.22618	1.18166
10398	0.946132	0.698924
10399	0.946132	1.25832
10400	0.946132	0.720748
10401	0.946131	0.74021
10402	0.946132	0.935883
10403	0.946132	0.835169
10404	0.946131	2.1
10405	0.94613	2.1
10406	0.946131	2.1
10407	0.946133	2.1
10408	0.946129	2.1
10409	0.946131	2.1
10410	0.946131	2.1
10411	0.810945	0.695744
10412	0.810943	0.998918
10413	2.50715	0.986246
10414	1.3517	0.784225
10415	1.08133	0.733837
10416	1.08132	0.947656
10417	1.08132	0.516536
10418	0.810932	0.688204
10419	1.07811	1.02673
10420	0.94613	0.716882
10421	0.946128	1.10876
10422	1.25358	0.566971
10423	0.946131	0.751141
10424	1.25358	0.733152
10425	0.94613	0.761072
10426	0.946129	2.1
10427	1.09197	2.1
10428	0.946131	2.1
10429	1.09197	2.1
10430	1.09532	2.1
10431	1.09532	2.1
10432	0.946131	2.1
10433	1.58788	2.1
10434	1.34255	2.1
10435	0.594912	2.1
10436	0.58894	2.1
10437	0.946129	2.1

10438	1.16679	2.1
10439	1.04071	2.1
10440	0.535418	2.1
10441	0.946131	2.1
10442	0.946128	2.1
10443	0.946131	1.98629
10444	1.20221	2.1
10445	0.946128	2.1
10446	0.94613	2.1
10447	1.20221	2.1
10448	0.946132	2.1
10449	0.946133	2.1
10450	0.946132	2.1
10451	0.946132	2.1
10452	0.946133	2.1
10453	0.946133	2.1
10454	0.946132	2.1
10455	0.946132	0.819774
10456	0.946132	1.01298
10457	0.946133	1.20421
10458	0.946132	0.875344
10459	1.411	1.08992
10460	0.946132	0.968627
10461	0.946133	1.05956
10462	1.05575	0.20054
10463	0.563734	0.197298
10464	1.05575	0.214405
10465	0.773833	0.140697
10466	0.751686	0.15737
10467	0.75884	0.206703
10468	0.815612	0.149328
10469	0.793465	0.170771
10470	0.768563	0.248523
10471	0.946129	0.258969
10472	0.946125	0.224022
10473	0.946126	0.316057
10474	0.94613	0.228417
10475	0.946124	0.333916
10476	1.31678	0.195826
10477	0.946128	0.236156
10478	0.946132	0.46686
10479	1.29914	0.384399
10480	0.946132	0.381234
10481	0.946132	0.410203
10482	0.946132	0.47106
10483	1.29914	0.260892
10484	0.946132	0.421551
10485	0.810946	0.453808
10486	0.810943	0.447203
10487	2.21082	0.287485
10488	1.35168	0.338149
10489	1.08132	0.393692
10490	1.08132	0.400368
10491	1.0813	0.443705
10492	0.81096	0.436749
10493	0.625813	0.0682121
10494	0.946132	0.0748018
10495	0.946131	0.0890598

10496	0.946132	0.0812662
10497	0.946131	0.079451
10498	1.39301	0.0894446
10499	0.625812	0.0789589
10500	0.810946	1.19695
10501	0.810945	2.1
10502	0.810931	2.01468
10503	2.70043	2.1
10504	1.3517	2.08755
10505	1.08131	1.27098
10506	1.08131	2.1
10507	1.08133	1.32943
10508	0.810943	2.1
10509	0.810944	2.1
10510	0.810958	2.1
10511	2.44802	2.1
10512	1.35168	2.1
10513	1.08132	2.1
10514	1.08132	2.1
10515	1.0813	2.1
10516	0.946131	0.413383
10517	0.946129	0.429365
10518	0.946128	0.404753
10519	0.946131	0.406937
10520	1.3492	0.406373
10521	1.3492	0.415134
10522	0.94613	0.408111
10523	0.946132	0.488979
10524	0.946132	0.434048
10525	0.946133	0.487013
10526	0.946133	0.410688
10527	0.946132	0.553067
10528	0.946132	0.40786
10529	0.946132	0.532535
10530	0.946129	0.346827
10531	0.946133	0.367352
10532	0.946131	0.403273
10533	0.94613	0.390424
10534	0.946131	0.385572
10535	0.946131	0.302922
10536	0.946131	0.354843
10537	0.946119	0.300106
10538	0.946119	0.326045
10539	0.946119	0.351255
10540	0.946125	0.292062
10541	0.94612	0.325313
10542	0.946132	0.294246
10543	0.946107	0.297966
10544	0.946133	2.1
10545	0.946132	2.1
10546	0.946132	2.1
10547	0.946132	2.1
10548	0.946132	2.1
10549	0.946133	2.1
10550	1.3868	2.1
10551	0.810947	2.1
10552	0.810946	2.1
10553	0.81096	2.1

10554	2.66094	2.1
10555	1.35168	2.1
10556	1.08132	2.1
10557	1.08132	2.1
10558	1.0813	2.1
10559	0.810946	2.1
10560	0.810946	2.1
10561	0.810933	2.1
10562	2.75737	2.1
10563	1.3517	2.1
10564	1.08132	2.1
10565	1.08132	2.1
10566	1.08133	2.1
10567	0.946133	2.1
10568	0.946132	2.1
10569	0.946132	0.995036
10570	0.946132	2.1
10571	0.946133	1.01032
10572	0.946132	2.1
10573	0.946133	2.1
10574	0.946133	2.1
10575	0.946133	2.1
10576	0.946132	2.1
10577	0.946132	2.1
10578	0.946133	2.1
10579	0.946132	2.1
10580	1.28569	2.1
10581	0.946132	1.15278
10582	0.946133	2.1
10583	0.946133	2.1
10584	0.946132	1.66477
10585	0.946132	2.1
10586	0.946132	1.98853
10587	0.946133	1.8857
10588	0.946132	0.215184
10589	0.946131	0.218571
10590	0.94613	0.196631
10591	0.946133	0.170145
10592	0.946128	0.241357
10593	0.946131	0.16238
10594	0.946131	0.247763
10595	0.946131	1.97515
10596	0.94613	1.49493
10597	0.946131	2.1
10598	0.94613	1.55007
10599	1.41833	0.991381
10600	0.946131	0.891444
10601	0.946131	1.93503
10602	1.26155	0.0556238
10603	0.946131	0.0783701
10604	0.946131	0.0626034
10605	0.946132	0.0522865
10606	0.946132	0.0606258
10607	0.946131	0.0614118
10608	0.946132	0.0534833
10609	0.810946	2.1
10610	0.810946	2.1
10611	0.810959	2.1

10612	2.47938	2.1
10613	1.35168	2.1
10614	1.08132	2.1
10615	1.08132	2.1
10616	1.08131	2.1
10617	0.946131	0.471921
10618	0.94613	0.425482
10619	0.94613	0.658979
10620	0.946132	0.382496
10621	0.946128	0.831544
10622	0.94613	0.594547
10623	0.94613	0.622038
10624	0.94613	0.0619244
10625	0.946131	0.0656897
10626	0.94613	0.097399
10627	0.946129	0.0775274
10628	0.946132	0.0781715
10629	0.94613	0.0788996
10630	0.946131	0.0758683
10631	0.946132	2.1
10632	0.946131	2.1
10633	0.946128	2.1
10634	1.40756	2.1
10635	0.946131	2.1
10636	0.946129	2.1
10637	0.94613	2.1
10638	0.946131	1.00547
10639	0.946131	1.00593
10640	0.946131	1.62995
10641	0.946132	1.06487
10642	1.31947	1.03623
10643	0.946131	1.02956
10644	0.946131	1.17201
10645	0.946131	2.1
10646	1.25768	2.1
10647	0.946132	2.1
10648	0.946132	2.1
10649	0.946129	2.1
10650	0.946131	2.1
10651	0.94613	2.1
10652	0.946132	0.351189
10653	0.946129	0.33861
10654	0.946129	0.392757
10655	0.94613	0.36274
10656	1.31716	0.357517
10657	1.31716	0.352412
10658	0.946131	0.388299
10659	0.810943	1.03557
10660	0.810942	2.1
10661	0.81093	1.81648
10662	2.37052	2.1
10663	1.3517	1.66738
10664	1.08132	0.879761
10665	1.08132	2.1
10666	1.08133	1.05557
10667	0.946128	0.963474
10668	1.10335	0.81906
10669	0.94613	0.733795

10670	0.946131	1.23382
10671	0.946131	0.657537
10672	1.10335	0.953129
10673	0.94613	1.08501
10674	0.946132	0.977411
10675	1.18818	0.853824
10676	0.946132	0.798748
10677	0.946132	1.01995
10678	0.946132	0.744855
10679	1.18818	0.912538
10680	0.946132	0.96949
10681	0.946125	2.1
10682	0.94612	2.1
10683	0.946119	2.1
10684	0.946119	2.1
10685	0.946119	2.1
10686	0.946132	2.1
10687	1.31522	2.1
10688	1.28803	1.58498
10689	0.946132	0.719707
10690	0.946132	2.06696
10691	0.946133	2.03609
10692	0.946133	0.601462
10693	0.946133	1.41743
10694	0.946132	1.03684
10695	0.946133	0.981801
10696	0.946132	1.36253
10697	0.946132	0.966963
10698	0.946132	0.788043
10699	0.946133	0.775916
10700	0.946132	0.770832
10701	1.37658	0.862316
10702	1.02239	1.62226
10703	0.946128	0.870043
10704	0.946132	1.3761
10705	1.02239	0.976584
10706	0.94613	0.819167
10707	0.96283	1.40815
10708	0.92943	0.834437
10709	0.946132	0.05
10710	0.946131	0.05
10711	0.946126	0.0561171
10712	0.946129	0.05
10713	0.946128	0.05
10714	0.946128	0.05
10715	0.946129	0.05
10716	0.810946	0.0658221
10717	0.810944	0.0512026
10718	2.22493	0.05
10719	1.21651	0.05
10720	1.0813	0.0505844
10721	0.946154	0.05
10722	0.979951	0.05
10723	0.70959	0.05
10724	0.946132	0.0801545
10725	0.946131	0.11199
10726	0.946131	0.0699818
10727	0.946133	0.0765613

10728	0.946131	0.106319
10729	0.946132	0.0874019
10730	0.946132	0.0976387
10731	0.946132	1.96914
10732	0.946131	1.69867
10733	0.946132	2.1
10734	0.946132	1.07158
10735	0.946132	0.794585
10736	0.946132	1.09623
10737	1.40924	1.73176
10738	0.946131	2.1
10739	0.946128	2.1
10740	0.946127	2.1
10741	0.94613	2.1
10742	1.41743	2.1
10743	0.946128	2.1
10744	0.946129	2.1
10745	1.37675	0.607705
10746	0.946128	0.679234
10747	0.94613	0.607934
10748	0.94613	0.868237
10749	0.94613	0.617855
10750	0.946129	0.660209
10751	0.94613	0.782474
10752	0.946132	0.405066
10753	0.946132	0.479884
10754	0.946132	0.556259
10755	1.35721	0.362114
10756	0.946132	0.709139
10757	0.946132	0.543992
10758	1.35721	0.500655
10759	0.946133	0.758691
10760	0.946133	0.643151
10761	0.946132	0.748957
10762	1.10003	0.580499
10763	0.946133	0.584881
10764	0.946132	0.698688
10765	0.946132	0.645176
10766	0.946132	0.211219
10767	0.946131	0.132693
10768	0.946131	0.164989
10769	0.946131	0.243921
10770	1.0616	0.138244
10771	1.0616	0.154674
10772	0.946132	0.243109
10773	0.946132	0.272146
10774	0.946131	0.168004
10775	0.946131	0.21195
10776	0.946131	0.314969
10777	1.09402	0.176411
10778	1.09402	0.199883
10779	0.946131	0.315674
10780	0.946132	0.0642221
10781	0.946126	0.054171
10782	1.24093	0.0595361
10783	0.946129	0.0739954
10784	0.946129	0.0682313
10785	0.946128	0.0717866

10786	0.946129	0.0672193
10787	0.946131	0.0904159
10788	0.946131	0.0811838
10789	1.2929	0.0876607
10790	0.946131	0.116393
10791	0.946131	0.114071
10792	0.946131	0.116996
10793	0.946131	0.0995548
10794	0.810946	0.05
10795	0.810945	0.05
10796	0.810959	0.05
10797	2.41233	0.05
10798	1.35168	0.05
10799	1.08132	0.05
10800	1.08132	0.05
10801	1.0813	0.05
10802	0.810946	1.14117
10803	0.810943	1.86006
10804	0.750103	1.54485
10805	2.22778	1.95541
10806	1.27059	1.68166
10807	1.00021	1.31962
10808	1.02049	1.9145
10809	1.08133	1.28056
10810	0.946128	1.46777
10811	0.946132	2.1
10812	0.946132	2.1
10813	0.94613	2.00595
10814	0.94613	2.1
10815	0.94613	2.1
10816	0.946131	2.09523
10817	0.946126	0.48765
10818	0.946131	0.467983
10819	0.946127	0.517825
10820	1.3324	0.443159
10821	0.946127	0.458983
10822	0.946128	0.491906
10823	0.946128	0.547541
10824	0.94613	2.1
10825	0.946131	2.1
10826	0.94613	2.1
10827	0.946128	2.1
10828	0.946133	2.09506
10829	0.946131	2.1
10830	0.946131	2.1
10831	0.946133	2.1
10832	0.946132	2.1
10833	0.946133	2.1
10834	0.946133	2.1
10835	0.946132	2.1
10836	0.946132	2.1
10837	1.36541	2.1
10838	0.946131	0.720157
10839	0.94613	2.1
10840	0.946131	0.938197
10841	0.94613	0.799755
10842	1.39509	1.28813
10843	0.946131	0.654812

10844	0.946131	1.56256
10845	0.946133	2.1
10846	0.946133	2.1
10847	0.946132	2.1
10848	0.946132	2.1
10849	0.946133	2.1
10850	0.946132	2.1
10851	1.29319	2.1
10852	1.01196	0.0964494
10853	0.946131	0.16005
10854	0.946132	0.0839415
10855	0.946132	0.111282
10856	0.946132	0.151168
10857	0.946132	0.141258
10858	0.946132	0.118644
10859	0.946112	2.1
10860	0.946118	2.08032
10861	0.946116	1.16843
10862	0.946117	1.51844
10863	0.946118	2.1
10864	0.94613	2.1
10865	0.946105	2.1
10866	1.28684	1.01373
10867	0.946131	1.04065
10868	0.946132	1.0687
10869	0.946132	1.34147
10870	0.946132	1.3266
10871	0.946132	1.09623
10872	0.946132	1.48993
10873	0.94613	2.01706
10874	0.94613	2.1
10875	0.946128	1.60978
10876	0.946131	1.40825
10877	0.946131	1.72655
10878	0.94613	1.64826
10879	1.10185	2.02327
10880	0.810947	0.05
10881	0.810945	0.05
10882	1.57348	0.05
10883	1.3517	0.05
10884	1.08131	0.05
10885	1.08132	0.05
10886	1.08133	0.05
10887	0.810932	0.05
10888	0.946119	0.432775
10889	0.946116	0.404954
10890	0.946117	0.430482
10891	0.946121	0.377424
10892	0.946118	0.389617
10893	0.94613	0.408903
10894	0.946105	0.394469
10895	0.94612	0.523025
10896	0.946122	0.411222
10897	0.946119	0.451114
10898	0.946119	0.529823
10899	1.20884	0.413444
10900	0.946132	0.487634
10901	0.946107	0.448438

10902	2.67849	0.909607
10903	1.17709	0.879349
10904	0.605418	1.05518
10905	0.286984	0.935397
10906	0.172654	0.965689
10907	0.810946	0.685101
10908	0.940878	0.644907
10909	0.941235	0.633804
10910	1.26696	0.674212
10911	1.56837	0.694314
10912	1.13737	0.662663
10913	1.34115	0.686754
10914	1.24889	0.644388
10915	0.946132	1.19603
10916	0.946129	0.939361
10917	0.946128	1.05952
10918	0.94613	0.972508
10919	0.94613	1.21883
10920	0.946131	1.09395
10921	0.94613	1.09516
10922	0.946116	2.1
10923	0.94612	2.1
10924	0.946118	2.1
10925	0.946123	2.1
10926	0.946118	2.1
10927	0.946131	2.1
10928	1.23836	2.1
10929	0.810946	0.05
10930	0.810946	0.05
10931	0.810959	0.05
10932	2.80677	0.05
10933	1.35168	0.05
10934	1.08132	0.05
10935	1.08132	0.05
10936	1.08131	0.05
10937	0.810947	0.430135
10938	0.810947	0.65393
10939	2.41574	0.635038
10940	1.21653	0.61107
10941	1.08133	0.517142
10942	0.946153	0.670968
10943	0.979949	0.55295
10944	0.709567	0.518904
10945	0.94613	2.1
10946	0.946131	2.1
10947	0.946128	2.1
10948	1.34782	2.1
10949	0.946128	2.1
10950	0.946129	2.1
10951	1.34782	2.1
10952	0.946132	1.68428
10953	0.946132	2.03787
10954	0.94613	2.1
10955	1.16023	1.98089
10956	0.946131	2.1
10957	0.946131	2.1
10958	1.16023	2.1
10959	0.946131	0.906747

10960	0.946127	2.1
10961	0.946127	1.84702
10962	0.946131	1.41105
10963	0.946128	2.1
10964	0.94613	1.01334
10965	0.946129	1.50617
10966	0.946133	0.41006
10967	0.946132	0.32468
10968	0.946132	0.371741
10969	0.946133	0.421491
10970	1.20045	0.322639
10971	1.20045	0.343395
10972	0.946132	0.42543
10973	0.918871	0.125535
10974	0.971961	0.107328
10975	1.37324	0.0632389
10976	1.78255	0.0616065
10977	1.25155	0.106146
10978	1.42172	0.0625428
10979	1.40554	0.0838044
10980	1.08862	0.0906814
10981	1.03803	0.0789474
10982	0.946132	0.0813847
10983	0.946126	0.0891541
10984	0.943647	0.0791119
10985	0.948611	0.0945538
10986	0.946129	0.0843571
10987	0.946129	0.0906599
10988	1.1699	0.67857
10989	0.946132	0.58989
10990	0.946133	0.82131
10991	0.946133	0.599802
10992	0.946132	0.617304
10993	0.946133	0.672938
10994	0.946132	0.771946
10995	0.946132	2.1
10996	0.946131	2.1
10997	0.946131	2.1
10998	0.946132	2.1
10999	0.946131	2.1
11000	0.946131	2.1
11001	0.946132	2.1
11002	0.946132	0.802727
11003	1.38623	0.726633
11004	0.946132	0.675685
11005	0.946132	0.669269
11006	0.946133	0.740948
11007	0.946132	0.627188
11008	0.946133	0.889654
11009	0.946132	2.1
11010	1.40098	2.1
11011	0.946131	2.1
11012	0.946131	2.1
11013	0.946132	2.1
11014	0.946132	2.1
11015	0.946132	2.1
11016	1.01777	0.662046
11017	0.946132	0.47781

11018	0.946133	0.926639
11019	1.68068	0.57639
11020	1.68068	1.19601
11021	1.41894	0.51596
11022	1.20787	0.438024
11023	0.946132	0.513545
11024	0.946133	2.1
11025	0.946133	2.1
11026	0.946132	2.1
11027	1.40199	2.1
11028	0.946133	2.1
11029	0.946132	2.1
11030	0.946132	2.1
11031	0.810946	1.04648
11032	0.810947	0.76355
11033	2.37827	1.2548
11034	1.3517	1.04473
11035	1.08132	1.05418
11036	1.08132	1.09555
11037	1.08133	1.01637
11038	0.810934	0.961339
11039	0.946124	2.00176
11040	0.94613	1.66957
11041	1.40248	1.0569
11042	0.946131	1.02846
11043	0.946125	0.738616
11044	0.946127	1.59765
11045	0.946128	0.795869
11046	1.25703	1.76211
11047	0.87821	1.02054
11048	1.18912	1.631
11049	0.946119	0.927886
11050	0.87825	1.06463
11051	1.1891	1.18208
11052	0.946107	1.68024
11053	0.946132	2.1
11054	1.28543	1.97726
11055	0.946132	1.70985
11056	0.946132	1.41016
11057	0.946131	0.961703
11058	0.946132	0.871031
11059	0.946132	0.854494
11060	0.946115	2.1
11061	0.946119	2.1
11062	0.946116	2.1
11063	0.946123	2.1
11064	0.946118	2.1
11065	1.41636	2.1
11066	0.946129	2.1
11067	0.946132	0.660004
11068	0.946132	1.67992
11069	0.946132	0.839524
11070	0.946131	2.1
11071	0.946131	1.01816
11072	1.41432	1.98345
11073	0.946132	0.971691
11074	0.810947	0.141954
11075	0.810947	0.1996

11076	2.44433	0.100326
11077	1.21654	0.0983435
11078	0.946154	0.177955
11079	0.97995	0.126977
11080	1.08133	0.114488
11081	0.810934	0.125127
11082	0.851542	0.0890414
11083	0.835698	0.117304
11084	1.22216	0.0874428
11085	0.946133	0.111706
11086	1.01217	0.106168
11087	1.09996	0.114798
11088	0.851529	0.130287
11089	0.946132	1.39331
11090	0.946132	1.87374
11091	0.946129	2.1
11092	0.94613	1.46457
11093	0.946131	2.1
11094	0.946131	1.95581
11095	1.2254	2.1
11096	0.800897	1.0793
11097	0.838029	2.01664
11098	0.838013	1.39391
11099	0.93679	0.936066
11100	1.20217	1.07775
11101	1.16923	1.20482
11102	0.94948	1.15326
11103	0.958118	1.3135
11104	0.36751	1.18968
11105	0.800535	1.60199
11106	0.272087	0.784671
11107	0.428084	0.987148
11108	0.986335	1.56411
11109	0.946132	0.370778
11110	0.946133	0.518811
11111	1.19601	0.376037
11112	0.946132	0.410541
11113	0.946132	0.442739
11114	0.946133	0.544859
11115	0.946133	0.336574
11116	0.946132	1.44648
11117	1.36956	2.1
11118	0.946132	2.1
11119	0.946132	1.20227
11120	0.946132	2.1
11121	0.946133	1.56313
11122	0.946132	1.94621
11123	0.946132	0.129688
11124	0.946131	0.555174
11125	0.946131	0.346489
11126	1.34268	0.187011
11127	0.946133	0.507099
11128	0.946132	0.362411
11129	0.946131	0.442491
11130	0.946132	1.89792
11131	0.946131	1.82595
11132	0.946131	2.06047
11133	0.946132	2.06812

11134	0.946132	1.85882
11135	0.946132	2.1
11136	0.946132	1.91749
11137	0.810946	0.939554
11138	0.810943	0.83882
11139	2.51472	0.929656
11140	1.3517	0.841581
11141	1.08132	0.812496
11142	1.08132	0.915158
11143	1.08133	0.876142
11144	0.810933	0.867177
11145	0.902316	0.690008
11146	1.04517	0.734054
11147	0.974924	0.48774
11148	0.530063	0.864166
11149	1.1272	0.77283
11150	0.68207	0.737914
11151	0.477061	0.831061
11152	0.810946	0.0898723
11153	0.810943	0.166438
11154	0.810959	0.139812
11155	1.99211	0.112608
11156	1.35168	0.101734
11157	1.08132	0.104416
11158	1.08132	0.10759
11159	1.08131	0.158435
11160	0.94613	0.166442
11161	0.946131	0.144247
11162	0.946132	0.144245
11163	0.946132	0.156982
11164	0.946129	0.251352
11165	0.946131	0.182236
11166	1.16699	0.186191
11167	0.810943	1.0434
11168	0.810942	0.978432
11169	1.6943	1.43821
11170	1.27059	1.49184
11171	1.00021	1.38939
11172	1.02049	1.20938
11173	1.08133	1.04545
11174	0.750103	1.0271
11175	0.957813	1.04504
11176	0.946128	1.14477
11177	0.946132	0.876184
11178	1.04832	1.19984
11179	0.870714	0.897089
11180	0.94613	0.930651
11181	0.94613	1.00696
11182	1.86374	2.07876
11183	2.0815	1.02725
11184	1.15918	0.71216
11185	0.946129	0.684171
11186	1.15918	0.495915
11187	0.946131	0.892576
11188	0.946131	0.483707
11189	0.946131	0.686682
11190	0.94613	0.712134
11191	0.946119	0.807755

11192	1.3612	0.889118
11193	0.946125	0.847877
11194	0.946119	0.78656
11195	0.94612	1.3186
11196	0.946132	0.872045
11197	0.946107	0.854766
11198	0.810947	0.354166
11199	0.810947	0.390117
11200	2.50398	0.345818
11201	1.3517	0.34128
11202	1.08132	0.340507
11203	1.08133	0.367782
11204	1.08132	0.336924
11205	0.810934	0.353222
11206	0.946132	2.1
11207	0.946132	2.1
11208	0.946132	2.1
11209	1.39754	2.1
11210	0.946132	2.1
11211	0.946132	2.1
11212	0.946131	2.1
11213	0.946129	2.1
11214	1.41732	2.1
11215	0.946129	2.1
11216	0.946131	2.1
11217	0.946131	2.1
11218	0.946131	2.1
11219	0.946131	2.1
11220	0.946132	1.02561
11221	1.25276	1.84379
11222	0.946131	2.1
11223	0.94613	1.01107
11224	0.94613	1.6103
11225	0.94613	1.0594
11226	0.946131	1.3438
11227	1.21261	0.865959
11228	0.94613	1.03577
11229	0.94613	1.54885
11230	0.946128	0.766085
11231	0.946131	1.2477
11232	0.94613	1.16025
11233	0.946129	1.30144
11234	0.946128	2.1
11235	0.946133	2.1
11236	0.946132	2.1
11237	0.946131	2.1
11238	0.94613	2.1
11239	0.946131	2.1
11240	1.39407	2.1
11241	0.946116	1.1561
11242	0.946119	1.12742
11243	0.946119	0.903382
11244	1.15369	1.14018
11245	0.946119	0.793227
11246	0.946131	1.01211
11247	0.946106	0.946244
11248	0.946131	1.92821
11249	0.946131	2.1

11250	0.946131	2.1
11251	0.946133	2.1
11252	0.946129	2.1
11253	0.946131	2.1
11254	0.946131	2.1
11255	1.19564	0.05
11256	0.498881	0.05
11257	1.33964	0.05
11258	0.498878	0.05
11259	0.923583	0.05
11260	0.831259	0.05
11261	0.553735	0.05
11262	0.946132	0.05
11263	0.946132	0.05
11264	0.946131	0.05
11265	0.946131	0.05
11266	0.946132	0.05
11267	0.946131	0.05
11268	1.28673	0.05
11269	0.946132	0.105256
11270	0.946132	0.104893
11271	1.33178	0.0941998
11272	0.946132	0.116645
11273	0.946132	0.0976487
11274	0.946131	0.113814
11275	0.946131	0.0998239
11276	0.946128	0.51362
11277	0.946132	0.45656
11278	0.946129	0.563154
11279	0.946131	0.252568
11280	0.946131	0.3201
11281	0.946129	0.47392
11282	0.94613	0.402539
11283	0.946131	2.1
11284	0.946131	2.1
11285	0.946129	2.1
11286	1.40525	2.1
11287	0.94613	2.1
11288	0.94613	2.1
11289	1.40525	2.1
11290	0.946131	2.1
11291	0.946126	2.1
11292	0.946127	2.1
11293	0.946127	2.1
11294	1.4073	2.1
11295	0.946129	2.1
11296	0.946129	2.1
11297	0.946131	0.185229
11298	0.946131	0.13481
11299	0.946132	0.215837
11300	0.946131	0.116028
11301	0.946131	0.123295
11302	1.05016	0.133832
11303	0.946132	0.206016
11304	0.946128	0.185352
11305	0.946132	0.143999
11306	0.946131	0.207376
11307	0.946129	0.124409

11308	0.94613	0.131676
11309	1.13398	0.147455
11310	0.94613	0.215862
11311	0.946132	0.05
11312	0.946132	0.05
11313	0.946131	0.0683497
11314	0.946131	0.0546744
11315	0.946132	0.0508779
11316	0.946131	0.0512932
11317	0.946132	0.052279
11318	0.946131	0.305668
11319	0.94613	0.19086
11320	0.946131	0.232896
11321	0.94613	0.357999
11322	1.38645	0.190732
11323	1.38645	0.206248
11324	0.946131	0.290613
11325	0.946131	0.470163
11326	0.946131	0.356833
11327	0.94613	0.486772
11328	1.23923	0.315388
11329	0.94613	0.319871
11330	0.946131	0.468022
11331	0.946131	0.516816
11332	0.946132	0.05
11333	0.946131	0.0571248
11334	0.946131	0.05
11335	0.946132	0.05
11336	0.946132	0.05
11337	0.946132	0.05
11338	1.40511	0.05
11339	0.946132	2.1
11340	0.946132	1.80888
11341	0.946132	2.1
11342	0.946132	1.82276
11343	0.946132	1.16607
11344	0.946131	2.1
11345	1.12968	2.1
11346	0.946132	1.30045
11347	0.946128	2.1
11348	0.946128	2.05411
11349	0.94613	1.63011
11350	0.94613	2.1
11351	0.94613	1.32578
11352	0.94613	1.94799
11353	0.946133	2.1
11354	0.946133	2.1
11355	0.946132	2.1
11356	0.946132	2.1
11357	0.946132	2.1
11358	0.946133	2.1
11359	1.31323	2.1
11360	0.94613	1.20396
11361	0.946131	0.629904
11362	0.946131	0.489614
11363	0.94613	0.355625
11364	0.946131	1.22346
11365	0.946131	0.794359

11366	0.946131	0.760081
11367	0.946132	0.579344
11368	0.946132	0.392066
11369	0.946132	0.598416
11370	1.36899	0.475293
11371	0.946132	0.346865
11372	0.946132	0.330817
11373	0.946133	0.413907
11374	1.03543	0.220352
11375	0.946131	0.287515
11376	0.946131	0.172818
11377	0.946132	0.178591
11378	0.94613	0.292216
11379	0.950987	0.148705
11380	0.941275	0.326394
11381	0.946131	0.131237
11382	0.94613	0.147002
11383	1.28952	0.161447
11384	0.946132	0.120369
11385	0.94613	0.206111
11386	0.946131	0.193973
11387	0.946131	0.137762
11388	0.94613	2.1
11389	1.35966	2.1
11390	0.946131	2.1
11391	0.946131	2.1
11392	0.946129	2.1
11393	0.94613	2.1
11394	0.94613	2.1
11395	0.810946	2.1
11396	0.810942	0.789734
11397	0.810932	2.02813
11398	2.6655	1.78135
11399	1.3517	1.1614
11400	1.08132	2.1
11401	1.08131	0.76589
11402	1.08133	1.68146
11403	0.94613	2.1
11404	0.946131	2.1
11405	0.94613	1.23595
11406	0.946128	2.1
11407	0.946131	1.81407
11408	0.94613	2.1
11409	0.94613	2.1
11410	0.946128	2.1
11411	0.946128	2.1
11412	0.946133	2.1
11413	1.25375	2.1
11414	0.94613	2.1
11415	0.946131	2.1
11416	1.25375	2.1
11417	0.94613	2.1
11418	0.94613	2.1
11419	0.946132	2.1
11420	0.946132	2.1
11421	0.946128	2.1
11422	0.94613	2.1
11423	1.31453	2.1

11424	0.946131	0.159476
11425	0.946129	0.106711
11426	0.94613	0.160908
11427	0.94613	0.0954316
11428	0.94613	0.104904
11429	1.12018	0.104562
11430	0.94613	0.161607
11431	0.946131	0.266392
11432	0.946129	0.172519
11433	0.946131	0.266936
11434	1.16373	0.152592
11435	0.94613	0.158224
11436	0.94613	0.241739
11437	0.946131	0.297393
11438	1.36811	0.486897
11439	0.946132	0.522178
11440	1.36811	0.461278
11441	0.946129	0.507318
11442	0.94613	0.525783
11443	0.946129	0.51014
11444	0.946129	0.498249
11445	0.946132	0.422459
11446	0.946133	0.319374
11447	0.946132	0.403221
11448	0.946133	0.429859
11449	1.35732	0.339424
11450	1.35732	0.367709
11451	0.946133	0.41405
11452	1.36399	0.582035
11453	0.946129	0.765803
11454	0.94613	0.614889
11455	0.946131	0.807001
11456	0.94613	1.28927
11457	0.946131	0.721185
11458	0.946131	0.874031
11459	0.946133	2.1
11460	0.946132	2.1
11461	0.946132	2.1
11462	1.38302	2.1
11463	0.946133	2.1
11464	0.946132	2.1
11465	0.946133	2.1
11466	0.810943	2.1
11467	0.810944	2.1
11468	0.810945	2.1
11469	2.80542	2.1
11470	1.35169	2.1
11471	1.08132	2.1
11472	1.08132	2.1
11473	1.08132	2.1
11474	1.30189	2.1
11475	0.946131	2.1
11476	0.946132	2.1
11477	0.946131	2.1
11478	0.946131	2.1
11479	0.946131	2.1
11480	0.946132	2.1
11481	0.810946	0.781108

11482	0.810947	0.644627
11483	2.33293	0.698238
11484	1.24357	0.678953
11485	1.08132	0.699524
11486	0.973183	0.74123
11487	1.00023	0.72237
11488	0.810934	0.726655
11489	1.06584	0.642265
11490	0.946129	0.699488
11491	0.84731	0.631606
11492	1.16646	0.684071
11493	0.946126	0.742335
11494	1.2831	0.648936
11495	0.851527	0.684759
11496	0.946131	2.1
11497	0.946128	2.1
11498	0.946128	2.1
11499	0.94613	2.1
11500	1.31961	2.1
11501	0.94613	2.1
11502	0.94613	2.1
11503	0.946131	2.1
11504	1.3605	2.1
11505	0.946133	2.1
11506	0.946132	2.1
11507	0.94613	2.1
11508	0.946131	2.1
11509	0.946131	2.1
11510	0.946128	1.84375
11511	0.946133	1.2904
11512	0.946132	2.1
11513	0.946131	1.07567
11514	0.946131	0.697434
11515	0.946131	1.21797
11516	1.30116	1.62964
11517	0.946132	0.0909863
11518	0.946129	0.120052
11519	0.94613	0.0829709
11520	1.3035	0.0888999
11521	0.946131	0.0675912
11522	0.94613	0.0895531
11523	0.946131	0.156046
11524	0.946132	2.1
11525	1.2051	2.1
11526	1.2051	2.1
11527	0.946132	2.1
11528	1.37869	2.1
11529	0.946132	2.1
11530	0.946131	2.1
11531	0.810946	2.1
11532	0.810945	2.1
11533	2.69247	2.1
11534	1.35169	2.1
11535	1.08132	2.1
11536	1.08132	2.1
11537	1.08132	2.1
11538	0.810946	2.1
11539	1.22993	2.1

11540	0.946131	2.1
11541	0.946131	2.1
11542	1.34623	2.1
11543	0.946131	2.1
11544	1.34623	2.1
11545	0.946131	2.1
11546	0.946131	0.0717062
11547	0.946132	0.098341
11548	0.946132	0.0759681
11549	0.946132	0.0658935
11550	0.946131	0.067243
11551	0.946132	0.087422
11552	1.32299	0.0648149
11553	0.94613	0.355498
11554	0.946131	0.379883
11555	0.946128	0.36102
11556	0.946132	0.26371
11557	0.946133	0.322121
11558	0.946131	0.374704
11559	0.946131	0.337052
11560	0.946132	2.1
11561	0.946129	2.1
11562	0.946128	2.1
11563	0.94613	2.1
11564	0.94613	2.1
11565	0.946131	2.1
11566	0.94613	2.1
11567	0.94613	2.1
11568	0.94613	2.1
11569	0.946131	2.1
11570	0.946132	2.1
11571	0.946129	2.1
11572	0.946131	2.1
11573	0.946131	2.1
11574	0.946132	1.28755
11575	0.946131	1.32031
11576	1.13731	1.09273
11577	0.94775	1.43356
11578	0.944514	1.69775
11579	0.946132	1.92551
11580	1.039	1.89209
11581	0.946131	1.21968
11582	0.946128	1.10478
11583	0.94613	0.933411
11584	0.946131	1.18369
11585	0.946128	1.36332
11586	0.946129	1.64851
11587	1.23726	1.57854
11588	0.946132	2.1
11589	0.946132	1.85899
11590	0.946129	2.1
11591	1.39214	2.1
11592	0.946131	1.94351
11593	0.946131	2.1
11594	0.94613	2.1
11595	0.946125	0.380284
11596	0.946119	0.24911
11597	0.946118	0.154989

11598	0.946118	0.248278
11599	0.94612	0.405851
11600	0.946106	0.175896
11601	0.946131	0.360586
11602	1.26893	0.609694
11603	0.946131	0.589802
11604	1.26893	0.689216
11605	0.94613	0.656135
11606	0.946131	0.723793
11607	0.946131	0.573334
11608	0.946131	0.83268
11609	0.946133	0.05
11610	0.946133	0.05
11611	0.946132	0.0606926
11612	1.28924	0.05
11613	0.946133	0.058279
11614	0.946132	0.0553243
11615	0.946132	0.050597
11616	0.946133	0.533667
11617	0.946132	0.545499
11618	0.946132	0.526077
11619	0.946133	0.499092
11620	0.946133	0.516303
11621	0.946132	0.53073
11622	0.946133	0.522016
11623	0.946132	0.67611
11624	0.946133	0.553283
11625	0.946133	0.661013
11626	0.946132	0.68191
11627	1.15388	0.629016
11628	0.946132	0.674393
11629	0.946133	0.652647
11630	0.946132	0.511943
11631	0.946133	0.497439
11632	0.946132	0.506143
11633	0.946133	0.46556
11634	0.946133	0.480276
11635	0.946132	0.498243
11636	0.946132	0.482303
11637	1.0644	0.994307
11638	1.12486	1.63382
11639	1.08674	1.81399
11640	0.946132	1.09885
11641	0.946132	1.7754
11642	0.946133	1.29703
11643	0.946132	1.21618
11644	0.808978	1.0244
11645	0.911537	1.48699
11646	0.932832	1.24049
11647	1.14604	1.7919
11648	1.56953	1.37386
11649	1.25866	1.13142
11650	1.18488	1.61074
11651	1.341	1.04927
11652	0.946131	0.351352
11653	0.946131	0.236092
11654	0.946131	0.339316
11655	0.94613	0.420124

11656	1.33922	0.191569
11657	0.946131	0.409088
11658	0.946131	0.354148
11659	0.946132	0.0894442
11660	0.946133	0.0782335
11661	0.946132	0.0899695
11662	0.946132	0.0742714
11663	0.946132	0.102244
11664	0.946133	0.0834925
11665	0.946132	0.0837255
11666	0.946129	0.736513
11667	0.946133	2.1
11668	0.946133	0.874227
11669	0.946131	1.0489
11670	1.40496	2.01074
11671	0.946131	0.7704
11672	0.946131	2.1
11673	0.94613	0.829196
11674	0.946127	0.59471
11675	0.946127	0.743406
11676	0.94613	0.85055
11677	1.38913	0.66043
11678	0.946128	0.783214
11679	0.946128	0.700429
11680	1.98863	0.05
11681	2.59915	0.0571391
11682	1.40338	0.05
11683	0.946131	0.05
11684	1.22266	0.05
11685	1.40338	0.05
11686	0.946132	0.05
11687	0.946132	0.05
11688	1.22266	0.05
11689	0.94613	0.0590883
11690	0.94613	0.0671735
11691	0.946124	0.120113
11692	1.21428	0.0601103
11693	0.946126	0.119842
11694	0.946127	0.106674
11695	0.946127	0.0790635
11696	1.03411	0.0964267
11697	0.946131	0.207042
11698	1.03411	0.0960038
11699	0.946129	0.205628
11700	1.14258	0.112673
11701	0.952143	0.182003
11702	0.940118	0.127983
11703	0.810943	2.1
11704	0.810945	2.1
11705	0.810958	2.1
11706	2.82603	2.1
11707	1.35168	2.1
11708	1.08132	2.1
11709	1.08132	2.1
11710	1.0813	2.1
11711	0.946133	0.317381
11712	0.946132	0.313295
11713	0.946132	0.318733

11714	0.946132	0.293716
11715	0.946132	0.292169
11716	1.11539	0.297828
11717	0.946133	0.318399
11718	0.946133	2.1
11719	0.946132	2.1
11720	0.946133	2.1
11721	0.946133	2.1
11722	0.946132	2.1
11723	1.41195	2.1
11724	0.946133	2.1
11725	0.946131	0.05
11726	0.946131	0.051698
11727	0.946131	0.05
11728	0.946131	0.05
11729	0.946131	0.05
11730	0.946131	0.05
11731	1.15968	0.05
11732	0.946131	1.11699
11733	0.94613	1.25249
11734	0.94613	2.1
11735	1.34386	1.33677
11736	0.946132	1.91754
11737	0.94613	2.06927
11738	1.34386	1.70271
11739	0.946131	1.09588
11740	0.935202	1.04154
11741	0.946131	1.84933
11742	1.25054	1.18386
11743	0.850155	1.63888
11744	0.946131	1.60939
11745	0.935205	1.41716
11746	0.946131	0.455948
11747	1.40193	0.354435
11748	0.946132	0.369599
11749	0.946132	0.384747
11750	0.946129	0.467086
11751	1.40193	0.290445
11752	0.946131	0.41681
11753	0.808779	0.332345
11754	0.561937	0.19752
11755	1.40193	0.245013
11756	1.40193	0.175583
11757	0.561936	0.266718
11758	0.561937	0.168062
11759	1.40193	0.211361
11760	0.946132	0.05
11761	0.946132	0.05
11762	0.946132	0.0563753
11763	0.946132	0.05
11764	0.946133	0.0569851
11765	0.946132	0.05
11766	0.946133	0.05
11767	0.946127	0.473667
11768	0.946131	0.451811
11769	0.946131	0.409162
11770	0.946127	0.475102
11771	1.19583	0.411724

11772	1.19583	0.418024
11773	0.946129	0.447117
11774	0.589428	0.535487
11775	1.08737	0.446045
11776	1.10961	0.533745
11777	0.872901	0.557411
11778	0.541327	0.464232
11779	0.541312	0.460878
11780	0.85153	0.523448
11781	0.946132	1.08399
11782	0.946131	2.1
11783	0.946131	2.1
11784	0.946132	1.35259
11785	0.946132	2.1
11786	0.946132	1.94314
11787	0.946132	1.9407
11788	0.946132	1.00673
11789	1.2778	2.1
11790	0.946131	2.1
11791	0.946131	1.03146
11792	0.94613	2.01237
11793	0.94613	1.08703
11794	0.94613	1.4634
11795	0.946118	0.736162
11796	0.946112	0.824596
11797	0.946118	0.685659
11798	0.946119	0.684941
11799	0.946117	0.743934
11800	1.35631	0.603782
11801	0.946105	0.606915
11802	0.946133	0.741503
11803	0.946133	1.86745
11804	0.946132	0.773309
11805	0.946132	1.82038
11806	0.946133	0.44544
11807	0.946132	0.709163
11808	0.946132	0.672521
11809	0.94613	1.51834
11810	0.946131	0.605418
11811	0.946128	0.615466
11812	0.946128	0.505392
11813	0.94613	1.25563
11814	0.946129	0.773909
11815	0.946129	0.731644
11816	0.946132	1.09496
11817	1.15139	1.09247
11818	0.946132	0.99298
11819	0.946132	1.18234
11820	0.946132	1.31991
11821	0.946132	1.32965
11822	0.946132	1.13899
11823	0.946133	2.1
11824	1.36151	2.1
11825	0.946132	2.1
11826	0.946132	2.1
11827	0.946132	1.7244
11828	0.946132	2.1
11829	0.946133	2.1

11830	0.946127	2.1
11831	1.38397	2.1
11832	0.94613	2.1
11833	0.946131	2.1
11834	0.946128	2.1
11835	0.94613	2.1
11836	0.946129	2.1
11837	0.946131	0.365396
11838	0.946127	0.418546
11839	0.946127	0.415012
11840	0.946132	0.368022
11841	0.946131	0.420409
11842	0.946128	0.358053
11843	0.946129	0.371359
11844	1.0268	0.05
11845	1.23823	0.05
11846	1.2171	0.05
11847	0.883446	0.05
11848	1.23827	0.05
11849	1.32614	0.05
11850	0.94613	0.05
11851	0.946132	2.1
11852	0.946129	2.1
11853	0.946129	2.1
11854	0.94613	2.1
11855	1.36523	2.1
11856	0.946131	2.1
11857	0.94613	2.1
11858	0.946132	0.221526
11859	0.946131	0.151965
11860	0.946132	0.244556
11861	0.946131	0.129059
11862	0.946131	0.137875
11863	1.01653	0.14898
11864	0.946131	0.272349
11865	0.94612	2.1
11866	0.946121	1.57975
11867	0.946119	1.9786
11868	0.946119	1.9157
11869	0.946119	2.1
11870	0.946132	2.1
11871	0.946107	2.1
11872	0.810942	0.757863
11873	0.810944	0.611593
11874	2.60324	0.824973
11875	1.21651	0.776511
11876	1.0813	0.742443
11877	0.946153	0.613957
11878	0.979948	2.1
11879	0.709588	0.894141
11880	1.3492	0.238722
11881	0.946132	0.255503
11882	0.946131	0.186786
11883	0.94613	0.255208
11884	0.946129	0.338799
11885	0.946129	0.259905
11886	0.94613	0.287763
11887	0.946131	0.281917

11888	0.94613	0.394756
11889	0.946128	0.31065
11890	0.946133	0.225294
11891	0.946133	0.336163
11892	0.946131	0.3646
11893	0.946131	0.287466
11894	1.23351	0.05
11895	0.946131	0.0558619
11896	0.946131	0.05
11897	0.946132	0.05
11898	0.946132	0.05
11899	0.946131	0.05
11900	0.946132	0.05
11901	1.35657	0.05
11902	0.946132	0.0724669
11903	0.946131	0.0540002
11904	0.946132	0.05
11905	0.946132	0.05
11906	0.946131	0.0520739
11907	0.946131	0.05
11908	0.946132	0.565513
11909	0.946132	0.469166
11910	0.946132	0.590286
11911	0.946131	0.316645
11912	0.946131	0.486733
11913	0.946132	0.491796
11914	0.946132	0.45563
11915	0.946133	0.447306
11916	0.946132	0.364367
11917	0.946133	0.498028
11918	0.946132	0.24114
11919	0.946132	0.405945
11920	0.946132	0.421665
11921	0.946133	0.381703
11922	0.946131	0.963234
11923	1.4101	1.88266
11924	0.946131	2.1
11925	0.946131	0.733535
11926	0.946131	1.12496
11927	0.946131	0.712396
11928	0.946131	1.92366
11929	0.810944	1.1988
11930	0.810939	1.13458
11931	0.709586	0.875713
11932	2.71261	0.936165
11933	1.21651	1.02607
11934	0.946151	0.83801
11935	0.979943	1.04764
11936	1.0813	1.30411
11937	0.946132	2.1
11938	0.946131	2.1
11939	0.946132	2.1
11940	0.946131	2.1
11941	0.946131	2.1
11942	1.37733	2.1
11943	0.946131	2.1
11944	0.946133	2.1
11945	0.946132	0.962065

11946	0.946132	2.1
11947	0.946133	2.1
11948	0.946133	2.1
11949	0.946132	1.56981
11950	1.32582	2.1
11951	0.946132	2.1
11952	0.946132	2.1
11953	0.946132	2.08624
11954	0.946132	2.1
11955	0.946132	1.64305
11956	0.946132	2.1
11957	0.946131	2.1
11958	0.946133	1.77299
11959	0.946132	1.15127
11960	0.946132	1.57266
11961	0.946132	1.07557
11962	0.946132	1.04779
11963	0.946133	1.08228
11964	1.3469	1.11168
11965	0.946126	0.722208
11966	0.94613	2.1
11967	0.946131	1.0366
11968	0.946127	0.865244
11969	1.41206	2.1
11970	0.946128	1.80078
11971	0.946128	2.1
11972	0.946127	2.1
11973	0.94613	2.06401
11974	0.946131	0.866832
11975	0.94613	2.1
11976	0.946128	0.898367
11977	0.946129	1.76547
11978	0.946129	1.72741
11979	0.946131	0.157759
11980	0.94613	0.131939
11981	1.24448	0.144789
11982	0.946129	0.241692
11983	0.946131	0.209425
11984	0.94613	0.217234
11985	0.946131	0.174791
11986	0.946132	0.247299
11987	0.946133	0.223555
11988	1.11015	0.227519
11989	0.946133	0.440704
11990	0.946132	0.392482
11991	0.946132	0.407434
11992	0.946132	0.286226
11993	1.38775	2.1
11994	0.946131	2.1
11995	0.946131	2.1
11996	0.946132	2.1
11997	0.946132	2.1
11998	0.946131	2.1
11999	0.946131	2.1
12000	0.946129	1.10262
12001	0.946129	2.0258
12002	0.946133	0.78126
12003	0.946131	2.02103

12004	0.946131	0.571394
12005	0.946131	0.873279
12006	0.946131	0.798761
12007	0.946132	2.1
12008	0.946133	2.1
12009	0.946132	2.1
12010	1.40774	2.1
12011	0.946132	2.1
12012	0.946133	2.1
12013	0.946132	2.1
12014	0.610968	0.05
12015	0.688233	0.05
12016	0.715226	0.05
12017	0.792409	0.05
12018	1.13418	0.05
12019	0.956667	0.05
12020	0.757321	0.05
12021	0.968845	0.05
12022	0.595679	0.05
12023	0.946133	1.24244
12024	1.28917	1.43312
12025	0.946131	1.91311
12026	0.94613	0.893411
12027	0.946131	1.21133
12028	0.946131	1.06953
12029	0.946131	1.59646
12030	0.946132	0.39944
12031	0.946128	0.418622
12032	0.94613	0.523277
12033	1.40668	0.429475
12034	0.946131	0.484677
12035	0.946131	0.432155
12036	0.94613	0.442544
12037	0.810946	2.1
12038	0.810945	2.1
12039	2.78163	2.1
12040	1.3517	2.1
12041	1.08132	2.1
12042	1.08133	2.1
12043	1.08132	2.1
12044	0.810933	2.1
12045	0.946131	2.1
12046	1.39081	2.1
12047	0.94613	2.1
12048	1.30339	2.1
12049	1.30339	2.1
12050	1.39081	2.1
12051	0.946131	2.1
12052	0.946127	1.20363
12053	0.821631	1.66924
12054	0.946131	0.840569
12055	0.946131	0.970246
12056	0.946128	0.901257
12057	0.996109	0.784494
12058	0.827876	0.767027
12059	0.946131	2.1
12060	0.946129	2.1
12061	0.946129	2.1

12062	0.94613	2.1
12063	0.94613	2.1
12064	0.94613	2.1
12065	0.94613	2.1
12066	0.946132	0.546765
12067	1.41723	0.582429
12068	0.946132	0.507784
12069	0.946133	0.417839
12070	0.946132	0.722257
12071	0.946132	0.619369
12072	0.946133	0.615345
12073	0.946133	0.509083
12074	0.946132	0.403696
12075	0.946133	0.367221
12076	0.946133	0.440374
12077	0.946132	0.637985
12078	0.946132	0.553906
12079	1.34254	0.519697
12080	1.26501	0.905299
12081	0.946132	1.01789
12082	0.946133	1.03237
12083	0.946133	1.33118
12084	0.946132	1.19725
12085	0.946133	1.02567
12086	0.946132	1.54419
12087	0.946131	0.150458
12088	0.946131	0.165262
12089	0.946131	0.179408
12090	0.946131	0.0886
12091	0.946131	0.115368
12092	0.946131	0.154559
12093	0.946131	0.133881
12094	0.878843	0.079315
12095	0.82561	0.0870817
12096	0.586974	0.0824388
12097	1.05343	0.086515
12098	0.80631	0.0575602
12099	0.782663	0.0814088
12100	0.789405	0.0623511
12101	0.902491	0.0578914
12102	1.05344	0.0740976
12103	0.94613	0.0695525
12104	0.946131	0.0832157
12105	0.946131	0.130053
12106	0.94613	0.101583
12107	0.946131	0.0879824
12108	0.946131	0.0960767
12109	0.946131	0.0886022
12110	0.946128	2.1
12111	0.946133	2.1
12112	0.946133	0.802413
12113	0.94613	2.1
12114	0.946131	0.968673
12115	0.946131	1.12435
12116	0.946131	1.29275
12117	0.946131	0.470485
12118	0.946131	0.462052
12119	0.94613	0.450987

12120	0.946128	0.452968
12121	0.946133	0.469066
12122	1.2996	0.426134
12123	0.946131	0.446001
12124	0.946132	2.1
12125	0.946132	2.1
12126	0.946132	1.04935
12127	0.946132	2.1
12128	0.946131	1.90261
12129	0.946132	2.1
12130	0.946132	2.1
12131	1.99211	0.0737895
12132	2.55024	0.0618059
12133	0.946131	0.0792124
12134	0.946131	0.0705779
12135	0.946131	0.0808158
12136	0.946131	0.0762485
12137	0.946131	0.0974552
12138	0.946131	0.0856213
12139	1.35726	0.0870453
12140	0.946128	2.1
12141	0.946132	1.5008
12142	0.946131	1.8344
12143	0.94613	1.57599
12144	0.94613	1.35895
12145	0.94613	2.1
12146	1.39852	2.09374
12147	1.31666	0.0778421
12148	0.946131	0.144231
12149	0.946128	0.0908498
12150	0.94613	0.0686851
12151	0.946128	0.088713
12152	0.946128	0.0910241
12153	0.946128	0.0889284
12154	1.32275	0.120215
12155	0.946131	0.257217
12156	0.946129	0.13934
12157	0.946128	0.136366
12158	0.946129	0.158166
12159	0.946128	0.13689
12160	0.946129	0.145738
12161	0.946132	2.1
12162	0.946131	2.1
12163	0.946131	2.1
12164	0.946132	2.1
12165	0.94613	2.1
12166	0.946131	2.1
12167	1.41125	2.1
12168	0.946132	2.1
12169	0.946132	2.1
12170	0.946131	2.1
12171	0.946132	2.1
12172	0.946132	2.1
12173	0.946132	2.1
12174	1.31904	2.1
12175	1.02425	0.224547
12176	0.946132	0.22055
12177	0.94613	0.147371

12178	0.946131	0.349829
12179	1.1534	0.16178
12180	0.961161	0.140576
12181	0.9311	0.350403
12182	1.40092	1.03957
12183	0.946133	1.05945
12184	0.946132	1.10978
12185	0.946131	1.3402
12186	0.946131	0.991341
12187	0.94613	1.09692
12188	0.946131	1.44995
12189	0.946113	1.44633
12190	0.946119	0.548547
12191	0.946117	1.53069
12192	1.40015	0.899415
12193	0.946118	0.545702
12194	0.946131	0.697995
12195	0.946105	0.612003
12196	0.946132	0.425054
12197	0.946133	0.415837
12198	0.946132	0.577316
12199	1.03547	0.436341
12200	0.946133	0.409737
12201	0.946133	0.468478
12202	0.946132	0.419834
12203	0.946133	2.1
12204	1.36694	2.1
12205	0.946133	2.1
12206	0.946132	2.1
12207	0.946133	2.1
12208	0.946133	2.1
12209	0.946132	2.1
12210	0.946132	0.321369
12211	0.946133	0.326602
12212	0.946132	0.325121
12213	0.946133	0.324252
12214	0.946133	0.320495
12215	0.946132	0.33121
12216	0.946132	0.326138
12217	0.491552	0.127741
12218	0.913078	0.247958
12219	0.946132	0.370808
12220	0.491552	0.114592
12221	0.946132	0.262047
12222	0.49154	0.320937
12223	1.53168	0.248357
12224	1.53168	0.148779
12225	0.946127	2.1
12226	1.38752	2.1
12227	0.946129	2.1
12228	0.94613	2.1
12229	0.946128	2.1
12230	0.946129	2.1
12231	0.946129	2.1
12232	0.946132	2.1
12233	0.946132	2.1
12234	0.946132	2.1
12235	0.946132	2.1

12236	0.946132	2.1
12237	0.946131	2.1
12238	0.946132	2.1
12239	0.94613	0.464901
12240	0.94613	0.42846
12241	0.94613	0.430441
12242	0.946131	0.43199
12243	0.946129	0.465321
12244	1.16834	0.325026
12245	0.94613	0.430491
12246	0.946127	0.473643
12247	1.35594	0.421953
12248	0.946131	0.431699
12249	0.946128	0.483105
12250	0.946127	0.319513
12251	0.946129	0.460669
12252	0.94613	0.436887
12253	0.94613	0.619934
12254	0.946131	0.397209
12255	0.946128	0.421931
12256	0.946128	0.359478
12257	0.946131	0.599222
12258	0.94613	0.478038
12259	0.94613	0.482725
12260	0.946132	0.422227
12261	0.946132	0.391842
12262	0.946129	0.43989
12263	1.39751	0.383868
12264	0.94613	0.467942
12265	0.946131	0.379818
12266	0.946131	0.390582
12267	0.946132	0.280823
12268	0.700041	0.292248
12269	0.700013	0.362874
12270	1.7697	0.171411
12271	1.7697	0.178426
12272	1.21134	0.219578
12273	0.946131	0.22863
12274	1.25837	0.217986
12275	0.946133	2.1
12276	0.946132	1.47722
12277	0.946132	1.24592
12278	0.946132	1.62382
12279	0.946132	2.05919
12280	0.946133	2.0623
12281	1.40583	1.87208
12282	0.946131	1.02878
12283	0.946131	1.59049
12284	0.946131	1.05128
12285	0.946131	2.1
12286	0.946131	1.65122
12287	0.827865	1.43417
12288	0.946131	1.41367
12289	0.946132	0.0654706
12290	0.946129	0.0592641
12291	0.946129	0.0728677
12292	0.946131	0.0628358
12293	0.94613	0.0790835

12294	1.21087	0.0571624
12295	0.946131	0.0620383
12296	0.94613	0.683122
12297	0.946131	0.936836
12298	0.94613	0.624469
12299	0.946128	0.665352
12300	0.946132	0.638242
12301	1.17452	0.611873
12302	0.94613	0.600023
12303	0.946119	0.563878
12304	0.946114	0.62325
12305	0.946119	0.53219
12306	0.946119	0.557196
12307	0.946119	0.538467
12308	1.27376	0.528819
12309	0.946106	0.526252
12310	0.946133	2.1
12311	1.39621	2.1
12312	0.946132	2.1
12313	0.946132	2.1
12314	0.946133	2.1
12315	0.946132	2.1
12316	0.946132	2.1
12317	0.946132	2.1
12318	0.946132	2.1
12319	0.946131	2.1
12320	0.946131	2.1
12321	0.946132	2.1
12322	0.946132	2.1
12323	1.34352	2.1
12324	0.946132	2.041
12325	1.41272	2.1
12326	0.946131	2.1
12327	0.94613	1.553
12328	0.94613	2.05012
12329	0.94613	1.58203
12330	0.94613	2.1
12331	0.946132	2.1
12332	0.946131	1.48323
12333	0.946132	2.1
12334	0.946132	1.25682
12335	0.946132	0.924125
12336	0.946132	2.01589
12337	1.23536	2.01552
12338	0.946132	2.1
12339	0.946131	2.1
12340	0.946131	2.1
12341	0.946132	2.1
12342	0.946132	2.1
12343	1.41131	2.1
12344	0.946131	2.1
12345	1.41894	1.1095
12346	0.946132	0.523075
12347	0.946133	0.5736
12348	0.946132	0.647718
12349	0.946132	0.630552
12350	0.946133	0.63589
12351	0.946132	0.581088

12352	0.946128	0.402394
12353	0.946133	0.666178
12354	0.946131	0.601635
12355	0.946129	2.08823
12356	0.94613	0.567495
12357	1.4099	0.72238
12358	0.946131	0.582977
12359	0.810947	2.1
12360	0.810945	0.881821
12361	0.810933	2.00065
12362	2.62883	2.09247
12363	1.3517	1.83016
12364	1.08132	2.1
12365	1.08132	0.848808
12366	1.08133	1.45572
12367	0.946131	0.990862
12368	0.946126	2.1
12369	0.946127	0.88336
12370	0.94613	1.06058
12371	0.946128	1.72801
12372	0.946129	0.706763
12373	0.946129	0.981152
12374	1.21347	2.1
12375	0.946129	2.1
12376	0.94613	2.1
12377	1.21347	2.1
12378	1.33064	2.1
12379	0.94613	2.1
12380	1.33064	2.1
12381	0.94613	0.05
12382	0.946129	0.05
12383	0.94613	0.05
12384	0.946132	0.05
12385	0.946126	0.05
12386	0.946129	0.05
12387	0.94613	0.05
12388	0.946132	0.90768
12389	1.37938	1.08507
12390	0.946131	1.37382
12391	0.94613	0.797639
12392	0.946131	1.5767
12393	0.94613	0.906506
12394	0.94613	1.05673
12395	0.946133	0.0647321
12396	0.946133	0.0594065
12397	1.2968	0.0606719
12398	0.946132	0.0670457
12399	0.946133	0.0819224
12400	1.2968	0.0562536
12401	0.946133	0.0665803
12402	1.43643	0.0644211
12403	0.946129	0.731756
12404	0.946133	0.650346
12405	0.946131	0.745815
12406	0.94613	0.488256
12407	0.946131	0.657737
12408	0.946131	0.662859
12409	0.946131	0.635636

12410	0.872447	1.08433
12411	0.946132	0.974118
12412	0.872447	0.912673
12413	0.827867	1.03842
12414	0.946132	1.19017
12415	0.827867	1.29178
12416	0.872447	1.30616
12417	0.946117	0.899809
12418	1.27213	0.874368
12419	0.946124	0.776149
12420	0.946119	0.87767
12421	0.946113	1.31562
12422	0.946129	0.993339
12423	0.946104	0.931355
12424	0.94613	0.565627
12425	0.946131	0.625407
12426	0.94613	0.530385
12427	0.946129	0.62009
12428	0.946132	0.185281
12429	0.946131	0.520183
12430	0.94613	0.344227
12431	0.810942	2.1
12432	0.810943	2.1
12433	2.80212	2.1
12434	1.3517	2.1
12435	1.08133	2.1
12436	1.08132	2.1
12437	1.08132	2.1
12438	0.810932	2.1
12439	1.29521	2.1
12440	0.946129	2.1
12441	0.946131	2.1
12442	1.40106	2.1
12443	0.94613	2.1
12444	1.40106	2.1
12445	0.946131	2.1
12446	0.946132	0.523157
12447	1.24596	0.45661
12448	0.94613	0.476179
12449	0.946131	0.509257
12450	0.946131	0.422449
12451	0.946131	0.509379
12452	0.946131	0.486433
12453	0.946131	2.1
12454	0.946131	2.1
12455	0.946131	2.1
12456	0.946128	2.1
12457	0.946133	2.1
12458	0.94613	2.1
12459	0.946131	2.1
12460	0.879548	0.727427
12461	1.21527	0.717981
12462	0.967506	0.781262
12463	0.946132	0.997716
12464	0.946133	0.618674
12465	0.967511	1.22446
12466	0.946132	1.0131
12467	0.946132	1.02828

12468	0.946132	0.764221
12469	0.974253	0.865042
12470	0.974253	1.3735
12471	0.91801	0.735843
12472	0.918009	0.812124
12473	0.946132	1.04097
12474	0.946132	0.118235
12475	0.946131	0.0959622
12476	0.946131	0.130979
12477	0.946133	0.081013
12478	0.946132	0.0872237
12479	1.28644	0.0932422
12480	0.946132	0.123795
12481	0.946131	0.131324
12482	0.946131	0.101752
12483	0.946131	0.144206
12484	0.946131	0.0841197
12485	0.946131	0.0921602
12486	1.40103	0.094575
12487	0.946131	0.133146
12488	0.946128	0.351917
12489	0.946132	0.246936
12490	0.946129	0.407628
12491	1.03327	0.205513
12492	0.946129	0.225627
12493	0.946129	0.341785
12494	0.94613	0.407948
12495	0.946134	0.39601
12496	0.946133	0.451491
12497	0.946132	0.258257
12498	0.946133	0.43853
12499	0.946132	0.19782
12500	0.946132	0.265097
12501	0.946133	0.104564
12502	0.856654	0.051632
12503	0.946131	0.0666753
12504	0.946131	0.05
12505	0.946132	0.053007
12506	0.856616	0.0573349
12507	1.03565	0.05
12508	1.03565	0.0602538
12509	1.15844	0.488821
12510	0.946132	0.518761
12511	0.946132	0.428722
12512	0.946132	0.48154
12513	0.946132	0.432223
12514	0.946132	0.481233
12515	0.946131	0.474712
12516	1.4582	2.1
12517	1.35067	2.1
12518	0.946131	2.1
12519	1.04106	2.1
12520	0.938748	2.1
12521	0.943015	2.1
12522	1.05017	2.1
12523	0.938793	2.1
12524	0.946131	0.05
12525	0.946132	0.0512231

12526	0.946131	0.0807672
12527	0.94613	0.0636779
12528	0.946133	0.0556025
12529	0.946131	0.0606458
12530	0.946131	0.0588437
12531	0.946132	0.514676
12532	0.946133	0.556102
12533	0.946132	0.509401
12534	0.946133	0.67945
12535	0.946133	0.651898
12536	0.946132	0.59504
12537	0.946133	0.660442
12538	0.946128	0.785028
12539	0.946132	0.965013
12540	0.946132	0.799954
12541	0.94613	0.820686
12542	1.23426	0.884443
12543	0.94613	0.863715
12544	0.946131	0.887932
12545	0.810943	0.46352
12546	0.810946	0.539931
12547	2.60388	0.53654
12548	1.3517	0.486596
12549	1.08133	0.454853
12550	1.08132	0.550383
12551	1.08132	0.465225
12552	0.810933	0.396903
12553	0.810945	0.593108
12554	0.810945	0.316156
12555	2.72377	0.920446
12556	1.3517	0.757207
12557	1.08133	0.580429
12558	1.08132	0.577446
12559	1.08132	1.88532
12560	0.810932	0.777092
12561	1.25104	0.260177
12562	0.946131	0.642927
12563	0.946131	0.452606
12564	1.36188	2.02794
12565	0.946131	0.705401
12566	1.36188	0.838896
12567	0.946132	0.851458
12568	0.946132	1.97411
12569	0.946132	1.99401
12570	0.946132	1.84368
12571	0.946132	2.01438
12572	0.946132	1.95775
12573	0.946132	2.1
12574	1.25488	2.1
12575	1.32132	0.0588655
12576	0.946126	0.0755748
12577	0.946127	0.05
12578	0.946128	0.066023
12579	0.946128	0.0706196
12580	0.946128	0.0664684
12581	0.946128	0.0662746
12582	0.946133	2.1
12583	1.41123	1.16741

12584	0.946134	1.33905
12585	0.946133	2.1
12586	0.946132	0.824004
12587	0.946133	1.41611
12588	0.946132	1.20122
12589	1.32163	0.219252
12590	0.946133	0.228593
12591	0.946133	0.210482
12592	0.946132	0.213333
12593	0.946132	0.136916
12594	0.946132	0.193146
12595	0.946133	0.22195
12596	0.946133	2.07579
12597	0.946133	2.1
12598	0.946132	1.96334
12599	0.946132	2.1
12600	0.946132	0.707543
12601	0.946133	1.93163
12602	0.946132	1.88673
12603	0.94613	0.362648
12604	0.946131	0.356858
12605	0.946128	0.362722
12606	0.946133	0.342535
12607	0.946133	0.344689
12608	0.946131	0.356819
12609	0.946131	0.34832
12610	0.946133	0.589812
12611	0.946132	0.478749
12612	0.946132	0.600951
12613	0.946134	0.504882
12614	0.946133	0.702432
12615	0.946133	0.548051
12616	0.946132	0.551302
12617	0.94613	1.64116
12618	0.946128	2.07768
12619	0.946127	1.27439
12620	0.94613	1.13652
12621	0.946131	1.55162
12622	0.946128	1.48812
12623	1.21099	1.85092
12624	0.946132	0.745305
12625	0.946131	0.702286
12626	0.946131	0.793066
12627	0.946133	0.417957
12628	0.946133	0.57915
12629	0.946131	0.705669
12630	0.946132	0.625856
12631	1.34794	0.742891
12632	0.517994	0.755971
12633	0.517965	0.64494
12634	0.946132	0.843405
12635	0.9198	1.39918
12636	0.946132	0.98914
12637	0.9198	0.961
12638	0.946132	0.926408
12639	0.94613	0.954084
12640	0.946131	0.614207
12641	0.946131	0.948676

12642	0.946131	1.26729
12643	0.946131	0.963528
12644	1.23224	0.796374
12645	0.946132	1.58732
12646	0.946132	1.8222
12647	0.946133	1.34063
12648	0.946132	0.88292
12649	0.946132	1.12799
12650	0.946133	1.31386
12651	1.37511	1.41975
12652	0.946133	0.709951
12653	0.946129	0.746063
12654	0.851104	0.991545
12655	0.946131	0.853104
12656	1.0412	0.943968
12657	1.0412	0.939666
12658	0.851066	0.861883
12659	1.31357	0.133129
12660	0.946131	0.309017
12661	0.94613	0.178557
12662	0.946131	0.260273
12663	0.94613	0.227649
12664	0.946131	0.200672
12665	0.946131	0.206722
12666	0.946133	0.831492
12667	1.38149	0.977869
12668	0.946132	1.04176
12669	0.946132	0.779392
12670	0.946133	1.62843
12671	0.946132	0.886893
12672	0.946133	0.928152
12673	0.810944	1.08262
12674	0.810945	2.1
12675	0.810958	2.08165
12676	2.65782	2.1
12677	1.35168	1.94093
12678	1.08132	1.06983
12679	1.08132	2.1
12680	1.0813	1.04817
12681	0.946113	0.05
12682	0.946119	0.05
12683	0.946118	0.0533485
12684	0.946118	0.05
12685	0.946119	0.05
12686	0.946131	0.05
12687	0.946106	0.05
12688	0.946132	0.05
12689	0.946132	0.0650102
12690	0.946128	0.0641046
12691	0.946131	0.0632599
12692	0.94613	0.0670704
12693	0.94613	0.0612677
12694	0.94613	0.0763434
12695	1.00099	0.0741949
12696	0.946132	0.122931
12697	1.17876	0.0666314
12698	0.982282	0.0839573
12699	0.909981	0.119298

12700	0.946132	0.110603
12701	0.946131	0.091489
12702	0.946132	0.979965
12703	1.35328	1.22592
12704	0.946132	1.77304
12705	0.946132	0.792467
12706	0.946133	1.51066
12707	0.946132	0.892785
12708	0.946133	1.3991
12709	0.946133	0.05
12710	0.946133	0.0530136
12711	0.946132	0.05
12712	0.946132	0.0536829
12713	0.946132	0.05
12714	0.946132	0.0514738
12715	0.946133	0.0523519
12716	0.900195	0.05
12717	0.946132	0.05
12718	0.900167	0.05
12719	1.41963	0.05
12720	0.900195	0.05
12721	0.946133	0.05
12722	1.35385	0.05
12723	0.946132	0.94586
12724	0.946129	1.47818
12725	0.946129	1.18888
12726	0.946131	0.906277
12727	0.946131	1.24782
12728	1.41585	0.970275
12729	0.94613	1.93231
12730	0.810946	2.1
12731	0.810946	1.66776
12732	2.68895	2.1
12733	1.3517	2.1
12734	1.08133	2.1
12735	1.08132	2.01059
12736	1.08132	2.1
12737	0.810933	2.1
12738	1.19886	1.78382
12739	0.946131	2.1
12740	0.946131	2.06166
12741	1.34447	2.1
12742	0.946131	2.1
12743	1.34447	2.1
12744	0.946131	2.1
12745	0.946133	0.0523349
12746	0.946133	0.0574697
12747	0.946132	0.0925531
12748	1.35065	0.0530423
12749	0.946132	0.0978767
12750	0.946133	0.0858569
12751	0.946132	0.0639783
12752	0.946131	0.674109
12753	0.946131	0.661117
12754	0.94613	0.71654
12755	0.946131	0.37992
12756	0.946131	0.495431
12757	0.946131	0.631675

12758	0.946131	0.55944
12759	0.946127	0.373749
12760	0.946131	0.376028
12761	0.946127	0.371036
12762	0.94613	0.322006
12763	0.946131	0.355154
12764	0.946128	0.372585
12765	0.946129	0.358127
12766	0.810946	0.05
12767	0.810947	0.05
12768	0.810959	0.05
12769	2.4391	0.05
12770	1.35168	0.05
12771	1.08132	0.05
12772	1.08132	0.05
12773	1.08131	0.05
12774	0.946132	0.05
12775	0.946132	0.05
12776	0.946132	0.05
12777	1.40837	0.05
12778	0.946132	0.05
12779	0.946132	0.05
12780	1.40837	0.05
12781	0.946127	1.88465
12782	0.946132	2.1
12783	0.946131	0.741806
12784	0.946127	2.0948
12785	0.946131	1.30235
12786	0.946129	0.962502
12787	0.94613	1.37873
12788	0.946133	1.72771
12789	0.946132	1.32701
12790	0.946132	1.28754
12791	0.946132	1.03508
12792	0.946132	1.44175
12793	0.946132	0.85708
12794	0.946133	2.1
12795	0.946132	0.795681
12796	1.24344	0.707157
12797	0.946132	0.628059
12798	0.946132	1.01571
12799	0.946132	0.625433
12800	1.24344	0.841908
12801	0.946132	0.987353
12802	0.946131	0.424756
12803	0.946128	0.411793
12804	0.946128	0.400166
12805	0.946131	0.422578
12806	1.41232	0.392946
12807	0.946129	0.420786
12808	0.94613	0.399495
12809	0.946128	2.1
12810	0.946133	1.95358
12811	0.946133	0.831537
12812	0.946131	2.1
12813	0.946131	0.910113
12814	0.946131	1.44493
12815	0.94613	1.22553

12816	0.946133	0.571715
12817	1.41556	0.551848
12818	0.946133	0.45862
12819	0.946133	0.469619
12820	0.946132	0.677966
12821	0.946132	0.59137
12822	0.946132	0.620841
12823	0.94612	0.541338
12824	1.41843	0.497263
12825	0.946114	0.499856
12826	0.94612	0.462545
12827	0.946119	0.623973
12828	1.41845	0.493614
12829	0.946107	0.59285
12830	0.946129	0.0670057
12831	0.946129	0.060306
12832	0.94613	0.0831493
12833	0.946132	0.0674005
12834	0.946126	0.0893073
12835	1.1509	0.057557
12836	0.94613	0.0654305
12837	0.946131	0.0505691
12838	0.946132	0.05
12839	0.946132	0.05812
12840	0.946132	0.0506099
12841	0.946132	0.0607985
12842	1.19263	0.05
12843	0.946132	0.0517611
12844	0.94613	0.554632
12845	0.946131	0.63077
12846	0.946128	0.547947
12847	0.946133	0.690647
12848	0.946133	0.642084
12849	0.946131	0.579999
12850	0.946131	0.645994
12851	0.946133	0.996765
12852	1.22848	1.31064
12853	1.00336	1.33591
12854	0.827866	1.03783
12855	0.946132	1.73885
12856	0.827865	1.29663
12857	0.946133	1.10632
12858	0.946131	0.604748
12859	0.94613	0.405903
12860	0.946127	0.597245
12861	1.41498	0.543278
12862	0.94613	0.415
12863	0.946128	0.501915
12864	0.946128	0.453235
12865	0.94613	0.05
12866	0.94613	0.05
12867	0.94613	0.05
12868	1.37358	0.05
12869	0.946129	0.05
12870	0.94613	0.05
12871	1.37357	0.05
12872	0.946111	0.05
12873	0.946118	0.05

12874	0.946118	0.05
12875	1.36568	0.05
12876	0.946116	0.05
12877	0.94613	0.05
12878	0.946105	0.05
12879	0.946132	0.478677
12880	0.946131	0.396149
12881	0.94613	0.524185
12882	1.23175	0.349625
12883	0.946131	0.343122
12884	0.946131	0.485189
12885	0.946131	0.505404
12886	0.946132	0.0561229
12887	0.946126	0.0541348
12888	0.946128	0.0590598
12889	0.946129	0.0562787
12890	0.946129	0.0651603
12891	0.946128	0.0588106
12892	1.25698	0.0592596
12893	0.810943	2.1
12894	0.810944	2.1
12895	2.81171	2.1
12896	1.3517	2.1
12897	1.08132	2.1
12898	1.08133	2.1
12899	1.08132	2.1
12900	0.810932	2.1
12901	0.946132	0.426235
12902	0.946131	0.435007
12903	0.946131	0.483695
12904	0.946132	0.195416
12905	0.946132	0.288527
12906	0.946132	0.42269
12907	0.946131	0.360274
12908	0.810936	0.746556
12909	0.810935	1.8989
12910	2.80914	1.11419
12911	1.35167	0.974562
12912	1.0813	0.909292
12913	1.0813	1.81708
12914	1.0813	0.708558
12915	0.810934	0.907581
12916	1.23851	2.1
12917	0.946132	0.815058
12918	0.946131	2.1
12919	1.40459	0.752935
12920	0.946132	1.14436
12921	1.40459	1.04311
12922	0.946132	1.1098
12923	1.19974	0.14573
12924	0.946131	0.401389
12925	1.19974	0.149019
12926	0.946132	0.406376
12927	0.946132	0.206296
12928	0.946132	0.396595
12929	0.946132	0.202726
12930	0.939141	0.927478
12931	1.14375	1.25003

12932	1.03306	1.34889
12933	0.946132	1.00753
12934	0.946126	1.50412
12935	0.946129	1.14892
12936	0.94613	1.04618
12937	0.946132	0.337737
12938	0.946131	0.342079
12939	0.946132	0.342341
12940	0.946132	0.335005
12941	0.946132	0.331089
12942	0.946132	0.349081
12943	0.946132	0.338161
12944	0.946131	2.1
12945	0.94613	2.1
12946	0.94613	2.1
12947	0.946131	2.1
12948	0.946129	2.1
12949	0.94613	2.1
12950	1.24512	2.1
12951	0.946133	0.266493
12952	0.946129	0.291794
12953	0.946131	0.322057
12954	0.946132	0.26038
12955	0.946131	0.276675
12956	0.946131	0.315759
12957	0.946131	0.279566
12958	0.946132	0.29145
12959	0.946133	0.307201
12960	0.946132	0.299499
12961	0.946132	0.278779
12962	0.946133	0.300439
12963	0.946132	0.316631
12964	0.946132	0.301667
12965	0.946132	0.55774
12966	1.41392	0.491039
12967	0.946132	0.48881
12968	0.946132	0.604639
12969	0.946132	0.447184
12970	0.946132	0.564534
12971	0.946132	0.517958
12972	0.946132	0.692015
12973	1.24333	0.615917
12974	0.946132	0.575653
12975	0.946133	0.796126
12976	0.946132	0.516384
12977	0.946132	0.759245
12978	0.946133	0.612423
12979	1.30649	0.229047
12980	0.94613	0.256248
12981	0.94613	0.207759
12982	0.946128	0.226372
12983	0.946126	0.2881
12984	0.946127	0.266206
12985	0.946127	0.257023
12986	0.709578	0.385264
12987	0.810943	0.384014
12988	2.53303	0.181218
12989	1.21651	0.19614

12990	0.979951	0.228941
12991	1.08132	0.207668
12992	0.946141	0.396979
12993	0.81096	0.294857
12994	0.946131	0.235069
12995	0.946131	0.289524
12996	0.946131	0.260936
12997	0.946131	0.139573
12998	0.946131	0.182348
12999	0.946131	0.28312
13000	0.946131	0.231052
13001	0.946132	0.32578
13002	0.946129	0.32324
13003	0.946129	0.328147
13004	0.94613	0.327548
13005	1.20634	0.324877
13006	1.20634	0.321272
13007	0.946131	0.331706
13008	0.946133	0.606647
13009	0.946132	0.593678
13010	0.946132	0.549102
13011	0.946132	0.638194
13012	0.946132	0.52585
13013	1.30107	0.618519
13014	0.946133	0.632921
13015	0.810946	2.1
13016	0.810944	2.1
13017	2.73936	2.1
13018	1.35168	2.1
13019	1.0813	2.1
13020	1.08132	2.1
13021	1.08132	2.1
13022	0.810958	2.1
13023	0.946129	1.53786
13024	0.946129	2.04819
13025	0.946133	0.814291
13026	1.30812	1.6477
13027	0.946131	0.937501
13028	0.946131	1.21132
13029	0.946131	1.06407
13030	1.00901	0.995508
13031	0.94613	1.21646
13032	0.94613	0.711189
13033	1.17001	1.07467
13034	0.917259	0.682223
13035	0.94613	0.873088
13036	0.94613	0.842846
13037	0.946126	2.1
13038	0.946127	2.1
13039	0.946131	2.1
13040	0.946127	2.1
13041	0.946131	2.1
13042	0.946129	2.1
13043	1.398	2.1
13044	0.946133	2.1
13045	1.3033	2.1
13046	0.946132	2.1
13047	0.946132	2.1

13048	0.946132	2.1
13049	0.946132	2.1
13050	0.946133	2.1
13051	0.946131	0.578097
13052	0.946131	0.669707
13053	1.09897	0.485942
13054	0.946129	0.834191
13055	0.946131	0.740884
13056	0.94613	0.772239
13057	0.94613	0.603478
13058	0.946132	2.1
13059	0.946131	2.1
13060	0.946131	2.1
13061	0.946132	2.1
13062	0.94613	2.1
13063	0.946131	2.1
13064	1.39858	2.1
13065	1.07117	0.707652
13066	1.08878	0.895068
13067	0.946119	1.06678
13068	1.08878	1.16453
13069	0.946119	0.605782
13070	0.946132	0.931263
13071	1.07116	0.724044
13072	0.946132	0.142327
13073	0.946132	0.119743
13074	0.946132	0.165447
13075	0.946132	0.097236
13076	0.946132	0.105772
13077	1.10733	0.119673
13078	0.946132	0.148052
13079	0.946128	0.159334
13080	0.946132	0.12111
13081	0.94613	0.186908
13082	0.946129	0.101787
13083	0.94613	0.110857
13084	1.3367	0.120909
13085	0.94613	0.165108
13086	0.810943	2.1
13087	0.810944	2.1
13088	2.70234	2.1
13089	1.21653	2.1
13090	1.08132	2.1
13091	0.946155	2.1
13092	0.979951	2.1
13093	0.709578	2.1
13094	1.27174	2.1
13095	0.946129	2.1
13096	0.94613	2.1
13097	1.35117	2.1
13098	0.94613	2.1
13099	1.35117	2.1
13100	0.94613	2.1
13101	0.946133	0.944604
13102	0.946133	0.903194
13103	0.946132	1.44076
13104	0.946133	0.847434
13105	0.946132	2.1

13106	0.946133	1.35956
13107	0.997296	1.31568
13108	0.810943	0.759025
13109	0.810944	1.42985
13110	0.810945	2.1
13111	2.78833	2.1
13112	1.35169	2.1
13113	1.08132	0.948708
13114	1.08132	2.1
13115	1.08132	2.1
13116	0.810946	2.1
13117	0.810943	0.93643
13118	0.810933	1.52175
13119	2.70049	2.0905
13120	1.3517	1.10681
13121	1.08132	2.03117
13122	1.08132	0.680241
13123	1.08133	0.948909
13124	0.946127	1.0314
13125	1.35469	0.995809
13126	0.94613	0.971248
13127	0.946131	1.05039
13128	0.946128	0.89794
13129	1.35469	1.10818
13130	0.946129	1.15199
13131	0.946131	0.422321
13132	1.2722	0.345339
13133	0.946132	0.337872
13134	0.946132	0.362944
13135	0.946129	0.447785
13136	1.2722	0.272032
13137	0.94613	0.37132
13138	0.946133	0.358674
13139	0.946132	0.358124
13140	0.946132	0.373866
13141	0.946132	0.366034
13142	1.41461	0.362612
13143	1.41461	0.357835
13144	0.946133	0.38001
13145	0.946131	0.814194
13146	1.30165	0.653098
13147	0.946132	0.591467
13148	0.946132	0.93796
13149	0.946129	0.652958
13150	1.30165	0.870045
13151	0.946131	0.967611
13152	0.946132	0.783529
13153	0.946129	1.07404
13154	0.94613	0.764593
13155	1.39356	1.43655
13156	0.946131	0.873005
13157	0.946131	0.854858
13158	0.94613	1.15984
13159	0.810946	1.58763
13160	0.810947	2.05479
13161	0.810934	2.1
13162	2.35003	2.1
13163	1.3517	2.1

13164	1.08132	2.1
13165	1.08132	2.1
13166	1.08133	2.1
13167	0.946131	0.055064
13168	0.946131	0.059316
13169	0.946125	0.0838048
13170	0.946127	0.0681444
13171	0.946129	0.0706103
13172	0.946128	0.0683291
13173	0.946128	0.0682208
13174	0.946131	0.058585
13175	0.946131	0.0655898
13176	0.946131	0.0952737
13177	0.946131	0.0759531
13178	0.946132	0.0677105
13179	0.946132	0.0744891
13180	0.946131	0.0687891
13181	0.946129	2.1
13182	0.946132	2.1
13183	0.946132	2.1
13184	0.94613	2.1
13185	1.30082	2.1
13186	0.94613	2.1
13187	0.946131	2.1
13188	1.20416	0.0997954
13189	0.946132	0.21884
13190	0.946131	0.133928
13191	0.946132	0.130908
13192	0.946131	0.127584
13193	0.946131	0.138258
13194	0.946132	0.109973
13195	1.18711	0.0846141
13196	0.946131	0.195106
13197	0.946131	0.126283
13198	0.946132	0.108124
13199	0.946131	0.116111
13200	0.946131	0.134995
13201	0.946132	0.120277
13202	0.946133	0.05
13203	0.946133	0.05
13204	0.946132	0.05
13205	1.30212	0.05
13206	0.946133	0.052717
13207	0.946132	0.05
13208	1.30212	0.05
13209	0.946131	0.31427
13210	0.946131	0.273754
13211	0.946131	0.319831
13212	0.946132	0.317811
13213	1.31467	0.297266
13214	1.31467	0.283739
13215	0.946131	0.324922
13216	0.946133	0.198749
13217	0.946132	0.158208
13218	0.946132	0.228654
13219	0.946132	0.161564
13220	1.25124	0.181314
13221	1.25124	0.159939

13222	0.946132	0.264544
13223	0.946132	2.1
13224	0.946132	2.1
13225	0.946132	2.1
13226	1.36422	2.1
13227	0.946132	2.1
13228	0.946131	2.1
13229	0.946132	2.1
13230	0.946131	1.23045
13231	1.41851	1.83909
13232	0.946132	2.1
13233	0.946132	0.992777
13234	0.946132	1.54063
13235	0.946132	0.998337
13236	0.946132	1.62903
13237	0.946133	0.203308
13238	0.946132	0.198955
13239	0.946132	0.190077
13240	0.946132	0.190319
13241	0.946132	0.200379
13242	1.37794	0.145931
13243	0.946132	0.202161
13244	0.946132	2.1
13245	0.946132	2.1
13246	0.946131	2.1
13247	0.946131	2.1
13248	0.946132	2.1
13249	0.946132	2.1
13250	1.4045	2.1
13251	0.946133	2.1
13252	0.946132	2.1
13253	0.946131	2.1
13254	0.946132	2.1
13255	0.946131	2.1
13256	0.946131	2.1
13257	0.946132	2.1
13258	0.946132	2.1
13259	0.946132	2.1
13260	0.946131	2.1
13261	0.946132	2.1
13262	0.946131	2.1
13263	0.946131	2.1
13264	0.946131	2.1
13265	0.946128	2.1
13266	1.31641	2.1
13267	0.946129	2.1
13268	0.94613	2.1
13269	0.94613	2.1
13270	0.94613	2.1
13271	0.94613	2.1
13272	0.946132	2.1
13273	0.946131	2.1
13274	0.946131	0.958426
13275	0.946132	2.1
13276	0.946132	1.18914
13277	0.946132	1.93986
13278	0.946132	2.1
13279	0.946132	2.1

13280	1.41507	2.1
13281	0.946132	2.1
13282	0.946132	2.1
13283	0.946133	2.1
13284	0.946133	2.1
13285	0.946132	2.1
13286	0.946132	2.1
13287	0.946131	2.1
13288	0.946131	2.1
13289	0.946133	2.1
13290	0.946132	2.1
13291	1.32608	2.1
13292	0.946132	2.1
13293	0.94613	1.88791
13294	0.94613	2.1
13295	0.94613	0.776705
13296	0.946131	2.0223
13297	0.946129	1.35783
13298	0.94613	0.865522
13299	0.94613	1.1627
13300	1.33728	0.721811
13301	0.946131	0.627649
13302	0.94613	0.504087
13303	0.946132	0.688655
13304	0.946132	0.891881
13305	0.946131	0.575019
13306	0.946131	0.602469
13307	0.946127	2.1
13308	1.40641	1.65787
13309	0.946129	2.03381
13310	0.94613	2.1
13311	0.946128	1.03947
13312	0.946129	1.6629
13313	0.946129	1.57664
13314	0.946129	2.1
13315	0.946128	2.1
13316	0.946133	2.1
13317	1.37467	2.1
13318	0.946131	2.1
13319	0.946131	2.1
13320	1.37467	2.1
13321	0.946131	2.1
13322	0.946131	2.1
13323	0.946132	2.1
13324	0.946132	2.1
13325	0.946129	2.1
13326	0.946131	2.1
13327	1.34912	2.1
13328	0.946132	2.1
13329	0.946133	2.1
13330	0.946133	2.1
13331	0.946132	2.1
13332	0.946132	2.1
13333	0.946133	2.1
13334	0.946132	2.1
13335	0.946127	2.1
13336	0.946131	1.22969
13337	0.946132	2.1

13338	0.946131	1.13271
13339	0.946127	0.967593
13340	0.94613	2.1
13341	1.33607	2.1
13342	0.946117	0.549751
13343	0.946117	0.723553
13344	0.946115	1.10014
13345	0.946124	2.05575
13346	0.946119	0.566581
13347	1.32662	0.793778
13348	0.94613	0.581473
13349	0.946119	2.1
13350	0.946114	2.1
13351	0.946118	2.1
13352	0.946114	2.1
13353	0.946118	2.1
13354	0.946104	2.1
13355	0.946129	2.1
13356	0.946132	1.2127
13357	0.946133	2.1
13358	0.946133	2.1
13359	0.946133	1.9268
13360	0.946132	2.1
13361	0.946132	2.1
13362	0.946133	2.1
13363	0.946133	2.02424
13364	0.946132	2.1
13365	0.946132	2.1
13366	0.946133	2.1
13367	0.946132	2.1
13368	0.946132	2.1
13369	0.946132	2.1
13370	0.946133	1.73202
13371	1.30881	2.1
13372	0.946132	2.1
13373	0.946131	1.14248
13374	0.946131	1.30963
13375	0.946131	0.99009
13376	0.946131	1.86715
13377	0.946128	2.1
13378	0.946128	2.1
13379	0.946132	2.1
13380	0.94613	2.1
13381	0.94613	2.1
13382	0.94613	2.1
13383	1.34652	2.1
13384	0.946133	0.861811
13385	0.946133	0.784939
13386	0.946132	1.49167
13387	0.946133	0.786835
13388	0.946132	1.97741
13389	0.946132	0.981378
13390	0.946132	1.18749
13391	0.946132	0.368994
13392	0.946128	0.344583
13393	0.94613	0.369922
13394	0.946131	0.344644
13395	0.94613	0.402386

13396	0.94613	0.350869
13397	0.94613	0.35694
13398	0.946128	0.51356
13399	0.946132	0.53545
13400	0.94613	0.512892
13401	0.94613	0.418386
13402	0.946131	0.532318
13403	0.94613	0.535222
13404	0.94613	0.485619
13405	0.946129	0.908121
13406	1.32056	1.35301
13407	0.946131	1.93775
13408	0.94613	0.858327
13409	0.946124	1.51968
13410	0.946128	0.923314
13411	0.946127	1.11164
13412	0.946129	0.909935
13413	1.20351	1.27384
13414	0.946132	1.67529
13415	0.946132	0.934312
13416	0.946126	1.63768
13417	0.946129	1.05765
13418	0.946129	1.04586
13419	0.946127	1.06965
13420	0.946127	1.53256
13421	0.946131	0.810359
13422	1.25592	1.14249
13423	0.946127	0.85711
13424	0.946129	0.959456
13425	0.94613	1.02327
13426	1.02332	1.75862
13427	0.946133	1.01208
13428	0.946132	1.10695
13429	1.02332	1.15277
13430	0.946132	0.806291
13431	0.96196	1.30515
13432	0.930306	0.900253
13433	0.946133	1.67319
13434	0.946133	1.83551
13435	0.946132	2.1
13436	1.20057	1.91702
13437	0.946132	2.1
13438	0.946133	2.1
13439	1.20057	2.1
13440	0.946128	1.60807
13441	0.946129	0.779849
13442	0.946129	1.26984
13443	1.13245	0.785607
13444	0.946126	0.659847
13445	0.946128	0.974594
13446	0.946129	0.776304
13447	0.946131	1.26737
13448	0.946131	1.67452
13449	0.94613	2.1
13450	1.25	1.56143
13451	0.946129	2.1
13452	0.946131	2.1
13453	1.25	2.1

13454	0.540425	1.67703
13455	0.748493	1.75274
13456	1.46266	2.1
13457	1.20046	2.02919
13458	0.600454	1.75811
13459	1.41283	2.08593
13460	0.582567	1.73468
13461	0.946131	1.43098
13462	0.946131	1.21833
13463	0.946132	1.23499
13464	0.946132	1.26246
13465	0.94613	1.15788
13466	0.946131	2.03083
13467	1.28439	1.91857
13468	0.946132	0.636216
13469	0.946131	0.607913
13470	0.946131	0.711305
13471	0.946132	0.58766
13472	0.946132	0.752716
13473	1.39335	0.546817
13474	0.946132	0.631232
13475	0.946131	0.424323
13476	0.946131	0.649217
13477	0.94613	0.314288
13478	0.946132	0.351795
13479	0.946131	0.535772
13480	0.946131	0.504628
13481	0.946131	0.423193
13482	0.946131	0.438496
13483	0.94613	0.432696
13484	0.946126	0.468618
13485	0.946131	0.399073
13486	0.946125	0.536104
13487	0.946127	0.403963
13488	0.946128	0.48314
13489	0.946131	0.0766028
13490	0.946131	0.112702
13491	0.946131	0.0768397
13492	0.946131	0.0700121
13493	0.946131	0.0732816
13494	0.946131	0.0951881
13495	1.39168	0.0726296
13496	0.946131	0.380281
13497	0.946126	0.346843
13498	0.946127	0.396796
13499	0.946131	0.363224
13500	0.94613	0.38464
13501	1.412	0.363388
13502	0.946128	0.368946
13503	0.948901	0.318847
13504	0.943364	0.352724
13505	1.03768	0.335292
13506	0.946132	0.557546
13507	0.946133	0.564099
13508	1.03767	0.560968
13509	0.946132	0.390773
13510	1.00902	0.681245
13511	0.88324	0.626014

13512	0.971584	0.536616
13513	0.946132	0.686763
13514	0.946129	0.885489
13515	0.946131	0.803122
13516	0.971572	0.814171
13517	0.946131	0.0624878
13518	0.946125	0.0540963
13519	1.33587	0.059129
13520	0.946126	0.0750494
13521	0.946127	0.063915
13522	0.946126	0.0682804
13523	0.946128	0.0656339
13524	0.946132	0.0786985
13525	0.946132	0.0706251
13526	1.27512	0.0655451
13527	0.946133	0.108733
13528	0.946132	0.0926949
13529	0.946133	0.0881567
13530	0.946133	0.0971686
13531	0.946128	0.223548
13532	0.946133	0.243697
13533	0.946131	0.27034
13534	0.94613	0.103651
13535	0.946131	0.160429
13536	0.94613	0.232453
13537	0.946131	0.191187
13538	0.946131	0.183077
13539	0.946131	0.191521
13540	0.946131	0.228964
13541	0.946131	0.0850247
13542	0.946131	0.134005
13543	0.946131	0.194047
13544	0.946131	0.159454
13545	0.94613	0.05
13546	0.946131	0.05
13547	0.946131	0.05
13548	0.94613	0.05
13549	0.946132	0.05
13550	0.946131	0.05
13551	1.35316	0.05
13552	0.94613	0.423817
13553	0.94613	0.384587
13554	0.946128	0.430416
13555	0.946132	0.394254
13556	0.946132	0.425466
13557	1.28727	0.400966
13558	0.94613	0.398789
13559	0.946131	0.249096
13560	0.946132	0.285107
13561	0.946132	0.223782
13562	1.39502	0.220823
13563	0.946132	0.190065
13564	0.946132	0.237284
13565	0.946132	0.318192
13566	0.946125	0.502355
13567	0.946131	0.447562
13568	0.94613	0.374502
13569	0.946129	0.506251

13570	0.946126	0.696703
13571	0.946127	0.626117
13572	1.36173	0.595421
13573	0.946129	1.19992
13574	1.02363	1.21542
13575	0.94613	0.936466
13576	0.946131	1.17896
13577	0.946131	0.728805
13578	1.02363	0.871873
13579	0.946131	0.918883
13580	0.946128	0.05
13581	1.24678	0.05
13582	0.94613	0.05
13583	0.94613	0.05
13584	0.94613	0.05
13585	0.946131	0.05
13586	0.94613	0.05
13587	0.946129	0.05
13588	1.25215	0.05
13589	0.946132	0.05
13590	0.946126	0.05
13591	0.946129	0.05
13592	0.94613	0.05
13593	0.946129	0.05
13594	0.946129	0.619993
13595	0.946133	0.69695
13596	0.946133	0.574048
13597	0.946131	0.577153
13598	0.946131	0.621741
13599	1.29689	0.514375
13600	0.946131	0.533762
13601	0.946128	0.206663
13602	0.946132	0.115082
13603	0.946132	0.157998
13604	0.94613	0.24317
13605	1.22716	0.1267
13606	1.22716	0.147656
13607	0.946131	0.228201
13608	0.946127	0.275394
13609	0.946131	0.165954
13610	0.946131	0.223395
13611	0.946128	0.314671
13612	1.30463	0.179701
13613	1.30463	0.199819
13614	0.946129	0.301934
13615	1.29958	0.0589716
13616	0.946132	0.0897733
13617	0.946132	0.0620367
13618	0.946132	0.05
13619	0.946132	0.0570194
13620	0.946132	0.0621957
13621	0.946133	0.0578945
13622	1.26201	0.0544325
13623	0.946133	0.0946953
13624	0.946132	0.0661298
13625	0.946132	0.0516065
13626	0.946132	0.0612856
13627	0.946133	0.0673989

13628	0.946132	0.0606235
13629	0.946133	0.181816
13630	0.946132	0.12892
13631	0.946132	0.204709
13632	0.946133	0.108265
13633	0.946132	0.117517
13634	1.40427	0.123775
13635	0.946133	0.191691
13636	0.946132	0.186716
13637	0.946132	0.126771
13638	0.946132	0.20246
13639	0.946132	0.108819
13640	0.946132	0.118033
13641	1.35123	0.117384
13642	0.946131	0.175345
13643	0.946128	0.356614
13644	0.946132	0.226868
13645	0.946132	0.284829
13646	0.946129	0.40434
13647	1.22144	0.229329
13648	1.22144	0.249966
13649	0.946131	0.371185
13650	0.946127	0.494488
13651	0.946132	0.486595
13652	0.946131	0.468381
13653	0.946127	0.477074
13654	0.946131	0.486879
13655	1.23351	0.44288
13656	0.94613	0.462495
13657	1.18119	0.110282
13658	0.946131	0.16282
13659	0.946132	0.0932751
13660	0.946132	0.127471
13661	0.94613	0.166814
13662	0.946131	0.148398
13663	0.946131	0.138681
13664	0.946132	0.432163
13665	0.946132	0.396291
13666	0.946133	0.455045
13667	1.30563	0.333818
13668	0.946132	0.36374
13669	0.946132	0.430831
13670	0.946133	0.468986
13671	1.20256	0.558639
13672	0.946132	0.992472
13673	0.946133	0.716592
13674	0.946132	0.565337
13675	0.946132	0.685895
13676	0.946132	0.773623
13677	0.946133	0.715562
13678	0.94613	0.699841
13679	0.94613	0.627072
13680	0.946128	0.846122
13681	1.33007	0.597054
13682	0.946129	0.636115
13683	0.94613	0.743093
13684	0.946131	0.818113
13685	0.946127	2.1

13686	0.946127	2.1
13687	0.946131	2.1
13688	1.30136	2.1
13689	0.946128	2.1
13690	0.946129	2.1
13691	1.30136	2.1
13692	0.810942	2.1
13693	0.810942	2.1
13694	0.81093	2.1
13695	2.3208	2.1
13696	1.3517	2.1
13697	1.08132	2.1
13698	1.08132	2.1
13699	1.08133	2.1
13700	0.946132	2.1
13701	0.946132	2.1
13702	0.946132	2.1
13703	0.946132	2.1
13704	0.946132	2.1
13705	1.39823	2.1
13706	0.946132	2.1
13707	1.37713	2.1
13708	0.946131	2.1
13709	0.946131	2.1
13710	0.946131	2.1
13711	0.946131	2.1
13712	0.946131	2.1
13713	0.946131	2.1
13714	0.946128	2.1
13715	0.946128	2.1
13716	0.946133	1.84934
13717	0.946131	2.1
13718	0.946131	0.893387
13719	0.946131	2.1
13720	0.94613	2.1
13721	0.946129	2.1
13722	0.946129	2.1
13723	0.946133	2.1
13724	0.946131	2.1
13725	0.946131	2.1
13726	0.946131	2.1
13727	0.946131	2.1
13728	0.946132	1.01085
13729	1.33694	2.1
13730	0.946133	2.1
13731	0.946133	0.901493
13732	0.946132	1.78759
13733	0.946132	0.925823
13734	0.946133	1.58242
13735	0.946131	1.43329
13736	0.94613	2.1
13737	0.946131	2.1
13738	0.946133	2.04117
13739	0.946129	2.1
13740	0.946131	2.1
13741	0.946131	2.1
13742	0.946132	1.80924
13743	1.35943	2.07223

13744	0.946132	2.1
13745	0.946132	1.05427
13746	0.946131	1.30607
13747	0.946132	0.952263
13748	0.946132	1.96215
13749	0.946132	0.0790109
13750	0.946132	0.119145
13751	0.946133	0.063995
13752	0.946133	0.0962328
13753	0.946132	0.0719254
13754	0.946132	0.0834927
13755	0.946132	0.0970022
13756	1.29322	0.385662
13757	0.946131	0.401936
13758	0.946132	0.355245
13759	0.946129	0.424562
13760	0.946131	0.381148
13761	0.946131	0.356317
13762	0.946131	0.419355
13763	0.946132	0.469392
13764	0.946132	0.705433
13765	0.946131	0.450524
13766	1.41492	0.206989
13767	0.946132	0.62409
13768	0.946132	0.403624
13769	0.946131	0.431904
13770	0.946132	2.1
13771	0.946132	2.1
13772	0.946132	2.1
13773	1.35028	2.1
13774	0.946132	2.1
13775	0.946132	2.1
13776	0.946132	2.1
13777	0.946132	1.11301
13778	0.946132	2.1
13779	0.946132	1.4672
13780	1.32461	1.37248
13781	0.946131	2.1
13782	0.946132	1.4616
13783	0.946132	2.1
13784	0.946131	2.1
13785	0.946131	2.1
13786	0.946131	2.1
13787	0.946132	2.1
13788	0.946129	2.1
13789	0.94613	2.1
13790	1.40369	2.1
13791	0.946132	0.294579
13792	0.946131	0.2006
13793	0.946131	0.338472
13794	0.979598	0.168064
13795	0.946131	0.187235
13796	0.946131	0.280201
13797	0.946132	0.319345
13798	0.946131	2.1
13799	1.41897	2.1
13800	0.946132	2.1
13801	0.946132	2.1

13802	0.94613	1.12508
13803	0.946131	2.1
13804	0.946131	2.1
13805	0.946131	2.1
13806	0.94613	2.1
13807	0.946132	2.1
13808	0.946132	2.1
13809	0.946129	2.1
13810	0.946131	2.1
13811	1.37791	2.1
13812	0.946127	2.1
13813	0.946131	2.1
13814	0.946131	2.1
13815	0.94613	2.1
13816	0.946128	2.1
13817	0.946129	2.1
13818	1.39384	2.1
13819	0.810945	2.1
13820	0.810944	1.37114
13821	0.810958	1.71136
13822	2.70852	2.01241
13823	1.35168	1.43924
13824	1.08132	1.88999
13825	1.08132	1.02087
13826	1.0813	1.33482
13827	1.3335	2.1
13828	0.946132	2.1
13829	0.94613	2.1
13830	0.946132	2.1
13831	0.946131	2.1
13832	0.94613	2.1
13833	0.946131	2.1
13834	0.946132	1.10948
13835	0.946131	0.503956
13836	0.94613	0.70947
13837	1.41192	0.709449
13838	0.946132	0.498877
13839	0.946131	0.639296
13840	0.94613	0.555532
13841	0.946133	0.636264
13842	0.946133	0.520606
13843	0.946132	0.579305
13844	1.39428	0.485946
13845	0.946132	0.431261
13846	0.946132	0.445693
13847	0.946133	0.480652
13848	0.94613	0.742636
13849	0.946129	2.1
13850	0.94613	0.899184
13851	0.946128	0.905451
13852	1.40684	2.1
13853	0.94613	0.870793
13854	0.94613	2.1
13855	0.946131	0.73497
13856	0.946131	2.1
13857	0.946131	1.04969
13858	0.946131	0.914641
13859	1.39021	2.1

13860	0.946131	1.1473
13861	0.946131	2.1
13862	0.946131	1.89451
13863	0.946131	2.1
13864	0.946131	1.09654
13865	0.946131	2.1
13866	0.946131	2.1
13867	0.946131	0.970642
13868	0.946131	1.208
13869	0.810946	2.03103
13870	0.810946	1.40983
13871	2.69264	2.1
13872	1.35168	2.1
13873	1.0813	2.1
13874	1.08132	1.99648
13875	1.08132	2.1
13876	0.810958	2.1
13877	1.16687	1.20882
13878	0.946131	2.07272
13879	0.946131	1.38874
13880	1.34632	2.1
13881	0.946131	2.1
13882	1.34632	2.1
13883	0.946131	2.1
13884	0.946128	1.12048
13885	0.946133	1.40689
13886	0.946133	0.752834
13887	0.946131	0.825567
13888	0.946131	0.856174
13889	1.3564	0.700641
13890	0.94613	0.708699
13891	0.946128	2.1
13892	0.946132	2.1
13893	0.94613	2.1
13894	0.94613	2.1
13895	0.946131	2.1
13896	1.35827	2.1
13897	0.94613	2.1
13898	0.946133	0.580941
13899	0.946132	0.705052
13900	0.946132	0.599147
13901	0.946133	0.71898
13902	0.946133	0.655329
13903	0.946132	0.639751
13904	0.946133	0.694129
13905	0.946132	2.1
13906	0.946132	2.1
13907	0.946132	0.798329
13908	0.946131	2.1
13909	0.946132	1.27957
13910	0.946132	1.17047
13911	0.946132	1.12995
13912	0.94613	0.631523
13913	0.94613	0.437742
13914	0.946128	0.647014
13915	0.946132	0.403598
13916	0.946132	0.670362
13917	0.94613	0.517362

13918	0.946131	0.49146
13919	0.946126	0.32567
13920	0.946131	0.263201
13921	0.946127	0.385979
13922	0.946129	0.23305
13923	0.94613	0.325921
13924	0.946128	0.284375
13925	0.946128	0.287173
13926	0.946131	0.326265
13927	0.946127	0.310282
13928	0.946127	0.3443
13929	0.946131	0.303792
13930	0.946131	0.3403
13931	0.946128	0.33212
13932	0.946129	0.330344
13933	0.94613	0.333601
13934	0.946131	0.32088
13935	0.946129	0.337973
13936	0.946132	0.317998
13937	0.946132	0.335348
13938	0.946131	0.333194
13939	0.94613	0.328461
13940	0.94613	0.351886
13941	0.946131	0.33562
13942	0.946129	0.368735
13943	0.946132	0.338656
13944	0.946132	0.349942
13945	1.32827	0.340283
13946	0.946131	0.348042
13947	0.946132	2.1
13948	0.946132	2.1
13949	0.946131	1.437
13950	0.946131	1.64915
13951	0.946132	2.1
13952	0.946131	2.1
13953	0.946131	2.1
13954	0.946132	0.331421
13955	0.946132	0.710776
13956	0.946131	0.419077
13957	1.38356	0.374609
13958	0.946133	0.661261
13959	0.946131	0.472515
13960	0.946131	0.541435
13961	0.94613	2.1
13962	0.946131	2.1
13963	0.946131	2.1
13964	0.946128	2.1
13965	0.946133	2.1
13966	0.946131	2.1
13967	1.36918	2.1
13968	0.946131	0.598484
13969	0.94613	1.28872
13970	0.946129	0.870832
13971	0.946133	2.1
13972	0.946132	0.766607
13973	1.3771	1.50137
13974	0.946131	0.755675
13975	1.30029	1.03037

13976	0.94613	0.689443
13977	0.946129	1.46905
13978	0.946132	1.46099
13979	0.946132	0.533735
13980	0.946131	0.926816
13981	0.94613	0.805246
13982	0.946133	0.053012
13983	0.946132	0.0671345
13984	0.946132	0.0528555
13985	0.946132	0.05
13986	0.946132	0.0504
13987	0.946132	0.0588099
13988	1.21174	0.052638
13989	0.810946	2.1
13990	0.810945	2.1
13991	2.63045	2.1
13992	1.35169	2.1
13993	1.08132	2.1
13994	1.08132	2.1
13995	1.08132	2.1
13996	0.810946	2.1
13997	1.1796	2.1
13998	0.946131	2.1
13999	0.946132	2.1
14000	1.31522	2.1
14001	0.946132	2.1
14002	1.31522	2.1
14003	0.946132	2.1
14004	0.946131	2.1
14005	1.40615	2.1
14006	0.946129	2.1
14007	0.946128	2.1
14008	0.946133	2.1
14009	0.946131	2.1
14010	0.94613	2.1
14011	0.810945	2.1
14012	0.810944	2.1
14013	2.80479	2.1
14014	1.35169	2.1
14015	1.08132	2.1
14016	1.08132	2.1
14017	1.08132	2.1
14018	0.810945	2.1
14019	1.35562	2.1
14020	0.94613	2.1
14021	1.35561	2.1
14022	0.946133	2.1
14023	0.946131	2.1
14024	1.40239	2.1
14025	0.946131	2.1
14026	0.946125	0.3921
14027	0.946131	0.595452
14028	0.946127	0.219216
14029	0.946129	0.382979
14030	0.946128	0.550069
14031	0.946129	0.586084
14032	0.946128	0.361838
14033	0.946119	2.1

14034	0.946113	2.1
14035	0.94612	2.1
14036	0.946119	2.1
14037	1.41915	2.1
14038	0.946107	2.1
14039	0.946132	2.1
14040	0.810946	1.11978
14041	0.810943	2.00257
14042	0.810934	1.60073
14043	2.31411	2.07357
14044	1.35171	1.87996
14045	1.08132	1.25878
14046	1.08132	2.06034
14047	1.08133	1.16214
14048	0.946127	1.97044
14049	0.94613	2.1
14050	0.94613	2.1
14051	0.946131	2.1
14052	0.946126	2.1
14053	0.946128	2.1
14054	0.946128	2.1
14055	0.810944	2.1
14056	0.810947	1.9158
14057	0.81096	2.1
14058	2.78019	2.1
14059	1.35168	2.1
14060	1.08132	2.1
14061	1.08132	2.04841
14062	1.08131	2.1
14063	0.946133	0.295243
14064	0.946132	0.313657
14065	0.946132	0.30665
14066	0.946133	0.280164
14067	0.946132	0.316702
14068	1.3495	0.240033
14069	0.946133	0.338391
14070	0.946133	2.1
14071	0.946132	2.1
14072	0.946132	2.1
14073	1.39529	2.1
14074	0.946133	2.1
14075	0.946132	2.1
14076	0.946133	2.1
14077	0.946133	2.1
14078	0.946132	2.1
14079	0.946132	2.1
14080	0.946132	2.1
14081	0.946132	1.92262
14082	0.946133	2.1
14083	0.946132	2.1
14084	0.810946	2.1
14085	0.810945	2.1
14086	2.67861	2.1
14087	1.35169	2.1
14088	1.08132	2.1
14089	1.08132	2.1
14090	1.08132	2.1
14091	0.810946	2.1

14092	1.24616	2.1
14093	0.946132	2.1
14094	0.946132	2.1
14095	1.33931	2.1
14096	0.946132	2.1
14097	1.33931	2.1
14098	0.946133	2.1
14099	0.946133	0.451832
14100	0.946133	0.410054
14101	0.946132	0.428862
14102	0.946133	0.456407
14103	1.3855	0.405182
14104	1.3855	0.412245
14105	0.946133	0.450161
14106	0.946128	0.817082
14107	0.946132	2.1
14108	0.946128	1.17735
14109	0.94613	2.1
14110	0.946131	2.1
14111	1.40225	2.1
14112	0.94613	2.06856
14113	1.40548	0.0534729
14114	0.946118	0.0750167
14115	0.946114	0.059417
14116	0.946112	0.05
14117	0.946117	0.0506816
14118	0.946128	0.0541978
14119	0.946103	0.0575559
14120	0.94613	2.1
14121	0.946132	2.1
14122	0.946131	2.1
14123	0.946132	2.1
14124	1.37811	2.1
14125	0.946131	2.1
14126	0.946131	2.1
14127	0.946132	2.1
14128	0.946133	2.1
14129	0.946133	2.1
14130	0.946132	2.1
14131	0.946133	2.1
14132	0.946132	2.1
14133	0.946132	2.1
14134	0.946118	1.02155
14135	0.946113	2.1
14136	0.946118	2.1
14137	0.946119	2.1
14138	0.946117	2.1
14139	0.946105	2.1
14140	0.946131	2.1
14141	0.946118	0.0767861
14142	0.946118	0.0718826
14143	0.946117	0.0822208
14144	0.946113	0.0561885
14145	0.94612	0.0605021
14146	1.41377	0.0650784
14147	0.946106	0.0754764
14148	0.946132	0.724812
14149	1.28436	0.624136

14150	0.946131	0.620079
14151	0.946131	0.738489
14152	0.946131	0.520698
14153	0.94613	0.76963
14154	0.946131	0.652439
14155	0.810947	0.463236
14156	0.810946	0.48936
14157	2.68675	0.415673
14158	1.35168	0.426672
14159	1.08132	0.457745
14160	1.08132	0.440447
14161	1.0813	0.469644
14162	0.81096	0.444259
14163	0.946132	0.4188
14164	1.34338	0.399515
14165	0.946132	0.409413
14166	1.34338	0.433338
14167	0.946132	0.43775
14168	1.34337	0.421448
14169	0.946132	0.416578
14170	0.946127	2.09085
14171	0.946131	1.93138
14172	0.946127	1.81741
14173	0.946131	1.45451
14174	0.946131	1.87221
14175	0.946128	1.66424
14176	1.30281	2.0655
14177	0.946132	0.586772
14178	0.946132	0.554476
14179	0.946132	0.545543
14180	0.946131	0.542271
14181	0.946132	0.593811
14182	1.37973	0.467282
14183	0.946132	0.515434
14184	0.946131	0.72109
14185	1.41798	0.653116
14186	0.946132	0.625198
14187	0.946132	0.655804
14188	0.946129	0.774099
14189	0.946131	0.654987
14190	0.946131	0.767548
14191	0.946118	0.655648
14192	1.41776	0.610235
14193	0.946113	0.61621
14194	0.946119	0.597327
14195	0.946117	0.704845
14196	1.41778	0.587035
14197	0.946105	0.711589
14198	0.946131	0.773013
14199	1.41798	0.818765
14200	0.946132	0.860579
14201	0.946132	0.733458
14202	0.94613	0.800005
14203	1.41799	0.696859
14204	0.946131	0.710651
14205	0.946133	2.1
14206	0.946132	2.1
14207	0.946132	0.889774

14208	0.946132	2.1
14209	0.946133	1.20461
14210	0.946132	1.9617
14211	0.946132	2.1
14212	0.946131	2.1
14213	0.946131	2.1
14214	0.94613	0.725801
14215	0.946129	2.1
14216	0.946132	0.976642
14217	0.94613	0.953183
14218	0.946131	1.83142
14219	0.94612	1.14847
14220	0.946126	2.1
14221	0.946119	1.03739
14222	0.94612	1.30322
14223	1.40766	1.927
14224	0.946132	0.889157
14225	0.946107	2.1
14226	0.946128	2.1
14227	1.31213	2.1
14228	0.946129	2.1
14229	0.94613	2.1
14230	0.94613	2.1
14231	0.94613	2.1
14232	0.94613	2.1
14233	0.946127	1.19201
14234	0.946131	2.09904
14235	0.94613	2.1
14236	0.94613	1.25887
14237	1.36653	2.02975
14238	0.946128	1.34377
14239	0.946128	1.98152
14240	0.946127	2.1
14241	0.94613	2.1
14242	0.946131	2.1
14243	0.94613	2.1
14244	0.946128	2.1
14245	0.946128	2.1
14246	0.946129	2.1
14247	0.946133	2.1
14248	1.40081	2.1
14249	0.946132	2.1
14250	0.946132	2.1
14251	0.946133	2.1
14252	0.946132	2.1
14253	0.946133	2.1
14254	0.946128	2.1
14255	1.32282	2.1
14256	0.946129	2.1
14257	0.94613	2.1
14258	0.946131	2.1
14259	0.946131	2.1
14260	0.946131	2.1
14261	0.810945	1.3578
14262	0.810943	2.1
14263	0.810933	2.1
14264	2.44097	2.1
14265	1.3517	2.1

14266	1.08132	1.40531
14267	1.08132	2.1
14268	1.08133	1.80379
14269	0.946131	2.1
14270	0.94613	2.1
14271	0.946133	2.1
14272	0.946133	2.1
14273	0.946128	2.1
14274	0.946131	2.1
14275	1.27676	2.1
14276	0.946126	0.656728
14277	0.946126	0.707674
14278	0.946131	0.590747
14279	0.946127	0.699207
14280	0.94613	0.426092
14281	0.946128	0.580456
14282	0.946128	0.506935
14283	0.946132	0.325602
14284	0.946133	0.332182
14285	0.946132	0.326299
14286	0.946132	0.324182
14287	0.946132	0.320882
14288	0.946133	0.330437
14289	0.946132	0.327433
14290	0.946128	0.332784
14291	0.946131	0.337799
14292	0.94613	0.335079
14293	0.946129	0.329614
14294	0.94613	0.32663
14295	0.94613	0.340194
14296	0.94613	0.333061
14297	0.946132	0.291677
14298	0.946128	0.315742
14299	0.94613	0.307333
14300	0.946131	0.287191
14301	0.94613	0.304444
14302	0.94613	0.318047
14303	0.946131	0.290678
14304	0.94612	0.31345
14305	0.946119	0.324968
14306	0.946119	0.310542
14307	0.946124	0.307434
14308	0.946119	0.306073
14309	0.946132	0.320717
14310	0.946107	0.311376
14311	0.946128	2.1
14312	0.946133	2.1
14313	0.946131	2.1
14314	0.94613	2.1
14315	0.946131	2.1
14316	1.37869	2.1
14317	0.946131	2.1
14318	1.35703	2.1
14319	0.946132	2.1
14320	0.946132	2.1
14321	0.946132	2.1
14322	0.946132	2.1
14323	0.946132	2.1

14324	0.946133	2.1
14325	0.946133	2.1
14326	0.946133	0.820912
14327	0.946132	2.1
14328	1.37017	2.1
14329	0.946133	1.10426
14330	0.946132	2.1
14331	0.946133	2.1
14332	0.946114	0.05
14333	0.946119	0.05
14334	0.946119	0.0529727
14335	0.94612	0.05
14336	0.946119	0.05
14337	0.946132	0.05
14338	0.946107	0.05
14339	0.946131	0.0667829
14340	0.946131	0.0714775
14341	0.946126	0.104972
14342	0.946127	0.089746
14343	0.94613	0.0761103
14344	0.946129	0.0884883
14345	0.946128	0.0813757
14346	0.946131	1.01943
14347	0.946131	0.936243
14348	0.946132	0.763143
14349	0.946132	1.00174
14350	0.94613	1.22779
14351	0.946131	1.29352
14352	1.3279	1.22231
14353	0.946132	2.1
14354	1.40932	2.1
14355	0.946132	2.1
14356	0.946132	2.1
14357	0.946132	2.1
14358	0.946131	2.1
14359	0.946132	2.1
14360	0.946131	0.112943
14361	0.946131	0.108713
14362	0.946132	0.117689
14363	0.946132	0.118542
14364	0.94613	0.144037
14365	0.946131	0.1497
14366	1.26868	0.137323
14367	0.810943	2.1
14368	0.810945	2.1
14369	0.810932	2.1
14370	2.82321	2.1
14371	1.3517	2.1
14372	1.08132	2.1
14373	1.08132	2.1
14374	1.08133	2.1
14375	0.810943	0.492356
14376	0.810944	0.419651
14377	2.63079	0.535645
14378	1.35168	0.46745
14379	1.0813	0.467167
14380	1.08132	0.358266
14381	1.08132	0.643496

14382	0.810957	0.515264
14383	1.17856	0.267487
14384	0.946132	0.450067
14385	0.946128	0.416157
14386	1.3154	0.671139
14387	0.94613	0.46249
14388	1.31539	0.528163
14389	0.94613	0.617984
14390	0.94613	1.36028
14391	0.94613	1.22822
14392	0.946131	1.09293
14393	0.946131	1.31383
14394	0.946129	1.38807
14395	0.94613	1.99802
14396	1.21997	1.92749
14397	0.946131	0.994319
14398	1.25083	0.827781
14399	0.946132	0.786486
14400	0.946132	1.05612
14401	0.946129	0.762401
14402	1.25083	1.05043
14403	0.946131	1.09866
14404	0.946127	1.34188
14405	1.41342	0.761182
14406	0.94613	1.10971
14407	0.946131	1.01784
14408	0.946127	0.710312
14409	0.946129	0.832692
14410	0.946129	0.845249
14411	0.946131	1.80238
14412	1.29149	1.88657
14413	0.946133	2.1
14414	0.946133	1.09648
14415	0.946128	1.54773
14416	0.946131	0.824452
14417	0.94613	1.30821
14418	0.94613	1.91502
14419	0.94613	1.10766
14420	0.946132	2.1
14421	0.946132	0.95571
14422	0.946128	0.752451
14423	0.94613	1.69256
14424	1.41022	1.82235
14425	0.946132	0.403381
14426	0.946132	0.342707
14427	0.946128	0.377958
14428	0.94613	0.321601
14429	0.94613	0.42282
14430	0.94613	0.299568
14431	0.946131	0.420721
14432	0.946129	0.967455
14433	1.37758	1.65721
14434	0.946131	2.1
14435	0.94613	0.771707
14436	0.946124	1.45928
14437	0.946127	0.807876
14438	0.946127	1.75195
14439	0.946129	0.96157

14440	1.38259	1.51629
14441	0.946132	2.08992
14442	0.946132	0.803713
14443	0.946126	1.54709
14444	0.946129	0.855323
14445	0.94613	1.47439
14446	0.946131	0.298865
14447	0.946132	0.314665
14448	0.946132	0.325974
14449	0.946132	0.308777
14450	0.946132	0.310927
14451	0.946132	0.32536
14452	0.946132	0.303314
14453	0.946131	0.11729
14454	0.94613	0.118344
14455	0.94613	0.0985237
14456	0.946133	0.117354
14457	0.946128	0.0984403
14458	1.25017	0.0881377
14459	0.946131	0.12236
14460	0.946131	2.1
14461	0.946131	2.1
14462	0.946131	2.1
14463	1.3937	2.1
14464	0.946133	2.1
14465	0.946131	2.1
14466	0.94613	2.1
14467	0.946131	0.592999
14468	0.946131	0.402652
14469	0.946131	0.43447
14470	0.94613	0.379474
14471	0.946133	0.579407
14472	0.946132	0.457984
14473	0.946131	0.448249
14474	0.946132	0.460928
14475	0.946132	0.430363
14476	0.946132	0.396352
14477	0.946132	0.412793
14478	0.946131	0.371438
14479	0.946132	0.348188
14480	0.946132	0.35193
14481	0.946129	0.181475
14482	0.94613	0.555645
14483	0.94613	0.498834
14484	0.946128	0.555383
14485	0.946132	0.18317
14486	0.946131	0.479153
14487	0.94613	0.47749
14488	0.946132	1.16122
14489	0.946132	0.559435
14490	0.946132	0.665691
14491	1.41619	0.761208
14492	0.946133	0.360188
14493	0.946133	0.639316
14494	0.946132	0.55432
14495	0.946132	2.1
14496	0.946132	1.31829
14497	0.946132	2.1

14498	1.38244	2.1
14499	0.946132	0.689379
14500	0.946131	2.1
14501	0.946132	2.08743
14502	0.810946	0.644949
14503	0.810943	0.853915
14504	0.810934	0.631336
14505	2.42741	0.485978
14506	1.35171	0.542019
14507	1.08132	0.592247
14508	1.08132	0.637098
14509	1.08133	0.627678
14510	0.810947	0.577982
14511	0.810943	0.867007
14512	2.44179	0.481406
14513	1.35171	0.555449
14514	1.08132	0.557086
14515	1.08132	0.557609
14516	1.08133	0.578096
14517	0.810934	0.554282
14518	0.946132	1.38815
14519	0.946132	1.38609
14520	0.946133	0.846581
14521	0.946133	1.36312
14522	0.946132	1.54523
14523	0.946132	1.38253
14524	1.12786	1.19326
14525	1.27806	0.70899
14526	0.946132	0.917132
14527	0.946133	0.796198
14528	0.946133	1.08371
14529	0.946132	1.54242
14530	0.946132	0.907844
14531	0.946133	1.39424
14532	0.946132	2.1
14533	0.946132	2.1
14534	0.946132	2.1
14535	1.37098	2.1
14536	0.946133	2.1
14537	0.946132	2.1
14538	1.37098	2.1
14539	0.946125	1.10885
14540	0.946129	0.984887
14541	0.946131	2.03484
14542	0.94613	0.833833
14543	0.946124	0.735344
14544	0.946127	0.846765
14545	1.39514	1.16629
14546	0.946132	2.1
14547	0.946131	2.1
14548	0.94613	2.1
14549	1.39909	2.1
14550	0.946132	0.694042
14551	0.946131	2.1
14552	0.94613	2.1
14553	0.946133	1.2672
14554	0.946133	0.463922
14555	0.946132	1.55649

14556	1.36835	0.922004
14557	0.946132	0.494646
14558	0.946133	0.755397
14559	0.946132	0.508503
14560	0.946132	1.88088
14561	0.946133	2.1
14562	0.946133	2.1
14563	0.946132	2.1
14564	1.39047	2.1
14565	0.946132	2.1
14566	0.946132	2.1
14567	1.33174	0.066451
14568	0.946128	0.0704821
14569	0.94613	0.0786443
14570	0.94613	0.068286
14571	0.94613	0.0597263
14572	0.94613	0.0686855
14573	0.946131	0.0695495
14574	0.946128	0.089423
14575	1.36004	0.0800279
14576	0.946129	0.0790677
14577	0.94613	0.0905848
14578	0.94613	0.103584
14579	1.36003	0.0652397
14580	0.94613	0.087134
14581	0.946124	2.1
14582	0.946119	2.1
14583	0.946119	2.1
14584	0.946119	2.1
14585	0.94612	2.1
14586	0.946132	2.1
14587	1.29062	2.1
14588	0.946128	2.1
14589	0.946128	2.1
14590	0.946133	2.1
14591	0.94613	2.1
14592	0.946131	2.1
14593	0.946131	2.1
14594	1.30584	2.1
14595	0.946131	2.01753
14596	0.946131	2.1
14597	0.946131	2.1
14598	0.946132	2.1
14599	1.39549	2.1
14600	0.946131	2.1
14601	0.946131	2.1
14602	0.913386	0.893943
14603	1.17466	0.816308
14604	1.00473	0.751724
14605	0.946132	0.832515
14606	0.946126	1.23344
14607	0.94613	0.962751
14608	0.946129	0.911317
14609	0.946132	0.704993
14610	1.38894	0.699428
14611	0.946132	0.66249
14612	0.946132	0.661807
14613	0.946132	0.889072

14614	0.946132	0.708484
14615	0.946133	0.724386
14616	0.946128	0.671857
14617	1.23865	0.667705
14618	0.946129	0.626214
14619	0.94613	0.630886
14620	0.94613	0.707554
14621	0.94613	0.642071
14622	0.94613	0.680253
14623	0.946126	0.885686
14624	1.40248	1.01025
14625	0.946129	0.948155
14626	0.94613	0.889901
14627	0.946124	1.64768
14628	0.946127	1.02132
14629	0.946128	0.965797
14630	0.946133	0.725708
14631	1.1391	0.788562
14632	0.946132	0.783162
14633	0.946132	0.678882
14634	0.946132	1.12007
14635	0.946133	0.744171
14636	0.946132	0.762877
14637	1.17156	1.01695
14638	0.946133	1.00161
14639	1.33893	0.712336
14640	0.827864	0.773865
14641	0.995232	0.674658
14642	0.827864	1.00824
14643	0.94613	0.634497
14644	1.21387	0.635124
14645	0.946132	0.653625
14646	0.946131	0.507396
14647	0.94613	0.577871
14648	0.94613	0.554809
14649	0.94613	0.606233
14650	0.94613	0.570139
14651	0.810943	0.413013
14652	0.810945	0.366661
14653	2.42463	0.370306
14654	1.3517	0.372549
14655	1.08132	0.398319
14656	1.08132	0.403054
14657	1.08133	0.378261
14658	0.810932	0.408785
14659	0.946133	0.528093
14660	0.946132	0.454605
14661	0.946131	0.505432
14662	1.36748	0.559476
14663	0.946133	0.524578
14664	0.946131	0.458928
14665	0.946132	0.367106
14666	1.35856	0.341199
14667	0.946131	0.352469
14668	0.94613	0.325437
14669	0.94613	0.358026
14670	0.946131	0.314726
14671	0.94613	0.328856

14672	0.94613	0.359393
14673	0.946132	1.13123
14674	0.946131	2.1
14675	0.946131	2.1
14676	0.946131	2.1
14677	1.41149	2.1
14678	1.41149	2.1
14679	0.946131	2.1
14680	0.946128	2.1
14681	0.946128	2.1
14682	0.946133	2.1
14683	0.946131	2.1
14684	0.946131	2.1
14685	0.946131	2.1
14686	0.94613	2.1
14687	1.41388	0.090634
14688	0.94613	0.12929
14689	0.946132	0.0747052
14690	0.946132	0.105574
14691	0.946129	0.136418
14692	0.946131	0.117834
14693	0.946131	0.11691
14694	1.02798	0.0959838
14695	0.946132	0.213293
14696	1.02798	0.0888541
14697	0.946133	0.196781
14698	1.14931	0.119412
14699	0.957746	0.183567
14700	0.93452	0.12979
14701	0.946129	2.1
14702	0.946134	2.1
14703	0.946133	2.1
14704	0.946131	2.1
14705	0.946131	2.1
14706	0.946131	2.1
14707	0.946131	2.1
14708	0.946133	1.21274
14709	0.946132	0.761461
14710	0.946132	1.02747
14711	0.946132	1.66344
14712	1.38773	0.801765
14713	0.946132	1.10816
14714	0.946133	0.887567
14715	0.946131	0.191143
14716	0.946131	0.207556
14717	1.41512	0.19725
14718	0.946133	0.280168
14719	0.946131	0.296799
14720	0.946131	0.294666
14721	0.946131	0.219534
14722	1.35381	0.744775
14723	0.946132	1.87435
14724	1.35381	0.730591
14725	0.946133	1.97775
14726	0.946132	0.857962
14727	0.946132	1.33641
14728	0.946133	0.918631
14729	1.3365	0.720529

14730	0.946132	1.01481
14731	1.3365	0.660004
14732	0.946129	0.868798
14733	0.94613	0.8149
14734	0.946128	0.945433
14735	0.946129	0.731519
14736	0.939895	1.25974
14737	1.14285	1.9665
14738	0.952366	1.79143
14739	0.946131	1.56417
14740	0.94613	1.6372
14741	0.946131	1.60433
14742	0.94613	1.63888
14743	0.946131	2.1
14744	0.946131	2.1
14745	0.946131	0.963986
14746	0.946129	2.1
14747	0.946133	0.979544
14748	0.946131	2.1
14749	0.946131	2.1
14750	0.946133	2.1
14751	0.946132	2.1
14752	0.946132	2.1
14753	0.946133	2.1
14754	0.946132	2.1
14755	0.946132	2.1
14756	0.946133	2.1
14757	0.946133	2.0771
14758	0.946132	1.18107
14759	0.946132	1.77516
14760	0.946133	1.38269
14761	0.946132	2.1
14762	0.946132	1.99201
14763	0.946132	1.46291
14764	0.946132	0.737768
14765	0.946131	2.1
14766	0.946131	0.790952
14767	0.946131	0.812421
14768	1.38969	2.1
14769	0.946131	1.3533
14770	0.946132	2.1
14771	0.946128	2.09496
14772	0.946133	1.54424
14773	0.946132	2.04234
14774	0.946131	1.42156
14775	0.94613	1.43202
14776	0.946131	1.85232
14777	1.28464	1.85477
14778	0.946119	0.771959
14779	1.37492	0.853538
14780	0.946125	0.972055
14781	0.946119	0.645012
14782	0.946119	1.25017
14783	0.946132	0.692445
14784	0.946107	0.880024
14785	0.810942	2.1
14786	0.810943	2.1
14787	0.709587	2.1

14788	2.64488	2.1
14789	1.21651	2.1
14790	0.946151	2.1
14791	0.979948	2.1
14792	1.0813	2.1
14793	0.946133	1.39304
14794	0.946132	2.09596
14795	0.946132	2.1
14796	0.946133	0.967859
14797	0.946132	0.73753
14798	0.946132	0.880152
14799	1.41134	1.27381
14800	0.946133	1.9804
14801	0.946132	1.66991
14802	0.946133	2.1
14803	0.946133	1.10054
14804	0.946132	0.778175
14805	0.946133	1.31351
14806	1.31338	1.68521
14807	2.07612	2.03147
14808	0.946102	0.05
14809	0.752448	0.05
14810	1.20617	0.05
14811	0.94613	0.05
14812	0.75242	0.05
14813	0.946131	0.05
14814	0.752419	0.05
14815	0.946132	0.947235
14816	1.25736	0.896387
14817	1.25736	0.842756
14818	0.946132	0.877544
14819	1.08054	0.774417
14820	1.25736	0.841981
14821	0.946132	1.0169
14822	0.946128	2.1
14823	1.1896	2.1
14824	1.18959	2.1
14825	0.946131	0.845945
14826	1.33271	1.71907
14827	0.946105	2.01929
14828	0.946156	1.87503
14829	0.741946	0.05
14830	1.33587	0.05
14831	0.741947	0.05
14832	1.21954	0.05
14833	0.94613	0.05
14834	0.741946	0.05
14835	1.81347	0.05
14836	1.81347	0.05
14837	0.653322	0.05
14838	1.36935	0.05
14839	0.661882	0.05
14840	1.04753	0.05
14841	0.507464	0.05
14842	0.945844	1.28443
14843	1.59507	2.1
14844	1.04043	1.05908
14845	0.946131	1.89754

14846	1.32891	1.58883
14847	0.946131	1.10022
14848	0.946131	1.06084
14849	1.28866	1.23943
14850	1.12682	0.322085
14851	0.851543	0.327875
14852	1.04072	0.321771
14853	0.946132	0.348144
14854	1.13437	0.300096
14855	1.04167	0.332058
14856	0.936652	0.327193
14857	1.258	2.1
14858	0.946131	2.1
14859	0.946132	2.1
14860	1.36968	2.1
14861	0.946132	2.1
14862	1.36968	2.1
14863	0.946131	2.1
14864	2.30046	0.336121
14865	2.82969	0.400124
14866	0.946132	1.30716
14867	1.35022	2.1
14868	1.12356	1.1833
14869	0.94613	2.1
14870	1.35022	2.1
14871	0.946131	2.02189
14872	0.94613	1.86107
14873	0.946131	2.1
14874	1.22401	2.1
14875	0.952456	2.1
14876	0.94613	2.1
14877	1.22401	2.1
14878	0.946131	2.1
14879	0.946131	2.1
14880	1.33047	2.1
14881	0.946132	2.1
14882	1.33047	2.1
14883	0.946131	2.1
14884	1.33047	2.1
14885	0.946132	2.1
14886	1.2107	2.1
14887	0.61957	1.48392
14888	1.80666	2.1
14889	1.53743	2.1
14890	0.619572	1.2603
14891	1.32547	2.1
14892	1.16166	2.0474
14893	1.04074	2.05742
14894	0.557621	2.1
14895	0.946128	0.742323
14896	1.36607	1.67083
14897	0.94613	1.17994
14898	1.36607	2.1
14899	1.39416	2.1
14900	0.94613	2.1
14901	0.94613	2.1
14902	1.23969	2.1
14903	0.946131	2.1

14904	0.946131	2.1
14905	1.13537	2.1
14906	1.05719	2.1
14907	0.806848	2.1
14908	1.30251	2.1
14909	0.851526	1.92147
14910	1.14176	2.1
14911	0.93666	1.2479
14912	0.946114	2.1
14913	1.15832	1.52801
14914	0.924624	1.53275
14915	0.967609	1.20663
14916	0.771764	0.197245
14917	1.26652	0.186384
14918	0.946133	0.204183
14919	1.17957	0.112775
14920	0.771735	0.153985
14921	0.946122	0.20807
14922	0.946133	0.15287
14923	0.924325	0.889175
14924	0.656739	0.950852
14925	0.637876	0.940446
14926	0.530092	0.918235
14927	0.914415	0.855494
14928	0.821311	1.17221
14929	0.630589	1.08078
14930	0.738823	1.06236
14931	0.839109	0.970318
14932	1.55029	0.05
14933	1.13537	0.367009
14934	0.946131	0.697096
14935	0.946128	0.571026
14936	1.30162	2.1
14937	0.952476	0.761427
14938	1.29289	0.90488
14939	0.939783	0.930326
14940	0.94612	2.1
14941	0.946131	2.1
14942	1.07998	2.1
14943	0.836985	2.1
14944	1.1604	2.1
14945	0.94613	2.1
14946	0.837016	2.1
14947	0.712461	0.582104
14948	1.36399	0.602971
14949	1.8724	0.54119
14950	1.8724	0.484233
14951	0.623426	0.885823
14952	1.36399	0.553346
14953	1.22089	0.543287
14954	0.946132	0.541716
14955	0.946131	2.1
14956	1.32241	2.1
14957	1.32241	2.1
14958	0.946133	2.1
14959	1.40271	2.1
14960	0.946131	2.1
14961	0.946131	2.1

14962	1.1989	1.14205
14963	0.954106	1.34922
14964	0.820068	1.00151
14965	0.563139	1.2471
14966	1.06448	1.32806
14967	0.892472	1.87881
14968	0.862598	1.09348
14969	0.745851	1.88043
14970	1.13336	1.27926
14971	0.946129	2.1
14972	1.28254	2.1
14973	1.28254	2.1
14974	0.946131	2.1
14975	1.41302	2.1
14976	0.946131	2.1
14977	0.946131	2.1
14978	0.851543	1.2639
14979	1.21961	2.1
14980	0.851484	1.1899
14981	0.851543	2.1
14982	1.15705	1.94454
14983	0.946133	1.72381
14984	0.909417	1.65928
14985	1.13808	0.612105
14986	0.946133	0.423053
14987	0.946131	0.619079
14988	1.30194	0.428274
14989	0.946131	0.481264
14990	1.30194	0.409184
14991	0.946131	0.437286
14992	0.930941	1.0862
14993	1.20185	0.846162
14994	1.02404	0.879507
14995	0.946124	1.18394
14996	1.3563	0.953877
14997	0.946127	1.26771
14998	0.946128	1.04774
14999	0.946116	2.1
15000	1.3144	2.09819
15001	1.13088	2.1
15002	0.946118	0.764836
15003	1.31438	1.55069
15004	0.946105	1.51764
15005	0.946131	1.31178
15006	1.76972	0.337557
15007	1.42091	0.227458
15008	0.946128	0.334526
15009	0.946101	0.384104
15010	0.863552	0.387555
15011	0.94613	0.301647
15012	1.21232	0.366477
15013	0.94613	0.392115
15014	1.2137	0.50456
15015	1.23224	0.695562
15016	1.86512	0.568938
15017	1.86512	0.48096
15018	0.580825	1.04557
15019	1.2137	0.731768

15020	0.946133	0.725736
15021	0.580822	0.669315
15022	0.946128	2.1
15023	1.40586	2.1
15024	0.94613	2.1
15025	1.3719	2.1
15026	1.3719	2.1
15027	1.40586	2.1
15028	0.94613	2.1
15029	0.946132	2.1
15030	1.35025	2.06804
15031	1.1794	2.05583
15032	0.946132	0.775873
15033	1.35025	1.11413
15034	0.946131	1.16946
15035	0.946132	0.988001
15036	0.946133	2.1
15037	1.60149	2.1
15038	1.67058	2.1
15039	0.851542	2.1
15040	1.20296	2.1
15041	1.17501	2.1
15042	0.946132	2.1
15043	1.04993	2.1
15044	0.851541	1.11249
15045	1.48219	2.1
15046	0.874565	1.05108
15047	0.946129	2.1
15048	1.22049	2.1
15049	0.946131	2.1
15050	0.946131	1.84311
15051	0.94613	2.1
15052	0.848719	2.1
15053	1.17195	2.1
15054	0.946129	1.2331
15055	1.35426	1.31703
15056	0.94613	1.61418
15057	0.94613	1.78534
15058	0.946133	2.1
15059	1.14342	2.1
15060	1.14342	2.1
15061	0.946133	2.1
15062	1.39009	2.1
15063	0.946132	2.1
15064	0.946132	2.1
15065	1.38819	2.1
15066	0.946133	2.1
15067	1.38819	2.1
15068	0.946131	2.1
15069	1.4116	2.1
15070	0.946131	2.1
15071	1.38819	2.1
15072	0.946127	2.1
15073	1.32244	2.1
15074	1.2176	2.1
15075	0.946131	2.1
15076	1.32244	2.1
15077	0.94613	2.1

15078	0.946129	2.1
15079	1.32415	2.1
15080	1.11239	2.1
15081	0.984099	2.1
15082	1.65532	2.1
15083	1.53082	2.1
15084	1.32068	2.1
15085	1.32068	2.1
15086	1.08172	2.1
15087	3.76293	0.0808783
15088	1.36335	0.05
15089	0.953925	2.1
15090	0.989497	2.1
15091	0.98949	2.1
15092	1.06341	2.1
15093	1.27875	2.1
15094	1.04598	2.1
15095	0.603154	2.1
15096	0.582457	2.1
15097	0.582457	2.1
15098	0.568994	2.1
15099	1.5791	2.1
15100	1.09506	2.1
15101	1.45327	2.08996
15102	1.88406	2.1
15103	1.88404	2.1
15104	1.27389	1.83509
15105	1.14206	2.1
15106	1.00232	2.1
15107	1.00231	2.1
15108	1.68436	2.1
15109	1.4677	0.0551406
15110	1.28828	0.0776987
15111	1.28828	0.0501839
15112	1.46932	0.0561596
15113	1.28228	2.1
15114	1.64268	0.918213
15115	0.959473	2.1
15116	1.10101	2.1
15117	1.07976	2.1
15118	1.2873	1.01506
15119	1.70303	0.0916886
15120	1.40066	0.165151
15121	1.5563	0.135949
15122	1.13727	0.186645
15123	1.03529	2.1
15124	1.11285	2.1
15125	1.03529	2.1
15126	1.28667	2.1
15127	1.32119	2.1
15128	1.44777	2.1
15129	1.44778	2.1
15130	1.61121	2.1
15131	1.09372	0.08102
15132	1.17664	0.0956183
15133	1.17664	0.0805045
15134	0.872931	0.0617982
15135	1.02415	2.1

15136	1.28228	2.1
15137	2.14692	2.1
15138	3.90469	2.1
15139	1.00509	2.1
15140	1.28228	2.1
15141	3.32936	0.05
15142	1.1333	0.05
15143	1.11048	2.1
15144	0.979854	2.1
15145	1.65815	2.1
15146	1.00185	2.1
15147	1.84887	2.1
15148	1.59597	2.1
15149	1.78468	2.1
15150	1.87508	2.1
15151	1.82716	2.1
15152	1.33429	2.1
15153	1.34626	2.1
15154	0.797142	2.1
15155	1.00007	0.897545
15156	1.02051	2.1
15157	1.02051	0.934671
15158	1.03373	2.01039
15159	1.05786	2.1
15160	0.887631	2.1
15161	0.992773	2.1
15162	1.28462	2.1
15163	2.35274	1.76621
15164	1.82533	0.989452
15165	1.82533	1.50802
15166	1.19496	0.777145
15167	1.28597	0.05
15168	1.09725	2.1
15169	1.60948	2.1
15170	1.20421	2.1
15171	1.16566	2.1
15172	1.88522	2.1
15173	1.81948	0.501429
15174	1.14042	0.394844
15175	1.21558	0.620326
15176	1.56876	0.303049
15177	1.6697	2.1
15178	1.81655	2.1
15179	1.81655	2.1
15180	1.5956	2.1
15181	0.906059	2.1
15182	1.63045	2.1
15183	0.9533	0.987108
15184	1.1475	0.893789
15185	1.72231	2.03135
15186	1.94936	1.15613
15187	1.06826	2.1
15188	1.08576	2.1
15189	1.06826	2.1
15190	0.973559	2.1
15191	1.62737	0.05
15192	0.965676	0.05
15193	1.45055	0.05

15194	1.17631	0.05
15195	1.92535	0.0713789
15196	1.9503	0.122636
15197	1.99828	0.0694621
15198	1.70302	0.120646
15199	1.65139	0.119955
15200	1.26784	0.134564
15201	1.35923	0.186093
15202	1.70302	0.196029
15203	1.33033	2.1
15204	1.12481	2.1
15205	1.29452	2.1
15206	1.25399	2.1
15207	1.12481	2.1
15208	0.918358	2.1
15209	1.39332	2.1
15210	1.32368	2.1
15211	1.11712	0.91619
15212	1.07999	1.00154
15213	1.07999	0.768434
15214	1.05619	0.94871
15215	1.1766	2.1
15216	1.35735	2.1
15217	1.42456	0.0738101
15218	1.089	0.10102
15219	1.11081	0.05
15220	2.88521	0.05
15221	1.09754	0.05
15222	1.85479	0.05
15223	1.27922	0.669515
15224	1.03556	0.628956
15225	1.07508	1.30299
15226	1.09795	1.89936
15227	0.967672	0.891368
15228	1.07508	1.17356
15229	1.03933	1.22836
15230	1.02975	2.1
15231	1.02975	1.16932
15232	1.01121	2.1
15233	1.24526	0.05
15234	1.72368	0.05
15235	1.89607	0.05
15236	2.13512	0.05
15237	1.34591	0.520895
15238	1.14721	0.549285
15239	1.15539	0.888616
15240	1.90938	0.724026
15241	1.90941	2.08242
15242	1.89242	1.3698
15243	1.18907	2.1
15244	1.20408	2.1
15245	1.20409	2.1
15246	1.22655	2.1
15247	0.701447	2.1
15248	1.57567	2.1
15249	1.38806	2.1
15250	2.09407	2.1
15251	1.07612	1.02016

15252	0.990019	1.08444
15253	1.15564	1.60371
15254	1.01243	1.27136
15255	1.28956	0.32771
15256	1.35146	0.323153
15257	1.35145	0.327518
15258	1.22811	0.329072
15259	1.09662	2.1
15260	1.42486	2.1
15261	1.10106	0.05
15262	1.90814	0.05
15263	1.3946	0.05
15264	1.11926	0.05
15265	0.725839	2.1
15266	0.925068	2.1
15267	0.925074	2.1
15268	1.43282	2.1
15269	1.91756	0.900984
15270	1.74321	0.932216
15271	1.74321	0.803917
15272	1.63413	0.842686
15273	1.9194	0.294583
15274	1.62246	0.217534
15275	1.90456	0.180534
15276	1.06578	0.208109
15277	1.46019	0.225666
15278	1.17036	0.147236
15279	1.70233	1.15847
15280	1.53515	1.05798
15281	2.03494	2.1
15282	1.78441	2.1
15283	1.75992	2.1
15284	1.94351	2.1
15285	1.51057	0.0797752
15286	0.991435	0.0785165
15287	1.98068	0.107993
15288	1.64283	0.105457
15289	2.96126	0.095613
15290	2.20692	0.0968452
15291	1.95054	0.0642863
15292	1.21325	0.0632508
15293	0.996828	0.277687
15294	1.01976	0.216991
15295	1.17129	2.1
15296	1.13664	2.1
15297	1.13664	2.1
15298	1.09627	2.1
15299	2.65123	2.1
15300	3.23062	2.1
15301	3.23062	2.1
15302	3.92038	2.1
15303	1.89019	0.383249
15304	1.79109	0.452988
15305	1.79109	0.282397
15306	1.54197	0.320089
15307	3.41824	0.913459
15308	1.93553	0.201541
15309	2.26215	0.861025

15310	2.0103	0.243241
15311	1.51569	1.03049
15312	1.01683	0.952567
15313	1.78595	2.1
15314	1.66537	2.1
15315	3.73426	2.1
15316	3.53905	2.1
15317	1.66537	2.1
15318	1.52903	2.1
15319	3.53905	2.1
15320	3.3837	2.1
15321	1.19901	0.920788
15322	1.35002	1.61807
15323	1.35002	0.672488
15324	1.11683	0.691827
15325	1.75326	1.9087
15326	0.712404	1.45583
15327	2.97768	2.1
15328	2.98949	2.1
15329	1.2832	2.1
15330	1.39518	2.1
15331	2.98949	2.1
15332	2.99339	2.1
15333	1.39517	2.1
15334	1.46124	2.1
15335	1.28534	2.1
15336	1.03391	2.1
15337	1.75366	0.942248
15338	1.26247	1.14623
15339	1.13621	1.72998
15340	2.43994	2.1
15341	1.47904	0.0791205
15342	1.80085	0.0843863
15343	1.80085	0.0687424
15344	1.64656	0.116442
15345	2.87331	2.1
15346	2.82863	2.1
15347	2.82859	2.1
15348	2.84673	2.1
15349	1.0961	0.364823
15350	1.39448	0.422494
15351	1.21366	0.401452
15352	1.20143	0.349921
15353	1.85795	0.167848
15354	1.36768	0.14001
15355	1.36769	0.123225
15356	1.45789	0.104421
15357	1.32605	0.05
15358	1.27148	0.05
15359	1.70218	0.107693
15360	1.3063	0.202943
15361	1.45603	0.141675
15362	1.89226	0.220176
15363	1.2742	0.146511
15364	2.10112	0.0964272
15365	1.10748	0.31564
15366	1.1893	0.318861
15367	1.04921	2.1

15368	1.22927	2.1
15369	1.22927	2.1
15370	1.46568	2.1
15371	0.811186	2.1
15372	0.792162	2.1
15373	0.792162	2.1
15374	0.758749	2.1
15375	1.03581	2.1
15376	0.737481	2.1
15377	2.24071	2.1
15378	1.34868	2.1
15379	1.47203	0.810641
15380	1.40967	0.864453
15381	1.40967	0.904997
15382	1.27916	0.907528
15383	1.17418	0.777318
15384	1.04921	2.1
15385	1.28291	1.1141
15386	1.3213	0.487777
15387	1.46812	0.913943
15388	2.42522	1.2284
15389	1.17879	0.761566
15390	1.28291	0.793966
15391	1.28291	0.755971
15392	1.13438	0.776614
15393	1.75004	2.1
15394	1.78247	2.1
15395	1.78247	2.1
15396	1.76421	2.1
15397	2.25975	0.05
15398	3.66111	0.05
15399	3.6611	0.05
15400	3.44043	0.05
15401	1.13339	2.1
15402	1.13322	2.1
15403	1.13323	2.1
15404	1.20761	2.1
15405	1.34337	0.735074
15406	1.90912	2.09953
15407	1.04399	1.93457
15408	2.00578	2.1
15409	1.04973	2.1
15410	1.22615	1.48687
15411	1.93384	2.1
15412	2.62816	0.692679
15413	1.13127	2.1
15414	1.47642	0.922614
15415	2.62816	2.1
15416	3.31755	0.70136
15417	1.47642	2.1
15418	1.80454	0.86001
15419	0.964782	1.80911
15420	0.949135	2.1
15421	0.949134	1.85269
15422	0.949134	2.1
15423	1.10476	0.514472
15424	0.982858	0.310473
15425	1.10938	0.434387

15426	0.982858	0.437557
15427	1.01012	0.05
15428	0.974081	0.05
15429	1.82643	0.05
15430	1.29585	0.05
15431	1.00763	0.926804
15432	1.3294	0.798861
15433	1.3294	0.622427
15434	1.30001	0.973134
15435	1.59151	2.1
15436	1.99443	2.1
15437	1.07369	2.1
15438	1.30584	2.1
15439	1.05071	2.1
15440	0.948676	2.1
15441	1.13842	2.1
15442	1.42884	2.1
15443	1.59019	0.05
15444	1.38212	0.0521924
15445	1.34102	0.05
15446	1.02663	0.05
15447	0.797011	2.1
15448	0.81301	2.1
15449	0.81301	2.1
15450	0.811187	2.1
15451	0.991869	0.354124
15452	1.23868	0.352103
15453	1.67201	2.1
15454	1.61201	2.1
15455	1.61202	2.1
15456	1.57627	2.1
15457	0.94994	2.1
15458	1.77867	2.1
15459	0.854945	2.1
15460	0.973905	2.1
15461	1.64193	2.1
15462	1.68893	2.1
15463	1.68893	2.1
15464	1.70002	2.1
15465	2.08149	0.438781
15466	1.69456	0.346498
15467	1.67399	0.05
15468	1.83922	0.693366
15469	1.91703	2.1
15470	0.967492	2.1
15471	1.13317	2.1
15472	0.980766	2.1
15473	2.06158	0.265772
15474	0.986498	0.176215
15475	1.76289	0.222756
15476	1.17248	0.147147
15477	1.51794	0.158295
15478	1.30266	0.193723
15479	1.03165	0.390141
15480	0.947692	0.445679
15481	1.20473	0.313155
15482	0.947693	0.242646
15483	1.54357	2.1

15484	0.980841	2.1
15485	1.34124	0.47887
15486	1.39818	0.590255
15487	1.39818	0.418497
15488	1.48035	0.531307
15489	1.03932	2.1
15490	1.2065	2.1
15491	1.20651	2.1
15492	1.12717	2.1
15493	1.09483	2.1
15494	0.994293	2.1
15495	0.994293	2.1
15496	0.97356	2.1
15497	0.995252	0.71432
15498	1.66294	0.702546
15499	1.82775	0.571264
15500	1.42762	0.526438
15501	1.04713	0.388857
15502	1.05475	0.370871
15503	1.29002	0.223679
15504	1.05475	0.244728
15505	1.17585	2.1
15506	1.39223	2.1
15507	1.39223	2.1
15508	1.71255	2.1
15509	1.56166	0.8038
15510	1.37762	0.726403
15511	1.16858	0.924488
15512	1.0361	2.1
15513	1.23966	0.05
15514	1.10718	0.05
15515	1.99957	0.05
15516	0.658392	0.05
15517	1.36904	0.05
15518	1.12524	0.05
15519	1.04321	1.11725
15520	1.04627	1.1199
15521	1.91351	0.700842
15522	1.63379	0.797792
15523	1.79718	0.849043
15524	0.59634	0.930953
15525	0.958257	2.1
15526	1.06457	2.1
15527	0.958255	2.1
15528	1.03933	2.1
15529	1.14195	0.565407
15530	1.49438	0.491311
15531	1.49438	0.541172
15532	1.31259	1.44043
15533	1.23036	2.1
15534	1.2016	2.1
15535	1.2016	2.1
15536	1.17129	2.1
15537	1.05724	0.781671
15538	1.04291	0.99384
15539	1.12341	2.1
15540	2.32525	2.1
15541	3.39038	2.1

15542	2.09374	2.1
15543	1.76185	0.715119
15544	1.7934	0.820926
15545	1.65809	0.812621
15546	1.54852	1.21687
15547	1.38766	2.1
15548	1.28765	2.1
15549	1.26599	2.1
15550	1.21397	2.1
15551	1.28765	2.1
15552	1.20045	2.1
15553	1.21398	2.1
15554	1.15234	2.1
15555	0.953634	0.148912
15556	1.39132	0.1695
15557	1.17285	2.1
15558	1.21408	2.1
15559	1.21408	2.1
15560	0.982848	2.1
15561	2.00101	2.1
15562	2.00973	2.1
15563	2.20314	2.1
15564	1.89318	2.1
15565	1.23267	1.12786
15566	0.947921	2.1
15567	1.18872	2.1
15568	0.740752	2.1
15569	1.92269	0.443061
15570	1.53584	0.405755
15571	1.53584	0.441798
15572	1.21067	0.359177
15573	1.40707	0.0597511
15574	1.50437	0.0579184
15575	1.50435	0.055045
15576	1.45446	0.0575409
15577	1.47954	0.624233
15578	2.08687	0.897904
15579	2.08687	1.66061
15580	1.1233	2.1
15581	1.04025	0.668052
15582	1.36005	1.31154
15583	1.47606	0.354242
15584	1.3407	0.35859
15585	0.758743	2.1
15586	0.716917	2.1
15587	0.716922	2.1
15588	0.672964	2.1
15589	2.40014	2.1
15590	1.96848	1.0509
15591	1.96848	2.1
15592	1.53555	0.827048
15593	1.06191	2.1
15594	1.29579	2.1
15595	1.29579	2.1
15596	1.71289	2.1
15597	1.78584	2.1
15598	2.15211	2.1
15599	2.00427	2.1

15600	3.41615	2.1
15601	1.66408	2.1
15602	1.73954	2.1
15603	2.91663	2.1
15604	3.65818	2.1
15605	1.93286	2.1
15606	1.08831	2.1
15607	1.31242	0.0607738
15608	1.11712	0.0649795
15609	1.26311	0.0647317
15610	1.31242	0.0683016
15611	1.10378	2.1
15612	0.968594	2.1
15613	1.48389	0.05
15614	1.49613	0.05
15615	1.04349	0.542343
15616	1.26409	0.435779
15617	1.26409	0.415651
15618	1.05724	0.520356
15619	1.65723	0.348545
15620	1.83116	0.34427
15621	2.06284	0.356021
15622	2.00512	0.356309
15623	1.83116	0.348277
15624	2.03139	0.340593
15625	2.20564	0.357495
15626	1.90048	0.352857
15627	0.963392	2.1
15628	1.01346	0.702833
15629	2.98092	2.1
15630	2.70295	2.1
15631	2.70295	2.1
15632	2.48828	2.1
15633	1.63661	2.1
15634	1.64158	2.1
15635	1.64158	2.1
15636	1.6488	2.1
15637	1.17506	0.320625
15638	1.09667	0.257396
15639	2.00539	0.494876
15640	1.09667	0.403772
15641	1.54946	2.1
15642	1.43577	2.1
15643	1.26103	2.1
15644	1.49808	2.1
15645	1.089	0.0790534
15646	1.22586	0.0793779
15647	2.35027	0.436457
15648	1.37549	0.322271
15649	3.33706	0.320221
15650	1.69894	0.270503
15651	2.00539	0.390528
15652	1.53798	0.29044
15653	1.28938	2.1
15654	1.20969	2.1
15655	1.20969	2.1
15656	1.15864	2.1
15657	1.7986	2.1

15658	1.75869	2.1
15659	1.58281	2.1
15660	1.7392	2.1
15661	1.30733	0.840057
15662	1.5419	1.17627
15663	1.50883	0.948646
15664	1.85439	0.885028
15665	1.73531	0.848879
15666	1.70225	0.777596
15667	1.22176	0.38753
15668	1.59492	0.454148
15669	1.59492	0.406672
15670	2.15542	0.436534
15671	3.24379	2.1
15672	2.64775	2.1
15673	3.16707	2.1
15674	1.93032	2.1
15675	1.50738	2.1
15676	1.24128	2.1
15677	1.24128	2.1
15678	0.994293	2.1
15679	2.90808	0.05
15680	2.45797	0.05
15681	1.85501	0.05
15682	2.90809	0.05
15683	0.892835	1.50673
15684	1.55871	1.93619
15685	1.04615	1.23503
15686	1.66643	0.725533
15687	1.70044	2.1
15688	1.80224	2.1
15689	1.70186	0.842267
15690	3.30123	0.77284
15691	1.74789	2.1
15692	1.77446	2.1
15693	1.77901	2.1
15694	1.57995	2.1
15695	1.81518	2.1
15696	2.17716	2.1
15697	2.17716	2.1
15698	2.65123	2.1
15699	2.01899	1.32502
15700	1.77479	1.15945
15701	1.19122	0.753363
15702	1.46724	0.531499
15703	1.70944	0.05
15704	1.08929	0.05
15705	1.68454	0.05
15706	1.08929	0.05
15707	1.51067	0.921898
15708	1.06729	0.784438
15709	1.37762	0.784745
15710	1.13992	0.730757
15711	1.26397	2.1
15712	1.04083	2.1
15713	1.00278	2.1
15714	1.04084	2.1
15715	1.1016	0.0801202

15716	1.23477	0.0833201
15717	1.23477	0.0525121
15718	1.64985	0.0589866
15719	1.11117	2.1
15720	1.13006	2.1
15721	1.13006	2.1
15722	1.14721	2.1
15723	1.21946	2.1
15724	1.0186	2.1
15725	1.13178	2.1
15726	1.00133	2.1
15727	1.67046	0.459666
15728	0.986036	0.508471
15729	1.67046	0.551857
15730	1.34857	1.52561
15731	2.46016	2.1
15732	2.07161	2.1
15733	2.10732	2.1
15734	2.08371	2.1
15735	1.35602	2.1
15736	0.829065	2.1
15737	1.81667	2.1
15738	0.99406	2.1
15739	1.81667	2.1
15740	1.68073	2.1
15741	2.05736	2.1
15742	2.97311	1.73316
15743	2.14011	2.1
15744	1.23411	2.1
15745	1.13632	0.156388
15746	1.40862	0.149049
15747	1.28055	0.128105
15748	1.14305	0.140633
15749	1.12035	0.05
15750	1.15483	0.05
15751	1.94099	0.05
15752	1.82126	0.05
15753	1.15481	0.05
15754	0.998957	0.05
15755	2.10078	0.05
15756	1.81617	0.05
15757	1.27581	2.1
15758	0.968536	2.1
15759	1.33916	2.1
15760	1.27581	2.1
15761	1.32784	2.1
15762	1.4259	2.1
15763	1.70377	2.1
15764	1.54231	2.1
15765	3.77569	2.1
15766	3.42809	2.1
15767	1.54231	2.1
15768	1.51298	2.1
15769	3.55866	2.1
15770	2.86136	2.1
15771	1.83301	0.887999
15772	1.26371	0.745636
15773	1.98301	0.911074

15774	2.0609	0.799864
15775	2.26047	0.651803
15776	2.00537	0.587736
15777	1.7913	0.589777
15778	1.48864	0.503966
15779	1.09504	2.1
15780	1.39827	2.1
15781	2.28084	2.1
15782	1.05983	2.1
15783	1.33593	0.05
15784	1.31508	0.05
15785	1.31508	0.05
15786	1.09599	0.05
15787	1.30647	2.1
15788	1.28846	2.1
15789	1.28846	2.1
15790	1.26923	2.1
15791	1.2955	2.1
15792	1.09044	2.1
15793	2.12209	2.1
15794	1.26438	2.1
15795	0.911819	2.1
15796	1.38752	2.1
15797	2.57704	2.1
15798	2.71736	2.1
15799	1.42284	2.1
15800	1.41562	2.1
15801	2.69131	2.1
15802	2.36178	2.1
15803	1.41562	2.1
15804	1.39564	2.1
15805	1.32368	2.1
15806	0.908053	2.1
15807	0.908053	2.1
15808	0.629213	2.1
15809	1.8758	1.39158
15810	1.27362	0.890685
15811	1.3756	0.618267
15812	2.28749	0.937301
15813	1.84577	0.872912
15814	2.17765	0.859287
15815	1.38969	0.75698
15816	1.84578	0.836725
15817	1.32801	0.628826
15818	1.76377	0.640346
15819	1.22845	0.936983
15820	1.57766	0.690596
15821	1.57766	2.08548
15822	1.39067	1.53721
15823	1.15743	0.170044
15824	1.37721	0.126846
15825	1.69055	0.260748
15826	1.14858	0.290136
15827	1.38333	1.08303
15828	1.10575	0.530048
15829	1.22418	2.1
15830	1.10574	0.973337
15831	1.91509	0.231599

15832	1.02001	0.149136
15833	1.03074	0.481708
15834	1.19122	0.221266
15835	1.67887	2.1
15836	1.67597	2.1
15837	1.84984	2.1
15838	1.77775	2.1
15839	1.73571	2.1
15840	1.27921	2.1
15841	2.11851	1.53231
15842	1.27921	2.1
15843	1.42486	2.1
15844	1.12503	2.1
15845	1.12503	2.1
15846	1.30116	2.1
15847	0.568995	2.1
15848	0.557348	2.1
15849	0.557348	2.1
15850	0.53907	2.1
15851	0.633575	2.1
15852	1.33756	0.804345
15853	1.51074	2.1
15854	1.93384	0.711875
15855	0.655202	2.1
15856	0.731934	2.1
15857	0.731934	2.1
15858	1.09061	2.1
15859	0.966979	2.1
15860	1.31502	2.1
15861	1.31502	2.1
15862	1.72116	2.1
15863	1.70002	2.1
15864	1.67969	2.1
15865	1.67969	2.1
15866	1.62408	2.1
15867	1.01531	2.1
15868	1.66018	2.1
15869	1.01531	2.1
15870	0.886968	2.1
15871	1.22585	0.0803264
15872	1.20674	0.0781818
15873	1.20672	0.0715273
15874	1.02962	0.0755804
15875	0.956467	1.0874
15876	2.00551	0.791527
15877	0.553801	2.1
15878	0.617006	2.1
15879	1.26923	2.1
15880	1.23614	2.1
15881	1.23614	2.1
15882	1.16817	2.1
15883	2.14482	2.1
15884	1.72637	1.91182
15885	1.09368	2.1
15886	1.26195	2.1
15887	0.760546	2.1
15888	1.84094	2.1
15889	2.62868	2.1

15890	2.79784	2.1
15891	2.79784	2.1
15892	3.00129	2.1
15893	1.27593	0.731404
15894	1.38877	1.50154
15895	1.06486	0.381742
15896	1.2474	0.21891
15897	1.76234	0.05
15898	1.8685	0.05
15899	1.69863	0.05
15900	1.29666	0.05
15901	1.91646	2.1
15902	1.8582	2.1
15903	1.8582	2.1
15904	1.7986	2.1
15905	3.44043	0.05
15906	1.94764	0.05
15907	1.94764	0.05
15908	1.36307	0.05
15909	1.32075	0.05
15910	1.12971	0.05
15911	2.06319	0.0561332
15912	1.94982	0.05
15913	1.77255	0.0647022
15914	0.979297	0.05
15915	1.59997	2.1
15916	1.93506	2.1
15917	1.94409	2.1
15918	1.9597	2.1
15919	0.57899	2.1
15920	0.953036	2.1
15921	0.953049	2.1
15922	1.53503	2.1
15923	1.39615	0.232973
15924	1.06474	0.259836
15925	0.947564	0.299775
15926	1.45506	0.281899
15927	1.17799	0.506424
15928	1.21067	0.432606
15929	1.35945	0.353517
15930	2.05648	0.475978
15931	1.36208	0.391175
15932	2.15229	0.28479
15933	2.89288	0.280126
15934	1.95719	0.304998
15935	2.25169	0.190946
15936	1.52756	0.238039
15937	1.19322	0.468752
15938	1.23146	0.268428
15939	2.45024	0.648595
15940	0.794122	0.362822
15941	1.6343	0.336269
15942	1.34161	0.21066
15943	1.05353	0.460909
15944	1.50795	0.189284
15945	1.05353	0.159725
15946	1.20669	0.198794
15947	1.59289	2.1

15948	0.989543	2.1
15949	1.40588	2.1
15950	0.991427	2.1
15951	0.921822	1.61723
15952	1.46903	0.759636
15953	1.46903	2.1
15954	1.8214	0.612305
15955	1.39702	2.1
15956	1.25103	2.1
15957	1.25103	2.1
15958	1.10378	2.1
15959	1.71813	2.1
15960	1.39595	2.1
15961	1.386 2.1	
15962	1.13008	2.1
15963	1.31711	0.05
15964	1.70191	0.05
15965	0.893249	0.05
15966	1.42669	0.05
15967	1.23943	0.719251
15968	1.1483	0.667857
15969	1.04598	2.1
15970	1.1483	0.743964
15971	1.00381	0.522637
15972	1.11433	0.473446
15973	0.973501	0.897327
15974	1.0756	0.946449
15975	1.20669	0.242579
15976	1.18508	0.250334
15977	1.18508	0.26793
15978	0.983027	0.286398
15979	1.43687	2.1
15980	1.71059	2.1
15981	1.71059	2.1
15982	1.66762	2.1
15983	2.8415	2.1
15984	2.40555	2.1
15985	1.91546	2.1
15986	1.61385	2.1
15987	2.01569	2.1
15988	1.33183	2.1
15989	1.23218	2.1
15990	1.23999	2.1
15991	1.01335	0.72146
15992	1.12088	0.835132
15993	1.25346	1.45892
15994	1.15644	2.1
15995	1.3764	0.892461
15996	1.27558	0.728043
15997	1.2064	1.81975
15998	1.15067	2.1
15999	1.01595	2.1
16000	1.15067	2.1
16001	1.41902	0.735516
16002	1.30272	2.1
16003	1.30272	0.683325
16004	1.19548	2.1
16005	2.99339	2.1

16006	2.98041	2.1
16007	1.46124	2.1
16008	1.42105	2.1
16009	2.98041	2.1
16010	2.86339	2.1
16011	1.42105	2.1
16012	1.42284	2.1
16013	2.31301	0.874939
16014	1.80273	0.847257
16015	2.17765	0.933118
16016	2.31301	0.805183
16017	1.76378	0.662522
16018	2.10275	0.620333
16019	2.10275	0.663896
16020	2.26047	0.624244
16021	0.97541	2.1
16022	1.40863	2.1
16023	1.40863	2.1
16024	1.30751	2.1
16025	1.20143	0.356193
16026	1.19756	0.333091
16027	1.43707	0.329232
16028	1.28954	0.320253
16029	3.34254	0.0832357
16030	1.92916	0.05
16031	1.80089	0.0644338
16032	1.1323	0.0501529
16033	1.47321	0.0849754
16034	1.23756	0.085121
16035	1.87291	2.1
16036	1.53642	2.1
16037	1.53642	2.1
16038	1.5791	2.1
16039	1.0446	2.1
16040	0.992674	2.1
16041	1.12324	0.05
16042	1.29163	0.05
16043	1.29163	0.05
16044	1.15371	0.05
16045	0.996831	0.122318
16046	1.00192	0.135581
16047	1.0019	0.109101
16048	0.958937	0.128645
16049	1.07283	2.1
16050	1.25294	0.862194
16051	1.25294	0.947336
16052	1.04788	0.931735
16053	1.14496	2.1
16054	1.07652	2.1
16055	1.07652	2.1
16056	0.991406	2.1
16057	1.60976	2.1
16058	1.37	2.1
16059	1.37	2.0537
16060	1.14195	2.1
16061	1.26593	1.41521
16062	1.75366	2.1
16063	1.57621	0.387815

16064	1.24538	0.405048
16065	0.969118	2.1
16066	1.04147	2.1
16067	0.969118	2.1
16068	1.22615	2.1
16069	0.98084	2.1
16070	1.01843	2.1
16071	1.09435	2.1
16072	1.50876	2.1
16073	1.5976	2.1
16074	1.84322	2.1
16075	1.84324	2.1
16076	1.70377	2.1
16077	1.20709	2.1
16078	2.54729	2.1
16079	0.974716	0.139511
16080	1.54468	0.250067
16081	1.39021	0.130084
16082	1.70303	0.220038
16083	1.22852	1.80277
16084	1.578	1.05897
16085	1.578	1.79348
16086	1.93852	0.801496
16087	2.2506	2.1
16088	1.39302	1.4672
16089	1.5478	2.1
16090	1.2269	2.1
16091	1.70048	2.1
16092	1.45477	0.860136
16093	1.30929	2.1
16094	1.15069	0.879242
16095	1.00255	1.58583
16096	1.05481	0.583579
16097	1.05481	2.01783
16098	1.1032	0.606469
16099	0.993888	2.1
16100	1.19294	2.1
16101	1.01121	0.323569
16102	0.991866	0.337113
16103	2.20052	0.692827
16104	1.7409	2.1
16105	1.75142	1.63612
16106	1.46811	2.1
16107	1.7409	0.935525
16108	1.23267	2.1
16109	1.46811	2.1
16110	1.18872	2.1
16111	1.12665	2.1
16112	1.50045	2.1
16113	1.50045	2.1
16114	1.37704	2.1
16115	1.35308	0.945851
16116	1.29983	0.547831
16117	1.29983	1.03118
16118	0.970212	0.587525
16119	1.82272	2.1
16120	1.62784	2.1
16121	1.62784	2.1

16122	1.87691	2.1
16123	3.65294	2.1
16124	2.33374	2.1
16125	2.12888	2.1
16126	1.62229	2.1
16127	1.41496	2.1
16128	1.32707	0.654125
16129	2.18871	0.572542
16130	1.4235	0.239313
16131	1.46015	0.0995393
16132	1.25635	0.113025
16133	1.45501	0.331166
16134	1.3543	0.270603
16135	1.38139	2.1
16136	1.98159	2.1
16137	2.15868	2.1
16138	4.04308	2.1
16139	1.5362	2.1
16140	1.56328	2.1
16141	1.56327	2.1
16142	1.58163	2.1
16143	1.88987	0.246836
16144	1.08761	0.162772
16145	0.862154	0.184487
16146	1.77688	0.167251
16147	1.14238	1.8288
16148	1.11691	2.1
16149	1.01537	1.3653
16150	1.02923	2.1
16151	0.977425	0.0995042
16152	1.15211	0.0600526
16153	2.0815	2.1
16154	1.18621	2.1
16155	1.01485	2.1
16156	1.17054	2.1
16157	1.17054	2.1
16158	1.12665	2.1
16159	1.87213	0.05
16160	1.82244	0.05
16161	1.45039	0.05
16162	1.7226	0.05
16163	1.841	0.05
16164	1.79011	0.05
16165	1.8792	0.05
16166	1.45053	0.05
16167	1.08947	2.1
16168	1.5008	2.1
16169	1.5008	2.1
16170	1.83186	2.1
16171	1.04159	0.05
16172	1.2489	0.05
16173	1.2489	0.05
16174	1.33592	0.05
16175	1.51973	0.351279
16176	0.980547	0.37507
16177	1.51974	0.407894
16178	1.15991	0.45189
16179	1.34965	2.1

16180	1.2724	2.1
16181	1.2724	2.1
16182	1.38438	2.1
16183	1.02923	1.52389
16184	1.06521	2.1
16185	1.06521	1.78765
16186	1.0752	2.1
16187	2.03139	0.342386
16188	1.92951	0.332937
16189	1.74852	0.351883
16190	1.62772	0.345811
16191	1.73655	0.335183
16192	1.76809	0.326848
16193	1.62772	0.345711
16194	1.1432	0.335747
16195	1.09235	2.1
16196	1.48081	2.1
16197	1.46712	2.1
16198	1.89321	2.1
16199	1.94548	2.1
16200	1.5636	2.1
16201	1.5636	2.1
16202	1.21124	2.1
16203	1.52314	2.1
16204	2.0219	2.1
16205	1.51113	2.1
16206	2.0219	2.1
16207	0.987247	0.476014
16208	1.18347	0.24862
16209	1.89226	0.530992
16210	1.61441	0.335729
16211	1.42884	2.1
16212	1.10961	2.1
16213	1.56991	2.1
16214	1.05337	2.1
16215	1.05337	2.1
16216	1.91547	2.1
16217	1.05931	2.1
16218	1.1729	2.1
16219	1.34526	2.1
16220	1.0593	2.1
16221	1.0856	2.1
16222	1.90854	2.1
16223	1.91674	2.1
16224	1.11345	2.1
16225	1.75067	2.1
16226	1.50022	2.1
16227	1.50022	2.1
16228	1.53619	2.1
16229	1.4748	2.1
16230	1.74431	1.82842
16231	2.02581	1.73049
16232	1.25288	1.66336
16233	1.01814	0.74217
16234	1.2881	0.542639
16235	1.2881	0.594695
16236	1.54433	0.514592
16237	1.19999	0.577283

16238	1.44056	0.443371
16239	1.02011	0.304313
16240	1.17129	0.440029
16241	1.13775	1.9672
16242	3.34815	2.04259
16243	1.60428	1.72314
16244	1.85928	1.85948
16245	1.75592	2.1
16246	1.25989	2.1
16247	1.75592	2.1
16248	1.71549	2.1
16249	1.37634	2.1
16250	1.64693	2.1
16251	1.64693	2.1
16252	1.64193	2.1
16253	1.18351	0.0619568
16254	2.19398	0.0578988
16255	2.16272	1.04739
16256	1.19837	0.993739
16257	1.04973	2.1
16258	1.20149	2.1
16259	1.60574	2.1
16260	1.11164	0.700493
16261	1.87823	0.726933
16262	1.11164	0.824381
16263	0.772882	2.1
16264	1.4714	2.1
16265	2.14242	0.718526
16266	1.12336	2.1
16267	2.8386	0.675085
16268	2.13346	2.1
16269	1.06788	0.300613
16270	0.947662	0.265584
16271	1.05297	0.210143
16272	1.42907	0.236806
16273	2.17465	2.1
16274	2.18394	2.1
16275	1.14808	2.1
16276	1.21292	2.1
16277	1.21292	2.1
16278	1.27229	2.1
16279	1.20859	0.739045
16280	1.21073	0.824139
16281	1.21073	0.666255
16282	1.22857	0.833115
16283	1.79158	2.1
16284	1.73899	2.1
16285	1.34012	2.1
16286	1.39991	2.1
16287	1.73898	2.1
16288	1.61245	2.1
16289	1.40136	2.1
16290	1.57234	2.1
16291	1.82162	2.1
16292	3.09875	2.1
16293	1.10264	2.1
16294	1.02339	2.1
16295	1.02339	2.1

16296	1.23042	2.1
16297	2.82266	0.656012
16298	1.05698	0.512246
16299	1.96384	0.654286
16300	1.10244	0.616966
16301	0.929061	2.1
16302	1.47007	2.1
16303	0.985303	2.1
16304	1.24554	2.1
16305	1.05993	2.1
16306	2.02074	2.1
16307	2.10106	1.02499
16308	1.18171	0.898656
16309	1.68111	0.981468
16310	1.84226	0.73316
16311	1.82131	0.202742
16312	1.32371	0.155849
16313	1.41902	0.108947
16314	1.00205	0.0953058
16315	1.65532	2.1
16316	1.18977	2.1
16317	1.15471	2.1
16318	0.993889	2.1
16319	2.08521	2.1
16320	2.09885	2.1
16321	2.09497	2.1
16322	2.10604	2.1
16323	0.881028	1.36177
16324	1.52828	0.896266
16325	1.40574	0.05
16326	1.09599	0.05
16327	1.45506	2.1
16328	1.50524	2.1
16329	1.50524	2.1
16330	1.54711	2.1
16331	1.41469	2.1
16332	2.40442	2.1
16333	1.40794	2.1
16334	1.89226	2.1
16335	1.31346	2.1
16336	1.51067	2.1
16337	1.31363	2.1
16338	1.11833	2.1
16339	2.3373	2.1
16340	2.19994	2.1
16341	2.19994	2.1
16342	1.09616	2.1
16343	1.63719	2.1
16344	1.52204	2.1
16345	1.52205	2.1
16346	1.40118	2.1
16347	2.5946	2.1
16348	4.07065	2.1
16349	1.33916	2.1
16350	2.05217	2.1
16351	1.67023	2.1
16352	2.05217	2.1
16353	1.59458	0.558508

16354	1.02247	0.514765
16355	1.68108	0.556207
16356	1.03079	0.493751
16357	1.1643	0.325317
16358	1.85795	0.313195
16359	1.70302	0.273022
16360	1.42076	0.193932
16361	1.91351	0.225754
16362	1.01148	0.197225
16363	2.52402	0.05
16364	0.752625	0.05
16365	0.886968	2.1
16366	1.28845	2.1
16367	1.28845	2.1
16368	1.27766	2.1
16369	1.82268	2.1
16370	1.87118	2.1
16371	1.87119	2.1
16372	1.91704	2.1
16373	3.13938	2.1
16374	2.97839	2.1
16375	2.97839	2.1
16376	2.87331	2.1
16377	1.73443	0.83388
16378	1.59457	0.68718
16379	2.01827	2.1
16380	2.04586	2.1
16381	2.04586	2.1
16382	2.00865	2.1
16383	1.19901	1.71043
16384	1.16661	2.1
16385	1.13215	0.398827
16386	1.96256	0.28841
16387	2.34043	0.05
16388	2.04154	0.05
16389	1.58991	0.05
16390	1.45893	0.05
16391	2.56818	0.05
16392	1.70532	0.05
16393	1.17798	0.445273
16394	1.25796	0.542144
16395	1.25797	0.54977
16396	1.10719	0.709287
16397	1.51067	2.1
16398	1.60707	2.1
16399	2.06946	1.73568
16400	3.19218	2.1
16401	2.19965	2.1
16402	1.30521	2.1
16403	2.23684	1.02465
16404	1.43363	1.5841
16405	1.51007	2.1
16406	2.46356	2.1
16407	1.90428	2.1
16408	1.13618	2.1
16409	2.13276	0.324405
16410	0.998345	0.29915
16411	1.2861	0.333259

16412	1.20457	0.328498
16413	1.51789	2.1
16414	1.49872	2.1
16415	1.49872	2.1
16416	1.47712	2.1
16417	1.87129	0.391137
16418	0.949364	0.388511
16419	1.83142	0.383161
16420	1.55063	0.37856
16421	1.03298	0.376892
16422	0.980664	0.56903
16423	1.51696	0.327046
16424	1.34927	0.502269
16425	1.70302	0.496626
16426	1.65952	0.306914
16427	1.76658	0.238888
16428	0.986848	0.179812
16429	2.13757	1.23663
16430	1.98142	0.59326
16431	1.98142	0.968484
16432	1.65411	0.645746
16433	1.1323	0.0954945
16434	1.46015	0.0961838
16435	2.15376	1.86991
16436	1.64622	1.8783
16437	0.991867	0.479027
16438	1.58074	0.41644
16439	1.69368	2.1
16440	1.76403	2.1
16441	1.76403	2.1
16442	1.83318	2.1
16443	1.28418	0.552641
16444	1.16851	0.433525
16445	1.1685	0.425502
16446	1.1726	0.396771
16447	1.06455	2.1
16448	1.18773	2.1
16449	0.98511	2.1
16450	1.18774	2.1
16451	1.76476	2.1
16452	1.79825	2.1
16453	1.79825	2.1
16454	0.961895	2.1
16455	1.59589	0.05
16456	2.25975	0.05
16457	2.76339	2.1
16458	3.0551	2.1
16459	1.68761	2.1
16460	1.59561	2.1
16461	2.17257	2.1
16462	1.05845	2.1
16463	1.03512	2.1
16464	2.19011	2.1
16465	1.44859	2.1
16466	1.35897	0.841864
16467	1.35897	2.1
16468	1.17822	1.30569
16469	1.13641	2.1

16470	1.18301	2.1
16471	1.18301	2.1
16472	1.25173	2.1
16473	2.41297	2.1
16474	1.36463	2.1
16475	1.54711	2.1
16476	1.5735	2.1
16477	1.5735	2.1
16478	1.58713	2.1
16479	1.69044	0.05
16480	1.57979	0.05
16481	1.57979	0.05
16482	1.14387	0.05
16483	1.04199	2.1
16484	1.96491	2.1
16485	1.04199	2.1
16486	1.46892	2.1
16487	0.983724	2.1
16488	1.70579	2.1
16489	1.88522	2.1
16490	1.23914	2.1
16491	1.79424	2.1
16492	1.52851	2.1
16493	1.04921	2.1
16494	1.11285	2.1
16495	1.25334	0.836673
16496	1.07496	0.661984
16497	1.07497	2.1
16498	1.34852	0.727753
16499	1.27391	2.1
16500	2.20307	2.1
16501	1.81311	2.1
16502	2.10305	2.1
16503	1.21059	0.426644
16504	1.44253	0.452537
16505	1.9194	0.261905
16506	1.21159	0.321796
16507	0.947979	2.1
16508	1.01225	0.542052
16509	0.947979	2.1
16510	1.01225	0.824399
16511	1.00255	2.1
16512	1.01225	2.1
16513	1.01225	2.1
16514	1.06561	0.894183
16515	3.50562	2.1
16516	3.61336	2.1
16517	3.61336	2.1
16518	3.73635	2.1
16519	1.61245	2.1
16520	1.52301	2.1
16521	1.57612	2.1
16522	1.83035	2.1
16523	1.52301	2.1
16524	1.24789	2.1
16525	1.85156	2.1
16526	1.89072	2.1
16527	0.995735	2.1

16528	1.57738	2.1
16529	1.87141	0.106918
16530	1.73388	0.138401
16531	1.61344	0.133943
16532	0.922676	0.176647
16533	1.56614	2.1
16534	0.956294	2.1
16535	1.82589	2.1
16536	1.63719	2.1
16537	1.12399	0.05
16538	1.50103	0.05
16539	1.56976	0.05
16540	1.03567	0.05
16541	1.21776	0.05
16542	1.89708	0.05
16543	1.16323	0.05
16544	1.63533	0.05
16545	2.29397	0.05
16546	3.01596	0.05
16547	1.80145	0.05
16548	1.7666	0.05
16549	1.91621	0.75288
16550	1.10719	0.542882
16551	1.57828	1.09897
16552	1.17105	1.20862
16553	1.15806	2.1
16554	1.31565	2.1
16555	1.31565	2.1
16556	1.46643	2.1
16557	1.47507	2.1
16558	1.87858	2.1
16559	0.958661	2.1
16560	1.87858	2.1
16561	1.44293	0.094735
16562	1.43213	0.153631
16563	1.43213	0.0939802
16564	1.10476	0.133585
16565	1.03235	2.02285
16566	1.31	0.568488
16567	1.31	2.1
16568	1.50108	0.822581
16569	1.32215	2.1
16570	1.10611	2.1
16571	1.10611	2.1
16572	0.904978	2.1
16573	1.06486	0.38039
16574	1.18347	0.243898
16575	1.70535	2.1
16576	1.94779	2.1
16577	1.77071	2.1
16578	1.82268	2.1
16579	0.61701	2.1
16580	0.672495	2.1
16581	0.672494	2.1
16582	0.713174	2.1
16583	1.40395	2.1
16584	1.34245	2.1
16585	1.34243	2.1

16586	1.30646	2.1
16587	1.06204	0.412
16588	0.632691	0.484446
16589	1.75766	0.481921
16590	1.14287	0.675624
16591	1.52101	0.351077
16592	1.71414	0.362587
16593	1.86411	0.358828
16594	1.6253	0.365666
16595	1.04355	0.366863
16596	1.0443	0.374238
16597	1.34852	2.1
16598	1.56016	0.635909
16599	1.56016	0.674509
16600	1.03235	0.634063
16601	1.2966	0.0598218
16602	1.82354	0.05
16603	1.2966	0.05
16604	1.67687	0.05
16605	1.19669	1.24531
16606	1.82979	1.66634
16607	1.58972	1.2516
16608	1.09219	1.27841
16609	1.42077	0.942378
16610	1.89226	0.819251
16611	1.19547	0.679487
16612	1.10187	2.1
16613	1.10188	0.693945
16614	1.01479	2.1
16615	1.81663	2.1
16616	1.81533	2.1
16617	1.83247	2.1
16618	1.51511	2.1
16619	0.983148	2.1
16620	1.7513	2.1
16621	1.85017	2.1
16622	1.26103	2.1
16623	1.23152	2.1
16624	1.65508	2.1
16625	1.23152	2.1
16626	2.15082	2.1
16627	1.57974	2.1
16628	1.07289	2.1
16629	0.81534	2.1
16630	1.51007	2.1
16631	1.00898	0.0548858
16632	1.18959	0.057234
16633	1.32178	0.05
16634	1.25929	0.05
16635	2.17892	0.407713
16636	1.22938	0.293341
16637	3.02933	0.547137
16638	2.34619	0.455358
16639	1.47679	0.363478
16640	1.0819	0.386193
16641	3.10558	2.1
16642	3.31457	2.1
16643	0.985109	2.1

16644	1.10667	2.1
16645	3.31457	2.1
16646	3.54443	2.1
16647	1.10667	2.1
16648	1.25987	2.1
16649	1.77063	0.995783
16650	1.51065	0.82754
16651	1.24688	1.96208
16652	1.37652	2.1
16653	1.37652	1.22557
16654	1.54168	2.1
16655	1.17042	0.914454
16656	1.13092	1.11839
16657	1.13092	1.59135
16658	1.52938	2.1
16659	1.11787	0.408282
16660	1.00613	0.565991
16661	0.781373	0.402828
16662	1.6885	0.379755
16663	2.49439	0.332928
16664	1.42657	0.3693
16665	1.77903	1.78774
16666	2.63606	2.1
16667	2.10425	2.1
16668	1.21425	1.04282
16669	1.31253	1.29092
16670	1.28418	1.67676
16671	1.28418	1.04799
16672	1.58972	1.17278
16673	0.858948	2.1
16674	1.90196	2.1
16675	1.81024	2.1
16676	1.90194	2.1
16677	1.4263	0.771577
16678	0.947921	0.613156
16679	1.22852	0.882235
16680	1.09194	0.762724
16681	1.60578	0.0724285
16682	1.14721	0.0779506
16683	1.06336	0.108356
16684	1.14721	0.0833828
16685	1.11924	0.05
16686	1.53814	0.05
16687	1.18421	0.05
16688	1.8458	0.05
16689	1.7565	2.06956
16690	1.09344	1.2421
16691	1.09344	0.91249
16692	1.16971	1.95797
16693	1.71465	0.05
16694	1.19412	0.05
16695	1.64985	0.0885831
16696	1.05417	0.0728076
16697	1.2127	0.05
16698	1.48867	0.05
16699	1.27806	0.05
16700	1.21269	0.05
16701	1.37813	1.00485

16702	1.0417	1.07555
16703	1.88626	0.681355
16704	1.14977	0.932602
16705	1.78803	1.6729
16706	1.33793	1.12234
16707	1.42266	1.5363
16708	1.26865	1.53279
16709	1.09518	2.1
16710	1.36844	2.1
16711	1.20455	2.1
16712	1.36844	2.1
16713	1.22297	0.959178
16714	1.11256	1.05563
16715	1.11256	0.902402
16716	1.03217	0.970355
16717	1.18422	0.05
16718	1.4282	0.05
16719	1.04159	0.05
16720	1.4282	0.05
16721	0.988805	2.1
16722	1.05481	2.1
16723	1.52938	0.780027
16724	1.26593	1.13663
16725	1.53082	2.1
16726	1.09847	2.1
16727	1.75211	0.625589
16728	1.22222	1.61183
16729	1.75211	0.512731
16730	1.51592	1.55073
16731	1.63413	0.707959
16732	1.26662	0.723378
16733	1.42495	0.583694
16734	1.4356	0.61791
16735	1.29389	1.58433
16736	1.04164	2.1
16737	1.40574	1.2867
16738	1.04164	1.07565
16739	2.22344	0.60727
16740	1.45945	0.352201
16741	0.987248	0.479391
16742	1.2474	0.294063
16743	0.974702	0.795233
16744	1.43777	0.623754
16745	1.71289	2.1
16746	1.91529	2.1
16747	1.91529	2.1
16748	2.01827	2.1
16749	1.87111	1.02504
16750	1.00864	0.604322
16751	1.26865	0.682828
16752	1.18769	0.931984
16753	1.24167	1.94893
16754	1.36133	0.677713
16755	2.28168	2.1
16756	3.98524	2.1
16757	1.36133	2.03406
16758	1.69436	0.801772
16759	2.21926	1.126

16760	1.70302	1.96751
16761	1.50732	0.138798
16762	1.42237	0.141697
16763	1.18659	0.172617
16764	1.50732	0.182606
16765	1.19785	0.0697058
16766	1.40969	0.0540313
16767	1.28339	0.083122
16768	1.40882	0.0734321
16769	0.968595	0.811666
16770	1.41903	0.73999
16771	1.12567	2.1
16772	1.15898	2.1
16773	1.27241	2.1
16774	0.995796	2.1
16775	1.89983	0.05
16776	1.05417	0.05
16777	0.571999	0.05
16778	0.743597	0.05
16779	1.89353	0.05
16780	0.924068	0.05
16781	1.52851	2.1
16782	1.26969	2.1
16783	1.07763	0.44018
16784	1.82301	0.373913
16785	1.07763	0.238234
16786	1.17506	0.297779
16787	1.20045	2.1
16788	1.12486	2.1
16789	1.15234	2.1
16790	1.11598	2.1
16791	1.12486	2.1
16792	1.0491	2.1
16793	1.11598	2.1
16794	1.1047	2.1
16795	1.05902	2.1
16796	1.14361	2.1
16797	1.14361	2.1
16798	1.26851	2.1
16799	1.29389	1.05291
16800	0.988456	1.05272
16801	1.83688	0.05
16802	1.70302	0.05
16803	1.49386	0.05
16804	1.83151	0.05
16805	1.01346	0.683946
16806	0.983607	0.715307
16807	0.983596	2.1
16808	1.08332	0.784044
16809	2.36848	0.333608
16810	2.97066	0.31192
16811	1.12352	0.301113
16812	2.5311	0.241616
16813	1.09062	2.1
16814	0.962182	2.1
16815	0.962181	2.1
16816	1.2258	2.1
16817	0.974888	2.1

16818	1.06312	2.1
16819	1.73583	0.0545005
16820	1.72399	0.0524289
16821	1.724	0.0781961
16822	1.50951	0.0720026
16823	1.26755	2.1
16824	1.03824	2.1
16825	1.27875	2.1
16826	1.03824	2.1
16827	1.18902	2.1
16828	1.534	2.1
16829	1.11764	2.1
16830	1.534	2.1
16831	1.52008	2.1
16832	1.34406	2.1
16833	1.34406	2.1
16834	1.2187	2.1
16835	0.963571	0.849674
16836	1.19612	0.786383
16837	0.963571	1.55757
16838	1.00007	1.82584
16839	0.903333	2.1
16840	1.12724	2.1
16841	1.23771	2.1
16842	1.17683	2.1
16843	1.20059	0.681891
16844	0.772023	0.464409
16845	1.14749	0.77414
16846	1.2802	0.754779
16847	1.85091	1.15756
16848	2.11193	0.937923
16849	1.2802	0.69551
16850	1.42913	0.707576
16851	2.11193	0.854219
16852	1.27737	0.653622
16853	1.30584	2.1
16854	1.60936	2.1
16855	1.3413	0.122403
16856	1.53336	0.0648243
16857	2.60679	0.0855778
16858	1.3413	0.0678152
16859	1.35877	1.66669
16860	1.11024	1.83145
16861	1.27839	2.1
16862	1.08171	2.1
16863	1.27841	2.1
16864	1.0577	2.1
16865	1.71255	2.1
16866	1.10611	2.1
16867	0.960106	2.1
16868	1.40921	2.1
16869	1.90705	0.445997
16870	2.79564	0.41237
16871	1.45321	0.447105
16872	1.90705	0.438591
16873	0.974718	0.292373
16874	1.53671	0.264715
16875	1.69039	0.272321

16876	1.78229	0.246585
16877	1.0101	2.1
16878	1.3167	2.1
16879	1.19699	2.1
16880	1.3212	2.1
16881	1.60811	0.397249
16882	1.43224	0.34685
16883	1.35401	0.354577
16884	1.17464	0.296155
16885	1.27696	2.1
16886	1.05025	2.1
16887	1.05025	2.1
16888	1.18076	2.1
16889	1.37224	1.4196
16890	1.13438	1.33168
16891	1.76788	1.15926
16892	1.73444	1.12911
16893	1.37704	2.1
16894	1.77042	2.1
16895	1.77042	2.1
16896	1.09778	2.1
16897	1.28667	2.1
16898	1.28228	2.1
16899	1.30646	0.746564
16900	1.68867	0.865042
16901	1.30645	1.09845
16902	0.986494	2.06426
16903	1.6253	0.361015
16904	1.48676	0.365018
16905	1.48676	0.36263
16906	1.32974	0.361676
16907	0.634347	0.05
16908	1.15142	0.05
16909	1.11395	0.05
16910	1.18068	0.05
16911	1.36904	0.05
16912	0.764563	0.05
16913	2.09213	0.05
16914	1.56631	2.1
16915	1.08011	2.1
16916	1.80283	2.1
16917	1.50738	2.1
16918	1.44951	0.315887
16919	1.50102	0.312608
16920	1.50102	0.278463
16921	1.39615	0.273272
16922	1.02653	0.450409
16923	2.98258	0.466765
16924	2.0815	0.05
16925	1.39525	0.05
16926	1.82176	0.05
16927	0.977607	0.05
16928	1.96133	1.25185
16929	1.27189	1.05273
16930	0.826032	0.90329
16931	2.12831	0.869839
16932	1.77798	2.00092
16933	1.02969	1.39498

16934	1.06463	1.05848
16935	1.49219	2.1
16936	1.10003	2.1
16937	1.21223	2.1
16938	1.00513	1.11445
16939	1.43788	1.602
16940	1.47134	0.609331
16941	0.968249	0.646963
16942	3.22585	0.076914
16943	3.36933	0.0642264
16944	3.36932	0.0882824
16945	3.03865	0.05
16946	1.66295	0.10251
16947	1.65583	0.0812258
16948	1.65583	0.139019
16949	1.4732	0.0665869
16950	1.73704	0.05
16951	1.76476	0.723948
16952	2.13345	0.750858
16953	1.55816	2.1
16954	1.55816	0.823557
16955	1.02601	2.1
16956	1.08039	1.49281
16957	1.05498	0.879758
16958	1.08039	2.1
16959	1.2873	2.1
16960	1.01121	2.08412
16961	0.987703	2.1
16962	0.98771	1.97815
16963	0.964782	2.1
16964	1.35719	2.1
16965	2.61092	2.1
16966	2.6607	2.1
16967	3.41693	2.1
16968	1.23125	2.1
16969	1.73772	2.1
16970	0.99845	0.630033
16971	1.5176	0.686649
16972	1.13271	0.0912529
16973	1.332	0.067661
16974	1.63159	2.1
16975	1.61342	2.1
16976	1.03679	2.1
16977	1.19087	2.1
16978	1.97172	2.1
16979	2.68634	2.1
16980	1.19086	2.1
16981	1.50091	2.1
16982	2.84677	2.1
16983	2.77894	2.1
16984	2.77894	2.1
16985	2.74206	2.1
16986	1.3284	2.1
16987	1.83524	2.1
16988	1.65853	2.1
16989	1.56741	2.09945
16990	1.00509	2.1
16991	1.45246	2.1

16992	1.26193	2.1
16993	1.45244	2.1
16994	1.61963	0.131987
16995	1.68189	0.621256
16996	1.68189	0.812005
16997	1.49155	0.655876
16998	1.58054	2.1
16999	1.59169	2.1
17000	1.59169	2.1
17001	1.60497	2.1
17002	0.743595	0.05
17003	0.702359	0.05
17004	1.15317	0.05
17005	2.24367	0.05
17006	1.15252	0.05
17007	2.60354	0.05
17008	1.45158	2.1
17009	1.56004	2.1
17010	1.64527	2.1
17011	1.00371	2.1
17012	1.38287	0.491676
17013	0.893024	0.588154
17014	1.23527	0.620107
17015	1.23706	0.580292
17016	2.20993	0.48744
17017	1.54858	1.04515
17018	0.798666	0.368579
17019	1.22487	0.379331
17020	1.33158	0.358512
17021	1.85902	0.371546
17022	1.08334	2.1
17023	1.17885	0.948687
17024	1.17885	0.754454
17025	1.07283	0.887262
17026	1.30723	0.05
17027	1.62507	0.05
17028	1.62507	0.05
17029	1.08854	0.05
17030	1.59475	2.1
17031	1.58874	2.1
17032	1.58875	2.1
17033	1.35787	2.1
17034	1.04147	2.1
17035	1.15564	1.64504
17036	1.78301	2.1
17037	1.90618	2.1
17038	1.71555	2.1
17039	2.13143	2.1
17040	1.10817	2.1
17041	1.032	2.1
17042	1.032	2.1
17043	0.953636	2.1
17044	1.27916	1.01133
17045	1.11938	0.924502
17046	1.11937	0.918299
17047	1.9186	1.13068
17048	1.19967	0.166817
17049	1.63489	0.192587

17050	1.00763	0.245802
17051	1.81656	0.258458
17052	1.31814	0.15175
17053	2.98183	0.199134
17054	1.20149	2.1
17055	1.29129	2.1
17056	1.29129	2.1
17057	1.37138	2.1
17058	1.23104	0.990243
17059	1.75236	1.32881
17060	1.6	0.55676
17061	1.60565	0.821355
17062	1.60565	0.748655
17063	1.40013	0.840031
17064	1.23042	2.1
17065	1.18278	2.1
17066	1.10853	2.1
17067	1.04724	2.1
17068	1.1766	2.1
17069	1.00319	2.1
17070	1.29435	2.1
17071	1.00319	2.1
17072	1.05655	1.34408
17073	1.01606	1.41042
17074	1.80391	2.1
17075	2.7699	2.1
17076	1.52073	2.1
17077	2.06835	2.1
17078	2.00202	2.1
17079	2.04906	2.1
17080	0.968536	2.1
17081	1.02461	2.1
17082	1.59784	2.1
17083	1.78249	2.1
17084	1.81409	2.1
17085	1.79844	2.00362
17086	1.77093	1.77451
17087	2.02356	1.35851
17088	1.32449	2.1
17089	1.10003	2.1
17090	1.16915	0.674553
17091	1.05937	0.512578
17092	1.28096	2.1
17093	1.34696	2.1
17094	1.34695	2.1
17095	1.36457	2.1
17096	1.06478	2.1
17097	1.06239	2.1
17098	1.06239	2.1
17099	1.09629	2.1
17100	0.798209	1.93633
17101	1.8532	2.1
17102	2.10112	0.114994
17103	2.8704	0.101027
17104	2.8704	0.101004
17105	2.92457	0.0927922
17106	1.0752	2.01041
17107	1.07745	2.1

17108	1.07745	2.09946
17109	1.08911	2.1
17110	1.40182	2.1
17111	1.00133	2.1
17112	1.40182	2.1
17113	1.24509	2.1
17114	2.22243	0.05
17115	1.07303	0.05
17116	1.29736	0.05
17117	1.73584	0.05
17118	2.0815	0.586033
17119	1.07999	0.528424
17120	2.1692	2.1
17121	1.99992	2.1
17122	1.99992	2.1
17123	1.78327	2.1
17124	1.52661	0.05
17125	0.956003	0.05
17126	1.53172	0.05
17127	0.955991	0.05
17128	1.35025	0.757862
17129	1.51084	2.1
17130	1.51084	0.826286
17131	1.67115	2.1
17132	2.00977	2.1
17133	1.63632	2.1
17134	1.63632	2.1
17135	2.66128	2.1
17136	1.13525	2.1
17137	2.37566	2.1
17138	1.25987	2.1
17139	1.4472	2.1
17140	1.4472	2.1
17141	1.66018	2.1
17142	0.994001	1.00003
17143	1.83151	0.707439
17144	1.83151	0.508229
17145	1.57621	0.558709
17146	1.40915	0.190535
17147	1.24653	0.504841
17148	1.24653	0.0920748
17149	1.10609	0.669373
17150	1.61121	2.1
17151	1.74472	2.1
17152	1.74472	2.1
17153	1.0368	2.1
17154	1.04788	2.1
17155	1.28522	2.09079
17156	1.28522	2.1
17157	1.54196	1.1511
17158	1.23387	2.1
17159	0.896519	2.1
17160	1.60428	2.1
17161	1.08467	2.1
17162	1.56875	0.572758
17163	1.39072	0.215876
17164	1.39073	0.363149
17165	1.6	0.68402

17166	1.01346	1.91461
17167	1.06838	0.964007
17168	0.968463	0.95881
17169	1.06837	0.972466
17170	3.22433	0.43369
17171	2.05993	1.34777
17172	2.01836	0.434716
17173	1.66154	1.41927
17174	1.20871	1.06373
17175	1.10586	2.1
17176	1.10586	0.931456
17177	1.03853	2.1
17178	1.12076	2.1
17179	1.03899	2.1
17180	1.03899	2.1
17181	0.963973	2.1
17182	0.987132	2.1
17183	1.0894	2.1
17184	2.37823	2.03424
17185	1.75647	1.42313
17186	1.64625	2.1
17187	1.56195	2.1
17188	1.41995	2.1
17189	1.15171	2.1
17190	1.36457	2.1
17191	1.33847	1.912
17192	1.33847	2.1
17193	0.869601	1.55069
17194	1.42255	2.1
17195	1.3868	2.1
17196	1.3868	2.1
17197	1.33962	2.1
17198	1.05481	2.1
17199	1.08074	2.1
17200	1.08074	2.1
17201	0.857557	2.1
17202	1.13537	1.85176
17203	1.09194	0.820011
17204	1.01179	1.95217
17205	1.13537	0.679421
17206	1.15645	1.98094
17207	1.56409	0.373845
17208	1.56409	0.907576
17209	1.16239	1.0535
17210	1.10746	0.44331
17211	1.47952	0.523394
17212	1.14733	1.74323
17213	3.77068	2.1
17214	1.38497	2.1
17215	1.54599	2.1
17216	1.54601	2.1
17217	1.27697	2.1
17218	1.01393	1.84085
17219	1.10558	1.82971
17220	0.995012	1.02051
17221	1.17624	1.4382
17222	1.60497	2.1
17223	1.61588	2.1

17224	1.61587	2.1
17225	1.62182	2.1
17226	1.48866	0.05
17227	1.65977	0.05
17228	1.65977	0.05
17229	1.69044	0.05
17230	0.961892	2.1
17231	1.00659	2.1
17232	1.0066	2.1
17233	1.06226	2.1
17234	1.15171	2.1
17235	1.50853	0.861624
17236	1.50853	2.1
17237	1.2955	2.1
17238	1.3204	0.171047
17239	2.13295	0.102435
17240	2.75524	0.0836995
17241	1.18156	0.0572374
17242	1.73729	2.1
17243	1.20356	2.1
17244	1.62483	2.1
17245	1.52008	2.1
17246	1.19964	2.1
17247	1.0577	2.1
17248	1.19964	2.1
17249	1.07156	2.1
17250	1.98241	2.1
17251	1.43687	2.04689
17252	1.27863	2.01792
17253	1.19915	1.17574
17254	0.97269	1.30447
17255	1.0739	1.01985
17256	1.1427	2.09636
17257	1.22476	0.923025
17258	1.06584	1.36492
17259	1.22626	1.05456
17260	1.31388	2.1
17261	1.16932	2.1
17262	1.28819	2.1
17263	1.0499	2.1
17264	1.55894	2.1
17265	1.78709	2.1
17266	1.75986	2.1
17267	1.764 2.1	
17268	1.44801	2.1
17269	1.28186	2.1
17270	1.20797	1.9242
17271	1.06821	1.99429
17272	1.87287	0.855149
17273	2.08014	1.01537
17274	1.63225	1.06517
17275	1.39501	1.30623
17276	1.20132	0.05
17277	1.16182	0.05
17278	1.08848	0.0537531
17279	1.20132	0.0566409
17280	1.23475	1.01533
17281	1.16661	1.56988

17282	2.31317	2.1
17283	2.50145	2.1
17284	1.04367	2.1
17285	2.23875	2.1
17286	0.953632	0.124655
17287	1.38646	0.150776
17288	1.38646	0.145708
17289	1.61963	0.11927
17290	1.62934	1.07599
17291	1.88626	0.958713
17292	1.0775	0.703442
17293	1.119	0.625618
17294	1.08133	0.460355
17295	1.48312	0.327464
17296	1.57125	0.203707
17297	1.74098	0.185031
17298	1.94372	1.00023
17299	1.22237	1.03809
17300	1.18549	0.390941
17301	1.01294	0.472746
17302	1.01294	0.559426
17303	1.2064	0.606177
17304	1.14599	1.40758
17305	1.00065	0.574685
17306	1.76357	2.1
17307	2.23468	2.1
17308	2.21735	2.1
17309	3.32769	2.1
17310	1.19647	0.908117
17311	1.35307	0.578738
17312	1.19645	0.626048
17313	1.43778	0.558604
17314	1.34412	0.422102
17315	1.1315	1.08277
17316	1.1315	0.462563
17317	1.23943	0.420147
17318	1.57829	2.1
17319	1.22537	2.1
17320	1.14357	2.1
17321	1.32772	2.04006
17322	1.4259	2.1
17323	1.34245	2.1
17324	1.47953	2.1
17325	1.70303	2.1
17326	2.21127	0.0769969
17327	1.47904	0.0691591
17328	1.18793	0.075628
17329	2.21126	0.0810888
17330	1.1032	1.53409
17331	1.14986	0.702692
17332	1.14986	2.04983
17333	1.19611	0.754704
17334	1.91883	2.1
17335	1.83019	2.1
17336	1.83019	2.1
17337	1.82462	2.1
17338	1.27362	0.280008
17339	1.27421	0.198958

17340	1.27421	0.104154
17341	1.10736	0.0972463
17342	1.3003	0.573671
17343	1.31415	1.03029
17344	1.31415	0.588035
17345	1.32201	1.1948
17346	1.21124	2.1
17347	0.81568	2.1
17348	0.81568	2.1
17349	0.551816	2.1
17350	1.67119	2.1
17351	1.63384	2.1
17352	1.63384	2.1
17353	1.63661	2.1
17354	1.48734	2.1
17355	1.52245	2.1
17356	1.52245	2.1
17357	1.50714	2.1
17358	0.996778	0.05
17359	1.56091	0.05
17360	1.56091	0.0519783
17361	1.90457	0.0584116
17362	1.1181	2.1
17363	1.06543	2.1
17364	2.66828	2.1
17365	2.76932	2.1
17366	1.06543	2.1
17367	1.01474	2.1
17368	2.76932	2.1
17369	2.86704	2.1
17370	1.29724	0.350168
17371	1.96938	0.315119
17372	1.82462	2.1
17373	1.84553	2.1
17374	1.05556	2.1
17375	1.25363	2.1
17376	1.84552	2.1
17377	1.53208	2.1
17378	1.12826	2.1
17379	1.39075	2.1
17380	0.9702	0.984447
17381	1.16481	0.650509
17382	1.08894	0.883493
17383	1.91756	0.969359
17384	1.50865	0.698861
17385	1.06012	0.534313
17386	1.5456	0.295121
17387	1.37427	0.311854
17388	1.1211	0.132999
17389	1.5456	0.172368
17390	1.33135	0.171689
17391	1.07956	0.198433
17392	2.23255	0.187142
17393	1.21031	0.1491
17394	1.52903	2.1
17395	1.35977	2.1
17396	3.3837	2.1
17397	3.20581	2.1

17398	1.35977	2.1
17399	1.15414	2.1
17400	3.20582	2.1
17401	2.07596	2.1
17402	1.6812	0.101545
17403	1.03939	0.0911967
17404	1.30047	2.1
17405	1.5586	2.1
17406	1.5586	2.1
17407	1.84214	2.1
17408	1.31825	0.549138
17409	1.31923	1.0021
17410	1.31924	0.552165
17411	1.33026	0.798767
17412	1.15818	0.847722
17413	1.17822	1.08622
17414	1.15818	2.1
17415	1.28096	1.35865
17416	1.06848	0.566688
17417	1.89639	0.561367
17418	1.9186	0.948976
17419	1.77065	0.865234
17420	1.59103	0.805256
17421	1.56164	0.72614
17422	1.09697	0.05
17423	2.38719	0.05
17424	2.22123	0.05
17425	2.46059	0.05
17426	1.45506	0.332828
17427	1.03645	0.339131
17428	1.30347	2.1
17429	1.2627	2.1
17430	1.2627	2.1
17431	1.23036	2.1
17432	1.41469	2.1
17433	1.45257	2.1
17434	1.46907	0.0789709
17435	1.70302	0.0853363
17436	1.49196	0.0534233
17437	1.36307	0.0611392
17438	1.05677	2.1
17439	0.978698	2.1
17440	0.978699	2.1
17441	2.01822	2.1
17442	1.47446	2.1
17443	1.55432	2.1
17444	1.55432	2.1
17445	1.06053	2.1
17446	1.86813	1.12599
17447	1.21892	1.6073
17448	1.53064	0.948617
17449	1.30664	1.00949
17450	1.09629	2.1
17451	1.19244	2.1
17452	1.19244	2.1
17453	1.37635	2.1
17454	1.49358	0.450605
17455	1.57702	0.359392

17456	2.09374	2.1
17457	1.88175	2.1
17458	1.88175	2.1
17459	1.51085	2.1
17460	1.10938	0.344392
17461	1.02478	0.465933
17462	1.33061	0.114177
17463	1.13272	0.0633794
17464	1.75505	2.1
17465	1.78529	2.1
17466	1.6353	2.1
17467	1.59307	2.1
17468	1.60017	0.672627
17469	1.35608	0.8206
17470	1.35608	0.542895
17471	1.04713	0.555087
17472	1.01479	0.493946
17473	1.3868	0.455006
17474	1.3868	0.453992
17475	1.28418	0.709959
17476	2.15949	2.1
17477	2.90429	2.1
17478	1.51902	2.1
17479	1.54755	2.1
17480	2.90429	2.1
17481	3.49966	2.1
17482	1.70842	2.1
17483	1.97526	2.1
17484	0.949712	0.345772
17485	1.92784	0.283434
17486	1.34093	0.328587
17487	2.53426	0.383288
17488	1.92783	0.279054
17489	3.89373	0.210439
17490	2.53426	0.358529
17491	2.35961	0.369045
17492	1.68108	0.49553
17493	1.15558	0.47791
17494	1.67265	0.468123
17495	1.27608	0.45164
17496	1.42249	2.1
17497	1.39708	2.1
17498	1.39708	2.1
17499	1.39327	2.1
17500	1.97046	0.108544
17501	1.11688	0.0802488
17502	1.28418	0.429486
17503	1.17302	0.678747
17504	1.17302	0.451499
17505	1.04349	0.671848
17506	0.979853	2.1
17507	1.26016	2.1
17508	1.18901	2.1
17509	1.26016	2.1
17510	1.22857	0.628993
17511	1.25848	0.770303
17512	1.25848	0.604149
17513	1.2858	0.714065

17514	1.09154	2.1
17515	2.01915	2.1
17516	1.23013	2.1
17517	1.55698	2.1
17518	1.55698	2.1
17519	1.8575	2.1
17520	2.01854	2.1
17521	1.20686	2.1
17522	1.20684	1.64158
17523	1.77796	2.1
17524	1.46129	2.1
17525	1.04193	2.1
17526	1.10051	2.1
17527	0.983723	2.1
17528	1.1729	2.1
17529	0.98505	2.1
17530	0.985052	2.1
17531	1.03951	2.1
17532	1.03949	2.1
17533	1.17583	2.1
17534	1.42866	2.1
17535	1.97261	2.1
17536	1.73274	2.1
17537	1.53629	2.1
17538	1.98182	2.1
17539	1.72511	2.1
17540	1.42174	2.1
17541	1.44472	2.1
17542	1.72511	2.1
17543	1.45411	2.1
17544	1.44472	2.1
17545	1.43836	2.1
17546	0.962703	0.474461
17547	1.0171	0.594726
17548	1.53336	0.0812727
17549	1.40274	0.0816901
17550	1.40272	0.081269
17551	1.00213	0.0813103
17552	1.50892	0.0892951
17553	1.64984	0.0852055
17554	1.67464	0.0952601
17555	1.67481	0.0934528
17556	1.08212	2.1
17557	1.28095	2.1
17558	1.46956	2.1
17559	1.70303	2.1
17560	1.2925	0.05
17561	1.84716	0.05
17562	1.48676	0.330181
17563	1.48797	0.323136
17564	1.33917	0.3235
17565	1.22691	0.309168
17566	0.528744	2.1
17567	1.13728	2.1
17568	1.13728	2.1
17569	1.99843	2.1
17570	1.17464	0.25874
17571	1.0372	0.164366

17572	2.0815	0.092891
17573	0.933462	0.084969
17574	1.54931	2.1
17575	1.5485	2.1
17576	1.4077	2.1
17577	1.29931	2.1
17578	1.14931	0.490675
17579	2.02223	0.479055
17580	1.04482	0.330518
17581	0.94971	0.422906
17582	1.54632	1.23086
17583	1.15693	1.34793
17584	1.66763	2.1
17585	1.41238	2.1
17586	1.41238	2.1
17587	1.48068	2.1
17588	1.0171	0.437734
17589	1.1211	0.574952
17590	1.1211	0.362121
17591	1.28418	0.548358
17592	0.993021	2.1
17593	1.764 2.1	
17594	0.993023	2.1
17595	1.05787	2.1
17596	1.90914	0.453587
17597	1.12229	1.25987
17598	1.02025	0.566564
17599	0.585368	1.63197
17600	1.99323	2.1
17601	2.34988	2.1
17602	2.34988	2.1
17603	2.36987	2.1
17604	1.23107	0.0904131
17605	1.90664	0.134067
17606	1.53496	0.0861487
17607	1.70302	0.148555
17608	1.98859	2.1
17609	1.61057	2.1
17610	1.79844	2.1
17611	2.10811	2.1
17612	1.4618	2.1
17613	1.96722	2.1
17614	1.95191	2.1
17615	2.13418	2.1
17616	1.50368	0.0525598
17617	1.61417	0.05
17618	1.15008	0.05
17619	1.19685	0.05
17620	1.90456	0.057855
17621	1.90899	0.050352
17622	2.08864	0.0552016
17623	2.10447	0.05
17624	1.16653	2.1
17625	1.23017	2.1
17626	1.0752	0.455515
17627	1.34412	0.390815
17628	1.06226	2.1
17629	1.09252	2.1

17630	1.09252	2.1
17631	0.88579	2.1
17632	1.56178	0.223481
17633	1.14305	0.194542
17634	1.56175	0.177616
17635	1.08211	0.14329
17636	1.32813	2.1
17637	1.32461	2.1
17638	1.32461	2.1
17639	1.32455	2.1
17640	1.42729	0.190776
17641	1.18716	0.186554
17642	1.47169	0.342787
17643	1.24687	0.249032
17644	1.09627	2.1
17645	1.04759	2.1
17646	1.04759	2.1
17647	0.987132	2.1
17648	1.11181	1.3135
17649	1.80662	2.1
17650	1.8066	2.1
17651	1.06461	1.68975
17652	1.56091	2.1
17653	1.9842	2.1
17654	1.73525	0.269409
17655	1.40557	0.216166
17656	2.26906	0.278122
17657	1.31248	0.406628
17658	1.66832	0.244977
17659	1.32634	0.171851
17660	1.03314	0.05
17661	1.24682	0.05
17662	1.38536	0.05
17663	1.52661	0.05
17664	1.177	0.911907
17665	1.36405	0.773504
17666	1.19837	0.853134
17667	1.17701	0.431943
17668	0.993831	0.355923
17669	1.0823	0.344741
17670	1.0823	0.338778
17671	1.01121	0.337788
17672	1.49653	0.136474
17673	1.89583	0.239102
17674	0.67242	0.118161
17675	1.36047	0.21601
17676	1.00366	0.228915
17677	1.24728	0.289931
17678	1.13387	0.05
17679	1.60867	0.05
17680	1.30572	0.0764824
17681	1.54982	0.0546937
17682	0.788592	0.05
17683	1.82961	0.05
17684	1.54982	0.0660025
17685	1.82961	0.05
17686	1.26062	0.05
17687	1.64002	0.05

17688	1.12035	0.05
17689	1.64002	0.05
17690	1.1673	2.1
17691	1.75367	2.1
17692	0.964034	1.98061
17693	1.0305	1.50895
17694	1.30896	1.80953
17695	1.52776	1.74398
17696	1.52776	2.1
17697	1.17989	1.97471
17698	1.16759	2.1
17699	1.8575	2.1
17700	1.16759	2.1
17701	1.60936	2.1
17702	1.10747	0.317184
17703	1.47688	0.311237
17704	1.40794	2.1
17705	1.95729	1.20499
17706	1.95729	2.1
17707	3.18156	1.61865
17708	1.87598	0.845922
17709	1.94343	2.1
17710	1.94343	0.831635
17711	2.00551	2.1
17712	1.20067	0.980211
17713	1.70744	1.79692
17714	1.87244	1.6275
17715	1.32075	1.19056
17716	1.64955	2.1
17717	1.44221	2.1
17718	1.31109	2.1
17719	1.14206	2.1
17720	1.60452	1.61243
17721	1.12111	0.706285
17722	1.00832	2.1
17723	0.974137	2.1
17724	0.974137	2.1
17725	0.98505	2.1
17726	1.02368	2.1
17727	0.985051	2.1
17728	0.995734	0.386151
17729	1.31993	0.384089
17730	1.31993	0.367217
17731	1.18549	0.498496
17732	1.5176	0.64686
17733	1.33622	0.601977
17734	1.33621	0.606717
17735	1.02782	0.576494
17736	1.66774	2.1
17737	1.85305	2.1
17738	1.85305	2.1
17739	2.07088	2.1
17740	1.78873	2.1
17741	1.47533	2.1
17742	1.23152	2.1
17743	0.99922	2.1
17744	1.48493	2.1
17745	1.1394	2.1

17746	1.19179	2.1
17747	1.64289	2.1
17748	1.47858	2.1
17749	1.89687	2.1
17750	0.972464	2.1
17751	1.79473	2.1
17752	1.06971	2.1
17753	1.21089	2.1
17754	1.12572	2.1
17755	1.30566	2.1
17756	1.30566	2.1
17757	1.52724	2.1
17758	2.32237	0.374014
17759	2.20248	0.321286
17760	2.79564	0.430117
17761	2.32237	0.391411
17762	1.59354	0.245731
17763	3.098	0.232039
17764	3.09801	0.219349
17765	3.45132	0.209422
17766	1.24001	2.03813
17767	1.41822	0.719777
17768	1.41821	2.1
17769	1.44859	0.866082
17770	1.34539	2.1
17771	1.06991	2.07335
17772	1.18076	2.1
17773	0.978586	2.1
17774	0.999189	0.148588
17775	2.41076	0.267503
17776	0.968475	2.02028
17777	1.02074	2.1
17778	1.02074	1.85461
17779	1.07866	2.1
17780	0.817702	0.105139
17781	1.5082	0.119237
17782	1.48376	0.15483
17783	1.18947	0.117753
17784	1.10638	2.1
17785	1.86766	2.1
17786	1.19785	2.1
17787	1.34141	2.1
17788	1.34141	2.1
17789	0.991985	2.1
17790	2.35516	0.702122
17791	1.24815	0.74874
17792	1.80454	2.1
17793	1.22633	2.1
17794	0.696255	2.1
17795	1.19379	0.776465
17796	1.14721	2.1
17797	1.16153	2.1
17798	1.16153	2.1
17799	1.17261	2.1
17800	1.20814	2.1
17801	1.54791	2.1
17802	1.54791	2.1
17803	1.60477	2.1

17804	1.29931	2.1
17805	1.19098	2.1
17806	1.19098	2.1
17807	1.13341	2.1
17808	1.00503	2.00759
17809	1.25334	0.653242
17810	1.22655	2.1
17811	1.26282	2.1
17812	1.26282	2.1
17813	0.999489	1.25516
17814	1.54197	0.20752
17815	1.17152	0.239659
17816	1.17152	0.225186
17817	1.49627	0.201443
17818	1.36405	0.797463
17819	0.976334	0.63618
17820	1.12495	2.1
17821	1.54096	2.1
17822	1.75263	2.1
17823	1.49114	2.1
17824	0.842998	1.94865
17825	1.60244	0.911204
17826	1.48911	1.61096
17827	2.18685	0.977363
17828	1.75236	1.22582
17829	1.54143	1.54304
17830	1.40013	0.782254
17831	0.946646	0.887743
17832	1.50714	2.1
17833	1.46055	2.1
17834	1.46055	2.1
17835	1.42249	2.1
17836	2.12839	0.116502
17837	1.75211	0.110006
17838	1.76297	0.0860619
17839	1.70736	0.0715455
17840	1.19952	0.270042
17841	1.56475	0.170477
17842	1.74312	0.145092
17843	1.48973	0.100389
17844	1.91518	0.269161
17845	1.16534	0.252783
17846	1.53556	2.1
17847	1.56253	2.1
17848	1.56253	2.1
17849	1.58054	2.1
17850	1.27806	0.05
17851	1.44593	0.05
17852	1.44593	0.05
17853	1.3946	0.05
17854	1.34857	0.490657
17855	1.03818	1.65667
17856	1.38969	0.563933
17857	1.85548	0.622437
17858	1.03108	0.559728
17859	1.85548	0.602804
17860	1.49346	1.75898
17861	1.83192	2.1

17862	1.75291	1.38487
17863	1.35435	1.90516
17864	0.921851	0.546521
17865	1.09646	0.520315
17866	1.2183	0.513768
17867	1.36717	0.459688
17868	2.14179	0.408607
17869	1.79805	0.463518
17870	1.3428	0.449594
17871	2.30493	0.442356
17872	1.81545	0.440104
17873	1.92269	0.441104
17874	2.30492	0.46602
17875	1.3663	0.518596
17876	1.22418	1.71497
17877	0.956698	0.665975
17878	1.4706	1.85924
17879	0.953295	2.1
17880	1.47059	0.949729
17881	1.26929	2.1
17882	1.24538	0.309216
17883	1.4468	0.243078
17884	2.86762	2.1
17885	2.32911	2.1
17886	2.10864	2.1
17887	1.94548	2.1
17888	1.83134	2.1
17889	2.19519	2.1
17890	2.19853	2.1
17891	2.63855	2.1
17892	1.08213	0.146907
17893	1.46389	0.219814
17894	1.62235	0.33753
17895	0.980549	0.356335
17896	1.08195	1.91755
17897	0.99002	2.03015
17898	1.2269	2.1
17899	1.0838	2.1
17900	0.889491	2.1
17901	1.19587	2.05498
17902	1.18801	0.0815056
17903	1.57125	0.0835723
17904	1.47712	2.1
17905	1.45182	2.1
17906	1.45182	2.1
17907	1.42255	2.1
17908	1.17816	0.0577592
17909	1.00982	0.0627717
17910	1.21056	0.05
17911	1.17816	0.05
17912	3.80902	0.641969
17913	0.976991	0.574523
17914	1.09962	2.1
17915	1.60976	2.08668
17916	1.87029	2.1
17917	2.33806	2.1
17918	2.12549	2.1
17919	2.21472	2.1

17920	2.16221	0.143229
17921	1.99528	0.119878
17922	1.99528	0.115154
17923	1.97969	0.10768
17924	3.60854	2.1
17925	4.00847	2.1
17926	2.70372	2.1
17927	1.253	2.1
17928	1.65739	2.1
17929	0.994294	2.1
17930	1.36203	2.1
17931	1.00965	2.1
17932	2.33374	2.1
17933	1.36209	2.1
17934	1.64983	0.0674176
17935	1.36416	0.0672959
17936	2.19766	0.0599457
17937	1.36416	0.0755946
17938	2.15871	0.626709
17939	1.81454	0.537775
17940	1.54607	0.399517
17941	1.57702	0.505274
17942	0.948748	0.05
17943	1.04522	0.05
17944	1.60994	0.05
17945	1.90382	0.05
17946	1.90382	0.05
17947	1.57999	0.05
17948	1.73905	0.64287
17949	1.26535	0.967601
17950	2.12552	0.896916
17951	1.06891	0.79587
17952	1.13999	0.82874
17953	2.29812	0.899683
17954	2.83329	0.790543
17955	2.16272	0.722469
17956	1.71277	2.1
17957	1.68401	2.1
17958	1.68401	2.1
17959	1.69368	2.1
17960	1.06472	2.1
17961	1.56017	2.1
17962	1.2765	2.1
17963	1.99541	2.1
17964	1.46041	0.05
17965	2.04817	0.05
17966	2.04817	0.05
17967	2.33181	0.05
17968	2.06353	2.1
17969	1.35449	2.1
17970	1.52045	2.1
17971	1.45437	2.1
17972	1.04405	0.05
17973	1.85945	0.05
17974	1.85945	0.05
17975	2.22679	0.05
17976	1.33339	2.1
17977	1.19376	2.1

17978	1.33266	2.1
17979	1.44244	2.1
17980	1.20455	2.1
17981	1.52852	2.1
17982	1.03882	2.1
17983	1.52852	2.1
17984	1.44801	2.09481
17985	1.00775	2.04105
17986	0.53907	2.1
17987	0.506124	2.1
17988	2.39045	2.1
17989	0.973794	2.1
17990	1.11193	2.1
17991	1.32784	2.1
17992	1.31595	2.1
17993	0.966115	2.1
17994	1.25953	2.1
17995	1.38086	2.1
17996	1.38086	2.1
17997	1.04101	2.1
17998	1.99319	0.0887506
17999	1.23107	0.104642
18000	3.3606	0.102099
18001	1.23112	0.116548
18002	1.40239	2.1
18003	1.26091	2.1
18004	1.26091	2.1
18005	1.05776	2.1
18006	1.23768	0.537742
18007	1.0142	1.29165
18008	1.11435	1.01663
18009	1.01422	0.795245
18010	1.24001	0.677997
18011	1.42186	0.97944
18012	1.42186	0.736637
18013	1.47204	0.827716
18014	1.27361	2.1
18015	1.56642	2.1
18016	1.71676	2.1
18017	2.16815	2.1
18018	1.07855	1.09823
18019	1.07612	1.15779
18020	1.703	0.0944026
18021	1.46853	0.082652
18022	1.45448	0.0578099
18023	1.27778	0.0604954
18024	0.991986	2.1
18025	1.1192	2.1
18026	1.1192	2.1
18027	1.25953	2.1
18028	2.08506	2.1
18029	1.75162	0.982512
18030	1.94523	2.1
18031	1.88405	2.03149
18032	1.15506	0.05
18033	1.1233	0.05
18034	2.09736	2.1
18035	2.98303	2.1

18036	2.71184	2.1
18037	3.51614	2.1
18038	1.18769	2.1
18039	1.24053	2.1
18040	1.24053	2.1
18041	1.45327	2.1
18042	1.26865	1.5639
18043	1.1744	1.31047
18044	1.1744	0.916031
18045	1.11712	1.06995
18046	1.15841	0.399218
18047	1.02247	0.417262
18048	1.93649	2.1
18049	1.81191	2.1
18050	1.64718	2.1
18051	1.47521	2.1
18052	1.59654	2.1
18053	1.57823	2.1
18054	1.57823	2.1
18055	1.55768	2.1
18056	1.00665	0.326573
18057	1.28239	0.320837
18058	1.28239	0.324059
18059	1.44951	0.31336
18060	1.22083	2.1
18061	0.957622	2.1
18062	0.887896	2.1
18063	1.27387	2.1
18064	1.00521	0.961899
18065	1.00503	1.01292
18066	1.95844	2.1
18067	1.05042	2.1
18068	2.78662	2.1
18069	2.22399	2.1
18070	2.3356	0.0527622
18071	1.49543	0.0696713
18072	1.96854	0.05
18073	1.21681	0.05
18074	2.17803	1.59434
18075	1.50998	1.95099
18076	2.35239	1.40205
18077	2.26987	1.08888
18078	1.03824	0.322377
18079	1.51396	0.431963
18080	1.51396	0.384648
18081	1.48693	0.369283
18082	1.08784	2.1
18083	1.36865	2.0558
18084	0.712405	2.1
18085	1.08784	2.1
18086	2.02522	2.1
18087	1.6738	2.1
18088	1.35175	2.1
18089	1.50094	2.1
18090	1.6738	2.1
18091	1.62871	2.1
18092	1.35084	2.1
18093	1.34276	2.1

18094	1.18041	2.1
18095	1.43652	2.1
18096	0.974196	2.1
18097	1.28037	2.1
18098	1.28037	2.1
18099	1.05071	2.1
18100	2.44737	0.05
18101	1.4672	0.05
18102	1.22883	0.0537925
18103	2.08149	0.0622854
18104	1.82208	1.88174
18105	1.59826	1.82348
18106	1.59826	1.31049
18107	1.01393	2.1
18108	1.42757	2.1
18109	0.996775	2.1
18110	1.42757	2.1
18111	1.26397	2.1
18112	1.02461	2.1
18113	1.46129	2.1
18114	1.11833	2.1
18115	1.335	2.1
18116	2.07087	2.1
18117	1.24041	2.1
18118	1.335	2.1
18119	1.55398	2.1
18120	1.21171	0.0943022
18121	1.48589	0.0951075
18122	1.32886	0.0771953
18123	1.8027	0.0778866
18124	1.53515	1.49476
18125	1.60428	1.49372
18126	1.00035	0.05
18127	1.03534	0.0674145
18128	1.03535	0.640657
18129	1.09962	1.54329
18130	0.62132	1.08817
18131	1.21033	2.02015
18132	1.30326	1.47533
18133	2.6823	2.1
18134	0.991407	1.77667
18135	1.29729	0.926037
18136	1.29729	0.883217
18137	1.16858	2.1
18138	1.66137	0.387449
18139	1.23012	0.18256
18140	1.66139	0.400242
18141	1.41821	0.237407
18142	1.56043	0.592436
18143	1.06848	1.02421
18144	1.56043	1.71511
18145	1.90911	1.34907
18146	0.982847	2.1
18147	1.05863	2.1
18148	1.05863	2.1
18149	1.22352	2.1
18150	1.23019	1.31767
18151	1.60509	1.89131

18152	1.19122	2.1
18153	1.47769	2.1
18154	2.18868	2.1
18155	1.40984	1.45777
18156	3.55197	2.1
18157	3.57227	2.1
18158	3.57227	2.1
18159	3.48926	2.1
18160	1.4375	0.389839
18161	1.11314	0.390913
18162	1.62408	2.1
18163	1.74438	2.1
18164	1.72387	2.1
18165	1.50018	2.1
18166	1.1486	2.1
18167	1.18474	2.1
18168	1.18474	2.1
18169	1.2295	2.1
18170	1.28754	2.1
18171	1.36838	2.1
18172	1.36838	2.1
18173	1.21703	2.1
18174	2.74206	2.1
18175	2.70795	2.1
18176	2.70795	2.1
18177	2.55918	2.1
18178	1.35065	0.802378
18179	1.65411	0.57163
18180	1.48572	0.723132
18181	1.28393	0.676315
18182	1.25265	0.05
18183	1.70944	0.05
18184	1.31259	0.569171
18185	1.14945	1.14527
18186	1.14945	0.519181
18187	0.995725	1.32385
18188	1.00307	0.395609
18189	2.05786	0.408316
18190	1.56357	0.368488
18191	1.10907	0.395695
18192	1.27558	1.27837
18193	1.05175	0.876561
18194	1.2685	2.1
18195	1.41369	2.1
18196	1.4137	2.1
18197	1.49654	2.1
18198	1.21489	2.1
18199	1.14605	1.62404
18200	1.22476	2.1
18201	1.21489	1.0951
18202	2.51472	1.09361
18203	2.52091	1.05967
18204	2.52091	0.915846
18205	1.79171	0.879126
18206	1.03373	1.83292
18207	1.04037	2.1
18208	1.04038	1.7513
18209	1.03933	2.1

18210	1.74123	0.1302
18211	1.41902	0.109272
18212	1.89777	0.135591
18213	1.89899	0.199657
18214	1.24728	0.185807
18215	1.30394	0.218229
18216	1.30394	0.13264
18217	1.16535	0.105541
18218	1.09914	2.1
18219	1.54259	2.1
18220	1.40233	2.1
18221	1.69858	2.1
18222	1.53555	2.1
18223	1.09859	0.784994
18224	1.09859	2.1
18225	0.725879	0.670819
18226	1.07213	2.1
18227	1.31589	2.1
18228	1.36904	2.1
18229	1.69711	2.1
18230	1.51028	0.0635212
18231	0.993598	0.103214
18232	0.61563	0.0654312
18233	1.26311	0.0717254
18234	1.19496	2.1
18235	1.65925	2.1
18236	1.65925	2.1
18237	1.48162	2.1
18238	2.75738	1.3362
18239	1.24699	1.23687
18240	4.08729	1.81389
18241	2.35271	1.51598
18242	1.55063	0.405665
18243	1.34563	0.359086
18244	1.34563	0.372977
18245	1.08189	0.342935
18246	1.09662	0.295515
18247	2.35961	0.360272
18248	0.986952	0.194017
18249	0.813251	0.161528
18250	1.17897	0.0545757
18251	1.67685	0.05
18252	1.00573	1.18842
18253	1.08542	0.835026
18254	1.00573	2.1
18255	1.12572	2.1
18256	1.05499	2.1
18257	1.00521	1.01698
18258	2.22679	0.05
18259	2.86746	0.05
18260	2.86746	0.05
18261	1.99958	0.05
18262	0.996517	2.1
18263	1.01169	2.1
18264	1.01169	2.1
18265	1.26346	2.1
18266	1.68274	2.1
18267	1.75505	2.1

18268	2.28748	1.6311
18269	1.36555	1.51201
18270	3.47888	0.05
18271	0.692788	0.0550771
18272	1.0685	0.47237
18273	1.14547	0.568633
18274	1.19017	2.1
18275	1.24976	2.1
18276	1.24976	2.1
18277	1.30682	2.1
18278	2.2153	2.1
18279	1.85457	2.1
18280	2.03267	2.1
18281	1.51042	2.1
18282	1.05619	0.70136
18283	1.05793	0.83918
18284	1.05793	0.682564
18285	1.00763	0.834922
18286	1.77677	2.1
18287	1.02524	2.1
18288	1.02524	2.1
18289	1.18803	2.1
18290	1.68669	2.1
18291	1.71162	2.1
18292	1.556 2.1	
18293	1.39523	2.1
18294	0.960288	0.692145
18295	1.24201	0.850399
18296	1.24202	2.1
18297	1.3592	2.1
18298	2.14045	0.152607
18299	1.62054	0.135423
18300	2.20248	0.295136
18301	2.34699	0.250373
18302	3.45132	0.162709
18303	3.38241	0.165899
18304	3.38241	0.13062
18305	2.96127	0.130757
18306	2.02612	2.1
18307	2.09351	2.1
18308	2.09351	2.1
18309	2.17465	2.1
18310	1.07445	2.1
18311	1.14808	2.1
18312	1.39067	2.07138
18313	1.20958	1.313
18314	1.20958	1.7852
18315	1.00382	1.29608
18316	0.986496	2.1
18317	1.27142	2.1
18318	1.39857	2.1
18319	2.26482	2.0214
18320	2.24367	0.05
18321	1.9234	0.05
18322	1.59076	0.05
18323	1.185 0.05	
18324	3.86179	0.05
18325	3.0638	0.05

18326	2.7574	0.05
18327	1.83266	0.05
18328	2.59418	2.1
18329	2.23048	2.1
18330	1.39564	2.1
18331	1.36763	2.1
18332	2.23048	2.1
18333	2.02522	2.1
18334	1.36763	2.1
18335	1.35175	2.1
18336	1.16181	0.05
18337	0.97096	0.05
18338	2.05665	0.17405
18339	0.483583	0.192182
18340	0.601928	0.0909116
18341	3.88545	0.234553
18342	1.04909	2.1
18343	0.983143	2.1
18344	1.10469	2.1
18345	1.10516	2.1
18346	1.10517	2.1
18347	1.09951	2.1
18348	2.33985	2.1
18349	2.35026	2.1
18350	2.35025	2.1
18351	1.19462	2.1
18352	1.57627	2.1
18353	1.5493	2.1
18354	1.5493	2.1
18355	1.53556	2.1
18356	1.42763	0.517798
18357	1.24682	0.515337
18358	1.24682	0.467104
18359	1.00307	0.484894
18360	1.37392	2.1
18361	1.45408	2.1
18362	1.45408	2.1
18363	1.54249	2.1
18364	1.24474	1.35155
18365	1.07467	1.41445
18366	1.37813	1.71488
18367	1.24474	1.65949
18368	1.06341	2.1
18369	1.09194	2.1
18370	1.09194	2.1
18371	1.11117	2.1
18372	1.1658	2.1
18373	1.93707	2.1
18374	1.93707	2.1
18375	1.47729	2.1
18376	1.25669	0.638409
18377	1.07519	2.1
18378	1.65417	2.1
18379	1.50956	2.1
18380	1.37231	2.1
18381	1.27285	2.1
18382	1.23612	2.1
18383	1.1899	2.1

18384	1.08172	2.1
18385	1.20357	2.1
18386	1.5664	2.1
18387	1.7472	2.1
18388	1.48434	2.1
18389	1.23989	2.1
18390	1.58836	2.1
18391	1.38766	2.1
18392	1.23989	2.1
18393	1.26599	2.1
18394	2.56248	2.1
18395	2.08673	0.741452
18396	1.87216	0.808284
18397	1.60476	0.234432
18398	1.84355	2.1
18399	1.25971	0.710333
18400	1.44141	0.706812
18401	1.25521	0.192675
18402	1.19837	0.05
18403	2.31936	0.05
18404	1.38153	2.1
18405	2.41734	2.1
18406	2.70856	2.1
18407	3.48385	2.1
18408	1.89226	1.11188
18409	1.64617	1.40503
18410	1.89112	1.17446
18411	1.23135	0.539185
18412	1.23134	0.60644
18413	1.34927	0.568708
18414	3.60096	0.05
18415	1.68103	0.0521894
18416	2.25644	2.1
18417	1.91955	2.1
18418	1.91955	2.1
18419	1.47688	2.1
18420	1.67375	2.1
18421	1.50033	2.1
18422	1.5098	2.1
18423	1.46908	2.1
18424	1.69055	0.196586
18425	1.53463	0.178468
18426	1.53463	0.107695
18427	1.41943	0.112269
18428	1.19022	1.76326
18429	1.95981	1.34616
18430	1.15009	0.902999
18431	1.31612	0.912715
18432	1.31611	0.632845
18433	1.10735	0.321426
18434	1.18601	0.318836
18435	1.3499	0.315179
18436	1.3499	0.315093
18437	1.29001	0.313677
18438	1.22083	2.1
18439	1.06191	2.1
18440	1.348	1.00166
18441	1.05758	1.49453

18442	1.34799	2.04327
18443	1.18647	1.65206
18444	2.92457	0.0920379
18445	1.99182	0.0890637
18446	2.25313	0.0887861
18447	1.30859	0.0932189
18448	1.30682	2.1
18449	1.38957	2.1
18450	1.38957	2.1
18451	1.47446	2.1
18452	0.953638	1.17749
18453	1.03408	1.759
18454	1.17885	0.520391
18455	1.3663	0.463892
18456	1.08265	0.682169
18457	1.71948	0.816747
18458	1.29501	0.939015
18459	0.955093	0.866377
18460	0.955081	0.680612
18461	1.88345	0.653565
18462	1.2243	2.1
18463	1.23169	2.1
18464	2.04726	1.75041
18465	0.842869	1.83091
18466	3.18156	2.1
18467	3.60786	1.31558
18468	3.60786	2.1
18469	2.38566	1.02672
18470	1.24526	0.05
18471	1.59019	0.05
18472	1.16776	0.309373
18473	1.05042	0.98811
18474	1.17418	1.74086
18475	1.05042	2.1
18476	0.949135	2.1
18477	0.973448	2.1
18478	0.973447	2.1
18479	1.02958	2.1
18480	3.57768	2.1
18481	3.04246	2.1
18482	3.04246	2.1
18483	2.47824	2.1
18484	1.35879	2.1
18485	1.10407	1.02153
18486	1.14599	0.586987
18487	1.10407	0.608641
18488	1.87794	1.50971
18489	1.53429	1.23756
18490	1.12717	2.1
18491	1.38634	2.1
18492	1.38634	2.1
18493	1.13127	2.1
18494	1.04546	2.1
18495	1.06007	2.1
18496	1.06007	2.1
18497	1.19785	2.1
18498	1.43652	2.1
18499	1.71741	2.05013

18500	1.70921	0.936954
18501	1.06994	0.932936
18502	0.960106	2.1
18503	1.48077	2.1
18504	1.48077	2.1
18505	2.5946	2.1
18506	2.03045	1.43419
18507	1.76788	1.86804
18508	2.03045	2.1
18509	0.633098	2.1
18510	1.06756	2.1
18511	1.38667	2.1
18512	0.966979	2.1
18513	1.38669	2.1
18514	1.07156	2.1
18515	0.986095	2.1
18516	1.32813	2.1
18517	0.986095	2.1
18518	1.98697	1.16004
18519	1.12016	2.1
18520	1.24463	1.46414
18521	1.1967	2.04289
18522	1.30001	0.558853
18523	1.29175	1.12743
18524	1.29175	0.572842
18525	1.3003	1.21501
18526	1.5606	0.05
18527	2.32098	0.05
18528	2.57888	0.05
18529	2.97463	0.05
18530	1.24555	2.1
18531	1.46101	2.1
18532	1.90723	2.1
18533	2.29126	2.1
18534	1.46101	2.1
18535	1.84089	2.1
18536	2.5519	2.1
18537	2.91663	2.1
18538	1.10476	0.0977099
18539	1.44103	0.0882345
18540	1.44103	0.0870083
18541	1.53336	0.0847405
18542	0.672964	2.1
18543	0.633884	2.1
18544	0.633884	2.1
18545	0.603154	2.1
18546	2.29663	2.1
18547	2.06892	2.1
18548	1.3685	2.1
18549	1.39067	2.1
18550	2.06892	2.1
18551	1.78208	2.1
18552	1.39067	2.1
18553	1.42174	2.1
18554	1.01335	1.80527
18555	1.08195	1.01249
18556	1.86463	1.50992
18557	1.39827	1.67219

18558	0.861515	2.07677
18559	2.10074	2.1
18560	2.09511	0.587688
18561	2.04853	0.534316
18562	2.21488	0.554365
18563	1.38287	0.675248
18564	0.974717	0.668118
18565	0.998451	0.63027
18566	1.42281	0.95221
18567	1.82595	0.884013
18568	1.50458	0.748169
18569	1.09408	0.644466
18570	1.23498	1.22387
18571	1.05654	1.17897
18572	1.17261	2.1
18573	1.18058	2.1
18574	1.18059	2.1
18575	1.18907	2.1
18576	2.38998	2.1
18577	2.77437	2.1
18578	1.04546	2.1
18579	1.16817	2.1
18580	2.77437	2.1
18581	2.97768	2.1
18582	1.16817	2.1
18583	1.2832	2.1
18584	1.75766	0.339485
18585	1.4934	0.749262
18586	1.4934	0.402222
18587	1.20324	0.930807
18588	1.07236	0.62074
18589	1.37641	0.546423
18590	1.42913	0.568251
18591	1.93373	0.612437
18592	1.42629	0.560279
18593	1.00636	0.470299
18594	0.933959	1.96494
18595	2.22873	0.758738
18596	2.22873	1.05754
18597	2.13757	0.589455
18598	1.10517	2.1
18599	1.86737	2.1
18600	0.811 2.1	
18601	1.19179	2.1
18602	1.18647	2.1
18603	0.988453	2.1
18604	1.12457	0.108556
18605	1.23851	0.0908647
18606	0.92492	2.1
18607	1.45656	2.1
18608	1.61251	0.746958
18609	1.56696	0.770017
18610	1.90245	0.625357
18611	1.46046	0.618218
18612	1.60329	0.0598629
18613	1.38247	0.0582438
18614	1.38247	0.0509575
18615	1.23198	0.0528219

18616	1.09518	2.1
18617	1.28159	2.1
18618	0.977777	2.1
18619	1.80778	2.1
18620	1.80777	2.1
18621	1.16655	2.1
18622	1.3515	2.1
18623	0.956573	2.1
18624	1.08022	2.1
18625	1.03437	2.1
18626	1.03437	2.1
18627	0.975409	2.1
18628	1.47369	0.0851109
18629	1.31167	0.156701
18630	1.01847	0.08675
18631	2.75562	0.0894671
18632	1.13443	2.1
18633	1.32709	2.1
18634	1.01051	0.224348
18635	0.958947	0.152494
18636	1.7211	2.1
18637	1.922 2.1	
18638	1.922 2.1	
18639	0.991113	2.1
18640	1.00636	0.424458
18641	1.31658	0.525064
18642	1.44627	0.470018
18643	1.03824	0.420051
18644	1.21223	2.1
18645	1.01242	2.1
18646	1.01243	2.1
18647	0.956698	1.41326
18648	1.23167	2.1
18649	1.06935	2.1
18650	1.29215	0.05
18651	0.998971	0.05
18652	1.50103	0.05
18653	1.44836	0.05
18654	3.02513	2.1
18655	3.7335	2.1
18656	2.49988	2.1
18657	1.24948	2.1
18658	0.963972	2.1
18659	1.1822	2.1
18660	1.1822	2.1
18661	1.10817	2.1
18662	1.30237	2.1
18663	1.41908	2.1
18664	1.41908	2.1
18665	1.48734	2.1
18666	1.31127	0.598514
18667	1.40822	0.562841
18668	1.40822	0.482907
18669	1.22176	0.470048
18670	1.82525	2.1
18671	1.59394	2.1
18672	1.22731	0.0734784
18673	0.981584	0.0583276

18674	1.1427	0.658666
18675	1.44091	0.906762
18676	1.49154	0.675476
18677	1.07526	0.643243
18678	1.07527	0.444937
18679	0.96029	0.66527
18680	2.40568	0.98042
18681	3.46486	0.866569
18682	2.417	0.951054
18683	1.52054	0.733354
18684	1.51638	0.336808
18685	1.04786	0.251032
18686	1.51404	0.304501
18687	1.3461	0.208957
18688	1.27696	0.193093
18689	0.736457	0.11197
18690	1.16817	2.1
18691	1.04263	2.1
18692	1.13449	2.1
18693	1.50524	2.1
18694	2.1182	2.1
18695	2.49001	2.1
18696	1.11764	2.1
18697	1.39292	2.1
18698	2.49001	2.1
18699	2.76339	2.1
18700	1.39292	2.1
18701	1.68761	2.1
18702	1.25173	2.1
18703	1.34701	2.1
18704	1.02415	2.1
18705	1.34701	1.98293
18706	1.332	0.0564356
18707	0.989764	0.05
18708	0.963167	0.05
18709	1.21056	0.05
18710	1.67375	2.1
18711	1.17965	2.1
18712	1.09154	2.1
18713	1.52314	2.1
18714	1.54433	0.507722
18715	1.81969	0.443282
18716	1.3598	0.443016
18717	1.71041	0.388864
18718	1.70934	0.39646
18719	1.33871	0.376231
18720	1.79485	0.380917
18721	1.51404	0.309215
18722	1.30751	2.1
18723	1.20969	2.1
18724	1.20969	2.1
18725	1.12076	2.1
18726	1.49808	2.1
18727	1.50201	2.1
18728	1.50201	2.1
18729	1.31594	2.1
18730	1.2187	2.1
18731	1.11545	2.1

18732	1.227	2.1
18733	1.09645	2.1
18734	1.05902	0.05
18735	1.41595	0.237299
18736	1.35696	2.05365
18737	1.29446	0.64663
18738	0.857957	2.1
18739	1.21125	0.05
18740	2.43115	2.1
18741	2.1151	0.49116
18742	2.0815	1.1427
18743	1.1723	1.99932
18744	1.48035	0.411229
18745	1.59971	0.524835
18746	1.33668	0.455981
18747	1.0961	0.413757
18748	1.46235	2.1
18749	0.882195	2.1
18750	2.46624	2.1
18751	2.72161	2.1
18752	2.96094	2.1
18753	2.25644	2.1
18754	1.25923	0.05
18755	0.976218	0.05
18756	1.10319	0.05
18757	1.82583	0.05
18758	0.888955	2.1
18759	1.30817	2.1
18760	1.30817	2.1
18761	1.60787	2.1
18762	0.988805	1.40074
18763	1.54168	0.726577
18764	1.19496	0.742438
18765	1.12224	1.48091
18766	1.93597	1.09389
18767	1.31113	1.02602
18768	1.17879	0.952558
18769	1.07468	0.98799
18770	2.0523	2.1
18771	2.41385	2.1
18772	2.41385	2.1
18773	3.0355	2.1
18774	1.36717	0.456641
18775	1.45535	0.291365
18776	1.45535	0.284793
18777	1.49906	0.197144
18778	1.37785	0.803467
18779	1.04413	1.99127
18780	1.06729	0.743907
18781	1.54305	0.687757
18782	1.07444	2.1
18783	1.52724	0.910156
18784	1.49151	2.1
18785	1.36814	2.1
18786	1.36814	2.1
18787	1.15541	2.1
18788	1.19379	2.1
18789	1.78657	0.684815

18790	1.6079	2.1
18791	2.59469	0.698831
18792	1.35545	0.617258
18793	1.23602	0.784856
18794	1.23452	2.1
18795	1.56842	0.810266
18796	1.15864	2.1
18797	1.11955	2.1
18798	1.11955	2.1
18799	1.08022	2.1
18800	1.64268	1.98778
18801	1.26247	2.1
18802	1.16912	2.1
18803	2.1095	0.917581
18804	1.75549	0.0612014
18805	1.7491	0.0796055
18806	1.59833	0.090027
18807	1.68338	0.105487
18808	1.14846	0.0665611
18809	1.06515	0.0691193
18810	1.2721	0.163766
18811	1.32639	0.122904
18812	1.32639	0.332042
18813	1.19323	0.159184
18814	1.81034	2.1
18815	1.9034	2.1
18816	2.08245	2.1
18817	1.84887	2.1
18818	1.41448	2.1
18819	1.77326	2.1
18820	1.76872	2.1
18821	1.86766	2.1
18822	1.46643	2.1
18823	1.58995	2.1
18824	1.58995	2.1
18825	1.13854	2.1
18826	1.23654	2.1
18827	1.04442	2.1
18828	1.23654	2.1
18829	1.08576	2.1
18830	0.966979	2.1
18831	1.41758	2.1
18832	1.03108	0.424178
18833	1.94412	0.46945
18834	1.32455	2.1
18835	1.32328	2.1
18836	1.32328	2.1
18837	1.34008	2.1
18838	1.60861	2.1
18839	1.69586	2.1
18840	1.69586	2.1
18841	1.78301	2.1
18842	1.7513	2.1
18843	1.67906	2.1
18844	1.67906	2.1
18845	1.60635	2.1
18846	1.68073	2.1
18847	1.57234	2.1

18848	1.57233	2.1
18849	1.47012	2.1
18850	1.22811	0.33426
18851	1.79683	0.326909
18852	1.53417	0.326883
18853	1.08732	0.334899
18854	1.8903	1.04371
18855	1.20247	0.968995
18856	1.12087	1.59743
18857	1.54699	1.87442
18858	1.43635	2.1
18859	1.02368	2.1
18860	1.43635	2.1
18861	1.23007	2.1
18862	1.035	2.1
18863	1.07889	2.1
18864	3.0355	2.1
18865	3.78805	1.96617
18866	2.57183	1.25459
18867	1.27835	0.934932
18868	1.29813	0.345548
18869	1.42965	0.346182
18870	1.69832	0.350511
18871	2.18359	0.355686
18872	1.42964	0.347584
18873	1.65723	0.34608
18874	1.98508	0.353622
18875	2.06284	0.356357
18876	1.50705	0.713671
18877	1.51153	0.457624
18878	1.36037	0.473857
18879	1.09767	0.449933
18880	1.70048	1.45652
18881	1.15177	1.20439
18882	0.713174	2.1
18883	0.763575	2.1
18884	0.763575	2.1
18885	0.797011	2.1
18886	1.01179	0.776213
18887	1.27737	0.76595
18888	3.16531	0.23755
18889	1.0141	0.147974
18890	1.47354	0.427738
18891	1.62495	0.353131
18892	1.62495	0.380393
18893	1.39296	0.560814
18894	0.998682	2.1
18895	1.93528	2.1
18896	2.71383	2.1
18897	1.13987	2.1
18898	1.93528	2.1
18899	1.78595	2.1
18900	3.98933	2.1
18901	3.73426	2.1
18902	1.02438	2.1
18903	1.12376	2.1
18904	1.12375	2.1
18905	1.19016	2.1

18906	1.37157	0.129984
18907	1.60814	0.117314
18908	1.70303	0.188981
18909	1.01219	0.163897
18910	1.06757	1.32285
18911	1.23475	2.1
18912	1.2295	2.1
18913	1.28014	2.1
18914	1.28014	2.1
18915	1.33339	2.1
18916	1.3201	2.1
18917	1.2679	2.1
18918	1.2679	2.1
18919	1.23504	2.1
18920	1.50108	2.1
18921	1.3715	2.1
18922	1.3715	2.1
18923	1.25075	2.1
18924	1.99222	1.17649
18925	1.4155	1.01889
18926	1.4777	2.1
18927	1.52613	2.1
18928	1.91031	2.1
18929	1.03968	2.1
18930	2.04289	0.374568
18931	0.967192	0.284011
18932	1.03043	0.185529
18933	1.65452	0.412316
18934	1.21564	2.1
18935	1.16938	2.1
18936	2.47056	2.1
18937	2.56853	2.1
18938	1.16938	2.1
18939	1.1181	2.1
18940	2.56853	2.1
18941	2.66828	2.1
18942	1.0465	2.1
18943	1.44709	2.1
18944	1.44709	2.1
18945	1.96491	2.1
18946	1.01976	0.422612
18947	0.961779	0.346261
18948	1.11764	0.405064
18949	0.961779	0.353031
18950	1.21171	0.0738036
18951	1.35007	0.106392
18952	1.03102	0.541865
18953	1.42446	0.540408
18954	1.42446	0.488914
18955	1.03166	0.473168
18956	1.2951	0.226951
18957	1.33575	0.284762
18958	1.33575	0.30881
18959	1.20473	0.29618
18960	1.10049	0.736007
18961	0.99747	0.722464
18962	1.54249	2.1
18963	1.65677	2.1

18964	1.65677	2.1
18965	1.77677	2.1
18966	1.73525	0.30715
18967	1.05689	0.415347
18968	1.26051	0.269938
18969	1.16259	0.186084
18970	1.34009	2.1
18971	1.398	2.1
18972	1.398	2.1
18973	1.50437	2.1
18974	2.3245	2.1
18975	1.18237	2.1
18976	1.24509	2.1
18977	1.01595	2.1
18978	3.29047	2.1
18979	2.79168	2.1
18980	1.06561	2.1
18981	1.11077	1.30904
18982	1.11077	0.808909
18983	0.953927	1.05412
18984	1.30509	0.444485
18985	1.04685	0.488806
18986	1.04685	0.317104
18987	0.834593	0.364254
18988	1.0775	0.449795
18989	1.27362	0.513813
18990	1.71762	0.70368
18991	2.5736	0.614609
18992	2.04717	0.748376
18993	1.45521	0.643213
18994	1.33962	2.1
18995	1.27956	2.1
18996	1.27956	2.1
18997	1.20478	2.1
18998	3.65841	0.0552507
18999	1.18656	0.05
19000	1.72272	0.0721874
19001	1.6812	0.062119
19002	1.09951	2.1
19003	1.08231	2.1
19004	1.08231	2.1
19005	1.06478	2.1
19006	2.16196	1.29366
19007	1.00387	0.850067
19008	1.00387	0.859529
19009	0.993999	0.662496
19010	1.04101	2.1
19011	1.27119	2.1
19012	1.27119	2.1
19013	1.51748	2.1
19014	3.00129	2.1
19015	3.27303	2.1
19016	3.27303	2.1
19017	3.60855	2.1
19018	2.22399	2.1
19019	1.71995	2.1
19020	1.54793	2.1
19021	1.227	2.1

19022	1.07624	2.1
19023	1.264 2.1	
19024	1.19323	1.06231
19025	1.21286	0.981702
19026	1.21286	0.84692
19027	1.20859	0.854509
19028	1.60578	0.109611
19029	1.27778	0.075262
19030	1.0525	2.1
19031	1.05758	1.10782
19032	1.0525	0.87204
19033	1.17042	1.28237
19034	1.62536	0.05
19035	3.71662	0.05
19036	1.78169	0.0944801
19037	1.91434	0.100971
19038	2.12331	0.108907
19039	1.33406	0.103495
19040	1.38437	2.1
19041	1.51829	2.1
19042	1.51829	2.1
19043	1.6697	2.1
19044	0.947114	2.1
19045	1.13335	2.1
19046	1.13335	2.1
19047	1.00965	2.1
19048	1.47521	2.1
19049	1.27679	2.1
19050	1.14911	2.1
19051	0.93841	2.1
19052	1.68437	2.1
19053	1.53822	2.1
19054	1.53822	2.1
19055	1.39702	2.1
19056	0.726453	2.1
19057	0.754101	2.1
19058	0.754101	2.1
19059	0.69776	2.1
19060	0.964033	2.1
19061	1.62397	2.02374
19062	1.70302	1.7144
19063	1.78637	1.39104
19064	3.68158	0.0613336
19065	2.11685	0.0572801
19066	1.33125	2.1
19067	1.15471	2.1
19068	1.0465	2.1
19069	1.33125	2.1
19070	1.17683	2.1
19071	1.25889	2.1
19072	1.25889	2.1
19073	1.34239	2.1
19074	1.40591	0.34405
19075	0.79348	0.248338
19076	1.04019	0.17266
19077	0.999187	0.138149
19078	1.30218	0.145967
19079	1.83339	0.158682

19080	1.88574	0.0934928
19081	1.5177	0.0814554
19082	1.23504	2.1
19083	1.19583	2.1
19084	1.19583	2.1
19085	1.14496	2.1
19086	1.62183	2.1
19087	1.62316	2.1
19088	1.62316	2.1
19089	1.62241	2.1
19090	1.02305	0.496124
19091	1.21136	0.396103
19092	0.993832	0.297967
19093	1.21137	0.395241
19094	1.12064	2.1
19095	1.24965	2.1
19096	1.24965	2.1
19097	1.41077	2.1
19098	1.00052	0.137945
19099	1.49842	0.106844
19100	1.49843	0.0907529
19101	1.10801	0.0625924
19102	1.54196	0.80845
19103	1.18041	0.940984
19104	1.73571	1.51105
19105	1.37224	1.93859
19106	1.4375	0.274859
19107	1.09529	0.278098
19108	1.44421	0.194741
19109	1.0953	0.225503
19110	1.0753	2.1
19111	0.815365	2.1
19112	0.815365	2.1
19113	0.597537	2.1
19114	1.03609	0.551988
19115	1.18952	0.561767
19116	1.54283	0.417455
19117	1.16719	0.423842
19118	1.16719	0.551122
19119	1.89226	0.503154
19120	2.55919	2.1
19121	2.37492	2.1
19122	2.37492	2.1
19123	2.07435	2.1
19124	1.36016	0.394115
19125	1.70302	0.38947
19126	1.85252	0.373122
19127	1.2111	0.481542
19128	1.62905	2.1
19129	1.49692	2.1
19130	1.49692	2.1
19131	1.40395	2.1
19132	2.19083	2.1
19133	1.30542	2.1
19134	1.58736	2.1
19135	2.16579	2.1
19136	2.0815	2.1
19137	1.17935	2.1

19138	1.53803	1.07619
19139	1.09148	0.65589
19140	1.81301	0.947824
19141	1.38333	0.561381
19142	1.04412	2.1
19143	1.44437	2.1
19144	1.4675	0.318348
19145	1.00665	0.322589
19146	1.50951	0.127746
19147	0.983105	0.0988939
19148	1.80252	0.161538
19149	0.540005	0.100229
19150	1.2545	0.355528
19151	2.14514	0.275231
19152	1.32051	0.198801
19153	1.82302	0.288012
19154	2.71057	2.1
19155	2.51107	2.1
19156	1.34817	2.1
19157	1.3551	2.1
19158	2.51107	2.1
19159	2.29663	2.1
19160	1.3551	2.1
19161	1.3685	2.1
19162	1.51748	2.1
19163	1.23389	2.1
19164	0.901928	0.0779782
19165	1.70302	0.10703
19166	1.66787	0.0715378
19167	1.92535	0.115619
19168	1.13571	0.286532
19169	1.02836	0.34552
19170	1.55398	2.1
19171	1.54179	2.1
19172	1.89608	2.1
19173	1.07167	2.1
19174	3.3488	0.211764
19175	0.550044	0.160547
19176	0.962877	0.371847
19177	1.00065	0.410823
19178	1.00613	0.405693
19179	1.07497	0.427453
19180	1.00486	0.05
19181	1.00486	0.05
19182	0.726453	2.1
19183	1.32634	0.20614
19184	1.40008	0.324847
19185	1.40008	0.194022
19186	2.42267	0.184719
19187	1.9216	0.0611528
19188	1.28066	0.05
19189	1.79102	0.748508
19190	1.18219	0.410099
19191	1.18219	0.693573
19192	0.709123	0.443878
19193	1.07866	2.1
19194	1.15038	2.1
19195	1.15038	2.0006

19196	1.24687	2.1
19197	1.83613	1.84268
19198	1.99157	1.89239
19199	1.45789	0.0721495
19200	1.1706	0.0713257
19201	1.74746	0.05
19202	1.1706	0.05
19203	1.96383	0.0763833
19204	1.67727	0.0772629
19205	1.67727	0.0651678
19206	1.60329	0.0627491
19207	1.18803	2.1
19208	1.3227	2.1
19209	1.3227	2.1
19210	1.40844	2.1
19211	1.07889	2.1
19212	1.07721	2.1
19213	1.07721	2.1
19214	1.00832	2.1
19215	0.953523	0.715423
19216	1.62875	0.687258
19217	1.07427	0.680816
19218	1.19722	0.582087
19219	1.1972	0.511078
19220	1.1599	0.507304
19221	2.97463	0.05
19222	1.9477	0.05
19223	1.95222	0.05
19224	1.75083	0.05
19225	3.46787	2.1
19226	3.02896	2.1
19227	3.02896	2.1
19228	2.46145	2.1
19229	0.970962	0.05
19230	1.0227	0.05
19231	1.12328	0.05
19232	1.02269	0.05
19233	0.904978	2.1
19234	0.725887	2.1
19235	0.725887	2.1
19236	0.601592	2.1
19237	0.956294	2.1
19238	1.13179	2.1
19239	0.956294	2.1
19240	1.13179	2.1
19241	1.05556	2.1
19242	1.13179	2.1
19243	1.38859	2.1
19244	1.13179	2.1
19245	1.06659	2.00473
19246	1.05776	2.1
19247	1.06659	2.1
19248	1.04291	0.666194
19249	1.54699	0.994644
19250	1.42307	1.05076
19251	1.5654	1.62816
19252	1.97906	2.1
19253	1.15354	0.05

19254	1.15505	0.0602722
19255	1.26455	0.0545924
19256	2.50042	0.05628
19257	2.50042	0.0757394
19258	3.22585	0.072465
19259	1.19258	0.0723741
19260	1.34562	0.0957435
19261	1.49515	0.146349
19262	1.66295	0.110739
19263	1.02395	2.1
19264	1.51067	2.1
19265	1.35821	1.86715
19266	2.10671	2.1
19267	1.0305	1.27969
19268	1.07855	1.2309
19269	1.20761	2.1
19270	0.98488	2.1
19271	1.4162	2.1
19272	1.14825	2.1
19273	1.29415	2.1
19274	1.6067	2.1
19275	1.23851	0.149873
19276	1.09651	0.118601
19277	1.60636	2.1
19278	1.53521	2.1
19279	1.53521	2.1
19280	1.46279	2.1
19281	1.28044	0.368264
19282	1.64147	0.363201
19283	1.18422	0.289405
19284	1.68208	0.405455
19285	1.09726	2.1
19286	1.234 2.1	
19287	1.234 2.1	
19288	1.09483	2.1
19289	1.89318	2.1
19290	1.98448	2.1
19291	1.98448	2.1
19292	2.02612	2.1
19293	1.22026	0.786557
19294	1.23527	0.652865
19295	1.09044	0.680995
19296	0.993366	0.472565
19297	1.22026	0.889522
19298	0.993368	1.06131
19299	2.61357	2.1
19300	2.48208	2.1
19301	2.48208	2.1
19302	2.62868	2.1
19303	1.85439	0.809275
19304	1.81776	0.706222
19305	1.81776	0.651862
19306	2.1587	0.694588
19307	1.8914	0.778257
19308	2.23961	0.715357
19309	1.49357	0.409307
19310	2.23961	0.669187
19311	1.78014	0.575628

19312	1.67635	0.553695
19313	1.66219	0.513678
19314	1.56036	0.501051
19315	1.14721	0.503493
19316	2.22495	0.526979
19317	1.88509	0.342496
19318	1.95249	0.33392
19319	2.11959	0.32734
19320	1.37427	0.313496
19321	1.39379	0.601437
19322	1.17614	0.566562
19323	1.39379	0.943231
19324	1.14397	0.285187
19325	1.09651	2.1
19326	1.42843	1.84706
19327	1.7069	2.1
19328	2.31293	2.03023
19329	1.56839	2.1
19330	1.78252	0.703399
19331	1.78254	0.656567
19332	1.09142	0.792926
19333	0.99829	0.0854351
19334	1.09372	0.0898371
19335	1.88175	0.167265
19336	0.898452	0.119747
19337	1.06936	2.1
19338	1.4162	2.1
19339	1.17989	2.1
19340	1.47411	1.55808
19341	1.25376	1.85347
19342	1.00757	0.733212
19343	1.4185	0.40743
19344	1.02011	0.43825
19345	2.22495	0.456502
19346	1.79542	0.435665
19347	1.79542	0.298833
19348	1.19352	0.302289
19349	2.38566	2.1
19350	1.83255	1.23016
19351	1.62667	2.1
19352	1.17879	1.42682
19353	2.79168	2.1
19354	2.28162	2.1
19355	2.28162	2.1
19356	1.90905	2.1
19357	1.40117	2.1
19358	1.28268	2.1
19359	1.41096	2.1
19360	1.22978	2.1
19361	3.20034	2.1
19362	3.42052	2.1
19363	3.42052	2.1
19364	3.50562	2.1
19365	1.46279	2.1
19366	1.3864	2.1
19367	1.3864	2.1
19368	1.3201	2.1
19369	1.35561	0.05

19370	0.974077	0.05
19371	0.967673	0.856136
19372	1.22482	0.728849
19373	1.02305	0.411733
19374	1.22482	0.64263
19375	0.999492	2.1
19376	1.14657	2.1
19377	1.14657	2.1
19378	1.31346	2.1
19379	1.84417	0.0773778
19380	1.31777	0.0535556
19381	1.19796	0.05
19382	0.691742	0.05
19383	1.16725	2.1
19384	1.9679	2.1
19385	1.08848	0.0616319
19386	1.15356	0.0557228
19387	1.58713	2.1
19388	1.58742	2.1
19389	1.58742	2.1
19390	1.49662	2.1
19391	1.32201	0.56189
19392	1.32097	0.911168
19393	1.32097	0.564604
19394	1.31825	0.985305
19395	1.12028	2.1
19396	1.15852	1.97951
19397	1.15852	2.1
19398	1.16239	1.5219
19399	1.07624	2.1
19400	1.30047	2.1
19401	1.44847	0.760038
19402	1.55292	0.74309
19403	1.21125	0.05
19404	1.15262	2.1
19405	1.05025	2.1
19406	1.55351	2.1
19407	1.5329	0.0808916
19408	2.26758	0.0574996
19409	1.94968	0.112438
19410	1.65747	0.1049
19411	1.42549	0.126335
19412	1.57656	0.123766
19413	2.11157	0.484868
19414	1.56531	0.497255
19415	1.69279	0.454447
19416	1.06204	0.529901
19417	0.947863	1.01734
19418	1.19854	1.25617
19419	0.947862	0.802468
19420	1.31126	0.716238
19421	1.3425	1.35281
19422	1.29976	1.03784
19423	1.05129	0.212753
19424	1.51026	0.229387
19425	1.05129	0.191261
19426	1.2951	0.211388
19427	1.7155	2.1

19428	1.72196	2.1
19429	1.72196	2.1
19430	1.77867	2.1
19431	0.964755	2.1
19432	1.14142	2.1
19433	1.03766	2.1
19434	0.986723	2.1
19435	1.28072	0.606202
19436	1.10609	0.769811
19437	1.28072	0.49798
19438	1.16776	1.1529
19439	1.61687	2.1
19440	1.60965	2.1
19441	1.60965	2.1
19442	1.59654	2.1
19443	1.26512	2.1
19444	1.23964	2.1
19445	2.07997	2.1
19446	2.60048	2.1
19447	1.23732	2.1
19448	1.3372	2.1
19449	2.62253	2.1
19450	2.47056	2.1
19451	0.92782	0.17539
19452	1.40335	0.232808
19453	1.26301	0.237916
19454	1.52668	0.231157
19455	0.99818	0.348656
19456	1.51165	0.361252
19457	1.66213	0.344861
19458	1.55246	0.353622
19459	2.31255	2.1
19460	1.34366	2.1
19461	1.90577	2.1
19462	0.969739	2.1
19463	3.4245	2.1
19464	2.8415	2.1
19465	1.97559	2.1
19466	1.82362	2.1
19467	1.48162	2.1
19468	1.23204	2.1
19469	1.23204	2.1
19470	0.95947	2.1
19471	1.05267	2.1
19472	1.44091	0.984839
19473	1.05267	1.40632
19474	1.5795	2.04289
19475	0.738711	2.1
19476	1.28549	2.1
19477	1.15694	2.1
19478	1.72061	2.1
19479	1.69858	2.1
19480	0.954563	2.1
19481	0.859087	2.1
19482	0.788886	2.1
19483	1.08732	0.33891
19484	1.3357	0.332076
19485	1.3357	0.335594

19486	1.48676	0.3292
19487	2.15079	2.1
19488	2.12414	2.1
19489	2.12414	2.1
19490	2.04664	2.1
19491	1.98859	2.1
19492	0.992159	2.1
19493	1.48064	2.1
19494	1.2163	2.1
19495	1.26337	2.1
19496	1.63159	2.1
19497	1.00366	0.489563
19498	1.65452	0.461235
19499	1.10408	2.1
19500	1.37704	2.1
19501	1.50224	2.1
19502	1.42866	2.1
19503	1.43939	2.1
19504	1.02784	2.1
19505	1.00763	0.212059
19506	1.23746	0.198488
19507	1.23747	0.212104
19508	1.13642	0.254616
19509	1.49906	0.204082
19510	1.46506	0.148352
19511	1.46506	0.166062
19512	1.33061	0.107184
19513	1.08166	0.159162
19514	1.01052	0.130899
19515	1.02836	0.15756
19516	1.08166	0.153252
19517	1.25334	0.217951
19518	1.23749	0.19448
19519	1.37501	0.320889
19520	1.47354	0.307152
19521	1.30167	2.1
19522	1.29897	2.1
19523	1.51042	2.1
19524	1.07684	2.1
19525	1.07684	2.1
19526	0.765121	2.1
19527	1.5535	0.667546
19528	1.40602	2.1
19529	1.40603	0.714858
19530	1.25669	2.1
19531	1.08276	2.1
19532	1.60948	2.1
19533	1.26929	1.12321
19534	1.09847	2.1
19535	0.827082	0.405326
19536	1.15841	0.413703
19537	1.25063	0.380756
19538	1.14397	0.376372
19539	1.25063	0.534757
19540	1.10476	0.299072
19541	1.14872	0.0730471
19542	1.3659	0.0875345
19543	1.36589	0.0660521

19544	1.4677	0.0895587
19545	0.590354	2.1
19546	0.655202	2.1
19547	3.48926	2.1
19548	3.32709	2.1
19549	3.32709	2.1
19550	3.13938	2.1
19551	1.24458	0.215758
19552	1.29681	0.286239
19553	1.40915	0.378812
19554	1.17891	0.481665
19555	1.33171	0.05
19556	1.37681	0.471198
19557	1.37681	2.1
19558	1.4516	2.1
19559	2.68241	0.122455
19560	1.61031	0.0755328
19561	1.6488	2.1
19562	1.6585	2.1
19563	1.65848	2.1
19564	1.67061	2.1
19565	1.15152	2.1
19566	1.32548	2.1
19567	1.43677	2.1
19568	1.10264	2.1
19569	1.01472	2.1
19570	0.966964	2.1
19571	2.867	2.1
19572	2.94897	2.1
19573	1.65815	2.1
19574	1.4502	2.1
19575	1.4502	2.1
19576	1.26755	2.1
19577	1.37138	2.1
19578	1.45026	2.1
19579	1.45026	2.1
19580	1.53446	2.1
19581	0.973502	1.8808
19582	1.08542	1.75414
19583	1.13387	0.0532438
19584	1.71031	0.05
19585	1.08911	2.1
19586	1.10748	2.1
19587	1.21823	2.1
19588	1.25005	2.1
19589	0.657014	2.05652
19590	1.38139	2.1
19591	1.01485	2.1
19592	1.26045	2.1
19593	1.12468	2.1
19594	1.26045	2.1
19595	1.89278	0.643918
19596	1.06433	0.566197
19597	1.87861	0.584686
19598	0.967544	0.455162
19599	1.50319	2.1
19600	3.47245	1.66373
19601	2.2354	0.559609

19602	1.16701	0.467538
19603	1.3592	2.1
19604	1.3305	0.734485
19605	1.3305	1.38488
19606	1.15008	0.685401
19607	1.12457	0.0615756
19608	1.40707	0.0598138
19609	1.01778	2.1
19610	1.35733	2.1
19611	1.01779	2.1
19612	1.12064	2.1
19613	1.30694	1.11067
19614	1.42716	1.20033
19615	1.42717	1.21565
19616	1.04321	1.18732
19617	1.19352	0.231806
19618	1.32634	0.411246
19619	1.14605	2.1
19620	1.0417	1.96292
19621	1.19676	0.359848
19622	1.03804	0.358958
19623	2.09455	0.358186
19624	1.15338	0.366603
19625	2.21119	2.1
19626	1.26097	2.06554
19627	1.88394	0.487679
19628	1.24106	0.232841
19629	2.98143	2.1
19630	2.56248	0.929108
19631	1.88394	1.15263
19632	1.68703	0.171875
19633	1.5846	2.1
19634	1.94799	2.1
19635	1.20091	1.99525
19636	1.40393	2.1
19637	3.09031	2.1
19638	2.90243	2.1
19639	1.27227	2.1
19640	1.32592	2.1
19641	2.90247	2.1
19642	2.71057	2.1
19643	1.32594	2.1
19644	1.34817	2.1
19645	1.4092	2.1
19646	1.44859	2.1
19647	1.52008	2.1
19648	1.401	2.1
19649	1.40099	2.1
19650	1.28936	2.1
19651	1.56002	0.05
19652	1.08811	0.05
19653	1.46041	0.05
19654	1.56002	0.05
19655	1.50524	2.1
19656	1.21304	2.1
19657	1.21304	2.1
19658	0.947115	2.1
19659	1.60665	2.06798

19660	1.85179	1.528
19661	1.85178	1.6339
19662	2.06277	0.905165
19663	2.17534	2.1
19664	1.23007	2.1
19665	2.17533	2.1
19666	1.66635	1.84767
19667	2.00869	2.1
19668	0.986724	2.1
19669	2.00475	2.1
19670	1.99394	2.1
19671	1.67244	0.276543
19672	1.39604	0.243975
19673	1.39604	0.272211
19674	0.954046	0.187691
19675	0.988863	2.1
19676	1.9864	2.1
19677	1.9864	2.1
19678	3.38645	2.1
19679	1.46932	0.05
19680	1.50847	0.0520967
19681	1.50848	0.05
19682	1.42705	0.0507622
19683	1.50108	2.1
19684	1.51621	0.745628
19685	1.94317	0.776804
19686	1.18382	0.560076
19687	2.05091	0.0634002
19688	1.88593	0.05
19689	1.54299	0.05
19690	0.987265	0.05
19691	1.27766	2.1
19692	1.5178	2.1
19693	1.5178	2.1
19694	1.81518	2.1
19695	1.04626	1.04057
19696	1.31057	1.05952
19697	1.31057	1.03419
19698	1.22297	1.06255
19699	1.67844	0.895206
19700	1.62862	2.1
19701	1.62112	0.855916
19702	1.87598	2.1
19703	1.17844	2.1
19704	1.37063	2.1
19705	1.37063	2.1
19706	1.68723	2.1
19707	1.99943	0.0724158
19708	1.17631	0.05
19709	1.4263	0.9306
19710	1.31023	2.1
19711	1.31023	0.884231
19712	1.20871	2.1
19713	1.50356	2.1
19714	1.69311	2.1
19715	1.69599	2.1
19716	1.6801	2.1
19717	1.5843	0.05

19718	0.840233	0.05
19719	1.95358	0.05
19720	1.40216	0.05
19721	1.39078	2.01933
19722	1.18948	1.68452
19723	1.01606	1.25899
19724	1.18948	1.51583
19725	1.63823	2.1
19726	1.67687	2.1
19727	1.67687	2.1
19728	1.52855	2.1
19729	1.11048	2.1
19730	1.25346	2.1
19731	2.42984	1.89028
19732	3.41731	0.932687
19733	1.2258	2.1
19734	1.2966	2.1
19735	1.2966	2.1
19736	1.37392	2.1
19737	1.41758	2.1
19738	1.35504	2.1
19739	1.35504	2.1
19740	1.30347	2.1
19741	1.50877	2.1
19742	1.11193	2.1
19743	1.40984	1.9933
19744	1.54537	1.62836
19745	1.71638	2.1
19746	1.77795	1.27176
19747	2.07593	2.1
19748	1.93387	2.1
19749	1.9339	2.1
19750	1.75669	2.1
19751	1.80299	1.03062
19752	1.31251	0.898229
19753	1.31249	0.759278
19754	1.13411	0.695207
19755	1.06313	2.1
19756	1.04921	2.1
19757	1.55766	2.1
19758	1.53728	2.1
19759	1.53729	2.1
19760	1.51789	2.1
19761	0.993021	0.550099
19762	1.02478	0.564737
19763	1.03408	0.555622
19764	1.09056	0.560567
19765	0.697755	2.1
19766	0.632901	2.1
19767	1.34072	0.340021
19768	1.80841	0.343522
19769	1.80841	0.340816
19770	1.17793	0.348627
19771	1.75032	1.822
19772	1.13076	2.1
19773	2.11851	2.1
19774	1.73255	2.1
19775	1.73255	2.1

19776	1.11181	2.1
19777	1.43772	2.1
19778	1.67381	2.1
19779	1.52164	2.1
19780	1.60861	2.1
19781	1.30588	0.114701
19782	1.75241	0.119811
19783	1.33026	0.836074
19784	0.962705	0.708688
19785	0.99678	0.0630167
19786	1.30572	0.0530505
19787	1.41943	0.0800194
19788	1.2683	0.072571
19789	1.14145	0.055422
19790	1.01361	0.0500361
19791	1.29943	2.1
19792	1.3483	2.1
19793	1.34832	2.1
19794	1.83488	2.1
19795	1.71065	2.1
19796	1.66178	2.1
19797	1.39076	2.1
19798	1.37456	2.1
19799	1.66126	2.1
19800	1.562 2.1	
19801	1.37456	2.1
19802	1.36065	2.1
19803	1.04026	0.448865
19804	1.51898	0.470331
19805	1.67088	0.399312
19806	1.9726	0.42173
19807	1.74852	1.29128
19808	1.95512	1.3882
19809	1.16165	0.834866
19810	1.06584	0.672284
19811	1.39327	2.1
19812	1.41394	2.1
19813	1.41394	2.1
19814	1.45506	2.1
19815	1.39523	2.1
19816	1.21119	2.1
19817	1.09005	2.1
19818	0.921311	2.1
19819	1.58164	2.1
19820	1.77574	2.1
19821	1.73184	2.1
19822	1.59423	2.1
19823	1.8214	2.1
19824	3.10544	0.971457
19825	1.01243	1.94971
19826	1.30896	0.829223
19827	2.16956	1.06946
19828	1.29608	0.792011
19829	1.38617	1.8924
19830	1.49536	1.5358
19831	2.35239	1.19132
19832	1.24613	1.52017
19833	1.94351	2.1

19834	2.05361	2.1
19835	2.05358	2.1
19836	2.38994	2.1
19837	1.13533	2.1
19838	1.08276	2.1
19839	1.55611	0.0834183
19840	1.33957	0.0807941
19841	1.33957	0.0741928
19842	1.69011	0.12319
19843	1.5515	0.75841
19844	1.59106	0.740226
19845	1.02958	2.1
19846	1.12266	2.1
19847	1.12266	2.1
19848	1.24925	2.1
19849	1.80223	2.1
19850	1.20242	0.980679
19851	2.13665	0.619057
19852	1.3924	0.631582
19853	0.904894	0.981054
19854	1.82633	2.1
19855	1.94138	0.05
19856	1.34009	0.05
19857	1.15096	2.1
19858	0.994292	2.1
19859	1.10101	2.1
19860	1.15096	2.1
19861	1.50438	2.1
19862	1.67825	2.1
19863	1.67825	2.1
19864	1.91894	2.1
19865	2.04664	2.1
19866	1.92063	2.1
19867	1.72857	2.1
19868	1.89757	2.1
19869	1.61843	0.0520975
19870	1.49431	0.0507895
19871	2.33925	2.1
19872	1.80683	2.1
19873	1.64257	2.1
19874	1.50108	2.1
19875	2.82561	2.1
19876	2.97847	2.1
19877	2.97848	2.1
19878	3.20034	2.1
19879	0.66055	2.1
19880	1.95919	2.1
19881	1.07976	2.1
19882	1.30237	2.1
19883	1.99943	0.05
19884	1.31802	0.0518367
19885	1.53336	0.0868324
19886	1.31802	0.0545354
19887	1.27737	0.733892
19888	1.44848	0.72237
19889	2.35499	0.0765419
19890	1.30041	0.0773803
19891	2.35499	0.0559847

19892	1.65381	0.05
19893	0.996083	2.1
19894	1.28754	2.1
19895	0.996081	2.1
19896	1.44859	2.1
19897	1.24763	1.55944
19898	1.03697	1.79339
19899	0.951273	1.30852
19900	1.60018	0.948574
19901	2.0815	1.39468
19902	1.20488	1.89986
19903	2.95785	2.1
19904	2.52245	1.29866
19905	1.68455	0.0506718
19906	1.12971	0.05
19907	1.1952	0.443593
19908	2.0815	0.522222
19909	1.3963	2.1
19910	1.4934	2.1
19911	1.47973	2.1
19912	1.25559	2.1
19913	1.1233	2.1
19914	1.15009	2.1
19915	1.15009	2.1
19916	0.977775	2.1
19917	1.16482	0.922559
19918	1.29313	0.582952
19919	1.29313	0.679655
19920	1.294	0.5844
19921	1.48693	0.36776
19922	1.47608	0.365019
19923	1.36005	1.6371
19924	1.47109	2.1
19925	1.47109	2.1
19926	1.3515	2.1
19927	2.48828	2.1
19928	2.32378	2.1
19929	2.32378	2.1
19930	2.1692	2.1
19931	1.03217	0.817629
19932	0.953521	0.885519
19933	1.4515	0.0622279
19934	3.72406	0.0599864
19935	0.963395	1.47376
19936	1.01346	0.975061
19937	1.62241	2.1
19938	1.62027	2.1
19939	1.62027	2.1
19940	1.61687	2.1
19941	1.2593	0.05
19942	0.981586	0.05
19943	0.721961	2.1
19944	0.888955	2.1
19945	1.22031	0.05
19946	1.27566	2.1
19947	2.50066	2.1
19948	1.19814	2.1
19949	1.42278	0.05

19950	2.02657	0.05
19951	1.0756	0.706028
19952	1.10049	0.693833
19953	2.26155	2.1
19954	2.15634	2.1
19955	2.15634	2.1
19956	2.01613	2.1
19957	1.94233	1.15261
19958	1.18127	1.81848
19959	1.36865	1.8924
19960	1.75755	1.65535
19961	2.01613	2.1
19962	1.78958	2.1
19963	1.97995	2.1
19964	1.79198	2.1
19965	1.20841	2.1
19966	1.6519	2.1
19967	1.80207	2.1
19968	1.66774	2.1
19969	1.17935	0.0886631
19970	1.88071	0.15571
19971	1.58723	0.130069
19972	1.7361	0.119316
19973	0.794165	2.1
19974	1.21327	2.1
19975	1.21327	2.1
19976	1.34268	2.1
19977	1.34497	0.05
19978	0.793303	0.05
19979	1.12323	0.05
19980	1.34496	0.05
19981	0.928226	0.385279
19982	1.89804	0.405744
19983	2.60141	0.05
19984	2.60142	2.1
19985	2.60772	0.567604
19986	0.99747	2.05274
19987	1.27096	0.769057
19988	1.27096	0.670924
19989	1.03472	0.784647
19990	1.09962	0.567098
19991	1.21148	0.879413
19992	1.21148	0.700955
19993	1.35025	2.1
19994	1.09795	2.1
19995	1.00775	2.1
19996	1.07162	2.1
19997	1.60665	2.02388
19998	1.52984	2.1
19999	1.61179	2.1
20000	1.70057	2.1
20001	1.76063	2.1
20002	1.14547	0.431453
20003	0.964782	0.394249
20004	1.17208	0.385351
20005	0.964769	0.332408
20006	1.04268	2.1
20007	1.57459	2.1

20008	1.57459	2.1
20009	1.32449	2.1
20010	2.07527	0.810773
20011	1.00013	0.674459
20012	0.813711	0.05
20013	1.05677	1.72858
20014	1.30664	1.19867
20015	1.96011	2.01095
20016	1.7151	1.01292
20017	1.61309	1.04893
20018	1.18954	0.571379
20019	1.05447	1.14255
20020	1.05446	0.413169
20021	1.11787	0.406889
20022	1.44437	2.1
20023	1.84739	2.03996
20024	1.84739	1.6379
20025	1.06203	2.1
20026	1.78391	2.1
20027	1.89267	2.1
20028	1.9212	2.1
20029	2.02997	2.1
20030	1.32051	0.417256
20031	1.47169	0.317876
20032	1.20691	0.0521166
20033	1.72271	0.05
20034	1.2858	0.531021
20035	1.31011	0.703615
20036	1.31011	0.490275
20037	1.34124	0.675183
20038	1.294	0.738805
20039	1.03079	0.594395
20040	1.83192	2.02726
20041	1.81979	2.1
20042	1.81979	2.08032
20043	1.57829	2.1
20044	1.35435	1.77793
20045	1.19023	1.98644
20046	0.991115	2.1
20047	1.27725	2.1
20048	1.38204	2.1
20049	1.48013	2.1
20050	0.983493	2.1
20051	2.26155	2.1
20052	1.02311	0.695551
20053	1.55485	0.631899
20054	1.71033	0.624858
20055	2.07319	0.558491
20056	2.29074	0.638831
20057	1.50822	0.73299
20058	2.08147	1.37705
20059	1.1585	2.1
20060	3.74092	0.865615
20061	2.40799	2.1
20062	2.05349	1.09871
20063	1.92656	2.1
20064	1.09634	0.170871
20065	1.45502	0.255006

20066	1.17261	0.334829
20067	1.29383	0.318271
20068	1.29383	0.329424
20069	1.47689	0.326834
20070	1.85717	0.535566
20071	1.53798	0.459783
20072	1.9493	2.1
20073	2.0371	1.30882
20074	1.8519	1.88419
20075	1.92119	1.09104
20076	1.00764	0.11935
20077	1.0302	0.10889
20078	1.0302	0.09195
20079	0.90047	0.0943142
20080	1.49155	0.218169
20081	2.26403	0.109503
20082	2.06158	0.32762
20083	1.09506	0.326542
20084	1.9212	1.17186
20085	1.99586	0.919738
20086	1.99586	0.998962
20087	2.07527	0.888544
20088	1.20478	2.1
20089	1.09662	2.1
20090	1.55961	2.1
20091	1.03472	2.1
20092	1.54983	2.1
20093	2.02953	2.1
20094	1.84502	2.1
20095	2.1221	2.1
20096	1.02969	1.50207
20097	2.23523	1.98504
20098	1.2377	0.47514
20099	1.03818	0.513839
20100	1.978	2.1
20101	1.78159	2.1
20102	1.45762	2.1
20103	1.34234	2.1
20104	2.01624	2.1
20105	2.52072	2.1
20106	1.23013	2.1
20107	2.5593	2.1
20108	3.06464	1.14241
20109	2.63697	0.748884
20110	2.63697	0.931465
20111	1.87111	0.647473
20112	1.04574	0.157466
20113	2.13428	0.135413
20114	1.61715	2.1
20115	1.44137	2.1
20116	1.44867	2.1
20117	1.18283	2.1
20118	1.50584	0.1374
20119	1.45466	0.148692
20120	1.4959	0.188623
20121	1.93489	0.204072
20122	2.60772	2.1
20123	2.68868	2.1

20124	2.68868	2.1
20125	2.82561	2.1
20126	1.49654	2.1
20127	1.38672	2.1
20128	1.36813	2.1
20129	1.98396	2.1
20130	1.93852	1.46662
20131	2.14237	0.938179
20132	1.44421	0.142481
20133	1.21749	0.111018
20134	1.09572	0.123921
20135	1.11081	0.0715007
20136	2.32363	2.1
20137	1.24375	2.1
20138	3.79242	2.1
20139	3.46787	2.1
20140	1.19854	1.36829
20141	1.35487	1.36665
20142	1.35486	1.56013
20143	1.38616	1.52705
20144	1.67265	0.418418
20145	0.909487	0.390884
20146	2.53562	2.1
20147	1.09564	2.1
20148	2.9068	2.1
20149	3.57307	0.648675
20150	2.50369	1.05492
20151	1.47348	2.1
20152	1.24925	2.1
20153	1.3991	2.1
20154	1.3991	2.1
20155	1.52984	2.1
20156	1.60428	2.1
20157	1.50097	2.1
20158	1.50097	2.1
20159	1.40239	2.1
20160	2.19621	2.1
20161	2.35003	2.1
20162	1.57932	0.081725
20163	1.61194	0.0542278
20164	2.0815	0.05
20165	1.18539	0.05
20166	1.5795	0.868218
20167	1.53064	1.02811
20168	1.89227	0.05
20169	3.01037	0.05
20170	2.08149	1.6326
20171	2.06678	1.53454
20172	1.89226	0.05
20173	3.47888	0.05
20174	1.89226	0.108035
20175	3.65842	0.0639834
20176	3.88546	0.196591
20177	1.89226	0.404918
20178	1.11239	2.1
20179	1.29133	0.0638967
20180	0.889309	0.0561434
20181	0.881934	0.0826272

20182	1.88406	2.0984
20183	0.636946	1.97657
20184	0.636945	1.23487
20185	1.28073	2.1
20186	1.28075	1.99562
20187	0.942007	2.00272
20188	1.68436	2.1
20189	1.4677	0.0523738
20190	0.851542	0.109234
20191	1.39063	0.0780195
20192	0.851539	0.0914854
20193	1.11597	0.0997629
20194	1.35403	0.0830612
20195	0.959473	2.1
20196	1.07976	2.1
20197	0.946129	1.06957
20198	0.946129	0.387593
20199	1.38464	0.562804
20200	1.36991	0.463195
20201	0.946131	0.486457
20202	0.94613	0.787196
20203	1.34493	0.418537
20204	1.36991	0.397766
20205	1.36991	0.330836
20206	0.946131	0.378527
20207	0.78392	0.829837
20208	0.80282	0.800992
20209	1.10351	0.824331
20210	0.80846	0.888432
20211	0.838026	0.809322
20212	1.08558	0.816242
20213	0.718203	0.879438
20214	0.501866	0.69977
20215	0.85426	0.90134
20216	1.24249	0.151716
20217	1.37052	0.257173
20218	0.851541	0.270866
20219	1.0092	0.135714
20220	0.851554	0.244599
20221	1.49826	0.148926
20222	1.13727	0.0776235
20223	0.778149	0.290057
20224	1.20257	0.535771
20225	0.946133	0.552527
20226	0.778149	0.261347
20227	0.77815	0.481764
20228	0.946132	2.1
20229	1.17602	2.1
20230	0.946132	2.1
20231	1.31257	2.1
20232	1.17602	2.1
20233	1.35821	0.407527
20234	1.19993	0.349027
20235	0.946131	0.364514
20236	0.946131	0.399822
20237	1.08848	0.40419
20238	1.00509	2.1
20239	1.19629	2.1

20240	0.867593	2.1
20241	0.757921	2.1
20242	0.729116	0.0601296
20243	0.89162	0.0701971
20244	1.16811	0.0576222
20245	1.3676	2.1
20246	1.2638	0.852348
20247	0.946132	2.1
20248	0.946132	2.1
20249	1.2638	2.1
20250	1.00185	2.1
20251	1.60948	2.1
20252	0.602075	2.1
20253	0.942611	2.1
20254	0.946132	2.1
20255	0.942611	2.1
20256	1.11822	2.1
20257	1.21558	0.277209
20258	0.607788	0.395648
20259	0.827029	0.296543
20260	1.00691	0.666169
20261	0.607789	0.399955
20262	0.607775	0.599606
20263	1.23248	0.865643
20264	0.902653	0.989832
20265	0.639143	1.02571
20266	0.851545	0.892202
20267	0.605153	0.871655
20268	1.06826	2.1
20269	1.29365	2.1
20270	1.41232	2.1
20271	0.946131	2.1
20272	1.20854	2.1
20273	0.946131	2.1
20274	0.909005	0.0544516
20275	1.16672	0.05
20276	0.810959	0.0509143
20277	0.946131	0.12527
20278	1.40104	0.0868943
20279	0.946131	0.111509
20280	1.35403	0.107958
20281	1.40104	0.0914628
20282	1.92535	0.0696723
20283	2.14534	0.0662102
20284	1.77532	0.113079
20285	0.851541	0.19479
20286	1.05585	0.237005
20287	1.27098	0.156003
20288	0.870925	0.205676
20289	1.15547	0.202022
20290	1.47583	0.152412
20291	1.35007	0.080276
20292	0.740095	0.05
20293	0.644912	0.05
20294	0.819489	0.05
20295	0.983028	0.325485
20296	0.861849	0.05
20297	1.40511	0.05

20298	0.86182	0.05
20299	0.9705	0.05
20300	1.40511	0.05
20301	1.15539	2.1
20302	0.946119	1.31747
20303	0.736382	2.1
20304	0.736384	2.1
20305	1.41561	2.1
20306	0.785651	2.1
20307	1.34479	2.1
20308	1.38885	2.1
20309	0.94613	2.1
20310	1.29461	2.1
20311	0.94613	2.1
20312	0.776179	1.53423
20313	0.858611	2.1
20314	0.946128	1.60858
20315	1.04073	2.1
20316	1.16399	2.1
20317	0.990019	2.1
20318	1.15564	2.1
20319	1.28956	0.322433
20320	1.35145	0.327883
20321	1.09662	2.1
20322	1.8155	0.05
20323	0.811459	0.05
20324	0.731982	0.05
20325	0.900554	0.05
20326	0.946127	0.742876
20327	0.817066	0.762923
20328	0.817064	0.751195
20329	1.41798	0.783911
20330	0.871606	0.771159
20331	1.30265	0.15182
20332	0.849402	1.62298
20333	0.868582	1.14236
20334	0.909137	1.27903
20335	0.807016	1.23072
20336	0.83393	0.658252
20337	0.9034	0.945366
20338	1.53515	0.935416
20339	1.17665	0.404134
20340	0.946132	0.582503
20341	0.946129	0.364687
20342	1.35977	0.337736
20343	1.34254	0.309068
20344	1.14241	0.242258
20345	0.946132	0.391563
20346	0.946132	0.224938
20347	1.27376	0.219811
20348	1.35978	0.201752
20349	1.01976	0.186843
20350	1.23498	0.832667
20351	1.35002	0.909934
20352	1.11683	1.05851
20353	0.88359	1.57032
20354	0.751374	1.20346
20355	0.791714	1.20608

20356	1.6067	2.1
20357	0.923782	2.1
20358	1.44689	2.1
20359	0.94613	2.1
20360	0.946131	2.1
20361	1.5261	2.1
20362	1.26247	2.1
20363	1.80085	0.0746941
20364	1.7693	0.058101
20365	0.900422	0.0716478
20366	1.31357	0.126458
20367	0.94613	0.112495
20368	0.900424	0.101139
20369	0.900397	0.118333
20370	1.39448	0.334072
20371	1.36769	0.0624737
20372	0.713524	0.146285
20373	0.713554	0.0764353
20374	0.946124	0.117827
20375	1.24979	0.0840327
20376	1.04765	0.125609
20377	0.70054	0.05
20378	0.776991	0.05
20379	0.738905	0.05
20380	1.32605	0.05
20381	0.784957	0.05
20382	0.803955	0.05
20383	0.819486	0.05
20384	1.79306	0.122459
20385	1.18275	0.147667
20386	1.25626	0.10815
20387	0.936806	0.0887728
20388	0.653124	0.157081
20389	0.946132	0.136036
20390	0.653151	0.151136
20391	1.10748	0.316907
20392	1.95244	2.1
20393	1.40967	1.90881
20394	1.27916	1.66549
20395	0.946132	0.51297
20396	0.946132	0.693556
20397	1.30555	0.754966
20398	1.38651	0.722964
20399	0.946132	0.650448
20400	1.23571	0.766028
20401	1.21261	1.12971
20402	1.34225	0.979244
20403	0.946132	1.37731
20404	0.734059	0.577874
20405	0.734084	0.978318
20406	0.78438	0.413559
20407	1.13748	0.679985
20408	0.946131	0.848537
20409	0.78438	0.418488
20410	0.784392	0.681703
20411	0.626495	2.1
20412	0.694635	2.1
20413	0.946125	2.1

20414	0.694632	2.1
20415	1.38521	2.1
20416	0.795469	2.1
20417	0.850877	2.1
20418	1.37098	2.1
20419	0.946131	2.1
20420	0.79547	2.1
20421	0.946132	0.818916
20422	1.30448	1.3335
20423	0.628361	0.684845
20424	0.686964	0.721801
20425	0.493261	0.763289
20426	1.90912	1.96948
20427	0.946127	1.18189
20428	1.36604	0.641309
20429	0.671687	0.775631
20430	0.946128	0.90447
20431	0.671699	0.713514
20432	1.04973	2.1
20433	0.982858	0.341065
20434	0.974081	0.05
20435	1.69993	0.05
20436	1.00763	0.650588
20437	0.755566	2.1
20438	0.946129	2.1
20439	1.04184	2.1
20440	1.13509	2.1
20441	0.755566	2.1
20442	1.114 2.1	
20443	0.810946	2.1
20444	0.786259	2.1
20445	1.43455	2.1
20446	1.05071	2.1
20447	0.746095	2.1
20448	0.82737	2.1
20449	0.946126	1.94966
20450	1.04071	2.1
20451	1.0852	2.1
20452	1.59019	0.05
20453	0.621972	0.0620951
20454	1.14475	0.0629523
20455	0.691062	0.0676806
20456	1.20057	0.0534354
20457	0.846868	0.0606894
20458	1.26062	0.05
20459	0.795093	0.0523224
20460	1.20056	0.0512322
20461	0.795065	0.0576424
20462	1.18203	0.05
20463	1.20057	0.0503119
20464	0.991869	0.344592
20465	0.841088	2.1
20466	0.946131	2.1
20467	0.869097	2.1
20468	0.946131	2.1
20469	0.841089	2.1
20470	1.77867	2.1
20471	1.99296	0.183031

20472	1.91703	2.1
20473	0.651334	0.283306
20474	1.31467	0.291899
20475	0.651332	0.251253
20476	0.651332	0.274868
20477	1.03079	0.299895
20478	0.808519	0.129007
20479	1.41417	0.0930305
20480	1.03165	0.358865
20481	1.43577	2.1
20482	1.39053	2.1
20483	1.07006	2.1
20484	0.946132	2.1
20485	0.946133	2.1
20486	1.07006	2.1
20487	1.12717	2.1
20488	0.994293	2.1
20489	0.946132	2.1
20490	1.31912	2.1
20491	0.946131	2.1
20492	1.40571	2.1
20493	1.31912	2.1
20494	1.12292	0.355384
20495	1.07755	0.440941
20496	0.851539	0.405568
20497	0.946131	0.276692
20498	0.929834	0.208215
20499	1.83919	0.549597
20500	1.04713	0.387735
20501	0.929726	0.32242
20502	1.50985	0.292014
20503	0.946127	0.334192
20504	1.0227	0.313307
20505	1.2582	0.309273
20506	1.1391	0.709765
20507	0.780831	0.832428
20508	0.78086	0.728534
20509	0.946133	0.828958
20510	0.780831	0.696325
20511	1.37762	0.759634
20512	0.946116	1.29409
20513	1.22882	2.1
20514	1.40015	0.84884
20515	1.40017	2.1
20516	0.946105	2.1
20517	0.553567	0.05
20518	1.19263	0.05
20519	0.946132	0.05
20520	0.553594	0.05
20521	1.19263	0.05
20522	1.18237	0.05
20523	0.946133	0.05
20524	0.978467	0.05
20525	0.56262	0.05
20526	0.946132	0.05
20527	0.562633	0.05
20528	0.900175	0.05
20529	1.04627	1.01512

20530	0.549928	0.651449
20531	1.17765	0.769019
20532	1.11479	0.833014
20533	0.398261	0.762984
20534	0.927608	0.866427
20535	1.38467	0.670965
20536	1.04824	0.587274
20537	1.33893	0.797615
20538	1.55775	0.753856
20539	0.816895	0.784994
20540	1.96057	0.866643
20541	1.35798	2.1
20542	1.29262	2.1
20543	0.94613	2.1
20544	0.94613	2.1
20545	1.29261	2.1
20546	1.18067	2.1
20547	0.946131	2.1
20548	0.946132	2.1
20549	1.27809	2.1
20550	1.35799	2.1
20551	1.03933	2.1
20552	1.49438	1.81122
20553	0.946132	2.1
20554	1.13537	2.1
20555	1.41666	1.53793
20556	1.74237	2.1
20557	0.946132	2.1
20558	1.04291	0.40338
20559	1.63802	2.1
20560	1.24714	1.14495
20561	0.842013	1.66711
20562	0.810959	2.1
20563	0.767923	0.430567
20564	1.19583	0.436733
20565	0.767921	0.418951
20566	0.767921	0.427523
20567	0.961348	0.459072
20568	2.08687	1.2574
20569	0.739729	0.569671
20570	1.04342	0.52226
20571	0.739757	0.543583
20572	0.946129	0.577898
20573	1.04341	0.740455
20574	1.38683	0.386052
20575	1.34153	0.377147
20576	0.946129	0.389451
20577	0.946131	0.381506
20578	1.02028	0.376458
20579	1.47606	0.344515
20580	1.20739	0.366463
20581	0.957684	0.351048
20582	0.946116	0.375961
20583	0.851528	0.379722
20584	0.957685	0.37339
20585	1.04504	2.1
20586	0.902938	2.1
20587	0.946132	2.1

20588	1.07566	2.1
20589	1.4558	2.1
20590	1.93286	2.1
20591	1.27241	2.1
20592	0.828671	2.1
20593	0.810933	2.1
20594	1.31335	0.05
20595	1.40305	0.05
20596	0.912698	0.05
20597	0.810946	0.05
20598	1.11608	0.05
20599	1.26409	0.484889
20600	0.946132	0.483741
20601	1.27376	0.508701
20602	0.946132	0.488749
20603	1.03333	0.510907
20604	1.27376	0.510602
20605	0.94613	0.427077
20606	0.812384	0.417424
20607	0.687744	0.329786
20608	0.946132	0.437312
20609	0.687758	0.416831
20610	1.09667	0.34246
20611	1.56977	2.1
20612	0.851544	2.1
20613	1.01136	2.1
20614	0.851481	1.72152
20615	0.946131	2.1
20616	0.705409	2.1
20617	1.32449	0.0928264
20618	1.13508	0.0770293
20619	0.94613	0.130238
20620	0.94613	0.113357
20621	0.965822	0.116111
20622	1.95301	0.223411
20623	0.851538	0.465806
20624	1.29262	0.446335
20625	0.95417	0.40003
20626	0.851541	0.479892
20627	0.94613	0.449819
20628	1.02727	0.751028
20629	1.37675	1.21226
20630	0.955646	0.653858
20631	0.95114	0.760801
20632	0.851528	0.747346
20633	1.76513	1.11153
20634	0.87155	2.1
20635	0.94613	2.1
20636	0.871519	2.1
20637	0.966631	2.1
20638	1.37791	2.1
20639	1.19122	0.531929
20640	0.818971	1.035
20641	0.628799	0.447099
20642	0.861027	0.857526
20643	0.800034	0.0607779
20644	0.91846	0.0557614
20645	0.765645	0.0565916

20646	1.13709	0.73259
20647	1.29054	0.810302
20648	0.755336	0.887052
20649	0.755334	0.812981
20650	0.946133	0.924437
20651	0.755336	0.693673
20652	1.14444	0.81118
20653	0.872823	0.834058
20654	0.533645	0.776504
20655	0.946132	0.746902
20656	0.533645	0.598036
20657	1.02311	0.698115
20658	1.00278	2.1
20659	1.1016	0.053805
20660	0.629615	0.0551912
20661	1.12628	0.0724719
20662	0.946131	0.069988
20663	0.629615	0.0540758
20664	0.629641	0.065662
20665	1.67046	1.43282
20666	1.81667	2.1
20667	1.7145	2.1
20668	0.94613	2.1
20669	1.29322	2.1
20670	1.00365	2.1
20671	0.946131	2.1
20672	1.29322	2.1
20673	1.27581	2.1
20674	1.33916	2.1
20675	1.32784	2.1
20676	1.74787	2.1
20677	2.28084	2.1
20678	2.98092	2.1
20679	0.946132	0.712362
20680	1.2435	2.1
20681	0.946132	1.84937
20682	1.4099	0.75541
20683	1.2435	1.14597
20684	1.26438	2.1
20685	0.745287	2.1
20686	0.696827	2.1
20687	0.696781	2.1
20688	1.41195	2.1
20689	0.946131	2.1
20690	1.8758	0.884342
20691	0.832979	0.570635
20692	0.75054	0.498483
20693	0.810958	0.504185
20694	0.922889	0.829162
20695	0.694844	0.583568
20696	0.694814	0.703044
20697	0.946131	0.64843
20698	0.694844	0.65693
20699	1.02369	2.1
20700	1.4468	0.121565
20701	0.946125	0.399531
20702	0.93487	0.362272
20703	1.23608	0.457132

20704	1.34257	0.297044
20705	0.851525	0.349242
20706	1.03298	0.341565
20707	1.22418	0.760223
20708	0.946131	1.46157
20709	1.41833	1.0051
20710	1.23452	0.82342
20711	0.946131	0.635076
20712	1.41833	0.752238
20713	1.13077	0.271259
20714	0.942874	1.47792
20715	0.946132	1.16165
20716	0.504366	1.42816
20717	0.593647	2.1
20718	0.898829	1.5664
20719	1.27921	2.1
20720	1.12503	2.1
20721	1.22539	2.1
20722	1.10332	0.860948
20723	0.851541	2.1
20724	0.94613	2.1
20725	0.945109	2.1
20726	0.607962	2.1
20727	0.946133	2.1
20728	0.607961	2.1
20729	0.661956	2.1
20730	1.35048	2.1
20731	1.66018	2.1
20732	0.956467	2.1
20733	0.851542	2.1
20734	0.890734	2.1
20735	0.802455	2.1
20736	1.35943	2.1
20737	1.04798	2.1
20738	1.26426	0.569262
20739	1.3689	0.474415
20740	0.946133	0.494842
20741	0.946133	0.589976
20742	1.09729	0.562068
20743	0.765259	2.1
20744	0.810945	2.1
20745	0.810957	2.1
20746	1.10284	1.68959
20747	1.09368	2.1
20748	0.772601	2.1
20749	0.788011	2.1
20750	0.810934	2.1
20751	0.694357	0.820895
20752	1.22641	0.887765
20753	0.946132	1.44495
20754	0.946132	0.872624
20755	0.946133	0.742514
20756	1.42754	1.44571
20757	0.776414	0.68771
20758	0.806525	1.08136
20759	0.575339	0.763192
20760	0.97154	0.660802
20761	0.8217	0.895021

20762	0.838039	1.23897
20763	0.94613	0.843512
20764	0.946132	0.671154
20765	1.39575	0.463804
20766	1.39575	0.604562
20767	1.12818	0.610925
20768	0.946132	1.05461
20769	0.946133	0.836617
20770	1.06879	0.557202
20771	1.06879	0.739179
20772	0.970358	0.711052
20773	1.2474	0.394249
20774	1.01919	0.0655217
20775	0.94613	0.0729329
20776	1.28952	0.0799276
20777	1.00809	0.0617832
20778	0.849344	0.0638707
20779	0.640304	0.0864725
20780	1.35065	0.0602737
20781	0.946132	0.0590359
20782	0.640333	0.0566257
20783	0.946132	0.0840967
20784	0.685489	2.1
20785	0.94613	2.1
20786	0.68546	2.1
20787	0.874264	2.1
20788	1.27676	2.1
20789	0.946129	0.315791
20790	1.09024	0.30022
20791	0.714563	0.313119
20792	1.12335	0.305085
20793	1.07931	0.301605
20794	1.17799	0.406224
20795	1.8795	0.241372
20796	2.15229	0.266548
20797	1.13859	0.538045
20798	0.946132	0.972574
20799	1.11374	0.658043
20800	1.1386	0.708222
20801	0.615713	0.761519
20802	1.19322	0.302617
20803	1.3377	0.205639
20804	0.670801	0.237948
20805	0.94613	0.744515
20806	0.66617	0.551581
20807	1.36173	0.557646
20808	0.980473	0.463554
20809	1.39132	0.106344
20810	0.864653	0.05
20811	1.23837	0.05
20812	0.810959	0.05
20813	1.1483	0.165384
20814	1.00381	1.46061
20815	0.946128	1.54938
20816	1.39696	1.11536
20817	0.946131	0.985336
20818	0.946131	1.06242
20819	1.38613	1.10517

20820	0.973501	1.64226
20821	0.946127	0.959868
20822	1.35968	1.00568
20823	0.946131	0.871103
20824	0.946129	1.1449
20825	1.39696	1.06705
20826	1.12088	2.1
20827	1.41122	1.93929
20828	1.14464	0.738766
20829	0.946131	2.04695
20830	0.946131	1.5618
20831	1.14464	1.81473
20832	1.33266	1.58073
20833	1.07341	0.882558
20834	0.827287	1.52722
20835	0.946132	1.36791
20836	0.827287	2.05786
20837	1.15644	1.21689
20838	1.27558	0.562972
20839	1.15067	0.535055
20840	0.946131	1.87597
20841	1.29143	0.511923
20842	1.36281	2.00241
20843	1.36281	0.67749
20844	0.946131	0.699219
20845	1.40863	2.1
20846	1.20143	0.327136
20847	1.19755	0.323575
20848	0.566153	0.0696891
20849	0.946131	0.172206
20850	1.07979	0.0687348
20851	1.00463	0.158749
20852	0.566153	0.258672
20853	0.768212	2.1
20854	1.32461	2.05227
20855	1.13268	2.1
20856	1.13268	1.44458
20857	0.946133	1.33991
20858	1.53642	2.1
20859	1.5791	2.1
20860	0.726635	1.95974
20861	1.2334	1.33036
20862	1.29198	1.66631
20863	1.29198	0.990832
20864	0.946131	0.85021
20865	0.956585	2.1
20866	1.25294	2.1
20867	0.946133	2.1
20868	1.35287	2.1
20869	1.23877	2.09643
20870	0.946131	2.1
20871	1.35287	2.1
20872	1.01843	2.1
20873	3.77569	2.1
20874	0.86707	2.1
20875	0.867066	2.1
20876	1.38639	2.1
20877	1.38639	2.1

20878	0.896168	2.1
20879	0.974716	0.199661
20880	1.09411	0.242384
20881	0.663171	0.241303
20882	0.663171	0.320125
20883	0.946132	0.301363
20884	0.66317	0.291097
20885	1.47104	0.162892
20886	1.13629	0.167298
20887	0.772342	0.169097
20888	0.772312	0.218406
20889	0.94613	0.189786
20890	0.77234	0.198956
20891	1.43154	2.1
20892	1.37704	2.1
20893	1.40025	2.1
20894	1.3063	2.1
20895	0.946132	2.1
20896	1.04075	2.1
20897	0.946131	2.1
20898	0.946118	1.10623
20899	0.750011	0.383317
20900	1.0971	0.657277
20901	0.889667	0.479161
20902	0.709598	0.679958
20903	2.39737	0.655017
20904	1.19974	0.0995741
20905	0.94613	0.289515
20906	1.25809	0.137141
20907	1.19974	0.229666
20908	0.73005	0.386321
20909	1.50764	0.06621
20910	1.25809	0.102789
20911	0.946133	0.278456
20912	1.10357	0.151823
20913	1.25809	0.199604
20914	0.628177	0.316506
20915	1.3543	0.0917283
20916	0.793711	0.256681
20917	1.18455	0.264577
20918	1.03966	0.255261
20919	0.946131	0.30452
20920	0.767453	0.18315
20921	0.612292	0.301289
20922	0.946131	0.248032
20923	1.24128	0.30266
20924	1.08761	0.133069
20925	0.762059	0.0591869
20926	1.005 2.1	
20927	1.17054	2.1
20928	1.12665	2.1
20929	0.750368	2.1
20930	0.851542	2.1
20931	0.975436	2.1
20932	0.975442	2.1
20933	0.675367	2.1
20934	1.51973	0.394136
20935	0.94613	0.394169

20936	1.38954	0.410095
20937	0.946131	0.402999
20938	1.07912	0.415699
20939	1.38954	0.408329
20940	2.0219	2.1
20941	1.18347	0.239604
20942	0.946132	0.575469
20943	0.94613	0.458733
20944	1.00202	0.405554
20945	1.00201	0.411764
20946	0.946132	0.472153
20947	0.946132	0.658476
20948	1.37981	0.519979
20949	1.26874	0.470583
20950	1.26874	0.465698
20951	0.946132	0.504636
20952	2.8687	0.383712
20953	1.42884	2.1
20954	1.05188	2.1
20955	0.625307	2.1
20956	0.810956	2.1
20957	0.526656	2.1
20958	0.526684	2.1
20959	0.724122	2.1
20960	0.946132	2.1
20961	0.526684	2.1
20962	1.05337	2.1
20963	1.0593	2.1
20964	1.47981	2.1
20965	1.74431	2.00419
20966	0.885612	2.07206
20967	0.810946	1.32421
20968	0.845185	1.57898
20969	1.38622	0.687898
20970	0.723194	0.625336
20971	0.554302	0.734334
20972	0.709597	0.582142
20973	0.909507	0.621449
20974	0.912754	0.476436
20975	1.15174	0.434976
20976	0.946131	0.483993
20977	0.912753	0.478477
20978	1.15174	0.482634
20979	1.02011	0.242739
20980	0.792754	1.77559
20981	0.946129	2.1
20982	0.792754	1.67721
20983	1.27575	1.58901
20984	0.94613	1.55089
20985	1.60132	1.99276
20986	0.922455	2.05537
20987	1.18713	1.32754
20988	0.926957	1.55579
20989	1.66525	1.70941
20990	0.946132	2.1
20991	0.851541	2.1
20992	0.961191	2.1
20993	1.27534	2.1

20994	0.851554	2.1
20995	1.0499	2.1
20996	1.01776	0.0676863
20997	0.97622	0.0560698
20998	0.810934	0.0847845
20999	2.6802	0.0606186
21000	1.31485	0.0650313
21001	1.09699	0.05
21002	0.681649	0.05
21003	0.790369	0.05
21004	0.946131	0.0529686
21005	0.838026	1.34716
21006	0.567833	0.695243
21007	0.891492	1.12488
21008	0.986984	0.516905
21009	0.946132	1.08104
21010	1.19028	0.679501
21011	0.986984	0.655862
21012	0.595623	0.843418
21013	0.946131	2.1
21014	1.36502	2.1
21015	1.08109	2.1
21016	0.946129	2.1
21017	1.36503	2.1
21018	0.773897	1.97237
21019	0.946133	1.06656
21020	0.613447	2.1
21021	0.613449	1.51894
21022	0.946131	1.85981
21023	1.11163	1.98553
21024	0.966114	2.1
21025	0.735699	2.1
21026	0.662147	2.1
21027	1.04072	2.1
21028	0.946131	2.1
21029	0.862978	2.1
21030	1.48839	0.807226
21031	0.936141	0.716757
21032	0.946131	0.883689
21033	0.936142	1.99933
21034	1.40595	1.23642
21035	0.946131	0.944498
21036	1.05297	0.222841
21037	3.09035	2.1
21038	1.19952	2.1
21039	0.907198	2.1
21040	0.849144	2.1
21041	0.612469	0.542421
21042	0.551237	0.59103
21043	0.985256	0.674046
21044	0.551209	0.608707
21045	0.85153	0.744901
21046	0.624712	2.1
21047	0.900018	2.1
21048	0.851542	2.1
21049	0.864787	2.1
21050	1.27669	2.1
21051	0.946711	0.741207

21052	0.810947	0.804951
21053	0.841547	0.835624
21054	0.764114	0.775375
21055	1.26692	1.51474
21056	0.946132	0.842482
21057	1.37675	1.0853
21058	1.26692	0.856929
21059	1.37545	1.32318
21060	1.49637	0.205377
21061	0.709598	0.274376
21062	0.751252	0.23213
21063	1.36811	0.238824
21064	0.635625	0.248893
21065	1.31713	0.109073
21066	1.18977	2.1
21067	0.893377	1.57617
21068	0.707344	2.1
21069	0.946131	2.1
21070	1.20221	2.1
21071	1.20221	2.1
21072	0.707345	2.1
21073	1.26786	2.1
21074	0.988048	2.1
21075	0.751293	2.04588
21076	0.632008	1.81655
21077	1.51067	2.1
21078	1.13534	2.1
21079	0.946133	2.1
21080	0.946131	2.1
21081	1.25716	2.1
21082	1.3199	2.1
21083	1.09616	2.1
21084	1.36677	1.0788
21085	1.34752	1.17717
21086	0.838041	0.943604
21087	1.67023	2.1
21088	1.02247	0.481231
21089	1.03079	0.459565
21090	0.928982	0.323453
21091	1.13635	0.254186
21092	0.946132	0.346168
21093	0.92898	0.264801
21094	1.13635	0.295576
21095	1.1643	0.147282
21096	0.851541	0.380379
21097	1.39939	0.280106
21098	0.946129	0.389875
21099	1.07775	0.322777
21100	1.14503	0.328805
21101	1.78573	0.177629
21102	1.29827	0.189008
21103	0.872373	0.0682094
21104	1.01162	0.0737581
21105	0.83804	0.0622851
21106	1.48851	0.0684291
21107	1.47385	0.0610414
21108	0.843858	0.0913855
21109	0.938626	0.100749

21110	0.89567	0.0753451
21111	1.59457	0.623929
21112	2.09666	0.185566
21113	0.775132	0.05
21114	0.927005	0.05
21115	0.854224	0.05
21116	0.775142	0.05
21117	0.946106	0.05
21118	1.80784	2.1
21119	2.04593	2.1
21120	1.32282	2.1
21121	0.952138	2.1
21122	0.946132	2.1
21123	0.946132	2.1
21124	0.952138	2.1
21125	1.46988	2.1
21126	0.80465	0.319669
21127	1.14155	0.320814
21128	0.810931	0.320456
21129	1.66203	0.389899
21130	0.674605	0.521945
21131	0.946132	0.573492
21132	1.26706	0.612905
21133	0.674633	0.519765
21134	0.946132	0.624375
21135	0.980664	0.596354
21136	1.34927	0.556933
21137	0.758454	0.369607
21138	0.946133	0.340355
21139	0.985371	0.268791
21140	1.28632	0.346849
21141	0.946133	0.39955
21142	1.46868	0.255425
21143	1.7522	0.0649058
21144	0.737431	2.1
21145	0.663679	2.1
21146	1.16541	1.64236
21147	1.3179	1.90261
21148	1.04081	1.87742
21149	0.887866	1.60103
21150	0.806961	1.16614
21151	0.929061	1.48529
21152	1.28418	0.376675
21153	1.38752	0.384741
21154	1.33038	0.383799
21155	0.946132	0.353784
21156	0.946132	0.335166
21157	1.14897	0.364927
21158	1.1685	0.326378
21159	1.18774	2.1
21160	0.961899	2.1
21161	1.48841	2.1
21162	1.79105	2.1
21163	3.51614	2.1
21164	0.682342	2.1
21165	0.783184	2.1
21166	0.946132	2.1
21167	0.868396	2.1

21168	1.21813	2.1
21169	0.848305	0.591671
21170	0.94559	0.647499
21171	0.805691	0.716547
21172	0.752217	2.1
21173	0.946132	2.1
21174	0.839123	2.1
21175	0.946131	2.1
21176	0.752187	2.1
21177	1.96491	2.1
21178	0.520997	2.1
21179	0.946132	2.1
21180	0.73446	2.1
21181	0.946133	2.1
21182	0.520995	2.1
21183	1.88522	2.1
21184	1.8361	2.1
21185	1.11285	2.1
21186	1.25334	1.0877
21187	2.00756	2.1
21188	1.44252	0.353491
21189	1.00255	0.83185
21190	1.64516	2.1
21191	1.12399	0.05
21192	1.13542	0.0507343
21193	1.09038	0.0613419
21194	0.746903	0.0704863
21195	0.672241	0.0525374
21196	0.871774	0.0597507
21197	1.73513	1.05486
21198	0.949995	2.1
21199	1.17244	2.03471
21200	0.946127	2.1
21201	1.3028	1.90144
21202	1.17244	1.75179
21203	0.774101	2.1
21204	0.946127	2.1
21205	0.939289	2.1
21206	0.946129	2.1
21207	0.774101	2.1
21208	1.87858	2.1
21209	1.44293	0.106753
21210	1.43213	0.0956166
21211	0.946131	2.1
21212	0.716614	2.1
21213	0.716644	2.1
21214	1.35828	2.1
21215	0.790935	2.1
21216	0.730157	2.1
21217	1.41149	2.1
21218	0.730186	2.1
21219	0.730186	2.1
21220	0.946131	2.1
21221	0.946132	1.05476
21222	0.946132	0.851603
21223	0.990713	0.564833
21224	1.40779	0.696433
21225	0.946132	0.760294

21226	1.06486	0.439119
21227	0.946131	0.843158
21228	0.946133	0.616921
21229	1.40779	0.546287
21230	1.26874	0.6067
21231	0.946131	0.610152
21232	1.02588	0.518517
21233	0.826073	0.372238
21234	1.41233	0.369642
21235	0.861269	0.370095
21236	1.04073	0.395317
21237	1.27111	0.403232
21238	1.56016	1.517
21239	1.60866	0.05
21240	0.94613	0.0716677
21241	0.946129	0.0777789
21242	1.36376	0.0560458
21243	1.22187	0.0760365
21244	0.94613	0.0751042
21245	1.82581	1.26093
21246	1.19705	1.04329
21247	1.7513	2.1
21248	1.85017	2.1
21249	1.72116	2.1
21250	1.12058	2.1
21251	0.899991	2.1
21252	0.616288	2.1
21253	1.07289	2.1
21254	1.7092	0.494795
21255	0.946132	0.441647
21256	0.946133	0.199593
21257	1.18717	0.319312
21258	1.40029	0.465845
21259	0.946132	0.320672
21260	0.94613	0.353899
21261	1.18547	0.346663
21262	0.960326	0.35242
21263	0.960345	0.342367
21264	0.942557	0.357343
21265	1.18601	0.324583
21266	0.946132	0.33387
21267	1.30307	0.330544
21268	1.08169	0.330874
21269	1.08169	0.326063
21270	1.31716	0.337358
21271	1.51065	0.792182
21272	0.877642	0.0547829
21273	0.617384	0.05
21274	0.575741	0.052852
21275	0.972448	0.05
21276	1.11334	0.0517811
21277	1.13092	2.1
21278	0.946127	0.531571
21279	1.31724	0.586261
21280	1.39751	0.382853
21281	1.39751	0.467247
21282	0.946128	0.483178
21283	0.946132	0.387036

21284	0.713284	0.370315
21285	1.40668	0.423246
21286	1.03547	0.367543
21287	0.713298	0.380157
21288	1.88774	0.342619
21289	0.830164	1.87536
21290	1.05211	2.1
21291	0.946119	0.899001
21292	0.946107	2.1
21293	1.283	2.1
21294	2.63609	2.1
21295	1.28418	0.948095
21296	0.946128	0.941091
21297	1.10335	0.844051
21298	0.598377	1.25111
21299	1.18818	0.867157
21300	1.10335	0.821643
21301	1.58972	0.996446
21302	1.90194	2.1
21303	0.946118	2.1
21304	0.946115	2.1
21305	0.950969	2.1
21306	1.36918	2.1
21307	0.950955	2.1
21308	1.4263	2.1
21309	1.11978	0.801098
21310	0.946132	1.13596
21311	0.946133	1.35494
21312	1.37658	0.815215
21313	1.2364	0.902702
21314	1.05496	0.783882
21315	0.946132	1.45869
21316	0.946132	1.01425
21317	1.21858	0.753632
21318	1.37658	0.939264
21319	1.09194	1.53926
21320	1.14721	0.0630659
21321	1.53814	0.05
21322	0.546751	0.737337
21323	0.492063	1.08172
21324	1.16889	1.74786
21325	1.00349	1.75609
21326	0.863559	0.876405
21327	1.38102	1.42664
21328	0.597449	0.895382
21329	0.537748	0.717474
21330	1.16297	1.2532
21331	0.999366	1.10779
21332	0.896554	0.82742
21333	0.566962	0.05
21334	0.946129	0.05
21335	0.452729	0.05
21336	0.696552	0.05
21337	0.852524	0.05
21338	1.57992	0.05
21339	1.64985	0.053557
21340	0.946128	2.1
21341	1.21847	2.1

21342	0.946129	2.1
21343	1.41131	2.1
21344	1.21847	2.1
21345	1.21269	0.05
21346	1.0417	2.1
21347	1.15821	0.895493
21348	0.946132	0.86559
21349	0.946132	1.68819
21350	1.37967	0.65517
21351	0.851529	0.683929
21352	1.20455	2.1
21353	1.4282	0.05
21354	1.26593	2.1
21355	0.838026	0.106762
21356	0.985096	0.0904739
21357	0.83804	0.100217
21358	0.822395	0.929493
21359	0.420272	0.568253
21360	0.946131	2.1
21361	1.17461	2.1
21362	0.94613	2.1
21363	1.40225	2.1
21364	1.17461	2.1
21365	1.09847	2.1
21366	1.22222	0.832155
21367	1.41798	0.638099
21368	0.791658	0.700698
21369	0.791656	0.709519
21370	0.946129	0.722621
21371	0.817064	0.698574
21372	0.946114	0.634438
21373	0.75824	0.633395
21374	0.75821	0.642777
21375	1.41778	0.611313
21376	0.791635	0.622888
21377	1.04164	1.93809
21378	0.946131	0.762853
21379	1.11172	0.641287
21380	1.30076	0.532475
21381	1.35721	0.577989
21382	1.11172	0.575197
21383	1.87218	0.342989
21384	0.987248	0.364473
21385	0.729695	0.600822
21386	0.974389	0.459443
21387	1.18002	0.44966
21388	0.822612	0.43059
21389	1.35721	0.445735
21390	0.634295	0.658692
21391	0.634326	0.664911
21392	1.13245	1.00767
21393	1.13245	0.84561
21394	0.937961	0.615467
21395	1.18769	0.764898
21396	0.854949	0.573893
21397	0.967357	0.632722
21398	0.810934	0.564491
21399	3.91082	2.1

21400	0.946132	2.1
21401	0.946132	2.1
21402	1.05383	2.1
21403	1.05383	2.1
21404	0.986498	2.1
21405	1.50732	0.12605
21406	0.753659	0.192179
21407	1.22144	0.230384
21408	1.30463	0.165748
21409	0.753659	0.204889
21410	1.30463	0.195175
21411	0.886189	0.193206
21412	1.04321	0.184204
21413	0.909928	0.153264
21414	1.18659	0.151265
21415	1.19785	0.0510628
21416	1.40969	0.0753637
21417	1.24094	0.0549581
21418	0.946126	0.0677515
21419	1.2929	0.0535361
21420	1.24093	0.0594689
21421	0.867944	0.0542444
21422	1.41903	2.1
21423	2.47881	0.05
21424	1.12943	0.05
21425	0.764229	2.1
21426	0.764256	2.1
21427	0.94613	2.1
21428	0.94613	2.1
21429	1.1995	2.1
21430	1.83423	2.1
21431	0.634816	2.04248
21432	0.877837	2.1
21433	0.94613	2.04714
21434	1.04073	2.1
21435	1.06485	2.1
21436	0.94613	0.382069
21437	0.62725	0.332239
21438	0.798212	0.382204
21439	1.33241	0.253965
21440	0.62725	0.338261
21441	1.82301	0.236943
21442	1.40615	2.1
21443	0.782504	2.1
21444	0.748635	2.1
21445	0.946131	2.1
21446	0.748607	2.1
21447	0.988456	2.1
21448	1.64833	0.05
21449	1.35657	0.0515321
21450	1.26155	0.0528915
21451	0.915755	0.0578886
21452	0.915755	0.0511054
21453	0.946132	0.0542385
21454	1.49386	0.05
21455	1.01346	2.1
21456	0.946131	0.68899
21457	0.946132	2.08313

21458	1.38651	2.1
21459	1.39523	2.1
21460	0.946132	1.88572
21461	1.06312	2.1
21462	0.946131	2.1
21463	0.946129	2.1
21464	1.39523	2.1
21465	1.37372	2.1
21466	0.946131	2.1
21467	1.2929	0.0784035
21468	0.94613	0.117541
21469	1.24448	0.0744546
21470	1.2929	0.0885247
21471	0.862011	0.0815155
21472	1.73583	0.05
21473	1.724	0.0565275
21474	1.03824	2.1
21475	1.534	2.1
21476	0.725492	1.64444
21477	0.771967	1.82325
21478	0.61731	1.02226
21479	0.475743	1.33391
21480	0.719694	1.57299
21481	1.14878	0.977261
21482	0.946117	1.6117
21483	0.94613	1.75241
21484	0.946105	1.7095
21485	0.482666	1.00706
21486	0.609172	1.01941
21487	0.946131	2.1
21488	1.39509	0.660074
21489	1.07439	2.0077
21490	0.946131	0.885916
21491	1.39509	0.994629
21492	0.963571	1.14857
21493	0.591011	0.517249
21494	1.1357	0.419522
21495	2.11193	0.908567
21496	1.60936	2.1
21497	1.34118	0.317582
21498	1.27696	0.34122
21499	0.94613	0.331765
21500	0.94613	0.310412
21501	1.04257	0.278857
21502	1.53336	0.0526226
21503	1.0631	0.0726964
21504	1.04072	0.139794
21505	0.670678	0.0923298
21506	0.709531	0.11655
21507	0.891275	0.120899
21508	0.683583	0.179055
21509	0.902478	0.105933
21510	0.807671	0.111512
21511	1.11024	2.1
21512	1.08171	2.1
21513	1.16725	2.1
21514	0.946128	2.1
21515	1.13537	2.1

21516	1.40525	2.1
21517	1.28365	2.1
21518	1.04523	2.1
21519	0.960106	2.1
21520	0.946128	2.1
21521	1.23452	2.1
21522	0.946129	2.1
21523	1.3704	2.1
21524	1.23452	2.1
21525	1.16906	0.784872
21526	0.946131	1.38397
21527	0.867246	0.860116
21528	0.867217	1.67044
21529	0.946131	1.01741
21530	1.13438	1.38441
21531	1.28684	1.44634
21532	0.946132	1.55154
21533	0.883935	1.15
21534	0.883938	2.1
21535	0.946133	1.30036
21536	1.73444	0.994563
21537	1.21835	2.1
21538	1.28228	2.1
21539	1.8643	1.11143
21540	0.743379	0.379905
21541	1.34921	0.378369
21542	0.743381	0.379158
21543	0.743381	0.375705
21544	0.946131	0.391489
21545	0.915528	2.1
21546	0.982742	2.1
21547	0.975965	2.1
21548	0.891132	0.05
21549	1.27533	0.05
21550	0.946132	0.05
21551	1.25215	0.05
21552	0.590343	0.05
21553	0.946133	0.05
21554	0.590331	0.05
21555	1.62375	2.1
21556	0.687769	0.570448
21557	1.14374	0.543832
21558	1.20885	0.506966
21559	0.946132	0.577304
21560	1.14374	0.649599
21561	1.09308	0.40945
21562	1.02055	0.395541
21563	0.810934	0.374406
21564	0.917609	1.00517
21565	1.13094	0.98354
21566	0.70959	0.947848
21567	1.11223	2.1
21568	0.83051	1.02556
21569	0.810946	1.26494
21570	0.810959	0.973789
21571	1.5773	0.05
21572	1.96133	0.910595
21573	1.0214	0.9555

21574	0.762783	1.33157
21575	0.838036	0.855554
21576	0.63591	1.51948
21577	0.967411	0.841475
21578	0.946132	2.03932
21579	0.946132	1.93131
21580	1.39699	1.4182
21581	1.61642	1.91431
21582	0.860954	0.830505
21583	0.957411	1.49921
21584	0.660856	0.986107
21585	1.10003	2.1
21586	0.968249	2.1
21587	0.910184	1.0903
21588	1.30029	0.619764
21589	0.754119	0.620877
21590	0.754121	0.847213
21591	0.94613	1.03108
21592	1.08039	2.1
21593	0.810942	0.183036
21594	1.15153	0.178803
21595	1.06968	0.149431
21596	1.38456	2.1
21597	1.04118	2.1
21598	0.838027	2.1
21599	1.04073	2.1
21600	0.754209	2.1
21601	1.332	0.0572985
21602	1.26193	2.1
21603	1.15317	0.05
21604	3.86179	0.05
21605	1.56005	2.1
21606	0.683035	0.458894
21607	0.931463	0.432054
21608	0.556019	0.638362
21609	0.929172	0.537175
21610	0.756874	0.414889
21611	1.23527	0.576501
21612	2.05961	0.628792
21613	0.946132	2.1
21614	1.16684	2.1
21615	1.3937	2.1
21616	1.3937	2.1
21617	0.946132	2.1
21618	1.17885	2.1
21619	1.62507	0.05
21620	1.58874	2.1
21621	0.819953	2.1
21622	1.16023	2.1
21623	1.34782	2.1
21624	1.16023	2.1
21625	0.819955	2.1
21626	0.79437	2.1
21627	0.794373	2.1
21628	1.20056	1.88148
21629	1.25	2.1
21630	1.20056	2.1
21631	1.35787	2.1

21632	0.946131	2.03998
21633	1.38585	1.92159
21634	0.946128	0.7699
21635	0.946129	1.19558
21636	1.35714	1.20523
21637	1.04147	2.1
21638	0.946133	1.45227
21639	1.30382	1.13039
21640	0.946131	0.621916
21641	0.946131	0.986278
21642	1.38585	1.12627
21643	1.22305	1.34197
21644	1.00763	0.201607
21645	0.813199	0.323695
21646	0.810948	0.312246
21647	1.15928	0.266263
21648	0.944166	0.0853649
21649	0.94613	0.123544
21650	0.946133	0.0871021
21651	1.4179	0.105724
21652	0.946132	0.0902728
21653	1.36965	0.056087
21654	1.05444	0.0768785
21655	0.690302	0.110967
21656	0.838038	0.0705618
21657	0.802821	0.673163
21658	1.40944	0.817047
21659	0.946126	1.05985
21660	0.799998	0.372556
21661	0.800035	0.686792
21662	1.1766	2.1
21663	1.00319	2.1
21664	1.01606	1.44404
21665	0.872158	2.1
21666	1.04884	1.97672
21667	0.679494	2.1
21668	0.946131	2.1
21669	1.37609	2.1
21670	0.94613	2.1
21671	0.946131	2.1
21672	1.18486	2.1
21673	0.726287	2.1
21674	0.946131	2.1
21675	0.726286	2.1
21676	1.30318	2.1
21677	1.01712	2.1
21678	0.946132	2.1
21679	1.04404	2.1
21680	1.37017	2.1
21681	1.37017	2.1
21682	0.946133	2.1
21683	0.992084	0.936695
21684	0.946131	0.865407
21685	0.529655	1.62445
21686	0.529686	0.916759
21687	0.946131	0.791292
21688	1.05937	1.10576
21689	1.14622	0.628647

21690	0.946131	0.74022
21691	0.584575	1.06087
21692	0.584575	0.663826
21693	0.946129	0.608103
21694	0.709714	2.1
21695	0.946132	2.1
21696	0.709714	2.1
21697	1.37199	2.1
21698	0.82899	2.1
21699	1.00133	2.1
21700	0.778424	0.05
21701	0.946132	0.05
21702	1.24094	0.05
21703	1.00691	0.05
21704	0.64868	0.05
21705	1.29736	0.05
21706	1.24767	0.553764
21707	1.28953	0.712739
21708	1.34612	0.731327
21709	0.946133	0.576905
21710	1.14187	1.02892
21711	0.946132	0.638127
21712	0.946129	0.584602
21713	1.4099	1.74241
21714	0.946131	0.63636
21715	1.34612	0.543563
21716	1.4099	0.552322
21717	0.956003	0.05
21718	1.40572	0.05
21719	3.54443	2.1
21720	0.994001	0.490062
21721	1.03679	2.1
21722	1.08467	2.1
21723	1.56875	0.310616
21724	0.968463	1.99817
21725	0.974889	2.1
21726	0.946131	2.1
21727	1.30203	2.1
21728	1.40199	2.1
21729	1.40199	2.1
21730	0.946132	2.1
21731	0.946132	2.1
21732	1.36994	2.1
21733	1.37372	2.1
21734	1.37372	2.1
21735	0.946132	2.1
21736	0.928979	0.913077
21737	0.565509	1.07283
21738	1.60196	0.646302
21739	1.16239	0.763259
21740	1.10746	0.437318
21741	1.15412	2.1
21742	1.15171	2.1
21743	0.946133	2.1
21744	0.940877	2.1
21745	0.940878	2.1
21746	1.41432	2.1
21747	1.04687	2.1

21748	2.75524	0.0822071
21749	0.946132	0.102938
21750	0.766452	0.0717713
21751	0.766427	0.10086
21752	1.36004	0.0607367
21753	1.06648	0.0929556
21754	1.62483	2.1
21755	1.0577	2.1
21756	1.39158	2.1
21757	0.94255	2.1
21758	1.05029	2.1
21759	1.03735	1.87135
21760	0.931055	2.1
21761	1.03797	1.97682
21762	1.43687	1.89121
21763	1.28765	1.5183
21764	1.1811	0.967021
21765	0.946132	1.19023
21766	0.946132	1.20927
21767	1.1811	1.17188
21768	0.67805	0.744831
21769	1.50998	1.88196
21770	1.16661	2.1
21771	1.36749	2.1
21772	0.810945	0.896748
21773	1.14745	1.16692
21774	0.865588	0.640902
21775	1.36665	0.771476
21776	0.506964	1.20776
21777	0.506966	0.847705
21778	0.552786	0.79819
21779	0.946133	0.622024
21780	1.21336	0.678048
21781	0.552786	0.835044
21782	0.552757	1.33407
21783	0.653468	0.76168
21784	1.36665	1.53551
21785	1.27922	0.377164
21786	1.08133	0.323388
21787	1.05348	0.141745
21788	0.946132	0.273968
21789	1.19601	0.171653
21790	1.13276	0.175402
21791	0.7856	0.175964
21792	1.57125	0.164207
21793	1.19601	0.258289
21794	0.946131	0.743407
21795	1.34534	0.585066
21796	1.19601	0.35709
21797	0.870489	0.40461
21798	1.49404	2.1
21799	1.35943	1.06806
21800	1.10427	1.35991
21801	0.94613	1.00242
21802	0.946131	1.09577
21803	1.10427	1.14675
21804	1.2064	0.185593
21805	0.946132	0.411589

21806	1.11288	0.538624
21807	1.34268	0.335073
21808	1.34268	0.368095
21809	0.946132	0.380376
21810	0.946128	2.1
21811	0.946128	0.582367
21812	1.30835	1.41824
21813	1.38463	0.727121
21814	0.946129	0.74622
21815	1.43778	0.790225
21816	1.23943	0.461143
21817	1.4748	2.1
21818	0.71295	2.1
21819	0.712951	2.1
21820	0.946132	2.1
21821	0.946133	2.1
21822	1.13976	2.1
21823	1.71109	2.1
21824	1.32426	2.1
21825	0.671255	2.1
21826	1.29123	2.1
21827	0.946132	2.1
21828	0.946132	2.05317
21829	1.25603	2.09094
21830	2.21126	0.0754774
21831	0.892889	2.1
21832	0.946129	2.1
21833	0.892888	2.1
21834	0.92872	2.1
21835	1.34352	2.1
21836	0.622784	0.521921
21837	1.02947	0.357312
21838	0.810957	0.325914
21839	1.91756	0.999317
21840	1.06012	0.855149
21841	1.09569	0.471909
21842	1.5456	0.178451
21843	1.1211	0.0815472
21844	1.21031	0.116633
21845	1.03939	0.0927922
21846	0.519697	0.0961735
21847	0.933889	0.223294
21848	0.519726	0.200805
21849	1.16699	0.174681
21850	0.933889	0.194127
21851	0.840599	0.0680603
21852	0.94613	0.168448
21853	0.840568	0.117552
21854	1.26868	0.114473
21855	1.16699	0.103461
21856	1.17822	2.1
21857	0.922044	0.418689
21858	1.29322	0.439358
21859	0.946132	0.40236
21860	0.995178	0.486439
21861	0.922056	0.443406
21862	1.76232	0.694757
21863	1.77065	1.00004

21864	1.38149	0.775742
21865	0.959301	0.998419
21866	0.946133	0.836487
21867	0.946132	0.934993
21868	0.959301	0.877205
21869	1.3612	0.787309
21870	0.885314	0.971416
21871	0.885309	0.820114
21872	0.94613	0.934911
21873	0.8853	0.767826
21874	1.56164	0.841449
21875	1.45506	0.335775
21876	1.45257	2.1
21877	0.851541	0.0964911
21878	1.29018	0.0595782
21879	0.851541	0.0789948
21880	1.21087	0.0645359
21881	1.24979	0.0804306
21882	1.56983	0.0501888
21883	1.60151	0.05
21884	1.78952	2.1
21885	0.638164	2.1
21886	0.638163	2.1
21887	1.39754	2.1
21888	1.39754	2.1
21889	0.681589	2.1
21890	1.34993	2.1
21891	1.04525	2.1
21892	0.946133	2.1
21893	1.04072	2.1
21894	0.857805	2.1
21895	1.06053	2.1
21896	1.49066	2.1
21897	1.4185	0.919728
21898	0.934059	1.6539
21899	0.93406	0.913585
21900	0.94613	1.34714
21901	0.93406	1.03393
21902	1.25277	1.06892
21903	1.00792	2.00829
21904	0.609466	1.05275
21905	0.946132	1.55838
21906	0.609463	1.68029
21907	1.30664	2.1
21908	0.810943	1.56519
21909	1.05741	1.23605
21910	1.3868	0.738161
21911	1.15558	0.410194
21912	1.13537	2.1
21913	0.946132	2.1
21914	0.946132	2.1
21915	1.08266	2.1
21916	1.54934	2.1
21917	1.26016	2.1
21918	2.01915	2.1
21919	0.60346	1.05366
21920	0.651529	1.15089
21921	1.15581	1.27813

21922	1.13037	0.972035
21923	1.04071	0.908174
21924	1.77796	1.78638
21925	0.532308	1.06735
21926	0.532308	0.98834
21927	1.15369	1.73693
21928	1.1537	1.53643
21929	0.903288	1.17719
21930	0.804766	2.1
21931	0.804739	2.1
21932	0.946128	2.1
21933	0.946131	2.1
21934	1.16745	2.1
21935	1.04193	2.1
21936	0.98505	2.1
21937	1.40724	0.563363
21938	1.14216	0.504419
21939	0.946131	0.431417
21940	0.946131	0.480365
21941	1.10941	0.489634
21942	0.81268	0.05
21943	0.946131	0.05
21944	1.28673	0.05
21945	1.28673	0.05
21946	0.812681	0.05
21947	1.84716	0.05
21948	1.84716	0.05
21949	0.92361	0.05
21950	0.94613	0.05
21951	0.923582	0.05
21952	1.26227	0.05
21953	0.923581	0.05
21954	0.747394	2.1
21955	1.32582	2.1
21956	0.747365	2.1
21957	0.97587	2.1
21958	1.32582	2.1
21959	1.04182	0.0758851
21960	0.961743	0.05
21961	0.946126	0.0715165
21962	1.47463	0.0567096
21963	1.12183	0.0572866
21964	0.851552	0.0556216
21965	1.33177	0.0760466
21966	0.946131	0.131778
21967	1.41512	0.128762
21968	1.33178	0.0927508
21969	0.946131	0.0928873
21970	0.927735	0.576202
21971	0.97206	0.434559
21972	0.515566	0.52607
21973	0.946129	0.483643
21974	0.515538	0.48648
21975	1.04482	0.372207
21976	0.975535	0.369515
21977	0.522411	0.353722
21978	0.522382	0.437691
21979	0.925346	0.437217

21980	0.575817	0.42248
21981	0.946129	2.1
21982	1.14256	2.1
21983	1.32054	2.1
21984	1.40525	2.1
21985	1.14256	2.1
21986	1.59475	2.1
21987	0.773813	2.1
21988	1.4073	2.1
21989	0.773813	2.1
21990	0.773813	2.1
21991	1.4073	2.1
21992	0.993021	2.1
21993	1.50466	0.08706
21994	0.851539	0.163712
21995	1.20299	0.110978
21996	0.946131	0.131361
21997	1.54464	0.138267
21998	1.20309	0.120624
21999	2.00701	0.0877534
22000	0.810946	0.172425
22001	1.07678	0.166791
22002	0.900128	0.136495
22003	1.26842	2.1
22004	1.11206	2.1
22005	0.946132	2.1
22006	1.04074	2.1
22007	0.948809	2.1
22008	0.946131	0.05
22009	0.929617	0.0548273
22010	1.24057	0.05
22011	1.36485	0.05
22012	0.851554	0.05
22013	1.96823	0.05
22014	0.914836	0.05
22015	0.94613	0.0675691
22016	1.38911	0.05
22017	0.914805	0.05
22018	0.946131	0.05
22019	0.946131	0.0776821
22020	1.04072	0.0710495
22021	1.26837	0.0554785
22022	0.970054	0.0713566
22023	0.923958	0.0688721
22024	1.16653	2.1
22025	1.34412	1.13948
22026	0.851541	0.139961
22027	1.38645	0.183513
22028	0.891637	0.140684
22029	0.851487	0.149864
22030	0.890871	0.164713
22031	1.56178	0.155036
22032	0.946128	0.05
22033	1.26868	0.05
22034	0.946131	0.05
22035	1.18789	0.0689818
22036	1.26868	0.05
22037	1.26868	0.05

22038	0.946128	0.05
22039	1.18716	0.116308
22040	0.946131	0.279178
22041	0.59358	0.16442
22042	1.23923	0.297071
22043	1.00024	0.16789
22044	0.593593	0.243215
22045	0.946131	0.335801
22046	0.713645	0.237886
22047	1.23175	0.367386
22048	1.23923	0.189038
22049	0.713633	0.278918
22050	1.24687	0.135116
22051	1.80662	2.1
22052	2.15762	2.1
22053	0.780455	2.1
22054	0.946131	2.1
22055	0.780455	2.1
22056	1.40583	2.1
22057	1.39852	2.1
22058	0.565619	2.1
22059	1.1428	2.1
22060	0.663168	0.432231
22061	1.02163	0.336049
22062	1.04072	0.437351
22063	0.663169	0.406476
22064	1.13511	0.421815
22065	1.85277	0.218307
22066	1.17701	0.76487
22067	0.993831	0.311918
22068	1.0823	0.321528
22069	0.795761	0.362642
22070	1.24596	0.264082
22071	0.566075	0.360966
22072	0.94613	0.398079
22073	0.56605	0.383374
22074	0.774911	0.0552632
22075	0.946131	0.0744929
22076	1.24417	0.0519586
22077	0.774911	0.0518856
22078	0.946132	0.0612959
22079	1.54982	0.0507388
22080	1.82961	0.05
22081	1.12035	0.05
22082	0.876835	2.1
22083	0.946131	1.91577
22084	0.876836	2.1
22085	1.12968	2.1
22086	1.23536	2.1
22087	2.15082	2.1
22088	0.58365	2.1
22089	0.946133	2.1
22090	0.583653	2.1
22091	0.992097	2.1
22092	1.12968	2.1
22093	1.0305	2.1
22094	0.82613	0.243048
22095	0.879644	0.207726

22096	1.00688	0.191304
22097	1.52776	2.1
22098	0.851537	1.59508
22099	1.00851	1.46111
22100	0.946128	0.894012
22101	0.851552	1.13357
22102	1.4895	1.60926
22103	0.761574	2.1
22104	0.946132	2.1
22105	1.01095	2.1
22106	1.01095	2.1
22107	0.761572	2.1
22108	1.8575	2.1
22109	1.19984	0.314876
22110	1.25797	0.320542
22111	0.946132	0.32146
22112	0.946132	0.316378
22113	0.95332	0.319474
22114	0.946131	2.1
22115	0.851542	1.40299
22116	1.03172	2.1
22117	1.22889	2.1
22118	1.0313	2.1
22119	1.15977	1.15574
22120	0.918875	0.892727
22121	0.8654	1.74336
22122	0.865374	0.86785
22123	0.946132	0.892251
22124	0.985051	2.1
22125	1.31993	0.501838
22126	1.33622	0.644851
22127	0.959992	0.587184
22128	0.926499	2.1
22129	1.03544	2.1
22130	0.946132	2.1
22131	0.946132	2.1
22132	1.40335	2.1
22133	0.772299	2.1
22134	0.851529	2.1
22135	0.857243	2.1
22136	1.38155	2.1
22137	1.05462	2.1
22138	0.832138	2.1
22139	0.946132	2.1
22140	0.832137	2.1
22141	0.959414	2.1
22142	1.36541	2.1
22143	1.91894	2.1
22144	1.04661	0.862523
22145	1.28355	2.1
22146	0.877353	0.744597
22147	0.877353	0.840323
22148	0.94613	0.779809
22149	1.20537	0.204025
22150	1.09914	0.108098
22151	0.94613	0.142224
22152	0.499593	0.170043
22153	0.499606	0.15662

22154	1.0959	0.0980253
22155	0.838027	0.0910474
22156	0.883644	0.131725
22157	0.95019	0.112057
22158	1.10638	2.1
22159	0.946132	2.1
22160	1.41744	2.1
22161	1.05842	2.1
22162	0.94613	2.1
22163	1.41744	2.1
22164	0.991985	2.1
22165	3.31755	1.17879
22166	1.05556	2.1
22167	0.624075	0.92513
22168	0.814554	0.919986
22169	0.946131	1.09917
22170	0.814554	1.13297
22171	1.31857	1.65315
22172	1.26282	2.1
22173	0.946132	2.1
22174	1.14302	2.1
22175	0.946132	2.1
22176	0.946132	2.1
22177	1.40372	2.1
22178	1.24688	0.182752
22179	0.89889	2.1
22180	1.17286	2.1
22181	0.71741	2.1
22182	0.491096	0.639169
22183	1.05432	0.488917
22184	0.719531	2.1
22185	1.25145	2.1
22186	0.864686	2.1
22187	0.867974	2.1
22188	1.21734	2.1
22189	0.405517	0.674084
22190	0.818878	1.2085
22191	0.502457	0.0858991
22192	1.05868	0.117569
22193	0.912018	0.0960927
22194	1.40591	0.291051
22195	0.957584	0.290346
22196	0.946131	0.537873
22197	1.11612	0.423839
22198	1.3365	0.419353
22199	0.946128	0.569415
22200	1.16534	0.241175
22201	1.38969	0.589465
22202	0.891597	1.36351
22203	0.640032	1.93188
22204	0.956758	1.44735
22205	1.3428	0.43018
22206	0.953295	2.1
22207	0.946131	0.399802
22208	1.16167	0.410819
22209	0.94613	0.415131
22210	0.951675	0.410826
22211	1.16167	0.414427

22212	1.24538	0.162482
22213	1.13482	2.1
22214	1.38775	2.1
22215	0.934061	2.1
22216	0.91408	2.1
22217	1.06099	2.1
22218	0.980549	0.23111
22219	0.946132	0.308398
22220	1.10321	0.375383
22221	0.99126	0.305442
22222	0.991261	0.340791
22223	1.35732	0.322898
22224	1.1952	1.94123
22225	0.946132	1.09617
22226	0.946132	2.1
22227	1.32819	1.19875
22228	1.30171	0.942887
22229	1.29667	0.078366
22230	1.00982	0.0557247
22231	0.407589	0.564113
22232	1.18266	1.0958
22233	1.04037	0.579028
22234	0.956694	0.696893
22235	0.552639	0.574918
22236	0.810943	1.81966
22237	1.43144	0.716968
22238	0.918074	0.716574
22239	1.60976	2.1
22240	4.00847	2.1
22241	2.3995	0.121046
22242	1.36203	2.1
22243	0.919619	2.1
22244	1.30188	2.1
22245	0.681073	2.1
22246	0.681043	2.1
22247	1.04687	2.1
22248	3.32085	2.1
22249	1.80396	0.0571379
22250	0.773007	0.43786
22251	0.773034	0.401456
22252	1.22141	0.455936
22253	1.22141	0.447796
22254	0.946131	0.50056
22255	2.02071	0.377663
22256	0.951912	0.05
22257	1.39027	0.0559251
22258	0.94613	0.059445
22259	0.829922	0.05
22260	0.829946	0.0502592
22261	1.24902	1.17997
22262	2.8333	0.91421
22263	0.946131	0.05
22264	0.929725	0.05
22265	1.1134	0.05
22266	1.17056	0.05
22267	0.929713	0.05
22268	0.749096	2.1
22269	1.31961	2.1

22270	0.870191	2.1
22271	1.04071	2.1
22272	1.1877	2.1
22273	1.03882	2.1
22274	1.44801	2.09083
22275	3.55198	2.1
22276	1.11193	2.1
22277	0.663922	2.1
22278	0.663922	2.1
22279	0.946129	2.1
22280	0.946131	2.1
22281	0.97079	2.1
22282	1.36049	2.1
22283	1.06105	2.1
22284	0.92251	2.1
22285	0.94613	2.1
22286	0.922511	2.1
22287	1.04101	2.1
22288	0.810944	0.125811
22289	0.994109	0.117338
22290	0.623143	0.106471
22291	0.864244	0.133788
22292	1.14627	0.125833
22293	0.64586	0.11839
22294	0.721422	0.0768913
22295	0.870885	0.0549765
22296	0.855175	0.0840836
22297	0.946131	0.79917
22298	1.38157	2.1
22299	1.33266	0.652047
22300	0.946131	1.56642
22301	1.38158	0.899327
22302	0.946132	1.22937
22303	1.3268	2.1
22304	0.946132	0.878729
22305	0.946132	2.1
22306	1.38158	2.1
22307	1.42186	2.02717
22308	1.47204	1.92061
22309	1.14665	2.07855
22310	0.946132	1.03276
22311	0.94613	2.1
22312	1.30116	1.73653
22313	1.32819	1.12058
22314	1.25388	2.1
22315	0.887808	1.05038
22316	0.946132	2.1
22317	1.23536	1.99874
22318	1.34309	1.92771
22319	1.07612	2.1
22320	0.728982	0.0912164
22321	1.3157	0.0841408
22322	0.946131	0.14626
22323	0.686176	0.121112
22324	1.3035	0.12835
22325	0.686139	0.124628
22326	0.906806	0.119883
22327	0.794122	0.102024

22328	1.17173	0.111382
22329	1.8194	0.0686833
22330	0.851543	2.1
22331	1.19345	2.1
22332	0.946133	2.1
22333	0.946132	2.1
22334	1.56179	2.1
22335	0.620263	1.98276
22336	1.30226	1.61671
22337	1.2334	1.04821
22338	1.2334	0.84714
22339	0.946133	0.892516
22340	1.24053	2.1
22341	1.4828	2.1
22342	0.957622	2.1
22343	1.00521	1.4113
22344	1.49463	2.1
22345	1.39621	2.1
22346	1.09861	2.1
22347	0.946131	2.1
22348	0.946131	2.1
22349	1.09861	2.1
22350	2.16987	0.0501501
22351	0.572182	0.0546872
22352	1.15802	0.0585037
22353	0.63574	0.0594358
22354	1.18897	0.0500979
22355	0.996733	0.0556939
22356	1.82402	1.55945
22357	0.969602	1.25332
22358	0.794777	1.71519
22359	1.02626	1.39266
22360	1.51396	0.307856
22361	1.40574	0.395709
22362	1.38683	0.392196
22363	0.946126	0.397528
22364	0.946128	0.359562
22365	1.07138	0.290635
22366	0.946131	2.1
22367	1.41331	2.1
22368	0.94613	2.1
22369	0.94613	2.1
22370	1.27451	2.1
22371	0.974196	2.1
22372	1.2049	0.0518855
22373	1.42757	2.1
22374	1.02461	2.1
22375	2.07087	2.1
22376	1.92515	1.29153
22377	0.802138	1.25507
22378	0.946128	1.66678
22379	0.802107	1.2379
22380	1.23726	1.19405
22381	1.21997	1.15297
22382	0.767577	0.906282
22383	0.946131	1.48729
22384	0.767577	1.06244
22385	1.3279	1.05847

22386	1.23726	0.988055
22387	1.29729	2.1
22388	0.946128	2.1
22389	1.14186	2.1
22390	1.39214	2.1
22391	1.39214	2.1
22392	0.946129	2.1
22393	1.23012	0.177583
22394	0.653228	0.473014
22395	0.946133	1.47786
22396	1.15918	1.53646
22397	1.26893	0.652108
22398	0.653228	1.25272
22399	1.06848	1.51162
22400	0.615121	2.1
22401	0.841488	1.09995
22402	0.946131	2.1
22403	1.04071	2.1
22404	0.965717	2.1
22405	1.77893	1.26162
22406	1.11314	0.331424
22407	1.65411	0.755094
22408	0.85472	0.0552722
22409	1.1604	0.05
22410	1.19141	0.0505208
22411	1.19141	0.05
22412	0.94613	0.0539371
22413	1.70944	0.05
22414	1.89082	0.354577
22415	1.05175	0.778462
22416	0.815276	2.1
22417	0.843283	2.1
22418	0.946131	2.1
22419	0.843284	2.1
22420	1.38897	2.1
22421	1.01019	0.630026
22422	1.05197	0.596538
22423	0.810959	0.579096
22424	1.33037	0.331293
22425	1.3415	0.34146
22426	0.946118	0.325268
22427	0.946131	0.313024
22428	1.09803	0.32936
22429	0.930281	2.1
22430	1.3335	2.1
22431	0.784151	1.81768
22432	0.78415	2.1
22433	0.946132	2.1
22434	1.6299	0.0621502
22435	0.995795	2.1
22436	1.84846	1.3263
22437	0.929873	1.56345
22438	0.889245	1.57735
22439	0.808072	1.77549
22440	0.851541	1.49157
22441	1.51956	1.27532
22442	0.94613	1.20672
22443	0.851553	1.326

22444	0.946787	1.24534
22445	1.15544	1.56728
22446	1.30289	0.210124
22447	1.28323	0.222257
22448	0.85024	0.218446
22449	1.09662	0.292921
22450	1.67687	0.05
22451	0.946127	2.1
22452	1.40496	2.1
22453	1.22442	1.37573
22454	0.946128	1.7265
22455	1.40496	1.71777
22456	1.00573	2.1
22457	0.636837	1.46389
22458	0.988642	1.78878
22459	0.573141	1.03461
22460	0.946128	1.62442
22461	0.786538	1.98793
22462	2.28748	1.19487
22463	0.682805	2.1
22464	0.946132	2.1
22465	0.682776	2.08923
22466	0.946132	2.1
22467	0.79775	2.1
22468	1.05403	0.0587547
22469	0.888273	0.0562637
22470	0.876373	0.0516694
22471	0.576083	0.0531278
22472	0.946132	0.0749368
22473	0.576057	0.05
22474	0.576058	0.0580487
22475	0.946131	0.060555
22476	1.41724	0.512729
22477	1.31678	0.746785
22478	0.946132	0.731883
22479	0.946132	0.687171
22480	1.31678	0.658813
22481	0.935738	2.1
22482	0.946132	2.1
22483	0.935765	2.1
22484	1.32949	2.1
22485	0.946131	2.1
22486	1.02525	2.1
22487	0.946128	2.1
22488	1.41207	2.1
22489	1.04675	2.1
22490	0.946131	2.1
22491	1.41207	2.1
22492	0.946131	2.1
22493	0.946132	2.1
22494	1.41125	2.1
22495	1.41125	2.1
22496	1.27087	2.1
22497	0.924071	0.388162
22498	0.608893	0.265267
22499	0.528907	0.383821
22500	1.78249	0.356907
22501	0.720094	0.338564

22502	0.781667	2.1
22503	0.781638	2.1
22504	1.40756	2.1
22505	1.40756	2.1
22506	0.797635	2.1
22507	1.11946	0.05
22508	1.32068	0.05
22509	0.88757	0.0858917
22510	1.27891	0.0756745
22511	0.810957	0.0768901
22512	0.914656	0.106716
22513	0.640819	0.0658288
22514	0.745643	0.100798
22515	0.865108	0.118283
22516	1.06883	0.103929
22517	0.806001	0.114556
22518	1.19462	2.1
22519	1.42763	0.503015
22520	1.24682	0.446835
22521	0.78392	0.312052
22522	1.02874	0.30482
22523	0.884512	0.298461
22524	1.1658	2.1
22525	0.663026	0.05
22526	1.21174	0.05
22527	0.663056	0.0535319
22528	1.15968	0.05
22529	1.21174	0.05
22530	0.993976	0.05
22531	0.945495	0.05
22532	0.671202	0.05
22533	1.37312	2.1
22534	1.28499	2.1
22535	0.946133	2.1
22536	0.946132	2.1
22537	1.28499	2.1
22538	1.25081	2.1
22539	0.946132	2.1
22540	0.946132	2.1
22541	1.27985	2.1
22542	1.37312	2.1
22543	0.830638	2.1
22544	1.05358	2.1
22545	1.22209	2.1
22546	1.09874	2.1
22547	0.74758	2.1
22548	2.50011	1.71117
22549	0.6157	0.257883
22550	0.674634	0.490209
22551	0.946128	0.925385
22552	0.674633	0.561104
22553	0.946131	0.767441
22554	1.96192	1.87105
22555	1.31612	0.279483
22556	1.10735	0.227516
22557	0.936676	0.286433
22558	0.808779	0.350877
22559	0.808808	0.370835

22560	1.00245	0.355922
22561	1.26179	0.276084
22562	1.05758	2.1
22563	1.03408	2.1
22564	0.946132	0.0589614
22565	0.914368	0.0550169
22566	1.20524	0.05
22567	1.12743	0.0536512
22568	0.851555	0.0520607
22569	1.17885	0.553109
22570	0.867881	0.470857
22571	1.00122	0.471313
22572	0.867883	0.450847
22573	0.867881	0.472122
22574	1.19583	0.490767
22575	1.17743	1.56391
22576	0.878684	1.12155
22577	0.687087	2.07954
22578	0.955079	0.599381
22579	0.672977	0.639094
22580	0.735937	0.658563
22581	0.851531	0.665115
22582	0.672957	0.653965
22583	0.923974	0.634455
22584	1.2243	2.1
22585	0.808463	0.916668
22586	0.971078	0.998461
22587	1.06161	1.02264
22588	0.810947	0.98083
22589	0.886074	1.14283
22590	0.786829	1.21707
22591	0.55062	0.832846
22592	0.574458	1.73214
22593	0.946129	0.946329
22594	1.42526	1.08893
22595	1.15962	0.970679
22596	1.24526	0.05
22597	0.94613	0.422046
22598	0.946128	0.483492
22599	1.23279	0.568485
22600	1.38645	0.546948
22601	0.94613	0.493166
22602	1.05042	1.19753
22603	0.946128	2.1
22604	0.946129	1.23593
22605	1.24286	2.1
22606	1.39659	2.1
22607	0.94613	2.1
22608	1.10407	0.987908
22609	1.32809	1.61096
22610	0.993999	2.07325
22611	0.810959	1.35557
22612	0.983643	1.52774
22613	0.869552	2.02312
22614	0.747801	1.36521
22615	1.53429	1.05789
22616	1.13127	2.1
22617	1.38397	2.1

22618	1.08366	2.1
22619	0.946129	2.1
22620	0.946131	2.1
22621	1.08366	2.1
22622	1.06007	2.1
22623	1.79641	2.1
22624	0.758799	2.1
22625	1.28442	2.1
22626	1.06957	2.1
22627	0.946133	2.1
22628	1.28442	2.1
22629	1.76788	1.87692
22630	1.38669	2.1
22631	0.986095	2.1
22632	1.38478	2.02192
22633	0.946132	0.935571
22634	1.24344	0.770565
22635	0.622283	1.62633
22636	1.10335	0.825438
22637	1.24344	0.879486
22638	1.1967	1.34138
22639	0.946131	0.0742183
22640	0.901005	0.0674248
22641	1.05751	0.0700504
22642	1.37771	0.066491
22643	0.851527	0.0687064
22644	1.26643	1.04007
22645	1.08992	1.43064
22646	0.946132	1.32678
22647	0.946132	0.990561
22648	1.08992	1.51292
22649	1.53534	2.1
22650	1.58825	1.17646
22651	1.0447	1.87156
22652	0.593818	1.24594
22653	0.89883	1.51313
22654	0.734088	1.78481
22655	0.998451	0.751605
22656	1.64156	0.774408
22657	1.05654	1.09695
22658	1.37641	0.444337
22659	1.74553	0.537392
22660	1.31112	0.502759
22661	0.74518	0.578848
22662	0.851539	0.466416
22663	0.851554	0.301274
22664	0.851475	0.219863
22665	0.946132	0.05
22666	1.28895	0.05
22667	1.11356	0.05
22668	0.946132	0.05
22669	0.946132	0.05
22670	1.84214	2.1
22671	1.23851	0.0870771
22672	1.25866	0.41154
22673	1.28655	0.755853
22674	0.946131	0.689859
22675	0.946132	0.653553

22676	1.28655	0.621625
22677	1.11929	0.582372
22678	0.946132	0.710893
22679	0.946132	0.552613
22680	1.34254	0.516584
22681	1.25866	0.484041
22682	0.946132	2.1
22683	1.27451	2.1
22684	1.19803	2.1
22685	0.94613	2.1
22686	1.27451	2.1
22687	0.946131	2.1
22688	1.36523	2.1
22689	1.14599	2.1
22690	0.946129	2.1
22691	1.36523	2.1
22692	1.09518	2.1
22693	0.977777	2.1
22694	0.583237	2.1
22695	0.583267	2.1
22696	0.903872	2.1
22697	0.946129	2.1
22698	0.583258	2.1
22699	1.58179	0.0858328
22700	0.946131	0.136618
22701	1.35123	0.113071
22702	0.655837	0.128609
22703	1.12018	0.130827
22704	1.35123	0.116596
22705	0.62967	0.117412
22706	0.860497	0.17841
22707	0.816639	0.137228
22708	0.746716	2.1
22709	0.810947	2.1
22710	0.810959	2.1
22711	0.653422	2.06726
22712	0.289127	1.13736
22713	0.475716	1.99493
22714	0.980651	1.16478
22715	0.946119	2.1
22716	0.946132	2.1
22717	1.08299	2.1
22718	1.34539	2.1
22719	1.28753	0.0956937
22720	0.946132	0.22141
22721	0.946132	0.178786
22722	1.19719	0.175381
22723	0.946133	0.184775
22724	0.958947	0.110436
22725	1.3492	0.124703
22726	0.946132	0.272833
22727	0.946128	0.253914
22728	1.22588	0.231935
22729	0.946129	0.241321
22730	0.991113	2.1
22731	1.35966	2.1
22732	0.961 2.1	
22733	0.946129	2.1

22734	0.946131	2.1
22735	0.961001	2.1
22736	0.727096	1.72776
22737	0.810946	1.03987
22738	0.810959	1.30015
22739	1.31658	0.381561
22740	1.23167	2.1
22741	1.44836	0.05
22742	1.50103	0.05
22743	1.23351	0.05
22744	1.35657	0.05
22745	0.851541	0.0516455
22746	0.93668	0.05
22747	0.946131	0.05
22748	3.7335	2.1
22749	1.1822	2.1
22750	1.31127	0.544014
22751	1.3689	0.437922
22752	1.35822	0.357178
22753	0.946133	0.373786
22754	0.946132	0.439712
22755	1.14926	0.428987
22756	1.40822	0.424702
22757	1.04935	0.468989
22758	0.704112	0.428988
22759	0.704112	0.466333
22760	0.946131	0.512166
22761	0.704112	0.484027
22762	1.37721	0.0704058
22763	0.85154	2.1
22764	1.06949	2.1
22765	0.851541	2.1
22766	0.946128	2.1
22767	1.1175	2.1
22768	1.44091	2.1
22769	0.96029	0.663201
22770	3.46486	1.01166
22771	0.603286	0.318313
22772	0.432283	0.142852
22773	0.851541	0.276304
22774	1.09355	0.149135
22775	0.851534	0.309936
22776	1.04263	2.1
22777	0.851541	2.1
22778	0.965604	2.1
22779	0.94613	2.1
22780	1.23953	2.1
22781	0.869615	2.1
22782	1.02415	2.1
22783	0.983032	2.1
22784	1.35385	1.47766
22785	1.59971	0.385011
22786	0.915178	2.1
22787	0.962435	2.1
22788	1.3116	2.1
22789	1.38302	2.1
22790	1.13227	2.1
22791	1.25923	0.05

22792	0.988805	2.1
22793	0.611486	0.83439
22794	1.1019	0.903992
22795	1.11124	1.32997
22796	0.716713	0.828319
22797	0.946131	1.13912
22798	1.22297	1.02758
22799	1.12873	1.3957
22800	0.946131	1.38186
22801	0.946132	1.41501
22802	1.3469	1.67611
22803	1.25366	1.89763
22804	1.1611	0.89437
22805	0.946132	1.17692
22806	0.946132	0.934522
22807	1.2854	1.17578
22808	1.3469	1.32179
22809	1.07468	1.00834
22810	1.79317	2.1
22811	1.07444	2.1
22812	1.69436	1.81007
22813	0.851539	2.1
22814	0.870925	1.93844
22815	0.851541	2.1
22816	0.870951	2.1
22817	1.26097	2.1
22818	1.26247	2.1
22819	0.946132	1.87018
22820	1.24041	2.1
22821	0.946132	1.44042
22822	0.946132	2.1
22823	1.40274	2.1
22824	0.946131	0.0564827
22825	0.797945	0.0542002
22826	1.12988	0.0549051
22827	1.00681	0.0536268
22828	0.797932	0.0555993
22829	0.977483	0.243142
22830	0.943362	0.604482
22831	0.9891	0.269902
22832	1.1703	0.335792
22833	0.663171	0.373278
22834	1.40882	0.11639
22835	1.32639	0.184797
22836	1.24448	0.116189
22837	0.946132	0.220269
22838	1.11015	0.116633
22839	1.24448	0.147127
22840	0.754768	0.138687
22841	1.13854	2.1
22842	1.23654	2.1
22843	0.714101	0.527492
22844	0.851538	0.575379
22845	0.79261	1.09733
22846	1.30598	0.705275
22847	1.05338	0.622107
22848	1.41758	2.1
22849	0.83317	0.333868

22850	1.00058	0.414906
22851	0.810958	0.409123
22852	1.63791	0.334429
22853	1.55116	2.1
22854	1.29735	1.12537
22855	0.945147	1.9842
22856	0.94515	1.13053
22857	0.94613	1.54582
22858	0.945149	1.52758
22859	1.02368	2.1
22860	1.12787	2.1
22861	1.04007	2.1
22862	3.78805	2.1
22863	0.816891	0.138513
22864	0.943996	0.139634
22865	0.870002	0.129287
22866	0.564533	0.190247
22867	0.564536	0.395264
22868	0.946128	0.722325
22869	0.564535	0.457281
22870	0.946128	0.623904
22871	1.15177	2.1
22872	1.16806	0.873287
22873	0.999083	0.996538
22874	0.946131	0.976839
22875	0.946131	0.949512
22876	0.999084	1.14405
22877	1.28918	0.807355
22878	1.0383	1.00223
22879	0.946133	0.922241
22880	0.946132	0.954941
22881	1.0383	1.03946
22882	1.27737	1.49228
22883	1.39296	0.45752
22884	0.998682	2.1
22885	3.98933	2.1
22886	1.05328	0.150803
22887	1.23475	2.1
22888	0.900856	1.75676
22889	0.94001	0.899999
22890	0.707748	0.753724
22891	0.707751	1.2905
22892	0.946128	0.807109
22893	0.70775	1.01277
22894	0.996109	0.941843
22895	1.68551	2.1
22896	0.851539	2.1
22897	0.962688	1.46305
22898	0.94613	0.848584
22899	1.04073	2.0916
22900	1.44824	2.1
22901	0.794313	0.437868
22902	0.838031	0.44071
22903	1.11076	0.409763
22904	1.38813	2.1
22905	1.17204	2.1
22906	0.946132	2.1
22907	0.946131	2.1

22908	1.17204	2.1
22909	0.961779	0.289671
22910	1.0685	0.532086
22911	0.810947	1.82263
22912	1.2899	0.887924
22913	0.890404	0.882496
22914	0.909981	2.02852
22915	0.810946	1.07963
22916	0.890402	0.923092
22917	0.522844	1.66733
22918	0.593846	1.58325
22919	1.30226	1.01667
22920	1.30226	0.954247
22921	0.946132	1.19513
22922	1.03537	0.121091
22923	1.50035	0.0897467
22924	0.940356	0.161507
22925	0.946131	0.12966
22926	1.03441	0.144413
22927	1.21171	0.0807963
22928	1.35169	0.10581
22929	1.32449	0.0845818
22930	0.946131	0.139079
22931	0.946131	0.126021
22932	1.06627	0.124701
22933	1.03102	0.528251
22934	0.488712	0.0665617
22935	0.946131	0.101728
22936	1.23146	0.0788666
22937	1.13242	0.0950846
22938	0.946131	0.0816133
22939	0.409199	0.05
22940	0.429959	0.0590526
22941	1.08357	0.0871351
22942	0.80926	0.0927103
22943	0.879791	0.0723296
22944	0.946129	1.00747
22945	1.28828	1.44193
22946	0.946131	1.00907
22947	0.946131	1.61194
22948	1.35969	1.57452
22949	1.10049	1.54879
22950	0.923724	0.982932
22951	0.923695	2.0632
22952	0.94613	0.977736
22953	0.94613	2.0448
22954	1.28828	1.99274
22955	1.73525	0.257435
22956	0.656239	0.396331
22957	1.2996	0.318898
22958	0.812939	0.421673
22959	0.65624	0.365721
22960	1.2996	0.387999
22961	1.67372	2.1
22962	0.946131	2.1
22963	0.946132	2.1
22964	1.05978	2.1
22965	1.31049	2.1

22966	0.946132	2.1
22967	1.11077	2.1
22968	0.579725	0.520318
22969	0.373532	0.376249
22970	1.13537	0.527735
22971	1.20726	0.473114
22972	0.946131	0.479207
22973	1.45694	0.616872
22974	0.750367	0.0558027
22975	0.946126	0.0660977
22976	0.750365	0.0570589
22977	1.25698	0.0537571
22978	1.35726	0.0549491
22979	1.6812	0.0610464
22980	1.78665	1.52663
22981	1.46568	2.1
22982	1.29606	2.1
22983	1.25298	2.1
22984	0.946115	2.1
22985	0.946129	2.1
22986	1.25296	2.1
22987	1.27778	0.0616589
22988	1.17042	2.1
22989	1.25858	0.05
22990	0.93106	0.05
22991	0.810959	0.05
22992	1.38905	2.1
22993	1.19216	2.1
22994	0.946131	2.1
22995	0.946131	2.1
22996	1.19216	2.1
22997	1.58339	2.1
22998	0.81094	0.0658824
22999	0.845129	0.0833428
23000	0.918535	0.0702776
23001	0.946132	2.1
23002	0.946132	2.1
23003	1.31904	2.1
23004	1.31904	2.1
23005	1.21275	2.1
23006	1.15471	2.1
23007	1.33125	2.1
23008	0.702954	0.235172
23009	0.942973	0.19728
23010	0.94613	0.334629
23011	0.641772	0.189641
23012	0.702942	0.320126
23013	0.991865	0.389223
23014	0.639952	0.377776
23015	1.17333	0.22879
23016	0.946132	0.340708
23017	0.490975	0.300493
23018	0.495934	0.393527
23019	1.19904	0.222914
23020	1.30488	0.0858001
23021	1.02305	0.340537
23022	1.49842	0.0874514
23023	1.54196	2.1

23024	1.37224	1.99178
23025	1.0953	0.16513
23026	1.18952	1.18104
23027	1.68851	0.444758
23028	0.946131	0.412092
23029	1.12491	0.381242
23030	0.583591	0.370716
23031	1.28727	0.42835
23032	1.12491	0.403838
23033	0.884641	0.505594
23034	0.810944	0.475975
23035	0.917973	0.47709
23036	0.846671	0.46815
23037	0.596968	0.395317
23038	0.716347	0.460419
23039	1.36255	0.373974
23040	1.11077	2.1
23041	0.946132	2.1
23042	0.946132	2.1
23043	1.26267	2.1
23044	1.26441	2.1
23045	1.85238	2.1
23046	1.36693	2.1
23047	0.616123	2.1
23048	0.481844	2.1
23049	0.709599	2.1
23050	0.815889	2.1
23051	1.1502	2.1
23052	1.63937	0.635403
23053	1.25797	0.318944
23054	1.22878	0.325639
23055	0.946132	0.327018
23056	0.946132	0.322351
23057	0.965737	0.324814
23058	1.1893	0.322456
23059	1.22877	0.322712
23060	0.735217	0.328814
23061	0.85154	0.330842
23062	0.851553	0.326319
23063	0.851473	0.328467
23064	1.58916	0.0968595
23065	1.25545	0.140817
23066	0.810942	0.347759
23067	0.919314	0.373333
23068	0.928633	0.30073
23069	2.14514	0.198297
23070	1.23389	2.1
23071	0.946128	0.119746
23072	0.969504	0.0825191
23073	0.851539	0.100259
23074	1.41503	0.100874
23075	0.958517	0.0905694
23076	0.990615	0.0772004
23077	1.7943	0.0716057
23078	0.946133	0.507211
23079	1.27581	0.521501
23080	0.946132	0.515416
23081	1.36362	0.465751

23082	1.36936	0.45565
23083	1.71903	2.1
23084	0.751639	2.1
23085	0.77464	2.1
23086	0.946133	2.1
23087	1.04073	2.1
23088	1.23551	2.1
23089	0.94613	2.1
23090	1.18682	2.1
23091	0.946131	2.1
23092	0.94613	2.1
23093	1.40638	2.1
23094	0.483346	0.303839
23095	0.432527	0.201373
23096	0.946132	0.418929
23097	0.483312	0.289351
23098	0.946132	0.334668
23099	0.878798	0.412647
23100	0.810944	0.416763
23101	1.14604	0.353867
23102	0.946129	0.376455
23103	1.35594	0.291281
23104	0.538378	0.285845
23105	0.946131	0.399209
23106	0.538378	0.374732
23107	1.00065	0.5519
23108	0.962878	0.410793
23109	1.32634	0.240762
23110	1.28066	0.05
23111	0.785651	2.1
23112	1.20327	1.79526
23113	1.47058	1.83887
23114	0.551834	0.978283
23115	1.04072	1.9764
23116	0.506719	1.24026
23117	1.12786	1.44025
23118	1.45789	0.05
23119	0.681532	0.0550018
23120	1.21087	0.05
23121	0.94613	0.05
23122	0.681534	0.05
23123	1.21087	0.0523282
23124	1.98618	0.718878
23125	1.71742	0.873249
23126	1.07427	0.707811
23127	1.1972	0.549702
23128	0.933474	0.547058
23129	1.1745	0.623187
23130	0.94612	0.560139
23131	0.933485	0.616155
23132	1.17449	0.603963
23133	0.851541	0.0522293
23134	1.12774	0.05
23135	0.946129	0.0513911
23136	0.851486	0.0597965
23137	1.12517	0.0530619
23138	1.12328	0.05
23139	1.13179	2.1

23140	1.02438	2.1
23141	1.05776	2.1
23142	1.41273	1.06986
23143	1.1229	2.1
23144	0.875912	1.04471
23145	0.946132	2.06491
23146	0.875912	1.52468
23147	1.42307	2.1
23148	1.15354	0.05
23149	0.848382	0.05
23150	1.2685	1.54967
23151	0.938104	1.14182
23152	0.793166	2.1
23153	1.07855	2.1
23154	0.98488	2.1
23155	1.4162	2.1
23156	1.09651	0.113642
23157	1.98502	0.387657
23158	1.41168	0.317096
23159	1.33879	0.312785
23160	0.946127	0.432581
23161	1.13528	0.278807
23162	0.946128	0.325698
23163	1.09726	2.1
23164	1.22026	0.756532
23165	1.09044	2.1
23166	2.23961	0.698218
23167	1.14721	0.388335
23168	1.17614	0.889283
23169	0.615065	2.1
23170	0.946131	2.1
23171	0.778488	2.1
23172	0.94613	2.1
23173	0.615066	2.1
23174	1.78254	2.01548
23175	0.898452	0.0896906
23176	1.47411	2.1
23177	0.59676	0.451022
23178	0.59676	0.312942
23179	0.94613	0.45797
23180	1.13511	0.423076
23181	0.946131	0.419766
23182	0.916275	2.1
23183	0.946132	2.00986
23184	0.916274	1.16524
23185	1.36182	1.76359
23186	0.946131	1.82422
23187	0.857202	0.05
23188	0.941918	0.05
23189	0.943349	0.05
23190	1.2493	0.05
23191	0.946133	0.05
23192	1.01814	0.05
23193	0.927881	0.05
23194	1.11845	0.05
23195	1.2493	0.05
23196	1.11845	0.05
23197	0.946129	0.05

23198	0.967673	0.657788
23199	1.01332	0.0603706
23200	0.946133	0.0645783
23201	0.81938	0.0626548
23202	0.819377	0.059288
23203	0.946133	0.0677378
23204	1.22474	0.392817
23205	1.15321	0.385661
23206	0.797489	0.396585
23207	0.946131	0.416648
23208	0.797461	0.405312
23209	1.35328	0.777651
23210	1.03318	1.0749
23211	0.946131	0.880054
23212	0.94613	0.93086
23213	1.03319	0.984559
23214	1.37938	0.779377
23215	1.00512	1.08763
23216	0.946133	0.854634
23217	0.946132	0.939409
23218	1.00512	0.920167
23219	1.5515	1.15852
23220	1.55351	2.1
23221	0.94261	0.0823865
23222	0.828156	0.0766824
23223	0.916495	0.0795659
23224	3.06309	0.0525589
23225	0.619832	0.0526928
23226	0.705903	0.0607979
23227	1.2968	0.0505481
23228	0.946133	0.0558671
23229	0.705904	0.05
23230	1.2968	0.0609994
23231	1.01971	0.0501778
23232	0.60302	0.05
23233	1.07242	0.716344
23234	1.26426	0.676429
23235	0.946131	0.679584
23236	0.946131	0.744612
23237	0.963109	0.728018
23238	0.947862	0.696372
23239	1.40248	0.98827
23240	0.767143	1.09282
23241	0.767114	1.04191
23242	0.946129	1.10838
23243	0.767143	0.920224
23244	1.22637	0.960249
23245	1.2281	0.978181
23246	0.590909	1.1274
23247	0.490832	0.993761
23248	0.804174	1.12657
23249	0.747368	0.899226
23250	1.05129	0.17831
23251	1.10609	0.576162
23252	1.3639	0.351058
23253	0.776117	0.359898
23254	0.752329	0.355861
23255	1.04073	0.364717

23256	0.67713	0.360164
23257	3.4245	2.1
23258	1.18526	2.1
23259	1.2161	1.07867
23260	0.876834	1.61071
23261	0.876831	0.97504
23262	0.946132	1.60108
23263	0.876834	1.1413
23264	0.954563	2.1
23265	0.924132	2.1
23266	1.25075	2.1
23267	1.01652	2.1
23268	0.92413	2.1
23269	1.37579	2.1
23270	0.992159	2.1
23271	1.26092	2.1
23272	0.929435	2.1
23273	0.844958	2.1
23274	0.79598	2.1
23275	0.852559	2.1
23276	0.946131	0.55573
23277	1.28435	0.474761
23278	0.827261	0.527469
23279	0.946128	0.549222
23280	0.827298	0.533824
23281	1.65452	0.299098
23282	1.79929	2.1
23283	0.791947	1.79239
23284	0.902072	1.07912
23285	0.882672	1.41227
23286	1.21703	2.1
23287	0.900338	2.1
23288	1.37712	2.1
23289	0.782591	2.1
23290	0.782562	2.1
23291	0.94613	2.1
23292	1.34749	2.1
23293	0.61359	2.1
23294	1.14397	0.499138
23295	1.02963	0.0670347
23296	1.36589	0.0610385
23297	0.946132	0.130957
23298	1.28644	0.0885037
23299	0.946131	0.104829
23300	1.40104	0.106874
23301	1.28644	0.0934008
23302	0.810946	0.240591
23303	1.09178	0.227607
23304	1.26956	0.181586
23305	0.810943	0.227111
23306	0.885633	0.227192
23307	1.00844	0.19707
23308	0.825671	0.247299
23309	0.792093	0.204711
23310	0.925037	0.21222
23311	0.946131	0.590497
23312	0.946131	0.125318
23313	1.18052	0.307035

23314	1.40265	0.312725
23315	0.946132	0.479102
23316	1.17891	0.283587
23317	0.734248	0.05
23318	0.810943	0.05
23319	0.731621	0.05
23320	0.799857	0.478849
23321	0.834507	0.563965
23322	0.810932	0.483202
23323	1.01316	0.10601
23324	1.23884	0.143318
23325	1.88438	2.1
23326	0.946129	2.07892
23327	1.38614	2.00463
23328	1.34245	1.01438
23329	0.946131	1.16806
23330	1.38614	1.09189
23331	0.946132	0.05
23332	0.910733	0.0544771
23333	1.24726	0.05
23334	1.13742	0.05
23335	0.851555	0.05
23336	1.50368	0.05
23337	0.94613	0.589953
23338	1.08998	1.83196
23339	1.36899	0.212978
23340	1.36898	0.673284
23341	0.946131	0.65094
23342	1.26045	2.1
23343	1.06432	0.710456
23344	1.89016	0.569396
23345	0.83514	1.28613
23346	1.36653	2.03076
23347	1.23426	1.5284
23348	0.94613	1.15591
23349	0.835112	1.89277
23350	1.14567	0.942467
23351	1.12096	0.870944
23352	0.881106	1.01633
23353	1.17787	0.445321
23354	1.29738	0.759129
23355	0.940472	0.521024
23356	1.03452	0.707249
23357	0.946131	0.560712
23358	2.7489	0.519132
23359	1.01778	2.1
23360	1.04321	0.899041
23361	1.19352	0.200253
23362	1.16912	1.44131
23363	0.946132	1.12408
23364	0.946132	1.77278
23365	1.40924	1.73903
23366	1.22927	1.9932
23367	2.09455	0.344238
23368	3.00575	2.1
23369	1.08928	0.660442
23370	1.94799	2.1
23371	1.4092	2.1

23372	0.946132	2.1
23373	1.16	2.1
23374	1.01985	2.1
23375	0.946133	2.1
23376	1.16	2.1
23377	1.15371	0.05
23378	1.56002	0.05
23379	1.23007	2.0618
23380	1.9992	2.1
23381	0.652605	1.94867
23382	0.937903	2.1
23383	0.946132	1.73875
23384	0.937902	2.1
23385	1.228	2.1
23386	1.33694	1.09717
23387	1.00191	2.1
23388	0.716814	1.06207
23389	0.94613	1.91336
23390	0.716816	1.91366
23391	1.70388	1.6968
23392	0.946129	0.576955
23393	1.09362	0.619872
23394	0.946131	0.617712
23395	1.4185	0.635802
23396	1.09362	0.589798
23397	1.31057	1.05339
23398	1.17631	0.05
23399	1.19404	0.05
23400	0.94613	0.0878724
23401	0.946129	0.0667767
23402	0.99972	0.0722147
23403	0.94613	0.0740586
23404	0.835199	0.05
23405	1.23351	0.05
23406	0.582785	0.05
23407	0.701079	0.05
23408	0.946132	0.05
23409	2.17385	0.05
23410	1.18948	1.93289
23411	1.25346	2.1
23412	1.18586	0.733471
23413	0.880876	0.546132
23414	0.68632	0.785422
23415	0.946118	2.1
23416	0.966936	2.1
23417	0.946117	2.1
23418	1.41636	2.1
23419	1.03798	2.1
23420	0.729178	0.631733
23421	0.721888	0.915867
23422	0.946132	0.666718
23423	0.656286	0.817453
23424	1.13898	0.734402
23425	1.02351	0.186786
23426	1.48803	0.25558
23427	0.946131	0.196398
23428	1.04921	2.1
23429	1.02478	0.201264

23430	0.993023	2.1
23431	1.80841	0.338202
23432	1.29215	0.331617
23433	0.904206	0.333781
23434	0.904207	0.341994
23435	0.946132	0.340958
23436	0.904207	0.336253
23437	1.07347	2.1
23438	0.555875	1.07418
23439	0.555904	1.25597
23440	1.30841	1.7568
23441	1.30841	1.88447
23442	0.91951	1.74456
23443	1.33026	0.538243
23444	1.43289	0.05
23445	1.15703	1.51522
23446	0.886594	2.03572
23447	1.05129	1.72665
23448	1.08421	0.647623
23449	1.35946	0.660505
23450	0.882955	0.764624
23451	0.946131	0.755958
23452	0.882985	0.718812
23453	0.583501	0.628953
23454	0.532921	0.643249
23455	0.94613	1.39114
23456	0.532892	1.02384
23457	1.23224	1.03552
23458	1.12786	0.949598
23459	0.946131	0.646457
23460	1.23732	0.35849
23461	1.40609	0.576662
23462	1.40609	0.297516
23463	0.946131	0.440472
23464	0.910672	1.25758
23465	1.08478	1.17958
23466	0.94613	0.649246
23467	0.94613	1.03461
23468	1.30382	0.999574
23469	3.10544	1.98746
23470	0.679509	1.91708
23471	1.15966	1.4202
23472	0.75499	2.02231
23473	1.37511	1.59492
23474	1.01776	1.39317
23475	2.35239	1.18501
23476	0.92406	2.1
23477	0.946118	2.1
23478	0.92406	2.1
23479	1.07979	2.1
23480	1.29604	2.1
23481	1.29897	2.1
23482	0.902645	0.662172
23483	0.729493	1.27531
23484	0.906844	0.911747
23485	0.739518	0.081317
23486	1.10564	0.178479
23487	0.946131	0.185902

23488	0.739519	0.105363
23489	0.739506	0.144962
23490	1.79907	0.0831172
23491	1.59106	0.924738
23492	1.11067	0.703978
23493	0.795516	0.865099
23494	0.795519	0.749382
23495	0.946132	0.84442
23496	0.795506	0.731878
23497	1.80223	2.1
23498	1.95572	1.90465
23499	1.16237	0.850493
23500	1.07518	1.61278
23501	0.696199	0.815396
23502	0.94613	1.01492
23503	0.696199	1.02561
23504	1.6379	0.05
23505	0.946118	0.05
23506	0.851529	0.05
23507	1.08716	0.05
23508	0.973118	0.05
23509	0.851528	0.05
23510	1.15096	2.1
23511	0.65023	0.0836604
23512	0.94613	0.073789
23513	0.650205	0.113552
23514	0.650205	0.0786459
23515	0.811233	0.128702
23516	0.778721	1.91854
23517	0.810945	0.879225
23518	0.997518	0.874014
23519	0.838028	0.0768431
23520	0.952893	0.0620776
23521	0.838037	0.0704369
23522	1.58166	0.0500162
23523	1.44848	1.38063
23524	1.30041	0.0594155
23525	1.03697	0.684499
23526	0.946132	1.22862
23527	1.0277	1.27744
23528	0.946131	1.33011
23529	1.00501	1.12508
23530	1.0277	1.00974
23531	1.64988	2.1
23532	0.946131	1.90865
23533	0.94613	0.545506
23534	1.27702	1.50866
23535	1.55163	0.584709
23536	0.897905	0.590234
23537	1.12971	0.05
23538	1.35715	0.466725
23539	0.881884	0.670116
23540	0.664007	0.470825
23541	0.663978	0.553466
23542	0.94613	0.520301
23543	0.664006	0.541567
23544	1.48693	0.348192
23545	1.29289	0.327356

23546	1.32081	0.332862
23547	0.946119	0.335238
23548	0.946132	0.325951
23549	1.02972	0.317032
23550	0.946131	2.1
23551	0.946127	2.1
23552	1.23513	2.1
23553	1.38154	2.1
23554	0.94613	2.1
23555	1.25933	0.061189
23556	0.917954	0.0706089
23557	0.868589	0.0766466
23558	3.0957	2.1
23559	0.711391	0.05
23560	0.711391	0.05
23561	0.991303	0.05
23562	1.37322	0.05
23563	0.729145	0.05
23564	2.55648	0.05
23565	1.0756	1.7825
23566	0.760299	1.19823
23567	0.763592	0.6465
23568	0.946126	0.707749
23569	0.76359	0.613862
23570	1.35862	1.07273
23571	0.946133	2.1
23572	1.00807	2.1
23573	0.946132	2.1
23574	1.39823	2.1
23575	1.07817	2.1
23576	1.44379	1.29851
23577	1.17934	0.114247
23578	1.13451	0.416384
23579	0.94613	0.668245
23580	1.03974	2.1
23581	1.36835	0.669834
23582	1.36835	0.887425
23583	0.94613	1.18208
23584	1.27096	1.88552
23585	0.853042	2.1
23586	0.946131	2.1
23587	1.13658	2.1
23588	1.06997	2.1
23589	0.94613	2.1
23590	1.41556	0.521003
23591	1.21708	0.657359
23592	0.94613	0.67112
23593	0.946131	0.62229
23594	1.21708	0.6128
23595	0.964782	0.531527
23596	0.946119	0.614946
23597	1.23447	0.604034
23598	0.946118	0.617593
23599	1.41844	0.590769
23600	1.23446	0.617264
23601	1.1357	0.396054
23602	0.619856	0.0689106
23603	1.1509	0.05

23604	0.946126	0.0564268
23605	1.19262	0.05
23606	1.1509	0.0627446
23607	1.04268	2.1
23608	2.07527	0.827016
23609	1.38259	0.857098
23610	0.980054	1.31411
23611	0.946124	0.90116
23612	0.946127	1.07043
23613	0.980056	0.969624
23614	1.62916	1.99436
23615	0.946131	2.1
23616	1.40571	2.1
23617	0.946131	2.1
23618	1.38885	2.1
23619	1.40571	2.1
23620	1.11787	0.568715
23621	0.946129	0.560311
23622	1.28831	1.4414
23623	1.41498	0.473357
23624	1.41498	0.68007
23625	0.946131	0.627403
23626	0.850964	0.05
23627	0.921299	0.05
23628	1.37358	0.05
23629	0.946132	0.05
23630	0.850964	0.05
23631	0.823988	0.05
23632	0.823984	0.05
23633	1.36568	0.05
23634	1.36569	0.05
23635	0.921275	0.05
23636	1.84739	2.1
23637	1.47169	0.183083
23638	0.946131	0.431055
23639	0.735844	0.38222
23640	1.11819	0.468869
23641	1.10904	0.288709
23642	0.735831	0.396104
23643	0.946131	2.1
23644	0.946132	2.1
23645	1.23487	2.1
23646	1.3281	2.1
23647	0.946131	2.1
23648	1.72271	0.05
23649	0.861359	0.0550169
23650	0.946131	0.103329
23651	0.861357	0.0751189
23652	1.1369	0.0756932
23653	1.26868	0.0717278
23654	0.789173	2.04515
23655	0.789143	2.1
23656	0.946133	1.50265
23657	1.34386	1.85175
23658	0.789144	1.84345
23659	0.983493	2.1
23660	1.18944	0.678745
23661	1.05121	0.666372

23662	0.777398	0.701645
23663	0.946132	0.745613
23664	0.777423	0.807434
23665	1.09841	0.272993
23666	0.963919	0.242938
23667	0.474855	0.377029
23668	0.946131	0.363917
23669	0.474855	0.381875
23670	3.40074	1.02674
23671	1.29453	2.1
23672	1.01139	0.562073
23673	0.946133	0.807834
23674	1.35381	0.416836
23675	1.18322	0.699973
23676	0.946133	0.877387
23677	1.45502	0.0785062
23678	0.970088	1.09922
23679	0.960333	1.22973
23680	0.838036	1.31244
23681	1.30927	0.337231
23682	0.828617	0.351649
23683	0.714795	0.347064
23684	0.946132	0.340642
23685	0.714824	0.34354
23686	0.946131	2.1
23687	0.851542	2.1
23688	1.38204	2.1
23689	1.24512	2.1
23690	1.04086	2.1
23691	0.723573	2.1
23692	0.946131	2.1
23693	0.982454	2.1
23694	0.982454	2.1
23695	0.723544	2.1
23696	1.17261	0.281414
23697	1.34153	0.298097
23698	1.41383	0.316594
23699	0.946132	0.312124
23700	0.946133	0.294835
23701	1.12818	0.313941
23702	1.29383	0.288979
23703	0.946133	0.511077
23704	1.0027	0.501193
23705	0.768989	0.45052
23706	0.946133	0.538049
23707	0.768977	0.508063
23708	0.946132	0.564538
23709	1.24333	0.490502
23710	0.928583	0.544487
23711	0.946132	0.578652
23712	0.928583	0.574168
23713	1.53798	0.345129
23714	2.95436	0.0920612
23715	1.30649	0.121993
23716	0.809789	0.249702
23717	0.745744	0.217509
23718	0.745774	0.200134
23719	0.946128	0.195838

23720	1.00777	0.183678
23721	1.40271	0.149465
23722	0.768387	0.238789
23723	0.946132	0.218562
23724	0.922045	0.215754
23725	0.946132	2.1
23726	1.39021	2.1
23727	1.10046	2.1
23728	0.946132	2.1
23729	1.39021	2.1
23730	2.06158	0.298424
23731	0.913967	0.556852
23732	1.30107	0.770914
23733	0.946132	0.563137
23734	0.913966	0.607669
23735	1.30107	0.589184
23736	1.64117	2.1
23737	0.514872	1.27851
23738	0.514846	0.918478
23739	1.30811	1.42516
23740	1.30812	0.902824
23741	0.888989	0.977351
23742	1.99279	1.28319
23743	0.910271	0.824158
23744	0.838027	0.894238
23745	0.928444	0.90017
23746	1.2377	1.14986
23747	0.851537	1.59834
23748	0.899142	2.1
23749	0.810075	2.1
23750	1.3771	2.1
23751	1.03281	2.1
23752	0.719877	2.1
23753	0.901838	2.1
23754	1.01748	2.1
23755	1.12315	2.1
23756	0.64787	2.1
23757	1.173 2.1	
23758	0.876903	2.1
23759	0.597337	2.1
23760	1.00291	2.1
23761	0.597309	2.1
23762	1.09897	0.381373
23763	0.946132	0.832434
23764	1.1386	0.384166
23765	1.09897	0.516915
23766	0.596652	0.602676
23767	2.5593	2.1
23768	0.946133	0.670932
23769	1.29172	0.627004
23770	1.11337	0.486899
23771	0.946131	0.527357
23772	0.946131	0.4657
23773	0.96926	0.740703
23774	0.946132	0.919563
23775	0.946132	0.972406
23776	1.07118	0.639323
23777	1.29698	0.664716

23778	1.37867	0.834507
23779	0.946119	1.27646
23780	0.94612	1.77166
23781	1.33154	0.835374
23782	0.946107	1.03633
23783	2.17757	1.32384
23784	0.946131	0.139462
23785	0.68393	0.0810991
23786	0.68396	0.108288
23787	1.33671	0.116978
23788	1.10733	0.112828
23789	2.31367	2.1
23790	0.989326	1.45084
23791	0.689952	2.1
23792	0.854787	1.33047
23793	3.73635	2.1
23794	1.41489	2.1
23795	1.25544	0.812771
23796	0.946128	1.92909
23797	0.946129	1.63821
23798	1.13536	2.03657
23799	2.10284	1.09432
23800	0.851542	2.1
23801	0.828523	2.1
23802	0.946132	2.1
23803	0.946132	2.1
23804	1.50873	2.1
23805	0.989048	2.1
23806	0.925055	2.1
23807	0.838041	2.1
23808	0.807714	0.919074
23809	0.656831	0.362951
23810	0.997603	0.803676
23811	0.946131	0.927604
23812	1.02602	0.814861
23813	0.946129	0.958853
23814	1.35469	0.809887
23815	1.25083	0.807771
23816	0.851541	0.388505
23817	0.851483	0.37536
23818	0.851539	0.407481
23819	1.19785	0.342253
23820	1.07615	0.322754
23821	0.714507	0.350001
23822	1.41461	0.350939
23823	0.714507	0.349031
23824	0.714507	0.346389
23825	0.946133	0.358088
23826	0.946131	0.84497
23827	0.972232	0.745308
23828	0.94613	1.09664
23829	1.25083	0.720238
23830	1.30165	0.779815
23831	1.05332	2.1
23832	0.574768	2.1
23833	1.94541	0.05269
23834	0.789633	0.0666874
23835	0.946127	0.0807111

23836	1.33798	0.0660002
23837	1.22903	0.0742657
23838	0.946128	0.0704556
23839	0.946128	2.1
23840	1.11882	2.1
23841	0.996487	2.1
23842	0.94613	2.1
23843	1.11883	2.1
23844	0.946127	2.1
23845	1.30082	2.1
23846	1.02608	2.1
23847	0.946128	2.1
23848	1.30081	2.1
23849	1.3507	1.40499
23850	1.19583	0.609235
23851	0.946127	1.72097
23852	0.94613	1.19387
23853	1.19583	1.40601
23854	0.477652	0.0552933
23855	1.20416	0.114443
23856	0.946131	0.0987123
23857	0.477652	0.0666551
23858	0.477691	0.101606
23859	1.34536	0.05
23860	0.758971	0.19338
23861	1.25124	0.191587
23862	1.31467	0.157278
23863	0.758971	0.173083
23864	1.31467	0.188775
23865	0.946131	2.1
23866	1.14184	2.1
23867	1.35028	2.1
23868	1.35028	2.1
23869	0.946131	2.1
23870	1.13537	2.06942
23871	0.946132	1.094
23872	0.946132	2.1
23873	1.23031	1.87694
23874	1.27555	1.83546
23875	1.53064	2.1
23876	1.28421	1.01764
23877	1.07707	0.931142
23878	0.946125	0.933349
23879	0.946127	1.00678
23880	1.07707	1.0094
23881	0.946133	0.196141
23882	1.3495	0.137494
23883	0.946132	0.223611
23884	1.05704	0.167414
23885	1.3495	0.17504
23886	0.946132	0.0948778
23887	1.37794	0.079141
23888	0.946132	0.10927
23889	1.01367	0.100397
23890	1.37794	0.0945245
23891	0.928516	2.1
23892	0.946133	2.1
23893	0.928518	2.1

23894	1.14105	2.1
23895	1.26406	2.1
23896	0.797664	2.1
23897	0.946132	2.1
23898	0.797663	2.1
23899	1.34285	2.1
23900	0.929875	2.1
23901	0.946131	1.23478
23902	1.21139	0.430808
23903	1.41492	0.449408
23904	1.41492	0.389718
23905	0.946131	0.386136
23906	0.946131	2.1
23907	0.689388	2.1
23908	0.94365	2.1
23909	0.777345	2.1
23910	0.709599	2.1
23911	0.774968	2.1
23912	0.946132	2.1
23913	0.77497	2.1
23914	0.868145	2.1
23915	1.28401	2.1
23916	0.94613	0.533169
23917	1.19655	0.725473
23918	1.39428	0.433886
23919	1.39428	0.548023
23920	0.946131	0.554099
23921	1.1848	2.1
23922	1.07591	2.1
23923	0.946131	2.1
23924	0.946131	2.1
23925	0.874788	2.1
23926	1.35703	2.1
23927	1.35357	2.1
23928	0.946132	2.1
23929	1.26022	2.1
23930	0.946132	2.1
23931	0.946131	2.1
23932	0.966426	2.1
23933	0.946133	2.1
23934	0.946131	2.1
23935	1.3484	2.1
23936	1.26441	2.1
23937	1.2079	2.1
23938	0.946133	2.1
23939	0.946132	2.1
23940	1.2079	2.1
23941	1.2736	2.1
23942	1.25774	2.1
23943	0.946133	2.1
23944	0.851555	2.1
23945	0.96172	2.1
23946	0.946128	2.1
23947	1.06879	2.1
23948	0.94613	2.1
23949	1.32608	2.1
23950	1.06879	2.1
23951	0.946132	2.1

23952	1.32608	2.1
23953	0.946131	2.1
23954	1.40488	2.1
23955	1.32608	2.1
23956	0.94613	0.903318
23957	1.19234	0.709951
23958	0.946129	0.857838
23959	0.94613	1.07481
23960	1.31649	1.23075
23961	0.912059	0.842542
23962	1.04514	0.917395
23963	0.946132	0.865369
23964	0.946132	1.47111
23965	1.34646	1.55902
23966	1.01187	0.474423
23967	1.33728	0.57965
23968	0.761974	0.451095
23969	0.761974	0.562929
23970	0.94613	0.478894
23971	1.40641	1.50419
23972	0.955752	0.803393
23973	0.736738	1.62227
23974	0.94613	0.930109
23975	0.736739	1.01868
23976	0.861471	2.1
23977	1.34556	2.1
23978	0.810957	2.1
23979	0.946131	2.1
23980	1.37811	2.1
23981	1.17949	2.1
23982	0.946132	2.1
23983	1.37811	2.1
23984	0.946131	2.1
23985	1.40447	2.1
23986	0.946132	2.1
23987	0.946132	2.1
23988	1.39826	2.1
23989	0.946131	2.1
23990	1.283 2.1	
23991	0.94613	1.78995
23992	0.946131	2.1
23993	1.40447	2.1
23994	1.20541	2.1
23995	0.946131	1.59574
23996	0.946127	2.1
23997	1.33607	2.1
23998	1.41021	2.1
23999	1.01271	2.1
24000	0.946131	2.1
24001	0.920606	2.1
24002	1.13078	2.1
24003	1.60331	2.1
24004	0.946118	0.753809
24005	1.09718	1.93279
24006	0.946116	0.951502
24007	1.32662	0.798923
24008	1.0972	0.841859
24009	0.94613	0.770677

24010	1.32666	1.20849
24011	0.946128	0.710464
24012	1.31381	0.670465
24013	1.32666	0.611865
24014	0.94612	1.24454
24015	0.940748	2.1
24016	1.03561	2.1
24017	1.35111	2.1
24018	0.851527	2.0779
24019	0.607156	1.43235
24020	0.789158	2.1
24021	0.946129	0.915844
24022	1.04071	1.96788
24023	1.13144	1.9882
24024	0.946133	2.1
24025	1.35663	2.1
24026	0.946132	2.1
24027	0.946133	2.1
24028	1.34237	2.1
24029	0.919455	2.1
24030	1.01143	2.1
24031	0.946128	2.05499
24032	0.946131	2.1
24033	1.62796	2.1
24034	1.17796	1.33774
24035	1.23024	2.0724
24036	0.946132	0.988022
24037	0.85153	1.5783
24038	0.986867	1.41358
24039	0.889308	2.1
24040	0.94613	2.1
24041	0.889338	2.1
24042	0.946131	2.1
24043	0.889337	2.1
24044	1.18396	1.0289
24045	0.94613	0.867133
24046	0.946125	1.59877
24047	1.39514	1.04904
24048	1.30809	1.21638
24049	0.803322	2.1
24050	0.925893	1.64841
24051	1.36183	1.23927
24052	0.925892	1.34819
24053	0.946133	1.26938
24054	1.38354	0.384408
24055	1.16009	0.38073
24056	0.946132	0.361944
24057	0.946132	0.362428
24058	1.01462	0.34728
24059	1.18854	0.891169
24060	0.888265	1.63272
24061	0.745095	0.995221
24062	0.709597	1.43034
24063	0.936541	1.23652
24064	1.24142	0.669858
24065	1.35059	0.580909
24066	0.946128	0.44559
24067	0.94613	0.617279

24068	1.16623	0.600324
24069	1.24945	0.332333
24070	1.33035	0.325836
24071	0.946127	0.311206
24072	0.946128	0.31082
24073	1.13343	0.32732
24074	0.637995	0.812486
24075	1.10455	0.912701
24076	1.22641	1.20469
24077	1.22641	1.75051
24078	0.946132	1.17231
24079	1.3665	0.328837
24080	1.28507	0.321958
24081	0.946127	0.3249
24082	0.946129	0.325502
24083	1.2051	0.328167
24084	0.797374	2.1
24085	0.797376	2.1
24086	1.13113	2.1
24087	1.20057	2.1
24088	1.12049	2.1
24089	0.59387	0.720262
24090	1.13245	0.747514
24091	0.966134	0.882768
24092	0.593842	0.693982
24093	0.946128	0.865746
24094	0.641455	0.753956
24095	0.916507	0.657916
24096	1.04072	1.12955
24097	0.415585	0.643794
24098	0.507244	1.02898
24099	0.678938	2.1
24100	1.24154	2.1
24101	1.25	1.54219
24102	1.24154	1.86382
24103	1.25	1.76041
24104	0.851009	1.46747
24105	0.946131	1.78624
24106	0.851539	1.39606
24107	1.21997	1.22136
24108	1.45109	1.24081
24109	0.804877	0.810121
24110	1.00062	0.473524
24111	0.851536	0.695041
24112	0.909673	0.478715
24113	0.946129	0.715726
24114	1.20494	0.258919
24115	1.09088	0.298339
24116	0.887634	0.33749
24117	0.81027	0.175755
24118	0.900294	0.217302
24119	0.747713	0.0802972
24120	0.975467	0.0854632
24121	0.747712	0.0847857
24122	1.39168	0.0686598
24123	0.975467	0.0798992
24124	0.94613	0.360054
24125	1.32827	0.34484

24126	0.946128	0.338313
24127	1.41201	0.372633
24128	1.32827	0.34905
24129	0.981121	0.140952
24130	0.936311	0.26744
24131	1.00715	0.142068
24132	1.15914	0.171743
24133	0.704409	0.169685
24134	0.713444	0.920525
24135	0.954538	0.744081
24136	0.944508	0.769567
24137	0.851165	0.963376
24138	0.946131	1.27302
24139	0.851192	0.902025
24140	0.946131	0.909308
24141	1.3279	0.852718
24142	1.33587	0.05
24143	0.946126	0.0599038
24144	1.27512	0.0581823
24145	1.33587	0.0565336
24146	0.748059	0.0530724
24147	1.17451	0.137218
24148	1.04299	0.140131
24149	0.851542	0.193666
24150	0.946131	0.16972
24151	0.892109	0.165807
24152	0.946131	0.05
24153	0.851541	0.05
24154	1.2178	0.05
24155	1.2179	0.05
24156	0.946793	0.05
24157	0.709599	0.247804
24158	0.987426	0.159232
24159	1.39502	0.2348
24160	1.13624	0.148208
24161	0.73176	0.221507
24162	1.36697	0.0648734
24163	1.33393	0.057756
24164	0.946131	0.0965428
24165	0.946132	0.0827825
24166	1.23845	0.0831086
24167	0.50702	0.832267
24168	1.06442	0.816901
24169	0.946131	0.941818
24170	1.02363	0.962302
24171	1.13537	0.0601673
24172	1.25049	0.0502016
24173	0.94613	0.0615841
24174	1.14993	0.05
24175	1.38056	0.05
24176	0.946126	0.05
24177	1.24678	0.05
24178	0.684521	0.05
24179	0.946124	0.05
24180	0.684518	0.05
24181	0.851542	0.56372
24182	0.925459	0.551837
24183	0.946131	0.577804

24184	0.851474	0.611664
24185	0.923305	0.613113
24186	1.4055	0.05
24187	1.32077	0.0513339
24188	0.946127	0.0680397
24189	1.21757	0.0543268
24190	0.946129	0.0550733
24191	0.711214	0.129262
24192	1.30463	0.178718
24193	0.711185	0.14069
24194	0.711185	0.152181
24195	1.22716	0.147044
24196	1.29958	0.05
24197	1.26201	0.0583907
24198	0.568927	0.0620961
24199	0.568927	0.05
24200	0.946132	0.0581366
24201	0.851542	0.168414
24202	1.44968	0.115318
24203	0.851543	0.135514
24204	1.35123	0.14832
24205	1.40427	0.122773
24206	0.593295	0.244354
24207	0.946129	0.274149
24208	1.22144	0.192509
24209	0.908278	0.282474
24210	1.22144	0.233311
24211	0.867593	0.445328
24212	1.23351	0.397049
24213	0.946131	0.446366
24214	1.05516	0.435878
24215	1.23351	0.43045
24216	1.15564	0.0590609
24217	0.946131	0.115008
24218	0.766652	0.0832239
24219	0.766681	0.0731418
24220	0.946131	0.0954543
24221	0.946131	0.308599
24222	0.897851	0.263259
24223	1.1751	0.354538
24224	1.00273	0.268478
24225	0.851554	0.2727
24226	0.883272	0.126841
24227	1.17111	0.368797
24228	0.851541	0.277149
24229	1.01665	0.230235
24230	0.851555	0.231515
24231	0.946131	0.521349
24232	0.911508	0.483232
24233	1.08054	0.546056
24234	1.33007	0.406983
24235	0.911471	0.476553
24236	1.26787	1.87709
24237	1.16641	1.4322
24238	0.946132	1.41301
24239	0.946133	1.14558
24240	1.16641	1.66637
24241	0.946131	1.77649

24242	1.25037	0.104191
24243	1.34733	0.706644
24244	1.34733	0.458998
24245	0.946131	0.562742
24246	0.946131	0.0769502
24247	1.41377	0.0643273
24248	0.94613	0.0762247
24249	1.30541	0.0664479
24250	1.41377	0.064899
24251	1.3473	2.1
24252	1.11804	1.61816
24253	0.94613	2.1
24254	0.946131	2.1
24255	1.11805	2.1
24256	1.32068	2.1
24257	0.946131	2.1
24258	0.946128	2.1
24259	1.39384	2.1
24260	1.4181	2.1
24261	1.12913	2.1
24262	0.94613	2.1
24263	0.946129	2.1
24264	1.20328	2.1
24265	1.39385	2.1
24266	1.3335	2.1
24267	1.40987	2.1
24268	0.94613	2.1
24269	1.21474	2.1
24270	0.94613	2.1
24271	0.946132	0.725121
24272	1.15203	2.1
24273	1.41192	0.712547
24274	1.41192	2.09262
24275	0.946132	2.1
24276	0.946131	2.1
24277	1.40684	2.1
24278	1.2597	1.59785
24279	0.946131	1.87522
24280	1.40684	0.963509
24281	0.946131	1.88716
24282	1.39113	2.1
24283	1.34049	0.897811
24284	0.946131	1.20972
24285	1.39113	0.923992
24286	0.946132	1.65916
24287	1.31649	0.902252
24288	0.946132	0.868314
24289	0.946132	1.04441
24290	1.34297	1.07755
24291	0.946131	0.78278
24292	1.41766	0.764758
24293	0.954559	1.55254
24294	0.946131	0.67192
24295	0.946131	0.571541
24296	1.25447	0.363614
24297	1.35559	0.338645
24298	0.946127	0.309707
24299	0.946128	0.354635

24300	1.14874	0.380759
24301	1.33035	0.332676
24302	1.3665	0.32173
24303	0.946129	0.321682
24304	0.94613	0.326358
24305	1.20732	0.329463
24306	0.946131	2.1
24307	0.946131	1.22208
24308	1.32949	2.1
24309	1.34921	2.1
24310	0.946131	2.1
24311	0.946132	0.638118
24312	1.25479	0.619444
24313	1.38357	0.481833
24314	1.38357	0.399856
24315	0.946132	0.407335
24316	0.946131	1.17057
24317	1.31034	0.410292
24318	1.37127	0.660828
24319	1.37127	0.445254
24320	0.946131	0.542462
24321	1.23923	0.233311
24322	1.41169	0.286328
24323	0.94613	0.393997
24324	1.12703	0.191319
24325	0.946131	0.252661
24326	0.946119	2.1
24327	1.41915	2.1
24328	1.02679	2.1
24329	0.946107	2.1
24330	1.41917	2.1
24331	0.946133	2.1
24332	1.36581	2.1
24333	0.946131	2.1
24334	1.36581	2.1
24335	0.946131	2.1
24336	0.946132	2.1
24337	1.04384	2.1
24338	1.34973	2.1
24339	1.34967	2.1
24340	0.851555	2.1
24341	0.946132	0.328618
24342	1.3855	0.406832
24343	1.08603	0.305932
24344	1.08602	0.35166
24345	1.16234	0.381224
24346	0.946133	0.410095
24347	1.29348	0.44548
24348	1.20111	0.394454
24349	1.20112	0.432045
24350	1.3855	0.434736
24351	0.946128	0.893545
24352	1.30798	2.1
24353	0.946129	2.1
24354	1.34828	1.96926
24355	1.30797	2.08421
24356	1.24932	0.05
24357	1.40549	0.05

24358	0.946117	0.0574305
24359	1.19615	0.05
24360	0.946106	0.05
24361	0.946131	2.1
24362	1.29926	2.1
24363	1.25144	2.1
24364	0.946131	2.1
24365	1.29925	2.1
24366	0.946132	2.1
24367	1.39826	2.1
24368	0.946133	2.1
24369	0.946133	2.1
24370	1.29926	2.1
24371	0.946119	0.0972935
24372	1.33614	0.0742653
24373	0.946118	0.0872635
24374	1.41377	0.0750862
24375	1.33612	0.0769645
24376	0.946133	1.30678
24377	1.3933	1.97882
24378	1.34999	0.899212
24379	0.946133	1.3184
24380	1.3933	1.10506
24381	0.946131	1.32503
24382	1.36139	2.1
24383	0.946132	1.04031
24384	0.946131	1.7776
24385	1.39329	1.025
24386	1.37449	1.28562
24387	1.27925	0.643822
24388	0.919796	0.684264
24389	1.01176	0.82365
24390	0.94613	0.928595
24391	0.946133	1.49164
24392	1.27679	2.1
24393	0.946133	1.09833
24394	0.946132	2.1
24395	1.39731	1.2375
24396	0.946119	2.1
24397	1.40765	1.35671
24398	1.1981	1.63445
24399	0.946131	1.07873
24400	1.40764	1.77277
24401	0.946127	2.1
24402	1.3557	2.1
24403	0.946127	2.1
24404	0.946128	2.1
24405	1.13485	2.1
24406	1.4008	2.1
24407	1.1587	2.1
24408	0.946131	2.1
24409	0.946131	2.1
24410	1.1587	2.1
24411	1.32082	0.330088
24412	1.29215	0.33597
24413	0.94613	0.338427
24414	0.946131	0.334078
24415	1.02366	0.332276

24416	1.27697	0.320618
24417	1.2929	0.33555
24418	0.946132	0.333233
24419	0.946132	0.307516
24420	1.01451	0.286586
24421	0.946132	2.1
24422	1.27252	2.1
24423	0.946132	2.1
24424	1.37869	2.1
24425	1.27252	2.1
24426	1.15627	2.1
24427	1.35703	2.1
24428	0.946132	2.1
24429	1.03783	2.1
24430	0.946132	2.1
24431	0.946128	2.1
24432	1.37869	2.1
24433	0.946131	2.1
24434	1.35357	2.1
24435	1.37869	2.1
24436	0.946132	2.1
24437	0.946131	2.1
24438	1.33471	2.1
24439	0.946131	2.1
24440	1.36373	2.1
24441	1.41342	0.837595
24442	1.06832	0.942684
24443	0.946128	1.45212
24444	0.94613	0.704783
24445	1.06832	1.28156
24446	1.26929	2.1
24447	0.946131	0.934794
24448	0.946128	2.1
24449	1.41022	1.25416
24450	1.37034	0.879351
24451	1.37759	0.898012
24452	1.07612	1.02954
24453	0.946126	0.877331
24454	0.946129	1.01359
24455	1.07612	0.947373
24456	0.946131	0.655411
24457	0.94613	0.389796
24458	1.23848	0.474376
24459	1.38897	0.448163
24460	0.946131	0.478346
24461	0.946132	0.431452
24462	0.946133	0.378227
24463	1.34369	0.339238
24464	1.40332	0.385961
24465	0.946132	0.34316
24466	0.946131	2.1
24467	1.24023	2.1
24468	1.38244	2.1
24469	1.38244	2.1
24470	0.946131	2.1
24471	0.946132	0.743834
24472	1.33468	2.1
24473	1.41619	0.809633

24474	1.41619	1.98454
24475	0.946131	2.01124
24476	1.1908	1.06252
24477	0.946128	0.928154
24478	0.946126	1.31407
24479	1.26654	1.54802
24480	1.39514	1.94139
24481	0.93608	2.01295
24482	1.04105	2.1
24483	1.39551	2.1
24484	1.52471	2.1
24485	1.04072	2.1
24486	1.33174	0.05
24487	0.946128	0.05
24488	0.94613	0.05
24489	1.17801	0.05
24490	0.94613	0.05
24491	0.946132	2.1
24492	0.946132	2.1
24493	1.29065	2.1
24494	1.29065	2.1
24495	0.975266	2.1
24496	0.946131	2.1
24497	1.26025	2.1
24498	0.955601	2.1
24499	0.946131	2.1
24500	1.26025	2.1
24501	0.94613	0.342677
24502	1.23146	0.330221
24503	0.946127	0.332224
24504	1.32827	0.347293
24505	1.23146	0.334277
24506	1.25008	0.694007
24507	1.05018	0.732197
24508	0.94613	0.687456
24509	0.932744	0.802633
24510	1.04207	0.739695
24511	0.946132	0.445841
24512	1.10023	0.643406
24513	1.36748	0.406905
24514	1.36748	0.531602
24515	0.946133	0.491244
24516	1.06023	0.304227
24517	1.08019	0.295102
24518	0.94613	0.355709
24519	1.06023	0.322524
24520	0.94613	0.32369
24521	1.16727	0.374917
24522	1.35856	0.382585
24523	0.946132	0.425602
24524	1.16727	0.37794
24525	0.946132	0.363113
24526	0.946133	2.1
24527	0.946131	2.1
24528	1.21691	2.1
24529	1.39558	2.1
24530	0.946131	2.1
24531	1.09905	0.334811

24532	0.946132	0.630689
24533	1.3365	0.390403
24534	1.35381	0.505889
24535	0.946132	0.689504
24536	0.94613	0.401427
24537	0.946128	0.490309
24538	1.40332	0.441275
24539	1.39041	0.220543
24540	0.94613	0.449083
24541	0.946132	0.529991
24542	1.3659	0.70812
24543	1.39041	0.501571
24544	1.39041	0.59134
24545	0.946132	0.637155
24546	0.840292	2.01163
24547	0.946133	1.41436
24548	0.851541	2.03911
24549	1.28464	1.73406
24550	1.28806	1.60486
24551	1.12591	0.721174
24552	1.05033	0.831243
24553	0.946132	0.764747
24554	0.946132	0.877173
24555	1.13537	0.851488
24556	1.21177	1.05535
24557	0.946132	1.16381
24558	0.946132	2.1
24559	1.41134	1.13656
24560	1.27079	1.24214
24561	0.946131	0.0830757
24562	1.35403	0.0809738
24563	0.94613	0.118267
24564	0.94613	0.104212
24565	0.946131	0.0904785
24566	0.946131	0.280335
24567	0.946132	0.0935411
24568	0.94613	0.408725
24569	1.37052	0.200051
24570	0.946131	0.289674
24571	0.946133	0.152945
24572	0.946132	0.419589
24573	0.946133	0.132456
24574	1.24249	0.151417
24575	0.946132	0.206845
24576	1.20608	2.1
24577	0.946132	2.1
24578	0.946131	1.80795
24579	0.946132	2.1
24580	0.946132	2.1
24581	0.946131	2.1
24582	0.946128	2.1
24583	0.946129	2.1
24584	1.41232	2.1
24585	0.94613	2.1
24586	0.946132	0.103473
24587	1.32449	0.116119
24588	0.946131	0.152718
24589	0.946131	0.132587

24590	1.32449	0.11997
24591	0.946116	0.813004
24592	1.41563	1.95751
24593	0.946118	1.20517
24594	0.946105	1.61649
24595	0.946131	0.76088
24596	1.26563	2.1
24597	1.32819	1.83659
24598	0.946131	0.668281
24599	0.946131	2.05499
24600	0.946131	0.961154
24601	0.946131	1.88163
24602	0.946133	1.13084
24603	1.30382	2.1
24604	0.946131	1.08428
24605	0.946131	1.03238
24606	1.30382	1.86082
24607	0.946133	0.05
24608	1.15803	0.05
24609	1.28895	0.05
24610	0.954071	0.05
24611	0.946132	0.05
24612	1.0264	0.646304
24613	0.946132	1.39789
24614	0.946132	1.02663
24615	0.946131	1.24159
24616	0.946132	1.07638
24617	1.35977	0.184727
24618	0.946132	0.416481
24619	0.946129	0.37031
24620	0.94613	0.348759
24621	0.946129	0.320824
24622	0.876631	0.53264
24623	0.946133	0.667018
24624	0.946132	1.08767
24625	0.946132	1.12115
24626	0.876606	0.949485
24627	0.946131	0.168947
24628	1.24979	0.0838359
24629	0.94613	0.133082
24630	0.94613	0.0898989
24631	0.94613	0.119613
24632	0.921791	0.0594319
24633	0.946131	0.0529667
24634	0.921762	0.0574887
24635	0.946131	0.05
24636	0.946131	0.0501037
24637	0.810944	0.180605
24638	0.810945	0.270918
24639	0.810959	0.234654
24640	0.946133	0.317674
24641	1.25797	0.32078
24642	0.946132	0.323106
24643	0.946132	0.317087
24644	1.25797	0.318467
24645	1.13748	0.521748
24646	0.851512	0.708685
24647	0.94613	0.980521

24648	1.02373	0.540501
24649	0.946131	0.801452
24650	0.946122	2.1
24651	1.3852	2.1
24652	0.946124	2.1
24653	0.946118	2.1
24654	1.38521	2.1
24655	0.946131	0.656581
24656	0.946131	0.962281
24657	1.30448	0.865365
24658	0.946129	0.649016
24659	0.94613	0.650275
24660	0.946133	2.1
24661	0.94613	2.1
24662	0.946131	1.71197
24663	0.946131	1.61616
24664	1.36503	2.1
24665	0.810947	0.05
24666	0.810947	0.05
24667	0.810934	0.05
24668	3.34066	0.05
24669	0.946133	0.775859
24670	1.4185	0.615452
24671	0.946132	0.556813
24672	0.946133	0.638852
24673	0.946132	0.656678
24674	0.94613	2.1
24675	0.946128	2.1
24676	1.03925	2.1
24677	0.944743	2.1
24678	0.947518	2.1
24679	0.946132	2.1
24680	1.20573	2.1
24681	0.94613	2.1
24682	0.94613	2.1
24683	1.20573	2.1
24684	1.20056	0.05
24685	0.946132	0.0557784
24686	0.946131	0.0661281
24687	0.946131	0.0542585
24688	0.946131	0.0550642
24689	0.94613	2.1
24690	0.946128	2.1
24691	1.38302	2.1
24692	0.94613	2.1
24693	0.94613	2.1
24694	0.827866	0.272173
24695	0.913552	0.20846
24696	0.716526	0.308808
24697	0.82784	0.222852
24698	1.20634	0.305192
24699	0.946131	0.261743
24700	0.94613	0.278108
24701	1.39502	0.159045
24702	0.88146	0.269765
24703	0.946132	2.1
24704	0.946132	2.1
24705	0.946132	2.1

24706	0.946132	2.1
24707	1.39053	2.1
24708	0.946131	2.1
24709	1.40571	2.1
24710	0.946131	2.1
24711	0.946131	2.1
24712	0.946132	2.1
24713	0.94613	0.390154
24714	1.2582	0.454896
24715	0.946127	0.396161
24716	0.946129	0.428206
24717	0.946129	0.405914
24718	0.946129	0.899963
24719	0.946132	0.931786
24720	0.946131	0.797093
24721	0.946131	1.01726
24722	1.29054	0.780983
24723	0.810944	0.05
24724	0.810941	0.05
24725	0.810931	0.05
24726	0.946132	0.679234
24727	0.946132	0.613606
24728	0.946132	1.0527
24729	0.946132	0.71566
24730	0.927608	0.669732
24731	0.946128	2.1
24732	0.946133	2.1
24733	0.946131	2.1
24734	0.94613	2.1
24735	1.38813	2.1
24736	0.94613	0.584624
24737	0.946128	2.1
24738	0.946132	0.523637
24739	1.41666	0.939144
24740	0.94613	0.598505
24741	0.946118	0.36234
24742	1.34151	0.34734
24743	0.946116	0.372081
24744	0.946131	0.362829
24745	1.34149	0.358211
24746	0.946132	2.1
24747	1.31889	2.1
24748	0.946132	2.1
24749	0.946132	2.1
24750	1.31889	2.1
24751	0.864997	0.05
24752	0.928981	0.05
24753	0.872219	0.05
24754	0.946127	0.554507
24755	0.946131	0.508023
24756	1.37709	0.47049
24757	0.946131	0.563574
24758	0.946129	0.508711
24759	1.21882	0.05
24760	0.938168	0.05
24761	0.938161	0.05
24762	1.06327	0.05
24763	1.33457	0.05

24764	0.946133	0.868224
24765	0.946132	0.857425
24766	0.946133	0.793613
24767	0.946132	1.12444
24768	1.14444	0.736046
24769	1.12628	0.0734461
24770	0.946132	0.102205
24771	0.946131	0.125805
24772	1.12628	0.0726625
24773	0.946131	0.0840149
24774	0.946132	2.1
24775	0.946132	2.1
24776	0.946133	2.1
24777	0.946132	2.1
24778	1.29322	2.1
24779	0.946131	2.1
24780	0.946132	2.1
24781	0.94613	2.1
24782	0.946131	2.1
24783	1.28441	2.1
24784	0.946118	1.66
24785	0.946119	1.64289
24786	0.946117	2.1
24787	1.28075	2.1
24788	0.946105	2.08224
24789	0.810945	2.1
24790	0.810942	2.1
24791	0.81093	2.1
24792	0.946127	2.1
24793	0.946127	2.1
24794	1.28228	2.1
24795	0.946129	2.1
24796	0.946132	2.1
24797	1.14042	2.1
24798	0.946132	2.1
24799	0.946133	2.1
24800	1.14042	2.1
24801	0.946133	0.358943
24802	0.946132	0.337109
24803	1.28632	0.260733
24804	1.28632	0.156287
24805	0.946133	0.311377
24806	0.946131	1.13725
24807	0.946131	0.950221
24808	0.946131	1.04983
24809	1.31817	1.15754
24810	0.946131	0.966074
24811	1.35048	2.1
24812	0.946132	2.1
24813	0.946132	2.1
24814	0.946132	2.1
24815	0.946131	2.1
24816	0.946131	2.1
24817	1.35943	2.1
24818	0.94613	2.1
24819	0.946132	2.1
24820	0.946131	2.1
24821	0.990713	2.1

24822	0.946132	2.1
24823	0.946132	2.1
24824	0.946133	2.1
24825	0.946132	2.1
24826	0.946133	0.955495
24827	1.01433	0.996869
24828	0.877931	0.983046
24829	0.96573	0.784901
24830	0.946131	0.779311
24831	0.946131	0.902713
24832	0.946133	0.7471
24833	1.39575	0.579475
24834	0.946131	0.628862
24835	0.94613	0.626239
24836	0.810943	0.539932
24837	0.810942	0.545221
24838	0.709589	0.487484
24839	0.924071	0.46903
24840	1.04293	0.392345
24841	1.15747	0.239106
24842	0.946131	0.473125
24843	1.04071	0.449441
24844	0.810946	0.55147
24845	0.810943	0.460846
24846	0.810959	0.405428
24847	0.946128	1.17201
24848	1.39696	1.73307
24849	0.94613	0.840446
24850	0.94613	0.882958
24851	1.39696	1.33588
24852	0.946131	0.798005
24853	0.946131	1.67474
24854	0.946131	1.36813
24855	0.946131	0.979982
24856	1.29735	1.65985
24857	0.946128	1.13828
24858	0.946133	0.698074
24859	0.94613	2.1
24860	0.946131	1.16433
24861	1.33266	0.890256
24862	0.946131	2.1
24863	0.94613	0.229547
24864	0.946131	1.61332
24865	1.40609	0.528461
24866	0.946131	0.708661
24867	0.946131	1.08848
24868	0.946133	1.21059
24869	0.946129	1.22814
24870	1.23339	1.43891
24871	0.946131	1.10556
24872	0.946132	1.67689
24873	0.946133	1.50626
24874	0.946132	2.1
24875	1.29198	2.04491
24876	0.946132	1.18124
24877	0.946131	2.1
24878	1.07297	2.1
24879	0.946127	2.1

24880	0.94613	2.1
24881	1.07297	2.1
24882	0.946125	2.1
24883	0.946126	2.1
24884	0.946126	2.1
24885	1.3864	2.1
24886	0.946119	2.1
24887	0.946128	0.274501
24888	1.09411	0.279159
24889	0.946131	0.379728
24890	0.946131	0.326414
24891	1.09411	0.309783
24892	0.946131	0.216797
24893	1.25837	0.226469
24894	0.946131	0.326755
24895	0.946131	0.278376
24896	1.25837	0.261979
24897	0.810945	2.1
24898	0.810943	2.1
24899	0.810933	2.1
24900	0.94613	0.288801
24901	0.946131	0.576592
24902	0.946132	0.407357
24903	0.946129	0.188828
24904	0.946131	0.515047
24905	0.946131	0.307096
24906	1.28911	0.290994
24907	0.946129	0.332462
24908	0.946133	0.306715
24909	0.946131	0.314659
24910	0.94613	0.439254
24911	1.38954	0.462856
24912	0.946132	0.432804
24913	0.94613	0.452805
24914	0.946131	0.441517
24915	0.946131	0.685166
24916	0.946131	0.542846
24917	0.946131	0.498118
24918	1.37981	0.498179
24919	0.946131	0.523091
24920	0.946131	1.23343
24921	1.26958	2.1
24922	0.946127	1.81422
24923	0.946129	2.1
24924	1.26957	2.1
24925	0.94613	2.1
24926	1.16889	2.1
24927	0.946126	2.1
24928	0.946128	2.1
24929	1.16889	2.1
24930	0.946133	0.480897
24931	1.13511	0.435095
24932	0.946132	0.490124
24933	0.946133	0.448877
24934	0.946132	0.448881
24935	1.27575	1.2168
24936	0.946129	2.02329
24937	0.946131	1.49811

24938	0.94613	2.07015
24939	0.946131	1.81856
24940	0.946131	2.1
24941	0.946128	2.1
24942	1.41712	2.1
24943	1.41712	2.1
24944	0.94613	2.1
24945	0.946129	0.0678224
24946	0.946132	0.056185
24947	0.946131	0.0579462
24948	0.946131	0.0801493
24949	0.946131	0.0589181
24950	0.979045	1.37536
24951	0.946131	0.874451
24952	0.617549	1.72459
24953	0.555844	1.59202
24954	1.04071	0.89716
24955	0.946132	1.19491
24956	0.946132	0.987118
24957	0.946131	1.8666
24958	1.22618	0.885611
24959	0.946132	0.755817
24960	0.946131	2.1
24961	1.13976	2.1
24962	0.946129	2.1
24963	0.946131	2.1
24964	1.13976	2.1
24965	0.810945	0.598334
24966	0.810943	0.546025
24967	0.810932	0.743565
24968	1.25358	0.945422
24969	0.94613	0.607102
24970	0.946128	0.620619
24971	1.25358	1.69822
24972	1.07811	0.744115
24973	0.946129	2.1
24974	1.09197	2.1
24975	0.946131	2.1
24976	0.946131	2.1
24977	1.09197	2.1
24978	0.594912	2.1
24979	0.993703	2.1
24980	1.10173	2.1
24981	0.9916	2.1
24982	0.946131	2.1
24983	0.946128	2.1
24984	0.946131	2.1
24985	0.94613	2.1
24986	0.946129	2.1
24987	0.946132	2.1
24988	1.40335	2.1
24989	0.946133	2.1
24990	0.946133	2.1
24991	1.40335	2.1
24992	0.946132	0.89052
24993	0.946132	1.14608
24994	0.946132	0.938106
24995	0.946132	1.01826

24996	1.411	1.1385
24997	0.611675	0.212089
24998	0.946129	0.359883
24999	1.31678	0.192418
25000	0.946124	0.359176
25001	0.946127	0.23617
25002	0.946128	0.356845
25003	0.946132	0.490031
25004	0.946131	0.424914
25005	0.946132	0.492032
25006	0.946132	0.444865
25007	0.946132	0.435244
25008	0.810946	0.462236
25009	0.810943	0.463575
25010	0.81096	0.435196
25011	1.39301	0.0860294
25012	0.946132	0.0838384
25013	0.625812	0.0709383
25014	0.946132	0.0818178
25015	0.625812	0.0839669
25016	0.810946	0.95467
25017	0.810945	1.109
25018	0.810931	2.01276
25019	0.810943	2.1
25020	0.810944	2.1
25021	0.810958	2.1
25022	0.946131	0.421057
25023	0.946131	0.404316
25024	0.946128	0.42564
25025	0.946129	0.409276
25026	0.94613	0.412208
25027	0.946133	0.475604
25028	0.946133	0.508849
25029	0.98537	0.413889
25030	0.985371	0.243261
25031	0.946132	0.547406
25032	0.946129	0.376842
25033	1.38752	0.333569
25034	0.946131	0.382137
25035	0.946131	0.319303
25036	1.38752	0.35857
25037	0.946119	0.316129
25038	1.33036	0.279902
25039	0.946119	0.342683
25040	0.946132	0.267448
25041	1.33035	0.297161
25042	1.3868	2.1
25043	0.946132	2.1
25044	0.946132	2.1
25045	0.946133	2.1
25046	0.946132	2.1
25047	0.810947	2.1
25048	0.810946	2.1
25049	0.81096	2.1
25050	0.810946	2.1
25051	0.810946	2.1
25052	0.810933	2.1
25053	0.946133	2.1

25054	1.35342	2.1
25055	0.946133	2.03149
25056	0.946132	2.1
25057	1.35342	2.1
25058	0.946132	2.1
25059	0.946132	2.1
25060	1.28569	2.1
25061	0.946132	2.1
25062	0.946133	2.1
25063	0.946132	1.20593
25064	1.32547	2.1
25065	0.946132	1.36191
25066	0.946132	1.54725
25067	1.32547	2.1
25068	0.94613	0.216084
25069	0.946133	0.214029
25070	1.39664	0.18949
25071	1.39664	0.149058
25072	0.946131	0.210356
25073	0.946131	0.992078
25074	0.94613	2.1
25075	0.946132	0.767973
25076	0.946131	1.25792
25077	1.41833	1.17823
25078	0.946131	0.0593027
25079	0.946131	0.0587301
25080	0.946131	0.0634695
25081	1.26155	0.0572204
25082	0.946132	0.061301
25083	0.810946	2.1
25084	0.810946	2.1
25085	0.810959	2.1
25086	0.94613	0.892878
25087	0.946132	0.651583
25088	1.40779	0.497932
25089	1.40779	0.584202
25090	0.94613	0.636959
25091	0.94613	0.0794622
25092	0.946129	0.095471
25093	1.36376	0.0668853
25094	1.36376	0.0709601
25095	0.946131	0.0794337
25096	0.946128	2.1
25097	0.946127	2.1
25098	0.946131	2.1
25099	1.40756	2.1
25100	0.94613	2.1
25101	0.946131	1.005
25102	0.946132	1.57969
25103	0.94613	0.99048
25104	0.946131	0.964415
25105	1.31947	1.28932
25106	0.946131	2.1
25107	0.946131	2.1
25108	0.946129	2.1
25109	0.946131	2.1
25110	1.25768	2.1
25111	0.946132	0.368481

25112	0.94613	0.384692
25113	0.946131	0.36038
25114	0.946131	0.3522
25115	0.946131	0.389639
25116	0.810943	0.845753
25117	0.810942	0.963388
25118	0.81093	1.79509
25119	0.946128	0.890207
25120	0.946133	0.729092
25121	0.946131	0.922994
25122	0.946131	0.869713
25123	0.94613	0.940658
25124	0.946132	0.910261
25125	0.946131	0.764711
25126	0.946132	0.942646
25127	0.946132	0.912177
25128	0.946132	0.977259
25129	0.946119	2.1
25130	0.946119	2.1
25131	1.31523	2.1
25132	0.946132	2.1
25133	0.946107	2.1
25134	0.946133	1.68869
25135	0.946132	0.731639
25136	0.946132	0.703819
25137	1.28803	1.11403
25138	0.946132	1.02902
25139	1.37658	0.977025
25140	0.946132	0.879548
25141	0.946133	1.41775
25142	0.946132	1.05815
25143	0.946132	1.25426
25144	0.946132	0.879645
25145	0.94613	0.972585
25146	0.94613	1.23186
25147	1.15541	0.861064
25148	0.92943	1.07107
25149	0.946126	0.05
25150	0.946129	0.0545049
25151	1.33849	0.05
25152	1.33849	0.05
25153	0.946129	0.05
25154	0.810946	0.05
25155	0.810944	0.0565914
25156	0.810959	0.05
25157	1.39027	0.0710754
25158	0.946131	0.0999575
25159	0.946131	0.129997
25160	1.39027	0.0736937
25161	0.946131	0.0842279
25162	1.40924	1.93435
25163	0.946131	1.08475
25164	0.946132	1.91033
25165	0.946132	0.96557
25166	0.946132	1.51051
25167	0.946131	2.1
25168	0.94613	2.1
25169	0.946128	2.1

25170	0.946128	2.1
25171	1.41744	2.1
25172	0.946132	0.572679
25173	0.946128	0.585257
25174	0.94613	0.812762
25175	1.37675	0.600457
25176	0.94613	0.852993
25177	0.946132	0.759008
25178	0.946132	0.547125
25179	0.946132	0.479022
25180	0.946132	0.535593
25181	0.946131	0.523324
25182	0.946132	0.751935
25183	0.946132	0.61648
25184	0.946133	1.10807
25185	1.10003	0.565388
25186	0.946132	0.837492
25187	0.946132	0.183849
25188	0.946131	0.217618
25189	0.946132	0.148805
25190	0.946131	0.204332
25191	0.946132	0.169506
25192	0.946132	0.241962
25193	0.946131	0.277903
25194	0.946132	0.190905
25195	0.946132	0.263396
25196	0.946131	0.216909
25197	1.24094	0.0522874
25198	0.946126	0.0705279
25199	0.946128	0.058289
25200	0.946129	0.0743547
25201	0.946129	0.0623564
25202	1.2929	0.0730641
25203	0.946131	0.113243
25204	0.946131	0.0903183
25205	0.946131	0.105926
25206	0.946131	0.0957845
25207	0.810946	0.05
25208	0.810945	0.05
25209	0.810959	0.05
25210	0.810946	1.20386
25211	0.810943	1.19284
25212	0.810933	1.47136
25213	0.946128	1.96911
25214	1.18315	2.1
25215	0.94613	1.9994
25216	0.94613	1.93018
25217	1.18316	2.1
25218	0.946126	0.488922
25219	1.3324	0.44022
25220	0.946127	0.500965
25221	0.94613	0.438696
25222	0.946128	0.465775
25223	0.94613	2.1
25224	0.946128	2.1
25225	1.39523	2.1
25226	1.39523	2.1
25227	0.946131	2.1

25228	1.36541	2.1
25229	0.946132	2.1
25230	0.946132	2.1
25231	0.946132	2.1
25232	0.946132	2.1
25233	0.946131	2.01384
25234	0.94613	0.960881
25235	0.946132	1.27032
25236	0.946131	0.641289
25237	1.39509	1.12605
25238	0.946132	2.1
25239	0.946132	2.1
25240	1.29319	2.1
25241	0.946132	2.1
25242	0.946133	2.1
25243	0.946132	0.102086
25244	0.946131	0.174712
25245	0.946132	0.115293
25246	1.01196	0.113972
25247	0.946132	0.153312
25248	0.946116	2.1
25249	0.946117	0.935257
25250	1.38153	2.1
25251	1.38155	2.1
25252	0.946105	2.1
25253	0.946132	1.71365
25254	0.946131	1.65107
25255	0.946132	1.44826
25256	1.28684	1.53364
25257	0.946132	1.15092
25258	1.10185	1.41721
25259	0.94613	1.55164
25260	0.946128	2.1
25261	0.94613	2.04741
25262	0.94613	1.51796
25263	0.810947	0.05
25264	0.810945	0.05
25265	0.810932	0.05
25266	0.946119	0.405297
25267	1.16717	0.375598
25268	0.946117	0.412148
25269	0.94613	0.382682
25270	1.16715	0.379597
25271	0.94612	0.469976
25272	0.946119	0.464048
25273	0.946119	0.445644
25274	0.946132	0.403744
25275	1.20882	0.419663
25276	1.07043	0.649668
25277	0.823268	0.651987
25278	0.810959	0.608087
25279	0.946132	1.57903
25280	1.14879	0.814356
25281	0.94613	1.29589
25282	0.946131	1.46183
25283	1.14879	0.891859
25284	1.23837	2.1
25285	0.94612	2.1

25286	0.946118	2.1
25287	0.946131	2.1
25288	0.946105	2.1
25289	0.810946	0.05
25290	0.810946	0.05
25291	0.810959	0.05
25292	0.810947	0.924302
25293	0.810947	0.463122
25294	0.810934	0.444607
25295	0.946128	2.1
25296	0.946131	2.1
25297	0.946128	2.1
25298	0.946129	2.1
25299	0.94613	2.1
25300	0.94613	2.1
25301	0.946131	2.1
25302	0.946131	1.9636
25303	0.946131	2.1
25304	0.946131	2.1
25305	0.946131	1.43109
25306	1.38585	2.1
25307	0.946128	0.99156
25308	0.94613	1.31194
25309	1.38585	1.6076
25310	0.946133	0.404859
25311	0.946133	0.410684
25312	0.946132	0.34904
25313	0.946132	0.404636
25314	0.946132	0.375262
25315	0.918871	0.0967031
25316	0.810248	0.123188
25317	0.838037	0.109118
25318	0.946126	0.125607
25319	0.943647	0.0881779
25320	1.13836	0.080981
25321	1.03802	0.0878811
25322	0.946129	0.0873514
25323	0.946133	0.915121
25324	0.946132	0.78582
25325	0.946132	1.65589
25326	1.1699	0.73214
25327	0.946132	0.6699
25328	0.946131	2.1
25329	0.946132	2.1
25330	1.37199	2.1
25331	1.37199	2.1
25332	0.946132	2.1
25333	0.946132	0.847014
25334	0.946133	0.678366
25335	0.946133	0.980737
25336	0.946132	0.707354
25337	1.38623	0.711052
25338	0.946132	2.1
25339	0.946132	2.1
25340	0.946132	2.1
25341	0.946132	2.1
25342	1.40098	2.1
25343	1.20787	0.60816

25344	1.41894	0.579353
25345	0.946133	0.463478
25346	1.01777	0.644308
25347	0.946132	2.1
25348	0.946132	2.1
25349	0.946133	2.1
25350	1.40199	2.1
25351	0.946132	2.1
25352	0.810946	0.905856
25353	0.810947	1.29128
25354	0.810934	1.00347
25355	0.946126	0.828969
25356	0.94613	1.14399
25357	0.946125	1.08855
25358	1.40247	0.906858
25359	0.946128	0.865879
25360	1.25703	1.19465
25361	0.87821	0.893795
25362	0.878238	0.864927
25363	0.946119	1.5221
25364	0.946132	2.06012
25365	0.946132	0.767853
25366	0.946132	0.792194
25367	0.946131	1.21587
25368	0.946132	0.892076
25369	1.28543	0.752576
25370	0.946115	2.1
25371	1.41638	2.1
25372	0.946116	2.1
25373	0.946104	2.1
25374	0.946129	2.1
25375	0.946132	1.21482
25376	1.41432	2.1
25377	0.946132	1.70375
25378	0.946132	2.07916
25379	0.946132	0.98446
25380	0.810947	0.192949
25381	0.810947	0.190384
25382	0.709567	0.20371
25383	0.851542	0.137448
25384	1.01217	0.0925836
25385	0.697993	0.0781092
25386	0.946133	0.10893
25387	0.851488	0.0761492
25388	0.946129	2.05146
25389	0.94613	2.1
25390	1.2254	1.52884
25391	0.946131	2.1
25392	0.946131	1.64366
25393	0.700785	2.1
25394	0.915893	1.46975
25395	0.838013	1.34577
25396	0.760188	1.11976
25397	1.19601	0.237325
25398	0.946133	0.420929
25399	0.946132	0.488028
25400	0.946132	0.34803
25401	0.946132	0.466834

25402	0.946132	0.838131
25403	0.946133	2.1
25404	0.946132	1.08772
25405	0.946133	1.66228
25406	1.36956	1.89894
25407	0.946131	0.626583
25408	0.94613	0.248765
25409	0.946133	0.555361
25410	1.34268	0.278117
25411	0.946131	0.433496
25412	0.946132	2.05937
25413	1.25603	1.7986
25414	0.946132	2.06099
25415	0.946132	2.02882
25416	1.25603	2.0817
25417	0.810946	0.908039
25418	0.810943	0.926106
25419	0.810933	0.882327
25420	0.496545	0.455501
25421	0.805767	0.866534
25422	0.936671	0.780537
25423	0.530063	0.728309
25424	1.05954	0.744606
25425	0.810946	0.154213
25426	0.810943	0.153451
25427	0.810959	0.120981
25428	1.16699	0.102541
25429	0.946131	0.221073
25430	0.946129	0.206935
25431	0.946131	0.175844
25432	0.946131	0.171253
25433	0.810943	1.0148
25434	0.810942	1.0893
25435	0.810933	1.01456
25436	0.946132	0.976122
25437	1.04832	0.979683
25438	0.870714	1.02521
25439	0.957819	1.19148
25440	0.94613	0.88415
25441	1.82931	2.1
25442	0.946132	0.754348
25443	0.946129	0.696972
25444	0.94613	0.768483
25445	0.946131	0.74615
25446	0.94613	0.627709
25447	0.946119	0.870019
25448	0.94612	0.978969
25449	0.94612	0.7926
25450	0.946132	0.941007
25451	1.36118	0.820166
25452	0.810947	0.349311
25453	0.810947	0.341317
25454	0.810934	0.34091
25455	0.946132	2.1
25456	0.946132	2.1
25457	0.946132	2.1
25458	1.39754	2.1
25459	0.946131	2.1

25460	0.946129	2.1
25461	0.946133	2.1
25462	0.946131	2.1
25463	0.946131	2.1
25464	1.41732	2.1
25465	0.946132	0.804456
25466	0.946128	1.54493
25467	0.94613	0.915496
25468	0.94613	1.00491
25469	1.25277	1.30167
25470	0.94613	1.255
25471	0.946128	1.76532
25472	1.21261	0.905123
25473	0.94613	1.30223
25474	0.94613	1.00455
25475	1.39407	2.1
25476	0.946133	2.1
25477	0.94613	2.1
25478	0.946131	2.1
25479	0.946131	2.1
25480	0.946119	0.892345
25481	0.946119	1.10539
25482	0.946119	1.1083
25483	1.1537	1.10581
25484	0.946106	0.845026
25485	0.946131	2.1
25486	1.11822	2.1
25487	0.946129	2.1
25488	0.946131	2.1
25489	1.11822	2.1
25490	1.46806	0.05
25491	0.498881	0.05
25492	0.996658	0.05
25493	1.2179	0.05
25494	0.991519	0.05
25495	0.946131	0.05
25496	0.946131	0.05
25497	1.28673	0.05
25498	0.946131	0.05
25499	0.946131	0.05
25500	1.33178	0.0791743
25501	0.946132	0.107801
25502	0.946132	0.0980815
25503	0.946132	0.111697
25504	0.946132	0.105899
25505	0.946128	0.34599
25506	0.972061	0.33594
25507	0.946129	0.476291
25508	0.946129	0.432517
25509	0.972059	0.401909
25510	0.946129	2.1
25511	0.94613	2.1
25512	0.94613	2.1
25513	0.94613	2.1
25514	0.94613	2.1
25515	0.946131	2.1
25516	0.946127	2.1
25517	0.946131	2.1

25518	0.946129	2.1
25519	1.4073	2.1
25520	0.946131	0.154437
25521	1.05016	0.111776
25522	0.946132	0.170089
25523	0.946131	0.17331
25524	0.946132	0.138592
25525	0.946128	0.15307
25526	1.13398	0.120892
25527	0.946131	0.184489
25528	0.946131	0.177761
25529	0.94613	0.147801
25530	0.946131	0.05
25531	0.946131	0.0664035
25532	1.38911	0.05
25533	1.38911	0.0510961
25534	0.946132	0.0525468
25535	0.946131	0.253727
25536	0.94613	0.274309
25537	0.946131	0.216445
25538	0.946131	0.266799
25539	0.946131	0.242217
25540	0.946131	0.429972
25541	1.23923	0.323324
25542	0.94613	0.444275
25543	0.946131	0.333593
25544	0.946131	0.361128
25545	1.40511	0.05
25546	0.946131	0.05
25547	0.946131	0.0577531
25548	0.946132	0.05
25549	0.946131	0.05
25550	1.12968	2.1
25551	0.946132	1.48021
25552	0.946132	2.1
25553	0.946131	2.07189
25554	0.946132	2.1
25555	0.946132	1.30245
25556	1.4172	2.1
25557	0.94613	1.01378
25558	0.94613	1.444
25559	1.4172	1.94837
25560	0.946132	2.1
25561	0.946132	2.1
25562	1.31323	2.1
25563	0.946133	2.1
25564	0.946132	2.1
25565	0.946131	2.04323
25566	0.94613	0.407008
25567	1.36546	0.970332
25568	1.36546	0.798043
25569	0.946131	0.758581
25570	0.946132	0.47379
25571	0.946133	0.564329
25572	0.946132	0.358589
25573	1.36899	0.408164
25574	0.946133	0.405243
25575	0.946131	0.242832

25576	0.946132	0.204886
25577	1.03543	0.209577
25578	1.1412	0.145147
25579	0.941275	0.267103
25580	1.28952	0.121402
25581	0.94613	0.14981
25582	0.946129	0.159043
25583	0.946132	0.116786
25584	0.94613	0.133704
25585	0.94613	2.1
25586	0.94613	2.1
25587	0.946129	2.1
25588	0.94613	2.1
25589	1.35966	2.1
25590	0.810946	2.0081
25591	0.810942	2.1
25592	0.810932	1.73691
25593	0.94613	1.9919
25594	1.40372	2.1
25595	0.946131	1.26899
25596	0.94613	2.1
25597	1.40372	2.1
25598	0.946133	2.1
25599	0.946131	2.1
25600	0.94613	2.1
25601	0.946131	2.1
25602	0.946131	2.1
25603	1.31453	2.1
25604	0.94613	2.1
25605	0.946128	2.1
25606	0.94613	2.1
25607	0.94613	2.1
25608	0.946131	0.111503
25609	1.12018	0.0985388
25610	0.94613	0.125347
25611	0.94613	0.145268
25612	0.94613	0.110637
25613	0.946131	0.163611
25614	1.16373	0.155297
25615	0.946131	0.23829
25616	0.94613	0.16384
25617	0.946131	0.178251
25618	0.946126	0.483432
25619	0.946132	0.521498
25620	0.946128	0.494988
25621	0.946129	0.525287
25622	0.946129	0.559384
25623	0.946132	0.38107
25624	0.946133	0.417345
25625	0.946132	0.362219
25626	0.946132	0.402544
25627	0.946133	0.403403
25628	0.946132	0.830743
25629	0.946129	1.23542
25630	0.94613	0.758761
25631	1.36399	0.888336
25632	0.946131	0.792432
25633	0.946132	2.1

25634	0.946132	2.1
25635	0.946133	2.1
25636	1.38302	2.1
25637	0.946133	2.1
25638	0.810943	2.1
25639	0.810944	2.1
25640	0.810945	2.1
25641	0.946132	2.1
25642	0.946131	2.1
25643	0.946132	2.1
25644	1.30188	2.1
25645	0.946132	2.1
25646	0.810946	0.718725
25647	0.72985	1.24292
25648	0.810934	0.842999
25649	0.856243	0.672545
25650	0.946129	0.641003
25651	0.946132	0.681799
25652	1.19686	0.613296
25653	0.851527	0.74673
25654	0.946131	2.1
25655	0.94613	2.1
25656	0.94613	2.1
25657	0.94613	2.1
25658	1.31961	2.1
25659	0.946131	2.1
25660	0.946131	2.1
25661	0.94613	2.1
25662	0.946131	2.1
25663	1.3605	2.1
25664	1.30116	2.1
25665	0.946133	0.76501
25666	0.946131	2.05933
25667	0.946131	1.09662
25668	0.94613	1.78494
25669	0.946132	0.126644
25670	1.3035	0.076991
25671	0.94613	0.131196
25672	0.946131	0.0972767
25673	0.946131	0.130451
25674	0.946132	2.1
25675	1.37869	2.1
25676	1.2051	2.1
25677	0.946132	2.1
25678	1.2051	2.1
25679	0.810946	2.1
25680	0.810945	2.1
25681	0.810946	2.1
25682	1.34623	2.1
25683	0.946131	2.1
25684	0.946131	2.1
25685	1.34623	2.1
25686	1.22993	2.1
25687	1.32299	0.0654211
25688	0.946132	0.085902
25689	0.946132	0.0979667
25690	0.946132	0.074768
25691	0.946131	0.0834334

25692	0.94613	0.295301
25693	1.40574	0.2664
25694	0.946128	0.375791
25695	0.946131	0.387968
25696	1.40574	0.368666
25697	0.946132	2.1
25698	1.41331	2.1
25699	0.94613	2.1
25700	0.946131	2.1
25701	1.41331	2.1
25702	0.94613	2.1
25703	1.37609	2.1
25704	0.946129	2.1
25705	0.946131	2.1
25706	1.37609	2.1
25707	1.039	0.946941
25708	0.946131	1.79669
25709	0.944514	1.26546
25710	0.946132	1.66751
25711	0.946132	1.43159
25712	1.23726	0.928008
25713	0.946128	1.39732
25714	0.946128	1.18696
25715	0.946129	1.45672
25716	0.94613	1.26836
25717	0.946129	2.00244
25718	0.946131	2.1
25719	0.946131	1.88449
25720	1.39214	2.1
25721	0.94613	2.1
25722	0.946118	0.604727
25723	0.946118	0.397419
25724	1.41078	0.273707
25725	1.41076	0.119618
25726	0.946131	0.359283
25727	0.946131	0.762692
25728	0.946131	0.648574
25729	0.946132	0.692319
25730	0.94613	0.605681
25731	0.946131	0.574614
25732	0.946132	0.0662579
25733	0.946132	0.0502732
25734	0.946133	0.05
25735	1.28924	0.05
25736	0.946132	0.0549891
25737	0.946133	0.518414
25738	1.09812	0.390608
25739	0.946132	0.537999
25740	0.946132	0.54296
25741	1.09812	0.555299
25742	0.946132	0.684467
25743	0.946132	0.656817
25744	0.946132	0.66966
25745	0.946132	0.655652
25746	1.15388	0.686518
25747	0.946132	0.49135
25748	0.974688	0.430521
25749	0.946132	0.497769

25750	0.946132	0.489533
25751	0.974687	0.502814
25752	1.0644	1.07696
25753	0.946133	1.75143
25754	0.946132	1.0644
25755	0.946133	1.14295
25756	1.26547	1.25959
25757	1.02905	0.975701
25758	0.911537	1.0187
25759	0.838038	1.196
25760	0.946131	0.290416
25761	0.94613	0.344478
25762	0.946132	0.283031
25763	0.946131	0.33489
25764	1.33922	0.331905
25765	0.946132	0.111469
25766	0.946132	0.0809737
25767	1.40926	0.0874537
25768	1.40927	0.0856871
25769	0.946132	0.0820775
25770	0.946129	1.98894
25771	0.946131	1.86688
25772	0.946131	1.08093
25773	0.946131	0.884902
25774	1.40496	1.41167
25775	0.94613	0.779243
25776	0.94613	0.74374
25777	0.946127	0.742826
25778	0.946128	0.672579
25779	1.38914	0.701738
25780	2.675	0.05
25781	1.22266	0.05
25782	0.946131	0.05
25783	0.946132	0.05
25784	0.946132	0.05
25785	1.22266	0.05
25786	0.946124	0.132508
25787	0.946128	0.0736054
25788	0.946126	0.0644194
25789	1.21428	0.0694141
25790	0.946127	0.112637
25791	0.946131	0.105228
25792	0.946131	0.232645
25793	0.946132	0.119431
25794	0.946129	0.0948552
25795	0.940118	0.185479
25796	0.810943	2.1
25797	0.810945	2.1
25798	0.810958	2.1
25799	0.946133	0.309171
25800	1.11539	0.286693
25801	0.946132	0.32184
25802	0.946132	0.315743
25803	0.946133	0.311512
25804	0.946133	2.1
25805	1.41195	2.1
25806	0.946133	2.1
25807	0.946132	2.1

25808	0.946133	2.1
25809	1.15968	0.05
25810	0.946131	0.05
25811	0.946131	0.0527765
25812	0.946131	0.05
25813	0.946131	0.05
25814	0.94613	2.08777
25815	0.946128	2.1
25816	0.946132	1.22252
25817	0.94613	1.94632
25818	0.94613	1.54065
25819	0.946131	1.81914
25820	1.04211	1.80735
25821	0.850155	1.09438
25822	0.946131	1.51874
25823	0.946131	1.28194
25824	0.946131	0.484031
25825	0.94613	0.407299
25826	0.946129	0.486345
25827	0.946131	0.434561
25828	0.946131	0.419115
25829	1.40193	0.213126
25830	1.19756	0.254485
25831	0.561936	0.296544
25832	0.808778	0.296389
25833	1.19756	0.272271
25834	0.946132	0.0589108
25835	0.946132	0.0546245
25836	1.33916	0.05
25837	1.33916	0.05
25838	0.946133	0.0512944
25839	0.946127	0.471837
25840	0.946127	0.415187
25841	0.946131	0.459819
25842	0.946128	0.438039
25843	0.946129	0.433072
25844	0.589428	0.562288
25845	0.851501	0.430267
25846	0.868924	0.518242
25847	0.877703	0.523608
25848	0.85153	0.501727
25849	0.946132	1.01118
25850	1.25372	2.1
25851	0.946132	1.13573
25852	0.946132	1.63567
25853	1.25372	2.06497
25854	0.946132	0.761184
25855	0.946128	1.9434
25856	0.94613	0.907842
25857	0.94613	1.03762
25858	1.2778	1.51959
25859	0.946118	0.692476
25860	1.35629	0.707945
25861	0.946117	0.713779
25862	0.946131	0.680091
25863	0.946105	0.622541
25864	0.946132	0.525047
25865	0.946132	0.676497

25866	1.38646	0.67731
25867	1.38646	0.711738
25868	0.946132	0.730683
25869	0.946128	2.09194
25870	0.946128	0.60485
25871	1.39659	0.945667
25872	1.39659	0.768556
25873	0.946129	0.731867
25874	0.946132	1.58869
25875	0.946131	1.28119
25876	0.946132	1.30246
25877	0.946132	1.35554
25878	1.15139	1.18689
25879	0.946133	2.1
25880	0.946132	1.84664
25881	0.946132	2.1
25882	0.946132	2.1
25883	1.36151	2.1
25884	0.946127	2.1
25885	0.946131	2.1
25886	0.946128	2.1
25887	0.94613	2.1
25888	1.38397	2.1
25889	0.946131	0.357241
25890	1.31112	0.321778
25891	0.946127	0.437921
25892	0.946128	0.483557
25893	1.31112	0.373721
25894	1.29778	0.05
25895	0.883417	0.05
25896	1.32613	0.05
25897	0.94613	0.05
25898	0.94613	0.05
25899	0.946132	2.1
25900	0.94613	2.1
25901	0.946131	2.1
25902	0.946131	2.1
25903	1.36523	2.1
25904	0.946132	0.162056
25905	1.01653	0.129319
25906	0.946132	0.232441
25907	0.946131	0.211626
25908	0.946131	0.157599
25909	0.94612	2.1
25910	1.08301	1.31363
25911	0.946119	2.1
25912	0.946132	2.1
25913	1.08299	1.77686
25914	0.810942	2.1
25915	0.810944	0.768761
25916	0.810957	0.899466
25917	0.946126	0.294286
25918	0.946132	0.329237
25919	0.946129	0.295464
25920	1.3492	0.259405
25921	0.94613	0.288249
25922	0.946131	0.273101
25923	1.24766	0.213776

25924	0.946128	0.402167
25925	0.946131	0.436543
25926	1.24766	0.346039
25927	0.946132	0.05
25928	0.946131	0.05
25929	0.946131	0.0507095
25930	1.23351	0.05
25931	0.946132	0.05
25932	0.946131	0.0506788
25933	0.946132	0.05
25934	0.946131	0.0617891
25935	1.35657	0.05
25936	0.946131	0.0511696
25937	0.946132	0.501437
25938	1.3689	0.399435
25939	0.946132	0.485737
25940	0.946132	0.492309
25941	1.3689	0.453399
25942	0.946133	0.412235
25943	1.35822	0.33029
25944	0.946133	0.376291
25945	0.946132	0.399249
25946	1.35822	0.371377
25947	0.946131	0.671531
25948	0.946131	1.13491
25949	0.946131	1.06312
25950	0.946131	0.794197
25951	1.4101	1.39039
25952	0.810944	1.46383
25953	0.810939	1.47737
25954	0.810955	1.08536
25955	0.946132	2.1
25956	1.37733	2.1
25957	0.946132	2.1
25958	0.946131	2.1
25959	0.946131	2.1
25960	1.32582	2.1
25961	0.946132	1.63781
25962	0.946132	0.966836
25963	0.946132	1.89135
25964	0.946132	2.1
25965	0.946132	1.88376
25966	0.946132	2.07677
25967	1.31401	2.1
25968	1.31401	2.1
25969	0.946131	2.1
25970	1.3469	1.24469
25971	0.946132	1.48581
25972	0.946132	1.68163
25973	0.946133	0.915918
25974	0.946132	1.07673
25975	0.946126	2.1
25976	0.946127	2.1
25977	0.94613	2.1
25978	0.946128	2.1
25979	1.41206	2.1
25980	0.946127	1.11503
25981	1.40109	1.93052

25982	0.946128	1.14671
25983	0.946129	1.82687
25984	1.40109	1.67709
25985	1.24448	0.107077
25986	0.94613	0.227483
25987	0.946132	0.151218
25988	0.946129	0.20591
25989	0.946131	0.175481
25990	1.11015	0.144865
25991	0.946133	0.421961
25992	0.946132	0.264113
25993	0.946133	0.323218
25994	0.946132	0.317974
25995	0.946132	2.1
25996	0.946131	2.1
25997	0.946131	2.1
25998	1.38775	2.1
25999	0.946131	2.1
26000	0.946133	0.621049
26001	0.946131	0.673937
26002	1.30598	0.995219
26003	1.30598	0.858152
26004	0.946131	0.838783
26005	0.946132	2.1
26006	0.946132	2.1
26007	0.946132	2.1
26008	1.40774	2.1
26009	0.946132	2.1
26010	0.798396	0.05
26011	0.946133	0.774541
26012	0.946128	1.35114
26013	0.946131	1.02393
26014	0.946131	1.00897
26015	1.28918	1.06981
26016	0.946132	0.4061
26017	1.40668	0.385834
26018	0.94613	0.389794
26019	0.94613	0.386011
26020	0.94613	0.389448
26021	0.810946	2.1
26022	0.810945	2.1
26023	0.810933	2.1
26024	0.946131	2.1
26025	1.30338	2.1
26026	0.94613	2.1
26027	1.30339	2.1
26028	0.946131	2.1
26029	0.946127	0.830843
26030	0.87161	1.25846
26031	0.946128	0.904634
26032	0.946129	1.07734
26033	0.827876	0.824186
26034	0.946131	2.1
26035	1.40796	2.1
26036	0.94613	2.1
26037	0.94613	2.1
26038	1.40796	2.1
26039	0.946132	0.670721

26040	0.946132	0.7545
26041	0.946132	0.659041
26042	0.946132	0.626046
26043	1.41724	0.590511
26044	1.34254	0.281035
26045	0.946132	0.622775
26046	0.946132	0.563409
26047	0.946132	0.536626
26048	0.946133	0.496809
26049	0.946133	1.71668
26050	0.946132	1.23055
26051	0.946132	1.24245
26052	1.26501	1.682
26053	0.946132	1.14073
26054	0.946131	0.117748
26055	1.35169	0.12469
26056	0.946131	0.164059
26057	0.946131	0.139833
26058	1.35169	0.127662
26059	0.676844	0.075109
26060	0.946131	0.0884926
26061	0.94613	0.114463
26062	1.13242	0.069405
26063	1.13242	0.0879146
26064	0.946131	0.0937338
26065	0.946128	0.953211
26066	1.28828	1.81575
26067	0.946131	1.04859
26068	0.946131	1.11166
26069	1.28828	1.40487
26070	0.946131	0.478403
26071	1.2996	0.447256
26072	0.946133	0.479346
26073	0.946131	0.459731
26074	0.946131	0.454284
26075	0.946132	0.899569
26076	1.40638	2.1
26077	0.946131	0.784046
26078	0.946132	2.1
26079	1.40638	2.1
26080	2.84909	0.0592876
26081	1.35726	0.0645753
26082	0.946131	0.0911517
26083	0.946131	0.0901173
26084	0.946131	0.0796483
26085	0.946131	0.083435
26086	1.39852	2.02871
26087	0.946132	1.70335
26088	0.94613	2.1
26089	0.94613	2.09503
26090	0.94613	2.00729
26091	0.946126	0.0847489
26092	0.946131	0.0798758
26093	0.946128	0.109816
26094	1.31666	0.087182
26095	0.946128	0.0917661
26096	0.946126	0.132354
26097	0.946131	0.107493

26098	0.946129	0.172658
26099	1.32275	0.103521
26100	0.946129	0.14075
26101	0.946131	2.1
26102	0.946132	2.1
26103	1.41125	2.1
26104	0.946131	2.1
26105	0.946131	2.1
26106	0.946131	2.1
26107	0.946132	2.1
26108	1.31904	2.1
26109	0.946132	2.1
26110	0.946132	2.1
26111	0.946129	0.273612
26112	0.946132	0.251345
26113	0.94613	0.253229
26114	1.02426	0.165097
26115	0.9311	0.317273
26116	0.946128	1.67497
26117	0.946133	1.07895
26118	0.946131	1.37216
26119	1.40092	1.51158
26120	0.946131	1.19243
26121	0.946117	0.554419
26122	0.946118	1.41834
26123	0.946118	0.540175
26124	1.40017	0.684264
26125	0.946105	0.6095
26126	0.946132	0.418202
26127	1.03547	0.386555
26128	0.946132	0.36857
26129	0.946133	0.439715
26130	0.946132	0.405587
26131	0.946133	2.1
26132	0.946132	2.1
26133	0.946133	2.1
26134	0.946133	2.1
26135	1.36694	2.1
26136	0.946132	0.321741
26137	1.22878	0.32582
26138	0.946132	0.327207
26139	0.946132	0.322489
26140	1.22878	0.32429
26141	1.11014	0.148442
26142	0.913078	0.17428
26143	1.11015	0.250862
26144	0.49154	0.249753
26145	0.946127	2.1
26146	0.946131	2.1
26147	0.946128	2.1
26148	0.946129	2.1
26149	1.38752	2.1
26150	0.946132	2.1
26151	1.37277	2.1
26152	0.946132	2.1
26153	0.946131	2.1
26154	1.37277	2.1
26155	0.94613	0.484146

26156	1.16834	0.414604
26157	0.946129	0.487735
26158	0.94613	0.441321
26159	0.94613	0.435089
26160	0.946127	0.491977
26161	0.946131	0.438662
26162	1.35594	0.41883
26163	0.946128	0.498898
26164	0.94613	0.445574
26165	0.946128	0.831322
26166	0.946128	0.400889
26167	1.38464	0.551708
26168	1.38463	0.472377
26169	0.94613	0.483953
26170	0.946129	0.82694
26171	0.946131	0.417412
26172	0.94613	0.397505
26173	1.39751	0.35538
26174	0.946131	0.437886
26175	1.25837	0.190603
26176	1.21134	0.16693
26177	0.700013	0.250581
26178	0.700041	0.239527
26179	1.40583	1.56158
26180	0.946132	2.1
26181	0.946132	2.1
26182	0.946133	1.98672
26183	0.946132	1.76445
26184	0.827865	2.06271
26185	0.946131	2.1
26186	0.946131	1.62003
26187	0.946131	1.90671
26188	0.946131	1.50161
26189	0.946132	0.0817134
26190	1.21087	0.0619422
26191	0.94613	0.0768978
26192	0.94613	0.0635951
26193	0.946131	0.0713134
26194	0.94613	0.617578
26195	1.17452	0.767845
26196	0.946132	0.623883
26197	0.94613	0.700326
26198	0.94613	0.608365
26199	0.946119	0.537022
26200	1.27374	0.583556
26201	0.946119	0.538319
26202	0.946131	0.559067
26203	0.946106	0.528111
26204	0.946133	2.1
26205	0.946132	2.1
26206	0.946133	2.1
26207	0.946132	2.1
26208	1.39621	2.1
26209	1.34352	2.1
26210	0.946132	2.1
26211	0.946132	2.1
26212	0.946132	2.1
26213	0.946132	2.1

26214	0.946132	0.74765
26215	0.946128	2.06677
26216	0.94613	1.09347
26217	0.94613	1.5869
26218	1.41273	1.93262
26219	1.23536	2.1
26220	0.946131	1.06866
26221	0.946132	2.1
26222	0.946132	1.81181
26223	0.946132	2.05132
26224	0.946132	2.1
26225	1.41131	2.1
26226	0.946131	2.1
26227	0.946131	2.1
26228	0.946131	2.1
26229	0.946133	0.569919
26230	0.946132	1.64404
26231	0.946133	0.556292
26232	1.41894	0.524913
26233	0.946132	0.482467
26234	0.946128	0.626582
26235	1.4099	0.916444
26236	0.946131	0.681756
26237	0.946131	1.93232
26238	0.946131	0.550465
26239	0.810947	0.997792
26240	0.810945	1.08055
26241	0.810933	1.80173
26242	0.946131	0.714457
26243	1.25765	2.09998
26244	0.946128	1.04337
26245	0.946129	0.684081
26246	1.25765	1.02723
26247	1.21347	2.1
26248	0.946129	2.1
26249	0.94613	2.1
26250	1.21347	2.1
26251	0.94613	2.1
26252	0.94613	0.05
26253	0.946132	0.05
26254	1.2493	0.05
26255	1.2493	0.05
26256	0.94613	0.05
26257	0.946132	0.81561
26258	0.946128	1.19472
26259	0.946131	0.820153
26260	0.94613	0.972154
26261	1.37938	0.908188
26262	0.946133	0.0875276
26263	0.946132	0.0790962
26264	0.946133	0.0831131
26265	0.946132	0.0589083
26266	0.946133	0.0866368
26267	0.946129	0.666871
26268	1.26426	0.577039
26269	0.946131	0.656208
26270	0.946131	0.674912
26271	1.26426	0.635525

26272	0.872447	1.85112
26273	0.946132	1.20577
26274	0.946132	1.45472
26275	0.946132	1.70355
26276	0.946133	1.30503
26277	0.946117	1.19592
26278	0.946116	1.03611
26279	0.946113	0.974308
26280	0.946129	1.14354
26281	1.27211	0.881378
26282	0.94613	0.429579
26283	0.946129	0.335211
26284	1.40029	0.462871
26285	1.40028	0.451609
26286	0.94613	0.495275
26287	0.810942	2.1
26288	0.810943	2.1
26289	0.810932	2.1
26290	1.29521	2.1
26291	0.946129	2.1
26292	0.946131	2.1
26293	1.29521	2.1
26294	1.29521	2.1
26295	0.946132	0.535676
26296	0.946129	0.46197
26297	1.24596	0.459727
26298	0.946131	0.534932
26299	0.946131	0.479718
26300	0.946131	2.1
26301	1.35616	2.1
26302	0.946133	2.1
26303	0.94613	2.1
26304	1.35616	2.1
26305	0.879548	0.735924
26306	1.01272	0.783315
26307	0.946133	0.800762
26308	0.946133	0.684932
26309	0.946132	0.797814
26310	0.946132	0.909829
26311	0.918009	0.669522
26312	0.91801	1.08919
26313	0.946132	0.833872
26314	0.946132	0.975207
26315	0.946132	0.0931468
26316	1.28644	0.0799696
26317	0.946131	0.118708
26318	0.946131	0.110035
26319	0.946132	0.0977445
26320	0.946131	0.0904834
26321	1.40103	0.0867192
26322	0.946131	0.13001
26323	0.946131	0.121412
26324	0.946131	0.104131
26325	0.946128	0.278312
26326	1.03327	0.206991
26327	0.946129	0.314791
26328	0.946131	0.217008
26329	0.94613	0.250632

26330	0.946132	0.419259
26331	0.946133	0.0852219
26332	1.40265	0.329168
26333	1.40265	0.237533
26334	0.946133	0.325004
26335	0.946131	0.0560571
26336	0.946131	0.0614814
26337	0.946131	0.0682722
26338	0.856655	0.05
26339	0.856616	0.0515031
26340	0.946132	0.459199
26341	0.946132	0.482779
26342	0.946132	0.580505
26343	1.15844	0.430579
26344	0.946131	0.552039
26345	0.946131	2.1
26346	1.4031	2.1
26347	1.40304	2.1
26348	0.851554	2.1
26349	0.946131	0.0546091
26350	0.94613	0.0801236
26351	1.24417	0.05
26352	1.24416	0.0540898
26353	0.946131	0.0596741
26354	0.946132	0.621586
26355	1.20992	0.658821
26356	0.946132	0.549979
26357	0.946132	0.693555
26358	1.20992	0.699059
26359	0.946128	0.838769
26360	0.94613	0.93053
26361	0.94613	0.857956
26362	0.94613	0.880543
26363	1.23426	0.948061
26364	0.810943	0.531146
26365	0.810946	0.383541
26366	0.810933	0.408417
26367	0.810945	2.1
26368	0.810945	0.565728
26369	0.810932	0.773741
26370	1.25104	0.968504
26371	0.946131	2.1
26372	0.946131	0.633116
26373	1.25104	0.943014
26374	1.25104	0.692561
26375	1.25488	1.89216
26376	0.946132	2.05298
26377	0.946132	1.89198
26378	0.946132	2.1
26379	0.946131	2.1
26380	0.946131	0.0577499
26381	0.946126	0.0800996
26382	0.946128	0.0583004
26383	1.32131	0.0648651
26384	0.946128	0.0713493
26385	0.946133	2.01813
26386	0.946132	0.835322
26387	0.946132	2.1

26388	0.946133	1.90485
26389	1.41123	1.04333
26390	0.946132	0.199929
26391	0.946133	0.214555
26392	0.946132	0.292983
26393	1.32163	0.16678
26394	0.946133	0.263401
26395	0.946132	0.725339
26396	0.946132	1.70252
26397	1.38651	1.9907
26398	1.38651	1.94499
26399	0.946132	1.9847
26400	0.94613	0.350691
26401	1.3639	0.342787
26402	0.946128	0.359789
26403	0.946131	0.347199
26404	1.3639	0.34199
26405	0.946133	0.680174
26406	1.40724	0.529218
26407	0.946132	0.4817
26408	0.946133	0.55476
26409	1.40724	0.552002
26410	1.21099	1.14736
26411	0.946128	1.41786
26412	0.946127	2.09961
26413	0.946128	1.90412
26414	0.946129	1.2907
26415	0.946132	0.59397
26416	1.35946	0.568339
26417	0.946131	0.707255
26418	0.946131	0.68998
26419	1.35946	0.633641
26420	1.34794	0.643453
26421	0.517994	1.21413
26422	0.946132	0.701744
26423	0.946132	0.855495
26424	0.517965	0.917967
26425	1.23224	0.695967
26426	0.94613	1.64412
26427	0.946131	1.01785
26428	0.946131	0.865755
26429	0.946131	0.814742
26430	1.37511	0.949672
26431	0.946132	1.06389
26432	0.946133	1.81211
26433	0.946133	1.56158
26434	0.946132	1.06574
26435	0.851106	0.629869
26436	0.946129	0.661816
26437	0.946131	0.836215
26438	0.946131	1.01063
26439	0.851066	0.85747
26440	0.946131	0.168243
26441	0.946131	0.359873
26442	0.94613	0.24145
26443	1.31357	0.135945
26444	0.946131	0.273643
26445	0.946133	0.8619

26446	0.946132	1.13872
26447	0.946133	0.80062
26448	0.946132	0.964633
26449	1.38149	0.860449
26450	0.810944	0.787637
26451	0.810945	1.06181
26452	0.810958	2.02215
26453	0.946118	0.05
26454	0.946118	0.0522893
26455	1.37835	0.05
26456	1.37837	0.05
26457	0.946106	0.05
26458	0.946128	0.0760974
26459	0.946131	0.0711975
26460	1.34831	0.0719478
26461	1.34831	0.0523936
26462	0.94613	0.0943314
26463	0.946132	0.0782982
26464	0.946132	0.136091
26465	0.909981	0.0844974
26466	1.00099	0.088114
26467	0.946131	0.112148
26468	0.946132	0.74703
26469	0.946133	1.27905
26470	0.946133	0.86783
26471	0.946132	0.955968
26472	1.35328	0.953341
26473	0.946132	0.0555061
26474	0.946132	0.0504613
26475	1.35386	0.0679538
26476	1.35385	0.05
26477	0.946133	0.0660058
26478	0.946121	0.0513861
26479	0.946132	0.05
26480	0.900167	0.05
26481	1.01189	0.05
26482	0.946132	0.0506781
26483	0.946132	2.1
26484	1.41585	1.1166
26485	0.946131	1.14863
26486	0.946131	1.0937
26487	0.94613	1.06218
26488	0.810946	2.1
26489	0.810946	2.1
26490	0.810933	2.1
26491	1.34447	2.1
26492	0.946131	2.1
26493	0.946131	2.1
26494	1.34447	2.1
26495	1.19886	2.1
26496	0.946132	0.10163
26497	0.946132	0.0593641
26498	0.946132	0.0622436
26499	1.35065	0.0570562
26500	0.946132	0.0949206
26501	0.946131	0.516158
26502	1.3022	0.518355
26503	0.94613	0.672654

26504	0.946131	0.622187
26505	1.3022	0.561494
26506	0.946127	0.354019
26507	1.38683	0.328971
26508	0.946127	0.374677
26509	0.946128	0.375123
26510	1.38683	0.371278
26511	0.810946	0.05
26512	0.810947	0.05
26513	0.810959	0.05
26514	0.946132	0.05
26515	0.946131	0.05
26516	0.946132	0.05
26517	0.946132	0.05
26518	0.946132	0.05
26519	0.946127	1.00993
26520	1.35968	1.75428
26521	0.946131	0.946161
26522	0.946129	0.919116
26523	1.35969	1.53499
26524	0.946133	2.09144
26525	1.35862	1.50635
26526	0.946132	2.1
26527	0.946132	0.993943
26528	1.35862	1.39113
26529	0.946132	0.898498
26530	0.946132	0.790788
26531	0.946132	0.980464
26532	0.946131	0.701234
26533	0.946132	0.766944
26534	0.946131	0.413602
26535	0.946131	0.40241
26536	0.946128	0.418785
26537	0.946129	0.377867
26538	1.41232	0.392154
26539	0.946128	0.949838
26540	1.29331	1.69408
26541	0.946131	1.01152
26542	0.946131	1.33867
26543	1.29331	1.25431
26544	0.946133	0.642337
26545	0.946132	0.680782
26546	0.946132	0.642227
26547	0.946132	0.612306
26548	1.41556	0.587876
26549	0.94612	0.608551
26550	0.946119	0.598262
26551	0.946119	0.608996
26552	0.946132	0.56167
26553	0.946107	0.571967
26554	0.946129	0.0950899
26555	1.1509	0.063681
26556	0.946126	0.0865587
26557	0.946129	0.0633813
26558	0.94613	0.0865194
26559	0.946131	0.0684428
26560	1.19263	0.0513758
26561	0.946132	0.0598271

26562	0.946132	0.05
26563	0.946132	0.0581215
26564	0.94613	0.595815
26565	1.36939	0.700649
26566	0.946128	0.622695
26567	0.946131	0.68365
26568	1.36939	0.663938
26569	0.946133	0.875837
26570	0.827865	1.61252
26571	0.946132	0.904011
26572	0.946132	1.1723
26573	1.40398	1.15692
26574	0.946127	0.449381
26575	0.946127	0.551253
26576	0.94613	0.409781
26577	1.41498	0.482518
26578	0.946128	0.473271
26579	0.94613	0.05
26580	0.946131	0.05
26581	0.946129	0.05
26582	0.94613	0.05
26583	0.94613	0.05
26584	0.946118	0.053269
26585	0.946119	0.05
26586	0.946116	0.05
26587	1.36569	0.05
26588	0.946105	0.05
26589	0.946132	0.419223
26590	1.23175	0.365563
26591	0.94613	0.416406
26592	0.946133	0.360525
26593	0.946131	0.394408
26594	1.25698	0.05
26595	0.946126	0.0629596
26596	0.946129	0.06085
26597	0.946128	0.0555991
26598	0.946129	0.0582317
26599	0.810943	2.1
26600	0.810944	2.1
26601	0.810932	2.1
26602	0.946132	0.307157
26603	1.09841	0.308927
26604	0.946131	0.444263
26605	0.946132	0.386951
26606	1.09841	0.37669
26607	0.810936	0.739585
26608	0.810935	0.659137
26609	0.810934	0.964759
26610	1.23851	2.02732
26611	0.946132	0.781012
26612	0.946131	0.804197
26613	1.23851	2.1
26614	1.23851	1.12919
26615	0.946132	0.17016
26616	0.946131	0.473563
26617	0.946132	0.193588
26618	0.946132	0.313007
26619	0.946132	0.526757

26620	0.939141	1.02798
26621	0.953118	1.4871
26622	0.946126	0.956662
26623	0.946129	1.04913
26624	1.03306	1.12892
26625	0.946132	0.333599
26626	1.30927	0.336631
26627	0.946132	0.343778
26628	0.946132	0.335107
26629	1.30928	0.334734
26630	0.94613	2.1
26631	0.946131	2.1
26632	1.24511	2.1
26633	0.94613	2.1
26634	0.94613	2.1
26635	0.946133	0.274642
26636	1.34152	0.262952
26637	0.946131	0.308242
26638	0.946131	0.244817
26639	1.34152	0.269899
26640	0.946132	0.30465
26641	1.41383	0.295539
26642	0.946132	0.313061
26643	0.946132	0.288382
26644	1.41383	0.291907
26645	0.946132	0.605922
26646	0.946131	0.525325
26647	1.41392	0.484763
26648	0.946132	0.615883
26649	0.946132	0.533542
26650	0.946132	0.721932
26651	0.946132	0.649868
26652	1.24333	0.588482
26653	0.946133	0.794663
26654	0.946133	0.681321
26655	0.946124	0.265214
26656	0.94613	0.286481
26657	0.946126	0.322501
26658	1.3065	0.236436
26659	0.946127	0.309396
26660	0.810946	0.42325
26661	0.810943	0.304554
26662	0.81096	0.385217
26663	0.946131	0.187907
26664	1.40271	0.216721
26665	0.946131	0.295588
26666	0.946131	0.239051
26667	1.40271	0.22949
26668	0.946132	0.322701
26669	0.94613	0.332304
26670	0.946131	0.323084
26671	0.94613	0.325
26672	0.946131	0.328596
26673	0.946133	0.574167
26674	1.30107	0.592987
26675	0.946132	0.570189
26676	0.946132	0.595641
26677	0.946133	0.608758

26678	0.810946	2.1
26679	0.810944	2.1
26680	0.810958	2.1
26681	0.946133	1.05333
26682	0.946131	0.949235
26683	0.946131	2.1
26684	1.30812	1.31187
26685	0.946131	0.953341
26686	0.94613	0.79108
26687	0.975002	0.915062
26688	0.917259	1.64577
26689	1.00902	1.09669
26690	0.94613	0.727536
26691	0.946131	2.1
26692	0.946127	2.1
26693	1.398 2.1	
26694	0.946129	2.1
26695	0.946129	2.1
26696	0.946133	2.1
26697	0.946132	2.1
26698	0.946132	2.1
26699	0.946132	2.1
26700	1.3033	2.1
26701	1.09897	0.268892
26702	0.946131	0.816765
26703	0.946132	0.688672
26704	0.946129	0.661596
26705	0.946131	0.737757
26706	0.946131	2.1
26707	0.946132	2.1
26708	1.39858	2.1
26709	0.946131	2.1
26710	0.946131	2.1
26711	0.946119	0.654582
26712	1.08878	0.698856
26713	0.946119	1.2013
26714	1.0888	0.699711
26715	0.946107	1.14367
26716	0.946132	0.110797
26717	1.10733	0.0920178
26718	0.946132	0.14447
26719	0.946131	0.133426
26720	0.946132	0.12115
26721	0.946128	0.121572
26722	1.33671	0.0991899
26723	0.94613	0.142389
26724	0.94613	0.146247
26725	0.94613	0.125245
26726	0.810943	2.1
26727	0.810944	2.1
26728	0.810945	2.1
26729	1.35117	2.1
26730	0.946129	2.1
26731	0.94613	2.1
26732	1.35117	2.1
26733	1.27174	2.1
26734	0.946132	2.07563
26735	0.946133	1.96373

26736	0.997296	1.07308
26737	0.946133	1.40495
26738	0.946132	1.20219
26739	0.810943	2.1
26740	0.810944	0.95986
26741	0.810945	2.1
26742	0.810946	0.885602
26743	0.810943	1.81559
26744	0.810933	1.27471
26745	0.946127	1.03576
26746	0.946131	0.918483
26747	0.946128	1.02384
26748	0.94613	1.07319
26749	0.946129	1.12794
26750	0.946131	0.461884
26751	0.946131	0.394865
26752	0.946129	0.469827
26753	0.946131	0.402446
26754	0.94613	0.395929
26755	0.946133	0.364579
26756	0.946132	0.376577
26757	0.946132	0.363827
26758	0.946132	0.36072
26759	0.946133	0.37807
26760	0.946131	0.849704
26761	0.94613	0.729383
26762	0.946129	0.865558
26763	0.946131	0.750834
26764	0.946131	0.856023
26765	0.946132	0.82134
26766	0.946129	1.68955
26767	0.94613	1.05704
26768	0.946131	1.05426
26769	1.39356	1.27879
26770	0.810946	2.1
26771	0.810947	2.1
26772	0.810934	2.08038
26773	0.946125	0.0767086
26774	0.946127	0.0825277
26775	1.33798	0.0558775
26776	1.33797	0.0651682
26777	0.946128	0.0699911
26778	0.946131	0.0647837
26779	0.946131	0.0822041
26780	1.23146	0.0561852
26781	1.23146	0.0730271
26782	0.946131	0.0733654
26783	0.946129	2.1
26784	0.94613	2.1
26785	0.946131	2.1
26786	0.94613	2.1
26787	1.30082	2.1
26788	0.946131	0.111573
26789	0.946132	0.126206
26790	0.946131	0.144608
26791	1.20416	0.09389
26792	0.946132	0.139061
26793	0.946132	0.0959926

26794	0.946131	0.0988174
26795	0.946131	0.160547
26796	1.18711	0.0845104
26797	0.946132	0.125592
26798	0.946132	0.0516498
26799	0.946132	0.05
26800	0.946133	0.0502003
26801	0.946132	0.05
26802	0.946133	0.05
26803	0.946131	0.315214
26804	0.946132	0.31994
26805	0.94613	0.309345
26806	0.946131	0.31215
26807	0.946131	0.322752
26808	0.946133	0.21296
26809	0.946132	0.255543
26810	0.946133	0.19534
26811	0.946132	0.192474
26812	0.946132	0.233805
26813	0.946132	2.1
26814	0.946132	2.1
26815	0.946132	2.1
26816	1.36422	2.1
26817	0.946132	2.1
26818	0.946131	0.76566
26819	0.946132	1.47069
26820	0.946132	0.942277
26821	0.946132	0.975
26822	1.4185	1.37862
26823	0.946133	0.254827
26824	1.37794	0.158598
26825	0.946132	0.258385
26826	0.946133	0.17903
26827	0.946132	0.200441
26828	1.4045	2.1
26829	0.946132	2.1
26830	0.946132	2.1
26831	0.946132	2.1
26832	0.946132	2.1
26833	0.946131	2.1
26834	0.946132	2.1
26835	1.34285	2.1
26836	1.34285	2.1
26837	0.946132	2.1
26838	0.946131	2.1
26839	0.946132	2.1
26840	1.40739	2.1
26841	1.40739	2.1
26842	0.946131	2.1
26843	0.946128	2.1
26844	0.946132	2.1
26845	0.94613	2.1
26846	0.94613	2.1
26847	1.3164	2.1
26848	0.946132	1.08685
26849	1.3484	2.1
26850	0.946132	0.978324
26851	0.946132	1.98907

26852	1.3484	2.04837
26853	0.946132	2.1
26854	0.946132	2.1
26855	0.946133	2.1
26856	0.946133	2.1
26857	1.41507	2.1
26858	0.946132	2.1
26859	1.32608	2.1
26860	0.946131	2.1
26861	0.946131	2.1
26862	0.946132	2.1
26863	0.94613	0.873749
26864	1.34646	1.96073
26865	0.946129	0.927972
26866	0.94613	0.845609
26867	1.34646	1.31515
26868	0.946131	0.492984
26869	0.946131	1.85324
26870	0.94613	0.641631
26871	1.33728	0.522791
26872	0.946131	0.57619
26873	0.946127	1.47293
26874	0.946131	1.00279
26875	0.946128	1.99557
26876	0.946129	1.98657
26877	1.4064	1.36845
26878	0.946133	2.1
26879	0.946131	2.1
26880	0.946131	2.1
26881	0.946131	2.1
26882	0.94613	2.1
26883	1.34912	2.1
26884	0.946131	2.1
26885	0.946129	2.1
26886	0.946131	2.1
26887	0.94613	2.1
26888	0.946132	2.1
26889	1.40447	2.1
26890	0.946132	2.1
26891	0.946133	2.1
26892	1.40447	2.1
26893	1.33607	2.1
26894	0.946131	0.984059
26895	0.946127	2.1
26896	0.94613	2.1
26897	0.946129	2.1
26898	0.946117	0.661384
26899	1.32664	0.980326
26900	0.946115	0.750865
26901	0.946105	1.33839
26902	0.94613	0.542547
26903	0.946118	2.1
26904	0.946114	2.1
26905	1.35886	2.1
26906	1.35884	2.1
26907	0.946129	2.1
26908	0.946132	1.97927
26909	1.2571	2.1

26910	0.946132	2.1
26911	0.946132	2.1
26912	1.2571	2.1
26913	0.946133	2.1
26914	1.35663	2.1
26915	0.946132	2.1
26916	0.946132	2.1
26917	1.35663	2.1
26918	0.946133	0.798537
26919	0.946129	1.4918
26920	0.946131	1.09982
26921	0.946131	0.98334
26922	1.30881	1.82091
26923	0.946132	2.1
26924	0.94613	2.1
26925	1.34652	2.1
26926	0.94613	2.1
26927	0.94613	2.1
26928	0.946132	2.06622
26929	0.946133	1.32042
26930	1.36183	0.898871
26931	1.36183	0.982974
26932	0.946132	1.16583
26933	0.946132	0.393799
26934	1.38353	0.351451
26935	0.94613	0.347096
26936	0.94613	0.372299
26937	1.38354	0.360594
26938	0.946128	0.514615
26939	1.24887	0.347125
26940	0.94613	0.520194
26941	0.94613	0.563543
26942	1.24887	0.557853
26943	0.946129	0.823144
26944	0.946126	1.32643
26945	0.946124	0.861212
26946	0.946128	0.920651
26947	1.32056	1.07234
26948	0.946129	0.893928
26949	0.946128	1.45106
26950	0.946126	0.885471
26951	0.946129	1.0163
26952	1.20351	1.07598
26953	0.946131	0.965763
26954	0.946131	1.05193
26955	0.946127	1.2292
26956	1.25591	1.25352
26957	0.94613	0.818873
26958	0.946132	1.09545
26959	0.946133	1.03934
26960	0.946132	1.27466
26961	1.15436	0.958249
26962	0.930306	0.891685
26963	0.946132	2.1
26964	0.946132	2.1
26965	0.946132	1.75786
26966	0.946133	2.1
26967	0.946132	2.1

26968	0.946129	1.10651
26969	0.946132	0.842927
26970	0.946126	1.22075
26971	1.13245	0.721957
26972	0.946129	0.846221
26973	0.94613	2.1
26974	0.946132	2.1
26975	0.946129	1.60643
26976	0.946131	2.1
26977	0.946131	1.98917
26978	1.41279	1.90101
26979	1.76254	2.1
26980	0.600454	1.70956
26981	0.858991	1.68829
26982	0.940722	1.81336
26983	1.28439	1.26663
26984	0.946131	1.2169
26985	0.94613	1.36259
26986	0.946131	1.81967
26987	0.946131	1.77227
26988	0.946132	0.891536
26989	1.39335	0.54916
26990	0.946132	0.79547
26991	0.946132	0.585252
26992	0.946132	0.752867
26993	1.33879	0.29592
26994	0.946131	0.474249
26995	0.94613	0.67044
26996	1.33879	0.298301
26997	0.946131	0.49617
26998	0.946126	0.461542
26999	0.946131	0.459167
27000	1.37342	0.434301
27001	1.37343	0.418517
27002	0.946128	0.549152
27003	1.39168	0.0690322
27004	0.946131	0.0973803
27005	0.946131	0.11582
27006	0.946131	0.081695
27007	0.946131	0.0923918
27008	0.946131	0.388889
27009	1.412	0.35183
27010	0.946127	0.340369
27011	0.946128	0.362675
27012	0.946128	0.361566
27013	1.1387	0.182382
27014	0.943364	0.581374
27015	0.946132	0.39781
27016	0.946132	0.395567
27017	0.946133	0.438854
27018	1.21085	0.364506
27019	0.88324	0.867928
27020	0.946129	0.723656
27021	0.946131	0.779843
27022	0.946131	0.695369
27023	1.33587	0.0521669
27024	0.946125	0.0696644
27025	0.946129	0.0575857

27026	0.946126	0.072302
27027	0.946127	0.0619081
27028	1.27512	0.0718301
27029	0.946132	0.107938
27030	0.946132	0.116666
27031	0.946133	0.0996791
27032	0.946132	0.0809357
27033	0.946128	0.167377
27034	1.30498	0.172901
27035	0.946131	0.245624
27036	0.94613	0.207041
27037	1.30497	0.190457
27038	0.946131	0.137065
27039	1.25626	0.139444
27040	0.946131	0.190787
27041	0.946131	0.166965
27042	1.25626	0.156375
27043	0.946131	0.05
27044	0.94613	0.05
27045	1.35316	0.05
27046	0.946131	0.05
27047	0.946131	0.05
27048	0.94613	0.43644
27049	1.28727	0.408978
27050	0.946128	0.367095
27051	0.946131	0.40455
27052	0.94613	0.396462
27053	0.946131	0.236866
27054	1.39502	0.191192
27055	0.946132	0.322201
27056	0.946132	0.236853
27057	0.946132	0.298159
27058	1.36173	0.241512
27059	0.946131	0.68373
27060	0.946126	0.572505
27061	0.946127	0.605723
27062	0.946128	0.51201
27063	0.946129	0.863785
27064	0.946133	0.750892
27065	0.946131	0.886195
27066	0.946131	1.01116
27067	0.946131	0.933783
27068	0.946128	0.05
27069	0.946132	0.05
27070	1.24678	0.05
27071	0.94613	0.05
27072	0.94613	0.05
27073	0.946129	0.05
27074	0.94613	0.05
27075	1.25215	0.05
27076	0.946126	0.05
27077	0.946129	0.05
27078	0.946129	0.619757
27079	1.29689	0.604414
27080	0.946131	0.630405
27081	0.946131	0.58742
27082	0.946131	0.546362
27083	0.946128	0.180407

27084	0.94613	0.205407
27085	0.946131	0.143656
27086	0.94613	0.192887
27087	0.946131	0.165578
27088	0.946127	0.233639
27089	0.946128	0.292141
27090	0.94613	0.192953
27091	0.946129	0.260047
27092	0.946129	0.226772
27093	0.946133	0.05
27094	0.946132	0.0549122
27095	0.946132	0.0805452
27096	1.29957	0.0526363
27097	0.946133	0.059244
27098	0.946132	0.0588544
27099	0.946133	0.0560342
27100	0.946132	0.0757046
27101	1.26201	0.0597663
27102	0.946132	0.0638175
27103	0.946133	0.128679
27104	1.40427	0.108763
27105	0.946132	0.16066
27106	0.946132	0.165085
27107	0.946133	0.13387
27108	0.946132	0.115888
27109	1.35123	0.112212
27110	0.946132	0.144492
27111	0.946132	0.167185
27112	0.946131	0.130742
27113	0.946128	0.299335
27114	0.946129	0.356157
27115	0.946131	0.251356
27116	0.94613	0.340172
27117	0.946131	0.28803
27118	0.946127	0.498791
27119	1.23351	0.470448
27120	0.946131	0.501816
27121	0.946129	0.474446
27122	0.94613	0.468641
27123	0.946131	0.124571
27124	0.946131	0.182556
27125	0.94613	0.133885
27126	1.18119	0.126836
27127	0.946131	0.152897
27128	0.946132	0.424575
27129	1.30563	0.334256
27130	0.946133	0.439196
27131	0.946132	0.353913
27132	0.946133	0.399476
27133	0.946132	0.561633
27134	0.946132	0.240136
27135	0.946133	0.746783
27136	1.20256	0.348302
27137	0.946133	0.644364
27138	0.94613	0.652408
27139	1.33007	0.597469
27140	0.946128	0.691526
27141	0.946132	0.576129

27142	0.946131	0.606365
27143	0.946131	2.1
27144	0.946131	2.1
27145	0.946128	2.1
27146	0.946129	2.1
27147	0.94613	2.1
27148	0.810942	2.1
27149	0.810942	2.1
27150	0.81093	2.1
27151	0.946132	2.1
27152	1.39823	2.1
27153	0.946132	2.1
27154	0.946131	2.1
27155	0.946132	2.1
27156	0.946131	2.1
27157	0.946131	2.1
27158	0.946131	2.1
27159	1.37713	2.1
27160	0.946131	2.1
27161	0.946133	0.919966
27162	0.946131	1.74269
27163	1.40594	2.1
27164	1.40594	2.1
27165	0.94613	2.1
27166	0.946133	2.1
27167	0.946131	2.1
27168	1.38155	2.1
27169	1.38155	2.1
27170	0.946131	2.1
27171	0.946132	0.779161
27172	0.946133	1.7427
27173	0.946132	0.922523
27174	0.946132	0.900614
27175	1.33694	1.53027
27176	0.946131	1.92507
27177	1.228	2.1
27178	0.946129	2.08945
27179	0.946131	2.1
27180	1.228	2.1
27181	0.946132	0.989937
27182	0.946132	1.41646
27183	0.946131	1.83281
27184	0.946132	1.04484
27185	1.35943	1.79103
27186	0.946133	0.0898999
27187	0.946133	0.0780673
27188	1.02377	0.12794
27189	1.02377	0.0770321
27190	0.946132	0.0921659
27191	0.94613	0.373232
27192	0.946131	0.380782
27193	0.946132	0.417607
27194	1.29322	0.356443
27195	0.946131	0.407052
27196	0.946131	0.683599
27197	0.946131	0.20118
27198	0.946132	0.691099
27199	1.41492	0.173702

27200	0.946131	0.602963
27201	0.946132	2.1
27202	0.946131	2.1
27203	0.946132	2.1
27204	1.35028	2.1
27205	0.946132	2.1
27206	0.946132	2.1
27207	0.946132	2.01226
27208	0.946131	1.94148
27209	1.32461	2.09946
27210	0.946132	1.77027
27211	0.946131	2.1
27212	0.946132	2.1
27213	1.40369	2.1
27214	0.94613	2.1
27215	0.946131	2.1
27216	0.946132	0.193134
27217	0.979597	0.171848
27218	0.946131	0.236174
27219	0.946132	0.180561
27220	0.946132	0.203662
27221	0.946131	2.1
27222	0.946131	1.12026
27223	0.94613	2.1
27224	0.946131	2.1
27225	1.41897	2.1
27226	1.37791	2.1
27227	0.94613	2.1
27228	0.946129	2.1
27229	0.946131	2.1
27230	0.946131	2.1
27231	1.39384	2.1
27232	0.946131	2.1
27233	0.946128	2.1
27234	0.946129	2.1
27235	0.946129	2.1
27236	0.810945	1.31537
27237	0.810944	2.1
27238	0.810958	1.58452
27239	0.946128	2.1
27240	0.946132	2.1
27241	0.94613	2.1
27242	1.3335	2.1
27243	0.946131	2.1
27244	0.94613	0.498492
27245	0.946129	0.701381
27246	0.946132	0.368151
27247	1.41192	0.635998
27248	0.94613	0.408019
27249	0.946132	0.615945
27250	0.946133	0.559844
27251	0.946132	0.453527
27252	1.39428	0.448492
27253	0.946133	0.439615
27254	0.94613	1.56089
27255	0.946128	1.82857
27256	0.946132	0.920462
27257	0.94613	1.62288

27258	1.40684	1.78557
27259	0.946131	2.1
27260	0.946131	2.1
27261	0.946131	1.94797
27262	0.946131	1.53298
27263	1.39021	2.1
27264	0.946131	1.00165
27265	1.31649	2.0053
27266	0.946131	0.864945
27267	0.946131	0.894307
27268	1.31649	1.25858
27269	0.810946	2.1
27270	0.810946	1.7232
27271	0.810958	2.1
27272	1.16687	2.1
27273	0.946131	2.1
27274	0.946131	1.84106
27275	1.16687	2.1
27276	1.16687	2.1
27277	0.946128	0.86075
27278	1.3564	0.915688
27279	0.946131	0.905218
27280	0.946131	0.97759
27281	0.94613	0.740347
27282	0.946128	2.1
27283	1.35828	2.1
27284	0.94613	2.1
27285	0.946131	2.1
27286	0.94613	2.1
27287	0.946133	0.625815
27288	1.36477	0.71434
27289	0.946132	0.690047
27290	0.946132	0.713548
27291	1.36477	0.719406
27292	0.946132	0.878173
27293	1.31857	1.82198
27294	0.946132	1.08282
27295	0.946132	1.2551
27296	1.31857	1.23412
27297	0.94613	0.673974
27298	1.35059	0.448868
27299	0.946128	0.459585
27300	0.94613	0.535482
27301	1.35059	0.491987
27302	0.946126	0.338121
27303	1.35559	0.266876
27304	0.946127	0.29416
27305	0.946128	0.260259
27306	1.35559	0.275494
27307	0.946131	0.34052
27308	1.33035	0.315382
27309	0.946127	0.318999
27310	0.946128	0.325672
27311	1.33035	0.32365
27312	0.94613	0.337591
27313	1.3665	0.321491
27314	0.946129	0.323579
27315	0.946131	0.330897

27316	1.3665	0.324794
27317	0.94613	0.354997
27318	1.32828	0.336678
27319	0.946129	0.332227
27320	0.94613	0.343877
27321	0.946131	0.343645
27322	0.946131	2.1
27323	0.946131	0.988858
27324	1.32949	2.1
27325	1.32949	2.1
27326	0.946131	2.1
27327	0.946131	1.25692
27328	0.94613	0.372562
27329	0.946133	0.649148
27330	1.38357	0.450359
27331	0.946131	0.559675
27332	0.946131	2.1
27333	0.946128	2.1
27334	1.36919	2.1
27335	0.946131	2.1
27336	0.94613	2.1
27337	0.946131	0.782869
27338	1.3771	2.1
27339	0.946129	1.62228
27340	0.94613	1.6734
27341	0.946131	0.752281
27342	0.946131	1.13132
27343	0.94613	0.764913
27344	0.946129	0.59244
27345	1.30029	0.831441
27346	0.94613	0.749396
27347	1.21174	0.05
27348	0.946132	0.0589718
27349	0.946132	0.0674315
27350	0.946132	0.0550071
27351	0.946133	0.0569366
27352	0.810946	2.1
27353	0.810945	2.1
27354	0.810946	2.1
27355	1.1796	2.1
27356	0.946131	2.1
27357	0.946132	2.1
27358	1.1796	2.1
27359	1.1796	2.1
27360	0.946131	2.1
27361	0.946131	2.1
27362	0.946133	2.1
27363	0.946131	2.1
27364	1.40615	2.1
27365	0.810945	2.1
27366	0.810944	2.1
27367	0.810945	2.1
27368	1.40239	2.1
27369	0.94613	2.1
27370	1.35561	2.1
27371	0.946133	2.1
27372	1.35562	2.1
27373	1.41169	0.27394

27374	0.946131	0.451783
27375	0.946127	0.674907
27376	1.41169	0.301929
27377	0.946128	0.500661
27378	0.946119	2.1
27379	0.946119	2.1
27380	0.946119	2.1
27381	0.946107	2.1
27382	1.41917	2.1
27383	0.810946	0.971976
27384	0.810943	1.03188
27385	0.810934	1.60775
27386	0.946127	2.1
27387	1.1995	2.1
27388	0.946126	2.1
27389	0.946128	2.1
27390	1.1995	2.1
27391	0.810944	2.1
27392	0.810947	2.1
27393	0.81096	2.1
27394	0.946133	0.382257
27395	1.3495	0.248914
27396	0.946132	0.352873
27397	0.946132	0.281766
27398	0.946133	0.320821
27399	0.946132	2.1
27400	0.946132	2.1
27401	0.946133	2.1
27402	1.39529	2.1
27403	0.946133	2.1
27404	0.946133	2.1
27405	1.28525	2.1
27406	0.946132	2.1
27407	0.946133	2.1
27408	1.28525	2.1
27409	0.810946	2.1
27410	0.810945	2.1
27411	0.810946	2.1
27412	1.33931	2.1
27413	0.946132	2.1
27414	0.946132	2.1
27415	1.33931	2.1
27416	1.24616	2.1
27417	0.946133	0.431041
27418	0.946133	0.445267
27419	0.946132	0.423429
27420	0.946132	0.42893
27421	0.946133	0.436094
27422	0.946128	2.1
27423	1.40225	2.1
27424	0.946128	2.1
27425	0.94613	2.1
27426	0.94613	2.07718
27427	0.946113	0.0630965
27428	0.946118	0.0540472
27429	0.946114	0.0671147
27430	1.40551	0.0538486
27431	0.946103	0.0548355

27432	0.94613	2.1
27433	0.946132	2.1
27434	0.946129	2.1
27435	0.946131	2.1
27436	1.37811	2.1
27437	0.946132	2.1
27438	1.39826	2.1
27439	0.946133	2.1
27440	0.946132	2.1
27441	1.39826	2.1
27442	0.946118	2.1
27443	1.28298	2.1
27444	0.946117	2.1
27445	0.946105	2.1
27446	1.283 2.1	
27447	0.946118	0.0539856
27448	1.41375	0.0579546
27449	0.946117	0.0868895
27450	0.946131	0.0723765
27451	0.946106	0.0684999
27452	0.946132	0.736132
27453	0.946129	0.627509
27454	1.28436	0.62472
27455	0.946131	0.740787
27456	0.946131	0.654766
27457	0.810947	0.472038
27458	0.810946	0.490783
27459	0.81096	0.463046
27460	0.946132	0.429247
27461	1.34338	0.421946
27462	0.961435	0.389028
27463	0.961435	0.416022
27464	0.946132	0.424394
27465	1.3028	1.53324
27466	0.946131	1.45122
27467	0.946127	1.9101
27468	0.946128	2.06461
27469	0.946129	1.50939
27470	0.946132	0.616122
27471	1.37973	0.525552
27472	0.946132	0.626114
27473	0.946132	0.535654
27474	0.946132	0.524005
27475	0.946131	0.790541
27476	0.94613	0.716134
27477	0.946129	0.791543
27478	0.946131	0.718821
27479	1.41798	0.696998
27480	0.946118	0.712105
27481	0.946118	0.639533
27482	0.946117	0.713649
27483	0.946131	0.649029
27484	0.946105	0.645136
27485	0.946131	0.843552
27486	0.946131	0.757363
27487	0.94613	0.840155
27488	0.946131	0.759245
27489	0.946131	0.716581

27490	0.946133	1.0054
27491	1.40274	2.1
27492	0.946133	0.808605
27493	0.946132	1.81865
27494	1.40274	1.75503
27495	0.946131	1.1196
27496	1.39731	1.99389
27497	0.946132	0.857362
27498	0.94613	1.02249
27499	1.39731	1.45266
27500	0.94612	2.00872
27501	0.94612	1.36268
27502	0.946119	1.29531
27503	0.946132	1.42231
27504	1.40764	1.82317
27505	0.946128	2.1
27506	0.946132	2.1
27507	0.94613	2.1
27508	0.94613	2.1
27509	1.31213	2.1
27510	0.946127	1.21177
27511	0.94613	2.1
27512	0.946127	1.23256
27513	0.946128	1.27105
27514	1.36653	1.99556
27515	0.946127	2.1
27516	1.3557	2.1
27517	0.946128	2.1
27518	0.946128	2.1
27519	1.3557	2.1
27520	0.946133	2.1
27521	0.946132	2.1
27522	0.946133	2.1
27523	0.946132	2.1
27524	1.40081	2.1
27525	0.946128	2.1
27526	0.946133	2.1
27527	0.946131	2.1
27528	0.946131	2.1
27529	1.32282	2.1
27530	0.810945	1.36996
27531	0.810943	1.39142
27532	0.810933	2.1
27533	1.27676	2.1
27534	0.94613	2.09152
27535	0.946128	2.1
27536	0.946131	2.1
27537	0.946131	2.1
27538	0.946131	0.506167
27539	0.946127	0.489234
27540	1.41328	0.605772
27541	1.41327	0.574396
27542	0.946128	0.553313
27543	0.946132	0.32129
27544	1.32083	0.325926
27545	0.946132	0.332332
27546	0.946133	0.327388
27547	1.32082	0.326991

27548	0.946128	0.328371
27549	1.29215	0.331295
27550	0.94613	0.338628
27551	0.94613	0.331818
27552	1.29215	0.331252
27553	0.946132	0.284837
27554	1.27696	0.291113
27555	0.94613	0.315954
27556	0.94613	0.320309
27557	1.27696	0.304742
27558	0.94612	0.300774
27559	1.29289	0.309886
27560	0.946119	0.324113
27561	0.946132	0.322322
27562	1.29287	0.317713
27563	0.946128	2.1
27564	1.37869	2.1
27565	0.946131	2.1
27566	0.94613	2.1
27567	0.946131	2.1
27568	0.946133	2.1
27569	0.946132	2.1
27570	0.946132	2.1
27571	1.35703	2.1
27572	0.946133	2.1
27573	0.946132	1.4154
27574	0.946132	2.1
27575	0.946133	0.806829
27576	1.37017	2.1
27577	0.946133	2.1
27578	0.946119	0.05
27579	0.94612	0.0530104
27580	1.38583	0.05
27581	1.38585	0.05
27582	0.946107	0.05
27583	0.946126	0.0799478
27584	0.946127	0.105049
27585	1.22904	0.0679601
27586	1.22904	0.0685756
27587	0.946128	0.0797752
27588	1.3279	0.750861
27589	0.946131	1.23102
27590	0.94613	1.02082
27591	0.946131	1.19455
27592	0.946131	1.01911
27593	0.946132	2.1
27594	0.946132	2.1
27595	0.946132	2.1
27596	0.946131	2.1
27597	1.40932	2.1
27598	1.26868	0.0787894
27599	0.946131	0.138049
27600	0.94613	0.125255
27601	0.946131	0.119217
27602	0.946131	0.119241
27603	0.810943	2.1
27604	0.810945	2.1
27605	0.810932	2.1

27606	0.810943	0.729183
27607	0.810944	0.48661
27608	0.810957	0.44637
27609	1.31539	0.531879
27610	0.946132	0.86905
27611	0.946128	0.326083
27612	1.3154	0.607966
27613	1.17856	0.448613
27614	1.21997	1.09509
27615	0.94613	1.47129
27616	0.946129	1.28937
27617	0.94613	1.74908
27618	0.94613	1.56385
27619	0.946131	0.963969
27620	0.946131	0.794103
27621	0.946129	1.00056
27622	0.94613	0.969337
27623	0.946131	1.08379
27624	0.946127	0.834047
27625	0.946131	0.617826
27626	0.946127	1.60093
27627	0.946129	0.965691
27628	1.41342	0.735643
27629	0.946131	0.742714
27630	0.946131	1.36966
27631	0.946128	1.622
27632	0.946131	0.821083
27633	1.29149	1.23863
27634	1.41022	2.1
27635	0.94613	0.774305
27636	0.946128	2.06832
27637	0.94613	1.78498
27638	0.94613	2.09594
27639	0.946128	0.313032
27640	0.94613	0.406211
27641	1.27864	0.299154
27642	1.27864	0.239384
27643	0.946131	0.407973
27644	0.946129	0.743413
27645	0.946125	1.43326
27646	0.946124	1.02018
27647	0.946127	0.860402
27648	1.37759	1.23955
27649	0.946129	0.783418
27650	0.94613	1.39093
27651	0.946126	0.904551
27652	0.946129	0.89533
27653	1.38259	1.11169
27654	0.946131	0.288516
27655	1.34118	0.294868
27656	0.946132	0.316002
27657	0.946132	0.324762
27658	1.34118	0.31363
27659	0.946131	0.128017
27660	1.25017	0.104446
27661	0.946128	0.125125
27662	0.946131	0.104824
27663	0.946131	0.10339

27664	0.946131	2.1
27665	0.946128	2.1
27666	0.946133	2.1
27667	1.3937	2.1
27668	0.94613	2.1
27669	0.946131	0.663068
27670	0.94613	0.417365
27671	1.40404	0.51797
27672	1.40404	0.439072
27673	0.946131	0.459661
27674	0.946132	0.496121
27675	0.946132	0.372323
27676	1.38897	0.362281
27677	1.38897	0.335926
27678	0.946132	0.361918
27679	0.94613	0.409375
27680	0.946128	0.489546
27681	1.40332	0.405253
27682	1.40331	0.448508
27683	0.94613	0.487745
27684	0.946132	0.485862
27685	0.946132	0.6656
27686	0.946133	0.5664
27687	1.41619	0.651021
27688	0.946132	0.555577
27689	0.946132	0.696551
27690	0.946132	2.1
27691	0.946132	1.31806
27692	1.38244	2.1
27693	0.946132	2.04995
27694	0.810946	0.943859
27695	0.810943	0.660639
27696	0.810934	0.59783
27697	0.810947	0.836986
27698	0.810943	0.565529
27699	0.810934	0.580299
27700	1.12786	1.01512
27701	0.946132	1.90753
27702	0.946132	1.45835
27703	0.946132	1.19243
27704	0.946133	1.14934
27705	0.946132	1.4629
27706	0.946132	1.53314
27707	0.946132	1.08408
27708	1.27806	1.52292
27709	0.946133	1.04877
27710	0.946132	2.1
27711	0.946132	2.1
27712	0.946133	2.1
27713	0.946132	2.1
27714	0.946133	2.1
27715	1.39514	1.51329
27716	0.946129	0.916876
27717	0.946124	1.23935
27718	0.946127	0.811128
27719	0.946127	1.34826
27720	0.94613	2.048
27721	0.946129	2.1

27722	0.946132	2.1
27723	1.3991	2.1
27724	0.94613	2.1
27725	0.946132	0.457207
27726	0.946133	1.00374
27727	0.946132	1.11751
27728	1.36835	0.614763
27729	0.946132	0.527212
27730	0.946132	2.1
27731	0.946132	2.1
27732	0.946133	2.1
27733	0.946132	2.1
27734	1.39047	2.1
27735	0.946132	0.0691373
27736	0.946128	0.0672324
27737	0.94613	0.0817353
27738	1.33174	0.0628338
27739	0.946131	0.0813364
27740	0.946128	0.109057
27741	0.946131	0.0950864
27742	0.94613	0.11715
27743	0.94613	0.0786379
27744	0.94613	0.119513
27745	0.946119	2.1
27746	0.946119	2.1
27747	1.29064	2.1
27748	0.946132	2.1
27749	0.946107	2.1
27750	0.946133	2.1
27751	0.94613	2.1
27752	1.30584	2.1
27753	0.946131	2.1
27754	0.946131	2.1
27755	0.946131	2.1
27756	0.946132	2.1
27757	0.94613	2.1
27758	0.946131	2.1
27759	1.39549	2.1
27760	0.913386	1.11407
27761	0.978873	0.967507
27762	0.946126	0.9569
27763	0.94613	1.16846
27764	1.00473	0.811485
27765	0.946132	0.722978
27766	0.946133	0.72684
27767	0.946132	0.704451
27768	0.946132	0.81078
27769	1.38894	0.658271
27770	0.946128	0.688186
27771	0.946131	0.665047
27772	0.94613	0.675655
27773	0.94613	0.688322
27774	1.23865	0.636614
27775	0.946126	1.18365
27776	0.946129	1.22835
27777	0.946124	0.948055
27778	0.946127	1.13903
27779	1.40248	0.931307

27780	0.946133	0.762488
27781	0.946132	0.834923
27782	0.946132	0.716256
27783	0.946133	0.840954
27784	1.1391	0.71427
27785	0.827862	0.651793
27786	0.946133	0.691893
27787	0.946131	0.829844
27788	0.946131	0.703897
27789	0.94613	0.678986
27790	0.946128	0.554959
27791	0.946132	0.642514
27792	0.94613	0.692317
27793	1.21388	0.507202
27794	0.94613	0.658296
27795	0.810943	0.407811
27796	0.810945	0.448954
27797	0.810932	0.433546
27798	0.946131	0.533178
27799	0.946131	0.50644
27800	0.946133	0.536195
27801	1.36748	0.391377
27802	0.946132	0.30941
27803	0.946128	0.334953
27804	0.946131	0.338534
27805	0.94613	0.358598
27806	1.35856	0.286388
27807	0.94613	0.349884
27808	0.946132	2.1
27809	0.946131	2.1
27810	0.946133	2.1
27811	0.946132	2.1
27812	0.946131	2.1
27813	0.946133	2.1
27814	0.946131	2.1
27815	1.39558	2.1
27816	1.39558	2.1
27817	0.94613	2.1
27818	0.946131	0.103952
27819	0.94613	0.147944
27820	0.946129	0.114625
27821	1.41388	0.0981731
27822	0.946131	0.13057
27823	0.946133	0.104736
27824	0.946132	0.239628
27825	0.946132	0.121548
27826	0.946133	0.116046
27827	0.93452	0.202199
27828	0.946129	2.1
27829	1.38897	2.1
27830	0.946131	2.1
27831	0.946131	2.1
27832	1.38897	2.1
27833	0.946133	1.40215
27834	0.946132	1.02197
27835	0.946133	1.19113
27836	0.946132	0.681197
27837	1.38773	0.853027

27838	1.41512	0.140764
27839	0.946131	0.300684
27840	0.946129	0.2213
27841	0.946133	0.22583
27842	0.946131	0.23664
27843	0.946132	0.757524
27844	0.946132	2.1
27845	0.946132	0.827271
27846	0.946133	0.939138
27847	0.946133	2.1
27848	0.946126	0.672901
27849	0.946132	0.947837
27850	0.946129	0.757851
27851	0.946129	0.772245
27852	0.946129	0.95553
27853	0.939895	1.64821
27854	1.03386	2.01143
27855	0.94613	1.57738
27856	0.946131	1.26656
27857	1.03386	1.53041
27858	0.946131	0.955281
27859	1.39309	2.1
27860	0.946133	0.885419
27861	0.946131	2.1
27862	1.39309	2.1
27863	0.946133	2.1
27864	1.24853	2.1
27865	0.946132	2.1
27866	0.946132	2.1
27867	1.24853	2.1
27868	0.946133	1.94279
27869	1.39699	0.880355
27870	0.946132	1.53694
27871	0.946132	2.1
27872	1.39699	1.11293
27873	0.946132	2.1
27874	0.946131	0.862016
27875	0.946133	2.1
27876	0.946131	2.08021
27877	1.38969	2.1
27878	1.28464	2.00283
27879	0.946133	1.95999
27880	0.94613	2.04593
27881	0.946131	1.80986
27882	0.946131	1.80024
27883	0.946119	0.758457
27884	0.94612	0.846193
27885	0.946119	0.766303
27886	0.946132	0.819159
27887	1.3749	0.70796
27888	0.810942	2.1
27889	0.810943	2.1
27890	0.810957	2.1
27891	1.41134	2.03963
27892	0.946132	0.83861
27893	0.946132	1.69339
27894	0.946132	0.939212
27895	0.946132	1.7444

27896	1.31338	2.0649
27897	0.946132	0.98389
27898	0.946132	1.98197
27899	0.946133	1.25871
27900	0.946132	1.78829
27901	0.946103	0.05
27902	0.752448	0.05
27903	0.946132	0.05
27904	0.946131	0.05
27905	1.20617	0.05
27906	0.946132	0.893346
27907	1.08054	0.770869
27908	1.25736	0.891758
27909	0.946132	0.902187
27910	0.946131	0.876068
27911	0.946128	1.48352
27912	1.33275	0.835465
27913	1.1896	1.50285
27914	0.946131	1.68895
27915	1.18962	1.77362
27916	1.21955	0.05
27917	0.946131	0.05
27918	1.33587	0.05
27919	0.741946	0.05
27920	1.07387	0.05
27921	0.945844	0.815617
27922	1.3292	2.07469
27923	1.59472	1.62813
27924	0.946131	0.898533
27925	1.04043	1.11193
27926	1.13529	0.29418
27927	0.946132	0.323196
27928	1.24893	0.298889
27929	1.12675	0.325881
27930	0.936652	0.320424
27931	1.36968	2.1
27932	0.946131	2.1
27933	0.946132	2.1
27934	1.36968	2.1
27935	1.258 2.1	
27936	3.17458	0.153793
27937	0.946132	1.04548
27938	1.35022	2.1
27939	1.35022	2.1
27940	0.94613	1.14374
27941	1.12356	1.95098
27942	0.946131	2.1
27943	1.22401	2.1
27944	1.22401	2.1
27945	0.94613	2.1
27946	0.952455	2.1
27947	1.2107	2.1
27948	0.946132	2.1
27949	1.33047	2.1
27950	0.946131	2.1
27951	0.946132	2.1
27952	0.61957	1.69602
27953	0.8636	2.1

27954	0.946132	1.58115
27955	1.19294	2.1
27956	0.946128	2.1
27957	1.36607	2.1
27958	0.94613	1.22815
27959	1.39416	2.1
27960	1.36607	2.1
27961	1.21474	2.1
27962	0.946131	2.1
27963	0.946131	2.1
27964	1.16288	2.1
27965	1.08542	2.1
27966	0.851526	0.933959
27967	1.30374	2.09172
27968	1.01708	2.07697
27969	0.946114	0.917982
27970	0.96976	1.3142
27971	0.946132	0.0936516
27972	1.49997	0.136669
27973	0.771735	0.104071
27974	0.771764	0.154885
27975	0.946133	0.174223
27976	0.700863	0.97498
27977	1.42222	1.0229
27978	0.946131	2.1
27979	0.946128	0.701446
27980	1.42509	1.9349
27981	1.14298	0.764784
27982	0.837014	2.1
27983	0.946131	2.1
27984	1.29426	2.1
27985	0.836985	2.1
27986	0.946129	2.1
27987	1.22089	0.483683
27988	0.946132	0.847307
27989	0.712486	0.540127
27990	0.6234	0.518488
27991	0.946131	2.1
27992	1.40271	2.1
27993	1.32241	2.1
27994	0.946133	2.1
27995	1.32241	2.1
27996	0.769373	1.13278
27997	0.946129	2.1
27998	1.41302	2.1
27999	1.28254	2.1
28000	0.946131	2.1
28001	1.28254	2.1
28002	0.851543	1.16036
28003	1.23056	2.1
28004	1.24356	2.1
28005	0.851543	1.23976
28006	0.909417	1.61737
28007	1.13808	0.468224
28008	0.946133	0.516184
28009	0.946131	0.415782
28010	1.13808	0.788706
28011	1.13808	0.475176

28012	0.930941	1.144
28013	1.37808	0.958738
28014	1.17573	0.799622
28015	0.946124	1.12962
28016	1.02404	0.873492
28017	0.946116	1.84051
28018	1.3144	0.759581
28019	1.3144	1.81815
28020	0.946118	2.05933
28021	1.13089	1.3086
28022	0.946128	0.322272
28023	0.946101	0.273042
28024	1.2606	0.352842
28025	1.26055	0.308109
28026	0.580823	0.649251
28027	0.946132	0.944812
28028	0.580825	0.669515
28029	1.23224	0.702279
28030	0.946128	2.1
28031	1.3719	2.1
28032	0.94613	2.1
28033	1.3719	2.1
28034	0.94613	2.1
28035	0.946132	1.03815
28036	1.35025	0.730423
28037	1.35025	0.925493
28038	0.946132	1.32207
28039	1.1794	1.02535
28040	0.946133	2.1
28041	1.25598	2.1
28042	1.29249	2.1
28043	1.38154	2.1
28044	0.851541	1.5965
28045	1.31508	2.1
28046	1.37558	2.1
28047	0.946129	1.31916
28048	0.867663	1.87147
28049	0.94613	1.20751
28050	1.35426	1.0627
28051	0.84872	1.09615
28052	0.946129	0.947618
28053	1.17195	1.84637
28054	0.946133	2.1
28055	1.39009	2.1
28056	1.14342	2.1
28057	0.946133	2.1
28058	1.14343	2.1
28059	1.38819	2.1
28060	0.946133	2.1
28061	1.4116	2.1
28062	0.946131	2.1
28063	0.946131	2.1
28064	0.946127	2.1
28065	1.32244	2.1
28066	1.32244	2.1
28067	0.946131	2.1
28068	1.2176	2.1
28069	1.75823	2.1

28070	1.11239	2.1
28071	2.17979	2.1
28072	1.65533	2.1
28073	3.41356	2.1
28074	1.94841	2.1
28075	1.91767	2.1
28076	1.76726	2.1
28077	1.70302	2.1
28078	3.76137	2.1
28079	1.94841	2.1
28080	1.33962	2.1
28081	1.31365	2.1
28082	1.70302	2.1
28083	3.2123	2.1
28084	1.76726	2.1
28085	1.70302	2.1
28086	1.47581	2.1
28087	1.7457	2.1
28088	3.22764	2.1
28089	1.20478	2.1
28090	1.70302	2.1
28091	1.27956	2.1
28092	1.18569	2.1
28093	1.63979	2.1
28094	1.51949	2.1
28095	1.53082	2.1
28096	1.92911	2.1
28097	1.32068	2.1
28098	1.74454	2.1
28099	3.27536	2.1
28100	1.08172	2.1
28101	1.74454	2.1
28102	1.52603	2.1
28103	1.52603	2.1
28104	1.32068	2.1
28105	1.32068	2.1
28106	1.89226	2.1
28107	2.37364	2.1
28108	1.89226	2.1
28109	2.42769	2.1
28110	4.31995	2.1
28111	2.37365	2.1
28112	1.5362	2.1
28113	2.4277	2.1
28114	1.56328	2.1
28115	3.9639	2.1
28116	1.89225	2.1
28117	2.42768	2.1
28118	1.89213	2.1
28119	1.89225	2.1
28120	2.42756	2.1
28121	2.42768	2.1
28122	2.42768	2.1
28123	1.58163	2.1
28124	1.56315	2.1
28125	1.56327	2.1
28126	2.42757	2.1
28127	2.42768	2.1

28128	1.01641	2.1
28129	2.15005	2.1
28130	1.00099	2.1
28131	2.15114	2.1
28132	3.15104	2.1
28133	0.994813	2.1
28134	2.15114	2.1
28135	2.17159	2.1
28136	2.17159	2.1
28137	1.00099	2.1
28138	1.00099	2.1
28139	1.29608	2.1
28140	1.29608	2.1
28141	0.942035	1.83382
28142	0.942035	1.13973
28143	1.29198	1.76625
28144	0.942033	2.1
28145	0.946132	2.1
28146	0.946132	1.42355
28147	0.946132	1.08821
28148	0.942033	1.41674
28149	0.946133	0.938548
28150	1.29198	1.19114
28151	1.99985	2.1
28152	1.14207	2.1
28153	1.83584	2.1
28154	1.00232	2.1
28155	3.00217	2.1
28156	1.99867	0.0538185
28157	2.0815	0.0715041
28158	1.56806	0.0580227
28159	1.28828	0.0701635
28160	3.58519	0.0713436
28161	1.89226	0.0914785
28162	1.89226	0.119931
28163	1.89226	0.100584
28164	1.89226	0.117725
28165	1.89226	0.0961241
28166	1.89226	0.116403
28167	1.89226	0.0744046
28168	1.89226	0.101233
28169	2.23193	0.0778842
28170	2.48978	0.131371
28171	4.38204	0.0801167
28172	1.85293	0.673907
28173	1.64279	0.379326
28174	1.76268	0.612989
28175	1.54214	0.369547
28176	3.39507	0.46654
28177	1.6428	0.395932
28178	1.17302	0.504437
28179	1.17302	0.712947
28180	1.28419	0.494861
28181	1.54214	0.386975
28182	1.65331	0.477293
28183	1.76268	0.684009
28184	1.54214	0.427426
28185	1.65324	0.593518

28186	1.42491	0.383252
28187	3.19538	0.454919
28188	0.651043	0.58066
28189	0.651043	0.562566
28190	0.647925	0.445895
28191	0.647925	0.585533
28192	0.521747	0.484826
28193	0.58651	0.500344
28194	0.77107	0.426325
28195	0.712457	0.43177
28196	0.712457	0.412264
28197	0.521747	0.429045
28198	0.58651	0.40912
28199	0.77107	0.430473
28200	1.2873	2.1
28201	1.48751	2.1
28202	1.59659	2.1
28203	1.77596	1.34663
28204	3.06326	2.1
28205	1.07976	2.1
28206	1.59659	2.1
28207	1.30237	2.1
28208	1.77723	2.1
28209	2.89897	2.1
28210	1.77723	2.1
28211	1.77596	1.36772
28212	1.82614	2.1
28213	1.59659	2.1
28214	2.0055	2.1
28215	1.77596	2.1
28216	2.64523	0.643244
28217	2.73982	0.387208
28218	2.50057	0.607791
28219	2.61606	0.389581
28220	5.26129	0.468257
28221	2.73982	0.376041
28222	2.68987	0.424806
28223	2.61607	0.415554
28224	2.57611	0.401492
28225	5.30593	0.32876
28226	1.89226	0.469471
28227	1.89226	0.69426
28228	1.89226	0.6071
28229	2.02178	0.619536
28230	3.78452	0.647734
28231	1.89226	0.399104
28232	1.89226	0.457356
28233	1.89226	0.990343
28234	1.89226	0.309165
28235	1.89226	1.10291
28236	1.89226	0.252465
28237	2.35204	2.1
28238	1.50108	2.1
28239	1.3715	2.1
28240	2.18469	2.1
28241	3.68577	2.1
28242	2.62514	2.1
28243	2.1847	2.1

28244	2.0487	2.1
28245	2.45213	2.1
28246	4.63682	2.1
28247	1.25075	2.1
28248	2.18469	2.1
28249	1.3715	2.1
28250	1.3715	2.1
28251	2.0487	2.1
28252	2.0487	2.1
28253	1.28667	2.1
28254	1.11285	2.1
28255	1.03529	2.1
28256	1.03529	2.1
28257	1.30711	2.1
28258	1.30711	2.1
28259	2.35053	2.1
28260	1.89225	2.1
28261	1.89225	2.1
28262	2.51216	2.1
28263	4.24278	2.1
28264	1.89225	2.1
28265	1.89225	2.1
28266	1.89225	2.1
28267	1.89225	2.1
28268	3.7845	2.1
28269	1.92025	2.1
28270	1.32119	2.1
28271	1.44777	2.1
28272	1.33834	2.1
28273	1.92025	2.1
28274	1.81082	2.1
28275	1.89226	2.1
28276	2.67272	2.1
28277	2.51218	2.1
28278	2.51218	2.1
28279	1.89226	2.1
28280	1.89226	2.1
28281	1.89226	2.1
28282	1.89226	2.1
28283	1.89226	2.1
28284	1.89226	2.1
28285	3.78452	2.1
28286	1.92027	2.1
28287	1.44778	2.1
28288	1.61121	2.1
28289	2.01555	2.1
28290	3.53147	2.1
28291	1.90727	0.367243
28292	2.29851	0.422027
28293	2.71643	0.436669
28294	2.17695	0.332625
28295	4.47547	0.338642
28296	1.90728	0.392604
28297	1.56339	0.331925
28298	1.58143	0.343612
28299	2.17696	0.38532
28300	3.74035	0.375364
28301	1.91368	0.348764

28302	2.17696	0.389769
28303	2.39986	0.396775
28304	1.75904	0.344723
28305	3.936	0.348307
28306	1.18793	0.0826783
28307	2.74744	0.126857
28308	1.38716	0.113168
28309	2.57465	0.0883386
28310	3.93537	0.116918
28311	1.3148	0.110703
28312	2.74744	0.179085
28313	2.70805	0.107425
28314	1.38716	0.116712
28315	2.70805	0.188973
28316	1.38716	0.146765
28317	1.20608	2.1
28318	0.975871	2.1
28319	0.946132	2.1
28320	0.946132	2.1
28321	0.946132	2.1
28322	0.946132	2.1
28323	0.975871	2.1
28324	0.975871	2.1
28325	1.20608	2.1
28326	0.946132	2.1
28327	0.946133	2.1
28328	0.975871	2.1
28329	2.8198	0.7903
28330	2.29967	1.52747
28331	2.09542	2.1
28332	2.63784	0.683433
28333	4.93751	1.61597
28334	2.09542	2.1
28335	1.39523	2.1
28336	1.21119	2.1
28337	1.39523	2.1
28338	2.11563	2.1
28339	2.29967	1.21318
28340	1.88584	2.1
28341	2.63784	0.613907
28342	2.11262	1.01764
28343	1.91132	0.863338
28344	4.02153	1.00681
28345	1.88584	2.1
28346	1.21119	2.1
28347	1.12606	2.1
28348	1.91132	0.844208
28349	3.1225	2.1
28350	2.18077	2.00031
28351	1.78167	1.95863
28352	2.2947	0.832944
28353	1.93139	1.34187
28354	4.07637	0.935211
28355	1.5709	1.19787
28356	1.03235	2.00228
28357	2.05933	1.78299
28358	1.78168	0.994114
28359	1.31	0.708547

28360	1.03235	0.788986
28361	2.2947	1.04344
28362	2.31785	2.03683
28363	2.40627	1.97474
28364	2.08288	0.911692
28365	4.37758	2.07119
28366	1.95737	1.6353
28367	1.31	2.00147
28368	2.08289	0.696453
28369	1.50108	0.878464
28370	3.39289	0.826941
28371	2.49676	2.1
28372	2.24781	1.97684
28373	2.5276	0.971079
28374	2.7352	2.1
28375	5.02436	2.00512
28376	2.49676	1.81746
28377	2.43549	2.1
28378	2.6398	2.1
28379	2.7352	2.1
28380	5.13656	2.1
28381	1.89226	2.1
28382	2.7352	2.1
28383	1.89226	1.32442
28384	2.6398	2.1
28385	1.89226	1.92979
28386	2.6398	2.1
28387	2.16843	0.05
28388	1.00185	0.05
28389	1.90039	2.1
28390	3.1547	0.05
28391	2.54064	0.05
28392	2.46752	2.1
28393	1.90039	2.1
28394	2.26044	2.1
28395	4.36791	0.495321
28396	1.65815	2.1
28397	2.48652	2.1
28398	1.65815	2.1
28399	1.9004	2.1
28400	2.21711	2.1
28401	2.5034	2.1
28402	2.37167	2.1
28403	2.59965	2.1
28404	2.62812	2.1
28405	2.40308	2.1
28406	4.99979	2.1
28407	2.50137	2.1
28408	2.59965	2.1
28409	2.62812	2.1
28410	2.5214	2.1
28411	5.12948	2.1
28412	2.62814	2.1
28413	2.4031	2.1
28414	2.43509	2.1
28415	2.65066	2.1
28416	5.06323	2.1
28417	2.62814	2.1

28418	2.52142	2.1
28419	2.53008	2.1
28420	2.65066	2.1
28421	5.15822	2.1
28422	2.0815	2.1
28423	1.92067	2.1
28424	1.59611	2.1
28425	1.89226	2.1
28426	1.62453	2.1
28427	1.92067	2.1
28428	1.89226	2.1
28429	1.89226	2.1
28430	1.89226	2.1
28431	1.89226	2.1
28432	3.78453	2.1
28433	0.946131	2.1
28434	0.946131	2.1
28435	0.929501	2.1
28436	0.910761	2.1
28437	0.9461	2.1
28438	0.9461	2.1
28439	0.910792	2.1
28440	0.910792	2.1
28441	0.946131	2.1
28442	0.929501	2.1
28443	0.910761	2.1
28444	0.946131	2.1
28445	2.0815	2.1
28446	1.81661	2.1
28447	2.1341	2.1
28448	2.0815	2.1
28449	1.86921	2.1
28450	1.81661	2.1
28451	1.89226	2.1
28452	1.89226	2.1
28453	1.89226	2.1
28454	1.89226	2.1
28455	1.89226	2.1
28456	1.89226	2.1
28457	0.946131	2.1
28458	0.946131	2.1
28459	0.949104	2.1
28460	0.9295	2.1
28461	0.946132	2.1
28462	0.946132	2.1
28463	0.929499	2.1
28464	0.929499	2.1
28465	0.946131	2.1
28466	0.949105	2.1
28467	0.946131	2.1
28468	0.929501	2.1
28469	1.66104	2.1
28470	2.34044	2.1
28471	2.08889	2.1
28472	1.33264	2.1
28473	3.67308	2.1
28474	2.0889	2.1
28475	1.08011	2.1

28476	1.08011	2.1
28477	1.33264	2.1
28478	1.87598	2.1
28479	2.12851	2.1
28480	2.27472	2.1
28481	1.89226	1.04239
28482	2.27703	1.70555
28483	1.89226	0.773645
28484	4.16699	0.811261
28485	1.89226	1.99742
28486	1.89226	1.27831
28487	1.89226	1.35359
28488	1.89226	1.01822
28489	3.78452	1.7656
28490	1.00007	1.53731
28491	1.32316	0.958127
28492	1.29417	0.886899
28493	1.29417	1.41531
28494	1.02051	0.86681
28495	1.02051	1.11183
28496	2.2846	1.98842
28497	1.89226	0.896968
28498	2.27703	0.830398
28499	2.27703	2.1
28500	1.89226	0.620764
28501	1.89226	2.1
28502	1.89226	1.8715
28503	1.89226	1.64451
28504	1.89226	0.974162
28505	1.89226	0.67991
28506	3.78452	0.883471
28507	1.02051	2.1
28508	1.32316	1.5662
28509	1.03373	0.956418
28510	1.34066	0.950397
28511	2.35689	1.92342
28512	1.703	2.1
28513	1.89224	2.1
28514	1.52266	2.1
28515	1.89224	2.1
28516	1.23344	2.1
28517	1.53282	2.1
28518	1.89223	2.1
28519	1.89223	1.98412
28520	1.89223	2.1
28521	1.89223	2.1
28522	3.78447	2.1
28523	0.796845	2.1
28524	0.796845	2.1
28525	0.812146	2.1
28526	0.812146	2.1
28527	1.08006	2.1
28528	1.02679	2.1
28529	0.551538	2.1
28530	0.528929	2.1
28531	0.551529	2.1
28532	1.08005	2.1
28533	0.528935	2.1

28534	1.0268	2.1
28535	1.5001	2.1
28536	1.70302	2.1
28537	2.0726	2.1
28538	1.70302	2.1
28539	1.99601	2.1
28540	1.70303	2.1
28541	1.89226	2.1
28542	1.89226	2.1
28543	1.89226	2.1
28544	1.89226	2.1
28545	1.89226	2.1
28546	1.89226	1.67234
28547	1.17285	2.1
28548	2.16015	2.1
28549	2.23563	2.1
28550	2.23563	2.1
28551	1.10309	2.1
28552	1.10309	2.1
28553	0.946128	2.1
28554	0.946128	2.1
28555	1.21847	0.600763
28556	1.2884	2.1
28557	0.94613	2.1
28558	0.94613	2.1
28559	1.21847	2.1
28560	1.21847	0.591416
28561	0.946131	2.1
28562	1.21847	2.1
28563	0.94613	2.1
28564	1.2884	2.1
28565	1.83922	0.654073
28566	2.5768	2.1
28567	1.83488	2.1
28568	1.82846	0.05
28569	2.43693	2.1
28570	2.43693	1.23372
28571	2.36134	2.1
28572	1.81656	2.1
28573	1.86454	2.1
28574	1.6697	2.1
28575	2.55618	2.1
28576	2.36134	2.1
28577	2.55618	2.1
28578	1.9323	2.1
28579	1.9302	2.1
28580	1.81656	2.1
28581	2.66983	2.1
28582	2.55618	2.1
28583	1.89226	0.785353
28584	1.70302	0.742956
28585	1.94935	0.910843
28586	1.70302	0.73547
28587	1.95569	0.779417
28588	1.70302	0.600934
28589	2.82465	2.1
28590	2.41707	2.1
28591	2.24481	2.1

28592	2.68958	2.1
28593	5.10665	2.1
28594	1.41232	2.1
28595	1.34479	2.1
28596	0.946129	2.1
28597	0.946129	2.1
28598	0.94613	2.1
28599	0.94613	2.1
28600	1.41232	2.1
28601	1.41232	2.1
28602	1.34479	2.1
28603	0.94613	2.1
28604	0.946131	2.1
28605	1.41232	2.1
28606	1.86859	0.05
28607	1.89226	0.0538141
28608	1.89226	0.0558863
28609	1.89226	0.05
28610	1.45875	0.0523835
28611	1.45875	0.05
28612	2.1769	0.575986
28613	2.50259	0.519155
28614	1.94955	0.418658
28615	2.25959	0.632409
28616	4.45214	0.677023
28617	1.89226	0.568863
28618	1.89226	0.600475
28619	1.89226	0.485415
28620	1.89226	0.589763
28621	3.78452	0.479465
28622	1.89226	0.64975
28623	1.89226	0.800124
28624	1.89226	0.590181
28625	1.89226	0.737249
28626	3.78452	1.16415
28627	2.50259	0.563375
28628	2.49445	0.673646
28629	2.25959	0.535574
28630	2.20843	0.559321
28631	4.71102	0.509744
28632	1.94955	0.426044
28633	2.25959	0.445687
28634	1.24689	0.352036
28635	1.79906	0.413006
28636	3.74861	0.38364
28637	1.89226	0.501299
28638	1.89226	0.515081
28639	1.89226	0.403403
28640	1.89226	0.467396
28641	3.78452	0.57991
28642	1.89226	0.522006
28643	1.89226	0.569387
28644	1.89226	0.447076
28645	1.89226	0.466979
28646	3.78452	0.440757
28647	2.25959	0.422134
28648	2.20843	0.466341
28649	1.79906	0.399573

28650	1.98391	0.478396
28651	4.00748	0.45399
28652	1.89226	2.1
28653	1.89226	2.1
28654	1.89226	2.1
28655	1.89226	2.1
28656	3.78452	2.1
28657	1.89226	2.1
28658	1.89226	2.1
28659	1.89226	2.1
28660	1.89226	2.1
28661	3.78452	2.1
28662	1.64718	2.1
28663	1.23085	2.1
28664	1.46028	2.1
28665	1.39564	2.1
28666	1.68372	2.1
28667	1.64718	2.1
28668	1.64718	2.1
28669	1.64981	2.1
28670	1.70303	2.1
28671	1.6991	2.1
28672	3.3199	2.1
28673	1.89226	2.1
28674	1.89226	2.1
28675	1.89226	2.1
28676	1.89226	2.1
28677	3.78452	2.1
28678	1.89226	2.1
28679	1.89226	2.1
28680	1.89226	2.1
28681	1.89226	2.1
28682	3.78452	2.1
28683	0.666701	2.1
28684	0.65896	2.1
28685	0.920797	2.1
28686	0.895441	2.1
28687	0.775871	2.1
28688	0.687493	2.1
28689	0.777393	2.1
28690	0.777393	2.1
28691	0.677856	2.1
28692	0.90095	2.1
28693	0.621435	2.1
28694	0.858124	2.1
28695	1.70303	2.1
28696	1.70731	2.1
28697	1.79663	2.1
28698	1.68783	2.1
28699	3.17337	2.1
28700	1.22724	2.1
28701	2.0021	2.1
28702	1.90427	2.1
28703	1.08698	2.1
28704	3.08907	2.1
28705	2.41689	2.1
28706	2.00209	2.1
28707	1.90427	2.1

28708	2.35245	2.1
28709	4.35454	2.1
28710	1.90427	2.1
28711	0.956294	2.1
28712	0.956294	2.1
28713	1.08698	2.1
28714	1.81488	2.1
28715	1.94556	2.1
28716	1.90427	2.1
28717	2.35245	2.1
28718	2.28979	2.1
28719	1.81488	2.1
28720	4.16733	2.1
28721	1.6272	2.1
28722	1.70302	2.1
28723	1.70303	2.1
28724	1.70302	2.1
28725	1.91167	2.1
28726	1.91167	2.1
28727	1.49063	2.1
28728	1.89226	2.1
28729	1.89226	2.1
28730	1.89226	2.1
28731	1.6272	2.1
28732	1.6272	2.1
28733	2.25988	1.00689
28734	1.89226	0.49644
28735	1.89226	0.922354
28736	2.23152	0.751247
28737	4.12379	0.665663
28738	2.25988	0.777341
28739	1.16361	0.934245
28740	1.15853	2.07005
28741	2.23153	0.674755
28742	3.39513	1.01696
28743	1.89226	0.892642
28744	2.23153	0.638891
28745	2.21022	1.07343
28746	2.23153	0.762483
28747	1.87095	0.648719
28748	1.89226	0.502454
28749	1.16239	1.48756
28750	2.23153	0.578721
28751	1.15853	1.05818
28752	1.15853	0.632573
28753	2.21022	1.13948
28754	2.21022	0.644283
28755	1.78381	0.0764991
28756	1.70302	0.0991589
28757	1.15453	0.106209
28758	1.35007	0.120022
28759	1.82021	0.0723375
28760	2.12851	0.0833931
28761	1.89226	0.1127
28762	1.89226	0.163674
28763	1.89226	0.161078
28764	1.89226	0.196496
28765	1.89226	0.095375

28766	1.89226	0.122455
28767	1.89258	0.0764728
28768	1.089	0.104129
28769	1.22586	0.0746097
28770	1.79877	0.0993434
28771	2.88777	0.0775349
28772	1.91564	0.0737258
28773	1.79877	0.104464
28774	1.96356	0.105149
28775	1.70302	0.114511
28776	2.37828	0.074236
28777	2.04859	0.0807432
28778	1.89226	0.10905
28779	1.89226	0.156571
28780	1.89226	0.103973
28781	1.89226	0.175991
28782	3.78453	0.11442
28783	1.89226	0.0986873
28784	1.89226	0.149807
28785	1.89226	0.103779
28786	1.89226	0.16614
28787	1.89226	0.0921123
28788	1.89226	0.141961
28789	2.28593	2.1
28790	1.89226	1.27213
28791	2.2731	2.1
28792	1.89226	0.762428
28793	4.17819	0.965002
28794	1.89226	2.1
28795	1.89226	2.02507
28796	1.89226	1.15999
28797	1.89226	1.00871
28798	3.78452	1.38876
28799	1.03933	1.9272
28800	1.34355	0.947385
28801	1.35048	0.901013
28802	1.35048	1.8443
28803	1.02975	0.797211
28804	1.02975	1.2453
28805	2.25105	2.1
28806	1.89226	1.06905
28807	2.27311	1.25868
28808	2.27311	2.1
28809	1.89226	0.793912
28810	1.89226	2.1
28811	1.89226	2.0933
28812	1.89226	1.74295
28813	1.89226	1.22762
28814	1.89226	0.620811
28815	3.78452	0.959859
28816	1.02975	2.1
28817	1.34355	1.63973
28818	1.01121	1.08579
28819	1.33079	0.893122
28820	2.35476	2.03215
28821	1.24526	0.05
28822	2.36406	0.05
28823	2.81021	0.05

28824	1.7237	0.05
28825	4.08776	0.05
28826	1.89226	2.1
28827	1.89226	0.918112
28828	1.89226	2.1
28829	1.89226	0.830372
28830	3.78453	2.1
28831	1.79028	2.1
28832	1.89238	0.919156
28833	1.65533	2.1
28834	1.77821	1.93263
28835	3.5477	2.1
28836	1.89238	0.930056
28837	1.7465	0.294659
28838	1.77821	1.92724
28839	1.63557	0.511484
28840	3.52795	0.690844
28841	1.89226	0.918124
28842	1.89226	0.485491
28843	1.89226	0.844225
28844	1.89226	0.317143
28845	3.78452	0.615071
28846	1.89226	2.1
28847	1.89226	0.864395
28848	1.89226	2.1
28849	1.89226	1.28326
28850	3.78452	2.1
28851	1.65532	2.1
28852	1.77821	1.9151
28853	1.53278	2.1
28854	1.67571	1.36676
28855	3.33103	2.1
28856	1.77821	1.93957
28857	1.63557	0.535829
28858	1.67571	1.38207
28859	1.52615	0.23018
28860	3.31128	0.681301
28861	1.89226	0.885253
28862	1.89226	0.334365
28863	1.89226	1.39864
28864	1.89226	0.427032
28865	3.78452	0.690984
28866	2.83129	2.08389
28867	1.89226	0.537345
28868	1.89226	0.802925
28869	2.6491	1.00919
28870	4.54136	0.730617
28871	2.83129	2.1
28872	1.47278	2.1
28873	1.3909	2.1
28874	2.6491	0.994664
28875	4.12188	2.1
28876	1.89225	2.1
28877	2.65393	2.1
28878	1.89225	2.07364
28879	2.69591	2.1
28880	4.58816	2.1
28881	1.91703	2.1

28882	2.00209	2.1
28883	2.65393	2.1
28884	2.65393	2.1
28885	2.69591	2.1
28886	2.00209	2.1
28887	1.89226	2.1
28888	2.69593	2.1
28889	1.93967	2.1
28890	1.89226	2.1
28891	2.74334	2.1
28892	2.69593	2.0715
28893	2.74335	2.1
28894	2.0021	2.1
28895	2.76367	2.1
28896	2.69593	2.1
28897	2.06984	2.1
28898	2.0021	2.1
28899	2.24481	2.1
28900	2.58921	2.1
28901	2.68958	2.1
28902	2.68958	2.1
28903	2.11984	2.1
28904	2.11984	2.1
28905	2.11984	2.1
28906	2.58921	2.1
28907	2.46488	2.1
28908	1.98339	2.1
28909	4.5726	2.1
28910	2.7777	2.1
28911	2.58921	2.1
28912	2.46488	2.1
28913	2.70401	2.1
28914	5.29322	2.1
28915	2.17741	2.1
28916	2.54197	2.1
28917	2.54018	2.1
28918	2.18608	2.1
28919	4.72805	2.1
28920	2.17742	2.1
28921	1.58054	2.1
28922	1.59169	2.1
28923	2.18608	2.1
28924	3.76662	2.1
28925	2.54018	2.1
28926	2.18608	2.1
28927	2.19104	2.1
28928	2.5392	2.1
28929	4.72528	2.1
28930	1.60497	2.1
28931	2.18608	2.1
28932	1.59169	2.1
28933	1.59169	2.1
28934	2.19105	2.1
28935	2.19105	2.1
28936	2.58661	0.965244
28937	1.06203	2.1
28938	2.7043	2.1
28939	2.21397	2.1

28940	1.55236	2.1
28941	1.06203	2.0996
28942	1.89226	1.21971
28943	1.89226	1.84328
28944	1.89226	0.754508
28945	1.89226	1.9602
28946	3.78453	1.34894
28947	1.08195	1.4562
28948	0.990019	2.1
28949	1.31976	1.86069
28950	1.4539	0.867801
28951	2.40171	2.07083
28952	1.89226	0.978292
28953	1.89226	2.1
28954	1.89226	1.02161
28955	1.89226	0.690091
28956	3.78453	1.14362
28957	1.89226	1.40711
28958	1.89226	1.23346
28959	1.89226	0.862259
28960	1.89226	2.1
28961	3.78453	1.13766
28962	1.43895	1.96872
28963	1.31976	1.9952
28964	1.70066	0.810362
28965	1.33783	2.1
28966	1.43895	2.1
28967	1.07612	2.1
28968	0.65988	1.86647
28969	0.850329	1.34067
28970	0.919598	0.923932
28971	0.726952	1.64204
28972	0.788106	0.815018
28973	0.788106	2.1
28974	0.789175	2.04665
28975	0.789175	0.670509
28976	0.850329	0.982404
28977	0.919598	0.94418
28978	0.659879	1.57547
28979	0.726952	1.31429
28980	1.34389	2.1
28981	1.41279	1.68725
28982	1.15564	2.1
28983	1.15564	2.1
28984	1.55998	1.16676
28985	1.55998	2.00057
28986	1.89226	1.21239
28987	1.89226	1.96935
28988	1.89226	2.1
28989	1.89226	1.00217
28990	1.89226	2.1
28991	1.89226	1.02708
28992	1.55998	2.1
28993	1.41279	1.975
28994	1.72901	1.29905
28995	1.62414	2.1
28996	3.1418	1.85308
28997	1.89226	1.12111

28998	1.89226	2.1
28999	1.89226	1.37438
29000	1.89226	0.863602
29001	3.78452	1.72496
29002	1.89226	0.745054
29003	1.89226	2.1
29004	1.89226	1.20081
29005	1.89226	2.00414
29006	1.89226	0.877999
29007	1.89226	1.08926
29008	1.62414	1.93017
29009	1.01243	2.1
29010	1.70932	2.1
29011	1.41279	1.29008
29012	1.30896	1.55015
29013	1.01243	1.13556
29014	1.9719	2.1
29015	1.10725	2.1
29016	1.12411	2.1
29017	1.98691	2.1
29018	3.09601	2.1
29019	1.97189	2.1
29020	2.47932	2.1
29021	2.49092	2.1
29022	1.98691	2.1
29023	4.46282	2.1
29024	1.1486	2.1
29025	1.98691	2.1
29026	1.12411	2.1
29027	1.12411	2.1
29028	2.01602	2.1
29029	2.01602	2.1
29030	2.49093	2.1
29031	1.98691	2.1
29032	2.01601	2.1
29033	2.51651	2.1
29034	4.50694	2.1
29035	1.93147	0.327795
29036	2.51593	0.320157
29037	1.35146	0.324145
29038	1.35146	0.320001
29039	2.48665	0.322907
29040	1.90664	0.314549
29041	1.63979	2.1
29042	1.7457	2.1
29043	1.66046	2.1
29044	1.56558	2.1
29045	3.31128	2.1
29046	0.819895	2.1
29047	0.782792	2.1
29048	0.60239	2.1
29049	0.54831	2.1
29050	0.688347	2.1
29051	0.688347	2.1
29052	0.679857	2.1
29053	0.679857	2.1
29054	0.782792	2.1
29055	0.54831	2.1

29056	0.60239	2.1
29057	0.819895	2.1
29058	1.66046	2.1
29059	1.42485	2.1
29060	1.56558	2.1
29061	1.56558	2.1
29062	1.52995	2.1
29063	1.52995	2.1
29064	2.58101	0.526648
29065	2.09803	0.791922
29066	2.408	0.238081
29067	1.86893	1.26117
29068	4.44994	0.645598
29069	2.36062	0.749352
29070	1.25104	2.1
29071	1.25104	1.95835
29072	1.39703	2.1
29073	1.86893	1.36956
29074	2.01492	2.1
29075	2.408	0.464683
29076	1.86893	0.763529
29077	2.22575	0.27068
29078	1.70677	0.75451
29079	4.09468	0.647336
29080	0.737605	2.1
29081	0.737605	0.773863
29082	0.741302	0.807161
29083	0.741302	2.1
29084	0.55189	2.1
29085	0.625517	2.1
29086	0.934468	0.757341
29087	0.853389	0.742989
29088	0.853389	2.1
29089	0.55189	2.1
29090	0.625517	2.1
29091	0.934467	2.1
29092	2.35053	2.1
29093	1.09615	2.1
29094	1.25998	2.1
29095	2.51216	2.1
29096	3.61051	2.1
29097	1.33636	2.1
29098	1.25609	2.1
29099	0.698455	2.1
29100	0.629995	2.1
29101	0.986092	2.1
29102	0.986092	2.1
29103	0.980263	2.1
29104	0.980263	2.1
29105	1.33636	2.1
29106	0.698455	2.1
29107	0.629995	2.1
29108	1.25609	2.1
29109	1.89226	0.861127
29110	1.89226	0.908118
29111	1.89226	0.964536
29112	1.89226	0.887827
29113	3.78452	0.860069

29114	1.74321	0.830486
29115	1.89226	0.842076
29116	1.91755	0.88902
29117	1.91755	0.835029
29118	1.89226	0.88208
29119	1.89226	0.833767
29120	0.946132	0.849725
29121	0.946132	0.803118
29122	0.949798	0.723457
29123	1.08054	0.831748
29124	1.08054	0.771384
29125	1.08054	0.828392
29126	0.946132	0.807935
29127	0.946132	0.830462
29128	0.946132	0.807719
29129	0.949798	0.716967
29130	1.08054	0.778542
29131	0.946131	0.915853
29132	1.89226	0.931268
29133	1.89226	0.811078
29134	1.89226	0.783263
29135	1.89226	0.793826
29136	1.89226	0.865251
29137	1.89226	0.880529
29138	2.39378	0.300702
29139	2.29234	0.129886
29140	2.29234	0.266817
29141	2.7933	0.200508
29142	1.18422	0.185376
29143	1.68518	0.198272
29144	2.79329	0.139529
29145	1.89226	0.146801
29146	2.29233	0.117964
29147	1.89226	0.103317
29148	4.18459	0.0926585
29149	0.857046	0.0621753
29150	0.857037	0.12052
29151	0.94613	0.0846838
29152	0.94613	0.154841
29153	0.851539	0.109833
29154	0.851539	0.0829878
29155	1.0315	0.0740566
29156	0.952867	0.0909558
29157	0.851486	0.0641477
29158	0.952869	0.0755012
29159	1.03156	0.105413
29160	0.851528	0.0987523
29161	1.61981	2.1
29162	1.93096	2.1
29163	2.00776	2.1
29164	1.62218	2.1
29165	3.62757	2.1
29166	2.00776	2.1
29167	1.68541	2.1
29168	1.83215	2.1
29169	1.62218	2.1
29170	2.21773	2.1
29171	2.00776	2.1

29172	1.89188	0.0816457
29173	1.70303	0.100727
29174	1.89226	0.0957341
29175	1.72465	0.0903149
29176	1.89226	0.0985236
29177	1.70552	0.0978937
29178	2.48659	0.112452
29179	2.0815	0.156467
29180	1.89226	0.161889
29181	2.08111	0.137777
29182	1.89226	0.154472
29183	2.08111	0.145777
29184	2.35332	0.346016
29185	1.83284	0.165428
29186	2.28483	0.207889
29187	2.71955	0.170912
29188	4.63815	0.17234
29189	1.89227	2.1
29190	1.89227	2.1
29191	1.89227	2.1
29192	1.89227	2.1
29193	3.78453	2.1
29194	1.33506	2.1
29195	1.17129	2.1
29196	1.33506	2.1
29197	1.38415	2.1
29198	1.13664	2.1
29199	1.18573	2.1
29200	1.89226	2.1
29201	2.68785	2.1
29202	1.89226	2.1
29203	2.6692	2.1
29204	4.58011	2.1
29205	1.89226	2.1
29206	1.89226	2.1
29207	1.89226	2.1
29208	1.89226	2.1
29209	3.78453	2.1
29210	1.33506	2.1
29211	1.13664	2.1
29212	1.27575	2.1
29213	1.09627	2.1
29214	2.43133	2.1
29215	1.89226	2.1
29216	2.65349	2.1
29217	2.66919	2.1
29218	2.66919	2.1
29219	1.89226	2.1
29220	1.89226	2.1
29221	1.89226	2.1
29222	1.89226	2.1
29223	1.89226	2.1
29224	1.89226	2.1
29225	3.78453	2.1
29226	1.16834	2.1
29227	1.16834	2.1
29228	0.946132	2.1
29229	0.946132	2.1

29230	1.26978	2.1
29231	1.16834	2.1
29232	0.94613	2.1
29233	0.94613	2.1
29234	0.94613	2.1
29235	1.26978	2.1
29236	0.94613	2.1
29237	1.16834	2.1
29238	2.14698	2.1
29239	1.89226	2.1
29240	1.89226	2.1
29241	1.89226	2.1
29242	2.40719	2.1
29243	2.40719	2.1
29244	1.89226	2.1
29245	1.89226	2.1
29246	1.89226	2.1
29247	1.89226	2.1
29248	3.78453	2.1
29249	1.26977	2.1
29250	1.26977	2.1
29251	0.946133	2.1
29252	0.946133	2.1
29253	1.37467	2.1
29254	1.26978	2.1
29255	0.946131	2.1
29256	0.946131	2.1
29257	1.37467	2.1
29258	0.94613	2.1
29259	0.946131	2.1
29260	1.26978	2.1
29261	1.2036	2.1
29262	1.34912	2.1
29263	0.946131	2.1
29264	0.946131	2.1
29265	1.34912	2.1
29266	1.34912	2.1
29267	0.946129	2.1
29268	0.946129	2.1
29269	1.34912	2.1
29270	0.94613	2.1
29271	0.946131	2.1
29272	1.2036	2.1
29273	2.47164	0.560663
29274	1.89019	0.30494
29275	1.79109	0.550213
29276	2.41632	0.360582
29277	4.26273	0.550406
29278	1.54197	0.486343
29279	2.41632	0.261679
29280	1.79109	0.421424
29281	1.79109	0.490227
29282	2.20929	0.31235
29283	2.20929	0.414977
29284	1.70302	0.938341
29285	1.48104	1.07807
29286	1.99365	1.12755
29287	1.32897	0.924063

29288	1.70302	1.1184
29289	1.14034	0.911192
29290	1.70302	0.922399
29291	1.99365	0.960389
29292	1.99297	1.01071
29293	2.36337	0.79263
29294	4.04804	0.858602
29295	1.51569	0.835612
29296	2.49093	0.835551
29297	1.6026	0.921177
29298	1.43229	0.731388
29299	2.0815	0.941575
29300	1.83402	0.749363
29301	2.54665	2.1
29302	2.34044	2.1
29303	2.08889	2.1
29304	2.37653	2.1
29305	4.71697	2.1
29306	2.54665	2.1
29307	2.45212	2.1
29308	2.31012	2.1
29309	2.37653	2.1
29310	4.82865	2.1
29311	2.08889	2.1
29312	2.37653	2.1
29313	2.23366	2.1
29314	1.87598	2.1
29315	4.25251	2.1
29316	2.31012	2.1
29317	2.37653	2.1
29318	2.23367	2.1
29319	2.17384	2.1
29320	4.55037	2.1
29321	0.946131	2.1
29322	0.946131	2.1
29323	1.30338	2.1
29324	1.23186	2.1
29325	0.94613	2.1
29326	0.94613	2.1
29327	1.23187	2.1
29328	1.23187	2.1
29329	0.946131	2.1
29330	1.23186	2.1
29331	1.30338	2.1
29332	0.946131	2.1
29333	1.89226	2.1
29334	2.46373	2.1
29335	2.34725	2.1
29336	2.46373	2.1
29337	1.77579	2.1
29338	1.89226	2.1
29339	2.61901	2.1
29340	2.45899	2.1
29341	2.64067	2.1
29342	2.52708	2.1
29343	5.14609	2.1
29344	2.41795	2.1
29345	2.0815	2.1

29346	2.52708	2.1
29347	1.92277	2.1
29348	4.31368	2.1
29349	2.64067	2.1
29350	2.52708	2.1
29351	2.58638	2.1
29352	2.51039	2.1
29353	5.11346	2.1
29354	2.52708	2.1
29355	2.13642	2.1
29356	2.51039	2.1
29357	2.16633	2.1
29358	4.69341	2.1
29359	1.61028	1.2448
29360	1.65712	0.704287
29361	1.35002	0.600011
29362	1.35002	1.27005
29363	1.88637	0.72657
29364	1.88637	2.04658
29365	2.57663	0.881605
29366	2.24227	2.1
29367	2.48354	0.864351
29368	2.14317	2.1
29369	4.7198	2.1
29370	2.24227	2.1
29371	1.77353	2.1
29372	2.14317	2.1
29373	1.69956	2.1
29374	3.9167	2.1
29375	2.48353	0.81095
29376	2.14317	2.1
29377	2.39282	1.20936
29378	1.79824	2.1
29379	4.53599	2.1
29380	2.14317	2.1
29381	1.69956	2.1
29382	1.91427	2.1
29383	1.6347	2.1
29384	3.77787	2.1
29385	1.89226	0.862632
29386	1.89226	1.11217
29387	1.89226	1.15542
29388	1.89226	1.05342
29389	3.78453	1.67928
29390	1.89226	0.799558
29391	1.89226	0.768472
29392	1.89226	0.914172
29393	1.89226	0.923676
29394	3.78452	0.632592
29395	1.89226	0.795144
29396	1.89652	1.64822
29397	1.89226	0.672348
29398	1.89226	0.941264
29399	1.19352	0.970962
29400	1.19352	2.08564
29401	1.89226	0.960104
29402	1.89653	0.698704
29403	1.89226	1.57658

29404	1.89226	1.24422
29405	1.89226	0.830691
29406	1.89226	0.908118
29407	1.89226	1.51674
29408	1.75326	0.812899
29409	1.89226	2.1
29410	1.12948	1.74337
29411	1.89226	0.654089
29412	1.12948	0.583561
29413	2.61064	2.1
29414	1.89226	2.1
29415	1.89226	2.1
29416	1.89226	2.1
29417	2.6085	2.1
29418	2.6085	2.1
29419	2.6085	2.1
29420	1.89226	2.1
29421	1.89226	2.1
29422	1.89226	2.1
29423	2.62161	2.1
29424	2.62161	2.1
29425	2.16535	2.1
29426	1.44603	2.1
29427	2.79784	2.1
29428	1.84756	2.1
29429	4.01292	2.1
29430	2.65262	2.1
29431	1.03391	2.1
29432	2.20239	2.1
29433	1.74416	2.1
29434	1.49213	2.1
29435	1.03391	2.1
29436	2.29066	2.1
29437	2.49174	2.1
29438	2.56502	2.1
29439	2.33802	2.1
29440	4.85568	2.1
29441	2.39663	2.1
29442	2.49174	2.1
29443	2.56502	2.1
29444	2.45929	2.1
29445	4.96165	2.1
29446	2.56502	2.1
29447	2.33802	2.1
29448	2.37168	2.1
29449	2.59967	2.1
29450	4.9367	2.1
29451	2.56502	2.1
29452	2.45929	2.1
29453	2.50138	2.1
29454	2.59967	2.1
29455	5.0664	2.1
29456	2.27009	0.900394
29457	1.26247	2.1
29458	1.86812	2.07032
29459	2.837	1.22066
29460	4.13822	2.06868
29461	1.89226	0.600952

29462	1.89226	2.1
29463	1.89226	1.062
29464	1.89226	1.76738
29465	3.78453	1.62022
29466	1.75835	2.1
29467	1.72866	2.1
29468	1.65313	0.841579
29469	1.64822	2.1
29470	3.3818	2.1
29471	1.72866	2.1
29472	1.58401	2.1
29473	1.64822	2.1
29474	1.52441	2.1
29475	3.25307	2.1
29476	1.89226	2.1
29477	1.89226	2.1
29478	1.89226	1.85181
29479	1.89226	2.1
29480	3.78452	2.1
29481	1.89226	1.19114
29482	1.89226	1.88326
29483	1.89226	0.619406
29484	1.89226	2.1
29485	3.78453	1.33744
29486	1.65313	1.03907
29487	1.64822	2.1
29488	1.55779	2.1
29489	1.57968	2.1
29490	3.23281	2.1
29491	1.64822	2.1
29492	1.52441	2.1
29493	1.57967	2.1
29494	1.474 2.1	
29495	3.10408	2.1
29496	1.89226	1.92308
29497	1.89226	2.1
29498	1.89226	2.1
29499	1.89226	2.1
29500	3.78452	2.1
29501	1.86067	1.1471
29502	1.58407	2.1
29503	2.07965	0.818039
29504	1.83123	1.5529
29505	3.66372	1.00999
29506	1.89226	0.881467
29507	1.89226	2.1
29508	1.89226	1.02955
29509	1.89226	1.50311
29510	3.78452	1.21686
29511	1.89226	0.854717
29512	1.89226	1.96896
29513	1.89226	0.785657
29514	1.89226	2.1
29515	1.89226	0.713176
29516	1.89226	2.1
29517	1.83123	0.853215
29518	1.03472	2.07026
29519	1.91068	1.6451

29520	1.58406	0.9807
29521	1.36133	1.04398
29522	1.03472	0.829473
29523	2.07965	0.964333
29524	1.83123	1.67633
29525	2.28385	1.0304
29526	2.09114	1.28131
29527	4.1708	1.54041
29528	1.89226	1.26563
29529	1.89226	1.93038
29530	1.89226	0.816692
29531	1.89226	1.74689
29532	3.78452	1.03374
29533	1.89226	0.896151
29534	1.89226	2.1
29535	1.89226	0.959181
29536	1.89226	2.00495
29537	3.78452	1.12679
29538	1.83123	0.840749
29539	1.36133	2.1
29540	2.09114	0.824564
29541	1.69436	1.65262
29542	3.45248	1.04362
29543	1.89226	2.1
29544	1.89226	1.26032
29545	1.89226	2.1
29546	1.89226	0.782849
29547	3.78453	2.1
29548	1.53278	2.1
29549	1.67571	1.34963
29550	1.40182	2.1
29551	1.55686	1.95821
29552	3.07752	2.1
29553	1.67571	1.46076
29554	1.52615	0.269253
29555	1.55686	1.9518
29556	1.38963	0.134855
29557	3.06534	0.661062
29558	1.89226	1.47252
29559	1.89226	0.505898
29560	1.89226	0.799605
29561	1.89226	0.159553
29562	3.78452	0.623442
29563	1.89226	2.1
29564	1.89226	0.893654
29565	1.89226	2.1
29566	1.89226	1.52332
29567	3.78453	2.1
29568	1.40182	2.1
29569	1.55686	1.85773
29570	1.24509	2.1
29571	1.38426	1.72294
29572	2.78608	2.1
29573	1.55686	1.9701
29574	1.38963	0.422344
29575	1.2064	1.60714
29576	1.2064	0.439057
29577	1.38426	1.68883

29578	1.56749	0.608919
29579	0.810946	0.82278
29580	0.810946	0.470618
29581	0.810945	0.857437
29582	0.810945	1.82462
29583	0.810945	0.774341
29584	0.810945	0.724424
29585	1.89226	0.516412
29586	1.35169	0.622597
29587	1.08132	1.92085
29588	1.08132	0.228386
29589	1.08132	0.669823
29590	1.28418	0.516795
29591	1.59382	0.548488
29592	1.78289	0.640652
29593	1.51021	0.371891
29594	3.10403	0.448738
29595	1.76897	0.939815
29596	1.59382	0.411112
29597	1.78289	0.664773
29598	1.94302	0.713916
29599	3.53684	0.756797
29600	1.89226	0.395726
29601	1.89226	0.589117
29602	1.89226	0.603817
29603	1.89226	0.283729
29604	3.78453	0.372272
29605	1.89227	0.538737
29606	1.89227	0.358683
29607	1.89227	0.293545
29608	1.89227	0.444589
29609	1.89227	0.393906
29610	1.89227	0.513097
29611	1.78286	0.650258
29612	1.51019	0.322276
29613	1.79979	0.436035
29614	2.02511	0.485995
29615	3.5353	0.534536
29616	1.78286	0.666127
29617	1.94299	0.686623
29618	2.17831	0.886765
29619	2.02511	0.452811
29620	3.9681	0.520413
29621	1.89224	0.570808
29622	1.89224	0.33117
29623	1.89224	0.377585
29624	1.89224	0.602316
29625	3.78448	0.527351
29626	1.89224	0.406707
29627	1.89224	0.355022
29628	1.89224	0.317424
29629	1.89224	0.385227
29630	1.89224	0.431899
29631	1.89224	0.489924
29632	1.83198	0.424387
29633	1.0961	0.317193
29634	1.39448	0.383607
29635	1.99568	0.388103

29636	3.09178	0.380715
29637	1.25503	0.34655
29638	1.99568	0.360168
29639	2.3661	0.419136
29640	1.70302	0.343068
29641	3.55833	0.329266
29642	2.69899	0.176235
29643	1.85796	0.159406
29644	2.11407	0.157791
29645	1.36769	0.123589
29646	3.97203	0.115921
29647	1.89226	0.242516
29648	1.89226	0.217292
29649	1.89226	0.207263
29650	1.89226	0.138662
29651	3.78452	0.25898
29652	1.89225	0.160205
29653	2.63355	0.155643
29654	2.09529	0.144944
29655	2.09529	0.183992
29656	1.89225	0.167782
29657	1.89225	0.187671
29658	1.89226	0.184171
29659	1.89226	0.218674
29660	1.89226	0.159784
29661	1.89226	0.327486
29662	1.89226	0.123218
29663	1.89226	0.242249
29664	2.12204	0.138314
29665	1.89226	0.211622
29666	2.05798	0.122915
29667	2.01954	0.151688
29668	1.70302	0.178411
29669	1.3865	0.220765
29670	1.28326	0.406525
29671	1.33326	0.576514
29672	1.19052	0.141692
29673	1.25369	0.542764
29674	2.53694	0.502275
29675	1.33327	0.49958
29676	1.27339	0.744948
29677	1.25369	0.527563
29678	1.19733	1.71876
29679	2.52708	0.623775
29680	1.19052	0.275154
29681	1.25369	0.54321
29682	1.10938	0.35623
29683	1.17614	0.601406
29684	2.36307	0.475247
29685	1.25369	0.523952
29686	1.19733	1.9624
29687	1.17614	0.607305
29688	1.11868	1.28306
29689	2.37237	0.738795
29690	1.10747	0.310134
29691	1.47688	0.3034
29692	1.97732	0.318722
29693	1.62206	0.316717

29694	3.09894	0.316377
29695	1.89226	0.308832
29696	1.89226	0.321101
29697	1.89226	0.318483
29698	1.89226	0.317784
29699	1.89226	0.315274
29700	1.89226	0.313132
29701	1.62206	0.316301
29702	1.70302	0.319723
29703	1.22962	0.320776
29704	1.1893	0.319501
29705	2.15177	0.318931
29706	2.08121	0.316081
29707	1.97732	0.32033
29708	2.08121	0.316916
29709	1.62206	0.318494
29710	1.62206	0.318256
29711	2.41464	0.317852
29712	2.41464	0.316808
29713	1.89226	0.317117
29714	1.89226	0.324177
29715	1.89226	0.316772
29716	1.89226	0.322701
29717	3.78452	0.317208
29718	0.946133	0.318381
29719	0.946133	0.319659
29720	0.946132	0.324076
29721	0.946132	0.323906
29722	0.946132	0.324688
29723	0.946132	0.321909
29724	0.946133	0.318783
29725	0.946133	0.318575
29726	0.946132	0.319958
29727	0.946132	0.331775
29728	0.946132	0.323134
29729	0.946132	0.315932
29730	1.83157	0.740943
29731	2.19989	0.733276
29732	2.20664	0.578072
29733	1.81487	0.715628
29734	4.03146	0.760564
29735	2.41055	2.1
29736	2.19989	1.32724
29737	2.20665	1.21623
29738	2.38023	1.18709
29739	4.61044	1.02601
29740	2.19989	1.57945
29741	2.22558	1.74883
29742	2.38023	1.35414
29743	2.07065	0.82
29744	4.42547	1.1189
29745	2.19988	0.812634
29746	1.70303	0.318008
29747	1.89939	0.770144
29748	1.86357	0.692739
29749	3.78536	0.690532
29750	1.04921	2.1
29751	1.30711	2.1

29752	1.53076	2.1
29753	1.22927	2.1
29754	2.57997	2.1
29755	1.53076	2.1
29756	1.46568	2.1
29757	1.56056	2.1
29758	1.22927	2.1
29759	1.86206	2.1
29760	1.53076	2.1
29761	1.18353	2.1
29762	2.28593	2.1
29763	1.16979	2.1
29764	2.2731	1.86904
29765	3.45571	2.1
29766	0.854776	1.50621
29767	0.854776	2.1
29768	0.855641	2.1
29769	0.855641	2.1
29770	1.12552	2.08871
29771	1.13655	2.1
29772	0.57216	2.1
29773	0.584893	2.1
29774	0.57216	2.1
29775	1.12552	2.1
29776	0.584893	2.1
29777	1.13655	2.1
29778	1.54462	2.1
29779	2.76309	2.1
29780	1.34869	2.1
29781	2.48573	2.1
29782	4.03035	2.1
29783	1.35328	0.968412
29784	1.0383	0.85107
29785	1.03318	1.6179
29786	0.704834	1.35469
29787	1.0257	1.37117
29788	1.0257	0.900854
29789	1.03241	0.760786
29790	1.03241	1.69219
29791	1.35328	1.08308
29792	1.03319	1.75053
29793	1.0383	1.42729
29794	0.704835	1.21832
29795	2.27126	0.401156
29796	2.77291	1.24987
29797	1.81771	0.652854
29798	2.6111	0.552143
29799	4.88236	0.663563
29800	2.77291	0.871102
29801	1.89227	0.668864
29802	1.89227	0.589559
29803	1.89227	1.80525
29804	2.6111	0.507737
29805	2.6111	1.92275
29806	2.22061	0.611313
29807	2.41522	0.576802
29808	2.41522	0.491513
29809	2.6111	0.872007

29810	1.75765	0.49013
29811	1.95353	1.27613
29812	2.27502	0.763874
29813	1.89227	1.42606
29814	1.89227	0.75986
29815	2.8189	0.602608
29816	4.71116	0.682885
29817	1.89226	0.602523
29818	1.89226	1.36606
29819	1.89226	0.821852
29820	1.89226	0.658462
29821	1.89226	1.13281
29822	1.89226	0.902621
29823	1.89226	1.63461
29824	1.89226	1.57945
29825	1.89226	1.21966
29826	1.89226	0.969105
29827	3.78453	1.62773
29828	1.89226	1.45779
29829	2.57079	0.731668
29830	2.5932	0.986685
29831	1.89226	0.958125
29832	4.48546	0.874926
29833	0.589396	1.01463
29834	0.641454	0.811233
29835	0.814062	1.31695
29836	0.726518	1.07612
29837	0.675126	1.25302
29838	0.675125	1.07112
29839	0.692847	1.26695
29840	0.692847	1.03542
29841	0.641454	0.886141
29842	0.814062	1.16657
29843	0.726518	1.36644
29844	0.589396	1.059
29845	1.89226	1.30849
29846	1.89226	0.973929
29847	1.89226	1.29478
29848	1.89226	1.69676
29849	1.89226	1.09407
29850	1.89226	1.64716
29851	1.16906	0.592882
29852	1.2966	0.928692
29853	0.946132	0.894297
29854	0.946132	1.5192
29855	1.16906	0.932358
29856	1.16906	0.663504
29857	0.946132	0.961291
29858	0.946132	1.07593
29859	1.16906	1.29308
29860	0.946131	1.1082
29861	1.2966	0.956279
29862	0.946132	1.63029
29863	0.641455	1.03731
29864	0.567191	0.801738
29865	0.807594	1.35091
29866	0.814062	1.1168
29867	0.673735	1.44353

29868	0.673734	0.896299
29869	0.707519	1.05225
29870	0.707519	1.41343
29871	0.807594	1.23679
29872	0.56719	1.65968
29873	0.641455	1.26033
29874	0.814063	1.71194
29875	1.89225	2.1
29876	1.89225	2.1
29877	1.89225	2.1
29878	1.89225	2.1
29879	3.7845	2.1
29880	1.89225	2.1
29881	1.89225	2.1
29882	1.89225	2.1
29883	1.89225	2.1
29884	1.89225	2.1
29885	1.89225	2.1
29886	1.89226	2.1
29887	1.89226	2.1
29888	1.89226	2.1
29889	1.89226	2.1
29890	3.78452	2.1
29891	1.89226	2.1
29892	1.89226	2.1
29893	1.89226	2.1
29894	1.89226	2.1
29895	1.89226	2.1
29896	1.89226	2.1
29897	1.89226	2.1
29898	1.89226	2.1
29899	1.89226	2.1
29900	1.89226	2.1
29901	3.78452	2.1
29902	1.89226	2.1
29903	1.38928	2.1
29904	1.89226	2.1
29905	1.51378	2.1
29906	1.89061	2.1
29907	1.89226	2.1
29908	1.89226	2.1
29909	1.89226	1.50508
29910	1.89226	1.51504
29911	1.89226	2.1
29912	3.78453	2.1
29913	1.04572	2.1
29914	0.994213	2.1
29915	0.912466	1.66999
29916	0.912466	2.00691
29917	0.947743	1.77219
29918	1.00388	2.1
29919	0.900913	1.43841
29920	0.900913	2.1
29921	0.900915	2.1
29922	0.946131	2.1
29923	0.946131	2.1
29924	0.999196	2.1
29925	1.89226	1.36774

29926	1.89226	2.1
29927	1.89226	2.1
29928	1.89226	1.65274
29929	3.78453	2.1
29930	0.946161	2.1
29931	1.1354	2.1
29932	0.850847	2.1
29933	0.850847	2.1
29934	1.01445	2.1
29935	1.02106	2.1
29936	0.946131	2.1
29937	0.946131	2.1
29938	1.13537	2.1
29939	1.01444	2.1
29940	0.850877	2.1
29941	0.946131	2.1
29942	1.80183	2.1
29943	1.89226	1.74566
29944	1.89226	1.6841
29945	1.89226	2.1
29946	1.79606	1.62088
29947	1.79606	2.1
29948	1.24001	2.00492
29949	1.41822	1.66502
29950	1.95665	0.821485
29951	1.77342	1.43253
29952	3.19666	1.12076
29953	1.95665	1.09192
29954	1.44859	1.99384
29955	1.41821	1.81043
29956	1.41821	1.79537
29957	1.95543	1.11258
29958	1.95543	1.47129
29959	1.95665	0.969839
29960	1.77342	1.53944
29961	2.33611	1.23848
29962	2.49694	0.801235
29963	4.29276	1.63405
29964	1.95665	1.42773
29965	1.95544	1.56427
29966	2.4728	0.711419
29967	2.49693	1.30602
29968	4.42945	1.04964
29969	2.24937	2.1
29970	2.49053	2.1
29971	2.16323	2.1
29972	2.36377	2.1
29973	4.61314	2.1
29974	2.49053	2.1
29975	2.58639	2.1
29976	2.36377	2.1
29977	2.47395	2.1
29978	4.95016	2.1
29979	2.45054	2.1
29980	2.36377	2.1
29981	1.92911	2.1
29982	2.181 2.1	
29983	4.29288	2.1

29984	2.36377	2.1
29985	2.47395	2.1
29986	2.181 2.1	
29987	2.29881	2.1
29988	4.66258	2.1
29989	1.57885	2.1
29990	1.13339	2.1
29991	1.48594	2.1
29992	1.13322	2.1
29993	2.71207	2.1
29994	1.48596	2.1
29995	1.20761	2.1
29996	1.20761	2.1
29997	1.13323	2.1
29998	1.46568	2.1
29999	1.3913	2.1
30000	1.89226	0.531445
30001	1.89226	1.11303
30002	1.89226	0.619765
30003	1.89226	0.928749
30004	1.89226	0.496265
30005	1.89226	0.671901
30006	1.11712	0.898768
30007	0.946129	1.08577
30008	0.686934	0.683454
30009	0.946133	0.694193
30010	0.9461	0.876192
30011	1.10729	2.1
30012	0.686963	1.13621
30013	0.686963	0.532761
30014	1.10945	0.97107
30015	0.80397	2.1
30016	0.946131	0.696283
30017	0.946131	1.22036
30018	1.89226	2.1
30019	1.89226	1.16308
30020	1.89226	0.909436
30021	1.89226	1.04178
30022	3.78452	1.08687
30023	1.22615	2.1
30024	1.3439	2.1
30025	1.47868	2.1
30026	1.55998	0.980121
30027	2.78613	2.1
30028	1.04973	2.1
30029	1.47868	2.1
30030	1.20149	2.1
30031	1.61721	2.1
30032	2.68017	2.1
30033	1.89226	2.1
30034	1.89226	2.1
30035	1.89226	2.1
30036	1.89226	0.706592
30037	3.78452	2.1
30038	0.946133	0.687154
30039	0.946133	1.61074
30040	0.946128	2.1
30041	0.946128	2.1

30042	0.946131	0.654029
30043	0.946131	1.14354
30044	0.946131	2.08791
30045	0.946131	0.768133
30046	0.94613	1.65625
30047	0.94613	1.02054
30048	0.946131	1.26173
30049	0.946131	0.994928
30050	0.798126	2.1
30051	0.798126	1.98706
30052	0.805719	2.1
30053	0.805719	2.1
30054	0.779992	2.1
30055	0.864507	1.98815
30056	0.739338	2.1
30057	0.808607	2.1
30058	0.808606	2.1
30059	0.864506	2.1
30060	0.739339	2.1
30061	0.779993	2.1
30062	0.946128	2.1
30063	0.946128	2.1
30064	1.18959	0.905116
30065	1.00304	2.1
30066	1.00304	2.1
30067	1.00304	1.88234
30068	0.94613	2.0641
30069	0.94613	0.930851
30070	0.94613	2.1
30071	1.18959	2.06274
30072	1.00304	1.92422
30073	0.94613	2.06008
30074	2.19163	2.1
30075	1.89226	1.80714
30076	2.16541	2.1
30077	1.89226	0.972477
30078	4.08389	1.39669
30079	1.89226	2.1
30080	1.89226	1.316
30081	1.89226	1.83839
30082	1.89226	0.979806
30083	3.78452	1.81061
30084	0.964782	2.1
30085	1.30151	0.922813
30086	1.30318	0.933402
30087	1.30318	2.1
30088	0.949134	0.829764
30089	0.949134	1.92216
30090	2.15005	2.1
30091	1.89226	1.11001
30092	2.16541	1.8181
30093	2.16541	2.1
30094	1.89226	0.860915
30095	1.89226	2.1
30096	1.89226	2.02829
30097	1.89226	1.87062
30098	1.89226	1.69044
30099	1.89226	0.673196

30100	3.78452	1.13586
30101	0.949134	2.1
30102	1.30151	1.7127
30103	0.949134	1.52373
30104	1.32143	0.951343
30105	2.25064	2.1
30106	0.982859	0.347462
30107	1.28326	0.405817
30108	1.19052	0.362985
30109	1.19052	0.42655
30110	1.10476	0.343371
30111	1.10476	0.43097
30112	1.37265	0.657238
30113	1.38247	0.844354
30114	1.36636	0.994208
30115	1.3294	0.628839
30116	2.71187	0.738597
30117	1.89226	0.87255
30118	1.89226	0.578232
30119	1.89226	0.65333
30120	1.89226	0.74902
30121	1.89226	0.653492
30122	1.89226	0.778044
30123	1.36636	1.24082
30124	1.3294	0.617006
30125	1.30001	0.753799
30126	1.36416	0.984428
30127	2.69356	0.855561
30128	1.89226	1.03049
30129	1.89226	0.583229
30130	1.89226	0.690322
30131	1.89226	0.81437
30132	1.89226	0.625379
30133	1.89226	0.731785
30134	2.83701	0.659197
30135	1.89226	0.678305
30136	1.89226	0.966168
30137	2.79312	0.549885
30138	4.68539	0.639306
30139	1.44288	2.1
30140	0.948677	2.1
30141	1.84076	2.1
30142	1.42884	2.1
30143	2.78943	2.1
30144	1.89226	1.88685
30145	1.89226	2.1
30146	1.89226	2.1
30147	1.89226	1.91305
30148	3.78452	2.1
30149	0.909402	1.65138
30150	0.909402	2.03663
30151	1.3063	2.1
30152	1.56757	2.1
30153	1.00038	1.33325
30154	1.5235	2.1
30155	0.946127	2.1
30156	0.946127	1.47273
30157	0.946128	2.1

30158	1.26957	2.1
30159	1.00038	2.1
30160	0.946129	2.1
30161	1.17256	2.1
30162	2.2846	2.05823
30163	1.1844	2.1
30164	2.28904	0.923128
30165	3.469 2.1	
30166	0.867886	0.891587
30167	0.867886	2.1
30168	0.867277	2.1
30169	0.867277	2.1
30170	1.14296	1.17142
30171	1.14452	2.1
30172	0.591766	2.1
30173	0.592199	2.1
30174	0.591766	2.1
30175	1.14296	2.1
30176	0.5922	2.1
30177	1.14452	2.1
30178	1.27408	0.330487
30179	1.56339	0.36253
30180	1.52522	0.369772
30181	1.34447	0.328281
30182	2.90786	0.33623
30183	1.27408	0.344616
30184	1.01121	0.322046
30185	0.991868	0.330101
30186	1.34447	0.351137
30187	2.35569	0.336537
30188	0.944818	0.386298
30189	0.883008	0.379147
30190	0.66957	0.338187
30191	0.616883	0.336915
30192	0.755679	0.340594
30193	0.755679	0.371939
30194	0.791912	0.409774
30195	0.701943	0.332048
30196	0.901541	0.363369
30197	0.634847	0.372837
30198	0.82781	0.359784
30199	0.655405	0.357429
30200	1.73819	2.1
30201	1.70303	2.1
30202	1.70303	2.1
30203	1.70303	2.1
30204	1.94516	2.1
30205	1.94517	2.1
30206	0.94994	2.1
30207	2.07469	2.1
30208	1.08506	2.1
30209	2.0815	2.1
30210	3.12902	2.1
30211	0.946131	2.1
30212	0.946131	2.1
30213	0.946131	2.1
30214	0.946131	2.1
30215	0.946128	2.1

30216	0.946128	2.1
30217	0.946133	2.1
30218	0.946133	2.1
30219	0.946131	2.1
30220	0.946131	2.1
30221	0.94613	2.1
30222	0.94613	2.1
30223	2.51796	2.1
30224	1.64193	2.1
30225	2.51172	2.1
30226	1.68893	2.1
30227	4.20689	2.1
30228	2.51172	2.1
30229	1.70002	2.1
30230	1.70002	2.1
30231	1.68893	2.1
30232	2.48689	2.1
30233	2.4758	2.1
30234	2.27963	0.05
30235	2.21582	0.05
30236	2.60486	0.05
30237	2.5105	0.05
30238	4.79013	0.05
30239	2.21582	0.05
30240	2.02069	0.05
30241	2.5105	0.05
30242	2.28615	0.05
30243	4.53119	0.05
30244	2.60486	0.05
30245	2.5105	0.05
30246	2.55791	0.05
30247	2.47141	0.05
30248	5.06842	0.05
30249	2.5105	0.05
30250	2.28615	0.05
30251	2.47141	0.05
30252	2.23337	0.05
30253	4.74387	0.05
30254	2.06984	2.1
30255	0.967499	2.1
30256	2.12435	2.1
30257	2.0021	2.1
30258	1.08975	2.1
30259	0.967499	2.1
30260	1.89226	0.323393
30261	1.89226	0.32014
30262	1.89226	0.323176
30263	1.89226	0.322509
30264	1.89226	0.318065
30265	1.89226	0.317192
30266	1.89226	1.73537
30267	1.89226	2.1
30268	1.89226	2.1
30269	1.89226	2.1
30270	3.78452	2.1
30271	1.89227	2.1
30272	1.89227	2.1
30273	1.89227	2.1

30274	1.89227	2.1
30275	3.78453	2.1
30276	0.55882	2.1
30277	0.696462	2.1
30278	1.07003	2.1
30279	0.937179	2.1
30280	0.818077	2.1
30281	0.818077	2.1
30282	0.815565	2.1
30283	0.815565	2.1
30284	0.696462	2.1
30285	1.07003	2.1
30286	0.93718	2.1
30287	0.55882	2.1
30288	2.53857	2.1
30289	1.87436	2.1
30290	2.27541	2.1
30291	2.27541	2.1
30292	2.14005	2.1
30293	2.14005	2.1
30294	1.89226	2.1
30295	1.89226	2.0558
30296	1.89226	0.960359
30297	1.89226	2.1
30298	3.78452	2.1
30299	1.89227	2.1
30300	1.89227	2.1
30301	1.89227	2.1
30302	1.89227	2.1
30303	3.78453	2.1
30304	1.39292	2.1
30305	2.14006	2.1
30306	2.41083	2.1
30307	1.68761	2.1
30308	3.80376	2.1
30309	2.53857	2.1
30310	2.14005	2.1
30311	2.41083	2.1
30312	2.82043	2.1
30313	4.9494	2.1
30314	1.90439	0.428942
30315	1.51835	0.424776
30316	1.20473	0.368375
30317	0.947693	0.437701
30318	2.85208	0.429162
30319	2.586	2.1
30320	1.46262	2.1
30321	2.14009	2.1
30322	2.78101	2.1
30323	4.72609	2.1
30324	1.89224	2.1
30325	2.78101	2.1
30326	2.586	2.1
30327	2.586	2.1
30328	1.89224	2.1
30329	1.89224	2.1
30330	0.994293	2.1
30331	1.19	2.1

30332	1.09483	2.1
30333	1.09483	2.1
30334	1.06827	2.1
30335	1.06827	2.1
30336	1.89226	2.1
30337	1.89226	2.1
30338	1.89226	2.1
30339	1.89226	2.1
30340	3.78452	2.1
30341	0.946132	2.1
30342	0.946132	2.1
30343	1.31912	2.1
30344	1.3572	2.1
30345	0.946131	2.1
30346	0.946131	2.1
30347	1.31912	2.1
30348	1.31912	2.1
30349	0.946131	2.1
30350	1.31912	2.1
30351	1.35721	2.1
30352	0.946131	2.1
30353	2.24577	0.561476
30354	1.09408	0.313942
30355	1.60239	0.512438
30356	1.09408	0.532255
30357	2.39404	0.480645
30358	2.07382	0.33755
30359	1.70303	0.362946
30360	1.3216	0.409161
30361	1.23104	0.345098
30362	1.05475	0.390541
30363	2.60408	0.371206
30364	1.89226	0.426711
30365	1.89226	0.436447
30366	1.89226	0.381578
30367	1.89226	0.442824
30368	3.78452	0.441236
30369	1.89226	0.342017
30370	2.3845	0.347317
30371	2.6875	0.348029
30372	1.89226	0.34947
30373	2.6875	0.334094
30374	1.89226	0.338809
30375	1.46759	2.1
30376	1.17585	2.1
30377	1.85189	2.1
30378	1.39223	2.1
30379	2.85981	2.1
30380	1.85189	2.1
30381	1.71255	2.1
30382	1.71255	2.1
30383	1.39223	2.1
30384	2.28511	2.1
30385	1.96479	2.1
30386	1.89227	0.776672
30387	1.89227	0.898196
30388	1.89227	0.994596
30389	1.89227	0.722533

30390	1.89227	1.47217
30391	1.89227	0.818918
30392	1.89226	0.818724
30393	1.89226	0.866991
30394	1.89226	1.02389
30395	1.89226	0.716815
30396	1.89226	1.51522
30397	1.89226	0.796031
30398	1.89226	0.793773
30399	1.89226	0.951313
30400	1.89226	0.971213
30401	1.89226	0.706419
30402	1.89226	1.6181
30403	1.89226	0.828079
30404	1.89226	0.710846
30405	2.77788	0.766661
30406	2.21062	0.834145
30407	2.21062	0.664595
30408	1.89226	0.973088
30409	1.89226	0.714604
30410	0.868548	0.713667
30411	0.610104	0.776065
30412	0.796678	0.711178
30413	0.865021	0.660995
30414	1.0883	0.689653
30415	0.904938	0.758106
30416	2.21062	0.724004
30417	1.49548	0.699609
30418	1.2068	0.854275
30419	1.54764	0.708527
30420	0.790005	0.662336
30421	1.89226	0.919922
30422	2.67457	2.1
30423	1.89226	1.25019
30424	2.56352	2.1
30425	4.56683	2.1
30426	2.67456	2.1
30427	2.20011	2.1
30428	2.56351	2.1
30429	2.11083	2.1
30430	4.78539	2.1
30431	2.56348	2.1
30432	2.1108	2.1
30433	2.45763	2.1
30434	2.00986	2.1
30435	4.56843	2.1
30436	0.946133	0.05
30437	0.946133	0.05
30438	0.946132	0.05
30439	0.946132	0.05
30440	0.946132	0.05
30441	0.946132	0.05
30442	0.946132	0.05
30443	0.946132	0.05
30444	0.946132	0.05
30445	0.946132	0.05
30446	0.946133	0.05
30447	0.946133	0.05

30448	1.45927	0.797702
30449	1.25133	1.11061
30450	1.00082	1.02194
30451	1.41689	1.08485
30452	1.0432	0.864836
30453	1.45927	1.02307
30454	1.41689	0.92687
30455	1.29579	1.29007
30456	1.34874	1.09308
30457	1.25133	1.08912
30458	2.76563	1.38307
30459	0.675555	1.19447
30460	0.674371	1.13141
30461	0.625664	1.03518
30462	0.523133	1.05867
30463	0.676509	1.12358
30464	0.676509	1.30618
30465	0.62471	1.12072
30466	0.62471	0.955995
30467	0.675556	0.975699
30468	0.523132	0.894498
30469	0.674373	0.903475
30470	0.625663	0.970208
30471	1.89226	0.713278
30472	1.89226	0.711507
30473	1.89226	0.704768
30474	1.89226	0.731324
30475	3.78453	0.955293
30476	0.851517	1.10018
30477	0.946133	0.75755
30478	0.946132	0.820964
30479	0.946132	1.06473
30480	0.946133	0.668966
30481	0.851517	1.63574
30482	0.946132	0.765314
30483	0.946132	0.69538
30484	0.851516	0.763827
30485	0.851516	0.685125
30486	0.946133	1.01714
30487	0.946133	0.75926
30488	0.569995	0.755282
30489	0.816895	0.900325
30490	1.33893	0.975996
30491	1.18795	0.844612
30492	1.18795	0.7889
30493	0.569995	1.13111
30494	0.816895	0.822151
30495	1.33893	0.952241
30496	0.859572	1.06004
30497	0.859572	1.00061
30498	1.04936	0.815399
30499	1.04936	0.720662
30500	1.89226	2.1
30501	1.89226	2.1
30502	2.55618	2.1
30503	2.55618	2.1
30504	2.66983	2.1
30505	2.00591	2.1

30506	1.89226	0.601173
30507	2.64586	0.899941
30508	2.83331	2.03197
30509	2.83331	0.581586
30510	1.89226	2.07144
30511	1.89226	0.57042
30512	2.64586	1.74075
30513	2.0815	2.1
30514	1.99985	2.1
30515	1.94005	2.1
30516	2.83332	2.01102
30517	2.74859	2.1
30518	1.98061	0.723112
30519	1.68356	2.1
30520	1.83879	0.774142
30521	1.55987	2.1
30522	3.54048	2.1
30523	1.68356	2.1
30524	1.20969	2.1
30525	1.20969	2.1
30526	1.30751	2.1
30527	1.55987	2.1
30528	1.65769	2.1
30529	1.83879	0.79889
30530	1.55987	2.1
30531	1.68443	0.732386
30532	1.4449	2.1
30533	3.2443	2.1
30534	0.660368	2.1
30535	0.660368	2.1
30536	0.666922	2.1
30537	0.666922	2.1
30538	0.560377	2.1
30539	0.604842	2.1
30540	0.779935	2.1
30541	0.722448	2.1
30542	0.722446	2.1
30543	0.560377	2.1
30544	0.604844	2.1
30545	0.779934	2.1
30546	1.89227	2.1
30547	1.89227	2.1
30548	1.89227	2.1
30549	1.89227	2.1
30550	3.78453	2.1
30551	1.42896	2.1
30552	1.23037	2.1
30553	1.42896	2.1
30554	1.474	2.1
30555	1.2016	2.1
30556	1.24665	2.1
30557	1.89226	2.1
30558	2.7188	2.1
30559	1.89226	2.1
30560	2.70702	2.1
30561	4.61106	2.1
30562	1.89226	2.1
30563	1.89226	2.1

30564	1.89226	2.1
30565	1.89226	2.1
30566	3.78453	2.1
30567	0.644603	2.1
30568	0.644603	2.1
30569	0.648272	2.1
30570	0.648272	2.1
30571	0.585644	2.1
30572	0.600802	2.1
30573	0.692073	2.1
30574	0.714478	2.1
30575	0.585644	2.1
30576	0.692073	2.1
30577	0.714478	2.1
30578	0.600802	2.1
30579	1.35351	2.1
30580	1.35351	2.1
30581	0.946131	2.1
30582	0.946131	2.1
30583	1.34392	2.1
30584	1.35351	2.1
30585	0.946132	2.1
30586	0.946132	2.1
30587	0.946132	2.1
30588	1.34392	2.1
30589	0.946132	2.1
30590	1.35351	2.1
30591	1.87609	2.1
30592	1.79155	2.1
30593	1.79155	2.1
30594	1.89226	2.1
30595	1.89226	2.1
30596	1.99211	2.1
30597	1.89226	2.1
30598	1.71706	2.1
30599	1.71706	2.1
30600	1.79155	2.1
30601	1.89226	2.1
30602	1.96675	2.1
30603	1.46869	2.1
30604	1.89226	2.1
30605	1.89226	2.1
30606	1.89226	2.1
30607	1.5001	2.1
30608	1.5001	2.1
30609	1.89226	2.1
30610	1.89226	2.1
30611	1.89226	2.1
30612	1.89226	2.1
30613	1.89226	2.1
30614	1.89226	1.0964
30615	1.21408	2.1
30616	2.23563	2.1
30617	2.34021	2.1
30618	2.34021	2.1
30619	1.17285	2.1
30620	1.17285	2.1
30621	1.89226	2.1

30622	1.89226	2.1
30623	1.47937	2.1
30624	1.89226	2.1
30625	1.14822	2.1
30626	1.46869	1.5103
30627	1.89226	2.06036
30628	1.89226	2.1
30629	1.89226	2.1
30630	1.89226	2.1
30631	1.89226	2.1
30632	1.89226	0.963936
30633	1.25716	2.1
30634	2.34021	2.1
30635	2.4542	2.1
30636	2.4542	2.1
30637	1.21408	2.1
30638	1.21408	2.1
30639	1.66824	2.1
30640	2.44883	2.00738
30641	1.93003	2.1
30642	2.24795	2.1
30643	3.88907	2.1
30644	1.89319	2.1
30645	2.24795	2.1
30646	2.82724	2.1
30647	2.31318	2.1
30648	2.30868	2.1
30649	1.93003	2.1
30650	1.89226	0.909725
30651	1.42589	2.1
30652	1.42589	1.38472
30653	1.80501	2.1
30654	1.89226	1.20736
30655	2.27137	2.1
30656	1.21067	0.397561
30657	2.39166	0.505517
30658	1.73577	0.438786
30659	1.73577	0.444504
30660	1.53584	0.438917
30661	1.53584	0.421354
30662	1.35025	1.07341
30663	2.0599	0.7602
30664	1.51084	0.878503
30665	2.27218	0.510843
30666	3.57074	0.873709
30667	2.0599	0.674078
30668	2.51818	0.9291
30669	2.27218	0.482612
30670	2.66532	0.811755
30671	4.72522	0.646514
30672	1.67115	0.948274
30673	2.27218	0.703167
30674	2.39605	0.934488
30675	2.39605	0.677826
30676	1.51084	0.861994
30677	1.51084	0.839343
30678	2.27218	0.614011
30679	2.66532	1.13356

30680	2.39605	0.588704
30681	2.76829	0.806743
30682	5.06137	0.669515
30683	2.08684	0.724823
30684	2.36033	1.0352
30685	1.89224	0.662409
30686	1.89224	0.954173
30687	4.25257	0.728941
30688	2.41473	0.37767
30689	1.00307	0.400314
30690	2.01365	0.385648
30691	1.00307	0.410724
30692	3.05113	0.373966
30693	2.04056	0.414118
30694	1.11099	2.1
30695	2.25103	2.1
30696	2.22227	2.1
30697	2.22227	2.1
30698	1.14431	2.1
30699	1.14431	2.1
30700	1.07485	2.1
30701	2.22229	2.1
30702	2.19163	2.1
30703	2.19163	2.1
30704	1.111 2.1	
30705	1.111 2.1	
30706	1.0547	1.46987
30707	1.19886	2.1
30708	0.946131	2.1
30709	0.946131	2.1
30710	0.946131	2.1
30711	0.946131	1.65092
30712	1.19886	2.1
30713	1.19886	2.1
30714	1.0547	2.1
30715	0.946131	2.1
30716	0.946132	2.1
30717	1.19886	2.1
30718	1.89417	1.766
30719	2.64424	0.442576
30720	1.76585	1.06777
30721	2.47695	0.619177
30722	4.37112	0.748584
30723	1.89226	2.00923
30724	1.89226	0.687578
30725	1.89226	2.1
30726	1.89226	0.624736
30727	3.78453	0.825058
30728	0.930281	1.03123
30729	1.0547	2.1
30730	0.946131	2.1
30731	0.946131	2.1
30732	0.930309	2.1
30733	0.930309	1.11941
30734	1.07052	2.1
30735	1.07052	2.1
30736	0.93028	2.1
30737	0.946131	2.1

30738	1.0547	2.1
30739	0.946131	2.1
30740	1.00015	0.19173
30741	1.00015	0.832346
30742	1.01918	0.989428
30743	1.01918	0.585239
30744	1.17437	0.599576
30745	1.23847	0.216427
30746	0.882926	0.98573
30747	0.780859	0.821719
30748	0.780859	0.60293
30749	1.17438	0.651397
30750	0.882925	0.687999
30751	1.23848	0.6884
30752	1.89226	2.1
30753	1.89226	0.596562
30754	1.89226	0.712588
30755	1.89226	1.37114
30756	1.89226	0.698563
30757	1.89226	1.80896
30758	1.22083	2.1
30759	1.29579	2.1
30760	1.26236	2.1
30761	1.06191	2.1
30762	1.42128	2.1
30763	1.22083	2.1
30764	1.42128	2.1
30765	1.45806	2.1
30766	1.43502	2.1
30767	1.29579	2.1
30768	1.5605	2.1
30769	1.42128	2.1
30770	1.02813	2.1
30771	1.12121	2.1
30772	1.40557	2.1
30773	1.35298	2.1
30774	1.1314	2.1
30775	1.28679	2.1
30776	1.1715	2.1
30777	1.1715	2.1
30778	0.975656	2.1
30779	1.24908	2.1
30780	0.98956	2.1
30781	1.2681	2.1
30782	0.946132	2.1
30783	0.946132	2.1
30784	1.31889	2.1
30785	1.31889	2.1
30786	1.16546	2.1
30787	1.31889	2.1
30788	0.946133	2.1
30789	0.946133	2.1
30790	0.946133	2.1
30791	1.31889	2.1
30792	0.946132	2.1
30793	1.16546	2.1
30794	2.51518	0.0584804
30795	2.37584	0.0702666

30796	1.11712	0.0596193
30797	1.31242	0.0793101
30798	3.68826	0.0691972
30799	2.51518	0.0633074
30800	1.26311	0.0828438
30801	2.48977	0.0659234
30802	2.48977	0.0740737
30803	1.31242	0.064402
30804	1.31242	0.0759928
30805	1.89226	0.455648
30806	1.89226	0.680973
30807	1.89226	0.413532
30808	1.89226	0.66429
30809	3.78452	0.520882
30810	2.04737	0.392833
30811	1.77122	0.672605
30812	1.94498	0.411485
30813	1.65174	0.60284
30814	3.71621	0.504153
30815	1.41077	2.0137
30816	1.29729	1.07907
30817	1.77123	0.552459
30818	1.77123	0.835712
30819	1.65175	0.599506
30820	1.29129	0.61516
30821	1.89226	0.583004
30822	1.89226	2.03765
30823	1.89226	2.1
30824	1.89226	0.553876
30825	1.89226	1.94165
30826	1.89226	0.64587
30827	1.89226	0.407101
30828	1.89226	0.665308
30829	1.89226	0.410499
30830	1.89226	0.587463
30831	3.78451	0.487041
30832	1.94498	0.400811
30833	1.65174	0.615334
30834	1.7968	0.43903
30835	1.49831	0.574094
30836	3.44329	0.504123
30837	1.49831	0.57302
30838	1.29729	1.22288
30839	1.49831	2.00716
30840	1.65175	0.58638
30841	1.16858	1.12583
30842	1.32201	0.622625
30843	1.89226	0.518206
30844	1.89226	1.58188
30845	1.89226	0.572456
30846	1.89226	1.78205
30847	3.78452	0.663711
30848	1.89226	0.05
30849	1.89226	0.0507955
30850	1.89226	0.0501831
30851	1.89226	0.05
30852	1.89226	0.05
30853	1.89226	0.05

30854	1.65324	0.631539
30855	1.26409	0.370585
30856	1.42491	0.372402
30857	1.42491	0.646187
30858	1.52268	0.4176
30859	1.52268	0.639398
30860	1.52268	0.961145
30861	1.26409	0.415587
30862	1.32697	0.588789
30863	1.05724	0.370465
30864	2.57991	0.504146
30865	1.89226	0.34712
30866	1.96548	0.360397
30867	2.0178	0.367047
30868	1.96548	0.357667
30869	1.94458	0.352897
30870	1.89226	0.347244
30871	1.99366	0.344431
30872	2.0178	0.361118
30873	1.89226	0.364512
30874	2.0178	0.356573
30875	1.75999	0.352389
30876	1.89226	0.345768
30877	0.946126	2.1
30878	0.946126	2.1
30879	1.28228	2.1
30880	1.19444	2.1
30881	0.946127	2.1
30882	0.946127	2.1
30883	1.19444	2.1
30884	1.19444	2.1
30885	0.946128	2.1
30886	1.19444	2.1
30887	1.28228	2.1
30888	0.946128	2.1
30889	1.89226	2.1
30890	1.89226	2.1
30891	1.89226	2.1
30892	1.89226	1.33704
30893	3.78452	2.1
30894	2.14104	2.1
30895	1.89226	2.1
30896	2.19948	2.1
30897	1.89226	2.1
30898	1.89226	2.1
30899	2.19948	2.1
30900	1.89226	2.1
30901	2.38888	2.1
30902	2.23932	2.1
30903	2.38888	2.1
30904	1.74269	2.1
30905	1.89226	2.1
30906	1.89226	2.1
30907	1.89226	1.02788
30908	1.89226	2.1
30909	1.89226	2.1
30910	3.78452	2.1
30911	0.946132	2.1

30912	0.946132	2.1
30913	1.07052	2.1
30914	1.07052	2.1
30915	0.946132	2.1
30916	0.946132	2.1
30917	1.05132	2.1
30918	1.07052	2.1
30919	1.05132	2.1
30920	0.946132	2.1
30921	1.07052	2.1
30922	0.946133	2.1
30923	2.40171	2.1
30924	1.89226	2.1
30925	1.89226	2.1
30926	1.89226	2.1
30927	2.42723	2.1
30928	2.42723	2.1
30929	1.89226	2.1
30930	1.89226	1.1254
30931	1.89226	2.1
30932	1.89226	1.6728
30933	3.78452	2.07483
30934	1.63661	2.1
30935	2.3532	1.04727
30936	2.33287	0.844143
30937	2.33287	2.1
30938	1.64158	0.787814
30939	1.64158	2.1
30940	2.38918	2.1
30941	1.89226	2.1
30942	1.89226	2.1
30943	1.89226	2.1
30944	2.40171	2.1
30945	2.40171	2.1
30946	1.89226	2.1
30947	1.89226	1.88986
30948	1.89226	2.1
30949	1.89226	1.43153
30950	1.89226	2.1
30951	1.89226	2.01314
30952	1.64158	2.1
30953	2.3532	1.25155
30954	1.6488	2.1
30955	2.37364	1.72561
30956	4.002 2.1	
30957	2.54752	0.511601
30958	1.42445	0.533475
30959	1.86697	0.552096
30960	1.86697	0.508201
30961	2.06666	0.552306
30962	2.06666	0.51422
30963	2.50872	0.442599
30964	2.06666	0.477145
30965	1.90439	0.45844
30966	1.51835	0.457211
30967	3.97105	0.450603
30968	0.755161	0.479395
30969	0.755161	0.47131

30970	0.793994	0.458264
30971	0.793994	0.473209
30972	0.712225	0.473344
30973	0.515826	0.471199
30974	1.03333	0.477203
30975	0.759175	0.465263
30976	0.759173	0.459737
30977	0.515827	0.434915
30978	0.712226	0.457421
30979	1.03333	0.466209
30980	1.69633	0.428092
30981	1.89226	0.422924
30982	1.62477	0.395042
30983	1.89226	0.416522
30984	1.62477	0.426817
30985	1.89226	0.436835
30986	1.89226	0.50435
30987	1.89226	0.544287
30988	1.89226	0.532634
30989	1.89226	0.535622
30990	3.78452	0.533756
30991	2.28622	0.430255
30992	1.09667	0.329811
30993	1.17506	0.318671
30994	2.24411	0.371211
30995	1.17506	0.441478
30996	2.24411	0.467391
30997	2.24741	2.1
30998	1.70303	2.1
30999	2.49168	2.1
31000	1.57936	2.1
31001	2.53729	2.1
31002	1.70303	2.1
31003	2.24741	2.1
31004	2.47557	2.1
31005	1.89227	2.1
31006	1.89227	2.1
31007	4.36784	2.1
31008	2.27016	0.08653
31009	1.10476	0.129607
31010	1.44103	0.0922221
31011	1.10476	0.176129
31012	2.26791	0.0917872
31013	1.93165	0.137916
31014	2.27016	0.0796724
31015	1.89226	0.143238
31016	1.89226	0.0956835
31017	2.4065	0.121971
31018	4.29876	0.123324
31019	2.27016	0.0788406
31020	1.44103	0.110707
31021	1.53336	0.0820046
31022	2.4065	0.116229
31023	3.84753	0.0839991
31024	2.7542	0.467174
31025	1.89226	0.485107
31026	2.35027	0.481394
31027	1.89226	0.482539

31028	2.35027	0.470504
31029	1.89226	0.471328
31030	2.3301	2.1
31031	2.36071	1.31935
31032	2.07629	2.1
31033	2.01105	2.1
31034	4.37176	2.1
31035	1.86512	2.1
31036	2.01105	2.1
31037	2.01105	2.1
31038	2.3301	2.1
31039	1.57459	2.1
31040	1.89364	2.1
31041	2.52671	2.1
31042	2.01105	2.1
31043	1.78251	2.1
31044	1.70302	0.989703
31045	3.98362	2.1
31046	1.227 2.1	
31047	2.01105	2.1
31048	1.45869	2.1
31049	1.41713	2.1
31050	1.70302	2.1
31051	1.64994	2.1
31052	2.00989	2.1
31053	1.28938	2.1
31054	1.8769	2.1
31055	1.20969	2.1
31056	3.21958	2.1
31057	1.8769	2.1
31058	1.15864	2.1
31059	1.15864	2.1
31060	1.20969	2.1
31061	1.77353	2.1
31062	1.82458	2.1
31063	1.7986	2.1
31064	2.48717	2.1
31065	2.50161	2.1
31066	1.75869	2.1
31067	4.30021	2.1
31068	2.50161	2.1
31069	1.75251	2.1
31070	1.81678	2.1
31071	1.75869	2.1
31072	2.5597	2.1
31073	2.50161	2.1
31074	1.91334	0.60517
31075	1.85439	0.754598
31076	1.91334	0.646369
31077	1.70303	0.738558
31078	1.70303	0.570642
31079	1.49692	0.603777
31080	2.39986	0.357106
31081	1.89226	0.43598
31082	1.89226	0.433836
31083	2.50756	0.373863
31084	4.39982	0.386439
31085	2.39986	0.405088

31086	1.70303	0.356297
31087	1.75564	0.350466
31088	1.75904	0.389447
31089	2.50272	0.383168
31090	2.50756	0.406066
31091	1.89226	0.431836
31092	2.50756	0.378506
31093	2.47857	0.401008
31094	2.50756	0.436708
31095	1.86327	0.366761
31096	1.89226	0.410518
31097	1.22241	0.420132
31098	1.20827	0.434478
31099	0.772416	0.369198
31100	0.555208	0.393331
31101	1.004	0.370866
31102	1.004	0.403552
31103	0.93504	0.469123
31104	0.744364	0.379423
31105	1.2089	0.413456
31106	0.654935	0.422174
31107	0.773927	0.396729
31108	1.03618	0.415581
31109	1.89227	0.05
31110	1.89227	0.05
31111	1.89227	0.05
31112	1.89227	0.05
31113	3.78453	0.05
31114	1.89226	0.05
31115	1.89226	0.05
31116	1.89226	0.05
31117	1.89226	0.05
31118	3.78452	0.05
31119	1.82643	0.05
31120	2.7084	0.05
31121	2.12654	0.05
31122	2.65614	0.05
31123	1.82643	0.05
31124	2.35603	0.05
31125	1.29385	0.05
31126	0.946133	0.05
31127	1.05492	0.05
31128	0.946132	0.05
31129	1.05492	0.05
31130	1.29385	0.05
31131	0.946133	0.05
31132	0.946133	0.05
31133	1.29385	0.05
31134	1.29385	0.05
31135	0.946132	0.05
31136	0.946132	0.05
31137	0.946132	0.05
31138	1.27329	0.05
31139	1.29385	0.05
31140	0.946129	0.05
31141	1.29385	0.05
31142	1.27328	0.05
31143	0.946131	0.05

31144	0.946131	0.05
31145	1.27328	0.05
31146	1.27328	0.05
31147	0.94613	0.05
31148	0.94613	0.05
31149	1.89226	1.56389
31150	1.89226	1.86392
31151	1.89226	0.837144
31152	1.89226	1.14983
31153	3.78453	1.24122
31154	1.89226	1.32523
31155	1.89226	1.82886
31156	1.89226	0.787289
31157	1.89226	1.49454
31158	3.78453	1.01637
31159	1.21488	1.80207
31160	1.14605	2.1
31161	1.62974	1.32741
31162	1.52399	1.11018
31163	2.84463	1.91175
31164	0.577078	1.1722
31165	0.81251	2.1
31166	0.821003	1.54949
31167	0.543413	2.05794
31168	0.683929	1.26217
31169	0.722433	2.1
31170	0.730674	1.97129
31171	0.730674	1.09605
31172	0.857766	1.56346
31173	0.862119	1.22095
31174	0.600938	1.93857
31175	0.596094	1.57601
31176	1.89226	0.972121
31177	1.89226	1.60888
31178	1.89226	0.781221
31179	1.89226	1.26815
31180	3.78453	1.02556
31181	1.89226	0.897557
31182	1.89226	2.01761
31183	1.89226	0.89537
31184	1.89226	1.10916
31185	3.78453	1.28366
31186	1.62974	1.92967
31187	1.56958	2.06899
31188	2.2362	0.975331
31189	2.11424	1.54241
31190	3.86595	1.46401
31191	2.2362	1.0791
31192	1.85414	2.1
31193	2.28211	0.809893
31194	1.78367	0.935119
31195	2.2362	1.78334
31196	1.73256	2.04953
31197	0.851539	1.55312
31198	0.851539	0.723135
31199	1.14454	1.02091
31200	0.938988	2.00585
31201	0.851485	1.44421

31202	0.920593	0.785137
31203	0.851541	0.517042
31204	0.851541	0.843139
31205	1.04073	0.678917
31206	1.16342	0.735006
31207	0.825315	0.929297
31208	0.825316	0.770845
31209	1.89226	1.79149
31210	1.89226	2.1
31211	1.89226	2.1
31212	1.89226	1.80671
31213	3.78453	2.1
31214	2.57016	2.1
31215	1.74789	2.1
31216	2.57016	2.1
31217	2.522	2.1
31218	1.61727	2.1
31219	1.56911	2.1
31220	1.28075	2.1
31221	1.406	2.1
31222	0.946131	1.96337
31223	0.946131	1.63764
31224	1.28075	1.98754
31225	1.00333	2.1
31226	0.94613	2.1
31227	0.94613	2.00653
31228	1.041	2.09861
31229	1.041	2.0995
31230	0.943484	1.98328
31231	1.27717	2.1
31232	1.89226	2.1
31233	1.89226	1.63539
31234	1.89226	1.71217
31235	1.89226	2.1
31236	1.89226	1.7836
31237	1.89226	2.1
31238	2.57016	2.1
31239	1.61727	2.1
31240	2.74196	2.1
31241	1.59094	2.1
31242	4.16109	2.1
31243	1.89226	1.65256
31244	2.0815	2.1
31245	2.2034	2.1
31246	1.82493	1.40914
31247	1.87847	2.1
31248	2.16142	2.1
31249	1.89226	2.1
31250	1.89226	2.1
31251	1.89226	2.1
31252	1.89226	2.1
31253	3.78453	2.1
31254	0.985252	2.1
31255	0.985252	2.1
31256	0.946132	2.1
31257	0.946132	2.1
31258	1.0655	2.1
31259	0.985255	2.1

31260	0.94613	2.1
31261	0.94613	2.1
31262	0.94613	2.1
31263	1.0655	2.1
31264	0.94613	2.1
31265	0.985255	2.1
31266	1.89226	2.1
31267	1.89226	2.1
31268	1.89226	2.1
31269	1.89226	2.1
31270	3.78453	2.1
31271	1.06549	2.1
31272	1.06549	2.1
31273	0.946133	2.1
31274	0.946133	2.1
31275	1.16834	2.1
31276	1.0655	2.1
31277	0.946131	2.1
31278	0.946131	2.1
31279	0.946131	2.1
31280	1.16834	2.1
31281	0.94613	2.1
31282	1.0655	2.1
31283	0.966631	2.1
31284	1.07349	2.1
31285	0.946131	2.1
31286	0.946131	2.1
31287	1.07349	2.1
31288	1.07349	2.1
31289	0.946129	2.1
31290	0.946129	2.1
31291	1.07349	2.1
31292	0.946131	2.1
31293	0.94613	2.1
31294	0.966631	2.1
31295	2.38055	0.813871
31296	1.89226	1.35284
31297	1.89226	1.18404
31298	1.46724	0.386409
31299	3.35949	0.882058
31300	1.42249	2.1
31301	2.05499	2.1
31302	1.39708	2.1
31303	2.09033	2.1
31304	3.45207	2.1
31305	2.05499	2.1
31306	2.57425	2.1
31307	2.51287	2.1
31308	2.47753	2.1
31309	2.09033	2.1
31310	2.05499	1.53331
31311	1.39327	2.1
31312	2.09033	2.1
31313	2.16217	2.1
31314	2.16217	2.1
31315	1.39708	2.1
31316	1.39708	2.1
31317	2.16217	2.1

31318	2.57426	2.1
31319	2.73006	2.1
31320	2.73006	2.1
31321	2.09033	2.1
31322	2.09033	2.1
31323	1.89226	0.0836574
31324	1.89226	0.05
31325	1.89226	0.0580052
31326	1.89226	0.079639
31327	3.78452	0.0794806
31328	0.838749	0.05
31329	0.838749	0.05
31330	0.85824	0.05
31331	0.85824	0.0505473
31332	0.544644	0.05
31333	0.854718	0.05
31334	0.842271	0.05
31335	1.19141	0.05
31336	0.842272	0.05
31337	0.544643	0.05
31338	0.854718	0.05
31339	1.19141	0.05
31340	1.34214	0.05
31341	1.34214	0.05
31342	0.893236	0.0627165
31343	0.893236	0.0543066
31344	0.893265	0.0592114
31345	1.28924	0.05
31346	0.946133	0.05
31347	0.946133	0.0542991
31348	0.946132	0.0509919
31349	0.893265	0.0575923
31350	0.946133	0.0525863
31351	1.28924	0.05
31352	2.7013	0.0534596
31353	1.89226	0.072852
31354	1.9216	0.0773361
31355	1.9216	0.05
31356	1.89226	0.0765448
31357	1.89226	0.05
31358	1.26397	2.1
31359	1.52995	2.1
31360	1.30116	2.1
31361	1.04084	2.1
31362	2.57079	2.1
31363	1.47013	2.1
31364	2.17459	2.1
31365	2.10668	2.1
31366	1.38328	2.1
31367	3.55787	2.1
31368	2.55006	2.1
31369	2.17459	2.1
31370	2.10668	2.1
31371	2.49168	2.1
31372	4.66626	2.1
31373	0.613622	2.1
31374	0.691639	2.1
31375	1.00105	2.1

31376	1.05334	2.1
31377	0.839874	2.1
31378	0.839874	2.1
31379	0.852814	2.1
31380	0.852814	2.1
31381	0.613622	2.1
31382	1.00105	2.1
31383	1.05334	2.1
31384	0.691639	2.1
31385	1.00105	2.1
31386	1.05334	2.1
31387	1.20844	2.1
31388	1.24584	2.1
31389	1.12717	2.1
31390	1.12717	2.1
31391	1.13462	2.1
31392	1.13462	2.1
31393	1.20845	2.1
31394	1.00105	2.1
31395	1.05334	2.1
31396	1.24584	2.1
31397	2.22558	1.40982
31398	1.85981	1.14283
31399	2.18943	1.15587
31400	1.89142	0.604004
31401	4.0854	1.03852
31402	2.02641	2.1
31403	1.85981	1.03266
31404	2.18943	1.11806
31405	1.67346	1.04889
31406	3.88622	0.892849
31407	1.85981	0.437036
31408	1.10736	0.252192
31409	1.27421	0.545768
31410	1.67346	0.270904
31411	1.27421	0.212262
31412	1.67346	0.858741
31413	1.85981	0.527194
31414	1.27362	0.622359
31415	1.89142	0.725331
31416	1.27421	0.375867
31417	3.13343	0.494004
31418	1.81893	0.093672
31419	1.64985	0.121065
31420	1.23477	0.0631073
31421	2.25405	0.132104
31422	3.48882	0.129329
31423	2.23933	0.0601085
31424	1.89226	0.0844424
31425	1.89226	0.087774
31426	2.78053	0.0807528
31427	1.89226	0.0644745
31428	2.78053	0.0584013
31429	0.946131	0.102162
31430	0.946131	0.0987544
31431	0.946132	0.114609
31432	0.946132	0.171642
31433	0.946131	0.181154

31434	0.946131	0.162803
31435	0.946132	0.0976145
31436	0.946132	0.179915
31437	0.946131	0.110674
31438	0.946131	0.13904
31439	0.946132	0.0922167
31440	0.946132	0.158727
31441	1.89226	2.1
31442	2.48596	2.1
31443	1.89226	2.1
31444	2.51506	2.1
31445	4.40732	2.1
31446	2.48596	2.1
31447	1.63823	2.1
31448	2.51507	2.1
31449	1.67687	2.1
31450	4.1533	2.1
31451	1.89226	2.1
31452	2.51506	2.1
31453	1.92044	2.1
31454	1.89226	2.1
31455	2.54325	2.1
31456	2.51506	2.1
31457	2.51507	2.1
31458	1.5704	2.1
31459	1.70505	2.1
31460	1.67687	2.1
31461	2.54325	2.1
31462	2.51507	2.1
31463	2.0815	2.1
31464	1.70303	2.1
31465	1.70303	2.1
31466	1.80354	2.1
31467	1.98172	2.1
31468	2.09868	2.1
31469	1.27944	2.1
31470	1.79028	2.1
31471	1.13179	2.1
31472	1.65533	2.1
31473	2.93477	2.1
31474	1.89226	2.1
31475	1.89226	2.1
31476	1.89226	2.1
31477	1.89226	2.1
31478	1.89226	2.1
31479	1.89226	2.1
31480	1.98171	2.1
31481	1.89226	2.1
31482	1.89226	2.1
31483	1.89226	2.1
31484	1.65798	2.1
31485	1.65797	2.1
31486	1.00133	2.1
31487	1.65532	2.1
31488	1.53278	2.1
31489	1.53278	2.1
31490	1.13179	2.1
31491	1.13179	2.1

31492	0.810946	2.1
31493	0.810946	2.1
31494	0.810944	2.1
31495	0.810944	2.1
31496	0.810946	2.1
31497	0.810946	2.1
31498	1.89226	2.1
31499	1.35169	2.1
31500	1.08132	2.1
31501	1.08132	2.1
31502	1.08132	2.1
31503	1.99528	0.163813
31504	1.70303	0.216881
31505	2.08152	0.190794
31506	1.70303	0.322495
31507	2.33877	0.13559
31508	1.93967	0.199923
31509	1.89226	0.199659
31510	1.89226	0.383449
31511	1.89226	0.233525
31512	1.89226	0.346043
31513	3.78452	0.351022
31514	1.26259	0.199179
31515	2.17205	0.283741
31516	2.32468	0.217337
31517	2.32468	0.228237
31518	1.28055	0.209064
31519	1.28055	0.224294
31520	0.946131	0.197623
31521	0.946131	0.156688
31522	0.997642	0.180756
31523	0.997642	0.128851
31524	0.946131	0.203388
31525	0.946131	0.243746
31526	0.989846	0.155394
31527	0.997642	0.115562
31528	0.946132	0.163978
31529	0.989846	0.144002
31530	0.997641	0.12765
31531	0.946131	0.178881
31532	1.89226	0.230322
31533	1.89226	0.336054
31534	1.89226	0.210065
31535	1.89226	0.389006
31536	3.78452	0.226746
31537	1.28055	0.158029
31538	2.17205	0.273743
31539	1.14305	0.179276
31540	1.92737	0.237786
31541	3.3151	0.241199
31542	0.954908	0.05
31543	0.9705	0.05
31544	0.946131	0.05
31545	0.946131	0.05
31546	0.946131	0.05
31547	0.946131	0.05
31548	0.9705	0.05
31549	0.9705	0.05

31550	0.954908	0.05
31551	0.946131	0.05
31552	0.946131	0.05
31553	0.9705	0.05
31554	0.835187	0.05
31555	0.954896	0.05
31556	0.946118	0.05
31557	0.946118	0.05
31558	0.835186	0.05
31559	0.835186	0.05
31560	1.06583	0.05
31561	1.06583	0.05
31562	0.835198	0.05
31563	0.946131	0.05
31564	0.954883	0.05
31565	0.946106	0.05
31566	1.89227	2.1
31567	1.89227	2.1
31568	1.89227	2.1
31569	1.89227	2.1
31570	1.89227	2.1
31571	1.89227	1.89839
31572	0.885265	2.1
31573	0.885265	2.1
31574	0.871467	2.1
31575	0.871467	2.1
31576	1.11883	2.1
31577	0.787875	2.1
31578	0.996487	2.1
31579	0.637907	2.1
31580	0.996487	2.1
31581	1.11883	2.1
31582	0.787875	2.1
31583	0.637906	2.1
31584	1.89226	2.1
31585	2.39258	2.1
31586	2.39258	2.1
31587	2.54729	2.1
31588	1.89226	2.1
31589	2.04697	2.1
31590	1.89226	2.1
31591	2.0815	2.1
31592	2.0815	2.1
31593	2.48371	2.1
31594	2.0815	2.1
31595	2.51469	2.1
31596	1.00035	0.05
31597	1.55755	0.0533493
31598	1.03534	0.144532
31599	1.61333	0.0970901
31600	2.59289	0.05
31601	1.55755	0.0533493
31602	1.85217	0.810877
31603	1.61333	0.173878
31604	1.92875	0.666657
31605	3.48629	0.176253
31606	1.09962	0.468887
31607	1.61334	0.266536

31608	1.70868	0.612372
31609	1.70868	0.533718
31610	1.03535	0.709396
31611	1.03535	0.425728
31612	1.61334	0.175033
31613	1.92876	0.645734
31614	1.70868	0.168482
31615	2.05274	0.754451
31616	3.63744	0.317552
31617	1.82233	0.527067
31618	2.05768	0.75866
31619	1.89226	0.832183
31620	1.65406	0.574532
31621	1.89226	0.776825
31622	1.65406	0.5731
31623	2.26047	0.583633
31624	2.0815	0.897248
31625	1.89226	0.876104
31626	2.01156	0.608139
31627	1.89226	0.811212
31628	2.01156	0.59686
31629	1.89226	2.06412
31630	1.89226	2.1
31631	1.89226	2.1
31632	1.89226	1.66197
31633	3.78453	1.88513
31634	2.70055	2.1
31635	2.28367	2.1
31636	2.56456	2.1
31637	2.33565	2.1
31638	4.84823	2.1
31639	2.28367	2.1
31640	1.5791	2.1
31641	1.96966	2.1
31642	1.09506	2.1
31643	3.37873	2.1
31644	1.89226	2.1
31645	1.89226	1.39085
31646	1.89226	1.56819
31647	1.89226	2.1
31648	3.78452	2.1
31649	0.94612	2.1
31650	0.94612	2.1
31651	0.946119	1.99586
31652	0.946119	1.59622
31653	0.946119	1.38992
31654	0.946119	2.1
31655	0.946119	2.1
31656	0.946119	1.87419
31657	0.946132	2.1
31658	0.946132	2.1
31659	0.946107	2.1
31660	0.946107	2.1
31661	2.56452	2.1
31662	1.57307	2.1
31663	2.10299	2.1
31664	2.1115	2.1
31665	2.52196	2.1

31666	2.52196	2.1
31667	1.89226	2.1
31668	1.89226	0.847198
31669	1.89226	2.1
31670	1.89226	2.1
31671	3.78452	2.1
31672	2.37665	2.1
31673	1.89226	2.1
31674	2.28084	2.1
31675	1.89226	2.1
31676	4.26891	2.1
31677	0.946129	0.872832
31678	0.946129	2.1
31679	0.946133	2.1
31680	0.946133	2.1
31681	0.946131	2.1
31682	0.946131	0.841538
31683	0.946131	2.1
31684	0.946131	2.1
31685	0.946131	2.1
31686	0.946131	2.1
31687	0.946131	2.1
31688	0.946131	2.1
31689	1.7207	0.05
31690	1.65815	0.05
31691	1.33593	0.05
31692	1.31508	0.05
31693	2.99408	0.05
31694	0.676047	0.05
31695	0.676047	0.05
31696	0.684362	0.05
31697	0.684362	0.05
31698	0.547994	0.05
31699	0.702871	0.05
31700	0.829076	0.05
31701	0.657538	0.05
31702	0.657538	0.05
31703	0.547994	0.05
31704	0.829077	0.05
31705	0.702871	0.05
31706	1.9518	2.1
31707	1.30648	2.1
31708	1.28846	2.1
31709	1.92033	2.1
31710	3.22681	2.1
31711	1.89226	2.1
31712	1.89226	2.1
31713	1.89226	2.1
31714	1.89226	2.1
31715	3.78453	2.1
31716	0.946128	2.1
31717	0.946128	2.1
31718	1.10113	2.1
31719	1.09587	2.1
31720	0.946131	2.1
31721	0.946131	2.1
31722	1.09587	2.1
31723	1.09587	2.1

31724	0.946131	2.1
31725	1.09587	2.1
31726	1.10112	2.1
31727	0.94613	2.1
31728	1.26923	2.1
31729	1.92033	2.1
31730	1.28846	2.1
31731	1.28846	2.1
31732	1.89452	2.1
31733	1.89452	2.1
31734	1.89226	2.1
31735	1.89226	2.1
31736	1.89226	2.1
31737	1.89226	2.1
31738	3.78453	2.1
31739	1.89226	2.1
31740	2.19174	2.1
31741	2.15616	2.1
31742	2.19174	2.1
31743	1.85669	2.1
31744	1.89226	2.1
31745	1.89226	2.1
31746	1.89226	2.1
31747	1.89226	2.1
31748	1.89226	2.1
31749	3.78452	2.1
31750	1.89226	2.1
31751	1.89226	2.1
31752	1.89226	2.1
31753	1.89226	2.1
31754	3.78452	2.1
31755	1.51223	2.1
31756	1.41562	2.1
31757	1.5139	2.1
31758	1.42284	2.1
31759	1.6033	2.1
31760	1.51223	2.1
31761	1.51223	2.1
31762	1.25953	2.1
31763	1.38085	2.1
31764	1.6033	2.1
31765	2.89309	2.1
31766	0.946131	2.1
31767	0.946131	2.1
31768	0.946131	2.1
31769	0.946131	2.1
31770	0.946132	2.1
31771	0.946132	2.1
31772	0.946129	2.1
31773	0.946129	2.1
31774	0.946131	2.1
31775	0.946131	2.1
31776	0.94613	2.1
31777	0.94613	2.1
31778	1.89226	2.1
31779	1.89226	2.1
31780	1.89226	2.1
31781	1.89226	2.1

31782	3.78452	2.1
31783	0.707806	2.1
31784	0.697816	2.1
31785	0.823593	2.1
31786	0.801649	2.1
31787	0.74182	2.1
31788	0.74182	2.1
31789	0.757645	2.1
31790	0.757645	2.1
31791	0.823592	2.1
31792	0.697818	2.1
31793	0.707807	2.1
31794	0.801647	2.1
31795	1.52534	2.1
31796	1.6033	2.1
31797	1.64719	2.1
31798	1.38085	2.1
31799	1.64719	2.1
31800	1.38085	2.1
31801	2.67041	2.1
31802	1.89226	0.679541
31803	1.89226	1.88993
31804	1.89226	0.962718
31805	2.487	1.57667
31806	2.487	1.17432
31807	2.70097	2.1
31808	1.70303	2.1
31809	1.71163	2.1
31810	2.487	1.86017
31811	4.46723	2.1
31812	1.14984	1.70983
31813	1.2435	2.1
31814	0.778	2.1
31815	0.697616	2.1
31816	0.967211	2.1
31817	0.967211	0.987866
31818	0.973907	2.1
31819	0.973907	2.1
31820	0.697616	2.1
31821	1.14984	2.1
31822	0.778	2.1
31823	1.2435	2.1
31824	2.4158	2.1
31825	1.26438	2.1
31826	1.676	2.1
31827	2.77505	2.1
31828	4.0918	2.1
31829	2.58258	2.1
31830	1.89226	2.1
31831	1.89226	2.1
31832	1.89226	2.1
31833	2.47032	2.1
31834	2.47032	2.1
31835	1.23516	2.1
31836	1.18734	2.1
31837	0.946131	2.1
31838	0.946131	2.1
31839	1.18734	2.1

31840	1.18734	2.1
31841	0.94613	2.1
31842	0.94613	2.1
31843	1.18734	2.1
31844	0.946131	2.1
31845	0.946131	2.1
31846	1.23516	2.1
31847	2.8239	2.1
31848	1.39361	2.1
31849	1.12342	2.1
31850	2.57275	2.1
31851	3.96637	2.1
31852	0.937929	0.725894
31853	0.937929	0.657099
31854	1.39734	0.571412
31855	1.39734	0.569449
31856	1.38914	0.602716
31857	0.94613	0.664069
31858	0.937902	0.68845
31859	0.94613	0.481403
31860	0.94613	0.572287
31861	0.94613	0.608371
31862	1.38914	0.620038
31863	0.937902	0.592843
31864	1.89226	0.55353
31865	2.17765	0.856985
31866	1.84577	0.787502
31867	1.84577	0.631321
31868	1.89226	0.716183
31869	1.89226	0.573874
31870	2.11262	0.81912
31871	1.57766	0.939277
31872	2.22689	2.1
31873	1.91132	0.540088
31874	2.19624	1.61499
31875	1.79689	0.490905
31876	1.99656	0.385103
31877	1.57766	0.842057
31878	1.39067	2.1
31879	1.73802	0.692108
31880	3.31568	0.892501
31881	1.89226	0.485552
31882	1.89226	0.377162
31883	1.89226	0.414453
31884	1.89226	0.55607
31885	1.89226	0.274527
31886	1.89226	0.445854
31887	1.84936	0.596049
31888	2.0815	0.291774
31889	2.0815	0.405409
31890	2.47217	0.498527
31891	1.04528	0.203519
31892	1.62774	0.470082
31893	1.89226	0.426638
31894	1.89226	0.343946
31895	1.89226	0.386719
31896	1.89226	0.455582
31897	1.89226	0.303644

31898	1.89226	0.408926
31899	1.69055	0.197025
31900	2.68518	0.335881
31901	1.69055	0.200137
31902	2.25055	0.361496
31903	2.39587	0.150877
31904	3.01801	0.337244
31905	1.89226	0.338188
31906	1.89226	0.368802
31907	1.89226	0.221363
31908	1.89226	0.194468
31909	3.78452	0.323224
31910	1.24499	0.849226
31911	1.70302	0.913856
31912	1.41207	2.1
31913	1.10574	0.618574
31914	2.66056	0.921828
31915	1.70303	1.27798
31916	2.39605	0.937198
31917	2.72674	0.964127
31918	2.46904	0.707932
31919	1.84557	1.05423
31920	1.67115	0.922681
31921	2.46904	0.604998
31922	2.76829	1.38648
31923	2.83666	0.525544
31924	2.83666	0.77569
31925	2.39605	0.691204
31926	2.39605	1.34673
31927	1.07323	0.431392
31928	1.07323	1.91199
31929	1.06715	1.80983
31930	1.18052	0.803482
31931	1.30067	0.609499
31932	1.26542	0.678182
31933	0.914516	0.965995
31934	0.839633	1.11869
31935	0.936958	0.858586
31936	1.26751	0.840671
31937	0.847764	0.791462
31938	1.35395	0.899104
31939	2.69069	0.575202
31940	1.03074	0.495838
31941	1.74098	0.173079
31942	1.97397	0.401465
31943	3.71495	0.395961
31944	2.44554	2.1
31945	1.89226	2.1
31946	1.89226	2.1
31947	1.89226	2.1
31948	2.40645	2.1
31949	2.40645	2.1
31950	1.89226	2.1
31951	1.89226	1.04297
31952	1.89226	2.1
31953	1.89226	0.910387
31954	3.78452	2.1
31955	1.82117	2.1

31956	2.55786	0.90398
31957	2.51431	2.1
31958	2.51431	2.1
31959	1.85054	1.97174
31960	1.84375	2.1
31961	2.38467	2.1
31962	1.89226	2.1
31963	1.89226	2.1
31964	1.89226	2.1
31965	2.44554	2.1
31966	2.44554	2.1
31967	1.89226	2.1
31968	1.89226	1.89057
31969	1.89226	2.1
31970	1.89226	1.73709
31971	1.89226	2.1
31972	1.89226	1.93356
31973	2.02397	2.1
31974	2.55786	1.77714
31975	1.77775	2.1
31976	2.61404	2.08013
31977	4.33561	2.1
31978	1.89226	1.20342
31979	1.89226	1.158
31980	1.89226	0.870981
31981	1.89226	1.02716
31982	3.78453	1.13253
31983	1.89226	1.25848
31984	2.80184	1.6077
31985	1.89226	1.05037
31986	1.89226	1.41051
31987	2.03045	1.48696
31988	2.03045	1.91886
31989	2.52263	1.20811
31990	1.27921	2.1
31991	1.58429	2.1
31992	1.73571	2.09582
31993	2.37121	1.57081
31994	2.52263	1.3242
31995	0.764974	2.1
31996	0.650577	2.1
31997	0.712428	2.1
31998	0.562516	2.1
31999	0.67264	2.1
32000	0.67264	2.1
32001	0.654851	2.1
32002	0.654851	2.1
32003	0.562515	2.1
32004	0.650579	2.1
32005	0.712427	2.1
32006	0.764976	2.1
32007	1.89227	2.1
32008	1.89227	2.1
32009	1.89227	2.1
32010	1.89227	2.1
32011	3.78453	2.1
32012	1.89226	2.1
32013	1.89226	2.1

32014	1.89226	2.1
32015	1.89226	2.1
32016	3.78453	2.1
32017	1.49248	2.1
32018	1.24959	2.1
32019	1.28211	2.1
32020	1.26432	2.1
32021	1.51027	2.1
32022	1.49248	2.1
32023	1.49248	2.1
32024	1.19017	2.1
32025	1.24977	2.1
32026	1.51027	2.1
32027	2.74225	2.1
32028	1.89226	2.1
32029	1.89226	2.1
32030	1.89226	2.1
32031	1.89226	2.1
32032	3.78453	2.1
32033	1.89226	2.1
32034	1.89226	2.1
32035	1.89226	2.1
32036	1.89226	2.1
32037	3.78453	2.1
32038	0.624796	2.1
32039	0.618472	2.1
32040	0.770147	2.1
32041	0.755132	2.1
32042	0.681445	2.1
32043	0.681445	2.1
32044	0.69216	2.1
32045	0.69216	2.1
32046	0.618472	2.1
32047	0.770147	2.1
32048	0.624796	2.1
32049	0.755132	2.1
32050	1.54029	2.1
32051	1.51027	2.1
32052	1.54029	2.1
32053	1.30682	2.1
32054	1.24977	2.1
32055	1.24977	2.1
32056	0.994293	2.1
32057	2.17159	2.1
32058	2.21149	2.1
32059	2.21149	2.1
32060	0.994814	2.1
32061	0.994814	2.1
32062	0.990481	2.1
32063	2.21149	2.1
32064	2.2671	2.1
32065	2.2671	2.1
32066	0.994293	2.1
32067	0.994293	2.1
32068	2.41579	2.1
32069	1.09847	2.1
32070	1.70302	0.909388
32071	2.45069	2.1

32072	3.61169	2.1
32073	2.48689	2.1
32074	1.67969	2.1
32075	1.67969	2.1
32076	1.70002	2.1
32077	2.43826	2.1
32078	2.45859	2.1
32079	2.43827	2.1
32080	1.62408	2.1
32081	1.62408	2.1
32082	1.67969	2.1
32083	2.36729	2.1
32084	2.4229	2.1
32085	2.47199	2.1
32086	1.0823	2.1
32087	1.0607	2.1
32088	2.44276	2.1
32089	3.52506	2.1
32090	2.47199	2.1
32091	1.89226	2.1
32092	1.89226	2.1
32093	2.44277	2.1
32094	4.33503	2.1
32095	1.89226	2.1
32096	1.89226	2.1
32097	1.89226	2.1
32098	1.89226	2.1
32099	3.78453	2.1
32100	1.89226	2.1
32101	1.89226	2.1
32102	1.89226	2.1
32103	1.89226	2.1
32104	1.89226	2.1
32105	1.89226	2.1
32106	1.02986	2.1
32107	2.44276	2.1
32108	1.0607	2.1
32109	1.0607	2.1
32110	2.40055	2.1
32111	2.40055	2.1
32112	1.89226	2.1
32113	2.44277	2.1
32114	2.40056	2.1
32115	2.44277	2.1
32116	1.85005	2.1
32117	1.89226	2.1
32118	1.89226	2.1
32119	1.89226	2.1
32120	1.89226	2.1
32121	1.89226	2.1
32122	3.78453	2.1
32123	1.89226	2.1
32124	1.89226	2.1
32125	1.89226	2.1
32126	1.89226	2.1
32127	3.78452	2.1
32128	2.79502	0.396916
32129	2.63448	0.573591

32130	2.68986	0.384105
32131	2.53868	0.548359
32132	5.32434	0.457549
32133	2.63449	0.519175
32134	2.39282	1.86565
32135	2.53869	0.530621
32136	2.3085	0.790534
32137	4.94299	0.626276
32138	2.68986	0.379378
32139	2.53868	0.539749
32140	2.5761	0.40867
32141	2.43404	0.522639
32142	5.1239	0.457233
32143	2.53869	0.512612
32144	2.3085	0.78689
32145	2.43405	0.443668
32146	2.22575	0.760905
32147	4.74255	0.615703
32148	1.89226	2.1
32149	1.89226	2.1
32150	1.89226	2.1
32151	1.89226	2.1
32152	3.78453	2.1
32153	1.32391	2.1
32154	1.44951	2.1
32155	1.44951	2.1
32156	1.89226	2.1
32157	1.89226	2.1
32158	2.20203	2.1
32159	0.507656	2.1
32160	0.578945	2.1
32161	1.05568	2.1
32162	0.959007	2.1
32163	0.76264	2.1
32164	0.76264	2.1
32165	0.775313	2.1
32166	0.775313	2.1
32167	0.578945	2.1
32168	1.05568	2.1
32169	0.959007	2.1
32170	0.507656	2.1
32171	1.89226	0.089626
32172	2.4065	0.120249
32173	2.36561	0.0895193
32174	2.4065	0.139071
32175	1.85138	0.0737514
32176	1.89226	0.11741
32177	1.40274	0.0798184
32178	2.4065	0.114805
32179	1.53336	0.0807091
32180	1.53336	0.0838347
32181	2.36562	0.101643
32182	2.36562	0.106758
32183	0.946115	0.107294
32184	0.946115	0.0799596
32185	1.06496	0.072905
32186	1.18279	0.0930231
32187	0.946117	0.108884

32188	0.946117	0.123128
32189	1.06496	0.0868612
32190	1.06496	0.0704333
32191	0.94613	0.0966764
32192	1.06497	0.0826863
32193	0.946105	0.0818161
32194	1.18278	0.0955406
32195	1.00213	0.079852
32196	2.36559	0.104315
32197	1.40272	0.0775673
32198	1.40272	0.0803013
32199	2.12992	0.0970643
32200	2.12992	0.101498
32201	0.95646	2.1
32202	2.07201	1.00021
32203	1.01808	2.1
32204	2.14876	0.822996
32205	3.09009	2.1
32206	1.89226	2.1
32207	1.89226	0.812033
32208	1.89226	2.1
32209	1.89226	2.1
32210	3.78453	2.1
32211	1.89227	2.1
32212	1.89227	2.1
32213	1.89227	2.1
32214	1.89227	2.1
32215	1.89227	2.1
32216	1.89227	2.1
32217	2.71886	2.1
32218	1.60497	2.1
32219	1.43935	2.1
32220	2.55151	2.1
32221	4.15648	2.1
32222	2.71886	2.1
32223	1.89226	2.1
32224	1.89226	2.1
32225	2.55151	2.1
32226	4.44377	2.1
32227	1.89226	2.1
32228	1.89226	2.1
32229	1.89226	2.1
32230	1.89226	0.842346
32231	3.78453	2.1
32232	1.89226	2.1
32233	1.89226	2.1
32234	1.89226	2.1
32235	1.89226	2.1
32236	3.78453	2.1
32237	1.58942	0.558563
32238	1.92622	0.655705
32239	2.52852	0.689597
32240	2.19457	0.510318
32241	4.12079	0.504952
32242	1.92649	0.548116
32243	1.44426	0.33827
32244	1.87529	0.371574
32245	2.19457	0.522224

32246	3.63883	0.510458
32247	1.87529	0.386145
32248	2.19457	0.496394
32249	2.7378	0.52913
32250	2.29852	0.362578
32251	4.49309	0.354929
32252	1.35976	2.1
32253	1.89452	2.1
32254	1.39616	2.1
32255	1.1423	2.1
32256	1.8996	2.1
32257	1.52068	2.1
32258	1.89226	2.1
32259	1.89226	2.1
32260	1.89226	2.1
32261	1.89226	2.1
32262	1.89226	2.1
32263	1.89226	2.1
32264	1.89226	2.1
32265	2.15617	2.1
32266	2.0953	2.1
32267	2.15617	2.1
32268	1.83139	2.1
32269	1.89226	2.1
32270	1.16817	2.1
32271	1.8996	2.1
32272	1.23614	2.1
32273	1.48338	2.1
32274	1.80218	2.1
32275	2.16263	2.1
32276	1.89226	2.1
32277	1.89226	2.1
32278	1.89226	2.1
32279	1.89226	2.1
32280	1.89226	2.1
32281	1.89226	2.1
32282	1.89226	2.1
32283	2.0953	2.1
32284	2.03074	2.1
32285	2.0953	2.1
32286	1.8277	2.1
32287	1.89226	2.1
32288	1.74455	2.1
32289	2.181 2.1	
32290	1.74455	2.1
32291	1.92911	2.1
32292	1.98195	2.1
32293	2.16651	2.1
32294	2.181 2.1	
32295	2.29881	2.1
32296	1.98195	2.1
32297	1.80729	2.1
32298	4.28075	2.1
32299	1.74455	2.1
32300	1.98195	2.1
32301	1.52603	2.1
32302	1.76603	2.1
32303	3.51058	2.1

32304	1.76603	2.1
32305	1.94506	2.1
32306	1.76603	2.1
32307	1.98195	2.1
32308	1.86795	2.1
32309	2.08387	2.1
32310	0.984305	2.1
32311	1.9	2.1
32312	2.86619	2.1
32313	1.86189	2.1
32314	3.76189	2.1
32315	1.4621	2.1
32316	1.89226	2.1
32317	2.81374	2.1
32318	1.89226	2.1
32319	3.35437	2.1
32320	0.946133	2.09174
32321	0.946133	2.1
32322	0.946132	2.02234
32323	0.946132	2.1
32324	0.946132	2.1
32325	0.946132	2.1
32326	0.946133	2.1
32327	0.946133	2.1
32328	0.946132	2.1
32329	0.946132	2.02781
32330	0.946132	2.1
32331	0.946132	2.1
32332	1.64268	1.38712
32333	1.93673	2.1
32334	2.25284	2.1
32335	1.97548	2.1
32336	3.89552	2.1
32337	1.86206	2.1
32338	1.93673	2.1
32339	2.25284	2.1
32340	2.20075	2.1
32341	4.1149	2.1
32342	2.25284	2.1
32343	2.24781	2.1
32344	1.97548	1.2913
32345	1.97548	2.1
32346	2.49676	1.64407
32347	2.49676	2.1
32348	2.25284	2.1
32349	2.43549	2.1
32350	2.44467	2.1
32351	2.20075	2.1
32352	2.49676	2.1
32353	2.25284	2.1
32354	2.57928	2.1
32355	2.32606	2.1
32356	2.59522	2.1
32357	2.39022	2.1
32358	4.9695	2.1
32359	2.32606	2.1
32360	1.77677	2.1
32361	2.39022	2.1

32362	2.28669	2.1
32363	4.16699	2.1
32364	2.59522	2.1
32365	2.39022	2.1
32366	2.61901	2.1
32367	2.45899	2.1
32368	5.00923	2.1
32369	2.39022	2.1
32370	2.11575	2.1
32371	2.41795	2.1
32372	2.0815	2.1
32373	4.67085	2.1
32374	1.89226	2.1
32375	2.24723	2.1
32376	1.95405	2.1
32377	1.89226	2.1
32378	2.30902	2.1
32379	2.24723	2.1
32380	1.89226	2.1
32381	1.89226	2.1
32382	1.89226	2.1
32383	1.89226	2.1
32384	3.78453	2.1
32385	0.946131	2.1
32386	0.946131	2.1
32387	1.14643	2.1
32388	1.10673	2.1
32389	1.10673	2.1
32390	1.10673	2.1
32391	0.946133	2.1
32392	0.946133	2.1
32393	0.946131	2.1
32394	1.14643	2.1
32395	1.10673	2.1
32396	0.946131	2.1
32397	1.89226	2.1
32398	2.30902	2.1
32399	1.96635	2.1
32400	1.89226	2.1
32401	2.38311	2.1
32402	2.30902	2.1
32403	1.89226	2.1
32404	1.89226	2.1
32405	1.89226	2.1
32406	1.89226	2.1
32407	1.89226	2.1
32408	1.89226	2.1
32409	0.946131	2.1
32410	0.946131	2.1
32411	1.19156	2.1
32412	1.14643	2.1
32413	1.14643	2.1
32414	1.14643	2.1
32415	0.946133	2.1
32416	0.946133	2.1
32417	0.946131	2.1
32418	1.19156	2.1
32419	0.946131	2.1

32420	1.14643	2.1
32421	1.89226	1.01044
32422	1.89226	0.721789
32423	1.89226	0.643547
32424	1.89226	0.957079
32425	1.89226	0.715409
32426	1.89226	1.52207
32427	1.89226	0.753944
32428	1.89226	0.740412
32429	1.89226	0.703047
32430	1.9645	0.796809
32431	3.78452	0.921454
32432	2.39489	2.1
32433	2.15715	2.1
32434	2.07821	2.1
32435	2.33541	2.1
32436	4.49256	2.1
32437	2.39489	2.1
32438	2.34898	2.1
32439	2.30729	2.1
32440	2.33541	2.1
32441	4.6844	2.1
32442	2.07821	2.1
32443	2.33541	2.1
32444	2.26415	2.1
32445	1.99632	2.1
32446	4.33174	2.1
32447	2.30729	2.1
32448	2.33542	2.1
32449	2.26416	2.1
32450	2.26618	2.1
32451	4.60159	2.1
32452	1.19837	1.28104
32453	2.79151	0.45694
32454	2.25636	0.90606
32455	2.25636	0.714581
32456	1.94072	0.804252
32457	1.94072	0.748109
32458	2.53725	0.079886
32459	1.16699	0.0797523
32460	1.69864	0.0568115
32461	2.0815	0.0797459
32462	3.58273	0.0728474
32463	1.91646	2.1
32464	2.51686	2.1
32465	2.49872	2.1
32466	1.85819	2.1
32467	4.41518	2.1
32468	0.929098	2.1
32469	0.8993	2.1
32470	1.24359	2.1
32471	1.24936	2.1
32472	1.06832	2.1
32473	1.06832	2.1
32474	1.08034	2.1
32475	1.08034	2.1
32476	0.899298	2.1
32477	1.24359	2.1

32478	1.24936	2.1
32479	0.929097	2.1
32480	2.0815	0.0893543
32481	1.89226	0.05
32482	2.0815	0.0673165
32483	1.82811	0.0551314
32484	1.94982	0.0718679
32485	1.71246	0.0565181
32486	2.3256	2.1
32487	1.89226	2.1
32488	1.89226	2.1
32489	1.89226	2.1
32490	2.38467	2.1
32491	2.38467	2.1
32492	1.89226	2.1
32493	1.89226	2.1
32494	1.89226	2.1
32495	1.89226	2.07715
32496	1.89226	2.1
32497	1.89226	2.1
32498	1.89763	2.1
32499	2.61405	2.1
32500	2.15035	2.1
32501	2.68796	2.1
32502	1.42219	2.1
32503	1.77775	2.1
32504	2.26306	2.1
32505	1.89226	2.1
32506	1.89226	2.1
32507	1.89226	2.1
32508	2.3256	2.1
32509	2.3256	2.1
32510	1.89226	2.1
32511	1.89226	2.1
32512	1.89226	2.1
32513	1.89226	2.1
32514	1.89226	2.1
32515	1.89226	2.0372
32516	1.92887	2.1
32517	2.41915	2.1
32518	2.50142	2.1
32519	2.50142	2.1
32520	1.90388	2.1
32521	1.89763	2.1
32522	1.89224	2.1
32523	1.89224	2.1
32524	1.89224	2.1
32525	1.89224	2.1
32526	3.78448	2.1
32527	2.25315	2.1
32528	1.05843	2.1
32529	2.38995	2.1
32530	1.31541	2.1
32531	3.56856	2.1
32532	0.517552	2.1
32533	0.68548	2.1
32534	1.27675	2.1
32535	1.13076	2.1

32536	0.913611	2.1
32537	0.913612	2.1
32538	0.902633	2.1
32539	0.902633	2.1
32540	0.68547	2.1
32541	1.27673	2.1
32542	1.13078	2.1
32543	0.517559	2.1
32544	1.89226	2.1
32545	1.89226	2.1
32546	1.89226	2.1
32547	1.89226	2.1
32548	3.78453	2.1
32549	0.962936	2.1
32550	0.962936	2.1
32551	0.996141	2.1
32552	0.996141	2.1
32553	0.830609	2.1
32554	0.657714	2.1
32555	1.30136	2.1
32556	1.19499	2.1
32557	1.30136	2.1
32558	0.830609	2.1
32559	1.19499	2.1
32560	0.657713	2.1
32561	1.60057	0.310742
32562	1.39615	0.319544
32563	1.06474	0.320799
32564	1.39615	0.330592
32565	1.64655	0.282359
32566	2.04424	0.320511
32567	1.89226	0.320081
32568	1.89226	0.341818
32569	1.89226	0.332634
32570	1.89226	0.332892
32571	1.89226	0.325543
32572	1.89226	0.328862
32573	2.56322	0.313169
32574	1.89226	0.325128
32575	1.89226	0.320538
32576	1.89226	0.320005
32577	2.18047	0.316479
32578	2.18047	0.318212
32579	1.89226	0.657448
32580	1.89226	0.674718
32581	1.89226	0.757147
32582	1.65573	0.545918
32583	1.89226	0.772569
32584	1.65573	0.555109
32585	1.05353	0.270299
32586	1.83158	0.300883
32587	1.50282	0.190685
32588	1.20669	0.207298
32589	2.88511	0.191589
32590	1.44807	2.1
32591	1.93592	2.1
32592	1.80703	2.1
32593	1.27806	2.1

32594	3.21398	2.1
32595	2.0815	2.1
32596	1.87465	2.1
32597	1.87465	2.1
32598	1.50566	2.1
32599	2.0815	2.1
32600	1.69263	2.1
32601	1.89226	2.1
32602	1.89226	2.1
32603	1.89226	2.1
32604	1.89226	2.1
32605	1.89226	2.1
32606	1.89226	2.1
32607	1.62632	2.1
32608	0.994294	2.1
32609	0.994294	2.1
32610	1.27806	2.1
32611	1.81921	2.1
32612	2.10776	2.1
32613	2.0815	2.1
32614	1.70303	2.1
32615	1.70303	2.1
32616	1.85749	2.1
32617	1.90451	2.1
32618	2.07724	2.1
32619	1.89226	2.1
32620	1.89226	2.1
32621	1.89226	2.1
32622	1.89226	2.1
32623	1.89226	2.1
32624	1.89226	2.1
32625	2.69773	2.1
32626	1.88129	2.1
32627	2.55196	2.1
32628	2.26469	2.1
32629	4.94367	2.1
32630	1.88129	2.1
32631	1.25889	2.1
32632	1.26614	2.1
32633	1.05914	2.1
32634	2.26469	2.1
32635	1.88129	2.1
32636	2.55196	2.1
32637	2.0588	2.1
32638	2.6536	2.1
32639	2.15287	2.1
32640	4.70484	2.1
32641	0.8516	2.1
32642	0.8516	2.1
32643	0.849002	2.1
32644	0.849002	2.1
32645	0.629445	2.1
32646	0.671197	2.1
32647	1.0294	2.1
32648	1.07644	2.1
32649	1.07644	2.1
32650	0.671197	2.1
32651	0.629446	2.1

32652	1.0294	2.1
32653	2.02115	1.98434
32654	1.18382	2.07715
32655	2.29107	0.632301
32656	1.46903	1.41081
32657	3.47488	0.904216
32658	2.60763	1.05862
32659	2.29107	1.39397
32660	1.82141	1.2535
32661	1.82141	2.09832
32662	1.46903	1.87121
32663	2.29107	1.91166
32664	1.64007	2.1
32665	2.2024	2.1
32666	1.44044	2.1
32667	2.02634	2.1
32668	3.6664	2.1
32669	1.25565	2.1
32670	2.02635	2.1
32671	1.87228	2.1
32672	1.87228	2.1
32673	1.44045	2.1
32674	1.44045	2.1
32675	1.89226	0.05
32676	1.78503	0.05
32677	1.78503	0.05
32678	1.40048	0.05
32679	2.0815	0.05
32680	1.62972	0.05
32681	1.27875	2.1
32682	1.1483	0.576972
32683	1.40014	0.670289
32684	1.40014	2.1
32685	1.04598	0.69949
32686	1.04598	2.1
32687	2.63443	1.08615
32688	2.26444	2.02218
32689	2.79392	0.693025
32690	2.4863	2.1
32691	5.12073	1.41877
32692	1.83158	0.614559
32693	1.18508	0.412246
32694	1.20669	0.426443
32695	1.81488	0.552557
32696	1.20669	0.324785
32697	1.81488	0.484073
32698	1.08134	0.388463
32699	1.70303	0.536572
32700	1.24027	0.468808
32701	1.30359	0.307053
32702	1.89939	0.576422
32703	1.70303	0.536615
32704	1.89226	2.1
32705	2.69027	2.1
32706	1.89226	2.1
32707	2.2078	2.1
32708	1.89226	2.1
32709	2.2078	2.1

32710	1.89226	2.1
32711	2.78977	2.1
32712	1.89226	2.1
32713	2.69027	2.1
32714	1.89226	2.1
32715	2.69027	2.1
32716	1.89226	2.1
32717	1.89226	2.08584
32718	1.89226	1.45756
32719	1.89226	2.1
32720	3.78452	2.08171
32721	1.43687	2.01965
32722	1.71059	2.1
32723	2.53014	2.09572
32724	2.6521	2.1
32725	4.24073	2.1
32726	2.6521	2.1
32727	1.66762	2.1
32728	2.50299	2.1
32729	2.50299	2.1
32730	1.71059	2.1
32731	1.71059	2.1
32732	1.89226	2.1
32733	1.89226	2.1
32734	1.89226	2.1
32735	1.89226	1.98241
32736	1.89226	2.1
32737	1.89226	2.0171
32738	2.10662	0.586082
32739	2.77417	0.578801
32740	2.78873	0.851154
32741	2.11661	0.551835
32742	4.89078	0.593219
32743	2.10662	0.69594
32744	1.22857	0.498778
32745	1.25849	0.730135
32746	2.11661	0.617451
32747	3.34518	0.635283
32748	2.78873	0.71928
32749	2.11661	0.512504
32750	2.12475	0.587016
32751	2.79029	0.596465
32752	4.9069	0.584615
32753	1.2858	0.633163
32754	2.11661	0.558371
32755	1.25849	0.590983
32756	1.25849	0.625213
32757	2.12475	0.577293
32758	2.12475	0.608374
32759	1.13537	2.1
32760	1.20714	2.1
32761	0.946129	2.1
32762	0.946129	2.1
32763	0.946131	2.1
32764	0.946131	2.1
32765	1.29521	2.1
32766	1.20713	2.1
32767	1.13537	2.1

32768	0.946131	2.1
32769	0.94613	2.1
32770	1.29521	2.1
32771	1.00533	2.1
32772	0.876055	2.1
32773	0.946128	2.1
32774	0.946128	2.1
32775	0.913908	2.1
32776	0.913908	2.1
32777	1.17081	2.1
32778	0.896413	2.1
32779	0.955905	2.1
32780	0.893715	2.1
32781	1.20327	2.1
32782	0.998544	2.1
32783	1.89226	1.65453
32784	1.89226	1.95328
32785	1.89226	0.760729
32786	1.89226	1.26529
32787	3.78452	0.948715
32788	0.540975	1.21189
32789	0.726949	2.1
32790	0.712198	2.09006
32791	0.506675	1.10592
32792	0.611916	1.17618
32793	0.611916	2.1
32794	0.621708	1.79839
32795	0.621708	1.09943
32796	0.726951	1.37605
32797	0.712196	1.16301
32798	0.506674	1.56684
32799	0.540976	1.3351
32800	1.89226	1.0788
32801	1.89226	2.1
32802	1.89226	1.09621
32803	1.89226	1.17378
32804	3.78453	1.66038
32805	1.89226	0.989273
32806	1.89226	1.14985
32807	1.89226	0.780837
32808	1.89226	2.01449
32809	1.89226	0.817315
32810	1.89226	2.1
32811	0.919594	1.06207
32812	0.726949	1.84154
32813	0.898318	1.12911
32814	0.712198	1.81722
32815	0.817559	0.927326
32816	0.817559	2.1
32817	0.814234	0.914243
32818	0.814234	1.77414
32819	0.919597	0.963265
32820	0.898316	0.836059
32821	0.726951	1.86172
32822	0.712196	1.36346
32823	1.79663	0.881775
32824	1.12088	2.1
32825	1.54699	1.14375

32826	1.17475	1.20711
32827	1.79663	1.98323
32828	1.42439	2.0947
32829	2.28928	1.03862
32830	1.23077	1.40986
32831	1.65458	0.951685
32832	2.66533	1.86236
32833	3.94385	1.19769
32834	1.89226	2.1
32835	1.89226	1.66766
32836	1.89226	2.1
32837	1.89226	0.905569
32838	3.78452	2.1
32839	1.38426	1.89237
32840	1.15067	2.1
32841	1.38426	2.1
32842	1.01595	2.1
32843	1.24509	2.1
32844	1.01595	2.1
32845	2.62097	2.1
32846	1.89226	0.923263
32847	2.72561	2.1
32848	2.72561	2.05827
32849	1.89226	2.1
32850	1.89226	2.1
32851	1.90103	0.404835
32852	1.41902	0.737345
32853	1.77249	0.392184
32854	1.30272	0.973654
32855	3.20375	0.622959
32856	1.77249	0.469593
32857	1.19548	0.732372
32858	1.19548	0.54901
32859	1.30272	0.677068
32860	1.65815	0.355784
32861	1.76539	0.695009
32862	2.62161	2.1
32863	1.89226	2.1
32864	1.89226	2.1
32865	1.89226	2.1
32866	2.6446	2.1
32867	2.6446	2.1
32868	2.64459	2.1
32869	1.89226	2.1
32870	1.89226	2.1
32871	1.89226	2.1
32872	2.58257	2.1
32873	2.58257	2.1
32874	1.89226	0.569151
32875	1.89226	0.946153
32876	2.31301	0.910525
32877	2.76309	0.635837
32878	1.89226	0.842901
32879	2.26047	0.611153
32880	1.89226	0.565546
32881	2.31301	0.933114
32882	2.17765	0.84591
32883	2.17765	0.62956

32884	1.89226	0.790585
32885	1.89226	0.587754
32886	2.18516	2.1
32887	1.85196	2.1
32888	1.78595	2.1
32889	2.14606	2.1
32890	3.99802	2.1
32891	2.18516	2.1
32892	2.21189	2.1
32893	2.18937	2.1
32894	2.14606	2.1
32895	4.35796	2.1
32896	1.60735	2.1
32897	2.14606	2.1
32898	2.15714	2.1
32899	1.70302	2.1
32900	3.70055	2.1
32901	2.18937	2.1
32902	2.14606	2.1
32903	2.0927	2.1
32904	2.162 2.1	
32905	4.30806	2.1
32906	2.17326	2.1
32907	2.58517	2.1
32908	2.55318	2.1
32909	2.1665	2.1
32910	4.75167	2.1
32911	2.17326	2.1
32912	1.53556	2.1
32913	1.56253	2.1
32914	2.1665	2.1
32915	3.70206	2.1
32916	2.55317	2.1
32917	2.1665	2.1
32918	2.17741	2.1
32919	2.54197	2.1
32920	4.70847	2.1
32921	2.17742	2.1
32922	2.1665	2.1
32923	2.18452	2.1
32924	1.58054	2.1
32925	1.56253	2.1
32926	1.56253	2.1
32927	1.71051	0.318752
32928	1.89807	0.31683
32929	1.19755	0.295669
32930	1.19755	0.295662
32931	2.25632	0.327837
32932	2.25632	0.313622
32933	0.876046	0.117339
32934	1.27293	0.0981078
32935	0.946132	0.139734
32936	0.946132	0.143527
32937	0.946131	0.122844
32938	0.869312	0.114148
32939	1.12767	0.123077
32940	0.946132	0.135699
32941	1.22225	0.132317

32942	1.37968	0.101611
32943	0.851555	0.151112
32944	0.851555	0.119616
32945	0.946133	1.1376
32946	0.946133	2.1
32947	0.946132	1.26587
32948	0.946132	0.911744
32949	0.946133	1.11112
32950	0.946133	1.20353
32951	0.946132	2.07597
32952	0.946132	1.20036
32953	0.946132	1.91346
32954	0.946132	1.23338
32955	0.946133	1.1941
32956	0.946133	1.67415
32957	1.35152	2.1
32958	0.948936	2.1
32959	1.90166	2.1
32960	1.70303	2.1
32961	2.90858	2.1
32962	1.89226	2.1
32963	1.89226	2.1
32964	1.89226	2.03795
32965	1.89226	2.1
32966	3.78452	2.1
32967	1.90166	2.1
32968	1.70303	2.1
32969	2.52454	1.16472
32970	2.13919	2.1
32971	3.99529	2.1
32972	1.89226	0.99515
32973	1.89226	1.70616
32974	1.89226	2.1
32975	1.89226	2.1
32976	3.78452	2.1
32977	1.89226	2.1
32978	1.89226	2.1
32979	1.89226	2.1
32980	1.89226	2.1
32981	1.89226	0.855181
32982	1.89226	2.02405
32983	2.13918	2.1
32984	0.992674	2.1
32985	2.3397	2.1
32986	1.8023	2.1
32987	1.47769	2.1
32988	0.992674	2.1
32989	1.89226	0.334424
32990	1.89226	0.34124
32991	1.89226	0.490428
32992	1.89226	0.224672
32993	3.78452	0.34212
32994	1.89226	0.331689
32995	2.54752	0.145036
32996	2.63974	0.235511
32997	1.89226	0.219697
32998	4.53201	0.210716
32999	0.498412	0.218608

33000	0.500955	0.184368
33001	0.57955	0.313278
33002	0.563873	0.225829
33003	0.529107	0.206694
33004	0.529107	0.178302
33005	0.53572	0.332778
33006	0.53572	0.199234
33007	0.500956	0.206871
33008	0.579548	0.206249
33009	0.563871	0.21399
33010	0.498414	0.212757
33011	1.89223	0.405683
33012	1.89223	0.223831
33013	1.89223	0.298225
33014	1.89223	0.357096
33015	1.89223	0.286336
33016	1.89223	0.339031
33017	1.28752	0.150429
33018	1.31985	0.202807
33019	0.946119	0.19553
33020	0.946119	0.271271
33021	1.28752	0.198605
33022	1.28752	0.157456
33023	0.946119	0.187353
33024	0.946119	0.41005
33025	1.28753	0.189231
33026	0.946132	0.18279
33027	1.31984	0.21037
33028	0.946107	0.221256
33029	0.500948	0.186321
33030	0.479465	0.167568
33031	0.562335	0.261522
33032	0.579542	0.192949
33033	0.528436	0.181072
33034	0.528436	0.166036
33035	0.530571	0.180688
33036	0.530571	0.267358
33037	0.56234	0.185005
33038	0.479473	0.188816
33039	0.500943	0.192372
33040	0.579533	0.191556
33041	1.89226	2.1
33042	1.89226	2.1
33043	1.89226	2.1
33044	1.89226	2.1
33045	3.78452	2.1
33046	1.50767	2.1
33047	1.14496	2.1
33048	1.50767	2.1
33049	1.58539	2.1
33050	1.07653	2.1
33051	1.15425	2.1
33052	1.89226	2.1
33053	2.43826	2.1
33054	2.43826	2.1
33055	2.48688	2.1
33056	1.89226	2.1
33057	1.94088	2.1

33058	1.89226	1.94252
33059	1.89226	2.1
33060	1.89226	2.03936
33061	1.89226	2.1
33062	3.78452	2.1
33063	1.50767	1.90255
33064	1.07653	2.1
33065	1.41077	1.17102
33066	0.991407	2.1
33067	2.49907	2.1
33068	1.21913	2.1
33069	1.21913	2.1
33070	0.946128	2.1
33071	0.946128	2.1
33072	1.21913	2.1
33073	1.18364	2.1
33074	0.946131	2.1
33075	0.946131	2.1
33076	1.18364	2.1
33077	0.946129	2.1
33078	1.21913	2.1
33079	0.94613	2.1
33080	1.89226	0.634632
33081	1.89226	2.05684
33082	1.89226	0.640988
33083	1.89226	2.1
33084	3.78452	0.748454
33085	1.93823	0.636099
33086	1.60976	2.1
33087	1.93823	2.1
33088	2.20045	0.600183
33089	1.36999	2.1
33090	1.63222	0.690357
33091	1.38302	2.1
33092	1.38302	2.1
33093	0.946132	2.1
33094	0.946132	2.1
33095	1.27258	2.1
33096	1.38302	2.1
33097	0.946133	2.1
33098	0.946133	2.1
33099	0.946133	2.1
33100	1.27258	2.1
33101	0.946132	2.1
33102	1.38302	2.1
33103	1.89226	0.587241
33104	1.89226	2.1
33105	1.89226	0.634203
33106	1.89226	2.1
33107	3.78452	0.783347
33108	1.93823	0.582944
33109	1.36999	2.01941
33110	1.70423	0.556974
33111	1.14195	2.1
33112	3.08018	0.724681
33113	1.27258	2.1
33114	1.27258	2.1
33115	0.946132	2.1

33116	0.946132	2.1
33117	1.17233	2.1
33118	1.27258	2.1
33119	0.946132	2.1
33120	0.946132	2.05926
33121	1.17233	2.1
33122	0.946132	2.1
33123	0.946132	2.1
33124	1.27258	2.1
33125	2.70575	2.1
33126	1.89226	0.828684
33127	2.71429	2.1
33128	2.71429	1.76303
33129	1.89226	1.51005
33130	1.89226	0.859582
33131	0.946133	0.750549
33132	0.946133	2.1
33133	1.28713	2.1
33134	1.28713	2.1
33135	0.946131	2.1
33136	0.946131	0.739145
33137	1.28713	2.1
33138	1.36503	1.10349
33139	0.946131	2.1
33140	1.36503	2.04789
33141	1.28713	2.1
33142	0.946131	2.1
33143	1.89226	2.1
33144	1.89226	1.23227
33145	1.89226	2.1
33146	1.89226	1.0093
33147	1.89226	2.1
33148	1.89226	1.08758
33149	1.22615	2.1
33150	1.04147	2.1
33151	1.3439	2.1
33152	1.3439	2.1
33153	0.969118	2.1
33154	0.969118	2.0899
33155	0.980841	2.1
33156	0.992505	2.1
33157	1.34289	2.1
33158	1.70303	2.1
33159	2.56081	2.1
33160	1.89225	2.1
33161	2.66785	2.1
33162	2.79506	2.1
33163	2.79506	2.1
33164	1.89225	2.1
33165	1.89225	2.1
33166	0.978778	2.1
33167	0.978778	2.1
33168	0.993303	2.1
33169	0.993303	2.1
33170	0.603538	2.1
33171	0.638158	2.1
33172	1.33393	2.1
33173	1.39753	2.1

33174	1.33391	2.1
33175	0.603535	2.1
33176	0.638164	2.1
33177	1.39754	2.1
33178	1.33393	2.1
33179	1.33393	2.1
33180	0.946132	2.1
33181	0.946132	2.1
33182	1.33393	2.1
33183	1.27365	2.1
33184	0.946132	2.1
33185	0.946132	2.1
33186	0.946132	2.1
33187	1.27365	2.1
33188	1.33393	2.1
33189	0.946131	2.1
33190	1.89226	2.1
33191	1.89226	2.1
33192	1.89226	2.1
33193	1.89226	2.1
33194	3.78452	2.1
33195	1.89226	2.1
33196	1.89226	2.1
33197	1.89226	2.1
33198	1.89226	2.1
33199	3.78453	2.1
33200	0.979364	2.1
33201	0.979364	2.1
33202	0.89618	2.1
33203	0.89618	2.1
33204	0.896178	2.1
33205	0.929411	2.1
33206	0.946133	2.1
33207	0.946133	2.1
33208	0.946131	2.1
33209	0.89618	2.1
33210	0.946131	2.1
33211	0.929413	2.1
33212	1.89226	2.1
33213	1.89226	2.1
33214	1.89226	2.1
33215	1.89226	2.1
33216	1.89226	2.1
33217	1.89226	2.1
33218	1.89225	2.1
33219	1.89225	2.1
33220	1.89225	2.1
33221	1.89225	2.1
33222	3.7845	2.1
33223	1.89225	2.1
33224	1.89225	2.1
33225	1.89225	2.1
33226	1.89225	2.1
33227	1.89225	2.1
33228	1.89225	2.1
33229	0.946128	0.386771
33230	0.946128	0.264887
33231	0.946132	0.378139

33232	0.946132	0.26279
33233	0.94613	0.377082
33234	0.94613	0.427294
33235	0.946129	0.249383
33236	0.946129	0.184159
33237	0.94613	0.313093
33238	0.94613	0.324407
33239	0.94613	0.305756
33240	0.94613	0.280227
33241	1.89227	1.20954
33242	1.89227	1.18382
33243	1.89227	0.898157
33244	1.89227	0.831068
33245	3.78453	0.918822
33246	1.89226	1.12186
33247	2.43717	1.79513
33248	2.63259	1.00883
33249	1.89226	0.744269
33250	4.52485	1.2397
33251	0.616808	0.99127
33252	0.792277	1.34617
33253	1.04986	0.885122
33254	0.72303	1.29975
33255	0.702675	0.586832
33256	0.76862	2.09747
33257	0.809154	1.62831
33258	0.809154	0.927506
33259	0.808757	1.29174
33260	1.07789	0.74847
33261	0.772568	0.901549
33262	0.598878	1.44345
33263	1.89226	1.1869
33264	1.89226	1.08778
33265	1.89226	0.922447
33266	1.89226	0.867382
33267	3.78453	1.12952
33268	1.36428	1.91843
33269	1.31629	1.03319
33270	0.946132	1.09193
33271	0.946132	0.965918
33272	1.36428	0.787472
33273	1.36428	1.91083
33274	0.946132	1.16464
33275	0.946132	1.61763
33276	1.36428	1.83478
33277	0.946132	0.881106
33278	1.31629	1.42714
33279	0.946132	0.747162
33280	1.578	1.3594
33281	2.85336	0.753959
33282	2.11368	0.651214
33283	2.14375	0.989273
33284	1.93852	1.00055
33285	1.93852	1.56901
33286	2.34806	0.504492
33287	1.42913	0.371164
33288	1.61539	0.553917
33289	2.45322	0.473828

33290	3.88235	0.489439
33291	2.34806	0.454829
33292	1.89226	0.410583
33293	1.89226	0.533561
33294	2.45322	0.430751
33295	4.34548	0.438206
33296	1.89226	0.404915
33297	1.89226	0.362197
33298	1.89226	0.420391
33299	1.89226	0.395858
33300	3.78452	0.40061
33301	1.89226	0.436744
33302	1.89226	0.374686
33303	1.89226	0.465535
33304	1.89226	0.455742
33305	3.78452	0.427853
33306	1.25972	0.451295
33307	2.45322	0.39934
33308	2.58823	0.517934
33309	1.79398	0.443734
33310	4.2472	0.446753
33311	1.89226	0.469165
33312	2.45322	0.376464
33313	2.58823	0.429464
33314	2.45322	0.419984
33315	2.02727	0.406563
33316	1.89226	0.403782
33317	1.89226	0.368238
33318	1.89226	0.360063
33319	1.89226	0.416931
33320	1.89226	0.365355
33321	3.78452	0.365095
33322	1.89226	0.403439
33323	1.89226	0.395668
33324	1.89226	0.32945
33325	1.89226	0.211256
33326	1.89226	0.447004
33327	1.89226	0.382407
33328	1.70048	1.50966
33329	2.1572	0.307967
33330	1.45477	0.85029
33331	1.93973	0.361608
33332	3.64021	0.721559
33333	1.17614	0.882636
33334	1.93973	0.484469
33335	1.69661	1.03042
33336	1.69661	0.452925
33337	1.45477	1.04887
33338	1.45477	0.573564
33339	1.94343	1.93852
33340	2.53782	1.10227
33341	2.59903	1.58457
33342	2.59903	0.756113
33343	1.87598	1.18733
33344	1.87598	0.815457
33345	0.946131	0.811083
33346	0.946131	1.0933
33347	1.26891	1.89224

33348	1.26891	0.589186
33349	0.94613	1.19365
33350	0.94613	0.981417
33351	1.26891	1.16485
33352	1.29952	0.819386
33353	1.29952	0.628392
33354	0.946131	0.580544
33355	1.26891	0.849333
33356	0.946131	0.85721
33357	2.00551	1.8871
33358	2.59903	1.40665
33359	2.65811	1.74879
33360	2.65811	0.887691
33361	1.94343	1.0901
33362	1.94343	0.8976
33363	2.65811	0.829009
33364	1.89226	1.54759
33365	1.89226	0.751653
33366	1.89226	1.17089
33367	2.59903	0.858073
33368	2.59903	1.80634
33369	1.89227	2.1
33370	1.89227	2.1
33371	1.89227	2.1
33372	1.89227	2.1
33373	3.78453	2.1
33374	1.15471	2.1
33375	0.993889	2.1
33376	1.78919	2.1
33377	1.77325	2.1
33378	2.78308	2.1
33379	1.89226	2.1
33380	1.89226	2.1
33381	1.89226	2.1
33382	1.89226	2.1
33383	3.78453	2.1
33384	1.78919	2.1
33385	1.77325	2.1
33386	2.45969	2.1
33387	2.70835	2.1
33388	4.49753	2.1
33389	0.939314	1.96737
33390	0.939315	2.1
33391	1.01133	2.1
33392	1.01133	2.1
33393	0.596471	2.1
33394	1.20813	2.1
33395	0.886619	2.1
33396	1.35417	2.1
33397	1.35417	2.1
33398	1.20813	2.1
33399	0.886621	2.1
33400	0.59647	2.1
33401	0.946131	2.1
33402	0.946131	2.1
33403	0.94613	2.1
33404	0.94613	2.1
33405	0.946133	2.1

33406	0.946133	2.1
33407	0.946128	2.1
33408	0.946128	2.1
33409	0.946131	2.1
33410	0.946131	2.1
33411	0.946131	2.1
33412	0.946131	2.1
33413	1.03939	2.1
33414	1.03939	2.1
33415	0.946132	0.893749
33416	0.946132	0.956808
33417	0.946132	1.02375
33418	0.946132	2.1
33419	1.23851	0.89625
33420	1.03939	2.1
33421	1.03939	1.38731
33422	0.946132	1.52035
33423	0.946133	1.47271
33424	1.23851	1.43272
33425	0.902503	2.1
33426	0.946129	2.1
33427	0.946132	0.895107
33428	0.946132	1.17069
33429	0.902472	1.17805
33430	0.902472	2.1
33431	1.08736	0.905125
33432	0.994093	2.1
33433	0.946133	1.06074
33434	0.902504	1.28228
33435	1.03939	1.25247
33436	0.946132	1.3299
33437	1.35306	0.995788
33438	1.29982	0.656823
33439	1.7216	0.647332
33440	1.89183	0.617097
33441	3.07466	0.658378
33442	0.485098	0.660588
33443	0.582401	0.685352
33444	0.649908	0.830712
33445	0.8608	0.696308
33446	0.587739	0.550684
33447	0.58774	0.758004
33448	0.644567	0.68518
33449	0.644567	1.31291
33450	0.58241	0.807102
33451	0.86081	0.576853
33452	0.649898	1.02152
33453	0.485093	0.684907
33454	2.25936	2.1
33455	2.71441	2.1
33456	2.63824	2.1
33457	2.20652	2.1
33458	4.92092	2.1
33459	2.25936	2.1
33460	1.57627	2.1
33461	1.5493	2.1
33462	2.20652	2.1
33463	3.78279	2.1

33464	2.63824	2.1
33465	2.20652	2.1
33466	2.17326	2.1
33467	2.58517	2.1
33468	4.79169	2.1
33469	2.17326	2.1
33470	2.20652	2.1
33471	2.19278	2.1
33472	1.53556	2.1
33473	1.5493	2.1
33474	1.5493	2.1
33475	1.97465	2.1
33476	2.13925	2.1
33477	2.04483	2.1
33478	1.62784	2.1
33479	3.76709	2.1
33480	1.89226	2.1
33481	1.89226	2.1
33482	1.89226	2.1
33483	1.89226	2.1
33484	1.89226	2.1
33485	1.89226	2.1
33486	0.724037	2.1
33487	0.81392	2.1
33488	0.96796	2.1
33489	1.02242	2.1
33490	0.882091	2.1
33491	0.882091	2.1
33492	0.899789	2.1
33493	0.899789	2.1
33494	0.724036	2.1
33495	0.96796	2.1
33496	1.02242	2.1
33497	0.81392	2.1
33498	1.13537	2.1
33499	1.63054	2.1
33500	0.946131	2.1
33501	0.946131	2.1
33502	0.946131	2.1
33503	0.946131	2.1
33504	1.258	2.1
33505	1.63054	2.1
33506	1.22632	2.1
33507	1.22632	2.1
33508	0.946131	2.1
33509	1.258	2.1
33510	1.89226	2.1
33511	1.89226	2.1
33512	1.89226	2.1
33513	1.89226	2.1
33514	1.89226	2.1
33515	1.89226	2.1
33516	0.809739	2.1
33517	0.862674	2.1
33518	1.1995	2.1
33519	1.33899	2.1
33520	1.06324	2.1
33521	1.06324	2.1

33522	0.981932	2.1
33523	1.19563	2.1
33524	0.809727	2.1
33525	1.14092	2.1
33526	1.33902	2.1
33527	0.863494	2.1
33528	2.23742	2.1
33529	1.84902	2.1
33530	1.88088	2.1
33531	2.20392	2.1
33532	4.08644	2.1
33533	1.3592	2.1
33534	1.84902	2.1
33535	1.88088	2.1
33536	1.3305	2.1
33537	1.88088	2.1
33538	1.3305	2.1
33539	1.84902	2.1
33540	1.15008	0.508023
33541	1.3305	1.40424
33542	1.70539	2.1
33543	2.9991	2.1
33544	1.84901	2.1
33545	2.0264	2.1
33546	2.20391	2.1
33547	1.70539	2.1
33548	3.87541	2.1
33549	1.03852	2.04518
33550	1.38247	0.708996
33551	1.36635	0.816033
33552	1.01537	1.20956
33553	2.39784	1.02456
33554	2.0815	1.10475
33555	1.91744	0.869187
33556	1.91744	0.857937
33557	2.06496	2.1
33558	1.89227	0.831333
33559	2.03474	2.1
33560	1.89226	0.725304
33561	1.89226	0.906125
33562	1.89226	0.689418
33563	1.89226	1.41356
33564	1.89226	0.678567
33565	1.89226	1.31487
33566	1.36635	0.930873
33567	1.02923	1.76515
33568	1.02923	0.865397
33569	1.01537	1.80692
33570	1.36416	0.792729
33571	1.3503	2.07766
33572	1.89226	1.12938
33573	1.89359	2.08318
33574	1.89359	1.02755
33575	1.91744	2.1
33576	1.89226	0.981339
33577	1.91611	2.1
33578	0.946128	1.63721
33579	0.946128	1.04559

33580	0.946133	0.816494
33581	0.946133	0.679842
33582	0.94613	0.683804
33583	0.94613	0.987586
33584	0.946131	1.85382
33585	0.946131	0.743324
33586	0.946131	0.879722
33587	0.946131	0.871517
33588	0.946131	0.914638
33589	0.946131	0.918348
33590	2.689 2.1	
33591	2.0815	2.1
33592	2.72786	2.1
33593	2.48295	2.1
33594	5.02397	2.1
33595	2.72786	2.1
33596	1.86424	2.1
33597	2.7188	2.1
33598	2.08981	2.1
33599	4.81768	2.1
33600	2.08982	2.1
33601	0.986987	2.1
33602	2.08982	2.1
33603	1.87249	2.1
33604	1.01964	2.1
33605	0.906115	2.1
33606	1.44247	2.1
33607	1.43872	2.1
33608	1.17054	2.1
33609	1.17054	2.1
33610	1.43872	2.1
33611	1.26045	2.1
33612	2.0815	0.05
33613	2.0815	0.05
33614	2.0815	0.0512058
33615	1.81468	0.05
33616	1.89065	0.05
33617	1.55625	0.05
33618	1.89062	0.05
33619	1.89224	0.0510802
33620	1.89224	0.0512864
33621	2.27073	0.05
33622	1.86856	0.05
33623	2.24232	0.05
33624	1.21419	2.1
33625	2.79716	2.1
33626	2.50005	2.1
33627	2.50005	2.1
33628	1.5008	2.1
33629	1.5008	2.1
33630	1.60139	0.05
33631	1.7103	0.05
33632	1.04159	0.05
33633	1.2489	0.05
33634	2.75189	0.05
33635	0.732812	0.05
33636	0.732812	0.05
33637	0.751986	0.05

33638	0.751986	0.05
33639	0.860346	0.05
33640	0.667961	0.05
33641	0.85515	0.05
33642	0.624452	0.05
33643	0.62445	0.05
33644	0.667962	0.05
33645	0.855148	0.05
33646	0.860348	0.05
33647	1.89226	0.471759
33648	2.50872	0.482099
33649	2.77909	0.467836
33650	1.89226	0.483057
33651	2.77909	0.468957
33652	1.89226	0.484604
33653	2.15825	0.439136
33654	2.50872	0.437689
33655	1.90439	0.384515
33656	1.90439	0.454229
33657	2.77909	0.412377
33658	2.77909	0.461436
33659	1.12468	2.1
33660	1.43872	2.1
33661	1.64926	2.1
33662	1.2724	2.1
33663	2.77395	2.1
33664	1.64926	2.1
33665	1.38438	2.1
33666	1.47244	2.1
33667	1.2724	2.1
33668	1.8493	2.1
33669	1.64926	2.1
33670	1.36416	0.864
33671	1.0652	1.92023
33672	1.0652	0.922166
33673	1.02923	1.96162
33674	1.37496	0.851475
33675	1.33898	2.1
33676	1.89227	1.39894
33677	1.90433	2.1
33678	1.90433	1.30981
33679	1.89359	2.1
33680	1.89227	1.1857
33681	1.88153	2.1
33682	0.946129	1.89044
33683	0.946129	1.36539
33684	0.946133	0.895761
33685	0.946133	0.746135
33686	0.946131	0.750148
33687	0.946131	1.19264
33688	0.946131	1.98729
33689	0.946131	0.793472
33690	0.946131	0.965161
33691	0.946131	0.974386
33692	0.946131	1.03432
33693	0.946131	1.0212
33694	1.37496	0.886137
33695	1.07519	2.0388

33696	1.07519	0.936313
33697	1.0652	2.04649
33698	1.39725	0.870686
33699	1.38726	2.1
33700	1.89227	1.69487
33701	1.9052	2.1
33702	1.9052	1.54778
33703	1.90433	2.1
33704	1.89227	1.34929
33705	1.8914	2.1
33706	0.946129	2.01885
33707	0.946129	1.49497
33708	0.946133	0.945084
33709	0.946133	0.757899
33710	0.946131	0.764195
33711	0.946131	1.39974
33712	0.946131	2.07268
33713	0.946131	0.816707
33714	0.946131	1.00656
33715	0.946131	1.04442
33716	0.946131	1.07396
33717	0.946131	1.08953
33718	1.91316	0.339274
33719	1.89226	0.358731
33720	1.89226	0.355289
33721	1.89226	0.351165
33722	1.99366	0.345535
33723	1.99366	0.341513
33724	1.93083	0.329933
33725	1.89226	0.351817
33726	1.51379	0.341586
33727	1.89226	0.342472
33728	1.53051	0.336433
33729	1.91316	0.335766
33730	2.16731	2.1
33731	1.21373	2.1
33732	1.47804	2.1
33733	2.37601	2.1
33734	3.64535	2.1
33735	1.7211	2.1
33736	2.37601	2.1
33737	1.47804	2.1
33738	1.47804	2.1
33739	2.56843	2.1
33740	2.56843	2.1
33741	2.58731	2.1
33742	1.59094	2.1
33743	1.59094	2.1
33744	1.76816	2.1
33745	2.41707	2.1
33746	2.5943	2.1
33747	0.718346	2.1
33748	0.795468	2.1
33749	1.20854	2.1
33750	1.1224	2.1
33751	0.961203	2.1
33752	0.961203	2.1
33753	0.95667	2.1

33754	0.95667	2.1
33755	0.718346	2.1
33756	1.1224	2.1
33757	0.795469	2.1
33758	1.20854	2.1
33759	1.84076	2.1
33760	1.42884	2.1
33761	2.24274	2.1
33762	1.85415	2.1
33763	3.69491	2.1
33764	1.89226	2.1
33765	1.89226	1.24009
33766	1.89226	1.62691
33767	1.89226	2.1
33768	3.78452	2.1
33769	2.24273	2.1
33770	2.24412	2.1
33771	1.85415	2.1
33772	1.85415	2.1
33773	2.68473	2.1
33774	2.68473	2.1
33775	1.89226	1.09619
33776	1.89226	2.1
33777	1.89226	2.1
33778	1.89226	0.983048
33779	1.89226	2.1
33780	1.89226	0.986235
33781	1.89226	0.997143
33782	1.89226	2.1
33783	1.89226	2.1
33784	1.89226	1.2415
33785	3.78451	2.1
33786	1.85415	2.1
33787	1.10961	2.1
33788	2.24411	2.1
33789	1.51067	2.1
33790	3.35373	2.1
33791	1.70302	2.1
33792	1.89226	2.1
33793	1.76399	2.1
33794	1.43577	2.1
33795	2.08149	2.1
33796	1.70302	2.1
33797	1.89226	2.1
33798	1.89226	2.1
33799	1.89226	2.1
33800	1.89226	2.1
33801	1.89226	2.04799
33802	1.89226	2.1
33803	1.0593	2.1
33804	1.17289	2.1
33805	1.5379	2.1
33806	1.46757	2.1
33807	2.71079	2.1
33808	0.931235	2.1
33809	1.1151	2.1
33810	0.975236	2.1
33811	0.975236	2.1

33812	1.31147	2.1
33813	0.762882	2.1
33814	1.13152	2.1
33815	0.505799	2.1
33816	1.31149	2.1
33817	1.07629	2.1
33818	0.884817	2.1
33819	0.505793	2.1
33820	2.69027	2.1
33821	1.60441	2.1
33822	2.69027	2.1
33823	1.64771	2.1
33824	2.78977	2.1
33825	1.72731	2.1
33826	0.77878	0.05
33827	0.843226	0.05
33828	0.845997	0.05
33829	0.926091	0.05
33830	0.77878	0.05
33831	0.926093	0.05
33832	1.3922	1.34671
33833	1.3922	1.39707
33834	0.872156	2.05858
33835	0.872156	1.91765
33836	0.872157	2.1
33837	0.946132	1.31093
33838	0.946129	1.0417
33839	1.31822	2.1
33840	0.94613	1.79721
33841	0.94613	1.89051
33842	1.31823	1.67205
33843	0.872157	1.87618
33844	1.81534	0.59569
33845	1.89226	0.604375
33846	1.59602	0.696262
33847	1.59602	0.774343
33848	1.89226	0.463557
33849	1.89226	0.514423
33850	1.89227	0.55094
33851	1.89227	0.569597
33852	1.89227	0.600963
33853	1.89227	0.520117
33854	3.78453	0.516058
33855	1.89226	0.569767
33856	1.89226	0.471955
33857	1.89226	0.520593
33858	1.89226	0.521826
33859	3.78453	0.581568
33860	2.30348	0.429578
33861	1.4185	0.451745
33862	1.82551	0.473191
33863	1.02011	0.420669
33864	3.24401	0.426541
33865	2.46702	0.400323
33866	1.17129	0.435652
33867	1.82551	0.394341
33868	1.82551	0.422508
33869	1.73525	0.403361

33870	1.73525	0.435754
33871	1.89227	0.531568
33872	1.89227	0.466339
33873	1.89227	0.466898
33874	1.89227	0.480778
33875	1.89227	0.515174
33876	1.89227	0.528609
33877	2.80034	1.0458
33878	2.45767	2.1
33879	2.67994	0.835717
33880	2.35395	2.1
33881	5.1376	2.1
33882	2.45766	2.1
33883	1.8769	2.1
33884	2.00989	2.1
33885	2.00989	2.1
33886	2.35395	2.1
33887	2.35395	2.1
33888	2.57662	1.0666
33889	2.35395	2.1
33890	2.57662	2.1
33891	2.67993	1.77074
33892	2.24227	2.1
33893	2.34558	2.09435
33894	1.02874	2.1
33895	1.02874	2.1
33896	1.03084	2.1
33897	1.03084	2.1
33898	0.938448	2.1
33899	0.886762	2.1
33900	1.17698	2.1
33901	1.12114	2.1
33902	1.12113	2.1
33903	0.886764	2.1
33904	0.93845	2.1
33905	1.17697	2.1
33906	1.91969	2.1
33907	2.5508	2.1
33908	1.88273	2.1
33909	2.76609	2.1
33910	4.68578	2.1
33911	2.83423	2.1
33912	1.89226	2.1
33913	2.76609	2.1
33914	1.89226	2.1
33915	4.65835	2.1
33916	1.56685	0.676958
33917	1.11712	0.641067
33918	1.07999	1.0872
33919	1.51448	0.736153
33920	2.6316	0.772121
33921	1.56685	0.56269
33922	1.67652	0.886474
33923	1.5955	1.04147
33924	1.51448	0.643526
33925	3.19099	0.788331
33926	1.89226	0.904005
33927	1.89226	0.513936

33928	1.89226	0.667737
33929	1.89226	0.747635
33930	3.78453	0.745577
33931	1.89226	0.759748
33932	1.89226	0.569913
33933	1.89226	0.889022
33934	1.89226	0.86818
33935	1.89226	0.592749
33936	1.89226	0.56503
33937	1.0562	0.829311
33938	1.51448	0.646874
33939	1.07999	0.722179
33940	1.07999	0.663405
33941	1.46441	0.797751
33942	1.46441	0.68927
33943	1.5955	1.07949
33944	1.51448	0.6214
33945	1.46441	0.685162
33946	1.52909	0.830549
33947	3.04357	0.752233
33948	1.89226	0.640553
33949	1.89226	0.735497
33950	1.89226	0.849539
33951	1.89226	0.559203
33952	3.78453	0.633121
33953	1.89227	0.667865
33954	1.89227	0.517826
33955	1.89227	0.837959
33956	1.89227	0.651421
33957	3.78453	0.6915
33958	1.89994	0.593525
33959	1.70303	0.48081
33960	1.68576	0.209048
33961	1.89608	0.263514
33962	3.8237	0.38508
33963	1.89994	0.576457
33964	2.31116	0.827058
33965	1.9386	0.671716
33966	1.88653	0.978979
33967	3.74885	0.737613
33968	2.31117	0.886906
33969	2.20665	1.10423
33970	2.09617	0.886141
33971	2.41055	2.1
33972	2.09617	1.04031
33973	2.41055	2.1
33974	1.10777	0.288504
33975	1.0523	0.525369
33976	0.912098	0.375995
33977	0.757533	0.404262
33978	0.89287	0.36365
33979	1.07571	0.192207
33980	0.77485	0.291187
33981	0.98645	0.233456
33982	0.989171	0.19993
33983	0.875089	0.232123
33984	0.979686	0.309947
33985	0.979686	0.413834

33986	2.52442	2.1
33987	1.49998	2.1
33988	2.51692	2.1
33989	1.56957	2.1
33990	4.09399	2.1
33991	1.01298	2.1
33992	1.01298	2.1
33993	1.03079	2.1
33994	1.03079	2.1
33995	0.820964	2.1
33996	0.784786	2.1
33997	1.25898	2.1
33998	1.25846	2.1
33999	1.25898	2.1
34000	0.820964	2.1
34001	1.25846	2.1
34002	0.784786	2.1
34003	1.89226	0.0607228
34004	1.89226	0.0569567
34005	2.6297	0.0512703
34006	1.89226	0.0539777
34007	3.78452	0.0586001
34008	1.89226	0.05
34009	1.89226	0.05
34010	1.89226	0.05
34011	1.89226	0.0560206
34012	1.89226	0.05
34013	1.89226	0.0623428
34014	2.19397	0.05
34015	1.89226	0.05
34016	2.34112	0.05
34017	2.34112	0.05
34018	1.89226	0.05
34019	1.89226	0.05
34020	2.23366	2.1
34021	1.87598	2.1
34022	1.64716	2.1
34023	2.06048	2.1
34024	3.93645	2.1
34025	2.23367	2.1
34026	2.17384	2.1
34027	2.04818	2.1
34028	2.06048	2.1
34029	4.23432	2.1
34030	1.9855	2.1
34031	2.06048	2.1
34032	1.93163	2.1
34033	1.50737	2.1
34034	3.56785	2.1
34035	2.04818	2.1
34036	2.06048	2.1
34037	2.27465	2.1
34038	1.92264	2.1
34039	3.98312	2.1
34040	2.08149	0.543487
34041	1.89226	1.56093
34042	1.89226	1.18535
34043	1.33153	0.498004

34044	1.68038	1.74126
34045	1.18244	0.765541
34046	1.89226	0.913843
34047	1.89226	1.61545
34048	1.89226	1.45428
34049	1.89226	0.779189
34050	3.78452	0.979787
34051	2.69069	0.624092
34052	1.89226	0.861097
34053	1.89226	0.866478
34054	1.97397	0.643667
34055	1.89226	0.836636
34056	1.97397	0.614496
34057	1.89226	1.62298
34058	2.38056	0.809692
34059	1.89226	0.794792
34060	1.89226	1.19157
34061	3.78452	1.06522
34062	0.904435	2.1
34063	0.904435	2.1
34064	0.925315	2.1
34065	0.925315	2.1
34066	1.13312	2.1
34067	1.08109	2.1
34068	0.70697	2.1
34069	0.696633	2.1
34070	0.70697	2.1
34071	1.13312	2.1
34072	0.696633	2.1
34073	1.08109	2.1
34074	1.45506	2.1
34075	2.26623	2.1
34076	2.38363	2.1
34077	2.38363	2.1
34078	1.41394	2.1
34079	1.41394	2.1
34080	0.946131	1.6955
34081	0.946131	2.1
34082	1.13311	2.1
34083	1.13311	2.1
34084	0.946127	1.089
34085	0.946127	2.1
34086	1.13312	2.1
34087	1.19182	2.1
34088	1.19181	2.1
34089	0.946129	2.1
34090	1.13312	2.1
34091	0.94613	2.1
34092	1.89226	0.699154
34093	1.89226	1.58537
34094	1.89226	0.75564
34095	1.89226	1.41643
34096	1.89226	0.783255
34097	1.89226	1.48052
34098	1.05168	2.1
34099	0.696527	1.90389
34100	0.907442	0.894874
34101	0.907442	1.01596

34102	0.953145	0.829331
34103	0.782285	2.1
34104	0.773928	2.07405
34105	0.773928	0.913932
34106	0.696538	0.980713
34107	0.851554	1.01791
34108	1.04071	1.09874
34109	1.12335	1.08091
34110	1.89226	0.735204
34111	1.89226	0.535088
34112	1.89226	0.516255
34113	1.89226	0.450506
34114	3.78453	0.46748
34115	1.89226	0.653412
34116	1.89226	0.530111
34117	1.89226	0.51995
34118	1.89226	0.479995
34119	3.78453	0.60822
34120	2.50912	0.496274
34121	1.89226	0.472693
34122	1.89226	0.463059
34123	2.63195	0.442251
34124	4.40139	0.43897
34125	2.50912	0.499296
34126	1.3598	0.451863
34127	1.70303	0.426149
34128	2.36874	0.368069
34129	3.82055	0.470328
34130	1.89227	0.506867
34131	1.89227	0.44902
34132	1.89227	0.422822
34133	1.89227	0.384686
34134	3.78453	0.488806
34135	1.89227	0.512433
34136	1.89227	0.466294
34137	1.89227	0.432916
34138	1.89227	0.39898
34139	3.78453	0.411758
34140	1.89226	0.448708
34141	2.67088	0.33215
34142	2.63195	0.334828
34143	2.63195	0.428055
34144	1.89226	0.374337
34145	1.89226	0.461126
34146	1.30202	0.385573
34147	1.28304	0.41194
34148	0.809902	0.385451
34149	0.888801	0.394448
34150	1.13262	0.219574
34151	0.968276	0.297402
34152	1.0733	0.401075
34153	1.0733	0.389521
34154	1.35419	0.33981
34155	0.801222	0.254699
34156	0.899732	0.418213
34157	1.23967	0.328101
34158	1.31595	2.1
34159	0.869501	2.1

34160	2.65473	2.1
34161	1.88782	2.1
34162	3.20377	2.1
34163	1.89227	2.1
34164	1.89227	2.1
34165	1.89227	2.1
34166	1.89227	2.1
34167	3.78453	2.1
34168	2.65473	2.1
34169	1.89226	2.1
34170	1.88781	2.1
34171	1.88782	2.1
34172	2.0815	2.1
34173	2.0815	2.1
34174	1.89226	1.75372
34175	1.89226	2.1
34176	1.89226	2.04919
34177	1.89226	1.99025
34178	1.89226	1.85556
34179	1.89226	1.7604
34180	1.89226	1.79327
34181	1.89226	1.88998
34182	1.89226	2.1
34183	1.89226	2.1
34184	1.89226	2.00424
34185	1.89226	1.87102
34186	2.39057	1.65751
34187	1.89226	0.833301
34188	1.89226	0.715284
34189	1.89226	2.0852
34190	2.14243	0.683132
34191	2.14243	2.1
34192	1.89226	0.540952
34193	1.89226	1.10142
34194	1.89226	0.660708
34195	1.89226	0.750261
34196	1.89226	0.757595
34197	1.89226	1.18004
34198	1.89226	0.599409
34199	1.89226	1.30735
34200	1.89226	0.64803
34201	1.89226	0.783242
34202	1.89226	0.70846
34203	1.89226	1.34236
34204	2.56323	0.272218
34205	1.36324	0.315896
34206	1.05296	0.281909
34207	2.18048	0.315144
34208	3.54372	0.281611
34209	0.946128	1.58557
34210	0.946128	2.1
34211	1.08733	2.1
34212	1.08733	2.1
34213	0.946131	2.1
34214	0.946131	2.05953
34215	1.09197	2.1
34216	1.08732	2.1
34217	0.946131	2.1

34218	1.09197	2.1
34219	1.08732	2.1
34220	0.946131	2.1
34221	1.35111	1.03127
34222	1.51327	1.01209
34223	1.64493	0.996288
34224	1.31058	1.06775
34225	2.99605	1.19758
34226	1.8124	0.978736
34227	1.51327	1.10113
34228	1.64493	1.1419
34229	1.31358	0.988411
34230	1.8124	1.09725
34231	1.48105	0.935064
34232	1.64493	0.987234
34233	1.31058	1.04515
34234	1.22297	0.977364
34235	1.70101	0.977243
34236	2.8679	0.892367
34237	1.70101	1.02851
34238	1.99297	0.949315
34239	1.64493	1.06839
34240	1.64493	0.908543
34241	1.99297	1.05808
34242	1.8124	0.851947
34243	2.0815	2.1
34244	1.80258	2.1
34245	1.80258	2.1
34246	1.83506	2.1
34247	2.0815	2.1
34248	2.1212	2.1
34249	2.0815	2.1
34250	1.82129	2.1
34251	1.82129	2.1
34252	1.80445	2.1
34253	2.0815	2.1
34254	2.06092	2.1
34255	1.89224	2.1
34256	1.703 2.1	
34257	1.97839	2.1
34258	1.703 2.1	
34259	1.97402	2.1
34260	1.703 2.1	
34261	1.89226	0.68337
34262	1.89226	0.855891
34263	1.89226	0.677385
34264	2.75351	0.764827
34265	3.78453	0.78204
34266	1.89226	0.691649
34267	3.02887	1.08764
34268	2.03149	0.646471
34269	2.03149	0.715755
34270	1.89226	0.827166
34271	1.89226	1.02633
34272	2.17979	2.1
34273	1.65533	2.1
34274	2.57212	2.1
34275	2.20346	2.1

34276	4.22745	2.1
34277	1.89227	2.1
34278	2.20347	2.1
34279	1.89227	2.1
34280	2.57212	2.1
34281	1.89227	2.1
34282	2.57212	2.1
34283	2.05274	0.681693
34284	2.16783	2.1
34285	2.24429	0.642411
34286	2.38404	2.1
34287	4.41212	0.766072
34288	2.16783	1.96373
34289	2.05967	2.1
34290	2.38404	2.1
34291	2.24695	2.1
34292	4.41478	2.1
34293	2.24429	0.625856
34294	2.38404	2.1
34295	2.51819	0.668577
34296	2.60723	2.1
34297	4.85152	1.00412
34298	2.38404	2.1
34299	2.24695	2.1
34300	2.60723	1.96834
34301	2.45247	2.1
34302	4.85418	2.1
34303	1.89227	2.1
34304	1.89227	2.1
34305	1.89227	2.1
34306	1.89227	2.1
34307	3.78453	2.1
34308	1.89226	2.1
34309	1.89226	2.1
34310	1.89226	2.1
34311	1.89226	2.1
34312	3.78453	2.1
34313	0.618473	2.1
34314	0.619945	2.1
34315	0.797692	2.1
34316	0.770147	2.1
34317	0.68852	2.1
34318	0.68852	2.1
34319	0.701573	2.1
34320	0.701573	2.1
34321	0.619945	2.1
34322	0.797691	2.1
34323	0.618473	2.1
34324	0.770147	2.1
34325	0.797692	2.1
34326	0.770148	2.1
34327	0.694787	2.1
34328	0.653411	2.1
34329	0.722102	2.1
34330	0.722102	2.1
34331	0.729001	2.1
34332	0.729001	2.1
34333	0.797692	2.1

34334	0.694787	2.1
34335	0.770148	2.1
34336	0.653412	2.1
34337	1.89227	2.1
34338	1.89227	2.1
34339	1.89227	2.1
34340	1.89227	2.1
34341	3.78453	2.1
34342	1.89226	2.1
34343	1.89226	2.1
34344	1.89226	2.1
34345	1.89226	2.1
34346	3.78453	2.1
34347	1.23989	2.1
34348	1.59538	2.1
34349	1.66647	2.1
34350	1.26599	2.1
34351	2.90636	2.1
34352	1.66647	2.1
34353	1.38957	2.1
34354	1.47446	2.1
34355	1.40337	2.1
34356	1.66647	2.1
34357	1.59538	2.1
34358	2.80619	2.1
34359	2.0815	2.1
34360	2.0815	2.1
34361	2.0815	2.1
34362	2.73721	2.1
34363	2.74192	2.1
34364	2.73721	2.1
34365	2.0815	2.1
34366	2.0815	2.1
34367	2.0815	2.1
34368	2.689	2.1
34369	2.69166	2.1
34370	2.62369	2.1
34371	2.19717	2.1
34372	2.60405	2.1
34373	2.20566	2.1
34374	4.80122	2.1
34375	2.19717	2.1
34376	1.37392	2.1
34377	2.20566	2.1
34378	1.45408	2.1
34379	3.65125	2.1
34380	2.60405	2.1
34381	2.20566	2.1
34382	2.57466	2.1
34383	2.21715	2.1
34384	4.8212	2.1
34385	2.20566	2.1
34386	1.54249	2.1
34387	1.54249	2.1
34388	1.45408	2.1
34389	2.21715	2.1
34390	2.12874	2.1
34391	1.60647	1.68398

34392	1.31346	2.1
34393	1.82649	1.50947
34394	1.51067	2.1
34395	3.13995	2.1
34396	1.89226	1.08948
34397	1.89226	2.1
34398	1.89226	1.51628
34399	1.89226	2.1
34400	3.78452	2.1
34401	1.89226	2.1
34402	2.6096	2.1
34403	2.8067	2.1
34404	2.8067	2.1
34405	1.89226	2.1
34406	1.89226	2.1
34407	1.82649	1.12075
34408	1.3596	2.1
34409	2.17708	1.50396
34410	1.70302	2.1
34411	3.40243	2.1
34412	1.89226	1.11744
34413	1.89226	2.1
34414	1.89226	0.964027
34415	1.89226	2.1
34416	3.78452	1.99094
34417	2.27067	2.1
34418	2.43549	2.1
34419	2.63979	2.1
34420	2.41828	2.1
34421	2.27067	2.1
34422	2.04916	2.1
34423	1.30047	2.1
34424	2.04916	2.1
34425	2.27068	2.1
34426	1.5586	2.1
34427	3.57115	2.1
34428	2.27068	2.1
34429	1.84214	2.1
34430	1.80224	2.1
34431	1.5586	2.1
34432	2.51432	2.1
34433	2.27068	2.1
34434	1.63719	2.1
34435	2.15714	2.1
34436	2.07821	2.1
34437	1.52204	2.1
34438	3.67919	2.1
34439	2.07821	2.1
34440	1.40118	2.1
34441	1.40118	2.1
34442	1.52204	2.1
34443	1.99632	2.1
34444	2.11718	2.1
34445	0.946131	1.74128
34446	0.946131	1.33316
34447	1.06957	2.1
34448	1.06957	2.1
34449	0.946132	2.1

34450	0.946132	0.986788
34451	1.411	1.67242
34452	1.06957	2.1
34453	0.946132	1.63753
34454	1.411	1.93385
34455	1.06957	2.04967
34456	0.946132	1.96852
34457	1.99875	0.677401
34458	2.57079	0.658537
34459	2.59319	0.975938
34460	1.9105	0.544114
34461	4.59194	0.714576
34462	1.99875	0.650427
34463	1.02247	0.457235
34464	1.07017	0.50122
34465	1.15841	0.497009
34466	1.91051	0.4889
34467	1.99875	0.499579
34468	2.59319	0.899587
34469	1.9105	0.568447
34470	2.33812	0.671585
34471	2.59319	0.647521
34472	1.59457	0.740657
34473	1.59457	0.711264
34474	2.50906	0.623308
34475	2.0467	0.494399
34476	2.03357	0.556392
34477	2.53176	0.500593
34478	4.54263	0.526252
34479	1.294	0.657901
34480	1.66636	0.567438
34481	2.03357	0.525395
34482	1.03079	0.496301
34483	3.32757	0.548561
34484	1.68108	0.518467
34485	2.53176	0.492992
34486	2.36417	0.456189
34487	2.71666	0.456533
34488	1.68108	0.644273
34489	2.03357	0.606115
34490	1.89226	0.398313
34491	2.59302	0.4092
34492	2.2727	0.402674
34493	2.2727	0.386707
34494	1.89226	0.433087
34495	1.89226	0.398783
34496	1.89226	0.471765
34497	1.89226	0.447815
34498	1.89226	0.429056
34499	1.89226	0.35411
34500	3.78452	0.471056
34501	1.89225	0.497042
34502	1.89225	0.25449
34503	1.89225	0.356737
34504	1.89225	0.40532
34505	1.89225	0.373164
34506	1.89225	0.407881
34507	0.934114	0.417866

34508	0.934114	0.32414
34509	0.944544	0.394502
34510	0.944544	0.385464
34511	0.721493	0.311843
34512	0.58215	0.291527
34513	1.2965	0.392359
34514	1.13634	0.333807
34515	1.13635	0.316543
34516	0.582148	0.299519
34517	1.29651	0.386487
34518	0.721491	0.344019
34519	1.89226	0.372914
34520	1.89226	0.313838
34521	1.89226	0.301082
34522	1.89226	0.241407
34523	3.78452	0.24378
34524	0.946131	0.495081
34525	0.946131	0.503085
34526	0.946131	0.473097
34527	0.946131	0.497027
34528	0.946131	0.473475
34529	0.946131	0.484768
34530	0.946131	0.499135
34531	0.946131	0.522539
34532	0.946131	0.490531
34533	0.946131	0.488477
34534	0.946131	0.490393
34535	0.946131	0.489626
34536	1.89226	0.0847083
34537	1.89226	0.0905275
34538	2.78602	0.0817015
34539	1.89226	0.118146
34540	3.78453	0.0843588
34541	1.52601	2.1
34542	1.76601	2.1
34543	1.27839	2.1
34544	1.56095	2.1
34545	3.0444	2.1
34546	1.76601	2.1
34547	1.86793	2.1
34548	1.56095	2.1
34549	1.68152	2.1
34550	3.44753	2.1
34551	1.27841	2.1
34552	1.56097	2.1
34553	1.0577	2.1
34554	1.36861	2.1
34555	2.64702	2.1
34556	1.56097	2.1
34557	1.68154	2.1
34558	1.36861	2.1
34559	1.53296	2.1
34560	3.05015	2.1
34561	1.89226	2.1
34562	1.89226	2.1
34563	1.89226	2.1
34564	1.89226	2.1
34565	3.78453	2.1

34566	0.983075	2.1
34567	0.788361	2.1
34568	0.724726	2.1
34569	0.724726	2.1
34570	0.983027	2.1
34571	0.724734	2.1
34572	0.788364	2.1
34573	0.788364	2.1
34574	0.788335	2.1
34575	0.94613	2.1
34576	0.724757	2.1
34577	0.946131	2.1
34578	1.15789	2.1
34579	2.11135	2.1
34580	2.25826	2.1
34581	1.28845	2.1
34582	3.41615	2.1
34583	1.89226	2.1
34584	1.89226	2.1
34585	1.89226	2.1
34586	1.89226	2.1
34587	3.78453	2.1
34588	1.57667	2.1
34589	1.70002	2.1
34590	1.70002	2.1
34591	1.89226	2.1
34592	1.89226	2.1
34593	2.05244	2.1
34594	2.25826	2.1
34595	1.42214	2.1
34596	1.43675	2.1
34597	1.28845	2.1
34598	2.40656	2.1
34599	2.25826	2.1
34600	1.58066	2.1
34601	1.74247	2.1
34602	1.85097	2.1
34603	1.61155	2.1
34604	3.43163	2.1
34605	0.785612	2.1
34606	0.789638	2.1
34607	0.882561	2.1
34608	0.763529	2.1
34609	0.686921	2.1
34610	0.86048	2.1
34611	0.871812	2.1
34612	0.871812	2.1
34613	0.830183	2.1
34614	0.882572	2.1
34615	0.902315	2.1
34616	0.785602	2.1
34617	1.01972	0.05
34618	0.936657	0.05
34619	0.946131	0.05
34620	0.946131	0.05
34621	0.927018	0.05
34622	0.927018	0.05
34623	1.1376	0.05

34624	0.953663	0.05
34625	1.1257	0.05
34626	1.04073	0.05
34627	1.00358	0.05
34628	0.851528	0.05
34629	1.73576	0.461445
34630	2.00244	0.640351
34631	1.17798	0.477689
34632	1.25796	0.486799
34633	2.99373	0.448212
34634	2.00244	0.620934
34635	1.10719	0.531551
34636	2.03236	0.683431
34637	2.03236	0.58937
34638	1.25797	0.640685
34639	1.25797	0.525315
34640	1.89226	2.1
34641	1.89226	2.1
34642	1.89226	2.1
34643	1.89226	2.1
34644	3.78452	2.1
34645	2.3174	2.1
34646	1.50005	2.1
34647	1.90428	2.1
34648	2.64564	2.1
34649	4.22168	2.1
34650	1.89226	2.1
34651	1.89226	2.1
34652	1.89226	2.1
34653	1.89226	2.1
34654	1.89226	2.1
34655	1.89226	2.1
34656	1.32749	0.322049
34657	1.70302	0.33661
34658	2.08149	0.330445
34659	1.70302	0.331408
34660	2.19634	0.326079
34661	1.93083	0.326622
34662	1.89226	1.23724
34663	1.89226	1.97735
34664	1.89226	0.746979
34665	1.89226	2.1
34666	3.78453	1.0018
34667	1.89226	1.20886
34668	1.89226	1.92851
34669	1.89226	0.921813
34670	1.89226	1.41918
34671	3.78452	1.69207
34672	2.17499	1.01532
34673	1.79663	1.90271
34674	2.1538	0.750069
34675	1.818	1.0455
34676	2.17499	1.49612
34677	1.83919	2.1
34678	0.898316	2.03431
34679	1.0769	1.13202
34680	0.968276	2.07924
34681	0.773495	1.34241

34682	0.921126	1.77707
34683	0.921126	2.05737
34684	0.92927	1.66877
34685	0.92927	1.62645
34686	1.0769	1.54386
34687	0.968276	1.16558
34688	0.898316	2.0113
34689	0.773495	1.34715
34690	1.89226	0.893468
34691	1.89226	2.1
34692	1.89226	1.13427
34693	1.89226	1.07372
34694	3.78453	1.95773
34695	1.89226	1.17419
34696	1.89226	2.1
34697	1.89226	0.807351
34698	1.89226	1.55862
34699	3.78452	1.05258
34700	2.17499	1.77586
34701	2.1538	2.1
34702	2.53574	1.05947
34703	2.54157	0.939704
34704	4.71073	1.78492
34705	1.0769	1.4335
34706	1.26787	1.02609
34707	1.16641	1.22377
34708	0.968276	2.00263
34709	1.11625	1.30029
34710	1.11625	1.69314
34711	1.11989	1.82295
34712	1.11989	0.83101
34713	1.26787	1.10888
34714	1.16641	2.1
34715	1.0769	2.1
34716	0.968277	1.72228
34717	1.89226	0.492669
34718	1.89226	0.480554
34719	1.89226	0.463056
34720	1.89226	0.468196
34721	3.78452	0.488059
34722	1.91739	0.439784
34723	2.20045	0.440826
34724	1.79005	0.43029
34725	2.33802	0.419446
34726	4.25539	0.420268
34727	2.52754	0.401168
34728	1.89226	0.421386
34729	2.69841	0.403633
34730	2.69841	0.42482
34731	1.89226	0.394777
34732	1.89226	0.416024
34733	1.89226	0.485278
34734	1.89226	0.508489
34735	1.89226	0.461245
34736	1.89226	0.50788
34737	3.78452	0.46805
34738	1.93544	0.470144
34739	2.33802	0.448133

34740	1.55063	0.4095
34741	2.1769	0.420659
34742	3.88865	0.46024
34743	2.20929	0.359871
34744	2.55162	0.233679
34745	2.27807	0.414514
34746	1.48831	0.241971
34747	4.48736	0.252822
34748	2.27807	0.426864
34749	2.72723	0.230306
34750	2.55162	0.334551
34751	2.55162	0.399272
34752	2.43157	0.253589
34753	2.43157	0.342808
34754	2.27807	0.307454
34755	1.51612	0.196573
34756	1.96048	0.180303
34757	1.73022	0.183878
34758	1.70302	0.35265
34759	1.61016	0.336954
34760	2.27807	0.316996
34761	2.08048	0.17712
34762	1.98522	0.197263
34763	2.43157	0.252099
34764	1.83798	0.201824
34765	2.27807	0.262148
34766	1.70302	0.387373
34767	2.68507	0.261522
34768	2.23856	0.339415
34769	2.52174	0.234462
34770	2.35331	0.449795
34771	2.65099	0.333273
34772	1.70303	0.391492
34773	0.986848	0.202035
34774	2.41566	0.179689
34775	1.70303	0.165132
34776	1.88139	0.19681
34777	1.64955	0.196447
34778	1.89226	2.1
34779	2.34978	1.64548
34780	1.89226	2.05327
34781	2.68773	1.30234
34782	1.89226	2.1
34783	2.68773	1.54024
34784	1.89226	2.1
34785	1.89226	1.31919
34786	1.89226	1.39425
34787	1.89226	2.00848
34788	3.78453	1.87576
34789	1.00825	1.05242
34790	1.09847	1.27879
34791	0.939987	1.1225
34792	0.783516	1.71138
34793	0.838041	1.38021
34794	0.838041	1.77784
34795	1.49693	1.83656
34796	1.26602	1.36093
34797	1.10875	1.15036

34798	1.05187	2.08219
34799	1.28743	1.42388
34800	0.946132	1.09258
34801	0.946132	1.35351
34802	0.946132	2.1
34803	0.946132	1.89915
34804	0.946132	1.02364
34805	1.31823	2.1
34806	0.946132	1.31234
34807	0.946132	1.94949
34808	0.946131	1.99743
34809	0.946131	2.1
34810	0.946132	1.70019
34811	1.31823	1.93619
34812	1.51688	0.300971
34813	1.79978	0.43181
34814	1.1685	0.274403
34815	1.1685	0.373617
34816	1.85857	0.359868
34817	1.51018	0.408381
34818	0.946116	0.31599
34819	0.946116	0.432562
34820	0.94612	0.318592
34821	0.94612	0.294299
34822	0.946118	0.271088
34823	0.946118	0.415962
34824	0.946118	0.253195
34825	0.946118	0.427776
34826	0.946131	0.279581
34827	0.946131	0.408421
34828	0.946105	0.357079
34829	0.946105	0.373181
34830	1.89224	2.1
34831	1.89224	2.1
34832	1.89224	2.1
34833	1.89224	2.1
34834	1.89224	2.1
34835	1.89224	2.1
34836	1.38678	2.1
34837	1.3349	2.1
34838	0.946119	2.1
34839	0.946119	2.1
34840	1.38678	2.1
34841	1.38678	2.1
34842	0.946119	2.1
34843	0.946119	2.1
34844	1.38676	2.1
34845	0.946107	2.1
34846	0.946132	2.1
34847	1.33491	2.1
34848	1.06455	2.1
34849	1.03932	2.1
34850	1.20649	2.1
34851	1.18773	2.1
34852	2.27105	2.1
34853	1.89226	2.1
34854	1.89226	2.1
34855	1.89226	2.1

34856	1.89226	2.1
34857	1.89226	2.1
34858	1.89226	2.1
34859	1.36918	2.1
34860	1.18774	2.1
34861	1.31144	2.1
34862	1.20651	2.1
34863	1.31144	2.1
34864	1.20651	2.1
34865	2.29066	2.1
34866	1.76476	2.1
34867	1.79825	2.1
34868	2.33801	2.1
34869	4.08891	2.1
34870	1.79825	2.1
34871	2.33801	2.1
34872	2.37168	2.1
34873	1.49325	2.1
34874	4.16993	2.1
34875	1.13641	2.1
34876	1.43213	0.821245
34877	1.44085	1.05619
34878	1.18301	2.1
34879	2.61513	1.88087
34880	1.89226	1.89881
34881	2.23875	2.1
34882	2.23875	1.49842
34883	2.10287	2.1
34884	1.89226	1.50888
34885	1.75639	2.1
34886	1.89226	0.845647
34887	1.89226	1.7088
34888	1.89226	0.854511
34889	1.89226	2.08236
34890	1.89226	0.828495
34891	1.89226	2.1
34892	1.44085	1.21531
34893	1.25173	2.08855
34894	1.25173	0.946127
34895	1.18301	2.1
34896	1.4617	0.913403
34897	1.39298	2.1
34898	1.89227	1.76474
34899	2.23875	2.1
34900	2.42504	2.1
34901	1.89227	1.08721
34902	4.13101	2.1
34903	1.89226	0.847662
34904	1.89226	1.0507
34905	1.89226	0.784146
34906	1.89226	1.92327
34907	1.89226	0.78105
34908	1.89226	2.06227
34909	1.89226	2.1
34910	2.41297	2.1
34911	2.70685	1.96104
34912	2.70685	2.1
34913	1.89226	1.96897

34914	1.89226	2.1
34915	1.89226	2.1
34916	1.89226	2.1
34917	1.89226	2.1
34918	1.89226	2.1
34919	1.89226	2.1
34920	1.89226	2.1
34921	1.89226	2.05352
34922	1.89226	2.1
34923	1.89226	2.1
34924	1.89226	2.1
34925	3.78453	2.1
34926	1.89226	2.1
34927	1.89226	2.1
34928	1.89226	2.1
34929	1.89226	2.1
34930	1.89226	2.1
34931	1.89226	2.1
34932	1.69044	0.05
34933	1.57979	0.05
34934	2.27963	0.05
34935	2.21582	0.05
34936	3.85942	0.05
34937	2.21582	0.05
34938	1.30722	0.05
34939	2.02069	0.05
34940	2.02069	0.05
34941	1.57979	0.05
34942	1.57979	0.05
34943	1.67825	2.1
34944	1.89226	2.1
34945	1.89226	2.1
34946	1.89226	2.1
34947	1.91894	2.1
34948	1.91894	2.1
34949	0.946133	2.1
34950	0.946133	2.1
34951	0.946132	2.1
34952	0.946132	2.1
34953	0.946132	2.1
34954	0.946132	2.1
34955	0.946133	2.1
34956	0.946133	2.1
34957	0.946133	2.1
34958	0.946133	2.1
34959	0.946132	2.1
34960	0.946132	2.1
34961	0.942609	1.74176
34962	0.942609	2.04025
34963	1.329 2.1	
34964	1.329 2.1	
34965	1.32547	2.1
34966	0.94261	2.1
34967	0.946132	2.00912
34968	0.946132	2.1
34969	0.946132	2.08731
34970	1.32547	2.1
34971	0.946132	2.1

34972	0.94261	2.1
34973	1.55831	2.03701
34974	1.07496	2.1
34975	1.80327	0.740169
34976	1.34851	0.880084
34977	2.87824	0.977467
34978	2.14346	0.537763
34979	2.77937	0.526072
34980	2.79173	0.68009
34981	2.17129	0.476981
34982	4.95066	0.520349
34983	2.14346	0.59193
34984	1.34124	0.424134
34985	1.39818	0.520993
34986	2.1713	0.512425
34987	3.51253	0.505918
34988	2.79173	0.602344
34989	2.21882	0.459361
34990	2.17129	0.48789
34991	2.17129	0.503434
34992	2.81448	0.50582
34993	2.81448	0.519124
34994	1.10941	0.480326
34995	1.08565	0.527405
34996	0.699089	0.422864
34997	0.740176	0.470409
34998	0.908594	0.424837
34999	0.908594	0.462546
35000	0.91723	0.574705
35001	0.91723	0.441779
35002	1.10941	0.475399
35003	0.740176	0.468949
35004	0.699089	0.462764
35005	1.08565	0.454668
35006	1.89227	0.439402
35007	1.89227	0.424223
35008	1.89227	0.339575
35009	1.89227	0.343242
35010	3.78453	0.40541
35011	2.40032	0.555461
35012	2.19526	0.396434
35013	1.12907	0.260179
35014	1.44253	0.477636
35015	3.32433	0.39223
35016	2.19526	0.346726
35017	2.71093	0.431191
35018	1.29826	0.418885
35019	2.0815	0.288038
35020	4.18911	0.41097
35021	1.89226	0.364578
35022	1.89226	0.363361
35023	1.89226	0.20989
35024	1.89226	0.276955
35025	3.78452	0.238602
35026	2.30822	0.318596
35027	1.89226	0.340893
35028	2.79329	0.207795
35029	2.79329	0.14934

35030	1.89226	0.412977
35031	1.89226	0.348255
35032	0.946131	1.88217
35033	0.946131	0.760144
35034	1.38414	2.1
35035	1.38414	0.514976
35036	0.94613	2.1
35037	0.94613	1.82181
35038	1.38415	0.829906
35039	1.41833	0.60227
35040	0.946132	0.890544
35041	1.41833	0.867274
35042	1.38414	0.917608
35043	0.946131	1.12963
35044	1.89226	0.925711
35045	2.6536	2.1
35046	1.89226	0.792125
35047	2.72238	2.1
35048	4.61465	2.1
35049	0.946132	2.1
35050	0.946132	1.14222
35051	1.38657	0.901699
35052	1.38657	2.1
35053	1.38657	2.1
35054	1.36119	2.1
35055	0.946131	0.798363
35056	0.946131	1.55432
35057	0.946132	2.1
35058	1.38657	2.1
35059	0.946132	1.64653
35060	1.36119	2.1
35061	1.89226	2.1
35062	2.4717	2.1
35063	2.5026	2.1
35064	2.4717	2.1
35065	1.92316	2.1
35066	1.89226	2.1
35067	1.89226	2.1
35068	1.89226	1.40414
35069	1.89226	2.1
35070	1.89226	0.724601
35071	3.78453	2.1
35072	0.946128	2.1
35073	0.946128	0.716953
35074	1.3419	1.00276
35075	1.3419	2.1
35076	0.946131	0.85073
35077	0.946131	0.840496
35078	1.36607	2.1
35079	1.3419	2.1
35080	0.946131	2.1
35081	1.36607	2.1
35082	1.3419	2.1
35083	0.94613	2.1
35084	1.89226	2.1
35085	2.50259	2.1
35086	2.53563	2.1
35087	2.50259	2.1

35088	1.92529	2.1
35089	1.89226	2.1
35090	0.946132	0.796186
35091	0.946132	2.1
35092	0.946131	1.12274
35093	0.946131	2.1
35094	0.946132	0.900027
35095	0.946132	1.76507
35096	0.946131	2.1
35097	0.946131	2.1
35098	0.946131	2.1
35099	0.946131	2.1
35100	0.946131	2.1
35101	0.946131	2.1
35102	2.0815	2.1
35103	1.86506	2.1
35104	1.86506	2.1
35105	1.82567	2.1
35106	2.0815	2.1
35107	2.03335	2.1
35108	2.0815	2.1
35109	2.0815	2.1
35110	2.0815	2.1
35111	1.88668	2.1
35112	1.83221	2.1
35113	1.57275	2.1
35114	2.34061	0.102857
35115	1.89226	0.207197
35116	2.23254	0.0936275
35117	1.89226	0.101881
35118	2.23254	0.190202
35119	1.89226	0.217721
35120	1.8619	0.129885
35121	1.89226	0.289396
35122	2.34061	0.126757
35123	1.89226	0.129299
35124	2.34061	0.242464
35125	1.89226	0.255417
35126	1.89227	0.261748
35127	1.89227	0.140179
35128	1.89227	0.205066
35129	1.89227	0.272062
35130	1.89227	0.138298
35131	1.89227	0.21713
35132	1.89227	0.199815
35133	1.89227	0.108658
35134	1.89227	0.15702
35135	1.89227	0.205104
35136	1.89227	0.118656
35137	1.89227	0.168784
35138	1.06365	0.141573
35139	0.503305	0.13799
35140	0.794912	0.0937279
35141	1.42752	0.113257
35142	1.34826	0.137151
35143	1.07163	0.167408
35144	0.533113	0.149433
35145	0.761277	0.137802

35146	0.848403	0.143907
35147	0.876191	0.095665
35148	0.963372	0.201051
35149	0.963372	0.150731
35150	1.55571	0.0792333
35151	2.76574	0.129999
35152	2.92453	0.0920321
35153	1.70301	0.104528
35154	2.33089	0.112618
35155	1.06905	0.0976224
35156	2.28979	2.1
35157	1.81488	2.1
35158	1.63722	2.1
35159	2.22627	2.1
35160	4.04115	2.1
35161	1.98246	2.1
35162	2.15715	2.1
35163	2.22627	2.1
35164	2.22627	2.1
35165	1.63719	2.1
35166	1.63719	2.1
35167	2.1644	0.05
35168	1.903	0.05
35169	1.23639	0.05
35170	1.42704	0.0588697
35171	3.61546	0.05
35172	1.89226	0.061571
35173	1.89226	0.0848969
35174	1.89226	0.0742189
35175	1.89226	0.07804
35176	3.78451	0.0752496
35177	2.61343	0.0659254
35178	1.89226	0.068662
35179	2.08149	0.0675263
35180	1.74357	0.0531829
35181	2.08149	0.0825577
35182	1.74357	0.0705262
35183	1.63483	0.05
35184	1.8631	0.05
35185	1.8631	0.05
35186	1.70301	0.05
35187	2.0815	0.05
35188	1.94319	0.05
35189	2.0815	0.05
35190	1.89226	0.05
35191	1.89226	0.05
35192	2.05236	0.05
35193	2.23014	0.05
35194	2.41882	0.05
35195	2.14993	2.1
35196	1.87217	1.98379
35197	2.34488	2.1
35198	2.5254	1.49945
35199	4.39757	1.57033
35200	0.949995	2.1
35201	0.630972	2.1
35202	0.936084	2.1
35203	1.17244	2.1

35204	0.922394	2.1
35205	0.922394	2.1
35206	0.88102	2.1
35207	0.88102	2.1
35208	0.949998	2.1
35209	1.17244	2.1
35210	0.936081	2.1
35211	0.630973	2.1
35212	1.89226	2.1
35213	1.89226	2.04511
35214	1.89226	2.1
35215	1.89226	2.1
35216	3.78452	2.1
35217	1.89226	1.24952
35218	1.89226	1.67386
35219	1.89226	1.18903
35220	1.89226	1.66689
35221	1.89226	1.17749
35222	1.89226	1.63827
35223	2.34488	1.70641
35224	2.5254	1.27686
35225	1.89226	1.33089
35226	1.89226	1.79826
35227	4.41766	1.99599
35228	1.89226	1.88151
35229	1.89226	2.1
35230	1.89226	1.84002
35231	1.89226	2.00159
35232	3.78452	1.4498
35233	1.89226	1.13438
35234	1.89226	1.74453
35235	1.89226	1.14864
35236	1.89226	1.37138
35237	1.89226	1.12786
35238	1.89226	1.36521
35239	1.5605	2.1
35240	1.15806	2.1
35241	1.31565	2.1
35242	1.65642	2.1
35243	2.87615	2.1
35244	1.46643	2.1
35245	1.65642	2.1
35246	1.31565	2.1
35247	1.31565	2.1
35248	1.74246	2.1
35249	1.74246	2.1
35250	1.89226	2.1
35251	1.89226	2.1
35252	1.89226	2.1
35253	1.89226	2.1
35254	1.89226	2.1
35255	1.89226	2.1
35256	1.15099	2.1
35257	1.15099	2.1
35258	0.939316	2.1
35259	0.939316	2.1
35260	0.946127	2.1
35261	0.946127	2.1

35262	1.14418	2.1
35263	0.939289	2.1
35264	1.14417	2.1
35265	0.946129	2.1
35266	0.939287	2.1
35267	0.94613	2.1
35268	1.89226	2.1
35269	1.89226	2.1
35270	1.89226	2.1
35271	1.89226	2.04228
35272	3.78452	2.1
35273	1.93164	0.130281
35274	2.64898	0.0915956
35275	1.43213	0.137215
35276	1.43213	0.172476
35277	2.63206	0.0926445
35278	2.13254	0.143802
35279	1.89226	2.1
35280	1.89226	2.1
35281	1.89226	2.1
35282	1.89226	2.1
35283	3.78452	2.1
35284	2.71655	2.1
35285	1.43323	2.1
35286	1.28413	2.1
35287	2.53614	2.1
35288	3.96937	2.1
35289	1.89226	2.1
35290	1.89226	2.1
35291	1.89226	2.1
35292	1.89226	2.1
35293	1.89226	2.1
35294	1.89226	2.1
35295	1.33356	2.1
35296	2.82297	2.1
35297	2.771 2.1	
35298	2.771 2.1	
35299	1.46037	2.1
35300	1.46037	2.1
35301	2.56034	2.1
35302	1.18474	2.1
35303	1.15113	2.1
35304	2.53724	2.1
35305	3.72198	2.1
35306	2.56034	2.1
35307	1.89226	2.1
35308	1.89226	2.1
35309	2.53725	2.1
35310	4.42951	2.1
35311	1.89226	2.1
35312	1.89226	2.1
35313	1.89226	2.1
35314	1.89226	2.1
35315	3.78453	2.1
35316	1.89226	2.1
35317	1.89226	2.1
35318	1.89226	2.1
35319	1.89226	2.1

35320	3.78452	2.1
35321	1.1229	2.1
35322	2.53724	2.1
35323	1.15113	2.1
35324	1.15113	2.1
35325	2.51559	2.1
35326	2.51559	2.1
35327	1.89226	2.1
35328	2.53724	2.1
35329	2.51559	2.1
35330	2.53724	2.1
35331	1.87061	2.1
35332	1.89226	2.1
35333	1.89226	2.1
35334	1.89226	2.1
35335	1.89226	2.1
35336	1.89226	2.1
35337	3.78453	2.1
35338	1.89226	2.1
35339	1.89226	2.1
35340	1.89226	2.1
35341	1.89226	2.1
35342	3.78452	2.1
35343	1.98143	1.04934
35344	2.81559	0.462443
35345	1.65411	0.619046
35346	2.26675	0.856082
35347	4.24818	0.695735
35348	0.810656	0.712165
35349	0.810656	2.1
35350	0.821888	2.1
35351	0.821888	0.991293
35352	1.1235	0.800012
35353	1.07439	1.39821
35354	0.535693	2.1
35355	0.509042	2.1
35356	0.535693	2.1
35357	1.1235	2.1
35358	0.509043	2.1
35359	1.07439	2.1
35360	1.10378	2.1
35361	2.247	1.31876
35362	2.27472	2.1
35363	2.27472	1.63016
35364	1.07139	2.1
35365	1.07139	2.1
35366	2.08081	2.1
35367	1.40395	2.1
35368	1.34245	2.1
35369	2.00239	2.1
35370	3.40634	2.1
35371	1.89226	2.1
35372	1.89226	2.1
35373	1.89226	2.1
35374	1.89226	2.1
35375	3.78453	2.1
35376	0.946128	2.1
35377	0.946128	2.1

35378	1.13785	2.1
35379	1.11496	2.1
35380	0.946131	2.1
35381	0.946131	2.1
35382	1.11495	2.1
35383	1.11495	2.1
35384	0.94613	2.1
35385	1.11495	2.1
35386	1.13785	2.1
35387	0.946131	2.1
35388	0.975888	2.1
35389	1.00118	2.1
35390	0.671218	2.1
35391	0.653229	2.1
35392	0.825372	2.1
35393	0.825372	2.1
35394	0.829038	2.1
35395	0.829038	2.1
35396	0.975901	2.1
35397	0.653238	2.1
35398	0.671209	2.1
35399	1.00117	2.1
35400	1.89224	2.1
35401	1.89224	2.1
35402	1.89224	2.1
35403	1.89224	2.1
35404	3.78448	2.1
35405	0.946115	2.1
35406	0.946115	2.1
35407	1.11494	2.1
35408	1.10111	2.1
35409	0.946118	2.1
35410	0.946118	2.1
35411	1.10111	2.1
35412	1.10111	2.1
35413	1.10112	2.1
35414	0.94613	2.1
35415	0.946105	2.1
35416	1.11492	2.1
35417	1.04028	0.482665
35418	2.27126	0.430609
35419	2.0815	0.440606
35420	1.82249	0.595266
35421	1.33723	0.445458
35422	1.18684	0.571261
35423	0.812616	0.38168
35424	0.812616	0.380726
35425	0.997777	0.376116
35426	1.08122	0.384731
35427	0.94613	0.37607
35428	0.94613	0.389218
35429	0.812648	0.380596
35430	0.932779	0.377062
35431	0.812648	0.365394
35432	0.94613	0.379171
35433	0.849335	0.366603
35434	0.94613	0.37064
35435	1.80327	0.934095

35436	1.56016	0.880174
35437	1.57731	1.80792
35438	1.34851	1.73703
35439	2.03207	0.992774
35440	1.80327	1.96899
35441	2.03207	1.49469
35442	1.56016	1.50997
35443	2.18076	1.0314
35444	1.78167	1.02835
35445	3.81374	1.55607
35446	1.37597	1.75014
35447	1.60058	2.09014
35448	1.70302	0.953222
35449	1.85448	1.11833
35450	3.15028	1.09081
35451	1.60058	0.917129
35452	1.4054	2.04639
35453	1.66202	0.946517
35454	1.70302	1.83979
35455	3.09979	1.26501
35456	1.70302	1.46013
35457	1.85448	1.3868
35458	2.21317	1.10186
35459	2.0961	1.07688
35460	4.01757	1.58048
35461	1.66202	1.09237
35462	1.70302	1.87591
35463	2.09611	0.83856
35464	2.00892	1.62324
35465	3.6468	1.06044
35466	1.2966	0.05839
35467	2.25495	0.05
35468	1.2966	0.0731496
35469	2.21117	0.05
35470	1.67687	0.0671649
35471	2.6167	0.05
35472	1.89227	0.0903769
35473	1.89227	0.0586889
35474	1.89227	0.072442
35475	1.89227	0.119267
35476	3.78453	0.0800208
35477	2.44375	0.0691302
35478	1.89227	0.0956119
35479	2.81853	0.0975998
35480	2.81853	0.0869202
35481	1.89227	0.076872
35482	1.89227	0.0550208
35483	1.89226	0.903733
35484	1.75853	1.08379
35485	2.37636	0.879348
35486	2.37636	1.1093
35487	1.89226	0.794083
35488	1.89226	0.98531
35489	1.65814	0.489375
35490	1.10187	0.717328
35491	1.10187	0.551094
35492	1.19547	0.679772
35493	1.56263	0.318175

35494	1.65624	0.703506
35495	1.56264	0.467735
35496	1.01479	0.725131
35497	1.01479	0.591117
35498	1.10187	0.694723
35499	1.47561	0.318988
35500	1.5627	0.729588
35501	1.89225	2.1
35502	1.89225	2.1
35503	1.89225	2.1
35504	1.89225	2.1
35505	3.7845	2.1
35506	2.54386	2.1
35507	1.91546	2.1
35508	2.46008	2.1
35509	1.82815	2.1
35510	4.37554	2.1
35511	1.91546	2.1
35512	1.04909	2.1
35513	1.82815	2.1
35514	0.983141	2.1
35515	2.8986	2.1
35516	1.89225	2.1
35517	1.89225	2.1
35518	1.89225	2.1
35519	1.89225	2.1
35520	3.78449	2.1
35521	1.89226	2.1
35522	1.89226	2.1
35523	1.89226	2.1
35524	1.89226	2.1
35525	1.89226	2.1
35526	1.89226	2.1
35527	2.46009	2.1
35528	1.7513	2.1
35529	1.82817	2.1
35530	1.82817	2.1
35531	2.38127	2.1
35532	2.38127	2.1
35533	2.55006	2.1
35534	2.64569	2.1
35535	2.60128	2.1
35536	2.49168	2.1
35537	5.13737	2.1
35538	2.51605	2.1
35539	2.64569	2.1
35540	2.60128	2.1
35541	2.48891	2.1
35542	5.1346	2.1
35543	2.60128	2.1
35544	2.49168	2.1
35545	2.41689	2.1
35546	2.55248	2.1
35547	5.04416	2.1
35548	2.60128	2.1
35549	2.48891	2.1
35550	2.45922	2.1
35551	2.55249	2.1

35552	5.04139	2.1
35553	1.89227	1.41736
35554	1.89227	2.0649
35555	1.89227	1.92386
35556	1.89227	1.75121
35557	1.89227	1.70022
35558	1.89227	1.54744
35559	0.96063	2.1
35560	0.96063	2.1
35561	0.958806	2.1
35562	0.958806	2.1
35563	1.28892	2.1
35564	0.749042	2.1
35565	1.17409	2.1
35566	0.630516	2.1
35567	1.17409	2.1
35568	1.28892	2.1
35569	0.630515	2.1
35570	0.749042	2.1
35571	0.92511	1.55788
35572	0.92511	1.72629
35573	1.19511	1.70644
35574	1.19511	1.9922
35575	0.946132	1.87637
35576	1.17409	1.66353
35577	0.925081	1.45095
35578	0.946129	1.85546
35579	0.946131	1.15878
35580	0.946131	1.43408
35581	1.17409	1.64318
35582	0.925083	1.28965
35583	1.89226	0.709947
35584	1.89226	0.84808
35585	1.89226	0.781074
35586	1.89226	0.766246
35587	1.89226	0.7762
35588	1.89226	0.764916
35589	1.89226	0.804469
35590	1.89226	0.914992
35591	1.89226	0.8758
35592	1.89226	0.85116
35593	3.78453	0.847841
35594	0.946132	2.1
35595	0.946132	2.03483
35596	1.28892	2.1
35597	1.28892	2.1
35598	0.946132	2.1
35599	1.28892	2.1
35600	0.946133	2.1
35601	1.17409	1.99423
35602	0.946132	1.97145
35603	0.946132	2.1
35604	1.17409	2.1
35605	1.28892	2.1
35606	0.625229	2.1
35607	0.890321	2.1
35608	1.21636	2.1
35609	1.13022	2.1

35610	1.07094	2.1
35611	1.03541	2.1
35612	0.942207	2.1
35613	0.942207	2.1
35614	0.898766	2.1
35615	1.26626	2.1
35616	1.0853	2.1
35617	0.600358	2.1
35618	0.719012	2.1
35619	0.719012	2.1
35620	1.17957	2.1
35621	1.17957	2.1
35622	0.719012	2.1
35623	0.952456	2.1
35624	0.946129	2.1
35625	0.946129	2.1
35626	0.946131	2.1
35627	0.952454	2.1
35628	0.946131	2.1
35629	0.719011	2.1
35630	0.94613	2.1
35631	0.94613	2.1
35632	0.94613	2.1
35633	0.94613	2.1
35634	0.946131	2.1
35635	0.946131	2.1
35636	0.946129	2.1
35637	0.946129	2.1
35638	0.94613	2.1
35639	0.94613	2.1
35640	0.94613	2.1
35641	0.94613	2.1
35642	1.1587	2.1
35643	0.973158	2.1
35644	0.750023	2.1
35645	0.536443	2.1
35646	0.84054	2.1
35647	0.84054	2.1
35648	0.854602	2.1
35649	0.854602	2.1
35650	1.1587	2.1
35651	0.750023	2.1
35652	0.973158	2.1
35653	0.536443	2.1
35654	1.89227	0.708248
35655	1.89227	2.1
35656	1.89227	1.15977
35657	1.89227	0.832567
35658	3.78453	1.00625
35659	1.89226	2.1
35660	1.89226	1.19125
35661	1.89226	0.93597
35662	1.89226	2.1
35663	3.78453	1.73304
35664	1.7632	1.24608
35665	1.61149	2.1
35666	1.98749	2.1
35667	2.11615	2.1

35668	3.75068	2.1
35669	1.7632	2.1
35670	1.61149	1.66121
35671	1.97733	0.796182
35672	2.11615	2.1
35673	3.74052	1.40194
35674	1.89226	1.04331
35675	1.89226	0.910385
35676	1.89226	0.723923
35677	1.89226	1.89323
35678	3.78453	0.955369
35679	1.89226	0.837045
35680	1.89226	2.1
35681	1.89226	1.94221
35682	1.89226	1.17462
35683	3.78453	1.97897
35684	1.98749	2.1
35685	2.11615	2.1
35686	2.43151	1.21011
35687	2.27541	2.1
35688	4.41899	2.1
35689	1.97733	0.833399
35690	2.11615	2.1
35691	2.43151	1.97658
35692	2.33986	0.775288
35693	4.40883	1.72109
35694	2.17892	0.381625
35695	2.80057	0.594452
35696	1.88349	0.503462
35697	2.63062	0.438845
35698	4.80954	0.476417
35699	1.89226	0.799247
35700	1.89226	0.417518
35701	1.89226	0.623941
35702	1.89226	0.395535
35703	3.78452	0.507834
35704	2.0155	0.696225
35705	1.97652	0.419745
35706	1.8247	0.61587
35707	1.80645	0.368755
35708	3.80122	0.475845
35709	1.97652	0.45223
35710	1.79681	0.474999
35711	1.80645	0.391
35712	1.59244	0.449414
35713	3.56896	0.372284
35714	1.89226	0.461531
35715	1.89226	0.695342
35716	1.89226	0.435586
35717	1.89226	0.455203
35718	3.78452	0.402553
35719	1.89223	0.618853
35720	1.89223	0.431726
35721	1.89223	0.679617
35722	1.89223	0.376228
35723	3.78447	0.48172
35724	1.82467	0.61276
35725	1.80643	0.379958

35726	1.55159	0.581086
35727	1.53969	0.36973
35728	3.36437	0.459025
35729	1.80643	0.407827
35730	1.59242	0.51151
35731	1.5397	0.403489
35732	1.40538	0.442121
35733	3.13211	0.389828
35734	1.89224	0.476102
35735	1.89224	0.524185
35736	1.89224	0.408356
35737	1.89224	0.517556
35738	3.78447	0.419528
35739	1.37427	0.321316
35740	2.37086	0.324487
35741	2.37972	0.3197
35742	1.94703	0.328459
35743	2.19299	0.324299
35744	1.91813	0.3337
35745	1.07566	2.1
35746	1.12954	2.1
35747	0.946131	2.1
35748	0.946131	2.1
35749	1.12954	2.1
35750	1.12954	2.1
35751	0.946131	2.1
35752	0.946131	2.1
35753	1.12954	2.1
35754	0.946131	2.1
35755	0.946131	2.1
35756	1.07566	2.1
35757	2.25908	2.1
35758	1.89226	2.1
35759	1.89226	2.1
35760	1.89226	2.1
35761	2.37567	2.1
35762	2.37567	2.1
35763	1.37597	1.5662
35764	1.70302	0.803386
35765	1.24162	1.46601
35766	1.12219	1.90021
35767	1.37072	0.987168
35768	1.70302	2.1
35769	1.37072	2.1
35770	1.70302	1.12108
35771	1.54168	1.22479
35772	2.21318	1.02799
35773	3.39238	1.31243
35774	1.89226	0.100877
35775	1.89226	0.0960011
35776	1.89226	0.0688832
35777	1.89226	0.062809
35778	3.78452	0.0682616
35779	1.89227	0.0986415
35780	1.89227	0.0919538
35781	1.89227	0.0651441
35782	1.89227	0.0772354
35783	3.78453	0.0908692

35784	1.89226	0.0704315
35785	1.89226	0.081219
35786	1.89226	0.0620106
35787	1.89226	0.0564196
35788	3.78453	0.0631906
35789	1.89226	0.0736627
35790	2.21654	0.0546227
35791	2.72007	0.0586085
35792	2.72007	0.0699832
35793	1.89226	0.053093
35794	1.89226	0.0643504
35795	0.946128	0.0514973
35796	0.946128	0.0590482
35797	0.946132	0.05
35798	0.946132	0.0587928
35799	0.94613	0.0507787
35800	0.94613	0.0556572
35801	0.946131	0.0535756
35802	0.946131	0.0581903
35803	0.94613	0.05
35804	0.94613	0.0584166
35805	0.94613	0.0704541
35806	0.94613	0.0626346
35807	1.89226	0.0608691
35808	1.89226	0.0648017
35809	1.89226	0.05
35810	1.89226	0.05
35811	3.78453	0.052946
35812	1.33457	0.05
35813	0.946131	0.05
35814	1.08045	0.05
35815	0.946132	0.05
35816	1.08045	0.05
35817	1.08045	0.05
35818	0.946132	0.0534
35819	0.946132	0.05
35820	1.33457	0.05
35821	1.08045	0.05
35822	0.946132	0.0550054
35823	0.946132	0.0526084
35824	1.53347	1.03504
35825	1.13092	2.1
35826	1.52938	1.72878
35827	1.93568	1.61667
35828	3.06285	2.02563
35829	1.89226	0.427563
35830	2.72959	0.560677
35831	2.82996	0.559571
35832	2.82996	0.423669
35833	1.89226	0.61203
35834	1.89226	0.424141
35835	2.7296	0.534501
35836	2.57663	1.2996
35837	2.48354	0.45431
35838	2.48354	0.870709
35839	2.82996	0.41239
35840	2.82996	0.963055
35841	2.63449	0.554066

35842	2.7296	0.561265
35843	2.39282	0.567349
35844	2.39282	1.75986
35845	2.48354	1.0345
35846	2.72521	0.882795
35847	1.89224	2.1
35848	1.89224	1.60815
35849	1.89224	0.804215
35850	1.89224	2.08991
35851	3.78447	1.02751
35852	1.89226	1.64943
35853	2.10425	2.1
35854	2.5142	2.1
35855	2.5142	2.1
35856	1.89226	2.1
35857	1.89226	1.61776
35858	0.946133	0.898817
35859	0.946133	1.23277
35860	0.946128	2.1
35861	0.946128	2.1
35862	0.946131	2.04784
35863	0.946131	0.852988
35864	0.94613	0.613937
35865	0.94613	2.1
35866	0.946131	1.3325
35867	0.946131	1.47732
35868	0.946131	1.18736
35869	0.946131	1.26004
35870	1.89226	0.696831
35871	1.89226	0.925133
35872	1.89226	0.760278
35873	1.89226	0.962843
35874	1.89226	0.727528
35875	1.89226	0.887198
35876	1.94446	0.769056
35877	2.50167	1.22395
35878	1.28418	0.878368
35879	1.94446	0.928725
35880	2.05205	0.953901
35881	2.05205	1.05951
35882	1.27835	2.1
35883	1.08341	2.1
35884	0.946132	2.1
35885	0.946132	2.1
35886	0.851542	2.1
35887	0.851542	2.1
35888	0.977356	2.1
35889	1.18368	2.1
35890	1.18378	2.1
35891	0.851555	2.1
35892	0.977346	2.1
35893	0.851476	2.1
35894	1.07369	2.1
35895	2.5813	2.1
35896	1.95053	2.1
35897	1.95053	2.1
35898	1.90196	2.1
35899	1.90196	2.1

35900	1.89226	2.1
35901	1.89226	2.1
35902	1.89226	2.1
35903	1.89226	2.1
35904	3.78452	2.1
35905	1.89224	2.1
35906	1.89224	2.1
35907	1.89224	2.1
35908	1.89224	2.1
35909	1.89224	2.1
35910	1.89224	2.1
35911	1.97004	0.58027
35912	1.8072	1.31629
35913	1.5866	1.11945
35914	1.79478	0.637608
35915	3.60198	0.912777
35916	1.23267	2.0675
35917	1.8072	0.835339
35918	1.58661	0.731404
35919	0.947921	1.53337
35920	2.75512	1.15831
35921	1.89226	0.830024
35922	1.89226	2.06297
35923	1.89226	0.742911
35924	1.89226	1.18608
35925	1.89226	0.727214
35926	1.89226	1.76374
35927	1.89226	1.29773
35928	1.89226	0.594026
35929	1.89226	0.658346
35930	1.89226	1.07278
35931	3.78452	0.805929
35932	1.4263	1.37698
35933	1.79478	0.689099
35934	1.67651	0.76385
35935	1.79478	1.40677
35936	1.46834	0.61443
35937	1.5866	0.893258
35938	1.89226	0.708796
35939	1.89226	0.960638
35940	1.89226	0.624445
35941	1.89226	0.901916
35942	1.89226	0.660462
35943	1.89226	1.43852
35944	2.23956	1.48731
35945	1.81847	1.79828
35946	2.10991	0.942319
35947	2.75317	1.07368
35948	4.34947	1.35074
35949	1.60578	0.0924711
35950	1.94974	0.0958394
35951	1.68062	0.148689
35952	1.14721	0.0838476
35953	3.2864	0.0935947
35954	1.3946	0.05
35955	1.692 0.05	
35956	2.0154	0.05
35957	2.0154	0.05

35958	1.11926	0.05
35959	1.11926	0.05
35960	1.90347	0.05
35961	1.83626	0.05
35962	1.84583	0.05
35963	1.89434	0.05
35964	3.79781	0.05
35965	1.89226	1.12294
35966	1.89226	0.928439
35967	1.89226	0.753508
35968	1.89226	1.11127
35969	3.78452	0.905759
35970	1.20986	1.26682
35971	1.20986	1.20711
35972	0.912635	1.06017
35973	0.912635	1.61284
35974	0.912667	0.863099
35975	1.17637	1.5206
35976	0.946125	1.45892
35977	0.946125	1.62735
35978	0.946128	1.7534
35979	0.912664	1.05473
35980	0.946127	1.99705
35981	1.17637	1.14857
35982	1.89226	0.05
35983	1.65573	0.0519876
35984	1.65573	0.0623428
35985	1.89226	0.05
35986	1.89226	0.0594799
35987	2.12879	0.05
35988	1.89226	0.0558939
35989	1.89226	0.0569264
35990	1.89226	0.0827752
35991	1.89226	0.05
35992	1.89226	0.0796094
35993	1.89226	0.05
35994	2.40928	0.05
35995	1.89226	0.0523768
35996	1.95959	0.0508747
35997	1.70302	0.05
35998	3.47338	0.0514472
35999	1.89226	0.0560099
36000	1.89226	0.0543616
36001	1.89226	0.0506654
36002	1.89226	0.0771154
36003	3.78452	0.050227
36004	1.64985	0.131885
36005	2.47846	0.192506
36006	2.25405	0.123572
36007	1.80768	0.103893
36008	4.06173	0.11195
36009	1.98846	0.0844721
36010	0.912729	0.101797
36011	1.05417	0.0598634
36012	1.70303	0.107258
36013	2.82107	0.106682
36014	2.47846	0.20415
36015	1.70303	0.170594

36016	2.86918	0.251874
36017	2.05973	0.236833
36018	2.51808	0.169381
36019	1.80768	0.154682
36020	0.946131	0.153254
36021	0.946131	0.136643
36022	0.946132	0.132172
36023	0.946132	0.23172
36024	0.946131	0.253555
36025	0.946131	0.212404
36026	0.946133	0.156369
36027	0.946133	0.141462
36028	0.946131	0.183865
36029	0.946131	0.20603
36030	0.946132	0.189538
36031	0.946132	0.210104
36032	0.946133	0.0936674
36033	0.946133	0.0915719
36034	0.946132	0.115767
36035	0.946132	0.125607
36036	0.946133	0.129011
36037	0.946133	0.125112
36038	0.946132	0.0818788
36039	0.946132	0.150399
36040	0.946132	0.096732
36041	0.946132	0.117442
36042	0.946133	0.0912527
36043	0.946133	0.125823
36044	1.89227	0.244813
36045	1.89227	0.307714
36046	1.89227	0.287481
36047	1.89227	0.222775
36048	1.89227	0.364124
36049	1.89227	0.311833
36050	1.1656	2.1
36051	1.21847	2.1
36052	0.836006	2.1
36053	0.806007	2.1
36054	1.00653	2.1
36055	1.00653	2.1
36056	1.01794	2.1
36057	1.01794	2.1
36058	1.1656	2.1
36059	0.806007	2.1
36060	0.836006	2.1
36061	1.21847	2.1
36062	2.82263	2.1
36063	2.3312	2.1
36064	2.25936	2.1
36065	2.71441	2.1
36066	5.0456	2.1
36067	2.25936	2.1
36068	1.61201	2.1
36069	1.57627	2.1
36070	1.61201	2.1
36071	2.29545	2.1
36072	2.3312	2.1
36073	1.21269	0.05

36074	1.48867	0.05
36075	1.68264	0.05
36076	1.9898	0.05
36077	3.17131	0.05
36078	2.0487	2.1
36079	2.45212	2.1
36080	2.31012	2.1
36081	1.94095	2.1
36082	4.39307	2.1
36083	1.02435	2.1
36084	0.970469	2.1
36085	0.625375	2.1
36086	0.567166	2.1
36087	0.796854	2.1
36088	0.796854	2.1
36089	0.79466	2.1
36090	0.79466	2.1
36091	0.970471	2.1
36092	0.567165	2.1
36093	0.625374	2.1
36094	1.02435	2.1
36095	2.31012	2.1
36096	1.94095	2.1
36097	1.4825	2.1
36098	2.17384	2.1
36099	4.11479	2.1
36100	0.946187	2.1
36101	0.803763	2.1
36102	0.581357	2.1
36103	0.510356	2.1
36104	0.745632	2.1
36105	0.745632	2.1
36106	0.734566	2.1
36107	0.734563	2.1
36108	0.896142	2.1
36109	0.510366	2.1
36110	0.946173	2.1
36111	0.552965	2.1
36112	1.89226	0.896999
36113	1.89226	1.43709
36114	1.89226	1.677
36115	1.89226	1.77626
36116	1.89226	0.9706
36117	1.89226	1.45073
36118	1.50743	1.36944
36119	1.0417	2.1
36120	1.50743	2.00742
36121	1.62974	1.0072
36122	1.14605	0.993118
36123	1.14605	2.1
36124	1.89226	0.822175
36125	1.89226	1.80409
36126	1.89226	0.727424
36127	1.89226	1.31341
36128	1.89226	0.774043
36129	1.89226	1.62025
36130	1.89226	1.60295
36131	1.89226	1.22471

36132	1.89226	0.71781
36133	1.89226	1.80923
36134	3.78453	1.02416
36135	1.77186	1.53786
36136	1.35668	2.02502
36137	1.70254	0.913455
36138	1.38756	1.96599
36139	1.77186	1.76398
36140	1.37814	2.1
36141	0.734857	2.03368
36142	1.00549	1.60259
36143	0.835261	0.989307
36144	1.08357	2.01286
36145	0.97665	1.37774
36146	0.795559	2.1
36147	0.891385	1.55638
36148	0.891385	0.739824
36149	0.874231	1.03211
36150	1.08356	1.24465
36151	0.734863	1.26322
36152	0.79449	1.62772
36153	1.89226	1.14846
36154	1.89226	0.564866
36155	2.42671	0.674547
36156	1.98783	1.03233
36157	4.31897	0.68671
36158	0.579854	0.725699
36159	0.965582	0.809734
36160	0.634403	0.878478
36161	1.17796	0.696328
36162	1.00592	0.704888
36163	0.797259	1.8198
36164	0.905246	1.0446
36165	0.905246	0.629462
36166	0.695715	0.724232
36167	0.637106	1.01243
36168	1.24374	0.87837
36169	0.95617	0.94735
36170	2.0815	0.685402
36171	1.60521	1.10848
36172	1.29556	0.981991
36173	2.32205	1.36951
36174	1.28445	0.645308
36175	2.0815	0.966018
36176	2.3085	0.78034
36177	2.21147	2.1
36178	2.3085	2.1
36179	2.39282	1.53469
36180	1.98899	2.1
36181	2.0733	2.00605
36182	2.3275	2.1
36183	1.63471	2.1
36184	1.98899	2.1
36185	1.56801	2.1
36186	3.62369	2.1
36187	2.3085	0.751747
36188	1.98899	2.1
36189	2.22575	0.803572

36190	1.90502	2.1
36191	4.21353	2.1
36192	1.90502	2.1
36193	1.56801	2.1
36194	1.90502	2.1
36195	1.98899	2.1
36196	1.49733	2.1
36197	1.58129	2.1
36198	1.89226	0.912511
36199	2.6491	0.886925
36200	2.49266	2.1
36201	2.6491	0.844725
36202	1.73582	0.86324
36203	1.89226	0.563028
36204	1.31854	2.1
36205	2.6491	0.8783
36206	1.3909	2.1
36207	1.3909	1.45732
36208	2.49266	2.1
36209	2.49266	1.96255
36210	1.89226	0.996844
36211	2.49266	0.817813
36212	2.38634	2.1
36213	2.49266	0.797925
36214	1.78595	0.852741
36215	1.89226	0.570383
36216	1.23042	2.1
36217	2.49266	0.774035
36218	1.31854	2.1
36219	1.31854	0.925914
36220	2.38635	2.1
36221	2.38635	0.99441
36222	2.83487	2.1
36223	1.89226	2.1
36224	2.594	2.1
36225	2.594	2.1
36226	1.89226	2.1
36227	1.89226	2.1
36228	1.89226	2.1
36229	1.89226	2.1
36230	1.89226	2.1
36231	1.89226	2.1
36232	3.78452	2.1
36233	1.09518	2.1
36234	1.28158	2.1
36235	1.36844	2.1
36236	1.59972	2.1
36237	2.65003	2.1
36238	1.89226	2.1
36239	1.89226	2.1
36240	1.89226	2.1
36241	1.89226	2.1
36242	3.78452	2.1
36243	1.36844	2.1
36244	1.59972	2.1
36245	1.67352	2.1
36246	1.94291	2.1
36247	3.31135	2.1

36248	1.52938	2.08469
36249	1.93568	1.99378
36250	2.36221	0.809916
36251	1.94723	2.1
36252	3.89158	1.5729
36253	1.94724	1.00975
36254	1.26593	2.1
36255	1.75367	2.03625
36256	2.4322	1.24149
36257	3.7009	2.1
36258	2.36221	0.941421
36259	2.43219	1.97785
36260	1.94723	1.24081
36261	1.94723	1.51211
36262	2.8202	1.48986
36263	2.8202	1.85995
36264	2.51604	2.1
36265	2.18533	2.1
36266	2.17228	2.1
36267	2.4889	2.1
36268	4.67423	2.1
36269	2.17228	2.1
36270	1.62183	2.1
36271	1.62316	2.1
36272	1.62183	2.1
36273	2.18666	2.1
36274	2.18533	2.1
36275	2.17227	2.1
36276	2.4889	2.1
36277	2.45922	2.1
36278	2.15686	2.1
36279	4.64576	2.1
36280	2.17228	2.1
36281	1.62316	2.1
36282	1.62241	2.1
36283	2.15686	2.1
36284	3.78002	2.1
36285	1.89226	0.846891
36286	1.89226	0.820322
36287	1.89226	0.7443
36288	1.89226	0.68593
36289	3.78453	1.1566
36290	1.89226	0.772333
36291	1.89226	0.734272
36292	1.89226	0.652827
36293	1.89226	0.515042
36294	3.78452	0.61056
36295	1.89226	0.797327
36296	1.89226	0.588207
36297	1.89226	0.740284
36298	1.89226	0.72117
36299	1.89226	0.630947
36300	1.89226	0.648164
36301	1.89226	0.788582
36302	1.89226	0.83145
36303	2.53413	0.53301
36304	1.89226	0.526281
36305	3.78453	0.567419

36306	1.38048	0.612936
36307	1.38048	0.643507
36308	0.490332	0.587472
36309	0.490332	0.570724
36310	0.924679	0.366896
36311	0.946131	1.19065
36312	0.946131	0.888655
36313	0.490332	0.251546
36314	0.924678	0.428269
36315	0.490332	0.337958
36316	0.946131	0.717629
36317	0.946131	0.582486
36318	1.89227	0.487979
36319	1.89227	0.69608
36320	1.89227	0.559122
36321	1.89227	0.785558
36322	1.89227	0.491084
36323	1.89227	0.618596
36324	1.30803	2.1
36325	2.55151	2.1
36326	1.43935	2.1
36327	1.43935	2.1
36328	2.34922	2.1
36329	2.34922	2.1
36330	0.946131	2.08
36331	0.946131	2.1
36332	1.27575	2.1
36333	1.17461	2.1
36334	0.94613	2.1
36335	0.94613	2.1
36336	1.17461	2.1
36337	1.17461	2.1
36338	0.946131	2.1
36339	1.17461	2.1
36340	0.946132	2.1
36341	1.27575	2.1
36342	1.89226	2.1
36343	1.89226	0.799271
36344	1.89226	2.1
36345	1.89226	2.1
36346	1.89226	1.87217
36347	1.89226	2.1
36348	0.946133	2.1
36349	0.946133	2.1
36350	0.946132	2.1
36351	0.946132	2.1
36352	0.946133	2.1
36353	0.946133	1.63177
36354	0.946132	2.1
36355	0.946132	2.1
36356	0.946132	2.1
36357	0.946132	2.1
36358	0.946133	2.1
36359	0.946133	2.1
36360	1.07043	2.1
36361	1.17461	2.1
36362	0.654016	2.1
36363	0.5829	2.1

36364	0.870481	2.1
36365	0.870481	2.1
36366	0.887027	2.1
36367	0.887027	2.1
36368	1.07043	2.1
36369	0.5829	2.1
36370	0.654016	2.1
36371	1.17461	2.1
36372	1.89226	2.1
36373	1.89226	0.772732
36374	1.89226	2.1
36375	1.89226	1.73556
36376	3.78453	2.1
36377	1.89226	2.1
36378	1.89226	2.1
36379	1.89226	2.1
36380	1.89226	2.0627
36381	3.78453	2.1
36382	1.26929	1.84779
36383	2.19313	2.1
36384	1.09847	2.1
36385	1.69916	2.1
36386	2.96844	2.1
36387	1.84124	2.1
36388	2.24937	2.1
36389	1.69915	2.1
36390	1.75691	2.1
36391	3.94852	2.1
36392	1.53082	2.1
36393	2.04422	2.1
36394	1.53082	2.1
36395	1.69915	2.1
36396	1.92911	2.1
36397	2.09744	2.1
36398	1.39263	0.690633
36399	1.0171	0.470329
36400	1.1211	0.556939
36401	1.46482	0.607916
36402	2.48192	0.587202
36403	1.39263	0.688751
36404	1.57962	0.770232
36405	1.65238	0.982312
36406	1.46482	0.614136
36407	3.04443	0.692111
36408	1.89226	0.769274
36409	1.89226	0.454666
36410	1.89226	0.569414
36411	1.89226	0.673301
36412	3.78453	0.665185
36413	1.89227	0.482434
36414	1.89227	0.528158
36415	1.89227	0.60568
36416	1.89227	0.4074
36417	3.78453	0.456781
36418	1.28418	0.539288
36419	1.46482	0.594367
36420	1.1211	0.456696
36421	1.1211	0.675648

36422	1.59382	0.4523
36423	1.59382	0.661829
36424	1.76897	0.9232
36425	1.46482	0.578901
36426	1.59382	0.69771
36427	1.46482	0.764998
36428	1.78138	0.653573
36429	1.65238	0.724235
36430	1.89226	0.521192
36431	1.89226	0.625082
36432	1.89226	0.496926
36433	1.89226	0.732834
36434	1.89226	0.371138
36435	1.89226	0.745054
36436	1.89226	0.580509
36437	1.89226	0.318573
36438	1.89226	0.336582
36439	1.89226	0.528095
36440	1.89226	0.434288
36441	1.89226	0.542557
36442	1.96832	1.0289
36443	2.40858	0.879578
36444	1.75211	0.614264
36445	2.20797	2.1
36446	4.1607	0.814236
36447	1.75211	0.627071
36448	2.20797	2.1
36449	1.51592	1.25735
36450	2.02064	0.852735
36451	3.77275	1.40919
36452	2.71793	0.559326
36453	2.67099	0.467593
36454	2.55105	0.492905
36455	2.71297	0.451304
36456	5.26898	0.486494
36457	2.36417	0.559835
36458	2.67098	0.479656
36459	2.55104	0.498664
36460	1.83418	0.417726
36461	4.91522	0.478453
36462	2.55105	0.48521
36463	2.71297	0.454205
36464	2.58032	0.461461
36465	2.28922	0.407006
36466	5.13137	0.464263
36467	2.55104	0.489587
36468	2.23787	0.420817
36469	1.67265	0.425189
36470	2.28922	0.418811
36471	4.22369	0.428814
36472	0.851542	0.766291
36473	0.851542	0.751869
36474	0.981419	0.683333
36475	0.854774	0.737393
36476	0.949431	0.696145
36477	0.753763	0.739912
36478	0.946131	0.75513
36479	0.946131	0.765446

36480	1.04073	0.745868
36481	0.981433	0.649675
36482	0.851528	0.811435
36483	0.854828	0.731979
36484	1.89226	0.872951
36485	1.89226	0.740261
36486	1.89226	0.731068
36487	1.89226	0.739307
36488	1.89226	0.842183
36489	1.89226	0.85409
36490	0.814227	0.66613
36491	0.814227	0.669597
36492	0.946117	0.62049
36493	1.07285	0.626025
36494	0.879132	0.613464
36495	0.923293	0.620182
36496	0.946119	0.671274
36497	0.946119	0.67724
36498	0.946131	0.652037
36499	0.814236	0.574971
36500	0.946106	0.701611
36501	0.870405	0.652665
36502	1.89224	0.780679
36503	1.89224	0.654159
36504	1.89224	0.656965
36505	1.89224	0.666459
36506	1.89224	0.756969
36507	1.89224	0.76839
36508	1.29388	1.14567
36509	1.04164	2.1
36510	1.64834	1.86837
36511	1.82955	1.04291
36512	2.94222	1.94601
36513	1.4934	0.636301
36514	2.41522	0.526284
36515	2.20387	0.490108
36516	2.20387	0.888514
36517	1.75765	0.537099
36518	1.75765	0.858235
36519	2.41522	0.452679
36520	2.77303	2.1
36521	2.20387	0.993928
36522	2.58627	0.736376
36523	5.00149	1.01184
36524	1.22222	0.679398
36525	2.20387	0.617275
36526	1.96831	0.530657
36527	1.96831	0.990393
36528	1.4934	0.576604
36529	1.4934	1.04872
36530	1.96832	1.24743
36531	2.58627	0.745216
36532	2.40858	0.619393
36533	2.40858	2.08903
36534	2.20387	0.743188
36535	2.20387	2.1
36536	1.5605	2.1
36537	1.53775	2.1

36538	1.55398	2.1
36539	1.45806	2.1
36540	1.65642	2.1
36541	1.5605	2.1
36542	0.768876	2.1
36543	0.79033	2.1
36544	0.828211	2.1
36545	0.871233	2.1
36546	0.803843	2.1
36547	0.803843	2.1
36548	0.814698	2.1
36549	0.814698	2.1
36550	0.79033	2.1
36551	0.871233	2.1
36552	0.768876	2.1
36553	0.828212	2.1
36554	1.80252	2.1
36555	2.09715	0.669856
36556	1.9394	1.55645
36557	1.50853	1.55997
36558	3.60567	1.78391
36559	1.96416	0.637372
36560	2.09715	0.704965
36561	1.88484	1.39657
36562	1.57916	0.577108
36563	3.67631	0.751496
36564	1.89226	0.676682
36565	1.89226	0.40538
36566	1.89226	0.589809
36567	1.89226	0.684636
36568	3.78453	0.540461
36569	1.89226	0.642817
36570	1.89226	0.740962
36571	1.89226	2.1
36572	1.89226	0.355203
36573	1.89226	1.54399
36574	1.89226	0.222032
36575	1.5852	1.28209
36576	1.50853	1.05554
36577	1.2955	2.1
36578	1.44472	0.667683
36579	2.95325	1.06446
36580	1.53065	1.13587
36581	1.57916	0.503193
36582	1.44472	0.676645
36583	1.78861	0.732992
36584	1.23527	0.742271
36585	1.23527	0.706762
36586	1.89227	0.505148
36587	1.89227	0.550057
36588	1.89227	0.750116
36589	1.89227	0.569748
36590	3.78453	0.571716
36591	1.89226	0.674258
36592	1.89226	0.686464
36593	1.89226	2.05919
36594	1.89226	0.653144
36595	1.89226	0.844231

36596	1.89226	0.332923
36597	1.89227	0.938555
36598	1.89227	0.701531
36599	1.89227	0.703993
36600	1.89227	0.685287
36601	3.78453	0.679253
36602	0.943708	0.702112
36603	0.752219	0.642372
36604	0.946132	0.663209
36605	0.946132	0.699811
36606	0.946133	0.556932
36607	0.946133	1.06902
36608	0.752218	0.86199
36609	0.752218	0.728631
36610	0.752218	0.69387
36611	0.946132	0.608577
36612	0.946133	0.831866
36613	0.943708	0.718095
36614	0.943992	2.1
36615	0.943992	1.13276
36616	1.23507	0.836274
36617	1.48209	1.91055
36618	1.0384	2.1
36619	1.47953	1.90376
36620	0.946128	0.813535
36621	0.946128	1.06417
36622	0.946129	1.86928
36623	1.23293	1.74076
36624	1.0384	1.63329
36625	0.946129	1.56808
36626	1.89226	1.34776
36627	2.46586	1.87434
36628	2.80218	0.807253
36629	2.80218	2.1
36630	1.89226	0.804384
36631	1.89226	2.1
36632	1.32455	2.1
36633	1.89128	2.1
36634	1.32328	2.1
36635	1.97299	2.1
36636	3.29754	2.1
36637	1.89128	2.1
36638	2.4255	2.1
36639	1.973 2.1	
36640	2.63807	2.1
36641	4.3985	2.1
36642	1.34008	2.1
36643	1.97299	2.1
36644	2.10766	2.1
36645	2.10766	2.1
36646	1.32328	2.1
36647	1.32328	2.1
36648	2.41055	2.1
36649	2.37491	2.1
36650	2.40754	2.1
36651	2.38023	2.1
36652	4.78546	2.1
36653	2.23743	2.1

36654	2.37491	2.1
36655	2.40754	2.1
36656	2.20392	2.1
36657	4.61234	2.1
36658	2.37491	2.1
36659	2.02641	2.1
36660	2.20392	2.1
36661	2.18943	2.1
36662	4.40132	2.1
36663	2.37491	2.1
36664	2.22558	2.1
36665	2.38023	2.1
36666	2.18943	2.1
36667	4.60049	2.1
36668	1.89226	0.217236
36669	1.89226	0.400229
36670	1.89226	0.332788
36671	1.89226	0.319029
36672	1.89226	0.256741
36673	1.89226	0.24645
36674	1.03599	0.104418
36675	0.706698	0.0931253
36676	1.41141	0.121026
36677	1.17573	0.107476
36678	1.11957	0.0815898
36679	0.765034	0.0847988
36680	1.05913	0.0760683
36681	1.45054	0.125863
36682	1.04847	0.0883559
36683	1.04847	0.10076
36684	1.15323	0.111704
36685	1.05825	0.0864384
36686	1.89226	0.129401
36687	1.89226	0.119653
36688	1.89226	0.123298
36689	1.89226	0.106697
36690	1.89226	0.140096
36691	1.89226	0.125151
36692	2.22575	0.497873
36693	1.70677	0.754575
36694	1.90157	0.264044
36695	1.5534	0.877001
36696	3.77914	0.643709
36697	1.70678	0.741678
36698	0.968594	2.1
36699	0.968594	1.88503
36700	1.10378	2.1
36701	1.5534	0.907028
36702	1.68859	2.1
36703	2.31429	0.41882
36704	1.5534	1.02581
36705	1.90103	0.275556
36706	1.41902	0.75584
36707	3.45443	0.645309
36708	2.53709	0.05
36709	1.209 0.05	
36710	1.05417	0.05
36711	2.6513	0.05

36712	3.70547	0.05
36713	1.02675	0.05
36714	1.16384	0.05
36715	2.22062	0.05
36716	2.48862	0.05
36717	3.38446	0.05
36718	1.89226	0.05
36719	1.89226	0.05
36720	1.89226	0.05
36721	1.89226	0.05
36722	1.89226	0.0540318
36723	1.89226	0.05
36724	1.89226	0.887116
36725	1.89226	1.65306
36726	1.89226	1.76217
36727	1.89226	1.05817
36728	1.89226	1.39841
36729	1.89226	1.01136
36730	1.89226	0.816889
36731	1.89226	1.29059
36732	1.89226	1.40423
36733	1.89226	0.928844
36734	1.89226	1.30374
36735	1.89226	0.911489
36736	1.89227	0.970531
36737	1.89227	1.49674
36738	1.75032	1.58459
36739	1.75032	1.07649
36740	1.89227	1.39692
36741	1.89227	1.0304
36742	1.89226	0.4927
36743	1.89226	0.523404
36744	1.89226	0.496692
36745	1.89226	0.508044
36746	1.89226	0.514838
36747	1.89226	0.516761
36748	0.946132	0.405421
36749	0.946132	0.407905
36750	0.798215	0.391383
36751	0.848163	0.374738
36752	0.848163	0.421409
36753	0.946131	0.415936
36754	0.946132	0.377049
36755	0.798216	0.377189
36756	0.996105	0.412622
36757	0.996105	0.360824
36758	0.79819	0.385224
36759	0.79819	0.433443
36760	1.07763	0.415582
36761	2.28622	0.401621
36762	2.16109	0.370281
36763	1.17506	0.382982
36764	3.36385	0.402234
36765	1.89226	2.1
36766	1.6663	2.1
36767	1.6663	2.1
36768	1.71706	2.1
36769	1.89226	2.1

36770	1.94302	2.1
36771	1.89226	2.1
36772	1.62801	2.1
36773	1.62801	2.1
36774	1.6663	2.1
36775	1.89226	2.1
36776	1.93055	2.1
36777	1.48734	2.1
36778	1.99609	2.1
36779	1.52245	2.1
36780	2.04137	2.1
36781	3.51854	2.1
36782	1.99609	2.1
36783	2.28563	2.1
36784	2.04136	2.1
36785	2.35019	1.48705
36786	4.34628	2.1
36787	1.50714	2.1
36788	2.04137	2.1
36789	2.04945	2.1
36790	2.04945	2.1
36791	1.52245	2.1
36792	1.52245	2.1
36793	2.04136	2.1
36794	2.35019	1.62031
36795	2.04945	2.1
36796	2.38681	2.1
36797	4.39964	2.1
36798	2.50924	0.05
36799	1.38305	2.1
36800	2.56918	2.1
36801	1.44535	2.1
36802	3.95222	2.1
36803	1.89226	2.1
36804	1.89226	2.1
36805	1.89226	2.1
36806	1.89226	2.1
36807	3.78453	2.1
36808	1.40615	2.1
36809	1.38851	2.1
36810	0.748607	2.1
36811	0.724468	2.1
36812	1.06362	2.1
36813	1.06362	2.1
36814	1.067 2.1	
36815	1.067 2.1	
36816	1.40615	2.1
36817	0.748607	2.1
36818	0.724468	2.1
36819	1.38851	2.1
36820	2.56918	2.1
36821	1.53377	2.1
36822	1.53706	2.1
36823	1.44535	2.1
36824	2.66088	2.1
36825	2.56918	2.1
36826	1.89226	2.1
36827	1.89226	2.1

36828	1.89226	2.1
36829	1.89226	2.1
36830	3.78453	2.1
36831	1.40239	2.1
36832	1.68466	2.03406
36833	1.26091	2.1
36834	1.54641	1.49694
36835	2.94881	2.1
36836	1.68466	2.1
36837	1.65324	0.568521
36838	1.54641	1.68037
36839	1.52268	0.488091
36840	3.19965	0.733977
36841	1.05776	2.1
36842	1.54641	1.66303
36843	1.36595	2.1
36844	1.36595	1.95143
36845	1.26091	2.1
36846	1.26091	2.07854
36847	1.54641	1.96723
36848	1.32697	0.562567
36849	1.52268	0.554581
36850	1.52268	2.1
36851	1.36595	0.494233
36852	1.36595	2.1
36853	1.18647	1.38632
36854	0.988454	2.1
36855	1.4867	1.91556
36856	1.63892	1.6111
36857	2.67317	2.1
36858	1.4867	2.1
36859	1.63892	1.78894
36860	2.09605	0.787468
36861	1.97645	2.03959
36862	3.58275	1.31851
36863	1.29389	2.0426
36864	1.97645	1.62469
36865	1.4867	1.3554
36866	1.4867	2.1
36867	1.82955	0.995761
36868	1.82955	2.1
36869	1.13454	0.977431
36870	0.983594	2.1
36871	1.16809	2.1
36872	1.06836	1.49921
36873	1.08332	1.60434
36874	0.983594	1.10971
36875	1.48751	2.1
36876	1.48047	1.58451
36877	1.77596	1.45921
36878	1.75506	1.68146
36879	3.25643	1.84102
36880	1.48047	1.47411
36881	1.25334	2.1
36882	1.75505	1.28923
36883	1.55831	0.764219
36884	3.03878	1.74183
36885	1.77596	1.47795

36886	1.75506	2.07754
36887	2.00551	2.08834
36888	2.00083	0.904686
36889	3.77679	1.98355
36890	1.75506	1.83193
36891	1.80328	0.808235
36892	1.55831	1.76457
36893	1.55831	2.0657
36894	2.00083	0.879559
36895	2.00083	1.16898
36896	2.58627	0.720963
36897	2.79047	2.1
36898	2.40858	2.1
36899	2.64748	2.09125
36900	5.23375	2.1
36901	1.26755	2.1
36902	1.71717	2.1
36903	1.03824	2.1
36904	1.48901	2.1
36905	2.75656	2.1
36906	1.89226	2.1
36907	1.89226	2.1
36908	1.89226	2.1
36909	1.89226	2.1
36910	1.89226	2.1
36911	1.89226	2.1
36912	1.36541	2.1
36913	1.22156	2.1
36914	0.946132	2.1
36915	0.946132	2.1
36916	1.36541	2.1
36917	1.36541	2.1
36918	0.946132	2.1
36919	0.946132	2.1
36920	1.36541	2.1
36921	0.946133	2.1
36922	0.946132	2.1
36923	1.22156	2.1
36924	1.18901	2.1
36925	1.61149	2.1
36926	1.98748	2.1
36927	1.534 2.1	
36928	3.1765	2.1
36929	1.89226	2.1
36930	1.89226	2.1
36931	1.89226	1.82397
36932	1.89226	2.1
36933	3.78452	2.1
36934	1.98748	2.1
36935	1.534 2.1	
36936	2.27541	2.1
36937	1.98748	2.1
36938	1.87436	2.1
36939	1.87436	2.1
36940	2.2988	2.1
36941	2.2604	2.1
36942	2.20831	2.1
36943	2.02802	2.1

36944	4.32683	2.1
36945	2.26041	2.1
36946	1.98957	2.1
36947	2.02803	2.1
36948	1.75021	2.1
36949	4.0176	2.1
36950	2.34607	2.1
36951	2.02803	2.1
36952	1.86795	2.1
36953	1.81448	2.1
36954	3.89597	2.1
36955	2.02803	2.1
36956	1.75021	2.1
36957	1.81448	2.1
36958	1.57887	2.1
36959	3.6069	2.1
36960	0.946131	0.476327
36961	0.946131	2.1
36962	1.1235	1.69951
36963	1.1235	2.08926
36964	0.946131	1.009
36965	0.946131	0.750215
36966	1.1235	2.05078
36967	1.13736	2.1
36968	1.13736	0.726462
36969	0.946131	1.01611
36970	1.1235	1.52536
36971	0.946131	1.11747
36972	1.89226	2.02962
36973	1.89226	2.1
36974	1.89226	0.960316
36975	1.89226	0.80274
36976	3.78452	0.760436
36977	0.563266	2.1
36978	0.563266	1.05257
36979	0.565606	0.704519
36980	0.565606	2.04772
36981	0.647086	1.65683
36982	0.624219	1.39067
36983	0.500035	1.00684
36984	0.481785	1.00098
36985	0.500034	0.68159
36986	0.647087	0.72066
36987	0.624219	1.22237
36988	0.481785	0.925036
36989	0.915788	0.401109
36990	0.915788	0.461895
36991	1.02732	0.645973
36992	1.02732	0.531371
36993	0.660831	0.569937
36994	1.11155	0.42658
36995	0.713295	0.425179
36996	1.26874	0.615985
36997	0.713295	0.513265
36998	0.660831	0.500885
36999	1.11155	0.509923
37000	1.26874	0.499963
37001	1.89226	0.694858

37002	1.85091	0.600473
37003	2.11193	0.740484
37004	1.85091	0.647291
37005	2.15328	0.647474
37006	1.89226	0.562482
37007	0.946131	0.471382
37008	0.946131	0.575907
37009	1.18275	0.463241
37010	1.05596	0.632888
37011	0.946127	0.505903
37012	0.946127	0.209823
37013	1.18275	0.614338
37014	1.18275	0.632479
37015	0.946128	0.63201
37016	1.18275	0.424753
37017	0.946129	0.584301
37018	1.05596	0.418562
37019	1.60936	2.1
37020	2.35846	2.1
37021	2.06424	2.1
37022	2.57651	2.1
37023	4.42269	2.1
37024	1.89226	2.1
37025	1.89226	2.1
37026	1.89226	2.1
37027	1.89226	2.1
37028	3.78453	2.1
37029	1.89226	2.1
37030	1.89226	2.1
37031	1.89226	2.1
37032	1.89226	2.1
37033	1.89226	2.1
37034	1.89226	2.1
37035	1.95053	2.1
37036	2.06424	2.1
37037	1.95053	2.1
37038	1.30584	2.1
37039	2.5	2.1
37040	1.8553	2.1
37041	2.49999	2.1
37042	2.57651	2.1
37043	2.49999	2.1
37044	2.06423	2.1
37045	2.80738	2.1
37046	2.37162	2.1
37047	1.89226	2.1
37048	1.89226	2.1
37049	1.89226	2.1
37050	1.89226	2.1
37051	1.89226	2.1
37052	1.89226	2.1
37053	1.49295	0.34284
37054	1.03824	0.246132
37055	1.51396	0.404454
37056	2.08514	0.371145
37057	3.12338	0.363635
37058	1.49798	0.352845
37059	2.32017	0.19678

37060	2.68236	0.40316
37061	2.08514	0.357023
37062	4.4053	0.313505
37063	1.89226	0.266174
37064	1.89226	0.29792
37065	1.89226	0.352342
37066	1.89226	0.335379
37067	3.78452	0.334695
37068	1.89226	0.376738
37069	1.89226	0.250644
37070	1.89226	0.368134
37071	1.89226	0.331464
37072	1.89226	0.276901
37073	1.89226	0.20691
37074	1.51396	0.330755
37075	2.08514	0.239564
37076	2.55393	0.374591
37077	2.02901	0.35341
37078	4.11415	0.313739
37079	1.89226	0.306071
37080	1.89226	0.258524
37081	1.89226	0.333439
37082	1.89226	0.332571
37083	3.78451	0.281854
37084	1.89226	0.247562
37085	1.89226	0.352561
37086	1.89226	0.235025
37087	1.89226	0.222947
37088	1.89226	0.353187
37089	1.89226	0.342122
37090	0.946131	0.156434
37091	0.946131	0.17323
37092	0.946132	0.333126
37093	0.946132	0.198612
37094	0.946132	0.242104
37095	0.946132	0.185553
37096	0.946132	0.230595
37097	0.946132	0.286137
37098	0.946132	0.198833
37099	0.946132	0.233033
37100	0.946132	0.241837
37101	0.946132	0.191131
37102	1.89226	2.1
37103	1.89226	1.0238
37104	1.89226	2.1
37105	1.89226	0.885188
37106	3.78452	2.1
37107	1.28367	2.1
37108	1.82707	1.14324
37109	1.11025	2.1
37110	1.6365	0.861093
37111	2.92016	2.1
37112	1.82707	1.70819
37113	1.8247	0.51767
37114	1.8247	1.85849
37115	2.01549	0.622084
37116	1.6365	0.870322
37117	1.82729	0.471966

37118	1.89226	1.77565
37119	1.89226	0.571693
37120	1.89226	0.455018
37121	1.89226	1.53461
37122	1.89226	0.392782
37123	1.89226	1.489
37124	1.63648	2.07037
37125	1.82467	0.51822
37126	1.35877	1.378
37127	1.55159	0.445985
37128	3.18345	0.738421
37129	1.89223	1.86835
37130	1.89223	0.547451
37131	1.89223	1.58236
37132	1.89223	0.578916
37133	3.78447	0.76402
37134	1.59972	2.1
37135	1.28037	2.1
37136	1.63702	2.1
37137	1.28158	2.1
37138	1.63581	2.1
37139	1.28037	2.1
37140	1.89226	2.1
37141	1.89226	2.1
37142	1.89226	2.1
37143	1.89226	2.1
37144	1.89226	2.1
37145	1.89226	2.1
37146	1.89226	2.1
37147	1.89226	2.1
37148	1.89226	2.1
37149	1.89226	2.1
37150	3.78451	2.1
37151	1.28037	2.1
37152	1.05071	2.1
37153	1.63581	2.1
37154	1.44287	2.1
37155	2.72324	2.1
37156	1.59972	2.1
37157	1.63581	2.1
37158	1.94291	2.1
37159	2.00118	2.1
37160	3.57872	2.1
37161	1.89226	2.1
37162	1.89226	2.1
37163	1.89226	2.1
37164	1.89226	2.01599
37165	3.78452	2.1
37166	0.946131	1.62734
37167	0.946131	2.08842
37168	0.946127	2.1
37169	0.946127	2.1
37170	0.94613	1.23215
37171	0.94613	2.1
37172	0.946128	2.1
37173	0.946128	1.84334
37174	0.946128	2.1
37175	0.946128	2.1

37176	0.946129	2.1
37177	0.946129	2.1
37178	0.865056	2.1
37179	0.865056	2.1
37180	0.873224	2.1
37181	0.873224	2.1
37182	0.721437	2.1
37183	0.920378	2.1
37184	0.817903	2.1
37185	1.00059	2.1
37186	1.00059	2.1
37187	0.920376	2.1
37188	0.817904	2.1
37189	0.721437	2.1
37190	3.09157	2.1
37191	1.71255	2.1
37192	2.0815	2.1
37193	1.9918	2.1
37194	2.51364	2.1
37195	2.39396	2.1
37196	2.0815	2.1
37197	1.05051	2.1
37198	2.56724	2.1
37199	1.63991	2.1
37200	3.47929	2.1
37201	1.89226	0.247567
37202	2.2973	0.502095
37203	1.90705	0.467823
37204	1.90705	0.337003
37205	1.89226	0.408636
37206	1.89226	0.305094
37207	1.89226	0.269619
37208	1.90705	0.495879
37209	1.45321	0.495976
37210	1.45321	0.363619
37211	1.89226	0.418564
37212	1.89226	0.30939
37213	0.901146	2.1
37214	0.901146	2.1
37215	1.15227	2.1
37216	1.21279	2.1
37217	0.991265	2.1
37218	1.15003	2.1
37219	0.946132	2.1
37220	0.946132	2.1
37221	0.946132	2.1
37222	1.09997	2.1
37223	0.991266	2.1
37224	0.946132	2.1
37225	1.89226	2.1
37226	1.89226	2.1
37227	1.89226	2.1
37228	1.89226	2.1
37229	1.89226	2.1
37230	1.89226	2.1
37231	1.62229	2.1
37232	1.06053	2.1
37233	1.19698	2.1

37234	1.70106	2.1
37235	2.81928	2.1
37236	0.946132	2.1
37237	0.946132	2.1
37238	1.17527	2.1
37239	1.09997	2.1
37240	1.09997	2.1
37241	1.09997	2.1
37242	0.946133	2.1
37243	0.946133	2.1
37244	1.17527	2.1
37245	0.946132	2.1
37246	0.946133	2.1
37247	1.09997	2.1
37248	1.89226	2.1
37249	1.89226	2.1
37250	1.89226	2.1
37251	1.89226	2.1
37252	1.89226	2.1
37253	1.89226	2.1
37254	0.905416	2.1
37255	0.850527	2.1
37256	0.660601	2.1
37257	0.598494	2.1
37258	0.750137	2.1
37259	0.750137	2.1
37260	0.753774	2.1
37261	0.753774	2.1
37262	0.660599	2.1
37263	0.905418	2.1
37264	0.598493	2.1
37265	0.850529	2.1
37266	2.43509	2.1
37267	2.65066	2.1
37268	2.66602	2.1
37269	2.46339	2.1
37270	5.10111	2.1
37271	2.53008	2.1
37272	2.65066	2.1
37273	2.66602	2.1
37274	2.52852	2.1
37275	5.1961	2.1
37276	2.66602	2.1
37277	2.46339	2.1
37278	2.47932	2.1
37279	2.67393	2.1
37280	5.14535	2.1
37281	2.66602	2.1
37282	2.52853	2.1
37283	2.51686	2.1
37284	2.67393	2.1
37285	5.18288	2.1
37286	1.89226	0.709541
37287	1.89226	0.404427
37288	1.89226	0.629004
37289	1.89226	0.405469
37290	3.78452	0.505809
37291	1.55161	0.625304

37292	1.53972	0.399766
37293	1.32259	0.624828
37294	1.35082	0.375414
37295	2.8623	0.477991
37296	1.53972	0.438794
37297	1.4054	0.475047
37298	1.35083	0.395548
37299	1.24913	0.431426
37300	2.78885	0.392263
37301	1.89226	0.44347
37302	1.89226	0.67607
37303	1.89226	0.456921
37304	1.89226	0.429365
37305	3.78452	0.400031
37306	1.89226	0.578287
37307	1.89226	0.438786
37308	1.89226	0.69827
37309	1.89226	0.435317
37310	1.89226	0.619213
37311	1.89226	0.442056
37312	1.14599	0.576901
37313	1.35082	0.396908
37314	1.14599	0.478444
37315	1.32259	0.644557
37316	1.19993	0.393721
37317	1.37652	0.645916
37318	1.35083	0.415178
37319	1.24913	0.542837
37320	1.19993	0.458258
37321	1.11787	0.419859
37322	2.44906	0.383653
37323	1.89226	0.504799
37324	1.89226	0.538593
37325	1.89226	0.458413
37326	1.89226	0.429284
37327	3.78452	0.410038
37328	1.51697	0.196359
37329	2.57264	0.322041
37330	1.30203	0.136925
37331	2.41701	0.221689
37332	3.93398	0.253434
37333	1.17464	0.104362
37334	2.41701	0.206861
37335	2.32462	0.177172
37336	2.32462	0.16279
37337	1.30203	0.163775
37338	1.30203	0.148546
37339	2.08149	2.1
37340	1.9839	2.05832
37341	1.71398	2.1
37342	1.99182	0.543004
37343	3.71121	0.9243
37344	2.16061	1.72649
37345	2.01399	0.38917
37346	1.99182	0.571032
37347	1.83895	0.510148
37348	4.00581	0.518166
37349	1.90444	2.1

37350	1.99182	0.587743
37351	1.71717	2.1
37352	1.805	0.650162
37353	3.70899	1.89189
37354	1.99182	0.601771
37355	1.83896	0.413603
37356	1.805	0.754412
37357	1.65243	0.433186
37358	3.64425	0.504599
37359	1.18076	2.1
37360	1.27697	2.1
37361	1.05025	2.1
37362	1.27697	2.1
37363	1.25	2.1
37364	1.47671	2.1
37365	1.89226	2.1
37366	1.89226	1.17133
37367	1.89226	2.1
37368	1.89226	2.1
37369	3.78452	2.1
37370	2.64124	2.1
37371	1.89226	2.1
37372	1.89226	2.1
37373	1.89226	2.1
37374	2.46904	2.1
37375	2.46904	2.1
37376	1.89226	2.1
37377	1.89226	2.1
37378	2.7408	2.1
37379	2.82858	1.8513
37380	4.72084	2.1
37381	1.87875	2.1
37382	1.37704	2.1
37383	1.77042	2.1
37384	2.27801	2.1
37385	3.64916	2.1
37386	1.77042	2.1
37387	2.27801	2.1
37388	2.58523	2.1
37389	1.90178	2.1
37390	4.35565	2.1
37391	1.28667	2.1
37392	1.30711	2.1
37393	1.53077	2.1
37394	1.55635	2.1
37395	2.81743	2.1
37396	1.55635	2.1
37397	1.28228	2.1
37398	1.64268	1.42196
37399	1.93673	2.1
37400	3.19903	2.1
37401	1.53077	2.1
37402	1.93673	2.1
37403	1.55635	2.1
37404	1.55635	2.1
37405	1.86206	2.1
37406	1.86206	2.1
37407	1.64892	2.1

37408	1.60532	2.1
37409	1.85282	2.1
37410	1.96716	2.1
37411	3.61608	2.1
37412	1.60531	2.1
37413	1.32414	2.1
37414	1.96716	2.1
37415	1.75823	2.1
37416	3.2913	2.1
37417	1.91219	2.1
37418	1.96716	2.1
37419	2.0815	2.1
37420	2.51396	2.1
37421	4.30843	2.1
37422	1.96716	2.1
37423	2.17979	2.1
37424	1.75823	2.1
37425	1.75823	2.1
37426	2.28541	2.1
37427	2.28541	2.1
37428	1.89226	1.10818
37429	1.89226	1.87715
37430	1.89226	1.14002
37431	1.89226	1.02747
37432	3.78453	1.19624
37433	1.89226	1.36505
37434	1.89226	1.37326
37435	1.89226	1.12613
37436	1.89226	1.49136
37437	1.89226	1.20111
37438	1.89226	1.69134
37439	1.61016	1.16205
37440	1.45303	1.8097
37441	1.62812	1.28349
37442	1.86379	1.27071
37443	3.23828	1.3144
37444	1.61016	1.58824
37445	1.78716	1.92272
37446	2.0815	0.96266
37447	1.6774	1.75946
37448	3.54454	1.65056
37449	1.89226	1.41391
37450	1.89226	1.27218
37451	1.89226	0.912461
37452	1.89226	1.54485
37453	3.78453	1.18207
37454	1.89226	1.50519
37455	1.89226	1.52797
37456	1.89226	1.2297
37457	1.89226	1.98095
37458	1.89226	0.911934
37459	1.89226	1.15601
37460	0.807591	1.61138
37461	0.814059	1.2445
37462	1.00217	1.68128
37463	0.931896	1.33204
37464	0.850586	1.38539
37465	0.850586	1.92557

37466	0.8889	1.11322
37467	0.8889	1.53561
37468	0.807592	0.96258
37469	1.00217	1.63307
37470	0.814061	1.34931
37471	0.931894	1.94768
37472	2.0815	1.12604
37473	1.6774	2.07655
37474	2.00435	1.22292
37475	2.36104	1.20321
37476	3.86809	1.26588
37477	1.00532	0.370683
37478	1.00532	0.378175
37479	1.00183	0.378466
37480	1.00183	0.375538
37481	1.26377	0.374957
37482	1.34921	0.377485
37483	0.664872	0.377885
37484	0.743381	0.381475
37485	0.664872	0.371238
37486	1.26377	0.374607
37487	1.34921	0.381363
37488	0.743381	0.381291
37489	1.26826	0.05
37490	0.946131	2.06279
37491	1.02404	1.77054
37492	0.94613	2.1
37493	1.30281	1.96657
37494	0.930941	2.1
37495	0.930941	2.04675
37496	1.318	1.98382
37497	1.58161	1.81034
37498	0.94613	1.97624
37499	0.94613	1.58816
37500	1.56338	1.75185
37501	1.02404	1.70297
37502	2.81374	1.73721
37503	1.89226	2.1
37504	1.89226	2.06952
37505	1.89226	2.09515
37506	3.78453	2.1
37507	0.946133	2.1
37508	0.946133	1.81726
37509	0.946132	1.87395
37510	0.946132	2.1
37511	0.946132	2.1
37512	0.946132	1.91935
37513	0.946133	2.1
37514	0.946133	1.82222
37515	0.946132	2.1
37516	0.946132	1.83631
37517	0.946133	2.1
37518	0.946133	1.93364
37519	1.89226	1.37686
37520	1.89226	2.1
37521	1.89226	1.55471
37522	1.89226	1.80132
37523	3.78452	1.90408

37524	1.89226	2.09693
37525	1.89226	1.98495
37526	1.89226	2.1
37527	1.89226	1.55534
37528	1.89226	2.1
37529	1.89226	1.58152
37530	0.946133	1.60552
37531	0.946133	2.1
37532	0.946132	1.51633
37533	0.946132	2.06261
37534	0.946132	2.06063
37535	0.946132	1.95681
37536	0.946133	2.05384
37537	0.946133	1.63797
37538	0.946132	2.07032
37539	0.946132	1.76634
37540	0.946132	2.1
37541	0.946132	1.80588
37542	2.56953	2.1
37543	2.64148	2.1
37544	2.48631	2.1
37545	2.62213	2.1
37546	5.19166	2.1
37547	2.64148	2.1
37548	2.57929	2.1
37549	2.62213	2.1
37550	2.59522	2.1
37551	5.20142	2.1
37552	2.48631	2.1
37553	2.62213	2.1
37554	2.41176	2.1
37555	2.59262	2.1
37556	5.03389	2.1
37557	2.62213	2.1
37558	2.59522	2.1
37559	2.59262	2.1
37560	2.61901	2.1
37561	5.24114	2.1
37562	0.946132	0.05
37563	0.946132	0.05
37564	0.946133	0.05
37565	0.946133	0.05
37566	0.946132	0.05
37567	0.946132	0.05
37568	0.946133	0.05
37569	0.946133	0.05
37570	0.946132	0.05
37571	0.946132	0.05
37572	0.946133	0.05
37573	0.946133	0.05
37574	1.5681	0.05
37575	1.01248	0.05
37576	1.10749	0.05
37577	0.570913	0.05
37578	1.15589	0.05
37579	1.15588	0.05
37580	0.977549	0.05
37581	0.977549	0.05

37582	1.01249	0.05
37583	0.654445	0.05
37584	1.36795	0.05
37585	1.10748	0.05
37586	1.73883	0.05
37587	1.87638	0.05
37588	1.85219	0.05
37589	1.93113	1.53386
37590	3.7407	0.05
37591	1.87638	0.05
37592	1.73883	2.1
37593	1.93113	1.24249
37594	1.80426	2.1
37595	3.66996	2.1
37596	2.02912	0.321287
37597	1.44951	0.325232
37598	1.50103	0.321521
37599	2.02248	0.328021
37600	3.47199	0.321011
37601	1.89226	0.325195
37602	1.89226	0.334074
37603	1.89226	0.32902
37604	1.89226	0.339237
37605	3.78453	0.33275
37606	2.57881	0.323019
37607	1.89226	0.330966
37608	1.89226	0.329622
37609	1.89226	0.327378
37610	2.6428	0.323538
37611	2.6428	0.322775
37612	1.01379	0.323242
37613	0.985937	0.323482
37614	0.769295	0.320964
37615	0.715547	0.316608
37616	0.853042	0.32397
37617	0.853042	0.324327
37618	0.864117	0.326908
37619	0.864114	0.295889
37620	0.715556	0.330095
37621	1.03047	0.324453
37622	0.731721	0.323195
37623	0.985921	0.324773
37624	1.89227	0.324638
37625	1.89227	0.332774
37626	1.89227	0.325192
37627	1.89227	0.350579
37628	3.78453	0.326133
37629	0.946129	0.32384
37630	0.946129	0.321243
37631	1.28161	0.317519
37632	1.3214	0.322146
37633	0.94613	0.327926
37634	0.94613	0.326928
37635	1.28161	0.31917
37636	1.28161	0.311691
37637	1.28161	0.318464
37638	0.94613	0.322416
37639	0.946131	0.326666

37640	1.3214	0.32098
37641	0.946132	0.487476
37642	0.946132	0.465117
37643	0.706045	0.433086
37644	1.16718	0.423323
37645	0.706017	0.426339
37646	0.706017	0.525641
37647	1.4073	0.423701
37648	1.4073	0.41148
37649	0.94613	0.444503
37650	1.16719	0.445782
37651	0.706046	0.466538
37652	0.946131	0.453888
37653	1.89226	0.513091
37654	1.89226	0.481061
37655	1.89226	0.428338
37656	1.89226	0.554561
37657	1.89226	0.424287
37658	1.89226	0.554237
37659	0.946119	0.471217
37660	0.946119	0.445451
37661	0.946115	0.468778
37662	0.946115	0.420642
37663	0.946117	0.468965
37664	0.946117	0.505855
37665	0.946117	0.432117
37666	0.946117	0.415171
37667	0.94613	0.423332
37668	0.94613	0.469202
37669	0.946105	0.463822
37670	0.946105	0.434508
37671	0.946132	0.711504
37672	0.946132	0.672257
37673	0.946131	0.738995
37674	0.946131	0.635608
37675	0.946132	0.689823
37676	0.946132	0.747256
37677	0.946132	0.661977
37678	0.827865	0.659207
37679	0.946132	0.66335
37680	0.946132	0.690048
37681	0.946132	0.673718
37682	0.827865	0.680765
37683	1.89226	1.23443
37684	1.89226	1.01365
37685	1.89226	1.33133
37686	1.89226	0.919246
37687	1.89226	1.83417
37688	1.89226	1.12189
37689	0.93094	0.894712
37690	0.93094	0.998694
37691	1.04649	0.9086
37692	0.614468	0.745274
37693	0.950358	0.800405
37694	0.799012	0.774431
37695	0.682742	0.925176
37696	0.682742	0.900813
37697	1.04073	1.43614

37698	1.15596	1.13466
37699	0.614478	1.14024
37700	0.851524	0.992489
37701	2.22575	0.775819
37702	1.90502	2.1
37703	2.10893	0.747087
37704	1.80258	2.1
37705	4.01395	2.1
37706	1.90502	2.1
37707	1.40863	2.1
37708	1.49733	2.1
37709	1.49733	2.1
37710	1.80258	2.1
37711	1.80258	2.1
37712	2.10893	0.716462
37713	1.68356	2.1
37714	1.80258	2.1
37715	1.80258	1.38071
37716	1.98061	2.1
37717	1.98061	1.40382
37718	1.80258	2.1
37719	1.40863	2.1
37720	1.68356	2.1
37721	1.30751	2.1
37722	3.11009	2.1
37723	1.35374	1.34427
37724	1.35374	1.72419
37725	0.746096	1.05473
37726	0.746096	1.0398
37727	1.1537	1.06231
37728	0.746095	1.362
37729	0.946132	1.12723
37730	0.946132	0.897248
37731	0.946132	0.870717
37732	0.746095	0.981565
37733	0.946131	0.844678
37734	1.1537	0.915816
37735	2.15247	2.1
37736	1.70303	0.993556
37737	1.41207	2.1
37738	1.43052	2.1
37739	3.22793	2.1
37740	0.940246	2.1
37741	0.728442	2.1
37742	0.650037	2.1
37743	0.539874	2.1
37744	0.694756	2.1
37745	0.694756	2.1
37746	0.776346	2.1
37747	0.656772	2.1
37748	0.733146	2.1
37749	0.563769	2.1
37750	0.66316	2.1
37751	0.899692	2.1
37752	1.21223	2.1
37753	1.22418	2.1
37754	1.43052	2.1
37755	1.43052	1.11226

37756	1.24353	2.1
37757	1.46181	1.45966
37758	1.89226	0.240497
37759	2.39894	0.129169
37760	1.89226	0.355614
37761	2.37417	0.164066
37762	1.89226	0.263876
37763	2.37417	0.112433
37764	1.12767	0.0925751
37765	1.19947	0.167416
37766	0.94613	0.252097
37767	0.94613	0.122783
37768	1.12766	0.0819648
37769	0.946128	0.0892013
37770	1.19947	0.135831
37771	0.946132	0.264941
37772	1.19947	0.218907
37773	1.19947	0.0959044
37774	0.94613	0.221878
37775	0.94613	0.0955809
37776	2.22621	0.05
37777	1.14533	2.1
37778	1.1131	2.1
37779	0.88238	2.1
37780	0.868521	0.86662
37781	1.01153	2.1
37782	1.01153	2.1
37783	1.00232	2.1
37784	1.00232	2.1
37785	1.14533	2.1
37786	0.882378	2.1
37787	0.868519	2.1
37788	1.11311	2.1
37789	0.910183	1.38365
37790	1.07811	0.983494
37791	0.94613	0.575174
37792	0.94613	0.667271
37793	0.910212	0.67204
37794	0.910212	1.62454
37795	1.11403	0.585948
37796	1.11403	1.75883
37797	0.910183	0.775989
37798	0.94613	0.851851
37799	0.94613	0.959113
37800	1.07811	0.963405
37801	1.87228	0.780707
37802	2.81189	2.1
37803	1.6197	1.94898
37804	2.56716	1.55229
37805	4.43944	1.86743
37806	0.946132	0.949012
37807	0.946132	2.04206
37808	0.946132	0.615651
37809	0.946132	0.694297
37810	0.946131	0.749504
37811	0.946131	2.06045
37812	0.946132	0.630962
37813	0.946132	1.06645

37814	0.946132	0.794428
37815	0.946132	0.811938
37816	0.946132	0.919583
37817	0.946132	0.994577
37818	1.28724	2.1
37819	2.56716	1.10647
37820	2.3014	1.00264
37821	2.3014	2.1
37822	1.6197	1.20175
37823	1.6197	2.1
37824	0.946132	1.78715
37825	0.946132	1.32561
37826	0.946132	0.651139
37827	0.946132	0.761436
37828	0.946132	0.787923
37829	0.946132	1.93142
37830	0.946133	2.1
37831	0.946133	0.663586
37832	0.946133	0.891656
37833	0.946133	0.961391
37834	0.946132	0.94349
37835	0.946132	0.950423
37836	1.08039	2.1
37837	1.24445	1.70487
37838	1.2873	2.04664
37839	1.48751	2.08465
37840	2.53175	2.1
37841	0.946125	0.720637
37842	0.946125	2.1
37843	1.12552	1.95203
37844	1.12552	2.1
37845	0.946124	1.3267
37846	0.946124	1.57667
37847	1.12552	2.1
37848	1.11114	2.1
37849	1.11113	1.55539
37850	0.946118	1.96204
37851	1.12552	2.0506
37852	0.946131	1.88685
37853	1.89225	0.969214
37854	1.89225	0.999945
37855	1.89225	1.79875
37856	1.89225	2.1
37857	1.89225	0.785891
37858	1.89225	2.08656
37859	0.987703	0.922886
37860	1.33078	1.43338
37861	1.31565	1.08529
37862	1.31565	1.71834
37863	1.01121	1.02699
37864	1.01121	1.99977
37865	2.19163	2.1
37866	1.89226	1.66129
37867	1.89226	2.1
37868	1.89226	1.31656
37869	2.22229	2.1
37870	2.22229	1.09172
37871	1.89226	2.1

37872	1.89226	0.680495
37873	1.89226	0.687053
37874	1.89226	2.06112
37875	1.89226	0.767726
37876	1.89226	1.96333
37877	0.57141	2.0169
37878	0.57141	0.81675
37879	0.574036	1.03218
37880	0.574036	2.1
37881	0.651591	1.75789
37882	0.657827	1.05196
37883	0.482391	1.13877
37884	0.493855	1.41053
37885	0.482391	0.755343
37886	0.651591	0.850935
37887	0.493855	1.30012
37888	0.657828	1.30561
37889	1.88428	2.1
37890	2.03975	2.1
37891	1.97316	2.1
37892	1.8266	2.1
37893	3.86635	2.1
37894	2.23053	2.1
37895	1.42255	2.1
37896	1.3868	2.1
37897	1.8266	2.1
37898	3.24915	2.1
37899	1.97316	2.1
37900	1.8266	2.1
37901	1.77128	2.1
37902	1.91767	2.1
37903	3.74427	2.1
37904	1.77128	2.1
37905	1.8266	2.1
37906	1.77942	2.1
37907	1.33962	2.1
37908	1.3868	2.1
37909	1.3868	2.1
37910	0.944111	2.1
37911	0.944111	2.1
37912	1.26036	2.1
37913	1.16968	2.1
37914	1.03853	2.1
37915	1.16646	2.1
37916	0.946129	2.1
37917	0.946129	2.1
37918	0.94613	2.1
37919	1.25768	2.1
37920	1.03853	2.1
37921	0.946131	2.1
37922	1.82441	0.110679
37923	1.33061	0.105941
37924	1.13272	0.0780912
37925	1.60249	0.0931297
37926	2.95712	0.106504
37927	1.82441	0.119361
37928	2.08757	0.115946
37929	1.81292	0.0930846

37930	1.60249	0.0865383
37931	3.63733	0.102799
37932	1.81292	0.0836449
37933	1.332	0.0611539
37934	1.60249	0.0664466
37935	1.60249	0.0856879
37936	1.5344	0.0595191
37937	1.5344	0.0781357
37938	1.10107	2.1
37939	2.51559	2.1
37940	1.1229	2.1
37941	1.1229	2.1
37942	2.49456	2.1
37943	2.49456	2.1
37944	1.89226	2.1
37945	2.51559	2.1
37946	2.49457	2.1
37947	2.51559	2.1
37948	1.87124	2.1
37949	1.89226	2.1
37950	1.89226	2.1
37951	1.89226	2.1
37952	1.89226	2.1
37953	1.89226	2.1
37954	1.89226	2.1
37955	1.89226	2.1
37956	1.89226	2.1
37957	1.89226	2.1
37958	1.89226	2.1
37959	1.89226	2.1
37960	1.89226	2.1
37961	1.89226	2.1
37962	1.23599	2.1
37963	1.24728	2.1
37964	0.550534	2.1
37965	0.541151	2.1
37966	0.893754	2.1
37967	0.893754	2.1
37968	0.894678	2.1
37969	0.894678	2.1
37970	1.23599	2.1
37971	0.541149	2.1
37972	0.550533	2.1
37973	1.24728	2.1
37974	0.946132	2.1
37975	0.946132	2.1
37976	1.23599	2.1
37977	1.24728	2.1
37978	0.946132	2.1
37979	0.946132	2.1
37980	1.23599	2.1
37981	1.23599	2.1
37982	1.23599	2.1
37983	0.946132	2.1
37984	0.946132	2.1
37985	1.24728	2.1
37986	1.89226	2.1
37987	1.89226	2.1

37988	1.89226	2.1
37989	1.89226	2.1
37990	1.89226	2.1
37991	1.89226	2.1
37992	0.946128	2.1
37993	0.946128	2.1
37994	0.946132	2.1
37995	0.946132	2.1
37996	0.94613	2.1
37997	0.94613	2.1
37998	0.946129	2.1
37999	0.946129	2.1
38000	0.94613	2.1
38001	0.94613	2.1
38002	0.94613	2.1
38003	0.94613	2.1
38004	2.58696	0.420132
38005	2.57073	0.475132
38006	2.12585	0.432462
38007	2.07607	0.484874
38008	4.69658	0.469333
38009	1.89226	0.474872
38010	1.89226	0.497976
38011	1.89226	0.452696
38012	1.89226	0.519724
38013	3.78452	0.467665
38014	1.89226	0.500917
38015	1.89226	0.571983
38016	1.89226	0.525512
38017	1.89226	0.553388
38018	3.78453	0.550056
38019	2.57074	0.477655
38020	2.32635	0.553821
38021	2.07608	0.490047
38022	1.73623	0.51912
38023	4.30697	0.487301
38024	2.12582	0.422234
38025	1.51971	0.433077
38026	1.57255	0.413814
38027	2.07604	0.448112
38028	1.62233	0.403011
38029	2.12582	0.437433
38030	1.89223	0.439571
38031	1.89223	0.503955
38032	1.89223	0.475361
38033	1.89223	0.465495
38034	1.89223	0.470071
38035	1.89223	0.461396
38036	0.946119	0.526817
38037	0.946119	0.504112
38038	0.946119	0.466569
38039	0.946119	0.523065
38040	0.946119	0.529915
38041	0.946119	0.530013
38042	0.94612	0.509278
38043	0.94612	0.481507
38044	0.946132	0.507088
38045	0.946132	0.490315

38046	0.946107	0.513132
38047	0.946107	0.508883
38048	0.811456	0.459437
38049	0.811456	0.51183
38050	0.806521	0.434696
38051	0.806521	0.510642
38052	0.868104	0.508404
38053	0.579949	0.499023
38054	0.759859	0.4362
38055	1.03802	0.447525
38056	0.579957	0.468603
38057	0.759868	0.466666
38058	1.03801	0.484803
38059	0.868093	0.490802
38060	2.7408	2.1
38061	2.82858	1.70254
38062	2.57368	2.1
38063	2.52633	2.1
38064	5.3549	2.1
38065	2.7408	2.1
38066	2.31353	2.1
38067	2.14063	2.1
38068	2.52632	2.1
38069	4.83985	2.1
38070	2.57368	2.1
38071	2.52633	2.1
38072	2.3301	2.1
38073	2.36071	1.68947
38074	4.88704	2.1
38075	1.86512	2.1
38076	2.52632	2.1
38077	2.14063	2.1
38078	2.14063	2.1
38079	2.3301	2.1
38080	2.3301	2.1
38081	1.86431	2.1
38082	1.89227	2.1
38083	1.68992	2.1
38084	1.68992	2.1
38085	1.89227	2.1
38086	1.89227	2.1
38087	2.0815	2.1
38088	1.89226	2.1
38089	2.04022	2.1
38090	1.86431	2.1
38091	2.07081	2.1
38092	1.89226	2.1
38093	1.91686	1.10766
38094	1.65683	1.10506
38095	1.86871	2.1
38096	1.65683	1.0167
38097	2.13075	1.33941
38098	1.89226	0.671295
38099	1.89226	1.10842
38100	1.89226	0.609447
38101	1.89226	0.762812
38102	1.89226	1.18481
38103	3.78452	0.802908

38104	0.851541	2.1
38105	0.851541	1.75322
38106	0.952803	2.1
38107	0.851573	2.1
38108	0.946129	2.1
38109	0.946129	0.913666
38110	0.929184	2.1
38111	0.757482	2.1
38112	0.836274	2.1
38113	0.851528	2.1
38114	0.952816	2.1
38115	1.04073	2.1
38116	1.70303	0.988033
38117	1.70302	0.85662
38118	1.5544	1.64675
38119	1.70302	0.839633
38120	1.74957	0.95459
38121	1.91686	0.643371
38122	1.89226	0.711001
38123	1.89226	0.865477
38124	1.89226	1.235
38125	1.89226	0.755843
38126	1.89226	0.774515
38127	1.89226	0.550512
38128	1.89226	1.83521
38129	1.89226	2.1
38130	2.03472	2.1
38131	1.89226	1.87022
38132	1.99827	2.1
38133	1.85836	0.999375
38134	1.00509	2.1
38135	1.55998	2.1
38136	1.83347	2.1
38137	1.45246	2.1
38138	3.01244	2.1
38139	1.23839	2.1
38140	0.884251	2.1
38141	0.946133	2.1
38142	0.946133	2.1
38143	0.884226	2.1
38144	0.884226	2.1
38145	0.946158	2.1
38146	0.946159	2.1
38147	1.23839	2.1
38148	0.946131	2.1
38149	0.946131	2.1
38150	0.884254	2.1
38151	1.89226	2.1
38152	1.89226	1.36607
38153	1.89226	2.04778
38154	1.89226	2.1
38155	1.89226	2.1
38156	1.89226	2.1
38157	2.1499	2.1
38158	1.45244	2.1
38159	1.83345	2.09622
38160	1.83345	2.1
38161	1.87214	2.05995

38162	1.87214	2.1
38163	1.89224	2.1
38164	1.89224	1.07529
38165	1.89224	1.77654
38166	1.89224	2.01205
38167	3.78448	2.1
38168	1.49155	0.742342
38169	2.0815	0.870513
38170	1.77515	0.986816
38171	1.72377	2.1
38172	3.50659	0.931528
38173	1.69876	0.184185
38174	2.0815	1.69964
38175	1.9133	0.745036
38176	1.68189	0.351752
38177	2.17628	1.81724
38178	1.94335	1.57972
38179	2.65129	0.05
38180	1.15317	0.05
38181	2.57085	0.05
38182	1.209	0.05
38183	2.57085	0.05
38184	1.209	0.05
38185	1.89226	0.05
38186	1.89226	0.05
38187	1.89226	0.05
38188	1.89226	0.05
38189	3.78452	0.05
38190	2.48861	0.05
38191	1.15252	0.05
38192	2.60515	0.05
38193	2.60515	0.05
38194	1.16384	0.05
38195	1.16384	0.05
38196	1.59903	0.757462
38197	1.26865	0.706417
38198	1.30486	1.2816
38199	1.70302	0.879597
38200	2.98573	0.852714
38201	1.59903	0.766275
38202	1.79478	0.837465
38203	1.79478	0.798876
38204	1.97005	0.887373
38205	1.50365	0.694349
38206	1.64192	1.24784
38207	1.89226	0.626324
38208	1.89226	0.717467
38209	1.89226	0.85533
38210	1.89226	0.591806
38211	3.78453	0.60038
38212	1.89226	0.7828
38213	1.89226	0.682641
38214	1.89226	0.782552
38215	1.89226	0.620741
38216	1.89226	0.808762
38217	1.89226	0.959167
38218	0.761672	0.652657
38219	0.896406	0.739587

38220	0.603509	0.728338
38221	0.574071	0.910846
38222	0.695159	0.931816
38223	0.695159	0.594264
38224	0.762564	1.35278
38225	0.770159	0.7082
38226	0.803021	0.882109
38227	0.572529	1.1086
38228	0.696466	0.622089
38229	0.899733	0.660218
38230	1.66034	0.5472
38231	1.79478	0.8721
38232	1.56685	0.649395
38233	1.68512	1.39456
38234	1.67652	0.708192
38235	1.67652	0.892793
38236	1.89226	0.888076
38237	1.89226	0.636628
38238	1.89226	0.766516
38239	1.89226	0.798065
38240	1.89226	0.787981
38241	1.89226	0.824697
38242	0.946133	0.595868
38243	0.946133	0.721149
38244	0.946132	0.880495
38245	0.946132	0.715516
38246	0.946132	0.770348
38247	0.946132	0.518234
38248	0.946133	1.44813
38249	0.946133	0.663363
38250	0.946133	0.752574
38251	0.946133	0.760218
38252	0.946132	0.597511
38253	0.946132	0.58239
38254	1.80424	2.1
38255	1.45158	2.1
38256	1.9098	2.1
38257	1.56003	2.1
38258	3.36138	2.1
38259	1.90982	2.1
38260	1.71606	2.1
38261	2.15314	2.1
38262	1.70302	2.1
38263	3.89051	2.1
38264	1.96416	0.691638
38265	1.23706	0.702531
38266	1.00053	0.600271
38267	1.38287	0.532534
38268	1.57916	0.5397
38269	1.9615	0.61743
38270	0.827865	0.539735
38271	0.946132	0.49881
38272	0.946132	0.456746
38273	0.946132	0.555101
38274	0.946132	0.619219
38275	0.946132	0.547242
38276	0.946131	0.666788
38277	0.946131	0.540359

38278	0.827865	0.723163
38279	0.946132	0.463755
38280	0.946132	0.428714
38281	0.946132	0.483244
38282	1.89227	2.1
38283	1.89227	2.1
38284	1.89227	2.1
38285	1.89227	2.1
38286	3.78453	2.1
38287	2.3819	2.1
38288	1.58089	2.1
38289	1.58089	2.1
38290	1.6287	2.1
38291	2.33368	2.1
38292	2.3815	2.1
38293	0.946133	2.1
38294	0.946133	2.1
38295	0.946132	2.1
38296	0.946132	2.1
38297	0.946132	2.1
38298	0.946132	2.1
38299	0.946132	2.1
38300	0.946132	2.1
38301	0.946133	2.1
38302	0.946133	2.1
38303	0.946132	2.1
38304	0.946132	2.1
38305	1.89226	2.1
38306	1.89226	2.1
38307	1.89226	2.1
38308	1.89226	2.1
38309	3.78453	2.1
38310	2.7874	2.1
38311	2.33368	2.1
38312	2.71897	2.1
38313	2.27495	2.1
38314	5.05266	2.1
38315	0.970991	2.1
38316	0.970991	2.1
38317	0.956929	2.1
38318	0.956929	2.1
38319	0.732928	2.1
38320	0.790444	2.1
38321	1.16684	2.1
38322	1.13748	2.1
38323	1.13748	2.1
38324	0.732928	2.1
38325	0.790444	2.1
38326	1.16684	2.1
38327	0.946132	2.1
38328	0.946132	2.1
38329	0.946131	2.1
38330	0.946131	2.1
38331	0.946132	2.1
38332	0.946132	2.1
38333	0.946132	2.1
38334	0.946132	2.1
38335	0.946132	2.1

38336	0.946132	2.1
38337	0.946132	2.1
38338	0.946132	2.1
38339	1.10747	0.396014
38340	2.30331	0.435285
38341	1.47954	0.388787
38342	2.63789	0.384192
38343	3.78285	0.397173
38344	1.65215	0.376307
38345	2.63789	0.378451
38346	2.82465	0.399828
38347	2.82465	0.400516
38348	1.47954	0.371085
38349	1.47954	0.377911
38350	1.13456	1.74927
38351	1.08334	2.1
38352	1.20674	0.845487
38353	1.17885	2.04108
38354	2.29008	1.50141
38355	1.20674	1.36685
38356	1.17885	2.1
38357	1.28649	0.95973
38358	1.28516	1.72848
38359	2.4919	1.50375
38360	1.89226	2.08886
38361	1.89226	2.1
38362	1.89226	2.1
38363	1.89226	2.02905
38364	1.89226	2.1
38365	1.89226	1.94329
38366	2.50288	1.33304
38367	2.02115	2.02639
38368	2.7717	1.31615
38369	2.29107	0.821393
38370	4.79395	2.05119
38371	1.27339	0.738609
38372	1.10817	2.1
38373	1.19733	1.75512
38374	1.032 2.1	
38375	2.30539	2.1
38376	1.19733	1.96584
38377	0.953637	2.1
38378	0.953637	2.08778
38379	1.032 2.1	
38380	1.11868	1.33855
38381	1.19704	2.1
38382	2.01024	0.888943
38383	1.11938	1.01628
38384	1.9186	1.36035
38385	2.76298	0.874702
38386	3.92884	1.06331
38387	0.851541	0.332155
38388	0.851541	0.290834
38389	0.957577	0.256802
38390	0.848952	0.260053
38391	1.08043	0.336826
38392	0.851541	0.35951
38393	0.851484	0.262665

38394	0.957578	0.209179
38395	1.20045	0.325297
38396	0.726289	0.194666
38397	0.946131	0.263386
38398	0.946131	0.344117
38399	1.50524	2.1
38400	2.38363	2.1
38401	2.49018	2.1
38402	2.49018	2.1
38403	1.45506	2.1
38404	1.45506	2.1
38405	2.49017	2.1
38406	1.89226	2.1
38407	1.89226	2.1
38408	1.89226	2.1
38409	2.38363	2.1
38410	2.38363	2.1
38411	1.54711	2.1
38412	2.49018	2.1
38413	2.56975	2.1
38414	2.56975	2.1
38415	1.50524	2.1
38416	1.50524	2.1
38417	2.56975	2.1
38418	1.89226	2.1
38419	1.89226	2.1
38420	1.89226	2.1
38421	2.49017	2.1
38422	2.49017	2.1
38423	1.89226	0.124986
38424	1.89226	0.155119
38425	1.89226	0.233131
38426	1.89226	0.101282
38427	1.89226	0.236858
38428	1.89226	0.0958547
38429	1.89226	0.157089
38430	1.89226	0.146295
38431	1.89226	0.367548
38432	1.89226	0.108814
38433	1.89226	0.368102
38434	1.89226	0.113296
38435	1.89226	0.116265
38436	1.89226	0.106924
38437	2.8358	0.0888546
38438	1.89226	0.324695
38439	3.78453	0.0872052
38440	1.60565	0.268545
38441	1.89226	1.31746
38442	1.89226	0.985228
38443	1.40013	0.709249
38444	1.89226	1.13452
38445	1.40013	0.864246
38446	1.1766	2.1
38447	1.29435	2.1
38448	1.35735	2.1
38449	1.53509	2.1
38450	2.65169	2.1
38451	0.69701	2.1

38452	0.69701	2.1
38453	0.711303	2.1
38454	0.711303	2.1
38455	0.761138	2.1
38456	0.612149	2.1
38457	0.647174	2.1
38458	0.767548	2.1
38459	0.767548	2.1
38460	0.761139	2.1
38461	0.612149	2.1
38462	0.647174	2.1
38463	1.29764	1.17595
38464	1.29579	1.35083
38465	1.34875	1.23807
38466	1.06717	1.44963
38467	1.29764	1.14624
38468	1.01606	1.18534
38469	0.756635	1.1299
38470	0.648821	1.14609
38471	0.675557	1.27143
38472	0.674374	1.19217
38473	0.74214	1.30564
38474	0.74214	1.2118
38475	0.688868	1.08963
38476	0.688868	1.15144
38477	0.675557	0.98655
38478	0.756634	1.0605
38479	0.648821	0.951213
38480	0.674374	1.02899
38481	1.51327	1.26218
38482	1.48105	1.06873
38483	1.29764	1.28287
38484	1.29764	1.17511
38485	1.48105	1.24648
38486	1.05655	1.04512
38487	1.13537	2.1
38488	1.07326	2.1
38489	0.94613	2.1
38490	0.94613	2.1
38491	0.946132	2.1
38492	0.946132	2.1
38493	1.13537	2.1
38494	1.08927	2.1
38495	1.13537	2.1
38496	0.946131	2.1
38497	0.946131	2.1
38498	0.95947	2.1
38499	1.46892	2.1
38500	1.89226	2.1
38501	1.89226	2.1
38502	1.89226	2.1
38503	1.88031	2.1
38504	1.88031	2.1
38505	1.89226	2.1
38506	1.89226	2.1
38507	1.89226	2.1
38508	1.89226	2.1
38509	3.78453	2.1

38510	2.18254	2.1
38511	1.89226	2.1
38512	2.50005	2.1
38513	1.89226	2.1
38514	4.39231	2.1
38515	1.88031	2.1
38516	2.0815	2.1
38517	2.0815	2.1
38518	1.92078	2.1
38519	2.0815	2.1
38520	1.9218	2.1
38521	1.89227	2.1
38522	1.89227	2.1
38523	1.89227	2.1
38524	1.89227	2.1
38525	1.89227	2.1
38526	1.89227	2.1
38527	1.98693	2.1
38528	1.82857	2.1
38529	2.32	2.1
38530	2.36972	2.1
38531	4.14857	2.1
38532	1.82857	2.1
38533	1.58688	2.1
38534	2.36971	2.1
38535	2.19093	2.1
38536	4.0195	2.1
38537	0.946132	2.1
38538	0.946132	2.1
38539	1.18486	2.1
38540	1.18486	2.1
38541	1.18486	2.1
38542	0.946132	2.1
38543	0.946132	2.1
38544	1.16	2.1
38545	0.946133	2.1
38546	0.946133	2.1
38547	1.16	2.1
38548	1.18486	2.1
38549	1.07798	1.38494
38550	1.08101	1.13624
38551	0.910786	2.1
38552	0.910786	1.90866
38553	0.851537	1.63191
38554	0.901377	1.15241
38555	1.14652	1.6507
38556	1.04072	2.1
38557	0.946129	1.6867
38558	0.946129	1.80325
38559	1.0019	1.4596
38560	1.04263	1.50757
38561	1.89226	2.1
38562	1.89226	2.1
38563	1.89226	2.1
38564	1.89226	2.1
38565	3.78452	2.1
38566	1.89226	2.1
38567	2.30146	2.1

38568	1.89226	2.1
38569	2.18412	2.1
38570	4.19372	2.1
38571	2.30146	2.1
38572	1.39962	2.1
38573	2.18411	2.1
38574	1.28765	2.1
38575	3.5891	2.1
38576	1.89226	2.1
38577	1.89226	2.1
38578	1.89226	2.1
38579	1.89226	2.1
38580	1.89226	2.1
38581	1.89226	2.1
38582	1.89226	2.1
38583	1.89226	2.1
38584	1.89226	2.1
38585	1.89226	2.1
38586	3.78452	2.1
38587	2.18412	2.1
38588	1.20045	2.1
38589	1.20045	2.1
38590	1.28765	2.1
38591	2.08808	2.1
38592	2.17528	2.1
38593	0.946131	2.1
38594	0.946131	2.1
38595	0.946128	2.1
38596	0.946128	2.1
38597	0.946127	2.1
38598	0.946127	2.1
38599	0.946131	2.1
38600	0.946131	2.1
38601	0.946129	2.1
38602	0.946129	2.1
38603	0.94613	2.1
38604	0.94613	2.1
38605	1.89226	0.630018
38606	1.89226	1.0582
38607	1.89226	1.06798
38608	1.89226	0.660512
38609	3.78452	0.571944
38610	1.89227	0.644024
38611	1.89227	1.4743
38612	1.89227	0.575915
38613	1.89227	0.953698
38614	1.89227	0.621591
38615	1.89227	1.15832
38616	1.89226	1.31786
38617	1.89226	0.675242
38618	1.89226	0.681305
38619	2.29245	0.697581
38620	3.78452	0.930586
38621	1.89226	1.10656
38622	1.89226	0.67173
38623	1.89226	0.674526
38624	1.89226	0.846666
38625	1.89226	0.539598

38626	1.89226	0.597698
38627	1.28095	1.70946
38628	1.81846	2.02442
38629	2.10991	0.926936
38630	1.34695	1.74169
38631	3.39086	1.83221
38632	2.10991	1.07967
38633	1.36457	1.786
38634	1.67421	2.04085
38635	1.34695	1.83733
38636	2.43717	1.28596
38637	2.10991	1.45605
38638	2.69541	2.1
38639	1.48572	2.1
38640	2.63553	2.1
38641	1.45385	2.1
38642	4.14926	2.1
38643	2.63553	2.1
38644	1.44207	2.1
38645	1.44207	2.1
38646	1.45385	2.1
38647	2.588	2.1
38648	2.59978	2.1
38649	1.89227	2.1
38650	1.89227	2.1
38651	1.89227	2.1
38652	1.89227	2.1
38653	3.78453	2.1
38654	1.89226	2.1
38655	1.89226	2.1
38656	1.89226	2.1
38657	1.89226	2.1
38658	3.78453	2.1
38659	2.15929	2.1
38660	1.38276	2.1
38661	1.43617	2.1
38662	1.35614	2.1
38663	2.23932	2.1
38664	2.15929	2.1
38665	2.15929	2.1
38666	2.25168	2.1
38667	2.3558	2.1
38668	2.23932	2.1
38669	4.51509	2.1
38670	1.89226	2.1
38671	1.89226	2.1
38672	1.89226	2.1
38673	1.89226	2.1
38674	3.78453	2.1
38675	1.89226	2.1
38676	1.89226	2.1
38677	1.89226	2.1
38678	1.89226	2.1
38679	1.89226	2.1
38680	1.89226	2.1
38681	2.23932	2.1
38682	1.43854	2.1
38683	1.49173	2.1

38684	1.38276	2.1
38685	2.34829	2.1
38686	2.23932	2.1
38687	2.3558	2.1
38688	2.34829	2.1
38689	2.40685	2.1
38690	2.23932	2.1
38691	2.52332	2.1
38692	2.3558	2.1
38693	1.89226	0.111585
38694	1.87361	0.146584
38695	2.25082	0.107308
38696	1.87361	0.184771
38697	2.26947	0.0915525
38698	1.89226	0.142816
38699	1.89226	0.150716
38700	1.89226	0.213827
38701	1.89226	0.154218
38702	1.89226	0.304331
38703	1.89226	0.122198
38704	1.89226	0.236118
38705	2.41314	0.129951
38706	1.89226	0.189499
38707	1.89226	0.139413
38708	1.89226	0.262873
38709	2.12204	0.101084
38710	2.12204	0.189223
38711	1.89226	0.107351
38712	2.25082	0.131152
38713	2.28189	0.100682
38714	2.25082	0.185354
38715	1.92333	0.0786291
38716	1.89226	0.14202
38717	1.89226	0.15565
38718	1.89226	0.18558
38719	1.89226	0.154593
38720	1.89226	0.298539
38721	1.89226	0.118179
38722	1.89226	0.211572
38723	2.42521	0.123336
38724	1.89226	0.16325
38725	1.89226	0.123072
38726	1.89226	0.224136
38727	2.41314	0.094914
38728	2.41314	0.15829
38729	1.39725	0.98996
38730	1.07745	2.1
38731	1.07745	0.925652
38732	1.07519	2.1
38733	1.41336	0.858914
38734	1.41111	2.1
38735	1.89226	1.86745
38736	1.92039	2.1
38737	1.92039	1.47378
38738	1.9052	2.1
38739	1.89226	1.26039
38740	1.87708	2.1
38741	0.946128	2.08938

38742	0.946128	1.39438
38743	0.946133	0.912521
38744	0.946133	0.785585
38745	0.94613	0.790344
38746	0.94613	1.29984
38747	0.946131	2.1
38748	0.946131	0.812874
38749	0.946131	0.993855
38750	0.946131	0.997854
38751	0.946131	1.0405
38752	0.946131	1.07499
38753	1.41336	0.92
38754	1.08911	2.1
38755	1.08911	0.997044
38756	1.07745	2.1
38757	1.42381	0.930824
38758	1.41215	2.1
38759	1.89227	2.01722
38760	1.95833	2.1
38761	1.95833	1.72884
38762	1.92039	2.1
38763	1.89227	1.48382
38764	1.85433	2.1
38765	0.946129	2.1
38766	0.946129	1.58166
38767	0.946133	1.03979
38768	0.946133	0.799943
38769	0.946131	0.799837
38770	0.946131	1.43518
38771	0.946131	2.1
38772	0.946131	0.881519
38773	0.946131	1.07065
38774	0.946131	1.07984
38775	0.946131	1.15539
38776	0.946131	1.17454
38777	1.41943	2.1
38778	2.74398	2.1
38779	1.18238	2.1
38780	2.53897	2.1
38781	3.9584	2.1
38782	2.74398	2.1
38783	1.89226	2.1
38784	1.89226	2.1
38785	1.89226	2.1
38786	2.53897	2.1
38787	2.53897	2.1
38788	1.89227	0.901927
38789	1.89227	0.70254
38790	1.89227	0.868119
38791	1.89227	0.644724
38792	3.78453	0.707653
38793	1.89226	0.677181
38794	2.08134	0.576512
38795	2.00632	0.556752
38796	2.00632	0.598051
38797	1.89226	0.61038
38798	1.89226	0.667338
38799	1.89227	0.744923

38800	1.89227	0.630078
38801	1.89227	0.665599
38802	1.89227	0.547911
38803	3.78453	0.687971
38804	1.89227	0.836716
38805	1.89227	0.54228
38806	1.89227	0.538281
38807	1.89227	0.633754
38808	1.89227	0.658193
38809	1.89227	0.864902
38810	2.50912	0.510009
38811	1.89226	0.590078
38812	1.89226	0.476508
38813	2.31261	0.548687
38814	2.31261	0.560131
38815	1.89226	0.631365
38816	2.50912	0.513651
38817	1.19999	0.500343
38818	1.44056	0.461138
38819	1.24405	0.495756
38820	2.50912	0.512734
38821	2.31261	0.552448
38822	2.69224	0.726788
38823	2.28373	0.603491
38824	1.94788	1.21243
38825	2.4106	0.608067
38826	4.69434	0.843322
38827	2.69223	0.525208
38828	2.63783	0.693913
38829	2.34737	1.03772
38830	2.4106	0.657785
38831	5.04843	0.735518
38832	1.89226	2.1
38833	1.99753	2.1
38834	1.87366	2.1
38835	1.99753	2.1
38836	1.76839	2.1
38837	1.89226	2.1
38838	1.89226	2.1
38839	1.89226	0.812503
38840	1.89226	2.1
38841	1.89226	2.1
38842	3.78452	2.1
38843	0.946132	2.1
38844	0.946132	2.1
38845	1.02744	2.1
38846	1.02744	2.1
38847	0.946132	2.1
38848	0.946132	2.1
38849	1.00989	2.1
38850	1.02744	2.1
38851	1.00989	2.1
38852	0.946133	2.1
38853	1.02744	2.1
38854	0.946132	2.1
38855	1.70302	2.1
38856	1.87366	2.1
38857	1.71618	2.1

38858	1.87366	2.1
38859	1.73105	2.1
38860	1.89226	2.0289
38861	1.89226	2.07013
38862	1.89226	2.1
38863	1.89226	2.1
38864	1.89226	2.1
38865	1.89226	2.1
38866	1.89226	0.798488
38867	1.96624	2.1
38868	1.89226	2.1
38869	1.89226	2.1
38870	1.89226	2.1
38871	2.01977	2.1
38872	2.01977	2.1
38873	1.1032	1.1374
38874	1.15114	2.1
38875	1.14986	0.520447
38876	1.19242	2.1
38877	2.301	1.53046
38878	1.89226	1.24327
38879	1.89226	2.1
38880	1.89226	0.588052
38881	1.89226	1.62949
38882	3.78453	0.984687
38883	0.946132	2.1
38884	0.946132	1.70587
38885	0.946132	0.842766
38886	0.946132	2.1
38887	0.946133	2.1
38888	0.946133	2.1
38889	0.946132	1.31292
38890	0.946132	1.0198
38891	0.946132	2.1
38892	0.946132	2.1
38893	0.946133	2.1
38894	0.946133	2.1
38895	1.19243	1.55676
38896	0.953928	2.1
38897	1.18671	2.1
38898	1.15114	1.3012
38899	0.9895	2.1
38900	0.953928	2.02787
38901	1.19611	0.586261
38902	1.19242	2.1
38903	1.24693	0.56276
38904	1.24693	1.70383
38905	1.14986	0.712322
38906	1.14986	2.1
38907	1.89225	0.628003
38908	1.89225	2.1
38909	1.89225	2.1
38910	1.89225	0.848806
38911	1.89225	1.2345
38912	1.89225	0.942807
38913	1.89225	1.11392
38914	1.89225	2.1
38915	1.89225	2.1

38916	1.89225	2.1
38917	1.89225	0.775219
38918	1.89225	2.1
38919	0.561532	2.1
38920	0.561532	1.06485
38921	0.566382	0.953982
38922	0.566382	2.1
38923	0.494747	2.1
38924	0.531705	2.1
38925	0.596208	1.4295
38926	0.623464	0.832566
38927	0.623469	2.1
38928	0.531708	2.1
38929	0.596205	2.1
38930	0.494743	2.1
38931	1.25742	2.1
38932	1.18783	2.1
38933	0.946131	2.1
38934	0.946131	2.1
38935	1.25742	2.1
38936	1.25742	2.1
38937	0.946131	2.1
38938	0.946131	2.1
38939	1.25742	2.1
38940	0.946131	2.1
38941	0.946131	2.1
38942	1.18783	2.1
38943	1.35048	2.1
38944	1.25742	2.1
38945	0.946132	2.1
38946	0.946132	2.1
38947	1.35048	2.1
38948	1.35048	2.1
38949	0.946132	2.1
38950	0.946132	2.1
38951	1.35044	2.1
38952	0.946106	2.1
38953	1.25745	2.1
38954	0.946158	2.1
38955	1.13525	2.1
38956	2.70095	2.1
38957	2.51483	2.1
38958	2.51483	2.1
38959	1.21593	2.1
38960	1.21593	2.1
38961	1.93142	0.536065
38962	2.8053	0.14886
38963	1.559	0.475649
38964	2.60359	0.21487
38965	4.53501	0.335541
38966	2.80531	0.299751
38967	1.89226	0.226497
38968	2.60359	0.350303
38969	1.89226	0.515182
38970	4.49586	0.215314
38971	1.22938	0.453742
38972	2.60359	0.166155
38973	2.37434	0.53722

38974	2.37434	0.412846
38975	1.55901	0.537631
38976	1.55901	0.161091
38977	1.18717	0.508758
38978	1.18717	0.147722
38979	0.946132	0.261554
38980	0.946132	0.398334
38981	0.946133	0.408523
38982	0.946133	0.506679
38983	1.3018	0.262532
38984	1.18717	0.510547
38985	1.18714	0.402495
38986	0.946107	0.415305
38987	1.30183	0.367628
38988	0.946158	0.159997
38989	1.89226	2.1
38990	2.80196	2.1
38991	2.67272	2.1
38992	2.67272	2.1
38993	1.89226	2.1
38994	1.89226	2.1
38995	1.89226	2.1
38996	1.89226	2.1
38997	1.89226	2.1
38998	1.89226	2.1
38999	1.89226	2.1
39000	1.89226	2.1
39001	1.74471	2.1
39002	2.01555	2.1
39003	1.61121	2.1
39004	1.61121	2.1
39005	2.13878	2.1
39006	2.13878	2.1
39007	1.89226	2.1
39008	1.89226	2.1
39009	1.89226	2.1
39010	1.89226	2.1
39011	1.89226	2.1
39012	1.89226	2.1
39013	1.09766	2.1
39014	1.04263	2.1
39015	0.881794	2.1
39016	0.894188	2.1
39017	0.795402	2.1
39018	0.99524	2.1
39019	1.00647	2.1
39020	1.00647	2.1
39021	1.15401	2.1
39022	0.881789	2.1
39023	0.850526	2.1
39024	1.04263	2.1
39025	1.4054	0.979956
39026	1.04788	2.1
39027	1.70302	1.00548
39028	1.41375	2.1
39029	2.82706	1.4258
39030	1.70302	0.94418
39031	1.38776	2.1

39032	1.59457	1.33725
39033	1.41375	2.1
39034	1.77261	0.890432
39035	1.70302	2.1
39036	1.89226	2.1
39037	1.89226	2.1
39038	1.89226	2.1
39039	1.89226	2.1
39040	3.78453	2.1
39041	2.18042	2.1
39042	1.74738	2.1
39043	2.08433	2.1
39044	1.66347	2.1
39045	3.83171	2.1
39046	1.74738	2.1
39047	1.1181	2.1
39048	1.66347	2.1
39049	1.06543	2.1
39050	2.81281	2.1
39051	1.89226	2.1
39052	1.89226	2.1
39053	1.89226	2.1
39054	1.89226	2.1
39055	3.78453	2.1
39056	1.89227	2.1
39057	1.89227	2.1
39058	1.89227	2.1
39059	1.89227	2.1
39060	3.78453	2.1
39061	2.08433	2.1
39062	1.66347	2.1
39063	1.97092	2.1
39064	1.57431	2.1
39065	3.65864	2.1
39066	1.66347	2.1
39067	1.01474	2.1
39068	1.01474	2.1
39069	1.06544	2.1
39070	1.57431	2.1
39071	1.625 2.1	
39072	1.89226	2.1
39073	1.89226	2.1
39074	1.89226	2.1
39075	1.89226	2.1
39076	1.89226	2.1
39077	1.89226	2.1
39078	1.08467	2.1
39079	1.87228	2.1
39080	1.73305	2.1
39081	1.73305	2.1
39082	1.25565	2.1
39083	1.25565	2.1
39084	0.695361	0.506053
39085	0.799996	0.662959
39086	1.13751	0.782319
39087	1.40945	0.802483
39088	1.40944	0.899745
39089	0.799999	0.225269

39090	1.13751	0.66703
39091	0.695363	0.367239
39092	1.01059	0.52731
39093	1.01059	0.592361
39094	0.926908	0.939435
39095	0.926909	0.389138
39096	1.05481	2.1
39097	1.78069	1.95581
39098	1.08074	2.1
39099	1.83284	2.1
39100	2.86143	2.1
39101	1.78069	2.1
39102	2.22044	1.38756
39103	1.83284	2.1
39104	2.28696	0.931365
39105	4.06766	1.47137
39106	1.07196	2.1
39107	1.64955	2.1
39108	2.02665	2.1
39109	2.0284	2.1
39110	1.08074	2.1
39111	1.08074	2.1
39112	1.64955	2.1
39113	2.28696	1.15618
39114	1.92033	2.1
39115	2.1004	1.70362
39116	3.74988	1.97386
39117	2.83787	0.592636
39118	1.70303	1.09223
39119	1.70303	0.557873
39120	1.83195	0.565982
39121	2.64075	0.553033
39122	2.70231	0.965301
39123	1.2087	1.67496
39124	1.52909	0.712325
39125	1.43063	0.696893
39126	1.10586	1.22375
39127	2.63494	0.869698
39128	1.89227	0.828652
39129	2.10732	2.1
39130	2.10732	0.926271
39131	2.2365	2.1
39132	1.89227	0.882388
39133	2.02145	2.1
39134	1.89226	0.724967
39135	1.89226	1.05911
39136	1.89226	0.713975
39137	1.89226	1.03638
39138	1.89226	0.678117
39139	1.89226	1.39484
39140	0.51926	1.22051
39141	0.552927	1.37749
39142	0.691235	0.785776
39143	0.715314	0.667107
39144	0.619684	0.694571
39145	0.619684	1.6594
39146	0.624477	2.0158
39147	0.624477	0.706438

39148	0.519262	0.875985
39149	0.691234	0.921724
39150	0.715313	0.930817
39151	0.552928	0.917578
39152	1.89226	0.931624
39153	1.87321	2.1
39154	1.87321	1.2753
39155	1.89658	2.1
39156	2.0815	0.935257
39157	2.14789	2.1
39158	0.946128	1.43729
39159	0.946128	1.07058
39160	0.946133	0.673498
39161	0.946133	0.656725
39162	0.94613	0.641361
39163	0.94613	1.00665
39164	0.946131	0.723489
39165	0.946131	1.24287
39166	0.946131	0.758039
39167	0.946131	0.763514
39168	0.946131	0.858393
39169	0.946131	0.854473
39170	1.99297	0.975113
39171	2.19514	0.704222
39172	2.46153	0.85429
39173	2.187	0.839948
39174	4.45451	0.859392
39175	2.0815	0.777176
39176	2.33402	0.728871
39177	2.46154	0.881199
39178	2.83046	0.631034
39179	4.94496	0.73755
39180	2.46153	0.808409
39181	2.187	0.76232
39182	2.27351	0.8696
39183	2.63356	0.667571
39184	4.73504	0.772577
39185	2.46154	0.833983
39186	2.83447	0.580314
39187	2.74513	0.709707
39188	2.5731	0.791989
39189	2.63357	0.679253
39190	2.46154	0.742438
39191	2.80398	2.1
39192	2.60406	2.1
39193	2.73988	2.1
39194	2.57466	2.1
39195	5.34394	2.1
39196	1.89226	0.986046
39197	1.89226	0.750904
39198	1.89226	0.59779
39199	1.89226	1.09891
39200	1.89226	0.661903
39201	1.89226	1.67969
39202	1.89224	1.12712
39203	1.89224	0.77798
39204	1.89224	0.681593
39205	1.89224	1.34856

39206	1.89224	0.767322
39207	1.89224	1.74647
39208	1.40245	1.01466
39209	1.40246	1.72862
39210	0.946117	1.12368
39211	0.946117	1.81794
39212	1.17635	0.86962
39213	1.40246	1.74645
39214	0.946112	0.815081
39215	0.946112	1.56546
39216	0.946127	1.15407
39217	1.17637	0.864051
39218	0.946102	1.77411
39219	1.40244	1.02823
39220	2.53269	2.1
39221	1.70302	2.1
39222	1.56194	2.1
39223	2.17516	2.1
39224	4.19807	2.1
39225	1.89226	2.1
39226	1.89226	0.780087
39227	1.89226	2.1
39228	1.89226	1.87943
39229	3.78453	2.1
39230	2.57276	2.1
39231	1.89226	2.1
39232	1.89226	2.1
39233	1.89226	2.1
39234	2.32525	2.1
39235	2.32525	2.1
39236	1.15171	2.1
39237	2.17517	2.1
39238	1.41995	2.1
39239	1.41995	2.1
39240	1.80252	2.1
39241	1.80252	2.1
39242	1.89226	2.1
39243	1.89226	2.1
39244	1.89226	2.1
39245	1.89226	0.692258
39246	3.78453	2.1
39247	0.946133	2.1
39248	0.946133	2.1
39249	1.16263	2.1
39250	1.04687	2.1
39251	0.946133	2.1
39252	0.946133	2.02483
39253	1.04687	2.1
39254	1.04687	2.1
39255	0.946133	2.1
39256	1.04687	2.1
39257	0.946132	2.1
39258	1.16263	2.1
39259	0.682281	1.66745
39260	0.669233	2.04858
39261	1.21859	2.08971
39262	1.31629	1.30645
39263	0.916158	1.14137

39264	0.916158	2.1
39265	0.971658	1.63324
39266	0.971658	1.7485
39267	0.669234	1.77689
39268	1.31629	1.36966
39269	0.682283	1.6747
39270	1.21858	1.87715
39271	0.669232	1.32954
39272	0.623495	1.82619
39273	1.36428	1.62005
39274	1.31629	1.00542
39275	0.946475	0.924985
39276	0.946476	2.07464
39277	0.99331	1.49945
39278	0.99331	1.46182
39279	1.36428	1.47604
39280	0.623497	1.09695
39281	0.669234	1.46762
39282	1.31629	1.17285
39283	2.02069	0.05
39284	1.46253	0.05
39285	2.51479	0.05
39286	1.87136	0.05
39287	3.89203	0.05
39288	1.46253	0.05
39289	1.38536	0.05
39290	1.38536	0.05
39291	1.05579	0.05
39292	1.73281	0.05
39293	1.46916	0.05
39294	2.51479	0.05
39295	1.87136	0.05
39296	2.23338	0.05
39297	2.15603	0.05
39298	4.55146	0.05
39299	0.822553	0.05
39300	0.822563	0.05
39301	0.842935	0.05
39302	0.842935	0.05
39303	0.744203	0.05
39304	0.675346	0.05
39305	0.874795	0.05
39306	1.00453	0.05
39307	1.00454	0.05
39308	0.782417	0.05
39309	0.675337	0.05
39310	0.858007	0.05
39311	0.946132	1.22598
39312	1.24847	0.809215
39313	0.946132	1.05745
39314	1.16806	1.28477
39315	1.24847	0.715686
39316	1.24847	1.80461
39317	0.946133	0.683904
39318	0.946133	1.5919
39319	0.946133	0.941888
39320	0.946133	0.737198
39321	1.16805	1.34782

39322	1.24847	0.834943
39323	1.2364	1.31282
39324	0.946129	0.933411
39325	0.946133	1.29972
39326	1.24847	0.923263
39327	1.2364	0.859609
39328	1.2364	1.5894
39329	0.946131	0.725923
39330	0.946131	1.91197
39331	0.946131	1.10378
39332	0.946131	0.861
39333	1.2364	1.60554
39334	1.24847	0.929634
39335	1.89226	1.31685
39336	1.89226	1.20397
39337	1.89226	0.785876
39338	1.89226	0.868093
39339	1.89226	1.17435
39340	1.89226	1.50163
39341	1.89226	1.1206
39342	1.89226	0.884492
39343	1.89226	0.776637
39344	1.89226	1.2408
39345	1.89226	0.857181
39346	1.89226	1.37454
39347	1.69014	0.981941
39348	1.09194	1.33372
39349	1.13537	1.49036
39350	1.13537	1.36614
39351	1.58481	0.805058
39352	1.58481	0.950845
39353	1.58147	0.902139
39354	1.13537	1.01016
39355	1.69015	1.09747
39356	1.01179	0.782736
39357	1.69015	1.01365
39358	1.01179	1.34628
39359	0.946132	1.83963
39360	0.946132	1.11505
39361	1.28543	0.757276
39362	1.07341	1.81767
39363	1.07341	1.62978
39364	1.07341	0.636307
39365	0.946132	0.973296
39366	0.946132	0.807388
39367	0.946133	0.839922
39368	1.28543	0.846986
39369	1.07341	0.842775
39370	0.946132	0.995122
39371	1.89226	0.904361
39372	1.89226	0.729008
39373	1.89226	0.6691
39374	1.89226	1.64368
39375	1.89226	0.676448
39376	1.89226	2.1
39377	0.805744	2.089
39378	0.988659	0.986935
39379	0.782044	0.685635

39380	0.578223	0.99657
39381	0.77823	0.738969
39382	0.778231	2.1
39383	0.788653	0.648142
39384	0.788653	1.26762
39385	0.988662	0.99529
39386	0.782042	0.949566
39387	0.805745	1.07201
39388	0.578222	0.965154
39389	1.89226	0.930364
39390	1.89226	0.919783
39391	1.89226	0.697678
39392	1.89226	1.81236
39393	1.89226	0.635909
39394	1.89226	2.1
39395	1.97732	1.23433
39396	1.97819	0.81283
39397	1.56408	0.716385
39398	1.56408	1.58517
39399	2.33986	0.827457
39400	2.33986	2.03003
39401	1.84516	2.1
39402	2.21152	2.1
39403	1.90702	2.1
39404	1.54599	2.1
39405	3.75751	2.1
39406	1.89224	2.1
39407	1.89224	2.1
39408	1.89224	2.1
39409	1.89224	2.06242
39410	1.89224	2.1
39411	1.89224	1.53251
39412	1.47672	2.1
39413	1.54601	2.1
39414	1.27697	2.1
39415	1.54601	2.1
39416	1.638	2.1
39417	1.90705	2.1
39418	0.946132	1.14205
39419	0.946132	2.1
39420	0.946131	2.1
39421	0.946131	2.1
39422	0.946131	2.1
39423	0.946131	1.30189
39424	0.946132	2.1
39425	0.946132	2.1
39426	0.946132	2.1
39427	0.946132	2.1
39428	0.946131	2.1
39429	0.946131	2.1
39430	0.946128	2.1
39431	0.946128	2.1
39432	1.32062	2.1
39433	1.4164	2.1
39434	0.946129	2.1
39435	0.946129	2.1
39436	1.32062	2.1
39437	1.32062	2.1

39438	1.32062	2.1
39439	0.94613	2.1
39440	1.41639	2.1
39441	0.946129	2.1
39442	1.98979	0.05
39443	1.65977	0.05
39444	2.21772	0.05
39445	2.21772	0.05
39446	1.48866	0.05
39447	1.48866	0.05
39448	0.973129	0.05
39449	0.973129	0.05
39450	0.980954	0.05
39451	0.980954	0.05
39452	0.845218	0.05
39453	1.13981	0.05
39454	0.829886	0.05
39455	1.10886	0.05
39456	1.10886	0.05
39457	1.13982	0.05
39458	0.845219	0.05
39459	0.829886	0.05
39460	0.946132	0.790314
39461	0.946132	1.65625
39462	1.3352	2.1
39463	1.41432	2.1
39464	0.946133	2.1
39465	0.946133	0.646323
39466	1.3352	1.52295
39467	1.3352	2.1
39468	1.3352	1.78371
39469	0.946132	1.92828
39470	1.41432	1.83834
39471	0.946133	1.92801
39472	2.70097	2.1
39473	1.88175	2.1
39474	1.51482	2.1
39475	1.88175	2.1
39476	2.37784	2.1
39477	2.82864	2.1
39478	1.89227	0.248471
39479	1.89227	0.185565
39480	1.89227	0.151586
39481	1.89227	0.125054
39482	3.78453	0.124555
39483	1.89226	0.149414
39484	2.13295	0.0946417
39485	2.43203	0.11623
39486	2.43203	0.147007
39487	1.89226	0.101489
39488	1.89226	0.140027
39489	0.946133	0.0964773
39490	0.946133	0.138951
39491	0.946132	0.0812237
39492	0.946132	0.124235
39493	0.946132	0.0841291
39494	0.946132	0.0936611
39495	0.946133	0.0931623

39496	0.946133	0.12153
39497	0.946132	0.0857742
39498	0.946132	0.111293
39499	0.946132	0.153676
39500	0.946132	0.136278
39501	1.54987	2.1
39502	1.24128	2.1
39503	1.24128	2.1
39504	1.50738	2.1
39505	1.75702	2.1
39506	2.02312	2.1
39507	1.89289	2.1
39508	1.92264	2.1
39509	1.72994	2.1
39510	1.75702	2.1
39511	3.67966	2.1
39512	1.75702	2.1
39513	0.994291	2.1
39514	0.994291	2.1
39515	1.24128	2.1
39516	1.4964	2.1
39517	1.74339	2.1
39518	1.72993	2.1
39519	1.49641	2.1
39520	1.49641	2.1
39521	1.75702	2.1
39522	1.3939	2.1
39523	1.65452	2.1
39524	1.19964	2.1
39525	1.53296	2.1
39526	1.19964	2.1
39527	1.36861	2.1
39528	1.42624	2.1
39529	1.59521	2.1
39530	1.19964	2.1
39531	1.42624	2.1
39532	1.07156	2.1
39533	1.36226	2.1
39534	2.5619	2.1
39535	2.14058	1.07171
39536	1.93569	2.1
39537	2.36221	1.2788
39538	2.5753	1.26242
39539	4.50279	2.01314
39540	2.14057	1.6829
39541	2.20831	2.1
39542	2.64725	0.968728
39543	2.57529	1.73431
39544	4.78782	1.60998
39545	2.64725	1.12448
39546	2.57529	1.92821
39547	1.89226	0.987708
39548	1.89226	0.840572
39549	4.53951	1.51471
39550	2.0815	2.1
39551	1.86137	2.1
39552	1.86137	2.1
39553	1.86159	2.1

39554	2.0815	2.1
39555	2.10235	2.1
39556	1.33552	2.1
39557	2.32417	2.1
39558	1.18872	2.1
39559	1.93101	2.1
39560	3.26653	2.1
39561	1.89226	2.1
39562	1.89226	2.1
39563	1.89226	2.1
39564	1.89226	2.1
39565	1.89226	2.1
39566	1.89226	2.1
39567	0.955191	2.1
39568	1.03372	2.1
39569	0.946131	2.1
39570	0.946131	2.1
39571	0.930654	2.1
39572	0.930654	2.1
39573	1.04233	2.1
39574	1.05247	2.1
39575	0.955341	2.1
39576	0.946131	2.1
39577	1.02375	2.1
39578	1.13537	2.1
39579	1.1549	2.1
39580	1.93101	2.1
39581	1.91108	2.1
39582	1.52487	2.1
39583	1.3076	2.1
39584	1.06985	2.1
39585	1.89226	2.1
39586	1.89226	2.1
39587	1.89226	2.1
39588	1.89226	2.1
39589	1.89226	2.1
39590	1.89226	2.1
39591	1.58713	2.1
39592	2.6845	2.1
39593	1.58742	2.1
39594	2.74201	2.1
39595	4.27191	2.1
39596	2.68449	2.1
39597	1.89226	2.1
39598	2.74201	2.1
39599	1.89226	2.1
39600	4.57675	2.1
39601	1.57523	2.1
39602	2.74201	2.1
39603	2.8146	2.1
39604	2.8146	2.1
39605	1.58742	2.1
39606	1.58742	2.1
39607	0.946131	1.97759
39608	0.946131	2.1
39609	1.371	2.1
39610	1.371	2.1
39611	0.946127	2.1

39612	0.946127	1.24105
39613	1.37101	2.1
39614	1.4073	2.1
39615	0.94613	2.1
39616	1.4073	2.1
39617	1.371	2.1
39618	0.946129	2.1
39619	1.44801	1.94087
39620	1.06821	1.68443
39621	2.00542	1.77659
39622	2.27703	2.0121
39623	3.34524	1.56942
39624	1.70301	0.428252
39625	1.89226	0.618351
39626	1.65809	0.463966
39627	1.89226	0.577657
39628	1.62758	0.468221
39629	1.85744	0.558465
39630	1.89226	0.415926
39631	1.89226	0.53107
39632	1.89226	0.491704
39633	1.89226	0.47488
39634	3.78452	0.49203
39635	1.89226	0.534772
39636	1.89226	0.462334
39637	1.89226	0.47102
39638	1.89226	0.606514
39639	3.78452	0.501204
39640	0.851541	0.631262
39641	0.851541	0.610792
39642	0.91193	0.526174
39643	0.824837	0.551461
39644	0.854559	0.538565
39645	0.851534	0.782273
39646	0.81851	0.61222
39647	0.717219	0.456455
39648	0.871743	0.604669
39649	0.837985	0.543244
39650	0.82902	0.540465
39651	0.82902	0.629137
39652	1.89226	0.56218
39653	1.92659	0.563203
39654	2.03235	0.575285
39655	1.89226	0.670407
39656	2.03235	0.485668
39657	1.89226	0.552676
39658	0.585557	0.745556
39659	0.585557	0.6373
39660	1.22612	0.626798
39661	1.09436	0.737241
39662	0.851537	0.845295
39663	0.526987	0.681442
39664	0.983879	0.57332
39665	1.04072	0.679993
39666	0.585526	0.649193
39667	0.792325	0.644325
39668	0.946129	0.671246
39669	0.946129	0.681203

39670	1.68152	2.1
39671	1.81445	2.1
39672	1.68152	2.1
39673	1.86792	2.1
39674	1.65888	2.1
39675	1.84529	2.1
39676	1.81445	2.1
39677	1.57885	2.1
39678	1.65889	2.1
39679	1.48594	2.1
39680	3.23773	2.1
39681	1.68154	2.1
39682	1.65891	2.1
39683	1.53296	2.1
39684	1.56299	2.1
39685	3.24453	2.1
39686	1.56299	2.1
39687	1.48596	2.1
39688	1.56299	2.1
39689	1.65891	2.1
39690	1.46568	2.1
39691	1.5616	2.1
39692	2.68785	2.1
39693	2.08831	2.1
39694	2.66919	2.1
39695	2.1024	2.1
39696	4.79025	2.1
39697	2.08832	2.1
39698	1.15478	2.1
39699	1.15478	2.1
39700	1.09062	2.1
39701	2.10241	2.1
39702	2.03825	2.1
39703	2.65348	2.1
39704	2.1024	2.1
39705	2.12313	2.1
39706	2.12313	2.1
39707	2.66919	2.1
39708	2.66919	2.1
39709	0.813169	2.1
39710	0.813169	2.1
39711	0.825788	2.1
39712	0.825788	2.1
39713	0.612902	2.1
39714	0.577387	2.1
39715	1.0512	2.1
39716	1.06157	2.1
39717	1.06157	2.1
39718	0.612902	2.1
39719	0.577387	2.1
39720	1.0512	2.1
39721	1.27227	2.1
39722	1.91724	2.1
39723	1.32591	2.1
39724	1.99837	2.1
39725	3.24315	2.1
39726	1.89223	2.1
39727	1.89223	2.1

39728	1.89223	2.1
39729	1.89223	1.19135
39730	3.78446	2.1
39731	1.89224	2.1
39732	1.89224	0.907372
39733	1.89224	1.88429
39734	1.89224	1.83708
39735	3.78447	1.78095
39736	1.91724	2.1
39737	2.321	2.1
39738	1.99838	2.1
39739	2.4149	1.73695
39740	4.33214	2.1
39741	1.34817	2.1
39742	1.9984	2.1
39743	2.06238	2.1
39744	2.06238	2.1
39745	1.32593	2.1
39746	1.32593	2.1
39747	1.89226	2.1
39748	1.89226	1.06036
39749	1.89226	2.1
39750	1.89226	2.1
39751	1.89226	2.1
39752	1.89226	2.1
39753	1.89226	1.80012
39754	1.89226	1.94402
39755	1.89226	2.1
39756	1.89226	0.865179
39757	3.78452	1.87872
39758	1.9984	2.1
39759	2.41494	1.84658
39760	2.06239	2.1
39761	2.5194	2.1
39762	4.47732	2.1
39763	1.19901	0.931233
39764	1.16661	2.1
39765	1.56859	2.1
39766	1.61029	1.15634
39767	2.7676	2.01796
39768	1.56859	2.1
39769	1.88637	0.776195
39770	1.61029	0.691714
39771	1.61029	1.98572
39772	1.856	0.70023
39773	1.856	1.98534
39774	1.856	0.772689
39775	1.57916	2.1
39776	1.56859	0.859834
39777	1.56859	2.1
39778	1.57916	1.3526
39779	1.23475	2.1
39780	0.890924	0.328131
39781	1.07608	0.599927
39782	0.72877	0.570703
39783	0.910106	0.606496
39784	1.00196	0.476498
39785	1.09519	0.784855

39786	0.839967	0.583601
39787	0.719043	0.374726
39788	0.893859	0.365462
39789	0.942408	0.73731
39790	0.873497	0.600736
39791	0.873497	0.834665
39792	1.53046	0.21221
39793	1.89994	0.649324
39794	1.049	0.229663
39795	1.049	0.223446
39796	1.52512	0.231056
39797	1.89994	0.406093
39798	1.65573	0.7935
39799	1.89226	0.640403
39800	1.89226	0.665357
39801	2.73329	1.04893
39802	3.548	0.908151
39803	1.65573	0.662095
39804	2.42671	0.811572
39805	2.73329	0.625279
39806	1.89226	0.575203
39807	2.73329	1.00843
39808	1.89226	0.926738
39809	0.941078	0.621703
39810	1.01599	0.667946
39811	0.818936	0.584118
39812	0.651554	0.618052
39813	0.904729	0.635194
39814	0.665756	0.631849
39815	0.843423	0.713936
39816	0.918649	0.743118
39817	0.855806	0.801259
39818	0.814384	0.452762
39819	0.844437	0.699897
39820	0.844437	0.663084
39821	2.22558	1.02886
39822	1.95785	0.676321
39823	2.07065	0.795064
39824	1.89142	0.739069
39825	2.07065	1.63245
39826	1.89142	1.31838
39827	0.640813	0.734385
39828	0.951654	0.865065
39829	0.797442	0.600138
39830	0.535364	0.65787
39831	0.525269	0.703934
39832	0.620874	0.664637
39833	0.916953	0.794026
39834	0.969374	0.775738
39835	0.788925	1.13564
39836	0.69596	0.371111
39837	0.743154	0.706622
39838	0.743154	0.760504
39839	1.89226	1.69458
39840	1.89226	1.38952
39841	1.89226	0.794157
39842	1.89226	2.1
39843	3.78452	1.00975

39844	1.32541	1.49091
39845	1.13722	1.07053
39846	0.91103	2.1
39847	0.881123	2.02342
39848	1.03363	2.1
39849	1.03364	1.73232
39850	1.1505	1.54118
39851	1.1505	2.07112
39852	1.32539	1.86738
39853	1.07662	2.1
39854	0.881121	1.93696
39855	0.962303	1.61398
39856	1.89226	0.898687
39857	1.89226	2.1
39858	1.89226	1.96422
39859	1.89226	1.02901
39860	3.78453	1.88558
39861	1.89226	0.763503
39862	1.89226	2.0853
39863	1.89226	1.21444
39864	1.89226	1.10596
39865	1.89226	1.31435
39866	1.89226	1.45993
39867	1.27606	1.62778
39868	1.07664	1.11922
39869	0.905895	2.1
39870	0.696099	2.1
39871	0.984902	2.1
39872	0.984905	1.42919
39873	1.01878	2.1
39874	1.01878	0.811052
39875	1.27604	1.98118
39876	1.06044	2.1
39877	1.13192	2.00217
39878	0.696111	1.98483
39879	1.89226	0.514839
39880	1.89226	0.154698
39881	1.89226	0.182098
39882	1.89226	0.518988
39883	1.89226	0.228574
39884	1.89226	0.506233
39885	1.52615	0.532938
39886	1.01294	0.128651
39887	1.01294	0.621703
39888	1.18549	0.343691
39889	1.38963	0.441466
39890	1.56218	0.24134
39891	1.89226	0.410605
39892	2.40801	0.475925
39893	2.58101	0.526429
39894	2.58101	0.401168
39895	1.89226	0.538666
39896	1.89226	0.366743
39897	2.24071	2.1
39898	2.79317	1.00498
39899	1.99136	2.1
39900	2.6167	0.952363
39901	4.85741	2.1

39902	2.79317	1.27059
39903	1.89226	0.318842
39904	1.89226	2.1
39905	1.89226	0.635369
39906	2.6167	1.25817
39907	2.6167	0.604479
39908	1.08354	1.06675
39909	1.08354	2.1
39910	1.10222	2.1
39911	1.10222	1.53018
39912	1.2275	1.34575
39913	1.30835	1.05712
39914	0.995678	2.1
39915	0.877412	2.1
39916	0.877411	2.1
39917	1.2275	2.1
39918	0.995678	2.1
39919	1.30835	2.1
39920	1.05793	0.751432
39921	1.46441	0.652678
39922	1.0562	0.668025
39923	1.0562	0.715686
39924	1.41787	0.729821
39925	1.41787	0.717629
39926	1.41787	0.665464
39927	1.52909	0.873879
39928	1.46441	0.622031
39929	1.46441	0.883245
39930	1.43063	0.611958
39931	1.43063	0.836596
39932	1.89226	0.888309
39933	1.89226	0.570581
39934	1.89226	0.633953
39935	1.89226	0.866446
39936	3.78453	0.735911
39937	1.89227	0.744432
39938	1.89227	0.637292
39939	1.89227	0.627632
39940	1.89227	0.709277
39941	1.89227	0.619388
39942	1.89227	0.680917
39943	1.00763	0.644516
39944	1.41787	0.71558
39945	1.05793	0.629202
39946	1.05793	0.791978
39947	1.37265	0.657991
39948	1.37265	0.766489
39949	1.37265	0.655729
39950	1.43063	0.773065
39951	1.41787	0.634558
39952	1.41787	0.963047
39953	1.38247	0.612978
39954	1.38247	0.881225
39955	0.946132	0.767256
39956	0.946132	0.697155
39957	0.946132	0.632906
39958	0.946132	0.554685
39959	0.946131	0.557752

39960	0.946131	0.696764
39961	0.946132	0.805417
39962	0.946132	0.652864
39963	0.946132	0.663707
39964	0.946132	0.673836
39965	0.946132	0.654449
39966	0.946132	0.625131
39967	1.89226	0.673886
39968	1.89226	0.649813
39969	1.89226	0.607873
39970	1.89226	0.776761
39971	1.89226	0.596771
39972	1.89226	0.728925
39973	1.19645	0.901456
39974	1.89183	0.573073
39975	1.91106	0.746556
39976	1.35306	0.614428
39977	1.75446	0.765915
39978	1.19645	0.618926
39979	2.37576	0.506296
39980	1.90139	0.76419
39981	2.1845	2.1
39982	1.86568	0.629155
39983	4.24145	0.741814
39984	2.29435	0.656247
39985	1.40602	1.93926
39986	1.40602	0.563135
39987	1.55351	0.696908
39988	1.86568	0.73148
39989	2.01317	2.1
39990	2.18452	1.78346
39991	1.8657	0.405371
39992	2.01399	0.521829
39993	1.70141	1.4698
39994	3.87969	0.53129
39995	0.774999	2.1
39996	0.774999	0.541039
39997	0.778721	0.679214
39998	0.778721	2.04997
39999	0.628348	1.399
40000	0.703016	0.768733
40001	0.932848	0.573869
40002	0.850704	0.62972
40003	0.850704	0.7125
40004	0.628348	0.725355
40005	0.703015	0.588145
40006	0.932848	0.60865
40007	1.65243	0.426995
40008	1.34412	0.392768
40009	1.45569	0.500532
40010	1.1315	0.564307
40011	2.78393	0.414552
40012	1.48901	2.1
40013	1.805 0.636923	
40014	1.48901	0.667239
40015	1.71717	2.1
40016	1.60635	0.601338
40017	1.83451	2.1

40018	1.805	0.677136
40019	1.65243	0.417145
40020	1.60635	0.645688
40021	1.45569	0.458506
40022	3.25879	0.511365
40023	1.27875	2.1
40024	1.60635	0.646406
40025	1.27875	0.728238
40026	1.48901	2.1
40027	1.40014	0.653801
40028	1.6104	2.1
40029	0.715199	0.502253
40030	0.715199	0.708966
40031	0.712714	0.694835
40032	0.712714	0.432947
40033	0.727843	0.422536
40034	0.619713	0.430493
40035	0.803177	0.671093
40036	0.70007	0.653719
40037	0.700069	0.548537
40038	0.619713	0.54189
40039	0.803177	0.511415
40040	0.727844	0.511557
40041	1.14357	2.1
40042	2.3506	2.07836
40043	1.14357	2.1
40044	2.68773	2.01618
40045	1.57829	2.1
40046	2.68773	2.01097
40047	2.00551	2.1
40048	2.65811	1.16778
40049	2.07203	2.1
40050	2.72117	0.487295
40051	4.72667	1.19633
40052	0.946131	0.778583
40053	0.946131	1.93025
40054	1.32905	1.97668
40055	1.32905	0.696195
40056	0.94613	1.09151
40057	0.94613	1.30455
40058	1.32905	1.25106
40059	1.36058	1.06429
40060	1.36058	0.632998
40061	0.946131	0.59647
40062	1.32905	0.946396
40063	0.946131	1.43537
40064	1.21649	0.471088
40065	1.21649	2.1
40066	1.21459	2.0363
40067	1.21459	1.97334
40068	1.39508	0.927905
40069	1.36057	0.749199
40070	1.03601	1.54755
40071	1.07438	2.08705
40072	1.07439	0.985094
40073	1.39509	1.02316
40074	1.036	1.10088
40075	1.36056	0.928251

40076	2.79015	0.772618
40077	2.72114	2.1
40078	1.89225	0.818222
40079	1.89225	1.90821
40080	1.89225	1.33168
40081	2.72114	0.801959
40082	0.876258	2.1
40083	0.892889	2.1
40084	1.34352	2.1
40085	1.27985	2.1
40086	1.07462	2.1
40087	1.07462	2.1
40088	1.09812	2.1
40089	1.09812	2.1
40090	0.892888	2.1
40091	1.34352	2.1
40092	0.876256	2.1
40093	1.27985	2.1
40094	1.39725	1.60833
40095	1.30029	0.654674
40096	1.31415	0.778026
40097	1.41336	1.08889
40098	2.71366	0.89575
40099	1.89226	0.672408
40100	1.89226	0.819162
40101	1.89226	1.21109
40102	1.89226	0.634829
40103	3.78453	0.737438
40104	0.946133	0.846362
40105	0.946133	0.693736
40106	1.38709	0.53733
40107	1.39437	0.725303
40108	0.946132	0.5412
40109	0.946132	0.65814
40110	1.39437	0.912165
40111	1.39437	0.589276
40112	0.946132	0.652082
40113	1.39437	0.622629
40114	1.38709	0.63681
40115	0.946132	0.634553
40116	0.706681	1.27438
40117	0.711908	1.08882
40118	0.657077	0.678426
40119	0.661003	0.861413
40120	0.684188	0.685525
40121	0.684188	1.06104
40122	0.683496	1.79938
40123	0.683496	0.727809
40124	0.711908	0.875174
40125	0.661003	0.925284
40126	0.657076	0.912977
40127	0.706681	0.900035
40128	0.946132	1.14997
40129	0.946132	0.81872
40130	0.946132	0.711795
40131	0.946132	0.610491
40132	0.946132	0.615246
40133	0.946132	0.832005

40134	0.946133	0.735907
40135	0.946133	1.20895
40136	0.946133	0.760665
40137	0.946133	0.748239
40138	0.946132	0.783629
40139	0.946132	0.789804
40140	2.79029	0.536218
40141	1.89227	0.862097
40142	2.78873	0.634109
40143	2.78873	0.624844
40144	1.89383	0.67566
40145	1.89227	0.650083
40146	1.43669	2.1
40147	2.11984	2.1
40148	2.24481	2.1
40149	2.24481	2.1
40150	1.20311	2.1
40151	1.20311	2.1
40152	0.49703	2.1
40153	0.601553	2.1
40154	1.05992	2.1
40155	0.991694	2.1
40156	0.787558	2.1
40157	0.787558	2.1
40158	0.805689	2.1
40159	0.805689	2.1
40160	0.991694	2.1
40161	0.49703	2.1
40162	1.05992	2.1
40163	0.601553	2.1
40164	2.45905	2.1
40165	1.89226	2.1
40166	1.89226	2.1
40167	1.89226	2.0318
40168	2.47395	2.1
40169	2.47395	2.1
40170	1.89226	2.1
40171	1.89226	1.3582
40172	1.89226	2.1
40173	1.89226	1.02593
40174	3.78452	1.92061
40175	1.38427	2.1
40176	2.31203	0.930255
40177	2.28743	0.834467
40178	2.28743	2.1
40179	1.63384	0.777787
40180	1.63384	2.1
40181	2.42723	2.1
40182	1.89226	2.1
40183	1.89226	2.1
40184	1.89226	2.1
40185	2.45905	2.1
40186	2.45905	1.95249
40187	0.946131	0.674731
40188	0.946131	2.1
40189	0.946131	2.1
40190	0.946131	1.29783
40191	0.94613	1.11172

40192	0.94613	1.60656
40193	0.946132	2.1
40194	0.946132	1.61028
40195	0.946131	1.254
40196	0.946131	1.92496
40197	0.946131	1.69989
40198	0.946131	2.1
40199	0.989441	2.1
40200	0.989441	1.44594
40201	0.993918	2.1
40202	0.993918	1.87426
40203	1.16644	1.68193
40204	1.15601	0.948325
40205	0.818308	2.1
40206	0.816922	2.1
40207	0.818307	2.05125
40208	1.16644	2.1
40209	1.15601	2.1
40210	0.816922	1.34399
40211	0.996778	0.056605
40212	2.44375	0.053139
40213	0.996778	0.0812089
40214	1.92547	0.0512227
40215	1.56091	0.0825141
40216	2.4896	0.0515427
40217	1.56091	0.0806516
40218	2.44375	0.0522299
40219	1.90456	0.077032
40220	1.90456	0.0848243
40221	2.81853	0.058191
40222	2.47487	0.0580804
40223	1.32143	1.63055
40224	1.43294	2.09535
40225	1.37444	0.880649
40226	1.51691	1.85676
40227	2.80738	1.49968
40228	1.89226	1.27003
40229	1.89226	2.1
40230	1.89226	0.965261
40231	1.89226	1.42107
40232	3.78453	1.22825
40233	1.89226	1.18376
40234	1.89226	2.1
40235	1.89226	1.00428
40236	1.89226	2.1
40237	3.78453	1.28584
40238	1.43294	1.18906
40239	1.3268	2.1
40240	1.51691	1.19106
40241	1.44091	2.1
40242	2.87385	2.08104
40243	1.37444	2.07736
40244	1.51691	2.08443
40245	1.4714	0.854111
40246	1.65434	1.41672
40247	3.02878	1.44009
40248	1.89226	2.1
40249	1.89226	1.77643

40250	1.89226	1.12815
40251	1.89226	1.44629
40252	3.78452	1.42586
40253	1.89226	1.13658
40254	1.89226	2.1
40255	1.89226	1.0362
40256	1.89226	2.1
40257	3.78453	1.86127
40258	1.51691	1.37058
40259	1.44091	2.1
40260	1.65434	1.05913
40261	1.60647	2.1
40262	3.09526	1.91453
40263	1.05013	2.1
40264	1.05013	2.1
40265	0.94613	2.1
40266	0.94613	2.1
40267	1.04959	2.1
40268	1.05014	2.1
40269	0.946129	2.1
40270	0.946129	2.1
40271	0.946131	2.1
40272	1.04958	2.1
40273	0.946131	2.1
40274	1.05013	2.1
40275	1.04959	2.1
40276	1.04959	2.1
40277	0.946131	2.1
40278	0.946131	2.1
40279	1.04765	2.1
40280	1.04959	2.1
40281	0.94613	2.1
40282	0.94613	2.1
40283	0.946131	2.1
40284	1.04765	2.1
40285	0.946131	2.1
40286	1.04959	2.1
40287	1.85744	2.1
40288	1.89226	2.1
40289	1.89226	2.1
40290	1.89226	2.1
40291	1.94839	2.1
40292	1.94839	2.1
40293	0.97419	2.1
40294	0.822054	2.1
40295	0.946123	2.1
40296	0.946123	2.1
40297	1.04202	2.1
40298	0.84898	2.1
40299	0.933307	2.1
40300	0.933307	2.1
40301	0.933285	2.1
40302	1.05018	2.1
40303	0.851528	2.1
40304	0.876787	2.1
40305	1.87528	0.420018
40306	1.21137	0.25072
40307	1.57893	0.336432

40308	1.21137	0.455985
40309	1.81182	0.31657
40310	1.44426	0.4176
40311	0.993833	0.313805
40312	1.21137	0.359783
40313	1.57893	0.410022
40314	1.31774	0.31375
40315	2.5291	0.311265
40316	1.87528	0.440145
40317	1.57893	0.283982
40318	1.90728	0.337853
40319	2.29851	0.416474
40320	3.87744	0.413196
40321	1.90728	0.387295
40322	1.31774	0.332116
40323	1.56339	0.341552
40324	1.31774	0.39061
40325	1.82458	0.34232
40326	1.57893	0.385418
40327	1.89226	0.809262
40328	1.89226	0.949429
40329	1.89226	0.907128
40330	1.89226	0.947442
40331	1.89226	0.930318
40332	1.89226	0.973847
40333	1.89226	0.896467
40334	1.89226	1.01101
40335	1.89226	1.07587
40336	1.89226	0.931559
40337	3.78452	0.989941
40338	1.89226	0.942874
40339	2.17251	0.982874
40340	2.45575	0.899073
40341	2.45575	0.948327
40342	1.89226	0.880967
40343	1.89226	0.934719
40344	1.08625	1.0245
40345	1.22788	0.911262
40346	0.54447	0.853826
40347	0.794861	0.94233
40348	0.967729	1.01565
40349	0.967729	1.02845
40350	0.913386	0.904407
40351	0.913386	0.864432
40352	1.08625	0.988772
40353	0.544469	0.9146
40354	0.794862	0.984737
40355	1.22788	0.954868
40356	0.946128	0.965956
40357	0.946128	1.03526
40358	0.946132	0.92785
40359	0.946132	0.89888
40360	0.94613	0.924121
40361	0.94613	0.950387
40362	0.94613	1.04774
40363	0.94613	0.864285
40364	0.94613	0.953046
40365	0.94613	0.947847

40366	0.94613	0.949119
40367	0.94613	0.959051
40368	0.946127	0.895063
40369	0.946127	0.881432
40370	1.08625	0.864991
40371	0.958779	0.956635
40372	1.08625	0.894125
40373	1.08625	0.930681
40374	0.946128	0.862579
40375	0.946128	0.883344
40376	0.946129	0.901285
40377	0.958777	0.910695
40378	1.08625	0.889123
40379	0.946129	0.8949
40380	2.16338	0.286673
40381	1.94002	0.232755
40382	1.98836	0.260802
40383	2.60614	0.296072
40384	1.5456	0.228355
40385	2.16338	0.24818
40386	1.19952	0.203691
40387	2.34061	0.201868
40388	1.21031	0.185387
40389	2.1711	0.151027
40390	3.54013	0.180349
40391	1.13624	2.1
40392	1.70302	2.1
40393	1.8267	2.1
40394	1.11545	2.1
40395	2.85455	2.1
40396	1.82671	2.1
40397	1.91767	2.1
40398	1.81563	2.1
40399	1.91767	2.1
40400	1.71362	2.1
40401	1.83277	2.1
40402	1.66064	2.1
40403	0.996776	2.1
40404	0.996776	2.1
40405	1.11545	2.1
40406	1.54278	2.1
40407	1.66145	2.1
40408	1.66064	2.1
40409	1.7457	2.1
40410	1.81563	2.1
40411	1.81563	2.1
40412	1.54278	2.1
40413	1.54278	2.1
40414	2.71672	2.1
40415	2.70402	2.1
40416	2.64996	2.1
40417	2.69443	2.1
40418	5.39845	2.1
40419	2.71672	2.1
40420	2.54198	2.1
40421	2.54019	2.1
40422	2.69443	2.1
40423	5.23641	2.1

40424	2.64996	2.1
40425	2.69443	2.1
40426	2.68213	2.1
40427	2.61243	2.1
40428	5.30686	2.1
40429	2.54019	2.1
40430	2.69443	2.1
40431	2.68213	2.1
40432	2.5392	2.1
40433	5.23363	2.1
40434	1.89226	2.1
40435	2.34725	2.1
40436	2.22414	2.1
40437	2.34725	2.1
40438	1.76914	2.1
40439	1.89226	2.1
40440	0.946131	2.1
40441	0.946131	2.1
40442	1.11207	2.1
40443	1.03798	2.1
40444	0.94613	2.1
40445	0.94613	2.1
40446	1.03798	2.1
40447	1.03798	2.1
40448	0.946131	2.1
40449	1.03798	2.1
40450	0.946131	2.1
40451	1.11207	2.1
40452	1.89226	0.257881
40453	1.89226	0.227847
40454	1.89226	0.257546
40455	1.89226	0.195122
40456	1.89226	0.29906
40457	1.89226	0.231251
40458	1.89226	0.252602
40459	1.89226	0.277437
40460	1.89226	0.313513
40461	1.89226	0.215652
40462	1.89226	0.342443
40463	1.89226	0.23943
40464	1.89226	0.148125
40465	1.89226	0.13476
40466	1.89226	0.149162
40467	1.89226	0.127734
40468	1.89226	0.167773
40469	1.89226	0.145193
40470	1.44085	1.25024
40471	1.31825	0.646175
40472	1.31923	0.810942
40473	1.31825	1.01781
40474	1.43311	0.737044
40475	1.43213	0.880255
40476	1.89226	0.656306
40477	1.89226	0.853209
40478	1.89226	0.635064
40479	1.89226	0.882983
40480	1.89226	0.618154
40481	1.89226	0.981855

40482	0.946133	0.736335
40483	0.946133	0.637499
40484	1.38969	0.515395
40485	1.39587	0.625696
40486	0.946132	0.515662
40487	0.946132	0.613174
40488	1.39587	0.754253
40489	1.39587	0.559608
40490	0.946132	0.589596
40491	1.39587	0.576489
40492	1.38969	0.591965
40493	0.946132	0.593339
40494	1.44085	1.52252
40495	1.31923	0.622
40496	1.33026	0.762572
40497	1.4617	0.965901
40498	2.78093	0.879829
40499	1.89226	0.633294
40500	1.89226	0.794984
40501	1.89226	0.614837
40502	1.89226	0.876772
40503	1.89226	0.600884
40504	1.89226	0.965952
40505	0.946133	0.688805
40506	0.946133	0.60778
40507	1.39587	0.564177
40508	1.40724	0.486643
40509	0.946132	0.492812
40510	0.946132	0.593322
40511	1.40724	0.700923
40512	1.40724	0.562782
40513	0.946132	0.563893
40514	1.40724	0.566023
40515	0.946133	0.630353
40516	1.39587	0.595237
40517	1.15818	1.82625
40518	1.81846	1.78202
40519	1.52782	0.974846
40520	1.52782	2.1
40521	1.28095	0.847091
40522	1.28095	2.07371
40523	2.56709	2.1
40524	1.4349	0.464875
40525	1.4349	0.688111
40526	1.75471	0.737466
40527	2.29909	0.678919
40528	2.61889	0.840674
40529	2.5671	2.1
40530	2.82858	1.46351
40531	2.57368	2.1
40532	2.29909	0.70393
40533	5.12767	2.04762
40534	2.29909	1.83281
40535	1.09148	0.417208
40536	1.09148	0.68874
40537	1.4349	0.651581
40538	1.99776	0.64157
40539	2.34119	0.724837

40540	2.57368	2.1
40541	1.99777	0.676061
40542	1.99777	1.46244
40543	2.29909	1.96073
40544	2.36071	1.34473
40545	2.66203	2.1
40546	1.89226	0.741917
40547	1.89226	0.867182
40548	1.89226	0.818391
40549	1.89226	0.742216
40550	3.78453	0.737132
40551	0.946128	0.869147
40552	0.946128	1.24204
40553	0.946133	0.809096
40554	0.946133	0.884755
40555	0.946131	0.920169
40556	0.946131	0.983062
40557	0.946131	1.10754
40558	0.946131	0.912866
40559	0.94613	1.13659
40560	0.94613	0.94003
40561	0.946131	0.893899
40562	0.946131	1.00483
40563	1.89226	0.894163
40564	1.89226	0.997778
40565	1.89226	0.762978
40566	1.89226	0.996789
40567	3.78453	1.01233
40568	1.89226	0.921595
40569	1.89226	0.823938
40570	1.89226	0.728778
40571	1.89226	0.84763
40572	3.78453	0.884677
40573	1.89226	0.851797
40574	2.61682	1.18912
40575	1.89226	1.09666
40576	1.89226	1.11628
40577	1.89226	0.855851
40578	1.89226	1.03883
40579	1.89226	0.526815
40580	1.89226	0.522186
40581	1.89226	0.60374
40582	1.89226	0.627503
40583	3.78452	0.68711
40584	1.89226	0.521297
40585	1.89226	0.892686
40586	1.89226	0.5459
40587	1.89226	0.642685
40588	1.89226	0.690793
40589	1.89226	0.861124
40590	1.89227	0.53395
40591	1.89227	0.460632
40592	1.89227	0.716332
40593	1.89227	0.621587
40594	1.89227	0.545719
40595	1.89227	0.527105
40596	0.80447	0.910505
40597	0.534241	0.898685

40598	0.995175	0.969327
40599	0.92204	0.707738
40600	0.922042	0.541217
40601	0.534241	0.545753
40602	0.804468	0.871233
40603	0.995176	0.454747
40604	0.71543	0.709454
40605	0.71543	0.582754
40606	0.813988	0.543602
40607	0.813988	0.460727
40608	0.922045	0.434496
40609	0.942899	0.570931
40610	0.946132	0.479428
40611	0.946132	0.459535
40612	1.05169	0.374052
40613	0.936661	0.531323
40614	0.922045	0.50129
40615	0.946133	0.427754
40616	1.01665	0.427212
40617	1.13008	0.388819
40618	0.851529	0.443892
40619	0.851529	0.474282
40620	1.41908	2.1
40621	1.77723	2.1
40622	1.90485	2.1
40623	1.90485	2.1
40624	1.30237	2.1
40625	1.30237	2.1
40626	1.77723	2.1
40627	2.0055	1.57733
40628	1.90485	2.1
40629	2.17158	2.1
40630	3.91035	2.1
40631	0.850906	1.87982
40632	0.850906	2.1
40633	0.85668	2.1
40634	0.85668	2.1
40635	0.998045	2.1
40636	0.952425	2.1
40637	0.74367	2.1
40638	0.709541	2.1
40639	0.74367	2.1
40640	0.998045	2.1
40641	0.709541	2.1
40642	0.952425	2.1
40643	1.90485	2.1
40644	2.28563	2.1
40645	2.26282	2.1
40646	2.17158	1.74003
40647	1.99609	2.1
40648	1.90485	0.985941
40649	2.00098	0.330284
40650	1.90462	0.324404
40651	1.60057	0.332169
40652	1.79224	0.354392
40653	3.69686	0.329836
40654	2.65654	0.348931
40655	1.79224	0.347575

40656	2.22332	0.345218
40657	2.22332	0.348757
40658	2.24163	0.347683
40659	2.24163	0.351375
40660	1.89226	0.350653
40661	1.89226	0.341796
40662	1.89226	0.347761
40663	1.89226	0.389581
40664	3.78452	0.351325
40665	1.89226	0.326156
40666	1.89226	0.355203
40667	1.89226	0.33949
40668	1.89226	0.352111
40669	1.89226	0.355747
40670	1.89226	0.354804
40671	2.24163	0.367947
40672	1.03645	0.367982
40673	1.79224	0.370243
40674	1.79224	0.362525
40675	1.54283	0.377507
40676	1.54283	0.371345
40677	0.946132	0.368749
40678	0.946132	0.365981
40679	0.946129	0.366193
40680	0.946129	0.360397
40681	0.94613	0.370574
40682	0.94613	0.36451
40683	0.946131	0.370825
40684	0.946131	0.364079
40685	0.94613	0.377407
40686	0.94613	0.377338
40687	0.946131	0.356737
40688	0.946131	0.357537
40689	1.89226	2.1
40690	1.89226	2.1
40691	1.89226	2.1
40692	1.89226	2.1
40693	3.78453	2.1
40694	1.52441	2.1
40695	1.30347	2.1
40696	1.52441	2.1
40697	1.58401	2.1
40698	1.2627	2.1
40699	1.3223	2.1
40700	1.89226	2.1
40701	2.72787	2.1
40702	2.72787	2.1
40703	2.74762	2.1
40704	1.89226	2.1
40705	1.91202	2.1
40706	1.89227	2.1
40707	1.89227	2.1
40708	1.89227	2.1
40709	1.89227	2.1
40710	3.78453	2.1
40711	0.56414	2.1
40712	0.561557	2.1
40713	0.527284	2.1

40714	0.541143	2.1
40715	0.527276	2.1
40716	0.631685	2.1
40717	1.52441	2.1
40718	1.00232	2.1
40719	0.784495	2.1
40720	0.842304	2.1
40721	0.721569	2.1
40722	1.36393	2.1
40723	1.36393	2.1
40724	0.946132	2.1
40725	0.946132	2.1
40726	1.36393	2.1
40727	1.3594	2.1
40728	0.946132	2.1
40729	0.946132	2.1
40730	0.946107	2.1
40731	1.35936	2.1
40732	1.36397	2.1
40733	0.946158	2.1
40734	1.89226	2.1
40735	1.89226	2.1
40736	1.89226	2.1
40737	1.89226	2.1
40738	3.78453	2.1
40739	2.20802	2.1
40740	1.05677	2.1
40741	2.30479	2.1
40742	0.978699	2.1
40743	3.18672	2.1
40744	0.94613	2.1
40745	0.94613	2.1
40746	0.669866	2.1
40747	0.669866	2.1
40748	0.681588	2.1
40749	0.669865	2.1
40750	0.946133	2.1
40751	0.946133	2.1
40752	0.94613	2.1
40753	0.681589	2.1
40754	0.946131	2.1
40755	0.669867	2.1
40756	2.73514	2.1
40757	1.78301	2.1
40758	1.90618	2.1
40759	2.83464	2.1
40760	4.64132	2.1
40761	2.73514	2.1
40762	1.89226	2.1
40763	1.89226	2.1
40764	2.83464	2.1
40765	4.6274	2.1
40766	2.03915	2.1
40767	2.26616	2.1
40768	2.23549	2.1
40769	2.01704	2.1
40770	4.2832	2.1
40771	2.03916	2.1

40772	1.55766	2.1
40773	1.53728	2.1
40774	2.01704	2.1
40775	3.5747	2.1
40776	2.23551	2.1
40777	2.01705	2.1
40778	1.99823	2.1
40779	2.21189	2.1
40780	4.22894	2.1
40781	1.51788	2.1
40782	2.01705	2.1
40783	1.53729	2.1
40784	1.53729	2.1
40785	1.99823	2.1
40786	1.99823	2.1
40787	2.588	2.1
40788	1.45581	2.1
40789	1.45581	2.1
40790	1.44207	2.1
40791	2.54949	2.1
40792	2.53574	2.1
40793	0.98641	2.1
40794	0.98641	2.1
40795	1.00371	2.1
40796	1.00371	2.1
40797	0.749992	2.1
40798	0.727904	2.1
40799	1.26221	2.1
40800	1.27474	2.1
40801	1.26221	2.1
40802	0.749993	2.1
40803	0.727904	2.1
40804	1.27474	2.1
40805	1.23105	0.829015
40806	1.35435	1.72734
40807	1.89226	1.20944
40808	1.89226	1.09638
40809	1.89226	1.5616
40810	1.7043	1.27367
40811	1.89226	1.89729
40812	1.89226	1.1285
40813	1.89226	0.753673
40814	1.89226	1.7773
40815	3.78453	1.35405
40816	1.89226	0.618765
40817	1.89226	1.7554
40818	1.89226	0.961076
40819	1.89226	1.02149
40820	1.89226	1.27722
40821	1.89226	1.37321
40822	0.946131	0.803892
40823	1.40945	0.717749
40824	0.94613	0.792034
40825	0.94613	1.40173
40826	0.946129	1.43351
40827	0.946129	0.96642
40828	0.946132	1.58043
40829	1.40945	0.679982

40830	0.94613	0.930619
40831	0.94613	0.878767
40832	0.946131	0.915408
40833	0.94613	0.86952
40834	0.946133	0.958435
40835	0.946133	0.862286
40836	0.946132	1.2201
40837	0.946132	1.65167
40838	0.946132	1.62178
40839	0.946132	0.981527
40840	0.946133	1.25507
40841	0.946133	0.87181
40842	0.946132	1.18294
40843	0.946132	1.12623
40844	0.946133	1.01326
40845	0.946133	1.01187
40846	1.89226	0.974306
40847	1.89226	1.94259
40848	1.89226	1.31241
40849	1.89226	1.1887
40850	1.89226	1.62336
40851	1.89226	1.42602
40852	1.89226	2.1
40853	1.89226	2.1
40854	1.89226	2.1
40855	1.89226	2.1
40856	1.89226	2.1
40857	1.89226	2.1
40858	1.89226	2.1
40859	1.89226	2.1
40860	1.89226	2.1
40861	1.89226	2.1
40862	3.78453	2.1
40863	1.66647	2.1
40864	1.15399	2.1
40865	1.25443	2.1
40866	1.26599	2.1
40867	1.58697	2.1
40868	1.66647	2.1
40869	1.66647	2.1
40870	1.47446	2.1
40871	1.55433	2.1
40872	1.74241	2.1
40873	3.2208	2.1
40874	1.89227	2.1
40875	1.89227	2.1
40876	1.89227	2.1
40877	1.89227	2.1
40878	3.78453	2.1
40879	0.946132	2.1
40880	0.946132	2.1
40881	0.946132	2.1
40882	0.946132	2.1
40883	0.946132	2.1
40884	0.946132	2.1
40885	0.946133	2.1
40886	0.946133	2.1
40887	0.946132	2.1

40888	0.946132	2.1
40889	0.946133	2.1
40890	0.946133	2.1
40891	0.625067	2.1
40892	0.61907	2.1
40893	0.896913	2.1
40894	0.946199	2.1
40895	0.806287	2.1
40896	0.677685	2.1
40897	0.750657	2.1
40898	0.750657	2.1
40899	0.646004	2.1
40900	0.890236	2.1
40901	0.61049	2.1
40902	0.888979	2.1
40903	1.6223	2.1
40904	1.98034	2.1
40905	1.75003	2.1
40906	1.55433	2.1
40907	1.75003	2.1
40908	1.55433	2.1
40909	1.88602	2.1
40910	2.08149	2.1
40911	2.07991	2.1
40912	1.88473	2.1
40913	2.08149	2.1
40914	1.88602	2.1
40915	1.88458	2.1
40916	2.08149	2.1
40917	1.7069	2.1
40918	1.56284	2.1
40919	2.08149	2.1
40920	1.88458	2.1
40921	1.5955	2.1
40922	1.89226	2.1
40923	1.91975	2.1
40924	1.62299	2.1
40925	1.89226	2.06948
40926	1.5955	2.1
40927	1.89226	1.98128
40928	1.89226	2.1
40929	1.89226	1.64614
40930	1.89226	2.1
40931	1.89226	0.998994
40932	1.89226	2.1
40933	1.89226	2.1
40934	1.89226	2.1
40935	1.89226	2.1
40936	1.89226	2.1
40937	1.89226	1.677
40938	1.89226	2.1
40939	1.62299	2.1
40940	1.89226	2.1
40941	1.71752	2.1
40942	1.44824	2.1
40943	1.89226	2.1
40944	1.62299	2.1
40945	1.89226	0.72919

40946	2.8317	0.723255
40947	1.89226	0.76336
40948	2.64055	0.550755
40949	4.53281	0.734954
40950	2.8317	0.866796
40951	1.83151	0.53481
40952	2.01001	0.69009
40953	2.01001	0.8478
40954	2.64055	0.554994
40955	2.64055	0.628065
40956	0.946132	0.481916
40957	0.946132	0.538
40958	1.16167	0.551499
40959	1.16167	0.584871
40960	1.16167	0.549858
40961	1.32027	0.474898
40962	0.946131	0.490976
40963	0.946131	0.612161
40964	0.94613	0.494215
40965	1.16167	0.47042
40966	0.946131	0.515534
40967	1.32027	0.505504
40968	2.32335	0.617402
40969	1.83151	0.495739
40970	2.38525	0.542353
40971	2.64055	0.528775
40972	1.57621	0.484713
40973	1.83151	0.481004
40974	2.25677	2.1
40975	1.88158	2.1
40976	1.86856	2.1
40977	2.0815	2.1
40978	1.70303	2.1
40979	1.88158	2.1
40980	2.50381	2.1
40981	1.89227	2.1
40982	2.25677	2.1
40983	2.25677	2.1
40984	1.89227	2.1
40985	1.89227	2.1
40986	1.89226	0.335265
40987	1.50899	0.353102
40988	2.13659	0.353287
40989	1.50899	0.468638
40990	2.51986	0.298357
40991	1.89226	0.411506
40992	0.946131	0.405287
40993	0.946131	0.298009
40994	1.06829	0.326791
40995	0.895375	0.22836
40996	0.94613	0.331719
40997	0.94613	0.43791
40998	1.33922	0.2895
40999	0.895376	0.203976
41000	0.946131	0.289713
41001	1.33922	0.281276
41002	0.946131	0.253874
41003	1.06829	0.325333

41004	0.841467	0.477719
41005	0.578428	0.50477
41006	1.18079	0.465974
41007	0.920237	0.499708
41008	0.772317	0.44104
41009	0.856555	0.533891
41010	0.910271	0.479005
41011	0.910271	0.55143
41012	0.975752	0.477037
41013	0.536929	0.450552
41014	0.81952	0.542753
41015	1.15252	0.467592
41016	1.89226	2.1
41017	2.51431	2.1
41018	1.89226	2.1
41019	1.89226	2.1
41020	2.78815	2.1
41021	2.78815	2.1
41022	2.0815	2.1
41023	2.0516	2.1
41024	2.0815	2.1
41025	3.06699	2.1
41026	2.76576	2.1
41027	2.8002	2.1
41028	1.94112	2.1
41029	1.89226	2.1
41030	2.20358	2.1
41031	1.89226	2.1
41032	4.09584	2.1
41033	1.09154	2.1
41034	2.62647	2.1
41035	2.20358	2.1
41036	2.20358	2.1
41037	1.52314	2.1
41038	1.52314	2.1
41039	1.31323	2.1
41040	1.31323	2.1
41041	0.946132	2.1
41042	0.946132	2.1
41043	0.946132	2.1
41044	0.946132	2.1
41045	1.31323	2.1
41046	1.10179	2.1
41047	1.31323	2.1
41048	0.946132	2.1
41049	1.10179	2.1
41050	0.946133	2.1
41051	1.89226	0.72928
41052	1.89226	1.00588
41053	1.89226	0.958167
41054	1.89226	1.04911
41055	1.89226	0.722577
41056	1.89226	0.901279
41057	1.35126	1.34037
41058	1.35126	2.1
41059	0.903285	1.08198
41060	0.903285	1.28766
41061	1.30841	1.28171

41062	0.903313	1.91171
41063	0.946132	1.14352
41064	0.946132	0.980731
41065	0.946132	1.19048
41066	0.903312	1.18631
41067	1.30841	1.10503
41068	0.946133	1.03528
41069	2.62097	2.1
41070	2.72561	1.98253
41071	2.39016	2.1
41072	2.51062	2.1
41073	5.11577	2.1
41074	2.51062	2.1
41075	2.58286	0.551451
41076	2.40107	1.72212
41077	2.40107	0.607598
41078	2.72561	1.63402
41079	2.72561	0.622772
41080	2.39016	2.1
41081	2.51062	2.1
41082	2.20242	2.1
41083	2.34107	1.50311
41084	4.73124	2.1
41085	2.51062	2.1
41086	2.40107	0.541983
41087	2.34107	1.5455
41088	2.25197	0.434897
41089	4.74215	0.693674
41090	2.75218	2.1
41091	1.89226	2.1
41092	2.3349	2.1
41093	2.3349	2.1
41094	1.89226	2.1
41095	1.89226	2.1
41096	1.46129	2.1
41097	0.996308	2.1
41098	1.88649	2.1
41099	1.70302	2.1
41100	2.95419	2.1
41101	1.89227	2.1
41102	1.89227	1.92939
41103	1.89227	2.1
41104	1.89227	2.1
41105	3.78453	2.1
41106	0.646558	2.1
41107	0.646557	2.1
41108	0.60697	2.1
41109	0.60697	2.1
41110	0.587917	2.1
41111	0.519747	2.1
41112	0.586443	2.1
41113	0.733782	2.1
41114	0.733793	2.1
41115	0.587924	2.1
41116	0.519739	2.1
41117	0.586436	2.1
41118	2.1642	2.1
41119	1.42867	2.1

41120	2.28847	2.1
41121	1.68299	2.1
41122	3.71714	2.1
41123	1.89226	2.1
41124	1.89226	2.1
41125	1.89226	2.1
41126	1.89226	2.1
41127	3.78453	2.1
41128	0.946131	2.1
41129	0.946131	2.1
41130	1.24994	2.1
41131	1.24994	2.1
41132	1.11582	2.1
41133	1.24994	2.1
41134	0.946133	2.1
41135	0.946133	2.1
41136	0.946131	2.1
41137	1.24994	2.1
41138	0.94613	2.1
41139	1.11582	2.1
41140	2.28847	2.1
41141	1.53629	2.1
41142	1.57498	2.1
41143	1.47417	2.1
41144	2.42221	2.1
41145	2.28847	2.1
41146	1.89226	2.1
41147	1.89226	2.1
41148	1.89226	2.1
41149	1.89226	2.1
41150	3.78453	2.1
41151	1.89226	2.1
41152	2.49988	2.1
41153	2.71233	2.1
41154	2.71233	2.1
41155	1.89226	2.1
41156	1.89226	2.1
41157	0.924534	2.1
41158	0.924534	2.1
41159	0.990395	2.1
41160	0.990395	2.1
41161	0.946131	2.1
41162	0.946131	2.1
41163	0.924505	2.1
41164	0.968797	2.1
41165	0.924505	2.1
41166	0.946131	2.1
41167	0.968797	2.1
41168	0.946131	2.1
41169	0.924533	2.1
41170	0.924533	2.1
41171	0.948264	2.1
41172	0.945345	2.1
41173	0.851541	2.1
41174	0.851541	2.1
41175	0.958934	2.1
41176	1.02617	2.1
41177	0.936668	2.1

41178	1.04829	2.1
41179	0.924505	2.1
41180	0.946131	2.1
41181	2.28431	0.526648
41182	1.48035	0.394302
41183	1.59971	0.45346
41184	1.48035	0.467777
41185	2.33818	0.434327
41186	2.21882	0.44006
41187	2.28431	0.473107
41188	1.89226	0.466642
41189	1.89226	0.548904
41190	2.3968	0.423908
41191	4.28906	0.45929
41192	2.28431	0.494774
41193	1.70303	0.383457
41194	1.5428	0.389001
41195	1.59971	0.415001
41196	2.31152	0.426369
41197	2.3968	0.438158
41198	1.99823	2.1
41199	2.21189	2.1
41200	2.18937	2.1
41201	1.979 2.1	
41202	4.19089	2.1
41203	0.999114	2.1
41204	0.9895	2.1
41205	0.758942	2.1
41206	0.749358	2.1
41207	0.874236	2.1
41208	0.874236	2.1
41209	0.874236	2.1
41210	0.874236	2.1
41211	0.9895	2.1
41212	0.749358	2.1
41213	0.758942	2.1
41214	0.999114	2.1
41215	2.18937	2.1
41216	1.979 2.1	
41217	1.95416	2.1
41218	2.162 2.1	
41219	4.14099	2.1
41220	1.95416	2.1
41221	1.49871	2.1
41222	1.47712	2.1
41223	1.49871	2.1
41224	1.9574	2.1
41225	1.979 2.1	
41226	1.68508	0.0806462
41227	1.64984	0.116481
41228	2.67711	0.0612193
41229	2.76407	0.113646
41230	4.41391	0.113178
41231	1.84227	0.0865513
41232	2.76407	0.110231
41233	2.76407	0.127382
41234	1.85007	0.0885907
41235	2.72769	0.119673

41236	1.80812	0.0825433
41237	1.94516	2.1
41238	1.89226	2.1
41239	1.89226	2.1
41240	1.89226	2.1
41241	1.98853	2.1
41242	1.98852	2.1
41243	1.28095	2.1
41244	2.0815	2.1
41245	2.29123	2.1
41246	2.06833	2.1
41247	1.08212	2.1
41248	0.976848	2.1
41249	1.89226	2.1
41250	1.89226	2.1
41251	1.89226	2.1
41252	1.89226	2.1
41253	1.89226	2.1
41254	1.89226	2.1
41255	1.13537	2.1
41256	1.18233	2.1
41257	0.946132	2.1
41258	0.946132	2.1
41259	0.946132	2.1
41260	0.946132	2.1
41261	0.994264	2.1
41262	1.18233	2.1
41263	1.13537	2.1
41264	0.946131	2.1
41265	0.946132	2.1
41266	0.994264	2.1
41267	0.953204	2.1
41268	0.953204	2.1
41269	0.966515	2.1
41270	0.966515	2.1
41271	1.30583	2.1
41272	1.14561	2.1
41273	0.640475	2.1
41274	0.774103	2.1
41275	1.30584	2.1
41276	0.774101	2.1
41277	0.640474	2.1
41278	1.14562	2.1
41279	0.946131	2.1
41280	0.946131	2.1
41281	0.946131	2.1
41282	0.946131	2.1
41283	0.946128	2.1
41284	0.946128	2.1
41285	0.946133	2.1
41286	0.946133	2.1
41287	0.94613	2.1
41288	0.94613	2.1
41289	0.946131	2.1
41290	0.946131	2.1
41291	0.946132	0.05
41292	0.946132	0.05
41293	0.946131	0.05

41294	0.946131	0.05
41295	0.946131	0.05
41296	0.946131	0.05
41297	0.946132	0.05
41298	0.946132	0.05
41299	0.946131	0.05
41300	0.946131	0.05
41301	0.946132	0.05
41302	0.946132	0.05
41303	0.946131	0.05
41304	0.946131	0.05
41305	0.946131	0.05
41306	0.946131	0.05
41307	0.94613	0.05
41308	0.94613	0.05
41309	0.946132	0.05
41310	0.946132	0.05
41311	0.946131	0.05
41312	0.946131	0.05
41313	0.946131	0.05
41314	0.946131	0.05
41315	2.57882	0.321618
41316	1.48676	0.32791
41317	1.48797	0.32308
41318	2.64281	0.323335
41319	4.12957	0.323127
41320	2.56323	0.29691
41321	2.64281	0.320355
41322	2.51807	0.312158
41323	1.36324	0.32206
41324	1.48797	0.323996
41325	1.48797	0.320651
41326	1.04367	2.1
41327	2.42504	2.1
41328	2.65164	2.1
41329	1.49479	2.1
41330	3.91983	2.1
41331	2.15622	1.02041
41332	1.70539	2.1
41333	2.02369	2.1
41334	2.39039	1.31244
41335	4.17992	2.1
41336	0.966979	2.1
41337	1.7054	2.1
41338	2.0237	2.1
41339	1.31502	2.1
41340	2.99067	2.1
41341	2.02369	2.1
41342	2.65637	1.49834
41343	2.39039	1.02102
41344	2.39039	2.1
41345	2.29332	0.997741
41346	2.29332	2.1
41347	2.0237	2.04181
41348	1.72116	2.1
41349	1.58465	2.1
41350	1.31502	2.1
41351	2.29332	1.92867

41352	2.0237	2.1
41353	1.0372	0.0777592
41354	2.32462	0.159257
41355	2.2291	0.110588
41356	2.2291	0.126501
41357	1.17464	0.100517
41358	1.17464	0.104625
41359	2.68289	0.057844
41360	1.07805	0.0632571
41361	1.70302	0.0518541
41362	2.06653	0.0611408
41363	3.5779	0.0608076
41364	1.89226	0.145848
41365	1.89226	0.245909
41366	1.89226	0.178637
41367	1.89226	0.0952174
41368	3.78453	0.149825
41369	1.89226	0.0752026
41370	1.89226	0.0912471
41371	1.89226	0.0904052
41372	1.89226	0.087175
41373	1.89226	0.0802655
41374	1.89226	0.0764733
41375	1.89225	0.0819379
41376	1.89225	0.0764401
41377	1.89225	0.0812716
41378	1.89225	0.0753994
41379	1.89225	0.0874465
41380	1.89225	0.0827026
41381	0.94613	0.113063
41382	0.94613	0.155661
41383	0.94613	0.233642
41384	0.94613	0.170549
41385	0.946128	0.13727
41386	0.946128	0.148365
41387	0.946132	0.259861
41388	0.946132	0.104237
41389	0.94613	0.127566
41390	0.94613	0.16095
41391	0.94613	0.158008
41392	0.94613	0.117547
41393	1.29417	1.52999
41394	1.32732	2.1
41395	1.32316	0.85265
41396	1.34973	2.1
41397	2.65048	1.65703
41398	1.89226	1.08641
41399	1.89226	2.1
41400	1.89226	0.86501
41401	1.89226	2.08475
41402	3.78453	1.17069
41403	1.89226	1.15656
41404	1.89226	2.1
41405	1.89226	0.89106
41406	1.89226	2.1
41407	3.78453	2.1
41408	1.32732	1.54264
41409	1.13006	2.1

41410	1.13358	2.1
41411	1.11117	2.1
41412	1.34973	1.26213
41413	1.32732	2.1
41414	1.32316	1.31492
41415	1.34973	2.1
41416	1.34066	0.884093
41417	1.36601	2.1
41418	2.68918	1.75747
41419	1.89226	1.28789
41420	1.89226	2.1
41421	1.89226	0.657491
41422	1.89226	2.1
41423	3.78453	1.35753
41424	1.89226	0.977278
41425	1.89226	2.1
41426	1.89226	2.1
41427	1.89226	2.1
41428	1.89226	0.888582
41429	1.89226	2.1
41430	0.624134	2.1
41431	0.624134	1.98374
41432	0.624334	0.856366
41433	0.624334	2.1
41434	0.56503	2.1
41435	0.573605	2.1
41436	0.674864	1.02342
41437	0.683007	1.0602
41438	0.683007	2.1
41439	0.573605	2.1
41440	0.565029	2.1
41441	0.674864	2.1
41442	2.16633	2.1
41443	1.40846	2.1
41444	2.13977	2.1
41445	1.40771	2.1
41446	3.57404	2.1
41447	2.13977	2.1
41448	1.29932	2.1
41449	1.29932	2.1
41450	1.40771	2.1
41451	1.98957	2.1
41452	2.09796	2.1
41453	2.6044	0.500338
41454	1.85548	0.571171
41455	1.89226	0.377078
41456	1.89226	0.645059
41457	3.74774	0.551552
41458	1.89226	0.350468
41459	1.89226	0.644867
41460	1.89226	0.421217
41461	1.89226	0.538015
41462	3.78453	0.496032
41463	1.89226	2.1
41464	2.64107	2.1
41465	1.89226	2.1
41466	2.78977	2.1
41467	1.89226	2.1

41468	2.78977	2.1
41469	1.89226	2.1
41470	1.89226	2.1
41471	1.89226	2.1
41472	1.89226	2.1
41473	1.89226	2.1
41474	1.89226	2.1
41475	2.43173	2.1
41476	1.41238	2.1
41477	2.0815	2.1
41478	1.98048	2.1
41479	1.62014	2.1
41480	1.50086	2.1
41481	0.946129	0.510176
41482	0.946129	0.467681
41483	1.27636	0.465607
41484	1.1984	0.391102
41485	0.946131	0.377899
41486	0.946131	0.456399
41487	1.27636	0.510996
41488	1.27636	0.438602
41489	0.946131	0.452116
41490	1.27636	0.4127
41491	0.946131	0.447938
41492	1.1984	0.448061
41493	1.99569	0.376061
41494	2.55272	0.481817
41495	1.77403	0.360961
41496	1.70303	0.40244
41497	3.05772	0.403343
41498	2.55121	0.407639
41499	0.946128	0.441108
41500	0.946128	0.494825
41501	1.38752	0.452902
41502	1.27636	0.336059
41503	0.94613	0.300318
41504	0.94613	0.44584
41505	1.38752	0.494611
41506	1.38752	0.422835
41507	0.946131	0.416353
41508	1.38752	0.422091
41509	0.946131	0.438843
41510	1.27636	0.422125
41511	2.77504	0.454108
41512	1.99569	0.357776
41513	2.29794	0.394944
41514	1.99569	0.336782
41515	2.85497	0.382034
41516	2.55272	0.350428
41517	1.53284	2.1
41518	1.89226	2.1
41519	1.89226	2.1
41520	1.89226	2.1
41521	1.54763	2.1
41522	1.54763	2.1
41523	0.946131	2.01856
41524	0.946131	2.1
41525	0.946127	2.1

41526	0.946127	2.1
41527	0.946127	2.1
41528	0.946127	1.62269
41529	0.946131	2.1
41530	0.946131	2.1
41531	0.94613	2.1
41532	0.94613	2.1
41533	0.946129	2.1
41534	0.946129	2.1
41535	0.751754	2.1
41536	0.751754	2.1
41537	0.771562	2.1
41538	0.771561	2.1
41539	1.0268	2.1
41540	0.95482	2.1
41541	0.528937	2.1
41542	0.496512	2.1
41543	0.528936	2.1
41544	1.0268	2.1
41545	0.954821	2.1
41546	0.49651	2.1
41547	2.64075	0.54672
41548	1.35181	0.96116
41549	1.35181	0.499592
41550	1.83823	0.632749
41551	2.28373	0.616021
41552	2.88566	1.23449
41553	0.493018	0.966543
41554	0.675903	0.856619
41555	1.14187	0.571398
41556	0.973937	0.513573
41557	0.821166	0.568751
41558	0.821166	1.29098
41559	0.828674	2.0659
41560	0.828674	0.518805
41561	0.973937	0.694483
41562	0.493018	0.710785
41563	0.675903	0.652069
41564	1.14187	0.733259
41565	1.89226	2.1
41566	2.11667	2.1
41567	1.91184	2.1
41568	1.89226	2.1
41569	2.13625	2.1
41570	2.11667	2.1
41571	1.89226	2.1
41572	1.89226	2.1
41573	1.89226	2.1
41574	1.89226	2.1
41575	3.78453	2.1
41576	0.907729	2.1
41577	0.907729	2.1
41578	1.04459	2.1
41579	0.890955	2.1
41580	0.988181	2.1
41581	0.841856	2.1
41582	0.946133	2.1
41583	0.946133	2.1

41584	1.04072	2.1
41585	1.1064	2.1
41586	0.89099	2.1
41587	0.851541	2.1
41588	1.89225	2.1
41589	2.13623	2.1
41590	1.91044	2.1
41591	1.89225	2.1
41592	2.15442	2.1
41593	2.13623	2.1
41594	1.89225	2.1
41595	1.89225	2.1
41596	1.89225	2.1
41597	1.89225	2.1
41598	1.89225	2.1
41599	1.89225	2.1
41600	0.907723	2.1
41601	0.907723	2.1
41602	1.07536	2.1
41603	1.07814	2.1
41604	0.998499	2.1
41605	1.03575	2.1
41606	0.946126	2.1
41607	0.946126	2.1
41608	1.04072	2.1
41609	1.14025	2.1
41610	0.851528	2.1
41611	0.898284	2.1
41612	1.89226	0.132806
41613	1.89226	0.320185
41614	1.89226	0.204527
41615	1.89226	0.206938
41616	1.89226	0.176047
41617	1.89226	0.178382
41618	2.53008	2.1
41619	1.94534	2.1
41620	1.98702	2.1
41621	1.98858	2.1
41622	2.52852	2.1
41623	2.53008	2.1
41624	1.03267	2.1
41625	1.12591	2.1
41626	1.2959	2.1
41627	1.28992	2.1
41628	1.10851	2.1
41629	1.33078	2.1
41630	1.10661	2.1
41631	1.10661	2.1
41632	0.982208	2.1
41633	1.28992	2.1
41634	1.03267	2.1
41635	1.23262	2.1
41636	2.2712	2.1
41637	1.89226	2.1
41638	1.95954	2.1
41639	1.95954	2.1
41640	1.89226	2.1
41641	1.89226	2.1

41642	1.89226	2.1
41643	1.89226	2.1
41644	1.89226	2.1
41645	1.89226	2.1
41646	3.78453	2.1
41647	1.33887	2.1
41648	1.25395	2.1
41649	0.88327	2.1
41650	0.676665	2.1
41651	0.84881	2.1
41652	0.987503	2.1
41653	1.13243	2.1
41654	1.13243	2.1
41655	1.15338	2.1
41656	0.849966	2.1
41657	0.793074	2.1
41658	1.11957	2.1
41659	0.946129	2.1
41660	0.946129	2.1
41661	1.28254	2.1
41662	1.1356	2.1
41663	1.1356	2.1
41664	1.1356	2.1
41665	0.946131	2.1
41666	0.946131	2.1
41667	0.946131	2.1
41668	1.28254	2.1
41669	1.1356	2.1
41670	0.946131	2.1
41671	1.89227	2.1
41672	1.89227	2.1
41673	1.89227	2.1
41674	1.89227	2.1
41675	3.78453	2.1
41676	0.936287	0.05
41677	1.01399	0.05
41678	0.959415	0.0500754
41679	0.959415	0.0528692
41680	0.910786	0.0511838
41681	1.38301	0.05
41682	1.0286	0.05
41683	0.535742	0.05
41684	0.505202	0.05
41685	0.937685	0.05
41686	1.11066	0.05
41687	1.46907	0.05
41688	1.00546	0.0585414
41689	1.01524	0.0539048
41690	0.94613	0.0841021
41691	0.94613	0.0759911
41692	1.05018	0.0802736
41693	0.914025	0.0519666
41694	0.913512	0.0777167
41695	0.851542	0.0847154
41696	1.05385	0.0792806
41697	1.05494	0.0520138
41698	0.91404	0.0776722
41699	0.91404	0.061958

41700	2.03253	0.676928
41701	1.82325	0.381319
41702	1.93938	0.64611
41703	1.73756	0.366725
41704	3.77009	0.476114
41705	1.82325	0.377447
41706	1.47562	0.600858
41707	1.73756	0.377921
41708	1.3868	0.485037
41709	3.21317	0.397305
41710	1.93938	0.657987
41711	1.73756	0.412774
41712	1.85293	0.630337
41713	1.64279	0.372099
41714	3.59049	0.469109
41715	1.73756	0.400864
41716	1.3868	0.528613
41717	1.64279	0.394066
41718	1.28419	0.572741
41719	3.02174	0.430244
41720	1.35048	1.93251
41721	1.37999	2.1
41722	1.34355	0.931153
41723	1.37854	2.1
41724	2.72354	1.39171
41725	1.89226	1.06362
41726	1.89226	2.1
41727	1.89226	0.871187
41728	1.89226	1.32754
41729	3.78452	1.02048
41730	1.89226	1.13754
41731	1.89226	2.1
41732	1.89226	0.800795
41733	1.89226	2.1
41734	3.78453	2.04689
41735	1.37999	1.20736
41736	1.18059	2.1
41737	1.17117	2.1
41738	1.17262	2.1
41739	1.37855	1.09674
41740	1.37999	2.1
41741	1.34355	1.52677
41742	1.37854	2.1
41743	1.33079	0.923165
41744	1.3741	2.1
41745	2.71765	1.51515
41746	1.89226	1.30122
41747	1.89226	2.04443
41748	1.89226	0.800454
41749	1.89226	2.1
41750	3.78453	1.45025
41751	1.89226	0.957682
41752	1.89226	2.1
41753	1.89226	1.05202
41754	1.89226	2.1
41755	3.78453	1.8348
41756	0.640305	2.1
41757	0.640305	1.46318

41758	0.643507	0.752443
41759	0.643507	2.1
41760	0.590294	2.1
41761	0.594539	2.1
41762	0.689273	0.978844
41763	0.687048	1.54199
41764	0.687048	2.1
41765	0.594538	1.12013
41766	0.590294	2.1
41767	0.689273	2.1
41768	2.01399	0.410783
41769	1.70141	0.462423
41770	1.83896	0.603065
41771	1.52482	0.614568
41772	3.53881	0.480096
41773	1.70141	0.520177
41774	1.0752	0.789709
41775	1.0752	0.506842
41776	1.25669	0.632462
41777	1.52482	0.574057
41778	1.70632	1.2522
41779	1.83896	0.463697
41780	1.52482	0.427125
41781	1.65244	0.459314
41782	1.34412	0.532565
41783	3.17725	0.454753
41784	1.26782	0.129233
41785	2.90353	0.142888
41786	2.54203	0.12407
41787	2.41979	0.18826
41788	1.77007	0.10396
41789	1.703	0.142917
41790	0.946118	0.271689
41791	0.946118	0.220821
41792	1.38643	0.201527
41793	1.38644	0.148021
41794	0.946117	0.271956
41795	0.946117	0.296342
41796	1.38644	0.14539
41797	1.15545	0.186361
41798	0.946131	0.242955
41799	1.15546	0.183232
41800	1.38642	0.203004
41801	0.946105	0.25927
41802	2.42256	0.46831
41803	2.63056	0.438521
41804	2.43174	0.420216
41805	2.25035	0.414646
41806	4.8543	0.420814
41807	2.58031	0.46213
41808	2.63056	0.446753
41809	2.43174	0.420141
41810	2.32127	0.407446
41811	5.01205	0.453408
41812	2.43174	0.408625
41813	2.25035	0.347766
41814	2.11465	0.31765
41815	2.13042	0.308221

41816	4.54639	0.33999
41817	2.43174	0.411515
41818	2.32127	0.371646
41819	1.92997	0.326274
41820	2.13041	0.324224
41821	4.36171	0.365787
41822	1.89227	2.1
41823	1.89227	2.1
41824	1.89227	2.1
41825	1.89227	2.1
41826	3.78453	2.1
41827	0.568724	2.1
41828	0.568724	2.1
41829	0.57678	2.1
41830	0.57678	2.1
41831	0.523796	2.1
41832	0.548137	2.1
41833	0.597368	2.1
41834	0.637877	2.1
41835	0.597367	2.1
41836	0.523796	2.1
41837	0.637877	2.1
41838	0.548137	2.1
41839	1.32674	2.1
41840	1.32674	2.1
41841	0.946132	2.1
41842	0.946132	2.1
41843	1.3195	2.1
41844	1.32674	2.1
41845	0.946132	2.1
41846	0.946132	2.1
41847	0.946132	2.1
41848	1.3195	2.1
41849	1.32674	2.1
41850	0.946133	2.1
41851	1.89227	2.1
41852	1.89227	2.1
41853	1.89227	2.1
41854	1.89227	2.1
41855	3.78453	2.1
41856	1.19473	2.1
41857	0.987132	2.1
41858	0.987132	2.1
41859	1.04759	2.1
41860	1.0894	2.1
41861	1.14986	2.1
41862	1.3195	2.1
41863	1.3195	2.1
41864	0.946132	2.1
41865	0.946132	2.1
41866	1.31185	2.1
41867	1.3195	2.1
41868	0.946132	2.1
41869	0.946132	2.1
41870	1.31185	2.1
41871	0.946133	2.1
41872	0.946132	2.1
41873	1.3195	2.1

41874	1.11256	0.920047
41875	1.70101	0.897496
41876	1.22297	0.927237
41877	1.22297	0.923478
41878	1.7252	0.961944
41879	1.7252	0.950985
41880	1.99298	0.914978
41881	1.70101	0.941404
41882	1.7252	0.953453
41883	2.18701	0.81392
41884	3.71818	0.851519
41885	1.03218	0.87527
41886	1.7252	0.83199
41887	1.11256	0.890826
41888	1.11256	0.862109
41889	1.7457	0.90618
41890	1.7457	0.874937
41891	1.7457	0.879005
41892	2.187	0.739673
41893	1.7252	0.839566
41894	1.7252	0.84621
41895	2.27351	0.836908
41896	2.27351	0.844708
41897	1.95544	0.935642
41898	1.35897	2.09713
41899	1.44859	1.40574
41900	1.44859	1.73457
41901	1.80709	1.04495
41902	1.80709	1.66837
41903	1.80709	0.865471
41904	1.17822	1.98282
41905	1.35897	1.39223
41906	1.35897	1.93666
41907	1.52782	0.865244
41908	1.52782	1.5771
41909	1.11978	1.65188
41910	0.903546	0.994772
41911	0.977719	1.78863
41912	1.2364	1.0083
41913	1.03812	0.863776
41914	1.03812	2.06629
41915	1.05938	0.815628
41916	1.05938	1.79709
41917	1.11978	1.18328
41918	1.2364	0.828726
41919	0.97772	1.43616
41920	0.903546	1.13494
41921	1.80709	1.79332
41922	1.81847	1.40356
41923	1.96029	1.84331
41924	1.52782	1.5919
41925	2.23956	1.0816
41926	1.80709	1.41278
41927	1.89226	0.472845
41928	2.32962	0.407208
41929	2.5992	0.413032
41930	1.89226	0.423974
41931	2.5992	0.464645

41932	1.89226	0.468017
41933	2.55791	0.05
41934	2.47141	0.05
41935	2.13515	0.05
41936	2.10939	0.05
41937	4.66731	0.05
41938	2.47141	0.05
41939	2.23337	0.05
41940	2.10939	0.05
41941	2.03438	0.05
41942	4.34276	0.05
41943	2.13512	0.05
41944	1.71027	0.05
41945	1.71027	0.05
41946	2.10936	0.05
41947	1.60137	0.05
41948	2.00046	0.05
41949	1.71027	0.05
41950	2.03435	0.05
41951	1.71027	0.05
41952	2.10936	0.05
41953	1.72067	0.05
41954	2.11976	0.05
41955	1.177	0.513247
41956	1.36405	0.808396
41957	2.25635	0.863241
41958	2.60151	0.451944
41959	3.6204	0.701694
41960	0.658869	0.35336
41961	0.781697	0.360452
41962	0.637037	0.322172
41963	0.541148	0.322901
41964	0.654682	0.320887
41965	0.654682	0.351279
41966	0.641224	0.374498
41967	0.641224	0.310914
41968	0.637039	0.343728
41969	0.781696	0.341163
41970	0.658867	0.341646
41971	0.541149	0.339048
41972	1.89226	0.463736
41973	1.59152	0.309225
41974	1.89226	0.3485
41975	1.82125	0.325437
41976	1.89226	0.441049
41977	1.82125	0.42806
41978	2.18967	0.0592626
41979	1.89227	0.0837685
41980	2.72752	0.0864616
41981	2.72752	0.0830935
41982	1.89227	0.0588947
41983	1.89227	0.05
41984	2.18967	0.0543148
41985	1.92547	0.0989911
41986	1.92547	0.0711635
41987	2.72752	0.0595193
41988	1.30572	0.0673769
41989	2.10777	0.0541414

41990	2.39663	2.1
41991	1.92293	2.1
41992	1.96889	2.1
41993	2.45928	2.1
41994	4.36552	2.1
41995	1.02605	2.1
41996	1.92293	2.1
41997	1.9689	2.1
41998	1.04679	2.1
41999	2.99495	2.1
42000	1.96889	2.1
42001	2.45928	2.1
42002	2.50138	2.1
42003	1.99782	2.1
42004	4.47027	2.1
42005	1.9689	2.1
42006	1.03449	2.1
42007	1.07572	2.1
42008	1.04679	2.1
42009	1.99783	2.1
42010	1.9689	2.1
42011	1.72162	0.801761
42012	1.89186	0.626258
42013	2.29771	0.678711
42014	1.80149	0.503736
42015	4.01933	0.627382
42016	1.16482	0.835934
42017	1.97003	0.550457
42018	1.72162	0.508886
42019	1.72162	0.610281
42020	1.29313	0.627272
42021	1.29313	0.790827
42022	2.29771	0.717541
42023	2.84159	0.457635
42024	2.19622	0.53902
42025	1.97366	0.532762
42026	2.50906	0.58724
42027	2.29771	0.612135
42028	1.29313	0.771903
42029	2.4483	0.531348
42030	2.14223	0.524841
42031	2.14228	0.599835
42032	1.294	0.596075
42033	1.294	0.693533
42034	1.21495	0.05
42035	1.70303	0.0543152
42036	1.7924	0.05
42037	1.64002	0.05
42038	3.11963	0.05
42039	2.81021	0.05
42040	1.89226	0.0572898
42041	1.89226	0.05
42042	1.89226	0.05
42043	2.36406	0.05
42044	2.36406	0.05
42045	1.89226	0.05
42046	1.89226	0.0707654
42047	1.89226	0.05

42048	1.89226	0.0538491
42049	1.89226	0.0545125
42050	1.89226	0.060574
42051	2.29976	0.05
42052	1.64002	0.0501805
42053	1.8959	0.05
42054	1.90873	0.05
42055	1.85594	0.05
42056	1.85594	0.05
42057	1.89226	0.0517417
42058	1.89226	0.0713363
42059	1.89226	0.0522883
42060	1.89226	0.0565723
42061	3.78453	0.0524303
42062	1.89226	0.837964
42063	2.52823	2.1
42064	2.83441	1.54151
42065	2.83441	2.1
42066	1.89226	1.21025
42067	1.89226	1.59843
42068	1.04522	1.72937
42069	1.04522	1.40921
42070	1.12831	1.8603
42071	1.18364	2.1
42072	0.640525	1.67548
42073	0.924882	1.74942
42074	1.27877	1.94627
42075	1.43363	0.913191
42076	0.93474	1.75019
42077	1.58926	1.36875
42078	1.29573	1.85267
42079	0.664247	1.92493
42080	2.02191	2.1
42081	1.89226	2.1
42082	1.89226	2.1
42083	1.89226	2.1
42084	2.58639	2.1
42085	2.58639	2.1
42086	1.16759	2.1
42087	2.11828	2.1
42088	1.60936	2.1
42089	2.35846	2.1
42090	3.52605	2.1
42091	1.89226	2.1
42092	1.89226	2.1
42093	1.89226	2.1
42094	1.89226	2.1
42095	3.78453	2.1
42096	2.82765	0.317075
42097	1.89809	0.30821
42098	2.15971	0.316235
42099	1.89809	0.301081
42100	2.29233	0.311398
42101	2.03071	0.264819
42102	1.90664	0.323758
42103	2.15971	0.321342
42104	1.28956	0.314564
42105	1.28956	0.308328

42106	2.64414	0.316843
42107	2.02706	0.295587
42108	2.82765	0.290543
42109	1.89226	0.316091
42110	2.39968	0.310154
42111	2.39968	0.305728
42112	1.89226	0.313102
42113	1.89226	0.305998
42114	0.978643	2.1
42115	0.703968	2.1
42116	0.946129	2.1
42117	0.946129	2.1
42118	0.703939	2.1
42119	0.703939	2.1
42120	0.946159	2.1
42121	0.946159	2.1
42122	0.978642	2.1
42123	0.94613	2.1
42124	0.94613	2.1
42125	0.703968	2.1
42126	1.89226	2.1
42127	2.17309	1.01657
42128	2.17309	2.1
42129	1.47475	1.69984
42130	1.89226	2.02679
42131	1.19393	1.16098
42132	1.89226	2.1
42133	1.89226	2.1
42134	1.89226	2.1
42135	1.89226	1.94919
42136	3.78452	2.1
42137	1.17426	2.1
42138	0.978644	2.1
42139	0.946129	2.1
42140	0.946129	2.1
42141	0.946131	2.1
42142	0.946131	2.1
42143	0.978643	2.1
42144	0.978642	2.1
42145	1.17426	2.1
42146	0.946131	2.1
42147	0.978642	2.1
42148	0.94613	2.1
42149	1.89226	2.1
42150	1.89226	1.01515
42151	1.89226	2.05966
42152	2.17309	1.13331
42153	2.56346	1.97112
42154	2.94389	1.39266
42155	0.946131	2.1
42156	0.946131	1.99353
42157	0.946128	2.1
42158	0.946128	2.1
42159	0.946131	2.1
42160	0.946131	1.9797
42161	0.946128	2.1
42162	0.946128	2.1
42163	0.94613	2.1

42164	0.94613	2.1
42165	0.946129	2.1
42166	0.946129	2.1
42167	1.89226	0.758433
42168	1.89226	0.695165
42169	1.89226	0.897839
42170	1.89226	0.850199
42171	3.78453	0.615914
42172	1.89226	0.896049
42173	1.89226	0.697513
42174	1.89226	1.05912
42175	1.89226	0.9481
42176	1.89226	0.717042
42177	1.89226	0.665366
42178	1.89226	0.721608
42179	1.30733	0.830163
42180	1.89226	1.04706
42181	1.92403	0.960184
42182	3.38635	0.672432
42183	1.89226	0.718999
42184	3.02887	1.06821
42185	2.0815	0.58842
42186	1.88422	0.734068
42187	2.0815	0.88555
42188	1.88422	1.60541
42189	1.89226	1.24831
42190	1.70221	0.862475
42191	1.89226	1.78604
42192	1.20068	1.22141
42193	1.89226	0.888496
42194	1.20068	0.7221
42195	1.01393	1.05736
42196	1.20067	0.832802
42197	1.89226	1.55815
42198	2.44958	1.61127
42199	1.01393	0.695467
42200	1.89226	0.673652
42201	1.89226	2.1
42202	1.89226	1.53399
42203	1.89226	2.1
42204	1.89226	2.1
42205	1.89226	2.1
42206	1.89226	2.1
42207	1.89226	2.1
42208	1.89226	0.560824
42209	1.89226	0.616668
42210	1.89226	1.93559
42211	1.89226	0.678009
42212	1.89226	2.02446
42213	1.89226	2.1
42214	1.89226	1.25623
42215	1.89226	2.1
42216	1.89226	2.1
42217	1.89226	2.1
42218	1.89226	2.1
42219	1.91161	2.1
42220	2.45782	0.919074
42221	1.39653	2.1

42222	2.44652	1.03843
42223	4.35812	2.1
42224	2.73092	0.905254
42225	1.89226	0.441573
42226	1.89226	2.01601
42227	1.89226	0.506961
42228	2.44652	1.3853
42229	2.44652	0.565569
42230	1.89226	2.1
42231	1.89226	0.650002
42232	1.89226	0.702558
42233	1.89226	1.79624
42234	1.89226	0.699161
42235	1.89226	1.81289
42236	2.64924	2.1
42237	1.31109	2.1
42238	1.31109	2.1
42239	1.49958	2.1
42240	1.95039	2.1
42241	2.14451	2.1
42242	0.825157	2.1
42243	0.634856	2.1
42244	0.816836	2.1
42245	0.816836	2.1
42246	0.556743	2.1
42247	0.639142	2.1
42248	0.985106	2.1
42249	0.795045	2.1
42250	0.967461	2.1
42251	0.552494	2.1
42252	0.676829	2.1
42253	0.98803	2.1
42254	2.28564	2.1
42255	2.34084	1.17139
42256	2.3502	1.27147
42257	2.42908	2.1
42258	4.69104	1.67162
42259	2.34084	1.78054
42260	2.18077	2.1
42261	2.42908	2.1
42262	2.2947	0.920803
42263	4.63554	1.9314
42264	2.3502	1.42797
42265	2.42908	2.1
42266	2.38681	2.1
42267	2.50438	0.96589
42268	4.85458	2.1
42269	2.42908	2.1
42270	2.2947	1.24788
42271	2.50438	0.950143
42272	2.40627	2.1
42273	4.79908	1.08926
42274	1.89226	0.698986
42275	2.20711	1.11542
42276	1.89226	0.694084
42277	1.89226	1.39803
42278	2.02693	0.717316
42279	2.02693	1.4671

42280	1.2269	1.97198
42281	2.20711	1.05403
42282	2.0815	0.975869
42283	1.11506	2.1
42284	3.22109	1.61434
42285	0.545802	1.38253
42286	0.547489	1.84909
42287	0.85914	1.0698
42288	1.11641	1.1099
42289	0.899974	1.16894
42290	0.849893	1.98754
42291	0.747885	1.45651
42292	0.747885	0.965575
42293	0.557184	1.77612
42294	0.887039	1.76539
42295	1.08948	1.63003
42296	0.59815	1.71996
42297	0.974139	2.1
42298	1.33385	2.1
42299	1.02368	2.1
42300	1.02368	2.1
42301	1.00833	2.1
42302	1.00833	2.1
42303	1.89226	0.509263
42304	1.89226	0.371714
42305	1.89226	0.523921
42306	1.89226	0.342815
42307	3.78453	0.398506
42308	1.74651	0.551538
42309	1.44605	0.352813
42310	1.63557	0.520259
42311	1.31993	0.0589633
42312	3.08162	0.403809
42313	1.89226	0.539322
42314	1.89226	0.390309
42315	1.89226	0.504434
42316	1.89226	0.0943791
42317	3.78452	0.36672
42318	1.63557	0.537452
42319	1.31992	0.256404
42320	1.52615	0.540826
42321	1.18549	0.0803268
42322	2.82106	0.323824
42323	1.89226	0.358727
42324	2.58101	0.496869
42325	2.73797	0.536425
42326	2.73797	0.389054
42327	1.89226	0.553957
42328	1.89226	0.362579
42329	1.91762	0.592914
42330	1.44354	0.572792
42331	1.33622	0.562657
42332	1.33622	0.556886
42333	2.20248	0.624115
42334	2.20248	0.607671
42335	1.11712	2.1
42336	1.11712	2.1
42337	1.11421	2.1

42338	1.11421	2.1
42339	0.833871	2.1
42340	0.926528	2.1
42341	1.3048	2.1
42342	1.40335	2.1
42343	0.926527	2.1
42344	1.40335	2.1
42345	1.3048	2.1
42346	0.833871	2.1
42347	0.879657	2.1
42348	0.937695	2.1
42349	0.946129	2.1
42350	0.946129	2.1
42351	0.851541	2.1
42352	0.851541	2.1
42353	1.1126	2.1
42354	1.04519	2.1
42355	0.851486	2.1
42356	0.980501	2.1
42357	0.898251	2.1
42358	0.851528	2.1
42359	1.37756	2.1
42360	2.73081	2.1
42361	2.44311	2.1
42362	2.44311	2.1
42363	1.66428	2.1
42364	1.66428	2.1
42365	1.89226	2.1
42366	1.89226	1.5891
42367	1.89226	2.1
42368	1.89226	1.57698
42369	3.78452	2.1
42370	1.65781	2.1
42371	1.89837	1.79933
42372	1.46713	2.1
42373	1.98027	1.32482
42374	3.63808	2.1
42375	2.29054	1.92429
42376	2.26369	0.584166
42377	1.98027	1.50491
42378	2.15617	0.251249
42379	4.24396	0.738923
42380	1.89226	1.98127
42381	1.89226	0.584064
42382	1.89226	1.76439
42383	1.89226	0.574177
42384	3.78452	0.776958
42385	0.946131	1.1185
42386	0.946131	2.1
42387	0.946128	2.1
42388	0.946128	1.77382
42389	0.946129	1.25458
42390	0.946129	1.44348
42391	0.94613	2.1
42392	0.94613	2.1
42393	0.94613	2.1
42394	0.94613	2.1
42395	0.94613	2.1

42396	0.94613	2.1
42397	0.812186	1.49237
42398	0.812186	2.1
42399	0.819781	1.52469
42400	0.819781	2.1
42401	0.913532	1.13755
42402	0.990134	1.19146
42403	0.733564	2.1
42404	0.641833	2.1
42405	0.641832	2.1
42406	0.913533	2.1
42407	0.733564	2.1
42408	0.990134	2.1
42409	0.994262	0.347336
42410	0.994262	1.98423
42411	0.997354	1.00937
42412	0.997354	0.584836
42413	1.00775	0.407388
42414	1.07808	0.531828
42415	0.913533	1.45652
42416	0.990135	1.41207
42417	0.913532	0.720473
42418	1.00775	0.719589
42419	0.990134	0.701326
42420	1.07808	0.704303
42421	0.946131	2.01398
42422	0.946131	0.59326
42423	0.946128	1.32743
42424	0.946128	0.593624
42425	0.946128	0.56261
42426	0.946128	0.545825
42427	0.94613	1.52717
42428	0.94613	1.70629
42429	0.946129	0.794757
42430	0.946129	0.7973
42431	0.946129	0.69901
42432	0.946129	0.712206
42433	0.816232	2.1
42434	0.760038	2.1
42435	0.845999	2.1
42436	0.845999	2.1
42437	1.0631	2.1
42438	1.28131	2.1
42439	0.474065	2.1
42440	0.591905	2.1
42441	0.601756	2.1
42442	1.23903	2.1
42443	0.988592	2.1
42444	0.49051	2.1
42445	2.57021	2.1
42446	1.89226	2.09294
42447	2.68172	2.1
42448	1.89226	0.960751
42449	4.46247	1.72778
42450	1.89226	2.1
42451	1.89226	1.74198
42452	1.89226	1.37632
42453	1.89226	1.28221

42454	3.78452	1.57051
42455	1.12573	2.1
42456	1.65504	1.18647
42457	1.38848	1.81831
42458	1.38848	2.1
42459	1.30567	0.940511
42460	1.30567	1.92342
42461	2.82413	2.1
42462	1.89226	1.02254
42463	2.68172	2.1
42464	2.68172	2.1
42465	1.89226	1.56976
42466	1.89226	2.1
42467	1.89226	1.42939
42468	1.89226	1.67109
42469	1.89226	2.1
42470	1.89226	0.923662
42471	3.78452	1.4273
42472	1.30567	2.1
42473	1.65504	1.46523
42474	1.52724	2.1
42475	1.89249	1.89213
42476	3.18228	2.1
42477	2.57386	0.203278
42478	1.89226	0.437683
42479	1.89226	0.408048
42480	2.66753	0.263907
42481	1.89226	0.350422
42482	2.66753	0.273361
42483	1.89226	0.193598
42484	1.89226	0.488557
42485	2.2973	0.450131
42486	3.12479	0.296455
42487	1.89226	0.403581
42488	2.57385	0.296208
42489	0.489293	2.1
42490	0.672698	2.1
42491	0.59038	2.1
42492	0.738357	2.1
42493	0.62269	2.1
42494	0.62269	2.1
42495	0.640388	2.1
42496	0.640388	2.1
42497	0.59038	2.1
42498	0.489293	2.1
42499	0.738357	2.1
42500	0.672698	2.1
42501	1.89226	2.1
42502	1.89226	0.979207
42503	1.89226	2.1
42504	1.89226	0.692439
42505	3.78452	1.68521
42506	2.82858	1.57319
42507	1.89226	0.748619
42508	2.5671	2.1
42509	1.89226	2.1
42510	2.5671	2.1
42511	1.89226	1.01181

42512	2.06581	0.05
42513	2.47308	0.05
42514	1.89433	0.05
42515	1.67571	0.05
42516	3.87274	0.05
42517	2.55791	0.05
42518	2.10276	0.05
42519	2.47308	0.05
42520	2.47308	0.05
42521	1.83415	0.05
42522	1.90922	0.05
42523	1.18421	0.05
42524	2.03277	0.05
42525	1.18421	0.05
42526	2.01275	0.05
42527	1.42818	0.05
42528	2.25673	0.05
42529	0.882904	0.05
42530	0.958492	0.05
42531	0.905756	0.05
42532	0.905756	0.05
42533	1.06352	0.05
42534	0.797654	0.05
42535	1.11319	0.05
42536	0.716795	0.05
42537	0.820721	0.05
42538	0.731963	0.05
42539	1.13143	0.05
42540	1.10528	0.05
42541	1.89226	0.268427
42542	1.89226	0.165716
42543	1.89226	0.185356
42544	1.89226	0.312658
42545	1.89226	0.124829
42546	1.89226	0.269089
42547	1.01241	0.332012
42548	0.703929	0.35824
42549	0.946133	0.239207
42550	0.946133	0.276523
42551	1.04072	0.275138
42552	1.163	0.172999
42553	0.639952	0.33878
42554	0.851543	0.137691
42555	0.946132	0.108701
42556	0.920761	0.242628
42557	0.711038	0.335754
42558	0.711038	0.379179
42559	1.89226	0.225052
42560	1.89226	0.121559
42561	1.89226	0.132005
42562	1.89226	0.264676
42563	1.89226	0.106412
42564	1.89226	0.233894
42565	1.89226	0.100385
42566	1.89226	0.132671
42567	2.41075	0.092062
42568	1.89226	0.0689343
42569	3.78451	0.119311

42570	0.968476	1.61986
42571	1.20674	0.903431
42572	1.13456	0.898446
42573	1.13456	2.08377
42574	1.02074	0.825261
42575	1.02074	1.50753
42576	1.02074	2.1
42577	1.20674	1.59451
42578	1.07866	1.12786
42579	1.28649	0.97154
42580	2.2854	1.78062
42581	1.94221	0.442323
42582	1.36717	0.422294
42583	1.45535	0.434045
42584	1.93771	0.35159
42585	3.39756	0.446923
42586	1.94222	0.478015
42587	2.25035	0.406735
42588	2.41806	0.440616
42589	2.42256	0.398563
42590	1.93772	0.452323
42591	1.94222	0.431154
42592	1.49906	0.28286
42593	1.93771	0.288925
42594	1.45535	0.376707
42595	1.45535	0.404064
42596	1.91484	0.209447
42597	1.91484	0.224168
42598	1.91484	0.296411
42599	2.25035	0.347964
42600	1.93772	0.36835
42601	1.93772	0.378745
42602	2.11465	0.232638
42603	2.11465	0.235259
42604	2.61735	0.295891
42605	1.5082	0.18582
42606	2.36481	0.358541
42607	1.58597	0.310253
42608	2.36481	0.272011
42609	1.46653	0.207836
42610	2.36482	0.39072
42611	1.08133	0.462467
42612	1.5082	0.174187
42613	2.01868	0.39718
42614	3.52688	0.422694
42615	0.94613	0.270779
42616	0.94613	0.277705
42617	0.946131	0.336173
42618	0.946131	0.426869
42619	0.946129	0.408043
42620	0.946129	0.306338
42621	0.946132	0.301275
42622	0.946132	0.359497
42623	0.94613	0.340112
42624	0.94613	0.289309
42625	0.946131	0.32688
42626	0.946131	0.333635
42627	2.53725	0.144815

42628	1.89227	0.180625
42629	1.89227	0.180697
42630	2.27073	0.185671
42631	1.89227	0.148833
42632	2.27073	0.151473
42633	1.89227	0.273349
42634	1.89227	0.416374
42635	1.89227	0.442972
42636	1.89227	0.342845
42637	1.89227	0.356502
42638	1.89227	0.270403
42639	1.86766	2.1
42640	2.83487	2.1
42641	2.84318	2.1
42642	2.59401	2.1
42643	2.11684	2.1
42644	1.86766	2.1
42645	1.10638	2.1
42646	2.11684	2.1
42647	1.29111	2.1
42648	2.37439	2.1
42649	3.40796	2.1
42650	2.56843	2.1
42651	1.89226	2.1
42652	1.89226	2.1
42653	2.71932	2.1
42654	4.46069	2.1
42655	1.89226	2.1
42656	1.89226	2.1
42657	1.89226	2.1
42658	1.89226	2.1
42659	3.78452	2.1
42660	0.6416	2.1
42661	0.697585	2.1
42662	0.670706	2.1
42663	0.598927	2.1
42664	0.644313	2.1
42665	0.644313	2.1
42666	0.652199	2.1
42667	0.652199	2.1
42668	0.697587	2.1
42669	0.670705	2.1
42670	0.598926	2.1
42671	0.641601	2.1
42672	1.89226	2.1
42673	1.89226	2.1
42674	1.89226	2.1
42675	1.89226	2.1
42676	1.89226	2.1
42677	1.89226	2.1
42678	0.697585	2.1
42679	0.730617	2.1
42680	0.712082	2.1
42681	0.670706	2.1
42682	0.69854	2.1
42683	0.69854	2.1
42684	0.702783	2.1
42685	0.702783	2.1

42686	0.730618	2.1
42687	0.712081	2.1
42688	0.697586	2.1
42689	0.670705	2.1
42690	1.89226	1.1482
42691	1.89226	1.50355
42692	1.89226	0.826917
42693	1.89226	2.1
42694	1.89226	0.789818
42695	1.89226	2.1
42696	2.0815	2.07013
42697	1.12076	2.1
42698	1.38662	1.01786
42699	2.01244	2.1
42700	3.41508	2.1
42701	1.89226	2.1
42702	2.54324	2.1
42703	1.91981	2.1
42704	1.89226	2.1
42705	2.57079	2.1
42706	2.54324	2.1
42707	2.54325	2.1
42708	1.94779	2.1
42709	1.76879	2.1
42710	1.73811	2.1
42711	2.5708	2.1
42712	2.54325	2.1
42713	1.89226	2.1
42714	2.57079	2.1
42715	1.91894	2.1
42716	1.89226	2.1
42717	2.59747	2.1
42718	2.57079	2.1
42719	2.5708	2.1
42720	1.82268	2.1
42721	1.79738	2.1
42722	1.77071	2.1
42723	2.59747	2.1
42724	2.5708	2.1
42725	1.89226	0.476431
42726	1.89226	0.636117
42727	1.89226	0.425532
42728	1.89226	0.682286
42729	3.78451	0.524143
42730	2.21582	0.424749
42731	1.94412	0.622138
42732	2.13428	0.420559
42733	1.86292	0.574623
42734	4.07839	0.495823
42735	1.94412	0.500843
42736	1.5854	2.1
42737	1.86293	0.529002
42738	1.50767	2.01342
42739	3.45179	0.737435
42740	1.89226	0.515403
42741	1.89226	2.1
42742	1.89226	0.614402
42743	1.89226	2.04567

42744	3.78452	0.728168
42745	1.89226	0.423086
42746	1.89226	0.783315
42747	1.89226	0.427599
42748	1.89226	0.624037
42749	3.78452	0.521325
42750	2.13428	0.413785
42751	1.86293	0.550382
42752	2.04737	0.405089
42753	1.77122	0.615164
42754	3.9055	0.483975
42755	1.86293	0.408133
42756	1.50767	2.04207
42757	1.41077	0.585477
42758	1.41077	0.813383
42759	1.77123	0.588068
42760	1.86813	0.863624
42761	0.810943	0.577372
42762	0.810943	2.1
42763	0.810945	0.541801
42764	0.810945	0.591117
42765	0.810958	0.703748
42766	0.810958	0.693701
42767	1.89226	2.06449
42768	1.35168	0.7407
42769	1.08132	0.595826
42770	1.08132	1.97643
42771	1.0813	0.708566
42772	0.745979	2.1
42773	0.745979	2.1
42774	0.778781	2.1
42775	0.778781	2.1
42776	0.59549	2.1
42777	0.649658	2.1
42778	0.994784	2.1
42779	0.875102	2.1
42780	0.875102	2.1
42781	0.59549	2.1
42782	0.649658	2.1
42783	0.994784	2.1
42784	1.57887	2.1
42785	1.75021	2.1
42786	1.13341	2.1
42787	1.13341	2.1
42788	1.19098	2.1
42789	1.63644	2.1
42790	2.5761	0.39596
42791	2.43404	0.517253
42792	2.44813	0.379906
42793	2.31751	0.544072
42794	4.88218	0.455723
42795	2.43405	0.45048
42796	2.22575	0.777541
42797	2.31751	0.439855
42798	2.10893	0.735794
42799	4.54298	0.614216
42800	2.44813	0.368821
42801	2.31751	0.533036

42802	2.30908	0.354291
42803	2.19815	0.517122
42804	4.64628	0.443985
42805	2.31751	0.446979
42806	2.10893	0.73445
42807	2.19815	0.464389
42808	1.98061	1.66867
42809	4.30708	0.610487
42810	0.94613	2.1
42811	0.94613	1.58059
42812	1.40372	2.01663
42813	1.40372	2.1
42814	1.40372	2.1
42815	1.37167	2.1
42816	0.946132	0.987413
42817	0.946132	2.1
42818	0.946131	2.1
42819	1.40372	2.1
42820	0.94613	2.1
42821	1.37167	2.1
42822	2.80744	2.1
42823	2.06984	2.1
42824	2.83418	2.1
42825	2.74335	2.1
42826	2.16067	2.1
42827	2.06984	2.1
42828	1.077	0.338383
42829	0.809116	0.269966
42830	0.600419	0.445169
42831	0.790277	0.291245
42832	0.927647	0.23903
42833	0.758598	0.243669
42834	0.85749	0.365842
42835	0.85749	0.402764
42836	1.02417	0.280326
42837	0.600411	0.324748
42838	0.751701	0.314645
42839	1.07702	0.276052
42840	1.89226	2.1
42841	1.89226	2.1
42842	1.89226	2.1
42843	1.89226	2.1
42844	1.89226	2.1
42845	1.89226	2.1
42846	1.89226	2.1
42847	1.89226	2.06262
42848	1.89226	2.0529
42849	1.89226	2.1
42850	3.78453	2.1
42851	1.89226	2.1
42852	1.89226	2.1
42853	1.89226	2.1
42854	1.89226	2.1
42855	1.89226	2.1
42856	1.89226	2.1
42857	0.946131	2.1
42858	0.946131	2.1
42859	0.946131	2.1

42860	0.946131	2.05129
42861	0.946133	2.1
42862	0.946133	1.96
42863	0.946128	2.1
42864	0.946128	2.1
42865	0.946131	2.1
42866	0.946131	2.1
42867	0.94613	2.1
42868	0.94613	2.1
42869	2.17159	2.1
42870	2.00083	1.87641
42871	2.21305	2.1
42872	2.00551	1.28376
42873	2.20837	2.1
42874	2.00083	1.3799
42875	2.20837	1.47634
42876	1.80328	1.04062
42877	2.22962	2.07968
42878	2.00083	1.31504
42879	2.03207	0.973363
42880	1.80328	0.936326
42881	2.28564	2.1
42882	2.20837	1.31987
42883	2.30406	2.1
42884	2.17159	1.97882
42885	2.34084	1.90117
42886	2.20837	1.94252
42887	1.09523	0.839957
42888	1.09523	1.81083
42889	1.09934	1.97876
42890	1.09934	2.1
42891	1.01603	0.797773
42892	1.09038	1.91794
42893	1.10419	0.909309
42894	1.17042	2.1
42895	1.17042	1.99023
42896	1.09038	1.44403
42897	1.10419	1.06356
42898	1.01603	2.01038
42899	2.60152	0.50034
42900	0.976334	0.643433
42901	2.22344	0.680633
42902	2.22344	0.519447
42903	1.36405	0.737426
42904	1.36405	0.391136
42905	1.89226	2.00273
42906	1.70302	2.1
42907	1.69019	2.1
42908	1.70302	1.85509
42909	1.73933	2.1
42910	1.70303	0.865927
42911	1.89226	2.1
42912	1.89226	0.672619
42913	1.89226	0.957699
42914	1.89226	1.6101
42915	3.78452	1.03141
42916	1.89226	1.17818
42917	1.59036	1.7792

42918	1.65683	2.1
42919	1.59036	1.17588
42920	1.95873	2.0934
42921	1.89226	0.74574
42922	0.946131	0.650847
42923	0.946131	0.746771
42924	0.946127	1.68627
42925	0.946127	1.08936
42926	0.946127	1.11778
42927	0.946127	0.584236
42928	0.946131	1.55122
42929	0.946131	0.769144
42930	0.946129	0.846219
42931	0.946129	0.854997
42932	0.94613	0.88137
42933	0.94613	0.853296
42934	0.851541	2.1
42935	0.851541	2.1
42936	0.851483	2.1
42937	1.15713	2.1
42938	0.851539	2.1
42939	0.851539	1.00333
42940	0.942127	2.1
42941	1.14455	2.1
42942	0.838683	2.1
42943	0.946131	2.1
42944	0.957503	2.1
42945	0.957504	2.1
42946	1.89226	0.736185
42947	1.89226	0.287571
42948	1.89226	0.574792
42949	1.89226	1.35085
42950	3.78452	0.421442
42951	1.89226	0.125364
42952	1.89226	0.352135
42953	1.89226	0.428024
42954	1.89226	0.334726
42955	1.89226	0.124487
42956	1.89226	0.0933911
42957	1.89226	0.326256
42958	1.89226	0.196187
42959	1.89226	0.298219
42960	1.89226	0.232004
42961	3.78453	0.258022
42962	1.89226	0.276948
42963	1.89226	2.1
42964	1.89226	1.54447
42965	1.89226	0.996523
42966	3.78453	1.36453
42967	1.22575	0.588113
42968	1.05438	0.533273
42969	0.946131	0.578998
42970	0.946131	0.563148
42971	0.946129	1.1022
42972	1.22575	0.445477
42973	1.05438	0.550344
42974	0.946132	0.536847
42975	1.05438	0.563782

42976	1.05438	0.756467
42977	0.946131	0.557582
42978	0.946131	0.634229
42979	2.4515	0.561967
42980	1.13726	0.124047
42981	2.10875	0.219664
42982	1.49515	0.172296
42983	2.10875	0.198818
42984	1.49515	0.127756
42985	0.831477	0.214441
42986	0.747575	0.267065
42987	1.22575	0.451307
42988	1.28174	0.169834
42989	1.28174	0.393854
42990	0.831476	0.282994
42991	1.22575	0.340294
42992	0.747575	0.0946368
42993	1.03561	0.332478
42994	1.03561	0.448982
42995	1.02162	0.244751
42996	1.02162	0.271107
42997	1.89227	1.19994
42998	2.56348	0.750916
42999	1.89227	2.1
43000	2.45151	0.613568
43001	1.89227	2.1
43002	2.45151	0.692016
43003	1.40013	0.711883
43004	1.89226	1.55549
43005	1.89226	0.989123
43006	0.946643	0.536314
43007	1.89226	1.28164
43008	0.946643	0.915719
43009	1.89226	0.0996338
43010	1.89226	0.163811
43011	1.89226	0.132353
43012	1.89226	0.168953
43013	3.78453	0.172468
43014	1.89226	0.241399
43015	1.89226	0.286354
43016	1.89226	0.230583
43017	1.89226	0.408366
43018	3.78453	0.225589
43019	0.946131	0.343009
43020	0.946131	0.301059
43021	0.946128	0.196605
43022	0.946128	0.219407
43023	0.94613	0.313615
43024	0.94613	0.345679
43025	0.946129	0.250979
43026	0.946129	0.204261
43027	0.94613	0.281311
43028	0.94613	0.16652
43029	0.94613	0.241329
43030	0.94613	0.318869
43031	1.89226	0.0995322
43032	1.89226	0.117444
43033	1.17244	0.105911

43034	1.89226	0.118761
43035	0.975291	0.109209
43036	1.57408	0.123826
43037	0.946132	0.278395
43038	0.946132	0.145518
43039	0.946131	0.301936
43040	0.946131	0.187809
43041	0.946132	0.244216
43042	0.946132	0.272377
43043	0.946131	0.187512
43044	0.946131	0.198273
43045	0.946131	0.244254
43046	0.946131	0.237786
43047	0.946132	0.205239
43048	0.946132	0.238974
43049	2.74744	0.219412
43050	1.89226	0.401156
43051	2.70805	0.257697
43052	1.89226	0.309083
43053	2.70805	0.279134
43054	1.89226	0.361015
43055	2.74744	0.156007
43056	1.89226	0.275597
43057	2.57465	0.252774
43058	1.89226	0.386792
43059	4.6397	0.318505
43060	1.89226	0.349233
43061	1.89226	0.548841
43062	1.89226	0.543494
43063	1.89226	0.729882
43064	3.78452	0.511527
43065	1.89226	0.492434
43066	1.89226	0.62571
43067	1.89226	0.449901
43068	1.89226	0.350091
43069	1.89226	0.73782
43070	1.89226	0.670788
43071	2.16206	0.119385
43072	2.67861	0.147916
43073	3.11996	0.142264
43074	1.70303	0.122405
43075	2.89015	0.135295
43076	1.486	0.118856
43077	2.12839	0.13243
43078	2.83631	0.293447
43079	3.08096	0.165123
43080	1.84157	0.140631
43081	3.08096	0.203813
43082	1.84157	0.167582
43083	1.89225	0.857314
43084	1.89225	0.585093
43085	1.89225	0.69975
43086	1.89225	0.897494
43087	1.89225	0.663351
43088	1.89225	0.838136
43089	1.89226	0.558523
43090	1.89226	0.588714
43091	1.89226	0.907662

43092	1.89226	0.537387
43093	3.78452	0.545941
43094	1.89226	0.483655
43095	2.49041	0.485229
43096	1.89226	0.48214
43097	1.89226	0.314638
43098	2.27073	0.297046
43099	2.27073	0.46931
43100	1.09904	0.225872
43101	0.677147	0.187062
43102	1.3365	0.16953
43103	0.95759	0.276322
43104	0.957591	0.205902
43105	0.677148	0.255631
43106	1.09904	0.257529
43107	1.3365	0.211941
43108	0.996015	0.204055
43109	0.996015	0.202421
43110	1.01764	0.312767
43111	1.01764	0.211147
43112	2.77349	0.387994
43113	2.72723	0.197692
43114	2.43156	0.421535
43115	2.4564	0.207565
43116	5.20505	0.371551
43117	2.77349	0.336043
43118	2.68508	0.180172
43119	2.33761	0.293564
43120	2.4564	0.194163
43121	5.1111	0.217308
43122	2.43156	0.260202
43123	2.70208	0.180298
43124	1.85779	0.334938
43125	2.10695	0.167073
43126	4.77572	0.180346
43127	2.33761	0.211811
43128	2.70208	0.169729
43129	1.85779	0.256214
43130	1.61597	0.136615
43131	4.19542	0.201667
43132	0.946132	1.18283
43133	0.946132	1.56528
43134	0.746729	1.41958
43135	0.746729	1.86589
43136	0.946132	1.91829
43137	0.915958	1.51308
43138	0.946133	1.1651
43139	0.74673	2.02543
43140	0.946133	1.52521
43141	0.946133	1.52584
43142	0.915958	1.53021
43143	0.746729	1.60171
43144	1.50743	2.1
43145	2.45501	1.4523
43146	2.29118	2.1
43147	2.29118	1.8253
43148	1.75482	2.1
43149	1.75482	1.81517

43150	2.45501	1.91762
43151	2.76927	0.573187
43152	2.29118	1.73233
43153	2.64523	0.405443
43154	5.10024	0.773267
43155	1.25559	2.1
43156	2.29118	1.73789
43157	2.0815	2.1
43158	2.26387	1.80898
43159	1.35668	2.1
43160	1.47667	1.84836
43161	2.28481	1.31583
43162	2.64523	0.603774
43163	2.50057	1.45285
43164	2.50057	0.586263
43165	2.29118	1.55753
43166	2.29118	0.617766
43167	0.946129	0.432543
43168	0.946129	0.41463
43169	1.23929	0.387332
43170	1.15246	0.394108
43171	0.946131	0.40148
43172	0.946131	0.437426
43173	1.15246	0.411625
43174	1.15246	0.381284
43175	0.946131	0.41271
43176	1.15246	0.421121
43177	0.946131	0.381595
43178	1.23928	0.412519
43179	0.946128	0.450104
43180	0.946128	0.440967
43181	1.15246	0.400012
43182	0.942814	0.451181
43183	0.946131	0.407592
43184	0.946131	0.450093
43185	0.961347	0.43209
43186	0.942811	0.425768
43187	0.946131	0.41552
43188	0.961347	0.40428
43189	0.946131	0.347921
43190	1.15246	0.418082
43191	2.32334	0.437894
43192	1.57621	0.383263
43193	1.90335	0.393385
43194	1.24538	0.37212
43195	3.47956	0.362263
43196	0.965762	0.236752
43197	0.965762	0.332636
43198	1.00245	0.24201
43199	1.00245	0.258822
43200	0.92786	0.282865
43201	0.705297	0.189113
43202	0.856414	0.293711
43203	1.28968	0.246106
43204	1.00306	0.237756
43205	0.705303	0.224545
43206	1.28967	0.269035
43207	0.975478	0.302473

43208	3.05308	2.1
43209	1.76816	2.1
43210	1.76816	2.1
43211	1.69129	2.1
43212	2.58731	2.1
43213	2.51043	2.1
43214	1.89226	0.263558
43215	2.71464	0.323848
43216	2.34627	0.316242
43217	1.89226	0.35719
43218	2.34627	0.236629
43219	1.89226	0.279399
43220	2.34627	0.219389
43221	2.71464	0.230085
43222	1.98252	0.194141
43223	1.98252	0.313826
43224	1.51026	0.189707
43225	2.34627	0.236519
43226	2.38698	1.00384
43227	2.35142	2.1
43228	2.60343	1.15116
43229	2.6636	1.14094
43230	4.99041	1.19192
43231	1.07811	1.99098
43232	1.1952	2.1
43233	1.17571	1.67108
43234	1.30171	0.938998
43235	1.18322	0.892107
43236	1.18322	2.1
43237	1.18769	1.86889
43238	1.18769	0.977628
43239	1.1952	2.08811
43240	1.30171	1.82821
43241	1.17571	1.83737
43242	1.07811	1.62241
43243	1.89226	1.2419
43244	2.6636	1.72574
43245	1.89226	0.714328
43246	2.60343	1.27467
43247	1.89226	1.1692
43248	2.60343	2.1
43249	1.5344	0.0558428
43250	1.4516	0.0636299
43251	1.17816	0.0539028
43252	1.21056	0.0517036
43253	2.71257	0.0639693
43254	1.89226	1.82121
43255	1.89226	0.704116
43256	1.89226	0.898474
43257	1.89226	1.0282
43258	3.78452	0.834086
43259	1.89226	0.968841
43260	1.89226	0.533456
43261	1.89226	0.705775
43262	1.89226	0.630467
43263	1.65573	0.83144
43264	1.65573	0.685657
43265	1.89226	0.85167

43266	1.89226	1.07585
43267	1.89226	1.99854
43268	2.64212	0.696291
43269	3.78452	1.20597
43270	1.89226	0.72161
43271	1.89226	2.1
43272	1.89226	0.765347
43273	1.89226	2.1
43274	3.78452	0.860917
43275	2.7367	0.484963
43276	2.07947	2.1
43277	2.47319	0.764697
43278	1.85057	2.1
43279	4.55267	1.74658
43280	2.07948	2.1
43281	1.3294	2.1
43282	1.85057	2.1
43283	1.09962	2.1
43284	3.1791	2.1
43285	1.89226	2.1
43286	1.89226	2.1
43287	1.89226	2.1
43288	1.89226	2.1
43289	3.78453	2.1
43290	0.94613	2.1
43291	0.94613	0.669554
43292	0.94613	0.730596
43293	0.94613	2.1
43294	0.946128	2.1
43295	0.946128	2.1
43296	0.946132	0.659265
43297	0.946132	0.671471
43298	0.94613	1.17101
43299	0.94613	1.43257
43300	0.946131	1.49043
43301	0.946131	1.27937
43302	1.00875	2.1
43303	1.00875	0.627073
43304	1.01676	2.1
43305	1.01676	0.637841
43306	0.925282	2.1
43307	0.804877	2.1
43308	1.2366	0.672532
43309	1.10023	0.671959
43310	0.804878	0.964779
43311	1.10023	0.990583
43312	1.2366	0.807691
43313	0.925284	0.771988
43314	1.89226	2.1
43315	1.89226	2.1
43316	1.89226	2.1
43317	1.89226	2.1
43318	3.78453	2.1
43319	1.89226	2.1
43320	1.89226	2.1
43321	1.89226	2.1
43322	1.89226	2.1
43323	1.89226	2.1

43324	1.89226	2.1
43325	1.09766	2.1
43326	1.14842	2.1
43327	1.02909	2.1
43328	1.07659	2.1
43329	1.16718	2.1
43330	0.954189	2.1
43331	1.06653	2.1
43332	1.06653	2.1
43333	1.20738	2.1
43334	1.02909	2.1
43335	1.09767	2.1
43336	0.815951	2.1
43337	2.00793	2.1
43338	1.14048	2.1
43339	1.19086	2.1
43340	1.13723	2.1
43341	2.00793	2.1
43342	2.11772	2.1
43343	1.89226	2.1
43344	1.89226	2.1
43345	1.89226	2.1
43346	1.89226	2.1
43347	1.89226	2.1
43348	1.89226	2.1
43349	1.89226	2.1
43350	1.89226	2.1
43351	1.89226	2.1
43352	1.89226	2.1
43353	1.89226	2.1
43354	1.89226	2.1
43355	1.1779	2.1
43356	1.26166	2.1
43357	1.0875	2.1
43358	1.00397	2.1
43359	1.13286	2.1
43360	1.13286	2.1
43361	1.13277	2.1
43362	1.13277	2.1
43363	1.26166	2.1
43364	1.08749	2.1
43365	1.1779	2.1
43366	1.00396	2.1
43367	1.0875	2.1
43368	1.00397	2.1
43369	0.685749	2.1
43370	0.595432	2.1
43371	0.83976	2.1
43372	0.83976	2.1
43373	0.843169	2.1
43374	0.843169	2.1
43375	1.0875	2.1
43376	0.685749	2.1
43377	1.00397	2.1
43378	0.595432	2.1
43379	1.89226	2.1
43380	1.86443	2.1
43381	2.4988	2.1

43382	2.0815	2.1
43383	2.28173	2.1
43384	1.86443	2.1
43385	1.89226	2.1
43386	1.89226	2.1
43387	1.89226	2.1
43388	1.89226	2.1
43389	1.89226	2.1
43390	1.89226	2.1
43391	0.946131	2.1
43392	0.946131	2.1
43393	0.969491	2.1
43394	0.949105	2.1
43395	0.949103	2.1
43396	0.949103	2.1
43397	0.946133	2.1
43398	0.946133	2.1
43399	0.946131	2.1
43400	0.969492	2.1
43401	0.946131	2.1
43402	0.949105	2.1
43403	1.89226	2.1
43404	2.07428	2.1
43405	1.93464	2.1
43406	1.89226	2.1
43407	2.11667	2.1
43408	2.07428	2.1
43409	0.946132	2.1
43410	0.946132	2.1
43411	0.946131	2.1
43412	0.946131	2.1
43413	0.946132	2.1
43414	0.946132	2.1
43415	0.946132	2.1
43416	0.946132	2.1
43417	0.946132	2.1
43418	0.946132	2.1
43419	0.946132	2.1
43420	0.946132	2.1
43421	1.97992	2.1
43422	1.89226	2.1
43423	1.89226	2.1
43424	1.93898	2.1
43425	1.93898	2.1
43426	1.89226	2.1
43427	2.12475	0.535727
43428	2.79029	0.580209
43429	2.7803	0.789327
43430	2.13052	0.510579
43431	4.92081	0.556113
43432	1.06238	0.695993
43433	1.06526	0.548832
43434	0.642901	0.501109
43435	0.655055	0.583696
43436	0.856391	0.521636
43437	0.856391	0.517726
43438	0.86104	0.871085
43439	0.86104	0.504594

43440	1.06526	0.567265
43441	0.655055	0.560188
43442	0.642901	0.550776
43443	1.06238	0.529915
43444	2.7803	0.676384
43445	2.13053	0.457814
43446	2.14346	0.553725
43447	2.77937	0.559892
43448	4.9099	0.555383
43449	2.14346	0.620141
43450	1.31011	0.447857
43451	1.34124	0.547718
43452	1.31011	0.594744
43453	2.16166	0.494292
43454	2.13053	0.511517
43455	1.89226	2.1
43456	2.58176	2.1
43457	2.01422	2.1
43458	1.89226	2.1
43459	2.70373	2.1
43460	2.58176	2.1
43461	1.89226	2.1
43462	1.89226	2.1
43463	1.89226	2.1
43464	1.89226	2.1
43465	1.89226	2.1
43466	1.89226	2.1
43467	0.946128	2.1
43468	0.946128	2.1
43469	1.38521	2.1
43470	1.38521	2.1
43471	1.38521	2.1
43472	1.35186	2.1
43473	0.946131	2.1
43474	0.946131	2.1
43475	0.94613	2.1
43476	1.38521	2.1
43477	0.946131	2.1
43478	1.35186	2.1
43479	0.946133	2.1
43480	0.946133	2.1
43481	0.946132	2.1
43482	0.946132	2.1
43483	0.946132	2.1
43484	0.946132	2.1
43485	0.946132	2.1
43486	0.946132	2.1
43487	0.946132	2.1
43488	0.946132	2.1
43489	0.946133	2.1
43490	0.946133	2.1
43491	2.33374	2.1
43492	1.89226	2.1
43493	1.89226	2.1
43494	1.89226	2.1
43495	2.60377	2.1
43496	2.60377	2.1
43497	2.6771	0.0660306

43498	1.36416	0.0903795
43499	1.64983	0.0746778
43500	1.64983	0.0682229
43501	2.44131	0.0830412
43502	2.44131	0.0799473
43503	2.58321	0.461065
43504	1.89226	0.482498
43505	2.44283	0.392745
43506	2.44283	0.417901
43507	1.89226	0.59303
43508	1.89226	0.748586
43509	2.58321	0.556294
43510	2.44283	0.435155
43511	1.49358	0.379527
43512	1.57702	0.443702
43513	3.93641	0.405117
43514	0.951913	0.0547139
43515	0.81556	0.0508846
43516	0.946132	0.0672625
43517	0.946132	0.0644839
43518	1.04072	0.0666439
43519	1.12182	0.0658319
43520	0.856745	0.0522493
43521	0.851542	0.0528426
43522	0.923163	0.0540126
43523	0.923163	0.0707838
43524	1.04304	0.0646407
43525	0.85098	0.05
43526	2.70125	1.01693
43527	2.37734	0.956657
43528	2.63495	0.837824
43529	2.30539	2.01806
43530	5.01229	1.6752
43531	1.89226	1.32851
43532	1.89226	1.42147
43533	1.89226	1.07456
43534	1.89226	1.99167
43535	3.78452	0.846401
43536	1.89226	1.95384
43537	1.89226	2.1
43538	1.89226	1.82848
43539	1.89226	2.1
43540	3.78452	2.1
43541	2.37734	1.03185
43542	1.79346	2.1
43543	2.53594	1.61444
43544	1.70302	1.87892
43545	4.27682	1.58205
43546	2.63495	0.722554
43547	2.30539	1.18269
43548	2.49445	0.874873
43549	2.12089	0.745294
43550	4.75584	0.76187
43551	1.89226	0.871994
43552	1.89226	1.37191
43553	1.89226	0.864184
43554	1.89226	1.43877
43555	3.78452	2.02951

43556	1.89226	1.23638
43557	1.89226	1.66056
43558	1.89226	1.45383
43559	1.89226	1.66567
43560	1.89226	0.96382
43561	1.89226	1.00942
43562	2.53594	0.988638
43563	1.60017	0.991017
43564	1.57079	1.20336
43565	1.70302	0.863068
43566	2.15772	1.02821
43567	2.33299	0.748157
43568	2.09114	2.1
43569	2.34898	2.1
43570	2.30729	2.1
43571	2.06556	2.1
43572	4.41454	2.1
43573	2.09114	2.1
43574	1.59654	2.1
43575	1.57823	2.1
43576	2.06556	2.1
43577	3.6621	2.1
43578	2.30729	2.1
43579	2.06556	2.1
43580	2.03917	2.1
43581	2.26617	2.1
43582	4.33173	2.1
43583	2.03917	2.1
43584	2.06556	2.1
43585	2.045 2.1	
43586	1.55767	2.1
43587	1.57823	2.1
43588	1.57823	2.1
43589	1.89225	1.57597
43590	1.09726	2.1
43591	1.61196	2.1
43592	1.28597	2.1
43593	1.28597	0.05
43594	1.45633	2.1
43595	1.45633	2.1
43596	1.9544	0.05
43597	1.61196	2.1
43598	1.45633	2.1
43599	1.53735	2.1
43600	3.14931	2.1
43601	1.89226	2.1
43602	1.89226	2.1
43603	1.89226	2.1
43604	1.89226	2.1
43605	3.78453	2.1
43606	0.810946	2.1
43607	0.810946	2.1
43608	0.810947	2.1
43609	0.810947	2.1
43610	0.810933	2.1
43611	0.810933	2.1
43612	1.89226	2.1
43613	1.3517	2.1

43614	1.08132	2.1
43615	1.08132	2.1
43616	1.08133	2.1
43617	0.851539	2.1
43618	0.851539	2.1
43619	0.972053	2.1
43620	1.05632	2.1
43621	0.94613	2.1
43622	0.94613	1.42859
43623	1.0279	2.1
43624	0.94991	2.1
43625	1.0279	2.1
43626	0.946131	2.1
43627	0.865643	2.1
43628	0.94613	2.1
43629	1.89226	2.05973
43630	1.89226	2.1
43631	1.89226	2.1
43632	1.89226	1.81882
43633	3.78452	2.1
43634	1.03882	2.1
43635	1.91121	2.1
43636	1.41833	2.1
43637	2.34547	2.1
43638	3.32954	2.1
43639	2.3979	2.07918
43640	1.89226	2.1
43641	1.89226	2.1
43642	1.89226	2.1
43643	2.0558	2.1
43644	2.0558	1.15484
43645	1.89226	2.1
43646	1.89226	0.885765
43647	1.89226	1.78998
43648	1.89226	2.1
43649	3.78452	2.09671
43650	1.05816	2.1
43651	1.05816	2.1
43652	0.842045	2.1
43653	1.05252	2.1
43654	1.36057	2.1
43655	1.14339	2.1
43656	0.873397	2.1
43657	0.585073	2.1
43658	0.873388	2.1
43659	1.15129	2.1
43660	0.69143	2.1
43661	1.1434	2.1
43662	1.93338	2.1
43663	1.85883	2.1
43664	1.51714	2.1
43665	1.85883	2.1
43666	1.54795	2.1
43667	1.89226	2.1
43668	1.89226	2.1
43669	1.89226	2.1
43670	1.89226	2.1
43671	1.89226	2.1

43672	3.78452	2.1
43673	0.844726	2.1
43674	0.844726	2.1
43675	0.960524	2.1
43676	1.16288	2.1
43677	0.851541	2.1
43678	0.851541	2.1
43679	0.929147	2.1
43680	1.15533	2.1
43681	0.844697	2.1
43682	0.946131	2.1
43683	0.851632	2.1
43684	0.946131	2.1
43685	1.89226	2.1
43686	1.70303	2.1
43687	1.74006	2.1
43688	1.70303	2.1
43689	1.99471	2.1
43690	1.93338	2.1
43691	0.946131	2.1
43692	0.946131	2.1
43693	0.946131	2.1
43694	0.946131	2.1
43695	0.94613	2.1
43696	0.94613	2.1
43697	0.946132	2.1
43698	0.946132	2.1
43699	0.946131	2.1
43700	0.946131	2.1
43701	0.946131	2.1
43702	0.946131	2.1
43703	1.58158	2.1
43704	1.89226	2.1
43705	1.89226	2.1
43706	1.89226	2.1
43707	1.68939	2.1
43708	1.68939	2.1
43709	0.946132	0.05
43710	0.946132	0.0504766
43711	1.12988	0.05
43712	1.30783	0.05
43713	1.30783	0.05
43714	0.946133	0.05
43715	0.946132	0.05
43716	1.12988	0.05
43717	1.30783	0.05
43718	1.30783	0.05
43719	0.946133	0.05
43720	0.946133	0.05
43721	1.89227	0.05
43722	1.89227	0.05
43723	1.89227	0.05
43724	1.89227	0.0632596
43725	1.89227	0.05
43726	1.89227	0.0606432
43727	1.89226	0.05
43728	1.89226	0.05
43729	1.89226	0.05

43730	1.89226	0.05
43731	1.89226	0.05
43732	1.89226	0.05
43733	0.946131	0.05
43734	0.946131	0.05
43735	1.30783	0.05
43736	1.37715	0.05
43737	1.37715	0.05
43738	0.946133	0.05
43739	1.30783	0.05
43740	0.946129	0.05
43741	1.37715	0.05
43742	1.37715	0.05
43743	0.946131	0.05
43744	0.946131	0.05
43745	1.17056	0.05
43746	0.591757	0.0656853
43747	0.522025	0.05
43748	0.929727	0.05
43749	0.929728	0.05
43750	1.17056	0.05
43751	0.591756	0.05
43752	0.522025	0.05
43753	0.889054	0.05
43754	0.889054	0.05
43755	0.803535	0.0505856
43756	0.803535	0.05
43757	1.49814	2.1
43758	2.37439	2.1
43759	2.63923	2.1
43760	2.63923	2.1
43761	1.29111	2.1
43762	1.29111	2.1
43763	0.946132	2.1
43764	0.946132	2.1
43765	1.18719	2.1
43766	1.18719	2.1
43767	0.94613	2.1
43768	0.94613	1.90834
43769	1.1872	2.1
43770	1.31961	2.1
43771	0.946131	2.1
43772	1.31961	2.1
43773	1.1872	2.1
43774	0.94613	2.1
43775	2.49445	0.577857
43776	2.12089	0.631672
43777	2.20843	0.517708
43778	1.82805	0.697527
43779	4.32932	0.609735
43780	1.89226	0.748426
43781	1.89226	1.05832
43782	1.89226	0.666236
43783	1.89226	0.664134
43784	3.78452	0.597194
43785	0.94613	0.708563
43786	0.94613	0.669997
43787	0.946128	0.624013

43788	0.946128	0.813886
43789	0.946131	0.781065
43790	0.946131	0.812594
43791	0.946127	0.811725
43792	0.946127	0.619547
43793	0.946129	0.707202
43794	0.946129	0.6493
43795	0.946129	0.730842
43796	0.946129	0.730926
43797	1.82804	0.642492
43798	1.60017	0.752327
43799	1.87679	0.791527
43800	2.12088	0.569249
43801	1.35608	0.820723
43802	1.60017	0.656327
43803	2.20843	0.43713
43804	1.3216	0.465901
43805	1.44426	0.45134
43806	1.82805	0.471635
43807	1.80399	0.434722
43808	2.20843	0.46058
43809	1.89226	0.526482
43810	1.89226	0.502834
43811	1.89226	0.681581
43812	1.89226	0.543314
43813	1.89226	0.578846
43814	1.89226	0.497835
43815	1.89226	0.651075
43816	1.89226	0.504793
43817	1.89226	0.546154
43818	1.89226	0.538185
43819	1.89226	0.648517
43820	1.89226	0.477358
43821	0.627705	0.437707
43822	0.634941	0.554477
43823	0.629317	0.443188
43824	0.783422	0.437734
43825	0.629328	0.467434
43826	0.448764	0.478508
43827	1.35608	0.502814
43828	1.00805	0.511118
43829	0.814295	0.4354
43830	0.598375	0.549803
43831	1.04461	0.492352
43832	1.09795	1.75272
43833	1.00775	1.67816
43834	1.61202	1.95553
43835	1.63419	1.4354
43836	2.64194	1.34954
43837	1.44801	1.58821
43838	1.61201	1.82921
43839	2.35614	1.74882
43840	2.27703	1.75503
43841	3.88904	1.81289
43842	2.35615	1.53356
43843	1.63419	1.20395
43844	2.29852	1.34621
43845	1.63419	1.27283

43846	2.27634	1.39216
43847	1.61202	1.58163
43848	0.973793	2.1
43849	2.2671	2.1
43850	2.32981	2.1
43851	2.32981	2.1
43852	0.990481	2.1
43853	0.990481	2.1
43854	1.50877	2.1
43855	1.11193	2.1
43856	2.29667	2.1
43857	1.94158	2.1
43858	3.45035	2.1
43859	2.29667	2.1
43860	1.89227	2.1
43861	1.94158	2.1
43862	1.94158	2.1
43863	1.89227	2.1
43864	1.89227	2.1
43865	2.51379	2.1
43866	1.30026	2.1
43867	1.84502	2.1
43868	2.72099	2.1
43869	4.35882	2.1
43870	1.89226	2.1
43871	2.721	2.1
43872	2.5138	2.1
43873	2.5138	2.1
43874	1.89226	2.1
43875	1.89226	2.1
43876	1.89226	0.129642
43877	1.89226	0.15773
43878	2.59101	0.118735
43879	1.89226	0.230023
43880	3.78453	0.126381
43881	0.851541	0.141855
43882	0.851541	0.117182
43883	1.42502	0.126908
43884	0.948679	0.102605
43885	0.946132	0.156027
43886	0.946132	0.166449
43887	0.853617	0.128651
43888	0.853618	0.0899801
43889	0.851475	0.139815
43890	0.948666	0.167342
43891	1.166	0.132415
43892	0.851554	0.153378
43893	2.51818	0.666159
43894	2.60723	2.1
43895	2.66532	0.657791
43896	2.76315	2.1
43897	5.27255	0.778728
43898	2.60723	1.15741
43899	2.45247	2.1
43900	2.76315	1.88372
43901	2.55197	2.1
43902	5.1592	2.1
43903	1.23768	0.642625

43904	1.11433	0.733218
43905	1.32037	1.37519
43906	1.32037	0.790425
43907	1.0142	0.918233
43908	1.0142	0.669601
43909	0.886711	0.923877
43910	1.16806	0.870044
43911	0.999083	1.92452
43912	0.710926	1.16962
43913	0.937757	1.41938
43914	0.937757	1.04862
43915	0.941224	0.705479
43916	0.941224	1.94647
43917	1.16805	1.0688
43918	0.999084	2.03124
43919	0.886709	1.64648
43920	0.710928	1.29013
43921	2.29332	1.18807
43922	1.54904	2.1
43923	2.0815	2.1
43924	2.44199	2.04497
43925	4.00418	2.1
43926	1.53873	0.402936
43927	1.53422	0.573208
43928	1.39661	0.363469
43929	1.42387	0.539799
43930	2.9626	0.496538
43931	1.53423	0.446433
43932	1.44091	0.697765
43933	1.42387	0.502398
43934	1.35215	1.83565
43935	2.86479	0.635499
43936	1.39661	0.428565
43937	1.42387	0.537607
43938	1.28325	0.407315
43939	1.33326	0.5845
43940	2.70713	0.476877
43941	1.42387	0.538675
43942	1.35215	1.83773
43943	1.33327	0.540194
43944	1.27339	0.742801
43945	2.69726	0.678874
43946	2.58442	2.1
43947	1.1468	2.1
43948	1.18775	2.1
43949	2.60047	2.1
43950	3.74728	2.1
43951	2.58442	2.1
43952	1.89226	2.1
43953	1.89226	2.1
43954	2.60048	2.1
43955	4.49274	2.1
43956	1.89226	2.1
43957	1.89226	1.94964
43958	1.89226	2.1
43959	1.89226	2.1
43960	3.78453	2.1
43961	1.89226	2.1

43962	1.89226	1.70632
43963	1.89226	2.1
43964	1.89226	0.732014
43965	3.78452	2.1
43966	1.22262	2.1
43967	2.60047	2.1
43968	1.18775	2.1
43969	1.18775	2.1
43970	2.61433	2.1
43971	2.61433	2.1
43972	1.89226	2.1
43973	2.60048	2.1
43974	2.61434	2.1
43975	2.60048	2.1
43976	1.90612	2.1
43977	1.89226	2.1
43978	1.89226	2.1
43979	1.89226	2.00216
43980	1.89226	2.1
43981	1.89226	1.73339
43982	3.78453	2.1
43983	1.89226	2.1
43984	1.89226	2.04639
43985	1.89226	2.1
43986	1.89226	1.43009
43987	3.78452	2.1
43988	2.12556	2.1
43989	0.991113	2.1
43990	1.27725	2.1
43991	2.31081	2.1
43992	3.40281	2.1
43993	1.89226	2.1
43994	2.31082	2.1
43995	2.12556	2.1
43996	2.12556	2.1
43997	1.89226	2.1
43998	1.89226	2.1
43999	1.22544	2.1
44000	1.12649	2.1
44001	0.775254	2.1
44002	0.643133	2.1
44003	0.917924	2.1
44004	0.934955	2.1
44005	0.975579	2.1
44006	0.975579	2.1
44007	1.23113	2.1
44008	0.685272	2.1
44009	0.651719	2.1
44010	1.1791	2.1
44011	1.89226	2.1
44012	2.5138	2.1
44013	2.31082	2.1
44014	2.31082	2.1
44015	1.89226	2.1
44016	1.89226	2.1
44017	1.89226	2.1
44018	1.89226	2.1
44019	1.89226	2.1

44020	1.89226	2.1
44021	3.78453	2.1
44022	0.922224	2.1
44023	0.922224	2.1
44024	1.23555	2.1
44025	1.19715	2.1
44026	1.01445	2.1
44027	1.16061	2.1
44028	0.946132	2.1
44029	0.946132	2.1
44030	0.946131	2.1
44031	1.2051	2.1
44032	1.13537	2.1
44033	1.01445	2.1
44034	0.946133	2.1
44035	0.946133	2.1
44036	1.20648	2.1
44037	1.20648	2.1
44038	1.06454	2.1
44039	1.20648	2.1
44040	0.946133	2.1
44041	0.946133	2.1
44042	1.20649	2.1
44043	0.946133	2.1
44044	1.06454	2.1
44045	0.946132	2.1
44046	0.946132	2.1
44047	0.946132	2.1
44048	0.946132	2.1
44049	0.946132	2.1
44050	0.946132	2.1
44051	0.946132	2.1
44052	0.946133	2.1
44053	0.946133	2.1
44054	0.946133	2.1
44055	0.946133	2.1
44056	0.946132	2.1
44057	0.946132	2.1
44058	1.33078	1.50248
44059	1.37409	2.1
44060	1.31565	0.72329
44061	1.37114	2.1
44062	2.68973	1.64499
44063	1.89225	1.74489
44064	1.89225	2.1
44065	1.89225	0.724918
44066	1.89225	1.70358
44067	3.7845	1.10292
44068	1.89225	1.0504
44069	1.89225	2.1
44070	1.89225	0.937297
44071	1.89225	2.1
44072	3.7845	1.51076
44073	1.37409	1.12607
44074	1.20408	2.1
44075	1.18612	2.1
44076	1.18907	2.1
44077	1.37114	0.938946

44078	1.37409	2.1
44079	1.31566	1.9288
44080	1.37115	2.1
44081	1.30318	0.704915
44082	1.37433	1.63481
44083	2.68998	1.28283
44084	1.89226	1.98275
44085	1.89226	1.99997
44086	1.89226	0.773809
44087	1.89226	1.70209
44088	3.78453	1.16123
44089	1.89226	1.0195
44090	1.89226	2.1
44091	1.89226	2.1
44092	1.89226	2.1
44093	1.89226	0.909333
44094	1.89226	2.1
44095	0.647001	2.1
44096	0.647001	1.79774
44097	0.651851	0.792803
44098	0.651851	2.1
44099	0.602046	2.1
44100	0.613277	2.1
44101	0.685576	0.956129
44102	0.687164	1.03287
44103	0.687164	2.1
44104	0.613276	1.96612
44105	0.602046	2.08978
44106	0.685576	2.1
44107	0.946128	0.860757
44108	0.946128	0.560447
44109	1.15307	1.47784
44110	1.19317	0.891812
44111	0.94613	1.07264
44112	0.94613	0.300595
44113	1.15307	1.14309
44114	1.15307	1.53747
44115	0.946131	0.927842
44116	1.15307	0.851541
44117	0.946131	0.793749
44118	1.19317	0.693884
44119	1.18278	2.1
44120	2.38635	0.721667
44121	1.23042	2.1
44122	1.23042	0.91116
44123	2.30614	2.1
44124	2.30614	0.983029
44125	0.946129	0.749972
44126	0.946129	0.628441
44127	1.12994	1.01281
44128	1.15307	0.803471
44129	0.946131	0.954719
44130	0.946131	0.438749
44131	1.12994	0.781128
44132	1.12994	0.883484
44133	1.12994	0.859018
44134	0.946131	0.899744
44135	0.946131	0.723022

44136	1.15307	0.673753
44137	2.25988	1.02464
44138	2.30614	0.613623
44139	2.28696	1.53327
44140	1.16361	2.06631
44141	1.18278	1.81619
44142	1.18278	0.805115
44143	2.3771	2.1
44144	1.90121	2.1
44145	1.81191	2.1
44146	2.46904	2.1
44147	4.37025	2.1
44148	1.15676	2.1
44149	1.23452	2.1
44150	0.823592	2.1
44151	0.737606	2.1
44152	0.988113	2.1
44153	0.988113	2.1
44154	0.984014	2.1
44155	0.984014	2.1
44156	1.15676	2.1
44157	0.737605	2.1
44158	0.823592	2.1
44159	1.23452	2.1
44160	1.30318	2.09637
44161	1.37433	2.1
44162	1.30151	0.938205
44163	1.39067	2.02872
44164	2.67584	1.33564
44165	1.89226	1.48924
44166	1.89226	2.1
44167	1.89226	0.914927
44168	1.89226	1.31801
44169	3.78453	1.08401
44170	1.89226	0.84078
44171	1.89226	2.1
44172	1.89226	0.753632
44173	1.89226	2.1
44174	1.89226	0.899038
44175	1.89226	2.1
44176	0.656819	2.1
44177	0.656819	0.841893
44178	0.661755	0.897056
44179	0.661755	2.1
44180	0.613277	2.1
44181	0.63141	2.1
44182	0.687164	0.872875
44183	0.695336	1.19467
44184	0.695337	2.07029
44185	0.63141	1.87426
44186	0.687164	2.1
44187	0.613276	2.1
44188	1.30151	1.75209
44189	1.39067	2.1
44190	1.32143	0.926761
44191	1.43294	2.03842
44192	2.73445	1.8324
44193	1.89226	1.59531

44194	1.89226	1.90203
44195	1.89226	0.808554
44196	1.89226	2.1
44197	3.78452	1.33795
44198	1.89226	1.14835
44199	1.89226	1.01129
44200	1.89226	0.878649
44201	1.89226	2.1
44202	1.89226	2.1
44203	1.89226	2.1
44204	1.43294	1.13302
44205	1.26282	2.1
44206	1.3268	0.845107
44207	1.3268	2.1
44208	1.39067	0.982812
44209	1.39067	2.1
44210	1.5511	0.320501
44211	1.00665	0.324954
44212	1.28239	0.320564
44213	1.88631	0.321321
44214	2.89296	0.32063
44215	1.89227	0.32117
44216	1.89227	0.328552
44217	1.89227	0.333522
44218	1.89227	0.331842
44219	1.89227	0.324578
44220	1.89227	0.32325
44221	2.08848	0.326791
44222	1.89226	0.32662
44223	1.89226	0.321191
44224	2.37947	0.334438
44225	4.27173	0.326346
44226	1.01456	0.32235
44227	0.943157	0.321898
44228	0.641198	0.32322
44229	0.724757	0.322653
44230	0.830923	0.324599
44231	0.830923	0.323771
44232	0.836991	0.322806
44233	0.836991	0.318245
44234	1.01456	0.325015
44235	0.724757	0.332447
44236	0.641197	0.3231
44237	0.943157	0.324191
44238	1.89227	0.323444
44239	1.89227	0.331253
44240	1.89227	0.325927
44241	1.89227	0.332566
44242	3.78453	0.325792
44243	0.946129	0.325402
44244	0.946129	0.323865
44245	1.28941	0.32762
44246	1.18974	0.328034
44247	0.94613	0.331493
44248	0.94613	0.327541
44249	1.28941	0.321862
44250	1.28941	0.324755
44251	1.28941	0.323127

44252	0.946131	0.326327
44253	0.94613	0.324184
44254	1.18974	0.327607
44255	0.710636	2.1
44256	0.780249	2.1
44257	0.579033	2.1
44258	0.478812	2.1
44259	0.621822	2.1
44260	0.621822	2.1
44261	0.63724	2.1
44262	0.63724	2.1
44263	0.780251	2.1
44264	0.579032	2.1
44265	0.710637	2.1
44266	0.478811	2.1
44267	1.05499	2.00552
44268	1.00521	2.04791
44269	1.24445	1.54163
44270	1.23082	0.933544
44271	2.28581	1.96774
44272	1.48751	1.89253
44273	1.23083	1.39814
44274	1.4941	1.99401
44275	1.24445	1.2366
44276	1.48047	1.21694
44277	1.23083	1.19278
44278	0.621185	1.80989
44279	0.621185	1.95868
44280	0.620899	1.12688
44281	0.620899	2.1
44282	0.502515	1.59472
44283	0.626672	1.16555
44284	0.615412	0.96148
44285	0.740234	2.07159
44286	0.740233	0.99706
44287	0.626672	0.902391
44288	0.615413	1.10308
44289	0.502516	1.35533
44290	1.95844	2.1
44291	2.6562	2.1
44292	1.95844	2.1
44293	2.20883	2.1
44294	2.38225	2.1
44295	2.63264	2.1
44296	1.89226	2.1
44297	1.89226	2.1
44298	1.89226	2.1
44299	1.89226	2.1
44300	3.78453	2.1
44301	1.95844	2.1
44302	2.38225	2.1
44303	1.71082	2.1
44304	2.14647	2.1
44305	4.1049	2.1
44306	0.946131	2.1
44307	0.946131	2.1
44308	0.946131	2.1
44309	0.946131	2.1

44310	0.946131	2.1
44311	0.946131	2.1
44312	0.946131	2.1
44313	0.946131	2.1
44314	0.946131	2.1
44315	0.946131	2.1
44316	0.946131	2.1
44317	0.946131	2.1
44318	2.12597	2.1
44319	1.2295	2.1
44320	1.28014	2.1
44321	2.19723	2.1
44322	3.40611	2.1
44323	2.12597	2.1
44324	2.79242	2.1
44325	2.73375	2.1
44326	2.66249	2.1
44327	2.19723	2.1
44328	2.12597	2.1
44329	1.13716	2.1
44330	1.09861	2.1
44331	0.666692	2.1
44332	0.640071	2.1
44333	0.891617	2.1
44334	0.891617	2.1
44335	0.885614	2.1
44336	0.885614	2.1
44337	1.13716	2.1
44338	0.666692	2.1
44339	0.640071	2.1
44340	1.09861	2.1
44341	1.89226	0.0654541
44342	1.70303	0.067279
44343	1.89226	0.05
44344	2.64598	0.076768
44345	1.81091	0.05
44346	2.53222	0.0721837
44347	2.32422	0.0581136
44348	1.89226	0.0709782
44349	2.78336	0.0834006
44350	2.78336	0.0784157
44351	1.89226	0.053792
44352	1.89226	0.05
44353	0.946132	0.0560889
44354	0.946132	0.0507881
44355	0.946131	0.051701
44356	0.946131	0.067361
44357	0.946132	0.0727618
44358	0.946132	0.0678837
44359	0.946131	0.0533414
44360	0.946131	0.0717587
44361	0.946131	0.0588985
44362	0.946131	0.0603474
44363	0.946131	0.0652484
44364	0.946131	0.062697
44365	0.946131	0.0764489
44366	0.946131	0.0998009
44367	0.946132	0.0748429

44368	0.946132	0.101102
44369	0.946132	0.104634
44370	0.946132	0.100439
44371	0.946131	0.0712478
44372	0.946131	0.0712619
44373	0.946131	0.0764807
44374	0.946131	0.0921907
44375	0.946131	0.0940126
44376	0.946131	0.0799934
44377	2.24323	1.05901
44378	1.89226	1.8091
44379	2.75021	1.25305
44380	2.75021	1.45872
44381	1.89226	0.919044
44382	1.89226	1.10383
44383	2.30908	0.391881
44384	2.19815	0.514557
44385	2.1609	0.382126
44386	1.83796	0.571589
44387	4.35904	0.458751
44388	2.19815	0.508787
44389	2.02268	0.737836
44390	2.17869	0.755314
44391	1.9559	0.550661
44392	2.0815	0.744805
44393	1.83424	0.547617
44394	2.1609	0.38684
44395	1.88877	0.572816
44396	1.98413	0.349923
44397	1.87355	0.533519
44398	4.03445	0.483439
44399	1.87355	0.501558
44400	2.02268	0.794898
44401	1.87355	0.783154
44402	2.10488	0.566701
44403	1.68443	0.734178
44404	1.89448	0.592454
44405	0.946131	0.228168
44406	0.946131	0.343012
44407	1.40574	0.410753
44408	1.12373	0.321582
44409	0.946129	0.2793
44410	0.946129	0.218409
44411	1.40574	0.407072
44412	1.40574	0.333342
44413	0.946131	0.360692
44414	1.40574	0.402216
44415	1.12373	0.310531
44416	0.946131	0.239032
44417	2.14277	0.454157
44418	2.81148	0.457338
44419	1.42762	0.345601
44420	1.42762	0.378471
44421	2.96261	0.379799
44422	2.24746	0.344642
44423	1.89226	0.747724
44424	1.89226	0.82267
44425	1.89226	0.781875

44426	1.89226	0.992337
44427	3.78453	1.23254
44428	1.89226	0.619945
44429	1.89226	0.787694
44430	1.89226	0.79661
44431	1.89226	0.932882
44432	3.78452	0.684581
44433	0.68433	0.727675
44434	0.946127	1.04499
44435	0.543921	1.23836
44436	0.946131	0.836296
44437	0.946131	0.883994
44438	0.946131	2.1
44439	0.543918	0.939755
44440	0.543918	1.03774
44441	0.684332	0.942807
44442	0.54392	2.1
44443	0.946129	0.653473
44444	0.946129	0.99821
44445	1.89226	0.85279
44446	1.08784	1.09667
44447	1.19352	0.563599
44448	1.89226	0.959275
44449	1.19352	2.06465
44450	1.89226	1.7132
44451	0.635711	0.6937
44452	0.946132	0.98752
44453	0.946132	1.36461
44454	0.56474	0.851016
44455	0.750045	0.936678
44456	1.25657	2.1
44457	0.635682	0.879975
44458	0.635682	0.656228
44459	0.891954	0.809246
44460	1.32751	2.1
44461	0.564766	0.716497
44462	0.564766	1.46608
44463	0.858345	0.882013
44464	0.946128	1.20391
44465	0.635712	0.918834
44466	0.946133	0.876215
44467	0.977198	1.03077
44468	0.944521	2.1
44469	0.635682	1.16422
44470	0.635682	0.523511
44471	1.02924	0.994167
44472	0.705625	2.1
44473	0.851528	0.553643
44474	0.851528	1.82911
44475	2.18903	2.1
44476	2.10182	2.1
44477	2.10182	2.1
44478	1.89226	2.1
44479	1.89226	2.1
44480	1.64983	2.1
44481	1.89226	2.1
44482	1.83867	2.1
44483	1.83867	2.1

44484	1.89164	2.1
44485	2.0815	2.1
44486	2.17638	2.1
44487	2.57957	0.920317
44488	2.3506	1.55976
44489	2.69056	0.744748
44490	2.49439	1.51566
44491	5.04116	0.882391
44492	2.3506	0.910623
44493	1.8911	1.81891
44494	2.49439	0.980783
44495	2.06689	2.1
44496	4.41748	1.64922
44497	2.69056	0.894461
44498	2.49439	1.57013
44499	2.77227	0.768195
44500	2.63443	1.32762
44501	5.32499	1.54241
44502	2.49439	0.988704
44503	2.06689	2.1
44504	2.63443	0.738796
44505	2.26444	2.1
44506	4.70131	1.28889
44507	2.46327	2.1
44508	2.54347	2.1
44509	2.52869	2.1
44510	2.16534	2.1
44511	2.82661	2.1
44512	2.46327	2.1
44513	0.946131	2.1
44514	0.946131	2.1
44515	1.27174	2.1
44516	1.27174	2.1
44517	1.27174	2.1
44518	0.94613	2.1
44519	0.946128	2.1
44520	1.4133	2.1
44521	0.946129	2.1
44522	0.946129	2.1
44523	1.27174	2.1
44524	1.4133	2.1
44525	1.42757	2.1
44526	1.7457	2.1
44527	1.66047	2.1
44528	1.7457	2.1
44529	1.45754	2.1
44530	1.54278	2.1
44531	1.42757	2.1
44532	1.52996	2.1
44533	1.66047	2.1
44534	1.66047	2.1
44535	1.26397	2.1
44536	1.26397	2.1
44537	0.968536	2.1
44538	1.02461	2.1
44539	1.57575	2.1
44540	1.62391	2.1
44541	2.59245	2.1

44542	0.946131	2.1
44543	0.946131	2.1
44544	0.946132	2.1
44545	0.946132	2.1
44546	0.946132	2.1
44547	0.946132	2.0585
44548	0.946132	2.1
44549	0.946132	2.1
44550	0.946132	2.1
44551	0.946132	2.1
44552	0.946132	2.1
44553	0.946132	2.1
44554	2.23765	2.1
44555	1.62391	2.1
44556	2.22154	2.1
44557	1.57575	2.1
44558	2.2697	2.1
44559	1.62391	2.1
44560	1.89226	2.1
44561	1.89226	2.1
44562	1.89226	2.1
44563	1.89226	2.1
44564	1.89226	2.1
44565	1.89226	1.7443
44566	1.89226	2.1
44567	1.89226	2.1
44568	1.89226	2.1
44569	1.89226	2.1
44570	1.89226	2.1
44571	1.89226	1.51557
44572	2.2697	2.1
44573	1.46129	2.1
44574	2.25873	2.1
44575	1.62391	2.1
44576	2.09611	2.1
44577	1.46129	2.1
44578	1.89226	2.1
44579	2.07088	2.1
44580	1.89226	2.1
44581	2.31884	2.1
44582	4.21111	2.1
44583	1.89226	2.1
44584	2.31884	2.1
44585	2.11713	2.1
44586	1.89226	2.1
44587	2.54371	2.1
44588	2.31884	2.1
44589	2.54371	2.1
44590	1.24041	2.1
44591	2.55208	2.1
44592	2.31884	2.1
44593	1.23712	2.1
44594	1.00388	2.1
44595	2.72769	0.103423
44596	2.57507	0.16258
44597	1.89226	0.0877937
44598	1.89226	0.155562
44599	4.46733	0.105351

44600	1.89226	0.154544
44601	2.57506	0.171846
44602	1.89226	0.09933
44603	2.30076	0.161937
44604	1.89226	0.0988376
44605	2.30076	0.174601
44606	1.21171	0.079013
44607	1.48589	0.108441
44608	2.09166	0.0749228
44609	1.78381	0.0935991
44610	3.2697	0.0944813
44611	2.09166	0.0722621
44612	1.70302	0.101238
44613	1.48589	0.0840655
44614	1.18027	0.0726054
44615	2.26571	0.0922094
44616	1.79969	0.0851347
44617	1.89226	0.0921264
44618	1.89226	0.165547
44619	1.89226	0.142813
44620	1.89226	0.111855
44621	1.89226	0.15571
44622	1.89226	0.138596
44623	0.946131	0.115555
44624	0.946131	0.228854
44625	0.946128	0.173635
44626	0.946128	0.133391
44627	0.946129	0.166863
44628	0.946129	0.171266
44629	0.94613	0.102846
44630	0.94613	0.101828
44631	0.94613	0.122189
44632	0.94613	0.13619
44633	0.94613	0.141383
44634	0.94613	0.132046
44635	1.89226	1.44991
44636	1.89226	1.54903
44637	1.89226	1.9899
44638	1.89226	1.22054
44639	1.89226	1.90691
44640	1.89226	1.17179
44641	1.89226	1.48227
44642	1.89226	1.4607
44643	1.89226	1.88448
44644	1.89226	1.20188
44645	1.89226	1.80939
44646	1.89226	1.17213
44647	1.89226	1.2571
44648	1.89226	1.33202
44649	1.89226	1.71889
44650	1.89226	1.02675
44651	1.89226	1.68173
44652	1.89226	1.01199
44653	1.79103	0.824247
44654	0.968249	2.1
44655	1.44807	1.29972
44656	2.17984	0.655399
44657	3.2391	1.90158

44658	1.29735	1.47077
44659	1.08992	1.29975
44660	0.945147	1.89622
44661	0.724033	2.1
44662	1.00723	2.1
44663	1.00723	1.55734
44664	1.01416	0.736221
44665	1.01416	1.73277
44666	1.29735	1.4318
44667	0.94515	1.9743
44668	0.724034	1.65064
44669	1.08992	1.99831
44670	1.39214	1.17363
44671	1.39214	2.1
44672	0.946129	1.97943
44673	0.946129	2.1
44674	1.33728	2.1
44675	1.39214	2.1
44676	0.94613	1.34603
44677	0.94613	1.63431
44678	0.94613	2.1
44679	1.33728	2.1
44680	0.94613	2.1
44681	1.39214	2.1
44682	2.78428	2.1
44683	2.28373	2.1
44684	2.67456	2.1
44685	2.20011	2.1
44686	4.95829	2.1
44687	1.90165	0.0853384
44688	2.24206	0.28368
44689	1.66137	0.202506
44690	2.07108	0.264162
44691	3.90343	0.0892615
44692	1.89224	0.412038
44693	1.89224	0.19125
44694	1.89224	0.141495
44695	1.89224	0.335653
44696	3.78448	0.24281
44697	2.82159	0.282263
44698	1.89226	0.414305
44699	2.50138	0.511427
44700	1.89226	0.136114
44701	4.39365	0.234663
44702	1.66139	0.190358
44703	1.86399	0.30188
44704	1.56005	0.147649
44705	1.90324	0.208591
44706	3.56463	0.164889
44707	0.946133	0.144933
44708	0.946133	0.287493
44709	0.946132	0.182103
44710	0.946132	0.392351
44711	0.946133	0.207851
44712	0.946133	0.269956
44713	0.946132	0.17961
44714	0.946132	0.271941
44715	0.946133	0.0988669

44716	0.946133	0.244687
44717	0.946132	0.160782
44718	0.946132	0.358156
44719	1.5176	0.691564
44720	1.97964	0.583176
44721	2.32889	0.62014
44722	1.91762	0.636708
44723	3.84649	0.603691
44724	2.3376	0.615012
44725	1.97963	0.602013
44726	2.32889	0.665947
44727	2.58303	0.553454
44728	4.66649	0.648881
44729	2.32889	0.597114
44730	2.20248	0.559425
44731	1.91762	0.545951
44732	1.91762	0.557792
44733	2.5615	0.613335
44734	2.5615	0.629864
44735	2.32889	0.640063
44736	2.58303	0.532705
44737	2.71793	0.565199
44738	2.56149	0.537491
44739	5.04681	0.549324
44740	1.89226	0.621692
44741	2.53785	1.90616
44742	1.89226	0.68428
44743	2.60895	0.580555
44744	1.89226	0.903147
44745	2.60895	0.698226
44746	0.493248	0.702106
44747	1.30448	1.13621
44748	1.26893	1.8576
44749	0.653226	1.73263
44750	0.653227	0.917229
44751	0.493247	2.1
44752	1.26893	2.09998
44753	1.30448	0.681488
44754	0.832232	2.03137
44755	0.832233	2.08607
44756	0.929944	0.562136
44757	0.929944	0.923589
44758	1.89226	2.1
44759	1.47937	2.1
44760	1.50143	2.1
44761	1.47937	2.1
44762	1.91432	2.1
44763	1.89226	1.1589
44764	1.89226	1.74591
44765	1.89226	2.1
44766	1.89226	2.1
44767	1.89226	1.96843
44768	1.89226	2.1
44769	1.89226	0.856658
44770	1.33419	2.1
44771	2.4542	2.1
44772	2.59834	2.1
44773	2.59834	2.1

44774	1.25716	2.1
44775	1.25716	2.1
44776	1.70303	2.1
44777	1.50143	2.1
44778	1.60878	2.1
44779	1.50143	2.1
44780	1.93599	2.1
44781	1.89226	0.974767
44782	0.946131	0.827364
44783	0.946131	1.02026
44784	0.946127	2.1
44785	0.946127	2.1
44786	0.946127	2.1
44787	0.946127	0.711178
44788	0.946131	2.1
44789	0.946131	1.04345
44790	0.94613	1.38453
44791	0.94613	1.46521
44792	0.946129	1.82943
44793	0.946129	1.62632
44794	1.43912	2.1
44795	2.59834	2.1
44796	2.78093	2.1
44797	2.78093	2.1
44798	1.33419	2.1
44799	1.33419	2.1
44800	1.89226	2.1
44801	2.26322	1.21453
44802	2.55682	2.1
44803	2.55682	2.1
44804	1.89226	2.1
44805	1.89226	2.1
44806	2.58032	0.464927
44807	2.28922	0.41096
44808	1.88424	0.407532
44809	2.32127	0.412349
44810	4.46456	0.412791
44811	1.67265	0.453205
44812	2.28922	0.423408
44813	1.88424	0.386718
44814	1.11314	0.385831
44815	3.55689	0.400976
44816	1.43751	0.363452
44817	2.32127	0.391961
44818	1.92998	0.287963
44819	2.37671	0.340836
44820	1.43751	0.422202
44821	1.88424	0.429923
44822	2.36729	2.1
44823	1.56714	2.1
44824	1.56714	2.1
44825	1.62408	2.1
44826	2.28373	2.1
44827	2.34067	2.1
44828	0.937268	2.1
44829	0.937268	2.1
44830	0.94636	2.1
44831	0.94636	2.1

44832	0.76004	2.1
44833	0.783572	2.1
44834	1.14187	2.1
44835	1.10006	2.1
44836	1.10006	2.1
44837	0.76004	2.1
44838	0.783572	2.1
44839	1.14187	2.1
44840	1.28753	2.1
44841	1.36838	2.1
44842	1.62617	2.1
44843	1.82857	2.1
44844	3.1161	2.1
44845	1.82856	2.1
44846	1.17996	2.1
44847	1.53417	2.1
44848	1.56618	2.1
44849	1.36838	2.1
44850	1.36838	2.1
44851	1.35065	0.801775
44852	1.42659	0.443539
44853	2.26675	0.613137
44854	2.26675	0.582582
44855	1.62137	0.5582
44856	2.53747	0.622875
44857	1.89226	0.0820133
44858	1.89226	0.05
44859	1.89226	0.0599601
44860	1.89226	0.0737739
44861	3.78452	0.0729372
44862	1.36569	0.05
44863	1.36569	0.05
44864	0.946131	0.05
44865	0.946131	0.0588171
44866	1.16041	0.05
44867	1.36569	0.05
44868	0.946129	0.05
44869	0.946129	0.0596676
44870	1.1604	0.05
44871	0.94613	0.05
44872	0.946131	0.05
44873	1.36569	0.05
44874	2.73139	0.05
44875	1.25265	0.05
44876	1.25265	0.05
44877	1.64799	0.05
44878	2.32081	0.05
44879	2.71615	0.05
44880	0.946133	0.05
44881	0.946133	0.0505851
44882	0.946132	0.0719094
44883	0.946132	0.0613398
44884	0.946132	0.0543067
44885	0.946132	0.0633634
44886	0.946133	0.0514563
44887	0.946133	0.0605727
44888	0.946132	0.0551121
44889	0.946132	0.0607565

44890	0.946133	0.0581322
44891	0.946133	0.0540358
44892	0.946131	0.0513516
44893	0.946131	0.0551285
44894	0.94613	0.10716
44895	0.94613	0.0667887
44896	0.946132	0.0847339
44897	0.946132	0.0630257
44898	0.946129	0.0731111
44899	0.946129	0.0986171
44900	0.946131	0.0728296
44901	0.946131	0.0583313
44902	0.946131	0.0583121
44903	0.946131	0.0685547
44904	1.89226	0.594053
44905	2.48503	0.855791
44906	2.64586	1.86417
44907	2.64586	0.596297
44908	1.89226	1.85215
44909	1.89226	0.499535
44910	2.48504	1.69326
44911	1.99985	2.1
44912	1.83584	2.1
44913	1.83584	2.1
44914	2.64586	1.9913
44915	2.64586	2.1
44916	1.89225	0.587178
44917	2.33095	0.869798
44918	2.48501	1.73112
44919	2.48501	0.567824
44920	1.89225	1.78678
44921	1.89225	0.552514
44922	2.33096	1.27882
44923	1.83583	2.1
44924	1.68436	2.1
44925	1.68436	2.1
44926	2.48502	1.77664
44927	2.48502	2.1
44928	1.14807	2.1
44929	1.76545	2.1
44930	1.21292	2.1
44931	1.83936	2.1
44932	2.97837	2.1
44933	1.89226	2.1
44934	1.89226	2.1
44935	1.89226	2.1
44936	1.89226	1.1125
44937	3.78451	2.1
44938	1.89226	2.1
44939	1.89226	1.03541
44940	1.89226	1.63003
44941	1.89226	1.27737
44942	3.78452	1.624
44943	1.76545	2.1
44944	2.12608	2.1
44945	1.83937	2.1
44946	2.22529	1.16173
44947	3.99074	2.1

44948	0.780222	2.1
44949	0.780222	2.1
44950	0.784871	2.1
44951	0.784871	2.1
44952	0.95863	2.1
44953	0.919681	2.1
44954	0.636145	2.1
44955	0.606461	2.1
44956	0.636144	2.1
44957	0.958631	2.1
44958	0.60646	2.1
44959	0.919683	2.1
44960	1.89226	2.1
44961	1.89226	1.08772
44962	1.89226	2.1
44963	1.89226	2.1
44964	3.78452	2.1
44965	1.89226	1.66716
44966	1.89226	1.53946
44967	1.89226	2.1
44968	1.89226	0.86949
44969	3.78452	1.41554
44970	1.03789	1.87656
44971	1.03789	2.1
44972	1.04231	2.1
44973	1.04231	2.1
44974	1.16052	2.1
44975	1.11265	0.850527
44976	0.958634	2.1
44977	0.919685	2.1
44978	0.958634	2.1
44979	1.16052	2.1
44980	0.919685	2.1
44981	1.11265	2.1
44982	1.43837	2.1
44983	2.57085	2.1
44984	1.39332	2.1
44985	2.58228	2.1
44986	3.96417	2.1
44987	1.89226	2.1
44988	1.89226	2.1
44989	1.89226	2.1
44990	1.89226	2.1
44991	1.89226	2.1
44992	1.89226	2.1
44993	1.89226	2.1
44994	1.89226	0.865861
44995	1.89226	2.1
44996	1.89226	2.1
44997	3.78452	2.1
44998	2.57085	2.1
44999	1.89226	2.1
45000	2.58229	2.1
45001	1.89226	2.1
45002	4.46312	2.1
45003	1.32368	2.1
45004	2.58228	2.1
45005	2.55352	2.1

45006	2.55352	2.1
45007	1.39332	2.1
45008	1.39332	2.1
45009	1.89226	2.1
45010	1.89226	2.1
45011	1.89226	2.1
45012	1.89226	2.1
45013	1.89226	2.1
45014	1.89226	2.1
45015	1.89226	2.1
45016	1.89226	2.1
45017	1.89226	2.1
45018	1.89226	2.1
45019	1.89226	2.1
45020	1.89226	1.12143
45021	2.55353	2.1
45022	1.89226	2.1
45023	2.58228	2.1
45024	1.89226	2.1
45025	1.89226	2.1
45026	2.58228	2.1
45027	1.40574	1.34965
45028	1.05175	1.08202
45029	1.71613	1.50979
45030	1.99961	1.05099
45031	3.12187	1.3066
45032	1.71613	1.49619
45033	2.50733	1.10297
45034	1.99961	1.63136
45035	1.99961	1.54787
45036	2.25746	1.04152
45037	2.25746	1.06295
45038	2.25746	0.970282
45039	1.89526	0.981545
45040	1.71613	1.32545
45041	1.71613	0.773491
45042	1.89526	1.03286
45043	1.27558	0.779573
45044	1.07536	2.1
45045	1.07536	2.1
45046	1.07034	2.1
45047	1.07034	2.1
45048	0.766879	2.1
45049	0.81527	2.1
45050	1.38896	2.1
45051	1.33043	2.1
45052	0.815264	2.1
45053	1.38895	2.1
45054	0.766884	2.1
45055	1.33044	2.1
45056	1.89225	2.1
45057	1.89225	2.1
45058	1.89225	2.1
45059	1.89225	2.1
45060	1.89225	2.1
45061	1.89225	2.1
45062	1.64059	2.1
45063	1.89225	2.1

45064	1.565	2.1
45065	1.565	2.1
45066	1.89225	2.1
45067	1.89225	2.1
45068	1.89226	2.1
45069	1.89226	2.1
45070	1.89226	2.1
45071	1.89226	2.1
45072	1.89226	2.1
45073	1.89226	2.1
45074	1.67906	2.1
45075	1.89226	2.1
45076	1.6406	2.1
45077	1.6406	2.1
45078	1.89226	2.1
45079	1.89226	2.1
45080	2.18106	1.44373
45081	2.20831	2.1
45082	2.64725	0.982992
45083	2.63102	1.37469
45084	4.82831	1.5827
45085	2.18106	1.18633
45086	2.09605	2.1
45087	2.5601	1.35998
45088	2.63102	1.40315
45089	4.74116	2.033
45090	2.64725	1.13677
45091	1.89226	1.30912
45092	1.87603	2.1
45093	2.63102	1.15936
45094	1.89226	1.58946
45095	2.64725	0.9577
45096	0.946132	1.44935
45097	1.28005	1.1164
45098	0.946132	2.05242
45099	1.31551	0.985694
45100	1.28005	1.50026
45101	1.28005	2.01774
45102	0.946132	1.00542
45103	0.946132	1.28407
45104	0.946132	1.04643
45105	0.946132	1.19784
45106	1.28005	1.44941
45107	1.31551	1.69521
45108	2.0815	2.1
45109	1.59318	2.1
45110	2.0815	2.1
45111	1.40161	2.1
45112	2.49782	2.1
45113	1.68553	2.1
45114	0.819885	2.1
45115	0.935082	2.1
45116	0.867363	2.1
45117	0.867363	2.1
45118	0.715431	2.1
45119	0.902777	2.1
45120	0.738933	2.1
45121	0.980053	2.1

45122	0.936716	2.1
45123	0.917651	2.1
45124	0.775853	2.1
45125	0.74037	2.1
45126	2.78977	2.1
45127	2.38239	2.1
45128	2.78977	2.1
45129	1.96468	2.1
45130	2.64107	2.1
45131	1.80733	2.1
45132	1.80141	2.1
45133	2.28512	2.1
45134	2.28512	2.1
45135	1.8519	2.1
45136	2.64107	2.1
45137	2.20785	2.1
45138	0.946133	0.576303
45139	0.946133	0.536854
45140	0.946132	0.569896
45141	0.946132	0.548636
45142	0.946132	0.567611
45143	0.946132	0.564896
45144	0.946132	0.532213
45145	0.946132	0.540229
45146	0.946132	0.558779
45147	0.946132	0.557244
45148	0.946133	0.550225
45149	0.946133	0.56489
45150	1.89226	0.44445
45151	1.89226	0.455822
45152	1.89226	0.378987
45153	1.89226	0.451744
45154	1.89226	0.434463
45155	1.89226	0.472719
45156	1.89226	0.459085
45157	1.89226	0.485045
45158	1.89226	0.459857
45159	1.89226	0.497349
45160	3.78453	0.478646
45161	1.89226	0.562946
45162	1.89226	0.513713
45163	1.89226	0.485977
45164	1.89226	0.561019
45165	1.89226	0.509885
45166	1.89226	0.552753
45167	2.12925	0.0612936
45168	2.51397	0.0890565
45169	2.22488	0.0622942
45170	1.86113	0.0858624
45171	4.3751	0.0849718
45172	2.82754	0.0576788
45173	2.51397	0.0863678
45174	2.22489	0.0557768
45175	2.47291	0.0903562
45176	4.98688	0.0583579
45177	1.89226	0.0959543
45178	1.89226	0.150277
45179	1.89226	0.10628

45180	1.89226	0.147137
45181	3.78453	0.144566
45182	1.89226	0.0985472
45183	1.89226	0.147558
45184	1.89226	0.0948227
45185	1.89226	0.150586
45186	3.78452	0.1
45187	0.90848	0.0750018
45188	1.11244	0.0595005
45189	0.930566	0.0650737
45190	0.73385	0.0804685
45191	0.921298	0.0777341
45192	0.921298	0.0836165
45193	0.924994	0.0601568
45194	0.924994	0.0600435
45195	0.908478	0.0724097
45196	0.733851	0.0599393
45197	1.11244	0.0629876
45198	0.930567	0.0703188
45199	2.22489	0.0555167
45200	2.0815	0.0764379
45201	2.0815	0.0777661
45202	2.30674	0.0637663
45203	1.99867	0.0724673
45204	2.33358	0.0570082
45205	1.89226	0.0988368
45206	1.89226	0.139782
45207	1.89226	0.102682
45208	1.89226	0.121744
45209	1.89226	0.100722
45210	1.89226	0.116404
45211	0.946131	0.153402
45212	0.946131	0.122512
45213	0.946128	0.148662
45214	0.946128	0.10071
45215	0.946129	0.136027
45216	0.946129	0.152079
45217	0.94613	0.0884512
45218	0.94613	0.0936326
45219	0.94613	0.120479
45220	0.94613	0.0922593
45221	0.94613	0.106821
45222	0.94613	0.121544
45223	2.28904	2.1
45224	1.89226	0.921798
45225	2.2846	1.09398
45226	2.2846	2.1
45227	1.89226	0.695884
45228	1.89226	2.1
45229	1.89226	1.12352
45230	1.89226	1.05908
45231	1.89226	2.1
45232	1.89226	2.1
45233	1.89226	0.794926
45234	1.89226	1.72127
45235	1.04037	0.928541
45236	1.34066	1.47571
45237	1.34955	0.932357

45238	1.34955	1.75335
45239	1.03373	1.0291
45240	1.03373	2.1
45241	0.946131	0.596581
45242	0.946131	2.1
45243	1.14452	1.79109
45244	1.14452	2.1
45245	0.946131	0.966831
45246	0.946131	0.916016
45247	1.14296	1.48055
45248	1.14452	2.1
45249	1.14296	0.781724
45250	0.946131	1.06127
45251	1.14452	1.34116
45252	0.946132	1.06676
45253	1.89226	1.04333
45254	1.89226	1.76707
45255	1.89226	1.41042
45256	1.89226	2.1
45257	1.89226	0.738926
45258	1.89226	2.02375
45259	0.597454	1.00347
45260	0.597454	1.50949
45261	0.597972	0.926
45262	0.597972	2.1
45263	0.675238	1.75323
45264	0.674776	1.06598
45265	0.519667	1.04235
45266	0.520188	1.1933
45267	0.519667	0.77041
45268	0.675238	0.866964
45269	0.520188	1.18189
45270	0.674777	1.1706
45271	1.89226	0.444585
45272	1.82125	0.290481
45273	1.89226	0.355194
45274	1.84314	0.274005
45275	1.89226	0.480927
45276	1.84314	0.455933
45277	1.89226	0.282417
45278	1.84314	0.336678
45279	1.89226	0.277685
45280	1.65794	0.250889
45281	1.89226	0.282034
45282	1.65794	0.25165
45283	1.348	1.97805
45284	1.43485	2.1
45285	1.79929	0.981359
45286	1.75309	1.82252
45287	3.14728	1.80192
45288	1.348	1.23555
45289	1.63892	1.96101
45290	1.59157	2.1
45291	1.18647	1.96586
45292	1.75309	1.37138
45293	1.348	1.75199
45294	1.79929	1.52026
45295	1.75309	2.1

45296	2.18106	1.21044
45297	2.20831	1.27037
45298	3.98034	1.89168
45299	2.18106	1.05288
45300	1.63892	2.05181
45301	1.75309	1.0872
45302	1.75309	2.09523
45303	2.09605	1.02321
45304	2.09605	2.1
45305	2.66076	0.39266
45306	1.70302	0.295413
45307	2.41575	0.351629
45308	1.70302	0.317074
45309	3.08598	0.330508
45310	2.3661	0.280897
45311	1.71053	0.321555
45312	2.41575	0.374621
45313	1.20143	0.276702
45314	1.20143	0.306346
45315	2.2123	0.301401
45316	1.66739	0.299402
45317	2.25633	0.319867
45318	2.68302	0.352321
45319	1.71051	0.281166
45320	1.71051	0.306841
45321	2.74192	0.3118
45322	2.1961	0.256087
45323	1.32553	0.782084
45324	2.34875	0.556269
45325	2.19139	0.9536
45326	2.19139	0.375458
45327	1.56172	0.910217
45328	1.56172	0.284548
45329	1.89226	1.99455
45330	1.89226	0.559749
45331	1.89226	0.978623
45332	1.89226	0.669778
45333	3.78453	0.779573
45334	2.667 2.1	
45335	1.26097	0.887049
45336	1.26097	2.1
45337	1.5683	2.1
45338	2.42949	2.1
45339	2.73682	2.1
45340	1.13213	0.728936
45341	2.19139	0.469128
45342	2.0815	0.851181
45343	1.77615	0.39881
45344	1.37767	0.834543
45345	1.19297	0.243522
45346	0.946132	0.577065
45347	0.946132	0.934164
45348	0.946131	1.7842
45349	0.946131	0.672129
45350	0.946131	0.628692
45351	0.946131	0.347386
45352	0.946132	0.904953
45353	0.946132	1.93976

45354	0.946132	0.817473
45355	0.946132	0.807264
45356	0.946131	0.750063
45357	0.946131	0.777194
45358	2.02064	0.90177
45359	2.3606	2.1
45360	1.47478	2.1
45361	2.22997	2.1
45362	4.25061	2.1
45363	2.36059	2.1
45364	2.56952	2.1
45365	2.22997	2.1
45366	2.48631	2.1
45367	4.79949	2.1
45368	1.84261	2.1
45369	2.22997	2.1
45370	1.65706	1.41828
45371	1.78837	2.1
45372	3.88703	2.1
45373	2.22997	2.1
45374	2.48631	2.1
45375	2.19528	2.1
45376	2.41175	2.1
45377	4.64172	2.1
45378	1.6466	2.1
45379	1.89226	2.1
45380	1.35972	2.1
45381	1.35972	2.1
45382	1.89226	2.1
45383	1.89226	2.1
45384	1.89226	2.1
45385	1.89226	2.1
45386	1.89226	2.1
45387	1.89226	2.1
45388	3.78453	2.1
45389	2.44797	2.1
45390	1.07214	2.1
45391	1.31589	2.1
45392	1.1211	2.1
45393	2.44797	2.1
45394	2.25318	2.1
45395	1.95954	2.1
45396	1.89226	2.1
45397	1.6466	2.1
45398	1.6466	2.1
45399	1.89226	2.1
45400	1.89226	2.1
45401	0.946133	2.1
45402	0.946133	2.1
45403	0.946132	2.1
45404	0.946132	2.1
45405	0.946132	2.1
45406	0.946132	2.1
45407	0.946133	2.1
45408	0.946133	2.1
45409	0.946132	2.1
45410	0.946132	2.1
45411	0.946133	2.1

45412	0.946133	2.1
45413	2.44797	2.1
45414	1.36904	2.1
45415	1.70303	2.1
45416	2.37586	2.1
45417	4.00507	2.1
45418	0.631553	0.084976
45419	1.24489	0.0649467
45420	1.11597	0.0702098
45421	0.496799	0.0922527
45422	0.872272	0.0907003
45423	0.872272	0.0830861
45424	0.869415	0.0626966
45425	0.869415	0.0679828
45426	0.4968	0.0796131
45427	0.631553	0.0792929
45428	1.11597	0.0693376
45429	1.24489	0.0833021
45430	1.84253	2.1
45431	2.17384	2.1
45432	2.04818	2.1
45433	1.65925	2.1
45434	3.83309	2.1
45435	2.04818	2.1
45436	1.65925	2.1
45437	1.48162	2.1
45438	1.92264	2.1
45439	3.58189	2.1
45440	2.3305	0.377478
45441	1.89226	0.408168
45442	2.52753	0.391262
45443	2.52753	0.413574
45444	1.89226	0.382259
45445	1.89226	0.405535
45446	1.89226	0.407648
45447	1.89226	0.523541
45448	1.89226	0.474663
45449	1.89226	0.491405
45450	1.89226	0.442838
45451	1.89226	0.486671
45452	1.34563	0.380763
45453	2.1769	0.435287
45454	1.94955	0.390392
45455	1.94955	0.496905
45456	1.55063	0.400877
45457	1.55063	0.496876
45458	2.09455	0.355261
45459	1.89226	0.397755
45460	2.3305	0.368916
45461	2.3305	0.383216
45462	1.89226	0.360512
45463	1.89226	0.377039
45464	1.89226	0.382304
45465	1.89226	0.406295
45466	1.89226	0.457154
45467	1.89226	0.388373
45468	1.89226	0.400995
45469	1.89226	0.451427

45470	0.773392	0.344468
45471	0.612765	0.800467
45472	0.79504	0.405946
45473	0.575323	0.531795
45474	1.0819	0.356121
45475	1.06106	0.401316
45476	1.04675	0.35089
45477	0.86858	0.425837
45478	0.796873	0.384209
45479	0.557469	0.398003
45480	0.807663	0.411161
45481	2.2024	2.1
45482	2.34106	1.57041
45483	2.02634	2.1
45484	2.20125	2.1
45485	4.36739	2.1
45486	2.34106	1.83259
45487	2.25195	0.610407
45488	2.20125	2.1
45489	2.13132	0.535499
45490	4.47237	0.767115
45491	2.02635	2.1
45492	2.22465	2.1
45493	1.68504	2.1
45494	2.0815	1.79623
45495	3.92673	2.1
45496	2.22465	2.1
45497	2.13133	0.56128
45498	2.0815	1.85004
45499	2.23579	0.580717
45500	4.44862	0.739274
45501	1.89226	0.923948
45502	1.89226	1.30903
45503	1.89226	1.71214
45504	1.89226	0.997604
45505	1.89226	1.55816
45506	1.89226	0.961736
45507	1.93967	0.150795
45508	1.70302	0.244133
45509	1.21883	0.208968
45510	1.70302	0.324239
45511	1.19889	0.148487
45512	1.70302	0.252338
45513	0.946131	0.337021
45514	0.946131	0.267293
45515	0.946131	0.234202
45516	0.946131	0.295254
45517	0.94613	0.327153
45518	0.94613	0.388048
45519	0.946131	0.198997
45520	0.946131	0.259817
45521	0.946131	0.228205
45522	0.946131	0.264781
45523	0.946131	0.28712
45524	0.946131	0.203265
45525	0.883128	0.271123
45526	0.883128	0.335834
45527	0.827519	0.231907

45528	0.82752	0.285057
45529	1.16234	0.276097
45530	1.19063	0.313975
45531	0.631294	0.245879
45532	0.548311	0.22265
45533	0.631293	0.224874
45534	1.16234	0.258089
45535	1.19063	0.280638
45536	0.54831	0.326284
45537	1.89226	0.0606726
45538	1.89226	0.0989582
45539	1.89226	0.126339
45540	1.89226	0.0738732
45541	3.78453	0.0781137
45542	2.33328	0.05
45543	1.87991	0.057348
45544	1.87991	0.070527
45545	2.82938	0.05
45546	1.84457	0.0686904
45547	2.79403	0.05
45548	2.82937	0.05
45549	2.70881	0.0530106
45550	1.8799	0.0625688
45551	1.82739	0.05
45552	4.58874	0.05
45553	1.89226	0.111237
45554	1.89226	0.0579896
45555	1.89226	0.0808251
45556	1.89226	0.110538
45557	3.78452	0.10272
45558	1.17897	0.0599082
45559	1.82739	0.05
45560	1.17897	0.0528988
45561	1.76199	0.05
45562	1.23885	0.0572426
45563	1.80989	0.05
45564	0.946131	0.07168
45565	0.946131	0.0680896
45566	0.946131	0.0907301
45567	0.946131	0.0889324
45568	0.946129	0.104991
45569	0.946129	0.0623904
45570	0.946133	0.0835882
45571	0.946133	0.0918977
45572	0.946131	0.0837611
45573	0.946131	0.0635447
45574	0.946131	0.0718416
45575	0.946131	0.0710291
45576	0.946126	0.6299
45577	0.946126	2.1
45578	1.24887	2.1
45579	1.24887	2.1
45580	0.946127	1.92327
45581	0.946127	0.70697
45582	1.24887	2.1
45583	1.28511	1.42389
45584	1.2851	1.37633
45585	0.946128	1.7646

45586	1.24887	1.85948
45587	0.946128	2.02757
45588	1.89226	2.1
45589	1.89226	1.65913
45590	1.89226	1.76536
45591	1.89226	0.787614
45592	3.78452	1.30721
45593	0.592142	1.83282
45594	0.592142	1.67602
45595	0.60496	2.1
45596	0.60496	2.1
45597	0.694237	2.01443
45598	0.608714	0.987656
45599	0.562863	1.61681
45600	0.502864	2.1
45601	0.562863	1.82314
45602	0.694237	1.88984
45603	0.608714	2.1
45604	0.502864	1.66446
45605	1.89226	0.629473
45606	1.89226	2.1
45607	1.89226	1.38868
45608	1.89226	1.76226
45609	3.78453	1.68988
45610	1.55779	2.1
45611	1.57968	2.1
45612	1.47186	0.898546
45613	1.51725	2.1
45614	3.05154	2.1
45615	1.57967	2.1
45616	1.474 2.1	
45617	1.51725	2.1
45618	1.42895	2.1
45619	3.00863	2.1
45620	1.89226	2.1
45621	1.89226	2.1
45622	1.89226	1.855
45623	1.89226	2.1
45624	3.78452	2.1
45625	1.89226	1.0397
45626	1.89226	1.88853
45627	1.89226	0.622291
45628	1.89226	2.1
45629	3.78453	1.3093
45630	1.47186	0.848069
45631	1.51725	2.1
45632	1.38859	2.1
45633	1.45332	2.1
45634	2.92519	2.1
45635	1.51725	2.1
45636	1.42895	2.1
45637	1.45332	2.1
45638	1.38414	2.1
45639	2.88228	2.1
45640	1.89226	1.94678
45641	1.89226	2.1
45642	1.89226	2.1
45643	1.89226	2.1

45644	3.78452	2.1
45645	1.34497	0.05
45646	1.36747	0.05
45647	1.18919	0.05
45648	2.08149	0.05
45649	1.23556	0.05
45650	2.13702	0.05
45651	1.10106	0.05
45652	2.08001	0.05
45653	1.34497	0.05
45654	2.31605	0.05
45655	1.34497	0.05
45656	2.31605	0.05
45657	2.31605	0.05
45658	2.0815	0.05
45659	1.9419	0.05
45660	2.46665	0.05
45661	4.52922	0.05
45662	1.20349	0.05
45663	2.08149	0.05
45664	1.36948	0.05
45665	1.34737	0.05
45666	2.08149	0.05
45667	2.08149	0.05
45668	0.946132	0.05
45669	0.946132	0.05
45670	1.37715	0.05
45671	0.823199	0.05
45672	1.21598	0.05
45673	0.946132	0.05
45674	1.37715	0.05
45675	0.946132	0.05
45676	1.21598	0.05
45677	0.823198	0.05
45678	0.946133	0.05
45679	0.946133	0.05
45680	1.89226	0.05
45681	1.89226	0.05
45682	1.89226	0.05
45683	1.89226	0.05
45684	3.78453	0.05
45685	1.89227	0.05
45686	1.89227	0.05
45687	1.89227	0.05
45688	1.89227	0.05
45689	3.78453	0.05
45690	0.946132	0.05
45691	0.946132	0.05
45692	1.21599	0.05
45693	1.21599	0.05
45694	0.966749	0.05
45695	0.946133	0.05
45696	1.21598	0.05
45697	0.946132	0.05
45698	0.966748	0.05
45699	1.21598	0.05
45700	0.946133	0.05
45701	0.946133	0.05

45702	0.946133	0.05
45703	0.946133	0.05
45704	1.1134	0.05
45705	1.11555	0.05
45706	1.11555	0.05
45707	0.946132	0.05
45708	0.946132	0.05
45709	1.1134	0.05
45710	1.11555	0.05
45711	1.11555	0.05
45712	0.946132	0.05
45713	0.946132	0.05
45714	0.946133	0.05
45715	0.946133	0.05
45716	1.11555	0.05
45717	0.978467	0.05
45718	0.978468	0.05
45719	0.946133	0.05
45720	1.11555	0.05
45721	0.946132	0.05
45722	0.978468	0.05
45723	0.978468	0.05
45724	0.946132	0.05
45725	0.946132	0.05
45726	0.851542	2.1
45727	0.851542	2.1
45728	1.14168	2.1
45729	1.27897	2.1
45730	1.02817	2.1
45731	1.13536	2.1
45732	0.946131	2.1
45733	0.861207	2.08429
45734	0.94613	2.08009
45735	0.94613	2.1
45736	0.868751	2.1
45737	1.15181	2.1
45738	0.946132	1.12439
45739	0.946132	1.10332
45740	0.946128	1.18931
45741	0.946128	0.82772
45742	0.94613	2.1
45743	0.94613	1.16122
45744	0.946131	0.996065
45745	0.946131	0.813273
45746	0.94613	0.751242
45747	0.94613	1.10035
45748	0.94613	0.972367
45749	0.94613	1.70202
45750	2.53273	0.0561683
45751	1.89226	0.072009
45752	1.89226	0.066785
45753	1.89226	0.0720287
45754	1.89226	0.0521542
45755	1.89226	0.0582858
45756	1.89226	2.1
45757	1.89226	2.1
45758	1.89226	2.1
45759	1.89226	2.1

45760	3.78452	2.1
45761	1.89226	2.1
45762	2.54394	2.1
45763	1.89226	2.1
45764	2.42221	2.1
45765	4.43621	2.1
45766	2.54394	2.1
45767	1.62743	2.1
45768	2.42221	2.1
45769	1.5437	2.1
45770	4.08764	2.1
45771	1.89226	2.1
45772	1.89226	2.1
45773	1.89226	2.1
45774	1.89226	2.1
45775	3.78452	2.1
45776	1.89226	2.1
45777	1.89226	2.1
45778	1.89226	2.1
45779	1.89226	2.1
45780	3.78452	2.1
45781	1.89226	2.1
45782	2.30146	2.1
45783	2.42221	2.1
45784	1.89226	2.1
45785	1.89226	2.1
45786	2.42221	2.1
45787	2.30146	2.1
45788	2.42221	2.1
45789	1.39962	2.1
45790	1.39962	2.1
45791	1.5437	2.1
45792	2.44553	2.1
45793	1.89226	2.1
45794	1.89226	2.1
45795	1.89226	2.1
45796	1.89226	2.1
45797	3.78452	2.1
45798	1.62196	2.1
45799	2.00695	2.1
45800	1.95288	2.1
45801	1.70303	2.1
45802	3.53316	2.1
45803	1.80703	2.1
45804	2.00695	2.1
45805	1.92991	2.1
45806	2.00695	2.1
45807	1.85888	2.1
45808	1.93592	2.1
45809	1.89226	2.1
45810	1.89226	2.1
45811	1.89226	2.1
45812	1.89226	2.1
45813	1.89226	2.1
45814	1.89226	2.1
45815	1.89226	2.1
45816	1.89226	2.1
45817	1.89226	2.1

45818	1.89226	2.1
45819	3.78453	2.1
45820	1.95288	2.1
45821	1.50524	2.1
45822	1.83564	2.1
45823	1.70303	2.1
45824	2.00749	2.1
45825	1.84671	2.1
45826	1.62632	2.1
45827	1.82498	2.1
45828	1.92991	2.1
45829	1.92991	2.1
45830	1.47928	2.1
45831	1.47751	2.1
45832	0.946128	2.1
45833	0.946128	2.1
45834	0.946132	2.1
45835	0.946132	2.1
45836	0.94613	2.1
45837	0.94613	2.1
45838	0.946129	2.1
45839	0.946129	2.1
45840	0.94613	2.1
45841	0.94613	2.1
45842	0.94613	2.1
45843	0.94613	2.1
45844	1.89226	2.1
45845	1.89226	2.1
45846	1.89226	2.1
45847	1.89226	2.1
45848	1.89226	2.1
45849	1.89226	2.1
45850	2.27351	0.811949
45851	2.74068	0.637878
45852	2.72278	0.705152
45853	2.63356	0.763964
45854	2.36273	0.692093
45855	2.27351	0.746288
45856	2.36273	0.73883
45857	2.83112	0.594515
45858	2.81212	0.66502
45859	2.74068	0.700346
45860	2.43416	0.653899
45861	2.36273	0.685716
45862	1.89227	0.678096
45863	2.83112	0.562953
45864	2.74069	0.666376
45865	2.74069	0.537275
45866	1.89227	0.716171
45867	1.89227	0.62129
45868	2.40887	2.1
45869	1.4494	2.1
45870	1.52164	2.1
45871	2.47534	2.1
45872	3.93051	2.1
45873	1.89226	2.1
45874	2.47534	2.1
45875	2.40887	2.1

45876	2.40887	2.1
45877	1.89226	2.1
45878	1.89226	2.1
45879	1.60861	2.1
45880	2.47534	2.1
45881	1.52164	2.1
45882	1.52164	2.1
45883	2.55301	2.1
45884	2.55301	2.1
45885	0.946128	2.1
45886	0.946128	2.1
45887	1.23767	2.1
45888	1.2765	2.1
45889	1.23767	2.1
45890	1.23767	2.1
45891	0.946131	2.1
45892	0.946131	2.1
45893	0.946131	2.1
45894	1.2765	2.1
45895	1.23767	2.1
45896	0.946131	2.1
45897	1.89226	2.1
45898	1.87511	2.1
45899	1.87511	2.1
45900	1.95852	2.1
45901	2.0815	2.1
45902	2.22169	2.1
45903	1.89226	2.1
45904	1.89226	2.1
45905	1.89226	2.1
45906	1.89226	2.1
45907	1.89226	2.1
45908	1.89226	2.1
45909	2.0815	2.1
45910	1.80067	2.1
45911	1.80067	2.1
45912	2.05518	2.1
45913	1.89226	2.1
45914	2.16509	2.1
45915	1.87153	2.1
45916	2.65897	2.1
45917	1.58724	2.1
45918	2.38924	2.1
45919	4.26077	2.1
45920	1.89226	2.1
45921	1.89226	2.1
45922	1.89226	2.1
45923	1.89226	2.1
45924	1.89226	2.1
45925	1.89226	2.1
45926	1.89226	0.839334
45927	2.21022	0.585196
45928	2.19769	0.951706
45929	2.21022	0.700835
45930	1.87973	0.641669
45931	1.89226	0.508252
45932	1.18376	1.15181
45933	2.21022	0.523805

45934	1.16239	1.00846
45935	1.16239	0.577857
45936	2.19769	1.02655
45937	2.19769	0.573018
45938	0.946128	0.670582
45939	0.946128	0.60278
45940	1.09362	0.853695
45941	1.09884	0.627549
45942	0.94613	0.682591
45943	0.94613	0.509329
45944	1.09362	0.76807
45945	1.09362	0.668315
45946	0.946131	0.706625
45947	1.09362	0.718423
45948	0.946131	0.577384
45949	1.09884	0.531694
45950	1.19323	1.01447
45951	2.19769	0.489789
45952	1.18376	0.887098
45953	1.18376	0.59317
45954	2.18724	0.902087
45955	2.18724	0.608686
45956	2.0815	2.1
45957	0.922714	2.1
45958	2.0815	2.1
45959	1.84882	2.1
45960	1.20787	2.1
45961	1.08321	2.1
45962	2.6238	0.135143
45963	1.89226	0.242326
45964	1.89226	0.215185
45965	2.4866	0.162394
45966	1.89226	0.198054
45967	2.4866	0.169591
45968	2.66753	0.177899
45969	1.89226	0.352507
45970	1.89226	0.328988
45971	2.6238	0.224965
45972	1.89226	0.291711
45973	2.6238	0.244231
45974	0.946129	1.58516
45975	0.946129	2.1
45976	1.04676	2.1
45977	1.04676	2.1
45978	0.946131	2.1
45979	0.946131	2.1
45980	1.08732	2.1
45981	1.04676	2.1
45982	1.08732	2.1
45983	0.946131	2.1
45984	1.04676	2.1
45985	0.946131	2.1
45986	1.20958	1.68403
45987	1.73802	0.686878
45988	1.39067	0.811038
45989	1.39067	0.643182
45990	1.50969	0.895919
45991	1.50969	0.950896

45992	1.00382	1.10383
45993	1.50969	0.491498
45994	1.20958	0.951935
45995	1.20958	0.557328
45996	1.32039	1.21372
45997	1.32039	0.705266
45998	2.0815	2.1
45999	2.51396	2.1
46000	2.19826	2.1
46001	2.54175	2.1
46002	4.79599	2.1
46003	2.54174	2.1
46004	2.17978	2.1
46005	2.54174	2.1
46006	2.2854	2.1
46007	2.57212	2.1
46008	2.31578	2.1
46009	2.27103	2.1
46010	2.54175	2.1
46011	2.4255	2.1
46012	2.82251	2.1
46013	4.96725	2.1
46014	2.60614	0.270239
46015	2.75374	0.219504
46016	1.94002	0.200841
46017	2.08392	0.278435
46018	4.69006	0.225407
46019	2.75373	0.196974
46020	2.6875	0.293358
46021	2.08392	0.21068
46022	1.99944	0.240718
46023	4.77142	0.249853
46024	1.1211	0.0802352
46025	2.08392	0.155643
46026	1.17787	0.124063
46027	1.94002	0.101637
46028	1.32178	0.111736
46029	2.08392	0.112276
46030	2.08392	0.100216
46031	1.99944	0.113834
46032	1.32178	0.0745174
46033	1.25929	0.0968691
46034	3.34321	0.0912213
46035	1.22266	0.05
46036	0.779473	0.05
46037	0.94613	0.05
46038	0.94613	0.05
46039	1.04072	0.05
46040	1.1407	0.05
46041	1.10042	0.05
46042	0.851542	0.05
46043	0.942046	0.05
46044	0.942046	0.05
46045	1.04114	0.05
46046	0.826602	0.05
46047	2.37468	2.1
46048	1.89226	2.1
46049	1.89226	2.1

46050	1.89226	2.1
46051	2.27917	2.1
46052	2.27917	2.1
46053	1.13959	2.1
46054	1.09451	2.1
46055	0.946131	2.1
46056	0.946131	2.1
46057	1.09451	2.1
46058	1.09451	2.1
46059	0.94613	2.1
46060	0.94613	2.1
46061	1.09451	2.1
46062	0.946131	2.1
46063	0.946131	2.1
46064	1.13959	2.1
46065	2.52454	0.741262
46066	2.12221	2.1
46067	2.67624	0.95563
46068	2.33865	1.39831
46069	4.86319	0.926103
46070	1.89226	1.06374
46071	1.89226	2.1
46072	1.89226	1.22158
46073	1.89226	2.1
46074	3.78452	2.1
46075	1.89226	2.1
46076	1.89226	2.1
46077	1.89226	2.1
46078	1.89226	2.1
46079	3.78452	2.1
46080	2.12221	2.1
46081	1.47769	2.1
46082	2.33865	2.1
46083	1.62898	2.1
46084	3.81634	2.1
46085	2.67624	2.08774
46086	2.37734	1.11777
46087	2.33865	2.1
46088	2.33865	1.75691
46089	2.70125	2.1
46090	2.70125	2.1
46091	1.89226	1.73788
46092	1.89226	2.1
46093	1.89226	2.1
46094	1.89226	2.1
46095	1.89226	0.919966
46096	1.89226	1.10653
46097	1.89226	2.1
46098	1.89226	2.1
46099	1.89226	2.1
46100	1.89226	2.1
46101	1.89226	2.1
46102	1.89226	2.1
46103	2.37733	1.52694
46104	1.95927	2.1
46105	1.82516	1.05377
46106	1.82516	2.1
46107	2.33865	2.1

46108	2.33865	2.1
46109	2.57466	2.1
46110	2.21715	2.1
46111	2.55653	2.1
46112	2.24775	2.1
46113	4.77367	2.1
46114	0.937095	2.1
46115	0.937095	2.1
46116	0.958028	2.1
46117	0.958028	2.1
46118	0.828386	2.1
46119	0.771244	2.1
46120	1.10858	2.1
46121	1.12388	2.1
46122	1.12388	2.1
46123	0.828386	2.1
46124	0.771244	2.1
46125	1.10858	2.1
46126	2.55653	2.1
46127	2.32606	2.1
46128	2.24775	2.1
46129	2.24775	2.1
46130	2.57928	2.1
46131	2.57928	2.1
46132	0.990481	2.1
46133	0.990481	2.1
46134	1.00094	2.1
46135	1.00094	2.1
46136	0.888385	2.1
46137	0.828386	2.1
46138	1.16303	2.1
46139	1.12388	2.1
46140	1.16303	2.1
46141	0.888385	2.1
46142	1.12388	2.1
46143	0.828387	2.1
46144	0.962524	2.1
46145	0.962524	2.1
46146	0.7976	2.1
46147	0.7976	2.1
46148	0.797628	2.1
46149	0.814 2.1	
46150	0.946125	2.1
46151	0.946125	2.1
46152	0.946118	2.1
46153	0.797623	2.1
46154	0.814006	2.1
46155	0.946131	2.1
46156	1.8978	0.05
46157	2.23892	0.05
46158	1.71469	0.05
46159	4.13672	0.05
46160	2.64136	0.05
46161	2.23892	0.260065
46162	2.37578	0.495337
46163	1.77489	1.9812
46164	4.6147	0.392764
46165	1.02284	2.1

46166	0.898796	0.530755
46167	0.948554	0.704284
46168	0.948554	2.1
46169	0.878797	1.58723
46170	0.796197	2.1
46171	1.09145	0.582813
46172	0.963109	0.596002
46173	1.12114	0.883447
46174	0.782739	0.787914
46175	1.11084	0.676705
46176	0.850742	0.676705
46177	2.48024	2.1
46178	2.405 2.1	
46179	2.24585	2.1
46180	2.34795	2.1
46181	4.75295	2.1
46182	1.93794	2.1
46183	2.405 2.1	
46184	2.24585	2.1
46185	1.67594	1.60752
46186	4.08094	2.1
46187	1.89226	2.1
46188	1.89226	0.615904
46189	1.89226	1.5581
46190	1.89226	0.804149
46191	3.78452	0.964925
46192	1.89226	2.1
46193	1.89226	1.72505
46194	1.89226	2.1
46195	1.89226	0.826615
46196	3.78452	2.1
46197	2.24585	2.1
46198	2.34795	2.1
46199	2.21155	2.1
46200	2.06626	2.1
46201	4.4142	2.1
46202	2.24585	2.1
46203	1.41498	1.15504
46204	1.41498	2.1
46205	1.67594	2.1
46206	2.06625	2.1
46207	2.32721	2.1
46208	1.89226	1.11379
46209	1.89226	0.795145
46210	1.89226	2.1
46211	1.89226	0.696574
46212	1.89226	2.1
46213	1.89226	0.636145
46214	1.89226	2.1
46215	1.89226	0.811458
46216	1.89226	2.1
46217	1.89226	1.3972
46218	3.78452	2.1
46219	1.89226	0.135046
46220	1.89226	0.234583
46221	1.89226	0.323054
46222	1.89226	0.118025
46223	3.78452	0.124699

46224	1.89226	0.15837
46225	2.53655	0.0820585
46226	1.89226	0.0750248
46227	1.89226	0.0807081
46228	1.89226	0.163034
46229	1.89226	0.171584
46230	1.89227	2.1
46231	1.89227	2.1
46232	1.89227	2.1
46233	1.89227	2.1
46234	1.89227	2.1
46235	1.89227	2.1
46236	0.91339	2.1
46237	0.91339	2.1
46238	1.2158	2.1
46239	1.20629	2.1
46240	1.00473	2.1
46241	1.15749	2.1
46242	0.946132	2.1
46243	0.946132	2.1
46244	0.946133	2.1
46245	1.17513	2.1
46246	1.00473	2.1
46247	0.946132	2.1
46248	1.89226	2.1
46249	1.89226	2.1
46250	1.89226	2.1
46251	1.89226	2.1
46252	1.89226	2.1
46253	1.89226	2.1
46254	1.89226	2.1
46255	2.6066	2.1
46256	2.35026	2.1
46257	2.35026	2.1
46258	1.89226	2.1
46259	1.89226	2.1
46260	2.04056	0.432197
46261	2.77366	0.421875
46262	1.24682	0.387208
46263	1.24682	0.439161
46264	2.93651	0.408506
46265	2.14277	0.438285
46266	2.68524	1.63286
46267	2.81847	1.53949
46268	1.89226	0.939133
46269	1.89226	1.61021
46270	4.57751	1.11456
46271	2.68524	1.23352
46272	1.89226	1.79714
46273	1.89226	1.84787
46274	2.50732	1.39662
46275	1.89226	1.62422
46276	2.50732	1.78703
46277	1.89226	1.02584
46278	1.89226	1.83481
46279	1.89226	1.43787
46280	1.89226	1.82074
46281	1.89226	1.09488

46282	1.89226	1.69466
46283	1.89226	1.77329
46284	1.89226	1.35302
46285	1.89226	1.36979
46286	1.89226	2.01984
46287	1.89226	0.92484
46288	1.89226	1.135
46289	1.61016	1.13542
46290	1.07467	1.50692
46291	1.24474	1.0426
46292	1.24474	1.8569
46293	1.45303	1.14792
46294	1.45303	1.88229
46295	1.61016	1.52477
46296	1.24474	2.1
46297	1.61016	1.64223
46298	1.77186	1.25148
46299	1.37813	1.24227
46300	1.37813	1.67251
46301	1.73305	2.1
46302	2.0815	1.906
46303	1.97658	2.1
46304	1.75791	2.1
46305	1.87228	2.1
46306	1.66515	2.1
46307	2.0815	2.03759
46308	1.93938	0.512721
46309	2.03253	0.412349
46310	2.2142	2.1
46311	1.97658	0.43645
46312	2.15324	2.1
46313	1.73305	2.1
46314	1.97658	1.8941
46315	1.60428	2.1
46316	1.8589	2.1
46317	3.58085	2.1
46318	1.97657	1.92199
46319	1.93938	0.60154
46320	1.51316	2.1
46321	1.85293	0.57528
46322	3.82951	0.767339
46323	1.70303	0.290588
46324	1.96087	0.32703
46325	1.42308	0.322068
46326	1.96087	0.321667
46327	1.25023	0.303402
46328	1.70303	0.316868
46329	0.946132	0.323318
46330	0.946132	0.316353
46331	0.946131	0.317716
46332	0.946131	0.321367
46333	0.946132	0.329384
46334	0.946132	0.326788
46335	0.946132	0.316108
46336	0.946132	0.315074
46337	0.946132	0.323394
46338	0.946132	0.314954
46339	0.946132	0.319288

46340	0.946132	0.325973
46341	1.89226	0.328872
46342	1.89226	0.360933
46343	1.89226	0.336486
46344	1.89226	0.35434
46345	3.78452	0.338271
46346	1.89226	0.320819
46347	1.89226	0.342771
46348	1.89226	0.335659
46349	1.89226	0.337442
46350	1.89226	0.325175
46351	1.89226	0.325604
46352	1.89226	0.32484
46353	1.89226	0.336719
46354	1.89226	0.343287
46355	1.89226	0.333942
46356	1.89226	0.327297
46357	1.89226	0.321639
46358	1.89226	0.313803
46359	1.89226	0.329934
46360	2.48255	0.317934
46361	1.89226	0.319553
46362	3.78452	0.318196
46363	1.89226	2.1
46364	2.42758	2.1
46365	1.9209	2.1
46366	1.89226	2.1
46367	2.45622	2.1
46368	2.42758	2.1
46369	2.42758	2.1
46370	1.49987	2.1
46371	1.61028	2.1
46372	1.58164	2.1
46373	2.45622	2.1
46374	2.42758	2.1
46375	0.94613	2.1
46376	0.94613	2.1
46377	1.24298	2.1
46378	1.24298	2.1
46379	1.24298	2.1
46380	1.22811	2.1
46381	0.946131	2.1
46382	0.946131	2.1
46383	0.94613	2.1
46384	1.24298	2.1
46385	0.94613	2.1
46386	1.22811	2.1
46387	2.48596	2.1
46388	2.45622	2.1
46389	1.63823	2.1
46390	1.4304	2.1
46391	1.45739	2.1
46392	2.45622	2.1
46393	2.4169	2.1
46394	2.55249	2.1
46395	2.51125	2.1
46396	2.35245	2.1
46397	4.90494	2.1

46398	2.45922	2.1
46399	2.55249	2.1
46400	2.51126	2.1
46401	2.43226	2.1
46402	4.98474	2.1
46403	2.51125	2.1
46404	2.35245	2.1
46405	2.28979	2.1
46406	2.47228	2.1
46407	4.82473	2.1
46408	2.51126	2.1
46409	2.43226	2.1
46410	2.40668	2.1
46411	2.47228	2.1
46412	4.90453	2.1
46413	2.8045	2.1
46414	1.93707	2.1
46415	2.14087	2.1
46416	2.14087	2.1
46417	2.61595	2.1
46418	2.61595	2.08471
46419	1.89226	2.1
46420	1.89226	2.1
46421	1.89226	2.1
46422	1.89226	0.872514
46423	1.89226	2.1
46424	1.89226	0.768646
46425	1.22368	1.78786
46426	1.32339	2.1
46427	0.957125	2.1
46428	0.750074	2.1
46429	1.08438	2.1
46430	1.08438	1.9225
46431	1.13094	2.1
46432	0.970797	2.1
46433	1.17568	2.1
46434	0.764392	2.1
46435	0.944286	2.1
46436	1.27523	2.1
46437	1.89226	2.1
46438	1.89226	2.1
46439	1.89226	2.1
46440	1.89226	0.804984
46441	3.78453	2.1
46442	2.8239	2.1
46443	1.89227	2.1
46444	2.57276	2.1
46445	2.57276	2.1
46446	1.89227	2.1
46447	1.89227	2.1
46448	1.18139	2.1
46449	1.85195	2.1
46450	1.78595	2.1
46451	1.08172	2.1
46452	2.93367	2.1
46453	1.60735	2.1
46454	1.20356	2.1
46455	0.967034	2.1

46456	1.08172	2.1
46457	1.67324	2.1
46458	1.77293	2.1
46459	1.89226	2.1
46460	1.89226	2.1
46461	1.8213	2.1
46462	1.82129	2.1
46463	2.14171	2.1
46464	2.40192	2.1
46465	0.810943	2.1
46466	0.810943	2.1
46467	0.798469	2.1
46468	0.788336	2.1
46469	0.804015	2.1
46470	0.810945	2.1
46471	1.947	2.1
46472	1.36506	2.1
46473	1.08686	2.1
46474	1.07208	2.1
46475	1.08132	2.1
46476	1.13537	0.229009
46477	1.14754	0.792311
46478	0.946131	0.609477
46479	0.946131	0.94737
46480	0.94613	0.586362
46481	0.94613	0.28271
46482	1.25104	0.999784
46483	1.14754	0.740551
46484	1.13537	0.650708
46485	0.946131	0.659366
46486	0.946131	0.697032
46487	1.25104	0.684169
46488	0.96542	0.151558
46489	0.922284	0.680157
46490	0.946131	0.946205
46491	0.946131	0.62851
46492	0.946131	0.619845
46493	0.946131	0.182758
46494	1.13537	0.960803
46495	0.924824	0.721373
46496	0.859476	0.586449
46497	0.842305	0.625895
46498	1.25996	0.631379
46499	1.04996	0.640555
46500	1.71007	0.05
46501	2.73283	0.05
46502	2.73283	0.05
46503	1.89226	0.05
46504	1.89226	0.05
46505	1.13262	0.05
46506	0.946131	0.05
46507	0.946131	0.05
46508	0.946131	0.05
46509	0.946131	0.0598928
46510	0.946132	0.0614963
46511	0.946132	0.0567762
46512	0.946131	0.05
46513	0.946131	0.0624617

46514	0.946131	0.05
46515	0.946131	0.0503813
46516	0.946132	0.0555758
46517	0.946132	0.05
46518	0.946131	0.05
46519	1.36642	0.05
46520	0.946125	0.05
46521	0.946125	0.05
46522	0.946128	0.05
46523	0.946128	0.05
46524	0.946126	0.05
46525	0.946128	0.05
46526	0.946128	0.05
46527	0.946128	0.05
46528	1.36641	0.05
46529	0.946128	0.05
46530	1.89226	0.05
46531	1.89226	0.0656177
46532	1.89226	0.0500214
46533	1.89226	0.0500467
46534	1.89226	0.0553392
46535	1.89226	0.0553251
46536	2.69784	2.1
46537	2.51651	2.1
46538	2.56999	2.1
46539	2.74623	2.1
46540	5.26783	2.1
46541	2.69784	2.1
46542	2.48718	2.1
46543	2.50161	2.1
46544	2.74623	2.1
46545	5.19945	2.1
46546	1.89226	2.1
46547	1.89226	2.1
46548	1.89226	2.1
46549	1.89226	2.1
46550	3.78453	2.1
46551	0.874264	2.1
46552	1.09879	2.1
46553	0.946131	2.1
46554	0.946131	2.1
46555	1.17062	2.1
46556	1.17062	2.1
46557	0.874293	2.1
46558	0.874293	2.1
46559	1.09879	2.1
46560	0.94613	2.1
46561	0.946131	2.1
46562	0.874265	2.1
46563	1.89226	2.1
46564	1.89226	2.1
46565	1.89226	2.1
46566	1.89226	2.1
46567	3.78453	2.1
46568	0.977382	2.1
46569	1.16424	2.1
46570	0.946133	2.1
46571	0.946133	2.1

46572	1.33012	2.1
46573	1.25589	2.1
46574	0.888498	2.1
46575	0.888498	2.1
46576	1.25375	2.1
46577	0.946131	2.1
46578	0.946131	2.1
46579	0.977384	2.1
46580	1.89226	2.1
46581	2.19757	2.1
46582	2.62906	2.1
46583	1.89226	2.1
46584	1.89226	2.1
46585	2.62906	2.1
46586	1.27944	1.16582
46587	1.27731	2.07678
46588	0.615701	1.9615
46589	0.615701	1.24844
46590	0.615673	0.233602
46591	0.946133	1.73166
46592	0.948264	0.697046
46593	0.946132	1.15719
46594	0.94752	0.727435
46595	0.61706	0.496894
46596	0.946132	0.843369
46597	0.946132	0.840863
46598	2.76604	2.1
46599	1.66728	2.1
46600	1.66728	2.1
46601	1.83036	2.1
46602	2.54515	2.1
46603	2.70823	2.1
46604	2.54515	2.1
46605	1.49958	2.1
46606	1.49958	2.1
46607	1.66728	2.1
46608	2.34465	2.1
46609	2.51235	2.1
46610	1.21128	2.1
46611	1.21128	2.1
46612	0.946131	2.1
46613	0.946131	2.1
46614	0.946131	2.01342
46615	0.946131	2.1
46616	1.31401	2.1
46617	1.21128	2.1
46618	1.21128	2.1
46619	0.946131	2.1
46620	0.946131	2.1
46621	1.31401	2.1
46622	1.67374	2.1
46623	2.29586	2.1
46624	1.50033	2.1
46625	2.16598	2.1
46626	3.83972	2.1
46627	1.89226	2.1
46628	1.89226	2.1
46629	1.89226	2.1

46630	1.89226	2.1
46631	3.78452	2.1
46632	1.13537	2.1
46633	1.02188	2.1
46634	0.946132	2.1
46635	0.946132	2.1
46636	0.946132	2.1
46637	0.946132	2.1
46638	1.21128	2.1
46639	1.02188	2.1
46640	1.04672	2.1
46641	1.04672	2.1
46642	1.21128	2.1
46643	0.946132	2.1
46644	0.815411	2.1
46645	0.870464	2.1
46646	0.885437	2.1
46647	0.885437	2.1
46648	1.06395	2.1
46649	1.00483	2.1
46650	0.757125	2.1
46651	0.673956	2.1
46652	0.684461	2.1
46653	1.06979	2.1
46654	0.731403	2.1
46655	1.0559	2.1
46656	1.89226	2.1
46657	1.89226	2.1
46658	1.89226	2.1
46659	1.89226	2.1
46660	1.89226	2.1
46661	1.89226	2.1
46662	1.76897	0.97892
46663	1.76932	0.963816
46664	1.97455	1.74
46665	1.94302	0.736099
46666	3.71233	0.892334
46667	1.83347	2.1
46668	1.76932	0.980052
46669	1.97455	1.81776
46670	1.76932	1.89689
46671	1.76522	1.91898
46672	1.55999	2.03578
46673	1.89227	0.979796
46674	1.89227	1.13616
46675	1.89227	1.91302
46676	1.89227	0.845432
46677	3.78453	0.954905
46678	1.89226	0.964509
46679	1.89226	0.543806
46680	1.89226	0.618657
46681	1.89226	0.819416
46682	3.78452	0.847572
46683	1.97452	1.47907
46684	1.94299	0.671631
46685	2.17831	0.901057
46686	2.23195	0.889715
46687	4.17495	1.00412

46688	2.23196	0.936207
46689	2.1499	2.1
46690	1.97452	1.49253
46691	1.97452	1.27294
46692	2.09088	1.77562
46693	1.83345	1.52024
46694	0.94612	1.04438
46695	0.94612	1.3277
46696	0.946119	0.856506
46697	0.946119	0.820029
46698	0.946119	0.820161
46699	0.946119	1.03104
46700	0.94612	0.92055
46701	0.94612	1.3385
46702	0.946132	0.955524
46703	0.946132	0.909947
46704	0.946107	0.984221
46705	0.946107	0.976666
46706	1.89224	0.548627
46707	1.89224	0.782066
46708	1.89224	0.831947
46709	1.89224	0.510791
46710	3.78447	0.533871
46711	1.70302	0.562678
46712	1.056	0.539549
46713	1.21829	0.575054
46714	1.89906	0.529246
46715	3.07892	0.542732
46716	2.20248	0.555664
46717	1.56498	0.529967
46718	1.46539	0.517695
46719	1.48787	0.521222
46720	2.37625	0.570312
46721	2.37625	0.567685
46722	1.14104	0.513402
46723	1.00675	0.513146
46724	0.700689	0.5033
46725	0.624389	0.521414
46726	0.782149	0.430277
46727	0.940197	0.507524
46728	0.803437	0.483608
46729	0.803437	0.515248
46730	0.995423	0.486867
46731	0.700699	0.462551
46732	0.593895	0.558001
46733	1.00673	0.510692
46734	1.18097	0.507553
46735	1.15839	0.49139
46736	0.995407	0.509752
46737	0.865932	0.492372
46738	1.17299	0.478683
46739	0.957054	0.510757
46740	1.08016	0.491234
46741	1.08016	0.534315
46742	0.995425	0.504828
46743	1.24161	0.464114
46744	0.796771	0.488005
46745	1.15837	0.536574

46746	2.57264	0.141553
46747	1.89226	0.22869
46748	2.41701	0.147541
46749	1.89226	0.119053
46750	4.30928	0.122168
46751	1.69055	0.18225
46752	2.39587	0.186604
46753	1.53463	0.106731
46754	2.2053	0.104015
46755	3.89585	0.22058
46756	0.946129	0.15464
46757	0.946129	0.130896
46758	0.94613	0.13938
46759	0.94613	0.172799
46760	0.946132	0.143505
46761	0.946132	0.253507
46762	0.946126	0.182703
46763	0.946126	0.155231
46764	0.94613	0.125699
46765	0.94613	0.167642
46766	0.946129	0.163706
46767	0.946129	0.195997
46768	2.41701	0.134089
46769	1.89226	0.0937779
46770	1.89226	0.0762692
46771	1.89226	0.101861
46772	2.32462	0.117161
46773	2.32462	0.171966
46774	1.41942	0.0638706
46775	2.2053	0.0988749
46776	2.02097	0.0777035
46777	2.02097	0.0802954
46778	1.53463	0.0714188
46779	1.53463	0.0705317
46780	1.89226	0.0813413
46781	1.89226	0.132351
46782	1.89226	0.090662
46783	1.89226	0.117463
46784	1.89226	0.100806
46785	1.89226	0.116276
46786	1.89226	1.07466
46787	1.89226	1.98551
46788	1.89226	1.92136
46789	1.89226	1.28047
46790	1.89226	1.69329
46791	1.89226	1.16223
46792	1.02816	1.15676
46793	1.02816	1.49152
46794	0.595141	1.45555
46795	0.595141	1.71978
46796	0.946132	1.34253
46797	0.595113	1.78475
46798	0.677174	1.69548
46799	0.946132	1.11929
46800	0.946132	1.4513
46801	0.946132	1.44086
46802	0.595113	1.41678
46803	0.677174	1.54157

46804	1.89226	1.16886
46805	1.89226	2.0864
46806	1.89226	1.69317
46807	1.89226	1.2625
46808	1.89226	1.91567
46809	1.89226	1.39293
46810	0.946133	1.14842
46811	0.946133	0.895246
46812	0.946132	1.66601
46813	0.946132	1.77267
46814	0.946132	1.74611
46815	0.946132	1.14602
46816	0.946132	1.43142
46817	0.946132	0.899553
46818	0.946132	1.33499
46819	0.946132	1.48231
46820	0.946133	1.20701
46821	0.946133	1.18526
46822	0.946131	1.39803
46823	0.946131	1.33359
46824	0.946131	2.0511
46825	0.946131	2.04437
46826	0.946128	2.09992
46827	0.946128	1.40265
46828	0.946133	2.07074
46829	0.946133	1.28948
46830	0.94613	1.68988
46831	0.94613	1.90507
46832	0.946131	1.72364
46833	0.946131	1.56047
46834	2.0264	1.06863
46835	1.67345	1.05761
46836	2.0264	0.791207
46837	1.70539	0.624799
46838	1.31611	0.924756
46839	1.31611	0.786356
46840	1.89226	0.524538
46841	1.89226	0.521532
46842	1.89226	0.601804
46843	1.89226	0.489647
46844	3.78452	0.49354
46845	1.89226	0.582703
46846	1.89226	0.555373
46847	1.89226	0.550169
46848	1.89226	0.606512
46849	1.89226	0.566507
46850	1.89226	0.633931
46851	1.02645	0.329007
46852	0.810942	0.404281
46853	0.882781	0.426592
46854	0.882781	0.408211
46855	2.80386	0.347809
46856	1.56719	0.405739
46857	1.22499	0.335849
46858	1.08132	0.43106
46859	1.36865	0.384134
46860	0.810957	0.415127
46861	0.810957	0.388175

46862	0.946132	0.494031
46863	0.946132	0.51235
46864	0.946129	0.445071
46865	0.946129	0.461534
46866	0.946131	0.445407
46867	0.946131	0.456982
46868	0.946131	0.505384
46869	0.946131	0.48106
46870	0.94613	0.498975
46871	0.94613	0.491303
46872	0.946131	0.546462
46873	0.946131	0.482867
46874	1.89226	0.510595
46875	1.89226	0.468143
46876	1.89226	0.556639
46877	1.89226	0.461612
46878	3.78453	0.466028
46879	1.30462	0.328458
46880	1.79906	0.373702
46881	1.01299	0.383362
46882	1.44185	0.349909
46883	1.3499	0.346848
46884	1.79906	0.342638
46885	1.89226	0.356096
46886	1.89226	0.409157
46887	1.89226	0.341488
46888	1.89226	0.382988
46889	1.89226	0.393228
46890	1.89226	0.4593
46891	1.89226	0.395885
46892	1.89226	0.395972
46893	1.89226	0.414971
46894	1.89226	0.398405
46895	1.89226	0.403005
46896	1.89226	0.390467
46897	1.3499	0.335238
46898	1.79905	0.364462
46899	1.29001	0.348828
46900	1.36005	0.372802
46901	1.88294	0.373536
46902	1.79905	0.359236
46903	1.89226	0.341436
46904	2.75374	0.32415
46905	2.60614	0.339183
46906	1.89226	0.340204
46907	2.60614	0.321613
46908	1.89226	0.324414
46909	0.946131	0.334071
46910	0.946131	0.337811
46911	1.37687	0.343651
46912	1.37687	0.351708
46913	0.946131	0.344876
46914	1.34375	0.352464
46915	1.37686	0.332315
46916	0.946128	0.33125
46917	1.37687	0.334262
46918	1.34375	0.337176
46919	0.94613	0.345134

46920	0.94613	0.34186
46921	1.44247	2.1
46922	1.12665	2.1
46923	1.50044	2.1
46924	1.73651	2.1
46925	2.94291	2.1
46926	1.73652	2.1
46927	1.64926	2.1
46928	1.44247	2.1
46929	1.44247	2.1
46930	1.64926	2.1
46931	1.43872	2.1
46932	1.50044	2.1
46933	1.73651	2.1
46934	2.00955	2.1
46935	1.87875	2.1
46936	3.50999	2.1
46937	1.64926	2.1
46938	1.73652	2.1
46939	2.00955	2.1
46940	1.8493	2.1
46941	3.65881	2.1
46942	1.89226	0.0978887
46943	2.28188	0.121328
46944	1.99182	0.0990778
46945	2.28188	0.170903
46946	1.6022	0.0731847
46947	1.89226	0.129903
46948	1.89226	0.153611
46949	1.89226	0.182294
46950	1.89226	0.157016
46951	1.89226	0.293804
46952	1.89226	0.115772
46953	1.89226	0.197331
46954	1.89226	0.116937
46955	1.89226	0.138149
46956	2.19578	0.110414
46957	1.89226	0.231765
46958	2.81422	0.0952411
46959	2.42521	0.147847
46960	1.89224	0.0932711
46961	1.99179	0.110208
46962	1.454	0.100754
46963	1.99179	0.13768
46964	1.35444	0.0753757
46965	1.89224	0.106457
46966	1.89224	0.132697
46967	1.89224	0.169524
46968	1.89224	0.148288
46969	1.89224	0.249958
46970	1.89224	0.111242
46971	1.89224	0.170785
46972	1.703	0.110735
46973	2.19575	0.13241
46974	1.9911	0.114375
46975	2.19575	0.185659
46976	1.67811	0.077855
46977	1.89223	0.127538

46978	1.69586	2.1
46979	2.55301	2.1
46980	1.60861	2.1
46981	1.60861	2.1
46982	2.65233	2.1
46983	2.65233	2.1
46984	0.946129	2.1
46985	0.946129	2.1
46986	1.2765	2.1
46987	1.32617	2.1
46988	1.2765	2.1
46989	1.2765	2.1
46990	0.946131	2.1
46991	0.946131	2.1
46992	0.946131	2.1
46993	1.32616	2.1
46994	0.946131	2.1
46995	1.2765	2.1
46996	1.36757	2.1
46997	1.32617	2.1
46998	0.891503	2.1
46999	0.847932	2.1
47000	1.10722	2.1
47001	1.10722	2.1
47002	1.10829	2.1
47003	1.10829	2.1
47004	1.36757	2.1
47005	0.891504	2.1
47006	0.847932	2.1
47007	1.32617	2.1
47008	0.946129	2.1
47009	0.946129	2.1
47010	1.36757	2.1
47011	1.32617	2.1
47012	1.32616	2.1
47013	1.32616	2.1
47014	0.946131	2.1
47015	0.946131	2.1
47016	1.36757	2.1
47017	0.946131	2.1
47018	0.946131	2.1
47019	1.32616	2.1
47020	1.89226	0.0790286
47021	1.89226	0.0569805
47022	1.89226	0.0582498
47023	1.89226	0.0903274
47024	3.78452	0.0672241
47025	2.36336	0.05
47026	1.89226	0.0570163
47027	2.67831	0.0583781
47028	2.67831	0.0514674
47029	1.89226	0.0519485
47030	1.89226	0.05
47031	1.89227	0.0572239
47032	1.89227	0.09688
47033	1.89227	0.0911217
47034	1.89227	0.0625695
47035	3.78453	0.0746065

47036	1.89226	0.0532638
47037	1.89226	0.091162
47038	1.89226	0.0991507
47039	1.89226	0.0680858
47040	3.78452	0.0760366
47041	2.36336	0.05
47042	2.0815	0.0673346
47043	2.0815	0.0586134
47044	2.41048	0.05
47045	1.66267	0.0630231
47046	2.13452	0.05
47047	1.89226	0.0919031
47048	1.89226	0.0673915
47049	1.89226	0.0886372
47050	1.89226	0.0566226
47051	1.89226	0.102143
47052	1.89226	0.0734528
47053	1.89226	0.416493
47054	1.89226	0.493086
47055	1.89226	0.41649
47056	1.89226	0.487081
47057	1.89226	0.408998
47058	1.89226	0.47854
47059	0.946128	0.546185
47060	0.946128	0.528489
47061	1.00122	0.464122
47062	1.00122	0.466707
47063	0.94613	0.566876
47064	1.01618	0.502286
47065	0.946131	0.504625
47066	1.00122	0.448848
47067	1.00122	0.457411
47068	1.01618	0.490823
47069	0.946131	0.541303
47070	0.946131	0.498272
47071	1.89226	1.15595
47072	1.89226	0.793151
47073	1.89226	0.755613
47074	1.89226	1.61716
47075	3.78452	0.869321
47076	1.89226	0.920766
47077	1.89226	2.06486
47078	1.89226	2.08443
47079	1.89226	1.04934
47080	1.89226	1.84163
47081	1.89226	1.04676
47082	1.89226	0.67041
47083	1.89226	1.4806
47084	1.89226	1.12124
47085	1.89226	0.763794
47086	1.89226	1.14184
47087	1.89226	0.77842
47088	1.89226	0.777164
47089	1.89226	1.98257
47090	1.89226	1.75108
47091	1.89226	0.910533
47092	1.89226	1.66284
47093	1.89226	0.972687

47094	2.71279	0.888633
47095	1.4155	0.768345
47096	2.28488	0.701441
47097	1.06122	0.827127
47098	3.70038	0.704833
47099	1.89226	0.940275
47100	1.89226	0.867301
47101	1.89226	0.791594
47102	1.89226	0.776312
47103	3.78452	1.28304
47104	0.851542	0.730394
47105	0.851542	0.69522
47106	0.851484	0.734139
47107	0.865567	0.898849
47108	0.981616	0.729359
47109	0.864767	0.777609
47110	0.851542	0.794255
47111	0.851542	0.719522
47112	0.851477	0.756203
47113	0.851477	0.812302
47114	0.865635	0.744845
47115	0.816472	0.703128
47116	1.68443	0.708214
47117	1.44489	2.1
47118	1.54924	0.853267
47119	1.34389	2.1
47120	3.02832	2.1
47121	1.44489	2.1
47122	1.03899	2.1
47123	1.03899	2.1
47124	1.12075	2.1
47125	1.34389	2.1
47126	1.42566	2.1
47127	1.54924	0.83777
47128	1.34389	2.1
47129	1.44091	0.772156
47130	1.25866	2.1
47131	2.7848	2.1
47132	0.573141	2.1
47133	0.573141	2.1
47134	0.575681	2.1
47135	0.575681	2.1
47136	0.481985	2.1
47137	0.519492	2.1
47138	0.671948	2.1
47139	0.629331	2.1
47140	0.629329	2.1
47141	0.481986	2.1
47142	0.519494	2.1
47143	0.671947	2.1
47144	1.89226	0.652417
47145	1.89226	1.06817
47146	1.89226	0.80266
47147	2.31925	0.80529
47148	3.78452	0.990572
47149	1.30916	2.1
47150	1.17426	2.1
47151	0.946129	2.1

47152	0.946129	2.1
47153	0.94613	2.1
47154	0.94613	2.1
47155	1.17426	2.1
47156	1.17426	2.1
47157	1.30916	2.1
47158	0.94613	2.1
47159	1.17426	2.1
47160	0.94613	2.1
47161	2.56346	2.1
47162	1.89226	1.03819
47163	1.89226	2.03048
47164	1.89226	1.20716
47165	2.6074	1.99348
47166	2.6074	1.21566
47167	1.89226	2.1
47168	1.89226	2.1
47169	1.89226	2.1
47170	1.89226	1.98921
47171	3.78452	2.1
47172	1.33064	2.1
47173	1.84121	2.1
47174	0.946129	2.1
47175	0.946129	2.1
47176	0.946131	2.1
47177	0.946131	2.1
47178	1.30916	2.1
47179	1.8412	2.1
47180	1.33064	2.1
47181	0.94613	2.1
47182	1.30916	2.1
47183	0.946131	2.1
47184	1.19283	1.04853
47185	1.19283	0.93841
47186	0.946131	2.1
47187	0.946131	2.00925
47188	0.946132	2.05808
47189	0.946132	1.02368
47190	1.3037	2.1
47191	1.19283	0.964342
47192	1.19283	1.54955
47193	0.946131	1.53583
47194	0.946131	1.45
47195	1.3037	1.62681
47196	1.89226	2.1
47197	1.89226	2.00132
47198	1.89226	2.1
47199	1.89226	2.1
47200	1.89226	2.1
47201	1.89226	2.1
47202	2.17708	1.15347
47203	1.70302	2.1
47204	2.28188	1.17814
47205	2.13621	2.1
47206	4.28253	2.09968
47207	1.89226	0.934679
47208	1.89226	2.1
47209	1.89226	1.21285

47210	1.89226	2.1
47211	3.78452	2.1
47212	0.946132	2.1
47213	0.946132	2.1
47214	0.946132	2.1
47215	0.946132	2.1
47216	0.946131	2.1
47217	0.946131	2.1
47218	0.946132	2.1
47219	0.946132	2.1
47220	0.946132	2.1
47221	0.946132	2.1
47222	0.946132	2.1
47223	0.946132	2.1
47224	1.74933	2.1
47225	1.11833	2.1
47226	1.90074	2.1
47227	1.669	2.1
47228	1.335	2.1
47229	1.11833	2.1
47230	2.28188	1.03535
47231	2.31804	2.1
47232	2.42758	1.19153
47233	2.14895	2.1
47234	4.43083	2.1
47235	1.89226	1.04772
47236	1.89226	2.1
47237	1.89226	1.00503
47238	1.89226	2.1
47239	3.78452	1.49826
47240	1.89226	2.1
47241	1.89226	2.1
47242	1.89226	2.1
47243	1.89226	2.1
47244	1.89226	2.1
47245	1.89226	2.1
47246	0.864133	2.1
47247	0.864133	2.1
47248	0.967939	2.1
47249	0.793739	2.1
47250	0.650797	2.1
47251	0.757546	2.1
47252	1.08941	2.1
47253	1.10136	2.1
47254	1.10138	2.1
47255	0.796445	2.1
47256	0.866907	2.1
47257	0.650788	2.1
47258	2.63062	0.550849
47259	1.89226	0.507637
47260	1.89226	0.487282
47261	1.89226	0.659315
47262	2.46557	0.443963
47263	2.46557	0.690233
47264	1.16777	0.494559
47265	1.38859	2.1
47266	1.05042	0.85701
47267	1.28909	1.35658

47268	2.45686	1.21696
47269	1.89227	0.590842
47270	1.89227	0.641582
47271	1.89227	0.487054
47272	1.89227	1.80122
47273	3.78453	0.580774
47274	2.15114	2.1
47275	1.89226	1.86352
47276	2.15005	2.01178
47277	2.15005	2.1
47278	1.89226	0.93723
47279	1.89226	2.1
47280	1.89226	2.1
47281	1.89226	0.959001
47282	1.89226	2.1
47283	1.89226	2.1
47284	1.89226	0.81182
47285	1.89226	2.07425
47286	0.973447	1.74983
47287	1.32143	1.83615
47288	1.37444	1.89793
47289	1.37444	1.8198
47290	0.949134	1.98481
47291	0.949134	2.1
47292	0.946131	0.802341
47293	0.946131	2.1
47294	1.07557	1.9923
47295	1.07557	2.1
47296	0.946131	1.46426
47297	0.946131	1.89797
47298	1.07557	2.1
47299	1.08579	2.1
47300	1.08579	2.1
47301	0.946131	2.1
47302	1.07557	2.1
47303	0.946131	2.1
47304	0.946131	0.688682
47305	0.946131	2.1
47306	0.946131	1.41518
47307	0.946131	1.60373
47308	0.94613	0.954093
47309	0.94613	1.19742
47310	0.946131	1.72957
47311	0.946131	2.06011
47312	0.946131	1.23572
47313	0.946131	1.25977
47314	0.946131	1.29249
47315	0.946131	1.50293
47316	0.606115	1.70328
47317	0.606115	1.52765
47318	0.616308	2.1
47319	0.616308	2.1
47320	0.68722	0.976623
47321	0.735699	1.85557
47322	0.514789	1.47184
47323	0.486724	2.06475
47324	0.514788	1.1993
47325	0.735699	1.29185

47326	0.486723	1.84052
47327	0.687221	1.55719
47328	2.33761	0.687767
47329	2.51749	0.530212
47330	2.68092	0.581298
47331	2.58304	0.564243
47332	5.01853	0.554215
47333	2.50906	0.623505
47334	2.51749	0.553251
47335	2.68091	0.599492
47336	2.53176	0.48087
47337	5.18997	0.582536
47338	2.68092	0.582632
47339	2.58304	0.540969
47340	2.71793	0.582707
47341	2.67098	0.478034
47342	5.39885	0.559021
47343	2.68091	0.601061
47344	2.53175	0.475011
47345	2.36417	0.534731
47346	2.67098	0.491944
47347	5.04508	0.510022
47348	2.76309	2.1
47349	1.89226	1.40873
47350	1.89226	2.1
47351	1.89226	2.05293
47352	2.48573	2.1
47353	2.48573	1.71921
47354	1.35879	2.1
47355	1.55161	0.59608
47356	1.10407	1.12576
47357	1.32259	0.581379
47358	2.68138	0.811128
47359	1.89226	2.1
47360	1.89226	0.592192
47361	1.89226	1.47541
47362	1.89226	0.572734
47363	3.78452	0.739457
47364	1.19612	1.62308
47365	1.24694	2.1
47366	1.24844	0.60828
47367	1.29747	2.1
47368	2.49358	1.55044
47369	1.89226	1.75167
47370	1.89226	2.1
47371	1.89226	0.58966
47372	1.89226	2.1
47373	3.78453	1.23588
47374	1.89226	1.03802
47375	1.89226	2.1
47376	1.89226	2.1
47377	1.89226	2.1
47378	1.89226	1.02152
47379	1.89226	2.1
47380	0.587463	2.1
47381	0.587463	2.1
47382	0.581977	0.935732
47383	0.581977	2.1

47384	0.531709	2.1
47385	0.545972	2.1
47386	0.623469	1.53255
47387	0.648733	1.5275
47388	0.648733	2.1
47389	0.545971	2.1
47390	0.623469	2.1
47391	0.531709	2.1
47392	1.29417	0.697197
47393	1.29747	2.1
47394	1.27829	2.1
47395	1.24844	0.891751
47396	1.32732	1.36564
47397	1.29747	0.84428
47398	1.89226	0.728904
47399	1.89226	2.1
47400	1.89226	0.623584
47401	1.89226	0.899389
47402	1.89226	1.05109
47403	1.89226	2.1
47404	1.89226	2.1
47405	1.89226	2.1
47406	1.89226	0.858539
47407	1.89226	2.1
47408	1.89226	2.07557
47409	1.89226	0.806092
47410	0.60346	2.1
47411	0.60346	1.23244
47412	0.60086	0.725996
47413	0.60086	2.1
47414	0.545971	2.1
47415	0.555587	2.1
47416	0.648733	1.04448
47417	0.663661	0.999696
47418	0.663661	2.1
47419	0.555587	1.62559
47420	0.648733	2.1
47421	0.545971	2.1
47422	1.34066	1.29294
47423	1.36601	2.1
47424	1.34955	0.645095
47425	1.37623	2.1
47426	2.71556	1.48667
47427	1.89226	1.02194
47428	1.89226	2.1
47429	1.89226	0.638313
47430	1.89226	1.58337
47431	3.78452	0.9808
47432	0.946132	2.1
47433	0.946132	1.1058
47434	0.946132	0.693685
47435	0.946132	2.1
47436	0.946132	2.1
47437	0.946132	2.1
47438	0.946131	0.903425
47439	0.946131	0.886389
47440	0.946132	2.1
47441	0.946132	1.94402

47442	0.946132	2.0842
47443	0.946132	2.08506
47444	0.631351	0.756584
47445	0.631351	2.1
47446	0.632421	0.951775
47447	0.632421	2.1
47448	0.573604	2.1
47449	0.580765	2.1
47450	0.683006	0.758832
47451	0.688118	1.15224
47452	0.688118	2.1
47453	0.580765	2.1
47454	0.683007	2.1
47455	0.573604	2.1
47456	1.34955	1.54604
47457	1.37624	2.1
47458	1.35048	0.671505
47459	1.37999	2.1
47460	2.72954	1.25378
47461	0.946131	1.5774
47462	0.946131	1.01356
47463	0.946131	2.08503
47464	0.946131	2.1
47465	0.94613	2.1
47466	0.94613	1.19878
47467	0.946133	0.911577
47468	0.946133	1.26439
47469	0.946131	1.59406
47470	0.946131	0.947054
47471	0.946132	1.17542
47472	0.946132	1.076
47473	1.89226	1.13274
47474	1.89226	1.05801
47475	1.89226	1.29985
47476	1.89226	2.1
47477	1.89226	2.1
47478	1.89226	2.1
47479	0.532189	0.795115
47480	0.531141	2.1
47481	0.497784	2.1
47482	0.502536	2.1
47483	1.37999	1.23743
47484	0.92433	2.1
47485	0.670081	2.03449
47486	0.727155	2.1
47487	0.786438	0.859846
47488	0.589798	2.1
47489	0.497784	2.1
47490	1.89226	2.1
47491	2.72301	2.1
47492	2.4158	2.07094
47493	2.4158	2.1
47494	1.89226	2.06875
47495	1.89226	2.1
47496	1.89226	2.1
47497	1.89226	2.1
47498	1.89226	2.1
47499	1.89226	2.1

47500	1.89226	2.1
47501	1.89226	2.1
47502	1.36919	2.1
47503	1.12717	2.1
47504	1.38634	2.1
47505	1.56541	2.1
47506	2.75552	2.1
47507	1.89226	2.1
47508	1.89226	2.1
47509	1.89226	2.1
47510	1.89226	2.1
47511	1.89226	2.1
47512	1.89226	2.1
47513	0.907036	2.1
47514	0.782706	2.1
47515	0.838808	2.1
47516	0.693168	2.1
47517	0.794747	2.1
47518	0.794747	2.1
47519	0.805458	2.1
47520	0.805458	2.1
47521	0.907036	2.1
47522	0.838808	2.1
47523	0.693169	2.1
47524	0.782707	2.1
47525	0.94613	2.1
47526	0.94613	2.1
47527	1.188	2.1
47528	1.28421	2.1
47529	1.18801	2.1
47530	1.18801	2.1
47531	0.946129	2.1
47532	0.946129	2.1
47533	1.28421	2.1
47534	0.94613	2.1
47535	1.188	2.1
47536	0.94613	2.1
47537	1.89226	2.1
47538	1.89226	2.1
47539	1.89226	2.1
47540	1.89226	2.1
47541	1.89226	2.1
47542	1.89226	2.1
47543	1.16817	2.1
47544	1.06007	2.1
47545	1.19785	2.1
47546	1.2832	2.1
47547	2.36602	2.1
47548	0.960106	2.1
47549	2.0073	2.1
47550	1.51754	2.1
47551	2.56883	2.1
47552	3.52484	2.1
47553	1.06756	2.1
47554	1.57913	2.02861
47555	1.87532	1.21792
47556	1.38666	2.1
47557	2.94287	2.1

47558	2.15622	0.957377
47559	1.38668	2.1
47560	1.87534	2.1
47561	1.87534	2.1
47562	1.70539	1.9748
47563	1.70539	2.1
47564	0.986095	2.1
47565	1.36226	2.1
47566	1.3331	2.1
47567	1.3331	2.1
47568	1.07156	2.1
47569	1.07156	2.1
47570	0.682081	1.17823
47571	0.68748	0.985259
47572	0.650003	0.651222
47573	0.645873	0.811493
47574	0.666344	0.660163
47575	0.666344	0.960356
47576	0.66161	1.68831
47577	0.66161	0.700747
47578	0.68748	0.822812
47579	0.645873	0.83998
47580	0.650004	0.83592
47581	0.682082	0.825005
47582	0.946132	0.985306
47583	0.946132	0.781334
47584	0.946132	0.685406
47585	0.946132	0.576756
47586	0.946132	0.580106
47587	0.946132	0.780351
47588	0.946133	0.712466
47589	0.946133	1.00601
47590	0.946132	0.730039
47591	0.946132	0.723924
47592	0.946133	0.74365
47593	0.946133	0.73875
47594	1.89227	0.919078
47595	2.79312	0.534035
47596	2.77591	0.689628
47597	2.79312	0.701365
47598	1.87505	0.6053
47599	1.89227	0.633329
47600	0.68748	1.22299
47601	0.698626	0.999299
47602	0.645873	0.678216
47603	0.650148	0.810027
47604	0.670561	0.686836
47605	0.670561	1.01431
47606	0.667067	1.69895
47607	0.667067	0.682495
47608	0.698627	0.847987
47609	0.650148	0.894911
47610	0.68748	0.858403
47611	0.645873	0.885773
47612	0.946132	1.03782
47613	0.946132	0.785885
47614	0.946132	0.719978
47615	0.946132	0.615332

47616	0.946132	0.615887
47617	0.946132	0.792924
47618	0.946133	0.739829
47619	0.946133	1.08686
47620	0.946133	0.752167
47621	0.946133	0.751418
47622	0.946132	0.780718
47623	0.946132	0.769552
47624	0.946133	0.781476
47625	0.946133	0.653488
47626	1.38709	0.721643
47627	1.38795	0.539267
47628	0.946132	0.546215
47629	0.946132	0.656965
47630	1.38709	0.845603
47631	1.38709	0.59774
47632	1.38709	0.672959
47633	0.946133	0.673424
47634	0.946132	0.656479
47635	1.38795	0.631527
47636	1.70301	0.0924832
47637	2.07659	0.0682218
47638	1.89226	0.0797504
47639	1.89226	0.0963047
47640	1.76332	0.065774
47641	1.76331	0.0776032
47642	1.89226	0.141951
47643	1.89226	0.0912383
47644	1.89226	0.124568
47645	1.89226	0.149977
47646	1.89226	0.0907604
47647	1.89226	0.10801
47648	1.89226	0.124287
47649	1.89226	0.138465
47650	1.89226	0.077719
47651	1.89226	0.091725
47652	3.78452	0.119682
47653	1.89226	0.0746066
47654	2.12492	0.0653522
47655	1.89226	0.0825194
47656	1.89226	0.0857287
47657	2.07659	0.0636654
47658	2.07659	0.0643567
47659	0.94613	0.0503393
47660	0.94613	0.0694147
47661	1.17498	0.0513249
47662	1.32446	0.05
47663	1.32446	0.05
47664	0.94613	0.0524907
47665	0.946127	0.0542111
47666	1.17498	0.0517423
47667	1.32446	0.05
47668	1.32446	0.05
47669	0.946128	0.0656918
47670	0.946128	0.0566762
47671	1.89226	2.1
47672	1.86472	2.1
47673	2.11049	2.1

47674	1.89226	2.1
47675	2.08295	2.1
47676	1.86472	2.1
47677	1.89226	2.1
47678	2.08295	2.1
47679	2.14023	2.1
47680	1.89226	2.1
47681	2.33091	2.1
47682	2.08295	2.1
47683	0.809215	2.1
47684	0.809215	1.76351
47685	0.810915	2.1
47686	0.810915	2.1
47687	1.09581	2.1
47688	1.08271	2.1
47689	0.520996	2.1
47690	0.537426	2.1
47691	0.520997	2.1
47692	1.08271	2.1
47693	0.537426	2.1
47694	1.09581	2.1
47695	0.79674	2.1
47696	0.79674	2.1
47697	0.799279	2.1
47698	0.799279	2.1
47699	1.07502	2.1
47700	1.0827	2.1
47701	0.508206	2.1
47702	0.520996	2.1
47703	0.508206	2.1
47704	1.07502	2.1
47705	0.520996	2.1
47706	1.0827	2.1
47707	2.15714	2.1
47708	1.70302	2.1
47709	1.78732	2.1
47710	2.02219	2.1
47711	3.98971	2.1
47712	2.0927	2.1
47713	2.162	2.1
47714	2.11563	2.1
47715	2.02219	2.1
47716	4.18419	2.1
47717	1.62483	2.1
47718	2.02219	2.1
47719	1.93617	2.1
47720	1.52008	2.1
47721	3.54227	2.1
47722	2.11563	2.1
47723	2.02219	2.1
47724	2.29218	2.1
47725	2.03975	2.1
47726	4.06194	2.1
47727	2.007	2.1
47728	1.89226	2.1
47729	1.89226	2.1
47730	1.89226	2.1
47731	2.06776	2.1

47732	2.06776	2.1
47733	0.946129	2.1
47734	0.946129	2.1
47735	1.0035	2.1
47736	1.0035	2.1
47737	0.946131	2.1
47738	0.946131	2.1
47739	0.968797	2.1
47740	1.0035	2.1
47741	0.968771	2.1
47742	0.946105	2.1
47743	1.00353	2.1
47744	0.946157	2.1
47745	1.79103	2.04396
47746	2.53286	0.710451
47747	2.60781	2.07702
47748	2.219	0.847018
47749	2.17985	1.52429
47750	1.79103	0.678785
47751	2.219	1.53423
47752	2.66359	1.56476
47753	2.70084	2.1
47754	2.38698	1.19041
47755	2.53285	0.962601
47756	2.219	0.834721
47757	1.33179	1.36885
47758	0.946128	0.88656
47759	0.946133	1.09529
47760	1.26643	1.903
47761	1.3318	1.01929
47762	1.3318	1.79533
47763	0.946131	0.851514
47764	0.946131	1.24833
47765	0.946131	0.830689
47766	0.946131	0.729052
47767	1.33179	1.03653
47768	1.26643	1.97694
47769	2.28979	2.1
47770	2.47227	2.1
47771	2.43451	2.1
47772	2.22627	2.1
47773	4.69855	2.1
47774	2.40667	2.1
47775	2.47228	2.1
47776	2.43451	2.1
47777	2.38069	2.1
47778	4.85296	2.1
47779	2.43451	2.1
47780	2.15715	2.1
47781	2.15715	2.1
47782	2.22627	2.1
47783	2.39489	2.1
47784	2.46402	2.1
47785	2.43451	2.1
47786	2.34899	2.1
47787	2.38069	2.1
47788	2.38069	2.1
47789	2.3949	2.1

47790	2.3949	2.1
47791	2.85425	0.839166
47792	1.70302	0.492805
47793	1.70302	0.533255
47794	1.52254	0.530172
47795	2.34181	0.540383
47796	2.08499	0.595558
47797	1.89226	0.54106
47798	1.89226	0.53119
47799	1.89226	0.456865
47800	1.89226	0.467633
47801	1.89226	0.429322
47802	1.89226	0.588497
47803	2.34181	0.645725
47804	1.38287	0.613943
47805	1.38287	0.599899
47806	1.84132	0.542241
47807	1.96416	0.531615
47808	2.4907	0.577521
47809	0.931432	0.718357
47810	0.946135	0.455724
47811	0.946133	0.421555
47812	0.946133	0.43793
47813	0.931464	0.503805
47814	0.931464	0.72695
47815	1.15573	0.433136
47816	0.96378	0.890358
47817	0.931434	0.550245
47818	0.946131	0.454953
47819	0.946131	0.448468
47820	1.13808	0.433982
47821	0.946132	0.637676
47822	0.946132	0.505415
47823	0.946132	0.58055
47824	0.946132	0.433137
47825	0.946133	0.596233
47826	0.946133	0.465786
47827	0.946132	0.662784
47828	0.946132	0.552361
47829	0.946133	0.511759
47830	0.946133	0.650518
47831	0.946132	0.452926
47832	0.946132	0.396094
47833	1.68074	2.1
47834	2.29459	2.1
47835	2.23361	2.1
47836	1.57234	2.1
47837	3.86693	2.1
47838	2.61243	2.1
47839	2.29458	2.1
47840	2.2336	2.1
47841	2.58234	2.1
47842	4.87692	2.1
47843	0.73506	2.1
47844	0.786166	2.1
47845	1.08729	2.1
47846	1.11679	2.1
47847	0.93134	2.1

47848	0.93134	2.1
47849	0.942113	2.1
47850	0.942113	2.1
47851	0.735065	2.1
47852	1.08729	2.1
47853	1.11679	2.1
47854	0.78616	2.1
47855	1.08729	2.1
47856	1.11679	2.1
47857	1.27502	2.1
47858	1.29116	2.1
47859	1.19259	2.1
47860	1.19259	2.1
47861	1.19923	2.1
47862	1.19923	2.1
47863	1.27503	2.1
47864	1.08729	2.1
47865	1.11679	2.1
47866	1.29115	2.1
47867	0.974715	0.822125
47868	1.43779	0.635674
47869	1.93915	0.656309
47870	1.53908	0.682897
47871	2.91387	0.661991
47872	1.53908	0.642327
47873	0.99845	0.661557
47874	1.5176	0.797023
47875	1.97963	0.606247
47876	3.05668	0.673173
47877	1.93915	0.639477
47878	1.97963	0.623739
47879	1.53908	0.623924
47880	1.53908	0.559324
47881	2.3376	0.766629
47882	2.3376	0.639502
47883	1.89226	2.1
47884	2.59747	2.1
47885	1.91802	2.1
47886	1.89226	2.1
47887	2.62322	2.1
47888	2.59747	2.1
47889	2.59747	2.1
47890	1.87119	2.1
47891	1.84844	2.1
47892	1.82268	2.1
47893	2.62323	2.1
47894	2.59747	2.1
47895	0.94613	2.1
47896	0.94613	2.1
47897	1.32697	2.1
47898	1.32697	2.1
47899	1.32697	2.1
47900	1.31161	2.1
47901	0.946132	2.1
47902	0.946132	1.83628
47903	0.94613	2.1
47904	1.32697	2.1
47905	0.946131	2.1

47906	1.31161	2.1
47907	2.62323	2.1
47908	1.91704	2.1
47909	1.90191	2.1
47910	1.87119	2.1
47911	2.65395	2.1
47912	2.62323	2.1
47913	1.89226	2.1
47914	1.89226	2.1
47915	2.15958	2.1
47916	2.15958	2.1
47917	2.46558	2.1
47918	2.19826	2.1
47919	2.46558	2.1
47920	1.89226	2.1
47921	1.89226	2.1
47922	1.89226	2.1
47923	2.61064	2.1
47924	2.61064	2.1
47925	1.81759	0.66857
47926	1.89526	0.719963
47927	2.32219	0.930135
47928	1.881	0.603636
47929	1.81759	0.782861
47930	1.3764	0.570635
47931	1.81759	0.683554
47932	2.57079	0.658124
47933	2.32219	1.09171
47934	2.32219	0.632977
47935	1.99875	0.766422
47936	1.99875	0.54102
47937	0.536182	0.500406
47938	0.579206	0.435078
47939	0.908796	0.669545
47940	0.999375	0.557812
47941	0.732091	0.590439
47942	0.732091	0.443221
47943	0.755911	0.492406
47944	0.755911	0.604129
47945	0.999376	0.53599
47946	0.579206	0.574548
47947	0.908796	0.562885
47948	0.536182	0.550537
47949	0.94613	0.454282
47950	0.94613	0.455406
47951	1.31112	0.529405
47952	1.18275	0.356527
47953	0.946126	0.409581
47954	0.946126	0.211365
47955	1.31112	0.521743
47956	1.31112	0.426434
47957	0.946128	0.449505
47958	1.31112	0.482249
47959	0.946128	0.431546
47960	1.18275	0.236232
47961	2.64617	0.683232
47962	1.27737	0.465931
47963	1.83076	0.668392

47964	1.27737	0.621208
47965	3.03764	0.674535
47966	2.3655	0.456669
47967	1.12991	0.05
47968	1.22375	0.05
47969	1.35931	0.05
47970	1.04971	0.05
47971	1.0419	0.05
47972	1.15237	0.05
47973	1.19593	0.05
47974	1.30306	0.05
47975	1.12937	0.05
47976	1.31934	0.05
47977	1.15203	0.05
47978	1.15203	0.05
47979	2.65227	0.05
47980	1.46041	0.05
47981	2.22712	0.05
47982	2.04816	0.05
47983	2.22712	0.05
47984	2.04816	0.05
47985	1.89226	0.177839
47986	1.89226	0.581823
47987	1.89226	0.437325
47988	1.89226	0.462384
47989	3.78452	0.493964
47990	1.24653	0.452733
47991	1.66554	0.552984
47992	1.53538	0.305636
47993	1.53538	0.652626
47994	1.40915	0.301649
47995	1.40915	0.667064
47996	1.66555	0.560013
47997	1.75835	2.1
47998	1.53538	0.739811
47999	1.65313	0.859987
48000	3.31868	0.890087
48001	1.89226	0.571578
48002	1.89226	0.599451
48003	1.89226	0.467912
48004	1.89226	1.17664
48005	3.78453	0.554309
48006	1.89226	0.314003
48007	1.89226	0.475401
48008	1.89226	0.355815
48009	1.89226	0.647046
48010	3.78452	0.25559
48011	0.658778	1.12069
48012	0.658778	0.312248
48013	0.661956	0.467028
48014	0.661956	0.621055
48015	0.703822	0.607449
48016	0.767689	0.883865
48017	0.623266	0.311938
48018	0.553046	0.601864
48019	0.703804	0.605401
48020	0.55303	0.609267
48021	0.623282	0.482988

48022	0.767711	0.534803
48023	1.53538	0.725554
48024	1.65313	1.03559
48025	1.40765	0.601674
48026	1.55779	2.1
48027	3.06078	0.721089
48028	1.89227	0.448286
48029	1.89227	1.24241
48030	1.89227	0.626285
48031	1.89227	0.617807
48032	3.78453	0.664157
48033	1.10517	2.1
48034	2.16532	2.1
48035	1.94374	2.1
48036	1.94001	2.1
48037	1.37756	2.1
48038	1.37756	2.1
48039	1.7453	0.0943002
48040	1.83284	0.137061
48041	2.28483	0.182707
48042	1.77804	0.102101
48043	1.7453	0.248564
48044	1.23851	0.113583
48045	1.7453	0.136846
48046	2.54752	0.113834
48047	2.28483	0.131891
48048	2.28483	0.109677
48049	2.0189	0.151328
48050	2.0189	0.128931
48051	1.12457	0.101409
48052	2.0189	0.0801692
48053	1.7453	0.0956952
48054	1.7453	0.0836657
48055	1.40707	0.115236
48056	1.40707	0.103315
48057	2.41176	2.1
48058	2.59262	2.1
48059	2.34085	2.1
48060	2.55827	2.1
48061	4.97003	2.1
48062	2.59262	2.1
48063	2.61901	2.1
48064	2.55827	2.1
48065	2.64067	2.1
48066	5.17728	2.1
48067	2.34084	2.1
48068	2.55827	2.1
48069	2.24937	2.1
48070	2.49053	2.1
48071	4.80764	2.1
48072	2.55826	2.1
48073	2.64067	2.1
48074	2.49053	2.1
48075	2.58639	2.1
48076	5.14465	2.1
48077	2.27918	0.0652641
48078	2.44132	0.088284
48079	1.89226	0.0549441

48080	1.89226	0.0652514
48081	4.33359	0.0582324
48082	1.89226	0.0629775
48083	2.44132	0.0920347
48084	2.6771	0.0837766
48085	2.6771	0.0736144
48086	1.89226	0.0895126
48087	1.89226	0.08588
48088	1.60329	0.0564862
48089	1.38246	0.070366
48090	1.98182	0.0525487
48091	2.2217	0.0663265
48092	3.60417	0.0669544
48093	2.05362	0.05
48094	1.02663	0.0568838
48095	1.43255	0.0508206
48096	1.52072	0.05
48097	1.70302	0.0537429
48098	1.7685	0.0545065
48099	0.946131	0.0636276
48100	0.946131	0.0546339
48101	0.946131	0.050547
48102	0.946131	0.0753729
48103	0.946131	0.0799144
48104	0.946131	0.0758024
48105	0.946131	0.0570741
48106	0.946131	0.0570886
48107	0.946131	0.0642563
48108	0.946131	0.0676183
48109	0.946131	0.0666424
48110	0.946131	0.0686943
48111	1.89226	0.0667282
48112	1.89226	0.101708
48113	1.89226	0.0859881
48114	1.89226	0.073179
48115	1.89226	0.0961988
48116	1.89226	0.0913555
48117	1.92645	0.672756
48118	2.83045	0.648865
48119	1.92225	0.737957
48120	2.01873	0.548782
48121	2.51732	0.766575
48122	2.51732	0.60858
48123	2.51732	0.494769
48124	2.23857	0.478406
48125	1.92645	0.590352
48126	1.92645	0.41036
48127	2.23857	0.698098
48128	1.50865	0.36842
48129	2.02687	2.1
48130	2.39604	2.1
48131	2.29198	2.1
48132	2.73046	2.1
48133	4.68803	2.1
48134	1.21148	0.550195
48135	1.87957	0.589182
48136	2.03555	0.547733
48137	2.03555	0.48972

48138	1.09962	0.516419
48139	1.09962	0.585194
48140	1.92558	0.430189
48141	2.05274	0.730695
48142	2.2443	0.473113
48143	2.24429	0.680064
48144	1.70869	0.264375
48145	1.70868	0.799194
48146	0.818397	0.435769
48147	0.818397	0.760947
48148	0.827911	0.688316
48149	0.827908	0.655291
48150	1.00418	0.555207
48151	1.02054	0.537924
48152	0.692018	0.635528
48153	0.620899	0.611371
48154	0.65823	0.497499
48155	1.00418	0.380054
48156	0.620899	0.523736
48157	1.0262	0.478948
48158	1.92558	0.625812
48159	2.51818	0.819998
48160	1.94816	0.492493
48161	2.01985	0.744556
48162	1.85389	0.439746
48163	1.92558	0.869351
48164	0.903844	2.1
48165	0.903844	2.1
48166	0.946148	2.1
48167	0.946151	2.1
48168	0.946118	2.1
48169	0.903876	2.1
48170	0.946118	2.1
48171	1.10235	2.1
48172	0.946131	2.1
48173	0.946131	2.1
48174	1.10234	2.1
48175	0.903863	2.1
48176	1.63471	2.1
48177	1.08022	2.1
48178	1.56801	2.1
48179	1.03437	2.1
48180	2.66908	2.1
48181	1.56801	2.1
48182	0.97541	2.1
48183	0.97541	2.1
48184	1.03437	2.1
48185	1.49733	2.1
48186	1.55629	2.1
48187	1.89226	0.135008
48188	1.89226	0.295132
48189	1.89226	0.202532
48190	1.89226	0.263642
48191	3.78452	0.284545
48192	0.946131	0.182477
48193	1.16373	0.174501
48194	0.946131	0.221455
48195	0.734637	0.163966

48196	0.946132	0.191803
48197	0.946132	0.203082
48198	1.16373	0.141759
48199	0.734636	0.136714
48200	0.946093	0.187799
48201	0.946093	0.174226
48202	0.734675	0.160419
48203	0.734675	0.180614
48204	1.98413	0.405195
48205	1.87355	0.532405
48206	1.72774	0.378062
48207	1.67865	0.571218
48208	3.60128	0.452489
48209	1.87355	0.50445
48210	1.54924	0.790206
48211	1.68443	0.714461
48212	1.68443	0.541146
48213	1.67866	0.724677
48214	1.67866	0.545094
48215	1.72774	0.378617
48216	1.53422	0.567774
48217	1.67865	0.519239
48218	1.67865	0.406893
48219	1.53873	0.51868
48220	1.53873	0.407458
48221	1.67866	0.515871
48222	1.54924	0.844067
48223	1.53423	0.425059
48224	1.44091	0.771317
48225	3.11957	0.641098
48226	1.89226	2.1
48227	1.89226	1.34589
48228	1.65573	1.65506
48229	1.65573	1.95494
48230	1.89226	1.86965
48231	1.89226	2.1
48232	1.89226	2.0977
48233	1.89614	1.1752
48234	1.7508	1.36377
48235	1.72666	1.50712
48236	1.89226	1.511
48237	1.89226	1.92077
48238	1.89223	2.01681
48239	1.89223	1.23155
48240	1.89223	1.23308
48241	1.89223	1.61623
48242	1.89223	1.37753
48243	1.89223	2.03687
48244	1.89224	1.91473
48245	1.89224	0.963379
48246	1.89224	1.23561
48247	1.89224	1.26841
48248	1.89224	1.42883
48249	1.89224	1.75589
48250	2.09773	2.1
48251	2.06623	2.1
48252	2.43268	2.1
48253	2.33713	2.1

48254	1.703	2.1
48255	1.61372	2.1
48256	0.646929	0.999356
48257	0.689776	2.1
48258	1.05897	2.1
48259	0.956364	2.1
48260	0.905594	2.1
48261	0.904315	1.0339
48262	0.727014	2.1
48263	0.850757	2.1
48264	0.798445	2.1
48265	0.654101	2.1
48266	0.717539	2.1
48267	1.0478	2.1
48268	1.89223	0.985266
48269	1.89223	0.785607
48270	1.89223	2.1
48271	1.6557	0.378412
48272	3.54794	0.82612
48273	0.946118	0.894574
48274	0.946118	2.1
48275	0.946118	2.1
48276	0.946118	2.03212
48277	0.946117	2.0178
48278	0.946117	0.765053
48279	0.946119	2.1
48280	0.946119	2.1
48281	0.946105	2.05311
48282	0.946105	2.1
48283	0.946131	2.06912
48284	0.946131	2.07918
48285	1.47671	2.1
48286	1.70303	2.1
48287	1.90705	2.1
48288	1.98945	2.1
48289	1.3454	2.1
48290	1.40353	2.1
48291	1.89226	2.1
48292	1.89226	1.36362
48293	1.89226	2.1
48294	1.89226	0.823583
48295	1.89226	2.1
48296	1.89226	0.738269
48297	2.39438	0.15347
48298	2.69842	0.131719
48299	2.45177	0.228416
48300	2.28563	0.116455
48301	4.84615	0.153991
48302	1.28421	2.1
48303	1.35966	2.1
48304	0.961001	2.1
48305	0.860549	2.1
48306	1.1039	2.1
48307	1.1039	2.1
48308	1.11631	2.1
48309	1.11631	2.1
48310	1.35966	2.1
48311	0.961001	2.1

48312	0.860549	2.1
48313	1.28421	2.1
48314	1.89226	1.31861
48315	1.89226	1.90119
48316	1.89226	2.08827
48317	1.89226	1.09968
48318	3.78452	1.34564
48319	1.48702	0.957018
48320	1.40656	1.05317
48321	0.626442	1.58311
48322	0.626442	1.39425
48323	0.626442	1.73032
48324	0.946132	1.03622
48325	1.05489	0.748727
48326	1.05012	1.88075
48327	0.946131	1.34716
48328	0.946131	1.38023
48329	0.626442	1.40039
48330	1.05012	1.28698
48331	1.89226	1.89374
48332	1.97212	1.11775
48333	1.89226	1.09715
48334	1.89226	1.45274
48335	3.78453	1.53722
48336	1.89226	2.05534
48337	1.89226	1.10317
48338	1.89226	1.2093
48339	1.89226	1.71421
48340	3.78452	1.64952
48341	1.89226	1.05662
48342	1.89226	1.41889
48343	1.89226	1.69817
48344	1.89226	0.867194
48345	1.89226	1.7146
48346	1.89226	0.887122
48347	0.946161	0.796603
48348	0.946161	1.06912
48349	0.932156	1.4378
48350	0.932156	1.13936
48351	0.932184	1.35604
48352	0.946132	0.632923
48353	0.946133	1.41769
48354	0.946133	0.863357
48355	0.946132	0.98029
48356	0.946132	1.03815
48357	0.932185	1.00133
48358	0.946133	0.89141
48359	0.946132	1.05296
48360	0.946132	0.876188
48361	0.946132	2.08451
48362	0.946132	1.44999
48363	0.946132	1.37904
48364	0.946132	1.01975
48365	0.946133	1.10082
48366	0.946133	1.6267
48367	0.946132	1.29753
48368	0.946132	1.49086
48369	0.946133	1.04794

48370	0.946133	1.18233
48371	1.79397	0.444287
48372	1.00636	0.248619
48373	1.31658	0.504503
48374	2.02924	0.427796
48375	3.0356	0.438319
48376	1.89226	0.426104
48377	1.89226	0.217124
48378	1.89226	0.411341
48379	1.89226	0.325322
48380	1.89226	0.319215
48381	1.89226	0.227136
48382	0.946131	0.22022
48383	0.946131	0.354733
48384	1.24766	0.47832
48385	1.03691	0.272994
48386	0.946129	0.257438
48387	0.946129	0.172309
48388	1.24766	0.468174
48389	1.24766	0.343958
48390	0.946131	0.345028
48391	1.24766	0.437066
48392	1.03691	0.283872
48393	0.946131	0.22875
48394	1.44824	0.357148
48395	2.02924	0.275308
48396	1.7065	0.415402
48397	1.38604	0.455174
48398	2.32017	0.379754
48399	2.32017	0.374676
48400	1.89226	0.273927
48401	1.89226	0.365328
48402	1.89226	0.196895
48403	1.89226	0.151631
48404	1.89226	0.394451
48405	1.89226	0.361534
48406	0.478349	0.682265
48407	0.612092	1.79401
48408	0.606115	2.1
48409	0.506213	2.1
48410	0.550677	2.1
48411	0.550677	0.673406
48412	0.567628	2.1
48413	0.567628	2.1
48414	0.506213	1.77028
48415	0.478349	1.91826
48416	0.606116	1.63572
48417	0.612092	2.04046
48418	1.52226	2.1
48419	1.54241	2.1
48420	1.23167	2.1
48421	1.23167	2.1
48422	1.75555	2.1
48423	1.75555	2.1
48424	1.75557	2.1
48425	1.54243	2.1
48426	1.9943	2.1
48427	1.85871	2.1

48428	3.53673	2.1
48429	1.85871	2.1
48430	1.06936	2.1
48431	1.88926	2.1
48432	1.54243	2.1
48433	1.41619	2.1
48434	1.06936	2.1
48435	1.28649	1.51836
48436	1.28516	2.1
48437	1.38986	0.735707
48438	1.42399	1.58586
48439	2.67502	1.28936
48440	1.42399	0.861763
48441	1.07283	2.1
48442	1.46528	2.1
48443	1.28517	0.95491
48444	1.25294	2.03038
48445	1.07283	0.87469
48446	1.38986	1.96917
48447	1.42399	2.09838
48448	1.52885	0.941891
48449	1.60058	1.19194
48450	2.99043	1.25891
48451	0.715316	2.1
48452	0.715316	1.33864
48453	0.719126	0.663455
48454	0.719126	2.1
48455	0.722447	1.93229
48456	0.626469	2.07081
48457	0.711995	0.865245
48458	0.800291	0.950348
48459	0.80029	1.34993
48460	0.722447	1.114
48461	0.711995	1.42693
48462	0.62647	1.45673
48463	2.05632	0.05
48464	1.70303	0.05
48465	2.3923	0.05
48466	2.61277	0.05
48467	1.44836	0.05
48468	1.58184	0.05
48469	1.89226	0.05
48470	1.89226	0.0646821
48471	1.89226	0.0539054
48472	1.89226	0.0548242
48473	3.78453	0.0555265
48474	1.44091	0.69834
48475	1.1822	2.1
48476	1.25866	2.1
48477	1.25866	1.61634
48478	1.35215	2.1
48479	1.35215	1.60032
48480	1.27338	0.75072
48481	1.1822	2.1
48482	1.27338	2.1
48483	1.35215	1.66578
48484	1.10817	2.1
48485	1.18694	2.1

48486	0.946132	0.535463
48487	0.946132	0.462467
48488	0.946132	0.410344
48489	0.946132	0.369573
48490	0.946133	0.404969
48491	0.946133	0.535536
48492	0.946132	0.455187
48493	0.946132	0.301927
48494	0.946132	0.447903
48495	0.946132	0.465759
48496	0.946133	0.445407
48497	0.946133	0.431073
48498	2.6875	0.208992
48499	2.40558	0.161628
48500	2.1994	0.168993
48501	1.70303	0.202378
48502	4.68305	0.183767
48503	0.915523	0.148921
48504	0.915523	0.185021
48505	0.861057	0.12536
48506	0.946796	0.145696
48507	0.838652	0.164659
48508	0.654182	0.12744
48509	1.14044	0.119476
48510	0.80252	0.160216
48511	0.836435	0.137939
48512	0.608659	0.144343
48513	1.22198	0.146229
48514	0.863472	0.184611
48515	2.1994	0.0708985
48516	0.981586	0.0736588
48517	1.59619	0.0795086
48518	1.70303	0.0797741
48519	1.24816	0.0687147
48520	1.38523	0.0639196
48521	2.71141	2.1
48522	1.70302	2.1
48523	2.56527	2.1
48524	1.88649	2.1
48525	2.41279	2.1
48526	1.70302	2.1
48527	1.89226	1.43456
48528	1.89226	2.1
48529	1.89226	2.1
48530	1.89226	1.13626
48531	3.78453	2.1
48532	1.89226	1.41593
48533	1.89226	2.1
48534	1.89226	2.1
48535	1.89226	2.1
48536	1.89226	2.1
48537	1.89226	1.26408
48538	2.18867	2.1
48539	0.983723	2.1
48540	2.42263	2.1
48541	1.8014	2.1
48542	1.62992	2.1
48543	0.983723	2.1

48544	2.71141	2.1
48545	1.89226	2.1
48546	2.31157	2.1
48547	2.31157	2.1
48548	1.89226	2.1
48549	1.89226	2.1
48550	1.89227	1.98095
48551	1.89227	0.955404
48552	1.89227	0.948358
48553	1.89227	1.7935
48554	3.78453	1.13636
48555	1.89226	1.04278
48556	1.89226	2.1
48557	1.89226	2.08477
48558	1.89226	1.05815
48559	1.89226	1.95173
48560	1.89226	1.05916
48561	1.89226	1.30451
48562	1.89226	1.1509
48563	1.89226	2.1
48564	1.89226	0.842434
48565	1.89226	1.21727
48566	1.89226	0.666887
48567	1.1427	1.84991
48568	1.14956	2.1
48569	1.70302	1.13908
48570	1.81267	1.98231
48571	2.88983	1.91117
48572	1.89226	0.831624
48573	1.89226	1.99553
48574	1.89226	0.861506
48575	1.89226	1.15009
48576	3.78453	1.21179
48577	1.89226	1.26765
48578	1.89226	1.64662
48579	1.89226	0.719613
48580	1.89226	1.22697
48581	3.78452	0.974969
48582	0.884197	2.09138
48583	1.1125	1.25137
48584	1.13273	1.30125
48585	0.844557	1.73833
48586	0.981137	1.06585
48587	1.13971	1.88683
48588	0.980432	1.81988
48589	0.980432	1.10214
48590	1.15859	1.16698
48591	1.12206	0.882652
48592	0.898619	2.04102
48593	0.94426	1.37908
48594	2.21016	1.08777
48595	1.44091	2.1
48596	1.98408	1.14379
48597	1.4218	1.0132
48598	2.21016	2.03236
48599	1.64788	2.06851
48600	1.07526	0.423729
48601	1.91531	2.1

48602	1.49155	1.80174
48603	1.58551	0.764346
48604	1.49155	0.675195
48605	1.58551	1.44421
48606	1.98979	0.05
48607	2.21773	0.05
48608	2.30533	0.05
48609	2.55895	0.05
48610	4.52305	0.05
48611	2.55896	0.05
48612	2.27963	0.05
48613	2.60486	0.05
48614	2.60486	0.05
48615	2.21773	0.05
48616	2.21773	0.05
48617	2.30533	0.05
48618	2.55895	0.05
48619	2.29539	0.05
48620	2.47308	0.05
48621	4.7784	0.05
48622	2.55896	0.05
48623	2.55792	0.05
48624	2.55792	0.05
48625	2.60486	0.05
48626	2.47308	0.05
48627	2.52003	0.05
48628	0.946133	1.55763
48629	0.946133	1.8083
48630	1.20851	1.19073
48631	1.20851	1.13652
48632	0.967411	1.13898
48633	1.20851	0.953574
48634	0.946132	1.97401
48635	0.946132	1.45507
48636	0.946132	1.53875
48637	1.20851	1.1352
48638	0.967412	1.18338
48639	0.946133	1.46992
48640	1.89226	1.66141
48641	1.89226	1.05674
48642	1.89226	1.04892
48643	1.89226	1.07997
48644	1.89226	1.64346
48645	1.89226	1.82272
48646	1.89226	1.40741
48647	2.41701	1.44269
48648	2.71724	1.03601
48649	2.71724	1.34903
48650	1.89226	1.75062
48651	1.89226	2.0553
48652	0.946131	1.78195
48653	0.946131	2.1
48654	0.946128	1.37819
48655	0.946128	1.64751
48656	0.94613	0.94795
48657	0.94613	1.09884
48658	0.94613	1.78974
48659	0.94613	1.97922

48660	0.94613	1.48161
48661	0.94613	1.03157
48662	0.94613	1.24913
48663	0.94613	2.02096
48664	0.851541	0.322753
48665	0.851541	0.383708
48666	0.851481	0.361313
48667	0.997219	0.38189
48668	0.997112	0.282418
48669	0.992853	0.254993
48670	0.85154	0.318902
48671	0.85154	0.366447
48672	0.894824	0.250834
48673	0.894824	0.359667
48674	0.94613	0.386235
48675	0.906485	0.265648
48676	0.802947	2.1
48677	0.845556	2.1
48678	0.582339	2.1
48679	0.519756	2.1
48680	0.714508	2.1
48681	0.714508	2.1
48682	0.628932	2.1
48683	0.708518	2.1
48684	0.801358	2.1
48685	0.476016	2.1
48686	0.569477	2.1
48687	0.878553	2.1
48688	1.89226	2.1
48689	1.89226	2.1
48690	1.89226	2.1
48691	1.89226	2.1
48692	1.89226	2.1
48693	1.89226	2.1
48694	0.946128	2.1
48695	0.946128	2.1
48696	1.01537	2.1
48697	0.9662	2.1
48698	0.946131	2.1
48699	0.946131	2.1
48700	0.966198	2.1
48701	0.966198	2.1
48702	0.94613	2.1
48703	0.966198	2.1
48704	0.946131	2.1
48705	1.01537	2.1
48706	1.89226	2.1
48707	1.89226	2.1
48708	1.91883	2.1
48709	1.91883	2.1
48710	2.15905	2.1
48711	2.13249	2.1
48712	1.07953	2.1
48713	1.2107	2.1
48714	0.946132	2.1
48715	0.946132	2.1
48716	1.2107	2.1
48717	1.2107	2.1

48718	0.946132	2.1
48719	0.946132	2.1
48720	1.2107	2.1
48721	0.946133	2.1
48722	0.946132	2.1
48723	1.07953	2.1
48724	2.46752	2.1
48725	2.53733	2.1
48726	2.26044	2.1
48727	2.33222	0.789984
48728	4.79974	2.1
48729	2.53733	2.1
48730	2.37576	0.459383
48731	2.33222	0.948756
48732	2.1845	1.6662
48733	4.70798	0.935977
48734	2.48652	2.1
48735	2.33224	0.779647
48736	2.08149	2.1
48737	1.9839	2.1
48738	4.45623	2.1
48739	2.33223	0.817295
48740	2.18452	0.949219
48741	2.16061	2.1
48742	2.01399	0.473426
48743	4.34623	0.70401
48744	1.4617	0.941477
48745	1.34701	2.05765
48746	1.34701	0.933392
48747	1.25173	2.1
48748	1.5117	0.89307
48749	1.41642	2.1
48750	2.65164	2.1
48751	1.89227	1.10147
48752	1.89227	2.09628
48753	1.89227	2.1
48754	2.42504	1.87101
48755	2.42504	2.1
48756	1.89226	0.827011
48757	1.89226	1.07051
48758	1.89226	2.08459
48759	1.89226	0.717528
48760	3.78452	0.882804
48761	1.5117	0.991406
48762	1.34701	1.43414
48763	1.47001	2.1
48764	1.57961	0.812806
48765	2.92663	1.09708
48766	1.89226	1.49118
48767	1.89226	0.707666
48768	1.89226	0.810857
48769	1.89226	0.977599
48770	3.78452	0.94181
48771	0.412773	0.05
48772	0.570839	0.0525453
48773	0.50721	0.05
48774	0.513638	0.0628953
48775	0.518805	0.05

48776	0.41278	0.05
48777	1.5344	0.0617751
48778	0.939282	0.0544544
48779	0.720051	0.05
48780	0.691767	0.0553761
48781	0.761152	0.0569662
48782	1.89226	1.39588
48783	1.89226	2.1
48784	1.89226	1.9581
48785	1.89226	2.1
48786	1.89226	1.66406
48787	1.89226	2.1
48788	1.24618	2.1
48789	2.10585	2.1
48790	1.08134	2.1
48791	1.93032	2.1
48792	3.1765	2.1
48793	1.98474	2.1
48794	2.57777	2.1
48795	1.93031	2.1
48796	2.19041	2.1
48797	3.99251	2.1
48798	1.89226	2.01035
48799	1.89226	2.1
48800	1.89226	2.1
48801	1.89226	1.98425
48802	3.78452	2.1
48803	1.67374	2.1
48804	2.19042	2.1
48805	1.67374	2.1
48806	2.09769	2.1
48807	2.29586	2.1
48808	2.7198	2.1
48809	1.89226	2.1
48810	1.89226	2.1
48811	1.89226	2.1
48812	1.89226	2.1
48813	1.89226	1.96877
48814	1.89226	2.1
48815	0.946132	0.552766
48816	0.946132	0.493073
48817	0.947763	0.498881
48818	0.909304	0.522974
48819	0.946127	0.470696
48820	0.947831	0.434749
48821	0.907673	0.44463
48822	0.907673	0.52363
48823	0.94613	0.453505
48824	0.947834	0.441654
48825	0.907671	0.583607
48826	0.94613	0.466819
48827	0.906519	0.41625
48828	0.906519	0.390086
48829	0.946097	0.406885
48830	0.946097	0.422386
48831	0.947834	0.409161
48832	0.906485	0.386105
48833	0.946131	0.389151

48834	0.946131	0.421621
48835	0.94613	0.421324
48836	0.906485	0.372686
48837	0.946131	0.428325
48838	0.947835	0.421929
48839	2.58638	2.1
48840	2.51039	2.1
48841	2.47395	2.1
48842	2.41539	2.1
48843	5.00178	2.1
48844	2.51039	2.1
48845	2.16633	2.1
48846	2.4154	2.1
48847	2.13977	2.1
48848	4.58173	2.1
48849	2.47395	2.1
48850	2.41539	2.1
48851	2.2988	2.1
48852	2.2604	2.1
48853	4.7142	2.1
48854	2.4154	2.1
48855	2.13977	2.1
48856	2.26041	2.1
48857	1.98957	2.1
48858	4.40497	2.1
48859	2.15721	0.513471
48860	2.31843	0.403882
48861	1.93973	0.581838
48862	2.13243	0.201254
48863	4.25816	0.426738
48864	2.31844	0.403457
48865	2.30908	0.402776
48866	2.13243	0.361701
48867	2.1609	0.381358
48868	4.47934	0.35618
48869	1.93973	0.575852
48870	2.13243	0.360163
48871	1.69661	0.554618
48872	1.9339	0.290413
48873	3.87363	0.445656
48874	2.13243	0.341062
48875	2.1609	0.389923
48876	1.9339	0.373777
48877	1.98414	0.4215
48878	4.0948	0.335426
48879	1.88857	0.05
48880	2.34107	0.05
48881	0.502431	0.05
48882	0.71416	0.05
48883	0.50243	0.05
48884	0.944284	0.05
48885	1.88856	2.1
48886	2.34107	2.1
48887	2.39662	2.1
48888	1.92293	2.1
48889	4.28519	2.1
48890	1.00486	2.1
48891	1.92293	2.1

48892	1.88857	2.1
48893	1.88857	2.1
48894	1.02605	2.1
48895	1.02605	2.1
48896	2.53728	2.1
48897	1.70303	2.1
48898	2.03734	2.1
48899	1.3624	2.1
48900	2.54706	2.1
48901	1.70303	2.1
48902	2.54706	2.1
48903	1.50876	2.1
48904	2.74067	2.1
48905	1.85391	2.1
48906	2.29667	2.1
48907	1.50876	2.1
48908	0.946133	2.1
48909	0.946133	2.1
48910	1.24191	2.1
48911	1.24191	2.1
48912	0.946132	2.1
48913	1.24191	2.1
48914	1.23779	2.1
48915	0.946132	2.1
48916	0.946132	2.1
48917	0.946132	2.1
48918	1.23779	2.1
48919	1.24191	2.1
48920	0.946131	2.1
48921	0.946131	2.1
48922	1.14833	2.1
48923	1.14833	2.1
48924	1.14834	2.1
48925	0.946132	2.1
48926	0.94613	2.1
48927	1.24191	2.1
48928	0.946131	2.1
48929	0.946131	2.1
48930	1.24191	2.1
48931	1.14833	2.1
48932	2.08149	2.1
48933	2.31116	2.1
48934	1.9701	1.10711
48935	2.18328	2.1
48936	4.49563	1.80806
48937	2.08149	2.1
48938	2.14059	2.1
48939	1.72377	2.1
48940	2.18328	2.1
48941	3.88931	2.1
48942	2.14059	2.1
48943	2.40755	2.1
48944	2.31145	2.1
48945	2.23743	2.1
48946	2.31145	2.1
48947	2.23743	2.1
48948	2.31117	2.1
48949	2.40754	2.1

48950	2.41055	2.1
48951	2.31145	2.1
48952	2.41055	1.84207
48953	2.31145	2.1
48954	2.5615	0.541571
48955	2.37625	0.504919
48956	2.20248	0.495041
48957	2.20248	0.507865
48958	2.65967	0.54852
48959	2.65967	0.571266
48960	2.71793	0.544774
48961	2.65966	0.486578
48962	2.56149	0.466574
48963	2.56149	0.486181
48964	2.71296	0.514731
48965	2.71296	0.552637
48966	1.31528	0.48871
48967	1.32983	0.467159
48968	1.21128	0.497397
48969	1.18812	0.47787
48970	1.24232	0.467062
48971	1.24232	0.483962
48972	1.26109	0.472542
48973	1.26109	0.521491
48974	1.21128	0.495823
48975	1.31528	0.455202
48976	1.18812	0.473507
48977	1.32983	0.522
48978	2.63056	0.457291
48979	2.58032	0.459387
48980	2.65966	0.419515
48981	2.65966	0.466581
48982	2.58032	0.501025
48983	2.71296	0.520287
48984	1.92039	2.1
48985	1.06226	2.1
48986	1.09252	2.1
48987	1.96052	2.1
48988	3.01291	2.1
48989	1.92039	2.1
48990	2.46338	2.1
48991	2.47522	2.1
48992	2.43508	2.1
48993	1.96052	2.1
48994	1.92039	2.1
48995	0.985949	2.1
48996	0.980261	2.1
48997	0.553625	2.1
48998	0.546262	2.1
48999	0.765671	2.1
49000	0.765671	2.1
49001	0.766539	2.1
49002	0.766539	2.1
49003	0.985949	2.1
49004	0.553625	2.1
49005	0.546261	2.1
49006	0.980261	2.1
49007	1.96052	2.1

49008	2.46338	2.1
49009	1.97189	2.1
49010	1.96052	2.1
49011	2.47932	2.1
49012	2.47932	2.1
49013	0.964608	0.05
49014	0.691524	0.05
49015	1.25462	0.05
49016	0.691524	0.05
49017	1.89226	0.05
49018	1.89226	0.05
49019	1.44894	0.05
49020	2.77655	0.05
49021	1.4237	0.05
49022	2.77701	0.05
49023	2.77701	0.05
49024	1.38847	2.1
49025	1.46031	1.74098
49026	1.65504	1.20258
49027	1.75396	1.19626
49028	3.11535	1.39316
49029	1.89226	1.59923
49030	1.89226	1.94249
49031	1.89226	1.31421
49032	1.89226	0.950115
49033	3.78452	1.68149
49034	1.89226	1.37504
49035	1.89226	2.04531
49036	1.89226	0.782917
49037	1.89226	1.73117
49038	3.78452	0.974742
49039	1.46031	1.14571
49040	1.34851	2.00413
49041	1.75395	1.08105
49042	1.62287	1.25088
49043	3.08318	1.72925
49044	1.65504	1.48115
49045	1.75396	1.78771
49046	1.89249	1.52178
49047	1.9857	1.09698
49048	3.64073	1.77303
49049	1.89226	1.69847
49050	1.89226	1.51392
49051	1.89226	1.01958
49052	1.89226	1.24369
49053	3.78452	1.10967
49054	1.89226	1.00658
49055	1.89226	2.07319
49056	1.89226	1.16035
49057	1.89226	1.46417
49058	3.78452	1.86699
49059	1.75395	1.53557
49060	1.62287	1.69098
49061	1.98569	0.970307
49062	1.86067	1.19263
49063	3.60856	1.11977
49064	1.35697	1.17043
49065	2.02064	0.946805

49066	1.89989	0.746903
49067	1.49571	2.1
49068	1.66753	0.838081
49069	1.36431	2.1
49070	0.744004	2.1
49071	0.743997	1.18913
49072	0.717972	0.762918
49073	0.717972	1.49473
49074	0.997982	2.1
49075	0.807796	1.67306
49076	0.632235	0.810086
49077	0.488575	2.1
49078	0.849275	1.61835
49079	0.488581	1.77279
49080	0.724729	1.73118
49081	0.997967	1.77234
49082	2.5276	1.37857
49083	1.70791	1.33426
49084	1.82872	0.823776
49085	1.56362	2.1
49086	2.5276	1.00791
49087	2.24781	2.1
49088	1.37591	2.1
49089	1.23217	1.10214
49090	1.00442	1.63705
49091	0.945282	0.908167
49092	1.15446	1.04789
49093	1.09123	2.1
49094	1.21506	1.36543
49095	1.21506	1.03
49096	1.39452	1.15035
49097	1.13108	1.68211
49098	1.27876	1.04323
49099	0.929006	0.920796
49100	1.46505	0.219393
49101	1.91484	0.217567
49102	1.49906	0.261208
49103	1.49906	0.284459
49104	1.90196	0.157301
49105	1.90196	0.166913
49106	1.90196	0.220884
49107	2.11465	0.247016
49108	1.91484	0.285128
49109	1.91484	0.315914
49110	2.04016	0.176169
49111	2.04016	0.194044
49112	0.912201	0.155597
49113	0.950978	0.167765
49114	0.665307	0.12574
49115	0.732528	0.177242
49116	0.829512	0.0972627
49117	0.829512	0.150774
49118	0.815217	0.147354
49119	0.815217	0.147734
49120	0.912202	0.126108
49121	0.665305	0.131799
49122	0.732527	0.229806
49123	0.95098	0.145656

49124	1.90196	0.203358
49125	2.08757	0.142479
49126	1.9626	0.156262
49127	2.04016	0.144132
49128	1.82441	0.162255
49129	1.90196	0.164335
49130	1.13048	2.1
49131	0.821162	2.1
49132	0.946131	2.1
49133	0.946131	2.1
49134	1.04567	2.1
49135	0.882347	2.1
49136	0.897276	2.1
49137	0.897276	2.1
49138	0.863109	2.1
49139	1.01055	2.1
49140	0.940305	2.1
49141	0.942166	2.1
49142	2.23933	0.05
49143	0.976217	0.05
49144	2.78053	0.05
49145	2.78053	0.05
49146	0.976217	0.0522117
49147	1.65984	0.05
49148	1.54168	2.1
49149	2.01197	1.47908
49150	1.69136	2.1
49151	2.13284	0.876523
49152	3.67452	1.6031
49153	0.988805	2.1
49154	1.78069	1.40025
49155	1.69136	1.50538
49156	1.69136	2.1
49157	1.05481	1.17309
49158	1.05481	2.1
49159	1.69136	2.1
49160	2.13284	1.13482
49161	1.78069	1.99299
49162	2.22044	1.09904
49163	3.91353	1.86856
49164	2.69379	0.868668
49165	2.32219	0.941497
49166	2.25746	1.6737
49167	2.25746	0.828734
49168	2.32219	0.978903
49169	1.89526	0.772794
49170	1.4556	2.1
49171	1.85028	2.1
49172	2.4909	0.765864
49173	2.03799	2.1
49174	2.73495	1.00613
49175	1.85028	1.39528
49176	2.71938	0.783259
49177	1.85028	2.1
49178	3.36051	2.1
49179	2.73495	1.32437
49180	2.47584	1.72349
49181	1.85028	0.958322

49182	2.47584	1.10269
49183	1.04413	2.1
49184	2.1461	2.1
49185	1.74586	2.08087
49186	1.44437	2.1
49187	1.04413	1.95728
49188	1.89226	2.1
49189	1.89226	1.11658
49190	1.89226	1.24167
49191	1.89226	1.17179
49192	3.78452	1.27215
49193	1.52724	2.1
49194	1.89249	2.1
49195	1.68143	2.1
49196	2.02433	1.09812
49197	3.55157	2.1
49198	1.07444	2.1
49199	1.76545	2.1
49200	1.68142	2.1
49201	1.68142	2.1
49202	1.14807	2.1
49203	1.14807	2.1
49204	1.89226	2.1
49205	1.89226	2.1
49206	1.89226	2.1
49207	1.89226	0.931288
49208	1.89226	2.1
49209	1.89226	0.773832
49210	1.89226	2.1
49211	1.89226	1.29774
49212	1.89226	2.1
49213	1.89226	0.86607
49214	3.78452	1.20344
49215	1.68143	2.1
49216	2.02433	1.22627
49217	1.76545	2.1
49218	2.12608	2.1
49219	3.78978	2.1
49220	2.62871	1.91737
49221	1.49149	2.1
49222	1.36814	2.1
49223	2.44761	0.983415
49224	3.9391	2.1
49225	1.89226	2.1
49226	1.89226	0.636032
49227	1.89226	0.98217
49228	1.89226	1.96861
49229	3.78452	0.952453
49230	0.851542	2.1
49231	0.851542	1.30263
49232	1.00897	2.1
49233	0.931224	2.1
49234	0.838952	2.1
49235	0.838952	0.985219
49236	0.956848	2.1
49237	0.942474	2.1
49238	0.946132	2.1
49239	0.838952	2.1

49240	0.878743	2.1
49241	0.946132	2.1
49242	1.15541	2.1
49243	2.44761	0.769683
49244	1.36814	2.1
49245	1.36814	1.20611
49246	2.19439	2.1
49247	2.19439	2.1
49248	1.89226	0.763384
49249	1.89226	1.39622
49250	1.89226	2.1
49251	1.89226	0.720193
49252	3.78452	1.18027
49253	1.89226	2.1
49254	1.67791	2.1
49255	1.5713	2.1
49256	1.67791	2.1
49257	1.78566	2.1
49258	1.89226	1.57527
49259	1.89226	0.692812
49260	1.62911	1.96993
49261	2.24602	1.71793
49262	1.89226	1.63239
49263	1.98287	0.979379
49264	1.62911	0.926146
49265	1.89226	1.27621
49266	1.89226	1.68336
49267	1.89226	0.899526
49268	1.89226	2.1
49269	3.78452	1.10135
49270	1.26688	2.1
49271	1.11804	2.1
49272	0.852467	0.940574
49273	0.693314	2.1
49274	0.977459	1.7429
49275	0.977459	2.1
49276	0.982741	0.895486
49277	0.982741	2.09718
49278	1.26689	2.1
49279	0.852464	2.1
49280	0.693312	2.1
49281	1.11804	2.1
49282	0.946131	2.1
49283	0.946131	0.799894
49284	1.1794	0.806459
49285	1.1794	2.07253
49286	0.991434	2.1
49287	1.1794	0.950852
49288	0.946131	0.840369
49289	0.946131	0.793947
49290	0.946131	1.69846
49291	1.1794	1.34873
49292	0.946131	1.50165
49293	0.991434	1.07936
49294	1.89226	0.770236
49295	1.89226	2.1
49296	1.89226	0.868168
49297	1.89226	1.11446

49298	3.78452	1.10933
49299	2.53377	2.1
49300	1.53443	2.05255
49301	2.0815	0.853784
49302	2.82979	2.1
49303	4.35462	2.07823
49304	1.96192	0.751331
49305	1.23602	2.00434
49306	2.16506	0.86384
49307	1.56841	1.57325
49308	3.40108	1.21002
49309	1.89226	0.997651
49310	1.89226	1.98397
49311	1.89226	0.897483
49312	1.89226	2.1
49313	3.78452	1.11281
49314	0.946129	2.1
49315	0.946129	1.83306
49316	1.13067	0.877847
49317	1.12952	2.1
49318	0.946129	2.1
49319	1.13089	1.98582
49320	0.944978	0.86338
49321	0.944978	1.85725
49322	0.946131	2.1
49323	1.13089	2.1
49324	0.944977	2.1
49325	0.946131	2.1
49326	0.710208	2.1
49327	0.710208	2.1
49328	0.718894	2.1
49329	0.718894	2.1
49330	0.559773	2.1
49331	0.579321	2.1
49332	0.886767	2.1
49333	0.849781	2.1
49334	0.849781	2.1
49335	0.559774	2.1
49336	0.579321	2.1
49337	0.886766	2.1
49338	1.63471	2.1
49339	1.69956	2.1
49340	1.08022	2.1
49341	1.08022	2.1
49342	1.11955	2.1
49343	1.67403	2.1
49344	1.03766	2.1
49345	2.02825	2.1
49346	2.02794	2.1
49347	2.0348	2.1
49348	1.07196	2.1
49349	1.07196	2.1
49350	1.9276	2.1
49351	2.1004	2.1
49352	2.1306	2.1
49353	2.1306	1.99985
49354	1.92178	2.1
49355	1.92032	1.60762

49356	0.986724	2.1
49357	2.03064	2.1
49358	2.01385	2.1
49359	2.02404	2.1
49360	1.03766	2.1
49361	1.03766	2.1
49362	1.92027	2.1
49363	2.1306	2.1
49364	2.15388	2.1
49365	2.15388	2.1
49366	1.92614	2.1
49367	1.9276	2.06228
49368	1.09578	2.1
49369	0.963018	2.1
49370	0.782956	1.08424
49371	0.529693	2.1
49372	0.6994	1.0924
49373	0.862532	2.1
49374	0.898605	2.1
49375	0.898605	1.1944
49376	0.776756	2.01204
49377	1.14805	2.07528
49378	0.966521	2.04941
49379	0.617676	1.34335
49380	2.67185	1.19562
49381	2.099 2.1	
49382	2.80548	1.10885
49383	2.2727	2.1
49384	4.94455	2.1
49385	1.89226	1.19916
49386	1.89226	2.1
49387	1.89226	1.47572
49388	1.89226	2.1
49389	3.78453	2.1
49390	1.89226	2.1
49391	1.89226	2.1
49392	1.89226	2.1
49393	1.89226	2.1
49394	1.89226	2.1
49395	1.89226	2.1
49396	0.884801	2.1
49397	0.884801	2.1
49398	0.895199	2.1
49399	0.895199	2.1
49400	0.622774	2.1
49401	0.730501	2.1
49402	1.0495	2.1
49403	1.13635	2.1
49404	1.13635	2.1
49405	0.730503	2.1
49406	1.0495	2.1
49407	0.622775	2.1
49408	1.89226	0.975977
49409	1.89226	2.1
49410	1.89226	1.78634
49411	1.89226	2.1
49412	1.89226	1.08346
49413	1.89226	2.1

49414	1.89226	2.1
49415	1.89226	2.1
49416	1.89226	2.1
49417	1.89226	2.1
49418	1.89226	2.1
49419	1.89226	2.1
49420	2.2727	2.1
49421	1.89586	2.1
49422	1.66913	2.1
49423	1.46101	2.1
49424	2.48082	2.1
49425	2.2727	2.1
49426	1.89226	1.44035
49427	1.89226	2.1
49428	1.84641	2.1
49429	1.89226	1.93397
49430	1.30868	2.1
49431	1.34118	0.961243
49432	1.89226	0.780531
49433	1.89226	0.865907
49434	1.89226	0.959743
49435	1.89226	0.773742
49436	1.89226	0.839003
49437	1.89226	0.675751
49438	1.89226	0.876765
49439	1.89226	0.576697
49440	1.89226	0.719755
49441	1.89226	0.854882
49442	3.78452	0.769216
49443	0.946132	1.22474
49444	0.946132	1.00551
49445	0.946131	2.04611
49446	0.946131	2.1
49447	0.946132	2.1
49448	0.946132	0.852631
49449	0.946132	2.1
49450	0.946132	1.33096
49451	0.946132	1.89356
49452	0.946132	1.74061
49453	0.946132	2.0816
49454	0.946132	1.96497
49455	1.89227	1.08113
49456	1.84641	1.08181
49457	2.29517	1.83197
49458	1.84641	0.979006
49459	2.34102	1.10426
49460	1.89227	0.822322
49461	1.89226	0.683558
49462	1.89226	0.699784
49463	1.89226	0.808806
49464	1.89226	0.659264
49465	1.89226	0.707191
49466	1.89226	0.575951
49467	1.89226	0.611744
49468	1.89226	0.70021
49469	1.89226	0.75247
49470	1.89226	0.627509
49471	1.89226	0.666558

49472	1.89226	0.556543
49473	1.89226	1.14511
49474	1.89226	0.888899
49475	1.89226	1.35886
49476	1.89226	0.810465
49477	1.89226	1.00525
49478	1.89226	0.80702
49479	0.851544	0.0547248
49480	0.757542	0.0915838
49481	1.13421	0.0534877
49482	1.04072	0.0633591
49483	0.946132	0.0525407
49484	0.946132	0.055112
49485	0.841686	0.0643902
49486	0.834428	0.0554212
49487	1.13649	0.0533896
49488	0.937687	0.0522368
49489	0.841713	0.0664139
49490	0.841713	0.0748919
49491	1.89226	0.385355
49492	1.89226	0.451332
49493	1.89226	0.470811
49494	1.89226	0.328632
49495	1.89226	0.495538
49496	1.89226	0.347777
49497	2.31988	1.08545
49498	2.00082	1.90298
49499	2.4594	0.839443
49500	2.17638	1.94902
49501	4.46022	1.05622
49502	2.00082	0.90926
49503	1.41829	2.1
49504	1.61207	1.09741
49505	1.43652	2.1
49506	2.17637	0.862534
49507	2.00082	2.1
49508	2.4594	0.814367
49509	2.17638	2.1
49510	2.57957	0.841778
49511	2.3506	1.7763
49512	4.81	1.1857
49513	2.3506	0.998131
49514	1.57368	2.1
49515	1.89111	0.660912
49516	1.89111	2.1
49517	2.17638	0.696135
49518	2.17638	2.1
49519	1.32813	2.1
49520	1.7237	2.1
49521	1.32461	2.1
49522	1.81656	2.1
49523	3.14468	2.1
49524	1.55132	2.1
49525	2.0815	2.1
49526	1.81656	2.1
49527	2.19826	2.1
49528	3.65818	2.1
49529	0.775951	2.1

49530	0.775951	2.1
49531	0.794601	2.1
49532	0.794601	2.1
49533	0.945641	2.1
49534	0.90828	2.1
49535	0.662302	2.1
49536	0.662273	2.1
49537	0.662274	2.1
49538	0.945639	2.1
49539	0.662304	2.1
49540	0.908279	2.1
49541	1.03062	2.1
49542	1.03062	2.1
49543	1.05054	2.1
49544	1.05054	2.1
49545	1.13551	2.1
49546	1.21275	2.1
49547	0.945641	2.1
49548	0.90828	2.1
49549	0.945641	2.1
49550	1.21275	2.1
49551	0.90828	2.1
49552	1.13552	2.1
49553	1.89226	2.1
49554	1.79883	2.1
49555	1.95341	2.1
49556	1.89226	2.1
49557	1.85998	2.1
49558	1.79883	2.1
49559	1.89226	2.1
49560	1.89226	2.1
49561	1.89226	2.1
49562	1.89226	2.1
49563	3.78453	2.1
49564	0.851541	2.1
49565	0.851541	2.1
49566	0.970398	2.1
49567	0.954365	2.1
49568	0.855356	2.1
49569	0.855357	2.1
49570	0.946133	2.1
49571	0.946133	2.1
49572	1.04072	2.1
49573	0.970397	2.1
49574	0.950003	2.1
49575	0.851538	2.1
49576	1.89226	2.1
49577	1.85998	2.1
49578	1.95296	2.1
49579	1.89226	2.1
49580	1.92067	2.1
49581	1.85998	2.1
49582	0.946133	2.1
49583	0.946133	2.1
49584	0.946132	2.1
49585	0.946132	2.1
49586	0.946132	2.1
49587	0.946132	2.1

49588	0.946132	2.1
49589	0.946132	2.1
49590	0.946132	2.1
49591	0.946132	2.1
49592	0.946133	2.1
49593	0.946133	2.1
49594	1.82152	2.1
49595	1.89226	2.1
49596	1.89226	2.1
49597	2.11152	2.1
49598	1.7472	2.1
49599	1.89226	2.1
49600	2.02687	2.1
49601	2.29199	2.1
49602	1.2734	2.1
49603	1.41077	2.1
49604	1.47381	2.1
49605	2.29199	2.1
49606	1.46889	2.1
49607	2.29199	2.1
49608	1.86766	2.1
49609	2.59401	2.1
49610	4.15965	2.1
49611	1.58995	2.1
49612	1.74246	2.1
49613	1.46643	2.1
49614	1.46643	2.1
49615	1.85097	2.1
49616	1.85097	2.1
49617	1.58995	2.1
49618	1.66584	2.1
49619	1.89214	2.1
49620	1.91787	2.1
49621	3.48208	2.1
49622	1.18474	2.1
49623	1.8144	2.1
49624	1.1486	2.1
49625	1.1486	2.1
49626	2.13071	2.1
49627	2.1257	2.1
49628	2.0815	2.1
49629	2.51651	2.1
49630	1.73681	2.1
49631	2.01601	2.1
49632	2.21406	2.1
49633	2.56998	2.1
49634	1.19995	2.1
49635	1.06256	2.1
49636	0.620677	2.1
49637	0.598084	2.1
49638	0.831426	2.1
49639	0.946056	2.1
49640	0.827692	2.1
49641	0.827692	2.1
49642	1.08958	2.1
49643	0.630132	2.1
49644	0.577547	2.1
49645	1.06104	2.1

49646	2.0815	2.1
49647	2.66249	2.1
49648	2.78966	2.1
49649	2.47108	2.1
49650	2.33858	2.1
49651	2.0815	2.1
49652	1.53735	2.1
49653	1.42278	2.1
49654	1.23654	2.1
49655	1.34678	2.1
49656	2.76955	2.1
49657	1.89226	2.1
49658	1.89226	2.1
49659	1.89226	2.1
49660	1.89226	2.1
49661	3.78452	2.1
49662	1.23654	2.1
49663	1.34678	2.1
49664	1.19 2.1	
49665	1.08576	2.1
49666	2.43254	2.1
49667	1.38776	2.1
49668	1.29366	2.1
49669	0.946131	2.1
49670	0.946131	2.1
49671	0.946131	2.1
49672	0.946131	2.1
49673	1.38775	2.1
49674	1.38775	2.1
49675	1.29366	2.1
49676	0.946132	2.1
49677	0.946131	2.1
49678	1.38775	2.1
49679	0.946131	2.1
49680	0.946131	2.1
49681	0.946129	2.1
49682	0.946129	2.1
49683	0.94613	2.1
49684	0.94613	2.1
49685	0.946131	2.1
49686	0.946131	2.1
49687	0.946131	2.1
49688	0.946131	2.1
49689	0.94613	2.1
49690	0.94613	2.1
49691	1.89226	0.464368
49692	1.89226	0.953278
49693	1.89226	0.571563
49694	1.89226	0.635392
49695	1.89226	0.63636
49696	1.89226	0.936266
49697	1.89226	0.528765
49698	1.89226	0.961327
49699	1.89226	0.551882
49700	1.89226	0.642648
49701	1.89226	0.591999
49702	1.89226	0.911187
49703	1.89226	0.54105

49704	1.89226	1.01328
49705	1.89226	0.600569
49706	1.89226	0.822697
49707	1.89226	0.554161
49708	1.89226	0.984097
49709	0.92338	2.1
49710	0.92338	0.750896
49711	0.94428	0.738246
49712	0.94428	0.588421
49713	1.19528	0.793412
49714	1.30598	2.1
49715	0.714073	0.630469
49716	0.561679	1.19733
49717	1.19528	0.81703
49718	0.561678	0.824495
49719	0.714072	1.02698
49720	1.30598	0.973784
49721	2.61197	1.30166
49722	1.89226	0.840719
49723	1.89226	0.819957
49724	1.89226	2.03743
49725	2.39056	0.732517
49726	2.39056	2.04505
49727	0.946132	0.995474
49728	0.946132	0.78764
49729	0.946132	0.489718
49730	0.946132	0.593082
49731	0.946131	0.601722
49732	0.946131	1.01854
49733	0.946132	0.858194
49734	0.946132	0.551781
49735	0.946132	0.68932
49736	0.946132	0.654014
49737	0.946132	0.71401
49738	0.946132	0.767263
49739	1.89224	2.1
49740	1.89224	2.1
49741	1.89224	2.1
49742	1.89224	2.1
49743	3.78448	2.1
49744	1.97089	2.1
49745	1.57428	2.1
49746	1.84991	2.1
49747	1.49078	2.1
49748	3.42419	2.1
49749	0.621927	2.1
49750	0.621927	2.1
49751	0.630824	2.1
49752	0.630824	2.1
49753	0.483483	2.1
49754	0.507361	2.1
49755	0.787142	2.1
49756	0.745391	2.1
49757	0.745381	2.1
49758	0.483476	2.1
49759	0.507368	2.1
49760	0.787152	2.1
49761	1.89224	2.1

49762	1.89224	2.1
49763	1.89224	2.1
49764	1.89224	2.1
49765	1.89224	2.1
49766	1.89224	2.1
49767	1.89227	2.1
49768	1.89227	2.1
49769	1.89227	2.1
49770	1.89227	2.1
49771	1.89227	2.1
49772	1.89227	2.1
49773	1.84994	2.1
49774	1.41758	2.1
49775	1.4908	2.1
49776	1.4908	2.1
49777	1.74391	2.1
49778	1.74391	2.1
49779	1.89226	2.1
49780	1.89226	2.1
49781	1.89226	2.1
49782	1.89226	2.1
49783	3.78452	2.1
49784	1.01323	2.1
49785	1.01323	2.1
49786	1.01445	2.1
49787	1.01445	2.1
49788	0.839531	2.1
49789	0.875652	2.1
49790	1.19063	2.1
49791	1.15203	2.1
49792	1.15203	2.1
49793	0.839531	2.1
49794	0.875652	2.1
49795	1.19063	2.1
49796	1.89226	2.1
49797	2.75587	2.1
49798	2.81512	2.1
49799	2.81512	2.1
49800	1.89226	2.1
49801	1.89226	2.1
49802	1.89226	2.1
49803	1.89226	2.1
49804	1.89226	2.1
49805	1.89226	2.1
49806	3.78452	2.1
49807	0.974831	2.1
49808	0.974831	2.1
49809	0.976681	2.1
49810	0.976681	2.1
49811	0.803179	2.1
49812	0.83953	2.1
49813	1.11198	2.1
49814	1.15203	2.1
49815	1.11198	2.1
49816	0.803179	2.1
49817	1.15203	2.1
49818	0.83953	2.1
49819	1.37794	2.1

49820	1.37794	2.1
49821	0.946128	2.1
49822	0.946128	2.1
49823	1.3477	2.1
49824	1.37793	2.1
49825	0.946131	2.1
49826	0.946131	2.1
49827	0.946129	2.1
49828	1.3477	2.1
49829	1.37793	2.1
49830	0.946129	2.1
49831	2.38577	1.05334
49832	1.20247	2.1
49833	1.75182	2.1
49834	2.82546	1.15962
49835	4.1376	2.1
49836	2.82546	2.1
49837	1.89226	0.787031
49838	2.38577	1.54904
49839	2.38577	1.55384
49840	1.89226	1.80942
49841	1.89226	1.29424
49842	1.035 2.1	
49843	2.15429	2.1
49844	0.970998	2.1
49845	2.0815	2.1
49846	2.89672	2.1
49847	0.946131	0.360235
49848	0.946131	0.353136
49849	0.853249	0.357624
49850	1.02715	0.360324
49851	0.851537	0.359
49852	0.851537	0.360996
49853	1.02663	0.348234
49854	1.13139	0.347085
49855	0.946128	0.345663
49856	0.932039	0.360122
49857	0.843985	0.340638
49858	0.946129	0.347734
49859	1.89226	0.34652
49860	1.86408	0.358739
49861	1.96548	0.365414
49862	1.86408	0.36013
49863	1.99366	0.350536
49864	1.89226	0.349756
49865	2.70702	2.1
49866	2.08981	2.1
49867	2.08693	2.1
49868	2.08693	2.1
49869	2.7188	2.1
49870	2.7188	2.1
49871	2.08982	2.1
49872	1.01964	2.1
49873	2.08693	2.1
49874	1.04615	2.1
49875	3.13597	2.1
49876	2.70702	2.1
49877	2.08693	2.1

49878	2.68784	2.1
49879	2.08831	2.1
49880	4.77477	2.1
49881	0.778233	2.1
49882	0.778233	2.1
49883	0.789001	2.1
49884	0.789001	2.1
49885	0.545307	2.1
49886	0.523075	2.1
49887	1.04347	2.1
49888	1.04416	2.1
49889	1.04416	2.1
49890	0.545308	2.1
49891	1.04347	2.1
49892	0.523075	2.1
49893	1.89226	0.584714
49894	1.89226	0.565184
49895	1.89226	0.528776
49896	1.89226	0.397914
49897	3.78453	0.449442
49898	1.39237	0.603726
49899	1.17933	1.5777
49900	0.755796	0.81027
49901	0.755796	1.29024
49902	0.755767	0.375052
49903	0.94613	1.00544
49904	1.15918	0.480227
49905	0.94613	0.696826
49906	1.11632	0.516884
49907	0.92595	0.402102
49908	0.94613	0.626158
49909	0.94613	0.602944
49910	1.89226	0.571609
49911	1.89226	0.463863
49912	1.89226	0.387385
49913	1.89226	0.417441
49914	3.78452	0.444944
49915	0.950067	0.410153
49916	0.75071	0.469381
49917	0.906516	0.363009
49918	0.906516	0.33872
49919	0.81586	0.322396
49920	0.851541	0.479677
49921	0.962101	0.267072
49922	0.851476	0.503114
49923	0.962105	0.258279
49924	0.815808	0.327023
49925	0.851529	0.509879
49926	0.851529	0.404582
49927	1.05032	0.409717
49928	0.972326	0.441658
49929	0.906515	0.355145
49930	0.906515	0.355005
49931	1.12245	0.313469
49932	1.04072	0.375431
49933	0.815857	0.264925
49934	0.851539	0.451511
49935	1.04451	0.254924

49936	0.815853	0.418614
49937	0.919795	0.411277
49938	0.919795	0.421588
49939	1.5855	2.1
49940	2.14058	2.1
49941	1.9153	2.1
49942	1.79922	2.1
49943	3.72608	2.1
49944	1.58551	1.03391
49945	1.24201	1.17303
49946	0.960287	0.933074
49947	1.79922	2.1
49948	2.82752	2.1
49949	0.679602	2.1
49950	0.621009	1.96629
49951	0.899614	2.1
49952	0.940441	2.1
49953	0.679602	2.1
49954	0.94044	2.1
49955	0.621009	2.1
49956	0.899615	2.1
49957	0.794027	2.1
49958	0.794027	2.1
49959	0.785189	2.1
49960	0.785189	0.758149
49961	2.14058	2.1
49962	1.79922	2.1
49963	2.23742	2.1
49964	2.23742	2.1
49965	1.78404	2.1
49966	1.88088	2.1
49967	1.15177	2.1
49968	2.0815	1.37972
49969	1.93765	2.1
49970	1.78342	1.40021
49971	1.39511	2.1
49972	1.28406	1.47024
49973	1.97719	1.51472
49974	2.50057	0.592789
49975	2.35349	1.02688
49976	2.35349	0.550447
49977	2.31686	1.20739
49978	2.32857	0.60381
49979	1.93765	1.31598
49980	2.35349	0.606108
49981	1.70048	1.04828
49982	2.15721	0.242368
49983	4.05397	0.721827
49984	0.850388	0.803319
49985	0.850388	2.1
49986	0.840019	2.1
49987	0.840019	2.1
49988	1.13736	1.91911
49989	1.13852	1.12074
49990	0.573661	2.1
49991	0.551891	2.1
49992	0.573661	2.1
49993	1.13852	2.1

49994	0.551892	2.1
49995	1.13736	2.1
49996	0.860203	0.909659
49997	0.860203	2.1
49998	0.855758	2.1
49999	0.855758	2.1
50000	1.1423	1.22847
50001	1.13852	2.1
50002	0.58628	2.1
50003	0.573661	2.1
50004	0.586281	2.1
50005	1.1423	2.1
50006	0.573661	2.1
50007	1.13852	2.1
50008	1.28918	0.989255
50009	0.999086	0.864461
50010	1.0383	1.82084
50011	0.736019	1.2787
50012	1.00954	1.34333
50013	1.00954	0.926002
50014	1.01566	0.723299
50015	1.01566	1.7943
50016	1.28918	1.05471
50017	1.0383	1.91743
50018	0.999086	1.51507
50019	0.736019	1.24668
50020	2.20797	2.1
50021	2.64748	2.1
50022	2.50375	0.946294
50023	2.50375	2.1
50024	2.40858	1.70885
50025	2.40858	2.1
50026	2.64748	2.1
50027	2.74744	2.1
50028	2.50375	2.1
50029	2.6521	2.1
50030	5.29958	2.1
50031	2.20797	2.1
50032	2.50375	2.1
50033	2.02064	0.813344
50034	2.3606	2.1
50035	4.52439	2.1
50036	2.36059	2.1
50037	2.6521	2.1
50038	2.56952	2.1
50039	2.56952	2.1
50040	2.50375	2.1
50041	2.50375	2.1
50042	2.49439	0.332545
50043	1.89226	0.358969
50044	2.81337	0.373295
50045	1.89226	0.382126
50046	4.38665	0.366181
50047	1.89226	0.376884
50048	1.89226	0.440304
50049	1.89226	0.511886
50050	1.89226	0.496008
50051	3.78452	0.43661

50052	1.89226	0.361613
50053	1.89226	0.38486
50054	1.89226	0.389145
50055	1.89226	0.428833
50056	3.78452	0.389134
50057	1.93948	0.583865
50058	1.98874	0.411837
50059	1.62494	0.557716
50060	2.14218	0.666607
50061	1.62494	0.374864
50062	2.14218	0.408377
50063	1.47354	0.431181
50064	1.98874	0.376106
50065	2.01865	0.434207
50066	1.62495	0.366453
50067	2.01865	0.483522
50068	1.62495	0.369123
50069	1.89226	0.615853
50070	1.89226	2.1
50071	1.89226	0.817827
50072	1.89226	1.83272
50073	3.78453	1.50792
50074	2.05257	2.1
50075	1.94441	2.1
50076	1.88487	0.773933
50077	1.82441	2.1
50078	3.82928	2.1
50079	1.94441	2.1
50080	1.74391	2.1
50081	1.82441	2.1
50082	1.65636	2.1
50083	3.60077	2.1
50084	1.89226	2.1
50085	1.89226	2.1
50086	1.89226	1.88994
50087	1.89226	2.1
50088	3.78452	2.1
50089	1.89226	0.865331
50090	1.89226	1.9178
50091	1.89226	0.607784
50092	1.89226	2.1
50093	3.78453	1.03065
50094	1.88487	0.827444
50095	1.82441	2.1
50096	1.75835	2.1
50097	1.72866	2.1
50098	3.61354	2.1
50099	1.82441	2.1
50100	1.65636	2.1
50101	1.72866	2.1
50102	1.58401	2.1
50103	3.38503	2.1
50104	1.89226	1.91955
50105	1.89226	2.1
50106	1.89226	2.1
50107	1.89226	2.1
50108	3.78452	2.1
50109	2.27432	2.1

50110	1.33339	2.1
50111	1.38905	2.1
50112	2.34471	2.1
50113	3.66337	2.1
50114	1.89226	2.1
50115	2.34471	2.1
50116	2.27432	2.1
50117	2.27432	2.1
50118	1.89226	2.1
50119	1.89226	2.1
50120	1.20443	2.1
50121	1.17235	2.1
50122	0.724695	2.1
50123	0.694522	2.1
50124	0.949994	2.1
50125	0.949993	2.1
50126	0.948955	2.1
50127	0.948955	2.1
50128	1.20443	2.1
50129	0.7247	2.1
50130	0.694517	2.1
50131	1.17234	2.1
50132	1.89225	2.1
50133	2.40885	2.1
50134	2.3447	2.1
50135	2.3447	2.1
50136	1.89225	2.1
50137	1.89225	2.1
50138	1.37138	2.1
50139	1.82597	2.1
50140	1.45027	2.1
50141	1.92772	2.1
50142	3.27624	2.1
50143	1.89226	2.1
50144	1.89226	2.1
50145	1.89226	2.1
50146	1.89226	1.60435
50147	3.78452	2.1
50148	1.89226	2.1
50149	1.89226	0.822261
50150	1.89226	1.40206
50151	1.89226	0.993952
50152	3.78452	1.93984
50153	1.82597	2.1
50154	2.02312	0.723818
50155	1.92772	2.1
50156	2.15888	2.1
50157	3.98485	1.15445
50158	1.53446	2.1
50159	1.92772	2.1
50160	2.03975	2.1
50161	2.03975	2.1
50162	1.45027	2.1
50163	1.45027	2.1
50164	1.89226	2.1
50165	1.89226	1.74591
50166	1.89226	2.1
50167	1.89226	2.1

50168	1.89226	2.1
50169	1.89226	2.1
50170	1.89226	1.51651
50171	1.89226	2.1
50172	1.89226	2.1
50173	1.89226	0.861517
50174	3.78452	1.79066
50175	1.92772	2.1
50176	2.15888	2.1
50177	2.03975	2.1
50178	2.3111	2.1
50179	4.19863	2.1
50180	1.89226	2.1
50181	1.89226	2.1
50182	1.89226	2.1
50183	1.89226	2.1
50184	3.78452	2.1
50185	1.78376	2.1
50186	1.3201	2.1
50187	1.78376	2.1
50188	1.86102	2.1
50189	1.2679	2.1
50190	1.34517	2.1
50191	1.89226	2.1
50192	2.52442	2.1
50193	1.89226	2.1
50194	2.51691	2.1
50195	4.41667	2.1
50196	1.89226	2.1
50197	1.89226	2.1
50198	1.89226	2.1
50199	1.89226	2.1
50200	3.78452	2.1
50201	1.78376	2.1
50202	1.2679	2.1
50203	1.71839	2.1
50204	1.23504	2.1
50205	3.0188	2.1
50206	1.89226	2.1
50207	2.51795	2.1
50208	2.51691	2.1
50209	2.51691	2.1
50210	1.89226	2.1
50211	1.89226	2.1
50212	1.89226	0.797646
50213	1.89226	1.30998
50214	1.89226	0.923024
50215	1.89226	0.88436
50216	3.78452	0.850431
50217	1.89227	0.811704
50218	1.89227	0.85818
50219	3.26088	1.07243
50220	2.79543	0.893081
50221	2.20733	0.857075
50222	1.89227	0.81214
50223	1.89226	1.03098
50224	1.89226	0.855006
50225	1.89226	0.773399

50226	1.89226	0.862364
50227	1.89226	0.886117
50228	1.89226	1.12426
50229	1.89227	0.802983
50230	2.20733	0.81909
50231	1.46973	1.18243
50232	1.89227	0.798214
50233	1.7848	1.04653
50234	2.20733	0.764784
50235	1.89226	2.1
50236	2.55682	2.1
50237	2.81593	2.1
50238	2.81593	2.1
50239	1.89226	2.1
50240	1.89226	2.1
50241	1.0607	2.1
50242	1.0607	2.1
50243	1.12617	2.1
50244	0.986957	2.1
50245	0.699328	1.97906
50246	0.785567	1.5691
50247	1.28855	2.1
50248	1.41913	2.1
50249	0.766737	0.983416
50250	1.34711	2.1
50251	1.24643	2.1
50252	0.687289	1.45445
50253	3.11063	0.447413
50254	1.89226	0.478822
50255	1.96536	0.46454
50256	1.89226	0.468125
50257	1.96536	0.453669
50258	1.89226	0.45541
50259	1.89226	0.637315
50260	1.89226	0.500872
50261	1.89226	0.551293
50262	1.89226	0.540093
50263	3.78452	0.629657
50264	1.89226	0.471652
50265	1.96256	0.353328
50266	1.89226	0.422961
50267	1.89226	0.481647
50268	2.49192	0.475971
50269	2.49192	0.398725
50270	1.85716	0.465855
50271	2.56871	0.469625
50272	1.03043	0.423931
50273	1.03043	0.436997
50274	2.48125	0.335752
50275	1.65452	0.459849
50276	1.89226	2.1
50277	2.09622	2.1
50278	2.09622	2.1
50279	2.08508	2.1
50280	1.89226	2.1
50281	1.88112	2.1
50282	1.04811	2.1
50283	1.04811	2.1

50284	0.946131	2.1
50285	0.946131	2.1
50286	1.05014	2.1
50287	1.04811	2.1
50288	0.94613	2.1
50289	0.94613	2.1
50290	0.946131	2.1
50291	1.05013	2.1
50292	1.04811	2.1
50293	0.946131	2.1
50294	1.398	2.1
50295	2.10766	2.1
50296	2.3018	2.1
50297	2.3018	2.1
50298	1.34008	2.1
50299	1.34008	2.1
50300	1.1509	2.1
50301	1.1509	2.1
50302	0.946132	2.1
50303	0.946132	2.1
50304	0.946132	2.1
50305	0.946132	2.1
50306	1.05383	2.1
50307	1.1509	2.1
50308	1.1509	2.1
50309	0.946133	2.1
50310	0.946132	2.1
50311	1.05383	2.1
50312	0.931141	2.1
50313	0.931141	2.1
50314	0.971946	2.1
50315	0.971946	2.1
50316	1.28569	2.1
50317	1.1509	2.1
50318	0.699	2.1
50319	0.752185	2.1
50320	1.28568	2.1
50321	0.752186	2.1
50322	0.699001	2.1
50323	1.1509	2.1
50324	2.30181	2.1
50325	1.89226	2.1
50326	1.89226	2.1
50327	1.89226	2.1
50328	2.57137	2.1
50329	2.57137	2.1
50330	2.0815	2.1
50331	1.21962	2.1
50332	1.55173	2.1
50333	2.57849	2.1
50334	3.79001	2.1
50335	1.38779	2.1
50336	1.2923	2.1
50337	1.03974	2.1
50338	1.28832	2.1
50339	1.33566	2.1
50340	1.21116	2.1
50341	1.22343	2.1

50342	1.22343	2.1
50343	1.42288	2.1
50344	1.20138	2.1
50345	1.26025	2.1
50346	1.00644	2.1
50347	1.26066	2.1
50348	1.14271	2.1
50349	0.886635	2.1
50350	0.795277	2.1
50351	1.02966	2.1
50352	1.02967	2.1
50353	1.05216	2.1
50354	1.05216	2.1
50355	1.26068	2.1
50356	0.790042	2.1
50357	0.795269	2.1
50358	1.20135	2.1
50359	1.89226	0.622951
50360	1.89226	0.570044
50361	1.89226	0.63862
50362	1.89226	0.482446
50363	1.89226	0.67226
50364	1.89226	0.572669
50365	1.89227	0.527748
50366	1.89227	0.471293
50367	1.89227	0.544867
50368	1.89227	0.378237
50369	1.89227	0.607527
50370	1.89227	0.479056
50371	0.563873	0.318401
50372	0.50988	0.244339
50373	0.598693	0.446547
50374	0.558819	0.336278
50375	0.564855	0.303323
50376	0.564855	0.265376
50377	0.557838	0.405241
50378	0.557838	0.350443
50379	0.563873	0.34183
50380	0.598694	0.334232
50381	0.558819	0.312407
50382	0.50988	0.325594
50383	0.598693	0.423411
50384	0.558819	0.309764
50385	0.572737	0.561961
50386	0.534248	0.459508
50387	0.566788	0.431677
50388	0.566788	0.371074
50389	0.566153	0.512815
50390	0.566153	0.453007
50391	0.598694	0.447376
50392	0.572737	0.456629
50393	0.534248	0.42207
50394	0.558819	0.402622
50395	1.85217	0.810877
50396	1.93111	2.06895
50397	1.92875	0.699681
50398	2.02391	2.1
50399	3.85986	2.1

50400	1.93112	1.99324
50401	1.80425	2.1
50402	2.02392	2.1
50403	1.90981	2.1
50404	3.84092	2.1
50405	1.92876	0.640515
50406	2.02393	2.1
50407	2.05274	0.749342
50408	2.16783	2.1
50409	4.09659	1.39987
50410	2.02393	2.1
50411	1.90982	2.1
50412	2.16784	2.1
50413	2.05967	2.1
50414	4.07766	2.1
50415	1.89226	1.12864
50416	1.89226	1.01246
50417	1.89226	1.14898
50418	1.89226	1.08551
50419	3.78453	0.887879
50420	1.89226	0.875996
50421	1.89226	1.21965
50422	1.89226	1.80145
50423	2.44829	0.819649
50424	3.78453	1.67357
50425	1.89226	1.08528
50426	1.89226	1.24754
50427	1.89226	0.955789
50428	2.60452	0.900246
50429	3.78453	1.74687
50430	1.58723	0.103501
50431	1.88071	0.190681
50432	2.97376	0.101233
50433	2.13255	0.140284
50434	4.43982	0.138887
50435	2.20641	0.290351
50436	1.33575	0.413732
50437	2.15824	0.347283
50438	2.15824	0.344196
50439	1.2951	0.340059
50440	1.2951	0.351769
50441	0.825383	0.424525
50442	0.825383	0.385492
50443	0.856104	0.347928
50444	0.856104	0.34913
50445	0.952196	0.398596
50446	0.602363	0.355347
50447	0.667877	0.419791
50448	1.07913	0.358978
50449	0.602363	0.407696
50450	0.667876	0.412447
50451	1.07913	0.42002
50452	0.952197	0.386202
50453	2.57657	0.896991
50454	1.44437	2.1
50455	2.65378	2.1
50456	2.25076	1.4288
50457	1.84739	2.09792

50458	1.44437	1.03013
50459	2.0189	0.127448
50460	2.54752	0.130996
50461	2.63975	0.186392
50462	2.31049	0.0935814
50463	4.65865	0.131328
50464	1.40707	0.0880392
50465	1.89605	0.0787731
50466	2.0189	0.0874438
50467	2.0189	0.0759352
50468	1.50438	0.0985389
50469	1.50438	0.0864676
50470	2.63971	0.15996
50471	2.38246	0.0971864
50472	2.13402	0.114809
50473	2.57503	0.102543
50474	4.77373	0.11163
50475	1.50436	0.0811564
50476	1.96803	0.0796081
50477	2.13402	0.105583
50478	1.45446	0.0665097
50479	3.63838	0.0822308
50480	2.30793	0.394216
50481	2.11032	0.324234
50482	1.24082	0.32212
50483	1.05689	0.374559
50484	3.36482	0.38177
50485	1.89226	0.448314
50486	1.89226	0.473361
50487	1.89226	0.475008
50488	1.89226	0.452202
50489	3.78453	0.455435
50490	0.812967	0.401849
50491	0.812967	0.416084
50492	1.00181	0.403373
50493	1.08141	0.385489
50494	0.946132	0.369106
50495	0.812938	0.416062
50496	0.932815	0.427617
50497	1.05577	0.389355
50498	0.812938	0.402081
50499	0.932812	0.415994
50500	0.946133	0.420933
50501	0.946133	0.409661
50502	2.53897	2.1
50503	1.89226	2.1
50504	1.89226	2.1
50505	1.89226	2.1
50506	2.3245	2.1
50507	2.3245	2.1
50508	1.00255	0.896917
50509	1.06561	2.1
50510	1.05481	0.529922
50511	1.11077	2.1
50512	2.12042	1.26817
50513	1.89226	1.19728
50514	1.89226	2.1
50515	1.89226	0.614831

50516	1.89226	2.06111
50517	3.78453	1.00104
50518	1.89226	0.928356
50519	2.77314	2.1
50520	2.81276	0.805752
50521	2.81276	2.1
50522	1.89226	0.995837
50523	1.89226	2.1
50524	1.11077	2.1
50525	1.15114	2.09003
50526	1.10274	0.784828
50527	1.1032	1.22928
50528	1.05481	1.23597
50529	1.1032	0.868376
50530	1.89226	0.982868
50531	1.89226	2.07398
50532	1.89226	0.507742
50533	1.89226	2.1
50534	3.78452	0.989039
50535	0.721957	0.565227
50536	0.721957	0.568103
50537	0.946103	0.566804
50538	0.946103	0.526832
50539	0.814237	0.536588
50540	0.721929	0.498741
50541	0.946132	0.574713
50542	0.946132	0.582577
50543	0.721928	0.596153
50544	0.946131	0.6144
50545	0.946132	0.632632
50546	0.814237	0.612512
50547	1.89226	0.703184
50548	1.89226	0.573488
50549	1.89226	0.576369
50550	1.89226	0.58631
50551	1.89226	0.669159
50552	1.89226	0.68138
50553	1.37196	0.559142
50554	2.83556	0.447126
50555	1.5165	0.535639
50556	1.5165	0.569088
50557	2.74923	0.496223
50558	2.74923	0.559544
50559	0.946128	0.55271
50560	0.946128	0.575737
50561	0.827866	0.510213
50562	0.946132	0.540034
50563	0.94613	0.501898
50564	0.94613	0.516409
50565	0.94613	0.579606
50566	0.94613	0.579606
50567	0.94613	0.589346
50568	0.827864	0.619489
50569	0.94613	0.585413
50570	0.94613	0.678039
50571	1.36208	0.488861
50572	2.74923	0.33407
50573	1.37196	0.444663

50574	1.37196	0.485061
50575	2.53256	0.352942
50576	2.53256	0.410717
50577	1.07705	2.1
50578	1.07705	2.1
50579	1.08253	2.1
50580	1.08253	2.1
50581	0.762985	2.1
50582	0.781638	2.1
50583	1.37794	2.1
50584	1.40756	2.1
50585	1.37794	2.1
50586	0.762986	2.1
50587	0.781638	2.1
50588	1.40756	2.1
50589	2.69541	2.1
50590	2.75587	2.1
50591	1.48572	2.1
50592	1.48572	2.1
50593	1.52597	2.1
50594	2.73566	2.1
50595	2.0554	1.3848
50596	1.00387	0.825623
50597	2.01001	0.699901
50598	0.994002	0.691455
50599	3.01389	0.616413
50600	1.89226	2.1
50601	2.41579	2.1
50602	2.12209	2.1
50603	2.12209	2.1
50604	1.89226	2.1
50605	1.89226	2.1
50606	1.89226	2.1
50607	1.89226	2.1
50608	1.89226	2.1
50609	1.89226	2.1
50610	1.89226	2.1
50611	1.89226	2.1
50612	0.88209	2.1
50613	0.899471	2.1
50614	0.651501	2.1
50615	0.533531	2.1
50616	0.724105	2.1
50617	0.711243	2.1
50618	0.692622	2.1
50619	0.692622	2.1
50620	0.890668	2.1
50621	0.738066	2.1
50622	0.778223	2.1
50623	0.531121	2.1
50624	1.89226	2.1
50625	2.77504	2.1
50626	2.4158	2.1
50627	2.4158	2.1
50628	1.89226	2.1
50629	1.89226	2.1
50630	1.89226	2.1
50631	1.89226	2.1

50632	1.89226	2.1
50633	1.89226	2.1
50634	1.89226	2.1
50635	1.89226	2.1
50636	1.70303	2.1
50637	1.39832	2.1
50638	1.51749	2.1
50639	1.68783	2.1
50640	3.24844	2.1
50641	1.89226	2.1
50642	2.38311	2.1
50643	1.98321	2.1
50644	1.89226	2.1
50645	2.47406	2.1
50646	2.38311	2.1
50647	1.89226	2.1
50648	1.89226	2.1
50649	1.89226	2.1
50650	1.89226	2.1
50651	1.89226	2.1
50652	1.89226	2.1
50653	0.946131	2.1
50654	0.946131	2.1
50655	1.25167	2.1
50656	1.19156	2.1
50657	1.19156	2.1
50658	1.19156	2.1
50659	0.946133	2.1
50660	0.946133	2.1
50661	0.946131	2.1
50662	1.25167	2.1
50663	0.946131	2.1
50664	1.19156	2.1
50665	1.89226	2.1
50666	2.47406	2.1
50667	1.99996	2.1
50668	1.89226	2.1
50669	2.58176	2.1
50670	2.47406	2.1
50671	1.89226	2.1
50672	1.89226	2.1
50673	1.89226	2.1
50674	1.89226	2.1
50675	1.89226	2.1
50676	1.89226	2.1
50677	0.946131	2.1
50678	0.946131	2.1
50679	1.32241	2.1
50680	1.25167	2.1
50681	1.25167	2.1
50682	1.25167	2.1
50683	0.946133	2.1
50684	0.946133	2.1
50685	0.946131	2.1
50686	1.32241	2.1
50687	0.946131	2.1
50688	1.25167	2.1
50689	1.65151	2.1

50690	1.92471	2.1
50691	1.81834	2.1
50692	2.13272	2.1
50693	3.74306	2.1
50694	1.92472	2.1
50695	1.9943	2.1
50696	2.13273	2.1
50697	2.24475	2.1
50698	4.16947	2.1
50699	1.81834	2.1
50700	2.13272	2.1
50701	2.02687	2.1
50702	2.39605	2.1
50703	4.21439	2.1
50704	2.13273	2.1
50705	2.24475	2.1
50706	2.39605	2.1
50707	2.54903	2.1
50708	4.6408	2.1
50709	1.10534	2.1
50710	1.22993	2.1
50711	0.946132	2.1
50712	0.946132	2.1
50713	0.946132	2.1
50714	0.946132	2.1
50715	1.22993	2.1
50716	1.22993	2.1
50717	1.10534	2.1
50718	0.946131	2.1
50719	0.946132	2.1
50720	1.22993	2.1
50721	1.47221	2.1
50722	2.14647	2.1
50723	1.95567	2.1
50724	1.95567	2.1
50725	1.71082	2.1
50726	1.71082	2.1
50727	0.946131	2.1
50728	0.946131	2.1
50729	0.946131	2.1
50730	0.946131	2.1
50731	0.946132	2.1
50732	0.946132	2.1
50733	0.946131	2.1
50734	0.946131	2.1
50735	0.946131	2.1
50736	0.946131	2.1
50737	0.946132	2.1
50738	0.946132	2.1
50739	0.998086	2.1
50740	1.09429	2.1
50741	0.946125	2.1
50742	0.946125	2.1
50743	0.90732	2.1
50744	0.90732	2.1
50745	1.14414	2.1
50746	1.16553	2.1
50747	1.05018	2.1

50748	0.907332	2.1
50749	0.99482	2.1
50750	0.851529	2.1
50751	0.80534	2.1
50752	0.80534	2.1
50753	0.812206	2.1
50754	0.812206	2.1
50755	0.895134	2.1
50756	0.977828	2.1
50757	0.736099	2.1
50758	0.639718	2.1
50759	0.639722	2.1
50760	0.89514	2.1
50761	0.736095	2.1
50762	0.977821	2.1
50763	0.946125	2.1
50764	0.946125	2.1
50765	0.946125	2.1
50766	0.946125	2.1
50767	0.946125	2.1
50768	0.946125	2.1
50769	0.946124	2.1
50770	0.946124	2.1
50771	0.946131	2.1
50772	0.946131	2.1
50773	0.946118	2.1
50774	0.946118	2.1
50775	2.22622	0.05
50776	2.43347	0.05
50777	2.34108	0.05
50778	1.24587	2.1
50779	1.21674	2.1
50780	1.14533	2.1
50781	1.11311	2.1
50782	1.17874	2.1
50783	1.17874	2.1
50784	1.18024	2.1
50785	1.18024	2.1
50786	1.14533	2.1
50787	1.24587	2.1
50788	1.21674	2.1
50789	1.11311	2.1
50790	2.43347	2.1
50791	2.34107	2.1
50792	2.39663	2.1
50793	2.49174	2.1
50794	4.8301	2.1
50795	1.86206	2.1
50796	1.41058	2.1
50797	1.70302	2.1
50798	2.42083	2.1
50799	3.68129	2.1
50800	1.70302	2.1
50801	2.43549	2.1
50802	2.20075	2.1
50803	2.50358	2.1
50804	2.04916	2.1
50805	2.33113	2.1

50806	1.07624	2.1
50807	2.12621	2.1
50808	1.57297	2.1
50809	1.60383	2.1
50810	1.30047	2.1
50811	1.30047	2.1
50812	2.33379	2.08223
50813	2.56149	1.1299
50814	2.36734	1.11918
50815	2.62796	1.1057
50816	4.92883	1.24029
50817	2.5615	1.21883
50818	2.57957	1.32234
50819	2.62796	1.09536
50820	2.69056	1.04574
50821	5.25206	1.56774
50822	2.39322	1.24885
50823	2.62796	1.78135
50824	2.6849	1.9892
50825	2.6849	1.64654
50826	2.36734	1.95969
50827	2.36734	1.73968
50828	2.62796	1.55393
50829	2.69056	1.65861
50830	2.6849	0.88214
50831	2.77227	0.794471
50832	5.37546	0.972116
50833	1.89226	0.05
50834	1.89226	0.0503144
50835	1.89226	0.05
50836	1.89226	0.05
50837	1.89226	0.05
50838	1.89226	0.05
50839	1.06974	0.05
50840	1.06974	0.05
50841	1.02299	0.05
50842	1.02299	0.05
50843	1.35299	0.05
50844	1.26227	0.05
50845	0.646252	0.05
50846	0.830464	0.05
50847	0.830463	0.05
50848	1.35299	0.05
50849	1.26227	0.05
50850	0.646252	0.05
50851	2.52454	0.05
50852	1.89226	0.05
50853	2.70598	0.05
50854	1.89226	0.05
50855	4.59824	0.05
50856	1.89226	0.05
50857	1.89226	0.0580891
50858	1.89226	0.05
50859	1.89226	0.05
50860	1.89226	0.0512614
50861	1.89226	0.05
50862	1.89226	0.05
50863	1.89226	0.05

50864	1.89226	0.05
50865	1.89226	0.05
50866	1.89226	0.05
50867	1.89226	0.05
50868	1.7666	0.05
50869	2.70598	0.05
50870	2.65378	0.05
50871	2.65378	0.05
50872	1.66093	0.05
50873	1.66093	0.05
50874	2.70598	0.05
50875	1.89226	0.05
50876	1.89226	0.05
50877	1.89226	0.05
50878	2.65377	0.05
50879	2.65377	0.05
50880	0.946132	0.05
50881	0.946132	0.05
50882	0.946131	0.05
50883	0.946131	0.0529892
50884	0.946131	0.0515645
50885	0.946131	0.05
50886	0.946132	0.0514795
50887	0.946132	0.05
50888	0.946132	0.05
50889	0.946132	0.05
50890	0.946131	0.05
50891	0.946131	0.05
50892	1.69858	2.1
50893	2.18238	2.1
50894	2.506	2.1
50895	1.93586	2.1
50896	4.20458	2.1
50897	2.21772	2.1
50898	2.18238	2.1
50899	2.506	2.1
50900	2.59215	2.1
50901	4.72372	2.1
50902	2.50596	2.1
50903	1.93584	2.1
50904	2.16728	2.1
50905	2.7679	2.1
50906	4.67325	2.1
50907	1.53296	2.1
50908	1.56299	2.1
50909	1.42624	2.1
50910	1.52961	2.1
50911	2.98923	2.1
50912	1.56299	2.1
50913	1.46568	2.1
50914	1.52961	2.1
50915	1.51916	2.1
50916	3.08215	2.1
50917	1.36226	2.1
50918	1.52961	2.1
50919	1.36226	2.1
50920	1.42624	2.1
50921	1.55369	2.1

50922	1.61768	2.1
50923	1.52961	2.1
50924	1.51916	2.1
50925	1.55369	2.1
50926	1.64892	2.1
50927	3.07285	2.1
50928	2.13405	0.104273
50929	2.57507	0.107952
50930	2.39438	0.15577
50931	1.93408	0.0886251
50932	4.52843	0.108682
50933	2.13405	0.0959734
50934	1.27778	0.0681741
50935	1.2545	0.0790647
50936	1.45448	0.0852006
50937	1.93408	0.0744804
50938	2.13405	0.0799804
50939	2.39438	0.136845
50940	1.93407	0.088945
50941	1.60578	0.104623
50942	1.87985	0.101561
50943	4.00016	0.108022
50944	1.0525	2.1
50945	1.05758	2.1
50946	1.43484	0.923631
50947	1.38506	1.87036
50948	2.48734	1.93303
50949	1.20455	2.1
50950	1.67352	2.1
50951	1.52852	2.1
50952	2.05164	2.1
50953	3.20203	2.1
50954	1.89226	2.09917
50955	1.89226	2.1
50956	1.89226	2.1
50957	1.89226	2.1
50958	3.78452	2.1
50959	1.89226	2.1
50960	1.89226	2.1
50961	1.89226	2.1
50962	1.89226	2.1
50963	1.89226	2.1
50964	1.89226	1.9042
50965	2.05165	2.1
50966	1.94291	2.1
50967	2.09398	2.1
50968	1.67352	2.1
50969	2.36337	2.1
50970	1.94291	2.1
50971	1.52851	2.1
50972	2.52049	2.1
50973	2.43433	2.1
50974	2.05164	2.1
50975	1.9112	2.1
50976	1.52851	2.1
50977	1.89226	2.1
50978	1.89226	1.31771
50979	1.89226	2.1

50980	1.89226	2.1
50981	3.78451	2.1
50982	1.89226	1.70698
50983	1.89226	2.1
50984	1.89226	2.1
50985	1.89226	1.11796
50986	3.78452	2.1
50987	2.5205	2.1
50988	2.36337	2.1
50989	2.4605	2.1
50990	2.05165	2.1
50991	2.77222	2.1
50992	2.36337	2.1
50993	1.89226	2.1
50994	1.89226	2.1
50995	1.89226	2.1
50996	1.89226	2.1
50997	1.89226	2.1
50998	1.89226	2.1
50999	1.89226	2.1
51000	1.89226	2.1
51001	1.89226	2.1
51002	1.89226	2.1
51003	3.78452	2.1
51004	1.38905	2.1
51005	1.26166	2.1
51006	1.19216	2.1
51007	1.0875	2.1
51008	1.24393	2.1
51009	1.24393	2.1
51010	1.23261	2.1
51011	1.23261	2.1
51012	1.38905	2.1
51013	1.19216	2.1
51014	1.26166	2.1
51015	1.0875	2.1
51016	1.16233	2.1
51017	1.06028	2.1
51018	0.777395	2.1
51019	0.705508	2.1
51020	0.940018	2.1
51021	0.936465	2.1
51022	0.954216	2.1
51023	0.954216	2.1
51024	1.16125	2.1
51025	0.685505	2.1
51026	1.1157	2.1
51027	0.703534	2.1
51028	1.89226	2.1
51029	1.89226	2.1
51030	1.89226	2.1
51031	1.89226	2.1
51032	1.89226	2.1
51033	1.89226	2.1
51034	1.89226	2.1
51035	1.89226	2.1
51036	1.89226	2.1
51037	1.89226	2.1

51038	1.89226	2.1
51039	1.89226	2.1
51040	2.38433	2.1
51041	1.43456	2.1
51042	1.93881	2.1
51043	2.36952	2.1
51044	4.32313	2.1
51045	1.8493	2.1
51046	1.5927	2.1
51047	1.45535	2.1
51048	1.38438	2.1
51049	1.89657	2.1
51050	1.8493	2.1
51051	2.0815	2.1
51052	1.6697	2.1
51053	2.0657	2.1
51054	1.5927	2.1
51055	2.59749	2.1
51056	2.0815	2.1
51057	2.76597	2.1
51058	1.89226	2.1
51059	1.89226	2.1
51060	1.89226	2.1
51061	2.6126	2.1
51062	2.6126	2.1
51063	2.62513	2.1
51064	1.89226	2.1
51065	1.89226	2.1
51066	1.89226	2.1
51067	2.76598	2.1
51068	2.76598	2.1
51069	2.54665	2.1
51070	2.6126	2.1
51071	2.76598	2.1
51072	2.76598	2.1
51073	2.34044	2.1
51074	2.34044	2.1
51075	2.62514	2.1
51076	2.76598	2.1
51077	2.54666	2.1
51078	2.45212	2.1
51079	5.2181	2.1
51080	2.14063	2.1
51081	1.47521	2.1
51082	1.27679	2.1
51083	1.47521	2.1
51084	2.11511	2.1
51085	2.31353	2.1
51086	0.93256	2.1
51087	1.07032	2.1
51088	0.638396	2.1
51089	0.521342	2.1
51090	0.790674	2.1
51091	0.790674	2.1
51092	0.80098	2.1
51093	0.800981	2.1
51094	0.932535	2.1
51095	0.521328	2.1

51096	1.07034	2.1
51097	0.638414	2.1
51098	1.89226	2.1
51099	1.89226	1.21454
51100	1.89226	1.28288
51101	1.89226	2.1
51102	3.78453	1.53651
51103	1.89226	2.09762
51104	1.9842	2.1
51105	1.89226	1.93037
51106	1.89226	2.1
51107	2.79705	2.07108
51108	2.79705	2.1
51109	0.469955	2.1
51110	0.791665	2.1
51111	0.989115	1.42319
51112	0.763235	2.09851
51113	0.776356	1.79188
51114	0.805461	2.1
51115	0.776759	1.70333
51116	0.776759	2.1
51117	0.799151	2.06511
51118	0.940869	2.1
51119	0.78449	1.99404
51120	0.489635	2.00625
51121	1.89226	1.14606
51122	1.89226	1.98677
51123	1.89226	1.16883
51124	1.89226	2.09666
51125	1.89226	1.10236
51126	1.89226	2.1
51127	2.46058	1.79652
51128	2.28991	2.1
51129	1.8525	1.87223
51130	1.87697	2.1
51131	2.28991	1.71197
51132	1.62397	2.1
51133	0.946131	0.0695251
51134	0.974033	0.0668195
51135	1.31667	0.0757358
51136	0.946131	0.0811352
51137	0.885497	0.07719
51138	0.885497	0.0735932
51139	1.0468	0.0706261
51140	1.04678	0.0697815
51141	1.31667	0.0770595
51142	0.974019	0.0670086
51143	0.946131	0.0727642
51144	0.946131	0.0681416
51145	1.89226	0.0860399
51146	1.89226	0.158888
51147	1.89226	0.147659
51148	1.89226	0.118479
51149	3.78452	0.147111
51150	0.946125	0.166793
51151	0.946125	0.154121
51152	0.946131	0.175495
51153	0.946131	0.194728

51154	0.946128	0.193634
51155	0.946128	0.171064
51156	0.946128	0.130436
51157	0.946128	0.157577
51158	0.946128	0.171857
51159	0.946128	0.165867
51160	0.946128	0.170422
51161	0.946128	0.169686
51162	0.946131	0.0882133
51163	0.98453	0.0840399
51164	1.32276	0.120987
51165	0.946131	0.127842
51166	0.946132	0.110934
51167	0.946132	0.103252
51168	0.98453	0.0982374
51169	1.32276	0.0965492
51170	1.32276	0.113257
51171	1.32276	0.0799939
51172	0.946132	0.103387
51173	0.946132	0.0818519
51174	1.89227	2.1
51175	1.89227	2.1
51176	1.89227	2.1
51177	1.89227	2.1
51178	1.89227	2.1
51179	1.89227	2.1
51180	1.89226	2.1
51181	1.89226	2.1
51182	1.89226	2.1
51183	1.89226	2.1
51184	1.89226	2.1
51185	1.89226	2.1
51186	1.05556	2.1
51187	1.38859	2.1
51188	1.46996	2.1
51189	1.25364	2.1
51190	2.52552	2.1
51191	0.694296	2.1
51192	0.73498	2.1
51193	0.561881	2.1
51194	0.512189	2.1
51195	0.621304	2.1
51196	0.621304	2.1
51197	0.625865	2.1
51198	0.625865	2.1
51199	0.734979	2.1
51200	0.561881	2.1
51201	0.51219	2.1
51202	0.694296	2.1
51203	0.946126	2.1
51204	0.946126	2.1
51205	0.946126	2.1
51206	0.946126	2.1
51207	0.946126	2.1
51208	0.946126	2.1
51209	0.946126	2.1
51210	0.946126	2.1
51211	0.946132	2.1

51212	0.946132	2.1
51213	0.94612	2.1
51214	0.94612	2.1
51215	1.89225	2.1
51216	1.89225	2.1
51217	1.89225	2.1
51218	1.89225	2.1
51219	1.89225	2.1
51220	1.89225	2.1
51221	0.626814	2.1
51222	0.632155	2.1
51223	0.746234	2.1
51224	0.734974	2.1
51225	0.68202	2.1
51226	0.68202	2.1
51227	0.685108	2.1
51228	0.685108	2.1
51229	0.746239	2.1
51230	0.632159	2.1
51231	0.734969	2.1
51232	0.62681	2.1
51233	0.746235	2.1
51234	0.734975	2.1
51235	0.595081	2.1
51236	0.561877	2.1
51237	0.648584	2.1
51238	0.648584	2.1
51239	0.659528	2.1
51240	0.659528	2.1
51241	0.746239	2.1
51242	0.595085	2.1
51243	0.734969	2.1
51244	0.561874	2.1
51245	1.89249	2.1
51246	2.2137	2.1
51247	1.33125	2.1
51248	1.06499	2.1
51249	2.12571	2.1
51250	1.78918	2.1
51251	0.946131	2.1
51252	0.946131	2.1
51253	0.94613	2.1
51254	0.94613	2.1
51255	0.946132	2.1
51256	0.946132	2.1
51257	0.946129	2.1
51258	0.946129	2.1
51259	0.946131	2.1
51260	0.946131	2.1
51261	0.946131	2.1
51262	0.946131	2.1
51263	1.89227	2.1
51264	1.89227	2.1
51265	1.89227	2.1
51266	1.89227	2.1
51267	3.78453	2.1
51268	1.89226	2.1
51269	1.89226	2.1

51270	1.89226	2.1
51271	1.89226	2.1
51272	1.89226	2.1
51273	1.89226	2.1
51274	2.01388	2.1
51275	1.38757	2.1
51276	1.24229	2.1
51277	1.3309	2.1
51278	1.88011	2.1
51279	2.01388	2.1
51280	2.01388	2.1
51281	2.01555	2.1
51282	2.13879	2.1
51283	1.80766	2.1
51284	4.15267	2.1
51285	0.946133	2.1
51286	0.946133	2.1
51287	0.946132	2.1
51288	0.946132	2.1
51289	0.946132	2.1
51290	0.946132	2.1
51291	0.946133	2.1
51292	0.946133	2.1
51293	0.946132	2.1
51294	0.946132	2.1
51295	0.946132	2.1
51296	0.946132	2.1
51297	1.89226	2.1
51298	1.89226	2.1
51299	1.89226	2.1
51300	1.89226	2.1
51301	1.89226	2.1
51302	1.89226	2.1
51303	0.676745	2.1
51304	0.68382	2.1
51305	1.07048	2.1
51306	1.1327	2.1
51307	0.929324	2.1
51308	0.871438	2.1
51309	0.867362	2.1
51310	0.867362	2.1
51311	1.10666	2.1
51312	0.695036	2.1
51313	0.744098	2.1
51314	1.10144	2.1
51315	2.25168	2.1
51316	2.22499	2.1
51317	2.15929	2.1
51318	2.13879	2.1
51319	2.15929	2.1
51320	2.13879	2.1
51321	2.15314	2.1
51322	1.70302	2.1
51323	2.24694	2.1
51324	1.65245	2.1
51325	3.82722	2.1
51326	1.65245	2.1
51327	1.00371	2.1

51328	1.7437	2.1
51329	1.65748	2.1
51330	1.10459	2.1
51331	1.00371	2.1
51332	2.24694	2.1
51333	1.65246	2.1
51334	2.69773	2.1
51335	1.88129	2.1
51336	4.12824	2.1
51337	1.65246	2.1
51338	1.10459	2.1
51339	1.88129	2.1
51340	1.05914	2.1
51341	2.76496	2.1
51342	1.89226	2.1
51343	1.89226	2.1
51344	1.89226	2.1
51345	1.89226	2.1
51346	3.78452	2.1
51347	0.718577	2.1
51348	0.718577	2.1
51349	0.725249	2.1
51350	0.725249	2.1
51351	0.597916	2.1
51352	0.61752	2.1
51353	0.826306	2.1
51354	0.859194	2.1
51355	0.826306	2.1
51356	0.597916	2.1
51357	0.859194	2.1
51358	0.61752	2.1
51359	1.25898	2.1
51360	1.25898	2.1
51361	0.946128	2.1
51362	0.946128	2.1
51363	1.25897	2.1
51364	1.25585	2.1
51365	0.946131	2.1
51366	0.946131	2.1
51367	0.946129	2.1
51368	1.25586	2.1
51369	1.25898	2.1
51370	0.946129	2.1
51371	1.89226	2.04671
51372	1.89226	2.1
51373	1.89226	2.1
51374	1.89226	2.1
51375	3.78452	2.1
51376	0.693258	2.1
51377	0.693258	1.86723
51378	0.697356	2.09375
51379	0.697356	2.1
51380	0.572479	2.1
51381	0.597916	2.1
51382	0.792698	1.95468
51383	0.826306	2.08155
51384	0.572478	2.1
51385	0.792697	2.1

51386	0.826307	2.1
51387	0.597917	2.1
51388	1.25586	2.1
51389	1.25586	2.1
51390	0.946128	2.1
51391	0.946128	2.1
51392	1.25585	2.1
51393	1.24344	2.1
51394	0.946131	2.1
51395	0.946131	2.1
51396	0.946129	2.1
51397	1.24344	2.1
51398	0.94613	2.1
51399	1.25586	2.1
51400	1.65151	2.1
51401	1.81835	2.1
51402	1.12064	2.1
51403	1.12064	2.1
51404	1.24965	2.1
51405	1.81835	2.1
51406	1.24965	2.1
51407	1.81835	2.1
51408	1.41077	2.1
51409	2.02687	2.1
51410	3.22911	2.1
51411	1.89122	1.01053
51412	1.38776	2.02962
51413	2.37014	0.888167
51414	1.80818	2.1
51415	3.6994	1.66128
51416	2.15466	0.873175
51417	2.00083	2.02209
51418	1.80818	1.31936
51419	1.80818	0.906061
51420	2.31988	1.69673
51421	2.31988	0.915412
51422	1.80818	0.768875
51423	1.18041	2.1
51424	2.00082	1.05036
51425	1.43652	2.1
51426	3.18123	1.86876
51427	1.89226	0.942589
51428	1.89226	1.53012
51429	1.89226	1.03759
51430	1.89226	0.940352
51431	3.78453	1.02572
51432	1.89226	1.46877
51433	1.89226	1.48906
51434	1.89226	1.01122
51435	1.89226	1.06769
51436	3.78452	1.61589
51437	0.80759	1.20881
51438	0.686119	1.569
51439	0.972147	1.28242
51440	1.00217	1.49105
51441	0.821334	1.90573
51442	0.821334	1.57244
51443	0.866957	1.22061

51444	0.866957	1.21896
51445	0.972145	1.67288
51446	0.686121	2.1
51447	0.807592	1.77063
51448	1.00217	2.1
51449	0.972146	1.27858
51450	1.00217	1.50295
51451	1.26654	1.71091
51452	1.1908	1.28978
51453	1.05501	1.46377
51454	1.05501	2.1
51455	1.10794	1.2176
51456	1.10794	0.865898
51457	0.972146	0.894279
51458	1.26654	1.50988
51459	1.00217	1.26371
51460	1.1908	1.95898
51461	1.89226	1.18912
51462	1.89226	0.873962
51463	1.89226	0.940236
51464	1.89226	1.71308
51465	1.89226	0.786649
51466	1.89226	1.0896
51467	0.972147	1.38314
51468	0.867854	1.54533
51469	1.26132	1.09903
51470	1.26654	1.48799
51471	1.0424	1.69753
51472	1.0424	2.05422
51473	1.092	0.841864
51474	1.092	1.37154
51475	1.26132	1.52682
51476	0.867854	2.1
51477	0.972147	1.82715
51478	1.26654	2.1
51479	1.4375	0.368457
51480	1.92998	0.314899
51481	1.66953	0.184399
51482	1.0953	0.283845
51483	3.10703	0.242987
51484	1.89226	2.1
51485	1.89226	2.1
51486	1.89226	2.1
51487	1.89226	2.1
51488	3.78452	2.1
51489	2.74034	2.1
51490	2.08808	2.1
51491	2.63859	2.1
51492	2.00343	2.1
51493	4.72667	2.1
51494	0.799825	2.1
51495	0.799825	2.1
51496	0.80211	2.1
51497	0.80211	2.1
51498	0.562428	2.1
51499	0.600223	2.1
51500	1.04404	2.1
51501	1.00171	2.1

51502	1.00171	2.1
51503	0.56243	2.1
51504	0.600225	2.1
51505	1.04404	2.1
51506	0.94613	2.1
51507	0.94613	2.1
51508	0.946128	2.1
51509	0.946128	2.1
51510	0.946127	2.1
51511	0.946127	2.1
51512	0.946131	2.1
51513	0.946131	2.1
51514	0.946129	2.1
51515	0.946129	2.1
51516	0.946129	2.1
51517	0.946129	2.1
51518	1.89226	2.1
51519	1.89226	2.1
51520	1.89226	2.1
51521	1.89226	2.1
51522	3.78452	2.1
51523	2.63859	2.1
51524	2.00343	2.1
51525	2.54388	2.1
51526	1.91548	2.1
51527	4.55407	2.1
51528	1.91548	2.1
51529	2.00342	2.1
51530	1.04909	2.1
51531	1.04909	2.1
51532	1.12486	2.1
51533	1.99124	2.1
51534	0.810942	2.1
51535	0.810942	2.1
51536	0.810942	2.1
51537	0.810942	2.1
51538	1.89226	2.1
51539	1.35169	2.1
51540	1.08131	2.1
51541	1.08132	2.1
51542	1.08132	2.1
51543	0.810944	2.1
51544	0.810944	2.1
51545	1.89226	0.443734
51546	1.89226	0.619713
51547	1.89226	0.412735
51548	1.89226	0.684557
51549	3.78452	0.501281
51550	1.7968	0.407207
51551	1.49831	0.600486
51552	1.59244	0.407618
51553	1.3395	0.546638
51554	3.09074	0.490894
51555	1.49831	0.570026
51556	1.16858	1.5364
51557	1.33951	0.421325
51558	1.0361	0.920103
51559	2.53442	0.660937

51560	1.89226	0.589251
51561	1.89226	0.569261
51562	1.89226	0.606687
51563	1.89226	1.84062
51564	1.89226	1.31101
51565	1.89226	1.17849
51566	1.89223	0.411572
51567	1.89223	0.584023
51568	1.89223	0.498367
51569	1.89223	0.605917
51570	1.89223	0.415468
51571	1.89223	0.565617
51572	1.59241	0.399405
51573	1.18952	0.572208
51574	1.33949	0.542887
51575	1.33949	0.422091
51576	1.40538	0.553718
51577	1.40538	0.408225
51578	1.89226	0.354397
51579	1.89226	0.442091
51580	1.89226	0.383528
51581	1.89226	0.426116
51582	1.89226	0.412026
51583	1.89226	0.459935
51584	1.89226	2.1
51585	1.89226	2.1
51586	1.89226	2.1
51587	1.89226	2.1
51588	3.78452	2.1
51589	1.89226	2.1
51590	1.89226	2.1
51591	1.89226	2.1
51592	1.89226	2.1
51593	3.78452	2.1
51594	2.01867	2.1
51595	1.46955	2.1
51596	1.58476	2.1
51597	1.38189	2.1
51598	2.22153	2.1
51599	2.01867	2.1
51600	2.01867	2.1
51601	2.52881	2.1
51602	2.21732	2.1
51603	2.21732	2.1
51604	2.22154	2.1
51605	2.22154	2.1
51606	1.89226	2.1
51607	1.89226	2.1
51608	1.89226	2.1
51609	1.89226	2.1
51610	1.89226	2.1
51611	1.89226	2.1
51612	1.89226	2.1
51613	1.89226	2.1
51614	1.89226	2.1
51615	1.89226	2.1
51616	1.89226	2.1
51617	1.89226	2.1

51618	2.22153	2.1
51619	1.63361	2.1
51620	1.77336	2.1
51621	1.46955	2.1
51622	2.52534	2.1
51623	2.22153	2.1
51624	2.34431	2.1
51625	1.62905	2.1
51626	1.49693	2.1
51627	2.19197	2.1
51628	3.82103	2.1
51629	1.89226	2.1
51630	1.89226	2.1
51631	1.89226	2.1
51632	1.89226	2.1
51633	3.78453	2.1
51634	0.946128	2.1
51635	0.946128	2.1
51636	1.22372	2.1
51637	1.17331	2.1
51638	0.946131	2.1
51639	0.946131	2.1
51640	1.17331	2.1
51641	1.17331	2.1
51642	0.946131	2.1
51643	1.17331	2.1
51644	1.22372	2.1
51645	0.94613	2.1
51646	1.04041	2.1
51647	1.09599	2.1
51648	0.748462	2.1
51649	0.701976	2.1
51650	0.896701	2.1
51651	0.896701	2.1
51652	0.901262	2.1
51653	0.901262	2.1
51654	1.04041	2.1
51655	0.701977	2.1
51656	0.748463	2.1
51657	1.09599	2.1
51658	1.89226	2.1
51659	1.89226	2.1
51660	1.89226	2.1
51661	1.89226	2.1
51662	3.78453	2.1
51663	0.946128	2.1
51664	0.946128	2.1
51665	1.17331	2.1
51666	1.13785	2.1
51667	0.94613	2.1
51668	0.94613	2.1
51669	1.13785	2.1
51670	1.13785	2.1
51671	1.13785	2.1
51672	0.94613	2.1
51673	0.94613	2.1
51674	1.17331	2.1
51675	2.50381	2.1

51676	1.89226	2.1
51677	1.89226	2.1
51678	2.73387	2.1
51679	4.39607	2.1
51680	1.80045	2.1
51681	1.99777	0.670869
51682	2.45134	1.13395
51683	2.12463	1.56823
51684	1.70302	1.15125
51685	1.42655	2.1
51686	2.20231	2.1
51687	1.70302	0.893548
51688	1.93128	2.1
51689	1.66768	1.39358
51690	1.24499	0.671396
51691	1.70302	0.667866
51692	2.55572	0.323476
51693	1.22811	0.331374
51694	1.82584	0.330536
51695	1.22811	0.328002
51696	2.62764	0.328039
51697	1.93147	0.326103
51698	2.14513	0.3335
51699	1.89226	0.46162
51700	2.66481	0.347417
51701	1.89226	0.360115
51702	2.66481	0.473073
51703	1.89226	0.46996
51704	2.11649	2.1
51705	1.89226	2.1
51706	1.89226	2.1
51707	1.89226	2.1
51708	2.15426	2.1
51709	2.15426	2.1
51710	2.06776	2.1
51711	1.89226	2.1
51712	1.89226	2.1
51713	1.89226	2.1
51714	2.11649	2.1
51715	2.11649	2.1
51716	1.89226	2.1
51717	1.89226	2.1
51718	1.89226	2.1
51719	1.89226	2.1
51720	1.89226	2.1
51721	1.89226	2.1
51722	0.998134	2.1
51723	0.918904	2.1
51724	0.894708	2.1
51725	0.758742	2.1
51726	0.864276	2.1
51727	0.864276	2.1
51728	0.8926	2.1
51729	0.8926	2.1
51730	0.998134	2.1
51731	0.894709	2.1
51732	0.758742	2.1
51733	0.918904	2.1

51734	1.89226	2.1
51735	1.89226	2.1
51736	1.89226	2.1
51737	1.89226	2.1
51738	1.89226	2.1
51739	1.89226	2.1
51740	1.89226	2.1
51741	1.89226	2.1
51742	1.89226	2.1
51743	1.89226	2.1
51744	1.89226	2.1
51745	1.89226	2.1
51746	1.99627	2.1
51747	1.61047	2.1
51748	2.0815	2.1
51749	2.07569	2.1
51750	3.75063	2.1
51751	0.848077	2.1
51752	1.07748	2.1
51753	0.802498	2.1
51754	0.626808	2.1
51755	0.875552	2.1
51756	0.737527	2.1
51757	0.835602	2.1
51758	0.835602	2.1
51759	0.983245	2.1
51760	0.781108	2.1
51761	0.872306	2.1
51762	0.601497	2.1
51763	2.74745	2.1
51764	2.73988	2.1
51765	2.6521	2.1
51766	2.67792	2.1
51767	5.39198	2.1
51768	2.73988	2.1
51769	2.57466	2.1
51770	2.67791	2.1
51771	2.55653	2.1
51772	5.29641	2.1
51773	2.6521	2.1
51774	2.64148	2.1
51775	2.67792	2.1
51776	2.67792	2.1
51777	2.56953	2.1
51778	2.56953	2.1
51779	2.64148	2.1
51780	2.55653	2.1
51781	2.57929	2.1
51782	2.57929	2.1
51783	2.67792	2.1
51784	2.67792	2.1
51785	1.89226	0.511261
51786	1.89226	0.441355
51787	1.89226	0.598738
51788	1.89226	0.117663
51789	3.78453	0.455327
51790	1.68703	0.510541
51791	2.50385	0.30584

51792	1.45958	0.227264
51793	2.0815	0.14509
51794	3.93806	0.321925
51795	2.50385	0.296841
51796	2.57414	0.387271
51797	2.0815	0.353998
51798	2.39921	0.398535
51799	4.91181	0.268497
51800	1.89226	0.445002
51801	1.89226	0.384142
51802	1.89226	0.209505
51803	1.89226	0.349418
51804	3.78453	0.367064
51805	1.89226	0.61683
51806	1.89226	0.259089
51807	1.89226	0.448499
51808	1.89226	0.457348
51809	1.89226	0.212725
51810	1.89226	0.522134
51811	1.23013	0.212451
51812	2.0815	0.311468
51813	1.90167	0.472902
51814	1.71243	0.377831
51815	1.45887	0.309024
51816	1.31369	0.255744
51817	2.0815	0.356645
51818	2.39921	0.313023
51819	1.7115	0.0734311
51820	2.2421	0.360286
51821	4.09829	0.366286
51822	1.89227	0.323245
51823	1.89227	0.356137
51824	1.89227	0.416133
51825	1.89227	0.268558
51826	3.78453	0.304649
51827	2.39697	0.05
51828	1.85594	0.05
51829	1.91161	0.05
51830	2.49867	0.05
51831	4.35461	0.05
51832	1.12035	0.05
51833	1.85594	0.05
51834	1.91161	0.05
51835	1.15483	0.05
51836	3.01077	0.05
51837	1.89226	0.05
51838	1.89226	0.0546612
51839	1.89226	0.05
51840	1.89226	0.05
51841	1.89226	0.05
51842	1.89226	0.05
51843	1.89226	0.0511603
51844	1.89226	0.0590579
51845	1.89226	0.0565891
51846	1.89226	0.05
51847	1.89226	0.0623466
51848	1.89226	0.056912
51849	0.936516	0.05

51850	1.03496	0.05
51851	1.22607	0.0523592
51852	1.28058	0.05
51853	1.06317	0.05
51854	1.06317	0.05
51855	1.19735	0.05
51856	1.0813	0.05
51857	0.858168	0.05
51858	1.32278	0.05
51859	0.957674	0.05
51860	1.25178	0.05
51861	1.92659	0.05
51862	0.998957	0.05
51863	0.998957	0.05
51864	1.15481	0.05
51865	1.6031	0.05
51866	1.74459	0.05
51867	0.946119	0.05
51868	0.946119	0.05
51869	0.946118	0.05
51870	0.946118	0.05
51871	0.946118	0.05
51872	0.946118	0.05
51873	0.946119	0.05
51874	0.946119	0.05
51875	0.946131	0.05
51876	0.946131	0.05
51877	0.946106	0.05
51878	0.946106	0.05
51879	0.946118	0.0515256
51880	0.946118	0.059654
51881	0.946119	0.0516199
51882	0.946119	0.0593871
51883	0.946118	0.0603502
51884	0.946118	0.0580383
51885	0.946119	0.0515147
51886	0.946119	0.0501878
51887	0.946131	0.0529411
51888	0.946131	0.0528266
51889	0.946106	0.0546337
51890	0.946106	0.0529484
51891	2.47164	0.565599
51892	2.83689	0.380014
51893	2.73872	0.573061
51894	2.41632	0.379264
51895	5.21036	0.392885
51896	0.946133	0.520183
51897	0.946133	0.56872
51898	1.41845	0.411992
51899	1.36936	0.566285
51900	1.41845	0.369152
51901	1.41845	0.349802
51902	0.946132	0.565952
51903	0.946132	0.576529
51904	0.946132	0.532412
51905	1.36936	0.425063
51906	1.41845	0.481666
51907	0.946133	0.550424

51908	1.36936	0.225857
51909	1.27581	0.449024
51910	1.20816	0.335445
51911	1.10464	0.52676
51912	1.24454	0.273187
51913	1.24454	0.278164
51914	1.23943	0.483388
51915	1.23943	0.520364
51916	1.10465	0.444998
51917	1.27581	0.411264
51918	1.36936	0.446665
51919	1.20816	0.376848
51920	0.946131	2.1
51921	0.946131	2.1
51922	1.37277	2.1
51923	1.37277	2.1
51924	1.37277	2.1
51925	1.27186	2.1
51926	0.946131	2.1
51927	0.946131	2.1
51928	0.946131	2.1
51929	1.37277	2.1
51930	0.946131	2.1
51931	1.27186	2.1
51932	2.74554	2.1
51933	1.23712	2.1
51934	2.80992	2.1
51935	2.54371	2.1
51936	1.50333	2.1
51937	1.23712	2.1
51938	2.77314	2.1
51939	2.33316	2.1
51940	2.28748	2.1
51941	2.28748	2.1
51942	2.81275	2.1
51943	2.81275	2.1
51944	2.28749	2.1
51945	1.45887	2.1
51946	2.33317	2.1
51947	1.50022	2.1
51948	3.79204	2.1
51949	2.37364	2.1
51950	2.33317	2.1
51951	1.5362	2.1
51952	1.5362	2.1
51953	1.50022	2.1
51954	2.33317	2.1
51955	1.89226	0.53854
51956	1.89226	0.531505
51957	1.89226	0.518156
51958	1.89226	0.550449
51959	1.89226	0.528066
51960	1.89226	0.570574
51961	0.946128	0.454917
51962	0.946128	0.468154
51963	1.0644	0.429839
51964	0.946133	0.452226
51965	1.0644	0.425254

51966	0.94613	0.440299
51967	0.946131	0.475495
51968	0.946131	0.456479
51969	1.0644	0.448497
51970	1.0644	0.453616
51971	0.946131	0.453562
51972	0.946131	0.445346
51973	1.89226	0.758557
51974	1.89226	0.441004
51975	1.89226	0.617661
51976	1.89226	0.365141
51977	3.78452	0.472679
51978	1.14599	0.751366
51979	1.19993	0.397138
51980	1.00065	0.596055
51981	1.07497	0.36211
51982	2.22096	0.517568
51983	0.546551	0.468213
51984	0.546551	0.466736
51985	0.54987	0.424806
51986	0.54987	0.624341
51987	0.503067	0.418757
51988	0.558937	0.417087
51989	0.599966	0.363116
51990	0.537484	0.420401
51991	0.503067	0.349407
51992	0.537485	0.349674
51993	0.599966	0.389168
51994	0.558936	0.365827
51995	0.946132	0.419473
51996	0.946132	0.518191
51997	0.94613	0.408323
51998	0.94613	0.591156
51999	0.946131	0.449012
52000	0.946131	0.448171
52001	0.946131	0.430463
52002	0.946131	0.433568
52003	0.946131	0.385914
52004	0.946131	0.379673
52005	0.946131	0.435591
52006	0.946131	0.468727
52007	2.19104	2.1
52008	2.5392	2.1
52009	2.53302	2.1
52010	2.19226	2.1
52011	4.73146	2.1
52012	1.09552	2.1
52013	1.09613	2.1
52014	0.802484	2.1
52015	0.807941	2.1
52016	0.95052	2.1
52017	0.95052	2.1
52018	0.952944	2.1
52019	0.952944	2.1
52020	1.09613	2.1
52021	0.807941	2.1
52022	0.802484	2.1
52023	1.09552	2.1

52024	2.533	2.1
52025	2.18531	2.1
52026	2.19224	2.1
52027	2.19224	2.1
52028	2.51603	2.1
52029	2.51603	2.1
52030	1.09266	2.1
52031	1.09612	2.1
52032	0.807935	2.1
52033	0.81091	2.1
52034	0.951897	2.1
52035	0.951897	2.1
52036	0.955134	2.1
52037	0.955134	2.1
52038	0.810915	2.1
52039	1.09266	2.1
52040	1.09611	2.1
52041	0.80793	2.1
52042	2.12741	0.63088
52043	2.79312	0.612171
52044	2.77591	0.941803
52045	2.10714	0.589458
52046	4.90026	0.591647
52047	2.12741	0.639807
52048	1.20859	0.542657
52049	1.21073	0.940644
52050	2.10714	0.641623
52051	3.31573	0.689426
52052	1.38708	0.769085
52053	1.38795	0.587196
52054	1.05331	0.629339
52055	1.05357	0.553828
52056	1.22049	0.574776
52057	1.22049	0.625138
52058	1.22078	1.03014
52059	1.22078	0.541489
52060	1.05331	0.654556
52061	1.38709	0.670932
52062	1.38795	0.568288
52063	1.05357	0.600543
52064	2.10662	0.795869
52065	2.10714	0.539634
52066	2.12499	0.575863
52067	1.22857	0.743503
52068	1.21073	0.622422
52069	1.21073	0.530625
52070	1.82014	0.12814
52071	2.08757	0.11907
52072	1.81292	0.0915558
52073	1.67485	0.115083
52074	3.63306	0.100475
52075	1.82013	0.12812
52076	1.21748	0.132433
52077	1.29891	0.13773
52078	1.4442	0.153419
52079	1.67484	0.114731
52080	1.82013	0.139499
52081	1.81292	0.0825984

52082	1.4516	0.0726916
52083	1.39633	0.0725006
52084	1.67485	0.0794509
52085	1.5344	0.0698797
52086	1.81292	0.0806425
52087	1.67484	0.0789182
52088	1.00982	0.0803215
52089	0.994235	0.0856454
52090	1.21748	0.094543
52091	1.45159	0.0723522
52092	1.67484	0.0906054
52093	1.55216	2.1
52094	1.34407	2.1
52095	1.34407	2.1
52096	1.52008	2.1
52097	1.81783	2.1
52098	1.99384	2.1
52099	1.90817	2.1
52100	2.03975	2.1
52101	1.97317	2.1
52102	1.81783	2.1
52103	3.85758	2.1
52104	0.599049	2.1
52105	0.660673	2.1
52106	0.785063	2.1
52107	0.924278	2.1
52108	0.760416	2.1
52109	0.760416	2.1
52110	0.719088	2.1
52111	0.694532	2.1
52112	0.522441	2.1
52113	0.796789	2.1
52114	0.655207	2.1
52115	0.886155	2.1
52116	1.97317	2.1
52117	1.81783	2.1
52118	1.65098	2.1
52119	1.91767	2.1
52120	3.7355	2.1
52121	1.15038	1.11266
52122	1.28649	1.65488
52123	1.38986	1.2418
52124	1.38986	1.96432
52125	1.07866	1.32908
52126	1.07866	2.09162
52127	0.664498	0.677965
52128	0.664498	2.1
52129	0.675122	1.69745
52130	0.675122	2.1
52131	0.69493	0.876229
52132	0.764429	1.40638
52133	0.623438	1.20067
52134	0.575191	1.93483
52135	0.764429	1.01007
52136	0.623438	0.944386
52137	0.575191	1.83835
52138	0.69493	1.62611
52139	1.89226	0.534543

52140	1.89226	0.384495
52141	1.89226	0.486296
52142	1.89226	0.40092
52143	3.78453	0.320124
52144	2.50075	0.508284
52145	2.15276	0.391151
52146	2.24902	0.488495
52147	2.30422	0.266129
52148	4.40178	0.279458
52149	1.70424	0.560129
52150	1.34494	0.569969
52151	2.15276	0.348118
52152	2.15276	0.56144
52153	2.0793	0.319334
52154	1.63078	0.468603
52155	1.89226	0.327238
52156	1.89226	0.557493
52157	1.89226	0.630747
52158	1.89226	0.38767
52159	1.89226	0.585091
52160	1.89226	0.469595
52161	1.89226	0.504374
52162	1.89226	0.443717
52163	1.89226	0.456038
52164	1.89226	0.274521
52165	3.78452	0.353746
52166	2.24902	0.509656
52167	1.91497	0.345271
52168	1.97135	0.543086
52169	1.73957	0.355587
52170	3.98859	0.292004
52171	1.73957	0.246755
52172	1.34494	0.549462
52173	1.73957	0.656314
52174	1.69006	0.305056
52175	1.3126	0.574121
52176	1.34848	0.460596
52177	0.946132	0.365122
52178	0.946132	0.584843
52179	0.946132	0.433621
52180	0.946132	0.632727
52181	0.946132	0.600726
52182	0.946132	0.569792
52183	0.946131	0.373073
52184	0.946131	0.334558
52185	0.946132	0.429498
52186	0.946132	0.43509
52187	0.946132	0.478908
52188	0.946132	0.493723
52189	1.89226	2.1
52190	1.89226	1.41503
52191	1.89226	2.00744
52192	1.89226	1.65147
52193	3.78453	1.83396
52194	1.89226	1.97572
52195	2.82624	1.39013
52196	1.89226	1.77606
52197	2.02536	1.56516

52198	1.89226	1.70883
52199	2.02536	1.37363
52200	2.25027	0.05
52201	1.1706	0.0524823
52202	2.02733	0.0507496
52203	2.02733	0.05457
52204	1.61462	0.05
52205	1.69832	0.0507259
52206	1.89226	0.0863386
52207	1.89226	0.0740344
52208	1.89226	0.0662836
52209	1.89226	0.0654578
52210	3.78452	0.0585983
52211	0.946131	0.05
52212	0.946131	0.05
52213	1.18182	0.05
52214	1.18182	0.05
52215	0.973819	0.05
52216	0.94613	0.05
52217	1.18182	0.05
52218	0.94613	0.05
52219	0.973819	0.05
52220	1.18182	0.05
52221	0.94613	0.0500338
52222	0.94613	0.05
52223	2.23944	2.1
52224	1.52903	2.1
52225	1.35977	2.1
52226	2.04847	2.1
52227	3.5775	2.1
52228	2.23944	2.1
52229	2.34795	2.1
52230	2.34795	2.1
52231	2.48025	2.1
52232	2.04847	2.1
52233	2.18077	2.1
52234	1.89226	2.1
52235	1.89226	1.84766
52236	1.89226	2.1
52237	1.89226	2.1
52238	3.78452	2.1
52239	0.946127	2.1
52240	0.946127	2.1
52241	0.946131	2.1
52242	0.946131	2.1
52243	0.946128	2.1
52244	0.946128	2.1
52245	0.94613	2.1
52246	0.94613	2.1
52247	0.946129	2.1
52248	0.946129	2.1
52249	0.946129	2.1
52250	0.946129	2.1
52251	1.26956	2.1
52252	2.04847	2.1
52253	1.49576	2.1
52254	1.22377	2.1
52255	1.91172	2.1

52256	1.50198	2.1
52257	1.48568	2.1
52258	2.34795	2.1
52259	2.04847	2.1
52260	2.04847	2.1
52261	2.21155	2.1
52262	2.21155	2.1
52263	1.89226	2.1
52264	1.89226	2.1
52265	1.89226	2.1
52266	1.89226	1.93557
52267	1.89226	2.1
52268	1.89226	1.19339
52269	0.810943	2.1
52270	0.810943	2.1
52271	0.810944	2.1
52272	0.810944	2.1
52273	0.810957	2.1
52274	0.810957	2.1
52275	1.89226	2.1
52276	1.35168	2.1
52277	1.08132	2.1
52278	1.08132	2.1
52279	1.0813	2.1
52280	0.946132	0.083013
52281	1.33855	0.119906
52282	0.946131	0.0633551
52283	1.38204	0.118267
52284	0.946131	0.122779
52285	0.946131	0.11833
52286	1.38204	0.0783188
52287	1.38204	0.0736641
52288	0.946131	0.08543
52289	0.946131	0.100169
52290	1.33855	0.0793201
52291	1.38204	0.110155
52292	1.89226	0.131954
52293	2.76407	0.145229
52294	1.89226	0.0840611
52295	2.72769	0.141305
52296	1.89226	0.0830828
52297	2.72769	0.141855
52298	2.03914	0.0633434
52299	1.67727	0.0908124
52300	1.94831	0.0766846
52301	1.81591	0.0662467
52302	2.32098	0.0838457
52303	2.32098	0.0792717
52304	0.801647	0.0517856
52305	1.11086	0.0642472
52306	0.838634	0.0751151
52307	1.16049	0.0585183
52308	0.977891	0.0779216
52309	0.977891	0.076858
52310	0.984246	0.0521081
52311	0.984246	0.0650807
52312	1.11086	0.0624172
52313	1.16049	0.0702059

52314	0.801647	0.06876
52315	0.838634	0.0686937
52316	0.946132	0.0870174
52317	0.946132	0.134146
52318	0.946131	0.0685214
52319	0.946131	0.121335
52320	0.946131	0.128525
52321	0.946131	0.121496
52322	0.946132	0.0786539
52323	0.946132	0.0775179
52324	0.946131	0.088972
52325	0.946131	0.0941286
52326	0.946131	0.0913099
52327	0.946131	0.10749
52328	0.946131	0.0960168
52329	0.946131	0.173178
52330	0.946131	0.121995
52331	0.946131	0.0803817
52332	0.946131	0.129452
52333	0.946131	0.132063
52334	0.946131	0.0864509
52335	0.946131	0.0871534
52336	0.946131	0.0975816
52337	0.946131	0.107675
52338	0.946131	0.118045
52339	0.946131	0.121542
52340	2.0815	2.1
52341	1.20787	2.1
52342	1.92277	2.1
52343	1.3227	2.1
52344	3.29401	2.1
52345	2.16633	2.1
52346	2.13642	2.1
52347	1.40846	2.1
52348	1.40846	2.1
52349	1.3227	2.1
52350	2.08058	2.1
52351	0.938106	2.1
52352	0.790258	2.1
52353	0.742975	2.1
52354	0.742975	2.1
52355	0.874389	2.1
52356	1.17364	2.1
52357	0.525948	2.1
52358	0.52513	2.1
52359	0.553732	2.1
52360	0.876996	2.1
52361	0.530954	2.1
52362	1.095 2.1	
52363	1.00833	2.1
52364	1.70608	2.1
52365	1.33385	2.1
52366	1.33385	2.1
52367	1.07722	2.1
52368	1.07722	2.1
52369	0.953523	0.826691
52370	1.7457	0.773791
52371	1.03218	0.821627

52372	1.03218	0.793579
52373	1.79675	0.829368
52374	1.79675	0.800398
52375	1.13676	0.798241
52376	1.18136	0.693505
52377	0.87285	0.776874
52378	0.898375	0.807384
52379	1.03191	0.769129
52380	1.03191	0.689131
52381	1.0223	0.783224
52382	1.0223	0.811389
52383	1.18136	0.756508
52384	0.898374	0.733547
52385	0.87285	0.7577
52386	1.13676	0.758051
52387	2.36273	0.72428
52388	1.46965	0.678849
52389	1.79675	0.699938
52390	1.79675	0.632289
52391	2.43417	0.734303
52392	2.43417	0.667095
52393	1.4714	2.1
52394	1.65434	1.80521
52395	1.62322	0.94653
52396	1.86068	1.62319
52397	3.27756	1.41451
52398	1.89226	2.1
52399	1.89226	1.81095
52400	1.89226	0.985339
52401	1.89226	1.13417
52402	3.78453	1.17215
52403	1.89226	1.1697
52404	1.89226	2.1
52405	1.89226	0.931233
52406	1.89226	2.1
52407	3.78453	1.29882
52408	1.65434	1.20226
52409	1.60647	2.1
52410	1.86068	1.0726
52411	1.82649	2.1
52412	3.48083	1.95493
52413	1.62322	2.1
52414	1.86068	1.77214
52415	1.84368	0.921836
52416	2.18862	1.24197
52417	3.81184	1.36137
52418	1.89226	2.1
52419	1.89226	1.6632
52420	1.89226	0.887513
52421	1.89226	1.28911
52422	3.78453	1.3421
52423	1.89226	1.20749
52424	1.89226	2.1
52425	1.89226	1.02317
52426	1.89226	2.1
52427	3.78453	1.85829
52428	1.86068	1.07522
52429	1.82649	2.1

52430	2.18862	1.00435
52431	2.1128	2.1
52432	4.01511	1.27892
52433	1.74351	2.1
52434	1.13854	2.1
52435	1.40233	2.1
52436	1.922 2.1	
52437	3.14584	2.1
52438	1.922 2.1	
52439	2.00775	2.1
52440	1.7435	2.1
52441	2.09224	2.1
52442	2.00775	2.1
52443	2.09295	2.1
52444	1.69858	2.1
52445	1.922 2.1	
52446	1.40233	2.1
52447	1.40233	2.1
52448	2.18238	2.1
52449	2.18238	2.1
52450	1.00387	2.1
52451	1.10886	2.1
52452	0.961001	2.1
52453	1.09119	2.1
52454	1.02868	2.1
52455	1.02868	2.1
52456	1.04118	2.1
52457	1.04118	2.1
52458	1.10886	2.1
52459	1.09119	2.1
52460	0.960999	2.1
52461	1.00388	2.1
52462	0.885958	0.586131
52463	0.885958	0.53799
52464	0.875826	0.540174
52465	0.875826	0.702235
52466	0.913965	0.709408
52467	0.598609	0.564693
52468	1.16317	0.557206
52469	0.868115	0.529673
52470	0.868115	0.593007
52471	0.598609	0.57879
52472	1.16317	0.590134
52473	0.913965	0.607282
52474	0.946131	0.600067
52475	0.946131	0.573088
52476	0.946131	0.6372
52477	0.946131	0.575442
52478	0.946131	0.62175
52479	0.946131	0.610115
52480	0.946131	0.576629
52481	0.946131	0.566546
52482	0.946131	0.602397
52483	0.946131	0.608503
52484	0.946131	0.59267
52485	0.946131	0.594276
52486	0.946133	0.060822
52487	0.946133	0.0602405

52488	1.06246	0.0604275
52489	1.06246	0.0621855
52490	0.973851	0.0631789
52491	0.946133	0.0635247
52492	1.06246	0.0539798
52493	0.946132	0.0725312
52494	0.97385	0.0639116
52495	1.06246	0.0586488
52496	0.946133	0.0574752
52497	0.946133	0.0676611
52498	1.89226	0.094717
52499	1.89226	0.0846748
52500	1.89226	0.0887587
52501	1.89226	0.103088
52502	1.89226	0.0781808
52503	1.89226	0.0875846
52504	0.946131	0.0774088
52505	0.946131	0.0749141
52506	0.946127	0.0766865
52507	0.946127	0.0756308
52508	0.946131	0.0928802
52509	0.946131	0.0667475
52510	0.946127	0.0730924
52511	0.946127	0.0769256
52512	0.946128	0.0717034
52513	0.946128	0.0735653
52514	0.946129	0.0741114
52515	0.946129	0.0834065
52516	0.946132	0.05
52517	0.946132	0.0503581
52518	1.32446	0.05
52519	0.794912	0.05
52520	1.32429	0.05
52521	0.946131	0.05
52522	1.32446	0.05
52523	0.94613	0.05
52524	1.32429	0.05
52525	0.794915	0.05
52526	0.94613	0.05
52527	0.94613	0.05
52528	1.89226	0.05
52529	2.36365	0.05
52530	2.64857	0.05
52531	1.89226	0.05
52532	2.64857	0.05
52533	1.89226	0.05
52534	1.18463	2.1
52535	1.27174	2.1
52536	0.946129	2.1
52537	0.946129	2.1
52538	0.946131	2.1
52539	0.946131	2.1
52540	1.27174	2.1
52541	1.27174	2.1
52542	1.18463	2.1
52543	0.94613	2.1
52544	0.946131	2.1
52545	1.27174	2.1

52546	2.57997	2.1
52547	1.89226	2.1
52548	1.89226	2.1
52549	1.89226	2.1
52550	2.41915	2.1
52551	2.41915	2.1
52552	1.89226	2.1
52553	1.89226	2.1
52554	1.89226	2.1
52555	1.89226	2.1
52556	3.78452	2.1
52557	1.08807	2.1
52558	1.18463	2.1
52559	0.946129	2.1
52560	0.946129	2.1
52561	0.946131	2.1
52562	0.946131	2.1
52563	1.18463	2.1
52564	1.18463	2.1
52565	0.946131	2.1
52566	1.08807	2.1
52567	1.18463	2.1
52568	0.94613	2.1
52569	1.07017	2.1
52570	1.07017	2.1
52571	0.946131	2.1
52572	0.946131	2.1
52573	0.946132	2.1
52574	0.946132	2.1
52575	1.20957	2.1
52576	1.07017	2.1
52577	1.07017	2.1
52578	0.946132	2.1
52579	0.946131	2.1
52580	1.20957	2.1
52581	1.89226	2.1
52582	1.89226	2.1
52583	1.89226	2.1
52584	1.89226	2.1
52585	1.89226	2.1
52586	1.89226	2.1
52587	1.16181	0.05
52588	1.0227	0.05
52589	1.0227	0.0533548
52590	0.970959	0.05
52591	1.24295	0.05
52592	1.19121	0.05
52593	1.12076	2.1
52594	2.53614	2.1
52595	1.28413	2.1
52596	1.28413	2.1
52597	2.36423	2.1
52598	2.36423	2.1
52599	1.89226	2.1
52600	1.89226	2.1
52601	1.89226	2.1
52602	1.89226	2.1
52603	3.78452	2.1

52604	1.22557	2.1
52605	2.771	2.1
52606	2.73012	2.1
52607	2.73012	2.1
52608	1.33356	2.1
52609	1.33356	2.1
52610	0.998682	2.1
52611	2.36423	2.1
52612	1.12076	2.1
52613	1.12076	2.1
52614	2.13757	2.1
52615	2.13757	2.1
52616	1.89226	2.1
52617	1.89226	2.1
52618	1.89226	2.1
52619	1.89226	2.1
52620	1.89226	2.1
52621	1.89226	2.1
52622	1.13987	2.1
52623	2.73011	2.1
52624	2.70875	2.1
52625	2.70875	2.1
52626	1.22557	2.1
52627	1.22557	2.1
52628	1.89227	2.1
52629	2.79243	2.1
52630	2.6625	2.1
52631	2.6625	2.1
52632	1.89227	2.1
52633	1.89227	2.1
52634	1.89226	2.1
52635	2.55971	2.1
52636	1.89226	2.1
52637	1.89226	2.1
52638	2.68704	2.1
52639	2.68704	2.1
52640	2.22043	1.5991
52641	2.38975	1.63662
52642	2.28696	1.10648
52643	2.48682	0.906117
52644	4.67671	1.0756
52645	2.38976	1.02136
52646	2.31988	1.57542
52647	2.48683	0.988003
52648	2.4594	1.30167
52649	4.84915	1.3006
52650	2.28696	1.43999
52651	2.48682	1.33833
52652	2.33379	1.53281
52653	2.56149	0.812434
52654	4.84845	1.29551
52655	2.48683	1.52976
52656	2.4594	1.37169
52657	2.5615	0.865306
52658	2.57957	1.1079
52659	5.0209	0.989521
52660	1.06659	1.75503
52661	1.04291	0.632428

52662	1.36595	0.605393
52663	1.36595	1.95802
52664	1.02761	2.1
52665	1.32697	0.599145
52666	0.946132	1.46462
52667	0.946132	1.22383
52668	1.36956	2.1
52669	1.1229	2.1
52670	1.1229	1.16791
52671	1.1229	2.1
52672	0.946132	0.831624
52673	0.946132	2.09031
52674	0.946132	1.76217
52675	1.36956	1.83514
52676	1.1229	1.78447
52677	0.946133	1.69631
52678	0.946133	0.973087
52679	0.946133	0.894865
52680	0.946128	2.1
52681	0.946128	1.86388
52682	0.946131	1.93604
52683	0.946131	1.30074
52684	0.946131	0.66507
52685	0.946131	2.07121
52686	0.94613	1.73469
52687	0.94613	1.58501
52688	0.946131	1.35414
52689	0.946131	1.26969
52690	0.966427	1.16497
52691	0.802265	2.04769
52692	0.803714	1.96015
52693	0.808476	1.47261
52694	1.94371	1.9999
52695	1.36851	2.05983
52696	0.853864	2.1
52697	1.26635	1.41371
52698	1.09085	1.2375
52699	0.857653	2.04062
52700	0.630243	1.53179
52701	1.20132	0.05
52702	1.24295	0.0515383
52703	1.24295	0.0563482
52704	1.16182	0.05
52705	1.28465	0.0523331
52706	1.20351	0.05
52707	0.600658	0.05
52708	0.642323	0.0550457
52709	0.54424	0.0563966
52710	0.576779	0.05
52711	0.590985	0.0588411
52712	0.590985	0.061449
52713	0.586452	0.05
52714	0.586452	0.0535608
52715	0.642323	0.0555026
52716	0.576779	0.0534289
52717	0.600658	0.0570902
52718	0.54424	0.0554521
52719	1.28463	0.05

52720	1.24294	0.0512481
52721	1.12328	0.05
52722	1.15505	0.0518287
52723	2.39798	0.05
52724	1.98431	0.0716808
52725	2.18735	0.0798073
52726	1.98431	0.0931887
52727	1.70303	0.0625115
52728	1.70302	0.107579
52729	1.37788	0.068806
52730	1.89226	0.13623
52731	2.37417	0.146222
52732	1.89226	0.18623
52733	2.18735	0.117602
52734	1.89226	0.221514
52735	2.18735	0.139998
52736	1.89226	0.187682
52737	1.89226	0.539475
52738	1.89226	0.229163
52739	1.89226	0.456094
52740	3.78452	0.452622
52741	1.8613	0.303362
52742	2.03576	0.534423
52743	1.61652	0.197376
52744	1.83082	0.708904
52745	3.65228	0.253609
52746	2.03576	0.487333
52747	2.05257	2.1
52748	1.83082	0.684938
52749	1.88487	0.770674
52750	3.92064	0.788051
52751	1.89226	0.486349
52752	1.89226	0.619031
52753	1.89226	0.394681
52754	1.89226	0.821111
52755	3.78453	0.545582
52756	0.946131	0.532434
52757	0.946131	0.168544
52758	0.94613	0.0924845
52759	0.94613	0.476986
52760	0.946129	0.338158
52761	0.946129	0.552191
52762	0.946132	0.498907
52763	0.946132	0.0833094
52764	0.94613	0.348136
52765	0.94613	0.288433
52766	0.946131	0.127679
52767	0.946131	0.344382
52768	0.804708	0.910756
52769	0.804708	0.196917
52770	0.815274	0.563303
52771	0.815274	0.315956
52772	0.83277	0.526122
52773	0.915407	0.806978
52774	0.80826	0.328773
52775	0.704574	0.226238
52776	0.832771	0.450593
52777	0.704573	0.477139

52778	0.808259	0.454846
52779	0.915409	0.398825
52780	1.83082	0.700599
52781	1.88487	0.823694
52782	1.66555	0.555244
52783	1.75835	2.1
52784	3.55042	0.687276
52785	0.810947	0.288611
52786	0.810947	0.615759
52787	0.810947	0.382016
52788	0.810947	0.54288
52789	0.810934	0.564044
52790	0.810934	0.568669
52791	1.89227	0.869161
52792	1.3517	0.640199
52793	1.08132	0.576507
52794	1.08132	0.607572
52795	1.08133	0.577633
52796	1.89226	1.15967
52797	1.70303	1.49951
52798	1.89226	2.1
52799	2.24712	2.1
52800	2.07363	2.1
52801	1.74617	1.339
52802	1.89226	2.1
52803	1.89226	1.21421
52804	1.89226	0.992818
52805	1.89226	2.1
52806	3.78452	1.19692
52807	1.50108	2.1
52808	1.43894	2.07819
52809	1.8322	0.903684
52810	1.40967	2.1
52811	1.50108	2.1
52812	1.07854	2.1
52813	1.89226	0.93215
52814	1.89226	2.1
52815	1.89226	1.97195
52816	1.89226	0.771321
52817	3.78452	1.79774
52818	1.89226	2.1
52819	1.89226	1.13341
52820	1.89226	1.09893
52821	1.89226	2.1
52822	3.78453	1.19466
52823	1.54457	2.1
52824	1.35097	1.6889
52825	1.70754	0.927921
52826	1.29629	2.1
52827	1.54457	2.05964
52828	1.0305	2.1
52829	0.731763	2.1
52830	1.00043	1.84443
52831	1.13883	1.12849
52832	0.939026	1.94395
52833	1.04003	1.13584
52834	0.853868	2.1
52835	0.927188	2.1

52836	0.927188	0.754782
52837	0.861565	1.32806
52838	1.13882	1.82002
52839	0.731769	1.72527
52840	0.89319	1.98458
52841	1.46568	2.1
52842	1.20761	2.1
52843	1.51916	2.1
52844	1.36261	2.1
52845	2.72677	2.1
52846	1.51916	2.1
52847	1.60532	2.1
52848	1.36261	2.1
52849	1.36261	2.1
52850	1.64892	2.1
52851	1.64892	2.1
52852	1.3626	2.1
52853	0.98488	2.1
52854	1.60532	2.1
52855	1.32414	2.1
52856	2.68675	2.1
52857	1.78711	2.1
52858	1.14825	2.1
52859	2.16535	2.1
52860	1.6067	2.1
52861	3.3136	2.1
52862	1.89226	2.1
52863	1.89226	2.1
52864	1.89226	2.1
52865	1.89226	2.1
52866	3.78452	2.1
52867	2.34552	2.1
52868	1.53521	2.1
52869	1.53521	2.1
52870	1.60636	2.1
52871	1.92797	2.1
52872	1.99589	2.1
52873	1.34771	2.1
52874	1.34771	2.1
52875	0.946128	2.1
52876	0.946128	2.1
52877	1.3477	2.1
52878	1.31776	2.1
52879	0.946131	2.1
52880	0.946131	2.1
52881	0.94613	2.1
52882	1.31776	2.1
52883	0.946129	2.1
52884	1.3477	2.1
52885	1.89226	2.1
52886	1.89226	2.1
52887	1.89226	2.1
52888	1.89226	2.1
52889	3.78452	2.1
52890	0.92843	2.1
52891	0.672114	2.1
52892	0.89514	2.1
52893	0.89514	2.1

52894	0.720104	2.1
52895	0.755753	2.1
52896	0.789158	2.1
52897	0.967302	2.1
52898	1.06565	2.1
52899	0.7397	2.1
52900	0.758893	2.1
52901	0.94672	2.1
52902	1.89226	2.1
52903	2.588	2.1
52904	2.588	2.1
52905	2.63552	2.1
52906	1.89226	2.1
52907	1.93978	2.1
52908	1.49347	0.135528
52909	2.67757	0.201859
52910	1.80061	0.129134
52911	2.27056	0.157813
52912	4.07117	0.123039
52913	1.1016	0.0940742
52914	2.27057	0.116183
52915	1.49348	0.167617
52916	1.81893	0.17976
52917	1.49348	0.0941142
52918	1.81893	0.0861373
52919	1.81893	0.153377
52920	2.82339	0.237883
52921	2.27057	0.134371
52922	2.25405	0.127314
52923	4.52462	0.109871
52924	1.89226	0.145335
52925	1.89226	0.269451
52926	1.89226	0.256027
52927	1.89226	0.185918
52928	1.89226	0.263795
52929	1.89226	0.228665
52930	1.89226	0.118364
52931	1.89226	0.200358
52932	1.89226	0.104226
52933	1.89226	0.125595
52934	3.78453	0.100671
52935	1.89226	0.230975
52936	1.89226	0.296062
52937	1.89226	0.130189
52938	1.89226	0.304768
52939	3.78452	0.332887
52940	1.89226	0.305824
52941	1.89226	0.556868
52942	1.89226	0.258472
52943	1.89226	0.332062
52944	3.78451	0.257713
52945	1.45633	2.1
52946	1.34678	2.1
52947	1.34678	2.1
52948	1.53735	2.1
52949	1.234	2.1
52950	1.42457	2.1
52951	1.89226	2.1

52952	1.89226	2.1
52953	1.89226	2.1
52954	1.89226	2.1
52955	1.89226	2.1
52956	1.89226	2.1
52957	1.09483	2.1
52958	1.34678	2.1
52959	1.234	2.1
52960	1.234	2.1
52961	1.19	2.1
52962	1.19	2.1
52963	0.946131	2.1
52964	0.946131	2.1
52965	0.946131	2.1
52966	0.946131	2.1
52967	0.946131	2.1
52968	0.946131	2.1
52969	0.946132	2.1
52970	0.946132	2.1
52971	0.946132	2.1
52972	0.946132	2.1
52973	0.946131	2.1
52974	0.946131	2.1
52975	2.82263	2.1
52976	1.89226	2.1
52977	2.71441	2.1
52978	2.71441	2.1
52979	1.89226	2.1
52980	1.89226	2.1
52981	1.1412	0.91233
52982	1.1412	2.1
52983	1.14626	2.1
52984	1.14626	2.1
52985	1.28511	1.37714
52986	1.34086	2.1
52987	0.992242	2.1
52988	0.946593	2.1
52989	0.992242	2.1
52990	1.34086	2.1
52991	0.946594	2.1
52992	1.28511	2.1
52993	1.18954	1.87023
52994	1.18954	2.1
52995	1.21477	2.1
52996	1.21477	2.1
52997	1.41207	2.1
52998	1.34086	2.1
52999	1.01306	2.1
53000	0.992242	2.1
53001	1.01306	2.1
53002	1.41207	2.1
53003	0.992242	2.1
53004	1.34086	2.1
53005	1.09044	2.1
53006	1.44472	0.614898
53007	1.2955	1.09581
53008	1.2955	0.743916
53009	1.22025	1.1929

53010	1.22025	0.87695
53011	0.810944	0.648401
53012	0.810944	1.96781
53013	0.810946	0.738084
53014	0.810946	0.431712
53015	0.810959	0.702159
53016	0.810959	0.637867
53017	1.89226	0.620648
53018	1.35167	0.704542
53019	1.08132	2.00212
53020	1.08132	0.435571
53021	1.0813	0.692957
53022	1.41894	0.631964
53023	1.28953	0.48153
53024	0.946132	0.510783
53025	0.946132	1.21743
53026	0.946133	0.792919
53027	0.946133	0.534624
53028	1.41894	0.693202
53029	1.41894	1.06075
53030	1.28953	0.570815
53031	0.946132	0.753553
53032	0.946132	0.697702
53033	1.41894	0.543084
53034	1.89226	2.1
53035	2.15444	2.1
53036	1.93239	2.1
53037	1.89226	2.1
53038	2.19457	2.1
53039	2.15444	2.1
53040	1.89226	2.1
53041	1.89226	2.1
53042	1.89226	2.1
53043	1.89226	2.1
53044	1.89226	2.1
53045	1.89226	2.1
53046	0.938507	2.1
53047	0.938507	2.1
53048	1.07871	2.1
53049	1.15801	2.1
53050	1.03236	2.1
53051	1.14766	2.1
53052	0.946133	2.1
53053	0.946133	2.1
53054	0.946131	2.1
53055	1.07009	2.1
53056	0.946131	2.1
53057	1.03237	2.1
53058	1.89226	2.1
53059	2.19457	2.1
53060	1.94492	2.1
53061	1.89226	2.1
53062	2.24723	2.1
53063	2.19457	2.1
53064	0.946132	2.1
53065	0.946132	2.1
53066	0.946131	2.1
53067	0.946131	2.1

53068	0.946132	2.1
53069	0.946132	2.1
53070	0.946132	2.1
53071	0.946132	2.1
53072	0.946132	2.1
53073	0.946132	2.1
53074	0.946132	2.1
53075	0.946132	2.1
53076	2.21345	2.1
53077	1.89226	2.1
53078	1.89226	2.1
53079	2.14017	2.1
53080	2.14017	2.1
53081	1.89226	2.1
53082	1.89226	0.505595
53083	1.89226	0.759579
53084	1.89226	0.556935
53085	1.85439	0.708871
53086	2.23961	0.481092
53087	2.19479	0.553513
53088	1.2916	0.51651
53089	1.2916	0.502436
53090	0.946128	0.497143
53091	0.946128	0.504897
53092	1.2916	0.456132
53093	0.946131	0.501628
53094	0.946126	0.699831
53095	1.1198	0.433989
53096	1.2916	0.45278
53097	1.1198	0.574212
53098	0.946128	0.618371
53099	0.946128	0.555715
53100	1.89226	0.645192
53101	2.71262	0.576574
53102	1.89226	0.5877
53103	2.49959	0.598924
53104	4.39185	0.697262
53105	2.71262	0.57258
53106	1.34592	0.565749
53107	2.49959	0.587166
53108	1.14721	0.545262
53109	3.84551	0.527095
53110	0.946131	0.551535
53111	0.946131	0.563607
53112	1.11247	0.525776
53113	1.11247	0.519433
53114	1.24979	0.523874
53115	1.11247	0.528347
53116	0.946129	0.575183
53117	0.946129	0.549981
53118	0.946131	0.50731
53119	1.11247	0.464746
53120	0.946131	0.482835
53121	1.24979	0.504
53122	0.946131	2.1
53123	0.946131	2.1
53124	0.94613	2.1
53125	0.94613	2.1

53126	0.946132	2.1
53127	0.946132	2.1
53128	0.946129	2.1
53129	0.946129	2.1
53130	0.946131	2.1
53131	0.946131	2.1
53132	0.946131	2.1
53133	0.946131	2.1
53134	2.77816	2.1
53135	1.89226	2.1
53136	1.89226	2.1
53137	1.89226	2.1
53138	3.78453	2.1
53139	1.89226	2.1
53140	1.89226	2.1
53141	1.89226	2.1
53142	1.89226	2.1
53143	1.89226	2.1
53144	1.89226	2.1
53145	1.89226	2.1
53146	1.89226	2.1
53147	1.89226	2.1
53148	1.89226	2.1
53149	3.78452	2.1
53150	1.55698	2.1
53151	1.89226	2.1
53152	1.89226	2.1
53153	1.89226	2.1
53154	1.8575	2.1
53155	1.8575	2.1
53156	1.89226	2.1
53157	1.89226	2.1
53158	1.89226	2.1
53159	1.89226	2.1
53160	1.89226	2.1
53161	1.89226	2.1
53162	1.89227	2.1
53163	1.89227	2.1
53164	1.89227	2.1
53165	1.89227	2.1
53166	1.89227	2.1
53167	1.89227	2.1
53168	1.35509	2.1
53169	2.06238	2.1
53170	2.12244	2.1
53171	2.12244	2.1
53172	1.34817	2.1
53173	1.34817	2.1
53174	0.946126	0.946857
53175	0.946126	2.1
53176	0.946131	2.1
53177	0.946131	2.1
53178	0.946127	2.1
53179	0.946127	1.03634
53180	0.94613	2.1
53181	0.94613	2.1
53182	0.946128	2.1
53183	0.946128	2.1

53184	0.946128	2.1
53185	0.946128	2.1
53186	1.89226	2.1
53187	1.89226	0.905809
53188	1.89226	1.57941
53189	1.89226	2.1
53190	1.89226	0.935451
53191	1.89226	2.1
53192	1.16742	2.1
53193	1.16742	1.75657
53194	1.18122	2.1
53195	1.18122	2.1
53196	1.31745	2.1
53197	1.2597	0.888435
53198	1.03119	2.1
53199	1.06122	2.1
53200	1.06122	2.1
53201	1.31745	2.1
53202	1.03119	2.1
53203	1.2597	2.1
53204	1.36849	2.1
53205	2.12244	2.1
53206	2.20092	2.1
53207	2.20092	2.1
53208	1.35509	2.1
53209	1.35509	2.1
53210	1.89226	2.1
53211	1.89226	2.1
53212	1.89226	2.1
53213	1.89226	2.1
53214	1.89226	2.1
53215	1.89226	2.1
53216	1.89226	2.1
53217	1.89226	0.836543
53218	1.89226	1.89581
53219	1.89226	1.20827
53220	3.78452	1.94792
53221	1.21731	2.1
53222	1.21731	0.99137
53223	1.23412	2.1
53224	1.23412	2.1
53225	1.39021	2.1
53226	1.31745	1.17404
53227	1.06122	2.1
53228	1.10046	2.1
53229	1.10046	2.1
53230	1.39021	2.1
53231	1.06122	2.1
53232	1.31745	2.1
53233	2.16503	0.787352
53234	1.78252	1.10301
53235	1.77293	0.894244
53236	1.56839	2.1
53237	2.36957	0.59445
53238	2.16503	2.1
53239	1.89223	0.814399
53240	1.89223	2.1
53241	1.89223	0.743554

53242	1.89223	1.05674
53243	3.78447	1.35541
53244	2.3696	1.06136
53245	1.78254	1.56472
53246	2.63298	0.655226
53247	2.07312	1.88402
53248	4.44272	1.29559
53249	1.89226	1.06876
53250	1.89226	1.58666
53251	1.89226	1.01167
53252	1.89226	2.1
53253	1.89226	0.643864
53254	1.89226	2.1
53255	1.89226	0.636842
53256	1.89226	0.447673
53257	1.89226	0.649754
53258	1.89226	0.371559
53259	3.78452	0.472393
53260	2.26369	0.636147
53261	2.2168	0.392554
53262	2.15617	0.575164
53263	2.10864	0.363866
53264	4.37234	0.461156
53265	2.2168	0.401215
53266	1.94499	0.455034
53267	1.94499	0.515971
53268	2.04737	0.469253
53269	2.10864	0.367202
53270	2.21103	0.447082
53271	1.89226	0.588502
53272	1.89226	0.446322
53273	1.89226	0.478307
53274	1.89226	0.467072
53275	1.89226	0.568346
53276	1.89226	0.489808
53277	1.89226	0.606969
53278	1.89226	0.43229
53279	1.89226	0.489054
53280	1.89226	0.654283
53281	1.89226	0.389547
53282	1.89226	0.675125
53283	1.02995	0.391731
53284	1.02995	0.629342
53285	1.03212	0.426119
53286	1.03212	0.580145
53287	0.988256	0.387516
53288	1.05432	0.373062
53289	1.00775	0.554174
53290	1.07809	0.567539
53291	1.00775	0.453785
53292	0.988257	0.469949
53293	1.07808	0.477679
53294	1.05432	0.48721
53295	2.10864	0.460242
53296	1.94499	0.488227
53297	1.97652	0.412528
53298	1.79681	0.439576
53299	3.9215	0.386076

53300	0.810943	0.472344
53301	0.810943	0.651515
53302	0.810943	0.438446
53303	0.810943	0.459061
53304	0.810958	0.369751
53305	0.810958	0.425821
53306	1.89226	0.479998
53307	1.35168	0.404823
53308	1.08132	0.459911
53309	1.08132	0.490259
53310	1.0813	0.425833
53311	0.657403	0.141797
53312	1.35403	0.138018
53313	1.24038	0.132415
53314	0.499144	0.12946
53315	1.24039	0.156156
53316	1.35403	0.1428
53317	0.657402	0.131934
53318	0.499144	0.128173
53319	0.915415	0.147958
53320	0.915415	0.150011
53321	0.937755	0.135053
53322	0.937755	0.142117
53323	1.72526	2.1
53324	1.17989	2.0115
53325	1.96035	2.1
53326	1.47412	2.1
53327	3.14024	2.1
53328	1.89226	1.05975
53329	1.89226	2.1
53330	1.89226	1.2345
53331	1.89226	0.828437
53332	3.78452	1.70783
53333	0.946131	1.60931
53334	0.946131	1.23432
53335	1.25764	1.60284
53336	1.25764	2.1
53337	1.07087	1.13156
53338	1.25765	1.11027
53339	0.946128	1.08298
53340	0.946128	0.679967
53341	0.94613	0.936338
53342	1.25765	1.07745
53343	1.07086	1.32665
53344	0.946129	1.25477
53345	1.96035	2.1
53346	1.32668	2.1
53347	2.29301	2.1
53348	1.70303	1.65152
53349	3.56428	2.1
53350	1.89226	1.15934
53351	1.89226	1.22843
53352	1.89226	0.622365
53353	1.89226	2.03521
53354	3.78452	0.596388
53355	0.897738	0.493545
53356	0.897738	0.49723
53357	0.946102	0.466459

53358	0.946102	0.43538
53359	1.11247	0.466842
53360	0.89771	0.424337
53361	0.94613	0.473404
53362	0.94613	0.503554
53363	0.897709	0.5042
53364	0.946131	0.516677
53365	0.946131	0.549767
53366	1.11247	0.498402
53367	1.89226	2.1
53368	1.89226	1.95441
53369	1.89226	2.1
53370	1.89226	2.1
53371	1.89226	2.1
53372	1.89226	2.1
53373	2.15429	2.1
53374	1.89226	2.1
53375	1.89226	2.1
53376	1.89226	2.1
53377	2.27074	2.1
53378	2.27074	2.1
53379	0.993311	1.20693
53380	0.993311	1.53147
53381	0.904322	2.1
53382	0.904322	1.94931
53383	0.803324	2.1
53384	1.36183	1.2098
53385	0.916274	2.1
53386	0.535808	1.379
53387	0.535808	1.57905
53388	0.803322	1.72655
53389	0.916276	1.55514
53390	1.36183	1.78337
53391	1.89226	2.1
53392	1.89226	1.62053
53393	1.89226	2.1
53394	1.89226	2.1
53395	1.89226	2.1
53396	1.89226	2.1
53397	2.11955	2.1
53398	2.62097	2.1
53399	1.85542	2.1
53400	2.39016	2.1
53401	4.50971	2.1
53402	2.39016	2.1
53403	2.20242	2.1
53404	2.39016	2.1
53405	1.64008	2.1
53406	1.85542	2.1
53407	1.64008	2.1
53408	1.79667	2.1
53409	1.28267	2.1
53410	1.28267	2.1
53411	1.40117	2.1
53412	1.74514	2.1
53413	1.83994	2.1
53414	0.575914	2.1
53415	0.625292	2.1

53416	0.949144	2.1
53417	0.882782	2.1
53418	0.773697	2.1
53419	0.773697	2.1
53420	0.786497	2.1
53421	0.786494	2.1
53422	0.605477	2.1
53423	0.949148	2.1
53424	0.625291	2.1
53425	0.862374	2.1
53426	1.89226	2.1
53427	2.44918	2.1
53428	2.4616	2.1
53429	2.44918	2.1
53430	1.90468	2.1
53431	1.89226	2.1
53432	1.89226	2.1
53433	1.89226	0.929026
53434	1.89226	2.1
53435	1.89226	0.717735
53436	3.78453	2.1
53437	0.946128	2.1
53438	0.946128	0.690933
53439	1.23631	1.0263
53440	1.23631	2.1
53441	0.946131	0.915174
53442	0.946131	0.957909
53443	1.3147	2.1
53444	1.23631	2.1
53445	1.3147	2.1
53446	0.946131	2.1
53447	1.23631	2.1
53448	0.94613	2.1
53449	1.89226	2.1
53450	2.4616	2.1
53451	2.4717	2.1
53452	2.4616	2.1
53453	1.90237	2.1
53454	1.89226	2.1
53455	0.946132	0.755414
53456	0.946132	2.1
53457	0.946131	2.1
53458	0.946131	0.891657
53459	0.946132	0.816756
53460	0.946132	1.80385
53461	0.946131	2.1
53462	0.946131	2.1
53463	0.946131	2.1
53464	0.946131	2.1
53465	0.946131	2.1
53466	0.946131	2.1
53467	0.946128	2.1
53468	0.946128	1.40905
53469	1.3147	0.793217
53470	1.3147	2.1
53471	0.94613	0.773702
53472	0.94613	1.25616
53473	1.3419	2.1

53474	1.3147	2.1
53475	1.3419	2.1
53476	0.94613	2.1
53477	1.3147	2.05005
53478	0.94613	2.1
53479	1.89226	2.1
53480	1.89226	2.1
53481	1.89226	2.1
53482	1.89226	2.1
53483	3.78452	2.1
53484	2.3333	2.1
53485	1.3864	2.1
53486	1.3864	2.1
53487	1.4628	2.1
53488	1.95763	2.1
53489	2.03403	2.1
53490	1.294 2.1	
53491	1.294 2.1	
53492	0.946128	2.1
53493	0.946128	2.1
53494	1.294 2.1	
53495	1.27474	2.1
53496	0.946131	2.1
53497	0.946131	2.1
53498	0.946129	2.1
53499	1.27474	2.1
53500	1.294 2.1	
53501	0.94613	2.1
53502	1.89226	2.1
53503	1.89226	2.1
53504	1.89226	2.1
53505	1.89226	2.1
53506	1.89226	2.1
53507	1.89226	2.1
53508	0.808088	2.1
53509	0.808088	2.1
53510	0.815623	2.1
53511	0.815623	2.1
53512	0.660053	2.1
53513	0.693199	2.1
53514	0.930512	2.1
53515	0.978817	2.1
53516	0.660053	2.1
53517	0.930513	2.1
53518	0.693198	2.1
53519	0.978817	2.1
53520	1.27474	2.1
53521	1.27474	2.1
53522	0.946128	2.1
53523	0.946128	2.1
53524	1.26221	2.1
53525	1.27474	2.1
53526	0.946131	2.1
53527	0.946131	2.1
53528	0.94613	2.1
53529	1.26221	2.1
53530	0.946129	2.1
53531	1.27474	2.1

53532	0.90207	0.05
53533	0.90207	0.05
53534	0.895719	0.05
53535	0.895719	0.05
53536	0.927855	0.05
53537	1.2493	0.05
53538	0.85723	0.05
53539	0.548484	0.05
53540	0.548485	0.05
53541	0.927855	0.05
53542	0.85723	0.05
53543	1.2493	0.05
53544	1.41319	0.736633
53545	1.22482	0.454572
53546	1.46629	0.577024
53547	1.92622	0.702157
53548	3.15104	0.693586
53549	1.02305	0.412852
53550	1.22482	0.495988
53551	1.80336	0.566378
53552	1.44426	0.368593
53553	2.66908	0.372954
53554	1.3268	2.1
53555	0.999489	2.1
53556	1.44091	1.97553
53557	1.14657	2.1
53558	2.4404	2.1
53559	1.89226	1.06441
53560	1.89226	2.1
53561	1.89226	1.96923
53562	1.89226	2.1
53563	3.78452	2.1
53564	1.89226	2.1
53565	2.28605	2.1
53566	2.43157	2.1
53567	2.43157	2.1
53568	1.89226	2.1
53569	1.89226	2.1
53570	1.60647	2.1
53571	1.44091	1.58202
53572	1.31346	2.1
53573	1.31346	2.1
53574	1.14657	2.1
53575	1.44091	2.1
53576	1.89226	1.52535
53577	1.89226	2.1
53578	1.89226	1.50363
53579	1.89226	2.1
53580	3.78452	2.1
53581	0.946132	2.1
53582	0.946132	2.1
53583	1.3048	2.1
53584	1.3048	2.1
53585	1.3048	2.1
53586	1.21578	2.1
53587	0.946132	2.1
53588	0.946132	2.1
53589	1.3048	2.1

53590	0.946132	2.1
53591	0.946132	2.1
53592	1.21578	2.1
53593	1.89227	0.0683961
53594	1.89227	0.0785669
53595	1.89227	0.05
53596	1.89227	0.0619811
53597	3.78453	0.0746248
53598	1.6671	0.05
53599	1.87338	0.0622543
53600	1.6671	0.0583879
53601	2.02098	0.0776401
53602	1.38328	0.05
53603	1.73715	0.0561446
53604	1.89226	0.05
53605	1.89226	0.0569728
53606	1.89226	0.05
53607	1.89226	0.05
53608	3.78453	0.05
53609	1.6671	0.05
53610	1.38328	0.05
53611	1.35562	0.05
53612	1.01012	0.05
53613	2.67722	0.05
53614	0.819377	0.05
53615	0.647925	0.05
53616	1.17802	0.05
53617	1.33174	0.05
53618	0.98543	0.05
53619	0.98543	0.0518018
53620	0.994237	0.05
53621	0.994237	0.05
53622	1.17802	0.05
53623	0.647926	0.05
53624	1.33174	0.05
53625	0.819377	0.05
53626	1.27806	0.05
53627	1.68264	0.05
53628	1.44593	0.05
53629	1.878 0.05	
53630	3.15606	0.05
53631	1.878 0.05	
53632	1.9898	0.05
53633	1.878 0.05	
53634	1.68264	0.05
53635	2.30533	0.05
53636	2.10997	0.05
53637	0.814587	0.05
53638	0.814596	0.05
53639	0.82775	0.05
53640	0.82775	0.05
53641	1.0107	0.05
53642	0.91549	0.05
53643	0.741058	0.05
53644	0.714748	0.05
53645	0.714758	0.05
53646	1.03225	0.05
53647	0.704864	0.05

53648	0.915488	0.05
53649	1.90347	0.05
53650	2.06584	0.05
53651	1.878	0.05
53652	1.50236	0.05
53653	2.05749	0.05
53654	2.30533	0.05
53655	2.24475	2.1
53656	1.85871	2.1
53657	2.13711	2.1
53658	2.13711	2.1
53659	1.9943	2.1
53660	1.9943	2.1
53661	1.85871	2.1
53662	1.7871	2.1
53663	1.41619	2.1
53664	1.41619	2.1
53665	2.13711	2.1
53666	2.13711	2.1
53667	2.24475	2.1
53668	2.13711	2.1
53669	2.54902	2.1
53670	2.46327	2.1
53671	4.70802	2.1
53672	1.06913	2.1
53673	1.06913	2.1
53674	1.0821	2.1
53675	1.0821	2.1
53676	0.893552	2.1
53677	1.08267	2.1
53678	1.06855	2.1
53679	1.23163	2.1
53680	1.23163	2.1
53681	1.08267	2.1
53682	0.893551	2.1
53683	1.06855	2.1
53684	1.67046	0.874737
53685	2.4106	0.515568
53686	2.05835	0.999816
53687	2.4106	1.08764
53688	1.59562	0.584989
53689	1.94788	0.572554
53690	2.34737	0.800322
53691	2.4106	0.534016
53692	2.05834	1.08484
53693	1.99655	0.967367
53694	4.40715	0.756655
53695	1.67046	0.700689
53696	2.05835	0.517166
53697	1.588	0.883676
53698	1.34857	1.30387
53699	3.40692	0.686464
53700	1.73802	0.674876
53701	1.89668	0.850802
53702	1.99655	1.83196
53703	1.99655	0.568433
53704	1.79981	0.754176
53705	2.05834	0.493047

53706	1.89226	1.11243
53707	1.89226	0.894599
53708	1.89226	0.649525
53709	1.89226	1.79543
53710	1.89226	0.661854
53711	1.89226	2.1
53712	1.89226	0.905528
53713	1.89226	2.1
53714	1.89226	1.44033
53715	1.89226	0.689433
53716	3.78453	1.49159
53717	0.719473	2.1
53718	0.916103	1.43901
53719	1.02744	1.01042
53720	0.850328	2.02271
53721	0.888094	0.96412
53722	0.888094	2.1
53723	0.878337	2.05837
53724	0.878337	0.667601
53725	0.916103	1.13668
53726	1.02744	1.25431
53727	0.719473	1.63524
53728	0.850328	1.86405
53729	1.02744	1.18158
53730	0.850329	2.05784
53731	1.0875	1.05234
53732	0.919597	1.31199
53733	0.975841	0.812201
53734	0.975841	2.1
53735	0.971196	0.787075
53736	0.971196	1.88213
53737	1.02744	1.56933
53738	1.0875	1.02216
53739	0.850328	1.90776
53740	0.919598	1.60538
53741	1.89226	1.71907
53742	1.89226	1.33304
53743	1.89226	0.764459
53744	1.89226	1.58519
53745	3.78453	1.1959
53746	1.89226	1.25215
53747	1.89226	0.887478
53748	1.89226	0.681691
53749	1.89226	1.87181
53750	1.89226	0.644365
53751	1.89226	2.1
53752	1.11103	1.4057
53753	0.916103	2.1
53754	1.21177	1.1412
53755	1.02744	1.2319
53756	1.07188	0.860421
53757	1.07188	2.1
53758	1.06659	0.8266
53759	1.06659	1.98449
53760	1.11103	1.93908
53761	1.21177	1.43087
53762	0.916104	1.87421
53763	1.02744	1.99648

53764	2.54158	0.975567
53765	2.42354	1.27318
53766	2.17499	0.773103
53767	2.17499	2.1
53768	2.42354	1.63112
53769	2.05488	2.1
53770	1.89226	0.0999463
53771	1.89226	0.155907
53772	1.89226	0.118812
53773	1.89226	0.167125
53774	3.78453	0.114438
53775	1.89226	0.0862399
53776	1.89226	0.14262
53777	1.89226	0.0989789
53778	1.89226	0.125902
53779	1.89226	0.106366
53780	1.89226	0.140961
53781	1.89226	0.21034
53782	1.89226	0.23403
53783	1.89226	0.202303
53784	1.89226	0.139678
53785	1.89226	0.329833
53786	1.89226	0.247466
53787	1.89226	0.234554
53788	1.89226	0.298213
53789	1.89226	0.238796
53790	1.89226	0.343136
53791	3.78452	0.301164
53792	0.802544	0.114607
53793	0.946132	0.0782648
53794	0.946132	0.0952254
53795	0.787038	0.132452
53796	0.787039	0.105002
53797	0.787039	0.111511
53798	0.961637	0.0977935
53799	0.961637	0.0967924
53800	0.787038	0.108016
53801	0.802544	0.107928
53802	0.946132	0.0978495
53803	0.946132	0.0964953
53804	1.89226	0.0761226
53805	1.60509	0.107933
53806	1.89226	0.0803583
53807	1.89226	0.0939663
53808	1.3905	0.0899798
53809	1.3905	0.10617
53810	0.946132	0.247569
53811	0.946132	0.104239
53812	0.946131	0.183487
53813	0.946131	0.203111
53814	0.946132	0.202371
53815	0.946132	0.203614
53816	0.946131	0.172184
53817	0.946131	0.177794
53818	0.946131	0.179596
53819	0.946131	0.192726
53820	0.946132	0.180757
53821	0.946132	0.162138

53822	0.946131	0.236504
53823	0.946131	0.263604
53824	0.946131	0.309097
53825	0.946131	0.16262
53826	0.946132	0.24709
53827	0.946132	0.26432
53828	0.946131	0.186093
53829	0.946131	0.183637
53830	0.946131	0.237066
53831	0.946131	0.145855
53832	0.946132	0.194694
53833	0.946132	0.193766
53834	1.42382	1.75564
53835	1.32201	0.662108
53836	1.32096	0.786354
53837	1.42866	1.17318
53838	2.75067	0.908146
53839	1.89226	1.44638
53840	1.89226	0.578507
53841	1.89226	0.738996
53842	1.89226	0.838517
53843	1.89226	0.6725
53844	1.89226	0.765476
53845	1.89226	0.961556
53846	2.79029	0.531833
53847	2.7803	0.632541
53848	2.79029	0.66601
53849	1.88227	0.579291
53850	1.89226	0.619951
53851	0.716065	0.970312
53852	0.714333	1.56093
53853	0.660483	0.682738
53854	0.659126	0.843847
53855	0.687538	0.680967
53856	0.687538	0.928248
53857	0.685921	1.9694
53858	0.685921	0.728753
53859	0.659126	0.823432
53860	0.716065	0.835806
53861	0.660483	0.879413
53862	0.714333	0.873315
53863	1.89226	0.93765
53864	1.89226	0.579749
53865	1.89226	0.709128
53866	1.89226	0.808474
53867	3.78453	0.784442
53868	0.946133	0.614725
53869	0.946133	0.716316
53870	1.39015	0.592568
53871	1.38969	0.496256
53872	0.946132	0.502894
53873	0.946132	0.606537
53874	1.38969	0.741935
53875	1.38969	0.597794
53876	1.38969	0.591279
53877	0.946132	0.593072
53878	0.946133	0.667059
53879	1.39015	0.605721

53880	2.72157	0.445425
53881	1.22176	0.392827
53882	1.59492	0.413524
53883	1.22176	0.483467
53884	2.40743	0.391946
53885	2.03426	0.471755
53886	0.946131	0.495997
53887	0.946131	0.410698
53888	1.36079	0.394813
53889	1.01713	0.437321
53890	0.94613	0.428547
53891	0.94613	0.51504
53892	1.36079	0.388919
53893	1.36079	0.360099
53894	0.946131	0.450445
53895	1.36079	0.472589
53896	1.01713	0.445451
53897	0.946131	0.454425
53898	1.89226	0.449291
53899	1.89226	0.388193
53900	1.89226	0.349905
53901	1.89226	0.557148
53902	3.78453	0.439042
53903	1.89226	0.404469
53904	1.89226	0.374709
53905	1.89226	0.379342
53906	1.89226	0.485553
53907	1.89226	0.290564
53908	1.89226	0.435396
53909	0.946131	0.480841
53910	0.946131	0.399199
53911	0.946131	0.430824
53912	1.36079	0.361015
53913	0.94613	0.429158
53914	0.94613	0.511955
53915	0.946133	0.391229
53916	0.946133	0.190275
53917	0.946131	0.415522
53918	0.946131	0.468893
53919	0.946132	0.292449
53920	1.36079	0.408777
53921	1.89226	0.379463
53922	1.89226	0.542598
53923	1.89226	0.395183
53924	1.89226	0.473525
53925	1.89226	0.411006
53926	1.89226	0.48623
53927	1.89227	0.43043
53928	1.89227	0.337523
53929	1.89227	0.392071
53930	1.89227	0.482263
53931	1.89227	0.284058
53932	1.89227	0.403957
53933	1.60428	2.1
53934	2.2367	2.1
53935	1.50097	2.1
53936	1.80125	0.90861
53937	3.40553	2.1

53938	1.89095	2.1
53939	1.85293	0.575977
53940	1.80125	1.03316
53941	1.76268	0.574984
53942	3.65418	0.766214
53943	0.803177	2.1
53944	0.803177	2.1
53945	0.798644	2.1
53946	0.798644	1.97292
53947	0.84233	1.88254
53948	0.900624	2.05053
53949	0.750483	2.1
53950	0.701197	2.1
53951	0.701197	2.1
53952	0.84233	2.1
53953	0.750483	2.1
53954	0.900625	2.1
53955	0.860925	0.45331
53956	0.860925	2.1
53957	0.862743	1.92887
53958	0.862743	0.634495
53959	0.881338	0.590909
53960	0.826621	0.570959
53961	0.84233	2.03289
53962	0.900625	2.1
53963	0.84233	0.721176
53964	0.826621	0.730451
53965	0.900624	0.757417
53966	0.881339	0.734045
53967	1.37938	0.93673
53968	1.03319	0.911504
53969	1.00512	1.25801
53970	0.639582	1.33679
53971	1.00463	1.25458
53972	1.00463	0.88265
53973	1.01434	0.834652
53974	1.01434	1.44514
53975	1.37938	1.05216
53976	1.00512	1.40939
53977	1.03319	1.20431
53978	0.639582	1.14918
53979	0.728713	0.104213
53980	0.728713	0.102562
53981	0.73425	0.492265
53982	0.73425	0.590083
53983	0.525122	2.1
53984	0.605622	0.0803927
53985	0.948901	0.0803927
53986	0.857342	0.0803927
53987	0.85734	0.0810225
53988	0.525123	0.583809
53989	0.948899	0.0810225
53990	0.605624	0.0810225
53991	1.75449	0.656713
53992	1.4378	0.701426
53993	1.93916	0.78374
53994	2.22512	0.560605
53995	3.69364	0.714509

53996	1.75448	0.816968
53997	1.89186	0.579384
53998	2.29771	0.640155
53999	2.22512	0.58437
54000	4.05219	0.62576
54001	1.93916	0.76688
54002	2.51749	0.524643
54003	2.62357	0.614201
54004	2.22512	0.567424
54005	2.33761	0.714386
54006	1.93916	0.632529
54007	2.29771	0.672919
54008	2.51749	0.545684
54009	2.22512	0.542253
54010	2.22512	0.512619
54011	2.50905	0.700989
54012	2.50905	0.632034
54013	0.87382	0.0704814
54014	1.33662	0.106264
54015	1.20756	0.0887664
54016	0.890467	0.155586
54017	1.08859	0.154298
54018	1.08859	0.144435
54019	1.13084	0.0709701
54020	1.08793	0.105471
54021	1.34722	0.104767
54022	1.27184	0.124518
54023	0.917678	0.119231
54024	0.97821	0.11876
54025	1.28396	0.10024
54026	1.70302	0.191567
54027	2.30076	0.143165
54028	1.20417	0.107296
54029	2.57506	0.148005
54030	2.2656	0.131668
54031	1.03642	0.816889
54032	1.03642	0.409759
54033	1.07029	0.375417
54034	1.07029	0.438751
54035	1.31531	0.705916
54036	1.23279	0.450021
54037	0.941743	0.403307
54038	0.791399	0.60929
54039	0.791399	0.454116
54040	1.23279	0.45513
54041	0.941744	0.453572
54042	1.31531	0.457224
54043	0.937354	0.877847
54044	0.937354	0.397925
54045	0.954794	0.394246
54046	0.954794	0.449511
54047	1.13563	0.444108
54048	1.23279	0.711842
54049	0.791398	0.414798
54050	0.659359	0.584784
54051	1.13563	0.453525
54052	0.659359	0.458557
54053	0.791399	0.448913

54054	1.23279	0.45232
54055	2.29852	0.878287
54056	1.89227	0.825745
54057	2.14485	0.748365
54058	2.14485	0.988186
54059	1.89227	0.785912
54060	1.89227	1.01946
54061	0.947863	0.823029
54062	1.19854	0.961878
54063	2.16841	0.922037
54064	1.76597	0.836948
54065	2.96451	0.799344
54066	1.89227	0.758793
54067	1.89227	0.750297
54068	1.89227	0.694102
54069	1.89227	0.920932
54070	1.89227	0.698673
54071	1.89227	0.914599
54072	2.46489	2.1
54073	1.98339	2.1
54074	1.81667	2.1
54075	2.36914	2.1
54076	4.35253	2.1
54077	2.46488	2.1
54078	2.70401	2.1
54079	2.64996	2.1
54080	2.36914	2.1
54081	5.07315	2.1
54082	1.81667	2.1
54083	2.29459	2.1
54084	2.36914	2.1
54085	2.36914	2.1
54086	1.68073	2.1
54087	1.68073	2.1
54088	2.64996	2.1
54089	2.29458	2.1
54090	2.29458	2.1
54091	2.36914	2.1
54092	2.61243	2.1
54093	2.68698	2.1
54094	1.89226	1.18092
54095	1.89226	0.980858
54096	1.89226	1.41529
54097	1.89226	1.01915
54098	1.89226	1.57148
54099	1.89226	1.03942
54100	1.89224	1.46342
54101	1.89224	0.946825
54102	1.65571	1.15191
54103	1.65571	0.942693
54104	1.89224	1.3469
54105	1.89224	1.079
54106	1.89224	1.17174
54107	1.89224	1.04156
54108	1.89224	1.32392
54109	1.89224	0.915126
54110	1.89224	1.66558
54111	1.89224	1.00676

54112	1.98252	0.242466
54113	2.20641	0.270246
54114	1.05129	0.213603
54115	1.2951	0.33425
54116	3.27762	0.236941
54117	1.85598	2.01447
54118	1.88635	1.06158
54119	2.12346	0.949637
54120	2.10838	2.1
54121	3.97943	1.24232
54122	2.10838	1.16521
54123	1.87532	2.1
54124	1.85597	0.761161
54125	1.85597	2.1
54126	1.87532	1.20852
54127	1.57914	2.1
54128	2.12348	1.07183
54129	2.35141	1.91074
54130	2.37191	1.19621
54131	2.10841	1.39613
54132	2.38698	0.984787
54133	2.12348	1.56424
54134	1.07811	1.87659
54135	0.937672	2.1
54136	1.17571	1.74541
54137	1.05421	0.801292
54138	1.07087	1.06641
54139	1.07087	2.1
54140	1.06145	2.0264
54141	1.06145	0.944909
54142	1.17571	1.98307
54143	1.07811	1.86303
54144	1.05421	1.82605
54145	0.937672	1.8762
54146	1.67334	2.1
54147	2.76609	2.1
54148	2.74144	2.1
54149	2.74144	2.1
54150	1.71157	2.1
54151	1.71157	2.1
54152	2.76609	2.1
54153	1.89226	2.1
54154	1.89226	2.1
54155	1.89226	2.1
54156	2.74143	2.1
54157	2.74143	2.1
54158	1.10393	2.1
54159	1.10393	2.1
54160	1.10788	2.1
54161	1.10788	2.1
54162	1.38302	2.1
54163	1.37072	2.1
54164	0.836672	2.1
54165	0.841089	2.1
54166	1.38302	2.1
54167	0.84109	2.1
54168	0.836671	2.1
54169	1.37072	2.1

54170	1.38302	2.1
54171	1.38302	2.1
54172	0.946131	2.1
54173	0.946131	2.1
54174	0.946128	2.1
54175	0.946128	2.1
54176	1.38302	2.1
54177	1.37072	2.1
54178	1.38302	2.1
54179	0.946131	2.1
54180	0.94613	2.1
54181	1.37072	2.1
54182	1.28072	0.617704
54183	1.55779	2.1
54184	1.28072	1.2467
54185	1.40765	0.706762
54186	1.47186	1.37203
54187	1.59879	0.896951
54188	1.89227	0.642919
54189	1.89227	0.625392
54190	1.89227	0.512558
54191	1.89227	1.16272
54192	1.89227	0.451518
54193	1.89227	0.903832
54194	2.80057	0.54892
54195	1.89226	0.532959
54196	1.89226	0.503098
54197	1.89226	0.640392
54198	2.63061	0.426621
54199	2.63061	0.682902
54200	1.28072	0.580505
54201	1.38859	2.1
54202	1.47186	1.11468
54203	1.47186	0.727817
54204	1.16777	1.37443
54205	1.16777	0.793442
54206	1.89226	0.597198
54207	1.89226	0.620273
54208	1.89226	0.551193
54209	1.89226	1.29851
54210	1.89226	0.495944
54211	1.89226	1.72056
54212	1.83221	2.1
54213	2.0815	2.1
54214	2.0815	2.1
54215	2.08153	2.1
54216	1.86703	2.1
54217	1.87402	2.1
54218	1.86703	2.1
54219	2.08508	2.1
54220	2.08508	2.1
54221	2.29001	2.1
54222	1.89226	2.1
54223	2.08048	2.1
54224	2.7278	0.346906
54225	1.10909	0.363038
54226	1.50472	0.359276
54227	1.10909	0.373803

54228	2.69881	0.341657
54229	2.30319	0.368303
54230	1.23204	2.1
54231	1.92264	2.1
54232	1.48162	2.1
54233	1.48162	2.1
54234	1.72994	2.1
54235	1.72994	2.1
54236	0.864968	2.1
54237	0.696952	2.1
54238	0.61602	2.1
54239	0.479736	2.1
54240	0.66444	2.1
54241	0.66444	2.1
54242	0.680264	2.1
54243	0.680264	2.1
54244	0.696952	2.1
54245	0.479736	2.1
54246	0.61602	2.1
54247	0.864968	2.1
54248	1.89226	0.643305
54249	2.43219	1.96153
54250	1.89226	1.11905
54251	2.82019	1.17891
54252	1.89226	1.61482
54253	2.82019	1.30622
54254	1.89226	0.725857
54255	2.83701	2.08867
54256	2.2701	1.16093
54257	2.2701	0.990732
54258	1.89226	1.29415
54259	1.89226	1.21287
54260	1.89226	1.53069
54261	1.89226	0.721481
54262	1.89226	0.848612
54263	1.89226	1.18356
54264	1.89226	1.12185
54265	1.89226	1.11196
54266	1.5795	1.66847
54267	1.44091	1.70253
54268	1.05267	1.97052
54269	1.05267	1.25107
54270	1.98408	1.70853
54271	1.98408	1.14756
54272	2.20624	2.1
54273	1.08831	2.1
54274	2.49053	2.1
54275	1.42486	2.1
54276	3.57883	2.1
54277	1.89226	2.1
54278	1.89226	2.1
54279	1.89226	2.1
54280	1.89226	2.1
54281	3.78453	2.1
54282	0.946132	2.1
54283	0.946132	2.1
54284	0.853591	2.1
54285	0.642207	2.1

54286	0.94613	2.1
54287	0.94613	2.1
54288	0.642209	2.1
54289	0.642209	2.1
54290	0.946131	2.1
54291	0.853591	2.1
54292	0.642207	2.1
54293	0.946132	2.1
54294	1.0506	2.1
54295	1.0506	2.1
54296	1.0879	2.1
54297	0.960424	2.1
54298	0.708687	2.1
54299	0.782927	2.1
54300	1.40041	2.1
54301	1.2518	2.1
54302	0.767748	2.1
54303	1.31801	2.1
54304	1.21412	2.1
54305	0.69461	2.1
54306	1.89226	2.1
54307	1.89226	2.1
54308	1.89226	2.1
54309	1.89226	2.1
54310	1.89226	2.1
54311	1.89226	2.1
54312	0.922223	2.1
54313	0.922223	2.1
54314	1.04548	2.1
54315	0.768229	2.1
54316	0.948441	2.1
54317	0.806483	2.1
54318	0.85362	2.1
54319	0.85362	2.1
54320	1.05942	2.1
54321	0.922187	2.1
54322	0.851554	2.1
54323	0.768264	2.1
54324	2.16728	2.1
54325	0.95455	2.1
54326	1.21372	2.1
54327	0.98227	2.1
54328	2.16728	2.1
54329	1.93584	2.1
54330	2.08849	0.326457
54331	1.08732	0.33549
54332	1.3357	0.330553
54333	2.37948	0.329839
54334	3.4668	0.329925
54335	2.57882	0.322585
54336	2.37948	0.327416
54337	2.53055	0.327715
54338	1.48676	0.332747
54339	1.3357	0.333458
54340	1.3357	0.327587
54341	1.89226	2.1
54342	1.89226	2.1
54343	2.17471	2.1

54344	1.89226	2.1
54345	2.60085	2.1
54346	2.26306	2.1
54347	0.946131	1.02651
54348	0.946131	2.1
54349	0.946131	2.01133
54350	0.946131	2.1
54351	0.94613	2.1
54352	0.94613	2.1
54353	0.946132	2.1
54354	0.946132	2.1
54355	0.946131	2.1
54356	0.946131	2.1
54357	0.946131	2.1
54358	0.946131	2.1
54359	1.89226	2.1
54360	2.17471	2.1
54361	2.08196	2.1
54362	2.17471	2.1
54363	1.79952	2.1
54364	1.89226	2.1
54365	1.89226	2.1
54366	1.89226	2.1
54367	1.89226	2.1
54368	1.89226	2.1
54369	1.89226	2.1
54370	1.89226	2.1
54371	1.86016	2.1
54372	1.89226	2.1
54373	1.89226	2.1
54374	1.89226	2.1
54375	1.84826	2.1
54376	1.84826	2.1
54377	2.50136	2.1
54378	1.99781	2.1
54379	1.99729	2.1
54380	2.5214	2.1
54381	4.49865	2.1
54382	0.51724	2.1
54383	0.496076	2.1
54384	0.998906	2.1
54385	0.998645	2.1
54386	0.742249	2.1
54387	0.742249	2.1
54388	0.752734	2.1
54389	0.752734	2.1
54390	0.998639	2.1
54391	0.496073	2.1
54392	0.517243	2.1
54393	0.998913	2.1
54394	0.998653	2.1
54395	0.994293	2.1
54396	1.26071	2.1
54397	1.26504	2.1
54398	1.12529	2.1
54399	1.12529	2.1
54400	1.12971	2.1
54401	1.12971	2.1

54402	1.26504	2.1
54403	0.994292	2.1
54404	0.998651	2.1
54405	1.26071	2.1
54406	1.89226	2.1
54407	1.89226	2.1
54408	1.89226	2.1
54409	1.89226	2.1
54410	3.78452	2.1
54411	1.89226	2.1
54412	1.89226	2.1
54413	1.89226	2.1
54414	1.89226	2.1
54415	3.78452	2.1
54416	0.675872	2.1
54417	0.675641	2.1
54418	0.943072	2.1
54419	0.898317	2.1
54420	0.775717	2.1
54421	0.775717	2.1
54422	0.79824	2.1
54423	0.79824	2.1
54424	0.675642	2.1
54425	0.94307	2.1
54426	0.675874	2.1
54427	0.898315	2.1
54428	0.943071	2.1
54429	0.898316	2.1
54430	0.998133	2.1
54431	0.918903	2.1
54432	0.922369	2.1
54433	0.922369	2.1
54434	0.939606	2.1
54435	0.939606	2.1
54436	0.943071	2.1
54437	0.998133	2.1
54438	0.898316	2.1
54439	0.918903	2.1
54440	0.946131	2.1
54441	0.946131	2.1
54442	0.946131	2.1
54443	0.946131	2.1
54444	0.946132	2.1
54445	0.946132	2.1
54446	0.946129	2.1
54447	0.946129	2.1
54448	0.94613	2.1
54449	0.94613	2.1
54450	0.946131	2.1
54451	0.946131	2.1
54452	1.89226	2.1
54453	1.89226	2.1
54454	1.89226	2.1
54455	1.89226	2.1
54456	1.89226	2.1
54457	1.89226	2.1
54458	1.35129	2.1
54459	1.88614	2.1

54460	2.01867	2.1
54461	1.38189	2.1
54462	3.36995	2.1
54463	0.943071	2.1
54464	1.00934	2.1
54465	0.998134	2.1
54466	1.10866	2.1
54467	0.992617	2.1
54468	0.992617	2.1
54469	1.01485	2.1
54470	1.01485	2.1
54471	1.00934	2.1
54472	1.10866	2.1
54473	0.943072	2.1
54474	0.998134	2.1
54475	2.67272	2.1
54476	1.61859	2.1
54477	1.70303	2.1
54478	2.52175	2.1
54479	4.5326	2.1
54480	2.00954	2.1
54481	2.27801	2.1
54482	1.87875	2.1
54483	1.87875	2.1
54484	2.37837	2.1
54485	2.37837	2.1
54486	2.00955	2.1
54487	1.78	2.1
54488	2.21813	2.1
54489	1.8493	2.1
54490	2.37838	2.1
54491	2.00955	2.1
54492	2.37838	2.1
54493	2.27801	2.1
54494	2.58523	2.1
54495	2.71597	2.1
54496	4.9636	2.1
54497	2.37838	2.1
54498	2.71597	2.1
54499	1.72709	2.1
54500	2.0815	2.1
54501	2.59749	2.1
54502	2.78034	2.1
54503	0.94613	0.725072
54504	0.94613	0.758706
54505	0.946128	0.631386
54506	0.946128	0.603071
54507	0.94613	0.61351
54508	0.94613	0.64773
54509	0.946128	0.755559
54510	0.946128	0.563879
54511	0.946129	0.702486
54512	0.946129	0.682289
54513	0.946129	0.657246
54514	0.946129	0.669336
54515	1.89226	0.867505
54516	1.89226	0.79797
54517	1.89226	0.863607

54518	1.89226	0.701334
54519	3.78452	0.721176
54520	0.946133	0.600865
54521	0.946133	0.612487
54522	0.946132	0.504791
54523	0.946132	0.516502
54524	0.946132	0.544368
54525	0.946132	0.532184
54526	0.946133	0.601106
54527	0.946133	0.582187
54528	0.946132	0.577371
54529	0.946132	0.437132
54530	0.946132	0.523363
54531	0.946132	0.538176
54532	1.89226	0.660196
54533	2.10495	0.518847
54534	1.89226	0.515333
54535	2.30932	0.51229
54536	1.89226	0.736618
54537	2.30932	0.727273
54538	2.30932	0.533705
54539	1.00366	0.361142
54540	2.10496	0.435121
54541	1.24728	0.47412
54542	3.31298	0.475348
54543	1.87557	2.1
54544	1.07167	2.1
54545	2.03045	2.1
54546	1.25185	2.1
54547	3.10212	2.1
54548	1.89226	2.1
54549	1.89226	2.1
54550	1.89226	2.1
54551	1.89226	2.1
54552	3.78453	2.1
54553	0.851541	2.1
54554	0.851541	2.1
54555	1.12236	2.1
54556	1.05783	2.1
54557	0.870663	2.1
54558	0.951856	2.1
54559	0.946132	2.1
54560	0.946132	2.1
54561	1.04073	2.1
54562	1.1296	2.1
54563	0.776062	2.1
54564	0.851527	2.1
54565	0.859395	2.1
54566	0.859395	2.1
54567	0.870165	2.1
54568	0.870165	2.1
54569	0.625924	2.1
54570	0.714333	2.1
54571	1.0821	2.1
54572	1.01523	2.1
54573	1.0821	2.1
54574	0.714333	2.1
54575	1.01523	2.1

54576	0.625924	2.1
54577	1.89226	2.1
54578	1.89226	2.1
54579	1.89226	2.1
54580	1.89226	2.1
54581	3.78453	2.1
54582	1.89226	2.1
54583	1.89226	2.1
54584	2.23164	2.1
54585	2.67802	2.1
54586	2.03663	2.1
54587	1.89226	2.1
54588	2.42221	2.1
54589	1.61933	2.1
54590	2.80896	2.1
54591	1.87043	2.1
54592	4.29262	2.1
54593	1.89226	1.8622
54594	1.89226	2.1
54595	1.89226	2.1
54596	1.89226	2.1
54597	1.89226	2.1
54598	1.89226	2.1
54599	2.80892	2.1
54600	1.87041	2.1
54601	2.67182	1.66509
54602	2.30891	2.1
54603	5.07665	2.1
54604	1.89224	2.05139
54605	1.89224	2.1
54606	1.89224	1.62591
54607	1.89224	2.1
54608	3.78448	2.1
54609	0.946118	2.1
54610	0.946118	2.1
54611	0.946118	2.1
54612	0.946118	2.1
54613	0.946115	2.1
54614	0.946115	2.1
54615	0.94612	2.1
54616	0.94612	2.1
54617	0.94613	2.1
54618	0.94613	2.1
54619	0.946105	2.1
54620	0.946105	2.1
54621	2.3089	2.1
54622	1.02783	2.1
54623	1.98533	2.1
54624	1.76762	2.1
54625	1.24553	2.1
54626	1.02783	2.1
54627	2.2049	0.284471
54628	2.34673	0.266251
54629	1.00763	0.246649
54630	1.23746	0.344621
54631	3.44236	0.272432
54632	1.89226	0.413877
54633	1.89226	0.450541

54634	1.89226	0.422298
54635	1.89226	0.441325
54636	3.78452	0.445783
54637	1.89226	0.454134
54638	1.89226	0.440559
54639	1.89226	0.443471
54640	1.89226	0.46717
54641	3.78453	0.448866
54642	0.891618	0.358142
54643	0.891618	0.357407
54644	0.902158	0.27815
54645	0.902158	0.298739
54646	1.15396	0.35704
54647	0.620408	0.309513
54648	1.17337	0.274042
54649	0.618733	0.353476
54650	0.620408	0.342027
54651	0.618733	0.297885
54652	1.17337	0.329418
54653	1.15396	0.351334
54654	0.69978	0.348483
54655	0.69978	0.33585
54656	0.946102	0.35199
54657	0.946102	0.355472
54658	0.831702	0.366498
54659	0.946129	0.386501
54660	0.699752	0.325618
54661	0.946132	0.30895
54662	0.831703	0.36049
54663	0.699751	0.299162
54664	0.946131	0.316824
54665	0.946131	0.360434
54666	0.831675	0.393549
54667	0.831675	0.39182
54668	0.946163	0.384115
54669	0.864397	0.385924
54670	0.853471	0.419046
54671	0.962623	0.380427
54672	0.748555	0.368015
54673	0.851543	0.389758
54674	0.864714	0.391555
54675	0.760411	0.377853
54676	0.946133	0.391075
54677	0.946133	0.400308
54678	1.89226	0.492502
54679	1.89226	0.744694
54680	1.89226	0.650441
54681	1.89226	0.60374
54682	3.78452	0.6598
54683	1.89226	0.690435
54684	1.89226	0.59518
54685	1.89226	0.583882
54686	1.89226	0.823774
54687	3.78452	0.628132
54688	0.946132	0.789863
54689	0.946132	0.870309
54690	0.946132	0.671333
54691	0.946132	0.718869

54692	0.946132	0.663424
54693	0.946132	1.04473
54694	0.946133	0.582453
54695	0.946133	0.837735
54696	0.946133	0.703363
54697	0.946133	0.667431
54698	0.946132	0.733247
54699	0.946132	0.86891
54700	1.89226	0.751362
54701	2.39715	0.756905
54702	1.89226	0.900822
54703	1.89226	0.994033
54704	1.89226	0.613731
54705	1.89226	0.674657
54706	0.94613	0.810622
54707	0.94613	0.75495
54708	0.94613	0.682437
54709	0.94613	0.646314
54710	0.946131	0.685431
54711	0.946131	0.919837
54712	0.946129	0.754944
54713	0.946129	0.641339
54714	0.94613	0.644409
54715	0.94613	0.707609
54716	0.94613	0.745571
54717	0.94613	0.752818
54718	2.54204	2.1
54719	2.3148	2.1
54720	2.55328	2.1
54721	2.39646	2.1
54722	4.71126	2.1
54723	1.62611	2.1
54724	2.3148	2.1
54725	2.16986	2.1
54726	1.43745	2.1
54727	3.75225	2.1
54728	1.89226	2.1
54729	1.89226	2.1
54730	1.89226	2.1
54731	1.89226	2.1
54732	3.78453	2.1
54733	1.89226	2.1
54734	1.89226	2.1
54735	1.89226	2.1
54736	1.89226	2.1
54737	3.78453	2.1
54738	2.12125	2.1
54739	2.39642	2.1
54740	2.2941	2.1
54741	1.9933	2.1
54742	4.38972	2.1
54743	1.73784	2.1
54744	1.26892	2.1
54745	1.26892	2.1
54746	1.43743	2.1
54747	1.9933	2.1
54748	2.1618	2.1
54749	0.946118	2.1

54750	0.946118	2.1
54751	0.946118	2.1
54752	0.946118	2.1
54753	0.946119	2.1
54754	0.946119	2.1
54755	0.946118	2.1
54756	0.946118	2.1
54757	0.946131	2.1
54758	0.946131	2.1
54759	0.946106	2.1
54760	0.946106	2.1
54761	1.89224	2.1
54762	1.89224	2.1
54763	1.89224	2.1
54764	1.89224	2.1
54765	3.78448	2.1
54766	2.45922	2.1
54767	2.15686	2.1
54768	2.14259	2.1
54769	2.43225	2.1
54770	4.58911	2.1
54771	1.62027	2.1
54772	2.15686	2.1
54773	1.62241	2.1
54774	1.62241	2.1
54775	2.1426	2.1
54776	2.1426	2.1
54777	2.14259	2.1
54778	2.43225	2.1
54779	2.40667	2.1
54780	2.1281	2.1
54781	4.56035	2.1
54782	1.0713	2.1
54783	1.06405	2.1
54784	0.810136	2.1
54785	0.808433	2.1
54786	0.938508	2.1
54787	0.938508	2.1
54788	0.941223	2.1
54789	0.941223	2.1
54790	1.06405	2.1
54791	0.808433	2.1
54792	0.810135	2.1
54793	1.0713	2.1
54794	1.89226	0.561892
54795	1.89226	0.404212
54796	1.89226	0.538614
54797	1.89226	0.461083
54798	3.78453	0.465262
54799	1.89227	0.437412
54800	1.89227	0.395297
54801	1.89227	0.528449
54802	1.89227	0.31791
54803	3.78453	0.432553
54804	1.19	0.291686
54805	1.12469	0.180049
54806	1.01052	0.248541
54807	1.08166	0.209425

54808	2.20051	0.203838
54809	0.594999	0.306566
54810	0.540832	0.260091
54811	0.514182	0.379663
54812	0.567857	0.255033
54813	0.554259	0.235459
54814	0.554259	0.221906
54815	0.55443	0.30821
54816	0.55443	0.321938
54817	0.540832	0.278282
54818	0.514182	0.22735
54819	0.567857	0.257416
54820	0.594999	0.250175
54821	1.38674	0.392531
54822	0.946132	0.468706
54823	1.36361	0.374099
54824	0.946132	0.448986
54825	1.36362	0.367864
54826	1.36362	0.273263
54827	0.946131	0.467407
54828	0.946131	0.505673
54829	1.38674	0.413384
54830	1.36361	0.327342
54831	0.946132	0.451036
54832	0.946132	0.423058
54833	1.89226	0.426656
54834	2.68508	0.25705
54835	2.77349	0.313868
54836	1.89226	0.358898
54837	2.77349	0.323515
54838	1.89226	0.361359
54839	2.15315	0.296669
54840	1.96254	0.322397
54841	1.51377	0.317578
54842	1.89226	0.317852
54843	1.47799	0.304695
54844	1.89226	0.30196
54845	2.49439	0.323306
54846	1.89226	0.349311
54847	2.15315	0.341524
54848	1.89226	0.333526
54849	2.15315	0.341717
54850	1.89226	0.327962
54851	1.89226	0.353221
54852	1.89226	0.36669
54853	1.89226	0.350491
54854	1.89226	0.336937
54855	1.89226	0.399859
54856	1.89226	0.377089
54857	1.89226	0.340174
54858	1.89226	0.334638
54859	1.89226	0.326518
54860	1.89226	0.335006
54861	1.89226	0.322388
54862	1.89226	0.325305
54863	1.375	0.327039
54864	2.01865	0.354351
54865	2.12047	0.332935

54866	1.47354	0.32482
54867	2.12047	0.362686
54868	1.47354	0.346674
54869	0.687499	0.235961
54870	1.06023	0.265286
54871	1.08019	0.255052
54872	0.604229	0.214793
54873	1.08018	0.259001
54874	1.06023	0.279479
54875	0.687502	0.222523
54876	0.60423	0.218429
54877	0.80646	0.238014
54878	0.806461	0.243585
54879	0.858001	0.268888
54880	0.858001	0.232865
54881	2.26414	2.1
54882	1.87342	2.1
54883	1.87342	2.1
54884	1.79667	2.1
54885	2.44045	2.1
54886	2.31924	2.1
54887	2.26414	2.1
54888	2.26616	2.1
54889	2.2355	2.1
54890	2.21858	2.1
54891	4.48474	2.1
54892	2.18516	2.1
54893	1.87344	2.1
54894	2.21859	2.1
54895	2.66233	2.1
54896	1.85196	2.1
54897	2.22236	2.1
54898	2.23551	2.1
54899	2.21859	2.1
54900	2.18516	2.1
54901	2.21189	2.1
54902	4.43049	2.1
54903	1.17171	2.1
54904	2.38924	2.1
54905	2.0815	2.1
54906	2.33348	2.1
54907	1.42851	2.1
54908	1.5959	2.1
54909	1.89226	2.1
54910	1.89226	2.1
54911	1.89226	2.1
54912	1.89226	2.1
54913	3.78452	2.1
54914	2.75425	2.1
54915	1.34366	2.1
54916	1.34366	2.1
54917	1.56512	2.1
54918	2.54186	2.1
54919	2.76332	2.1
54920	1.07751	2.1
54921	2.0815	2.1
54922	1.97259	2.1
54923	1.76455	2.1

54924	1.30191	2.1
54925	1.16461	2.1
54926	0.946131	2.1
54927	0.946131	2.1
54928	0.946129	2.1
54929	0.946129	2.1
54930	0.946131	2.1
54931	0.946131	2.1
54932	0.94613	2.1
54933	0.94613	2.1
54934	0.94613	2.1
54935	0.94613	2.1
54936	0.946131	2.1
54937	0.946131	2.1
54938	1.89226	0.415126
54939	1.89226	0.370693
54940	1.89226	0.341771
54941	1.89226	0.262388
54942	3.78453	0.277858
54943	1.89226	0.422515
54944	1.89226	0.391566
54945	1.89226	0.360859
54946	1.89226	0.253633
54947	3.78453	0.408456
54948	0.946132	0.311605
54949	0.946132	0.389349
54950	1.30327	0.253681
54951	1.33544	0.33581
54952	1.33544	0.253955
54953	1.33544	0.219285
54954	0.946132	0.290241
54955	0.946132	0.274196
54956	0.946132	0.225552
54957	1.30327	0.196259
54958	1.33544	0.31909
54959	0.946132	0.358585
54960	1.30327	0.247419
54961	1.33544	0.301779
54962	0.709426	0.228199
54963	0.790129	0.27553
54964	1.05886	0.166202
54965	1.05886	0.215308
54966	1.03454	0.332084
54967	1.03454	0.267465
54968	1.30327	0.221429
54969	0.709426	0.169276
54970	1.33544	0.335747
54971	0.790128	0.266552
54972	0.946132	0.201193
54973	0.946132	0.294923
54974	0.827866	0.156465
54975	0.946133	0.263777
54976	0.946132	0.151235
54977	0.946132	0.176617
54978	0.946133	0.20351
54979	0.946133	0.288998
54980	0.946132	0.15491
54981	0.827866	0.265086

54982	0.946133	0.31748
54983	0.946133	0.279981
54984	1.89227	0.338831
54985	1.89227	0.17879
54986	1.89227	0.220254
54987	1.89227	0.257284
54988	1.89227	0.22313
54989	1.89227	0.262447
54990	0.946133	0.18764
54991	0.946133	0.266127
54992	1.21602	0.154447
54993	1.30327	0.225465
54994	1.30327	0.162659
54995	1.30327	0.149669
54996	0.946132	0.17609
54997	0.946132	0.185973
54998	0.946132	0.148552
54999	1.21602	0.133463
55000	0.946133	0.231863
55001	1.30327	0.203019
55002	1.04227	0.13813
55003	1.11706	0.211183
55004	0.968413	0.11373
55005	0.945482	0.152454
55006	1.3204	0.163916
55007	1.23215	0.172239
55008	0.810791	0.185358
55009	1.38978	0.123559
55010	1.07728	0.186874
55011	1.11704	0.20157
55012	0.608052	0.183398
55013	1.89226	0.134054
55014	1.89226	0.0765167
55015	1.89226	0.0895795
55016	1.89226	0.109068
55017	3.78453	0.102523
55018	1.89226	0.0628693
55019	1.89226	0.0678428
55020	1.89226	0.0840388
55021	1.89226	0.052876
55022	3.78451	0.0640608
55023	2.58476	0.064216
55024	1.56598	0.05
55025	1.55045	0.0561948
55026	1.5124	0.056913
55027	2.62281	0.0509633
55028	2.58476	0.0514568
55029	2.58477	0.07217
55030	1.89226	0.0878365
55031	1.89226	0.0866575
55032	1.89226	0.0742102
55033	2.62282	0.0852283
55034	2.62282	0.0730456
55035	1.89224	0.0918243
55036	1.89224	0.110365
55037	1.89224	0.152443
55038	1.89224	0.0810548
55039	3.78448	0.0940087

55040	1.89223	0.0860799
55041	1.89223	0.0547455
55042	1.89223	0.0666085
55043	1.89223	0.0654923
55044	1.89223	0.0709557
55045	1.89223	0.0701917
55046	2.62278	0.0666499
55047	1.45053	0.05
55048	1.49678	0.051276
55049	1.56596	0.0564033
55050	2.5536	0.05
55051	2.62278	0.0526847
55052	1.31139	0.0709124
55053	1.2768	0.06087
55054	0.946118	0.118622
55055	0.946118	0.0843539
55056	1.2768	0.0846916
55057	1.2768	0.077116
55058	0.946116	0.0994436
55059	0.946116	0.0694386
55060	1.27682	0.0662306
55061	0.94613	0.0893523
55062	0.946105	0.0852431
55063	1.31138	0.0679203
55064	1.38506	1.59446
55065	1.53348	2.1
55066	1.17042	1.55148
55067	1.17042	1.98061
55068	1.73669	1.61712
55069	1.73669	2.00049
55070	1.73669	1.35766
55071	1.79928	0.888123
55072	1.38506	1.1957
55073	1.38506	1.98156
55074	1.79928	1.89187
55075	1.43484	2.01488
55076	1.73669	1.12769
55077	1.93568	1.91791
55078	1.53347	1.0828
55079	1.53347	1.8881
55080	2.14058	1.14784
55081	2.14058	2.02483
55082	0.89964	0.875834
55083	1.10415	2.1
55084	0.868346	1.23976
55085	1.07029	2.09005
55086	0.98687	1.44765
55087	0.98687	2.1
55088	0.985627	0.700069
55089	0.985627	2.00231
55090	1.07029	1.74485
55091	1.10415	1.40194
55092	0.868344	1.89089
55093	0.899642	1.90769
55094	2.40667	2.1
55095	2.1281	2.1
55096	2.11176	2.1
55097	2.38069	2.1

55098	4.50878	2.1
55099	2.11176	2.1
55100	1.61687	2.1
55101	1.60965	2.1
55102	1.61687	2.1
55103	2.12088	2.1
55104	2.1281	2.1
55105	2.11176	2.1
55106	2.34898	2.1
55107	2.34898	2.1
55108	2.38069	2.1
55109	2.09114	2.1
55110	2.12285	2.1
55111	1.05588	2.1
55112	1.04557	2.1
55113	0.804824	2.1
55114	0.798269	2.1
55115	0.92615	2.1
55116	0.92615	2.1
55117	0.927999	2.1
55118	0.927999	2.1
55119	1.04557	2.1
55120	0.798269	2.1
55121	1.05588	2.1
55122	0.804824	2.1
55123	1.43548	0.362116
55124	1.53873	0.425401
55125	1.25063	0.37804
55126	1.39662	0.445019
55127	2.78937	0.362763
55128	1.25063	0.371961
55129	1.39662	0.440701
55130	1.10476	0.356787
55131	1.28326	0.403839
55132	2.53389	0.376882
55133	1.51147	0.0659149
55134	1.86114	0.0964811
55135	1.86114	0.0904433
55136	2.12926	0.0780378
55137	1.3659	0.0785322
55138	1.63401	0.0655448
55139	1.89226	0.0894994
55140	1.89226	0.145555
55141	1.89226	0.114506
55142	1.89226	0.0951782
55143	1.89226	0.146502
55144	1.89226	0.147035
55145	1.89226	0.203578
55146	1.89226	0.288798
55147	2.46286	0.180041
55148	1.89226	0.352183
55149	3.78452	0.184008
55150	1.89226	0.302578
55151	1.89226	0.464216
55152	1.89226	0.426287
55153	1.89226	0.425391
55154	1.89226	0.314257
55155	1.89226	0.316392

55156	1.89226	0.241482
55157	1.89226	0.310758
55158	2.28992	0.220378
55159	1.89226	0.380302
55160	3.78452	0.230424
55161	1.13537	0.134072
55162	0.886595	0.338195
55163	0.946131	0.299953
55164	0.946131	0.274549
55165	0.94613	0.427122
55166	0.94613	0.157665
55167	1.25069	0.308385
55168	0.841855	0.502007
55169	1.27436	0.185631
55170	1.06196	0.233005
55171	1.09758	0.165305
55172	0.830306	0.11642
55173	0.826931	0.578068
55174	0.826933	0.087333
55175	0.764631	0.125342
55176	0.764631	0.3531
55177	1.00427	0.373101
55178	0.788034	0.194417
55179	0.726852	0.112243
55180	0.604206	0.428141
55181	0.828475	0.198405
55182	0.604198	0.191001
55183	0.8332	0.153625
55184	1.00428	0.170609
55185	1.89226	0.419908
55186	1.89226	0.282924
55187	1.89226	0.093264
55188	1.89226	0.328783
55189	1.89226	0.188026
55190	1.89226	0.420741
55191	0.768789	0.595725
55192	0.849132	0.05
55193	0.666423	0.585668
55194	0.688993	0.4956
55195	0.868671	0.05
55196	0.89117	0.569851
55197	0.705632	2.1
55198	1.80425	2.1
55199	1.73883	2.1
55200	1.45159	2.1
55201	1.45159	2.1
55202	1.37681	2.1
55203	1.73883	2.1
55204	1.39679	0.05
55205	2.08149	0.0647882
55206	1.89226	0.0504392
55207	2.32763	0.05
55208	1.89226	0.05
55209	2.31274	0.05
55210	1.89226	0.05
55211	1.89226	0.098033
55212	1.89226	0.0653452
55213	1.89226	0.0514572

55214	1.89226	0.0805281
55215	1.89226	0.0620111
55216	1.89226	0.0518843
55217	1.89226	0.07602
55218	1.89226	0.0577854
55219	1.89226	0.0537079
55220	1.89226	0.0629157
55221	1.89226	0.0581691
55222	1.95181	0.05
55223	1.89226	0.0565898
55224	1.89226	0.05
55225	1.89226	0.05
55226	3.78451	0.05
55227	0.946131	0.05
55228	0.946131	0.05
55229	0.946125	0.05
55230	0.946125	0.0570883
55231	0.946127	0.0592696
55232	0.946127	0.0573134
55233	0.946128	0.0579054
55234	0.946128	0.05
55235	0.946128	0.05
55236	0.946128	0.052187
55237	0.946128	0.05
55238	0.946128	0.0555407
55239	1.89226	0.0545406
55240	1.89226	0.0953241
55241	1.89226	0.0676758
55242	1.89226	0.064329
55243	1.89226	0.0747676
55244	1.89226	0.0712265
55245	1.89226	0.651534
55246	1.89226	0.629668
55247	1.89226	0.544968
55248	1.89226	0.517774
55249	3.78453	0.578825
55250	1.89226	0.676637
55251	1.89226	0.603852
55252	1.89226	0.595057
55253	1.89226	0.579704
55254	3.78452	0.594433
55255	1.21387	0.577749
55256	0.8164	0.510917
55257	0.946133	0.551999
55258	0.946133	0.613882
55259	0.946102	0.480533
55260	0.946102	0.65899
55261	0.816431	0.69861
55262	0.816431	0.58813
55263	0.816401	0.549254
55264	0.94613	0.500189
55265	0.946131	0.834449
55266	1.21388	0.588023
55267	0.946132	0.478092
55268	0.946132	0.50615
55269	0.946132	0.542987
55270	0.946132	0.552341
55271	0.946132	0.461726

55272	0.946132	0.536125
55273	0.946132	0.60515
55274	0.946132	0.506067
55275	0.946131	0.453548
55276	0.946131	0.516064
55277	0.946132	0.608806
55278	0.946132	0.541669
55279	1.89226	0.591647
55280	1.89226	0.445079
55281	1.89226	0.438936
55282	1.89226	0.61267
55283	1.89226	0.477857
55284	1.89226	0.763635
55285	1.89226	0.652628
55286	1.89226	0.619198
55287	2.15871	0.495012
55288	2.15871	0.649687
55289	1.81454	0.465941
55290	1.54809	0.705956
55291	1.89226	0.861175
55292	1.89226	1.42247
55293	1.89226	0.895301
55294	1.89226	0.810753
55295	3.78453	1.08599
55296	1.89226	1.08933
55297	1.89226	0.751672
55298	1.89226	0.708234
55299	1.89226	1.0221
55300	1.89226	1.45698
55301	1.89226	1.13545
55302	2.29205	1.02968
55303	1.42633	1.0145
55304	1.69015	1.24813
55305	1.69015	1.22801
55306	2.10364	0.685661
55307	2.05344	0.721875
55308	2.29205	0.799262
55309	1.58147	0.943732
55310	2.16529	0.705668
55311	1.56339	0.643769
55312	2.29205	1.34469
55313	1.69015	1.18568
55314	1.89226	1.13193
55315	1.89226	0.958801
55316	1.89226	0.756923
55317	1.89226	0.928922
55318	3.78453	0.818679
55319	1.89226	0.882149
55320	1.89226	1.36567
55321	1.89226	0.993441
55322	1.89226	0.781418
55323	3.78452	1.31946
55324	2.29205	1.33807
55325	2.85335	0.618627
55326	2.55409	1.04034
55327	2.04345	0.735796
55328	2.76338	0.927194
55329	2.29205	0.813833

55330	2.29205	0.946012
55331	2.16529	1.18857
55332	2.72418	0.907559
55333	2.76338	0.570868
55334	5.01623	0.952706
55335	1.89227	0.217269
55336	1.89227	0.176524
55337	1.89227	0.105539
55338	1.89227	0.15306
55339	3.78453	0.126681
55340	2.39588	0.117276
55341	2.14918	0.121927
55342	2.14918	0.133019
55343	2.37907	0.202828
55344	2.20531	0.0730594
55345	2.4352	0.116226
55346	1.89226	0.137281
55347	2.64327	0.179783
55348	2.36175	0.122634
55349	1.89226	0.110616
55350	4.25402	0.179742
55351	1.89226	0.1113
55352	1.89226	0.08597
55353	1.89226	0.0824697
55354	1.89226	0.114389
55355	1.89226	0.0897695
55356	1.89226	0.129824
55357	1.05395	0.0607378
55358	1.05395	0.0755462
55359	1.03114	0.0778059
55360	1.03114	0.0866794
55361	0.936689	0.0703907
55362	1.07459	0.105772
55363	1.10265	0.0759449
55364	1.01049	0.0814353
55365	0.93669	0.0636682
55366	1.01049	0.0716097
55367	1.07459	0.0990123
55368	1.10265	0.0711282
55369	1.18088	0.0939704
55370	0.776509	0.0796657
55371	0.946132	0.0744696
55372	0.946132	0.0898659
55373	1.04225	0.0840124
55374	0.830392	0.141815
55375	0.931352	0.0931925
55376	0.931352	0.0971943
55377	1.13006	0.0765389
55378	1.04074	0.0787804
55379	1.0628	0.0940932
55380	0.851529	0.110263
55381	2.40032	2.1
55382	1.89226	2.1
55383	1.89226	2.1
55384	1.89226	2.1
55385	2.38918	2.1
55386	2.38918	2.1
55387	1.89226	2.1

55388	1.89226	1.91446
55389	1.89226	2.1
55390	1.89226	1.47861
55391	1.89226	2.1
55392	1.89226	1.91585
55393	1.6585	2.1
55394	2.37364	2.05175
55395	2.39622	2.1
55396	2.39622	2.00539
55397	1.6488	2.1
55398	1.6488	2.0367
55399	2.44603	2.1
55400	1.89224	2.1
55401	1.89224	2.1
55402	1.89224	2.1
55403	2.40029	2.1
55404	2.40029	2.1
55405	1.89224	2.1
55406	1.89224	1.42291
55407	1.89224	2.1
55408	1.89224	1.17593
55409	1.89224	2.1
55410	1.89224	1.20232
55411	1.01946	1.70344
55412	1.01946	2.1
55413	1.02497	2.1
55414	1.02497	2.1
55415	1.21519	1.41446
55416	1.19809	1.16903
55417	0.835303	2.1
55418	0.82924	2.1
55419	0.835314	2.1
55420	1.2152	2.1
55421	0.829228	2.1
55422	1.19808	2.1
55423	1.91027	2.1
55424	1.05809	2.1
55425	1.00659	2.1
55426	2.06727	2.1
55427	3.11819	2.1
55428	2.37166	2.1
55429	2.06726	2.1
55430	1.66994	2.1
55431	1.67623	2.1
55432	2.40308	2.1
55433	2.40308	2.1
55434	0.960194	2.1
55435	0.939664	2.1
55436	0.531131	2.1
55437	0.503298	2.1
55438	0.729927	2.1
55439	0.729927	2.1
55440	0.733565	2.1
55441	0.733565	2.1
55442	0.960195	2.1
55443	0.531132	2.1
55444	0.503297	2.1
55445	0.939663	2.1

55446	1.20154	2.1
55447	1.21754	2.1
55448	0.960195	2.1
55449	0.939665	2.1
55450	1.07745	2.1
55451	1.07745	2.1
55452	1.07976	2.1
55453	1.07976	2.1
55454	0.960194	2.1
55455	1.21754	2.1
55456	0.939662	2.1
55457	1.20155	2.1
55458	1.89224	2.1
55459	1.89224	2.1
55460	2.08609	2.1
55461	2.08609	2.1
55462	2.09527	2.1
55463	1.90142	2.1
55464	1.65815	2.1
55465	1.71398	2.1
55466	2.2001	2.1
55467	2.23791	2.1
55468	1.4502	2.1
55469	1.4502	2.1
55470	0.793587	2.1
55471	0.793587	2.1
55472	0.792408	2.1
55473	0.792408	2.1
55474	0.952221	2.1
55475	0.858585	2.1
55476	0.725101	2.1
55477	0.633774	2.1
55478	0.633775	2.1
55479	0.858585	2.1
55480	0.952221	2.1
55481	0.725101	2.1
55482	1.5735	2.1
55483	2.56975	2.1
55484	2.63166	2.1
55485	2.63166	2.1
55486	1.54711	2.1
55487	1.54711	2.1
55488	0.946131	2.1
55489	0.946131	2.1
55490	1.28487	2.1
55491	1.28487	2.1
55492	0.946127	2.03275
55493	0.946127	2.1
55494	1.28488	2.1
55495	1.31583	2.1
55496	1.31583	2.1
55497	0.94613	2.1
55498	1.28487	2.1
55499	0.946129	2.1
55500	1.0596	2.1
55501	1.0596	2.1
55502	1.06939	2.1
55503	1.06939	2.1

55504	1.34225	2.1
55505	1.31583	2.1
55506	0.793563	2.1
55507	0.786748	2.1
55508	0.793563	2.1
55509	1.34225	2.1
55510	0.786748	2.1
55511	1.31583	2.1
55512	0.946131	2.1
55513	0.946131	2.1
55514	1.31582	2.1
55515	1.31582	2.1
55516	0.946127	2.1
55517	0.946127	2.1
55518	1.34225	2.1
55519	1.31583	2.1
55520	1.34225	2.1
55521	0.946129	2.1
55522	1.31583	2.1
55523	0.946129	2.1
55524	1.89226	2.1
55525	1.89226	2.1
55526	1.89226	2.1
55527	1.89226	2.1
55528	1.89226	2.1
55529	1.89226	2.1
55530	1.89226	2.1
55531	1.89226	2.1
55532	1.89226	2.1
55533	1.89226	2.1
55534	1.89226	2.1
55535	1.89226	2.1
55536	1.26992	2.1
55537	1.75003	2.1
55538	1.79883	2.1
55539	1.26732	2.1
55540	3.06875	2.1
55541	0.875017	2.1
55542	0.899414	2.1
55543	0.811147	2.1
55544	0.85053	2.1
55545	0.851513	2.1
55546	0.851513	2.1
55547	0.859048	2.1
55548	0.859048	2.1
55549	0.899413	2.1
55550	0.85053	2.1
55551	0.811147	2.1
55552	0.875016	2.1
55553	1.89227	2.1
55554	1.89227	2.1
55555	1.89227	2.1
55556	1.89227	2.1
55557	1.89227	2.1
55558	1.89227	2.1
55559	1.89226	2.1
55560	1.89226	2.1
55561	1.89226	2.1

55562	1.89226	2.1
55563	1.89226	2.1
55564	1.89226	2.1
55565	0.633661	2.1
55566	0.642005	2.1
55567	0.93285	2.1
55568	0.899413	2.1
55569	0.764487	2.1
55570	0.764487	2.1
55571	0.776931	2.1
55572	0.776931	2.1
55573	0.642004	2.1
55574	0.93285	2.1
55575	0.899414	2.1
55576	0.633661	2.1
55577	0.932851	2.1
55578	0.899414	2.1
55579	0.905419	2.1
55580	0.85053	2.1
55581	0.886336	2.1
55582	0.886336	2.1
55583	0.897045	2.1
55584	0.897045	2.1
55585	0.93285	2.1
55586	0.905419	2.1
55587	0.899414	2.1
55588	0.850531	2.1
55589	2.39322	2.1
55590	2.74149	0.791568
55591	2.70944	2.1
55592	2.6849	0.833026
55593	2.41776	2.1
55594	2.39322	0.65837
55595	2.41776	2.1
55596	2.80992	1.79695
55597	2.77256	2.1
55598	2.74149	1.07876
55599	2.44882	2.1
55600	2.41776	0.751215
55601	2.80992	2.01906
55602	1.89226	1.69956
55603	2.74149	1.5981
55604	2.74149	2.05493
55605	1.89226	0.978128
55606	1.89226	1.36303
55607	1.89226	0.0607283
55608	1.89226	0.0560239
55609	1.89226	0.0891538
55610	1.89226	0.05
55611	1.89226	0.080231
55612	1.89226	0.05
55613	2.38796	0.05
55614	2.0815	0.0573744
55615	2.0815	0.05
55616	2.49452	0.05
55617	1.55994	0.0521588
55618	2.14721	0.05
55619	1.89227	0.0702041

55620	1.89227	0.0592005
55621	1.89227	0.0936134
55622	1.89227	0.05
55623	1.89227	0.0883432
55624	1.89227	0.0523106
55625	1.13387	0.0528337
55626	2.27494	0.05
55627	1.13387	0.0719083
55628	2.19489	0.05
55629	1.60867	0.0647785
55630	2.6994	0.05
55631	1.89226	0.071737
55632	1.89226	0.0537518
55633	1.89226	0.0576523
55634	1.89226	0.105539
55635	3.78453	0.0601883
55636	1.42381	1.04772
55637	1.10747	2.1
55638	1.10747	0.933834
55639	1.08911	2.1
55640	1.42866	0.895998
55641	1.4103	2.1
55642	1.89226	2.1
55643	1.88181	2.1
55644	1.88181	1.83064
55645	1.76249	2.1
55646	2.0815	1.57184
55647	1.97539	2.1
55648	0.946128	2.1
55649	0.946128	1.64071
55650	0.946133	0.991991
55651	0.946133	0.750082
55652	0.94613	0.756176
55653	0.94613	1.48141
55654	0.946131	2.1
55655	0.946131	0.855911
55656	0.946131	1.04328
55657	0.946131	1.0688
55658	0.946131	1.1159
55659	0.946131	1.13105
55660	1.42866	0.959133
55661	1.43213	0.917795
55662	1.10748	1.8429
55663	1.10748	2.1
55664	1.13641	2.1
55665	1.43213	2.1
55666	2.0815	2.1
55667	2.10287	2.1
55668	2.10287	1.79046
55669	2.0921	2.1
55670	1.89227	1.68043
55671	1.83938	2.1
55672	0.810944	0.874083
55673	0.810944	0.758395
55674	0.810945	0.772308
55675	0.810945	1.48754
55676	0.810933	0.968681
55677	0.810933	0.979738

55678	1.89226	2.1
55679	1.3517	1.25288
55680	1.08132	0.966991
55681	1.08132	1.84091
55682	1.08133	1
55683	2.33097	1.08866
55684	1.53822	2.1
55685	1.53822	1.98171
55686	1.68437	2.1
55687	2.17996	1.95696
55688	2.32612	2.1
55689	0.99189	1.10498
55690	1.18948	0.525668
55691	1.19756	0.280608
55692	1.19756	1.83463
55693	0.978851	1.25478
55694	0.926995	0.751751
55695	1.26924	0.251788
55696	1.34642	0.533355
55697	1.3228	0.639338
55698	0.990899	0.638215
55699	1.0627	0.655503
55700	1.33473	0.652024
55701	1.96196	0.756426
55702	1.39702	2.1
55703	1.39702	1.71778
55704	1.53822	2.1
55705	2.17372	1.44121
55706	2.31011	2.1
55707	1.89226	0.629233
55708	1.89226	0.370671
55709	1.89226	0.499561
55710	1.89226	0.344986
55711	3.78453	0.396709
55712	2.38475	0.573088
55713	1.73957	0.386561
55714	1.88452	0.49898
55715	1.58695	0.1462
55716	3.6241	0.40925
55717	1.73958	0.37751
55718	1.3126	0.582006
55719	1.58695	0.257242
55720	1.14946	0.565009
55721	2.88903	0.452224
55722	1.89226	0.365108
55723	1.89226	0.609621
55724	1.89226	0.283734
55725	1.89226	0.6003
55726	3.78453	0.474765
55727	1.89225	0.507698
55728	1.89225	0.387073
55729	1.89225	0.479491
55730	1.89225	0.384318
55731	3.7845	0.310234
55732	1.88451	0.506392
55733	1.58694	0.22142
55734	1.74649	0.53779
55735	1.44604	0.354031

55736	3.33055	0.317603
55737	1.58694	0.326
55738	0.995727	0.575008
55739	0.995727	0.489197
55740	1.14945	0.507625
55741	1.44604	0.349559
55742	1.59976	0.53838
55743	1.89225	0.370259
55744	1.89225	0.562731
55745	1.89225	0.588139
55746	1.89225	0.409258
55747	1.89225	0.597721
55748	1.89225	0.375648
55749	2.01197	1.56023
55750	2.0961	1.47159
55751	2.13283	1.06087
55752	2.25399	0.846096
55753	4.22894	1.24327
55754	2.09611	0.975657
55755	2.37014	1.05879
55756	2.29031	1.39028
55757	2.10084	1.818
55758	2.25399	0.832592
55759	2.09611	1.81211
55760	2.13283	1.45689
55761	2.25399	2.00802
55762	2.22044	1.19964
55763	2.38975	0.873092
55764	4.52258	1.30177
55765	2.38976	0.707105
55766	2.15466	1.15281
55767	2.31988	0.664092
55768	2.31988	1.52155
55769	2.25399	0.74849
55770	2.25399	2.1
55771	2.58286	0.593159
55772	2.29193	0.373353
55773	2.40107	0.631297
55774	2.13815	0.286802
55775	4.72101	0.444422
55776	2.29193	0.371863
55777	1.90104	0.509898
55778	2.13815	0.310282
55779	1.77249	0.483849
55780	4.03919	0.402575
55781	2.25197	0.604327
55782	2.13815	0.380586
55783	2.00625	0.558119
55784	2.00625	0.377783
55785	2.40107	0.520678
55786	2.40107	0.421992
55787	2.13815	0.36214
55788	1.77249	0.487005
55789	2.00625	0.409376
55790	1.65815	0.521493
55791	3.7963	0.404218
55792	2.43417	0.666957
55793	2.06653	0.601126

55794	1.64587	0.611648
55795	1.65284	0.555351
55796	2.46898	0.67281
55797	2.46898	0.619241
55798	2.46895	0.60815
55799	1.89016	0.468713
55800	1.87861	0.502254
55801	1.87861	0.406761
55802	2.47161	0.613134
55803	2.47161	0.557539
55804	0.946132	0.739572
55805	0.946132	0.726003
55806	0.863311	0.847209
55807	1.34768	0.82278
55808	0.851542	0.838433
55809	0.851542	0.656721
55810	1.30451	0.781065
55811	1.45304	0.819583
55812	0.946132	0.746487
55813	1.20992	0.781021
55814	0.855162	0.832897
55815	0.946132	0.764028
55816	1.89226	0.670316
55817	1.89226	0.838015
55818	1.89226	0.833588
55819	1.89226	0.664025
55820	1.89226	0.86199
55821	1.89226	0.683393
55822	0.946132	0.604391
55823	0.946132	0.531064
55824	0.946133	0.625467
55825	0.946133	0.515593
55826	0.946132	0.621677
55827	0.946132	0.445416
55828	0.946132	0.548185
55829	0.946132	0.582887
55830	0.946132	0.646868
55831	0.946132	0.589726
55832	0.946133	0.515977
55833	0.946133	0.601121
55834	2.35574	0.733854
55835	1.89226	0.548035
55836	1.89226	1.03191
55837	1.89226	0.532031
55838	2.67456	0.760946
55839	2.67456	0.195584
55840	0.583505	0.415626
55841	0.761972	0.467465
55842	1.17787	0.667838
55843	1.33728	0.443656
55844	0.965128	0.618146
55845	0.965128	0.43894
55846	0.974714	0.528924
55847	0.974715	0.652372
55848	1.17787	0.477173
55849	0.583506	0.638503
55850	1.33728	0.426489
55851	0.761974	0.496047

55852	1.89226	0.623151
55853	1.89226	0.469112
55854	1.89226	0.427352
55855	1.89226	0.389186
55856	1.89226	0.403035
55857	1.89226	0.550371
55858	1.89226	0.531683
55859	1.89226	0.348609
55860	1.89226	0.498032
55861	1.89226	0.455267
55862	3.78453	0.444187
55863	0.946132	0.399275
55864	0.946132	0.438962
55865	0.946132	0.517938
55866	0.946132	0.410942
55867	0.946133	0.500258
55868	0.946133	0.42347
55869	0.946132	0.55343
55870	0.946132	0.507651
55871	0.946133	0.462288
55872	0.946133	0.594676
55873	0.946132	0.431842
55874	0.946132	0.506846
55875	1.01779	2.1
55876	1.50685	2.1
55877	1.12064	2.1
55878	1.65151	2.1
55879	2.6275	2.1
55880	1.35733	2.1
55881	1.53507	2.1
55882	1.50683	2.1
55883	1.73626	2.1
55884	3.09359	2.1
55885	1.73626	2.1
55886	1.52226	2.1
55887	1.75555	2.1
55888	1.75555	2.1
55889	1.53507	2.1
55890	1.53507	2.1
55891	1.65151	2.1
55892	1.73628	2.1
55893	1.69528	2.1
55894	1.50685	2.1
55895	1.92471	2.1
55896	1.73628	2.1
55897	1.73629	2.1
55898	1.75558	2.1
55899	1.9943	2.1
55900	1.9943	2.1
55901	1.92471	2.1
55902	1.73629	2.1
55903	2.5601	1.02724
55904	1.97645	2.02242
55905	2.09605	0.941777
55906	2.09605	1.80714
55907	2.45853	1.06623
55908	2.45853	2.06889
55909	2.33824	1.64336

55910	1.97646	1.59257
55911	2.45853	0.918126
55912	1.94984	2.05579
55913	2.33824	1.71582
55914	1.82955	2.1
55915	1.22926	2.0634
55916	0.946128	1.95747
55917	0.946133	2.1
55918	1.28005	1.26311
55919	1.22927	1.36958
55920	1.22926	1.79329
55921	0.946131	1.63916
55922	0.946131	1.31938
55923	0.946131	1.38823
55924	0.946131	1.23713
55925	1.28005	1.46335
55926	1.22926	1.36993
55927	2.35349	0.591357
55928	2.61606	0.403275
55929	2.48209	0.590577
55930	2.48209	0.404379
55931	2.50057	0.57838
55932	2.50057	0.416279
55933	2.61607	0.423152
55934	2.57611	0.398736
55935	2.4821	0.415446
55936	2.44814	0.373483
55937	5.06421	0.347105
55938	2.15721	0.513783
55939	2.48209	0.41175
55940	2.31844	0.611639
55941	2.31844	0.387976
55942	2.35349	0.600617
55943	2.35349	0.39019
55944	2.44814	0.378749
55945	2.30908	0.407327
55946	2.44814	0.386295
55947	2.31844	0.392636
55948	2.4821	0.386879
55949	2.31844	0.36074
55950	0.91766	0.360264
55951	0.91766	0.372245
55952	0.912463	0.362341
55953	0.912463	0.376192
55954	1.16525	0.368364
55955	1.26377	0.368254
55956	0.576692	0.371031
55957	0.664871	0.373912
55958	1.16525	0.359309
55959	0.576692	0.362904
55960	0.664871	0.377805
55961	1.26377	0.376307
55962	1.72901	1.98807
55963	1.62414	2.1
55964	1.88971	2.1
55965	1.8411	1.20721
55966	3.57011	2.1
55967	1.89226	2.09448

55968	1.89226	1.16528
55969	1.89226	0.796985
55970	1.89226	1.66284
55971	3.78452	0.861696
55972	1.89226	0.913566
55973	1.89226	2.1
55974	1.89226	1.32676
55975	1.89226	1.01324
55976	3.78452	2.1
55977	1.62414	2.1
55978	1.30896	2.1
55979	1.8411	0.965556
55980	1.52776	2.06639
55981	3.15006	1.2218
55982	1.51448	2.02244
55983	1.8411	1.84454
55984	2.02311	1.8022
55985	1.99309	0.802595
55986	3.86421	1.23097
55987	1.89226	0.805295
55988	1.89226	2.1
55989	1.89226	1.43041
55990	1.89226	0.916759
55991	3.78452	2.06041
55992	1.89226	2.1
55993	1.89226	1.31175
55994	1.89226	1.07202
55995	1.89226	2.1
55996	3.78452	1.60893
55997	1.8411	1.29604
55998	1.52776	2.1
55999	1.99309	2.1
56000	1.72526	2.0199
56001	3.56636	2.1
56002	1.89226	2.1
56003	1.89226	1.30619
56004	1.89226	1.50855
56005	1.89226	2.1
56006	3.78453	1.52271
56007	1.0424	2.1
56008	1.12935	2.1
56009	0.929383	2.1
56010	0.929383	2.1
56011	1.24941	2.1
56012	1.12686	2.1
56013	0.946132	2.1
56014	0.946132	2.1
56015	1.12944	2.08961
56016	0.851554	2.04892
56017	1.05017	2.09095
56018	0.929315	2.1
56019	0.898029	2.1
56020	0.973994	2.1
56021	0.946131	1.34771
56022	0.946131	1.69196
56023	1.0221	1.68301
56024	1.0221	2.1
56025	0.898028	1.27173

56026	0.898028	2.1
56027	0.973994	2.1
56028	0.946131	2.1
56029	0.946131	2.1
56030	0.898029	2.1
56031	1.89226	1.49651
56032	1.89226	1.81127
56033	1.89226	1.27261
56034	1.89226	1.8257
56035	1.89226	1.34018
56036	1.89226	1.90123
56037	1.13537	1.68479
56038	0.879308	2.1
56039	0.946131	1.41114
56040	0.946131	1.45434
56041	1.13537	1.4998
56042	0.85187	2.1
56043	0.94613	1.71385
56044	0.94613	1.23375
56045	1.05777	1.8551
56046	1.05777	2.06431
56047	0.799318	1.88233
56048	0.776452	1.96975
56049	0.946116	2.1
56050	0.946116	2.1
56051	1.09531	2.1
56052	1.09531	2.1
56053	0.946118	2.1
56054	0.946118	2.1
56055	1.09135	2.1
56056	1.09531	2.1
56057	1.09134	2.1
56058	0.946105	2.1
56059	1.09532	2.1
56060	0.946131	2.1
56061	0.946129	2.1
56062	0.946129	2.1
56063	1.09137	2.1
56064	1.09137	2.1
56065	0.946131	2.1
56066	0.946131	2.1
56067	1.07713	2.1
56068	1.09137	2.1
56069	0.946131	2.1
56070	1.07713	2.1
56071	1.09137	2.1
56072	0.946131	2.1
56073	0.938916	2.1
56074	0.938916	2.1
56075	0.936995	2.1
56076	1.00447	2.1
56077	0.979819	2.1
56078	1.16821	2.1
56079	1.01263	2.1
56080	0.719167	2.1
56081	1.04536	2.1
56082	1.18901	2.1
56083	0.758057	2.1

56084	0.924111	2.1
56085	1.00532	2.1
56086	1.00532	2.1
56087	0.9925	2.1
56088	0.9925	2.1
56089	1.29322	2.1
56090	1.01985	2.1
56091	1.00365	2.1
56092	0.704601	2.1
56093	1.00365	2.1
56094	1.29322	2.1
56095	1.01985	2.1
56096	0.704602	2.1
56097	2.20011	2.1
56098	1.401	2.1
56099	1.401	2.1
56100	1.52008	2.1
56101	2.11084	2.1
56102	2.22991	2.1
56103	0.854068	2.1
56104	0.854068	2.1
56105	0.851357	2.1
56106	0.851357	2.1
56107	0.644681	2.1
56108	0.700493	2.1
56109	1.0554	2.1
56110	1.00493	2.1
56111	1.00495	2.1
56112	0.64469	2.1
56113	0.700483	2.1
56114	1.05539	2.1
56115	2.46665	0.05
56116	2.22713	0.05
56117	1.85877	0.05
56118	1.8967	0.05
56119	1.56002	0.05
56120	2.22713	0.05
56121	1.89225	2.1
56122	1.89225	2.1
56123	1.89225	2.1
56124	1.89225	2.1
56125	1.89225	2.1
56126	1.89225	2.1
56127	1.89225	2.1
56128	1.89225	2.1
56129	1.89225	2.1
56130	1.89225	2.1
56131	1.89225	2.1
56132	1.89225	2.1
56133	1.284	2.1
56134	1.86569	2.1
56135	1.95398	2.1
56136	1.31091	2.1
56137	3.23798	2.1
56138	0.932844	2.1
56139	0.976992	2.1
56140	0.960128	2.1
56141	0.905413	2.1

56142	0.938531	2.1
56143	0.938531	2.1
56144	0.943874	2.1
56145	0.943874	2.1
56146	0.976985	2.1
56147	0.960122	2.1
56148	0.93285	2.1
56149	0.905419	2.1
56150	1.89227	2.1
56151	1.89227	2.1
56152	1.89227	2.1
56153	1.89227	2.1
56154	3.78453	2.1
56155	1.89226	2.1
56156	1.89226	2.1
56157	1.89226	2.1
56158	1.89226	2.1
56159	1.89226	2.1
56160	1.89226	2.1
56161	0.65546	2.1
56162	0.665451	2.1
56163	1.00694	2.1
56164	0.976998	2.1
56165	0.816261	2.1
56166	0.816261	2.1
56167	0.826189	2.1
56168	0.826189	2.1
56169	1.00694	2.1
56170	0.665451	2.1
56171	0.976998	2.1
56172	0.65546	2.1
56173	2.01555	2.1
56174	2.01388	2.1
56175	1.92027	2.1
56176	1.92027	2.1
56177	2.01388	2.1
56178	1.954 2.1	
56179	2.75466	2.1
56180	1.89226	2.1
56181	1.89226	2.1
56182	2.54931	2.1
56183	4.44158	2.1
56184	2.47917	2.1
56185	1.92772	2.1
56186	1.80682	2.1
56187	2.54931	2.1
56188	4.47702	2.1
56189	0.946132	2.1
56190	0.946132	2.1
56191	1.17602	2.1
56192	1.27466	2.1
56193	0.946132	2.1
56194	0.946132	2.1
56195	1.17602	2.1
56196	1.17602	2.1
56197	0.946132	2.1
56198	1.17602	2.1
56199	0.946131	2.1

56200	1.27466	2.1
56201	2.35204	2.1
56202	2.54931	2.1
56203	2.40783	2.1
56204	1.50108	2.1
56205	1.64256	2.1
56206	1.64256	2.1
56207	1.89226	2.1
56208	1.89226	0.919134
56209	2.31012	1.67188
56210	2.17534	1.9457
56211	4.0676	1.80207
56212	2.17533	1.93766
56213	1.89226	0.828021
56214	2.17533	0.988762
56215	1.89226	0.933273
56216	1.66635	1.76528
56217	1.38328	1.74475
56218	1.92027	2.1
56219	2.17597	2.06648
56220	2.14619	2.1
56221	2.15388	1.99473
56222	1.91087	2.1
56223	1.92027	0.857592
56224	1.08622	0.88519
56225	1.08622	2.1
56226	0.971184	2.1
56227	0.972761	2.1
56228	1.14845	1.31502
56229	1.16321	2.1
56230	0.894769	2.1
56231	0.894967	2.1
56232	0.894775	2.1
56233	1.28563	2.1
56234	1.14844	2.1
56235	1.0167	2.1
56236	1.89226	0.882462
56237	1.89226	2.1
56238	1.89226	2.0539
56239	1.89226	0.96828
56240	1.89226	2.04844
56241	1.89226	1.02276
56242	0.937933	1.49812
56243	0.937933	1.87205
56244	1.26192	2.1
56245	1.26192	2.1
56246	0.937903	1.27062
56247	1.25372	2.1
56248	0.946132	1.51198
56249	0.946132	2.1
56250	0.946133	2.1
56251	1.25372	2.1
56252	0.946132	2.1
56253	0.937902	2.1
56254	0.946132	0.923303
56255	0.946132	0.780919
56256	0.946128	2.06846
56257	0.946128	2.00277

56258	0.94613	1.93161
56259	0.94613	0.90522
56260	0.94613	0.709702
56261	0.94613	1.68832
56262	0.946131	1.11076
56263	0.946131	1.25877
56264	0.94613	1.08424
56265	0.94613	1.07
56266	0.946132	1.08811
56267	0.946132	0.911187
56268	1.2778	2.1
56269	1.00191	2.1
56270	1.00191	1.02906
56271	1.00191	2.1
56272	0.94613	2.1
56273	0.94613	1.00276
56274	0.946131	1.50958
56275	1.2778	1.77851
56276	1.00191	1.33528
56277	0.94613	1.2692
56278	1.50847	0.0528028
56279	2.0815	0.0646552
56280	1.56806	0.0526893
56281	2.0815	0.0590963
56282	1.52043	0.0525404
56283	1.9224	0.0579917
56284	1.06882	0.05
56285	0.69567	0.0624135
56286	0.773112	0.05
56287	1.09473	0.0522592
56288	0.8941	0.0581558
56289	0.8941	0.0615764
56290	0.937064	0.0513568
56291	0.981131	0.05
56292	0.763762	0.0552191
56293	0.722535	0.0551259
56294	1.2153	0.0525752
56295	1.07279	0.0543233
56296	1.89226	0.0855766
56297	1.89226	0.0950963
56298	1.89226	0.0813907
56299	1.89226	0.0717647
56300	1.89226	0.113519
56301	1.89226	0.103226
56302	1.89226	0.0842483
56303	1.89226	0.113528
56304	1.89226	0.0941116
56305	1.89226	0.123612
56306	3.78453	0.110703
56307	2.51518	0.0768232
56308	1.89226	0.084476
56309	1.89226	0.074841
56310	1.89226	0.0658564
56311	2.37584	0.0952284
56312	2.37584	0.0871542
56313	1.25759	0.0881901
56314	0.946131	0.0938497
56315	0.946131	0.0777581

56316	1.24489	0.11445
56317	0.946131	0.0907523
56318	0.946131	0.0978193
56319	1.25759	0.0825306
56320	1.25759	0.0785319
56321	1.25759	0.0889013
56322	1.24489	0.0872796
56323	0.946131	0.0845304
56324	0.946131	0.0875929
56325	1.08028	0.621044
56326	1.09362	0.691771
56327	0.596616	0.681812
56328	0.606432	0.754845
56329	0.844265	0.767999
56330	0.844265	0.456704
56331	0.855786	0.975182
56332	0.855786	0.682794
56333	1.08028	0.78355
56334	0.606433	0.667361
56335	0.596616	0.636466
56336	1.09362	0.6177
56337	2.837	0.790495
56338	2.16055	0.532117
56339	2.12741	0.693909
56340	2.79312	0.65617
56341	4.95367	0.675758
56342	2.12741	0.744757
56343	1.21286	0.569444
56344	1.20859	0.834548
56345	1.21286	0.88635
56346	2.15628	0.654337
56347	2.16056	0.505478
56348	1.89226	2.1
56349	1.89226	2.1
56350	1.89226	2.1
56351	1.89226	2.1
56352	3.78453	2.1
56353	0.946102	2.1
56354	0.946102	2.1
56355	0.850039	2.1
56356	0.850039	2.1
56357	0.914891	2.1
56358	0.85001	2.1
56359	0.946131	2.1
56360	0.946131	2.1
56361	0.94613	2.1
56362	0.91489	2.1
56363	0.850011	2.1
56364	0.946131	2.1
56365	0.71107	2.1
56366	0.784813	2.1
56367	1.28453	2.1
56368	1.20328	2.1
56369	0.992128	2.1
56370	0.992128	2.1
56371	0.995965	2.1
56372	0.995965	2.1
56373	0.784813	2.1

56374	1.28453	2.1
56375	0.711071	2.1
56376	1.20328	2.1
56377	1.89226	2.1
56378	1.89226	2.1
56379	1.89226	2.1
56380	1.89226	2.1
56381	3.78453	2.1
56382	0.946132	2.1
56383	0.946132	2.1
56384	0.914889	2.1
56385	0.914889	2.1
56386	0.985255	2.1
56387	0.914891	2.1
56388	0.946131	2.1
56389	0.946131	2.1
56390	0.946131	2.1
56391	0.985255	2.1
56392	0.94613	2.1
56393	0.91489	2.1
56394	0.784813	2.1
56395	0.871551	2.1
56396	1.37791	2.1
56397	1.28453	2.1
56398	1.07644	2.1
56399	1.07644	2.1
56400	1.07964	2.1
56401	1.07964	2.1
56402	0.871551	2.1
56403	1.37791	2.1
56404	0.784813	2.1
56405	1.28453	2.1
56406	1.73075	1.17384
56407	2.10582	0.843566
56408	1.89319	0.814353
56409	1.38766	1.62083
56410	3.27777	0.82931
56411	1.08715	1.36968
56412	1.54185	1.2446
56413	1.73074	1.14597
56414	1.73074	1.80409
56415	1.18948	0.949999
56416	1.18948	1.31724
56417	1.89319	0.776461
56418	1.41689	1.40433
56419	1.11619	0.939189
56420	1.38766	1.16851
56421	1.6052	0.830265
56422	1.89319	1.10069
56423	0.647894	1.26113
56424	0.594738	1.39899
56425	0.708445	1.22122
56426	0.770926	1.03901
56427	0.738326	1.22812
56428	0.738326	1.71089
56429	0.680494	1.2094
56430	0.680494	0.892126
56431	0.708443	0.979707

56432	0.647895	1.00317
56433	0.770925	1.04175
56434	0.59474	0.871975
56435	1.89111	0.868492
56436	1.06994	2.1
56437	2.06689	1.64037
56438	1.37063	2.1
56439	3.13683	2.1
56440	2.06689	1.03009
56441	1.39295	2.1
56442	1.56818	1.45007
56443	1.37063	2.1
56444	2.26444	1.06355
56445	2.06689	2.1
56446	1.49808	2.1
56447	1.50201	2.1
56448	2.57784	2.1
56449	2.58615	2.1
56450	4.08424	2.1
56451	0.946133	1.89242
56452	0.946133	1.61397
56453	0.946132	2.1
56454	0.946132	2.1
56455	0.946132	2.1
56456	0.946132	1.8233
56457	0.946132	2.1
56458	0.946132	1.62858
56459	0.946133	1.7769
56460	0.946133	2.1
56461	0.946132	2.1
56462	0.946132	2.1
56463	1.89226	1.85715
56464	1.89226	2.1
56465	1.89226	2.1
56466	1.89226	2.1
56467	3.78452	2.1
56468	0.977201	2.1
56469	0.977201	2.1
56470	0.980489	2.1
56471	0.980489	2.1
56472	0.657973	2.1
56473	1.20669	2.1
56474	0.751002	2.1
56475	1.29307	2.1
56476	1.29308	2.1
56477	1.20669	2.1
56478	0.751003	2.1
56479	0.657972	2.1
56480	0.946131	2.1
56481	0.946131	2.1
56482	1.29308	2.1
56483	1.29308	2.1
56484	0.946132	2.1
56485	1.29308	2.1
56486	0.946132	2.1
56487	1.28892	2.1
56488	0.946132	2.1
56489	0.946132	2.1

56490	1.28892	2.1
56491	1.29308	2.1
56492	0.946132	1.02476
56493	0.946132	0.843518
56494	0.946131	1.35839
56495	0.946131	1.04202
56496	0.946132	1.35141
56497	0.946132	1.50701
56498	0.946132	0.937979
56499	0.946132	0.844823
56500	0.946132	0.907398
56501	0.946132	1.02231
56502	0.946132	0.985994
56503	0.946132	1.48114
56504	1.89226	1.27707
56505	1.89226	1.72591
56506	1.89226	1.99028
56507	1.89226	1.20174
56508	1.89226	1.93794
56509	1.89226	1.14869
56510	0.94613	2.1
56511	0.94613	2.1
56512	1.20669	2.1
56513	1.20669	2.1
56514	1.20669	2.1
56515	0.946132	2.1
56516	0.946129	2.1
56517	1.29307	2.1
56518	0.946131	2.1
56519	0.946131	2.1
56520	1.29308	2.1
56521	1.20669	2.1
56522	1.89226	0.15256
56523	1.89226	0.0792692
56524	1.89226	0.0947751
56525	1.89226	0.132132
56526	3.78453	0.127992
56527	0.946132	0.0595152
56528	0.946132	0.0658277
56529	0.946126	0.0680733
56530	0.946126	0.100188
56531	0.946129	0.0720557
56532	0.946129	0.0647612
56533	0.946129	0.0747352
56534	0.946129	0.0959632
56535	0.946128	0.0667166
56536	0.946128	0.0722983
56537	0.946129	0.0683549
56538	0.946129	0.0713912
56539	0.725277	0.0501787
56540	0.588155	0.05
56541	1.19403	0.0665516
56542	1.27681	0.0510603
56543	0.918903	0.0514794
56544	0.918903	0.05
56545	0.946068	0.0554425
56546	0.946068	0.05
56547	1.19404	0.053163

56548	0.588153	0.0528173
56549	0.725275	0.0523111
56550	1.27682	0.0508529
56551	1.19404	0.0716546
56552	1.27682	0.0539992
56553	0.946131	0.0979305
56554	0.946131	0.0787995
56555	1.19404	0.0806169
56556	1.19404	0.0736061
56557	0.946129	0.0703684
56558	0.946129	0.0765215
56559	1.19404	0.0734972
56560	0.94613	0.0743984
56561	1.27682	0.0630157
56562	0.946131	0.0822673
56563	1.89226	0.0827534
56564	1.89226	0.139271
56565	1.89226	0.149606
56566	1.89226	0.101817
56567	1.89226	0.11955
56568	1.89226	0.0782158
56569	1.4263	1.51763
56570	1.67651	0.640395
56571	1.5955	0.756841
56572	1.31022	1.56286
56573	2.98674	0.935141
56574	1.18824	2.1
56575	1.28802	2.1
56576	0.946132	0.911207
56577	0.946132	0.891552
56578	0.946132	0.891385
56579	0.946132	2.1
56580	1.28802	0.774162
56581	1.28802	2.1
56582	1.18824	1.19107
56583	0.946132	1.31414
56584	0.946132	1.47678
56585	1.28802	1.32083
56586	1.89226	0.65399
56587	1.89226	0.969864
56588	1.89226	1.51084
56589	1.89226	0.59761
56590	3.78452	0.769248
56591	1.5955	0.807232
56592	1.52909	0.671397
56593	1.31022	0.823339
56594	1.31022	1.58475
56595	1.2087	1.32421
56596	1.52909	1.40158
56597	1.11825	2.1
56598	1.18824	2.1
56599	0.946132	0.998316
56600	0.946132	0.873036
56601	0.946132	0.916349
56602	0.946132	2.1
56603	1.18824	0.841405
56604	1.18824	2.1
56605	0.946132	0.932832

56606	1.11825	1.13994
56607	1.18824	1.33549
56608	0.946133	1.5359
56609	0.810943	1.1474
56610	0.810943	0.926656
56611	0.810945	0.659537
56612	0.810945	0.992516
56613	1.89226	0.686417
56614	1.35168	0.776967
56615	1.08132	0.632155
56616	1.0813	0.851129
56617	1.08132	1.13508
56618	0.810959	0.795665
56619	0.810959	0.831694
56620	1.89226	0.434921
56621	1.89226	0.189129
56622	1.89226	0.242919
56623	1.89226	0.385172
56624	3.78453	0.411669
56625	1.89226	0.11638
56626	1.89226	0.149818
56627	1.89226	0.161895
56628	1.89226	0.0766232
56629	1.89226	0.158774
56630	1.89226	0.0790229
56631	0.946131	0.0834494
56632	0.946131	0.08688
56633	0.946126	0.158853
56634	0.946126	0.103841
56635	0.946128	0.194346
56636	0.946128	0.101184
56637	0.946128	0.157022
56638	0.946128	0.116403
56639	0.946128	0.116266
56640	0.946128	0.120298
56641	0.946128	0.108533
56642	0.946128	0.103182
56643	1.89226	0.409808
56644	1.89226	0.11252
56645	1.89226	0.25108
56646	1.89226	0.236374
56647	1.89226	0.199949
56648	1.89226	0.18854
56649	1.89226	0.184365
56650	1.89226	0.329709
56651	1.89226	0.578477
56652	1.89226	0.205909
56653	3.78453	0.185674
56654	1.07176	0.0871833
56655	1.07176	0.106718
56656	0.946126	0.156886
56657	0.946126	0.134667
56658	1.07176	0.124222
56659	0.946129	0.0770641
56660	1.27474	0.133808
56661	0.94613	0.15912
56662	1.27474	0.132729
56663	1.07176	0.0520508

56664	0.94613	0.0976925
56665	0.94613	0.0948954
56666	0.723486	0.0684489
56667	0.94613	0.0700613
56668	0.946126	0.0973284
56669	0.946126	0.0893906
56670	0.946129	0.108875
56671	0.723484	0.0718235
56672	1.07176	0.0916421
56673	0.946129	0.0583654
56674	1.32398	0.0921063
56675	1.19835	0.0718452
56676	0.723471	0.0752733
56677	0.723471	0.0832196
56678	0.810946	0.441185
56679	0.810946	0.208627
56680	0.810946	0.431825
56681	0.810946	0.314752
56682	0.810933	0.101201
56683	0.810933	0.325494
56684	1.89226	0.0821462
56685	1.3517	0.290499
56686	1.08132	0.124034
56687	1.08132	0.280339
56688	1.08133	0.243996
56689	2.39923	2.1
56690	1.89226	2.1
56691	1.89226	2.1
56692	1.89226	2.1
56693	2.44606	2.1
56694	2.44606	2.1
56695	1.89226	2.1
56696	1.89226	2.1
56697	1.89226	2.1
56698	1.89226	1.95969
56699	1.89226	2.1
56700	1.89226	2.05728
56701	1.70302	2.1
56702	2.43041	2.1
56703	2.25275	2.1
56704	2.4747	1.90852
56705	1.52079	2.1
56706	1.67063	2.1
56707	2.40644	2.1
56708	1.89226	2.1
56709	1.89226	2.1
56710	1.89226	2.1
56711	2.39923	2.1
56712	2.39923	2.1
56713	0.946131	0.808864
56714	0.946131	2.1
56715	0.946131	2.1
56716	0.946131	1.87879
56717	0.94613	1.3677
56718	0.94613	1.25444
56719	0.946131	2.1
56720	0.946131	2.1
56721	0.946131	2.1

56722	0.946131	2.1
56723	0.946131	2.1
56724	0.946131	2.1
56725	1.04938	2.1
56726	1.04938	2.02411
56727	1.111	2.1
56728	1.06978	2.1
56729	1.2748	2.0602
56730	1.25472	1.09191
56731	0.88782	2.1
56732	0.882987	2.1
56733	0.973779	2.1
56734	1.31997	2.1
56735	0.913905	2.1
56736	1.31312	2.1
56737	2.6536	2.1
56738	2.15287	2.1
56739	2.72238	2.1
56740	2.22887	2.1
56741	4.87525	2.1
56742	2.15288	2.1
56743	1.40719	2.1
56744	1.41839	2.1
56745	1.34239	2.1
56746	2.22887	2.1
56747	2.15288	2.1
56748	2.72237	2.1
56749	2.22887	2.1
56750	2.77314	2.1
56751	2.28748	2.1
56752	5.00985	2.1
56753	0.922802	2.1
56754	0.922802	2.1
56755	0.92107	2.1
56756	0.92107	2.1
56757	0.703593	2.1
56758	0.729436	2.1
56759	1.11444	2.1
56760	1.14374	2.1
56761	1.14374	2.1
56762	0.729436	2.1
56763	0.703593	2.1
56764	1.11444	2.1
56765	1.89226	2.06847
56766	1.89226	2.1
56767	1.89226	1.11535
56768	1.89226	2.1
56769	1.89226	1.53436
56770	1.89226	2.1
56771	0.489927	2.1
56772	0.63008	2.1
56773	0.697385	2.1
56774	0.55524	2.1
56775	0.592141	2.1
56776	0.592141	2.1
56777	0.593179	2.08451
56778	0.593179	2.1
56779	0.63008	2.1

56780	0.697385	2.1
56781	0.55524	2.1
56782	0.489927	2.1
56783	0.946133	2.1
56784	0.946133	1.70954
56785	0.946132	1.23848
56786	0.946132	0.824779
56787	0.946133	1.0249
56788	0.946133	2.1
56789	0.946132	1.95222
56790	0.946132	0.91544
56791	0.946132	1.28562
56792	0.946132	1.09314
56793	0.946132	0.946105
56794	0.946132	1.03293
56795	1.89226	2.1
56796	1.89226	1.60011
56797	1.89226	1.04754
56798	1.89226	2.1
56799	1.89226	1.0667
56800	1.89226	2.1
56801	0.805746	2.1
56802	0.63008	2.1
56803	0.881598	2.01382
56804	0.697385	2.1
56805	0.749417	2.1
56806	0.749417	2.1
56807	0.753714	1.63718
56808	0.753714	2.1
56809	0.881598	2.1
56810	0.805746	2.1
56811	0.63008	2.1
56812	0.697385	2.1
56813	0.881599	2.1
56814	0.697385	2.1
56815	0.805746	1.49146
56816	0.626731	0.991491
56817	0.755479	1.24234
56818	0.755479	2.1
56819	0.752851	0.975617
56820	0.752851	2.1
56821	0.881598	2.1
56822	0.805747	2.1
56823	0.697385	2.1
56824	0.626731	2.1
56825	2.65348	2.1
56826	2.12314	2.1
56827	2.63899	2.1
56828	2.13898	2.1
56829	4.76213	2.1
56830	0.763518	2.1
56831	0.765889	2.1
56832	0.84086	2.1
56833	0.84086	2.1
56834	0.632078	2.1
56835	0.597565	2.1
56836	1.08813	2.1
56837	0.985643	2.1

56838	0.982464	2.1
56839	0.66404	2.1
56840	1.0358	2.1
56841	0.598023	2.1
56842	1.29293	2.1
56843	1.29628	2.1
56844	1.19266	2.1
56845	1.19266	2.1
56846	1.07109	2.1
56847	1.13093	2.1
56848	1.34467	2.1
56849	1.35251	2.1
56850	1.34369	2.1
56851	1.12525	2.1
56852	1.28746	2.1
56853	1.13511	2.1
56854	2.19717	2.1
56855	2.16547	2.1
56856	1.37392	2.1
56857	1.37392	2.1
56858	1.2966	2.1
56859	2.11985	2.1
56860	1.89226	2.1
56861	1.89226	2.1
56862	1.89226	2.1
56863	1.89226	2.1
56864	1.89226	2.1
56865	1.89226	2.1
56866	1.65637	2.1
56867	1.41758	2.1
56868	1.65637	2.1
56869	1.74391	2.1
56870	1.35504	2.1
56871	1.44258	2.1
56872	1.40774	2.1
56873	1.40774	2.1
56874	0.946131	2.1
56875	0.946131	2.1
56876	1.40774	2.1
56877	1.38882	2.1
56878	0.946132	2.1
56879	0.946132	2.1
56880	0.946132	2.1
56881	1.38882	2.1
56882	0.946132	2.1
56883	1.40774	2.1
56884	1.89227	2.1
56885	1.89227	2.1
56886	1.89227	2.1
56887	1.89227	2.1
56888	3.78453	2.1
56889	0.603148	2.1
56890	0.600177	2.1
56891	0.558615	2.1
56892	0.580714	2.1
56893	0.558606	2.1
56894	0.67883	2.1
56895	1.65637	2.1

56896	1.07933	2.1
56897	0.842649	2.1
56898	0.905168	2.1
56899	0.774334	2.1
56900	1.38882	2.1
56901	1.38882	2.1
56902	0.946132	2.1
56903	0.946132	2.1
56904	1.38882	2.1
56905	1.37381	2.1
56906	0.946132	2.1
56907	0.946132	2.1
56908	0.946132	2.1
56909	1.37381	2.1
56910	0.946133	2.1
56911	1.38882	2.1
56912	2.3771	2.1
56913	1.9339	2.1
56914	1.70782	2.1
56915	1.9339	2.1
56916	2.42693	2.1
56917	2.83279	2.1
56918	2.34901	0.631679
56919	1.45833	0.651625
56920	1.86695	0.584889
56921	1.03101	0.67992
56922	3.32528	0.610638
56923	1.89226	0.396738
56924	1.89226	0.399656
56925	1.89226	0.327458
56926	1.89226	0.272076
56927	3.78453	0.384518
56928	2.71753	0.306845
56929	1.89226	0.39267
56930	2.76228	0.204805
56931	2.76228	0.327095
56932	1.89226	0.401496
56933	1.89226	0.434715
56934	0.946131	0.31987
56935	0.946131	0.310766
56936	0.946132	0.294362
56937	0.946132	0.326743
56938	0.946132	0.383705
56939	0.946132	0.252451
56940	0.946132	0.377719
56941	0.946132	0.241798
56942	0.946132	0.243151
56943	0.946132	0.284853
56944	0.946132	0.373121
56945	0.946132	0.339413
56946	1.89227	0.362211
56947	1.89227	0.162704
56948	1.89227	0.235155
56949	1.89227	0.333206
56950	1.89227	0.147843
56951	1.89227	0.242531
56952	2.71753	0.275347
56953	2.37907	0.129186

56954	2.37907	0.21235
56955	2.76228	0.312192
56956	2.39588	0.0995043
56957	2.77909	0.199937
56958	1.6571	0.862969
56959	1.11682	1.58466
56960	1.47132	0.989341
56961	1.93763	0.59236
56962	3.12842	1.25178
56963	1.93762	0.701375
56964	2.12345	1.06775
56965	1.65709	0.636925
56966	1.65709	1.63273
56967	2.12345	0.962
56968	1.88635	2.03913
56969	1.47134	1.87433
56970	2.219	0.705865
56971	2.25734	2.0982
56972	1.93765	0.783112
56973	1.79103	1.20122
56974	1.47134	0.695349
56975	1.06174	0.921453
56976	1.19349	1.99511
56977	1.1095	1.73172
56978	0.968827	0.824212
56979	1.07892	0.786888
56980	1.07892	2.1
56981	1.0834	0.874476
56982	1.0834	1.01741
56983	1.1095	1.14376
56984	1.19349	1.00097
56985	0.968825	1.05345
56986	1.06174	1.05441
56987	2.40627	2.1
56988	2.08288	1.17491
56989	2.5444	0.829926
56990	2.29285	1.42458
56991	4.62729	0.961656
56992	2.08289	0.836278
56993	1.65703	0.991474
56994	1.71105	2.1
56995	1.50108	1.73034
56996	2.29286	1.82688
56997	2.08289	1.86739
56998	2.5444	0.984631
56999	2.29285	2.1
57000	2.71429	1.95114
57001	2.50288	0.77128
57002	5.04728	2.09731
57003	2.50288	0.796537
57004	2.00927	1.96683
57005	2.02116	1.07676
57006	2.02116	2.1
57007	2.29286	0.964462
57008	2.29286	1.53564
57009	1.89226	1.482
57010	1.89226	2.09863
57011	1.89226	0.649737

57012	1.89226	2.1
57013	3.78453	1.42031
57014	1.17418	1.29108
57015	1.29741	2.1
57016	1.04921	2.1
57017	1.19352	2.1
57018	2.3677	2.1
57019	1.29741	2.1
57020	1.27575	2.1
57021	1.19352	2.1
57022	1.19473	2.1
57023	2.46927	2.1
57024	1.89226	2.1
57025	1.89226	2.1
57026	1.89226	2.1
57027	1.89226	2.1
57028	1.89226	2.1
57029	1.89226	2.1
57030	0.561332	2.1
57031	0.561332	2.1
57032	0.567597	2.1
57033	0.567597	2.1
57034	0.597365	2.1
57035	0.544699	2.1
57036	0.596761	2.1
57037	0.531564	2.1
57038	0.5447	2.1
57039	0.531563	2.1
57040	0.596759	2.1
57041	0.597366	2.1
57042	0.946131	2.1
57043	0.946131	2.1
57044	0.946131	1.79933
57045	0.946131	2.1
57046	0.946129	2.1
57047	0.946129	2.1
57048	0.946133	2.1
57049	0.946133	1.90297
57050	0.946131	2.1
57051	0.946131	2.1
57052	0.946131	2.1
57053	0.946131	2.1
57054	1.10938	0.398934
57055	1.17614	0.592143
57056	1.02478	0.193956
57057	1.09056	0.557367
57058	2.19994	0.526646
57059	1.17614	0.59293
57060	1.11868	1.18763
57061	1.03408	0.562562
57062	1.03408	1.68899
57063	1.09056	0.558386
57064	1.17516	1.95698
57065	1.89226	0.594799
57066	1.89226	0.543731
57067	1.89226	0.599386
57068	1.89226	0.466705
57069	1.89226	0.632937

57070	1.89226	0.554766
57071	1.89226	0.459719
57072	1.89226	0.552719
57073	1.89226	0.513259
57074	1.89226	0.375471
57075	1.89226	0.603584
57076	1.89226	0.358401
57077	0.579551	0.312997
57078	0.563873	0.223633
57079	0.611341	0.414033
57080	0.598693	0.307044
57081	0.589856	0.281131
57082	0.589856	0.25284
57083	0.588387	0.372485
57084	0.588387	0.333062
57085	0.57955	0.317994
57086	0.611341	0.302013
57087	0.563873	0.300778
57088	0.598693	0.293999
57089	0.611341	0.397939
57090	0.598693	0.291596
57091	0.586048	0.383573
57092	0.572737	0.432899
57093	0.591824	0.381942
57094	0.591824	0.342453
57095	0.592254	0.457904
57096	0.592254	0.420989
57097	0.611341	0.403213
57098	0.586048	0.374989
57099	0.598693	0.379845
57100	0.572737	0.389474
57101	0.946119	0.491493
57102	0.946119	0.578374
57103	0.946119	0.499163
57104	0.946119	0.589665
57105	0.94612	0.491779
57106	0.94612	0.454888
57107	0.946119	0.574591
57108	0.946119	0.609667
57109	0.946132	0.546522
57110	0.946132	0.54089
57111	0.946107	0.561276
57112	0.946107	0.565328
57113	1.89224	0.489497
57114	1.89224	0.426774
57115	1.89224	0.479857
57116	1.89224	0.33095
57117	1.89224	0.571882
57118	1.89224	0.469013
57119	0.562335	0.269647
57120	0.579543	0.198754
57121	0.59499	0.335427
57122	0.611333	0.258826
57123	0.586619	0.240615
57124	0.586619	0.225028
57125	0.587049	0.305206
57126	0.587049	0.280338
57127	0.594999	0.282632

57128	0.562342	0.278972
57129	0.579535	0.275668
57130	0.611325	0.262354
57131	0.59499	0.319
57132	0.611333	0.245667
57133	0.567849	0.305467
57134	0.58604	0.351965
57135	0.590978	0.299447
57136	0.590978	0.278157
57137	0.590052	0.372112
57138	0.590052	0.343171
57139	0.567857	0.342099
57140	0.594998	0.332813
57141	0.611325	0.343547
57142	0.586032	0.320719
57143	1.69661	0.559042
57144	1.9339	0.387904
57145	1.39379	0.572014
57146	1.69107	0.177647
57147	3.32768	0.463275
57148	1.9339	0.37121
57149	1.98414	0.418478
57150	1.69107	0.357054
57151	1.72774	0.383737
57152	3.66164	0.352383
57153	1.39379	0.621358
57154	1.69107	0.358189
57155	1.14397	0.50536
57156	1.43548	0.252576
57157	2.82927	0.458458
57158	1.69107	0.339874
57159	1.72774	0.380519
57160	1.53873	0.36651
57161	1.53873	0.408127
57162	1.43548	0.34842
57163	1.62449	0.44214
57164	2.58431	0.332976
57165	1.34072	0.34352
57166	1.80841	0.34058
57167	1.34072	0.344995
57168	2.51501	0.334422
57169	2.04732	0.341722
57170	1.89226	0.34348
57171	1.89226	0.357267
57172	1.89226	0.352271
57173	1.89226	0.361452
57174	1.89226	0.344467
57175	1.89226	0.355416
57176	0.946131	0.362362
57177	0.946131	0.352071
57178	1.3639	0.35083
57179	1.1516	0.362593
57180	0.946129	0.363756
57181	0.946129	0.366781
57182	1.36391	0.349468
57183	1.36391	0.344018
57184	0.946131	0.362249
57185	1.3639	0.356401

57186	1.1516	0.353795
57187	0.946131	0.357416
57188	0.946131	0.351589
57189	0.946131	0.342723
57190	0.94613	0.357367
57191	0.94613	0.343368
57192	0.946128	0.361823
57193	0.946128	0.351521
57194	0.946133	0.340846
57195	0.946133	0.342293
57196	0.946131	0.348162
57197	0.946131	0.362105
57198	0.946131	0.343802
57199	0.946131	0.346236
57200	1.89226	1.03679
57201	2.63634	1.93289
57202	1.89226	1.08251
57203	1.83902	1.19084
57204	3.73128	1.04922
57205	1.47059	1.69844
57206	2.02928	2.1
57207	1.47059	2.1
57208	1.65706	2.1
57209	1.98628	2.1
57210	2.17274	2.1
57211	2.43618	2.1
57212	2.41176	2.1
57213	1.98627	2.1
57214	2.34084	2.1
57215	4.39803	2.1
57216	1.47059	1.56579
57217	1.82453	2.1
57218	1.98627	2.1
57219	1.98627	2.1
57220	1.26929	2.1
57221	1.26929	2.1
57222	1.98627	2.1
57223	2.34084	2.1
57224	1.47264	2.1
57225	2.24937	2.1
57226	4.23564	2.1
57227	1.33026	0.715287
57228	1.51171	0.913397
57229	1.51171	0.671632
57230	1.4617	1.00615
57231	1.35382	0.668982
57232	1.30382	1.42488
57233	1.89226	0.630706
57234	1.89226	0.704591
57235	1.89226	0.592783
57236	1.89226	0.848854
57237	1.89226	0.5863
57238	1.89226	0.906976
57239	1.39263	0.718808
57240	0.962704	0.477382
57241	1.0171	0.570739
57242	0.962704	0.641465
57243	1.40822	0.535724

57244	1.35382	0.58741
57245	0.755853	1.10357
57246	0.789808	0.792284
57247	0.696315	0.71894
57248	0.676912	0.607067
57249	0.729751	0.604158
57250	0.729751	0.774438
57251	0.722417	1.44137
57252	0.722417	0.57596
57253	0.696315	0.760697
57254	0.789808	0.746232
57255	0.755853	0.758267
57256	0.676912	0.757138
57257	0.946132	0.700022
57258	0.946132	0.768423
57259	0.946132	0.623718
57260	0.946132	0.547381
57261	0.946131	0.549353
57262	0.946131	0.696459
57263	0.946132	0.617243
57264	0.946132	0.76974
57265	0.946132	0.6203
57266	0.946132	0.635805
57267	0.946132	0.643637
57268	0.946132	0.643178
57269	0.946133	0.60497
57270	0.946133	0.534685
57271	0.946132	0.470035
57272	0.946132	0.532209
57273	0.946132	0.469595
57274	0.946132	0.534953
57275	0.946133	0.504726
57276	0.946133	0.617276
57277	0.946132	0.541185
57278	0.946132	0.541545
57279	0.946132	0.531516
57280	0.946132	0.536727
57281	1.89227	1.6926
57282	2.53014	1.71072
57283	1.89227	2.1
57284	1.98241	1.43947
57285	1.89227	2.1
57286	1.98241	1.50823
57287	1.89226	1.25794
57288	1.89226	2.1
57289	1.89226	1.67581
57290	1.89226	1.56623
57291	1.89226	2.1
57292	1.89226	2.0927
57293	1.89226	1.18342
57294	1.89226	2.09991
57295	1.89226	1.4957
57296	1.89226	1.4768
57297	3.78452	1.59009
57298	2.45304	1.08943
57299	1.89226	1.90599
57300	1.89226	1.46437
57301	1.89226	1.41618

57302	1.89226	1.26104
57303	1.89226	1.24036
57304	1.89227	1.80034
57305	1.89227	1.56931
57306	1.89227	2.09432
57307	1.89227	1.26691
57308	1.89227	2.1
57309	1.89227	1.30927
57310	1.89226	1.38827
57311	1.89226	2.1
57312	1.89226	1.66828
57313	1.89226	1.68127
57314	1.89226	2.0666
57315	1.89226	2.0555
57316	2.09871	0.598058
57317	2.71891	0.673588
57318	1.31126	0.596268
57319	1.31126	0.682575
57320	2.55341	0.596988
57321	1.76597	0.79159
57322	1.89226	0.732552
57323	1.89226	0.72868
57324	1.89226	0.617345
57325	1.89226	0.895296
57326	1.89226	0.610916
57327	1.89226	0.898092
57328	0.946128	0.729124
57329	0.946128	0.602053
57330	0.946133	0.577175
57331	0.946133	0.513486
57332	0.946131	0.567797
57333	0.946131	0.689935
57334	0.94613	0.497561
57335	0.94613	0.619741
57336	0.946131	0.604735
57337	0.946131	0.610396
57338	0.946131	0.60663
57339	0.946131	0.603619
57340	2.71891	0.56363
57341	1.89226	0.551051
57342	2.09871	0.497875
57343	2.09871	0.694512
57344	1.89226	0.424309
57345	1.89226	0.675848
57346	1.89227	0.700473
57347	1.89227	0.689945
57348	1.89227	0.620304
57349	1.89227	0.844411
57350	1.89227	0.620492
57351	1.89227	0.842472
57352	0.946129	0.692939
57353	0.946129	0.614638
57354	0.946133	0.556708
57355	0.946133	0.485381
57356	0.946131	0.55133
57357	0.946131	0.665549
57358	0.946131	0.473672
57359	0.946131	0.627217

57360	0.946131	0.576543
57361	0.946131	0.581788
57362	0.946131	0.567718
57363	0.946131	0.586018
57364	2.32462	0.0929063
57365	1.89226	0.058018
57366	1.89226	0.0508927
57367	1.89226	0.0618137
57368	2.2291	0.0858474
57369	2.2291	0.108908
57370	0.847669	0.0556816
57371	0.847669	0.0583795
57372	0.796966	0.0551728
57373	0.796966	0.0744959
57374	0.833552	0.05
57375	1.01049	0.06617
57376	0.709709	0.0523364
57377	0.634147	0.0532677
57378	0.634148	0.05
57379	0.833549	0.063836
57380	0.709711	0.0577547
57381	1.01049	0.069448
57382	1.89226	0.05
57383	1.89226	0.097875
57384	1.89226	0.0603547
57385	1.89226	0.0888392
57386	1.89226	0.0571849
57387	1.89226	0.0756328
57388	1.03611	0.0524614
57389	0.936655	0.0564114
57390	0.946132	0.0559424
57391	0.946132	0.0695803
57392	0.941918	0.05
57393	0.941918	0.054612
57394	1.00308	0.0573198
57395	1.04117	0.05
57396	1.1406	0.05
57397	1.04074	0.05
57398	0.851529	0.0562075
57399	1.00311	0.0803498
57400	1.01814	0.05
57401	1.6671	0.058858
57402	1.35561	0.05
57403	1.35561	0.05
57404	1.2683	0.05
57405	1.2683	0.05
57406	0.946128	0.0592682
57407	0.946128	0.05
57408	0.946129	0.05
57409	0.946129	0.0607266
57410	0.946132	0.059618
57411	0.946132	0.05
57412	0.946126	0.05
57413	0.946126	0.0523629
57414	0.946129	0.05
57415	0.946129	0.05
57416	0.946129	0.05
57417	0.946129	0.0610491

57418	2.23338	0.05
57419	1.96	0.05
57420	2.03438	0.05
57421	1.87835	0.05
57422	4.11172	0.05
57423	1.96	0.05
57424	1.52661	0.05
57425	1.87834	0.05
57426	1.53175	0.05
57427	3.40495	0.05
57428	2.03435	0.05
57429	1.65813	0.05
57430	1.65813	0.05
57431	1.87832	0.05
57432	1.72067	0.05
57433	1.94086	0.05
57434	1.65813	0.05
57435	1.40572	0.05
57436	1.87832	0.05
57437	1.87832	0.05
57438	1.31154	0.05
57439	1.53173	0.05
57440	1.86662	2.1
57441	2.0815	2.1
57442	2.0815	2.1
57443	2.0815	2.1
57444	1.87246	2.1
57445	1.87362	2.1
57446	1.0273	2.1
57447	0.996347	2.1
57448	0.946129	2.1
57449	0.946129	2.1
57450	1.04427	2.1
57451	1.04196	2.1
57452	0.910674	2.1
57453	0.910674	2.1
57454	1.04072	2.1
57455	1.10931	2.1
57456	1.03132	2.1
57457	0.933698	2.1
57458	2.63789	0.434908
57459	1.89226	0.522466
57460	2.3033	0.436138
57461	2.3033	0.521451
57462	1.89226	0.429833
57463	1.89226	0.526654
57464	1.89226	0.560126
57465	1.89226	0.621462
57466	1.89226	0.546257
57467	1.89226	0.58769
57468	3.78452	0.61292
57469	1.51898	0.51363
57470	1.36006	0.59675
57471	2.03143	0.635288
57472	2.03143	0.58869
57473	1.04026	0.556165
57474	1.04026	0.545516
57475	0.94613	0.426537

57476	0.94613	0.456125
57477	1.31894	0.403784
57478	1.31894	0.42476
57479	0.94613	0.398013
57480	0.94613	0.477684
57481	1.31894	0.421917
57482	1.41232	0.455611
57483	0.946129	0.428906
57484	1.41232	0.434305
57485	1.31895	0.426412
57486	0.946129	0.430166
57487	1.89226	0.472996
57488	1.89226	0.54709
57489	1.89226	0.455759
57490	1.89226	0.551098
57491	1.89226	0.438178
57492	1.89226	0.54964
57493	1.89804	0.447823
57494	2.03143	0.505231
57495	2.46286	0.522215
57496	2.46287	0.483515
57497	1.51898	0.460421
57498	1.51898	0.447233
57499	1.89226	2.1
57500	1.89226	2.1
57501	1.89226	2.1
57502	1.89226	2.1
57503	3.78452	2.1
57504	1.3864	2.1
57505	1.3864	2.1
57506	0.946132	2.1
57507	0.946132	2.1
57508	1.32905	2.1
57509	1.3864	2.1
57510	0.946132	2.1
57511	0.946132	2.1
57512	0.946132	2.1
57513	1.32905	2.1
57514	0.946133	2.1
57515	1.3864	2.1
57516	2.77279	2.1
57517	1.67883	2.1
57518	1.67883	2.1
57519	1.73415	2.1
57520	2.65811	2.1
57521	2.71343	2.1
57522	0.946131	2.1
57523	0.946131	2.1
57524	0.946128	2.1
57525	0.946128	2.1
57526	0.946127	2.1
57527	0.946127	2.1
57528	0.946131	2.1
57529	0.946131	2.1
57530	0.946129	2.1
57531	0.946129	2.1
57532	0.94613	2.1
57533	0.94613	2.1

57534	1.89226	2.1
57535	1.89226	2.1
57536	1.89226	2.1
57537	1.89226	2.1
57538	3.78452	2.1
57539	1.89226	2.1
57540	2.54394	2.1
57541	2.54394	2.1
57542	2.65811	2.1
57543	1.89226	2.1
57544	2.00643	2.1
57545	2.54394	2.1
57546	2.6581	2.1
57547	1.62743	2.1
57548	1.62743	2.1
57549	1.67883	2.1
57550	2.59533	2.1
57551	1.89226	2.1
57552	1.89226	2.1
57553	1.89226	2.1
57554	1.89226	2.1
57555	1.89226	2.1
57556	1.89226	2.1
57557	1.89226	1.25412
57558	1.89226	1.09294
57559	1.89226	1.9501
57560	1.89226	0.871304
57561	1.89226	1.64541
57562	1.89226	0.834478
57563	1.89226	1.25352
57564	1.89226	1.08419
57565	1.89226	1.91269
57566	1.89226	0.973545
57567	1.89226	1.56875
57568	1.89226	0.890707
57569	1.89226	0.856167
57570	1.89226	0.672947
57571	1.89226	1.21785
57572	1.89226	0.637074
57573	1.89226	1.09974
57574	1.89226	0.623591
57575	2.68537	0.37163
57576	2.22575	0.528841
57577	2.47464	0.311133
57578	1.79093	0.479325
57579	4.70039	0.414773
57580	2.81217	0.599792
57581	2.47464	0.369863
57582	2.58286	0.541822
57583	2.29193	0.258448
57584	5.0575	0.413064
57585	2.47464	0.354511
57586	2.20365	0.481983
57587	2.29193	0.375411
57588	1.90104	0.516019
57589	4.37568	0.388585
57590	1.89226	0.664624
57591	2.16957	2.1

57592	2.52823	1.03551
57593	2.52823	2.1
57594	1.89226	0.860806
57595	1.89226	1.37574
57596	1.36226	2.1
57597	1.55369	2.1
57598	1.33309	2.1
57599	1.62593	2.1
57600	2.98819	2.1
57601	0.802281	2.1
57602	0.802281	2.1
57603	0.835143	2.1
57604	0.835143	2.1
57605	0.824458	2.1
57606	0.92641	2.1
57607	0.776847	2.1
57608	0.812967	2.1
57609	0.812967	2.1
57610	0.926411	2.1
57611	0.776847	2.1
57612	0.824458	2.1
57613	1.33309	2.1
57614	1.62593	2.1
57615	1.32813	2.1
57616	1.7237	2.1
57617	2.95406	2.1
57618	0.814544	2.1
57619	0.875312	2.1
57620	0.910502	2.1
57621	0.910502	2.1
57622	0.886633	2.1
57623	0.975739	2.1
57624	0.792623	2.1
57625	0.840283	2.1
57626	0.796372	2.1
57627	1.06159	2.1
57628	0.81948	2.1
57629	0.933833	2.1
57630	2.63997	2.1
57631	1.29694	2.1
57632	1.28141	2.1
57633	2.62623	2.1
57634	3.92317	2.1
57635	2.63998	2.1
57636	1.89226	2.1
57637	1.89226	2.1
57638	2.62623	2.1
57639	4.5185	2.1
57640	1.89226	2.1
57641	1.89226	2.1
57642	1.89226	2.1
57643	1.89226	1.89266
57644	3.78453	2.1
57645	1.89226	2.1
57646	1.89226	2.1
57647	1.89226	2.1
57648	1.89226	2.1
57649	1.89226	2.1

57650	1.89226	2.1
57651	1.25468	2.1
57652	2.62623	2.1
57653	1.28141	2.1
57654	1.28141	2.1
57655	2.60665	2.1
57656	2.60665	2.1
57657	1.89226	2.1
57658	2.62623	2.1
57659	2.60666	2.1
57660	2.62623	2.1
57661	1.87269	2.1
57662	1.89226	2.1
57663	1.89226	2.1
57664	1.89226	2.1
57665	1.89226	2.1
57666	1.89226	2.1
57667	3.78453	2.1
57668	1.89226	2.1
57669	1.89226	1.78889
57670	1.89226	2.1
57671	1.89226	2.1
57672	1.89226	2.1
57673	1.89226	2.06898
57674	2.30147	1.23437
57675	2.27703	1.87816
57676	1.89227	1.11575
57677	1.89227	1.55425
57678	4.1693	1.45608
57679	1.38617	1.19744
57680	1.24613	1.33538
57681	2.59886	1.09657
57682	2.6238	1.38783
57683	3.86993	1.09984
57684	0.946133	1.37489
57685	0.946133	0.929068
57686	0.946132	0.931239
57687	0.946132	1.87536
57688	0.946133	1.84178
57689	0.946133	1.52839
57690	0.946132	0.915783
57691	0.946132	1.05101
57692	0.946133	1.22648
57693	0.946133	1.17212
57694	0.946132	1.05943
57695	0.946132	1.07449
57696	2.21773	2.1
57697	2.59216	2.1
57698	1.68541	2.1
57699	1.68541	2.1
57700	1.84814	2.1
57701	2.38046	2.1
57702	1.89966	2.1
57703	1.70303	2.1
57704	1.2028	2.1
57705	1.29897	2.1
57706	2.02873	2.1
57707	2.19093	2.1

57708	2.75218	2.1
57709	1.70303	2.1
57710	3.10254	2.1
57711	2.24783	2.1
57712	2.56839	2.1
57713	1.70303	2.1
57714	2.56839	2.1
57715	1.08276	2.1
57716	2.24061	2.1
57717	1.67491	2.1
57718	1.60948	2.1
57719	1.08276	2.1
57720	1.89226	0.879766
57721	1.89226	1.0272
57722	1.89226	0.906368
57723	1.89226	0.855764
57724	3.78453	0.726923
57725	1.89226	0.802476
57726	1.89226	0.972444
57727	1.89226	0.836936
57728	1.89226	0.843692
57729	3.78452	1.48942
57730	1.89226	0.940124
57731	2.7535	0.655241
57732	1.89226	1.14409
57733	1.89226	0.983464
57734	1.89226	0.761303
57735	1.89226	0.782193
57736	1.89226	0.98461
57737	1.89226	0.823927
57738	1.89226	0.643095
57739	1.89226	0.979894
57740	1.89226	0.794916
57741	1.89226	1.46719
57742	1.89227	0.996405
57743	1.89227	0.876297
57744	1.89227	0.890885
57745	1.89227	0.816351
57746	3.78453	1.68034
57747	1.89226	0.877702
57748	2.53412	0.567677
57749	1.89226	1.67202
57750	1.89226	0.69538
57751	1.89226	0.813278
57752	1.89226	0.649523
57753	2.21126	0.159654
57754	1.89226	0.23058
57755	2.57464	0.233838
57756	1.89226	0.227297
57757	2.57464	0.154836
57758	1.89226	0.162077
57759	1.89226	0.298752
57760	1.89226	0.321869
57761	1.89226	0.705722
57762	1.89226	0.365179
57763	1.89226	0.719157
57764	1.89226	0.271166
57765	1.1418	0.104262

57766	0.653021	0.0701258
57767	1.37282	0.0834265
57768	0.931386	0.114102
57769	1.34568	0.157823
57770	1.17669	0.0876358
57771	0.666595	0.0943833
57772	0.786895	0.103587
57773	0.933982	0.107779
57774	1.05351	0.124073
57775	1.00792	0.0885913
57776	1.00792	0.0966847
57777	1.89226	2.1
57778	1.89226	2.1
57779	1.89226	2.1
57780	1.89226	2.1
57781	3.78452	2.1
57782	1.89226	2.1
57783	1.89226	2.1
57784	1.89226	2.1
57785	1.89226	2.1
57786	1.89226	2.1
57787	1.89226	2.1
57788	1.65642	2.1
57789	1.30248	2.1
57790	1.38888	2.1
57791	1.25987	2.1
57792	1.70611	2.1
57793	1.65642	2.1
57794	1.65642	2.1
57795	1.81407	2.1
57796	2.19428	2.1
57797	1.92555	2.1
57798	3.8507	2.1
57799	1.89226	2.1
57800	1.89226	2.1
57801	1.89226	2.1
57802	1.89226	2.1
57803	3.78452	2.1
57804	1.89226	2.1
57805	1.89226	2.1
57806	1.89226	2.1
57807	1.89226	2.1
57808	1.89226	2.1
57809	1.89226	2.1
57810	0.705495	2.1
57811	0.809317	2.1
57812	1.1655	2.1
57813	1.09605	2.1
57814	0.983375	2.1
57815	0.797301	2.1
57816	0.906633	2.1
57817	0.906633	2.1
57818	0.85085	2.1
57819	1.16548	2.1
57820	0.705501	2.1
57821	1.00314	2.1
57822	2.19428	2.1
57823	2.31862	2.1

57824	2.27408	2.1
57825	2.45893	2.1
57826	4.46836	2.1
57827	1.89226	0.758742
57828	1.89226	1.01241
57829	1.89226	0.922945
57830	1.89226	0.748638
57831	1.89226	1.37712
57832	1.89226	0.970634
57833	2.0815	0.757427
57834	2.22136	0.855936
57835	1.5515	0.793439
57836	1.86323	0.687014
57837	2.74986	1.05024
57838	3.30238	0.787336
57839	1.89224	0.766086
57840	1.89224	0.940253
57841	1.89224	0.957045
57842	1.89224	0.729738
57843	1.89224	1.47049
57844	1.89224	0.873835
57845	1.89224	0.759083
57846	1.89224	1.11673
57847	1.89224	0.854455
57848	1.89224	0.713168
57849	1.89224	1.37122
57850	1.89224	0.914077
57851	1.17846	0.697696
57852	0.810935	0.703095
57853	0.93347	1.15573
57854	0.93347	0.719118
57855	0.810932	0.85888
57856	0.810932	0.751718
57857	2.22133	0.779352
57858	1.71919	0.769837
57859	1.32629	0.683671
57860	1.0813	0.85957
57861	1.57137	0.758187
57862	2.21149	2.1
57863	1.89226	2.04321
57864	1.89226	2.1
57865	1.89226	1.62974
57866	2.17159	2.1
57867	2.17159	1.9007
57868	0.946131	0.668089
57869	0.946131	2.1
57870	0.946131	1.76175
57871	0.946131	2.1
57872	0.94613	1.00483
57873	0.94613	1.74508
57874	0.946132	2.1
57875	0.946132	1.98242
57876	0.946131	1.22645
57877	0.946131	1.1507
57878	0.946131	1.49832
57879	0.946131	1.44143
57880	1.12266	1.64655
57881	1.4714	1.74061

57882	1.62322	1.53921
57883	1.62322	1.54427
57884	1.02958	1.82295
57885	1.02958	2.1
57886	2.2671	2.1
57887	1.89226	2.09204
57888	1.89226	2.1
57889	1.89226	1.85707
57890	2.21149	2.1
57891	2.21149	2.1
57892	1.89226	1.86167
57893	1.89226	1.4061
57894	1.89226	1.6767
57895	1.89226	1.17986
57896	1.89226	2.02396
57897	1.89226	1.84741
57898	0.736914	1.66099
57899	0.736914	1.87723
57900	0.822839	2.03
57901	0.822836	2.1
57902	0.791298	0.909174
57903	1.00003	1.83582
57904	0.640254	1.85649
57905	0.575379	2.05779
57906	0.609001	1.2591
57907	1.00005	1.29281
57908	0.575371	1.86988
57909	0.831908	1.33345
57910	1.89226	2.1
57911	1.89226	2.1
57912	1.89226	2.1
57913	1.89226	2.1
57914	1.89226	2.1
57915	1.89226	2.1
57916	1.89226	2.1
57917	1.89226	2.1
57918	1.89226	2.1
57919	1.89226	2.1
57920	1.89226	2.1
57921	1.89226	2.1
57922	0.730617	2.1
57923	0.71052	2.1
57924	0.7329	2.1
57925	0.712083	2.1
57926	0.701051	2.1
57927	0.701051	2.1
57928	0.721552	2.1
57929	0.721552	2.1
57930	0.710521	2.1
57931	0.732898	2.1
57932	0.730619	2.1
57933	0.712081	2.1
57934	0.7329	2.1
57935	0.712082	2.1
57936	0.559601	2.1
57937	0.495993	2.1
57938	0.603718	2.1
57939	0.603718	2.1

57940	0.625174	2.1
57941	0.625174	2.1
57942	0.732899	2.1
57943	0.559601	2.1
57944	0.712082	2.1
57945	0.495993	2.1
57946	0.94613	2.1
57947	0.94613	2.1
57948	0.94613	2.1
57949	0.94613	2.1
57950	0.946131	2.1
57951	0.946131	2.1
57952	0.946129	2.1
57953	0.946129	2.1
57954	0.94613	2.1
57955	0.94613	2.1
57956	0.94613	2.1
57957	0.94613	2.1
57958	1.89226	2.1
57959	1.89226	2.1
57960	1.89226	2.1
57961	1.89226	2.1
57962	1.89226	2.1
57963	1.89226	2.1
57964	1.42104	2.1
57965	1.4658	2.1
57966	1.51223	2.1
57967	1.42283	2.1
57968	2.93327	2.1
57969	0.732899	2.1
57970	0.756115	2.1
57971	0.629763	2.1
57972	0.559601	2.1
57973	0.646134	2.1
57974	0.646134	2.1
57975	0.669582	2.1
57976	0.669582	2.1
57977	0.756115	2.1
57978	0.629763	2.1
57979	0.732899	2.1
57980	0.559601	2.1
57981	2.566	2.1
57982	1.20242	1.54908
57983	2.64084	2.1
57984	2.18297	2.1
57985	1.66029	2.1
57986	1.20242	1.3348
57987	0.946133	1.11539
57988	0.946133	1.38819
57989	0.946129	2.1
57990	0.946129	2.1
57991	0.946131	2.1
57992	0.946131	1.04052
57993	0.946131	0.595379
57994	0.946131	2.04681
57995	0.946131	0.856266
57996	0.946131	1.5513
57997	0.946131	1.24767

57998	0.946131	1.31365
57999	1.89226	0.05
58000	1.89226	0.0500137
58001	1.89226	0.05
58002	1.89226	0.05
58003	3.78453	0.05
58004	1.03073	0.05
58005	1.03073	0.05
58006	1.04066	0.05
58007	1.04066	0.05
58008	1.20802	0.05
58009	1.32689	0.05
58010	0.883301	0.05
58011	0.744506	0.05
58012	1.20802	0.05
58013	0.744506	0.05
58014	0.883301	0.05
58015	1.32689	0.05
58016	1.20802	0.05
58017	1.20802	0.05
58018	0.946131	0.05
58019	0.946131	0.05
58020	0.94613	0.05
58021	0.94613	0.05
58022	1.20802	0.05
58023	1.32689	0.05
58024	1.20802	0.05
58025	0.946131	0.05
58026	0.946131	0.05
58027	1.32689	0.05
58028	1.89226	0.05
58029	1.89226	0.0574514
58030	1.89226	0.05
58031	1.89226	0.05
58032	1.89226	0.05
58033	1.89226	0.05
58034	0.946119	0.05
58035	0.946119	0.05
58036	0.946118	0.0527469
58037	0.946118	0.0618599
58038	0.946118	0.0601243
58039	0.946118	0.05
58040	0.946119	0.05
58041	0.946119	0.0508011
58042	0.946131	0.0505981
58043	0.946131	0.0509076
58044	0.946106	0.05
58045	0.946106	0.05
58046	1.3939	2.1
58047	1.49641	2.1
58048	1.15096	2.1
58049	1.10101	2.1
58050	2.59742	2.1
58051	1.22106	2.1
58052	2.60663	2.1
58053	1.25467	2.1
58054	1.25467	2.1
58055	2.58377	2.1

58056	2.58377	2.1
58057	1.89225	2.1
58058	2.60664	2.1
58059	2.58377	2.1
58060	2.60664	2.1
58061	1.86938	2.1
58062	1.89225	2.1
58063	1.89225	2.1
58064	1.89225	2.1
58065	1.89225	2.1
58066	1.89225	2.1
58067	1.89225	2.1
58068	1.89225	2.1
58069	0.946121	2.1
58070	0.946121	2.1
58071	0.946126	2.1
58072	0.946126	2.1
58073	0.946124	1.80131
58074	0.946124	1.81737
58075	0.946123	2.1
58076	0.946123	2.1
58077	0.946117	2.1
58078	0.946117	2.1
58079	0.94613	2.1
58080	0.94613	2.1
58081	1.28017	2.1
58082	1.29189	2.1
58083	0.610534	2.1
58084	0.592373	2.1
58085	0.943764	2.1
58086	0.943764	2.1
58087	0.940501	2.1
58088	0.940501	2.1
58089	1.28017	2.1
58090	0.592371	2.1
58091	0.610533	2.1
58092	1.29189	2.1
58093	0.946133	2.1
58094	0.946133	2.1
58095	1.28017	2.1
58096	1.29189	2.1
58097	0.946132	2.1
58098	0.946132	2.1
58099	1.28017	2.1
58100	1.28017	2.1
58101	1.28017	2.1
58102	0.946132	2.1
58103	0.946133	2.1
58104	1.2919	2.1
58105	1.89226	2.1
58106	1.89226	2.1
58107	1.89226	2.1
58108	1.89226	2.1
58109	1.89226	2.1
58110	1.89226	2.1
58111	1.89226	2.1
58112	1.89226	2.1
58113	1.89226	2.1

58114	1.89226	2.1
58115	3.78452	2.1
58116	1.89226	2.1
58117	2.08196	2.1
58118	1.97854	2.1
58119	2.08196	2.1
58120	1.78884	2.1
58121	1.89226	2.1
58122	1.89226	2.1
58123	1.89226	2.1
58124	1.89226	2.1
58125	1.89226	2.1
58126	1.89226	2.1
58127	1.89226	2.1
58128	1.83688	2.1
58129	1.89226	2.1
58130	1.89226	2.1
58131	1.89226	2.1
58132	1.86015	2.1
58133	1.86015	2.1
58134	1.89226	2.1
58135	1.97854	2.1
58136	1.85883	2.1
58137	1.97854	2.1
58138	1.77255	2.1
58139	1.89226	2.1
58140	0.946131	1.96008
58141	0.946131	2.1
58142	0.946131	2.1
58143	0.946131	2.1
58144	0.94613	2.1
58145	0.94613	2.1
58146	0.946132	2.1
58147	0.946132	2.1
58148	0.946131	2.1
58149	0.946131	2.1
58150	0.946131	2.1
58151	0.946131	2.1
58152	0.918412	2.1
58153	0.918412	2.1
58154	0.94893	2.1
58155	0.750211	2.1
58156	0.851541	2.1
58157	0.851541	2.1
58158	0.970566	2.1
58159	0.826567	2.1
58160	0.970554	2.1
58161	1.04071	2.1
58162	0.826632	2.1
58163	0.851554	2.1
58164	1.89226	0.108056
58165	1.89226	0.112253
58166	1.89226	0.0803671
58167	1.89226	0.10837
58168	3.78453	0.112437
58169	0.946131	0.0795039
58170	0.946131	0.138428
58171	0.811232	0.139403

58172	0.811232	0.102084
58173	0.94613	0.150874
58174	0.94613	0.12306
58175	0.959701	0.118543
58176	0.811233	0.167956
58177	0.94613	0.0714679
58178	0.811233	0.114009
58179	0.959702	0.102875
58180	0.946131	0.126249
58181	1.89226	0.0923199
58182	1.89226	0.105731
58183	1.89226	0.126754
58184	1.89226	0.0863908
58185	1.89226	0.0785361
58186	1.89226	0.0657721
58187	1.89227	0.0974429
58188	1.89227	0.11409
58189	1.89227	0.120925
58190	1.89227	0.101109
58191	1.89227	0.0998899
58192	1.89227	0.0881141
58193	1.89226	0.0719371
58194	1.89226	0.0870965
58195	1.89226	0.0655728
58196	1.89226	0.0535784
58197	3.78453	0.0619415
58198	1.89226	0.0746115
58199	1.89226	0.0608552
58200	1.89226	0.0615471
58201	1.89226	0.0727907
58202	1.89226	0.0620397
58203	1.89226	0.0733343
58204	1.89226	2.1
58205	2.36204	2.1
58206	2.39853	2.1
58207	2.36204	2.1
58208	1.92876	2.1
58209	1.89226	2.1
58210	1.89226	2.1
58211	1.89226	0.923324
58212	1.89226	2.1
58213	1.89226	0.63145
58214	3.78453	2.1
58215	0.946128	2.1
58216	0.946128	0.635687
58217	1.13182	0.827268
58218	1.13182	2.1
58219	0.94613	0.788374
58220	0.94613	0.933452
58221	1.1749	2.1
58222	1.13182	2.1
58223	1.1749	2.1
58224	0.94613	2.04168
58225	1.13182	1.94378
58226	0.94613	2.1
58227	1.89226	2.1
58228	2.39853	2.1
58229	2.44918	2.1

58230	2.39853	2.1
58231	1.94291	2.1
58232	1.89226	2.1
58233	0.946132	0.707935
58234	0.946132	2.1
58235	0.946131	2.1
58236	0.946131	0.955935
58237	0.946132	0.867364
58238	0.946132	1.56564
58239	0.946131	2.1
58240	0.946131	2.1
58241	0.946131	2.1
58242	0.946131	2.1
58243	0.946132	2.1
58244	0.946132	2.1
58245	0.946128	2.1
58246	0.946128	1.81081
58247	1.1749	0.87931
58248	1.1749	2.1
58249	0.946131	0.869443
58250	0.946131	0.95701
58251	1.23631	2.1
58252	1.1749	2.1
58253	1.23631	2.1
58254	0.94613	2.1
58255	1.1749	2.01703
58256	0.946131	0.963075
58257	1.89226	0.641243
58258	1.89226	2.1
58259	1.89226	1.6142
58260	1.89226	1.70794
58261	3.78453	1.92846
58262	1.38859	2.1
58263	1.45333	2.1
58264	1.28909	1.17912
58265	1.38091	2.1
58266	2.74242	2.1
58267	1.45332	2.1
58268	1.38414	2.1
58269	1.38091	2.1
58270	1.33506	2.1
58271	2.78838	2.1
58272	1.89226	2.1
58273	1.89226	2.1
58274	1.89226	1.78494
58275	1.89226	2.1
58276	3.78452	2.1
58277	1.89226	1.94559
58278	1.89226	1.87262
58279	1.89226	0.725166
58280	1.89226	2.1
58281	1.89226	0.761957
58282	1.89226	2.1
58283	1.17418	1.81413
58284	1.38091	2.1
58285	1.17418	2.1
58286	1.28909	2.1
58287	1.29741	2.1

58288	1.41232	2.1
58289	1.38091	2.1
58290	1.33506	2.1
58291	1.27575	2.1
58292	1.27575	2.1
58293	1.29741	2.1
58294	1.35671	2.1
58295	1.89226	1.9302
58296	1.89226	2.1
58297	1.89226	2.1
58298	1.89226	2.1
58299	3.78452	2.1
58300	1.92737	0.226424
58301	2.40223	0.325789
58302	1.56178	0.216406
58303	1.97605	0.360436
58304	3.90343	0.22922
58305	1.89226	0.297637
58306	1.89226	0.433427
58307	1.89226	0.355644
58308	1.89226	0.416103
58309	3.78452	0.418053
58310	1.89226	0.434806
58311	1.89226	0.452003
58312	1.89226	0.439202
58313	1.89226	0.473424
58314	1.89226	0.419007
58315	1.89226	0.451248
58316	1.13641	0.419612
58317	1.13641	0.37665
58318	1.12763	0.342385
58319	1.12763	0.417381
58320	1.29348	0.417613
58321	1.06293	0.435607
58322	1.20111	0.320771
58323	0.988028	0.37932
58324	0.988028	0.389291
58325	1.06293	0.401655
58326	1.29348	0.400002
58327	1.20111	0.36598
58328	1.56176	0.191422
58329	1.46387	0.311912
58330	1.97603	0.261315
58331	1.97603	0.23369
58332	1.08211	0.238568
58333	1.08211	0.209268
58334	1.89224	0.304371
58335	1.89224	0.387872
58336	1.89224	0.368168
58337	1.89224	0.411484
58338	1.89224	0.276726
58339	1.89224	0.339087
58340	1.89224	0.414798
58341	1.89224	0.438795
58342	1.89224	0.396837
58343	1.89224	0.455817
58344	3.78448	0.413107
58345	0.820185	0.267053

58346	0.818947	0.399228
58347	0.911041	0.416935
58348	0.695265	0.401721
58349	1.46388	0.328623
58350	1.23607	0.356337
58351	0.927068	0.34735
58352	1.02691	0.421276
58353	1.12918	0.282062
58354	0.846843	0.343701
58355	0.911039	0.383615
58356	1.29129	2.1
58357	1.45547	2.1
58358	1.67327	2.1
58359	1.66698	2.1
58360	1.20149	2.1
58361	1.20149	2.1
58362	0.946133	0.778618
58363	0.946133	2.1
58364	0.946129	2.1
58365	0.946129	2.1
58366	0.946131	2.05426
58367	0.946131	0.772951
58368	0.946131	2.1
58369	0.946131	1.66563
58370	0.946131	2.1
58371	0.946131	2.1
58372	0.946131	1.99228
58373	0.946131	2.1
58374	1.89226	1.80558
58375	1.89226	1.50093
58376	1.89226	2.1
58377	1.89226	0.811059
58378	3.78452	1.18798
58379	0.86875	1.2808
58380	0.86875	2.1
58381	0.899002	2.1
58382	0.966245	2.1
58383	0.849364	0.878736
58384	1.01918	2.1
58385	0.764737	2.1
58386	0.89249	2.1
58387	0.764624	2.1
58388	0.967869	2.1
58389	0.808606	2.08956
58390	0.864506	1.76082
58391	0.773929	2.1
58392	0.773929	2.1
58393	0.781991	2.1
58394	0.836559	2.1
58395	0.983761	2.1
58396	0.834687	2.1
58397	0.680545	2.1
58398	0.640799	2.1
58399	0.70286	2.1
58400	0.935844	2.1
58401	0.629479	2.1
58402	0.823655	2.1
58403	0.946133	0.874237

58404	0.946133	2.1
58405	0.946128	2.1
58406	0.946128	2.1
58407	0.94613	2.1
58408	0.94613	0.835582
58409	0.946131	2.1
58410	0.946131	2.07631
58411	0.946131	2.1
58412	0.946131	2.1
58413	0.946131	2.1
58414	0.946131	2.1
58415	1.89226	2.1
58416	1.89226	0.819963
58417	1.89226	1.48064
58418	1.89226	1.32747
58419	1.89226	1.00667
58420	1.89226	1.24351
58421	0.931638	2.1
58422	0.931638	1.04997
58423	1.03559	2.1
58424	0.977442	1.93836
58425	0.826487	1.32414
58426	0.993902	1.15975
58427	1.04922	2.1
58428	0.894324	2.1
58429	0.912984	2.1
58430	1.01156	2.1
58431	0.944692	1.76672
58432	1.15456	1.92903
58433	1.89226	0.0796517
58434	1.89226	0.123995
58435	1.89226	0.177399
58436	1.89226	0.0766468
58437	3.78453	0.0803487
58438	1.89226	0.0775835
58439	1.89226	0.0750437
58440	2.64262	0.0769947
58441	2.64262	0.0587644
58442	1.78052	0.0506224
58443	1.03015	0.0814536
58444	0.999721	0.0597833
58445	0.659011	0.0535774
58446	1.15564	0.114079
58447	1.41388	0.0672414
58448	1.01583	0.0732651
58449	1.01583	0.0596328
58450	1.05706	0.106625
58451	1.05706	0.0710886
58452	1.15564	0.0691033
58453	0.65901	0.0643372
58454	1.41388	0.0691213
58455	0.999721	0.0641076
58456	1.89226	2.1
58457	1.89226	2.1
58458	1.89226	2.1
58459	1.89226	2.1
58460	3.78453	2.1
58461	2.39022	2.1

58462	1.90734	2.1
58463	2.27634	2.1
58464	1.82666	2.1
58465	4.18368	2.1
58466	1.90734	2.1
58467	1.21564	2.1
58468	1.82666	2.1
58469	1.16938	2.1
58470	3.07672	2.1
58471	1.89226	2.1
58472	1.89226	2.1
58473	1.89226	2.1
58474	1.89226	2.1
58475	1.89226	2.1
58476	1.89226	2.1
58477	1.89227	2.1
58478	1.89227	2.1
58479	1.89227	2.1
58480	1.89227	2.1
58481	3.78453	2.1
58482	2.27634	2.1
58483	1.82666	2.1
58484	2.18042	2.1
58485	1.74738	2.1
58486	4.02372	2.1
58487	1.74738	2.1
58488	1.82666	2.1
58489	1.1181	2.1
58490	1.1181	2.1
58491	1.16938	2.1
58492	1.79865	2.1
58493	1.89226	2.1
58494	1.89226	2.1
58495	1.89226	2.1
58496	1.89226	2.1
58497	1.89226	2.1
58498	1.89226	2.1
58499	1.89226	1.33075
58500	1.89226	0.73073
58501	1.89226	0.849489
58502	1.89226	0.89345
58503	1.89226	1.38101
58504	1.89226	0.910552
58505	0.790736	0.922874
58506	1.08265	0.806465
58507	0.938909	1.23633
58508	0.638683	0.896262
58509	0.858588	0.966198
58510	0.858588	0.782118
58511	0.862743	1.33881
58512	0.862743	1.01391
58513	1.08265	1.20674
58514	0.938911	0.758812
58515	0.790735	1.18549
58516	0.638685	0.776344
58517	1.89226	0.757263
58518	1.89226	1.19109
58519	1.89226	0.876896

58520	1.89226	0.769134
58521	1.89226	1.34052
58522	1.89226	1.14978
58523	1.89226	0.804019
58524	1.89226	1.25616
58525	1.89226	0.787922
58526	1.89226	0.816789
58527	1.89226	1.04492
58528	1.89226	1.22645
58529	1.08265	0.767312
58530	1.36209	0.71868
58531	1.25233	0.775586
58532	0.938909	0.887473
58533	1.14195	0.726016
58534	1.14195	0.677698
58535	1.15904	1.22394
58536	1.15904	0.829886
58537	1.36209	0.968407
58538	1.25233	0.626156
58539	1.08265	0.960563
58540	0.938911	0.693215
58541	1.22077	0.704259
58542	0.915244	0.757511
58543	0.967835	0.977388
58544	0.742495	0.927581
58545	0.78648	0.91809
58546	0.985041	0.747239
58547	0.989098	1.4029
58548	0.989098	0.832062
58549	1.03206	0.801462
58550	0.967897	1.09857
58551	0.915404	1.06355
58552	0.706105	0.797304
58553	2.69662	0.05
58554	1.89226	0.0632008
58555	2.35499	0.05
58556	1.89226	0.05
58557	4.24725	0.05
58558	1.89227	0.0501642
58559	1.89227	0.0500153
58560	1.89227	0.05
58561	1.89227	0.05
58562	3.78453	0.05
58563	2.35499	0.05
58564	1.70302	0.05
58565	1.70302	0.05
58566	1.703	0.05
58567	1.97374	0.05
58568	1.97372	0.05
58569	0.946133	0.05
58570	0.946133	0.05
58571	0.946132	0.05
58572	0.946132	0.05
58573	0.946133	0.05
58574	0.946133	0.05
58575	0.946132	0.05
58576	0.946132	0.05
58577	0.946133	0.05

58578	0.946133	0.05
58579	0.946132	0.05
58580	0.946132	0.05
58581	0.6794	2.1
58582	0.6794	2.1
58583	0.670812	2.1
58584	0.62182	2.1
58585	0.648326	2.1
58586	0.792154	2.1
58587	0.719167	2.1
58588	0.494515	2.1
58589	0.649421	2.1
58590	0.804869	2.1
58591	0.485585	2.1
58592	0.627666	2.1
58593	1.79347	2.1
58594	1.03968	1.31018
58595	1.70303	1.72387
58596	0.933265	1.25564
58597	2.62068	1.1109
58598	1.89226	2.1
58599	1.89226	2.1
58600	1.89226	2.1
58601	1.89226	1.02164
58602	3.78452	2.1
58603	1.89226	1.29246
58604	2.24604	2.1
58605	2.0554	1.99807
58606	2.0554	1.48004
58607	1.89226	2.1
58608	1.89226	2.09425
58609	1.89226	2.1
58610	1.89226	0.579824
58611	1.89226	0.684644
58612	1.89226	1.76649
58613	1.89226	0.69415
58614	1.89226	1.8698
58615	2.0815	2.03765
58616	2.64424	0.209037
58617	2.80807	0.444509
58618	3.232	1.11693
58619	1.89417	0.452804
58620	2.18012	1.14186
58621	0.946131	2.03473
58622	0.946131	0.414509
58623	0.946131	2.1
58624	0.946131	0.733461
58625	0.94613	0.697365
58626	0.94613	0.600789
58627	0.946132	2.08277
58628	0.946132	2.1
58629	0.946131	0.842644
58630	0.946131	0.890137
58631	0.946131	0.83721
58632	0.946131	0.829503
58633	1.89227	0.0597349
58634	1.89227	0.0850241
58635	1.89227	0.112913

58636	1.89227	0.0625577
58637	3.78453	0.0647614
58638	2.70881	0.0553198
58639	2.68687	0.05
58640	2.03046	0.05
58641	1.9682	0.0546337
58642	4.67701	0.0510624
58643	1.08518	0.05
58644	1.08518	0.05
58645	1.09033	0.0543876
58646	1.09033	0.05
58647	0.842273	0.05
58648	1.19141	0.05
58649	1.34343	0.05
58650	0.9841	0.05
58651	0.9841	0.05
58652	0.842273	0.05
58653	1.34344	0.05
58654	1.19141	0.05
58655	0.946133	0.0628121
58656	0.946133	0.052781
58657	0.946132	0.11465
58658	0.946132	0.0712852
58659	0.946132	0.0615193
58660	0.946132	0.0525815
58661	0.946132	0.109821
58662	0.946132	0.0826536
58663	0.946132	0.0541093
58664	0.946132	0.0792993
58665	0.946133	0.0825909
58666	0.946133	0.064083
58667	1.89227	0.102543
58668	1.89227	0.0543844
58669	1.89227	0.0866992
58670	1.89227	0.0610763
58671	1.89227	0.0874044
58672	1.89227	0.0605218
58673	2.03046	0.05
58674	1.9682	0.05
58675	1.23886	0.0641833
58676	1.12971	0.05
58677	3.20706	0.05
58678	1.89226	0.537342
58679	1.89226	0.848775
58680	1.89226	0.48451
58681	1.89226	0.702823
58682	3.78452	0.630442
58683	2.45569	0.421591
58684	1.89226	0.499289
58685	2.22922	0.448544
58686	2.0815	0.594408
58687	1.85074	0.261268
58688	1.70303	0.508838
58689	2.72856	0.406461
58690	1.89226	0.627016
58691	1.89226	0.344162
58692	1.89226	0.548064
58693	3.78452	0.51906

58694	1.89226	0.56229
58695	1.89226	0.739511
58696	1.89226	0.490403
58697	1.89226	0.743056
58698	3.78452	0.611052
58699	0.946131	0.739985
58700	0.946131	0.478611
58701	0.946131	0.645492
58702	0.946131	0.408017
58703	0.94613	0.618355
58704	0.94613	0.677036
58705	0.946132	0.447986
58706	0.946132	0.491837
58707	0.946131	0.561642
58708	0.946131	0.574495
58709	0.946131	0.57374
58710	0.946131	0.568964
58711	2.0595	0.353491
58712	2.58581	0.355929
58713	1.48694	0.293946
58714	1.48694	0.318583
58715	2.60157	0.343862
58716	2.02901	0.304733
58717	1.89226	0.306511
58718	1.89226	0.361427
58719	1.89226	0.278921
58720	1.89226	0.299285
58721	1.89226	0.351995
58722	1.89226	0.353112
58723	2.04728	0.350759
58724	2.64161	0.340283
58725	1.47606	0.329187
58726	1.47606	0.340615
58727	2.63069	0.339125
58728	2.05947	0.332949
58729	1.89224	0.333051
58730	1.89224	0.348647
58731	1.89224	0.339989
58732	1.89224	0.342049
58733	1.89224	0.335641
58734	1.89224	0.311833
58735	0.946114	0.340869
58736	0.946114	0.338306
58737	0.946118	0.361189
58738	0.946118	0.338441
58739	0.946114	0.364428
58740	0.946114	0.354088
58741	0.946118	0.348696
58742	0.946118	0.340364
58743	0.946128	0.358167
58744	0.946128	0.361036
58745	0.946103	0.346224
58746	0.946103	0.337538
58747	1.1233	2.1
58748	1.15009	2.1
58749	2.36036	2.1
58750	2.38225	2.1
58751	3.50554	2.1

58752	2.38224	2.1
58753	0.977775	2.1
58754	2.20473	2.1
58755	2.20473	2.1
58756	1.15009	2.1
58757	1.15009	2.1
58758	0.946119	1.99431
58759	0.946119	1.99071
58760	1.19111	2.08741
58761	1.19111	1.9237
58762	0.946118	2.00428
58763	1.19111	2.1
58764	1.18016	1.96442
58765	0.946118	0.905487
58766	0.94613	0.603062
58767	0.94613	1.74789
58768	1.18015	1.83342
58769	1.19109	1.89033
58770	0.946118	2.1
58771	0.946118	2.1
58772	1.10235	2.1
58773	1.10235	2.1
58774	0.946118	2.1
58775	1.10235	2.1
58776	0.946115	1.97939
58777	1.1911	2.1
58778	0.946129	1.97794
58779	0.946129	2.1
58780	1.19109	2.1
58781	1.10234	2.1
58782	1.89226	2.1
58783	2.23932	2.1
58784	2.11568	2.1
58785	2.23932	2.1
58786	1.76862	2.1
58787	1.89226	2.1
58788	1.89226	2.1
58789	1.89226	2.1
58790	1.89226	2.1
58791	1.89226	2.1
58792	1.89226	2.1
58793	1.89226	0.984951
58794	2.07838	2.1
58795	1.89226	2.1
58796	1.89226	2.1
58797	1.89226	2.1
58798	2.10264	2.1
58799	2.10264	2.1
58800	1.89226	2.1
58801	2.11568	2.1
58802	1.99753	2.1
58803	2.11568	2.1
58804	1.77411	2.1
58805	1.89226	2.1
58806	0.946128	0.80663
58807	0.946128	2.1
58808	0.946133	2.1
58809	0.946133	2.1

58810	0.94613	2.1
58811	0.94613	0.792411
58812	0.946131	2.1
58813	0.946131	2.1
58814	0.946131	2.1
58815	0.946131	2.1
58816	0.946131	2.1
58817	0.946131	2.1
58818	0.946132	2.1
58819	0.946132	2.1
58820	1.03919	2.1
58821	1.03919	2.1
58822	0.946131	2.1
58823	0.946131	2.1
58824	1.02744	2.1
58825	1.03919	2.1
58826	1.02744	2.1
58827	0.946132	2.1
58828	1.03919	2.1
58829	0.946132	2.1
58830	1.64834	1.96423
58831	1.82955	1.66998
58832	2.33824	1.05875
58833	2.19301	2.03743
58834	3.98658	1.51655
58835	1.40574	2.03767
58836	2.19301	1.4498
58837	1.64834	1.55525
58838	1.64834	1.83484
58839	1.99961	1.09107
58840	1.99961	1.41628
58841	2.33824	1.16039
58842	2.19301	2.0256
58843	2.68524	1.59536
58844	2.81847	1.39342
58845	5.02349	1.73492
58846	1.99961	1.99782
58847	2.19301	1.27692
58848	2.68524	1.23747
58849	2.50732	1.70974
58850	4.68485	1.41389
58851	1.89226	2.1
58852	1.89226	1.00468
58853	1.89226	1.00359
58854	1.89226	2.1
58855	3.78453	1.61212
58856	1.75142	2.1
58857	2.47026	2.1
58858	1.46811	2.1
58859	2.26652	2.1
58860	4.01794	2.1
58861	1.89226	1.09065
58862	1.89226	2.1
58863	1.89226	1.72877
58864	1.89226	2.1
58865	1.89226	1.46464
58866	1.89226	2.1
58867	1.89226	1.25466

58868	1.89226	2.1
58869	1.89226	1.71681
58870	1.89226	2.1
58871	1.89226	1.10806
58872	1.89226	2.1
58873	0.812082	2.1
58874	0.878759	2.1
58875	0.882782	2.1
58876	0.882782	2.1
58877	1.05572	2.1
58878	1.04037	2.1
58879	0.73849	2.1
58880	0.626857	2.1
58881	0.638677	2.1
58882	1.05581	2.1
58883	0.715695	2.1
58884	1.10492	2.1
58885	1.17058	2.1
58886	1.07863	2.1
58887	1.21113	2.1
58888	1.21113	2.1
58889	1.2811	2.1
58890	1.37319	2.1
58891	1.01788	2.1
58892	0.999492	2.1
58893	1.04352	2.1
58894	1.32112	2.1
58895	1.10492	2.1
58896	1.34699	2.1
58897	1.89226	1.62711
58898	1.89226	2.1
58899	1.89226	2.1
58900	1.89226	2.1
58901	1.89226	1.6629
58902	1.89226	2.1
58903	1.19813	0.05
58904	1.19813	0.05
58905	2.43432	0.05
58906	1.21716	0.05
58907	0.946124	0.05
58908	1.21715	0.05
58909	0.946118	0.05
58910	2.56627	2.1
58911	1.89226	2.1
58912	1.89226	2.1
58913	1.89226	2.1
58914	2.43434	2.1
58915	2.43434	2.1
58916	1.89227	0.05
58917	1.89227	0.0502721
58918	1.89227	0.05
58919	1.89227	0.05
58920	1.89227	0.05
58921	1.89227	0.05
58922	1.89226	0.056133
58923	1.89226	0.075788
58924	1.89226	0.0671601
58925	1.89226	0.05

58926	3.78453	0.0587743
58927	1.89226	0.05
58928	2.0764	0.05
58929	1.89226	0.05
58930	2.81674	0.05
58931	1.89226	0.05
58932	2.81674	0.05
58933	0.946132	0.0506085
58934	0.946132	0.05
58935	0.946131	0.0717432
58936	0.946131	0.0552196
58937	0.946132	0.0503605
58938	0.946132	0.0513058
58939	0.946132	0.0769516
58940	0.946132	0.05
58941	0.946132	0.0519321
58942	0.946132	0.0567482
58943	0.946132	0.0544292
58944	0.946132	0.0514961
58945	0.578684	1.30035
58946	0.578684	2.1
58947	0.589487	1.39525
58948	0.589487	2.1
58949	0.625461	2.03324
58950	0.5378	1.96803
58951	0.608714	2.1
58952	0.54271	1.29553
58953	0.608714	1.40352
58954	0.625461	1.0373
58955	0.54271	1.59641
58956	0.537801	1.68306
58957	0.946128	1.06429
58958	0.946128	1.96784
58959	0.946133	0.982609
58960	0.946133	2.1
58961	0.946131	1.23325
58962	0.946131	1.03579
58963	0.946131	0.986774
58964	0.946131	1.28805
58965	0.94613	1.16772
58966	0.94613	0.919902
58967	0.946131	1.15009
58968	0.946131	1.30407
58969	1.21743	1.84167
58970	1.25092	2.1
58971	1.38847	1.06647
58972	1.46031	1.02834
58973	2.67774	1.4805
58974	0.946128	1.00958
58975	0.946128	2.06327
58976	0.946132	2.06485
58977	0.946132	1.0834
58978	0.94613	1.66725
58979	0.94613	1.01551
58980	0.94613	1.37209
58981	0.94613	1.07612
58982	0.94613	1.00646
58983	0.94613	0.936661

58984	0.94613	1.39891
58985	0.94613	1.04578
58986	1.89226	0.962479
58987	1.89226	1.0422
58988	1.89226	0.741141
58989	1.89226	2.1
58990	1.89226	2.1
58991	1.89226	1.93477
58992	0.539566	1.00098
58993	0.534046	2.1
58994	0.577914	1.6372
58995	0.471623	1.55888
58996	1.46031	0.822679
58997	0.945865	1.05419
58998	0.770603	0.871674
58999	0.737201	2.0394
59000	0.714829	1.09862
59001	0.536085	1.34264
59002	0.471617	1.19877
59003	1.89226	2.02274
59004	1.89226	0.874626
59005	1.89226	0.870807
59006	1.89226	1.19247
59007	1.89226	1.20768
59008	1.89226	2.04226
59009	1.89225	1.44794
59010	1.89225	0.723644
59011	1.65572	0.736178
59012	1.65572	0.949963
59013	1.89225	0.940675
59014	1.89225	1.63515
59015	1.89226	1.23006
59016	1.89226	0.987816
59017	1.89226	0.987533
59018	1.89226	0.56755
59019	3.78453	1.31913
59020	1.89226	1.76981
59021	1.89226	0.710677
59022	1.89226	0.735923
59023	1.89226	1.0423
59024	1.89226	0.986051
59025	1.89226	1.93498
59026	1.89225	1.17936
59027	1.89225	0.568673
59028	1.89225	0.616848
59029	1.89225	0.815584
59030	1.89225	0.730629
59031	1.89225	1.31774
59032	0.763592	0.723255
59033	0.763592	1.14167
59034	0.987417	0.852037
59035	0.987417	0.882817
59036	0.763592	0.637766
59037	0.804881	0.580292
59038	0.946126	0.877588
59039	0.946126	0.669053
59040	0.804879	0.559055
59041	0.94613	0.761872

59042	0.946129	0.939864
59043	0.76359	0.66195
59044	1.46055	2.1
59045	2.04945	2.1
59046	2.04587	2.1
59047	2.04587	2.1
59048	1.50714	2.1
59049	1.50714	2.1
59050	2.04587	2.1
59051	2.38681	2.1
59052	2.42082	2.1
59053	2.42082	2.1
59054	2.04945	2.1
59055	2.04945	2.1
59056	0.872964	2.1
59057	0.872964	2.1
59058	0.884806	2.1
59059	0.884806	2.1
59060	1.0275	2.1
59061	1.02293	2.1
59062	0.711243	2.1
59063	0.730273	2.1
59064	0.711244	2.1
59065	1.0275	2.1
59066	0.730273	2.1
59067	1.02293	2.1
59068	2.04587	2.1
59069	2.47753	2.1
59070	2.42994	2.1
59071	2.42082	2.1
59072	2.05499	2.1
59073	2.04587	1.4137
59074	0.946132	2.1
59075	0.946132	2.1
59076	1.13078	2.1
59077	1.07817	2.1
59078	0.946132	2.1
59079	0.946132	2.1
59080	1.07817	2.1
59081	1.07817	2.1
59082	0.946132	2.1
59083	1.07817	2.1
59084	1.13078	2.1
59085	0.946132	2.1
59086	1.89226	2.1
59087	1.89226	2.1
59088	1.89226	2.1
59089	1.89226	2.1
59090	1.89226	2.1
59091	1.89226	2.1
59092	1.89226	2.1
59093	1.89226	2.1
59094	1.89226	2.1
59095	1.89226	2.1
59096	1.89226	2.1
59097	1.89226	2.1
59098	1.89226	2.1
59099	1.89226	2.1

59100	1.8173	2.1
59101	2.66885	2.1
59102	2.7438	2.1
59103	2.7438	2.1
59104	1.89226	0.761988
59105	1.89226	0.683179
59106	1.89226	0.685534
59107	1.89226	0.983979
59108	3.78453	0.738214
59109	1.89226	0.580529
59110	1.89226	0.741902
59111	1.89226	0.641848
59112	1.89226	1.21964
59113	1.89226	0.493965
59114	1.89226	0.721081
59115	1.89226	0.620548
59116	1.97385	0.988856
59117	1.7531	0.762157
59118	1.89226	1.35976
59119	1.7531	0.526055
59120	1.89226	0.748222
59121	0.946131	1.08567
59122	0.840904	0.721679
59123	0.684333	0.772577
59124	0.94613	0.861383
59125	0.972337	0.859927
59126	0.946278	1.84794
59127	0.684304	0.657466
59128	0.684304	0.92313
59129	0.999838	0.749114
59130	0.759605	1.89727
59131	0.851528	0.704595
59132	0.851528	1.20135
59133	1.89226	0.661168
59134	2.23818	1.26698
59135	1.79771	0.791914
59136	1.89226	1.19265
59137	1.79771	0.590119
59138	1.89226	0.793753
59139	2.79647	2.1
59140	2.01613	2.1
59141	1.46826	2.1
59142	2.54504	2.1
59143	4.56117	2.1
59144	1.89226	2.1
59145	1.89226	2.1
59146	1.89226	2.1
59147	1.89226	2.1
59148	1.89226	2.1
59149	1.89226	2.1
59150	1.89226	2.1
59151	2.66884	2.1
59152	2.56022	2.1
59153	2.66884	2.1
59154	1.78364	2.1
59155	1.89226	2.1
59156	1.14282	2.1
59157	1.24068	2.1

59158	0.880748	2.1
59159	0.834916	2.1
59160	1.05539	2.1
59161	1.05539	2.1
59162	1.04312	2.1
59163	1.04313	2.1
59164	1.2015	2.1
59165	0.83492	2.1
59166	0.789149	2.1
59167	1.24066	2.1
59168	1.89226	2.1
59169	1.89226	2.1
59170	1.89226	2.1
59171	1.89226	2.1
59172	3.78453	2.1
59173	0.946128	2.1
59174	0.946128	2.1
59175	1.28011	2.1
59176	1.22372	2.1
59177	0.946131	2.1
59178	0.946131	2.1
59179	1.22372	2.1
59180	1.22372	2.1
59181	1.22372	2.1
59182	0.94613	2.1
59183	0.946131	2.1
59184	1.28011	2.1
59185	2.28604	2.1
59186	1.34268	2.1
59187	2.43157	2.1
59188	1.50172	2.1
59189	3.77425	2.1
59190	2.6096	2.1
59191	2.43157	2.1
59192	1.66774	2.1
59193	1.66774	2.1
59194	1.50172	2.1
59195	2.43157	2.1
59196	2.16067	2.1
59197	1.08975	2.1
59198	2.19336	2.1
59199	2.06984	2.1
59200	1.21327	2.1
59201	1.08975	2.1
59202	2.28604	2.1
59203	1.21327	2.1
59204	2.29008	2.1
59205	2.16067	2.1
59206	1.34268	2.1
59207	1.21327	2.1
59208	1.89226	0.467179
59209	1.89226	0.454796
59210	1.89226	0.450094
59211	1.89226	0.468487
59212	3.78452	0.446729
59213	1.89804	0.442689
59214	2.21656	0.470552
59215	1.70537	0.456501

59216	2.50023	0.417189
59217	3.8982	0.420831
59218	1.03136	0.406951
59219	1.89486	0.39247
59220	0.949364	0.398956
59221	1.82283	0.388917
59222	2.84422	0.401643
59223	1.89226	0.396788
59224	1.89226	0.41529
59225	1.89226	0.421286
59226	1.89226	0.410102
59227	3.78452	0.420661
59228	0.946132	0.477933
59229	0.946132	0.460272
59230	0.946129	0.498868
59231	0.946129	0.444731
59232	0.94613	0.444994
59233	0.94613	0.532515
59234	0.946131	0.45731
59235	0.946131	0.470174
59236	0.946131	0.487122
59237	0.946131	0.479111
59238	0.946131	0.42652
59239	0.946131	0.533526
59240	1.09466	0.44495
59241	1.09466	0.405247
59242	0.866512	0.435482
59243	1.08352	0.444978
59244	1.19189	0.433936
59245	1.21883	0.439756
59246	0.971139	0.444165
59247	0.873213	0.419383
59248	0.873202	0.420139
59249	1.00855	0.439507
59250	0.923735	0.43643
59251	1.21885	0.41773
59252	1.89225	0.05
59253	2.40019	0.05
59254	1.89225	0.0783972
59255	1.89225	0.0623537
59256	1.89225	0.0539704
59257	1.89225	0.170243
59258	1.89225	0.0783972
59259	1.89225	0.0911607
59260	2.05088	0.055042
59261	1.89225	0.05
59262	1.89225	0.05
59263	1.89225	0.0979446
59264	2.03956	0.055042
59265	2.03956	0.0623537
59266	1.89226	2.1
59267	2.40021	1.46079
59268	2.38473	0.554083
59269	2.40021	2.1
59270	1.87679	0.633263
59271	1.89226	1.76026
59272	1.89226	1.88355
59273	1.89226	0.532519

59274	1.89226	1.33179
59275	1.89226	0.310116
59276	1.89226	0.758215
59277	1.89226	0.383808
59278	2.0692	0.836793
59279	1.89226	0.540091
59280	1.89226	1.99406
59281	1.89226	0.529623
59282	2.05089	2.02774
59283	2.05089	0.51667
59284	1.89226	0.852695
59285	1.89226	2.1
59286	1.89226	1.03608
59287	1.89226	2.1
59288	3.78452	2.01766
59289	1.89226	0.649471
59290	2.50051	2.1
59291	1.89226	1.10882
59292	2.30076	2.1
59293	4.39277	2.1
59294	2.50052	2.1
59295	1.70377	2.1
59296	2.30076	2.1
59297	1.54231	2.1
59298	4.04283	2.1
59299	1.89226	2.1
59300	1.89226	2.1
59301	1.89226	2.1
59302	1.89226	2.1
59303	1.89226	2.1
59304	1.89226	2.1
59305	1.89226	0.679678
59306	1.89226	2.1
59307	1.89226	0.807324
59308	1.89226	2.1
59309	3.78452	2.1
59310	2.07948	2.1
59311	2.30076	2.1
59312	1.3294	2.1
59313	1.3294	2.1
59314	1.54232	2.1
59315	2.29239	2.1
59316	0.810947	2.1
59317	0.810947	2.1
59318	0.810946	2.1
59319	0.810946	2.1
59320	1.89226	2.1
59321	1.35169	2.1
59322	1.08132	2.1
59323	1.08132	2.1
59324	1.08132	2.1
59325	0.810946	2.1
59326	0.810946	2.1
59327	1.34851	1.04202
59328	0.997471	2.1
59329	1.62287	0.844267
59330	1.27096	1.6467
59331	2.62034	1.08301

59332	1.89226	0.946477
59333	1.89226	2.1
59334	1.89226	1.04907
59335	1.89226	2.05571
59336	3.78452	1.23581
59337	0.946128	2.1
59338	0.946128	1.31947
59339	1.29331	0.791724
59340	1.29331	2.01674
59341	1.29331	1.20442
59342	1.10699	2.1
59343	0.946131	0.76375
59344	0.946131	1.1269
59345	0.94613	1.37921
59346	1.29331	1.40518
59347	1.10699	1.20569
59348	0.946131	1.12823
59349	1.62287	0.94106
59350	1.27096	1.8838
59351	1.86067	0.925951
59352	1.58407	1.82814
59353	3.20694	1.4803
59354	1.89226	1.19066
59355	1.89226	1.93399
59356	1.89226	0.893195
59357	1.89226	1.89749
59358	3.78452	1.18257
59359	2.62761	0.707936
59360	2.55849	0.962965
59361	2.12261	1.32747
59362	2.22939	0.744936
59363	4.78788	0.82566
59364	0.772522	1.51152
59365	0.839588	1.16514
59366	1.31126	0.648938
59367	1.08787	0.664024
59368	1.01318	0.698205
59369	1.01318	1.80941
59370	0.900815	1.97703
59371	1.10799	0.55407
59372	0.772511	0.890394
59373	1.03473	0.943628
59374	1.31126	0.949768
59375	0.841464	1.05101
59376	1.89227	0.662819
59377	1.89227	1.18466
59378	1.89227	0.712034
59379	1.89227	1.25009
59380	1.89227	0.674153
59381	1.89227	1.17543
59382	1.89226	0.731662
59383	1.89226	0.843356
59384	1.89226	0.6787
59385	1.89226	0.977534
59386	1.89226	0.601076
59387	1.89226	0.82892
59388	2.12261	1.12323
59389	2.22939	0.625144

59390	1.97004	0.666858
59391	1.8072	1.08003
59392	4.03658	0.756573
59393	1.5847	1.69661
59394	1.8072	0.720136
59395	2.12262	0.87582
59396	2.12262	1.51599
59397	1.23267	0.781066
59398	1.23267	1.4477
59399	1.89227	0.719602
59400	1.89227	1.64978
59401	1.89227	0.73706
59402	1.89227	1.32407
59403	1.89227	0.70214
59404	1.89227	1.34839
59405	1.89226	0.63754
59406	1.89226	0.85179
59407	1.89226	1.07608
59408	1.89226	0.58258
59409	3.78452	0.71588
59410	2.49093	2.1
59411	2.67393	2.1
59412	2.47932	2.1
59413	2.47932	2.1
59414	2.67994	2.1
59415	2.67994	2.1
59416	2.49873	2.1
59417	2.67393	2.1
59418	2.67993	2.1
59419	2.69807	2.1
59420	2.49873	2.1
59421	2.51686	2.1
59422	2.49093	2.1
59423	2.67994	2.1
59424	2.69784	2.1
59425	2.51651	2.1
59426	5.18877	2.1
59427	1.24359	2.1
59428	1.24936	2.1
59429	1.33997	2.1
59430	1.34892	2.1
59431	1.28805	2.1
59432	1.28805	2.1
59433	1.2955	2.1
59434	1.2955	2.1
59435	1.34892	2.1
59436	1.24359	2.1
59437	1.24936	2.1
59438	1.33997	2.1
59439	1.15506	1.47607
59440	1.15506	2.1
59441	0.946128	2.1
59442	0.946128	2.1
59443	0.946131	2.1
59444	1.15506	1.5609
59445	1.06997	2.1
59446	0.946128	2.1
59447	1.06997	2.08771

59448	1.15506	2.1
59449	0.946129	2.1
59450	0.946129	2.1
59451	1.70608	2.1
59452	2.13994	2.1
59453	1.33384	2.1
59454	1.43635	2.1
59455	3.14243	2.1
59456	2.13994	2.1
59457	2.31012	2.1
59458	1.43635	2.1
59459	1.23007	2.1
59460	3.74646	2.1
59461	2.46361	2.1
59462	1.89226	1.99791
59463	1.89226	2.1
59464	1.89226	1.81317
59465	2.39045	2.1
59466	2.39045	2.1
59467	1.89226	2.1
59468	1.89226	1.43424
59469	1.89226	2.06895
59470	1.89226	0.97656
59471	3.78452	1.89072
59472	1.52984	2.1
59473	2.25682	0.909554
59474	2.20831	0.792273
59475	2.20831	2.1
59476	1.61178	0.731487
59477	1.61178	2.1
59478	2.47395	2.1
59479	1.89226	2.1
59480	1.89226	2.1
59481	1.89226	2.1
59482	2.46362	2.1
59483	2.46362	2.0801
59484	0.946131	0.694253
59485	0.946131	2.1
59486	0.946131	2.1
59487	0.946131	1.29357
59488	0.94613	1.13211
59489	0.94613	1.85695
59490	0.946132	2.1
59491	0.946132	1.79961
59492	0.946131	1.06078
59493	0.946131	1.55151
59494	0.946131	1.80947
59495	0.946131	2.1
59496	0.982398	2.1
59497	0.982398	1.3905
59498	0.931703	2.1
59499	0.97549	1.71636
59500	1.11509	1.62556
59501	1.10017	0.961498
59502	0.774576	2.1
59503	0.789859	2.1
59504	0.716502	1.74523
59505	1.12793	1.97022

59506	1.14398	1.98899
59507	0.817011	1.20925
59508	1.82499	2.1
59509	1.50524	2.1
59510	1.21304	2.1
59511	1.54191	2.1
59512	3.04715	2.1
59513	1.54191	2.1
59514	1.49823	2.1
59515	1.82499	2.1
59516	1.82499	2.1
59517	1.21147	2.1
59518	1.48977	2.1
59519	1.89226	2.1
59520	1.89226	2.1
59521	1.89226	2.1
59522	1.89226	2.1
59523	3.78452	2.1
59524	1.89226	2.1
59525	1.89226	2.1
59526	1.89226	2.1
59527	1.89226	2.1
59528	1.89226	2.1
59529	1.89226	2.1
59530	0.566674	2.1
59531	0.770957	2.1
59532	0.60652	2.1
59533	0.473557	2.1
59534	0.604441	2.1
59535	0.604441	2.1
59536	0.640073	2.1
59537	0.640073	2.1
59538	0.473557	2.1
59539	0.566674	2.1
59540	0.60652	2.1
59541	0.770956	2.1
59542	1.36203	2.1
59543	1.13335	2.1
59544	1.54191	2.1
59545	1.54191	2.1
59546	1.00965	2.1
59547	1.00965	2.1
59548	1.89226	2.1
59549	1.89226	2.1
59550	1.89226	2.1
59551	1.89226	2.1
59552	1.89226	2.1
59553	1.89226	2.1
59554	0.946131	2.1
59555	0.946131	2.1
59556	0.946131	2.1
59557	0.946131	2.1
59558	0.946132	2.1
59559	0.946132	2.1
59560	0.946131	2.1
59561	0.946131	2.1
59562	0.946132	2.1
59563	0.946132	2.1

59564	0.946131	2.1
59565	0.946131	2.1
59566	0.946132	0.0730767
59567	0.946132	0.0933223
59568	0.946133	0.0643608
59569	0.946133	0.0922254
59570	0.946132	0.069317
59571	0.946132	0.0711764
59572	0.946132	0.0749882
59573	0.946132	0.0908339
59574	0.946132	0.0721394
59575	0.946132	0.0819987
59576	0.946133	0.091901
59577	0.946133	0.0735949
59578	1.89226	0.113999
59579	1.89226	0.0957637
59580	1.89226	0.0855858
59581	1.89226	0.0723958
59582	3.78453	0.0689348
59583	1.89226	0.0995706
59584	1.89226	0.0646545
59585	1.89226	0.0829816
59586	1.89226	0.0907554
59587	1.89226	0.0791109
59588	1.89226	0.088101
59589	0.946132	0.0542797
59590	0.946132	0.0643561
59591	0.946131	0.05
59592	0.946131	0.0649652
59593	0.946132	0.0565334
59594	0.946132	0.0555876
59595	0.946132	0.0541365
59596	0.946132	0.0661623
59597	0.946132	0.0637741
59598	0.946132	0.0552572
59599	0.946132	0.0747457
59600	0.946132	0.0669309
59601	0.946129	0.0521917
59602	0.946129	0.0682709
59603	0.946128	0.05
59604	0.946128	0.0606069
59605	0.946132	0.0509508
59606	0.946132	0.0536524
59607	0.946126	0.0648092
59608	0.946126	0.0624529
59609	0.946129	0.0512538
59610	0.946129	0.0599829
59611	0.946129	0.0642734
59612	0.946129	0.0761404
59613	1.20674	0.0748207
59614	1.79877	0.0961193
59615	1.22586	0.0754818
59616	1.22586	0.0754389
59617	1.7919	0.0909553
59618	1.7919	0.0932278
59619	2.27622	0.0728169
59620	1.79877	0.101056
59621	1.7919	0.0720333

59622	2.27102	0.103313
59623	4.06979	0.0743091
59624	1.89226	0.100896
59625	1.89226	0.174905
59626	1.89226	0.104415
59627	1.89226	0.161521
59628	3.78452	0.157808
59629	1.89226	0.0957822
59630	1.89226	0.127042
59631	1.89226	0.0917917
59632	1.89226	0.154316
59633	3.78453	0.0964849
59634	1.13259	0.071932
59635	1.79187	0.0897211
59636	1.32741	0.0704984
59637	1.18784	0.0728165
59638	1.703	0.0842422
59639	1.49575	0.0888532
59640	1.46201	0.0678109
59641	2.27099	0.0992418
59642	1.79187	0.0696793
59643	1.79187	0.0705241
59644	2.12923	0.0952468
59645	2.12923	0.0991664
59646	1.89224	0.0998485
59647	1.89224	0.153348
59648	1.89224	0.0976303
59649	1.89224	0.152096
59650	3.78447	0.103635
59651	0.946119	0.165967
59652	0.946119	0.118351
59653	0.946118	0.135401
59654	0.946118	0.099305
59655	0.946118	0.137744
59656	0.946118	0.150041
59657	0.946119	0.0906838
59658	0.946119	0.0836821
59659	0.946131	0.116587
59660	0.946131	0.0935557
59661	0.946106	0.107871
59662	0.946106	0.12047
59663	2.07526	0.584876
59664	1.89226	0.468985
59665	1.89226	0.685711
59666	2.13445	0.644348
59667	4.02671	0.537993
59668	1.89226	0.465356
59669	1.89226	0.518197
59670	1.89226	0.561604
59671	1.89226	0.39875
59672	1.89226	0.664314
59673	1.89226	0.449104
59674	2.13446	0.605442
59675	1.00013	0.624513
59676	1.1475	0.890066
59677	2.20046	0.622114
59678	3.20059	0.696728
59679	0.946133	0.523894

59680	0.946133	0.58072
59681	1.06722	0.576497
59682	1.10023	0.511146
59683	0.946131	0.539983
59684	0.946131	0.416187
59685	1.10023	0.623719
59686	1.10023	0.553428
59687	0.946131	0.604062
59688	1.10023	0.50788
59689	0.94613	0.542183
59690	1.06723	0.495737
59691	1.89226	0.425051
59692	1.89226	0.451555
59693	1.89226	0.557891
59694	1.89226	0.456897
59695	3.78452	0.485195
59696	1.89226	0.619076
59697	1.89226	0.556061
59698	1.89226	0.631299
59699	1.89226	0.560149
59700	1.89226	0.585398
59701	1.89226	0.512821
59702	1.89226	2.1
59703	1.89226	0.339803
59704	1.89226	0.511664
59705	1.89226	0.333115
59706	1.89226	2.1
59707	0.828588	0.05
59708	0.828588	0.05
59709	0.841316	0.05
59710	0.841316	1.89663
59711	0.528387	2.1
59712	0.565893	0.05
59713	1.10401	0.05
59714	1.10401	1.89663
59715	0.528387	2.1
59716	1.89226	2.1
59717	1.36047	0.511664
59718	1.33973	2.1
59719	1.33973	2.1
59720	1.89226	0.05
59721	2.28951	0.322383
59722	1.89226	0.914627
59723	2.80796	1.92423
59724	2.01584	1.76561
59725	2.01584	1.07342
59726	1.89226	1.57523
59727	1.89226	1.04561
59728	1.89226	0.768909
59729	1.89226	1.57198
59730	1.89226	1.19911
59731	1.89226	0.886503
59732	1.89226	1.28502
59733	1.89226	0.935553
59734	2.15223	0.891169
59735	1.30664	2.04193
59736	1.96011	1.87117
59737	2.76516	0.93983

59738	4.11234	1.90346
59739	1.89226	0.727153
59740	1.89226	1.60613
59741	1.89226	1.10291
59742	1.89226	0.840729
59743	1.89226	1.20213
59744	1.89226	0.889321
59745	2.81143	2.1
59746	2.58517	2.1
59747	2.55318	2.1
59748	2.7542	2.1
59749	5.33937	2.1
59750	2.7777	2.1
59751	2.7542	2.1
59752	2.71672	2.1
59753	2.70401	2.1
59754	5.45821	2.1
59755	2.55318	2.1
59756	2.7542	2.1
59757	2.71672	2.1
59758	2.54198	2.1
59759	5.29618	2.1
59760	1.95416	2.1
59761	2.162 2.1	
59762	2.11563	2.1
59763	1.91813	2.1
59764	4.08013	2.1
59765	1.95417	2.1
59766	1.47712	2.1
59767	1.45182	2.1
59768	1.91813	2.1
59769	3.39525	2.1
59770	2.11562	2.1
59771	1.91813	2.1
59772	1.50979	2.1
59773	2.03974	2.1
59774	3.95787	2.1
59775	1.85605	2.1
59776	1.91813	2.1
59777	1.88885	2.1
59778	1.42255	2.1
59779	1.45182	2.1
59780	1.45182	2.1
59781	0.94613	0.60909
59782	0.94613	0.43307
59783	0.946128	0.405376
59784	0.946128	0.688679
59785	0.946127	0.567089
59786	0.946127	0.580663
59787	0.946131	0.405057
59788	0.946131	0.393485
59789	0.946129	0.470545
59790	0.946129	0.48936
59791	0.946128	0.511607
59792	0.946128	0.507756
59793	0.543052	0.59648
59794	0.54608	0.422357
59795	0.602293	0.391297

59796	0.535325	0.405545
59797	1.05446	0.583371
59798	0.850496	0.506652
59799	0.699838	0.436115
59800	0.679751	0.578044
59801	0.713797	0.488201
59802	0.509794	0.443379
59803	0.602304	0.43569
59804	1.40017	0.570561
59805	1.40017	1.19093
59806	0.94613	0.350895
59807	0.94613	1.53304
59808	1.40017	1.20727
59809	1.33997	0.844215
59810	0.946131	0.525962
59811	0.946131	0.496641
59812	1.33997	0.647541
59813	0.946131	0.651875
59814	0.946131	0.65873
59815	1.40017	0.652895
59816	1.89226	0.0536457
59817	1.89226	0.0749425
59818	1.89226	0.0754258
59819	1.89226	0.05
59820	3.78452	0.0556811
59821	0.946133	0.05
59822	0.946133	0.0514231
59823	0.946132	0.0855054
59824	0.946132	0.0557694
59825	0.946132	0.0528498
59826	0.946132	0.0584615
59827	0.946133	0.0812825
59828	0.946133	0.0525656
59829	0.946132	0.0505173
59830	0.946132	0.0612334
59831	0.946132	0.0555189
59832	0.946132	0.0565184
59833	1.89224	0.0764577
59834	1.89224	0.0512813
59835	1.89224	0.0579748
59836	1.89224	0.0597777
59837	1.89224	0.0667189
59838	1.89224	0.0690368
59839	0.94612	0.05
59840	0.94612	0.0507509
59841	0.946119	0.0764722
59842	0.946119	0.0568089
59843	0.946119	0.0548939
59844	0.946119	0.0602322
59845	0.94612	0.0554534
59846	0.94612	0.0716399
59847	0.946132	0.055386
59848	0.946132	0.051222
59849	0.946107	0.05
59850	0.946107	0.059161
59851	2.63939	2.1
59852	1.2862	2.1
59853	1.29891	2.1

59854	2.64419	2.1
59855	3.93039	2.1
59856	2.6394	2.1
59857	1.89226	2.1
59858	1.89226	2.1
59859	2.64419	2.1
59860	4.53646	2.1
59861	1.89226	2.1
59862	1.89226	2.1
59863	1.89226	2.1
59864	1.89226	1.81614
59865	3.78453	2.1
59866	1.89226	2.1
59867	1.89226	2.06024
59868	1.89226	2.1
59869	1.89226	2.1
59870	1.89226	2.1
59871	1.89226	2.1
59872	1.31998	2.1
59873	1.32209	2.1
59874	0.649456	2.1
59875	0.648474	2.1
59876	0.984995	2.1
59877	0.984995	2.1
59878	0.985573	2.1
59879	0.985573	2.1
59880	1.31999	2.1
59881	0.648472	2.1
59882	0.649455	2.1
59883	1.32209	2.1
59884	0.946133	2.1
59885	0.946133	2.1
59886	1.31999	2.1
59887	1.3221	2.1
59888	0.946132	2.1
59889	0.946132	2.1
59890	1.31999	2.1
59891	1.31999	2.1
59892	1.31999	2.1
59893	0.946132	2.1
59894	0.946133	2.1
59895	1.3221	2.1
59896	0.946132	1.8421
59897	0.946132	2.1
59898	0.946131	2.1
59899	0.946131	2.1
59900	0.946132	1.70545
59901	0.946132	2.1
59902	0.946131	2.1
59903	0.946131	2.1
59904	0.946131	2.1
59905	0.946131	2.1
59906	0.946132	2.1
59907	0.946132	2.1
59908	0.946128	2.1
59909	0.946128	2.1
59910	0.946132	1.74161
59911	0.946132	2.1

59912	0.946131	1.81169
59913	0.946131	1.90322
59914	0.94613	2.1
59915	0.94613	2.1
59916	0.94613	2.1
59917	0.94613	2.1
59918	0.946131	2.1
59919	0.946131	2.1
59920	1.89226	0.402961
59921	1.89226	0.60681
59922	1.89226	0.57695
59923	1.89226	0.561404
59924	1.89226	0.478352
59925	1.89226	0.474125
59926	1.89226	0.518443
59927	1.89226	0.77914
59928	1.89226	0.624618
59929	1.89226	0.741957
59930	3.78453	0.792521
59931	0.946133	0.535031
59932	0.946133	0.541466
59933	0.946132	0.509301
59934	0.946132	0.496362
59935	0.946133	0.582126
59936	0.946133	0.581886
59937	0.946132	0.536083
59938	0.946132	0.457661
59939	0.946132	0.538944
59940	0.946132	0.604986
59941	0.946132	0.508574
59942	0.946132	0.674369
59943	2.23638	0.411131
59944	1.89226	0.495256
59945	2.66014	0.587073
59946	2.66014	0.610616
59947	1.89226	0.590501
59948	1.89226	0.516533
59949	1.11819	0.338322
59950	1.33007	0.351671
59951	0.660255	0.381541
59952	0.91151	0.373476
59953	0.911511	0.44668
59954	1.33007	0.409583
59955	1.11819	0.268833
59956	0.660255	0.341484
59957	0.985327	0.320818
59958	0.985327	0.420249
59959	1.005	0.352639
59960	1.005	0.40925
59961	1.89226	2.1
59962	1.89226	2.1
59963	1.89226	2.1
59964	1.89226	2.1
59965	3.78453	2.1
59966	1.59533	2.1
59967	2.68571	2.1
59968	1.28291	2.1
59969	2.46974	2.1

59970	4.06506	2.1
59971	2.68571	2.1
59972	1.89226	2.1
59973	1.89226	2.1
59974	1.89226	2.1
59975	2.46973	2.1
59976	2.46973	2.1
59977	1.89226	2.1
59978	1.89226	2.1
59979	1.89226	2.1
59980	1.89226	2.1
59981	1.89226	2.1
59982	1.89226	2.1
59983	1.89226	2.1
59984	1.89226	2.1
59985	1.89226	2.1
59986	1.89226	2.1
59987	1.89226	2.1
59988	1.89226	2.1
59989	0.883594	2.1
59990	0.883594	2.1
59991	0.876484	2.1
59992	0.876484	2.1
59993	1.10442	2.1
59994	1.23487	2.1
59995	0.641457	2.1
59996	0.52521	2.1
59997	1.10442	2.1
59998	0.52521	2.1
59999	0.641457	2.1
60000	1.23487	2.1
60001	0.946131	2.1
60002	0.946131	2.1
60003	0.946131	2.1
60004	0.946131	2.1
60005	0.946131	2.1
60006	0.946131	2.1
60007	0.946131	2.1
60008	0.946131	2.1
60009	0.946131	2.1
60010	0.946131	2.1
60011	0.946131	2.1
60012	0.946131	2.1
60013	2.04016	0.222551
60014	2.13042	0.251386
60015	2.11465	0.283172
60016	2.11465	0.365005
60017	1.95908	0.167651
60018	1.95908	0.213517
60019	1.66953	0.212885
60020	2.13041	0.263919
60021	1.95908	0.165026
60022	2.21952	0.220803
60023	1.66953	0.309241
60024	1.92997	0.351186
60025	2.04016	0.197596
60026	1.95908	0.187403
60027	1.82014	0.123682

60028	2.08757	0.146752
60029	3.86029	0.164815
60030	0.834764	0.223634
60031	0.722102	0.16259
60032	0.979537	0.189084
60033	0.910065	0.135487
60034	0.840053	0.139746
60035	0.840052	0.268551
60036	0.861587	0.17768
60037	0.861587	0.184554
60038	0.910067	0.155814
60039	0.722101	0.128946
60040	0.834763	0.208068
60041	0.979539	0.182826
60042	1.89226	0.0918196
60043	1.89226	0.0878474
60044	1.89226	0.0906347
60045	1.89226	0.0806815
60046	1.89226	0.103342
60047	1.89226	0.0935499
60048	1.89226	0.0955696
60049	1.89226	0.113552
60050	1.89226	0.148511
60051	1.89226	0.0725837
60052	3.78453	0.0995824
60053	1.89226	0.0636091
60054	1.89226	0.0646145
60055	1.89226	0.0663447
60056	1.89226	0.0589539
60057	1.89226	0.0726691
60058	1.89226	0.0656572
60059	1.20691	0.0533651
60060	2.27379	0.0533584
60061	2.58476	0.0610593
60062	1.5124	0.05
60063	3.79167	0.0555367
60064	1.89226	0.110483
60065	2.2738	0.0664537
60066	2.58477	0.0683097
60067	1.89226	0.083527
60068	4.47703	0.0795397
60069	0.952225	1.39633
60070	0.952225	1.77523
60071	0.909865	1.71993
60072	0.909865	1.97901
60073	0.909895	2.1
60074	0.946132	1.4784
60075	0.946132	1.77303
60076	0.915958	1.39662
60077	0.946132	1.73683
60078	0.946132	1.6972
60079	0.909894	1.65994
60080	0.915958	1.80787
60081	0.983493	2.1
60082	2.40055	2.1
60083	1.02986	2.1
60084	1.02986	2.1
60085	2.34217	2.1

60086	2.34217	2.1
60087	1.89226	2.1
60088	2.40056	2.1
60089	2.34217	2.1
60090	2.40056	2.1
60091	1.83388	2.1
60092	1.89226	2.1
60093	1.89226	2.1
60094	1.89226	2.1
60095	1.89226	2.1
60096	1.89226	2.1
60097	1.89226	2.1
60098	1.89226	2.1
60099	1.89226	2.1
60100	1.89226	2.1
60101	1.89226	2.1
60102	1.89226	2.1
60103	1.89226	2.1
60104	1.89226	2.1
60105	0.946133	2.1
60106	0.946133	2.1
60107	1.17109	2.1
60108	1.13078	2.1
60109	0.946132	2.1
60110	0.946132	2.1
60111	1.13078	2.1
60112	1.13078	2.1
60113	1.13078	2.1
60114	0.946133	2.1
60115	0.946132	2.1
60116	1.17109	2.1
60117	1.89226	2.1
60118	1.89226	2.1
60119	1.89226	2.1
60120	1.89226	2.1
60121	1.89226	2.1
60122	1.89226	2.1
60123	2.33963	1.06217
60124	1.89227	0.731664
60125	1.74565	0.794959
60126	1.74565	0.852018
60127	1.89227	0.64997
60128	1.89227	0.745798
60129	1.89227	0.870877
60130	1.89227	0.757017
60131	1.89227	1.32318
60132	1.89227	0.807218
60133	3.78453	0.789747
60134	1.31398	0.69179
60135	1.10392	0.720189
60136	0.796878	0.701356
60137	0.524356	0.66732
60138	0.870614	0.678178
60139	0.945739	0.644161
60140	0.89208	0.737233
60141	0.89208	0.838047
60142	1.23425	0.659313
60143	0.774658	0.815218

60144	1.03182	0.669615
60145	0.557289	0.620514
60146	2.33963	0.969458
60147	1.89226	0.637904
60148	1.89226	0.9349
60149	2.77245	0.710705
60150	4.23189	0.840184
60151	1.89226	0.970905
60152	1.89226	0.72302
60153	1.89226	1.08682
60154	1.89226	0.991077
60155	1.89226	0.743043
60156	1.89226	0.712684
60157	1.89226	0.412746
60158	1.89226	0.487013
60159	1.89226	0.400461
60160	1.89226	0.591069
60161	1.89226	0.345332
60162	1.89226	0.570214
60163	1.89226	0.36883
60164	1.89226	0.413116
60165	1.89226	0.355453
60166	1.89226	0.524321
60167	1.89226	0.248721
60168	1.89226	0.468257
60169	0.946128	0.304467
60170	0.946128	0.218342
60171	0.963918	0.299556
60172	1.37543	0.180886
60173	0.946129	0.285292
60174	0.946129	0.366038
60175	1.37542	0.199986
60176	1.37542	0.166946
60177	0.94613	0.259531
60178	1.37542	0.314806
60179	0.963916	0.29534
60180	0.94613	0.295001
60181	0.946132	0.405304
60182	0.946132	0.295986
60183	0.946131	0.377254
60184	0.946131	0.269789
60185	0.946131	0.375335
60186	0.946131	0.448942
60187	0.946132	0.283655
60188	0.946132	0.183286
60189	0.946131	0.320629
60190	0.946131	0.350865
60191	0.946131	0.327789
60192	0.946131	0.285075
60193	1.89226	0.445494
60194	1.89226	0.502827
60195	1.89226	0.340051
60196	1.89226	0.566034
60197	3.78452	0.452568
60198	0.946127	0.506962
60199	0.946127	0.343388
60200	0.946131	0.431668
60201	0.946131	0.326326

60202	0.946128	0.430137
60203	0.946128	0.495431
60204	0.94613	0.258771
60205	0.94613	0.342123
60206	0.946129	0.358208
60207	0.946129	0.40606
60208	0.946129	0.326019
60209	0.946129	0.401984
60210	1.89226	0.716956
60211	1.89226	0.792561
60212	1.89226	1.74829
60213	1.89226	0.585372
60214	3.78453	0.602065
60215	1.89226	0.651075
60216	1.89226	0.160507
60217	1.89226	0.435732
60218	1.89226	0.555198
60219	3.78453	0.645456
60220	0.946131	0.388074
60221	0.946131	0.47447
60222	0.946132	0.923062
60223	0.946132	0.592854
60224	0.946132	0.808965
60225	0.946132	0.557381
60226	0.946132	0.811993
60227	0.946132	0.641034
60228	0.946132	0.659918
60229	0.946132	0.730841
60230	0.946132	0.777409
60231	0.946132	0.607562
60232	0.548169	0.19854
60233	0.736603	0.123249
60234	1.18322	0.189027
60235	1.01139	0.234058
60236	1.01139	0.172736
60237	0.548168	0.144631
60238	0.736603	0.115378
60239	1.18322	0.111381
60240	0.861589	0.113265
60241	0.861589	0.194575
60242	0.869798	0.169626
60243	0.869798	0.198051
60244	1.16037	0.213922
60245	0.942012	0.158489
60246	0.942012	0.177656
60247	0.62356	0.214271
60248	0.891932	0.167142
60249	0.900255	0.193961
60250	2.02279	0.237693
60251	1.48677	0.177963
60252	1.54724	0.155455
60253	0.831456	0.156602
60254	1.19775	0.243666
60255	1.89226	1.13052
60256	2.76291	1.24384
60257	1.89226	1.54382
60258	1.89226	1.05268
60259	3.78452	1.33225

60260	1.89226	0.86005
60261	1.89226	1.24624
60262	1.89226	1.59122
60263	1.89226	0.916038
60264	1.89226	1.57586
60265	1.89226	0.917419
60266	1.89226	1.52162
60267	1.89226	0.946028
60268	1.89226	1.15692
60269	1.89226	1.10364
60270	3.78452	1.02974
60271	1.89226	0.817804
60272	1.89226	1.38097
60273	1.89226	1.42768
60274	1.89226	0.883263
60275	3.78453	1.15808
60276	0.946128	0.906125
60277	0.946128	1.09137
60278	0.946129	1.43516
60279	0.946129	1.16254
60280	0.946132	0.83097
60281	0.946132	1.22908
60282	0.946126	1.25198
60283	0.946126	0.885666
60284	0.946129	1.15432
60285	0.946129	1.07308
60286	0.946129	1.02572
60287	0.946129	0.973829
60288	2.80495	1.13438
60289	1.89225	0.942448
60290	1.89225	1.36707
60291	1.87794	1.33992
60292	1.89225	1.04169
60293	1.87794	1.03456
60294	2.25195	0.628853
60295	2.00624	0.440378
60296	2.13132	0.582685
60297	1.90239	0.279145
60298	4.13756	0.443224
60299	0.804661	0.507454
60300	0.804663	0.378478
60301	0.889591	0.368706
60302	0.889591	0.551719
60303	0.808324	0.486373
60304	0.761765	0.503121
60305	1.02822	0.358167
60306	0.879624	0.342773
60307	0.879617	0.418693
60308	0.800864	0.409149
60309	0.978023	0.428002
60310	0.808329	0.414936
60311	2.13133	0.613451
60312	2.00558	0.283113
60313	2.09316	0.254795
60314	2.09316	0.648322
60315	2.03253	0.376287
60316	2.03253	0.706145
60317	0.84577	0.513752

60318	0.845767	0.435554
60319	0.843917	0.377836
60320	0.843917	0.619065
60321	0.76177	0.461057
60322	0.719345	0.506284
60323	0.934435	0.341865
60324	0.884197	0.342599
60325	0.934439	0.373592
60326	0.75627	0.401771
60327	0.849424	0.387865
60328	0.76177	0.411258
60329	2.61855	0.334819
60330	1.17793	0.345928
60331	1.42965	0.349515
60332	1.17793	0.345719
60333	2.50057	0.339494
60334	2.24885	0.341963
60335	0.946133	0.339361
60336	0.946133	0.334377
60337	1.30928	0.340262
60338	1.12443	0.343286
60339	0.946132	0.345058
60340	0.946132	0.337548
60341	1.30928	0.329768
60342	1.30928	0.333749
60343	0.946132	0.33954
60344	1.30928	0.33661
60345	1.12443	0.33401
60346	0.946133	0.336137
60347	1.89226	0.337113
60348	1.89226	0.356533
60349	1.89226	0.348659
60350	1.89226	0.357101
60351	3.78452	0.353721
60352	1.89226	0.365245
60353	1.89226	0.345284
60354	1.89226	0.34718
60355	1.89226	0.354274
60356	1.89226	0.346373
60357	1.89226	0.34854
60358	1.89226	0.344134
60359	1.89226	0.350745
60360	1.89226	0.341002
60361	1.89226	0.369561
60362	3.78452	0.345555
60363	0.94613	0.34731
60364	0.94613	0.355156
60365	0.946131	0.359686
60366	0.946131	0.358962
60367	0.946128	0.359848
60368	0.946128	0.356282
60369	0.946133	0.344094
60370	0.946133	0.346491
60371	0.946131	0.348505
60372	0.946131	0.362954
60373	0.946131	0.355632
60374	0.946131	0.346896
60375	1.89226	2.1

60376	1.89226	2.1
60377	1.89226	2.1
60378	1.89226	2.1
60379	3.78453	2.1
60380	1.0465	2.1
60381	2.73926	2.1
60382	2.03721	2.1
60383	2.03721	2.1
60384	1.44709	2.1
60385	1.44709	2.1
60386	0.946131	2.1
60387	0.946131	2.1
60388	0.946131	2.1
60389	0.946131	2.1
60390	0.946132	2.1
60391	0.946132	2.1
60392	0.94613	2.1
60393	0.94613	2.1
60394	0.946131	2.1
60395	0.946131	2.1
60396	0.946131	2.1
60397	0.946131	2.1
60398	0.946132	2.1
60399	0.946132	2.1
60400	0.946131	2.1
60401	0.946131	2.1
60402	0.946132	2.1
60403	0.946132	2.1
60404	0.946132	2.1
60405	0.946132	2.1
60406	0.946132	2.1
60407	0.946132	2.1
60408	0.946132	2.1
60409	0.946132	2.1
60410	1.89226	2.1
60411	1.89226	2.1
60412	1.89226	2.1
60413	1.89226	2.1
60414	3.78452	2.1
60415	1.61969	0.30886
60416	1.79374	0.278394
60417	1.29382	0.253347
60418	1.29382	0.299571
60419	2.05759	0.291714
60420	2.05759	0.299414
60421	1.89226	0.278994
60422	1.89226	0.307707
60423	1.89226	0.255957
60424	1.89226	0.321676
60425	1.89226	0.265888
60426	1.89226	0.299443
60427	1.89226	0.615242
60428	1.89226	0.657135
60429	1.89226	0.62613
60430	1.89226	0.633445
60431	1.89226	0.589507
60432	1.89226	0.595242
60433	1.89226	1.04394

60434	1.89226	0.744048
60435	1.89226	0.85152
60436	1.89226	0.923087
60437	3.78453	1.04292
60438	0.946131	0.700011
60439	0.946131	0.725667
60440	0.946131	0.641695
60441	0.946131	0.707309
60442	0.946131	0.647262
60443	0.946131	0.624577
60444	0.946131	0.726838
60445	0.946131	0.790199
60446	0.946131	0.683703
60447	0.946131	0.713014
60448	0.946131	0.711236
60449	0.946131	0.665077
60450	2.45177	0.197992
60451	2.68507	0.157918
60452	2.3376	0.256831
60453	2.22086	0.143253
60454	4.78937	0.193438
60455	2.45178	0.165923
60456	2.35552	0.112386
60457	1.68062	0.171284
60458	1.81902	0.125683
60459	4.13239	0.135113
60460	2.3376	0.18452
60461	1.55612	0.122687
60462	1.69811	0.127372
60463	2.31527	0.157991
60464	1.79553	0.126347
60465	2.3376	0.156843
60466	1.55613	0.113702
60467	1.06336	0.115696
60468	1.97516	0.117289
60469	1.88966	0.104333
60470	1.06336	0.186443
60471	1.68062	0.0988457
60472	1.89226	0.904897
60473	1.91634	0.780644
60474	2.03709	1.21535
60475	1.91634	0.800109
60476	1.83185	0.897219
60477	1.70303	0.58836
60478	1.89226	0.683361
60479	1.89226	0.694156
60480	1.89226	0.988317
60481	1.89226	0.68323
60482	1.89226	0.712735
60483	1.89226	0.523792
60484	1.89226	1.24882
60485	2.03472	1.499
60486	2.22183	2.1
60487	2.03472	0.942809
60488	2.07936	1.76004
60489	1.89226	0.73908
60490	1.89226	0.867677
60491	1.8519	0.696154

60492	1.92119	1.00607
60493	1.8519	0.731752
60494	1.96156	0.754928
60495	1.89226	0.562507
60496	1.89226	0.642382
60497	1.89226	0.660863
60498	1.89226	0.905277
60499	1.89226	0.665359
60500	1.89226	0.649317
60501	1.89226	0.493223
60502	1.89226	1.02326
60503	2.22183	0.965838
60504	2.39541	1.77714
60505	2.22183	0.833604
60506	2.06585	1.04515
60507	1.89226	0.708174
60508	1.47001	2.1
60509	1.57962	0.778076
60510	1.65238	0.989382
60511	1.60502	1.13805
60512	3.18464	1.14428
60513	1.02415	2.1
60514	1.60503	1.37807
60515	1.47001	2.1
60516	1.47001	2.1
60517	1.28228	2.1
60518	1.28228	2.1
60519	0.946133	2.1
60520	0.946133	1.99032
60521	0.946132	1.16132
60522	0.946132	0.923718
60523	0.946132	0.92173
60524	0.946132	1.90527
60525	0.946133	1.53257
60526	0.946133	2.1
60527	0.946132	1.62169
60528	0.946132	1.19711
60529	0.946133	1.72823
60530	0.946133	1.89306
60531	1.89226	0.750408
60532	1.89226	0.873267
60533	1.89226	1.13147
60534	1.89226	0.664829
60535	3.78452	0.773065
60536	1.76897	0.966815
60537	1.60502	1.0244
60538	1.65238	0.808743
60539	1.65238	1.78817
60540	1.76932	0.782628
60541	1.76932	1.56347
60542	1.55998	2.1
60543	1.60503	1.08321
60544	1.76932	1.99708
60545	1.60503	2.1
60546	1.44657	2.05665
60547	1.28228	2.1
60548	0.946133	1.50399
60549	0.946133	2.1

60550	0.946132	1.22722
60551	0.946132	0.843619
60552	0.946132	0.832217
60553	0.946132	1.27401
60554	0.946133	1.0843
60555	0.946133	2.1
60556	0.946132	1.44027
60557	0.946132	1.40365
60558	0.946132	1.74637
60559	0.946132	1.42124
60560	0.946128	1.04901
60561	0.946128	0.730868
60562	0.946133	0.543326
60563	0.946133	0.735828
60564	0.94613	0.544925
60565	0.94613	0.793259
60566	0.946131	1.05103
60567	0.946131	0.787416
60568	0.946131	0.770442
60569	0.946131	0.761222
60570	0.946131	0.789285
60571	0.946131	0.807089
60572	1.85779	0.188818
60573	1.00763	0.1472
60574	1.74874	0.148256
60575	1.74873	0.137585
60576	1.14467	0.216405
60577	1.14466	0.192052
60578	1.85779	0.141096
60579	1.61597	0.116193
60580	1.00053	0.150895
60581	1.03018	0.107318
60582	2.65819	0.11375
60583	1.89226	0.529411
60584	1.89226	0.432708
60585	1.89226	0.536263
60586	1.89226	0.509082
60587	3.78451	0.521119
60588	1.89226	0.528109
60589	1.89226	0.222471
60590	1.89226	0.388617
60591	1.89226	0.416214
60592	1.89226	0.327118
60593	1.89226	0.35323
60594	1.11983	0.187486
60595	0.810945	0.218571
60596	0.913906	0.170999
60597	0.913906	0.202055
60598	2.23303	0.136332
60599	1.66057	0.146673
60600	1.28724	0.14793
60601	1.08132	0.155887
60602	1.49316	0.192674
60603	0.810958	0.187418
60604	0.810958	0.173301
60605	0.827861	0.422464
60606	0.946126	0.453005
60607	0.946132	0.503479

60608	0.946132	0.402361
60609	0.827863	0.436988
60610	0.827863	0.44124
60611	0.946129	0.465563
60612	0.946129	0.410192
60613	0.827863	0.439377
60614	0.946128	0.458583
60615	0.946129	0.337732
60616	0.946129	0.447933
60617	0.974718	0.154522
60618	1.53671	0.300834
60619	2.80542	0.165427
60620	2.27259	0.223645
60621	3.8093	0.213581
60622	1.89226	0.284361
60623	1.89226	0.503518
60624	1.89226	0.23118
60625	1.89226	0.45244
60626	3.78453	0.414122
60627	0.946132	0.251869
60628	0.946132	0.387694
60629	0.946131	0.41717
60630	0.946131	0.319363
60631	0.946132	0.417993
60632	0.946132	0.464787
60633	0.946131	0.233851
60634	0.946131	0.239476
60635	0.946132	0.34098
60636	0.946132	0.423016
60637	0.946131	0.342593
60638	0.946131	0.346449
60639	2.80542	0.1981
60640	1.89226	0.242114
60641	2.27259	0.248741
60642	2.27259	0.349004
60643	1.89226	0.117787
60644	1.89226	0.199278
60645	1.89226	0.224978
60646	1.89226	0.479665
60647	1.89226	0.260828
60648	1.89226	0.472327
60649	1.89226	0.263794
60650	1.89226	0.481417
60651	0.946132	0.482503
60652	0.946132	0.269527
60653	0.946131	0.460129
60654	0.946131	0.333939
60655	0.946132	0.458793
60656	0.946132	0.460067
60657	0.946131	0.23985
60658	0.946131	0.261946
60659	0.946131	0.325955
60660	0.946131	0.370638
60661	0.946131	0.27912
60662	0.946131	0.364523
60663	2.68213	2.1
60664	2.58234	2.1
60665	2.58234	2.1

60666	2.61243	2.1
60667	2.67035	2.1
60668	2.70044	2.1
60669	2.68213	2.1
60670	2.53921	2.1
60671	2.53303	2.1
60672	2.67035	2.1
60673	5.20956	2.1
60674	2.67033	2.1
60675	2.58232	2.1
60676	2.55004	2.1
60677	2.64567	2.1
60678	5.228	2.1
60679	2.53301	2.1
60680	2.64567	2.1
60681	2.64567	2.1
60682	2.67033	2.1
60683	2.51603	2.1
60684	2.54069	2.1
60685	0.907497	2.1
60686	0.907497	2.1
60687	0.926641	2.1
60688	0.926641	2.1
60689	1.14989	2.1
60690	1.10046	2.1
60691	0.695334	2.1
60692	0.684247	2.1
60693	0.695333	2.1
60694	1.14989	2.1
60695	0.684246	2.1
60696	1.10046	2.1
60697	0.946126	0.851644
60698	0.946126	2.1
60699	0.946131	2.1
60700	0.946131	2.1
60701	0.946127	2.1
60702	0.946127	0.959012
60703	0.94613	2.1
60704	0.94613	2.1
60705	0.946128	2.1
60706	0.946128	2.1
60707	0.946128	2.1
60708	0.946128	2.1
60709	1.89226	2.07471
60710	1.89226	1.16354
60711	1.89226	2.1
60712	1.89226	0.815141
60713	1.89226	2.1
60714	1.89226	0.71663
60715	1.42174	2.1
60716	2.29978	2.1
60717	2.41233	2.1
60718	2.41233	2.1
60719	1.39067	2.1
60720	1.39067	2.1
60721	1.89226	2.1
60722	1.89226	2.1
60723	1.89226	2.1

60724	1.89226	2.08606
60725	3.78452	2.1
60726	1.89226	2.1
60727	1.89226	2.09682
60728	1.89226	2.1
60729	1.89226	1.80813
60730	1.89226	2.1
60731	1.89226	0.967352
60732	2.41234	2.1
60733	1.89226	2.09503
60734	2.29979	2.1
60735	1.89226	2.1
60736	1.89226	2.1
60737	2.29979	2.1
60738	1.09506	0.331122
60739	2.82921	0.342008
60740	2.53251	0.333198
60741	2.53251	0.337761
60742	1.42901	0.333479
60743	1.42901	0.33889
60744	0.946132	0.33715
60745	0.946132	0.335758
60746	0.946128	0.33403
60747	0.946128	0.342248
60748	0.94613	0.345043
60749	0.94613	0.344979
60750	0.946131	0.339461
60751	0.946131	0.333882
60752	0.94613	0.336419
60753	0.94613	0.332738
60754	0.94613	0.344246
60755	0.94613	0.347841
60756	1.89226	0.352414
60757	1.89226	0.368192
60758	1.89226	0.352041
60759	1.89226	0.360961
60760	3.78452	0.351598
60761	2.53251	0.33697
60762	1.89226	0.353958
60763	2.82921	0.340723
60764	1.89226	0.339968
60765	1.89226	0.363848
60766	2.82921	0.36599
60767	1.89226	0.342693
60768	1.89226	0.33263
60769	1.89226	0.341473
60770	1.89226	0.343052
60771	1.89226	0.333903
60772	1.89226	0.337739
60773	2.53251	0.330703
60774	1.89226	0.334359
60775	2.06158	0.325149
60776	1.89226	0.326386
60777	4.42477	0.324001
60778	1.23296	2.1
60779	2.55352	2.1
60780	2.47476	2.1
60781	2.47476	2.1

60782	1.32368	2.1
60783	1.32368	2.1
60784	1.89226	2.1
60785	1.89226	2.1
60786	1.89226	2.1
60787	1.89226	2.1
60788	1.89226	2.1
60789	1.89226	2.1
60790	1.89226	2.1
60791	1.89226	2.1
60792	1.89226	2.1
60793	1.89226	2.1
60794	1.89226	2.1
60795	1.89226	0.960451
60796	2.47476	2.1
60797	1.89226	2.1
60798	1.89226	2.1
60799	1.89226	2.1
60800	2.55353	2.1
60801	2.55353	2.1
60802	1.05983	2.1
60803	2.47476	2.1
60804	2.37664	2.1
60805	2.37664	2.1
60806	1.23296	2.1
60807	1.23296	2.1
60808	0.946127	2.05025
60809	0.946127	2.1
60810	0.946131	2.1
60811	0.946131	2.1
60812	0.946127	2.1
60813	0.946127	2.04311
60814	0.946131	2.1
60815	0.946131	2.1
60816	0.946129	2.1
60817	0.946129	2.1
60818	0.946128	2.1
60819	0.946128	2.1
60820	1.89226	2.1
60821	1.89226	2.1
60822	1.89226	2.1
60823	1.89226	0.842374
60824	3.78452	2.1
60825	2.37665	2.1
60826	1.89226	2.1
60827	1.89226	2.1
60828	1.89226	2.1
60829	2.47476	2.1
60830	2.47476	2.1
60831	1.89226	0.753975
60832	1.92119	0.640404
60833	1.99586	0.897265
60834	1.92119	0.715056
60835	1.96693	0.645608
60836	1.89226	0.525288
60837	1.89226	0.626646
60838	1.89226	0.595901
60839	1.89226	0.806361

60840	1.89226	0.610761
60841	1.89226	0.602194
60842	1.89226	0.464167
60843	1.89226	0.872096
60844	2.39541	0.872687
60845	2.57183	1.33559
60846	2.39541	0.76633
60847	2.06868	0.822621
60848	1.89226	0.628278
60849	0.946133	0.586167
60850	0.946133	0.606828
60851	0.997928	0.635956
60852	1.03763	0.685232
60853	0.946131	0.689409
60854	0.946131	0.438632
60855	1.03763	0.644534
60856	1.03763	0.62391
60857	1.03763	0.652896
60858	0.946131	0.627762
60859	0.94613	0.598685
60860	0.99793	0.545182
60861	1.89226	0.465734
60862	1.89226	0.610814
60863	1.89226	0.65917
60864	1.89226	0.43186
60865	1.89226	0.788732
60866	1.89226	0.478131
60867	0.946131	0.607102
60868	0.946131	0.632855
60869	1.36939	0.729355
60870	1.28592	0.850469
60871	0.946129	0.779809
60872	0.946129	0.351531
60873	1.36939	0.66933
60874	1.36939	0.786259
60875	0.946131	0.628487
60876	1.36939	0.645585
60877	0.946131	0.69121
60878	1.28592	0.641776
60879	1.89226	0.527828
60880	1.89226	0.660017
60881	1.89226	0.587832
60882	1.89226	0.575955
60883	3.78453	0.59925
60884	1.89226	0.663981
60885	1.89226	0.665218
60886	1.89226	0.582622
60887	1.89226	0.669873
60888	3.78452	0.621365
60889	1.91005	0.726565
60890	1.89226	0.774042
60891	2.60215	0.599782
60892	2.60215	0.705702
60893	1.89226	0.640333
60894	1.89226	0.829839
60895	0.946132	0.640208
60896	0.946132	0.605094
60897	0.946132	0.64921

60898	0.946132	0.580892
60899	0.946132	0.636335
60900	0.946132	0.664218
60901	0.946132	0.601607
60902	0.946132	0.560482
60903	0.946132	0.607073
60904	0.946132	0.608943
60905	0.946131	0.584824
60906	0.946131	0.606196
60907	1.89226	0.639967
60908	1.89226	0.691132
60909	1.89226	0.880645
60910	1.89226	0.67006
60911	1.89226	0.627666
60912	1.89226	0.834497
60913	0.780483	0.731807
60914	0.78928	1.00147
60915	0.818566	0.735706
60916	0.460387	0.947813
60917	1.82793	0.600548
60918	1.32039	0.780159
60919	0.613889	0.664512
60920	1.0592	0.781404
60921	1.48698	0.835731
60922	0.818555	0.74981
60923	1.11515	0.814227
60924	1.2802	0.76397
60925	2.20046	0.508907
60926	1.1475	0.683471
60927	1.1475	0.530329
60928	2.27062	0.655796
60929	2.27062	0.51024
60930	1.89226	0.606626
60931	2.20046	0.468143
60932	2.27062	0.581334
60933	2.20046	0.53868
60934	1.96242	0.49576
60935	1.89226	0.472596
60936	1.89226	0.465983
60937	1.89226	0.401673
60938	1.89226	0.507007
60939	1.89226	0.455911
60940	3.78452	0.442076
60941	1.89226	0.510345
60942	1.89226	0.418043
60943	1.89226	0.609703
60944	1.89226	0.486673
60945	3.78452	0.515189
60946	1.17403	0.509324
60947	1.13531	0.571432
60948	0.6401	0.529211
60949	0.714566	0.55842
60950	0.915987	0.556005
60951	0.915987	0.352941
60952	0.933891	0.654219
60953	0.933891	0.559655
60954	1.17403	0.609016
60955	0.714566	0.543804

60956	0.6401	0.573679
60957	1.13531	0.515512
60958	0.946133	0.466155
60959	0.946133	0.514226
60960	1.17403	0.453107
60961	1.13531	0.478769
60962	0.946131	0.467692
60963	0.946131	0.413418
60964	1.17403	0.548302
60965	1.17403	0.461152
60966	1.17403	0.478254
60967	0.94613	0.469174
60968	0.946131	0.485162
60969	1.13531	0.447705
60970	1.89226	0.409359
60971	1.89226	0.41976
60972	1.89226	0.425331
60973	1.89226	0.368962
60974	1.89226	0.486737
60975	1.89226	0.402922
60976	0.946131	0.438841
60977	0.946131	0.447182
60978	0.946131	0.477615
60979	0.946131	0.513792
60980	0.946129	0.483609
60981	0.946129	0.395819
60982	0.946133	0.597498
60983	0.946133	0.434862
60984	0.946131	0.467705
60985	0.946131	0.458399
60986	0.946131	0.428107
60987	0.946131	0.444306
60988	2.29413	2.1
60989	1.99332	2.1
60990	1.84774	2.1
60991	2.21582	2.1
60992	4.20915	2.1
60993	0.517359	2.1
60994	0.634469	2.1
60995	0.996661	2.1
60996	0.92387	2.1
60997	0.768098	2.1
60998	0.768098	2.1
60999	0.790242	2.1
61000	0.790242	2.1
61001	0.923871	2.1
61002	0.517359	2.1
61003	0.634469	2.1
61004	0.996661	2.1
61005	1.89226	2.1
61006	1.89226	2.1
61007	1.89226	2.1
61008	1.89226	2.1
61009	3.78453	2.1
61010	0.946132	2.1
61011	0.946132	2.1
61012	0.946131	2.1
61013	0.946131	2.1

61014	0.946132	2.1
61015	0.946132	2.1
61016	0.946131	2.1
61017	0.946131	2.1
61018	0.946132	2.1
61019	0.946132	2.1
61020	0.946131	2.1
61021	0.946131	2.1
61022	1.62708	2.1
61023	2.21582	2.1
61024	2.13925	2.1
61025	2.21582	2.1
61026	1.77117	2.1
61027	1.84774	2.1
61028	0.946132	2.1
61029	0.946132	2.1
61030	0.946131	2.1
61031	0.946131	2.1
61032	0.946132	2.1
61033	0.946132	2.1
61034	0.946131	2.1
61035	0.946131	2.1
61036	0.946131	2.1
61037	0.946131	2.1
61038	0.946131	2.1
61039	0.946131	2.1
61040	1.89226	1.25503
61041	1.89226	0.701045
61042	1.89226	0.695014
61043	1.89226	0.877254
61044	3.78453	0.989228
61045	1.89226	0.694055
61046	1.89226	1.06644
61047	1.89226	1.01674
61048	1.89226	1.63631
61049	1.89226	0.651015
61050	1.89226	0.884968
61051	1.89226	0.700417
61052	1.89226	1.48253
61053	1.89226	0.640735
61054	1.89226	1.13391
61055	1.89226	0.700248
61056	1.89226	1.56607
61057	0.914369	0.894088
61058	0.914369	0.977446
61059	0.810945	1.96074
61060	0.810945	0.921706
61061	2.26581	2.1
61062	1.66199	1.70156
61063	1.49503	0.982579
61064	1.28816	2.1
61065	1.08133	1.14849
61066	0.810933	1.14068
61067	1.1212	1.12618
61068	1.89227	0.613692
61069	1.89227	0.597997
61070	1.89227	0.523955
61071	1.89227	0.692234

61072	3.78453	0.558395
61073	1.89226	0.535638
61074	1.89226	0.704075
61075	1.89226	0.651749
61076	1.89226	0.59282
61077	3.78453	0.642399
61078	1.89226	0.712923
61079	1.89226	0.63438
61080	1.89226	0.596832
61081	1.89226	0.823158
61082	3.78453	0.637858
61083	1.89226	0.608078
61084	1.89226	0.872805
61085	1.89226	0.69852
61086	2.25318	0.689768
61087	3.78453	0.810285
61088	1.89226	0.672428
61089	1.89226	0.836962
61090	1.89226	0.662597
61091	1.9637	0.67223
61092	3.78453	0.76228
61093	1.89226	0.693818
61094	1.89226	0.686055
61095	1.89226	0.589413
61096	1.89226	0.760679
61097	3.78453	0.636515
61098	1.89226	0.694947
61099	1.89226	1.01828
61100	1.89226	0.765599
61101	2.53384	0.76428
61102	3.78453	0.884898
61103	1.55405	0.686231
61104	1.03818	1.26147
61105	1.03818	0.607035
61106	1.34857	0.706811
61107	1.46338	0.587932
61108	1.77377	1.11861
61109	1.86273	0.594892
61110	1.73802	0.678729
61111	1.50969	1.68894
61112	1.46337	0.94322
61113	3.20139	0.718551
61114	1.2377	1.01731
61115	1.46337	0.641334
61116	1.27408	0.735656
61117	1.32039	1.61789
61118	1.50969	0.868356
61119	1.50969	0.696416
61120	1.89226	1.7713
61121	1.89226	2.1
61122	1.89226	2.1
61123	1.89226	2.1
61124	1.89226	2.1
61125	1.89226	0.802358
61126	1.89169	2.1
61127	1.89226	2.1
61128	1.89226	2.1
61129	1.89226	2.1

61130	1.96624	2.1
61131	1.96624	2.1
61132	2.62871	2.1
61133	1.6201	2.1
61134	1.49149	2.1
61135	1.6201	2.1
61136	2.62559	2.1
61137	2.75419	1.01366
61138	0.946129	0.669456
61139	0.946129	1.6322
61140	0.946133	2.1
61141	0.946133	2.1
61142	0.946131	2.01743
61143	0.946131	0.633044
61144	0.946131	2.1
61145	0.946131	1.74533
61146	0.946131	1.40852
61147	0.946131	1.55328
61148	0.946131	1.85039
61149	0.946131	1.27527
61150	0.851543	2.1
61151	0.851543	2.1
61152	1.04043	2.1
61153	0.997939	2.1
61154	0.946132	2.1
61155	0.946132	1.10159
61156	0.894185	2.1
61157	0.893901	2.1
61158	0.998267	2.1
61159	1.04074	2.1
61160	0.85127	2.1
61161	0.85153	2.1
61162	1.89226	2.1
61163	1.89226	2.08797
61164	1.89226	2.1
61165	1.89226	2.1
61166	1.89226	2.1
61167	1.89226	2.1
61168	0.928691	2.1
61169	0.928691	2.1
61170	0.935332	2.1
61171	0.935332	2.1
61172	1.398	2.1
61173	1.14418	2.1
61174	0.479331	2.1
61175	0.719848	2.1
61176	1.398	2.1
61177	0.719848	2.1
61178	1.14417	2.1
61179	0.47933	2.1
61180	2.28835	2.1
61181	1.89226	2.1
61182	1.89226	2.1
61183	1.89226	2.1
61184	2.796	2.1
61185	2.796	2.1
61186	0.946131	2.1
61187	0.946131	2.1

61188	0.946131	2.1
61189	0.946131	1.7893
61190	0.946128	1.8124
61191	0.946128	2.1
61192	0.946133	2.09405
61193	0.946133	2.1
61194	0.946131	2.1
61195	0.946131	2.1
61196	0.94613	2.1
61197	0.94613	2.1
61198	0.946131	2.1
61199	0.946131	2.1
61200	0.946131	2.1
61201	0.946131	1.94028
61202	0.946131	1.964
61203	0.946131	2.1
61204	0.946131	2.1
61205	0.946131	2.1
61206	0.946131	2.1
61207	0.946131	2.1
61208	0.946131	2.1
61209	0.946131	2.1
61210	1.89226	2.1
61211	1.89226	1.36738
61212	1.89226	2.05511
61213	1.89226	2.1
61214	3.78452	2.1
61215	2.05207	0.588692
61216	1.70302	0.721489
61217	1.33794	1.66564
61218	1.98194	0.763366
61219	3.36873	0.815236
61220	2.62761	0.695546
61221	1.98194	0.781982
61222	2.28009	1.25796
61223	2.28009	0.614988
61224	2.22939	1.09087
61225	2.22939	0.675402
61226	1.89226	0.789401
61227	1.89226	0.59014
61228	1.89226	0.672729
61229	1.89226	0.608351
61230	1.89226	0.745778
61231	1.89226	0.709997
61232	1.89227	0.768752
61233	1.89227	0.700519
61234	1.89227	1.71784
61235	1.89227	0.93168
61236	1.89227	0.621394
61237	1.89227	0.452881
61238	0.888355	0.590088
61239	0.990973	0.926126
61240	0.71133	0.796824
61241	0.634324	1.04871
61242	0.806209	0.952957
61243	0.806209	0.489841
61244	0.819087	1.57944
61245	0.819087	0.976807

61246	0.888355	1.04406
61247	0.634324	1.11048
61248	0.71133	0.806143
61249	0.990972	0.83359
61250	1.11469	0.982974
61251	0.985023	0.840769
61252	0.888354	0.624984
61253	0.990972	1.04882
61254	0.994754	0.841369
61255	0.994754	0.751788
61256	1.0083	1.19123
61257	1.0083	0.671862
61258	0.888354	0.672096
61259	0.985024	0.815164
61260	0.990971	0.662188
61261	1.11469	0.608721
61262	0.946132	0.955135
61263	0.946132	0.649933
61264	0.946132	0.693996
61265	0.946132	0.665027
61266	0.946132	0.662656
61267	0.946132	0.616793
61268	0.946133	1.30307
61269	0.946133	0.743089
61270	0.946133	0.759642
61271	0.946133	0.710489
61272	0.946132	0.639418
61273	0.946132	0.675923
61274	0.810944	0.717817
61275	0.810944	0.584449
61276	0.810947	0.841966
61277	0.810947	0.45736
61278	1.89227	1.43793
61279	1.35168	0.76354
61280	1.08132	0.73098
61281	1.08131	0.8236
61282	1.08132	0.566853
61283	0.810959	0.569577
61284	0.810959	0.603352
61285	1.70303	2.1
61286	2.03975	2.1
61287	1.99324	2.1
61288	2.17615	2.1
61289	1.40549	2.1
61290	1.53446	2.1
61291	0.946133	1.87227
61292	0.946133	2.1
61293	0.946129	2.1
61294	0.946129	2.1
61295	0.946131	2.1
61296	0.946131	0.932714
61297	0.946131	2.1
61298	0.946131	2.1
61299	0.946131	2.1
61300	0.946131	2.1
61301	0.946131	2.1
61302	0.946131	2.1
61303	1.89226	2.1

61304	1.89226	1.34767
61305	1.89226	2.1
61306	1.89226	1.12584
61307	1.89226	2.1
61308	1.89226	1.73228
61309	2.17615	2.1
61310	2.3111	2.1
61311	2.50288	2.1
61312	2.50288	2.1
61313	2.03975	2.1
61314	2.03975	2.1
61315	1.76401	2.1
61316	1.95852	2.1
61317	2.32134	2.1
61318	2.12306	2.1
61319	1.8513	2.1
61320	1.70303	2.1
61321	1.89226	2.1
61322	1.89226	2.1
61323	1.89226	2.1
61324	1.89226	1.23762
61325	3.78452	2.1
61326	1.89226	2.1
61327	1.89226	0.712135
61328	1.89226	1.28239
61329	1.89226	2.1
61330	3.78452	2.1
61331	1.2243	2.1
61332	1.2243	2.1
61333	1.24189	2.1
61334	1.24189	2.1
61335	1.37811	2.1
61336	1.25144	2.006
61337	1.08807	2.1
61338	1.17949	2.1
61339	1.17949	2.1
61340	1.37811	2.1
61341	1.08807	2.1
61342	1.25144	2.1
61343	1.89226	2.1
61344	1.89226	2.1
61345	1.89226	2.1
61346	1.89226	2.1
61347	1.89226	2.1
61348	1.89226	2.1
61349	1.89227	2.1
61350	1.89227	2.1
61351	1.89227	2.1
61352	1.89227	2.1
61353	1.89227	2.1
61354	1.89227	2.1
61355	1.89224	2.1
61356	1.89224	2.1
61357	1.89224	2.1
61358	1.89224	2.1
61359	3.78447	2.1
61360	1.89224	2.1
61361	1.89224	2.1

61362	1.89224	2.1
61363	1.89224	2.1
61364	1.89224	2.1
61365	1.89224	2.1
61366	1.89224	2.1
61367	1.89224	2.1
61368	1.89224	2.1
61369	1.89224	2.1
61370	3.78448	2.1
61371	1.89224	2.1
61372	1.703 2.1	
61373	1.89224	2.1
61374	1.72991	2.1
61375	1.07643	2.1
61376	1.36657	2.1
61377	1.44472	2.1
61378	2.41233	2.1
61379	2.51628	2.1
61380	2.51628	2.1
61381	1.42174	2.1
61382	1.42174	2.1
61383	1.89226	2.1
61384	1.89226	1.90711
61385	1.89226	2.1
61386	1.89226	2.1
61387	1.89226	2.1
61388	1.89226	2.1
61389	1.89226	2.1
61390	1.89226	2.1
61391	1.89226	2.1
61392	1.89226	0.794704
61393	3.78452	2.1
61394	2.51628	2.1
61395	1.89226	2.1
61396	1.89226	2.1
61397	1.89226	2.1
61398	2.41234	2.1
61399	2.41234	2.1
61400	0.996254	2.1
61401	0.996254	2.1
61402	1.01153	2.1
61403	1.01153	2.1
61404	1.28542	2.1
61405	1.25814	2.1
61406	0.719184	2.1
61407	0.72236	2.1
61408	0.719183	2.1
61409	1.28542	2.1
61410	0.722359	2.1
61411	1.25814	2.1
61412	0.946126	1.824
61413	0.946126	2.1
61414	0.946131	2.1
61415	0.946131	2.1
61416	0.946127	2.1
61417	0.946127	1.77922
61418	0.94613	2.1
61419	0.94613	2.1

61420	0.946128	2.1
61421	0.946128	2.1
61422	0.946128	2.1
61423	0.946128	2.1
61424	1.89226	2.1
61425	1.89226	0.905143
61426	1.89226	2.1
61427	1.89226	2.1
61428	1.89226	1.40719
61429	1.89226	2.1
61430	0.946132	2.1
61431	0.946132	2.1
61432	1.25814	2.1
61433	1.25814	2.1
61434	0.946131	2.1
61435	0.946131	1.3241
61436	1.28543	2.1
61437	1.25814	2.1
61438	1.28543	2.1
61439	0.946132	2.1
61440	1.25814	2.1
61441	0.946132	2.1
61442	1.89226	1.23155
61443	1.89226	0.701101
61444	1.89226	1.12132
61445	1.89226	0.969524
61446	1.89226	0.851116
61447	1.89226	0.750261
61448	1.89226	1.11465
61449	2.27719	0.683275
61450	1.89226	0.705547
61451	1.89226	0.855394
61452	3.78452	0.817722
61453	1.89225	1.28251
61454	1.89225	0.740792
61455	1.89225	0.89183
61456	1.89225	1.03852
61457	3.78451	1.00973
61458	1.89226	0.621668
61459	1.89226	0.741176
61460	1.89226	0.886009
61461	1.89226	0.459701
61462	3.78452	0.653913
61463	0.946132	0.388551
61464	0.946132	0.663516
61465	0.946132	0.7494
61466	0.946132	0.652447
61467	0.946131	0.584675
61468	0.946131	0.47371
61469	0.946132	0.737817
61470	0.946132	0.455152
61471	0.946132	0.5051
61472	0.946132	0.578712
61473	0.946132	0.578008
61474	0.946132	0.377744
61475	0.946126	0.693271
61476	0.946126	0.534383
61477	0.946132	1.22477

61478	0.946132	0.826132
61479	0.946128	0.658552
61480	0.946128	0.777193
61481	0.946129	0.739292
61482	0.946129	1.01168
61483	0.946129	0.843961
61484	0.946129	0.913481
61485	0.946129	0.740878
61486	0.946129	0.749155
61487	1.39858	2.1
61488	1.39858	2.1
61489	0.94613	2.1
61490	0.94613	2.1
61491	0.946132	2.1
61492	0.946132	2.1
61493	1.39858	2.1
61494	1.25002	2.1
61495	1.39858	2.1
61496	0.946131	2.1
61497	1.25002	2.1
61498	0.946131	2.1
61499	2.0815	2.1
61500	1.89226	2.1
61501	1.89226	2.1
61502	2.05298	2.1
61503	2.5593	2.1
61504	2.77667	2.1
61505	0.946133	2.1
61506	0.946133	2.1
61507	0.946132	2.1
61508	0.946132	2.1
61509	0.946132	2.1
61510	0.946132	2.1
61511	0.946133	2.1
61512	0.946133	2.1
61513	0.946132	2.1
61514	0.946132	2.1
61515	0.946132	2.1
61516	0.946132	2.1
61517	1.38908	2.1
61518	1.38908	2.1
61519	0.946132	2.1
61520	0.946132	2.1
61521	0.946132	2.1
61522	0.946132	2.1
61523	1.38908	2.1
61524	1.27965	2.1
61525	1.38908	2.1
61526	0.946132	2.1
61527	0.946133	2.1
61528	1.27965	2.1
61529	0.946133	2.1
61530	0.946133	2.1
61531	0.946132	2.1
61532	0.946132	2.1
61533	0.946132	2.1
61534	0.946132	2.1
61535	0.946133	2.1

61536	0.946133	2.1
61537	0.946132	2.1
61538	0.946132	2.1
61539	0.946133	2.1
61540	0.946133	2.1
61541	1.89227	1.19853
61542	1.89227	1.39707
61543	1.89227	0.887846
61544	1.89227	0.647364
61545	3.78453	0.922092
61546	1.33154	0.924334
61547	1.33154	1.07115
61548	0.946132	1.06265
61549	0.946132	0.848946
61550	1.19095	0.743648
61551	1.33154	1.1312
61552	0.946133	1.04535
61553	0.946133	1.14602
61554	0.946133	1.8658
61555	1.19095	0.812618
61556	0.946132	1.61781
61557	1.33154	0.923067
61558	1.0888	0.819696
61559	1.04745	0.81653
61560	0.946132	0.790341
61561	0.946132	0.81948
61562	1.04745	0.635505
61563	1.04745	1.13588
61564	0.946132	1.45321
61565	0.946132	0.653525
61566	1.04745	1.34525
61567	0.946133	0.684573
61568	0.946132	1.26072
61569	1.0888	0.824654
61570	1.89226	1.80027
61571	1.89226	0.630371
61572	1.89226	0.607953
61573	1.89226	0.779611
61574	1.89226	0.721557
61575	1.89226	0.992369
61576	1.89226	1.43012
61577	1.87592	0.594429
61578	1.87592	0.672476
61579	2.3819	0.815001
61580	1.89226	0.968123
61581	2.39825	1.20235
61582	1.89226	0.6653
61583	2.0949	0.991853
61584	1.88741	0.569623
61585	1.89226	0.590256
61586	1.89226	0.649077
61587	1.88741	1.04066
61588	1.07508	1.08952
61589	1.63419	1.29242
61590	1.63419	1.13717
61591	1.09795	1.44555
61592	1.56137	1.0654
61593	1.02513	1.75285

61594	0.967672	0.847821
61595	1.56137	0.982254
61596	1.56137	0.83905
61597	1.07508	1.02649
61598	1.41319	0.793444
61599	0.926902	1.14614
61600	1.56137	1.04339
61601	1.63419	1.11582
61602	2.29852	1.35079
61603	2.14485	0.93033
61604	3.77904	0.912881
61605	1.41319	0.748562
61606	1.92622	0.714583
61607	1.56137	0.676108
61608	1.56137	1.02935
61609	2.14485	0.931854
61610	1.92622	0.811682
61611	1.89227	2.1
61612	1.89227	2.1
61613	1.89227	2.1
61614	1.89227	2.1
61615	3.78453	2.1
61616	2.56618	2.1
61617	2.08612	2.1
61618	2.48048	2.1
61619	1.99644	2.1
61620	4.5666	2.1
61621	2.08612	2.1
61622	1.30659	2.1
61623	1.99644	2.1
61624	1.25404	2.1
61625	3.34016	2.1
61626	1.89226	2.1
61627	1.89226	2.1
61628	1.89226	2.1
61629	1.89226	2.1
61630	1.89226	2.1
61631	1.89226	2.1
61632	1.89227	2.1
61633	1.89227	2.1
61634	1.89227	2.1
61635	1.89227	2.1
61636	3.78453	2.1
61637	2.48048	2.1
61638	1.99644	2.1
61639	2.39022	2.1
61640	1.90734	2.1
61641	4.38782	2.1
61642	1.90734	2.1
61643	1.99644	2.1
61644	1.21564	2.1
61645	1.21564	2.1
61646	1.25404	2.1
61647	1.94574	2.1
61648	0.810946	2.1
61649	0.810946	2.1
61650	0.810947	2.1
61651	0.810947	2.1

61652	1.89226	2.1
61653	1.3517	2.1
61654	1.08133	2.1
61655	1.08132	2.1
61656	1.08132	2.1
61657	0.810934	2.1
61658	0.810934	2.1
61659	1.89226	2.1
61660	1.89226	0.695848
61661	1.89226	1.01139
61662	1.89226	2.03026
61663	3.78453	1.68859
61664	1.89226	0.226872
61665	1.89226	0.171696
61666	1.89226	0.982651
61667	1.89226	0.421114
61668	1.89226	0.790702
61669	1.89226	0.142333
61670	0.946131	0.583264
61671	0.946131	0.342073
61672	0.94613	0.704867
61673	0.94613	0.619996
61674	0.946128	0.495086
61675	0.946128	0.360312
61676	0.946133	0.133683
61677	0.946133	0.788624
61678	0.946131	0.531641
61679	0.946131	0.527555
61680	0.946131	0.630182
61681	0.946131	0.466078
61682	1.89226	2.1
61683	1.89226	0.626013
61684	1.89226	0.933052
61685	1.89226	0.855309
61686	1.89226	0.804661
61687	1.89226	0.743098
61688	2.56348	0.755011
61689	1.89226	1.2194
61690	1.89226	2.1
61691	2.53234	0.930774
61692	4.45574	0.762856
61693	2.53234	0.252287
61694	1.65583	0.0867516
61695	2.56348	0.218331
61696	2.56348	0.356996
61697	1.66295	0.109778
61698	1.65583	0.136289
61699	0.736602	0.178019
61700	0.827916	0.156742
61701	1.26617	0.186255
61702	1.18322	0.0900513
61703	0.7366	0.150986
61704	1.18321	0.154824
61705	1.26617	0.132082
61706	0.827918	0.118679
61707	0.99929	0.13624
61708	0.99929	0.164608
61709	1.00348	0.15639

61710	1.00348	0.191966
61711	1.26617	0.673416
61712	1.18322	0.635634
61713	0.946131	1.99658
61714	0.946131	0.66883
61715	0.946129	0.679882
61716	1.18321	0.523623
61717	0.946133	1.49095
61718	1.26617	1.03618
61719	1.26617	1.02444
61720	1.26617	0.232125
61721	0.946131	1.22305
61722	0.946131	0.815517
61723	2.23267	0.178412
61724	1.89226	0.270727
61725	2.07099	0.22827
61726	1.89226	0.257758
61727	2.07099	0.232169
61728	1.89226	0.248162
61729	0.946132	0.37815
61730	1.35403	0.211326
61731	1.24038	0.228643
61732	0.946131	0.248945
61733	0.946131	0.333906
61734	0.946131	0.35281
61735	1.24039	0.237341
61736	1.35403	0.246891
61737	1.24038	0.316575
61738	1.24038	0.333863
61739	0.946131	0.26028
61740	0.946131	0.442285
61741	1.89226	0.46479
61742	1.89226	0.586082
61743	1.89226	0.439798
61744	1.89226	0.415182
61745	1.89226	0.656382
61746	1.89226	0.630576
61747	1.89226	0.32257
61748	1.89226	0.559288
61749	1.89226	0.472237
61750	1.89226	0.509073
61751	3.78452	0.542953
61752	1.56971	0.248169
61753	2.80086	0.475604
61754	2.59806	0.31871
61755	1.93489	0.303516
61756	2.59806	0.474386
61757	1.93489	0.368297
61758	0.827467	0.259125
61759	1.26652	0.299634
61760	1.13679	0.290866
61761	0.639035	0.284635
61762	1.11547	0.375686
61763	1.30656	0.393784
61764	0.869062	0.258623
61765	0.696608	0.253666
61766	1.07205	0.29486
61767	0.953656	0.373105

61768	0.970744	0.275443
61769	0.970744	0.356566
61770	2.38681	2.1
61771	2.50438	1.07195
61772	2.42082	1.67752
61773	2.59106	2.1
61774	4.92521	2.03427
61775	2.50438	1.01978
61776	2.40627	2.1
61777	2.59106	2.1
61778	2.5444	0.947612
61779	5.04879	2.1
61780	2.42082	2.1
61781	2.70575	2.1
61782	2.64778	2.1
61783	2.59106	1.49686
61784	2.47753	2.1
61785	2.42082	0.890402
61786	2.70575	2.1
61787	2.5444	1.22306
61788	2.76095	2.1
61789	2.59106	1.61487
61790	2.71429	2.1
61791	2.5444	1.40251
61792	1.89226	2.1
61793	2.38473	2.1
61794	2.36689	2.1
61795	2.38473	2.1
61796	1.87442	2.1
61797	1.89226	2.1
61798	1.89226	2.1
61799	1.89226	0.677572
61800	1.89226	0.913786
61801	1.89226	0.671874
61802	1.89226	1.64946
61803	1.89226	0.697956
61804	2.14952	0.943402
61805	1.89226	0.58893
61806	1.89226	0.990534
61807	1.89226	0.581658
61808	2.0692	2.1
61809	2.0692	0.713103
61810	1.89226	2.1
61811	2.36689	2.1
61812	2.36204	2.1
61813	2.36689	2.1
61814	1.88742	2.1
61815	1.89226	2.1
61816	0.946132	0.603264
61817	0.946132	2.1
61818	0.946131	2.1
61819	0.946131	1.35625
61820	0.946132	0.847637
61821	0.946132	1.59209
61822	0.946131	2.1
61823	0.946131	2.1
61824	0.946132	1.61432
61825	0.946132	2.1

61826	0.946131	1.15
61827	0.946131	2.1
61828	0.946128	2.1
61829	0.946128	1.87058
61830	1.07476	0.851335
61831	1.07476	2.1
61832	0.946131	0.930409
61833	0.946131	1.83447
61834	1.13182	2.1
61835	1.07476	2.1
61836	1.13182	1.38923
61837	0.946131	1.58407
61838	1.07476	1.08318
61839	0.94613	0.758022
61840	1.89227	0.43764
61841	1.89227	0.526077
61842	1.89227	0.65827
61843	1.89227	0.607862
61844	3.78453	0.511845
61845	1.89226	0.462472
61846	1.89226	0.523389
61847	1.89226	0.471128
61848	1.89226	0.760226
61849	3.78452	0.492036
61850	1.89226	0.51819
61851	1.89226	0.610393
61852	1.89226	0.881541
61853	1.89226	0.828832
61854	3.78452	0.558896
61855	1.89226	0.52135
61856	1.89226	0.62927
61857	1.89226	0.570771
61858	1.89226	0.580674
61859	1.89226	0.661465
61860	1.89226	0.637561
61861	2.58344	0.902072
61862	2.00267	0.450793
61863	2.14218	0.806498
61864	2.22674	1.16973
61865	2.14218	0.400345
61866	2.22674	0.455354
61867	2.83531	1.23464
61868	1.56043	0.591748
61869	2.22674	1.12753
61870	1.90912	1.69259
61871	2.22674	0.447067
61872	1.90912	0.535622
61873	0.780217	0.633689
61874	1.11337	0.679697
61875	1.00133	1.09426
61876	0.804472	0.751795
61877	0.804472	0.911783
61878	0.780217	0.544774
61879	1.00133	0.447892
61880	1.11337	0.902341
61881	0.856668	0.847768
61882	0.856668	1.58231
61883	0.924883	0.536909

61884	0.924883	0.486569
61885	1.93107	2.1
61886	1.68657	2.1
61887	1.7237	2.1
61888	1.89226	2.1
61889	1.53436	2.1
61890	1.68657	2.1
61891	1.89226	2.1
61892	1.89226	2.1
61893	1.89226	2.1
61894	1.89226	2.1
61895	3.78453	2.1
61896	0.946131	2.1
61897	0.946131	2.1
61898	0.855092	2.1
61899	0.83953	2.1
61900	0.946131	2.1
61901	0.946131	2.1
61902	0.839531	2.1
61903	0.839531	2.1
61904	0.946131	2.1
61905	0.855093	2.1
61906	0.839531	2.1
61907	0.946131	2.1
61908	1.89226	2.1
61909	1.70302	2.1
61910	2.21419	2.1
61911	1.93107	2.1
61912	1.97872	2.1
61913	1.70302	2.1
61914	0.946133	2.1
61915	0.946133	2.1
61916	0.946132	2.1
61917	0.946132	2.1
61918	0.946132	2.1
61919	0.946132	2.1
61920	0.946132	2.1
61921	0.946132	2.1
61922	0.946133	2.1
61923	0.946133	2.1
61924	0.946132	2.1
61925	0.946132	2.1
61926	1.90081	2.1
61927	1.89226	2.1
61928	1.51379	2.1
61929	1.36813	2.1
61930	1.71019	2.1
61931	1.89226	2.1
61932	1.89227	1.24658
61933	1.89227	0.728505
61934	1.89227	0.719645
61935	1.89227	0.961533
61936	1.89227	1.32076
61937	1.89227	1.78296
61938	1.36428	1.08682
61939	1.37869	0.940461
61940	0.946132	0.785172
61941	0.946132	0.994316

61942	1.37869	0.73322
61943	1.37869	1.9994
61944	0.946133	1.15197
61945	0.946133	1.35938
61946	1.37869	0.735517
61947	0.946132	0.904032
61948	1.36428	1.15014
61949	0.946133	0.63185
61950	0.94612	0.953303
61951	0.94612	0.884404
61952	0.946119	0.912434
61953	0.946119	1.0093
61954	0.946119	0.656459
61955	0.946119	1.64299
61956	0.94612	1.26141
61957	0.94612	1.62625
61958	0.946132	0.899255
61959	0.946132	0.6809
61960	0.946107	0.707911
61961	0.946107	1.15724
61962	1.89225	0.05
61963	1.89225	2.1
61964	1.63564	0.05
61965	1.42278	2.1
61966	1.42278	2.1
61967	1.57534	0.05
61968	1.53735	2.1
61969	1.68991	0.05
61970	1.57535	0.05
61971	1.04442	2.1
61972	1.04442	2.1
61973	1.22031	0.05
61974	1.42278	2.1
61975	1.59868	0.05
61976	0.946132	2.1
61977	0.946132	2.1
61978	0.946131	2.1
61979	0.946131	2.1
61980	0.946131	2.1
61981	0.946131	2.1
61982	0.946132	2.1
61983	0.946132	2.1
61984	0.946132	2.1
61985	0.946132	2.1
61986	0.946131	2.1
61987	0.946131	2.1
61988	1.89226	2.1
61989	1.89226	2.1
61990	1.89226	2.1
61991	1.89226	2.1
61992	1.89226	2.1
61993	1.89226	2.1
61994	2.03143	0.684149
61995	1.47111	1.08418
61996	2.28685	1.12211
61997	2.28685	0.949045
61998	1.36006	0.793414
61999	1.36006	0.761554

62000	1.89226	0.649642
62001	1.89226	0.982113
62002	1.89226	0.916243
62003	1.89226	0.961694
62004	1.89226	0.644684
62005	1.89226	0.735165
62006	1.89226	0.946145
62007	1.89226	2.1
62008	1.89226	2.1
62009	1.89226	2.1
62010	1.89226	0.999194
62011	1.89226	1.86761
62012	0.902821	2.1
62013	0.902821	1.56238
62014	0.889218	2.1
62015	0.889218	2.1
62016	0.675759	2.1
62017	1.05649	2.1
62018	0.735554	2.08937
62019	1.14342	1.99704
62020	1.14343	0.956528
62021	1.05649	2.1
62022	0.735554	2.1
62023	0.675758	2.1
62024	2.46286	0.544112
62025	2.28685	0.705708
62026	2.67843	0.737878
62027	2.67843	0.612435
62028	2.03143	0.622556
62029	2.03143	0.555944
62030	1.89226	0.591156
62031	1.89226	0.667122
62032	1.89226	0.600321
62033	1.89226	0.614819
62034	3.78452	0.671156
62035	0.94613	0.969621
62036	0.94613	0.891409
62037	0.946128	1.16617
62038	0.946128	0.76637
62039	0.94613	2.1
62040	0.94613	0.786844
62041	0.946128	0.628374
62042	0.946128	0.82849
62043	0.946129	0.54206
62044	0.946129	0.893204
62045	0.946129	0.611578
62046	0.946129	2.03411
62047	2.67843	0.661797
62048	2.11297	1.20167
62049	2.52453	1.15246
62050	2.52453	1.36829
62051	2.28685	0.83319
62052	2.28685	0.768251
62053	1.89226	0.473536
62054	1.89226	0.769394
62055	1.89226	0.439679
62056	1.89226	0.776541
62057	3.78451	0.570481

62058	2.39524	0.414673
62059	2.10188	0.739459
62060	2.29839	0.432631
62061	2.02242	0.632681
62062	4.40028	0.530145
62063	1.71839	2.1
62064	1.65261	2.1
62065	2.10189	0.603068
62066	2.10189	1.29656
62067	2.02243	0.632148
62068	1.63893	0.636493
62069	0.946132	2.1
62070	0.946132	0.650701
62071	0.946129	0.640709
62072	0.946129	2.1
62073	0.94613	2.1
62074	0.94613	2.1
62075	0.946131	0.628645
62076	0.946131	0.627469
62077	0.946131	0.76177
62078	0.946131	0.770211
62079	0.946131	0.817618
62080	0.946131	0.825765
62081	1.89226	0.43125
62082	1.89226	0.843225
62083	1.89226	0.402793
62084	1.89226	0.639247
62085	3.78452	0.529879
62086	2.2984	0.420646
62087	2.02242	0.661065
62088	2.21582	0.304072
62089	1.94412	0.62275
62090	4.24251	0.522319
62091	1.94412	0.584606
62092	1.5854	1.89216
62093	2.02243	2.1
62094	2.02243	0.638405
62095	1.57431	0.628986
62096	1.65261	2.08754
62097	0.810943	0.645092
62098	0.810943	2.1
62099	0.810945	2.06118
62100	0.810945	2.1
62101	1.89226	0.597886
62102	1.3517	0.731723
62103	1.08133	0.773581
62104	1.08132	2.1
62105	1.08132	0.505382
62106	0.810933	0.783059
62107	0.810933	0.784502
62108	1.89226	0.10265
62109	1.89226	0.216308
62110	1.89226	0.173172
62111	1.89226	0.153412
62112	1.89226	0.143236
62113	1.89226	0.127948
62114	1.89226	0.169174
62115	1.89226	0.332686

62116	1.89226	0.224606
62117	1.89226	0.300437
62118	3.78452	0.315525
62119	1.89226	0.145635
62120	1.89226	0.273823
62121	1.89226	0.209006
62122	1.89226	0.193216
62123	1.89226	0.182552
62124	1.89226	0.168832
62125	0.946128	0.259276
62126	0.946128	0.181752
62127	0.946132	0.176296
62128	0.946132	0.227694
62129	0.94613	0.286889
62130	0.94613	0.312724
62131	0.946129	0.211942
62132	0.946129	0.164
62133	0.94613	0.170227
62134	0.94613	0.230862
62135	0.94613	0.187581
62136	0.94613	0.23224
62137	1.89226	0.111324
62138	1.89226	0.237208
62139	1.89226	0.153494
62140	1.89226	0.154533
62141	1.89226	0.230252
62142	1.89226	0.234875
62143	2.81478	2.1
62144	1.89226	2.1
62145	2.65557	2.1
62146	1.89226	2.1
62147	4.54783	2.1
62148	1.89226	2.1
62149	1.89226	2.1
62150	1.89226	2.1
62151	1.89226	2.1
62152	3.78452	2.1
62153	2.65557	2.1
62154	1.89226	2.1
62155	1.89226	2.1
62156	1.89226	2.1
62157	2.57997	2.1
62158	2.57997	2.1
62159	0.946131	2.1
62160	0.946131	2.1
62161	0.946128	2.1
62162	0.946128	2.1
62163	0.94613	2.1
62164	0.94613	2.1
62165	0.946129	2.1
62166	0.946129	2.1
62167	0.94613	2.1
62168	0.94613	2.1
62169	0.94613	2.1
62170	0.94613	2.1
62171	1.89226	0.974726
62172	2.27703	1.54822
62173	2.35615	1.64353

62174	2.35615	1.06155
62175	1.89226	1.59587
62176	1.89226	1.03764
62177	0.946132	0.936617
62178	1.17807	1.69135
62179	1.14926	0.89371
62180	0.946132	1.08693
62181	0.946133	1.07691
62182	0.946133	1.17152
62183	1.14926	0.995057
62184	1.14926	0.975835
62185	0.946132	1.12859
62186	0.946132	1.0067
62187	1.17807	1.21106
62188	1.14926	0.977389
62189	2.16841	0.94877
62190	1.35487	1.1298
62191	1.19854	0.999686
62192	1.19854	1.00552
62193	2.46078	1.00126
62194	2.46078	1.0265
62195	0.69308	1.00703
62196	1.3119	1.2343
62197	0.677433	1.22265
62198	1.23039	1.0843
62199	0.978178	1.22377
62200	0.978178	1.23852
62201	0.945294	1.06998
62202	0.945294	1.09043
62203	1.3119	1.12109
62204	1.23039	1.06866
62205	0.693081	1.0668
62206	0.677432	1.07385
62207	1.89226	0.885871
62208	1.89226	1.74928
62209	1.89226	1.51747
62210	1.89226	0.9607
62211	1.89226	1.44601
62212	1.89226	0.954427
62213	0.946132	0.860468
62214	0.946132	1.60166
62215	0.946132	0.775293
62216	0.946132	0.990181
62217	0.946133	0.982438
62218	0.946133	1.13642
62219	0.946132	0.877596
62220	0.946132	0.815913
62221	0.946133	0.964436
62222	0.946133	0.879461
62223	0.946132	0.995301
62224	0.946132	0.901511
62225	2.60637	1.76676
62226	1.89226	2.1
62227	1.89226	2.1
62228	1.89226	1.18103
62229	3.78452	2.1
62230	1.89226	0.828056
62231	1.8396	1.34605

62232	1.89226	1.55882
62233	1.89226	0.847326
62234	1.60993	2.04038
62235	1.60993	0.879034
62236	0.946131	1.0264
62237	0.946131	1.33359
62238	0.946128	2.1
62239	0.946128	1.61775
62240	0.946131	1.0297
62241	0.946131	1.41866
62242	0.946127	2.1
62243	0.946127	1.25548
62244	0.946129	1.5978
62245	0.946129	1.58583
62246	0.94613	1.43656
62247	0.94613	1.46614
62248	1.89226	2.1
62249	1.89226	1.28428
62250	1.89226	2.1
62251	1.89226	2.00163
62252	1.89226	2.0701
62253	1.89226	1.96553
62254	1.89226	0.81119
62255	1.89226	1.1713
62256	1.89226	1.52904
62257	1.89226	0.772912
62258	1.89226	1.7849
62259	1.89226	0.751246
62260	0.946131	1.02964
62261	0.946131	1.40824
62262	0.946128	2.1
62263	0.946128	1.43979
62264	0.946131	1.02535
62265	0.946131	1.31292
62266	0.946127	1.38807
62267	0.946127	2.1
62268	0.94613	1.53003
62269	0.94613	1.56442
62270	0.946129	1.42173
62271	0.946129	1.50894
62272	0.946133	2.1
62273	0.946133	2.1
62274	1.28153	2.1
62275	1.26781	2.1
62276	0.946132	2.1
62277	0.946132	2.1
62278	1.28153	2.1
62279	1.28153	2.1
62280	0.946132	2.1
62281	1.28153	2.1
62282	0.946133	2.1
62283	1.26781	2.1
62284	1.89226	2.1
62285	1.89226	0.771709
62286	1.89226	2.1
62287	1.89226	2.1
62288	1.89226	2.1
62289	1.89226	0.9139

62290	2.5844	2.1
62291	1.09563	2.1
62292	1.1468	2.1
62293	1.09563	2.1
62294	2.6142	2.1
62295	2.56304	2.1
62296	0.946126	2.1
62297	0.946126	2.1
62298	1.2922	2.1
62299	1.28152	2.1
62300	0.946126	2.1
62301	0.946126	2.1
62302	1.2922	2.1
62303	1.2922	2.1
62304	1.29221	2.1
62305	0.946132	2.1
62306	0.946119	2.1
62307	1.28151	2.1
62308	1.89225	2.1
62309	1.89225	1.36228
62310	1.89225	2.1
62311	1.89225	2.1
62312	1.89225	2.1
62313	1.89225	1.04639
62314	0.946121	0.904745
62315	0.946121	2.1
62316	0.946126	0.911902
62317	0.946126	2.1
62318	0.946124	0.854178
62319	0.946124	2.1
62320	0.946123	2.1
62321	0.946123	2.1
62322	0.94613	2.1
62323	0.94613	2.1
62324	0.946117	2.1
62325	0.946117	2.1
62326	1.89226	0.887929
62327	1.89226	0.964898
62328	1.89226	0.810618
62329	1.89226	2.1
62330	1.89226	0.644072
62331	1.89226	2.1
62332	1.89226	1.4541
62333	1.89226	1.28471
62334	1.89226	0.748191
62335	1.89226	2.1
62336	1.89226	0.63271
62337	1.89226	2.1
62338	1.89226	1.63496
62339	2.81281	2.09724
62340	2.50369	0.881057
62341	2.50369	2.1
62342	1.89226	0.935956
62343	1.89226	2.1
62344	1.89226	2.1
62345	1.89226	2.1
62346	1.89226	1.5084
62347	1.89226	2.1

62348	1.89226	1.6259
62349	1.89226	2.1
62350	1.89226	2.1
62351	1.89226	2.1
62352	1.89226	2.1
62353	1.89226	2.1
62354	1.89226	2.1
62355	1.89226	2.1
62356	0.907036	2.1
62357	1.09714	2.1
62358	0.994609	2.1
62359	0.838808	2.1
62360	0.976536	2.1
62361	0.976536	2.1
62362	0.959412	2.1
62363	0.959412	2.1
62364	1.09714	2.1
62365	0.994609	2.1
62366	0.907036	2.1
62367	0.838808	2.1
62368	0.99461	2.1
62369	0.838808	2.1
62370	0.738212	2.1
62371	0.565634	2.1
62372	0.775921	2.1
62373	0.775921	2.1
62374	0.784322	2.1
62375	0.784322	2.1
62376	0.994609	2.1
62377	0.738212	2.1
62378	0.838808	2.1
62379	0.565635	2.1
62380	1.89226	2.1
62381	1.89226	2.1
62382	1.89226	1.07248
62383	1.89226	2.1
62384	1.89226	0.917256
62385	1.89226	2.1
62386	1.89226	2.1
62387	1.89226	2.1
62388	1.89226	2.1
62389	1.89226	2.1
62390	1.89226	2.1
62391	1.89226	2.1
62392	2.19428	2.1
62393	1.98922	2.1
62394	2.27322	2.1
62395	2.45893	2.1
62396	4.4675	2.1
62397	0.99461	2.1
62398	1.13661	2.1
62399	0.902272	2.1
62400	0.738212	2.1
62401	0.931874	2.1
62402	0.931874	2.1
62403	0.942953	2.1
62404	0.942953	2.1
62405	1.13658	2.1

62406	0.902248	2.1
62407	0.994637	2.1
62408	0.738233	2.1
62409	1.89226	1.67142
62410	1.89226	1.24536
62411	1.89226	1.86814
62412	1.89226	1.69256
62413	1.89226	1.60954
62414	1.89226	1.3878
62415	1.89227	1.88136
62416	2.58246	1.03836
62417	1.89227	1.28724
62418	1.89227	1.58149
62419	3.78453	1.20476
62420	1.89226	2.1
62421	1.89226	1.34721
62422	1.65573	1.73593
62423	1.65573	1.84792
62424	1.89226	1.84652
62425	1.89226	1.97712
62426	1.89226	1.19673
62427	1.89226	1.21043
62428	1.89226	1.20134
62429	1.89226	1.1986
62430	1.89226	1.17806
62431	1.89226	1.18784
62432	1.89226	1.21883
62433	1.89226	1.19902
62434	1.89226	1.19198
62435	1.89226	1.25657
62436	1.89226	1.18577
62437	1.89226	1.30942
62438	0.946132	1.90178
62439	0.946132	1.77829
62440	1.01023	1.11057
62441	1.13651	1.48324
62442	1.01023	1.08236
62443	0.946133	1.37133
62444	1.0644	1.43018
62445	1.0644	1.8505
62446	0.946132	1.91679
62447	0.946132	1.48905
62448	1.13651	1.28479
62449	0.946132	1.42001
62450	1.89226	0.786142
62451	1.89226	1.57471
62452	1.89226	1.13239
62453	1.89226	0.63274
62454	3.78451	0.862225
62455	1.00934	0.564969
62456	0.741561	0.477755
62457	0.946131	0.75837
62458	0.946131	0.884215
62459	0.946131	1.13549
62460	0.741561	0.232767
62461	1.00934	0.363283
62462	0.946131	0.938181
62463	0.741586	0.639142

62464	0.741586	0.617634
62465	0.946106	0.751655
62466	0.946106	0.501917
62467	1.14895	0.758324
62468	0.957449	0.883668
62469	0.934806	1.26673
62470	0.934806	1.16128
62471	1.13536	0.54261
62472	1.02829	1.55171
62473	0.946129	1.66415
62474	0.946129	0.736809
62475	0.946127	1.05395
62476	0.946127	1.12967
62477	0.946127	0.997884
62478	1.0283	1.10605
62479	1.89226	1.48692
62480	1.89226	0.82494
62481	1.89226	0.793543
62482	1.89226	1.30241
62483	3.78452	1.09836
62484	1.89226	0.624386
62485	1.89226	0.950457
62486	1.89226	0.860212
62487	1.89226	0.537893
62488	3.78453	0.671843
62489	1.89226	0.949323
62490	2.69068	0.624694
62491	1.89226	0.557314
62492	1.89226	0.854145
62493	3.78452	0.795472
62494	1.89226	0.824528
62495	1.89226	1.46768
62496	1.89226	1.42825
62497	1.89226	0.953841
62498	1.89226	1.26395
62499	1.89226	0.851346
62500	1.89226	2.1
62501	1.89226	2.1
62502	1.89226	2.1
62503	1.89226	2.1
62504	1.89226	2.1
62505	1.89226	2.1
62506	1.89226	2.1
62507	1.89226	2.1
62508	1.89226	2.1
62509	1.89226	2.1
62510	1.89226	2.1
62511	1.89226	2.1
62512	0.985109	2.1
62513	1.31144	2.1
62514	1.45864	2.1
62515	1.10667	2.1
62516	2.44375	2.1
62517	0.655718	2.1
62518	0.729316	2.1
62519	0.684594	2.1
62520	0.782707	2.1
62521	0.700849	2.1

62522	0.700849	2.1
62523	0.71306	2.1
62524	0.71306	2.1
62525	0.729318	2.1
62526	0.782705	2.1
62527	0.684592	2.1
62528	0.655719	2.1
62529	0.946128	2.1
62530	0.946128	2.1
62531	0.946133	2.1
62532	0.946133	2.1
62533	0.946131	2.1
62534	0.946131	2.1
62535	0.946131	2.1
62536	0.946131	2.1
62537	0.94613	2.1
62538	0.94613	2.1
62539	0.946131	2.1
62540	0.946131	2.1
62541	1.89226	2.1
62542	1.89226	2.1
62543	1.89226	2.1
62544	1.89226	2.1
62545	1.89226	2.1
62546	1.89226	2.1
62547	0.553334	2.1
62548	0.629936	2.1
62549	0.828211	2.1
62550	0.729319	2.1
62551	0.674025	2.1
62552	0.674025	2.1
62553	0.68523	2.1
62554	0.68523	2.1
62555	0.828211	2.1
62556	0.629935	2.1
62557	0.72932	2.1
62558	0.553334	2.1
62559	0.828208	2.1
62560	0.729316	2.1
62561	0.907037	2.1
62562	0.782707	2.1
62563	0.799048	2.1
62564	0.799048	2.1
62565	0.811867	2.1
62566	0.811867	2.1
62567	0.828209	2.1
62568	0.907035	2.1
62569	0.729318	2.1
62570	0.782706	2.1
62571	2.32981	2.1
62572	1.89226	2.1
62573	1.89226	2.1
62574	1.89226	1.90723
62575	2.2671	2.1
62576	2.2671	2.1
62577	1.89226	2.00936
62578	1.89226	1.99946
62579	1.89226	2.02471

62580	1.89226	1.29438
62581	1.89226	2.06904
62582	1.89226	1.87822
62583	1.3991	1.86837
62584	1.81793	1.63262
62585	2.07226	1.44227
62586	2.07226	1.27822
62587	1.24924	2.02514
62588	1.24924	2.08725
62589	0.946131	0.77448
62590	0.946131	2.1
62591	1.16491	2.1
62592	1.16491	2.1
62593	0.946131	2.1
62594	0.946131	1.01112
62595	1.16491	2.1
62596	1.19523	2.1
62597	0.946132	2.1
62598	1.19523	2.1
62599	1.16491	2.1
62600	0.946131	2.1
62601	1.89226	2.1
62602	1.89226	1.25546
62603	1.89226	2.1
62604	1.89226	0.734122
62605	3.78452	1.06311
62606	0.901204	1.34947
62607	0.901204	2.1
62608	0.902504	1.98225
62609	0.902504	2.08444
62610	1.10416	1.59607
62611	1.03613	0.951195
62612	0.764922	2.1
62613	0.699552	2.1
62614	0.764921	1.41193
62615	1.10416	1.67367
62616	0.699551	1.7366
62617	1.03613	1.22924
62618	1.89226	0.78602
62619	1.89226	0.965683
62620	1.89226	0.976355
62621	1.89226	0.82233
62622	3.78453	0.921409
62623	1.89226	0.930789
62624	1.89226	1.05303
62625	1.89226	0.920471
62626	1.89226	1.20338
62627	1.89226	0.929727
62628	1.89226	1.21567
62629	0.946131	0.981149
62630	0.946131	0.987077
62631	0.946131	0.873074
62632	0.946131	0.839668
62633	0.946131	0.86612
62634	0.946131	0.99141
62635	0.946131	1.00666
62636	0.946131	0.792344
62637	0.946131	0.867381

62638	0.946131	0.864294
62639	0.946131	0.858924
62640	0.946131	0.885302
62641	1.89226	0.93028
62642	1.89226	1.06229
62643	1.89226	0.950882
62644	1.89226	1.11462
62645	1.89226	0.951081
62646	1.89226	1.11656
62647	0.946127	0.979531
62648	0.946127	0.976351
62649	1.22788	0.92439
62650	1.35469	0.963771
62651	1.35469	1.00319
62652	1.35469	0.971501
62653	0.946128	0.982835
62654	0.946128	0.886878
62655	0.946129	0.941522
62656	1.22788	0.968021
62657	1.35469	0.962123
62658	0.946129	0.928151
62659	1.22788	0.9887
62660	1.35469	1.03343
62661	1.02602	0.863649
62662	0.794861	0.97371
62663	1.15301	1.02224
62664	1.15301	1.05005
62665	1.10089	0.850429
62666	1.10089	0.963976
62667	0.794861	0.932705
62668	1.22788	1.01211
62669	1.02602	1.00053
62670	1.35469	1.00254
62671	1.89226	0.453895
62672	1.89226	0.629247
62673	1.89226	1.00999
62674	1.89226	0.494153
62675	3.78452	0.570701
62676	1.13536	1.32503
62677	0.942524	0.571419
62678	0.946131	0.575166
62679	0.946131	0.475359
62680	0.946127	0.591342
62681	0.946127	1.3603
62682	1.26282	0.475749
62683	0.917471	1.15651
62684	1.21023	0.685016
62685	1.00851	0.681654
62686	1.17955	0.64925
62687	0.883746	0.619671
62688	1.89226	0.541413
62689	1.89226	1.09492
62690	1.89226	0.670357
62691	1.89226	0.791609
62692	1.89226	0.671814
62693	1.89226	0.899016
62694	1.89226	0.478663
62695	1.89226	0.728867

62696	1.89226	0.537047
62697	1.89226	0.588215
62698	1.89226	0.533497
62699	1.89226	0.70041
62700	0.94613	0.798503
62701	0.94613	0.583983
62702	0.94613	0.470232
62703	0.94613	0.550138
62704	0.946128	0.54576
62705	0.946128	0.838068
62706	0.946131	0.99324
62707	0.946131	0.442814
62708	0.94613	0.614714
62709	0.94613	0.649172
62710	0.94613	0.61764
62711	0.94613	0.593061
62712	0.883758	1.10776
62713	1.03041	0.807462
62714	0.946131	0.639112
62715	0.946131	0.511856
62716	0.851537	0.613105
62717	0.851537	1.12497
62718	1.23942	0.503433
62719	1.14906	1.35575
62720	0.851484	0.62205
62721	0.985518	0.593482
62722	1.0156	0.6799
62723	0.851526	0.684349
62724	1.89226	0.566621
62725	1.89226	1.63742
62726	1.89226	0.668883
62727	1.89226	0.826733
62728	1.89226	0.680805
62729	1.89226	0.951367
62730	1.89226	0.530889
62731	1.89226	0.738421
62732	1.89226	0.578096
62733	1.89226	0.688106
62734	1.89226	0.544609
62735	1.89226	0.723602
62736	1.89227	0.388659
62737	1.89227	0.439624
62738	1.89227	0.431226
62739	1.89227	0.532694
62740	3.78453	0.4245
62741	1.89226	0.441108
62742	1.89226	0.441744
62743	1.89226	0.501566
62744	1.89226	0.646658
62745	3.78452	0.508637
62746	0.946132	0.5427
62747	0.946132	0.569789
62748	0.946128	0.705316
62749	0.946128	0.57014
62750	0.94613	0.511224
62751	0.94613	0.583717
62752	0.94613	0.436238
62753	0.94613	0.614752

62754	0.946131	0.551355
62755	0.946131	0.730865
62756	0.94613	0.472877
62757	0.94613	0.5103
62758	1.89226	0.54914
62759	1.89226	0.495665
62760	1.89226	0.544636
62761	1.89226	0.454694
62762	1.89226	0.590634
62763	1.89226	0.473048
62764	1.98874	0.378166
62765	2.33455	0.409294
62766	2.01865	0.491208
62767	2.07024	0.495697
62768	4.32329	0.507018
62769	1.98874	0.41599
62770	2.00266	0.455524
62771	2.14218	0.657867
62772	2.07024	0.790727
62773	3.9914	0.449681
62774	1.00133	0.563818
62775	0.804471	0.462404
62776	1.03512	0.645305
62777	0.995176	0.595386
62778	0.80447	1.12066
62779	0.995178	0.478499
62780	1.00133	0.511122
62781	1.03512	0.927533
62782	0.880587	0.896055
62783	0.880587	0.735156
62784	0.959002	0.422477
62785	0.959002	0.466194
62786	2.33455	0.417421
62787	1.99036	0.435044
62788	2.07024	0.50388
62789	2.58645	0.524986
62790	4.32491	0.393702
62791	1.89226	0.584237
62792	1.89226	0.555898
62793	1.89226	0.605307
62794	1.89226	0.519517
62795	3.78453	0.518226
62796	1.89226	0.70932
62797	1.89226	0.567391
62798	1.89226	0.632616
62799	1.89226	0.612859
62800	3.78453	0.709281
62801	1.89226	0.532463
62802	1.89226	0.490466
62803	1.89226	0.503571
62804	1.89226	0.526254
62805	1.89226	0.501049
62806	1.89226	0.526179
62807	1.89226	0.522021
62808	2.1523	0.251883
62809	2.53257	0.301771
62810	2.53257	0.289383
62811	1.89226	0.514316

62812	1.89226	0.508303
62813	0.946133	0.499812
62814	0.946133	0.534687
62815	0.946132	0.462432
62816	0.946132	0.47656
62817	0.946132	0.459922
62818	0.946132	0.471103
62819	0.946132	0.537187
62820	0.946132	0.478495
62821	0.946133	0.512423
62822	0.946133	0.517366
62823	0.946132	0.549836
62824	0.946132	0.509576
62825	0.946132	0.559288
62826	0.946132	0.606193
62827	0.946132	0.520309
62828	0.946132	0.55865
62829	0.946132	0.52043
62830	0.946132	0.533226
62831	0.946132	0.60812
62832	0.946132	0.56064
62833	0.946132	0.563397
62834	0.946132	0.564612
62835	0.946131	0.562823
62836	0.946131	0.559269
62837	1.62772	0.352331
62838	1.70303	0.370755
62839	1.70303	0.36684
62840	1.70303	0.36375
62841	1.90116	0.358754
62842	1.90116	0.355525
62843	1.89226	0.349652
62844	1.89226	0.381284
62845	1.89226	0.382274
62846	1.89226	0.386258
62847	1.89226	0.356641
62848	1.89226	0.35793
62849	1.89226	0.397919
62850	1.89226	0.414977
62851	1.89226	0.375633
62852	1.89226	0.41357
62853	3.78452	0.408098
62854	1.89226	0.39437
62855	1.89226	0.384185
62856	1.89226	0.378922
62857	1.89226	0.390705
62858	1.89226	0.376749
62859	1.89226	0.390577
62860	0.946132	0.352362
62861	0.946132	0.350639
62862	0.946129	0.357923
62863	0.946129	0.363009
62864	0.94613	0.360187
62865	0.94613	0.361746
62866	0.946131	0.356793
62867	0.946131	0.347675
62868	0.94613	0.348248
62869	0.94613	0.352226

62870	0.946131	0.345458
62871	0.946131	0.369261
62872	1.89226	0.367091
62873	1.89226	0.378434
62874	1.89226	0.406265
62875	1.89226	0.404598
62876	1.89226	0.361789
62877	1.89226	0.361921
62878	1.89226	0.557242
62879	1.89226	0.830332
62880	1.89226	0.802425
62881	1.89226	0.549916
62882	3.78452	0.658851
62883	1.89226	0.67519
62884	1.89226	0.822354
62885	1.89226	0.612387
62886	1.89226	0.870871
62887	1.89226	0.633123
62888	1.89226	0.955668
62889	1.30165	0.718972
62890	0.946127	0.809407
62891	1.09659	0.66399
62892	0.946131	0.698555
62893	1.09659	0.658999
62894	1.09659	1.15346
62895	0.946128	1.00981
62896	0.946128	0.574331
62897	1.30165	0.696747
62898	1.09659	1.0027
62899	0.946129	0.731674
62900	0.946129	0.926708
62901	0.946128	0.798784
62902	0.946128	0.848511
62903	0.946133	0.670709
62904	0.946133	0.750579
62905	0.946131	0.651674
62906	0.946131	1.06576
62907	0.946131	0.844406
62908	0.946131	0.598122
62909	0.94613	0.667064
62910	0.94613	0.627001
62911	0.946131	0.610656
62912	0.946131	0.765315
62913	0.946133	0.845342
62914	0.946133	0.887536
62915	0.946132	0.713589
62916	0.946132	0.763062
62917	0.946132	0.687031
62918	0.946132	0.964988
62919	0.946132	0.891902
62920	0.946132	0.690284
62921	0.946132	0.749933
62922	0.946132	0.75498
62923	0.946133	0.79012
62924	0.946133	0.838761
62925	1.30165	1.17917
62926	0.972228	1.05033
62927	1.09659	0.909569

62928	0.656268	1.38331
62929	1.06217	0.879232
62930	1.06217	1.75654
62931	1.00665	0.687882
62932	1.00665	1.17495
62933	0.97223	0.730797
62934	0.656267	0.701101
62935	1.30165	0.778774
62936	1.09659	0.953583
62937	0.946132	0.882949
62938	0.946132	1.16627
62939	1.19895	1.95393
62940	1.19895	2.1
62941	0.94613	2.04968
62942	0.94613	0.745641
62943	1.39356	1.40886
62944	1.19895	2.1
62945	0.94613	1.15152
62946	1.39356	1.23332
62947	1.19895	1.65382
62948	0.946131	1.07691
62949	1.89226	0.790896
62950	1.89226	1.77465
62951	1.89226	2.09365
62952	1.89226	0.770485
62953	1.89226	2.1
62954	1.89226	0.796694
62955	0.946132	0.716343
62956	0.946132	0.720639
62957	0.946128	0.947724
62958	0.946128	1.05528
62959	0.94613	0.952965
62960	0.94613	0.625332
62961	0.94613	0.753708
62962	0.94613	0.970724
62963	0.946131	0.75141
62964	0.946131	0.73893
62965	0.94613	0.788486
62966	0.94613	0.82241
62967	1.89226	0.07516
62968	1.89226	0.0901158
62969	1.89226	0.121703
62970	1.89226	0.0592557
62971	1.89226	0.125533
62972	1.89226	0.0588957
62973	1.29881	0.0504131
62974	1.09985	0.05
62975	0.946126	0.0821619
62976	0.946126	0.0561108
62977	0.851539	0.0816779
62978	1.20421	0.0659918
62979	0.851483	0.0557807
62980	0.999426	0.0770427
62981	1.47172	0.0649267
62982	0.999413	0.0527004
62983	0.851552	0.0610326
62984	0.851552	0.0605846
62985	1.89226	0.0990607

62986	1.89226	0.0982647
62987	1.89226	0.175182
62988	1.89226	0.080047
62989	1.89226	0.164388
62990	1.89226	0.0766631
62991	1.89226	0.0824758
62992	1.89226	0.095229
62993	1.89226	0.14221
62994	1.89226	0.0706881
62995	1.89226	0.145703
62996	1.89226	0.0681074
62997	1.33915	2.1
62998	1.99297	2.1
62999	2.05216	2.1
63000	2.60163	2.1
63001	4.04513	2.1
63002	1.89226	1.06995
63003	1.89226	2.1
63004	1.89226	2.1
63005	1.89226	1.23192
63006	3.78452	2.00263
63007	0.946132	1.46658
63008	0.946132	1.23545
63009	0.946133	2.1
63010	0.946133	2.1
63011	0.946132	2.1
63012	0.946132	0.945222
63013	0.946133	2.1
63014	0.946133	1.33689
63015	0.946132	2.05575
63016	0.946132	1.82859
63017	0.946132	2.1
63018	0.946132	2.1
63019	1.89226	1.11079
63020	1.89226	0.803682
63021	1.89226	0.89047
63022	1.89226	1.19564
63023	3.78452	0.962233
63024	1.89227	0.834473
63025	1.89227	1.13212
63026	1.89227	1.20187
63027	1.89227	0.795849
63028	1.89227	1.29663
63029	1.89227	0.82718
63030	2.29301	2.1
63031	1.70303	2.1
63032	2.46749	2.1
63033	2.30728	2.1
63034	4.53937	2.06129
63035	1.89226	0.805411
63036	1.89226	2.1
63037	1.89226	2.1
63038	1.89226	0.89319
63039	3.78452	2.1
63040	1.89226	0.682522
63041	1.89226	2.0936
63042	1.89226	0.965121
63043	1.89226	1.95435

63044	1.89226	0.786416
63045	1.89226	1.11009
63046	2.30728	2.1
63047	1.00757	1.14675
63048	2.0833	2.1
63049	1.65318	1.65929
63050	1.417	1.49754
63051	1.00757	1.94231
63052	2.46749	2.1
63053	2.0975	2.1
63054	2.79653	2.1
63055	2.44785	2.1
63056	4.91534	2.1
63057	1.89226	2.1
63058	1.89226	1.73448
63059	1.89226	0.700496
63060	1.89226	2.1
63061	3.78452	0.839685
63062	1.89226	0.971281
63063	1.89226	1.8207
63064	1.89226	2.1
63065	1.89226	1.47095
63066	1.89226	2.1
63067	1.89226	1.46803
63068	0.970559	1.26036
63069	0.970559	2.1
63070	0.979305	2.1
63071	0.979305	2.1
63072	0.708502	1.07864
63073	0.901117	2.1
63074	1.04875	2.1
63075	1.22392	0.90443
63076	1.22392	2.1
63077	0.901117	2.1
63078	1.04875	2.1
63079	0.7085	2.1
63080	1.89226	1.12876
63081	1.89226	0.732168
63082	1.89226	0.857615
63083	1.89226	0.924543
63084	3.78452	1.22521
63085	1.89226	0.981787
63086	1.89226	0.71446
63087	1.89226	0.903832
63088	1.89226	0.799028
63089	3.78452	0.778569
63090	1.89226	0.544588
63091	1.89226	0.403947
63092	1.89226	0.574893
63093	1.89226	0.537325
63094	3.78453	0.542073
63095	1.89226	0.600168
63096	1.89226	0.647114
63097	1.89226	0.54863
63098	1.89226	0.543754
63099	1.89226	0.536325
63100	1.89226	0.524496
63101	1.89226	0.649404

63102	2.35441	0.652943
63103	1.89226	0.884804
63104	2.30931	0.550927
63105	4.24667	0.596479
63106	1.11612	0.583889
63107	0.946131	0.566143
63108	1.1772	0.588295
63109	0.946131	0.653783
63110	1.17721	0.676637
63111	1.11612	0.600801
63112	0.94613	0.631332
63113	0.94613	0.625385
63114	1.11612	0.633894
63115	1.11612	0.594499
63116	0.946131	0.556402
63117	0.946131	0.591259
63118	1.11612	0.264058
63119	0.582675	0.251559
63120	1.17721	0.346508
63121	0.651969	0.261516
63122	0.651969	0.30584
63123	0.582674	0.215109
63124	1.17721	0.267268
63125	1.11612	0.246054
63126	0.877955	0.316981
63127	0.877955	0.331727
63128	0.881925	0.246625
63129	0.881925	0.266946
63130	1.17721	0.485774
63131	0.651969	0.416258
63132	1.15466	0.445957
63133	0.623642	0.442241
63134	0.623642	0.455332
63135	0.651969	0.3803
63136	1.17721	0.479581
63137	1.15466	0.468501
63138	0.904714	0.488677
63139	0.904714	0.404742
63140	0.901914	0.507205
63141	0.901914	0.443725
63142	1.89226	1.04945
63143	1.89226	0.948383
63144	1.89226	0.769797
63145	1.89226	1.21032
63146	1.89226	0.699455
63147	1.89226	2.03481
63148	1.89226	1.05301
63149	1.89226	0.705721
63150	1.89226	0.668272
63151	1.89226	2.07439
63152	1.89226	0.654516
63153	1.89226	2.1
63154	2.33986	1.72065
63155	1.97819	0.882444
63156	2.39166	0.632476
63157	2.70141	1.91767
63158	4.73151	0.862127
63159	0.989095	1.04096

63160	1.19583	0.726876
63161	0.851194	0.713301
63162	0.581197	0.76987
63163	0.872704	0.595556
63164	0.872704	1.15484
63165	0.904322	0.779859
63166	0.904322	0.873666
63167	1.19583	0.652654
63168	0.851193	0.695016
63169	0.989095	0.811138
63170	0.581197	0.695991
63171	1.89226	0.82312
63172	1.89226	1.00687
63173	1.89226	0.891785
63174	1.89226	1.33826
63175	1.89226	0.783245
63176	1.89226	2.03215
63177	1.89226	1.03571
63178	1.89226	0.674285
63179	1.89226	0.691027
63180	1.89226	2.01836
63181	1.89226	0.622057
63182	1.89226	2.1
63183	2.39166	0.816501
63184	1.70239	1.02462
63185	2.13665	0.793592
63186	2.82684	0.861934
63187	4.52831	0.819468
63188	1.89226	0.0913821
63189	1.89226	0.216411
63190	1.89226	0.171476
63191	1.89226	0.20342
63192	3.78453	0.204124
63193	1.89226	0.092804
63194	1.89226	0.125687
63195	1.89226	0.177319
63196	1.89226	0.0762952
63197	1.89226	0.199784
63198	1.89226	0.077831
63199	1.89226	0.137007
63200	1.89226	0.161118
63201	1.89226	0.231409
63202	1.89226	0.105781
63203	1.89226	0.236985
63204	1.89226	0.100649
63205	1.89226	0.227019
63206	1.89226	0.265635
63207	1.89226	0.524504
63208	1.89226	0.145594
63209	1.89226	0.549044
63210	1.89226	0.145838
63211	1.89226	0.169286
63212	1.89226	0.256177
63213	1.89226	0.381828
63214	1.89226	0.12644
63215	1.89226	0.492315
63216	1.89226	0.146309
63217	1.5169	0.398854

63218	1.61969	0.280482
63219	1.17262	0.246831
63220	1.17262	0.342942
63221	1.88868	0.301068
63222	1.88868	0.381291
63223	1.79981	0.444142
63224	1.88868	0.278584
63225	1.5169	0.2763
63226	1.5169	0.431118
63227	2.12891	0.337376
63228	2.12891	0.464317
63229	1.89226	0.428067
63230	1.89226	0.307656
63231	1.89226	0.282115
63232	1.89226	0.353393
63233	3.78452	0.347504
63234	0.946132	0.252808
63235	0.946132	0.363718
63236	0.946132	0.272017
63237	0.946132	0.258757
63238	0.946132	0.238088
63239	0.946132	0.359963
63240	0.946133	0.238677
63241	0.946133	0.363087
63242	0.946133	0.254331
63243	0.946133	0.303026
63244	0.946132	0.318257
63245	0.946132	0.27849
63246	1.88868	0.369568
63247	2.05759	0.258733
63248	1.61969	0.25728
63249	1.61969	0.347184
63250	2.29747	0.292224
63251	2.29747	0.339594
63252	2.12891	0.48044
63253	2.29747	0.269095
63254	1.88868	0.279872
63255	1.88868	0.363782
63256	2.50894	0.31523
63257	2.50894	0.400058
63258	1.89226	0.279287
63259	1.89226	0.338851
63260	1.89226	0.203847
63261	1.89226	0.396821
63262	1.89226	0.225179
63263	1.89226	0.430368
63264	0.946132	0.343607
63265	0.946132	0.232075
63266	0.946132	0.251374
63267	0.946132	0.259204
63268	0.946131	0.229641
63269	0.946131	0.372308
63270	0.946132	0.239755
63271	0.946132	0.342829
63272	0.946132	0.254935
63273	0.946132	0.311289
63274	0.946132	0.253181
63275	0.946132	0.330096

63276	2.42758	1.07473
63277	2.14895	2.1
63278	2.56975	1.05462
63279	2.28142	2.1
63280	4.71869	1.73336
63281	1.89226	1.00546
63282	1.89226	2.1
63283	1.89226	1.07222
63284	1.89226	2.1
63285	3.78452	2.0417
63286	1.89226	1.95325
63287	1.89226	2.1
63288	1.89226	1.76041
63289	1.89226	2.1
63290	1.89226	1.80832
63291	1.89226	2.1
63292	0.960916	2.1
63293	0.960916	1.73798
63294	0.978118	2.07354
63295	0.875674	2.1
63296	0.775661	2.1
63297	0.773613	2.1
63298	1.07631	1.91932
63299	1.14266	2.1
63300	1.16927	2.1
63301	0.898612	2.1
63302	1.04757	2.1
63303	0.757536	2.1
63304	2.56975	0.987981
63305	2.28142	2.1
63306	2.64384	1.09193
63307	2.40621	2.1
63308	4.97596	1.87313
63309	1.89226	1.00143
63310	1.89226	2.1
63311	1.89226	0.776397
63312	1.89226	2.1
63313	3.78452	1.14593
63314	1.89226	1.46063
63315	1.89226	2.1
63316	1.89226	2.1
63317	1.89226	2.1
63318	1.89226	1.78507
63319	1.89226	2.1
63320	1.03328	2.1
63321	1.03328	2.1
63322	1.04321	1.23729
63323	1.15424	2.1
63324	0.936183	2.1
63325	0.944491	2.1
63326	1.14266	1.67945
63327	1.20517	1.87038
63328	1.23323	2.1
63329	0.961268	2.1
63330	1.11215	2.1
63331	0.827247	2.1
63332	1.89226	2.1
63333	1.89226	0.743069

63334	1.89226	2.1
63335	1.89226	0.756869
63336	3.78453	2.09284
63337	2.14647	2.1
63338	2.20589	1.11112
63339	1.95567	2.1
63340	2.03224	0.754766
63341	4.17871	2.1
63342	2.06035	1.4137
63343	1.88452	0.50186
63344	1.88452	1.0995
63345	2.26997	0.276641
63346	2.03224	0.750387
63347	2.42272	0.578476
63348	1.89226	0.786355
63349	1.89226	0.306043
63350	1.89226	0.0906784
63351	1.89226	0.781251
63352	1.89226	0.166819
63353	1.89226	0.954221
63354	1.89225	2.1
63355	1.89225	1.18819
63356	1.89225	0.941175
63357	1.89225	2.1
63358	1.89225	0.804187
63359	1.89225	2.1
63360	0.952418	1.89219
63361	0.952418	2.1
63362	0.95883	1.14126
63363	0.95883	2.1
63364	0.946182	0.914569
63365	1.01611	1.23973
63366	0.895134	2.1
63367	0.977828	2.1
63368	0.89514	2.1
63369	0.946189	2.1
63370	0.977821	2.1
63371	1.01611	2.1
63372	2.03223	1.95413
63373	1.88451	0.515934
63374	1.89236	1.08291
63375	1.74649	0.257498
63376	3.77687	0.638531
63377	0.81094	1.18049
63378	0.81094	0.518095
63379	0.810939	1.04685
63380	0.810939	0.83089
63381	0.810945	0.68466
63382	0.810945	0.710007
63383	1.89225	0.19579
63384	1.35167	0.605276
63385	1.08131	1.2537
63386	1.08131	0.298294
63387	1.0813	0.64796
63388	1.89226	0.0505583
63389	1.89226	0.0608004
63390	1.89226	0.0576055
63391	1.89226	0.0535385

63392	1.89226	0.0561527
63393	1.89226	0.051872
63394	1.01842	0.05
63395	0.936656	0.05
63396	0.946131	0.05
63397	0.946131	0.05
63398	1.11157	0.05
63399	1.04281	0.05
63400	0.925803	0.05
63401	0.925803	0.05
63402	0.851553	0.05
63403	1.11158	0.05
63404	1.05017	0.05
63405	0.925798	0.05
63406	1.89226	0.0525187
63407	1.89226	0.0678533
63408	1.89226	0.0710994
63409	1.89226	0.059109
63410	1.89226	0.065322
63411	1.89226	0.052843
63412	1.23504	0.05
63413	1.23504	0.05
63414	0.946131	0.05
63415	0.946131	0.05
63416	1.37358	0.05
63417	1.23504	0.05
63418	0.946129	0.05
63419	0.946129	0.0503721
63420	1.37358	0.05
63421	0.946131	0.05
63422	0.94613	0.05
63423	1.23504	0.05
63424	1.00261	0.05
63425	1.00261	0.05
63426	1.02952	0.05
63427	1.02952	0.05
63428	0.850964	0.05
63429	0.658553	0.05
63430	1.37358	0.05
63431	1.23504	0.05
63432	1.37358	0.05
63433	0.850965	0.05
63434	1.23504	0.05
63435	0.658553	0.05
63436	0.946133	0.05
63437	0.946133	0.05
63438	0.946132	0.0745548
63439	0.946132	0.0509815
63440	0.946132	0.05
63441	0.946132	0.05
63442	0.946133	0.0585442
63443	0.946133	0.051681
63444	0.946133	0.0522906
63445	0.946133	0.0529903
63446	0.946132	0.0501321
63447	0.946132	0.0509579
63448	1.89226	0.328001
63449	1.89226	0.331947

63450	1.89226	0.320135
63451	1.89226	0.320975
63452	3.78452	0.331789
63453	1.89226	0.33435
63454	1.89226	0.338099
63455	1.89226	0.319928
63456	1.89226	0.323718
63457	3.78452	0.320381
63458	1.89227	0.325014
63459	1.89227	0.32604
63460	1.89227	0.316186
63461	1.89227	0.322915
63462	1.89227	0.318116
63463	1.89227	0.323375
63464	0.94613	0.239657
63465	0.94613	0.258339
63466	0.946127	0.249351
63467	0.946127	0.264605
63468	0.94613	0.252407
63469	0.94613	0.269647
63470	0.946127	0.228987
63471	0.946127	0.317765
63472	0.946128	0.22994
63473	0.946128	0.199284
63474	0.946128	0.279679
63475	0.946128	0.271661
63476	0.946132	0.29397
63477	0.946132	0.298078
63478	0.946128	0.288403
63479	0.946128	0.291756
63480	0.94613	0.285259
63481	0.94613	0.30939
63482	0.946131	0.266734
63483	0.946131	0.291969
63484	0.94613	0.268527
63485	0.94613	0.25487
63486	0.94613	0.304871
63487	0.94613	0.315648
63488	1.89226	2.1
63489	1.89226	1.81901
63490	1.89226	2.06991
63491	1.89226	2.1
63492	3.78453	2.1
63493	2.72844	2.1
63494	1.89226	2.1
63495	2.26536	2.1
63496	2.26536	2.1
63497	1.89226	2.1
63498	1.89226	2.1
63499	1.89226	2.1
63500	1.89226	1.21812
63501	1.89226	1.86151
63502	1.89226	2.06708
63503	1.89226	1.83969
63504	1.89226	2.02169
63505	1.89226	2.05747
63506	1.89226	2.1
63507	1.89226	2.1

63508	1.89226	2.1
63509	1.89226	1.74726
63510	1.89226	2.1
63511	2.72844	2.1
63512	2.26536	2.1
63513	2.28367	2.1
63514	1.5791	2.1
63515	4.54903	2.1
63516	0.810945	2.02526
63517	0.810945	1.37009
63518	0.810943	1.30434
63519	0.810943	2.1
63520	1.89226	2.1
63521	1.3517	1.75377
63522	1.08133	2.08733
63523	1.08132	2.03823
63524	1.08132	1.86391
63525	0.810933	2.1
63526	0.810933	1.90327
63527	1.89227	2.1
63528	1.89227	2.1
63529	1.89227	2.1
63530	1.89227	2.1
63531	3.78453	2.1
63532	2.71897	2.1
63533	2.27495	2.1
63534	2.648	2.1
63535	2.20832	2.1
63536	4.92729	2.1
63537	2.27495	2.1
63538	1.38455	2.1
63539	1.38455	2.1
63540	1.46586	2.1
63541	2.20831	2.1
63542	2.28962	2.1
63543	0.946133	2.1
63544	0.946133	2.1
63545	0.946132	2.1
63546	0.946132	2.1
63547	0.946132	2.1
63548	0.946132	2.1
63549	0.946132	2.1
63550	0.946132	2.1
63551	0.946132	2.1
63552	0.946132	2.1
63553	0.946133	2.1
63554	0.946133	2.1
63555	1.89226	2.1
63556	1.89226	2.1
63557	1.89226	2.1
63558	1.89226	2.1
63559	1.89226	2.1
63560	1.89226	2.1
63561	2.648	2.1
63562	2.08612	2.1
63563	2.08612	2.1
63564	2.20831	2.1
63565	2.56618	2.1

63566	2.68837	2.1
63567	2.08612	2.1
63568	2.20831	2.1
63569	1.30659	2.1
63570	1.30659	2.1
63571	1.38455	2.1
63572	2.16408	2.1
63573	0.810946	2.1
63574	0.810946	2.1
63575	0.810946	2.1
63576	0.810946	2.1
63577	1.89226	2.1
63578	1.3517	2.1
63579	1.08133	2.1
63580	1.08132	2.1
63581	1.08132	2.1
63582	0.810934	2.1
63583	0.810934	2.1
63584	1.89226	1.39749
63585	1.89226	1.21721
63586	1.89226	0.85209
63587	1.89226	2.07543
63588	3.78452	1.08703
63589	1.89226	0.926923
63590	1.89226	2.1
63591	1.89226	2.03229
63592	1.89226	1.09338
63593	3.78453	1.82485
63594	1.08733	1.99399
63595	0.856615	2.1
63596	1.25964	1.2794
63597	1.12378	1.30834
63598	1.23551	1.04052
63599	0.969894	2.1
63600	0.985977	2.09738
63601	0.985977	1.03347
63602	1.16169	1.42649
63603	1.21072	1.70771
63604	0.765175	1.56601
63605	0.863419	1.48964
63606	2.39934	1.97018
63607	2.44427	1.39767
63608	2.64003	0.907012
63609	2.39077	2.05688
63610	2.39934	1.74811
63611	2.10194	2.1
63612	1.89226	0.88473
63613	1.89226	2.1
63614	1.89226	1.35197
63615	1.89226	0.749805
63616	3.78452	1.4967
63617	0.946132	1.58737
63618	0.946132	1.97161
63619	0.946131	0.964064
63620	0.946131	0.984037
63621	0.946132	0.952134
63622	0.946132	2.1
63623	0.946132	1.69474

63624	0.946132	1.01495
63625	0.946132	1.21478
63626	0.946132	1.44621
63627	0.946132	1.22894
63628	0.946132	1.66893
63629	1.19967	2.01416
63630	0.946128	1.47112
63631	0.946133	1.00198
63632	1.32002	1.46933
63633	1.19967	1.95408
63634	1.19967	1.90334
63635	0.94613	1.98102
63636	0.94613	0.733895
63637	0.946131	1.1207
63638	0.946131	1.26127
63639	1.19967	1.46161
63640	1.32001	1.46993
63641	1.89226	0.530199
63642	1.89226	0.700933
63643	1.89226	0.45428
63644	1.89226	1.18737
63645	3.78451	0.601198
63646	2.6402	0.439089
63647	2.28552	0.69629
63648	2.50958	0.442838
63649	2.18948	0.655777
63650	4.79509	0.54627
63651	2.28552	0.638874
63652	1.86102	2.1
63653	2.18949	0.656615
63654	1.78376	2.1
63655	4.06928	0.860829
63656	1.89226	0.637784
63657	1.89226	2.1
63658	1.89226	0.690278
63659	1.89226	2.1
63660	3.78452	1.33067
63661	1.89226	0.451458
63662	1.89226	1.12633
63663	1.89226	0.388851
63664	1.89226	0.735999
63665	3.78452	0.568157
63666	2.50958	0.440529
63667	2.18948	0.605292
63668	2.39524	0.41668
63669	2.10188	0.724483
63670	4.61146	0.516656
63671	2.18949	0.439446
63672	1.78376	2.1
63673	1.71839	0.663513
63674	1.71839	1.86895
63675	2.10189	0.670177
63676	2.16726	2.01011
63677	0.810943	0.682109
63678	0.810943	2.1
63679	0.810945	0.66689
63680	0.810945	0.651605
63681	0.810959	0.792584

63682	0.810959	0.81435
63683	1.89226	2.1
63684	1.35168	0.939363
63685	1.08132	0.663945
63686	1.08132	2.1
63687	1.0813	0.805453
63688	1.89226	0.751943
63689	1.89226	1.63152
63690	1.89226	0.949488
63691	1.89226	1.01278
63692	1.89226	1.13638
63693	1.89226	1.66688
63694	1.28421	1.50133
63695	0.992041	0.810129
63696	1.07707	2.1
63697	0.789746	1.55213
63698	1.03815	1.91183
63699	1.03815	1.65454
63700	1.03581	1.71368
63701	1.03581	1.18737
63702	1.28421	1.41116
63703	1.07707	1.93839
63704	0.789748	1.65938
63705	0.992038	1.13814
63706	1.89226	0.758303
63707	1.89226	1.73945
63708	1.89226	1.05173
63709	1.89226	0.92104
63710	1.89226	1.17489
63711	1.89226	1.08865
63712	1.89226	0.744479
63713	1.89226	1.16786
63714	1.89226	0.842823
63715	1.89226	0.931802
63716	1.89226	0.966013
63717	1.89226	1.35682
63718	0.92236	2.08511
63719	0.655971	1.72813
63720	0.916772	1.94472
63721	0.913834	1.2333
63722	1.18074	1.69737
63723	0.922346	2.07843
63724	2.15414	0.900073
63725	1.53528	1.71067
63726	1.22748	1.87378
63727	1.57442	0.98853
63728	0.874683	1.72202
63729	1.25935	2.1
63730	2.61433	2.1
63731	1.22262	2.1
63732	1.22262	2.1
63733	2.62813	2.1
63734	2.62813	2.1
63735	1.89226	2.1
63736	2.61434	2.1
63737	2.62814	2.1
63738	2.61434	2.1
63739	1.90607	2.1

63740	1.89226	2.1
63741	1.89226	2.1
63742	1.89226	2.1
63743	1.89226	2.1
63744	1.89226	2.1
63745	1.89226	2.1
63746	1.89226	2.1
63747	1.89226	0.813923
63748	1.89226	1.96504
63749	1.89226	2.1
63750	1.89226	2.1
63751	1.89226	2.1
63752	1.89226	2.1
63753	1.31969	2.1
63754	1.31406	2.1
63755	0.629677	2.1
63756	0.643104	2.1
63757	0.976621	2.1
63758	0.976621	2.1
63759	0.98055	2.1
63760	0.98055	2.1
63761	1.3197	2.1
63762	0.643102	2.1
63763	0.629675	2.1
63764	1.31407	2.1
63765	0.946133	2.1
63766	0.946133	2.1
63767	1.3197	2.1
63768	1.31407	2.1
63769	0.946132	2.1
63770	0.946132	2.1
63771	1.3197	2.1
63772	1.3197	2.1
63773	1.3197	2.1
63774	0.946133	2.1
63775	0.946132	2.1
63776	1.31407	2.1
63777	1.89226	2.1
63778	1.89226	1.7026
63779	1.89226	2.1
63780	1.89226	2.1
63781	1.89226	2.1
63782	1.89226	2.02288
63783	1.89226	2.1
63784	1.89226	1.0511
63785	1.89226	2.1
63786	1.89226	2.1
63787	1.89226	2.1
63788	1.89226	1.23372
63789	1.89226	0.331847
63790	1.89226	0.245714
63791	1.89226	0.232052
63792	1.89226	0.202585
63793	3.78452	0.215308
63794	1.89226	0.255621
63795	1.89226	0.173041
63796	1.89226	0.179053
63797	1.89226	0.193385

63798	1.89226	0.212533
63799	1.89226	0.235668
63800	0.946131	0.123969
63801	0.946131	0.13026
63802	0.946128	0.123929
63803	0.946128	0.123922
63804	0.94613	0.113919
63805	0.94613	0.123164
63806	0.94613	0.100506
63807	0.94613	0.161222
63808	0.94613	0.141108
63809	0.94613	0.137968
63810	0.94613	0.149576
63811	0.94613	0.137604
63812	1.89226	0.126708
63813	1.89226	0.143102
63814	1.89226	0.129031
63815	1.89226	0.0979588
63816	1.89226	0.113992
63817	1.89226	0.135336
63818	0.924614	0.0931426
63819	0.914275	0.088956
63820	0.906003	0.0854576
63821	0.62479	0.0821533
63822	2.02733	0.0914702
63823	1.48332	0.0770466
63824	0.833106	0.0780519
63825	1.19364	0.0816319
63826	1.57482	0.0886658
63827	1.18103	0.0780836
63828	0.905988	0.0757874
63829	1.89226	2.1
63830	1.89226	2.1
63831	1.89226	2.1
63832	1.89226	2.1
63833	3.78452	2.1
63834	1.89226	2.1
63835	1.89226	2.1
63836	1.89226	2.1
63837	1.89226	2.1
63838	1.89226	2.1
63839	1.89226	2.1
63840	0.816805	2.1
63841	0.928516	2.1
63842	1.4045	2.1
63843	1.26268	2.1
63844	1.0881	2.1
63845	1.0881	2.1
63846	1.10309	2.1
63847	1.10309	2.1
63848	0.928518	2.1
63849	1.4045	2.1
63850	0.816807	2.1
63851	1.26267	2.1
63852	1.89226	2.1
63853	2.52535	2.1
63854	1.89226	2.1
63855	1.89226	2.1

63856	2.809	2.1
63857	2.809	2.1
63858	1.89226	2.1
63859	1.89226	2.1
63860	1.89226	2.1
63861	1.89226	2.1
63862	3.78452	2.1
63863	0.946127	2.1
63864	0.946127	2.1
63865	0.946131	2.1
63866	0.946131	2.1
63867	0.94613	2.1
63868	0.94613	2.1
63869	0.946128	2.1
63870	0.946128	2.1
63871	0.946129	2.1
63872	0.946129	2.1
63873	0.946129	2.1
63874	0.946129	2.1
63875	1.89224	0.610352
63876	1.89224	2.1
63877	1.89224	0.762529
63878	1.89224	2.026
63879	3.78448	1.25241
63880	2.39244	1.98424
63881	2.27036	2.1
63882	2.25945	0.722832
63883	2.27398	2.1
63884	4.52981	2.1
63885	2.27036	2.1
63886	1.97088	2.1
63887	1.86302	2.1
63888	1.84991	2.1
63889	4.12027	2.1
63890	1.89224	2.1
63891	1.89224	2.1
63892	1.89224	2.07275
63893	1.89224	2.1
63894	3.78447	2.1
63895	1.89226	0.787995
63896	1.89226	1.94191
63897	1.89226	0.610649
63898	1.89226	2.1
63899	3.78453	1.03259
63900	2.25948	0.762731
63901	2.33611	2.1
63902	2.05257	2.1
63903	1.94441	2.1
63904	4.20389	2.1
63905	1.92514	2.1
63906	1.84993	2.1
63907	1.94441	2.1
63908	1.74391	2.1
63909	3.79434	2.1
63910	1.89226	1.98603
63911	1.89226	2.1
63912	1.89226	2.1
63913	1.89226	2.1

63914	3.78452	2.1
63915	15.1381	0.449997
63916	15.1381	0.467001
63917	15.1381	0.443467
63918	15.1381	0.788516
63919	30.2762	0.439823
63920	15.1381	0.408776
63921	15.1381	0.613665
63922	15.1381	0.466138
63923	15.1381	0.589851
63924	30.2762	0.564733
63925	15.1381	1.74631
63926	15.1381	1.94173
63927	15.1381	1.29824
63928	15.1381	2.1
63929	30.2762	1.95138
63930	15.1381	0.370418
63931	15.1381	0.484265
63932	15.1381	0.453992
63933	15.1381	0.458075
63934	30.2762	0.374947
63935	1.89225	1.25913
63936	1.89225	2.1
63937	1.89225	1.85332
63938	1.89225	2.1
63939	3.78449	2.1
63940	1.39909	2.1
63941	1.39909	2.1
63942	0.946123	2.1
63943	0.946123	2.1
63944	1.3291	2.1
63945	1.39908	2.1
63946	0.946126	2.1
63947	0.946126	1.72881
63948	0.946118	2.1
63949	1.32909	2.1
63950	0.94613	2.1
63951	1.3991	2.1
63952	3.07801	2.1
63953	1.84322	2.1
63954	1.84322	2.1
63955	1.68788	2.1
63956	2.6582	2.1
63957	2.50287	2.1
63958	1.89225	2.1
63959	1.89225	2.1
63960	1.89225	2.1
63961	1.89225	2.1
63962	3.7845	2.1
63963	1.89226	1.37765
63964	1.89226	2.1
63965	1.89226	0.973594
63966	1.89226	2.1
63967	3.78452	2.1
63968	1.89226	1.07431
63969	2.50051	2.1
63970	2.50051	0.807629
63971	2.65822	2.1

63972	1.89226	1.14693
63973	2.04997	2.1
63974	2.50052	2.1
63975	2.65822	2.1
63976	1.70377	2.1
63977	1.70377	2.1
63978	1.84324	2.1
63979	2.63998	2.1
63980	0.810947	2.1
63981	0.810947	2.1
63982	0.810946	2.1
63983	0.810946	2.1
63984	1.89226	2.1
63985	1.35169	2.1
63986	1.08132	2.1
63987	1.08132	2.1
63988	1.08132	2.1
63989	0.810946	2.1
63990	0.810946	2.1
63991	1.89226	2.1
63992	1.89226	2.1
63993	1.89226	2.1
63994	1.89226	2.1
63995	3.78453	2.1
63996	0.946164	2.1
63997	0.946164	2.1
63998	0.929842	2.1
63999	0.929842	2.1
64000	0.946132	2.1
64001	0.946132	2.1
64002	1.04101	2.1
64003	0.929874	2.1
64004	0.946132	2.1
64005	0.929874	2.1
64006	1.04101	2.1
64007	0.946132	2.1
64008	1.89226	2.1
64009	1.89226	2.1
64010	1.89226	2.1
64011	1.89226	2.1
64012	3.78453	2.1
64013	1.89226	2.1
64014	1.89226	2.1
64015	1.89226	2.1
64016	1.89226	2.1
64017	1.89226	2.1
64018	1.89226	2.1
64019	1.89226	2.1
64020	1.89226	2.1
64021	1.89226	2.1
64022	1.89226	2.1
64023	1.89226	2.1
64024	1.89226	2.1
64025	0.946131	2.1
64026	0.946131	2.1
64027	0.946131	2.1
64028	0.946131	2.1
64029	0.946132	2.1

64030	0.946132	2.1
64031	0.946131	2.1
64032	0.946131	2.1
64033	0.946131	2.1
64034	0.946131	2.1
64035	0.946132	2.1
64036	0.946132	2.1
64037	1.89226	0.702082
64038	1.89226	0.515567
64039	1.89226	0.432195
64040	1.89226	0.53976
64041	3.78453	0.52403
64042	2.73497	0.402171
64043	1.93823	0.628225
64044	1.93823	0.441687
64045	2.20046	0.61718
64046	2.42279	0.454367
64047	2.68501	0.874041
64048	1.89226	0.437513
64049	1.89226	0.628369
64050	1.89226	0.705764
64051	1.89226	0.438058
64052	1.89226	0.689985
64053	1.89226	0.315411
64054	1.89226	0.448764
64055	1.89226	0.385892
64056	1.89226	0.40516
64057	1.89226	0.811085
64058	1.89226	0.414097
64059	1.89226	0.438908
64060	2.82984	0.467431
64061	2.15276	0.387707
64062	2.15276	0.545842
64063	2.42278	0.32541
64064	2.50074	0.331516
64065	2.77077	0.379253
64066	2.42279	0.38403
64067	1.93823	0.588603
64068	1.70423	0.285009
64069	1.70423	0.561373
64070	2.15276	0.300982
64071	2.38676	0.594584
64072	0.810946	0.431588
64073	0.810946	0.657171
64074	0.810944	0.384836
64075	0.810944	0.382177
64076	0.810946	0.456458
64077	0.810946	0.460424
64078	1.89226	0.588295
64079	1.35169	0.487512
64080	1.08132	0.414167
64081	1.08132	0.609493
64082	1.08132	0.493964
64083	1.89226	2.1
64084	1.89226	2.1
64085	1.89226	2.1
64086	1.89226	2.1
64087	3.78452	2.1

64088	1.89226	2.1
64089	1.89226	2.1
64090	1.89226	2.1
64091	1.89226	2.1
64092	3.78453	2.1
64093	1.04144	2.1
64094	1.04144	2.1
64095	0.938972	2.1
64096	0.938972	2.1
64097	0.969661	2.1
64098	1.03285	2.1
64099	0.946133	2.1
64100	0.946133	2.1
64101	0.946131	2.1
64102	0.969663	2.1
64103	0.946131	2.1
64104	1.03284	2.1
64105	0.946131	2.1
64106	0.946131	2.1
64107	0.946128	2.1
64108	0.946128	2.1
64109	0.946127	2.1
64110	0.946127	2.1
64111	0.946131	2.1
64112	0.946131	2.1
64113	0.94613	2.1
64114	0.94613	2.1
64115	0.946129	2.1
64116	0.946129	2.1
64117	1.89226	2.1
64118	1.89226	2.1
64119	1.89226	2.1
64120	1.89226	2.1
64121	3.78452	2.1
64122	1.89226	2.1
64123	1.89226	2.1
64124	1.89226	2.1
64125	1.89226	2.1
64126	3.78453	2.1
64127	1.89226	2.1
64128	1.89226	2.1
64129	1.85882	2.1
64130	1.85882	2.1
64131	1.93932	2.1
64132	1.97276	2.1
64133	0.94613	2.1
64134	0.94613	2.1
64135	0.946128	2.1
64136	0.946128	2.1
64137	0.946127	2.1
64138	0.946127	2.1
64139	0.946131	2.1
64140	0.946131	2.1
64141	0.946129	2.1
64142	0.946129	2.1
64143	0.946129	2.1
64144	0.946129	2.1
64145	1.89225	2.1

64146	1.89225	2.1
64147	1.89225	2.1
64148	1.89225	2.1
64149	3.7845	2.1
64150	1.0086	2.1
64151	1.09028	2.1
64152	0.946125	2.1
64153	0.946125	2.1
64154	0.916906	2.1
64155	0.916906	2.1
64156	1.13051	2.1
64157	1.1505	2.1
64158	1.11557	2.1
64159	1.04071	2.1
64160	0.851542	2.1
64161	0.991196	2.1
64162	1.89225	2.1
64163	1.89225	2.1
64164	1.89225	2.1
64165	1.89225	2.1
64166	3.7845	2.1
64167	1.89225	2.1
64168	1.89225	2.1
64169	1.89225	2.1
64170	1.89225	2.1
64171	1.89225	2.1
64172	1.89225	2.1
64173	1.89226	2.1
64174	1.89226	2.1
64175	1.89226	2.1
64176	1.89226	2.1
64177	3.78452	2.1
64178	0.890962	2.1
64179	0.952547	2.1
64180	0.946132	2.1
64181	0.946132	2.1
64182	0.851543	2.1
64183	0.851543	2.1
64184	1.11558	2.1
64185	1.06304	2.1
64186	0.994315	2.1
64187	1.04072	2.1
64188	0.900943	2.1
64189	0.851542	2.1
64190	1.89226	2.1
64191	1.89226	2.1
64192	1.89226	2.1
64193	1.89226	2.1
64194	1.89226	2.1
64195	1.89226	2.1
64196	1.89226	2.1
64197	1.89226	2.1
64198	1.89226	2.1
64199	1.89226	2.1
64200	3.78452	2.1
64201	1.89226	2.1
64202	1.89226	2.1
64203	1.89226	2.1

64204	1.89226	2.1
64205	3.78452	2.1
64206	0.782015	2.1
64207	0.929718	2.1
64208	0.946132	2.1
64209	0.946132	2.1
64210	0.782016	2.1
64211	0.782016	2.1
64212	1.06706	2.1
64213	1.11785	2.1
64214	0.782015	2.1
64215	0.946133	2.1
64216	0.878931	2.1
64217	0.946132	2.1
64218	1.89226	2.1
64219	1.89226	2.1
64220	1.89226	2.1
64221	1.89226	2.1
64222	1.89226	2.1
64223	1.89226	2.1
64224	0.946131	2.1
64225	0.946131	2.1
64226	0.946129	2.1
64227	0.946129	2.1
64228	0.946131	2.1
64229	0.946131	2.1
64230	0.94613	2.1
64231	0.94613	2.1
64232	0.946131	2.1
64233	0.946131	2.1
64234	0.94613	2.1
64235	0.94613	2.1
64236	0.827866	2.1
64237	0.946131	2.1
64238	0.946129	2.1
64239	0.946129	2.1
64240	0.94613	2.1
64241	0.94613	2.1
64242	0.94613	2.1
64243	0.94613	2.1
64244	0.827865	2.1
64245	0.94613	2.1
64246	0.94613	2.1
64247	0.94613	2.1
64248	1.89226	2.1
64249	1.89226	2.1
64250	1.89226	2.1
64251	1.89226	2.1
64252	3.78452	2.1
64253	1.89226	2.1
64254	1.89226	2.1
64255	1.89226	2.1
64256	1.89226	2.1
64257	1.89226	2.1
64258	1.89226	2.1
64259	1.89226	2.1
64260	1.89226	2.1
64261	1.89226	2.1

64262	1.89226	2.1
64263	3.78453	2.1
64264	1.89226	2.1
64265	1.89226	2.1
64266	1.89226	2.1
64267	1.89226	2.1
64268	3.78452	2.1
64269	0.744377	2.1
64270	0.541919	2.1
64271	0.652022	2.1
64272	0.544198	2.1
64273	0.764633	2.1
64274	0.673254	2.1
64275	1.89226	2.1
64276	1.31149	2.1
64277	1.05423	2.1
64278	1.02189	2.1
64279	0.870602	2.1
64280	1.89226	2.1
64281	1.89226	2.1
64282	1.89226	2.1
64283	1.89226	2.1
64284	1.89226	2.1
64285	1.89226	2.1
64286	1.89226	2.1
64287	1.89226	2.1
64288	1.89226	2.1
64289	1.89226	2.1
64290	1.89226	2.1
64291	1.89226	2.1
64292	1.89226	2.1
64293	1.89226	2.1
64294	1.89226	2.1
64295	1.89226	2.1
64296	1.89226	2.1
64297	1.89226	2.1
64298	0.815314	2.1
64299	0.815313	2.1
64300	0.714029	2.1
64301	0.782951	2.1
64302	0.810944	2.1
64303	0.782954	2.1
64304	1.89226	2.1
64305	1.30999	2.1
64306	1.08132	2.1
64307	1.03962	2.1
64308	0.95209	2.1
64309	1.89226	2.1
64310	1.89226	2.1
64311	1.89226	2.1
64312	1.89226	2.1
64313	1.89226	2.1
64314	1.89226	2.1
64315	1.89226	2.1
64316	1.89226	2.1
64317	1.89226	2.1
64318	1.89226	2.1
64319	1.89226	2.1

64320	1.89226	2.1
64321	1.89226	2.1
64322	1.89226	2.1
64323	1.89226	2.1
64324	1.89226	2.1
64325	1.89226	2.1
64326	1.89226	2.1
64327	0.871558	2.1
64328	0.713078	2.1
64329	0.756858	2.1
64330	0.703674	2.1
64331	0.838025	2.1
64332	0.862196	2.1
64333	1.89226	2.1
64334	1.33194	2.1
64335	1.10834	2.1
64336	1.06163	2.1
64337	1.01785	2.1
64338	1.89226	2.1
64339	1.89226	2.1
64340	1.89226	2.1
64341	1.89226	2.1
64342	3.78452	2.1
64343	1.89226	2.1
64344	1.89226	2.1
64345	1.89226	2.1
64346	1.89226	2.1
64347	1.89226	2.1
64348	1.89226	2.1
64349	1.89226	2.1
64350	1.89226	2.1
64351	1.89226	2.1
64352	1.89226	2.1
64353	1.89226	2.1
64354	1.89226	2.1
64355	0.958406	2.1
64356	1.1383	2.1
64357	0.865636	2.1
64358	1.10093	2.1
64359	0.838024	2.1
64360	0.975154	2.1
64361	1.89226	2.1
64362	1.38553	2.1
64363	1.10834	2.1
64364	1.11522	2.1
64365	1.15271	2.1
64366	1.89227	2.1
64367	1.89227	2.1
64368	1.89227	2.1
64369	1.89227	2.1
64370	3.78453	2.1
64371	0.946132	2.1
64372	0.946132	2.1
64373	0.946132	2.1
64374	0.946132	2.1
64375	0.946132	2.1
64376	0.946132	2.1
64377	0.946133	2.1

64378	0.946133	2.1
64379	0.946132	2.1
64380	0.946132	2.1
64381	0.946133	2.1
64382	0.946133	2.1
64383	0.719271	2.1
64384	0.774968	2.1
64385	1.28401	2.1
64386	1.17415	2.1
64387	0.960999	2.1
64388	0.960999	2.1
64389	0.988115	2.1
64390	0.988115	2.1
64391	0.774968	2.1
64392	1.28401	2.1
64393	0.719271	2.1
64394	1.17415	2.1
64395	2.77811	2.1
64396	2.34829	2.1
64397	2.56802	2.1
64398	2.52333	2.1
64399	2.56802	2.1
64400	2.52333	2.1
64401	1.89227	2.1
64402	1.89227	2.1
64403	1.89227	2.1
64404	1.89227	2.1
64405	3.78453	2.1
64406	1.89226	2.1
64407	1.89226	2.1
64408	1.89226	2.1
64409	1.89226	2.1
64410	1.89226	2.1
64411	1.89226	2.1
64412	1.89226	2.1
64413	2.77811	2.1
64414	1.89226	2.1
64415	2.56802	2.1
64416	1.89226	2.1
64417	2.56802	2.1
64418	1.89226	0.481685
64419	1.89226	1.6333
64420	1.89226	0.462453
64421	1.89226	0.77832
64422	3.78452	0.585953
64423	2.41802	0.656002
64424	1.95764	2.1
64425	1.95764	0.696276
64426	2.27727	2.1
64427	2.39311	0.695146
64428	2.73961	2.1
64429	0.946132	2.1
64430	0.946132	0.696635
64431	0.94613	0.72542
64432	0.94613	2.1
64433	0.946131	2.1
64434	0.946131	2.1
64435	0.946131	0.714148

64436	0.946131	0.688917
64437	0.946131	0.888791
64438	0.946131	0.9073
64439	0.946131	0.787104
64440	0.946131	0.803869
64441	1.89226	0.518717
64442	1.89226	0.707239
64443	1.89226	0.787644
64444	1.89226	0.506528
64445	1.89226	1.03648
64446	1.89226	0.51284
64447	2.78856	0.435732
64448	2.28552	0.697791
64449	2.28552	0.489605
64450	2.3931	0.631872
64451	2.6402	0.461818
64452	2.74778	0.614059
64453	2.39311	0.602384
64454	1.95764	2.1
64455	2.28552	0.654198
64456	1.86103	2.1
64457	4.24316	0.873292
64458	1.89226	0.652223
64459	1.89226	2.1
64460	1.89226	0.68794
64461	1.89226	2.1
64462	1.89226	0.690798
64463	1.89226	2.1
64464	1.47412	1.95571
64465	2.18862	1.46936
64466	2.07226	1.02867
64467	2.33645	1.18051
64468	4.26088	1.18433
64469	1.89226	0.942003
64470	1.89226	1.51403
64471	1.89226	1.08704
64472	1.89226	1.76441
64473	1.89226	1.33231
64474	1.89226	2.1
64475	1.89226	1.10835
64476	1.89226	2.1
64477	1.89226	0.616945
64478	1.89226	2.1
64479	3.78453	1.15151
64480	2.18862	1.09667
64481	2.1128	2.1
64482	2.33645	0.944366
64483	2.28188	2.1
64484	4.47051	1.76352
64485	2.07226	2.1
64486	2.33645	1.47788
64487	2.20831	0.914338
64488	2.4646	0.984859
64489	4.53686	1.11131
64490	0.946131	0.848397
64491	0.946131	2.1
64492	0.946131	2.1
64493	0.946131	1.16547

64494	0.94613	2.1
64495	0.94613	1.08077
64496	0.946133	2.01395
64497	0.946133	2.1
64498	0.946132	0.858577
64499	0.946132	0.807569
64500	0.946131	1.84576
64501	0.946131	1.34237
64502	1.89226	1.56607
64503	1.89226	2.1
64504	1.89226	0.881778
64505	1.89226	2.05893
64506	3.78453	1.39091
64507	2.33646	1.06831
64508	2.28189	2.1
64509	2.4646	0.854462
64510	2.42758	2.1
64511	4.74648	1.19964
64512	1.89226	2.1
64513	1.89226	2.1
64514	1.89226	2.1
64515	1.89226	2.1
64516	1.89226	2.1
64517	1.89226	2.1
64518	1.89226	2.1
64519	1.89226	2.1
64520	1.89226	2.1
64521	1.89226	2.1
64522	1.89226	2.1
64523	1.89226	2.1
64524	1.89226	2.1
64525	1.89226	2.1
64526	1.89226	2.1
64527	1.89226	2.1
64528	3.78453	2.1
64529	1.89226	2.1
64530	1.89226	2.1
64531	1.89226	2.1
64532	1.89226	2.1
64533	1.89226	2.1
64534	1.89226	2.1
64535	1.89226	2.1
64536	1.89226	2.1
64537	1.89226	2.1
64538	1.89226	2.1
64539	3.78452	2.1
64540	0.877238	2.1
64541	0.877238	2.1
64542	1.24746	2.1
64543	1.04764	2.1
64544	0.964969	2.1
64545	1.1521	2.1
64546	0.946132	2.1
64547	0.946132	2.1
64548	0.946132	2.1
64549	1.12659	2.1
64550	0.964969	2.1
64551	0.946131	2.1

64552	1.89226	2.1
64553	1.89226	2.1
64554	1.89226	2.1
64555	1.89226	2.1
64556	1.89226	2.1
64557	1.89226	2.1
64558	1.89226	2.1
64559	1.89226	2.1
64560	1.89226	2.1
64561	1.89226	2.1
64562	3.78453	2.1
64563	0.868144	2.1
64564	0.992762	2.1
64565	0.946131	2.1
64566	0.946131	2.1
64567	1.07072	2.1
64568	1.07072	2.1
64569	0.868171	2.1
64570	0.868171	2.1
64571	0.992762	2.1
64572	0.946131	2.1
64573	0.946131	2.1
64574	0.868144	2.1
64575	0.946132	2.1
64576	0.946132	2.1
64577	0.946131	2.1
64578	0.946131	2.1
64579	0.946132	2.1
64580	0.946132	2.1
64581	0.946132	2.1
64582	0.946132	2.1
64583	0.946132	2.1
64584	0.946132	2.1
64585	0.946132	2.1
64586	0.946132	2.1
64587	1.89227	2.1
64588	1.89227	2.1
64589	1.89227	2.1
64590	1.89227	2.1
64591	1.89227	2.1
64592	1.89227	2.1
64593	1.89226	2.1
64594	1.89226	2.1
64595	1.89226	2.1
64596	1.89226	2.1
64597	1.89226	2.1
64598	1.89226	2.1
64599	1.98552	2.1
64600	2.22298	2.1
64601	2.22298	2.1
64602	1.89226	2.1
64603	1.89226	2.1
64604	1.54525	2.1
64605	0.810946	2.1
64606	0.810946	2.1
64607	0.810947	2.1
64608	0.810947	2.1
64609	0.81096	2.1

64610	0.81096	2.1
64611	1.89226	2.1
64612	1.35168	2.1
64613	1.08132	2.1
64614	1.08132	2.1
64615	1.0813	2.1
64616	1.89226	2.1
64617	1.89226	1.75943
64618	1.89226	2.1
64619	1.89226	1.23721
64620	3.78452	2.1
64621	1.7753	2.1
64622	2.39703	1.76977
64623	1.90577	2.1
64624	2.03445	2.01525
64625	3.82335	2.1
64626	2.39703	1.82785
64627	2.47949	0.594915
64628	2.26051	2.03701
64629	2.36891	0.345306
64630	4.76594	0.773726
64631	1.89226	1.89243
64632	1.89226	0.577275
64633	1.89226	1.44328
64634	1.89226	0.499766
64635	3.78452	0.710321
64636	1.89226	2.1
64637	1.89226	1.88075
64638	1.89226	2.1
64639	1.89226	1.70656
64640	1.89226	2.1
64641	1.89226	1.60828
64642	0.883745	1.48389
64643	0.973143	2.1
64644	0.988173	2.1
64645	0.988173	1.18454
64646	0.941927	0.956781
64647	1.02971	1.59264
64648	0.854065	2.1
64649	0.82702	2.1
64650	0.847835	2.1
64651	0.981126	2.1
64652	1.02996	2.1
64653	1.10704	2.1
64654	2.28031	2.1
64655	2.26369	0.582488
64656	2.26369	2.1
64657	2.36891	0.619359
64658	2.30788	0.943787
64659	2.42455	0.60954
64660	1.89226	2.04229
64661	1.89226	0.58231
64662	1.89226	0.565173
64663	1.89226	1.94083
64664	1.89226	0.572125
64665	1.89226	1.80517
64666	2.71406	2.1
64667	1.83244	2.1

64668	1.83244	2.1
64669	2.07567	2.1
64670	2.52044	2.1
64671	2.76367	2.1
64672	1.89226	2.1
64673	1.89226	2.1
64674	1.89226	2.1
64675	1.89226	2.1
64676	3.78453	2.1
64677	1.89226	2.1
64678	1.89226	2.1
64679	1.89226	2.1
64680	1.89226	2.1
64681	1.89226	2.1
64682	1.89226	2.1
64683	2.70714	2.1
64684	2.3148	2.1
64685	2.3148	2.1
64686	2.52044	2.1
64687	2.54204	2.1
64688	2.74768	2.1
64689	2.52044	2.1
64690	2.3148	2.1
64691	1.83244	2.1
64692	1.83244	2.1
64693	1.62611	2.1
64694	2.3148	2.1
64695	0.946131	2.1
64696	0.946131	2.1
64697	0.946131	2.1
64698	0.946131	2.1
64699	0.946131	2.1
64700	0.946131	2.1
64701	0.946131	2.1
64702	0.946131	2.1
64703	0.946131	2.1
64704	0.946131	2.1
64705	0.946131	2.1
64706	0.946131	2.1
64707	1.89226	2.1
64708	1.89226	2.1
64709	1.89226	2.1
64710	1.89226	2.1
64711	3.78453	2.1
64712	1.89226	2.1
64713	1.89226	2.1
64714	1.89226	2.1
64715	1.89226	2.1
64716	1.89226	2.1
64717	1.89226	2.1
64718	1.89227	2.1
64719	1.89227	2.1
64720	1.89227	2.1
64721	1.89227	2.1
64722	3.78453	2.1
64723	1.89226	2.1
64724	1.89226	2.1
64725	1.89226	2.1

64726	1.89226	2.1
64727	3.78453	2.1
64728	0.915781	2.1
64729	0.863524	2.1
64730	0.717056	2.1
64731	0.830182	2.1
64732	0.865094	2.1
64733	0.918003	2.1
64734	1.89226	2.1
64735	1.29933	2.1
64736	1.11215	2.1
64737	1.05227	2.1
64738	0.966269	2.1
64739	1.89226	2.1
64740	1.89226	2.1
64741	1.89226	2.1
64742	1.89226	2.1
64743	1.89226	2.1
64744	1.89226	2.1
64745	1.89226	2.1
64746	1.89226	2.1
64747	1.89226	2.1
64748	1.89226	2.1
64749	1.89226	2.1
64750	1.89226	2.1
64751	1.89226	2.1
64752	1.89226	2.1
64753	1.89226	2.1
64754	1.89226	2.1
64755	1.89226	2.1
64756	1.89226	2.1
64757	1.01725	2.1
64758	0.997048	2.1
64759	0.818657	2.1
64760	0.960724	2.1
64761	0.838013	2.1
64762	1.04859	2.1
64763	1.89226	2.1
64764	1.41754	2.1
64765	1.17625	2.1
64766	1.0793	2.1
64767	1.18822	2.1
64768	2.16402	2.1
64769	1.89452	2.1
64770	1.50084	2.1
64771	2.09288	2.1
64772	3.9874	2.1
64773	2.16402	2.1
64774	2.13925	2.1
64775	2.04483	2.1
64776	2.09288	2.1
64777	4.23212	2.1
64778	1.89226	2.1
64779	1.89226	2.1
64780	1.89226	2.1
64781	1.89226	2.1
64782	3.78452	2.1
64783	1.89226	2.1

64784	1.89226	2.1
64785	1.89226	2.1
64786	1.89226	2.1
64787	1.89226	2.1
64788	1.89226	2.1
64789	1.9824	2.1
64790	2.09288	2.1
64791	1.69889	2.1
64792	1.6817	2.1
64793	2.00695	2.1
64794	2.00695	2.1
64795	1.93592	2.1
64796	2.09288	2.1
64797	2.00695	2.1
64798	2.09288	2.1
64799	1.9589	2.1
64800	2.04483	2.1
64801	0.946128	2.1
64802	0.946128	2.1
64803	0.946132	2.1
64804	0.946132	2.1
64805	0.94613	2.1
64806	0.94613	2.1
64807	0.946129	2.1
64808	0.946129	2.1
64809	0.94613	2.1
64810	0.94613	2.1
64811	0.94613	2.1
64812	0.94613	2.1
64813	0.946131	2.1
64814	0.946131	2.1
64815	0.946131	2.1
64816	0.946131	2.1
64817	0.946131	2.1
64818	0.946131	2.1
64819	0.946131	2.1
64820	0.946131	2.1
64821	0.946131	2.1
64822	0.946131	2.1
64823	0.946131	2.1
64824	0.946131	2.1
64825	1.89226	1.06572
64826	2.48083	2.1
64827	2.6968	0.945743
64828	2.6968	2.1
64829	1.89226	0.980658
64830	1.89226	2.1
64831	1.89226	1.01051
64832	1.89226	2.1
64833	1.89226	1.08762
64834	1.89226	2.1
64835	3.78453	2.1
64836	1.89226	2.1
64837	1.89226	2.1
64838	1.89226	2.1
64839	1.89226	2.1
64840	1.89226	2.1
64841	1.89226	2.1

64842	1.1017	2.1
64843	1.1017	2.1
64844	1.10605	2.1
64845	1.01647	2.1
64846	0.868255	2.1
64847	0.883424	2.1
64848	1.24624	2.1
64849	1.35473	2.1
64850	0.942231	2.1
64851	1.31464	2.1
64852	0.910064	2.1
64853	1.27147	2.1
64854	1.89226	1.05941
64855	1.89226	2.1
64856	1.89226	0.745883
64857	1.89226	2.1
64858	3.78453	2.06415
64859	1.89226	2.1
64860	1.89226	2.1
64861	1.89226	2.1
64862	1.89226	2.1
64863	1.89226	2.1
64864	1.89226	2.1
64865	1.89226	2.1
64866	1.89226	2.1
64867	1.89226	2.1
64868	1.89226	2.1
64869	3.78453	2.1
64870	1.89226	2.1
64871	2.70015	2.1
64872	1.89226	2.1
64873	2.61064	2.1
64874	4.59241	2.1
64875	2.70015	2.1
64876	2.3372	2.1
64877	2.61064	2.1
64878	1.85184	2.1
64879	4.55198	2.1
64880	1.89226	2.1
64881	1.89226	2.1
64882	1.89226	2.1
64883	1.89226	2.1
64884	1.89226	2.1
64885	1.89226	2.1
64886	1.89227	2.1
64887	1.89227	2.1
64888	1.89227	2.1
64889	1.89227	2.1
64890	3.78453	2.1
64891	1.89226	2.1
64892	2.52194	2.1
64893	2.61064	2.1
64894	2.61064	2.1
64895	1.89226	2.1
64896	1.89226	2.1
64897	1.095 2.1	
64898	1.095 2.1	
64899	1.09189	2.1

64900	1.09189	2.1
64901	0.875334	2.1
64902	0.925919	2.1
64903	1.26097	2.1
64904	1.30532	2.1
64905	1.26097	2.1
64906	0.875334	2.1
64907	1.30532	2.1
64908	0.92592	2.1
64909	1.89226	2.1
64910	1.89226	2.1
64911	1.89226	2.1
64912	1.89226	2.1
64913	1.89226	2.1
64914	1.89226	2.1
64915	1.89226	2.1
64916	1.89226	2.1
64917	1.89226	2.1
64918	1.89226	2.1
64919	3.78453	2.1
64920	1.26097	2.1
64921	1.26097	2.1
64922	0.946132	2.1
64923	0.946132	2.1
64924	1.22176	2.1
64925	1.26097	2.1
64926	0.946132	2.1
64927	0.946132	2.1
64928	0.946132	2.1
64929	1.22176	2.1
64930	1.26097	2.1
64931	0.946132	2.1
64932	2.52194	2.1
64933	1.67381	2.1
64934	1.67381	2.1
64935	1.75067	2.1
64936	2.44352	2.1
64937	2.52038	2.1
64938	1.89226	2.1
64939	1.89226	2.1
64940	1.89226	2.1
64941	1.89226	2.1
64942	1.89226	2.1
64943	1.89226	2.1
64944	1.89227	2.1
64945	1.89227	2.1
64946	1.89227	2.1
64947	1.89227	2.1
64948	1.89227	2.1
64949	1.89227	2.1
64950	1.22176	2.1
64951	1.22176	2.1
64952	0.946132	2.1
64953	0.946132	2.1
64954	1.19095	2.1
64955	1.22176	2.1
64956	0.946133	2.1
64957	0.946133	2.1

64958	1.19095	2.1
64959	0.946132	2.1
64960	0.946133	2.1
64961	1.22176	2.1
64962	2.44352	2.1
64963	1.6287	2.1
64964	1.6287	2.1
64965	1.67381	2.1
64966	2.3819	2.1
64967	2.427	2.1
64968	1.89226	2.1
64969	1.89226	2.1
64970	1.89226	2.1
64971	1.89226	2.1
64972	1.89226	2.1
64973	1.89226	2.1
64974	1.89226	1.78225
64975	1.89226	2.1
64976	1.89226	1.68842
64977	1.89226	2.1
64978	3.78453	2.1
64979	1.89226	2.02646
64980	1.89226	2.1
64981	1.89226	2.0179
64982	1.89226	2.1
64983	1.89226	1.25105
64984	1.89226	2.1
64985	1.89226	2.1
64986	1.89226	2.1
64987	1.89226	2.1
64988	1.89226	2.1
64989	1.89226	2.1
64990	1.89226	2.1
64991	0.851542	2.1
64992	0.851542	2.1
64993	1.1736	2.1
64994	1.29261	2.1
64995	0.852708	2.1
64996	1.18843	2.1
64997	0.946132	2.1
64998	0.946132	2.1
64999	0.946132	2.1
65000	1.07901	2.1
65001	1.04208	2.1
65002	0.936669	2.1
65003	1.89227	1.0374
65004	1.89227	2.1
65005	1.89227	1.15149
65006	1.89227	2.1
65007	3.78453	2.1
65008	1.89226	1.85901
65009	1.89226	2.1
65010	1.89226	1.28809
65011	1.89226	2.1
65012	3.78453	2.1
65013	1.89226	2.1
65014	1.89226	2.1
65015	1.89226	2.1

65016	1.89226	2.1
65017	1.89226	2.1
65018	1.89226	2.1
65019	0.946133	2.1
65020	0.946133	2.1
65021	1.28525	2.1
65022	1.28525	2.1
65023	1.07901	2.1
65024	1.28525	2.1
65025	0.946132	2.1
65026	0.946132	2.09899
65027	0.946132	2.1
65028	1.28525	2.1
65029	0.946133	2.1
65030	1.07901	2.1
65031	1.89226	2.1
65032	1.89226	2.1
65033	1.89226	2.1
65034	1.89226	2.1
65035	1.89226	2.1
65036	1.89226	2.1
65037	1.89226	2.1
65038	1.89226	2.1
65039	1.89226	2.1
65040	1.89226	2.1
65041	1.89226	2.1
65042	1.89226	2.1
65043	1.24419	2.1
65044	1.09093	2.1
65045	1.22722	2.1
65046	1.18154	2.1
65047	1.27455	2.1
65048	1.08473	2.1
65049	1.15541	2.1
65050	1.15541	2.1
65051	1.29607	2.1
65052	1.23814	2.1
65053	1.13024	2.1
65054	1.12096	2.1
65055	1.18858	2.1
65056	1.13181	2.1
65057	0.988718	2.1
65058	0.802498	2.1
65059	0.994034	2.1
65060	1.15773	2.1
65061	1.0029	2.1
65062	1.0029	2.1
65063	1.23814	2.1
65064	0.997523	2.1
65065	1.11498	2.1
65066	0.899532	2.1
65067	1.89226	2.1
65068	1.89226	2.1
65069	1.89226	2.1
65070	1.89226	2.1
65071	1.89226	2.1
65072	1.89226	2.1
65073	1.89226	2.1

65074	1.89226	2.1
65075	1.89226	2.1
65076	1.89226	2.1
65077	1.89226	2.1
65078	1.89226	2.1
65079	0.993139	2.1
65080	0.834108	2.1
65081	1.01147	2.1
65082	1.01358	2.1
65083	1.20067	2.1
65084	0.993156	2.1
65085	2.4158	2.1
65086	1.69956	2.1
65087	1.3544	2.1
65088	1.60095	2.1
65089	1.11219	2.1
65090	1.89226	2.1
65091	1.89226	2.1
65092	1.89226	2.05393
65093	1.89226	2.1
65094	3.78452	2.1
65095	1.89226	2.1
65096	1.89226	2.1
65097	1.89226	2.1
65098	1.89226	2.1
65099	1.89226	2.1
65100	1.89226	2.1
65101	1.89226	2.1
65102	2.83014	2.1
65103	2.32404	2.1
65104	2.32404	2.1
65105	1.89226	2.1
65106	1.89226	2.1
65107	1.37966	2.1
65108	1.13294	2.1
65109	0.881723	2.1
65110	0.582304	2.1
65111	0.977944	2.1
65112	0.977948	2.1
65113	1.0264	2.1
65114	1.0264	2.1
65115	1.37967	2.1
65116	1.03896	2.1
65117	1.19108	2.1
65118	0.582298	2.1
65119	1.89226	1.50461
65120	1.89226	2.1
65121	1.89226	2.1
65122	1.89226	2.1
65123	3.78452	2.1
65124	0.946131	2.1
65125	0.946131	2.1
65126	0.946131	2.1
65127	0.946131	2.1
65128	0.946132	2.1
65129	0.946132	2.1
65130	0.946129	2.1
65131	0.946129	2.1

65132	0.946131	2.1
65133	0.946131	2.1
65134	0.94613	2.1
65135	0.94613	2.1
65136	1.89226	2.1
65137	1.89226	1.84887
65138	1.89226	2.1
65139	1.89226	2.1
65140	3.78453	2.1
65141	1.89226	2.1
65142	1.89226	2.1
65143	1.89226	2.1
65144	1.89226	2.1
65145	1.89226	2.1
65146	1.89226	2.1
65147	2.65216	2.1
65148	1.93528	2.1
65149	1.78595	2.1
65150	2.48544	2.1
65151	4.42072	2.1
65152	1.89226	2.1
65153	1.89226	2.1
65154	1.89226	2.1
65155	1.89226	2.1
65156	1.89226	2.1
65157	1.89226	2.1
65158	1.89226	2.1
65159	1.89226	2.1
65160	1.89226	2.1
65161	1.89226	2.1
65162	3.78452	2.1
65163	1.66537	2.1
65164	2.48544	2.1
65165	1.78595	2.1
65166	1.78595	2.1
65167	2.36197	2.1
65168	2.36197	2.1
65169	2.80975	2.1
65170	2.48544	2.1
65171	2.36198	2.1
65172	2.65135	2.1
65173	5.13679	2.1
65174	1.89226	2.1
65175	1.89226	2.1
65176	1.89226	2.1
65177	1.89226	2.1
65178	1.89226	2.1
65179	1.89226	2.1
65180	0.946127	2.1
65181	0.946127	2.1
65182	0.946131	2.1
65183	0.946131	2.1
65184	0.946128	2.1
65185	0.946128	2.1
65186	0.94613	2.1
65187	0.94613	2.1
65188	0.946129	2.1
65189	0.946129	2.1

65190	0.946129	2.1
65191	0.946129	2.1
65192	1.11972	2.1
65193	1.18099	2.1
65194	0.832687	2.1
65195	0.764517	2.1
65196	0.974426	2.1
65197	0.974426	2.1
65198	0.971078	2.1
65199	0.971078	2.1
65200	1.11972	2.1
65201	0.764515	2.1
65202	0.832686	2.1
65203	1.18099	2.1
65204	2.36198	2.1
65205	2.48025	2.1
65206	2.48025	2.1
65207	2.65135	2.1
65208	2.23944	2.1
65209	2.41055	2.1
65210	0.946132	1.07822
65211	0.946132	2.1
65212	0.946131	2.1
65213	0.946131	2.1
65214	0.946131	2.1
65215	0.946131	1.67973
65216	0.946132	2.1
65217	0.946132	2.1
65218	0.946131	2.1
65219	0.946131	2.1
65220	0.946132	2.1
65221	0.946132	2.1
65222	0.810942	2.1
65223	0.810942	2.1
65224	0.810944	2.1
65225	0.810944	2.1
65226	1.89226	2.1
65227	1.3517	2.1
65228	1.08132	2.1
65229	1.08133	2.1
65230	1.08132	2.1
65231	0.810932	2.1
65232	0.810932	2.1
65233	1.89249	2.08212
65234	1.9857	1.61781
65235	2.02433	1.3077
65236	2.15807	1.31035
65237	4.01003	1.43247
65238	1.89226	1.25096
65239	1.89226	1.9266
65240	1.89226	1.60265
65241	1.89226	0.961616
65242	3.78452	1.88995
65243	1.89226	1.72195
65244	1.89226	1.67535
65245	1.89226	0.809052
65246	1.89226	1.49206
65247	3.78452	0.991915

65248	1.98569	1.29367
65249	1.86067	1.65406
65250	2.15807	1.17579
65251	2.07965	1.13219
65252	4.06534	1.88109
65253	2.02433	1.49765
65254	2.15807	1.97499
65255	2.12608	2.1
65256	2.3025	1.01828
65257	4.32683	1.95393
65258	1.89226	1.74049
65259	1.89226	1.36137
65260	1.89226	0.98118
65261	1.89226	1.11378
65262	3.78452	1.09676
65263	1.89226	1.01326
65264	1.89226	2.00622
65265	1.89226	1.12127
65266	1.89226	1.10311
65267	3.78452	1.78197
65268	2.15807	1.76432
65269	2.07965	1.59232
65270	2.3025	0.979894
65271	2.28384	1.23738
65272	4.38215	1.05371
65273	1.89226	0.679567
65274	1.89226	0.397431
65275	1.89226	0.555708
65276	1.89226	0.418569
65277	3.78453	0.49355
65278	2.16905	0.437951
65279	2.56369	0.393171
65280	1.88394	0.533921
65281	2.41938	0.173056
65282	4.44763	0.424673
65283	2.56369	0.369524
65284	2.80663	0.459873
65285	2.41938	0.20691
65286	2.69045	0.382202
65287	5.25414	0.37377
65288	1.89226	0.369659
65289	1.89226	0.369952
65290	1.89226	0.415231
65291	1.89226	0.388575
65292	3.78453	0.339801
65293	1.89226	0.524889
65294	1.89226	0.418121
65295	1.89226	0.652927
65296	1.89226	0.419356
65297	1.89226	0.617106
65298	1.89226	0.388162
65299	1.01988	0.15971
65300	1.01988	0.355704
65301	1.03333	0.492251
65302	1.03333	0.410874
65303	1.13811	0.333957
65304	1.20969	0.249455
65305	0.941974	0.499654

65306	0.843515	0.272664
65307	0.843514	0.231125
65308	1.13811	0.207547
65309	0.941973	0.390621
65310	1.20969	0.363055
65311	2.41938	0.222153
65312	2.57414	0.379191
65313	2.57414	0.431234
65314	2.69045	0.458666
65315	2.27623	0.221658
65316	2.39253	0.498669
65317	1.89227	0.40525
65318	1.89227	0.38375
65319	1.89227	0.349703
65320	1.89227	0.35935
65321	1.89227	0.35038
65322	1.89227	0.365613
65323	2.28385	1.3555
65324	2.09114	1.53638
65325	2.46968	0.814657
65326	2.32438	1.3482
65327	4.56082	0.895441
65328	1.89226	0.889688
65329	1.89226	1.96061
65330	1.89226	1.02408
65331	1.89226	1.35885
65332	3.78452	1.42915
65333	1.89226	0.957316
65334	1.89226	1.96525
65335	1.89226	0.901353
65336	1.89226	1.42382
65337	3.78452	1.09333
65338	2.09114	0.828721
65339	1.5249	1.91361
65340	2.55684	0.848851
65341	1.86899	1.57337
65342	3.96011	1.2202
65343	2.46968	0.995505
65344	2.32439	1.72302
65345	2.62768	1.02506
65346	2.506	1.30881
65347	4.97568	1.89267
65348	1.89226	1.31358
65349	1.89226	1.4193
65350	1.89226	0.691682
65351	1.89226	1.40134
65352	3.78452	0.928602
65353	1.89226	0.889612
65354	1.89226	1.44245
65355	1.89226	1.02895
65356	1.89226	2.1
65357	1.89226	0.709311
65358	1.89226	2.1
65359	2.55684	1.02913
65360	2.16506	1.31378
65361	1.86901	1.6751
65362	1.86899	1.0016
65363	2.75664	1.16888

65364	2.75662	0.879218
65365	1.89224	0.11195
65366	1.89224	0.446779
65367	1.89224	0.200594
65368	1.89224	0.553966
65369	3.78447	0.247298
65370	2.24206	0.227522
65371	2.38862	0.643362
65372	2.07107	0.219912
65373	2.23097	0.508888
65374	4.47303	0.445881
65375	2.38863	0.646505
65376	2.25945	0.713649
65377	2.25945	0.546203
65378	2.39244	1.98889
65379	2.23098	0.512062
65380	2.36397	2.1
65381	1.89224	0.347104
65382	1.89224	0.75642
65383	1.89224	0.613255
65384	1.89224	0.544694
65385	1.89224	0.591803
65386	1.89224	0.54803
65387	1.89226	0.191302
65388	1.89226	0.507297
65389	1.89226	0.481126
65390	1.89226	0.422363
65391	1.89226	0.292305
65392	1.89226	0.360411
65393	0.920975	0.750111
65394	0.920979	0.257036
65395	1.03015	0.517276
65396	1.03015	0.141824
65397	0.992407	0.428866
65398	1.08759	0.681083
65399	0.854346	0.237474
65400	0.87241	0.194378
65401	0.872397	0.428355
65402	1.04334	0.417531
65403	1.00965	0.380419
65404	1.0876	0.361653
65405	2.23101	0.635248
65406	2.25948	0.756731
65407	2.03577	0.478996
65408	2.05257	2.1
65409	4.29524	0.661051
65410	0.810947	0.259527
65411	0.810947	0.601537
65412	0.810947	0.496993
65413	0.810947	0.385697
65414	0.810934	0.533964
65415	0.810934	0.546431
65416	1.89227	0.786681
65417	1.3517	0.619624
65418	1.08132	0.555477
65419	1.08132	0.606036
65420	1.08133	0.552816
65421	1.89226	1.08385

65422	1.89226	1.7188
65423	1.89226	0.892464
65424	1.89226	2.1
65425	1.89226	0.666486
65426	1.89226	2.1
65427	1.89226	1.15232
65428	1.89226	1.44499
65429	1.89226	0.89649
65430	1.89226	2.1
65431	1.89226	0.853638
65432	1.89226	2.1
65433	0.879432	1.97094
65434	0.879432	1.01218
65435	0.900223	0.648328
65436	0.900223	2.09613
65437	0.54571	1.88925
65438	0.743092	1.25727
65439	1.03656	1.94183
65440	1.19234	0.781727
65441	1.19234	1.23736
65442	0.743092	1.56358
65443	1.03656	1.61884
65444	0.545711	1.22228
65445	1.89226	0.76774
65446	2.38467	1.70452
65447	2.69293	0.77887
65448	2.69293	2.02563
65449	1.89226	0.681566
65450	1.89226	1.55927
65451	1.89226	0.913254
65452	1.89226	1.59413
65453	1.89226	0.793549
65454	1.89226	1.84731
65455	3.78452	1.15256
65456	0.946129	2.1
65457	0.946129	1.58082
65458	0.946133	0.730573
65459	0.946133	2.1
65460	0.946131	1.04132
65461	0.946131	2.1
65462	0.946131	0.705903
65463	0.946131	1.14657
65464	0.946131	1.2131
65465	0.946131	1.25391
65466	0.946131	1.0913
65467	0.946131	1.03102
65468	1.04846	0.674233
65469	1.04846	1.79746
65470	1.05594	0.707523
65471	1.05594	2.06133
65472	0.743092	1.63598
65473	0.912061	1.09005
65474	1.19234	0.728987
65475	1.34647	0.883433
65476	0.912061	0.748631
65477	1.34646	1.01388
65478	1.19234	0.893508
65479	0.743093	0.908228

65480	1.89226	0.878511
65481	1.89226	1.79931
65482	1.89226	0.664877
65483	1.89226	1.00432
65484	3.78452	0.983155
65485	0.946128	2.1
65486	0.946128	1.0154
65487	0.946133	1.40358
65488	0.946133	0.764555
65489	0.94613	2.1
65490	0.94613	1.95531
65491	0.946131	1.17226
65492	0.946131	0.822032
65493	0.946131	1.68115
65494	0.946131	1.64557
65495	0.946131	1.03079
65496	0.946131	1.07256
65497	1.89226	1.12886
65498	1.89226	2.1
65499	1.89226	0.866997
65500	1.89226	1.29764
65501	3.78452	1.50618
65502	1.89226	1.01823
65503	1.89226	1.2189
65504	1.89226	0.941853
65505	1.89226	2.1
65506	1.89226	0.616323
65507	1.89226	2.1
65508	1.89226	0.981054
65509	1.89226	1.39068
65510	1.89226	0.843095
65511	1.89226	2.1
65512	1.89226	0.751057
65513	1.89226	2.1
65514	0.946132	2.1
65515	0.946132	1.04862
65516	1.17758	0.765572
65517	1.17759	2.1
65518	1.04514	2.07706
65519	1.17758	1.02482
65520	0.946133	0.737967
65521	0.946133	0.922748
65522	0.946107	1.06258
65523	1.17755	1.07938
65524	1.04517	1.12207
65525	0.946158	1.03346
65526	1.89226	2.1
65527	2.10171	2.1
65528	1.95064	2.1
65529	2.10171	2.1
65530	1.7412	2.1
65531	1.89226	2.1
65532	1.89226	2.1
65533	1.89226	2.1
65534	1.89226	2.1
65535	1.89226	2.1
65536	1.89226	2.1
65537	1.89226	2.1

65538	1.89226	2.1
65539	1.89226	0.775142
65540	1.89226	2.1
65541	1.89226	1.90001
65542	3.78453	2.1
65543	0.946133	2.1
65544	0.946133	1.47625
65545	0.946132	2.1
65546	0.946132	2.1
65547	0.946133	2.1
65548	0.946133	1.91694
65549	0.946132	2.1
65550	0.946132	2.1
65551	0.946132	2.1
65552	0.946132	2.1
65553	0.946132	2.1
65554	0.946132	2.1
65555	1.70303	2.1
65556	1.95064	2.1
65557	1.74039	2.1
65558	1.95064	2.1
65559	1.69368	2.1
65560	1.89226	2.1
65561	1.89226	2.1
65562	1.89226	2.1
65563	1.89226	2.1
65564	1.89226	2.1
65565	3.78453	2.1
65566	0.946132	0.736685
65567	0.946132	2.1
65568	0.946132	2.1
65569	0.946132	1.988
65570	0.946133	1.65036
65571	0.946133	0.779305
65572	0.946132	2.1
65573	0.946132	2.1
65574	0.946107	2.1
65575	0.946107	2.1
65576	0.946158	2.1
65577	0.946158	2.1
65578	0.946133	2.1
65579	0.946133	2.1
65580	0.946132	2.1
65581	0.946132	2.1
65582	0.946133	2.1
65583	0.946133	1.38461
65584	0.946132	2.1
65585	0.946132	2.1
65586	0.946107	2.1
65587	0.946107	2.1
65588	0.946158	2.1
65589	0.946158	2.1
65590	1.89226	2.1
65591	1.89226	2.1
65592	1.89226	1.17035
65593	1.89226	2.1
65594	1.89226	1.08174
65595	1.89226	2.1

65596	1.89226	2.1
65597	1.89226	2.1
65598	1.89226	2.1
65599	1.89226	2.1
65600	1.89226	2.09982
65601	1.89226	2.1
65602	2.5198	2.1
65603	2.45893	2.1
65604	2.27322	2.1
65605	2.27322	2.1
65606	2.63576	2.1
65607	2.63576	2.1
65608	1.24045	2.1
65609	1.11906	2.1
65610	1.13578	2.1
65611	0.916205	2.1
65612	1.12539	2.1
65613	1.08404	2.1
65614	1.08484	2.1
65615	1.08484	2.1
65616	1.22836	2.1
65617	0.919363	2.1
65618	1.16507	2.1
65619	0.924864	2.1
65620	1.89226	2.1
65621	1.89226	1.86194
65622	1.89226	0.909146
65623	1.89226	2.1
65624	1.89226	0.845775
65625	1.89226	2.1
65626	1.89226	2.1
65627	1.89226	2.1
65628	1.89226	2.1
65629	1.89226	2.1
65630	1.89226	2.04326
65631	1.89226	2.1
65632	2.6946	2.1
65633	2.63576	2.1
65634	2.5198	2.1
65635	2.5198	2.1
65636	2.8362	2.1
65637	2.8362	2.1
65638	1.3265	2.1
65639	1.24045	2.1
65640	0.983223	1.63901
65641	0.954429	2.1
65642	1.06046	2.1
65643	1.10594	2.1
65644	1.1915	1.51138
65645	1.1915	2.1
65646	1.31357	2.1
65647	1.09005	2.1
65648	1.29145	2.1
65649	0.995825	2.1
65650	1.89226	2.1
65651	1.89226	2.1
65652	1.89226	2.1
65653	1.89226	2.1

65654	3.78452	2.1
65655	1.89226	2.1
65656	1.89226	2.1
65657	1.89226	2.1
65658	1.89226	2.1
65659	3.78453	2.1
65660	1.91802	2.1
65661	2.27409	2.1
65662	2.48048	2.1
65663	2.22264	2.1
65664	1.91802	2.1
65665	1.66018	2.1
65666	1.13704	2.1
65667	1.24023	2.1
65668	1.22947	2.1
65669	1.31788	2.1
65670	1.23859	2.1
65671	1.23859	2.1
65672	1.23111	2.1
65673	1.23111	2.1
65674	1.24024	2.1
65675	1.31788	2.1
65676	1.13704	2.1
65677	1.22947	2.1
65678	1.89226	2.1
65679	1.89226	2.1
65680	1.89226	2.1
65681	1.89226	2.1
65682	1.89226	2.1
65683	1.89226	2.1
65684	1.89226	2.1
65685	1.89226	2.1
65686	1.89226	2.1
65687	1.89226	2.1
65688	3.78453	2.1
65689	1.05568	2.1
65690	0.959008	2.1
65691	1.32068	2.1
65692	1.24024	2.1
65693	1.15206	2.1
65694	1.15206	2.1
65695	1.14386	2.1
65696	1.14386	2.1
65697	1.05568	2.1
65698	1.32068	2.1
65699	0.959008	2.1
65700	1.24024	2.1
65701	1.32068	2.1
65702	1.24023	2.1
65703	1.31788	2.1
65704	1.4181	2.1
65705	1.31436	2.1
65706	1.31436	2.1
65707	1.3242	2.1
65708	1.3242	2.1
65709	1.32068	2.1
65710	1.4181	2.1
65711	1.24024	2.1

65712	1.31788	2.1
65713	1.89226	0.859261
65714	1.89226	0.441339
65715	1.89226	0.61082
65716	1.89226	0.61062
65717	1.89226	0.601333
65718	1.89226	0.658521
65719	1.02925	0.32939
65720	1.16678	0.471748
65721	0.946132	0.439765
65722	0.946132	0.743262
65723	0.935677	0.4923
65724	0.935677	0.371689
65725	1.18902	0.624867
65726	1.21562	0.567568
65727	1.13433	0.433258
65728	1.04071	0.449744
65729	0.851553	0.505996
65730	1.06075	0.45631
65731	1.89226	0.4918
65732	1.89226	0.440704
65733	1.89226	0.399953
65734	1.89226	0.331065
65735	1.89226	0.41237
65736	1.89226	0.542625
65737	1.89226	0.631294
65738	1.89226	0.222495
65739	1.89226	0.458069
65740	1.89226	0.447479
65741	3.78453	0.423478
65742	0.946128	0.536693
65743	0.946128	0.467392
65744	0.946133	0.440004
65745	0.946133	0.394398
65746	0.946131	0.398346
65747	0.946131	0.457147
65748	0.94613	0.507903
65749	0.94613	0.439426
65750	0.946131	0.469675
65751	0.946131	0.418728
65752	0.946131	0.355642
65753	0.946131	0.369572
65754	0.946132	0.581161
65755	0.946132	0.418645
65756	0.946132	0.621083
65757	0.946132	0.433942
65758	0.946132	0.480921
65759	0.946132	0.293148
65760	0.946131	0.521977
65761	0.946131	0.600463
65762	0.946132	0.490896
65763	0.946132	0.595616
65764	0.946132	0.336982
65765	0.946132	0.485841
65766	0.946131	2.03298
65767	0.946131	1.0355
65768	0.946131	2.1
65769	0.946131	0.768939

65770	0.946131	0.891267
65771	0.946131	1.85646
65772	0.946131	0.876819
65773	0.946131	0.72954
65774	0.946131	1.00482
65775	0.946131	1.00658
65776	0.946131	1.09086
65777	0.946131	1.06552
65778	1.89226	0.923681
65779	1.89226	1.16596
65780	1.89226	0.792562
65781	1.89226	2.1
65782	1.89226	0.724848
65783	1.89226	2.1
65784	1.89226	0.898917
65785	1.89226	0.917952
65786	1.89226	0.956266
65787	1.89226	1.96275
65788	1.89226	0.769364
65789	1.89226	2.1
65790	1.89226	0.994545
65791	1.89226	0.923945
65792	1.89226	0.734208
65793	1.89226	2.1
65794	1.89226	0.627685
65795	1.89226	2.1
65796	1.89226	1.39625
65797	1.89226	2.1
65798	1.89226	0.802157
65799	1.89226	2.1
65800	3.78452	1.37541
65801	0.946131	1.97843
65802	0.946131	0.810225
65803	1.17195	0.771339
65804	1.17195	2.1
65805	0.958489	2.1
65806	1.17195	1.33632
65807	0.946129	0.791322
65808	0.946129	1.13368
65809	0.946131	1.0886
65810	1.17195	1.10526
65811	0.958487	1.16155
65812	0.94613	1.13672
65813	2.69656	2.1
65814	2.17516	2.09566
65815	2.41885	2.1
65816	2.41885	1.17094
65817	2.46476	2.1
65818	2.46476	0.808152
65819	2.69657	1.93107
65820	2.30233	0.599883
65821	2.30233	1.24026
65822	2.59476	0.769908
65823	2.46477	1.19136
65824	2.7572	0.818551
65825	1.89226	1.60445
65826	1.89226	0.460734
65827	1.89226	0.524201

65828	1.89226	0.581021
65829	1.89226	0.502069
65830	1.89226	0.636836
65831	1.89226	1.25296
65832	1.89226	0.748415
65833	1.89226	1.88189
65834	1.89226	0.504238
65835	3.78452	0.805749
65836	2.46477	1.86509
65837	2.17516	2.01412
65838	1.80252	2.1
65839	2.09715	0.653208
65840	4.27231	1.99958
65841	2.46477	1.90082
65842	2.09715	0.675386
65843	2.30233	0.78679
65844	2.30233	0.753539
65845	1.96416	0.519573
65846	2.09715	0.520794
65847	1.89227	0.660507
65848	1.89227	0.41459
65849	1.89227	0.514668
65850	1.89227	0.516317
65851	1.89227	0.614259
65852	1.89227	0.718337
65853	1.89226	1.65269
65854	1.89226	0.425507
65855	1.89226	0.636621
65856	1.89226	0.651054
65857	3.78452	0.643786
65858	0.946132	2.1
65859	0.946132	2.1
65860	0.946132	2.1
65861	0.946132	2.1
65862	0.946132	2.1
65863	0.946132	2.1
65864	0.946132	2.1
65865	0.946132	2.1
65866	0.946132	2.1
65867	0.946132	2.1
65868	0.946131	2.1
65869	0.946131	2.1
65870	1.89226	2.1
65871	1.89226	2.1
65872	1.89226	2.1
65873	1.89226	2.1
65874	3.78453	2.1
65875	1.89226	2.1
65876	1.89226	2.1
65877	1.89226	2.1
65878	1.89226	2.1
65879	1.89226	2.1
65880	1.89226	2.1
65881	1.89226	2.1
65882	1.63707	2.1
65883	1.63707	2.1
65884	1.38859	2.1
65885	1.89226	2.1

65886	1.64377	2.1
65887	0.94613	2.1
65888	0.94613	2.1
65889	0.94613	2.1
65890	0.94613	2.1
65891	0.946131	2.1
65892	0.946131	2.1
65893	0.946129	2.1
65894	0.946129	2.1
65895	0.94613	2.1
65896	0.94613	2.1
65897	0.94613	2.1
65898	0.94613	2.1
65899	2.15888	2.1
65900	1.99309	2.1
65901	2.16841	2.1
65902	2.16841	2.1
65903	2.02311	0.780948
65904	2.02311	0.714834
65905	1.89226	0.815008
65906	1.89226	1.02692
65907	1.89226	2.1
65908	1.89226	0.897981
65909	1.89226	1.81963
65910	1.89226	0.690739
65911	1.89226	1.42783
65912	1.89226	2.1
65913	1.89226	2.1
65914	1.89226	1.16389
65915	1.89226	2.1
65916	1.89226	1.61027
65917	1.99309	2.1
65918	1.96035	2.1
65919	1.72526	2.1
65920	1.72526	1.65142
65921	2.16841	2.1
65922	2.16841	2.1
65923	2.15888	2.1
65924	2.16841	2.1
65925	2.3111	2.1
65926	2.35938	2.1
65927	4.51826	2.1
65928	1.89226	1.90773
65929	1.89226	0.785739
65930	1.89226	0.689826
65931	1.89226	0.349919
65932	3.78452	1.9626
65933	1.89226	1.1626
65934	1.89226	2.1
65935	1.89226	2.1
65936	1.89226	0.576444
65937	3.78452	2.1
65938	2.16841	2.1
65939	1.96035	2.1
65940	2.35938	2.1
65941	2.18493	2.1
65942	4.31973	2.1
65943	1.89226	2.1

65944	1.89226	2.1
65945	1.89226	2.1
65946	1.89226	2.1
65947	3.78453	2.1
65948	1.89226	2.1
65949	1.89226	2.1
65950	1.89226	2.1
65951	1.89226	2.1
65952	3.78452	2.1
65953	0.946158	2.1
65954	0.946158	2.1
65955	0.818509	2.1
65956	0.818509	2.1
65957	0.933975	2.1
65958	0.818539	2.1
65959	0.946128	2.1
65960	0.946128	2.1
65961	0.94613	2.1
65962	0.933974	2.1
65963	0.818537	2.1
65964	0.946129	2.1
65965	0.946131	2.1
65966	0.946131	2.1
65967	0.946131	2.1
65968	0.946131	2.1
65969	0.946132	2.1
65970	0.946132	2.1
65971	0.946129	2.1
65972	0.946129	2.1
65973	0.946131	2.1
65974	0.946131	2.1
65975	0.94613	2.1
65976	0.94613	2.1
65977	1.89226	2.1
65978	1.89226	2.1
65979	1.89226	2.1
65980	1.89226	2.1
65981	3.78453	2.1
65982	1.89226	2.1
65983	1.89226	2.1
65984	1.89226	2.1
65985	1.89226	2.1
65986	3.78452	2.1
65987	1.89226	2.1
65988	1.8706	2.1
65989	1.8706	2.1
65990	1.68112	2.1
65991	2.0815	2.1
65992	1.88715	2.1
65993	0.94613	2.1
65994	0.94613	2.1
65995	0.94613	2.1
65996	0.94613	2.1
65997	0.946131	2.1
65998	0.946131	2.1
65999	0.946129	2.1
66000	0.946129	2.1
66001	0.94613	2.1

66002	0.94613	2.1
66003	0.94613	2.1
66004	0.94613	2.1
66005	1.76401	2.1
66006	2.60983	2.1
66007	2.50461	2.1
66008	2.35897	2.1
66009	1.90965	2.1
66010	1.76401	2.1
66011	1.89226	2.1
66012	1.89226	0.997605
66013	1.89226	2.1
66014	1.89226	2.1
66015	3.78452	2.1
66016	1.89226	0.95109
66017	1.89226	2.1
66018	1.89226	2.1
66019	1.89226	0.730049
66020	1.89226	2.1
66021	1.89226	0.779496
66022	1.90965	2.1
66023	2.83834	2.1
66024	2.7538	2.1
66025	2.60983	2.1
66026	2.05361	2.1
66027	1.90965	2.1
66028	1.89226	2.1
66029	1.89226	2.1
66030	1.89226	2.1
66031	1.89226	1.02099
66032	1.89226	2.1
66033	1.89226	1.04629
66034	1.89226	1.46459
66035	1.89226	2.1
66036	1.89226	2.1
66037	1.89226	0.742744
66038	3.78452	2.1
66039	2.83834	2.1
66040	1.89226	2.1
66041	2.60983	2.1
66042	2.60983	2.1
66043	1.89226	2.1
66044	1.89226	2.1
66045	1.89226	2.04398
66046	1.89226	2.06357
66047	1.89226	2.1
66048	1.89226	1.29754
66049	1.89226	2.1
66050	1.89226	1.08074
66051	1.89226	1.06394
66052	1.89226	2.1
66053	1.89226	2.1
66054	1.89226	1.06754
66055	1.89226	2.1
66056	1.89226	1.25269
66057	2.44785	2.1
66058	1.80223	2.1
66059	2.80894	2.1

66060	2.18296	1.47632
66061	4.63081	2.1
66062	1.89226	1.37504
66063	1.89226	2.1
66064	1.89226	2.1
66065	1.89226	0.958483
66066	1.89226	2.1
66067	1.89226	0.990913
66068	0.946131	0.927919
66069	0.946131	1.22908
66070	0.946127	1.69705
66071	0.946127	2.1
66072	0.946131	1.08122
66073	0.946131	2.1
66074	0.946128	2.1
66075	0.946128	1.36962
66076	0.94613	1.90322
66077	0.94613	1.9264
66078	0.946129	1.91364
66079	0.946129	1.76404
66080	1.89226	0.941067
66081	1.89226	2.1
66082	1.89226	2.1
66083	1.89226	1.11636
66084	3.78452	2.1
66085	1.89226	2.1
66086	1.89226	2.1
66087	1.89226	2.1
66088	1.89226	2.1
66089	3.78453	2.1
66090	2.41083	2.1
66091	2.93937	2.1
66092	1.68761	2.1
66093	1.68761	2.1
66094	1.67248	2.1
66095	2.3957	2.1
66096	1.89226	2.1
66097	1.89226	0.993397
66098	1.89226	0.85754
66099	1.89226	2.1
66100	1.89226	0.857967
66101	1.89226	2.1
66102	1.89227	2.1
66103	1.89227	2.1
66104	1.89227	2.1
66105	1.89227	2.1
66106	1.89227	2.1
66107	1.89227	2.1
66108	1.89224	0.670428
66109	1.89224	0.520839
66110	1.89224	0.878552
66111	1.89224	0.669327
66112	3.78448	0.578465
66113	1.89224	0.910382
66114	1.89224	0.616071
66115	1.89224	0.878512
66116	1.89224	0.55827
66117	1.89224	1.10388

66118	1.89224	0.758078
66119	1.11152	0.577647
66120	1.29346	0.84921
66121	0.978596	0.822042
66122	0.918324	1.09402
66123	1.06821	1.0479
66124	1.06821	0.522179
66125	0.985654	1.54202
66126	1.15278	1.0088
66127	1.07693	1.02531
66128	0.902432	1.17542
66129	0.968939	1.28496
66130	1.34644	0.787365
66131	0.946132	0.597033
66132	0.946132	0.708988
66133	0.946132	0.745826
66134	0.946132	0.597874
66135	0.946131	0.625861
66136	0.946131	0.523959
66137	0.946132	0.909082
66138	0.946132	0.60335
66139	0.946132	0.681372
66140	0.946132	0.63373
66141	0.946132	0.535431
66142	0.946132	0.539281
66143	0.946133	0.743615
66144	0.946133	0.535134
66145	0.946132	0.698416
66146	0.946132	0.916413
66147	0.946132	0.768517
66148	0.946132	0.435781
66149	0.946133	1.55856
66150	0.946133	0.584136
66151	0.946132	0.84499
66152	0.946132	0.726958
66153	0.946132	0.572652
66154	0.946132	0.549452
66155	1.89224	0.69824
66156	1.89224	2.1
66157	1.89224	0.835691
66158	1.89224	1.55778
66159	1.89224	0.858068
66160	1.89224	2.1
66161	1.89224	0.843808
66162	1.89224	2.1
66163	1.89224	1.25588
66164	1.89224	2.1
66165	1.89224	1.05855
66166	1.89224	2.1
66167	1.89226	0.803413
66168	1.89226	2.1
66169	1.89226	2.1
66170	1.89226	0.896577
66171	3.78453	1.73782
66172	1.11056	2.1
66173	1.21595	2.1
66174	1.12763	2.1
66175	1.12763	2.00108

66176	1.23339	2.1
66177	1.35697	2.1
66178	1.03581	2.1
66179	0.95954	2.1
66180	0.89761	2.1
66181	1.26602	2.1
66182	1.12749	2.1
66183	1.45307	2.1
66184	2.71775	2.1
66185	1.89226	2.1
66186	1.89226	2.1
66187	1.89226	2.1
66188	2.47025	2.1
66189	2.47025	2.1
66190	0.94613	2.1
66191	0.94613	2.1
66192	0.94613	0.909439
66193	0.94613	1.67217
66194	0.946128	1.23344
66195	0.946128	2.1
66196	0.946132	2.1
66197	0.946132	1.06367
66198	0.94613	2.1
66199	0.94613	2.1
66200	0.94613	2.1
66201	0.94613	2.1
66202	1.89226	0.813403
66203	1.89226	2.08506
66204	1.89226	2.1
66205	1.89226	0.992146
66206	1.89226	2.1
66207	1.89226	1.1206
66208	1.89226	0.936765
66209	1.89226	2.06325
66210	1.89226	1.94464
66211	1.89226	0.949007
66212	1.89226	1.88991
66213	1.89226	1.18296
66214	0.946132	1.057
66215	0.946132	1.36564
66216	0.946133	2.1
66217	0.946133	2.1
66218	0.946133	2.1
66219	0.946133	0.983794
66220	0.946132	0.898577
66221	0.946132	2.1
66222	0.946133	2.1
66223	0.946133	2.1
66224	0.946132	2.1
66225	0.946132	2.1
66226	1.89226	0.869614
66227	1.89226	2.1
66228	1.89226	2.1
66229	1.89226	1.13281
66230	1.89226	2.1
66231	1.89226	1.16066
66232	0.946131	1.29911
66233	0.946131	0.942389

66234	0.946127	1.92318
66235	0.946127	2.1
66236	0.946131	1.2072
66237	0.946131	2.1
66238	0.946127	2.1
66239	0.946127	1.13443
66240	0.94613	1.58757
66241	0.94613	1.64127
66242	0.946129	1.50451
66243	0.946129	1.55065
66244	1.94291	2.1
66245	2.00118	2.1
66246	2.36337	2.1
66247	2.41217	2.1
66248	4.35508	2.1
66249	1.89226	2.1
66250	1.89226	1.416
66251	1.89226	2.1
66252	1.89226	2.1
66253	3.78452	2.1
66254	1.89226	1.63943
66255	1.89226	2.1
66256	1.89226	2.1
66257	1.89226	1.43694
66258	3.78452	2.1
66259	1.06209	2.1
66260	1.06209	2.1
66261	1.05987	2.1
66262	1.05987	2.1
66263	0.920378	2.1
66264	1.12137	2.1
66265	1.00059	2.1
66266	1.20608	2.1
66267	1.20608	2.1
66268	1.12137	2.1
66269	1.00059	2.1
66270	0.920376	2.1
66271	2.77222	2.1
66272	2.41216	2.1
66273	2.77002	2.1
66274	2.36337	2.1
66275	2.81882	2.1
66276	2.41216	2.1
66277	1.89226	1.41577
66278	1.89226	2.1
66279	1.89226	2.1
66280	1.89226	2.1
66281	1.89226	2.1
66282	1.89226	1.07306
66283	1.89226	2.1
66284	1.89226	1.06189
66285	1.89226	1.33337
66286	1.89226	2.1
66287	3.78451	2.0222
66288	2.41216	2.1
66289	2.24273	2.1
66290	2.81882	2.1
66291	2.68473	2.1

66292	5.09689	2.1
66293	1.89226	2.1
66294	1.89226	2.1
66295	1.89226	2.1
66296	1.89226	2.1
66297	3.78453	2.1
66298	2.17614	2.1
66299	2.65326	2.1
66300	1.93101	2.1
66301	2.58552	2.1
66302	4.58427	2.1
66303	2.65326	2.1
66304	1.89226	2.1
66305	1.89226	2.1
66306	1.89226	2.1
66307	2.58552	2.1
66308	2.58552	2.1
66309	1.89226	2.1
66310	1.89226	2.1
66311	1.89226	2.1
66312	1.89226	2.1
66313	3.78453	2.1
66314	1.89226	2.1
66315	1.89226	2.1
66316	1.89226	2.1
66317	1.89226	2.1
66318	3.78453	2.1
66319	1.93101	2.1
66320	2.58552	2.1
66321	1.46507	2.1
66322	2.52893	2.1
66323	4.45994	2.1
66324	1.26446	2.1
66325	1.26446	2.1
66326	0.946132	2.1
66327	0.946132	2.1
66328	0.94613	2.1
66329	0.94613	2.1
66330	1.29276	2.1
66331	1.26446	2.1
66332	1.26446	2.1
66333	0.94613	2.1
66334	0.94613	2.1
66335	1.29276	2.1
66336	1.89226	2.1
66337	1.89226	2.1
66338	1.89226	2.1
66339	1.89226	2.1
66340	3.78453	2.1
66341	1.89226	2.1
66342	1.89226	2.1
66343	1.89226	2.1
66344	1.89226	2.1
66345	3.78453	2.1
66346	1.83378	2.1
66347	2.52893	2.1
66348	1.75592	2.1
66349	2.49844	2.1

66350	4.28485	2.1
66351	1.24922	2.1
66352	1.24922	2.1
66353	0.946133	2.1
66354	0.946133	2.1
66355	0.946131	2.1
66356	0.946131	2.1
66357	1.26446	2.1
66358	1.24922	2.1
66359	1.24922	2.1
66360	0.946131	2.1
66361	1.26446	2.1
66362	0.94613	2.1
66363	0.946132	2.1
66364	0.946132	2.1
66365	0.946132	2.1
66366	0.946132	2.1
66367	0.946132	2.1
66368	0.946132	2.1
66369	0.946132	2.1
66370	0.946132	2.1
66371	0.946132	2.1
66372	0.946132	2.1
66373	0.946131	2.1
66374	0.946131	2.1
66375	1.89226	2.1
66376	1.89226	2.1
66377	1.89226	2.1
66378	1.89226	2.1
66379	1.89226	2.1
66380	1.89226	2.1
66381	1.0465	2.1
66382	1.0465	2.1
66383	1.06047	2.1
66384	1.06047	2.1
66385	1.25687	2.1
66386	1.24922	2.1
66387	0.87796	2.1
66388	0.857749	2.1
66389	0.857749	2.1
66390	1.25687	2.1
66391	0.877959	2.1
66392	1.24922	2.1
66393	2.49843	2.1
66394	1.89226	2.1
66395	2.51374	2.1
66396	1.89226	2.1
66397	4.406 2.1	
66398	1.89226	2.1
66399	1.89226	2.1
66400	1.89226	2.1
66401	1.89226	2.1
66402	1.89226	2.1
66403	1.89226	2.1
66404	1.89226	0.894982
66405	1.89226	2.1
66406	1.89226	2.1
66407	1.89226	0.993294

66408	1.89226	2.1
66409	1.89226	1.04489
66410	1.89226	2.1
66411	1.89226	1.06504
66412	1.89226	2.1
66413	1.89226	1.02492
66414	1.89226	2.1
66415	1.89226	1.08077
66416	1.03835	2.1
66417	1.03835	2.1
66418	1.15388	2.1
66419	0.94267	2.1
66420	0.736433	1.92461
66421	0.849239	2.1
66422	1.15014	2.1
66423	1.39058	2.1
66424	0.838639	2.1
66425	1.39056	2.1
66426	1.09399	2.1
66427	0.736444	2.1
66428	1.89226	0.846244
66429	1.89226	1.96973
66430	1.89226	2.06633
66431	1.89226	0.870136
66432	1.89226	1.96904
66433	1.89226	0.905244
66434	0.94613	0.933464
66435	0.94613	0.906697
66436	0.946127	2.1
66437	0.946127	1.93005
66438	0.946131	2.04748
66439	0.946131	0.901182
66440	0.946126	2.1
66441	0.946126	0.938265
66442	0.946128	1.61893
66443	0.946128	1.5667
66444	0.946128	1.14769
66445	0.946128	1.47336
66446	1.89226	1.77903
66447	1.89226	0.9999
66448	1.89226	0.721064
66449	1.89226	2.1
66450	3.78453	0.993507
66451	0.946133	1.5438
66452	0.946133	0.703645
66453	0.946128	1.80343
66454	0.946128	1.22943
66455	0.946131	1.42674
66456	0.946131	1.30837
66457	0.946131	0.583163
66458	0.946131	1.24895
66459	0.946131	1.04793
66460	0.946131	1.0176
66461	0.94613	1.1076
66462	0.94613	1.18221
66463	1.89226	0.791825
66464	1.89226	2.1
66465	1.89226	1.57457

66466	1.89226	0.955955
66467	1.89226	1.53516
66468	1.89226	1.10125
66469	1.89226	0.77038
66470	1.89226	1.60912
66471	1.89226	1.09244
66472	1.89226	0.946926
66473	1.89226	1.17623
66474	1.89226	1.30989
66475	1.89226	0.790571
66476	1.89226	1.93442
66477	1.89226	1.07357
66478	1.89226	1.2103
66479	1.89226	1.13437
66480	1.89226	1.65826
66481	1.89226	0.860906
66482	2.67388	2.1
66483	2.23684	1.82607
66484	2.23684	1.26667
66485	1.89226	1.89952
66486	1.89226	1.5348
66487	1.89226	2.1
66488	1.89226	2.1
66489	1.89226	2.1
66490	1.89226	2.1
66491	1.89226	2.1
66492	1.89226	2.1
66493	1.72197	2.1
66494	2.51374	2.1
66495	2.58004	2.1
66496	2.58004	2.1
66497	1.7155	2.1
66498	1.7155	2.1
66499	2.51374	2.1
66500	1.89226	2.1
66501	1.89226	2.1
66502	1.89226	2.1
66503	2.58003	2.1
66504	2.58003	2.1
66505	0.946132	2.1
66506	0.946132	2.1
66507	0.946132	2.1
66508	0.946132	2.1
66509	0.946132	2.08762
66510	0.946132	2.1
66511	0.946132	2.1
66512	0.946132	2.1
66513	0.946131	2.1
66514	0.946131	2.1
66515	0.946132	2.1
66516	0.946132	2.1
66517	1.89226	2.1
66518	1.89226	2.1
66519	1.89226	2.1
66520	1.89226	2.1
66521	3.78453	2.1
66522	1.08262	2.1
66523	1.08262	2.1

66524	1.09674	2.1
66525	1.09674	2.1
66526	1.34652	2.1
66527	1.29002	2.1
66528	0.860984	2.1
66529	0.889338	2.1
66530	1.34652	2.1
66531	0.889338	2.1
66532	0.860983	2.1
66533	1.29002	2.1
66534	1.34652	2.1
66535	1.34652	2.1
66536	0.946133	2.1
66537	0.946133	2.1
66538	0.946131	2.1
66539	0.946131	2.1
66540	1.34652	2.1
66541	1.29002	2.1
66542	1.34652	2.1
66543	0.946131	2.1
66544	0.94613	2.1
66545	1.29002	2.1
66546	1.89226	2.1
66547	1.89226	2.1
66548	1.89226	2.1
66549	1.89226	2.1
66550	1.89226	2.1
66551	1.89226	2.1
66552	1.89226	2.1
66553	1.89226	2.1
66554	1.89226	2.1
66555	1.89226	2.1
66556	1.89226	2.1
66557	1.89226	2.1
66558	0.946131	2.1
66559	0.946131	2.1
66560	0.946131	2.1
66561	0.946131	1.90989
66562	0.946131	1.87268
66563	0.946131	2.1
66564	0.946131	2.1
66565	0.946131	2.1
66566	0.946131	2.1
66567	0.946131	2.1
66568	0.946131	2.1
66569	0.946131	2.1
66570	1.89226	2.1
66571	1.89226	2.1
66572	1.89226	2.1
66573	1.89226	2.1
66574	1.89226	2.1
66575	1.89226	2.1
66576	1.04103	2.1
66577	1.13849	2.1
66578	0.943014	2.1
66579	0.943014	2.1
66580	0.943591	2.1
66581	0.943591	2.1

66582	1.03757	2.1
66583	1.04098	2.1
66584	1.03761	2.1
66585	0.946131	2.1
66586	0.943041	2.1
66587	0.946131	2.1
66588	0.946128	2.1
66589	0.946128	2.1
66590	0.946133	2.1
66591	0.946133	2.1
66592	0.946131	2.1
66593	0.946131	2.1
66594	0.946131	2.1
66595	0.946131	2.1
66596	0.946131	2.1
66597	0.946131	2.1
66598	0.94613	2.1
66599	0.94613	2.1
66600	0.946132	2.1
66601	0.946132	2.1
66602	0.946132	2.1
66603	0.946132	1.95086
66604	0.946132	1.95018
66605	0.946132	2.1
66606	0.946132	2.1
66607	0.946132	2.1
66608	0.946132	2.1
66609	0.946132	2.1
66610	0.946131	2.1
66611	0.946131	2.1
66612	1.89226	0.839012
66613	1.89226	1.97974
66614	1.89226	0.940925
66615	1.89226	0.928159
66616	3.78452	1.22374
66617	0.946132	1.11918
66618	0.946132	1.33084
66619	0.946131	0.935959
66620	0.946131	0.882392
66621	0.946132	0.930847
66622	0.946132	2.1
66623	0.946132	1.13999
66624	0.946132	1.06029
66625	0.946132	1.03496
66626	0.946132	1.05438
66627	0.946132	1.12538
66628	0.946132	1.24196
66629	0.935482	1.74326
66630	1.08999	1.50949
66631	1.24315	1.20935
66632	1.12559	1.66249
66633	1.21343	1.16101
66634	0.889397	2.1
66635	1.01265	0.811159
66636	1.01265	1.81432
66637	1.07332	1.01653
66638	1.34292	1.48572
66639	0.865649	1.49225

66640	0.840024	1.92914
66641	1.15433	1.39324
66642	1.02858	1.86684
66643	1.34082	1.32966
66644	1.08357	1.35907
66645	1.10938	0.936426
66646	1.33349	2.1
66647	1.12232	1.80348
66648	1.12232	0.827396
66649	1.21357	1.37504
66650	1.3408	1.57113
66651	1.02859	1.58834
66652	1.24211	1.92342
66653	1.89226	1.12506
66654	1.89226	0.711843
66655	1.89226	0.842943
66656	1.89226	2.03481
66657	1.89226	0.691919
66658	1.89226	1.26836
66659	1.89226	0.824917
66660	1.89226	1.00821
66661	1.89226	0.828653
66662	1.89226	1.66009
66663	1.89226	1.05527
66664	1.89226	1.68981
66665	2.0815	1.42282
66666	2.79029	1.3217
66667	2.36793	1.0816
66668	2.58428	2.1
66669	2.38161	0.790152
66670	2.59922	1.67897
66671	1.89226	2.1
66672	1.89226	1.26239
66673	1.89226	1.77511
66674	1.89226	1.91755
66675	1.89226	1.73543
66676	1.89226	1.95227
66677	1.89226	1.36021
66678	1.89226	0.613811
66679	1.89226	0.782754
66680	1.89226	0.910505
66681	3.78452	0.830785
66682	1.89226	1.86437
66683	1.89226	0.854221
66684	1.89226	1.42939
66685	1.89226	1.34318
66686	1.89226	1.27966
66687	1.89226	1.34441
66688	0.946132	0.955359
66689	0.946132	1.7207
66690	0.94613	2.1
66691	0.94613	1.46599
66692	0.946131	1.9277
66693	0.946131	0.946923
66694	0.946131	1.65343
66695	0.946131	2.1
66696	0.946131	1.57656
66697	0.946131	1.76254

66698	0.946131	1.78716
66699	0.946131	1.69894
66700	1.85178	1.71396
66701	1.89226	0.866389
66702	2.0815	0.713765
66703	2.27054	1.21007
66704	1.70303	0.600452
66705	1.5217	0.698923
66706	1.89226	1.93542
66707	1.89226	0.812924
66708	1.89226	1.14126
66709	1.89226	0.986113
66710	1.89226	1.80638
66711	1.89226	1.21676
66712	2.58823	0.461038
66713	2.02924	0.390315
66714	1.79398	0.377686
66715	1.79398	0.266451
66716	2.76708	0.457943
66717	2.76708	0.398653
66718	0.946133	0.39615
66719	0.946133	0.431001
66720	1.29411	0.407163
66721	1.38353	0.363929
66722	0.946131	0.367764
66723	0.946131	0.374321
66724	1.38354	0.442871
66725	1.38354	0.393356
66726	0.946131	0.379229
66727	1.38354	0.38449
66728	0.94613	0.399624
66729	1.29411	0.388026
66730	1.89226	0.370605
66731	1.89226	0.326407
66732	1.89226	0.365234
66733	1.89226	0.357905
66734	3.78452	0.354647
66735	0.946131	0.302883
66736	0.946131	0.370051
66737	0.946131	0.364375
66738	0.946131	0.304298
66739	0.946129	0.32497
66740	0.946129	0.255661
66741	0.946133	0.39068
66742	0.946133	0.368493
66743	0.946131	0.386786
66744	0.946131	0.364987
66745	0.946131	0.231317
66746	0.946131	0.354153
66747	1.89226	0.325134
66748	1.89226	0.332482
66749	1.89226	0.36532
66750	1.89226	0.329593
66751	3.78452	0.329174
66752	0.94613	0.334291
66753	0.94613	0.340099
66754	0.946131	0.31029
66755	0.946131	0.336657

66756	0.946128	0.338786
66757	0.946128	0.319106
66758	0.946133	0.333026
66759	0.946133	0.339989
66760	0.946131	0.353745
66761	0.946131	0.336286
66762	0.946131	0.337397
66763	0.946131	0.277154
66764	0.946129	0.687168
66765	0.946129	0.653168
66766	1.13486	0.667784
66767	0.859753	0.715665
66768	0.851541	0.704098
66769	0.851541	0.605715
66770	1.34346	0.668261
66771	1.26212	0.680718
66772	0.851553	0.620719
66773	1.12403	0.646964
66774	0.851474	0.44289
66775	0.956163	0.656286
66776	0.946132	0.496209
66777	0.946132	0.465256
66778	0.946133	0.51798
66779	0.946133	0.269049
66780	0.946132	0.454476
66781	0.946132	0.229372
66782	0.946133	0.404319
66783	0.946133	0.486562
66784	0.946133	0.52256
66785	0.946133	0.396796
66786	0.946132	0.24509
66787	0.946132	0.443959
66788	1.89226	0.369359
66789	1.89226	0.537465
66790	1.89226	0.575219
66791	1.89226	0.508806
66792	3.78453	0.480152
66793	0.946128	0.664255
66794	0.946128	0.640344
66795	0.946132	0.696145
66796	0.946132	0.697654
66797	0.94613	0.689839
66798	0.94613	0.631854
66799	0.94613	0.737205
66800	0.94613	0.628403
66801	0.946131	0.704182
66802	0.946131	0.673677
66803	0.94613	0.656592
66804	0.94613	0.666954
66805	0.946132	0.446926
66806	0.946132	0.29654
66807	0.946133	0.433712
66808	0.946133	0.205393
66809	0.946132	0.312403
66810	0.946132	0.213967
66811	0.946133	0.314633
66812	0.946133	0.237089
66813	0.946132	0.427926

66814	0.946132	0.355834
66815	0.946132	0.286273
66816	0.946132	0.21079
66817	1.89226	0.465245
66818	1.89226	0.335494
66819	1.89226	0.507131
66820	1.89226	0.225022
66821	1.89226	0.497385
66822	1.89226	0.358057
66823	1.89226	0.584974
66824	1.89226	0.526472
66825	1.89226	0.450655
66826	1.89226	0.478629
66827	1.89226	0.518902
66828	1.89226	0.531104
66829	1.89226	2.1
66830	1.89226	2.1
66831	1.89226	2.1
66832	1.89226	2.1
66833	3.78452	2.1
66834	1.07652	2.1
66835	1.07652	2.1
66836	0.928749	2.1
66837	0.928749	2.1
66838	0.946132	2.1
66839	0.946132	2.1
66840	1.05914	2.1
66841	0.928746	2.1
66842	1.05914	2.1
66843	0.946131	2.1
66844	0.94613	2.1
66845	0.928747	2.1
66846	1.89226	2.1
66847	1.89226	2.1
66848	1.89226	2.1
66849	1.89226	2.1
66850	1.89226	2.1
66851	1.89226	2.1
66852	1.89227	2.1
66853	1.89227	2.1
66854	1.89227	2.1
66855	1.89227	2.1
66856	1.89227	2.1
66857	1.89227	2.1
66858	1.89226	2.1
66859	1.89226	2.1
66860	1.89226	2.1
66861	1.89226	2.1
66862	1.89226	2.1
66863	1.89226	2.1
66864	2.11828	2.1
66865	1.89226	2.1
66866	2.35845	2.1
66867	1.89226	2.1
66868	4.25071	2.1
66869	1.89226	2.1
66870	1.89226	2.1
66871	1.89226	2.1

66872	1.89226	2.1
66873	3.78453	2.1
66874	1.89226	2.1
66875	1.89226	2.1
66876	1.89226	2.1
66877	1.89226	2.1
66878	1.89226	2.1
66879	1.89226	2.1
66880	1.89226	0.800392
66881	1.89226	1.67359
66882	1.65573	1.44628
66883	1.65573	0.878727
66884	1.89226	1.42727
66885	1.89226	0.888651
66886	1.89226	0.708481
66887	1.89226	1.40841
66888	1.89226	1.04349
66889	1.89226	0.819366
66890	1.89226	1.14485
66891	1.89226	0.859623
66892	1.89226	0.765154
66893	1.89226	1.50698
66894	1.89226	1.28046
66895	1.89226	0.85136
66896	1.89226	1.37415
66897	1.89226	0.873739
66898	1.89226	0.693413
66899	1.89226	1.46889
66900	1.89226	1.02214
66901	1.89226	0.788882
66902	1.89226	1.10651
66903	1.89226	0.826362
66904	0.946128	0.871144
66905	0.946128	0.75711
66906	0.946125	1.20941
66907	0.946125	1.18385
66908	0.94613	0.834945
66909	0.94613	2.02378
66910	0.946124	1.53993
66911	0.946124	0.822001
66912	0.946127	0.969799
66913	0.946127	0.967463
66914	0.946127	1.00226
66915	0.946127	1.06449
66916	1.89226	0.728237
66917	1.89226	1.0272
66918	1.89226	0.724217
66919	1.89226	0.654152
66920	3.78452	0.759642
66921	1.89226	0.948094
66922	1.89226	0.974239
66923	1.89226	0.761469
66924	1.89226	0.982373
66925	3.78453	0.925817
66926	1.05926	1.37606
66927	0.891358	2.1
66928	0.946129	1.09218
66929	0.946129	1.65135

66930	0.946101	1.08529
66931	0.946101	2.1
66932	0.891386	1.13152
66933	0.891386	1.33385
66934	0.891357	1.41463
66935	0.946129	1.18138
66936	0.94613	1.33808
66937	1.05926	1.25925
66938	0.946126	0.848112
66939	0.946126	1.0154
66940	0.94613	0.830299
66941	0.94613	1.05141
66942	0.946131	0.938556
66943	0.946131	1.0511
66944	0.946125	1.04822
66945	0.946125	0.656676
66946	0.946127	0.861344
66947	0.946127	0.83615
66948	0.946128	1.03913
66949	0.946128	0.948276
66950	1.89226	0.799529
66951	1.89226	0.685251
66952	1.89226	0.61372
66953	1.89226	0.957464
66954	1.89226	0.660264
66955	1.89226	1.10968
66956	1.89226	0.838864
66957	1.89226	1.10728
66958	1.89226	0.980559
66959	1.89226	1.08179
66960	1.89226	0.834895
66961	1.89226	1.04186
66962	1.16109	1.73437
66963	1.23243	2.1
66964	0.659819	1.18307
66965	0.659819	1.86707
66966	0.697965	0.972648
66967	1.00087	2.1
66968	0.891386	1.66887
66969	0.891386	1.19428
66970	0.946129	1.63127
66971	0.65979	1.09683
66972	0.891357	1.53569
66973	0.946129	1.29363
66974	1.89225	0.722259
66975	1.89225	0.974309
66976	1.89225	1.14721
66977	1.89225	0.654764
66978	1.89225	1.80525
66979	1.89225	0.845172
66980	1.79981	0.444007
66981	2.02514	0.475161
66982	2.33247	0.646031
66983	2.12891	0.315766
66984	4.15405	0.364791
66985	2.48284	0.827962
66986	2.02514	0.441665
66987	2.33247	0.585187

66988	2.02514	0.695757
66989	2.48567	0.585087
66990	2.17834	0.682547
66991	1.89226	0.371679
66992	1.89226	0.593692
66993	1.89226	0.662245
66994	1.89226	0.343873
66995	3.78453	0.434798
66996	1.89227	0.400384
66997	1.89227	0.26542
66998	1.89227	0.251384
66999	1.89227	0.454321
67000	1.89227	0.306779
67001	1.89227	0.489192
67002	2.50894	0.288979
67003	2.70118	0.573756
67004	2.12891	0.347366
67005	2.12891	0.468
67006	2.7125	0.399486
67007	2.33247	0.433255
67008	1.89226	0.609551
67009	1.89226	0.363302
67010	1.89226	0.369881
67011	1.89226	0.558167
67012	1.89226	0.340924
67013	1.89226	0.532486
67014	0.946133	0.367236
67015	0.946133	0.441371
67016	0.946132	0.251143
67017	0.946132	0.347922
67018	0.946132	0.233245
67019	0.946132	0.495562
67020	0.946133	0.245864
67021	0.946133	0.470878
67022	0.946132	0.286955
67023	0.946132	0.370672
67024	0.946132	0.374344
67025	0.946132	0.334283
67026	1.90098	0.369322
67027	1.36463	0.386107
67028	1.56656	0.381955
67029	1.36463	0.395478
67030	2.04386	0.358212
67031	1.89227	0.387377
67032	1.89226	0.374864
67033	1.89226	0.386937
67034	1.89226	0.385769
67035	1.89226	0.40096
67036	1.89226	0.366533
67037	1.89226	0.388682
67038	1.89226	0.404203
67039	1.89226	0.422163
67040	1.89226	0.426088
67041	1.89226	0.450202
67042	1.89226	0.392178
67043	1.89226	0.42727
67044	1.89226	0.402154
67045	1.89226	0.418734

67046	1.89226	0.430174
67047	1.89226	0.438093
67048	1.89226	0.392392
67049	1.89226	0.418202
67050	1.89226	0.358128
67051	1.70303	0.374095
67052	2.1292	0.376744
67053	1.70303	0.38126
67054	2.32302	0.35298
67055	1.90098	0.370377
67056	1.89226	0.363984
67057	1.89226	0.378937
67058	1.89226	0.378478
67059	1.89226	0.388497
67060	1.89226	0.357162
67061	1.89226	0.375674
67062	1.89226	0.392068
67063	1.89226	0.409926
67064	1.89226	0.415005
67065	1.89226	0.422061
67066	1.89226	0.383217
67067	1.89226	0.402011
67068	1.89226	0.38732
67069	1.89226	0.40675
67070	1.89226	0.410122
67071	1.89226	0.413305
67072	1.89226	0.379116
67073	1.89226	0.394544
67074	1.89226	0.698268
67075	1.89226	0.937445
67076	1.89226	0.914746
67077	1.89226	0.720381
67078	3.78453	0.677932
67079	1.89226	0.876652
67080	1.89226	1.17999
67081	1.89226	0.754251
67082	1.89226	1.2536
67083	1.89226	0.894155
67084	1.89226	1.93221
67085	1.89226	0.932727
67086	1.89226	0.735188
67087	1.89226	0.711912
67088	1.89226	0.818565
67089	3.78452	0.903982
67090	1.89224	0.913406
67091	1.89224	0.683837
67092	1.89224	0.764211
67093	1.89224	0.979388
67094	1.89224	0.700613
67095	1.89224	0.83731
67096	1.89224	0.87747
67097	1.89224	1.3509
67098	1.89224	1.02324
67099	1.89224	2.07256
67100	1.89224	0.74106
67101	1.89224	1.27772
67102	1.31307	1.87194
67103	1.31307	1.91836

67104	0.888948	1.06154
67105	0.888948	0.947453
67106	1.2559	1.1008
67107	0.888979	1.93254
67108	0.946114	1.31473
67109	0.946114	0.903604
67110	0.946129	0.823893
67111	0.888989	1.00355
67112	0.946104	0.752602
67113	1.25588	0.889225
67114	1.89224	0.673273
67115	1.89224	1.02461
67116	1.89224	0.7114
67117	1.89224	0.971226
67118	1.89224	0.817358
67119	1.89224	1.30911
67120	1.79375	0.313542
67121	1.47689	0.292454
67122	1.97732	0.310759
67123	2.26687	0.31388
67124	3.74375	0.302966
67125	2.05759	0.335959
67126	2.26686	0.293913
67127	1.79374	0.282482
67128	1.79374	0.315877
67129	2.49889	0.321384
67130	2.49889	0.325822
67131	1.89226	0.297445
67132	1.89226	0.312271
67133	1.89226	0.296297
67134	1.89226	0.32182
67135	3.78452	0.31747
67136	1.89227	0.31665
67137	1.89227	0.31231
67138	1.89227	0.312287
67139	1.89227	0.318062
67140	1.89227	0.29667
67141	1.89227	0.278881
67142	1.97732	0.313006
67143	2.26687	0.310669
67144	2.6607	0.331253
67145	2.41464	0.318157
67146	4.68151	0.317787
67147	1.89226	0.294285
67148	1.89226	0.33473
67149	1.89226	0.343599
67150	1.89226	0.326775
67151	3.78452	0.302478
67152	1.89226	0.311885
67153	1.89226	0.321327
67154	1.89226	0.323521
67155	1.89226	0.33465
67156	1.89226	0.316184
67157	1.89226	0.321685
67158	1.89226	0.685262
67159	1.89226	1.00024
67160	1.89226	0.788936
67161	1.89226	0.719205

67162	3.78452	0.718064
67163	1.89226	0.751095
67164	1.89226	1.15426
67165	1.89226	0.7672
67166	1.89226	0.938932
67167	1.89226	0.804925
67168	1.89226	1.13437
67169	1.89226	1.02887
67170	1.89226	0.73123
67171	1.89226	0.673135
67172	1.89226	1.01168
67173	1.89226	0.710684
67174	1.89226	1.08445
67175	1.89226	0.786948
67176	1.89226	0.714483
67177	1.89226	0.644188
67178	1.89226	0.953673
67179	1.89226	0.682534
67180	1.89226	1.18218
67181	1.89226	0.752573
67182	2.45282	0.986653
67183	1.89226	1.04955
67184	1.89226	1.22138
67185	1.89226	0.735771
67186	1.89226	0.899987
67187	1.89226	0.728355
67188	1.89226	0.992764
67189	1.89226	0.717425
67190	1.89226	0.786318
67191	1.89226	1.13063
67192	1.89226	1.70773
67193	1.87308	0.32131
67194	1.88631	0.322455
67195	1.63908	0.32231
67196	1.70302	0.322481
67197	2.08745	0.320331
67198	2.16916	0.319831
67199	2.41019	0.321318
67200	2.733	0.32537
67201	2.08121	0.321396
67202	2.08121	0.32268
67203	2.74362	0.319536
67204	2.41464	0.319771
67205	1.89226	0.32087
67206	1.89226	0.326822
67207	1.89226	0.319062
67208	1.89226	0.322772
67209	1.89226	0.326512
67210	1.89226	0.329752
67211	0.946133	0.323346
67212	0.946133	0.320912
67213	0.946132	0.327409
67214	0.946132	0.32558
67215	0.946132	0.328745
67216	0.946132	0.32875
67217	0.946133	0.321414
67218	0.946133	0.322184
67219	0.946132	0.323653

67220	0.946132	0.338878
67221	0.946133	0.320725
67222	0.946133	0.328001
67223	2.02912	0.325912
67224	2.57016	0.324301
67225	1.88631	0.326087
67226	1.88631	0.328173
67227	2.55301	0.320946
67228	2.4102	0.323223
67229	1.89226	0.324254
67230	1.89226	0.336351
67231	1.89226	0.335786
67232	1.89226	0.335434
67233	3.78452	0.330927
67234	1.89227	0.325941
67235	1.89227	0.336921
67236	1.89227	0.333491
67237	1.89227	0.336438
67238	1.89227	0.326323
67239	1.89227	0.326533
67240	1.89226	2.1
67241	1.89226	2.1
67242	1.89226	2.0275
67243	1.89226	2.1
67244	3.78452	2.1
67245	1.2515	1.96397
67246	1.34788	2.1
67247	0.946132	2.1
67248	0.946132	2.1
67249	0.973043	2.1
67250	1.16766	2.1
67251	0.919222	1.6702
67252	1.2159	2.1
67253	0.946132	2.1
67254	0.946132	2.1
67255	1.33746	2.1
67256	1.13537	2.1
67257	1.89226	2.1
67258	1.89226	2.1
67259	1.89226	2.1
67260	1.89226	2.1
67261	1.89226	2.1
67262	1.89226	2.1
67263	1.89226	2.03302
67264	1.89226	2.1
67265	1.89226	2.1
67266	1.89226	2.05924
67267	3.78452	2.1
67268	1.89227	2.1
67269	1.89227	1.5035
67270	1.89227	2.1
67271	1.89227	1.82683
67272	1.89227	2.1
67273	1.89227	1.7957
67274	0.946132	2.08583
67275	0.946132	2.06044
67276	0.946129	2.1
67277	0.946129	2.1

67278	0.946131	2.1
67279	0.946131	2.1
67280	0.946131	2.1
67281	0.946131	2.06779
67282	0.946131	2.1
67283	0.946131	2.1
67284	0.94613	2.1
67285	0.94613	2.1
67286	1.89226	0.921903
67287	1.89226	0.884399
67288	1.89226	0.93535
67289	1.89226	0.772383
67290	3.78453	0.818978
67291	0.946132	1.57366
67292	0.946132	0.851646
67293	0.946132	1.0273
67294	0.946132	0.875584
67295	0.946132	0.698427
67296	0.946132	1.57424
67297	0.946132	0.884599
67298	0.946132	0.916596
67299	0.946131	1.76652
67300	0.946131	0.788889
67301	0.946132	1.09631
67302	0.946132	0.840533
67303	1.89226	1.08577
67304	1.89226	0.681323
67305	1.89226	0.642211
67306	1.89226	0.780616
67307	1.89226	0.8632
67308	1.89226	1.39338
67309	1.89226	1.2985
67310	1.89226	0.743571
67311	1.89226	0.669532
67312	1.89226	0.74887
67313	3.78453	0.767453
67314	0.96614	0.902124
67315	0.96614	0.81633
67316	0.946131	0.613295
67317	0.946131	0.743008
67318	0.946131	0.790055
67319	0.96614	0.755901
67320	1.37736	0.893457
67321	0.946131	1.03071
67322	1.37736	1.30729
67323	0.96614	0.719208
67324	0.946131	1.14545
67325	0.946131	0.694398
67326	0.946129	1.32143
67327	0.946129	0.805511
67328	0.946128	0.861615
67329	0.946128	1.24699
67330	0.946132	0.652534
67331	0.946132	1.71702
67332	0.946126	0.892279
67333	0.946126	0.79137
67334	0.946129	0.681287
67335	0.946129	0.746189

67336	0.946129	0.681325
67337	0.946129	0.877675
67338	1.89226	0.333171
67339	1.65723	0.347969
67340	1.83117	0.351819
67341	1.65723	0.345982
67342	2.0662	0.339651
67343	1.89226	0.337417
67344	1.89226	0.329567
67345	1.89226	0.346867
67346	1.89226	0.350618
67347	1.89226	0.347068
67348	1.89226	0.333401
67349	1.89226	0.332999
67350	1.89226	0.343231
67351	1.89226	0.366908
67352	1.89226	0.373675
67353	1.89226	0.371421
67354	1.89226	0.344235
67355	1.89226	0.344154
67356	1.89226	0.34419
67357	1.89226	0.358667
67358	1.89226	0.368418
67359	1.89226	0.365474
67360	1.89226	0.346885
67361	1.89226	0.347313
67362	1.89226	0.331969
67363	1.83117	0.347385
67364	1.92807	0.34862
67365	1.83117	0.342891
67366	1.98916	0.33675
67367	1.89226	0.333484
67368	1.89226	0.326766
67369	1.89226	0.346949
67370	1.89226	0.335929
67371	1.89226	0.341726
67372	3.78453	0.33501
67373	1.89226	0.344522
67374	1.89226	0.369872
67375	1.89226	0.37556
67376	1.89226	0.376719
67377	1.89226	0.342838
67378	1.89226	0.343006
67379	1.89226	0.340723
67380	1.89226	0.36412
67381	1.89226	0.368389
67382	1.89226	0.364532
67383	1.89226	0.345348
67384	1.89226	0.343577
67385	1.88037	0.360671
67386	1.89227	0.374251
67387	1.89227	0.378446
67388	1.89227	0.371368
67389	1.98599	0.366564
67390	1.98599	0.360743
67391	1.89226	0.360189
67392	1.89226	0.381184
67393	1.89226	0.387958

67394	1.89226	0.386246
67395	1.89226	0.36377
67396	1.89226	0.364221
67397	1.89226	0.395869
67398	1.89226	0.405831
67399	1.89226	0.390508
67400	1.89226	0.430345
67401	3.78452	0.396439
67402	1.89226	0.390674
67403	1.89226	0.403822
67404	1.89226	0.396803
67405	1.89226	0.393328
67406	1.89226	0.393925
67407	1.89226	0.392787
67408	1.90116	0.357726
67409	1.89227	0.373526
67410	1.89226	0.374621
67411	1.89227	0.368198
67412	1.88036	0.366408
67413	1.88037	0.358475
67414	1.89226	0.35811
67415	1.89226	0.38409
67416	1.89226	0.383801
67417	1.89226	0.378883
67418	1.89226	0.36315
67419	1.89226	0.360585
67420	0.946132	0.404753
67421	0.946132	0.398528
67422	0.946128	0.406891
67423	0.946128	0.405056
67424	0.94613	0.40775
67425	0.94613	0.414607
67426	0.94613	0.398334
67427	0.94613	0.394763
67428	0.946131	0.404169
67429	0.946131	0.409065
67430	0.94613	0.388615
67431	0.94613	0.425652
67432	1.89226	0.399033
67433	1.89226	0.398141
67434	1.89226	0.387786
67435	1.89226	0.397523
67436	1.89226	0.388356
67437	1.89226	0.397459
67438	1.89226	0.697323
67439	1.89226	1.27859
67440	2.35239	1.24429
67441	2.27873	0.786657
67442	1.89226	1.18415
67443	1.83301	0.7861
67444	2.68449	0.877703
67445	1.89226	1.91704
67446	1.89226	0.952188
67447	1.89226	1.08027
67448	3.78453	0.991302
67449	0.946132	1.73755
67450	0.946132	0.954707
67451	0.946129	0.849346

67452	0.946129	1.65698
67453	0.946131	1.65376
67454	0.946131	1.19718
67455	0.94613	0.947406
67456	0.94613	1.08994
67457	0.946131	1.23583
67458	0.946131	1.00661
67459	0.946131	1.0662
67460	0.946131	1.31662
67461	1.89226	0.691631
67462	1.89226	1.03665
67463	1.89226	1.23222
67464	1.89226	0.73022
67465	1.89226	1.1785
67466	1.89226	0.733222
67467	1.70303	0.705151
67468	1.89226	1.17577
67469	1.89226	0.682597
67470	1.89226	0.824076
67471	3.78453	0.757614
67472	1.89227	1.03267
67473	1.89227	1.14882
67474	1.89227	0.772383
67475	1.89227	1.54716
67476	3.78453	1.10249
67477	1.89226	0.92206
67478	1.89226	1.73246
67479	1.89226	1.0002
67480	1.89226	1.34059
67481	3.78452	1.22224
67482	1.89226	2.03618
67483	1.89226	2.1
67484	1.89226	2.1
67485	1.89226	1.95444
67486	3.78452	1.94973
67487	0.946132	1.54139
67488	0.946132	1.96662
67489	0.946129	2.1
67490	0.946129	2.1
67491	0.94613	2.1
67492	0.94613	1.77027
67493	0.946131	2.1
67494	0.946131	1.91027
67495	0.946131	1.9254
67496	0.946131	2.1
67497	0.946131	2.04789
67498	0.946131	1.69271
67499	1.89226	2.1
67500	1.89226	1.68389
67501	1.89226	1.92361
67502	1.89226	2.1
67503	3.78452	2.1
67504	1.89226	1.37342
67505	1.89226	2.09615
67506	1.89226	2.1
67507	1.89226	1.46284
67508	1.89226	2.1
67509	1.89226	1.40647

67510	1.89227	2.07567
67511	2.48308	1.31337
67512	1.89227	1.46744
67513	1.89227	1.89524
67514	3.78453	2.05429
67515	1.89226	1.63311
67516	1.89226	2.05123
67517	1.89226	2.1
67518	1.89226	1.55455
67519	1.89226	2.0979
67520	1.89226	1.43268
67521	1.89226	1.36942
67522	1.89226	1.4488
67523	1.89226	1.8522
67524	1.89226	1.35177
67525	3.78453	1.93441
67526	0.946131	1.58612
67527	0.946131	1.87954
67528	0.946131	1.83097
67529	0.946131	2.1
67530	0.946131	1.88253
67531	0.946131	1.77731
67532	0.946131	1.91108
67533	0.946131	1.91367
67534	0.946131	1.73615
67535	0.946131	2.01021
67536	0.946131	1.92915
67537	0.946131	1.62837
67538	1.23719	1.82687
67539	1.28439	1.51406
67540	0.94613	1.3001
67541	0.94613	1.43429
67542	1.28439	1.57729
67543	1.6431	1.92958
67544	0.946129	1.44209
67545	0.946129	1.26522
67546	1.28439	1.18019
67547	0.946131	1.12043
67548	1.21038	1.08351
67549	1.21038	1.16715
67550	1.89226	1.72522
67551	1.89226	1.23961
67552	1.89226	1.38782
67553	1.89226	1.45449
67554	1.89226	1.28932
67555	1.89226	1.38266
67556	1.89226	1.59542
67557	1.89226	1.77733
67558	1.89226	2.03942
67559	1.89226	1.43856
67560	1.89226	1.89299
67561	1.89226	1.31882
67562	1.89226	0.980915
67563	1.89226	0.55898
67564	1.89226	0.59561
67565	1.89226	0.726535
67566	1.89226	0.710593
67567	1.89226	1.0165

67568	0.946128	0.580011
67569	0.946128	0.740846
67570	0.946125	0.455831
67571	0.946125	0.591359
67572	0.94613	0.508267
67573	0.94613	0.607707
67574	0.946124	0.643314
67575	0.946124	0.752263
67576	0.946127	0.507213
67577	0.946127	0.474132
67578	0.946127	0.64722
67579	0.946127	0.737562
67580	1.89226	0.44526
67581	2.78671	0.489337
67582	2.59302	0.477442
67583	2.59302	0.466184
67584	1.89226	0.537639
67585	1.89226	0.484425
67586	1.89226	0.524346
67587	1.89226	0.568462
67588	1.89226	0.507586
67589	1.89226	0.494562
67590	1.89226	0.622877
67591	1.89226	0.561616
67592	1.89225	0.478171
67593	1.89225	0.393856
67594	1.89225	0.571057
67595	1.89225	0.4517
67596	1.89225	0.575756
67597	1.89225	0.453202
67598	0.979536	0.442316
67599	0.816597	0.448067
67600	1.15446	0.436247
67601	1.07423	0.395099
67602	1.08452	0.424675
67603	0.603522	0.412438
67604	1.70302	0.412222
67605	1.29344	0.396
67606	1.21806	0.406045
67607	0.723758	0.395944
67608	1.37562	0.446997
67609	1.89226	0.325598
67610	1.92807	0.343836
67611	1.90843	0.340246
67612	1.92807	0.339103
67613	1.87263	0.331038
67614	1.89226	0.32952
67615	0.946132	0.33351
67616	0.946132	0.328961
67617	0.946131	0.333485
67618	0.946131	0.337147
67619	0.946132	0.341689
67620	0.946132	0.339397
67621	0.946132	0.327126
67622	0.946132	0.324788
67623	0.946132	0.330943
67624	0.946132	0.350067
67625	0.946132	0.332211

67626	0.946132	0.340036
67627	1.89226	0.338708
67628	1.89226	0.370399
67629	1.89226	0.350253
67630	1.89226	0.367685
67631	3.78452	0.351782
67632	1.89226	0.336869
67633	1.89226	0.36378
67634	1.89226	0.361615
67635	1.89226	0.35627
67636	1.89226	0.341396
67637	1.89226	0.33792
67638	1.70303	0.321528
67639	1.90843	0.335466
67640	1.77002	0.332571
67641	1.90843	0.331943
67642	1.75709	0.324892
67643	1.89226	0.324266
67644	1.89226	0.319983
67645	1.89226	0.33923
67646	1.89226	0.326646
67647	1.89226	0.334716
67648	3.78453	0.326315
67649	1.89226	0.342746
67650	1.89226	0.362539
67651	1.89226	0.33727
67652	1.89226	0.375563
67653	3.78452	0.358968
67654	0.946131	0.333846
67655	0.946131	0.33892
67656	0.946131	0.338725
67657	0.946131	0.345474
67658	0.946129	0.349191
67659	0.946129	0.347579
67660	0.946133	0.335927
67661	0.946133	0.332271
67662	0.946131	0.352818
67663	0.946131	0.33627
67664	0.946131	0.333252
67665	0.946131	0.349688
67666	1.89226	0.405501
67667	2.10275	0.674035
67668	1.76377	0.638505
67669	1.76377	0.514651
67670	1.89226	0.573654
67671	1.89226	0.463782
67672	1.89226	0.397023
67673	1.89226	0.68812
67674	2.10275	0.657418
67675	2.55889	0.493554
67676	1.89226	0.605565
67677	2.30274	0.469599
67678	1.89226	0.560876
67679	1.89226	0.920944
67680	1.89226	0.881044
67681	1.89226	0.598231
67682	1.89226	0.810429
67683	1.89226	0.577209

67684	1.89226	0.528116
67685	1.89226	0.920157
67686	1.89226	0.828196
67687	1.89226	0.603074
67688	1.89226	0.765354
67689	1.89226	0.578248
67690	1.89226	0.367505
67691	1.89226	0.647258
67692	1.89226	0.613832
67693	1.89226	0.465643
67694	1.89226	0.562414
67695	1.89226	0.449115
67696	1.89226	0.374823
67697	1.89226	0.665455
67698	1.89226	0.628796
67699	1.89226	0.47816
67700	1.89226	0.58737
67701	1.89226	0.465172
67702	1.89226	0.561077
67703	1.89226	0.879957
67704	1.89226	0.825268
67705	1.89226	0.607886
67706	1.89226	0.785391
67707	1.89226	0.595537
67708	1.89226	0.754579
67709	1.89226	0.855573
67710	1.89226	0.543527
67711	1.89226	0.610456
67712	1.89226	0.548686
67713	1.89226	0.777463
67714	0.946131	0.372216
67715	0.976399	0.325418
67716	1.10124	0.22078
67717	0.946131	0.575841
67718	0.887633	0.544938
67719	0.887633	0.446509
67720	1.16933	0.370038
67721	1.23163	0.326849
67722	0.946131	0.420943
67723	0.946131	0.383261
67724	0.976399	0.405096
67725	1.14992	0.409072
67726	1.89226	0.499094
67727	1.89226	0.830632
67728	1.89226	0.457947
67729	1.89226	0.573368
67730	3.78452	0.48032
67731	1.89226	0.349588
67732	1.89226	0.588944
67733	1.89226	0.283336
67734	1.89226	0.424039
67735	3.78453	0.328472
67736	0.946132	0.227216
67737	0.946132	0.477069
67738	0.946131	0.33445
67739	0.946131	0.469873
67740	0.946131	0.460534
67741	0.946131	0.380441

67742	0.946132	0.193604
67743	0.946132	0.218763
67744	0.946131	0.276809
67745	0.946131	0.310547
67746	0.946132	0.319221
67747	0.946132	0.242218
67748	0.946132	0.562873
67749	0.946132	0.357066
67750	0.946131	0.372192
67751	0.946131	0.628854
67752	0.946131	0.630672
67753	0.946131	0.535443
67754	0.946132	0.406387
67755	0.946132	0.333476
67756	0.946131	0.457322
67757	0.946131	0.499725
67758	0.946132	0.492008
67759	0.946132	0.45215
67760	1.89226	0.572577
67761	1.89226	0.668078
67762	1.89226	0.408681
67763	1.89226	0.720572
67764	3.78452	0.645149
67765	1.89226	0.712163
67766	1.89226	0.659443
67767	1.89226	0.58857
67768	1.89226	0.525097
67769	3.78453	0.562908
67770	1.89226	0.509432
67771	1.89226	0.688673
67772	1.89226	0.599484
67773	1.89226	0.714604
67774	1.89226	0.506555
67775	1.89226	0.571523
67776	1.89225	0.471287
67777	1.89225	0.56214
67778	1.89225	0.536882
67779	1.89225	0.674276
67780	1.89225	0.443332
67781	1.89225	0.507161
67782	1.89226	0.513059
67783	1.89226	0.603593
67784	1.89226	0.559404
67785	1.89226	0.650025
67786	1.89226	0.471127
67787	1.89226	0.516856
67788	1.89226	0.709811
67789	1.89226	0.533996
67790	1.89226	0.456216
67791	1.89226	0.439348
67792	3.78453	0.482711
67793	1.39491	0.490603
67794	1.39491	0.490069
67795	0.924649	0.501365
67796	0.924649	0.505562
67797	0.946131	0.522108
67798	1.37343	0.426246
67799	0.924678	0.225309

67800	0.946131	0.782317
67801	0.924678	0.484399
67802	1.37343	0.4294
67803	0.946131	0.613954
67804	0.946131	0.580656
67805	1.89226	0.525546
67806	1.89226	0.408164
67807	1.89226	0.465205
67808	1.89226	0.507049
67809	1.89226	0.410813
67810	1.89226	0.444163
67811	0.946126	0.0923622
67812	0.975461	0.0894875
67813	0.946132	0.067347
67814	1.12674	0.119254
67815	0.946129	0.123549
67816	0.946129	0.114154
67817	1.12673	0.0931056
67818	1.12673	0.0890892
67819	0.946129	0.0978641
67820	0.946129	0.106845
67821	0.975464	0.108876
67822	1.12673	0.105813
67823	1.89226	0.114635
67824	1.89226	0.0949997
67825	1.89226	0.141748
67826	1.89226	0.103136
67827	1.89226	0.121943
67828	1.89226	0.100749
67829	0.946126	0.113376
67830	0.946126	0.136496
67831	0.946131	0.0954167
67832	0.946131	0.205289
67833	0.946129	0.213255
67834	0.946129	0.1871
67835	0.946128	0.125093
67836	0.946128	0.116593
67837	0.946129	0.153075
67838	0.946129	0.180392
67839	0.946128	0.164252
67840	0.946128	0.162555
67841	1.89226	0.100101
67842	1.89226	0.185855
67843	1.89226	0.0886177
67844	1.89226	0.106939
67845	3.78451	0.0908026
67846	1.89226	0.137096
67847	1.89226	0.135882
67848	1.89226	0.0810489
67849	1.89226	0.142472
67850	3.78452	0.146115
67851	0.946125	0.122703
67852	0.946125	0.155715
67853	0.946131	0.118487
67854	0.946131	0.225816
67855	0.946128	0.239958
67856	0.946128	0.219742
67857	0.946128	0.16565

67858	0.946128	0.121114
67859	0.946128	0.158344
67860	0.946128	0.187578
67861	0.946128	0.155772
67862	0.946128	0.184172
67863	1.89226	0.33417
67864	1.89226	0.386578
67865	1.89226	0.394992
67866	1.89226	0.350536
67867	3.78452	0.357749
67868	1.89226	0.337985
67869	1.89226	0.385195
67870	1.89226	0.361011
67871	1.89226	0.383875
67872	1.89226	0.352812
67873	1.89226	0.373715
67874	1.12492	0.402674
67875	1.41201	0.406893
67876	1.12082	0.367532
67877	0.771416	0.365825
67878	1.10725	0.373155
67879	1.10725	0.380294
67880	1.07618	0.475829
67881	1.07618	0.345441
67882	1.12492	0.39221
67883	0.771415	0.403537
67884	1.12082	0.373324
67885	1.41201	0.385759
67886	2.82401	0.359883
67887	1.89226	0.422708
67888	1.89226	0.363431
67889	1.89226	0.388803
67890	2.24983	0.380365
67891	2.24983	0.401035
67892	0.946133	0.40625
67893	0.946133	0.400509
67894	0.946129	0.37142
67895	0.946129	0.371382
67896	0.946131	0.386362
67897	0.946131	0.411161
67898	0.946131	0.415689
67899	0.946131	0.348159
67900	0.946131	0.379185
67901	0.946131	0.393887
67902	0.946131	0.377524
67903	0.946131	0.389888
67904	0.946132	0.394482
67905	0.946132	0.388593
67906	0.946133	0.373571
67907	0.946133	0.370523
67908	0.946132	0.383052
67909	0.946132	0.391715
67910	0.946133	0.378996
67911	0.946133	0.383287
67912	0.946132	0.390348
67913	0.946132	0.375555
67914	0.946133	0.38459
67915	0.946133	0.373319

67916	1.89227	0.429165
67917	1.89227	0.564864
67918	1.89227	0.577414
67919	1.89227	0.398023
67920	1.89227	0.593307
67921	1.89227	0.414179
67922	1.42982	0.144818
67923	1.19944	0.0960117
67924	0.936311	0.239891
67925	0.936311	0.14645
67926	0.946132	0.168398
67927	1.02993	0.172761
67928	1.41513	0.194673
67929	0.946133	0.217904
67930	0.946133	0.162838
67931	0.946133	0.228298
67932	1.02993	0.210856
67933	1.18181	0.153766
67934	1.89226	0.481425
67935	1.89226	0.663329
67936	1.89226	0.742305
67937	1.89226	0.346625
67938	3.78453	0.510603
67939	1.89226	0.200412
67940	1.89226	0.29451
67941	1.89226	0.301926
67942	1.89226	0.222951
67943	1.89226	0.269907
67944	1.89226	0.19613
67945	1.89226	0.212664
67946	1.89226	0.259691
67947	1.89226	0.288853
67948	1.89226	0.211147
67949	1.89226	0.280458
67950	1.89226	0.204216
67951	0.946131	0.356251
67952	0.946131	0.497921
67953	0.946132	0.654001
67954	0.946132	0.572249
67955	0.946132	0.422979
67956	0.946132	0.525809
67957	0.946132	0.678599
67958	0.946132	0.368001
67959	0.946132	0.447953
67960	0.946132	0.50046
67961	0.946132	0.495617
67962	0.946132	0.365332
67963	1.89226	1.00678
67964	1.89226	1.06928
67965	1.89226	1.29194
67966	1.89226	0.865158
67967	1.89226	1.28893
67968	1.89226	0.861095
67969	1.89226	1.24236
67970	2.22749	0.723785
67971	1.89226	0.786727
67972	1.89226	0.877742
67973	3.78453	0.892

67974	1.89226	1.0455
67975	1.89226	1.14774
67976	1.89226	1.31911
67977	1.89226	0.822552
67978	3.78453	0.953157
67979	1.89226	0.818393
67980	1.89226	0.876007
67981	1.89226	1.03414
67982	1.89226	0.711421
67983	1.89226	1.06366
67984	1.89226	0.732867
67985	0.94613	0.687266
67986	0.94613	0.956154
67987	0.94613	1.21816
67988	0.94613	0.880783
67989	0.946131	0.818353
67990	0.946131	0.69971
67991	0.946129	1.13462
67992	0.946129	0.815989
67993	0.94613	0.840821
67994	0.94613	0.874794
67995	0.94613	0.825295
67996	0.94613	0.781407
67997	1.89226	0.111823
67998	1.89226	0.111719
67999	1.89226	0.112683
68000	1.89226	0.100789
68001	1.89226	0.118912
68002	1.89226	0.107222
68003	1.89226	0.122612
68004	1.89226	0.195426
68005	1.89226	0.172531
68006	1.89226	0.0879992
68007	3.78452	0.129912
68008	1.89225	0.0758191
68009	1.89225	0.0747408
68010	1.89225	0.0746332
68011	1.89225	0.0677513
68012	1.89225	0.0847888
68013	1.89225	0.0786199
68014	1.89226	0.0635732
68015	1.89226	0.0614871
68016	1.89226	0.067503
68017	1.89226	0.0616739
68018	1.89226	0.0697881
68019	1.89226	0.0649149
68020	0.946132	0.0920779
68021	0.946132	0.131168
68022	0.946132	0.191201
68023	0.946132	0.121373
68024	0.946131	0.102336
68025	0.946131	0.0944054
68026	0.946132	0.200146
68027	0.946132	0.0903709
68028	0.946132	0.100077
68029	0.946132	0.116468
68030	0.946132	0.114733
68031	0.946132	0.100299

68032	1.89226	0.242893
68033	1.89226	0.37187
68034	1.89226	0.242667
68035	1.89226	0.440876
68036	1.89226	0.215649
68037	1.89226	0.409173
68038	1.89226	0.305308
68039	1.89226	0.223428
68040	1.89226	0.275909
68041	1.89226	0.387892
68042	1.89226	0.141693
68043	1.89226	0.332142
68044	1.89226	0.144156
68045	1.89226	0.214032
68046	1.89226	0.20199
68047	1.89226	0.264446
68048	1.89226	0.114988
68049	1.89226	0.160892
68050	1.89226	0.248594
68051	1.89226	0.369847
68052	1.89226	0.212893
68053	1.89226	0.410775
68054	3.78452	0.260997
68055	1.89226	0.208944
68056	1.89226	0.275071
68057	1.89226	0.212612
68058	1.89226	0.418305
68059	1.89226	0.169861
68060	1.89226	0.350731
68061	1.89226	0.05
68062	1.89226	0.0524731
68063	1.89226	0.05
68064	1.89226	0.05
68065	3.78453	0.05
68066	1.89226	0.05
68067	2.48862	0.05
68068	1.89226	0.05
68069	2.22062	0.05
68070	1.89226	0.05
68071	2.22062	0.05
68072	1.89227	0.05
68073	1.89227	0.0597337
68074	1.89227	0.05
68075	1.89227	0.05
68076	1.89227	0.0546612
68077	1.89227	0.05
68078	0.946132	0.05
68079	0.946132	0.05
68080	0.946131	0.05
68081	0.946131	0.0532124
68082	0.94613	0.0537381
68083	0.94613	0.05
68084	0.946133	0.05
68085	0.946133	0.05
68086	0.946131	0.05
68087	0.946131	0.05
68088	0.946131	0.05
68089	0.946131	0.0501972

68090	1.89226	0.05
68091	1.89226	0.05
68092	1.89226	0.05
68093	1.89226	0.05
68094	3.78453	0.05
68095	1.89226	0.05
68096	1.89226	0.0672922
68097	1.89226	0.0542857
68098	1.89226	0.05
68099	3.78453	0.05
68100	1.89226	0.42852
68101	1.89226	0.393081
68102	1.89226	0.384088
68103	1.89226	0.435107
68104	1.89226	0.396717
68105	1.89226	0.439112
68106	1.89226	0.434136
68107	1.89226	0.390413
68108	1.89226	0.389736
68109	1.89226	0.467218
68110	3.78452	0.414153
68111	1.89226	0.358831
68112	1.89226	0.4227
68113	1.89226	0.427563
68114	1.89226	0.393129
68115	3.78453	0.394816
68116	2.57454	0.43317
68117	1.89226	0.458207
68118	1.3663	0.450403
68119	1.89226	0.435392
68120	3.25856	0.440173
68121	1.89226	0.439691
68122	1.89226	0.40811
68123	1.89226	0.403104
68124	1.89226	0.458806
68125	3.78451	0.421787
68126	1.89226	0.40498
68127	1.89226	0.499016
68128	1.89226	0.408585
68129	1.89226	0.451592
68130	1.89226	0.409227
68131	1.89226	0.451466
68132	0.810946	0.429119
68133	0.810946	0.420916
68134	0.810946	0.446821
68135	0.810946	0.479594
68136	0.810959	0.44023
68137	0.810959	0.417213
68138	1.89226	0.455197
68139	1.35168	0.436958
68140	1.08132	0.417817
68141	1.08132	0.480571
68142	1.0813	0.435884
68143	1.89226	0.358406
68144	1.89226	0.433299
68145	1.89226	0.568211
68146	1.89226	0.449856
68147	3.78453	0.423622

68148	1.89226	0.438112
68149	1.89226	0.403468
68150	1.89226	0.440722
68151	1.89226	0.556096
68152	1.89226	0.363969
68153	1.89226	0.417542
68154	1.89226	0.402609
68155	1.89226	0.463837
68156	1.89226	0.557289
68157	1.89226	0.456915
68158	3.78452	0.439806
68159	1.89226	0.431929
68160	1.89226	0.435989
68161	1.89226	0.456905
68162	1.89226	0.637695
68163	3.78452	0.463048
68164	2.142	0.647384
68165	1.93949	0.39467
68166	1.39296	0.606187
68167	2.55711	0.740802
68168	1.39296	0.382593
68169	2.55711	0.449056
68170	1.89226	0.466693
68171	2.5571	0.380461
68172	2.142	0.481251
68173	1.89226	0.742844
68174	4.44936	0.488217
68175	0.946131	0.496516
68176	0.946131	0.499645
68177	1.27855	0.5123
68178	1.29171	0.476805
68179	1.29171	0.538265
68180	0.946127	0.490102
68181	1.27855	0.380841
68182	0.946131	0.681968
68183	1.29172	0.542536
68184	1.29172	0.460359
68185	0.946129	0.420293
68186	0.946129	0.454952
68187	1.27855	0.540313
68188	1.29172	0.504586
68189	0.969743	0.569752
68190	1.07109	0.535486
68191	1.29172	0.907577
68192	1.07109	0.429571
68193	0.969745	0.393194
68194	1.27855	0.700429
68195	1.10873	0.668653
68196	1.10873	0.740027
68197	1.15273	0.433074
68198	1.15273	0.460695
68199	1.89227	0.318779
68200	1.89227	0.322453
68201	1.89227	0.328202
68202	1.89227	0.321137
68203	2.1288	0.317728
68204	2.1288	0.308289
68205	1.89227	0.331444

68206	1.89227	0.33746
68207	1.89227	0.324728
68208	1.89227	0.334383
68209	3.78453	0.331651
68210	1.89226	0.344815
68211	1.89226	0.355149
68212	1.89226	0.334985
68213	1.89226	0.342684
68214	3.78453	0.338312
68215	1.89226	0.328433
68216	1.89226	0.337032
68217	1.89226	0.324999
68218	1.89226	0.323573
68219	1.89226	0.331471
68220	1.89226	0.338473
68221	1.94432	0.219084
68222	1.89226	0.3213
68223	2.07911	0.228716
68224	1.89226	0.257606
68225	2.07911	0.252906
68226	1.89226	0.288022
68227	1.89226	0.323686
68228	1.89226	0.296097
68229	1.89226	0.33629
68230	1.89226	0.288476
68231	1.89226	0.326504
68232	1.89226	0.330837
68233	1.89226	0.681091
68234	1.89226	0.696601
68235	1.89226	0.787164
68236	1.89226	0.587356
68237	1.89226	0.811863
68238	1.89226	0.611454
68239	1.89226	0.44074
68240	1.89226	0.436101
68241	1.89226	0.535256
68242	1.89226	0.330057
68243	1.89226	0.614302
68244	1.89226	0.410682
68245	1.86778	0.321993
68246	2.72348	0.304624
68247	1.89226	0.414344
68248	1.89226	0.141464
68249	3.76004	0.328142
68250	1.89226	0.673892
68251	1.89226	0.703721
68252	1.89226	0.86091
68253	1.89226	0.561799
68254	3.78451	0.65805
68255	1.89226	0.471397
68256	1.89226	0.50688
68257	1.89226	0.576146
68258	1.89226	0.359411
68259	1.89226	0.630356
68260	1.89226	0.436397
68261	0.94613	0.253303
68262	0.94613	0.384693
68263	0.94613	0.601733

68264	0.94613	0.349068
68265	0.946131	0.401141
68266	0.946131	0.17502
68267	0.946129	0.464086
68268	0.946129	0.33859
68269	0.94613	0.34618
68270	0.94613	0.421378
68271	0.94613	0.331043
68272	0.94613	0.371642
68273	0.946124	0.549756
68274	0.946124	0.276295
68275	0.94613	0.703339
68276	0.94613	0.566127
68277	0.946128	0.430442
68278	0.946128	0.504378
68279	0.946126	0.700064
68280	0.946126	0.550548
68281	0.946127	0.55504
68282	0.946127	0.570511
68283	0.946127	0.531353
68284	0.946127	0.465386
68285	2.09166	0.0701826
68286	2.1285	0.0960191
68287	1.78381	0.0972907
68288	1.78381	0.108424
68289	2.47689	0.0708149
68290	2.47689	0.0804871
68291	2.09167	0.0676674
68292	2.26572	0.102794
68293	2.73393	0.0640344
68294	2.4769	0.095659
68295	4.74261	0.091899
68296	1.89226	0.0936086
68297	1.89226	0.167381
68298	1.89226	0.129141
68299	1.89226	0.10283
68300	1.89226	0.154148
68301	1.89226	0.136666
68302	0.946131	0.115394
68303	0.946131	0.202282
68304	0.946129	0.125866
68305	0.946129	0.177794
68306	0.94613	0.164527
68307	0.94613	0.169448
68308	0.946131	0.0975453
68309	0.946131	0.100501
68310	0.94613	0.120138
68311	0.94613	0.132461
68312	0.946131	0.122001
68313	0.946131	0.136266
68314	2.27622	0.0955895
68315	2.66786	0.0675236
68316	2.1285	0.0938766
68317	2.1285	0.103459
68318	2.62461	0.0686563
68319	2.47689	0.0963012
68320	1.89226	0.0977545
68321	1.89226	0.162217

68322	1.89226	0.0990869
68323	1.89226	0.155315
68324	3.78453	0.141322
68325	1.89226	0.100009
68326	1.89226	0.158342
68327	1.89226	0.147121
68328	1.89226	0.174161
68329	1.89226	0.0960581
68330	1.89226	0.119949
68331	1.89226	0.692341
68332	1.89226	0.886482
68333	1.89226	0.705915
68334	1.89226	0.833341
68335	1.89226	0.715836
68336	1.89226	0.849112
68337	1.89226	0.941515
68338	1.89226	0.738679
68339	1.89226	0.730134
68340	1.89226	0.992885
68341	3.78452	0.823046
68342	1.89227	0.730486
68343	1.89227	0.969567
68344	1.89227	0.927394
68345	1.89227	0.820952
68346	3.78453	0.87006
68347	0.946127	0.978946
68348	0.946127	0.869397
68349	1.06438	1.47163
68350	0.946131	0.800476
68351	1.06438	0.983797
68352	0.94613	1.03866
68353	0.946128	0.885829
68354	0.946128	0.763374
68355	1.06438	0.969455
68356	1.06438	1.37986
68357	0.946129	0.938699
68358	0.946129	0.899613
68359	1.89226	0.751864
68360	1.89226	0.934175
68361	1.89226	0.824911
68362	1.89226	0.91407
68363	1.89226	0.852509
68364	1.89226	0.952936
68365	0.946133	0.89113
68366	0.946133	0.832984
68367	1.39771	1.11014
68368	0.946132	0.787889
68369	1.39771	1.06233
68370	1.39772	0.821965
68371	0.946133	0.856301
68372	0.946133	0.767226
68373	0.946132	1.01312
68374	1.39772	1.12825
68375	0.946132	0.971801
68376	0.946132	0.860114
68377	1.98182	0.0537673
68378	2.2217	0.0731324
68379	2.79889	0.0507002

68380	2.50097	0.0620024
68381	4.72267	0.0544085
68382	2.05362	0.05
68383	1.70302	0.0557995
68384	2.75109	0.0538997
68385	2.138 0.05	
68386	2.0815	0.0576006
68387	1.84751	0.0560266
68388	0.946131	0.0789328
68389	0.946131	0.0614174
68390	0.946131	0.0544489
68391	0.946131	0.0751731
68392	0.946131	0.0801316
68393	0.946131	0.0742234
68394	0.946132	0.0551102
68395	0.946132	0.0584528
68396	0.946131	0.0631878
68397	0.946131	0.0636558
68398	0.946132	0.0651549
68399	0.946132	0.0688665
68400	1.89226	0.0735405
68401	1.89226	0.0998684
68402	1.89226	0.0630628
68403	1.89226	0.0978703
68404	3.78451	0.092747
68405	0.946126	0.0587129
68406	1.25048	0.05
68407	0.946131	0.05
68408	1.39945	0.0626042
68409	0.946127	0.0674019
68410	0.946127	0.0659834
68411	1.39944	0.05
68412	1.39944	0.05
68413	0.946129	0.0572273
68414	0.946129	0.0597354
68415	1.39944	0.0563321
68416	1.25048	0.0587763
68417	1.89226	0.0544111
68418	1.89226	0.086367
68419	1.89226	0.0582093
68420	1.89226	0.0683122
68421	1.89226	0.062287
68422	1.89226	0.0752567
68423	0.946126	0.101378
68424	0.946126	0.0756879
68425	0.946131	0.0656039
68426	0.946131	0.0903089
68427	0.946128	0.0972324
68428	0.946128	0.092488
68429	0.946129	0.0685421
68430	0.946129	0.0675688
68431	0.946129	0.0762694
68432	0.946129	0.0767071
68433	0.946128	0.0829849
68434	0.946128	0.083473
68435	1.89226	0.05
68436	1.89226	0.0593008
68437	1.89226	0.05

68438	1.89226	0.05
68439	1.89226	0.05
68440	1.89226	0.05
68441	1.01393	0.05
68442	0.775245	0.05
68443	0.946126	0.05
68444	0.946126	0.05
68445	0.78305	0.05
68446	0.78305	0.05
68447	0.946159	0.05
68448	0.929267	0.05
68449	1.14956	0.05
68450	1.04073	0.05
68451	0.851526	0.05
68452	0.704781	0.05
68453	1.89226	0.05
68454	1.89226	0.054918
68455	1.89226	0.05
68456	1.89226	0.05
68457	3.78451	0.05
68458	1.89226	0.05
68459	1.89226	0.0749724
68460	1.89226	0.0527343
68461	1.89226	0.05
68462	1.89226	0.0610774
68463	1.89226	0.0566547
68464	1.89226	0.05
68465	1.89226	0.0588736
68466	1.89226	0.05
68467	1.89226	0.05
68468	1.89226	0.05
68469	1.89226	0.0507407
68470	1.16317	0.05
68471	1.0292	0.05
68472	0.946126	0.05
68473	0.946126	0.05
68474	0.93562	0.05
68475	0.93562	0.05
68476	1.0418	0.05
68477	1.04181	0.05
68478	1.16317	0.05
68479	0.946129	0.05
68480	1.02919	0.05
68481	0.946128	0.05
68482	0.946131	0.05
68483	0.946131	0.05
68484	0.946126	0.05
68485	0.946126	0.0506899
68486	0.946129	0.050793
68487	0.946129	0.05
68488	0.946128	0.05
68489	0.946128	0.05
68490	0.946129	0.05
68491	0.946129	0.05
68492	0.946128	0.05
68493	0.946128	0.05
68494	1.89226	0.05
68495	1.89226	0.0753649

68496	1.89226	0.054362
68497	1.89226	0.0548694
68498	1.89226	0.0600292
68499	1.89226	0.0606536
68500	1.89226	0.0518954
68501	1.89226	0.05
68502	1.89226	0.05
68503	1.89226	0.05
68504	1.89226	0.05
68505	1.89226	0.05
68506	1.89226	0.0534976
68507	1.89226	0.05
68508	1.89226	0.05
68509	1.89226	0.05
68510	3.78453	0.05
68511	1.89226	0.05
68512	1.89226	0.0507694
68513	1.89226	0.05
68514	1.89226	0.05
68515	3.78452	0.05
68516	1.89225	0.0521995
68517	1.89225	0.051067
68518	1.89225	0.05
68519	1.89225	0.05
68520	1.89225	0.05
68521	1.89225	0.05
68522	0.946132	0.05
68523	0.946132	0.05
68524	0.946133	0.05
68525	0.946133	0.05
68526	0.946132	0.05
68527	0.946132	0.05
68528	0.946132	0.05
68529	0.946132	0.05
68530	0.946133	0.05
68531	0.946133	0.05
68532	0.946132	0.05
68533	0.946132	0.05
68534	1.89226	0.05
68535	1.89226	0.0560836
68536	1.89226	0.05
68537	1.89226	0.05
68538	3.78453	0.05
68539	1.13537	0.05
68540	1.09222	0.05
68541	0.946126	0.05
68542	0.946126	0.05
68543	0.946128	0.05
68544	0.946128	0.05
68545	1.16317	0.05
68546	1.13034	0.05
68547	1.30155	0.05
68548	1.08462	0.05
68549	0.992915	0.05
68550	0.807642	0.05
68551	0.946131	0.05
68552	0.946131	0.05
68553	0.946125	0.05

68554	0.946125	0.0506729
68555	0.946128	0.0501467
68556	0.946128	0.05
68557	0.946128	0.05
68558	0.946128	0.05
68559	0.946128	0.05
68560	0.946128	0.05
68561	0.946128	0.05
68562	0.946128	0.05
68563	1.89226	0.05
68564	1.89226	0.0723793
68565	1.89226	0.056021
68566	1.89226	0.0579273
68567	1.89226	0.0559237
68568	1.89226	0.0593509
68569	1.89224	0.05
68570	1.89224	0.05
68571	1.89224	0.0529554
68572	1.89224	0.05
68573	1.89224	0.0536696
68574	1.89224	0.05
68575	0.946086	0.05
68576	1.17919	0.05
68577	0.89738	0.05
68578	0.89738	0.05
68579	0.946116	0.05
68580	0.946116	0.05
68581	1.13048	0.05
68582	1.11844	0.05
68583	0.946128	0.05
68584	0.897362	0.05
68585	1.1282	0.05
68586	0.946103	0.05
68587	1.89223	0.05
68588	1.89223	0.0523458
68589	1.89223	0.05
68590	1.89223	0.05
68591	3.78446	0.05
68592	0.946118	0.0555683
68593	0.946118	0.05
68594	0.946118	0.059532
68595	0.946118	0.0707618
68596	0.946119	0.0689785
68597	0.946119	0.054455
68598	0.946118	0.0524746
68599	0.946118	0.0593684
68600	0.946131	0.0606171
68601	0.946131	0.0574151
68602	0.946106	0.0587777
68603	0.946106	0.058372
68604	1.89227	0.722776
68605	1.89227	0.786628
68606	1.89227	0.757083
68607	1.89227	0.681513
68608	3.78453	0.67252
68609	1.89226	0.79298
68610	1.89226	0.602623
68611	1.89226	0.594693

68612	1.89226	0.6245
68613	1.89226	0.735121
68614	1.89226	0.778249
68615	1.89226	0.53514
68616	2.59378	0.490864
68617	2.30348	0.481017
68618	2.30348	0.516641
68619	1.89226	0.528602
68620	1.89226	0.573869
68621	1.89226	0.694193
68622	1.89226	0.605979
68623	1.89226	0.618739
68624	1.89226	0.614216
68625	3.78453	0.697459
68626	1.89226	0.614642
68627	1.89226	0.550883
68628	1.89226	0.537482
68629	1.89226	0.580184
68630	1.89226	0.632118
68631	1.89226	0.689583
68632	0.904406	0.492448
68633	1.01667	0.509115
68634	1.11974	0.490742
68635	0.994417	0.513848
68636	1.02013	0.500221
68637	0.628203	0.528037
68638	1.70302	0.518498
68639	1.49898	0.496056
68640	1.14559	0.510085
68641	0.900571	0.501509
68642	1.58886	0.512507
68643	2.81102	0.05
68644	2.3923	0.05
68645	2.05632	0.05
68646	2.43514	0.0562595
68647	4.82744	0.05
68648	1.89226	0.0640048
68649	1.89226	0.0679827
68650	1.89226	0.0665206
68651	1.89226	0.0540916
68652	1.89226	0.0804123
68653	1.89226	0.0686788
68654	1.89226	0.0706053
68655	1.89226	0.0829001
68656	1.89226	0.0688666
68657	1.89226	0.0924518
[truncated: 615,113 more chars]
